# Supplementary material for: A genome-wide CRISPR knockout screen identified host genes essential for Brucella invasion and intracellular survival
Source: Emerg Microbes Infect. 2026 Aug 19;15(1):2713322. doi: 10.1080/22221751.2026.2713322 (PMC13491901; doi:10.1080/22221751.2026.2713322)
Supplement: Supplementary file 1 [file TEMI_A_2713322_SM6411.pdf]

**Table S1 SgRNAs enriched in the sorted RED-negative cells (1st)**

| gene | unique.id | count | Zscore | name | Description |
|---|---|---|---|---|---|
| ENSG00000166710 | HGLibA_03944 | 1342 | 12.20569027 | B2M | beta-2-microglobulin [Source:HGNC Symbol;Acc:HGNC:914] |
| ENSG00000172292 | HGLibA_09216 | 1336 | 12.14598404 | CERS6 | ceramide synthase 6 [Source:HGNC Symbol;Acc:HGNC:23826] |
| ENSG00000134460 | HGLibA_23119 | 1141 | 10.20553174 | IL2RA | interleukin 2 receptor subunit alpha [Source:HGNC Symbol;Acc:HGNC:6008] |
| ENSG00000172292 | HGLibA_09214 | 1139 | 10.18562967 | CERS6 | ceramide synthase 6 [Source:HGNC Symbol;Acc:HGNC:23826] |
| ENSG00000100079 | HGLibA_26361 | 1087 | 9.668175722 | LGALS2 | galectin 2 [Source:HGNC Symbol;Acc:HGNC:6562] |
| ENSG00000131828 | HGLibA_35861 | 993 | 8.732778204 | PDHA1 | pyruvate dehydrogenase E1 alpha 1 subunit [Source:HGNC Symbol;Acc:HGNC:8806] |
| ENSG00000241852 | HGLibA_06637 | 974 | 8.543708493 | C8orf58 | chromosome 8 open reading frame 58 [Source:HGNC Symbol;Acc:HGNC:32233] |
| ENSG00000262246 | HGLibA_10791 | 930 | 8.105862846 | CORO7 | coronin 7 [Source:HGNC Symbol;Acc:HGNC:26161] |
| ENSG00000165175 | HGLibA_29235 | 916 | 7.966548322 | MID1IP1 | MID1 interacting protein 1 [Source:HGNC Symbol;Acc:HGNC:20715] |
| ENSG00000198178 | HGLibA_09982 | 910 | 7.906842098 | CLEC4C | C-type lectin domain family 4 member C [Source:HGNC Symbol;Acc:HGNC:13258] |
| ENSG00000134108 | HGLibA_02999 | 881 | 7.618262012 | ARL8B | ADP ribosylation factor like GTPase 8B [Source:HGNC Symbol;Acc:HGNC:25564] |
| ENSG00000100335 | HGLibA_29242 | 853 | 7.339632964 | MIEF1 | mitochondrial elongation factor 1 [Source:HGNC Symbol;Acc:HGNC:25979] |
| ENSG00000090686 | HGLibA_53382 | 844 | 7.250073628 | USP48 | ubiquitin specific peptidase 48 [Source:HGNC Symbol;Acc:HGNC:18533] |
| ENSG00000138439 | HGLibA_16044 | 839 | 7.200318441 | FAM117B | family with sequence similarity 117 member B [Source:HGNC Symbol;Acc:HGNC:14440] |
| ENSG00000081377 | HGLibA_08512 | 829 | 7.100808066 | CDC14B | cell division cycle 14B [Source:HGNC Symbol;Acc:HGNC:1719] |
| ENSG00000138074 | HGLibA_45278 | 826 | 7.070954954 | SLC5A6 | solute carrier family 5 member 6 [Source:HGNC Symbol;Acc:HGNC:11041] |
| ENSG00000070182 | HGLibA_46877 | 808 | 6.89183628 | SPTB | erythrocytic [Source:HGNC Symbol;Acc:HGNC:11274]" |
| FAM109A | HGLibA_16005 | 808 | 6.89183628 | FAM109A | - |
| ENSG00000149743 | HGLibA_51779 | 806 | 6.871934206 | TRPT1 | tRNA phosphotransferase 1 [Source:HGNC Symbol;Acc:HGNC:20316] |
| ATHL1 | HGLibA_03508 | 793 | 6.742570719 | ATHL1 | - |
| ENSG00000054967 | HGLibA_40828 | 787 | 6.682864494 | RELT | TNF receptor [Source:HGNC Symbol;Acc:HGNC:13764]" |
| ENSG00000164808 | HGLibA_46663 | 768 | 6.493794783 | SPIDR | scaffold protein involved in DNA repair [Source:HGNC Symbol;Acc:HGNC:28971] |
| ENSG00000145107 | HGLibA_49698 | 758 | 6.394284409 | TM4SF19 | transmembrane 4 L six family member 19 [Source:HGNC Symbol;Acc:HGNC:25167] |
| ENSG00000187175 | HGLibA_25629 | 756 | 6.374382334 | KRTAP12-1 | keratin associated protein 12-1 [Source:HGNC Symbol;Acc:HGNC:20529] |
| ENSG00000101846 | HGLibA_47604 | 744 | 6.254969885 | STS | steroid sulfatase [Source:HGNC Symbol;Acc:HGNC:11425] |
| ENSG00000100744 | HGLibA_20433 | 740 | 6.215165736 | GSKIP | GSK3B interacting protein [Source:HGNC Symbol;Acc:HGNC:20343] |
| ENSG00000078900 | HGLibA_51095 | 732 | 6.135557436 | TP73 | tumor protein p73 [Source:HGNC Symbol;Acc:HGNC:12003] |
| hsa-mir-492 | HGLibA_61369 | 731 | 6.125606399 | hsa-mir-492 | - |
| ENSG00000114904 | HGLibA_31652 | 729 | 6.105704324 | NEK4 | NIMA related kinase 4 [Source:HGNC Symbol;Acc:HGNC:11399] |
| ENSG00000176142 | HGLibA_50385 | 729 | 6.105704324 | TMEM39A | transmembrane protein 39A [Source:HGNC Symbol;Acc:HGNC:25600] |
| ENSG00000154252 | HGLibA_18500 | 725 | 6.065900174 | GAL3ST2 | galactose-3-O-sulfotransferase 2 [Source:HGNC Symbol;Acc:HGNC:24869] |
| ENSG00000175084 | HGLibA_13005 | 723 | 6.045998099 | DES | desmin [Source:HGNC Symbol;Acc:HGNC:2770] |
| TMEM5 | HGLibA_50419 | 722 | 6.036047062 | TMEM5 | - |
| LOC147646 | HGLibA_26887 | 717 | 5.986291875 | LOC147646 | - |
| ENSG00000145861 | HGLibA_05939 | 704 | 5.856928388 | C1QTNF2 | C1q and TNF related 2 [Source:HGNC Symbol;Acc:HGNC:14325] |
| ENSG00000149599 | HGLibA_14146 | 702 | 5.837026313 | DUSP15 | dual specificity phosphatase 15 [Source:HGNC Symbol;Acc:HGNC:16236] |
| ENSG00000103494 | HGLibA_41840 | 687 | 5.687760752 | RPGRIP1L | RPGRIP1 like [Source:HGNC Symbol;Acc:HGNC:29168] |
| ENSG00000182853 | HGLibA_53691 | 687 | 5.687760752 | VMO1 | vitelline membrane outer layer 1 homolog [Source:HGNC Symbol;Acc:HGNC:30387] |
| ENSG00000197128 | HGLibA_56710 | 686 | 5.677809715 | ZNF772 | zinc finger protein 772 [Source:HGNC Symbol;Acc:HGNC:33106] |
| ENSG00000196154 | HGLibA_42556 | 683 | 5.647956602 | S100A4 | S100 calcium binding protein A4 [Source:HGNC Symbol;Acc:HGNC:10494] |
| ENSG00000214357 | HGLibA_31725 | 680 | 5.61810349 | NEURL1B | neuralized E3 ubiquitin protein ligase 1B [Source:HGNC Symbol;Acc:HGNC:35422] |
| ENSG00000107821 | HGLibA_24088 | 674 | 5.558397265 | KAZALD1 | Kazal type serine peptidase inhibitor domain 1 [Source:HGNC Symbol;Acc:HGNC:25460] |
| ENSG00000128422 | HGLibA_25435 | 673 | 5.548446228 | KRT17 | keratin 17 [Source:HGNC Symbol;Acc:HGNC:6427] |
| ENSG00000145012 | HGLibA_27110 | 672 | 5.538495191 | LPP | LIM domain containing preferred translocation partner in lipoma [Source:HGNC Symbol;Acc:HGNC:6679] |
| ENSG00000152359 | HGLibA_37450 | 672 | 5.538495191 | POC5 | POC5 centriolar protein [Source:HGNC Symbol;Acc:HGNC:26658] |
| ENSG00000006059 | HGLibA_25479 | 670 | 5.518593116 | KRT33A | keratin 33A [Source:HGNC Symbol;Acc:HGNC:6450] |
| ENSG00000173227 | HGLibA_48053 | 667 | 5.488740004 | SYT12 | synaptotagmin 12 [Source:HGNC Symbol;Acc:HGNC:18381] |
| ENSG00000179869 | HGLibA_00103 | 665 | 5.468837929 | ABCA13 | ATP binding cassette subfamily A member 13 [Source:HGNC Symbol;Acc:HGNC:14638] |
| ENSG00000184470 | HGLibA_52448 | 665 | 5.468837929 | TXNRD2 | thioredoxin reductase 2 [Source:HGNC Symbol;Acc:HGNC:18155] |
| ENSG00000148408 | HGLibA_06877 | 664 | 5.458886891 | CACNA1B | calcium voltage-gated channel subunit alpha1 B [Source:HGNC Symbol;Acc:HGNC:1389] |
| ENSG00000255582 | HGLibA_33487 | 663 | 5.448935854 | OR10G2 | olfactory receptor family 10 subfamily G member 2 [Source:HGNC Symbol;Acc:HGNC:8170] |
| ENSG00000105369 | HGLibA_08448 | 661 | 5.429033779 | CD79A | CD79a molecule [Source:HGNC Symbol;Acc:HGNC:1698] |
| ENSG00000181722 | HGLibA_54838 | 661 | 5.429033779 | ZBTB20 | zinc finger and BTB domain containing 20 [Source:HGNC Symbol;Acc:HGNC:13503] |
| ENSG00000185231 | HGLibA_28543 | 661 | 5.429033779 | MC2R | melanocortin 2 receptor [Source:HGNC Symbol;Acc:HGNC:6930] |
| ENSG00000155846 | HGLibA_37848 | 659 | 5.409131704 | PPARGC1B | PPARG coactivator 1 beta [Source:HGNC Symbol;Acc:HGNC:30022] |
| ENSG00000068976 | HGLibA_39690 | 658 | 5.399180667 | PYGM | muscle associated [Source:HGNC Symbol;Acc:HGNC:9726]" |
| ENSG00000105289 | HGLibA_49577 | 658 | 5.399180667 | TJP3 | tight junction protein 3 [Source:HGNC Symbol;Acc:HGNC:11829] |
| ENSG00000146072 | HGLibA_50778 | 656 | 5.379278592 | TNFRSF21 | TNF receptor superfamily member 21 [Source:HGNC Symbol;Acc:HGNC:13469] |
| ENSG00000083444 | HGLibA_37208 | 651 | 5.329523405 | PLOD1 | 2-oxoglutarate 5-dioxygenase 1 [Source:HGNC Symbol;Acc:HGNC:9081]" |
| ENSG00000126214 | HGLibA_25028 | 649 | 5.30962133 | KLC1 | kinesin light chain 1 [Source:HGNC Symbol;Acc:HGNC:6387] |
| ENSG00000163810 | HGLibA_49262 | 647 | 5.289719255 | TGM4 | transglutaminase 4 [Source:HGNC Symbol;Acc:HGNC:11780] |
| ENSG00000165023 | HGLibA_13258 | 644 | 5.259866143 | DIRAS2 | DIRAS family GTPase 2 [Source:HGNC Symbol;Acc:HGNC:19323] |
| ENSG00000144668 | HGLibA_23748 | 643 | 5.249915105 | ITGA9 | integrin subunit alpha 9 [Source:HGNC Symbol;Acc:HGNC:6145] |
| ENSG00000173542 | HGLibA_29587 | 643 | 5.249915105 | MOB1B | MOB kinase activator 1B [Source:HGNC Symbol;Acc:HGNC:29801] |
| ENSG00000127081 | HGLibA_56077 | 641 | 5.23001303 | ZNF484 | zinc finger protein 484 [Source:HGNC Symbol;Acc:HGNC:23385] |
| ENSG00000105699 | HGLibA_27567 | 640 | 5.220061993 | LSR | lipolysis stimulated lipoprotein receptor [Source:HGNC Symbol;Acc:HGNC:29572] |
| ENSG00000172497 | HGLibA_00472 | 637 | 5.190208881 | ACOT12 | acyl-CoA thioesterase 12 [Source:HGNC Symbol;Acc:HGNC:24436] |
| ENSG00000173457 | HGLibA_38039 | 632 | 5.140453694 | PPP1R14B | protein phosphatase 1 regulatory inhibitor subunit 14B [Source:HGNC Symbol;Acc:HGNC:9057] |
| FAM127C | HGLibA_16092 | 631 | 5.130502656 | FAM127C | - |
| hsa-mir-6084 | HGLibA_62598 | 631 | 5.130502656 | hsa-mir-6084 | - |
| ENSG00000154237 | HGLibA_27463 | 630 | 5.120551619 | LRRK1 | leucine rich repeat kinase 1 [Source:HGNC Symbol;Acc:HGNC:18608] |
| ENSG00000181830 | HGLibA_44971 | 626 | 5.080747469 | SLC35C1 | solute carrier family 35 member C1 [Source:HGNC Symbol;Acc:HGNC:20197] |
| ENSG00000100003 | HGLibA_43194 | 625 | 5.070796432 | SEC14L2 | SEC14 like lipid binding 2 [Source:HGNC Symbol;Acc:HGNC:10699] |
| ENSG00000114487 | HGLibA_29648 | 624 | 5.060845394 | MORC1 | MORC family CW-type zinc finger 1 [Source:HGNC Symbol;Acc:HGNC:7198] |
| ENSG00000173890 | HGLibA_19955 | 623 | 5.050894357 | GPR160 | G protein-coupled receptor 160 [Source:HGNC Symbol;Acc:HGNC:23693] |
| ENSG00000159399 | HGLibA_21581 | 621 | 5.030992282 | HK2 | hexokinase 2 [Source:HGNC Symbol;Acc:HGNC:4923] |
| ENSG00000132823 | HGLibA_34679 | 620 | 5.021041245 | OSER1 | oxidative stress responsive serine rich 1 [Source:HGNC Symbol;Acc:HGNC:16105] |
| ENSG00000177519 | HGLibA_42053 | 619 | 5.011090207 | RPRM | TP53 dependent G2 arrest mediator homolog [Source:HGNC Symbol;Acc:HGNC:24201]" |
| hsa-mir-5739 | HGLibA_62387 | 619 | 5.011090207 | hsa-mir-5739 | - |
| ENSG00000069509 | HGLibA_18163 | 617 | 4.991188132 | FUNDC1 | FUN14 domain containing 1 [Source:HGNC Symbol;Acc:HGNC:28746] |
| ENSG00000110203 | HGLibA_17790 | 617 | 4.991188132 | FOLR3 | folate receptor 3 [Source:HGNC Symbol;Acc:HGNC:3795] |
| ENSG00000187492 | HGLibA_08707 | 616 | 4.981237095 | CDHR4 | cadherin related family member 4 [Source:HGNC Symbol;Acc:HGNC:34527] |
| ENSG00000164379 | HGLibA_17942 | 615 | 4.971286057 | FOXQ1 | forkhead box Q1 [Source:HGNC Symbol;Acc:HGNC:20951] |
| ENSG00000134443 | HGLibA_20369 | 614 | 4.96133502 | GRP | gastrin releasing peptide [Source:HGNC Symbol;Acc:HGNC:4605] |
| ENSG00000173511 | HGLibA_53614 | 614 | 4.96133502 | VEGFB | vascular endothelial growth factor B [Source:HGNC Symbol;Acc:HGNC:12681] |
| ENSG00000011332 | HGLibA_13828 | 613 | 4.951383983 | DPF1 | double PHD fingers 1 [Source:HGNC Symbol;Acc:HGNC:20225] |
| ENSG00000131828 | HGLibA_35863 | 612 | 4.941432945 | PDHA1 | pyruvate dehydrogenase E1 alpha 1 subunit [Source:HGNC Symbol;Acc:HGNC:8806] |
| ENSG00000188886 | HGLibA_03378 | 611 | 4.931481908 | ASTL | astacin like metalloendopeptidase [Source:HGNC Symbol;Acc:HGNC:31704] |
| ENSG00000182752 | HGLibA_35145 | 609 | 4.911579833 | PAPPA | pappalysin 1 [Source:HGNC Symbol;Acc:HGNC:8602] |
| SLC22A20 | HGLibA_44569 | 609 | 4.911579833 | SLC22A20 | - |
| ENSG00000152217 | HGLibA_43634 | 608 | 4.901628795 | SETBP1 | SET binding protein 1 [Source:HGNC Symbol;Acc:HGNC:15573] |
| ENSG00000183145 | HGLibA_41334 | 608 | 4.901628795 | RIPPLY3 | ripply transcriptional repressor 3 [Source:HGNC Symbol;Acc:HGNC:3047] |
| ENSG00000105245 | HGLibA_33015 | 605 | 4.871775683 | NUMBL | endocytic adaptor protein [Source:HGNC Symbol;Acc:HGNC:8061]" |
| H1FNT | HGLibA_20737 | 605 | 4.871775683 | H1FNT | - |
| ENSG00000146360 | HGLibA_20058 | 604 | 4.861824646 | GPR6 | G protein-coupled receptor 6 [Source:HGNC Symbol;Acc:HGNC:4515] |
| ENSG00000197774 | HGLibA_15037 | 604 | 4.861824646 | EME2 | essential meiotic structure-specific endonuclease subunit 2 [Source:HGNC Symbol;Acc:HGNC:27289] |
| ENSG00000136840 | HGLibA_47273 | 602 | 4.841922571 | ST6GALNAC4 | 6-sialyltransferase 4 [Source:HGNC Symbol;Acc:HGNC:17846]" |
| ENSG00000165556 | HGLibA_08898 | 602 | 4.841922571 | CDX2 | caudal type homeobox 2 [Source:HGNC Symbol;Acc:HGNC:1806] |
| ENSG00000177138 | HGLibA_16822 | 602 | 4.841922571 | FAM9B | family with sequence similarity 9 member B [Source:HGNC Symbol;Acc:HGNC:18404] |
| hsa-mir-31 | HGLibA_58496 | 601 | 4.831971533 | hsa-mir-31 | - |
| ENSG00000205497 | HGLibA_34077 | 598 | 4.802118421 | OR51A4 | olfactory receptor family 51 subfamily A member 4 [Source:HGNC Symbol;Acc:HGNC:14795] |
| ENSG00000221938 | HGLibA_33724 | 598 | 4.802118421 | OR2A14 | olfactory receptor family 2 subfamily A member 14 [Source:HGNC Symbol;Acc:HGNC:15084] |
| ENSG00000135175 | HGLibA_33261 | 595 | 4.772265309 | OCM2 | oncomodulin 2 [Source:HGNC Symbol;Acc:HGNC:34396] |
| ENSG00000137460 | HGLibA_17475 | 595 | 4.772265309 | FHDC1 | FH2 domain containing 1 [Source:HGNC Symbol;Acc:HGNC:29363] |
| ENSG00000077092 | HGLibA_40275 | 594 | 4.762314272 | RARB | retinoic acid receptor beta [Source:HGNC Symbol;Acc:HGNC:9865] |
| ENSG00000089094 | HGLibA_24496 | 591 | 4.732461159 | KDM2B | lysine demethylase 2B [Source:HGNC Symbol;Acc:HGNC:13610] |
| ENSG00000196352 | HGLibA_08411 | 591 | 4.732461159 | CD55 | CD55 molecule (Cromer blood group) [Source:HGNC Symbol;Acc:HGNC:2665] |
| HIST3H3 | HGLibA_21561 | 591 | 4.732461159 | HIST3H3 | - |
| ENSG00000099866 | HGLibA_27854 | 590 | 4.722510122 | MADCAM1 | mucosal vascular addressin cell adhesion molecule 1 [Source:HGNC Symbol;Acc:HGNC:6765] |
| ENSG00000142319 | HGLibA_45324 | 590 | 4.722510122 | SLC6A3 | solute carrier family 6 member 3 [Source:HGNC Symbol;Acc:HGNC:11049] |
| ENSG00000173065 | HGLibA_16525 | 590 | 4.722510122 | FAM222B | family with sequence similarity 222 member B [Source:HGNC Symbol;Acc:HGNC:25563] |
| ENSG00000174600 | HGLibA_10196 | 590 | 4.722510122 | CMKLR1 | chemerin chemokine-like receptor 1 [Source:HGNC Symbol;Acc:HGNC:2121] |
| ENSG00000163104 | HGLibA_45615 | 589 | 4.712559084 | SMARCAD1 | containing DEAD/H box 1 [Source:HGNC Symbol;Acc:HGNC:18398]" |
| ENSG00000178996 | HGLibA_46050 | 587 | 4.69265701 | SNX18 | sorting nexin 18 [Source:HGNC Symbol;Acc:HGNC:19245] |
| ENSG00000204977 | HGLibA_51408 | 585 | 4.672754935 | TRIM13 | tripartite motif containing 13 [Source:HGNC Symbol;Acc:HGNC:9976] |
| ENSG00000140464 | HGLibA_37313 | 584 | 4.662803897 | PML | promyelocytic leukemia [Source:HGNC Symbol;Acc:HGNC:9113] |
| ENSG00000276747 | HGLibA_34982 | 584 | 4.662803897 | PADI6 | peptidyl arginine deiminase 6 [Source:HGNC Symbol;Acc:HGNC:20449] |
| ENSG00000152137 | HGLibA_22318 | 582 | 4.642901822 | HSPB8 | heat shock protein family B (small) member 8 [Source:HGNC Symbol;Acc:HGNC:30171] |
| ENSG00000106028 | HGLibA_47115 | 581 | 4.632950785 | SSBP1 | single stranded DNA binding protein 1 [Source:HGNC Symbol;Acc:HGNC:11317] |
| ENSG00000240891 | HGLibA_37025 | 580 | 4.622999748 | PLCXD2 | phosphatidylinositol specific phospholipase C X domain containing 2 [Source:HGNC Symbol;Acc:HGNC:26462] |
| ENSG00000163982 | HGLibA_34736 | 579 | 4.61304871 | OTOP1 | otopetrin 1 [Source:HGNC Symbol;Acc:HGNC:19656] |
| ENSG00000090971 | HGLibA_31171 | 578 | 4.603097673 | NAT14 | N-acetyltransferase 14 (putative) [Source:HGNC Symbol;Acc:HGNC:28918] |
| ENSG00000124313 | HGLibA_23544 | 576 | 4.583195598 | IQSEC2 | IQ motif and Sec7 domain 2 [Source:HGNC Symbol;Acc:HGNC:29059] |
| ENSG00000103253 | HGLibA_20817 | 574 | 4.563293523 | HAGHL | hydroxyacylglutathione hydrolase like [Source:HGNC Symbol;Acc:HGNC:14177] |
| ENSG00000134769 | HGLibA_14063 | 574 | 4.563293523 | DTNA | dystrobrevin alpha [Source:HGNC Symbol;Acc:HGNC:3057] |
| ENSG00000180329 | HGLibA_07786 | 573 | 4.553342486 | CCDC43 | coiled-coil domain containing 43 [Source:HGNC Symbol;Acc:HGNC:26472] |
| ENSG00000188315 | HGLibA_06257 | 573 | 4.553342486 | C3orf62 | chromosome 3 open reading frame 62 [Source:HGNC Symbol;Acc:HGNC:24771] |
| ENSG00000109180 | HGLibA_33247 | 572 | 4.543391448 | OCIAD1 | OCIA domain containing 1 [Source:HGNC Symbol;Acc:HGNC:16074] |
| ENSG00000155256 | HGLibA_55288 | 572 | 4.543391448 | ZFYVE27 | zinc finger FYVE-type containing 27 [Source:HGNC Symbol;Acc:HGNC:26559] |
| ENSG00000172073 | HGLibA_49106 | 572 | 4.543391448 | TEX37 | testis expressed 37 [Source:HGNC Symbol;Acc:HGNC:26341] |
| ENSG00000001626 | HGLibA_09294 | 571 | 4.533440411 | CFTR | cystic fibrosis transmembrane conductance regulator [Source:HGNC Symbol;Acc:HGNC:1884] |
| ENSG00000111305 | HGLibA_20420 | 571 | 4.533440411 | GSG1 | germ cell associated 1 [Source:HGNC Symbol;Acc:HGNC:19716] |
| hsa-mir-564 | HGLibA_62220 | 571 | 4.533440411 | hsa-mir-564 | - |

| | | | | | |
|---|---|---|---|---|---|
| ENSG00000081818 | HGLibA_35499 | 570 | 4.523489373 | PCDHB4 | protocadherin beta 4 [Source:HGNC Symbol;Acc:HGNC:8689] |
| ENSG00000154646 | HGLibA_50633 | 569 | 4.513538336 | TMPRSS15 | transmembrane serine protease 15 [Source:HGNC Symbol;Acc:HGNC:9490] |
| ENSG00000169174 | HGLibA_35689 | 569 | 4.513538336 | PCSK9 | proprotein convertase subtilisin/kexin type 9 [Source:HGNC Symbol;Acc:HGNC:20001] |
| hsa-mir-938 | HGLibA_64328 | 568 | 4.503587298 | hsa-mir-938 | - |
| hsa-mir-943 | HGLibA_64360 | 568 | 4.503587298 | hsa-mir-943 | - |
| ENSG00000080189 | HGLibA_44973 | 567 | 4.493636261 | SLC35C2 | solute carrier family 35 member C2 [Source:HGNC Symbol;Acc:HGNC:17117] |
| ENSG00000170279 | HGLibA_06536 | 567 | 4.493636261 | C7orf33 | chromosome 7 open reading frame 33 [Source:HGNC Symbol;Acc:HGNC:21724] |
| ENSG00000156345 | HGLibA_08758 | 566 | 4.483685224 | CDK20 | cyclin dependent kinase 20 [Source:HGNC Symbol;Acc:HGNC:21420] |
| ENSG00000137473 | HGLibA_52100 | 565 | 4.473734186 | TTC29 | tetratricopeptide repeat domain 29 [Source:HGNC Symbol;Acc:HGNC:29936] |
| ENSG00000179542 | HGLibA_45521 | 565 | 4.473734186 | SLITRK4 | SLIT and NTRK like family member 4 [Source:HGNC Symbol;Acc:HGNC:23502] |
| ENSG00000008083 | HGLibA_23936 | 564 | 4.463783149 | JARID2 | jumonji and AT-rich interaction domain containing 2 [Source:HGNC Symbol;Acc:HGNC:6196] |
| ENSG00000232070 | HGLibA_50325 | 564 | 4.463783149 | TMEM253 | transmembrane protein 253 [Source:HGNC Symbol;Acc:HGNC:32545] |
| ENSG00000169717 | HGLibA_00681 | 563 | 4.453832111 | ACTRT2 | actin related protein T2 [Source:HGNC Symbol;Acc:HGNC:24026] |
| ENSG00000257138 | HGLibA_48392 | 563 | 4.453832111 | TAS2R38 | taste 2 receptor member 38 [Source:HGNC Symbol;Acc:HGNC:9584] |
| ENSG00000197114 | HGLibA_55307 | 562 | 4.443881074 | ZGPAT | zinc finger CCCH-type and G-patch domain containing [Source:HGNC Symbol;Acc:HGNC:15948] |
| ENSG00000162951 | HGLibA_27484 | 561 | 4.433930036 | LRRTM1 | leucine rich repeat transmembrane neuronal 1 [Source:HGNC Symbol;Acc:HGNC:19408] |
| ENSG00000188738 | HGLibA_18094 | 561 | 4.433930036 | FSIP2 | fibrous sheath interacting protein 2 [Source:HGNC Symbol;Acc:HGNC:21675] |
| ENSG00000196233 | HGLibA_26193 | 561 | 4.433930036 | LCOR | ligand dependent nuclear receptor corepressor [Source:HGNC Symbol;Acc:HGNC:29503] |
| ENSG00000126653 | HGLibA_32779 | 559 | 4.414027962 | NSRP1 | nuclear speckle splicing regulatory protein 1 [Source:HGNC Symbol;Acc:HGNC:25305] |
| ENSG00000065559 | HGLibA_28147 | 558 | 4.404076924 | MAP2K4 | mitogen-activated protein kinase kinase 4 [Source:HGNC Symbol;Acc:HGNC:6844] |
| ENSG00000174123 | HGLibA_49642 | 558 | 4.404076924 | TLR10 | toll like receptor 10 [Source:HGNC Symbol;Acc:HGNC:15634] |
| ENSG00000214706 | HGLibA_22741 | 558 | 4.404076924 | IFRD2 | interferon related developmental regulator 2 [Source:HGNC Symbol;Acc:HGNC:5457] |
| ENSG00000170144 | HGLibA_21802 | 556 | 4.384174849 | HNRNPA3 | heterogeneous nuclear ribonucleoprotein A3 [Source:HGNC Symbol;Acc:HGNC:24941] |
| ENSG00000110697 | HGLibA_36750 | 555 | 4.374223812 | PITPNM1 | phosphatidylinositol transfer protein membrane associated 1 [Source:HGNC Symbol;Acc:HGNC:9003] |
| ENSG00000170949 | HGLibA_55514 | 555 | 4.374223812 | ZNF160 | zinc finger protein 160 [Source:HGNC Symbol;Acc:HGNC:12948] |
| ENSG00000181722 | HGLibA_54839 | 555 | 4.374223812 | ZBTB20 | zinc finger and BTB domain containing 20 [Source:HGNC Symbol;Acc:HGNC:13503] |
| ENSG00000110484 | HGLibA_42926 | 554 | 4.364272775 | SCGB2A2 | secretoglobin family 2A member 2 [Source:HGNC Symbol;Acc:HGNC:7050] |
| ENSG00000134339 | HGLibA_42612 | 553 | 4.354321737 | SAA2 | serum amyloid A2 [Source:HGNC Symbol;Acc:HGNC:10514] |
| ENSG00000105784 | HGLibA_42451 | 552 | 4.3443707 | RUNDC3B | RUN domain containing 3B [Source:HGNC Symbol;Acc:HGNC:30286] |
| ENSG00000128045 | HGLibA_40368 | 552 | 4.3443707 | RASL11B | RAS like family 11 member B [Source:HGNC Symbol;Acc:HGNC:23804] |
| ENSG00000165801 | HGLibA_02866 | 552 | 4.3443707 | ARHGEF40 | Rho guanine nucleotide exchange factor 40 [Source:HGNC Symbol;Acc:HGNC:25516] |
| ENSG00000156453 | HGLibA_35386 | 551 | 4.334419662 | PCDH1 | protocadherin 1 [Source:HGNC Symbol;Acc:HGNC:8655] |
| ENSG00000115226 | HGLibA_17753 | 550 | 4.324468625 | FNDC4 | fibronectin type III domain containing 4 [Source:HGNC Symbol;Acc:HGNC:20239] |
| ENSG00000143891 | HGLibA_18520 | 550 | 4.324468625 | GALM | galactose mutarotase [Source:HGNC Symbol;Acc:HGNC:24063] |
| ENSG00000165863 | HGLibA_05097 | 550 | 4.324468625 | C10orf82 | chromosome 10 open reading frame 82 [Source:HGNC Symbol;Acc:HGNC:28500] |
| ENSG00000196767 | HGLibA_37774 | 550 | 4.324468625 | POU3F4 | POU class 3 homeobox 4 [Source:HGNC Symbol;Acc:HGNC:9217] |
| ENSG00000151892 | HGLibA_18966 | 549 | 4.314517587 | GFRA1 | GDNF family receptor alpha 1 [Source:HGNC Symbol;Acc:HGNC:4243] |
| ENSG00000166948 | HGLibA_49267 | 549 | 4.314517587 | TGM6 | transglutaminase 6 [Source:HGNC Symbol;Acc:HGNC:16255] |
| ENSG00000197465 | HGLibA_20706 | 549 | 4.314517587 | GYPE | glycophorin E (MNS blood group) [Source:HGNC Symbol;Acc:HGNC:4705] |
| ENSG00000105426 | HGLibA_39536 | 548 | 4.30456655 | PTPRS | receptor type S [Source:HGNC Symbol;Acc:HGNC:9681]" |
| ENSG00000175591 | HGLibA_34885 | 548 | 4.30456655 | P2RY2 | purinergic receptor P2Y2 [Source:HGNC Symbol;Acc:HGNC:8541] |
| CCDC129 | HGLibA_07586 | 547 | 4.294615513 | CCDC129 | - |
| ENSG00000143595 | HGLibA_02581 | 547 | 4.294615513 | AQP10 | aquaporin 10 [Source:HGNC Symbol;Acc:HGNC:16029] |
| ENSG00000176293 | HGLibA_55471 | 547 | 4.294615513 | ZNF135 | zinc finger protein 135 [Source:HGNC Symbol;Acc:HGNC:12919] |
| ENSG00000155660 | HGLibA_35881 | 546 | 4.284664475 | PDIA4 | protein disulfide isomerase family A member 4 [Source:HGNC Symbol;Acc:HGNC:30167] |
| ENSG00000186193 | HGLibA_42735 | 546 | 4.284664475 | SAPCD2 | suppressor APC domain containing 2 [Source:HGNC Symbol;Acc:HGNC:28055] |
| ENSG00000187961 | HGLibA_25142 | 546 | 4.284664475 | KLHL17 | kelch like family member 17 [Source:HGNC Symbol;Acc:HGNC:24023] |
| ENSG00000139641 | HGLibA_15606 | 545 | 4.274713438 | ESYT1 | extended synaptotagmin 1 [Source:HGNC Symbol;Acc:HGNC:29534] |
| ENSG00000136630 | HGLibA_21659 | 544 | 4.2647624 | HLX | H2.0 like homeobox [Source:HGNC Symbol;Acc:HGNC:4978] |
| ENSG00000221946 | HGLibA_18245 | 544 | 4.2647624 | FXYD7 | FXYD domain containing ion transport regulator 7 [Source:HGNC Symbol;Acc:HGNC:4034] |
| ENSG00000182742 | HGLibA_21938 | 543 | 4.254811363 | HOXB4 | homeobox B4 [Source:HGNC Symbol;Acc:HGNC:5115] |
| ENSG00000180914 | HGLibA_34842 | 541 | 4.234909288 | OXTR | oxytocin receptor [Source:HGNC Symbol;Acc:HGNC:8529] |
| ENSG00000166140 | HGLibA_55275 | 540 | 4.224958251 | ZFYVE19 | zinc finger FYVE-type containing 19 [Source:HGNC Symbol;Acc:HGNC:20758] |
| ENSG00000181830 | HGLibA_44970 | 540 | 4.224958251 | SLC35C1 | solute carrier family 35 member C1 [Source:HGNC Symbol;Acc:HGNC:20197] |
| ENSG00000183283 | HGLibA_12367 | 539 | 4.215007213 | DAZAP2 | DAZ associated protein 2 [Source:HGNC Symbol;Acc:HGNC:2684] |
| ENSG00000141639 | HGLibA_28273 | 538 | 4.205056176 | MAPK4 | mitogen-activated protein kinase 4 [Source:HGNC Symbol;Acc:HGNC:6878] |
| ENSG00000157036 | HGLibA_15766 | 537 | 4.195105138 | EXOG | exo/endonuclease G [Source:HGNC Symbol;Acc:HGNC:3347] |
| ENSG00000184545 | HGLibA_14192 | 537 | 4.195105138 | DUSP8 | dual specificity phosphatase 8 [Source:HGNC Symbol;Acc:HGNC:3074] |
| hsa-mir-6836 | HGLibA_63521 | 537 | 4.195105138 | hsa-mir-6836 | - |
| ENSG00000125363 | HGLibA_01766 | 535 | 4.175203063 | AMELX | amelogenin X-linked [Source:HGNC Symbol;Acc:HGNC:461] |
| ENSG00000188959 | HGLibA_06689 | 535 | 4.175203063 | C9orf152 | chromosome 9 open reading frame 152 [Source:HGNC Symbol;Acc:HGNC:31455] |
| ENSG00000206384 | HGLibA_10609 | 535 | 4.175203063 | COL6A6 | collagen type VI alpha 6 chain [Source:HGNC Symbol;Acc:HGNC:27023] |
| C4orf32 | HGLibA_06321 | 534 | 4.165252026 | C4orf32 | - |
| hsa-mir-6868 | HGLibA_63657 | 534 | 4.165252026 | hsa-mir-6868 | - |
| ENSG00000126264 | HGLibA_20995 | 533 | 4.155300989 | HCST | hematopoietic cell signal transducer [Source:HGNC Symbol;Acc:HGNC:16977] |
| ENSG00000106992 | HGLibA_01375 | 532 | 4.145349951 | AK1 | adenylate kinase 1 [Source:HGNC Symbol;Acc:HGNC:361] |
| ENSG00000197706 | HGLibA_34406 | 532 | 4.145349951 | OR6C74 | olfactory receptor family 6 subfamily C member 74 [Source:HGNC Symbol;Acc:HGNC:31303] |
| ENSG00000259956 | HGLibA_40493 | 532 | 4.145349951 | RBM15B | RNA binding motif protein 15B [Source:HGNC Symbol;Acc:HGNC:24303] |
| ENSG00000170502 | HGLibA_32993 | 531 | 4.135398914 | NUDT9 | nudix hydrolase 9 [Source:HGNC Symbol;Acc:HGNC:8056] |
| ENSG00000204475 | HGLibA_31368 | 531 | 4.135398914 | NCR3 | natural cytotoxicity triggering receptor 3 [Source:HGNC Symbol;Acc:HGNC:19077] |
| ENSG00000100246 | HGLibA_13646 | 529 | 4.115496839 | DNAL4 | dynein axonemal light chain 4 [Source:HGNC Symbol;Acc:HGNC:2955] |
| hsa-mir-4800 | HGLibA_61306 | 529 | 4.115496839 | hsa-mir-4800 | - |
| ENSG00000139190 | HGLibA_53488 | 528 | 4.105545801 | VAMP1 | vesicle associated membrane protein 1 [Source:HGNC Symbol;Acc:HGNC:12642] |
| ENSG00000242173 | HGLibA_02800 | 528 | 4.105545801 | ARHGDIG | Rho GDP dissociation inhibitor gamma [Source:HGNC Symbol;Acc:HGNC:680] |
| ENSG00000005249 | HGLibA_38529 | 527 | 4.095594764 | PRKAR2B | protein kinase cAMP-dependent type II regulatory subunit beta [Source:HGNC Symbol;Acc:HGNC:9392] |
| ENSG00000151746 | HGLibA_04495 | 527 | 4.095594764 | BICD1 | BICD cargo adaptor 1 [Source:HGNC Symbol;Acc:HGNC:1049] |
| ENSG00000161509 | HGLibA_20304 | 527 | 4.095594764 | GRIN2C | glutamate ionotropic receptor NMDA type subunit 2C [Source:HGNC Symbol;Acc:HGNC:4587] |
| ENSG00000164180 | HGLibA_50027 | 527 | 4.095594764 | TMEM161B | transmembrane protein 161B [Source:HGNC Symbol;Acc:HGNC:28483] |
| ENSG00000168772 | HGLibA_11970 | 527 | 4.095594764 | CXXC4 | CXXC finger protein 4 [Source:HGNC Symbol;Acc:HGNC:24593] |
| C20orf166 | HGLibA_05993 | 526 | 4.085643727 | C20orf166 | - |
| ENSG00000122335 | HGLibA_43462 | 526 | 4.085643727 | SERAC1 | serine active site containing 1 [Source:HGNC Symbol;Acc:HGNC:21061] |
| ENSG00000151379 | HGLibA_30188 | 526 | 4.085643727 | MSGN1 | mesogenin 1 [Source:HGNC Symbol;Acc:HGNC:14907] |
| hsa-mir-3616 | HGLibA_59092 | 526 | 4.085643727 | hsa-mir-3616 | - |
| C12orf45 | HGLibA_05249 | 525 | 4.075692689 | C12orf45 | - |
| ENSG00000110436 | HGLibA_44510 | 525 | 4.075692689 | SLC1A2 | solute carrier family 1 member 2 [Source:HGNC Symbol;Acc:HGNC:10940] |
| ENSG00000163328 | HGLibA_19943 | 525 | 4.075692689 | GPR155 | G protein-coupled receptor 155 [Source:HGNC Symbol;Acc:HGNC:22951] |
| ENSG00000184330 | HGLibA_42568 | 525 | 4.075692689 | S100A7A | S100 calcium binding protein A7A [Source:HGNC Symbol;Acc:HGNC:21657] |
| LOC256021 | HGLibA_26905 | 525 | 4.075692689 | LOC256021 | - |
| ENSG00000198650 | HGLibA_48438 | 524 | 4.065741652 | TAT | tyrosine aminotransferase [Source:HGNC Symbol;Acc:HGNC:11573] |
| ENSG00000049323 | HGLibA_27590 | 523 | 4.055790614 | LTBP1 | latent transforming growth factor beta binding protein 1 [Source:HGNC Symbol;Acc:HGNC:6714] |
| ENSG00000108961 | HGLibA_40219 | 523 | 4.055790614 | RANGRF | RAN guanine nucleotide release factor [Source:HGNC Symbol;Acc:HGNC:17679] |
| ENSG00000136003 | HGLibA_23651 | 523 | 4.055790614 | ISCU | iron-sulfur cluster assembly enzyme [Source:HGNC Symbol;Acc:HGNC:29882] |
| ENSG00000184227 | HGLibA_00466 | 523 | 4.055790614 | ACOT1 | acyl-CoA thioesterase 1 [Source:HGNC Symbol;Acc:HGNC:33128] |
| ATP5L | HGLibA_03678 | 522 | 4.045839577 | ATP5L | - |
| ENSG00000108557 | HGLibA_40139 | 522 | 4.045839577 | RAI1 | retinoic acid induced 1 [Source:HGNC Symbol;Acc:HGNC:9834] |
| ENSG00000171773 | HGLibA_33144 | 522 | 4.045839577 | NXNL1 | nucleoredoxin like 1 [Source:HGNC Symbol;Acc:HGNC:25179] |
| ENSG00000180304 | HGLibA_33215 | 521 | 4.03588854 | OAZ2 | ornithine decarboxylase antizyme 2 [Source:HGNC Symbol;Acc:HGNC:8096] |
| ENSG00000185022 | HGLibA_27878 | 521 | 4.03588854 | MAFF | MAF bZIP transcription factor F [Source:HGNC Symbol;Acc:HGNC:6780] |
| hsa-mir-552 | HGLibA_62126 | 521 | 4.03588854 | hsa-mir-552 | - |
| ENSG00000140678 | HGLibA_23767 | 520 | 4.025937502 | ITGAX | integrin subunit alpha X [Source:HGNC Symbol;Acc:HGNC:6152] |
| ENSG00000001629 | HGLibA_01971 | 519 | 4.015986465 | ANKIB1 | ankyrin repeat and IBR domain containing 1 [Source:HGNC Symbol;Acc:HGNC:22215] |
| ENSG00000163635 | HGLibA_03849 | 519 | 4.015986465 | ATXN7 | ataxin 7 [Source:HGNC Symbol;Acc:HGNC:10560] |
| ENSG00000183775 | HGLibA_24432 | 519 | 4.015986465 | KCTD16 | potassium channel tetramerization domain containing 16 [Source:HGNC Symbol;Acc:HGNC:29244] |
| ENSG00000100354 | HGLibA_50917 | 516 | 3.986133352 | TNRC6B | trinucleotide repeat containing 6B [Source:HGNC Symbol;Acc:HGNC:29190] |
| ENSG00000140450 | HGLibA_03123 | 516 | 3.986133352 | ARRDC4 | arrestin domain containing 4 [Source:HGNC Symbol;Acc:HGNC:28087] |
| ENSG00000162065 | HGLibA_48512 | 516 | 3.986133352 | TBC1D24 | TBC1 domain family member 24 [Source:HGNC Symbol;Acc:HGNC:29203] |
| ENSG00000006704 | HGLibA_20569 | 515 | 3.976182315 | GTF2IRD1 | GTF2I repeat domain containing 1 [Source:HGNC Symbol;Acc:HGNC:4661] |
| ENSG00000113205 | HGLibA_35495 | 515 | 3.976182315 | PCDHB3 | protocadherin beta 3 [Source:HGNC Symbol;Acc:HGNC:8688] |
| ENSG00000171243 | HGLibA_46211 | 515 | 3.976182315 | SOSTDC1 | sclerostin domain containing 1 [Source:HGNC Symbol;Acc:HGNC:21748] |
| ENSG00000197993 | HGLibA_24550 | 515 | 3.976182315 | KEL | Kell metallo-endopeptidase (Kell blood group) [Source:HGNC Symbol;Acc:HGNC:6308] |
| ENSG00000198513 | HGLibA_03515 | 514 | 3.966231278 | ATL1 | atlastin GTPase 1 [Source:HGNC Symbol;Acc:HGNC:11231] |
| hsa-mir-7853 | HGLibA_64000 | 514 | 3.966231278 | hsa-mir-7853 | - |
| 12-Sep | HGLibA_43433 | 514 | 3.966231278 | 12-Sep | - |
| ENSG00000106012 | HGLibA_23500 | 513 | 3.95628024 | IQCE | IQ motif containing E [Source:HGNC Symbol;Acc:HGNC:29171] |
| ENSG00000160191 | HGLibA_35836 | 513 | 3.95628024 | PDE9A | phosphodiesterase 9A [Source:HGNC Symbol;Acc:HGNC:8795] |
| ENSG00000136603 | HGLibA_44281 | 512 | 3.946329203 | SKIL | SKI like proto-oncogene [Source:HGNC Symbol;Acc:HGNC:10897] |
| ENSG00000147127 | HGLibA_39942 | 512 | 3.946329203 | RAB41 | member RAS oncogene family [Source:HGNC Symbol;Acc:HGNC:18293]" |
| ENSG00000159363 | HGLibA_03570 | 512 | 3.946329203 | ATP13A2 | ATPase cation transporting 13A2 [Source:HGNC Symbol;Acc:HGNC:30213] |
| ENSG00000164251 | HGLibA_15866 | 512 | 3.946329203 | F2RL1 | F2R like trypsin receptor 1 [Source:HGNC Symbol;Acc:HGNC:3538] |
| C21orf59 | HGLibA_06045 | 511 | 3.936378165 | C21orf59 | - |
| hsa-mir-1179 | HGLibA_57211 | 511 | 3.936378165 | hsa-mir-1179 | - |
| ENSG00000010030 | HGLibA_15668 | 510 | 3.926427128 | ETV7 | ETS variant 7 [Source:HGNC Symbol;Acc:HGNC:18160] |
| ENSG00000196381 | HGLibA_56737 | 510 | 3.926427128 | ZNF781 | zinc finger protein 781 [Source:HGNC Symbol;Acc:HGNC:26745] |
| ENSG00000146938 | HGLibA_32077 | 509 | 3.91647609 | NLGN4X | neuroligin 4 X-linked [Source:HGNC Symbol;Acc:HGNC:14287] |
| C6orf106 | HGLibA_06433 | 508 | 3.906525053 | C6orf106 | - |
| ENSG00000248385 | HGLibA_48339 | 508 | 3.906525053 | TARM1 | activating receptor on myeloid cells 1 [Source:HGNC Symbol;Acc:HGNC:37250]" |
| ENSG00000156675 | HGLibA_39793 | 507 | 3.896574016 | RAB11FIP1 | RAB11 family interacting protein 1 [Source:HGNC Symbol;Acc:HGNC:30265] |
| ENSG00000258315 | HGLibA_05538 | 507 | 3.896574016 | C17orf49 | chromosome 17 open reading frame 49 [Source:HGNC Symbol;Acc:HGNC:28737] |
| CXorf48 | HGLibA_11929 | 506 | 3.886622978 | CXorf48 | - |
| ENSG00000076641 | HGLibA_35002 | 505 | 3.876671941 | PAG1 | phosphoprotein membrane anchor with glycosphingolipid microdomains 1 [Source:HGNC Symbol;Acc:HGNC:30043] |
| ENSG00000205832 | HGLibA_05511 | 505 | 3.876671941 | C16orf96 | chromosome 16 open reading frame 96 [Source:HGNC Symbol;Acc:HGNC:40031] |
| ENSG00000275410 | HGLibA_21778 | 505 | 3.876671941 | HNF1B | HNF1 homeobox B [Source:HGNC Symbol;Acc:HGNC:11630] |
| hsa-mir-4641 | HGLibA_60670 | 505 | 3.876671941 | hsa-mir-4641 | - |
| C16orf93 | HGLibA_05503 | 504 | 3.866720903 | C16orf93 | - |
| ENSG00000197774 | HGLibA_15038 | 503 | 3.856769866 | EME2 | essential meiotic structure-specific endonuclease subunit 2 [Source:HGNC Symbol;Acc:HGNC:27289] |

| | | | | | |
|---|---|---|---|---|---|
| ENSG00000064932 | HGLibA_42809 | 502 | 3.846818828 | SBNO2 | strawberry notch homolog 2 [Source:HGNC Symbol;Acc:HGNC:29158] |
| ENSG00000130640 | HGLibA_52309 | 502 | 3.846818828 | TUBGCP2 | tubulin gamma complex associated protein 2 [Source:HGNC Symbol;Acc:HGNC:18599] |
| ENSG00000132915 | HGLibA_35805 | 502 | 3.846818828 | PDE6A | phosphodiesterase 6A [Source:HGNC Symbol;Acc:HGNC:8785] |
| ENSG00000129152 | HGLibA_30881 | 501 | 3.836867791 | MYOD1 | myogenic differentiation 1 [Source:HGNC Symbol;Acc:HGNC:7611] |
| ENSG00000170144 | HGLibA_21801 | 501 | 3.836867791 | HNRNPA3 | heterogeneous nuclear ribonucleoprotein A3 [Source:HGNC Symbol;Acc:HGNC:24941] |
| ENSG00000269335 | HGLibA_22925 | 501 | 3.836867791 | IKBKG | inhibitor of nuclear factor kappa B kinase subunit gamma [Source:HGNC Symbol;Acc:HGNC:5961] |
| hsa-mir-8073 | HGLibA_64136 | 501 | 3.836867791 | hsa-mir-8073 | - |
| C15orf43 | HGLibA_05402 | 500 | 3.826916754 | C15orf43 | - |
| ENSG00000100711 | HGLibA_55283 | 500 | 3.826916754 | ZFYVE21 | zinc finger FYVE-type containing 21 [Source:HGNC Symbol;Acc:HGNC:20760] |
| ENSG00000105229 | HGLibA_36516 | 500 | 3.826916754 | PIAS4 | protein inhibitor of activated STAT 4 [Source:HGNC Symbol;Acc:HGNC:17002] |
| ENSG00000164736 | HGLibA_46246 | 500 | 3.826916754 | SOX17 | SRY-box 17 [Source:HGNC Symbol;Acc:HGNC:18122] |
| ENSG00000162391 | HGLibA_16162 | 499 | 3.816965716 | FAM151A | family with sequence similarity 151 member A [Source:HGNC Symbol;Acc:HGNC:25032] |
| ENSG00000165731 | HGLibA_40871 | 499 | 3.816965716 | RET | ret proto-oncogene [Source:HGNC Symbol;Acc:HGNC:9967] |
| ENSG00000154545 | HGLibA_27975 | 498 | 3.807014679 | MAGED4 | MAGE family member D4 [Source:HGNC Symbol;Acc:HGNC:23793] |
| ENSG00000255359 | HGLibA_07718 | 498 | 3.807014679 | CCDC179 | coiled-coil domain containing 179 [Source:HGNC Symbol;Acc:HGNC:44653] |
| ENSG00000136167 | HGLibA_26198 | 497 | 3.797063641 | LCP1 | lymphocyte cytosolic protein 1 [Source:HGNC Symbol;Acc:HGNC:6528] |
| ENSG00000139132 | HGLibA_17352 | 497 | 3.797063641 | FGD4 | RhoGEF and PH domain containing 4 [Source:HGNC Symbol;Acc:HGNC:19125]" |
| PGBD3 | HGLibA_36223 | 497 | 3.797063641 | PGBD3 | - |
| C12orf52 | HGLibA_05263 | 496 | 3.787112604 | C12orf52 | - |
| ENSG00000174996 | HGLibA_25030 | 496 | 3.787112604 | KLC2 | kinesin light chain 2 [Source:HGNC Symbol;Acc:HGNC:20716] |
| ENSG00000181631 | HGLibA_34879 | 496 | 3.787112604 | P2RY13 | purinergic receptor P2Y13 [Source:HGNC Symbol;Acc:HGNC:4537] |
| hsa-mir-4774 | HGLibA_61201 | 496 | 3.787112604 | hsa-mir-4774 | - |
| ENSG00000075426 | HGLibA_17805 | 495 | 3.777161566 | FOSL2 | AP-1 transcription factor subunit [Source:HGNC Symbol;Acc:HGNC:3798]" |
| ENSG00000197614 | HGLibA_29054 | 495 | 3.777161566 | MFAP5 | microfibril associated protein 5 [Source:HGNC Symbol;Acc:HGNC:29673] |
| hsa-mir-92b | HGLibA_64299 | 495 | 3.777161566 | hsa-mir-92b | - |
| PYCRL | HGLibA_39678 | 495 | 3.777161566 | PYCRL | - |
| ENSG00000171346 | HGLibA_25429 | 494 | 3.767210529 | KRT15 | keratin 15 [Source:HGNC Symbol;Acc:HGNC:6421] |
| ENSG00000188782 | HGLibA_07365 | 493 | 3.757259492 | CATSPER4 | cation channel sperm associated 4 [Source:HGNC Symbol;Acc:HGNC:23220] |
| TTC40 | HGLibA_52145 | 493 | 3.757259492 | TTC40 | - |
| ENSG00000102076 | HGLibA_33421 | 492 | 3.747308454 | OPN1LW | long wave sensitive [Source:HGNC Symbol;Acc:HGNC:9936]" |
| ENSG00000168757 | HGLibA_51966 | 492 | 3.747308454 | TSPY2 | testis specific protein Y-linked 2 [Source:HGNC Symbol;Acc:HGNC:23924] |
| ENSG00000171517 | HGLibA_27060 | 492 | 3.747308454 | LPAR3 | lysophosphatidic acid receptor 3 [Source:HGNC Symbol;Acc:HGNC:14298] |
| ENSG00000030582 | HGLibA_20366 | 491 | 3.737357417 | GRN | granulin precursor [Source:HGNC Symbol;Acc:HGNC:4601] |
| ENSG00000067177 | HGLibA_36403 | 491 | 3.737357417 | PHKA1 | phosphorylase kinase regulatory subunit alpha 1 [Source:HGNC Symbol;Acc:HGNC:8925] |
| ENSG00000136535 | HGLibA_48631 | 491 | 3.737357417 | TBR1 | brain 1 [Source:HGNC Symbol;Acc:HGNC:11590]" |
| ENSG00000154252 | HGLibA_18501 | 491 | 3.737357417 | GAL3ST2 | galactose-3-O-sulfotransferase 2 [Source:HGNC Symbol;Acc:HGNC:24869] |
| ENSG00000156735 | HGLibA_04084 | 491 | 3.737357417 | BAG4 | BCL2 associated athanogene 4 [Source:HGNC Symbol;Acc:HGNC:940] |
| ENSG00000157224 | HGLibA_09856 | 491 | 3.737357417 | CLDN12 | claudin 12 [Source:HGNC Symbol;Acc:HGNC:2034] |
| ENSG00000164078 | HGLibA_30258 | 491 | 3.737357417 | MST1R | macrophage stimulating 1 receptor [Source:HGNC Symbol;Acc:HGNC:7381] |
| ENSG00000172292 | HGLibA_09215 | 491 | 3.737357417 | CERS6 | ceramide synthase 6 [Source:HGNC Symbol;Acc:HGNC:23826] |
| ENSG00000131771 | HGLibA_38067 | 490 | 3.727406379 | PPP1R1B | protein phosphatase 1 regulatory inhibitor subunit 1B [Source:HGNC Symbol;Acc:HGNC:9287] |
| ENSG00000136235 | HGLibA_19834 | 490 | 3.727406379 | GPNMB | glycoprotein nmb [Source:HGNC Symbol;Acc:HGNC:4462] |
| ENSG00000244607 | HGLibA_07588 | 490 | 3.727406379 | CCDC13 | coiled-coil domain containing 13 [Source:HGNC Symbol;Acc:HGNC:26358] |
| ENSG00000100299 | HGLibA_03129 | 489 | 3.717455342 | ARSA | arylsulfatase A [Source:HGNC Symbol;Acc:HGNC:713] |
| ENSG00000047634 | HGLibA_42959 | 488 | 3.707504304 | SCML1 | Scm polycomb group protein like 1 [Source:HGNC Symbol;Acc:HGNC:10580] |
| ENSG00000087903 | HGLibA_40966 | 488 | 3.707504304 | RFX2 | regulatory factor X2 [Source:HGNC Symbol;Acc:HGNC:9983] |
| ENSG00000166523 | HGLibA_09989 | 488 | 3.707504304 | CLEC4E | C-type lectin domain family 4 member E [Source:HGNC Symbol;Acc:HGNC:14555] |
| ENSG00000143954 | HGLibA_40803 | 487 | 3.697553267 | REG3G | regenerating family member 3 gamma [Source:HGNC Symbol;Acc:HGNC:29595] |
| ENSG00000133661 | HGLibA_43778 | 486 | 3.68760223 | SFTPD | surfactant protein D [Source:HGNC Symbol;Acc:HGNC:10803] |
| TCEB3B | HGLibA_48742 | 486 | 3.68760223 | TCEB3B | - |
| ENSG00000117595 | HGLibA_23596 | 485 | 3.677651192 | IRF6 | interferon regulatory factor 6 [Source:HGNC Symbol;Acc:HGNC:6121] |
| ENSG00000029364 | HGLibA_45128 | 484 | 3.667700155 | SLC39A9 | solute carrier family 39 member 9 [Source:HGNC Symbol;Acc:HGNC:20182] |
| hsa-mir-718 | HGLibA_63882 | 484 | 3.667700155 | hsa-mir-718 | - |
| ENSG00000005108 | HGLibA_49406 | 483 | 3.657749117 | THSD7A | thrombospondin type 1 domain containing 7A [Source:HGNC Symbol;Acc:HGNC:22207] |
| ENSG00000109667 | HGLibA_44894 | 483 | 3.657749117 | SLC2A9 | solute carrier family 2 member 9 [Source:HGNC Symbol;Acc:HGNC:13446] |
| ENSG00000180901 | HGLibA_24444 | 483 | 3.657749117 | KCTD2 | potassium channel tetramerization domain containing 2 [Source:HGNC Symbol;Acc:HGNC:21294] |
| ENSG00000224659 | HGLibA_18460 | 483 | 3.657749117 | GAGE12J | G antigen 12J [Source:HGNC Symbol;Acc:HGNC:17778] |
| FBXO18 | HGLibA_17038 | 483 | 3.657749117 | FBXO18 | - |
| hsa-mir-1908 | HGLibA_58025 | 483 | 3.657749117 | hsa-mir-1908 | - |
| ENSG00000110328 | HGLibA_18552 | 482 | 3.64779808 | GALNT18 | polypeptide N-acetylgalactosaminyltransferase 18 [Source:HGNC Symbol;Acc:HGNC:30488] |
| ENSG00000113555 | HGLibA_35396 | 482 | 3.64779808 | PCDH12 | protocadherin 12 [Source:HGNC Symbol;Acc:HGNC:8657] |
| ENSG00000175718 | HGLibA_40609 | 482 | 3.64779808 | RBMXL3 | RBMX like 3 [Source:HGNC Symbol;Acc:HGNC:26859] |
| DUS2L | HGLibA_14118 | 481 | 3.637847043 | DUS2L | - |
| ENSG00000005812 | HGLibA_17005 | 481 | 3.637847043 | FBXL3 | F-box and leucine rich repeat protein 3 [Source:HGNC Symbol;Acc:HGNC:13599] |
| ENSG00000205420 | HGLibA_25514 | 481 | 3.637847043 | KRT6A | keratin 6A [Source:HGNC Symbol;Acc:HGNC:6443] |
| LOC554223 | HGLibA_26954 | 481 | 3.637847043 | LOC554223 | - |
| CCDC53 | HGLibA_07798 | 480 | 3.627896005 | CCDC53 | - |
| ENSG00000175267 | HGLibA_53902 | 480 | 3.627896005 | VWA3A | von Willebrand factor A domain containing 3A [Source:HGNC Symbol;Acc:HGNC:27088] |
| ENSG00000047849 | HGLibA_28208 | 479 | 3.617944968 | MAP4 | microtubule associated protein 4 [Source:HGNC Symbol;Acc:HGNC:6862] |
| ENSG00000123178 | HGLibA_46856 | 479 | 3.617944968 | SPRYD7 | SPRY domain containing 7 [Source:HGNC Symbol;Acc:HGNC:14297] |
| ENSG00000169155 | HGLibA_54890 | 479 | 3.617944968 | ZBTB43 | zinc finger and BTB domain containing 43 [Source:HGNC Symbol;Acc:HGNC:17908] |
| ENSG00000134516 | HGLibA_13740 | 478 | 3.60799393 | DOCK2 | dedicator of cytokinesis 2 [Source:HGNC Symbol;Acc:HGNC:2988] |
| ENSG00000142273 | HGLibA_07399 | 478 | 3.60799393 | CBLC | Cbl proto-oncogene C [Source:HGNC Symbol;Acc:HGNC:15961] |
| ENSG00000243130 | HGLibA_39038 | 478 | 3.60799393 | PSG11 | pregnancy specific beta-1-glycoprotein 11 [Source:HGNC Symbol;Acc:HGNC:9516] |
| ENSG00000102010 | HGLibA_04642 | 477 | 3.598042893 | BMX | BMX non-receptor tyrosine kinase [Source:HGNC Symbol;Acc:HGNC:1079] |
| ENSG00000132964 | HGLibA_08796 | 477 | 3.598042893 | CDK8 | cyclin dependent kinase 8 [Source:HGNC Symbol;Acc:HGNC:1779] |
| ENSG00000106993 | HGLibA_08547 | 476 | 3.588091855 | CDC37L1 | cell division cycle 37 like 1 [Source:HGNC Symbol;Acc:HGNC:17179] |
| ENSG00000133250 | HGLibA_55939 | 476 | 3.588091855 | ZNF414 | zinc finger protein 414 [Source:HGNC Symbol;Acc:HGNC:20630] |
| ENSG00000206561 | HGLibA_10646 | 476 | 3.588091855 | COLQ | collagen like tail subunit of asymmetric acetylcholinesterase [Source:HGNC Symbol;Acc:HGNC:2226] |
| ZAK | HGLibA_54771 | 476 | 3.588091855 | ZAK | - |
| ENSG00000114784 | HGLibA_14716 | 475 | 3.578140818 | EIF1B | eukaryotic translation initiation factor 1B [Source:HGNC Symbol;Acc:HGNC:30792] |
| ENSG00000119121 | HGLibA_51768 | 475 | 3.578140818 | TRPM6 | transient receptor potential cation channel subfamily M member 6 [Source:HGNC Symbol;Acc:HGNC:17995] |
| ENSG00000124535 | HGLibA_54413 | 475 | 3.578140818 | WRNIP1 | Werner helicase interacting protein 1 [Source:HGNC Symbol;Acc:HGNC:20876] |
| ENSG00000140836 | HGLibA_55173 | 475 | 3.578140818 | ZFHX3 | zinc finger homeobox 3 [Source:HGNC Symbol;Acc:HGNC:777] |
| ENSG00000163879 | HGLibA_13649 | 475 | 3.578140818 | DNALI1 | dynein axonemal light intermediate chain 1 [Source:HGNC Symbol;Acc:HGNC:14353] |
| ENSG00000165804 | HGLibA_55604 | 475 | 3.578140818 | ZNF219 | zinc finger protein 219 [Source:HGNC Symbol;Acc:HGNC:13011] |
| ENSG00000170801 | HGLibA_22414 | 475 | 3.578140818 | HTRA3 | HtrA serine peptidase 3 [Source:HGNC Symbol;Acc:HGNC:30406] |
| ENSG00000174562 | HGLibA_25248 | 475 | 3.578140818 | KLK15 | kallikrein related peptidase 15 [Source:HGNC Symbol;Acc:HGNC:20453] |
| FAM173B | HGLibA_16280 | 475 | 3.578140818 | FAM173B | - |
| ENSG00000100281 | HGLibA_21734 | 474 | 3.568189781 | HMGXB4 | HMG-box containing 4 [Source:HGNC Symbol;Acc:HGNC:5003] |
| ENSG00000103642 | HGLibA_25917 | 474 | 3.568189781 | LACTB | lactamase beta [Source:HGNC Symbol;Acc:HGNC:16468] |
| ENSG00000116678 | HGLibA_26313 | 474 | 3.568189781 | LEPR | leptin receptor [Source:HGNC Symbol;Acc:HGNC:6554] |
| ENSG00000130876 | HGLibA_45348 | 474 | 3.568189781 | SLC7A10 | solute carrier family 7 member 10 [Source:HGNC Symbol;Acc:HGNC:11058] |
| ENSG00000132702 | HGLibA_20845 | 474 | 3.568189781 | HAPLN2 | hyaluronan and proteoglycan link protein 2 [Source:HGNC Symbol;Acc:HGNC:17410] |
| ENSG00000138207 | HGLibA_40637 | 474 | 3.568189781 | RBP4 | retinol binding protein 4 [Source:HGNC Symbol;Acc:HGNC:9922] |
| ENSG00000155093 | HGLibA_39524 | 474 | 3.568189781 | PTPRN2 | receptor type N2 [Source:HGNC Symbol;Acc:HGNC:9677]" |
| ENSG00000167637 | HGLibA_55740 | 474 | 3.568189781 | ZNF283 | zinc finger protein 283 [Source:HGNC Symbol;Acc:HGNC:13077] |
| ENSG00000172403 | HGLibA_48021 | 474 | 3.568189781 | SYNPO2 | synaptopodin 2 [Source:HGNC Symbol;Acc:HGNC:17732] |
| ENSG00000057149 | HGLibA_43550 | 473 | 3.558238743 | SERPINB3 | serpin family B member 3 [Source:HGNC Symbol;Acc:HGNC:10569] |
| ENSG00000125144 | HGLibA_30289 | 473 | 3.558238743 | MT1G | metallothionein 1G [Source:HGNC Symbol;Acc:HGNC:7399] |
| ENSG00000143375 | HGLibA_09321 | 473 | 3.558238743 | CGN | cingulin [Source:HGNC Symbol;Acc:HGNC:17429] |
| ENSG00000169214 | HGLibA_34415 | 473 | 3.558238743 | OR6F1 | olfactory receptor family 6 subfamily F member 1 [Source:HGNC Symbol;Acc:HGNC:15027] |
| ENSG00000258227 | HGLibA_10000 | 473 | 3.558238743 | CLEC5A | C-type lectin domain containing 5A [Source:HGNC Symbol;Acc:HGNC:2054] |
| ENSG00000275410 | HGLibA_21777 | 473 | 3.558238743 | HNF1B | HNF1 homeobox B [Source:HGNC Symbol;Acc:HGNC:11630] |
| ENSG00000013364 | HGLibA_30602 | 472 | 3.548287706 | MVP | major vault protein [Source:HGNC Symbol;Acc:HGNC:7531] |
| ENSG00000110031 | HGLibA_27127 | 472 | 3.548287706 | LPXN | leupaxin [Source:HGNC Symbol;Acc:HGNC:14061] |
| ENSG00000159708 | HGLibA_27324 | 472 | 3.548287706 | LRRC36 | leucine rich repeat containing 36 [Source:HGNC Symbol;Acc:HGNC:25615] |
| ENSG00000165113 | HGLibA_19204 | 472 | 3.548287706 | GKAP1 | G kinase anchoring protein 1 [Source:HGNC Symbol;Acc:HGNC:17496] |
| ENSG00000080511 | HGLibA_40753 | 471 | 3.538336668 | RDH8 | retinol dehydrogenase 8 [Source:HGNC Symbol;Acc:HGNC:14423] |
| ENSG00000175066 | HGLibA_19202 | 471 | 3.538336668 | GK5 | glycerol kinase 5 [Source:HGNC Symbol;Acc:HGNC:28635] |
| ENSG00000185247 | HGLibA_27898 | 471 | 3.538336668 | MAGEA11 | MAGE family member A11 [Source:HGNC Symbol;Acc:HGNC:6798] |
| ENSG00000110675 | HGLibA_14938 | 470 | 3.528385631 | ELMOD1 | ELMO domain containing 1 [Source:HGNC Symbol;Acc:HGNC:25334] |
| ENSG00000135253 | HGLibA_24407 | 470 | 3.528385631 | KCP | kielin cysteine rich BMP regulator [Source:HGNC Symbol;Acc:HGNC:17585] |
| ENSG00000141574 | HGLibA_43270 | 470 | 3.528385631 | SECTM1 | secreted and transmembrane 1 [Source:HGNC Symbol;Acc:HGNC:10707] |
| ENSG00000236398 | HGLibA_48397 | 470 | 3.528385631 | TAS2R39 | taste 2 receptor member 39 [Source:HGNC Symbol;Acc:HGNC:18886] |
| hsa-mir-6837 | HGLibA_63523 | 470 | 3.528385631 | hsa-mir-6837 | - |
| ENSG00000089177 | HGLibA_24867 | 469 | 3.518434593 | KIF16B | kinesin family member 16B [Source:HGNC Symbol;Acc:HGNC:15869] |
| ENSG00000104055 | HGLibA_49265 | 469 | 3.518434593 | TGM5 | transglutaminase 5 [Source:HGNC Symbol;Acc:HGNC:11781] |
| ENSG00000132915 | HGLibA_35804 | 469 | 3.518434593 | PDE6A | phosphodiesterase 6A [Source:HGNC Symbol;Acc:HGNC:8785] |
| ENSG00000137106 | HGLibA_20258 | 469 | 3.518434593 | GRHPR | glyoxylate and hydroxypyruvate reductase [Source:HGNC Symbol;Acc:HGNC:4570] |
| ENSG00000140481 | HGLibA_07756 | 469 | 3.518434593 | CCDC33 | coiled-coil domain containing 33 [Source:HGNC Symbol;Acc:HGNC:26552] |
| ENSG00000176783 | HGLibA_42437 | 469 | 3.518434593 | RUFY1 | RUN and FYVE domain containing 1 [Source:HGNC Symbol;Acc:HGNC:19760] |
| ENSG00000203867 | HGLibA_40506 | 469 | 3.518434593 | RBM20 | RNA binding motif protein 20 [Source:HGNC Symbol;Acc:HGNC:27424] |
| ENSG00000100154 | HGLibA_52096 | 468 | 3.508483556 | TTC28 | tetratricopeptide repeat domain 28 [Source:HGNC Symbol;Acc:HGNC:29179] |
| ENSG00000112280 | HGLibA_10621 | 468 | 3.508483556 | COL9A1 | collagen type IX alpha 1 chain [Source:HGNC Symbol;Acc:HGNC:2217] |
| ENSG00000154803 | HGLibA_17607 | 468 | 3.508483556 | FLCN | folliculin [Source:HGNC Symbol;Acc:HGNC:27310] |
| ENSG00000258659 | HGLibA_51468 | 468 | 3.508483556 | TRIM34 | tripartite motif containing 34 [Source:HGNC Symbol;Acc:HGNC:10063] |
| C20orf195 | HGLibA_06002 | 467 | 3.498532519 | C20orf195 | - |
| ENSG00000104689 | HGLibA_50732 | 467 | 3.498532519 | TNFRSF10A | TNF receptor superfamily member 10a [Source:HGNC Symbol;Acc:HGNC:11904] |
| ENSG00000110844 | HGLibA_38736 | 467 | 3.498532519 | PRPF40B | pre-mRNA processing factor 40 homolog B [Source:HGNC Symbol;Acc:HGNC:25031] |
| ENSG00000125998 | HGLibA_16745 | 467 | 3.498532519 | FAM83C | family with sequence similarity 83 member C [Source:HGNC Symbol;Acc:HGNC:16121] |
| ENSG00000137267 | HGLibA_52277 | 467 | 3.498532519 | TUBB2A | tubulin beta 2A class IIa [Source:HGNC Symbol;Acc:HGNC:12412] |
| ENSG00000165533 | HGLibA_52155 | 467 | 3.498532519 | TTC8 | tetratricopeptide repeat domain 8 [Source:HGNC Symbol;Acc:HGNC:20087] |
| ENSG00000004809 | HGLibA_44556 | 466 | 3.488581481 | SLC22A16 | solute carrier family 22 member 16 [Source:HGNC Symbol;Acc:HGNC:20302] |
| ENSG00000090539 | HGLibA_09535 | 466 | 3.488581481 | CHRD | chordin [Source:HGNC Symbol;Acc:HGNC:1949] |

| | | | | | |
|---|---|---|---|---|---|
| ENSG00000104228 | HGLibA_51472 | 466 | 3.488581481 | TRIM35 | tripartite motif containing 35 [Source:HGNC Symbol;Acc:HGNC:16285] |
| ENSG00000123643 | HGLibA_45030 | 466 | 3.488581481 | SLC36A1 | solute carrier family 36 member 1 [Source:HGNC Symbol;Acc:HGNC:18761] |
| ENSG00000138100 | HGLibA_51544 | 466 | 3.488581481 | TRIM54 | tripartite motif containing 54 [Source:HGNC Symbol;Acc:HGNC:16008] |
| hsa-mir-885 | HGLibA_64222 | 466 | 3.488581481 | hsa-mir-885 | - |
| PLA2G16 | HGLibA_36878 | 466 | 3.488581481 | PLA2G16 | - |
| ENSG00000124098 | HGLibA_16456 | 465 | 3.478630444 | FAM210B | family with sequence similarity 210 member B [Source:HGNC Symbol;Acc:HGNC:16102] |
| ENSG00000111701 | HGLibA_02467 | 464 | 3.468679406 | APOBEC1 | apolipoprotein B mRNA editing enzyme catalytic subunit 1 [Source:HGNC Symbol;Acc:HGNC:604] |
| ENSG00000146674 | HGLibA_22820 | 464 | 3.468679406 | IGFBP3 | insulin like growth factor binding protein 3 [Source:HGNC Symbol;Acc:HGNC:5472] |
| ENSG00000165917 | HGLibA_40269 | 464 | 3.468679406 | RAPSN | receptor associated protein of the synapse [Source:HGNC Symbol;Acc:HGNC:9863] |
| ENSG00000197140 | HGLibA_00773 | 464 | 3.468679406 | ADAM32 | ADAM metallopeptidase domain 32 [Source:HGNC Symbol;Acc:HGNC:15479] |
| ENSG00000009830 | HGLibA_37671 | 463 | 3.458728369 | POMT2 | protein O-mannosyltransferase 2 [Source:HGNC Symbol;Acc:HGNC:19743] |
| ENSG00000176194 | HGLibA_09697 | 463 | 3.458728369 | CIDEA | cell death-inducing DFFA-like effector a [Source:HGNC Symbol;Acc:HGNC:1976] |
| ENSG00000188162 | HGLibA_34730 | 463 | 3.458728369 | OTOG | otogelin [Source:HGNC Symbol;Acc:HGNC:8516] |
| ENSG00000204439 | HGLibA_06488 | 463 | 3.458728369 | C6orf47 | chromosome 6 open reading frame 47 [Source:HGNC Symbol;Acc:HGNC:19076] |
| ENSG00000232125 | HGLibA_14288 | 463 | 3.458728369 | DYTN | dystrotelin [Source:HGNC Symbol;Acc:HGNC:23279] |
| hsa-mir-941-2 | HGLibA_64344 | 463 | 3.458728369 | hsa-mir-941-2 | - |
| ENSG00000103460 | HGLibA_51042 | 462 | 3.448777331 | TOX3 | TOX high mobility group box family member 3 [Source:HGNC Symbol;Acc:HGNC:11972] |
| ENSG00000143515 | HGLibA_03784 | 462 | 3.448777331 | ATP8B2 | ATPase phospholipid transporting 8B2 [Source:HGNC Symbol;Acc:HGNC:13534] |
| ENSG00000163295 | HGLibA_01696 | 462 | 3.448777331 | ALPI | intestinal [Source:HGNC Symbol;Acc:HGNC:437]" |
| ENSG00000164674 | HGLibA_48103 | 462 | 3.448777331 | SYTL3 | synaptotagmin like 3 [Source:HGNC Symbol;Acc:HGNC:15587] |
| ENSG00000196123 | HGLibA_24660 | 462 | 3.448777331 | KIAA0895L | KIAA0895 like [Source:HGNC Symbol;Acc:HGNC:34408] |
| hsa-mir-4712 | HGLibA_60950 | 462 | 3.448777331 | hsa-mir-4712 | - |
| PLAC1L | HGLibA_36935 | 462 | 3.448777331 | PLAC1L | - |
| PQLC3 | HGLibA_38258 | 462 | 3.448777331 | PQLC3 | - |
| ENSG00000171227 | HGLibA_50374 | 461 | 3.438826294 | TMEM37 | transmembrane protein 37 [Source:HGNC Symbol;Acc:HGNC:18216] |
| ENSG00000178217 | HGLibA_43900 | 461 | 3.438826294 | SH2D4B | SH2 domain containing 4B [Source:HGNC Symbol;Acc:HGNC:31440] |
| LOC100505841 | HGLibA_26856 | 461 | 3.438826294 | LOC100505841 | - |
| TARSL2 | HGLibA_48351 | 461 | 3.438826294 | TARSL2 | - |
| ENSG00000010379 | HGLibA_45298 | 460 | 3.428875257 | SLC6A13 | solute carrier family 6 member 13 [Source:HGNC Symbol;Acc:HGNC:11046] |
| ENSG00000155008 | HGLibA_02554 | 460 | 3.428875257 | APOOL | apolipoprotein O like [Source:HGNC Symbol;Acc:HGNC:24009] |
| hsa-mir-4634 | HGLibA_60640 | 460 | 3.428875257 | hsa-mir-4634 | - |
| hsa-mir-541 | HGLibA_61813 | 460 | 3.428875257 | hsa-mir-541 | - |
| hsa-mir-941-4 | HGLibA_64352 | 460 | 3.428875257 | hsa-mir-941-4 | - |
| TMEM194A | HGLibA_50153 | 460 | 3.428875257 | TMEM194A | - |
| ENSG00000049618 | HGLibA_02885 | 459 | 3.418924219 | ARID1B | AT-rich interaction domain 1B [Source:HGNC Symbol;Acc:HGNC:18040] |
| ENSG00000112655 | HGLibA_39389 | 459 | 3.418924219 | PTK7 | protein tyrosine kinase 7 (inactive) [Source:HGNC Symbol;Acc:HGNC:9618] |
| ENSG00000119335 | HGLibA_43630 | 459 | 3.418924219 | SET | SET nuclear proto-oncogene [Source:HGNC Symbol;Acc:HGNC:10760] |
| ENSG00000171444 | HGLibA_28561 | 459 | 3.418924219 | MCC | WNT signaling pathway regulator [Source:HGNC Symbol;Acc:HGNC:6935]" |
| ENSG00000173401 | HGLibA_19261 | 459 | 3.418924219 | GLIPR1L1 | GLIPR1 like 1 [Source:HGNC Symbol;Acc:HGNC:28392] |
| C17orf105 | HGLibA_05526 | 458 | 3.408973182 | C17orf105 | - |
| hsa-mir-6775 | HGLibA_63275 | 458 | 3.408973182 | hsa-mir-6775 | - |
| hsa-mir-941-3 | HGLibA_64348 | 458 | 3.408973182 | hsa-mir-941-3 | - |
| C17orf112 | HGLibA_05532 | 457 | 3.399022144 | C17orf112 | - |
| ENSG00000165185 | HGLibA_24828 | 457 | 3.399022144 | KIAA1958 | KIAA1958 [Source:HGNC Symbol;Acc:HGNC:23427] |
| ENSG00000226372 | HGLibA_12460 | 457 | 3.399022144 | DCAF8L1 | DDB1 and CUL4 associated factor 8 like 1 [Source:HGNC Symbol;Acc:HGNC:31810] |
| ENSG00000241973 | HGLibA_36498 | 457 | 3.399022144 | PI4KA | phosphatidylinositol 4-kinase alpha [Source:HGNC Symbol;Acc:HGNC:8983] |
| ENSG00000080845 | HGLibA_13348 | 456 | 3.389071107 | DLGAP4 | DLG associated protein 4 [Source:HGNC Symbol;Acc:HGNC:24476] |
| ENSG00000147155 | HGLibA_14374 | 456 | 3.389071107 | EBP | cholestenol delta-isomerase [Source:HGNC Symbol;Acc:HGNC:3133]" |
| ENSG00000164265 | HGLibA_42935 | 456 | 3.389071107 | SCGB3A2 | secretoglobin family 3A member 2 [Source:HGNC Symbol;Acc:HGNC:18391] |
| ENSG00000185201 | HGLibA_22644 | 456 | 3.389071107 | IFITM2 | interferon induced transmembrane protein 2 [Source:HGNC Symbol;Acc:HGNC:5413] |
| ENSG00000250151 | HGLibA_03090 | 456 | 3.389071107 | ARPC4-TTLL3 | ARPC4-TTLL3 readthrough [Source:HGNC Symbol;Acc:HGNC:38830] |
| ENSG00000090615 | HGLibA_19597 | 455 | 3.379120069 | GOLGA3 | golgin A3 [Source:HGNC Symbol;Acc:HGNC:4426] |
| ENSG00000139648 | HGLibA_25525 | 455 | 3.379120069 | KRT71 | keratin 71 [Source:HGNC Symbol;Acc:HGNC:28927] |
| ENSG00000162009 | HGLibA_47187 | 455 | 3.379120069 | SSTR5 | somatostatin receptor 5 [Source:HGNC Symbol;Acc:HGNC:11334] |
| ENSG00000171045 | HGLibA_51883 | 455 | 3.379120069 | TSNARE1 | t-SNARE domain containing 1 [Source:HGNC Symbol;Acc:HGNC:26437] |
| hsa-mir-6895 | HGLibA_63763 | 455 | 3.379120069 | hsa-mir-6895 | - |
| ENSG00000030582 | HGLibA_20364 | 454 | 3.369169032 | GRN | granulin precursor [Source:HGNC Symbol;Acc:HGNC:4601] |
| ENSG00000124222 | HGLibA_47625 | 454 | 3.369169032 | STX16 | syntaxin 16 [Source:HGNC Symbol;Acc:HGNC:11431] |
| ENSG00000151239 | HGLibA_52368 | 454 | 3.369169032 | TWF1 | twinfilin actin binding protein 1 [Source:HGNC Symbol;Acc:HGNC:9620] |
| ENSG00000176903 | HGLibA_37365 | 454 | 3.369169032 | PNMA1 | PNMA family member 1 [Source:HGNC Symbol;Acc:HGNC:9158] |
| hsa-mir-890 | HGLibA_64238 | 454 | 3.369169032 | hsa-mir-890 | - |
| ENSG00000089006 | HGLibA_46100 | 453 | 3.359217995 | SNX5 | sorting nexin 5 [Source:HGNC Symbol;Acc:HGNC:14969] |
| ENSG00000150048 | HGLibA_09955 | 453 | 3.359217995 | CLEC1A | C-type lectin domain family 1 member A [Source:HGNC Symbol;Acc:HGNC:24355] |
| ENSG00000218891 | HGLibA_56303 | 453 | 3.359217995 | ZNF579 | zinc finger protein 579 [Source:HGNC Symbol;Acc:HGNC:26646] |
| ENSG00000004399 | HGLibA_37283 | 452 | 3.349266957 | PLXND1 | plexin D1 [Source:HGNC Symbol;Acc:HGNC:9107] |
| ENSG00000129535 | HGLibA_32704 | 452 | 3.349266957 | NRL | neural retina leucine zipper [Source:HGNC Symbol;Acc:HGNC:8002] |
| hsa-mir-429 | HGLibA_59866 | 452 | 3.349266957 | hsa-mir-429 | - |
| VWA9 | HGLibA_53921 | 452 | 3.349266957 | VWA9 | - |
| ENSG00000111405 | HGLibA_15119 | 451 | 3.33931592 | ENDOU | poly(U) specific [Source:HGNC Symbol;Acc:HGNC:14369]" |
| ENSG00000132746 | HGLibA_01572 | 451 | 3.33931592 | ALDH3B2 | aldehyde dehydrogenase 3 family member B2 [Source:HGNC Symbol;Acc:HGNC:411] |
| ENSG00000137075 | HGLibA_41671 | 451 | 3.33931592 | RNF38 | ring finger protein 38 [Source:HGNC Symbol;Acc:HGNC:18052] |
| ENSG00000147799 | HGLibA_02770 | 451 | 3.33931592 | ARHGAP39 | Rho GTPase activating protein 39 [Source:HGNC Symbol;Acc:HGNC:29351] |
| ENSG00000171502 | HGLibA_10549 | 451 | 3.33931592 | COL24A1 | collagen type XXIV alpha 1 chain [Source:HGNC Symbol;Acc:HGNC:20821] |
| ENSG00000178460 | HGLibA_28635 | 451 | 3.33931592 | MCMDC2 | minichromosome maintenance domain containing 2 [Source:HGNC Symbol;Acc:HGNC:26368] |
| hsa-mir-4721 | HGLibA_60980 | 451 | 3.33931592 | hsa-mir-4721 | - |
| ENSG00000108242 | HGLibA_12116 | 450 | 3.329364882 | CYP2C18 | cytochrome P450 family 2 subfamily C member 18 [Source:HGNC Symbol;Acc:HGNC:2620] |
| ENSG00000109705 | HGLibA_32051 | 450 | 3.329364882 | NKX3-2 | NK3 homeobox 2 [Source:HGNC Symbol;Acc:HGNC:951] |
| ENSG00000175745 | HGLibA_32610 | 450 | 3.329364882 | NR2F1 | nuclear receptor subfamily 2 group F member 1 [Source:HGNC Symbol;Acc:HGNC:7975] |
| ENSG00000186847 | HGLibA_25427 | 450 | 3.329364882 | KRT14 | keratin 14 [Source:HGNC Symbol;Acc:HGNC:6416] |
| ENSG00000019485 | HGLibA_38369 | 449 | 3.319413845 | PRDM11 | PR/SET domain 11 [Source:HGNC Symbol;Acc:HGNC:13996] |
| ENSG00000174950 | HGLibA_08253 | 449 | 3.319413845 | CD164L2 | CD164 molecule like 2 [Source:HGNC Symbol;Acc:HGNC:32043] |
| ENSG00000184838 | HGLibA_38790 | 449 | 3.319413845 | PRR16 | proline rich 16 [Source:HGNC Symbol;Acc:HGNC:29654] |
| ENSG00000073008 | HGLibA_39616 | 448 | 3.309462808 | PVR | poliovirus receptor [Source:HGNC Symbol;Acc:HGNC:9705] |
| ENSG00000117713 | HGLibA_02881 | 448 | 3.309462808 | ARID1A | AT-rich interaction domain 1A [Source:HGNC Symbol;Acc:HGNC:11110] |
| ENSG00000120440 | HGLibA_52197 | 448 | 3.309462808 | TTLL2 | tubulin tyrosine ligase like 2 [Source:HGNC Symbol;Acc:HGNC:21211] |
| ENSG00000187867 | HGLibA_35076 | 448 | 3.309462808 | PALM3 | paralemmin 3 [Source:HGNC Symbol;Acc:HGNC:33274] |
| hsa-mir-1288 | HGLibA_57616 | 448 | 3.309462808 | hsa-mir-1288 | - |
| ENSG00000110680 | HGLibA_06993 | 447 | 3.29951177 | CALCA | calcitonin related polypeptide alpha [Source:HGNC Symbol;Acc:HGNC:1437] |
| ENSG00000115194 | HGLibA_44907 | 447 | 3.29951177 | SLC30A3 | solute carrier family 30 member 3 [Source:HGNC Symbol;Acc:HGNC:11014] |
| ENSG00000155876 | HGLibA_42225 | 447 | 3.29951177 | RRAGA | Ras related GTP binding A [Source:HGNC Symbol;Acc:HGNC:16963] |
| ENSG00000213694 | HGLibA_42600 | 447 | 3.29951177 | S1PR3 | sphingosine-1-phosphate receptor 3 [Source:HGNC Symbol;Acc:HGNC:3167] |
| ADSS | HGLibA_01076 | 446 | 3.289560733 | ADSS | - |
| ENSG00000067365 | HGLibA_28988 | 446 | 3.289560733 | METTL22 | methyltransferase like 22 [Source:HGNC Symbol;Acc:HGNC:28368] |
| ENSG00000070882 | HGLibA_34656 | 446 | 3.289560733 | OSBPL3 | oxysterol binding protein like 3 [Source:HGNC Symbol;Acc:HGNC:16370] |
| ENSG00000126217 | HGLibA_28579 | 446 | 3.289560733 | MCF2L | MCF.2 cell line derived transforming sequence like [Source:HGNC Symbol;Acc:HGNC:14576] |
| ENSG00000172803 | HGLibA_46091 | 446 | 3.289560733 | SNX32 | sorting nexin 32 [Source:HGNC Symbol;Acc:HGNC:26423] |
| ENSG00000223614 | HGLibA_56651 | 446 | 3.289560733 | ZNF735 | zinc finger protein 735 [Source:HGNC Symbol;Acc:HGNC:32466] |
| ENSG00000224531 | HGLibA_45729 | 446 | 3.289560733 | SMIM13 | small integral membrane protein 13 [Source:HGNC Symbol;Acc:HGNC:27356] |
| hsa-mir-3935 | HGLibA_59602 | 446 | 3.289560733 | hsa-mir-3935 | - |
| LOC554223 | HGLibA_26955 | 446 | 3.289560733 | LOC554223 | - |
| ENSG00000100154 | HGLibA_52095 | 445 | 3.279609695 | TTC28 | tetratricopeptide repeat domain 28 [Source:HGNC Symbol;Acc:HGNC:29179] |
| ENSG00000115042 | HGLibA_15958 | 445 | 3.279609695 | FAHD2A | fumarylacetoacetate hydrolase domain containing 2A [Source:HGNC Symbol;Acc:HGNC:24252] |
| ENSG00000123933 | HGLibA_30617 | 445 | 3.279609695 | MXD4 | MAX dimerization protein 4 [Source:HGNC Symbol;Acc:HGNC:13906] |
| ENSG00000154646 | HGLibA_50632 | 445 | 3.279609695 | TMPRSS15 | transmembrane serine protease 15 [Source:HGNC Symbol;Acc:HGNC:9490] |
| ENSG00000197977 | HGLibA_14959 | 445 | 3.279609695 | ELOVL2 | ELOVL fatty acid elongase 2 [Source:HGNC Symbol;Acc:HGNC:14416] |
| ENSG00000100312 | HGLibA_00530 | 444 | 3.269658658 | ACR | acrosin [Source:HGNC Symbol;Acc:HGNC:126] |
| ENSG00000162714 | HGLibA_56102 | 444 | 3.269658658 | ZNF496 | zinc finger protein 496 [Source:HGNC Symbol;Acc:HGNC:23713] |
| ENSG00000165140 | HGLibA_16964 | 444 | 3.269658658 | FBP1 | fructose-bisphosphatase 1 [Source:HGNC Symbol;Acc:HGNC:3606] |
| ENSG00000185238 | HGLibA_38626 | 444 | 3.269658658 | PRMT3 | protein arginine methyltransferase 3 [Source:HGNC Symbol;Acc:HGNC:30163] |
| ENSG00000197891 | HGLibA_44543 | 444 | 3.269658658 | SLC22A12 | solute carrier family 22 member 12 [Source:HGNC Symbol;Acc:HGNC:17989] |
| ENSG00000256394 | HGLibA_03311 | 444 | 3.269658658 | ASIC5 | acid sensing ion channel subunit family member 5 [Source:HGNC Symbol;Acc:HGNC:17537] |
| ENSG00000269858 | HGLibA_14641 | 444 | 3.269658658 | EGLN2 | egl-9 family hypoxia inducible factor 2 [Source:HGNC Symbol;Acc:HGNC:14660] |
| hsa-mir-105-1 | HGLibA_57177 | 444 | 3.269658658 | hsa-mir-105-1 | - |
| hsa-mir-4526 | HGLibA_60567 | 444 | 3.269658658 | hsa-mir-4526 | - |
| ENSG00000147257 | HGLibA_19767 | 443 | 3.25970762 | GPC3 | glypican 3 [Source:HGNC Symbol;Acc:HGNC:4451] |
| ENSG00000168334 | HGLibA_54511 | 443 | 3.25970762 | XIRP1 | xin actin binding repeat containing 1 [Source:HGNC Symbol;Acc:HGNC:14301] |
| ENSG00000180535 | HGLibA_04468 | 443 | 3.25970762 | BHLHA15 | basic helix-loop-helix family member a15 [Source:HGNC Symbol;Acc:HGNC:22265] |
| ENSG00000198732 | HGLibA_45794 | 443 | 3.25970762 | SMOC1 | SPARC related modular calcium binding 1 [Source:HGNC Symbol;Acc:HGNC:20318] |
| ENSG00000243244 | HGLibA_47544 | 443 | 3.25970762 | STON1 | stonin 1 [Source:HGNC Symbol;Acc:HGNC:17003] |
| ENSG00000018625 | HGLibA_03584 | 442 | 3.249756583 | ATP1A2 | ATPase Na+/K+ transporting subunit alpha 2 [Source:HGNC Symbol;Acc:HGNC:800] |
| ENSG00000063241 | HGLibA_23686 | 442 | 3.249756583 | ISOC2 | isochorismatase domain containing 2 [Source:HGNC Symbol;Acc:HGNC:26278] |
| ENSG00000162729 | HGLibA_22903 | 442 | 3.249756583 | IGSF8 | immunoglobulin superfamily member 8 [Source:HGNC Symbol;Acc:HGNC:17813] |
| ENSG00000120742 | HGLibA_43499 | 441 | 3.239805546 | SERP1 | stress associated endoplasmic reticulum protein 1 [Source:HGNC Symbol;Acc:HGNC:10759] |
| ENSG00000164304 | HGLibA_06982 | 441 | 3.239805546 | CAGE1 | cancer antigen 1 [Source:HGNC Symbol;Acc:HGNC:21622] |
| ENSG00000167210 | HGLibA_27035 | 441 | 3.239805546 | LOXHD1 | lipoxygenase homology domains 1 [Source:HGNC Symbol;Acc:HGNC:26521] |
| ENSG00000204140 | HGLibA_10107 | 441 | 3.239805546 | CLPSL1 | colipase like 1 [Source:HGNC Symbol;Acc:HGNC:21251] |
| ENSG00000253731 | HGLibA_35539 | 441 | 3.239805546 | PCDHGA6 | 6 [Source:HGNC Symbol;Acc:HGNC:8704]" |
| ENSG00000108839 | HGLibA_01677 | 440 | 3.229854508 | ALOX12 | 12S type [Source:HGNC Symbol;Acc:HGNC:429]" |
| ENSG00000150764 | HGLibA_13287 | 440 | 3.229854508 | DIXDC1 | DIX domain containing 1 [Source:HGNC Symbol;Acc:HGNC:23695] |
| ENSG00000165661 | HGLibA_39756 | 440 | 3.229854508 | QSOX2 | quiescin sulfhydryl oxidase 2 [Source:HGNC Symbol;Acc:HGNC:30249] |
| ENSG00000167766 | HGLibA_56829 | 440 | 3.229854508 | ZNF83 | zinc finger protein 83 [Source:HGNC Symbol;Acc:HGNC:13158] |
| ENSG00000169906 | HGLibA_42580 | 440 | 3.229854508 | S100G | S100 calcium binding protein G [Source:HGNC Symbol;Acc:HGNC:1436] |
| ENSG00000171169 | HGLibA_31077 | 440 | 3.229854508 | NAIF1 | nuclear apoptosis inducing factor 1 [Source:HGNC Symbol;Acc:HGNC:25446] |
| ENSG00000237521 | HGLibA_34479 | 440 | 3.229854508 | OR7E24 | olfactory receptor family 7 subfamily E member 24 [Source:HGNC Symbol;Acc:HGNC:8396] |
| hsa-mir-125a | HGLibA_57444 | 440 | 3.229854508 | hsa-mir-125a | - |

| | | | | | |
|---|---|---|---|---|---|
| hsa-mir-6790 | HGLibA_63337 | 440 | 3.229854508 | hsa-mir-6790 | - |
| LOC440335 | HGLibA_26944 | 440 | 3.229854508 | LOC440335 | - |
| C6orf48 | HGLibA_06490 | 439 | 3.219903471 | C6orf48 | - |
| ENSG00000105270 | HGLibA_10047 | 439 | 3.219903471 | CLIP3 | CAP-Gly domain containing linker protein 3 [Source:HGNC Symbol;Acc:HGNC:24314] |
| ENSG00000108448 | HGLibA_51419 | 439 | 3.219903471 | TRIM16L | tripartite motif containing 16 like [Source:HGNC Symbol;Acc:HGNC:32670] |
| ENSG00000125656 | HGLibA_10102 | 439 | 3.219903471 | CLPP | caseinolytic mitochondrial matrix peptidase proteolytic subunit [Source:HGNC Symbol;Acc:HGNC:2084] |
| ENSG00000164889 | HGLibA_45213 | 439 | 3.219903471 | SLC4A2 | solute carrier family 4 member 2 [Source:HGNC Symbol;Acc:HGNC:11028] |
| ENSG00000171723 | HGLibA_19804 | 439 | 3.219903471 | GPHN | gephyrin [Source:HGNC Symbol;Acc:HGNC:15465] |
| ENSG00000181009 | HGLibA_34201 | 439 | 3.219903471 | OR52N5 | olfactory receptor family 52 subfamily N member 5 [Source:HGNC Symbol;Acc:HGNC:15231] |
| hsa-mir-6728 | HGLibA_63064 | 439 | 3.219903471 | hsa-mir-6728 | - |
| ENSG00000105707 | HGLibA_22037 | 438 | 3.209952433 | HPN | hepsin [Source:HGNC Symbol;Acc:HGNC:5155] |
| ENSG00000110324 | HGLibA_22947 | 438 | 3.209952433 | IL10RA | interleukin 10 receptor subunit alpha [Source:HGNC Symbol;Acc:HGNC:5964] |
| ENSG00000124091 | HGLibA_18821 | 438 | 3.209952433 | GCNT7 | glucosaminyl (N-acetyl) transferase family member 7 [Source:HGNC Symbol;Acc:HGNC:16099] |
| ENSG00000133115 | HGLibA_47541 | 438 | 3.209952433 | STOML3 | stomatin like 3 [Source:HGNC Symbol;Acc:HGNC:19420] |
| ENSG00000140368 | HGLibA_39238 | 438 | 3.209952433 | PSTPIP1 | proline-serine-threonine phosphatase interacting protein 1 [Source:HGNC Symbol;Acc:HGNC:9580] |
| ENSG00000198753 | HGLibA_37275 | 438 | 3.209952433 | PLXNB3 | plexin B3 [Source:HGNC Symbol;Acc:HGNC:9105] |
| ENSG00000076242 | HGLibA_29380 | 437 | 3.200001396 | MLH1 | mutL homolog 1 [Source:HGNC Symbol;Acc:HGNC:7127] |
| ENSG00000128536 | HGLibA_08703 | 437 | 3.200001396 | CDHR3 | cadherin related family member 3 [Source:HGNC Symbol;Acc:HGNC:26308] |
| ENSG00000151276 | HGLibA_27995 | 437 | 3.200001396 | MAGI1 | WW and PDZ domain containing 1 [Source:HGNC Symbol;Acc:HGNC:946]" |
| ENSG00000151422 | HGLibA_17286 | 437 | 3.200001396 | FER | FER tyrosine kinase [Source:HGNC Symbol;Acc:HGNC:3655] |
| ENSG00000160323 | HGLibA_00799 | 437 | 3.200001396 | ADAMTS13 | ADAM metallopeptidase with thrombospondin type 1 motif 13 [Source:HGNC Symbol;Acc:HGNC:1366] |
| ENSG00000188157 | HGLibA_01250 | 437 | 3.200001396 | AGRN | agrin [Source:HGNC Symbol;Acc:HGNC:329] |
| ENSG00000214128 | HGLibA_50213 | 437 | 3.200001396 | TMEM213 | transmembrane protein 213 [Source:HGNC Symbol;Acc:HGNC:27220] |
| ENSG00000090863 | HGLibA_19244 | 436 | 3.190050358 | GLG1 | golgi glycoprotein 1 [Source:HGNC Symbol;Acc:HGNC:4316] |
| ENSG00000109906 | HGLibA_54827 | 436 | 3.190050358 | ZBTB16 | zinc finger and BTB domain containing 16 [Source:HGNC Symbol;Acc:HGNC:12930] |
| ENSG00000115705 | HGLibA_51157 | 436 | 3.190050358 | TPO | thyroid peroxidase [Source:HGNC Symbol;Acc:HGNC:12015] |
| ENSG00000131873 | HGLibA_09655 | 436 | 3.190050358 | CHSY1 | chondroitin sulfate synthase 1 [Source:HGNC Symbol;Acc:HGNC:17198] |
| ENSG00000154001 | HGLibA_38181 | 436 | 3.190050358 | PPP2R5E | protein phosphatase 2 regulatory subunit B'epsilon [Source:HGNC Symbol;Acc:HGNC:9313] |
| ENSG00000159363 | HGLibA_03569 | 436 | 3.190050358 | ATP13A2 | ATPase cation transporting 13A2 [Source:HGNC Symbol;Acc:HGNC:30213] |
| ENSG00000161091 | HGLibA_29090 | 436 | 3.190050358 | MFSD12 | major facilitator superfamily domain containing 12 [Source:HGNC Symbol;Acc:HGNC:28299] |
| H1FOO | HGLibA_20738 | 436 | 3.190050358 | H1FOO | - |
| HIST1H2BO | HGLibA_21454 | 436 | 3.190050358 | HIST1H2BO | - |
| ALPPL2 | HGLibA_01715 | 435 | 3.180099321 | ALPPL2 | - |
| C7orf43 | HGLibA_06544 | 435 | 3.180099321 | C7orf43 | - |
| ENSG00000100439 | HGLibA_00278 | 435 | 3.180099321 | ABHD4 | abhydrolase domain containing 4 [Source:HGNC Symbol;Acc:HGNC:20154] |
| ENSG00000101966 | HGLibA_54507 | 435 | 3.180099321 | XIAP | X-linked inhibitor of apoptosis [Source:HGNC Symbol;Acc:HGNC:592] |
| ENSG00000102908 | HGLibA_31769 | 435 | 3.180099321 | NFAT5 | nuclear factor of activated T cells 5 [Source:HGNC Symbol;Acc:HGNC:7774] |
| ENSG00000125089 | HGLibA_43979 | 435 | 3.180099321 | SH3TC1 | SH3 domain and tetratricopeptide repeats 1 [Source:HGNC Symbol;Acc:HGNC:26009] |
| ENSG00000125247 | HGLibA_50678 | 435 | 3.180099321 | TMTC4 | transmembrane and tetratricopeptide repeat containing 4 [Source:HGNC Symbol;Acc:HGNC:25904] |
| ENSG00000151948 | HGLibA_19331 | 435 | 3.180099321 | GLT1D1 | glycosyltransferase 1 domain containing 1 [Source:HGNC Symbol;Acc:HGNC:26483] |
| ENSG00000155252 | HGLibA_36492 | 435 | 3.180099321 | PI4K2A | phosphatidylinositol 4-kinase type 2 alpha [Source:HGNC Symbol;Acc:HGNC:30031] |
| hsa-mir-378d-2 | HGLibA_59441 | 435 | 3.180099321 | hsa-mir-378d-2 | - |
| ENSG00000090539 | HGLibA_09534 | 434 | 3.170148284 | CHRD | chordin [Source:HGNC Symbol;Acc:HGNC:1949] |
| ENSG00000135341 | HGLibA_28196 | 434 | 3.170148284 | MAP3K7 | mitogen-activated protein kinase kinase kinase 7 [Source:HGNC Symbol;Acc:HGNC:6859] |
| ENSG00000144481 | HGLibA_51772 | 434 | 3.170148284 | TRPM8 | transient receptor potential cation channel subfamily M member 8 [Source:HGNC Symbol;Acc:HGNC:17961] |
| ENSG00000186493 | HGLibA_06379 | 434 | 3.170148284 | C5orf38 | chromosome 5 open reading frame 38 [Source:HGNC Symbol;Acc:HGNC:24226] |
| ENSG00000198889 | HGLibA_12421 | 434 | 3.170148284 | DCAF12L1 | DDB1 and CUL4 associated factor 12 like 1 [Source:HGNC Symbol;Acc:HGNC:29395] |
| ENSG00000205426 | HGLibA_25558 | 434 | 3.170148284 | KRT81 | keratin 81 [Source:HGNC Symbol;Acc:HGNC:6458] |
| hsa-mir-5197 | HGLibA_61708 | 434 | 3.170148284 | hsa-mir-5197 | - |
| ENSG00000086289 | HGLibA_15274 | 433 | 3.160197246 | EPDR1 | ependymin related 1 [Source:HGNC Symbol;Acc:HGNC:17572] |
| ENSG00000090539 | HGLibA_09536 | 433 | 3.160197246 | CHRD | chordin [Source:HGNC Symbol;Acc:HGNC:1949] |
| ENSG00000149428 | HGLibA_22462 | 433 | 3.160197246 | HYOU1 | hypoxia up-regulated 1 [Source:HGNC Symbol;Acc:HGNC:16931] |
| ENSG00000152154 | HGLibA_50081 | 433 | 3.160197246 | TMEM178A | transmembrane protein 178A [Source:HGNC Symbol;Acc:HGNC:28517] |
| ENSG00000155016 | HGLibA_12145 | 433 | 3.160197246 | CYP2U1 | cytochrome P450 family 2 subfamily U member 1 [Source:HGNC Symbol;Acc:HGNC:20582] |
| ENSG00000157214 | HGLibA_47426 | 433 | 3.160197246 | STEAP2 | STEAP2 metalloreductase [Source:HGNC Symbol;Acc:HGNC:17885] |
| ENSG00000009413 | HGLibA_40888 | 432 | 3.150246209 | REV3L | DNA directed polymerase zeta catalytic subunit [Source:HGNC Symbol;Acc:HGNC:9968]" |
| ENSG00000121743 | HGLibA_19142 | 432 | 3.150246209 | GJA3 | gap junction protein alpha 3 [Source:HGNC Symbol;Acc:HGNC:4277] |
| ENSG00000125656 | HGLibA_10100 | 432 | 3.150246209 | CLPP | caseinolytic mitochondrial matrix peptidase proteolytic subunit [Source:HGNC Symbol;Acc:HGNC:2084] |
| ENSG00000126882 | HGLibA_16728 | 432 | 3.150246209 | FAM78A | family with sequence similarity 78 member A [Source:HGNC Symbol;Acc:HGNC:25465] |
| ENSG00000143867 | HGLibA_34700 | 432 | 3.150246209 | OSR1 | odd-skipped related transciption factor 1 [Source:HGNC Symbol;Acc:HGNC:8111] |
| ENSG00000144339 | HGLibA_49836 | 432 | 3.150246209 | TMEFF2 | transmembrane protein with EGF like and two follistatin like domains 2 [Source:HGNC Symbol;Acc:HGNC:11867] |
| ENSG00000146809 | HGLibA_03221 | 432 | 3.150246209 | ASB15 | ankyrin repeat and SOCS box containing 15 [Source:HGNC Symbol;Acc:HGNC:19767] |
| ENSG00000151914 | HGLibA_14044 | 432 | 3.150246209 | DST | dystonin [Source:HGNC Symbol;Acc:HGNC:1090] |
| ENSG00000175970 | HGLibA_52990 | 432 | 3.150246209 | UNC119B | unc-119 lipid binding chaperone B [Source:HGNC Symbol;Acc:HGNC:16488] |
| ENSG00000178719 | HGLibA_20316 | 432 | 3.150246209 | GRINA | glutamate ionotropic receptor NMDA type subunit associated protein 1 [Source:HGNC Symbol;Acc:HGNC:4589] |
| ENSG00000100626 | HGLibA_18547 | 431 | 3.140295171 | GALNT16 | polypeptide N-acetylgalactosaminyltransferase 16 [Source:HGNC Symbol;Acc:HGNC:23233] |
| ENSG00000124302 | HGLibA_09650 | 431 | 3.140295171 | CHST8 | carbohydrate sulfotransferase 8 [Source:HGNC Symbol;Acc:HGNC:15993] |
| ENSG00000127314 | HGLibA_40225 | 431 | 3.140295171 | RAP1B | member of RAS oncogene family [Source:HGNC Symbol;Acc:HGNC:9857]" |
| ENSG00000149133 | HGLibA_34286 | 431 | 3.140295171 | OR5F1 | olfactory receptor family 5 subfamily F member 1 [Source:HGNC Symbol;Acc:HGNC:8343] |
| ENSG00000186513 | HGLibA_34586 | 431 | 3.140295171 | OR9Q2 | olfactory receptor family 9 subfamily Q member 2 [Source:HGNC Symbol;Acc:HGNC:15328] |
| ENSG00000188211 | HGLibA_31372 | 431 | 3.140295171 | NCR3LG1 | natural killer cell cytotoxicity receptor 3 ligand 1 [Source:HGNC Symbol;Acc:HGNC:42400] |
| ENSG00000103035 | HGLibA_39180 | 430 | 3.130344134 | PSMD7 | non-ATPase 7 [Source:HGNC Symbol;Acc:HGNC:9565]" |
| ENSG00000126247 | HGLibA_07185 | 430 | 3.130344134 | CAPNS1 | calpain small subunit 1 [Source:HGNC Symbol;Acc:HGNC:1481] |
| ENSG00000184730 | HGLibA_02500 | 430 | 3.130344134 | APOBR | apolipoprotein B receptor [Source:HGNC Symbol;Acc:HGNC:24087] |
| C1orf64 | HGLibA_05885 | 429 | 3.120393096 | C1orf64 | - |
| ENSG00000134779 | HGLibA_51127 | 429 | 3.120393096 | TPGS2 | tubulin polyglutamylase complex subunit 2 [Source:HGNC Symbol;Acc:HGNC:24561] |
| ENSG00000156136 | HGLibA_12497 | 429 | 3.120393096 | DCK | deoxycytidine kinase [Source:HGNC Symbol;Acc:HGNC:2704] |
| ENSG00000160285 | HGLibA_27569 | 429 | 3.120393096 | LSS | lanosterol synthase [Source:HGNC Symbol;Acc:HGNC:6708] |
| ENSG00000189164 | HGLibA_56166 | 429 | 3.120393096 | ZNF527 | zinc finger protein 527 [Source:HGNC Symbol;Acc:HGNC:29385] |
| ENSG00000198538 | HGLibA_55720 | 429 | 3.120393096 | ZNF28 | zinc finger protein 28 [Source:HGNC Symbol;Acc:HGNC:13073] |
| ENSG00000198542 | HGLibA_23802 | 429 | 3.120393096 | ITGBL1 | integrin subunit beta like 1 [Source:HGNC Symbol;Acc:HGNC:6164] |
| ENSG00000203795 | HGLibA_16547 | 429 | 3.120393096 | FAM24A | family with sequence similarity 24 member A [Source:HGNC Symbol;Acc:HGNC:23470] |
| ENSG00000131849 | HGLibA_55461 | 428 | 3.110442059 | ZNF132 | zinc finger protein 132 [Source:HGNC Symbol;Acc:HGNC:12916] |
| ENSG00000164303 | HGLibA_15181 | 428 | 3.110442059 | ENPP6 | ectonucleotide pyrophosphatase/phosphodiesterase 6 [Source:HGNC Symbol;Acc:HGNC:23409] |
| ENSG00000167972 | HGLibA_00111 | 428 | 3.110442059 | ABCA3 | ATP binding cassette subfamily A member 3 [Source:HGNC Symbol;Acc:HGNC:33] |
| ENSG00000176922 | HGLibA_34130 | 428 | 3.110442059 | OR51S1 | olfactory receptor family 51 subfamily S member 1 [Source:HGNC Symbol;Acc:HGNC:15204] |
| ENSG00000180210 | HGLibA_15858 | 428 | 3.110442059 | F2 | thrombin [Source:HGNC Symbol;Acc:HGNC:3535]" |
| hsa-mir-7703 | HGLibA_63949 | 428 | 3.110442059 | hsa-mir-7703 | - |
| ENSG00000100234 | HGLibA_49541 | 427 | 3.100491022 | TIMP3 | TIMP metallopeptidase inhibitor 3 [Source:HGNC Symbol;Acc:HGNC:11822] |
| ENSG00000106853 | HGLibA_39351 | 427 | 3.100491022 | PTGR1 | prostaglandin reductase 1 [Source:HGNC Symbol;Acc:HGNC:18429] |
| ENSG00000130988 | HGLibA_41020 | 427 | 3.100491022 | RGN | regucalcin [Source:HGNC Symbol;Acc:HGNC:9989] |
| ENSG00000134030 | HGLibA_11633 | 427 | 3.100491022 | CTIF | cap binding complex dependent translation initiation factor [Source:HGNC Symbol;Acc:HGNC:23925] |
| ENSG00000163114 | HGLibA_35866 | 427 | 3.100491022 | PDHA2 | pyruvate dehydrogenase E1 alpha 2 subunit [Source:HGNC Symbol;Acc:HGNC:8807] |
| ENSG00000171401 | HGLibA_25422 | 427 | 3.100491022 | KRT13 | keratin 13 [Source:HGNC Symbol;Acc:HGNC:6415] |
| ENSG00000185090 | HGLibA_28093 | 427 | 3.100491022 | MANEAL | mannosidase endo-alpha like [Source:HGNC Symbol;Acc:HGNC:26452] |
| hsa-mir-7108 | HGLibA_63794 | 427 | 3.100491022 | hsa-mir-7108 | - |
| ENSG00000047597 | HGLibA_54517 | 426 | 3.090539984 | XK | X-linked Kx blood group [Source:HGNC Symbol;Acc:HGNC:12811] |
| ENSG00000078898 | HGLibA_04721 | 426 | 3.090539984 | BPIFB2 | BPI fold containing family B member 2 [Source:HGNC Symbol;Acc:HGNC:16177] |
| ENSG00000111206 | HGLibA_17905 | 426 | 3.090539984 | FOXM1 | forkhead box M1 [Source:HGNC Symbol;Acc:HGNC:3818] |
| ENSG00000138867 | HGLibA_20649 | 426 | 3.090539984 | GUCD1 | guanylyl cyclase domain containing 1 [Source:HGNC Symbol;Acc:HGNC:14237] |
| ENSG00000175325 | HGLibA_38686 | 426 | 3.090539984 | PROP1 | PROP paired-like homeobox 1 [Source:HGNC Symbol;Acc:HGNC:9455] |
| ENSG00000222014 | HGLibA_39977 | 426 | 3.090539984 | RAB6C | member RAS oncogene family [Source:HGNC Symbol;Acc:HGNC:16525]" |
| ENSG00000087111 | HGLibA_36586 | 425 | 3.080588947 | PIGS | phosphatidylinositol glycan anchor biosynthesis class S [Source:HGNC Symbol;Acc:HGNC:14937] |
| ENSG00000108947 | HGLibA_14602 | 425 | 3.080588947 | EFNB3 | ephrin B3 [Source:HGNC Symbol;Acc:HGNC:3228] |
| ENSG00000120057 | HGLibA_43745 | 425 | 3.080588947 | SFRP5 | secreted frizzled related protein 5 [Source:HGNC Symbol;Acc:HGNC:10779] |
| ENSG00000121314 | HGLibA_48430 | 425 | 3.080588947 | TAS2R8 | taste 2 receptor member 8 [Source:HGNC Symbol;Acc:HGNC:14915] |
| ENSG00000143793 | HGLibA_05856 | 425 | 3.080588947 | C1orf35 | chromosome 1 open reading frame 35 [Source:HGNC Symbol;Acc:HGNC:19032] |
| ENSG00000170558 | HGLibA_08660 | 425 | 3.080588947 | CDH2 | cadherin 2 [Source:HGNC Symbol;Acc:HGNC:1759] |
| ENSG00000174776 | HGLibA_54112 | 425 | 3.080588947 | WDR49 | WD repeat domain 49 [Source:HGNC Symbol;Acc:HGNC:26587] |
| ENSG00000182979 | HGLibA_30309 | 425 | 3.080588947 | MTA1 | metastasis associated 1 [Source:HGNC Symbol;Acc:HGNC:7410] |
| ENSG00000204472 | HGLibA_01330 | 425 | 3.080588947 | AIF1 | allograft inflammatory factor 1 [Source:HGNC Symbol;Acc:HGNC:352] |
| ENSG00000007080 | HGLibA_07574 | 424 | 3.070637909 | CCDC124 | coiled-coil domain containing 124 [Source:HGNC Symbol;Acc:HGNC:25171] |
| ENSG00000070501 | HGLibA_37489 | 424 | 3.070637909 | POLB | DNA polymerase beta [Source:HGNC Symbol;Acc:HGNC:9174] |
| ENSG00000129009 | HGLibA_23671 | 424 | 3.070637909 | ISLR | immunoglobulin superfamily containing leucine rich repeat [Source:HGNC Symbol;Acc:HGNC:6133] |
| ENSG00000184730 | HGLibA_02499 | 424 | 3.070637909 | APOBR | apolipoprotein B receptor [Source:HGNC Symbol;Acc:HGNC:24087] |
| ENSG00000198822 | HGLibA_20348 | 424 | 3.070637909 | GRM3 | glutamate metabotropic receptor 3 [Source:HGNC Symbol;Acc:HGNC:4595] |
| ENSG00000002330 | HGLibA_04071 | 423 | 3.060686872 | BAD | BCL2 associated agonist of cell death [Source:HGNC Symbol;Acc:HGNC:936] |
| ENSG00000143001 | HGLibA_50469 | 423 | 3.060686872 | TMEM61 | transmembrane protein 61 [Source:HGNC Symbol;Acc:HGNC:27296] |
| ENSG00000147804 | HGLibA_45112 | 423 | 3.060686872 | SLC39A4 | solute carrier family 39 member 4 [Source:HGNC Symbol;Acc:HGNC:17129] |
| ENSG00000155966 | HGLibA_01108 | 423 | 3.060686872 | AFF2 | AF4/FMR2 family member 2 [Source:HGNC Symbol;Acc:HGNC:3776] |
| ENSG00000162148 | HGLibA_38086 | 423 | 3.060686872 | PPP1R32 | protein phosphatase 1 regulatory subunit 32 [Source:HGNC Symbol;Acc:HGNC:28869] |
| ENSG00000170369 | HGLibA_11450 | 423 | 3.060686872 | CST2 | cystatin SA [Source:HGNC Symbol;Acc:HGNC:2474] |
| ENSG00000172081 | HGLibA_29593 | 423 | 3.060686872 | MOB3A | MOB kinase activator 3A [Source:HGNC Symbol;Acc:HGNC:29802] |
| ENSG00000182107 | HGLibA_50362 | 423 | 3.060686872 | TMEM30B | transmembrane protein 30B [Source:HGNC Symbol;Acc:HGNC:27254] |
| ENSG00000183395 | HGLibA_37295 | 423 | 3.060686872 | PMCH | pro-melanin concentrating hormone [Source:HGNC Symbol;Acc:HGNC:9109] |
| ENSG00000085563 | HGLibA_00130 | 422 | 3.050735834 | ABCB1 | ATP binding cassette subfamily B member 1 [Source:HGNC Symbol;Acc:HGNC:40] |
| ENSG00000117450 | HGLibA_38408 | 422 | 3.050735834 | PRDX1 | peroxiredoxin 1 [Source:HGNC Symbol;Acc:HGNC:9352] |
| ENSG00000120725 | HGLibA_44170 | 422 | 3.050735834 | SIL1 | SIL1 nucleotide exchange factor [Source:HGNC Symbol;Acc:HGNC:24624] |
| ENSG00000136694 | HGLibA_23145 | 422 | 3.050735834 | IL36A | interleukin 36 alpha [Source:HGNC Symbol;Acc:HGNC:15562] |
| ENSG00000164122 | HGLibA_03242 | 422 | 3.050735834 | ASB5 | ankyrin repeat and SOCS box containing 5 [Source:HGNC Symbol;Acc:HGNC:17180] |
| ENSG00000171714 | HGLibA_02182 | 422 | 3.050735834 | ANO5 | anoctamin 5 [Source:HGNC Symbol;Acc:HGNC:27337] |
| ENSG00000198945 | HGLibA_25901 | 422 | 3.050735834 | L3MBTL3 | histone methyl-lysine binding protein [Source:HGNC Symbol;Acc:HGNC:23035]" |
| hsa-mir-3649 | HGLibA_59140 | 422 | 3.050735834 | hsa-mir-3649 | - |
| ENSG00000075891 | HGLibA_35299 | 421 | 3.040784797 | PAX2 | paired box 2 [Source:HGNC Symbol;Acc:HGNC:8616] |
| ENSG00000110042 | HGLibA_14091 | 421 | 3.040784797 | DTX4 | deltex E3 ubiquitin ligase 4 [Source:HGNC Symbol;Acc:HGNC:29151] |

| | | | | | |
|---|---|---|---|---|---|
| ENSG00000111676 | HGLibA_03529 | 421 | 3.040784797 | ATN1 | atrophin 1 [Source:HGNC Symbol;Acc:HGNC:3033] |
| ENSG00000126705 | HGLibA_01296 | 421 | 3.040784797 | AHDC1 | AT-hook DNA binding motif containing 1 [Source:HGNC Symbol;Acc:HGNC:25230] |
| ENSG00000142634 | HGLibA_14578 | 421 | 3.040784797 | EFHD2 | EF-hand domain family member D2 [Source:HGNC Symbol;Acc:HGNC:28670] |
| ENSG00000166130 | HGLibA_22915 | 421 | 3.040784797 | IKBIP | IKBKB interacting protein [Source:HGNC Symbol;Acc:HGNC:26430] |
| hsa-mir-4304 | HGLibA_59929 | 421 | 3.040784797 | hsa-mir-4304 | - |
| ENSG00000116329 | HGLibA_33444 | 420 | 3.03083376 | OPRD1 | opioid receptor delta 1 [Source:HGNC Symbol;Acc:HGNC:8153] |
| ENSG00000131914 | HGLibA_26557 | 420 | 3.03083376 | LIN28A | lin-28 homolog A [Source:HGNC Symbol;Acc:HGNC:15986] |
| ENSG00000198168 | HGLibA_47889 | 420 | 3.03083376 | SVIP | small VCP interacting protein [Source:HGNC Symbol;Acc:HGNC:25238] |
| ENSG00000100519 | HGLibA_39144 | 419 | 3.020882722 | PSMC6 | ATPase 6 [Source:HGNC Symbol;Acc:HGNC:9553]" |
| ENSG00000112499 | HGLibA_44567 | 419 | 3.020882722 | SLC22A2 | solute carrier family 22 member 2 [Source:HGNC Symbol;Acc:HGNC:10966] |
| ENSG00000165282 | HGLibA_36573 | 419 | 3.020882722 | PIGO | phosphatidylinositol glycan anchor biosynthesis class O [Source:HGNC Symbol;Acc:HGNC:23215] |
| ENSG00000171853 | HGLibA_51305 | 419 | 3.020882722 | TRAPPC12 | trafficking protein particle complex 12 [Source:HGNC Symbol;Acc:HGNC:24284] |
| ENSG00000179588 | HGLibA_55251 | 419 | 3.020882722 | ZFPM1 | FOG family member 1 [Source:HGNC Symbol;Acc:HGNC:19762]" |
| ENSG00000011405 | HGLibA_36620 | 418 | 3.010931685 | PIK3C2A | phosphatidylinositol-4-phosphate 3-kinase catalytic subunit type 2 alpha [Source:HGNC Symbol;Acc:HGNC:8971] |
| ENSG00000113441 | HGLibA_26740 | 418 | 3.010931685 | LNPEP | leucyl and cystinyl aminopeptidase [Source:HGNC Symbol;Acc:HGNC:6656] |
| ENSG00000125864 | HGLibA_04456 | 418 | 3.010931685 | BFSP1 | beaded filament structural protein 1 [Source:HGNC Symbol;Acc:HGNC:1040] |
| ENSG00000152092 | HGLibA_03379 | 418 | 3.010931685 | ASTN1 | astrotactin 1 [Source:HGNC Symbol;Acc:HGNC:773] |
| ENSG00000156076 | HGLibA_54300 | 418 | 3.010931685 | WIF1 | WNT inhibitory factor 1 [Source:HGNC Symbol;Acc:HGNC:18081] |
| ENSG00000166349 | HGLibA_40132 | 418 | 3.010931685 | RAG1 | recombination activating 1 [Source:HGNC Symbol;Acc:HGNC:9831] |
| ENSG00000181191 | HGLibA_36782 | 418 | 3.010931685 | PJA1 | praja ring finger ubiquitin ligase 1 [Source:HGNC Symbol;Acc:HGNC:16648] |
| ENSG00000182557 | HGLibA_46738 | 418 | 3.010931685 | SPNS3 | sphingolipid transporter 3 (putative) [Source:HGNC Symbol;Acc:HGNC:28433] |
| ENSG00000247315 | HGLibA_55034 | 418 | 3.010931685 | ZCCHC3 | zinc finger CCHC-type containing 3 [Source:HGNC Symbol;Acc:HGNC:16230] |
| ENSG00000249992 | HGLibA_50015 | 418 | 3.010931685 | TMEM158 | transmembrane protein 158 (gene/pseudogene) [Source:HGNC Symbol;Acc:HGNC:30293] |
| ENSG00000065717 | HGLibA_49610 | 417 | 3.000980647 | TLE2 | transducin like enhancer of split 2 [Source:HGNC Symbol;Acc:HGNC:11838] |
| ENSG00000089050 | HGLibA_40447 | 417 | 3.000980647 | RBBP9 | serine hydrolase [Source:HGNC Symbol;Acc:HGNC:9892]" |
| ENSG00000124155 | HGLibA_36588 | 417 | 3.000980647 | PIGT | phosphatidylinositol glycan anchor biosynthesis class T [Source:HGNC Symbol;Acc:HGNC:14938] |
| ENSG00000135597 | HGLibA_40847 | 417 | 3.000980647 | REPS1 | RALBP1 associated Eps domain containing 1 [Source:HGNC Symbol;Acc:HGNC:15578] |
| ENSG00000137171 | HGLibA_25037 | 417 | 3.000980647 | KLC4 | kinesin light chain 4 [Source:HGNC Symbol;Acc:HGNC:21624] |
| ENSG00000172938 | HGLibA_29838 | 417 | 3.000980647 | MRGPRD | MAS related GPR family member D [Source:HGNC Symbol;Acc:HGNC:29626] |
| ENSG00000179542 | HGLibA_45522 | 417 | 3.000980647 | SLITRK4 | SLIT and NTRK like family member 4 [Source:HGNC Symbol;Acc:HGNC:23502] |
| hsa-mir-8082 | HGLibA_64171 | 417 | 3.000980647 | hsa-mir-8082 | - |
| ENSG00000118804 | HGLibA_47410 | 416 | 2.99102961 | STBD1 | starch binding domain 1 [Source:HGNC Symbol;Acc:HGNC:24854] |
| ENSG00000130584 | HGLibA_54898 | 416 | 2.99102961 | ZBTB46 | zinc finger and BTB domain containing 46 [Source:HGNC Symbol;Acc:HGNC:16094] |
| ENSG00000131389 | HGLibA_45334 | 416 | 2.99102961 | SLC6A6 | solute carrier family 6 member 6 [Source:HGNC Symbol;Acc:HGNC:11052] |
| ENSG00000137441 | HGLibA_17429 | 416 | 2.99102961 | FGFBP2 | fibroblast growth factor binding protein 2 [Source:HGNC Symbol;Acc:HGNC:29451] |
| ENSG00000163914 | HGLibA_41162 | 416 | 2.99102961 | RHO | rhodopsin [Source:HGNC Symbol;Acc:HGNC:10012] |
| ENSG00000171723 | HGLibA_19805 | 416 | 2.99102961 | GPHN | gephyrin [Source:HGNC Symbol;Acc:HGNC:15465] |
| ENSG00000185674 | HGLibA_27702 | 416 | 2.99102961 | LYG2 | lysozyme g2 [Source:HGNC Symbol;Acc:HGNC:29615] |
| FAM109B | HGLibA_16009 | 416 | 2.99102961 | FAM109B | - |
| FAM159A | HGLibA_16197 | 416 | 2.99102961 | FAM159A | - |
| ENSG00000075213 | HGLibA_43318 | 415 | 2.981078572 | SEMA3A | semaphorin 3A [Source:HGNC Symbol;Acc:HGNC:10723] |
| ENSG00000127328 | HGLibA_39928 | 415 | 2.981078572 | RAB3IP | RAB3A interacting protein [Source:HGNC Symbol;Acc:HGNC:16508] |
| ENSG00000130234 | HGLibA_00425 | 415 | 2.981078572 | ACE2 | angiotensin I converting enzyme 2 [Source:HGNC Symbol;Acc:HGNC:13557] |
| ENSG00000148602 | HGLibA_27185 | 415 | 2.981078572 | LRIT1 | Ig-like and transmembrane domains 1 [Source:HGNC Symbol;Acc:HGNC:23404]" |
| ENSG00000187672 | HGLibA_15427 | 415 | 2.981078572 | ERC2 | ELKS/RAB6-interacting/CAST family member 2 [Source:HGNC Symbol;Acc:HGNC:31922] |
| ENSG00000211584 | HGLibA_45199 | 415 | 2.981078572 | SLC48A1 | solute carrier family 48 member 1 [Source:HGNC Symbol;Acc:HGNC:26035] |
| ENSG00000121570 | HGLibA_13893 | 414 | 2.971127535 | DPPA4 | developmental pluripotency associated 4 [Source:HGNC Symbol;Acc:HGNC:19200] |
| ENSG00000127951 | HGLibA_17466 | 414 | 2.971127535 | FGL2 | fibrinogen like 2 [Source:HGNC Symbol;Acc:HGNC:3696] |
| ENSG00000134326 | HGLibA_10202 | 414 | 2.971127535 | CMPK2 | cytidine/uridine monophosphate kinase 2 [Source:HGNC Symbol;Acc:HGNC:27015] |
| ENSG00000137265 | HGLibA_23588 | 414 | 2.971127535 | IRF4 | interferon regulatory factor 4 [Source:HGNC Symbol;Acc:HGNC:6119] |
| ENSG00000146376 | HGLibA_02716 | 414 | 2.971127535 | ARHGAP18 | Rho GTPase activating protein 18 [Source:HGNC Symbol;Acc:HGNC:21035] |
| ENSG00000147889 | HGLibA_08831 | 414 | 2.971127535 | CDKN2A | cyclin dependent kinase inhibitor 2A [Source:HGNC Symbol;Acc:HGNC:1787] |
| ENSG00000156398 | HGLibA_43784 | 414 | 2.971127535 | SFXN2 | sideroflexin 2 [Source:HGNC Symbol;Acc:HGNC:16086] |
| ENSG00000172270 | HGLibA_04866 | 414 | 2.971127535 | BSG | basigin (Ok blood group) [Source:HGNC Symbol;Acc:HGNC:1116] |
| ENSG00000186862 | HGLibA_35981 | 414 | 2.971127535 | PDZD7 | PDZ domain containing 7 [Source:HGNC Symbol;Acc:HGNC:26257] |
| ENSG00000187118 | HGLibA_10185 | 414 | 2.971127535 | CMC1 | C-X9-C motif containing 1 [Source:HGNC Symbol;Acc:HGNC:28783] |
| hsa-mir-6730 | HGLibA_63072 | 414 | 2.971127535 | hsa-mir-6730 | - |
| ENSG00000085511 | HGLibA_28187 | 413 | 2.961176498 | MAP3K4 | mitogen-activated protein kinase kinase kinase 4 [Source:HGNC Symbol;Acc:HGNC:6856] |
| ENSG00000182253 | HGLibA_48016 | 413 | 2.961176498 | SYNM | synemin [Source:HGNC Symbol;Acc:HGNC:24466] |
| FAM134A | HGLibA_16122 | 413 | 2.961176498 | FAM134A | - |
| ENSG00000158062 | HGLibA_52779 | 412 | 2.95122546 | UBXN11 | UBX domain protein 11 [Source:HGNC Symbol;Acc:HGNC:30600] |
| ENSG00000160349 | HGLibA_26167 | 412 | 2.95122546 | LCN1 | lipocalin 1 [Source:HGNC Symbol;Acc:HGNC:6525] |
| hsa-mir-922 | HGLibA_64281 | 412 | 2.95122546 | hsa-mir-922 | - |
| ENSG00000006757 | HGLibA_37415 | 411 | 2.941274423 | PNPLA4 | patatin like phospholipase domain containing 4 [Source:HGNC Symbol;Acc:HGNC:24887] |
| ENSG00000091409 | HGLibA_23738 | 411 | 2.941274423 | ITGA6 | integrin subunit alpha 6 [Source:HGNC Symbol;Acc:HGNC:6142] |
| ENSG00000095370 | HGLibA_43894 | 411 | 2.941274423 | SH2D3C | SH2 domain containing 3C [Source:HGNC Symbol;Acc:HGNC:16884] |
| ENSG00000109758 | HGLibA_21244 | 411 | 2.941274423 | HGFAC | HGF activator [Source:HGNC Symbol;Acc:HGNC:4894] |
| ENSG00000125450 | HGLibA_33068 | 411 | 2.941274423 | NUP85 | nucleoporin 85 [Source:HGNC Symbol;Acc:HGNC:8734] |
| ENSG00000163964 | HGLibA_36600 | 411 | 2.941274423 | PIGX | phosphatidylinositol glycan anchor biosynthesis class X [Source:HGNC Symbol;Acc:HGNC:26046] |
| ENSG00000166562 | HGLibA_43184 | 411 | 2.941274423 | SEC11C | signal peptidase complex subunit [Source:HGNC Symbol;Acc:HGNC:23400]" |
| ENSG00000166736 | HGLibA_22380 | 411 | 2.941274423 | HTR3A | 5-hydroxytryptamine receptor 3A [Source:HGNC Symbol;Acc:HGNC:5297] |
| ENSG00000167670 | HGLibA_09347 | 411 | 2.941274423 | CHAF1A | chromatin assembly factor 1 subunit A [Source:HGNC Symbol;Acc:HGNC:1910] |
| ENSG00000184292 | HGLibA_48179 | 411 | 2.941274423 | TACSTD2 | tumor associated calcium signal transducer 2 [Source:HGNC Symbol;Acc:HGNC:11530] |
| ENSG00000185278 | HGLibA_54867 | 411 | 2.941274423 | ZBTB37 | zinc finger and BTB domain containing 37 [Source:HGNC Symbol;Acc:HGNC:28365] |
| ENSG00000224586 | HGLibA_20182 | 411 | 2.941274423 | GPX5 | glutathione peroxidase 5 [Source:HGNC Symbol;Acc:HGNC:4557] |
| hsa-mir-3659 | HGLibA_59182 | 411 | 2.941274423 | hsa-mir-3659 | - |
| ENSG00000105270 | HGLibA_10049 | 410 | 2.931323385 | CLIP3 | CAP-Gly domain containing linker protein 3 [Source:HGNC Symbol;Acc:HGNC:24314] |
| ENSG00000132623 | HGLibA_01953 | 410 | 2.931323385 | ANKEF1 | ankyrin repeat and EF-hand domain containing 1 [Source:HGNC Symbol;Acc:HGNC:15803] |
| ENSG00000146281 | HGLibA_37288 | 410 | 2.931323385 | PM20D2 | peptidase M20 domain containing 2 [Source:HGNC Symbol;Acc:HGNC:21408] |
| ENSG00000148400 | HGLibA_32340 | 410 | 2.931323385 | NOTCH1 | notch 1 [Source:HGNC Symbol;Acc:HGNC:7881] |
| ENSG00000153956 | HGLibA_06906 | 410 | 2.931323385 | CACNA2D1 | calcium voltage-gated channel auxiliary subunit alpha2delta 1 [Source:HGNC Symbol;Acc:HGNC:1399] |
| ENSG00000157483 | HGLibA_30831 | 410 | 2.931323385 | MYO1E | myosin IE [Source:HGNC Symbol;Acc:HGNC:7599] |
| ENSG00000173641 | HGLibA_22317 | 410 | 2.931323385 | HSPB7 | heat shock protein family B (small) member 7 [Source:HGNC Symbol;Acc:HGNC:5249] |
| ENSG00000183304 | HGLibA_16818 | 410 | 2.931323385 | FAM9A | family with sequence similarity 9 member A [Source:HGNC Symbol;Acc:HGNC:18403] |
| ENSG00000184486 | HGLibA_37769 | 410 | 2.931323385 | POU3F2 | POU class 3 homeobox 2 [Source:HGNC Symbol;Acc:HGNC:9215] |
| ENSG00000188580 | HGLibA_31992 | 410 | 2.931323385 | NKAIN2 | sodium/potassium transporting ATPase interacting 2 [Source:HGNC Symbol;Acc:HGNC:16443] |
| ENSG00000221852 | HGLibA_25589 | 410 | 2.931323385 | KRTAP1-5 | keratin associated protein 1-5 [Source:HGNC Symbol;Acc:HGNC:16777] |
| ENSG00000085788 | HGLibA_12606 | 409 | 2.921372348 | DDHD2 | DDHD domain containing 2 [Source:HGNC Symbol;Acc:HGNC:29106] |
| ENSG00000102390 | HGLibA_35327 | 409 | 2.921372348 | PBDC1 | polysaccharide biosynthesis domain containing 1 [Source:HGNC Symbol;Acc:HGNC:28790] |
| ENSG00000135211 | HGLibA_50465 | 409 | 2.921372348 | TMEM60 | transmembrane protein 60 [Source:HGNC Symbol;Acc:HGNC:21754] |
| ENSG00000136485 | HGLibA_12455 | 409 | 2.921372348 | DCAF7 | DDB1 and CUL4 associated factor 7 [Source:HGNC Symbol;Acc:HGNC:30915] |
| ENSG00000152467 | HGLibA_56984 | 409 | 2.921372348 | ZSCAN1 | zinc finger and SCAN domain containing 1 [Source:HGNC Symbol;Acc:HGNC:23712] |
| ENSG00000164946 | HGLibA_17984 | 409 | 2.921372348 | FREM1 | FRAS1 related extracellular matrix 1 [Source:HGNC Symbol;Acc:HGNC:23399] |
| PRAMEF21 | HGLibA_38312 | 409 | 2.921372348 | PRAMEF21 | - |
| ENSG00000060558 | HGLibA_19448 | 408 | 2.911421311 | GNA15 | G protein subunit alpha 15 [Source:HGNC Symbol;Acc:HGNC:4383] |
| ENSG00000066697 | HGLibA_30174 | 408 | 2.911421311 | MSANTD3 | Myb/SANT DNA binding domain containing 3 [Source:HGNC Symbol;Acc:HGNC:23370] |
| ENSG00000087495 | HGLibA_36304 | 408 | 2.911421311 | PHACTR3 | phosphatase and actin regulator 3 [Source:HGNC Symbol;Acc:HGNC:15833] |
| ENSG00000088002 | HGLibA_47762 | 408 | 2.911421311 | SULT2B1 | sulfotransferase family 2B member 1 [Source:HGNC Symbol;Acc:HGNC:11459] |
| ENSG00000130035 | HGLibA_18572 | 408 | 2.911421311 | GALNT8 | polypeptide N-acetylgalactosaminyltransferase 8 [Source:HGNC Symbol;Acc:HGNC:4130] |
| ENSG00000135205 | HGLibA_07627 | 408 | 2.911421311 | CCDC146 | coiled-coil domain containing 146 [Source:HGNC Symbol;Acc:HGNC:29296] |
| ENSG00000146166 | HGLibA_26417 | 408 | 2.911421311 | LGSN | lens protein with glutamine synthetase domain [Source:HGNC Symbol;Acc:HGNC:21016]" |
| ENSG00000184601 | HGLibA_05349 | 408 | 2.911421311 | C14orf180 | chromosome 14 open reading frame 180 [Source:HGNC Symbol;Acc:HGNC:33795] |
| ENSG00000198604 | HGLibA_04186 | 408 | 2.911421311 | BAZ1A | bromodomain adjacent to zinc finger domain 1A [Source:HGNC Symbol;Acc:HGNC:960] |
| ENSG00000244623 | HGLibA_33746 | 408 | 2.911421311 | OR2AE1 | olfactory receptor family 2 subfamily AE member 1 [Source:HGNC Symbol;Acc:HGNC:15087] |
| PRAC | HGLibA_38259 | 408 | 2.911421311 | PRAC | - |
| ENSG00000019582 | HGLibA_08445 | 407 | 2.901470273 | CD74 | CD74 molecule [Source:HGNC Symbol;Acc:HGNC:1697] |
| ENSG00000125611 | HGLibA_09375 | 407 | 2.901470273 | CHCHD5 | coiled-coil-helix-coiled-coil-helix domain containing 5 [Source:HGNC Symbol;Acc:HGNC:17840] |
| ENSG00000137285 | HGLibA_52279 | 407 | 2.901470273 | TUBB2B | tubulin beta 2B class IIb [Source:HGNC Symbol;Acc:HGNC:30829] |
| ENSG00000181541 | HGLibA_27823 | 407 | 2.901470273 | MAB21L2 | mab-21 like 2 [Source:HGNC Symbol;Acc:HGNC:6758] |
| hsa-mir-4269 | HGLibA_59782 | 407 | 2.901470273 | hsa-mir-4269 | - |
| hsa-mir-6126 | HGLibA_62653 | 407 | 2.901470273 | hsa-mir-6126 | - |
| KIAA1257 | HGLibA_24730 | 407 | 2.901470273 | KIAA1257 | - |
| ENSG00000035664 | HGLibA_12324 | 406 | 2.891519236 | DAPK2 | death associated protein kinase 2 [Source:HGNC Symbol;Acc:HGNC:2675] |
| ENSG00000115504 | HGLibA_14660 | 406 | 2.891519236 | EHBP1 | EH domain binding protein 1 [Source:HGNC Symbol;Acc:HGNC:29144] |
| ENSG00000115902 | HGLibA_44515 | 406 | 2.891519236 | SLC1A4 | solute carrier family 1 member 4 [Source:HGNC Symbol;Acc:HGNC:10942] |
| ENSG00000128881 | HGLibA_52042 | 406 | 2.891519236 | TTBK2 | tau tubulin kinase 2 [Source:HGNC Symbol;Acc:HGNC:19141] |
| ENSG00000135040 | HGLibA_30991 | 406 | 2.891519236 | NAA35 | NatC auxiliary subunit [Source:HGNC Symbol;Acc:HGNC:24340]" |
| ENSG00000136848 | HGLibA_12271 | 406 | 2.891519236 | DAB2IP | DAB2 interacting protein [Source:HGNC Symbol;Acc:HGNC:17294] |
| ENSG00000143013 | HGLibA_26712 | 406 | 2.891519236 | LMO4 | LIM domain only 4 [Source:HGNC Symbol;Acc:HGNC:6644] |
| ENSG00000153303 | HGLibA_18008 | 406 | 2.891519236 | FRMD1 | FERM domain containing 1 [Source:HGNC Symbol;Acc:HGNC:21240] |
| ENSG00000163082 | HGLibA_43853 | 406 | 2.891519236 | SGPP2 | sphingosine-1-phosphate phosphatase 2 [Source:HGNC Symbol;Acc:HGNC:19953] |
| ENSG00000178229 | HGLibA_56195 | 406 | 2.891519236 | ZNF543 | zinc finger protein 543 [Source:HGNC Symbol;Acc:HGNC:25281] |
| ENSG00000184164 | HGLibA_11128 | 406 | 2.891519236 | CRELD2 | cysteine rich with EGF like domains 2 [Source:HGNC Symbol;Acc:HGNC:28150] |
| ENSG00000186153 | HGLibA_54447 | 406 | 2.891519236 | WWOX | WW domain containing oxidoreductase [Source:HGNC Symbol;Acc:HGNC:12799] |
| ENSG00000196730 | HGLibA_12321 | 406 | 2.891519236 | DAPK1 | death associated protein kinase 1 [Source:HGNC Symbol;Acc:HGNC:2674] |
| ENSG00000239305 | HGLibA_41458 | 406 | 2.891519236 | RNF103 | ring finger protein 103 [Source:HGNC Symbol;Acc:HGNC:12859] |
| ENSG00000256043 | HGLibA_11724 | 406 | 2.891519236 | CTSO | cathepsin O [Source:HGNC Symbol;Acc:HGNC:2542] |
| hsa-mir-499a | HGLibA_61402 | 406 | 2.891519236 | hsa-mir-499a | - |
| LOC100505549 | HGLibA_26848 | 406 | 2.891519236 | LOC100505549 | - |
| C12orf61 | HGLibA_05275 | 405 | 2.881568198 | C12orf61 | - |
| ENSG00000076356 | HGLibA_37260 | 405 | 2.881568198 | PLXNA2 | plexin A2 [Source:HGNC Symbol;Acc:HGNC:9100] |
| ENSG00000109089 | HGLibA_08868 | 405 | 2.881568198 | CDR2L | cerebellar degeneration related protein 2 like [Source:HGNC Symbol;Acc:HGNC:29999] |
| ENSG00000112592 | HGLibA_48624 | 405 | 2.881568198 | TBP | TATA-box binding protein [Source:HGNC Symbol;Acc:HGNC:11588] |
| ENSG00000114487 | HGLibA_29647 | 405 | 2.881568198 | MORC1 | MORC family CW-type zinc finger 1 [Source:HGNC Symbol;Acc:HGNC:7198] |
| ENSG00000115488 | HGLibA_31714 | 405 | 2.881568198 | NEU2 | neuraminidase 2 [Source:HGNC Symbol;Acc:HGNC:7759] |

| | | | | | |
|---|---|---|---|---|---|
| ENSG00000163879 | HGLibA_13650 | 405 | 2.881568198 | DNALI1 | dynein axonemal light intermediate chain 1 [Source:HGNC Symbol;Acc:HGNC:14353] |
| ENSG00000164124 | HGLibA_49970 | 405 | 2.881568198 | TMEM144 | transmembrane protein 144 [Source:HGNC Symbol;Acc:HGNC:25633] |
| ENSG00000168646 | HGLibA_03923 | 405 | 2.881568198 | AXIN2 | axin 2 [Source:HGNC Symbol;Acc:HGNC:904] |
| ENSG00000169174 | HGLibA_35688 | 405 | 2.881568198 | PCSK9 | proprotein convertase subtilisin/kexin type 9 [Source:HGNC Symbol;Acc:HGNC:20001] |
| ENSG00000187026 | HGLibA_25712 | 405 | 2.881568198 | KRTAP21-2 | keratin associated protein 21-2 [Source:HGNC Symbol;Acc:HGNC:18946] |
| ENSG00000243708 | HGLibA_36902 | 405 | 2.881568198 | PLA2G4B | phospholipase A2 group IVB [Source:HGNC Symbol;Acc:HGNC:9036] |
| ENSG00000244694 | HGLibA_39283 | 405 | 2.881568198 | PTCHD4 | patched domain containing 4 [Source:HGNC Symbol;Acc:HGNC:21345] |
| GLTSCR1 | HGLibA_19352 | 405 | 2.881568198 | GLTSCR1 | - |
| ENSG00000099899 | HGLibA_51683 | 404 | 2.871617161 | TRMT2A | tRNA methyltransferase 2 homolog A [Source:HGNC Symbol;Acc:HGNC:24974] |
| ENSG00000104689 | HGLibA_50733 | 404 | 2.871617161 | TNFRSF10A | TNF receptor superfamily member 10a [Source:HGNC Symbol;Acc:HGNC:11904] |
| ENSG00000111707 | HGLibA_47711 | 404 | 2.871617161 | SUDS3 | SIN3A corepressor complex component [Source:HGNC Symbol;Acc:HGNC:29545]" |
| ENSG00000138380 | HGLibA_07244 | 404 | 2.871617161 | CARF | calcium responsive transcription factor [Source:HGNC Symbol;Acc:HGNC:14435] |
| ENSG00000148908 | HGLibA_41052 | 404 | 2.871617161 | RGS10 | regulator of G protein signaling 10 [Source:HGNC Symbol;Acc:HGNC:9992] |
| ENSG00000167676 | HGLibA_37179 | 404 | 2.871617161 | PLIN4 | perilipin 4 [Source:HGNC Symbol;Acc:HGNC:29393] |
| ENSG00000168743 | HGLibA_32497 | 404 | 2.871617161 | NPNT | nephronectin [Source:HGNC Symbol;Acc:HGNC:27405] |
| ENSG00000185475 | HGLibA_50091 | 404 | 2.871617161 | TMEM179B | transmembrane protein 179B [Source:HGNC Symbol;Acc:HGNC:33744] |
| ENSG00000198756 | HGLibA_10645 | 404 | 2.871617161 | COLGALT2 | collagen beta(1-O)galactosyltransferase 2 [Source:HGNC Symbol;Acc:HGNC:16790] |
| ENSG00000203859 | HGLibA_22208 | 404 | 2.871617161 | HSD3B2 | 3 beta- and steroid delta-isomerase 2 [Source:HGNC Symbol;Acc:HGNC:5218]" |
| ENSG00000205927 | HGLibA_33370 | 404 | 2.871617161 | OLIG2 | oligodendrocyte transcription factor 2 [Source:HGNC Symbol;Acc:HGNC:9398] |
| H1FX | HGLibA_20743 | 404 | 2.871617161 | H1FX | - |
| hsa-mir-4658 | HGLibA_60738 | 404 | 2.871617161 | hsa-mir-4658 | - |
| hsa-mir-604 | HGLibA_62509 | 404 | 2.871617161 | hsa-mir-604 | - |
| hsa-mir-6763 | HGLibA_63214 | 404 | 2.871617161 | hsa-mir-6763 | - |
| hsa-mir-941-1 | HGLibA_64340 | 404 | 2.871617161 | hsa-mir-941-1 | - |
| ENSG00000079393 | HGLibA_14139 | 403 | 2.861666123 | DUSP13 | dual specificity phosphatase 13 [Source:HGNC Symbol;Acc:HGNC:19681] |
| ENSG00000106031 | HGLibA_21902 | 403 | 2.861666123 | HOXA13 | homeobox A13 [Source:HGNC Symbol;Acc:HGNC:5102] |
| ENSG00000116337 | HGLibA_01825 | 403 | 2.861666123 | AMPD2 | adenosine monophosphate deaminase 2 [Source:HGNC Symbol;Acc:HGNC:469] |
| ENSG00000157168 | HGLibA_32676 | 403 | 2.861666123 | NRG1 | neuregulin 1 [Source:HGNC Symbol;Acc:HGNC:7997] |
| ENSG00000171827 | HGLibA_56275 | 403 | 2.861666123 | ZNF570 | zinc finger protein 570 [Source:HGNC Symbol;Acc:HGNC:26416] |
| ENSG00000172071 | HGLibA_14730 | 403 | 2.861666123 | EIF2AK3 | eukaryotic translation initiation factor 2 alpha kinase 3 [Source:HGNC Symbol;Acc:HGNC:3255] |
| ENSG00000183979 | HGLibA_32408 | 403 | 2.861666123 | NPB | neuropeptide B [Source:HGNC Symbol;Acc:HGNC:30099] |
| ENSG00000198125 | HGLibA_28455 | 403 | 2.861666123 | MB | myoglobin [Source:HGNC Symbol;Acc:HGNC:6915] |
| ENSG00000242252 | HGLibA_04462 | 403 | 2.861666123 | BGLAP | bone gamma-carboxyglutamate protein [Source:HGNC Symbol;Acc:HGNC:1043] |
| ENSG00000242265 | HGLibA_36028 | 403 | 2.861666123 | PEG10 | paternally expressed 10 [Source:HGNC Symbol;Acc:HGNC:14005] |
| hsa-mir-599 | HGLibA_62490 | 403 | 2.861666123 | hsa-mir-599 | - |
| TCEB3 | HGLibA_48740 | 403 | 2.861666123 | TCEB3 | - |
| ENSG00000175662 | HGLibA_50953 | 402 | 2.851715086 | TOM1L2 | target of myb1 like 2 membrane trafficking protein [Source:HGNC Symbol;Acc:HGNC:11984] |
| ENSG00000184635 | HGLibA_56910 | 402 | 2.851715086 | ZNF93 | zinc finger protein 93 [Source:HGNC Symbol;Acc:HGNC:13169] |
| ENSG00000106780 | HGLibA_28859 | 401 | 2.841764049 | MEGF9 | multiple EGF like domains 9 [Source:HGNC Symbol;Acc:HGNC:3234] |
| ENSG00000115919 | HGLibA_25880 | 401 | 2.841764049 | KYNU | kynureninase [Source:HGNC Symbol;Acc:HGNC:6469] |
| ENSG00000121898 | HGLibA_11039 | 401 | 2.841764049 | CPXM2 | M14 family member 2 [Source:HGNC Symbol;Acc:HGNC:26977]" |
| ENSG00000139668 | HGLibA_54008 | 401 | 2.841764049 | WDFY2 | WD repeat and FYVE domain containing 2 [Source:HGNC Symbol;Acc:HGNC:20482] |
| ENSG00000148985 | HGLibA_36210 | 401 | 2.841764049 | PGAP2 | post-GPI attachment to proteins 2 [Source:HGNC Symbol;Acc:HGNC:17893] |
| ENSG00000153531 | HGLibA_01029 | 401 | 2.841764049 | ADPRHL1 | ADP-ribosylhydrolase like 1 [Source:HGNC Symbol;Acc:HGNC:21303] |
| ENSG00000204366 | HGLibA_54821 | 401 | 2.841764049 | ZBTB12 | zinc finger and BTB domain containing 12 [Source:HGNC Symbol;Acc:HGNC:19066] |
| ENSG00000206102 | HGLibA_25684 | 401 | 2.841764049 | KRTAP19-8 | keratin associated protein 19-8 [Source:HGNC Symbol;Acc:HGNC:33898] |
| ENSG00000123119 | HGLibA_31583 | 400 | 2.831813011 | NECAB1 | N-terminal EF-hand calcium binding protein 1 [Source:HGNC Symbol;Acc:HGNC:20983] |
| ENSG00000156925 | HGLibA_55326 | 400 | 2.831813011 | ZIC3 | Zic family member 3 [Source:HGNC Symbol;Acc:HGNC:12874] |
| ENSG00000164736 | HGLibA_46245 | 400 | 2.831813011 | SOX17 | SRY-box 17 [Source:HGNC Symbol;Acc:HGNC:18122] |
| ENSG00000172752 | HGLibA_10605 | 400 | 2.831813011 | COL6A5 | collagen type VI alpha 5 chain [Source:HGNC Symbol;Acc:HGNC:26674] |
| ENSG00000183196 | HGLibA_09644 | 400 | 2.831813011 | CHST6 | carbohydrate sulfotransferase 6 [Source:HGNC Symbol;Acc:HGNC:6938] |
| ENSG00000184979 | HGLibA_53291 | 400 | 2.831813011 | USP18 | ubiquitin specific peptidase 18 [Source:HGNC Symbol;Acc:HGNC:12616] |
| ENSG00000185900 | HGLibA_37662 | 400 | 2.831813011 | POMK | protein-O-mannose kinase [Source:HGNC Symbol;Acc:HGNC:26267] |
| ENSG00000197921 | HGLibA_21190 | 400 | 2.831813011 | HES5 | hes family bHLH transcription factor 5 [Source:HGNC Symbol;Acc:HGNC:19764] |
| HIST1H1A | HGLibA_21357 | 400 | 2.831813011 | HIST1H1A | - |
| hsa-mir-1276 | HGLibA_57560 | 400 | 2.831813011 | hsa-mir-1276 | - |
| ENSG00000092969 | HGLibA_49214 | 399 | 2.821861974 | TGFB2 | transforming growth factor beta 2 [Source:HGNC Symbol;Acc:HGNC:11768] |
| ENSG00000106560 | HGLibA_19076 | 399 | 2.821861974 | GIMAP2 | IMAP family member 2 [Source:HGNC Symbol;Acc:HGNC:21789]" |
| ENSG00000115267 | HGLibA_22619 | 399 | 2.821861974 | IFIH1 | interferon induced with helicase C domain 1 [Source:HGNC Symbol;Acc:HGNC:18873] |
| ENSG00000117592 | HGLibA_38422 | 399 | 2.821861974 | PRDX6 | peroxiredoxin 6 [Source:HGNC Symbol;Acc:HGNC:16753] |
| ENSG00000148156 | HGLibA_00630 | 399 | 2.821861974 | ACTL7B | actin like 7B [Source:HGNC Symbol;Acc:HGNC:162] |
| ENSG00000148671 | HGLibA_00985 | 399 | 2.821861974 | ADIRF | adipogenesis regulatory factor [Source:HGNC Symbol;Acc:HGNC:24043] |
| ENSG00000162437 | HGLibA_40403 | 399 | 2.821861974 | RAVER2 | PTB binding 2 [Source:HGNC Symbol;Acc:HGNC:25577]" |
| ENSG00000198720 | HGLibA_02002 | 399 | 2.821861974 | ANKRD13B | ankyrin repeat domain 13B [Source:HGNC Symbol;Acc:HGNC:26363] |
| ENSG00000253797 | HGLibA_53438 | 399 | 2.821861974 | UTP14C | small subunit processome component [Source:HGNC Symbol;Acc:HGNC:20321]" |
| H2AFV | HGLibA_20758 | 399 | 2.821861974 | H2AFV | - |
| hsa-mir-6774 | HGLibA_63270 | 399 | 2.821861974 | hsa-mir-6774 | - |
| ATPIF1 | HGLibA_03806 | 398 | 2.811910936 | ATPIF1 | - |
| ENSG00000105122 | HGLibA_40317 | 398 | 2.811910936 | RASAL3 | RAS protein activator like 3 [Source:HGNC Symbol;Acc:HGNC:26129] |
| ENSG00000126016 | HGLibA_01813 | 398 | 2.811910936 | AMOT | angiomotin [Source:HGNC Symbol;Acc:HGNC:17810] |
| ENSG00000137757 | HGLibA_07317 | 398 | 2.811910936 | CASP5 | caspase 5 [Source:HGNC Symbol;Acc:HGNC:1506] |
| ENSG00000139719 | HGLibA_53763 | 398 | 2.811910936 | VPS33A | CORVET/HOPS core subunit [Source:HGNC Symbol;Acc:HGNC:18179]" |
| ENSG00000151332 | HGLibA_28497 | 398 | 2.811910936 | MBIP | MAP3K12 binding inhibitory protein 1 [Source:HGNC Symbol;Acc:HGNC:20427] |
| ENSG00000164871 | HGLibA_46349 | 398 | 2.811910936 | SPAG11B | sperm associated antigen 11B [Source:HGNC Symbol;Acc:HGNC:14534] |
| ENSG00000172936 | HGLibA_30686 | 398 | 2.811910936 | MYD88 | innate immune signal transduction adaptor [Source:HGNC Symbol;Acc:HGNC:7562]" |
| ENSG00000173660 | HGLibA_53122 | 398 | 2.811910936 | UQCRH | ubiquinol-cytochrome c reductase hinge protein [Source:HGNC Symbol;Acc:HGNC:12590] |
| ENSG00000188729 | HGLibA_34716 | 398 | 2.811910936 | OSTN | osteocrin [Source:HGNC Symbol;Acc:HGNC:29961] |
| ENSG00000004142 | HGLibA_37505 | 397 | 2.801959899 | POLDIP2 | DNA polymerase delta interacting protein 2 [Source:HGNC Symbol;Acc:HGNC:23781] |
| ENSG00000006659 | HGLibA_26353 | 397 | 2.801959899 | LGALS14 | galectin 14 [Source:HGNC Symbol;Acc:HGNC:30054] |
| ENSG00000099769 | HGLibA_22811 | 397 | 2.801959899 | IGFALS | insulin like growth factor binding protein acid labile subunit [Source:HGNC Symbol;Acc:HGNC:5468] |
| ENSG00000104442 | HGLibA_03006 | 397 | 2.801959899 | ARMC1 | armadillo repeat containing 1 [Source:HGNC Symbol;Acc:HGNC:17684] |
| ENSG00000156675 | HGLibA_39794 | 397 | 2.801959899 | RAB11FIP1 | RAB11 family interacting protein 1 [Source:HGNC Symbol;Acc:HGNC:30265] |
| ENSG00000166682 | HGLibA_50644 | 397 | 2.801959899 | TMPRSS5 | transmembrane serine protease 5 [Source:HGNC Symbol;Acc:HGNC:14908] |
| ENSG00000182575 | HGLibA_33168 | 397 | 2.801959899 | NXPH3 | neurexophilin 3 [Source:HGNC Symbol;Acc:HGNC:8077] |
| ENSG00000184349 | HGLibA_14593 | 397 | 2.801959899 | EFNA5 | ephrin A5 [Source:HGNC Symbol;Acc:HGNC:3225] |
| ENSG00000186897 | HGLibA_05934 | 397 | 2.801959899 | C1QL4 | complement C1q like 4 [Source:HGNC Symbol;Acc:HGNC:31416] |
| ENSG00000204363 | HGLibA_46414 | 397 | 2.801959899 | SPANXN5 | SPANX family member N5 [Source:HGNC Symbol;Acc:HGNC:33178] |
| ENSG00000256797 | HGLibA_25307 | 397 | 2.801959899 | KLRF2 | killer cell lectin like receptor F2 [Source:HGNC Symbol;Acc:HGNC:37646] |
| ENSG00000135973 | HGLibA_20045 | 396 | 2.792008861 | GPR45 | G protein-coupled receptor 45 [Source:HGNC Symbol;Acc:HGNC:4503] |
| ENSG00000156675 | HGLibA_39792 | 396 | 2.792008861 | RAB11FIP1 | RAB11 family interacting protein 1 [Source:HGNC Symbol;Acc:HGNC:30265] |
| ENSG00000186187 | HGLibA_56938 | 396 | 2.792008861 | ZNRF1 | zinc and ring finger 1 [Source:HGNC Symbol;Acc:HGNC:18452] |
| ENSG00000269190 | HGLibA_17036 | 396 | 2.792008861 | FBXO17 | F-box protein 17 [Source:HGNC Symbol;Acc:HGNC:18754] |
| ENSG00000276581 | HGLibA_46479 | 396 | 2.792008861 | SPATA31A5 | SPATA31 subfamily A member 5 [Source:HGNC Symbol;Acc:HGNC:32005] |
| H2AFY2 | HGLibA_20765 | 396 | 2.792008861 | H2AFY2 | - |
| ENSG00000089335 | HGLibA_55775 | 395 | 2.782057824 | ZNF302 | zinc finger protein 302 [Source:HGNC Symbol;Acc:HGNC:13848] |
| ENSG00000101850 | HGLibA_19915 | 395 | 2.782057824 | GPR143 | G protein-coupled receptor 143 [Source:HGNC Symbol;Acc:HGNC:20145] |
| ENSG00000108523 | HGLibA_41563 | 395 | 2.782057824 | RNF167 | ring finger protein 167 [Source:HGNC Symbol;Acc:HGNC:24544] |
| ENSG00000133065 | HGLibA_45140 | 395 | 2.782057824 | SLC41A1 | solute carrier family 41 member 1 [Source:HGNC Symbol;Acc:HGNC:19429] |
| ENSG00000137834 | HGLibA_45585 | 395 | 2.782057824 | SMAD6 | SMAD family member 6 [Source:HGNC Symbol;Acc:HGNC:6772] |
| ENSG00000149635 | HGLibA_33265 | 395 | 2.782057824 | OCSTAMP | osteoclast stimulatory transmembrane protein [Source:HGNC Symbol;Acc:HGNC:16116] |
| ENSG00000152454 | HGLibA_55678 | 395 | 2.782057824 | ZNF256 | zinc finger protein 256 [Source:HGNC Symbol;Acc:HGNC:13049] |
| ENSG00000159228 | HGLibA_07419 | 395 | 2.782057824 | CBR1 | carbonyl reductase 1 [Source:HGNC Symbol;Acc:HGNC:1548] |
| ENSG00000162065 | HGLibA_48511 | 395 | 2.782057824 | TBC1D24 | TBC1 domain family member 24 [Source:HGNC Symbol;Acc:HGNC:29203] |
| C1orf172 | HGLibA_05792 | 394 | 2.772106787 | C1orf172 | - |
| ENSG00000082701 | HGLibA_20431 | 394 | 2.772106787 | GSK3B | glycogen synthase kinase 3 beta [Source:HGNC Symbol;Acc:HGNC:4617] |
| ENSG00000088808 | HGLibA_38028 | 394 | 2.772106787 | PPP1R13B | protein phosphatase 1 regulatory subunit 13B [Source:HGNC Symbol;Acc:HGNC:14950] |
| ENSG00000095209 | HGLibA_50380 | 394 | 2.772106787 | TMEM38B | transmembrane protein 38B [Source:HGNC Symbol;Acc:HGNC:25535] |
| ENSG00000100139 | HGLibA_29214 | 394 | 2.772106787 | MICALL1 | MICAL like 1 [Source:HGNC Symbol;Acc:HGNC:29804] |
| ENSG00000109743 | HGLibA_04881 | 394 | 2.772106787 | BST1 | bone marrow stromal cell antigen 1 [Source:HGNC Symbol;Acc:HGNC:1118] |
| ENSG00000111962 | HGLibA_53424 | 394 | 2.772106787 | UST | uronyl 2-sulfotransferase [Source:HGNC Symbol;Acc:HGNC:17223] |
| ENSG00000133619 | HGLibA_25387 | 394 | 2.772106787 | KRBA1 | KRAB-A domain containing 1 [Source:HGNC Symbol;Acc:HGNC:22228] |
| ENSG00000165887 | HGLibA_02025 | 394 | 2.772106787 | ANKRD2 | ankyrin repeat domain 2 [Source:HGNC Symbol;Acc:HGNC:495] |
| ENSG00000168661 | HGLibA_55769 | 394 | 2.772106787 | ZNF30 | zinc finger protein 30 [Source:HGNC Symbol;Acc:HGNC:13090] |
| ENSG00000175485 | HGLibA_34207 | 394 | 2.772106787 | OR52W1 | olfactory receptor family 52 subfamily W member 1 [Source:HGNC Symbol;Acc:HGNC:15239] |
| ENSG00000178462 | HGLibA_52266 | 394 | 2.772106787 | TUBAL3 | tubulin alpha like 3 [Source:HGNC Symbol;Acc:HGNC:23534] |
| ENSG00000182050 | HGLibA_29146 | 394 | 2.772106787 | MGAT4C | MGAT4 family member C [Source:HGNC Symbol;Acc:HGNC:30871] |
| ENSG00000198752 | HGLibA_08559 | 394 | 2.772106787 | CDC42BPB | CDC42 binding protein kinase beta [Source:HGNC Symbol;Acc:HGNC:1738] |
| ENSG00000204941 | HGLibA_39048 | 394 | 2.772106787 | PSG5 | pregnancy specific beta-1-glycoprotein 5 [Source:HGNC Symbol;Acc:HGNC:9522] |
| ENSG00000100359 | HGLibA_43866 | 393 | 2.762155749 | SGSM3 | small G protein signaling modulator 3 [Source:HGNC Symbol;Acc:HGNC:25228] |
| ENSG00000113532 | HGLibA_47297 | 393 | 2.762155749 | ST8SIA4 | 8-sialyltransferase 4 [Source:HGNC Symbol;Acc:HGNC:10871]" |
| ENSG00000130876 | HGLibA_45349 | 393 | 2.762155749 | SLC7A10 | solute carrier family 7 member 10 [Source:HGNC Symbol;Acc:HGNC:11058] |
| ENSG00000135205 | HGLibA_07628 | 393 | 2.762155749 | CCDC146 | coiled-coil domain containing 146 [Source:HGNC Symbol;Acc:HGNC:29296] |
| ENSG00000138771 | HGLibA_44093 | 393 | 2.762155749 | SHROOM3 | shroom family member 3 [Source:HGNC Symbol;Acc:HGNC:30422] |
| ENSG00000140519 | HGLibA_41149 | 393 | 2.762155749 | RHCG | Rh family C glycoprotein [Source:HGNC Symbol;Acc:HGNC:18140] |
| ENSG00000168685 | HGLibA_23192 | 393 | 2.762155749 | IL7R | interleukin 7 receptor [Source:HGNC Symbol;Acc:HGNC:6024] |
| ENSG00000186715 | HGLibA_30255 | 393 | 2.762155749 | MST1L | macrophage stimulating 1 like [Source:HGNC Symbol;Acc:HGNC:7390] |
| ENSG00000206538 | HGLibA_53641 | 393 | 2.762155749 | VGLL3 | vestigial like family member 3 [Source:HGNC Symbol;Acc:HGNC:24327] |
| ENSG00000221986 | HGLibA_30661 | 393 | 2.762155749 | MYBPHL | myosin binding protein H like [Source:HGNC Symbol;Acc:HGNC:30434] |
| FAM86A | HGLibA_16771 | 393 | 2.762155749 | FAM86A | - |
| ENSG00000087237 | HGLibA_09243 | 392 | 2.752204712 | CETP | cholesteryl ester transfer protein [Source:HGNC Symbol;Acc:HGNC:1869] |
| ENSG00000111199 | HGLibA_51791 | 392 | 2.752204712 | TRPV4 | transient receptor potential cation channel subfamily V member 4 [Source:HGNC Symbol;Acc:HGNC:18083] |
| ENSG00000128422 | HGLibA_25436 | 392 | 2.752204712 | KRT17 | keratin 17 [Source:HGNC Symbol;Acc:HGNC:6427] |
| ENSG00000131015 | HGLibA_52964 | 392 | 2.752204712 | ULBP2 | UL16 binding protein 2 [Source:HGNC Symbol;Acc:HGNC:14894] |
| ENSG00000182636 | HGLibA_31399 | 392 | 2.752204712 | NDN | MAGE family member [Source:HGNC Symbol;Acc:HGNC:7675]" |
| ENSG00000184232 | HGLibA_33193 | 392 | 2.752204712 | OAF | out at first homolog [Source:HGNC Symbol;Acc:HGNC:28752] |

| | | | | | |
|---|---|---|---|---|---|
| ENSG00000198848 | HGLibA_09219 | 392 | 2.752204712 | CES1 | carboxylesterase 1 [Source:HGNC Symbol;Acc:HGNC:1863] |
| ENSG00000260238 | HGLibA_37305 | 392 | 2.752204712 | PMF1-BGLAP | PMF1-BGLAP readthrough [Source:HGNC Symbol;Acc:HGNC:42953] |
| hsa-mir-3663 | HGLibA_59205 | 392 | 2.752204712 | hsa-mir-3663 | - |
| ENSG00000084674 | HGLibA_02463 | 391 | 2.742253674 | APOB | apolipoprotein B [Source:HGNC Symbol;Acc:HGNC:603] |
| ENSG00000102226 | HGLibA_53197 | 391 | 2.742253674 | USP11 | ubiquitin specific peptidase 11 [Source:HGNC Symbol;Acc:HGNC:12609] |
| ENSG00000137760 | HGLibA_01666 | 391 | 2.742253674 | ALKBH8 | tRNA methyltransferase [Source:HGNC Symbol;Acc:HGNC:25189]" |
| ENSG00000153707 | HGLibA_39495 | 391 | 2.742253674 | PTPRD | receptor type D [Source:HGNC Symbol;Acc:HGNC:9668]" |
| ENSG00000157601 | HGLibA_30603 | 391 | 2.742253674 | MX1 | MX dynamin like GTPase 1 [Source:HGNC Symbol;Acc:HGNC:7532] |
| ENSG00000163785 | HGLibA_42518 | 391 | 2.742253674 | RYK | receptor-like tyrosine kinase [Source:HGNC Symbol;Acc:HGNC:10481] |
| ENSG00000165410 | HGLibA_09283 | 391 | 2.742253674 | CFL2 | cofilin 2 [Source:HGNC Symbol;Acc:HGNC:1875] |
| ENSG00000169372 | HGLibA_11059 | 391 | 2.742253674 | CRADD | CASP2 and RIPK1 domain containing adaptor with death domain [Source:HGNC Symbol;Acc:HGNC:2340] |
| ENSG00000178078 | HGLibA_47344 | 391 | 2.742253674 | STAP2 | signal transducing adaptor family member 2 [Source:HGNC Symbol;Acc:HGNC:30430] |
| ENSG00000179314 | HGLibA_54420 | 391 | 2.742253674 | WSCD1 | WSC domain containing 1 [Source:HGNC Symbol;Acc:HGNC:29060] |
| ENSG00000189280 | HGLibA_19170 | 391 | 2.742253674 | GJB5 | gap junction protein beta 5 [Source:HGNC Symbol;Acc:HGNC:4287] |
| ENSG00000196476 | HGLibA_06033 | 391 | 2.742253674 | C20orf96 | chromosome 20 open reading frame 96 [Source:HGNC Symbol;Acc:HGNC:16227] |
| C10orf128 | HGLibA_05048 | 390 | 2.732302637 | C10orf128 | - |
| C12orf66 | HGLibA_05281 | 390 | 2.732302637 | C12orf66 | - |
| ENSG00000079332 | HGLibA_42737 | 390 | 2.732302637 | SAR1A | secretion associated Ras related GTPase 1A [Source:HGNC Symbol;Acc:HGNC:10534] |
| ENSG00000160299 | HGLibA_35639 | 390 | 2.732302637 | PCNT | pericentrin [Source:HGNC Symbol;Acc:HGNC:16068] |
| ENSG00000169397 | HGLibA_41407 | 390 | 2.732302637 | RNASE3 | ribonuclease A family member 3 [Source:HGNC Symbol;Acc:HGNC:10046] |
| ENSG00000169740 | HGLibA_55791 | 390 | 2.732302637 | ZNF32 | zinc finger protein 32 [Source:HGNC Symbol;Acc:HGNC:13095] |
| ENSG00000186908 | HGLibA_55086 | 390 | 2.732302637 | ZDHHC17 | zinc finger DHHC-type containing 17 [Source:HGNC Symbol;Acc:HGNC:18412] |
| ENSG00000267631 | HGLibA_09301 | 390 | 2.732302637 | CGB1 | chorionic gonadotropin subunit beta 1 [Source:HGNC Symbol;Acc:HGNC:16721] |
| ADPRHL2 | HGLibA_01030 | 389 | 2.722351599 | ADPRHL2 | - |
| ENSG00000060138 | HGLibA_54649 | 389 | 2.722351599 | YBX3 | Y-box binding protein 3 [Source:HGNC Symbol;Acc:HGNC:2428] |
| ENSG00000095261 | HGLibA_39174 | 389 | 2.722351599 | PSMD5 | non-ATPase 5 [Source:HGNC Symbol;Acc:HGNC:9563]" |
| ENSG00000127526 | HGLibA_44985 | 389 | 2.722351599 | SLC35E1 | solute carrier family 35 member E1 [Source:HGNC Symbol;Acc:HGNC:20803] |
| ENSG00000130270 | HGLibA_03785 | 389 | 2.722351599 | ATP8B3 | ATPase phospholipid transporting 8B3 [Source:HGNC Symbol;Acc:HGNC:13535] |
| ENSG00000133030 | HGLibA_29778 | 389 | 2.722351599 | MPRIP | myosin phosphatase Rho interacting protein [Source:HGNC Symbol;Acc:HGNC:30321] |
| ENSG00000143815 | HGLibA_26081 | 389 | 2.722351599 | LBR | lamin B receptor [Source:HGNC Symbol;Acc:HGNC:6518] |
| ENSG00000157625 | HGLibA_48148 | 389 | 2.722351599 | TAB3 | TGF-beta activated kinase 1 (MAP3K7) binding protein 3 [Source:HGNC Symbol;Acc:HGNC:30681] |
| ENSG00000164465 | HGLibA_12469 | 389 | 2.722351599 | DCBLD1 | CUB and LCCL domain containing 1 [Source:HGNC Symbol;Acc:HGNC:21479]" |
| ENSG00000172007 | HGLibA_39884 | 389 | 2.722351599 | RAB33B | member RAS oncogene family [Source:HGNC Symbol;Acc:HGNC:16075]" |
| ENSG00000177108 | HGLibA_55105 | 389 | 2.722351599 | ZDHHC22 | zinc finger DHHC-type containing 22 [Source:HGNC Symbol;Acc:HGNC:20106] |
| ENSG00000205116 | HGLibA_50541 | 389 | 2.722351599 | TMEM88B | transmembrane protein 88B [Source:HGNC Symbol;Acc:HGNC:37099] |
| ENSG00000243678 | HGLibA_32156 | 389 | 2.722351599 | NME2 | NME/NM23 nucleoside diphosphate kinase 2 [Source:HGNC Symbol;Acc:HGNC:7850] |
| PVRL4 | HGLibA_39630 | 389 | 2.722351599 | PVRL4 | - |
| ENSG00000089685 | HGLibA_04521 | 388 | 2.712400562 | BIRC5 | baculoviral IAP repeat containing 5 [Source:HGNC Symbol;Acc:HGNC:593] |
| ENSG00000115297 | HGLibA_49677 | 388 | 2.712400562 | TLX2 | T cell leukemia homeobox 2 [Source:HGNC Symbol;Acc:HGNC:5057] |
| ENSG00000133958 | HGLibA_53029 | 388 | 2.712400562 | UNC79 | NALCN channel complex subunit [Source:HGNC Symbol;Acc:HGNC:19966]" |
| ENSG00000136155 | HGLibA_42891 | 388 | 2.712400562 | SCEL | sciellin [Source:HGNC Symbol;Acc:HGNC:10573] |
| ENSG00000146618 | HGLibA_17291 | 388 | 2.712400562 | FERD3L | Fer3 like bHLH transcription factor [Source:HGNC Symbol;Acc:HGNC:16660] |
| ENSG00000168454 | HGLibA_52420 | 388 | 2.712400562 | TXNDC2 | thioredoxin domain containing 2 [Source:HGNC Symbol;Acc:HGNC:16470] |
| ENSG00000169372 | HGLibA_11061 | 388 | 2.712400562 | CRADD | CASP2 and RIPK1 domain containing adaptor with death domain [Source:HGNC Symbol;Acc:HGNC:2340] |
| ENSG00000170289 | HGLibA_10277 | 388 | 2.712400562 | CNGB3 | cyclic nucleotide gated channel beta 3 [Source:HGNC Symbol;Acc:HGNC:2153] |
| ENSG00000171942 | HGLibA_33508 | 388 | 2.712400562 | OR10H2 | olfactory receptor family 10 subfamily H member 2 [Source:HGNC Symbol;Acc:HGNC:8173] |
| ENSG00000106785 | HGLibA_51412 | 387 | 2.702449525 | TRIM14 | tripartite motif containing 14 [Source:HGNC Symbol;Acc:HGNC:16283] |
| ENSG00000108950 | HGLibA_16444 | 387 | 2.702449525 | FAM20A | golgi associated secretory pathway pseudokinase [Source:HGNC Symbol;Acc:HGNC:23015]" |
| ENSG00000134107 | HGLibA_04478 | 387 | 2.702449525 | BHLHE40 | basic helix-loop-helix family member e40 [Source:HGNC Symbol;Acc:HGNC:1046] |
| ENSG00000137331 | HGLibA_22575 | 387 | 2.702449525 | IER3 | immediate early response 3 [Source:HGNC Symbol;Acc:HGNC:5392] |
| ENSG00000157514 | HGLibA_51824 | 387 | 2.702449525 | TSC22D3 | TSC22 domain family member 3 [Source:HGNC Symbol;Acc:HGNC:3051] |
| ENSG00000160293 | HGLibA_53550 | 387 | 2.702449525 | VAV2 | vav guanine nucleotide exchange factor 2 [Source:HGNC Symbol;Acc:HGNC:12658] |
| ENSG00000174125 | HGLibA_49641 | 387 | 2.702449525 | TLR1 | toll like receptor 1 [Source:HGNC Symbol;Acc:HGNC:11847] |
| ENSG00000176273 | HGLibA_45015 | 387 | 2.702449525 | SLC35G1 | solute carrier family 35 member G1 [Source:HGNC Symbol;Acc:HGNC:26607] |
| ENSG00000182854 | HGLibA_33990 | 387 | 2.702449525 | OR4F15 | olfactory receptor family 4 subfamily F member 15 [Source:HGNC Symbol;Acc:HGNC:15078] |
| ENSG00000185758 | HGLibA_09890 | 387 | 2.702449525 | CLDN24 | claudin 24 [Source:HGNC Symbol;Acc:HGNC:37200] |
| ENSG00000214814 | HGLibA_17287 | 387 | 2.702449525 | FER1L6 | fer-1 like family member 6 [Source:HGNC Symbol;Acc:HGNC:28065] |
| ENSG00000234224 | HGLibA_50249 | 387 | 2.702449525 | TMEM229A | transmembrane protein 229A [Source:HGNC Symbol;Acc:HGNC:37279] |
| C15orf54 | HGLibA_05414 | 386 | 2.692498487 | C15orf54 | - |
| ENSG00000042832 | HGLibA_49200 | 386 | 2.692498487 | TG | thyroglobulin [Source:HGNC Symbol;Acc:HGNC:11764] |
| ENSG00000072042 | HGLibA_40735 | 386 | 2.692498487 | RDH11 | retinol dehydrogenase 11 [Source:HGNC Symbol;Acc:HGNC:17964] |
| ENSG00000072135 | HGLibA_39444 | 386 | 2.692498487 | PTPN18 | non-receptor type 18 [Source:HGNC Symbol;Acc:HGNC:9649]" |
| ENSG00000075618 | HGLibA_18068 | 386 | 2.692498487 | FSCN1 | fascin actin-bundling protein 1 [Source:HGNC Symbol;Acc:HGNC:11148] |
| ENSG00000120068 | HGLibA_21951 | 386 | 2.692498487 | HOXB8 | homeobox B8 [Source:HGNC Symbol;Acc:HGNC:5119] |
| ENSG00000132286 | HGLibA_49495 | 386 | 2.692498487 | TIMM10B | translocase of inner mitochondrial membrane 10B [Source:HGNC Symbol;Acc:HGNC:4022] |
| ENSG00000140022 | HGLibA_47550 | 386 | 2.692498487 | STON2 | stonin 2 [Source:HGNC Symbol;Acc:HGNC:30652] |
| ENSG00000155249 | HGLibA_34015 | 386 | 2.692498487 | OR4K1 | olfactory receptor family 4 subfamily K member 1 [Source:HGNC Symbol;Acc:HGNC:14726] |
| ENSG00000166033 | HGLibA_22407 | 386 | 2.692498487 | HTRA1 | HtrA serine peptidase 1 [Source:HGNC Symbol;Acc:HGNC:9476] |
| ENSG00000183260 | HGLibA_00260 | 386 | 2.692498487 | ABHD16B | abhydrolase domain containing 16B [Source:HGNC Symbol;Acc:HGNC:16128] |
| ENSG00000243902 | HGLibA_14908 | 386 | 2.692498487 | ELFN2 | extracellular leucine rich repeat and fibronectin type III domain containing 2 [Source:NCBI gene;Acc:114794] |
| C14orf79 | HGLibA_05368 | 385 | 2.68254745 | C14orf79 | - |
| ENSG00000111012 | HGLibA_12098 | 385 | 2.68254745 | CYP27B1 | cytochrome P450 family 27 subfamily B member 1 [Source:HGNC Symbol;Acc:HGNC:2606] |
| ENSG00000151414 | HGLibA_31662 | 385 | 2.68254745 | NEK7 | NIMA related kinase 7 [Source:HGNC Symbol;Acc:HGNC:13386] |
| ENSG00000166033 | HGLibA_22406 | 385 | 2.68254745 | HTRA1 | HtrA serine peptidase 1 [Source:HGNC Symbol;Acc:HGNC:9476] |
| ENSG00000189280 | HGLibA_19169 | 385 | 2.68254745 | GJB5 | gap junction protein beta 5 [Source:HGNC Symbol;Acc:HGNC:4287] |
| hsa-mir-636 | HGLibA_62785 | 385 | 2.68254745 | hsa-mir-636 | - |
| hsa-mir-7152 | HGLibA_63835 | 385 | 2.68254745 | hsa-mir-7152 | - |
| hsa-mir-802 | HGLibA_64042 | 385 | 2.68254745 | hsa-mir-802 | - |
| ENSG00000079616 | HGLibA_24904 | 384 | 2.672596412 | KIF22 | kinesin family member 22 [Source:HGNC Symbol;Acc:HGNC:6391] |
| ENSG00000099721 | HGLibA_01768 | 384 | 2.672596412 | AMELY | amelogenin Y-linked [Source:HGNC Symbol;Acc:HGNC:462] |
| ENSG00000105948 | HGLibA_52089 | 384 | 2.672596412 | TTC26 | tetratricopeptide repeat domain 26 [Source:HGNC Symbol;Acc:HGNC:21882] |
| ENSG00000114841 | HGLibA_13468 | 384 | 2.672596412 | DNAH1 | dynein axonemal heavy chain 1 [Source:HGNC Symbol;Acc:HGNC:2940] |
| ENSG00000117594 | HGLibA_22159 | 384 | 2.672596412 | HSD11B1 | hydroxysteroid 11-beta dehydrogenase 1 [Source:HGNC Symbol;Acc:HGNC:5208] |
| ENSG00000122420 | HGLibA_39339 | 384 | 2.672596412 | PTGFR | prostaglandin F receptor [Source:HGNC Symbol;Acc:HGNC:9600] |
| ENSG00000147364 | HGLibA_17055 | 384 | 2.672596412 | FBXO25 | F-box protein 25 [Source:HGNC Symbol;Acc:HGNC:13596] |
| ENSG00000159433 | HGLibA_47379 | 384 | 2.672596412 | STARD9 | StAR related lipid transfer domain containing 9 [Source:HGNC Symbol;Acc:HGNC:19162] |
| ENSG00000163346 | HGLibA_35356 | 384 | 2.672596412 | PBXIP1 | PBX homeobox interacting protein 1 [Source:HGNC Symbol;Acc:HGNC:21199] |
| ENSG00000174373 | HGLibA_40155 | 384 | 2.672596412 | RALGAPA1 | Ral GTPase activating protein catalytic alpha subunit 1 [Source:HGNC Symbol;Acc:HGNC:17770] |
| ENSG00000197614 | HGLibA_29053 | 384 | 2.672596412 | MFAP5 | microfibril associated protein 5 [Source:HGNC Symbol;Acc:HGNC:29673] |
| ENSG00000256812 | HGLibA_07187 | 384 | 2.672596412 | CAPNS2 | calpain small subunit 2 [Source:HGNC Symbol;Acc:HGNC:16371] |
| hsa-mir-185 | HGLibA_58004 | 384 | 2.672596412 | hsa-mir-185 | - |
| hsa-mir-3178 | HGLibA_58786 | 384 | 2.672596412 | hsa-mir-3178 | - |
| C8orf31 | HGLibA_06606 | 383 | 2.662645375 | C8orf31 | - |
| ENSG00000073969 | HGLibA_32751 | 383 | 2.662645375 | NSF | vesicle fusing ATPase [Source:HGNC Symbol;Acc:HGNC:8016]" |
| ENSG00000103150 | HGLibA_29446 | 383 | 2.662645375 | MLYCD | malonyl-CoA decarboxylase [Source:HGNC Symbol;Acc:HGNC:7150] |
| ENSG00000106006 | HGLibA_21916 | 383 | 2.662645375 | HOXA6 | homeobox A6 [Source:HGNC Symbol;Acc:HGNC:5107] |
| ENSG00000131097 | HGLibA_21308 | 383 | 2.662645375 | HIGD1B | HIG1 hypoxia inducible domain family member 1B [Source:HGNC Symbol;Acc:HGNC:24318] |
| ENSG00000132639 | HGLibA_45877 | 383 | 2.662645375 | SNAP25 | synaptosome associated protein 25 [Source:HGNC Symbol;Acc:HGNC:11132] |
| ENSG00000137502 | HGLibA_39872 | 383 | 2.662645375 | RAB30 | member RAS oncogene family [Source:HGNC Symbol;Acc:HGNC:9770]" |
| ENSG00000181016 | HGLibA_27558 | 383 | 2.662645375 | LSMEM1 | leucine rich single-pass membrane protein 1 [Source:HGNC Symbol;Acc:HGNC:22036] |
| ENSG00000196408 | HGLibA_32384 | 383 | 2.662645375 | NOXO1 | NADPH oxidase organizer 1 [Source:HGNC Symbol;Acc:HGNC:19404] |
| ENSG00000197081 | HGLibA_22810 | 383 | 2.662645375 | IGF2R | insulin like growth factor 2 receptor [Source:HGNC Symbol;Acc:HGNC:5467] |
| ENSG00000206535 | HGLibA_26737 | 383 | 2.662645375 | LNP1 | leukemia NUP98 fusion partner 1 [Source:HGNC Symbol;Acc:HGNC:28014] |
| ENSG00000230522 | HGLibA_28474 | 383 | 2.662645375 | MBD3L2 | methyl-CpG binding domain protein 3 like 2 [Source:HGNC Symbol;Acc:HGNC:18532] |
| ENSG00000271303 | HGLibA_47097 | 383 | 2.662645375 | SRXN1 | sulfiredoxin 1 [Source:HGNC Symbol;Acc:HGNC:16132] |
| hsa-mir-6882 | HGLibA_63713 | 383 | 2.662645375 | hsa-mir-6882 | - |
| C19orf59 | HGLibA_05687 | 382 | 2.652694337 | C19orf59 | - |
| C9orf16 | HGLibA_06697 | 382 | 2.652694337 | C9orf16 | - |
| ENSG00000099810 | HGLibA_30319 | 382 | 2.652694337 | MTAP | methylthioadenosine phosphorylase [Source:HGNC Symbol;Acc:HGNC:7413] |
| ENSG00000101596 | HGLibA_45662 | 382 | 2.652694337 | SMCHD1 | structural maintenance of chromosomes flexible hinge domain containing 1 [Source:HGNC Symbol;Acc:HGNC:29090] |
| ENSG00000102239 | HGLibA_04844 | 382 | 2.652694337 | BRS3 | bombesin receptor subtype 3 [Source:HGNC Symbol;Acc:HGNC:1113] |
| ENSG00000116199 | HGLibA_16448 | 382 | 2.652694337 | FAM20B | glycosaminoglycan xylosylkinase [Source:HGNC Symbol;Acc:HGNC:23017]" |
| ENSG00000136261 | HGLibA_05016 | 382 | 2.652694337 | BZW2 | basic leucine zipper and W2 domains 2 [Source:HGNC Symbol;Acc:HGNC:18808] |
| ENSG00000142661 | HGLibA_30894 | 382 | 2.652694337 | MYOM3 | myomesin 3 [Source:HGNC Symbol;Acc:HGNC:26679] |
| ENSG00000149573 | HGLibA_29798 | 382 | 2.652694337 | MPZL2 | myelin protein zero like 2 [Source:HGNC Symbol;Acc:HGNC:3496] |
| ENSG00000151067 | HGLibA_06882 | 382 | 2.652694337 | CACNA1C | calcium voltage-gated channel subunit alpha1 C [Source:HGNC Symbol;Acc:HGNC:1390] |
| ENSG00000156427 | HGLibA_17384 | 382 | 2.652694337 | FGF18 | fibroblast growth factor 18 [Source:HGNC Symbol;Acc:HGNC:3674] |
| ENSG00000162068 | HGLibA_32857 | 382 | 2.652694337 | NTN3 | netrin 3 [Source:HGNC Symbol;Acc:HGNC:8030] |
| ENSG00000164047 | HGLibA_07102 | 382 | 2.652694337 | CAMP | cathelicidin antimicrobial peptide [Source:HGNC Symbol;Acc:HGNC:1472] |
| ENSG00000165730 | HGLibA_47553 | 382 | 2.652694337 | STOX1 | storkhead box 1 [Source:HGNC Symbol;Acc:HGNC:23508] |
| ENSG00000176788 | HGLibA_04169 | 382 | 2.652694337 | BASP1 | brain abundant membrane attached signal protein 1 [Source:HGNC Symbol;Acc:HGNC:957] |
| ENSG00000187116 | HGLibA_26501 | 382 | 2.652694337 | LILRA5 | leukocyte immunoglobulin like receptor A5 [Source:HGNC Symbol;Acc:HGNC:16309] |
| ENSG00000011566 | HGLibA_28218 | 381 | 2.6427433 | MAP4K3 | mitogen-activated protein kinase kinase kinase kinase 3 [Source:HGNC Symbol;Acc:HGNC:6865] |
| ENSG00000062485 | HGLibA_11301 | 381 | 2.6427433 | CS | citrate synthase [Source:HGNC Symbol;Acc:HGNC:2422] |
| ENSG00000066777 | HGLibA_02670 | 381 | 2.6427433 | ARFGEF1 | ADP ribosylation factor guanine nucleotide exchange factor 1 [Source:HGNC Symbol;Acc:HGNC:15772] |
| ENSG00000088298 | HGLibA_14456 | 381 | 2.6427433 | EDEM2 | ER degradation enhancing alpha-mannosidase like protein 2 [Source:HGNC Symbol;Acc:HGNC:15877] |
| ENSG00000104490 | HGLibA_31250 | 381 | 2.6427433 | NCALD | neurocalcin delta [Source:HGNC Symbol;Acc:HGNC:7655] |
| ENSG00000112425 | HGLibA_15334 | 381 | 2.6427433 | EPM2A | laforin glucan phosphatase [Source:HGNC Symbol;Acc:HGNC:3413]" |
| ENSG00000119715 | HGLibA_15597 | 381 | 2.6427433 | ESRRB | estrogen related receptor beta [Source:HGNC Symbol;Acc:HGNC:3473] |
| ENSG00000141179 | HGLibA_35690 | 381 | 2.6427433 | PCTP | phosphatidylcholine transfer protein [Source:HGNC Symbol;Acc:HGNC:8752] |
| ENSG00000144677 | HGLibA_11614 | 381 | 2.6427433 | CTDSPL | CTD small phosphatase like [Source:HGNC Symbol;Acc:HGNC:16890] |
| ENSG00000160223 | HGLibA_22519 | 381 | 2.6427433 | ICOSLG | inducible T cell costimulator ligand [Source:HGNC Symbol;Acc:HGNC:17087] |
| ENSG00000165935 | HGLibA_45664 | 381 | 2.6427433 | SMCO2 | single-pass membrane protein with coiled-coil domains 2 [Source:HGNC Symbol;Acc:HGNC:34448] |
| ENSG00000165953 | HGLibA_43515 | 381 | 2.6427433 | SERPINA12 | serpin family A member 12 [Source:HGNC Symbol;Acc:HGNC:18359] |
| ENSG00000171045 | HGLibA_51882 | 381 | 2.6427433 | TSNARE1 | t-SNARE domain containing 1 [Source:HGNC Symbol;Acc:HGNC:26437] |

| | | | | | |
|---|---|---|---|---|---|
| ENSG00000174839 | HGLibA_12964 | 381 | 2.6427433 | DENND6A | DENN domain containing 6A [Source:HGNC Symbol;Acc:HGNC:26635] |
| ENSG00000185483 | HGLibA_41769 | 381 | 2.6427433 | ROR1 | receptor tyrosine kinase like orphan receptor 1 [Source:HGNC Symbol;Acc:HGNC:10256] |
| ENSG00000196739 | HGLibA_10554 | 381 | 2.6427433 | COL27A1 | collagen type XXVII alpha 1 chain [Source:HGNC Symbol;Acc:HGNC:22986] |
| HIST1H4G | HGLibA_21504 | 381 | 2.6427433 | HIST1H4G | - |
| hsa-mir-4784 | HGLibA_61243 | 381 | 2.6427433 | hsa-mir-4784 | - |
| KIAA0101 | HGLibA_24589 | 381 | 2.6427433 | KIAA0101 | - |
| ARSE | HGLibA_03137 | 380 | 2.632792263 | ARSE | - |
| ENSG00000101421 | HGLibA_09490 | 380 | 2.632792263 | CHMP4B | charged multivesicular body protein 4B [Source:HGNC Symbol;Acc:HGNC:16171] |
| ENSG00000107833 | HGLibA_32492 | 380 | 2.632792263 | NPM3 | nucleophosmin/nucleoplasmin 3 [Source:HGNC Symbol;Acc:HGNC:7931] |
| ENSG00000109674 | HGLibA_31635 | 380 | 2.632792263 | NEIL3 | nei like DNA glycosylase 3 [Source:HGNC Symbol;Acc:HGNC:24573] |
| ENSG00000149658 | HGLibA_54730 | 380 | 2.632792263 | YTHDF1 | YTH N6-methyladenosine RNA binding protein 1 [Source:HGNC Symbol;Acc:HGNC:15867] |
| ENSG00000158769 | HGLibA_15846 | 380 | 2.632792263 | F11R | F11 receptor [Source:HGNC Symbol;Acc:HGNC:14685] |
| ENSG00000160051 | HGLibA_23492 | 380 | 2.632792263 | IQCC | IQ motif containing C [Source:HGNC Symbol;Acc:HGNC:25545] |
| ENSG00000161955 | HGLibA_50807 | 380 | 2.632792263 | TNFSF13 | TNF superfamily member 13 [Source:HGNC Symbol;Acc:HGNC:11928] |
| ENSG00000164692 | HGLibA_10534 | 380 | 2.632792263 | COL1A2 | collagen type I alpha 2 chain [Source:HGNC Symbol;Acc:HGNC:2198] |
| ENSG00000185940 | HGLibA_25800 | 380 | 2.632792263 | KRTAP5-5 | keratin associated protein 5-5 [Source:HGNC Symbol;Acc:HGNC:23601] |
| ENSG00000205476 | HGLibA_07898 | 380 | 2.632792263 | CCDC85C | coiled-coil domain containing 85C [Source:HGNC Symbol;Acc:HGNC:35459] |
| hsa-mir-34a | HGLibA_59034 | 380 | 2.632792263 | hsa-mir-34a | - |
| QTRTD1 | HGLibA_39762 | 380 | 2.632792263 | QTRTD1 | - |
| ENSG00000103375 | HGLibA_02607 | 379 | 2.622841225 | AQP8 | aquaporin 8 [Source:HGNC Symbol;Acc:HGNC:642] |
| ENSG00000141569 | HGLibA_51586 | 379 | 2.622841225 | TRIM65 | tripartite motif containing 65 [Source:HGNC Symbol;Acc:HGNC:27316] |
| ENSG00000148634 | HGLibA_21163 | 379 | 2.622841225 | HERC4 | HECT and RLD domain containing E3 ubiquitin protein ligase 4 [Source:HGNC Symbol;Acc:HGNC:24521] |
| ENSG00000149177 | HGLibA_39511 | 379 | 2.622841225 | PTPRJ | receptor type J [Source:HGNC Symbol;Acc:HGNC:9673]" |
| ENSG00000160691 | HGLibA_44000 | 379 | 2.622841225 | SHC1 | SHC adaptor protein 1 [Source:HGNC Symbol;Acc:HGNC:10840] |
| ENSG00000168878 | HGLibA_43771 | 379 | 2.622841225 | SFTPB | surfactant protein B [Source:HGNC Symbol;Acc:HGNC:10801] |
| ENSG00000174804 | HGLibA_18273 | 379 | 2.622841225 | FZD4 | frizzled class receptor 4 [Source:HGNC Symbol;Acc:HGNC:4042] |
| ENSG00000186812 | HGLibA_55917 | 379 | 2.622841225 | ZNF397 | zinc finger protein 397 [Source:HGNC Symbol;Acc:HGNC:18818] |
| ENSG00000198597 | HGLibA_56184 | 379 | 2.622841225 | ZNF536 | zinc finger protein 536 [Source:HGNC Symbol;Acc:HGNC:29025] |
| ENSG00000214046 | HGLibA_45768 | 379 | 2.622841225 | SMIM7 | small integral membrane protein 7 [Source:HGNC Symbol;Acc:HGNC:28419] |
| ENSG00000243566 | HGLibA_53081 | 379 | 2.622841225 | UPK3B | uroplakin 3B [Source:HGNC Symbol;Acc:HGNC:21444] |
| hsa-mir-3200 | HGLibA_58909 | 379 | 2.622841225 | hsa-mir-3200 | - |
| hsa-mir-500a | HGLibA_61444 | 379 | 2.622841225 | hsa-mir-500a | - |
| hsa-mir-598 | HGLibA_62485 | 379 | 2.622841225 | hsa-mir-598 | - |
| ENSG00000027869 | HGLibA_43889 | 378 | 2.612890188 | SH2D2A | SH2 domain containing 2A [Source:HGNC Symbol;Acc:HGNC:10821] |
| ENSG00000055163 | HGLibA_12035 | 378 | 2.612890188 | CYFIP2 | cytoplasmic FMR1 interacting protein 2 [Source:HGNC Symbol;Acc:HGNC:13760] |
| ENSG00000116194 | HGLibA_01910 | 378 | 2.612890188 | ANGPTL1 | angiopoietin like 1 [Source:HGNC Symbol;Acc:HGNC:489] |
| ENSG00000126583 | HGLibA_38547 | 378 | 2.612890188 | PRKCG | protein kinase C gamma [Source:HGNC Symbol;Acc:HGNC:9402] |
| ENSG00000140511 | HGLibA_20848 | 378 | 2.612890188 | HAPLN3 | hyaluronan and proteoglycan link protein 3 [Source:HGNC Symbol;Acc:HGNC:21446] |
| ENSG00000141485 | HGLibA_44396 | 378 | 2.612890188 | SLC13A5 | solute carrier family 13 member 5 [Source:HGNC Symbol;Acc:HGNC:23089] |
| ENSG00000145721 | HGLibA_26643 | 378 | 2.612890188 | LIX1 | limb and CNS expressed 1 [Source:HGNC Symbol;Acc:HGNC:18581] |
| ENSG00000151176 | HGLibA_36974 | 378 | 2.612890188 | PLBD2 | phospholipase B domain containing 2 [Source:HGNC Symbol;Acc:HGNC:27283] |
| ENSG00000159792 | HGLibA_39064 | 378 | 2.612890188 | PSKH1 | protein serine kinase H1 [Source:HGNC Symbol;Acc:HGNC:9529] |
| ENSG00000168955 | HGLibA_49699 | 378 | 2.612890188 | TM4SF20 | transmembrane 4 L six family member 20 [Source:HGNC Symbol;Acc:HGNC:26230] |
| ENSG00000173467 | HGLibA_01247 | 378 | 2.612890188 | AGR3 | protein disulphide isomerase family member [Source:HGNC Symbol;Acc:HGNC:24167]" |
| ENSG00000187010 | HGLibA_41151 | 378 | 2.612890188 | RHD | Rh blood group D antigen [Source:HGNC Symbol;Acc:HGNC:10009] |
| ENSG00000187860 | HGLibA_07660 | 378 | 2.612890188 | CCDC157 | coiled-coil domain containing 157 [Source:HGNC Symbol;Acc:HGNC:33854] |
| ENSG00000198930 | HGLibA_11306 | 378 | 2.612890188 | CSAG1 | chondrosarcoma associated gene 1 [Source:HGNC Symbol;Acc:HGNC:24294] |
| hsa-mir-1266 | HGLibA_57487 | 378 | 2.612890188 | hsa-mir-1266 | - |
| hsa-mir-4280 | HGLibA_59825 | 378 | 2.612890188 | hsa-mir-4280 | - |
| hsa-mir-595 | HGLibA_62471 | 378 | 2.612890188 | hsa-mir-595 | - |
| ENSG00000076555 | HGLibA_00349 | 377 | 2.60293915 | ACACB | acetyl-CoA carboxylase beta [Source:HGNC Symbol;Acc:HGNC:85] |
| ENSG00000081177 | HGLibA_15720 | 377 | 2.60293915 | EXD2 | exonuclease 3'-5' domain containing 2 [Source:HGNC Symbol;Acc:HGNC:20217] |
| ENSG00000088451 | HGLibA_49202 | 377 | 2.60293915 | TGDS | 6-dehydratase [Source:HGNC Symbol;Acc:HGNC:20324]" |
| ENSG00000095203 | HGLibA_15253 | 377 | 2.60293915 | EPB41L4B | erythrocyte membrane protein band 4.1 like 4B [Source:HGNC Symbol;Acc:HGNC:19818] |
| ENSG00000095261 | HGLibA_39175 | 377 | 2.60293915 | PSMD5 | non-ATPase 5 [Source:HGNC Symbol;Acc:HGNC:9563]" |
| ENSG00000126903 | HGLibA_44336 | 377 | 2.60293915 | SLC10A3 | solute carrier family 10 member 3 [Source:HGNC Symbol;Acc:HGNC:22979] |
| ENSG00000140557 | HGLibA_47291 | 377 | 2.60293915 | ST8SIA2 | 8-sialyltransferase 2 [Source:HGNC Symbol;Acc:HGNC:10870]" |
| ENSG00000152969 | HGLibA_23922 | 377 | 2.60293915 | JAKMIP1 | janus kinase and microtubule interacting protein 1 [Source:HGNC Symbol;Acc:HGNC:26460] |
| ENSG00000162433 | HGLibA_01384 | 377 | 2.60293915 | AK4 | adenylate kinase 4 [Source:HGNC Symbol;Acc:HGNC:363] |
| ENSG00000179981 | HGLibA_51862 | 377 | 2.60293915 | TSHZ1 | teashirt zinc finger homeobox 1 [Source:HGNC Symbol;Acc:HGNC:10669] |
| ENSG00000182578 | HGLibA_11324 | 377 | 2.60293915 | CSF1R | colony stimulating factor 1 receptor [Source:HGNC Symbol;Acc:HGNC:2433] |
| ENSG00000196743 | HGLibA_19389 | 377 | 2.60293915 | GM2A | GM2 ganglioside activator [Source:HGNC Symbol;Acc:HGNC:4367] |
| ENSG00000196748 | HGLibA_10111 | 377 | 2.60293915 | CLPSL2 | colipase like 2 [Source:HGNC Symbol;Acc:HGNC:21250] |
| ENSG00000205869 | HGLibA_25783 | 377 | 2.60293915 | KRTAP5-1 | keratin associated protein 5-1 [Source:HGNC Symbol;Acc:HGNC:23596] |
| ENSG00000277363 | HGLibA_46933 | 377 | 2.60293915 | SRCIN1 | SRC kinase signaling inhibitor 1 [Source:HGNC Symbol;Acc:HGNC:29506] |
| C14orf1 | HGLibA_05315 | 376 | 2.592988113 | C14orf1 | - |
| C9orf89 | HGLibA_06769 | 376 | 2.592988113 | C9orf89 | - |
| ENSG00000006638 | HGLibA_48680 | 376 | 2.592988113 | TBXA2R | thromboxane A2 receptor [Source:HGNC Symbol;Acc:HGNC:11608] |
| ENSG00000087088 | HGLibA_04182 | 376 | 2.592988113 | BAX | apoptosis regulator [Source:HGNC Symbol;Acc:HGNC:959]" |
| ENSG00000114670 | HGLibA_31644 | 376 | 2.592988113 | NEK11 | NIMA related kinase 11 [Source:HGNC Symbol;Acc:HGNC:18593] |
| ENSG00000132591 | HGLibA_15400 | 376 | 2.592988113 | ERAL1 | Era like 12S mitochondrial rRNA chaperone 1 [Source:HGNC Symbol;Acc:HGNC:3424] |
| ENSG00000139154 | HGLibA_01088 | 376 | 2.592988113 | AEBP2 | AE binding protein 2 [Source:HGNC Symbol;Acc:HGNC:24051] |
| ENSG00000140006 | HGLibA_54212 | 376 | 2.592988113 | WDR89 | WD repeat domain 89 [Source:HGNC Symbol;Acc:HGNC:20489] |
| ENSG00000145198 | HGLibA_53910 | 376 | 2.592988113 | VWA5B2 | von Willebrand factor A domain containing 5B2 [Source:HGNC Symbol;Acc:HGNC:25144] |
| ENSG00000169738 | HGLibA_12583 | 376 | 2.592988113 | DCXR | dicarbonyl and L-xylulose reductase [Source:HGNC Symbol;Acc:HGNC:18985] |
| ENSG00000204671 | HGLibA_23129 | 376 | 2.592988113 | IL31 | interleukin 31 [Source:HGNC Symbol;Acc:HGNC:19372] |
| ENSG00000242689 | HGLibA_10391 | 376 | 2.592988113 | CNTF | ciliary neurotrophic factor [Source:HGNC Symbol;Acc:HGNC:2169] |
| hsa-mir-3174 | HGLibA_58769 | 376 | 2.592988113 | hsa-mir-3174 | - |
| 8-Sep | HGLibA_43454 | 376 | 2.592988113 | 8-Sep | - |
| ENSG00000065361 | HGLibA_15420 | 375 | 2.583037076 | ERBB3 | erb-b2 receptor tyrosine kinase 3 [Source:HGNC Symbol;Acc:HGNC:3431] |
| ENSG00000070047 | HGLibA_36458 | 375 | 2.583037076 | PHRF1 | PHD and ring finger domains 1 [Source:HGNC Symbol;Acc:HGNC:24351] |
| ENSG00000110274 | HGLibA_09112 | 375 | 2.583037076 | CEP164 | centrosomal protein 164 [Source:HGNC Symbol;Acc:HGNC:29182] |
| ENSG00000123096 | HGLibA_47146 | 375 | 2.583037076 | SSPN | sarcospan [Source:HGNC Symbol;Acc:HGNC:11322] |
| ENSG00000153933 | HGLibA_13066 | 375 | 2.583037076 | DGKE | diacylglycerol kinase epsilon [Source:HGNC Symbol;Acc:HGNC:2852] |
| ENSG00000168077 | HGLibA_42863 | 375 | 2.583037076 | SCARA3 | scavenger receptor class A member 3 [Source:HGNC Symbol;Acc:HGNC:19000] |
| ENSG00000186867 | HGLibA_39738 | 375 | 2.583037076 | QRFPR | pyroglutamylated RFamide peptide receptor [Source:HGNC Symbol;Acc:HGNC:15565] |
| ENSG00000196531 | HGLibA_31031 | 375 | 2.583037076 | NACA | nascent polypeptide associated complex subunit alpha [Source:HGNC Symbol;Acc:HGNC:7629] |
| ENSG00000250067 | HGLibA_54692 | 375 | 2.583037076 | YJEFN3 | YjeF N-terminal domain containing 3 [Source:HGNC Symbol;Acc:HGNC:24785] |
| C12orf5 | HGLibA_05255 | 374 | 2.573086038 | C12orf5 | - |
| ENSG00000053371 | HGLibA_01498 | 374 | 2.573086038 | AKR7A2 | aldo-keto reductase family 7 member A2 [Source:HGNC Symbol;Acc:HGNC:389] |
| ENSG00000100344 | HGLibA_37410 | 374 | 2.573086038 | PNPLA3 | patatin like phospholipase domain containing 3 [Source:HGNC Symbol;Acc:HGNC:18590] |
| ENSG00000127948 | HGLibA_37706 | 374 | 2.573086038 | POR | cytochrome p450 oxidoreductase [Source:HGNC Symbol;Acc:HGNC:9208] |
| ENSG00000139209 | HGLibA_45070 | 374 | 2.573086038 | SLC38A4 | solute carrier family 38 member 4 [Source:HGNC Symbol;Acc:HGNC:14679] |
| ENSG00000149483 | HGLibA_49955 | 374 | 2.573086038 | TMEM138 | transmembrane protein 138 [Source:HGNC Symbol;Acc:HGNC:26944] |
| ENSG00000158258 | HGLibA_10138 | 374 | 2.573086038 | CLSTN2 | calsyntenin 2 [Source:HGNC Symbol;Acc:HGNC:17448] |
| ENSG00000158813 | HGLibA_14429 | 374 | 2.573086038 | EDA | ectodysplasin A [Source:HGNC Symbol;Acc:HGNC:3157] |
| ENSG00000176946 | HGLibA_49300 | 374 | 2.573086038 | THAP4 | THAP domain containing 4 [Source:HGNC Symbol;Acc:HGNC:23187] |
| ENSG00000182896 | HGLibA_50565 | 374 | 2.573086038 | TMEM95 | transmembrane protein 95 [Source:HGNC Symbol;Acc:HGNC:27898] |
| ENSG00000214688 | HGLibA_05021 | 374 | 2.573086038 | C10orf105 | chromosome 10 open reading frame 105 [Source:HGNC Symbol;Acc:HGNC:20304] |
| ENSG00000240583 | HGLibA_02578 | 374 | 2.573086038 | AQP1 | aquaporin 1 (Colton blood group) [Source:HGNC Symbol;Acc:HGNC:633] |
| hsa-mir-4330 | HGLibA_60047 | 374 | 2.573086038 | hsa-mir-4330 | - |
| ENSG00000040933 | HGLibA_23337 | 373 | 2.563135001 | INPP4A | inositol polyphosphate-4-phosphatase type I A [Source:HGNC Symbol;Acc:HGNC:6074] |
| ENSG00000100053 | HGLibA_11268 | 373 | 2.563135001 | CRYBB3 | crystallin beta B3 [Source:HGNC Symbol;Acc:HGNC:2400] |
| ENSG00000100097 | HGLibA_26346 | 373 | 2.563135001 | LGALS1 | galectin 1 [Source:HGNC Symbol;Acc:HGNC:6561] |
| ENSG00000101335 | HGLibA_30777 | 373 | 2.563135001 | MYL9 | myosin light chain 9 [Source:HGNC Symbol;Acc:HGNC:15754] |
| ENSG00000117054 | HGLibA_00367 | 373 | 2.563135001 | ACADM | acyl-CoA dehydrogenase medium chain [Source:HGNC Symbol;Acc:HGNC:89] |
| ENSG00000125170 | HGLibA_13776 | 373 | 2.563135001 | DOK4 | docking protein 4 [Source:HGNC Symbol;Acc:HGNC:19868] |
| ENSG00000128016 | HGLibA_55203 | 373 | 2.563135001 | ZFP36 | ZFP36 ring finger protein [Source:HGNC Symbol;Acc:HGNC:12862] |
| ENSG00000137080 | HGLibA_22676 | 373 | 2.563135001 | IFNA21 | interferon alpha 21 [Source:HGNC Symbol;Acc:HGNC:5424] |
| ENSG00000141179 | HGLibA_35692 | 373 | 2.563135001 | PCTP | phosphatidylcholine transfer protein [Source:HGNC Symbol;Acc:HGNC:8752] |
| ENSG00000168917 | HGLibA_45019 | 373 | 2.563135001 | SLC35G2 | solute carrier family 35 member G2 [Source:HGNC Symbol;Acc:HGNC:28480] |
| ENSG00000169871 | HGLibA_51550 | 373 | 2.563135001 | TRIM56 | tripartite motif containing 56 [Source:HGNC Symbol;Acc:HGNC:19028] |
| ENSG00000170927 | HGLibA_36813 | 373 | 2.563135001 | PKHD1 | fibrocystin/polyductin [Source:HGNC Symbol;Acc:HGNC:9016]" |
| ENSG00000178307 | HGLibA_49871 | 373 | 2.563135001 | TMEM11 | transmembrane protein 11 [Source:HGNC Symbol;Acc:HGNC:16823] |
| ENSG00000187193 | HGLibA_30298 | 373 | 2.563135001 | MT1X | metallothionein 1X [Source:HGNC Symbol;Acc:HGNC:7405] |
| ENSG00000198455 | HGLibA_57075 | 373 | 2.563135001 | ZXDB | zinc finger X-linked duplicated B [Source:HGNC Symbol;Acc:HGNC:13199] |
| hsa-mir-3141 | HGLibA_58624 | 373 | 2.563135001 | hsa-mir-3141 | - |
| LOC100507462 | HGLibA_26869 | 373 | 2.563135001 | LOC100507462 | - |
| TMEM110-MUSTN1 | HGLibA_49878 | 373 | 2.563135001 | TMEM110-MUSTN1 | - |
| ENSG00000075275 | HGLibA_09022 | 372 | 2.553183963 | CELSR1 | cadherin EGF LAG seven-pass G-type receptor 1 [Source:HGNC Symbol;Acc:HGNC:1850] |
| ENSG00000106829 | HGLibA_49616 | 372 | 2.553183963 | TLE4 | transducin like enhancer of split 4 [Source:HGNC Symbol;Acc:HGNC:11840] |
| ENSG00000112699 | HGLibA_19394 | 372 | 2.553183963 | GMDS | 6-dehydratase [Source:HGNC Symbol;Acc:HGNC:4369]" |
| ENSG00000136869 | HGLibA_49652 | 372 | 2.553183963 | TLR4 | toll like receptor 4 [Source:HGNC Symbol;Acc:HGNC:11850] |
| ENSG00000142784 | HGLibA_54231 | 372 | 2.553183963 | WDTC1 | WD and tetratricopeptide repeats 1 [Source:HGNC Symbol;Acc:HGNC:29175] |
| ENSG00000145901 | HGLibA_50836 | 372 | 2.553183963 | TNIP1 | TNFAIP3 interacting protein 1 [Source:HGNC Symbol;Acc:HGNC:16903] |
| ENSG00000161634 | HGLibA_12478 | 372 | 2.553183963 | DCD | dermcidin [Source:HGNC Symbol;Acc:HGNC:14669] |
| ENSG00000165816 | HGLibA_53900 | 372 | 2.553183963 | VWA2 | von Willebrand factor A domain containing 2 [Source:HGNC Symbol;Acc:HGNC:24709] |
| ENSG00000173409 | HGLibA_03172 | 372 | 2.553183963 | ARV1 | fatty acid homeostasis modulator [Source:HGNC Symbol;Acc:HGNC:29561]" |
| ENSG00000177951 | HGLibA_04435 | 372 | 2.553183963 | BET1L | Bet1 golgi vesicular membrane trafficking protein like [Source:HGNC Symbol;Acc:HGNC:19348] |
| ENSG00000188293 | HGLibA_22840 | 372 | 2.553183963 | IGFL1 | IGF like family member 1 [Source:HGNC Symbol;Acc:HGNC:24093] |
| ENSG00000204478 | HGLibA_38309 | 372 | 2.553183963 | PRAMEF20 | PRAME family member 20 [Source:HGNC Symbol;Acc:HGNC:25224] |
| ENSG00000215397 | HGLibA_43060 | 372 | 2.553183963 | SCRT2 | scratch family transcriptional repressor 2 [Source:HGNC Symbol;Acc:HGNC:15952] |
| ENSG00000283632 | HGLibA_15743 | 372 | 2.553183963 | EXOC3L2 | exocyst complex component 3 like 2 [Source:HGNC Symbol;Acc:HGNC:30162] |
| hsa-mir-4527 | HGLibA_60570 | 372 | 2.553183963 | hsa-mir-4527 | - |
| USMG5 | HGLibA_53186 | 372 | 2.553183963 | USMG5 | - |

| | | | | | |
|---|---|---|---|---|---|
| ENSG00000102383 | HGLibA_55081 | 371 | 2.543232926 | ZDHHC15 | zinc finger DHHC-type containing 15 [Source:HGNC Symbol;Acc:HGNC:20342] |
| ENSG00000142675 | HGLibA_10297 | 371 | 2.543232926 | CNKSR1 | connector enhancer of kinase suppressor of Ras 1 [Source:HGNC Symbol;Acc:HGNC:19700] |
| ENSG00000148204 | HGLibA_11071 | 371 | 2.543232926 | CRB2 | cell polarity complex component [Source:HGNC Symbol;Acc:HGNC:18688]" |
| ENSG00000149635 | HGLibA_33267 | 371 | 2.543232926 | OCSTAMP | osteoclast stimulatory transmembrane protein [Source:HGNC Symbol;Acc:HGNC:16116] |
| ENSG00000163866 | HGLibA_45725 | 371 | 2.543232926 | SMIM12 | small integral membrane protein 12 [Source:HGNC Symbol;Acc:HGNC:25154] |
| ENSG00000170323 | HGLibA_15918 | 371 | 2.543232926 | FABP4 | fatty acid binding protein 4 [Source:HGNC Symbol;Acc:HGNC:3559] |
| ENSG00000172830 | HGLibA_47138 | 371 | 2.543232926 | SSH3 | slingshot protein phosphatase 3 [Source:HGNC Symbol;Acc:HGNC:30581] |
| ENSG00000177489 | HGLibA_33795 | 371 | 2.543232926 | OR2G2 | olfactory receptor family 2 subfamily G member 2 [Source:HGNC Symbol;Acc:HGNC:15007] |
| ENSG00000185046 | HGLibA_02154 | 371 | 2.543232926 | ANKS1B | ankyrin repeat and sterile alpha motif domain containing 1B [Source:HGNC Symbol;Acc:HGNC:24600] |
| ENSG00000204424 | HGLibA_27671 | 371 | 2.543232926 | LY6G6F | lymphocyte antigen 6 family member G6F [Source:HGNC Symbol;Acc:HGNC:13933] |
| ENSG00000205177 | HGLibA_05210 | 371 | 2.543232926 | C11orf91 | chromosome 11 open reading frame 91 [Source:HGNC Symbol;Acc:HGNC:34444] |
| ENSG00000283597 | HGLibA_16255 | 371 | 2.543232926 | FAM169B | family with sequence similarity 169 member B [Source:HGNC Symbol;Acc:HGNC:26835] |
| hsa-mir-3170 | HGLibA_58758 | 371 | 2.543232926 | hsa-mir-3170 | - |
| LOC152586 | HGLibA_26894 | 371 | 2.543232926 | LOC152586 | - |
| ENSG00000000419 | HGLibA_13856 | 370 | 2.533281888 | DPM1 | catalytic [Source:HGNC Symbol;Acc:HGNC:3005]" |
| ENSG00000085741 | HGLibA_54351 | 370 | 2.533281888 | WNT11 | Wnt family member 11 [Source:HGNC Symbol;Acc:HGNC:12776] |
| ENSG00000099203 | HGLibA_49800 | 370 | 2.533281888 | TMED1 | transmembrane p24 trafficking protein 1 [Source:HGNC Symbol;Acc:HGNC:17291] |
| ENSG00000113790 | HGLibA_14680 | 370 | 2.533281888 | EHHADH | enoyl-CoA hydratase and 3-hydroxyacyl CoA dehydrogenase [Source:HGNC Symbol;Acc:HGNC:3247] |
| ENSG00000135424 | HGLibA_23742 | 370 | 2.533281888 | ITGA7 | integrin subunit alpha 7 [Source:HGNC Symbol;Acc:HGNC:6143] |
| ENSG00000177989 | HGLibA_33288 | 370 | 2.533281888 | ODF3B | outer dense fiber of sperm tails 3B [Source:HGNC Symbol;Acc:HGNC:34388] |
| ENSG00000183770 | HGLibA_17904 | 370 | 2.533281888 | FOXL2 | forkhead box L2 [Source:HGNC Symbol;Acc:HGNC:1092] |
| ENSG00000213445 | HGLibA_44190 | 370 | 2.533281888 | SIPA1 | signal-induced proliferation-associated 1 [Source:HGNC Symbol;Acc:HGNC:10885] |
| FAM213B | HGLibA_16475 | 370 | 2.533281888 | FAM213B | - |
| ENSG00000073150 | HGLibA_35112 | 369 | 2.523330851 | PANX2 | pannexin 2 [Source:HGNC Symbol;Acc:HGNC:8600] |
| ENSG00000105492 | HGLibA_44145 | 369 | 2.523330851 | SIGLEC6 | sialic acid binding Ig like lectin 6 [Source:HGNC Symbol;Acc:HGNC:10875] |
| ENSG00000105953 | HGLibA_33302 | 369 | 2.523330851 | OGDH | oxoglutarate dehydrogenase [Source:HGNC Symbol;Acc:HGNC:8124] |
| ENSG00000110442 | HGLibA_10680 | 369 | 2.523330851 | COMMD9 | COMM domain containing 9 [Source:HGNC Symbol;Acc:HGNC:25014] |
| ENSG00000119599 | HGLibA_12438 | 369 | 2.523330851 | DCAF4 | DDB1 and CUL4 associated factor 4 [Source:HGNC Symbol;Acc:HGNC:20229] |
| ENSG00000120738 | HGLibA_14647 | 369 | 2.523330851 | EGR1 | early growth response 1 [Source:HGNC Symbol;Acc:HGNC:3238] |
| ENSG00000124532 | HGLibA_30118 | 369 | 2.523330851 | MRS2 | magnesium transporter MRS2 [Source:HGNC Symbol;Acc:HGNC:13785] |
| ENSG00000143222 | HGLibA_52842 | 369 | 2.523330851 | UFC1 | ubiquitin-fold modifier conjugating enzyme 1 [Source:HGNC Symbol;Acc:HGNC:26941] |
| ENSG00000160856 | HGLibA_17231 | 369 | 2.523330851 | FCRL3 | Fc receptor like 3 [Source:HGNC Symbol;Acc:HGNC:18506] |
| ENSG00000164308 | HGLibA_15406 | 369 | 2.523330851 | ERAP2 | endoplasmic reticulum aminopeptidase 2 [Source:HGNC Symbol;Acc:HGNC:29499] |
| ENSG00000196581 | HGLibA_01370 | 369 | 2.523330851 | AJAP1 | adherens junctions associated protein 1 [Source:HGNC Symbol;Acc:HGNC:30801] |
| ENSG00000223496 | HGLibA_15786 | 369 | 2.523330851 | EXOSC6 | exosome component 6 [Source:HGNC Symbol;Acc:HGNC:19055] |
| ENSG00000233436 | HGLibA_04907 | 369 | 2.523330851 | BTBD18 | BTB domain containing 18 [Source:HGNC Symbol;Acc:HGNC:37214] |
| hsa-mir-3181 | HGLibA_58821 | 369 | 2.523330851 | hsa-mir-3181 | - |
| IMPAD1 | HGLibA_23252 | 369 | 2.523330851 | IMPAD1 | - |
| C15orf54 | HGLibA_05413 | 368 | 2.513379814 | C15orf54 | - |
| ENSG00000108691 | HGLibA_07987 | 368 | 2.513379814 | CCL2 | C-C motif chemokine ligand 2 [Source:HGNC Symbol;Acc:HGNC:10618] |
| ENSG00000111339 | HGLibA_03163 | 368 | 2.513379814 | ART4 | ADP-ribosyltransferase 4 (Dombrock blood group) [Source:HGNC Symbol;Acc:HGNC:726] |
| ENSG00000139644 | HGLibA_49746 | 368 | 2.513379814 | TMBIM6 | transmembrane BAX inhibitor motif containing 6 [Source:HGNC Symbol;Acc:HGNC:11723] |
| ENSG00000144821 | HGLibA_30718 | 368 | 2.513379814 | MYH15 | myosin heavy chain 15 [Source:HGNC Symbol;Acc:HGNC:31073] |
| ENSG00000170075 | HGLibA_20035 | 368 | 2.513379814 | GPR37L1 | G protein-coupled receptor 37 like 1 [Source:HGNC Symbol;Acc:HGNC:14923] |
| ENSG00000176971 | HGLibA_17501 | 368 | 2.513379814 | FIBIN | fin bud initiation factor homolog [Source:HGNC Symbol;Acc:HGNC:33747] |
| ENSG00000177910 | HGLibA_46492 | 368 | 2.513379814 | SPATA31C2 | SPATA31 subfamily C member 2 [Source:HGNC Symbol;Acc:HGNC:24508] |
| ENSG00000181085 | HGLibA_28264 | 368 | 2.513379814 | MAPK15 | mitogen-activated protein kinase 15 [Source:HGNC Symbol;Acc:HGNC:24667] |
| ENSG00000182372 | HGLibA_10084 | 368 | 2.513379814 | CLN8 | transmembrane ER and ERGIC protein [Source:HGNC Symbol;Acc:HGNC:2079]" |
| ENSG00000182809 | HGLibA_11154 | 368 | 2.513379814 | CRIP2 | cysteine rich protein 2 [Source:HGNC Symbol;Acc:HGNC:2361] |
| ENSG00000232399 | HGLibA_53223 | 368 | 2.513379814 | USP17L13 | ubiquitin specific peptidase 17-like family member 13 [Source:HGNC Symbol;Acc:HGNC:44441] |
| ENSG00000251192 | HGLibA_56510 | 368 | 2.513379814 | ZNF674 | zinc finger protein 674 [Source:HGNC Symbol;Acc:HGNC:17625] |
| EPRS | HGLibA_15365 | 368 | 2.513379814 | EPRS | - |
| hsa-mir-3144 | HGLibA_58634 | 368 | 2.513379814 | hsa-mir-3144 | - |
| hsa-mir-6840 | HGLibA_63535 | 368 | 2.513379814 | hsa-mir-6840 | - |
| hsa-mir-7153 | HGLibA_63840 | 368 | 2.513379814 | hsa-mir-7153 | - |
| ENSG00000062282 | HGLibA_13036 | 367 | 2.503428776 | DGAT2 | diacylglycerol O-acyltransferase 2 [Source:HGNC Symbol;Acc:HGNC:16940] |
| ENSG00000092201 | HGLibA_47804 | 367 | 2.503428776 | SUPT16H | facilitates chromatin remodeling subunit [Source:HGNC Symbol;Acc:HGNC:11465]" |
| ENSG00000100934 | HGLibA_43219 | 367 | 2.503428776 | SEC23A | coat complex II component [Source:HGNC Symbol;Acc:HGNC:10701]" |
| ENSG00000160783 | HGLibA_37303 | 367 | 2.503428776 | PMF1 | polyamine modulated factor 1 [Source:HGNC Symbol;Acc:HGNC:9112] |
| ENSG00000165392 | HGLibA_54408 | 367 | 2.503428776 | WRN | Werner syndrome RecQ like helicase [Source:HGNC Symbol;Acc:HGNC:12791] |
| ENSG00000169919 | HGLibA_20679 | 367 | 2.503428776 | GUSB | glucuronidase beta [Source:HGNC Symbol;Acc:HGNC:4696] |
| ENSG00000170579 | HGLibA_13340 | 367 | 2.503428776 | DLGAP1 | DLG associated protein 1 [Source:HGNC Symbol;Acc:HGNC:2905] |
| ENSG00000172752 | HGLibA_10606 | 367 | 2.503428776 | COL6A5 | collagen type VI alpha 5 chain [Source:HGNC Symbol;Acc:HGNC:26674] |
| ENSG00000179639 | HGLibA_17167 | 367 | 2.503428776 | FCER1A | Fc fragment of IgE receptor Ia [Source:HGNC Symbol;Acc:HGNC:3609] |
| ENSG00000179981 | HGLibA_51863 | 367 | 2.503428776 | TSHZ1 | teashirt zinc finger homeobox 1 [Source:HGNC Symbol;Acc:HGNC:10669] |
| ENSG00000182566 | HGLibA_09995 | 367 | 2.503428776 | CLEC4G | C-type lectin domain family 4 member G [Source:HGNC Symbol;Acc:HGNC:24591] |
| ENSG00000185149 | HGLibA_32552 | 367 | 2.503428776 | NPY2R | neuropeptide Y receptor Y2 [Source:HGNC Symbol;Acc:HGNC:7957] |
| ENSG00000204849 | HGLibA_46467 | 367 | 2.503428776 | SPATA31A1 | SPATA31 subfamily A member 1 [Source:HGNC Symbol;Acc:HGNC:23394] |
| ENSG00000213347 | HGLibA_30613 | 367 | 2.503428776 | MXD3 | MAX dimerization protein 3 [Source:HGNC Symbol;Acc:HGNC:14008] |
| hsa-mir-496 | HGLibA_61383 | 367 | 2.503428776 | hsa-mir-496 | - |
| ENSG00000101608 | HGLibA_30751 | 366 | 2.493477739 | MYL12A | myosin light chain 12A [Source:HGNC Symbol;Acc:HGNC:16701] |
| ENSG00000114544 | HGLibA_45146 | 366 | 2.493477739 | SLC41A3 | solute carrier family 41 member 3 [Source:HGNC Symbol;Acc:HGNC:31046] |
| ENSG00000131096 | HGLibA_39713 | 366 | 2.493477739 | PYY | peptide YY [Source:HGNC Symbol;Acc:HGNC:9748] |
| ENSG00000136854 | HGLibA_47662 | 366 | 2.493477739 | STXBP1 | syntaxin binding protein 1 [Source:HGNC Symbol;Acc:HGNC:11444] |
| ENSG00000165802 | HGLibA_32774 | 366 | 2.493477739 | NSMF | NMDA receptor synaptonuclear signaling and neuronal migration factor [Source:HGNC Symbol;Acc:HGNC:29843] |
| ENSG00000172731 | HGLibA_27282 | 366 | 2.493477739 | LRRC20 | leucine rich repeat containing 20 [Source:HGNC Symbol;Acc:HGNC:23421] |
| ENSG00000175193 | HGLibA_35203 | 366 | 2.493477739 | PARL | presenilin associated rhomboid like [Source:HGNC Symbol;Acc:HGNC:18253] |
| ENSG00000183318 | HGLibA_46593 | 366 | 2.493477739 | SPDYE4 | speedy/RINGO cell cycle regulator family member E4 [Source:HGNC Symbol;Acc:HGNC:35463] |
| ENSG00000205445 | HGLibA_25604 | 366 | 2.493477739 | KRTAP10-2 | keratin associated protein 10-2 [Source:HGNC Symbol;Acc:HGNC:22967] |
| ENSG00000217442 | HGLibA_47921 | 366 | 2.493477739 | SYCE3 | synaptonemal complex central element protein 3 [Source:HGNC Symbol;Acc:HGNC:35245] |
| ENSG00000269404 | HGLibA_46654 | 366 | 2.493477739 | SPIB | Spi-B transcription factor [Source:HGNC Symbol;Acc:HGNC:11242] |
| hsa-mir-4322 | HGLibA_60013 | 366 | 2.493477739 | hsa-mir-4322 | - |
| hsa-mir-6074 | HGLibA_62549 | 366 | 2.493477739 | hsa-mir-6074 | - |
| ENSG00000047249 | HGLibA_03765 | 365 | 2.483526701 | ATP6V1H | ATPase H+ transporting V1 subunit H [Source:HGNC Symbol;Acc:HGNC:18303] |
| ENSG00000066230 | HGLibA_45405 | 365 | 2.483526701 | SLC9A3 | solute carrier family 9 member A3 [Source:HGNC Symbol;Acc:HGNC:11073] |
| ENSG00000088881 | HGLibA_14357 | 365 | 2.483526701 | EBF4 | EBF family member 4 [Source:HGNC Symbol;Acc:HGNC:29278] |
| ENSG00000104067 | HGLibA_49572 | 365 | 2.483526701 | TJP1 | tight junction protein 1 [Source:HGNC Symbol;Acc:HGNC:11827] |
| ENSG00000104823 | HGLibA_14391 | 365 | 2.483526701 | ECH1 | enoyl-CoA hydratase 1 [Source:HGNC Symbol;Acc:HGNC:3149] |
| ENSG00000107018 | HGLibA_41351 | 365 | 2.483526701 | RLN1 | relaxin 1 [Source:HGNC Symbol;Acc:HGNC:10026] |
| ENSG00000110514 | HGLibA_27856 | 365 | 2.483526701 | MADD | MAP kinase activating death domain [Source:HGNC Symbol;Acc:HGNC:6766] |
| ENSG00000115353 | HGLibA_48172 | 365 | 2.483526701 | TACR1 | tachykinin receptor 1 [Source:HGNC Symbol;Acc:HGNC:11526] |
| ENSG00000131067 | HGLibA_19018 | 365 | 2.483526701 | GGT7 | gamma-glutamyltransferase 7 [Source:HGNC Symbol;Acc:HGNC:4259] |
| ENSG00000132840 | HGLibA_04489 | 365 | 2.483526701 | BHMT2 | betaine--homocysteine S-methyltransferase 2 [Source:HGNC Symbol;Acc:HGNC:1048] |
| ENSG00000134470 | HGLibA_22984 | 365 | 2.483526701 | IL15RA | interleukin 15 receptor subunit alpha [Source:HGNC Symbol;Acc:HGNC:5978] |
| ENSG00000151835 | HGLibA_42629 | 365 | 2.483526701 | SACS | sacsin molecular chaperone [Source:HGNC Symbol;Acc:HGNC:10519] |
| ENSG00000169967 | HGLibA_28181 | 365 | 2.483526701 | MAP3K2 | mitogen-activated protein kinase kinase kinase 2 [Source:HGNC Symbol;Acc:HGNC:6854] |
| ENSG00000187867 | HGLibA_35075 | 365 | 2.483526701 | PALM3 | paralemmin 3 [Source:HGNC Symbol;Acc:HGNC:33274] |
| ENSG00000198791 | HGLibA_10351 | 365 | 2.483526701 | CNOT7 | CCR4-NOT transcription complex subunit 7 [Source:HGNC Symbol;Acc:HGNC:14101] |
| ENSG00000214050 | HGLibA_17032 | 365 | 2.483526701 | FBXO16 | F-box protein 16 [Source:HGNC Symbol;Acc:HGNC:13618] |
| hsa-mir-4322 | HGLibA_60010 | 365 | 2.483526701 | hsa-mir-4322 | - |
| AIM1 | HGLibA_01350 | 364 | 2.473575664 | AIM1 | - |
| C14orf177 | HGLibA_05342 | 364 | 2.473575664 | C14orf177 | - |
| ENSG00000004848 | HGLibA_03180 | 364 | 2.473575664 | ARX | aristaless related homeobox [Source:HGNC Symbol;Acc:HGNC:18060] |
| ENSG00000051382 | HGLibA_36637 | 364 | 2.473575664 | PIK3CB | 5-bisphosphate 3-kinase catalytic subunit beta [Source:HGNC Symbol;Acc:HGNC:8976]" |
| ENSG00000059378 | HGLibA_35220 | 364 | 2.473575664 | PARP12 | poly(ADP-ribose) polymerase family member 12 [Source:HGNC Symbol;Acc:HGNC:21919] |
| ENSG00000068383 | HGLibA_23342 | 364 | 2.473575664 | INPP5A | inositol polyphosphate-5-phosphatase A [Source:HGNC Symbol;Acc:HGNC:6076] |
| ENSG00000101405 | HGLibA_34840 | 364 | 2.473575664 | OXT | oxytocin/neurophysin I prepropeptide [Source:HGNC Symbol;Acc:HGNC:8528] |
| ENSG00000108963 | HGLibA_13837 | 364 | 2.473575664 | DPH1 | diphthamide biosynthesis 1 [Source:HGNC Symbol;Acc:HGNC:3003] |
| ENSG00000112763 | HGLibA_04962 | 364 | 2.473575664 | BTN2A1 | butyrophilin subfamily 2 member A1 [Source:HGNC Symbol;Acc:HGNC:1136] |
| ENSG00000140545 | HGLibA_29061 | 364 | 2.473575664 | MFGE8 | milk fat globule-EGF factor 8 protein [Source:HGNC Symbol;Acc:HGNC:7036] |
| ENSG00000169071 | HGLibA_41772 | 364 | 2.473575664 | ROR2 | receptor tyrosine kinase like orphan receptor 2 [Source:HGNC Symbol;Acc:HGNC:10257] |
| ENSG00000172301 | HGLibA_10709 | 364 | 2.473575664 | COPRS | coordinator of PRMT5 and differentiation stimulator [Source:HGNC Symbol;Acc:HGNC:28848] |
| ENSG00000198142 | HGLibA_46218 | 364 | 2.473575664 | SOWAHC | sosondowah ankyrin repeat domain family member C [Source:HGNC Symbol;Acc:HGNC:26149] |
| ENSG00000205309 | HGLibA_32831 | 364 | 2.473575664 | NT5M | mitochondrial [Source:HGNC Symbol;Acc:HGNC:15769]" |
| MB21D1 | HGLibA_28457 | 364 | 2.473575664 | MB21D1 | - |
| C9orf3 | HGLibA_06721 | 363 | 2.463624626 | C9orf3 | - |
| ENSG00000002822 | HGLibA_27843 | 363 | 2.463624626 | MAD1L1 | mitotic arrest deficient 1 like 1 [Source:HGNC Symbol;Acc:HGNC:6762] |
| ENSG00000036530 | HGLibA_12168 | 363 | 2.463624626 | CYP46A1 | cytochrome P450 family 46 subfamily A member 1 [Source:HGNC Symbol;Acc:HGNC:2641] |
| ENSG00000077585 | HGLibA_19900 | 363 | 2.463624626 | GPR137B | G protein-coupled receptor 137B [Source:HGNC Symbol;Acc:HGNC:11862] |
| ENSG00000086065 | HGLibA_09497 | 363 | 2.463624626 | CHMP5 | charged multivesicular body protein 5 [Source:HGNC Symbol;Acc:HGNC:26942] |
| ENSG00000092068 | HGLibA_45381 | 363 | 2.463624626 | SLC7A8 | solute carrier family 7 member 8 [Source:HGNC Symbol;Acc:HGNC:11066] |
| ENSG00000101276 | HGLibA_45249 | 363 | 2.463624626 | SLC52A3 | solute carrier family 52 member 3 [Source:HGNC Symbol;Acc:HGNC:16187] |
| ENSG00000115221 | HGLibA_23794 | 363 | 2.463624626 | ITGB6 | integrin subunit beta 6 [Source:HGNC Symbol;Acc:HGNC:6161] |
| ENSG00000118965 | HGLibA_54072 | 363 | 2.463624626 | WDR35 | WD repeat domain 35 [Source:HGNC Symbol;Acc:HGNC:29250] |
| ENSG00000131409 | HGLibA_27381 | 363 | 2.463624626 | LRRC4B | leucine rich repeat containing 4B [Source:HGNC Symbol;Acc:HGNC:25042] |
| ENSG00000154175 | HGLibA_00298 | 363 | 2.463624626 | ABI3BP | ABI family member 3 binding protein [Source:HGNC Symbol;Acc:HGNC:17265] |
| ENSG00000157212 | HGLibA_35324 | 363 | 2.463624626 | PAXIP1 | PAX interacting protein 1 [Source:HGNC Symbol;Acc:HGNC:8624] |
| ENSG00000163637 | HGLibA_38473 | 363 | 2.463624626 | PRICKLE2 | prickle planar cell polarity protein 2 [Source:HGNC Symbol;Acc:HGNC:20340] |
| ENSG00000164749 | HGLibA_21783 | 363 | 2.463624626 | HNF4G | hepatocyte nuclear factor 4 gamma [Source:HGNC Symbol;Acc:HGNC:5026] |
| ENSG00000170484 | HGLibA_25533 | 363 | 2.463624626 | KRT74 | keratin 74 [Source:HGNC Symbol;Acc:HGNC:28929] |
| ENSG00000171303 | HGLibA_24320 | 363 | 2.463624626 | KCNK3 | potassium two pore domain channel subfamily K member 3 [Source:HGNC Symbol;Acc:HGNC:6278] |
| ENSG00000172175 | HGLibA_28035 | 363 | 2.463624626 | MALT1 | MALT1 paracaspase [Source:HGNC Symbol;Acc:HGNC:6819] |
| ENSG00000184814 | HGLibA_38825 | 363 | 2.463624626 | PRR23B | proline rich 23B [Source:HGNC Symbol;Acc:HGNC:33764] |
| ENSG00000185250 | HGLibA_37950 | 363 | 2.463624626 | PPIL6 | peptidylprolyl isomerase like 6 [Source:HGNC Symbol;Acc:HGNC:21557] |
| ENSG00000187583 | HGLibA_37147 | 363 | 2.463624626 | PLEKHN1 | pleckstrin homology domain containing N1 [Source:HGNC Symbol;Acc:HGNC:25284] |

| | | | | | |
|---|---|---|---|---|---|
| ENSG00000240505 | HGLibA_50753 | 363 | 2.463624626 | TNFRSF13B | TNF receptor superfamily member 13B [Source:HGNC Symbol;Acc:HGNC:18153] |
| ENSG00000258289 | HGLibA_09672 | 363 | 2.463624626 | CHURC1 | churchill domain containing 1 [Source:HGNC Symbol;Acc:HGNC:20099] |
| ENSG00000278889 | HGLibA_33848 | 363 | 2.463624626 | OR2S2 | olfactory receptor family 2 subfamily S member 2 (gene/pseudogene) [Source:HGNC Symbol;Acc:HGNC:8276] |
| hsa-mir-1-2 | HGLibA_57144 | 363 | 2.463624626 | hsa-mir-1-2 | - |
| ENSG00000042813 | HGLibA_56963 | 362 | 2.453673589 | ZPBP | zona pellucida binding protein [Source:HGNC Symbol;Acc:HGNC:15662] |
| ENSG00000092377 | HGLibA_48614 | 362 | 2.453673589 | TBL1Y | transducin beta like 1 Y-linked [Source:HGNC Symbol;Acc:HGNC:18502] |
| ENSG00000100591 | HGLibA_01314 | 362 | 2.453673589 | AHSA1 | activator of HSP90 ATPase activity 1 [Source:HGNC Symbol;Acc:HGNC:1189] |
| ENSG00000101605 | HGLibA_30890 | 362 | 2.453673589 | MYOM1 | myomesin 1 [Source:HGNC Symbol;Acc:HGNC:7613] |
| ENSG00000105767 | HGLibA_06973 | 362 | 2.453673589 | CADM4 | cell adhesion molecule 4 [Source:HGNC Symbol;Acc:HGNC:30825] |
| ENSG00000112164 | HGLibA_19293 | 362 | 2.453673589 | GLP1R | glucagon like peptide 1 receptor [Source:HGNC Symbol;Acc:HGNC:4324] |
| ENSG00000117971 | HGLibA_09598 | 362 | 2.453673589 | CHRNB4 | cholinergic receptor nicotinic beta 4 subunit [Source:HGNC Symbol;Acc:HGNC:1964] |
| ENSG00000126602 | HGLibA_51294 | 362 | 2.453673589 | TRAP1 | TNF receptor associated protein 1 [Source:HGNC Symbol;Acc:HGNC:16264] |
| ENSG00000130487 | HGLibA_25107 | 362 | 2.453673589 | KLHDC7B | kelch domain containing 7B [Source:HGNC Symbol;Acc:HGNC:25145] |
| ENSG00000144048 | HGLibA_14134 | 362 | 2.453673589 | DUSP11 | dual specificity phosphatase 11 [Source:HGNC Symbol;Acc:HGNC:3066] |
| ENSG00000145241 | HGLibA_09046 | 362 | 2.453673589 | CENPC | centromere protein C [Source:HGNC Symbol;Acc:HGNC:1854] |
| ENSG00000147408 | HGLibA_11346 | 362 | 2.453673589 | CSGALNACT1 | chondroitin sulfate N-acetylgalactosaminyltransferase 1 [Source:HGNC Symbol;Acc:HGNC:24290] |
| ENSG00000204618 | HGLibA_41672 | 362 | 2.453673589 | RNF39 | ring finger protein 39 [Source:HGNC Symbol;Acc:HGNC:18064] |
| ENSG00000288642 | HGLibA_08864 | 362 | 2.453673589 | CDR1 | - |
| hsa-mir-8068 | HGLibA_64111 | 362 | 2.453673589 | hsa-mir-8068 | - |
| KIAA1804 | HGLibA_24819 | 362 | 2.453673589 | KIAA1804 | - |
| ZNF812 | HGLibA_56804 | 362 | 2.453673589 | ZNF812 | - |
| ENSG00000072163 | HGLibA_26550 | 361 | 2.443722552 | LIMS2 | LIM zinc finger domain containing 2 [Source:HGNC Symbol;Acc:HGNC:16084] |
| ENSG00000072832 | HGLibA_11200 | 361 | 2.443722552 | CRMP1 | collapsin response mediator protein 1 [Source:HGNC Symbol;Acc:HGNC:2365] |
| ENSG00000087086 | HGLibA_18126 | 361 | 2.443722552 | FTL | ferritin light chain [Source:HGNC Symbol;Acc:HGNC:3999] |
| ENSG00000100478 | HGLibA_02350 | 361 | 2.443722552 | AP4S1 | adaptor related protein complex 4 subunit sigma 1 [Source:HGNC Symbol;Acc:HGNC:575] |
| ENSG00000100852 | HGLibA_02784 | 361 | 2.443722552 | ARHGAP5 | Rho GTPase activating protein 5 [Source:HGNC Symbol;Acc:HGNC:675] |
| ENSG00000101220 | HGLibA_06022 | 361 | 2.443722552 | C20orf27 | chromosome 20 open reading frame 27 [Source:HGNC Symbol;Acc:HGNC:15873] |
| ENSG00000101294 | HGLibA_21663 | 361 | 2.443722552 | HM13 | histocompatibility minor 13 [Source:HGNC Symbol;Acc:HGNC:16435] |
| ENSG00000101856 | HGLibA_36287 | 361 | 2.443722552 | PGRMC1 | progesterone receptor membrane component 1 [Source:HGNC Symbol;Acc:HGNC:16090] |
| ENSG00000109208 | HGLibA_45823 | 361 | 2.443722552 | SMR3A | submaxillary gland androgen regulated protein 3A [Source:HGNC Symbol;Acc:HGNC:19216] |
| ENSG00000126460 | HGLibA_38872 | 361 | 2.443722552 | PRRG2 | proline rich and Gla domain 2 [Source:HGNC Symbol;Acc:HGNC:9470] |
| ENSG00000136950 | HGLibA_03096 | 361 | 2.443722552 | ARPC5L | actin related protein 2/3 complex subunit 5 like [Source:HGNC Symbol;Acc:HGNC:23366] |
| ENSG00000144445 | HGLibA_24040 | 361 | 2.443722552 | KANSL1L | KAT8 regulatory NSL complex subunit 1 like [Source:HGNC Symbol;Acc:HGNC:26310] |
| ENSG00000152484 | HGLibA_53201 | 361 | 2.443722552 | USP12 | ubiquitin specific peptidase 12 [Source:HGNC Symbol;Acc:HGNC:20485] |
| ENSG00000188130 | HGLibA_28255 | 361 | 2.443722552 | MAPK12 | mitogen-activated protein kinase 12 [Source:HGNC Symbol;Acc:HGNC:6874] |
| ENSG00000189186 | HGLibA_12463 | 361 | 2.443722552 | DCAF8L2 | DDB1 and CUL4 associated factor 8 like 2 [Source:HGNC Symbol;Acc:HGNC:31811] |
| ENSG00000223547 | HGLibA_56861 | 361 | 2.443722552 | ZNF844 | zinc finger protein 844 [Source:HGNC Symbol;Acc:HGNC:25932] |
| hsa-let-7b | HGLibA_57110 | 361 | 2.443722552 | hsa-let-7b | - |
| C1QTNF9B-AS1 | HGLibA_05965 | 360 | 2.433771514 | C1QTNF9B-AS1 | - |
| ENSG00000025708 | HGLibA_52460 | 360 | 2.433771514 | TYMP | thymidine phosphorylase [Source:HGNC Symbol;Acc:HGNC:3148] |
| ENSG00000072858 | HGLibA_44113 | 360 | 2.433771514 | SIDT1 | SID1 transmembrane family member 1 [Source:HGNC Symbol;Acc:HGNC:25967] |
| ENSG00000139330 | HGLibA_24553 | 360 | 2.433771514 | KERA | keratocan [Source:HGNC Symbol;Acc:HGNC:6309] |
| ENSG00000139737 | HGLibA_44307 | 360 | 2.433771514 | SLAIN1 | SLAIN motif family member 1 [Source:HGNC Symbol;Acc:HGNC:26387] |
| ENSG00000142182 | HGLibA_13708 | 360 | 2.433771514 | DNMT3L | DNA methyltransferase 3 like [Source:HGNC Symbol;Acc:HGNC:2980] |
| ENSG00000151651 | HGLibA_00782 | 360 | 2.433771514 | ADAM8 | ADAM metallopeptidase domain 8 [Source:HGNC Symbol;Acc:HGNC:215] |
| ENSG00000154803 | HGLibA_17606 | 360 | 2.433771514 | FLCN | folliculin [Source:HGNC Symbol;Acc:HGNC:27310] |
| ENSG00000164081 | HGLibA_49084 | 360 | 2.433771514 | TEX264 | testis expressed 264 [Source:HGNC Symbol;Acc:HGNC:30247] |
| ENSG00000170909 | HGLibA_34671 | 360 | 2.433771514 | OSCAR | immunoglobulin-like receptor [Source:HGNC Symbol;Acc:HGNC:29960]" |
| ENSG00000175832 | HGLibA_15658 | 360 | 2.433771514 | ETV4 | ETS variant 4 [Source:HGNC Symbol;Acc:HGNC:3493] |
| ENSG00000175895 | HGLibA_37099 | 360 | 2.433771514 | PLEKHF2 | pleckstrin homology and FYVE domain containing 2 [Source:HGNC Symbol;Acc:HGNC:20757] |
| ENSG00000186153 | HGLibA_54448 | 360 | 2.433771514 | WWOX | WW domain containing oxidoreductase [Source:HGNC Symbol;Acc:HGNC:12799] |
| ENSG00000260903 | HGLibA_54530 | 360 | 2.433771514 | XKR7 | XK related 7 [Source:HGNC Symbol;Acc:HGNC:23062] |
| ENSG00000004455 | HGLibA_01380 | 359 | 2.423820477 | AK2 | adenylate kinase 2 [Source:HGNC Symbol;Acc:HGNC:362] |
| ENSG00000110651 | HGLibA_08455 | 359 | 2.423820477 | CD81 | CD81 molecule [Source:HGNC Symbol;Acc:HGNC:1701] |
| ENSG00000116685 | HGLibA_24839 | 359 | 2.423820477 | KIAA2013 | KIAA2013 [Source:HGNC Symbol;Acc:HGNC:28513] |
| ENSG00000136111 | HGLibA_48556 | 359 | 2.423820477 | TBC1D4 | TBC1 domain family member 4 [Source:HGNC Symbol;Acc:HGNC:19165] |
| ENSG00000163319 | HGLibA_30056 | 359 | 2.423820477 | MRPS18C | mitochondrial ribosomal protein S18C [Source:HGNC Symbol;Acc:HGNC:16633] |
| ENSG00000166405 | HGLibA_41231 | 359 | 2.423820477 | RIC3 | RIC3 acetylcholine receptor chaperone [Source:HGNC Symbol;Acc:HGNC:30338] |
| ENSG00000167996 | HGLibA_18115 | 359 | 2.423820477 | FTH1 | ferritin heavy chain 1 [Source:HGNC Symbol;Acc:HGNC:3976] |
| ENSG00000169710 | HGLibA_16894 | 359 | 2.423820477 | FASN | fatty acid synthase [Source:HGNC Symbol;Acc:HGNC:3594] |
| ENSG00000187650 | HGLibA_53689 | 359 | 2.423820477 | VMAC | vimentin type intermediate filament associated coiled-coil protein [Source:HGNC Symbol;Acc:HGNC:33803] |
| ENSG00000205730 | HGLibA_23869 | 359 | 2.423820477 | ITPRIPL2 | ITPRIP like 2 [Source:HGNC Symbol;Acc:HGNC:27257] |
| ENSG00000213809 | HGLibA_25314 | 359 | 2.423820477 | KLRK1 | killer cell lectin like receptor K1 [Source:HGNC Symbol;Acc:HGNC:18788] |
| hsa-mir-8058 | HGLibA_64071 | 359 | 2.423820477 | hsa-mir-8058 | - |
| C7orf73 | HGLibA_06591 | 358 | 2.413869439 | C7orf73 | - |
| ENSG00000014914 | HGLibA_30414 | 358 | 2.413869439 | MTMR11 | myotubularin related protein 11 [Source:HGNC Symbol;Acc:HGNC:24307] |
| ENSG00000026297 | HGLibA_41443 | 358 | 2.413869439 | RNASET2 | ribonuclease T2 [Source:HGNC Symbol;Acc:HGNC:21686] |
| ENSG00000101493 | HGLibA_56144 | 358 | 2.413869439 | ZNF516 | zinc finger protein 516 [Source:HGNC Symbol;Acc:HGNC:28990] |
| ENSG00000104805 | HGLibA_32910 | 358 | 2.413869439 | NUCB1 | nucleobindin 1 [Source:HGNC Symbol;Acc:HGNC:8043] |
| ENSG00000130487 | HGLibA_25109 | 358 | 2.413869439 | KLHDC7B | kelch domain containing 7B [Source:HGNC Symbol;Acc:HGNC:25145] |
| ENSG00000136026 | HGLibA_09759 | 358 | 2.413869439 | CKAP4 | cytoskeleton associated protein 4 [Source:HGNC Symbol;Acc:HGNC:16991] |
| ENSG00000149564 | HGLibA_15555 | 358 | 2.413869439 | ESAM | endothelial cell adhesion molecule [Source:HGNC Symbol;Acc:HGNC:17474] |
| ENSG00000151418 | HGLibA_03762 | 358 | 2.413869439 | ATP6V1G3 | ATPase H+ transporting V1 subunit G3 [Source:HGNC Symbol;Acc:HGNC:18265] |
| ENSG00000160161 | HGLibA_09713 | 358 | 2.413869439 | CILP2 | cartilage intermediate layer protein 2 [Source:HGNC Symbol;Acc:HGNC:24213] |
| ENSG00000163060 | HGLibA_48979 | 358 | 2.413869439 | TEKT4 | tektin 4 [Source:HGNC Symbol;Acc:HGNC:31012] |
| ENSG00000164440 | HGLibA_52394 | 358 | 2.413869439 | TXLNB | taxilin beta [Source:HGNC Symbol;Acc:HGNC:21617] |
| ENSG00000166342 | HGLibA_31704 | 358 | 2.413869439 | NETO1 | neuropilin and tolloid like 1 [Source:HGNC Symbol;Acc:HGNC:13823] |
| ENSG00000185962 | HGLibA_26129 | 358 | 2.413869439 | LCE3A | late cornified envelope 3A [Source:HGNC Symbol;Acc:HGNC:29461] |
| ENSG00000198087 | HGLibA_08326 | 358 | 2.413869439 | CD2AP | CD2 associated protein [Source:HGNC Symbol;Acc:HGNC:14258] |
| HEATR2 | HGLibA_21069 | 358 | 2.413869439 | HEATR2 | - |
| hsa-mir-6764 | HGLibA_63216 | 358 | 2.413869439 | hsa-mir-6764 | - |
| MTERFD1 | HGLibA_30339 | 358 | 2.413869439 | MTERFD1 | - |
| ENSG00000048545 | HGLibA_20634 | 357 | 2.403918402 | GUCA1A | guanylate cyclase activator 1A [Source:HGNC Symbol;Acc:HGNC:4678] |
| ENSG00000083857 | HGLibA_16914 | 357 | 2.403918402 | FAT1 | FAT atypical cadherin 1 [Source:HGNC Symbol;Acc:HGNC:3595] |
| ENSG00000115970 | HGLibA_49282 | 357 | 2.403918402 | THADA | armadillo repeat containing [Source:HGNC Symbol;Acc:HGNC:19217]" |
| ENSG00000116151 | HGLibA_29667 | 357 | 2.403918402 | MORN1 | MORN repeat containing 1 [Source:HGNC Symbol;Acc:HGNC:25852] |
| ENSG00000126091 | HGLibA_47243 | 357 | 2.403918402 | ST3GAL3 | 3-sialyltransferase 3 [Source:HGNC Symbol;Acc:HGNC:10866]" |
| ENSG00000138796 | HGLibA_20804 | 357 | 2.403918402 | HADH | hydroxyacyl-CoA dehydrogenase [Source:HGNC Symbol;Acc:HGNC:4799] |
| ENSG00000148344 | HGLibA_39324 | 357 | 2.403918402 | PTGES | prostaglandin E synthase [Source:HGNC Symbol;Acc:HGNC:9599] |
| ENSG00000161021 | HGLibA_28043 | 357 | 2.403918402 | MAML1 | mastermind like transcriptional coactivator 1 [Source:HGNC Symbol;Acc:HGNC:13632] |
| ENSG00000165819 | HGLibA_29006 | 357 | 2.403918402 | METTL3 | methyltransferase like 3 [Source:HGNC Symbol;Acc:HGNC:17563] |
| ENSG00000168116 | HGLibA_24788 | 357 | 2.403918402 | KIAA1586 | KIAA1586 [Source:HGNC Symbol;Acc:HGNC:21360] |
| ENSG00000175866 | HGLibA_04121 | 357 | 2.403918402 | BAIAP2 | BAI1 associated protein 2 [Source:HGNC Symbol;Acc:HGNC:947] |
| ENSG00000185304 | HGLibA_41027 | 357 | 2.403918402 | RGPD2 | RANBP2-like and GRIP domain containing 2 [Source:HGNC Symbol;Acc:HGNC:32415] |
| ENSG00000197245 | HGLibA_16022 | 357 | 2.403918402 | FAM110D | family with sequence similarity 110 member D [Source:HGNC Symbol;Acc:HGNC:25860] |
| ENSG00000214688 | HGLibA_05023 | 357 | 2.403918402 | C10orf105 | chromosome 10 open reading frame 105 [Source:HGNC Symbol;Acc:HGNC:20304] |
| ENSG00000237693 | HGLibA_23613 | 357 | 2.403918402 | IRGM | immunity related GTPase M [Source:HGNC Symbol;Acc:HGNC:29597] |
| PTPLAD2 | HGLibA_39421 | 357 | 2.403918402 | PTPLAD2 | - |
| ENSG00000060749 | HGLibA_39750 | 356 | 2.393967364 | QSER1 | glutamine and serine rich 1 [Source:HGNC Symbol;Acc:HGNC:26154] |
| ENSG00000102882 | HGLibA_28271 | 356 | 2.393967364 | MAPK3 | mitogen-activated protein kinase 3 [Source:HGNC Symbol;Acc:HGNC:6877] |
| ENSG00000113732 | HGLibA_03723 | 356 | 2.393967364 | ATP6V0E1 | ATPase H+ transporting V0 subunit e1 [Source:HGNC Symbol;Acc:HGNC:863] |
| ENSG00000117362 | HGLibA_02414 | 356 | 2.393967364 | APH1A | gamma-secretase subunit [Source:HGNC Symbol;Acc:HGNC:29509]" |
| ENSG00000135248 | HGLibA_16701 | 356 | 2.393967364 | FAM71F1 | family with sequence similarity 71 member F1 [Source:HGNC Symbol;Acc:HGNC:30704] |
| ENSG00000138039 | HGLibA_26423 | 356 | 2.393967364 | LHCGR | luteinizing hormone/choriogonadotropin receptor [Source:HGNC Symbol;Acc:HGNC:6585] |
| ENSG00000147145 | HGLibA_27061 | 356 | 2.393967364 | LPAR4 | lysophosphatidic acid receptor 4 [Source:HGNC Symbol;Acc:HGNC:4478] |
| ENSG00000152223 | HGLibA_15277 | 356 | 2.393967364 | EPG5 | ectopic P-granules autophagy protein 5 homolog [Source:HGNC Symbol;Acc:HGNC:29331] |
| ENSG00000161055 | HGLibA_42931 | 356 | 2.393967364 | SCGB3A1 | secretoglobin family 3A member 1 [Source:HGNC Symbol;Acc:HGNC:18384] |
| ENSG00000175520 | HGLibA_52739 | 356 | 2.393967364 | UBQLN3 | ubiquilin 3 [Source:HGNC Symbol;Acc:HGNC:12510] |
| ENSG00000177464 | HGLibA_20042 | 356 | 2.393967364 | GPR4 | G protein-coupled receptor 4 [Source:HGNC Symbol;Acc:HGNC:4497] |
| ENSG00000180628 | HGLibA_35600 | 356 | 2.393967364 | PCGF5 | polycomb group ring finger 5 [Source:HGNC Symbol;Acc:HGNC:28264] |
| ENSG00000183034 | HGLibA_34739 | 356 | 2.393967364 | OTOP2 | otopetrin 2 [Source:HGNC Symbol;Acc:HGNC:19657] |
| ENSG00000183873 | HGLibA_42998 | 356 | 2.393967364 | SCN5A | sodium voltage-gated channel alpha subunit 5 [Source:HGNC Symbol;Acc:HGNC:10593] |
| ENSG00000185736 | HGLibA_00875 | 356 | 2.393967364 | ADARB2 | RNA specific B2 (inactive) [Source:HGNC Symbol;Acc:HGNC:227]" |
| ENSG00000196605 | HGLibA_56867 | 356 | 2.393967364 | ZNF846 | zinc finger protein 846 [Source:HGNC Symbol;Acc:HGNC:27260] |
| ENSG00000204644 | HGLibA_55223 | 356 | 2.393967364 | ZFP57 | ZFP57 zinc finger protein [Source:HGNC Symbol;Acc:HGNC:18791] |
| ENSG00000259431 | HGLibA_49411 | 356 | 2.393967364 | THTPA | thiamine triphosphatase [Source:HGNC Symbol;Acc:HGNC:18987] |
| HIST1H4E | HGLibA_21497 | 356 | 2.393967364 | HIST1H4E | - |
| hsa-mir-3976 | HGLibA_59663 | 356 | 2.393967364 | hsa-mir-3976 | - |
| hsa-mir-548ay | HGLibA_61944 | 356 | 2.393967364 | hsa-mir-548ay | - |
| DDX26B | HGLibA_12684 | 355 | 2.384016327 | DDX26B | - |
| ENSG00000092036 | HGLibA_20882 | 355 | 2.384016327 | HAUS4 | HAUS augmin like complex subunit 4 [Source:HGNC Symbol;Acc:HGNC:20163] |
| ENSG00000101004 | HGLibA_31945 | 355 | 2.384016327 | NINL | ninein like [Source:HGNC Symbol;Acc:HGNC:29163] |
| ENSG00000101400 | HGLibA_45998 | 355 | 2.384016327 | SNTA1 | syntrophin alpha 1 [Source:HGNC Symbol;Acc:HGNC:11167] |
| ENSG00000101474 | HGLibA_02445 | 355 | 2.384016327 | APMAP | adipocyte plasma membrane associated protein [Source:HGNC Symbol;Acc:HGNC:13238] |
| ENSG00000102178 | HGLibA_52705 | 355 | 2.384016327 | UBL4A | ubiquitin like 4A [Source:HGNC Symbol;Acc:HGNC:12505] |
| ENSG00000125971 | HGLibA_14260 | 355 | 2.384016327 | DYNLRB1 | dynein light chain roadblock-type 1 [Source:HGNC Symbol;Acc:HGNC:15468] |
| ENSG00000139800 | HGLibA_55334 | 355 | 2.384016327 | ZIC5 | Zic family member 5 [Source:HGNC Symbol;Acc:HGNC:20322] |
| ENSG00000140391 | HGLibA_51925 | 355 | 2.384016327 | TSPAN3 | tetraspanin 3 [Source:HGNC Symbol;Acc:HGNC:17752] |
| ENSG00000160783 | HGLibA_37302 | 355 | 2.384016327 | PMF1 | polyamine modulated factor 1 [Source:HGNC Symbol;Acc:HGNC:9112] |
| ENSG00000188581 | HGLibA_25579 | 355 | 2.384016327 | KRTAP1-1 | keratin associated protein 1-1 [Source:HGNC Symbol;Acc:HGNC:16772] |
| ENSG00000197858 | HGLibA_19720 | 355 | 2.384016327 | GPAA1 | glycosylphosphatidylinositol anchor attachment 1 [Source:HGNC Symbol;Acc:HGNC:4446] |
| ENSG00000197859 | HGLibA_00850 | 355 | 2.384016327 | ADAMTSL2 | ADAMTS like 2 [Source:HGNC Symbol;Acc:HGNC:14631] |
| ENSG00000198286 | HGLibA_07220 | 355 | 2.384016327 | CARD11 | caspase recruitment domain family member 11 [Source:HGNC Symbol;Acc:HGNC:16393] |
| FAM57A | HGLibA_16644 | 355 | 2.384016327 | FAM57A | - |
| TMEM55A | HGLibA_50443 | 355 | 2.384016327 | TMEM55A | - |

| | | | | | |
|---|---|---|---|---|---|
| ENSG00000042304 | HGLibA_06194 | 354 | 2.37406529 | C2orf83 | chromosome 2 open reading frame 83 [Source:HGNC Symbol;Acc:HGNC:25344] |
| ENSG00000092200 | HGLibA_41837 | 354 | 2.37406529 | RPGRIP1 | RPGR interacting protein 1 [Source:HGNC Symbol;Acc:HGNC:13436] |
| ENSG00000103507 | HGLibA_04285 | 354 | 2.37406529 | BCKDK | branched chain ketoacid dehydrogenase kinase [Source:HGNC Symbol;Acc:HGNC:16902] |
| ENSG00000126602 | HGLibA_51293 | 354 | 2.37406529 | TRAP1 | TNF receptor associated protein 1 [Source:HGNC Symbol;Acc:HGNC:16264] |
| ENSG00000135363 | HGLibA_26706 | 354 | 2.37406529 | LMO2 | LIM domain only 2 [Source:HGNC Symbol;Acc:HGNC:6642] |
| ENSG00000135406 | HGLibA_38748 | 354 | 2.37406529 | PRPH | peripherin [Source:HGNC Symbol;Acc:HGNC:9461] |
| ENSG00000137745 | HGLibA_29489 | 354 | 2.37406529 | MMP13 | matrix metallopeptidase 13 [Source:HGNC Symbol;Acc:HGNC:7159] |
| ENSG00000143257 | HGLibA_32592 | 354 | 2.37406529 | NR1I3 | nuclear receptor subfamily 1 group I member 3 [Source:HGNC Symbol;Acc:HGNC:7969] |
| ENSG00000145423 | HGLibA_43738 | 354 | 2.37406529 | SFRP2 | secreted frizzled related protein 2 [Source:HGNC Symbol;Acc:HGNC:10777] |
| ENSG00000154678 | HGLibA_35776 | 354 | 2.37406529 | PDE1C | phosphodiesterase 1C [Source:HGNC Symbol;Acc:HGNC:8776] |
| ENSG00000159871 | HGLibA_27726 | 354 | 2.37406529 | LYPD5 | LY6/PLAUR domain containing 5 [Source:HGNC Symbol;Acc:HGNC:26397] |
| ENSG00000170624 | HGLibA_43803 | 354 | 2.37406529 | SGCD | sarcoglycan delta [Source:HGNC Symbol;Acc:HGNC:10807] |
| ENSG00000173581 | HGLibA_07524 | 354 | 2.37406529 | CCDC106 | coiled-coil domain containing 106 [Source:HGNC Symbol;Acc:HGNC:30181] |
| ENSG00000196350 | HGLibA_56640 | 354 | 2.37406529 | ZNF729 | zinc finger protein 729 [Source:HGNC Symbol;Acc:HGNC:32464] |
| ENSG00000197279 | HGLibA_55517 | 354 | 2.37406529 | ZNF165 | zinc finger protein 165 [Source:HGNC Symbol;Acc:HGNC:12953] |
| ENSG00000006071 | HGLibA_00186 | 353 | 2.364114252 | ABCC8 | ATP binding cassette subfamily C member 8 [Source:HGNC Symbol;Acc:HGNC:59] |
| ENSG00000100314 | HGLibA_06859 | 353 | 2.364114252 | CABP7 | calcium binding protein 7 [Source:HGNC Symbol;Acc:HGNC:20834] |
| ENSG00000100739 | HGLibA_04381 | 353 | 2.364114252 | BDKRB1 | bradykinin receptor B1 [Source:HGNC Symbol;Acc:HGNC:1029] |
| ENSG00000101438 | HGLibA_44932 | 353 | 2.364114252 | SLC32A1 | solute carrier family 32 member 1 [Source:HGNC Symbol;Acc:HGNC:11018] |
| ENSG00000103202 | HGLibA_32162 | 353 | 2.364114252 | NME4 | NME/NM23 nucleoside diphosphate kinase 4 [Source:HGNC Symbol;Acc:HGNC:7852] |
| ENSG00000103855 | HGLibA_08321 | 353 | 2.364114252 | CD276 | CD276 molecule [Source:HGNC Symbol;Acc:HGNC:19137] |
| ENSG00000105497 | HGLibA_55528 | 353 | 2.364114252 | ZNF175 | zinc finger protein 175 [Source:HGNC Symbol;Acc:HGNC:12964] |
| ENSG00000116478 | HGLibA_20996 | 353 | 2.364114252 | HDAC1 | histone deacetylase 1 [Source:HGNC Symbol;Acc:HGNC:4852] |
| ENSG00000116685 | HGLibA_24838 | 353 | 2.364114252 | KIAA2013 | KIAA2013 [Source:HGNC Symbol;Acc:HGNC:28513] |
| ENSG00000124839 | HGLibA_39819 | 353 | 2.364114252 | RAB17 | member RAS oncogene family [Source:HGNC Symbol;Acc:HGNC:16523]" |
| ENSG00000133789 | HGLibA_47897 | 353 | 2.364114252 | SWAP70 | switching B cell complex subunit SWAP70 [Source:HGNC Symbol;Acc:HGNC:17070] |
| ENSG00000135446 | HGLibA_08771 | 353 | 2.364114252 | CDK4 | cyclin dependent kinase 4 [Source:HGNC Symbol;Acc:HGNC:1773] |
| ENSG00000135503 | HGLibA_00688 | 353 | 2.364114252 | ACVR1B | activin A receptor type 1B [Source:HGNC Symbol;Acc:HGNC:172] |
| ENSG00000160767 | HGLibA_16358 | 353 | 2.364114252 | FAM189B | family with sequence similarity 189 member B [Source:HGNC Symbol;Acc:HGNC:1233] |
| ENSG00000160932 | HGLibA_27657 | 353 | 2.364114252 | LY6E | lymphocyte antigen 6 family member E [Source:HGNC Symbol;Acc:HGNC:6727] |
| ENSG00000166224 | HGLibA_43846 | 353 | 2.364114252 | SGPL1 | sphingosine-1-phosphate lyase 1 [Source:HGNC Symbol;Acc:HGNC:10817] |
| ENSG00000167528 | HGLibA_56442 | 353 | 2.364114252 | ZNF641 | zinc finger protein 641 [Source:HGNC Symbol;Acc:HGNC:31834] |
| ENSG00000171119 | HGLibA_32729 | 353 | 2.364114252 | NRTN | neurturin [Source:HGNC Symbol;Acc:HGNC:8007] |
| ENSG00000171813 | HGLibA_39643 | 353 | 2.364114252 | PWWP2B | PWWP domain containing 2B [Source:HGNC Symbol;Acc:HGNC:25150] |
| ENSG00000172487 | HGLibA_34539 | 353 | 2.364114252 | OR8J1 | olfactory receptor family 8 subfamily J member 1 [Source:HGNC Symbol;Acc:HGNC:14855] |
| ENSG00000178537 | HGLibA_44669 | 353 | 2.364114252 | SLC25A20 | solute carrier family 25 member 20 [Source:HGNC Symbol;Acc:HGNC:1421] |
| ENSG00000186352 | HGLibA_02090 | 353 | 2.364114252 | ANKRD37 | ankyrin repeat domain 37 [Source:HGNC Symbol;Acc:HGNC:29593] |
| ENSG00000212864 | HGLibA_41617 | 353 | 2.364114252 | RNF208 | ring finger protein 208 [Source:HGNC Symbol;Acc:HGNC:25420] |
| ENSG00000213973 | HGLibA_56917 | 353 | 2.364114252 | ZNF99 | zinc finger protein 99 [Source:HGNC Symbol;Acc:HGNC:13175] |
| hsa-mir-762 | HGLibA_63909 | 353 | 2.364114252 | hsa-mir-762 | - |
| ZBED1_Y | HGLibA_54790 | 353 | 2.364114252 | ZBED1_Y | - |
| CCDC135 | HGLibA_07602 | 352 | 2.354163215 | CCDC135 | - |
| ENSG00000100105 | HGLibA_35290 | 352 | 2.354163215 | PATZ1 | POZ/BTB and AT hook containing zinc finger 1 [Source:HGNC Symbol;Acc:HGNC:13071] |
| ENSG00000105173 | HGLibA_08083 | 352 | 2.354163215 | CCNE1 | cyclin E1 [Source:HGNC Symbol;Acc:HGNC:1589] |
| ENSG00000105366 | HGLibA_44150 | 352 | 2.354163215 | SIGLEC8 | sialic acid binding Ig like lectin 8 [Source:HGNC Symbol;Acc:HGNC:10877] |
| ENSG00000119227 | HGLibA_36605 | 352 | 2.354163215 | PIGZ | phosphatidylinositol glycan anchor biosynthesis class Z [Source:HGNC Symbol;Acc:HGNC:30596] |
| ENSG00000119922 | HGLibA_22628 | 352 | 2.354163215 | IFIT2 | interferon induced protein with tetratricopeptide repeats 2 [Source:HGNC Symbol;Acc:HGNC:5409] |
| ENSG00000122592 | HGLibA_21920 | 352 | 2.354163215 | HOXA7 | homeobox A7 [Source:HGNC Symbol;Acc:HGNC:5108] |
| ENSG00000125817 | HGLibA_09040 | 352 | 2.354163215 | CENPB | centromere protein B [Source:HGNC Symbol;Acc:HGNC:1852] |
| ENSG00000139083 | HGLibA_15666 | 352 | 2.354163215 | ETV6 | ETS variant 6 [Source:HGNC Symbol;Acc:HGNC:3495] |
| ENSG00000144035 | HGLibA_31181 | 352 | 2.354163215 | NAT8 | N-acetyltransferase 8 (putative) [Source:HGNC Symbol;Acc:HGNC:18069] |
| ENSG00000168772 | HGLibA_11971 | 352 | 2.354163215 | CXXC4 | CXXC finger protein 4 [Source:HGNC Symbol;Acc:HGNC:24593] |
| ENSG00000187094 | HGLibA_07952 | 352 | 2.354163215 | CCK | cholecystokinin [Source:HGNC Symbol;Acc:HGNC:1569] |
| hsa-mir-194-1 | HGLibA_58079 | 352 | 2.354163215 | hsa-mir-194-1 | - |
| PRR24 | HGLibA_38830 | 352 | 2.354163215 | PRR24 | - |
| ENSG00000011600 | HGLibA_52470 | 351 | 2.344212177 | TYROBP | TYRO protein tyrosine kinase binding protein [Source:HGNC Symbol;Acc:HGNC:12449] |
| ENSG00000085831 | HGLibA_52131 | 351 | 2.344212177 | TTC39A | tetratricopeptide repeat domain 39A [Source:HGNC Symbol;Acc:HGNC:18657] |
| ENSG00000101405 | HGLibA_34839 | 351 | 2.344212177 | OXT | oxytocin/neurophysin I prepropeptide [Source:HGNC Symbol;Acc:HGNC:8528] |
| ENSG00000102931 | HGLibA_02960 | 351 | 2.344212177 | ARL2BP | ADP ribosylation factor like GTPase 2 binding protein [Source:HGNC Symbol;Acc:HGNC:17146] |
| ENSG00000104848 | HGLibA_24143 | 351 | 2.344212177 | KCNA7 | potassium voltage-gated channel subfamily A member 7 [Source:HGNC Symbol;Acc:HGNC:6226] |
| ENSG00000135916 | HGLibA_23835 | 351 | 2.344212177 | ITM2C | integral membrane protein 2C [Source:HGNC Symbol;Acc:HGNC:6175] |
| ENSG00000146090 | HGLibA_40335 | 351 | 2.344212177 | RASGEF1C | RasGEF domain family member 1C [Source:HGNC Symbol;Acc:HGNC:27400] |
| ENSG00000154330 | HGLibA_36273 | 351 | 2.344212177 | PGM5 | phosphoglucomutase 5 [Source:HGNC Symbol;Acc:HGNC:8908] |
| ENSG00000159450 | HGLibA_48804 | 351 | 2.344212177 | TCHH | trichohyalin [Source:HGNC Symbol;Acc:HGNC:11791] |
| ENSG00000167904 | HGLibA_50496 | 351 | 2.344212177 | TMEM68 | transmembrane protein 68 [Source:HGNC Symbol;Acc:HGNC:26510] |
| ENSG00000182450 | HGLibA_24325 | 351 | 2.344212177 | KCNK4 | potassium two pore domain channel subfamily K member 4 [Source:HGNC Symbol;Acc:HGNC:6279] |
| ENSG00000183559 | HGLibA_05046 | 351 | 2.344212177 | C10orf120 | chromosome 10 open reading frame 120 [Source:HGNC Symbol;Acc:HGNC:25707] |
| ENSG00000185567 | HGLibA_01304 | 351 | 2.344212177 | AHNAK2 | AHNAK nucleoprotein 2 [Source:HGNC Symbol;Acc:HGNC:20125] |
| ENSG00000187699 | HGLibA_06196 | 351 | 2.344212177 | C2orf88 | chromosome 2 open reading frame 88 [Source:HGNC Symbol;Acc:HGNC:28191] |
| ENSG00000196119 | HGLibA_34490 | 351 | 2.344212177 | OR8A1 | olfactory receptor family 8 subfamily A member 1 [Source:HGNC Symbol;Acc:HGNC:8469] |
| ENSG00000206579 | HGLibA_54523 | 351 | 2.344212177 | XKR4 | XK related 4 [Source:HGNC Symbol;Acc:HGNC:29394] |
| ENSG00000223953 | HGLibA_05948 | 351 | 2.344212177 | C1QTNF5 | C1q and TNF related 5 [Source:HGNC Symbol;Acc:HGNC:14344] |
| hsa-mir-3944 | HGLibA_59637 | 351 | 2.344212177 | hsa-mir-3944 | - |
| hsa-mir-4646 | HGLibA_60688 | 351 | 2.344212177 | hsa-mir-4646 | - |
| hsa-mir-6775 | HGLibA_63273 | 351 | 2.344212177 | hsa-mir-6775 | - |
| ENSG00000013583 | HGLibA_21085 | 350 | 2.33426114 | HEBP1 | heme binding protein 1 [Source:HGNC Symbol;Acc:HGNC:17176] |
| ENSG00000055208 | HGLibA_48143 | 350 | 2.33426114 | TAB2 | TGF-beta activated kinase 1 (MAP3K7) binding protein 2 [Source:HGNC Symbol;Acc:HGNC:17075] |
| ENSG00000080910 | HGLibA_09266 | 350 | 2.33426114 | CFHR2 | complement factor H related 2 [Source:HGNC Symbol;Acc:HGNC:4890] |
| ENSG00000100994 | HGLibA_39684 | 350 | 2.33426114 | PYGB | glycogen phosphorylase B [Source:HGNC Symbol;Acc:HGNC:9723] |
| ENSG00000103154 | HGLibA_31586 | 350 | 2.33426114 | NECAB2 | N-terminal EF-hand calcium binding protein 2 [Source:HGNC Symbol;Acc:HGNC:23746] |
| ENSG00000103160 | HGLibA_22214 | 350 | 2.33426114 | HSDL1 | hydroxysteroid dehydrogenase like 1 [Source:HGNC Symbol;Acc:HGNC:16475] |
| ENSG00000126778 | HGLibA_44242 | 350 | 2.33426114 | SIX1 | SIX homeobox 1 [Source:HGNC Symbol;Acc:HGNC:10887] |
| ENSG00000130517 | HGLibA_36279 | 350 | 2.33426114 | PGPEP1 | pyroglutamyl-peptidase I [Source:HGNC Symbol;Acc:HGNC:13568] |
| ENSG00000131650 | HGLibA_25404 | 350 | 2.33426114 | KREMEN2 | kringle containing transmembrane protein 2 [Source:HGNC Symbol;Acc:HGNC:18797] |
| ENSG00000145362 | HGLibA_01936 | 350 | 2.33426114 | ANK2 | ankyrin 2 [Source:HGNC Symbol;Acc:HGNC:493] |
| ENSG00000148688 | HGLibA_42035 | 350 | 2.33426114 | RPP30 | ribonuclease P/MRP subunit p30 [Source:HGNC Symbol;Acc:HGNC:17688] |
| ENSG00000151116 | HGLibA_52840 | 350 | 2.33426114 | UEVLD | UEV and lactate/malate dehyrogenase domains [Source:HGNC Symbol;Acc:HGNC:30866] |
| ENSG00000183723 | HGLibA_10218 | 350 | 2.33426114 | CMTM4 | CKLF like MARVEL transmembrane domain containing 4 [Source:HGNC Symbol;Acc:HGNC:19175] |
| ENSG00000254415 | HGLibA_44134 | 350 | 2.33426114 | SIGLEC14 | sialic acid binding Ig like lectin 14 [Source:HGNC Symbol;Acc:HGNC:32926] |
| ENSG00000005884 | HGLibA_23731 | 349 | 2.324310102 | ITGA3 | integrin subunit alpha 3 [Source:HGNC Symbol;Acc:HGNC:6139] |
| ENSG00000063322 | HGLibA_28799 | 349 | 2.324310102 | MED29 | mediator complex subunit 29 [Source:HGNC Symbol;Acc:HGNC:23074] |
| ENSG00000088320 | HGLibA_40831 | 349 | 2.324310102 | REM1 | RRAD and GEM like GTPase 1 [Source:HGNC Symbol;Acc:HGNC:15922] |
| ENSG00000100197 | HGLibA_12126 | 349 | 2.324310102 | CYP2D6 | cytochrome P450 family 2 subfamily D member 6 [Source:HGNC Symbol;Acc:HGNC:2625] |
| ENSG00000100281 | HGLibA_21735 | 349 | 2.324310102 | HMGXB4 | HMG-box containing 4 [Source:HGNC Symbol;Acc:HGNC:5003] |
| ENSG00000103089 | HGLibA_15897 | 349 | 2.324310102 | FA2H | fatty acid 2-hydroxylase [Source:HGNC Symbol;Acc:HGNC:21197] |
| ENSG00000103404 | HGLibA_53333 | 349 | 2.324310102 | USP31 | ubiquitin specific peptidase 31 [Source:HGNC Symbol;Acc:HGNC:20060] |
| ENSG00000111907 | HGLibA_51113 | 349 | 2.324310102 | TPD52L1 | TPD52 like 1 [Source:HGNC Symbol;Acc:HGNC:12006] |
| ENSG00000140009 | HGLibA_15584 | 349 | 2.324310102 | ESR2 | estrogen receptor 2 [Source:HGNC Symbol;Acc:HGNC:3468] |
| ENSG00000145979 | HGLibA_48563 | 349 | 2.324310102 | TBC1D7 | TBC1 domain family member 7 [Source:HGNC Symbol;Acc:HGNC:21066] |
| ENSG00000147592 | HGLibA_25920 | 349 | 2.324310102 | LACTB2 | lactamase beta 2 [Source:HGNC Symbol;Acc:HGNC:18512] |
| ENSG00000149527 | HGLibA_37011 | 349 | 2.324310102 | PLCH2 | phospholipase C eta 2 [Source:HGNC Symbol;Acc:HGNC:29037] |
| ENSG00000167757 | HGLibA_25236 | 349 | 2.324310102 | KLK11 | kallikrein related peptidase 11 [Source:HGNC Symbol;Acc:HGNC:6359] |
| ENSG00000178750 | HGLibA_47632 | 349 | 2.324310102 | STX19 | syntaxin 19 [Source:HGNC Symbol;Acc:HGNC:19300] |
| ENSG00000184012 | HGLibA_50636 | 349 | 2.324310102 | TMPRSS2 | transmembrane serine protease 2 [Source:HGNC Symbol;Acc:HGNC:11876] |
| ENSG00000204694 | HGLibA_33564 | 349 | 2.324310102 | OR11A1 | olfactory receptor family 11 subfamily A member 1 [Source:HGNC Symbol;Acc:HGNC:8176] |
| ENSG00000212722 | HGLibA_25780 | 349 | 2.324310102 | KRTAP4-9 | keratin associated protein 4-9 [Source:HGNC Symbol;Acc:HGNC:18910] |
| ENSG00000271321 | HGLibA_11578 | 349 | 2.324310102 | CTAGE6 | CTAGE family member 6 [Source:HGNC Symbol;Acc:HGNC:28644] |
| hsa-mir-4286 | HGLibA_59852 | 349 | 2.324310102 | hsa-mir-4286 | - |
| hsa-mir-6126 | HGLibA_62650 | 349 | 2.324310102 | hsa-mir-6126 | - |
| ENSG00000084112 | HGLibA_47131 | 348 | 2.314359065 | SSH1 | slingshot protein phosphatase 1 [Source:HGNC Symbol;Acc:HGNC:30579] |
| ENSG00000088256 | HGLibA_19437 | 348 | 2.314359065 | GNA11 | G protein subunit alpha 11 [Source:HGNC Symbol;Acc:HGNC:4379] |
| ENSG00000104671 | HGLibA_12559 | 348 | 2.314359065 | DCTN6 | dynactin subunit 6 [Source:HGNC Symbol;Acc:HGNC:16964] |
| ENSG00000105131 | HGLibA_15328 | 348 | 2.314359065 | EPHX3 | epoxide hydrolase 3 [Source:HGNC Symbol;Acc:HGNC:23760] |
| ENSG00000125787 | HGLibA_19582 | 348 | 2.314359065 | GNRH2 | gonadotropin releasing hormone 2 [Source:HGNC Symbol;Acc:HGNC:4420] |
| ENSG00000125843 | HGLibA_02360 | 348 | 2.314359065 | AP5S1 | adaptor related protein complex 5 subunit sigma 1 [Source:HGNC Symbol;Acc:HGNC:15875] |
| ENSG00000133466 | HGLibA_05950 | 348 | 2.314359065 | C1QTNF6 | C1q and TNF related 6 [Source:HGNC Symbol;Acc:HGNC:14343] |
| ENSG00000137177 | HGLibA_24855 | 348 | 2.314359065 | KIF13A | kinesin family member 13A [Source:HGNC Symbol;Acc:HGNC:14566] |
| ENSG00000137273 | HGLibA_17866 | 348 | 2.314359065 | FOXF2 | forkhead box F2 [Source:HGNC Symbol;Acc:HGNC:3810] |
| ENSG00000141569 | HGLibA_51585 | 348 | 2.314359065 | TRIM65 | tripartite motif containing 65 [Source:HGNC Symbol;Acc:HGNC:27316] |
| ENSG00000141644 | HGLibA_28462 | 348 | 2.314359065 | MBD1 | methyl-CpG binding domain protein 1 [Source:HGNC Symbol;Acc:HGNC:6916] |
| ENSG00000143184 | HGLibA_54492 | 348 | 2.314359065 | XCL1 | X-C motif chemokine ligand 1 [Source:HGNC Symbol;Acc:HGNC:10645] |
| ENSG00000147394 | HGLibA_55550 | 348 | 2.314359065 | ZNF185 | zinc finger protein 185 with LIM domain [Source:HGNC Symbol;Acc:HGNC:12976] |
| ENSG00000148356 | HGLibA_27497 | 348 | 2.314359065 | LRSAM1 | leucine rich repeat and sterile alpha motif containing 1 [Source:HGNC Symbol;Acc:HGNC:25135] |
| ENSG00000163154 | HGLibA_50723 | 348 | 2.314359065 | TNFAIP8L2 | TNF alpha induced protein 8 like 2 [Source:HGNC Symbol;Acc:HGNC:26277] |
| ENSG00000171243 | HGLibA_46209 | 348 | 2.314359065 | SOSTDC1 | sclerostin domain containing 1 [Source:HGNC Symbol;Acc:HGNC:21748] |
| ENSG00000172139 | HGLibA_45439 | 348 | 2.314359065 | SLC9C1 | solute carrier family 9 member C1 [Source:HGNC Symbol;Acc:HGNC:31401] |
| ENSG00000183421 | HGLibA_41325 | 348 | 2.314359065 | RIPK4 | receptor interacting serine/threonine kinase 4 [Source:HGNC Symbol;Acc:HGNC:496] |
| ENSG00000184992 | HGLibA_04806 | 348 | 2.314359065 | BRI3BP | BRI3 binding protein [Source:HGNC Symbol;Acc:HGNC:14251] |
| ENSG00000196458 | HGLibA_56364 | 348 | 2.314359065 | ZNF605 | zinc finger protein 605 [Source:HGNC Symbol;Acc:HGNC:28068] |
| ENSG00000196642 | HGLibA_40039 | 348 | 2.314359065 | RABL6 | member RAS oncogene family like 6 [Source:HGNC Symbol;Acc:HGNC:24703]" |
| ENSG00000198483 | HGLibA_02085 | 348 | 2.314359065 | ANKRD35 | ankyrin repeat domain 35 [Source:HGNC Symbol;Acc:HGNC:26323] |
| hsa-mir-4304 | HGLibA_59927 | 348 | 2.314359065 | hsa-mir-4304 | - |
| ENSG00000070785 | HGLibA_14740 | 347 | 2.304408028 | EIF2B3 | eukaryotic translation initiation factor 2B subunit gamma [Source:HGNC Symbol;Acc:HGNC:3259] |
| ENSG00000078795 | HGLibA_36804 | 347 | 2.304408028 | PKD2L2 | transient receptor potential cation channel [Source:HGNC Symbol;Acc:HGNC:9012]" |
| ENSG00000105778 | HGLibA_03893 | 347 | 2.304408028 | AVL9 | AVL9 cell migration associated [Source:HGNC Symbol;Acc:HGNC:28994] |

| | | | | | |
|---|---|---|---|---|---|
| ENSG00000111481 | HGLibA_10737 | 347 | 2.304408028 | COPZ1 | coatomer protein complex subunit zeta 1 [Source:HGNC Symbol;Acc:HGNC:2243] |
| ENSG00000111642 | HGLibA_09396 | 347 | 2.304408028 | CHD4 | chromodomain helicase DNA binding protein 4 [Source:HGNC Symbol;Acc:HGNC:1919] |
| ENSG00000114480 | HGLibA_18719 | 347 | 2.304408028 | GBE1 | 4-alpha-glucan branching enzyme 1 [Source:HGNC Symbol;Acc:HGNC:4180]" |
| ENSG00000142798 | HGLibA_22340 | 347 | 2.304408028 | HSPG2 | heparan sulfate proteoglycan 2 [Source:HGNC Symbol;Acc:HGNC:5273] |
| ENSG00000156140 | HGLibA_00827 | 347 | 2.304408028 | ADAMTS3 | ADAM metallopeptidase with thrombospondin type 1 motif 3 [Source:HGNC Symbol;Acc:HGNC:219] |
| ENSG00000159921 | HGLibA_19504 | 347 | 2.304408028 | GNE | glucosamine (UDP-N-acetyl)-2-epimerase/N-acetylmannosamine kinase [Source:HGNC Symbol;Acc:HGNC:23657] |
| ENSG00000161277 | HGLibA_49313 | 347 | 2.304408028 | THAP8 | THAP domain containing 8 [Source:HGNC Symbol;Acc:HGNC:23191] |
| ENSG00000163736 | HGLibA_37854 | 347 | 2.304408028 | PPBP | pro-platelet basic protein [Source:HGNC Symbol;Acc:HGNC:9240] |
| ENSG00000165474 | HGLibA_19159 | 347 | 2.304408028 | GJB2 | gap junction protein beta 2 [Source:HGNC Symbol;Acc:HGNC:4284] |
| ENSG00000178685 | HGLibA_35213 | 347 | 2.304408028 | PARP10 | poly(ADP-ribose) polymerase family member 10 [Source:HGNC Symbol;Acc:HGNC:25895] |
| ENSG00000196867 | HGLibA_55196 | 347 | 2.304408028 | ZFP28 | ZFP28 zinc finger protein [Source:HGNC Symbol;Acc:HGNC:17801] |
| ENSG00000228607 | HGLibA_09893 | 347 | 2.304408028 | CLDN25 | claudin 25 [Source:HGNC Symbol;Acc:HGNC:37218] |
| hsa-mir-6868 | HGLibA_63658 | 347 | 2.304408028 | hsa-mir-6868 | - |
| C1orf186 | HGLibA_05806 | 346 | 2.29445699 | C1orf186 | - |
| ENSG00000113312 | HGLibA_52044 | 346 | 2.29445699 | TTC1 | tetratricopeptide repeat domain 1 [Source:HGNC Symbol;Acc:HGNC:12391] |
| ENSG00000115414 | HGLibA_17726 | 346 | 2.29445699 | FN1 | fibronectin 1 [Source:HGNC Symbol;Acc:HGNC:3778] |
| ENSG00000116954 | HGLibA_42229 | 346 | 2.29445699 | RRAGC | Ras related GTP binding C [Source:HGNC Symbol;Acc:HGNC:19902] |
| ENSG00000117528 | HGLibA_00197 | 346 | 2.29445699 | ABCD3 | ATP binding cassette subfamily D member 3 [Source:HGNC Symbol;Acc:HGNC:67] |
| ENSG00000121769 | HGLibA_15917 | 346 | 2.29445699 | FABP3 | fatty acid binding protein 3 [Source:HGNC Symbol;Acc:HGNC:3557] |
| ENSG00000122707 | HGLibA_40764 | 346 | 2.29445699 | RECK | reversion inducing cysteine rich protein with kazal motifs [Source:HGNC Symbol;Acc:HGNC:11345] |
| ENSG00000124782 | HGLibA_42246 | 346 | 2.29445699 | RREB1 | ras responsive element binding protein 1 [Source:HGNC Symbol;Acc:HGNC:10449] |
| ENSG00000128322 | HGLibA_22866 | 346 | 2.29445699 | IGLL1 | immunoglobulin lambda like polypeptide 1 [Source:HGNC Symbol;Acc:HGNC:5870] |
| ENSG00000132153 | HGLibA_13176 | 346 | 2.29445699 | DHX30 | DExH-box helicase 30 [Source:HGNC Symbol;Acc:HGNC:16716] |
| ENSG00000134551 | HGLibA_38465 | 346 | 2.29445699 | PRH2 | proline rich protein HaeIII subfamily 2 [Source:HGNC Symbol;Acc:HGNC:9367] |
| ENSG00000134940 | HGLibA_00539 | 346 | 2.29445699 | ACRV1 | acrosomal vesicle protein 1 [Source:HGNC Symbol;Acc:HGNC:127] |
| ENSG00000136425 | HGLibA_09686 | 346 | 2.29445699 | CIB2 | calcium and integrin binding family member 2 [Source:HGNC Symbol;Acc:HGNC:24579] |
| ENSG00000136738 | HGLibA_47331 | 346 | 2.29445699 | STAM | signal transducing adaptor molecule [Source:HGNC Symbol;Acc:HGNC:11357] |
| ENSG00000139291 | HGLibA_50137 | 346 | 2.29445699 | TMEM19 | transmembrane protein 19 [Source:HGNC Symbol;Acc:HGNC:25605] |
| ENSG00000139725 | HGLibA_41187 | 346 | 2.29445699 | RHOF | filopodia associated [Source:HGNC Symbol;Acc:HGNC:15703]" |
| ENSG00000143512 | HGLibA_21268 | 346 | 2.29445699 | HHIPL2 | HHIP like 2 [Source:HGNC Symbol;Acc:HGNC:25842] |
| ENSG00000151353 | HGLibA_50092 | 346 | 2.29445699 | TMEM18 | transmembrane protein 18 [Source:HGNC Symbol;Acc:HGNC:25257] |
| ENSG00000164181 | HGLibA_14975 | 346 | 2.29445699 | ELOVL7 | ELOVL fatty acid elongase 7 [Source:HGNC Symbol;Acc:HGNC:26292] |
| ENSG00000173253 | HGLibA_13419 | 346 | 2.29445699 | DMRT2 | doublesex and mab-3 related transcription factor 2 [Source:HGNC Symbol;Acc:HGNC:2935] |
| ENSG00000188386 | HGLibA_38198 | 346 | 2.29445699 | PPP3R2 | beta [Source:HGNC Symbol;Acc:HGNC:9318]" |
| ENSG00000232125 | HGLibA_14289 | 346 | 2.29445699 | DYTN | dystrotelin [Source:HGNC Symbol;Acc:HGNC:23279] |
| H1F0 | HGLibA_20732 | 346 | 2.29445699 | H1F0 | - |
| HIST1H4A | HGLibA_21487 | 346 | 2.29445699 | HIST1H4A | - |
| hsa-mir-346 | HGLibA_59030 | 346 | 2.29445699 | hsa-mir-346 | - |
| ENSG00000090339 | HGLibA_22493 | 345 | 2.284505953 | ICAM1 | intercellular adhesion molecule 1 [Source:HGNC Symbol;Acc:HGNC:5344] |
| ENSG00000100226 | HGLibA_20597 | 345 | 2.284505953 | GTPBP1 | GTP binding protein 1 [Source:HGNC Symbol;Acc:HGNC:4669] |
| ENSG00000117000 | HGLibA_41345 | 345 | 2.284505953 | RLF | rearranged L-myc fusion [Source:HGNC Symbol;Acc:HGNC:10025] |
| ENSG00000123454 | HGLibA_12383 | 345 | 2.284505953 | DBH | dopamine beta-hydroxylase [Source:HGNC Symbol;Acc:HGNC:2689] |
| ENSG00000130844 | HGLibA_55815 | 345 | 2.284505953 | ZNF331 | zinc finger protein 331 [Source:HGNC Symbol;Acc:HGNC:15489] |
| ENSG00000138756 | HGLibA_04602 | 345 | 2.284505953 | BMP2K | BMP2 inducible kinase [Source:HGNC Symbol;Acc:HGNC:18041] |
| ENSG00000143390 | HGLibA_40975 | 345 | 2.284505953 | RFX5 | regulatory factor X5 [Source:HGNC Symbol;Acc:HGNC:9986] |
| ENSG00000154783 | HGLibA_17355 | 345 | 2.284505953 | FGD5 | RhoGEF and PH domain containing 5 [Source:HGNC Symbol;Acc:HGNC:19117]" |
| ENSG00000162522 | HGLibA_24771 | 345 | 2.284505953 | KIAA1522 | KIAA1522 [Source:HGNC Symbol;Acc:HGNC:29301] |
| ENSG00000164733 | HGLibA_11691 | 345 | 2.284505953 | CTSB | cathepsin B [Source:HGNC Symbol;Acc:HGNC:2527] |
| ENSG00000164746 | HGLibA_06560 | 345 | 2.284505953 | C7orf57 | chromosome 7 open reading frame 57 [Source:HGNC Symbol;Acc:HGNC:22247] |
| ENSG00000164761 | HGLibA_50748 | 345 | 2.284505953 | TNFRSF11B | TNF receptor superfamily member 11b [Source:HGNC Symbol;Acc:HGNC:11909] |
| ENSG00000164978 | HGLibA_32966 | 345 | 2.284505953 | NUDT2 | nudix hydrolase 2 [Source:HGNC Symbol;Acc:HGNC:8049] |
| ENSG00000176083 | HGLibA_56533 | 345 | 2.284505953 | ZNF683 | zinc finger protein 683 [Source:HGNC Symbol;Acc:HGNC:28495] |
| ENSG00000176988 | HGLibA_17722 | 345 | 2.284505953 | FMR1NB | FMR1 neighbor [Source:HGNC Symbol;Acc:HGNC:26372] |
| ENSG00000183643 | HGLibA_05384 | 345 | 2.284505953 | C15orf32 | chromosome 15 open reading frame 32 [Source:HGNC Symbol;Acc:HGNC:26549] |
| ENSG00000183765 | HGLibA_09422 | 345 | 2.284505953 | CHEK2 | checkpoint kinase 2 [Source:HGNC Symbol;Acc:HGNC:16627] |
| ENSG00000211455 | HGLibA_47503 | 345 | 2.284505953 | STK38L | serine/threonine kinase 38 like [Source:HGNC Symbol;Acc:HGNC:17848] |
| ENSG00000256223 | HGLibA_55428 | 345 | 2.284505953 | ZNF10 | zinc finger protein 10 [Source:HGNC Symbol;Acc:HGNC:12879] |
| hsa-mir-3978 | HGLibA_59670 | 345 | 2.284505953 | hsa-mir-3978 | - |
| hsa-mir-4784 | HGLibA_61245 | 345 | 2.284505953 | hsa-mir-4784 | - |
| hsa-mir-6085 | HGLibA_62599 | 345 | 2.284505953 | hsa-mir-6085 | - |
| hsa-mir-6872 | HGLibA_63673 | 345 | 2.284505953 | hsa-mir-6872 | - |
| TARSL2 | HGLibA_48350 | 345 | 2.284505953 | TARSL2 | - |
| ENSG00000103460 | HGLibA_51041 | 344 | 2.274554915 | TOX3 | TOX high mobility group box family member 3 [Source:HGNC Symbol;Acc:HGNC:11972] |
| ENSG00000111879 | HGLibA_16326 | 344 | 2.274554915 | FAM184A | family with sequence similarity 184 member A [Source:HGNC Symbol;Acc:HGNC:20991] |
| ENSG00000132953 | HGLibA_54564 | 344 | 2.274554915 | XPO4 | exportin 4 [Source:HGNC Symbol;Acc:HGNC:17796] |
| ENSG00000136156 | HGLibA_23831 | 344 | 2.274554915 | ITM2B | integral membrane protein 2B [Source:HGNC Symbol;Acc:HGNC:6174] |
| ENSG00000139192 | HGLibA_48324 | 344 | 2.274554915 | TAPBPL | TAP binding protein like [Source:HGNC Symbol;Acc:HGNC:30683] |
| ENSG00000143753 | HGLibA_12921 | 344 | 2.274554915 | DEGS1 | sphingolipid 1 [Source:HGNC Symbol;Acc:HGNC:13709]" |
| ENSG00000154099 | HGLibA_13458 | 344 | 2.274554915 | DNAAF1 | dynein axonemal assembly factor 1 [Source:HGNC Symbol;Acc:HGNC:30539] |
| ENSG00000160190 | HGLibA_45043 | 344 | 2.274554915 | SLC37A1 | solute carrier family 37 member 1 [Source:HGNC Symbol;Acc:HGNC:11024] |
| ENSG00000162032 | HGLibA_46865 | 344 | 2.274554915 | SPSB3 | splA/ryanodine receptor domain and SOCS box containing 3 [Source:HGNC Symbol;Acc:HGNC:30629] |
| ENSG00000171401 | HGLibA_25423 | 344 | 2.274554915 | KRT13 | keratin 13 [Source:HGNC Symbol;Acc:HGNC:6415] |
| ENSG00000183742 | HGLibA_27830 | 344 | 2.274554915 | MACC1 | MET transcriptional regulator MACC1 [Source:HGNC Symbol;Acc:HGNC:30215] |
| ENSG00000184908 | HGLibA_09843 | 344 | 2.274554915 | CLCNKB | chloride voltage-gated channel Kb [Source:HGNC Symbol;Acc:HGNC:2027] |
| hsa-mir-1976 | HGLibA_58119 | 344 | 2.274554915 | hsa-mir-1976 | - |
| hsa-mir-3128 | HGLibA_58566 | 344 | 2.274554915 | hsa-mir-3128 | - |
| hsa-mir-378h | HGLibA_59458 | 344 | 2.274554915 | hsa-mir-378h | - |
| hsa-mir-5100 | HGLibA_61551 | 344 | 2.274554915 | hsa-mir-5100 | - |
| ALS2CR8 | HGLibA_01729 | 343 | 2.264603878 | ALS2CR8 | - |
| ENSG00000060762 | HGLibA_29703 | 343 | 2.264603878 | MPC1 | mitochondrial pyruvate carrier 1 [Source:HGNC Symbol;Acc:HGNC:21606] |
| ENSG00000076984 | HGLibA_28156 | 343 | 2.264603878 | MAP2K7 | mitogen-activated protein kinase kinase 7 [Source:HGNC Symbol;Acc:HGNC:6847] |
| ENSG00000091137 | HGLibA_44796 | 343 | 2.264603878 | SLC26A4 | solute carrier family 26 member 4 [Source:HGNC Symbol;Acc:HGNC:8818] |
| ENSG00000099977 | HGLibA_12643 | 343 | 2.264603878 | DDT | D-dopachrome tautomerase [Source:HGNC Symbol;Acc:HGNC:2732] |
| ENSG00000157077 | HGLibA_55294 | 343 | 2.264603878 | ZFYVE9 | zinc finger FYVE-type containing 9 [Source:HGNC Symbol;Acc:HGNC:6775] |
| ENSG00000157873 | HGLibA_50759 | 343 | 2.264603878 | TNFRSF14 | TNF receptor superfamily member 14 [Source:HGNC Symbol;Acc:HGNC:11912] |
| ENSG00000158480 | HGLibA_46445 | 343 | 2.264603878 | SPATA2 | spermatogenesis associated 2 [Source:HGNC Symbol;Acc:HGNC:14681] |
| ENSG00000163815 | HGLibA_09976 | 343 | 2.264603878 | CLEC3B | C-type lectin domain family 3 member B [Source:HGNC Symbol;Acc:HGNC:11891] |
| ENSG00000168806 | HGLibA_26162 | 343 | 2.264603878 | LCMT2 | leucine carboxyl methyltransferase 2 [Source:HGNC Symbol;Acc:HGNC:17558] |
| ENSG00000179094 | HGLibA_36055 | 343 | 2.264603878 | PER1 | period circadian regulator 1 [Source:HGNC Symbol;Acc:HGNC:8845] |
| ENSG00000179750 | HGLibA_02480 | 343 | 2.264603878 | APOBEC3B | apolipoprotein B mRNA editing enzyme catalytic subunit 3B [Source:HGNC Symbol;Acc:HGNC:17352] |
| ENSG00000189046 | HGLibA_01648 | 343 | 2.264603878 | ALKBH2 | alpha-ketoglutarate dependent dioxygenase [Source:HGNC Symbol;Acc:HGNC:32487]" |
| ENSG00000198336 | HGLibA_30763 | 343 | 2.264603878 | MYL4 | myosin light chain 4 [Source:HGNC Symbol;Acc:HGNC:7585] |
| ENSG00000213760 | HGLibA_03759 | 343 | 2.264603878 | ATP6V1G2 | ATPase H+ transporting V1 subunit G2 [Source:HGNC Symbol;Acc:HGNC:862] |
| ENSG00000248098 | HGLibA_04277 | 343 | 2.264603878 | BCKDHA | alpha polypeptide [Source:HGNC Symbol;Acc:HGNC:986]" |
| hsa-mir-551b | HGLibA_62124 | 343 | 2.264603878 | hsa-mir-551b | - |
| MURC | HGLibA_30570 | 343 | 2.264603878 | MURC | - |
| ENSG00000087494 | HGLibA_39376 | 342 | 2.25465284 | PTHLH | parathyroid hormone like hormone [Source:HGNC Symbol;Acc:HGNC:9607] |
| ENSG00000089472 | HGLibA_21145 | 342 | 2.25465284 | HEPH | hephaestin [Source:HGNC Symbol;Acc:HGNC:4866] |
| ENSG00000118200 | HGLibA_07110 | 342 | 2.25465284 | CAMSAP2 | calmodulin regulated spectrin associated protein family member 2 [Source:HGNC Symbol;Acc:HGNC:29188] |
| ENSG00000122852 | HGLibA_43767 | 342 | 2.25465284 | SFTPA1 | surfactant protein A1 [Source:HGNC Symbol;Acc:HGNC:10798] |
| ENSG00000133789 | HGLibA_47898 | 342 | 2.25465284 | SWAP70 | switching B cell complex subunit SWAP70 [Source:HGNC Symbol;Acc:HGNC:17070] |
| ENSG00000135899 | HGLibA_46287 | 342 | 2.25465284 | SP110 | SP110 nuclear body protein [Source:HGNC Symbol;Acc:HGNC:5401] |
| ENSG00000137343 | HGLibA_03422 | 342 | 2.25465284 | ATAT1 | alpha tubulin acetyltransferase 1 [Source:HGNC Symbol;Acc:HGNC:21186] |
| ENSG00000162814 | HGLibA_46434 | 342 | 2.25465284 | SPATA17 | spermatogenesis associated 17 [Source:HGNC Symbol;Acc:HGNC:25184] |
| ENSG00000167656 | HGLibA_27654 | 342 | 2.25465284 | LY6D | lymphocyte antigen 6 family member D [Source:HGNC Symbol;Acc:HGNC:13348] |
| ENSG00000174695 | HGLibA_50040 | 342 | 2.25465284 | TMEM167A | transmembrane protein 167A [Source:HGNC Symbol;Acc:HGNC:28330] |
| ENSG00000180061 | HGLibA_49995 | 342 | 2.25465284 | TMEM150B | transmembrane protein 150B [Source:HGNC Symbol;Acc:HGNC:34415] |
| ENSG00000182685 | HGLibA_04809 | 342 | 2.25465284 | BRICD5 | BRICHOS domain containing 5 [Source:HGNC Symbol;Acc:HGNC:28309] |
| ENSG00000187855 | HGLibA_03274 | 342 | 2.25465284 | ASCL4 | achaete-scute family bHLH transcription factor 4 [Source:HGNC Symbol;Acc:HGNC:24311] |
| ENSG00000204103 | HGLibA_27874 | 342 | 2.25465284 | MAFB | MAF bZIP transcription factor B [Source:HGNC Symbol;Acc:HGNC:6408] |
| ENSG00000257108 | HGLibA_31904 | 342 | 2.25465284 | NHLRC4 | NHL repeat containing 4 [Source:HGNC Symbol;Acc:HGNC:26700] |
| ENSG00000026103 | HGLibA_16888 | 341 | 2.244701803 | FAS | Fas cell surface death receptor [Source:HGNC Symbol;Acc:HGNC:11920] |
| ENSG00000099308 | HGLibA_28407 | 341 | 2.244701803 | MAST3 | microtubule associated serine/threonine kinase 3 [Source:HGNC Symbol;Acc:HGNC:19036] |
| ENSG00000099381 | HGLibA_43636 | 341 | 2.244701803 | SETD1A | SET domain containing 1A [Source:HGNC Symbol;Acc:HGNC:29010] |
| ENSG00000105662 | HGLibA_11227 | 341 | 2.244701803 | CRTC1 | CREB regulated transcription coactivator 1 [Source:HGNC Symbol;Acc:HGNC:16062] |
| ENSG00000113742 | HGLibA_10927 | 341 | 2.244701803 | CPEB4 | cytoplasmic polyadenylation element binding protein 4 [Source:HGNC Symbol;Acc:HGNC:21747] |
| ENSG00000115657 | HGLibA_00145 | 341 | 2.244701803 | ABCB6 | ATP binding cassette subfamily B member 6 (Langereis blood group) [Source:HGNC Symbol;Acc:HGNC:47] |
| ENSG00000129932 | HGLibA_13763 | 341 | 2.244701803 | DOHH | deoxyhypusine hydroxylase [Source:HGNC Symbol;Acc:HGNC:28662] |
| ENSG00000130385 | HGLibA_04597 | 341 | 2.244701803 | BMP15 | bone morphogenetic protein 15 [Source:HGNC Symbol;Acc:HGNC:1068] |
| ENSG00000130958 | HGLibA_44980 | 341 | 2.244701803 | SLC35D2 | solute carrier family 35 member D2 [Source:HGNC Symbol;Acc:HGNC:20799] |
| ENSG00000134996 | HGLibA_34711 | 341 | 2.244701803 | OSTF1 | osteoclast stimulating factor 1 [Source:HGNC Symbol;Acc:HGNC:8510] |
| ENSG00000135924 | HGLibA_13536 | 341 | 2.244701803 | DNAJB2 | DnaJ heat shock protein family (Hsp40) member B2 [Source:HGNC Symbol;Acc:HGNC:5228] |
| ENSG00000137090 | HGLibA_13417 | 341 | 2.244701803 | DMRT1 | doublesex and mab-3 related transcription factor 1 [Source:HGNC Symbol;Acc:HGNC:2934] |
| ENSG00000144802 | HGLibA_31835 | 341 | 2.244701803 | NFKBIZ | NFKB inhibitor zeta [Source:HGNC Symbol;Acc:HGNC:29805] |
| ENSG00000149571 | HGLibA_25007 | 341 | 2.244701803 | KIRREL3 | kirre like nephrin family adhesion molecule 3 [Source:HGNC Symbol;Acc:HGNC:23204] |
| ENSG00000182795 | HGLibA_05756 | 341 | 2.244701803 | C1orf116 | chromosome 1 open reading frame 116 [Source:HGNC Symbol;Acc:HGNC:28667] |
| ENSG00000197696 | HGLibA_32143 | 341 | 2.244701803 | NMB | neuromedin B [Source:HGNC Symbol;Acc:HGNC:7842] |
| ENSG00000198398 | HGLibA_50197 | 341 | 2.244701803 | TMEM207 | transmembrane protein 207 [Source:HGNC Symbol;Acc:HGNC:33705] |
| ENSG00000198569 | HGLibA_44944 | 341 | 2.244701803 | SLC34A3 | solute carrier family 34 member 3 [Source:HGNC Symbol;Acc:HGNC:20305] |
| ENSG00000279395 | HGLibA_34323 | 341 | 2.244701803 | OR5L1 | olfactory receptor family 5 subfamily L member 1 (gene/pseudogene) [Source:HGNC Symbol;Acc:HGNC:8350] |
| hsa-mir-4763 | HGLibA_61147 | 341 | 2.244701803 | hsa-mir-4763 | - |
| ENSG00000003096 | HGLibA_25133 | 340 | 2.234750766 | KLHL13 | kelch like family member 13 [Source:HGNC Symbol;Acc:HGNC:22931] |
| ENSG00000087266 | HGLibA_43929 | 340 | 2.234750766 | SH3BP2 | SH3 domain binding protein 2 [Source:HGNC Symbol;Acc:HGNC:10825] |
| ENSG00000106346 | HGLibA_53365 | 340 | 2.234750766 | USP42 | ubiquitin specific peptidase 42 [Source:HGNC Symbol;Acc:HGNC:20068] |
| ENSG00000108641 | HGLibA_04045 | 340 | 2.234750766 | B9D1 | B9 domain containing 1 [Source:HGNC Symbol;Acc:HGNC:24123] |

| | | | | | |
|---|---|---|---|---|---|
| ENSG00000134504 | HGLibA_24411 | 340 | 2.234750766 | KCTD1 | potassium channel tetramerization domain containing 1 [Source:HGNC Symbol;Acc:HGNC:18249] |
| ENSG00000149043 | HGLibA_48090 | 340 | 2.234750766 | SYT8 | synaptotagmin 8 [Source:HGNC Symbol;Acc:HGNC:19264] |
| ENSG00000160352 | HGLibA_56620 | 340 | 2.234750766 | ZNF714 | zinc finger protein 714 [Source:HGNC Symbol;Acc:HGNC:27124] |
| ENSG00000165806 | HGLibA_07321 | 340 | 2.234750766 | CASP7 | caspase 7 [Source:HGNC Symbol;Acc:HGNC:1508] |
| ENSG00000172487 | HGLibA_34537 | 340 | 2.234750766 | OR8J1 | olfactory receptor family 8 subfamily J member 1 [Source:HGNC Symbol;Acc:HGNC:14855] |
| ENSG00000179057 | HGLibA_22887 | 340 | 2.234750766 | IGSF22 | immunoglobulin superfamily member 22 [Source:HGNC Symbol;Acc:HGNC:26750] |
| ENSG00000179133 | HGLibA_05085 | 340 | 2.234750766 | C10orf67 | chromosome 10 open reading frame 67 [Source:HGNC Symbol;Acc:HGNC:28716] |
| ENSG00000180988 | HGLibA_34196 | 340 | 2.234750766 | OR52N2 | olfactory receptor family 52 subfamily N member 2 [Source:HGNC Symbol;Acc:HGNC:15228] |
| ENSG00000186814 | HGLibA_57016 | 340 | 2.234750766 | ZSCAN30 | zinc finger and SCAN domain containing 30 [Source:HGNC Symbol;Acc:HGNC:33517] |
| ENSG00000197576 | HGLibA_21912 | 340 | 2.234750766 | HOXA4 | homeobox A4 [Source:HGNC Symbol;Acc:HGNC:5105] |
| ENSG00000198040 | HGLibA_56851 | 340 | 2.234750766 | ZNF84 | zinc finger protein 84 [Source:HGNC Symbol;Acc:HGNC:13159] |
| ENSG00000198390 | HGLibA_25641 | 340 | 2.234750766 | KRTAP13-1 | keratin associated protein 13-1 [Source:HGNC Symbol;Acc:HGNC:18924] |
| hsa-mir-1915 | HGLibA_58064 | 340 | 2.234750766 | hsa-mir-1915 | - |
| hsa-mir-490 | HGLibA_61362 | 340 | 2.234750766 | hsa-mir-490 | - |
| USMG5 | HGLibA_53184 | 340 | 2.234750766 | USMG5 | - |
| CCDC58 | HGLibA_07804 | 339 | 2.224799728 | CCDC58 | - |
| ENSG00000074966 | HGLibA_52387 | 339 | 2.224799728 | TXK | TXK tyrosine kinase [Source:HGNC Symbol;Acc:HGNC:12434] |
| ENSG00000081818 | HGLibA_35500 | 339 | 2.224799728 | PCDHB4 | protocadherin beta 4 [Source:HGNC Symbol;Acc:HGNC:8689] |
| ENSG00000089639 | HGLibA_19410 | 339 | 2.224799728 | GMIP | GEM interacting protein [Source:HGNC Symbol;Acc:HGNC:24852] |
| ENSG00000110848 | HGLibA_08433 | 339 | 2.224799728 | CD69 | CD69 molecule [Source:HGNC Symbol;Acc:HGNC:1694] |
| ENSG00000121297 | HGLibA_51869 | 339 | 2.224799728 | TSHZ3 | teashirt zinc finger homeobox 3 [Source:HGNC Symbol;Acc:HGNC:30700] |
| ENSG00000127249 | HGLibA_03575 | 339 | 2.224799728 | ATP13A4 | ATPase 13A4 [Source:HGNC Symbol;Acc:HGNC:25422] |
| ENSG00000130363 | HGLibA_42331 | 339 | 2.224799728 | RSPH3 | radial spoke head 3 homolog [Source:HGNC Symbol;Acc:HGNC:21054] |
| ENSG00000131759 | HGLibA_40271 | 339 | 2.224799728 | RARA | retinoic acid receptor alpha [Source:HGNC Symbol;Acc:HGNC:9864] |
| ENSG00000132740 | HGLibA_22856 | 339 | 2.224799728 | IGHMBP2 | immunoglobulin mu DNA binding protein 2 [Source:HGNC Symbol;Acc:HGNC:5542] |
| ENSG00000135678 | HGLibA_10943 | 339 | 2.224799728 | CPM | carboxypeptidase M [Source:HGNC Symbol;Acc:HGNC:2311] |
| ENSG00000139915 | HGLibA_28680 | 339 | 2.224799728 | MDGA2 | MAM domain containing glycosylphosphatidylinositol anchor 2 [Source:HGNC Symbol;Acc:HGNC:19835] |
| ENSG00000147065 | HGLibA_30232 | 339 | 2.224799728 | MSN | moesin [Source:HGNC Symbol;Acc:HGNC:7373] |
| ENSG00000152082 | HGLibA_30945 | 339 | 2.224799728 | MZT2B | mitotic spindle organizing protein 2B [Source:HGNC Symbol;Acc:HGNC:25886] |
| ENSG00000153936 | HGLibA_22119 | 339 | 2.224799728 | HS2ST1 | heparan sulfate 2-O-sulfotransferase 1 [Source:HGNC Symbol;Acc:HGNC:5193] |
| ENSG00000155816 | HGLibA_17689 | 339 | 2.224799728 | FMN2 | formin 2 [Source:HGNC Symbol;Acc:HGNC:14074] |
| ENSG00000179915 | HGLibA_32734 | 339 | 2.224799728 | NRXN1 | neurexin 1 [Source:HGNC Symbol;Acc:HGNC:8008] |
| ENSG00000184083 | HGLibA_16064 | 339 | 2.224799728 | FAM120C | family with sequence similarity 120C [Source:HGNC Symbol;Acc:HGNC:16949] |
| ENSG00000197375 | HGLibA_44592 | 339 | 2.224799728 | SLC22A5 | solute carrier family 22 member 5 [Source:HGNC Symbol;Acc:HGNC:10969] |
| ENSG00000206503 | HGLibA_21595 | 339 | 2.224799728 | HLA-A | A [Source:HGNC Symbol;Acc:HGNC:4931]" |
| ENSG00000277632 | HGLibA_08019 | 339 | 2.224799728 | CCL3 | C-C motif chemokine ligand 3 [Source:HGNC Symbol;Acc:HGNC:10627] |
| GPR1 | HGLibA_19837 | 339 | 2.224799728 | GPR1 | - |
| HIST1H2BD | HGLibA_21419 | 339 | 2.224799728 | HIST1H2BD | - |
| hsa-mir-4279 | HGLibA_59821 | 339 | 2.224799728 | hsa-mir-4279 | - |
| hsa-mir-6861 | HGLibA_63626 | 339 | 2.224799728 | hsa-mir-6861 | - |
| AARS | HGLibA_00069 | 338 | 2.214848691 | AARS | - |
| C9orf89 | HGLibA_06771 | 338 | 2.214848691 | C9orf89 | - |
| ENSG00000021852 | HGLibA_06597 | 338 | 2.214848691 | C8B | complement C8 beta chain [Source:HGNC Symbol;Acc:HGNC:1353] |
| ENSG00000049860 | HGLibA_21204 | 338 | 2.214848691 | HEXB | hexosaminidase subunit beta [Source:HGNC Symbol;Acc:HGNC:4879] |
| ENSG00000073598 | HGLibA_17759 | 338 | 2.214848691 | FNDC8 | fibronectin type III domain containing 8 [Source:HGNC Symbol;Acc:HGNC:25286] |
| ENSG00000083817 | HGLibA_55945 | 338 | 2.214848691 | ZNF416 | zinc finger protein 416 [Source:HGNC Symbol;Acc:HGNC:20645] |
| ENSG00000088682 | HGLibA_10767 | 338 | 2.214848691 | COQ9 | coenzyme Q9 [Source:HGNC Symbol;Acc:HGNC:25302] |
| ENSG00000129451 | HGLibA_25233 | 338 | 2.214848691 | KLK10 | kallikrein related peptidase 10 [Source:HGNC Symbol;Acc:HGNC:6358] |
| ENSG00000137747 | HGLibA_50631 | 338 | 2.214848691 | TMPRSS13 | transmembrane serine protease 13 [Source:HGNC Symbol;Acc:HGNC:29808] |
| ENSG00000151414 | HGLibA_31661 | 338 | 2.214848691 | NEK7 | NIMA related kinase 7 [Source:HGNC Symbol;Acc:HGNC:13386] |
| ENSG00000160318 | HGLibA_09919 | 338 | 2.214848691 | CLDND2 | claudin domain containing 2 [Source:HGNC Symbol;Acc:HGNC:28511] |
| ENSG00000161849 | HGLibA_25568 | 338 | 2.214848691 | KRT84 | keratin 84 [Source:HGNC Symbol;Acc:HGNC:6461] |
| ENSG00000166368 | HGLibA_33782 | 338 | 2.214848691 | OR2D2 | olfactory receptor family 2 subfamily D member 2 [Source:HGNC Symbol;Acc:HGNC:8244] |
| ENSG00000167566 | HGLibA_31321 | 338 | 2.214848691 | NCKAP5L | NCK associated protein 5 like [Source:HGNC Symbol;Acc:HGNC:29321] |
| ENSG00000171928 | HGLibA_52361 | 338 | 2.214848691 | TVP23B | trans-golgi network vesicle protein 23 homolog B [Source:HGNC Symbol;Acc:HGNC:20399] |
| ENSG00000172421 | HGLibA_14537 | 338 | 2.214848691 | EFCAB3 | EF-hand calcium binding domain 3 [Source:HGNC Symbol;Acc:HGNC:26379] |
| ENSG00000178878 | HGLibA_02544 | 338 | 2.214848691 | APOLD1 | apolipoprotein L domain containing 1 [Source:HGNC Symbol;Acc:HGNC:25268] |
| ENSG00000179055 | HGLibA_33613 | 338 | 2.214848691 | OR13D1 | olfactory receptor family 13 subfamily D member 1 [Source:HGNC Symbol;Acc:HGNC:14695] |
| ENSG00000186635 | HGLibA_02624 | 338 | 2.214848691 | ARAP1 | ankyrin repeat and PH domain 1 [Source:HGNC Symbol;Acc:HGNC:16925]" |
| ENSG00000187045 | HGLibA_50649 | 338 | 2.214848691 | TMPRSS6 | transmembrane serine protease 6 [Source:HGNC Symbol;Acc:HGNC:16517] |
| ENSG00000196859 | HGLibA_25501 | 338 | 2.214848691 | KRT39 | keratin 39 [Source:HGNC Symbol;Acc:HGNC:32971] |
| ENSG00000258947 | HGLibA_52282 | 338 | 2.214848691 | TUBB3 | tubulin beta 3 class III [Source:HGNC Symbol;Acc:HGNC:20772] |
| hsa-mir-1303 | HGLibA_57729 | 338 | 2.214848691 | hsa-mir-1303 | - |
| WDR96 | HGLibA_54227 | 338 | 2.214848691 | WDR96 | - |
| ENSG00000103184 | HGLibA_43203 | 337 | 2.204897653 | SEC14L5 | SEC14 like lipid binding 5 [Source:HGNC Symbol;Acc:HGNC:29032] |
| ENSG00000106404 | HGLibA_09862 | 337 | 2.204897653 | CLDN15 | claudin 15 [Source:HGNC Symbol;Acc:HGNC:2036] |
| ENSG00000120289 | HGLibA_27951 | 337 | 2.204897653 | MAGEB4 | MAGE family member B4 [Source:HGNC Symbol;Acc:HGNC:6811] |
| ENSG00000132334 | HGLibA_39499 | 337 | 2.204897653 | PTPRE | receptor type E [Source:HGNC Symbol;Acc:HGNC:9669]" |
| ENSG00000141542 | HGLibA_39937 | 337 | 2.204897653 | RAB40B | member RAS oncogene family [Source:HGNC Symbol;Acc:HGNC:18284]" |
| ENSG00000153832 | HGLibA_17082 | 337 | 2.204897653 | FBXO36 | F-box protein 36 [Source:HGNC Symbol;Acc:HGNC:27020] |
| ENSG00000156671 | HGLibA_42696 | 337 | 2.204897653 | SAMD8 | sterile alpha motif domain containing 8 [Source:HGNC Symbol;Acc:HGNC:26320] |
| ENSG00000160410 | HGLibA_44056 | 337 | 2.204897653 | SHKBP1 | SH3KBP1 binding protein 1 [Source:HGNC Symbol;Acc:HGNC:19214] |
| ENSG00000160882 | HGLibA_12054 | 337 | 2.204897653 | CYP11B1 | cytochrome P450 family 11 subfamily B member 1 [Source:HGNC Symbol;Acc:HGNC:2591] |
| ENSG00000178363 | HGLibA_07030 | 337 | 2.204897653 | CALML3 | calmodulin like 3 [Source:HGNC Symbol;Acc:HGNC:1452] |
| ENSG00000182255 | HGLibA_24135 | 337 | 2.204897653 | KCNA4 | potassium voltage-gated channel subfamily A member 4 [Source:HGNC Symbol;Acc:HGNC:6222] |
| ENSG00000198821 | HGLibA_08309 | 337 | 2.204897653 | CD247 | CD247 molecule [Source:HGNC Symbol;Acc:HGNC:1677] |
| ENSG00000262655 | HGLibA_46756 | 337 | 2.204897653 | SPON1 | spondin 1 [Source:HGNC Symbol;Acc:HGNC:11252] |
| ENSG00000269058 | HGLibA_07049 | 337 | 2.204897653 | CALR3 | calreticulin 3 [Source:HGNC Symbol;Acc:HGNC:20407] |
| ZBED1_X | HGLibA_54787 | 337 | 2.204897653 | ZBED1_X | - |
| ENSG00000085514 | HGLibA_36669 | 336 | 2.194946616 | PILRA | paired immunoglobin like type 2 receptor alpha [Source:HGNC Symbol;Acc:HGNC:20396] |
| ENSG00000104472 | HGLibA_09531 | 336 | 2.194946616 | CHRAC1 | chromatin accessibility complex subunit 1 [Source:HGNC Symbol;Acc:HGNC:13544] |
| ENSG00000113073 | HGLibA_45233 | 336 | 2.194946616 | SLC4A9 | solute carrier family 4 member 9 [Source:HGNC Symbol;Acc:HGNC:11035] |
| ENSG00000142178 | HGLibA_44162 | 336 | 2.194946616 | SIK1 | salt inducible kinase 1 [Source:HGNC Symbol;Acc:HGNC:11142] |
| ENSG00000143632 | HGLibA_00596 | 336 | 2.194946616 | ACTA1 | skeletal muscle [Source:HGNC Symbol;Acc:HGNC:129]" |
| ENSG00000160803 | HGLibA_52742 | 336 | 2.194946616 | UBQLN4 | ubiquilin 4 [Source:HGNC Symbol;Acc:HGNC:1237] |
| ENSG00000171792 | HGLibA_41160 | 336 | 2.194946616 | RHNO1 | RAD9-HUS1-RAD1 interacting nuclear orphan 1 [Source:HGNC Symbol;Acc:HGNC:28206] |
| ENSG00000176771 | HGLibA_31318 | 336 | 2.194946616 | NCKAP5 | NCK associated protein 5 [Source:HGNC Symbol;Acc:HGNC:29847] |
| ENSG00000185988 | HGLibA_37201 | 336 | 2.194946616 | PLK5 | polo like kinase 5 [Source:HGNC Symbol;Acc:HGNC:27001] |
| ENSG00000204314 | HGLibA_38881 | 336 | 2.194946616 | PRRT1 | proline rich transmembrane protein 1 [Source:HGNC Symbol;Acc:HGNC:13943] |
| ENSG00000221821 | HGLibA_06483 | 336 | 2.194946616 | C6orf226 | chromosome 6 open reading frame 226 [Source:HGNC Symbol;Acc:HGNC:34431] |
| hsa-mir-3937 | HGLibA_59608 | 336 | 2.194946616 | hsa-mir-3937 | - |
| C2orf82 | HGLibA_06190 | 335 | 2.184995579 | C2orf82 | - |
| ENSG00000002587 | HGLibA_22120 | 335 | 2.184995579 | HS3ST1 | heparan sulfate-glucosamine 3-sulfotransferase 1 [Source:HGNC Symbol;Acc:HGNC:5194] |
| ENSG00000004142 | HGLibA_37503 | 335 | 2.184995579 | POLDIP2 | DNA polymerase delta interacting protein 2 [Source:HGNC Symbol;Acc:HGNC:23781] |
| ENSG00000040731 | HGLibA_08631 | 335 | 2.184995579 | CDH10 | cadherin 10 [Source:HGNC Symbol;Acc:HGNC:1749] |
| ENSG00000112078 | HGLibA_24447 | 335 | 2.184995579 | KCTD20 | potassium channel tetramerization domain containing 20 [Source:HGNC Symbol;Acc:HGNC:21052] |
| ENSG00000114784 | HGLibA_14717 | 335 | 2.184995579 | EIF1B | eukaryotic translation initiation factor 1B [Source:HGNC Symbol;Acc:HGNC:30792] |
| ENSG00000135926 | HGLibA_49739 | 335 | 2.184995579 | TMBIM1 | transmembrane BAX inhibitor motif containing 1 [Source:HGNC Symbol;Acc:HGNC:23410] |
| ENSG00000163376 | HGLibA_24118 | 335 | 2.184995579 | KBTBD8 | kelch repeat and BTB domain containing 8 [Source:HGNC Symbol;Acc:HGNC:30691] |
| ENSG00000164749 | HGLibA_21784 | 335 | 2.184995579 | HNF4G | hepatocyte nuclear factor 4 gamma [Source:HGNC Symbol;Acc:HGNC:5026] |
| ENSG00000169118 | HGLibA_11392 | 335 | 2.184995579 | CSNK1G1 | casein kinase 1 gamma 1 [Source:HGNC Symbol;Acc:HGNC:2454] |
| ENSG00000170099 | HGLibA_43525 | 335 | 2.184995579 | SERPINA6 | serpin family A member 6 [Source:HGNC Symbol;Acc:HGNC:1540] |
| ENSG00000171298 | HGLibA_18317 | 335 | 2.184995579 | GAA | acid [Source:HGNC Symbol;Acc:HGNC:4065]" |
| ENSG00000172578 | HGLibA_25218 | 335 | 2.184995579 | KLHL6 | kelch like family member 6 [Source:HGNC Symbol;Acc:HGNC:18653] |
| ENSG00000186118 | HGLibA_49110 | 335 | 2.184995579 | TEX38 | testis expressed 38 [Source:HGNC Symbol;Acc:HGNC:29589] |
| ENSG00000196092 | HGLibA_35306 | 335 | 2.184995579 | PAX5 | paired box 5 [Source:HGNC Symbol;Acc:HGNC:8619] |
| ENSG00000198563 | HGLibA_12699 | 335 | 2.184995579 | DDX39B | DExD-box helicase 39B [Source:HGNC Symbol;Acc:HGNC:13917] |
| ENSG00000204564 | HGLibA_06446 | 335 | 2.184995579 | C6orf136 | chromosome 6 open reading frame 136 [Source:HGNC Symbol;Acc:HGNC:21301] |
| ENSG00000285053 | HGLibA_48594 | 335 | 2.184995579 | TBCE | tubulin folding cofactor E [Source:NCBI gene;Acc:6905] |
| FAM46B | HGLibA_16598 | 335 | 2.184995579 | FAM46B | - |
| hsa-mir-6717 | HGLibA_63031 | 335 | 2.184995579 | hsa-mir-6717 | - |
| RRNAD1 | HGLibA_42264 | 335 | 2.184995579 | RRNAD1 | - |
| C20orf24 | HGLibA_06017 | 334 | 2.175044541 | C20orf24 | - |
| ENSG00000101445 | HGLibA_38057 | 334 | 2.175044541 | PPP1R16B | protein phosphatase 1 regulatory subunit 16B [Source:HGNC Symbol;Acc:HGNC:15850] |
| ENSG00000110711 | HGLibA_01362 | 334 | 2.175044541 | AIP | aryl hydrocarbon receptor interacting protein [Source:HGNC Symbol;Acc:HGNC:358] |
| ENSG00000121104 | HGLibA_16041 | 334 | 2.175044541 | FAM117A | family with sequence similarity 117 member A [Source:HGNC Symbol;Acc:HGNC:24179] |
| ENSG00000122254 | HGLibA_22124 | 334 | 2.175044541 | HS3ST2 | heparan sulfate-glucosamine 3-sulfotransferase 2 [Source:HGNC Symbol;Acc:HGNC:5195] |
| ENSG00000134247 | HGLibA_39342 | 334 | 2.175044541 | PTGFRN | prostaglandin F2 receptor inhibitor [Source:HGNC Symbol;Acc:HGNC:9601] |
| ENSG00000136237 | HGLibA_40256 | 334 | 2.175044541 | RAPGEF5 | Rap guanine nucleotide exchange factor 5 [Source:HGNC Symbol;Acc:HGNC:16862] |
| ENSG00000136754 | HGLibA_00290 | 334 | 2.175044541 | ABI1 | abl interactor 1 [Source:HGNC Symbol;Acc:HGNC:11320] |
| ENSG00000140416 | HGLibA_51141 | 334 | 2.175044541 | TPM1 | tropomyosin 1 [Source:HGNC Symbol;Acc:HGNC:12010] |
| ENSG00000145309 | HGLibA_06864 | 334 | 2.175044541 | CABS1 | spermatid associated 1 [Source:HGNC Symbol;Acc:HGNC:30710]" |
| ENSG00000162892 | HGLibA_23104 | 334 | 2.175044541 | IL24 | interleukin 24 [Source:HGNC Symbol;Acc:HGNC:11346] |
| ENSG00000169710 | HGLibA_16896 | 334 | 2.175044541 | FASN | fatty acid synthase [Source:HGNC Symbol;Acc:HGNC:3594] |
| ENSG00000176040 | HGLibA_50650 | 334 | 2.175044541 | TMPRSS7 | transmembrane serine protease 7 [Source:HGNC Symbol;Acc:HGNC:30846] |
| ENSG00000179059 | HGLibA_55219 | 334 | 2.175044541 | ZFP42 | ZFP42 zinc finger protein [Source:HGNC Symbol;Acc:HGNC:30949] |
| ENSG00000187026 | HGLibA_25710 | 334 | 2.175044541 | KRTAP21-2 | keratin associated protein 21-2 [Source:HGNC Symbol;Acc:HGNC:18946] |
| ENSG00000254636 | HGLibA_03059 | 334 | 2.175044541 | ARMS2 | age-related maculopathy susceptibility 2 [Source:HGNC Symbol;Acc:HGNC:32685] |
| GPR123 | HGLibA_19872 | 334 | 2.175044541 | GPR123 | - |
| GPR98 | HGLibA_20116 | 334 | 2.175044541 | GPR98 | - |
| hsa-mir-6070 | HGLibA_62533 | 334 | 2.175044541 | hsa-mir-6070 | - |
| hsa-mir-9-2 | HGLibA_64267 | 334 | 2.175044541 | hsa-mir-9-2 | - |
| ENSG00000009950 | HGLibA_29445 | 333 | 2.165093504 | MLXIPL | MLX interacting protein like [Source:HGNC Symbol;Acc:HGNC:12744] |
| ENSG00000068438 | HGLibA_18135 | 333 | 2.165093504 | FTSJ1 | FtsJ RNA methyltransferase homolog 1 [Source:HGNC Symbol;Acc:HGNC:13254] |
| ENSG00000074211 | HGLibA_38153 | 333 | 2.165093504 | PPP2R2C | protein phosphatase 2 regulatory subunit Bgamma [Source:HGNC Symbol;Acc:HGNC:9306] |

| | | | | | |
|---|---|---|---|---|---|
| ENSG00000085563 | HGLibA_00132 | 333 | 2.165093504 | ABCB1 | ATP binding cassette subfamily B member 1 [Source:HGNC Symbol;Acc:HGNC:40] |
| ENSG00000100997 | HGLibA_00240 | 333 | 2.165093504 | ABHD12 | abhydrolase domain containing 12 [Source:HGNC Symbol;Acc:HGNC:15868] |
| ENSG00000107831 | HGLibA_17421 | 333 | 2.165093504 | FGF8 | fibroblast growth factor 8 [Source:HGNC Symbol;Acc:HGNC:3686] |
| ENSG00000123584 | HGLibA_27923 | 333 | 2.165093504 | MAGEA9 | MAGE family member A9 [Source:HGNC Symbol;Acc:HGNC:6807] |
| ENSG00000130701 | HGLibA_40442 | 333 | 2.165093504 | RBBP8NL | RBBP8 N-terminal like [Source:HGNC Symbol;Acc:HGNC:16144] |
| ENSG00000141682 | HGLibA_37292 | 333 | 2.165093504 | PMAIP1 | phorbol-12-myristate-13-acetate-induced protein 1 [Source:HGNC Symbol;Acc:HGNC:9108] |
| ENSG00000144619 | HGLibA_10409 | 333 | 2.165093504 | CNTN4 | contactin 4 [Source:HGNC Symbol;Acc:HGNC:2174] |
| ENSG00000147378 | HGLibA_16922 | 333 | 2.165093504 | FATE1 | fetal and adult testis expressed 1 [Source:HGNC Symbol;Acc:HGNC:24683] |
| ENSG00000151962 | HGLibA_40562 | 333 | 2.165093504 | RBM46 | RNA binding motif protein 46 [Source:HGNC Symbol;Acc:HGNC:28401] |
| ENSG00000155368 | HGLibA_12385 | 333 | 2.165093504 | DBI | acyl-CoA binding protein [Source:HGNC Symbol;Acc:HGNC:2690]" |
| ENSG00000165682 | HGLibA_09958 | 333 | 2.165093504 | CLEC1B | C-type lectin domain family 1 member B [Source:HGNC Symbol;Acc:HGNC:24356] |
| ENSG00000168412 | HGLibA_30444 | 333 | 2.165093504 | MTNR1A | melatonin receptor 1A [Source:HGNC Symbol;Acc:HGNC:7463] |
| ENSG00000168517 | HGLibA_21214 | 333 | 2.165093504 | HEXIM2 | hexamethylene bisacetamide inducible 2 [Source:HGNC Symbol;Acc:HGNC:28591] |
| ENSG00000178093 | HGLibA_52022 | 333 | 2.165093504 | TSSK6 | testis specific serine kinase 6 [Source:HGNC Symbol;Acc:HGNC:30410] |
| ENSG00000188976 | HGLibA_32234 | 333 | 2.165093504 | NOC2L | NOC2 like nucleolar associated transcriptional repressor [Source:HGNC Symbol;Acc:HGNC:24517] |
| ENSG00000245680 | HGLibA_56322 | 333 | 2.165093504 | ZNF585B | zinc finger protein 585B [Source:HGNC Symbol;Acc:HGNC:30948] |
| hsa-mir-3674 | HGLibA_59250 | 333 | 2.165093504 | hsa-mir-3674 | - |
| hsa-mir-6889 | HGLibA_63740 | 333 | 2.165093504 | hsa-mir-6889 | - |
| ENSG00000067141 | HGLibA_31695 | 332 | 2.155142466 | NEO1 | neogenin 1 [Source:HGNC Symbol;Acc:HGNC:7754] |
| ENSG00000103037 | HGLibA_43654 | 332 | 2.155142466 | SETD6 | SET domain containing 6 [Source:HGNC Symbol;Acc:HGNC:26116] |
| ENSG00000108342 | HGLibA_11338 | 332 | 2.155142466 | CSF3 | colony stimulating factor 3 [Source:HGNC Symbol;Acc:HGNC:2438] |
| ENSG00000154957 | HGLibA_55533 | 332 | 2.155142466 | ZNF18 | zinc finger protein 18 [Source:HGNC Symbol;Acc:HGNC:12969] |
| ENSG00000158286 | HGLibA_41614 | 332 | 2.155142466 | RNF207 | ring finger protein 207 [Source:HGNC Symbol;Acc:HGNC:32947] |
| ENSG00000163950 | HGLibA_44328 | 332 | 2.155142466 | SLBP | stem-loop binding protein [Source:HGNC Symbol;Acc:HGNC:10904] |
| ENSG00000164237 | HGLibA_10183 | 332 | 2.155142466 | CMBL | carboxymethylenebutenolidase homolog [Source:HGNC Symbol;Acc:HGNC:25090] |
| ENSG00000167604 | HGLibA_31826 | 332 | 2.155142466 | NFKBID | NFKB inhibitor delta [Source:HGNC Symbol;Acc:HGNC:15671] |
| ENSG00000168077 | HGLibA_42862 | 332 | 2.155142466 | SCARA3 | scavenger receptor class A member 3 [Source:HGNC Symbol;Acc:HGNC:19000] |
| ENSG00000172031 | HGLibA_15333 | 332 | 2.155142466 | EPHX4 | epoxide hydrolase 4 [Source:HGNC Symbol;Acc:HGNC:23758] |
| ENSG00000177663 | HGLibA_23005 | 332 | 2.155142466 | IL17RA | interleukin 17 receptor A [Source:HGNC Symbol;Acc:HGNC:5985] |
| ENSG00000180305 | HGLibA_54248 | 332 | 2.155142466 | WFDC10A | WAP four-disulfide core domain 10A [Source:HGNC Symbol;Acc:HGNC:16139] |
| ENSG00000235863 | HGLibA_03959 | 332 | 2.155142466 | B3GALT4 | 3-galactosyltransferase 4 [Source:HGNC Symbol;Acc:HGNC:919]" |
| FAM101A | HGLibA_15974 | 332 | 2.155142466 | FAM101A | - |
| hsa-mir-1227 | HGLibA_57308 | 332 | 2.155142466 | hsa-mir-1227 | - |
| hsa-mir-4789 | HGLibA_61264 | 332 | 2.155142466 | hsa-mir-4789 | - |
| METTL12 | HGLibA_28952 | 332 | 2.155142466 | METTL12 | - |
| RAB7L1 | HGLibA_39981 | 332 | 2.155142466 | RAB7L1 | - |
| ENSG00000022567 | HGLibA_45181 | 331 | 2.145191429 | SLC45A4 | solute carrier family 45 member 4 [Source:HGNC Symbol;Acc:HGNC:29196] |
| ENSG00000100815 | HGLibA_51636 | 331 | 2.145191429 | TRIP11 | thyroid hormone receptor interactor 11 [Source:HGNC Symbol;Acc:HGNC:12305] |
| ENSG00000104915 | HGLibA_47616 | 331 | 2.145191429 | STX10 | syntaxin 10 [Source:HGNC Symbol;Acc:HGNC:11428] |
| ENSG00000105677 | HGLibA_49977 | 331 | 2.145191429 | TMEM147 | transmembrane protein 147 [Source:HGNC Symbol;Acc:HGNC:30414] |
| ENSG00000112303 | HGLibA_53713 | 331 | 2.145191429 | VNN2 | vanin 2 [Source:HGNC Symbol;Acc:HGNC:12706] |
| ENSG00000112394 | HGLibA_44419 | 331 | 2.145191429 | SLC16A10 | solute carrier family 16 member 10 [Source:HGNC Symbol;Acc:HGNC:17027] |
| ENSG00000112562 | HGLibA_45796 | 331 | 2.145191429 | SMOC2 | SPARC related modular calcium binding 2 [Source:HGNC Symbol;Acc:HGNC:20323] |
| ENSG00000112852 | HGLibA_35492 | 331 | 2.145191429 | PCDHB2 | protocadherin beta 2 [Source:HGNC Symbol;Acc:HGNC:8687] |
| ENSG00000115504 | HGLibA_14659 | 331 | 2.145191429 | EHBP1 | EH domain binding protein 1 [Source:HGNC Symbol;Acc:HGNC:29144] |
| ENSG00000121743 | HGLibA_19143 | 331 | 2.145191429 | GJA3 | gap junction protein alpha 3 [Source:HGNC Symbol;Acc:HGNC:4277] |
| ENSG00000131591 | HGLibA_05783 | 331 | 2.145191429 | C1orf159 | chromosome 1 open reading frame 159 [Source:HGNC Symbol;Acc:HGNC:26062] |
| ENSG00000134107 | HGLibA_04479 | 331 | 2.145191429 | BHLHE40 | basic helix-loop-helix family member e40 [Source:HGNC Symbol;Acc:HGNC:1046] |
| ENSG00000136295 | HGLibA_52238 | 331 | 2.145191429 | TTYH3 | tweety family member 3 [Source:HGNC Symbol;Acc:HGNC:22222] |
| ENSG00000148803 | HGLibA_18170 | 331 | 2.145191429 | FUOM | fucose mutarotase [Source:HGNC Symbol;Acc:HGNC:24733] |
| ENSG00000159200 | HGLibA_40667 | 331 | 2.145191429 | RCAN1 | regulator of calcineurin 1 [Source:HGNC Symbol;Acc:HGNC:3040] |
| ENSG00000164663 | HGLibA_53386 | 331 | 2.145191429 | USP49 | ubiquitin specific peptidase 49 [Source:HGNC Symbol;Acc:HGNC:20078] |
| ENSG00000167858 | HGLibA_48970 | 331 | 2.145191429 | TEKT1 | tektin 1 [Source:HGNC Symbol;Acc:HGNC:15534] |
| ENSG00000185869 | HGLibA_56828 | 331 | 2.145191429 | ZNF829 | zinc finger protein 829 [Source:HGNC Symbol;Acc:HGNC:34032] |
| ENSG00000196453 | HGLibA_56726 | 331 | 2.145191429 | ZNF777 | zinc finger protein 777 [Source:HGNC Symbol;Acc:HGNC:22213] |
| ENSG00000203879 | HGLibA_18882 | 331 | 2.145191429 | GDI1 | GDP dissociation inhibitor 1 [Source:HGNC Symbol;Acc:HGNC:4226] |
| ENSG00000204859 | HGLibA_54904 | 331 | 2.145191429 | ZBTB48 | zinc finger and BTB domain containing 48 [Source:HGNC Symbol;Acc:HGNC:4930] |
| ENSG00000241058 | HGLibA_32794 | 331 | 2.145191429 | NSUN6 | NOP2/Sun RNA methyltransferase family member 6 [Source:HGNC Symbol;Acc:HGNC:23529] |
| hsa-mir-1258 | HGLibA_57438 | 331 | 2.145191429 | hsa-mir-1258 | - |
| hsa-mir-197 | HGLibA_58103 | 331 | 2.145191429 | hsa-mir-197 | - |
| LOC149373 | HGLibA_26891 | 331 | 2.145191429 | LOC149373 | - |
| ENSG00000069020 | HGLibA_28410 | 330 | 2.135240391 | MAST4 | microtubule associated serine/threonine kinase family member 4 [Source:HGNC Symbol;Acc:HGNC:19037] |
| ENSG00000072786 | HGLibA_47451 | 330 | 2.135240391 | STK10 | serine/threonine kinase 10 [Source:HGNC Symbol;Acc:HGNC:11388] |
| ENSG00000075035 | HGLibA_54423 | 330 | 2.135240391 | WSCD2 | WSC domain containing 2 [Source:HGNC Symbol;Acc:HGNC:29117] |
| ENSG00000101162 | HGLibA_52273 | 330 | 2.135240391 | TUBB1 | tubulin beta 1 class VI [Source:HGNC Symbol;Acc:HGNC:16257] |
| ENSG00000112139 | HGLibA_28677 | 330 | 2.135240391 | MDGA1 | MAM domain containing glycosylphosphatidylinositol anchor 1 [Source:HGNC Symbol;Acc:HGNC:19267] |
| ENSG00000119242 | HGLibA_07929 | 330 | 2.135240391 | CCDC92 | coiled-coil domain containing 92 [Source:HGNC Symbol;Acc:HGNC:29563] |
| ENSG00000132357 | HGLibA_07234 | 330 | 2.135240391 | CARD6 | caspase recruitment domain family member 6 [Source:HGNC Symbol;Acc:HGNC:16394] |
| ENSG00000135838 | HGLibA_32482 | 330 | 2.135240391 | NPL | N-acetylneuraminate pyruvate lyase [Source:HGNC Symbol;Acc:HGNC:16781] |
| ENSG00000141380 | HGLibA_47101 | 330 | 2.135240391 | SS18 | nBAF chromatin remodeling complex subunit [Source:HGNC Symbol;Acc:HGNC:11340]" |
| ENSG00000141977 | HGLibA_09687 | 330 | 2.135240391 | CIB3 | calcium and integrin binding family member 3 [Source:HGNC Symbol;Acc:HGNC:24580] |
| ENSG00000154451 | HGLibA_18740 | 330 | 2.135240391 | GBP5 | guanylate binding protein 5 [Source:HGNC Symbol;Acc:HGNC:19895] |
| ENSG00000155026 | HGLibA_42326 | 330 | 2.135240391 | RSPH10B | radial spoke head 10 homolog B [Source:HGNC Symbol;Acc:HGNC:27362] |
| ENSG00000158220 | HGLibA_15612 | 330 | 2.135240391 | ESYT3 | extended synaptotagmin 3 [Source:HGNC Symbol;Acc:HGNC:24295] |
| ENSG00000166960 | HGLibA_07715 | 330 | 2.135240391 | CCDC178 | coiled-coil domain containing 178 [Source:HGNC Symbol;Acc:HGNC:29588] |
| ENSG00000173114 | HGLibA_27475 | 330 | 2.135240391 | LRRN3 | leucine rich repeat neuronal 3 [Source:HGNC Symbol;Acc:HGNC:17200] |
| ENSG00000182508 | HGLibA_26430 | 330 | 2.135240391 | LHFPL1 | LHFPL tetraspan subfamily member 1 [Source:HGNC Symbol;Acc:HGNC:6587] |
| ENSG00000186280 | HGLibA_24515 | 330 | 2.135240391 | KDM4D | lysine demethylase 4D [Source:HGNC Symbol;Acc:HGNC:25498] |
| ENSG00000198830 | HGLibA_21719 | 330 | 2.135240391 | HMGN2 | high mobility group nucleosomal binding domain 2 [Source:HGNC Symbol;Acc:HGNC:4986] |
| ENSG00000205076 | HGLibA_26373 | 330 | 2.135240391 | LGALS7 | galectin 7 [Source:HGNC Symbol;Acc:HGNC:6568] |
| ENSG00000221852 | HGLibA_25587 | 330 | 2.135240391 | KRTAP1-5 | keratin associated protein 1-5 [Source:HGNC Symbol;Acc:HGNC:16777] |
| hsa-mir-372 | HGLibA_59381 | 330 | 2.135240391 | hsa-mir-372 | - |
| hsa-mir-4302 | HGLibA_59918 | 330 | 2.135240391 | hsa-mir-4302 | - |
| hsa-mir-4515 | HGLibA_60503 | 330 | 2.135240391 | hsa-mir-4515 | - |
| hsa-mir-4800 | HGLibA_61305 | 330 | 2.135240391 | hsa-mir-4800 | - |
| hsa-mir-5706 | HGLibA_62365 | 330 | 2.135240391 | hsa-mir-5706 | - |
| hsa-mir-6795 | HGLibA_63359 | 330 | 2.135240391 | hsa-mir-6795 | - |
| C4orf40 | HGLibA_06328 | 329 | 2.125289354 | C4orf40 | - |
| ENSG00000084207 | HGLibA_20493 | 329 | 2.125289354 | GSTP1 | glutathione S-transferase pi 1 [Source:HGNC Symbol;Acc:HGNC:4638] |
| ENSG00000113492 | HGLibA_01273 | 329 | 2.125289354 | AGXT2 | alanine--glyoxylate aminotransferase 2 [Source:HGNC Symbol;Acc:HGNC:14412] |
| ENSG00000125148 | HGLibA_30302 | 329 | 2.125289354 | MT2A | metallothionein 2A [Source:HGNC Symbol;Acc:HGNC:7406] |
| ENSG00000136842 | HGLibA_50595 | 329 | 2.125289354 | TMOD1 | tropomodulin 1 [Source:HGNC Symbol;Acc:HGNC:11871] |
| ENSG00000150676 | HGLibA_07887 | 329 | 2.125289354 | CCDC83 | coiled-coil domain containing 83 [Source:HGNC Symbol;Acc:HGNC:28535] |
| ENSG00000170776 | HGLibA_01415 | 329 | 2.125289354 | AKAP13 | A-kinase anchoring protein 13 [Source:HGNC Symbol;Acc:HGNC:371] |
| ENSG00000178252 | HGLibA_54137 | 329 | 2.125289354 | WDR6 | WD repeat domain 6 [Source:HGNC Symbol;Acc:HGNC:12758] |
| ENSG00000179913 | HGLibA_03988 | 329 | 2.125289354 | B3GNT3 | 3-N-acetylglucosaminyltransferase 3 [Source:HGNC Symbol;Acc:HGNC:13528]" |
| ENSG00000204314 | HGLibA_38880 | 329 | 2.125289354 | PRRT1 | proline rich transmembrane protein 1 [Source:HGNC Symbol;Acc:HGNC:13943] |
| ENSG00000204514 | HGLibA_56809 | 329 | 2.125289354 | ZNF814 | zinc finger protein 814 [Source:HGNC Symbol;Acc:HGNC:33258] |
| ENSG00000204572 | HGLibA_25784 | 329 | 2.125289354 | KRTAP5-10 | keratin associated protein 5-10 [Source:HGNC Symbol;Acc:HGNC:23605] |
| ENSG00000205038 | HGLibA_36817 | 329 | 2.125289354 | PKHD1L1 | PKHD1 like 1 [Source:HGNC Symbol;Acc:HGNC:20313] |
| ENSG00000214706 | HGLibA_22740 | 329 | 2.125289354 | IFRD2 | interferon related developmental regulator 2 [Source:HGNC Symbol;Acc:HGNC:5457] |
| hsa-mir-198 | HGLibA_58126 | 329 | 2.125289354 | hsa-mir-198 | - |
| hsa-mir-4710 | HGLibA_60939 | 329 | 2.125289354 | hsa-mir-4710 | - |
| ENSG00000102128 | HGLibA_39934 | 328 | 2.115338317 | RAB40AL | RAB40A like [Source:HGNC Symbol;Acc:HGNC:25410] |
| ENSG00000103199 | HGLibA_56107 | 328 | 2.115338317 | ZNF500 | zinc finger protein 500 [Source:HGNC Symbol;Acc:HGNC:23716] |
| ENSG00000106682 | HGLibA_14849 | 328 | 2.115338317 | EIF4H | eukaryotic translation initiation factor 4H [Source:HGNC Symbol;Acc:HGNC:12741] |
| ENSG00000108700 | HGLibA_08041 | 328 | 2.115338317 | CCL8 | C-C motif chemokine ligand 8 [Source:HGNC Symbol;Acc:HGNC:10635] |
| ENSG00000114904 | HGLibA_31653 | 328 | 2.115338317 | NEK4 | NIMA related kinase 4 [Source:HGNC Symbol;Acc:HGNC:11399] |
| ENSG00000117154 | HGLibA_22883 | 328 | 2.115338317 | IGSF21 | immunoglobin superfamily member 21 [Source:HGNC Symbol;Acc:HGNC:28246] |
| ENSG00000146399 | HGLibA_48124 | 328 | 2.115338317 | TAAR1 | trace amine associated receptor 1 [Source:HGNC Symbol;Acc:HGNC:17734] |
| ENSG00000156103 | HGLibA_29498 | 328 | 2.115338317 | MMP16 | matrix metallopeptidase 16 [Source:HGNC Symbol;Acc:HGNC:7162] |
| ENSG00000169884 | HGLibA_54350 | 328 | 2.115338317 | WNT10B | Wnt family member 10B [Source:HGNC Symbol;Acc:HGNC:12775] |
| ENSG00000205476 | HGLibA_07899 | 328 | 2.115338317 | CCDC85C | coiled-coil domain containing 85C [Source:HGNC Symbol;Acc:HGNC:35459] |
| ENSG00000241360 | HGLibA_35964 | 328 | 2.115338317 | PDXP | pyridoxal phosphatase [Source:HGNC Symbol;Acc:HGNC:30259] |
| ENSG00000242802 | HGLibA_02361 | 328 | 2.115338317 | AP5Z1 | adaptor related protein complex 5 subunit zeta 1 [Source:HGNC Symbol;Acc:HGNC:22197] |
| hsa-mir-8085 | HGLibA_64181 | 328 | 2.115338317 | hsa-mir-8085 | - |
| LOC100130370 | HGLibA_26782 | 328 | 2.115338317 | LOC100130370 | - |
| MTERFD3 | HGLibA_30346 | 328 | 2.115338317 | MTERFD3 | - |
| ACN9 | HGLibA_00458 | 327 | 2.105387279 | ACN9 | - |
| ENSG00000018280 | HGLibA_44351 | 327 | 2.105387279 | SLC11A1 | solute carrier family 11 member 1 [Source:HGNC Symbol;Acc:HGNC:10907] |
| ENSG00000067082 | HGLibA_25077 | 327 | 2.105387279 | KLF6 | Kruppel like factor 6 [Source:HGNC Symbol;Acc:HGNC:2235] |
| ENSG00000094631 | HGLibA_21018 | 327 | 2.105387279 | HDAC6 | histone deacetylase 6 [Source:HGNC Symbol;Acc:HGNC:14064] |
| ENSG00000106723 | HGLibA_46667 | 327 | 2.105387279 | SPIN1 | spindlin 1 [Source:HGNC Symbol;Acc:HGNC:11243] |
| ENSG00000109133 | HGLibA_50369 | 327 | 2.105387279 | TMEM33 | transmembrane protein 33 [Source:HGNC Symbol;Acc:HGNC:25541] |
| ENSG00000139200 | HGLibA_36504 | 327 | 2.105387279 | PIANP | PILR alpha associated neural protein [Source:HGNC Symbol;Acc:HGNC:25338] |
| ENSG00000140798 | HGLibA_00166 | 327 | 2.105387279 | ABCC12 | ATP binding cassette subfamily C member 12 [Source:HGNC Symbol;Acc:HGNC:14640] |
| ENSG00000144645 | HGLibA_34642 | 327 | 2.105387279 | OSBPL10 | oxysterol binding protein like 10 [Source:HGNC Symbol;Acc:HGNC:16395] |
| ENSG00000155090 | HGLibA_25041 | 327 | 2.105387279 | KLF10 | Kruppel like factor 10 [Source:HGNC Symbol;Acc:HGNC:11810] |
| ENSG00000159251 | HGLibA_00609 | 327 | 2.105387279 | ACTC1 | cardiac muscle 1 [Source:HGNC Symbol;Acc:HGNC:143]" |
| ENSG00000171135 | HGLibA_23910 | 327 | 2.105387279 | JAGN1 | jagunal homolog 1 [Source:HGNC Symbol;Acc:HGNC:26926] |
| ENSG00000172985 | HGLibA_43975 | 327 | 2.105387279 | SH3RF3 | SH3 domain containing ring finger 3 [Source:HGNC Symbol;Acc:HGNC:24699] |
| ENSG00000173404 | HGLibA_23393 | 327 | 2.105387279 | INSM1 | INSM transcriptional repressor 1 [Source:HGNC Symbol;Acc:HGNC:6090] |
| ENSG00000184368 | HGLibA_28238 | 327 | 2.105387279 | MAP7D2 | MAP7 domain containing 2 [Source:HGNC Symbol;Acc:HGNC:25899] |
| ENSG00000255587 | HGLibA_39953 | 327 | 2.105387279 | RAB44 | member RAS oncogene family [Source:HGNC Symbol;Acc:HGNC:21068]" |
| ENSG00000272602 | HGLibA_56348 | 327 | 2.105387279 | ZNF595 | zinc finger protein 595 [Source:HGNC Symbol;Acc:HGNC:27196] |

| | | | | | |
|---|---|---|---|---|---|
| C19orf45 | HGLibA_05667 | 326 | 2.095436242 | C19orf45 | - |
| ENSG00000097033 | HGLibA_43954 | 326 | 2.095436242 | SH3GLB1 | endophilin B1 [Source:HGNC Symbol;Acc:HGNC:10833]" |
| ENSG00000103485 | HGLibA_39732 | 326 | 2.095436242 | QPRT | quinolinate phosphoribosyltransferase [Source:HGNC Symbol;Acc:HGNC:9755] |
| ENSG00000115318 | HGLibA_27045 | 326 | 2.095436242 | LOXL3 | lysyl oxidase like 3 [Source:HGNC Symbol;Acc:HGNC:13869] |
| ENSG00000127481 | HGLibA_52757 | 326 | 2.095436242 | UBR4 | ubiquitin protein ligase E3 component n-recognin 4 [Source:HGNC Symbol;Acc:HGNC:30313] |
| ENSG00000129911 | HGLibA_25061 | 326 | 2.095436242 | KLF16 | Kruppel like factor 16 [Source:HGNC Symbol;Acc:HGNC:16857] |
| ENSG00000130544 | HGLibA_56230 | 326 | 2.095436242 | ZNF557 | zinc finger protein 557 [Source:HGNC Symbol;Acc:HGNC:28632] |
| ENSG00000130684 | HGLibA_55829 | 326 | 2.095436242 | ZNF337 | zinc finger protein 337 [Source:HGNC Symbol;Acc:HGNC:15809] |
| ENSG00000130957 | HGLibA_16966 | 326 | 2.095436242 | FBP2 | fructose-bisphosphatase 2 [Source:HGNC Symbol;Acc:HGNC:3607] |
| ENSG00000133027 | HGLibA_36044 | 326 | 2.095436242 | PEMT | phosphatidylethanolamine N-methyltransferase [Source:HGNC Symbol;Acc:HGNC:8830] |
| ENSG00000133710 | HGLibA_46697 | 326 | 2.095436242 | SPINK5 | Kazal type 5 [Source:HGNC Symbol;Acc:HGNC:15464]" |
| ENSG00000139433 | HGLibA_19344 | 326 | 2.095436242 | GLTP | glycolipid transfer protein [Source:HGNC Symbol;Acc:HGNC:24867] |
| ENSG00000152670 | HGLibA_12708 | 326 | 2.095436242 | DDX4 | DEAD-box helicase 4 [Source:HGNC Symbol;Acc:HGNC:18700] |
| ENSG00000162236 | HGLibA_47651 | 326 | 2.095436242 | STX5 | syntaxin 5 [Source:HGNC Symbol;Acc:HGNC:11440] |
| ENSG00000166415 | HGLibA_54166 | 326 | 2.095436242 | WDR72 | WD repeat domain 72 [Source:HGNC Symbol;Acc:HGNC:26790] |
| ENSG00000167543 | HGLibA_51063 | 326 | 2.095436242 | TP53I13 | tumor protein p53 inducible protein 13 [Source:HGNC Symbol;Acc:HGNC:25102] |
| ENSG00000175182 | HGLibA_16105 | 326 | 2.095436242 | FAM131A | family with sequence similarity 131 member A [Source:HGNC Symbol;Acc:HGNC:28308] |
| ENSG00000183153 | HGLibA_19191 | 326 | 2.095436242 | GJD3 | gap junction protein delta 3 [Source:HGNC Symbol;Acc:HGNC:19147] |
| ENSG00000183153 | HGLibA_19190 | 326 | 2.095436242 | GJD3 | gap junction protein delta 3 [Source:HGNC Symbol;Acc:HGNC:19147] |
| ENSG00000188293 | HGLibA_22838 | 326 | 2.095436242 | IGFL1 | IGF like family member 1 [Source:HGNC Symbol;Acc:HGNC:24093] |
| ENSG00000188883 | HGLibA_25312 | 326 | 2.095436242 | KLRG2 | killer cell lectin like receptor G2 [Source:HGNC Symbol;Acc:HGNC:24778] |
| ENSG00000204421 | HGLibA_27664 | 326 | 2.095436242 | LY6G6C | lymphocyte antigen 6 family member G6C [Source:HGNC Symbol;Acc:HGNC:13936] |
| HIST1H3B | HGLibA_21458 | 326 | 2.095436242 | HIST1H3B | - |
| HIST1H3D | HGLibA_21464 | 326 | 2.095436242 | HIST1H3D | - |
| hsa-mir-133a-2 | HGLibA_57777 | 326 | 2.095436242 | hsa-mir-133a-2 | - |
| hsa-mir-3653 | HGLibA_59156 | 326 | 2.095436242 | hsa-mir-3653 | - |
| hsa-mir-3976 | HGLibA_59665 | 326 | 2.095436242 | hsa-mir-3976 | - |
| C14orf166B | HGLibA_05336 | 325 | 2.085485204 | C14orf166B | - |
| C7orf72 | HGLibA_06586 | 325 | 2.085485204 | C7orf72 | - |
| ENSG00000101276 | HGLibA_45251 | 325 | 2.085485204 | SLC52A3 | solute carrier family 52 member 3 [Source:HGNC Symbol;Acc:HGNC:16187] |
| ENSG00000113389 | HGLibA_32514 | 325 | 2.085485204 | NPR3 | natriuretic peptide receptor 3 [Source:HGNC Symbol;Acc:HGNC:7945] |
| ENSG00000117410 | HGLibA_03713 | 325 | 2.085485204 | ATP6V0B | ATPase H+ transporting V0 subunit b [Source:HGNC Symbol;Acc:HGNC:861] |
| ENSG00000132031 | HGLibA_28434 | 325 | 2.085485204 | MATN3 | matrilin 3 [Source:HGNC Symbol;Acc:HGNC:6909] |
| ENSG00000132854 | HGLibA_24034 | 325 | 2.085485204 | KANK4 | KN motif and ankyrin repeat domains 4 [Source:HGNC Symbol;Acc:HGNC:27263] |
| ENSG00000138286 | HGLibA_16152 | 325 | 2.085485204 | FAM149B1 | family with sequence similarity 149 member B1 [Source:HGNC Symbol;Acc:HGNC:29162] |
| ENSG00000138316 | HGLibA_00802 | 325 | 2.085485204 | ADAMTS14 | ADAM metallopeptidase with thrombospondin type 1 motif 14 [Source:HGNC Symbol;Acc:HGNC:14899] |
| ENSG00000141096 | HGLibA_13825 | 325 | 2.085485204 | DPEP3 | dipeptidase 3 [Source:HGNC Symbol;Acc:HGNC:23029] |
| ENSG00000157368 | HGLibA_23141 | 325 | 2.085485204 | IL34 | interleukin 34 [Source:HGNC Symbol;Acc:HGNC:28529] |
| ENSG00000157837 | HGLibA_46783 | 325 | 2.085485204 | SPPL3 | signal peptide peptidase like 3 [Source:HGNC Symbol;Acc:HGNC:30424] |
| ENSG00000171989 | HGLibA_26225 | 325 | 2.085485204 | LDHAL6B | lactate dehydrogenase A like 6B [Source:HGNC Symbol;Acc:HGNC:21481] |
| ENSG00000173285 | HGLibA_33530 | 325 | 2.085485204 | OR10K1 | olfactory receptor family 10 subfamily K member 1 [Source:HGNC Symbol;Acc:HGNC:14693] |
| ENSG00000179104 | HGLibA_50672 | 325 | 2.085485204 | TMTC2 | transmembrane and tetratricopeptide repeat containing 2 [Source:HGNC Symbol;Acc:HGNC:25440] |
| ENSG00000184937 | HGLibA_54426 | 325 | 2.085485204 | WT1 | Wilms tumor 1 [Source:HGNC Symbol;Acc:HGNC:12796] |
| ENSG00000196475 | HGLibA_19199 | 325 | 2.085485204 | GK2 | glycerol kinase 2 [Source:HGNC Symbol;Acc:HGNC:4291] |
| ENSG00000203784 | HGLibA_26282 | 325 | 2.085485204 | LELP1 | late cornified envelope like proline rich 1 [Source:HGNC Symbol;Acc:HGNC:32046] |
| HIST1H1T | HGLibA_21373 | 325 | 2.085485204 | HIST1H1T | - |
| hsa-mir-551b | HGLibA_62121 | 325 | 2.085485204 | hsa-mir-551b | - |
| MKL2 | HGLibA_29336 | 325 | 2.085485204 | MKL2 | - |
| MLLT4 | HGLibA_29421 | 325 | 2.085485204 | MLLT4 | - |
| ENSG00000004399 | HGLibA_37281 | 324 | 2.075534167 | PLXND1 | plexin D1 [Source:HGNC Symbol;Acc:HGNC:9107] |
| ENSG00000007933 | HGLibA_17708 | 324 | 2.075534167 | FMO3 | flavin containing monooxygenase 3 [Source:HGNC Symbol;Acc:HGNC:3771] |
| ENSG00000100644 | HGLibA_21298 | 324 | 2.075534167 | HIF1A | hypoxia inducible factor 1 subunit alpha [Source:HGNC Symbol;Acc:HGNC:4910] |
| ENSG00000101150 | HGLibA_51117 | 324 | 2.075534167 | TPD52L2 | TPD52 like 2 [Source:HGNC Symbol;Acc:HGNC:12007] |
| ENSG00000102048 | HGLibA_03254 | 324 | 2.075534167 | ASB9 | ankyrin repeat and SOCS box containing 9 [Source:HGNC Symbol;Acc:HGNC:17184] |
| ENSG00000107263 | HGLibA_40246 | 324 | 2.075534167 | RAPGEF1 | Rap guanine nucleotide exchange factor 1 [Source:HGNC Symbol;Acc:HGNC:4568] |
| ENSG00000129474 | HGLibA_01372 | 324 | 2.075534167 | AJUBA | ajuba LIM protein [Source:HGNC Symbol;Acc:HGNC:20250] |
| ENSG00000130755 | HGLibA_19405 | 324 | 2.075534167 | GMFG | glia maturation factor gamma [Source:HGNC Symbol;Acc:HGNC:4374] |
| ENSG00000133818 | HGLibA_42238 | 324 | 2.075534167 | RRAS2 | RAS related 2 [Source:HGNC Symbol;Acc:HGNC:17271] |
| ENSG00000136297 | HGLibA_29465 | 324 | 2.075534167 | MMD2 | monocyte to macrophage differentiation associated 2 [Source:HGNC Symbol;Acc:HGNC:30133] |
| ENSG00000145839 | HGLibA_23198 | 324 | 2.075534167 | IL9 | interleukin 9 [Source:HGNC Symbol;Acc:HGNC:6029] |
| ENSG00000162706 | HGLibA_06971 | 324 | 2.075534167 | CADM3 | cell adhesion molecule 3 [Source:HGNC Symbol;Acc:HGNC:17601] |
| ENSG00000162819 | HGLibA_04836 | 324 | 2.075534167 | BROX | BRO1 domain and CAAX motif containing [Source:HGNC Symbol;Acc:HGNC:26512] |
| ENSG00000163873 | HGLibA_20288 | 324 | 2.075534167 | GRIK3 | glutamate ionotropic receptor kainate type subunit 3 [Source:HGNC Symbol;Acc:HGNC:4581] |
| ENSG00000170390 | HGLibA_12502 | 324 | 2.075534167 | DCLK2 | doublecortin like kinase 2 [Source:HGNC Symbol;Acc:HGNC:19002] |
| ENSG00000181541 | HGLibA_27824 | 324 | 2.075534167 | MAB21L2 | mab-21 like 2 [Source:HGNC Symbol;Acc:HGNC:6758] |
| ENSG00000182732 | HGLibA_41100 | 324 | 2.075534167 | RGS6 | regulator of G protein signaling 6 [Source:HGNC Symbol;Acc:HGNC:10002] |
| ENSG00000187021 | HGLibA_37357 | 324 | 2.075534167 | PNLIPRP1 | pancreatic lipase related protein 1 [Source:HGNC Symbol;Acc:HGNC:9156] |
| ENSG00000196074 | HGLibA_47930 | 324 | 2.075534167 | SYCP2 | synaptonemal complex protein 2 [Source:HGNC Symbol;Acc:HGNC:11490] |
| ENSG00000196850 | HGLibA_38240 | 324 | 2.075534167 | PPTC7 | PTC7 protein phosphatase homolog [Source:HGNC Symbol;Acc:HGNC:30695] |
| ENSG00000206190 | HGLibA_03545 | 324 | 2.075534167 | ATP10A | ATPase phospholipid transporting 10A (putative) [Source:HGNC Symbol;Acc:HGNC:13542] |
| FOPNL | HGLibA_17791 | 324 | 2.075534167 | FOPNL | - |
| KIAA1644 | HGLibA_24797 | 324 | 2.075534167 | KIAA1644 | - |
| C1orf101 | HGLibA_05733 | 323 | 2.065583129 | C1orf101 | - |
| ENSG00000050165 | HGLibA_13299 | 323 | 2.065583129 | DKK3 | dickkopf WNT signaling pathway inhibitor 3 [Source:HGNC Symbol;Acc:HGNC:2893] |
| ENSG00000063127 | HGLibA_45306 | 323 | 2.065583129 | SLC6A16 | solute carrier family 6 member 16 [Source:HGNC Symbol;Acc:HGNC:13622] |
| ENSG00000095596 | HGLibA_12085 | 323 | 2.065583129 | CYP26A1 | cytochrome P450 family 26 subfamily A member 1 [Source:HGNC Symbol;Acc:HGNC:2603] |
| ENSG00000100055 | HGLibA_12245 | 323 | 2.065583129 | CYTH4 | cytohesin 4 [Source:HGNC Symbol;Acc:HGNC:9505] |
| ENSG00000101104 | HGLibA_34924 | 323 | 2.065583129 | PABPC1L | poly(A) binding protein cytoplasmic 1 like [Source:HGNC Symbol;Acc:HGNC:15797] |
| ENSG00000113805 | HGLibA_10406 | 323 | 2.065583129 | CNTN3 | contactin 3 [Source:HGNC Symbol;Acc:HGNC:2173] |
| ENSG00000119125 | HGLibA_18835 | 323 | 2.065583129 | GDA | guanine deaminase [Source:HGNC Symbol;Acc:HGNC:4212] |
| ENSG00000124479 | HGLibA_31409 | 323 | 2.065583129 | NDP | norrin cystine knot growth factor [Source:HGNC Symbol;Acc:HGNC:7678]" |
| ENSG00000125888 | HGLibA_04142 | 323 | 2.065583129 | BANF2 | barrier to autointegration factor 2 [Source:HGNC Symbol;Acc:HGNC:16172] |
| ENSG00000131480 | HGLibA_02269 | 323 | 2.065583129 | AOC2 | copper containing 2 [Source:HGNC Symbol;Acc:HGNC:549]" |
| ENSG00000134538 | HGLibA_45449 | 323 | 2.065583129 | SLCO1B1 | solute carrier organic anion transporter family member 1B1 [Source:HGNC Symbol;Acc:HGNC:10959] |
| ENSG00000134640 | HGLibA_30449 | 323 | 2.065583129 | MTNR1B | melatonin receptor 1B [Source:HGNC Symbol;Acc:HGNC:7464] |
| ENSG00000136286 | HGLibA_30838 | 323 | 2.065583129 | MYO1G | myosin IG [Source:HGNC Symbol;Acc:HGNC:13880] |
| ENSG00000137824 | HGLibA_41370 | 323 | 2.065583129 | RMDN3 | regulator of microtubule dynamics 3 [Source:HGNC Symbol;Acc:HGNC:25550] |
| ENSG00000141655 | HGLibA_50746 | 323 | 2.065583129 | TNFRSF11A | TNF receptor superfamily member 11a [Source:HGNC Symbol;Acc:HGNC:11908] |
| ENSG00000145907 | HGLibA_18299 | 323 | 2.065583129 | G3BP1 | G3BP stress granule assembly factor 1 [Source:HGNC Symbol;Acc:HGNC:30292] |
| ENSG00000148057 | HGLibA_22560 | 323 | 2.065583129 | IDNK | gluconokinase [Source:HGNC Symbol;Acc:HGNC:31367]" |
| ENSG00000157045 | HGLibA_32836 | 323 | 2.065583129 | NTAN1 | N-terminal asparagine amidase [Source:HGNC Symbol;Acc:HGNC:29909] |
| ENSG00000168875 | HGLibA_46241 | 323 | 2.065583129 | SOX14 | SRY-box 14 [Source:HGNC Symbol;Acc:HGNC:11193] |
| ENSG00000172742 | HGLibA_33984 | 323 | 2.065583129 | OR4D9 | olfactory receptor family 4 subfamily D member 9 [Source:HGNC Symbol;Acc:HGNC:15178] |
| ENSG00000174343 | HGLibA_09585 | 323 | 2.065583129 | CHRNA9 | cholinergic receptor nicotinic alpha 9 subunit [Source:HGNC Symbol;Acc:HGNC:14079] |
| ENSG00000178401 | HGLibA_13599 | 323 | 2.065583129 | DNAJC22 | DnaJ heat shock protein family (Hsp40) member C22 [Source:HGNC Symbol;Acc:HGNC:25802] |
| ENSG00000179152 | HGLibA_48690 | 323 | 2.065583129 | TCAIM | mitochondrial [Source:HGNC Symbol;Acc:HGNC:25241]" |
| ENSG00000203952 | HGLibA_07671 | 323 | 2.065583129 | CCDC160 | coiled-coil domain containing 160 [Source:HGNC Symbol;Acc:HGNC:37286] |
| hsa-mir-187 | HGLibA_58009 | 323 | 2.065583129 | hsa-mir-187 | - |
| hsa-mir-6858 | HGLibA_63606 | 323 | 2.065583129 | hsa-mir-6858 | - |
| ENSG00000100504 | HGLibA_39687 | 322 | 2.055632092 | PYGL | glycogen phosphorylase L [Source:HGNC Symbol;Acc:HGNC:9725] |
| ENSG00000101096 | HGLibA_31775 | 322 | 2.055632092 | NFATC2 | nuclear factor of activated T cells 2 [Source:HGNC Symbol;Acc:HGNC:7776] |
| ENSG00000102010 | HGLibA_04641 | 322 | 2.055632092 | BMX | BMX non-receptor tyrosine kinase [Source:HGNC Symbol;Acc:HGNC:1079] |
| ENSG00000104728 | HGLibA_02808 | 322 | 2.055632092 | ARHGEF10 | Rho guanine nucleotide exchange factor 10 [Source:HGNC Symbol;Acc:HGNC:14103] |
| ENSG00000109832 | HGLibA_12681 | 322 | 2.055632092 | DDX25 | DEAD-box helicase 25 [Source:HGNC Symbol;Acc:HGNC:18698] |
| ENSG00000110075 | HGLibA_38228 | 322 | 2.055632092 | PPP6R3 | protein phosphatase 6 regulatory subunit 3 [Source:HGNC Symbol;Acc:HGNC:1173] |
| ENSG00000120328 | HGLibA_35477 | 322 | 2.055632092 | PCDHB12 | protocadherin beta 12 [Source:HGNC Symbol;Acc:HGNC:8683] |
| ENSG00000121039 | HGLibA_40730 | 322 | 2.055632092 | RDH10 | retinol dehydrogenase 10 [Source:HGNC Symbol;Acc:HGNC:19975] |
| ENSG00000124357 | HGLibA_31065 | 322 | 2.055632092 | NAGK | N-acetylglucosamine kinase [Source:HGNC Symbol;Acc:HGNC:17174] |
| ENSG00000130055 | HGLibA_18892 | 322 | 2.055632092 | GDPD2 | glycerophosphodiester phosphodiesterase domain containing 2 [Source:HGNC Symbol;Acc:HGNC:25974] |
| ENSG00000131080 | HGLibA_14432 | 322 | 2.055632092 | EDA2R | ectodysplasin A2 receptor [Source:HGNC Symbol;Acc:HGNC:17756] |
| ENSG00000132879 | HGLibA_17105 | 322 | 2.055632092 | FBXO44 | F-box protein 44 [Source:HGNC Symbol;Acc:HGNC:24847] |
| ENSG00000134321 | HGLibA_42298 | 322 | 2.055632092 | RSAD2 | radical S-adenosyl methionine domain containing 2 [Source:HGNC Symbol;Acc:HGNC:30908] |
| ENSG00000136040 | HGLibA_37278 | 322 | 2.055632092 | PLXNC1 | plexin C1 [Source:HGNC Symbol;Acc:HGNC:9106] |
| ENSG00000136487 | HGLibA_19030 | 322 | 2.055632092 | GH2 | growth hormone 2 [Source:HGNC Symbol;Acc:HGNC:4262] |
| ENSG00000139687 | HGLibA_40413 | 322 | 2.055632092 | RB1 | RB transcriptional corepressor 1 [Source:HGNC Symbol;Acc:HGNC:9884] |
| ENSG00000140297 | HGLibA_18815 | 322 | 2.055632092 | GCNT3 | mucin type [Source:HGNC Symbol;Acc:HGNC:4205]" |
| ENSG00000158321 | HGLibA_03886 | 322 | 2.055632092 | AUTS2 | activator of transcription and developmental regulator [Source:HGNC Symbol;Acc:HGNC:14262]" |
| ENSG00000160219 | HGLibA_18326 | 322 | 2.055632092 | GAB3 | GRB2 associated binding protein 3 [Source:HGNC Symbol;Acc:HGNC:17515] |
| ENSG00000162989 | HGLibA_24277 | 322 | 2.055632092 | KCNJ3 | potassium voltage-gated channel subfamily J member 3 [Source:HGNC Symbol;Acc:HGNC:6264] |
| ENSG00000162998 | HGLibA_18059 | 322 | 2.055632092 | FRZB | frizzled related protein [Source:HGNC Symbol;Acc:HGNC:3959] |
| ENSG00000163686 | HGLibA_00284 | 322 | 2.055632092 | ABHD6 | abhydrolase domain containing 6 [Source:HGNC Symbol;Acc:HGNC:21398] |
| ENSG00000164512 | HGLibA_02126 | 322 | 2.055632092 | ANKRD55 | ankyrin repeat domain 55 [Source:HGNC Symbol;Acc:HGNC:25681] |
| ENSG00000164532 | HGLibA_48660 | 322 | 2.055632092 | TBX20 | T-box 20 [Source:HGNC Symbol;Acc:HGNC:11598] |
| ENSG00000164825 | HGLibA_12810 | 322 | 2.055632092 | DEFB1 | defensin beta 1 [Source:HGNC Symbol;Acc:HGNC:2766] |
| ENSG00000165502 | HGLibA_41960 | 322 | 2.055632092 | RPL36AL | ribosomal protein L36a like [Source:HGNC Symbol;Acc:HGNC:10346] |
| ENSG00000166501 | HGLibA_38536 | 322 | 2.055632092 | PRKCB | protein kinase C beta [Source:HGNC Symbol;Acc:HGNC:9395] |
| ENSG00000172346 | HGLibA_11313 | 322 | 2.055632092 | CSDC2 | cold shock domain containing C2 [Source:HGNC Symbol;Acc:HGNC:30359] |
| ENSG00000172530 | HGLibA_04149 | 322 | 2.055632092 | BANP | BTG3 associated nuclear protein [Source:HGNC Symbol;Acc:HGNC:13450] |
| ENSG00000173218 | HGLibA_53512 | 322 | 2.055632092 | VANGL1 | VANGL planar cell polarity protein 1 [Source:HGNC Symbol;Acc:HGNC:15512] |
| ENSG00000175348 | HGLibA_50576 | 322 | 2.055632092 | TMEM9B | TMEM9 domain family member B [Source:HGNC Symbol;Acc:HGNC:1168] |
| ENSG00000180304 | HGLibA_33217 | 322 | 2.055632092 | OAZ2 | ornithine decarboxylase antizyme 2 [Source:HGNC Symbol;Acc:HGNC:8096] |
| ENSG00000182771 | HGLibA_20273 | 322 | 2.055632092 | GRID1 | glutamate ionotropic receptor delta type subunit 1 [Source:HGNC Symbol;Acc:HGNC:4575] |
| ENSG00000187325 | HGLibA_48263 | 322 | 2.055632092 | TAF9B | TATA-box binding protein associated factor 9b [Source:HGNC Symbol;Acc:HGNC:17306] |
| ENSG00000197142 | HGLibA_00563 | 322 | 2.055632092 | ACSL5 | acyl-CoA synthetase long chain family member 5 [Source:HGNC Symbol;Acc:HGNC:16526] |
| ENSG00000198851 | HGLibA_08373 | 322 | 2.055632092 | CD3E | CD3e molecule [Source:HGNC Symbol;Acc:HGNC:1674] |

| | | | | | |
|---|---|---|---|---|---|
| ENSG00000215009 | HGLibA_00580 | 322 | 2.055632092 | ACSM4 | acyl-CoA synthetase medium chain family member 4 [Source:HGNC Symbol;Acc:HGNC:32016] |
| hsa-mir-6857 | HGLibA_63602 | 322 | 2.055632092 | hsa-mir-6857 | - |
| C7orf49 | HGLibA_06547 | 321 | 2.045681055 | C7orf49 | - |
| CECR6 | HGLibA_08983 | 321 | 2.045681055 | CECR6 | - |
| ENSG00000105583 | HGLibA_54197 | 321 | 2.045681055 | WDR83OS | WD repeat domain 83 opposite strand [Source:HGNC Symbol;Acc:HGNC:30203] |
| ENSG00000115255 | HGLibA_40791 | 321 | 2.045681055 | REEP6 | receptor accessory protein 6 [Source:HGNC Symbol;Acc:HGNC:30078] |
| ENSG00000115353 | HGLibA_48170 | 321 | 2.045681055 | TACR1 | tachykinin receptor 1 [Source:HGNC Symbol;Acc:HGNC:11526] |
| ENSG00000116221 | HGLibA_29966 | 321 | 2.045681055 | MRPL37 | mitochondrial ribosomal protein L37 [Source:HGNC Symbol;Acc:HGNC:14034] |
| ENSG00000119401 | HGLibA_51462 | 321 | 2.045681055 | TRIM32 | tripartite motif containing 32 [Source:HGNC Symbol;Acc:HGNC:16380] |
| ENSG00000138030 | HGLibA_24574 | 321 | 2.045681055 | KHK | ketohexokinase [Source:HGNC Symbol;Acc:HGNC:6315] |
| ENSG00000143258 | HGLibA_53302 | 321 | 2.045681055 | USP21 | ubiquitin specific peptidase 21 [Source:HGNC Symbol;Acc:HGNC:12620] |
| ENSG00000155719 | HGLibA_34723 | 321 | 2.045681055 | OTOA | otoancorin [Source:HGNC Symbol;Acc:HGNC:16378] |
| ENSG00000157837 | HGLibA_46784 | 321 | 2.045681055 | SPPL3 | signal peptide peptidase like 3 [Source:HGNC Symbol;Acc:HGNC:30424] |
| ENSG00000161682 | HGLibA_16268 | 321 | 2.045681055 | FAM171A2 | family with sequence similarity 171 member A2 [Source:HGNC Symbol;Acc:HGNC:30480] |
| ENSG00000174165 | HGLibA_55111 | 321 | 2.045681055 | ZDHHC24 | zinc finger DHHC-type containing 24 [Source:HGNC Symbol;Acc:HGNC:27387] |
| ENSG00000175029 | HGLibA_11587 | 321 | 2.045681055 | CTBP2 | C-terminal binding protein 2 [Source:HGNC Symbol;Acc:HGNC:2495] |
| ENSG00000182224 | HGLibA_11994 | 321 | 2.045681055 | CYB5D1 | cytochrome b5 domain containing 1 [Source:HGNC Symbol;Acc:HGNC:26516] |
| ENSG00000183230 | HGLibA_11644 | 321 | 2.045681055 | CTNNA3 | catenin alpha 3 [Source:HGNC Symbol;Acc:HGNC:2511] |
| ENSG00000197683 | HGLibA_25732 | 321 | 2.045681055 | KRTAP26-1 | keratin associated protein 26-1 [Source:HGNC Symbol;Acc:HGNC:33760] |
| ENSG00000198944 | HGLibA_46213 | 321 | 2.045681055 | SOWAHA | sosondowah ankyrin repeat domain family member A [Source:HGNC Symbol;Acc:HGNC:27033] |
| ENSG00000204967 | HGLibA_35446 | 321 | 2.045681055 | PCDHA4 | protocadherin alpha 4 [Source:HGNC Symbol;Acc:HGNC:8670] |
| ENSG00000258289 | HGLibA_09673 | 321 | 2.045681055 | CHURC1 | churchill domain containing 1 [Source:HGNC Symbol;Acc:HGNC:20099] |
| ENSG00000267978 | HGLibA_27925 | 321 | 2.045681055 | MAGEA9B | MAGE family member A9B [Source:HGNC Symbol;Acc:HGNC:31909] |
| ENSG00000087460 | HGLibA_19470 | 320 | 2.035730017 | GNAS | GNAS complex locus [Source:HGNC Symbol;Acc:HGNC:4392] |
| ENSG00000099849 | HGLibA_40392 | 320 | 2.035730017 | RASSF7 | Ras association domain family member 7 [Source:HGNC Symbol;Acc:HGNC:1166] |
| ENSG00000101464 | HGLibA_36591 | 320 | 2.035730017 | PIGU | phosphatidylinositol glycan anchor biosynthesis class U [Source:HGNC Symbol;Acc:HGNC:15791] |
| ENSG00000114933 | HGLibA_23328 | 320 | 2.035730017 | INO80D | INO80 complex subunit D [Source:HGNC Symbol;Acc:HGNC:25997] |
| ENSG00000120509 | HGLibA_35969 | 320 | 2.035730017 | PDZD11 | PDZ domain containing 11 [Source:HGNC Symbol;Acc:HGNC:28034] |
| ENSG00000124391 | HGLibA_22995 | 320 | 2.035730017 | IL17C | interleukin 17C [Source:HGNC Symbol;Acc:HGNC:5983] |
| ENSG00000130433 | HGLibA_06944 | 320 | 2.035730017 | CACNG6 | calcium voltage-gated channel auxiliary subunit gamma 6 [Source:HGNC Symbol;Acc:HGNC:13625] |
| ENSG00000130958 | HGLibA_44979 | 320 | 2.035730017 | SLC35D2 | solute carrier family 35 member D2 [Source:HGNC Symbol;Acc:HGNC:20799] |
| ENSG00000137709 | HGLibA_37762 | 320 | 2.035730017 | POU2F3 | POU class 2 homeobox 3 [Source:HGNC Symbol;Acc:HGNC:19864] |
| ENSG00000140743 | HGLibA_08866 | 320 | 2.035730017 | CDR2 | cerebellar degeneration related protein 2 [Source:HGNC Symbol;Acc:HGNC:1799] |
| ENSG00000141965 | HGLibA_17274 | 320 | 2.035730017 | FEM1A | fem-1 homolog A [Source:HGNC Symbol;Acc:HGNC:16934] |
| ENSG00000143507 | HGLibA_14129 | 320 | 2.035730017 | DUSP10 | dual specificity phosphatase 10 [Source:HGNC Symbol;Acc:HGNC:3065] |
| ENSG00000147160 | HGLibA_03916 | 320 | 2.035730017 | AWAT2 | acyl-CoA wax alcohol acyltransferase 2 [Source:HGNC Symbol;Acc:HGNC:23251] |
| ENSG00000147459 | HGLibA_13748 | 320 | 2.035730017 | DOCK5 | dedicator of cytokinesis 5 [Source:HGNC Symbol;Acc:HGNC:23476] |
| ENSG00000147804 | HGLibA_45111 | 320 | 2.035730017 | SLC39A4 | solute carrier family 39 member 4 [Source:HGNC Symbol;Acc:HGNC:17129] |
| ENSG00000148296 | HGLibA_47842 | 320 | 2.035730017 | SURF6 | surfeit 6 [Source:HGNC Symbol;Acc:HGNC:11478] |
| ENSG00000148602 | HGLibA_27184 | 320 | 2.035730017 | LRIT1 | Ig-like and transmembrane domains 1 [Source:HGNC Symbol;Acc:HGNC:23404]" |
| ENSG00000150676 | HGLibA_07886 | 320 | 2.035730017 | CCDC83 | coiled-coil domain containing 83 [Source:HGNC Symbol;Acc:HGNC:28535] |
| ENSG00000151327 | HGLibA_16294 | 320 | 2.035730017 | FAM177A1 | family with sequence similarity 177 member A1 [Source:HGNC Symbol;Acc:HGNC:19829] |
| ENSG00000162458 | HGLibA_16939 | 320 | 2.035730017 | FBLIM1 | filamin binding LIM protein 1 [Source:HGNC Symbol;Acc:HGNC:24686] |
| ENSG00000171860 | HGLibA_06206 | 320 | 2.035730017 | C3AR1 | complement C3a receptor 1 [Source:HGNC Symbol;Acc:HGNC:1319] |
| ENSG00000175077 | HGLibA_42421 | 320 | 2.035730017 | RTP1 | receptor transporter protein 1 [Source:HGNC Symbol;Acc:HGNC:28580] |
| ENSG00000176782 | HGLibA_12820 | 320 | 2.035730017 | DEFB104A | defensin beta 104A [Source:HGNC Symbol;Acc:HGNC:18115] |
| ENSG00000177599 | HGLibA_56093 | 320 | 2.035730017 | ZNF491 | zinc finger protein 491 [Source:HGNC Symbol;Acc:HGNC:23706] |
| ENSG00000182557 | HGLibA_46739 | 320 | 2.035730017 | SPNS3 | sphingolipid transporter 3 (putative) [Source:HGNC Symbol;Acc:HGNC:28433] |
| ENSG00000183569 | HGLibA_43482 | 320 | 2.035730017 | SERHL2 | serine hydrolase like 2 [Source:HGNC Symbol;Acc:HGNC:29446] |
| ENSG00000184497 | HGLibA_50334 | 320 | 2.035730017 | TMEM255B | transmembrane protein 255B [Source:HGNC Symbol;Acc:HGNC:28297] |
| ENSG00000186832 | HGLibA_25433 | 320 | 2.035730017 | KRT16 | keratin 16 [Source:HGNC Symbol;Acc:HGNC:6423] |
| ENSG00000203705 | HGLibA_48445 | 320 | 2.035730017 | TATDN3 | TatD DNase domain containing 3 [Source:HGNC Symbol;Acc:HGNC:27010] |
| ENSG00000204252 | HGLibA_21609 | 320 | 2.035730017 | HLA-DOA | DO alpha [Source:HGNC Symbol;Acc:HGNC:4936]" |
| ENSG00000254656 | HGLibA_42396 | 320 | 2.035730017 | RTL1 | retrotransposon Gag like 1 [Source:HGNC Symbol;Acc:HGNC:14665] |
| ENSG00000255582 | HGLibA_33489 | 320 | 2.035730017 | OR10G2 | olfactory receptor family 10 subfamily G member 2 [Source:HGNC Symbol;Acc:HGNC:8170] |
| FAM19A4 | HGLibA_16405 | 320 | 2.035730017 | FAM19A4 | - |
| hsa-mir-4666b | HGLibA_60783 | 320 | 2.035730017 | hsa-mir-4666b | - |
| hsa-mir-4701 | HGLibA_60911 | 320 | 2.035730017 | hsa-mir-4701 | - |
| hsa-mir-4717 | HGLibA_60969 | 320 | 2.035730017 | hsa-mir-4717 | - |
| hsa-mir-6808 | HGLibA_63409 | 320 | 2.035730017 | hsa-mir-6808 | - |
| ZNF812 | HGLibA_56802 | 320 | 2.035730017 | ZNF812 | - |
| C11orf35 | HGLibA_05132 | 319 | 2.02577898 | C11orf35 | - |
| ENSG00000076067 | HGLibA_40589 | 319 | 2.02577898 | RBMS2 | RNA binding motif single stranded interacting protein 2 [Source:HGNC Symbol;Acc:HGNC:9909] |
| ENSG00000105750 | HGLibA_56868 | 319 | 2.02577898 | ZNF85 | zinc finger protein 85 [Source:HGNC Symbol;Acc:HGNC:13160] |
| ENSG00000111581 | HGLibA_33017 | 319 | 2.02577898 | NUP107 | nucleoporin 107 [Source:HGNC Symbol;Acc:HGNC:29914] |
| ENSG00000119686 | HGLibA_17678 | 319 | 2.02577898 | FLVCR2 | feline leukemia virus subgroup C cellular receptor family member 2 [Source:HGNC Symbol;Acc:HGNC:20105] |
| ENSG00000119714 | HGLibA_20078 | 319 | 2.02577898 | GPR68 | G protein-coupled receptor 68 [Source:HGNC Symbol;Acc:HGNC:4519] |
| ENSG00000121270 | HGLibA_00164 | 319 | 2.02577898 | ABCC11 | ATP binding cassette subfamily C member 11 [Source:HGNC Symbol;Acc:HGNC:14639] |
| ENSG00000125538 | HGLibA_23039 | 319 | 2.02577898 | IL1B | interleukin 1 beta [Source:HGNC Symbol;Acc:HGNC:5992] |
| ENSG00000136928 | HGLibA_18345 | 319 | 2.02577898 | GABBR2 | gamma-aminobutyric acid type B receptor subunit 2 [Source:HGNC Symbol;Acc:HGNC:4507] |
| ENSG00000146757 | HGLibA_56907 | 319 | 2.02577898 | ZNF92 | zinc finger protein 92 [Source:HGNC Symbol;Acc:HGNC:13168] |
| ENSG00000147041 | HGLibA_48107 | 319 | 2.02577898 | SYTL5 | synaptotagmin like 5 [Source:HGNC Symbol;Acc:HGNC:15589] |
| ENSG00000147124 | HGLibA_55934 | 319 | 2.02577898 | ZNF41 | zinc finger protein 41 [Source:HGNC Symbol;Acc:HGNC:13107] |
| ENSG00000162391 | HGLibA_16163 | 319 | 2.02577898 | FAM151A | family with sequence similarity 151 member A [Source:HGNC Symbol;Acc:HGNC:25032] |
| ENSG00000162624 | HGLibA_26465 | 319 | 2.02577898 | LHX8 | LIM homeobox 8 [Source:HGNC Symbol;Acc:HGNC:28838] |
| ENSG00000162877 | HGLibA_37285 | 319 | 2.02577898 | PM20D1 | peptidase M20 domain containing 1 [Source:HGNC Symbol;Acc:HGNC:26518] |
| ENSG00000166289 | HGLibA_37095 | 319 | 2.02577898 | PLEKHF1 | pleckstrin homology and FYVE domain containing 1 [Source:HGNC Symbol;Acc:HGNC:20764] |
| ENSG00000169877 | HGLibA_01323 | 319 | 2.02577898 | AHSP | alpha hemoglobin stabilizing protein [Source:HGNC Symbol;Acc:HGNC:18075] |
| ENSG00000184313 | HGLibA_29876 | 319 | 2.02577898 | MROH7 | maestro heat like repeat family member 7 [Source:HGNC Symbol;Acc:HGNC:24802] |
| ENSG00000185823 | HGLibA_32390 | 319 | 2.02577898 | NPAP1 | nuclear pore associated protein 1 [Source:HGNC Symbol;Acc:HGNC:1190] |
| ENSG00000187122 | HGLibA_45505 | 319 | 2.02577898 | SLIT1 | slit guidance ligand 1 [Source:HGNC Symbol;Acc:HGNC:11085] |
| ENSG00000198417 | HGLibA_30285 | 319 | 2.02577898 | MT1F | metallothionein 1F [Source:HGNC Symbol;Acc:HGNC:7398] |
| ENSG00000198668 | HGLibA_07021 | 319 | 2.02577898 | CALM1 | calmodulin 1 [Source:HGNC Symbol;Acc:HGNC:1442] |
| ENSG00000213185 | HGLibA_16549 | 319 | 2.02577898 | FAM24B | family with sequence similarity 24 member B [Source:HGNC Symbol;Acc:HGNC:23475] |
| hsa-mir-485 | HGLibA_61331 | 319 | 2.02577898 | hsa-mir-485 | - |
| hsa-mir-542 | HGLibA_61820 | 319 | 2.02577898 | hsa-mir-542 | - |
| hsa-mir-758 | HGLibA_63893 | 319 | 2.02577898 | hsa-mir-758 | - |
| ENSG00000064115 | HGLibA_49717 | 318 | 2.015827942 | TM7SF3 | transmembrane 7 superfamily member 3 [Source:HGNC Symbol;Acc:HGNC:23049] |
| ENSG00000078900 | HGLibA_51096 | 318 | 2.015827942 | TP73 | tumor protein p73 [Source:HGNC Symbol;Acc:HGNC:12003] |
| ENSG00000100600 | HGLibA_26406 | 318 | 2.015827942 | LGMN | legumain [Source:HGNC Symbol;Acc:HGNC:9472] |
| ENSG00000100842 | HGLibA_14613 | 318 | 2.015827942 | EFS | embryonal Fyn-associated substrate [Source:HGNC Symbol;Acc:HGNC:16898] |
| ENSG00000101825 | HGLibA_30621 | 318 | 2.015827942 | MXRA5 | matrix remodeling associated 5 [Source:HGNC Symbol;Acc:HGNC:7539] |
| ENSG00000116871 | HGLibA_28234 | 318 | 2.015827942 | MAP7D1 | MAP7 domain containing 1 [Source:HGNC Symbol;Acc:HGNC:25514] |
| ENSG00000117069 | HGLibA_47277 | 318 | 2.015827942 | ST6GALNAC5 | 6-sialyltransferase 5 [Source:HGNC Symbol;Acc:HGNC:19342]" |
| ENSG00000123576 | HGLibA_15601 | 318 | 2.015827942 | ESX1 | ESX homeobox 1 [Source:HGNC Symbol;Acc:HGNC:14865] |
| ENSG00000142149 | HGLibA_22423 | 318 | 2.015827942 | HUNK | hormonally up-regulated Neu-associated kinase [Source:HGNC Symbol;Acc:HGNC:13326] |
| ENSG00000160271 | HGLibA_40165 | 318 | 2.015827942 | RALGDS | ral guanine nucleotide dissociation stimulator [Source:HGNC Symbol;Acc:HGNC:9842] |
| ENSG00000160345 | HGLibA_06664 | 318 | 2.015827942 | C9orf116 | chromosome 9 open reading frame 116 [Source:HGNC Symbol;Acc:HGNC:28435] |
| ENSG00000165495 | HGLibA_36848 | 318 | 2.015827942 | PKNOX2 | PBX/knotted 1 homeobox 2 [Source:HGNC Symbol;Acc:HGNC:16714] |
| ENSG00000167110 | HGLibA_19595 | 318 | 2.015827942 | GOLGA2 | golgin A2 [Source:HGNC Symbol;Acc:HGNC:4425] |
| ENSG00000176454 | HGLibA_27080 | 318 | 2.015827942 | LPCAT4 | lysophosphatidylcholine acyltransferase 4 [Source:HGNC Symbol;Acc:HGNC:30059] |
| ENSG00000177174 | HGLibA_33631 | 318 | 2.015827942 | OR14C36 | olfactory receptor family 14 subfamily C member 36 [Source:HGNC Symbol;Acc:HGNC:15026] |
| ENSG00000177628 | HGLibA_18709 | 318 | 2.015827942 | GBA | glucosylceramidase beta [Source:HGNC Symbol;Acc:HGNC:4177] |
| ENSG00000179774 | HGLibA_03537 | 318 | 2.015827942 | ATOH7 | atonal bHLH transcription factor 7 [Source:HGNC Symbol;Acc:HGNC:13907] |
| ENSG00000186925 | HGLibA_25678 | 318 | 2.015827942 | KRTAP19-6 | keratin associated protein 19-6 [Source:HGNC Symbol;Acc:HGNC:18941] |
| ENSG00000187021 | HGLibA_37358 | 318 | 2.015827942 | PNLIPRP1 | pancreatic lipase related protein 1 [Source:HGNC Symbol;Acc:HGNC:9156] |
| ENSG00000188620 | HGLibA_21765 | 318 | 2.015827942 | HMX3 | H6 family homeobox 3 [Source:HGNC Symbol;Acc:HGNC:5019] |
| ENSG00000196405 | HGLibA_15697 | 318 | 2.015827942 | EVL | Enah/Vasp-like [Source:HGNC Symbol;Acc:HGNC:20234] |
| FAM154B | HGLibA_16178 | 318 | 2.015827942 | FAM154B | - |
| HIST1H4E | HGLibA_21499 | 318 | 2.015827942 | HIST1H4E | - |
| hsa-mir-30a | HGLibA_58473 | 318 | 2.015827942 | hsa-mir-30a | - |
| WDR63 | HGLibA_54149 | 318 | 2.015827942 | WDR63 | - |
| ENSG00000058866 | HGLibA_13069 | 317 | 2.005876905 | DGKG | diacylglycerol kinase gamma [Source:HGNC Symbol;Acc:HGNC:2853] |
| ENSG00000083544 | HGLibA_48905 | 317 | 2.005876905 | TDRD3 | tudor domain containing 3 [Source:HGNC Symbol;Acc:HGNC:20612] |
| ENSG00000100417 | HGLibA_37314 | 317 | 2.005876905 | PMM1 | phosphomannomutase 1 [Source:HGNC Symbol;Acc:HGNC:9114] |
| ENSG00000107821 | HGLibA_24087 | 317 | 2.005876905 | KAZALD1 | Kazal type serine peptidase inhibitor domain 1 [Source:HGNC Symbol;Acc:HGNC:25460] |
| ENSG00000133246 | HGLibA_38272 | 317 | 2.005876905 | PRAM1 | PML-RARA regulated adaptor molecule 1 [Source:HGNC Symbol;Acc:HGNC:30091] |
| ENSG00000136938 | HGLibA_02201 | 317 | 2.005876905 | ANP32B | acidic nuclear phosphoprotein 32 family member B [Source:HGNC Symbol;Acc:HGNC:16677] |
| ENSG00000145040 | HGLibA_52826 | 317 | 2.005876905 | UCN2 | urocortin 2 [Source:HGNC Symbol;Acc:HGNC:18414] |
| ENSG00000148516 | HGLibA_55136 | 317 | 2.005876905 | ZEB1 | zinc finger E-box binding homeobox 1 [Source:HGNC Symbol;Acc:HGNC:11642] |
| ENSG00000162777 | HGLibA_12945 | 317 | 2.005876905 | DENND2D | DENN domain containing 2D [Source:HGNC Symbol;Acc:HGNC:26192] |
| ENSG00000163659 | HGLibA_49557 | 317 | 2.005876905 | TIPARP | TCDD inducible poly(ADP-ribose) polymerase [Source:HGNC Symbol;Acc:HGNC:23696] |
| ENSG00000163820 | HGLibA_18250 | 317 | 2.005876905 | FYCO1 | FYVE and coiled-coil domain containing 1 [Source:HGNC Symbol;Acc:HGNC:14673] |
| ENSG00000169704 | HGLibA_19713 | 317 | 2.005876905 | GP9 | glycoprotein IX platelet [Source:HGNC Symbol;Acc:HGNC:4444] |
| ENSG00000170464 | HGLibA_13588 | 317 | 2.005876905 | DNAJC18 | DnaJ heat shock protein family (Hsp40) member C18 [Source:HGNC Symbol;Acc:HGNC:28429] |
| ENSG00000182952 | HGLibA_21725 | 317 | 2.005876905 | HMGN4 | high mobility group nucleosomal binding domain 4 [Source:HGNC Symbol;Acc:HGNC:4989] |
| hsa-mir-1208 | HGLibA_57287 | 317 | 2.005876905 | hsa-mir-1208 | - |
| hsa-mir-5196 | HGLibA_61702 | 317 | 2.005876905 | hsa-mir-5196 | - |
| hsa-mir-561 | HGLibA_62209 | 317 | 2.005876905 | hsa-mir-561 | - |
| hsa-mir-6781 | HGLibA_63300 | 317 | 2.005876905 | hsa-mir-6781 | - |
| hsa-mir-6884 | HGLibA_63722 | 317 | 2.005876905 | hsa-mir-6884 | - |
| N6AMT2 | HGLibA_30968 | 317 | 2.005876905 | N6AMT2 | - |
| C7orf43 | HGLibA_06545 | 316 | 1.995925867 | C7orf43 | - |
| ENSG00000089012 | HGLibA_44212 | 316 | 1.995925867 | SIRPG | signal regulatory protein gamma [Source:HGNC Symbol;Acc:HGNC:15757] |
| ENSG00000103067 | HGLibA_15590 | 316 | 1.995925867 | ESRP2 | epithelial splicing regulatory protein 2 [Source:HGNC Symbol;Acc:HGNC:26152] |

| | | | | | |
|---|---|---|---|---|---|
| ENSG00000104267 | HGLibA_06803 | 316 | 1.995925867 | CA2 | carbonic anhydrase 2 [Source:HGNC Symbol;Acc:HGNC:1373] |
| ENSG00000106538 | HGLibA_40284 | 316 | 1.995925867 | RARRES2 | retinoic acid receptor responder 2 [Source:HGNC Symbol;Acc:HGNC:9868] |
| ENSG00000114124 | HGLibA_20337 | 316 | 1.995925867 | GRK7 | G protein-coupled receptor kinase 7 [Source:HGNC Symbol;Acc:HGNC:17031] |
| ENSG00000123096 | HGLibA_47148 | 316 | 1.995925867 | SSPN | sarcospan [Source:HGNC Symbol;Acc:HGNC:11322] |
| ENSG00000129170 | HGLibA_11442 | 316 | 1.995925867 | CSRP3 | cysteine and glycine rich protein 3 [Source:HGNC Symbol;Acc:HGNC:2472] |
| ENSG00000138964 | HGLibA_35262 | 316 | 1.995925867 | PARVG | parvin gamma [Source:HGNC Symbol;Acc:HGNC:14654] |
| ENSG00000160408 | HGLibA_47280 | 316 | 1.995925867 | ST6GALNAC6 | 6-sialyltransferase 6 [Source:HGNC Symbol;Acc:HGNC:23364]" |
| ENSG00000160447 | HGLibA_36844 | 316 | 1.995925867 | PKN3 | protein kinase N3 [Source:HGNC Symbol;Acc:HGNC:17999] |
| ENSG00000160654 | HGLibA_08377 | 316 | 1.995925867 | CD3G | CD3g molecule [Source:HGNC Symbol;Acc:HGNC:1675] |
| ENSG00000163083 | HGLibA_23305 | 316 | 1.995925867 | INHBB | inhibin subunit beta B [Source:HGNC Symbol;Acc:HGNC:6067] |
| ENSG00000166887 | HGLibA_53787 | 316 | 1.995925867 | VPS39 | HOPS complex subunit [Source:HGNC Symbol;Acc:HGNC:20593]" |
| ENSG00000175691 | HGLibA_56700 | 316 | 1.995925867 | ZNF77 | zinc finger protein 77 [Source:HGNC Symbol;Acc:HGNC:13150] |
| ENSG00000181192 | HGLibA_13164 | 316 | 1.995925867 | DHTKD1 | dehydrogenase E1 and transketolase domain containing 1 [Source:HGNC Symbol;Acc:HGNC:23537] |
| ENSG00000189077 | HGLibA_49895 | 316 | 1.995925867 | TMEM120A | transmembrane protein 120A [Source:HGNC Symbol;Acc:HGNC:21697] |
| ENSG00000204262 | HGLibA_10590 | 316 | 1.995925867 | COL5A2 | collagen type V alpha 2 chain [Source:HGNC Symbol;Acc:HGNC:2210] |
| ENSG00000240771 | HGLibA_02837 | 316 | 1.995925867 | ARHGEF25 | Rho guanine nucleotide exchange factor 25 [Source:HGNC Symbol;Acc:HGNC:30275] |
| ENSG00000255561 | HGLibA_17265 | 316 | 1.995925867 | FDXACB1 | ferredoxin-fold anticodon binding domain containing 1 [Source:HGNC Symbol;Acc:HGNC:25110] |
| ENSG00000260314 | HGLibA_29816 | 316 | 1.995925867 | MRC1 | mannose receptor C-type 1 [Source:HGNC Symbol;Acc:HGNC:7228] |
| ENSG00000269699 | HGLibA_55339 | 316 | 1.995925867 | ZIM2 | zinc finger imprinted 2 [Source:HGNC Symbol;Acc:HGNC:12875] |
| hsa-mir-6508 | HGLibA_62880 | 316 | 1.995925867 | hsa-mir-6508 | - |
| KIAA1967 | HGLibA_24832 | 316 | 1.995925867 | KIAA1967 | - |
| LOC100130880 | HGLibA_26798 | 316 | 1.995925867 | LOC100130880 | - |
| ENSG00000010327 | HGLibA_47307 | 315 | 1.98597483 | STAB1 | stabilin 1 [Source:HGNC Symbol;Acc:HGNC:18628] |
| ENSG00000034533 | HGLibA_03373 | 315 | 1.98597483 | ASTE1 | asteroid homolog 1 [Source:HGNC Symbol;Acc:HGNC:25021] |
| ENSG00000069188 | HGLibA_43155 | 315 | 1.98597483 | SDK2 | sidekick cell adhesion molecule 2 [Source:HGNC Symbol;Acc:HGNC:19308] |
| ENSG00000092969 | HGLibA_49213 | 315 | 1.98597483 | TGFB2 | transforming growth factor beta 2 [Source:HGNC Symbol;Acc:HGNC:11768] |
| ENSG00000102178 | HGLibA_52704 | 315 | 1.98597483 | UBL4A | ubiquitin like 4A [Source:HGNC Symbol;Acc:HGNC:12505] |
| ENSG00000109854 | HGLibA_22346 | 315 | 1.98597483 | HTATIP2 | HIV-1 Tat interactive protein 2 [Source:HGNC Symbol;Acc:HGNC:16637] |
| ENSG00000113140 | HGLibA_46418 | 315 | 1.98597483 | SPARC | secreted protein acidic and cysteine rich [Source:HGNC Symbol;Acc:HGNC:11219] |
| ENSG00000128342 | HGLibA_26475 | 315 | 1.98597483 | LIF | interleukin 6 family cytokine [Source:HGNC Symbol;Acc:HGNC:6596]" |
| ENSG00000133805 | HGLibA_01830 | 315 | 1.98597483 | AMPD3 | adenosine monophosphate deaminase 3 [Source:HGNC Symbol;Acc:HGNC:470] |
| ENSG00000135426 | HGLibA_49034 | 315 | 1.98597483 | TESPA1 | positive selection associated 1 [Source:HGNC Symbol;Acc:HGNC:29109]" |
| ENSG00000143333 | HGLibA_41067 | 315 | 1.98597483 | RGS16 | regulator of G protein signaling 16 [Source:HGNC Symbol;Acc:HGNC:9997] |
| ENSG00000146373 | HGLibA_41633 | 315 | 1.98597483 | RNF217 | ring finger protein 217 [Source:HGNC Symbol;Acc:HGNC:21487] |
| ENSG00000150281 | HGLibA_11619 | 315 | 1.98597483 | CTF1 | cardiotrophin 1 [Source:HGNC Symbol;Acc:HGNC:2499] |
| ENSG00000167604 | HGLibA_31827 | 315 | 1.98597483 | NFKBID | NFKB inhibitor delta [Source:HGNC Symbol;Acc:HGNC:15671] |
| ENSG00000168619 | HGLibA_00743 | 315 | 1.98597483 | ADAM18 | ADAM metallopeptidase domain 18 [Source:HGNC Symbol;Acc:HGNC:196] |
| ENSG00000169031 | HGLibA_10572 | 315 | 1.98597483 | COL4A3 | collagen type IV alpha 3 chain [Source:HGNC Symbol;Acc:HGNC:2204] |
| ENSG00000185875 | HGLibA_49358 | 315 | 1.98597483 | THNSL1 | threonine synthase like 1 [Source:HGNC Symbol;Acc:HGNC:26160] |
| ENSG00000197576 | HGLibA_21911 | 315 | 1.98597483 | HOXA4 | homeobox A4 [Source:HGNC Symbol;Acc:HGNC:5105] |
| ENSG00000204257 | HGLibA_21604 | 315 | 1.98597483 | HLA-DMA | DM alpha [Source:HGNC Symbol;Acc:HGNC:4934]" |
| ENSG00000255974 | HGLibA_12107 | 315 | 1.98597483 | CYP2A6 | cytochrome P450 family 2 subfamily A member 6 [Source:HGNC Symbol;Acc:HGNC:2610] |
| ENSG00000261210 | HGLibA_09953 | 315 | 1.98597483 | CLEC19A | C-type lectin domain containing 19A [Source:HGNC Symbol;Acc:HGNC:34522] |
| ENSG00000271858 | HGLibA_11986 | 315 | 1.98597483 | CYB561D2 | cytochrome b561 family member D2 [Source:NCBI gene;Acc:11068] |
| ENSG00000277149 | HGLibA_52484 | 315 | 1.98597483 | TYW1B | tRNA-yW synthesizing protein 1 homolog B [Source:HGNC Symbol;Acc:HGNC:33908] |
| ENSG00000279051 | HGLibA_34440 | 315 | 1.98597483 | OR6Q1 | olfactory receptor family 6 subfamily Q member 1 (gene/pseudogene) [Source:HGNC Symbol;Acc:HGNC:15302] |
| hsa-mir-194-2 | HGLibA_58083 | 315 | 1.98597483 | hsa-mir-194-2 | - |
| hsa-mir-5683 | HGLibA_62251 | 315 | 1.98597483 | hsa-mir-5683 | - |
| hsa-mir-6856 | HGLibA_63597 | 315 | 1.98597483 | hsa-mir-6856 | - |
| LOC100129361 | HGLibA_26759 | 315 | 1.98597483 | LOC100129361 | - |
| C1orf106 | HGLibA_05737 | 314 | 1.976023793 | C1orf106 | - |
| ENSG00000005108 | HGLibA_49405 | 314 | 1.976023793 | THSD7A | thrombospondin type 1 domain containing 7A [Source:HGNC Symbol;Acc:HGNC:22207] |
| ENSG00000006194 | HGLibA_55693 | 314 | 1.976023793 | ZNF263 | zinc finger protein 263 [Source:HGNC Symbol;Acc:HGNC:13056] |
| ENSG00000080224 | HGLibA_15301 | 314 | 1.976023793 | EPHA6 | EPH receptor A6 [Source:HGNC Symbol;Acc:HGNC:19296] |
| ENSG00000100197 | HGLibA_12127 | 314 | 1.976023793 | CYP2D6 | cytochrome P450 family 2 subfamily D member 6 [Source:HGNC Symbol;Acc:HGNC:2625] |
| ENSG00000105954 | HGLibA_32542 | 314 | 1.976023793 | NPVF | neuropeptide VF precursor [Source:HGNC Symbol;Acc:HGNC:13782] |
| ENSG00000112357 | HGLibA_36127 | 314 | 1.976023793 | PEX7 | peroxisomal biogenesis factor 7 [Source:HGNC Symbol;Acc:HGNC:8860] |
| ENSG00000115902 | HGLibA_44517 | 314 | 1.976023793 | SLC1A4 | solute carrier family 1 member 4 [Source:HGNC Symbol;Acc:HGNC:10942] |
| ENSG00000120129 | HGLibA_14128 | 314 | 1.976023793 | DUSP1 | dual specificity phosphatase 1 [Source:HGNC Symbol;Acc:HGNC:3064] |
| ENSG00000131845 | HGLibA_55776 | 314 | 1.976023793 | ZNF304 | zinc finger protein 304 [Source:HGNC Symbol;Acc:HGNC:13505] |
| ENSG00000133800 | HGLibA_27778 | 314 | 1.976023793 | LYVE1 | lymphatic vessel endothelial hyaluronan receptor 1 [Source:HGNC Symbol;Acc:HGNC:14687] |
| ENSG00000137266 | HGLibA_44573 | 314 | 1.976023793 | SLC22A23 | solute carrier family 22 member 23 [Source:HGNC Symbol;Acc:HGNC:21106] |
| ENSG00000140199 | HGLibA_44372 | 314 | 1.976023793 | SLC12A6 | solute carrier family 12 member 6 [Source:HGNC Symbol;Acc:HGNC:10914] |
| ENSG00000152463 | HGLibA_33340 | 314 | 1.976023793 | OLAH | oleoyl-ACP hydrolase [Source:HGNC Symbol;Acc:HGNC:25625] |
| ENSG00000165202 | HGLibA_33710 | 314 | 1.976023793 | OR1Q1 | olfactory receptor family 1 subfamily Q member 1 [Source:HGNC Symbol;Acc:HGNC:8223] |
| ENSG00000168348 | HGLibA_23398 | 314 | 1.976023793 | INSM2 | INSM transcriptional repressor 2 [Source:HGNC Symbol;Acc:HGNC:17539] |
| ENSG00000174482 | HGLibA_26589 | 314 | 1.976023793 | LINGO2 | leucine rich repeat and Ig domain containing 2 [Source:HGNC Symbol;Acc:HGNC:21207] |
| ENSG00000182858 | HGLibA_01613 | 314 | 1.976023793 | ALG12 | 6-mannosyltransferase [Source:HGNC Symbol;Acc:HGNC:19358]" |
| ENSG00000184384 | HGLibA_28047 | 314 | 1.976023793 | MAML2 | mastermind like transcriptional coactivator 2 [Source:HGNC Symbol;Acc:HGNC:16259] |
| ENSG00000185905 | HGLibA_05457 | 314 | 1.976023793 | C16orf54 | chromosome 16 open reading frame 54 [Source:HGNC Symbol;Acc:HGNC:26649] |
| ENSG00000198939 | HGLibA_55191 | 314 | 1.976023793 | ZFP2 | ZFP2 zinc finger protein [Source:HGNC Symbol;Acc:HGNC:26138] |
| ENSG00000237441 | HGLibA_41003 | 314 | 1.976023793 | RGL2 | ral guanine nucleotide dissociation stimulator like 2 [Source:HGNC Symbol;Acc:HGNC:9769] |
| hsa-mir-4284 | HGLibA_59844 | 314 | 1.976023793 | hsa-mir-4284 | - |
| hsa-mir-548y | HGLibA_62086 | 314 | 1.976023793 | hsa-mir-548y | - |
| hsa-mir-5704 | HGLibA_62356 | 314 | 1.976023793 | hsa-mir-5704 | - |
| C12orf61 | HGLibA_05277 | 313 | 1.966072755 | C12orf61 | - |
| C7orf63 | HGLibA_06572 | 313 | 1.966072755 | C7orf63 | - |
| ENSG00000013588 | HGLibA_20125 | 313 | 1.966072755 | GPRC5A | G protein-coupled receptor class C group 5 member A [Source:HGNC Symbol;Acc:HGNC:9836] |
| ENSG00000077044 | HGLibA_13063 | 313 | 1.966072755 | DGKD | diacylglycerol kinase delta [Source:HGNC Symbol;Acc:HGNC:2851] |
| ENSG00000100441 | HGLibA_24577 | 313 | 1.966072755 | KHNYN | KH and NYN domain containing [Source:HGNC Symbol;Acc:HGNC:20166] |
| ENSG00000104722 | HGLibA_31624 | 313 | 1.966072755 | NEFM | neurofilament medium [Source:HGNC Symbol;Acc:HGNC:7734] |
| ENSG00000107651 | HGLibA_43227 | 313 | 1.966072755 | SEC23IP | SEC23 interacting protein [Source:HGNC Symbol;Acc:HGNC:17018] |
| ENSG00000110680 | HGLibA_06992 | 313 | 1.966072755 | CALCA | calcitonin related polypeptide alpha [Source:HGNC Symbol;Acc:HGNC:1437] |
| ENSG00000110801 | HGLibA_39187 | 313 | 1.966072755 | PSMD9 | non-ATPase 9 [Source:HGNC Symbol;Acc:HGNC:9567]" |
| ENSG00000114631 | HGLibA_37462 | 313 | 1.966072755 | PODXL2 | podocalyxin like 2 [Source:HGNC Symbol;Acc:HGNC:17936] |
| ENSG00000128805 | HGLibA_02730 | 313 | 1.966072755 | ARHGAP22 | Rho GTPase activating protein 22 [Source:HGNC Symbol;Acc:HGNC:30320] |
| ENSG00000142224 | HGLibA_23035 | 313 | 1.966072755 | IL19 | interleukin 19 [Source:HGNC Symbol;Acc:HGNC:5990] |
| ENSG00000148331 | HGLibA_03245 | 313 | 1.966072755 | ASB6 | ankyrin repeat and SOCS box containing 6 [Source:HGNC Symbol;Acc:HGNC:17181] |
| ENSG00000160908 | HGLibA_55908 | 313 | 1.966072755 | ZNF394 | zinc finger protein 394 [Source:HGNC Symbol;Acc:HGNC:18832] |
| ENSG00000163374 | HGLibA_54761 | 313 | 1.966072755 | YY1AP1 | YY1 associated protein 1 [Source:HGNC Symbol;Acc:HGNC:30935] |
| ENSG00000163982 | HGLibA_34735 | 313 | 1.966072755 | OTOP1 | otopetrin 1 [Source:HGNC Symbol;Acc:HGNC:19656] |
| ENSG00000169213 | HGLibA_39910 | 313 | 1.966072755 | RAB3B | member RAS oncogene family [Source:HGNC Symbol;Acc:HGNC:9778]" |
| ENSG00000169744 | HGLibA_26215 | 313 | 1.966072755 | LDB2 | LIM domain binding 2 [Source:HGNC Symbol;Acc:HGNC:6533] |
| ENSG00000170876 | HGLibA_50403 | 313 | 1.966072755 | TMEM43 | transmembrane protein 43 [Source:HGNC Symbol;Acc:HGNC:28472] |
| ENSG00000172199 | HGLibA_34557 | 313 | 1.966072755 | OR8U1 | olfactory receptor family 8 subfamily U member 1 [Source:HGNC Symbol;Acc:HGNC:19611] |
| ENSG00000173578 | HGLibA_54499 | 313 | 1.966072755 | XCR1 | X-C motif chemokine receptor 1 [Source:HGNC Symbol;Acc:HGNC:1625] |
| ENSG00000180432 | HGLibA_12217 | 313 | 1.966072755 | CYP8B1 | cytochrome P450 family 8 subfamily B member 1 [Source:HGNC Symbol;Acc:HGNC:2653] |
| ENSG00000187135 | HGLibA_53864 | 313 | 1.966072755 | VSTM2B | V-set and transmembrane domain containing 2B [Source:HGNC Symbol;Acc:HGNC:33595] |
| ENSG00000197046 | HGLibA_44138 | 313 | 1.966072755 | SIGLEC15 | sialic acid binding Ig like lectin 15 [Source:HGNC Symbol;Acc:HGNC:27596] |
| ENSG00000197467 | HGLibA_10508 | 313 | 1.966072755 | COL13A1 | collagen type XIII alpha 1 chain [Source:HGNC Symbol;Acc:HGNC:2190] |
| ENSG00000204687 | HGLibA_28391 | 313 | 1.966072755 | MAS1L | G protein-coupled receptor [Source:HGNC Symbol;Acc:HGNC:13961]" |
| ENSG00000250317 | HGLibA_45747 | 313 | 1.966072755 | SMIM20 | small integral membrane protein 20 [Source:HGNC Symbol;Acc:HGNC:37260] |
| ENSG00000253958 | HGLibA_09887 | 313 | 1.966072755 | CLDN23 | claudin 23 [Source:HGNC Symbol;Acc:HGNC:17591] |
| hsa-mir-940 | HGLibA_64333 | 313 | 1.966072755 | hsa-mir-940 | - |
| C8orf4 | HGLibA_06618 | 312 | 1.956121718 | C8orf4 | - |
| ENSG00000029639 | HGLibA_49140 | 312 | 1.956121718 | TFB1M | mitochondrial [Source:HGNC Symbol;Acc:HGNC:17037]" |
| ENSG00000048462 | HGLibA_50764 | 312 | 1.956121718 | TNFRSF17 | TNF receptor superfamily member 17 [Source:HGNC Symbol;Acc:HGNC:11913] |
| ENSG00000071242 | HGLibA_42160 | 312 | 1.956121718 | RPS6KA2 | ribosomal protein S6 kinase A2 [Source:HGNC Symbol;Acc:HGNC:10431] |
| ENSG00000086730 | HGLibA_26061 | 312 | 1.956121718 | LAT2 | linker for activation of T cells family member 2 [Source:HGNC Symbol;Acc:HGNC:12749] |
| ENSG00000091527 | HGLibA_08892 | 312 | 1.956121718 | CDV3 | CDV3 homolog [Source:HGNC Symbol;Acc:HGNC:26928] |
| ENSG00000099904 | HGLibA_55128 | 312 | 1.956121718 | ZDHHC8 | zinc finger DHHC-type containing 8 [Source:HGNC Symbol;Acc:HGNC:18474] |
| ENSG00000105668 | HGLibA_53069 | 312 | 1.956121718 | UPK1A | uroplakin 1A [Source:HGNC Symbol;Acc:HGNC:12577] |
| ENSG00000109065 | HGLibA_31192 | 312 | 1.956121718 | NAT9 | N-acetyltransferase 9 (putative) [Source:HGNC Symbol;Acc:HGNC:23133] |
| ENSG00000111203 | HGLibA_23710 | 312 | 1.956121718 | ITFG2 | integrin alpha FG-GAP repeat containing 2 [Source:HGNC Symbol;Acc:HGNC:30879] |
| ENSG00000120685 | HGLibA_38694 | 312 | 1.956121718 | PROSER1 | proline and serine rich 1 [Source:HGNC Symbol;Acc:HGNC:20291] |
| ENSG00000124104 | HGLibA_46063 | 312 | 1.956121718 | SNX21 | sorting nexin family member 21 [Source:HGNC Symbol;Acc:HGNC:16154] |
| ENSG00000131089 | HGLibA_02879 | 312 | 1.956121718 | ARHGEF9 | Cdc42 guanine nucleotide exchange factor 9 [Source:HGNC Symbol;Acc:HGNC:14561] |
| ENSG00000131732 | HGLibA_55052 | 312 | 1.956121718 | ZCCHC9 | zinc finger CCHC-type containing 9 [Source:HGNC Symbol;Acc:HGNC:25424] |
| ENSG00000134020 | HGLibA_36016 | 312 | 1.956121718 | PEBP4 | phosphatidylethanolamine binding protein 4 [Source:HGNC Symbol;Acc:HGNC:28319] |
| ENSG00000136463 | HGLibA_48167 | 312 | 1.956121718 | TACO1 | translational activator of cytochrome c oxidase I [Source:HGNC Symbol;Acc:HGNC:24316] |
| ENSG00000137225 | HGLibA_07147 | 312 | 1.956121718 | CAPN11 | calpain 11 [Source:HGNC Symbol;Acc:HGNC:1478] |
| ENSG00000143452 | HGLibA_21887 | 312 | 1.956121718 | HORMAD1 | HORMA domain containing 1 [Source:HGNC Symbol;Acc:HGNC:25245] |
| ENSG00000163734 | HGLibA_11869 | 312 | 1.956121718 | CXCL3 | C-X-C motif chemokine ligand 3 [Source:HGNC Symbol;Acc:HGNC:4604] |
| ENSG00000167995 | HGLibA_04418 | 312 | 1.956121718 | BEST1 | bestrophin 1 [Source:HGNC Symbol;Acc:HGNC:12703] |
| ENSG00000169313 | HGLibA_34875 | 312 | 1.956121718 | P2RY12 | purinergic receptor P2Y12 [Source:HGNC Symbol;Acc:HGNC:18124] |
| ENSG00000170498 | HGLibA_25008 | 312 | 1.956121718 | KISS1 | KiSS-1 metastasis suppressor [Source:HGNC Symbol;Acc:HGNC:6341] |
| ENSG00000171459 | HGLibA_33695 | 312 | 1.956121718 | OR1L6 | olfactory receptor family 1 subfamily L member 6 [Source:HGNC Symbol;Acc:HGNC:8218] |
| ENSG00000175213 | HGLibA_55931 | 312 | 1.956121718 | ZNF408 | zinc finger protein 408 [Source:HGNC Symbol;Acc:HGNC:20041] |
| ENSG00000189051 | HGLibA_41644 | 312 | 1.956121718 | RNF222 | ring finger protein 222 [Source:HGNC Symbol;Acc:HGNC:34517] |
| ENSG00000250741 | HGLibA_32809 | 312 | 1.956121718 | NT5C1B-RDH14 | NT5C1B-RDH14 readthrough [Source:HGNC Symbol;Acc:HGNC:38831] |
| FAM198A | HGLibA_16388 | 312 | 1.956121718 | FAM198A | - |
| FAM207A | HGLibA_16430 | 312 | 1.956121718 | FAM207A | - |
| hsa-mir-6755 | HGLibA_63178 | 312 | 1.956121718 | hsa-mir-6755 | - |
| hsa-mir-8088 | HGLibA_64196 | 312 | 1.956121718 | hsa-mir-8088 | - |
| KIAA1841 | HGLibA_24824 | 312 | 1.956121718 | KIAA1841 | - |

| | | | | | |
|---|---|---|---|---|---|
| PRSS46 | HGLibA_38947 | 312 | 1.956121718 | PRSS46 | - |
| ENSG00000023697 | HGLibA_12992 | 311 | 1.94617068 | DERA | deoxyribose-phosphate aldolase [Source:HGNC Symbol;Acc:HGNC:24269] |
| ENSG00000033030 | HGLibA_55047 | 311 | 1.94617068 | ZCCHC8 | zinc finger CCHC-type containing 8 [Source:HGNC Symbol;Acc:HGNC:25265] |
| ENSG00000055732 | HGLibA_28644 | 311 | 1.94617068 | MCOLN3 | mucolipin 3 [Source:HGNC Symbol;Acc:HGNC:13358] |
| ENSG00000101624 | HGLibA_09165 | 311 | 1.94617068 | CEP76 | centrosomal protein 76 [Source:HGNC Symbol;Acc:HGNC:25727] |
| ENSG00000103855 | HGLibA_08322 | 311 | 1.94617068 | CD276 | CD276 molecule [Source:HGNC Symbol;Acc:HGNC:19137] |
| ENSG00000114349 | HGLibA_19471 | 311 | 1.94617068 | GNAT1 | G protein subunit alpha transducin 1 [Source:HGNC Symbol;Acc:HGNC:4393] |
| ENSG00000119640 | HGLibA_00710 | 311 | 1.94617068 | ACYP1 | acylphosphatase 1 [Source:HGNC Symbol;Acc:HGNC:179] |
| ENSG00000132623 | HGLibA_01951 | 311 | 1.94617068 | ANKEF1 | ankyrin repeat and EF-hand domain containing 1 [Source:HGNC Symbol;Acc:HGNC:15803] |
| ENSG00000136514 | HGLibA_42430 | 311 | 1.94617068 | RTP4 | receptor transporter protein 4 [Source:HGNC Symbol;Acc:HGNC:23992] |
| ENSG00000143801 | HGLibA_39029 | 311 | 1.94617068 | PSEN2 | presenilin 2 [Source:HGNC Symbol;Acc:HGNC:9509] |
| ENSG00000146083 | HGLibA_41687 | 311 | 1.94617068 | RNF44 | ring finger protein 44 [Source:HGNC Symbol;Acc:HGNC:19180] |
| ENSG00000147378 | HGLibA_16921 | 311 | 1.94617068 | FATE1 | fetal and adult testis expressed 1 [Source:HGNC Symbol;Acc:HGNC:24683] |
| ENSG00000165175 | HGLibA_29234 | 311 | 1.94617068 | MID1IP1 | MID1 interacting protein 1 [Source:HGNC Symbol;Acc:HGNC:20715] |
| ENSG00000169715 | HGLibA_30282 | 311 | 1.94617068 | MT1E | metallothionein 1E [Source:HGNC Symbol;Acc:HGNC:7397] |
| ENSG00000176046 | HGLibA_33086 | 311 | 1.94617068 | NUPR1 | transcriptional regulator [Source:HGNC Symbol;Acc:HGNC:29990]" |
| ENSG00000177151 | HGLibA_33880 | 311 | 1.94617068 | OR2T35 | olfactory receptor family 2 subfamily T member 35 [Source:HGNC Symbol;Acc:HGNC:31257] |
| ENSG00000182256 | HGLibA_18396 | 311 | 1.94617068 | GABRG3 | gamma-aminobutyric acid type A receptor gamma3 subunit [Source:HGNC Symbol;Acc:HGNC:4088] |
| ENSG00000197721 | HGLibA_11049 | 311 | 1.94617068 | CR1L | complement C3b/C4b receptor 1 like [Source:HGNC Symbol;Acc:HGNC:2335] |
| ENSG00000205710 | HGLibA_05528 | 311 | 1.94617068 | C17orf107 | chromosome 17 open reading frame 107 [Source:HGNC Symbol;Acc:HGNC:37238] |
| ENSG00000213699 | HGLibA_45013 | 311 | 1.94617068 | SLC35F6 | solute carrier family 35 member F6 [Source:HGNC Symbol;Acc:HGNC:26055] |
| ENSG00000223510 | HGLibA_08875 | 311 | 1.94617068 | CDRT15 | CMT1A duplicated region transcript 15 [Source:HGNC Symbol;Acc:HGNC:14395] |
| ENSG00000277481 | HGLibA_36794 | 311 | 1.94617068 | PKD1L3 | transient receptor potential channel interacting [Source:HGNC Symbol;Acc:HGNC:21716]" |
| ENSG00000277586 | HGLibA_31621 | 311 | 1.94617068 | NEFL | neurofilament light [Source:HGNC Symbol;Acc:HGNC:7739] |
| hsa-mir-4693 | HGLibA_60882 | 311 | 1.94617068 | hsa-mir-4693 | - |
| hsa-mir-5008 | HGLibA_61437 | 311 | 1.94617068 | hsa-mir-5008 | - |
| MINA | HGLibA_29273 | 311 | 1.94617068 | MINA | - |
| ENSG00000035681 | HGLibA_32764 | 310 | 1.936219643 | NSMAF | neutral sphingomyelinase activation associated factor [Source:HGNC Symbol;Acc:HGNC:8017] |
| ENSG00000057657 | HGLibA_38362 | 310 | 1.936219643 | PRDM1 | PR/SET domain 1 [Source:HGNC Symbol;Acc:HGNC:9346] |
| ENSG00000111981 | HGLibA_52959 | 310 | 1.936219643 | ULBP1 | UL16 binding protein 1 [Source:HGNC Symbol;Acc:HGNC:14893] |
| ENSG00000121351 | HGLibA_22469 | 310 | 1.936219643 | IAPP | islet amyloid polypeptide [Source:HGNC Symbol;Acc:HGNC:5329] |
| ENSG00000124089 | HGLibA_28548 | 310 | 1.936219643 | MC3R | melanocortin 3 receptor [Source:HGNC Symbol;Acc:HGNC:6931] |
| ENSG00000135040 | HGLibA_30990 | 310 | 1.936219643 | NAA35 | NatC auxiliary subunit [Source:HGNC Symbol;Acc:HGNC:24340]" |
| ENSG00000136147 | HGLibA_36335 | 310 | 1.936219643 | PHF11 | PHD finger protein 11 [Source:HGNC Symbol;Acc:HGNC:17024] |
| ENSG00000137802 | HGLibA_28311 | 310 | 1.936219643 | MAPKBP1 | mitogen-activated protein kinase binding protein 1 [Source:HGNC Symbol;Acc:HGNC:29536] |
| ENSG00000138028 | HGLibA_09326 | 310 | 1.936219643 | CGREF1 | cell growth regulator with EF-hand domain 1 [Source:HGNC Symbol;Acc:HGNC:16962] |
| ENSG00000158764 | HGLibA_23827 | 310 | 1.936219643 | ITLN2 | intelectin 2 [Source:HGNC Symbol;Acc:HGNC:20599] |
| ENSG00000161714 | HGLibA_36992 | 310 | 1.936219643 | PLCD3 | phospholipase C delta 3 [Source:HGNC Symbol;Acc:HGNC:9061] |
| ENSG00000163347 | HGLibA_09848 | 310 | 1.936219643 | CLDN1 | claudin 1 [Source:HGNC Symbol;Acc:HGNC:2032] |
| ENSG00000165626 | HGLibA_04415 | 310 | 1.936219643 | BEND7 | BEN domain containing 7 [Source:HGNC Symbol;Acc:HGNC:23514] |
| ENSG00000165923 | HGLibA_01169 | 310 | 1.936219643 | AGBL2 | ATP/GTP binding protein like 2 [Source:HGNC Symbol;Acc:HGNC:26296] |
| ENSG00000170542 | HGLibA_43567 | 310 | 1.936219643 | SERPINB9 | serpin family B member 9 [Source:HGNC Symbol;Acc:HGNC:8955] |
| ENSG00000170893 | HGLibA_51377 | 310 | 1.936219643 | TRH | thyrotropin releasing hormone [Source:HGNC Symbol;Acc:HGNC:12298] |
| ENSG00000175785 | HGLibA_38488 | 310 | 1.936219643 | PRIMA1 | proline rich membrane anchor 1 [Source:HGNC Symbol;Acc:HGNC:18319] |
| ENSG00000175906 | HGLibA_02972 | 310 | 1.936219643 | ARL4D | ADP ribosylation factor like GTPase 4D [Source:HGNC Symbol;Acc:HGNC:656] |
| ENSG00000176871 | HGLibA_54419 | 310 | 1.936219643 | WSB2 | WD repeat and SOCS box containing 2 [Source:HGNC Symbol;Acc:HGNC:19222] |
| ENSG00000180929 | HGLibA_20066 | 310 | 1.936219643 | GPR62 | G protein-coupled receptor 62 [Source:HGNC Symbol;Acc:HGNC:13301] |
| ENSG00000183186 | HGLibA_06100 | 310 | 1.936219643 | C2CD4C | C2 calcium dependent domain containing 4C [Source:HGNC Symbol;Acc:HGNC:29417] |
| ENSG00000197948 | HGLibA_17211 | 310 | 1.936219643 | FCHSD1 | FCH and double SH3 domains 1 [Source:HGNC Symbol;Acc:HGNC:25463] |
| HIST1H3F | HGLibA_21472 | 310 | 1.936219643 | HIST1H3F | - |
| hsa-mir-4790 | HGLibA_61268 | 310 | 1.936219643 | hsa-mir-4790 | - |
| C9orf91 | HGLibA_06777 | 309 | 1.926268605 | C9orf91 | - |
| ENSG00000060709 | HGLibA_41260 | 309 | 1.926268605 | RIMBP2 | RIMS binding protein 2 [Source:HGNC Symbol;Acc:HGNC:30339] |
| ENSG00000105370 | HGLibA_26521 | 309 | 1.926268605 | LIM2 | lens intrinsic membrane protein 2 [Source:HGNC Symbol;Acc:HGNC:6610] |
| ENSG00000115556 | HGLibA_36995 | 309 | 1.926268605 | PLCD4 | phospholipase C delta 4 [Source:HGNC Symbol;Acc:HGNC:9062] |
| ENSG00000122203 | HGLibA_24707 | 309 | 1.926268605 | KIAA1191 | KIAA1191 [Source:HGNC Symbol;Acc:HGNC:29209] |
| ENSG00000133256 | HGLibA_35808 | 309 | 1.926268605 | PDE6B | phosphodiesterase 6B [Source:HGNC Symbol;Acc:HGNC:8786] |
| ENSG00000133265 | HGLibA_22327 | 309 | 1.926268605 | HSPBP1 | HSPA (Hsp70) binding protein 1 [Source:HGNC Symbol;Acc:HGNC:24989] |
| ENSG00000133315 | HGLibA_27837 | 309 | 1.926268605 | MACROD1 | MACRO domain containing 1 [Source:HGNC Symbol;Acc:HGNC:29598] |
| ENSG00000136878 | HGLibA_53298 | 309 | 1.926268605 | USP20 | ubiquitin specific peptidase 20 [Source:HGNC Symbol;Acc:HGNC:12619] |
| ENSG00000136937 | HGLibA_31282 | 309 | 1.926268605 | NCBP1 | nuclear cap binding protein subunit 1 [Source:HGNC Symbol;Acc:HGNC:7658] |
| ENSG00000141905 | HGLibA_31807 | 309 | 1.926268605 | NFIC | nuclear factor I C [Source:HGNC Symbol;Acc:HGNC:7786] |
| ENSG00000152705 | HGLibA_07361 | 309 | 1.926268605 | CATSPER3 | cation channel sperm associated 3 [Source:HGNC Symbol;Acc:HGNC:20819] |
| ENSG00000154319 | HGLibA_16239 | 309 | 1.926268605 | FAM167A | family with sequence similarity 167 member A [Source:HGNC Symbol;Acc:HGNC:15549] |
| ENSG00000163964 | HGLibA_36601 | 309 | 1.926268605 | PIGX | phosphatidylinositol glycan anchor biosynthesis class X [Source:HGNC Symbol;Acc:HGNC:26046] |
| ENSG00000165105 | HGLibA_40326 | 309 | 1.926268605 | RASEF | RAS and EF-hand domain containing [Source:HGNC Symbol;Acc:HGNC:26464] |
| ENSG00000166557 | HGLibA_49807 | 309 | 1.926268605 | TMED3 | transmembrane p24 trafficking protein 3 [Source:HGNC Symbol;Acc:HGNC:28889] |
| ENSG00000166888 | HGLibA_47400 | 309 | 1.926268605 | STAT6 | signal transducer and activator of transcription 6 [Source:HGNC Symbol;Acc:HGNC:11368] |
| ENSG00000167461 | HGLibA_39985 | 309 | 1.926268605 | RAB8A | member RAS oncogene family [Source:HGNC Symbol;Acc:HGNC:7007]" |
| ENSG00000170962 | HGLibA_35851 | 309 | 1.926268605 | PDGFD | platelet derived growth factor D [Source:HGNC Symbol;Acc:HGNC:30620] |
| ENSG00000172465 | HGLibA_48704 | 309 | 1.926268605 | TCEAL1 | transcription elongation factor A like 1 [Source:HGNC Symbol;Acc:HGNC:11616] |
| ENSG00000214050 | HGLibA_17034 | 309 | 1.926268605 | FBXO16 | F-box protein 16 [Source:HGNC Symbol;Acc:HGNC:13618] |
| ENSG00000242220 | HGLibA_48837 | 309 | 1.926268605 | TCP10L | t-complex 10 like [Source:HGNC Symbol;Acc:HGNC:11657] |
| hsa-mir-5693 | HGLibA_62306 | 309 | 1.926268605 | hsa-mir-5693 | - |
| TCTEX1D4 | HGLibA_48869 | 309 | 1.926268605 | TCTEX1D4 | - |
| C11orf30 | HGLibA_05126 | 308 | 1.916317568 | C11orf30 | - |
| C12orf39 | HGLibA_05233 | 308 | 1.916317568 | C12orf39 | - |
| ENSG00000040933 | HGLibA_23336 | 308 | 1.916317568 | INPP4A | inositol polyphosphate-4-phosphatase type I A [Source:HGNC Symbol;Acc:HGNC:6074] |
| ENSG00000083454 | HGLibA_34858 | 308 | 1.916317568 | P2RX5 | purinergic receptor P2X 5 [Source:HGNC Symbol;Acc:HGNC:8536] |
| ENSG00000101888 | HGLibA_33176 | 308 | 1.916317568 | NXT2 | nuclear transport factor 2 like export factor 2 [Source:HGNC Symbol;Acc:HGNC:18151] |
| ENSG00000102468 | HGLibA_22371 | 308 | 1.916317568 | HTR2A | 5-hydroxytryptamine receptor 2A [Source:HGNC Symbol;Acc:HGNC:5293] |
| ENSG00000110881 | HGLibA_03300 | 308 | 1.916317568 | ASIC1 | acid sensing ion channel subunit 1 [Source:HGNC Symbol;Acc:HGNC:100] |
| ENSG00000125629 | HGLibA_23380 | 308 | 1.916317568 | INSIG2 | insulin induced gene 2 [Source:HGNC Symbol;Acc:HGNC:20452] |
| ENSG00000135245 | HGLibA_21317 | 308 | 1.916317568 | HILPDA | hypoxia inducible lipid droplet associated [Source:HGNC Symbol;Acc:HGNC:28859] |
| ENSG00000137801 | HGLibA_49321 | 308 | 1.916317568 | THBS1 | thrombospondin 1 [Source:HGNC Symbol;Acc:HGNC:11785] |
| ENSG00000142330 | HGLibA_07145 | 308 | 1.916317568 | CAPN10 | calpain 10 [Source:HGNC Symbol;Acc:HGNC:1477] |
| ENSG00000156467 | HGLibA_53109 | 308 | 1.916317568 | UQCRB | ubiquinol-cytochrome c reductase binding protein [Source:HGNC Symbol;Acc:HGNC:12582] |
| ENSG00000160310 | HGLibA_38624 | 308 | 1.916317568 | PRMT2 | protein arginine methyltransferase 2 [Source:HGNC Symbol;Acc:HGNC:5186] |
| ENSG00000163463 | HGLibA_25854 | 308 | 1.916317568 | KRTCAP2 | keratinocyte associated protein 2 [Source:HGNC Symbol;Acc:HGNC:28942] |
| ENSG00000169519 | HGLibA_28960 | 308 | 1.916317568 | METTL15 | methyltransferase like 15 [Source:HGNC Symbol;Acc:HGNC:26606] |
| ENSG00000170482 | HGLibA_44605 | 308 | 1.916317568 | SLC23A1 | solute carrier family 23 member 1 [Source:HGNC Symbol;Acc:HGNC:10974] |
| ENSG00000171466 | HGLibA_56249 | 308 | 1.916317568 | ZNF562 | zinc finger protein 562 [Source:HGNC Symbol;Acc:HGNC:25950] |
| ENSG00000172016 | HGLibA_40801 | 308 | 1.916317568 | REG3A | regenerating family member 3 alpha [Source:HGNC Symbol;Acc:HGNC:8601] |
| ENSG00000175198 | HGLibA_35380 | 308 | 1.916317568 | PCCA | propionyl-CoA carboxylase subunit alpha [Source:HGNC Symbol;Acc:HGNC:8653] |
| ENSG00000175489 | HGLibA_27289 | 308 | 1.916317568 | LRRC25 | leucine rich repeat containing 25 [Source:HGNC Symbol;Acc:HGNC:29806] |
| ENSG00000178821 | HGLibA_50431 | 308 | 1.916317568 | TMEM52 | transmembrane protein 52 [Source:HGNC Symbol;Acc:HGNC:27916] |
| ENSG00000180044 | HGLibA_06273 | 308 | 1.916317568 | C3orf80 | chromosome 3 open reading frame 80 [Source:HGNC Symbol;Acc:HGNC:40048] |
| ENSG00000181433 | HGLibA_42645 | 308 | 1.916317568 | SAGE1 | sarcoma antigen 1 [Source:HGNC Symbol;Acc:HGNC:30369] |
| ENSG00000182511 | HGLibA_17304 | 308 | 1.916317568 | FES | tyrosine kinase [Source:HGNC Symbol;Acc:HGNC:3657]" |
| ENSG00000188554 | HGLibA_31247 | 308 | 1.916317568 | NBR1 | autophagy cargo receptor [Source:HGNC Symbol;Acc:HGNC:6746]" |
| ENSG00000237136 | HGLibA_06343 | 308 | 1.916317568 | C4orf51 | chromosome 4 open reading frame 51 [Source:HGNC Symbol;Acc:HGNC:37264] |
| HIST3H2A | HGLibA_21556 | 308 | 1.916317568 | HIST3H2A | - |
| hsa-mir-148b | HGLibA_57882 | 308 | 1.916317568 | hsa-mir-148b | - |
| hsa-mir-4525 | HGLibA_60563 | 308 | 1.916317568 | hsa-mir-4525 | - |
| hsa-mir-7703 | HGLibA_63948 | 308 | 1.916317568 | hsa-mir-7703 | - |
| ENSG00000006788 | HGLibA_30713 | 307 | 1.906366531 | MYH13 | myosin heavy chain 13 [Source:HGNC Symbol;Acc:HGNC:7571] |
| ENSG00000073146 | HGLibA_29696 | 307 | 1.906366531 | MOV10L1 | Mov10 like RISC complex RNA helicase 1 [Source:HGNC Symbol;Acc:HGNC:7201] |
| ENSG00000077097 | HGLibA_50998 | 307 | 1.906366531 | TOP2B | DNA topoisomerase II beta [Source:HGNC Symbol;Acc:HGNC:11990] |
| ENSG00000088808 | HGLibA_38030 | 307 | 1.906366531 | PPP1R13B | protein phosphatase 1 regulatory subunit 13B [Source:HGNC Symbol;Acc:HGNC:14950] |
| ENSG00000101298 | HGLibA_45935 | 307 | 1.906366531 | SNPH | syntaphilin [Source:HGNC Symbol;Acc:HGNC:15931] |
| ENSG00000106771 | HGLibA_50299 | 307 | 1.906366531 | TMEM245 | transmembrane protein 245 [Source:HGNC Symbol;Acc:HGNC:1363] |
| ENSG00000114019 | HGLibA_01819 | 307 | 1.906366531 | AMOTL2 | angiomotin like 2 [Source:HGNC Symbol;Acc:HGNC:17812] |
| ENSG00000131236 | HGLibA_07132 | 307 | 1.906366531 | CAP1 | cyclase associated actin cytoskeleton regulatory protein 1 [Source:HGNC Symbol;Acc:HGNC:20040] |
| ENSG00000138639 | HGLibA_02734 | 307 | 1.906366531 | ARHGAP24 | Rho GTPase activating protein 24 [Source:HGNC Symbol;Acc:HGNC:25361] |
| ENSG00000144749 | HGLibA_27177 | 307 | 1.906366531 | LRIG1 | leucine rich repeats and immunoglobulin like domains 1 [Source:HGNC Symbol;Acc:HGNC:17360] |
| ENSG00000165694 | HGLibA_18025 | 307 | 1.906366531 | FRMD7 | FERM domain containing 7 [Source:HGNC Symbol;Acc:HGNC:8079] |
| ENSG00000177023 | HGLibA_12823 | 307 | 1.906366531 | DEFB104B | defensin beta 104B [Source:HGNC Symbol;Acc:HGNC:26165] |
| ENSG00000177303 | HGLibA_07291 | 307 | 1.906366531 | CASKIN2 | CASK interacting protein 2 [Source:HGNC Symbol;Acc:HGNC:18200] |
| ENSG00000178397 | HGLibA_16512 | 307 | 1.906366531 | FAM220A | family with sequence similarity 220 member A [Source:HGNC Symbol;Acc:HGNC:22422] |
| ENSG00000180638 | HGLibA_45196 | 307 | 1.906366531 | SLC47A2 | solute carrier family 47 member 2 [Source:HGNC Symbol;Acc:HGNC:26439] |
| ENSG00000182585 | HGLibA_15281 | 307 | 1.906366531 | EPGN | epithelial mitogen [Source:HGNC Symbol;Acc:HGNC:17470] |
| ENSG00000182938 | HGLibA_34742 | 307 | 1.906366531 | OTOP3 | otopetrin 3 [Source:HGNC Symbol;Acc:HGNC:19658] |
| ENSG00000184210 | HGLibA_13039 | 307 | 1.906366531 | DGAT2L6 | diacylglycerol O-acyltransferase 2 like 6 [Source:HGNC Symbol;Acc:HGNC:23250] |
| ENSG00000185565 | HGLibA_27511 | 307 | 1.906366531 | LSAMP | limbic system associated membrane protein [Source:HGNC Symbol;Acc:HGNC:6705] |
| ENSG00000187170 | HGLibA_26146 | 307 | 1.906366531 | LCE4A | late cornified envelope 4A [Source:HGNC Symbol;Acc:HGNC:16613] |
| ENSG00000188171 | HGLibA_56421 | 307 | 1.906366531 | ZNF626 | zinc finger protein 626 [Source:HGNC Symbol;Acc:HGNC:30461] |
| ENSG00000198707 | HGLibA_09130 | 307 | 1.906366531 | CEP290 | centrosomal protein 290 [Source:HGNC Symbol;Acc:HGNC:29021] |
| ENSG00000256660 | HGLibA_09931 | 307 | 1.906366531 | CLEC12B | C-type lectin domain family 12 member B [Source:HGNC Symbol;Acc:HGNC:31966] |
| TMEM8C | HGLibA_50553 | 307 | 1.906366531 | TMEM8C | - |
| C4orf6 | HGLibA_06346 | 306 | 1.896415493 | C4orf6 | - |
| ENSG00000072364 | HGLibA_01116 | 306 | 1.896415493 | AFF4 | AF4/FMR2 family member 4 [Source:HGNC Symbol;Acc:HGNC:17869] |
| ENSG00000072694 | HGLibA_17193 | 306 | 1.896415493 | FCGR2B | Fc fragment of IgG receptor IIb [Source:HGNC Symbol;Acc:HGNC:3618] |
| ENSG00000075239 | HGLibA_00393 | 306 | 1.896415493 | ACAT1 | acetyl-CoA acetyltransferase 1 [Source:HGNC Symbol;Acc:HGNC:93] |
| ENSG00000092841 | HGLibA_30769 | 306 | 1.896415493 | MYL6 | myosin light chain 6 [Source:HGNC Symbol;Acc:HGNC:7587] |
| ENSG00000100281 | HGLibA_21736 | 306 | 1.896415493 | HMGXB4 | HMG-box containing 4 [Source:HGNC Symbol;Acc:HGNC:5003] |

| | | | | | |
|---|---|---|---|---|---|
| ENSG00000119185 | HGLibA_23772 | 306 | 1.896415493 | ITGB1BP1 | integrin subunit beta 1 binding protein 1 [Source:HGNC Symbol;Acc:HGNC:23927] |
| ENSG00000120656 | HGLibA_48205 | 306 | 1.896415493 | TAF12 | TATA-box binding protein associated factor 12 [Source:HGNC Symbol;Acc:HGNC:11545] |
| ENSG00000121101 | HGLibA_49064 | 306 | 1.896415493 | TEX14 | intercellular bridge forming factor [Source:HGNC Symbol;Acc:HGNC:11737]" |
| ENSG00000130703 | HGLibA_34651 | 306 | 1.896415493 | OSBPL2 | oxysterol binding protein like 2 [Source:HGNC Symbol;Acc:HGNC:15761] |
| ENSG00000134046 | HGLibA_28467 | 306 | 1.896415493 | MBD2 | methyl-CpG binding domain protein 2 [Source:HGNC Symbol;Acc:HGNC:6917] |
| ENSG00000136279 | HGLibA_12396 | 306 | 1.896415493 | DBNL | drebrin like [Source:HGNC Symbol;Acc:HGNC:2696] |
| ENSG00000140320 | HGLibA_04109 | 306 | 1.896415493 | BAHD1 | bromo adjacent homology domain containing 1 [Source:HGNC Symbol;Acc:HGNC:29153] |
| ENSG00000147573 | HGLibA_51545 | 306 | 1.896415493 | TRIM55 | tripartite motif containing 55 [Source:HGNC Symbol;Acc:HGNC:14215] |
| ENSG00000151348 | HGLibA_15805 | 306 | 1.896415493 | EXT2 | exostosin glycosyltransferase 2 [Source:HGNC Symbol;Acc:HGNC:3513] |
| ENSG00000157103 | HGLibA_45288 | 306 | 1.896415493 | SLC6A1 | solute carrier family 6 member 1 [Source:HGNC Symbol;Acc:HGNC:11042] |
| ENSG00000158715 | HGLibA_45178 | 306 | 1.896415493 | SLC45A3 | solute carrier family 45 member 3 [Source:HGNC Symbol;Acc:HGNC:8642] |
| ENSG00000158748 | HGLibA_22401 | 306 | 1.896415493 | HTR6 | 5-hydroxytryptamine receptor 6 [Source:HGNC Symbol;Acc:HGNC:5301] |
| ENSG00000164930 | HGLibA_18279 | 306 | 1.896415493 | FZD6 | frizzled class receptor 6 [Source:HGNC Symbol;Acc:HGNC:4044] |
| ENSG00000165300 | HGLibA_45524 | 306 | 1.896415493 | SLITRK5 | SLIT and NTRK like family member 5 [Source:HGNC Symbol;Acc:HGNC:20295] |
| ENSG00000168124 | HGLibA_33664 | 306 | 1.896415493 | OR1F1 | olfactory receptor family 1 subfamily F member 1 [Source:HGNC Symbol;Acc:HGNC:8194] |
| ENSG00000169676 | HGLibA_13970 | 306 | 1.896415493 | DRD5 | dopamine receptor D5 [Source:HGNC Symbol;Acc:HGNC:3026] |
| ENSG00000174279 | HGLibA_15709 | 306 | 1.896415493 | EVX2 | even-skipped homeobox 2 [Source:HGNC Symbol;Acc:HGNC:3507] |
| ENSG00000182318 | HGLibA_57005 | 306 | 1.896415493 | ZSCAN22 | zinc finger and SCAN domain containing 22 [Source:HGNC Symbol;Acc:HGNC:4929] |
| ENSG00000183323 | HGLibA_07576 | 306 | 1.896415493 | CCDC125 | coiled-coil domain containing 125 [Source:HGNC Symbol;Acc:HGNC:28924] |
| ENSG00000196639 | HGLibA_22094 | 306 | 1.896415493 | HRH1 | histamine receptor H1 [Source:HGNC Symbol;Acc:HGNC:5182] |
| ENSG00000198911 | HGLibA_46952 | 306 | 1.896415493 | SREBF2 | sterol regulatory element binding transcription factor 2 [Source:HGNC Symbol;Acc:HGNC:11290] |
| ENSG00000204930 | HGLibA_16520 | 306 | 1.896415493 | FAM221B | family with sequence similarity 221 member B [Source:HGNC Symbol;Acc:HGNC:30762] |
| ENSG00000249581 | HGLibA_10124 | 306 | 1.896415493 | CLRN2 | clarin 2 [Source:HGNC Symbol;Acc:HGNC:33939] |
| ENSG00000256797 | HGLibA_25306 | 306 | 1.896415493 | KLRF2 | killer cell lectin like receptor F2 [Source:HGNC Symbol;Acc:HGNC:37646] |
| ENSG00000257949 | HGLibA_48988 | 306 | 1.896415493 | TEN1 | CST complex subunit [Source:HGNC Symbol;Acc:HGNC:37242]" |
| ENSG00000272573 | HGLibA_30581 | 306 | 1.896415493 | MUSTN1 | embryonic nuclear protein 1 [Source:HGNC Symbol;Acc:HGNC:22144]" |
| hsa-mir-1178 | HGLibA_57206 | 306 | 1.896415493 | hsa-mir-1178 | - |
| SEPW1 | HGLibA_43461 | 306 | 1.896415493 | SEPW1 | - |
| ENSG00000095713 | HGLibA_11219 | 305 | 1.886464456 | CRTAC1 | cartilage acidic protein 1 [Source:HGNC Symbol;Acc:HGNC:14882] |
| ENSG00000115415 | HGLibA_47382 | 305 | 1.886464456 | STAT1 | signal transducer and activator of transcription 1 [Source:HGNC Symbol;Acc:HGNC:11362] |
| ENSG00000115956 | HGLibA_37055 | 305 | 1.886464456 | PLEK | pleckstrin [Source:HGNC Symbol;Acc:HGNC:9070] |
| ENSG00000117834 | HGLibA_45287 | 305 | 1.886464456 | SLC5A9 | solute carrier family 5 member 9 [Source:HGNC Symbol;Acc:HGNC:22146] |
| ENSG00000126822 | HGLibA_37107 | 305 | 1.886464456 | PLEKHG3 | pleckstrin homology and RhoGEF domain containing G3 [Source:HGNC Symbol;Acc:HGNC:20364] |
| ENSG00000133216 | HGLibA_15314 | 305 | 1.886464456 | EPHB2 | EPH receptor B2 [Source:HGNC Symbol;Acc:HGNC:3393] |
| ENSG00000140993 | HGLibA_49481 | 305 | 1.886464456 | TIGD7 | tigger transposable element derived 7 [Source:HGNC Symbol;Acc:HGNC:18331] |
| ENSG00000146360 | HGLibA_20060 | 305 | 1.886464456 | GPR6 | G protein-coupled receptor 6 [Source:HGNC Symbol;Acc:HGNC:4515] |
| ENSG00000149577 | HGLibA_44117 | 305 | 1.886464456 | SIDT2 | SID1 transmembrane family member 2 [Source:HGNC Symbol;Acc:HGNC:24272] |
| ENSG00000150768 | HGLibA_13308 | 305 | 1.886464456 | DLAT | dihydrolipoamide S-acetyltransferase [Source:HGNC Symbol;Acc:HGNC:2896] |
| ENSG00000164626 | HGLibA_24328 | 305 | 1.886464456 | KCNK5 | potassium two pore domain channel subfamily K member 5 [Source:HGNC Symbol;Acc:HGNC:6280] |
| ENSG00000167904 | HGLibA_50494 | 305 | 1.886464456 | TMEM68 | transmembrane protein 68 [Source:HGNC Symbol;Acc:HGNC:26510] |
| ENSG00000168994 | HGLibA_39647 | 305 | 1.886464456 | PXDC1 | PX domain containing 1 [Source:HGNC Symbol;Acc:HGNC:21361] |
| ENSG00000169230 | HGLibA_38429 | 305 | 1.886464456 | PRELID1 | PRELI domain containing 1 [Source:HGNC Symbol;Acc:HGNC:30255] |
| ENSG00000169710 | HGLibA_16895 | 305 | 1.886464456 | FASN | fatty acid synthase [Source:HGNC Symbol;Acc:HGNC:3594] |
| ENSG00000174473 | HGLibA_18581 | 305 | 1.886464456 | GALNTL6 | polypeptide N-acetylgalactosaminyltransferase like 6 [Source:HGNC Symbol;Acc:HGNC:33844] |
| ENSG00000174776 | HGLibA_54111 | 305 | 1.886464456 | WDR49 | WD repeat domain 49 [Source:HGNC Symbol;Acc:HGNC:26587] |
| ENSG00000183695 | HGLibA_29849 | 305 | 1.886464456 | MRGPRX2 | MAS related GPR family member X2 [Source:HGNC Symbol;Acc:HGNC:17983] |
| ENSG00000184471 | HGLibA_05956 | 305 | 1.886464456 | C1QTNF8 | C1q and TNF related 8 [Source:HGNC Symbol;Acc:HGNC:31374] |
| ENSG00000184788 | HGLibA_42781 | 305 | 1.886464456 | SATL1 | spermidine/spermine N1-acetyl transferase like 1 [Source:HGNC Symbol;Acc:HGNC:27992] |
| ENSG00000196683 | HGLibA_50980 | 305 | 1.886464456 | TOMM7 | translocase of outer mitochondrial membrane 7 [Source:HGNC Symbol;Acc:HGNC:21648] |
| hsa-mir-4698 | HGLibA_60902 | 305 | 1.886464456 | hsa-mir-4698 | - |
| hsa-mir-597 | HGLibA_62480 | 305 | 1.886464456 | hsa-mir-597 | - |
| hsa-mir-676 | HGLibA_63196 | 305 | 1.886464456 | hsa-mir-676 | - |
| hsa-mir-6893 | HGLibA_63757 | 305 | 1.886464456 | hsa-mir-6893 | - |
| KIRREL | HGLibA_25001 | 305 | 1.886464456 | KIRREL | - |
| RSG1 | HGLibA_42314 | 305 | 1.886464456 | RSG1 | - |
| ENSG00000006327 | HGLibA_50750 | 304 | 1.876513418 | TNFRSF12A | TNF receptor superfamily member 12A [Source:HGNC Symbol;Acc:HGNC:18152] |
| ENSG00000006530 | HGLibA_01193 | 304 | 1.876513418 | AGK | acylglycerol kinase [Source:HGNC Symbol;Acc:HGNC:21869] |
| ENSG00000019582 | HGLibA_08444 | 304 | 1.876513418 | CD74 | CD74 molecule [Source:HGNC Symbol;Acc:HGNC:1697] |
| ENSG00000089091 | HGLibA_14296 | 304 | 1.876513418 | DZANK1 | double zinc ribbon and ankyrin repeat domains 1 [Source:HGNC Symbol;Acc:HGNC:15858] |
| ENSG00000101577 | HGLibA_27099 | 304 | 1.876513418 | LPIN2 | lipin 2 [Source:HGNC Symbol;Acc:HGNC:14450] |
| ENSG00000102977 | HGLibA_00419 | 304 | 1.876513418 | ACD | shelterin complex subunit and telomerase recruitment factor [Source:HGNC Symbol;Acc:HGNC:25070]" |
| ENSG00000107854 | HGLibA_50857 | 304 | 1.876513418 | TNKS2 | tankyrase 2 [Source:HGNC Symbol;Acc:HGNC:15677] |
| ENSG00000119969 | HGLibA_21113 | 304 | 1.876513418 | HELLS | lymphoid specific [Source:HGNC Symbol;Acc:HGNC:4861]" |
| ENSG00000133619 | HGLibA_25386 | 304 | 1.876513418 | KRBA1 | KRAB-A domain containing 1 [Source:HGNC Symbol;Acc:HGNC:22228] |
| ENSG00000140479 | HGLibA_35683 | 304 | 1.876513418 | PCSK6 | proprotein convertase subtilisin/kexin type 6 [Source:HGNC Symbol;Acc:HGNC:8569] |
| ENSG00000142303 | HGLibA_00795 | 304 | 1.876513418 | ADAMTS10 | ADAM metallopeptidase with thrombospondin type 1 motif 10 [Source:HGNC Symbol;Acc:HGNC:13201] |
| ENSG00000152266 | HGLibA_39363 | 304 | 1.876513418 | PTH | parathyroid hormone [Source:HGNC Symbol;Acc:HGNC:9606] |
| ENSG00000164651 | HGLibA_46316 | 304 | 1.876513418 | SP8 | Sp8 transcription factor [Source:HGNC Symbol;Acc:HGNC:19196] |
| ENSG00000166261 | HGLibA_55573 | 304 | 1.876513418 | ZNF202 | zinc finger protein 202 [Source:HGNC Symbol;Acc:HGNC:12994] |
| ENSG00000168028 | HGLibA_42198 | 304 | 1.876513418 | RPSA | ribosomal protein SA [Source:HGNC Symbol;Acc:HGNC:6502] |
| ENSG00000169169 | HGLibA_11023 | 304 | 1.876513418 | CPT1C | carnitine palmitoyltransferase 1C [Source:HGNC Symbol;Acc:HGNC:18540] |
| ENSG00000176428 | HGLibA_53784 | 304 | 1.876513418 | VPS37D | ESCRT-I subunit [Source:HGNC Symbol;Acc:HGNC:18287]" |
| ENSG00000185303 | HGLibA_43768 | 304 | 1.876513418 | SFTPA2 | surfactant protein A2 [Source:HGNC Symbol;Acc:HGNC:10799] |
| ENSG00000186451 | HGLibA_46425 | 304 | 1.876513418 | SPATA12 | spermatogenesis associated 12 [Source:HGNC Symbol;Acc:HGNC:23221] |
| ENSG00000188026 | HGLibA_41254 | 304 | 1.876513418 | RILPL1 | Rab interacting lysosomal protein like 1 [Source:HGNC Symbol;Acc:HGNC:26814] |
| ENSG00000188379 | HGLibA_22675 | 304 | 1.876513418 | IFNA2 | interferon alpha 2 [Source:HGNC Symbol;Acc:HGNC:5423] |
| ENSG00000197780 | HGLibA_48206 | 304 | 1.876513418 | TAF13 | TATA-box binding protein associated factor 13 [Source:HGNC Symbol;Acc:HGNC:11546] |
| ENSG00000197860 | HGLibA_43871 | 304 | 1.876513418 | SGTB | small glutamine rich tetratricopeptide repeat containing beta [Source:HGNC Symbol;Acc:HGNC:23567] |
| ENSG00000198589 | HGLibA_27134 | 304 | 1.876513418 | LRBA | LPS responsive beige-like anchor protein [Source:HGNC Symbol;Acc:HGNC:1742] |
| ENSG00000214376 | HGLibA_53871 | 304 | 1.876513418 | VSTM5 | V-set and transmembrane domain containing 5 [Source:HGNC Symbol;Acc:HGNC:34443] |
| ENSG00000233803 | HGLibA_51973 | 304 | 1.876513418 | TSPY4 | testis specific protein Y-linked 4 [Source:HGNC Symbol;Acc:HGNC:37287] |
| ENSG00000234616 | HGLibA_23992 | 304 | 1.876513418 | JRK | Jrk helix-turn-helix protein [Source:HGNC Symbol;Acc:HGNC:6199] |
| ENSG00000261509 | HGLibA_51082 | 304 | 1.876513418 | TP53TG3B | TP53 target 3B [Source:HGNC Symbol;Acc:HGNC:37202] |
| ENSG00000270601 | HGLibA_38319 | 304 | 1.876513418 | PRAMEF5 | PRAME family member 5 [Source:HGNC Symbol;Acc:HGNC:27995] |
| ENSG00000270806 | HGLibA_05541 | 304 | 1.876513418 | C17orf50 | chromosome 17 open reading frame 50 [Source:HGNC Symbol;Acc:HGNC:29581] |
| ENSG00000284609 | HGLibA_34500 | 304 | 1.876513418 | OR8B3 | olfactory receptor family 8 subfamily B member 3 [Source:HGNC Symbol;Acc:HGNC:8472] |
| ENSG00000015475 | HGLibA_04500 | 303 | 1.866562381 | BID | BH3 interacting domain death agonist [Source:HGNC Symbol;Acc:HGNC:1050] |
| ENSG00000021461 | HGLibA_12156 | 303 | 1.866562381 | CYP3A43 | cytochrome P450 family 3 subfamily A member 43 [Source:HGNC Symbol;Acc:HGNC:17450] |
| ENSG00000049656 | HGLibA_10116 | 303 | 1.866562381 | CLPTM1L | CLPTM1 like [Source:HGNC Symbol;Acc:HGNC:24308] |
| ENSG00000099804 | HGLibA_08541 | 303 | 1.866562381 | CDC34 | cell division cycle 34 [Source:HGNC Symbol;Acc:HGNC:1734] |
| ENSG00000102098 | HGLibA_42962 | 303 | 1.866562381 | SCML2 | Scm polycomb group protein like 2 [Source:HGNC Symbol;Acc:HGNC:10581] |
| ENSG00000106211 | HGLibA_22301 | 303 | 1.866562381 | HSPB1 | heat shock protein family B (small) member 1 [Source:HGNC Symbol;Acc:HGNC:5246] |
| ENSG00000109182 | HGLibA_11830 | 303 | 1.866562381 | CWH43 | cell wall biogenesis 43 C-terminal homolog [Source:HGNC Symbol;Acc:HGNC:26133] |
| ENSG00000116209 | HGLibA_50458 | 303 | 1.866562381 | TMEM59 | transmembrane protein 59 [Source:HGNC Symbol;Acc:HGNC:1239] |
| ENSG00000127329 | HGLibA_39488 | 303 | 1.866562381 | PTPRB | receptor type B [Source:HGNC Symbol;Acc:HGNC:9665]" |
| ENSG00000132958 | HGLibA_51216 | 303 | 1.866562381 | TPTE2 | transmembrane phosphoinositide 3-phosphatase and tensin homolog 2 [Source:HGNC Symbol;Acc:HGNC:17299] |
| ENSG00000135341 | HGLibA_28195 | 303 | 1.866562381 | MAP3K7 | mitogen-activated protein kinase kinase kinase 7 [Source:HGNC Symbol;Acc:HGNC:6859] |
| ENSG00000136731 | HGLibA_52866 | 303 | 1.866562381 | UGGT1 | UDP-glucose glycoprotein glucosyltransferase 1 [Source:HGNC Symbol;Acc:HGNC:15663] |
| ENSG00000142655 | HGLibA_36099 | 303 | 1.866562381 | PEX14 | peroxisomal biogenesis factor 14 [Source:HGNC Symbol;Acc:HGNC:8856] |
| ENSG00000155066 | HGLibA_38682 | 303 | 1.866562381 | PROM2 | prominin 2 [Source:HGNC Symbol;Acc:HGNC:20685] |
| ENSG00000157827 | HGLibA_17695 | 303 | 1.866562381 | FMNL2 | formin like 2 [Source:HGNC Symbol;Acc:HGNC:18267] |
| ENSG00000169469 | HGLibA_46805 | 303 | 1.866562381 | SPRR1B | small proline rich protein 1B [Source:HGNC Symbol;Acc:HGNC:11260] |
| ENSG00000169871 | HGLibA_51548 | 303 | 1.866562381 | TRIM56 | tripartite motif containing 56 [Source:HGNC Symbol;Acc:HGNC:19028] |
| ENSG00000174032 | HGLibA_44701 | 303 | 1.866562381 | SLC25A30 | solute carrier family 25 member 30 [Source:HGNC Symbol;Acc:HGNC:27371] |
| ENSG00000175287 | HGLibA_36471 | 303 | 1.866562381 | PHYHD1 | phytanoyl-CoA dioxygenase domain containing 1 [Source:HGNC Symbol;Acc:HGNC:23396] |
| ENSG00000176974 | HGLibA_44059 | 303 | 1.866562381 | SHMT1 | serine hydroxymethyltransferase 1 [Source:HGNC Symbol;Acc:HGNC:10850] |
| ENSG00000182952 | HGLibA_21726 | 303 | 1.866562381 | HMGN4 | high mobility group nucleosomal binding domain 4 [Source:HGNC Symbol;Acc:HGNC:4989] |
| ENSG00000183287 | HGLibA_07492 | 303 | 1.866562381 | CCBE1 | collagen and calcium binding EGF domains 1 [Source:HGNC Symbol;Acc:HGNC:29426] |
| ENSG00000185104 | HGLibA_15947 | 303 | 1.866562381 | FAF1 | Fas associated factor 1 [Source:HGNC Symbol;Acc:HGNC:3578] |
| ENSG00000185862 | HGLibA_15689 | 303 | 1.866562381 | EVI2B | ecotropic viral integration site 2B [Source:HGNC Symbol;Acc:HGNC:3500] |
| ENSG00000197125 | HGLibA_34504 | 303 | 1.866562381 | OR8B8 | olfactory receptor family 8 subfamily B member 8 [Source:HGNC Symbol;Acc:HGNC:8477] |
| ENSG00000241360 | HGLibA_35963 | 303 | 1.866562381 | PDXP | pyridoxal phosphatase [Source:HGNC Symbol;Acc:HGNC:30259] |
| hsa-mir-6751 | HGLibA_63161 | 303 | 1.866562381 | hsa-mir-6751 | - |
| C16orf11 | HGLibA_05437 | 302 | 1.856611344 | C16orf11 | - |
| C17orf47 | HGLibA_05533 | 302 | 1.856611344 | C17orf47 | - |
| C5orf30 | HGLibA_06374 | 302 | 1.856611344 | C5orf30 | - |
| ENSG00000001561 | HGLibA_15177 | 302 | 1.856611344 | ENPP4 | ectonucleotide pyrophosphatase/phosphodiesterase 4 [Source:HGNC Symbol;Acc:HGNC:3359] |
| ENSG00000008441 | HGLibA_31813 | 302 | 1.856611344 | NFIX | nuclear factor I X [Source:HGNC Symbol;Acc:HGNC:7788] |
| ENSG00000056972 | HGLibA_51252 | 302 | 1.856611344 | TRAF3IP2 | TRAF3 interacting protein 2 [Source:HGNC Symbol;Acc:HGNC:1343] |
| ENSG00000075142 | HGLibA_46989 | 302 | 1.856611344 | SRI | sorcin [Source:HGNC Symbol;Acc:HGNC:11292] |
| ENSG00000082556 | HGLibA_33447 | 302 | 1.856611344 | OPRK1 | opioid receptor kappa 1 [Source:HGNC Symbol;Acc:HGNC:8154] |
| ENSG00000100060 | HGLibA_29074 | 302 | 1.856611344 | MFNG | MFNG O-fucosylpeptide 3-beta-N-acetylglucosaminyltransferase [Source:HGNC Symbol;Acc:HGNC:7038] |
| ENSG00000100867 | HGLibA_13129 | 302 | 1.856611344 | DHRS2 | dehydrogenase/reductase 2 [Source:HGNC Symbol;Acc:HGNC:18349] |
| ENSG00000112394 | HGLibA_44420 | 302 | 1.856611344 | SLC16A10 | solute carrier family 16 member 10 [Source:HGNC Symbol;Acc:HGNC:17027] |
| ENSG00000116017 | HGLibA_02890 | 302 | 1.856611344 | ARID3A | AT-rich interaction domain 3A [Source:HGNC Symbol;Acc:HGNC:3031] |
| ENSG00000116213 | HGLibA_54403 | 302 | 1.856611344 | WRAP73 | antisense to TP73 [Source:HGNC Symbol;Acc:HGNC:12759]" |
| ENSG00000123983 | HGLibA_00556 | 302 | 1.856611344 | ACSL3 | acyl-CoA synthetase long chain family member 3 [Source:HGNC Symbol;Acc:HGNC:3570] |
| ENSG00000125813 | HGLibA_35294 | 302 | 1.856611344 | PAX1 | paired box 1 [Source:HGNC Symbol;Acc:HGNC:8615] |
| ENSG00000152192 | HGLibA_37776 | 302 | 1.856611344 | POU4F1 | POU class 4 homeobox 1 [Source:HGNC Symbol;Acc:HGNC:9218] |
| ENSG00000155542 | HGLibA_43665 | 302 | 1.856611344 | SETD9 | SET domain containing 9 [Source:HGNC Symbol;Acc:HGNC:28508] |
| ENSG00000157637 | HGLibA_45058 | 302 | 1.856611344 | SLC38A10 | solute carrier family 38 member 10 [Source:HGNC Symbol;Acc:HGNC:28237] |
| ENSG00000159409 | HGLibA_09009 | 302 | 1.856611344 | CELF3 | CUGBP Elav-like family member 3 [Source:HGNC Symbol;Acc:HGNC:11967] |
| ENSG00000162755 | HGLibA_25116 | 302 | 1.856611344 | KLHDC9 | kelch domain containing 9 [Source:HGNC Symbol;Acc:HGNC:28489] |
| ENSG00000163378 | HGLibA_15223 | 302 | 1.856611344 | EOGT | EGF domain specific O-linked N-acetylglucosamine transferase [Source:HGNC Symbol;Acc:HGNC:28526] |
| ENSG00000163739 | HGLibA_11842 | 302 | 1.856611344 | CXCL1 | C-X-C motif chemokine ligand 1 [Source:HGNC Symbol;Acc:HGNC:4602] |

| | | | | | |
|---|---|---|---|---|---|
| ENSG00000164776 | HGLibA_36411 | 302 | 1.856611344 | PHKG1 | phosphorylase kinase catalytic subunit gamma 1 [Source:HGNC Symbol;Acc:HGNC:8930] |
| ENSG00000175040 | HGLibA_09632 | 302 | 1.856611344 | CHST2 | carbohydrate sulfotransferase 2 [Source:HGNC Symbol;Acc:HGNC:1970] |
| ENSG00000176340 | HGLibA_10876 | 302 | 1.856611344 | COX8A | cytochrome c oxidase subunit 8A [Source:HGNC Symbol;Acc:HGNC:2294] |
| ENSG00000176532 | HGLibA_38786 | 302 | 1.856611344 | PRR15 | proline rich 15 [Source:HGNC Symbol;Acc:HGNC:22310] |
| ENSG00000177971 | HGLibA_23237 | 302 | 1.856611344 | IMP3 | U3 small nucleolar ribonucleoprotein [Source:HGNC Symbol;Acc:HGNC:14497]" |
| ENSG00000184454 | HGLibA_31333 | 302 | 1.856611344 | NCMAP | non-compact myelin associated protein [Source:HGNC Symbol;Acc:HGNC:29332] |
| ENSG00000204344 | HGLibA_47467 | 302 | 1.856611344 | STK19 | serine/threonine kinase 19 [Source:HGNC Symbol;Acc:HGNC:11398] |
| ENSG00000204610 | HGLibA_51413 | 302 | 1.856611344 | TRIM15 | tripartite motif containing 15 [Source:HGNC Symbol;Acc:HGNC:16284] |
| hsa-mir-4257 | HGLibA_59736 | 302 | 1.856611344 | hsa-mir-4257 | - |
| hsa-mir-548h-3 | HGLibA_62000 | 302 | 1.856611344 | hsa-mir-548h-3 | - |
| hsa-mir-574 | HGLibA_62390 | 302 | 1.856611344 | hsa-mir-574 | - |
| hsa-mir-646 | HGLibA_62828 | 302 | 1.856611344 | hsa-mir-646 | - |
| MUT | HGLibA_30583 | 302 | 1.856611344 | MUT | - |
| PAK7 | HGLibA_35054 | 302 | 1.856611344 | PAK7 | - |
| AQPEP | HGLibA_02615 | 301 | 1.846660306 | AQPEP | - |
| C20orf196 | HGLibA_06006 | 301 | 1.846660306 | C20orf196 | - |
| ENSG00000068137 | HGLibA_37132 | 301 | 1.846660306 | PLEKHH3 | MyTH4 and FERM domain containing H3 [Source:HGNC Symbol;Acc:HGNC:26105]" |
| ENSG00000079263 | HGLibA_46290 | 301 | 1.846660306 | SP140 | SP140 nuclear body protein [Source:HGNC Symbol;Acc:HGNC:17133] |
| ENSG00000100170 | HGLibA_45252 | 301 | 1.846660306 | SLC5A1 | solute carrier family 5 member 1 [Source:HGNC Symbol;Acc:HGNC:11036] |
| ENSG00000104518 | HGLibA_20414 | 301 | 1.846660306 | GSDMD | gasdermin D [Source:HGNC Symbol;Acc:HGNC:25697] |
| ENSG00000122729 | HGLibA_00461 | 301 | 1.846660306 | ACO1 | aconitase 1 [Source:HGNC Symbol;Acc:HGNC:117] |
| ENSG00000129151 | HGLibA_04203 | 301 | 1.846660306 | BBOX1 | gamma-butyrobetaine hydroxylase 1 [Source:HGNC Symbol;Acc:HGNC:964] |
| ENSG00000131069 | HGLibA_00590 | 301 | 1.846660306 | ACSS2 | acyl-CoA synthetase short chain family member 2 [Source:HGNC Symbol;Acc:HGNC:15814] |
| ENSG00000132004 | HGLibA_17160 | 301 | 1.846660306 | FBXW9 | F-box and WD repeat domain containing 9 [Source:HGNC Symbol;Acc:HGNC:28136] |
| ENSG00000135919 | HGLibA_43581 | 301 | 1.846660306 | SERPINE2 | serpin family E member 2 [Source:HGNC Symbol;Acc:HGNC:8951] |
| ENSG00000148826 | HGLibA_32059 | 301 | 1.846660306 | NKX6-2 | NK6 homeobox 2 [Source:HGNC Symbol;Acc:HGNC:19321] |
| ENSG00000156575 | HGLibA_38454 | 301 | 1.846660306 | PRG3 | pro eosinophil major basic protein 2 [Source:HGNC Symbol;Acc:HGNC:9363]" |
| ENSG00000157985 | HGLibA_01136 | 301 | 1.846660306 | AGAP1 | ankyrin repeat and PH domain 1 [Source:HGNC Symbol;Acc:HGNC:16922]" |
| ENSG00000164106 | HGLibA_43044 | 301 | 1.846660306 | SCRG1 | stimulator of chondrogenesis 1 [Source:HGNC Symbol;Acc:HGNC:17036] |
| ENSG00000165702 | HGLibA_18943 | 301 | 1.846660306 | GFI1B | growth factor independent 1B transcriptional repressor [Source:HGNC Symbol;Acc:HGNC:4238] |
| ENSG00000167112 | HGLibA_51806 | 301 | 1.846660306 | TRUB2 | TruB pseudouridine synthase family member 2 [Source:HGNC Symbol;Acc:HGNC:17170] |
| ENSG00000167972 | HGLibA_00109 | 301 | 1.846660306 | ABCA3 | ATP binding cassette subfamily A member 3 [Source:HGNC Symbol;Acc:HGNC:33] |
| ENSG00000172354 | HGLibA_19489 | 301 | 1.846660306 | GNB2 | G protein subunit beta 2 [Source:HGNC Symbol;Acc:HGNC:4398] |
| ENSG00000176979 | HGLibA_51564 | 301 | 1.846660306 | TRIM60 | tripartite motif containing 60 [Source:HGNC Symbol;Acc:HGNC:21162] |
| ENSG00000186458 | HGLibA_12901 | 301 | 1.846660306 | DEFB132 | defensin beta 132 [Source:HGNC Symbol;Acc:HGNC:33806] |
| ENSG00000186787 | HGLibA_46673 | 301 | 1.846660306 | SPIN2B | spindlin family member 2B [Source:HGNC Symbol;Acc:HGNC:33147] |
| ENSG00000186881 | HGLibA_33617 | 301 | 1.846660306 | OR13F1 | olfactory receptor family 13 subfamily F member 1 [Source:HGNC Symbol;Acc:HGNC:14723] |
| ENSG00000204991 | HGLibA_46728 | 301 | 1.846660306 | SPIRE2 | spire type actin nucleation factor 2 [Source:HGNC Symbol;Acc:HGNC:30623] |
| hsa-mir-4452 | HGLibA_60214 | 301 | 1.846660306 | hsa-mir-4452 | - |
| hsa-mir-454 | HGLibA_60621 | 301 | 1.846660306 | hsa-mir-454 | - |
| ENSG00000077150 | HGLibA_31817 | 300 | 1.836709269 | NFKB2 | nuclear factor kappa B subunit 2 [Source:HGNC Symbol;Acc:HGNC:7795] |
| ENSG00000078061 | HGLibA_02622 | 300 | 1.836709269 | ARAF | serine/threonine kinase [Source:HGNC Symbol;Acc:HGNC:646]" |
| ENSG00000106536 | HGLibA_37798 | 300 | 1.836709269 | POU6F2 | POU class 6 homeobox 2 [Source:HGNC Symbol;Acc:HGNC:21694] |
| ENSG00000112769 | HGLibA_25950 | 300 | 1.836709269 | LAMA4 | laminin subunit alpha 4 [Source:HGNC Symbol;Acc:HGNC:6484] |
| ENSG00000120437 | HGLibA_00395 | 300 | 1.836709269 | ACAT2 | acetyl-CoA acetyltransferase 2 [Source:HGNC Symbol;Acc:HGNC:94] |
| ENSG00000120896 | HGLibA_46180 | 300 | 1.836709269 | SORBS3 | sorbin and SH3 domain containing 3 [Source:HGNC Symbol;Acc:HGNC:30907] |
| ENSG00000121211 | HGLibA_29563 | 300 | 1.836709269 | MND1 | meiotic nuclear divisions 1 [Source:HGNC Symbol;Acc:HGNC:24839] |
| ENSG00000126218 | HGLibA_15842 | 300 | 1.836709269 | F10 | coagulation factor X [Source:HGNC Symbol;Acc:HGNC:3528] |
| ENSG00000136144 | HGLibA_40678 | 300 | 1.836709269 | RCBTB1 | RCC1 and BTB domain containing protein 1 [Source:HGNC Symbol;Acc:HGNC:18243] |
| ENSG00000145425 | HGLibA_42139 | 300 | 1.836709269 | RPS3A | ribosomal protein S3A [Source:HGNC Symbol;Acc:HGNC:10421] |
| ENSG00000150990 | HGLibA_13193 | 300 | 1.836709269 | DHX37 | DEAH-box helicase 37 [Source:HGNC Symbol;Acc:HGNC:17210] |
| ENSG00000158486 | HGLibA_13485 | 300 | 1.836709269 | DNAH3 | dynein axonemal heavy chain 3 [Source:HGNC Symbol;Acc:HGNC:2949] |
| ENSG00000162852 | HGLibA_10383 | 300 | 1.836709269 | CNST | connexin sorting protein [Source:HGNC Symbol;Acc:HGNC:26486]" |
| ENSG00000164077 | HGLibA_29638 | 300 | 1.836709269 | MON1A | secretory trafficking associated [Source:HGNC Symbol;Acc:HGNC:28207]" |
| ENSG00000164164 | HGLibA_34766 | 300 | 1.836709269 | OTUD4 | OTU deubiquitinase 4 [Source:HGNC Symbol;Acc:HGNC:24949] |
| ENSG00000164649 | HGLibA_08618 | 300 | 1.836709269 | CDCA7L | cell division cycle associated 7 like [Source:HGNC Symbol;Acc:HGNC:30777] |
| ENSG00000167880 | HGLibA_15701 | 300 | 1.836709269 | EVPL | envoplakin [Source:HGNC Symbol;Acc:HGNC:3503] |
| ENSG00000172348 | HGLibA_40670 | 300 | 1.836709269 | RCAN2 | regulator of calcineurin 2 [Source:HGNC Symbol;Acc:HGNC:3041] |
| ENSG00000179958 | HGLibA_12562 | 300 | 1.836709269 | DCTPP1 | dCTP pyrophosphatase 1 [Source:HGNC Symbol;Acc:HGNC:28777] |
| ENSG00000183840 | HGLibA_20038 | 300 | 1.836709269 | GPR39 | G protein-coupled receptor 39 [Source:HGNC Symbol;Acc:HGNC:4496] |
| ENSG00000184343 | HGLibA_47023 | 300 | 1.836709269 | SRPK3 | SRSF protein kinase 3 [Source:HGNC Symbol;Acc:HGNC:11402] |
| ENSG00000204525 | HGLibA_21600 | 300 | 1.836709269 | HLA-C | C [Source:HGNC Symbol;Acc:HGNC:4933]" |
| ENSG00000223601 | HGLibA_14365 | 300 | 1.836709269 | EBLN1 | endogenous Bornavirus like nucleoprotein 1 [Source:HGNC Symbol;Acc:HGNC:39430] |
| hsa-mir-516b-2 | HGLibA_61619 | 300 | 1.836709269 | hsa-mir-516b-2 | - |
| hsa-mir-6860 | HGLibA_63619 | 300 | 1.836709269 | hsa-mir-6860 | - |
| hsa-mir-6881 | HGLibA_63707 | 300 | 1.836709269 | hsa-mir-6881 | - |
| PGBD3 | HGLibA_36222 | 300 | 1.836709269 | PGBD3 | - |
| 3-Sep | HGLibA_43439 | 300 | 1.836709269 | 3-Sep | - |
| C5orf45 | HGLibA_06385 | 299 | 1.826758231 | C5orf45 | - |
| ENSG00000008382 | HGLibA_29741 | 299 | 1.826758231 | MPND | MPN domain containing [Source:HGNC Symbol;Acc:HGNC:25934] |
| ENSG00000013810 | HGLibA_48166 | 299 | 1.826758231 | TACC3 | transforming acidic coiled-coil containing protein 3 [Source:HGNC Symbol;Acc:HGNC:11524] |
| ENSG00000048140 | HGLibA_51912 | 299 | 1.826758231 | TSPAN17 | tetraspanin 17 [Source:HGNC Symbol;Acc:HGNC:13594] |
| ENSG00000090534 | HGLibA_49386 | 299 | 1.826758231 | THPO | thrombopoietin [Source:HGNC Symbol;Acc:HGNC:11795] |
| ENSG00000103522 | HGLibA_23085 | 299 | 1.826758231 | IL21R | interleukin 21 receptor [Source:HGNC Symbol;Acc:HGNC:6006] |
| ENSG00000104332 | HGLibA_43736 | 299 | 1.826758231 | SFRP1 | secreted frizzled related protein 1 [Source:HGNC Symbol;Acc:HGNC:10776] |
| ENSG00000109063 | HGLibA_30725 | 299 | 1.826758231 | MYH3 | myosin heavy chain 3 [Source:HGNC Symbol;Acc:HGNC:7573] |
| ENSG00000119953 | HGLibA_45788 | 299 | 1.826758231 | SMNDC1 | survival motor neuron domain containing 1 [Source:HGNC Symbol;Acc:HGNC:16900] |
| ENSG00000120256 | HGLibA_27202 | 299 | 1.826758231 | LRP11 | LDL receptor related protein 11 [Source:HGNC Symbol;Acc:HGNC:16936] |
| ENSG00000120784 | HGLibA_55202 | 299 | 1.826758231 | ZFP30 | ZFP30 zinc finger protein [Source:HGNC Symbol;Acc:HGNC:29555] |
| ENSG00000120820 | HGLibA_19340 | 299 | 1.826758231 | GLT8D2 | glycosyltransferase 8 domain containing 2 [Source:HGNC Symbol;Acc:HGNC:24890] |
| ENSG00000124232 | HGLibA_40650 | 299 | 1.826758231 | RBPJL | recombination signal binding protein for immunoglobulin kappa J region like [Source:HGNC Symbol;Acc:HGNC:13761] |
| ENSG00000135569 | HGLibA_48128 | 299 | 1.826758231 | TAAR5 | trace amine associated receptor 5 [Source:HGNC Symbol;Acc:HGNC:30236] |
| ENSG00000137880 | HGLibA_18784 | 299 | 1.826758231 | GCHFR | GTP cyclohydrolase I feedback regulator [Source:HGNC Symbol;Acc:HGNC:4194] |
| ENSG00000139364 | HGLibA_49934 | 299 | 1.826758231 | TMEM132B | transmembrane protein 132B [Source:HGNC Symbol;Acc:HGNC:29397] |
| ENSG00000142733 | HGLibA_28193 | 299 | 1.826758231 | MAP3K6 | mitogen-activated protein kinase kinase kinase 6 [Source:HGNC Symbol;Acc:HGNC:6858] |
| ENSG00000153246 | HGLibA_36926 | 299 | 1.826758231 | PLA2R1 | phospholipase A2 receptor 1 [Source:HGNC Symbol;Acc:HGNC:9042] |
| ENSG00000156983 | HGLibA_04839 | 299 | 1.826758231 | BRPF1 | bromodomain and PHD finger containing 1 [Source:HGNC Symbol;Acc:HGNC:14255] |
| ENSG00000161944 | HGLibA_03289 | 299 | 1.826758231 | ASGR2 | asialoglycoprotein receptor 2 [Source:HGNC Symbol;Acc:HGNC:743] |
| ENSG00000165215 | HGLibA_09894 | 299 | 1.826758231 | CLDN3 | claudin 3 [Source:HGNC Symbol;Acc:HGNC:2045] |
| ENSG00000169891 | HGLibA_40851 | 299 | 1.826758231 | REPS2 | RALBP1 associated Eps domain containing 2 [Source:HGNC Symbol;Acc:HGNC:9963] |
| ENSG00000177873 | HGLibA_56400 | 299 | 1.826758231 | ZNF619 | zinc finger protein 619 [Source:HGNC Symbol;Acc:HGNC:26910] |
| ENSG00000179292 | HGLibA_49999 | 299 | 1.826758231 | TMEM151A | transmembrane protein 151A [Source:HGNC Symbol;Acc:HGNC:28497] |
| ENSG00000185942 | HGLibA_31994 | 299 | 1.826758231 | NKAIN3 | sodium/potassium transporting ATPase interacting 3 [Source:HGNC Symbol;Acc:HGNC:26829] |
| ENSG00000186493 | HGLibA_06380 | 299 | 1.826758231 | C5orf38 | chromosome 5 open reading frame 38 [Source:HGNC Symbol;Acc:HGNC:24226] |
| ENSG00000187017 | HGLibA_15576 | 299 | 1.826758231 | ESPN | espin [Source:HGNC Symbol;Acc:HGNC:13281] |
| ENSG00000196498 | HGLibA_31360 | 299 | 1.826758231 | NCOR2 | nuclear receptor corepressor 2 [Source:HGNC Symbol;Acc:HGNC:7673] |
| ENSG00000197446 | HGLibA_12133 | 299 | 1.826758231 | CYP2F1 | cytochrome P450 family 2 subfamily F member 1 [Source:HGNC Symbol;Acc:HGNC:2632] |
| ENSG00000204979 | HGLibA_30137 | 299 | 1.826758231 | MS4A13 | membrane spanning 4-domains A13 [Source:HGNC Symbol;Acc:HGNC:16674] |
| ENSG00000241685 | HGLibA_03075 | 299 | 1.826758231 | ARPC1A | actin related protein 2/3 complex subunit 1A [Source:HGNC Symbol;Acc:HGNC:703] |
| hsa-mir-339 | HGLibA_59007 | 299 | 1.826758231 | hsa-mir-339 | - |
| hsa-mir-4444-1 | HGLibA_60177 | 299 | 1.826758231 | hsa-mir-4444-1 | - |
| C4orf27 | HGLibA_06311 | 298 | 1.816807194 | C4orf27 | - |
| ENSG00000035141 | HGLibA_16137 | 298 | 1.816807194 | FAM136A | family with sequence similarity 136 member A [Source:HGNC Symbol;Acc:HGNC:25911] |
| ENSG00000064687 | HGLibA_00123 | 298 | 1.816807194 | ABCA7 | ATP binding cassette subfamily A member 7 [Source:HGNC Symbol;Acc:HGNC:37] |
| ENSG00000099994 | HGLibA_47846 | 298 | 1.816807194 | SUSD2 | sushi domain containing 2 [Source:HGNC Symbol;Acc:HGNC:30667] |
| ENSG00000104960 | HGLibA_39399 | 298 | 1.816807194 | PTOV1 | prostate tumor overexpressed 1 [Source:HGNC Symbol;Acc:HGNC:9632] |
| ENSG00000108947 | HGLibA_14603 | 298 | 1.816807194 | EFNB3 | ephrin B3 [Source:HGNC Symbol;Acc:HGNC:3228] |
| ENSG00000114023 | HGLibA_16223 | 298 | 1.816807194 | FAM162A | family with sequence similarity 162 member A [Source:HGNC Symbol;Acc:HGNC:17865] |
| ENSG00000119686 | HGLibA_17677 | 298 | 1.816807194 | FLVCR2 | feline leukemia virus subgroup C cellular receptor family member 2 [Source:HGNC Symbol;Acc:HGNC:20105] |
| ENSG00000121060 | HGLibA_51442 | 298 | 1.816807194 | TRIM25 | tripartite motif containing 25 [Source:HGNC Symbol;Acc:HGNC:12932] |
| ENSG00000121570 | HGLibA_13894 | 298 | 1.816807194 | DPPA4 | developmental pluripotency associated 4 [Source:HGNC Symbol;Acc:HGNC:19200] |
| ENSG00000128567 | HGLibA_37459 | 298 | 1.816807194 | PODXL | podocalyxin like [Source:HGNC Symbol;Acc:HGNC:9171] |
| ENSG00000141741 | HGLibA_29250 | 298 | 1.816807194 | MIEN1 | migration and invasion enhancer 1 [Source:HGNC Symbol;Acc:HGNC:28230] |
| ENSG00000161649 | HGLibA_08344 | 298 | 1.816807194 | CD300LG | CD300 molecule like family member g [Source:HGNC Symbol;Acc:HGNC:30455] |
| ENSG00000163945 | HGLibA_53475 | 298 | 1.816807194 | UVSSA | UV stimulated scaffold protein A [Source:HGNC Symbol;Acc:HGNC:29304] |
| ENSG00000164221 | HGLibA_07545 | 298 | 1.816807194 | CCDC112 | coiled-coil domain containing 112 [Source:HGNC Symbol;Acc:HGNC:28599] |
| ENSG00000164889 | HGLibA_45215 | 298 | 1.816807194 | SLC4A2 | solute carrier family 4 member 2 [Source:HGNC Symbol;Acc:HGNC:11028] |
| ENSG00000166265 | HGLibA_12252 | 298 | 1.816807194 | CYYR1 | cysteine and tyrosine rich 1 [Source:HGNC Symbol;Acc:HGNC:16274] |
| ENSG00000171792 | HGLibA_41161 | 298 | 1.816807194 | RHNO1 | RAD9-HUS1-RAD1 interacting nuclear orphan 1 [Source:HGNC Symbol;Acc:HGNC:28206] |
| ENSG00000176390 | HGLibA_11196 | 298 | 1.816807194 | CRLF3 | cytokine receptor like factor 3 [Source:HGNC Symbol;Acc:HGNC:17177] |
| ENSG00000177663 | HGLibA_23004 | 298 | 1.816807194 | IL17RA | interleukin 17 receptor A [Source:HGNC Symbol;Acc:HGNC:5985] |
| ENSG00000183977 | HGLibA_37801 | 298 | 1.816807194 | PP2D1 | protein phosphatase 2C like domain containing 1 [Source:HGNC Symbol;Acc:HGNC:28406] |
| ENSG00000184500 | HGLibA_38689 | 298 | 1.816807194 | PROS1 | protein S [Source:HGNC Symbol;Acc:HGNC:9456] |
| ENSG00000189007 | HGLibA_00882 | 298 | 1.816807194 | ADAT2 | tRNA specific 2 [Source:HGNC Symbol;Acc:HGNC:21172]" |
| ENSG00000197651 | HGLibA_07942 | 298 | 1.816807194 | CCER1 | coiled-coil glutamate rich protein 1 [Source:HGNC Symbol;Acc:HGNC:28373] |
| ENSG00000277893 | HGLibA_46943 | 298 | 1.816807194 | SRD5A2 | steroid 5 alpha-reductase 2 [Source:HGNC Symbol;Acc:HGNC:11285] |
| LOC643669 | HGLibA_26969 | 298 | 1.816807194 | LOC643669 | - |
| SELV | HGLibA_43315 | 298 | 1.816807194 | SELV | - |
| C15orf60 | HGLibA_05425 | 297 | 1.806856156 | C15orf60 | - |
| ENSG00000064601 | HGLibA_11690 | 297 | 1.806856156 | CTSA | cathepsin A [Source:HGNC Symbol;Acc:HGNC:9251] |
| ENSG00000100129 | HGLibA_14799 | 297 | 1.806856156 | EIF3L | eukaryotic translation initiation factor 3 subunit L [Source:HGNC Symbol;Acc:HGNC:18138] |
| ENSG00000110074 | HGLibA_17952 | 297 | 1.806856156 | FOXRED1 | FAD dependent oxidoreductase domain containing 1 [Source:HGNC Symbol;Acc:HGNC:26927] |
| ENSG00000116830 | HGLibA_52171 | 297 | 1.806856156 | TTF2 | transcription termination factor 2 [Source:HGNC Symbol;Acc:HGNC:12398] |
| ENSG00000124067 | HGLibA_44365 | 297 | 1.806856156 | SLC12A4 | solute carrier family 12 member 4 [Source:HGNC Symbol;Acc:HGNC:10913] |
| ENSG00000153012 | HGLibA_26397 | 297 | 1.806856156 | LGI2 | leucine rich repeat LGI family member 2 [Source:HGNC Symbol;Acc:HGNC:18710] |

| | | | | | |
|---|---|---|---|---|---|
| ENSG00000161243 | HGLibA_17056 | 297 | 1.806856156 | FBXO27 | F-box protein 27 [Source:HGNC Symbol;Acc:HGNC:18753] |
| ENSG00000162949 | HGLibA_07153 | 297 | 1.806856156 | CAPN13 | calpain 13 [Source:HGNC Symbol;Acc:HGNC:16663] |
| ENSG00000166800 | HGLibA_26222 | 297 | 1.806856156 | LDHAL6A | lactate dehydrogenase A like 6A [Source:HGNC Symbol;Acc:HGNC:28335] |
| ENSG00000169410 | HGLibA_39482 | 297 | 1.806856156 | PTPN9 | non-receptor type 9 [Source:HGNC Symbol;Acc:HGNC:9661]" |
| ENSG00000173542 | HGLibA_29589 | 297 | 1.806856156 | MOB1B | MOB kinase activator 1B [Source:HGNC Symbol;Acc:HGNC:29801] |
| ENSG00000181031 | HGLibA_41846 | 297 | 1.806856156 | RPH3AL | rabphilin 3A like (without C2 domains) [Source:HGNC Symbol;Acc:HGNC:10296] |
| ENSG00000185156 | HGLibA_29111 | 297 | 1.806856156 | MFSD6L | major facilitator superfamily domain containing 6 like [Source:HGNC Symbol;Acc:HGNC:26656] |
| ENSG00000186119 | HGLibA_34283 | 297 | 1.806856156 | OR5D18 | olfactory receptor family 5 subfamily D member 18 [Source:HGNC Symbol;Acc:HGNC:15285] |
| ENSG00000198453 | HGLibA_56265 | 297 | 1.806856156 | ZNF568 | zinc finger protein 568 [Source:HGNC Symbol;Acc:HGNC:25392] |
| ENSG00000249242 | HGLibA_49996 | 297 | 1.806856156 | TMEM150C | transmembrane protein 150C [Source:HGNC Symbol;Acc:HGNC:37263] |
| hsa-mir-5685 | HGLibA_62258 | 297 | 1.806856156 | hsa-mir-5685 | - |
| hsa-mir-5699 | HGLibA_62329 | 297 | 1.806856156 | hsa-mir-5699 | - |
| hsa-mir-5703 | HGLibA_62353 | 297 | 1.806856156 | hsa-mir-5703 | - |
| hsa-mir-7152 | HGLibA_63838 | 297 | 1.806856156 | hsa-mir-7152 | - |
| 5-Sep | HGLibA_43445 | 297 | 1.806856156 | 5-Sep | - |
| ENSG00000089057 | HGLibA_44609 | 296 | 1.796905119 | SLC23A2 | solute carrier family 23 member 2 [Source:HGNC Symbol;Acc:HGNC:10973] |
| ENSG00000100577 | HGLibA_20504 | 296 | 1.796905119 | GSTZ1 | glutathione S-transferase zeta 1 [Source:HGNC Symbol;Acc:HGNC:4643] |
| ENSG00000105223 | HGLibA_37042 | 296 | 1.796905119 | PLD3 | phospholipase D family member 3 [Source:HGNC Symbol;Acc:HGNC:17158] |
| ENSG00000108849 | HGLibA_38244 | 296 | 1.796905119 | PPY | pancreatic polypeptide [Source:HGNC Symbol;Acc:HGNC:9327] |
| ENSG00000111906 | HGLibA_21034 | 296 | 1.796905119 | HDDC2 | HD domain containing 2 [Source:HGNC Symbol;Acc:HGNC:21078] |
| ENSG00000146385 | HGLibA_48136 | 296 | 1.796905119 | TAAR8 | trace amine associated receptor 8 [Source:HGNC Symbol;Acc:HGNC:14964] |
| ENSG00000159261 | HGLibA_09860 | 296 | 1.796905119 | CLDN14 | claudin 14 [Source:HGNC Symbol;Acc:HGNC:2035] |
| ENSG00000162951 | HGLibA_27485 | 296 | 1.796905119 | LRRTM1 | leucine rich repeat transmembrane neuronal 1 [Source:HGNC Symbol;Acc:HGNC:19408] |
| ENSG00000166181 | HGLibA_02419 | 296 | 1.796905119 | API5 | apoptosis inhibitor 5 [Source:HGNC Symbol;Acc:HGNC:594] |
| ENSG00000167291 | HGLibA_48484 | 296 | 1.796905119 | TBC1D16 | TBC1 domain family member 16 [Source:HGNC Symbol;Acc:HGNC:28356] |
| ENSG00000173805 | HGLibA_20837 | 296 | 1.796905119 | HAP1 | huntingtin associated protein 1 [Source:HGNC Symbol;Acc:HGNC:4812] |
| ENSG00000180269 | HGLibA_19905 | 296 | 1.796905119 | GPR139 | G protein-coupled receptor 139 [Source:HGNC Symbol;Acc:HGNC:19995] |
| ENSG00000182836 | HGLibA_37028 | 296 | 1.796905119 | PLCXD3 | phosphatidylinositol specific phospholipase C X domain containing 3 [Source:HGNC Symbol;Acc:HGNC:31822] |
| ENSG00000250298 | HGLibA_19093 | 296 | 1.796905119 | GIMD1 | GIMAP family P-loop NTPase domain containing 1 [Source:HGNC Symbol;Acc:HGNC:44141] |
| ENSG00000261210 | HGLibA_09951 | 296 | 1.796905119 | CLEC19A | C-type lectin domain containing 19A [Source:HGNC Symbol;Acc:HGNC:34522] |
| FAM45A | HGLibA_16594 | 296 | 1.796905119 | FAM45A | - |
| hsa-mir-3198-2 | HGLibA_58893 | 296 | 1.796905119 | hsa-mir-3198-2 | - |
| hsa-mir-3942 | HGLibA_59627 | 296 | 1.796905119 | hsa-mir-3942 | - |
| hsa-mir-624 | HGLibA_62735 | 296 | 1.796905119 | hsa-mir-624 | - |
| hsa-mir-6755 | HGLibA_63176 | 296 | 1.796905119 | hsa-mir-6755 | - |
| TUSC5 | HGLibA_52352 | 296 | 1.796905119 | TUSC5 | - |
| WHSC1 | HGLibA_54292 | 296 | 1.796905119 | WHSC1 | - |
| C2orf48 | HGLibA_06136 | 295 | 1.786954082 | C2orf48 | - |
| CCDC176 | HGLibA_07708 | 295 | 1.786954082 | CCDC176 | - |
| CNTD2 | HGLibA_10389 | 295 | 1.786954082 | CNTD2 | - |
| ENSG00000000938 | HGLibA_17469 | 295 | 1.786954082 | FGR | Src family tyrosine kinase [Source:HGNC Symbol;Acc:HGNC:3697]" |
| ENSG00000080189 | HGLibA_44974 | 295 | 1.786954082 | SLC35C2 | solute carrier family 35 member C2 [Source:HGNC Symbol;Acc:HGNC:17117] |
| ENSG00000101152 | HGLibA_13623 | 295 | 1.786954082 | DNAJC5 | DnaJ heat shock protein family (Hsp40) member C5 [Source:HGNC Symbol;Acc:HGNC:16235] |
| ENSG00000101443 | HGLibA_54261 | 295 | 1.786954082 | WFDC2 | WAP four-disulfide core domain 2 [Source:HGNC Symbol;Acc:HGNC:15939] |
| ENSG00000108106 | HGLibA_52656 | 295 | 1.786954082 | UBE2S | ubiquitin conjugating enzyme E2 S [Source:HGNC Symbol;Acc:HGNC:17895] |
| ENSG00000128463 | HGLibA_15015 | 295 | 1.786954082 | EMC4 | ER membrane protein complex subunit 4 [Source:HGNC Symbol;Acc:HGNC:28032] |
| ENSG00000134640 | HGLibA_30447 | 295 | 1.786954082 | MTNR1B | melatonin receptor 1B [Source:HGNC Symbol;Acc:HGNC:7464] |
| ENSG00000135506 | HGLibA_34633 | 295 | 1.786954082 | OS9 | endoplasmic reticulum lectin [Source:HGNC Symbol;Acc:HGNC:16994]" |
| ENSG00000139193 | HGLibA_08315 | 295 | 1.786954082 | CD27 | CD27 molecule [Source:HGNC Symbol;Acc:HGNC:11922] |
| ENSG00000140067 | HGLibA_16317 | 295 | 1.786954082 | FAM181A | family with sequence similarity 181 member A [Source:HGNC Symbol;Acc:HGNC:20491] |
| ENSG00000150054 | HGLibA_29765 | 295 | 1.786954082 | MPP7 | membrane palmitoylated protein 7 [Source:HGNC Symbol;Acc:HGNC:26542] |
| ENSG00000151364 | HGLibA_24425 | 295 | 1.786954082 | KCTD14 | potassium channel tetramerization domain containing 14 [Source:HGNC Symbol;Acc:HGNC:23295] |
| ENSG00000158553 | HGLibA_37652 | 295 | 1.786954082 | POM121L2 | POM121 transmembrane nucleoporin like 2 [Source:HGNC Symbol;Acc:HGNC:13973] |
| ENSG00000165370 | HGLibA_19840 | 295 | 1.786954082 | GPR101 | G protein-coupled receptor 101 [Source:HGNC Symbol;Acc:HGNC:14963] |
| ENSG00000171867 | HGLibA_38643 | 295 | 1.786954082 | PRNP | prion protein [Source:HGNC Symbol;Acc:HGNC:9449] |
| ENSG00000175426 | HGLibA_35668 | 295 | 1.786954082 | PCSK1 | proprotein convertase subtilisin/kexin type 1 [Source:HGNC Symbol;Acc:HGNC:8743] |
| ENSG00000179142 | HGLibA_12057 | 295 | 1.786954082 | CYP11B2 | cytochrome P450 family 11 subfamily B member 2 [Source:HGNC Symbol;Acc:HGNC:2592] |
| ENSG00000180758 | HGLibA_19948 | 295 | 1.786954082 | GPR157 | G protein-coupled receptor 157 [Source:HGNC Symbol;Acc:HGNC:23687] |
| ENSG00000183723 | HGLibA_10219 | 295 | 1.786954082 | CMTM4 | CKLF like MARVEL transmembrane domain containing 4 [Source:HGNC Symbol;Acc:HGNC:19175] |
| ENSG00000184378 | HGLibA_00682 | 295 | 1.786954082 | ACTRT3 | actin related protein T3 [Source:HGNC Symbol;Acc:HGNC:24022] |
| ENSG00000186716 | HGLibA_04367 | 295 | 1.786954082 | BCR | RhoGEF and GTPase activating protein [Source:HGNC Symbol;Acc:HGNC:1014]" |
| ENSG00000197838 | HGLibA_12103 | 295 | 1.786954082 | CYP2A13 | cytochrome P450 family 2 subfamily A member 13 [Source:HGNC Symbol;Acc:HGNC:2608] |
| ENSG00000205882 | HGLibA_12904 | 295 | 1.786954082 | DEFB134 | defensin beta 134 [Source:HGNC Symbol;Acc:HGNC:32399] |
| FAM129A | HGLibA_16097 | 295 | 1.786954082 | FAM129A | - |
| hsa-mir-199b | HGLibA_58138 | 295 | 1.786954082 | hsa-mir-199b | - |
| hsa-mir-4504 | HGLibA_60443 | 295 | 1.786954082 | hsa-mir-4504 | - |
| hsa-mir-6085 | HGLibA_62600 | 295 | 1.786954082 | hsa-mir-6085 | - |
| hsa-mir-8066 | HGLibA_64101 | 295 | 1.786954082 | hsa-mir-8066 | - |
| PHF17 | HGLibA_36354 | 295 | 1.786954082 | PHF17 | - |
| C1orf111 | HGLibA_05747 | 294 | 1.777003044 | C1orf111 | - |
| ENSG00000004846 | HGLibA_00142 | 294 | 1.777003044 | ABCB5 | ATP binding cassette subfamily B member 5 [Source:HGNC Symbol;Acc:HGNC:46] |
| ENSG00000006042 | HGLibA_50570 | 294 | 1.777003044 | TMEM98 | transmembrane protein 98 [Source:HGNC Symbol;Acc:HGNC:24529] |
| ENSG00000082458 | HGLibA_13330 | 294 | 1.777003044 | DLG3 | discs large MAGUK scaffold protein 3 [Source:HGNC Symbol;Acc:HGNC:2902] |
| ENSG00000095739 | HGLibA_04137 | 294 | 1.777003044 | BAMBI | BMP and activin membrane bound inhibitor [Source:HGNC Symbol;Acc:HGNC:30251] |
| ENSG00000100591 | HGLibA_01313 | 294 | 1.777003044 | AHSA1 | activator of HSP90 ATPase activity 1 [Source:HGNC Symbol;Acc:HGNC:1189] |
| ENSG00000105939 | HGLibA_54991 | 294 | 1.777003044 | ZC3HAV1 | antiviral 1 [Source:HGNC Symbol;Acc:HGNC:23721]" |
| ENSG00000108509 | HGLibA_07119 | 294 | 1.777003044 | CAMTA2 | calmodulin binding transcription activator 2 [Source:HGNC Symbol;Acc:HGNC:18807] |
| ENSG00000115944 | HGLibA_10863 | 294 | 1.777003044 | COX7A2L | cytochrome c oxidase subunit 7A2 like [Source:HGNC Symbol;Acc:HGNC:2289] |
| ENSG00000118242 | HGLibA_29824 | 294 | 1.777003044 | MREG | melanoregulin [Source:HGNC Symbol;Acc:HGNC:25478] |
| ENSG00000122694 | HGLibA_19269 | 294 | 1.777003044 | GLIPR2 | GLI pathogenesis related 2 [Source:HGNC Symbol;Acc:HGNC:18007] |
| ENSG00000136828 | HGLibA_40167 | 294 | 1.777003044 | RALGPS1 | Ral GEF with PH domain and SH3 binding motif 1 [Source:HGNC Symbol;Acc:HGNC:16851] |
| ENSG00000149930 | HGLibA_48309 | 294 | 1.777003044 | TAOK2 | TAO kinase 2 [Source:HGNC Symbol;Acc:HGNC:16835] |
| ENSG00000160293 | HGLibA_53552 | 294 | 1.777003044 | VAV2 | vav guanine nucleotide exchange factor 2 [Source:HGNC Symbol;Acc:HGNC:12658] |
| ENSG00000161634 | HGLibA_12479 | 294 | 1.777003044 | DCD | dermcidin [Source:HGNC Symbol;Acc:HGNC:14669] |
| ENSG00000163703 | HGLibA_11126 | 294 | 1.777003044 | CRELD1 | cysteine rich with EGF like domains 1 [Source:HGNC Symbol;Acc:HGNC:14630] |
| ENSG00000166049 | HGLibA_35264 | 294 | 1.777003044 | PASD1 | PAS domain containing repressor 1 [Source:HGNC Symbol;Acc:HGNC:20686] |
| ENSG00000167106 | HGLibA_15978 | 294 | 1.777003044 | FAM102A | family with sequence similarity 102 member A [Source:HGNC Symbol;Acc:HGNC:31419] |
| ENSG00000172116 | HGLibA_08473 | 294 | 1.777003044 | CD8B | CD8b molecule [Source:HGNC Symbol;Acc:HGNC:1707] |
| ENSG00000176895 | HGLibA_34078 | 294 | 1.777003044 | OR51A7 | olfactory receptor family 51 subfamily A member 7 [Source:HGNC Symbol;Acc:HGNC:15188] |
| ENSG00000181619 | HGLibA_19895 | 294 | 1.777003044 | GPR135 | G protein-coupled receptor 135 [Source:HGNC Symbol;Acc:HGNC:19991] |
| ENSG00000198074 | HGLibA_01474 | 294 | 1.777003044 | AKR1B10 | aldo-keto reductase family 1 member B10 [Source:HGNC Symbol;Acc:HGNC:382] |
| ENSG00000233013 | HGLibA_16196 | 294 | 1.777003044 | FAM157B | family with sequence similarity 157 member B [Source:HGNC Symbol;Acc:HGNC:34080] |
| FAM21A | HGLibA_16503 | 294 | 1.777003044 | FAM21A | - |
| HIST1H2BD | HGLibA_21420 | 294 | 1.777003044 | HIST1H2BD | - |
| hsa-mir-4769 | HGLibA_61171 | 294 | 1.777003044 | hsa-mir-4769 | - |
| hsa-mir-4780 | HGLibA_61229 | 294 | 1.777003044 | hsa-mir-4780 | - |
| RLTPR | HGLibA_41360 | 294 | 1.777003044 | RLTPR | - |
| C12orf68 | HGLibA_05286 | 293 | 1.767052007 | C12orf68 | - |
| C15orf56 | HGLibA_05418 | 293 | 1.767052007 | C15orf56 | - |
| DEFB131 | HGLibA_12897 | 293 | 1.767052007 | DEFB131 | - |
| ENSG00000000971 | HGLibA_09259 | 293 | 1.767052007 | CFH | complement factor H [Source:HGNC Symbol;Acc:HGNC:4883] |
| ENSG00000039537 | HGLibA_06425 | 293 | 1.767052007 | C6 | complement C6 [Source:HGNC Symbol;Acc:HGNC:1339] |
| ENSG00000047597 | HGLibA_54518 | 293 | 1.767052007 | XK | X-linked Kx blood group [Source:HGNC Symbol;Acc:HGNC:12811] |
| ENSG00000100083 | HGLibA_18980 | 293 | 1.767052007 | GGA1 | ARF binding protein 1 [Source:HGNC Symbol;Acc:HGNC:17842]" |
| ENSG00000104419 | HGLibA_31413 | 293 | 1.767052007 | NDRG1 | N-myc downstream regulated 1 [Source:HGNC Symbol;Acc:HGNC:7679] |
| ENSG00000124772 | HGLibA_10966 | 293 | 1.767052007 | CPNE5 | copine 5 [Source:HGNC Symbol;Acc:HGNC:2318] |
| ENSG00000126264 | HGLibA_20994 | 293 | 1.767052007 | HCST | hematopoietic cell signal transducer [Source:HGNC Symbol;Acc:HGNC:16977] |
| ENSG00000128739 | HGLibA_45994 | 293 | 1.767052007 | SNRPN | small nuclear ribonucleoprotein polypeptide N [Source:HGNC Symbol;Acc:HGNC:11164] |
| ENSG00000129244 | HGLibA_03596 | 293 | 1.767052007 | ATP1B2 | ATPase Na+/K+ transporting subunit beta 2 [Source:HGNC Symbol;Acc:HGNC:805] |
| ENSG00000131013 | HGLibA_37949 | 293 | 1.767052007 | PPIL4 | peptidylprolyl isomerase like 4 [Source:HGNC Symbol;Acc:HGNC:15702] |
| ENSG00000140525 | HGLibA_16854 | 293 | 1.767052007 | FANCI | FA complementation group I [Source:HGNC Symbol;Acc:HGNC:25568] |
| ENSG00000142515 | HGLibA_25254 | 293 | 1.767052007 | KLK3 | kallikrein related peptidase 3 [Source:HGNC Symbol;Acc:HGNC:6364] |
| ENSG00000143942 | HGLibA_09337 | 293 | 1.767052007 | CHAC2 | ChaC cation transport regulator homolog 2 [Source:HGNC Symbol;Acc:HGNC:32363] |
| ENSG00000144214 | HGLibA_27699 | 293 | 1.767052007 | LYG1 | lysozyme g1 [Source:HGNC Symbol;Acc:HGNC:27014] |
| ENSG00000147649 | HGLibA_30333 | 293 | 1.767052007 | MTDH | metadherin [Source:HGNC Symbol;Acc:HGNC:29608] |
| ENSG00000149260 | HGLibA_07169 | 293 | 1.767052007 | CAPN5 | calpain 5 [Source:HGNC Symbol;Acc:HGNC:1482] |
| ENSG00000152034 | HGLibA_28593 | 293 | 1.767052007 | MCHR2 | melanin concentrating hormone receptor 2 [Source:HGNC Symbol;Acc:HGNC:20867] |
| ENSG00000153563 | HGLibA_08472 | 293 | 1.767052007 | CD8A | CD8a molecule [Source:HGNC Symbol;Acc:HGNC:1706] |
| ENSG00000155363 | HGLibA_29692 | 293 | 1.767052007 | MOV10 | Mov10 RISC complex RNA helicase [Source:HGNC Symbol;Acc:HGNC:7200] |
| ENSG00000156009 | HGLibA_27920 | 293 | 1.767052007 | MAGEA8 | MAGE family member A8 [Source:HGNC Symbol;Acc:HGNC:6806] |
| ENSG00000157653 | HGLibA_06733 | 293 | 1.767052007 | C9orf43 | chromosome 9 open reading frame 43 [Source:HGNC Symbol;Acc:HGNC:23570] |
| ENSG00000159915 | HGLibA_55641 | 293 | 1.767052007 | ZNF233 | zinc finger protein 233 [Source:HGNC Symbol;Acc:HGNC:30946] |
| ENSG00000163141 | HGLibA_04661 | 293 | 1.767052007 | BNIPL | BCL2 interacting protein like [Source:HGNC Symbol;Acc:HGNC:16976] |
| ENSG00000166159 | HGLibA_27504 | 293 | 1.767052007 | LRTM2 | leucine rich repeats and transmembrane domains 2 [Source:HGNC Symbol;Acc:HGNC:32443] |
| ENSG00000168350 | HGLibA_12923 | 293 | 1.767052007 | DEGS2 | sphingolipid 2 [Source:HGNC Symbol;Acc:HGNC:20113]" |
| ENSG00000176720 | HGLibA_04676 | 293 | 1.767052007 | BOK | BCL2 family apoptosis regulator BOK [Source:HGNC Symbol;Acc:HGNC:1087] |
| ENSG00000181626 | HGLibA_02135 | 293 | 1.767052007 | ANKRD62 | ankyrin repeat domain 62 [Source:HGNC Symbol;Acc:HGNC:35241] |
| ENSG00000183706 | HGLibA_34048 | 293 | 1.767052007 | OR4N4 | olfactory receptor family 4 subfamily N member 4 [Source:HGNC Symbol;Acc:HGNC:15375] |
| ENSG00000204695 | HGLibA_33639 | 293 | 1.767052007 | OR14J1 | olfactory receptor family 14 subfamily J member 1 [Source:HGNC Symbol;Acc:HGNC:13971] |
| ENSG00000243708 | HGLibA_36904 | 293 | 1.767052007 | PLA2G4B | phospholipase A2 group IVB [Source:HGNC Symbol;Acc:HGNC:9036] |
| ENSG00000269858 | HGLibA_14642 | 293 | 1.767052007 | EGLN2 | egl-9 family hypoxia inducible factor 2 [Source:HGNC Symbol;Acc:HGNC:14660] |
| hsa-let-7f-1 | HGLibA_57125 | 293 | 1.767052007 | hsa-let-7f-1 | - |
| hsa-mir-1269a | HGLibA_57504 | 293 | 1.767052007 | hsa-mir-1269a | - |
| hsa-mir-378g | HGLibA_59455 | 293 | 1.767052007 | hsa-mir-378g | - |
| LDOC1L | HGLibA_26260 | 293 | 1.767052007 | LDOC1L | - |

| | | | | | |
|---|---|---|---|---|---|
| WBSCR16 | HGLibA_53989 | 293 | 1.767052007 | WBSCR16 | - |
| ENSG00000044574 | HGLibA_22289 | 292 | 1.757100969 | HSPA5 | heat shock protein family A (Hsp70) member 5 [Source:HGNC Symbol;Acc:HGNC:5238] |
| ENSG00000049768 | HGLibA_17935 | 292 | 1.757100969 | FOXP3 | forkhead box P3 [Source:HGNC Symbol;Acc:HGNC:6106] |
| ENSG00000071553 | HGLibA_03693 | 292 | 1.757100969 | ATP6AP1 | ATPase H+ transporting accessory protein 1 [Source:HGNC Symbol;Acc:HGNC:868] |
| ENSG00000075073 | HGLibA_48173 | 292 | 1.757100969 | TACR2 | tachykinin receptor 2 [Source:HGNC Symbol;Acc:HGNC:11527] |
| ENSG00000085644 | HGLibA_55591 | 292 | 1.757100969 | ZNF213 | zinc finger protein 213 [Source:HGNC Symbol;Acc:HGNC:13005] |
| ENSG00000104435 | HGLibA_47519 | 292 | 1.757100969 | STMN2 | stathmin 2 [Source:HGNC Symbol;Acc:HGNC:10577] |
| ENSG00000107521 | HGLibA_22046 | 292 | 1.757100969 | HPS1 | biogenesis of lysosomal organelles complex 3 subunit 1 [Source:HGNC Symbol;Acc:HGNC:5163]" |
| ENSG00000110324 | HGLibA_22948 | 292 | 1.757100969 | IL10RA | interleukin 10 receptor subunit alpha [Source:HGNC Symbol;Acc:HGNC:5964] |
| ENSG00000125386 | HGLibA_16364 | 292 | 1.757100969 | FAM193A | family with sequence similarity 193 member A [Source:HGNC Symbol;Acc:HGNC:16822] |
| ENSG00000130803 | HGLibA_55782 | 292 | 1.757100969 | ZNF317 | zinc finger protein 317 [Source:HGNC Symbol;Acc:HGNC:13507] |
| ENSG00000131507 | HGLibA_31391 | 292 | 1.757100969 | NDFIP1 | Nedd4 family interacting protein 1 [Source:HGNC Symbol;Acc:HGNC:17592] |
| ENSG00000134365 | HGLibA_09273 | 292 | 1.757100969 | CFHR4 | complement factor H related 4 [Source:HGNC Symbol;Acc:HGNC:16979] |
| ENSG00000135387 | HGLibA_07190 | 292 | 1.757100969 | CAPRIN1 | cell cycle associated protein 1 [Source:HGNC Symbol;Acc:HGNC:6743] |
| ENSG00000136531 | HGLibA_42980 | 292 | 1.757100969 | SCN2A | sodium voltage-gated channel alpha subunit 2 [Source:HGNC Symbol;Acc:HGNC:10588] |
| ENSG00000162415 | HGLibA_57049 | 292 | 1.757100969 | ZSWIM5 | zinc finger SWIM-type containing 5 [Source:HGNC Symbol;Acc:HGNC:29299] |
| ENSG00000162552 | HGLibA_54369 | 292 | 1.757100969 | WNT4 | Wnt family member 4 [Source:HGNC Symbol;Acc:HGNC:12783] |
| ENSG00000162779 | HGLibA_03918 | 292 | 1.757100969 | AXDND1 | axonemal dynein light chain domain containing 1 [Source:HGNC Symbol;Acc:HGNC:26564] |
| ENSG00000166856 | HGLibA_19986 | 292 | 1.757100969 | GPR182 | G protein-coupled receptor 182 [Source:HGNC Symbol;Acc:HGNC:13708] |
| ENSG00000170291 | HGLibA_14987 | 292 | 1.757100969 | ELP5 | elongator acetyltransferase complex subunit 5 [Source:HGNC Symbol;Acc:HGNC:30617] |
| ENSG00000170989 | HGLibA_42592 | 292 | 1.757100969 | S1PR1 | sphingosine-1-phosphate receptor 1 [Source:HGNC Symbol;Acc:HGNC:3165] |
| ENSG00000174080 | HGLibA_11704 | 292 | 1.757100969 | CTSF | cathepsin F [Source:HGNC Symbol;Acc:HGNC:2531] |
| ENSG00000176769 | HGLibA_48757 | 292 | 1.757100969 | TCERG1L | transcription elongation regulator 1 like [Source:HGNC Symbol;Acc:HGNC:23533] |
| ENSG00000177468 | HGLibA_33375 | 292 | 1.757100969 | OLIG3 | oligodendrocyte transcription factor 3 [Source:HGNC Symbol;Acc:HGNC:18003] |
| ENSG00000178988 | HGLibA_29830 | 292 | 1.757100969 | MRFAP1L1 | Morf4 family associated protein 1 like 1 [Source:HGNC Symbol;Acc:HGNC:28796] |
| ENSG00000179044 | HGLibA_15740 | 292 | 1.757100969 | EXOC3L1 | exocyst complex component 3 like 1 [Source:HGNC Symbol;Acc:HGNC:27540] |
| ENSG00000184530 | HGLibA_06501 | 292 | 1.757100969 | C6orf58 | chromosome 6 open reading frame 58 [Source:HGNC Symbol;Acc:HGNC:20960] |
| ENSG00000185247 | HGLibA_27900 | 292 | 1.757100969 | MAGEA11 | MAGE family member A11 [Source:HGNC Symbol;Acc:HGNC:6798] |
| ENSG00000186472 | HGLibA_35620 | 292 | 1.757100969 | PCLO | piccolo presynaptic cytomatrix protein [Source:HGNC Symbol;Acc:HGNC:13406] |
| ENSG00000186832 | HGLibA_25431 | 292 | 1.757100969 | KRT16 | keratin 16 [Source:HGNC Symbol;Acc:HGNC:6423] |
| ENSG00000203795 | HGLibA_16546 | 292 | 1.757100969 | FAM24A | family with sequence similarity 24 member A [Source:HGNC Symbol;Acc:HGNC:23470] |
| ENSG00000244045 | HGLibA_50164 | 292 | 1.757100969 | TMEM199 | transmembrane protein 199 [Source:HGNC Symbol;Acc:HGNC:18085] |
| hsa-mir-103b-2 | HGLibA_57172 | 292 | 1.757100969 | hsa-mir-103b-2 | - |
| hsa-mir-3941 | HGLibA_59624 | 292 | 1.757100969 | hsa-mir-3941 | - |
| hsa-mir-4725 | HGLibA_60996 | 292 | 1.757100969 | hsa-mir-4725 | - |
| CENPC1 | HGLibA_09047 | 291 | 1.747149932 | CENPC1 | - |
| ENSG00000086102 | HGLibA_31849 | 291 | 1.747149932 | NFX1 | X-box binding 1 [Source:HGNC Symbol;Acc:HGNC:7803]" |
| ENSG00000092607 | HGLibA_48648 | 291 | 1.747149932 | TBX15 | T-box 15 [Source:HGNC Symbol;Acc:HGNC:11594] |
| ENSG00000108984 | HGLibA_28155 | 291 | 1.747149932 | MAP2K6 | mitogen-activated protein kinase kinase 6 [Source:HGNC Symbol;Acc:HGNC:6846] |
| ENSG00000125648 | HGLibA_44678 | 291 | 1.747149932 | SLC25A23 | solute carrier family 25 member 23 [Source:HGNC Symbol;Acc:HGNC:19375] |
| ENSG00000127831 | HGLibA_53651 | 291 | 1.747149932 | VIL1 | villin 1 [Source:HGNC Symbol;Acc:HGNC:12690] |
| ENSG00000130340 | HGLibA_46110 | 291 | 1.747149932 | SNX9 | sorting nexin 9 [Source:HGNC Symbol;Acc:HGNC:14973] |
| ENSG00000133812 | HGLibA_42798 | 291 | 1.747149932 | SBF2 | SET binding factor 2 [Source:HGNC Symbol;Acc:HGNC:2135] |
| ENSG00000134571 | HGLibA_30656 | 291 | 1.747149932 | MYBPC3 | cardiac [Source:HGNC Symbol;Acc:HGNC:7551]" |
| ENSG00000137288 | HGLibA_53101 | 291 | 1.747149932 | UQCC2 | ubiquinol-cytochrome c reductase complex assembly factor 2 [Source:HGNC Symbol;Acc:HGNC:21237] |
| ENSG00000151552 | HGLibA_39720 | 291 | 1.747149932 | QDPR | quinoid dihydropteridine reductase [Source:HGNC Symbol;Acc:HGNC:9752] |
| ENSG00000152086 | HGLibA_52257 | 291 | 1.747149932 | TUBA3E | tubulin alpha 3e [Source:HGNC Symbol;Acc:HGNC:20765] |
| ENSG00000152939 | HGLibA_28381 | 291 | 1.747149932 | MARVELD2 | MARVEL domain containing 2 [Source:HGNC Symbol;Acc:HGNC:26401] |
| ENSG00000158481 | HGLibA_08271 | 291 | 1.747149932 | CD1C | CD1c molecule [Source:HGNC Symbol;Acc:HGNC:1636] |
| ENSG00000163785 | HGLibA_42519 | 291 | 1.747149932 | RYK | receptor-like tyrosine kinase [Source:HGNC Symbol;Acc:HGNC:10481] |
| ENSG00000166432 | HGLibA_55367 | 291 | 1.747149932 | ZMAT1 | zinc finger matrin-type 1 [Source:HGNC Symbol;Acc:HGNC:29377] |
| ENSG00000167747 | HGLibA_05672 | 291 | 1.747149932 | C19orf48 | chromosome 19 open reading frame 48 [Source:HGNC Symbol;Acc:HGNC:29667] |
| ENSG00000168140 | HGLibA_53537 | 291 | 1.747149932 | VASN | vasorin [Source:HGNC Symbol;Acc:HGNC:18517] |
| ENSG00000170054 | HGLibA_43533 | 291 | 1.747149932 | SERPINA9 | serpin family A member 9 [Source:HGNC Symbol;Acc:HGNC:15995] |
| ENSG00000171560 | HGLibA_17335 | 291 | 1.747149932 | FGA | fibrinogen alpha chain [Source:HGNC Symbol;Acc:HGNC:3661] |
| ENSG00000174915 | HGLibA_39291 | 291 | 1.747149932 | PTDSS2 | phosphatidylserine synthase 2 [Source:HGNC Symbol;Acc:HGNC:15463] |
| ENSG00000175664 | HGLibA_49079 | 291 | 1.747149932 | TEX26 | testis expressed 26 [Source:HGNC Symbol;Acc:HGNC:28622] |
| ENSG00000186652 | HGLibA_38451 | 291 | 1.747149932 | PRG2 | pro eosinophil major basic protein [Source:HGNC Symbol;Acc:HGNC:9362]" |
| ENSG00000204424 | HGLibA_27670 | 291 | 1.747149932 | LY6G6F | lymphocyte antigen 6 family member G6F [Source:HGNC Symbol;Acc:HGNC:13933] |
| ENSG00000205502 | HGLibA_06098 | 291 | 1.747149932 | C2CD4B | C2 calcium dependent domain containing 4B [Source:HGNC Symbol;Acc:HGNC:33628] |
| ENSG00000241839 | HGLibA_37153 | 291 | 1.747149932 | PLEKHO2 | pleckstrin homology domain containing O2 [Source:HGNC Symbol;Acc:HGNC:30026] |
| ENSG00000267855 | HGLibA_31471 | 291 | 1.747149932 | NDUFA7 | NADH:ubiquinone oxidoreductase subunit A7 [Source:HGNC Symbol;Acc:HGNC:7691] |
| ENSG00000279395 | HGLibA_34322 | 291 | 1.747149932 | OR5L1 | olfactory receptor family 5 subfamily L member 1 (gene/pseudogene) [Source:HGNC Symbol;Acc:HGNC:8350] |
| hsa-mir-4440 | HGLibA_60161 | 291 | 1.747149932 | hsa-mir-4440 | - |
| hsa-mir-6744 | HGLibA_63128 | 291 | 1.747149932 | hsa-mir-6744 | - |
| PTCHD2 | HGLibA_39276 | 291 | 1.747149932 | PTCHD2 | - |
| SELK | HGLibA_43291 | 291 | 1.747149932 | SELK | - |
| ENSG00000019169 | HGLibA_28360 | 290 | 1.737198894 | MARCO | macrophage receptor with collagenous structure [Source:HGNC Symbol;Acc:HGNC:6895] |
| ENSG00000090006 | HGLibA_27598 | 290 | 1.737198894 | LTBP4 | latent transforming growth factor beta binding protein 4 [Source:HGNC Symbol;Acc:HGNC:6717] |
| ENSG00000091428 | HGLibA_40253 | 290 | 1.737198894 | RAPGEF4 | Rap guanine nucleotide exchange factor 4 [Source:HGNC Symbol;Acc:HGNC:16626] |
| ENSG00000100290 | HGLibA_04503 | 290 | 1.737198894 | BIK | BCL2 interacting killer [Source:HGNC Symbol;Acc:HGNC:1051] |
| ENSG00000113966 | HGLibA_02984 | 290 | 1.737198894 | ARL6 | ADP ribosylation factor like GTPase 6 [Source:HGNC Symbol;Acc:HGNC:13210] |
| ENSG00000132563 | HGLibA_40778 | 290 | 1.737198894 | REEP2 | receptor accessory protein 2 [Source:HGNC Symbol;Acc:HGNC:17975] |
| ENSG00000140287 | HGLibA_21029 | 290 | 1.737198894 | HDC | histidine decarboxylase [Source:HGNC Symbol;Acc:HGNC:4855] |
| ENSG00000163013 | HGLibA_17097 | 290 | 1.737198894 | FBXO41 | F-box protein 41 [Source:HGNC Symbol;Acc:HGNC:29409] |
| ENSG00000163453 | HGLibA_22832 | 290 | 1.737198894 | IGFBP7 | insulin like growth factor binding protein 7 [Source:HGNC Symbol;Acc:HGNC:5476] |
| ENSG00000167291 | HGLibA_48485 | 290 | 1.737198894 | TBC1D16 | TBC1 domain family member 16 [Source:HGNC Symbol;Acc:HGNC:28356] |
| ENSG00000178498 | HGLibA_14084 | 290 | 1.737198894 | DTX3 | deltex E3 ubiquitin ligase 3 [Source:HGNC Symbol;Acc:HGNC:24457] |
| ENSG00000188051 | HGLibA_50236 | 290 | 1.737198894 | TMEM221 | transmembrane protein 221 [Source:HGNC Symbol;Acc:HGNC:21943] |
| ENSG00000196071 | HGLibA_33818 | 290 | 1.737198894 | OR2L13 | olfactory receptor family 2 subfamily L member 13 [Source:HGNC Symbol;Acc:HGNC:19578] |
| ENSG00000198283 | HGLibA_34266 | 290 | 1.737198894 | OR5B21 | olfactory receptor family 5 subfamily B member 21 [Source:HGNC Symbol;Acc:HGNC:19616] |
| ENSG00000198300 | HGLibA_36030 | 290 | 1.737198894 | PEG3 | paternally expressed 3 [Source:HGNC Symbol;Acc:HGNC:8826] |
| ENSG00000204308 | HGLibA_41691 | 290 | 1.737198894 | RNF5 | ring finger protein 5 [Source:HGNC Symbol;Acc:HGNC:10068] |
| ENSG00000254858 | HGLibA_29788 | 290 | 1.737198894 | MPV17L2 | MPV17 mitochondrial inner membrane protein like 2 [Source:HGNC Symbol;Acc:HGNC:28177] |
| ENSG00000255837 | HGLibA_48380 | 290 | 1.737198894 | TAS2R20 | taste 2 receptor member 20 [Source:HGNC Symbol;Acc:HGNC:19109] |
| ENSG00000267855 | HGLibA_31470 | 290 | 1.737198894 | NDUFA7 | NADH:ubiquinone oxidoreductase subunit A7 [Source:HGNC Symbol;Acc:HGNC:7691] |
| ENSG00000270011 | HGLibA_56240 | 290 | 1.737198894 | ZNF559-ZNF177 | ZNF559-ZNF177 readthrough [Source:HGNC Symbol;Acc:HGNC:42964] |
| hsa-mir-4253 | HGLibA_59718 | 290 | 1.737198894 | hsa-mir-4253 | - |
| hsa-mir-508 | HGLibA_61491 | 290 | 1.737198894 | hsa-mir-508 | - |
| hsa-mir-578 | HGLibA_62407 | 290 | 1.737198894 | hsa-mir-578 | - |
| hsa-mir-6717 | HGLibA_63030 | 290 | 1.737198894 | hsa-mir-6717 | - |
| LOC100505478 | HGLibA_26845 | 290 | 1.737198894 | LOC100505478 | - |
| PLCXD1_Y | HGLibA_37023 | 290 | 1.737198894 | PLCXD1_Y | - |
| AGPAT9 | HGLibA_01236 | 289 | 1.727247857 | AGPAT9 | - |
| ENSG00000011028 | HGLibA_29820 | 289 | 1.727247857 | MRC2 | mannose receptor C type 2 [Source:HGNC Symbol;Acc:HGNC:16875] |
| ENSG00000026652 | HGLibA_01226 | 289 | 1.727247857 | AGPAT4 | 1-acylglycerol-3-phosphate O-acyltransferase 4 [Source:HGNC Symbol;Acc:HGNC:20885] |
| ENSG00000068697 | HGLibA_26016 | 289 | 1.727247857 | LAPTM4A | lysosomal protein transmembrane 4 alpha [Source:HGNC Symbol;Acc:HGNC:6924] |
| ENSG00000073712 | HGLibA_17297 | 289 | 1.727247857 | FERMT2 | fermitin family member 2 [Source:HGNC Symbol;Acc:HGNC:15767] |
| ENSG00000075407 | HGLibA_55886 | 289 | 1.727247857 | ZNF37A | zinc finger protein 37A [Source:HGNC Symbol;Acc:HGNC:13102] |
| ENSG00000078269 | HGLibA_48005 | 289 | 1.727247857 | SYNJ2 | synaptojanin 2 [Source:HGNC Symbol;Acc:HGNC:11504] |
| ENSG00000082458 | HGLibA_13329 | 289 | 1.727247857 | DLG3 | discs large MAGUK scaffold protein 3 [Source:HGNC Symbol;Acc:HGNC:2902] |
| ENSG00000100997 | HGLibA_00238 | 289 | 1.727247857 | ABHD12 | abhydrolase domain containing 12 [Source:HGNC Symbol;Acc:HGNC:15868] |
| ENSG00000101844 | HGLibA_03488 | 289 | 1.727247857 | ATG4A | autophagy related 4A cysteine peptidase [Source:HGNC Symbol;Acc:HGNC:16489] |
| ENSG00000102531 | HGLibA_17747 | 289 | 1.727247857 | FNDC3A | fibronectin type III domain containing 3A [Source:HGNC Symbol;Acc:HGNC:20296] |
| ENSG00000114054 | HGLibA_35383 | 289 | 1.727247857 | PCCB | propionyl-CoA carboxylase subunit beta [Source:HGNC Symbol;Acc:HGNC:8654] |
| ENSG00000117525 | HGLibA_15875 | 289 | 1.727247857 | F3 | tissue factor [Source:HGNC Symbol;Acc:HGNC:3541]" |
| ENSG00000130052 | HGLibA_47375 | 289 | 1.727247857 | STARD8 | StAR related lipid transfer domain containing 8 [Source:HGNC Symbol;Acc:HGNC:19161] |
| ENSG00000133703 | HGLibA_25384 | 289 | 1.727247857 | KRAS | GTPase [Source:HGNC Symbol;Acc:HGNC:6407]" |
| ENSG00000136158 | HGLibA_46838 | 289 | 1.727247857 | SPRY2 | sprouty RTK signaling antagonist 2 [Source:HGNC Symbol;Acc:HGNC:11270] |
| ENSG00000137094 | HGLibA_13547 | 289 | 1.727247857 | DNAJB5 | DnaJ heat shock protein family (Hsp40) member B5 [Source:HGNC Symbol;Acc:HGNC:14887] |
| ENSG00000140955 | HGLibA_00723 | 289 | 1.727247857 | ADAD2 | adenosine deaminase domain containing 2 [Source:HGNC Symbol;Acc:HGNC:30714] |
| ENSG00000147100 | HGLibA_44435 | 289 | 1.727247857 | SLC16A2 | solute carrier family 16 member 2 [Source:HGNC Symbol;Acc:HGNC:10923] |
| ENSG00000159403 | HGLibA_05968 | 289 | 1.727247857 | C1R | complement C1r [Source:HGNC Symbol;Acc:HGNC:1246] |
| ENSG00000162817 | HGLibA_05753 | 289 | 1.727247857 | C1orf115 | chromosome 1 open reading frame 115 [Source:HGNC Symbol;Acc:HGNC:25873] |
| ENSG00000162891 | HGLibA_23072 | 289 | 1.727247857 | IL20 | interleukin 20 [Source:HGNC Symbol;Acc:HGNC:6002] |
| ENSG00000165471 | HGLibA_28500 | 289 | 1.727247857 | MBL2 | mannose binding lectin 2 [Source:HGNC Symbol;Acc:HGNC:6922] |
| ENSG00000165819 | HGLibA_29005 | 289 | 1.727247857 | METTL3 | methyltransferase like 3 [Source:HGNC Symbol;Acc:HGNC:17563] |
| ENSG00000166589 | HGLibA_08647 | 289 | 1.727247857 | CDH16 | cadherin 16 [Source:HGNC Symbol;Acc:HGNC:1755] |
| ENSG00000170743 | HGLibA_48094 | 289 | 1.727247857 | SYT9 | synaptotagmin 9 [Source:HGNC Symbol;Acc:HGNC:19265] |
| ENSG00000171643 | HGLibA_42591 | 289 | 1.727247857 | S100Z | S100 calcium binding protein Z [Source:HGNC Symbol;Acc:HGNC:30367] |
| ENSG00000172830 | HGLibA_47137 | 289 | 1.727247857 | SSH3 | slingshot protein phosphatase 3 [Source:HGNC Symbol;Acc:HGNC:30581] |
| ENSG00000196220 | HGLibA_46981 | 289 | 1.727247857 | SRGAP3 | SLIT-ROBO Rho GTPase activating protein 3 [Source:HGNC Symbol;Acc:HGNC:19744] |
| ENSG00000219200 | HGLibA_41435 | 289 | 1.727247857 | RNASEK | ribonuclease K [Source:HGNC Symbol;Acc:HGNC:33911] |
| ENSG00000248485 | HGLibA_35664 | 289 | 1.727247857 | PCP4L1 | Purkinje cell protein 4 like 1 [Source:HGNC Symbol;Acc:HGNC:20448] |
| hsa-mir-1231 | HGLibA_57320 | 289 | 1.727247857 | hsa-mir-1231 | - |
| hsa-mir-1915 | HGLibA_58066 | 289 | 1.727247857 | hsa-mir-1915 | - |
| hsa-mir-6828 | HGLibA_63490 | 289 | 1.727247857 | hsa-mir-6828 | - |
| hsa-mir-7-2 | HGLibA_63774 | 289 | 1.727247857 | hsa-mir-7-2 | - |
| CECR1 | HGLibA_08974 | 288 | 1.71729682 | CECR1 | - |
| ENSG00000010626 | HGLibA_27284 | 288 | 1.71729682 | LRRC23 | leucine rich repeat containing 23 [Source:HGNC Symbol;Acc:HGNC:19138] |
| ENSG00000020577 | HGLibA_42683 | 288 | 1.71729682 | SAMD4A | sterile alpha motif domain containing 4A [Source:HGNC Symbol;Acc:HGNC:23023] |
| ENSG00000064932 | HGLibA_42808 | 288 | 1.71729682 | SBNO2 | strawberry notch homolog 2 [Source:HGNC Symbol;Acc:HGNC:29158] |
| ENSG00000084072 | HGLibA_37928 | 288 | 1.71729682 | PPIE | peptidylprolyl isomerase E [Source:HGNC Symbol;Acc:HGNC:9258] |
| ENSG00000088320 | HGLibA_40830 | 288 | 1.71729682 | REM1 | RRAD and GEM like GTPase 1 [Source:HGNC Symbol;Acc:HGNC:15922] |
| ENSG00000108244 | HGLibA_25452 | 288 | 1.71729682 | KRT23 | keratin 23 [Source:HGNC Symbol;Acc:HGNC:6438] |

| | | | | | |
|---|---|---|---|---|---|
| ENSG00000122584 | HGLibA_33163 | 288 | 1.71729682 | NXPH1 | neurexophilin 1 [Source:HGNC Symbol;Acc:HGNC:20693] |
| ENSG00000132031 | HGLibA_28433 | 288 | 1.71729682 | MATN3 | matrilin 3 [Source:HGNC Symbol;Acc:HGNC:6909] |
| ENSG00000135740 | HGLibA_45419 | 288 | 1.71729682 | SLC9A5 | solute carrier family 9 member A5 [Source:HGNC Symbol;Acc:HGNC:11078] |
| ENSG00000142279 | HGLibA_54435 | 288 | 1.71729682 | WTIP | WT1 interacting protein [Source:HGNC Symbol;Acc:HGNC:20964] |
| ENSG00000148680 | HGLibA_22404 | 288 | 1.71729682 | HTR7 | 5-hydroxytryptamine receptor 7 [Source:HGNC Symbol;Acc:HGNC:5302] |
| ENSG00000148985 | HGLibA_36209 | 288 | 1.71729682 | PGAP2 | post-GPI attachment to proteins 2 [Source:HGNC Symbol;Acc:HGNC:17893] |
| ENSG00000159692 | HGLibA_11583 | 288 | 1.71729682 | CTBP1 | C-terminal binding protein 1 [Source:HGNC Symbol;Acc:HGNC:2494] |
| ENSG00000164929 | HGLibA_04051 | 288 | 1.71729682 | BAALC | MAP3K1 and KLF4 binding [Source:HGNC Symbol;Acc:HGNC:14333]" |
| ENSG00000170027 | HGLibA_54746 | 288 | 1.71729682 | YWHAG | tyrosine 3-monooxygenase/tryptophan 5-monooxygenase activation protein gamma [Source:HGNC Symbol;Acc:HGNC:12852] |
| ENSG00000172023 | HGLibA_40797 | 288 | 1.71729682 | REG1B | regenerating family member 1 beta [Source:HGNC Symbol;Acc:HGNC:9952] |
| ENSG00000177839 | HGLibA_35514 | 288 | 1.71729682 | PCDHB9 | protocadherin beta 9 [Source:HGNC Symbol;Acc:HGNC:8694] |
| ENSG00000197580 | HGLibA_04359 | 288 | 1.71729682 | BCO2 | beta-carotene oxygenase 2 [Source:HGNC Symbol;Acc:HGNC:18503] |
| ENSG00000241839 | HGLibA_37151 | 288 | 1.71729682 | PLEKHO2 | pleckstrin homology domain containing O2 [Source:HGNC Symbol;Acc:HGNC:30026] |
| ENSG00000275074 | HGLibA_32960 | 288 | 1.71729682 | NUDT18 | nudix hydrolase 18 [Source:HGNC Symbol;Acc:HGNC:26194] |
| hsa-mir-5572 | HGLibA_62153 | 288 | 1.71729682 | hsa-mir-5572 | - |
| hsa-mir-656 | HGLibA_62957 | 288 | 1.71729682 | hsa-mir-656 | - |
| ENSG00000001629 | HGLibA_01969 | 287 | 1.707345782 | ANKIB1 | ankyrin repeat and IBR domain containing 1 [Source:HGNC Symbol;Acc:HGNC:22215] |
| ENSG00000062096 | HGLibA_03141 | 287 | 1.707345782 | ARSF | arylsulfatase F [Source:HGNC Symbol;Acc:HGNC:721] |
| ENSG00000070748 | HGLibA_09357 | 287 | 1.707345782 | CHAT | choline O-acetyltransferase [Source:HGNC Symbol;Acc:HGNC:1912] |
| ENSG00000082146 | HGLibA_47575 | 287 | 1.707345782 | STRADB | STE20-related kinase adaptor beta [Source:HGNC Symbol;Acc:HGNC:13205] |
| ENSG00000092067 | HGLibA_08965 | 287 | 1.707345782 | CEBPE | CCAAT enhancer binding protein epsilon [Source:HGNC Symbol;Acc:HGNC:1836] |
| ENSG00000101945 | HGLibA_47859 | 287 | 1.707345782 | SUV39H1 | suppressor of variegation 3-9 homolog 1 [Source:HGNC Symbol;Acc:HGNC:11479] |
| ENSG00000105254 | HGLibA_48582 | 287 | 1.707345782 | TBCB | tubulin folding cofactor B [Source:HGNC Symbol;Acc:HGNC:1989] |
| ENSG00000107807 | HGLibA_49670 | 287 | 1.707345782 | TLX1 | T cell leukemia homeobox 1 [Source:HGNC Symbol;Acc:HGNC:5056] |
| ENSG00000116586 | HGLibA_25991 | 287 | 1.707345782 | LAMTOR2 | MAPK and MTOR activator 2 [Source:HGNC Symbol;Acc:HGNC:29796]" |
| ENSG00000123560 | HGLibA_37215 | 287 | 1.707345782 | PLP1 | proteolipid protein 1 [Source:HGNC Symbol;Acc:HGNC:9086] |
| ENSG00000125046 | HGLibA_47191 | 287 | 1.707345782 | SSUH2 | ssu-2 homolog (C. elegans) [Source:HGNC Symbol;Acc:HGNC:24809] |
| ENSG00000126759 | HGLibA_09291 | 287 | 1.707345782 | CFP | complement factor properdin [Source:HGNC Symbol;Acc:HGNC:8864] |
| ENSG00000137075 | HGLibA_41670 | 287 | 1.707345782 | RNF38 | ring finger protein 38 [Source:HGNC Symbol;Acc:HGNC:18052] |
| ENSG00000137343 | HGLibA_03421 | 287 | 1.707345782 | ATAT1 | alpha tubulin acetyltransferase 1 [Source:HGNC Symbol;Acc:HGNC:21186] |
| ENSG00000139344 | HGLibA_01759 | 287 | 1.707345782 | AMDHD1 | amidohydrolase domain containing 1 [Source:HGNC Symbol;Acc:HGNC:28577] |
| ENSG00000145850 | HGLibA_49488 | 287 | 1.707345782 | TIMD4 | T cell immunoglobulin and mucin domain containing 4 [Source:HGNC Symbol;Acc:HGNC:25132] |
| ENSG00000154589 | HGLibA_27692 | 287 | 1.707345782 | LY96 | lymphocyte antigen 96 [Source:HGNC Symbol;Acc:HGNC:17156] |
| ENSG00000160323 | HGLibA_00801 | 287 | 1.707345782 | ADAMTS13 | ADAM metallopeptidase with thrombospondin type 1 motif 13 [Source:HGNC Symbol;Acc:HGNC:1366] |
| ENSG00000166349 | HGLibA_40131 | 287 | 1.707345782 | RAG1 | recombination activating 1 [Source:HGNC Symbol;Acc:HGNC:9831] |
| ENSG00000166351 | HGLibA_37725 | 287 | 1.707345782 | POTED | POTE ankyrin domain family member D [Source:HGNC Symbol;Acc:HGNC:23822] |
| ENSG00000171711 | HGLibA_12913 | 287 | 1.707345782 | DEFB4A | defensin beta 4A [Source:HGNC Symbol;Acc:HGNC:2767] |
| ENSG00000173338 | HGLibA_24334 | 287 | 1.707345782 | KCNK7 | potassium two pore domain channel subfamily K member 7 [Source:HGNC Symbol;Acc:HGNC:6282] |
| ENSG00000183486 | HGLibA_30606 | 287 | 1.707345782 | MX2 | MX dynamin like GTPase 2 [Source:HGNC Symbol;Acc:HGNC:7533] |
| ENSG00000184486 | HGLibA_37768 | 287 | 1.707345782 | POU3F2 | POU class 3 homeobox 2 [Source:HGNC Symbol;Acc:HGNC:9215] |
| ENSG00000205356 | HGLibA_48942 | 287 | 1.707345782 | TECPR1 | tectonin beta-propeller repeat containing 1 [Source:HGNC Symbol;Acc:HGNC:22214] |
| ENSG00000206107 | HGLibA_25733 | 287 | 1.707345782 | KRTAP27-1 | keratin associated protein 27-1 [Source:HGNC Symbol;Acc:HGNC:33864] |
| ENSG00000214456 | HGLibA_37182 | 287 | 1.707345782 | PLIN5 | perilipin 5 [Source:HGNC Symbol;Acc:HGNC:33196] |
| ENSG00000216490 | HGLibA_22606 | 287 | 1.707345782 | IFI30 | lysosomal thiol reductase [Source:HGNC Symbol;Acc:HGNC:5398]" |
| hsa-mir-499b | HGLibA_61404 | 287 | 1.707345782 | hsa-mir-499b | - |
| hsa-mir-6499 | HGLibA_62842 | 287 | 1.707345782 | hsa-mir-6499 | - |
| SCXB | HGLibA_43082 | 287 | 1.707345782 | SCXB | - |
| UFD1L | HGLibA_52846 | 287 | 1.707345782 | UFD1L | - |
| DUS2L | HGLibA_14117 | 286 | 1.697394745 | DUS2L | - |
| ELMSAN1 | HGLibA_14949 | 286 | 1.697394745 | ELMSAN1 | - |
| ENSG00000008282 | HGLibA_48036 | 286 | 1.697394745 | SYPL1 | synaptophysin like 1 [Source:HGNC Symbol;Acc:HGNC:11507] |
| ENSG00000072786 | HGLibA_47450 | 286 | 1.697394745 | STK10 | serine/threonine kinase 10 [Source:HGNC Symbol;Acc:HGNC:11388] |
| ENSG00000100302 | HGLibA_40322 | 286 | 1.697394745 | RASD2 | RASD family member 2 [Source:HGNC Symbol;Acc:HGNC:18229] |
| ENSG00000104889 | HGLibA_41427 | 286 | 1.697394745 | RNASEH2A | ribonuclease H2 subunit A [Source:HGNC Symbol;Acc:HGNC:18518] |
| ENSG00000115828 | HGLibA_39726 | 286 | 1.697394745 | QPCT | glutaminyl-peptide cyclotransferase [Source:HGNC Symbol;Acc:HGNC:9753] |
| ENSG00000128311 | HGLibA_52025 | 286 | 1.697394745 | TST | thiosulfate sulfurtransferase [Source:HGNC Symbol;Acc:HGNC:12388] |
| ENSG00000134851 | HGLibA_50037 | 286 | 1.697394745 | TMEM165 | transmembrane protein 165 [Source:HGNC Symbol;Acc:HGNC:30760] |
| ENSG00000135502 | HGLibA_44784 | 286 | 1.697394745 | SLC26A10 | solute carrier family 26 member 10 [Source:HGNC Symbol;Acc:HGNC:14470] |
| ENSG00000135617 | HGLibA_38266 | 286 | 1.697394745 | PRADC1 | protease associated domain containing 1 [Source:HGNC Symbol;Acc:HGNC:16047] |
| ENSG00000138246 | HGLibA_13572 | 286 | 1.697394745 | DNAJC13 | DnaJ heat shock protein family (Hsp40) member C13 [Source:HGNC Symbol;Acc:HGNC:30343] |
| ENSG00000139835 | HGLibA_20384 | 286 | 1.697394745 | GRTP1 | growth hormone regulated TBC protein 1 [Source:HGNC Symbol;Acc:HGNC:20310] |
| ENSG00000143878 | HGLibA_41168 | 286 | 1.697394745 | RHOB | ras homolog family member B [Source:HGNC Symbol;Acc:HGNC:668] |
| ENSG00000149305 | HGLibA_22384 | 286 | 1.697394745 | HTR3B | 5-hydroxytryptamine receptor 3B [Source:HGNC Symbol;Acc:HGNC:5298] |
| ENSG00000156876 | HGLibA_42768 | 286 | 1.697394745 | SASS6 | SAS-6 centriolar assembly protein [Source:HGNC Symbol;Acc:HGNC:25403] |
| ENSG00000156976 | HGLibA_14808 | 286 | 1.697394745 | EIF4A2 | eukaryotic translation initiation factor 4A2 [Source:HGNC Symbol;Acc:HGNC:3284] |
| ENSG00000159339 | HGLibA_34979 | 286 | 1.697394745 | PADI4 | peptidyl arginine deiminase 4 [Source:HGNC Symbol;Acc:HGNC:18368] |
| ENSG00000163596 | HGLibA_22492 | 286 | 1.697394745 | ICA1L | islet cell autoantigen 1 like [Source:HGNC Symbol;Acc:HGNC:14442] |
| ENSG00000173068 | HGLibA_04648 | 286 | 1.697394745 | BNC2 | basonuclin 2 [Source:HGNC Symbol;Acc:HGNC:30988] |
| ENSG00000178187 | HGLibA_56031 | 286 | 1.697394745 | ZNF454 | zinc finger protein 454 [Source:HGNC Symbol;Acc:HGNC:21200] |
| ENSG00000178252 | HGLibA_54136 | 286 | 1.697394745 | WDR6 | WD repeat domain 6 [Source:HGNC Symbol;Acc:HGNC:12758] |
| ENSG00000178690 | HGLibA_14228 | 286 | 1.697394745 | DYNAP | dynactin associated protein [Source:HGNC Symbol;Acc:HGNC:26808] |
| ENSG00000179673 | HGLibA_42057 | 286 | 1.697394745 | RPRML | reprimo like [Source:HGNC Symbol;Acc:HGNC:32422] |
| ENSG00000179915 | HGLibA_32732 | 286 | 1.697394745 | NRXN1 | neurexin 1 [Source:HGNC Symbol;Acc:HGNC:8008] |
| ENSG00000183840 | HGLibA_20039 | 286 | 1.697394745 | GPR39 | G protein-coupled receptor 39 [Source:HGNC Symbol;Acc:HGNC:4496] |
| ENSG00000221880 | HGLibA_25582 | 286 | 1.697394745 | KRTAP1-3 | keratin associated protein 1-3 [Source:HGNC Symbol;Acc:HGNC:16771] |
| ENSG00000239998 | HGLibA_26492 | 286 | 1.697394745 | LILRA2 | leukocyte immunoglobulin like receptor A2 [Source:HGNC Symbol;Acc:HGNC:6603] |
| ENSG00000243137 | HGLibA_39046 | 286 | 1.697394745 | PSG4 | pregnancy specific beta-1-glycoprotein 4 [Source:HGNC Symbol;Acc:HGNC:9521] |
| ENSG00000259823 | HGLibA_27733 | 286 | 1.697394745 | LYPD8 | LY6/PLAUR domain containing 8 [Source:HGNC Symbol;Acc:HGNC:44208] |
| hsa-mir-3177 | HGLibA_58781 | 286 | 1.697394745 | hsa-mir-3177 | - |
| hsa-mir-3936 | HGLibA_59605 | 286 | 1.697394745 | hsa-mir-3936 | - |
| hsa-mir-4275 | HGLibA_59804 | 286 | 1.697394745 | hsa-mir-4275 | - |
| hsa-mir-7851 | HGLibA_63992 | 286 | 1.697394745 | hsa-mir-7851 | - |
| C1orf65 | HGLibA_05889 | 285 | 1.687443707 | C1orf65 | - |
| ENSG00000056487 | HGLibA_36371 | 285 | 1.687443707 | PHF21B | PHD finger protein 21B [Source:HGNC Symbol;Acc:HGNC:25161] |
| ENSG00000070018 | HGLibA_27229 | 285 | 1.687443707 | LRP6 | LDL receptor related protein 6 [Source:HGNC Symbol;Acc:HGNC:6698] |
| ENSG00000081181 | HGLibA_02688 | 285 | 1.687443707 | ARG2 | arginase 2 [Source:HGNC Symbol;Acc:HGNC:664] |
| ENSG00000091490 | HGLibA_43284 | 285 | 1.687443707 | SEL1L3 | SEL1L family member 3 [Source:HGNC Symbol;Acc:HGNC:29108] |
| ENSG00000100092 | HGLibA_43924 | 285 | 1.687443707 | SH3BP1 | SH3 domain binding protein 1 [Source:HGNC Symbol;Acc:HGNC:10824] |
| ENSG00000103154 | HGLibA_31587 | 285 | 1.687443707 | NECAB2 | N-terminal EF-hand calcium binding protein 2 [Source:HGNC Symbol;Acc:HGNC:23746] |
| ENSG00000105198 | HGLibA_26352 | 285 | 1.687443707 | LGALS13 | galectin 13 [Source:HGNC Symbol;Acc:HGNC:15449] |
| ENSG00000105379 | HGLibA_15622 | 285 | 1.687443707 | ETFB | electron transfer flavoprotein subunit beta [Source:HGNC Symbol;Acc:HGNC:3482] |
| ENSG00000110344 | HGLibA_52691 | 285 | 1.687443707 | UBE4A | ubiquitination factor E4A [Source:HGNC Symbol;Acc:HGNC:12499] |
| ENSG00000125084 | HGLibA_54344 | 285 | 1.687443707 | WNT1 | Wnt family member 1 [Source:HGNC Symbol;Acc:HGNC:12774] |
| ENSG00000126768 | HGLibA_49505 | 285 | 1.687443707 | TIMM17B | translocase of inner mitochondrial membrane 17B [Source:HGNC Symbol;Acc:HGNC:17310] |
| ENSG00000143393 | HGLibA_36501 | 285 | 1.687443707 | PI4KB | phosphatidylinositol 4-kinase beta [Source:HGNC Symbol;Acc:HGNC:8984] |
| ENSG00000145002 | HGLibA_16777 | 285 | 1.687443707 | FAM86B2 | family with sequence similarity 86 member B2 [Source:HGNC Symbol;Acc:HGNC:32222] |
| ENSG00000149262 | HGLibA_23422 | 285 | 1.687443707 | INTS4 | integrator complex subunit 4 [Source:HGNC Symbol;Acc:HGNC:25048] |
| ENSG00000162144 | HGLibA_11979 | 285 | 1.687443707 | CYB561A3 | cytochrome b561 family member A3 [Source:HGNC Symbol;Acc:HGNC:23014] |
| ENSG00000167755 | HGLibA_25265 | 285 | 1.687443707 | KLK6 | kallikrein related peptidase 6 [Source:HGNC Symbol;Acc:HGNC:6367] |
| ENSG00000180660 | HGLibA_27822 | 285 | 1.687443707 | MAB21L1 | mab-21 like 1 [Source:HGNC Symbol;Acc:HGNC:6757] |
| ENSG00000184117 | HGLibA_31970 | 285 | 1.687443707 | NIPSNAP1 | nipsnap homolog 1 [Source:HGNC Symbol;Acc:HGNC:7827] |
| ENSG00000188000 | HGLibA_34471 | 285 | 1.687443707 | OR7D2 | olfactory receptor family 7 subfamily D member 2 [Source:HGNC Symbol;Acc:HGNC:8378] |
| ENSG00000204428 | HGLibA_27663 | 285 | 1.687443707 | LY6G5C | lymphocyte antigen 6 family member G5C [Source:HGNC Symbol;Acc:HGNC:13932] |
| ENSG00000214944 | HGLibA_02842 | 285 | 1.687443707 | ARHGEF28 | Rho guanine nucleotide exchange factor 28 [Source:HGNC Symbol;Acc:HGNC:30322] |
| ENSG00000254505 | HGLibA_09488 | 285 | 1.687443707 | CHMP4A | charged multivesicular body protein 4A [Source:HGNC Symbol;Acc:HGNC:20274] |
| ENSG00000261934 | HGLibA_35547 | 285 | 1.687443707 | PCDHGA9 | 9 [Source:HGNC Symbol;Acc:HGNC:8707]" |
| hsa-mir-1178 | HGLibA_57205 | 285 | 1.687443707 | hsa-mir-1178 | - |
| hsa-mir-4318 | HGLibA_59992 | 285 | 1.687443707 | hsa-mir-4318 | - |
| hsa-mir-6768 | HGLibA_63232 | 285 | 1.687443707 | hsa-mir-6768 | - |
| C10orf131 | HGLibA_05054 | 284 | 1.67749267 | C10orf131 | - |
| C17orf82 | HGLibA_05585 | 284 | 1.67749267 | C17orf82 | - |
| ENSG00000006715 | HGLibA_53792 | 284 | 1.67749267 | VPS41 | HOPS complex subunit [Source:HGNC Symbol;Acc:HGNC:12713]" |
| ENSG00000089091 | HGLibA_14294 | 284 | 1.67749267 | DZANK1 | double zinc ribbon and ankyrin repeat domains 1 [Source:HGNC Symbol;Acc:HGNC:15858] |
| ENSG00000101365 | HGLibA_22548 | 284 | 1.67749267 | IDH3B | isocitrate dehydrogenase 3 (NAD(+)) beta [Source:HGNC Symbol;Acc:HGNC:5385] |
| ENSG00000105717 | HGLibA_35351 | 284 | 1.67749267 | PBX4 | PBX homeobox 4 [Source:HGNC Symbol;Acc:HGNC:13403] |
| ENSG00000125686 | HGLibA_28733 | 284 | 1.67749267 | MED1 | mediator complex subunit 1 [Source:HGNC Symbol;Acc:HGNC:9234] |
| ENSG00000128045 | HGLibA_40369 | 284 | 1.67749267 | RASL11B | RAS like family 11 member B [Source:HGNC Symbol;Acc:HGNC:23804] |
| ENSG00000129152 | HGLibA_30880 | 284 | 1.67749267 | MYOD1 | myogenic differentiation 1 [Source:HGNC Symbol;Acc:HGNC:7611] |
| ENSG00000132026 | HGLibA_42368 | 284 | 1.67749267 | RTBDN | retbindin [Source:HGNC Symbol;Acc:HGNC:30310] |
| ENSG00000134539 | HGLibA_25300 | 284 | 1.67749267 | KLRD1 | killer cell lectin like receptor D1 [Source:HGNC Symbol;Acc:HGNC:6378] |
| ENSG00000136160 | HGLibA_14480 | 284 | 1.67749267 | EDNRB | endothelin receptor type B [Source:HGNC Symbol;Acc:HGNC:3180] |
| ENSG00000138190 | HGLibA_15755 | 284 | 1.67749267 | EXOC6 | exocyst complex component 6 [Source:HGNC Symbol;Acc:HGNC:23196] |
| ENSG00000138685 | HGLibA_17389 | 284 | 1.67749267 | FGF2 | fibroblast growth factor 2 [Source:HGNC Symbol;Acc:HGNC:3676] |
| ENSG00000144331 | HGLibA_55900 | 284 | 1.67749267 | ZNF385B | zinc finger protein 385B [Source:HGNC Symbol;Acc:HGNC:26332] |
| ENSG00000149639 | HGLibA_46153 | 284 | 1.67749267 | SOGA1 | autophagy associated 1 [Source:HGNC Symbol;Acc:HGNC:16111]" |
| ENSG00000152404 | HGLibA_11827 | 284 | 1.67749267 | CWF19L2 | CWF19 like cell cycle control factor 2 [Source:HGNC Symbol;Acc:HGNC:26508] |
| ENSG00000153487 | HGLibA_23282 | 284 | 1.67749267 | ING1 | inhibitor of growth family member 1 [Source:HGNC Symbol;Acc:HGNC:6062] |
| ENSG00000156873 | HGLibA_36415 | 284 | 1.67749267 | PHKG2 | phosphorylase kinase catalytic subunit gamma 2 [Source:HGNC Symbol;Acc:HGNC:8931] |
| ENSG00000163440 | HGLibA_35750 | 284 | 1.67749267 | PDCL2 | phosducin like 2 [Source:HGNC Symbol;Acc:HGNC:29524] |
| ENSG00000163629 | HGLibA_39440 | 284 | 1.67749267 | PTPN13 | non-receptor type 13 [Source:HGNC Symbol;Acc:HGNC:9646]" |
| ENSG00000163812 | HGLibA_55114 | 284 | 1.67749267 | ZDHHC3 | zinc finger DHHC-type containing 3 [Source:HGNC Symbol;Acc:HGNC:18470] |
| ENSG00000164488 | HGLibA_12283 | 284 | 1.67749267 | DACT2 | dishevelled binding antagonist of beta catenin 2 [Source:HGNC Symbol;Acc:HGNC:21231] |
| ENSG00000167264 | HGLibA_14114 | 284 | 1.67749267 | DUS2 | dihydrouridine synthase 2 [Source:HGNC Symbol;Acc:HGNC:26014] |
| ENSG00000171017 | HGLibA_27439 | 284 | 1.67749267 | LRRC8E | leucine rich repeat containing 8 VRAC subunit E [Source:HGNC Symbol;Acc:HGNC:26272] |
| ENSG00000172150 | HGLibA_33644 | 284 | 1.67749267 | OR1A2 | olfactory receptor family 1 subfamily A member 2 [Source:HGNC Symbol;Acc:HGNC:8180] |

| | | | | | |
|---|---|---|---|---|---|
| ENSG00000172167 | HGLibA_30321 | 284 | 1.67749267 | MTBP | MDM2 binding protein [Source:HGNC Symbol;Acc:HGNC:7417] |
| ENSG00000176909 | HGLibA_28054 | 284 | 1.67749267 | MAMSTR | MEF2 activating motif and SAP domain containing transcriptional regulator [Source:HGNC Symbol;Acc:HGNC:26689] |
| ENSG00000181220 | HGLibA_56664 | 284 | 1.67749267 | ZNF746 | zinc finger protein 746 [Source:HGNC Symbol;Acc:HGNC:21948] |
| ENSG00000182168 | HGLibA_53021 | 284 | 1.67749267 | UNC5C | unc-5 netrin receptor C [Source:HGNC Symbol;Acc:HGNC:12569] |
| ENSG00000182333 | HGLibA_26609 | 284 | 1.67749267 | LIPF | gastric type [Source:HGNC Symbol;Acc:HGNC:6622]" |
| ENSG00000187954 | HGLibA_12041 | 284 | 1.67749267 | CYHR1 | cysteine and histidine rich 1 [Source:HGNC Symbol;Acc:HGNC:17806] |
| ENSG00000196406 | HGLibA_46392 | 284 | 1.67749267 | SPANXD | SPANX family member D [Source:HGNC Symbol;Acc:HGNC:14332] |
| ENSG00000197077 | HGLibA_24798 | 284 | 1.67749267 | KIAA1671 | KIAA1671 [Source:HGNC Symbol;Acc:HGNC:29345] |
| ENSG00000198898 | HGLibA_07207 | 284 | 1.67749267 | CAPZA2 | capping actin protein of muscle Z-line subunit alpha 2 [Source:HGNC Symbol;Acc:HGNC:1490] |
| ENSG00000203778 | HGLibA_16543 | 284 | 1.67749267 | FAM229B | family with sequence similarity 229 member B [Source:HGNC Symbol;Acc:HGNC:33858] |
| ENSG00000204010 | HGLibA_22626 | 284 | 1.67749267 | IFIT1B | interferon induced protein with tetratricopeptide repeats 1B [Source:HGNC Symbol;Acc:HGNC:23442] |
| ENSG00000204257 | HGLibA_21603 | 284 | 1.67749267 | HLA-DMA | DM alpha [Source:HGNC Symbol;Acc:HGNC:4934]" |
| ENSG00000204388 | HGLibA_22274 | 284 | 1.67749267 | HSPA1B | heat shock protein family A (Hsp70) member 1B [Source:HGNC Symbol;Acc:HGNC:5233] |
| ENSG00000234511 | HGLibA_06413 | 284 | 1.67749267 | C5orf58 | chromosome 5 open reading frame 58 [Source:HGNC Symbol;Acc:HGNC:37272] |
| ENSG00000240764 | HGLibA_35577 | 284 | 1.67749267 | PCDHGC5 | 5 [Source:HGNC Symbol;Acc:HGNC:8718]" |
| ENSG00000241322 | HGLibA_08871 | 284 | 1.67749267 | CDRT1 | CMT1A duplicated region transcript 1 [Source:HGNC Symbol;Acc:HGNC:14379] |
| ENSG00000260916 | HGLibA_08142 | 284 | 1.67749267 | CCPG1 | cell cycle progression 1 [Source:HGNC Symbol;Acc:HGNC:24227] |
| FAM46D | HGLibA_16606 | 284 | 1.67749267 | FAM46D | - |
| hsa-mir-138-2 | HGLibA_57818 | 284 | 1.67749267 | hsa-mir-138-2 | - |
| hsa-mir-212 | HGLibA_58243 | 284 | 1.67749267 | hsa-mir-212 | - |
| hsa-mir-6864 | HGLibA_63640 | 284 | 1.67749267 | hsa-mir-6864 | - |
| SPATA31A4 | HGLibA_46476 | 284 | 1.67749267 | SPATA31A4 | - |
| C19orf60 | HGLibA_05691 | 283 | 1.667541632 | C19orf60 | - |
| C7orf71 | HGLibA_06583 | 283 | 1.667541632 | C7orf71 | - |
| DARS | HGLibA_12340 | 283 | 1.667541632 | DARS | - |
| ENSG00000037280 | HGLibA_17672 | 283 | 1.667541632 | FLT4 | fms related tyrosine kinase 4 [Source:HGNC Symbol;Acc:HGNC:3767] |
| ENSG00000063127 | HGLibA_45308 | 283 | 1.667541632 | SLC6A16 | solute carrier family 6 member 16 [Source:HGNC Symbol;Acc:HGNC:13622] |
| ENSG00000066427 | HGLibA_03842 | 283 | 1.667541632 | ATXN3 | ataxin 3 [Source:HGNC Symbol;Acc:HGNC:7106] |
| ENSG00000083454 | HGLibA_34857 | 283 | 1.667541632 | P2RX5 | purinergic receptor P2X 5 [Source:HGNC Symbol;Acc:HGNC:8536] |
| ENSG00000100918 | HGLibA_40760 | 283 | 1.667541632 | REC8 | REC8 meiotic recombination protein [Source:HGNC Symbol;Acc:HGNC:16879] |
| ENSG00000101384 | HGLibA_23904 | 283 | 1.667541632 | JAG1 | jagged 1 [Source:HGNC Symbol;Acc:HGNC:6188] |
| ENSG00000114455 | HGLibA_21271 | 283 | 1.667541632 | HHLA2 | HERV-H LTR-associating 2 [Source:HGNC Symbol;Acc:HGNC:4905] |
| ENSG00000119946 | HGLibA_10314 | 283 | 1.667541632 | CNNM1 | cyclin and CBS domain divalent metal cation transport mediator 1 [Source:HGNC Symbol;Acc:HGNC:102] |
| ENSG00000125779 | HGLibA_35099 | 283 | 1.667541632 | PANK2 | pantothenate kinase 2 [Source:HGNC Symbol;Acc:HGNC:15894] |
| ENSG00000125816 | HGLibA_32039 | 283 | 1.667541632 | NKX2-4 | NK2 homeobox 4 [Source:HGNC Symbol;Acc:HGNC:7837] |
| ENSG00000129353 | HGLibA_45159 | 283 | 1.667541632 | SLC44A2 | solute carrier family 44 member 2 [Source:HGNC Symbol;Acc:HGNC:17292] |
| ENSG00000132530 | HGLibA_54462 | 283 | 1.667541632 | XAF1 | XIAP associated factor 1 [Source:HGNC Symbol;Acc:HGNC:30932] |
| ENSG00000136279 | HGLibA_12398 | 283 | 1.667541632 | DBNL | drebrin like [Source:HGNC Symbol;Acc:HGNC:2696] |
| ENSG00000136929 | HGLibA_21128 | 283 | 1.667541632 | HEMGN | hemogen [Source:HGNC Symbol;Acc:HGNC:17509] |
| ENSG00000137413 | HGLibA_48258 | 283 | 1.667541632 | TAF8 | TATA-box binding protein associated factor 8 [Source:HGNC Symbol;Acc:HGNC:17300] |
| ENSG00000144395 | HGLibA_07643 | 283 | 1.667541632 | CCDC150 | coiled-coil domain containing 150 [Source:HGNC Symbol;Acc:HGNC:26834] |
| ENSG00000144485 | HGLibA_21191 | 283 | 1.667541632 | HES6 | hes family bHLH transcription factor 6 [Source:HGNC Symbol;Acc:HGNC:18254] |
| ENSG00000159164 | HGLibA_47873 | 283 | 1.667541632 | SV2A | synaptic vesicle glycoprotein 2A [Source:HGNC Symbol;Acc:HGNC:20566] |
| ENSG00000160808 | HGLibA_30760 | 283 | 1.667541632 | MYL3 | myosin light chain 3 [Source:HGNC Symbol;Acc:HGNC:7584] |
| ENSG00000162191 | HGLibA_52773 | 283 | 1.667541632 | UBXN1 | UBX domain protein 1 [Source:HGNC Symbol;Acc:HGNC:18402] |
| ENSG00000165300 | HGLibA_45525 | 283 | 1.667541632 | SLITRK5 | SLIT and NTRK like family member 5 [Source:HGNC Symbol;Acc:HGNC:20295] |
| ENSG00000165997 | HGLibA_02979 | 283 | 1.667541632 | ARL5B | ADP ribosylation factor like GTPase 5B [Source:HGNC Symbol;Acc:HGNC:23052] |
| ENSG00000166664 | HGLibA_09545 | 283 | 1.667541632 | CHRFAM7A | CHRNA7 (exons 5-10) and FAM7A (exons A-E) fusion [Source:HGNC Symbol;Acc:HGNC:15781] |
| ENSG00000167220 | HGLibA_21053 | 283 | 1.667541632 | HDHD2 | haloacid dehalogenase like hydrolase domain containing 2 [Source:HGNC Symbol;Acc:HGNC:25364] |
| ENSG00000167775 | HGLibA_08352 | 283 | 1.667541632 | CD320 | CD320 molecule [Source:HGNC Symbol;Acc:HGNC:16692] |
| ENSG00000168769 | HGLibA_49039 | 283 | 1.667541632 | TET2 | tet methylcytosine dioxygenase 2 [Source:HGNC Symbol;Acc:HGNC:25941] |
| ENSG00000170322 | HGLibA_31840 | 283 | 1.667541632 | NFRKB | nuclear factor related to kappaB binding protein [Source:HGNC Symbol;Acc:HGNC:7802] |
| ENSG00000170961 | HGLibA_20865 | 283 | 1.667541632 | HAS2 | hyaluronan synthase 2 [Source:HGNC Symbol;Acc:HGNC:4819] |
| ENSG00000172663 | HGLibA_49946 | 283 | 1.667541632 | TMEM134 | transmembrane protein 134 [Source:HGNC Symbol;Acc:HGNC:26142] |
| ENSG00000174469 | HGLibA_10421 | 283 | 1.667541632 | CNTNAP2 | contactin associated protein like 2 [Source:HGNC Symbol;Acc:HGNC:13830] |
| ENSG00000180440 | HGLibA_43616 | 283 | 1.667541632 | SERTM1 | serine rich and transmembrane domain containing 1 [Source:HGNC Symbol;Acc:HGNC:33792] |
| ENSG00000182087 | HGLibA_50346 | 283 | 1.667541632 | TMEM259 | transmembrane protein 259 [Source:HGNC Symbol;Acc:HGNC:17039] |
| ENSG00000187446 | HGLibA_09516 | 283 | 1.667541632 | CHP1 | calcineurin like EF-hand protein 1 [Source:HGNC Symbol;Acc:HGNC:17433] |
| ENSG00000188643 | HGLibA_42547 | 283 | 1.667541632 | S100A16 | S100 calcium binding protein A16 [Source:HGNC Symbol;Acc:HGNC:20441] |
| ENSG00000197905 | HGLibA_48936 | 283 | 1.667541632 | TEAD4 | TEA domain transcription factor 4 [Source:HGNC Symbol;Acc:HGNC:11717] |
| ENSG00000198523 | HGLibA_37205 | 283 | 1.667541632 | PLN | phospholamban [Source:HGNC Symbol;Acc:HGNC:9080] |
| ENSG00000205084 | HGLibA_50257 | 283 | 1.667541632 | TMEM231 | transmembrane protein 231 [Source:HGNC Symbol;Acc:HGNC:37234] |
| ENSG00000206203 | HGLibA_52013 | 283 | 1.667541632 | TSSK2 | testis specific serine kinase 2 [Source:HGNC Symbol;Acc:HGNC:11401] |
| ZNF645 | HGLibA_56450 | 283 | 1.667541632 | ZNF645 | - |
| C7orf29 | HGLibA_06529 | 282 | 1.657590595 | C7orf29 | - |
| ENSG00000083307 | HGLibA_20250 | 282 | 1.657590595 | GRHL2 | grainyhead like transcription factor 2 [Source:HGNC Symbol;Acc:HGNC:2799] |
| ENSG00000094975 | HGLibA_47707 | 282 | 1.657590595 | SUCO | SUN domain containing ossification factor [Source:HGNC Symbol;Acc:HGNC:1240] |
| ENSG00000101347 | HGLibA_42704 | 282 | 1.657590595 | SAMHD1 | SAM and HD domain containing deoxynucleoside triphosphate triphosphohydrolase 1 [Source:HGNC Symbol;Acc:HGNC:15925] |
| ENSG00000104064 | HGLibA_18349 | 282 | 1.657590595 | GABPB1 | GA binding protein transcription factor subunit beta 1 [Source:HGNC Symbol;Acc:HGNC:4074] |
| ENSG00000111711 | HGLibA_19666 | 282 | 1.657590595 | GOLT1B | golgi transport 1B [Source:HGNC Symbol;Acc:HGNC:20175] |
| ENSG00000125510 | HGLibA_33450 | 282 | 1.657590595 | OPRL1 | opioid related nociceptin receptor 1 [Source:HGNC Symbol;Acc:HGNC:8155] |
| ENSG00000131398 | HGLibA_24167 | 282 | 1.657590595 | KCNC3 | potassium voltage-gated channel subfamily C member 3 [Source:HGNC Symbol;Acc:HGNC:6235] |
| ENSG00000138795 | HGLibA_26272 | 282 | 1.657590595 | LEF1 | lymphoid enhancer binding factor 1 [Source:HGNC Symbol;Acc:HGNC:6551] |
| ENSG00000141526 | HGLibA_44437 | 282 | 1.657590595 | SLC16A3 | solute carrier family 16 member 3 [Source:HGNC Symbol;Acc:HGNC:10924] |
| ENSG00000141574 | HGLibA_43272 | 282 | 1.657590595 | SECTM1 | secreted and transmembrane 1 [Source:HGNC Symbol;Acc:HGNC:10707] |
| ENSG00000142046 | HGLibA_50558 | 282 | 1.657590595 | TMEM91 | transmembrane protein 91 [Source:HGNC Symbol;Acc:HGNC:32393] |
| ENSG00000145491 | HGLibA_41766 | 282 | 1.657590595 | ROPN1L | rhophilin associated tail protein 1 like [Source:HGNC Symbol;Acc:HGNC:24060] |
| ENSG00000151498 | HGLibA_00359 | 282 | 1.657590595 | ACAD8 | acyl-CoA dehydrogenase family member 8 [Source:HGNC Symbol;Acc:HGNC:87] |
| ENSG00000161558 | HGLibA_49967 | 282 | 1.657590595 | TMEM143 | transmembrane protein 143 [Source:HGNC Symbol;Acc:HGNC:25603] |
| ENSG00000168000 | HGLibA_04860 | 282 | 1.657590595 | BSCL2 | seipin lipid droplet biogenesis associated [Source:HGNC Symbol;Acc:HGNC:15832]" |
| ENSG00000169604 | HGLibA_02214 | 282 | 1.657590595 | ANTXR1 | ANTXR cell adhesion molecule 1 [Source:HGNC Symbol;Acc:HGNC:21014] |
| ENSG00000170956 | HGLibA_08938 | 282 | 1.657590595 | CEACAM3 | carcinoembryonic antigen related cell adhesion molecule 3 [Source:HGNC Symbol;Acc:HGNC:1815] |
| ENSG00000172594 | HGLibA_45816 | 282 | 1.657590595 | SMPDL3A | sphingomyelin phosphodiesterase acid like 3A [Source:HGNC Symbol;Acc:HGNC:17389] |
| ENSG00000173212 | HGLibA_27826 | 282 | 1.657590595 | MAB21L3 | mab-21 like 3 [Source:HGNC Symbol;Acc:HGNC:26787] |
| ENSG00000175093 | HGLibA_46868 | 282 | 1.657590595 | SPSB4 | splA/ryanodine receptor domain and SOCS box containing 4 [Source:HGNC Symbol;Acc:HGNC:30630] |
| ENSG00000175485 | HGLibA_34208 | 282 | 1.657590595 | OR52W1 | olfactory receptor family 52 subfamily W member 1 [Source:HGNC Symbol;Acc:HGNC:15239] |
| ENSG00000175592 | HGLibA_17800 | 282 | 1.657590595 | FOSL1 | AP-1 transcription factor subunit [Source:HGNC Symbol;Acc:HGNC:13718]" |
| ENSG00000183779 | HGLibA_56582 | 282 | 1.657590595 | ZNF703 | zinc finger protein 703 [Source:HGNC Symbol;Acc:HGNC:25883] |
| FTSJD2 | HGLibA_18145 | 282 | 1.657590595 | FTSJD2 | - |
| hsa-mir-29a | HGLibA_58411 | 282 | 1.657590595 | hsa-mir-29a | - |
| ENSG00000091592 | HGLibA_32097 | 281 | 1.647639558 | NLRP1 | NLR family pyrin domain containing 1 [Source:HGNC Symbol;Acc:HGNC:14374] |
| ENSG00000101935 | HGLibA_01803 | 281 | 1.647639558 | AMMECR1 | midface hypoplasia and elliptocytosis chromosomal region gene 1 [Source:HGNC Symbol;Acc:HGNC:467]" |
| ENSG00000113758 | HGLibA_12388 | 281 | 1.647639558 | DBN1 | drebrin 1 [Source:HGNC Symbol;Acc:HGNC:2695] |
| ENSG00000122133 | HGLibA_34984 | 281 | 1.647639558 | PAEP | progestagen associated endometrial protein [Source:HGNC Symbol;Acc:HGNC:8573] |
| ENSG00000137171 | HGLibA_25035 | 281 | 1.647639558 | KLC4 | kinesin light chain 4 [Source:HGNC Symbol;Acc:HGNC:21624] |
| ENSG00000139914 | HGLibA_17539 | 281 | 1.647639558 | FITM1 | fat storage inducing transmembrane protein 1 [Source:HGNC Symbol;Acc:HGNC:33714] |
| ENSG00000145384 | HGLibA_15913 | 281 | 1.647639558 | FABP2 | fatty acid binding protein 2 [Source:HGNC Symbol;Acc:HGNC:3556] |
| ENSG00000149654 | HGLibA_08665 | 281 | 1.647639558 | CDH22 | cadherin 22 [Source:HGNC Symbol;Acc:HGNC:13251] |
| ENSG00000156958 | HGLibA_18519 | 281 | 1.647639558 | GALK2 | galactokinase 2 [Source:HGNC Symbol;Acc:HGNC:4119] |
| ENSG00000166257 | HGLibA_42988 | 281 | 1.647639558 | SCN3B | sodium voltage-gated channel beta subunit 3 [Source:HGNC Symbol;Acc:HGNC:20665] |
| ENSG00000166963 | HGLibA_28114 | 281 | 1.647639558 | MAP1A | microtubule associated protein 1A [Source:HGNC Symbol;Acc:HGNC:6835] |
| ENSG00000171501 | HGLibA_33707 | 281 | 1.647639558 | OR1N2 | olfactory receptor family 1 subfamily N member 2 [Source:HGNC Symbol;Acc:HGNC:15111] |
| ENSG00000171951 | HGLibA_42898 | 281 | 1.647639558 | SCG2 | secretogranin II [Source:HGNC Symbol;Acc:HGNC:10575] |
| ENSG00000181752 | HGLibA_34549 | 281 | 1.647639558 | OR8K5 | olfactory receptor family 8 subfamily K member 5 [Source:HGNC Symbol;Acc:HGNC:15315] |
| ENSG00000184916 | HGLibA_23908 | 281 | 1.647639558 | JAG2 | jagged 2 [Source:HGNC Symbol;Acc:HGNC:6189] |
| ENSG00000197584 | HGLibA_24344 | 281 | 1.647639558 | KCNMB2 | potassium calcium-activated channel subfamily M regulatory beta subunit 2 [Source:HGNC Symbol;Acc:HGNC:6286] |
| ENSG00000204965 | HGLibA_35448 | 281 | 1.647639558 | PCDHA5 | protocadherin alpha 5 [Source:HGNC Symbol;Acc:HGNC:8671] |
| ENSG00000205143 | HGLibA_02898 | 281 | 1.647639558 | ARID3C | AT-rich interaction domain 3C [Source:HGNC Symbol;Acc:HGNC:21209] |
| ENSG00000229549 | HGLibA_51977 | 281 | 1.647639558 | TSPY8 | testis specific protein Y-linked 8 [Source:HGNC Symbol;Acc:HGNC:37471] |
| ENSG00000234906 | HGLibA_02506 | 281 | 1.647639558 | APOC2 | apolipoprotein C2 [Source:HGNC Symbol;Acc:HGNC:609] |
| ENSG00000239713 | HGLibA_02492 | 281 | 1.647639558 | APOBEC3G | apolipoprotein B mRNA editing enzyme catalytic subunit 3G [Source:HGNC Symbol;Acc:HGNC:17357] |
| ENSG00000254245 | HGLibA_35531 | 281 | 1.647639558 | PCDHGA3 | 3 [Source:HGNC Symbol;Acc:HGNC:8701]" |
| ENSG00000275832 | HGLibA_02732 | 281 | 1.647639558 | ARHGAP23 | Rho GTPase activating protein 23 [Source:HGNC Symbol;Acc:HGNC:29293] |
| hsa-mir-4526 | HGLibA_60564 | 281 | 1.647639558 | hsa-mir-4526 | - |
| hsa-mir-492 | HGLibA_61370 | 281 | 1.647639558 | hsa-mir-492 | - |
| hsa-mir-549a | HGLibA_62093 | 281 | 1.647639558 | hsa-mir-549a | - |
| hsa-mir-6078 | HGLibA_62570 | 281 | 1.647639558 | hsa-mir-6078 | - |
| hsa-mir-675 | HGLibA_63155 | 281 | 1.647639558 | hsa-mir-675 | - |
| ENSG00000005981 | HGLibA_03238 | 280 | 1.63768852 | ASB4 | ankyrin repeat and SOCS box containing 4 [Source:HGNC Symbol;Acc:HGNC:16009] |
| ENSG00000072422 | HGLibA_41172 | 280 | 1.63768852 | RHOBTB1 | Rho related BTB domain containing 1 [Source:HGNC Symbol;Acc:HGNC:18738] |
| ENSG00000078401 | HGLibA_14470 | 280 | 1.63768852 | EDN1 | endothelin 1 [Source:HGNC Symbol;Acc:HGNC:3176] |
| ENSG00000086991 | HGLibA_32376 | 280 | 1.63768852 | NOX4 | NADPH oxidase 4 [Source:HGNC Symbol;Acc:HGNC:7891] |
| ENSG00000088888 | HGLibA_28445 | 280 | 1.63768852 | MAVS | mitochondrial antiviral signaling protein [Source:HGNC Symbol;Acc:HGNC:29233] |
| ENSG00000089639 | HGLibA_19408 | 280 | 1.63768852 | GMIP | GEM interacting protein [Source:HGNC Symbol;Acc:HGNC:24852] |
| ENSG00000102021 | HGLibA_27643 | 280 | 1.63768852 | LUZP4 | leucine zipper protein 4 [Source:HGNC Symbol;Acc:HGNC:24971] |
| ENSG00000105327 | HGLibA_04197 | 280 | 1.63768852 | BBC3 | BCL2 binding component 3 [Source:HGNC Symbol;Acc:HGNC:17868] |
| ENSG00000118997 | HGLibA_13495 | 280 | 1.63768852 | DNAH7 | dynein axonemal heavy chain 7 [Source:HGNC Symbol;Acc:HGNC:18661] |
| ENSG00000120251 | HGLibA_20264 | 280 | 1.63768852 | GRIA2 | glutamate ionotropic receptor AMPA type subunit 2 [Source:HGNC Symbol;Acc:HGNC:4572] |
| ENSG00000120254 | HGLibA_30378 | 280 | 1.63768852 | MTHFD1L | methylenetetrahydrofolate dehydrogenase (NADP+ dependent) 1 like [Source:HGNC Symbol;Acc:HGNC:21055] |
| ENSG00000125775 | HGLibA_43112 | 280 | 1.63768852 | SDCBP2 | syndecan binding protein 2 [Source:HGNC Symbol;Acc:HGNC:15756] |
| ENSG00000130997 | HGLibA_37542 | 280 | 1.63768852 | POLN | DNA polymerase nu [Source:HGNC Symbol;Acc:HGNC:18870] |
| ENSG00000149488 | HGLibA_49751 | 280 | 1.63768852 | TMC2 | transmembrane channel like 2 [Source:HGNC Symbol;Acc:HGNC:16527] |
| ENSG00000162769 | HGLibA_17676 | 280 | 1.63768852 | FLVCR1 | feline leukemia virus subgroup C cellular receptor 1 [Source:HGNC Symbol;Acc:HGNC:24682] |
| ENSG00000163393 | HGLibA_44553 | 280 | 1.63768852 | SLC22A15 | solute carrier family 22 member 15 [Source:HGNC Symbol;Acc:HGNC:20301] |
| ENSG00000167210 | HGLibA_27036 | 280 | 1.63768852 | LOXHD1 | lipoxygenase homology domains 1 [Source:HGNC Symbol;Acc:HGNC:26521] |

| | | | | | |
|---|---|---|---|---|---|
| ENSG00000174982 | HGLibA_34063 | 280 | 1.63768852 | OR4S2 | olfactory receptor family 4 subfamily S member 2 [Source:HGNC Symbol;Acc:HGNC:15183] |
| ENSG00000198146 | HGLibA_56705 | 280 | 1.63768852 | ZNF770 | zinc finger protein 770 [Source:HGNC Symbol;Acc:HGNC:26061] |
| ENSG00000215788 | HGLibA_50782 | 280 | 1.63768852 | TNFRSF25 | TNF receptor superfamily member 25 [Source:HGNC Symbol;Acc:HGNC:11910] |
| ENSG00000221818 | HGLibA_14351 | 280 | 1.63768852 | EBF2 | EBF transcription factor 2 [Source:HGNC Symbol;Acc:HGNC:19090] |
| ENSG00000254122 | HGLibA_35568 | 280 | 1.63768852 | PCDHGB7 | 7 [Source:HGNC Symbol;Acc:HGNC:8714]" |
| ENSG00000266560 | HGLibA_27897 | 280 | 1.63768852 | MAGEA10-MAGEA5 | MAGEA10-MAGEA5 readthrough [Source:NCBI gene;Acc:100533997] |
| FAM213A | HGLibA_16471 | 280 | 1.63768852 | FAM213A | - |
| CTSL2 | HGLibA_11722 | 279 | 1.627737483 | CTSL2 | - |
| ENSG00000007001 | HGLibA_53090 | 279 | 1.627737483 | UPP2 | uridine phosphorylase 2 [Source:HGNC Symbol;Acc:HGNC:23061] |
| ENSG00000012223 | HGLibA_27609 | 279 | 1.627737483 | LTF | lactotransferrin [Source:HGNC Symbol;Acc:HGNC:6720] |
| ENSG00000103160 | HGLibA_22213 | 279 | 1.627737483 | HSDL1 | hydroxysteroid dehydrogenase like 1 [Source:HGNC Symbol;Acc:HGNC:16475] |
| ENSG00000103942 | HGLibA_21868 | 279 | 1.627737483 | HOMER2 | homer scaffold protein 2 [Source:HGNC Symbol;Acc:HGNC:17513] |
| ENSG00000104626 | HGLibA_15479 | 279 | 1.627737483 | ERI1 | exoribonuclease 1 [Source:HGNC Symbol;Acc:HGNC:23994] |
| ENSG00000118194 | HGLibA_50885 | 279 | 1.627737483 | TNNT2 | cardiac type [Source:HGNC Symbol;Acc:HGNC:11949]" |
| ENSG00000125900 | HGLibA_44209 | 279 | 1.627737483 | SIRPD | signal regulatory protein delta [Source:HGNC Symbol;Acc:HGNC:16248] |
| ENSG00000131462 | HGLibA_52304 | 279 | 1.627737483 | TUBG1 | tubulin gamma 1 [Source:HGNC Symbol;Acc:HGNC:12417] |
| ENSG00000136444 | HGLibA_42295 | 279 | 1.627737483 | RSAD1 | radical S-adenosyl methionine domain containing 1 [Source:HGNC Symbol;Acc:HGNC:25634] |
| ENSG00000142279 | HGLibA_54437 | 279 | 1.627737483 | WTIP | WT1 interacting protein [Source:HGNC Symbol;Acc:HGNC:20964] |
| ENSG00000144043 | HGLibA_49081 | 279 | 1.627737483 | TEX261 | testis expressed 261 [Source:HGNC Symbol;Acc:HGNC:30712] |
| ENSG00000146966 | HGLibA_12939 | 279 | 1.627737483 | DENND2A | DENN domain containing 2A [Source:HGNC Symbol;Acc:HGNC:22212] |
| ENSG00000151577 | HGLibA_13964 | 279 | 1.627737483 | DRD3 | dopamine receptor D3 [Source:HGNC Symbol;Acc:HGNC:3024] |
| ENSG00000162882 | HGLibA_20790 | 279 | 1.627737483 | HAAO | 4-dioxygenase [Source:HGNC Symbol;Acc:HGNC:4796]" |
| ENSG00000162897 | HGLibA_17163 | 279 | 1.627737483 | FCAMR | Fc fragment of IgA and IgM receptor [Source:HGNC Symbol;Acc:HGNC:24692] |
| ENSG00000169688 | HGLibA_30280 | 279 | 1.627737483 | MT1B | metallothionein 1B [Source:HGNC Symbol;Acc:HGNC:7394] |
| ENSG00000170881 | HGLibA_41516 | 279 | 1.627737483 | RNF139 | ring finger protein 139 [Source:HGNC Symbol;Acc:HGNC:17023] |
| ENSG00000171855 | HGLibA_22702 | 279 | 1.627737483 | IFNB1 | interferon beta 1 [Source:HGNC Symbol;Acc:HGNC:5434] |
| ENSG00000172244 | HGLibA_06376 | 279 | 1.627737483 | C5orf34 | chromosome 5 open reading frame 34 [Source:HGNC Symbol;Acc:HGNC:24738] |
| ENSG00000177051 | HGLibA_17110 | 279 | 1.627737483 | FBXO46 | F-box protein 46 [Source:HGNC Symbol;Acc:HGNC:25069] |
| ENSG00000182083 | HGLibA_34376 | 279 | 1.627737483 | OR6B2 | olfactory receptor family 6 subfamily B member 2 [Source:HGNC Symbol;Acc:HGNC:15041] |
| ENSG00000183048 | HGLibA_44637 | 279 | 1.627737483 | SLC25A10 | solute carrier family 25 member 10 [Source:HGNC Symbol;Acc:HGNC:10980] |
| ENSG00000196313 | HGLibA_37643 | 279 | 1.627737483 | POM121 | POM121 transmembrane nucleoporin [Source:HGNC Symbol;Acc:HGNC:19702] |
| ENSG00000243056 | HGLibA_14834 | 279 | 1.627737483 | EIF4EBP3 | eukaryotic translation initiation factor 4E binding protein 3 [Source:HGNC Symbol;Acc:HGNC:3290] |
| ENSG00000243449 | HGLibA_06342 | 279 | 1.627737483 | C4orf48 | chromosome 4 open reading frame 48 [Source:HGNC Symbol;Acc:HGNC:34437] |
| ENSG00000253797 | HGLibA_53436 | 279 | 1.627737483 | UTP14C | small subunit processome component [Source:HGNC Symbol;Acc:HGNC:20321]" |
| hsa-mir-3175 | HGLibA_58775 | 279 | 1.627737483 | hsa-mir-3175 | - |
| hsa-mir-323b | HGLibA_58961 | 279 | 1.627737483 | hsa-mir-323b | - |
| hsa-mir-4480 | HGLibA_60340 | 279 | 1.627737483 | hsa-mir-4480 | - |
| hsa-mir-6080 | HGLibA_62580 | 279 | 1.627737483 | hsa-mir-6080 | - |
| hsa-mir-7108 | HGLibA_63793 | 279 | 1.627737483 | hsa-mir-7108 | - |
| LOR | HGLibA_27028 | 279 | 1.627737483 | LOR | - |
| C11orf83 | HGLibA_05194 | 278 | 1.617786445 | C11orf83 | - |
| ENSG00000079277 | HGLibA_29341 | 278 | 1.617786445 | MKNK1 | MAP kinase interacting serine/threonine kinase 1 [Source:HGNC Symbol;Acc:HGNC:7110] |
| ENSG00000080709 | HGLibA_24356 | 278 | 1.617786445 | KCNN2 | potassium calcium-activated channel subfamily N member 2 [Source:HGNC Symbol;Acc:HGNC:6291] |
| ENSG00000085276 | HGLibA_28723 | 278 | 1.617786445 | MECOM | MDS1 and EVI1 complex locus [Source:HGNC Symbol;Acc:HGNC:3498] |
| ENSG00000110328 | HGLibA_18550 | 278 | 1.617786445 | GALNT18 | polypeptide N-acetylgalactosaminyltransferase 18 [Source:HGNC Symbol;Acc:HGNC:30488] |
| ENSG00000132517 | HGLibA_45244 | 278 | 1.617786445 | SLC52A1 | solute carrier family 52 member 1 [Source:HGNC Symbol;Acc:HGNC:30225] |
| ENSG00000132950 | HGLibA_55402 | 278 | 1.617786445 | ZMYM5 | zinc finger MYM-type containing 5 [Source:HGNC Symbol;Acc:HGNC:13029] |
| ENSG00000133812 | HGLibA_42796 | 278 | 1.617786445 | SBF2 | SET binding factor 2 [Source:HGNC Symbol;Acc:HGNC:2135] |
| ENSG00000134072 | HGLibA_07057 | 278 | 1.617786445 | CAMK1 | calcium/calmodulin dependent protein kinase I [Source:HGNC Symbol;Acc:HGNC:1459] |
| ENSG00000135679 | HGLibA_28697 | 278 | 1.617786445 | MDM2 | MDM2 proto-oncogene [Source:HGNC Symbol;Acc:HGNC:6973] |
| ENSG00000137757 | HGLibA_07315 | 278 | 1.617786445 | CASP5 | caspase 5 [Source:HGNC Symbol;Acc:HGNC:1506] |
| ENSG00000140481 | HGLibA_07758 | 278 | 1.617786445 | CCDC33 | coiled-coil domain containing 33 [Source:HGNC Symbol;Acc:HGNC:26552] |
| ENSG00000143167 | HGLibA_19717 | 278 | 1.617786445 | GPA33 | glycoprotein A33 [Source:HGNC Symbol;Acc:HGNC:4445] |
| ENSG00000158457 | HGLibA_51933 | 278 | 1.617786445 | TSPAN33 | tetraspanin 33 [Source:HGNC Symbol;Acc:HGNC:28743] |
| ENSG00000161202 | HGLibA_14215 | 278 | 1.617786445 | DVL3 | dishevelled segment polarity protein 3 [Source:HGNC Symbol;Acc:HGNC:3087] |
| ENSG00000163283 | HGLibA_01711 | 278 | 1.617786445 | ALPP | placental [Source:HGNC Symbol;Acc:HGNC:439]" |
| ENSG00000163788 | HGLibA_45938 | 278 | 1.617786445 | SNRK | SNF related kinase [Source:HGNC Symbol;Acc:HGNC:30598] |
| ENSG00000165861 | HGLibA_55269 | 278 | 1.617786445 | ZFYVE1 | zinc finger FYVE-type containing 1 [Source:HGNC Symbol;Acc:HGNC:13180] |
| ENSG00000167207 | HGLibA_32246 | 278 | 1.617786445 | NOD2 | nucleotide binding oligomerization domain containing 2 [Source:HGNC Symbol;Acc:HGNC:5331] |
| ENSG00000167632 | HGLibA_51338 | 278 | 1.617786445 | TRAPPC9 | trafficking protein particle complex 9 [Source:HGNC Symbol;Acc:HGNC:30832] |
| ENSG00000169064 | HGLibA_54785 | 278 | 1.617786445 | ZBBX | zinc finger B-box domain containing [Source:HGNC Symbol;Acc:HGNC:26245] |
| ENSG00000171169 | HGLibA_31078 | 278 | 1.617786445 | NAIF1 | nuclear apoptosis inducing factor 1 [Source:HGNC Symbol;Acc:HGNC:25446] |
| ENSG00000173261 | HGLibA_36944 | 278 | 1.617786445 | PLAC8L1 | PLAC8 like 1 [Source:HGNC Symbol;Acc:HGNC:31746] |
| ENSG00000173598 | HGLibA_32979 | 278 | 1.617786445 | NUDT4 | nudix hydrolase 4 [Source:HGNC Symbol;Acc:HGNC:8051] |
| ENSG00000173714 | HGLibA_54283 | 278 | 1.617786445 | WFIKKN2 | kunitz and netrin domain containing 2 [Source:HGNC Symbol;Acc:HGNC:30916]" |
| ENSG00000174740 | HGLibA_34940 | 278 | 1.617786445 | PABPC5 | poly(A) binding protein cytoplasmic 5 [Source:HGNC Symbol;Acc:HGNC:13629] |
| ENSG00000176900 | HGLibA_34132 | 278 | 1.617786445 | OR51T1 | olfactory receptor family 51 subfamily T member 1 [Source:HGNC Symbol;Acc:HGNC:15205] |
| ENSG00000178229 | HGLibA_56194 | 278 | 1.617786445 | ZNF543 | zinc finger protein 543 [Source:HGNC Symbol;Acc:HGNC:25281] |
| ENSG00000179456 | HGLibA_54831 | 278 | 1.617786445 | ZBTB18 | zinc finger and BTB domain containing 18 [Source:HGNC Symbol;Acc:HGNC:13030] |
| ENSG00000180316 | HGLibA_37404 | 278 | 1.617786445 | PNPLA1 | patatin like phospholipase domain containing 1 [Source:HGNC Symbol;Acc:HGNC:21246] |
| ENSG00000181036 | HGLibA_17240 | 278 | 1.617786445 | FCRL6 | Fc receptor like 6 [Source:HGNC Symbol;Acc:HGNC:31910] |
| ENSG00000183864 | HGLibA_50938 | 278 | 1.617786445 | TOB2 | 2 [Source:HGNC Symbol;Acc:HGNC:11980]" |
| ENSG00000184557 | HGLibA_46130 | 278 | 1.617786445 | SOCS3 | suppressor of cytokine signaling 3 [Source:HGNC Symbol;Acc:HGNC:19391] |
| ENSG00000188938 | HGLibA_16056 | 278 | 1.617786445 | FAM120AOS | family with sequence similarity 120A opposite strand [Source:HGNC Symbol;Acc:HGNC:23389] |
| ENSG00000197134 | HGLibA_55681 | 278 | 1.617786445 | ZNF257 | zinc finger protein 257 [Source:HGNC Symbol;Acc:HGNC:13498] |
| ENSG00000204538 | HGLibA_39219 | 278 | 1.617786445 | PSORS1C2 | psoriasis susceptibility 1 candidate 2 [Source:HGNC Symbol;Acc:HGNC:17199] |
| ENSG00000205867 | HGLibA_25791 | 278 | 1.617786445 | KRTAP5-2 | keratin associated protein 5-2 [Source:HGNC Symbol;Acc:HGNC:23597] |
| FLJ44313 | HGLibA_17629 | 278 | 1.617786445 | FLJ44313 | - |
| hsa-mir-1264 | HGLibA_57480 | 278 | 1.617786445 | hsa-mir-1264 | - |
| hsa-mir-18a | HGLibA_58018 | 278 | 1.617786445 | hsa-mir-18a | - |
| hsa-mir-4492 | HGLibA_60385 | 278 | 1.617786445 | hsa-mir-4492 | - |
| hsa-mir-5092 | HGLibA_61526 | 278 | 1.617786445 | hsa-mir-5092 | - |
| hsa-mir-657 | HGLibA_62961 | 278 | 1.617786445 | hsa-mir-657 | - |
| DARC | HGLibA_12336 | 277 | 1.607835408 | DARC | - |
| ENSG00000060491 | HGLibA_33317 | 277 | 1.607835408 | OGFR | opioid growth factor receptor [Source:HGNC Symbol;Acc:HGNC:15768] |
| ENSG00000084453 | HGLibA_45444 | 277 | 1.607835408 | SLCO1A2 | solute carrier organic anion transporter family member 1A2 [Source:HGNC Symbol;Acc:HGNC:10956] |
| ENSG00000089693 | HGLibA_29377 | 277 | 1.607835408 | MLF2 | myeloid leukemia factor 2 [Source:HGNC Symbol;Acc:HGNC:7126] |
| ENSG00000100448 | HGLibA_11706 | 277 | 1.607835408 | CTSG | cathepsin G [Source:HGNC Symbol;Acc:HGNC:2532] |
| ENSG00000102393 | HGLibA_19215 | 277 | 1.607835408 | GLA | galactosidase alpha [Source:HGNC Symbol;Acc:HGNC:4296] |
| ENSG00000106554 | HGLibA_09367 | 277 | 1.607835408 | CHCHD3 | coiled-coil-helix-coiled-coil-helix domain containing 3 [Source:HGNC Symbol;Acc:HGNC:21906] |
| ENSG00000107404 | HGLibA_14209 | 277 | 1.607835408 | DVL1 | dishevelled segment polarity protein 1 [Source:HGNC Symbol;Acc:HGNC:3084] |
| ENSG00000113734 | HGLibA_04649 | 277 | 1.607835408 | BNIP1 | BCL2 interacting protein 1 [Source:HGNC Symbol;Acc:HGNC:1082] |
| ENSG00000116127 | HGLibA_01672 | 277 | 1.607835408 | ALMS1 | centrosome and basal body associated protein [Source:HGNC Symbol;Acc:HGNC:428]" |
| ENSG00000118432 | HGLibA_10373 | 277 | 1.607835408 | CNR1 | cannabinoid receptor 1 [Source:HGNC Symbol;Acc:HGNC:2159] |
| ENSG00000118432 | HGLibA_10374 | 277 | 1.607835408 | CNR1 | cannabinoid receptor 1 [Source:HGNC Symbol;Acc:HGNC:2159] |
| ENSG00000120088 | HGLibA_11142 | 277 | 1.607835408 | CRHR1 | corticotropin releasing hormone receptor 1 [Source:HGNC Symbol;Acc:HGNC:2357] |
| ENSG00000125447 | HGLibA_18985 | 277 | 1.607835408 | GGA3 | ARF binding protein 3 [Source:HGNC Symbol;Acc:HGNC:17079]" |
| ENSG00000131808 | HGLibA_18086 | 277 | 1.607835408 | FSHB | follicle stimulating hormone subunit beta [Source:HGNC Symbol;Acc:HGNC:3964] |
| ENSG00000133048 | HGLibA_09436 | 277 | 1.607835408 | CHI3L1 | chitinase 3 like 1 [Source:HGNC Symbol;Acc:HGNC:1932] |
| ENSG00000136158 | HGLibA_46839 | 277 | 1.607835408 | SPRY2 | sprouty RTK signaling antagonist 2 [Source:HGNC Symbol;Acc:HGNC:11270] |
| ENSG00000141448 | HGLibA_18679 | 277 | 1.607835408 | GATA6 | GATA binding protein 6 [Source:HGNC Symbol;Acc:HGNC:4174] |
| ENSG00000145431 | HGLibA_35846 | 277 | 1.607835408 | PDGFC | platelet derived growth factor C [Source:HGNC Symbol;Acc:HGNC:8801] |
| ENSG00000148377 | HGLibA_22557 | 277 | 1.607835408 | IDI2 | isopentenyl-diphosphate delta isomerase 2 [Source:HGNC Symbol;Acc:HGNC:23487] |
| ENSG00000150275 | HGLibA_35399 | 277 | 1.607835408 | PCDH15 | protocadherin related 15 [Source:HGNC Symbol;Acc:HGNC:14674] |
| ENSG00000153214 | HGLibA_50535 | 277 | 1.607835408 | TMEM87B | transmembrane protein 87B [Source:HGNC Symbol;Acc:HGNC:25913] |
| ENSG00000160551 | HGLibA_48307 | 277 | 1.607835408 | TAOK1 | TAO kinase 1 [Source:HGNC Symbol;Acc:HGNC:29259] |
| ENSG00000162496 | HGLibA_13133 | 277 | 1.607835408 | DHRS3 | dehydrogenase/reductase 3 [Source:HGNC Symbol;Acc:HGNC:17693] |
| ENSG00000163806 | HGLibA_46572 | 277 | 1.607835408 | SPDYA | speedy/RINGO cell cycle regulator family member A [Source:HGNC Symbol;Acc:HGNC:30613] |
| ENSG00000164066 | HGLibA_23439 | 277 | 1.607835408 | INTU | inturned planar cell polarity protein [Source:HGNC Symbol;Acc:HGNC:29239] |
| ENSG00000164754 | HGLibA_40064 | 277 | 1.607835408 | RAD21 | RAD21 cohesin complex component [Source:HGNC Symbol;Acc:HGNC:9811] |
| ENSG00000166482 | HGLibA_29051 | 277 | 1.607835408 | MFAP4 | microfibril associated protein 4 [Source:HGNC Symbol;Acc:HGNC:7035] |
| ENSG00000166619 | HGLibA_04540 | 277 | 1.607835408 | BLCAP | apoptosis inducing factor [Source:HGNC Symbol;Acc:HGNC:1055]" |
| ENSG00000169967 | HGLibA_28182 | 277 | 1.607835408 | MAP3K2 | mitogen-activated protein kinase kinase kinase 2 [Source:HGNC Symbol;Acc:HGNC:6854] |
| ENSG00000172007 | HGLibA_39882 | 277 | 1.607835408 | RAB33B | member RAS oncogene family [Source:HGNC Symbol;Acc:HGNC:16075]" |
| ENSG00000173110 | HGLibA_22292 | 277 | 1.607835408 | HSPA6 | heat shock protein family A (Hsp70) member 6 [Source:HGNC Symbol;Acc:HGNC:5239] |
| ENSG00000173114 | HGLibA_27477 | 277 | 1.607835408 | LRRN3 | leucine rich repeat neuronal 3 [Source:HGNC Symbol;Acc:HGNC:17200] |
| ENSG00000173269 | HGLibA_29549 | 277 | 1.607835408 | MMRN2 | multimerin 2 [Source:HGNC Symbol;Acc:HGNC:19888] |
| ENSG00000173662 | HGLibA_48355 | 277 | 1.607835408 | TAS1R1 | taste 1 receptor member 1 [Source:HGNC Symbol;Acc:HGNC:14448] |
| ENSG00000176531 | HGLibA_36432 | 277 | 1.607835408 | PHLDB3 | pleckstrin homology like domain family B member 3 [Source:HGNC Symbol;Acc:HGNC:30499] |
| ENSG00000184939 | HGLibA_55239 | 277 | 1.607835408 | ZFP90 | ZFP90 zinc finger protein [Source:HGNC Symbol;Acc:HGNC:23329] |
| ENSG00000275722 | HGLibA_27795 | 277 | 1.607835408 | LYZL6 | lysozyme like 6 [Source:HGNC Symbol;Acc:HGNC:29614] |
| hsa-mir-4480 | HGLibA_60337 | 277 | 1.607835408 | hsa-mir-4480 | - |
| hsa-mir-4644 | HGLibA_60679 | 277 | 1.607835408 | hsa-mir-4644 | - |
| N6AMT2 | HGLibA_30966 | 277 | 1.607835408 | N6AMT2 | - |
| PCNX | HGLibA_35642 | 277 | 1.607835408 | PCNX | - |
| SEPN1 | HGLibA_43417 | 277 | 1.607835408 | SEPN1 | - |
| C7orf73 | HGLibA_06589 | 276 | 1.59788437 | C7orf73 | - |
| ENSG00000033327 | HGLibA_18323 | 276 | 1.59788437 | GAB2 | GRB2 associated binding protein 2 [Source:HGNC Symbol;Acc:HGNC:14458] |
| ENSG00000105401 | HGLibA_08544 | 276 | 1.59788437 | CDC37 | cell division cycle 37 [Source:HGNC Symbol;Acc:HGNC:1735] |
| ENSG00000105737 | HGLibA_20292 | 276 | 1.59788437 | GRIK5 | glutamate ionotropic receptor kainate type subunit 5 [Source:HGNC Symbol;Acc:HGNC:4583] |
| ENSG00000109705 | HGLibA_32053 | 276 | 1.59788437 | NKX3-2 | NK3 homeobox 2 [Source:HGNC Symbol;Acc:HGNC:951] |
| ENSG00000123836 | HGLibA_36157 | 276 | 1.59788437 | PFKFB2 | 6-biphosphatase 2 [Source:HGNC Symbol;Acc:HGNC:8873]" |
| ENSG00000125245 | HGLibA_19980 | 276 | 1.59788437 | GPR18 | G protein-coupled receptor 18 [Source:HGNC Symbol;Acc:HGNC:4472] |
| ENSG00000125895 | HGLibA_50512 | 276 | 1.59788437 | TMEM74B | transmembrane protein 74B [Source:HGNC Symbol;Acc:HGNC:15893] |
| ENSG00000132670 | HGLibA_39483 | 276 | 1.59788437 | PTPRA | receptor type A [Source:HGNC Symbol;Acc:HGNC:9664]" |

| | | | | | |
|---|---|---|---|---|---|
| ENSG00000133317 | HGLibA_26349 | 276 | 1.59788437 | LGALS12 | galectin 12 [Source:HGNC Symbol;Acc:HGNC:15788] |
| ENSG00000134256 | HGLibA_08227 | 276 | 1.59788437 | CD101 | CD101 molecule [Source:HGNC Symbol;Acc:HGNC:5949] |
| ENSG00000135457 | HGLibA_49145 | 276 | 1.59788437 | TFCP2 | transcription factor CP2 [Source:HGNC Symbol;Acc:HGNC:11748] |
| ENSG00000136732 | HGLibA_20703 | 276 | 1.59788437 | GYPC | glycophorin C (Gerbich blood group) [Source:HGNC Symbol;Acc:HGNC:4704] |
| ENSG00000139547 | HGLibA_40747 | 276 | 1.59788437 | RDH16 | retinol dehydrogenase 16 [Source:HGNC Symbol;Acc:HGNC:29674] |
| ENSG00000143850 | HGLibA_37077 | 276 | 1.59788437 | PLEKHA6 | pleckstrin homology domain containing A6 [Source:HGNC Symbol;Acc:HGNC:17053] |
| ENSG00000146828 | HGLibA_44382 | 276 | 1.59788437 | SLC12A9 | solute carrier family 12 member 9 [Source:HGNC Symbol;Acc:HGNC:17435] |
| ENSG00000148795 | HGLibA_12062 | 276 | 1.59788437 | CYP17A1 | cytochrome P450 family 17 subfamily A member 1 [Source:HGNC Symbol;Acc:HGNC:2593] |
| ENSG00000157064 | HGLibA_32188 | 276 | 1.59788437 | NMNAT2 | nicotinamide nucleotide adenylyltransferase 2 [Source:HGNC Symbol;Acc:HGNC:16789] |
| ENSG00000161653 | HGLibA_31076 | 276 | 1.59788437 | NAGS | N-acetylglutamate synthase [Source:HGNC Symbol;Acc:HGNC:17996] |
| ENSG00000164808 | HGLibA_46665 | 276 | 1.59788437 | SPIDR | scaffold protein involved in DNA repair [Source:HGNC Symbol;Acc:HGNC:28971] |
| ENSG00000165606 | HGLibA_13981 | 276 | 1.59788437 | DRGX | dorsal root ganglia homeobox [Source:HGNC Symbol;Acc:HGNC:21536] |
| ENSG00000167619 | HGLibA_49974 | 276 | 1.59788437 | TMEM145 | transmembrane protein 145 [Source:HGNC Symbol;Acc:HGNC:26912] |
| ENSG00000167634 | HGLibA_32131 | 276 | 1.59788437 | NLRP7 | NLR family pyrin domain containing 7 [Source:HGNC Symbol;Acc:HGNC:22947] |
| ENSG00000171195 | HGLibA_30556 | 276 | 1.59788437 | MUC7 | secreted [Source:HGNC Symbol;Acc:HGNC:7518]" |
| ENSG00000171298 | HGLibA_18316 | 276 | 1.59788437 | GAA | acid [Source:HGNC Symbol;Acc:HGNC:4065]" |
| ENSG00000171853 | HGLibA_51306 | 276 | 1.59788437 | TRAPPC12 | trafficking protein particle complex 12 [Source:HGNC Symbol;Acc:HGNC:24284] |
| ENSG00000176170 | HGLibA_46643 | 276 | 1.59788437 | SPHK1 | sphingosine kinase 1 [Source:HGNC Symbol;Acc:HGNC:11240] |
| ENSG00000177669 | HGLibA_28522 | 276 | 1.59788437 | MBOAT4 | membrane bound O-acyltransferase domain containing 4 [Source:HGNC Symbol;Acc:HGNC:32311] |
| ENSG00000179813 | HGLibA_16485 | 276 | 1.59788437 | FAM216B | family with sequence similarity 216 member B [Source:HGNC Symbol;Acc:HGNC:26883] |
| ENSG00000179965 | HGLibA_56706 | 276 | 1.59788437 | ZNF771 | zinc finger protein 771 [Source:HGNC Symbol;Acc:HGNC:29653] |
| ENSG00000180992 | HGLibA_29904 | 276 | 1.59788437 | MRPL14 | mitochondrial ribosomal protein L14 [Source:HGNC Symbol;Acc:HGNC:14279] |
| ENSG00000182223 | HGLibA_54777 | 276 | 1.59788437 | ZAR1 | zygote arrest 1 [Source:HGNC Symbol;Acc:HGNC:20436] |
| ENSG00000196260 | HGLibA_43760 | 276 | 1.59788437 | SFTA2 | surfactant associated 2 [Source:HGNC Symbol;Acc:HGNC:18386] |
| ENSG00000197360 | HGLibA_56913 | 276 | 1.59788437 | ZNF98 | zinc finger protein 98 [Source:HGNC Symbol;Acc:HGNC:13174] |
| ENSG00000198242 | HGLibA_41907 | 276 | 1.59788437 | RPL23A | ribosomal protein L23a [Source:HGNC Symbol;Acc:HGNC:10317] |
| ENSG00000237330 | HGLibA_41647 | 276 | 1.59788437 | RNF223 | ring finger protein 223 [Source:HGNC Symbol;Acc:HGNC:40020] |
| ENSG00000244414 | HGLibA_09264 | 276 | 1.59788437 | CFHR1 | complement factor H related 1 [Source:HGNC Symbol;Acc:HGNC:4888] |
| ENSG00000249693 | HGLibA_49336 | 276 | 1.59788437 | THEGL | theg spermatid protein like [Source:HGNC Symbol;Acc:HGNC:43771] |
| hsa-mir-4736 | HGLibA_61040 | 276 | 1.59788437 | hsa-mir-4736 | - |
| hsa-mir-5698 | HGLibA_62325 | 276 | 1.59788437 | hsa-mir-5698 | - |
| hsa-mir-6773 | HGLibA_63265 | 276 | 1.59788437 | hsa-mir-6773 | - |
| hsa-mir-8080 | HGLibA_64161 | 276 | 1.59788437 | hsa-mir-8080 | - |
| C1orf168 | HGLibA_05789 | 275 | 1.587933333 | C1orf168 | - |
| ENSG00000006747 | HGLibA_42947 | 275 | 1.587933333 | SCIN | scinderin [Source:HGNC Symbol;Acc:HGNC:21695] |
| ENSG00000020256 | HGLibA_55229 | 275 | 1.587933333 | ZFP64 | ZFP64 zinc finger protein [Source:HGNC Symbol;Acc:HGNC:15940] |
| ENSG00000023191 | HGLibA_41711 | 275 | 1.587933333 | RNH1 | ribonuclease/angiogenin inhibitor 1 [Source:HGNC Symbol;Acc:HGNC:10074] |
| ENSG00000086666 | HGLibA_55163 | 275 | 1.587933333 | ZFAND6 | zinc finger AN1-type containing 6 [Source:HGNC Symbol;Acc:HGNC:30164] |
| ENSG00000105556 | HGLibA_29256 | 275 | 1.587933333 | MIER2 | MIER family member 2 [Source:HGNC Symbol;Acc:HGNC:29210] |
| ENSG00000106366 | HGLibA_43576 | 275 | 1.587933333 | SERPINE1 | serpin family E member 1 [Source:HGNC Symbol;Acc:HGNC:8583] |
| ENSG00000108798 | HGLibA_00297 | 275 | 1.587933333 | ABI3 | ABI family member 3 [Source:HGNC Symbol;Acc:HGNC:29859] |
| ENSG00000109189 | HGLibA_53377 | 275 | 1.587933333 | USP46 | ubiquitin specific peptidase 46 [Source:HGNC Symbol;Acc:HGNC:20075] |
| ENSG00000115904 | HGLibA_46202 | 275 | 1.587933333 | SOS1 | SOS Ras/Rac guanine nucleotide exchange factor 1 [Source:HGNC Symbol;Acc:HGNC:11187] |
| ENSG00000115935 | HGLibA_54303 | 275 | 1.587933333 | WIPF1 | WAS/WASL interacting protein family member 1 [Source:HGNC Symbol;Acc:HGNC:12736] |
| ENSG00000116032 | HGLibA_20314 | 275 | 1.587933333 | GRIN3B | glutamate ionotropic receptor NMDA type subunit 3B [Source:HGNC Symbol;Acc:HGNC:16768] |
| ENSG00000132405 | HGLibA_48479 | 275 | 1.587933333 | TBC1D14 | TBC1 domain family member 14 [Source:HGNC Symbol;Acc:HGNC:29246] |
| ENSG00000135324 | HGLibA_29809 | 275 | 1.587933333 | MRAP2 | melanocortin 2 receptor accessory protein 2 [Source:HGNC Symbol;Acc:HGNC:21232] |
| ENSG00000140835 | HGLibA_09638 | 275 | 1.587933333 | CHST4 | carbohydrate sulfotransferase 4 [Source:HGNC Symbol;Acc:HGNC:1972] |
| ENSG00000142494 | HGLibA_45192 | 275 | 1.587933333 | SLC47A1 | solute carrier family 47 member 1 [Source:HGNC Symbol;Acc:HGNC:25588] |
| ENSG00000148660 | HGLibA_07077 | 275 | 1.587933333 | CAMK2G | calcium/calmodulin dependent protein kinase II gamma [Source:HGNC Symbol;Acc:HGNC:1463] |
| ENSG00000153002 | HGLibA_10906 | 275 | 1.587933333 | CPB1 | carboxypeptidase B1 [Source:HGNC Symbol;Acc:HGNC:2299] |
| ENSG00000159674 | HGLibA_46759 | 275 | 1.587933333 | SPON2 | spondin 2 [Source:HGNC Symbol;Acc:HGNC:11253] |
| ENSG00000162849 | HGLibA_24919 | 275 | 1.587933333 | KIF26B | kinesin family member 26B [Source:HGNC Symbol;Acc:HGNC:25484] |
| ENSG00000163006 | HGLibA_07611 | 275 | 1.587933333 | CCDC138 | coiled-coil domain containing 138 [Source:HGNC Symbol;Acc:HGNC:26531] |
| ENSG00000164089 | HGLibA_15638 | 275 | 1.587933333 | ETNPPL | ethanolamine-phosphate phospho-lyase [Source:HGNC Symbol;Acc:HGNC:14404] |
| ENSG00000164687 | HGLibA_15922 | 275 | 1.587933333 | FABP5 | fatty acid binding protein 5 [Source:HGNC Symbol;Acc:HGNC:3560] |
| ENSG00000170271 | HGLibA_16931 | 275 | 1.587933333 | FAXDC2 | fatty acid hydroxylase domain containing 2 [Source:HGNC Symbol;Acc:HGNC:1334] |
| ENSG00000170310 | HGLibA_47660 | 275 | 1.587933333 | STX8 | syntaxin 8 [Source:HGNC Symbol;Acc:HGNC:11443] |
| ENSG00000170653 | HGLibA_03451 | 275 | 1.587933333 | ATF7 | activating transcription factor 7 [Source:HGNC Symbol;Acc:HGNC:792] |
| ENSG00000171121 | HGLibA_24349 | 275 | 1.587933333 | KCNMB3 | potassium calcium-activated channel subfamily M regulatory beta subunit 3 [Source:HGNC Symbol;Acc:HGNC:6287] |
| ENSG00000172487 | HGLibA_34538 | 275 | 1.587933333 | OR8J1 | olfactory receptor family 8 subfamily J member 1 [Source:HGNC Symbol;Acc:HGNC:14855] |
| ENSG00000172985 | HGLibA_43976 | 275 | 1.587933333 | SH3RF3 | SH3 domain containing ring finger 3 [Source:HGNC Symbol;Acc:HGNC:24699] |
| ENSG00000173264 | HGLibA_19898 | 275 | 1.587933333 | GPR137 | G protein-coupled receptor 137 [Source:HGNC Symbol;Acc:HGNC:24300] |
| ENSG00000177489 | HGLibA_33793 | 275 | 1.587933333 | OR2G2 | olfactory receptor family 2 subfamily G member 2 [Source:HGNC Symbol;Acc:HGNC:15007] |
| ENSG00000185176 | HGLibA_02586 | 275 | 1.587933333 | AQP12B | aquaporin 12B [Source:HGNC Symbol;Acc:HGNC:6096] |
| ENSG00000196361 | HGLibA_14885 | 275 | 1.587933333 | ELAVL3 | ELAV like RNA binding protein 3 [Source:HGNC Symbol;Acc:HGNC:3314] |
| ENSG00000204228 | HGLibA_22203 | 275 | 1.587933333 | HSD17B8 | hydroxysteroid 17-beta dehydrogenase 8 [Source:HGNC Symbol;Acc:HGNC:3554] |
| ENSG00000205858 | HGLibA_27422 | 275 | 1.587933333 | LRRC72 | leucine rich repeat containing 72 [Source:HGNC Symbol;Acc:HGNC:42972] |
| ENSG00000221937 | HGLibA_48401 | 275 | 1.587933333 | TAS2R40 | taste 2 receptor member 40 [Source:HGNC Symbol;Acc:HGNC:18885] |
| ENSG00000228927 | HGLibA_51971 | 275 | 1.587933333 | TSPY3 | testis specific protein Y-linked 3 [Source:HGNC Symbol;Acc:HGNC:33876] |
| ENSG00000243896 | HGLibA_33742 | 275 | 1.587933333 | OR2A7 | olfactory receptor family 2 subfamily A member 7 [Source:HGNC Symbol;Acc:HGNC:8234] |
| ENSG00000244752 | HGLibA_11265 | 275 | 1.587933333 | CRYBB2 | crystallin beta B2 [Source:HGNC Symbol;Acc:HGNC:2398] |
| ENSG00000259384 | HGLibA_19028 | 275 | 1.587933333 | GH1 | growth hormone 1 [Source:HGNC Symbol;Acc:HGNC:4261] |
| ENSG00000262655 | HGLibA_46758 | 275 | 1.587933333 | SPON1 | spondin 1 [Source:HGNC Symbol;Acc:HGNC:11252] |
| ENSG00000270885 | HGLibA_40362 | 275 | 1.587933333 | RASL10B | RAS like family 10 member B [Source:HGNC Symbol;Acc:HGNC:30295] |
| H1FX | HGLibA_20742 | 275 | 1.587933333 | H1FX | - |
| hsa-mir-1257 | HGLibA_57434 | 275 | 1.587933333 | hsa-mir-1257 | - |
| hsa-mir-1302-6 | HGLibA_57711 | 275 | 1.587933333 | hsa-mir-1302-6 | - |
| hsa-mir-181d | HGLibA_57978 | 275 | 1.587933333 | hsa-mir-181d | - |
| hsa-mir-6870 | HGLibA_63663 | 275 | 1.587933333 | hsa-mir-6870 | - |
| hsa-mir-6876 | HGLibA_63688 | 275 | 1.587933333 | hsa-mir-6876 | - |
| KIAA0146 | HGLibA_24595 | 275 | 1.587933333 | KIAA0146 | - |
| KIAA0226 | HGLibA_24604 | 275 | 1.587933333 | KIAA0226 | - |
| SCXA | HGLibA_43079 | 275 | 1.587933333 | SCXA | - |
| ENSG00000014216 | HGLibA_07143 | 274 | 1.577982296 | CAPN1 | calpain 1 [Source:HGNC Symbol;Acc:HGNC:1476] |
| ENSG00000079337 | HGLibA_40251 | 274 | 1.577982296 | RAPGEF3 | Rap guanine nucleotide exchange factor 3 [Source:HGNC Symbol;Acc:HGNC:16629] |
| ENSG00000089094 | HGLibA_24498 | 274 | 1.577982296 | KDM2B | lysine demethylase 2B [Source:HGNC Symbol;Acc:HGNC:13610] |
| ENSG00000095066 | HGLibA_21878 | 274 | 1.577982296 | HOOK2 | hook microtubule tethering protein 2 [Source:HGNC Symbol;Acc:HGNC:19885] |
| ENSG00000103534 | HGLibA_49761 | 274 | 1.577982296 | TMC5 | transmembrane channel like 5 [Source:HGNC Symbol;Acc:HGNC:22999] |
| ENSG00000108798 | HGLibA_00295 | 274 | 1.577982296 | ABI3 | ABI family member 3 [Source:HGNC Symbol;Acc:HGNC:29859] |
| ENSG00000109501 | HGLibA_54287 | 274 | 1.577982296 | WFS1 | wolframin ER transmembrane glycoprotein [Source:HGNC Symbol;Acc:HGNC:12762] |
| ENSG00000110315 | HGLibA_41524 | 274 | 1.577982296 | RNF141 | ring finger protein 141 [Source:HGNC Symbol;Acc:HGNC:21159] |
| ENSG00000114113 | HGLibA_40633 | 274 | 1.577982296 | RBP2 | retinol binding protein 2 [Source:HGNC Symbol;Acc:HGNC:9920] |
| ENSG00000116918 | HGLibA_51887 | 274 | 1.577982296 | TSNAX | translin associated factor X [Source:HGNC Symbol;Acc:HGNC:12380] |
| ENSG00000123560 | HGLibA_37217 | 274 | 1.577982296 | PLP1 | proteolipid protein 1 [Source:HGNC Symbol;Acc:HGNC:9086] |
| ENSG00000131094 | HGLibA_05923 | 274 | 1.577982296 | C1QL1 | complement C1q like 1 [Source:HGNC Symbol;Acc:HGNC:24182] |
| ENSG00000132911 | HGLibA_32218 | 274 | 1.577982296 | NMUR2 | neuromedin U receptor 2 [Source:HGNC Symbol;Acc:HGNC:16454] |
| ENSG00000132958 | HGLibA_51215 | 274 | 1.577982296 | TPTE2 | transmembrane phosphoinositide 3-phosphatase and tensin homolog 2 [Source:HGNC Symbol;Acc:HGNC:17299] |
| ENSG00000135100 | HGLibA_21773 | 274 | 1.577982296 | HNF1A | HNF1 homeobox A [Source:HGNC Symbol;Acc:HGNC:11621] |
| ENSG00000137831 | HGLibA_52504 | 274 | 1.577982296 | UACA | uveal autoantigen with coiled-coil domains and ankyrin repeats [Source:HGNC Symbol;Acc:HGNC:15947] |
| ENSG00000138767 | HGLibA_10347 | 274 | 1.577982296 | CNOT6L | CCR4-NOT transcription complex subunit 6 like [Source:HGNC Symbol;Acc:HGNC:18042] |
| ENSG00000144589 | HGLibA_47456 | 274 | 1.577982296 | STK11IP | serine/threonine kinase 11 interacting protein [Source:HGNC Symbol;Acc:HGNC:19184] |
| ENSG00000145934 | HGLibA_48998 | 274 | 1.577982296 | TENM2 | teneurin transmembrane protein 2 [Source:HGNC Symbol;Acc:HGNC:29943] |
| ENSG00000153498 | HGLibA_46338 | 274 | 1.577982296 | SPACA7 | sperm acrosome associated 7 [Source:HGNC Symbol;Acc:HGNC:29575] |
| ENSG00000157150 | HGLibA_49544 | 274 | 1.577982296 | TIMP4 | TIMP metallopeptidase inhibitor 4 [Source:HGNC Symbol;Acc:HGNC:11823] |
| ENSG00000160695 | HGLibA_53728 | 274 | 1.577982296 | VPS11 | CORVET/HOPS core subunit [Source:HGNC Symbol;Acc:HGNC:14583]" |
| ENSG00000162415 | HGLibA_57050 | 274 | 1.577982296 | ZSWIM5 | zinc finger SWIM-type containing 5 [Source:HGNC Symbol;Acc:HGNC:29299] |
| ENSG00000166948 | HGLibA_49269 | 274 | 1.577982296 | TGM6 | transglutaminase 6 [Source:HGNC Symbol;Acc:HGNC:16255] |
| ENSG00000169393 | HGLibA_14994 | 274 | 1.577982296 | ELSPBP1 | epididymal sperm binding protein 1 [Source:HGNC Symbol;Acc:HGNC:14417] |
| ENSG00000170961 | HGLibA_20864 | 274 | 1.577982296 | HAS2 | hyaluronan synthase 2 [Source:HGNC Symbol;Acc:HGNC:4819] |
| ENSG00000172146 | HGLibA_33642 | 274 | 1.577982296 | OR1A1 | olfactory receptor family 1 subfamily A member 1 [Source:HGNC Symbol;Acc:HGNC:8179] |
| ENSG00000172197 | HGLibA_28516 | 274 | 1.577982296 | MBOAT1 | membrane bound O-acyltransferase domain containing 1 [Source:HGNC Symbol;Acc:HGNC:21579] |
| ENSG00000176194 | HGLibA_09698 | 274 | 1.577982296 | CIDEA | cell death-inducing DFFA-like effector a [Source:HGNC Symbol;Acc:HGNC:1976] |
| ENSG00000176204 | HGLibA_27495 | 274 | 1.577982296 | LRRTM4 | leucine rich repeat transmembrane neuronal 4 [Source:HGNC Symbol;Acc:HGNC:19411] |
| ENSG00000179933 | HGLibA_05320 | 274 | 1.577982296 | C14orf119 | chromosome 14 open reading frame 119 [Source:HGNC Symbol;Acc:HGNC:20270] |
| ENSG00000184903 | HGLibA_23232 | 274 | 1.577982296 | IMMP2L | inner mitochondrial membrane peptidase subunit 2 [Source:HGNC Symbol;Acc:HGNC:14598] |
| ENSG00000187612 | HGLibA_34367 | 274 | 1.577982296 | OR5W2 | olfactory receptor family 5 subfamily W member 2 [Source:HGNC Symbol;Acc:HGNC:15299] |
| ENSG00000197849 | HGLibA_34518 | 274 | 1.577982296 | OR8G1 | olfactory receptor family 8 subfamily G member 1 (gene/pseudogene) [Source:HGNC Symbol;Acc:HGNC:8484] |
| ENSG00000198862 | HGLibA_27615 | 274 | 1.577982296 | LTN1 | listerin E3 ubiquitin protein ligase 1 [Source:HGNC Symbol;Acc:HGNC:13082] |
| ENSG00000213918 | HGLibA_13653 | 274 | 1.577982296 | DNASE1 | deoxyribonuclease 1 [Source:HGNC Symbol;Acc:HGNC:2956] |
| ENSG00000221838 | HGLibA_02346 | 274 | 1.577982296 | AP4M1 | adaptor related protein complex 4 subunit mu 1 [Source:HGNC Symbol;Acc:HGNC:574] |
| ENSG00000224940 | HGLibA_38891 | 274 | 1.577982296 | PRRT4 | proline rich transmembrane protein 4 [Source:HGNC Symbol;Acc:HGNC:37280] |
| ENSG00000244734 | HGLibA_20912 | 274 | 1.577982296 | HBB | hemoglobin subunit beta [Source:HGNC Symbol;Acc:HGNC:4827] |
| ENSG00000263761 | HGLibA_18863 | 274 | 1.577982296 | GDF2 | growth differentiation factor 2 [Source:HGNC Symbol;Acc:HGNC:4217] |
| hsa-mir-29b-1 | HGLibA_58414 | 274 | 1.577982296 | hsa-mir-29b-1 | - |
| hsa-mir-3151 | HGLibA_58665 | 274 | 1.577982296 | hsa-mir-3151 | - |
| hsa-mir-4738 | HGLibA_61048 | 274 | 1.577982296 | hsa-mir-4738 | - |
| hsa-mir-532 | HGLibA_61807 | 274 | 1.577982296 | hsa-mir-532 | - |
| hsa-mir-6720 | HGLibA_63041 | 274 | 1.577982296 | hsa-mir-6720 | - |
| hsa-mir-6818 | HGLibA_63448 | 274 | 1.577982296 | hsa-mir-6818 | - |
| C9orf96 | HGLibA_06782 | 273 | 1.568031258 | C9orf96 | - |
| ENSG00000007376 | HGLibA_42206 | 273 | 1.568031258 | RPUSD1 | RNA pseudouridine synthase domain containing 1 [Source:HGNC Symbol;Acc:HGNC:14173] |
| ENSG00000036530 | HGLibA_12169 | 273 | 1.568031258 | CYP46A1 | cytochrome P450 family 46 subfamily A member 1 [Source:HGNC Symbol;Acc:HGNC:2641] |
| ENSG00000055609 | HGLibA_25326 | 273 | 1.568031258 | KMT2C | lysine methyltransferase 2C [Source:HGNC Symbol;Acc:HGNC:13726] |
| ENSG00000065320 | HGLibA_32853 | 273 | 1.568031258 | NTN1 | netrin 1 [Source:HGNC Symbol;Acc:HGNC:8029] |

| | | | | | |
|---|---|---|---|---|---|
| ENSG00000099949 | HGLibA_27802 | 273 | 1.568031258 | LZTR1 | leucine zipper like transcription regulator 1 [Source:HGNC Symbol;Acc:HGNC:6742] |
| ENSG00000107669 | HGLibA_03428 | 273 | 1.568031258 | ATE1 | arginyltransferase 1 [Source:HGNC Symbol;Acc:HGNC:782] |
| ENSG00000108861 | HGLibA_14179 | 273 | 1.568031258 | DUSP3 | dual specificity phosphatase 3 [Source:HGNC Symbol;Acc:HGNC:3069] |
| ENSG00000113248 | HGLibA_35487 | 273 | 1.568031258 | PCDHB15 | protocadherin beta 15 [Source:HGNC Symbol;Acc:HGNC:8686] |
| ENSG00000121351 | HGLibA_22470 | 273 | 1.568031258 | IAPP | islet amyloid polypeptide [Source:HGNC Symbol;Acc:HGNC:5329] |
| ENSG00000124177 | HGLibA_09401 | 273 | 1.568031258 | CHD6 | chromodomain helicase DNA binding protein 6 [Source:HGNC Symbol;Acc:HGNC:19057] |
| ENSG00000131148 | HGLibA_15023 | 273 | 1.568031258 | EMC8 | ER membrane protein complex subunit 8 [Source:HGNC Symbol;Acc:HGNC:7864] |
| ENSG00000135480 | HGLibA_25522 | 273 | 1.568031258 | KRT7 | keratin 7 [Source:HGNC Symbol;Acc:HGNC:6445] |
| ENSG00000140564 | HGLibA_18172 | 273 | 1.568031258 | FURIN | paired basic amino acid cleaving enzyme [Source:HGNC Symbol;Acc:HGNC:8568]" |
| ENSG00000141096 | HGLibA_13824 | 273 | 1.568031258 | DPEP3 | dipeptidase 3 [Source:HGNC Symbol;Acc:HGNC:23029] |
| ENSG00000146409 | HGLibA_44496 | 273 | 1.568031258 | SLC18B1 | solute carrier family 18 member B1 [Source:HGNC Symbol;Acc:HGNC:21573] |
| ENSG00000148842 | HGLibA_10317 | 273 | 1.568031258 | CNNM2 | cyclin and CBS domain divalent metal cation transport mediator 2 [Source:HGNC Symbol;Acc:HGNC:103] |
| ENSG00000149300 | HGLibA_05150 | 273 | 1.568031258 | C11orf52 | chromosome 11 open reading frame 52 [Source:HGNC Symbol;Acc:HGNC:30531] |
| ENSG00000160185 | HGLibA_52559 | 273 | 1.568031258 | UBASH3A | ubiquitin associated and SH3 domain containing A [Source:HGNC Symbol;Acc:HGNC:12462] |
| ENSG00000163354 | HGLibA_12533 | 273 | 1.568031258 | DCST2 | DC-STAMP domain containing 2 [Source:HGNC Symbol;Acc:HGNC:26562] |
| ENSG00000166482 | HGLibA_29052 | 273 | 1.568031258 | MFAP4 | microfibril associated protein 4 [Source:HGNC Symbol;Acc:HGNC:7035] |
| ENSG00000166833 | HGLibA_31196 | 273 | 1.568031258 | NAV2 | neuron navigator 2 [Source:HGNC Symbol;Acc:HGNC:15997] |
| ENSG00000169933 | HGLibA_18037 | 273 | 1.568031258 | FRMPD4 | FERM and PDZ domain containing 4 [Source:HGNC Symbol;Acc:HGNC:29007] |
| ENSG00000171174 | HGLibA_40465 | 273 | 1.568031258 | RBKS | ribokinase [Source:HGNC Symbol;Acc:HGNC:30325] |
| ENSG00000171903 | HGLibA_12181 | 273 | 1.568031258 | CYP4F11 | cytochrome P450 family 4 subfamily F member 11 [Source:HGNC Symbol;Acc:HGNC:13265] |
| ENSG00000171988 | HGLibA_23952 | 273 | 1.568031258 | JMJD1C | jumonji domain containing 1C [Source:HGNC Symbol;Acc:HGNC:12313] |
| ENSG00000178852 | HGLibA_14529 | 273 | 1.568031258 | EFCAB13 | EF-hand calcium binding domain 13 [Source:HGNC Symbol;Acc:HGNC:26864] |
| ENSG00000180089 | HGLibA_50527 | 273 | 1.568031258 | TMEM86B | transmembrane protein 86B [Source:HGNC Symbol;Acc:HGNC:28448] |
| ENSG00000188833 | HGLibA_15219 | 273 | 1.568031258 | ENTPD8 | ectonucleoside triphosphate diphosphohydrolase 8 [Source:HGNC Symbol;Acc:HGNC:24860] |
| ENSG00000204128 | HGLibA_06175 | 273 | 1.568031258 | C2orf72 | chromosome 2 open reading frame 72 [Source:HGNC Symbol;Acc:HGNC:27418] |
| ENSG00000204913 | HGLibA_27347 | 273 | 1.568031258 | LRRC3C | leucine rich repeat containing 3C [Source:HGNC Symbol;Acc:HGNC:40034] |
| ENSG00000204963 | HGLibA_35453 | 273 | 1.568031258 | PCDHA7 | protocadherin alpha 7 [Source:HGNC Symbol;Acc:HGNC:8673] |
| ENSG00000205359 | HGLibA_45479 | 273 | 1.568031258 | SLCO6A1 | solute carrier organic anion transporter family member 6A1 [Source:HGNC Symbol;Acc:HGNC:23613] |
| ENSG00000205795 | HGLibA_12223 | 273 | 1.568031258 | CYS1 | cystin 1 [Source:HGNC Symbol;Acc:HGNC:18525] |
| ENSG00000205927 | HGLibA_33372 | 273 | 1.568031258 | OLIG2 | oligodendrocyte transcription factor 2 [Source:HGNC Symbol;Acc:HGNC:9398] |
| ENSG00000250120 | HGLibA_35427 | 273 | 1.568031258 | PCDHA10 | protocadherin alpha 10 [Source:HGNC Symbol;Acc:HGNC:8664] |
| ENSG00000257017 | HGLibA_22011 | 273 | 1.568031258 | HP | haptoglobin [Source:HGNC Symbol;Acc:HGNC:5141] |
| hsa-mir-105-2 | HGLibA_57183 | 273 | 1.568031258 | hsa-mir-105-2 | - |
| hsa-mir-4754 | HGLibA_61112 | 273 | 1.568031258 | hsa-mir-4754 | - |
| hsa-mir-658 | HGLibA_62964 | 273 | 1.568031258 | hsa-mir-658 | - |
| hsa-mir-6750 | HGLibA_63157 | 273 | 1.568031258 | hsa-mir-6750 | - |
| hsa-mir-6795 | HGLibA_63357 | 273 | 1.568031258 | hsa-mir-6795 | - |
| ZNF645 | HGLibA_56448 | 273 | 1.568031258 | ZNF645 | - |
| CCDC11 | HGLibA_07535 | 272 | 1.558080221 | CCDC11 | - |
| ENSG00000011454 | HGLibA_40008 | 272 | 1.558080221 | RABGAP1 | RAB GTPase activating protein 1 [Source:HGNC Symbol;Acc:HGNC:17155] |
| ENSG00000012211 | HGLibA_38475 | 272 | 1.558080221 | PRICKLE3 | prickle planar cell polarity protein 3 [Source:HGNC Symbol;Acc:HGNC:6645] |
| ENSG00000104826 | HGLibA_26419 | 272 | 1.558080221 | LHB | luteinizing hormone beta polypeptide [Source:HGNC Symbol;Acc:HGNC:6584] |
| ENSG00000104951 | HGLibA_23168 | 272 | 1.558080221 | IL4I1 | interleukin 4 induced 1 [Source:HGNC Symbol;Acc:HGNC:19094] |
| ENSG00000104967 | HGLibA_32368 | 272 | 1.558080221 | NOVA2 | NOVA alternative splicing regulator 2 [Source:HGNC Symbol;Acc:HGNC:7887] |
| ENSG00000106479 | HGLibA_56880 | 272 | 1.558080221 | ZNF862 | zinc finger protein 862 [Source:HGNC Symbol;Acc:HGNC:34519] |
| ENSG00000117643 | HGLibA_28067 | 272 | 1.558080221 | MAN1C1 | mannosidase alpha class 1C member 1 [Source:HGNC Symbol;Acc:HGNC:19080] |
| ENSG00000121335 | HGLibA_38341 | 272 | 1.558080221 | PRB2 | proline rich protein BstNI subfamily 2 [Source:HGNC Symbol;Acc:HGNC:9338] |
| ENSG00000124143 | HGLibA_02777 | 272 | 1.558080221 | ARHGAP40 | Rho GTPase activating protein 40 [Source:HGNC Symbol;Acc:HGNC:16226] |
| ENSG00000125898 | HGLibA_16012 | 272 | 1.558080221 | FAM110A | family with sequence similarity 110 member A [Source:HGNC Symbol;Acc:HGNC:16188] |
| ENSG00000129535 | HGLibA_32703 | 272 | 1.558080221 | NRL | neural retina leucine zipper [Source:HGNC Symbol;Acc:HGNC:8002] |
| ENSG00000135702 | HGLibA_09639 | 272 | 1.558080221 | CHST5 | carbohydrate sulfotransferase 5 [Source:HGNC Symbol;Acc:HGNC:1973] |
| ENSG00000137193 | HGLibA_36676 | 272 | 1.558080221 | PIM1 | serine/threonine kinase [Source:HGNC Symbol;Acc:HGNC:8986]" |
| ENSG00000137821 | HGLibA_27378 | 272 | 1.558080221 | LRRC49 | leucine rich repeat containing 49 [Source:HGNC Symbol;Acc:HGNC:25965] |
| ENSG00000141576 | HGLibA_41552 | 272 | 1.558080221 | RNF157 | ring finger protein 157 [Source:HGNC Symbol;Acc:HGNC:29402] |
| ENSG00000142233 | HGLibA_32862 | 272 | 1.558080221 | NTN5 | netrin 5 [Source:HGNC Symbol;Acc:HGNC:25208] |
| ENSG00000144230 | HGLibA_19963 | 272 | 1.558080221 | GPR17 | G protein-coupled receptor 17 [Source:HGNC Symbol;Acc:HGNC:4471] |
| ENSG00000150687 | HGLibA_38917 | 272 | 1.558080221 | PRSS23 | serine protease 23 [Source:HGNC Symbol;Acc:HGNC:14370] |
| ENSG00000152484 | HGLibA_53200 | 272 | 1.558080221 | USP12 | ubiquitin specific peptidase 12 [Source:HGNC Symbol;Acc:HGNC:20485] |
| ENSG00000159314 | HGLibA_02744 | 272 | 1.558080221 | ARHGAP27 | Rho GTPase activating protein 27 [Source:HGNC Symbol;Acc:HGNC:31813] |
| ENSG00000162735 | HGLibA_36104 | 272 | 1.558080221 | PEX19 | peroxisomal biogenesis factor 19 [Source:HGNC Symbol;Acc:HGNC:9713] |
| ENSG00000164167 | HGLibA_27548 | 272 | 1.558080221 | LSM6 | U6 small nuclear RNA and mRNA degradation associated [Source:HGNC Symbol;Acc:HGNC:17017]" |
| ENSG00000166140 | HGLibA_55277 | 272 | 1.558080221 | ZFYVE19 | zinc finger FYVE-type containing 19 [Source:HGNC Symbol;Acc:HGNC:20758] |
| ENSG00000170615 | HGLibA_44798 | 272 | 1.558080221 | SLC26A5 | solute carrier family 26 member 5 [Source:HGNC Symbol;Acc:HGNC:9359] |
| ENSG00000172171 | HGLibA_48966 | 272 | 1.558080221 | TEFM | mitochondrial [Source:HGNC Symbol;Acc:HGNC:26223]" |
| ENSG00000172476 | HGLibA_39932 | 272 | 1.558080221 | RAB40A | member RAS oncogene family [Source:HGNC Symbol;Acc:HGNC:18283]" |
| ENSG00000181847 | HGLibA_49485 | 272 | 1.558080221 | TIGIT | T cell immunoreceptor with Ig and ITIM domains [Source:HGNC Symbol;Acc:HGNC:26838] |
| ENSG00000184954 | HGLibA_34402 | 272 | 1.558080221 | OR6C70 | olfactory receptor family 6 subfamily C member 70 [Source:HGNC Symbol;Acc:HGNC:31299] |
| ENSG00000186517 | HGLibA_02752 | 272 | 1.558080221 | ARHGAP30 | Rho GTPase activating protein 30 [Source:HGNC Symbol;Acc:HGNC:27414] |
| ENSG00000188316 | HGLibA_15148 | 272 | 1.558080221 | ENO4 | enolase 4 [Source:HGNC Symbol;Acc:HGNC:31670] |
| ENSG00000197249 | HGLibA_43504 | 272 | 1.558080221 | SERPINA1 | serpin family A member 1 [Source:HGNC Symbol;Acc:HGNC:8941] |
| ENSG00000197893 | HGLibA_32644 | 272 | 1.558080221 | NRAP | nebulin related anchoring protein [Source:HGNC Symbol;Acc:HGNC:7988] |
| ENSG00000205476 | HGLibA_07897 | 272 | 1.558080221 | CCDC85C | coiled-coil domain containing 85C [Source:HGNC Symbol;Acc:HGNC:35459] |
| ENSG00000236125 | HGLibA_53279 | 272 | 1.558080221 | USP17L4 | ubiquitin specific peptidase 17-like family member 4 [Source:HGNC Symbol;Acc:HGNC:37176] |
| ENSG00000259224 | HGLibA_45028 | 272 | 1.558080221 | SLC35G6 | solute carrier family 35 member G6 [Source:HGNC Symbol;Acc:HGNC:31351] |
| ENSG00000262406 | HGLibA_29486 | 272 | 1.558080221 | MMP12 | matrix metallopeptidase 12 [Source:HGNC Symbol;Acc:HGNC:7158] |
| ERBB2IP | HGLibA_15417 | 272 | 1.558080221 | ERBB2IP | - |
| hsa-mir-3690-2 | HGLibA_59348 | 272 | 1.558080221 | hsa-mir-3690-2 | - |
| hsa-mir-4531 | HGLibA_60583 | 272 | 1.558080221 | hsa-mir-4531 | - |
| hsa-mir-6869 | HGLibA_63659 | 272 | 1.558080221 | hsa-mir-6869 | - |
| hsa-mir-6895 | HGLibA_63766 | 272 | 1.558080221 | hsa-mir-6895 | - |
| CCDC151 | HGLibA_07647 | 271 | 1.548129183 | CCDC151 | - |
| ENSG00000016864 | HGLibA_19336 | 271 | 1.548129183 | GLT8D1 | glycosyltransferase 8 domain containing 1 [Source:HGNC Symbol;Acc:HGNC:24870] |
| ENSG00000034510 | HGLibA_50657 | 271 | 1.548129183 | TMSB10 | thymosin beta 10 [Source:HGNC Symbol;Acc:HGNC:11879] |
| ENSG00000048545 | HGLibA_20635 | 271 | 1.548129183 | GUCA1A | guanylate cyclase activator 1A [Source:HGNC Symbol;Acc:HGNC:4678] |
| ENSG00000054148 | HGLibA_36457 | 271 | 1.548129183 | PHPT1 | phosphohistidine phosphatase 1 [Source:HGNC Symbol;Acc:HGNC:30033] |
| ENSG00000069188 | HGLibA_43153 | 271 | 1.548129183 | SDK2 | sidekick cell adhesion molecule 2 [Source:HGNC Symbol;Acc:HGNC:19308] |
| ENSG00000099219 | HGLibA_15509 | 271 | 1.548129183 | ERMP1 | endoplasmic reticulum metallopeptidase 1 [Source:HGNC Symbol;Acc:HGNC:23703] |
| ENSG00000099958 | HGLibA_13002 | 271 | 1.548129183 | DERL3 | derlin 3 [Source:HGNC Symbol;Acc:HGNC:14236] |
| ENSG00000104413 | HGLibA_15586 | 271 | 1.548129183 | ESRP1 | epithelial splicing regulatory protein 1 [Source:HGNC Symbol;Acc:HGNC:25966] |
| ENSG00000105205 | HGLibA_09801 | 271 | 1.548129183 | CLC | Charcot-Leyden crystal galectin [Source:HGNC Symbol;Acc:HGNC:2014] |
| ENSG00000106268 | HGLibA_32932 | 271 | 1.548129183 | NUDT1 | nudix hydrolase 1 [Source:HGNC Symbol;Acc:HGNC:8048] |
| ENSG00000114861 | HGLibA_17931 | 271 | 1.548129183 | FOXP1 | forkhead box P1 [Source:HGNC Symbol;Acc:HGNC:3823] |
| ENSG00000116005 | HGLibA_35694 | 271 | 1.548129183 | PCYOX1 | prenylcysteine oxidase 1 [Source:HGNC Symbol;Acc:HGNC:20588] |
| ENSG00000120093 | HGLibA_21934 | 271 | 1.548129183 | HOXB3 | homeobox B3 [Source:HGNC Symbol;Acc:HGNC:5114] |
| ENSG00000121741 | HGLibA_55393 | 271 | 1.548129183 | ZMYM2 | zinc finger MYM-type containing 2 [Source:HGNC Symbol;Acc:HGNC:12989] |
| ENSG00000124496 | HGLibA_51369 | 271 | 1.548129183 | TRERF1 | transcriptional regulating factor 1 [Source:HGNC Symbol;Acc:HGNC:18273] |
| ENSG00000125618 | HGLibA_35317 | 271 | 1.548129183 | PAX8 | paired box 8 [Source:HGNC Symbol;Acc:HGNC:8622] |
| ENSG00000126243 | HGLibA_27157 | 271 | 1.548129183 | LRFN3 | leucine rich repeat and fibronectin type III domain containing 3 [Source:HGNC Symbol;Acc:HGNC:28370] |
| ENSG00000129465 | HGLibA_41323 | 271 | 1.548129183 | RIPK3 | receptor interacting serine/threonine kinase 3 [Source:HGNC Symbol;Acc:HGNC:10021] |
| ENSG00000131059 | HGLibA_04717 | 271 | 1.548129183 | BPIFA3 | BPI fold containing family A member 3 [Source:HGNC Symbol;Acc:HGNC:16204] |
| ENSG00000143375 | HGLibA_09319 | 271 | 1.548129183 | CGN | cingulin [Source:HGNC Symbol;Acc:HGNC:17429] |
| ENSG00000144283 | HGLibA_36861 | 271 | 1.548129183 | PKP4 | plakophilin 4 [Source:HGNC Symbol;Acc:HGNC:9026] |
| ENSG00000166341 | HGLibA_12490 | 271 | 1.548129183 | DCHS1 | dachsous cadherin-related 1 [Source:HGNC Symbol;Acc:HGNC:13681] |
| ENSG00000168795 | HGLibA_54910 | 271 | 1.548129183 | ZBTB5 | zinc finger and BTB domain containing 5 [Source:HGNC Symbol;Acc:HGNC:23836] |
| ENSG00000168918 | HGLibA_23349 | 271 | 1.548129183 | INPP5D | inositol polyphosphate-5-phosphatase D [Source:HGNC Symbol;Acc:HGNC:6079] |
| ENSG00000170419 | HGLibA_53861 | 271 | 1.548129183 | VSTM2A | V-set and transmembrane domain containing 2A [Source:HGNC Symbol;Acc:HGNC:28499] |
| ENSG00000174004 | HGLibA_32722 | 271 | 1.548129183 | NRROS | negative regulator of reactive oxygen species [Source:HGNC Symbol;Acc:HGNC:24613] |
| ENSG00000174996 | HGLibA_25029 | 271 | 1.548129183 | KLC2 | kinesin light chain 2 [Source:HGNC Symbol;Acc:HGNC:20716] |
| ENSG00000179057 | HGLibA_22886 | 271 | 1.548129183 | IGSF22 | immunoglobulin superfamily member 22 [Source:HGNC Symbol;Acc:HGNC:26750] |
| ENSG00000182752 | HGLibA_35144 | 271 | 1.548129183 | PAPPA | pappalysin 1 [Source:HGNC Symbol;Acc:HGNC:8602] |
| ENSG00000183741 | HGLibA_07460 | 271 | 1.548129183 | CBX6 | chromobox 6 [Source:HGNC Symbol;Acc:HGNC:1556] |
| ENSG00000184302 | HGLibA_44259 | 271 | 1.548129183 | SIX6 | SIX homeobox 6 [Source:HGNC Symbol;Acc:HGNC:10892] |
| ENSG00000187513 | HGLibA_19144 | 271 | 1.548129183 | GJA4 | gap junction protein alpha 4 [Source:HGNC Symbol;Acc:HGNC:4278] |
| ENSG00000188215 | HGLibA_12572 | 271 | 1.548129183 | DCUN1D3 | defective in cullin neddylation 1 domain containing 3 [Source:HGNC Symbol;Acc:HGNC:28734] |
| ENSG00000188779 | HGLibA_44290 | 271 | 1.548129183 | SKOR1 | SKI family transcriptional corepressor 1 [Source:HGNC Symbol;Acc:HGNC:21326] |
| ENSG00000196535 | HGLibA_30811 | 271 | 1.548129183 | MYO18A | myosin XVIIIA [Source:HGNC Symbol;Acc:HGNC:31104] |
| ENSG00000204414 | HGLibA_11356 | 271 | 1.548129183 | CSHL1 | chorionic somatomammotropin hormone like 1 [Source:HGNC Symbol;Acc:HGNC:2442] |
| ENSG00000236980 | HGLibA_06279 | 271 | 1.548129183 | C3orf84 | chromosome 3 open reading frame 84 [Source:HGNC Symbol;Acc:HGNC:44666] |
| ENSG00000240204 | HGLibA_45775 | 271 | 1.548129183 | SMKR1 | small lysine rich protein 1 [Source:HGNC Symbol;Acc:HGNC:43561] |
| ENSG00000249581 | HGLibA_10125 | 271 | 1.548129183 | CLRN2 | clarin 2 [Source:HGNC Symbol;Acc:HGNC:33939] |
| ENSG00000259494 | HGLibA_29997 | 271 | 1.548129183 | MRPL46 | mitochondrial ribosomal protein L46 [Source:HGNC Symbol;Acc:HGNC:1192] |
| ENSG00000268988 | HGLibA_46404 | 271 | 1.548129183 | SPANXN2 | SPANX family member N2 [Source:HGNC Symbol;Acc:HGNC:33175] |
| hsa-mir-301a | HGLibA_58429 | 271 | 1.548129183 | hsa-mir-301a | - |
| hsa-mir-3650 | HGLibA_59145 | 271 | 1.548129183 | hsa-mir-3650 | - |
| hsa-mir-3928 | HGLibA_59590 | 271 | 1.548129183 | hsa-mir-3928 | - |
| hsa-mir-4260 | HGLibA_59749 | 271 | 1.548129183 | hsa-mir-4260 | - |
| hsa-mir-648 | HGLibA_62835 | 271 | 1.548129183 | hsa-mir-648 | - |
| hsa-mir-6799 | HGLibA_63374 | 271 | 1.548129183 | hsa-mir-6799 | - |
| LILRA3 | HGLibA_26496 | 271 | 1.548129183 | LILRA3 | - |
| PHF17 | HGLibA_36355 | 271 | 1.548129183 | PHF17 | - |
| C20orf196 | HGLibA_06005 | 270 | 1.538178146 | C20orf196 | - |
| ENSG00000005486 | HGLibA_41128 | 270 | 1.538178146 | RHBDD2 | rhomboid domain containing 2 [Source:HGNC Symbol;Acc:HGNC:23082] |
| ENSG00000040608 | HGLibA_42412 | 270 | 1.538178146 | RTN4R | reticulon 4 receptor [Source:HGNC Symbol;Acc:HGNC:18601] |
| ENSG00000046604 | HGLibA_14024 | 270 | 1.538178146 | DSG2 | desmoglein 2 [Source:HGNC Symbol;Acc:HGNC:3049] |
| ENSG00000048471 | HGLibA_46079 | 270 | 1.538178146 | SNX29 | sorting nexin 29 [Source:HGNC Symbol;Acc:HGNC:30542] |

| | | | | | |
|---|---|---|---|---|---|
| ENSG00000064490 | HGLibA_40987 | 270 | 1.538178146 | RFXANK | regulatory factor X associated ankyrin containing protein [Source:HGNC Symbol;Acc:HGNC:9987] |
| ENSG00000071909 | HGLibA_30848 | 270 | 1.538178146 | MYO3B | myosin IIIB [Source:HGNC Symbol;Acc:HGNC:15576] |
| ENSG00000072201 | HGLibA_26744 | 270 | 1.538178146 | LNX1 | ligand of numb-protein X 1 [Source:HGNC Symbol;Acc:HGNC:6657] |
| ENSG00000074370 | HGLibA_03610 | 270 | 1.538178146 | ATP2A3 | ATPase sarcoplasmic/endoplasmic reticulum Ca2+ transporting 3 [Source:HGNC Symbol;Acc:HGNC:813] |
| ENSG00000074966 | HGLibA_52386 | 270 | 1.538178146 | TXK | TXK tyrosine kinase [Source:HGNC Symbol;Acc:HGNC:12434] |
| ENSG00000084090 | HGLibA_47373 | 270 | 1.538178146 | STARD7 | StAR related lipid transfer domain containing 7 [Source:HGNC Symbol;Acc:HGNC:18063] |
| ENSG00000099250 | HGLibA_32716 | 270 | 1.538178146 | NRP1 | neuropilin 1 [Source:HGNC Symbol;Acc:HGNC:8004] |
| ENSG00000101986 | HGLibA_00190 | 270 | 1.538178146 | ABCD1 | ATP binding cassette subfamily D member 1 [Source:HGNC Symbol;Acc:HGNC:61] |
| ENSG00000104941 | HGLibA_42339 | 270 | 1.538178146 | RSPH6A | radial spoke head 6 homolog A [Source:HGNC Symbol;Acc:HGNC:14241] |
| ENSG00000113389 | HGLibA_32515 | 270 | 1.538178146 | NPR3 | natriuretic peptide receptor 3 [Source:HGNC Symbol;Acc:HGNC:7945] |
| ENSG00000118271 | HGLibA_52228 | 270 | 1.538178146 | TTR | transthyretin [Source:HGNC Symbol;Acc:HGNC:12405] |
| ENSG00000124260 | HGLibA_27894 | 270 | 1.538178146 | MAGEA10 | MAGE family member A10 [Source:HGNC Symbol;Acc:HGNC:6797] |
| ENSG00000126106 | HGLibA_50438 | 270 | 1.538178146 | TMEM53 | transmembrane protein 53 [Source:HGNC Symbol;Acc:HGNC:26186] |
| ENSG00000127324 | HGLibA_51950 | 270 | 1.538178146 | TSPAN8 | tetraspanin 8 [Source:HGNC Symbol;Acc:HGNC:11855] |
| ENSG00000128849 | HGLibA_09322 | 270 | 1.538178146 | CGNL1 | cingulin like 1 [Source:HGNC Symbol;Acc:HGNC:25931] |
| ENSG00000132326 | HGLibA_36058 | 270 | 1.538178146 | PER2 | period circadian regulator 2 [Source:HGNC Symbol;Acc:HGNC:8846] |
| ENSG00000133138 | HGLibA_48568 | 270 | 1.538178146 | TBC1D8B | TBC1 domain family member 8B [Source:HGNC Symbol;Acc:HGNC:24715] |
| ENSG00000140682 | HGLibA_49211 | 270 | 1.538178146 | TGFB1I1 | transforming growth factor beta 1 induced transcript 1 [Source:HGNC Symbol;Acc:HGNC:11767] |
| ENSG00000142675 | HGLibA_10296 | 270 | 1.538178146 | CNKSR1 | connector enhancer of kinase suppressor of Ras 1 [Source:HGNC Symbol;Acc:HGNC:19700] |
| ENSG00000143375 | HGLibA_09320 | 270 | 1.538178146 | CGN | cingulin [Source:HGNC Symbol;Acc:HGNC:17429] |
| ENSG00000151948 | HGLibA_19332 | 270 | 1.538178146 | GLT1D1 | glycosyltransferase 1 domain containing 1 [Source:HGNC Symbol;Acc:HGNC:26483] |
| ENSG00000153250 | HGLibA_40588 | 270 | 1.538178146 | RBMS1 | RNA binding motif single stranded interacting protein 1 [Source:HGNC Symbol;Acc:HGNC:9907] |
| ENSG00000161405 | HGLibA_22934 | 270 | 1.538178146 | IKZF3 | IKAROS family zinc finger 3 [Source:HGNC Symbol;Acc:HGNC:13178] |
| ENSG00000163630 | HGLibA_48028 | 270 | 1.538178146 | SYNPR | synaptoporin [Source:HGNC Symbol;Acc:HGNC:16507] |
| ENSG00000169239 | HGLibA_06815 | 270 | 1.538178146 | CA5B | carbonic anhydrase 5B [Source:HGNC Symbol;Acc:HGNC:1378] |
| ENSG00000169740 | HGLibA_55793 | 270 | 1.538178146 | ZNF32 | zinc finger protein 32 [Source:HGNC Symbol;Acc:HGNC:13095] |
| ENSG00000170017 | HGLibA_01532 | 270 | 1.538178146 | ALCAM | activated leukocyte cell adhesion molecule [Source:HGNC Symbol;Acc:HGNC:400] |
| ENSG00000171055 | HGLibA_17316 | 270 | 1.538178146 | FEZ2 | fasciculation and elongation protein zeta 2 [Source:HGNC Symbol;Acc:HGNC:3660] |
| ENSG00000172201 | HGLibA_22532 | 270 | 1.538178146 | ID4 | HLH protein [Source:HGNC Symbol;Acc:HGNC:5363]" |
| ENSG00000172789 | HGLibA_21970 | 270 | 1.538178146 | HOXC5 | homeobox C5 [Source:HGNC Symbol;Acc:HGNC:5127] |
| ENSG00000176029 | HGLibA_05119 | 270 | 1.538178146 | C11orf16 | chromosome 11 open reading frame 16 [Source:HGNC Symbol;Acc:HGNC:1169] |
| ENSG00000176900 | HGLibA_34133 | 270 | 1.538178146 | OR51T1 | olfactory receptor family 51 subfamily T member 1 [Source:HGNC Symbol;Acc:HGNC:15205] |
| ENSG00000182963 | HGLibA_19179 | 270 | 1.538178146 | GJC1 | gap junction protein gamma 1 [Source:HGNC Symbol;Acc:HGNC:4280] |
| ENSG00000188959 | HGLibA_06690 | 270 | 1.538178146 | C9orf152 | chromosome 9 open reading frame 152 [Source:HGNC Symbol;Acc:HGNC:31455] |
| ENSG00000196652 | HGLibA_55358 | 270 | 1.538178146 | ZKSCAN5 | zinc finger with KRAB and SCAN domains 5 [Source:HGNC Symbol;Acc:HGNC:12867] |
| ENSG00000196704 | HGLibA_01859 | 270 | 1.538178146 | AMZ2 | archaelysin family metallopeptidase 2 [Source:HGNC Symbol;Acc:HGNC:28041] |
| ENSG00000198837 | HGLibA_12954 | 270 | 1.538178146 | DENND4B | DENN domain containing 4B [Source:HGNC Symbol;Acc:HGNC:29044] |
| ENSG00000204969 | HGLibA_35438 | 270 | 1.538178146 | PCDHA2 | protocadherin alpha 2 [Source:HGNC Symbol;Acc:HGNC:8668] |
| ENSG00000205358 | HGLibA_30293 | 270 | 1.538178146 | MT1H | metallothionein 1H [Source:HGNC Symbol;Acc:HGNC:7400] |
| ENSG00000244025 | HGLibA_25669 | 270 | 1.538178146 | KRTAP19-3 | keratin associated protein 19-3 [Source:HGNC Symbol;Acc:HGNC:18938] |
| ENSG00000251664 | HGLibA_35432 | 270 | 1.538178146 | PCDHA12 | protocadherin alpha 12 [Source:HGNC Symbol;Acc:HGNC:8666] |
| ENSG00000257594 | HGLibA_18561 | 270 | 1.538178146 | GALNT4 | polypeptide N-acetylgalactosaminyltransferase 4 [Source:HGNC Symbol;Acc:HGNC:4126] |
| ENSG00000278662 | HGLibA_19623 | 270 | 1.538178146 | GOLGA6L10 | golgin A6 family-like 10 [Source:HGNC Symbol;Acc:HGNC:37228] |
| hsa-mir-3154 | HGLibA_58676 | 270 | 1.538178146 | hsa-mir-3154 | - |
| hsa-mir-3612 | HGLibA_59077 | 270 | 1.538178146 | hsa-mir-3612 | - |
| hsa-mir-4417 | HGLibA_60051 | 270 | 1.538178146 | hsa-mir-4417 | - |
| hsa-mir-4750 | HGLibA_61096 | 270 | 1.538178146 | hsa-mir-4750 | - |
| hsa-mir-4787 | HGLibA_61255 | 270 | 1.538178146 | hsa-mir-4787 | - |
| hsa-mir-5682 | HGLibA_62249 | 270 | 1.538178146 | hsa-mir-5682 | - |
| ENSG00000007341 | HGLibA_47284 | 269 | 1.528227108 | ST7L | suppression of tumorigenicity 7 like [Source:HGNC Symbol;Acc:HGNC:18441] |
| ENSG00000008441 | HGLibA_31812 | 269 | 1.528227108 | NFIX | nuclear factor I X [Source:HGNC Symbol;Acc:HGNC:7788] |
| ENSG00000068724 | HGLibA_52151 | 269 | 1.528227108 | TTC7A | tetratricopeptide repeat domain 7A [Source:HGNC Symbol;Acc:HGNC:19750] |
| ENSG00000087258 | HGLibA_19463 | 269 | 1.528227108 | GNAO1 | G protein subunit alpha o1 [Source:HGNC Symbol;Acc:HGNC:4389] |
| ENSG00000099260 | HGLibA_35080 | 269 | 1.528227108 | PALMD | palmdelphin [Source:HGNC Symbol;Acc:HGNC:15846] |
| ENSG00000099957 | HGLibA_34861 | 269 | 1.528227108 | P2RX6 | purinergic receptor P2X 6 [Source:HGNC Symbol;Acc:HGNC:8538] |
| ENSG00000101199 | HGLibA_02661 | 269 | 1.528227108 | ARFGAP1 | ADP ribosylation factor GTPase activating protein 1 [Source:HGNC Symbol;Acc:HGNC:15852] |
| ENSG00000105991 | HGLibA_21894 | 269 | 1.528227108 | HOXA1 | homeobox A1 [Source:HGNC Symbol;Acc:HGNC:5099] |
| ENSG00000116544 | HGLibA_13344 | 269 | 1.528227108 | DLGAP3 | DLG associated protein 3 [Source:HGNC Symbol;Acc:HGNC:30368] |
| ENSG00000130429 | HGLibA_03078 | 269 | 1.528227108 | ARPC1B | actin related protein 2/3 complex subunit 1B [Source:HGNC Symbol;Acc:HGNC:704] |
| ENSG00000134962 | HGLibA_25023 | 269 | 1.528227108 | KLB | klotho beta [Source:HGNC Symbol;Acc:HGNC:15527] |
| ENSG00000138068 | HGLibA_47766 | 269 | 1.528227108 | SULT6B1 | sulfotransferase family 6B member 1 [Source:HGNC Symbol;Acc:HGNC:33433] |
| ENSG00000140374 | HGLibA_15620 | 269 | 1.528227108 | ETFA | electron transfer flavoprotein subunit alpha [Source:HGNC Symbol;Acc:HGNC:3481] |
| ENSG00000146281 | HGLibA_37287 | 269 | 1.528227108 | PM20D2 | peptidase M20 domain containing 2 [Source:HGNC Symbol;Acc:HGNC:21408] |
| ENSG00000152377 | HGLibA_46748 | 269 | 1.528227108 | SPOCK1 | cwcv and kazal like domains proteoglycan 1 [Source:HGNC Symbol;Acc:HGNC:11251]" |
| ENSG00000157570 | HGLibA_51916 | 269 | 1.528227108 | TSPAN18 | tetraspanin 18 [Source:HGNC Symbol;Acc:HGNC:20660] |
| ENSG00000158517 | HGLibA_31296 | 269 | 1.528227108 | NCF1 | neutrophil cytosolic factor 1 [Source:HGNC Symbol;Acc:HGNC:7660] |
| ENSG00000164588 | HGLibA_20973 | 269 | 1.528227108 | HCN1 | hyperpolarization activated cyclic nucleotide gated potassium channel 1 [Source:HGNC Symbol;Acc:HGNC:4845] |
| ENSG00000164778 | HGLibA_15102 | 269 | 1.528227108 | EN2 | engrailed homeobox 2 [Source:HGNC Symbol;Acc:HGNC:3343] |
| ENSG00000166368 | HGLibA_33783 | 269 | 1.528227108 | OR2D2 | olfactory receptor family 2 subfamily D member 2 [Source:HGNC Symbol;Acc:HGNC:8244] |
| ENSG00000168703 | HGLibA_54257 | 269 | 1.528227108 | WFDC12 | WAP four-disulfide core domain 12 [Source:HGNC Symbol;Acc:HGNC:16115] |
| ENSG00000169306 | HGLibA_23054 | 269 | 1.528227108 | IL1RAPL1 | interleukin 1 receptor accessory protein like 1 [Source:HGNC Symbol;Acc:HGNC:5996] |
| ENSG00000171431 | HGLibA_25448 | 269 | 1.528227108 | KRT20 | keratin 20 [Source:HGNC Symbol;Acc:HGNC:20412] |
| ENSG00000171611 | HGLibA_39287 | 269 | 1.528227108 | PTCRA | pre T cell antigen receptor alpha [Source:HGNC Symbol;Acc:HGNC:21290] |
| ENSG00000172663 | HGLibA_49945 | 269 | 1.528227108 | TMEM134 | transmembrane protein 134 [Source:HGNC Symbol;Acc:HGNC:26142] |
| ENSG00000172766 | HGLibA_30980 | 269 | 1.528227108 | NAA16 | NatA auxiliary subunit [Source:HGNC Symbol;Acc:HGNC:26164]" |
| ENSG00000173349 | HGLibA_43757 | 269 | 1.528227108 | SFT2D3 | SFT2 domain containing 3 [Source:HGNC Symbol;Acc:HGNC:28767] |
| ENSG00000174307 | HGLibA_36422 | 269 | 1.528227108 | PHLDA3 | pleckstrin homology like domain family A member 3 [Source:HGNC Symbol;Acc:HGNC:8934] |
| ENSG00000177105 | HGLibA_41191 | 269 | 1.528227108 | RHOG | ras homolog family member G [Source:HGNC Symbol;Acc:HGNC:672] |
| ENSG00000182670 | HGLibA_52103 | 269 | 1.528227108 | TTC3 | tetratricopeptide repeat domain 3 [Source:HGNC Symbol;Acc:HGNC:12393] |
| ENSG00000183715 | HGLibA_33413 | 269 | 1.528227108 | OPCML | opioid binding protein/cell adhesion molecule like [Source:HGNC Symbol;Acc:HGNC:8143] |
| ENSG00000184022 | HGLibA_33854 | 269 | 1.528227108 | OR2T10 | olfactory receptor family 2 subfamily T member 10 [Source:HGNC Symbol;Acc:HGNC:19573] |
| ENSG00000186529 | HGLibA_12192 | 269 | 1.528227108 | CYP4F3 | cytochrome P450 family 4 subfamily F member 3 [Source:HGNC Symbol;Acc:HGNC:2646] |
| ENSG00000196262 | HGLibA_37893 | 269 | 1.528227108 | PPIA | peptidylprolyl isomerase A [Source:HGNC Symbol;Acc:HGNC:9253] |
| ENSG00000196961 | HGLibA_02304 | 269 | 1.528227108 | AP2A1 | adaptor related protein complex 2 subunit alpha 1 [Source:HGNC Symbol;Acc:HGNC:561] |
| ENSG00000205002 | HGLibA_00065 | 269 | 1.528227108 | AARD | alanine and arginine rich domain containing protein [Source:HGNC Symbol;Acc:HGNC:33842] |
| ENSG00000205208 | HGLibA_06334 | 269 | 1.528227108 | C4orf46 | chromosome 4 open reading frame 46 [Source:HGNC Symbol;Acc:HGNC:27320] |
| ENSG00000205457 | HGLibA_51085 | 269 | 1.528227108 | TP53TG3C | TP53 target 3C [Source:HGNC Symbol;Acc:HGNC:42962] |
| ENSG00000249158 | HGLibA_35429 | 269 | 1.528227108 | PCDHA11 | protocadherin alpha 11 [Source:HGNC Symbol;Acc:HGNC:8665] |
| ENSG00000249992 | HGLibA_50014 | 269 | 1.528227108 | TMEM158 | transmembrane protein 158 (gene/pseudogene) [Source:HGNC Symbol;Acc:HGNC:30293] |
| ENSG00000269437 | HGLibA_33132 | 269 | 1.528227108 | NXF2B | nuclear RNA export factor 2B [Source:HGNC Symbol;Acc:HGNC:23984] |
| ENSG00000276644 | HGLibA_12274 | 269 | 1.528227108 | DACH1 | dachshund family transcription factor 1 [Source:HGNC Symbol;Acc:HGNC:2663] |
| FAM21A | HGLibA_16505 | 269 | 1.528227108 | FAM21A | - |
| HIST1H2AI | HGLibA_21396 | 269 | 1.528227108 | HIST1H2AI | - |
| hsa-mir-4264 | HGLibA_59763 | 269 | 1.528227108 | hsa-mir-4264 | - |
| hsa-mir-6165 | HGLibA_62703 | 269 | 1.528227108 | hsa-mir-6165 | - |
| C1orf51 | HGLibA_05863 | 268 | 1.518276071 | C1orf51 | - |
| C2orf44 | HGLibA_06132 | 268 | 1.518276071 | C2orf44 | - |
| C5orf20 | HGLibA_06362 | 268 | 1.518276071 | C5orf20 | - |
| C9orf62 | HGLibA_06747 | 268 | 1.518276071 | C9orf62 | - |
| ENSG00000006194 | HGLibA_55692 | 268 | 1.518276071 | ZNF263 | zinc finger protein 263 [Source:HGNC Symbol;Acc:HGNC:13056] |
| ENSG00000061676 | HGLibA_31310 | 268 | 1.518276071 | NCKAP1 | NCK associated protein 1 [Source:HGNC Symbol;Acc:HGNC:7666] |
| ENSG00000065809 | HGLibA_16002 | 268 | 1.518276071 | FAM107B | family with sequence similarity 107 member B [Source:HGNC Symbol;Acc:HGNC:23726] |
| ENSG00000066294 | HGLibA_08465 | 268 | 1.518276071 | CD84 | CD84 molecule [Source:HGNC Symbol;Acc:HGNC:1704] |
| ENSG00000082269 | HGLibA_16132 | 268 | 1.518276071 | FAM135A | family with sequence similarity 135 member A [Source:HGNC Symbol;Acc:HGNC:21084] |
| ENSG00000085998 | HGLibA_37657 | 268 | 1.518276071 | POMGNT1 | 2-) [Source:HGNC Symbol;Acc:HGNC:19139]" |
| ENSG00000091136 | HGLibA_25955 | 268 | 1.518276071 | LAMB1 | laminin subunit beta 1 [Source:HGNC Symbol;Acc:HGNC:6486] |
| ENSG00000101082 | HGLibA_44302 | 268 | 1.518276071 | SLA2 | Src like adaptor 2 [Source:HGNC Symbol;Acc:HGNC:17329] |
| ENSG00000101435 | HGLibA_11475 | 268 | 1.518276071 | CST9L | cystatin 9 like [Source:HGNC Symbol;Acc:HGNC:16233] |
| ENSG00000102401 | HGLibA_03044 | 268 | 1.518276071 | ARMCX3 | armadillo repeat containing X-linked 3 [Source:HGNC Symbol;Acc:HGNC:24065] |
| ENSG00000105711 | HGLibA_42977 | 268 | 1.518276071 | SCN1B | sodium voltage-gated channel beta subunit 1 [Source:HGNC Symbol;Acc:HGNC:10586] |
| ENSG00000106526 | HGLibA_00664 | 268 | 1.518276071 | ACTR3C | ARP3 actin related protein 3 homolog C [Source:HGNC Symbol;Acc:HGNC:37282] |
| ENSG00000106603 | HGLibA_10444 | 268 | 1.518276071 | COA1 | cytochrome c oxidase assembly factor 1 homolog [Source:HGNC Symbol;Acc:HGNC:21868] |
| ENSG00000114770 | HGLibA_00178 | 268 | 1.518276071 | ABCC5 | ATP binding cassette subfamily C member 5 [Source:HGNC Symbol;Acc:HGNC:56] |
| ENSG00000126804 | HGLibA_54810 | 268 | 1.518276071 | ZBTB1 | zinc finger and BTB domain containing 1 [Source:HGNC Symbol;Acc:HGNC:20259] |
| ENSG00000138640 | HGLibA_16142 | 268 | 1.518276071 | FAM13A | family with sequence similarity 13 member A [Source:HGNC Symbol;Acc:HGNC:19367] |
| ENSG00000140941 | HGLibA_28125 | 268 | 1.518276071 | MAP1LC3B | microtubule associated protein 1 light chain 3 beta [Source:HGNC Symbol;Acc:HGNC:13352] |
| ENSG00000142166 | HGLibA_22696 | 268 | 1.518276071 | IFNAR1 | interferon alpha and beta receptor subunit 1 [Source:HGNC Symbol;Acc:HGNC:5432] |
| ENSG00000152475 | HGLibA_56844 | 268 | 1.518276071 | ZNF837 | zinc finger protein 837 [Source:HGNC Symbol;Acc:HGNC:25164] |
| ENSG00000156381 | HGLibA_02146 | 268 | 1.518276071 | ANKRD9 | ankyrin repeat domain 9 [Source:HGNC Symbol;Acc:HGNC:20096] |
| ENSG00000161202 | HGLibA_14213 | 268 | 1.518276071 | DVL3 | dishevelled segment polarity protein 3 [Source:HGNC Symbol;Acc:HGNC:3087] |
| ENSG00000162782 | HGLibA_48908 | 268 | 1.518276071 | TDRD5 | tudor domain containing 5 [Source:HGNC Symbol;Acc:HGNC:20614] |
| ENSG00000166024 | HGLibA_39769 | 268 | 1.518276071 | R3HCC1L | R3H domain and coiled-coil containing 1 like [Source:HGNC Symbol;Acc:HGNC:23512] |
| ENSG00000166439 | HGLibA_41567 | 268 | 1.518276071 | RNF169 | ring finger protein 169 [Source:HGNC Symbol;Acc:HGNC:26961] |
| ENSG00000169083 | HGLibA_02619 | 268 | 1.518276071 | AR | androgen receptor [Source:HGNC Symbol;Acc:HGNC:644] |
| ENSG00000171044 | HGLibA_54527 | 268 | 1.518276071 | XKR6 | XK related 6 [Source:HGNC Symbol;Acc:HGNC:27806] |
| ENSG00000174914 | HGLibA_34567 | 268 | 1.518276071 | OR9G1 | olfactory receptor family 9 subfamily G member 1 [Source:HGNC Symbol;Acc:HGNC:15319] |
| ENSG00000177000 | HGLibA_30387 | 268 | 1.518276071 | MTHFR | methylenetetrahydrofolate reductase [Source:HGNC Symbol;Acc:HGNC:7436] |
| ENSG00000181291 | HGLibA_49941 | 268 | 1.518276071 | TMEM132E | transmembrane protein 132E [Source:HGNC Symbol;Acc:HGNC:26991] |
| ENSG00000184602 | HGLibA_45932 | 268 | 1.518276071 | SNN | stannin [Source:HGNC Symbol;Acc:HGNC:11149] |
| ENSG00000186970 | HGLibA_25655 | 268 | 1.518276071 | KRTAP15-1 | keratin associated protein 15-1 [Source:HGNC Symbol;Acc:HGNC:18927] |
| ENSG00000188157 | HGLibA_01251 | 268 | 1.518276071 | AGRN | agrin [Source:HGNC Symbol;Acc:HGNC:329] |
| ENSG00000196967 | HGLibA_56321 | 268 | 1.518276071 | ZNF585A | zinc finger protein 585A [Source:HGNC Symbol;Acc:HGNC:26305] |
| ENSG00000197580 | HGLibA_04360 | 268 | 1.518276071 | BCO2 | beta-carotene oxygenase 2 [Source:HGNC Symbol;Acc:HGNC:18503] |
| ENSG00000198055 | HGLibA_20334 | 268 | 1.518276071 | GRK6 | G protein-coupled receptor kinase 6 [Source:HGNC Symbol;Acc:HGNC:4545] |
| ENSG00000198879 | HGLibA_43724 | 268 | 1.518276071 | SFMBT2 | Scm like with four mbt domains 2 [Source:HGNC Symbol;Acc:HGNC:20256] |

| | | | | | |
|---|---|---|---|---|---|
| ENSG00000203724 | HGLibA_05870 | 268 | 1.518276071 | C1orf53 | chromosome 1 open reading frame 53 [Source:HGNC Symbol;Acc:HGNC:30003] |
| ENSG00000223638 | HGLibA_40937 | 268 | 1.518276071 | RFPL4A | ret finger protein like 4A [Source:HGNC Symbol;Acc:HGNC:16449] |
| ENSG00000225190 | HGLibA_37136 | 268 | 1.518276071 | PLEKHM1 | pleckstrin homology and RUN domain containing M1 [Source:HGNC Symbol;Acc:HGNC:29017] |
| hsa-mir-133b | HGLibA_57779 | 268 | 1.518276071 | hsa-mir-133b | - |
| hsa-mir-3652 | HGLibA_59152 | 268 | 1.518276071 | hsa-mir-3652 | - |
| hsa-mir-887 | HGLibA_64225 | 268 | 1.518276071 | hsa-mir-887 | - |
| LOC286238 | HGLibA_26919 | 268 | 1.518276071 | LOC286238 | - |
| SMIM11 | HGLibA_45721 | 268 | 1.518276071 | SMIM11 | - |
| ENSG00000005448 | HGLibA_54123 | 267 | 1.508325034 | WDR54 | WD repeat domain 54 [Source:HGNC Symbol;Acc:HGNC:25770] |
| ENSG00000005884 | HGLibA_23730 | 267 | 1.508325034 | ITGA3 | integrin subunit alpha 3 [Source:HGNC Symbol;Acc:HGNC:6139] |
| ENSG00000106852 | HGLibA_26461 | 267 | 1.508325034 | LHX6 | LIM homeobox 6 [Source:HGNC Symbol;Acc:HGNC:21735] |
| ENSG00000114450 | HGLibA_19499 | 267 | 1.508325034 | GNB4 | G protein subunit beta 4 [Source:HGNC Symbol;Acc:HGNC:20731] |
| ENSG00000121410 | HGLibA_00003 | 267 | 1.508325034 | A1BG | alpha-1-B glycoprotein [Source:HGNC Symbol;Acc:HGNC:5] |
| ENSG00000144642 | HGLibA_40594 | 267 | 1.508325034 | RBMS3 | RNA binding motif single stranded interacting protein 3 [Source:HGNC Symbol;Acc:HGNC:13427] |
| ENSG00000146147 | HGLibA_29386 | 267 | 1.508325034 | MLIP | muscular LMNA interacting protein [Source:HGNC Symbol;Acc:HGNC:21355] |
| ENSG00000146414 | HGLibA_44080 | 267 | 1.508325034 | SHPRH | SNF2 histone linker PHD RING helicase [Source:HGNC Symbol;Acc:HGNC:19336] |
| ENSG00000148795 | HGLibA_12060 | 267 | 1.508325034 | CYP17A1 | cytochrome P450 family 17 subfamily A member 1 [Source:HGNC Symbol;Acc:HGNC:2593] |
| ENSG00000148948 | HGLibA_27384 | 267 | 1.508325034 | LRRC4C | leucine rich repeat containing 4C [Source:HGNC Symbol;Acc:HGNC:29317] |
| ENSG00000149609 | HGLibA_05991 | 267 | 1.508325034 | C20orf144 | chromosome 20 open reading frame 144 [Source:HGNC Symbol;Acc:HGNC:16137] |
| ENSG00000152582 | HGLibA_46612 | 267 | 1.508325034 | SPEF2 | sperm flagellar 2 [Source:HGNC Symbol;Acc:HGNC:26293] |
| ENSG00000154099 | HGLibA_13460 | 267 | 1.508325034 | DNAAF1 | dynein axonemal assembly factor 1 [Source:HGNC Symbol;Acc:HGNC:30539] |
| ENSG00000154611 | HGLibA_39090 | 267 | 1.508325034 | PSMA8 | proteasome subunit alpha 8 [Source:HGNC Symbol;Acc:HGNC:22985] |
| ENSG00000159733 | HGLibA_55292 | 267 | 1.508325034 | ZFYVE28 | zinc finger FYVE-type containing 28 [Source:HGNC Symbol;Acc:HGNC:29334] |
| ENSG00000163083 | HGLibA_23303 | 267 | 1.508325034 | INHBB | inhibin subunit beta B [Source:HGNC Symbol;Acc:HGNC:6067] |
| ENSG00000163288 | HGLibA_18374 | 267 | 1.508325034 | GABRB1 | gamma-aminobutyric acid type A receptor beta1 subunit [Source:HGNC Symbol;Acc:HGNC:4081] |
| ENSG00000163703 | HGLibA_11125 | 267 | 1.508325034 | CRELD1 | cysteine rich with EGF like domains 1 [Source:HGNC Symbol;Acc:HGNC:14630] |
| ENSG00000166913 | HGLibA_54739 | 267 | 1.508325034 | YWHAB | tyrosine 3-monooxygenase/tryptophan 5-monooxygenase activation protein beta [Source:HGNC Symbol;Acc:HGNC:12849] |
| ENSG00000170667 | HGLibA_40308 | 267 | 1.508325034 | RASA4B | RAS p21 protein activator 4B [Source:HGNC Symbol;Acc:HGNC:35202] |
| ENSG00000173198 | HGLibA_12226 | 267 | 1.508325034 | CYSLTR1 | cysteinyl leukotriene receptor 1 [Source:HGNC Symbol;Acc:HGNC:17451] |
| ENSG00000174529 | HGLibA_50520 | 267 | 1.508325034 | TMEM81 | transmembrane protein 81 [Source:HGNC Symbol;Acc:HGNC:32349] |
| ENSG00000177830 | HGLibA_09450 | 267 | 1.508325034 | CHID1 | chitinase domain containing 1 [Source:HGNC Symbol;Acc:HGNC:28474] |
| ENSG00000181191 | HGLibA_36784 | 267 | 1.508325034 | PJA1 | praja ring finger ubiquitin ligase 1 [Source:HGNC Symbol;Acc:HGNC:16648] |
| ENSG00000182533 | HGLibA_07381 | 267 | 1.508325034 | CAV3 | caveolin 3 [Source:HGNC Symbol;Acc:HGNC:1529] |
| ENSG00000184060 | HGLibA_00866 | 267 | 1.508325034 | ADAP2 | ArfGAP with dual PH domains 2 [Source:HGNC Symbol;Acc:HGNC:16487] |
| ENSG00000196119 | HGLibA_34491 | 267 | 1.508325034 | OR8A1 | olfactory receptor family 8 subfamily A member 1 [Source:HGNC Symbol;Acc:HGNC:8469] |
| ENSG00000196388 | HGLibA_23273 | 267 | 1.508325034 | INCA1 | cyclin A1 interacting protein 1 [Source:HGNC Symbol;Acc:HGNC:32224]" |
| ENSG00000198488 | HGLibA_03997 | 267 | 1.508325034 | B3GNT6 | 3-N-acetylglucosaminyltransferase 6 [Source:HGNC Symbol;Acc:HGNC:24141]" |
| ENSG00000217128 | HGLibA_17764 | 267 | 1.508325034 | FNIP1 | folliculin interacting protein 1 [Source:HGNC Symbol;Acc:HGNC:29418] |
| ENSG00000242441 | HGLibA_20523 | 267 | 1.508325034 | GTF2A1L | general transcription factor IIA subunit 1 like [Source:HGNC Symbol;Acc:HGNC:30727] |
| ENSG00000258947 | HGLibA_52281 | 267 | 1.508325034 | TUBB3 | tubulin beta 3 class III [Source:HGNC Symbol;Acc:HGNC:20772] |
| hsa-mir-1343 | HGLibA_57788 | 267 | 1.508325034 | hsa-mir-1343 | - |
| hsa-mir-34c | HGLibA_59044 | 267 | 1.508325034 | hsa-mir-34c | - |
| hsa-mir-378h | HGLibA_59456 | 267 | 1.508325034 | hsa-mir-378h | - |
| hsa-mir-6789 | HGLibA_63332 | 267 | 1.508325034 | hsa-mir-6789 | - |
| ENSG00000011105 | HGLibA_51952 | 266 | 1.498373996 | TSPAN9 | tetraspanin 9 [Source:HGNC Symbol;Acc:HGNC:21640] |
| ENSG00000054983 | HGLibA_18508 | 266 | 1.498373996 | GALC | galactosylceramidase [Source:HGNC Symbol;Acc:HGNC:4115] |
| ENSG00000067992 | HGLibA_35901 | 266 | 1.498373996 | PDK3 | pyruvate dehydrogenase kinase 3 [Source:HGNC Symbol;Acc:HGNC:8811] |
| ENSG00000079134 | HGLibA_49363 | 266 | 1.498373996 | THOC1 | THO complex 1 [Source:HGNC Symbol;Acc:HGNC:19070] |
| ENSG00000105486 | HGLibA_26480 | 266 | 1.498373996 | LIG1 | DNA ligase 1 [Source:HGNC Symbol;Acc:HGNC:6598] |
| ENSG00000113532 | HGLibA_47298 | 266 | 1.498373996 | ST8SIA4 | 8-sialyltransferase 4 [Source:HGNC Symbol;Acc:HGNC:10871]" |
| ENSG00000114204 | HGLibA_43601 | 266 | 1.498373996 | SERPINI2 | serpin family I member 2 [Source:HGNC Symbol;Acc:HGNC:8945] |
| ENSG00000123901 | HGLibA_20093 | 266 | 1.498373996 | GPR83 | G protein-coupled receptor 83 [Source:HGNC Symbol;Acc:HGNC:4523] |
| ENSG00000126453 | HGLibA_04311 | 266 | 1.498373996 | BCL2L12 | BCL2 like 12 [Source:HGNC Symbol;Acc:HGNC:13787] |
| ENSG00000133863 | HGLibA_49066 | 266 | 1.498373996 | TEX15 | meiosis and synapsis associated [Source:HGNC Symbol;Acc:HGNC:11738]" |
| ENSG00000134438 | HGLibA_40406 | 266 | 1.498373996 | RAX | retina and anterior neural fold homeobox [Source:HGNC Symbol;Acc:HGNC:18662] |
| ENSG00000134899 | HGLibA_15444 | 266 | 1.498373996 | ERCC5 | endonuclease [Source:HGNC Symbol;Acc:HGNC:3437]" |
| ENSG00000135248 | HGLibA_16702 | 266 | 1.498373996 | FAM71F1 | family with sequence similarity 71 member F1 [Source:HGNC Symbol;Acc:HGNC:30704] |
| ENSG00000139531 | HGLibA_47801 | 266 | 1.498373996 | SUOX | sulfite oxidase [Source:HGNC Symbol;Acc:HGNC:11460] |
| ENSG00000141744 | HGLibA_37391 | 266 | 1.498373996 | PNMT | phenylethanolamine N-methyltransferase [Source:HGNC Symbol;Acc:HGNC:9160] |
| ENSG00000143476 | HGLibA_14060 | 266 | 1.498373996 | DTL | denticleless E3 ubiquitin protein ligase homolog [Source:HGNC Symbol;Acc:HGNC:30288] |
| ENSG00000145348 | HGLibA_48599 | 266 | 1.498373996 | TBCK | TBC1 domain containing kinase [Source:HGNC Symbol;Acc:HGNC:28261] |
| ENSG00000153066 | HGLibA_52404 | 266 | 1.498373996 | TXNDC11 | thioredoxin domain containing 11 [Source:HGNC Symbol;Acc:HGNC:28030] |
| ENSG00000164600 | HGLibA_31744 | 266 | 1.498373996 | NEUROD6 | neuronal differentiation 6 [Source:HGNC Symbol;Acc:HGNC:13804] |
| ENSG00000167394 | HGLibA_56494 | 266 | 1.498373996 | ZNF668 | zinc finger protein 668 [Source:HGNC Symbol;Acc:HGNC:25821] |
| ENSG00000169057 | HGLibA_28728 | 266 | 1.498373996 | MECP2 | methyl-CpG binding protein 2 [Source:HGNC Symbol;Acc:HGNC:6990] |
| ENSG00000174343 | HGLibA_09587 | 266 | 1.498373996 | CHRNA9 | cholinergic receptor nicotinic alpha 9 subunit [Source:HGNC Symbol;Acc:HGNC:14079] |
| ENSG00000174939 | HGLibA_03354 | 266 | 1.498373996 | ASPHD1 | aspartate beta-hydroxylase domain containing 1 [Source:HGNC Symbol;Acc:HGNC:27380] |
| ENSG00000178722 | HGLibA_06422 | 266 | 1.498373996 | C5orf64 | chromosome 5 open reading frame 64 [Source:HGNC Symbol;Acc:HGNC:26744] |
| ENSG00000183034 | HGLibA_34737 | 266 | 1.498373996 | OTOP2 | otopetrin 2 [Source:HGNC Symbol;Acc:HGNC:19657] |
| ENSG00000183258 | HGLibA_12711 | 266 | 1.498373996 | DDX41 | DEAD-box helicase 41 [Source:HGNC Symbol;Acc:HGNC:18674] |
| ENSG00000183273 | HGLibA_07815 | 266 | 1.498373996 | CCDC60 | coiled-coil domain containing 60 [Source:HGNC Symbol;Acc:HGNC:28610] |
| ENSG00000186510 | HGLibA_09841 | 266 | 1.498373996 | CLCNKA | chloride voltage-gated channel Ka [Source:HGNC Symbol;Acc:HGNC:2026] |
| ENSG00000187049 | HGLibA_50221 | 266 | 1.498373996 | TMEM216 | transmembrane protein 216 [Source:HGNC Symbol;Acc:HGNC:25018] |
| ENSG00000187082 | HGLibA_12833 | 266 | 1.498373996 | DEFB106B | defensin beta 106B [Source:HGNC Symbol;Acc:HGNC:28879] |
| ENSG00000228198 | HGLibA_33836 | 266 | 1.498373996 | OR2M3 | olfactory receptor family 2 subfamily M member 3 [Source:HGNC Symbol;Acc:HGNC:8269] |
| FAM134C | HGLibA_16129 | 266 | 1.498373996 | FAM134C | - |
| hsa-mir-4531 | HGLibA_60585 | 266 | 1.498373996 | hsa-mir-4531 | - |
| hsa-mir-4634 | HGLibA_60641 | 266 | 1.498373996 | hsa-mir-4634 | - |
| hsa-mir-6791 | HGLibA_63341 | 266 | 1.498373996 | hsa-mir-6791 | - |
| hsa-mir-877 | HGLibA_64218 | 266 | 1.498373996 | hsa-mir-877 | - |
| hsa-mir-941-1 | HGLibA_64338 | 266 | 1.498373996 | hsa-mir-941-1 | - |
| SELV | HGLibA_43316 | 266 | 1.498373996 | SELV | - |
| SPRY3_Y | HGLibA_46845 | 266 | 1.498373996 | SPRY3_Y | - |
| TMEM246 | HGLibA_50302 | 266 | 1.498373996 | TMEM246 | - |
| DOPEY2 | HGLibA_13802 | 265 | 1.488422959 | DOPEY2 | - |
| ENSG00000029534 | HGLibA_01933 | 265 | 1.488422959 | ANK1 | ankyrin 1 [Source:HGNC Symbol;Acc:HGNC:492] |
| ENSG00000070886 | HGLibA_15307 | 265 | 1.488422959 | EPHA8 | EPH receptor A8 [Source:HGNC Symbol;Acc:HGNC:3391] |
| ENSG00000074071 | HGLibA_30095 | 265 | 1.488422959 | MRPS34 | mitochondrial ribosomal protein S34 [Source:HGNC Symbol;Acc:HGNC:16618] |
| ENSG00000096746 | HGLibA_21829 | 265 | 1.488422959 | HNRNPH3 | heterogeneous nuclear ribonucleoprotein H3 [Source:HGNC Symbol;Acc:HGNC:5043] |
| ENSG00000101440 | HGLibA_03315 | 265 | 1.488422959 | ASIP | agouti signaling protein [Source:HGNC Symbol;Acc:HGNC:745] |
| ENSG00000106624 | HGLibA_01085 | 265 | 1.488422959 | AEBP1 | AE binding protein 1 [Source:HGNC Symbol;Acc:HGNC:303] |
| ENSG00000114480 | HGLibA_18718 | 265 | 1.488422959 | GBE1 | 4-alpha-glucan branching enzyme 1 [Source:HGNC Symbol;Acc:HGNC:4180]" |
| ENSG00000127364 | HGLibA_48400 | 265 | 1.488422959 | TAS2R4 | taste 2 receptor member 4 [Source:HGNC Symbol;Acc:HGNC:14911] |
| ENSG00000135974 | HGLibA_06141 | 265 | 1.488422959 | C2orf49 | chromosome 2 open reading frame 49 [Source:HGNC Symbol;Acc:HGNC:28772] |
| ENSG00000137819 | HGLibA_35164 | 265 | 1.488422959 | PAQR5 | progestin and adipoQ receptor family member 5 [Source:HGNC Symbol;Acc:HGNC:29645] |
| ENSG00000141756 | HGLibA_17552 | 265 | 1.488422959 | FKBP10 | FK506 binding protein 10 [Source:HGNC Symbol;Acc:HGNC:18169] |
| ENSG00000143632 | HGLibA_00597 | 265 | 1.488422959 | ACTA1 | skeletal muscle [Source:HGNC Symbol;Acc:HGNC:129]" |
| ENSG00000145879 | HGLibA_46702 | 265 | 1.488422959 | SPINK7 | Kazal type 7 (putative) [Source:HGNC Symbol;Acc:HGNC:24643]" |
| ENSG00000147573 | HGLibA_51546 | 265 | 1.488422959 | TRIM55 | tripartite motif containing 55 [Source:HGNC Symbol;Acc:HGNC:14215] |
| ENSG00000149150 | HGLibA_45149 | 265 | 1.488422959 | SLC43A1 | solute carrier family 43 member 1 [Source:HGNC Symbol;Acc:HGNC:9225] |
| ENSG00000152082 | HGLibA_30947 | 265 | 1.488422959 | MZT2B | mitotic spindle organizing protein 2B [Source:HGNC Symbol;Acc:HGNC:25886] |
| ENSG00000152495 | HGLibA_07084 | 265 | 1.488422959 | CAMK4 | calcium/calmodulin dependent protein kinase IV [Source:HGNC Symbol;Acc:HGNC:1464] |
| ENSG00000156463 | HGLibA_43972 | 265 | 1.488422959 | SH3RF2 | SH3 domain containing ring finger 2 [Source:HGNC Symbol;Acc:HGNC:26299] |
| ENSG00000157578 | HGLibA_26093 | 265 | 1.488422959 | LCA5L | lebercilin like [Source:HGNC Symbol;Acc:HGNC:1255]" |
| ENSG00000162873 | HGLibA_25110 | 265 | 1.488422959 | KLHDC8A | kelch domain containing 8A [Source:HGNC Symbol;Acc:HGNC:25573] |
| ENSG00000163554 | HGLibA_46871 | 265 | 1.488422959 | SPTA1 | erythrocytic 1 [Source:HGNC Symbol;Acc:HGNC:11272]" |
| ENSG00000165985 | HGLibA_05931 | 265 | 1.488422959 | C1QL3 | complement C1q like 3 [Source:HGNC Symbol;Acc:HGNC:19359] |
| ENSG00000170638 | HGLibA_51229 | 265 | 1.488422959 | TRABD | TraB domain containing [Source:HGNC Symbol;Acc:HGNC:28805] |
| ENSG00000171433 | HGLibA_19290 | 265 | 1.488422959 | GLOD5 | glyoxalase domain containing 5 [Source:HGNC Symbol;Acc:HGNC:33358] |
| ENSG00000172171 | HGLibA_48964 | 265 | 1.488422959 | TEFM | mitochondrial [Source:HGNC Symbol;Acc:HGNC:26223]" |
| ENSG00000173404 | HGLibA_23395 | 265 | 1.488422959 | INSM1 | INSM transcriptional repressor 1 [Source:HGNC Symbol;Acc:HGNC:6090] |
| ENSG00000183665 | HGLibA_51676 | 265 | 1.488422959 | TRMT12 | tRNA methyltransferase 12 homolog [Source:HGNC Symbol;Acc:HGNC:26091] |
| ENSG00000185215 | HGLibA_50710 | 265 | 1.488422959 | TNFAIP2 | TNF alpha induced protein 2 [Source:HGNC Symbol;Acc:HGNC:11895] |
| ENSG00000186867 | HGLibA_39740 | 265 | 1.488422959 | QRFPR | pyroglutamylated RFamide peptide receptor [Source:HGNC Symbol;Acc:HGNC:15565] |
| ENSG00000186867 | HGLibA_39739 | 265 | 1.488422959 | QRFPR | pyroglutamylated RFamide peptide receptor [Source:HGNC Symbol;Acc:HGNC:15565] |
| ENSG00000187609 | HGLibA_15723 | 265 | 1.488422959 | EXD3 | exonuclease 3'-5' domain containing 3 [Source:HGNC Symbol;Acc:HGNC:26023] |
| ENSG00000198839 | HGLibA_55718 | 265 | 1.488422959 | ZNF277 | zinc finger protein 277 [Source:HGNC Symbol;Acc:HGNC:13070] |
| ENSG00000204116 | HGLibA_09444 | 265 | 1.488422959 | CHIC1 | cysteine rich hydrophobic domain 1 [Source:HGNC Symbol;Acc:HGNC:1934] |
| ENSG00000204514 | HGLibA_56808 | 265 | 1.488422959 | ZNF814 | zinc finger protein 814 [Source:HGNC Symbol;Acc:HGNC:33258] |
| ENSG00000204970 | HGLibA_35423 | 265 | 1.488422959 | PCDHA1 | protocadherin alpha 1 [Source:HGNC Symbol;Acc:HGNC:8663] |
| ENSG00000221859 | HGLibA_25595 | 265 | 1.488422959 | KRTAP10-10 | keratin associated protein 10-10 [Source:HGNC Symbol;Acc:HGNC:22972] |
| ENSG00000244462 | HGLibA_40478 | 265 | 1.488422959 | RBM12 | RNA binding motif protein 12 [Source:HGNC Symbol;Acc:HGNC:9898] |
| ENSG00000255587 | HGLibA_39951 | 265 | 1.488422959 | RAB44 | member RAS oncogene family [Source:HGNC Symbol;Acc:HGNC:21068]" |
| ENSG00000258992 | HGLibA_51964 | 265 | 1.488422959 | TSPY1 | testis specific protein Y-linked 1 [Source:HGNC Symbol;Acc:HGNC:12381] |
| ENSG00000259332 | HGLibA_47235 | 265 | 1.488422959 | ST20-MTHFS | ST20-MTHFS readthrough [Source:HGNC Symbol;Acc:HGNC:44655] |
| ENSG00000267909 | HGLibA_07711 | 265 | 1.488422959 | CCDC177 | coiled-coil domain containing 177 [Source:HGNC Symbol;Acc:HGNC:23243] |
| ENSG00000269405 | HGLibA_33130 | 265 | 1.488422959 | NXF2 | nuclear RNA export factor 2 [Source:HGNC Symbol;Acc:HGNC:8072] |
| HRSP12 | HGLibA_22111 | 265 | 1.488422959 | HRSP12 | - |
| hsa-mir-4675 | HGLibA_60818 | 265 | 1.488422959 | hsa-mir-4675 | - |
| hsa-mir-7977 | HGLibA_64036 | 265 | 1.488422959 | hsa-mir-7977 | - |
| LOC100506388 | HGLibA_26858 | 265 | 1.488422959 | LOC100506388 | - |
| PLAC1L | HGLibA_36936 | 265 | 1.488422959 | PLAC1L | - |
| TMEM8A | HGLibA_50546 | 265 | 1.488422959 | TMEM8A | - |
| C19orf80 | HGLibA_05710 | 264 | 1.478471921 | C19orf80 | - |
| ENSG00000097007 | HGLibA_00302 | 264 | 1.478471921 | ABL1 | non-receptor tyrosine kinase [Source:HGNC Symbol;Acc:HGNC:76]" |

| | | | | | |
|---|---|---|---|---|---|
| ENSG00000109519 | HGLibA_20370 | 264 | 1.478471921 | GRPEL1 | mitochondrial [Source:HGNC Symbol;Acc:HGNC:19696]" |
| ENSG00000112787 | HGLibA_16970 | 264 | 1.478471921 | FBRSL1 | fibrosin like 1 [Source:HGNC Symbol;Acc:HGNC:29308] |
| ENSG00000113946 | HGLibA_09864 | 264 | 1.478471921 | CLDN16 | claudin 16 [Source:HGNC Symbol;Acc:HGNC:2037] |
| ENSG00000115468 | HGLibA_14576 | 264 | 1.478471921 | EFHD1 | EF-hand domain family member D1 [Source:HGNC Symbol;Acc:HGNC:29556] |
| ENSG00000115468 | HGLibA_14575 | 264 | 1.478471921 | EFHD1 | EF-hand domain family member D1 [Source:HGNC Symbol;Acc:HGNC:29556] |
| ENSG00000130733 | HGLibA_54672 | 264 | 1.478471921 | YIPF2 | Yip1 domain family member 2 [Source:HGNC Symbol;Acc:HGNC:28476] |
| ENSG00000131668 | HGLibA_04165 | 264 | 1.478471921 | BARX1 | BARX homeobox 1 [Source:HGNC Symbol;Acc:HGNC:955] |
| ENSG00000147140 | HGLibA_32299 | 264 | 1.478471921 | NONO | non-POU domain containing octamer binding [Source:HGNC Symbol;Acc:HGNC:7871] |
| ENSG00000153303 | HGLibA_18009 | 264 | 1.478471921 | FRMD1 | FERM domain containing 1 [Source:HGNC Symbol;Acc:HGNC:21240] |
| ENSG00000153823 | HGLibA_36528 | 264 | 1.478471921 | PID1 | phosphotyrosine interaction domain containing 1 [Source:HGNC Symbol;Acc:HGNC:26084] |
| ENSG00000164114 | HGLibA_28243 | 264 | 1.478471921 | MAP9 | microtubule associated protein 9 [Source:HGNC Symbol;Acc:HGNC:26118] |
| ENSG00000164185 | HGLibA_56061 | 264 | 1.478471921 | ZNF474 | zinc finger protein 474 [Source:HGNC Symbol;Acc:HGNC:23245] |
| ENSG00000166949 | HGLibA_45574 | 264 | 1.478471921 | SMAD3 | SMAD family member 3 [Source:HGNC Symbol;Acc:HGNC:6769] |
| ENSG00000167985 | HGLibA_43138 | 264 | 1.478471921 | SDHAF2 | succinate dehydrogenase complex assembly factor 2 [Source:HGNC Symbol;Acc:HGNC:26034] |
| ENSG00000169071 | HGLibA_41773 | 264 | 1.478471921 | ROR2 | receptor tyrosine kinase like orphan receptor 2 [Source:HGNC Symbol;Acc:HGNC:10257] |
| ENSG00000176136 | HGLibA_28554 | 264 | 1.478471921 | MC5R | melanocortin 5 receptor [Source:HGNC Symbol;Acc:HGNC:6933] |
| ENSG00000179094 | HGLibA_36054 | 264 | 1.478471921 | PER1 | period circadian regulator 1 [Source:HGNC Symbol;Acc:HGNC:8845] |
| ENSG00000183638 | HGLibA_41791 | 264 | 1.478471921 | RP1L1 | RP1 like 1 [Source:HGNC Symbol;Acc:HGNC:15946] |
| ENSG00000187689 | HGLibA_01839 | 264 | 1.478471921 | AMTN | amelotin [Source:HGNC Symbol;Acc:HGNC:33188] |
| ENSG00000196793 | HGLibA_55655 | 264 | 1.478471921 | ZNF239 | zinc finger protein 239 [Source:HGNC Symbol;Acc:HGNC:13031] |
| ENSG00000221843 | HGLibA_06114 | 264 | 1.478471921 | C2orf16 | chromosome 2 open reading frame 16 [Source:HGNC Symbol;Acc:HGNC:25275] |
| ENSG00000239389 | HGLibA_35435 | 264 | 1.478471921 | PCDHA13 | protocadherin alpha 13 [Source:HGNC Symbol;Acc:HGNC:8667] |
| ENSG00000260691 | HGLibA_02026 | 264 | 1.478471921 | ANKRD20A1 | ankyrin repeat domain 20 family member A1 [Source:HGNC Symbol;Acc:HGNC:23665] |
| hsa-mir-135a-1 | HGLibA_57791 | 264 | 1.478471921 | hsa-mir-135a-1 | - |
| hsa-mir-3922 | HGLibA_59563 | 264 | 1.478471921 | hsa-mir-3922 | - |
| hsa-mir-4260 | HGLibA_59747 | 264 | 1.478471921 | hsa-mir-4260 | - |
| hsa-mir-4288 | HGLibA_59861 | 264 | 1.478471921 | hsa-mir-4288 | - |
| 10-Sep | HGLibA_43426 | 264 | 1.478471921 | 10-Sep | - |
| ENSG00000050165 | HGLibA_13301 | 263 | 1.468520884 | DKK3 | dickkopf WNT signaling pathway inhibitor 3 [Source:HGNC Symbol;Acc:HGNC:2893] |
| ENSG00000065609 | HGLibA_45888 | 263 | 1.468520884 | SNAP91 | synaptosome associated protein 91 [Source:HGNC Symbol;Acc:HGNC:14986] |
| ENSG00000074755 | HGLibA_57091 | 263 | 1.468520884 | ZZEF1 | zinc finger ZZ-type and EF-hand domain containing 1 [Source:HGNC Symbol;Acc:HGNC:29027] |
| ENSG00000090975 | HGLibA_36754 | 263 | 1.468520884 | PITPNM2 | phosphatidylinositol transfer protein membrane associated 2 [Source:HGNC Symbol;Acc:HGNC:21044] |
| ENSG00000103489 | HGLibA_54618 | 263 | 1.468520884 | XYLT1 | xylosyltransferase 1 [Source:HGNC Symbol;Acc:HGNC:15516] |
| ENSG00000109083 | HGLibA_22753 | 263 | 1.468520884 | IFT20 | intraflagellar transport 20 [Source:HGNC Symbol;Acc:HGNC:30989] |
| ENSG00000110218 | HGLibA_35108 | 263 | 1.468520884 | PANX1 | pannexin 1 [Source:HGNC Symbol;Acc:HGNC:8599] |
| ENSG00000112812 | HGLibA_38906 | 263 | 1.468520884 | PRSS16 | serine protease 16 [Source:HGNC Symbol;Acc:HGNC:9480] |
| ENSG00000119888 | HGLibA_15271 | 263 | 1.468520884 | EPCAM | epithelial cell adhesion molecule [Source:HGNC Symbol;Acc:HGNC:11529] |
| ENSG00000122299 | HGLibA_54982 | 263 | 1.468520884 | ZC3H7A | zinc finger CCCH-type containing 7A [Source:HGNC Symbol;Acc:HGNC:30959] |
| ENSG00000128791 | HGLibA_52383 | 263 | 1.468520884 | TWSG1 | twisted gastrulation BMP signaling modulator 1 [Source:HGNC Symbol;Acc:HGNC:12429] |
| ENSG00000133116 | HGLibA_25021 | 263 | 1.468520884 | KL | klotho [Source:HGNC Symbol;Acc:HGNC:6344] |
| ENSG00000135956 | HGLibA_49916 | 263 | 1.468520884 | TMEM127 | transmembrane protein 127 [Source:HGNC Symbol;Acc:HGNC:26038] |
| ENSG00000137699 | HGLibA_51454 | 263 | 1.468520884 | TRIM29 | tripartite motif containing 29 [Source:HGNC Symbol;Acc:HGNC:17274] |
| ENSG00000142233 | HGLibA_32861 | 263 | 1.468520884 | NTN5 | netrin 5 [Source:HGNC Symbol;Acc:HGNC:25208] |
| ENSG00000145309 | HGLibA_06863 | 263 | 1.468520884 | CABS1 | spermatid associated 1 [Source:HGNC Symbol;Acc:HGNC:30710]" |
| ENSG00000149305 | HGLibA_22382 | 263 | 1.468520884 | HTR3B | 5-hydroxytryptamine receptor 3B [Source:HGNC Symbol;Acc:HGNC:5298] |
| ENSG00000151468 | HGLibA_07751 | 263 | 1.468520884 | CCDC3 | coiled-coil domain containing 3 [Source:HGNC Symbol;Acc:HGNC:23813] |
| ENSG00000154553 | HGLibA_35912 | 263 | 1.468520884 | PDLIM3 | PDZ and LIM domain 3 [Source:HGNC Symbol;Acc:HGNC:20767] |
| ENSG00000163072 | HGLibA_32338 | 263 | 1.468520884 | NOSTRIN | nitric oxide synthase trafficking [Source:HGNC Symbol;Acc:HGNC:20203] |
| ENSG00000168026 | HGLibA_52069 | 263 | 1.468520884 | TTC21A | tetratricopeptide repeat domain 21A [Source:HGNC Symbol;Acc:HGNC:30761] |
| ENSG00000169926 | HGLibA_25051 | 263 | 1.468520884 | KLF13 | Kruppel like factor 13 [Source:HGNC Symbol;Acc:HGNC:13672] |
| ENSG00000174611 | HGLibA_25878 | 263 | 1.468520884 | KY | kyphoscoliosis peptidase [Source:HGNC Symbol;Acc:HGNC:26576] |
| ENSG00000175029 | HGLibA_11588 | 263 | 1.468520884 | CTBP2 | C-terminal binding protein 2 [Source:HGNC Symbol;Acc:HGNC:2495] |
| ENSG00000175482 | HGLibA_37500 | 263 | 1.468520884 | POLD4 | accessory subunit [Source:HGNC Symbol;Acc:HGNC:14106]" |
| ENSG00000177294 | HGLibA_17087 | 263 | 1.468520884 | FBXO39 | F-box protein 39 [Source:HGNC Symbol;Acc:HGNC:28565] |
| ENSG00000183395 | HGLibA_37294 | 263 | 1.468520884 | PMCH | pro-melanin concentrating hormone [Source:HGNC Symbol;Acc:HGNC:9109] |
| ENSG00000185634 | HGLibA_44008 | 263 | 1.468520884 | SHC4 | SHC adaptor protein 4 [Source:HGNC Symbol;Acc:HGNC:16743] |
| ENSG00000185924 | HGLibA_42415 | 263 | 1.468520884 | RTN4RL1 | reticulon 4 receptor like 1 [Source:HGNC Symbol;Acc:HGNC:21329] |
| ENSG00000188672 | HGLibA_41145 | 263 | 1.468520884 | RHCE | Rh blood group CcEe antigens [Source:HGNC Symbol;Acc:HGNC:10008] |
| ENSG00000188784 | HGLibA_36891 | 263 | 1.468520884 | PLA2G2E | phospholipase A2 group IIE [Source:HGNC Symbol;Acc:HGNC:13414] |
| ENSG00000217555 | HGLibA_09768 | 263 | 1.468520884 | CKLF | chemokine like factor [Source:HGNC Symbol;Acc:HGNC:13253] |
| ENSG00000259384 | HGLibA_19027 | 263 | 1.468520884 | GH1 | growth hormone 1 [Source:HGNC Symbol;Acc:HGNC:4261] |
| ENSG00000267041 | HGLibA_56871 | 263 | 1.468520884 | ZNF850 | zinc finger protein 850 [Source:HGNC Symbol;Acc:HGNC:27994] |
| hsa-mir-103a-2 | HGLibA_57164 | 263 | 1.468520884 | hsa-mir-103a-2 | - |
| hsa-mir-4655 | HGLibA_60729 | 263 | 1.468520884 | hsa-mir-4655 | - |
| hsa-mir-516b-1 | HGLibA_61615 | 263 | 1.468520884 | hsa-mir-516b-1 | - |
| hsa-mir-6749 | HGLibA_63150 | 263 | 1.468520884 | hsa-mir-6749 | - |
| hsa-mir-8075 | HGLibA_64141 | 263 | 1.468520884 | hsa-mir-8075 | - |
| KIAA1024 | HGLibA_24679 | 263 | 1.468520884 | KIAA1024 | - |
| C17orf72 | HGLibA_05568 | 262 | 1.458569847 | C17orf72 | - |
| ENSG00000010379 | HGLibA_45299 | 262 | 1.458569847 | SLC6A13 | solute carrier family 6 member 13 [Source:HGNC Symbol;Acc:HGNC:11046] |
| ENSG00000012660 | HGLibA_14970 | 262 | 1.458569847 | ELOVL5 | ELOVL fatty acid elongase 5 [Source:HGNC Symbol;Acc:HGNC:21308] |
| ENSG00000089063 | HGLibA_50254 | 262 | 1.458569847 | TMEM230 | transmembrane protein 230 [Source:HGNC Symbol;Acc:HGNC:15876] |
| ENSG00000103335 | HGLibA_36533 | 262 | 1.458569847 | PIEZO1 | piezo type mechanosensitive ion channel component 1 [Source:HGNC Symbol;Acc:HGNC:28993] |
| ENSG00000118507 | HGLibA_01443 | 262 | 1.458569847 | AKAP7 | A-kinase anchoring protein 7 [Source:HGNC Symbol;Acc:HGNC:377] |
| ENSG00000125877 | HGLibA_23837 | 262 | 1.458569847 | ITPA | inosine triphosphatase [Source:HGNC Symbol;Acc:HGNC:6176] |
| ENSG00000126759 | HGLibA_09290 | 262 | 1.458569847 | CFP | complement factor properdin [Source:HGNC Symbol;Acc:HGNC:8864] |
| ENSG00000130222 | HGLibA_18421 | 262 | 1.458569847 | GADD45G | growth arrest and DNA damage inducible gamma [Source:HGNC Symbol;Acc:HGNC:4097] |
| ENSG00000130368 | HGLibA_28387 | 262 | 1.458569847 | MAS1 | G protein-coupled receptor [Source:HGNC Symbol;Acc:HGNC:6899]" |
| ENSG00000132854 | HGLibA_24032 | 262 | 1.458569847 | KANK4 | KN motif and ankyrin repeat domains 4 [Source:HGNC Symbol;Acc:HGNC:27263] |
| ENSG00000134759 | HGLibA_14978 | 262 | 1.458569847 | ELP2 | elongator acetyltransferase complex subunit 2 [Source:HGNC Symbol;Acc:HGNC:18248] |
| ENSG00000135218 | HGLibA_08359 | 262 | 1.458569847 | CD36 | CD36 molecule [Source:HGNC Symbol;Acc:HGNC:1663] |
| ENSG00000135446 | HGLibA_08769 | 262 | 1.458569847 | CDK4 | cyclin dependent kinase 4 [Source:HGNC Symbol;Acc:HGNC:1773] |
| ENSG00000136560 | HGLibA_48304 | 262 | 1.458569847 | TANK | TRAF family member associated NFKB activator [Source:HGNC Symbol;Acc:HGNC:11562] |
| ENSG00000138078 | HGLibA_38440 | 262 | 1.458569847 | PREPL | prolyl endopeptidase like [Source:HGNC Symbol;Acc:HGNC:30228] |
| ENSG00000141562 | HGLibA_31143 | 262 | 1.458569847 | NARF | nuclear prelamin A recognition factor [Source:HGNC Symbol;Acc:HGNC:29916] |
| ENSG00000143437 | HGLibA_03063 | 262 | 1.458569847 | ARNT | aryl hydrocarbon receptor nuclear translocator [Source:HGNC Symbol;Acc:HGNC:700] |
| ENSG00000143537 | HGLibA_00738 | 262 | 1.458569847 | ADAM15 | ADAM metallopeptidase domain 15 [Source:HGNC Symbol;Acc:HGNC:193] |
| ENSG00000147059 | HGLibA_46670 | 262 | 1.458569847 | SPIN2A | spindlin family member 2A [Source:HGNC Symbol;Acc:HGNC:20694] |
| ENSG00000151632 | HGLibA_01483 | 262 | 1.458569847 | AKR1C2 | aldo-keto reductase family 1 member C2 [Source:HGNC Symbol;Acc:HGNC:385] |
| ENSG00000159200 | HGLibA_40669 | 262 | 1.458569847 | RCAN1 | regulator of calcineurin 1 [Source:HGNC Symbol;Acc:HGNC:3040] |
| ENSG00000160712 | HGLibA_23183 | 262 | 1.458569847 | IL6R | interleukin 6 receptor [Source:HGNC Symbol;Acc:HGNC:6019] |
| ENSG00000162510 | HGLibA_28428 | 262 | 1.458569847 | MATN1 | matrilin 1 [Source:HGNC Symbol;Acc:HGNC:6907] |
| ENSG00000162896 | HGLibA_36581 | 262 | 1.458569847 | PIGR | polymeric immunoglobulin receptor [Source:HGNC Symbol;Acc:HGNC:8968] |
| ENSG00000163071 | HGLibA_46439 | 262 | 1.458569847 | SPATA18 | spermatogenesis associated 18 [Source:HGNC Symbol;Acc:HGNC:29579] |
| ENSG00000166855 | HGLibA_10118 | 262 | 1.458569847 | CLPX | caseinolytic mitochondrial matrix peptidase chaperone subunit [Source:HGNC Symbol;Acc:HGNC:2088] |
| ENSG00000167716 | HGLibA_54186 | 262 | 1.458569847 | WDR81 | WD repeat domain 81 [Source:HGNC Symbol;Acc:HGNC:26600] |
| ENSG00000172927 | HGLibA_30690 | 262 | 1.458569847 | MYEOV | myeloma overexpressed [Source:HGNC Symbol;Acc:HGNC:7563] |
| ENSG00000175267 | HGLibA_53903 | 262 | 1.458569847 | VWA3A | von Willebrand factor A domain containing 3A [Source:HGNC Symbol;Acc:HGNC:27088] |
| ENSG00000176715 | HGLibA_00551 | 262 | 1.458569847 | ACSF3 | acyl-CoA synthetase family member 3 [Source:HGNC Symbol;Acc:HGNC:27288] |
| ENSG00000178860 | HGLibA_30183 | 262 | 1.458569847 | MSC | musculin [Source:HGNC Symbol;Acc:HGNC:7321] |
| ENSG00000182993 | HGLibA_05274 | 262 | 1.458569847 | C12orf60 | chromosome 12 open reading frame 60 [Source:HGNC Symbol;Acc:HGNC:28726] |
| ENSG00000183486 | HGLibA_30608 | 262 | 1.458569847 | MX2 | MX dynamin like GTPase 2 [Source:HGNC Symbol;Acc:HGNC:7533] |
| ENSG00000185238 | HGLibA_38625 | 262 | 1.458569847 | PRMT3 | protein arginine methyltransferase 3 [Source:HGNC Symbol;Acc:HGNC:30163] |
| ENSG00000186442 | HGLibA_25472 | 262 | 1.458569847 | KRT3 | keratin 3 [Source:HGNC Symbol;Acc:HGNC:6440] |
| ENSG00000186523 | HGLibA_16773 | 262 | 1.458569847 | FAM86B1 | family with sequence similarity 86 member B1 [Source:HGNC Symbol;Acc:HGNC:28268] |
| ENSG00000196092 | HGLibA_35307 | 262 | 1.458569847 | PAX5 | paired box 5 [Source:HGNC Symbol;Acc:HGNC:8619] |
| ENSG00000196361 | HGLibA_14884 | 262 | 1.458569847 | ELAVL3 | ELAV like RNA binding protein 3 [Source:HGNC Symbol;Acc:HGNC:3314] |
| ENSG00000197361 | HGLibA_17002 | 262 | 1.458569847 | FBXL22 | F-box and leucine rich repeat protein 22 [Source:HGNC Symbol;Acc:HGNC:27537] |
| ENSG00000197818 | HGLibA_45428 | 262 | 1.458569847 | SLC9A8 | solute carrier family 9 member A8 [Source:HGNC Symbol;Acc:HGNC:20728] |
| ENSG00000198910 | HGLibA_25883 | 262 | 1.458569847 | L1CAM | L1 cell adhesion molecule [Source:HGNC Symbol;Acc:HGNC:6470] |
| ENSG00000204962 | HGLibA_35457 | 262 | 1.458569847 | PCDHA8 | protocadherin alpha 8 [Source:HGNC Symbol;Acc:HGNC:8674] |
| ENSG00000221954 | HGLibA_33940 | 262 | 1.458569847 | OR4C12 | olfactory receptor family 4 subfamily C member 12 [Source:HGNC Symbol;Acc:HGNC:15168] |
| ENSG00000262664 | HGLibA_34789 | 262 | 1.458569847 | OVCA2 | serine hydrolase domain containing [Source:HGNC Symbol;Acc:HGNC:24203]" |
| ENSG00000273841 | HGLibA_48261 | 262 | 1.458569847 | TAF9 | TATA-box binding protein associated factor 9 [Source:HGNC Symbol;Acc:HGNC:11542] |
| FAM208B | HGLibA_16435 | 262 | 1.458569847 | FAM208B | - |
| GUCY1B3 | HGLibA_20658 | 262 | 1.458569847 | GUCY1B3 | - |
| hsa-mir-4323 | HGLibA_60014 | 262 | 1.458569847 | hsa-mir-4323 | - |
| hsa-mir-4428 | HGLibA_60100 | 262 | 1.458569847 | hsa-mir-4428 | - |
| hsa-mir-4504 | HGLibA_60444 | 262 | 1.458569847 | hsa-mir-4504 | - |
| hsa-mir-4519 | HGLibA_60518 | 262 | 1.458569847 | hsa-mir-4519 | - |
| OBFC1 | HGLibA_33222 | 262 | 1.458569847 | OBFC1 | - |
| OR9G9 | HGLibA_34574 | 262 | 1.458569847 | OR9G9 | - |
| ABP1 | HGLibA_00320 | 261 | 1.448618809 | ABP1 | - |
| BAGE | HGLibA_04093 | 261 | 1.448618809 | BAGE | - |
| DARC | HGLibA_12337 | 261 | 1.448618809 | DARC | - |
| ENSG00000005194 | HGLibA_09678 | 261 | 1.448618809 | CIAPIN1 | cytokine induced apoptosis inhibitor 1 [Source:HGNC Symbol;Acc:HGNC:28050] |
| ENSG00000059769 | HGLibA_13606 | 261 | 1.448618809 | DNAJC25 | DnaJ heat shock protein family (Hsp40) member C25 [Source:HGNC Symbol;Acc:HGNC:34187] |
| ENSG00000063180 | HGLibA_06792 | 261 | 1.448618809 | CA11 | carbonic anhydrase 11 [Source:HGNC Symbol;Acc:HGNC:1370] |
| ENSG00000072849 | HGLibA_12999 | 261 | 1.448618809 | DERL2 | derlin 2 [Source:HGNC Symbol;Acc:HGNC:17943] |
| ENSG00000085063 | HGLibA_08416 | 261 | 1.448618809 | CD59 | CD59 molecule (CD59 blood group) [Source:HGNC Symbol;Acc:HGNC:1689] |
| ENSG00000100341 | HGLibA_37418 | 261 | 1.448618809 | PNPLA5 | patatin like phospholipase domain containing 5 [Source:HGNC Symbol;Acc:HGNC:24888] |
| ENSG00000108417 | HGLibA_25495 | 261 | 1.448618809 | KRT37 | keratin 37 [Source:HGNC Symbol;Acc:HGNC:6455] |
| ENSG00000114993 | HGLibA_42389 | 261 | 1.448618809 | RTKN | rhotekin [Source:HGNC Symbol;Acc:HGNC:10466] |
| ENSG00000115165 | HGLibA_12246 | 261 | 1.448618809 | CYTIP | cytohesin 1 interacting protein [Source:HGNC Symbol;Acc:HGNC:9506] |
| ENSG00000120907 | HGLibA_01036 | 261 | 1.448618809 | ADRA1A | adrenoceptor alpha 1A [Source:HGNC Symbol;Acc:HGNC:277] |

| | | | | | |
|---|---|---|---|---|---|
| ENSG00000125430 | HGLibA_22129 | 261 | 1.448618809 | HS3ST3B1 | heparan sulfate-glucosamine 3-sulfotransferase 3B1 [Source:HGNC Symbol;Acc:HGNC:5198] |
| ENSG00000131116 | HGLibA_55970 | 261 | 1.448618809 | ZNF428 | zinc finger protein 428 [Source:HGNC Symbol;Acc:HGNC:20804] |
| ENSG00000135597 | HGLibA_40849 | 261 | 1.448618809 | REPS1 | RALBP1 associated Eps domain containing 1 [Source:HGNC Symbol;Acc:HGNC:15578] |
| ENSG00000135722 | HGLibA_17020 | 261 | 1.448618809 | FBXL8 | F-box and leucine rich repeat protein 8 [Source:HGNC Symbol;Acc:HGNC:17875] |
| ENSG00000136267 | HGLibA_13061 | 261 | 1.448618809 | DGKB | diacylglycerol kinase beta [Source:HGNC Symbol;Acc:HGNC:2850] |
| ENSG00000137393 | HGLibA_41528 | 261 | 1.448618809 | RNF144B | ring finger protein 144B [Source:HGNC Symbol;Acc:HGNC:21578] |
| ENSG00000137720 | HGLibA_05116 | 261 | 1.448618809 | C11orf1 | chromosome 11 open reading frame 1 [Source:HGNC Symbol;Acc:HGNC:1163] |
| ENSG00000140750 | HGLibA_02713 | 261 | 1.448618809 | ARHGAP17 | Rho GTPase activating protein 17 [Source:HGNC Symbol;Acc:HGNC:18239] |
| ENSG00000141401 | HGLibA_23247 | 261 | 1.448618809 | IMPA2 | inositol monophosphatase 2 [Source:HGNC Symbol;Acc:HGNC:6051] |
| ENSG00000141655 | HGLibA_50744 | 261 | 1.448618809 | TNFRSF11A | TNF receptor superfamily member 11a [Source:HGNC Symbol;Acc:HGNC:11908] |
| ENSG00000149591 | HGLibA_48270 | 261 | 1.448618809 | TAGLN | transgelin [Source:HGNC Symbol;Acc:HGNC:11553] |
| ENSG00000152076 | HGLibA_07866 | 261 | 1.448618809 | CCDC74B | coiled-coil domain containing 74B [Source:HGNC Symbol;Acc:HGNC:25267] |
| ENSG00000152102 | HGLibA_16250 | 261 | 1.448618809 | FAM168B | family with sequence similarity 168 member B [Source:HGNC Symbol;Acc:HGNC:27016] |
| ENSG00000153406 | HGLibA_32193 | 261 | 1.448618809 | NMRAL1 | NmrA like redox sensor 1 [Source:HGNC Symbol;Acc:HGNC:24987] |
| ENSG00000154764 | HGLibA_54383 | 261 | 1.448618809 | WNT7A | Wnt family member 7A [Source:HGNC Symbol;Acc:HGNC:12786] |
| ENSG00000154975 | HGLibA_06789 | 261 | 1.448618809 | CA10 | carbonic anhydrase 10 [Source:HGNC Symbol;Acc:HGNC:1369] |
| ENSG00000155744 | HGLibA_16083 | 261 | 1.448618809 | FAM126B | family with sequence similarity 126 member B [Source:HGNC Symbol;Acc:HGNC:28593] |
| ENSG00000160145 | HGLibA_24021 | 261 | 1.448618809 | KALRN | kalirin RhoGEF kinase [Source:HGNC Symbol;Acc:HGNC:4814] |
| ENSG00000160181 | HGLibA_49171 | 261 | 1.448618809 | TFF2 | trefoil factor 2 [Source:HGNC Symbol;Acc:HGNC:11756] |
| ENSG00000164104 | HGLibA_21692 | 261 | 1.448618809 | HMGB2 | high mobility group box 2 [Source:HGNC Symbol;Acc:HGNC:5000] |
| ENSG00000164794 | HGLibA_24401 | 261 | 1.448618809 | KCNV1 | potassium voltage-gated channel modifier subfamily V member 1 [Source:HGNC Symbol;Acc:HGNC:18861] |
| ENSG00000168703 | HGLibA_54255 | 261 | 1.448618809 | WFDC12 | WAP four-disulfide core domain 12 [Source:HGNC Symbol;Acc:HGNC:16115] |
| ENSG00000174527 | HGLibA_30841 | 261 | 1.448618809 | MYO1H | myosin IH [Source:HGNC Symbol;Acc:HGNC:13879] |
| ENSG00000185652 | HGLibA_32838 | 261 | 1.448618809 | NTF3 | neurotrophin 3 [Source:HGNC Symbol;Acc:HGNC:8023] |
| ENSG00000185775 | HGLibA_46482 | 261 | 1.448618809 | SPATA31A6 | SPATA31 subfamily A member 6 [Source:HGNC Symbol;Acc:HGNC:32006] |
| ENSG00000188393 | HGLibA_09962 | 261 | 1.448618809 | CLEC2A | C-type lectin domain family 2 member A [Source:HGNC Symbol;Acc:HGNC:24191] |
| ENSG00000188938 | HGLibA_16058 | 261 | 1.448618809 | FAM120AOS | family with sequence similarity 120A opposite strand [Source:HGNC Symbol;Acc:HGNC:23389] |
| ENSG00000197181 | HGLibA_36773 | 261 | 1.448618809 | PIWIL2 | piwi like RNA-mediated gene silencing 2 [Source:HGNC Symbol;Acc:HGNC:17644] |
| ENSG00000198252 | HGLibA_47687 | 261 | 1.448618809 | STYX | serine/threonine/tyrosine interacting protein [Source:HGNC Symbol;Acc:HGNC:11447] |
| ENSG00000204021 | HGLibA_26624 | 261 | 1.448618809 | LIPK | lipase family member K [Source:HGNC Symbol;Acc:HGNC:23444] |
| ENSG00000260325 | HGLibA_22321 | 261 | 1.448618809 | HSPB9 | heat shock protein family B (small) member 9 [Source:HGNC Symbol;Acc:HGNC:30589] |
| ENSG00000279301 | HGLibA_33856 | 261 | 1.448618809 | OR2T11 | olfactory receptor family 2 subfamily T member 11 (gene/pseudogene) [Source:HGNC Symbol;Acc:HGNC:19574] |
| FAM160A1 | HGLibA_16203 | 261 | 1.448618809 | FAM160A1 | - |
| hsa-mir-3938 | HGLibA_59613 | 261 | 1.448618809 | hsa-mir-3938 | - |
| hsa-mir-4455 | HGLibA_60226 | 261 | 1.448618809 | hsa-mir-4455 | - |
| hsa-mir-4665 | HGLibA_60772 | 261 | 1.448618809 | hsa-mir-4665 | - |
| hsa-mir-4668 | HGLibA_60790 | 261 | 1.448618809 | hsa-mir-4668 | - |
| hsa-mir-525 | HGLibA_61786 | 261 | 1.448618809 | hsa-mir-525 | - |
| hsa-mir-548i-2 | HGLibA_62016 | 261 | 1.448618809 | hsa-mir-548i-2 | - |
| hsa-mir-6754 | HGLibA_63172 | 261 | 1.448618809 | hsa-mir-6754 | - |
| hsa-mir-6797 | HGLibA_63365 | 261 | 1.448618809 | hsa-mir-6797 | - |
| LPPR3 | HGLibA_27118 | 261 | 1.448618809 | LPPR3 | - |
| PPP5D1 | HGLibA_38216 | 261 | 1.448618809 | PPP5D1 | - |
| ENSG00000071051 | HGLibA_31307 | 260 | 1.438667772 | NCK2 | NCK adaptor protein 2 [Source:HGNC Symbol;Acc:HGNC:7665] |
| ENSG00000073584 | HGLibA_45637 | 260 | 1.438667772 | SMARCE1 | member 1 [Source:HGNC Symbol;Acc:HGNC:11109]" |
| ENSG00000095777 | HGLibA_30845 | 260 | 1.438667772 | MYO3A | myosin IIIA [Source:HGNC Symbol;Acc:HGNC:7601] |
| ENSG00000099899 | HGLibA_51684 | 260 | 1.438667772 | TRMT2A | tRNA methyltransferase 2 homolog A [Source:HGNC Symbol;Acc:HGNC:24974] |
| ENSG00000100558 | HGLibA_37058 | 260 | 1.438667772 | PLEK2 | pleckstrin 2 [Source:HGNC Symbol;Acc:HGNC:19238] |
| ENSG00000100652 | HGLibA_44330 | 260 | 1.438667772 | SLC10A1 | solute carrier family 10 member 1 [Source:HGNC Symbol;Acc:HGNC:10905] |
| ENSG00000101098 | HGLibA_41287 | 260 | 1.438667772 | RIMS4 | regulating synaptic membrane exocytosis 4 [Source:HGNC Symbol;Acc:HGNC:16183] |
| ENSG00000101435 | HGLibA_11474 | 260 | 1.438667772 | CST9L | cystatin 9 like [Source:HGNC Symbol;Acc:HGNC:16233] |
| ENSG00000108666 | HGLibA_05574 | 260 | 1.438667772 | C17orf75 | chromosome 17 open reading frame 75 [Source:HGNC Symbol;Acc:HGNC:30173] |
| ENSG00000111679 | HGLibA_39474 | 260 | 1.438667772 | PTPN6 | non-receptor type 6 [Source:HGNC Symbol;Acc:HGNC:9658]" |
| ENSG00000114378 | HGLibA_22437 | 260 | 1.438667772 | HYAL1 | hyaluronidase 1 [Source:HGNC Symbol;Acc:HGNC:5320] |
| ENSG00000129219 | HGLibA_37038 | 260 | 1.438667772 | PLD2 | phospholipase D2 [Source:HGNC Symbol;Acc:HGNC:9068] |
| ENSG00000131738 | HGLibA_25484 | 260 | 1.438667772 | KRT33B | keratin 33B [Source:HGNC Symbol;Acc:HGNC:6451] |
| ENSG00000133687 | HGLibA_50670 | 260 | 1.438667772 | TMTC1 | transmembrane and tetratricopeptide repeat containing 1 [Source:HGNC Symbol;Acc:HGNC:24099] |
| ENSG00000133980 | HGLibA_53833 | 260 | 1.438667772 | VRTN | vertebrae development associated [Source:HGNC Symbol;Acc:HGNC:20223] |
| ENSG00000134779 | HGLibA_51125 | 260 | 1.438667772 | TPGS2 | tubulin polyglutamylase complex subunit 2 [Source:HGNC Symbol;Acc:HGNC:24561] |
| ENSG00000138413 | HGLibA_22539 | 260 | 1.438667772 | IDH1 | cytosolic [Source:HGNC Symbol;Acc:HGNC:5382]" |
| ENSG00000141503 | HGLibA_29275 | 260 | 1.438667772 | MINK1 | misshapen like kinase 1 [Source:HGNC Symbol;Acc:HGNC:17565] |
| ENSG00000143412 | HGLibA_02260 | 260 | 1.438667772 | ANXA9 | annexin A9 [Source:HGNC Symbol;Acc:HGNC:547] |
| ENSG00000150995 | HGLibA_23853 | 260 | 1.438667772 | ITPR1 | 5-trisphosphate receptor type 1 [Source:HGNC Symbol;Acc:HGNC:6180]" |
| ENSG00000156574 | HGLibA_32249 | 260 | 1.438667772 | NODAL | nodal growth differentiation factor [Source:HGNC Symbol;Acc:HGNC:7865] |
| ENSG00000163541 | HGLibA_47698 | 260 | 1.438667772 | SUCLG1 | succinate-CoA ligase alpha subunit [Source:HGNC Symbol;Acc:HGNC:11449] |
| ENSG00000165215 | HGLibA_09895 | 260 | 1.438667772 | CLDN3 | claudin 3 [Source:HGNC Symbol;Acc:HGNC:2045] |
| ENSG00000169926 | HGLibA_25050 | 260 | 1.438667772 | KLF13 | Kruppel like factor 13 [Source:HGNC Symbol;Acc:HGNC:13672] |
| ENSG00000170471 | HGLibA_40161 | 260 | 1.438667772 | RALGAPB | Ral GTPase activating protein non-catalytic beta subunit [Source:HGNC Symbol;Acc:HGNC:29221] |
| ENSG00000172893 | HGLibA_13094 | 260 | 1.438667772 | DHCR7 | 7-dehydrocholesterol reductase [Source:HGNC Symbol;Acc:HGNC:2860] |
| ENSG00000176182 | HGLibA_30913 | 260 | 1.438667772 | MYPOP | partner of profilin [Source:HGNC Symbol;Acc:HGNC:20178]" |
| ENSG00000185090 | HGLibA_28095 | 260 | 1.438667772 | MANEAL | mannosidase endo-alpha like [Source:HGNC Symbol;Acc:HGNC:26452] |
| ENSG00000188283 | HGLibA_55890 | 260 | 1.438667772 | ZNF383 | zinc finger protein 383 [Source:HGNC Symbol;Acc:HGNC:18609] |
| ENSG00000196502 | HGLibA_47732 | 260 | 1.438667772 | SULT1A1 | sulfotransferase family 1A member 1 [Source:HGNC Symbol;Acc:HGNC:11453] |
| ENSG00000197106 | HGLibA_45309 | 260 | 1.438667772 | SLC6A17 | solute carrier family 6 member 17 [Source:HGNC Symbol;Acc:HGNC:31399] |
| ENSG00000198919 | HGLibA_14305 | 260 | 1.438667772 | DZIP3 | DAZ interacting zinc finger protein 3 [Source:HGNC Symbol;Acc:HGNC:30938] |
| ENSG00000204967 | HGLibA_35444 | 260 | 1.438667772 | PCDHA4 | protocadherin alpha 4 [Source:HGNC Symbol;Acc:HGNC:8670] |
| ENSG00000234068 | HGLibA_35008 | 260 | 1.438667772 | PAGE2 | PAGE family member 2 [Source:HGNC Symbol;Acc:HGNC:31804] |
| ENSG00000255307 | HGLibA_34146 | 260 | 1.438667772 | OR52B2 | olfactory receptor family 52 subfamily B member 2 [Source:HGNC Symbol;Acc:HGNC:15207] |
| ENSG00000276231 | HGLibA_36664 | 260 | 1.438667772 | PIK3R6 | phosphoinositide-3-kinase regulatory subunit 6 [Source:HGNC Symbol;Acc:HGNC:27101] |
| ENSG00000284862 | HGLibA_07772 | 260 | 1.438667772 | CCDC39 | coiled-coil domain containing 39 [Source:NCBI gene;Acc:339829] |
| FAM188A | HGLibA_16345 | 260 | 1.438667772 | FAM188A | - |
| HIST1H2BM | HGLibA_21447 | 260 | 1.438667772 | HIST1H2BM | - |
| hsa-mir-26b | HGLibA_58372 | 260 | 1.438667772 | hsa-mir-26b | - |
| hsa-mir-3117 | HGLibA_58510 | 260 | 1.438667772 | hsa-mir-3117 | - |
| hsa-mir-3132 | HGLibA_58584 | 260 | 1.438667772 | hsa-mir-3132 | - |
| hsa-mir-361 | HGLibA_59067 | 260 | 1.438667772 | hsa-mir-361 | - |
| hsa-mir-4439 | HGLibA_60159 | 260 | 1.438667772 | hsa-mir-4439 | - |
| TMEM133 | HGLibA_49943 | 260 | 1.438667772 | TMEM133 | - |
| C10orf113 | HGLibA_05033 | 259 | 1.428716734 | C10orf113 | - |
| ENSG00000064393 | HGLibA_21342 | 259 | 1.428716734 | HIPK2 | homeodomain interacting protein kinase 2 [Source:HGNC Symbol;Acc:HGNC:14402] |
| ENSG00000074416 | HGLibA_29160 | 259 | 1.428716734 | MGLL | monoglyceride lipase [Source:HGNC Symbol;Acc:HGNC:17038] |
| ENSG00000078747 | HGLibA_23704 | 259 | 1.428716734 | ITCH | itchy E3 ubiquitin protein ligase [Source:HGNC Symbol;Acc:HGNC:13890] |
| ENSG00000090097 | HGLibA_35377 | 259 | 1.428716734 | PCBP4 | poly(rC) binding protein 4 [Source:HGNC Symbol;Acc:HGNC:8652] |
| ENSG00000107518 | HGLibA_03822 | 259 | 1.428716734 | ATRNL1 | attractin like 1 [Source:HGNC Symbol;Acc:HGNC:29063] |
| ENSG00000108576 | HGLibA_45329 | 259 | 1.428716734 | SLC6A4 | solute carrier family 6 member 4 [Source:HGNC Symbol;Acc:HGNC:11050] |
| ENSG00000111859 | HGLibA_31613 | 259 | 1.428716734 | NEDD9 | developmentally down-regulated 9 [Source:HGNC Symbol;Acc:HGNC:7733]" |
| ENSG00000112195 | HGLibA_51362 | 259 | 1.428716734 | TREML2 | triggering receptor expressed on myeloid cells like 2 [Source:HGNC Symbol;Acc:HGNC:21092] |
| ENSG00000112658 | HGLibA_46962 | 259 | 1.428716734 | SRF | serum response factor [Source:HGNC Symbol;Acc:HGNC:11291] |
| ENSG00000116031 | HGLibA_08292 | 259 | 1.428716734 | CD207 | CD207 molecule [Source:HGNC Symbol;Acc:HGNC:17935] |
| ENSG00000117362 | HGLibA_02413 | 259 | 1.428716734 | APH1A | gamma-secretase subunit [Source:HGNC Symbol;Acc:HGNC:29509]" |
| ENSG00000118454 | HGLibA_02007 | 259 | 1.428716734 | ANKRD13C | ankyrin repeat domain 13C [Source:HGNC Symbol;Acc:HGNC:25374] |
| ENSG00000120696 | HGLibA_24113 | 259 | 1.428716734 | KBTBD7 | kelch repeat and BTB domain containing 7 [Source:HGNC Symbol;Acc:HGNC:25266] |
| ENSG00000124198 | HGLibA_02674 | 259 | 1.428716734 | ARFGEF2 | ADP ribosylation factor guanine nucleotide exchange factor 2 [Source:HGNC Symbol;Acc:HGNC:15853] |
| ENSG00000127586 | HGLibA_09660 | 259 | 1.428716734 | CHTF18 | chromosome transmission fidelity factor 18 [Source:HGNC Symbol;Acc:HGNC:18435] |
| ENSG00000128815 | HGLibA_54013 | 259 | 1.428716734 | WDFY4 | WDFY family member 4 [Source:HGNC Symbol;Acc:HGNC:29323] |
| ENSG00000131508 | HGLibA_52584 | 259 | 1.428716734 | UBE2D2 | ubiquitin conjugating enzyme E2 D2 [Source:HGNC Symbol;Acc:HGNC:12475] |
| ENSG00000133169 | HGLibA_04439 | 259 | 1.428716734 | BEX1 | brain expressed X-linked 1 [Source:HGNC Symbol;Acc:HGNC:1036] |
| ENSG00000134470 | HGLibA_22983 | 259 | 1.428716734 | IL15RA | interleukin 15 receptor subunit alpha [Source:HGNC Symbol;Acc:HGNC:5978] |
| ENSG00000136159 | HGLibA_32950 | 259 | 1.428716734 | NUDT15 | nudix hydrolase 15 [Source:HGNC Symbol;Acc:HGNC:23063] |
| ENSG00000138433 | HGLibA_09719 | 259 | 1.428716734 | CIR1 | 1 [Source:HGNC Symbol;Acc:HGNC:24217]" |
| ENSG00000140297 | HGLibA_18814 | 259 | 1.428716734 | GCNT3 | mucin type [Source:HGNC Symbol;Acc:HGNC:4205]" |
| ENSG00000156869 | HGLibA_18041 | 259 | 1.428716734 | FRRS1 | ferric chelate reductase 1 [Source:HGNC Symbol;Acc:HGNC:27622] |
| ENSG00000159184 | HGLibA_21929 | 259 | 1.428716734 | HOXB13 | homeobox B13 [Source:HGNC Symbol;Acc:HGNC:5112] |
| ENSG00000166086 | HGLibA_23933 | 259 | 1.428716734 | JAM3 | junctional adhesion molecule 3 [Source:HGNC Symbol;Acc:HGNC:15532] |
| ENSG00000167800 | HGLibA_48645 | 259 | 1.428716734 | TBX10 | T-box 10 [Source:HGNC Symbol;Acc:HGNC:11593] |
| ENSG00000168282 | HGLibA_29135 | 259 | 1.428716734 | MGAT2 | 2-N-acetylglucosaminyltransferase [Source:HGNC Symbol;Acc:HGNC:7045]" |
| ENSG00000173482 | HGLibA_39516 | 259 | 1.428716734 | PTPRM | receptor type M [Source:HGNC Symbol;Acc:HGNC:9675]" |
| ENSG00000180592 | HGLibA_44279 | 259 | 1.428716734 | SKIDA1 | SKI/DACH domain containing 1 [Source:HGNC Symbol;Acc:HGNC:32697] |
| ENSG00000183798 | HGLibA_15054 | 259 | 1.428716734 | EMILIN3 | elastin microfibril interfacer 3 [Source:HGNC Symbol;Acc:HGNC:16123] |
| ENSG00000184261 | HGLibA_24300 | 259 | 1.428716734 | KCNK12 | potassium two pore domain channel subfamily K member 12 [Source:HGNC Symbol;Acc:HGNC:6274] |
| ENSG00000185950 | HGLibA_23623 | 259 | 1.428716734 | IRS2 | insulin receptor substrate 2 [Source:HGNC Symbol;Acc:HGNC:6126] |
| ENSG00000186280 | HGLibA_24514 | 259 | 1.428716734 | KDM4D | lysine demethylase 4D [Source:HGNC Symbol;Acc:HGNC:25498] |
| ENSG00000187021 | HGLibA_37356 | 259 | 1.428716734 | PNLIPRP1 | pancreatic lipase related protein 1 [Source:HGNC Symbol;Acc:HGNC:9156] |
| ENSG00000188340 | HGLibA_34434 | 259 | 1.428716734 | OR6N2 | olfactory receptor family 6 subfamily N member 2 [Source:HGNC Symbol;Acc:HGNC:15035] |
| ENSG00000196415 | HGLibA_38982 | 259 | 1.428716734 | PRTN3 | proteinase 3 [Source:HGNC Symbol;Acc:HGNC:9495] |
| ENSG00000196570 | HGLibA_36180 | 259 | 1.428716734 | PFN3 | profilin 3 [Source:HGNC Symbol;Acc:HGNC:18627] |
| ENSG00000214078 | HGLibA_10954 | 259 | 1.428716734 | CPNE1 | copine 1 [Source:HGNC Symbol;Acc:HGNC:2314] |
| ENSG00000221989 | HGLibA_33729 | 259 | 1.428716734 | OR2A2 | olfactory receptor family 2 subfamily A member 2 [Source:HGNC Symbol;Acc:HGNC:8230] |
| ENSG00000229474 | HGLibA_35287 | 259 | 1.428716734 | PATL2 | PAT1 homolog 2 [Source:HGNC Symbol;Acc:HGNC:33630] |
| ENSG00000236699 | HGLibA_02859 | 259 | 1.428716734 | ARHGEF38 | Rho guanine nucleotide exchange factor 38 [Source:HGNC Symbol;Acc:HGNC:25968] |
| ENSG00000255408 | HGLibA_35443 | 259 | 1.428716734 | PCDHA3 | protocadherin alpha 3 [Source:HGNC Symbol;Acc:HGNC:8669] |
| ENSG00000266094 | HGLibA_40386 | 259 | 1.428716734 | RASSF5 | Ras association domain family member 5 [Source:HGNC Symbol;Acc:HGNC:17609] |
| ENSG00000279000 | HGLibA_33474 | 259 | 1.428716734 | OR10A6 | olfactory receptor family 10 subfamily A member 6 (gene/pseudogene) [Source:HGNC Symbol;Acc:HGNC:15132] |
| ERO1LB | HGLibA_15520 | 259 | 1.428716734 | ERO1LB | - |
| hsa-mir-200b | HGLibA_58154 | 259 | 1.428716734 | hsa-mir-200b | - |

| | | | | | |
|---|---|---|---|---|---|
| hsa-mir-3671 | HGLibA_59242 | 259 | 1.428716734 | hsa-mir-3671 | - |
| hsa-mir-4310 | HGLibA_59955 | 259 | 1.428716734 | hsa-mir-4310 | - |
| hsa-mir-485 | HGLibA_61332 | 259 | 1.428716734 | hsa-mir-485 | - |
| hsa-mir-548at | HGLibA_61926 | 259 | 1.428716734 | hsa-mir-548at | - |
| hsa-mir-744 | HGLibA_63885 | 259 | 1.428716734 | hsa-mir-744 | - |
| ENSG00000065325 | HGLibA_19296 | 258 | 1.418765697 | GLP2R | glucagon like peptide 2 receptor [Source:HGNC Symbol;Acc:HGNC:4325] |
| ENSG00000070047 | HGLibA_36460 | 258 | 1.418765697 | PHRF1 | PHD and ring finger domains 1 [Source:HGNC Symbol;Acc:HGNC:24351] |
| ENSG00000100596 | HGLibA_46896 | 258 | 1.418765697 | SPTLC2 | serine palmitoyltransferase long chain base subunit 2 [Source:HGNC Symbol;Acc:HGNC:11278] |
| ENSG00000102409 | HGLibA_04446 | 258 | 1.418765697 | BEX4 | brain expressed X-linked 4 [Source:HGNC Symbol;Acc:HGNC:25475] |
| ENSG00000106245 | HGLibA_05001 | 258 | 1.418765697 | BUD31 | BUD31 homolog [Source:HGNC Symbol;Acc:HGNC:29629] |
| ENSG00000110944 | HGLibA_23097 | 258 | 1.418765697 | IL23A | interleukin 23 subunit alpha [Source:HGNC Symbol;Acc:HGNC:15488] |
| ENSG00000112658 | HGLibA_46961 | 258 | 1.418765697 | SRF | serum response factor [Source:HGNC Symbol;Acc:HGNC:11291] |
| ENSG00000112796 | HGLibA_15179 | 258 | 1.418765697 | ENPP5 | ectonucleotide pyrophosphatase/phosphodiesterase 5 (putative) [Source:HGNC Symbol;Acc:HGNC:13717] |
| ENSG00000114744 | HGLibA_10657 | 258 | 1.418765697 | COMMD2 | COMM domain containing 2 [Source:HGNC Symbol;Acc:HGNC:24993] |
| ENSG00000115825 | HGLibA_38571 | 258 | 1.418765697 | PRKD3 | protein kinase D3 [Source:HGNC Symbol;Acc:HGNC:9408] |
| ENSG00000117226 | HGLibA_18735 | 258 | 1.418765697 | GBP3 | guanylate binding protein 3 [Source:HGNC Symbol;Acc:HGNC:4184] |
| ENSG00000135414 | HGLibA_18857 | 258 | 1.418765697 | GDF11 | growth differentiation factor 11 [Source:HGNC Symbol;Acc:HGNC:4216] |
| ENSG00000135437 | HGLibA_40749 | 258 | 1.418765697 | RDH5 | retinol dehydrogenase 5 [Source:HGNC Symbol;Acc:HGNC:9940] |
| ENSG00000141540 | HGLibA_52233 | 258 | 1.418765697 | TTYH2 | tweety family member 2 [Source:HGNC Symbol;Acc:HGNC:13877] |
| ENSG00000153086 | HGLibA_00456 | 258 | 1.418765697 | ACMSD | aminocarboxymuconate semialdehyde decarboxylase [Source:HGNC Symbol;Acc:HGNC:19288] |
| ENSG00000153976 | HGLibA_22127 | 258 | 1.418765697 | HS3ST3A1 | heparan sulfate-glucosamine 3-sulfotransferase 3A1 [Source:HGNC Symbol;Acc:HGNC:5196] |
| ENSG00000155511 | HGLibA_20261 | 258 | 1.418765697 | GRIA1 | glutamate ionotropic receptor AMPA type subunit 1 [Source:HGNC Symbol;Acc:HGNC:4571] |
| ENSG00000155833 | HGLibA_12046 | 258 | 1.418765697 | CYLC2 | cylicin 2 [Source:HGNC Symbol;Acc:HGNC:2583] |
| ENSG00000164251 | HGLibA_15864 | 258 | 1.418765697 | F2RL1 | F2R like trypsin receptor 1 [Source:HGNC Symbol;Acc:HGNC:3538] |
| ENSG00000164828 | HGLibA_47787 | 258 | 1.418765697 | SUN1 | Sad1 and UNC84 domain containing 1 [Source:HGNC Symbol;Acc:HGNC:18587] |
| ENSG00000167100 | HGLibA_42673 | 258 | 1.418765697 | SAMD14 | sterile alpha motif domain containing 14 [Source:HGNC Symbol;Acc:HGNC:27312] |
| ENSG00000169255 | HGLibA_03949 | 258 | 1.418765697 | B3GALNT1 | 3-N-acetylgalactosaminyltransferase 1 (globoside blood group) [Source:HGNC Symbol;Acc:HGNC:918]" |
| ENSG00000170142 | HGLibA_52595 | 258 | 1.418765697 | UBE2E1 | ubiquitin conjugating enzyme E2 E1 [Source:HGNC Symbol;Acc:HGNC:12477] |
| ENSG00000170315 | HGLibA_52565 | 258 | 1.418765697 | UBB | ubiquitin B [Source:HGNC Symbol;Acc:HGNC:12463] |
| ENSG00000171724 | HGLibA_53544 | 258 | 1.418765697 | VAT1L | vesicle amine transport 1 like [Source:HGNC Symbol;Acc:HGNC:29315] |
| ENSG00000175229 | HGLibA_18502 | 258 | 1.418765697 | GAL3ST3 | galactose-3-O-sulfotransferase 3 [Source:HGNC Symbol;Acc:HGNC:24144] |
| ENSG00000181961 | HGLibA_33927 | 258 | 1.418765697 | OR4A16 | olfactory receptor family 4 subfamily A member 16 [Source:HGNC Symbol;Acc:HGNC:15153] |
| ENSG00000198478 | HGLibA_43920 | 258 | 1.418765697 | SH3BGRL2 | SH3 domain binding glutamate rich protein like 2 [Source:HGNC Symbol;Acc:HGNC:15567] |
| ENSG00000204572 | HGLibA_25785 | 258 | 1.418765697 | KRTAP5-10 | keratin associated protein 5-10 [Source:HGNC Symbol;Acc:HGNC:23605] |
| ENSG00000204642 | HGLibA_21644 | 258 | 1.418765697 | HLA-F | F [Source:HGNC Symbol;Acc:HGNC:4963]" |
| ENSG00000235718 | HGLibA_29078 | 258 | 1.418765697 | MFRP | membrane frizzled-related protein [Source:HGNC Symbol;Acc:HGNC:18121] |
| ENSG00000288596 | HGLibA_06624 | 258 | 1.418765697 | C8orf44 | - |
| GTF2H2D | HGLibA_20552 | 258 | 1.418765697 | GTF2H2D | - |
| hsa-mir-2682 | HGLibA_58360 | 258 | 1.418765697 | hsa-mir-2682 | - |
| hsa-mir-3606 | HGLibA_59059 | 258 | 1.418765697 | hsa-mir-3606 | - |
| hsa-mir-4484 | HGLibA_60356 | 258 | 1.418765697 | hsa-mir-4484 | - |
| hsa-mir-6763 | HGLibA_63215 | 258 | 1.418765697 | hsa-mir-6763 | - |
| hsa-mir-6852 | HGLibA_63580 | 258 | 1.418765697 | hsa-mir-6852 | - |
| hsa-mir-7848 | HGLibA_63980 | 258 | 1.418765697 | hsa-mir-7848 | - |
| LOC100287534 | HGLibA_26826 | 258 | 1.418765697 | LOC100287534 | - |
| NEURL | HGLibA_31723 | 258 | 1.418765697 | NEURL | - |
| SELT | HGLibA_43313 | 258 | 1.418765697 | SELT | - |
| ENSG00000029153 | HGLibA_03072 | 257 | 1.408814659 | ARNTL2 | aryl hydrocarbon receptor nuclear translocator like 2 [Source:HGNC Symbol;Acc:HGNC:18984] |
| ENSG00000046774 | HGLibA_27961 | 257 | 1.408814659 | MAGEC2 | MAGE family member C2 [Source:HGNC Symbol;Acc:HGNC:13574] |
| ENSG00000057252 | HGLibA_46113 | 257 | 1.408814659 | SOAT1 | sterol O-acyltransferase 1 [Source:HGNC Symbol;Acc:HGNC:11177] |
| ENSG00000103266 | HGLibA_47611 | 257 | 1.408814659 | STUB1 | STIP1 homology and U-box containing protein 1 [Source:HGNC Symbol;Acc:HGNC:11427] |
| ENSG00000105649 | HGLibA_39908 | 257 | 1.408814659 | RAB3A | member RAS oncogene family [Source:HGNC Symbol;Acc:HGNC:9777]" |
| ENSG00000109445 | HGLibA_55813 | 257 | 1.408814659 | ZNF330 | zinc finger protein 330 [Source:HGNC Symbol;Acc:HGNC:15462] |
| ENSG00000114638 | HGLibA_53071 | 257 | 1.408814659 | UPK1B | uroplakin 1B [Source:HGNC Symbol;Acc:HGNC:12578] |
| ENSG00000115386 | HGLibA_40793 | 257 | 1.408814659 | REG1A | regenerating family member 1 alpha [Source:HGNC Symbol;Acc:HGNC:9951] |
| ENSG00000115459 | HGLibA_14946 | 257 | 1.408814659 | ELMOD3 | ELMO domain containing 3 [Source:HGNC Symbol;Acc:HGNC:26158] |
| ENSG00000124159 | HGLibA_28437 | 257 | 1.408814659 | MATN4 | matrilin 4 [Source:HGNC Symbol;Acc:HGNC:6910] |
| ENSG00000126838 | HGLibA_39715 | 257 | 1.408814659 | PZP | alpha-2-macroglobulin like [Source:HGNC Symbol;Acc:HGNC:9750]" |
| ENSG00000131097 | HGLibA_21309 | 257 | 1.408814659 | HIGD1B | HIG1 hypoxia inducible domain family member 1B [Source:HGNC Symbol;Acc:HGNC:24318] |
| ENSG00000132003 | HGLibA_57047 | 257 | 1.408814659 | ZSWIM4 | zinc finger SWIM-type containing 4 [Source:HGNC Symbol;Acc:HGNC:25704] |
| ENSG00000134569 | HGLibA_27222 | 257 | 1.408814659 | LRP4 | LDL receptor related protein 4 [Source:HGNC Symbol;Acc:HGNC:6696] |
| ENSG00000136689 | HGLibA_23067 | 257 | 1.408814659 | IL1RN | interleukin 1 receptor antagonist [Source:HGNC Symbol;Acc:HGNC:6000] |
| ENSG00000137731 | HGLibA_18227 | 257 | 1.408814659 | FXYD2 | FXYD domain containing ion transport regulator 2 [Source:HGNC Symbol;Acc:HGNC:4026] |
| ENSG00000137752 | HGLibA_07295 | 257 | 1.408814659 | CASP1 | caspase 1 [Source:HGNC Symbol;Acc:HGNC:1499] |
| ENSG00000138356 | HGLibA_02275 | 257 | 1.408814659 | AOX1 | aldehyde oxidase 1 [Source:HGNC Symbol;Acc:HGNC:553] |
| ENSG00000140905 | HGLibA_18833 | 257 | 1.408814659 | GCSH | glycine cleavage system protein H [Source:HGNC Symbol;Acc:HGNC:4208] |
| ENSG00000142687 | HGLibA_24622 | 257 | 1.408814659 | KIAA0319L | KIAA0319 like [Source:HGNC Symbol;Acc:HGNC:30071] |
| ENSG00000154556 | HGLibA_46177 | 257 | 1.408814659 | SORBS2 | sorbin and SH3 domain containing 2 [Source:HGNC Symbol;Acc:HGNC:24098] |
| ENSG00000154589 | HGLibA_27691 | 257 | 1.408814659 | LY96 | lymphocyte antigen 96 [Source:HGNC Symbol;Acc:HGNC:17156] |
| ENSG00000157111 | HGLibA_50060 | 257 | 1.408814659 | TMEM171 | transmembrane protein 171 [Source:HGNC Symbol;Acc:HGNC:27031] |
| ENSG00000166813 | HGLibA_24960 | 257 | 1.408814659 | KIF7 | kinesin family member 7 [Source:HGNC Symbol;Acc:HGNC:30497] |
| ENSG00000167553 | HGLibA_52248 | 257 | 1.408814659 | TUBA1C | tubulin alpha 1c [Source:HGNC Symbol;Acc:HGNC:20768] |
| ENSG00000169964 | HGLibA_50398 | 257 | 1.408814659 | TMEM42 | transmembrane protein 42 [Source:HGNC Symbol;Acc:HGNC:28444] |
| ENSG00000174970 | HGLibA_33481 | 257 | 1.408814659 | OR10AG1 | olfactory receptor family 10 subfamily AG member 1 [Source:HGNC Symbol;Acc:HGNC:19607] |
| ENSG00000175197 | HGLibA_12616 | 257 | 1.408814659 | DDIT3 | DNA damage inducible transcript 3 [Source:HGNC Symbol;Acc:HGNC:2726] |
| ENSG00000176410 | HGLibA_13618 | 257 | 1.408814659 | DNAJC30 | DnaJ heat shock protein family (Hsp40) member C30 [Source:HGNC Symbol;Acc:HGNC:16410] |
| ENSG00000176986 | HGLibA_43235 | 257 | 1.408814659 | SEC24C | COPII coat complex component [Source:HGNC Symbol;Acc:HGNC:10705]" |
| ENSG00000178467 | HGLibA_34911 | 257 | 1.408814659 | P4HTM | transmembrane [Source:HGNC Symbol;Acc:HGNC:28858]" |
| ENSG00000179564 | HGLibA_27561 | 257 | 1.408814659 | LSMEM2 | leucine rich single-pass membrane protein 2 [Source:HGNC Symbol;Acc:HGNC:26781] |
| ENSG00000179600 | HGLibA_19801 | 257 | 1.408814659 | GPHB5 | glycoprotein hormone beta 5 [Source:HGNC Symbol;Acc:HGNC:18055] |
| ENSG00000184459 | HGLibA_04733 | 257 | 1.408814659 | BPIFC | BPI fold containing family C [Source:HGNC Symbol;Acc:HGNC:16503] |
| ENSG00000185926 | HGLibA_33958 | 257 | 1.408814659 | OR4C46 | olfactory receptor family 4 subfamily C member 46 [Source:HGNC Symbol;Acc:HGNC:31271] |
| ENSG00000187678 | HGLibA_46846 | 257 | 1.408814659 | SPRY4 | sprouty RTK signaling antagonist 4 [Source:HGNC Symbol;Acc:HGNC:15533] |
| ENSG00000197416 | HGLibA_15909 | 257 | 1.408814659 | FABP12 | fatty acid binding protein 12 [Source:HGNC Symbol;Acc:HGNC:34524] |
| ENSG00000198033 | HGLibA_52251 | 257 | 1.408814659 | TUBA3C | tubulin alpha 3c [Source:HGNC Symbol;Acc:HGNC:12408] |
| ENSG00000198090 | HGLibA_25769 | 257 | 1.408814659 | KRTAP4-6 | keratin associated protein 4-6 [Source:HGNC Symbol;Acc:HGNC:18909] |
| ENSG00000203730 | HGLibA_48958 | 257 | 1.408814659 | TEDDM1 | transmembrane epididymal protein 1 [Source:HGNC Symbol;Acc:HGNC:30233] |
| ENSG00000213996 | HGLibA_49713 | 257 | 1.408814659 | TM6SF2 | transmembrane 6 superfamily member 2 [Source:HGNC Symbol;Acc:HGNC:11861] |
| ENSG00000214517 | HGLibA_37999 | 257 | 1.408814659 | PPME1 | protein phosphatase methylesterase 1 [Source:HGNC Symbol;Acc:HGNC:30178] |
| ENSG00000222011 | HGLibA_16334 | 257 | 1.408814659 | FAM185A | family with sequence similarity 185 member A [Source:HGNC Symbol;Acc:HGNC:22412] |
| ENSG00000248383 | HGLibA_35463 | 257 | 1.408814659 | PCDHAC1 | 1 [Source:HGNC Symbol;Acc:HGNC:8676]" |
| FAM175B | HGLibA_16292 | 257 | 1.408814659 | FAM175B | - |
| hsa-mir-33a | HGLibA_59013 | 257 | 1.408814659 | hsa-mir-33a | - |
| hsa-mir-3682 | HGLibA_59283 | 257 | 1.408814659 | hsa-mir-3682 | - |
| hsa-mir-4256 | HGLibA_59732 | 257 | 1.408814659 | hsa-mir-4256 | - |
| hsa-mir-4312 | HGLibA_59964 | 257 | 1.408814659 | hsa-mir-4312 | - |
| hsa-mir-6890 | HGLibA_63746 | 257 | 1.408814659 | hsa-mir-6890 | - |
| hsa-mir-8075 | HGLibA_64143 | 257 | 1.408814659 | hsa-mir-8075 | - |
| OR9G9 | HGLibA_34573 | 257 | 1.408814659 | OR9G9 | - |
| BZRAP1 | HGLibA_05011 | 256 | 1.398863622 | BZRAP1 | - |
| C16orf45 | HGLibA_05447 | 256 | 1.398863622 | C16orf45 | - |
| CXorf57 | HGLibA_11946 | 256 | 1.398863622 | CXorf57 | - |
| ENSG00000066422 | HGLibA_54818 | 256 | 1.398863622 | ZBTB11 | zinc finger and BTB domain containing 11 [Source:HGNC Symbol;Acc:HGNC:16740] |
| ENSG00000081177 | HGLibA_15718 | 256 | 1.398863622 | EXD2 | exonuclease 3'-5' domain containing 2 [Source:HGNC Symbol;Acc:HGNC:20217] |
| ENSG00000090661 | HGLibA_09210 | 256 | 1.398863622 | CERS4 | ceramide synthase 4 [Source:HGNC Symbol;Acc:HGNC:23747] |
| ENSG00000102466 | HGLibA_17376 | 256 | 1.398863622 | FGF14 | fibroblast growth factor 14 [Source:HGNC Symbol;Acc:HGNC:3671] |
| ENSG00000106366 | HGLibA_43577 | 256 | 1.398863622 | SERPINE1 | serpin family E member 1 [Source:HGNC Symbol;Acc:HGNC:8583] |
| ENSG00000110057 | HGLibA_53038 | 256 | 1.398863622 | UNC93B1 | TLR signaling regulator [Source:HGNC Symbol;Acc:HGNC:13481]" |
| ENSG00000116815 | HGLibA_08415 | 256 | 1.398863622 | CD58 | CD58 molecule [Source:HGNC Symbol;Acc:HGNC:1688] |
| ENSG00000121578 | HGLibA_04033 | 256 | 1.398863622 | B4GALT4 | 4-galactosyltransferase 4 [Source:HGNC Symbol;Acc:HGNC:927]" |
| ENSG00000130559 | HGLibA_07105 | 256 | 1.398863622 | CAMSAP1 | calmodulin regulated spectrin associated protein 1 [Source:HGNC Symbol;Acc:HGNC:19946] |
| ENSG00000139433 | HGLibA_19342 | 256 | 1.398863622 | GLTP | glycolipid transfer protein [Source:HGNC Symbol;Acc:HGNC:24867] |
| ENSG00000145863 | HGLibA_18371 | 256 | 1.398863622 | GABRA6 | gamma-aminobutyric acid type A receptor alpha6 subunit [Source:HGNC Symbol;Acc:HGNC:4080] |
| ENSG00000148384 | HGLibA_23353 | 256 | 1.398863622 | INPP5E | inositol polyphosphate-5-phosphatase E [Source:HGNC Symbol;Acc:HGNC:21474] |
| ENSG00000150628 | HGLibA_46512 | 256 | 1.398863622 | SPATA4 | spermatogenesis associated 4 [Source:HGNC Symbol;Acc:HGNC:17333] |
| ENSG00000153721 | HGLibA_10301 | 256 | 1.398863622 | CNKSR3 | CNKSR family member 3 [Source:HGNC Symbol;Acc:HGNC:23034] |
| ENSG00000156265 | HGLibA_28199 | 256 | 1.398863622 | MAP3K7CL | MAP3K7 C-terminal like [Source:HGNC Symbol;Acc:HGNC:16457] |
| ENSG00000157551 | HGLibA_24263 | 256 | 1.398863622 | KCNJ15 | potassium voltage-gated channel subfamily J member 15 [Source:HGNC Symbol;Acc:HGNC:6261] |
| ENSG00000161031 | HGLibA_36251 | 256 | 1.398863622 | PGLYRP2 | peptidoglycan recognition protein 2 [Source:HGNC Symbol;Acc:HGNC:30013] |
| ENSG00000168334 | HGLibA_54510 | 256 | 1.398863622 | XIRP1 | xin actin binding repeat containing 1 [Source:HGNC Symbol;Acc:HGNC:14301] |
| ENSG00000168515 | HGLibA_42915 | 256 | 1.398863622 | SCGB1D1 | secretoglobin family 1D member 1 [Source:HGNC Symbol;Acc:HGNC:18395] |
| ENSG00000171608 | HGLibA_36638 | 256 | 1.398863622 | PIK3CD | 5-bisphosphate 3-kinase catalytic subunit delta [Source:HGNC Symbol;Acc:HGNC:8977]" |
| ENSG00000176678 | HGLibA_17900 | 256 | 1.398863622 | FOXL1 | forkhead box L1 [Source:HGNC Symbol;Acc:HGNC:3817] |
| ENSG00000176978 | HGLibA_13877 | 256 | 1.398863622 | DPP7 | dipeptidyl peptidase 7 [Source:HGNC Symbol;Acc:HGNC:14892] |
| ENSG00000177201 | HGLibA_33861 | 256 | 1.398863622 | OR2T12 | olfactory receptor family 2 subfamily T member 12 [Source:HGNC Symbol;Acc:HGNC:19592] |
| ENSG00000180875 | HGLibA_20245 | 256 | 1.398863622 | GREM2 | DAN family BMP antagonist [Source:HGNC Symbol;Acc:HGNC:17655]" |
| ENSG00000183134 | HGLibA_39306 | 256 | 1.398863622 | PTGDR2 | prostaglandin D2 receptor 2 [Source:HGNC Symbol;Acc:HGNC:4502] |
| ENSG00000185477 | HGLibA_20146 | 256 | 1.398863622 | GPRIN3 | GPRIN family member 3 [Source:HGNC Symbol;Acc:HGNC:27733] |
| ENSG00000197372 | HGLibA_56512 | 256 | 1.398863622 | ZNF675 | zinc finger protein 675 [Source:HGNC Symbol;Acc:HGNC:30768] |
| ENSG00000197951 | HGLibA_56608 | 256 | 1.398863622 | ZNF71 | zinc finger protein 71 [Source:HGNC Symbol;Acc:HGNC:13141] |
| ENSG00000198464 | HGLibA_56071 | 256 | 1.398863622 | ZNF480 | zinc finger protein 480 [Source:HGNC Symbol;Acc:HGNC:23305] |
| ENSG00000204371 | HGLibA_14688 | 256 | 1.398863622 | EHMT2 | euchromatic histone lysine methyltransferase 2 [Source:HGNC Symbol;Acc:HGNC:14129] |
| hsa-mir-1307 | HGLibA_57745 | 256 | 1.398863622 | hsa-mir-1307 | - |
| hsa-mir-4506 | HGLibA_60449 | 256 | 1.398863622 | hsa-mir-4506 | - |
| hsa-mir-4713 | HGLibA_60952 | 256 | 1.398863622 | hsa-mir-4713 | - |

| | | | | | |
|---|---|---|---|---|---|
| hsa-mir-676 | HGLibA_63199 | 256 | 1.398863622 | hsa-mir-676 | - |
| BAGE4 | HGLibA_04102 | 255 | 1.388912585 | BAGE4 | - |
| ENSG00000026025 | HGLibA_53655 | 255 | 1.388912585 | VIM | vimentin [Source:HGNC Symbol;Acc:HGNC:12692] |
| ENSG00000048462 | HGLibA_50762 | 255 | 1.388912585 | TNFRSF17 | TNF receptor superfamily member 17 [Source:HGNC Symbol;Acc:HGNC:11913] |
| ENSG00000066629 | HGLibA_15056 | 255 | 1.388912585 | EML1 | echinoderm microtubule associated protein like 1 [Source:HGNC Symbol;Acc:HGNC:3330] |
| ENSG00000102978 | HGLibA_37571 | 255 | 1.388912585 | POLR2C | RNA polymerase II subunit C [Source:HGNC Symbol;Acc:HGNC:9189] |
| ENSG00000104635 | HGLibA_45104 | 255 | 1.388912585 | SLC39A14 | solute carrier family 39 member 14 [Source:HGNC Symbol;Acc:HGNC:20858] |
| ENSG00000105088 | HGLibA_33347 | 255 | 1.388912585 | OLFM2 | olfactomedin 2 [Source:HGNC Symbol;Acc:HGNC:17189] |
| ENSG00000105229 | HGLibA_36517 | 255 | 1.388912585 | PIAS4 | protein inhibitor of activated STAT 4 [Source:HGNC Symbol;Acc:HGNC:17002] |
| ENSG00000110375 | HGLibA_53075 | 255 | 1.388912585 | UPK2 | uroplakin 2 [Source:HGNC Symbol;Acc:HGNC:12579] |
| ENSG00000112812 | HGLibA_38905 | 255 | 1.388912585 | PRSS16 | serine protease 16 [Source:HGNC Symbol;Acc:HGNC:9480] |
| ENSG00000116785 | HGLibA_09269 | 255 | 1.388912585 | CFHR3 | complement factor H related 3 [Source:HGNC Symbol;Acc:HGNC:16980] |
| ENSG00000119314 | HGLibA_39257 | 255 | 1.388912585 | PTBP3 | polypyrimidine tract binding protein 3 [Source:HGNC Symbol;Acc:HGNC:10253] |
| ENSG00000123353 | HGLibA_34628 | 255 | 1.388912585 | ORMDL2 | ORMDL sphingolipid biosynthesis regulator 2 [Source:HGNC Symbol;Acc:HGNC:16037] |
| ENSG00000125787 | HGLibA_19584 | 255 | 1.388912585 | GNRH2 | gonadotropin releasing hormone 2 [Source:HGNC Symbol;Acc:HGNC:4420] |
| ENSG00000125848 | HGLibA_17659 | 255 | 1.388912585 | FLRT3 | fibronectin leucine rich transmembrane protein 3 [Source:HGNC Symbol;Acc:HGNC:3762] |
| ENSG00000127515 | HGLibA_34458 | 255 | 1.388912585 | OR7A10 | olfactory receptor family 7 subfamily A member 10 [Source:HGNC Symbol;Acc:HGNC:8356] |
| ENSG00000130598 | HGLibA_50875 | 255 | 1.388912585 | TNNI2 | fast skeletal type [Source:HGNC Symbol;Acc:HGNC:11946]" |
| ENSG00000130811 | HGLibA_14784 | 255 | 1.388912585 | EIF3G | eukaryotic translation initiation factor 3 subunit G [Source:HGNC Symbol;Acc:HGNC:3274] |
| ENSG00000136883 | HGLibA_24854 | 255 | 1.388912585 | KIF12 | kinesin family member 12 [Source:HGNC Symbol;Acc:HGNC:21495] |
| ENSG00000143398 | HGLibA_36715 | 255 | 1.388912585 | PIP5K1A | phosphatidylinositol-4-phosphate 5-kinase type 1 alpha [Source:HGNC Symbol;Acc:HGNC:8994] |
| ENSG00000148154 | HGLibA_52860 | 255 | 1.388912585 | UGCG | UDP-glucose ceramide glucosyltransferase [Source:HGNC Symbol;Acc:HGNC:12524] |
| ENSG00000151287 | HGLibA_49094 | 255 | 1.388912585 | TEX30 | testis expressed 30 [Source:HGNC Symbol;Acc:HGNC:25188] |
| ENSG00000153885 | HGLibA_24429 | 255 | 1.388912585 | KCTD15 | potassium channel tetramerization domain containing 15 [Source:HGNC Symbol;Acc:HGNC:23297] |
| ENSG00000156218 | HGLibA_00855 | 255 | 1.388912585 | ADAMTSL3 | ADAMTS like 3 [Source:HGNC Symbol;Acc:HGNC:14633] |
| ENSG00000158815 | HGLibA_17380 | 255 | 1.388912585 | FGF17 | fibroblast growth factor 17 [Source:HGNC Symbol;Acc:HGNC:3673] |
| ENSG00000159322 | HGLibA_01021 | 255 | 1.388912585 | ADPGK | ADP dependent glucokinase [Source:HGNC Symbol;Acc:HGNC:25250] |
| ENSG00000159527 | HGLibA_36255 | 255 | 1.388912585 | PGLYRP3 | peptidoglycan recognition protein 3 [Source:HGNC Symbol;Acc:HGNC:30014] |
| ENSG00000161055 | HGLibA_42933 | 255 | 1.388912585 | SCGB3A1 | secretoglobin family 3A member 1 [Source:HGNC Symbol;Acc:HGNC:18384] |
| ENSG00000163884 | HGLibA_25057 | 255 | 1.388912585 | KLF15 | Kruppel like factor 15 [Source:HGNC Symbol;Acc:HGNC:14536] |
| ENSG00000172005 | HGLibA_28023 | 255 | 1.388912585 | MAL | T cell differentiation protein [Source:HGNC Symbol;Acc:HGNC:6817]" |
| ENSG00000172404 | HGLibA_13551 | 255 | 1.388912585 | DNAJB7 | DnaJ heat shock protein family (Hsp40) member B7 [Source:HGNC Symbol;Acc:HGNC:24986] |
| ENSG00000172940 | HGLibA_44547 | 255 | 1.388912585 | SLC22A13 | solute carrier family 22 member 13 [Source:HGNC Symbol;Acc:HGNC:8494] |
| ENSG00000176381 | HGLibA_38793 | 255 | 1.388912585 | PRR18 | proline rich 18 [Source:HGNC Symbol;Acc:HGNC:28574] |
| ENSG00000177468 | HGLibA_33373 | 255 | 1.388912585 | OLIG3 | oligodendrocyte transcription factor 3 [Source:HGNC Symbol;Acc:HGNC:18003] |
| ENSG00000177575 | HGLibA_08244 | 255 | 1.388912585 | CD163 | CD163 molecule [Source:HGNC Symbol;Acc:HGNC:1631] |
| ENSG00000179456 | HGLibA_54832 | 255 | 1.388912585 | ZBTB18 | zinc finger and BTB domain containing 18 [Source:HGNC Symbol;Acc:HGNC:13030] |
| ENSG00000184368 | HGLibA_28237 | 255 | 1.388912585 | MAP7D2 | MAP7 domain containing 2 [Source:HGNC Symbol;Acc:HGNC:25899] |
| ENSG00000184465 | HGLibA_54059 | 255 | 1.388912585 | WDR27 | WD repeat domain 27 [Source:HGNC Symbol;Acc:HGNC:21248] |
| ENSG00000186334 | HGLibA_45038 | 255 | 1.388912585 | SLC36A3 | solute carrier family 36 member 3 [Source:HGNC Symbol;Acc:HGNC:19659] |
| ENSG00000187840 | HGLibA_14829 | 255 | 1.388912585 | EIF4EBP1 | eukaryotic translation initiation factor 4E binding protein 1 [Source:HGNC Symbol;Acc:HGNC:3288] |
| ENSG00000188677 | HGLibA_35259 | 255 | 1.388912585 | PARVB | parvin beta [Source:HGNC Symbol;Acc:HGNC:14653] |
| ENSG00000204961 | HGLibA_35459 | 255 | 1.388912585 | PCDHA9 | protocadherin alpha 9 [Source:HGNC Symbol;Acc:HGNC:8675] |
| ENSG00000213463 | HGLibA_48010 | 255 | 1.388912585 | SYNJ2BP | synaptojanin 2 binding protein [Source:HGNC Symbol;Acc:HGNC:18955] |
| ENSG00000221923 | HGLibA_56893 | 255 | 1.388912585 | ZNF880 | zinc finger protein 880 [Source:HGNC Symbol;Acc:HGNC:37249] |
| ENSG00000241106 | HGLibA_21611 | 255 | 1.388912585 | HLA-DOB | DO beta [Source:HGNC Symbol;Acc:HGNC:4937]" |
| ENSG00000241644 | HGLibA_23315 | 255 | 1.388912585 | INMT | indolethylamine N-methyltransferase [Source:HGNC Symbol;Acc:HGNC:6069] |
| ENSG00000266967 | HGLibA_00074 | 255 | 1.388912585 | AARSD1 | alanyl-tRNA synthetase domain containing 1 [Source:HGNC Symbol;Acc:HGNC:28417] |
| hsa-mir-1973 | HGLibA_58117 | 255 | 1.388912585 | hsa-mir-1973 | - |
| hsa-mir-31 | HGLibA_58494 | 255 | 1.388912585 | hsa-mir-31 | - |
| hsa-mir-3974 | HGLibA_59656 | 255 | 1.388912585 | hsa-mir-3974 | - |
| hsa-mir-4686 | HGLibA_60856 | 255 | 1.388912585 | hsa-mir-4686 | - |
| hsa-mir-576 | HGLibA_62400 | 255 | 1.388912585 | hsa-mir-576 | - |
| hsa-mir-6748 | HGLibA_63147 | 255 | 1.388912585 | hsa-mir-6748 | - |
| hsa-mir-6808 | HGLibA_63410 | 255 | 1.388912585 | hsa-mir-6808 | - |
| C14orf183 | HGLibA_05354 | 254 | 1.378961547 | C14orf183 | - |
| ENSG00000011451 | HGLibA_54328 | 254 | 1.378961547 | WIZ | widely interspaced zinc finger motifs [Source:HGNC Symbol;Acc:HGNC:30917] |
| ENSG00000011485 | HGLibA_38212 | 254 | 1.378961547 | PPP5C | protein phosphatase 5 catalytic subunit [Source:HGNC Symbol;Acc:HGNC:9322] |
| ENSG00000053438 | HGLibA_32221 | 254 | 1.378961547 | NNAT | neuronatin [Source:HGNC Symbol;Acc:HGNC:7860] |
| ENSG00000068650 | HGLibA_03553 | 254 | 1.378961547 | ATP11A | ATPase phospholipid transporting 11A [Source:HGNC Symbol;Acc:HGNC:13552] |
| ENSG00000070526 | HGLibA_47263 | 254 | 1.378961547 | ST6GALNAC1 | 6-sialyltransferase 1 [Source:HGNC Symbol;Acc:HGNC:23614]" |
| ENSG00000072135 | HGLibA_39446 | 254 | 1.378961547 | PTPN18 | non-receptor type 18 [Source:HGNC Symbol;Acc:HGNC:9649]" |
| ENSG00000084090 | HGLibA_47371 | 254 | 1.378961547 | STARD7 | StAR related lipid transfer domain containing 7 [Source:HGNC Symbol;Acc:HGNC:18063] |
| ENSG00000091010 | HGLibA_37782 | 254 | 1.378961547 | POU4F3 | POU class 4 homeobox 3 [Source:HGNC Symbol;Acc:HGNC:9220] |
| ENSG00000091542 | HGLibA_01659 | 254 | 1.378961547 | ALKBH5 | RNA demethylase [Source:HGNC Symbol;Acc:HGNC:25996]" |
| ENSG00000096717 | HGLibA_44217 | 254 | 1.378961547 | SIRT1 | sirtuin 1 [Source:HGNC Symbol;Acc:HGNC:14929] |
| ENSG00000104714 | HGLibA_15487 | 254 | 1.378961547 | ERICH1 | glutamate rich 1 [Source:HGNC Symbol;Acc:HGNC:27234] |
| ENSG00000104728 | HGLibA_02806 | 254 | 1.378961547 | ARHGEF10 | Rho guanine nucleotide exchange factor 10 [Source:HGNC Symbol;Acc:HGNC:14103] |
| ENSG00000111275 | HGLibA_01560 | 254 | 1.378961547 | ALDH2 | aldehyde dehydrogenase 2 family member [Source:HGNC Symbol;Acc:HGNC:404] |
| ENSG00000115084 | HGLibA_45011 | 254 | 1.378961547 | SLC35F5 | solute carrier family 35 member F5 [Source:HGNC Symbol;Acc:HGNC:23617] |
| ENSG00000123329 | HGLibA_02793 | 254 | 1.378961547 | ARHGAP9 | Rho GTPase activating protein 9 [Source:HGNC Symbol;Acc:HGNC:14130] |
| ENSG00000128655 | HGLibA_35763 | 254 | 1.378961547 | PDE11A | phosphodiesterase 11A [Source:HGNC Symbol;Acc:HGNC:8773] |
| ENSG00000130822 | HGLibA_37342 | 254 | 1.378961547 | PNCK | pregnancy up-regulated nonubiquitous CaM kinase [Source:HGNC Symbol;Acc:HGNC:13415] |
| ENSG00000132334 | HGLibA_39500 | 254 | 1.378961547 | PTPRE | receptor type E [Source:HGNC Symbol;Acc:HGNC:9669]" |
| ENSG00000135750 | HGLibA_24295 | 254 | 1.378961547 | KCNK1 | potassium two pore domain channel subfamily K member 1 [Source:HGNC Symbol;Acc:HGNC:6272] |
| ENSG00000136247 | HGLibA_55117 | 254 | 1.378961547 | ZDHHC4 | zinc finger DHHC-type containing 4 [Source:HGNC Symbol;Acc:HGNC:18471] |
| ENSG00000139354 | HGLibA_18651 | 254 | 1.378961547 | GAS2L3 | growth arrest specific 2 like 3 [Source:HGNC Symbol;Acc:HGNC:27475] |
| ENSG00000145833 | HGLibA_12719 | 254 | 1.378961547 | DDX46 | DEAD-box helicase 46 [Source:HGNC Symbol;Acc:HGNC:18681] |
| ENSG00000146926 | HGLibA_03207 | 254 | 1.378961547 | ASB10 | ankyrin repeat and SOCS box containing 10 [Source:HGNC Symbol;Acc:HGNC:17185] |
| ENSG00000150722 | HGLibA_38072 | 254 | 1.378961547 | PPP1R1C | protein phosphatase 1 regulatory inhibitor subunit 1C [Source:HGNC Symbol;Acc:HGNC:14940] |
| ENSG00000155085 | HGLibA_01401 | 254 | 1.378961547 | AK9 | adenylate kinase 9 [Source:HGNC Symbol;Acc:HGNC:33814] |
| ENSG00000162992 | HGLibA_31734 | 254 | 1.378961547 | NEUROD1 | neuronal differentiation 1 [Source:HGNC Symbol;Acc:HGNC:7762] |
| ENSG00000171056 | HGLibA_46274 | 254 | 1.378961547 | SOX7 | SRY-box 7 [Source:HGNC Symbol;Acc:HGNC:18196] |
| ENSG00000172349 | HGLibA_22987 | 254 | 1.378961547 | IL16 | interleukin 16 [Source:HGNC Symbol;Acc:HGNC:5980] |
| ENSG00000180921 | HGLibA_16760 | 254 | 1.378961547 | FAM83H | family with sequence similarity 83 member H [Source:HGNC Symbol;Acc:HGNC:24797] |
| ENSG00000182393 | HGLibA_22719 | 254 | 1.378961547 | IFNL1 | interferon lambda 1 [Source:HGNC Symbol;Acc:HGNC:18363] |
| ENSG00000186881 | HGLibA_33616 | 254 | 1.378961547 | OR13F1 | olfactory receptor family 13 subfamily F member 1 [Source:HGNC Symbol;Acc:HGNC:14723] |
| ENSG00000188869 | HGLibA_49753 | 254 | 1.378961547 | TMC3 | transmembrane channel like 3 [Source:HGNC Symbol;Acc:HGNC:22995] |
| ENSG00000188906 | HGLibA_27468 | 254 | 1.378961547 | LRRK2 | leucine rich repeat kinase 2 [Source:HGNC Symbol;Acc:HGNC:18618] |
| ENSG00000197790 | HGLibA_34191 | 254 | 1.378961547 | OR52M1 | olfactory receptor family 52 subfamily M member 1 [Source:HGNC Symbol;Acc:HGNC:15225] |
| ENSG00000197885 | HGLibA_32015 | 254 | 1.378961547 | NKIRAS1 | NFKB inhibitor interacting Ras like 1 [Source:HGNC Symbol;Acc:HGNC:17899] |
| ENSG00000198055 | HGLibA_20336 | 254 | 1.378961547 | GRK6 | G protein-coupled receptor kinase 6 [Source:HGNC Symbol;Acc:HGNC:4545] |
| ENSG00000198633 | HGLibA_56183 | 254 | 1.378961547 | ZNF534 | zinc finger protein 534 [Source:HGNC Symbol;Acc:HGNC:26337] |
| ENSG00000204548 | HGLibA_12870 | 254 | 1.378961547 | DEFB121 | defensin beta 121 [Source:HGNC Symbol;Acc:HGNC:18101] |
| ENSG00000213203 | HGLibA_19071 | 254 | 1.378961547 | GIMAP1 | IMAP family member 1 [Source:HGNC Symbol;Acc:HGNC:23237]" |
| ENSG00000227471 | HGLibA_01477 | 254 | 1.378961547 | AKR1B15 | aldo-keto reductase family 1 member B15 [Source:HGNC Symbol;Acc:HGNC:37281] |
| ENSG00000257591 | HGLibA_56419 | 254 | 1.378961547 | ZNF625 | zinc finger protein 625 [Source:HGNC Symbol;Acc:HGNC:30571] |
| hsa-mir-302b | HGLibA_58440 | 254 | 1.378961547 | hsa-mir-302b | - |
| hsa-mir-4444-2 | HGLibA_60181 | 254 | 1.378961547 | hsa-mir-4444-2 | - |
| hsa-mir-5196 | HGLibA_61704 | 254 | 1.378961547 | hsa-mir-5196 | - |
| hsa-mir-7157 | HGLibA_63857 | 254 | 1.378961547 | hsa-mir-7157 | - |
| METTL20 | HGLibA_28974 | 254 | 1.378961547 | METTL20 | - |
| TCTE3 | HGLibA_48860 | 254 | 1.378961547 | TCTE3 | - |
| TMEM159 | HGLibA_50017 | 254 | 1.378961547 | TMEM159 | - |
| C15orf43 | HGLibA_05401 | 253 | 1.36901051 | C15orf43 | - |
| ENSG00000042980 | HGLibA_00765 | 253 | 1.36901051 | ADAM28 | ADAM metallopeptidase domain 28 [Source:HGNC Symbol;Acc:HGNC:206] |
| ENSG00000048028 | HGLibA_53319 | 253 | 1.36901051 | USP28 | ubiquitin specific peptidase 28 [Source:HGNC Symbol;Acc:HGNC:12625] |
| ENSG00000059145 | HGLibA_53051 | 253 | 1.36901051 | UNKL | unkempt family like zinc finger [Source:HGNC Symbol;Acc:HGNC:14184] |
| ENSG00000068001 | HGLibA_22441 | 253 | 1.36901051 | HYAL2 | hyaluronidase 2 [Source:HGNC Symbol;Acc:HGNC:5321] |
| ENSG00000078814 | HGLibA_30735 | 253 | 1.36901051 | MYH7B | myosin heavy chain 7B [Source:HGNC Symbol;Acc:HGNC:15906] |
| ENSG00000079432 | HGLibA_09695 | 253 | 1.36901051 | CIC | capicua transcriptional repressor [Source:HGNC Symbol;Acc:HGNC:14214] |
| ENSG00000102265 | HGLibA_49536 | 253 | 1.36901051 | TIMP1 | TIMP metallopeptidase inhibitor 1 [Source:HGNC Symbol;Acc:HGNC:11820] |
| ENSG00000102312 | HGLibA_37709 | 253 | 1.36901051 | PORCN | porcupine O-acyltransferase [Source:HGNC Symbol;Acc:HGNC:17652] |
| ENSG00000104866 | HGLibA_38095 | 253 | 1.36901051 | PPP1R37 | protein phosphatase 1 regulatory subunit 37 [Source:HGNC Symbol;Acc:HGNC:27607] |
| ENSG00000105738 | HGLibA_44197 | 253 | 1.36901051 | SIPA1L3 | signal induced proliferation associated 1 like 3 [Source:HGNC Symbol;Acc:HGNC:23801] |
| ENSG00000111450 | HGLibA_47643 | 253 | 1.36901051 | STX2 | syntaxin 2 [Source:HGNC Symbol;Acc:HGNC:3403] |
| ENSG00000114416 | HGLibA_18219 | 253 | 1.36901051 | FXR1 | FMR1 autosomal homolog 1 [Source:HGNC Symbol;Acc:HGNC:4023] |
| ENSG00000115211 | HGLibA_14743 | 253 | 1.36901051 | EIF2B4 | eukaryotic translation initiation factor 2B subunit delta [Source:HGNC Symbol;Acc:HGNC:3260] |
| ENSG00000130717 | HGLibA_52810 | 253 | 1.36901051 | UCK1 | uridine-cytidine kinase 1 [Source:HGNC Symbol;Acc:HGNC:14859] |
| ENSG00000132535 | HGLibA_13334 | 253 | 1.36901051 | DLG4 | discs large MAGUK scaffold protein 4 [Source:HGNC Symbol;Acc:HGNC:2903] |
| ENSG00000132781 | HGLibA_30586 | 253 | 1.36901051 | MUTYH | mutY DNA glycosylase [Source:HGNC Symbol;Acc:HGNC:7527] |
| ENSG00000133422 | HGLibA_29650 | 253 | 1.36901051 | MORC2 | MORC family CW-type zinc finger 2 [Source:HGNC Symbol;Acc:HGNC:23573] |
| ENSG00000148300 | HGLibA_40899 | 253 | 1.36901051 | REXO4 | 3'-5' exonuclease [Source:HGNC Symbol;Acc:HGNC:12820]" |
| ENSG00000155428 | HGLibA_51608 | 253 | 1.36901051 | TRIM74 | tripartite motif containing 74 [Source:HGNC Symbol;Acc:HGNC:17453] |
| ENSG00000162066 | HGLibA_01762 | 253 | 1.36901051 | AMDHD2 | amidohydrolase domain containing 2 [Source:HGNC Symbol;Acc:HGNC:24262] |
| ENSG00000164251 | HGLibA_15865 | 253 | 1.36901051 | F2RL1 | F2R like trypsin receptor 1 [Source:HGNC Symbol;Acc:HGNC:3538] |
| ENSG00000165985 | HGLibA_05929 | 253 | 1.36901051 | C1QL3 | complement C1q like 3 [Source:HGNC Symbol;Acc:HGNC:19359] |
| ENSG00000168256 | HGLibA_32019 | 253 | 1.36901051 | NKIRAS2 | NFKB inhibitor interacting Ras like 2 [Source:HGNC Symbol;Acc:HGNC:17898] |
| ENSG00000169855 | HGLibA_41732 | 253 | 1.36901051 | ROBO1 | roundabout guidance receptor 1 [Source:HGNC Symbol;Acc:HGNC:10249] |
| ENSG00000170525 | HGLibA_36158 | 253 | 1.36901051 | PFKFB3 | 6-biphosphatase 3 [Source:HGNC Symbol;Acc:HGNC:8874]" |
| ENSG00000177054 | HGLibA_55075 | 253 | 1.36901051 | ZDHHC13 | zinc finger DHHC-type containing 13 [Source:HGNC Symbol;Acc:HGNC:18413] |
| ENSG00000177098 | HGLibA_42996 | 253 | 1.36901051 | SCN4B | sodium voltage-gated channel beta subunit 4 [Source:HGNC Symbol;Acc:HGNC:10592] |
| ENSG00000181927 | HGLibA_34055 | 253 | 1.36901051 | OR4P4 | olfactory receptor family 4 subfamily P member 4 [Source:HGNC Symbol;Acc:HGNC:15180] |
| ENSG00000182782 | HGLibA_20950 | 253 | 1.36901051 | HCAR2 | hydroxycarboxylic acid receptor 2 [Source:HGNC Symbol;Acc:HGNC:24827] |
| ENSG00000183114 | HGLibA_16591 | 253 | 1.36901051 | FAM43B | family with sequence similarity 43 member B [Source:HGNC Symbol;Acc:HGNC:31791] |

| | | | | | |
|---|---|---|---|---|---|
| ENSG00000185158 | HGLibA_27334 | 253 | 1.36901051 | LRRC37B | leucine rich repeat containing 37B [Source:HGNC Symbol;Acc:HGNC:29070] |
| ENSG00000186119 | HGLibA_34282 | 253 | 1.36901051 | OR5D18 | olfactory receptor family 5 subfamily D member 18 [Source:HGNC Symbol;Acc:HGNC:15285] |
| ENSG00000186832 | HGLibA_25432 | 253 | 1.36901051 | KRT16 | keratin 16 [Source:HGNC Symbol;Acc:HGNC:6423] |
| ENSG00000196126 | HGLibA_21637 | 253 | 1.36901051 | HLA-DRB1 | DR beta 1 [Source:HGNC Symbol;Acc:HGNC:4948]" |
| ENSG00000197081 | HGLibA_22809 | 253 | 1.36901051 | IGF2R | insulin like growth factor 2 receptor [Source:HGNC Symbol;Acc:HGNC:5467] |
| ENSG00000205863 | HGLibA_05963 | 253 | 1.36901051 | C1QTNF9B | C1q and TNF related 9B [Source:HGNC Symbol;Acc:HGNC:34072] |
| ENSG00000214700 | HGLibA_05288 | 253 | 1.36901051 | C12orf71 | chromosome 12 open reading frame 71 [Source:HGNC Symbol;Acc:HGNC:34452] |
| ENSG00000234745 | HGLibA_21596 | 253 | 1.36901051 | HLA-B | B [Source:HGNC Symbol;Acc:HGNC:4932]" |
| ENSG00000234776 | HGLibA_05218 | 253 | 1.36901051 | C11orf94 | chromosome 11 open reading frame 94 [Source:HGNC Symbol;Acc:HGNC:37213] |
| ENSG00000234965 | HGLibA_44052 | 253 | 1.36901051 | SHISA8 | shisa family member 8 [Source:HGNC Symbol;Acc:HGNC:18351] |
| ENSG00000243509 | HGLibA_50786 | 253 | 1.36901051 | TNFRSF6B | TNF receptor superfamily member 6b [Source:HGNC Symbol;Acc:HGNC:11921] |
| ENSG00000259803 | HGLibA_44584 | 253 | 1.36901051 | SLC22A31 | solute carrier family 22 member 31 [Source:HGNC Symbol;Acc:HGNC:27091] |
| GIF | HGLibA_19061 | 253 | 1.36901051 | GIF | - |
| hsa-mir-433 | HGLibA_60045 | 253 | 1.36901051 | hsa-mir-433 | - |
| hsa-mir-4488 | HGLibA_60370 | 253 | 1.36901051 | hsa-mir-4488 | - |
| hsa-mir-5191 | HGLibA_61684 | 253 | 1.36901051 | hsa-mir-5191 | - |
| LOC284385 | HGLibA_26915 | 253 | 1.36901051 | LOC284385 | - |
| LOC646498 | HGLibA_26976 | 253 | 1.36901051 | LOC646498 | - |
| ATP5B | HGLibA_03640 | 252 | 1.359059472 | ATP5B | - |
| C14orf159 | HGLibA_05331 | 252 | 1.359059472 | C14orf159 | - |
| C20orf112 | HGLibA_05983 | 252 | 1.359059472 | C20orf112 | - |
| C8orf87 | HGLibA_06655 | 252 | 1.359059472 | C8orf87 | - |
| CXorf22 | HGLibA_11905 | 252 | 1.359059472 | CXorf22 | - |
| ENSG00000000938 | HGLibA_17468 | 252 | 1.359059472 | FGR | Src family tyrosine kinase [Source:HGNC Symbol;Acc:HGNC:3697]" |
| ENSG00000008405 | HGLibA_11239 | 252 | 1.359059472 | CRY1 | cryptochrome circadian regulator 1 [Source:HGNC Symbol;Acc:HGNC:2384] |
| ENSG00000083099 | HGLibA_27749 | 252 | 1.359059472 | LYRM2 | LYR motif containing 2 [Source:HGNC Symbol;Acc:HGNC:25229] |
| ENSG00000083782 | HGLibA_15396 | 252 | 1.359059472 | EPYC | epiphycan [Source:HGNC Symbol;Acc:HGNC:3053] |
| ENSG00000090447 | HGLibA_49137 | 252 | 1.359059472 | TFAP4 | transcription factor AP-4 [Source:HGNC Symbol;Acc:HGNC:11745] |
| ENSG00000100271 | HGLibA_52185 | 252 | 1.359059472 | TTLL1 | tubulin tyrosine ligase like 1 [Source:HGNC Symbol;Acc:HGNC:1312] |
| ENSG00000101182 | HGLibA_39087 | 252 | 1.359059472 | PSMA7 | proteasome subunit alpha 7 [Source:HGNC Symbol;Acc:HGNC:9536] |
| ENSG00000102837 | HGLibA_33353 | 252 | 1.359059472 | OLFM4 | olfactomedin 4 [Source:HGNC Symbol;Acc:HGNC:17190] |
| ENSG00000119321 | HGLibA_17560 | 252 | 1.359059472 | FKBP15 | FK506 binding protein 15 [Source:HGNC Symbol;Acc:HGNC:23397] |
| ENSG00000119650 | HGLibA_22757 | 252 | 1.359059472 | IFT43 | intraflagellar transport 43 [Source:HGNC Symbol;Acc:HGNC:29669] |
| ENSG00000127831 | HGLibA_53649 | 252 | 1.359059472 | VIL1 | villin 1 [Source:HGNC Symbol;Acc:HGNC:12690] |
| ENSG00000128645 | HGLibA_21982 | 252 | 1.359059472 | HOXD1 | homeobox D1 [Source:HGNC Symbol;Acc:HGNC:5132] |
| ENSG00000128710 | HGLibA_21987 | 252 | 1.359059472 | HOXD10 | homeobox D10 [Source:HGNC Symbol;Acc:HGNC:5133] |
| ENSG00000129187 | HGLibA_12540 | 252 | 1.359059472 | DCTD | dCMP deaminase [Source:HGNC Symbol;Acc:HGNC:2710] |
| ENSG00000129514 | HGLibA_17807 | 252 | 1.359059472 | FOXA1 | forkhead box A1 [Source:HGNC Symbol;Acc:HGNC:5021] |
| ENSG00000132256 | HGLibA_51534 | 252 | 1.359059472 | TRIM5 | tripartite motif containing 5 [Source:HGNC Symbol;Acc:HGNC:16276] |
| ENSG00000133454 | HGLibA_30815 | 252 | 1.359059472 | MYO18B | myosin XVIIIB [Source:HGNC Symbol;Acc:HGNC:18150] |
| ENSG00000136160 | HGLibA_14481 | 252 | 1.359059472 | EDNRB | endothelin receptor type B [Source:HGNC Symbol;Acc:HGNC:3180] |
| ENSG00000138381 | HGLibA_03337 | 252 | 1.359059472 | ASNSD1 | asparagine synthetase domain containing 1 [Source:HGNC Symbol;Acc:HGNC:24910] |
| ENSG00000138670 | HGLibA_40332 | 252 | 1.359059472 | RASGEF1B | RasGEF domain family member 1B [Source:HGNC Symbol;Acc:HGNC:24881] |
| ENSG00000142303 | HGLibA_00794 | 252 | 1.359059472 | ADAMTS10 | ADAM metallopeptidase with thrombospondin type 1 motif 10 [Source:HGNC Symbol;Acc:HGNC:13201] |
| ENSG00000150779 | HGLibA_49525 | 252 | 1.359059472 | TIMM8B | translocase of inner mitochondrial membrane 8 homolog B [Source:HGNC Symbol;Acc:HGNC:11818] |
| ENSG00000158985 | HGLibA_08583 | 252 | 1.359059472 | CDC42SE2 | CDC42 small effector 2 [Source:HGNC Symbol;Acc:HGNC:18547] |
| ENSG00000163394 | HGLibA_07955 | 252 | 1.359059472 | CCKAR | cholecystokinin A receptor [Source:HGNC Symbol;Acc:HGNC:1570] |
| ENSG00000163827 | HGLibA_27277 | 252 | 1.359059472 | LRRC2 | leucine rich repeat containing 2 [Source:HGNC Symbol;Acc:HGNC:14676] |
| ENSG00000165521 | HGLibA_15067 | 252 | 1.359059472 | EML5 | echinoderm microtubule associated protein like 5 [Source:HGNC Symbol;Acc:HGNC:18197] |
| ENSG00000166407 | HGLibA_26703 | 252 | 1.359059472 | LMO1 | LIM domain only 1 [Source:HGNC Symbol;Acc:HGNC:6641] |
| ENSG00000167011 | HGLibA_31174 | 252 | 1.359059472 | NAT16 | N-acetyltransferase 16 (putative) [Source:HGNC Symbol;Acc:HGNC:22030] |
| ENSG00000167178 | HGLibA_23673 | 252 | 1.359059472 | ISLR2 | immunoglobulin superfamily containing leucine rich repeat 2 [Source:HGNC Symbol;Acc:HGNC:29286] |
| ENSG00000169762 | HGLibA_48327 | 252 | 1.359059472 | TAPT1 | transmembrane anterior posterior transformation 1 [Source:HGNC Symbol;Acc:HGNC:26887] |
| ENSG00000171621 | HGLibA_46859 | 252 | 1.359059472 | SPSB1 | splA/ryanodine receptor domain and SOCS box containing 1 [Source:HGNC Symbol;Acc:HGNC:30628] |
| ENSG00000172673 | HGLibA_49350 | 252 | 1.359059472 | THEMIS | thymocyte selection associated [Source:HGNC Symbol;Acc:HGNC:21569] |
| ENSG00000172752 | HGLibA_10604 | 252 | 1.359059472 | COL6A5 | collagen type VI alpha 5 chain [Source:HGNC Symbol;Acc:HGNC:26674] |
| ENSG00000174038 | HGLibA_06677 | 252 | 1.359059472 | C9orf131 | chromosome 9 open reading frame 131 [Source:HGNC Symbol;Acc:HGNC:31418] |
| ENSG00000174226 | HGLibA_46086 | 252 | 1.359059472 | SNX31 | sorting nexin 31 [Source:HGNC Symbol;Acc:HGNC:28605] |
| ENSG00000174502 | HGLibA_44810 | 252 | 1.359059472 | SLC26A9 | solute carrier family 26 member 9 [Source:HGNC Symbol;Acc:HGNC:14469] |
| ENSG00000175336 | HGLibA_02521 | 252 | 1.359059472 | APOF | apolipoprotein F [Source:HGNC Symbol;Acc:HGNC:615] |
| ENSG00000179403 | HGLibA_53895 | 252 | 1.359059472 | VWA1 | von Willebrand factor A domain containing 1 [Source:HGNC Symbol;Acc:HGNC:30910] |
| ENSG00000182103 | HGLibA_16321 | 252 | 1.359059472 | FAM181B | family with sequence similarity 181 member B [Source:HGNC Symbol;Acc:HGNC:28512] |
| ENSG00000196123 | HGLibA_24661 | 252 | 1.359059472 | KIAA0895L | KIAA0895 like [Source:HGNC Symbol;Acc:HGNC:34408] |
| ENSG00000198488 | HGLibA_03996 | 252 | 1.359059472 | B3GNT6 | 3-N-acetylglucosaminyltransferase 6 [Source:HGNC Symbol;Acc:HGNC:24141]" |
| ENSG00000213886 | HGLibA_52571 | 252 | 1.359059472 | UBD | ubiquitin D [Source:HGNC Symbol;Acc:HGNC:18795] |
| ENSG00000234511 | HGLibA_06414 | 252 | 1.359059472 | C5orf58 | chromosome 5 open reading frame 58 [Source:HGNC Symbol;Acc:HGNC:37272] |
| ENSG00000241404 | HGLibA_14631 | 252 | 1.359059472 | EGFL8 | EGF like domain multiple 8 [Source:HGNC Symbol;Acc:HGNC:13944] |
| ENSG00000243073 | HGLibA_38317 | 252 | 1.359059472 | PRAMEF4 | PRAME family member 4 [Source:HGNC Symbol;Acc:HGNC:31971] |
| ENSG00000270011 | HGLibA_56238 | 252 | 1.359059472 | ZNF559-ZNF177 | ZNF559-ZNF177 readthrough [Source:HGNC Symbol;Acc:HGNC:42964] |
| ENSG00000288784 | HGLibA_11572 | 252 | 1.359059472 | CTAGE4 | - |
| HIST1H4A | HGLibA_21485 | 252 | 1.359059472 | HIST1H4A | - |
| hsa-mir-197 | HGLibA_58104 | 252 | 1.359059472 | hsa-mir-197 | - |
| hsa-mir-212 | HGLibA_58245 | 252 | 1.359059472 | hsa-mir-212 | - |
| hsa-mir-370 | HGLibA_59360 | 252 | 1.359059472 | hsa-mir-370 | - |
| hsa-mir-4284 | HGLibA_59842 | 252 | 1.359059472 | hsa-mir-4284 | - |
| hsa-mir-4669 | HGLibA_60792 | 252 | 1.359059472 | hsa-mir-4669 | - |
| hsa-mir-4749 | HGLibA_61095 | 252 | 1.359059472 | hsa-mir-4749 | - |
| hsa-mir-7106 | HGLibA_63786 | 252 | 1.359059472 | hsa-mir-7106 | - |
| TMEM173 | HGLibA_50063 | 252 | 1.359059472 | TMEM173 | - |
| EMR2 | HGLibA_15087 | 251 | 1.349108435 | EMR2 | - |
| ENSG00000007384 | HGLibA_41133 | 251 | 1.349108435 | RHBDF1 | rhomboid 5 homolog 1 [Source:HGNC Symbol;Acc:HGNC:20561] |
| ENSG00000016391 | HGLibA_09417 | 251 | 1.349108435 | CHDH | choline dehydrogenase [Source:HGNC Symbol;Acc:HGNC:24288] |
| ENSG00000064042 | HGLibA_26529 | 251 | 1.349108435 | LIMCH1 | LIM and calponin homology domains 1 [Source:HGNC Symbol;Acc:HGNC:29191] |
| ENSG00000085721 | HGLibA_42259 | 251 | 1.349108435 | RRN3 | RNA polymerase I transcription factor [Source:HGNC Symbol;Acc:HGNC:30346]" |
| ENSG00000101940 | HGLibA_54031 | 251 | 1.349108435 | WDR13 | WD repeat domain 13 [Source:HGNC Symbol;Acc:HGNC:14352] |
| ENSG00000105289 | HGLibA_49578 | 251 | 1.349108435 | TJP3 | tight junction protein 3 [Source:HGNC Symbol;Acc:HGNC:11829] |
| ENSG00000106605 | HGLibA_04576 | 251 | 1.349108435 | BLVRA | biliverdin reductase A [Source:HGNC Symbol;Acc:HGNC:1062] |
| ENSG00000108176 | HGLibA_13571 | 251 | 1.349108435 | DNAJC12 | DnaJ heat shock protein family (Hsp40) member C12 [Source:HGNC Symbol;Acc:HGNC:28908] |
| ENSG00000108244 | HGLibA_25453 | 251 | 1.349108435 | KRT23 | keratin 23 [Source:HGNC Symbol;Acc:HGNC:6438] |
| ENSG00000108840 | HGLibA_21015 | 251 | 1.349108435 | HDAC5 | histone deacetylase 5 [Source:HGNC Symbol;Acc:HGNC:14068] |
| ENSG00000110274 | HGLibA_09114 | 251 | 1.349108435 | CEP164 | centrosomal protein 164 [Source:HGNC Symbol;Acc:HGNC:29182] |
| ENSG00000114026 | HGLibA_33324 | 251 | 1.349108435 | OGG1 | 8-oxoguanine DNA glycosylase [Source:HGNC Symbol;Acc:HGNC:8125] |
| ENSG00000114374 | HGLibA_53419 | 251 | 1.349108435 | USP9Y | ubiquitin specific peptidase 9 Y-linked [Source:HGNC Symbol;Acc:HGNC:12633] |
| ENSG00000119509 | HGLibA_23441 | 251 | 1.349108435 | INVS | inversin [Source:HGNC Symbol;Acc:HGNC:17870] |
| ENSG00000121797 | HGLibA_08178 | 251 | 1.349108435 | CCRL2 | C-C motif chemokine receptor like 2 [Source:HGNC Symbol;Acc:HGNC:1612] |
| ENSG00000125255 | HGLibA_44334 | 251 | 1.349108435 | SLC10A2 | solute carrier family 10 member 2 [Source:HGNC Symbol;Acc:HGNC:10906] |
| ENSG00000125999 | HGLibA_04718 | 251 | 1.349108435 | BPIFB1 | BPI fold containing family B member 1 [Source:HGNC Symbol;Acc:HGNC:16108] |
| ENSG00000127585 | HGLibA_16986 | 251 | 1.349108435 | FBXL16 | F-box and leucine rich repeat protein 16 [Source:HGNC Symbol;Acc:HGNC:14150] |
| ENSG00000128872 | HGLibA_50598 | 251 | 1.349108435 | TMOD2 | tropomodulin 2 [Source:HGNC Symbol;Acc:HGNC:11872] |
| ENSG00000129515 | HGLibA_46103 | 251 | 1.349108435 | SNX6 | sorting nexin 6 [Source:HGNC Symbol;Acc:HGNC:14970] |
| ENSG00000134463 | HGLibA_14399 | 251 | 1.349108435 | ECHDC3 | enoyl-CoA hydratase domain containing 3 [Source:HGNC Symbol;Acc:HGNC:23489] |
| ENSG00000135569 | HGLibA_48129 | 251 | 1.349108435 | TAAR5 | trace amine associated receptor 5 [Source:HGNC Symbol;Acc:HGNC:30236] |
| ENSG00000149269 | HGLibA_35036 | 251 | 1.349108435 | PAK1 | p21 (RAC1) activated kinase 1 [Source:HGNC Symbol;Acc:HGNC:8590] |
| ENSG00000154485 | HGLibA_29514 | 251 | 1.349108435 | MMP21 | matrix metallopeptidase 21 [Source:HGNC Symbol;Acc:HGNC:14357] |
| ENSG00000157992 | HGLibA_25858 | 251 | 1.349108435 | KRTCAP3 | keratinocyte associated protein 3 [Source:HGNC Symbol;Acc:HGNC:28943] |
| ENSG00000163157 | HGLibA_50604 | 251 | 1.349108435 | TMOD4 | tropomodulin 4 [Source:HGNC Symbol;Acc:HGNC:11874] |
| ENSG00000163673 | HGLibA_12504 | 251 | 1.349108435 | DCLK3 | doublecortin like kinase 3 [Source:HGNC Symbol;Acc:HGNC:19005] |
| ENSG00000163818 | HGLibA_27799 | 251 | 1.349108435 | LZTFL1 | leucine zipper transcription factor like 1 [Source:HGNC Symbol;Acc:HGNC:6741] |
| ENSG00000164708 | HGLibA_36197 | 251 | 1.349108435 | PGAM2 | phosphoglycerate mutase 2 [Source:HGNC Symbol;Acc:HGNC:8889] |
| ENSG00000166166 | HGLibA_51698 | 251 | 1.349108435 | TRMT61A | tRNA methyltransferase 61A [Source:HGNC Symbol;Acc:HGNC:23790] |
| ENSG00000167065 | HGLibA_14152 | 251 | 1.349108435 | DUSP18 | dual specificity phosphatase 18 [Source:HGNC Symbol;Acc:HGNC:18484] |
| ENSG00000172009 | HGLibA_49382 | 251 | 1.349108435 | THOP1 | thimet oligopeptidase 1 [Source:HGNC Symbol;Acc:HGNC:11793] |
| ENSG00000172457 | HGLibA_34570 | 251 | 1.349108435 | OR9G4 | olfactory receptor family 9 subfamily G member 4 [Source:HGNC Symbol;Acc:HGNC:15322] |
| ENSG00000173572 | HGLibA_32109 | 251 | 1.349108435 | NLRP13 | NLR family pyrin domain containing 13 [Source:HGNC Symbol;Acc:HGNC:22937] |
| ENSG00000174276 | HGLibA_56926 | 251 | 1.349108435 | ZNHIT2 | zinc finger HIT-type containing 2 [Source:HGNC Symbol;Acc:HGNC:1177] |
| ENSG00000176567 | HGLibA_34067 | 251 | 1.349108435 | OR4X1 | olfactory receptor family 4 subfamily X member 1 (gene/pseudogene) [Source:HGNC Symbol;Acc:HGNC:14854] |
| ENSG00000179636 | HGLibA_51168 | 251 | 1.349108435 | TPPP2 | tubulin polymerization promoting protein family member 2 [Source:HGNC Symbol;Acc:HGNC:19293] |
| ENSG00000181072 | HGLibA_09549 | 251 | 1.349108435 | CHRM2 | cholinergic receptor muscarinic 2 [Source:HGNC Symbol;Acc:HGNC:1951] |
| ENSG00000186510 | HGLibA_09840 | 251 | 1.349108435 | CLCNKA | chloride voltage-gated channel Ka [Source:HGNC Symbol;Acc:HGNC:2026] |
| ENSG00000189079 | HGLibA_02889 | 251 | 1.349108435 | ARID2 | AT-rich interaction domain 2 [Source:HGNC Symbol;Acc:HGNC:18037] |
| ENSG00000196954 | HGLibA_07313 | 251 | 1.349108435 | CASP4 | caspase 4 [Source:HGNC Symbol;Acc:HGNC:1505] |
| ENSG00000206474 | HGLibA_33484 | 251 | 1.349108435 | OR10C1 | olfactory receptor family 10 subfamily C member 1 (gene/pseudogene) [Source:HGNC Symbol;Acc:HGNC:8165] |
| ENSG00000215183 | HGLibA_30228 | 251 | 1.349108435 | MSMP | prostate associated [Source:HGNC Symbol;Acc:HGNC:29663]" |
| ENSG00000228836 | HGLibA_11509 | 251 | 1.349108435 | CT45A5 | cancer/testis antigen family 45 member A5 [Source:HGNC Symbol;Acc:HGNC:33270] |
| ENSG00000243232 | HGLibA_35466 | 251 | 1.349108435 | PCDHAC2 | 2 [Source:HGNC Symbol;Acc:HGNC:8677]" |
| ENSG00000243678 | HGLibA_32157 | 251 | 1.349108435 | NME2 | NME/NM23 nucleoside diphosphate kinase 2 [Source:HGNC Symbol;Acc:HGNC:7850] |
| HIST1H4L | HGLibA_21518 | 251 | 1.349108435 | HIST1H4L | - |
| hsa-mir-27a | HGLibA_58377 | 251 | 1.349108435 | hsa-mir-27a | - |
| hsa-mir-4255 | HGLibA_59729 | 251 | 1.349108435 | hsa-mir-4255 | - |
| hsa-mir-548aw | HGLibA_61937 | 251 | 1.349108435 | hsa-mir-548aw | - |
| ANXA8L2 | HGLibA_02259 | 250 | 1.339157397 | ANXA8L2 | - |
| ENSG00000004776 | HGLibA_22313 | 250 | 1.339157397 | HSPB6 | heat shock protein family B (small) member 6 [Source:HGNC Symbol;Acc:HGNC:26511] |
| ENSG00000012504 | HGLibA_32585 | 250 | 1.339157397 | NR1H4 | nuclear receptor subfamily 1 group H member 4 [Source:HGNC Symbol;Acc:HGNC:7967] |
| ENSG00000018408 | HGLibA_54457 | 250 | 1.339157397 | WWTR1 | WW domain containing transcription regulator 1 [Source:HGNC Symbol;Acc:HGNC:24042] |
| ENSG00000033122 | HGLibA_27414 | 250 | 1.339157397 | LRRC7 | leucine rich repeat containing 7 [Source:HGNC Symbol;Acc:HGNC:18531] |

| | | | | | |
|---|---|---|---|---|---|
| ENSG00000075151 | HGLibA_14846 | 250 | 1.339157397 | EIF4G3 | eukaryotic translation initiation factor 4 gamma 3 [Source:HGNC Symbol;Acc:HGNC:3298] |
| ENSG00000090659 | HGLibA_08293 | 250 | 1.339157397 | CD209 | CD209 molecule [Source:HGNC Symbol;Acc:HGNC:1641] |
| ENSG00000095951 | HGLibA_21567 | 250 | 1.339157397 | HIVEP1 | human immunodeficiency virus type I enhancer binding protein 1 [Source:HGNC Symbol;Acc:HGNC:4920] |
| ENSG00000100813 | HGLibA_00440 | 250 | 1.339157397 | ACIN1 | apoptotic chromatin condensation inducer 1 [Source:HGNC Symbol;Acc:HGNC:17066] |
| ENSG00000101076 | HGLibA_21781 | 250 | 1.339157397 | HNF4A | hepatocyte nuclear factor 4 alpha [Source:HGNC Symbol;Acc:HGNC:5024] |
| ENSG00000101670 | HGLibA_26613 | 250 | 1.339157397 | LIPG | endothelial type [Source:HGNC Symbol;Acc:HGNC:6623]" |
| ENSG00000105011 | HGLibA_03283 | 250 | 1.339157397 | ASF1B | anti-silencing function 1B histone chaperone [Source:HGNC Symbol;Acc:HGNC:20996] |
| ENSG00000106290 | HGLibA_48247 | 250 | 1.339157397 | TAF6 | TATA-box binding protein associated factor 6 [Source:HGNC Symbol;Acc:HGNC:11540] |
| ENSG00000107758 | HGLibA_38188 | 250 | 1.339157397 | PPP3CB | protein phosphatase 3 catalytic subunit beta [Source:HGNC Symbol;Acc:HGNC:9315] |
| ENSG00000108239 | HGLibA_48474 | 250 | 1.339157397 | TBC1D12 | TBC1 domain family member 12 [Source:HGNC Symbol;Acc:HGNC:29082] |
| ENSG00000110448 | HGLibA_08401 | 250 | 1.339157397 | CD5 | CD5 molecule [Source:HGNC Symbol;Acc:HGNC:1685] |
| ENSG00000110931 | HGLibA_07091 | 250 | 1.339157397 | CAMKK2 | calcium/calmodulin dependent protein kinase kinase 2 [Source:HGNC Symbol;Acc:HGNC:1470] |
| ENSG00000116649 | HGLibA_46994 | 250 | 1.339157397 | SRM | spermidine synthase [Source:HGNC Symbol;Acc:HGNC:11296] |
| ENSG00000122711 | HGLibA_46693 | 250 | 1.339157397 | SPINK4 | Kazal type 4 [Source:HGNC Symbol;Acc:HGNC:16646]" |
| ENSG00000123607 | HGLibA_52071 | 250 | 1.339157397 | TTC21B | tetratricopeptide repeat domain 21B [Source:HGNC Symbol;Acc:HGNC:25660] |
| ENSG00000124159 | HGLibA_28436 | 250 | 1.339157397 | MATN4 | matrilin 4 [Source:HGNC Symbol;Acc:HGNC:6910] |
| ENSG00000126545 | HGLibA_11373 | 250 | 1.339157397 | CSN1S1 | casein alpha s1 [Source:HGNC Symbol;Acc:HGNC:2445] |
| ENSG00000138061 | HGLibA_12072 | 250 | 1.339157397 | CYP1B1 | cytochrome P450 family 1 subfamily B member 1 [Source:HGNC Symbol;Acc:HGNC:2597] |
| ENSG00000144560 | HGLibA_53645 | 250 | 1.339157397 | VGLL4 | vestigial like family member 4 [Source:HGNC Symbol;Acc:HGNC:28966] |
| ENSG00000149743 | HGLibA_51780 | 250 | 1.339157397 | TRPT1 | tRNA phosphotransferase 1 [Source:HGNC Symbol;Acc:HGNC:20316] |
| ENSG00000155307 | HGLibA_42711 | 250 | 1.339157397 | SAMSN1 | SH3 domain and nuclear localization signals 1 [Source:HGNC Symbol;Acc:HGNC:10528]" |
| ENSG00000161249 | HGLibA_13407 | 250 | 1.339157397 | DMKN | dermokine [Source:HGNC Symbol;Acc:HGNC:25063] |
| ENSG00000165188 | HGLibA_41590 | 250 | 1.339157397 | RNF183 | ring finger protein 183 [Source:HGNC Symbol;Acc:HGNC:28721] |
| ENSG00000165886 | HGLibA_52766 | 250 | 1.339157397 | UBTD1 | ubiquitin domain containing 1 [Source:HGNC Symbol;Acc:HGNC:25683] |
| ENSG00000166529 | HGLibA_57002 | 250 | 1.339157397 | ZSCAN21 | zinc finger and SCAN domain containing 21 [Source:HGNC Symbol;Acc:HGNC:13104] |
| ENSG00000168397 | HGLibA_03492 | 250 | 1.339157397 | ATG4B | autophagy related 4B cysteine peptidase [Source:HGNC Symbol;Acc:HGNC:20790] |
| ENSG00000170044 | HGLibA_56967 | 250 | 1.339157397 | ZPLD1 | zona pellucida like domain containing 1 [Source:HGNC Symbol;Acc:HGNC:27022] |
| ENSG00000170324 | HGLibA_18034 | 250 | 1.339157397 | FRMPD2 | FERM and PDZ domain containing 2 [Source:HGNC Symbol;Acc:HGNC:28572] |
| ENSG00000171561 | HGLibA_33760 | 250 | 1.339157397 | OR2AT4 | olfactory receptor family 2 subfamily AT member 4 [Source:HGNC Symbol;Acc:HGNC:19620] |
| ENSG00000175309 | HGLibA_36480 | 250 | 1.339157397 | PHYKPL | 5-phosphohydroxy-L-lysine phospho-lyase [Source:HGNC Symbol;Acc:HGNC:28249] |
| ENSG00000176396 | HGLibA_14697 | 250 | 1.339157397 | EID2 | EP300 interacting inhibitor of differentiation 2 [Source:HGNC Symbol;Acc:HGNC:28292] |
| ENSG00000179820 | HGLibA_30631 | 250 | 1.339157397 | MYADM | myeloid associated differentiation marker [Source:HGNC Symbol;Acc:HGNC:7544] |
| ENSG00000180875 | HGLibA_20246 | 250 | 1.339157397 | GREM2 | DAN family BMP antagonist [Source:HGNC Symbol;Acc:HGNC:17655]" |
| ENSG00000182158 | HGLibA_11097 | 250 | 1.339157397 | CREB3L2 | cAMP responsive element binding protein 3 like 2 [Source:HGNC Symbol;Acc:HGNC:23720] |
| ENSG00000184571 | HGLibA_36777 | 250 | 1.339157397 | PIWIL3 | piwi like RNA-mediated gene silencing 3 [Source:HGNC Symbol;Acc:HGNC:18443] |
| ENSG00000196961 | HGLibA_02305 | 250 | 1.339157397 | AP2A1 | adaptor related protein complex 2 subunit alpha 1 [Source:HGNC Symbol;Acc:HGNC:561] |
| ENSG00000204695 | HGLibA_33637 | 250 | 1.339157397 | OR14J1 | olfactory receptor family 14 subfamily J member 1 [Source:HGNC Symbol;Acc:HGNC:13971] |
| ENSG00000204969 | HGLibA_35440 | 250 | 1.339157397 | PCDHA2 | protocadherin alpha 2 [Source:HGNC Symbol;Acc:HGNC:8668] |
| ENSG00000205060 | HGLibA_44967 | 250 | 1.339157397 | SLC35B4 | solute carrier family 35 member B4 [Source:HGNC Symbol;Acc:HGNC:20584] |
| ENSG00000205362 | HGLibA_30278 | 250 | 1.339157397 | MT1A | metallothionein 1A [Source:HGNC Symbol;Acc:HGNC:7393] |
| ENSG00000212933 | HGLibA_25640 | 250 | 1.339157397 | KRTAP12-4 | keratin associated protein 12-4 [Source:HGNC Symbol;Acc:HGNC:20532] |
| ENSG00000240403 | HGLibA_24994 | 250 | 1.339157397 | KIR3DL2 | three Ig domains and long cytoplasmic tail 2 [Source:HGNC Symbol;Acc:HGNC:6339]" |
| ENSG00000254550 | HGLibA_33390 | 250 | 1.339157397 | OMP | olfactory marker protein [Source:HGNC Symbol;Acc:HGNC:8136] |
| hsa-mir-124-1 | HGLibA_57345 | 250 | 1.339157397 | hsa-mir-124-1 | - |
| hsa-mir-599 | HGLibA_62488 | 250 | 1.339157397 | hsa-mir-599 | - |
| C1orf228 | HGLibA_05839 | 249 | 1.32920636 | C1orf228 | - |
| C22orf24 | HGLibA_06060 | 249 | 1.32920636 | C22orf24 | - |
| ENSG00000006576 | HGLibA_36466 | 249 | 1.32920636 | PHTF2 | putative homeodomain transcription factor 2 [Source:HGNC Symbol;Acc:HGNC:13411] |
| ENSG00000008513 | HGLibA_47236 | 249 | 1.32920636 | ST3GAL1 | 3-sialyltransferase 1 [Source:HGNC Symbol;Acc:HGNC:10862]" |
| ENSG00000068354 | HGLibA_48515 | 249 | 1.32920636 | TBC1D25 | TBC1 domain family member 25 [Source:HGNC Symbol;Acc:HGNC:8092] |
| ENSG00000088888 | HGLibA_28446 | 249 | 1.32920636 | MAVS | mitochondrial antiviral signaling protein [Source:HGNC Symbol;Acc:HGNC:29233] |
| ENSG00000099917 | HGLibA_28757 | 249 | 1.32920636 | MED15 | mediator complex subunit 15 [Source:HGNC Symbol;Acc:HGNC:14248] |
| ENSG00000100314 | HGLibA_06861 | 249 | 1.32920636 | CABP7 | calcium binding protein 7 [Source:HGNC Symbol;Acc:HGNC:20834] |
| ENSG00000105784 | HGLibA_42452 | 249 | 1.32920636 | RUNDC3B | RUN domain containing 3B [Source:HGNC Symbol;Acc:HGNC:30286] |
| ENSG00000106077 | HGLibA_00236 | 249 | 1.32920636 | ABHD11 | abhydrolase domain containing 11 [Source:HGNC Symbol;Acc:HGNC:16407] |
| ENSG00000106484 | HGLibA_28923 | 249 | 1.32920636 | MEST | mesoderm specific transcript [Source:HGNC Symbol;Acc:HGNC:7028] |
| ENSG00000108774 | HGLibA_39967 | 249 | 1.32920636 | RAB5C | member RAS oncogene family [Source:HGNC Symbol;Acc:HGNC:9785]" |
| ENSG00000111331 | HGLibA_33203 | 249 | 1.32920636 | OAS3 | 2'-5'-oligoadenylate synthetase 3 [Source:HGNC Symbol;Acc:HGNC:8088] |
| ENSG00000112406 | HGLibA_21091 | 249 | 1.32920636 | HECA | cell cycle regulator [Source:HGNC Symbol;Acc:HGNC:21041]" |
| ENSG00000121764 | HGLibA_20988 | 249 | 1.32920636 | HCRTR1 | hypocretin receptor 1 [Source:HGNC Symbol;Acc:HGNC:4848] |
| ENSG00000125879 | HGLibA_34743 | 249 | 1.32920636 | OTOR | otoraplin [Source:HGNC Symbol;Acc:HGNC:8517] |
| ENSG00000132874 | HGLibA_44403 | 249 | 1.32920636 | SLC14A2 | solute carrier family 14 member 2 [Source:HGNC Symbol;Acc:HGNC:10919] |
| ENSG00000134853 | HGLibA_35853 | 249 | 1.32920636 | PDGFRA | platelet derived growth factor receptor alpha [Source:HGNC Symbol;Acc:HGNC:8803] |
| ENSG00000136933 | HGLibA_40005 | 249 | 1.32920636 | RABEPK | Rab9 effector protein with kelch motifs [Source:HGNC Symbol;Acc:HGNC:16896] |
| ENSG00000140400 | HGLibA_28082 | 249 | 1.32920636 | MAN2C1 | mannosidase alpha class 2C member 1 [Source:HGNC Symbol;Acc:HGNC:6827] |
| ENSG00000140553 | HGLibA_53005 | 249 | 1.32920636 | UNC45A | unc-45 myosin chaperone A [Source:HGNC Symbol;Acc:HGNC:30594] |
| ENSG00000144152 | HGLibA_16951 | 249 | 1.32920636 | FBLN7 | fibulin 7 [Source:HGNC Symbol;Acc:HGNC:26740] |
| ENSG00000145990 | HGLibA_18952 | 249 | 1.32920636 | GFOD1 | glucose-fructose oxidoreductase domain containing 1 [Source:HGNC Symbol;Acc:HGNC:21096] |
| ENSG00000146872 | HGLibA_49624 | 249 | 1.32920636 | TLK2 | tousled like kinase 2 [Source:HGNC Symbol;Acc:HGNC:11842] |
| ENSG00000147536 | HGLibA_19113 | 249 | 1.32920636 | GINS4 | GINS complex subunit 4 [Source:HGNC Symbol;Acc:HGNC:28226] |
| ENSG00000151240 | HGLibA_13254 | 249 | 1.32920636 | DIP2C | disco interacting protein 2 homolog C [Source:HGNC Symbol;Acc:HGNC:29150] |
| ENSG00000156976 | HGLibA_14807 | 249 | 1.32920636 | EIF4A2 | eukaryotic translation initiation factor 4A2 [Source:HGNC Symbol;Acc:HGNC:3284] |
| ENSG00000157542 | HGLibA_24285 | 249 | 1.32920636 | KCNJ6 | potassium voltage-gated channel subfamily J member 6 [Source:HGNC Symbol;Acc:HGNC:6267] |
| ENSG00000158481 | HGLibA_08270 | 249 | 1.32920636 | CD1C | CD1c molecule [Source:HGNC Symbol;Acc:HGNC:1636] |
| ENSG00000161638 | HGLibA_23736 | 249 | 1.32920636 | ITGA5 | integrin subunit alpha 5 [Source:HGNC Symbol;Acc:HGNC:6141] |
| ENSG00000163126 | HGLibA_02042 | 249 | 1.32920636 | ANKRD23 | ankyrin repeat domain 23 [Source:HGNC Symbol;Acc:HGNC:24470] |
| ENSG00000163348 | HGLibA_39696 | 249 | 1.32920636 | PYGO2 | pygopus family PHD finger 2 [Source:HGNC Symbol;Acc:HGNC:30257] |
| ENSG00000163517 | HGLibA_21003 | 249 | 1.32920636 | HDAC11 | histone deacetylase 11 [Source:HGNC Symbol;Acc:HGNC:19086] |
| ENSG00000164334 | HGLibA_16257 | 249 | 1.32920636 | FAM170A | family with sequence similarity 170 member A [Source:HGNC Symbol;Acc:HGNC:27963] |
| ENSG00000166833 | HGLibA_31198 | 249 | 1.32920636 | NAV2 | neuron navigator 2 [Source:HGNC Symbol;Acc:HGNC:15997] |
| ENSG00000169618 | HGLibA_38670 | 249 | 1.32920636 | PROKR1 | prokineticin receptor 1 [Source:HGNC Symbol;Acc:HGNC:4524] |
| ENSG00000170054 | HGLibA_43531 | 249 | 1.32920636 | SERPINA9 | serpin family A member 9 [Source:HGNC Symbol;Acc:HGNC:15995] |
| ENSG00000171291 | HGLibA_55995 | 249 | 1.32920636 | ZNF439 | zinc finger protein 439 [Source:HGNC Symbol;Acc:HGNC:20873] |
| ENSG00000173511 | HGLibA_53613 | 249 | 1.32920636 | VEGFB | vascular endothelial growth factor B [Source:HGNC Symbol;Acc:HGNC:12681] |
| ENSG00000175548 | HGLibA_01607 | 249 | 1.32920636 | ALG10B | 2-glucosyltransferase [Source:HGNC Symbol;Acc:HGNC:31088]" |
| ENSG00000175894 | HGLibA_51956 | 249 | 1.32920636 | TSPEAR | thrombospondin type laminin G domain and EAR repeats [Source:HGNC Symbol;Acc:HGNC:1268] |
| ENSG00000178075 | HGLibA_20199 | 249 | 1.32920636 | GRAMD1C | GRAM domain containing 1C [Source:HGNC Symbol;Acc:HGNC:25252] |
| ENSG00000178950 | HGLibA_18490 | 249 | 1.32920636 | GAK | cyclin G associated kinase [Source:HGNC Symbol;Acc:HGNC:4113] |
| ENSG00000179058 | HGLibA_06739 | 249 | 1.32920636 | C9orf50 | chromosome 9 open reading frame 50 [Source:HGNC Symbol;Acc:HGNC:23677] |
| ENSG00000180855 | HGLibA_56012 | 249 | 1.32920636 | ZNF443 | zinc finger protein 443 [Source:HGNC Symbol;Acc:HGNC:20878] |
| ENSG00000182107 | HGLibA_50364 | 249 | 1.32920636 | TMEM30B | transmembrane protein 30B [Source:HGNC Symbol;Acc:HGNC:27254] |
| ENSG00000182718 | HGLibA_02232 | 249 | 1.32920636 | ANXA2 | annexin A2 [Source:HGNC Symbol;Acc:HGNC:537] |
| ENSG00000183605 | HGLibA_43790 | 249 | 1.32920636 | SFXN4 | sideroflexin 4 [Source:HGNC Symbol;Acc:HGNC:16088] |
| ENSG00000184731 | HGLibA_16017 | 249 | 1.32920636 | FAM110C | family with sequence similarity 110 member C [Source:HGNC Symbol;Acc:HGNC:33340] |
| ENSG00000185753 | HGLibA_11919 | 249 | 1.32920636 | CXorf38 | chromosome X open reading frame 38 [Source:HGNC Symbol;Acc:HGNC:28589] |
| ENSG00000186866 | HGLibA_37472 | 249 | 1.32920636 | POFUT2 | protein O-fucosyltransferase 2 [Source:HGNC Symbol;Acc:HGNC:14683] |
| ENSG00000196422 | HGLibA_38080 | 249 | 1.32920636 | PPP1R26 | protein phosphatase 1 regulatory subunit 26 [Source:HGNC Symbol;Acc:HGNC:29089] |
| ENSG00000204071 | HGLibA_48720 | 249 | 1.32920636 | TCEAL6 | transcription elongation factor A like 6 [Source:HGNC Symbol;Acc:HGNC:24553] |
| ENSG00000204671 | HGLibA_23131 | 249 | 1.32920636 | IL31 | interleukin 31 [Source:HGNC Symbol;Acc:HGNC:19372] |
| ENSG00000205329 | HGLibA_34387 | 249 | 1.32920636 | OR6C3 | olfactory receptor family 6 subfamily C member 3 [Source:HGNC Symbol;Acc:HGNC:15437] |
| ENSG00000205642 | HGLibA_53590 | 249 | 1.32920636 | VCX3B | variable charge X-linked 3B [Source:HGNC Symbol;Acc:HGNC:31838] |
| ENSG00000214309 | HGLibA_28502 | 249 | 1.32920636 | MBLAC1 | metallo-beta-lactamase domain containing 1 [Source:HGNC Symbol;Acc:HGNC:22180] |
| ENSG00000234906 | HGLibA_02507 | 249 | 1.32920636 | APOC2 | apolipoprotein C2 [Source:HGNC Symbol;Acc:HGNC:609] |
| ENSG00000240303 | HGLibA_00355 | 249 | 1.32920636 | ACAD11 | acyl-CoA dehydrogenase family member 11 [Source:HGNC Symbol;Acc:HGNC:30211] |
| hsa-mir-139 | HGLibA_57820 | 249 | 1.32920636 | hsa-mir-139 | - |
| hsa-mir-139 | HGLibA_57822 | 249 | 1.32920636 | hsa-mir-139 | - |
| hsa-mir-219b | HGLibA_58284 | 249 | 1.32920636 | hsa-mir-219b | - |
| hsa-mir-32 | HGLibA_58905 | 249 | 1.32920636 | hsa-mir-32 | - |
| hsa-mir-3679 | HGLibA_59266 | 249 | 1.32920636 | hsa-mir-3679 | - |
| hsa-mir-5189 | HGLibA_61647 | 249 | 1.32920636 | hsa-mir-5189 | - |
| 12-Sep | HGLibA_43434 | 249 | 1.32920636 | 12-Sep | - |
| ENSG00000004846 | HGLibA_00144 | 248 | 1.319255323 | ABCB5 | ATP binding cassette subfamily B member 5 [Source:HGNC Symbol;Acc:HGNC:46] |
| ENSG00000042493 | HGLibA_07140 | 248 | 1.319255323 | CAPG | gelsolin like [Source:HGNC Symbol;Acc:HGNC:1474]" |
| ENSG00000044446 | HGLibA_36405 | 248 | 1.319255323 | PHKA2 | phosphorylase kinase regulatory subunit alpha 2 [Source:HGNC Symbol;Acc:HGNC:8926] |
| ENSG00000046651 | HGLibA_33299 | 248 | 1.319255323 | OFD1 | centriole and centriolar satellite protein [Source:HGNC Symbol;Acc:HGNC:2567]" |
| ENSG00000070190 | HGLibA_12333 | 248 | 1.319255323 | DAPP1 | dual adaptor of phosphotyrosine and 3-phosphoinositides 1 [Source:HGNC Symbol;Acc:HGNC:16500] |
| ENSG00000076928 | HGLibA_02804 | 248 | 1.319255323 | ARHGEF1 | Rho guanine nucleotide exchange factor 1 [Source:HGNC Symbol;Acc:HGNC:681] |
| ENSG00000085382 | HGLibA_20800 | 248 | 1.319255323 | HACE1 | HECT domain and ankyrin repeat containing E3 ubiquitin protein ligase 1 [Source:HGNC Symbol;Acc:HGNC:21033] |
| ENSG00000088992 | HGLibA_49026 | 248 | 1.319255323 | TESC | tescalcin [Source:HGNC Symbol;Acc:HGNC:26065] |
| ENSG00000104044 | HGLibA_33239 | 248 | 1.319255323 | OCA2 | OCA2 melanosomal transmembrane protein [Source:HGNC Symbol;Acc:HGNC:8101] |
| ENSG00000104133 | HGLibA_46630 | 248 | 1.319255323 | SPG11 | spatacsin vesicle trafficking associated [Source:HGNC Symbol;Acc:HGNC:11226]" |
| ENSG00000104755 | HGLibA_00750 | 248 | 1.319255323 | ADAM2 | ADAM metallopeptidase domain 2 [Source:HGNC Symbol;Acc:HGNC:198] |
| ENSG00000104901 | HGLibA_13307 | 248 | 1.319255323 | DKKL1 | dickkopf like acrosomal protein 1 [Source:HGNC Symbol;Acc:HGNC:16528] |
| ENSG00000107890 | HGLibA_02048 | 248 | 1.319255323 | ANKRD26 | ankyrin repeat domain 26 [Source:HGNC Symbol;Acc:HGNC:29186] |
| ENSG00000110092 | HGLibA_08073 | 248 | 1.319255323 | CCND1 | cyclin D1 [Source:HGNC Symbol;Acc:HGNC:1582] |
| ENSG00000121236 | HGLibA_51557 | 248 | 1.319255323 | TRIM6 | tripartite motif containing 6 [Source:HGNC Symbol;Acc:HGNC:16277] |
| ENSG00000121578 | HGLibA_04032 | 248 | 1.319255323 | B4GALT4 | 4-galactosyltransferase 4 [Source:HGNC Symbol;Acc:HGNC:927]" |
| ENSG00000122375 | HGLibA_33438 | 248 | 1.319255323 | OPN4 | opsin 4 [Source:HGNC Symbol;Acc:HGNC:14449] |
| ENSG00000124299 | HGLibA_36051 | 248 | 1.319255323 | PEPD | peptidase D [Source:HGNC Symbol;Acc:HGNC:8840] |
| ENSG00000124374 | HGLibA_35034 | 248 | 1.319255323 | PAIP2B | poly(A) binding protein interacting protein 2B [Source:HGNC Symbol;Acc:HGNC:29200] |
| ENSG00000127362 | HGLibA_48385 | 248 | 1.319255323 | TAS2R3 | taste 2 receptor member 3 [Source:HGNC Symbol;Acc:HGNC:14910] |
| ENSG00000127743 | HGLibA_22993 | 248 | 1.319255323 | IL17B | interleukin 17B [Source:HGNC Symbol;Acc:HGNC:5982] |
| ENSG00000129749 | HGLibA_09565 | 248 | 1.319255323 | CHRNA10 | cholinergic receptor nicotinic alpha 10 subunit [Source:HGNC Symbol;Acc:HGNC:13800] |
| ENSG00000139112 | HGLibA_18334 | 248 | 1.319255323 | GABARAPL1 | GABA type A receptor associated protein like 1 [Source:HGNC Symbol;Acc:HGNC:4068] |
| ENSG00000143183 | HGLibA_49781 | 248 | 1.319255323 | TMCO1 | transmembrane and coiled-coil domains 1 [Source:HGNC Symbol;Acc:HGNC:18188] |

| | | | | | |
|---|---|---|---|---|---|
| ENSG00000143382 | HGLibA_00858 | 248 | 1.319255323 | ADAMTSL4 | ADAMTS like 4 [Source:HGNC Symbol;Acc:HGNC:19706] |
| ENSG00000145244 | HGLibA_10771 | 248 | 1.319255323 | CORIN | serine peptidase [Source:HGNC Symbol;Acc:HGNC:19012]" |
| ENSG00000149262 | HGLibA_23421 | 248 | 1.319255323 | INTS4 | integrator complex subunit 4 [Source:HGNC Symbol;Acc:HGNC:25048] |
| ENSG00000151500 | HGLibA_49427 | 248 | 1.319255323 | THYN1 | thymocyte nuclear protein 1 [Source:HGNC Symbol;Acc:HGNC:29560] |
| ENSG00000151914 | HGLibA_14043 | 248 | 1.319255323 | DST | dystonin [Source:HGNC Symbol;Acc:HGNC:1090] |
| ENSG00000156097 | HGLibA_20062 | 248 | 1.319255323 | GPR61 | G protein-coupled receptor 61 [Source:HGNC Symbol;Acc:HGNC:13300] |
| ENSG00000161395 | HGLibA_36212 | 248 | 1.319255323 | PGAP3 | post-GPI attachment to proteins 3 [Source:HGNC Symbol;Acc:HGNC:23719] |
| ENSG00000163207 | HGLibA_23879 | 248 | 1.319255323 | IVL | involucrin [Source:HGNC Symbol;Acc:HGNC:6187] |
| ENSG00000168903 | HGLibA_04977 | 248 | 1.319255323 | BTNL3 | butyrophilin like 3 [Source:HGNC Symbol;Acc:HGNC:1143] |
| ENSG00000169221 | HGLibA_48466 | 248 | 1.319255323 | TBC1D10B | TBC1 domain family member 10B [Source:HGNC Symbol;Acc:HGNC:24510] |
| ENSG00000169488 | HGLibA_34025 | 248 | 1.319255323 | OR4K15 | olfactory receptor family 4 subfamily K member 15 [Source:HGNC Symbol;Acc:HGNC:15353] |
| ENSG00000171942 | HGLibA_33510 | 248 | 1.319255323 | OR10H2 | olfactory receptor family 10 subfamily H member 2 [Source:HGNC Symbol;Acc:HGNC:8173] |
| ENSG00000172201 | HGLibA_22533 | 248 | 1.319255323 | ID4 | HLH protein [Source:HGNC Symbol;Acc:HGNC:5363]" |
| ENSG00000173585 | HGLibA_08172 | 248 | 1.319255323 | CCR9 | C-C motif chemokine receptor 9 [Source:HGNC Symbol;Acc:HGNC:1610] |
| ENSG00000174306 | HGLibA_55318 | 248 | 1.319255323 | ZHX3 | zinc fingers and homeoboxes 3 [Source:HGNC Symbol;Acc:HGNC:15935] |
| ENSG00000177963 | HGLibA_41235 | 248 | 1.319255323 | RIC8A | RIC8 guanine nucleotide exchange factor A [Source:HGNC Symbol;Acc:HGNC:29550] |
| ENSG00000180287 | HGLibA_37046 | 248 | 1.319255323 | PLD5 | phospholipase D family member 5 [Source:HGNC Symbol;Acc:HGNC:26879] |
| ENSG00000180483 | HGLibA_12867 | 248 | 1.319255323 | DEFB119 | defensin beta 119 [Source:HGNC Symbol;Acc:HGNC:18099] |
| ENSG00000182325 | HGLibA_17014 | 248 | 1.319255323 | FBXL6 | F-box and leucine rich repeat protein 6 [Source:HGNC Symbol;Acc:HGNC:13603] |
| ENSG00000183304 | HGLibA_16817 | 248 | 1.319255323 | FAM9A | family with sequence similarity 9 member A [Source:HGNC Symbol;Acc:HGNC:18403] |
| ENSG00000197437 | HGLibA_33619 | 248 | 1.319255323 | OR13G1 | olfactory receptor family 13 subfamily G member 1 [Source:HGNC Symbol;Acc:HGNC:14999] |
| ENSG00000197706 | HGLibA_34405 | 248 | 1.319255323 | OR6C74 | olfactory receptor family 6 subfamily C member 74 [Source:HGNC Symbol;Acc:HGNC:31303] |
| ENSG00000213619 | HGLibA_31550 | 248 | 1.319255323 | NDUFS3 | NADH:ubiquinone oxidoreductase core subunit S3 [Source:HGNC Symbol;Acc:HGNC:7710] |
| ENSG00000232258 | HGLibA_49880 | 248 | 1.319255323 | TMEM114 | transmembrane protein 114 [Source:HGNC Symbol;Acc:HGNC:33227] |
| ENSG00000279051 | HGLibA_34438 | 248 | 1.319255323 | OR6Q1 | olfactory receptor family 6 subfamily Q member 1 (gene/pseudogene) [Source:HGNC Symbol;Acc:HGNC:15302] |
| FAM21C | HGLibA_16509 | 248 | 1.319255323 | FAM21C | - |
| hsa-mir-3689d-2 | HGLibA_59329 | 248 | 1.319255323 | hsa-mir-3689d-2 | - |
| hsa-mir-3939 | HGLibA_59618 | 248 | 1.319255323 | hsa-mir-3939 | - |
| hsa-mir-4523 | HGLibA_60550 | 248 | 1.319255323 | hsa-mir-4523 | - |
| hsa-mir-545 | HGLibA_61831 | 248 | 1.319255323 | hsa-mir-545 | - |
| SPRY3_X | HGLibA_46842 | 248 | 1.319255323 | SPRY3_X | - |
| ATP5J2-PTCD1 | HGLibA_03675 | 247 | 1.309304285 | ATP5J2-PTCD1 | - |
| BAGE5 | HGLibA_04105 | 247 | 1.309304285 | BAGE5 | - |
| ENSG00000038427 | HGLibA_53570 | 247 | 1.309304285 | VCAN | versican [Source:HGNC Symbol;Acc:HGNC:2464] |
| ENSG00000079101 | HGLibA_10165 | 247 | 1.309304285 | CLUL1 | clusterin like 1 [Source:HGNC Symbol;Acc:HGNC:2096] |
| ENSG00000084676 | HGLibA_31336 | 247 | 1.309304285 | NCOA1 | nuclear receptor coactivator 1 [Source:HGNC Symbol;Acc:HGNC:7668] |
| ENSG00000104371 | HGLibA_13303 | 247 | 1.309304285 | DKK4 | dickkopf WNT signaling pathway inhibitor 4 [Source:HGNC Symbol;Acc:HGNC:2894] |
| ENSG00000108671 | HGLibA_39154 | 247 | 1.309304285 | PSMD11 | non-ATPase 11 [Source:HGNC Symbol;Acc:HGNC:9556]" |
| ENSG00000116717 | HGLibA_18416 | 247 | 1.309304285 | GADD45A | growth arrest and DNA damage inducible alpha [Source:HGNC Symbol;Acc:HGNC:4095] |
| ENSG00000122420 | HGLibA_39340 | 247 | 1.309304285 | PTGFR | prostaglandin F receptor [Source:HGNC Symbol;Acc:HGNC:9600] |
| ENSG00000124205 | HGLibA_14476 | 247 | 1.309304285 | EDN3 | endothelin 3 [Source:HGNC Symbol;Acc:HGNC:3178] |
| ENSG00000124302 | HGLibA_09649 | 247 | 1.309304285 | CHST8 | carbohydrate sulfotransferase 8 [Source:HGNC Symbol;Acc:HGNC:15993] |
| ENSG00000130783 | HGLibA_07820 | 247 | 1.309304285 | CCDC62 | coiled-coil domain containing 62 [Source:HGNC Symbol;Acc:HGNC:30723] |
| ENSG00000135409 | HGLibA_01788 | 247 | 1.309304285 | AMHR2 | anti-Mullerian hormone receptor type 2 [Source:HGNC Symbol;Acc:HGNC:465] |
| ENSG00000137449 | HGLibA_10919 | 247 | 1.309304285 | CPEB2 | cytoplasmic polyadenylation element binding protein 2 [Source:HGNC Symbol;Acc:HGNC:21745] |
| ENSG00000141858 | HGLibA_42659 | 247 | 1.309304285 | SAMD1 | sterile alpha motif domain containing 1 [Source:HGNC Symbol;Acc:HGNC:17958] |
| ENSG00000143869 | HGLibA_18876 | 247 | 1.309304285 | GDF7 | growth differentiation factor 7 [Source:HGNC Symbol;Acc:HGNC:4222] |
| ENSG00000146926 | HGLibA_03205 | 247 | 1.309304285 | ASB10 | ankyrin repeat and SOCS box containing 10 [Source:HGNC Symbol;Acc:HGNC:17185] |
| ENSG00000148516 | HGLibA_55135 | 247 | 1.309304285 | ZEB1 | zinc finger E-box binding homeobox 1 [Source:HGNC Symbol;Acc:HGNC:11642] |
| ENSG00000149043 | HGLibA_48089 | 247 | 1.309304285 | SYT8 | synaptotagmin 8 [Source:HGNC Symbol;Acc:HGNC:19264] |
| ENSG00000159210 | HGLibA_45927 | 247 | 1.309304285 | SNF8 | ESCRT-II complex subunit [Source:HGNC Symbol;Acc:HGNC:17028]" |
| ENSG00000159884 | HGLibA_07527 | 247 | 1.309304285 | CCDC107 | coiled-coil domain containing 107 [Source:HGNC Symbol;Acc:HGNC:28465] |
| ENSG00000161921 | HGLibA_11859 | 247 | 1.309304285 | CXCL16 | C-X-C motif chemokine ligand 16 [Source:HGNC Symbol;Acc:HGNC:16642] |
| ENSG00000168899 | HGLibA_53499 | 247 | 1.309304285 | VAMP5 | vesicle associated membrane protein 5 [Source:HGNC Symbol;Acc:HGNC:12646] |
| ENSG00000169218 | HGLibA_42344 | 247 | 1.309304285 | RSPO1 | R-spondin 1 [Source:HGNC Symbol;Acc:HGNC:21679] |
| ENSG00000169313 | HGLibA_34876 | 247 | 1.309304285 | P2RY12 | purinergic receptor P2Y12 [Source:HGNC Symbol;Acc:HGNC:18124] |
| ENSG00000177311 | HGLibA_54872 | 247 | 1.309304285 | ZBTB38 | zinc finger and BTB domain containing 38 [Source:HGNC Symbol;Acc:HGNC:26636] |
| ENSG00000177627 | HGLibA_05265 | 247 | 1.309304285 | C12orf54 | chromosome 12 open reading frame 54 [Source:HGNC Symbol;Acc:HGNC:28553] |
| ENSG00000180772 | HGLibA_01264 | 247 | 1.309304285 | AGTR2 | angiotensin II receptor type 2 [Source:HGNC Symbol;Acc:HGNC:338] |
| ENSG00000181192 | HGLibA_13162 | 247 | 1.309304285 | DHTKD1 | dehydrogenase E1 and transketolase domain containing 1 [Source:HGNC Symbol;Acc:HGNC:23537] |
| ENSG00000185561 | HGLibA_49599 | 247 | 1.309304285 | TLCD2 | TLC domain containing 2 [Source:HGNC Symbol;Acc:HGNC:33522] |
| ENSG00000187950 | HGLibA_34792 | 247 | 1.309304285 | OVCH1 | ovochymase 1 [Source:HGNC Symbol;Acc:HGNC:23080] |
| ENSG00000205883 | HGLibA_12908 | 247 | 1.309304285 | DEFB135 | defensin beta 135 [Source:HGNC Symbol;Acc:HGNC:32400] |
| ENSG00000214193 | HGLibA_43943 | 247 | 1.309304285 | SH3D21 | SH3 domain containing 21 [Source:HGNC Symbol;Acc:HGNC:26236] |
| ENSG00000269556 | HGLibA_50121 | 247 | 1.309304285 | TMEM185A | transmembrane protein 185A [Source:HGNC Symbol;Acc:HGNC:17125] |
| ENSG00000284701 | HGLibA_50305 | 247 | 1.309304285 | TMEM247 | transmembrane protein 247 [Source:HGNC Symbol;Acc:HGNC:42967] |
| FAM203A | HGLibA_16416 | 247 | 1.309304285 | FAM203A | - |
| FAM73B | HGLibA_16718 | 247 | 1.309304285 | FAM73B | - |
| hsa-mir-1244-2 | HGLibA_57366 | 247 | 1.309304285 | hsa-mir-1244-2 | - |
| hsa-mir-1538 | HGLibA_57917 | 247 | 1.309304285 | hsa-mir-1538 | - |
| hsa-mir-3714 | HGLibA_59371 | 247 | 1.309304285 | hsa-mir-3714 | - |
| hsa-mir-4311 | HGLibA_59959 | 247 | 1.309304285 | hsa-mir-4311 | - |
| hsa-mir-758 | HGLibA_63894 | 247 | 1.309304285 | hsa-mir-758 | - |
| LOC100506688 | HGLibA_26865 | 247 | 1.309304285 | LOC100506688 | - |
| ENSG00000011143 | HGLibA_29358 | 246 | 1.299353248 | MKS1 | type 1 [Source:HGNC Symbol;Acc:HGNC:7121]" |
| ENSG00000050820 | HGLibA_04244 | 246 | 1.299353248 | BCAR1 | Cas family scaffold protein [Source:HGNC Symbol;Acc:HGNC:971]" |
| ENSG00000054938 | HGLibA_09542 | 246 | 1.299353248 | CHRDL2 | chordin like 2 [Source:HGNC Symbol;Acc:HGNC:24168] |
| ENSG00000089327 | HGLibA_18237 | 246 | 1.299353248 | FXYD5 | FXYD domain containing ion transport regulator 5 [Source:HGNC Symbol;Acc:HGNC:4029] |
| ENSG00000099840 | HGLibA_23901 | 246 | 1.299353248 | IZUMO4 | IZUMO family member 4 [Source:HGNC Symbol;Acc:HGNC:26950] |
| ENSG00000100225 | HGLibA_17125 | 246 | 1.299353248 | FBXO7 | F-box protein 7 [Source:HGNC Symbol;Acc:HGNC:13586] |
| ENSG00000100461 | HGLibA_40513 | 246 | 1.299353248 | RBM23 | RNA binding motif protein 23 [Source:HGNC Symbol;Acc:HGNC:20155] |
| ENSG00000101916 | HGLibA_49665 | 246 | 1.299353248 | TLR8 | toll like receptor 8 [Source:HGNC Symbol;Acc:HGNC:15632] |
| ENSG00000104728 | HGLibA_02807 | 246 | 1.299353248 | ARHGEF10 | Rho guanine nucleotide exchange factor 10 [Source:HGNC Symbol;Acc:HGNC:14103] |
| ENSG00000106692 | HGLibA_17600 | 246 | 1.299353248 | FKTN | fukutin [Source:HGNC Symbol;Acc:HGNC:3622] |
| ENSG00000111335 | HGLibA_33202 | 246 | 1.299353248 | OAS2 | 2'-5'-oligoadenylate synthetase 2 [Source:HGNC Symbol;Acc:HGNC:8087] |
| ENSG00000112137 | HGLibA_36297 | 246 | 1.299353248 | PHACTR1 | phosphatase and actin regulator 1 [Source:HGNC Symbol;Acc:HGNC:20990] |
| ENSG00000113520 | HGLibA_23165 | 246 | 1.299353248 | IL4 | interleukin 4 [Source:HGNC Symbol;Acc:HGNC:6014] |
| ENSG00000115705 | HGLibA_51155 | 246 | 1.299353248 | TPO | thyroid peroxidase [Source:HGNC Symbol;Acc:HGNC:12015] |
| ENSG00000116857 | HGLibA_50554 | 246 | 1.299353248 | TMEM9 | transmembrane protein 9 [Source:HGNC Symbol;Acc:HGNC:18823] |
| ENSG00000121058 | HGLibA_10495 | 246 | 1.299353248 | COIL | coilin [Source:HGNC Symbol;Acc:HGNC:2184] |
| ENSG00000124098 | HGLibA_16457 | 246 | 1.299353248 | FAM210B | family with sequence similarity 210 member B [Source:HGNC Symbol;Acc:HGNC:16102] |
| ENSG00000126254 | HGLibA_40552 | 246 | 1.299353248 | RBM42 | RNA binding motif protein 42 [Source:HGNC Symbol;Acc:HGNC:28117] |
| ENSG00000128052 | HGLibA_24543 | 246 | 1.299353248 | KDR | kinase insert domain receptor [Source:HGNC Symbol;Acc:HGNC:6307] |
| ENSG00000131196 | HGLibA_31772 | 246 | 1.299353248 | NFATC1 | nuclear factor of activated T cells 1 [Source:HGNC Symbol;Acc:HGNC:7775] |
| ENSG00000137040 | HGLibA_40210 | 246 | 1.299353248 | RANBP6 | RAN binding protein 6 [Source:HGNC Symbol;Acc:HGNC:9851] |
| ENSG00000139190 | HGLibA_53489 | 246 | 1.299353248 | VAMP1 | vesicle associated membrane protein 1 [Source:HGNC Symbol;Acc:HGNC:12642] |
| ENSG00000141371 | HGLibA_05558 | 246 | 1.299353248 | C17orf64 | chromosome 17 open reading frame 64 [Source:HGNC Symbol;Acc:HGNC:26990] |
| ENSG00000141582 | HGLibA_07454 | 246 | 1.299353248 | CBX4 | chromobox 4 [Source:HGNC Symbol;Acc:HGNC:1554] |
| ENSG00000144118 | HGLibA_40151 | 246 | 1.299353248 | RALB | RAS like proto-oncogene B [Source:HGNC Symbol;Acc:HGNC:9840] |
| ENSG00000148156 | HGLibA_00629 | 246 | 1.299353248 | ACTL7B | actin like 7B [Source:HGNC Symbol;Acc:HGNC:162] |
| ENSG00000152332 | HGLibA_52941 | 246 | 1.299353248 | UHMK1 | U2AF homology motif kinase 1 [Source:HGNC Symbol;Acc:HGNC:19683] |
| ENSG00000153113 | HGLibA_07347 | 246 | 1.299353248 | CAST | calpastatin [Source:HGNC Symbol;Acc:HGNC:1515] |
| ENSG00000154654 | HGLibA_31257 | 246 | 1.299353248 | NCAM2 | neural cell adhesion molecule 2 [Source:HGNC Symbol;Acc:HGNC:7657] |
| ENSG00000157927 | HGLibA_40113 | 246 | 1.299353248 | RADIL | Rap associating with DIL domain [Source:HGNC Symbol;Acc:HGNC:22226] |
| ENSG00000159335 | HGLibA_39395 | 246 | 1.299353248 | PTMS | parathymosin [Source:HGNC Symbol;Acc:HGNC:9629] |
| ENSG00000166321 | HGLibA_32944 | 246 | 1.299353248 | NUDT13 | nudix hydrolase 13 [Source:HGNC Symbol;Acc:HGNC:18827] |
| ENSG00000166762 | HGLibA_07357 | 246 | 1.299353248 | CATSPER2 | cation channel sperm associated 2 [Source:HGNC Symbol;Acc:HGNC:18810] |
| ENSG00000168685 | HGLibA_23193 | 246 | 1.299353248 | IL7R | interleukin 7 receptor [Source:HGNC Symbol;Acc:HGNC:6024] |
| ENSG00000172046 | HGLibA_53293 | 246 | 1.299353248 | USP19 | ubiquitin specific peptidase 19 [Source:HGNC Symbol;Acc:HGNC:12617] |
| ENSG00000172289 | HGLibA_33551 | 246 | 1.299353248 | OR10V1 | olfactory receptor family 10 subfamily V member 1 [Source:HGNC Symbol;Acc:HGNC:15136] |
| ENSG00000172349 | HGLibA_22985 | 246 | 1.299353248 | IL16 | interleukin 16 [Source:HGNC Symbol;Acc:HGNC:5980] |
| ENSG00000173769 | HGLibA_51007 | 246 | 1.299353248 | TOPAZ1 | testis and ovary specific PAZ domain containing 1 [Source:HGNC Symbol;Acc:HGNC:24746] |
| ENSG00000174473 | HGLibA_18582 | 246 | 1.299353248 | GALNTL6 | polypeptide N-acetylgalactosaminyltransferase like 6 [Source:HGNC Symbol;Acc:HGNC:33844] |
| ENSG00000176410 | HGLibA_13619 | 246 | 1.299353248 | DNAJC30 | DnaJ heat shock protein family (Hsp40) member C30 [Source:HGNC Symbol;Acc:HGNC:16410] |
| ENSG00000177425 | HGLibA_35293 | 246 | 1.299353248 | PAWR | pro-apoptotic WT1 regulator [Source:HGNC Symbol;Acc:HGNC:8614] |
| ENSG00000181544 | HGLibA_16832 | 246 | 1.299353248 | FANCB | FA complementation group B [Source:HGNC Symbol;Acc:HGNC:3583] |
| ENSG00000183486 | HGLibA_30607 | 246 | 1.299353248 | MX2 | MX dynamin like GTPase 2 [Source:HGNC Symbol;Acc:HGNC:7533] |
| ENSG00000183918 | HGLibA_43884 | 246 | 1.299353248 | SH2D1A | SH2 domain containing 1A [Source:HGNC Symbol;Acc:HGNC:10820] |
| ENSG00000184571 | HGLibA_36776 | 246 | 1.299353248 | PIWIL3 | piwi like RNA-mediated gene silencing 3 [Source:HGNC Symbol;Acc:HGNC:18443] |
| ENSG00000185561 | HGLibA_49598 | 246 | 1.299353248 | TLCD2 | TLC domain containing 2 [Source:HGNC Symbol;Acc:HGNC:33522] |
| ENSG00000187912 | HGLibA_09941 | 246 | 1.299353248 | CLEC17A | C-type lectin domain containing 17A [Source:HGNC Symbol;Acc:HGNC:34520] |
| ENSG00000198113 | HGLibA_51033 | 246 | 1.299353248 | TOR4A | torsin family 4 member A [Source:HGNC Symbol;Acc:HGNC:25981] |
| ENSG00000198125 | HGLibA_28454 | 246 | 1.299353248 | MB | myoglobin [Source:HGNC Symbol;Acc:HGNC:6915] |
| ENSG00000204382 | HGLibA_54468 | 246 | 1.299353248 | XAGE1B | X antigen family member 1B [Source:HGNC Symbol;Acc:HGNC:25400] |
| ENSG00000257103 | HGLibA_27530 | 246 | 1.299353248 | LSM14A | mRNA processing body assembly factor [Source:HGNC Symbol;Acc:HGNC:24489]" |
| ENSG00000269313 | HGLibA_28004 | 246 | 1.299353248 | MAGIX | X-linked [Source:HGNC Symbol;Acc:HGNC:30006]" |
| hsa-mir-3689a | HGLibA_59312 | 246 | 1.299353248 | hsa-mir-3689a | - |
| hsa-mir-429 | HGLibA_59868 | 246 | 1.299353248 | hsa-mir-429 | - |
| hsa-mir-589 | HGLibA_62453 | 246 | 1.299353248 | hsa-mir-589 | - |
| TXNRD3NB | HGLibA_52452 | 246 | 1.299353248 | TXNRD3NB | - |
| C16orf58 | HGLibA_05463 | 245 | 1.28940221 | C16orf58 | - |
| ENSG00000009950 | HGLibA_29443 | 245 | 1.28940221 | MLXIPL | MLX interacting protein like [Source:HGNC Symbol;Acc:HGNC:12744] |
| ENSG00000015413 | HGLibA_13817 | 245 | 1.28940221 | DPEP1 | dipeptidase 1 [Source:HGNC Symbol;Acc:HGNC:3002] |
| ENSG00000100991 | HGLibA_51738 | 245 | 1.28940221 | TRPC4AP | transient receptor potential cation channel subfamily C member 4 associated protein [Source:HGNC Symbol;Acc:HGNC:16181] |
| ENSG00000101294 | HGLibA_21662 | 245 | 1.28940221 | HM13 | histocompatibility minor 13 [Source:HGNC Symbol;Acc:HGNC:16435] |

| | | | | | |
|---|---|---|---|---|---|
| ENSG00000101307 | HGLibA_44205 | 245 | 1.28940221 | SIRPB1 | signal regulatory protein beta 1 [Source:HGNC Symbol;Acc:HGNC:15928] |
| ENSG00000102678 | HGLibA_17423 | 245 | 1.28940221 | FGF9 | fibroblast growth factor 9 [Source:HGNC Symbol;Acc:HGNC:3687] |
| ENSG00000105509 | HGLibA_20863 | 245 | 1.28940221 | HAS1 | hyaluronan synthase 1 [Source:HGNC Symbol;Acc:HGNC:4818] |
| ENSG00000107443 | HGLibA_08109 | 245 | 1.28940221 | CCNJ | cyclin J [Source:HGNC Symbol;Acc:HGNC:23434] |
| ENSG00000107551 | HGLibA_40382 | 245 | 1.28940221 | RASSF4 | Ras association domain family member 4 [Source:HGNC Symbol;Acc:HGNC:20793] |
| ENSG00000107821 | HGLibA_24086 | 245 | 1.28940221 | KAZALD1 | Kazal type serine peptidase inhibitor domain 1 [Source:HGNC Symbol;Acc:HGNC:25460] |
| ENSG00000110013 | HGLibA_44102 | 245 | 1.28940221 | SIAE | sialic acid acetylesterase [Source:HGNC Symbol;Acc:HGNC:18187] |
| ENSG00000123572 | HGLibA_32701 | 245 | 1.28940221 | NRK | Nik related kinase [Source:HGNC Symbol;Acc:HGNC:25391] |
| ENSG00000124493 | HGLibA_20349 | 245 | 1.28940221 | GRM4 | glutamate metabotropic receptor 4 [Source:HGNC Symbol;Acc:HGNC:4596] |
| ENSG00000125508 | HGLibA_46996 | 245 | 1.28940221 | SRMS | src-related kinase lacking C-terminal regulatory tyrosine and N-terminal myristylation sites [Source:HGNC Symbol;Acc:HGNC:11298] |
| ENSG00000127366 | HGLibA_48416 | 245 | 1.28940221 | TAS2R5 | taste 2 receptor member 5 [Source:HGNC Symbol;Acc:HGNC:14912] |
| ENSG00000129515 | HGLibA_46102 | 245 | 1.28940221 | SNX6 | sorting nexin 6 [Source:HGNC Symbol;Acc:HGNC:14970] |
| ENSG00000136011 | HGLibA_47309 | 245 | 1.28940221 | STAB2 | stabilin 2 [Source:HGNC Symbol;Acc:HGNC:18629] |
| ENSG00000136352 | HGLibA_32030 | 245 | 1.28940221 | NKX2-1 | NK2 homeobox 1 [Source:HGNC Symbol;Acc:HGNC:11825] |
| ENSG00000138185 | HGLibA_15196 | 245 | 1.28940221 | ENTPD1 | ectonucleoside triphosphate diphosphohydrolase 1 [Source:HGNC Symbol;Acc:HGNC:3363] |
| ENSG00000138772 | HGLibA_02237 | 245 | 1.28940221 | ANXA3 | annexin A3 [Source:HGNC Symbol;Acc:HGNC:541] |
| ENSG00000144674 | HGLibA_19600 | 245 | 1.28940221 | GOLGA4 | golgin A4 [Source:HGNC Symbol;Acc:HGNC:4427] |
| ENSG00000146463 | HGLibA_55399 | 245 | 1.28940221 | ZMYM4 | zinc finger MYM-type containing 4 [Source:HGNC Symbol;Acc:HGNC:13055] |
| ENSG00000147044 | HGLibA_07286 | 245 | 1.28940221 | CASK | calcium/calmodulin dependent serine protein kinase [Source:HGNC Symbol;Acc:HGNC:1497] |
| ENSG00000147869 | HGLibA_09188 | 245 | 1.28940221 | CER1 | DAN family BMP antagonist [Source:HGNC Symbol;Acc:HGNC:1862]" |
| ENSG00000147872 | HGLibA_37172 | 245 | 1.28940221 | PLIN2 | perilipin 2 [Source:HGNC Symbol;Acc:HGNC:248] |
| ENSG00000153048 | HGLibA_07246 | 245 | 1.28940221 | CARHSP1 | calcium regulated heat stable protein 1 [Source:HGNC Symbol;Acc:HGNC:17150] |
| ENSG00000153993 | HGLibA_43328 | 245 | 1.28940221 | SEMA3D | semaphorin 3D [Source:HGNC Symbol;Acc:HGNC:10726] |
| ENSG00000157119 | HGLibA_25207 | 245 | 1.28940221 | KLHL40 | kelch like family member 40 [Source:HGNC Symbol;Acc:HGNC:30372] |
| ENSG00000157978 | HGLibA_26253 | 245 | 1.28940221 | LDLRAP1 | low density lipoprotein receptor adaptor protein 1 [Source:HGNC Symbol;Acc:HGNC:18640] |
| ENSG00000159685 | HGLibA_09376 | 245 | 1.28940221 | CHCHD6 | coiled-coil-helix-coiled-coil-helix domain containing 6 [Source:HGNC Symbol;Acc:HGNC:28184] |
| ENSG00000160013 | HGLibA_39346 | 245 | 1.28940221 | PTGIR | prostaglandin I2 receptor [Source:HGNC Symbol;Acc:HGNC:9602] |
| ENSG00000162595 | HGLibA_13263 | 245 | 1.28940221 | DIRAS3 | DIRAS family GTPase 3 [Source:HGNC Symbol;Acc:HGNC:687] |
| ENSG00000165650 | HGLibA_35985 | 245 | 1.28940221 | PDZD8 | PDZ domain containing 8 [Source:HGNC Symbol;Acc:HGNC:26974] |
| ENSG00000169515 | HGLibA_07873 | 245 | 1.28940221 | CCDC8 | coiled-coil domain containing 8 [Source:HGNC Symbol;Acc:HGNC:25367] |
| ENSG00000175513 | HGLibA_51851 | 245 | 1.28940221 | TSGA10IP | testis specific 10 interacting protein [Source:HGNC Symbol;Acc:HGNC:26555] |
| ENSG00000177943 | HGLibA_28039 | 245 | 1.28940221 | MAMDC4 | MAM domain containing 4 [Source:HGNC Symbol;Acc:HGNC:24083] |
| ENSG00000178904 | HGLibA_13912 | 245 | 1.28940221 | DPY19L3 | dpy-19 like C-mannosyltransferase 3 [Source:HGNC Symbol;Acc:HGNC:27120] |
| ENSG00000182111 | HGLibA_56623 | 245 | 1.28940221 | ZNF716 | zinc finger protein 716 [Source:HGNC Symbol;Acc:HGNC:32458] |
| ENSG00000185201 | HGLibA_22645 | 245 | 1.28940221 | IFITM2 | interferon induced transmembrane protein 2 [Source:HGNC Symbol;Acc:HGNC:5413] |
| ENSG00000185518 | HGLibA_47878 | 245 | 1.28940221 | SV2B | synaptic vesicle glycoprotein 2B [Source:HGNC Symbol;Acc:HGNC:16874] |
| ENSG00000186603 | HGLibA_22029 | 245 | 1.28940221 | HPDL | 4-hydroxyphenylpyruvate dioxygenase like [Source:HGNC Symbol;Acc:HGNC:28242] |
| ENSG00000187079 | HGLibA_48927 | 245 | 1.28940221 | TEAD1 | TEA domain transcription factor 1 [Source:HGNC Symbol;Acc:HGNC:11714] |
| ENSG00000187902 | HGLibA_44048 | 245 | 1.28940221 | SHISA7 | shisa family member 7 [Source:HGNC Symbol;Acc:HGNC:35409] |
| ENSG00000187961 | HGLibA_25141 | 245 | 1.28940221 | KLHL17 | kelch like family member 17 [Source:HGNC Symbol;Acc:HGNC:24023] |
| ENSG00000197343 | HGLibA_56469 | 245 | 1.28940221 | ZNF655 | zinc finger protein 655 [Source:HGNC Symbol;Acc:HGNC:30899] |
| ENSG00000198108 | HGLibA_09659 | 245 | 1.28940221 | CHSY3 | chondroitin sulfate synthase 3 [Source:HGNC Symbol;Acc:HGNC:24293] |
| ENSG00000203737 | HGLibA_20051 | 245 | 1.28940221 | GPR52 | G protein-coupled receptor 52 [Source:HGNC Symbol;Acc:HGNC:4508] |
| hsa-mir-372 | HGLibA_59383 | 245 | 1.28940221 | hsa-mir-372 | - |
| hsa-mir-4787 | HGLibA_61256 | 245 | 1.28940221 | hsa-mir-4787 | - |
| hsa-mir-676 | HGLibA_63197 | 245 | 1.28940221 | hsa-mir-676 | - |
| LINS | HGLibA_26596 | 245 | 1.28940221 | LINS | - |
| LOC100129520 | HGLibA_26766 | 245 | 1.28940221 | LOC100129520 | - |
| SSPO | HGLibA_47149 | 245 | 1.28940221 | SSPO | - |
| CCDC42B | HGLibA_07785 | 244 | 1.279451173 | CCDC42B | - |
| ENSG00000004838 | HGLibA_55410 | 244 | 1.279451173 | ZMYND10 | zinc finger MYND-type containing 10 [Source:HGNC Symbol;Acc:HGNC:19412] |
| ENSG00000020633 | HGLibA_42463 | 244 | 1.279451173 | RUNX3 | runt related transcription factor 3 [Source:HGNC Symbol;Acc:HGNC:10473] |
| ENSG00000048649 | HGLibA_42310 | 244 | 1.279451173 | RSF1 | remodeling and spacing factor 1 [Source:HGNC Symbol;Acc:HGNC:18118] |
| ENSG00000100365 | HGLibA_31303 | 244 | 1.279451173 | NCF4 | neutrophil cytosolic factor 4 [Source:HGNC Symbol;Acc:HGNC:7662] |
| ENSG00000101473 | HGLibA_00490 | 244 | 1.279451173 | ACOT8 | acyl-CoA thioesterase 8 [Source:HGNC Symbol;Acc:HGNC:15919] |
| ENSG00000105428 | HGLibA_56948 | 244 | 1.279451173 | ZNRF4 | zinc and ring finger 4 [Source:HGNC Symbol;Acc:HGNC:17726] |
| ENSG00000108100 | HGLibA_08133 | 244 | 1.279451173 | CCNY | cyclin Y [Source:HGNC Symbol;Acc:HGNC:23354] |
| ENSG00000109101 | HGLibA_17908 | 244 | 1.279451173 | FOXN1 | forkhead box N1 [Source:HGNC Symbol;Acc:HGNC:12765] |
| ENSG00000111087 | HGLibA_19247 | 244 | 1.279451173 | GLI1 | GLI family zinc finger 1 [Source:HGNC Symbol;Acc:HGNC:4317] |
| ENSG00000113083 | HGLibA_27032 | 244 | 1.279451173 | LOX | lysyl oxidase [Source:HGNC Symbol;Acc:HGNC:6664] |
| ENSG00000113300 | HGLibA_10344 | 244 | 1.279451173 | CNOT6 | CCR4-NOT transcription complex subunit 6 [Source:HGNC Symbol;Acc:HGNC:14099] |
| ENSG00000119335 | HGLibA_43632 | 244 | 1.279451173 | SET | SET nuclear proto-oncogene [Source:HGNC Symbol;Acc:HGNC:10760] |
| ENSG00000121281 | HGLibA_00927 | 244 | 1.279451173 | ADCY7 | adenylate cyclase 7 [Source:HGNC Symbol;Acc:HGNC:238] |
| ENSG00000127481 | HGLibA_52756 | 244 | 1.279451173 | UBR4 | ubiquitin protein ligase E3 component n-recognin 4 [Source:HGNC Symbol;Acc:HGNC:30313] |
| ENSG00000127578 | HGLibA_54281 | 244 | 1.279451173 | WFIKKN1 | kunitz and netrin domain containing 1 [Source:HGNC Symbol;Acc:HGNC:30912]" |
| ENSG00000128203 | HGLibA_03356 | 244 | 1.279451173 | ASPHD2 | aspartate beta-hydroxylase domain containing 2 [Source:HGNC Symbol;Acc:HGNC:30437] |
| ENSG00000132109 | HGLibA_51430 | 244 | 1.279451173 | TRIM21 | tripartite motif containing 21 [Source:HGNC Symbol;Acc:HGNC:11312] |
| ENSG00000133561 | HGLibA_19084 | 244 | 1.279451173 | GIMAP6 | IMAP family member 6 [Source:HGNC Symbol;Acc:HGNC:21918]" |
| ENSG00000134326 | HGLibA_10203 | 244 | 1.279451173 | CMPK2 | cytidine/uridine monophosphate kinase 2 [Source:HGNC Symbol;Acc:HGNC:27015] |
| ENSG00000136869 | HGLibA_49651 | 244 | 1.279451173 | TLR4 | toll like receptor 4 [Source:HGNC Symbol;Acc:HGNC:11850] |
| ENSG00000139194 | HGLibA_40642 | 244 | 1.279451173 | RBP5 | retinol binding protein 5 [Source:HGNC Symbol;Acc:HGNC:15847] |
| ENSG00000141200 | HGLibA_24928 | 244 | 1.279451173 | KIF2B | kinesin family member 2B [Source:HGNC Symbol;Acc:HGNC:29443] |
| ENSG00000142178 | HGLibA_44161 | 244 | 1.279451173 | SIK1 | salt inducible kinase 1 [Source:HGNC Symbol;Acc:HGNC:11142] |
| ENSG00000147419 | HGLibA_07738 | 244 | 1.279451173 | CCDC25 | coiled-coil domain containing 25 [Source:HGNC Symbol;Acc:HGNC:25591] |
| ENSG00000153714 | HGLibA_27634 | 244 | 1.279451173 | LURAP1L | leucine rich adaptor protein 1 like [Source:HGNC Symbol;Acc:HGNC:31452] |
| ENSG00000160013 | HGLibA_39347 | 244 | 1.279451173 | PTGIR | prostaglandin I2 receptor [Source:HGNC Symbol;Acc:HGNC:9602] |
| ENSG00000163513 | HGLibA_49227 | 244 | 1.279451173 | TGFBR2 | transforming growth factor beta receptor 2 [Source:HGNC Symbol;Acc:HGNC:11773] |
| ENSG00000163754 | HGLibA_20688 | 244 | 1.279451173 | GYG1 | glycogenin 1 [Source:HGNC Symbol;Acc:HGNC:4699] |
| ENSG00000163873 | HGLibA_20286 | 244 | 1.279451173 | GRIK3 | glutamate ionotropic receptor kainate type subunit 3 [Source:HGNC Symbol;Acc:HGNC:4581] |
| ENSG00000164756 | HGLibA_44919 | 244 | 1.279451173 | SLC30A8 | solute carrier family 30 member 8 [Source:HGNC Symbol;Acc:HGNC:20303] |
| ENSG00000169021 | HGLibA_53119 | 244 | 1.279451173 | UQCRFS1 | Rieske iron-sulfur polypeptide 1 [Source:HGNC Symbol;Acc:HGNC:12587]" |
| ENSG00000173846 | HGLibA_37193 | 244 | 1.279451173 | PLK3 | polo like kinase 3 [Source:HGNC Symbol;Acc:HGNC:2154] |
| ENSG00000177879 | HGLibA_02336 | 244 | 1.279451173 | AP3S1 | adaptor related protein complex 3 subunit sigma 1 [Source:HGNC Symbol;Acc:HGNC:2013] |
| ENSG00000179477 | HGLibA_01680 | 244 | 1.279451173 | ALOX12B | 12R type [Source:HGNC Symbol;Acc:HGNC:430]" |
| ENSG00000180773 | HGLibA_45041 | 244 | 1.279451173 | SLC36A4 | solute carrier family 36 member 4 [Source:HGNC Symbol;Acc:HGNC:19660] |
| ENSG00000180879 | HGLibA_47163 | 244 | 1.279451173 | SSR4 | signal sequence receptor subunit 4 [Source:HGNC Symbol;Acc:HGNC:11326] |
| ENSG00000181472 | HGLibA_54834 | 244 | 1.279451173 | ZBTB2 | zinc finger and BTB domain containing 2 [Source:HGNC Symbol;Acc:HGNC:20868] |
| ENSG00000183840 | HGLibA_20037 | 244 | 1.279451173 | GPR39 | G protein-coupled receptor 39 [Source:HGNC Symbol;Acc:HGNC:4496] |
| ENSG00000186007 | HGLibA_26286 | 244 | 1.279451173 | LEMD1 | LEM domain containing 1 [Source:HGNC Symbol;Acc:HGNC:18725] |
| ENSG00000186977 | HGLibA_25676 | 244 | 1.279451173 | KRTAP19-5 | keratin associated protein 19-5 [Source:HGNC Symbol;Acc:HGNC:18940] |
| ENSG00000187391 | HGLibA_27999 | 244 | 1.279451173 | MAGI2 | WW and PDZ domain containing 2 [Source:HGNC Symbol;Acc:HGNC:18957]" |
| ENSG00000188243 | HGLibA_10671 | 244 | 1.279451173 | COMMD6 | COMM domain containing 6 [Source:HGNC Symbol;Acc:HGNC:24015] |
| ENSG00000196465 | HGLibA_30773 | 244 | 1.279451173 | MYL6B | myosin light chain 6B [Source:HGNC Symbol;Acc:HGNC:29823] |
| ENSG00000197223 | HGLibA_05719 | 244 | 1.279451173 | C1D | C1D nuclear receptor corepressor [Source:HGNC Symbol;Acc:HGNC:29911] |
| ENSG00000198373 | HGLibA_54453 | 244 | 1.279451173 | WWP2 | WW domain containing E3 ubiquitin protein ligase 2 [Source:HGNC Symbol;Acc:HGNC:16804] |
| ENSG00000198648 | HGLibA_47507 | 244 | 1.279451173 | STK39 | serine/threonine kinase 39 [Source:HGNC Symbol;Acc:HGNC:17717] |
| ENSG00000221989 | HGLibA_33728 | 244 | 1.279451173 | OR2A2 | olfactory receptor family 2 subfamily A member 2 [Source:HGNC Symbol;Acc:HGNC:8230] |
| ENSG00000227500 | HGLibA_42846 | 244 | 1.279451173 | SCAMP4 | secretory carrier membrane protein 4 [Source:HGNC Symbol;Acc:HGNC:30385] |
| ENSG00000275023 | HGLibA_29423 | 244 | 1.279451173 | MLLT6 | PHD finger containing [Source:HGNC Symbol;Acc:HGNC:7138]" |
| hsa-mir-219a-1 | HGLibA_58275 | 244 | 1.279451173 | hsa-mir-219a-1 | - |
| hsa-mir-3195 | HGLibA_58877 | 244 | 1.279451173 | hsa-mir-3195 | - |
| hsa-mir-4486 | HGLibA_60361 | 244 | 1.279451173 | hsa-mir-4486 | - |
| hsa-mir-4699 | HGLibA_60906 | 244 | 1.279451173 | hsa-mir-4699 | - |
| hsa-mir-5088 | HGLibA_61499 | 244 | 1.279451173 | hsa-mir-5088 | - |
| hsa-mir-5187 | HGLibA_61638 | 244 | 1.279451173 | hsa-mir-5187 | - |
| hsa-mir-6752 | HGLibA_63166 | 244 | 1.279451173 | hsa-mir-6752 | - |
| hsa-mir-7846 | HGLibA_63972 | 244 | 1.279451173 | hsa-mir-7846 | - |
| RGAG4 | HGLibA_40996 | 244 | 1.279451173 | RGAG4 | - |
| ENSG00000063245 | HGLibA_15342 | 243 | 1.269500135 | EPN1 | epsin 1 [Source:HGNC Symbol;Acc:HGNC:21604] |
| ENSG00000099994 | HGLibA_47847 | 243 | 1.269500135 | SUSD2 | sushi domain containing 2 [Source:HGNC Symbol;Acc:HGNC:30667] |
| ENSG00000104660 | HGLibA_26330 | 243 | 1.269500135 | LEPROTL1 | leptin receptor overlapping transcript like 1 [Source:HGNC Symbol;Acc:HGNC:6555] |
| ENSG00000105700 | HGLibA_25874 | 243 | 1.269500135 | KXD1 | KxDL motif containing 1 [Source:HGNC Symbol;Acc:HGNC:28420] |
| ENSG00000106153 | HGLibA_09366 | 243 | 1.269500135 | CHCHD2 | coiled-coil-helix-coiled-coil-helix domain containing 2 [Source:HGNC Symbol;Acc:HGNC:21645] |
| ENSG00000109684 | HGLibA_10088 | 243 | 1.269500135 | CLNK | cytokine dependent hematopoietic cell linker [Source:HGNC Symbol;Acc:HGNC:17438] |
| ENSG00000116478 | HGLibA_20998 | 243 | 1.269500135 | HDAC1 | histone deacetylase 1 [Source:HGNC Symbol;Acc:HGNC:4852] |
| ENSG00000120937 | HGLibA_32502 | 243 | 1.269500135 | NPPB | natriuretic peptide B [Source:HGNC Symbol;Acc:HGNC:7940] |
| ENSG00000132122 | HGLibA_46523 | 243 | 1.269500135 | SPATA6 | spermatogenesis associated 6 [Source:HGNC Symbol;Acc:HGNC:18309] |
| ENSG00000135355 | HGLibA_19140 | 243 | 1.269500135 | GJA10 | gap junction protein alpha 10 [Source:HGNC Symbol;Acc:HGNC:16995] |
| ENSG00000138185 | HGLibA_15198 | 243 | 1.269500135 | ENTPD1 | ectonucleoside triphosphate diphosphohydrolase 1 [Source:HGNC Symbol;Acc:HGNC:3363] |
| ENSG00000138964 | HGLibA_35261 | 243 | 1.269500135 | PARVG | parvin gamma [Source:HGNC Symbol;Acc:HGNC:14654] |
| ENSG00000139567 | HGLibA_00701 | 243 | 1.269500135 | ACVRL1 | activin A receptor like type 1 [Source:HGNC Symbol;Acc:HGNC:175] |
| ENSG00000139651 | HGLibA_56661 | 243 | 1.269500135 | ZNF740 | zinc finger protein 740 [Source:HGNC Symbol;Acc:HGNC:27465] |
| ENSG00000147364 | HGLibA_17054 | 243 | 1.269500135 | FBXO25 | F-box protein 25 [Source:HGNC Symbol;Acc:HGNC:13596] |
| ENSG00000151116 | HGLibA_52839 | 243 | 1.269500135 | UEVLD | UEV and lactate/malate dehyrogenase domains [Source:HGNC Symbol;Acc:HGNC:30866] |
| ENSG00000152430 | HGLibA_04693 | 243 | 1.269500135 | BOLL | RNA binding protein [Source:HGNC Symbol;Acc:HGNC:14273]" |
| ENSG00000155052 | HGLibA_10434 | 243 | 1.269500135 | CNTNAP5 | contactin associated protein like 5 [Source:HGNC Symbol;Acc:HGNC:18748] |
| ENSG00000161267 | HGLibA_04373 | 243 | 1.269500135 | BDH1 | 3-hydroxybutyrate dehydrogenase 1 [Source:HGNC Symbol;Acc:HGNC:1027] |
| ENSG00000162365 | HGLibA_12176 | 243 | 1.269500135 | CYP4A22 | cytochrome P450 family 4 subfamily A member 22 [Source:HGNC Symbol;Acc:HGNC:20575] |
| ENSG00000163251 | HGLibA_18274 | 243 | 1.269500135 | FZD5 | frizzled class receptor 5 [Source:HGNC Symbol;Acc:HGNC:4043] |
| ENSG00000163683 | HGLibA_45730 | 243 | 1.269500135 | SMIM14 | small integral membrane protein 14 [Source:HGNC Symbol;Acc:HGNC:27321] |
| ENSG00000166856 | HGLibA_19988 | 243 | 1.269500135 | GPR182 | G protein-coupled receptor 182 [Source:HGNC Symbol;Acc:HGNC:13708] |
| ENSG00000166912 | HGLibA_30412 | 243 | 1.269500135 | MTMR10 | myotubularin related protein 10 [Source:HGNC Symbol;Acc:HGNC:25999] |
| ENSG00000167769 | HGLibA_00427 | 243 | 1.269500135 | ACER1 | alkaline ceramidase 1 [Source:HGNC Symbol;Acc:HGNC:18356] |
| ENSG00000168040 | HGLibA_15932 | 243 | 1.269500135 | FADD | Fas associated via death domain [Source:HGNC Symbol;Acc:HGNC:3573] |
| ENSG00000168918 | HGLibA_23348 | 243 | 1.269500135 | INPP5D | inositol polyphosphate-5-phosphatase D [Source:HGNC Symbol;Acc:HGNC:6079] |

| | | | | | |
|---|---|---|---|---|---|
| ENSG00000168970 | HGLibA_23963 | 243 | 1.269500135 | JMJD7-PLA2G4B | JMJD7-PLA2G4B readthrough [Source:HGNC Symbol;Acc:HGNC:34449] |
| ENSG00000169154 | HGLibA_19693 | 243 | 1.269500135 | GOT1L1 | glutamic-oxaloacetic transaminase 1 like 1 [Source:HGNC Symbol;Acc:HGNC:28487] |
| ENSG00000174516 | HGLibA_36040 | 243 | 1.269500135 | PELI3 | pellino E3 ubiquitin protein ligase family member 3 [Source:HGNC Symbol;Acc:HGNC:30010] |
| ENSG00000178343 | HGLibA_44036 | 243 | 1.269500135 | SHISA3 | shisa family member 3 [Source:HGNC Symbol;Acc:HGNC:25159] |
| ENSG00000180739 | HGLibA_42606 | 243 | 1.269500135 | S1PR5 | sphingosine-1-phosphate receptor 5 [Source:HGNC Symbol;Acc:HGNC:14299] |
| ENSG00000181090 | HGLibA_14684 | 243 | 1.269500135 | EHMT1 | euchromatic histone lysine methyltransferase 1 [Source:HGNC Symbol;Acc:HGNC:24650] |
| ENSG00000183476 | HGLibA_43909 | 243 | 1.269500135 | SH2D7 | SH2 domain containing 7 [Source:HGNC Symbol;Acc:HGNC:34549] |
| ENSG00000188981 | HGLibA_30168 | 243 | 1.269500135 | MSANTD1 | Myb/SANT DNA binding domain containing 1 [Source:HGNC Symbol;Acc:HGNC:33741] |
| ENSG00000197721 | HGLibA_11048 | 243 | 1.269500135 | CR1L | complement C3b/C4b receptor 1 like [Source:HGNC Symbol;Acc:HGNC:2335] |
| ENSG00000205978 | HGLibA_33186 | 243 | 1.269500135 | NYNRIN | NYN domain and retroviral integrase containing [Source:HGNC Symbol;Acc:HGNC:20165] |
| ENSG00000213654 | HGLibA_20161 | 243 | 1.269500135 | GPSM3 | G protein signaling modulator 3 [Source:HGNC Symbol;Acc:HGNC:13945] |
| ENSG00000239697 | HGLibA_50803 | 243 | 1.269500135 | TNFSF12 | TNF superfamily member 12 [Source:HGNC Symbol;Acc:HGNC:11927] |
| ENSG00000260097 | HGLibA_46599 | 243 | 1.269500135 | SPDYE6 | speedy/RINGO cell cycle regulator family member E6 [Source:HGNC Symbol;Acc:HGNC:35465] |
| ENSG00000261011 | HGLibA_01139 | 243 | 1.269500135 | AGAP11 | ankyrin repeat and PH domain family (AGAP) pseudogene" |
| ENSG00000261594 | HGLibA_51103 | 243 | 1.269500135 | TPBGL | trophoblast glycoprotein like [Source:HGNC Symbol;Acc:HGNC:44159] |
| ENSG00000272636 | HGLibA_13728 | 243 | 1.269500135 | DOC2B | double C2 domain beta [Source:HGNC Symbol;Acc:HGNC:2986] |
| ENSG00000276409 | HGLibA_07969 | 243 | 1.269500135 | CCL14 | C-C motif chemokine ligand 14 [Source:HGNC Symbol;Acc:HGNC:10612] |
| FAM196B | HGLibA_16385 | 243 | 1.269500135 | FAM196B | - |
| FAM26D | HGLibA_16561 | 243 | 1.269500135 | FAM26D | - |
| hsa-mir-3140 | HGLibA_58620 | 243 | 1.269500135 | hsa-mir-3140 | - |
| hsa-mir-487a | HGLibA_61345 | 243 | 1.269500135 | hsa-mir-487a | - |
| hsa-mir-5090 | HGLibA_61520 | 243 | 1.269500135 | hsa-mir-5090 | - |
| hsa-mir-644a | HGLibA_62821 | 243 | 1.269500135 | hsa-mir-644a | - |
| KIAA0922 | HGLibA_24666 | 243 | 1.269500135 | KIAA0922 | - |
| LOC728392 | HGLibA_26990 | 243 | 1.269500135 | LOC728392 | - |
| BCMO1 | HGLibA_04356 | 242 | 1.259549098 | BCMO1 | - |
| ENSG00000008118 | HGLibA_07064 | 242 | 1.259549098 | CAMK1G | calcium/calmodulin dependent protein kinase IG [Source:HGNC Symbol;Acc:HGNC:14585] |
| ENSG00000063438 | HGLibA_01310 | 242 | 1.259549098 | AHRR | aryl-hydrocarbon receptor repressor [Source:HGNC Symbol;Acc:HGNC:346] |
| ENSG00000075290 | HGLibA_54390 | 242 | 1.259549098 | WNT8B | Wnt family member 8B [Source:HGNC Symbol;Acc:HGNC:12789] |
| ENSG00000100504 | HGLibA_39688 | 242 | 1.259549098 | PYGL | glycogen phosphorylase L [Source:HGNC Symbol;Acc:HGNC:9725] |
| ENSG00000101665 | HGLibA_45586 | 242 | 1.259549098 | SMAD7 | SMAD family member 7 [Source:HGNC Symbol;Acc:HGNC:6773] |
| ENSG00000102302 | HGLibA_17343 | 242 | 1.259549098 | FGD1 | RhoGEF and PH domain containing 1 [Source:HGNC Symbol;Acc:HGNC:3663]" |
| ENSG00000104880 | HGLibA_02828 | 242 | 1.259549098 | ARHGEF18 | Rho/Rac guanine nucleotide exchange factor 18 [Source:HGNC Symbol;Acc:HGNC:17090] |
| ENSG00000105369 | HGLibA_08447 | 242 | 1.259549098 | CD79A | CD79a molecule [Source:HGNC Symbol;Acc:HGNC:1698] |
| ENSG00000105643 | HGLibA_03115 | 242 | 1.259549098 | ARRDC2 | arrestin domain containing 2 [Source:HGNC Symbol;Acc:HGNC:25225] |
| ENSG00000105825 | HGLibA_49186 | 242 | 1.259549098 | TFPI2 | tissue factor pathway inhibitor 2 [Source:HGNC Symbol;Acc:HGNC:11761] |
| ENSG00000107104 | HGLibA_24023 | 242 | 1.259549098 | KANK1 | KN motif and ankyrin repeat domains 1 [Source:HGNC Symbol;Acc:HGNC:19309] |
| ENSG00000107854 | HGLibA_50855 | 242 | 1.259549098 | TNKS2 | tankyrase 2 [Source:HGNC Symbol;Acc:HGNC:15677] |
| ENSG00000116014 | HGLibA_25013 | 242 | 1.259549098 | KISS1R | KISS1 receptor [Source:HGNC Symbol;Acc:HGNC:4510] |
| ENSG00000116685 | HGLibA_24837 | 242 | 1.259549098 | KIAA2013 | KIAA2013 [Source:HGNC Symbol;Acc:HGNC:28513] |
| ENSG00000117625 | HGLibA_40716 | 242 | 1.259549098 | RCOR3 | REST corepressor 3 [Source:HGNC Symbol;Acc:HGNC:25594] |
| ENSG00000119125 | HGLibA_18836 | 242 | 1.259549098 | GDA | guanine deaminase [Source:HGNC Symbol;Acc:HGNC:4212] |
| ENSG00000124160 | HGLibA_31346 | 242 | 1.259549098 | NCOA5 | nuclear receptor coactivator 5 [Source:HGNC Symbol;Acc:HGNC:15909] |
| ENSG00000126777 | HGLibA_25873 | 242 | 1.259549098 | KTN1 | kinectin 1 [Source:HGNC Symbol;Acc:HGNC:6467] |
| ENSG00000138442 | HGLibA_54028 | 242 | 1.259549098 | WDR12 | WD repeat domain 12 [Source:HGNC Symbol;Acc:HGNC:14098] |
| ENSG00000140009 | HGLibA_15585 | 242 | 1.259549098 | ESR2 | estrogen receptor 2 [Source:HGNC Symbol;Acc:HGNC:3468] |
| ENSG00000142252 | HGLibA_18924 | 242 | 1.259549098 | GEMIN7 | gem nuclear organelle associated protein 7 [Source:HGNC Symbol;Acc:HGNC:20045] |
| ENSG00000143344 | HGLibA_41001 | 242 | 1.259549098 | RGL1 | ral guanine nucleotide dissociation stimulator like 1 [Source:HGNC Symbol;Acc:HGNC:30281] |
| ENSG00000144010 | HGLibA_51505 | 242 | 1.259549098 | TRIM43B | tripartite motif containing 43B [Source:HGNC Symbol;Acc:HGNC:37146] |
| ENSG00000145246 | HGLibA_03552 | 242 | 1.259549098 | ATP10D | ATPase phospholipid transporting 10D (putative) [Source:HGNC Symbol;Acc:HGNC:13549] |
| ENSG00000154330 | HGLibA_36272 | 242 | 1.259549098 | PGM5 | phosphoglucomutase 5 [Source:HGNC Symbol;Acc:HGNC:8908] |
| ENSG00000157856 | HGLibA_13956 | 242 | 1.259549098 | DRC1 | dynein regulatory complex subunit 1 [Source:HGNC Symbol;Acc:HGNC:24245] |
| ENSG00000158220 | HGLibA_15610 | 242 | 1.259549098 | ESYT3 | extended synaptotagmin 3 [Source:HGNC Symbol;Acc:HGNC:24295] |
| ENSG00000158286 | HGLibA_41613 | 242 | 1.259549098 | RNF207 | ring finger protein 207 [Source:HGNC Symbol;Acc:HGNC:32947] |
| ENSG00000162366 | HGLibA_35993 | 242 | 1.259549098 | PDZK1IP1 | PDZK1 interacting protein 1 [Source:HGNC Symbol;Acc:HGNC:16887] |
| ENSG00000171435 | HGLibA_25865 | 242 | 1.259549098 | KSR2 | kinase suppressor of ras 2 [Source:HGNC Symbol;Acc:HGNC:18610] |
| ENSG00000171492 | HGLibA_27436 | 242 | 1.259549098 | LRRC8D | leucine rich repeat containing 8 VRAC subunit D [Source:HGNC Symbol;Acc:HGNC:16992] |
| ENSG00000171631 | HGLibA_34891 | 242 | 1.259549098 | P2RY6 | pyrimidinergic receptor P2Y6 [Source:HGNC Symbol;Acc:HGNC:8543] |
| ENSG00000175221 | HGLibA_28761 | 242 | 1.259549098 | MED16 | mediator complex subunit 16 [Source:HGNC Symbol;Acc:HGNC:17556] |
| ENSG00000176022 | HGLibA_03966 | 242 | 1.259549098 | B3GALT6 | 3-galactosyltransferase 6 [Source:HGNC Symbol;Acc:HGNC:17978]" |
| ENSG00000178372 | HGLibA_07037 | 242 | 1.259549098 | CALML5 | calmodulin like 5 [Source:HGNC Symbol;Acc:HGNC:18180] |
| ENSG00000180425 | HGLibA_05177 | 242 | 1.259549098 | C11orf71 | chromosome 11 open reading frame 71 [Source:HGNC Symbol;Acc:HGNC:25937] |
| ENSG00000183137 | HGLibA_09150 | 242 | 1.259549098 | CEP57L1 | centrosomal protein 57 like 1 [Source:HGNC Symbol;Acc:HGNC:21561] |
| ENSG00000196159 | HGLibA_16918 | 242 | 1.259549098 | FAT4 | FAT atypical cadherin 4 [Source:HGNC Symbol;Acc:HGNC:23109] |
| ENSG00000231396 | HGLibA_53215 | 242 | 1.259549098 | USP17L10 | ubiquitin specific peptidase 17-like family member 10 [Source:HGNC Symbol;Acc:HGNC:44438] |
| ENSG00000243335 | HGLibA_24465 | 242 | 1.259549098 | KCTD7 | potassium channel tetramerization domain containing 7 [Source:HGNC Symbol;Acc:HGNC:21957] |
| ENSG00000244362 | HGLibA_25682 | 242 | 1.259549098 | KRTAP19-7 | keratin associated protein 19-7 [Source:HGNC Symbol;Acc:HGNC:18942] |
| EPT1 | HGLibA_15390 | 242 | 1.259549098 | EPT1 | - |
| ERO1L | HGLibA_15518 | 242 | 1.259549098 | ERO1L | - |
| FAM19A1 | HGLibA_16397 | 242 | 1.259549098 | FAM19A1 | - |
| FAM5C | HGLibA_16653 | 242 | 1.259549098 | FAM5C | - |
| hsa-mir-23c | HGLibA_58335 | 242 | 1.259549098 | hsa-mir-23c | - |
| hsa-mir-608 | HGLibA_62578 | 242 | 1.259549098 | hsa-mir-608 | - |
| CTSL2 | HGLibA_11721 | 241 | 1.249598061 | CTSL2 | - |
| ENSG00000010165 | HGLibA_28955 | 241 | 1.249598061 | METTL13 | eEF1A lysine and N-terminal methyltransferase [Source:HGNC Symbol;Acc:HGNC:24248] |
| ENSG00000023909 | HGLibA_18798 | 241 | 1.249598061 | GCLM | glutamate-cysteine ligase modifier subunit [Source:HGNC Symbol;Acc:HGNC:4312] |
| ENSG00000043039 | HGLibA_04166 | 241 | 1.249598061 | BARX2 | BARX homeobox 2 [Source:HGNC Symbol;Acc:HGNC:956] |
| ENSG00000055957 | HGLibA_23804 | 241 | 1.249598061 | ITIH1 | inter-alpha-trypsin inhibitor heavy chain 1 [Source:HGNC Symbol;Acc:HGNC:6166] |
| ENSG00000068654 | HGLibA_37548 | 241 | 1.249598061 | POLR1A | RNA polymerase I subunit A [Source:HGNC Symbol;Acc:HGNC:17264] |
| ENSG00000070159 | HGLibA_39465 | 241 | 1.249598061 | PTPN3 | non-receptor type 3 [Source:HGNC Symbol;Acc:HGNC:9655]" |
| ENSG00000081087 | HGLibA_34714 | 241 | 1.249598061 | OSTM1 | osteoclastogenesis associated transmembrane protein 1 [Source:HGNC Symbol;Acc:HGNC:21652] |
| ENSG00000082153 | HGLibA_05012 | 241 | 1.249598061 | BZW1 | basic leucine zipper and W2 domains 1 [Source:HGNC Symbol;Acc:HGNC:18380] |
| ENSG00000084731 | HGLibA_24940 | 241 | 1.249598061 | KIF3C | kinesin family member 3C [Source:HGNC Symbol;Acc:HGNC:6321] |
| ENSG00000100450 | HGLibA_20723 | 241 | 1.249598061 | GZMH | granzyme H [Source:HGNC Symbol;Acc:HGNC:4710] |
| ENSG00000103023 | HGLibA_38958 | 241 | 1.249598061 | PRSS54 | serine protease 54 [Source:HGNC Symbol;Acc:HGNC:26336] |
| ENSG00000104324 | HGLibA_10989 | 241 | 1.249598061 | CPQ | carboxypeptidase Q [Source:HGNC Symbol;Acc:HGNC:16910] |
| ENSG00000105427 | HGLibA_10261 | 241 | 1.249598061 | CNFN | cornifelin [Source:HGNC Symbol;Acc:HGNC:30183] |
| ENSG00000105605 | HGLibA_06947 | 241 | 1.249598061 | CACNG7 | calcium voltage-gated channel auxiliary subunit gamma 7 [Source:HGNC Symbol;Acc:HGNC:13626] |
| ENSG00000107447 | HGLibA_13717 | 241 | 1.249598061 | DNTT | DNA nucleotidylexotransferase [Source:HGNC Symbol;Acc:HGNC:2983] |
| ENSG00000107566 | HGLibA_15493 | 241 | 1.249598061 | ERLIN1 | ER lipid raft associated 1 [Source:HGNC Symbol;Acc:HGNC:16947] |
| ENSG00000110917 | HGLibA_29370 | 241 | 1.249598061 | MLEC | malectin [Source:HGNC Symbol;Acc:HGNC:28973] |
| ENSG00000111196 | HGLibA_28009 | 241 | 1.249598061 | MAGOHB | exon junction complex subunit [Source:HGNC Symbol;Acc:HGNC:25504]" |
| ENSG00000112640 | HGLibA_38179 | 241 | 1.249598061 | PPP2R5D | protein phosphatase 2 regulatory subunit B'delta [Source:HGNC Symbol;Acc:HGNC:9312] |
| ENSG00000113368 | HGLibA_26695 | 241 | 1.249598061 | LMNB1 | lamin B1 [Source:HGNC Symbol;Acc:HGNC:6637] |
| ENSG00000120645 | HGLibA_23548 | 241 | 1.249598061 | IQSEC3 | IQ motif and Sec7 domain 3 [Source:HGNC Symbol;Acc:HGNC:29193] |
| ENSG00000122417 | HGLibA_33280 | 241 | 1.249598061 | ODF2L | outer dense fiber of sperm tails 2 like [Source:HGNC Symbol;Acc:HGNC:29225] |
| ENSG00000127527 | HGLibA_15371 | 241 | 1.249598061 | EPS15L1 | epidermal growth factor receptor pathway substrate 15 like 1 [Source:HGNC Symbol;Acc:HGNC:24634] |
| ENSG00000130479 | HGLibA_28132 | 241 | 1.249598061 | MAP1S | microtubule associated protein 1S [Source:HGNC Symbol;Acc:HGNC:15715] |
| ENSG00000133466 | HGLibA_05952 | 241 | 1.249598061 | C1QTNF6 | C1q and TNF related 6 [Source:HGNC Symbol;Acc:HGNC:14343] |
| ENSG00000134504 | HGLibA_24410 | 241 | 1.249598061 | KCTD1 | potassium channel tetramerization domain containing 1 [Source:HGNC Symbol;Acc:HGNC:18249] |
| ENSG00000136161 | HGLibA_40681 | 241 | 1.249598061 | RCBTB2 | RCC1 and BTB domain containing protein 2 [Source:HGNC Symbol;Acc:HGNC:1914] |
| ENSG00000137392 | HGLibA_10103 | 241 | 1.249598061 | CLPS | colipase [Source:HGNC Symbol;Acc:HGNC:2085] |
| ENSG00000140612 | HGLibA_43182 | 241 | 1.249598061 | SEC11A | signal peptidase complex subunit [Source:HGNC Symbol;Acc:HGNC:17718]" |
| ENSG00000142192 | HGLibA_02559 | 241 | 1.249598061 | APP | amyloid beta precursor protein [Source:HGNC Symbol;Acc:HGNC:620] |
| ENSG00000142867 | HGLibA_04286 | 241 | 1.249598061 | BCL10 | B cell CLL/lymphoma 10 [Source:HGNC Symbol;Acc:HGNC:989] |
| ENSG00000143322 | HGLibA_00304 | 241 | 1.249598061 | ABL2 | non-receptor tyrosine kinase [Source:HGNC Symbol;Acc:HGNC:77]" |
| ENSG00000149452 | HGLibA_44601 | 241 | 1.249598061 | SLC22A8 | solute carrier family 22 member 8 [Source:HGNC Symbol;Acc:HGNC:10972] |
| ENSG00000153815 | HGLibA_10195 | 241 | 1.249598061 | CMIP | c-Maf inducing protein [Source:HGNC Symbol;Acc:HGNC:24319] |
| ENSG00000154146 | HGLibA_32687 | 241 | 1.249598061 | NRGN | neurogranin [Source:HGNC Symbol;Acc:HGNC:8000] |
| ENSG00000154274 | HGLibA_06298 | 241 | 1.249598061 | C4orf19 | chromosome 4 open reading frame 19 [Source:HGNC Symbol;Acc:HGNC:25618] |
| ENSG00000158525 | HGLibA_10897 | 241 | 1.249598061 | CPA5 | carboxypeptidase A5 [Source:HGNC Symbol;Acc:HGNC:15722] |
| ENSG00000163825 | HGLibA_42429 | 241 | 1.249598061 | RTP3 | receptor transporter protein 3 [Source:HGNC Symbol;Acc:HGNC:15572] |
| ENSG00000165269 | HGLibA_02604 | 241 | 1.249598061 | AQP7 | aquaporin 7 [Source:HGNC Symbol;Acc:HGNC:640] |
| ENSG00000165752 | HGLibA_47490 | 241 | 1.249598061 | STK32C | serine/threonine kinase 32C [Source:HGNC Symbol;Acc:HGNC:21332] |
| ENSG00000166145 | HGLibA_46712 | 241 | 1.249598061 | SPINT1 | Kunitz type 1 [Source:HGNC Symbol;Acc:HGNC:11246]" |
| ENSG00000166188 | HGLibA_55788 | 241 | 1.249598061 | ZNF319 | zinc finger protein 319 [Source:HGNC Symbol;Acc:HGNC:13644] |
| ENSG00000167232 | HGLibA_56904 | 241 | 1.249598061 | ZNF91 | zinc finger protein 91 [Source:HGNC Symbol;Acc:HGNC:13166] |
| ENSG00000167306 | HGLibA_30853 | 241 | 1.249598061 | MYO5B | myosin VB [Source:HGNC Symbol;Acc:HGNC:7603] |
| ENSG00000167380 | HGLibA_55623 | 241 | 1.249598061 | ZNF226 | zinc finger protein 226 [Source:HGNC Symbol;Acc:HGNC:13019] |
| ENSG00000168038 | HGLibA_52974 | 241 | 1.249598061 | ULK4 | unc-51 like kinase 4 [Source:HGNC Symbol;Acc:HGNC:15784] |
| ENSG00000169688 | HGLibA_30279 | 241 | 1.249598061 | MT1B | metallothionein 1B [Source:HGNC Symbol;Acc:HGNC:7394] |
| ENSG00000170074 | HGLibA_16168 | 241 | 1.249598061 | FAM153A | family with sequence similarity 153 member A [Source:HGNC Symbol;Acc:HGNC:29940] |
| ENSG00000173153 | HGLibA_15594 | 241 | 1.249598061 | ESRRA | estrogen related receptor alpha [Source:HGNC Symbol;Acc:HGNC:3471] |
| ENSG00000180929 | HGLibA_20065 | 241 | 1.249598061 | GPR62 | G protein-coupled receptor 62 [Source:HGNC Symbol;Acc:HGNC:13301] |
| ENSG00000181195 | HGLibA_36047 | 241 | 1.249598061 | PENK | proenkephalin [Source:HGNC Symbol;Acc:HGNC:8831] |
| ENSG00000182141 | HGLibA_56602 | 241 | 1.249598061 | ZNF708 | zinc finger protein 708 [Source:HGNC Symbol;Acc:HGNC:12945] |
| ENSG00000184507 | HGLibA_33101 | 241 | 1.249598061 | NUTM1 | NUT midline carcinoma family member 1 [Source:HGNC Symbol;Acc:HGNC:29919] |
| ENSG00000185722 | HGLibA_01957 | 241 | 1.249598061 | ANKFY1 | ankyrin repeat and FYVE domain containing 1 [Source:HGNC Symbol;Acc:HGNC:20763] |
| ENSG00000187244 | HGLibA_04233 | 241 | 1.249598061 | BCAM | basal cell adhesion molecule (Lutheran blood group) [Source:HGNC Symbol;Acc:HGNC:6722] |
| ENSG00000188032 | HGLibA_05696 | 241 | 1.249598061 | C19orf67 | chromosome 19 open reading frame 67 [Source:HGNC Symbol;Acc:HGNC:34354] |
| ENSG00000197496 | HGLibA_44854 | 241 | 1.249598061 | SLC2A10 | solute carrier family 2 member 10 [Source:HGNC Symbol;Acc:HGNC:13444] |
| ENSG00000197622 | HGLibA_08580 | 241 | 1.249598061 | CDC42SE1 | CDC42 small effector 1 [Source:HGNC Symbol;Acc:HGNC:17719] |
| ENSG00000204379 | HGLibA_54466 | 241 | 1.249598061 | XAGE1A | X antigen family member 1A [Source:HGNC Symbol;Acc:HGNC:4111] |
| ENSG00000204583 | HGLibA_27148 | 241 | 1.249598061 | LRCOL1 | leucine rich colipase like 1 [Source:HGNC Symbol;Acc:HGNC:44160] |
| ENSG00000213445 | HGLibA_44188 | 241 | 1.249598061 | SIPA1 | signal-induced proliferation-associated 1 [Source:HGNC Symbol;Acc:HGNC:10885] |

| | | | | | |
|---|---|---|---|---|---|
| ENSG00000213462 | HGLibA_15537 | 241 | 1.249598061 | ERV3-1 | envelope [Source:HGNC Symbol;Acc:HGNC:3454]" |
| ENSG00000241360 | HGLibA_35965 | 241 | 1.249598061 | PDXP | pyridoxal phosphatase [Source:HGNC Symbol;Acc:HGNC:30259] |
| ENSG00000254737 | HGLibA_33494 | 241 | 1.249598061 | OR10G4 | olfactory receptor family 10 subfamily G member 4 [Source:HGNC Symbol;Acc:HGNC:14809] |
| HIST1H2AD | HGLibA_21383 | 241 | 1.249598061 | HIST1H2AD | - |
| HIST1H4H | HGLibA_21507 | 241 | 1.249598061 | HIST1H4H | - |
| hsa-mir-1909 | HGLibA_58029 | 241 | 1.249598061 | hsa-mir-1909 | - |
| hsa-mir-3201 | HGLibA_58912 | 241 | 1.249598061 | hsa-mir-3201 | - |
| hsa-mir-4259 | HGLibA_59742 | 241 | 1.249598061 | hsa-mir-4259 | - |
| hsa-mir-4301 | HGLibA_59916 | 241 | 1.249598061 | hsa-mir-4301 | - |
| hsa-mir-4493 | HGLibA_60392 | 241 | 1.249598061 | hsa-mir-4493 | - |
| hsa-mir-4654 | HGLibA_60723 | 241 | 1.249598061 | hsa-mir-4654 | - |
| hsa-mir-4743 | HGLibA_61068 | 241 | 1.249598061 | hsa-mir-4743 | - |
| hsa-mir-5190 | HGLibA_61680 | 241 | 1.249598061 | hsa-mir-5190 | - |
| hsa-mir-6069 | HGLibA_62524 | 241 | 1.249598061 | hsa-mir-6069 | - |
| hsa-mir-609 | HGLibA_62624 | 241 | 1.249598061 | hsa-mir-609 | - |
| hsa-mir-6767 | HGLibA_63228 | 241 | 1.249598061 | hsa-mir-6767 | - |
| hsa-mir-6798 | HGLibA_63370 | 241 | 1.249598061 | hsa-mir-6798 | - |
| hsa-mir-6879 | HGLibA_63701 | 241 | 1.249598061 | hsa-mir-6879 | - |
| hsa-mir-921 | HGLibA_64279 | 241 | 1.249598061 | hsa-mir-921 | - |
| METTL12 | HGLibA_28951 | 241 | 1.249598061 | METTL12 | - |
| PPP2R3B_X | HGLibA_38157 | 241 | 1.249598061 | PPP2R3B_X | - |
| SPANXE | HGLibA_46395 | 241 | 1.249598061 | SPANXE | - |
| TMEM55A | HGLibA_50445 | 241 | 1.249598061 | TMEM55A | - |
| C16orf55 | HGLibA_05460 | 240 | 1.239647023 | C16orf55 | - |
| ENSG00000007392 | HGLibA_27619 | 240 | 1.239647023 | LUC7L | LUC7 like [Source:HGNC Symbol;Acc:HGNC:6723] |
| ENSG00000015592 | HGLibA_47526 | 240 | 1.239647023 | STMN4 | stathmin 4 [Source:HGNC Symbol;Acc:HGNC:16078] |
| ENSG00000026508 | HGLibA_08390 | 240 | 1.239647023 | CD44 | CD44 molecule (Indian blood group) [Source:HGNC Symbol;Acc:HGNC:1681] |
| ENSG00000054392 | HGLibA_21253 | 240 | 1.239647023 | HHAT | hedgehog acyltransferase [Source:HGNC Symbol;Acc:HGNC:18270] |
| ENSG00000054793 | HGLibA_03791 | 240 | 1.239647023 | ATP9A | ATPase phospholipid transporting 9A (putative) [Source:HGNC Symbol;Acc:HGNC:13540] |
| ENSG00000069696 | HGLibA_13967 | 240 | 1.239647023 | DRD4 | dopamine receptor D4 [Source:HGNC Symbol;Acc:HGNC:3025] |
| ENSG00000074181 | HGLibA_32349 | 240 | 1.239647023 | NOTCH3 | notch 3 [Source:HGNC Symbol;Acc:HGNC:7883] |
| ENSG00000087301 | HGLibA_52415 | 240 | 1.239647023 | TXNDC16 | thioredoxin domain containing 16 [Source:HGNC Symbol;Acc:HGNC:19965] |
| ENSG00000090020 | HGLibA_45401 | 240 | 1.239647023 | SLC9A1 | solute carrier family 9 member A1 [Source:HGNC Symbol;Acc:HGNC:11071] |
| ENSG00000095777 | HGLibA_30844 | 240 | 1.239647023 | MYO3A | myosin IIIA [Source:HGNC Symbol;Acc:HGNC:7601] |
| ENSG00000108511 | HGLibA_21945 | 240 | 1.239647023 | HOXB6 | homeobox B6 [Source:HGNC Symbol;Acc:HGNC:5117] |
| ENSG00000108700 | HGLibA_08043 | 240 | 1.239647023 | CCL8 | C-C motif chemokine ligand 8 [Source:HGNC Symbol;Acc:HGNC:10635] |
| ENSG00000116161 | HGLibA_06958 | 240 | 1.239647023 | CACYBP | calcyclin binding protein [Source:HGNC Symbol;Acc:HGNC:30423] |
| ENSG00000124103 | HGLibA_16437 | 240 | 1.239647023 | FAM209A | family with sequence similarity 209 member A [Source:HGNC Symbol;Acc:HGNC:16100] |
| ENSG00000131730 | HGLibA_09784 | 240 | 1.239647023 | CKMT2 | mitochondrial 2 [Source:HGNC Symbol;Acc:HGNC:1996]" |
| ENSG00000133318 | HGLibA_42403 | 240 | 1.239647023 | RTN3 | reticulon 3 [Source:HGNC Symbol;Acc:HGNC:10469] |
| ENSG00000135414 | HGLibA_18858 | 240 | 1.239647023 | GDF11 | growth differentiation factor 11 [Source:HGNC Symbol;Acc:HGNC:4216] |
| ENSG00000138755 | HGLibA_11879 | 240 | 1.239647023 | CXCL9 | C-X-C motif chemokine ligand 9 [Source:HGNC Symbol;Acc:HGNC:7098] |
| ENSG00000141503 | HGLibA_29276 | 240 | 1.239647023 | MINK1 | misshapen like kinase 1 [Source:HGNC Symbol;Acc:HGNC:17565] |
| ENSG00000144426 | HGLibA_31210 | 240 | 1.239647023 | NBEAL1 | neurobeachin like 1 [Source:HGNC Symbol;Acc:HGNC:20681] |
| ENSG00000145649 | HGLibA_20717 | 240 | 1.239647023 | GZMA | granzyme A [Source:HGNC Symbol;Acc:HGNC:4708] |
| ENSG00000151470 | HGLibA_06323 | 240 | 1.239647023 | C4orf33 | chromosome 4 open reading frame 33 [Source:HGNC Symbol;Acc:HGNC:27025] |
| ENSG00000154007 | HGLibA_03228 | 240 | 1.239647023 | ASB17 | ankyrin repeat and SOCS box containing 17 [Source:HGNC Symbol;Acc:HGNC:19769] |
| ENSG00000156471 | HGLibA_39288 | 240 | 1.239647023 | PTDSS1 | phosphatidylserine synthase 1 [Source:HGNC Symbol;Acc:HGNC:9587] |
| ENSG00000159714 | HGLibA_55066 | 240 | 1.239647023 | ZDHHC1 | zinc finger DHHC-type containing 1 [Source:HGNC Symbol;Acc:HGNC:17916] |
| ENSG00000162399 | HGLibA_04873 | 240 | 1.239647023 | BSND | barttin CLCNK type accessory beta subunit [Source:HGNC Symbol;Acc:HGNC:16512] |
| ENSG00000163694 | HGLibA_40567 | 240 | 1.239647023 | RBM47 | RNA binding motif protein 47 [Source:HGNC Symbol;Acc:HGNC:30358] |
| ENSG00000164414 | HGLibA_44947 | 240 | 1.239647023 | SLC35A1 | solute carrier family 35 member A1 [Source:HGNC Symbol;Acc:HGNC:11021] |
| ENSG00000164683 | HGLibA_21216 | 240 | 1.239647023 | HEY1 | hes related family bHLH transcription factor with YRPW motif 1 [Source:HGNC Symbol;Acc:HGNC:4880] |
| ENSG00000165802 | HGLibA_32775 | 240 | 1.239647023 | NSMF | NMDA receptor synaptonuclear signaling and neuronal migration factor [Source:HGNC Symbol;Acc:HGNC:29843] |
| ENSG00000167173 | HGLibA_05392 | 240 | 1.239647023 | C15orf39 | chromosome 15 open reading frame 39 [Source:HGNC Symbol;Acc:HGNC:24497] |
| ENSG00000168386 | HGLibA_17531 | 240 | 1.239647023 | FILIP1L | filamin A interacting protein 1 like [Source:HGNC Symbol;Acc:HGNC:24589] |
| ENSG00000172458 | HGLibA_22997 | 240 | 1.239647023 | IL17D | interleukin 17D [Source:HGNC Symbol;Acc:HGNC:5984] |
| ENSG00000173237 | HGLibA_05201 | 240 | 1.239647023 | C11orf86 | chromosome 11 open reading frame 86 [Source:HGNC Symbol;Acc:HGNC:34442] |
| ENSG00000174963 | HGLibA_55331 | 240 | 1.239647023 | ZIC4 | Zic family member 4 [Source:HGNC Symbol;Acc:HGNC:20393] |
| ENSG00000176402 | HGLibA_19185 | 240 | 1.239647023 | GJC3 | gap junction protein gamma 3 [Source:HGNC Symbol;Acc:HGNC:17495] |
| ENSG00000178372 | HGLibA_07036 | 240 | 1.239647023 | CALML5 | calmodulin like 5 [Source:HGNC Symbol;Acc:HGNC:18180] |
| ENSG00000178473 | HGLibA_52828 | 240 | 1.239647023 | UCN3 | urocortin 3 [Source:HGNC Symbol;Acc:HGNC:17781] |
| ENSG00000182132 | HGLibA_24233 | 240 | 1.239647023 | KCNIP1 | potassium voltage-gated channel interacting protein 1 [Source:HGNC Symbol;Acc:HGNC:15521] |
| ENSG00000182359 | HGLibA_24104 | 240 | 1.239647023 | KBTBD3 | kelch repeat and BTB domain containing 3 [Source:HGNC Symbol;Acc:HGNC:22934] |
| ENSG00000184986 | HGLibA_49902 | 240 | 1.239647023 | TMEM121 | transmembrane protein 121 [Source:HGNC Symbol;Acc:HGNC:20511] |
| ENSG00000188158 | HGLibA_31914 | 240 | 1.239647023 | NHS | NHS actin remodeling regulator [Source:HGNC Symbol;Acc:HGNC:7820] |
| ENSG00000197646 | HGLibA_35723 | 240 | 1.239647023 | PDCD1LG2 | programmed cell death 1 ligand 2 [Source:HGNC Symbol;Acc:HGNC:18731] |
| ENSG00000198033 | HGLibA_52253 | 240 | 1.239647023 | TUBA3C | tubulin alpha 3c [Source:HGNC Symbol;Acc:HGNC:12408] |
| ENSG00000204305 | HGLibA_01180 | 240 | 1.239647023 | AGER | advanced glycosylation end-product specific receptor [Source:HGNC Symbol;Acc:HGNC:320] |
| ENSG00000236446 | HGLibA_11552 | 240 | 1.239647023 | CT47B1 | member B1 [Source:HGNC Symbol;Acc:HGNC:33293]" |
| ENSG00000261456 | HGLibA_52293 | 240 | 1.239647023 | TUBB8 | tubulin beta 8 class VIII [Source:HGNC Symbol;Acc:HGNC:20773] |
| ENSG00000268104 | HGLibA_45300 | 240 | 1.239647023 | SLC6A14 | solute carrier family 6 member 14 [Source:HGNC Symbol;Acc:HGNC:11047] |
| ENSG00000275385 | HGLibA_07981 | 240 | 1.239647023 | CCL18 | C-C motif chemokine ligand 18 [Source:HGNC Symbol;Acc:HGNC:10616] |
| ENSG00000278057 | HGLibA_49087 | 240 | 1.239647023 | TEX28 | testis expressed 28 [Source:HGNC Symbol;Acc:HGNC:2563] |
| hsa-mir-218-1 | HGLibA_58269 | 240 | 1.239647023 | hsa-mir-218-1 | - |
| hsa-mir-4522 | HGLibA_60545 | 240 | 1.239647023 | hsa-mir-4522 | - |
| hsa-mir-499a | HGLibA_61399 | 240 | 1.239647023 | hsa-mir-499a | - |
| hsa-mir-548ag-1 | HGLibA_61873 | 240 | 1.239647023 | hsa-mir-548ag-1 | - |
| hsa-mir-6736 | HGLibA_63096 | 240 | 1.239647023 | hsa-mir-6736 | - |
| hsa-mir-8063 | HGLibA_64090 | 240 | 1.239647023 | hsa-mir-8063 | - |
| hsa-mir-944 | HGLibA_64362 | 240 | 1.239647023 | hsa-mir-944 | - |
| CCDC36 | HGLibA_07764 | 239 | 1.229695986 | CCDC36 | - |
| ENSG00000005206 | HGLibA_46779 | 239 | 1.229695986 | SPPL2B | signal peptide peptidase like 2B [Source:HGNC Symbol;Acc:HGNC:30627] |
| ENSG00000006652 | HGLibA_22737 | 239 | 1.229695986 | IFRD1 | interferon related developmental regulator 1 [Source:HGNC Symbol;Acc:HGNC:5456] |
| ENSG00000049541 | HGLibA_40905 | 239 | 1.229695986 | RFC2 | replication factor C subunit 2 [Source:HGNC Symbol;Acc:HGNC:9970] |
| ENSG00000076513 | HGLibA_02001 | 239 | 1.229695986 | ANKRD13A | ankyrin repeat domain 13A [Source:HGNC Symbol;Acc:HGNC:21268] |
| ENSG00000086598 | HGLibA_49804 | 239 | 1.229695986 | TMED2 | transmembrane p24 trafficking protein 2 [Source:HGNC Symbol;Acc:HGNC:16996] |
| ENSG00000087995 | HGLibA_28999 | 239 | 1.229695986 | METTL2A | methyltransferase like 2A [Source:HGNC Symbol;Acc:HGNC:25755] |
| ENSG00000099849 | HGLibA_40391 | 239 | 1.229695986 | RASSF7 | Ras association domain family member 7 [Source:HGNC Symbol;Acc:HGNC:1166] |
| ENSG00000101180 | HGLibA_22101 | 239 | 1.229695986 | HRH3 | histamine receptor H3 [Source:HGNC Symbol;Acc:HGNC:5184] |
| ENSG00000101470 | HGLibA_50867 | 239 | 1.229695986 | TNNC2 | fast skeletal type [Source:HGNC Symbol;Acc:HGNC:11944]" |
| ENSG00000105717 | HGLibA_35353 | 239 | 1.229695986 | PBX4 | PBX homeobox 4 [Source:HGNC Symbol;Acc:HGNC:13403] |
| ENSG00000109762 | HGLibA_46071 | 239 | 1.229695986 | SNX25 | sorting nexin 25 [Source:HGNC Symbol;Acc:HGNC:21883] |
| ENSG00000111752 | HGLibA_36319 | 239 | 1.229695986 | PHC1 | polyhomeotic homolog 1 [Source:HGNC Symbol;Acc:HGNC:3182] |
| ENSG00000113812 | HGLibA_00674 | 239 | 1.229695986 | ACTR8 | ARP8 actin related protein 8 homolog [Source:HGNC Symbol;Acc:HGNC:14672] |
| ENSG00000115457 | HGLibA_22819 | 239 | 1.229695986 | IGFBP2 | insulin like growth factor binding protein 2 [Source:HGNC Symbol;Acc:HGNC:5471] |
| ENSG00000117308 | HGLibA_18513 | 239 | 1.229695986 | GALE | UDP-galactose-4-epimerase [Source:HGNC Symbol;Acc:HGNC:4116] |
| ENSG00000117500 | HGLibA_49813 | 239 | 1.229695986 | TMED5 | transmembrane p24 trafficking protein 5 [Source:HGNC Symbol;Acc:HGNC:24251] |
| ENSG00000119685 | HGLibA_52206 | 239 | 1.229695986 | TTLL5 | tubulin tyrosine ligase like 5 [Source:HGNC Symbol;Acc:HGNC:19963] |
| ENSG00000120251 | HGLibA_20262 | 239 | 1.229695986 | GRIA2 | glutamate ionotropic receptor AMPA type subunit 2 [Source:HGNC Symbol;Acc:HGNC:4572] |
| ENSG00000124232 | HGLibA_40651 | 239 | 1.229695986 | RBPJL | recombination signal binding protein for immunoglobulin kappa J region like [Source:HGNC Symbol;Acc:HGNC:13761] |
| ENSG00000130561 | HGLibA_42640 | 239 | 1.229695986 | SAG | S-antigen visual arrestin [Source:HGNC Symbol;Acc:HGNC:10521] |
| ENSG00000130988 | HGLibA_41018 | 239 | 1.229695986 | RGN | regucalcin [Source:HGNC Symbol;Acc:HGNC:9989] |
| ENSG00000135341 | HGLibA_28197 | 239 | 1.229695986 | MAP3K7 | mitogen-activated protein kinase kinase kinase 7 [Source:HGNC Symbol;Acc:HGNC:6859] |
| ENSG00000138161 | HGLibA_11808 | 239 | 1.229695986 | CUZD1 | CUB and zona pellucida like domains 1 [Source:HGNC Symbol;Acc:HGNC:17937] |
| ENSG00000142182 | HGLibA_13707 | 239 | 1.229695986 | DNMT3L | DNA methyltransferase 3 like [Source:HGNC Symbol;Acc:HGNC:2980] |
| ENSG00000144730 | HGLibA_23013 | 239 | 1.229695986 | IL17RD | interleukin 17 receptor D [Source:HGNC Symbol;Acc:HGNC:17616] |
| ENSG00000145708 | HGLibA_11137 | 239 | 1.229695986 | CRHBP | corticotropin releasing hormone binding protein [Source:HGNC Symbol;Acc:HGNC:2356] |
| ENSG00000145863 | HGLibA_18370 | 239 | 1.229695986 | GABRA6 | gamma-aminobutyric acid type A receptor alpha6 subunit [Source:HGNC Symbol;Acc:HGNC:4080] |
| ENSG00000146411 | HGLibA_44862 | 239 | 1.229695986 | SLC2A12 | solute carrier family 2 member 12 [Source:HGNC Symbol;Acc:HGNC:18067] |
| ENSG00000151012 | HGLibA_45351 | 239 | 1.229695986 | SLC7A11 | solute carrier family 7 member 11 [Source:HGNC Symbol;Acc:HGNC:11059] |
| ENSG00000151838 | HGLibA_07705 | 239 | 1.229695986 | CCDC175 | coiled-coil domain containing 175 [Source:HGNC Symbol;Acc:HGNC:19847] |
| ENSG00000157219 | HGLibA_22399 | 239 | 1.229695986 | HTR5A | 5-hydroxytryptamine receptor 5A [Source:HGNC Symbol;Acc:HGNC:5300] |
| ENSG00000158092 | HGLibA_31304 | 239 | 1.229695986 | NCK1 | NCK adaptor protein 1 [Source:HGNC Symbol;Acc:HGNC:7664] |
| ENSG00000160285 | HGLibA_27568 | 239 | 1.229695986 | LSS | lanosterol synthase [Source:HGNC Symbol;Acc:HGNC:6708] |
| ENSG00000162402 | HGLibA_53307 | 239 | 1.229695986 | USP24 | ubiquitin specific peptidase 24 [Source:HGNC Symbol;Acc:HGNC:12623] |
| ENSG00000164111 | HGLibA_02244 | 239 | 1.229695986 | ANXA5 | annexin A5 [Source:HGNC Symbol;Acc:HGNC:543] |
| ENSG00000166478 | HGLibA_55491 | 239 | 1.229695986 | ZNF143 | zinc finger protein 143 [Source:HGNC Symbol;Acc:HGNC:12928] |
| ENSG00000169925 | HGLibA_04775 | 239 | 1.229695986 | BRD3 | bromodomain containing 3 [Source:HGNC Symbol;Acc:HGNC:1104] |
| ENSG00000171067 | HGLibA_05123 | 239 | 1.229695986 | C11orf24 | chromosome 11 open reading frame 24 [Source:HGNC Symbol;Acc:HGNC:1174] |
| ENSG00000173272 | HGLibA_30944 | 239 | 1.229695986 | MZT2A | mitotic spindle organizing protein 2A [Source:HGNC Symbol;Acc:HGNC:33187] |
| ENSG00000173482 | HGLibA_39517 | 239 | 1.229695986 | PTPRM | receptor type M [Source:HGNC Symbol;Acc:HGNC:9675]" |
| ENSG00000180287 | HGLibA_37048 | 239 | 1.229695986 | PLD5 | phospholipase D family member 5 [Source:HGNC Symbol;Acc:HGNC:26879] |
| ENSG00000184697 | HGLibA_09904 | 239 | 1.229695986 | CLDN6 | claudin 6 [Source:HGNC Symbol;Acc:HGNC:2048] |
| ENSG00000186280 | HGLibA_24516 | 239 | 1.229695986 | KDM4D | lysine demethylase 4D [Source:HGNC Symbol;Acc:HGNC:25498] |
| ENSG00000186971 | HGLibA_25652 | 239 | 1.229695986 | KRTAP13-4 | keratin associated protein 13-4 [Source:HGNC Symbol;Acc:HGNC:18926] |
| ENSG00000239779 | HGLibA_53967 | 239 | 1.229695986 | WBP1 | WW domain binding protein 1 [Source:HGNC Symbol;Acc:HGNC:12737] |
| ENSG00000254827 | HGLibA_44565 | 239 | 1.229695986 | SLC22A18AS | solute carrier family 22 member 18 antisense [Source:HGNC Symbol;Acc:HGNC:10965] |
| ENSG00000258986 | HGLibA_50088 | 239 | 1.229695986 | TMEM179 | transmembrane protein 179 [Source:HGNC Symbol;Acc:HGNC:20137] |
| ENSG00000265107 | HGLibA_19149 | 239 | 1.229695986 | GJA5 | gap junction protein alpha 5 [Source:HGNC Symbol;Acc:HGNC:4279] |
| ENSG00000268089 | HGLibA_18401 | 239 | 1.229695986 | GABRQ | gamma-aminobutyric acid type A receptor theta subunit [Source:HGNC Symbol;Acc:HGNC:14454] |
| FAM46D | HGLibA_16605 | 239 | 1.229695986 | FAM46D | - |
| hsa-mir-4294 | HGLibA_59887 | 239 | 1.229695986 | hsa-mir-4294 | - |
| hsa-mir-4440 | HGLibA_60163 | 239 | 1.229695986 | hsa-mir-4440 | - |
| hsa-mir-4450 | HGLibA_60205 | 239 | 1.229695986 | hsa-mir-4450 | - |

| | | | | | |
|---|---|---|---|---|---|
| hsa-mir-4645 | HGLibA_60683 | 239 | 1.229695986 | hsa-mir-4645 | - |
| hsa-mir-4792 | HGLibA_61276 | 239 | 1.229695986 | hsa-mir-4792 | - |
| hsa-mir-500b | HGLibA_61446 | 239 | 1.229695986 | hsa-mir-500b | - |
| hsa-mir-6816 | HGLibA_63441 | 239 | 1.229695986 | hsa-mir-6816 | - |
| hsa-mir-7973-2 | HGLibA_64020 | 239 | 1.229695986 | hsa-mir-7973-2 | - |
| UQCC | HGLibA_53096 | 239 | 1.229695986 | UQCC | - |
| C18orf42 | HGLibA_05615 | 238 | 1.219744948 | C18orf42 | - |
| CCDC129 | HGLibA_07585 | 238 | 1.219744948 | CCDC129 | - |
| ENSG00000051128 | HGLibA_21870 | 238 | 1.219744948 | HOMER3 | homer scaffold protein 3 [Source:HGNC Symbol;Acc:HGNC:17514] |
| ENSG00000060749 | HGLibA_39752 | 238 | 1.219744948 | QSER1 | glutamine and serine rich 1 [Source:HGNC Symbol;Acc:HGNC:26154] |
| ENSG00000064601 | HGLibA_11688 | 238 | 1.219744948 | CTSA | cathepsin A [Source:HGNC Symbol;Acc:HGNC:9251] |
| ENSG00000086967 | HGLibA_30652 | 238 | 1.219744948 | MYBPC2 | fast type [Source:HGNC Symbol;Acc:HGNC:7550]" |
| ENSG00000088726 | HGLibA_50389 | 238 | 1.219744948 | TMEM40 | transmembrane protein 40 [Source:HGNC Symbol;Acc:HGNC:25620] |
| ENSG00000101447 | HGLibA_16748 | 238 | 1.219744948 | FAM83D | family with sequence similarity 83 member D [Source:HGNC Symbol;Acc:HGNC:16122] |
| ENSG00000101639 | HGLibA_09125 | 238 | 1.219744948 | CEP192 | centrosomal protein 192 [Source:HGNC Symbol;Acc:HGNC:25515] |
| ENSG00000104320 | HGLibA_31218 | 238 | 1.219744948 | NBN | nibrin [Source:HGNC Symbol;Acc:HGNC:7652] |
| ENSG00000109113 | HGLibA_39885 | 238 | 1.219744948 | RAB34 | member RAS oncogene family [Source:HGNC Symbol;Acc:HGNC:16519]" |
| ENSG00000112218 | HGLibA_20069 | 238 | 1.219744948 | GPR63 | G protein-coupled receptor 63 [Source:HGNC Symbol;Acc:HGNC:13302] |
| ENSG00000113657 | HGLibA_13930 | 238 | 1.219744948 | DPYSL3 | dihydropyrimidinase like 3 [Source:HGNC Symbol;Acc:HGNC:3015] |
| ENSG00000125657 | HGLibA_50829 | 238 | 1.219744948 | TNFSF9 | TNF superfamily member 9 [Source:HGNC Symbol;Acc:HGNC:11939] |
| ENSG00000129159 | HGLibA_24162 | 238 | 1.219744948 | KCNC1 | potassium voltage-gated channel subfamily C member 1 [Source:HGNC Symbol;Acc:HGNC:6233] |
| ENSG00000134278 | HGLibA_46724 | 238 | 1.219744948 | SPIRE1 | spire type actin nucleation factor 1 [Source:HGNC Symbol;Acc:HGNC:30622] |
| ENSG00000135144 | HGLibA_14079 | 238 | 1.219744948 | DTX1 | deltex E3 ubiquitin ligase 1 [Source:HGNC Symbol;Acc:HGNC:3060] |
| ENSG00000138303 | HGLibA_03257 | 238 | 1.219744948 | ASCC1 | activating signal cointegrator 1 complex subunit 1 [Source:HGNC Symbol;Acc:HGNC:24268] |
| ENSG00000140263 | HGLibA_46193 | 238 | 1.219744948 | SORD | sorbitol dehydrogenase [Source:HGNC Symbol;Acc:HGNC:11184] |
| ENSG00000147789 | HGLibA_56569 | 238 | 1.219744948 | ZNF7 | zinc finger protein 7 [Source:HGNC Symbol;Acc:HGNC:13139] |
| ENSG00000152214 | HGLibA_41340 | 238 | 1.219744948 | RIT2 | Ras like without CAAX 2 [Source:HGNC Symbol;Acc:HGNC:10017] |
| ENSG00000158710 | HGLibA_48272 | 238 | 1.219744948 | TAGLN2 | transgelin 2 [Source:HGNC Symbol;Acc:HGNC:11554] |
| ENSG00000162188 | HGLibA_19524 | 238 | 1.219744948 | GNG3 | G protein subunit gamma 3 [Source:HGNC Symbol;Acc:HGNC:4405] |
| ENSG00000162390 | HGLibA_00469 | 238 | 1.219744948 | ACOT11 | acyl-CoA thioesterase 11 [Source:HGNC Symbol;Acc:HGNC:18156] |
| ENSG00000169957 | HGLibA_56697 | 238 | 1.219744948 | ZNF768 | zinc finger protein 768 [Source:HGNC Symbol;Acc:HGNC:26273] |
| ENSG00000171509 | HGLibA_42494 | 238 | 1.219744948 | RXFP1 | relaxin family peptide receptor 1 [Source:HGNC Symbol;Acc:HGNC:19718] |
| ENSG00000172818 | HGLibA_34802 | 238 | 1.219744948 | OVOL1 | ovo like transcriptional repressor 1 [Source:HGNC Symbol;Acc:HGNC:8525] |
| ENSG00000174500 | HGLibA_18826 | 238 | 1.219744948 | GCSAM | germinal center associated signaling and motility [Source:HGNC Symbol;Acc:HGNC:20253] |
| ENSG00000176920 | HGLibA_18189 | 238 | 1.219744948 | FUT2 | fucosyltransferase 2 [Source:HGNC Symbol;Acc:HGNC:4013] |
| ENSG00000179262 | HGLibA_40070 | 238 | 1.219744948 | RAD23A | nucleotide excision repair protein [Source:HGNC Symbol;Acc:HGNC:9812]" |
| ENSG00000180332 | HGLibA_24456 | 238 | 1.219744948 | KCTD4 | potassium channel tetramerization domain containing 4 [Source:HGNC Symbol;Acc:HGNC:23227] |
| ENSG00000182870 | HGLibA_18574 | 238 | 1.219744948 | GALNT9 | polypeptide N-acetylgalactosaminyltransferase 9 [Source:HGNC Symbol;Acc:HGNC:4131] |
| ENSG00000198300 | HGLibA_36029 | 238 | 1.219744948 | PEG3 | paternally expressed 3 [Source:HGNC Symbol;Acc:HGNC:8826] |
| ENSG00000203784 | HGLibA_26283 | 238 | 1.219744948 | LELP1 | late cornified envelope like proline rich 1 [Source:HGNC Symbol;Acc:HGNC:32046] |
| ENSG00000205328 | HGLibA_34397 | 238 | 1.219744948 | OR6C65 | olfactory receptor family 6 subfamily C member 65 [Source:HGNC Symbol;Acc:HGNC:31295] |
| ENSG00000241484 | HGLibA_02789 | 238 | 1.219744948 | ARHGAP8 | Rho GTPase activating protein 8 [Source:HGNC Symbol;Acc:HGNC:677] |
| ENSG00000271601 | HGLibA_26644 | 238 | 1.219744948 | LIX1L | limb and CNS expressed 1 like [Source:HGNC Symbol;Acc:HGNC:28715] |
| ENSG00000274736 | HGLibA_08001 | 238 | 1.219744948 | CCL23 | C-C motif chemokine ligand 23 [Source:HGNC Symbol;Acc:HGNC:10622] |
| FAM122A | HGLibA_16065 | 238 | 1.219744948 | FAM122A | - |
| FAM212A | HGLibA_16466 | 238 | 1.219744948 | FAM212A | - |
| GAREML | HGLibA_18630 | 238 | 1.219744948 | GAREML | - |
| hsa-mir-4787 | HGLibA_61254 | 238 | 1.219744948 | hsa-mir-4787 | - |
| hsa-mir-502 | HGLibA_61464 | 238 | 1.219744948 | hsa-mir-502 | - |
| hsa-mir-634 | HGLibA_62775 | 238 | 1.219744948 | hsa-mir-634 | - |
| KDELC2 | HGLibA_24475 | 238 | 1.219744948 | KDELC2 | - |
| C14orf79 | HGLibA_05369 | 237 | 1.209793911 | C14orf79 | - |
| C3orf83 | HGLibA_06276 | 237 | 1.209793911 | C3orf83 | - |
| ENSG00000074266 | HGLibA_14486 | 237 | 1.209793911 | EED | embryonic ectoderm development [Source:HGNC Symbol;Acc:HGNC:3188] |
| ENSG00000087448 | HGLibA_25212 | 237 | 1.209793911 | KLHL42 | kelch like family member 42 [Source:HGNC Symbol;Acc:HGNC:29252] |
| ENSG00000088179 | HGLibA_39469 | 237 | 1.209793911 | PTPN4 | non-receptor type 4 [Source:HGNC Symbol;Acc:HGNC:9656]" |
| ENSG00000089057 | HGLibA_44608 | 237 | 1.209793911 | SLC23A2 | solute carrier family 23 member 2 [Source:HGNC Symbol;Acc:HGNC:10973] |
| ENSG00000096696 | HGLibA_14038 | 237 | 1.209793911 | DSP | desmoplakin [Source:HGNC Symbol;Acc:HGNC:3052] |
| ENSG00000101180 | HGLibA_22100 | 237 | 1.209793911 | HRH3 | histamine receptor H3 [Source:HGNC Symbol;Acc:HGNC:5184] |
| ENSG00000102290 | HGLibA_35391 | 237 | 1.209793911 | PCDH11X | protocadherin 11 X-linked [Source:HGNC Symbol;Acc:HGNC:8656] |
| ENSG00000103353 | HGLibA_52697 | 237 | 1.209793911 | UBFD1 | ubiquitin family domain containing 1 [Source:HGNC Symbol;Acc:HGNC:30565] |
| ENSG00000103502 | HGLibA_08717 | 237 | 1.209793911 | CDIPT | CDP-diacylglycerol--inositol 3-phosphatidyltransferase [Source:HGNC Symbol;Acc:HGNC:1769] |
| ENSG00000104635 | HGLibA_45102 | 237 | 1.209793911 | SLC39A14 | solute carrier family 39 member 14 [Source:HGNC Symbol;Acc:HGNC:20858] |
| ENSG00000112531 | HGLibA_39724 | 237 | 1.209793911 | QKI | KH domain containing RNA binding [Source:HGNC Symbol;Acc:HGNC:21100]" |
| ENSG00000134049 | HGLibA_22579 | 237 | 1.209793911 | IER3IP1 | immediate early response 3 interacting protein 1 [Source:HGNC Symbol;Acc:HGNC:18550] |
| ENSG00000135253 | HGLibA_24408 | 237 | 1.209793911 | KCP | kielin cysteine rich BMP regulator [Source:HGNC Symbol;Acc:HGNC:17585] |
| ENSG00000136068 | HGLibA_17642 | 237 | 1.209793911 | FLNB | filamin B [Source:HGNC Symbol;Acc:HGNC:3755] |
| ENSG00000136856 | HGLibA_44890 | 237 | 1.209793911 | SLC2A8 | solute carrier family 2 member 8 [Source:HGNC Symbol;Acc:HGNC:13812] |
| ENSG00000141522 | HGLibA_02796 | 237 | 1.209793911 | ARHGDIA | Rho GDP dissociation inhibitor alpha [Source:HGNC Symbol;Acc:HGNC:678] |
| ENSG00000147488 | HGLibA_47228 | 237 | 1.209793911 | ST18 | C2H2C-type zinc finger [Source:HGNC Symbol;Acc:HGNC:18695]" |
| ENSG00000151575 | HGLibA_49111 | 237 | 1.209793911 | TEX9 | testis expressed 9 [Source:HGNC Symbol;Acc:HGNC:29585] |
| ENSG00000154359 | HGLibA_27020 | 237 | 1.209793911 | LONRF1 | LON peptidase N-terminal domain and ring finger 1 [Source:HGNC Symbol;Acc:HGNC:26302] |
| ENSG00000162616 | HGLibA_13544 | 237 | 1.209793911 | DNAJB4 | DnaJ heat shock protein family (Hsp40) member B4 [Source:HGNC Symbol;Acc:HGNC:14886] |
| ENSG00000165140 | HGLibA_16963 | 237 | 1.209793911 | FBP1 | fructose-bisphosphatase 1 [Source:HGNC Symbol;Acc:HGNC:3606] |
| ENSG00000168356 | HGLibA_42972 | 237 | 1.209793911 | SCN11A | sodium voltage-gated channel alpha subunit 11 [Source:HGNC Symbol;Acc:HGNC:10583] |
| ENSG00000168661 | HGLibA_55768 | 237 | 1.209793911 | ZNF30 | zinc finger protein 30 [Source:HGNC Symbol;Acc:HGNC:13090] |
| ENSG00000169554 | HGLibA_55139 | 237 | 1.209793911 | ZEB2 | zinc finger E-box binding homeobox 2 [Source:HGNC Symbol;Acc:HGNC:14881] |
| ENSG00000170369 | HGLibA_11451 | 237 | 1.209793911 | CST2 | cystatin SA [Source:HGNC Symbol;Acc:HGNC:2474] |
| ENSG00000170584 | HGLibA_32926 | 237 | 1.209793911 | NUDCD2 | NudC domain containing 2 [Source:HGNC Symbol;Acc:HGNC:30535] |
| ENSG00000171777 | HGLibA_40353 | 237 | 1.209793911 | RASGRP4 | RAS guanyl releasing protein 4 [Source:HGNC Symbol;Acc:HGNC:18958] |
| ENSG00000171798 | HGLibA_25339 | 237 | 1.209793911 | KNDC1 | kinase non-catalytic C-lobe domain containing 1 [Source:HGNC Symbol;Acc:HGNC:29374] |
| ENSG00000173369 | HGLibA_05916 | 237 | 1.209793911 | C1QB | complement C1q B chain [Source:HGNC Symbol;Acc:HGNC:1242] |
| ENSG00000174292 | HGLibA_50845 | 237 | 1.209793911 | TNK1 | tyrosine kinase non receptor 1 [Source:HGNC Symbol;Acc:HGNC:11940] |
| ENSG00000178343 | HGLibA_44035 | 237 | 1.209793911 | SHISA3 | shisa family member 3 [Source:HGNC Symbol;Acc:HGNC:25159] |
| ENSG00000179008 | HGLibA_05367 | 237 | 1.209793911 | C14orf39 | chromosome 14 open reading frame 39 [Source:HGNC Symbol;Acc:HGNC:19849] |
| ENSG00000179873 | HGLibA_32104 | 237 | 1.209793911 | NLRP11 | NLR family pyrin domain containing 11 [Source:HGNC Symbol;Acc:HGNC:22945] |
| ENSG00000180530 | HGLibA_32690 | 237 | 1.209793911 | NRIP1 | nuclear receptor interacting protein 1 [Source:HGNC Symbol;Acc:HGNC:8001] |
| ENSG00000181856 | HGLibA_44877 | 237 | 1.209793911 | SLC2A4 | solute carrier family 2 member 4 [Source:HGNC Symbol;Acc:HGNC:11009] |
| ENSG00000182334 | HGLibA_34348 | 237 | 1.209793911 | OR5P3 | olfactory receptor family 5 subfamily P member 3 [Source:HGNC Symbol;Acc:HGNC:14784] |
| ENSG00000182853 | HGLibA_53693 | 237 | 1.209793911 | VMO1 | vitelline membrane outer layer 1 homolog [Source:HGNC Symbol;Acc:HGNC:30387] |
| ENSG00000184611 | HGLibA_24227 | 237 | 1.209793911 | KCNH7 | potassium voltage-gated channel subfamily H member 7 [Source:HGNC Symbol;Acc:HGNC:18863] |
| ENSG00000185624 | HGLibA_34909 | 237 | 1.209793911 | P4HB | prolyl 4-hydroxylase subunit beta [Source:HGNC Symbol;Acc:HGNC:8548] |
| ENSG00000186081 | HGLibA_25509 | 237 | 1.209793911 | KRT5 | keratin 5 [Source:HGNC Symbol;Acc:HGNC:6442] |
| ENSG00000186854 | HGLibA_51230 | 237 | 1.209793911 | TRABD2A | TraB domain containing 2A [Source:HGNC Symbol;Acc:HGNC:27013] |
| ENSG00000187630 | HGLibA_13141 | 237 | 1.209793911 | DHRS4L2 | dehydrogenase/reductase 4 like 2 [Source:HGNC Symbol;Acc:HGNC:19731] |
| ENSG00000187942 | HGLibA_26245 | 237 | 1.209793911 | LDLRAD2 | low density lipoprotein receptor class A domain containing 2 [Source:HGNC Symbol;Acc:HGNC:32071] |
| ENSG00000188739 | HGLibA_40536 | 237 | 1.209793911 | RBM34 | RNA binding motif protein 34 [Source:HGNC Symbol;Acc:HGNC:28965] |
| ENSG00000196368 | HGLibA_32938 | 237 | 1.209793911 | NUDT11 | nudix hydrolase 11 [Source:HGNC Symbol;Acc:HGNC:18011] |
| ENSG00000196581 | HGLibA_01369 | 237 | 1.209793911 | AJAP1 | adherens junctions associated protein 1 [Source:HGNC Symbol;Acc:HGNC:30801] |
| ENSG00000197479 | HGLibA_35476 | 237 | 1.209793911 | PCDHB11 | protocadherin beta 11 [Source:HGNC Symbol;Acc:HGNC:8682] |
| ENSG00000205078 | HGLibA_47917 | 237 | 1.209793911 | SYCE1L | synaptonemal complex central element protein 1 like [Source:HGNC Symbol;Acc:HGNC:37236] |
| ENSG00000205495 | HGLibA_34177 | 237 | 1.209793911 | OR52J3 | olfactory receptor family 52 subfamily J member 3 [Source:HGNC Symbol;Acc:HGNC:14799] |
| ENSG00000231887 | HGLibA_38460 | 237 | 1.209793911 | PRH1 | proline rich protein HaeIII subfamily 1 [Source:HGNC Symbol;Acc:HGNC:9366] |
| ENSG00000243335 | HGLibA_24463 | 237 | 1.209793911 | KCTD7 | potassium channel tetramerization domain containing 7 [Source:HGNC Symbol;Acc:HGNC:21957] |
| FAM196B | HGLibA_16384 | 237 | 1.209793911 | FAM196B | - |
| FAM63B | HGLibA_16663 | 237 | 1.209793911 | FAM63B | - |
| hsa-mir-134 | HGLibA_57786 | 237 | 1.209793911 | hsa-mir-134 | - |
| hsa-mir-2278 | HGLibA_58318 | 237 | 1.209793911 | hsa-mir-2278 | - |
| hsa-mir-4710 | HGLibA_60940 | 237 | 1.209793911 | hsa-mir-4710 | - |
| hsa-mir-4718 | HGLibA_60971 | 237 | 1.209793911 | hsa-mir-4718 | - |
| hsa-mir-6771 | HGLibA_63259 | 237 | 1.209793911 | hsa-mir-6771 | - |
| hsa-mir-7158 | HGLibA_63859 | 237 | 1.209793911 | hsa-mir-7158 | - |
| KIAA1467 | HGLibA_24767 | 237 | 1.209793911 | KIAA1467 | - |
| LEPREL2 | HGLibA_26321 | 237 | 1.209793911 | LEPREL2 | - |
| TMEM8C | HGLibA_50552 | 237 | 1.209793911 | TMEM8C | - |
| C14orf183 | HGLibA_05353 | 236 | 1.199842873 | C14orf183 | - |
| CHDC2 | HGLibA_09413 | 236 | 1.199842873 | CHDC2 | - |
| ENSG00000026652 | HGLibA_01227 | 236 | 1.199842873 | AGPAT4 | 1-acylglycerol-3-phosphate O-acyltransferase 4 [Source:HGNC Symbol;Acc:HGNC:20885] |
| ENSG00000039560 | HGLibA_40142 | 236 | 1.199842873 | RAI14 | retinoic acid induced 14 [Source:HGNC Symbol;Acc:HGNC:14873] |
| ENSG00000064666 | HGLibA_10309 | 236 | 1.199842873 | CNN2 | calponin 2 [Source:HGNC Symbol;Acc:HGNC:2156] |
| ENSG00000066855 | HGLibA_30362 | 236 | 1.199842873 | MTFR1 | mitochondrial fission regulator 1 [Source:HGNC Symbol;Acc:HGNC:29510] |
| ENSG00000079819 | HGLibA_15245 | 236 | 1.199842873 | EPB41L2 | erythrocyte membrane protein band 4.1 like 2 [Source:HGNC Symbol;Acc:HGNC:3379] |
| ENSG00000086475 | HGLibA_43411 | 236 | 1.199842873 | SEPHS1 | selenophosphate synthetase 1 [Source:HGNC Symbol;Acc:HGNC:19685] |
| ENSG00000100242 | HGLibA_47790 | 236 | 1.199842873 | SUN2 | Sad1 and UNC84 domain containing 2 [Source:HGNC Symbol;Acc:HGNC:14210] |
| ENSG00000102753 | HGLibA_25359 | 236 | 1.199842873 | KPNA3 | karyopherin subunit alpha 3 [Source:HGNC Symbol;Acc:HGNC:6396] |
| ENSG00000106333 | HGLibA_35653 | 236 | 1.199842873 | PCOLCE | procollagen C-endopeptidase enhancer [Source:HGNC Symbol;Acc:HGNC:8738] |
| ENSG00000114654 | HGLibA_14557 | 236 | 1.199842873 | EFCC1 | EF-hand and coiled-coil domain containing 1 [Source:HGNC Symbol;Acc:HGNC:25692] |
| ENSG00000118680 | HGLibA_30755 | 236 | 1.199842873 | MYL12B | myosin light chain 12B [Source:HGNC Symbol;Acc:HGNC:29827] |
| ENSG00000118762 | HGLibA_36798 | 236 | 1.199842873 | PKD2 | transient receptor potential cation channel [Source:HGNC Symbol;Acc:HGNC:9009]" |
| ENSG00000119703 | HGLibA_54944 | 236 | 1.199842873 | ZC2HC1C | zinc finger C2HC-type containing 1C [Source:HGNC Symbol;Acc:HGNC:20354] |
| ENSG00000122386 | HGLibA_55575 | 236 | 1.199842873 | ZNF205 | zinc finger protein 205 [Source:HGNC Symbol;Acc:HGNC:12996] |
| ENSG00000122778 | HGLibA_24779 | 236 | 1.199842873 | KIAA1549 | KIAA1549 [Source:HGNC Symbol;Acc:HGNC:22219] |
| ENSG00000124233 | HGLibA_43380 | 236 | 1.199842873 | SEMG1 | semenogelin 1 [Source:HGNC Symbol;Acc:HGNC:10742] |
| ENSG00000124772 | HGLibA_10965 | 236 | 1.199842873 | CPNE5 | copine 5 [Source:HGNC Symbol;Acc:HGNC:2318] |
| ENSG00000125249 | HGLibA_40235 | 236 | 1.199842873 | RAP2A | member of RAS oncogene family [Source:HGNC Symbol;Acc:HGNC:9861]" |

| | | | | | |
|---|---|---|---|---|---|
| ENSG00000125903 | HGLibA_12890 | 236 | 1.199842873 | DEFB129 | defensin beta 129 [Source:HGNC Symbol;Acc:HGNC:16218] |
| ENSG00000125975 | HGLibA_05997 | 236 | 1.199842873 | C20orf173 | chromosome 20 open reading frame 173 [Source:HGNC Symbol;Acc:HGNC:16166] |
| ENSG00000126785 | HGLibA_41196 | 236 | 1.199842873 | RHOJ | ras homolog family member J [Source:HGNC Symbol;Acc:HGNC:688] |
| ENSG00000127324 | HGLibA_51948 | 236 | 1.199842873 | TSPAN8 | tetraspanin 8 [Source:HGNC Symbol;Acc:HGNC:11855] |
| ENSG00000131142 | HGLibA_08005 | 236 | 1.199842873 | CCL25 | C-C motif chemokine ligand 25 [Source:HGNC Symbol;Acc:HGNC:10624] |
| ENSG00000137802 | HGLibA_28309 | 236 | 1.199842873 | MAPKBP1 | mitogen-activated protein kinase binding protein 1 [Source:HGNC Symbol;Acc:HGNC:29536] |
| ENSG00000139211 | HGLibA_01795 | 236 | 1.199842873 | AMIGO2 | adhesion molecule with Ig like domain 2 [Source:HGNC Symbol;Acc:HGNC:24073] |
| ENSG00000142973 | HGLibA_12178 | 236 | 1.199842873 | CYP4B1 | cytochrome P450 family 4 subfamily B member 1 [Source:HGNC Symbol;Acc:HGNC:2644] |
| ENSG00000144118 | HGLibA_40149 | 236 | 1.199842873 | RALB | RAS like proto-oncogene B [Source:HGNC Symbol;Acc:HGNC:9840] |
| ENSG00000157191 | HGLibA_31595 | 236 | 1.199842873 | NECAP2 | NECAP endocytosis associated 2 [Source:HGNC Symbol;Acc:HGNC:25528] |
| ENSG00000157578 | HGLibA_26094 | 236 | 1.199842873 | LCA5L | lebercilin like [Source:HGNC Symbol;Acc:HGNC:1255]" |
| ENSG00000159495 | HGLibA_49272 | 236 | 1.199842873 | TGM7 | transglutaminase 7 [Source:HGNC Symbol;Acc:HGNC:30790] |
| ENSG00000160446 | HGLibA_55072 | 236 | 1.199842873 | ZDHHC12 | zinc finger DHHC-type containing 12 [Source:HGNC Symbol;Acc:HGNC:19159] |
| ENSG00000160888 | HGLibA_22573 | 236 | 1.199842873 | IER2 | immediate early response 2 [Source:HGNC Symbol;Acc:HGNC:28871] |
| ENSG00000162599 | HGLibA_31801 | 236 | 1.199842873 | NFIA | nuclear factor I A [Source:HGNC Symbol;Acc:HGNC:7784] |
| ENSG00000163154 | HGLibA_50724 | 236 | 1.199842873 | TNFAIP8L2 | TNF alpha induced protein 8 like 2 [Source:HGNC Symbol;Acc:HGNC:26277] |
| ENSG00000164418 | HGLibA_20284 | 236 | 1.199842873 | GRIK2 | glutamate ionotropic receptor kainate type subunit 2 [Source:HGNC Symbol;Acc:HGNC:4580] |
| ENSG00000166016 | HGLibA_00338 | 236 | 1.199842873 | ABTB2 | ankyrin repeat and BTB domain containing 2 [Source:HGNC Symbol;Acc:HGNC:23842] |
| ENSG00000169583 | HGLibA_10026 | 236 | 1.199842873 | CLIC3 | chloride intracellular channel 3 [Source:HGNC Symbol;Acc:HGNC:2064] |
| ENSG00000169826 | HGLibA_11349 | 236 | 1.199842873 | CSGALNACT2 | chondroitin sulfate N-acetylgalactosaminyltransferase 2 [Source:HGNC Symbol;Acc:HGNC:24292] |
| ENSG00000170619 | HGLibA_10668 | 236 | 1.199842873 | COMMD5 | COMM domain containing 5 [Source:HGNC Symbol;Acc:HGNC:17902] |
| ENSG00000171136 | HGLibA_41358 | 236 | 1.199842873 | RLN3 | relaxin 3 [Source:HGNC Symbol;Acc:HGNC:17135] |
| ENSG00000171368 | HGLibA_51164 | 236 | 1.199842873 | TPPP | tubulin polymerization promoting protein [Source:HGNC Symbol;Acc:HGNC:24164] |
| ENSG00000171649 | HGLibA_55335 | 236 | 1.199842873 | ZIK1 | zinc finger protein interacting with K protein 1 [Source:HGNC Symbol;Acc:HGNC:33104] |
| ENSG00000178537 | HGLibA_44670 | 236 | 1.199842873 | SLC25A20 | solute carrier family 25 member 20 [Source:HGNC Symbol;Acc:HGNC:1421] |
| ENSG00000178802 | HGLibA_29732 | 236 | 1.199842873 | MPI | mannose phosphate isomerase [Source:HGNC Symbol;Acc:HGNC:7216] |
| ENSG00000179119 | HGLibA_46908 | 236 | 1.199842873 | SPTY2D1 | SPT2 chromatin protein domain containing 1 [Source:HGNC Symbol;Acc:HGNC:26818] |
| ENSG00000182768 | HGLibA_31884 | 236 | 1.199842873 | NGRN | neurite outgrowth associated [Source:HGNC Symbol;Acc:HGNC:18077]" |
| ENSG00000184178 | HGLibA_42897 | 236 | 1.199842873 | SCFD2 | sec1 family domain containing 2 [Source:HGNC Symbol;Acc:HGNC:30676] |
| ENSG00000185070 | HGLibA_17656 | 236 | 1.199842873 | FLRT2 | fibronectin leucine rich transmembrane protein 2 [Source:HGNC Symbol;Acc:HGNC:3761] |
| ENSG00000185127 | HGLibA_06441 | 236 | 1.199842873 | C6orf120 | chromosome 6 open reading frame 120 [Source:HGNC Symbol;Acc:HGNC:21247] |
| ENSG00000187243 | HGLibA_27977 | 236 | 1.199842873 | MAGED4B | MAGE family member D4B [Source:HGNC Symbol;Acc:HGNC:22880] |
| ENSG00000188340 | HGLibA_34432 | 236 | 1.199842873 | OR6N2 | olfactory receptor family 6 subfamily N member 2 [Source:HGNC Symbol;Acc:HGNC:15035] |
| ENSG00000196418 | HGLibA_55456 | 236 | 1.199842873 | ZNF124 | zinc finger protein 124 [Source:HGNC Symbol;Acc:HGNC:12907] |
| ENSG00000197122 | HGLibA_46929 | 236 | 1.199842873 | SRC | non-receptor tyrosine kinase [Source:HGNC Symbol;Acc:HGNC:11283]" |
| ENSG00000197555 | HGLibA_44191 | 236 | 1.199842873 | SIPA1L1 | signal induced proliferation associated 1 like 1 [Source:HGNC Symbol;Acc:HGNC:20284] |
| ENSG00000198157 | HGLibA_21728 | 236 | 1.199842873 | HMGN5 | high mobility group nucleosome binding domain 5 [Source:HGNC Symbol;Acc:HGNC:8013] |
| ENSG00000198729 | HGLibA_38041 | 236 | 1.199842873 | PPP1R14C | protein phosphatase 1 regulatory inhibitor subunit 14C [Source:HGNC Symbol;Acc:HGNC:14952] |
| ENSG00000203909 | HGLibA_13896 | 236 | 1.199842873 | DPPA5 | developmental pluripotency associated 5 [Source:HGNC Symbol;Acc:HGNC:19201] |
| ENSG00000205030 | HGLibA_34326 | 236 | 1.199842873 | OR5L2 | olfactory receptor family 5 subfamily L member 2 [Source:HGNC Symbol;Acc:HGNC:8351] |
| ENSG00000205856 | HGLibA_06077 | 236 | 1.199842873 | C22orf42 | chromosome 22 open reading frame 42 [Source:HGNC Symbol;Acc:HGNC:27160] |
| ENSG00000229544 | HGLibA_32027 | 236 | 1.199842873 | NKX1-2 | NK1 homeobox 2 [Source:HGNC Symbol;Acc:HGNC:31652] |
| ENSG00000231887 | HGLibA_38462 | 236 | 1.199842873 | PRH1 | proline rich protein HaeIII subfamily 1 [Source:HGNC Symbol;Acc:HGNC:9366] |
| ENSG00000233412 | HGLibA_34296 | 236 | 1.199842873 | OR5H15 | olfactory receptor family 5 subfamily H member 15 [Source:HGNC Symbol;Acc:HGNC:31287] |
| GRASP | HGLibA_20221 | 236 | 1.199842873 | GRASP | - |
| hsa-mir-370 | HGLibA_59363 | 236 | 1.199842873 | hsa-mir-370 | - |
| hsa-mir-3936 | HGLibA_59606 | 236 | 1.199842873 | hsa-mir-3936 | - |
| hsa-mir-4253 | HGLibA_59721 | 236 | 1.199842873 | hsa-mir-4253 | - |
| hsa-mir-6747 | HGLibA_63142 | 236 | 1.199842873 | hsa-mir-6747 | - |
| LOC728819 | HGLibA_26992 | 236 | 1.199842873 | LOC728819 | - |
| XAGE1C | HGLibA_54471 | 236 | 1.199842873 | XAGE1C | - |
| ENSG00000070610 | HGLibA_18713 | 235 | 1.189891836 | GBA2 | glucosylceramidase beta 2 [Source:HGNC Symbol;Acc:HGNC:18986] |
| ENSG00000083828 | HGLibA_56326 | 235 | 1.189891836 | ZNF586 | zinc finger protein 586 [Source:HGNC Symbol;Acc:HGNC:25949] |
| ENSG00000102225 | HGLibA_08743 | 235 | 1.189891836 | CDK16 | cyclin dependent kinase 16 [Source:HGNC Symbol;Acc:HGNC:8749] |
| ENSG00000106351 | HGLibA_01186 | 235 | 1.189891836 | AGFG2 | ArfGAP with FG repeats 2 [Source:HGNC Symbol;Acc:HGNC:5177] |
| ENSG00000108691 | HGLibA_07989 | 235 | 1.189891836 | CCL2 | C-C motif chemokine ligand 2 [Source:HGNC Symbol;Acc:HGNC:10618] |
| ENSG00000114125 | HGLibA_41696 | 235 | 1.189891836 | RNF7 | ring finger protein 7 [Source:HGNC Symbol;Acc:HGNC:10070] |
| ENSG00000116721 | HGLibA_38278 | 235 | 1.189891836 | PRAMEF1 | PRAME family member 1 [Source:HGNC Symbol;Acc:HGNC:28840] |
| ENSG00000119640 | HGLibA_00711 | 235 | 1.189891836 | ACYP1 | acylphosphatase 1 [Source:HGNC Symbol;Acc:HGNC:179] |
| ENSG00000119973 | HGLibA_38601 | 235 | 1.189891836 | PRLHR | prolactin releasing hormone receptor [Source:HGNC Symbol;Acc:HGNC:4464] |
| ENSG00000124243 | HGLibA_04261 | 235 | 1.189891836 | BCAS4 | breast carcinoma amplified sequence 4 [Source:HGNC Symbol;Acc:HGNC:14367] |
| ENSG00000126259 | HGLibA_25003 | 235 | 1.189891836 | KIRREL2 | kirre like nephrin family adhesion molecule 2 [Source:HGNC Symbol;Acc:HGNC:18816] |
| ENSG00000133740 | HGLibA_14320 | 235 | 1.189891836 | E2F5 | E2F transcription factor 5 [Source:HGNC Symbol;Acc:HGNC:3119] |
| ENSG00000134369 | HGLibA_31193 | 235 | 1.189891836 | NAV1 | neuron navigator 1 [Source:HGNC Symbol;Acc:HGNC:15989] |
| ENSG00000145730 | HGLibA_35082 | 235 | 1.189891836 | PAM | peptidylglycine alpha-amidating monooxygenase [Source:HGNC Symbol;Acc:HGNC:8596] |
| ENSG00000147437 | HGLibA_19579 | 235 | 1.189891836 | GNRH1 | gonadotropin releasing hormone 1 [Source:HGNC Symbol;Acc:HGNC:4419] |
| ENSG00000154096 | HGLibA_49425 | 235 | 1.189891836 | THY1 | Thy-1 cell surface antigen [Source:HGNC Symbol;Acc:HGNC:11801] |
| ENSG00000154263 | HGLibA_00098 | 235 | 1.189891836 | ABCA10 | ATP binding cassette subfamily A member 10 [Source:HGNC Symbol;Acc:HGNC:30] |
| ENSG00000155876 | HGLibA_42224 | 235 | 1.189891836 | RRAGA | Ras related GTP binding A [Source:HGNC Symbol;Acc:HGNC:16963] |
| ENSG00000160224 | HGLibA_01367 | 235 | 1.189891836 | AIRE | autoimmune regulator [Source:HGNC Symbol;Acc:HGNC:360] |
| ENSG00000161217 | HGLibA_35700 | 235 | 1.189891836 | PCYT1A | alpha [Source:HGNC Symbol;Acc:HGNC:8754]" |
| ENSG00000161558 | HGLibA_49966 | 235 | 1.189891836 | TMEM143 | transmembrane protein 143 [Source:HGNC Symbol;Acc:HGNC:25603] |
| ENSG00000161860 | HGLibA_47919 | 235 | 1.189891836 | SYCE2 | synaptonemal complex central element protein 2 [Source:HGNC Symbol;Acc:HGNC:27411] |
| ENSG00000162191 | HGLibA_52775 | 235 | 1.189891836 | UBXN1 | UBX domain protein 1 [Source:HGNC Symbol;Acc:HGNC:18402] |
| ENSG00000164749 | HGLibA_21782 | 235 | 1.189891836 | HNF4G | hepatocyte nuclear factor 4 gamma [Source:HGNC Symbol;Acc:HGNC:5026] |
| ENSG00000165509 | HGLibA_27965 | 235 | 1.189891836 | MAGEC3 | MAGE family member C3 [Source:HGNC Symbol;Acc:HGNC:23798] |
| ENSG00000166272 | HGLibA_53975 | 235 | 1.189891836 | WBP1L | WW domain binding protein 1 like [Source:HGNC Symbol;Acc:HGNC:23510] |
| ENSG00000168594 | HGLibA_00766 | 235 | 1.189891836 | ADAM29 | ADAM metallopeptidase domain 29 [Source:HGNC Symbol;Acc:HGNC:207] |
| ENSG00000168803 | HGLibA_00724 | 235 | 1.189891836 | ADAL | adenosine deaminase like [Source:HGNC Symbol;Acc:HGNC:31853] |
| ENSG00000169718 | HGLibA_14113 | 235 | 1.189891836 | DUS1L | dihydrouridine synthase 1 like [Source:HGNC Symbol;Acc:HGNC:30086] |
| ENSG00000173175 | HGLibA_00921 | 235 | 1.189891836 | ADCY5 | adenylate cyclase 5 [Source:HGNC Symbol;Acc:HGNC:236] |
| ENSG00000174808 | HGLibA_04928 | 235 | 1.189891836 | BTC | betacellulin [Source:HGNC Symbol;Acc:HGNC:1121] |
| ENSG00000178796 | HGLibA_41247 | 235 | 1.189891836 | RIIAD1 | regulatory subunit of type II PKA R-subunit (RIIa) domain containing 1 [Source:HGNC Symbol;Acc:HGNC:26686] |
| ENSG00000181518 | HGLibA_34515 | 235 | 1.189891836 | OR8D4 | olfactory receptor family 8 subfamily D member 4 [Source:HGNC Symbol;Acc:HGNC:14840] |
| ENSG00000183117 | HGLibA_11363 | 235 | 1.189891836 | CSMD1 | CUB and Sushi multiple domains 1 [Source:HGNC Symbol;Acc:HGNC:14026] |
| ENSG00000185069 | HGLibA_25539 | 235 | 1.189891836 | KRT76 | keratin 76 [Source:HGNC Symbol;Acc:HGNC:24430] |
| ENSG00000188677 | HGLibA_35258 | 235 | 1.189891836 | PARVB | parvin beta [Source:HGNC Symbol;Acc:HGNC:14653] |
| ENSG00000196576 | HGLibA_37272 | 235 | 1.189891836 | PLXNB2 | plexin B2 [Source:HGNC Symbol;Acc:HGNC:9104] |
| ENSG00000198663 | HGLibA_06512 | 235 | 1.189891836 | C6orf89 | chromosome 6 open reading frame 89 [Source:HGNC Symbol;Acc:HGNC:21114] |
| ENSG00000203985 | HGLibA_26241 | 235 | 1.189891836 | LDLRAD1 | low density lipoprotein receptor class A domain containing 1 [Source:HGNC Symbol;Acc:HGNC:32069] |
| ENSG00000204779 | HGLibA_17852 | 235 | 1.189891836 | FOXD4L5 | forkhead box D4 like 5 [Source:HGNC Symbol;Acc:HGNC:18522] |
| ENSG00000205857 | HGLibA_31094 | 235 | 1.189891836 | NANOGNB | NANOG neighbor homeobox [Source:HGNC Symbol;Acc:HGNC:24958] |
| ENSG00000213145 | HGLibA_11151 | 235 | 1.189891836 | CRIP1 | cysteine rich protein 1 [Source:HGNC Symbol;Acc:HGNC:2360] |
| ENSG00000260238 | HGLibA_37307 | 235 | 1.189891836 | PMF1-BGLAP | PMF1-BGLAP readthrough [Source:HGNC Symbol;Acc:HGNC:42953] |
| ENSG00000266265 | HGLibA_25054 | 235 | 1.189891836 | KLF14 | Kruppel like factor 14 [Source:HGNC Symbol;Acc:HGNC:23025] |
| ENSG00000273111 | HGLibA_27721 | 235 | 1.189891836 | LYPD4 | LY6/PLAUR domain containing 4 [Source:HGNC Symbol;Acc:HGNC:28659] |
| H2AFJ | HGLibA_20755 | 235 | 1.189891836 | H2AFJ | - |
| HIST1H4I | HGLibA_21510 | 235 | 1.189891836 | HIST1H4I | - |
| hsa-mir-3690-1 | HGLibA_59347 | 235 | 1.189891836 | hsa-mir-3690-1 | - |
| hsa-mir-573 | HGLibA_62381 | 235 | 1.189891836 | hsa-mir-573 | - |
| hsa-mir-665 | HGLibA_63003 | 235 | 1.189891836 | hsa-mir-665 | - |
| hsa-mir-671 | HGLibA_63012 | 235 | 1.189891836 | hsa-mir-671 | - |
| PLCXD1_X | HGLibA_37020 | 235 | 1.189891836 | PLCXD1_X | - |
| SRCRB4D | HGLibA_46937 | 235 | 1.189891836 | SRCRB4D | - |
| C1orf172 | HGLibA_05791 | 234 | 1.179940799 | C1orf172 | - |
| ENSG00000076864 | HGLibA_40227 | 234 | 1.179940799 | RAP1GAP | RAP1 GTPase activating protein [Source:HGNC Symbol;Acc:HGNC:9858] |
| ENSG00000080618 | HGLibA_10908 | 234 | 1.179940799 | CPB2 | carboxypeptidase B2 [Source:HGNC Symbol;Acc:HGNC:2300] |
| ENSG00000080845 | HGLibA_13349 | 234 | 1.179940799 | DLGAP4 | DLG associated protein 4 [Source:HGNC Symbol;Acc:HGNC:24476] |
| ENSG00000093144 | HGLibA_14395 | 234 | 1.179940799 | ECHDC1 | ethylmalonyl-CoA decarboxylase 1 [Source:HGNC Symbol;Acc:HGNC:21489] |
| ENSG00000100568 | HGLibA_53890 | 234 | 1.179940799 | VTI1B | vesicle transport through interaction with t-SNAREs 1B [Source:HGNC Symbol;Acc:HGNC:17793] |
| ENSG00000102218 | HGLibA_41792 | 234 | 1.179940799 | RP2 | ARL3 GTPase activating protein [Source:HGNC Symbol;Acc:HGNC:10274]" |
| ENSG00000105492 | HGLibA_44143 | 234 | 1.179940799 | SIGLEC6 | sialic acid binding Ig like lectin 6 [Source:HGNC Symbol;Acc:HGNC:10875] |
| ENSG00000109991 | HGLibA_34851 | 234 | 1.179940799 | P2RX3 | purinergic receptor P2X 3 [Source:HGNC Symbol;Acc:HGNC:8534] |
| ENSG00000131409 | HGLibA_27380 | 234 | 1.179940799 | LRRC4B | leucine rich repeat containing 4B [Source:HGNC Symbol;Acc:HGNC:25042] |
| ENSG00000132259 | HGLibA_10272 | 234 | 1.179940799 | CNGA4 | cyclic nucleotide gated channel alpha 4 [Source:HGNC Symbol;Acc:HGNC:2152] |
| ENSG00000135312 | HGLibA_22358 | 234 | 1.179940799 | HTR1B | 5-hydroxytryptamine receptor 1B [Source:HGNC Symbol;Acc:HGNC:5287] |
| ENSG00000135773 | HGLibA_07181 | 234 | 1.179940799 | CAPN9 | calpain 9 [Source:HGNC Symbol;Acc:HGNC:1486] |
| ENSG00000136928 | HGLibA_18344 | 234 | 1.179940799 | GABBR2 | gamma-aminobutyric acid type B receptor subunit 2 [Source:HGNC Symbol;Acc:HGNC:4507] |
| ENSG00000138081 | HGLibA_17027 | 234 | 1.179940799 | FBXO11 | F-box protein 11 [Source:HGNC Symbol;Acc:HGNC:13590] |
| ENSG00000139974 | HGLibA_45075 | 234 | 1.179940799 | SLC38A6 | solute carrier family 38 member 6 [Source:HGNC Symbol;Acc:HGNC:19863] |
| ENSG00000140939 | HGLibA_32264 | 234 | 1.179940799 | NOL3 | nucleolar protein 3 [Source:HGNC Symbol;Acc:HGNC:7869] |
| ENSG00000144119 | HGLibA_05927 | 234 | 1.179940799 | C1QL2 | complement C1q like 2 [Source:HGNC Symbol;Acc:HGNC:24181] |
| ENSG00000148180 | HGLibA_20436 | 234 | 1.179940799 | GSN | gelsolin [Source:HGNC Symbol;Acc:HGNC:4620] |
| ENSG00000155970 | HGLibA_29227 | 234 | 1.179940799 | MICU3 | mitochondrial calcium uptake family member 3 [Source:HGNC Symbol;Acc:HGNC:27820] |
| ENSG00000156219 | HGLibA_03162 | 234 | 1.179940799 | ART3 | ADP-ribosyltransferase 3 [Source:HGNC Symbol;Acc:HGNC:725] |
| ENSG00000156413 | HGLibA_18199 | 234 | 1.179940799 | FUT6 | fucosyltransferase 6 [Source:HGNC Symbol;Acc:HGNC:4017] |
| ENSG00000159763 | HGLibA_36701 | 234 | 1.179940799 | PIP | prolactin induced protein [Source:HGNC Symbol;Acc:HGNC:8993] |
| ENSG00000162738 | HGLibA_53516 | 234 | 1.179940799 | VANGL2 | VANGL planar cell polarity protein 2 [Source:HGNC Symbol;Acc:HGNC:15511] |
| ENSG00000163002 | HGLibA_33047 | 234 | 1.179940799 | NUP35 | nucleoporin 35 [Source:HGNC Symbol;Acc:HGNC:29797] |
| ENSG00000163482 | HGLibA_47499 | 234 | 1.179940799 | STK36 | serine/threonine kinase 36 [Source:HGNC Symbol;Acc:HGNC:17209] |
| ENSG00000163751 | HGLibA_10890 | 234 | 1.179940799 | CPA3 | carboxypeptidase A3 [Source:HGNC Symbol;Acc:HGNC:2298] |
| ENSG00000163818 | HGLibA_27801 | 234 | 1.179940799 | LZTFL1 | leucine zipper transcription factor like 1 [Source:HGNC Symbol;Acc:HGNC:6741] |
| ENSG00000163866 | HGLibA_45726 | 234 | 1.179940799 | SMIM12 | small integral membrane protein 12 [Source:HGNC Symbol;Acc:HGNC:25154] |
| ENSG00000164096 | HGLibA_06317 | 234 | 1.179940799 | C4orf3 | chromosome 4 open reading frame 3 [Source:HGNC Symbol;Acc:HGNC:19225] |
| ENSG00000164989 | HGLibA_07694 | 234 | 1.179940799 | CCDC171 | coiled-coil domain containing 171 [Source:HGNC Symbol;Acc:HGNC:29828] |

| | | | | | |
|---|---|---|---|---|---|
| ENSG00000165264 | HGLibA_31524 | 234 | 1.179940799 | NDUFB6 | NADH:ubiquinone oxidoreductase subunit B6 [Source:HGNC Symbol;Acc:HGNC:7701] |
| ENSG00000165417 | HGLibA_20521 | 234 | 1.179940799 | GTF2A1 | general transcription factor IIA subunit 1 [Source:HGNC Symbol;Acc:HGNC:4646] |
| ENSG00000166272 | HGLibA_53974 | 234 | 1.179940799 | WBP1L | WW domain binding protein 1 like [Source:HGNC Symbol;Acc:HGNC:23510] |
| ENSG00000166923 | HGLibA_20242 | 234 | 1.179940799 | GREM1 | DAN family BMP antagonist [Source:HGNC Symbol;Acc:HGNC:2001]" |
| ENSG00000172772 | HGLibA_33555 | 234 | 1.179940799 | OR10W1 | olfactory receptor family 10 subfamily W member 1 [Source:HGNC Symbol;Acc:HGNC:15139] |
| ENSG00000176165 | HGLibA_17869 | 234 | 1.179940799 | FOXG1 | forkhead box G1 [Source:HGNC Symbol;Acc:HGNC:3811] |
| ENSG00000176239 | HGLibA_34090 | 234 | 1.179940799 | OR51B6 | olfactory receptor family 51 subfamily B member 6 [Source:HGNC Symbol;Acc:HGNC:19600] |
| ENSG00000177910 | HGLibA_46493 | 234 | 1.179940799 | SPATA31C2 | SPATA31 subfamily C member 2 [Source:HGNC Symbol;Acc:HGNC:24508] |
| ENSG00000178522 | HGLibA_01748 | 234 | 1.179940799 | AMBN | ameloblastin [Source:HGNC Symbol;Acc:HGNC:452] |
| ENSG00000182175 | HGLibA_41013 | 234 | 1.179940799 | RGMA | repulsive guidance molecule BMP co-receptor a [Source:HGNC Symbol;Acc:HGNC:30308] |
| ENSG00000182307 | HGLibA_06607 | 234 | 1.179940799 | C8orf33 | chromosome 8 open reading frame 33 [Source:HGNC Symbol;Acc:HGNC:26104] |
| ENSG00000183020 | HGLibA_02308 | 234 | 1.179940799 | AP2A2 | adaptor related protein complex 2 subunit alpha 2 [Source:HGNC Symbol;Acc:HGNC:562] |
| ENSG00000185821 | HGLibA_34411 | 234 | 1.179940799 | OR6C76 | olfactory receptor family 6 subfamily C member 76 [Source:HGNC Symbol;Acc:HGNC:31305] |
| ENSG00000186222 | HGLibA_04567 | 234 | 1.179940799 | BLOC1S4 | biogenesis of lysosomal organelles complex 1 subunit 4 [Source:HGNC Symbol;Acc:HGNC:24206] |
| ENSG00000186326 | HGLibA_41115 | 234 | 1.179940799 | RGS9BP | regulator of G protein signaling 9 binding protein [Source:HGNC Symbol;Acc:HGNC:30304] |
| ENSG00000188177 | HGLibA_54980 | 234 | 1.179940799 | ZC3H6 | zinc finger CCCH-type containing 6 [Source:HGNC Symbol;Acc:HGNC:24762] |
| ENSG00000197857 | HGLibA_55998 | 234 | 1.179940799 | ZNF44 | zinc finger protein 44 [Source:HGNC Symbol;Acc:HGNC:13110] |
| ENSG00000198157 | HGLibA_21729 | 234 | 1.179940799 | HMGN5 | high mobility group nucleosome binding domain 5 [Source:HGNC Symbol;Acc:HGNC:8013] |
| ENSG00000198692 | HGLibA_14714 | 234 | 1.179940799 | EIF1AY | eukaryotic translation initiation factor 1A Y-linked [Source:HGNC Symbol;Acc:HGNC:3252] |
| ENSG00000198889 | HGLibA_12420 | 234 | 1.179940799 | DCAF12L1 | DDB1 and CUL4 associated factor 12 like 1 [Source:HGNC Symbol;Acc:HGNC:29395] |
| ENSG00000215853 | HGLibA_42200 | 234 | 1.179940799 | RPTN | repetin [Source:HGNC Symbol;Acc:HGNC:26809] |
| ENSG00000221859 | HGLibA_25594 | 234 | 1.179940799 | KRTAP10-10 | keratin associated protein 10-10 [Source:HGNC Symbol;Acc:HGNC:22972] |
| ENSG00000237388 | HGLibA_33930 | 234 | 1.179940799 | OR4A47 | olfactory receptor family 4 subfamily A member 47 [Source:HGNC Symbol;Acc:HGNC:31266] |
| ENSG00000253910 | HGLibA_35554 | 234 | 1.179940799 | PCDHGB2 | 2 [Source:HGNC Symbol;Acc:HGNC:8709]" |
| ENSG00000273706 | HGLibA_26446 | 234 | 1.179940799 | LHX1 | LIM homeobox 1 [Source:HGNC Symbol;Acc:HGNC:6593] |
| hsa-mir-1257 | HGLibA_57436 | 234 | 1.179940799 | hsa-mir-1257 | - |
| hsa-mir-335 | HGLibA_58997 | 234 | 1.179940799 | hsa-mir-335 | - |
| hsa-mir-3689c | HGLibA_59321 | 234 | 1.179940799 | hsa-mir-3689c | - |
| hsa-mir-3934 | HGLibA_59595 | 234 | 1.179940799 | hsa-mir-3934 | - |
| hsa-mir-4462 | HGLibA_60254 | 234 | 1.179940799 | hsa-mir-4462 | - |
| hsa-mir-548al | HGLibA_61899 | 234 | 1.179940799 | hsa-mir-548al | - |
| hsa-mir-6741 | HGLibA_63119 | 234 | 1.179940799 | hsa-mir-6741 | - |
| hsa-mir-711 | HGLibA_63799 | 234 | 1.179940799 | hsa-mir-711 | - |
| SGK223 | HGLibA_43828 | 234 | 1.179940799 | SGK223 | - |
| WDR78 | HGLibA_54185 | 234 | 1.179940799 | WDR78 | - |
| XAGE1E | HGLibA_54478 | 234 | 1.179940799 | XAGE1E | - |
| EMR2 | HGLibA_15085 | 233 | 1.169989761 | EMR2 | - |
| ENSG00000015153 | HGLibA_54628 | 233 | 1.169989761 | YAF2 | YY1 associated factor 2 [Source:HGNC Symbol;Acc:HGNC:17363] |
| ENSG00000067836 | HGLibA_41750 | 233 | 1.169989761 | ROGDI | rogdi homolog [Source:HGNC Symbol;Acc:HGNC:29478] |
| ENSG00000073067 | HGLibA_12147 | 233 | 1.169989761 | CYP2W1 | cytochrome P450 family 2 subfamily W member 1 [Source:HGNC Symbol;Acc:HGNC:20243] |
| ENSG00000081842 | HGLibA_35450 | 233 | 1.169989761 | PCDHA6 | protocadherin alpha 6 [Source:HGNC Symbol;Acc:HGNC:8672] |
| ENSG00000090013 | HGLibA_04578 | 233 | 1.169989761 | BLVRB | biliverdin reductase B [Source:HGNC Symbol;Acc:HGNC:1063] |
| ENSG00000101224 | HGLibA_08530 | 233 | 1.169989761 | CDC25B | cell division cycle 25B [Source:HGNC Symbol;Acc:HGNC:1726] |
| ENSG00000107341 | HGLibA_52654 | 233 | 1.169989761 | UBE2R2 | ubiquitin conjugating enzyme E2 R2 [Source:HGNC Symbol;Acc:HGNC:19907] |
| ENSG00000109084 | HGLibA_50566 | 233 | 1.169989761 | TMEM97 | transmembrane protein 97 [Source:HGNC Symbol;Acc:HGNC:28106] |
| ENSG00000110900 | HGLibA_51894 | 233 | 1.169989761 | TSPAN11 | tetraspanin 11 [Source:HGNC Symbol;Acc:HGNC:30795] |
| ENSG00000111319 | HGLibA_43014 | 233 | 1.169989761 | SCNN1A | sodium channel epithelial 1 alpha subunit [Source:HGNC Symbol;Acc:HGNC:10599] |
| ENSG00000111481 | HGLibA_10738 | 233 | 1.169989761 | COPZ1 | coatomer protein complex subunit zeta 1 [Source:HGNC Symbol;Acc:HGNC:2243] |
| ENSG00000113456 | HGLibA_40054 | 233 | 1.169989761 | RAD1 | RAD1 checkpoint DNA exonuclease [Source:HGNC Symbol;Acc:HGNC:9806] |
| ENSG00000113460 | HGLibA_04825 | 233 | 1.169989761 | BRIX1 | biogenesis of ribosomes [Source:HGNC Symbol;Acc:HGNC:24170]" |
| ENSG00000113580 | HGLibA_32620 | 233 | 1.169989761 | NR3C1 | nuclear receptor subfamily 3 group C member 1 [Source:HGNC Symbol;Acc:HGNC:7978] |
| ENSG00000113719 | HGLibA_15467 | 233 | 1.169989761 | ERGIC1 | endoplasmic reticulum-golgi intermediate compartment 1 [Source:HGNC Symbol;Acc:HGNC:29205] |
| ENSG00000114487 | HGLibA_29649 | 233 | 1.169989761 | MORC1 | MORC family CW-type zinc finger 1 [Source:HGNC Symbol;Acc:HGNC:7198] |
| ENSG00000121900 | HGLibA_50441 | 233 | 1.169989761 | TMEM54 | transmembrane protein 54 [Source:HGNC Symbol;Acc:HGNC:24143] |
| ENSG00000122188 | HGLibA_26070 | 233 | 1.169989761 | LAX1 | lymphocyte transmembrane adaptor 1 [Source:HGNC Symbol;Acc:HGNC:26005] |
| ENSG00000123685 | HGLibA_04180 | 233 | 1.169989761 | BATF3 | basic leucine zipper ATF-like transcription factor 3 [Source:HGNC Symbol;Acc:HGNC:28915] |
| ENSG00000124942 | HGLibA_01301 | 233 | 1.169989761 | AHNAK | AHNAK nucleoprotein [Source:HGNC Symbol;Acc:HGNC:347] |
| ENSG00000125834 | HGLibA_47496 | 233 | 1.169989761 | STK35 | serine/threonine kinase 35 [Source:HGNC Symbol;Acc:HGNC:16254] |
| ENSG00000128274 | HGLibA_00016 | 233 | 1.169989761 | A4GALT | 4-galactosyltransferase (P blood group) [Source:HGNC Symbol;Acc:HGNC:18149]" |
| ENSG00000132394 | HGLibA_14514 | 233 | 1.169989761 | EEFSEC | selenocysteine-tRNA specific [Source:HGNC Symbol;Acc:HGNC:24614]" |
| ENSG00000132669 | HGLibA_41292 | 233 | 1.169989761 | RIN2 | Ras and Rab interactor 2 [Source:HGNC Symbol;Acc:HGNC:18750] |
| ENSG00000136867 | HGLibA_44928 | 233 | 1.169989761 | SLC31A2 | solute carrier family 31 member 2 [Source:HGNC Symbol;Acc:HGNC:11017] |
| ENSG00000137449 | HGLibA_10921 | 233 | 1.169989761 | CPEB2 | cytoplasmic polyadenylation element binding protein 2 [Source:HGNC Symbol;Acc:HGNC:21745] |
| ENSG00000145781 | HGLibA_10653 | 233 | 1.169989761 | COMMD10 | COMM domain containing 10 [Source:HGNC Symbol;Acc:HGNC:30201] |
| ENSG00000148803 | HGLibA_18171 | 233 | 1.169989761 | FUOM | fucose mutarotase [Source:HGNC Symbol;Acc:HGNC:24733] |
| ENSG00000151224 | HGLibA_28415 | 233 | 1.169989761 | MAT1A | methionine adenosyltransferase 1A [Source:HGNC Symbol;Acc:HGNC:6903] |
| ENSG00000153253 | HGLibA_42987 | 233 | 1.169989761 | SCN3A | sodium voltage-gated channel alpha subunit 3 [Source:HGNC Symbol;Acc:HGNC:10590] |
| ENSG00000154945 | HGLibA_02096 | 233 | 1.169989761 | ANKRD40 | ankyrin repeat domain 40 [Source:HGNC Symbol;Acc:HGNC:28233] |
| ENSG00000168411 | HGLibA_40960 | 233 | 1.169989761 | RFWD3 | ring finger and WD repeat domain 3 [Source:HGNC Symbol;Acc:HGNC:25539] |
| ENSG00000171617 | HGLibA_15110 | 233 | 1.169989761 | ENC1 | ectodermal-neural cortex 1 [Source:HGNC Symbol;Acc:HGNC:3345] |
| ENSG00000172551 | HGLibA_30560 | 233 | 1.169989761 | MUCL1 | mucin like 1 [Source:HGNC Symbol;Acc:HGNC:30588] |
| ENSG00000172572 | HGLibA_35782 | 233 | 1.169989761 | PDE3A | phosphodiesterase 3A [Source:HGNC Symbol;Acc:HGNC:8778] |
| ENSG00000172824 | HGLibA_09226 | 233 | 1.169989761 | CES4A | carboxylesterase 4A [Source:HGNC Symbol;Acc:HGNC:26741] |
| ENSG00000174885 | HGLibA_32126 | 233 | 1.169989761 | NLRP6 | NLR family pyrin domain containing 6 [Source:HGNC Symbol;Acc:HGNC:22944] |
| ENSG00000177675 | HGLibA_08247 | 233 | 1.169989761 | CD163L1 | CD163 molecule like 1 [Source:HGNC Symbol;Acc:HGNC:30375] |
| ENSG00000178394 | HGLibA_22357 | 233 | 1.169989761 | HTR1A | 5-hydroxytryptamine receptor 1A [Source:HGNC Symbol;Acc:HGNC:5286] |
| ENSG00000179958 | HGLibA_12563 | 233 | 1.169989761 | DCTPP1 | dCTP pyrophosphatase 1 [Source:HGNC Symbol;Acc:HGNC:28777] |
| ENSG00000184058 | HGLibA_48640 | 233 | 1.169989761 | TBX1 | T-box 1 [Source:HGNC Symbol;Acc:HGNC:11592] |
| ENSG00000196184 | HGLibA_33520 | 233 | 1.169989761 | OR10J1 | olfactory receptor family 10 subfamily J member 1 [Source:HGNC Symbol;Acc:HGNC:8175] |
| ENSG00000197965 | HGLibA_29795 | 233 | 1.169989761 | MPZL1 | myelin protein zero like 1 [Source:HGNC Symbol;Acc:HGNC:7226] |
| ENSG00000197969 | HGLibA_53731 | 233 | 1.169989761 | VPS13A | vacuolar protein sorting 13 homolog A [Source:HGNC Symbol;Acc:HGNC:1908] |
| ENSG00000198732 | HGLibA_45795 | 233 | 1.169989761 | SMOC1 | SPARC related modular calcium binding 1 [Source:HGNC Symbol;Acc:HGNC:20318] |
| ENSG00000198816 | HGLibA_55871 | 233 | 1.169989761 | ZNF358 | zinc finger protein 358 [Source:HGNC Symbol;Acc:HGNC:16838] |
| ENSG00000203907 | HGLibA_33402 | 233 | 1.169989761 | OOEP | oocyte expressed protein [Source:HGNC Symbol;Acc:HGNC:21382] |
| ENSG00000213563 | HGLibA_06650 | 233 | 1.169989761 | C8orf82 | chromosome 8 open reading frame 82 [Source:HGNC Symbol;Acc:HGNC:33826] |
| ENSG00000238243 | HGLibA_33905 | 233 | 1.169989761 | OR2W3 | olfactory receptor family 2 subfamily W member 3 [Source:HGNC Symbol;Acc:HGNC:15021] |
| ENSG00000254245 | HGLibA_35533 | 233 | 1.169989761 | PCDHGA3 | 3 [Source:HGNC Symbol;Acc:HGNC:8701]" |
| ENSG00000280314 | HGLibA_34546 | 233 | 1.169989761 | OR8K3 | olfactory receptor family 8 subfamily K member 3 (gene/pseudogene) [Source:HGNC Symbol;Acc:HGNC:15313] |
| FAM92B | HGLibA_16800 | 233 | 1.169989761 | FAM92B | - |
| hsa-mir-4456 | HGLibA_60229 | 233 | 1.169989761 | hsa-mir-4456 | - |
| hsa-mir-490 | HGLibA_61359 | 233 | 1.169989761 | hsa-mir-490 | - |
| hsa-mir-518c | HGLibA_61662 | 233 | 1.169989761 | hsa-mir-518c | - |
| hsa-mir-6746 | HGLibA_63138 | 233 | 1.169989761 | hsa-mir-6746 | - |
| C11orf84 | HGLibA_05195 | 232 | 1.160038724 | C11orf84 | - |
| C1orf204 | HGLibA_05822 | 232 | 1.160038724 | C1orf204 | - |
| C6orf10 | HGLibA_06430 | 232 | 1.160038724 | C6orf10 | - |
| C9orf117 | HGLibA_06669 | 232 | 1.160038724 | C9orf117 | - |
| ENSG00000011454 | HGLibA_40009 | 232 | 1.160038724 | RABGAP1 | RAB GTPase activating protein 1 [Source:HGNC Symbol;Acc:HGNC:17155] |
| ENSG00000014216 | HGLibA_07141 | 232 | 1.160038724 | CAPN1 | calpain 1 [Source:HGNC Symbol;Acc:HGNC:1476] |
| ENSG00000054179 | HGLibA_15199 | 232 | 1.160038724 | ENTPD2 | ectonucleoside triphosphate diphosphohydrolase 2 [Source:HGNC Symbol;Acc:HGNC:3364] |
| ENSG00000062194 | HGLibA_19757 | 232 | 1.160038724 | GPBP1 | GC-rich promoter binding protein 1 [Source:HGNC Symbol;Acc:HGNC:29520] |
| ENSG00000072121 | HGLibA_55286 | 232 | 1.160038724 | ZFYVE26 | zinc finger FYVE-type containing 26 [Source:HGNC Symbol;Acc:HGNC:20761] |
| ENSG00000072736 | HGLibA_31781 | 232 | 1.160038724 | NFATC3 | nuclear factor of activated T cells 3 [Source:HGNC Symbol;Acc:HGNC:7777] |
| ENSG00000077380 | HGLibA_14237 | 232 | 1.160038724 | DYNC1I2 | dynein cytoplasmic 1 intermediate chain 2 [Source:HGNC Symbol;Acc:HGNC:2964] |
| ENSG00000087095 | HGLibA_32081 | 232 | 1.160038724 | NLK | nemo like kinase [Source:HGNC Symbol;Acc:HGNC:29858] |
| ENSG00000101417 | HGLibA_39661 | 232 | 1.160038724 | PXMP4 | peroxisomal membrane protein 4 [Source:HGNC Symbol;Acc:HGNC:15920] |
| ENSG00000106004 | HGLibA_21914 | 232 | 1.160038724 | HOXA5 | homeobox A5 [Source:HGNC Symbol;Acc:HGNC:5106] |
| ENSG00000109920 | HGLibA_17740 | 232 | 1.160038724 | FNBP4 | formin binding protein 4 [Source:HGNC Symbol;Acc:HGNC:19752] |
| ENSG00000112983 | HGLibA_04784 | 232 | 1.160038724 | BRD8 | bromodomain containing 8 [Source:HGNC Symbol;Acc:HGNC:19874] |
| ENSG00000122145 | HGLibA_48664 | 232 | 1.160038724 | TBX22 | T-box 22 [Source:HGNC Symbol;Acc:HGNC:11600] |
| ENSG00000124496 | HGLibA_51368 | 232 | 1.160038724 | TRERF1 | transcriptional regulating factor 1 [Source:HGNC Symbol;Acc:HGNC:18273] |
| ENSG00000128218 | HGLibA_53725 | 232 | 1.160038724 | VPREB3 | V-set pre-B cell surrogate light chain 3 [Source:HGNC Symbol;Acc:HGNC:12710] |
| ENSG00000135299 | HGLibA_02128 | 232 | 1.160038724 | ANKRD6 | ankyrin repeat domain 6 [Source:HGNC Symbol;Acc:HGNC:17280] |
| ENSG00000137747 | HGLibA_50629 | 232 | 1.160038724 | TMPRSS13 | transmembrane serine protease 13 [Source:HGNC Symbol;Acc:HGNC:29808] |
| ENSG00000139053 | HGLibA_35819 | 232 | 1.160038724 | PDE6H | phosphodiesterase 6H [Source:HGNC Symbol;Acc:HGNC:8790] |
| ENSG00000149289 | HGLibA_54957 | 232 | 1.160038724 | ZC3H12C | zinc finger CCCH-type containing 12C [Source:HGNC Symbol;Acc:HGNC:29362] |
| ENSG00000154025 | HGLibA_45256 | 232 | 1.160038724 | SLC5A10 | solute carrier family 5 member 10 [Source:HGNC Symbol;Acc:HGNC:23155] |
| ENSG00000154760 | HGLibA_45491 | 232 | 1.160038724 | SLFN13 | schlafen family member 13 [Source:HGNC Symbol;Acc:HGNC:26481] |
| ENSG00000160469 | HGLibA_04848 | 232 | 1.160038724 | BRSK1 | BR serine/threonine kinase 1 [Source:HGNC Symbol;Acc:HGNC:18994] |
| ENSG00000160602 | HGLibA_31665 | 232 | 1.160038724 | NEK8 | NIMA related kinase 8 [Source:HGNC Symbol;Acc:HGNC:13387] |
| ENSG00000163431 | HGLibA_26717 | 232 | 1.160038724 | LMOD1 | leiomodin 1 [Source:HGNC Symbol;Acc:HGNC:6647] |
| ENSG00000163749 | HGLibA_07665 | 232 | 1.160038724 | CCDC158 | coiled-coil domain containing 158 [Source:HGNC Symbol;Acc:HGNC:26374] |
| ENSG00000165637 | HGLibA_53602 | 232 | 1.160038724 | VDAC2 | voltage dependent anion channel 2 [Source:HGNC Symbol;Acc:HGNC:12672] |
| ENSG00000166159 | HGLibA_27503 | 232 | 1.160038724 | LRTM2 | leucine rich repeats and transmembrane domains 2 [Source:HGNC Symbol;Acc:HGNC:32443] |
| ENSG00000166927 | HGLibA_30162 | 232 | 1.160038724 | MS4A7 | membrane spanning 4-domains A7 [Source:HGNC Symbol;Acc:HGNC:13378] |
| ENSG00000167280 | HGLibA_15127 | 232 | 1.160038724 | ENGASE | endo-beta-N-acetylglucosaminidase [Source:HGNC Symbol;Acc:HGNC:24622] |
| ENSG00000169918 | HGLibA_34777 | 232 | 1.160038724 | OTUD7A | OTU deubiquitinase 7A [Source:HGNC Symbol;Acc:HGNC:20718] |
| ENSG00000170175 | HGLibA_09590 | 232 | 1.160038724 | CHRNB1 | cholinergic receptor nicotinic beta 1 subunit [Source:HGNC Symbol;Acc:HGNC:1961] |
| ENSG00000175946 | HGLibA_25202 | 232 | 1.160038724 | KLHL38 | kelch like family member 38 [Source:HGNC Symbol;Acc:HGNC:34435] |
| ENSG00000176160 | HGLibA_22232 | 232 | 1.160038724 | HSF5 | heat shock transcription factor 5 [Source:HGNC Symbol;Acc:HGNC:26862] |
| ENSG00000177697 | HGLibA_08236 | 232 | 1.160038724 | CD151 | CD151 molecule (Raph blood group) [Source:HGNC Symbol;Acc:HGNC:1630] |
| ENSG00000178385 | HGLibA_37143 | 232 | 1.160038724 | PLEKHM3 | pleckstrin homology domain containing M3 [Source:HGNC Symbol;Acc:HGNC:34006] |
| ENSG00000178467 | HGLibA_34913 | 232 | 1.160038724 | P4HTM | transmembrane [Source:HGNC Symbol;Acc:HGNC:28858]" |
| ENSG00000178921 | HGLibA_36135 | 232 | 1.160038724 | PFAS | phosphoribosylformylglycinamidine synthase [Source:HGNC Symbol;Acc:HGNC:8863] |
| ENSG00000179213 | HGLibA_44155 | 232 | 1.160038724 | SIGLECL1 | SIGLEC family like 1 [Source:HGNC Symbol;Acc:HGNC:26856] |

| | | | | | |
|---|---|---|---|---|---|
| ENSG00000179580 | HGLibA_41546 | 232 | 1.160038724 | RNF151 | ring finger protein 151 [Source:HGNC Symbol;Acc:HGNC:23235] |
| ENSG00000180697 | HGLibA_06220 | 232 | 1.160038724 | C3orf22 | chromosome 3 open reading frame 22 [Source:HGNC Symbol;Acc:HGNC:28534] |
| ENSG00000182272 | HGLibA_04020 | 232 | 1.160038724 | B4GALNT4 | 4-N-acetyl-galactosaminyltransferase 4 [Source:HGNC Symbol;Acc:HGNC:26315]" |
| ENSG00000184361 | HGLibA_46507 | 232 | 1.160038724 | SPATA32 | spermatogenesis associated 32 [Source:HGNC Symbol;Acc:HGNC:26349] |
| ENSG00000185760 | HGLibA_24377 | 232 | 1.160038724 | KCNQ5 | potassium voltage-gated channel subfamily Q member 5 [Source:HGNC Symbol;Acc:HGNC:6299] |
| ENSG00000187240 | HGLibA_14246 | 232 | 1.160038724 | DYNC2H1 | dynein cytoplasmic 2 heavy chain 1 [Source:HGNC Symbol;Acc:HGNC:2962] |
| ENSG00000196236 | HGLibA_54558 | 232 | 1.160038724 | XPNPEP3 | X-prolyl aminopeptidase 3 [Source:HGNC Symbol;Acc:HGNC:28052] |
| ENSG00000198169 | HGLibA_55668 | 232 | 1.160038724 | ZNF251 | zinc finger protein 251 [Source:HGNC Symbol;Acc:HGNC:13045] |
| ENSG00000198689 | HGLibA_45421 | 232 | 1.160038724 | SLC9A6 | solute carrier family 9 member A6 [Source:HGNC Symbol;Acc:HGNC:11079] |
| ENSG00000205268 | HGLibA_35822 | 232 | 1.160038724 | PDE7A | phosphodiesterase 7A [Source:HGNC Symbol;Acc:HGNC:8791] |
| ENSG00000224440 | HGLibA_11937 | 232 | 1.160038724 | CXorf51A | chromosome X open reading frame 51A [Source:HGNC Symbol;Acc:HGNC:30533] |
| ENSG00000269743 | HGLibA_44772 | 232 | 1.160038724 | SLC25A53 | solute carrier family 25 member 53 [Source:HGNC Symbol;Acc:HGNC:31894] |
| ENSG00000280090 | HGLibA_34502 | 232 | 1.160038724 | OR8B4 | olfactory receptor family 8 subfamily B member 4 (gene/pseudogene) [Source:HGNC Symbol;Acc:HGNC:8473] |
| hsa-mir-1236 | HGLibA_57335 | 232 | 1.160038724 | hsa-mir-1236 | - |
| hsa-mir-3178 | HGLibA_58785 | 232 | 1.160038724 | hsa-mir-3178 | - |
| hsa-mir-4499 | HGLibA_60413 | 232 | 1.160038724 | hsa-mir-4499 | - |
| hsa-mir-584 | HGLibA_62433 | 232 | 1.160038724 | hsa-mir-584 | - |
| hsa-mir-585 | HGLibA_62438 | 232 | 1.160038724 | hsa-mir-585 | - |
| hsa-mir-6885 | HGLibA_63724 | 232 | 1.160038724 | hsa-mir-6885 | - |
| hsa-mir-8085 | HGLibA_64184 | 232 | 1.160038724 | hsa-mir-8085 | - |
| hsa-mir-941-3 | HGLibA_64345 | 232 | 1.160038724 | hsa-mir-941-3 | - |
| LPPR2 | HGLibA_27116 | 232 | 1.160038724 | LPPR2 | - |
| PPAPDC3 | HGLibA_37833 | 232 | 1.160038724 | PPAPDC3 | - |
| ENSG00000026297 | HGLibA_41442 | 231 | 1.150087686 | RNASET2 | ribonuclease T2 [Source:HGNC Symbol;Acc:HGNC:21686] |
| ENSG00000032444 | HGLibA_37420 | 231 | 1.150087686 | PNPLA6 | patatin like phospholipase domain containing 6 [Source:HGNC Symbol;Acc:HGNC:16268] |
| ENSG00000065243 | HGLibA_36840 | 231 | 1.150087686 | PKN2 | protein kinase N2 [Source:HGNC Symbol;Acc:HGNC:9406] |
| ENSG00000065308 | HGLibA_51287 | 231 | 1.150087686 | TRAM2 | translocation associated membrane protein 2 [Source:HGNC Symbol;Acc:HGNC:16855] |
| ENSG00000082701 | HGLibA_20432 | 231 | 1.150087686 | GSK3B | glycogen synthase kinase 3 beta [Source:HGNC Symbol;Acc:HGNC:4617] |
| ENSG00000094796 | HGLibA_25473 | 231 | 1.150087686 | KRT31 | keratin 31 [Source:HGNC Symbol;Acc:HGNC:6448] |
| ENSG00000096264 | HGLibA_31365 | 231 | 1.150087686 | NCR2 | natural cytotoxicity triggering receptor 2 [Source:HGNC Symbol;Acc:HGNC:6732] |
| ENSG00000106069 | HGLibA_09507 | 231 | 1.150087686 | CHN2 | chimerin 2 [Source:HGNC Symbol;Acc:HGNC:1944] |
| ENSG00000125834 | HGLibA_47494 | 231 | 1.150087686 | STK35 | serine/threonine kinase 35 [Source:HGNC Symbol;Acc:HGNC:16254] |
| ENSG00000125970 | HGLibA_40173 | 231 | 1.150087686 | RALY | RALY heterogeneous nuclear ribonucleoprotein [Source:HGNC Symbol;Acc:HGNC:15921] |
| ENSG00000132359 | HGLibA_40231 | 231 | 1.150087686 | RAP1GAP2 | RAP1 GTPase activating protein 2 [Source:HGNC Symbol;Acc:HGNC:29176] |
| ENSG00000132429 | HGLibA_37701 | 231 | 1.150087686 | POPDC3 | popeye domain containing 3 [Source:HGNC Symbol;Acc:HGNC:17649] |
| ENSG00000134265 | HGLibA_31134 | 231 | 1.150087686 | NAPG | NSF attachment protein gamma [Source:HGNC Symbol;Acc:HGNC:7642] |
| ENSG00000137522 | HGLibA_41483 | 231 | 1.150087686 | RNF121 | ring finger protein 121 [Source:HGNC Symbol;Acc:HGNC:21070] |
| ENSG00000138135 | HGLibA_09333 | 231 | 1.150087686 | CH25H | cholesterol 25-hydroxylase [Source:HGNC Symbol;Acc:HGNC:1907] |
| ENSG00000138686 | HGLibA_04224 | 231 | 1.150087686 | BBS7 | Bardet-Biedl syndrome 7 [Source:HGNC Symbol;Acc:HGNC:18758] |
| ENSG00000139192 | HGLibA_48325 | 231 | 1.150087686 | TAPBPL | TAP binding protein like [Source:HGNC Symbol;Acc:HGNC:30683] |
| ENSG00000141425 | HGLibA_42044 | 231 | 1.150087686 | RPRD1A | regulation of nuclear pre-mRNA domain containing 1A [Source:HGNC Symbol;Acc:HGNC:25560] |
| ENSG00000142459 | HGLibA_15696 | 231 | 1.150087686 | EVI5L | ecotropic viral integration site 5 like [Source:HGNC Symbol;Acc:HGNC:30464] |
| ENSG00000143819 | HGLibA_15323 | 231 | 1.150087686 | EPHX1 | epoxide hydrolase 1 [Source:HGNC Symbol;Acc:HGNC:3401] |
| ENSG00000144648 | HGLibA_00444 | 231 | 1.150087686 | ACKR2 | atypical chemokine receptor 2 [Source:HGNC Symbol;Acc:HGNC:1565] |
| ENSG00000145365 | HGLibA_49456 | 231 | 1.150087686 | TIFA | TRAF interacting protein with forkhead associated domain [Source:HGNC Symbol;Acc:HGNC:19075] |
| ENSG00000153132 | HGLibA_10015 | 231 | 1.150087686 | CLGN | calmegin [Source:HGNC Symbol;Acc:HGNC:2060] |
| ENSG00000160991 | HGLibA_34593 | 231 | 1.150087686 | ORAI2 | ORAI calcium release-activated calcium modulator 2 [Source:HGNC Symbol;Acc:HGNC:21667] |
| ENSG00000164651 | HGLibA_46314 | 231 | 1.150087686 | SP8 | Sp8 transcription factor [Source:HGNC Symbol;Acc:HGNC:19196] |
| ENSG00000165168 | HGLibA_12020 | 231 | 1.150087686 | CYBB | cytochrome b-245 beta chain [Source:HGNC Symbol;Acc:HGNC:2578] |
| ENSG00000167139 | HGLibA_48501 | 231 | 1.150087686 | TBC1D21 | TBC1 domain family member 21 [Source:HGNC Symbol;Acc:HGNC:28536] |
| ENSG00000168237 | HGLibA_19383 | 231 | 1.150087686 | GLYCTK | glycerate kinase [Source:HGNC Symbol;Acc:HGNC:24247] |
| ENSG00000168539 | HGLibA_09548 | 231 | 1.150087686 | CHRM1 | cholinergic receptor muscarinic 1 [Source:HGNC Symbol;Acc:HGNC:1950] |
| ENSG00000171467 | HGLibA_55785 | 231 | 1.150087686 | ZNF318 | zinc finger protein 318 [Source:HGNC Symbol;Acc:HGNC:13578] |
| ENSG00000172137 | HGLibA_06989 | 231 | 1.150087686 | CALB2 | calbindin 2 [Source:HGNC Symbol;Acc:HGNC:1435] |
| ENSG00000172289 | HGLibA_33550 | 231 | 1.150087686 | OR10V1 | olfactory receptor family 10 subfamily V member 1 [Source:HGNC Symbol;Acc:HGNC:15136] |
| ENSG00000172354 | HGLibA_19491 | 231 | 1.150087686 | GNB2 | G protein subunit beta 2 [Source:HGNC Symbol;Acc:HGNC:4398] |
| ENSG00000173762 | HGLibA_08434 | 231 | 1.150087686 | CD7 | CD7 molecule [Source:HGNC Symbol;Acc:HGNC:1695] |
| ENSG00000176566 | HGLibA_12444 | 231 | 1.150087686 | DCAF4L2 | DDB1 and CUL4 associated factor 4 like 2 [Source:HGNC Symbol;Acc:HGNC:26657] |
| ENSG00000176720 | HGLibA_04677 | 231 | 1.150087686 | BOK | BCL2 family apoptosis regulator BOK [Source:HGNC Symbol;Acc:HGNC:1087] |
| ENSG00000180772 | HGLibA_01265 | 231 | 1.150087686 | AGTR2 | angiotensin II receptor type 2 [Source:HGNC Symbol;Acc:HGNC:338] |
| ENSG00000181577 | HGLibA_06479 | 231 | 1.150087686 | C6orf223 | chromosome 6 open reading frame 223 [Source:HGNC Symbol;Acc:HGNC:28692] |
| ENSG00000181577 | HGLibA_06478 | 231 | 1.150087686 | C6orf223 | chromosome 6 open reading frame 223 [Source:HGNC Symbol;Acc:HGNC:28692] |
| ENSG00000184502 | HGLibA_18661 | 231 | 1.150087686 | GAST | gastrin [Source:HGNC Symbol;Acc:HGNC:4164] |
| ENSG00000185610 | HGLibA_12410 | 231 | 1.150087686 | DBX2 | developing brain homeobox 2 [Source:HGNC Symbol;Acc:HGNC:33186] |
| ENSG00000188707 | HGLibA_54806 | 231 | 1.150087686 | ZBED6CL | ZBED6 C-terminal like [Source:HGNC Symbol;Acc:HGNC:21720] |
| ENSG00000189375 | HGLibA_48520 | 231 | 1.150087686 | TBC1D28 | TBC1 domain family member 28 [Source:HGNC Symbol;Acc:HGNC:26858] |
| ENSG00000197580 | HGLibA_04358 | 231 | 1.150087686 | BCO2 | beta-carotene oxygenase 2 [Source:HGNC Symbol;Acc:HGNC:18503] |
| ENSG00000198538 | HGLibA_55719 | 231 | 1.150087686 | ZNF28 | zinc finger protein 28 [Source:HGNC Symbol;Acc:HGNC:13073] |
| ENSG00000206140 | HGLibA_50148 | 231 | 1.150087686 | TMEM191C | transmembrane protein 191C [Source:HGNC Symbol;Acc:HGNC:33601] |
| ENSG00000223638 | HGLibA_40938 | 231 | 1.150087686 | RFPL4A | ret finger protein like 4A [Source:HGNC Symbol;Acc:HGNC:16449] |
| ENSG00000233493 | HGLibA_50279 | 231 | 1.150087686 | TMEM238 | transmembrane protein 238 [Source:HGNC Symbol;Acc:HGNC:40042] |
| ENSG00000240204 | HGLibA_45777 | 231 | 1.150087686 | SMKR1 | small lysine rich protein 1 [Source:HGNC Symbol;Acc:HGNC:43561] |
| ENSG00000241186 | HGLibA_48884 | 231 | 1.150087686 | TDGF1 | teratocarcinoma-derived growth factor 1 [Source:HGNC Symbol;Acc:HGNC:11701] |
| ENSG00000244731 | HGLibA_06281 | 231 | 1.150087686 | C4A | complement C4A (Rodgers blood group) [Source:HGNC Symbol;Acc:HGNC:1323] |
| ENSG00000254997 | HGLibA_25811 | 231 | 1.150087686 | KRTAP5-9 | keratin associated protein 5-9 [Source:HGNC Symbol;Acc:HGNC:23604] |
| ENSG00000261678 | HGLibA_43059 | 231 | 1.150087686 | SCRT1 | scratch family transcriptional repressor 1 [Source:HGNC Symbol;Acc:HGNC:15950] |
| ENSG00000275183 | HGLibA_26303 | 231 | 1.150087686 | LENG9 | leukocyte receptor cluster member 9 [Source:HGNC Symbol;Acc:HGNC:16306] |
| FAM196A | HGLibA_16381 | 231 | 1.150087686 | FAM196A | - |
| hsa-mir-100 | HGLibA_57148 | 231 | 1.150087686 | hsa-mir-100 | - |
| hsa-mir-1203 | HGLibA_57269 | 231 | 1.150087686 | hsa-mir-1203 | - |
| hsa-mir-3689c | HGLibA_59320 | 231 | 1.150087686 | hsa-mir-3689c | - |
| hsa-mir-4434 | HGLibA_60125 | 231 | 1.150087686 | hsa-mir-4434 | - |
| hsa-mir-4458 | HGLibA_60238 | 231 | 1.150087686 | hsa-mir-4458 | - |
| hsa-mir-4530 | HGLibA_60578 | 231 | 1.150087686 | hsa-mir-4530 | - |
| hsa-mir-6068 | HGLibA_62520 | 231 | 1.150087686 | hsa-mir-6068 | - |
| hsa-mir-6072 | HGLibA_62541 | 231 | 1.150087686 | hsa-mir-6072 | - |
| hsa-mir-7155 | HGLibA_63849 | 231 | 1.150087686 | hsa-mir-7155 | - |
| LOC100506688 | HGLibA_26863 | 231 | 1.150087686 | LOC100506688 | - |
| CCDC151 | HGLibA_07646 | 230 | 1.140136649 | CCDC151 | - |
| DSCR3 | HGLibA_14007 | 230 | 1.140136649 | DSCR3 | - |
| ENSG00000003989 | HGLibA_45361 | 230 | 1.140136649 | SLC7A2 | solute carrier family 7 member 2 [Source:HGNC Symbol;Acc:HGNC:11060] |
| ENSG00000005022 | HGLibA_44762 | 230 | 1.140136649 | SLC25A5 | solute carrier family 25 member 5 [Source:HGNC Symbol;Acc:HGNC:10991] |
| ENSG00000056277 | HGLibA_55729 | 230 | 1.140136649 | ZNF280C | zinc finger protein 280C [Source:HGNC Symbol;Acc:HGNC:25955] |
| ENSG00000060982 | HGLibA_04264 | 230 | 1.140136649 | BCAT1 | branched chain amino acid transaminase 1 [Source:HGNC Symbol;Acc:HGNC:976] |
| ENSG00000065802 | HGLibA_03204 | 230 | 1.140136649 | ASB1 | ankyrin repeat and SOCS box containing 1 [Source:HGNC Symbol;Acc:HGNC:16011] |
| ENSG00000069702 | HGLibA_49230 | 230 | 1.140136649 | TGFBR3 | transforming growth factor beta receptor 3 [Source:HGNC Symbol;Acc:HGNC:11774] |
| ENSG00000081148 | HGLibA_23266 | 230 | 1.140136649 | IMPG2 | interphotoreceptor matrix proteoglycan 2 [Source:HGNC Symbol;Acc:HGNC:18362] |
| ENSG00000082126 | HGLibA_29756 | 230 | 1.140136649 | MPP4 | membrane palmitoylated protein 4 [Source:HGNC Symbol;Acc:HGNC:13680] |
| ENSG00000089116 | HGLibA_26459 | 230 | 1.140136649 | LHX5 | LIM homeobox 5 [Source:HGNC Symbol;Acc:HGNC:14216] |
| ENSG00000100813 | HGLibA_00441 | 230 | 1.140136649 | ACIN1 | apoptotic chromatin condensation inducer 1 [Source:HGNC Symbol;Acc:HGNC:17066] |
| ENSG00000106128 | HGLibA_19047 | 230 | 1.140136649 | GHRHR | growth hormone releasing hormone receptor [Source:HGNC Symbol;Acc:HGNC:4266] |
| ENSG00000106477 | HGLibA_09137 | 230 | 1.140136649 | CEP41 | centrosomal protein 41 [Source:HGNC Symbol;Acc:HGNC:12370] |
| ENSG00000106483 | HGLibA_43742 | 230 | 1.140136649 | SFRP4 | secreted frizzled related protein 4 [Source:HGNC Symbol;Acc:HGNC:10778] |
| ENSG00000106688 | HGLibA_44507 | 230 | 1.140136649 | SLC1A1 | solute carrier family 1 member 1 [Source:HGNC Symbol;Acc:HGNC:10939] |
| ENSG00000107614 | HGLibA_51345 | 230 | 1.140136649 | TRDMT1 | tRNA aspartic acid methyltransferase 1 [Source:HGNC Symbol;Acc:HGNC:2977] |
| ENSG00000107890 | HGLibA_02047 | 230 | 1.140136649 | ANKRD26 | ankyrin repeat domain 26 [Source:HGNC Symbol;Acc:HGNC:29186] |
| ENSG00000109610 | HGLibA_46151 | 230 | 1.140136649 | SOD3 | superoxide dismutase 3 [Source:HGNC Symbol;Acc:HGNC:11181] |
| ENSG00000115592 | HGLibA_38518 | 230 | 1.140136649 | PRKAG3 | protein kinase AMP-activated non-catalytic subunit gamma 3 [Source:HGNC Symbol;Acc:HGNC:9387] |
| ENSG00000118298 | HGLibA_06800 | 230 | 1.140136649 | CA14 | carbonic anhydrase 14 [Source:HGNC Symbol;Acc:HGNC:1372] |
| ENSG00000118640 | HGLibA_53508 | 230 | 1.140136649 | VAMP8 | vesicle associated membrane protein 8 [Source:HGNC Symbol;Acc:HGNC:12647] |
| ENSG00000119457 | HGLibA_45188 | 230 | 1.140136649 | SLC46A2 | solute carrier family 46 member 2 [Source:HGNC Symbol;Acc:HGNC:16055] |
| ENSG00000119703 | HGLibA_54942 | 230 | 1.140136649 | ZC2HC1C | zinc finger C2HC-type containing 1C [Source:HGNC Symbol;Acc:HGNC:20354] |
| ENSG00000120500 | HGLibA_03105 | 230 | 1.140136649 | ARR3 | arrestin 3 [Source:HGNC Symbol;Acc:HGNC:710] |
| ENSG00000120519 | HGLibA_44348 | 230 | 1.140136649 | SLC10A7 | solute carrier family 10 member 7 [Source:HGNC Symbol;Acc:HGNC:23088] |
| ENSG00000121680 | HGLibA_36101 | 230 | 1.140136649 | PEX16 | peroxisomal biogenesis factor 16 [Source:HGNC Symbol;Acc:HGNC:8857] |
| ENSG00000124818 | HGLibA_33439 | 230 | 1.140136649 | OPN5 | opsin 5 [Source:HGNC Symbol;Acc:HGNC:19992] |
| ENSG00000126226 | HGLibA_35608 | 230 | 1.140136649 | PCID2 | PCI domain containing 2 [Source:HGNC Symbol;Acc:HGNC:25653] |
| ENSG00000127418 | HGLibA_17453 | 230 | 1.140136649 | FGFRL1 | fibroblast growth factor receptor like 1 [Source:HGNC Symbol;Acc:HGNC:3693] |
| ENSG00000128652 | HGLibA_21998 | 230 | 1.140136649 | HOXD3 | homeobox D3 [Source:HGNC Symbol;Acc:HGNC:5137] |
| ENSG00000131050 | HGLibA_04713 | 230 | 1.140136649 | BPIFA2 | BPI fold containing family A member 2 [Source:HGNC Symbol;Acc:HGNC:16203] |
| ENSG00000131263 | HGLibA_41348 | 230 | 1.140136649 | RLIM | LIM domain interacting [Source:HGNC Symbol;Acc:HGNC:13429]" |
| ENSG00000132612 | HGLibA_53797 | 230 | 1.140136649 | VPS4A | vacuolar protein sorting 4 homolog A [Source:HGNC Symbol;Acc:HGNC:13488] |
| ENSG00000136147 | HGLibA_36336 | 230 | 1.140136649 | PHF11 | PHD finger protein 11 [Source:HGNC Symbol;Acc:HGNC:17024] |
| ENSG00000137203 | HGLibA_49120 | 230 | 1.140136649 | TFAP2A | transcription factor AP-2 alpha [Source:HGNC Symbol;Acc:HGNC:11742] |
| ENSG00000139219 | HGLibA_10559 | 230 | 1.140136649 | COL2A1 | collagen type II alpha 1 chain [Source:HGNC Symbol;Acc:HGNC:2200] |
| ENSG00000143951 | HGLibA_54020 | 230 | 1.140136649 | WDPCP | WD repeat containing planar cell polarity effector [Source:HGNC Symbol;Acc:HGNC:28027] |
| ENSG00000148482 | HGLibA_45097 | 230 | 1.140136649 | SLC39A12 | solute carrier family 39 member 12 [Source:HGNC Symbol;Acc:HGNC:20860] |
| ENSG00000152661 | HGLibA_19135 | 230 | 1.140136649 | GJA1 | gap junction protein alpha 1 [Source:HGNC Symbol;Acc:HGNC:4274] |
| ENSG00000153037 | HGLibA_47003 | 230 | 1.140136649 | SRP19 | signal recognition particle 19 [Source:HGNC Symbol;Acc:HGNC:11300] |
| ENSG00000154237 | HGLibA_27464 | 230 | 1.140136649 | LRRK1 | leucine rich repeat kinase 1 [Source:HGNC Symbol;Acc:HGNC:18608] |
| ENSG00000160299 | HGLibA_35641 | 230 | 1.140136649 | PCNT | pericentrin [Source:HGNC Symbol;Acc:HGNC:16068] |
| ENSG00000160714 | HGLibA_52646 | 230 | 1.140136649 | UBE2Q1 | ubiquitin conjugating enzyme E2 Q1 [Source:HGNC Symbol;Acc:HGNC:15698] |
| ENSG00000160961 | HGLibA_55819 | 230 | 1.140136649 | ZNF333 | zinc finger protein 333 [Source:HGNC Symbol;Acc:HGNC:15624] |
| ENSG00000162779 | HGLibA_03919 | 230 | 1.140136649 | AXDND1 | axonemal dynein light chain domain containing 1 [Source:HGNC Symbol;Acc:HGNC:26564] |
| ENSG00000163479 | HGLibA_47157 | 230 | 1.140136649 | SSR2 | signal sequence receptor subunit 2 [Source:HGNC Symbol;Acc:HGNC:11324] |

| | | | | | |
|---|---|---|---|---|---|
| ENSG00000164309 | HGLibA_10239 | 230 | 1.140136649 | CMYA5 | cardiomyopathy associated 5 [Source:HGNC Symbol;Acc:HGNC:14305] |
| ENSG00000166143 | HGLibA_38045 | 230 | 1.140136649 | PPP1R14D | protein phosphatase 1 regulatory inhibitor subunit 14D [Source:HGNC Symbol;Acc:HGNC:14953] |
| ENSG00000167131 | HGLibA_07513 | 230 | 1.140136649 | CCDC103 | coiled-coil domain containing 103 [Source:HGNC Symbol;Acc:HGNC:32700] |
| ENSG00000167766 | HGLibA_56830 | 230 | 1.140136649 | ZNF83 | zinc finger protein 83 [Source:HGNC Symbol;Acc:HGNC:13158] |
| ENSG00000167964 | HGLibA_39853 | 230 | 1.140136649 | RAB26 | member RAS oncogene family [Source:HGNC Symbol;Acc:HGNC:14259]" |
| ENSG00000168273 | HGLibA_45758 | 230 | 1.140136649 | SMIM4 | small integral membrane protein 4 [Source:HGNC Symbol;Acc:HGNC:37257] |
| ENSG00000168394 | HGLibA_48315 | 230 | 1.140136649 | TAP1 | ATP binding cassette subfamily B member [Source:HGNC Symbol;Acc:HGNC:43]" |
| ENSG00000170948 | HGLibA_28471 | 230 | 1.140136649 | MBD3L1 | methyl-CpG binding domain protein 3 like 1 [Source:HGNC Symbol;Acc:HGNC:15774] |
| ENSG00000176182 | HGLibA_30914 | 230 | 1.140136649 | MYPOP | partner of profilin [Source:HGNC Symbol;Acc:HGNC:20178]" |
| ENSG00000176472 | HGLibA_56289 | 230 | 1.140136649 | ZNF575 | zinc finger protein 575 [Source:HGNC Symbol;Acc:HGNC:27606] |
| ENSG00000178852 | HGLibA_14528 | 230 | 1.140136649 | EFCAB13 | EF-hand calcium binding domain 13 [Source:HGNC Symbol;Acc:HGNC:26864] |
| ENSG00000179071 | HGLibA_07917 | 230 | 1.140136649 | CCDC89 | coiled-coil domain containing 89 [Source:HGNC Symbol;Acc:HGNC:26762] |
| ENSG00000179163 | HGLibA_18154 | 230 | 1.140136649 | FUCA1 | alpha-L-fucosidase 1 [Source:HGNC Symbol;Acc:HGNC:4006] |
| ENSG00000182325 | HGLibA_17016 | 230 | 1.140136649 | FBXL6 | F-box and leucine rich repeat protein 6 [Source:HGNC Symbol;Acc:HGNC:13603] |
| ENSG00000182919 | HGLibA_05156 | 230 | 1.140136649 | C11orf54 | chromosome 11 open reading frame 54 [Source:HGNC Symbol;Acc:HGNC:30204] |
| ENSG00000183230 | HGLibA_11643 | 230 | 1.140136649 | CTNNA3 | catenin alpha 3 [Source:HGNC Symbol;Acc:HGNC:2511] |
| ENSG00000183260 | HGLibA_00261 | 230 | 1.140136649 | ABHD16B | abhydrolase domain containing 16B [Source:HGNC Symbol;Acc:HGNC:16128] |
| ENSG00000188559 | HGLibA_40159 | 230 | 1.140136649 | RALGAPA2 | Ral GTPase activating protein catalytic alpha subunit 2 [Source:HGNC Symbol;Acc:HGNC:16207] |
| ENSG00000196924 | HGLibA_17639 | 230 | 1.140136649 | FLNA | filamin A [Source:HGNC Symbol;Acc:HGNC:3754] |
| ENSG00000197057 | HGLibA_14057 | 230 | 1.140136649 | DTHD1 | death domain containing 1 [Source:HGNC Symbol;Acc:HGNC:37261] |
| ENSG00000198169 | HGLibA_55669 | 230 | 1.140136649 | ZNF251 | zinc finger protein 251 [Source:HGNC Symbol;Acc:HGNC:13045] |
| ENSG00000198542 | HGLibA_23801 | 230 | 1.140136649 | ITGBL1 | integrin subunit beta like 1 [Source:HGNC Symbol;Acc:HGNC:6164] |
| ENSG00000198914 | HGLibA_37771 | 230 | 1.140136649 | POU3F3 | POU class 3 homeobox 3 [Source:HGNC Symbol;Acc:HGNC:9216] |
| ENSG00000204590 | HGLibA_19543 | 230 | 1.140136649 | GNL1 | G protein nucleolar 1 (putative) [Source:HGNC Symbol;Acc:HGNC:4413] |
| ENSG00000231396 | HGLibA_53214 | 230 | 1.140136649 | USP17L10 | ubiquitin specific peptidase 17-like family member 10 [Source:HGNC Symbol;Acc:HGNC:44438] |
| ENSG00000247596 | HGLibA_52371 | 230 | 1.140136649 | TWF2 | twinfilin actin binding protein 2 [Source:HGNC Symbol;Acc:HGNC:9621] |
| ENSG00000256235 | HGLibA_45754 | 230 | 1.140136649 | SMIM3 | small integral membrane protein 3 [Source:HGNC Symbol;Acc:HGNC:30248] |
| ENSG00000279111 | HGLibA_33556 | 230 | 1.140136649 | OR10X1 | olfactory receptor family 10 subfamily X member 1 (gene/pseudogene) [Source:HGNC Symbol;Acc:HGNC:14995] |
| FAM105A | HGLibA_15994 | 230 | 1.140136649 | FAM105A | - |
| hsa-mir-150 | HGLibA_57893 | 230 | 1.140136649 | hsa-mir-150 | - |
| hsa-mir-3915 | HGLibA_59535 | 230 | 1.140136649 | hsa-mir-3915 | - |
| hsa-mir-4533 | HGLibA_60591 | 230 | 1.140136649 | hsa-mir-4533 | - |
| hsa-mir-6504 | HGLibA_62867 | 230 | 1.140136649 | hsa-mir-6504 | - |
| hsa-mir-6871 | HGLibA_63670 | 230 | 1.140136649 | hsa-mir-6871 | - |
| LOC158434 | HGLibA_26899 | 230 | 1.140136649 | LOC158434 | - |
| LOC649330 | HGLibA_26980 | 230 | 1.140136649 | LOC649330 | - |
| PPAP2C | HGLibA_37822 | 230 | 1.140136649 | PPAP2C | - |
| ENSG00000005893 | HGLibA_25979 | 229 | 1.130185612 | LAMP2 | lysosomal associated membrane protein 2 [Source:HGNC Symbol;Acc:HGNC:6501] |
| ENSG00000024526 | HGLibA_12975 | 229 | 1.130185612 | DEPDC1 | DEP domain containing 1 [Source:HGNC Symbol;Acc:HGNC:22949] |
| ENSG00000042088 | HGLibA_48889 | 229 | 1.130185612 | TDP1 | tyrosyl-DNA phosphodiesterase 1 [Source:HGNC Symbol;Acc:HGNC:18884] |
| ENSG00000058453 | HGLibA_11211 | 229 | 1.130185612 | CROCC | rootletin [Source:HGNC Symbol;Acc:HGNC:21299]" |
| ENSG00000059915 | HGLibA_39013 | 229 | 1.130185612 | PSD | pleckstrin and Sec7 domain containing [Source:HGNC Symbol;Acc:HGNC:9507] |
| ENSG00000065911 | HGLibA_30383 | 229 | 1.130185612 | MTHFD2 | methenyltetrahydrofolate cyclohydrolase [Source:HGNC Symbol;Acc:HGNC:7434]" |
| ENSG00000070601 | HGLibA_18033 | 229 | 1.130185612 | FRMPD1 | FERM and PDZ domain containing 1 [Source:HGNC Symbol;Acc:HGNC:29159] |
| ENSG00000086159 | HGLibA_02603 | 229 | 1.130185612 | AQP6 | aquaporin 6 [Source:HGNC Symbol;Acc:HGNC:639] |
| ENSG00000101017 | HGLibA_08383 | 229 | 1.130185612 | CD40 | CD40 molecule [Source:HGNC Symbol;Acc:HGNC:11919] |
| ENSG00000102543 | HGLibA_08501 | 229 | 1.130185612 | CDADC1 | cytidine and dCMP deaminase domain containing 1 [Source:HGNC Symbol;Acc:HGNC:20299] |
| ENSG00000110046 | HGLibA_03478 | 229 | 1.130185612 | ATG2A | autophagy related 2A [Source:HGNC Symbol;Acc:HGNC:29028] |
| ENSG00000111716 | HGLibA_26228 | 229 | 1.130185612 | LDHB | lactate dehydrogenase B [Source:HGNC Symbol;Acc:HGNC:6541] |
| ENSG00000113851 | HGLibA_11078 | 229 | 1.130185612 | CRBN | cereblon [Source:HGNC Symbol;Acc:HGNC:30185] |
| ENSG00000115207 | HGLibA_20584 | 229 | 1.130185612 | GTF3C2 | general transcription factor IIIC subunit 2 [Source:HGNC Symbol;Acc:HGNC:4665] |
| ENSG00000117148 | HGLibA_00633 | 229 | 1.130185612 | ACTL8 | actin like 8 [Source:HGNC Symbol;Acc:HGNC:24018] |
| ENSG00000118816 | HGLibA_08101 | 229 | 1.130185612 | CCNI | cyclin I [Source:HGNC Symbol;Acc:HGNC:1595] |
| ENSG00000119943 | HGLibA_39707 | 229 | 1.130185612 | PYROXD2 | pyridine nucleotide-disulphide oxidoreductase domain 2 [Source:HGNC Symbol;Acc:HGNC:23517] |
| ENSG00000125888 | HGLibA_04144 | 229 | 1.130185612 | BANF2 | barrier to autointegration factor 2 [Source:HGNC Symbol;Acc:HGNC:16172] |
| ENSG00000126091 | HGLibA_47242 | 229 | 1.130185612 | ST3GAL3 | 3-sialyltransferase 3 [Source:HGNC Symbol;Acc:HGNC:10866]" |
| ENSG00000130270 | HGLibA_03787 | 229 | 1.130185612 | ATP8B3 | ATPase phospholipid transporting 8B3 [Source:HGNC Symbol;Acc:HGNC:13535] |
| ENSG00000130830 | HGLibA_29746 | 229 | 1.130185612 | MPP1 | membrane palmitoylated protein 1 [Source:HGNC Symbol;Acc:HGNC:7219] |
| ENSG00000132535 | HGLibA_13332 | 229 | 1.130185612 | DLG4 | discs large MAGUK scaffold protein 4 [Source:HGNC Symbol;Acc:HGNC:2903] |
| ENSG00000132825 | HGLibA_38108 | 229 | 1.130185612 | PPP1R3D | protein phosphatase 1 regulatory subunit 3D [Source:HGNC Symbol;Acc:HGNC:9294] |
| ENSG00000140497 | HGLibA_42839 | 229 | 1.130185612 | SCAMP2 | secretory carrier membrane protein 2 [Source:HGNC Symbol;Acc:HGNC:10564] |
| ENSG00000143127 | HGLibA_23718 | 229 | 1.130185612 | ITGA10 | integrin subunit alpha 10 [Source:HGNC Symbol;Acc:HGNC:6135] |
| ENSG00000148730 | HGLibA_14831 | 229 | 1.130185612 | EIF4EBP2 | eukaryotic translation initiation factor 4E binding protein 2 [Source:HGNC Symbol;Acc:HGNC:3289] |
| ENSG00000151726 | HGLibA_00553 | 229 | 1.130185612 | ACSL1 | acyl-CoA synthetase long chain family member 1 [Source:HGNC Symbol;Acc:HGNC:3569] |
| ENSG00000153207 | HGLibA_01282 | 229 | 1.130185612 | AHCTF1 | AT-hook containing transcription factor 1 [Source:HGNC Symbol;Acc:HGNC:24618] |
| ENSG00000155367 | HGLibA_37985 | 229 | 1.130185612 | PPM1J | Mg2+/Mn2+ dependent 1J [Source:HGNC Symbol;Acc:HGNC:20785]" |
| ENSG00000156194 | HGLibA_37871 | 229 | 1.130185612 | PPEF2 | protein phosphatase with EF-hand domain 2 [Source:HGNC Symbol;Acc:HGNC:9244] |
| ENSG00000156234 | HGLibA_11855 | 229 | 1.130185612 | CXCL13 | C-X-C motif chemokine ligand 13 [Source:HGNC Symbol;Acc:HGNC:10639] |
| ENSG00000158292 | HGLibA_19939 | 229 | 1.130185612 | GPR153 | G protein-coupled receptor 153 [Source:HGNC Symbol;Acc:HGNC:23618] |
| ENSG00000162664 | HGLibA_55806 | 229 | 1.130185612 | ZNF326 | zinc finger protein 326 [Source:HGNC Symbol;Acc:HGNC:14104] |
| ENSG00000162704 | HGLibA_03093 | 229 | 1.130185612 | ARPC5 | actin related protein 2/3 complex subunit 5 [Source:HGNC Symbol;Acc:HGNC:708] |
| ENSG00000163827 | HGLibA_27279 | 229 | 1.130185612 | LRRC2 | leucine rich repeat containing 2 [Source:HGNC Symbol;Acc:HGNC:14676] |
| ENSG00000164440 | HGLibA_52393 | 229 | 1.130185612 | TXLNB | taxilin beta [Source:HGNC Symbol;Acc:HGNC:21617] |
| ENSG00000166428 | HGLibA_37043 | 229 | 1.130185612 | PLD4 | phospholipase D family member 4 [Source:HGNC Symbol;Acc:HGNC:23792] |
| ENSG00000166529 | HGLibA_57001 | 229 | 1.130185612 | ZSCAN21 | zinc finger and SCAN domain containing 21 [Source:HGNC Symbol;Acc:HGNC:13104] |
| ENSG00000168010 | HGLibA_03477 | 229 | 1.130185612 | ATG16L2 | autophagy related 16 like 2 [Source:HGNC Symbol;Acc:HGNC:25464] |
| ENSG00000169562 | HGLibA_19157 | 229 | 1.130185612 | GJB1 | gap junction protein beta 1 [Source:HGNC Symbol;Acc:HGNC:4283] |
| ENSG00000170153 | HGLibA_41543 | 229 | 1.130185612 | RNF150 | ring finger protein 150 [Source:HGNC Symbol;Acc:HGNC:23138] |
| ENSG00000170837 | HGLibA_20010 | 229 | 1.130185612 | GPR27 | G protein-coupled receptor 27 [Source:HGNC Symbol;Acc:HGNC:4482] |
| ENSG00000171462 | HGLibA_13357 | 229 | 1.130185612 | DLK2 | delta like non-canonical Notch ligand 2 [Source:HGNC Symbol;Acc:HGNC:21113] |
| ENSG00000172732 | HGLibA_30574 | 229 | 1.130185612 | MUS81 | MUS81 structure-specific endonuclease subunit [Source:HGNC Symbol;Acc:HGNC:29814] |
| ENSG00000184454 | HGLibA_31331 | 229 | 1.130185612 | NCMAP | non-compact myelin associated protein [Source:HGNC Symbol;Acc:HGNC:29332] |
| ENSG00000185129 | HGLibA_39586 | 229 | 1.130185612 | PURA | purine rich element binding protein A [Source:HGNC Symbol;Acc:HGNC:9701] |
| ENSG00000186393 | HGLibA_25462 | 229 | 1.130185612 | KRT26 | keratin 26 [Source:HGNC Symbol;Acc:HGNC:30840] |
| ENSG00000188037 | HGLibA_09820 | 229 | 1.130185612 | CLCN1 | chloride voltage-gated channel 1 [Source:HGNC Symbol;Acc:HGNC:2019] |
| ENSG00000196296 | HGLibA_03606 | 229 | 1.130185612 | ATP2A1 | ATPase sarcoplasmic/endoplasmic reticulum Ca2+ transporting 1 [Source:HGNC Symbol;Acc:HGNC:811] |
| ENSG00000213714 | HGLibA_16440 | 229 | 1.130185612 | FAM209B | family with sequence similarity 209 member B [Source:HGNC Symbol;Acc:HGNC:16101] |
| ENSG00000214860 | HGLibA_15704 | 229 | 1.130185612 | EVPLL | envoplakin like [Source:HGNC Symbol;Acc:HGNC:35236] |
| ENSG00000238269 | HGLibA_35011 | 229 | 1.130185612 | PAGE2B | PAGE family member 2B [Source:HGNC Symbol;Acc:HGNC:31805] |
| hsa-mir-514b | HGLibA_61599 | 229 | 1.130185612 | hsa-mir-514b | - |
| hsa-mir-941-4 | HGLibA_64349 | 229 | 1.130185612 | hsa-mir-941-4 | - |
| MFSD4 | HGLibA_29103 | 229 | 1.130185612 | MFSD4 | - |
| MYEOV2 | HGLibA_30694 | 229 | 1.130185612 | MYEOV2 | - |
| ENSG00000002746 | HGLibA_21103 | 228 | 1.120234574 | HECW1 | C2 and WW domain containing E3 ubiquitin protein ligase 1 [Source:HGNC Symbol;Acc:HGNC:22195]" |
| ENSG00000016602 | HGLibA_09811 | 228 | 1.120234574 | CLCA4 | chloride channel accessory 4 [Source:HGNC Symbol;Acc:HGNC:2018] |
| ENSG00000054282 | HGLibA_43118 | 228 | 1.120234574 | SDCCAG8 | serologically defined colon cancer antigen 8 [Source:HGNC Symbol;Acc:HGNC:10671] |
| ENSG00000064270 | HGLibA_03628 | 228 | 1.120234574 | ATP2C2 | ATPase secretory pathway Ca2+ transporting 2 [Source:HGNC Symbol;Acc:HGNC:29103] |
| ENSG00000069482 | HGLibA_18495 | 228 | 1.120234574 | GAL | galanin and GMAP prepropeptide [Source:HGNC Symbol;Acc:HGNC:4114] |
| ENSG00000075413 | HGLibA_28369 | 228 | 1.120234574 | MARK3 | microtubule affinity regulating kinase 3 [Source:HGNC Symbol;Acc:HGNC:6897] |
| ENSG00000100376 | HGLibA_16049 | 228 | 1.120234574 | FAM118A | family with sequence similarity 118 member A [Source:HGNC Symbol;Acc:HGNC:1313] |
| ENSG00000101109 | HGLibA_47510 | 228 | 1.120234574 | STK4 | serine/threonine kinase 4 [Source:HGNC Symbol;Acc:HGNC:11408] |
| ENSG00000102128 | HGLibA_39933 | 228 | 1.120234574 | RAB40AL | RAB40A like [Source:HGNC Symbol;Acc:HGNC:25410] |
| ENSG00000104936 | HGLibA_13415 | 228 | 1.120234574 | DMPK | DM1 protein kinase [Source:HGNC Symbol;Acc:HGNC:2933] |
| ENSG00000110375 | HGLibA_53073 | 228 | 1.120234574 | UPK2 | uroplakin 2 [Source:HGNC Symbol;Acc:HGNC:12579] |
| ENSG00000111652 | HGLibA_10729 | 228 | 1.120234574 | COPS7A | COP9 signalosome subunit 7A [Source:HGNC Symbol;Acc:HGNC:16758] |
| ENSG00000115593 | HGLibA_45852 | 228 | 1.120234574 | SMYD1 | SET and MYND domain containing 1 [Source:HGNC Symbol;Acc:HGNC:20986] |
| ENSG00000120332 | HGLibA_50862 | 228 | 1.120234574 | TNN | tenascin N [Source:HGNC Symbol;Acc:HGNC:22942] |
| ENSG00000121064 | HGLibA_43039 | 228 | 1.120234574 | SCPEP1 | serine carboxypeptidase 1 [Source:HGNC Symbol;Acc:HGNC:29507] |
| ENSG00000122852 | HGLibA_43766 | 228 | 1.120234574 | SFTPA1 | surfactant protein A1 [Source:HGNC Symbol;Acc:HGNC:10798] |
| ENSG00000123388 | HGLibA_21959 | 228 | 1.120234574 | HOXC11 | homeobox C11 [Source:HGNC Symbol;Acc:HGNC:5123] |
| ENSG00000127249 | HGLibA_03574 | 228 | 1.120234574 | ATP13A4 | ATPase 13A4 [Source:HGNC Symbol;Acc:HGNC:25422] |
| ENSG00000128052 | HGLibA_24542 | 228 | 1.120234574 | KDR | kinase insert domain receptor [Source:HGNC Symbol;Acc:HGNC:6307] |
| ENSG00000130385 | HGLibA_04596 | 228 | 1.120234574 | BMP15 | bone morphogenetic protein 15 [Source:HGNC Symbol;Acc:HGNC:1068] |
| ENSG00000131089 | HGLibA_02878 | 228 | 1.120234574 | ARHGEF9 | Cdc42 guanine nucleotide exchange factor 9 [Source:HGNC Symbol;Acc:HGNC:14561] |
| ENSG00000132000 | HGLibA_37455 | 228 | 1.120234574 | PODNL1 | podocan like 1 [Source:HGNC Symbol;Acc:HGNC:26275] |
| ENSG00000132446 | HGLibA_18122 | 228 | 1.120234574 | FTHL17 | ferritin heavy chain like 17 [Source:HGNC Symbol;Acc:HGNC:3987] |
| ENSG00000135916 | HGLibA_23834 | 228 | 1.120234574 | ITM2C | integral membrane protein 2C [Source:HGNC Symbol;Acc:HGNC:6175] |
| ENSG00000139641 | HGLibA_15605 | 228 | 1.120234574 | ESYT1 | extended synaptotagmin 1 [Source:HGNC Symbol;Acc:HGNC:29534] |
| ENSG00000141161 | HGLibA_53007 | 228 | 1.120234574 | UNC45B | unc-45 myosin chaperone B [Source:HGNC Symbol;Acc:HGNC:14304] |
| ENSG00000141646 | HGLibA_45579 | 228 | 1.120234574 | SMAD4 | SMAD family member 4 [Source:HGNC Symbol;Acc:HGNC:6770] |
| ENSG00000141665 | HGLibA_17031 | 228 | 1.120234574 | FBXO15 | F-box protein 15 [Source:HGNC Symbol;Acc:HGNC:13617] |
| ENSG00000143036 | HGLibA_45164 | 228 | 1.120234574 | SLC44A3 | solute carrier family 44 member 3 [Source:HGNC Symbol;Acc:HGNC:28689] |
| ENSG00000143569 | HGLibA_52554 | 228 | 1.120234574 | UBAP2L | ubiquitin associated protein 2 like [Source:HGNC Symbol;Acc:HGNC:29877] |
| ENSG00000145283 | HGLibA_44345 | 228 | 1.120234574 | SLC10A6 | solute carrier family 10 member 6 [Source:HGNC Symbol;Acc:HGNC:30603] |
| ENSG00000147852 | HGLibA_53682 | 228 | 1.120234574 | VLDLR | very low density lipoprotein receptor [Source:HGNC Symbol;Acc:HGNC:12698] |
| ENSG00000150551 | HGLibA_27714 | 228 | 1.120234574 | LYPD1 | LY6/PLAUR domain containing 1 [Source:HGNC Symbol;Acc:HGNC:28431] |
| ENSG00000152137 | HGLibA_22320 | 228 | 1.120234574 | HSPB8 | heat shock protein family B (small) member 8 [Source:HGNC Symbol;Acc:HGNC:30171] |
| ENSG00000156535 | HGLibA_08230 | 228 | 1.120234574 | CD109 | CD109 molecule [Source:HGNC Symbol;Acc:HGNC:21685] |
| ENSG00000157005 | HGLibA_47170 | 228 | 1.120234574 | SST | somatostatin [Source:HGNC Symbol;Acc:HGNC:11329] |
| ENSG00000157703 | HGLibA_47894 | 228 | 1.120234574 | SVOPL | SVOP like [Source:HGNC Symbol;Acc:HGNC:27034] |
| ENSG00000160271 | HGLibA_40164 | 228 | 1.120234574 | RALGDS | ral guanine nucleotide dissociation stimulator [Source:HGNC Symbol;Acc:HGNC:9842] |
| ENSG00000165948 | HGLibA_22598 | 228 | 1.120234574 | IFI27L1 | interferon alpha inducible protein 27 like 1 [Source:HGNC Symbol;Acc:HGNC:19754] |
| ENSG00000166946 | HGLibA_08082 | 228 | 1.120234574 | CCNDBP1 | cyclin D1 binding protein 1 [Source:HGNC Symbol;Acc:HGNC:1587] |
| ENSG00000167377 | HGLibA_55634 | 228 | 1.120234574 | ZNF23 | zinc finger protein 23 [Source:HGNC Symbol;Acc:HGNC:13023] |
| ENSG00000168124 | HGLibA_33665 | 228 | 1.120234574 | OR1F1 | olfactory receptor family 1 subfamily F member 1 [Source:HGNC Symbol;Acc:HGNC:8194] |

| | | | | | |
|---|---|---|---|---|---|
| ENSG00000169813 | HGLibA_21818 | 228 | 1.120234574 | HNRNPF | heterogeneous nuclear ribonucleoprotein F [Source:HGNC Symbol;Acc:HGNC:5039] |
| ENSG00000170465 | HGLibA_25519 | 228 | 1.120234574 | KRT6C | keratin 6C [Source:HGNC Symbol;Acc:HGNC:20406] |
| ENSG00000171794 | HGLibA_53427 | 228 | 1.120234574 | UTF1 | undifferentiated embryonic cell transcription factor 1 [Source:HGNC Symbol;Acc:HGNC:12634] |
| ENSG00000172164 | HGLibA_46000 | 228 | 1.120234574 | SNTB1 | syntrophin beta 1 [Source:HGNC Symbol;Acc:HGNC:11168] |
| ENSG00000172273 | HGLibA_21321 | 228 | 1.120234574 | HINFP | histone H4 transcription factor [Source:HGNC Symbol;Acc:HGNC:17850] |
| ENSG00000174516 | HGLibA_36039 | 228 | 1.120234574 | PELI3 | pellino E3 ubiquitin protein ligase family member 3 [Source:HGNC Symbol;Acc:HGNC:30010] |
| ENSG00000175324 | HGLibA_27517 | 228 | 1.120234574 | LSM1 | mRNA degradation associated [Source:HGNC Symbol;Acc:HGNC:20472]" |
| ENSG00000175352 | HGLibA_32697 | 228 | 1.120234574 | NRIP3 | nuclear receptor interacting protein 3 [Source:HGNC Symbol;Acc:HGNC:1167] |
| ENSG00000177684 | HGLibA_12855 | 228 | 1.120234574 | DEFB114 | defensin beta 114 [Source:HGNC Symbol;Acc:HGNC:18095] |
| ENSG00000180964 | HGLibA_48725 | 228 | 1.120234574 | TCEAL8 | transcription elongation factor A like 8 [Source:HGNC Symbol;Acc:HGNC:28683] |
| ENSG00000182601 | HGLibA_22132 | 228 | 1.120234574 | HS3ST4 | heparan sulfate-glucosamine 3-sulfotransferase 4 [Source:HGNC Symbol;Acc:HGNC:5200] |
| ENSG00000183150 | HGLibA_19993 | 228 | 1.120234574 | GPR19 | G protein-coupled receptor 19 [Source:HGNC Symbol;Acc:HGNC:4473] |
| ENSG00000185271 | HGLibA_25189 | 228 | 1.120234574 | KLHL33 | kelch like family member 33 [Source:HGNC Symbol;Acc:HGNC:31952] |
| ENSG00000185385 | HGLibA_34461 | 228 | 1.120234574 | OR7A17 | olfactory receptor family 7 subfamily A member 17 [Source:HGNC Symbol;Acc:HGNC:8363] |
| ENSG00000197375 | HGLibA_44590 | 228 | 1.120234574 | SLC22A5 | solute carrier family 22 member 5 [Source:HGNC Symbol;Acc:HGNC:10969] |
| ENSG00000197405 | HGLibA_06353 | 228 | 1.120234574 | C5AR1 | complement C5a receptor 1 [Source:HGNC Symbol;Acc:HGNC:1338] |
| ENSG00000198398 | HGLibA_50199 | 228 | 1.120234574 | TMEM207 | transmembrane protein 207 [Source:HGNC Symbol;Acc:HGNC:33705] |
| ENSG00000203778 | HGLibA_16544 | 228 | 1.120234574 | FAM229B | family with sequence similarity 229 member B [Source:HGNC Symbol;Acc:HGNC:33858] |
| ENSG00000204519 | HGLibA_56214 | 228 | 1.120234574 | ZNF551 | zinc finger protein 551 [Source:HGNC Symbol;Acc:HGNC:25108] |
| ENSG00000222011 | HGLibA_16332 | 228 | 1.120234574 | FAM185A | family with sequence similarity 185 member A [Source:HGNC Symbol;Acc:HGNC:22412] |
| FAM129C | HGLibA_16103 | 228 | 1.120234574 | FAM129C | - |
| hsa-mir-125b-1 | HGLibA_57448 | 228 | 1.120234574 | hsa-mir-125b-1 | - |
| hsa-mir-378i | HGLibA_59460 | 228 | 1.120234574 | hsa-mir-378i | - |
| hsa-mir-4733 | HGLibA_61030 | 228 | 1.120234574 | hsa-mir-4733 | - |
| hsa-mir-4755 | HGLibA_61118 | 228 | 1.120234574 | hsa-mir-4755 | - |
| hsa-mir-671 | HGLibA_63013 | 228 | 1.120234574 | hsa-mir-671 | - |
| C1orf106 | HGLibA_05738 | 227 | 1.110283537 | C1orf106 | - |
| CCDC147 | HGLibA_07630 | 227 | 1.110283537 | CCDC147 | - |
| ENSG00000024048 | HGLibA_52749 | 227 | 1.110283537 | UBR2 | ubiquitin protein ligase E3 component n-recognin 2 [Source:HGNC Symbol;Acc:HGNC:21289] |
| ENSG00000047315 | HGLibA_37567 | 227 | 1.110283537 | POLR2B | RNA polymerase II subunit B [Source:HGNC Symbol;Acc:HGNC:9188] |
| ENSG00000089505 | HGLibA_10208 | 227 | 1.110283537 | CMTM1 | CKLF like MARVEL transmembrane domain containing 1 [Source:HGNC Symbol;Acc:HGNC:19172] |
| ENSG00000090615 | HGLibA_19599 | 227 | 1.110283537 | GOLGA3 | golgin A3 [Source:HGNC Symbol;Acc:HGNC:4426] |
| ENSG00000099250 | HGLibA_32714 | 227 | 1.110283537 | NRP1 | neuropilin 1 [Source:HGNC Symbol;Acc:HGNC:8004] |
| ENSG00000100445 | HGLibA_43163 | 227 | 1.110283537 | SDR39U1 | short chain dehydrogenase/reductase family 39U member 1 [Source:HGNC Symbol;Acc:HGNC:20275] |
| ENSG00000101958 | HGLibA_19300 | 227 | 1.110283537 | GLRA2 | glycine receptor alpha 2 [Source:HGNC Symbol;Acc:HGNC:4327] |
| ENSG00000105278 | HGLibA_55261 | 227 | 1.110283537 | ZFR2 | zinc finger RNA binding protein 2 [Source:HGNC Symbol;Acc:HGNC:29189] |
| ENSG00000107242 | HGLibA_36717 | 227 | 1.110283537 | PIP5K1B | phosphatidylinositol-4-phosphate 5-kinase type 1 beta [Source:HGNC Symbol;Acc:HGNC:8995] |
| ENSG00000110245 | HGLibA_02510 | 227 | 1.110283537 | APOC3 | apolipoprotein C3 [Source:HGNC Symbol;Acc:HGNC:610] |
| ENSG00000112031 | HGLibA_30470 | 227 | 1.110283537 | MTRF1L | mitochondrial translational release factor 1 like [Source:HGNC Symbol;Acc:HGNC:21051] |
| ENSG00000126549 | HGLibA_47403 | 227 | 1.110283537 | STATH | statherin [Source:HGNC Symbol;Acc:HGNC:11369] |
| ENSG00000129484 | HGLibA_35231 | 227 | 1.110283537 | PARP2 | poly(ADP-ribose) polymerase 2 [Source:HGNC Symbol;Acc:HGNC:272] |
| ENSG00000130294 | HGLibA_24884 | 227 | 1.110283537 | KIF1A | kinesin family member 1A [Source:HGNC Symbol;Acc:HGNC:888] |
| ENSG00000131844 | HGLibA_28569 | 227 | 1.110283537 | MCCC2 | methylcrotonoyl-CoA carboxylase 2 [Source:HGNC Symbol;Acc:HGNC:6937] |
| ENSG00000133433 | HGLibA_20503 | 227 | 1.110283537 | GSTT2B | glutathione S-transferase theta 2B (gene/pseudogene) [Source:HGNC Symbol;Acc:HGNC:33437] |
| ENSG00000135452 | HGLibA_51927 | 227 | 1.110283537 | TSPAN31 | tetraspanin 31 [Source:HGNC Symbol;Acc:HGNC:10539] |
| ENSG00000136155 | HGLibA_42889 | 227 | 1.110283537 | SCEL | sciellin [Source:HGNC Symbol;Acc:HGNC:10573] |
| ENSG00000138175 | HGLibA_02962 | 227 | 1.110283537 | ARL3 | ADP ribosylation factor like GTPase 3 [Source:HGNC Symbol;Acc:HGNC:694] |
| ENSG00000141431 | HGLibA_03395 | 227 | 1.110283537 | ASXL3 | ASXL transcriptional regulator 3 [Source:HGNC Symbol;Acc:HGNC:29357] |
| ENSG00000143756 | HGLibA_17060 | 227 | 1.110283537 | FBXO28 | F-box protein 28 [Source:HGNC Symbol;Acc:HGNC:29046] |
| ENSG00000144119 | HGLibA_05926 | 227 | 1.110283537 | C1QL2 | complement C1q like 2 [Source:HGNC Symbol;Acc:HGNC:24181] |
| ENSG00000144410 | HGLibA_10978 | 227 | 1.110283537 | CPO | carboxypeptidase O [Source:HGNC Symbol;Acc:HGNC:21011] |
| ENSG00000144746 | HGLibA_02989 | 227 | 1.110283537 | ARL6IP5 | ADP ribosylation factor like GTPase 6 interacting protein 5 [Source:HGNC Symbol;Acc:HGNC:16937] |
| ENSG00000152104 | HGLibA_39441 | 227 | 1.110283537 | PTPN14 | non-receptor type 14 [Source:HGNC Symbol;Acc:HGNC:9647]" |
| ENSG00000153132 | HGLibA_10014 | 227 | 1.110283537 | CLGN | calmegin [Source:HGNC Symbol;Acc:HGNC:2060] |
| ENSG00000155100 | HGLibA_34774 | 227 | 1.110283537 | OTUD6B | OTU domain containing 6B [Source:HGNC Symbol;Acc:HGNC:24281] |
| ENSG00000155897 | HGLibA_00929 | 227 | 1.110283537 | ADCY8 | adenylate cyclase 8 [Source:HGNC Symbol;Acc:HGNC:239] |
| ENSG00000158874 | HGLibA_02455 | 227 | 1.110283537 | APOA2 | apolipoprotein A2 [Source:HGNC Symbol;Acc:HGNC:601] |
| ENSG00000159216 | HGLibA_42454 | 227 | 1.110283537 | RUNX1 | runt related transcription factor 1 [Source:HGNC Symbol;Acc:HGNC:10471] |
| ENSG00000162747 | HGLibA_17197 | 227 | 1.110283537 | FCGR3B | Fc fragment of IgG receptor IIIb [Source:HGNC Symbol;Acc:HGNC:3620] |
| ENSG00000163479 | HGLibA_47155 | 227 | 1.110283537 | SSR2 | signal sequence receptor subunit 2 [Source:HGNC Symbol;Acc:HGNC:11324] |
| ENSG00000163817 | HGLibA_45321 | 227 | 1.110283537 | SLC6A20 | solute carrier family 6 member 20 [Source:HGNC Symbol;Acc:HGNC:30927] |
| ENSG00000164871 | HGLibA_46348 | 227 | 1.110283537 | SPAG11B | sperm associated antigen 11B [Source:HGNC Symbol;Acc:HGNC:14534] |
| ENSG00000165269 | HGLibA_02606 | 227 | 1.110283537 | AQP7 | aquaporin 7 [Source:HGNC Symbol;Acc:HGNC:640] |
| ENSG00000165349 | HGLibA_45363 | 227 | 1.110283537 | SLC7A3 | solute carrier family 7 member 3 [Source:HGNC Symbol;Acc:HGNC:11061] |
| ENSG00000166171 | HGLibA_13813 | 227 | 1.110283537 | DPCD | deleted in primary ciliary dyskinesia homolog (mouse) [Source:HGNC Symbol;Acc:HGNC:24542] |
| ENSG00000166821 | HGLibA_36085 | 227 | 1.110283537 | PEX11A | peroxisomal biogenesis factor 11 alpha [Source:HGNC Symbol;Acc:HGNC:8852] |
| ENSG00000167355 | HGLibA_34087 | 227 | 1.110283537 | OR51B5 | olfactory receptor family 51 subfamily B member 5 [Source:HGNC Symbol;Acc:HGNC:19599] |
| ENSG00000168591 | HGLibA_50686 | 227 | 1.110283537 | TMUB2 | transmembrane and ubiquitin like domain containing 2 [Source:HGNC Symbol;Acc:HGNC:28459] |
| ENSG00000168944 | HGLibA_09101 | 227 | 1.110283537 | CEP120 | centrosomal protein 120 [Source:HGNC Symbol;Acc:HGNC:26690] |
| ENSG00000169064 | HGLibA_54784 | 227 | 1.110283537 | ZBBX | zinc finger B-box domain containing [Source:HGNC Symbol;Acc:HGNC:26245] |
| ENSG00000171421 | HGLibA_29964 | 227 | 1.110283537 | MRPL36 | mitochondrial ribosomal protein L36 [Source:HGNC Symbol;Acc:HGNC:14490] |
| ENSG00000172794 | HGLibA_39895 | 227 | 1.110283537 | RAB37 | member RAS oncogene family [Source:HGNC Symbol;Acc:HGNC:30268]" |
| ENSG00000176136 | HGLibA_28553 | 227 | 1.110283537 | MC5R | melanocortin 5 receptor [Source:HGNC Symbol;Acc:HGNC:6933] |
| ENSG00000177025 | HGLibA_05633 | 227 | 1.110283537 | C19orf18 | chromosome 19 open reading frame 18 [Source:HGNC Symbol;Acc:HGNC:28642] |
| ENSG00000177879 | HGLibA_02335 | 227 | 1.110283537 | AP3S1 | adaptor related protein complex 3 subunit sigma 1 [Source:HGNC Symbol;Acc:HGNC:2013] |
| ENSG00000180423 | HGLibA_20852 | 227 | 1.110283537 | HARBI1 | harbinger transposase derived 1 [Source:HGNC Symbol;Acc:HGNC:26522] |
| ENSG00000184388 | HGLibA_34931 | 227 | 1.110283537 | PABPC1L2B | poly(A) binding protein cytoplasmic 1 like 2B [Source:HGNC Symbol;Acc:HGNC:31852] |
| ENSG00000184544 | HGLibA_13152 | 227 | 1.110283537 | DHRS7C | dehydrogenase/reductase 7C [Source:HGNC Symbol;Acc:HGNC:32423] |
| ENSG00000185515 | HGLibA_04768 | 227 | 1.110283537 | BRCC3 | BRCA1/BRCA2-containing complex subunit 3 [Source:HGNC Symbol;Acc:HGNC:24185] |
| ENSG00000188060 | HGLibA_39946 | 227 | 1.110283537 | RAB42 | member RAS oncogene family [Source:HGNC Symbol;Acc:HGNC:28702]" |
| ENSG00000198130 | HGLibA_21284 | 227 | 1.110283537 | HIBCH | 3-hydroxyisobutyryl-CoA hydrolase [Source:HGNC Symbol;Acc:HGNC:4908] |
| ENSG00000204920 | HGLibA_55503 | 227 | 1.110283537 | ZNF155 | zinc finger protein 155 [Source:HGNC Symbol;Acc:HGNC:12940] |
| ENSG00000226763 | HGLibA_47055 | 227 | 1.110283537 | SRRM5 | serine/arginine repetitive matrix 5 [Source:HGNC Symbol;Acc:HGNC:37248] |
| ENSG00000240021 | HGLibA_49101 | 227 | 1.110283537 | TEX35 | testis expressed 35 [Source:HGNC Symbol;Acc:HGNC:25366] |
| FAIM3 | HGLibA_15971 | 227 | 1.110283537 | FAIM3 | - |
| FAM96A | HGLibA_16803 | 227 | 1.110283537 | FAM96A | - |
| HIST1H2AL | HGLibA_21405 | 227 | 1.110283537 | HIST1H2AL | - |
| hsa-mir-4713 | HGLibA_60951 | 227 | 1.110283537 | hsa-mir-4713 | - |
| hsa-mir-593 | HGLibA_62468 | 227 | 1.110283537 | hsa-mir-593 | - |
| hsa-mir-6076 | HGLibA_62557 | 227 | 1.110283537 | hsa-mir-6076 | - |
| hsa-mir-619 | HGLibA_62714 | 227 | 1.110283537 | hsa-mir-619 | - |
| hsa-mir-6772 | HGLibA_63262 | 227 | 1.110283537 | hsa-mir-6772 | - |
| hsa-mir-876 | HGLibA_64213 | 227 | 1.110283537 | hsa-mir-876 | - |
| LYRM5 | HGLibA_27755 | 227 | 1.110283537 | LYRM5 | - |
| NADKD1 | HGLibA_31051 | 227 | 1.110283537 | NADKD1 | - |
| C10orf10 | HGLibA_05018 | 226 | 1.100332499 | C10orf10 | - |
| C12orf65 | HGLibA_05278 | 226 | 1.100332499 | C12orf65 | - |
| ENSG00000026950 | HGLibA_04967 | 226 | 1.100332499 | BTN3A1 | butyrophilin subfamily 3 member A1 [Source:HGNC Symbol;Acc:HGNC:1138] |
| ENSG00000048342 | HGLibA_07482 | 226 | 1.100332499 | CC2D2A | coiled-coil and C2 domain containing 2A [Source:HGNC Symbol;Acc:HGNC:29253] |
| ENSG00000067208 | HGLibA_15693 | 226 | 1.100332499 | EVI5 | ecotropic viral integration site 5 [Source:HGNC Symbol;Acc:HGNC:3501] |
| ENSG00000067829 | HGLibA_22552 | 226 | 1.100332499 | IDH3G | isocitrate dehydrogenase 3 (NAD(+)) gamma [Source:HGNC Symbol;Acc:HGNC:5386] |
| ENSG00000073910 | HGLibA_18054 | 226 | 1.100332499 | FRY | FRY microtubule binding protein [Source:HGNC Symbol;Acc:HGNC:20367] |
| ENSG00000076201 | HGLibA_39464 | 226 | 1.100332499 | PTPN23 | non-receptor type 23 [Source:HGNC Symbol;Acc:HGNC:14406]" |
| ENSG00000076555 | HGLibA_00351 | 226 | 1.100332499 | ACACB | acetyl-CoA carboxylase beta [Source:HGNC Symbol;Acc:HGNC:85] |
| ENSG00000085999 | HGLibA_40102 | 226 | 1.100332499 | RAD54L | RAD54 like [Source:HGNC Symbol;Acc:HGNC:9826] |
| ENSG00000088836 | HGLibA_45208 | 226 | 1.100332499 | SLC4A11 | solute carrier family 4 member 11 [Source:HGNC Symbol;Acc:HGNC:16438] |
| ENSG00000105650 | HGLibA_35793 | 226 | 1.100332499 | PDE4C | phosphodiesterase 4C [Source:HGNC Symbol;Acc:HGNC:8782] |
| ENSG00000106263 | HGLibA_14765 | 226 | 1.100332499 | EIF3B | eukaryotic translation initiation factor 3 subunit B [Source:HGNC Symbol;Acc:HGNC:3280] |
| ENSG00000113905 | HGLibA_22090 | 226 | 1.100332499 | HRG | histidine rich glycoprotein [Source:HGNC Symbol;Acc:HGNC:5181] |
| ENSG00000116132 | HGLibA_38893 | 226 | 1.100332499 | PRRX1 | paired related homeobox 1 [Source:HGNC Symbol;Acc:HGNC:9142] |
| ENSG00000119231 | HGLibA_43394 | 226 | 1.100332499 | SENP5 | SUMO specific peptidase 5 [Source:HGNC Symbol;Acc:HGNC:28407] |
| ENSG00000121281 | HGLibA_00925 | 226 | 1.100332499 | ADCY7 | adenylate cyclase 7 [Source:HGNC Symbol;Acc:HGNC:238] |
| ENSG00000127824 | HGLibA_52261 | 226 | 1.100332499 | TUBA4A | tubulin alpha 4a [Source:HGNC Symbol;Acc:HGNC:12407] |
| ENSG00000132024 | HGLibA_07475 | 226 | 1.100332499 | CC2D1A | coiled-coil and C2 domain containing 1A [Source:HGNC Symbol;Acc:HGNC:30237] |
| ENSG00000132359 | HGLibA_40229 | 226 | 1.100332499 | RAP1GAP2 | RAP1 GTPase activating protein 2 [Source:HGNC Symbol;Acc:HGNC:29176] |
| ENSG00000134873 | HGLibA_09851 | 226 | 1.100332499 | CLDN10 | claudin 10 [Source:HGNC Symbol;Acc:HGNC:2033] |
| ENSG00000136193 | HGLibA_43049 | 226 | 1.100332499 | SCRN1 | secernin 1 [Source:HGNC Symbol;Acc:HGNC:22192] |
| ENSG00000137055 | HGLibA_36930 | 226 | 1.100332499 | PLAA | phospholipase A2 activating protein [Source:HGNC Symbol;Acc:HGNC:9043] |
| ENSG00000137218 | HGLibA_18050 | 226 | 1.100332499 | FRS3 | fibroblast growth factor receptor substrate 3 [Source:HGNC Symbol;Acc:HGNC:16970] |
| ENSG00000137474 | HGLibA_30861 | 226 | 1.100332499 | MYO7A | myosin VIIA [Source:HGNC Symbol;Acc:HGNC:7606] |
| ENSG00000137713 | HGLibA_38142 | 226 | 1.100332499 | PPP2R1B | protein phosphatase 2 scaffold subunit Abeta [Source:HGNC Symbol;Acc:HGNC:9303] |
| ENSG00000139517 | HGLibA_26747 | 226 | 1.100332499 | LNX2 | ligand of numb-protein X 2 [Source:HGNC Symbol;Acc:HGNC:20421] |
| ENSG00000142494 | HGLibA_45193 | 226 | 1.100332499 | SLC47A1 | solute carrier family 47 member 1 [Source:HGNC Symbol;Acc:HGNC:25588] |
| ENSG00000143195 | HGLibA_23210 | 226 | 1.100332499 | ILDR2 | immunoglobulin like domain containing receptor 2 [Source:HGNC Symbol;Acc:HGNC:18131] |
| ENSG00000156564 | HGLibA_27155 | 226 | 1.100332499 | LRFN2 | leucine rich repeat and fibronectin type III domain containing 2 [Source:HGNC Symbol;Acc:HGNC:21226] |
| ENSG00000156966 | HGLibA_03999 | 226 | 1.100332499 | B3GNT7 | 3-N-acetylglucosaminyltransferase 7 [Source:HGNC Symbol;Acc:HGNC:18811]" |
| ENSG00000158022 | HGLibA_51574 | 226 | 1.100332499 | TRIM63 | tripartite motif containing 63 [Source:HGNC Symbol;Acc:HGNC:16007] |
| ENSG00000160310 | HGLibA_38622 | 226 | 1.100332499 | PRMT2 | protein arginine methyltransferase 2 [Source:HGNC Symbol;Acc:HGNC:5186] |
| ENSG00000161640 | HGLibA_44128 | 226 | 1.100332499 | SIGLEC11 | sialic acid binding Ig like lectin 11 [Source:HGNC Symbol;Acc:HGNC:15622] |
| ENSG00000161996 | HGLibA_54214 | 226 | 1.100332499 | WDR90 | WD repeat domain 90 [Source:HGNC Symbol;Acc:HGNC:26960] |
| ENSG00000163053 | HGLibA_44432 | 226 | 1.100332499 | SLC16A14 | solute carrier family 16 member 14 [Source:HGNC Symbol;Acc:HGNC:26417] |
| ENSG00000164111 | HGLibA_02243 | 226 | 1.100332499 | ANXA5 | annexin A5 [Source:HGNC Symbol;Acc:HGNC:543] |
| ENSG00000164366 | HGLibA_07582 | 226 | 1.100332499 | CCDC127 | coiled-coil domain containing 127 [Source:HGNC Symbol;Acc:HGNC:30520] |
| ENSG00000164828 | HGLibA_47788 | 226 | 1.100332499 | SUN1 | Sad1 and UNC84 domain containing 1 [Source:HGNC Symbol;Acc:HGNC:18587] |
| ENSG00000164951 | HGLibA_35925 | 226 | 1.100332499 | PDP1 | pyruvate dehyrogenase phosphatase catalytic subunit 1 [Source:HGNC Symbol;Acc:HGNC:9279] |

| | | | | | |
|---|---|---|---|---|---|
| ENSG00000166016 | HGLibA_00337 | 226 | 1.100332499 | ABTB2 | ankyrin repeat and BTB domain containing 2 [Source:HGNC Symbol;Acc:HGNC:23842] |
| ENSG00000167701 | HGLibA_20165 | 226 | 1.100332499 | GPT | glutamic--pyruvic transaminase [Source:HGNC Symbol;Acc:HGNC:4552] |
| ENSG00000169567 | HGLibA_21323 | 226 | 1.100332499 | HINT1 | histidine triad nucleotide binding protein 1 [Source:HGNC Symbol;Acc:HGNC:4912] |
| ENSG00000170423 | HGLibA_25547 | 226 | 1.100332499 | KRT78 | keratin 78 [Source:HGNC Symbol;Acc:HGNC:28926] |
| ENSG00000172638 | HGLibA_14565 | 226 | 1.100332499 | EFEMP2 | EGF containing fibulin extracellular matrix protein 2 [Source:HGNC Symbol;Acc:HGNC:3219] |
| ENSG00000176024 | HGLibA_56387 | 226 | 1.100332499 | ZNF613 | zinc finger protein 613 [Source:HGNC Symbol;Acc:HGNC:25827] |
| ENSG00000177150 | HGLibA_16454 | 226 | 1.100332499 | FAM210A | family with sequence similarity 210 member A [Source:HGNC Symbol;Acc:HGNC:28346] |
| ENSG00000178814 | HGLibA_33420 | 226 | 1.100332499 | OPLAH | ATP-hydrolysing [Source:HGNC Symbol;Acc:HGNC:8149]" |
| ENSG00000179361 | HGLibA_02893 | 226 | 1.100332499 | ARID3B | AT-rich interaction domain 3B [Source:HGNC Symbol;Acc:HGNC:14350] |
| ENSG00000179886 | HGLibA_49475 | 226 | 1.100332499 | TIGD5 | tigger transposable element derived 5 [Source:HGNC Symbol;Acc:HGNC:18336] |
| ENSG00000180929 | HGLibA_20064 | 226 | 1.100332499 | GPR62 | G protein-coupled receptor 62 [Source:HGNC Symbol;Acc:HGNC:13301] |
| ENSG00000182492 | HGLibA_04463 | 226 | 1.100332499 | BGN | biglycan [Source:HGNC Symbol;Acc:HGNC:1044] |
| ENSG00000183305 | HGLibA_27908 | 226 | 1.100332499 | MAGEA2B | MAGE family member A2B [Source:HGNC Symbol;Acc:HGNC:19340] |
| ENSG00000185585 | HGLibA_33360 | 226 | 1.100332499 | OLFML2A | olfactomedin like 2A [Source:HGNC Symbol;Acc:HGNC:27270] |
| ENSG00000186204 | HGLibA_12184 | 226 | 1.100332499 | CYP4F12 | cytochrome P450 family 4 subfamily F member 12 [Source:HGNC Symbol;Acc:HGNC:18857] |
| ENSG00000187210 | HGLibA_18809 | 226 | 1.100332499 | GCNT1 | core 2 [Source:HGNC Symbol;Acc:HGNC:4203]" |
| ENSG00000189050 | HGLibA_41702 | 226 | 1.100332499 | RNFT1 | transmembrane 1 [Source:HGNC Symbol;Acc:HGNC:30206]" |
| ENSG00000196209 | HGLibA_44208 | 226 | 1.100332499 | SIRPB2 | signal regulatory protein beta 2 [Source:HGNC Symbol;Acc:HGNC:16247] |
| ENSG00000198890 | HGLibA_38633 | 226 | 1.100332499 | PRMT6 | protein arginine methyltransferase 6 [Source:HGNC Symbol;Acc:HGNC:18241] |
| ENSG00000204300 | HGLibA_50245 | 226 | 1.100332499 | TMEM225 | transmembrane protein 225 [Source:HGNC Symbol;Acc:HGNC:32390] |
| ENSG00000204323 | HGLibA_45762 | 226 | 1.100332499 | SMIM5 | small integral membrane protein 5 [Source:HGNC Symbol;Acc:HGNC:40030] |
| ENSG00000204385 | HGLibA_45167 | 226 | 1.100332499 | SLC44A4 | solute carrier family 44 member 4 [Source:HGNC Symbol;Acc:HGNC:13941] |
| ENSG00000205784 | HGLibA_03126 | 226 | 1.100332499 | ARRDC5 | arrestin domain containing 5 [Source:HGNC Symbol;Acc:HGNC:31407] |
| ENSG00000214042 | HGLibA_22690 | 226 | 1.100332499 | IFNA7 | interferon alpha 7 [Source:HGNC Symbol;Acc:HGNC:5428] |
| ENSG00000232237 | HGLibA_03277 | 226 | 1.100332499 | ASCL5 | achaete-scute family bHLH transcription factor 5 [Source:HGNC Symbol;Acc:HGNC:33169] |
| ENSG00000243509 | HGLibA_50788 | 226 | 1.100332499 | TNFRSF6B | TNF receptor superfamily member 6b [Source:HGNC Symbol;Acc:HGNC:11921] |
| ENSG00000244411 | HGLibA_25807 | 226 | 1.100332499 | KRTAP5-7 | keratin associated protein 5-7 [Source:HGNC Symbol;Acc:HGNC:23602] |
| ENSG00000249709 | HGLibA_56253 | 226 | 1.100332499 | ZNF564 | zinc finger protein 564 [Source:HGNC Symbol;Acc:HGNC:31106] |
| ENSG00000256394 | HGLibA_03312 | 226 | 1.100332499 | ASIC5 | acid sensing ion channel subunit family member 5 [Source:HGNC Symbol;Acc:HGNC:17537] |
| ENSG00000276644 | HGLibA_12273 | 226 | 1.100332499 | DACH1 | dachshund family transcription factor 1 [Source:HGNC Symbol;Acc:HGNC:2663] |
| ENSG00000283706 | HGLibA_38953 | 226 | 1.100332499 | PRSS50 | serine protease 50 [Source:NCBI gene;Acc:29122] |
| HEXDC | HGLibA_21208 | 226 | 1.100332499 | HEXDC | - |
| hsa-mir-339 | HGLibA_59009 | 226 | 1.100332499 | hsa-mir-339 | - |
| hsa-mir-422a | HGLibA_59695 | 226 | 1.100332499 | hsa-mir-422a | - |
| hsa-mir-4292 | HGLibA_59878 | 226 | 1.100332499 | hsa-mir-4292 | - |
| hsa-mir-4799 | HGLibA_61302 | 226 | 1.100332499 | hsa-mir-4799 | - |
| hsa-mir-5011 | HGLibA_61460 | 226 | 1.100332499 | hsa-mir-5011 | - |
| hsa-mir-6867 | HGLibA_63651 | 226 | 1.100332499 | hsa-mir-6867 | - |
| hsa-mir-941-2 | HGLibA_64341 | 226 | 1.100332499 | hsa-mir-941-2 | - |
| WARS | HGLibA_53944 | 226 | 1.100332499 | WARS | - |
| ZNF664-FAM101A | HGLibA_56484 | 226 | 1.100332499 | ZNF664-FAM101A | - |
| DAK | HGLibA_12302 | 225 | 1.090381462 | DAK | - |
| ENSG00000040341 | HGLibA_47409 | 225 | 1.090381462 | STAU2 | staufen double-stranded RNA binding protein 2 [Source:HGNC Symbol;Acc:HGNC:11371] |
| ENSG00000068903 | HGLibA_44220 | 225 | 1.090381462 | SIRT2 | sirtuin 2 [Source:HGNC Symbol;Acc:HGNC:10886] |
| ENSG00000071967 | HGLibA_12023 | 225 | 1.090381462 | CYBRD1 | cytochrome b reductase 1 [Source:HGNC Symbol;Acc:HGNC:20797] |
| ENSG00000079337 | HGLibA_40250 | 225 | 1.090381462 | RAPGEF3 | Rap guanine nucleotide exchange factor 3 [Source:HGNC Symbol;Acc:HGNC:16629] |
| ENSG00000096996 | HGLibA_22966 | 225 | 1.090381462 | IL12RB1 | interleukin 12 receptor subunit beta 1 [Source:HGNC Symbol;Acc:HGNC:5971] |
| ENSG00000100266 | HGLibA_34962 | 225 | 1.090381462 | PACSIN2 | protein kinase C and casein kinase substrate in neurons 2 [Source:HGNC Symbol;Acc:HGNC:8571] |
| ENSG00000101194 | HGLibA_44484 | 225 | 1.090381462 | SLC17A9 | solute carrier family 17 member 9 [Source:HGNC Symbol;Acc:HGNC:16192] |
| ENSG00000102854 | HGLibA_30221 | 225 | 1.090381462 | MSLN | mesothelin [Source:HGNC Symbol;Acc:HGNC:7371] |
| ENSG00000103657 | HGLibA_21154 | 225 | 1.090381462 | HERC1 | HECT and RLD domain containing E3 ubiquitin protein ligase family member 1 [Source:HGNC Symbol;Acc:HGNC:4867] |
| ENSG00000104177 | HGLibA_30689 | 225 | 1.090381462 | MYEF2 | myelin expression factor 2 [Source:HGNC Symbol;Acc:HGNC:17940] |
| ENSG00000107719 | HGLibA_35061 | 225 | 1.090381462 | PALD1 | phosphatase domain containing paladin 1 [Source:HGNC Symbol;Acc:HGNC:23530] |
| ENSG00000111537 | HGLibA_22706 | 225 | 1.090381462 | IFNG | interferon gamma [Source:HGNC Symbol;Acc:HGNC:5438] |
| ENSG00000115129 | HGLibA_51065 | 225 | 1.090381462 | TP53I3 | tumor protein p53 inducible protein 3 [Source:HGNC Symbol;Acc:HGNC:19373] |
| ENSG00000115718 | HGLibA_38649 | 225 | 1.090381462 | PROC | inactivator of coagulation factors Va and VIIIa [Source:HGNC Symbol;Acc:HGNC:9451]" |
| ENSG00000118292 | HGLibA_05872 | 225 | 1.090381462 | C1orf54 | chromosome 1 open reading frame 54 [Source:HGNC Symbol;Acc:HGNC:26258] |
| ENSG00000122824 | HGLibA_32934 | 225 | 1.090381462 | NUDT10 | nudix hydrolase 10 [Source:HGNC Symbol;Acc:HGNC:17621] |
| ENSG00000123570 | HGLibA_39993 | 225 | 1.090381462 | RAB9B | member RAS oncogene family [Source:HGNC Symbol;Acc:HGNC:14090]" |
| ENSG00000124151 | HGLibA_31340 | 225 | 1.090381462 | NCOA3 | nuclear receptor coactivator 3 [Source:HGNC Symbol;Acc:HGNC:7670] |
| ENSG00000125107 | HGLibA_10327 | 225 | 1.090381462 | CNOT1 | CCR4-NOT transcription complex subunit 1 [Source:HGNC Symbol;Acc:HGNC:7877] |
| ENSG00000125821 | HGLibA_14051 | 225 | 1.090381462 | DTD1 | D-tyrosyl-tRNA deacylase 1 [Source:HGNC Symbol;Acc:HGNC:16219] |
| ENSG00000127720 | HGLibA_28997 | 225 | 1.090381462 | METTL25 | methyltransferase like 25 [Source:HGNC Symbol;Acc:HGNC:26228] |
| ENSG00000129194 | HGLibA_46243 | 225 | 1.090381462 | SOX15 | SRY-box 15 [Source:HGNC Symbol;Acc:HGNC:11196] |
| ENSG00000129480 | HGLibA_14056 | 225 | 1.090381462 | DTD2 | D-tyrosyl-tRNA deacylase 2 (putative) [Source:HGNC Symbol;Acc:HGNC:20277] |
| ENSG00000134278 | HGLibA_46723 | 225 | 1.090381462 | SPIRE1 | spire type actin nucleation factor 1 [Source:HGNC Symbol;Acc:HGNC:30622] |
| ENSG00000135119 | HGLibA_41706 | 225 | 1.090381462 | RNFT2 | transmembrane 2 [Source:HGNC Symbol;Acc:HGNC:25905]" |
| ENSG00000137869 | HGLibA_12065 | 225 | 1.090381462 | CYP19A1 | cytochrome P450 family 19 subfamily A member 1 [Source:HGNC Symbol;Acc:HGNC:2594] |
| ENSG00000138029 | HGLibA_20812 | 225 | 1.090381462 | HADHB | hydroxyacyl-CoA dehydrogenase trifunctional multienzyme complex subunit beta [Source:HGNC Symbol;Acc:HGNC:4803] |
| ENSG00000140107 | HGLibA_44756 | 225 | 1.090381462 | SLC25A47 | solute carrier family 25 member 47 [Source:HGNC Symbol;Acc:HGNC:20115] |
| ENSG00000140795 | HGLibA_30790 | 225 | 1.090381462 | MYLK3 | myosin light chain kinase 3 [Source:HGNC Symbol;Acc:HGNC:29826] |
| ENSG00000140932 | HGLibA_10211 | 225 | 1.090381462 | CMTM2 | CKLF like MARVEL transmembrane domain containing 2 [Source:HGNC Symbol;Acc:HGNC:19173] |
| ENSG00000142449 | HGLibA_16960 | 225 | 1.090381462 | FBN3 | fibrillin 3 [Source:HGNC Symbol;Acc:HGNC:18794] |
| ENSG00000144057 | HGLibA_47262 | 225 | 1.090381462 | ST6GAL2 | 6-sialyltransferase 2 [Source:HGNC Symbol;Acc:HGNC:10861]" |
| ENSG00000152689 | HGLibA_40349 | 225 | 1.090381462 | RASGRP3 | RAS guanyl releasing protein 3 [Source:HGNC Symbol;Acc:HGNC:14545] |
| ENSG00000156642 | HGLibA_32528 | 225 | 1.090381462 | NPTN | neuroplastin [Source:HGNC Symbol;Acc:HGNC:17867] |
| ENSG00000158869 | HGLibA_17172 | 225 | 1.090381462 | FCER1G | Fc fragment of IgE receptor Ig [Source:HGNC Symbol;Acc:HGNC:3611] |
| ENSG00000160446 | HGLibA_55071 | 225 | 1.090381462 | ZDHHC12 | zinc finger DHHC-type containing 12 [Source:HGNC Symbol;Acc:HGNC:19159] |
| ENSG00000162378 | HGLibA_57086 | 225 | 1.090381462 | ZYG11B | cell cycle regulator [Source:HGNC Symbol;Acc:HGNC:25820]" |
| ENSG00000163737 | HGLibA_36128 | 225 | 1.090381462 | PF4 | platelet factor 4 [Source:HGNC Symbol;Acc:HGNC:8861] |
| ENSG00000163964 | HGLibA_36599 | 225 | 1.090381462 | PIGX | phosphatidylinositol glycan anchor biosynthesis class X [Source:HGNC Symbol;Acc:HGNC:26046] |
| ENSG00000164363 | HGLibA_45313 | 225 | 1.090381462 | SLC6A18 | solute carrier family 6 member 18 [Source:HGNC Symbol;Acc:HGNC:26441] |
| ENSG00000164649 | HGLibA_08616 | 225 | 1.090381462 | CDCA7L | cell division cycle associated 7 like [Source:HGNC Symbol;Acc:HGNC:30777] |
| ENSG00000165775 | HGLibA_18167 | 225 | 1.090381462 | FUNDC2 | FUN14 domain containing 2 [Source:HGNC Symbol;Acc:HGNC:24925] |
| ENSG00000166025 | HGLibA_01816 | 225 | 1.090381462 | AMOTL1 | angiomotin like 1 [Source:HGNC Symbol;Acc:HGNC:17811] |
| ENSG00000167889 | HGLibA_29154 | 225 | 1.090381462 | MGAT5B | 6-mannosylglycoprotein 6-beta-N-acetylglucosaminyltransferase B [Source:HGNC Symbol;Acc:HGNC:24140]" |
| ENSG00000172031 | HGLibA_15331 | 225 | 1.090381462 | EPHX4 | epoxide hydrolase 4 [Source:HGNC Symbol;Acc:HGNC:23758] |
| ENSG00000173868 | HGLibA_36440 | 225 | 1.090381462 | PHOSPHO1 | phosphoethanolamine/phosphocholine phosphatase [Source:HGNC Symbol;Acc:HGNC:16815] |
| ENSG00000174279 | HGLibA_15711 | 225 | 1.090381462 | EVX2 | even-skipped homeobox 2 [Source:HGNC Symbol;Acc:HGNC:3507] |
| ENSG00000174914 | HGLibA_34568 | 225 | 1.090381462 | OR9G1 | olfactory receptor family 9 subfamily G member 1 [Source:HGNC Symbol;Acc:HGNC:15319] |
| ENSG00000175262 | HGLibA_05766 | 225 | 1.090381462 | C1orf127 | chromosome 1 open reading frame 127 [Source:HGNC Symbol;Acc:HGNC:26730] |
| ENSG00000175938 | HGLibA_34595 | 225 | 1.090381462 | ORAI3 | ORAI calcium release-activated calcium modulator 3 [Source:HGNC Symbol;Acc:HGNC:28185] |
| ENSG00000176495 | HGLibA_34240 | 225 | 1.090381462 | OR5AN1 | olfactory receptor family 5 subfamily AN member 1 [Source:HGNC Symbol;Acc:HGNC:15255] |
| ENSG00000176887 | HGLibA_46230 | 225 | 1.090381462 | SOX11 | SRY-box 11 [Source:HGNC Symbol;Acc:HGNC:11191] |
| ENSG00000180113 | HGLibA_48912 | 225 | 1.090381462 | TDRD6 | tudor domain containing 6 [Source:HGNC Symbol;Acc:HGNC:21339] |
| ENSG00000180543 | HGLibA_51987 | 225 | 1.090381462 | TSPYL5 | TSPY like 5 [Source:HGNC Symbol;Acc:HGNC:29367] |
| ENSG00000187624 | HGLibA_05597 | 225 | 1.090381462 | C17orf97 | chromosome 17 open reading frame 97 [Source:HGNC Symbol;Acc:HGNC:33800] |
| ENSG00000188257 | HGLibA_36884 | 225 | 1.090381462 | PLA2G2A | phospholipase A2 group IIA [Source:HGNC Symbol;Acc:HGNC:9031] |
| ENSG00000188822 | HGLibA_10378 | 225 | 1.090381462 | CNR2 | cannabinoid receptor 2 [Source:HGNC Symbol;Acc:HGNC:2160] |
| ENSG00000196944 | HGLibA_33885 | 225 | 1.090381462 | OR2T4 | olfactory receptor family 2 subfamily T member 4 [Source:HGNC Symbol;Acc:HGNC:15016] |
| ENSG00000197084 | HGLibA_26106 | 225 | 1.090381462 | LCE1C | late cornified envelope 1C [Source:HGNC Symbol;Acc:HGNC:29464] |
| ENSG00000197123 | HGLibA_56520 | 225 | 1.090381462 | ZNF679 | zinc finger protein 679 [Source:HGNC Symbol;Acc:HGNC:28650] |
| ENSG00000198208 | HGLibA_42186 | 225 | 1.090381462 | RPS6KL1 | ribosomal protein S6 kinase like 1 [Source:HGNC Symbol;Acc:HGNC:20222] |
| ENSG00000198643 | HGLibA_16585 | 225 | 1.090381462 | FAM3D | family with sequence similarity 3 member D [Source:HGNC Symbol;Acc:HGNC:18665] |
| ENSG00000204642 | HGLibA_21645 | 225 | 1.090381462 | HLA-F | F [Source:HGNC Symbol;Acc:HGNC:4963]" |
| ENSG00000204956 | HGLibA_35517 | 225 | 1.090381462 | PCDHGA1 | 1 [Source:HGNC Symbol;Acc:HGNC:8696]" |
| ENSG00000206536 | HGLibA_34317 | 225 | 1.090381462 | OR5K3 | olfactory receptor family 5 subfamily K member 3 [Source:HGNC Symbol;Acc:HGNC:31290] |
| ENSG00000213614 | HGLibA_21201 | 225 | 1.090381462 | HEXA | hexosaminidase subunit alpha [Source:HGNC Symbol;Acc:HGNC:4878] |
| ENSG00000221818 | HGLibA_14353 | 225 | 1.090381462 | EBF2 | EBF transcription factor 2 [Source:HGNC Symbol;Acc:HGNC:19090] |
| ENSG00000268606 | HGLibA_27904 | 225 | 1.090381462 | MAGEA2 | MAGE family member A2 [Source:HGNC Symbol;Acc:HGNC:6800] |
| GPR112 | HGLibA_19851 | 225 | 1.090381462 | GPR112 | - |
| hsa-mir-3183 | HGLibA_58829 | 225 | 1.090381462 | hsa-mir-3183 | - |
| hsa-mir-3909 | HGLibA_59499 | 225 | 1.090381462 | hsa-mir-3909 | - |
| hsa-mir-4274 | HGLibA_59798 | 225 | 1.090381462 | hsa-mir-4274 | - |
| hsa-mir-4436b-1 | HGLibA_60141 | 225 | 1.090381462 | hsa-mir-4436b-1 | - |
| hsa-mir-6830 | HGLibA_63496 | 225 | 1.090381462 | hsa-mir-6830 | - |
| hsa-mir-7154 | HGLibA_63846 | 225 | 1.090381462 | hsa-mir-7154 | - |
| LOC100862671 | HGLibA_26879 | 225 | 1.090381462 | LOC100862671 | - |
| CARS | HGLibA_07260 | 224 | 1.080430424 | CARS | - |
| ENSG00000002919 | HGLibA_46030 | 224 | 1.080430424 | SNX11 | sorting nexin 11 [Source:HGNC Symbol;Acc:HGNC:14975] |
| ENSG00000005020 | HGLibA_44273 | 224 | 1.080430424 | SKAP2 | src kinase associated phosphoprotein 2 [Source:HGNC Symbol;Acc:HGNC:15687] |
| ENSG00000067141 | HGLibA_31696 | 224 | 1.080430424 | NEO1 | neogenin 1 [Source:HGNC Symbol;Acc:HGNC:7754] |
| ENSG00000069764 | HGLibA_36868 | 224 | 1.080430424 | PLA2G10 | phospholipase A2 group X [Source:HGNC Symbol;Acc:HGNC:9029] |
| ENSG00000083812 | HGLibA_55801 | 224 | 1.080430424 | ZNF324 | zinc finger protein 324 [Source:HGNC Symbol;Acc:HGNC:14096] |
| ENSG00000092098 | HGLibA_41662 | 224 | 1.080430424 | RNF31 | ring finger protein 31 [Source:HGNC Symbol;Acc:HGNC:16031] |
| ENSG00000093144 | HGLibA_14393 | 224 | 1.080430424 | ECHDC1 | ethylmalonyl-CoA decarboxylase 1 [Source:HGNC Symbol;Acc:HGNC:21489] |
| ENSG00000106477 | HGLibA_09136 | 224 | 1.080430424 | CEP41 | centrosomal protein 41 [Source:HGNC Symbol;Acc:HGNC:12370] |
| ENSG00000106991 | HGLibA_15124 | 224 | 1.080430424 | ENG | endoglin [Source:HGNC Symbol;Acc:HGNC:3349] |
| ENSG00000108309 | HGLibA_42448 | 224 | 1.080430424 | RUNDC3A | RUN domain containing 3A [Source:HGNC Symbol;Acc:HGNC:16984] |
| ENSG00000109016 | HGLibA_13148 | 224 | 1.080430424 | DHRS7B | dehydrogenase/reductase 7B [Source:HGNC Symbol;Acc:HGNC:24547] |
| ENSG00000110002 | HGLibA_53908 | 224 | 1.080430424 | VWA5A | von Willebrand factor A domain containing 5A [Source:HGNC Symbol;Acc:HGNC:6658] |
| ENSG00000112238 | HGLibA_38373 | 224 | 1.080430424 | PRDM13 | PR/SET domain 13 [Source:HGNC Symbol;Acc:HGNC:13998] |
| ENSG00000115507 | HGLibA_34784 | 224 | 1.080430424 | OTX1 | orthodenticle homeobox 1 [Source:HGNC Symbol;Acc:HGNC:8521] |
| ENSG00000115827 | HGLibA_12435 | 224 | 1.080430424 | DCAF17 | DDB1 and CUL4 associated factor 17 [Source:HGNC Symbol;Acc:HGNC:25784] |
| ENSG00000116954 | HGLibA_42231 | 224 | 1.080430424 | RRAGC | Ras related GTP binding C [Source:HGNC Symbol;Acc:HGNC:19902] |

| | | | | | |
|---|---|---|---|---|---|
| ENSG00000119946 | HGLibA_10315 | 224 | 1.080430424 | CNNM1 | cyclin and CBS domain divalent metal cation transport mediator 1 [Source:HGNC Symbol;Acc:HGNC:102] |
| ENSG00000122126 | HGLibA_33263 | 224 | 1.080430424 | OCRL | inositol polyphosphate-5-phosphatase [Source:HGNC Symbol;Acc:HGNC:8108]" |
| ENSG00000123584 | HGLibA_27924 | 224 | 1.080430424 | MAGEA9 | MAGE family member A9 [Source:HGNC Symbol;Acc:HGNC:6807] |
| ENSG00000125818 | HGLibA_39201 | 224 | 1.080430424 | PSMF1 | proteasome inhibitor subunit 1 [Source:HGNC Symbol;Acc:HGNC:9571] |
| ENSG00000130294 | HGLibA_24882 | 224 | 1.080430424 | KIF1A | kinesin family member 1A [Source:HGNC Symbol;Acc:HGNC:888] |
| ENSG00000132837 | HGLibA_13404 | 224 | 1.080430424 | DMGDH | dimethylglycine dehydrogenase [Source:HGNC Symbol;Acc:HGNC:24475] |
| ENSG00000135624 | HGLibA_08212 | 224 | 1.080430424 | CCT7 | chaperonin containing TCP1 subunit 7 [Source:HGNC Symbol;Acc:HGNC:1622] |
| ENSG00000136379 | HGLibA_00270 | 224 | 1.080430424 | ABHD17C | abhydrolase domain containing 17C [Source:HGNC Symbol;Acc:HGNC:26925] |
| ENSG00000141736 | HGLibA_15413 | 224 | 1.080430424 | ERBB2 | erb-b2 receptor tyrosine kinase 2 [Source:HGNC Symbol;Acc:HGNC:3430] |
| ENSG00000142156 | HGLibA_10596 | 224 | 1.080430424 | COL6A1 | collagen type VI alpha 1 chain [Source:HGNC Symbol;Acc:HGNC:2211] |
| ENSG00000145293 | HGLibA_15153 | 224 | 1.080430424 | ENOPH1 | enolase-phosphatase 1 [Source:HGNC Symbol;Acc:HGNC:24599] |
| ENSG00000145839 | HGLibA_23200 | 224 | 1.080430424 | IL9 | interleukin 9 [Source:HGNC Symbol;Acc:HGNC:6029] |
| ENSG00000148356 | HGLibA_27496 | 224 | 1.080430424 | LRSAM1 | leucine rich repeat and sterile alpha motif containing 1 [Source:HGNC Symbol;Acc:HGNC:25135] |
| ENSG00000152457 | HGLibA_12515 | 224 | 1.080430424 | DCLRE1C | DNA cross-link repair 1C [Source:HGNC Symbol;Acc:HGNC:17642] |
| ENSG00000157500 | HGLibA_02566 | 224 | 1.080430424 | APPL1 | phosphotyrosine interacting with PH domain and leucine zipper 1 [Source:HGNC Symbol;Acc:HGNC:24035]" |
| ENSG00000160683 | HGLibA_11892 | 224 | 1.080430424 | CXCR5 | C-X-C motif chemokine receptor 5 [Source:HGNC Symbol;Acc:HGNC:1060] |
| ENSG00000162551 | HGLibA_01710 | 224 | 1.080430424 | ALPL | biomineralization associated [Source:HGNC Symbol;Acc:HGNC:438]" |
| ENSG00000163644 | HGLibA_37988 | 224 | 1.080430424 | PPM1K | Mg2+/Mn2+ dependent 1K [Source:HGNC Symbol;Acc:HGNC:25415]" |
| ENSG00000165694 | HGLibA_18027 | 224 | 1.080430424 | FRMD7 | FERM domain containing 7 [Source:HGNC Symbol;Acc:HGNC:8079] |
| ENSG00000170871 | HGLibA_24612 | 224 | 1.080430424 | KIAA0232 | KIAA0232 [Source:HGNC Symbol;Acc:HGNC:28992] |
| ENSG00000171657 | HGLibA_20090 | 224 | 1.080430424 | GPR82 | G protein-coupled receptor 82 [Source:HGNC Symbol;Acc:HGNC:4533] |
| ENSG00000171936 | HGLibA_33512 | 224 | 1.080430424 | OR10H3 | olfactory receptor family 10 subfamily H member 3 [Source:HGNC Symbol;Acc:HGNC:8174] |
| ENSG00000172318 | HGLibA_03953 | 224 | 1.080430424 | B3GALT1 | 3-galactosyltransferase 1 [Source:HGNC Symbol;Acc:HGNC:916]" |
| ENSG00000178385 | HGLibA_37142 | 224 | 1.080430424 | PLEKHM3 | pleckstrin homology domain containing M3 [Source:HGNC Symbol;Acc:HGNC:34006] |
| ENSG00000179094 | HGLibA_36053 | 224 | 1.080430424 | PER1 | period circadian regulator 1 [Source:HGNC Symbol;Acc:HGNC:8845] |
| ENSG00000184792 | HGLibA_34641 | 224 | 1.080430424 | OSBP2 | oxysterol binding protein 2 [Source:HGNC Symbol;Acc:HGNC:8504] |
| ENSG00000185774 | HGLibA_24243 | 224 | 1.080430424 | KCNIP4 | potassium voltage-gated channel interacting protein 4 [Source:HGNC Symbol;Acc:HGNC:30083] |
| ENSG00000187678 | HGLibA_46847 | 224 | 1.080430424 | SPRY4 | sprouty RTK signaling antagonist 4 [Source:HGNC Symbol;Acc:HGNC:15533] |
| ENSG00000189045 | HGLibA_01950 | 224 | 1.080430424 | ANKDD1B | ankyrin repeat and death domain containing 1B [Source:HGNC Symbol;Acc:HGNC:32525] |
| ENSG00000189337 | HGLibA_24091 | 224 | 1.080430424 | KAZN | periplakin interacting protein [Source:HGNC Symbol;Acc:HGNC:29173]" |
| ENSG00000196341 | HGLibA_34508 | 224 | 1.080430424 | OR8D1 | olfactory receptor family 8 subfamily D member 1 [Source:HGNC Symbol;Acc:HGNC:8481] |
| ENSG00000197887 | HGLibA_33717 | 224 | 1.080430424 | OR1S2 | olfactory receptor family 1 subfamily S member 2 [Source:HGNC Symbol;Acc:HGNC:15141] |
| ENSG00000204371 | HGLibA_14686 | 224 | 1.080430424 | EHMT2 | euchromatic histone lysine methyltransferase 2 [Source:HGNC Symbol;Acc:HGNC:14129] |
| ENSG00000224389 | HGLibA_06284 | 224 | 1.080430424 | C4B | complement C4B (Chido blood group) [Source:HGNC Symbol;Acc:HGNC:1324] |
| ENSG00000236279 | HGLibA_09970 | 224 | 1.080430424 | CLEC2L | C-type lectin domain family 2 member L [Source:HGNC Symbol;Acc:HGNC:21969] |
| ENSG00000256162 | HGLibA_45778 | 224 | 1.080430424 | SMLR1 | small leucine rich protein 1 [Source:HGNC Symbol;Acc:HGNC:44670] |
| ENSG00000259431 | HGLibA_49413 | 224 | 1.080430424 | THTPA | thiamine triphosphatase [Source:HGNC Symbol;Acc:HGNC:18987] |
| ENSG00000261115 | HGLibA_50084 | 224 | 1.080430424 | TMEM178B | transmembrane protein 178B [Source:HGNC Symbol;Acc:HGNC:44112] |
| FAM129A | HGLibA_16096 | 224 | 1.080430424 | FAM129A | - |
| FAM160B1 | HGLibA_16209 | 224 | 1.080430424 | FAM160B1 | - |
| hsa-mir-320d-2 | HGLibA_58948 | 224 | 1.080430424 | hsa-mir-320d-2 | - |
| hsa-mir-3690-1 | HGLibA_59344 | 224 | 1.080430424 | hsa-mir-3690-1 | - |
| hsa-mir-4315-1 | HGLibA_59976 | 224 | 1.080430424 | hsa-mir-4315-1 | - |
| hsa-mir-551a | HGLibA_62118 | 224 | 1.080430424 | hsa-mir-551a | - |
| hsa-mir-5787 | HGLibA_62409 | 224 | 1.080430424 | hsa-mir-5787 | - |
| hsa-mir-656 | HGLibA_62959 | 224 | 1.080430424 | hsa-mir-656 | - |
| SGK223 | HGLibA_43830 | 224 | 1.080430424 | SGK223 | - |
| TMEM155 | HGLibA_50010 | 224 | 1.080430424 | TMEM155 | - |
| CXXC11 | HGLibA_11969 | 223 | 1.070479387 | CXXC11 | - |
| ENSG00000042813 | HGLibA_56962 | 223 | 1.070479387 | ZPBP | zona pellucida binding protein [Source:HGNC Symbol;Acc:HGNC:15662] |
| ENSG00000044012 | HGLibA_20646 | 223 | 1.070479387 | GUCA2B | guanylate cyclase activator 2B [Source:HGNC Symbol;Acc:HGNC:4683] |
| ENSG00000065054 | HGLibA_45412 | 223 | 1.070479387 | SLC9A3R2 | SLC9A3 regulator 2 [Source:HGNC Symbol;Acc:HGNC:11076] |
| ENSG00000084234 | HGLibA_02444 | 223 | 1.070479387 | APLP2 | amyloid beta precursor like protein 2 [Source:HGNC Symbol;Acc:HGNC:598] |
| ENSG00000084674 | HGLibA_02464 | 223 | 1.070479387 | APOB | apolipoprotein B [Source:HGNC Symbol;Acc:HGNC:603] |
| ENSG00000086570 | HGLibA_16915 | 223 | 1.070479387 | FAT2 | FAT atypical cadherin 2 [Source:HGNC Symbol;Acc:HGNC:3596] |
| ENSG00000087589 | HGLibA_07344 | 223 | 1.070479387 | CASS4 | Cas scaffold protein family member 4 [Source:HGNC Symbol;Acc:HGNC:15878] |
| ENSG00000089220 | HGLibA_36013 | 223 | 1.070479387 | PEBP1 | phosphatidylethanolamine binding protein 1 [Source:HGNC Symbol;Acc:HGNC:8630] |
| ENSG00000104973 | HGLibA_28786 | 223 | 1.070479387 | MED25 | mediator complex subunit 25 [Source:HGNC Symbol;Acc:HGNC:28845] |
| ENSG00000107187 | HGLibA_26453 | 223 | 1.070479387 | LHX3 | LIM homeobox 3 [Source:HGNC Symbol;Acc:HGNC:6595] |
| ENSG00000107263 | HGLibA_40244 | 223 | 1.070479387 | RAPGEF1 | Rap guanine nucleotide exchange factor 1 [Source:HGNC Symbol;Acc:HGNC:4568] |
| ENSG00000108439 | HGLibA_37428 | 223 | 1.070479387 | PNPO | pyridoxamine 5'-phosphate oxidase [Source:HGNC Symbol;Acc:HGNC:30260] |
| ENSG00000112679 | HGLibA_14162 | 223 | 1.070479387 | DUSP22 | dual specificity phosphatase 22 [Source:HGNC Symbol;Acc:HGNC:16077] |
| ENSG00000116151 | HGLibA_29666 | 223 | 1.070479387 | MORN1 | MORN repeat containing 1 [Source:HGNC Symbol;Acc:HGNC:25852] |
| ENSG00000120952 | HGLibA_38304 | 223 | 1.070479387 | PRAMEF2 | PRAME family member 2 [Source:HGNC Symbol;Acc:HGNC:28841] |
| ENSG00000124370 | HGLibA_28575 | 223 | 1.070479387 | MCEE | methylmalonyl-CoA epimerase [Source:HGNC Symbol;Acc:HGNC:16732] |
| ENSG00000124444 | HGLibA_56293 | 223 | 1.070479387 | ZNF576 | zinc finger protein 576 [Source:HGNC Symbol;Acc:HGNC:28357] |
| ENSG00000125652 | HGLibA_01663 | 223 | 1.070479387 | ALKBH7 | alkB homolog 7 [Source:HGNC Symbol;Acc:HGNC:21306] |
| ENSG00000130066 | HGLibA_42769 | 223 | 1.070479387 | SAT1 | spermidine/spermine N1-acetyltransferase 1 [Source:HGNC Symbol;Acc:HGNC:10540] |
| ENSG00000134201 | HGLibA_20483 | 223 | 1.070479387 | GSTM5 | glutathione S-transferase mu 5 [Source:HGNC Symbol;Acc:HGNC:4637] |
| ENSG00000138696 | HGLibA_04633 | 223 | 1.070479387 | BMPR1B | bone morphogenetic protein receptor type 1B [Source:HGNC Symbol;Acc:HGNC:1077] |
| ENSG00000142765 | HGLibA_48095 | 223 | 1.070479387 | SYTL1 | synaptotagmin like 1 [Source:HGNC Symbol;Acc:HGNC:15584] |
| ENSG00000143093 | HGLibA_47588 | 223 | 1.070479387 | STRIP1 | striatin interacting protein 1 [Source:HGNC Symbol;Acc:HGNC:25916] |
| ENSG00000143543 | HGLibA_24000 | 223 | 1.070479387 | JTB | jumping translocation breakpoint [Source:HGNC Symbol;Acc:HGNC:6201] |
| ENSG00000144045 | HGLibA_13937 | 223 | 1.070479387 | DQX1 | DEAQ-box RNA dependent ATPase 1 [Source:HGNC Symbol;Acc:HGNC:20410] |
| ENSG00000144362 | HGLibA_36445 | 223 | 1.070479387 | PHOSPHO2 | orphan 2 [Source:HGNC Symbol;Acc:HGNC:28316]" |
| ENSG00000146267 | HGLibA_16927 | 223 | 1.070479387 | FAXC | failed axon connections homolog [Source:HGNC Symbol;Acc:HGNC:20742] |
| ENSG00000151092 | HGLibA_31880 | 223 | 1.070479387 | NGLY1 | N-glycanase 1 [Source:HGNC Symbol;Acc:HGNC:17646] |
| ENSG00000155846 | HGLibA_37849 | 223 | 1.070479387 | PPARGC1B | PPARG coactivator 1 beta [Source:HGNC Symbol;Acc:HGNC:30022] |
| ENSG00000157111 | HGLibA_50059 | 223 | 1.070479387 | TMEM171 | transmembrane protein 171 [Source:HGNC Symbol;Acc:HGNC:27031] |
| ENSG00000161682 | HGLibA_16267 | 223 | 1.070479387 | FAM171A2 | family with sequence similarity 171 member A2 [Source:HGNC Symbol;Acc:HGNC:30480] |
| ENSG00000161973 | HGLibA_07780 | 223 | 1.070479387 | CCDC42 | coiled-coil domain containing 42 [Source:HGNC Symbol;Acc:HGNC:26528] |
| ENSG00000163464 | HGLibA_11880 | 223 | 1.070479387 | CXCR1 | C-X-C motif chemokine receptor 1 [Source:HGNC Symbol;Acc:HGNC:6026] |
| ENSG00000163751 | HGLibA_10891 | 223 | 1.070479387 | CPA3 | carboxypeptidase A3 [Source:HGNC Symbol;Acc:HGNC:2298] |
| ENSG00000164744 | HGLibA_47793 | 223 | 1.070479387 | SUN3 | Sad1 and UNC84 domain containing 3 [Source:HGNC Symbol;Acc:HGNC:22429] |
| ENSG00000166206 | HGLibA_18380 | 223 | 1.070479387 | GABRB3 | gamma-aminobutyric acid type A receptor beta3 subunit [Source:HGNC Symbol;Acc:HGNC:4083] |
| ENSG00000166866 | HGLibA_30819 | 223 | 1.070479387 | MYO1A | myosin IA [Source:HGNC Symbol;Acc:HGNC:7595] |
| ENSG00000169136 | HGLibA_03443 | 223 | 1.070479387 | ATF5 | activating transcription factor 5 [Source:HGNC Symbol;Acc:HGNC:790] |
| ENSG00000171054 | HGLibA_33624 | 223 | 1.070479387 | OR13H1 | olfactory receptor family 13 subfamily H member 1 [Source:HGNC Symbol;Acc:HGNC:14755] |
| ENSG00000171703 | HGLibA_48699 | 223 | 1.070479387 | TCEA2 | transcription elongation factor A2 [Source:HGNC Symbol;Acc:HGNC:11614] |
| ENSG00000179934 | HGLibA_08168 | 223 | 1.070479387 | CCR8 | C-C motif chemokine receptor 8 [Source:HGNC Symbol;Acc:HGNC:1609] |
| ENSG00000181467 | HGLibA_40240 | 223 | 1.070479387 | RAP2B | member of RAS oncogene family [Source:HGNC Symbol;Acc:HGNC:9862]" |
| ENSG00000181499 | HGLibA_34444 | 223 | 1.070479387 | OR6T1 | olfactory receptor family 6 subfamily T member 1 [Source:HGNC Symbol;Acc:HGNC:14848] |
| ENSG00000188064 | HGLibA_54384 | 223 | 1.070479387 | WNT7B | Wnt family member 7B [Source:HGNC Symbol;Acc:HGNC:12787] |
| ENSG00000188522 | HGLibA_16758 | 223 | 1.070479387 | FAM83G | family with sequence similarity 83 member G [Source:HGNC Symbol;Acc:HGNC:32554] |
| ENSG00000189420 | HGLibA_55246 | 223 | 1.070479387 | ZFP92 | ZFP92 zinc finger protein [Source:HGNC Symbol;Acc:HGNC:12865] |
| ENSG00000198146 | HGLibA_56703 | 223 | 1.070479387 | ZNF770 | zinc finger protein 770 [Source:HGNC Symbol;Acc:HGNC:26061] |
| ENSG00000198829 | HGLibA_47705 | 223 | 1.070479387 | SUCNR1 | succinate receptor 1 [Source:HGNC Symbol;Acc:HGNC:4542] |
| ENSG00000204366 | HGLibA_54820 | 223 | 1.070479387 | ZBTB12 | zinc finger and BTB domain containing 12 [Source:HGNC Symbol;Acc:HGNC:19066] |
| ENSG00000204941 | HGLibA_39050 | 223 | 1.070479387 | PSG5 | pregnancy specific beta-1-glycoprotein 5 [Source:HGNC Symbol;Acc:HGNC:9522] |
| ENSG00000231213 | HGLibA_37244 | 223 | 1.070479387 | PLSCR5 | phospholipid scramblase family member 5 [Source:HGNC Symbol;Acc:HGNC:19952] |
| ENSG00000256294 | HGLibA_55622 | 223 | 1.070479387 | ZNF225 | zinc finger protein 225 [Source:HGNC Symbol;Acc:HGNC:13018] |
| FAM212A | HGLibA_16464 | 223 | 1.070479387 | FAM212A | - |
| hsa-mir-1227 | HGLibA_57305 | 223 | 1.070479387 | hsa-mir-1227 | - |
| hsa-mir-216b | HGLibA_58262 | 223 | 1.070479387 | hsa-mir-216b | - |
| hsa-mir-3201 | HGLibA_58910 | 223 | 1.070479387 | hsa-mir-3201 | - |
| hsa-mir-373 | HGLibA_59386 | 223 | 1.070479387 | hsa-mir-373 | - |
| hsa-mir-4635 | HGLibA_60647 | 223 | 1.070479387 | hsa-mir-4635 | - |
| hsa-mir-4783 | HGLibA_61241 | 223 | 1.070479387 | hsa-mir-4783 | - |
| hsa-mir-555 | HGLibA_62136 | 223 | 1.070479387 | hsa-mir-555 | - |
| hsa-mir-623 | HGLibA_62733 | 223 | 1.070479387 | hsa-mir-623 | - |
| hsa-mir-6777 | HGLibA_63283 | 223 | 1.070479387 | hsa-mir-6777 | - |
| MLL4 | HGLibA_29401 | 223 | 1.070479387 | MLL4 | - |
| ENSG00000012124 | HGLibA_08296 | 222 | 1.06052835 | CD22 | CD22 molecule [Source:HGNC Symbol;Acc:HGNC:1643] |
| ENSG00000071073 | HGLibA_29140 | 222 | 1.06052835 | MGAT4A | 3-mannosyl-glycoprotein 4-beta-N-acetylglucosaminyltransferase A [Source:HGNC Symbol;Acc:HGNC:7047]" |
| ENSG00000083093 | HGLibA_35057 | 222 | 1.06052835 | PALB2 | partner and localizer of BRCA2 [Source:HGNC Symbol;Acc:HGNC:26144] |
| ENSG00000090932 | HGLibA_13362 | 222 | 1.06052835 | DLL3 | delta like canonical Notch ligand 3 [Source:HGNC Symbol;Acc:HGNC:2909] |
| ENSG00000102466 | HGLibA_17374 | 222 | 1.06052835 | FGF14 | fibroblast growth factor 14 [Source:HGNC Symbol;Acc:HGNC:3671] |
| ENSG00000103044 | HGLibA_20867 | 222 | 1.06052835 | HAS3 | hyaluronan synthase 3 [Source:HGNC Symbol;Acc:HGNC:4820] |
| ENSG00000105370 | HGLibA_26522 | 222 | 1.06052835 | LIM2 | lens intrinsic membrane protein 2 [Source:HGNC Symbol;Acc:HGNC:6610] |
| ENSG00000106952 | HGLibA_50827 | 222 | 1.06052835 | TNFSF8 | TNF superfamily member 8 [Source:HGNC Symbol;Acc:HGNC:11938] |
| ENSG00000111752 | HGLibA_36318 | 222 | 1.06052835 | PHC1 | polyhomeotic homolog 1 [Source:HGNC Symbol;Acc:HGNC:3182] |
| ENSG00000115419 | HGLibA_19326 | 222 | 1.06052835 | GLS | glutaminase [Source:HGNC Symbol;Acc:HGNC:4331] |
| ENSG00000118508 | HGLibA_39877 | 222 | 1.06052835 | RAB32 | member RAS oncogene family [Source:HGNC Symbol;Acc:HGNC:9772]" |
| ENSG00000119508 | HGLibA_32632 | 222 | 1.06052835 | NR4A3 | nuclear receptor subfamily 4 group A member 3 [Source:HGNC Symbol;Acc:HGNC:7982] |
| ENSG00000125734 | HGLibA_19847 | 222 | 1.06052835 | GPR108 | G protein-coupled receptor 108 [Source:HGNC Symbol;Acc:HGNC:17829] |
| ENSG00000126562 | HGLibA_54340 | 222 | 1.06052835 | WNK4 | WNK lysine deficient protein kinase 4 [Source:HGNC Symbol;Acc:HGNC:14544] |
| ENSG00000133111 | HGLibA_40988 | 222 | 1.06052835 | RFXAP | regulatory factor X associated protein [Source:HGNC Symbol;Acc:HGNC:9988] |
| ENSG00000134769 | HGLibA_14065 | 222 | 1.06052835 | DTNA | dystrobrevin alpha [Source:HGNC Symbol;Acc:HGNC:3057] |
| ENSG00000139631 | HGLibA_11304 | 222 | 1.06052835 | CSAD | cysteine sulfinic acid decarboxylase [Source:HGNC Symbol;Acc:HGNC:18966] |
| ENSG00000141574 | HGLibA_43271 | 222 | 1.06052835 | SECTM1 | secreted and transmembrane 1 [Source:HGNC Symbol;Acc:HGNC:10707] |
| ENSG00000143105 | HGLibA_24127 | 222 | 1.06052835 | KCNA10 | potassium voltage-gated channel subfamily A member 10 [Source:HGNC Symbol;Acc:HGNC:6219] |
| ENSG00000144218 | HGLibA_01112 | 222 | 1.06052835 | AFF3 | AF4/FMR2 family member 3 [Source:HGNC Symbol;Acc:HGNC:6473] |
| ENSG00000147454 | HGLibA_44723 | 222 | 1.06052835 | SLC25A37 | solute carrier family 25 member 37 [Source:HGNC Symbol;Acc:HGNC:29786] |
| ENSG00000149187 | HGLibA_09004 | 222 | 1.06052835 | CELF1 | CUGBP Elav-like family member 1 [Source:HGNC Symbol;Acc:HGNC:2549] |
| ENSG00000156738 | HGLibA_30128 | 222 | 1.06052835 | MS4A1 | membrane spanning 4-domains A1 [Source:HGNC Symbol;Acc:HGNC:7315] |

| | | | | | |
|---|---|---|---|---|---|
| ENSG00000157214 | HGLibA_47425 | 222 | 1.06052835 | STEAP2 | STEAP2 metalloreductase [Source:HGNC Symbol;Acc:HGNC:17885] |
| ENSG00000157800 | HGLibA_45049 | 222 | 1.06052835 | SLC37A3 | solute carrier family 37 member 3 [Source:HGNC Symbol;Acc:HGNC:20651] |
| ENSG00000158901 | HGLibA_54274 | 222 | 1.06052835 | WFDC8 | WAP four-disulfide core domain 8 [Source:HGNC Symbol;Acc:HGNC:16163] |
| ENSG00000162542 | HGLibA_49789 | 222 | 1.06052835 | TMCO4 | transmembrane and coiled-coil domains 4 [Source:HGNC Symbol;Acc:HGNC:27393] |
| ENSG00000163013 | HGLibA_17096 | 222 | 1.06052835 | FBXO41 | F-box protein 41 [Source:HGNC Symbol;Acc:HGNC:29409] |
| ENSG00000165240 | HGLibA_03769 | 222 | 1.06052835 | ATP7A | ATPase copper transporting alpha [Source:HGNC Symbol;Acc:HGNC:869] |
| ENSG00000166478 | HGLibA_55493 | 222 | 1.06052835 | ZNF143 | zinc finger protein 143 [Source:HGNC Symbol;Acc:HGNC:12928] |
| ENSG00000168143 | HGLibA_16743 | 222 | 1.06052835 | FAM83B | family with sequence similarity 83 member B [Source:HGNC Symbol;Acc:HGNC:21357] |
| ENSG00000169228 | HGLibA_39846 | 222 | 1.06052835 | RAB24 | member RAS oncogene family [Source:HGNC Symbol;Acc:HGNC:9765]" |
| ENSG00000179165 | HGLibA_39668 | 222 | 1.06052835 | PXT1 | peroxisomal testis enriched protein 1 [Source:HGNC Symbol;Acc:HGNC:18312] |
| ENSG00000182389 | HGLibA_06925 | 222 | 1.06052835 | CACNB4 | calcium voltage-gated channel auxiliary subunit beta 4 [Source:HGNC Symbol;Acc:HGNC:1404] |
| ENSG00000182973 | HGLibA_10330 | 222 | 1.06052835 | CNOT10 | CCR4-NOT transcription complex subunit 10 [Source:HGNC Symbol;Acc:HGNC:23817] |
| ENSG00000183844 | HGLibA_16580 | 222 | 1.06052835 | FAM3B | family with sequence similarity 3 member B [Source:HGNC Symbol;Acc:HGNC:1253] |
| ENSG00000185024 | HGLibA_04797 | 222 | 1.06052835 | BRF1 | RNA polymerase III transcription initiation factor subunit [Source:HGNC Symbol;Acc:HGNC:11551]" |
| ENSG00000185513 | HGLibA_25896 | 222 | 1.06052835 | L3MBTL1 | histone methyl-lysine binding protein [Source:HGNC Symbol;Acc:HGNC:15905]" |
| ENSG00000186283 | HGLibA_51031 | 222 | 1.06052835 | TOR3A | torsin family 3 member A [Source:HGNC Symbol;Acc:HGNC:11997] |
| ENSG00000187037 | HGLibA_19910 | 222 | 1.06052835 | GPR141 | G protein-coupled receptor 141 [Source:HGNC Symbol;Acc:HGNC:19997] |
| ENSG00000187170 | HGLibA_26144 | 222 | 1.06052835 | LCE4A | late cornified envelope 4A [Source:HGNC Symbol;Acc:HGNC:16613] |
| ENSG00000188316 | HGLibA_15150 | 222 | 1.06052835 | ENO4 | enolase 4 [Source:HGNC Symbol;Acc:HGNC:31670] |
| ENSG00000188833 | HGLibA_15217 | 222 | 1.06052835 | ENTPD8 | ectonucleoside triphosphate diphosphohydrolase 8 [Source:HGNC Symbol;Acc:HGNC:24860] |
| ENSG00000188917 | HGLibA_51687 | 222 | 1.06052835 | TRMT2B | tRNA methyltransferase 2 homolog B [Source:HGNC Symbol;Acc:HGNC:25748] |
| ENSG00000197415 | HGLibA_53624 | 222 | 1.06052835 | VEPH1 | ventricular zone expressed PH domain containing 1 [Source:HGNC Symbol;Acc:HGNC:25735] |
| ENSG00000204518 | HGLibA_00039 | 222 | 1.06052835 | AADACL4 | arylacetamide deacetylase like 4 [Source:HGNC Symbol;Acc:HGNC:32038] |
| ENSG00000205133 | HGLibA_51652 | 222 | 1.06052835 | TRIQK | triple QxxK/R motif containing [Source:HGNC Symbol;Acc:HGNC:27828] |
| ENSG00000205693 | HGLibA_28102 | 222 | 1.06052835 | MANSC4 | MANSC domain containing 4 [Source:HGNC Symbol;Acc:HGNC:40023] |
| ENSG00000213401 | HGLibA_27901 | 222 | 1.06052835 | MAGEA12 | MAGE family member A12 [Source:HGNC Symbol;Acc:HGNC:6799] |
| ENSG00000233838 | HGLibA_13846 | 222 | 1.06052835 | DPH3P1 | diphthamide biosynthesis 3 pseudogene 1 [Source:HGNC Symbol;Acc:HGNC:16136] |
| ENSG00000240563 | HGLibA_25886 | 222 | 1.06052835 | L1TD1 | LINE1 type transposase domain containing 1 [Source:HGNC Symbol;Acc:HGNC:25595] |
| ENSG00000240871 | HGLibA_25774 | 222 | 1.06052835 | KRTAP4-7 | keratin associated protein 4-7 [Source:HGNC Symbol;Acc:HGNC:18898] |
| ENSG00000243729 | HGLibA_34363 | 222 | 1.06052835 | OR5V1 | olfactory receptor family 5 subfamily V member 1 [Source:HGNC Symbol;Acc:HGNC:13972] |
| ENSG00000243789 | HGLibA_23960 | 222 | 1.06052835 | JMJD7 | jumonji domain containing 7 [Source:HGNC Symbol;Acc:HGNC:34397] |
| hsa-mir-1244-1 | HGLibA_57361 | 222 | 1.06052835 | hsa-mir-1244-1 | - |
| hsa-mir-1471 | HGLibA_57869 | 222 | 1.06052835 | hsa-mir-1471 | - |
| hsa-mir-181d | HGLibA_57980 | 222 | 1.06052835 | hsa-mir-181d | - |
| hsa-mir-4635 | HGLibA_60645 | 222 | 1.06052835 | hsa-mir-4635 | - |
| hsa-mir-4722 | HGLibA_60985 | 222 | 1.06052835 | hsa-mir-4722 | - |
| hsa-mir-8059 | HGLibA_64073 | 222 | 1.06052835 | hsa-mir-8059 | - |
| MKL2 | HGLibA_29337 | 222 | 1.06052835 | MKL2 | - |
| TMEM180 | HGLibA_50096 | 222 | 1.06052835 | TMEM180 | - |
| ELMSAN1 | HGLibA_14947 | 221 | 1.050577312 | ELMSAN1 | - |
| ENSG00000002726 | HGLibA_02267 | 221 | 1.050577312 | AOC1 | copper containing 1 [Source:HGNC Symbol;Acc:HGNC:80]" |
| ENSG00000011028 | HGLibA_29818 | 221 | 1.050577312 | MRC2 | mannose receptor C type 2 [Source:HGNC Symbol;Acc:HGNC:16875] |
| ENSG00000034533 | HGLibA_03374 | 221 | 1.050577312 | ASTE1 | asteroid homolog 1 [Source:HGNC Symbol;Acc:HGNC:25021] |
| ENSG00000053328 | HGLibA_28995 | 221 | 1.050577312 | METTL24 | methyltransferase like 24 [Source:HGNC Symbol;Acc:HGNC:21566] |
| ENSG00000063660 | HGLibA_19761 | 221 | 1.050577312 | GPC1 | glypican 1 [Source:HGNC Symbol;Acc:HGNC:4449] |
| ENSG00000089234 | HGLibA_04754 | 221 | 1.050577312 | BRAP | BRCA1 associated protein [Source:HGNC Symbol;Acc:HGNC:1099] |
| ENSG00000101333 | HGLibA_36987 | 221 | 1.050577312 | PLCB4 | phospholipase C beta 4 [Source:HGNC Symbol;Acc:HGNC:9059] |
| ENSG00000103671 | HGLibA_51645 | 221 | 1.050577312 | TRIP4 | thyroid hormone receptor interactor 4 [Source:HGNC Symbol;Acc:HGNC:12310] |
| ENSG00000104901 | HGLibA_13305 | 221 | 1.050577312 | DKKL1 | dickkopf like acrosomal protein 1 [Source:HGNC Symbol;Acc:HGNC:16528] |
| ENSG00000105376 | HGLibA_22505 | 221 | 1.050577312 | ICAM5 | intercellular adhesion molecule 5 [Source:HGNC Symbol;Acc:HGNC:5348] |
| ENSG00000106624 | HGLibA_01084 | 221 | 1.050577312 | AEBP1 | AE binding protein 1 [Source:HGNC Symbol;Acc:HGNC:303] |
| ENSG00000108599 | HGLibA_01407 | 221 | 1.050577312 | AKAP10 | A-kinase anchoring protein 10 [Source:HGNC Symbol;Acc:HGNC:368] |
| ENSG00000109107 | HGLibA_01598 | 221 | 1.050577312 | ALDOC | fructose-bisphosphate C [Source:HGNC Symbol;Acc:HGNC:418]" |
| ENSG00000112186 | HGLibA_07137 | 221 | 1.050577312 | CAP2 | cyclase associated actin cytoskeleton regulatory protein 2 [Source:HGNC Symbol;Acc:HGNC:20039] |
| ENSG00000117153 | HGLibA_25130 | 221 | 1.050577312 | KLHL12 | kelch like family member 12 [Source:HGNC Symbol;Acc:HGNC:19360] |
| ENSG00000118217 | HGLibA_03446 | 221 | 1.050577312 | ATF6 | activating transcription factor 6 [Source:HGNC Symbol;Acc:HGNC:791] |
| ENSG00000118257 | HGLibA_32718 | 221 | 1.050577312 | NRP2 | neuropilin 2 [Source:HGNC Symbol;Acc:HGNC:8005] |
| ENSG00000125144 | HGLibA_30288 | 221 | 1.050577312 | MT1G | metallothionein 1G [Source:HGNC Symbol;Acc:HGNC:7399] |
| ENSG00000126581 | HGLibA_04395 | 221 | 1.050577312 | BECN1 | beclin 1 [Source:HGNC Symbol;Acc:HGNC:1034] |
| ENSG00000130304 | HGLibA_44814 | 221 | 1.050577312 | SLC27A1 | solute carrier family 27 member 1 [Source:HGNC Symbol;Acc:HGNC:10995] |
| ENSG00000130383 | HGLibA_18196 | 221 | 1.050577312 | FUT5 | fucosyltransferase 5 [Source:HGNC Symbol;Acc:HGNC:4016] |
| ENSG00000133401 | HGLibA_35974 | 221 | 1.050577312 | PDZD2 | PDZ domain containing 2 [Source:HGNC Symbol;Acc:HGNC:18486] |
| ENSG00000134198 | HGLibA_51922 | 221 | 1.050577312 | TSPAN2 | tetraspanin 2 [Source:HGNC Symbol;Acc:HGNC:20659] |
| ENSG00000134627 | HGLibA_36779 | 221 | 1.050577312 | PIWIL4 | piwi like RNA-mediated gene silencing 4 [Source:HGNC Symbol;Acc:HGNC:18444] |
| ENSG00000137486 | HGLibA_03106 | 221 | 1.050577312 | ARRB1 | arrestin beta 1 [Source:HGNC Symbol;Acc:HGNC:711] |
| ENSG00000139211 | HGLibA_01796 | 221 | 1.050577312 | AMIGO2 | adhesion molecule with Ig like domain 2 [Source:HGNC Symbol;Acc:HGNC:24073] |
| ENSG00000139354 | HGLibA_18650 | 221 | 1.050577312 | GAS2L3 | growth arrest specific 2 like 3 [Source:HGNC Symbol;Acc:HGNC:27475] |
| ENSG00000139636 | HGLibA_26671 | 221 | 1.050577312 | LMBR1L | limb development membrane protein 1 like [Source:HGNC Symbol;Acc:HGNC:18268] |
| ENSG00000140030 | HGLibA_20073 | 221 | 1.050577312 | GPR65 | G protein-coupled receptor 65 [Source:HGNC Symbol;Acc:HGNC:4517] |
| ENSG00000153558 | HGLibA_16996 | 221 | 1.050577312 | FBXL2 | F-box and leucine rich repeat protein 2 [Source:HGNC Symbol;Acc:HGNC:13598] |
| ENSG00000157890 | HGLibA_28850 | 221 | 1.050577312 | MEGF11 | multiple EGF like domains 11 [Source:HGNC Symbol;Acc:HGNC:29635] |
| ENSG00000158764 | HGLibA_23826 | 221 | 1.050577312 | ITLN2 | intelectin 2 [Source:HGNC Symbol;Acc:HGNC:20599] |
| ENSG00000162971 | HGLibA_52490 | 221 | 1.050577312 | TYW5 | tRNA-yW synthesizing protein 5 [Source:HGNC Symbol;Acc:HGNC:26754] |
| ENSG00000163599 | HGLibA_11636 | 221 | 1.050577312 | CTLA4 | cytotoxic T-lymphocyte associated protein 4 [Source:HGNC Symbol;Acc:HGNC:2505] |
| ENSG00000164128 | HGLibA_32550 | 221 | 1.050577312 | NPY1R | neuropeptide Y receptor Y1 [Source:HGNC Symbol;Acc:HGNC:7956] |
| ENSG00000168904 | HGLibA_27300 | 221 | 1.050577312 | LRRC28 | leucine rich repeat containing 28 [Source:HGNC Symbol;Acc:HGNC:28355] |
| ENSG00000169933 | HGLibA_18039 | 221 | 1.050577312 | FRMPD4 | FERM and PDZ domain containing 4 [Source:HGNC Symbol;Acc:HGNC:29007] |
| ENSG00000171557 | HGLibA_17456 | 221 | 1.050577312 | FGG | fibrinogen gamma chain [Source:HGNC Symbol;Acc:HGNC:3694] |
| ENSG00000171700 | HGLibA_41076 | 221 | 1.050577312 | RGS19 | regulator of G protein signaling 19 [Source:HGNC Symbol;Acc:HGNC:13735] |
| ENSG00000172987 | HGLibA_22064 | 221 | 1.050577312 | HPSE2 | heparanase 2 (inactive) [Source:HGNC Symbol;Acc:HGNC:18374] |
| ENSG00000180071 | HGLibA_02019 | 221 | 1.050577312 | ANKRD18A | ankyrin repeat domain 18A [Source:HGNC Symbol;Acc:HGNC:23643] |
| ENSG00000180090 | HGLibA_33914 | 221 | 1.050577312 | OR3A1 | olfactory receptor family 3 subfamily A member 1 [Source:HGNC Symbol;Acc:HGNC:8282] |
| ENSG00000183386 | HGLibA_17486 | 221 | 1.050577312 | FHL3 | four and a half LIM domains 3 [Source:HGNC Symbol;Acc:HGNC:3704] |
| ENSG00000187959 | HGLibA_11008 | 221 | 1.050577312 | CPSF4L | cleavage and polyadenylation specific factor 4 like [Source:HGNC Symbol;Acc:HGNC:33632] |
| ENSG00000188021 | HGLibA_52734 | 221 | 1.050577312 | UBQLN2 | ubiquilin 2 [Source:HGNC Symbol;Acc:HGNC:12509] |
| ENSG00000196465 | HGLibA_30772 | 221 | 1.050577312 | MYL6B | myosin light chain 6B [Source:HGNC Symbol;Acc:HGNC:29823] |
| ENSG00000196639 | HGLibA_22093 | 221 | 1.050577312 | HRH1 | histamine receptor H1 [Source:HGNC Symbol;Acc:HGNC:5182] |
| ENSG00000197121 | HGLibA_36207 | 221 | 1.050577312 | PGAP1 | post-GPI attachment to proteins 1 [Source:HGNC Symbol;Acc:HGNC:25712] |
| ENSG00000197647 | HGLibA_55988 | 221 | 1.050577312 | ZNF433 | zinc finger protein 433 [Source:HGNC Symbol;Acc:HGNC:20811] |
| ENSG00000206562 | HGLibA_29014 | 221 | 1.050577312 | METTL6 | methyltransferase like 6 [Source:HGNC Symbol;Acc:HGNC:28343] |
| ENSG00000221855 | HGLibA_48404 | 221 | 1.050577312 | TAS2R41 | taste 2 receptor member 41 [Source:HGNC Symbol;Acc:HGNC:18883] |
| ENSG00000244355 | HGLibA_27669 | 221 | 1.050577312 | LY6G6D | lymphocyte antigen 6 family member G6D [Source:HGNC Symbol;Acc:HGNC:13935] |
| ENSG00000249158 | HGLibA_35431 | 221 | 1.050577312 | PCDHA11 | protocadherin alpha 11 [Source:HGNC Symbol;Acc:HGNC:8665] |
| hsa-mir-3174 | HGLibA_58768 | 221 | 1.050577312 | hsa-mir-3174 | - |
| hsa-mir-4273 | HGLibA_59796 | 221 | 1.050577312 | hsa-mir-4273 | - |
| hsa-mir-4655 | HGLibA_60727 | 221 | 1.050577312 | hsa-mir-4655 | - |
| hsa-mir-4790 | HGLibA_61266 | 221 | 1.050577312 | hsa-mir-4790 | - |
| hsa-mir-6753 | HGLibA_63169 | 221 | 1.050577312 | hsa-mir-6753 | - |
| hsa-mir-6825 | HGLibA_63478 | 221 | 1.050577312 | hsa-mir-6825 | - |
| KIAA1045 | HGLibA_24689 | 221 | 1.050577312 | KIAA1045 | - |
| LOC100129480 | HGLibA_26763 | 221 | 1.050577312 | LOC100129480 | - |
| XAGE1D | HGLibA_54475 | 221 | 1.050577312 | XAGE1D | - |
| C2orf53 | HGLibA_06147 | 220 | 1.040626275 | C2orf53 | - |
| ENSG00000008438 | HGLibA_36249 | 220 | 1.040626275 | PGLYRP1 | peptidoglycan recognition protein 1 [Source:HGNC Symbol;Acc:HGNC:8904] |
| ENSG00000011454 | HGLibA_40010 | 220 | 1.040626275 | RABGAP1 | RAB GTPase activating protein 1 [Source:HGNC Symbol;Acc:HGNC:17155] |
| ENSG00000064393 | HGLibA_21341 | 220 | 1.040626275 | HIPK2 | homeodomain interacting protein kinase 2 [Source:HGNC Symbol;Acc:HGNC:14402] |
| ENSG00000065675 | HGLibA_38556 | 220 | 1.040626275 | PRKCQ | protein kinase C theta [Source:HGNC Symbol;Acc:HGNC:9410] |
| ENSG00000084207 | HGLibA_20492 | 220 | 1.040626275 | GSTP1 | glutathione S-transferase pi 1 [Source:HGNC Symbol;Acc:HGNC:4638] |
| ENSG00000088832 | HGLibA_17563 | 220 | 1.040626275 | FKBP1A | FK506 binding protein 1A [Source:HGNC Symbol;Acc:HGNC:3711] |
| ENSG00000095713 | HGLibA_11220 | 220 | 1.040626275 | CRTAC1 | cartilage acidic protein 1 [Source:HGNC Symbol;Acc:HGNC:14882] |
| ENSG00000100321 | HGLibA_47990 | 220 | 1.040626275 | SYNGR1 | synaptogyrin 1 [Source:HGNC Symbol;Acc:HGNC:11498] |
| ENSG00000108582 | HGLibA_10910 | 220 | 1.040626275 | CPD | carboxypeptidase D [Source:HGNC Symbol;Acc:HGNC:2301] |
| ENSG00000108813 | HGLibA_13381 | 220 | 1.040626275 | DLX4 | distal-less homeobox 4 [Source:HGNC Symbol;Acc:HGNC:2917] |
| ENSG00000116771 | HGLibA_01199 | 220 | 1.040626275 | AGMAT | agmatinase [Source:HGNC Symbol;Acc:HGNC:18407] |
| ENSG00000116906 | HGLibA_19562 | 220 | 1.040626275 | GNPAT | glyceronephosphate O-acyltransferase [Source:HGNC Symbol;Acc:HGNC:4416] |
| ENSG00000117971 | HGLibA_09599 | 220 | 1.040626275 | CHRNB4 | cholinergic receptor nicotinic beta 4 subunit [Source:HGNC Symbol;Acc:HGNC:1964] |
| ENSG00000119403 | HGLibA_36357 | 220 | 1.040626275 | PHF19 | PHD finger protein 19 [Source:HGNC Symbol;Acc:HGNC:24566] |
| ENSG00000120889 | HGLibA_50735 | 220 | 1.040626275 | TNFRSF10B | TNF receptor superfamily member 10b [Source:HGNC Symbol;Acc:HGNC:11905] |
| ENSG00000121797 | HGLibA_08177 | 220 | 1.040626275 | CCRL2 | C-C motif chemokine receptor like 2 [Source:HGNC Symbol;Acc:HGNC:1612] |
| ENSG00000122145 | HGLibA_48666 | 220 | 1.040626275 | TBX22 | T-box 22 [Source:HGNC Symbol;Acc:HGNC:11600] |
| ENSG00000122705 | HGLibA_10144 | 220 | 1.040626275 | CLTA | clathrin light chain A [Source:HGNC Symbol;Acc:HGNC:2090] |
| ENSG00000123119 | HGLibA_31584 | 220 | 1.040626275 | NECAB1 | N-terminal EF-hand calcium binding protein 1 [Source:HGNC Symbol;Acc:HGNC:20983] |
| ENSG00000124143 | HGLibA_02778 | 220 | 1.040626275 | ARHGAP40 | Rho GTPase activating protein 40 [Source:HGNC Symbol;Acc:HGNC:16226] |
| ENSG00000126790 | HGLibA_25893 | 220 | 1.040626275 | L3HYPDH | trans-L-3-hydroxyproline dehydratase [Source:HGNC Symbol;Acc:HGNC:20488] |
| ENSG00000127511 | HGLibA_44185 | 220 | 1.040626275 | SIN3B | SIN3 transcription regulator family member B [Source:HGNC Symbol;Acc:HGNC:19354] |
| ENSG00000130159 | HGLibA_14422 | 220 | 1.040626275 | ECSIT | ECSIT signalling integrator [Source:HGNC Symbol;Acc:HGNC:29548] |
| ENSG00000132153 | HGLibA_13175 | 220 | 1.040626275 | DHX30 | DExH-box helicase 30 [Source:HGNC Symbol;Acc:HGNC:16716] |
| ENSG00000133704 | HGLibA_23474 | 220 | 1.040626275 | IPO8 | importin 8 [Source:HGNC Symbol;Acc:HGNC:9853] |
| ENSG00000134324 | HGLibA_27094 | 220 | 1.040626275 | LPIN1 | lipin 1 [Source:HGNC Symbol;Acc:HGNC:13345] |
| ENSG00000135476 | HGLibA_15572 | 220 | 1.040626275 | ESPL1 | separase [Source:HGNC Symbol;Acc:HGNC:16856]" |
| ENSG00000138073 | HGLibA_38425 | 220 | 1.040626275 | PREB | prolactin regulatory element binding [Source:HGNC Symbol;Acc:HGNC:9356] |
| ENSG00000140379 | HGLibA_04298 | 220 | 1.040626275 | BCL2A1 | BCL2 related protein A1 [Source:HGNC Symbol;Acc:HGNC:991] |
| ENSG00000141293 | HGLibA_44270 | 220 | 1.040626275 | SKAP1 | src kinase associated phosphoprotein 1 [Source:HGNC Symbol;Acc:HGNC:15605] |
| ENSG00000143556 | HGLibA_42567 | 220 | 1.040626275 | S100A7 | S100 calcium binding protein A7 [Source:HGNC Symbol;Acc:HGNC:10497] |
| ENSG00000147454 | HGLibA_44724 | 220 | 1.040626275 | SLC25A37 | solute carrier family 25 member 37 [Source:HGNC Symbol;Acc:HGNC:29786] |

| | | | | | |
|---|---|---|---|---|---|
| ENSG00000151062 | HGLibA_06915 | 220 | 1.040626275 | CACNA2D4 | calcium voltage-gated channel auxiliary subunit alpha2delta 4 [Source:HGNC Symbol;Acc:HGNC:20202] |
| ENSG00000158125 | HGLibA_54501 | 220 | 1.040626275 | XDH | xanthine dehydrogenase [Source:HGNC Symbol;Acc:HGNC:12805] |
| ENSG00000161640 | HGLibA_44130 | 220 | 1.040626275 | SIGLEC11 | sialic acid binding Ig like lectin 11 [Source:HGNC Symbol;Acc:HGNC:15622] |
| ENSG00000164692 | HGLibA_10532 | 220 | 1.040626275 | COL1A2 | collagen type I alpha 2 chain [Source:HGNC Symbol;Acc:HGNC:2198] |
| ENSG00000165046 | HGLibA_26336 | 220 | 1.040626275 | LETM2 | leucine zipper and EF-hand containing transmembrane protein 2 [Source:HGNC Symbol;Acc:HGNC:14648] |
| ENSG00000169908 | HGLibA_49691 | 220 | 1.040626275 | TM4SF1 | transmembrane 4 L six family member 1 [Source:HGNC Symbol;Acc:HGNC:11853] |
| ENSG00000171936 | HGLibA_33513 | 220 | 1.040626275 | OR10H3 | olfactory receptor family 10 subfamily H member 3 [Source:HGNC Symbol;Acc:HGNC:8174] |
| ENSG00000171940 | HGLibA_55600 | 220 | 1.040626275 | ZNF217 | zinc finger protein 217 [Source:HGNC Symbol;Acc:HGNC:13009] |
| ENSG00000174175 | HGLibA_43304 | 220 | 1.040626275 | SELP | selectin P [Source:HGNC Symbol;Acc:HGNC:10721] |
| ENSG00000175463 | HGLibA_48470 | 220 | 1.040626275 | TBC1D10C | TBC1 domain family member 10C [Source:HGNC Symbol;Acc:HGNC:24702] |
| ENSG00000178226 | HGLibA_38931 | 220 | 1.040626275 | PRSS36 | serine protease 36 [Source:HGNC Symbol;Acc:HGNC:26906] |
| ENSG00000180316 | HGLibA_37405 | 220 | 1.040626275 | PNPLA1 | patatin like phospholipase domain containing 1 [Source:HGNC Symbol;Acc:HGNC:21246] |
| ENSG00000181035 | HGLibA_44742 | 220 | 1.040626275 | SLC25A42 | solute carrier family 25 member 42 [Source:HGNC Symbol;Acc:HGNC:28380] |
| ENSG00000186579 | HGLibA_12831 | 220 | 1.040626275 | DEFB106A | defensin beta 106A [Source:HGNC Symbol;Acc:HGNC:18088] |
| ENSG00000187017 | HGLibA_15575 | 220 | 1.040626275 | ESPN | espin [Source:HGNC Symbol;Acc:HGNC:13281] |
| ENSG00000187033 | HGLibA_42693 | 220 | 1.040626275 | SAMD7 | sterile alpha motif domain containing 7 [Source:HGNC Symbol;Acc:HGNC:25394] |
| ENSG00000187954 | HGLibA_12040 | 220 | 1.040626275 | CYHR1 | cysteine and histidine rich 1 [Source:HGNC Symbol;Acc:HGNC:17806] |
| ENSG00000188176 | HGLibA_45836 | 220 | 1.040626275 | SMTNL2 | smoothelin like 2 [Source:HGNC Symbol;Acc:HGNC:24764] |
| ENSG00000196083 | HGLibA_23053 | 220 | 1.040626275 | IL1RAP | interleukin 1 receptor accessory protein [Source:HGNC Symbol;Acc:HGNC:5995] |
| ENSG00000196639 | HGLibA_22095 | 220 | 1.040626275 | HRH1 | histamine receptor H1 [Source:HGNC Symbol;Acc:HGNC:5182] |
| ENSG00000197790 | HGLibA_34189 | 220 | 1.040626275 | OR52M1 | olfactory receptor family 52 subfamily M member 1 [Source:HGNC Symbol;Acc:HGNC:15225] |
| ENSG00000198168 | HGLibA_47890 | 220 | 1.040626275 | SVIP | small VCP interacting protein [Source:HGNC Symbol;Acc:HGNC:25238] |
| ENSG00000198171 | HGLibA_12639 | 220 | 1.040626275 | DDRGK1 | DDRGK domain containing 1 [Source:HGNC Symbol;Acc:HGNC:16110] |
| ENSG00000204946 | HGLibA_56744 | 220 | 1.040626275 | ZNF783 | zinc finger family member 783 [Source:HGNC Symbol;Acc:HGNC:27222] |
| ENSG00000214456 | HGLibA_37181 | 220 | 1.040626275 | PLIN5 | perilipin 5 [Source:HGNC Symbol;Acc:HGNC:33196] |
| ENSG00000244537 | HGLibA_25759 | 220 | 1.040626275 | KRTAP4-2 | keratin associated protein 4-2 [Source:HGNC Symbol;Acc:HGNC:18900] |
| ENSG00000262406 | HGLibA_29485 | 220 | 1.040626275 | MMP12 | matrix metallopeptidase 12 [Source:HGNC Symbol;Acc:HGNC:7158] |
| ENSG00000275385 | HGLibA_07983 | 220 | 1.040626275 | CCL18 | C-C motif chemokine ligand 18 [Source:HGNC Symbol;Acc:HGNC:10616] |
| ENSG00000278224 | HGLibA_38480 | 220 | 1.040626275 | PRICKLE4 | prickle planar cell polarity protein 4 [Source:HGNC Symbol;Acc:HGNC:16805] |
| hsa-mir-2276 | HGLibA_58309 | 220 | 1.040626275 | hsa-mir-2276 | - |
| hsa-mir-4431 | HGLibA_60109 | 220 | 1.040626275 | hsa-mir-4431 | - |
| hsa-mir-4652 | HGLibA_60715 | 220 | 1.040626275 | hsa-mir-4652 | - |
| hsa-mir-4786 | HGLibA_61250 | 220 | 1.040626275 | hsa-mir-4786 | - |
| hsa-mir-5000 | HGLibA_61408 | 220 | 1.040626275 | hsa-mir-5000 | - |
| hsa-mir-637 | HGLibA_62789 | 220 | 1.040626275 | hsa-mir-637 | - |
| hsa-mir-7515 | HGLibA_63889 | 220 | 1.040626275 | hsa-mir-7515 | - |
| KIAA0368 | HGLibA_24629 | 220 | 1.040626275 | KIAA0368 | - |
| KIAA1244 | HGLibA_24727 | 220 | 1.040626275 | KIAA1244 | - |
| MTERFD3 | HGLibA_30345 | 220 | 1.040626275 | MTERFD3 | - |
| SETD8 | HGLibA_43660 | 220 | 1.040626275 | SETD8 | - |
| TCEB3CL2 | HGLibA_48751 | 220 | 1.040626275 | TCEB3CL2 | - |
| C11orf88 | HGLibA_05207 | 219 | 1.030675237 | C11orf88 | - |
| C15orf53 | HGLibA_05411 | 219 | 1.030675237 | C15orf53 | - |
| C9orf171 | HGLibA_06710 | 219 | 1.030675237 | C9orf171 | - |
| ENSG00000036473 | HGLibA_34720 | 219 | 1.030675237 | OTC | ornithine carbamoyltransferase [Source:HGNC Symbol;Acc:HGNC:8512] |
| ENSG00000066629 | HGLibA_15055 | 219 | 1.030675237 | EML1 | echinoderm microtubule associated protein like 1 [Source:HGNC Symbol;Acc:HGNC:3330] |
| ENSG00000070366 | HGLibA_45704 | 219 | 1.030675237 | SMG6 | nonsense mediated mRNA decay factor [Source:HGNC Symbol;Acc:HGNC:17809]" |
| ENSG00000070540 | HGLibA_54313 | 219 | 1.030675237 | WIPI1 | phosphoinositide interacting 1 [Source:HGNC Symbol;Acc:HGNC:25471]" |
| ENSG00000071051 | HGLibA_31308 | 219 | 1.030675237 | NCK2 | NCK adaptor protein 2 [Source:HGNC Symbol;Acc:HGNC:7665] |
| ENSG00000073670 | HGLibA_00732 | 219 | 1.030675237 | ADAM11 | ADAM metallopeptidase domain 11 [Source:HGNC Symbol;Acc:HGNC:189] |
| ENSG00000083782 | HGLibA_15395 | 219 | 1.030675237 | EPYC | epiphycan [Source:HGNC Symbol;Acc:HGNC:3053] |
| ENSG00000086232 | HGLibA_14722 | 219 | 1.030675237 | EIF2AK1 | eukaryotic translation initiation factor 2 alpha kinase 1 [Source:HGNC Symbol;Acc:HGNC:24921] |
| ENSG00000096384 | HGLibA_22252 | 219 | 1.030675237 | HSP90AB1 | heat shock protein 90 alpha family class B member 1 [Source:HGNC Symbol;Acc:HGNC:5258] |
| ENSG00000099715 | HGLibA_35393 | 219 | 1.030675237 | PCDH11Y | protocadherin 11 Y-linked [Source:HGNC Symbol;Acc:HGNC:15813] |
| ENSG00000100577 | HGLibA_20506 | 219 | 1.030675237 | GSTZ1 | glutathione S-transferase zeta 1 [Source:HGNC Symbol;Acc:HGNC:4643] |
| ENSG00000102870 | HGLibA_56430 | 219 | 1.030675237 | ZNF629 | zinc finger protein 629 [Source:HGNC Symbol;Acc:HGNC:29008] |
| ENSG00000104067 | HGLibA_49570 | 219 | 1.030675237 | TJP1 | tight junction protein 1 [Source:HGNC Symbol;Acc:HGNC:11827] |
| ENSG00000104892 | HGLibA_25033 | 219 | 1.030675237 | KLC3 | kinesin light chain 3 [Source:HGNC Symbol;Acc:HGNC:20717] |
| ENSG00000105186 | HGLibA_02052 | 219 | 1.030675237 | ANKRD27 | ankyrin repeat domain 27 [Source:HGNC Symbol;Acc:HGNC:25310] |
| ENSG00000106771 | HGLibA_50301 | 219 | 1.030675237 | TMEM245 | transmembrane protein 245 [Source:HGNC Symbol;Acc:HGNC:1363] |
| ENSG00000107968 | HGLibA_28201 | 219 | 1.030675237 | MAP3K8 | mitogen-activated protein kinase kinase kinase 8 [Source:HGNC Symbol;Acc:HGNC:6860] |
| ENSG00000108688 | HGLibA_08040 | 219 | 1.030675237 | CCL7 | C-C motif chemokine ligand 7 [Source:HGNC Symbol;Acc:HGNC:10634] |
| ENSG00000109016 | HGLibA_13147 | 219 | 1.030675237 | DHRS7B | dehydrogenase/reductase 7B [Source:HGNC Symbol;Acc:HGNC:24547] |
| ENSG00000119547 | HGLibA_33396 | 219 | 1.030675237 | ONECUT2 | one cut homeobox 2 [Source:HGNC Symbol;Acc:HGNC:8139] |
| ENSG00000119917 | HGLibA_22631 | 219 | 1.030675237 | IFIT3 | interferon induced protein with tetratricopeptide repeats 3 [Source:HGNC Symbol;Acc:HGNC:5411] |
| ENSG00000123607 | HGLibA_52073 | 219 | 1.030675237 | TTC21B | tetratricopeptide repeat domain 21B [Source:HGNC Symbol;Acc:HGNC:25660] |
| ENSG00000125386 | HGLibA_16363 | 219 | 1.030675237 | FAM193A | family with sequence similarity 193 member A [Source:HGNC Symbol;Acc:HGNC:16822] |
| ENSG00000129194 | HGLibA_46244 | 219 | 1.030675237 | SOX15 | SRY-box 15 [Source:HGNC Symbol;Acc:HGNC:11196] |
| ENSG00000131323 | HGLibA_51247 | 219 | 1.030675237 | TRAF3 | TNF receptor associated factor 3 [Source:HGNC Symbol;Acc:HGNC:12033] |
| ENSG00000135632 | HGLibA_45862 | 219 | 1.030675237 | SMYD5 | SMYD family member 5 [Source:HGNC Symbol;Acc:HGNC:16258] |
| ENSG00000136732 | HGLibA_20704 | 219 | 1.030675237 | GYPC | glycophorin C (Gerbich blood group) [Source:HGNC Symbol;Acc:HGNC:4704] |
| ENSG00000139597 | HGLibA_30954 | 219 | 1.030675237 | N4BP2L1 | NEDD4 binding protein 2 like 1 [Source:HGNC Symbol;Acc:HGNC:25037] |
| ENSG00000141985 | HGLibA_43946 | 219 | 1.030675237 | SH3GL1 | endophilin A2 [Source:HGNC Symbol;Acc:HGNC:10830]" |
| ENSG00000143315 | HGLibA_36566 | 219 | 1.030675237 | PIGM | phosphatidylinositol glycan anchor biosynthesis class M [Source:HGNC Symbol;Acc:HGNC:18858] |
| ENSG00000147912 | HGLibA_17024 | 219 | 1.030675237 | FBXO10 | F-box protein 10 [Source:HGNC Symbol;Acc:HGNC:13589] |
| ENSG00000152254 | HGLibA_18309 | 219 | 1.030675237 | G6PC2 | glucose-6-phosphatase catalytic subunit 2 [Source:HGNC Symbol;Acc:HGNC:28906] |
| ENSG00000155366 | HGLibA_41181 | 219 | 1.030675237 | RHOC | ras homolog family member C [Source:HGNC Symbol;Acc:HGNC:669] |
| ENSG00000156009 | HGLibA_27921 | 219 | 1.030675237 | MAGEA8 | MAGE family member A8 [Source:HGNC Symbol;Acc:HGNC:6806] |
| ENSG00000158296 | HGLibA_44389 | 219 | 1.030675237 | SLC13A3 | solute carrier family 13 member 3 [Source:HGNC Symbol;Acc:HGNC:14430] |
| ENSG00000159461 | HGLibA_01782 | 219 | 1.030675237 | AMFR | autocrine motility factor receptor [Source:HGNC Symbol;Acc:HGNC:463] |
| ENSG00000164853 | HGLibA_53041 | 219 | 1.030675237 | UNCX | UNC homeobox [Source:HGNC Symbol;Acc:HGNC:33194] |
| ENSG00000166111 | HGLibA_47893 | 219 | 1.030675237 | SVOP | SV2 related protein [Source:HGNC Symbol;Acc:HGNC:25417] |
| ENSG00000168828 | HGLibA_33626 | 219 | 1.030675237 | OR13J1 | olfactory receptor family 13 subfamily J member 1 [Source:HGNC Symbol;Acc:HGNC:15108] |
| ENSG00000171827 | HGLibA_56276 | 219 | 1.030675237 | ZNF570 | zinc finger protein 570 [Source:HGNC Symbol;Acc:HGNC:26416] |
| ENSG00000172932 | HGLibA_02009 | 219 | 1.030675237 | ANKRD13D | ankyrin repeat domain 13D [Source:HGNC Symbol;Acc:HGNC:27880] |
| ENSG00000175556 | HGLibA_27026 | 219 | 1.030675237 | LONRF3 | LON peptidase N-terminal domain and ring finger 3 [Source:HGNC Symbol;Acc:HGNC:21152] |
| ENSG00000180957 | HGLibA_36743 | 219 | 1.030675237 | PITPNB | phosphatidylinositol transfer protein beta [Source:HGNC Symbol;Acc:HGNC:9002] |
| ENSG00000183048 | HGLibA_44635 | 219 | 1.030675237 | SLC25A10 | solute carrier family 25 member 10 [Source:HGNC Symbol;Acc:HGNC:10980] |
| ENSG00000184445 | HGLibA_25350 | 219 | 1.030675237 | KNTC1 | kinetochore associated 1 [Source:HGNC Symbol;Acc:HGNC:17255] |
| ENSG00000186625 | HGLibA_24073 | 219 | 1.030675237 | KATNA1 | katanin catalytic subunit A1 [Source:HGNC Symbol;Acc:HGNC:6216] |
| ENSG00000187824 | HGLibA_50235 | 219 | 1.030675237 | TMEM220 | transmembrane protein 220 [Source:HGNC Symbol;Acc:HGNC:33757] |
| ENSG00000188707 | HGLibA_54804 | 219 | 1.030675237 | ZBED6CL | ZBED6 C-terminal like [Source:HGNC Symbol;Acc:HGNC:21720] |
| ENSG00000196260 | HGLibA_43759 | 219 | 1.030675237 | SFTA2 | surfactant associated 2 [Source:HGNC Symbol;Acc:HGNC:18386] |
| ENSG00000198876 | HGLibA_12419 | 219 | 1.030675237 | DCAF12 | DDB1 and CUL4 associated factor 12 [Source:HGNC Symbol;Acc:HGNC:19911] |
| ENSG00000204140 | HGLibA_10106 | 219 | 1.030675237 | CLPSL1 | colipase like 1 [Source:HGNC Symbol;Acc:HGNC:21251] |
| ENSG00000206177 | HGLibA_20932 | 219 | 1.030675237 | HBM | hemoglobin subunit mu [Source:HGNC Symbol;Acc:HGNC:4826] |
| ENSG00000212993 | HGLibA_37789 | 219 | 1.030675237 | POU5F1B | POU class 5 homeobox 1B [Source:HGNC Symbol;Acc:HGNC:9223] |
| ENSG00000213218 | HGLibA_11353 | 219 | 1.030675237 | CSH2 | chorionic somatomammotropin hormone 2 [Source:HGNC Symbol;Acc:HGNC:2441] |
| ENSG00000221888 | HGLibA_33651 | 219 | 1.030675237 | OR1C1 | olfactory receptor family 1 subfamily C member 1 [Source:HGNC Symbol;Acc:HGNC:8182] |
| ENSG00000235699 | HGLibA_11941 | 219 | 1.030675237 | CXorf51B | chromosome X open reading frame 51B [Source:HGNC Symbol;Acc:HGNC:42787] |
| ENSG00000288547 | HGLibA_06235 | 219 | 1.030675237 | C3orf36 | - |
| hsa-mir-18b | HGLibA_58023 | 219 | 1.030675237 | hsa-mir-18b | - |
| hsa-mir-3176 | HGLibA_58776 | 219 | 1.030675237 | hsa-mir-3176 | - |
| hsa-mir-3714 | HGLibA_59369 | 219 | 1.030675237 | hsa-mir-3714 | - |
| hsa-mir-4315-2 | HGLibA_59980 | 219 | 1.030675237 | hsa-mir-4315-2 | - |
| hsa-mir-4323 | HGLibA_60016 | 219 | 1.030675237 | hsa-mir-4323 | - |
| hsa-mir-451a | HGLibA_60523 | 219 | 1.030675237 | hsa-mir-451a | - |
| hsa-mir-451b | HGLibA_60527 | 219 | 1.030675237 | hsa-mir-451b | - |
| hsa-mir-4667 | HGLibA_60786 | 219 | 1.030675237 | hsa-mir-4667 | - |
| hsa-mir-4715 | HGLibA_60960 | 219 | 1.030675237 | hsa-mir-4715 | - |
| hsa-mir-4757 | HGLibA_61126 | 219 | 1.030675237 | hsa-mir-4757 | - |
| hsa-mir-5094 | HGLibA_61533 | 219 | 1.030675237 | hsa-mir-5094 | - |
| hsa-mir-510 | HGLibA_61546 | 219 | 1.030675237 | hsa-mir-510 | - |
| hsa-mir-548h-4 | HGLibA_62004 | 219 | 1.030675237 | hsa-mir-548h-4 | - |
| hsa-mir-624 | HGLibA_62736 | 219 | 1.030675237 | hsa-mir-624 | - |
| hsa-mir-6852 | HGLibA_63582 | 219 | 1.030675237 | hsa-mir-6852 | - |
| ITFG3 | HGLibA_23712 | 219 | 1.030675237 | ITFG3 | - |
| KIAA1161 | HGLibA_24703 | 219 | 1.030675237 | KIAA1161 | - |
| MKL2 | HGLibA_29335 | 219 | 1.030675237 | MKL2 | - |
| PROSC | HGLibA_38691 | 219 | 1.030675237 | PROSC | - |
| SMCR9 | HGLibA_45687 | 219 | 1.030675237 | SMCR9 | - |
| C2orf43 | HGLibA_06129 | 218 | 1.0207242 | C2orf43 | - |
| C5orf28 | HGLibA_06371 | 218 | 1.0207242 | C5orf28 | - |
| ENSG00000012061 | HGLibA_15430 | 218 | 1.0207242 | ERCC1 | endonuclease non-catalytic subunit [Source:HGNC Symbol;Acc:HGNC:3433]" |
| ENSG00000017427 | HGLibA_22790 | 218 | 1.0207242 | IGF1 | insulin like growth factor 1 [Source:HGNC Symbol;Acc:HGNC:5464] |
| ENSG00000019485 | HGLibA_38367 | 218 | 1.0207242 | PRDM11 | PR/SET domain 11 [Source:HGNC Symbol;Acc:HGNC:13996] |
| ENSG00000076685 | HGLibA_32811 | 218 | 1.0207242 | NT5C2 | cytosolic II [Source:HGNC Symbol;Acc:HGNC:8022]" |
| ENSG00000084676 | HGLibA_31334 | 218 | 1.0207242 | NCOA1 | nuclear receptor coactivator 1 [Source:HGNC Symbol;Acc:HGNC:7668] |
| ENSG00000104059 | HGLibA_16351 | 218 | 1.0207242 | FAM189A1 | family with sequence similarity 189 member A1 [Source:HGNC Symbol;Acc:HGNC:29075] |
| ENSG00000105609 | HGLibA_26519 | 218 | 1.0207242 | LILRB5 | leukocyte immunoglobulin like receptor B5 [Source:HGNC Symbol;Acc:HGNC:6609] |
| ENSG00000105866 | HGLibA_46304 | 218 | 1.0207242 | SP4 | Sp4 transcription factor [Source:HGNC Symbol;Acc:HGNC:11209] |
| ENSG00000107554 | HGLibA_13696 | 218 | 1.0207242 | DNMBP | dynamin binding protein [Source:HGNC Symbol;Acc:HGNC:30373] |
| ENSG00000112855 | HGLibA_20859 | 218 | 1.0207242 | HARS2 | mitochondrial [Source:HGNC Symbol;Acc:HGNC:4817]" |
| ENSG00000116703 | HGLibA_35711 | 218 | 1.0207242 | PDC | phosducin [Source:HGNC Symbol;Acc:HGNC:8759] |
| ENSG00000116711 | HGLibA_36901 | 218 | 1.0207242 | PLA2G4A | phospholipase A2 group IVA [Source:HGNC Symbol;Acc:HGNC:9035] |

| | | | | | |
|---|---|---|---|---|---|
| ENSG00000120278 | HGLibA_37100 | 218 | 1.0207242 | PLEKHG1 | pleckstrin homology and RhoGEF domain containing G1 [Source:HGNC Symbol;Acc:HGNC:20884] |
| ENSG00000123454 | HGLibA_12382 | 218 | 1.0207242 | DBH | dopamine beta-hydroxylase [Source:HGNC Symbol;Acc:HGNC:2689] |
| ENSG00000124145 | HGLibA_43105 | 218 | 1.0207242 | SDC4 | syndecan 4 [Source:HGNC Symbol;Acc:HGNC:10661] |
| ENSG00000125498 | HGLibA_24982 | 218 | 1.0207242 | KIR2DL1 | two Ig domains and long cytoplasmic tail 1 [Source:HGNC Symbol;Acc:HGNC:6329]" |
| ENSG00000128487 | HGLibA_46603 | 218 | 1.0207242 | SPECC1 | sperm antigen with calponin homology and coiled-coil domains 1 [Source:HGNC Symbol;Acc:HGNC:30615] |
| ENSG00000135482 | HGLibA_54947 | 218 | 1.0207242 | ZC3H10 | zinc finger CCCH-type containing 10 [Source:HGNC Symbol;Acc:HGNC:25893] |
| ENSG00000136404 | HGLibA_49709 | 218 | 1.0207242 | TM6SF1 | transmembrane 6 superfamily member 1 [Source:HGNC Symbol;Acc:HGNC:11860] |
| ENSG00000137266 | HGLibA_44572 | 218 | 1.0207242 | SLC22A23 | solute carrier family 22 member 23 [Source:HGNC Symbol;Acc:HGNC:21106] |
| ENSG00000139190 | HGLibA_53487 | 218 | 1.0207242 | VAMP1 | vesicle associated membrane protein 1 [Source:HGNC Symbol;Acc:HGNC:12642] |
| ENSG00000139567 | HGLibA_00700 | 218 | 1.0207242 | ACVRL1 | activin A receptor like type 1 [Source:HGNC Symbol;Acc:HGNC:175] |
| ENSG00000141519 | HGLibA_07775 | 218 | 1.0207242 | CCDC40 | coiled-coil domain containing 40 [Source:HGNC Symbol;Acc:HGNC:26090] |
| ENSG00000150782 | HGLibA_23022 | 218 | 1.0207242 | IL18 | interleukin 18 [Source:HGNC Symbol;Acc:HGNC:5986] |
| ENSG00000153071 | HGLibA_12267 | 218 | 1.0207242 | DAB2 | clathrin adaptor protein [Source:HGNC Symbol;Acc:HGNC:2662]" |
| ENSG00000158859 | HGLibA_00829 | 218 | 1.0207242 | ADAMTS4 | ADAM metallopeptidase with thrombospondin type 1 motif 4 [Source:HGNC Symbol;Acc:HGNC:220] |
| ENSG00000160180 | HGLibA_49175 | 218 | 1.0207242 | TFF3 | trefoil factor 3 [Source:HGNC Symbol;Acc:HGNC:11757] |
| ENSG00000163217 | HGLibA_04594 | 218 | 1.0207242 | BMP10 | bone morphogenetic protein 10 [Source:HGNC Symbol;Acc:HGNC:20869] |
| ENSG00000163406 | HGLibA_44409 | 218 | 1.0207242 | SLC15A2 | solute carrier family 15 member 2 [Source:HGNC Symbol;Acc:HGNC:10921] |
| ENSG00000163618 | HGLibA_06976 | 218 | 1.0207242 | CADPS | calcium dependent secretion activator [Source:HGNC Symbol;Acc:HGNC:1426] |
| ENSG00000164885 | HGLibA_08773 | 218 | 1.0207242 | CDK5 | cyclin dependent kinase 5 [Source:HGNC Symbol;Acc:HGNC:1774] |
| ENSG00000164932 | HGLibA_11629 | 218 | 1.0207242 | CTHRC1 | collagen triple helix repeat containing 1 [Source:HGNC Symbol;Acc:HGNC:18831] |
| ENSG00000165029 | HGLibA_00094 | 218 | 1.0207242 | ABCA1 | ATP binding cassette subfamily A member 1 [Source:HGNC Symbol;Acc:HGNC:29] |
| ENSG00000166828 | HGLibA_43021 | 218 | 1.0207242 | SCNN1G | sodium channel epithelial 1 gamma subunit [Source:HGNC Symbol;Acc:HGNC:10602] |
| ENSG00000173432 | HGLibA_42607 | 218 | 1.0207242 | SAA1 | serum amyloid A1 [Source:HGNC Symbol;Acc:HGNC:10513] |
| ENSG00000174721 | HGLibA_17432 | 218 | 1.0207242 | FGFBP3 | fibroblast growth factor binding protein 3 [Source:HGNC Symbol;Acc:HGNC:23428] |
| ENSG00000175874 | HGLibA_11124 | 218 | 1.0207242 | CREG2 | cellular repressor of E1A stimulated genes 2 [Source:HGNC Symbol;Acc:HGNC:14272] |
| ENSG00000176340 | HGLibA_10875 | 218 | 1.0207242 | COX8A | cytochrome c oxidase subunit 8A [Source:HGNC Symbol;Acc:HGNC:2294] |
| ENSG00000177202 | HGLibA_46329 | 218 | 1.0207242 | SPACA4 | sperm acrosome associated 4 [Source:HGNC Symbol;Acc:HGNC:16441] |
| ENSG00000180878 | HGLibA_05138 | 218 | 1.0207242 | C11orf42 | chromosome 11 open reading frame 42 [Source:HGNC Symbol;Acc:HGNC:28541] |
| ENSG00000182718 | HGLibA_02230 | 218 | 1.0207242 | ANXA2 | annexin A2 [Source:HGNC Symbol;Acc:HGNC:537] |
| ENSG00000184113 | HGLibA_09902 | 218 | 1.0207242 | CLDN5 | claudin 5 [Source:HGNC Symbol;Acc:HGNC:2047] |
| ENSG00000186980 | HGLibA_25721 | 218 | 1.0207242 | KRTAP23-1 | keratin associated protein 23-1 [Source:HGNC Symbol;Acc:HGNC:18928] |
| ENSG00000197345 | HGLibA_29927 | 218 | 1.0207242 | MRPL21 | mitochondrial ribosomal protein L21 [Source:HGNC Symbol;Acc:HGNC:14479] |
| ENSG00000197702 | HGLibA_35255 | 218 | 1.0207242 | PARVA | parvin alpha [Source:HGNC Symbol;Acc:HGNC:14652] |
| ENSG00000198053 | HGLibA_44201 | 218 | 1.0207242 | SIRPA | signal regulatory protein alpha [Source:HGNC Symbol;Acc:HGNC:9662] |
| ENSG00000204219 | HGLibA_48701 | 218 | 1.0207242 | TCEA3 | transcription elongation factor A3 [Source:HGNC Symbol;Acc:HGNC:11615] |
| ENSG00000204779 | HGLibA_17851 | 218 | 1.0207242 | FOXD4L5 | forkhead box D4 like 5 [Source:HGNC Symbol;Acc:HGNC:18522] |
| ENSG00000232263 | HGLibA_25729 | 218 | 1.0207242 | KRTAP25-1 | keratin associated protein 25-1 [Source:HGNC Symbol;Acc:HGNC:34003] |
| ENSG00000254550 | HGLibA_33388 | 218 | 1.0207242 | OMP | olfactory marker protein [Source:HGNC Symbol;Acc:HGNC:8136] |
| ENSG00000279301 | HGLibA_33857 | 218 | 1.0207242 | OR2T11 | olfactory receptor family 2 subfamily T member 11 (gene/pseudogene) [Source:HGNC Symbol;Acc:HGNC:19574] |
| FAM212A | HGLibA_16465 | 218 | 1.0207242 | FAM212A | - |
| hsa-mir-1248 | HGLibA_57389 | 218 | 1.0207242 | hsa-mir-1248 | - |
| hsa-mir-2682 | HGLibA_58362 | 218 | 1.0207242 | hsa-mir-2682 | - |
| hsa-mir-4524a | HGLibA_60555 | 218 | 1.0207242 | hsa-mir-4524a | - |
| hsa-mir-603 | HGLibA_62506 | 218 | 1.0207242 | hsa-mir-603 | - |
| hsa-mir-6788 | HGLibA_63330 | 218 | 1.0207242 | hsa-mir-6788 | - |
| KIAA1009 | HGLibA_24677 | 218 | 1.0207242 | KIAA1009 | - |
| LOC100288814 | HGLibA_26838 | 218 | 1.0207242 | LOC100288814 | - |
| CXorf64 | HGLibA_11957 | 217 | 1.010773162 | CXorf64 | - |
| ENSG00000007968 | HGLibA_14309 | 217 | 1.010773162 | E2F2 | E2F transcription factor 2 [Source:HGNC Symbol;Acc:HGNC:3114] |
| ENSG00000011021 | HGLibA_09834 | 217 | 1.010773162 | CLCN6 | chloride voltage-gated channel 6 [Source:HGNC Symbol;Acc:HGNC:2024] |
| ENSG00000054598 | HGLibA_17823 | 217 | 1.010773162 | FOXC1 | forkhead box C1 [Source:HGNC Symbol;Acc:HGNC:3800] |
| ENSG00000064651 | HGLibA_44361 | 217 | 1.010773162 | SLC12A2 | solute carrier family 12 member 2 [Source:HGNC Symbol;Acc:HGNC:10911] |
| ENSG00000068079 | HGLibA_22609 | 217 | 1.010773162 | IFI35 | interferon induced protein 35 [Source:HGNC Symbol;Acc:HGNC:5399] |
| ENSG00000070031 | HGLibA_43065 | 217 | 1.010773162 | SCT | secretin [Source:HGNC Symbol;Acc:HGNC:10607] |
| ENSG00000070915 | HGLibA_44364 | 217 | 1.010773162 | SLC12A3 | solute carrier family 12 member 3 [Source:HGNC Symbol;Acc:HGNC:10912] |
| ENSG00000074211 | HGLibA_38151 | 217 | 1.010773162 | PPP2R2C | protein phosphatase 2 regulatory subunit Bgamma [Source:HGNC Symbol;Acc:HGNC:9306] |
| ENSG00000086159 | HGLibA_02601 | 217 | 1.010773162 | AQP6 | aquaporin 6 [Source:HGNC Symbol;Acc:HGNC:639] |
| ENSG00000101222 | HGLibA_46608 | 217 | 1.010773162 | SPEF1 | sperm flagellar 1 [Source:HGNC Symbol;Acc:HGNC:15874] |
| ENSG00000110700 | HGLibA_42071 | 217 | 1.010773162 | RPS13 | ribosomal protein S13 [Source:HGNC Symbol;Acc:HGNC:10386] |
| ENSG00000113552 | HGLibA_19565 | 217 | 1.010773162 | GNPDA1 | glucosamine-6-phosphate deaminase 1 [Source:HGNC Symbol;Acc:HGNC:4417] |
| ENSG00000115268 | HGLibA_42076 | 217 | 1.010773162 | RPS15 | ribosomal protein S15 [Source:HGNC Symbol;Acc:HGNC:10388] |
| ENSG00000116017 | HGLibA_02891 | 217 | 1.010773162 | ARID3A | AT-rich interaction domain 3A [Source:HGNC Symbol;Acc:HGNC:3031] |
| ENSG00000119866 | HGLibA_04291 | 217 | 1.010773162 | BCL11A | B cell CLL/lymphoma 11A [Source:HGNC Symbol;Acc:HGNC:13221] |
| ENSG00000121446 | HGLibA_41117 | 217 | 1.010773162 | RGSL1 | regulator of G protein signaling like 1 [Source:HGNC Symbol;Acc:HGNC:18636] |
| ENSG00000124491 | HGLibA_15852 | 217 | 1.010773162 | F13A1 | coagulation factor XIII A chain [Source:HGNC Symbol;Acc:HGNC:3531] |
| ENSG00000125454 | HGLibA_44664 | 217 | 1.010773162 | SLC25A19 | solute carrier family 25 member 19 [Source:HGNC Symbol;Acc:HGNC:14409] |
| ENSG00000129071 | HGLibA_28487 | 217 | 1.010773162 | MBD4 | DNA glycosylase [Source:HGNC Symbol;Acc:HGNC:6919]" |
| ENSG00000129103 | HGLibA_47774 | 217 | 1.010773162 | SUMF2 | sulfatase modifying factor 2 [Source:HGNC Symbol;Acc:HGNC:20415] |
| ENSG00000130383 | HGLibA_18197 | 217 | 1.010773162 | FUT5 | fucosyltransferase 5 [Source:HGNC Symbol;Acc:HGNC:4016] |
| ENSG00000130413 | HGLibA_47492 | 217 | 1.010773162 | STK33 | serine/threonine kinase 33 [Source:HGNC Symbol;Acc:HGNC:14568] |
| ENSG00000131844 | HGLibA_28568 | 217 | 1.010773162 | MCCC2 | methylcrotonoyl-CoA carboxylase 2 [Source:HGNC Symbol;Acc:HGNC:6937] |
| ENSG00000132561 | HGLibA_28429 | 217 | 1.010773162 | MATN2 | matrilin 2 [Source:HGNC Symbol;Acc:HGNC:6908] |
| ENSG00000132821 | HGLibA_53866 | 217 | 1.010773162 | VSTM2L | V-set and transmembrane domain containing 2 like [Source:HGNC Symbol;Acc:HGNC:16096] |
| ENSG00000133131 | HGLibA_29658 | 217 | 1.010773162 | MORC4 | MORC family CW-type zinc finger 4 [Source:HGNC Symbol;Acc:HGNC:23485] |
| ENSG00000137709 | HGLibA_37763 | 217 | 1.010773162 | POU2F3 | POU class 2 homeobox 3 [Source:HGNC Symbol;Acc:HGNC:19864] |
| ENSG00000147246 | HGLibA_22378 | 217 | 1.010773162 | HTR2C | 5-hydroxytryptamine receptor 2C [Source:HGNC Symbol;Acc:HGNC:5295] |
| ENSG00000156587 | HGLibA_52629 | 217 | 1.010773162 | UBE2L6 | ubiquitin conjugating enzyme E2 L6 [Source:HGNC Symbol;Acc:HGNC:12490] |
| ENSG00000157570 | HGLibA_51915 | 217 | 1.010773162 | TSPAN18 | tetraspanin 18 [Source:HGNC Symbol;Acc:HGNC:20660] |
| ENSG00000163319 | HGLibA_30055 | 217 | 1.010773162 | MRPS18C | mitochondrial ribosomal protein S18C [Source:HGNC Symbol;Acc:HGNC:16633] |
| ENSG00000163873 | HGLibA_20287 | 217 | 1.010773162 | GRIK3 | glutamate ionotropic receptor kainate type subunit 3 [Source:HGNC Symbol;Acc:HGNC:4581] |
| ENSG00000164399 | HGLibA_23127 | 217 | 1.010773162 | IL3 | interleukin 3 [Source:HGNC Symbol;Acc:HGNC:6011] |
| ENSG00000165672 | HGLibA_38414 | 217 | 1.010773162 | PRDX3 | peroxiredoxin 3 [Source:HGNC Symbol;Acc:HGNC:9354] |
| ENSG00000167635 | HGLibA_55494 | 217 | 1.010773162 | ZNF146 | zinc finger protein 146 [Source:HGNC Symbol;Acc:HGNC:12931] |
| ENSG00000167965 | HGLibA_29435 | 217 | 1.010773162 | MLST8 | LST8 homolog [Source:HGNC Symbol;Acc:HGNC:24825]" |
| ENSG00000171989 | HGLibA_26226 | 217 | 1.010773162 | LDHAL6B | lactate dehydrogenase A like 6B [Source:HGNC Symbol;Acc:HGNC:21481] |
| ENSG00000172238 | HGLibA_03533 | 217 | 1.010773162 | ATOH1 | atonal bHLH transcription factor 1 [Source:HGNC Symbol;Acc:HGNC:797] |
| ENSG00000172288 | HGLibA_08906 | 217 | 1.010773162 | CDY1 | chromodomain Y-linked 1 [Source:HGNC Symbol;Acc:HGNC:1809] |
| ENSG00000173208 | HGLibA_00194 | 217 | 1.010773162 | ABCD2 | ATP binding cassette subfamily D member 2 [Source:HGNC Symbol;Acc:HGNC:66] |
| ENSG00000174891 | HGLibA_42359 | 217 | 1.010773162 | RSRC1 | arginine and serine rich coiled-coil 1 [Source:HGNC Symbol;Acc:HGNC:24152] |
| ENSG00000175311 | HGLibA_02159 | 217 | 1.010773162 | ANKS4B | ankyrin repeat and sterile alpha motif domain containing 4B [Source:HGNC Symbol;Acc:HGNC:26795] |
| ENSG00000175606 | HGLibA_50502 | 217 | 1.010773162 | TMEM70 | transmembrane protein 70 [Source:HGNC Symbol;Acc:HGNC:26050] |
| ENSG00000176014 | HGLibA_52290 | 217 | 1.010773162 | TUBB6 | tubulin beta 6 class V [Source:HGNC Symbol;Acc:HGNC:20776] |
| ENSG00000182022 | HGLibA_09629 | 217 | 1.010773162 | CHST15 | carbohydrate sulfotransferase 15 [Source:HGNC Symbol;Acc:HGNC:18137] |
| ENSG00000185697 | HGLibA_30642 | 217 | 1.010773162 | MYBL1 | MYB proto-oncogene like 1 [Source:HGNC Symbol;Acc:HGNC:7547] |
| ENSG00000197555 | HGLibA_44193 | 217 | 1.010773162 | SIPA1L1 | signal induced proliferation associated 1 like 1 [Source:HGNC Symbol;Acc:HGNC:20284] |
| ENSG00000197587 | HGLibA_13397 | 217 | 1.010773162 | DMBX1 | diencephalon/mesencephalon homeobox 1 [Source:HGNC Symbol;Acc:HGNC:19026] |
| ENSG00000198920 | HGLibA_24648 | 217 | 1.010773162 | KIAA0753 | KIAA0753 [Source:HGNC Symbol;Acc:HGNC:29110] |
| ENSG00000213096 | HGLibA_55675 | 217 | 1.010773162 | ZNF254 | zinc finger protein 254 [Source:HGNC Symbol;Acc:HGNC:13047] |
| ENSG00000213341 | HGLibA_09670 | 217 | 1.010773162 | CHUK | conserved helix-loop-helix ubiquitous kinase [Source:HGNC Symbol;Acc:HGNC:1974] |
| ENSG00000232263 | HGLibA_25728 | 217 | 1.010773162 | KRTAP25-1 | keratin associated protein 25-1 [Source:HGNC Symbol;Acc:HGNC:34003] |
| ENSG00000237765 | HGLibA_16414 | 217 | 1.010773162 | FAM200B | family with sequence similarity 200 member B [Source:HGNC Symbol;Acc:HGNC:27740] |
| ENSG00000250506 | HGLibA_08766 | 217 | 1.010773162 | CDK3 | cyclin dependent kinase 3 [Source:HGNC Symbol;Acc:HGNC:1772] |
| ENSG00000259207 | HGLibA_23780 | 217 | 1.010773162 | ITGB3 | integrin subunit beta 3 [Source:HGNC Symbol;Acc:HGNC:6156] |
| hsa-mir-320e | HGLibA_58951 | 217 | 1.010773162 | hsa-mir-320e | - |
| hsa-mir-4284 | HGLibA_59843 | 217 | 1.010773162 | hsa-mir-4284 | - |
| hsa-mir-4700 | HGLibA_60908 | 217 | 1.010773162 | hsa-mir-4700 | - |
| hsa-mir-711 | HGLibA_63801 | 217 | 1.010773162 | hsa-mir-711 | - |
| MUT | HGLibA_30582 | 217 | 1.010773162 | MUT | - |
| TMEM27 | HGLibA_50358 | 217 | 1.010773162 | TMEM27 | - |
| CCDC37 | HGLibA_07765 | 216 | 1.000822125 | CCDC37 | - |
| ENSG00000004779 | HGLibA_31479 | 216 | 1.000822125 | NDUFAB1 | NADH:ubiquinone oxidoreductase subunit AB1 [Source:HGNC Symbol;Acc:HGNC:7694] |
| ENSG00000008853 | HGLibA_41174 | 216 | 1.000822125 | RHOBTB2 | Rho related BTB domain containing 2 [Source:HGNC Symbol;Acc:HGNC:18756] |
| ENSG00000012983 | HGLibA_28222 | 216 | 1.000822125 | MAP4K5 | mitogen-activated protein kinase kinase kinase kinase 5 [Source:HGNC Symbol;Acc:HGNC:6867] |
| ENSG00000017797 | HGLibA_40153 | 216 | 1.000822125 | RALBP1 | ralA binding protein 1 [Source:HGNC Symbol;Acc:HGNC:9841] |
| ENSG00000040275 | HGLibA_46571 | 216 | 1.000822125 | SPDL1 | spindle apparatus coiled-coil protein 1 [Source:HGNC Symbol;Acc:HGNC:26010] |
| ENSG00000049656 | HGLibA_10117 | 216 | 1.000822125 | CLPTM1L | CLPTM1 like [Source:HGNC Symbol;Acc:HGNC:24308] |
| ENSG00000054219 | HGLibA_27681 | 216 | 1.000822125 | LY75 | lymphocyte antigen 75 [Source:HGNC Symbol;Acc:HGNC:6729] |
| ENSG00000065809 | HGLibA_16003 | 216 | 1.000822125 | FAM107B | family with sequence similarity 107 member B [Source:HGNC Symbol;Acc:HGNC:23726] |
| ENSG00000074410 | HGLibA_06795 | 216 | 1.000822125 | CA12 | carbonic anhydrase 12 [Source:HGNC Symbol;Acc:HGNC:1371] |
| ENSG00000077238 | HGLibA_23172 | 216 | 1.000822125 | IL4R | interleukin 4 receptor [Source:HGNC Symbol;Acc:HGNC:6015] |
| ENSG00000090975 | HGLibA_36752 | 216 | 1.000822125 | PITPNM2 | phosphatidylinositol transfer protein membrane associated 2 [Source:HGNC Symbol;Acc:HGNC:21044] |
| ENSG00000101335 | HGLibA_30778 | 216 | 1.000822125 | MYL9 | myosin light chain 9 [Source:HGNC Symbol;Acc:HGNC:15754] |
| ENSG00000104626 | HGLibA_15478 | 216 | 1.000822125 | ERI1 | exoribonuclease 1 [Source:HGNC Symbol;Acc:HGNC:23994] |
| ENSG00000105507 | HGLibA_06856 | 216 | 1.000822125 | CABP5 | calcium binding protein 5 [Source:HGNC Symbol;Acc:HGNC:13714] |
| ENSG00000106689 | HGLibA_26449 | 216 | 1.000822125 | LHX2 | LIM homeobox 2 [Source:HGNC Symbol;Acc:HGNC:6594] |
| ENSG00000111843 | HGLibA_49984 | 216 | 1.000822125 | TMEM14C | transmembrane protein 14C [Source:HGNC Symbol;Acc:HGNC:20952] |
| ENSG00000113520 | HGLibA_23166 | 216 | 1.000822125 | IL4 | interleukin 4 [Source:HGNC Symbol;Acc:HGNC:6014] |
| ENSG00000113583 | HGLibA_06358 | 216 | 1.000822125 | C5orf15 | chromosome 5 open reading frame 15 [Source:HGNC Symbol;Acc:HGNC:20656] |
| ENSG00000114316 | HGLibA_53359 | 216 | 1.000822125 | USP4 | ubiquitin specific peptidase 4 [Source:HGNC Symbol;Acc:HGNC:12627] |
| ENSG00000114547 | HGLibA_41763 | 216 | 1.000822125 | ROPN1B | rhophilin associated tail protein 1B [Source:HGNC Symbol;Acc:HGNC:31927] |
| ENSG00000116001 | HGLibA_49431 | 216 | 1.000822125 | TIA1 | TIA1 cytotoxic granule associated RNA binding protein [Source:HGNC Symbol;Acc:HGNC:11802] |
| ENSG00000116691 | HGLibA_29266 | 216 | 1.000822125 | MIIP | migration and invasion inhibitory protein [Source:HGNC Symbol;Acc:HGNC:25715] |
| ENSG00000117411 | HGLibA_04027 | 216 | 1.000822125 | B4GALT2 | 4-galactosyltransferase 2 [Source:HGNC Symbol;Acc:HGNC:925]" |
| ENSG00000123810 | HGLibA_04047 | 216 | 1.000822125 | B9D2 | B9 domain containing 2 [Source:HGNC Symbol;Acc:HGNC:28636] |
| ENSG00000124253 | HGLibA_35612 | 216 | 1.000822125 | PCK1 | phosphoenolpyruvate carboxykinase 1 [Source:HGNC Symbol;Acc:HGNC:8724] |

| | | | | | |
|---|---|---|---|---|---|
| ENSG00000125414 | HGLibA_30720 | 216 | 1.000822125 | MYH2 | myosin heavy chain 2 [Source:HGNC Symbol;Acc:HGNC:7572] |
| ENSG00000127663 | HGLibA_24509 | 216 | 1.000822125 | KDM4B | lysine demethylase 4B [Source:HGNC Symbol;Acc:HGNC:29136] |
| ENSG00000130413 | HGLibA_47491 | 216 | 1.000822125 | STK33 | serine/threonine kinase 33 [Source:HGNC Symbol;Acc:HGNC:14568] |
| ENSG00000134013 | HGLibA_27041 | 216 | 1.000822125 | LOXL2 | lysyl oxidase like 2 [Source:HGNC Symbol;Acc:HGNC:6666] |
| ENSG00000139648 | HGLibA_25524 | 216 | 1.000822125 | KRT71 | keratin 71 [Source:HGNC Symbol;Acc:HGNC:28927] |
| ENSG00000142698 | HGLibA_05905 | 216 | 1.000822125 | C1orf94 | chromosome 1 open reading frame 94 [Source:HGNC Symbol;Acc:HGNC:28250] |
| ENSG00000152137 | HGLibA_22319 | 216 | 1.000822125 | HSPB8 | heat shock protein family B (small) member 8 [Source:HGNC Symbol;Acc:HGNC:30171] |
| ENSG00000157017 | HGLibA_19049 | 216 | 1.000822125 | GHRL | ghrelin and obestatin prepropeptide [Source:HGNC Symbol;Acc:HGNC:18129] |
| ENSG00000157450 | HGLibA_41467 | 216 | 1.000822125 | RNF111 | ring finger protein 111 [Source:HGNC Symbol;Acc:HGNC:17384] |
| ENSG00000160685 | HGLibA_54918 | 216 | 1.000822125 | ZBTB7B | zinc finger and BTB domain containing 7B [Source:HGNC Symbol;Acc:HGNC:18668] |
| ENSG00000164187 | HGLibA_26679 | 216 | 1.000822125 | LMBRD2 | LMBR1 domain containing 2 [Source:HGNC Symbol;Acc:HGNC:25287] |
| ENSG00000164400 | HGLibA_11326 | 216 | 1.000822125 | CSF2 | colony stimulating factor 2 [Source:HGNC Symbol;Acc:HGNC:2434] |
| ENSG00000165821 | HGLibA_42649 | 216 | 1.000822125 | SALL2 | spalt like transcription factor 2 [Source:HGNC Symbol;Acc:HGNC:10526] |
| ENSG00000166224 | HGLibA_43847 | 216 | 1.000822125 | SGPL1 | sphingosine-1-phosphate lyase 1 [Source:HGNC Symbol;Acc:HGNC:10817] |
| ENSG00000166923 | HGLibA_20241 | 216 | 1.000822125 | GREM1 | DAN family BMP antagonist [Source:HGNC Symbol;Acc:HGNC:2001]" |
| ENSG00000168938 | HGLibA_37922 | 216 | 1.000822125 | PPIC | peptidylprolyl isomerase C [Source:HGNC Symbol;Acc:HGNC:9256] |
| ENSG00000170291 | HGLibA_14986 | 216 | 1.000822125 | ELP5 | elongator acetyltransferase complex subunit 5 [Source:HGNC Symbol;Acc:HGNC:30617] |
| ENSG00000170324 | HGLibA_18035 | 216 | 1.000822125 | FRMPD2 | FERM and PDZ domain containing 2 [Source:HGNC Symbol;Acc:HGNC:28572] |
| ENSG00000171596 | HGLibA_32214 | 216 | 1.000822125 | NMUR1 | neuromedin U receptor 1 [Source:HGNC Symbol;Acc:HGNC:4518] |
| ENSG00000171621 | HGLibA_46860 | 216 | 1.000822125 | SPSB1 | splA/ryanodine receptor domain and SOCS box containing 1 [Source:HGNC Symbol;Acc:HGNC:30628] |
| ENSG00000172264 | HGLibA_27839 | 216 | 1.000822125 | MACROD2 | MACRO domain containing 2 [Source:HGNC Symbol;Acc:HGNC:16126] |
| ENSG00000172889 | HGLibA_14628 | 216 | 1.000822125 | EGFL7 | EGF like domain multiple 7 [Source:HGNC Symbol;Acc:HGNC:20594] |
| ENSG00000175229 | HGLibA_18504 | 216 | 1.000822125 | GAL3ST3 | galactose-3-O-sulfotransferase 3 [Source:HGNC Symbol;Acc:HGNC:24144] |
| ENSG00000176887 | HGLibA_46232 | 216 | 1.000822125 | SOX11 | SRY-box 11 [Source:HGNC Symbol;Acc:HGNC:11191] |
| ENSG00000182749 | HGLibA_35170 | 216 | 1.000822125 | PAQR7 | progestin and adipoQ receptor family member 7 [Source:HGNC Symbol;Acc:HGNC:23146] |
| ENSG00000186350 | HGLibA_42506 | 216 | 1.000822125 | RXRA | retinoid X receptor alpha [Source:HGNC Symbol;Acc:HGNC:10477] |
| ENSG00000187045 | HGLibA_50648 | 216 | 1.000822125 | TMPRSS6 | transmembrane serine protease 6 [Source:HGNC Symbol;Acc:HGNC:16517] |
| ENSG00000187608 | HGLibA_23654 | 216 | 1.000822125 | ISG15 | ISG15 ubiquitin-like modifier [Source:HGNC Symbol;Acc:HGNC:4053] |
| ENSG00000189108 | HGLibA_23057 | 216 | 1.000822125 | IL1RAPL2 | interleukin 1 receptor accessory protein like 2 [Source:HGNC Symbol;Acc:HGNC:5997] |
| ENSG00000196136 | HGLibA_43517 | 216 | 1.000822125 | SERPINA3 | serpin family A member 3 [Source:HGNC Symbol;Acc:HGNC:16] |
| ENSG00000197241 | HGLibA_44889 | 216 | 1.000822125 | SLC2A7 | solute carrier family 2 member 7 [Source:HGNC Symbol;Acc:HGNC:13445] |
| ENSG00000198049 | HGLibA_03906 | 216 | 1.000822125 | AVPR1B | arginine vasopressin receptor 1B [Source:HGNC Symbol;Acc:HGNC:896] |
| ENSG00000205609 | HGLibA_14771 | 216 | 1.000822125 | EIF3CL | eukaryotic translation initiation factor 3 subunit C like [Source:HGNC Symbol;Acc:HGNC:26347] |
| ENSG00000206107 | HGLibA_25735 | 216 | 1.000822125 | KRTAP27-1 | keratin associated protein 27-1 [Source:HGNC Symbol;Acc:HGNC:33864] |
| hsa-mir-302e | HGLibA_58454 | 216 | 1.000822125 | hsa-mir-302e | - |
| hsa-mir-3656 | HGLibA_59169 | 216 | 1.000822125 | hsa-mir-3656 | - |
| hsa-mir-4666a | HGLibA_60779 | 216 | 1.000822125 | hsa-mir-4666a | - |
| hsa-mir-521-2 | HGLibA_61770 | 216 | 1.000822125 | hsa-mir-521-2 | - |
| hsa-mir-6848 | HGLibA_63564 | 216 | 1.000822125 | hsa-mir-6848 | - |
| hsa-mir-7855 | HGLibA_64009 | 216 | 1.000822125 | hsa-mir-7855 | - |
| KIAA1033 | HGLibA_24686 | 216 | 1.000822125 | KIAA1033 | - |
| LOC389895 | HGLibA_26931 | 216 | 1.000822125 | LOC389895 | - |
| ENSG00000004864 | HGLibA_44646 | 215 | 0.990871088 | SLC25A13 | solute carrier family 25 member 13 [Source:HGNC Symbol;Acc:HGNC:10983] |
| ENSG00000011485 | HGLibA_38213 | 215 | 0.990871088 | PPP5C | protein phosphatase 5 catalytic subunit [Source:HGNC Symbol;Acc:HGNC:9322] |
| ENSG00000101412 | HGLibA_14307 | 215 | 0.990871088 | E2F1 | E2F transcription factor 1 [Source:HGNC Symbol;Acc:HGNC:3113] |
| ENSG00000103316 | HGLibA_11292 | 215 | 0.990871088 | CRYM | crystallin mu [Source:HGNC Symbol;Acc:HGNC:2418] |
| ENSG00000104886 | HGLibA_37134 | 215 | 0.990871088 | PLEKHJ1 | pleckstrin homology domain containing J1 [Source:HGNC Symbol;Acc:HGNC:18211] |
| ENSG00000130818 | HGLibA_55966 | 215 | 0.990871088 | ZNF426 | zinc finger protein 426 [Source:HGNC Symbol;Acc:HGNC:20725] |
| ENSG00000131196 | HGLibA_31773 | 215 | 0.990871088 | NFATC1 | nuclear factor of activated T cells 1 [Source:HGNC Symbol;Acc:HGNC:7775] |
| ENSG00000132518 | HGLibA_20665 | 215 | 0.990871088 | GUCY2D | retinal [Source:HGNC Symbol;Acc:HGNC:4689]" |
| ENSG00000132661 | HGLibA_33175 | 215 | 0.990871088 | NXT1 | nuclear transport factor 2 like export factor 1 [Source:HGNC Symbol;Acc:HGNC:15913] |
| ENSG00000133640 | HGLibA_27456 | 215 | 0.990871088 | LRRIQ1 | leucine rich repeats and IQ motif containing 1 [Source:HGNC Symbol;Acc:HGNC:25708] |
| ENSG00000134516 | HGLibA_13739 | 215 | 0.990871088 | DOCK2 | dedicator of cytokinesis 2 [Source:HGNC Symbol;Acc:HGNC:2988] |
| ENSG00000135318 | HGLibA_32829 | 215 | 0.990871088 | NT5E | 5'-nucleotidase ecto [Source:HGNC Symbol;Acc:HGNC:8021] |
| ENSG00000135604 | HGLibA_47617 | 215 | 0.990871088 | STX11 | syntaxin 11 [Source:HGNC Symbol;Acc:HGNC:11429] |
| ENSG00000136870 | HGLibA_55552 | 215 | 0.990871088 | ZNF189 | zinc finger protein 189 [Source:HGNC Symbol;Acc:HGNC:12980] |
| ENSG00000141086 | HGLibA_11685 | 215 | 0.990871088 | CTRL | chymotrypsin like [Source:HGNC Symbol;Acc:HGNC:2524] |
| ENSG00000143891 | HGLibA_18521 | 215 | 0.990871088 | GALM | galactose mutarotase [Source:HGNC Symbol;Acc:HGNC:24063] |
| ENSG00000144455 | HGLibA_47770 | 215 | 0.990871088 | SUMF1 | sulfatase modifying factor 1 [Source:HGNC Symbol;Acc:HGNC:20376] |
| ENSG00000144677 | HGLibA_11615 | 215 | 0.990871088 | CTDSPL | CTD small phosphatase like [Source:HGNC Symbol;Acc:HGNC:16890] |
| ENSG00000145536 | HGLibA_00809 | 215 | 0.990871088 | ADAMTS16 | ADAM metallopeptidase with thrombospondin type 1 motif 16 [Source:HGNC Symbol;Acc:HGNC:17108] |
| ENSG00000146072 | HGLibA_50779 | 215 | 0.990871088 | TNFRSF21 | TNF receptor superfamily member 21 [Source:HGNC Symbol;Acc:HGNC:13469] |
| ENSG00000147596 | HGLibA_38376 | 215 | 0.990871088 | PRDM14 | PR/SET domain 14 [Source:HGNC Symbol;Acc:HGNC:14001] |
| ENSG00000155792 | HGLibA_12989 | 215 | 0.990871088 | DEPTOR | DEP domain containing MTOR interacting protein [Source:HGNC Symbol;Acc:HGNC:22953] |
| ENSG00000156876 | HGLibA_42766 | 215 | 0.990871088 | SASS6 | SAS-6 centriolar assembly protein [Source:HGNC Symbol;Acc:HGNC:25403] |
| ENSG00000157350 | HGLibA_47239 | 215 | 0.990871088 | ST3GAL2 | 3-sialyltransferase 2 [Source:HGNC Symbol;Acc:HGNC:10863]" |
| ENSG00000157653 | HGLibA_06734 | 215 | 0.990871088 | C9orf43 | chromosome 9 open reading frame 43 [Source:HGNC Symbol;Acc:HGNC:23570] |
| ENSG00000159263 | HGLibA_44177 | 215 | 0.990871088 | SIM2 | SIM bHLH transcription factor 2 [Source:HGNC Symbol;Acc:HGNC:10883] |
| ENSG00000159961 | HGLibA_33919 | 215 | 0.990871088 | OR3A3 | olfactory receptor family 3 subfamily A member 3 [Source:HGNC Symbol;Acc:HGNC:8284] |
| ENSG00000160307 | HGLibA_42579 | 215 | 0.990871088 | S100B | S100 calcium binding protein B [Source:HGNC Symbol;Acc:HGNC:10500] |
| ENSG00000160469 | HGLibA_04849 | 215 | 0.990871088 | BRSK1 | BR serine/threonine kinase 1 [Source:HGNC Symbol;Acc:HGNC:18994] |
| ENSG00000161513 | HGLibA_17267 | 215 | 0.990871088 | FDXR | ferredoxin reductase [Source:HGNC Symbol;Acc:HGNC:3642] |
| ENSG00000165091 | HGLibA_49747 | 215 | 0.990871088 | TMC1 | transmembrane channel like 1 [Source:HGNC Symbol;Acc:HGNC:16513] |
| ENSG00000166523 | HGLibA_09988 | 215 | 0.990871088 | CLEC4E | C-type lectin domain family 4 member E [Source:HGNC Symbol;Acc:HGNC:14555] |
| ENSG00000168040 | HGLibA_15930 | 215 | 0.990871088 | FADD | Fas associated via death domain [Source:HGNC Symbol;Acc:HGNC:3573] |
| ENSG00000168763 | HGLibA_10319 | 215 | 0.990871088 | CNNM3 | cyclin and CBS domain divalent metal cation transport mediator 3 [Source:HGNC Symbol;Acc:HGNC:104] |
| ENSG00000169239 | HGLibA_06816 | 215 | 0.990871088 | CA5B | carbonic anhydrase 5B [Source:HGNC Symbol;Acc:HGNC:1378] |
| ENSG00000170175 | HGLibA_09589 | 215 | 0.990871088 | CHRNB1 | cholinergic receptor nicotinic beta 1 subunit [Source:HGNC Symbol;Acc:HGNC:1961] |
| ENSG00000174796 | HGLibA_49307 | 215 | 0.990871088 | THAP6 | THAP domain containing 6 [Source:HGNC Symbol;Acc:HGNC:23189] |
| ENSG00000175105 | HGLibA_56467 | 215 | 0.990871088 | ZNF654 | zinc finger protein 654 [Source:HGNC Symbol;Acc:HGNC:25612] |
| ENSG00000175463 | HGLibA_48469 | 215 | 0.990871088 | TBC1D10C | TBC1 domain family member 10C [Source:HGNC Symbol;Acc:HGNC:24702] |
| ENSG00000180116 | HGLibA_05238 | 215 | 0.990871088 | C12orf40 | chromosome 12 open reading frame 40 [Source:HGNC Symbol;Acc:HGNC:26846] |
| ENSG00000182308 | HGLibA_12443 | 215 | 0.990871088 | DCAF4L1 | DDB1 and CUL4 associated factor 4 like 1 [Source:HGNC Symbol;Acc:HGNC:27723] |
| ENSG00000183337 | HGLibA_04361 | 215 | 0.990871088 | BCOR | BCL6 corepressor [Source:HGNC Symbol;Acc:HGNC:20893] |
| ENSG00000184351 | HGLibA_25663 | 215 | 0.990871088 | KRTAP19-1 | keratin associated protein 19-1 [Source:HGNC Symbol;Acc:HGNC:18936] |
| ENSG00000184845 | HGLibA_13958 | 215 | 0.990871088 | DRD1 | dopamine receptor D1 [Source:HGNC Symbol;Acc:HGNC:3020] |
| ENSG00000186310 | HGLibA_31117 | 215 | 0.990871088 | NAP1L3 | nucleosome assembly protein 1 like 3 [Source:HGNC Symbol;Acc:HGNC:7639] |
| ENSG00000187498 | HGLibA_10567 | 215 | 0.990871088 | COL4A1 | collagen type IV alpha 1 chain [Source:HGNC Symbol;Acc:HGNC:2202] |
| ENSG00000196357 | HGLibA_56258 | 215 | 0.990871088 | ZNF565 | zinc finger protein 565 [Source:HGNC Symbol;Acc:HGNC:26726] |
| ENSG00000196371 | HGLibA_18193 | 215 | 0.990871088 | FUT4 | fucosyltransferase 4 [Source:HGNC Symbol;Acc:HGNC:4015] |
| ENSG00000197037 | HGLibA_57011 | 215 | 0.990871088 | ZSCAN25 | zinc finger and SCAN domain containing 25 [Source:HGNC Symbol;Acc:HGNC:21961] |
| ENSG00000197208 | HGLibA_44589 | 215 | 0.990871088 | SLC22A4 | solute carrier family 22 member 4 [Source:HGNC Symbol;Acc:HGNC:10968] |
| ENSG00000198018 | HGLibA_15214 | 215 | 0.990871088 | ENTPD7 | ectonucleoside triphosphate diphosphohydrolase 7 [Source:HGNC Symbol;Acc:HGNC:19745] |
| ENSG00000198551 | HGLibA_56424 | 215 | 0.990871088 | ZNF627 | zinc finger protein 627 [Source:HGNC Symbol;Acc:HGNC:30570] |
| ENSG00000213020 | HGLibA_56383 | 215 | 0.990871088 | ZNF611 | zinc finger protein 611 [Source:HGNC Symbol;Acc:HGNC:28766] |
| ENSG00000222046 | HGLibA_12488 | 215 | 0.990871088 | DCDC2B | doublecortin domain containing 2B [Source:HGNC Symbol;Acc:HGNC:32576] |
| ENSG00000244476 | HGLibA_15540 | 215 | 0.990871088 | ERVFRD-1 | envelope [Source:HGNC Symbol;Acc:HGNC:33823]" |
| ENSG00000255346 | HGLibA_32380 | 215 | 0.990871088 | NOX5 | NADPH oxidase 5 [Source:HGNC Symbol;Acc:HGNC:14874] |
| ENSG00000258644 | HGLibA_48013 | 215 | 0.990871088 | SYNJ2BP-COX16 | SYNJ2BP-COX16 readthrough [Source:HGNC Symbol;Acc:HGNC:48350] |
| ENSG00000262209 | HGLibA_35557 | 215 | 0.990871088 | PCDHGB3 | 3 [Source:HGNC Symbol;Acc:HGNC:8710]" |
| ENSG00000277363 | HGLibA_46934 | 215 | 0.990871088 | SRCIN1 | SRC kinase signaling inhibitor 1 [Source:HGNC Symbol;Acc:HGNC:29506] |
| hsa-mir-2392 | HGLibA_58323 | 215 | 0.990871088 | hsa-mir-2392 | - |
| hsa-mir-3196 | HGLibA_58883 | 215 | 0.990871088 | hsa-mir-3196 | - |
| hsa-mir-3657 | HGLibA_59175 | 215 | 0.990871088 | hsa-mir-3657 | - |
| hsa-mir-3713 | HGLibA_59365 | 215 | 0.990871088 | hsa-mir-3713 | - |
| hsa-mir-372 | HGLibA_59380 | 215 | 0.990871088 | hsa-mir-372 | - |
| hsa-mir-4255 | HGLibA_59726 | 215 | 0.990871088 | hsa-mir-4255 | - |
| hsa-mir-5704 | HGLibA_62358 | 215 | 0.990871088 | hsa-mir-5704 | - |
| PTPLA | HGLibA_39415 | 215 | 0.990871088 | PTPLA | - |
| C15orf52 | HGLibA_05409 | 214 | 0.98092005 | C15orf52 | - |
| ENSG00000002745 | HGLibA_54354 | 214 | 0.98092005 | WNT16 | Wnt family member 16 [Source:HGNC Symbol;Acc:HGNC:16267] |
| ENSG00000011451 | HGLibA_54329 | 214 | 0.98092005 | WIZ | widely interspaced zinc finger motifs [Source:HGNC Symbol;Acc:HGNC:30917] |
| ENSG00000057019 | HGLibA_12471 | 214 | 0.98092005 | DCBLD2 | CUB and LCCL domain containing 2 [Source:HGNC Symbol;Acc:HGNC:24627]" |
| ENSG00000067798 | HGLibA_31200 | 214 | 0.98092005 | NAV3 | neuron navigator 3 [Source:HGNC Symbol;Acc:HGNC:15998] |
| ENSG00000070778 | HGLibA_39456 | 214 | 0.98092005 | PTPN21 | non-receptor type 21 [Source:HGNC Symbol;Acc:HGNC:9651]" |
| ENSG00000072201 | HGLibA_26745 | 214 | 0.98092005 | LNX1 | ligand of numb-protein X 1 [Source:HGNC Symbol;Acc:HGNC:6657] |
| ENSG00000080709 | HGLibA_24357 | 214 | 0.98092005 | KCNN2 | potassium calcium-activated channel subfamily N member 2 [Source:HGNC Symbol;Acc:HGNC:6291] |
| ENSG00000084710 | HGLibA_14609 | 214 | 0.98092005 | EFR3B | EFR3 homolog B [Source:HGNC Symbol;Acc:HGNC:29155] |
| ENSG00000099331 | HGLibA_30871 | 214 | 0.98092005 | MYO9B | myosin IXB [Source:HGNC Symbol;Acc:HGNC:7609] |
| ENSG00000100031 | HGLibA_19010 | 214 | 0.98092005 | GGT1 | gamma-glutamyltransferase 1 [Source:HGNC Symbol;Acc:HGNC:4250] |
| ENSG00000105996 | HGLibA_21904 | 214 | 0.98092005 | HOXA2 | homeobox A2 [Source:HGNC Symbol;Acc:HGNC:5103] |
| ENSG00000106113 | HGLibA_11145 | 214 | 0.98092005 | CRHR2 | corticotropin releasing hormone receptor 2 [Source:HGNC Symbol;Acc:HGNC:2358] |
| ENSG00000107593 | HGLibA_36801 | 214 | 0.98092005 | PKD2L1 | transient receptor potential cation channel [Source:HGNC Symbol;Acc:HGNC:9011]" |
| ENSG00000112319 | HGLibA_15826 | 214 | 0.98092005 | EYA4 | EYA transcriptional coactivator and phosphatase 4 [Source:HGNC Symbol;Acc:HGNC:3522] |
| ENSG00000112530 | HGLibA_34948 | 214 | 0.98092005 | PACRG | parkin coregulated [Source:HGNC Symbol;Acc:HGNC:19152] |
| ENSG00000112651 | HGLibA_29921 | 214 | 0.98092005 | MRPL2 | mitochondrial ribosomal protein L2 [Source:HGNC Symbol;Acc:HGNC:14056] |
| ENSG00000114850 | HGLibA_47160 | 214 | 0.98092005 | SSR3 | signal sequence receptor subunit 3 [Source:HGNC Symbol;Acc:HGNC:11325] |
| ENSG00000115457 | HGLibA_22817 | 214 | 0.98092005 | IGFBP2 | insulin like growth factor binding protein 2 [Source:HGNC Symbol;Acc:HGNC:5471] |
| ENSG00000119048 | HGLibA_52576 | 214 | 0.98092005 | UBE2B | ubiquitin conjugating enzyme E2 B [Source:HGNC Symbol;Acc:HGNC:12473] |
| ENSG00000128590 | HGLibA_13558 | 214 | 0.98092005 | DNAJB9 | DnaJ heat shock protein family (Hsp40) member B9 [Source:HGNC Symbol;Acc:HGNC:6968] |
| ENSG00000132517 | HGLibA_45245 | 214 | 0.98092005 | SLC52A1 | solute carrier family 52 member 1 [Source:HGNC Symbol;Acc:HGNC:30225] |
| ENSG00000134809 | HGLibA_49494 | 214 | 0.98092005 | TIMM10 | translocase of inner mitochondrial membrane 10 [Source:HGNC Symbol;Acc:HGNC:11814] |
| ENSG00000136939 | HGLibA_33693 | 214 | 0.98092005 | OR1L4 | olfactory receptor family 1 subfamily L member 4 [Source:HGNC Symbol;Acc:HGNC:8216] |
| ENSG00000140612 | HGLibA_43181 | 214 | 0.98092005 | SEC11A | signal peptidase complex subunit [Source:HGNC Symbol;Acc:HGNC:17718]" |
| ENSG00000140941 | HGLibA_28124 | 214 | 0.98092005 | MAP1LC3B | microtubule associated protein 1 light chain 3 beta [Source:HGNC Symbol;Acc:HGNC:13352] |

| | | | | | |
|---|---|---|---|---|---|
| ENSG00000148090 | HGLibA_03865 | 214 | 0.98092005 | AUH | AU RNA binding methylglutaconyl-CoA hydratase [Source:HGNC Symbol;Acc:HGNC:890] |
| ENSG00000159433 | HGLibA_47378 | 214 | 0.98092005 | STARD9 | StAR related lipid transfer domain containing 9 [Source:HGNC Symbol;Acc:HGNC:19162] |
| ENSG00000160188 | HGLibA_42322 | 214 | 0.98092005 | RSPH1 | radial spoke head component 1 [Source:HGNC Symbol;Acc:HGNC:12371] |
| ENSG00000162620 | HGLibA_27457 | 214 | 0.98092005 | LRRIQ3 | leucine rich repeats and IQ motif containing 3 [Source:HGNC Symbol;Acc:HGNC:28318] |
| ENSG00000162706 | HGLibA_06972 | 214 | 0.98092005 | CADM3 | cell adhesion molecule 3 [Source:HGNC Symbol;Acc:HGNC:17601] |
| ENSG00000163126 | HGLibA_02041 | 214 | 0.98092005 | ANKRD23 | ankyrin repeat domain 23 [Source:HGNC Symbol;Acc:HGNC:24470] |
| ENSG00000163412 | HGLibA_14824 | 214 | 0.98092005 | EIF4E3 | eukaryotic translation initiation factor 4E family member 3 [Source:HGNC Symbol;Acc:HGNC:31837] |
| ENSG00000163618 | HGLibA_06977 | 214 | 0.98092005 | CADPS | calcium dependent secretion activator [Source:HGNC Symbol;Acc:HGNC:1426] |
| ENSG00000165966 | HGLibA_35999 | 214 | 0.98092005 | PDZRN4 | PDZ domain containing ring finger 4 [Source:HGNC Symbol;Acc:HGNC:30552] |
| ENSG00000165972 | HGLibA_07770 | 214 | 0.98092005 | CCDC38 | coiled-coil domain containing 38 [Source:HGNC Symbol;Acc:HGNC:26843] |
| ENSG00000166225 | HGLibA_18047 | 214 | 0.98092005 | FRS2 | fibroblast growth factor receptor substrate 2 [Source:HGNC Symbol;Acc:HGNC:16971] |
| ENSG00000167984 | HGLibA_32089 | 214 | 0.98092005 | NLRC3 | NLR family CARD domain containing 3 [Source:HGNC Symbol;Acc:HGNC:29889] |
| ENSG00000170099 | HGLibA_43527 | 214 | 0.98092005 | SERPINA6 | serpin family A member 6 [Source:HGNC Symbol;Acc:HGNC:1540] |
| ENSG00000170946 | HGLibA_13602 | 214 | 0.98092005 | DNAJC24 | DnaJ heat shock protein family (Hsp40) member C24 [Source:HGNC Symbol;Acc:HGNC:26979] |
| ENSG00000172716 | HGLibA_45480 | 214 | 0.98092005 | SLFN11 | schlafen family member 11 [Source:HGNC Symbol;Acc:HGNC:26633] |
| ENSG00000174226 | HGLibA_46088 | 214 | 0.98092005 | SNX31 | sorting nexin 31 [Source:HGNC Symbol;Acc:HGNC:28605] |
| ENSG00000174469 | HGLibA_10422 | 214 | 0.98092005 | CNTNAP2 | contactin associated protein like 2 [Source:HGNC Symbol;Acc:HGNC:13830] |
| ENSG00000175265 | HGLibA_19639 | 214 | 0.98092005 | GOLGA8A | golgin A8 family member A [Source:HGNC Symbol;Acc:HGNC:31972] |
| ENSG00000178287 | HGLibA_46346 | 214 | 0.98092005 | SPAG11A | sperm associated antigen 11A [Source:HGNC Symbol;Acc:HGNC:33342] |
| ENSG00000178988 | HGLibA_29832 | 214 | 0.98092005 | MRFAP1L1 | Morf4 family associated protein 1 like 1 [Source:HGNC Symbol;Acc:HGNC:28796] |
| ENSG00000181472 | HGLibA_54835 | 214 | 0.98092005 | ZBTB2 | zinc finger and BTB domain containing 2 [Source:HGNC Symbol;Acc:HGNC:20868] |
| ENSG00000182919 | HGLibA_05157 | 214 | 0.98092005 | C11orf54 | chromosome 11 open reading frame 54 [Source:HGNC Symbol;Acc:HGNC:30204] |
| ENSG00000183647 | HGLibA_56177 | 214 | 0.98092005 | ZNF530 | zinc finger protein 530 [Source:HGNC Symbol;Acc:HGNC:29297] |
| ENSG00000185811 | HGLibA_22930 | 214 | 0.98092005 | IKZF1 | IKAROS family zinc finger 1 [Source:HGNC Symbol;Acc:HGNC:13176] |
| ENSG00000186335 | HGLibA_45033 | 214 | 0.98092005 | SLC36A2 | solute carrier family 36 member 2 [Source:HGNC Symbol;Acc:HGNC:18762] |
| ENSG00000186930 | HGLibA_25817 | 214 | 0.98092005 | KRTAP6-2 | keratin associated protein 6-2 [Source:HGNC Symbol;Acc:HGNC:18932] |
| ENSG00000187391 | HGLibA_27997 | 214 | 0.98092005 | MAGI2 | WW and PDZ domain containing 2 [Source:HGNC Symbol;Acc:HGNC:18957]" |
| ENSG00000188729 | HGLibA_34718 | 214 | 0.98092005 | OSTN | osteocrin [Source:HGNC Symbol;Acc:HGNC:29961] |
| ENSG00000196338 | HGLibA_32073 | 214 | 0.98092005 | NLGN3 | neuroligin 3 [Source:HGNC Symbol;Acc:HGNC:14289] |
| ENSG00000196455 | HGLibA_36658 | 214 | 0.98092005 | PIK3R4 | phosphoinositide-3-kinase regulatory subunit 4 [Source:HGNC Symbol;Acc:HGNC:8982] |
| ENSG00000198125 | HGLibA_28453 | 214 | 0.98092005 | MB | myoglobin [Source:HGNC Symbol;Acc:HGNC:6915] |
| ENSG00000198759 | HGLibA_14623 | 214 | 0.98092005 | EGFL6 | EGF like domain multiple 6 [Source:HGNC Symbol;Acc:HGNC:3235] |
| ENSG00000204345 | HGLibA_08338 | 214 | 0.98092005 | CD300LD | CD300 molecule like family member d [Source:HGNC Symbol;Acc:HGNC:16848] |
| ENSG00000204386 | HGLibA_31710 | 214 | 0.98092005 | NEU1 | neuraminidase 1 [Source:HGNC Symbol;Acc:HGNC:7758] |
| ENSG00000205649 | HGLibA_22354 | 214 | 0.98092005 | HTN3 | histatin 3 [Source:HGNC Symbol;Acc:HGNC:5284] |
| ENSG00000206260 | HGLibA_38820 | 214 | 0.98092005 | PRR23A | proline rich 23A [Source:HGNC Symbol;Acc:HGNC:37172] |
| ENSG00000212807 | HGLibA_33736 | 214 | 0.98092005 | OR2A42 | olfactory receptor family 2 subfamily A member 42 [Source:HGNC Symbol;Acc:HGNC:31230] |
| ENSG00000213977 | HGLibA_48453 | 214 | 0.98092005 | TAX1BP3 | Tax1 binding protein 3 [Source:HGNC Symbol;Acc:HGNC:30684] |
| ENSG00000234602 | HGLibA_28594 | 214 | 0.98092005 | MCIDAS | multiciliate differentiation and DNA synthesis associated cell cycle protein [Source:HGNC Symbol;Acc:HGNC:40050] |
| ENSG00000235699 | HGLibA_11942 | 214 | 0.98092005 | CXorf51B | chromosome X open reading frame 51B [Source:HGNC Symbol;Acc:HGNC:42787] |
| ENSG00000241563 | HGLibA_10796 | 214 | 0.98092005 | CORT | cortistatin [Source:HGNC Symbol;Acc:HGNC:2257] |
| ENSG00000271447 | HGLibA_29530 | 214 | 0.98092005 | MMP28 | matrix metallopeptidase 28 [Source:HGNC Symbol;Acc:HGNC:14366] |
| FAM64A | HGLibA_16664 | 214 | 0.98092005 | FAM64A | - |
| H2BFM | HGLibA_20773 | 214 | 0.98092005 | H2BFM | - |
| hsa-mir-125a | HGLibA_57442 | 214 | 0.98092005 | hsa-mir-125a | - |
| hsa-mir-1292 | HGLibA_57644 | 214 | 0.98092005 | hsa-mir-1292 | - |
| hsa-mir-3660 | HGLibA_59193 | 214 | 0.98092005 | hsa-mir-3660 | - |
| hsa-mir-4293 | HGLibA_59882 | 214 | 0.98092005 | hsa-mir-4293 | - |
| hsa-mir-4297 | HGLibA_59900 | 214 | 0.98092005 | hsa-mir-4297 | - |
| hsa-mir-4299 | HGLibA_59908 | 214 | 0.98092005 | hsa-mir-4299 | - |
| hsa-mir-4792 | HGLibA_61274 | 214 | 0.98092005 | hsa-mir-4792 | - |
| hsa-mir-584 | HGLibA_62434 | 214 | 0.98092005 | hsa-mir-584 | - |
| hsa-mir-6826 | HGLibA_63481 | 214 | 0.98092005 | hsa-mir-6826 | - |
| MLL2 | HGLibA_29395 | 214 | 0.98092005 | MLL2 | - |
| UPK3BL | HGLibA_53084 | 214 | 0.98092005 | UPK3BL | - |
| AZI1 | HGLibA_03932 | 213 | 0.970969013 | AZI1 | - |
| ENSG00000049167 | HGLibA_15455 | 213 | 0.970969013 | ERCC8 | CSA ubiquitin ligase complex subunit [Source:HGNC Symbol;Acc:HGNC:3439]" |
| ENSG00000068028 | HGLibA_40373 | 213 | 0.970969013 | RASSF1 | Ras association domain family member 1 [Source:HGNC Symbol;Acc:HGNC:9882] |
| ENSG00000077454 | HGLibA_27145 | 213 | 0.970969013 | LRCH4 | leucine rich repeats and calponin homology domain containing 4 [Source:HGNC Symbol;Acc:HGNC:6691] |
| ENSG00000088053 | HGLibA_19710 | 213 | 0.970969013 | GP6 | glycoprotein VI platelet [Source:HGNC Symbol;Acc:HGNC:14388] |
| ENSG00000102174 | HGLibA_36328 | 213 | 0.970969013 | PHEX | phosphate regulating endopeptidase homolog X-linked [Source:HGNC Symbol;Acc:HGNC:8918] |
| ENSG00000104774 | HGLibA_28075 | 213 | 0.970969013 | MAN2B1 | mannosidase alpha class 2B member 1 [Source:HGNC Symbol;Acc:HGNC:6826] |
| ENSG00000105750 | HGLibA_56870 | 213 | 0.970969013 | ZNF85 | zinc finger protein 85 [Source:HGNC Symbol;Acc:HGNC:13160] |
| ENSG00000105855 | HGLibA_23798 | 213 | 0.970969013 | ITGB8 | integrin subunit beta 8 [Source:HGNC Symbol;Acc:HGNC:6163] |
| ENSG00000111254 | HGLibA_01430 | 213 | 0.970969013 | AKAP3 | A-kinase anchoring protein 3 [Source:HGNC Symbol;Acc:HGNC:373] |
| ENSG00000112175 | HGLibA_04610 | 213 | 0.970969013 | BMP5 | bone morphogenetic protein 5 [Source:HGNC Symbol;Acc:HGNC:1072] |
| ENSG00000116095 | HGLibA_37069 | 213 | 0.970969013 | PLEKHA3 | pleckstrin homology domain containing A3 [Source:HGNC Symbol;Acc:HGNC:14338] |
| ENSG00000122477 | HGLibA_27342 | 213 | 0.970969013 | LRRC39 | leucine rich repeat containing 39 [Source:HGNC Symbol;Acc:HGNC:28228] |
| ENSG00000124374 | HGLibA_35033 | 213 | 0.970969013 | PAIP2B | poly(A) binding protein interacting protein 2B [Source:HGNC Symbol;Acc:HGNC:29200] |
| ENSG00000129159 | HGLibA_24163 | 213 | 0.970969013 | KCNC1 | potassium voltage-gated channel subfamily C member 1 [Source:HGNC Symbol;Acc:HGNC:6233] |
| ENSG00000129646 | HGLibA_39746 | 213 | 0.970969013 | QRICH2 | glutamine rich 2 [Source:HGNC Symbol;Acc:HGNC:25326] |
| ENSG00000130193 | HGLibA_49345 | 213 | 0.970969013 | THEM6 | thioesterase superfamily member 6 [Source:HGNC Symbol;Acc:HGNC:29656] |
| ENSG00000134152 | HGLibA_24084 | 213 | 0.970969013 | KATNBL1 | katanin regulatory subunit B1 like 1 [Source:HGNC Symbol;Acc:HGNC:26199] |
| ENSG00000134986 | HGLibA_32669 | 213 | 0.970969013 | NREP | neuronal regeneration related protein [Source:HGNC Symbol;Acc:HGNC:16834] |
| ENSG00000138031 | HGLibA_00915 | 213 | 0.970969013 | ADCY3 | adenylate cyclase 3 [Source:HGNC Symbol;Acc:HGNC:234] |
| ENSG00000138271 | HGLibA_20102 | 213 | 0.970969013 | GPR87 | G protein-coupled receptor 87 [Source:HGNC Symbol;Acc:HGNC:4538] |
| ENSG00000140807 | HGLibA_32006 | 213 | 0.970969013 | NKD1 | naked cuticle homolog 1 [Source:HGNC Symbol;Acc:HGNC:17045] |
| ENSG00000143224 | HGLibA_38001 | 213 | 0.970969013 | PPOX | protoporphyrinogen oxidase [Source:HGNC Symbol;Acc:HGNC:9280] |
| ENSG00000144908 | HGLibA_01554 | 213 | 0.970969013 | ALDH1L1 | aldehyde dehydrogenase 1 family member L1 [Source:HGNC Symbol;Acc:HGNC:3978] |
| ENSG00000146842 | HGLibA_50204 | 213 | 0.970969013 | TMEM209 | transmembrane protein 209 [Source:HGNC Symbol;Acc:HGNC:21898] |
| ENSG00000156787 | HGLibA_48535 | 213 | 0.970969013 | TBC1D31 | TBC1 domain family member 31 [Source:HGNC Symbol;Acc:HGNC:30888] |
| ENSG00000161647 | HGLibA_29753 | 213 | 0.970969013 | MPP3 | membrane palmitoylated protein 3 [Source:HGNC Symbol;Acc:HGNC:7221] |
| ENSG00000162722 | HGLibA_51552 | 213 | 0.970969013 | TRIM58 | tripartite motif containing 58 [Source:HGNC Symbol;Acc:HGNC:24150] |
| ENSG00000162891 | HGLibA_23073 | 213 | 0.970969013 | IL20 | interleukin 20 [Source:HGNC Symbol;Acc:HGNC:6002] |
| ENSG00000163281 | HGLibA_19568 | 213 | 0.970969013 | GNPDA2 | glucosamine-6-phosphate deaminase 2 [Source:HGNC Symbol;Acc:HGNC:21526] |
| ENSG00000163577 | HGLibA_14858 | 213 | 0.970969013 | EIF5A2 | eukaryotic translation initiation factor 5A2 [Source:HGNC Symbol;Acc:HGNC:3301] |
| ENSG00000163687 | HGLibA_13661 | 213 | 0.970969013 | DNASE1L3 | deoxyribonuclease 1 like 3 [Source:HGNC Symbol;Acc:HGNC:2959] |
| ENSG00000164776 | HGLibA_36412 | 213 | 0.970969013 | PHKG1 | phosphorylase kinase catalytic subunit gamma 1 [Source:HGNC Symbol;Acc:HGNC:8930] |
| ENSG00000165588 | HGLibA_34786 | 213 | 0.970969013 | OTX2 | orthodenticle homeobox 2 [Source:HGNC Symbol;Acc:HGNC:8522] |
| ENSG00000168061 | HGLibA_42624 | 213 | 0.970969013 | SAC3D1 | SAC3 domain containing 1 [Source:HGNC Symbol;Acc:HGNC:30179] |
| ENSG00000170881 | HGLibA_41518 | 213 | 0.970969013 | RNF139 | ring finger protein 139 [Source:HGNC Symbol;Acc:HGNC:17023] |
| ENSG00000171873 | HGLibA_01042 | 213 | 0.970969013 | ADRA1D | adrenoceptor alpha 1D [Source:HGNC Symbol;Acc:HGNC:280] |
| ENSG00000176022 | HGLibA_03967 | 213 | 0.970969013 | B3GALT6 | 3-galactosyltransferase 6 [Source:HGNC Symbol;Acc:HGNC:17978]" |
| ENSG00000176153 | HGLibA_20172 | 213 | 0.970969013 | GPX2 | glutathione peroxidase 2 [Source:HGNC Symbol;Acc:HGNC:4554] |
| ENSG00000176209 | HGLibA_45743 | 213 | 0.970969013 | SMIM19 | small integral membrane protein 19 [Source:HGNC Symbol;Acc:HGNC:25166] |
| ENSG00000178623 | HGLibA_20029 | 213 | 0.970969013 | GPR35 | G protein-coupled receptor 35 [Source:HGNC Symbol;Acc:HGNC:4492] |
| ENSG00000182077 | HGLibA_39281 | 213 | 0.970969013 | PTCHD3 | patched domain containing 3 [Source:HGNC Symbol;Acc:HGNC:24776] |
| ENSG00000186272 | HGLibA_55523 | 213 | 0.970969013 | ZNF17 | zinc finger protein 17 [Source:HGNC Symbol;Acc:HGNC:12958] |
| ENSG00000186329 | HGLibA_50210 | 213 | 0.970969013 | TMEM212 | transmembrane protein 212 [Source:HGNC Symbol;Acc:HGNC:34295] |
| ENSG00000187944 | HGLibA_06161 | 213 | 0.970969013 | C2orf66 | chromosome 2 open reading frame 66 [Source:HGNC Symbol;Acc:HGNC:33809] |
| ENSG00000187997 | HGLibA_05604 | 213 | 0.970969013 | C17orf99 | chromosome 17 open reading frame 99 [Source:HGNC Symbol;Acc:HGNC:34490] |
| ENSG00000196358 | HGLibA_32867 | 213 | 0.970969013 | NTNG2 | netrin G2 [Source:HGNC Symbol;Acc:HGNC:14288] |
| ENSG00000198794 | HGLibA_42848 | 213 | 0.970969013 | SCAMP5 | secretory carrier membrane protein 5 [Source:HGNC Symbol;Acc:HGNC:30386] |
| ENSG00000204595 | HGLibA_13900 | 213 | 0.970969013 | DPRX | divergent-paired related homeobox [Source:HGNC Symbol;Acc:HGNC:32166] |
| ENSG00000266094 | HGLibA_40387 | 213 | 0.970969013 | RASSF5 | Ras association domain family member 5 [Source:HGNC Symbol;Acc:HGNC:17609] |
| ENSG00000288547 | HGLibA_06237 | 213 | 0.970969013 | C3orf36 | - |
| hsa-mir-302d | HGLibA_58447 | 213 | 0.970969013 | hsa-mir-302d | - |
| hsa-mir-3181 | HGLibA_58822 | 213 | 0.970969013 | hsa-mir-3181 | - |
| hsa-mir-323a | HGLibA_58954 | 213 | 0.970969013 | hsa-mir-323a | - |
| hsa-mir-3972 | HGLibA_59649 | 213 | 0.970969013 | hsa-mir-3972 | - |
| hsa-mir-6761 | HGLibA_63205 | 213 | 0.970969013 | hsa-mir-6761 | - |
| hsa-mir-7973-1 | HGLibA_64016 | 213 | 0.970969013 | hsa-mir-7973-1 | - |
| KIAA1407 | HGLibA_24749 | 213 | 0.970969013 | KIAA1407 | - |
| C20orf194 | HGLibA_05998 | 212 | 0.961017975 | C20orf194 | - |
| C21orf33 | HGLibA_06039 | 212 | 0.961017975 | C21orf33 | - |
| ENSG00000022355 | HGLibA_18356 | 212 | 0.961017975 | GABRA1 | gamma-aminobutyric acid type A receptor alpha1 subunit [Source:HGNC Symbol;Acc:HGNC:4075] |
| ENSG00000038295 | HGLibA_49629 | 212 | 0.961017975 | TLL1 | tolloid like 1 [Source:HGNC Symbol;Acc:HGNC:11843] |
| ENSG00000048740 | HGLibA_09008 | 212 | 0.961017975 | CELF2 | CUGBP Elav-like family member 2 [Source:HGNC Symbol;Acc:HGNC:2550] |
| ENSG00000049247 | HGLibA_53462 | 212 | 0.961017975 | UTS2 | urotensin 2 [Source:HGNC Symbol;Acc:HGNC:12636] |
| ENSG00000061938 | HGLibA_50848 | 212 | 0.961017975 | TNK2 | tyrosine kinase non receptor 2 [Source:HGNC Symbol;Acc:HGNC:19297] |
| ENSG00000070081 | HGLibA_32913 | 212 | 0.961017975 | NUCB2 | nucleobindin 2 [Source:HGNC Symbol;Acc:HGNC:8044] |
| ENSG00000072315 | HGLibA_51739 | 212 | 0.961017975 | TRPC5 | transient receptor potential cation channel subfamily C member 5 [Source:HGNC Symbol;Acc:HGNC:12337] |
| ENSG00000076944 | HGLibA_47665 | 212 | 0.961017975 | STXBP2 | syntaxin binding protein 2 [Source:HGNC Symbol;Acc:HGNC:11445] |
| ENSG00000095110 | HGLibA_33151 | 212 | 0.961017975 | NXPE1 | neurexophilin and PC-esterase domain family member 1 [Source:HGNC Symbol;Acc:HGNC:28527] |
| ENSG00000100664 | HGLibA_14852 | 212 | 0.961017975 | EIF5 | eukaryotic translation initiation factor 5 [Source:HGNC Symbol;Acc:HGNC:3299] |
| ENSG00000102359 | HGLibA_47036 | 212 | 0.961017975 | SRPX2 | sushi repeat containing protein X-linked 2 [Source:HGNC Symbol;Acc:HGNC:30668] |
| ENSG00000102977 | HGLibA_00420 | 212 | 0.961017975 | ACD | shelterin complex subunit and telomerase recruitment factor [Source:HGNC Symbol;Acc:HGNC:25070]" |
| ENSG00000105889 | HGLibA_47424 | 212 | 0.961017975 | STEAP1B | STEAP family member 1B [Source:HGNC Symbol;Acc:HGNC:41907] |
| ENSG00000106080 | HGLibA_17558 | 212 | 0.961017975 | FKBP14 | FK506 binding protein 14 [Source:HGNC Symbol;Acc:HGNC:18625] |
| ENSG00000106261 | HGLibA_55345 | 212 | 0.961017975 | ZKSCAN1 | zinc finger with KRAB and SCAN domains 1 [Source:HGNC Symbol;Acc:HGNC:13101] |
| ENSG00000108590 | HGLibA_28804 | 212 | 0.961017975 | MED31 | mediator complex subunit 31 [Source:HGNC Symbol;Acc:HGNC:24260] |
| ENSG00000108830 | HGLibA_41447 | 212 | 0.961017975 | RND2 | Rho family GTPase 2 [Source:HGNC Symbol;Acc:HGNC:18315] |
| ENSG00000109743 | HGLibA_04880 | 212 | 0.961017975 | BST1 | bone marrow stromal cell antigen 1 [Source:HGNC Symbol;Acc:HGNC:1118] |
| ENSG00000110934 | HGLibA_04508 | 212 | 0.961017975 | BIN2 | bridging integrator 2 [Source:HGNC Symbol;Acc:HGNC:1053] |
| ENSG00000112312 | HGLibA_19418 | 212 | 0.961017975 | GMNN | DNA replication inhibitor [Source:HGNC Symbol;Acc:HGNC:17493]" |

| | | | | | |
|---|---|---|---|---|---|
| ENSG00000120937 | HGLibA_32503 | 212 | 0.961017975 | NPPB | natriuretic peptide B [Source:HGNC Symbol;Acc:HGNC:7940] |
| ENSG00000122691 | HGLibA_52374 | 212 | 0.961017975 | TWIST1 | twist family bHLH transcription factor 1 [Source:HGNC Symbol;Acc:HGNC:12428] |
| ENSG00000122970 | HGLibA_22777 | 212 | 0.961017975 | IFT81 | intraflagellar transport 81 [Source:HGNC Symbol;Acc:HGNC:14313] |
| ENSG00000123594 | HGLibA_03845 | 212 | 0.961017975 | ATXN3L | ataxin 3 like [Source:HGNC Symbol;Acc:HGNC:24173] |
| ENSG00000124557 | HGLibA_04959 | 212 | 0.961017975 | BTN1A1 | butyrophilin subfamily 1 member A1 [Source:HGNC Symbol;Acc:HGNC:1135] |
| ENSG00000125651 | HGLibA_20539 | 212 | 0.961017975 | GTF2F1 | general transcription factor IIF subunit 1 [Source:HGNC Symbol;Acc:HGNC:4652] |
| ENSG00000134057 | HGLibA_08057 | 212 | 0.961017975 | CCNB1 | cyclin B1 [Source:HGNC Symbol;Acc:HGNC:1579] |
| ENSG00000134955 | HGLibA_45045 | 212 | 0.961017975 | SLC37A2 | solute carrier family 37 member 2 [Source:HGNC Symbol;Acc:HGNC:20644] |
| ENSG00000135074 | HGLibA_00745 | 212 | 0.961017975 | ADAM19 | ADAM metallopeptidase domain 19 [Source:HGNC Symbol;Acc:HGNC:197] |
| ENSG00000140939 | HGLibA_32265 | 212 | 0.961017975 | NOL3 | nucleolar protein 3 [Source:HGNC Symbol;Acc:HGNC:7869] |
| ENSG00000145293 | HGLibA_15152 | 212 | 0.961017975 | ENOPH1 | enolase-phosphatase 1 [Source:HGNC Symbol;Acc:HGNC:24599] |
| ENSG00000146247 | HGLibA_36400 | 212 | 0.961017975 | PHIP | pleckstrin homology domain interacting protein [Source:HGNC Symbol;Acc:HGNC:15673] |
| ENSG00000147905 | HGLibA_55045 | 212 | 0.961017975 | ZCCHC7 | zinc finger CCHC-type containing 7 [Source:HGNC Symbol;Acc:HGNC:26209] |
| ENSG00000149634 | HGLibA_46458 | 212 | 0.961017975 | SPATA25 | spermatogenesis associated 25 [Source:HGNC Symbol;Acc:HGNC:16158] |
| ENSG00000151692 | HGLibA_41525 | 212 | 0.961017975 | RNF144A | ring finger protein 144A [Source:HGNC Symbol;Acc:HGNC:20457] |
| ENSG00000157303 | HGLibA_47850 | 212 | 0.961017975 | SUSD3 | sushi domain containing 3 [Source:HGNC Symbol;Acc:HGNC:28391] |
| ENSG00000161647 | HGLibA_29754 | 212 | 0.961017975 | MPP3 | membrane palmitoylated protein 3 [Source:HGNC Symbol;Acc:HGNC:7221] |
| ENSG00000163806 | HGLibA_46573 | 212 | 0.961017975 | SPDYA | speedy/RINGO cell cycle regulator family member A [Source:HGNC Symbol;Acc:HGNC:30613] |
| ENSG00000164363 | HGLibA_45314 | 212 | 0.961017975 | SLC6A18 | solute carrier family 6 member 18 [Source:HGNC Symbol;Acc:HGNC:26441] |
| ENSG00000164919 | HGLibA_10853 | 212 | 0.961017975 | COX6C | cytochrome c oxidase subunit 6C [Source:HGNC Symbol;Acc:HGNC:2285] |
| ENSG00000164946 | HGLibA_17983 | 212 | 0.961017975 | FREM1 | FRAS1 related extracellular matrix 1 [Source:HGNC Symbol;Acc:HGNC:23399] |
| ENSG00000166900 | HGLibA_47646 | 212 | 0.961017975 | STX3 | syntaxin 3 [Source:HGNC Symbol;Acc:HGNC:11438] |
| ENSG00000169139 | HGLibA_52670 | 212 | 0.961017975 | UBE2V2 | ubiquitin conjugating enzyme E2 V2 [Source:HGNC Symbol;Acc:HGNC:12495] |
| ENSG00000169891 | HGLibA_40852 | 212 | 0.961017975 | REPS2 | RALBP1 associated Eps domain containing 2 [Source:HGNC Symbol;Acc:HGNC:9963] |
| ENSG00000171711 | HGLibA_12914 | 212 | 0.961017975 | DEFB4A | defensin beta 4A [Source:HGNC Symbol;Acc:HGNC:2767] |
| ENSG00000176697 | HGLibA_04385 | 212 | 0.961017975 | BDNF | brain derived neurotrophic factor [Source:HGNC Symbol;Acc:HGNC:1033] |
| ENSG00000177030 | HGLibA_12769 | 212 | 0.961017975 | DEAF1 | DEAF1 transcription factor [Source:HGNC Symbol;Acc:HGNC:14677] |
| ENSG00000179886 | HGLibA_49474 | 212 | 0.961017975 | TIGD5 | tigger transposable element derived 5 [Source:HGNC Symbol;Acc:HGNC:18336] |
| ENSG00000180383 | HGLibA_12877 | 212 | 0.961017975 | DEFB124 | defensin beta 124 [Source:HGNC Symbol;Acc:HGNC:18104] |
| ENSG00000181291 | HGLibA_49940 | 212 | 0.961017975 | TMEM132E | transmembrane protein 132E [Source:HGNC Symbol;Acc:HGNC:26991] |
| ENSG00000181449 | HGLibA_46253 | 212 | 0.961017975 | SOX2 | SRY-box 2 [Source:HGNC Symbol;Acc:HGNC:11195] |
| ENSG00000185414 | HGLibA_29947 | 212 | 0.961017975 | MRPL30 | mitochondrial ribosomal protein L30 [Source:HGNC Symbol;Acc:HGNC:14036] |
| ENSG00000186684 | HGLibA_12099 | 212 | 0.961017975 | CYP27C1 | cytochrome P450 family 27 subfamily C member 1 [Source:HGNC Symbol;Acc:HGNC:33480] |
| ENSG00000188783 | HGLibA_38435 | 212 | 0.961017975 | PRELP | proline and arginine rich end leucine rich repeat protein [Source:HGNC Symbol;Acc:HGNC:9357] |
| ENSG00000204548 | HGLibA_12871 | 212 | 0.961017975 | DEFB121 | defensin beta 121 [Source:HGNC Symbol;Acc:HGNC:18101] |
| ENSG00000204632 | HGLibA_21647 | 212 | 0.961017975 | HLA-G | G [Source:HGNC Symbol;Acc:HGNC:4964]" |
| ENSG00000214941 | HGLibA_57055 | 212 | 0.961017975 | ZSWIM7 | zinc finger SWIM-type containing 7 [Source:HGNC Symbol;Acc:HGNC:26993] |
| ENSG00000250423 | HGLibA_24712 | 212 | 0.961017975 | KIAA1210 | KIAA1210 [Source:HGNC Symbol;Acc:HGNC:29218] |
| ENSG00000276070 | HGLibA_08032 | 212 | 0.961017975 | CCL4L2 | C-C motif chemokine ligand 4 like 2 [Source:HGNC Symbol;Acc:HGNC:24066] |
| ENSG00000278023 | HGLibA_40756 | 212 | 0.961017975 | RDM1 | RAD52 motif containing 1 [Source:HGNC Symbol;Acc:HGNC:19950] |
| FAM154A | HGLibA_16173 | 212 | 0.961017975 | FAM154A | - |
| hsa-mir-4692 | HGLibA_60877 | 212 | 0.961017975 | hsa-mir-4692 | - |
| hsa-mir-6075 | HGLibA_62552 | 212 | 0.961017975 | hsa-mir-6075 | - |
| KIAA1919 | HGLibA_24825 | 212 | 0.961017975 | KIAA1919 | - |
| LHFP | HGLibA_26427 | 212 | 0.961017975 | LHFP | - |
| LOC100996485 | HGLibA_26884 | 212 | 0.961017975 | LOC100996485 | - |
| USMG5 | HGLibA_53185 | 212 | 0.961017975 | USMG5 | - |
| WBSCR16 | HGLibA_53988 | 212 | 0.961017975 | WBSCR16 | - |
| AGXT2L2 | HGLibA_01280 | 211 | 0.951066938 | AGXT2L2 | - |
| AZI1 | HGLibA_03933 | 211 | 0.951066938 | AZI1 | - |
| C12orf61 | HGLibA_05276 | 211 | 0.951066938 | C12orf61 | - |
| C14orf105 | HGLibA_05317 | 211 | 0.951066938 | C14orf105 | - |
| DAK | HGLibA_12301 | 211 | 0.951066938 | DAK | - |
| ENSG00000004799 | HGLibA_35905 | 211 | 0.951066938 | PDK4 | pyruvate dehydrogenase kinase 4 [Source:HGNC Symbol;Acc:HGNC:8812] |
| ENSG00000052126 | HGLibA_37073 | 211 | 0.951066938 | PLEKHA5 | pleckstrin homology domain containing A5 [Source:HGNC Symbol;Acc:HGNC:30036] |
| ENSG00000070388 | HGLibA_17398 | 211 | 0.951066938 | FGF22 | fibroblast growth factor 22 [Source:HGNC Symbol;Acc:HGNC:3679] |
| ENSG00000078142 | HGLibA_36631 | 211 | 0.951066938 | PIK3C3 | phosphatidylinositol 3-kinase catalytic subunit type 3 [Source:HGNC Symbol;Acc:HGNC:8974] |
| ENSG00000079385 | HGLibA_08922 | 211 | 0.951066938 | CEACAM1 | carcinoembryonic antigen related cell adhesion molecule 1 [Source:HGNC Symbol;Acc:HGNC:1814] |
| ENSG00000089022 | HGLibA_28307 | 211 | 0.951066938 | MAPKAPK5 | mitogen-activated protein kinase-activated protein kinase 5 [Source:HGNC Symbol;Acc:HGNC:6889] |
| ENSG00000099797 | HGLibA_48947 | 211 | 0.951066938 | TECR | 3-enoyl-CoA reductase [Source:HGNC Symbol;Acc:HGNC:4551]" |
| ENSG00000100346 | HGLibA_06898 | 211 | 0.951066938 | CACNA1I | calcium voltage-gated channel subunit alpha1 I [Source:HGNC Symbol;Acc:HGNC:1396] |
| ENSG00000100852 | HGLibA_02782 | 211 | 0.951066938 | ARHGAP5 | Rho GTPase activating protein 5 [Source:HGNC Symbol;Acc:HGNC:675] |
| ENSG00000105649 | HGLibA_39907 | 211 | 0.951066938 | RAB3A | member RAS oncogene family [Source:HGNC Symbol;Acc:HGNC:9777]" |
| ENSG00000107643 | HGLibA_28282 | 211 | 0.951066938 | MAPK8 | mitogen-activated protein kinase 8 [Source:HGNC Symbol;Acc:HGNC:6881] |
| ENSG00000108474 | HGLibA_36565 | 211 | 0.951066938 | PIGL | phosphatidylinositol glycan anchor biosynthesis class L [Source:HGNC Symbol;Acc:HGNC:8966] |
| ENSG00000114737 | HGLibA_09736 | 211 | 0.951066938 | CISH | cytokine inducible SH2 containing protein [Source:HGNC Symbol;Acc:HGNC:1984] |
| ENSG00000116191 | HGLibA_40170 | 211 | 0.951066938 | RALGPS2 | Ral GEF with PH domain and SH3 binding motif 2 [Source:HGNC Symbol;Acc:HGNC:30279] |
| ENSG00000117036 | HGLibA_15653 | 211 | 0.951066938 | ETV3 | ETS variant 3 [Source:HGNC Symbol;Acc:HGNC:3492] |
| ENSG00000120093 | HGLibA_21936 | 211 | 0.951066938 | HOXB3 | homeobox B3 [Source:HGNC Symbol;Acc:HGNC:5114] |
| ENSG00000120837 | HGLibA_31857 | 211 | 0.951066938 | NFYB | nuclear transcription factor Y subunit beta [Source:HGNC Symbol;Acc:HGNC:7805] |
| ENSG00000121316 | HGLibA_36972 | 211 | 0.951066938 | PLBD1 | phospholipase B domain containing 1 [Source:HGNC Symbol;Acc:HGNC:26215] |
| ENSG00000130147 | HGLibA_43930 | 211 | 0.951066938 | SH3BP4 | SH3 domain binding protein 4 [Source:HGNC Symbol;Acc:HGNC:10826] |
| ENSG00000132313 | HGLibA_29960 | 211 | 0.951066938 | MRPL35 | mitochondrial ribosomal protein L35 [Source:HGNC Symbol;Acc:HGNC:14489] |
| ENSG00000132906 | HGLibA_07331 | 211 | 0.951066938 | CASP9 | caspase 9 [Source:HGNC Symbol;Acc:HGNC:1511] |
| ENSG00000134470 | HGLibA_22982 | 211 | 0.951066938 | IL15RA | interleukin 15 receptor subunit alpha [Source:HGNC Symbol;Acc:HGNC:5978] |
| ENSG00000138279 | HGLibA_02249 | 211 | 0.951066938 | ANXA7 | annexin A7 [Source:HGNC Symbol;Acc:HGNC:545] |
| ENSG00000144199 | HGLibA_15961 | 211 | 0.951066938 | FAHD2B | fumarylacetoacetate hydrolase domain containing 2B [Source:HGNC Symbol;Acc:HGNC:25318] |
| ENSG00000147255 | HGLibA_22874 | 211 | 0.951066938 | IGSF1 | immunoglobulin superfamily member 1 [Source:HGNC Symbol;Acc:HGNC:5948] |
| ENSG00000148459 | HGLibA_35949 | 211 | 0.951066938 | PDSS1 | decaprenyl diphosphate synthase subunit 1 [Source:HGNC Symbol;Acc:HGNC:17759] |
| ENSG00000148935 | HGLibA_18644 | 211 | 0.951066938 | GAS2 | growth arrest specific 2 [Source:HGNC Symbol;Acc:HGNC:4167] |
| ENSG00000158445 | HGLibA_24157 | 211 | 0.951066938 | KCNB1 | potassium voltage-gated channel subfamily B member 1 [Source:HGNC Symbol;Acc:HGNC:6231] |
| ENSG00000159335 | HGLibA_39393 | 211 | 0.951066938 | PTMS | parathymosin [Source:HGNC Symbol;Acc:HGNC:9629] |
| ENSG00000159788 | HGLibA_41058 | 211 | 0.951066938 | RGS12 | regulator of G protein signaling 12 [Source:HGNC Symbol;Acc:HGNC:9994] |
| ENSG00000160439 | HGLibA_40741 | 211 | 0.951066938 | RDH13 | retinol dehydrogenase 13 [Source:HGNC Symbol;Acc:HGNC:19978] |
| ENSG00000160588 | HGLibA_29802 | 211 | 0.951066938 | MPZL3 | myelin protein zero like 3 [Source:HGNC Symbol;Acc:HGNC:27279] |
| ENSG00000160683 | HGLibA_11894 | 211 | 0.951066938 | CXCR5 | C-X-C motif chemokine receptor 5 [Source:HGNC Symbol;Acc:HGNC:1060] |
| ENSG00000162757 | HGLibA_05893 | 211 | 0.951066938 | C1orf74 | chromosome 1 open reading frame 74 [Source:HGNC Symbol;Acc:HGNC:26319] |
| ENSG00000162897 | HGLibA_17161 | 211 | 0.951066938 | FCAMR | Fc fragment of IgA and IgM receptor [Source:HGNC Symbol;Acc:HGNC:24692] |
| ENSG00000164122 | HGLibA_03243 | 211 | 0.951066938 | ASB5 | ankyrin repeat and SOCS box containing 5 [Source:HGNC Symbol;Acc:HGNC:17180] |
| ENSG00000164794 | HGLibA_24403 | 211 | 0.951066938 | KCNV1 | potassium voltage-gated channel modifier subfamily V member 1 [Source:HGNC Symbol;Acc:HGNC:18861] |
| ENSG00000165572 | HGLibA_24110 | 211 | 0.951066938 | KBTBD6 | kelch repeat and BTB domain containing 6 [Source:HGNC Symbol;Acc:HGNC:25340] |
| ENSG00000169896 | HGLibA_23760 | 211 | 0.951066938 | ITGAM | integrin subunit alpha M [Source:HGNC Symbol;Acc:HGNC:6149] |
| ENSG00000171402 | HGLibA_54483 | 211 | 0.951066938 | XAGE3 | X antigen family member 3 [Source:HGNC Symbol;Acc:HGNC:14618] |
| ENSG00000172733 | HGLibA_39593 | 211 | 0.951066938 | PURG | purine rich element binding protein G [Source:HGNC Symbol;Acc:HGNC:17930] |
| ENSG00000173852 | HGLibA_13904 | 211 | 0.951066938 | DPY19L1 | dpy-19 like C-mannosyltransferase 1 [Source:HGNC Symbol;Acc:HGNC:22205] |
| ENSG00000176231 | HGLibA_33515 | 211 | 0.951066938 | OR10H4 | olfactory receptor family 10 subfamily H member 4 [Source:HGNC Symbol;Acc:HGNC:15388] |
| ENSG00000178773 | HGLibA_10972 | 211 | 0.951066938 | CPNE7 | copine 7 [Source:HGNC Symbol;Acc:HGNC:2320] |
| ENSG00000179626 | HGLibA_34392 | 211 | 0.951066938 | OR6C4 | olfactory receptor family 6 subfamily C member 4 [Source:HGNC Symbol;Acc:HGNC:19632] |
| ENSG00000180016 | HGLibA_33658 | 211 | 0.951066938 | OR1E1 | olfactory receptor family 1 subfamily E member 1 [Source:HGNC Symbol;Acc:HGNC:8189] |
| ENSG00000180537 | HGLibA_41587 | 211 | 0.951066938 | RNF182 | ring finger protein 182 [Source:HGNC Symbol;Acc:HGNC:28522] |
| ENSG00000182255 | HGLibA_24134 | 211 | 0.951066938 | KCNA4 | potassium voltage-gated channel subfamily A member 4 [Source:HGNC Symbol;Acc:HGNC:6222] |
| ENSG00000182759 | HGLibA_27872 | 211 | 0.951066938 | MAFA | MAF bZIP transcription factor A [Source:HGNC Symbol;Acc:HGNC:23145] |
| ENSG00000183137 | HGLibA_09149 | 211 | 0.951066938 | CEP57L1 | centrosomal protein 57 like 1 [Source:HGNC Symbol;Acc:HGNC:21561] |
| ENSG00000186130 | HGLibA_54914 | 211 | 0.951066938 | ZBTB6 | zinc finger and BTB domain containing 6 [Source:HGNC Symbol;Acc:HGNC:16764] |
| ENSG00000187550 | HGLibA_42802 | 211 | 0.951066938 | SBK2 | SH3 domain binding kinase family member 2 [Source:HGNC Symbol;Acc:HGNC:34416] |
| ENSG00000187569 | HGLibA_13891 | 211 | 0.951066938 | DPPA3 | developmental pluripotency associated 3 [Source:HGNC Symbol;Acc:HGNC:19199] |
| ENSG00000189184 | HGLibA_35406 | 211 | 0.951066938 | PCDH18 | protocadherin 18 [Source:HGNC Symbol;Acc:HGNC:14268] |
| ENSG00000196236 | HGLibA_54559 | 211 | 0.951066938 | XPNPEP3 | X-prolyl aminopeptidase 3 [Source:HGNC Symbol;Acc:HGNC:28052] |
| ENSG00000198768 | HGLibA_02397 | 211 | 0.951066938 | APCDD1L | APC down-regulated 1 like [Source:HGNC Symbol;Acc:HGNC:26892] |
| ENSG00000203756 | HGLibA_50298 | 211 | 0.951066938 | TMEM244 | transmembrane protein 244 [Source:HGNC Symbol;Acc:HGNC:21571] |
| ENSG00000205269 | HGLibA_50057 | 211 | 0.951066938 | TMEM170B | transmembrane protein 170B [Source:HGNC Symbol;Acc:HGNC:34244] |
| ENSG00000212122 | HGLibA_52010 | 211 | 0.951066938 | TSSK1B | testis specific serine kinase 1B [Source:HGNC Symbol;Acc:HGNC:14968] |
| ENSG00000272325 | HGLibA_32976 | 211 | 0.951066938 | NUDT3 | nudix hydrolase 3 [Source:HGNC Symbol;Acc:HGNC:8050] |
| FAM96B | HGLibA_16805 | 211 | 0.951066938 | FAM96B | - |
| hsa-mir-1291 | HGLibA_57639 | 211 | 0.951066938 | hsa-mir-1291 | - |
| hsa-mir-3116-2 | HGLibA_58507 | 211 | 0.951066938 | hsa-mir-3116-2 | - |
| hsa-mir-4300 | HGLibA_59911 | 211 | 0.951066938 | hsa-mir-4300 | - |
| hsa-mir-4674 | HGLibA_60814 | 211 | 0.951066938 | hsa-mir-4674 | - |
| hsa-mir-6773 | HGLibA_63267 | 211 | 0.951066938 | hsa-mir-6773 | - |
| hsa-mir-6841 | HGLibA_63540 | 211 | 0.951066938 | hsa-mir-6841 | - |
| hsa-mir-7112-2 | HGLibA_63817 | 211 | 0.951066938 | hsa-mir-7112-2 | - |
| hsa-mir-8052 | HGLibA_64045 | 211 | 0.951066938 | hsa-mir-8052 | - |
| hsa-mir-8060 | HGLibA_64079 | 211 | 0.951066938 | hsa-mir-8060 | - |
| hsa-mir-933 | HGLibA_64306 | 211 | 0.951066938 | hsa-mir-933 | - |
| hsa-mir-942 | HGLibA_64356 | 211 | 0.951066938 | hsa-mir-942 | - |
| PRR21 | HGLibA_38814 | 211 | 0.951066938 | PRR21 | - |
| C14orf182 | HGLibA_05350 | 210 | 0.9411159 | C14orf182 | - |
| C17orf62 | HGLibA_05555 | 210 | 0.9411159 | C17orf62 | - |
| CRAMP1L | HGLibA_11064 | 210 | 0.9411159 | CRAMP1L | - |
| ENSG00000005961 | HGLibA_23727 | 210 | 0.9411159 | ITGA2B | integrin subunit alpha 2b [Source:HGNC Symbol;Acc:HGNC:6138] |
| ENSG00000016602 | HGLibA_09812 | 210 | 0.9411159 | CLCA4 | chloride channel accessory 4 [Source:HGNC Symbol;Acc:HGNC:2018] |
| ENSG00000071859 | HGLibA_16628 | 210 | 0.9411159 | FAM50A | family with sequence similarity 50 member A [Source:HGNC Symbol;Acc:HGNC:18786] |
| ENSG00000075711 | HGLibA_13323 | 210 | 0.9411159 | DLG1 | discs large MAGUK scaffold protein 1 [Source:HGNC Symbol;Acc:HGNC:2900] |
| ENSG00000099804 | HGLibA_08543 | 210 | 0.9411159 | CDC34 | cell division cycle 34 [Source:HGNC Symbol;Acc:HGNC:1734] |
| ENSG00000100121 | HGLibA_19025 | 210 | 0.9411159 | GGTLC2 | gamma-glutamyltransferase light chain 2 [Source:HGNC Symbol;Acc:HGNC:18596] |

| | | | | | |
|---|---|---|---|---|---|
| ENSG00000101019 | HGLibA_53099 | 210 | 0.9411159 | UQCC1 | ubiquinol-cytochrome c reductase complex assembly factor 1 [Source:HGNC Symbol;Acc:HGNC:15891] |
| ENSG00000101343 | HGLibA_11203 | 210 | 0.9411159 | CRNKL1 | crooked neck pre-mRNA splicing factor 1 [Source:HGNC Symbol;Acc:HGNC:15762] |
| ENSG00000101361 | HGLibA_32314 | 210 | 0.9411159 | NOP56 | NOP56 ribonucleoprotein [Source:HGNC Symbol;Acc:HGNC:15911] |
| ENSG00000101938 | HGLibA_09537 | 210 | 0.9411159 | CHRDL1 | chordin like 1 [Source:HGNC Symbol;Acc:HGNC:29861] |
| ENSG00000106477 | HGLibA_09138 | 210 | 0.9411159 | CEP41 | centrosomal protein 41 [Source:HGNC Symbol;Acc:HGNC:12370] |
| ENSG00000107262 | HGLibA_04073 | 210 | 0.9411159 | BAG1 | BCL2 associated athanogene 1 [Source:HGNC Symbol;Acc:HGNC:937] |
| ENSG00000109193 | HGLibA_47754 | 210 | 0.9411159 | SULT1E1 | sulfotransferase family 1E member 1 [Source:HGNC Symbol;Acc:HGNC:11377] |
| ENSG00000110108 | HGLibA_49867 | 210 | 0.9411159 | TMEM109 | transmembrane protein 109 [Source:HGNC Symbol;Acc:HGNC:28771] |
| ENSG00000110148 | HGLibA_07959 | 210 | 0.9411159 | CCKBR | cholecystokinin B receptor [Source:HGNC Symbol;Acc:HGNC:1571] |
| ENSG00000114166 | HGLibA_24055 | 210 | 0.9411159 | KAT2B | lysine acetyltransferase 2B [Source:HGNC Symbol;Acc:HGNC:8638] |
| ENSG00000115641 | HGLibA_17484 | 210 | 0.9411159 | FHL2 | four and a half LIM domains 2 [Source:HGNC Symbol;Acc:HGNC:3703] |
| ENSG00000121318 | HGLibA_48365 | 210 | 0.9411159 | TAS2R10 | taste 2 receptor member 10 [Source:HGNC Symbol;Acc:HGNC:14918] |
| ENSG00000125257 | HGLibA_00177 | 210 | 0.9411159 | ABCC4 | ATP binding cassette subfamily C member 4 [Source:HGNC Symbol;Acc:HGNC:55] |
| ENSG00000126070 | HGLibA_01212 | 210 | 0.9411159 | AGO3 | RISC catalytic component [Source:HGNC Symbol;Acc:HGNC:18421]" |
| ENSG00000139436 | HGLibA_19132 | 210 | 0.9411159 | GIT2 | GIT ArfGAP 2 [Source:HGNC Symbol;Acc:HGNC:4273] |
| ENSG00000140750 | HGLibA_02714 | 210 | 0.9411159 | ARHGAP17 | Rho GTPase activating protein 17 [Source:HGNC Symbol;Acc:HGNC:18239] |
| ENSG00000142319 | HGLibA_45325 | 210 | 0.9411159 | SLC6A3 | solute carrier family 6 member 3 [Source:HGNC Symbol;Acc:HGNC:11049] |
| ENSG00000142675 | HGLibA_10295 | 210 | 0.9411159 | CNKSR1 | connector enhancer of kinase suppressor of Ras 1 [Source:HGNC Symbol;Acc:HGNC:19700] |
| ENSG00000143512 | HGLibA_21266 | 210 | 0.9411159 | HHIPL2 | HHIP like 2 [Source:HGNC Symbol;Acc:HGNC:25842] |
| ENSG00000143862 | HGLibA_02996 | 210 | 0.9411159 | ARL8A | ADP ribosylation factor like GTPase 8A [Source:HGNC Symbol;Acc:HGNC:25192] |
| ENSG00000145242 | HGLibA_15300 | 210 | 0.9411159 | EPHA5 | EPH receptor A5 [Source:HGNC Symbol;Acc:HGNC:3389] |
| ENSG00000145945 | HGLibA_16631 | 210 | 0.9411159 | FAM50B | family with sequence similarity 50 member B [Source:HGNC Symbol;Acc:HGNC:18789] |
| ENSG00000146857 | HGLibA_47571 | 210 | 0.9411159 | STRA8 | stimulated by retinoic acid 8 [Source:HGNC Symbol;Acc:HGNC:30653] |
| ENSG00000146955 | HGLibA_39826 | 210 | 0.9411159 | RAB19 | member RAS oncogene family [Source:HGNC Symbol;Acc:HGNC:19982]" |
| ENSG00000156475 | HGLibA_38148 | 210 | 0.9411159 | PPP2R2B | protein phosphatase 2 regulatory subunit Bbeta [Source:HGNC Symbol;Acc:HGNC:9305] |
| ENSG00000158805 | HGLibA_55715 | 210 | 0.9411159 | ZNF276 | zinc finger protein 276 [Source:HGNC Symbol;Acc:HGNC:23330] |
| ENSG00000163738 | HGLibA_30385 | 210 | 0.9411159 | MTHFD2L | methylenetetrahydrofolate dehydrogenase (NADP+ dependent) 2 like [Source:HGNC Symbol;Acc:HGNC:31865] |
| ENSG00000165584 | HGLibA_47207 | 210 | 0.9411159 | SSX3 | SSX family member 3 [Source:HGNC Symbol;Acc:HGNC:11337] |
| ENSG00000166128 | HGLibA_39989 | 210 | 0.9411159 | RAB8B | member RAS oncogene family [Source:HGNC Symbol;Acc:HGNC:30273]" |
| ENSG00000167083 | HGLibA_19542 | 210 | 0.9411159 | GNGT2 | G protein subunit gamma transducin 2 [Source:HGNC Symbol;Acc:HGNC:4412] |
| ENSG00000169550 | HGLibA_30525 | 210 | 0.9411159 | MUC15 | cell surface associated [Source:HGNC Symbol;Acc:HGNC:14956]" |
| ENSG00000169714 | HGLibA_10249 | 210 | 0.9411159 | CNBP | CCHC-type zinc finger nucleic acid binding protein [Source:HGNC Symbol;Acc:HGNC:13164] |
| ENSG00000169908 | HGLibA_49692 | 210 | 0.9411159 | TM4SF1 | transmembrane 4 L six family member 1 [Source:HGNC Symbol;Acc:HGNC:11853] |
| ENSG00000170456 | HGLibA_12962 | 210 | 0.9411159 | DENND5B | DENN domain containing 5B [Source:HGNC Symbol;Acc:HGNC:28338] |
| ENSG00000170819 | HGLibA_04458 | 210 | 0.9411159 | BFSP2 | beaded filament structural protein 2 [Source:HGNC Symbol;Acc:HGNC:1041] |
| ENSG00000171606 | HGLibA_55712 | 210 | 0.9411159 | ZNF274 | zinc finger protein 274 [Source:HGNC Symbol;Acc:HGNC:13068] |
| ENSG00000171843 | HGLibA_29416 | 210 | 0.9411159 | MLLT3 | super elongation complex subunit [Source:HGNC Symbol;Acc:HGNC:7136]" |
| ENSG00000172167 | HGLibA_30322 | 210 | 0.9411159 | MTBP | MDM2 binding protein [Source:HGNC Symbol;Acc:HGNC:7417] |
| ENSG00000173275 | HGLibA_56024 | 210 | 0.9411159 | ZNF449 | zinc finger protein 449 [Source:HGNC Symbol;Acc:HGNC:21039] |
| ENSG00000183960 | HGLibA_24230 | 210 | 0.9411159 | KCNH8 | potassium voltage-gated channel subfamily H member 8 [Source:HGNC Symbol;Acc:HGNC:18864] |
| ENSG00000185800 | HGLibA_13449 | 210 | 0.9411159 | DMWD | WD repeat containing [Source:HGNC Symbol;Acc:HGNC:2936]" |
| ENSG00000188763 | HGLibA_18287 | 210 | 0.9411159 | FZD9 | frizzled class receptor 9 [Source:HGNC Symbol;Acc:HGNC:4047] |
| ENSG00000196159 | HGLibA_16920 | 210 | 0.9411159 | FAT4 | FAT atypical cadherin 4 [Source:HGNC Symbol;Acc:HGNC:23109] |
| ENSG00000197857 | HGLibA_56000 | 210 | 0.9411159 | ZNF44 | zinc finger protein 44 [Source:HGNC Symbol;Acc:HGNC:13110] |
| ENSG00000198586 | HGLibA_49623 | 210 | 0.9411159 | TLK1 | tousled like kinase 1 [Source:HGNC Symbol;Acc:HGNC:11841] |
| ENSG00000206190 | HGLibA_03546 | 210 | 0.9411159 | ATP10A | ATPase phospholipid transporting 10A (putative) [Source:HGNC Symbol;Acc:HGNC:13542] |
| ENSG00000211455 | HGLibA_47504 | 210 | 0.9411159 | STK38L | serine/threonine kinase 38 like [Source:HGNC Symbol;Acc:HGNC:17848] |
| ENSG00000213918 | HGLibA_13654 | 210 | 0.9411159 | DNASE1 | deoxyribonuclease 1 [Source:HGNC Symbol;Acc:HGNC:2956] |
| ENSG00000213949 | HGLibA_23715 | 210 | 0.9411159 | ITGA1 | integrin subunit alpha 1 [Source:HGNC Symbol;Acc:HGNC:6134] |
| ENSG00000221813 | HGLibA_34373 | 210 | 0.9411159 | OR6B1 | olfactory receptor family 6 subfamily B member 1 [Source:HGNC Symbol;Acc:HGNC:8354] |
| ENSG00000225968 | HGLibA_14905 | 210 | 0.9411159 | ELFN1 | extracellular leucine rich repeat and fibronectin type III domain containing 1 [Source:HGNC Symbol;Acc:HGNC:33154] |
| ENSG00000233198 | HGLibA_41649 | 210 | 0.9411159 | RNF224 | ring finger protein 224 [Source:HGNC Symbol;Acc:HGNC:41912] |
| ENSG00000234068 | HGLibA_35007 | 210 | 0.9411159 | PAGE2 | PAGE family member 2 [Source:HGNC Symbol;Acc:HGNC:31804] |
| ENSG00000239998 | HGLibA_26491 | 210 | 0.9411159 | LILRA2 | leukocyte immunoglobulin like receptor A2 [Source:HGNC Symbol;Acc:HGNC:6603] |
| ENSG00000243729 | HGLibA_34365 | 210 | 0.9411159 | OR5V1 | olfactory receptor family 5 subfamily V member 1 [Source:HGNC Symbol;Acc:HGNC:13972] |
| ENSG00000253293 | HGLibA_21897 | 210 | 0.9411159 | HOXA10 | homeobox A10 [Source:HGNC Symbol;Acc:HGNC:5100] |
| ENSG00000260027 | HGLibA_21948 | 210 | 0.9411159 | HOXB7 | homeobox B7 [Source:HGNC Symbol;Acc:HGNC:5118] |
| ENSG00000275832 | HGLibA_02733 | 210 | 0.9411159 | ARHGAP23 | Rho GTPase activating protein 23 [Source:HGNC Symbol;Acc:HGNC:29293] |
| hsa-mir-3180-5 | HGLibA_58816 | 210 | 0.9411159 | hsa-mir-3180-5 | - |
| hsa-mir-3674 | HGLibA_59248 | 210 | 0.9411159 | hsa-mir-3674 | - |
| hsa-mir-378c | HGLibA_59435 | 210 | 0.9411159 | hsa-mir-378c | - |
| hsa-mir-4314 | HGLibA_59970 | 210 | 0.9411159 | hsa-mir-4314 | - |
| hsa-mir-4670 | HGLibA_60797 | 210 | 0.9411159 | hsa-mir-4670 | - |
| hsa-mir-4796 | HGLibA_61289 | 210 | 0.9411159 | hsa-mir-4796 | - |
| hsa-mir-486 | HGLibA_61335 | 210 | 0.9411159 | hsa-mir-486 | - |
| hsa-mir-506 | HGLibA_61483 | 210 | 0.9411159 | hsa-mir-506 | - |
| hsa-mir-512-1 | HGLibA_61564 | 210 | 0.9411159 | hsa-mir-512-1 | - |
| hsa-mir-562 | HGLibA_62213 | 210 | 0.9411159 | hsa-mir-562 | - |
| hsa-mir-586 | HGLibA_62443 | 210 | 0.9411159 | hsa-mir-586 | - |
| hsa-mir-7106 | HGLibA_63783 | 210 | 0.9411159 | hsa-mir-7106 | - |
| LOC100505841 | HGLibA_26855 | 210 | 0.9411159 | LOC100505841 | - |
| OR5R1 | HGLibA_34351 | 210 | 0.9411159 | OR5R1 | - |
| ENSG00000065621 | HGLibA_20489 | 209 | 0.931164863 | GSTO2 | glutathione S-transferase omega 2 [Source:HGNC Symbol;Acc:HGNC:23064] |
| ENSG00000074657 | HGLibA_56179 | 209 | 0.931164863 | ZNF532 | zinc finger protein 532 [Source:HGNC Symbol;Acc:HGNC:30940] |
| ENSG00000075240 | HGLibA_20210 | 209 | 0.931164863 | GRAMD4 | GRAM domain containing 4 [Source:HGNC Symbol;Acc:HGNC:29113] |
| ENSG00000086300 | HGLibA_46027 | 209 | 0.931164863 | SNX10 | sorting nexin 10 [Source:HGNC Symbol;Acc:HGNC:14974] |
| ENSG00000093010 | HGLibA_10687 | 209 | 0.931164863 | COMT | catechol-O-methyltransferase [Source:HGNC Symbol;Acc:HGNC:2228] |
| ENSG00000101200 | HGLibA_03897 | 209 | 0.931164863 | AVP | arginine vasopressin [Source:HGNC Symbol;Acc:HGNC:894] |
| ENSG00000101849 | HGLibA_48607 | 209 | 0.931164863 | TBL1X | transducin beta like 1 X-linked [Source:HGNC Symbol;Acc:HGNC:11585] |
| ENSG00000102858 | HGLibA_29172 | 209 | 0.931164863 | MGRN1 | mahogunin ring finger 1 [Source:HGNC Symbol;Acc:HGNC:20254] |
| ENSG00000103653 | HGLibA_11360 | 209 | 0.931164863 | CSK | C-terminal Src kinase [Source:HGNC Symbol;Acc:HGNC:2444] |
| ENSG00000108509 | HGLibA_07117 | 209 | 0.931164863 | CAMTA2 | calmodulin binding transcription activator 2 [Source:HGNC Symbol;Acc:HGNC:18807] |
| ENSG00000110324 | HGLibA_22946 | 209 | 0.931164863 | IL10RA | interleukin 10 receptor subunit alpha [Source:HGNC Symbol;Acc:HGNC:5964] |
| ENSG00000111911 | HGLibA_21330 | 209 | 0.931164863 | HINT3 | histidine triad nucleotide binding protein 3 [Source:HGNC Symbol;Acc:HGNC:18468] |
| ENSG00000112115 | HGLibA_22989 | 209 | 0.931164863 | IL17A | interleukin 17A [Source:HGNC Symbol;Acc:HGNC:5981] |
| ENSG00000114124 | HGLibA_20338 | 209 | 0.931164863 | GRK7 | G protein-coupled receptor kinase 7 [Source:HGNC Symbol;Acc:HGNC:17031] |
| ENSG00000124116 | HGLibA_54266 | 209 | 0.931164863 | WFDC3 | WAP four-disulfide core domain 3 [Source:HGNC Symbol;Acc:HGNC:15957] |
| ENSG00000124588 | HGLibA_32566 | 209 | 0.931164863 | NQO2 | N-ribosyldihydronicotinamide:quinone reductase 2 [Source:HGNC Symbol;Acc:HGNC:7856] |
| ENSG00000130590 | HGLibA_42661 | 209 | 0.931164863 | SAMD10 | sterile alpha motif domain containing 10 [Source:HGNC Symbol;Acc:HGNC:16129] |
| ENSG00000133124 | HGLibA_23624 | 209 | 0.931164863 | IRS4 | insulin receptor substrate 4 [Source:HGNC Symbol;Acc:HGNC:6128] |
| ENSG00000134077 | HGLibA_49420 | 209 | 0.931164863 | THUMPD3 | THUMP domain containing 3 [Source:HGNC Symbol;Acc:HGNC:24493] |
| ENSG00000139174 | HGLibA_38471 | 209 | 0.931164863 | PRICKLE1 | prickle planar cell polarity protein 1 [Source:HGNC Symbol;Acc:HGNC:17019] |
| ENSG00000140323 | HGLibA_13285 | 209 | 0.931164863 | DISP2 | dispatched RND transporter family member 2 [Source:HGNC Symbol;Acc:HGNC:19712] |
| ENSG00000140990 | HGLibA_31507 | 209 | 0.931164863 | NDUFB10 | NADH:ubiquinone oxidoreductase subunit B10 [Source:HGNC Symbol;Acc:HGNC:7696] |
| ENSG00000144381 | HGLibA_22331 | 209 | 0.931164863 | HSPD1 | heat shock protein family D (Hsp60) member 1 [Source:HGNC Symbol;Acc:HGNC:5261] |
| ENSG00000145780 | HGLibA_17280 | 209 | 0.931164863 | FEM1C | fem-1 homolog C [Source:HGNC Symbol;Acc:HGNC:16933] |
| ENSG00000148824 | HGLibA_30371 | 209 | 0.931164863 | MTG1 | mitochondrial ribosome associated GTPase 1 [Source:HGNC Symbol;Acc:HGNC:32159] |
| ENSG00000149575 | HGLibA_42982 | 209 | 0.931164863 | SCN2B | sodium voltage-gated channel beta subunit 2 [Source:HGNC Symbol;Acc:HGNC:10589] |
| ENSG00000158792 | HGLibA_46461 | 209 | 0.931164863 | SPATA2L | spermatogenesis associated 2 like [Source:HGNC Symbol;Acc:HGNC:28393] |
| ENSG00000159256 | HGLibA_29653 | 209 | 0.931164863 | MORC3 | MORC family CW-type zinc finger 3 [Source:HGNC Symbol;Acc:HGNC:23572] |
| ENSG00000164120 | HGLibA_22030 | 209 | 0.931164863 | HPGD | 15-hydroxyprostaglandin dehydrogenase [Source:HGNC Symbol;Acc:HGNC:5154] |
| ENSG00000166439 | HGLibA_41569 | 209 | 0.931164863 | RNF169 | ring finger protein 169 [Source:HGNC Symbol;Acc:HGNC:26961] |
| ENSG00000168306 | HGLibA_00501 | 209 | 0.931164863 | ACOX2 | acyl-CoA oxidase 2 [Source:HGNC Symbol;Acc:HGNC:120] |
| ENSG00000169271 | HGLibA_22311 | 209 | 0.931164863 | HSPB3 | heat shock protein family B (small) member 3 [Source:HGNC Symbol;Acc:HGNC:5248] |
| ENSG00000171202 | HGLibA_49911 | 209 | 0.931164863 | TMEM126A | transmembrane protein 126A [Source:HGNC Symbol;Acc:HGNC:25382] |
| ENSG00000172935 | HGLibA_29839 | 209 | 0.931164863 | MRGPRF | MAS related GPR family member F [Source:HGNC Symbol;Acc:HGNC:24828] |
| ENSG00000173431 | HGLibA_41418 | 209 | 0.931164863 | RNASE8 | ribonuclease A family member 8 [Source:HGNC Symbol;Acc:HGNC:19277] |
| ENSG00000175182 | HGLibA_16106 | 209 | 0.931164863 | FAM131A | family with sequence similarity 131 member A [Source:HGNC Symbol;Acc:HGNC:28308] |
| ENSG00000177666 | HGLibA_37407 | 209 | 0.931164863 | PNPLA2 | patatin like phospholipase domain containing 2 [Source:HGNC Symbol;Acc:HGNC:30802] |
| ENSG00000178772 | HGLibA_10949 | 209 | 0.931164863 | CPN2 | carboxypeptidase N subunit 2 [Source:HGNC Symbol;Acc:HGNC:2313] |
| ENSG00000180353 | HGLibA_20969 | 209 | 0.931164863 | HCLS1 | hematopoietic cell-specific Lyn substrate 1 [Source:HGNC Symbol;Acc:HGNC:4844] |
| ENSG00000183423 | HGLibA_27192 | 209 | 0.931164863 | LRIT3 | Ig-like and transmembrane domains 3 [Source:HGNC Symbol;Acc:HGNC:24783]" |
| ENSG00000184428 | HGLibA_50992 | 209 | 0.931164863 | TOP1MT | DNA topoisomerase I mitochondrial [Source:HGNC Symbol;Acc:HGNC:29787] |
| ENSG00000186442 | HGLibA_25470 | 209 | 0.931164863 | KRT3 | keratin 3 [Source:HGNC Symbol;Acc:HGNC:6440] |
| ENSG00000187566 | HGLibA_31896 | 209 | 0.931164863 | NHLRC1 | NHL repeat containing E3 ubiquitin protein ligase 1 [Source:HGNC Symbol;Acc:HGNC:21576] |
| ENSG00000187714 | HGLibA_44493 | 209 | 0.931164863 | SLC18A3 | solute carrier family 18 member A3 [Source:HGNC Symbol;Acc:HGNC:10936] |
| ENSG00000188886 | HGLibA_03376 | 209 | 0.931164863 | ASTL | astacin like metalloendopeptidase [Source:HGNC Symbol;Acc:HGNC:31704] |
| ENSG00000198157 | HGLibA_21730 | 209 | 0.931164863 | HMGN5 | high mobility group nucleosome binding domain 5 [Source:HGNC Symbol;Acc:HGNC:8013] |
| ENSG00000198574 | HGLibA_43885 | 209 | 0.931164863 | SH2D1B | SH2 domain containing 1B [Source:HGNC Symbol;Acc:HGNC:30416] |
| ENSG00000203933 | HGLibA_11963 | 209 | 0.931164863 | CXorf66 | chromosome X open reading frame 66 [Source:HGNC Symbol;Acc:HGNC:33743] |
| ENSG00000221946 | HGLibA_18246 | 209 | 0.931164863 | FXYD7 | FXYD domain containing ion transport regulator 7 [Source:HGNC Symbol;Acc:HGNC:4034] |
| ENSG00000272804 | HGLibA_25618 | 209 | 0.931164863 | KRTAP10-7 | keratin associated protein 10-7 [Source:HGNC Symbol;Acc:HGNC:22970] |
| GPR56 | HGLibA_20056 | 209 | 0.931164863 | GPR56 | - |
| hsa-mir-222 | HGLibA_58296 | 209 | 0.931164863 | hsa-mir-222 | - |
| hsa-mir-3925 | HGLibA_59574 | 209 | 0.931164863 | hsa-mir-3925 | - |
| hsa-mir-4316 | HGLibA_59982 | 209 | 0.931164863 | hsa-mir-4316 | - |
| hsa-mir-4468 | HGLibA_60280 | 209 | 0.931164863 | hsa-mir-4468 | - |
| hsa-mir-4469 | HGLibA_60281 | 209 | 0.931164863 | hsa-mir-4469 | - |
| hsa-mir-4514 | HGLibA_60498 | 209 | 0.931164863 | hsa-mir-4514 | - |
| hsa-mir-541 | HGLibA_61815 | 209 | 0.931164863 | hsa-mir-541 | - |
| hsa-mir-548l | HGLibA_62037 | 209 | 0.931164863 | hsa-mir-548l | - |
| hsa-mir-5590 | HGLibA_62202 | 209 | 0.931164863 | hsa-mir-5590 | - |
| hsa-mir-6080 | HGLibA_62582 | 209 | 0.931164863 | hsa-mir-6080 | - |
| KIAA0146 | HGLibA_24596 | 209 | 0.931164863 | KIAA0146 | - |
| OR8G2 | HGLibA_34519 | 209 | 0.931164863 | OR8G2 | - |

| | | | | | |
|---|---|---|---|---|---|
| TMEM180 | HGLibA_50097 | 209 | 0.931164863 | TMEM180 | - |
| WIBG | HGLibA_54299 | 209 | 0.931164863 | WIBG | - |
| XRCC6BP1 | HGLibA_54600 | 209 | 0.931164863 | XRCC6BP1 | - |
| ZNF664-FAM101A | HGLibA_56485 | 209 | 0.931164863 | ZNF664-FAM101A | - |
| CHDC2 | HGLibA_09414 | 208 | 0.921213826 | CHDC2 | - |
| ENSG00000013441 | HGLibA_10053 | 208 | 0.921213826 | CLK1 | CDC like kinase 1 [Source:HGNC Symbol;Acc:HGNC:2068] |
| ENSG00000027075 | HGLibA_38552 | 208 | 0.921213826 | PRKCH | protein kinase C eta [Source:HGNC Symbol;Acc:HGNC:9403] |
| ENSG00000038427 | HGLibA_53569 | 208 | 0.921213826 | VCAN | versican [Source:HGNC Symbol;Acc:HGNC:2464] |
| ENSG00000077942 | HGLibA_16943 | 208 | 0.921213826 | FBLN1 | fibulin 1 [Source:HGNC Symbol;Acc:HGNC:3600] |
| ENSG00000081041 | HGLibA_11866 | 208 | 0.921213826 | CXCL2 | C-X-C motif chemokine ligand 2 [Source:HGNC Symbol;Acc:HGNC:4603] |
| ENSG00000084693 | HGLibA_01177 | 208 | 0.921213826 | AGBL5 | ATP/GTP binding protein like 5 [Source:HGNC Symbol;Acc:HGNC:26147] |
| ENSG00000100033 | HGLibA_38659 | 208 | 0.921213826 | PRODH | proline dehydrogenase 1 [Source:HGNC Symbol;Acc:HGNC:9453] |
| ENSG00000101199 | HGLibA_02660 | 208 | 0.921213826 | ARFGAP1 | ADP ribosylation factor GTPase activating protein 1 [Source:HGNC Symbol;Acc:HGNC:15852] |
| ENSG00000105855 | HGLibA_23800 | 208 | 0.921213826 | ITGB8 | integrin subunit beta 8 [Source:HGNC Symbol;Acc:HGNC:6163] |
| ENSG00000107833 | HGLibA_32493 | 208 | 0.921213826 | NPM3 | nucleophosmin/nucleoplasmin 3 [Source:HGNC Symbol;Acc:HGNC:7931] |
| ENSG00000109943 | HGLibA_11223 | 208 | 0.921213826 | CRTAM | cytotoxic and regulatory T cell molecule [Source:HGNC Symbol;Acc:HGNC:24313] |
| ENSG00000110844 | HGLibA_38737 | 208 | 0.921213826 | PRPF40B | pre-mRNA processing factor 40 homolog B [Source:HGNC Symbol;Acc:HGNC:25031] |
| ENSG00000111652 | HGLibA_10727 | 208 | 0.921213826 | COPS7A | COP9 signalosome subunit 7A [Source:HGNC Symbol;Acc:HGNC:16758] |
| ENSG00000115155 | HGLibA_34727 | 208 | 0.921213826 | OTOF | otoferlin [Source:HGNC Symbol;Acc:HGNC:8515] |
| ENSG00000115520 | HGLibA_10746 | 208 | 0.921213826 | COQ10B | coenzyme Q10B [Source:HGNC Symbol;Acc:HGNC:25819] |
| ENSG00000118298 | HGLibA_06799 | 208 | 0.921213826 | CA14 | carbonic anhydrase 14 [Source:HGNC Symbol;Acc:HGNC:1372] |
| ENSG00000119535 | HGLibA_11342 | 208 | 0.921213826 | CSF3R | colony stimulating factor 3 receptor [Source:HGNC Symbol;Acc:HGNC:2439] |
| ENSG00000121966 | HGLibA_11890 | 208 | 0.921213826 | CXCR4 | C-X-C motif chemokine receptor 4 [Source:HGNC Symbol;Acc:HGNC:2561] |
| ENSG00000122859 | HGLibA_31752 | 208 | 0.921213826 | NEUROG3 | neurogenin 3 [Source:HGNC Symbol;Acc:HGNC:13806] |
| ENSG00000123415 | HGLibA_45843 | 208 | 0.921213826 | SMUG1 | single-strand-selective monofunctional uracil-DNA glycosylase 1 [Source:HGNC Symbol;Acc:HGNC:17148] |
| ENSG00000128607 | HGLibA_25092 | 208 | 0.921213826 | KLHDC10 | kelch domain containing 10 [Source:HGNC Symbol;Acc:HGNC:22194] |
| ENSG00000128917 | HGLibA_13365 | 208 | 0.921213826 | DLL4 | delta like canonical Notch ligand 4 [Source:HGNC Symbol;Acc:HGNC:2910] |
| ENSG00000130167 | HGLibA_51910 | 208 | 0.921213826 | TSPAN16 | tetraspanin 16 [Source:HGNC Symbol;Acc:HGNC:30725] |
| ENSG00000130540 | HGLibA_47764 | 208 | 0.921213826 | SULT4A1 | sulfotransferase family 4A member 1 [Source:HGNC Symbol;Acc:HGNC:14903] |
| ENSG00000130812 | HGLibA_01926 | 208 | 0.921213826 | ANGPTL6 | angiopoietin like 6 [Source:HGNC Symbol;Acc:HGNC:23140] |
| ENSG00000133193 | HGLibA_15988 | 208 | 0.921213826 | FAM104A | family with sequence similarity 104 member A [Source:HGNC Symbol;Acc:HGNC:25918] |
| ENSG00000138085 | HGLibA_03813 | 208 | 0.921213826 | ATRAID | all-trans retinoic acid induced differentiation factor [Source:HGNC Symbol;Acc:HGNC:24090] |
| ENSG00000138617 | HGLibA_35228 | 208 | 0.921213826 | PARP16 | poly(ADP-ribose) polymerase family member 16 [Source:HGNC Symbol;Acc:HGNC:26040] |
| ENSG00000139330 | HGLibA_24554 | 208 | 0.921213826 | KERA | keratocan [Source:HGNC Symbol;Acc:HGNC:6309] |
| ENSG00000148290 | HGLibA_47833 | 208 | 0.921213826 | SURF1 | cytochrome c oxidase assembly factor [Source:HGNC Symbol;Acc:HGNC:11474]" |
| ENSG00000148339 | HGLibA_44684 | 208 | 0.921213826 | SLC25A25 | solute carrier family 25 member 25 [Source:HGNC Symbol;Acc:HGNC:20663] |
| ENSG00000149657 | HGLibA_27533 | 208 | 0.921213826 | LSM14B | LSM family member 14B [Source:HGNC Symbol;Acc:HGNC:15887] |
| ENSG00000151025 | HGLibA_19952 | 208 | 0.921213826 | GPR158 | G protein-coupled receptor 158 [Source:HGNC Symbol;Acc:HGNC:23689] |
| ENSG00000154099 | HGLibA_13459 | 208 | 0.921213826 | DNAAF1 | dynein axonemal assembly factor 1 [Source:HGNC Symbol;Acc:HGNC:30539] |
| ENSG00000155307 | HGLibA_42709 | 208 | 0.921213826 | SAMSN1 | SH3 domain and nuclear localization signals 1 [Source:HGNC Symbol;Acc:HGNC:10528]" |
| ENSG00000156414 | HGLibA_48917 | 208 | 0.921213826 | TDRD9 | tudor domain containing 9 [Source:HGNC Symbol;Acc:HGNC:20122] |
| ENSG00000158163 | HGLibA_14300 | 208 | 0.921213826 | DZIP1L | DAZ interacting zinc finger protein 1 like [Source:HGNC Symbol;Acc:HGNC:26551] |
| ENSG00000162545 | HGLibA_07079 | 208 | 0.921213826 | CAMK2N1 | calcium/calmodulin dependent protein kinase II inhibitor 1 [Source:HGNC Symbol;Acc:HGNC:24190] |
| ENSG00000162631 | HGLibA_32865 | 208 | 0.921213826 | NTNG1 | netrin G1 [Source:HGNC Symbol;Acc:HGNC:23319] |
| ENSG00000163956 | HGLibA_27236 | 208 | 0.921213826 | LRPAP1 | LDL receptor related protein associated protein 1 [Source:HGNC Symbol;Acc:HGNC:6701] |
| ENSG00000165929 | HGLibA_48685 | 208 | 0.921213826 | TC2N | nuclear [Source:HGNC Symbol;Acc:HGNC:19859]" |
| ENSG00000166224 | HGLibA_43848 | 208 | 0.921213826 | SGPL1 | sphingosine-1-phosphate lyase 1 [Source:HGNC Symbol;Acc:HGNC:10817] |
| ENSG00000166634 | HGLibA_43541 | 208 | 0.921213826 | SERPINB12 | serpin family B member 12 [Source:HGNC Symbol;Acc:HGNC:14220] |
| ENSG00000166823 | HGLibA_28915 | 208 | 0.921213826 | MESP1 | mesoderm posterior bHLH transcription factor 1 [Source:HGNC Symbol;Acc:HGNC:29658] |
| ENSG00000169231 | HGLibA_49329 | 208 | 0.921213826 | THBS3 | thrombospondin 3 [Source:HGNC Symbol;Acc:HGNC:11787] |
| ENSG00000170222 | HGLibA_01035 | 208 | 0.921213826 | ADPRM | manganese dependent [Source:HGNC Symbol;Acc:HGNC:30925]" |
| ENSG00000172531 | HGLibA_38004 | 208 | 0.921213826 | PPP1CA | protein phosphatase 1 catalytic subunit alpha [Source:HGNC Symbol;Acc:HGNC:9281] |
| ENSG00000172967 | HGLibA_54520 | 208 | 0.921213826 | XKR3 | XK related 3 [Source:HGNC Symbol;Acc:HGNC:28778] |
| ENSG00000173239 | HGLibA_26627 | 208 | 0.921213826 | LIPM | lipase family member M [Source:HGNC Symbol;Acc:HGNC:23455] |
| ENSG00000180921 | HGLibA_16761 | 208 | 0.921213826 | FAM83H | family with sequence similarity 83 member H [Source:HGNC Symbol;Acc:HGNC:24797] |
| ENSG00000181785 | HGLibA_34249 | 208 | 0.921213826 | OR5AS1 | olfactory receptor family 5 subfamily AS member 1 [Source:HGNC Symbol;Acc:HGNC:15261] |
| ENSG00000183576 | HGLibA_43646 | 208 | 0.921213826 | SETD3 | SET domain containing 3 [Source:HGNC Symbol;Acc:HGNC:20493] |
| ENSG00000184709 | HGLibA_27293 | 208 | 0.921213826 | LRRC26 | leucine rich repeat containing 26 [Source:HGNC Symbol;Acc:HGNC:31409] |
| ENSG00000187116 | HGLibA_26502 | 208 | 0.921213826 | LILRA5 | leukocyte immunoglobulin like receptor A5 [Source:HGNC Symbol;Acc:HGNC:16309] |
| ENSG00000187959 | HGLibA_11010 | 208 | 0.921213826 | CPSF4L | cleavage and polyadenylation specific factor 4 like [Source:HGNC Symbol;Acc:HGNC:33632] |
| ENSG00000188483 | HGLibA_22583 | 208 | 0.921213826 | IER5L | immediate early response 5 like [Source:HGNC Symbol;Acc:HGNC:23679] |
| ENSG00000188629 | HGLibA_55532 | 208 | 0.921213826 | ZNF177 | zinc finger protein 177 [Source:HGNC Symbol;Acc:HGNC:12966] |
| ENSG00000196277 | HGLibA_20358 | 208 | 0.921213826 | GRM7 | glutamate metabotropic receptor 7 [Source:HGNC Symbol;Acc:HGNC:4599] |
| ENSG00000196730 | HGLibA_12323 | 208 | 0.921213826 | DAPK1 | death associated protein kinase 1 [Source:HGNC Symbol;Acc:HGNC:2674] |
| ENSG00000197635 | HGLibA_13871 | 208 | 0.921213826 | DPP4 | dipeptidyl peptidase 4 [Source:HGNC Symbol;Acc:HGNC:3009] |
| ENSG00000197961 | HGLibA_55454 | 208 | 0.921213826 | ZNF121 | zinc finger protein 121 [Source:HGNC Symbol;Acc:HGNC:12904] |
| ENSG00000198331 | HGLibA_22458 | 208 | 0.921213826 | HYLS1 | centriolar and ciliogenesis associated [Source:HGNC Symbol;Acc:HGNC:26558]" |
| ENSG00000204175 | HGLibA_20144 | 208 | 0.921213826 | GPRIN2 | G protein regulated inducer of neurite outgrowth 2 [Source:HGNC Symbol;Acc:HGNC:23730] |
| ENSG00000204406 | HGLibA_28491 | 208 | 0.921213826 | MBD5 | methyl-CpG binding domain protein 5 [Source:HGNC Symbol;Acc:HGNC:20444] |
| ENSG00000221869 | HGLibA_08962 | 208 | 0.921213826 | CEBPD | CCAAT enhancer binding protein delta [Source:HGNC Symbol;Acc:HGNC:1835] |
| ENSG00000235711 | HGLibA_02080 | 208 | 0.921213826 | ANKRD34C | ankyrin repeat domain 34C [Source:HGNC Symbol;Acc:HGNC:33888] |
| ENSG00000259224 | HGLibA_45029 | 208 | 0.921213826 | SLC35G6 | solute carrier family 35 member G6 [Source:HGNC Symbol;Acc:HGNC:31351] |
| FAM45A | HGLibA_16593 | 208 | 0.921213826 | FAM45A | - |
| GATSL3 | HGLibA_18707 | 208 | 0.921213826 | GATSL3 | - |
| HIST1H4H | HGLibA_21508 | 208 | 0.921213826 | HIST1H4H | - |
| hsa-mir-577 | HGLibA_62402 | 208 | 0.921213826 | hsa-mir-577 | - |
| hsa-mir-583 | HGLibA_62432 | 208 | 0.921213826 | hsa-mir-583 | - |
| hsa-mir-6747 | HGLibA_63141 | 208 | 0.921213826 | hsa-mir-6747 | - |
| hsa-mir-6803 | HGLibA_63390 | 208 | 0.921213826 | hsa-mir-6803 | - |
| PPAP2C | HGLibA_37821 | 208 | 0.921213826 | PPAP2C | - |
| ENSG00000007168 | HGLibA_34989 | 207 | 0.911262788 | PAFAH1B1 | platelet activating factor acetylhydrolase 1b regulatory subunit 1 [Source:HGNC Symbol;Acc:HGNC:8574] |
| ENSG00000010295 | HGLibA_22588 | 207 | 0.911262788 | IFFO1 | intermediate filament family orphan 1 [Source:HGNC Symbol;Acc:HGNC:24970] |
| ENSG00000011347 | HGLibA_48087 | 207 | 0.911262788 | SYT7 | synaptotagmin 7 [Source:HGNC Symbol;Acc:HGNC:11514] |
| ENSG00000083844 | HGLibA_55697 | 207 | 0.911262788 | ZNF264 | zinc finger protein 264 [Source:HGNC Symbol;Acc:HGNC:13057] |
| ENSG00000089486 | HGLibA_08713 | 207 | 0.911262788 | CDIP1 | cell death inducing p53 target 1 [Source:HGNC Symbol;Acc:HGNC:13234] |
| ENSG00000099282 | HGLibA_51908 | 207 | 0.911262788 | TSPAN15 | tetraspanin 15 [Source:HGNC Symbol;Acc:HGNC:23298] |
| ENSG00000100104 | HGLibA_47043 | 207 | 0.911262788 | SRRD | SRR1 domain containing [Source:HGNC Symbol;Acc:HGNC:33910] |
| ENSG00000100354 | HGLibA_50915 | 207 | 0.911262788 | TNRC6B | trinucleotide repeat containing 6B [Source:HGNC Symbol;Acc:HGNC:29190] |
| ENSG00000100379 | HGLibA_24435 | 207 | 0.911262788 | KCTD17 | potassium channel tetramerization domain containing 17 [Source:HGNC Symbol;Acc:HGNC:25705] |
| ENSG00000100422 | HGLibA_09195 | 207 | 0.911262788 | CERK | ceramide kinase [Source:HGNC Symbol;Acc:HGNC:19256] |
| ENSG00000102879 | HGLibA_10773 | 207 | 0.911262788 | CORO1A | coronin 1A [Source:HGNC Symbol;Acc:HGNC:2252] |
| ENSG00000102921 | HGLibA_30949 | 207 | 0.911262788 | N4BP1 | NEDD4 binding protein 1 [Source:HGNC Symbol;Acc:HGNC:29850] |
| ENSG00000105173 | HGLibA_08084 | 207 | 0.911262788 | CCNE1 | cyclin E1 [Source:HGNC Symbol;Acc:HGNC:1589] |
| ENSG00000106178 | HGLibA_08003 | 207 | 0.911262788 | CCL24 | C-C motif chemokine ligand 24 [Source:HGNC Symbol;Acc:HGNC:10623] |
| ENSG00000109814 | HGLibA_52864 | 207 | 0.911262788 | UGDH | UDP-glucose 6-dehydrogenase [Source:HGNC Symbol;Acc:HGNC:12525] |
| ENSG00000109919 | HGLibA_30329 | 207 | 0.911262788 | MTCH2 | mitochondrial carrier 2 [Source:HGNC Symbol;Acc:HGNC:17587] |
| ENSG00000111696 | HGLibA_32826 | 207 | 0.911262788 | NT5DC3 | 5'-nucleotidase domain containing 3 [Source:HGNC Symbol;Acc:HGNC:30826] |
| ENSG00000112115 | HGLibA_22990 | 207 | 0.911262788 | IL17A | interleukin 17A [Source:HGNC Symbol;Acc:HGNC:5981] |
| ENSG00000113719 | HGLibA_15466 | 207 | 0.911262788 | ERGIC1 | endoplasmic reticulum-golgi intermediate compartment 1 [Source:HGNC Symbol;Acc:HGNC:29205] |
| ENSG00000117407 | HGLibA_03171 | 207 | 0.911262788 | ARTN | artemin [Source:HGNC Symbol;Acc:HGNC:727] |
| ENSG00000117724 | HGLibA_09055 | 207 | 0.911262788 | CENPF | centromere protein F [Source:HGNC Symbol;Acc:HGNC:1857] |
| ENSG00000120903 | HGLibA_09568 | 207 | 0.911262788 | CHRNA2 | cholinergic receptor nicotinic alpha 2 subunit [Source:HGNC Symbol;Acc:HGNC:1956] |
| ENSG00000123095 | HGLibA_04483 | 207 | 0.911262788 | BHLHE41 | basic helix-loop-helix family member e41 [Source:HGNC Symbol;Acc:HGNC:16617] |
| ENSG00000124678 | HGLibA_48843 | 207 | 0.911262788 | TCP11 | t-complex 11 [Source:HGNC Symbol;Acc:HGNC:11658] |
| ENSG00000129292 | HGLibA_36367 | 207 | 0.911262788 | PHF20L1 | PHD finger protein 20 like 1 [Source:HGNC Symbol;Acc:HGNC:24280] |
| ENSG00000129480 | HGLibA_14054 | 207 | 0.911262788 | DTD2 | D-tyrosyl-tRNA deacylase 2 (putative) [Source:HGNC Symbol;Acc:HGNC:20277] |
| ENSG00000132612 | HGLibA_53796 | 207 | 0.911262788 | VPS4A | vacuolar protein sorting 4 homolog A [Source:HGNC Symbol;Acc:HGNC:13488] |
| ENSG00000133112 | HGLibA_51210 | 207 | 0.911262788 | TPT1 | translationally-controlled 1 [Source:HGNC Symbol;Acc:HGNC:12022]" |
| ENSG00000133115 | HGLibA_47539 | 207 | 0.911262788 | STOML3 | stomatin like 3 [Source:HGNC Symbol;Acc:HGNC:19420] |
| ENSG00000134318 | HGLibA_41747 | 207 | 0.911262788 | ROCK2 | Rho associated coiled-coil containing protein kinase 2 [Source:HGNC Symbol;Acc:HGNC:10252] |
| ENSG00000135698 | HGLibA_29727 | 207 | 0.911262788 | MPHOSPH6 | M-phase phosphoprotein 6 [Source:HGNC Symbol;Acc:HGNC:7214] |
| ENSG00000136193 | HGLibA_43050 | 207 | 0.911262788 | SCRN1 | secernin 1 [Source:HGNC Symbol;Acc:HGNC:22192] |
| ENSG00000137501 | HGLibA_48099 | 207 | 0.911262788 | SYTL2 | synaptotagmin like 2 [Source:HGNC Symbol;Acc:HGNC:15585] |
| ENSG00000138684 | HGLibA_23081 | 207 | 0.911262788 | IL21 | interleukin 21 [Source:HGNC Symbol;Acc:HGNC:6005] |
| ENSG00000139624 | HGLibA_09211 | 207 | 0.911262788 | CERS5 | ceramide synthase 5 [Source:HGNC Symbol;Acc:HGNC:23749] |
| ENSG00000139725 | HGLibA_41188 | 207 | 0.911262788 | RHOF | filopodia associated [Source:HGNC Symbol;Acc:HGNC:15703]" |
| ENSG00000141469 | HGLibA_44399 | 207 | 0.911262788 | SLC14A1 | solute carrier family 14 member 1 (Kidd blood group) [Source:HGNC Symbol;Acc:HGNC:10918] |
| ENSG00000141480 | HGLibA_03109 | 207 | 0.911262788 | ARRB2 | arrestin beta 2 [Source:HGNC Symbol;Acc:HGNC:712] |
| ENSG00000141526 | HGLibA_44439 | 207 | 0.911262788 | SLC16A3 | solute carrier family 16 member 3 [Source:HGNC Symbol;Acc:HGNC:10924] |
| ENSG00000142784 | HGLibA_54233 | 207 | 0.911262788 | WDTC1 | WD and tetratricopeptide repeats 1 [Source:HGNC Symbol;Acc:HGNC:29175] |
| ENSG00000144895 | HGLibA_14719 | 207 | 0.911262788 | EIF2A | eukaryotic translation initiation factor 2A [Source:HGNC Symbol;Acc:HGNC:3254] |
| ENSG00000145824 | HGLibA_11858 | 207 | 0.911262788 | CXCL14 | C-X-C motif chemokine ligand 14 [Source:HGNC Symbol;Acc:HGNC:10640] |
| ENSG00000149600 | HGLibA_10673 | 207 | 0.911262788 | COMMD7 | COMM domain containing 7 [Source:HGNC Symbol;Acc:HGNC:16223] |
| ENSG00000150457 | HGLibA_26066 | 207 | 0.911262788 | LATS2 | large tumor suppressor kinase 2 [Source:HGNC Symbol;Acc:HGNC:6515] |
| ENSG00000151468 | HGLibA_07752 | 207 | 0.911262788 | CCDC3 | coiled-coil domain containing 3 [Source:HGNC Symbol;Acc:HGNC:23813] |
| ENSG00000154447 | HGLibA_43970 | 207 | 0.911262788 | SH3RF1 | SH3 domain containing ring finger 1 [Source:HGNC Symbol;Acc:HGNC:17650] |
| ENSG00000157224 | HGLibA_09857 | 207 | 0.911262788 | CLDN12 | claudin 12 [Source:HGNC Symbol;Acc:HGNC:2034] |
| ENSG00000163508 | HGLibA_15227 | 207 | 0.911262788 | EOMES | eomesodermin [Source:HGNC Symbol;Acc:HGNC:3372] |
| ENSG00000163932 | HGLibA_38539 | 207 | 0.911262788 | PRKCD | protein kinase C delta [Source:HGNC Symbol;Acc:HGNC:9399] |
| ENSG00000164081 | HGLibA_49085 | 207 | 0.911262788 | TEX264 | testis expressed 264 [Source:HGNC Symbol;Acc:HGNC:30247] |
| ENSG00000164086 | HGLibA_14191 | 207 | 0.911262788 | DUSP7 | dual specificity phosphatase 7 [Source:HGNC Symbol;Acc:HGNC:3073] |
| ENSG00000164620 | HGLibA_40821 | 207 | 0.911262788 | RELL2 | RELT like 2 [Source:HGNC Symbol;Acc:HGNC:26902] |
| ENSG00000165061 | HGLibA_55375 | 207 | 0.911262788 | ZMAT4 | zinc finger matrin-type 4 [Source:HGNC Symbol;Acc:HGNC:25844] |
| ENSG00000167702 | HGLibA_24972 | 207 | 0.911262788 | KIFC2 | kinesin family member C2 [Source:HGNC Symbol;Acc:HGNC:29530] |
| ENSG00000169598 | HGLibA_13021 | 207 | 0.911262788 | DFFB | DNA fragmentation factor subunit beta [Source:HGNC Symbol;Acc:HGNC:2773] |

| | | | | | |
|---|---|---|---|---|---|
| ENSG00000170290 | HGLibA_45542 | 207 | 0.911262788 | SLN | sarcolipin [Source:HGNC Symbol;Acc:HGNC:11089] |
| ENSG00000171735 | HGLibA_07114 | 207 | 0.911262788 | CAMTA1 | calmodulin binding transcription activator 1 [Source:HGNC Symbol;Acc:HGNC:18806] |
| ENSG00000172123 | HGLibA_45485 | 207 | 0.911262788 | SLFN12 | schlafen family member 12 [Source:HGNC Symbol;Acc:HGNC:25500] |
| ENSG00000173156 | HGLibA_41185 | 207 | 0.911262788 | RHOD | ras homolog family member D [Source:HGNC Symbol;Acc:HGNC:670] |
| ENSG00000174483 | HGLibA_04206 | 207 | 0.911262788 | BBS1 | Bardet-Biedl syndrome 1 [Source:HGNC Symbol;Acc:HGNC:966] |
| ENSG00000175414 | HGLibA_02924 | 207 | 0.911262788 | ARL10 | ADP ribosylation factor like GTPase 10 [Source:HGNC Symbol;Acc:HGNC:22042] |
| ENSG00000180530 | HGLibA_32692 | 207 | 0.911262788 | NRIP1 | nuclear receptor interacting protein 1 [Source:HGNC Symbol;Acc:HGNC:8001] |
| ENSG00000181449 | HGLibA_46252 | 207 | 0.911262788 | SOX2 | SRY-box 2 [Source:HGNC Symbol;Acc:HGNC:11195] |
| ENSG00000182324 | HGLibA_24261 | 207 | 0.911262788 | KCNJ14 | potassium voltage-gated channel subfamily J member 14 [Source:HGNC Symbol;Acc:HGNC:6260] |
| ENSG00000185875 | HGLibA_49357 | 207 | 0.911262788 | THNSL1 | threonine synthase like 1 [Source:HGNC Symbol;Acc:HGNC:26160] |
| ENSG00000187325 | HGLibA_48264 | 207 | 0.911262788 | TAF9B | TATA-box binding protein associated factor 9b [Source:HGNC Symbol;Acc:HGNC:17306] |
| ENSG00000196839 | HGLibA_00717 | 207 | 0.911262788 | ADA | adenosine deaminase [Source:HGNC Symbol;Acc:HGNC:186] |
| ENSG00000196862 | HGLibA_41033 | 207 | 0.911262788 | RGPD4 | RANBP2-like and GRIP domain containing 4 [Source:HGNC Symbol;Acc:HGNC:32417] |
| ENSG00000197162 | HGLibA_56748 | 207 | 0.911262788 | ZNF785 | zinc finger protein 785 [Source:HGNC Symbol;Acc:HGNC:26496] |
| ENSG00000198598 | HGLibA_29500 | 207 | 0.911262788 | MMP17 | matrix metallopeptidase 17 [Source:HGNC Symbol;Acc:HGNC:7163] |
| ENSG00000198931 | HGLibA_02572 | 207 | 0.911262788 | APRT | adenine phosphoribosyltransferase [Source:HGNC Symbol;Acc:HGNC:626] |
| ENSG00000198959 | HGLibA_49255 | 207 | 0.911262788 | TGM2 | transglutaminase 2 [Source:HGNC Symbol;Acc:HGNC:11778] |
| ENSG00000204510 | HGLibA_38325 | 207 | 0.911262788 | PRAMEF7 | PRAME family member 7 [Source:HGNC Symbol;Acc:HGNC:28415] |
| ENSG00000212807 | HGLibA_33738 | 207 | 0.911262788 | OR2A42 | olfactory receptor family 2 subfamily A member 42 [Source:HGNC Symbol;Acc:HGNC:31230] |
| ENSG00000253309 | HGLibA_43582 | 207 | 0.911262788 | SERPINE3 | serpin family E member 3 [Source:HGNC Symbol;Acc:HGNC:24774] |
| ENSG00000254986 | HGLibA_13868 | 207 | 0.911262788 | DPP3 | dipeptidyl peptidase 3 [Source:HGNC Symbol;Acc:HGNC:3008] |
| ENSG00000258588 | HGLibA_51560 | 207 | 0.911262788 | TRIM6-TRIM34 | TRIM6-TRIM34 readthrough [Source:HGNC Symbol;Acc:HGNC:33440] |
| FAM92A1 | HGLibA_16796 | 207 | 0.911262788 | FAM92A1 | - |
| hsa-mir-1269a | HGLibA_57502 | 207 | 0.911262788 | hsa-mir-1269a | - |
| hsa-mir-195 | HGLibA_58090 | 207 | 0.911262788 | hsa-mir-195 | - |
| hsa-mir-3972 | HGLibA_59646 | 207 | 0.911262788 | hsa-mir-3972 | - |
| hsa-mir-4436a | HGLibA_60139 | 207 | 0.911262788 | hsa-mir-4436a | - |
| hsa-mir-6794 | HGLibA_63354 | 207 | 0.911262788 | hsa-mir-6794 | - |
| hsa-mir-6832 | HGLibA_63504 | 207 | 0.911262788 | hsa-mir-6832 | - |
| hsa-mir-7112-1 | HGLibA_63813 | 207 | 0.911262788 | hsa-mir-7112-1 | - |
| KIAA1598 | HGLibA_24791 | 207 | 0.911262788 | KIAA1598 | - |
| PPP2R3B_Y | HGLibA_38160 | 207 | 0.911262788 | PPP2R3B_Y | - |
| C16orf70 | HGLibA_05472 | 206 | 0.901311751 | C16orf70 | - |
| C2orf40 | HGLibA_06123 | 206 | 0.901311751 | C2orf40 | - |
| ENSG00000001461 | HGLibA_31963 | 206 | 0.901311751 | NIPAL3 | NIPA like domain containing 3 [Source:HGNC Symbol;Acc:HGNC:25233] |
| ENSG00000054803 | HGLibA_07414 | 206 | 0.901311751 | CBLN4 | cerebellin 4 precursor [Source:HGNC Symbol;Acc:HGNC:16231] |
| ENSG00000060140 | HGLibA_47683 | 206 | 0.901311751 | STYK1 | serine/threonine/tyrosine kinase 1 [Source:HGNC Symbol;Acc:HGNC:18889] |
| ENSG00000068615 | HGLibA_40777 | 206 | 0.901311751 | REEP1 | receptor accessory protein 1 [Source:HGNC Symbol;Acc:HGNC:25786] |
| ENSG00000069482 | HGLibA_18494 | 206 | 0.901311751 | GAL | galanin and GMAP prepropeptide [Source:HGNC Symbol;Acc:HGNC:4114] |
| ENSG00000069702 | HGLibA_49228 | 206 | 0.901311751 | TGFBR3 | transforming growth factor beta receptor 3 [Source:HGNC Symbol;Acc:HGNC:11774] |
| ENSG00000078070 | HGLibA_28564 | 206 | 0.901311751 | MCCC1 | methylcrotonoyl-CoA carboxylase 1 [Source:HGNC Symbol;Acc:HGNC:6936] |
| ENSG00000079459 | HGLibA_17251 | 206 | 0.901311751 | FDFT1 | farnesyl-diphosphate farnesyltransferase 1 [Source:HGNC Symbol;Acc:HGNC:3629] |
| ENSG00000099715 | HGLibA_35395 | 206 | 0.901311751 | PCDH11Y | protocadherin 11 Y-linked [Source:HGNC Symbol;Acc:HGNC:15813] |
| ENSG00000100884 | HGLibA_10969 | 206 | 0.901311751 | CPNE6 | copine 6 [Source:HGNC Symbol;Acc:HGNC:2319] |
| ENSG00000101782 | HGLibA_41313 | 206 | 0.901311751 | RIOK3 | RIO kinase 3 [Source:HGNC Symbol;Acc:HGNC:11451] |
| ENSG00000102032 | HGLibA_40840 | 206 | 0.901311751 | RENBP | renin binding protein [Source:HGNC Symbol;Acc:HGNC:9959] |
| ENSG00000106302 | HGLibA_22447 | 206 | 0.901311751 | HYAL4 | hyaluronidase 4 [Source:HGNC Symbol;Acc:HGNC:5323] |
| ENSG00000106304 | HGLibA_46376 | 206 | 0.901311751 | SPAM1 | sperm adhesion molecule 1 [Source:HGNC Symbol;Acc:HGNC:11217] |
| ENSG00000106617 | HGLibA_38515 | 206 | 0.901311751 | PRKAG2 | protein kinase AMP-activated non-catalytic subunit gamma 2 [Source:HGNC Symbol;Acc:HGNC:9386] |
| ENSG00000110917 | HGLibA_29368 | 206 | 0.901311751 | MLEC | malectin [Source:HGNC Symbol;Acc:HGNC:28973] |
| ENSG00000111341 | HGLibA_29167 | 206 | 0.901311751 | MGP | matrix Gla protein [Source:HGNC Symbol;Acc:HGNC:7060] |
| ENSG00000114745 | HGLibA_19678 | 206 | 0.901311751 | GORASP1 | golgi reassembly stacking protein 1 [Source:HGNC Symbol;Acc:HGNC:16769] |
| ENSG00000115523 | HGLibA_19557 | 206 | 0.901311751 | GNLY | granulysin [Source:HGNC Symbol;Acc:HGNC:4414] |
| ENSG00000118160 | HGLibA_45392 | 206 | 0.901311751 | SLC8A2 | solute carrier family 8 member A2 [Source:HGNC Symbol;Acc:HGNC:11069] |
| ENSG00000123159 | HGLibA_19117 | 206 | 0.901311751 | GIPC1 | GIPC PDZ domain containing family member 1 [Source:HGNC Symbol;Acc:HGNC:1226] |
| ENSG00000126953 | HGLibA_49523 | 206 | 0.901311751 | TIMM8A | translocase of inner mitochondrial membrane 8A [Source:HGNC Symbol;Acc:HGNC:11817] |
| ENSG00000130758 | HGLibA_28163 | 206 | 0.901311751 | MAP3K10 | mitogen-activated protein kinase kinase kinase 10 [Source:HGNC Symbol;Acc:HGNC:6849] |
| ENSG00000136305 | HGLibA_09700 | 206 | 0.901311751 | CIDEB | cell death-inducing DFFA-like effector b [Source:HGNC Symbol;Acc:HGNC:1977] |
| ENSG00000139505 | HGLibA_30432 | 206 | 0.901311751 | MTMR6 | myotubularin related protein 6 [Source:HGNC Symbol;Acc:HGNC:7453] |
| ENSG00000140538 | HGLibA_32880 | 206 | 0.901311751 | NTRK3 | neurotrophic receptor tyrosine kinase 3 [Source:HGNC Symbol;Acc:HGNC:8033] |
| ENSG00000141040 | HGLibA_55757 | 206 | 0.901311751 | ZNF287 | zinc finger protein 287 [Source:HGNC Symbol;Acc:HGNC:13502] |
| ENSG00000146833 | HGLibA_51488 | 206 | 0.901311751 | TRIM4 | tripartite motif containing 4 [Source:HGNC Symbol;Acc:HGNC:16275] |
| ENSG00000147381 | HGLibA_27915 | 206 | 0.901311751 | MAGEA4 | MAGE family member A4 [Source:HGNC Symbol;Acc:HGNC:6802] |
| ENSG00000150907 | HGLibA_17921 | 206 | 0.901311751 | FOXO1 | forkhead box O1 [Source:HGNC Symbol;Acc:HGNC:3819] |
| ENSG00000152128 | HGLibA_50030 | 206 | 0.901311751 | TMEM163 | transmembrane protein 163 [Source:HGNC Symbol;Acc:HGNC:25380] |
| ENSG00000154655 | HGLibA_25906 | 206 | 0.901311751 | L3MBTL4 | histone methyl-lysine binding protein [Source:HGNC Symbol;Acc:HGNC:26677]" |
| ENSG00000159708 | HGLibA_27322 | 206 | 0.901311751 | LRRC36 | leucine rich repeat containing 36 [Source:HGNC Symbol;Acc:HGNC:25615] |
| ENSG00000161807 | HGLibA_34481 | 206 | 0.901311751 | OR7G1 | olfactory receptor family 7 subfamily G member 1 [Source:HGNC Symbol;Acc:HGNC:8465] |
| ENSG00000162889 | HGLibA_28301 | 206 | 0.901311751 | MAPKAPK2 | mitogen-activated protein kinase-activated protein kinase 2 [Source:HGNC Symbol;Acc:HGNC:6887] |
| ENSG00000163312 | HGLibA_21117 | 206 | 0.901311751 | HELQ | POLQ like [Source:HGNC Symbol;Acc:HGNC:18536]" |
| ENSG00000163623 | HGLibA_32055 | 206 | 0.901311751 | NKX6-1 | NK6 homeobox 1 [Source:HGNC Symbol;Acc:HGNC:7839] |
| ENSG00000163904 | HGLibA_43387 | 206 | 0.901311751 | SENP2 | SUMO specific peptidase 2 [Source:HGNC Symbol;Acc:HGNC:23116] |
| ENSG00000166006 | HGLibA_24165 | 206 | 0.901311751 | KCNC2 | potassium voltage-gated channel subfamily C member 2 [Source:HGNC Symbol;Acc:HGNC:6234] |
| ENSG00000166228 | HGLibA_35360 | 206 | 0.901311751 | PCBD1 | pterin-4 alpha-carbinolamine dehydratase 1 [Source:HGNC Symbol;Acc:HGNC:8646] |
| ENSG00000169418 | HGLibA_32509 | 206 | 0.901311751 | NPR1 | natriuretic peptide receptor 1 [Source:HGNC Symbol;Acc:HGNC:7943] |
| ENSG00000170006 | HGLibA_50005 | 206 | 0.901311751 | TMEM154 | transmembrane protein 154 [Source:HGNC Symbol;Acc:HGNC:26489] |
| ENSG00000170920 | HGLibA_34486 | 206 | 0.901311751 | OR7G3 | olfactory receptor family 7 subfamily G member 3 [Source:HGNC Symbol;Acc:HGNC:8467] |
| ENSG00000174607 | HGLibA_52940 | 206 | 0.901311751 | UGT8 | UDP glycosyltransferase 8 [Source:HGNC Symbol;Acc:HGNC:12555] |
| ENSG00000175449 | HGLibA_40918 | 206 | 0.901311751 | RFESD | Rieske Fe-S domain containing [Source:HGNC Symbol;Acc:HGNC:29587] |
| ENSG00000176261 | HGLibA_54931 | 206 | 0.901311751 | ZBTB8OS | zinc finger and BTB domain containing 8 opposite strand [Source:HGNC Symbol;Acc:HGNC:24094] |
| ENSG00000178567 | HGLibA_15337 | 206 | 0.901311751 | EPM2AIP1 | EPM2A interacting protein 1 [Source:HGNC Symbol;Acc:HGNC:19735] |
| ENSG00000178597 | HGLibA_39003 | 206 | 0.901311751 | PSAPL1 | prosaposin like 1 (gene/pseudogene) [Source:HGNC Symbol;Acc:HGNC:33131] |
| ENSG00000179761 | HGLibA_36727 | 206 | 0.901311751 | PIPOX | pipecolic acid and sarcosine oxidase [Source:HGNC Symbol;Acc:HGNC:17804] |
| ENSG00000182180 | HGLibA_30044 | 206 | 0.901311751 | MRPS16 | mitochondrial ribosomal protein S16 [Source:HGNC Symbol;Acc:HGNC:14048] |
| ENSG00000183808 | HGLibA_40483 | 206 | 0.901311751 | RBM12B | RNA binding motif protein 12B [Source:HGNC Symbol;Acc:HGNC:32310] |
| ENSG00000188428 | HGLibA_04568 | 206 | 0.901311751 | BLOC1S5 | biogenesis of lysosomal organelles complex 1 subunit 5 [Source:HGNC Symbol;Acc:HGNC:18561] |
| ENSG00000203668 | HGLibA_09468 | 206 | 0.901311751 | CHML | Rab escort protein 2 [Source:HGNC Symbol;Acc:HGNC:1941]" |
| ENSG00000204505 | HGLibA_38333 | 206 | 0.901311751 | PRAMEF9 | PRAME family member 9 [Source:HGNC Symbol;Acc:HGNC:27996] |
| ENSG00000206260 | HGLibA_38821 | 206 | 0.901311751 | PRR23A | proline rich 23A [Source:HGNC Symbol;Acc:HGNC:37172] |
| ENSG00000213316 | HGLibA_27604 | 206 | 0.901311751 | LTC4S | leukotriene C4 synthase [Source:HGNC Symbol;Acc:HGNC:6719] |
| ENSG00000213563 | HGLibA_06651 | 206 | 0.901311751 | C8orf82 | chromosome 8 open reading frame 82 [Source:HGNC Symbol;Acc:HGNC:33826] |
| ENSG00000231213 | HGLibA_37242 | 206 | 0.901311751 | PLSCR5 | phospholipid scramblase family member 5 [Source:HGNC Symbol;Acc:HGNC:19952] |
| ENSG00000232382 | HGLibA_34310 | 206 | 0.901311751 | OR5K1 | olfactory receptor family 5 subfamily K member 1 [Source:HGNC Symbol;Acc:HGNC:8349] |
| ENSG00000244057 | HGLibA_26135 | 206 | 0.901311751 | LCE3C | late cornified envelope 3C [Source:HGNC Symbol;Acc:HGNC:16612] |
| ENSG00000256453 | HGLibA_13672 | 206 | 0.901311751 | DND1 | DND microRNA-mediated repression inhibitor 1 [Source:HGNC Symbol;Acc:HGNC:23799] |
| ENSG00000258289 | HGLibA_09674 | 206 | 0.901311751 | CHURC1 | churchill domain containing 1 [Source:HGNC Symbol;Acc:HGNC:20099] |
| ENSG00000276547 | HGLibA_35561 | 206 | 0.901311751 | PCDHGB5 | 5 [Source:HGNC Symbol;Acc:HGNC:8712]" |
| FAM195B | HGLibA_16379 | 206 | 0.901311751 | FAM195B | - |
| FAM86A | HGLibA_16769 | 206 | 0.901311751 | FAM86A | - |
| hsa-mir-1249 | HGLibA_57394 | 206 | 0.901311751 | hsa-mir-1249 | - |
| hsa-mir-1285-2 | HGLibA_57604 | 206 | 0.901311751 | hsa-mir-1285-2 | - |
| hsa-mir-154 | HGLibA_57926 | 206 | 0.901311751 | hsa-mir-154 | - |
| hsa-mir-15b | HGLibA_57943 | 206 | 0.901311751 | hsa-mir-15b | - |
| hsa-mir-301a | HGLibA_58428 | 206 | 0.901311751 | hsa-mir-301a | - |
| hsa-mir-3612 | HGLibA_59079 | 206 | 0.901311751 | hsa-mir-3612 | - |
| hsa-mir-3940 | HGLibA_59622 | 206 | 0.901311751 | hsa-mir-3940 | - |
| hsa-mir-4283-2 | HGLibA_59838 | 206 | 0.901311751 | hsa-mir-4283-2 | - |
| hsa-mir-4318 | HGLibA_59991 | 206 | 0.901311751 | hsa-mir-4318 | - |
| hsa-mir-4516 | HGLibA_60508 | 206 | 0.901311751 | hsa-mir-4516 | - |
| hsa-mir-648 | HGLibA_62834 | 206 | 0.901311751 | hsa-mir-648 | - |
| hsa-mir-6796 | HGLibA_63363 | 206 | 0.901311751 | hsa-mir-6796 | - |
| hsa-mir-7113 | HGLibA_63820 | 206 | 0.901311751 | hsa-mir-7113 | - |
| hsa-mir-8081 | HGLibA_64165 | 206 | 0.901311751 | hsa-mir-8081 | - |
| hsa-mir-937 | HGLibA_64324 | 206 | 0.901311751 | hsa-mir-937 | - |
| C4orf29 | HGLibA_06313 | 205 | 0.891360713 | C4orf29 | - |
| CCDC151 | HGLibA_07645 | 205 | 0.891360713 | CCDC151 | - |
| ENSG00000005001 | HGLibA_38915 | 205 | 0.891360713 | PRSS22 | serine protease 22 [Source:HGNC Symbol;Acc:HGNC:14368] |
| ENSG00000007047 | HGLibA_28372 | 205 | 0.891360713 | MARK4 | microtubule affinity regulating kinase 4 [Source:HGNC Symbol;Acc:HGNC:13538] |
| ENSG00000007264 | HGLibA_28423 | 205 | 0.891360713 | MATK | megakaryocyte-associated tyrosine kinase [Source:HGNC Symbol;Acc:HGNC:6906] |
| ENSG00000064787 | HGLibA_04251 | 205 | 0.891360713 | BCAS1 | breast carcinoma amplified sequence 1 [Source:HGNC Symbol;Acc:HGNC:974] |
| ENSG00000068383 | HGLibA_23343 | 205 | 0.891360713 | INPP5A | inositol polyphosphate-5-phosphatase A [Source:HGNC Symbol;Acc:HGNC:6076] |
| ENSG00000070366 | HGLibA_45705 | 205 | 0.891360713 | SMG6 | nonsense mediated mRNA decay factor [Source:HGNC Symbol;Acc:HGNC:17809]" |
| ENSG00000076321 | HGLibA_25149 | 205 | 0.891360713 | KLHL20 | kelch like family member 20 [Source:HGNC Symbol;Acc:HGNC:25056] |
| ENSG00000081800 | HGLibA_44384 | 205 | 0.891360713 | SLC13A1 | solute carrier family 13 member 1 [Source:HGNC Symbol;Acc:HGNC:10916] |
| ENSG00000088833 | HGLibA_32753 | 205 | 0.891360713 | NSFL1C | NSFL1 cofactor [Source:HGNC Symbol;Acc:HGNC:15912] |
| ENSG00000099910 | HGLibA_25155 | 205 | 0.891360713 | KLHL22 | kelch like family member 22 [Source:HGNC Symbol;Acc:HGNC:25888] |
| ENSG00000103194 | HGLibA_53195 | 205 | 0.891360713 | USP10 | ubiquitin specific peptidase 10 [Source:HGNC Symbol;Acc:HGNC:12608] |
| ENSG00000104142 | HGLibA_53747 | 205 | 0.891360713 | VPS18 | CORVET/HOPS core subunit [Source:HGNC Symbol;Acc:HGNC:15972]" |
| ENSG00000106397 | HGLibA_37213 | 205 | 0.891360713 | PLOD3 | 2-oxoglutarate 5-dioxygenase 3 [Source:HGNC Symbol;Acc:HGNC:9083]" |
| ENSG00000107614 | HGLibA_51344 | 205 | 0.891360713 | TRDMT1 | tRNA aspartic acid methyltransferase 1 [Source:HGNC Symbol;Acc:HGNC:2977] |
| ENSG00000108381 | HGLibA_03342 | 205 | 0.891360713 | ASPA | aspartoacylase [Source:HGNC Symbol;Acc:HGNC:756] |
| ENSG00000109654 | HGLibA_51427 | 205 | 0.891360713 | TRIM2 | tripartite motif containing 2 [Source:HGNC Symbol;Acc:HGNC:15974] |
| ENSG00000110660 | HGLibA_45003 | 205 | 0.891360713 | SLC35F2 | solute carrier family 35 member F2 [Source:HGNC Symbol;Acc:HGNC:23615] |
| ENSG00000113430 | HGLibA_23638 | 205 | 0.891360713 | IRX4 | iroquois homeobox 4 [Source:HGNC Symbol;Acc:HGNC:6129] |
| ENSG00000117425 | HGLibA_39271 | 205 | 0.891360713 | PTCH2 | patched 2 [Source:HGNC Symbol;Acc:HGNC:9586] |

| | | | | | |
|---|---|---|---|---|---|
| ENSG00000118898 | HGLibA_37960 | 205 | 0.891360713 | PPL | periplakin [Source:HGNC Symbol;Acc:HGNC:9273] |
| ENSG00000120210 | HGLibA_23392 | 205 | 0.891360713 | INSL6 | insulin like 6 [Source:HGNC Symbol;Acc:HGNC:6089] |
| ENSG00000122008 | HGLibA_37533 | 205 | 0.891360713 | POLK | DNA polymerase kappa [Source:HGNC Symbol;Acc:HGNC:9183] |
| ENSG00000122254 | HGLibA_22123 | 205 | 0.891360713 | HS3ST2 | heparan sulfate-glucosamine 3-sulfotransferase 2 [Source:HGNC Symbol;Acc:HGNC:5195] |
| ENSG00000122585 | HGLibA_32547 | 205 | 0.891360713 | NPY | neuropeptide Y [Source:HGNC Symbol;Acc:HGNC:7955] |
| ENSG00000127311 | HGLibA_21110 | 205 | 0.891360713 | HELB | DNA helicase B [Source:HGNC Symbol;Acc:HGNC:17196] |
| ENSG00000130755 | HGLibA_19407 | 205 | 0.891360713 | GMFG | glia maturation factor gamma [Source:HGNC Symbol;Acc:HGNC:4374] |
| ENSG00000130779 | HGLibA_10041 | 205 | 0.891360713 | CLIP1 | CAP-Gly domain containing linker protein 1 [Source:HGNC Symbol;Acc:HGNC:10461] |
| ENSG00000131127 | HGLibA_55487 | 205 | 0.891360713 | ZNF141 | zinc finger protein 141 [Source:HGNC Symbol;Acc:HGNC:12926] |
| ENSG00000134987 | HGLibA_54076 | 205 | 0.891360713 | WDR36 | WD repeat domain 36 [Source:HGNC Symbol;Acc:HGNC:30696] |
| ENSG00000136002 | HGLibA_02864 | 205 | 0.891360713 | ARHGEF4 | Rho guanine nucleotide exchange factor 4 [Source:HGNC Symbol;Acc:HGNC:684] |
| ENSG00000136010 | HGLibA_01556 | 205 | 0.891360713 | ALDH1L2 | aldehyde dehydrogenase 1 family member L2 [Source:HGNC Symbol;Acc:HGNC:26777] |
| ENSG00000136449 | HGLibA_30670 | 205 | 0.891360713 | MYCBPAP | MYCBP associated protein [Source:HGNC Symbol;Acc:HGNC:19677] |
| ENSG00000137509 | HGLibA_38358 | 205 | 0.891360713 | PRCP | prolylcarboxypeptidase [Source:HGNC Symbol;Acc:HGNC:9344] |
| ENSG00000138131 | HGLibA_27048 | 205 | 0.891360713 | LOXL4 | lysyl oxidase like 4 [Source:HGNC Symbol;Acc:HGNC:17171] |
| ENSG00000138594 | HGLibA_50601 | 205 | 0.891360713 | TMOD3 | tropomodulin 3 [Source:HGNC Symbol;Acc:HGNC:11873] |
| ENSG00000138813 | HGLibA_06295 | 205 | 0.891360713 | C4orf17 | chromosome 4 open reading frame 17 [Source:HGNC Symbol;Acc:HGNC:25274] |
| ENSG00000142669 | HGLibA_43923 | 205 | 0.891360713 | SH3BGRL3 | SH3 domain binding glutamate rich protein like 3 [Source:HGNC Symbol;Acc:HGNC:15568] |
| ENSG00000151806 | HGLibA_20669 | 205 | 0.891360713 | GUF1 | GTPase [Source:HGNC Symbol;Acc:HGNC:25799]" |
| ENSG00000153317 | HGLibA_03193 | 205 | 0.891360713 | ASAP1 | ankyrin repeat and PH domain 1 [Source:HGNC Symbol;Acc:HGNC:2720]" |
| ENSG00000158008 | HGLibA_15807 | 205 | 0.891360713 | EXTL1 | exostosin like glycosyltransferase 1 [Source:HGNC Symbol;Acc:HGNC:3515] |
| ENSG00000159588 | HGLibA_07689 | 205 | 0.891360713 | CCDC17 | coiled-coil domain containing 17 [Source:HGNC Symbol;Acc:HGNC:26574] |
| ENSG00000160746 | HGLibA_02175 | 205 | 0.891360713 | ANO10 | anoctamin 10 [Source:HGNC Symbol;Acc:HGNC:25519] |
| ENSG00000162728 | HGLibA_24290 | 205 | 0.891360713 | KCNJ9 | potassium voltage-gated channel subfamily J member 9 [Source:HGNC Symbol;Acc:HGNC:6270] |
| ENSG00000164684 | HGLibA_56585 | 205 | 0.891360713 | ZNF704 | zinc finger protein 704 [Source:HGNC Symbol;Acc:HGNC:32291] |
| ENSG00000165457 | HGLibA_17785 | 205 | 0.891360713 | FOLR2 | folate receptor beta [Source:HGNC Symbol;Acc:HGNC:3793] |
| ENSG00000165832 | HGLibA_51803 | 205 | 0.891360713 | TRUB1 | TruB pseudouridine synthase family member 1 [Source:HGNC Symbol;Acc:HGNC:16060] |
| ENSG00000167034 | HGLibA_32050 | 205 | 0.891360713 | NKX3-1 | NK3 homeobox 1 [Source:HGNC Symbol;Acc:HGNC:7838] |
| ENSG00000167635 | HGLibA_55496 | 205 | 0.891360713 | ZNF146 | zinc finger protein 146 [Source:HGNC Symbol;Acc:HGNC:12931] |
| ENSG00000170209 | HGLibA_01973 | 205 | 0.891360713 | ANKK1 | ankyrin repeat and kinase domain containing 1 [Source:HGNC Symbol;Acc:HGNC:21027] |
| ENSG00000171103 | HGLibA_51702 | 205 | 0.891360713 | TRMT61B | tRNA methyltransferase 61B [Source:HGNC Symbol;Acc:HGNC:26070] |
| ENSG00000171405 | HGLibA_54486 | 205 | 0.891360713 | XAGE5 | X antigen family member 5 [Source:HGNC Symbol;Acc:HGNC:30930] |
| ENSG00000171503 | HGLibA_15626 | 205 | 0.891360713 | ETFDH | electron transfer flavoprotein dehydrogenase [Source:HGNC Symbol;Acc:HGNC:3483] |
| ENSG00000171989 | HGLibA_26227 | 205 | 0.891360713 | LDHAL6B | lactate dehydrogenase A like 6B [Source:HGNC Symbol;Acc:HGNC:21481] |
| ENSG00000172469 | HGLibA_28090 | 205 | 0.891360713 | MANEA | mannosidase endo-alpha [Source:HGNC Symbol;Acc:HGNC:21072] |
| ENSG00000173991 | HGLibA_48693 | 205 | 0.891360713 | TCAP | titin-cap [Source:HGNC Symbol;Acc:HGNC:11610] |
| ENSG00000178301 | HGLibA_02583 | 205 | 0.891360713 | AQP11 | aquaporin 11 [Source:HGNC Symbol;Acc:HGNC:19940] |
| ENSG00000179163 | HGLibA_18155 | 205 | 0.891360713 | FUCA1 | alpha-L-fucosidase 1 [Source:HGNC Symbol;Acc:HGNC:4006] |
| ENSG00000180988 | HGLibA_34195 | 205 | 0.891360713 | OR52N2 | olfactory receptor family 52 subfamily N member 2 [Source:HGNC Symbol;Acc:HGNC:15228] |
| ENSG00000181609 | HGLibA_34155 | 205 | 0.891360713 | OR52D1 | olfactory receptor family 52 subfamily D member 1 [Source:HGNC Symbol;Acc:HGNC:15212] |
| ENSG00000181786 | HGLibA_00635 | 205 | 0.891360713 | ACTL9 | actin like 9 [Source:HGNC Symbol;Acc:HGNC:28494] |
| ENSG00000183092 | HGLibA_04397 | 205 | 0.891360713 | BEGAIN | brain enriched guanylate kinase associated [Source:HGNC Symbol;Acc:HGNC:24163] |
| ENSG00000183579 | HGLibA_56945 | 205 | 0.891360713 | ZNRF3 | zinc and ring finger 3 [Source:HGNC Symbol;Acc:HGNC:18126] |
| ENSG00000186471 | HGLibA_01417 | 205 | 0.891360713 | AKAP14 | A-kinase anchoring protein 14 [Source:HGNC Symbol;Acc:HGNC:24061] |
| ENSG00000188334 | HGLibA_04875 | 205 | 0.891360713 | BSPH1 | binder of sperm protein homolog 1 [Source:HGNC Symbol;Acc:HGNC:33906] |
| ENSG00000188958 | HGLibA_53464 | 205 | 0.891360713 | UTS2B | urotensin 2B [Source:HGNC Symbol;Acc:HGNC:30894] |
| ENSG00000196466 | HGLibA_56777 | 205 | 0.891360713 | ZNF799 | zinc finger protein 799 [Source:HGNC Symbol;Acc:HGNC:28071] |
| ENSG00000196470 | HGLibA_44105 | 205 | 0.891360713 | SIAH1 | siah E3 ubiquitin protein ligase 1 [Source:HGNC Symbol;Acc:HGNC:10857] |
| ENSG00000196578 | HGLibA_34235 | 205 | 0.891360713 | OR5AC2 | olfactory receptor family 5 subfamily AC member 2 [Source:HGNC Symbol;Acc:HGNC:15431] |
| ENSG00000196935 | HGLibA_46968 | 205 | 0.891360713 | SRGAP1 | SLIT-ROBO Rho GTPase activating protein 1 [Source:HGNC Symbol;Acc:HGNC:17382] |
| ENSG00000197110 | HGLibA_22726 | 205 | 0.891360713 | IFNL3 | interferon lambda 3 [Source:HGNC Symbol;Acc:HGNC:18365] |
| ENSG00000197355 | HGLibA_52510 | 205 | 0.891360713 | UAP1L1 | UDP-N-acetylglucosamine pyrophosphorylase 1 like 1 [Source:HGNC Symbol;Acc:HGNC:28082] |
| ENSG00000204175 | HGLibA_20143 | 205 | 0.891360713 | GPRIN2 | G protein regulated inducer of neurite outgrowth 2 [Source:HGNC Symbol;Acc:HGNC:23730] |
| ENSG00000215595 | HGLibA_06015 | 205 | 0.891360713 | C20orf202 | chromosome 20 open reading frame 202 [Source:HGNC Symbol;Acc:HGNC:37254] |
| ENSG00000288784 | HGLibA_11573 | 205 | 0.891360713 | CTAGE4 | - |
| GPR97 | HGLibA_20114 | 205 | 0.891360713 | GPR97 | - |
| hsa-mir-378a | HGLibA_59426 | 205 | 0.891360713 | hsa-mir-378a | - |
| hsa-mir-3973 | HGLibA_59652 | 205 | 0.891360713 | hsa-mir-3973 | - |
| hsa-mir-4641 | HGLibA_60668 | 205 | 0.891360713 | hsa-mir-4641 | - |
| hsa-mir-5092 | HGLibA_61528 | 205 | 0.891360713 | hsa-mir-5092 | - |
| hsa-mir-548i-2 | HGLibA_62015 | 205 | 0.891360713 | hsa-mir-548i-2 | - |
| hsa-mir-6134 | HGLibA_62689 | 205 | 0.891360713 | hsa-mir-6134 | - |
| LRMP | HGLibA_27194 | 205 | 0.891360713 | LRMP | - |
| MLL5 | HGLibA_29404 | 205 | 0.891360713 | MLL5 | - |
| PRKCDBP | HGLibA_38542 | 205 | 0.891360713 | PRKCDBP | - |
| ADC | HGLibA_00888 | 204 | 0.881409676 | ADC | - |
| EFCAB4B | HGLibA_14545 | 204 | 0.881409676 | EFCAB4B | - |
| ENSG00000011105 | HGLibA_51951 | 204 | 0.881409676 | TSPAN9 | tetraspanin 9 [Source:HGNC Symbol;Acc:HGNC:21640] |
| ENSG00000018408 | HGLibA_54458 | 204 | 0.881409676 | WWTR1 | WW domain containing transcription regulator 1 [Source:HGNC Symbol;Acc:HGNC:24042] |
| ENSG00000038219 | HGLibA_04671 | 204 | 0.881409676 | BOD1L1 | biorientation of chromosomes in cell division 1 like 1 [Source:HGNC Symbol;Acc:HGNC:31792] |
| ENSG00000054282 | HGLibA_43119 | 204 | 0.881409676 | SDCCAG8 | serologically defined colon cancer antigen 8 [Source:HGNC Symbol;Acc:HGNC:10671] |
| ENSG00000066422 | HGLibA_54816 | 204 | 0.881409676 | ZBTB11 | zinc finger and BTB domain containing 11 [Source:HGNC Symbol;Acc:HGNC:16740] |
| ENSG00000090512 | HGLibA_17306 | 204 | 0.881409676 | FETUB | fetuin B [Source:HGNC Symbol;Acc:HGNC:3658] |
| ENSG00000100156 | HGLibA_44453 | 204 | 0.881409676 | SLC16A8 | solute carrier family 16 member 8 [Source:HGNC Symbol;Acc:HGNC:16270] |
| ENSG00000102387 | HGLibA_48255 | 204 | 0.881409676 | TAF7L | TATA-box binding protein associated factor 7 like [Source:HGNC Symbol;Acc:HGNC:11548] |
| ENSG00000102981 | HGLibA_35185 | 204 | 0.881409676 | PARD6A | par-6 family cell polarity regulator alpha [Source:HGNC Symbol;Acc:HGNC:15943] |
| ENSG00000104327 | HGLibA_06987 | 204 | 0.881409676 | CALB1 | calbindin 1 [Source:HGNC Symbol;Acc:HGNC:1434] |
| ENSG00000104408 | HGLibA_14777 | 204 | 0.881409676 | EIF3E | eukaryotic translation initiation factor 3 subunit E [Source:HGNC Symbol;Acc:HGNC:3277] |
| ENSG00000105204 | HGLibA_14275 | 204 | 0.881409676 | DYRK1B | dual specificity tyrosine phosphorylation regulated kinase 1B [Source:HGNC Symbol;Acc:HGNC:3092] |
| ENSG00000106066 | HGLibA_11029 | 204 | 0.881409676 | CPVL | vitellogenic like [Source:HGNC Symbol;Acc:HGNC:14399]" |
| ENSG00000106976 | HGLibA_13683 | 204 | 0.881409676 | DNM1 | dynamin 1 [Source:HGNC Symbol;Acc:HGNC:2972] |
| ENSG00000108651 | HGLibA_53456 | 204 | 0.881409676 | UTP6 | small subunit processome component [Source:HGNC Symbol;Acc:HGNC:18279]" |
| ENSG00000109787 | HGLibA_25069 | 204 | 0.881409676 | KLF3 | Kruppel like factor 3 [Source:HGNC Symbol;Acc:HGNC:16516] |
| ENSG00000111052 | HGLibA_26573 | 204 | 0.881409676 | LIN7A | crumbs cell polarity complex component [Source:HGNC Symbol;Acc:HGNC:17787]" |
| ENSG00000111215 | HGLibA_38838 | 204 | 0.881409676 | PRR4 | proline rich 4 [Source:HGNC Symbol;Acc:HGNC:18020] |
| ENSG00000112308 | HGLibA_06502 | 204 | 0.881409676 | C6orf62 | chromosome 6 open reading frame 62 [Source:HGNC Symbol;Acc:HGNC:20998] |
| ENSG00000114993 | HGLibA_42390 | 204 | 0.881409676 | RTKN | rhotekin [Source:HGNC Symbol;Acc:HGNC:10466] |
| ENSG00000116478 | HGLibA_20997 | 204 | 0.881409676 | HDAC1 | histone deacetylase 1 [Source:HGNC Symbol;Acc:HGNC:4852] |
| ENSG00000118526 | HGLibA_48772 | 204 | 0.881409676 | TCF21 | transcription factor 21 [Source:HGNC Symbol;Acc:HGNC:11632] |
| ENSG00000120075 | HGLibA_21940 | 204 | 0.881409676 | HOXB5 | homeobox B5 [Source:HGNC Symbol;Acc:HGNC:5116] |
| ENSG00000122126 | HGLibA_33264 | 204 | 0.881409676 | OCRL | inositol polyphosphate-5-phosphatase [Source:HGNC Symbol;Acc:HGNC:8108]" |
| ENSG00000122741 | HGLibA_12411 | 204 | 0.881409676 | DCAF10 | DDB1 and CUL4 associated factor 10 [Source:HGNC Symbol;Acc:HGNC:23686] |
| ENSG00000125363 | HGLibA_01765 | 204 | 0.881409676 | AMELX | amelogenin X-linked [Source:HGNC Symbol;Acc:HGNC:461] |
| ENSG00000129991 | HGLibA_50878 | 204 | 0.881409676 | TNNI3 | cardiac type [Source:HGNC Symbol;Acc:HGNC:11947]" |
| ENSG00000130640 | HGLibA_52308 | 204 | 0.881409676 | TUBGCP2 | tubulin gamma complex associated protein 2 [Source:HGNC Symbol;Acc:HGNC:18599] |
| ENSG00000133980 | HGLibA_53834 | 204 | 0.881409676 | VRTN | vertebrae development associated [Source:HGNC Symbol;Acc:HGNC:20223] |
| ENSG00000135312 | HGLibA_22359 | 204 | 0.881409676 | HTR1B | 5-hydroxytryptamine receptor 1B [Source:HGNC Symbol;Acc:HGNC:5287] |
| ENSG00000135835 | HGLibA_24792 | 204 | 0.881409676 | KIAA1614 | KIAA1614 [Source:HGNC Symbol;Acc:HGNC:29327] |
| ENSG00000137441 | HGLibA_17428 | 204 | 0.881409676 | FGFBP2 | fibroblast growth factor binding protein 2 [Source:HGNC Symbol;Acc:HGNC:29451] |
| ENSG00000139292 | HGLibA_26411 | 204 | 0.881409676 | LGR5 | leucine rich repeat containing G protein-coupled receptor 5 [Source:HGNC Symbol;Acc:HGNC:4504] |
| ENSG00000139921 | HGLibA_50688 | 204 | 0.881409676 | TMX1 | thioredoxin related transmembrane protein 1 [Source:HGNC Symbol;Acc:HGNC:15487] |
| ENSG00000143554 | HGLibA_44819 | 204 | 0.881409676 | SLC27A3 | solute carrier family 27 member 3 [Source:HGNC Symbol;Acc:HGNC:10997] |
| ENSG00000143753 | HGLibA_12919 | 204 | 0.881409676 | DEGS1 | sphingolipid 1 [Source:HGNC Symbol;Acc:HGNC:13709]" |
| ENSG00000145087 | HGLibA_47677 | 204 | 0.881409676 | STXBP5L | syntaxin binding protein 5 like [Source:HGNC Symbol;Acc:HGNC:30757] |
| ENSG00000146038 | HGLibA_12485 | 204 | 0.881409676 | DCDC2 | doublecortin domain containing 2 [Source:HGNC Symbol;Acc:HGNC:18141] |
| ENSG00000148841 | HGLibA_23862 | 204 | 0.881409676 | ITPRIP | 5-trisphosphate receptor interacting protein [Source:HGNC Symbol;Acc:HGNC:29370]" |
| ENSG00000150782 | HGLibA_23021 | 204 | 0.881409676 | IL18 | interleukin 18 [Source:HGNC Symbol;Acc:HGNC:5986] |
| ENSG00000155463 | HGLibA_34810 | 204 | 0.881409676 | OXA1L | mitochondrial inner membrane protein [Source:HGNC Symbol;Acc:HGNC:8526]" |
| ENSG00000157426 | HGLibA_00078 | 204 | 0.881409676 | AASDH | aminoadipate-semialdehyde dehydrogenase [Source:HGNC Symbol;Acc:HGNC:23993] |
| ENSG00000157657 | HGLibA_56398 | 204 | 0.881409676 | ZNF618 | zinc finger protein 618 [Source:HGNC Symbol;Acc:HGNC:29416] |
| ENSG00000158163 | HGLibA_14302 | 204 | 0.881409676 | DZIP1L | DAZ interacting zinc finger protein 1 like [Source:HGNC Symbol;Acc:HGNC:26551] |
| ENSG00000163214 | HGLibA_13201 | 204 | 0.881409676 | DHX57 | DExH-box helicase 57 [Source:HGNC Symbol;Acc:HGNC:20086] |
| ENSG00000164342 | HGLibA_49649 | 204 | 0.881409676 | TLR3 | toll like receptor 3 [Source:HGNC Symbol;Acc:HGNC:11849] |
| ENSG00000164610 | HGLibA_41796 | 204 | 0.881409676 | RP9 | pre-mRNA splicing factor [Source:HGNC Symbol;Acc:HGNC:10288]" |
| ENSG00000166428 | HGLibA_37044 | 204 | 0.881409676 | PLD4 | phospholipase D family member 4 [Source:HGNC Symbol;Acc:HGNC:23792] |
| ENSG00000167995 | HGLibA_04419 | 204 | 0.881409676 | BEST1 | bestrophin 1 [Source:HGNC Symbol;Acc:HGNC:12703] |
| ENSG00000168137 | HGLibA_43652 | 204 | 0.881409676 | SETD5 | SET domain containing 5 [Source:HGNC Symbol;Acc:HGNC:25566] |
| ENSG00000169194 | HGLibA_22971 | 204 | 0.881409676 | IL13 | interleukin 13 [Source:HGNC Symbol;Acc:HGNC:5973] |
| ENSG00000170153 | HGLibA_41544 | 204 | 0.881409676 | RNF150 | ring finger protein 150 [Source:HGNC Symbol;Acc:HGNC:23138] |
| ENSG00000170537 | HGLibA_49765 | 204 | 0.881409676 | TMC7 | transmembrane channel like 7 [Source:HGNC Symbol;Acc:HGNC:23000] |
| ENSG00000172260 | HGLibA_31625 | 204 | 0.881409676 | NEGR1 | neuronal growth regulator 1 [Source:HGNC Symbol;Acc:HGNC:17302] |
| ENSG00000174080 | HGLibA_11705 | 204 | 0.881409676 | CTSF | cathepsin F [Source:HGNC Symbol;Acc:HGNC:2531] |
| ENSG00000174574 | HGLibA_01456 | 204 | 0.881409676 | AKIRIN1 | akirin 1 [Source:HGNC Symbol;Acc:HGNC:25744] |
| ENSG00000176893 | HGLibA_34112 | 204 | 0.881409676 | OR51G2 | olfactory receptor family 51 subfamily G member 2 [Source:HGNC Symbol;Acc:HGNC:15198] |
| ENSG00000178562 | HGLibA_08323 | 204 | 0.881409676 | CD28 | CD28 molecule [Source:HGNC Symbol;Acc:HGNC:1653] |
| ENSG00000180974 | HGLibA_34161 | 204 | 0.881409676 | OR52E4 | olfactory receptor family 52 subfamily E member 4 [Source:HGNC Symbol;Acc:HGNC:15213] |
| ENSG00000181965 | HGLibA_31747 | 204 | 0.881409676 | NEUROG1 | neurogenin 1 [Source:HGNC Symbol;Acc:HGNC:7764] |
| ENSG00000182645 | HGLibA_07697 | 204 | 0.881409676 | CCDC172 | coiled-coil domain containing 172 [Source:HGNC Symbol;Acc:HGNC:30524] |
| ENSG00000184394 | HGLibA_34052 | 204 | 0.881409676 | OR4N5 | olfactory receptor family 4 subfamily N member 5 [Source:HGNC Symbol;Acc:HGNC:15358] |
| ENSG00000185359 | HGLibA_21246 | 204 | 0.881409676 | HGS | hepatocyte growth factor-regulated tyrosine kinase substrate [Source:HGNC Symbol;Acc:HGNC:4897] |
| ENSG00000186364 | HGLibA_32958 | 204 | 0.881409676 | NUDT17 | nudix hydrolase 17 [Source:HGNC Symbol;Acc:HGNC:26618] |
| ENSG00000187800 | HGLibA_36009 | 204 | 0.881409676 | PEAR1 | platelet endothelial aggregation receptor 1 [Source:HGNC Symbol;Acc:HGNC:33631] |
| ENSG00000197081 | HGLibA_22808 | 204 | 0.881409676 | IGF2R | insulin like growth factor 2 receptor [Source:HGNC Symbol;Acc:HGNC:5467] |
| ENSG00000204599 | HGLibA_51482 | 204 | 0.881409676 | TRIM39 | tripartite motif containing 39 [Source:HGNC Symbol;Acc:HGNC:10065] |
| ENSG00000205352 | HGLibA_38775 | 204 | 0.881409676 | PRR13 | proline rich 13 [Source:HGNC Symbol;Acc:HGNC:24528] |

| | | | | | |
|---|---|---|---|---|---|
| ENSG00000229453 | HGLibA_46705 | 204 | 0.881409676 | SPINK8 | Kazal type 8 (putative) [Source:HGNC Symbol;Acc:HGNC:33160]" |
| ENSG00000242114 | HGLibA_30359 | 204 | 0.881409676 | MTFP1 | mitochondrial fission process 1 [Source:HGNC Symbol;Acc:HGNC:26945] |
| ENSG00000242574 | HGLibA_21606 | 204 | 0.881409676 | HLA-DMB | DM beta [Source:HGNC Symbol;Acc:HGNC:4935]" |
| ENSG00000258484 | HGLibA_46627 | 204 | 0.881409676 | SPESP1 | sperm equatorial segment protein 1 [Source:HGNC Symbol;Acc:HGNC:15570] |
| ENSG00000278619 | HGLibA_29860 | 204 | 0.881409676 | MRM1 | mitochondrial rRNA methyltransferase 1 [Source:HGNC Symbol;Acc:HGNC:26202] |
| HIST1H1E | HGLibA_21369 | 204 | 0.881409676 | HIST1H1E | - |
| hsa-mir-23a | HGLibA_58328 | 204 | 0.881409676 | hsa-mir-23a | - |
| hsa-mir-3691 | HGLibA_59352 | 204 | 0.881409676 | hsa-mir-3691 | - |
| hsa-mir-3976 | HGLibA_59664 | 204 | 0.881409676 | hsa-mir-3976 | - |
| hsa-mir-494 | HGLibA_61376 | 204 | 0.881409676 | hsa-mir-494 | - |
| hsa-mir-494 | HGLibA_61377 | 204 | 0.881409676 | hsa-mir-494 | - |
| hsa-mir-512-2 | HGLibA_61568 | 204 | 0.881409676 | hsa-mir-512-2 | - |
| hsa-mir-5584 | HGLibA_62178 | 204 | 0.881409676 | hsa-mir-5584 | - |
| hsa-mir-7113 | HGLibA_63821 | 204 | 0.881409676 | hsa-mir-7113 | - |
| 8-Mar | HGLibA_28349 | 203 | 0.871458638 | 8-Mar | - |
| C12orf68 | HGLibA_05285 | 203 | 0.871458638 | C12orf68 | - |
| C17orf102 | HGLibA_05520 | 203 | 0.871458638 | C17orf102 | - |
| ENSG00000010219 | HGLibA_14283 | 203 | 0.871458638 | DYRK4 | dual specificity tyrosine phosphorylation regulated kinase 4 [Source:HGNC Symbol;Acc:HGNC:3095] |
| ENSG00000032219 | HGLibA_02901 | 203 | 0.871458638 | ARID4A | AT-rich interaction domain 4A [Source:HGNC Symbol;Acc:HGNC:9885] |
| ENSG00000069424 | HGLibA_24151 | 203 | 0.871458638 | KCNAB2 | potassium voltage-gated channel subfamily A regulatory beta subunit 2 [Source:HGNC Symbol;Acc:HGNC:6229] |
| ENSG00000070269 | HGLibA_50351 | 203 | 0.871458638 | TMEM260 | transmembrane protein 260 [Source:HGNC Symbol;Acc:HGNC:20185] |
| ENSG00000070718 | HGLibA_02331 | 203 | 0.871458638 | AP3M2 | adaptor related protein complex 3 subunit mu 2 [Source:HGNC Symbol;Acc:HGNC:570] |
| ENSG00000075975 | HGLibA_29352 | 203 | 0.871458638 | MKRN2 | makorin ring finger protein 2 [Source:HGNC Symbol;Acc:HGNC:7113] |
| ENSG00000076554 | HGLibA_51110 | 203 | 0.871458638 | TPD52 | tumor protein D52 [Source:HGNC Symbol;Acc:HGNC:12005] |
| ENSG00000077254 | HGLibA_53338 | 203 | 0.871458638 | USP33 | ubiquitin specific peptidase 33 [Source:HGNC Symbol;Acc:HGNC:20059] |
| ENSG00000079950 | HGLibA_47656 | 203 | 0.871458638 | STX7 | syntaxin 7 [Source:HGNC Symbol;Acc:HGNC:11442] |
| ENSG00000080839 | HGLibA_40468 | 203 | 0.871458638 | RBL1 | RB transcriptional corepressor like 1 [Source:HGNC Symbol;Acc:HGNC:9893] |
| ENSG00000101464 | HGLibA_36592 | 203 | 0.871458638 | PIGU | phosphatidylinositol glycan anchor biosynthesis class U [Source:HGNC Symbol;Acc:HGNC:15791] |
| ENSG00000104695 | HGLibA_38136 | 203 | 0.871458638 | PPP2CB | protein phosphatase 2 catalytic subunit beta [Source:HGNC Symbol;Acc:HGNC:9300] |
| ENSG00000112559 | HGLibA_28671 | 203 | 0.871458638 | MDFI | MyoD family inhibitor [Source:HGNC Symbol;Acc:HGNC:6967] |
| ENSG00000112742 | HGLibA_52179 | 203 | 0.871458638 | TTK | TTK protein kinase [Source:HGNC Symbol;Acc:HGNC:12401] |
| ENSG00000114098 | HGLibA_03031 | 203 | 0.871458638 | ARMC8 | armadillo repeat containing 8 [Source:HGNC Symbol;Acc:HGNC:24999] |
| ENSG00000115232 | HGLibA_23732 | 203 | 0.871458638 | ITGA4 | integrin subunit alpha 4 [Source:HGNC Symbol;Acc:HGNC:6140] |
| ENSG00000115539 | HGLibA_35753 | 203 | 0.871458638 | PDCL3 | phosducin like 3 [Source:HGNC Symbol;Acc:HGNC:28860] |
| ENSG00000115998 | HGLibA_06126 | 203 | 0.871458638 | C2orf42 | chromosome 2 open reading frame 42 [Source:HGNC Symbol;Acc:HGNC:26056] |
| ENSG00000119720 | HGLibA_32667 | 203 | 0.871458638 | NRDE2 | domain containing [Source:HGNC Symbol;Acc:HGNC:20186]" |
| ENSG00000120289 | HGLibA_27950 | 203 | 0.871458638 | MAGEB4 | MAGE family member B4 [Source:HGNC Symbol;Acc:HGNC:6811] |
| ENSG00000123989 | HGLibA_09523 | 203 | 0.871458638 | CHPF | chondroitin polymerizing factor [Source:HGNC Symbol;Acc:HGNC:24291] |
| ENSG00000124194 | HGLibA_18841 | 203 | 0.871458638 | GDAP1L1 | ganglioside induced differentiation associated protein 1 like 1 [Source:HGNC Symbol;Acc:HGNC:4213] |
| ENSG00000127743 | HGLibA_22992 | 203 | 0.871458638 | IL17B | interleukin 17B [Source:HGNC Symbol;Acc:HGNC:5982] |
| ENSG00000129911 | HGLibA_25060 | 203 | 0.871458638 | KLF16 | Kruppel like factor 16 [Source:HGNC Symbol;Acc:HGNC:16857] |
| ENSG00000132017 | HGLibA_12430 | 203 | 0.871458638 | DCAF15 | DDB1 and CUL4 associated factor 15 [Source:HGNC Symbol;Acc:HGNC:25095] |
| ENSG00000136928 | HGLibA_18343 | 203 | 0.871458638 | GABBR2 | gamma-aminobutyric acid type B receptor subunit 2 [Source:HGNC Symbol;Acc:HGNC:4507] |
| ENSG00000137809 | HGLibA_23721 | 203 | 0.871458638 | ITGA11 | integrin subunit alpha 11 [Source:HGNC Symbol;Acc:HGNC:6136] |
| ENSG00000140873 | HGLibA_00814 | 203 | 0.871458638 | ADAMTS18 | ADAM metallopeptidase with thrombospondin type 1 motif 18 [Source:HGNC Symbol;Acc:HGNC:17110] |
| ENSG00000140876 | HGLibA_32987 | 203 | 0.871458638 | NUDT7 | nudix hydrolase 7 [Source:HGNC Symbol;Acc:HGNC:8054] |
| ENSG00000143801 | HGLibA_39028 | 203 | 0.871458638 | PSEN2 | presenilin 2 [Source:HGNC Symbol;Acc:HGNC:9509] |
| ENSG00000148541 | HGLibA_16147 | 203 | 0.871458638 | FAM13C | family with sequence similarity 13 member C [Source:HGNC Symbol;Acc:HGNC:19371] |
| ENSG00000148600 | HGLibA_08698 | 203 | 0.871458638 | CDHR1 | cadherin related family member 1 [Source:HGNC Symbol;Acc:HGNC:14550] |
| ENSG00000151247 | HGLibA_14816 | 203 | 0.871458638 | EIF4E | eukaryotic translation initiation factor 4E [Source:HGNC Symbol;Acc:HGNC:3287] |
| ENSG00000164331 | HGLibA_01985 | 203 | 0.871458638 | ANKRA2 | ankyrin repeat family A member 2 [Source:HGNC Symbol;Acc:HGNC:13208] |
| ENSG00000164967 | HGLibA_42033 | 203 | 0.871458638 | RPP25L | ribonuclease P/MRP subunit p25 like [Source:HGNC Symbol;Acc:HGNC:19909] |
| ENSG00000165061 | HGLibA_55376 | 203 | 0.871458638 | ZMAT4 | zinc finger matrin-type 4 [Source:HGNC Symbol;Acc:HGNC:25844] |
| ENSG00000165312 | HGLibA_34759 | 203 | 0.871458638 | OTUD1 | OTU deubiquitinase 1 [Source:HGNC Symbol;Acc:HGNC:27346] |
| ENSG00000165841 | HGLibA_12119 | 203 | 0.871458638 | CYP2C19 | cytochrome P450 family 2 subfamily C member 19 [Source:HGNC Symbol;Acc:HGNC:2621] |
| ENSG00000166707 | HGLibA_55024 | 203 | 0.871458638 | ZCCHC18 | zinc finger CCHC-type containing 18 [Source:HGNC Symbol;Acc:HGNC:32459] |
| ENSG00000166716 | HGLibA_56338 | 203 | 0.871458638 | ZNF592 | zinc finger protein 592 [Source:HGNC Symbol;Acc:HGNC:28986] |
| ENSG00000167535 | HGLibA_06923 | 203 | 0.871458638 | CACNB3 | calcium voltage-gated channel auxiliary subunit beta 3 [Source:HGNC Symbol;Acc:HGNC:1403] |
| ENSG00000168092 | HGLibA_34992 | 203 | 0.871458638 | PAFAH1B2 | platelet activating factor acetylhydrolase 1b catalytic subunit 2 [Source:HGNC Symbol;Acc:HGNC:8575] |
| ENSG00000169035 | HGLibA_25268 | 203 | 0.871458638 | KLK7 | kallikrein related peptidase 7 [Source:HGNC Symbol;Acc:HGNC:6368] |
| ENSG00000169105 | HGLibA_09626 | 203 | 0.871458638 | CHST14 | carbohydrate sulfotransferase 14 [Source:HGNC Symbol;Acc:HGNC:24464] |
| ENSG00000170142 | HGLibA_52594 | 203 | 0.871458638 | UBE2E1 | ubiquitin conjugating enzyme E2 E1 [Source:HGNC Symbol;Acc:HGNC:12477] |
| ENSG00000172425 | HGLibA_52123 | 203 | 0.871458638 | TTC36 | tetratricopeptide repeat domain 36 [Source:HGNC Symbol;Acc:HGNC:33708] |
| ENSG00000174032 | HGLibA_44703 | 203 | 0.871458638 | SLC25A30 | solute carrier family 25 member 30 [Source:HGNC Symbol;Acc:HGNC:27371] |
| ENSG00000175206 | HGLibA_32499 | 203 | 0.871458638 | NPPA | natriuretic peptide A [Source:HGNC Symbol;Acc:HGNC:7939] |
| ENSG00000177186 | HGLibA_33846 | 203 | 0.871458638 | OR2M7 | olfactory receptor family 2 subfamily M member 7 [Source:HGNC Symbol;Acc:HGNC:19594] |
| ENSG00000177257 | HGLibA_12917 | 203 | 0.871458638 | DEFB4B | defensin beta 4B [Source:HGNC Symbol;Acc:HGNC:30193] |
| ENSG00000181544 | HGLibA_16833 | 203 | 0.871458638 | FANCB | FA complementation group B [Source:HGNC Symbol;Acc:HGNC:3583] |
| ENSG00000183513 | HGLibA_10451 | 203 | 0.871458638 | COA5 | cytochrome c oxidase assembly factor 5 [Source:HGNC Symbol;Acc:HGNC:33848] |
| ENSG00000183690 | HGLibA_14574 | 203 | 0.871458638 | EFHC2 | EF-hand domain containing 2 [Source:HGNC Symbol;Acc:HGNC:26233] |
| ENSG00000185104 | HGLibA_15945 | 203 | 0.871458638 | FAF1 | Fas associated factor 1 [Source:HGNC Symbol;Acc:HGNC:3578] |
| ENSG00000185339 | HGLibA_48823 | 203 | 0.871458638 | TCN2 | transcobalamin 2 [Source:HGNC Symbol;Acc:HGNC:11653] |
| ENSG00000187118 | HGLibA_10184 | 203 | 0.871458638 | CMC1 | C-X9-C motif containing 1 [Source:HGNC Symbol;Acc:HGNC:28783] |
| ENSG00000188039 | HGLibA_33122 | 203 | 0.871458638 | NWD1 | NACHT and WD repeat domain containing 1 [Source:HGNC Symbol;Acc:HGNC:27619] |
| ENSG00000189127 | HGLibA_02079 | 203 | 0.871458638 | ANKRD34B | ankyrin repeat domain 34B [Source:HGNC Symbol;Acc:HGNC:33736] |
| ENSG00000189319 | HGLibA_16639 | 203 | 0.871458638 | FAM53B | family with sequence similarity 53 member B [Source:HGNC Symbol;Acc:HGNC:28968] |
| ENSG00000189430 | HGLibA_31361 | 203 | 0.871458638 | NCR1 | natural cytotoxicity triggering receptor 1 [Source:HGNC Symbol;Acc:HGNC:6731] |
| ENSG00000196372 | HGLibA_03216 | 203 | 0.871458638 | ASB13 | ankyrin repeat and SOCS box containing 13 [Source:HGNC Symbol;Acc:HGNC:19765] |
| ENSG00000197256 | HGLibA_24028 | 203 | 0.871458638 | KANK2 | KN motif and ankyrin repeat domains 2 [Source:HGNC Symbol;Acc:HGNC:29300] |
| ENSG00000197265 | HGLibA_20536 | 203 | 0.871458638 | GTF2E2 | general transcription factor IIE subunit 2 [Source:HGNC Symbol;Acc:HGNC:4651] |
| ENSG00000198276 | HGLibA_52817 | 203 | 0.871458638 | UCKL1 | uridine-cytidine kinase 1 like 1 [Source:HGNC Symbol;Acc:HGNC:15938] |
| ENSG00000198546 | HGLibA_56129 | 203 | 0.871458638 | ZNF511 | zinc finger protein 511 [Source:HGNC Symbol;Acc:HGNC:28445] |
| ENSG00000204438 | HGLibA_19730 | 203 | 0.871458638 | GPANK1 | G-patch domain and ankyrin repeats 1 [Source:HGNC Symbol;Acc:HGNC:13920] |
| ENSG00000205085 | HGLibA_16703 | 203 | 0.871458638 | FAM71F2 | family with sequence similarity 71 member F2 [Source:HGNC Symbol;Acc:HGNC:27998] |
| ENSG00000213694 | HGLibA_42599 | 203 | 0.871458638 | S1PR3 | sphingosine-1-phosphate receptor 3 [Source:HGNC Symbol;Acc:HGNC:3167] |
| ENSG00000214827 | HGLibA_30332 | 203 | 0.871458638 | MTCP1 | mature T cell proliferation 1 [Source:HGNC Symbol;Acc:HGNC:7423] |
| ENSG00000237763 | HGLibA_01842 | 203 | 0.871458638 | AMY1A | alpha 1A (salivary) [Source:HGNC Symbol;Acc:HGNC:474]" |
| ENSG00000251692 | HGLibA_39573 | 203 | 0.871458638 | PTX4 | pentraxin 4 [Source:HGNC Symbol;Acc:HGNC:14171] |
| ENSG00000263639 | HGLibA_30223 | 203 | 0.871458638 | MSMB | microseminoprotein beta [Source:HGNC Symbol;Acc:HGNC:7372] |
| ENSG00000269226 | HGLibA_50663 | 203 | 0.871458638 | TMSB15B | thymosin beta 15B [Source:NCBI gene;Acc:286527] |
| FAM129A | HGLibA_16095 | 203 | 0.871458638 | FAM129A | - |
| FAM96A | HGLibA_16804 | 203 | 0.871458638 | FAM96A | - |
| HIATL1 | HGLibA_21280 | 203 | 0.871458638 | HIATL1 | - |
| hsa-mir-152 | HGLibA_57904 | 203 | 0.871458638 | hsa-mir-152 | - |
| hsa-mir-20b | HGLibA_58205 | 203 | 0.871458638 | hsa-mir-20b | - |
| hsa-mir-3923 | HGLibA_59567 | 203 | 0.871458638 | hsa-mir-3923 | - |
| hsa-mir-4438 | HGLibA_60155 | 203 | 0.871458638 | hsa-mir-4438 | - |
| hsa-mir-4502 | HGLibA_60435 | 203 | 0.871458638 | hsa-mir-4502 | - |
| hsa-mir-6509 | HGLibA_62885 | 203 | 0.871458638 | hsa-mir-6509 | - |
| hsa-mir-6807 | HGLibA_63405 | 203 | 0.871458638 | hsa-mir-6807 | - |
| MESDC2 | HGLibA_28913 | 203 | 0.871458638 | MESDC2 | - |
| MINA | HGLibA_29272 | 203 | 0.871458638 | MINA | - |
| TTC18 | HGLibA_52064 | 203 | 0.871458638 | TTC18 | - |
| ZCCHC5 | HGLibA_55040 | 203 | 0.871458638 | ZCCHC5 | - |
| C17orf66 | HGLibA_05562 | 202 | 0.861507601 | C17orf66 | - |
| C8orf87 | HGLibA_06657 | 202 | 0.861507601 | C8orf87 | - |
| DBC1 | HGLibA_12373 | 202 | 0.861507601 | DBC1 | - |
| ENSG00000003402 | HGLibA_09286 | 202 | 0.861507601 | CFLAR | CASP8 and FADD like apoptosis regulator [Source:HGNC Symbol;Acc:HGNC:1876] |
| ENSG00000007171 | HGLibA_32329 | 202 | 0.861507601 | NOS2 | nitric oxide synthase 2 [Source:HGNC Symbol;Acc:HGNC:7873] |
| ENSG00000019102 | HGLibA_53845 | 202 | 0.861507601 | VSIG2 | V-set and immunoglobulin domain containing 2 [Source:HGNC Symbol;Acc:HGNC:17149] |
| ENSG00000039139 | HGLibA_13489 | 202 | 0.861507601 | DNAH5 | dynein axonemal heavy chain 5 [Source:HGNC Symbol;Acc:HGNC:2950] |
| ENSG00000089327 | HGLibA_18236 | 202 | 0.861507601 | FXYD5 | FXYD domain containing ion transport regulator 5 [Source:HGNC Symbol;Acc:HGNC:4029] |
| ENSG00000099812 | HGLibA_29311 | 202 | 0.861507601 | MISP | mitotic spindle positioning [Source:HGNC Symbol;Acc:HGNC:27000] |
| ENSG00000100280 | HGLibA_02281 | 202 | 0.861507601 | AP1B1 | adaptor related protein complex 1 subunit beta 1 [Source:HGNC Symbol;Acc:HGNC:554] |
| ENSG00000102024 | HGLibA_37227 | 202 | 0.861507601 | PLS3 | plastin 3 [Source:HGNC Symbol;Acc:HGNC:9091] |
| ENSG00000105707 | HGLibA_22038 | 202 | 0.861507601 | HPN | hepsin [Source:HGNC Symbol;Acc:HGNC:5155] |
| ENSG00000107249 | HGLibA_19278 | 202 | 0.861507601 | GLIS3 | GLIS family zinc finger 3 [Source:HGNC Symbol;Acc:HGNC:28510] |
| ENSG00000107249 | HGLibA_19276 | 202 | 0.861507601 | GLIS3 | GLIS family zinc finger 3 [Source:HGNC Symbol;Acc:HGNC:28510] |
| ENSG00000109625 | HGLibA_11042 | 202 | 0.861507601 | CPZ | carboxypeptidase Z [Source:HGNC Symbol;Acc:HGNC:2333] |
| ENSG00000114125 | HGLibA_41698 | 202 | 0.861507601 | RNF7 | ring finger protein 7 [Source:HGNC Symbol;Acc:HGNC:10070] |
| ENSG00000115241 | HGLibA_37979 | 202 | 0.861507601 | PPM1G | Mg2+/Mn2+ dependent 1G [Source:HGNC Symbol;Acc:HGNC:9278]" |
| ENSG00000120498 | HGLibA_49051 | 202 | 0.861507601 | TEX11 | testis expressed 11 [Source:HGNC Symbol;Acc:HGNC:11733] |
| ENSG00000123595 | HGLibA_39991 | 202 | 0.861507601 | RAB9A | member RAS oncogene family [Source:HGNC Symbol;Acc:HGNC:9792]" |
| ENSG00000124226 | HGLibA_41478 | 202 | 0.861507601 | RNF114 | ring finger protein 114 [Source:HGNC Symbol;Acc:HGNC:13094] |
| ENSG00000124444 | HGLibA_56294 | 202 | 0.861507601 | ZNF576 | zinc finger protein 576 [Source:HGNC Symbol;Acc:HGNC:28357] |
| ENSG00000125944 | HGLibA_21842 | 202 | 0.861507601 | HNRNPR | heterogeneous nuclear ribonucleoprotein R [Source:HGNC Symbol;Acc:HGNC:5047] |
| ENSG00000129355 | HGLibA_08846 | 202 | 0.861507601 | CDKN2D | cyclin dependent kinase inhibitor 2D [Source:HGNC Symbol;Acc:HGNC:1790] |
| ENSG00000130701 | HGLibA_40444 | 202 | 0.861507601 | RBBP8NL | RBBP8 N-terminal like [Source:HGNC Symbol;Acc:HGNC:16144] |
| ENSG00000132932 | HGLibA_03779 | 202 | 0.861507601 | ATP8A2 | ATPase phospholipid transporting 8A2 [Source:HGNC Symbol;Acc:HGNC:13533] |
| ENSG00000132964 | HGLibA_08798 | 202 | 0.861507601 | CDK8 | cyclin dependent kinase 8 [Source:HGNC Symbol;Acc:HGNC:1779] |
| ENSG00000133878 | HGLibA_14169 | 202 | 0.861507601 | DUSP26 | dual specificity phosphatase 26 [Source:HGNC Symbol;Acc:HGNC:28161] |
| ENSG00000136099 | HGLibA_35419 | 202 | 0.861507601 | PCDH8 | protocadherin 8 [Source:HGNC Symbol;Acc:HGNC:8660] |
| ENSG00000137880 | HGLibA_18785 | 202 | 0.861507601 | GCHFR | GTP cyclohydrolase I feedback regulator [Source:HGNC Symbol;Acc:HGNC:4194] |
| ENSG00000138801 | HGLibA_35152 | 202 | 0.861507601 | PAPSS1 | 3'-phosphoadenosine 5'-phosphosulfate synthase 1 [Source:HGNC Symbol;Acc:HGNC:8603] |
| ENSG00000140543 | HGLibA_13014 | 202 | 0.861507601 | DET1 | COP1 ubiquitin ligase partner [Source:HGNC Symbol;Acc:HGNC:25477]" |

| | | | | | |
|---|---|---|---|---|---|
| ENSG00000140575 | HGLibA_23532 | 202 | 0.861507601 | IQGAP1 | IQ motif containing GTPase activating protein 1 [Source:HGNC Symbol;Acc:HGNC:6110] |
| ENSG00000141101 | HGLibA_32233 | 202 | 0.861507601 | NOB1 | NIN1 (RPN12) binding protein 1 homolog [Source:HGNC Symbol;Acc:HGNC:29540] |
| ENSG00000143164 | HGLibA_12450 | 202 | 0.861507601 | DCAF6 | DDB1 and CUL4 associated factor 6 [Source:HGNC Symbol;Acc:HGNC:30002] |
| ENSG00000146192 | HGLibA_17344 | 202 | 0.861507601 | FGD2 | RhoGEF and PH domain containing 2 [Source:HGNC Symbol;Acc:HGNC:3664]" |
| ENSG00000147642 | HGLibA_47909 | 202 | 0.861507601 | SYBU | syntabulin [Source:HGNC Symbol;Acc:HGNC:26011] |
| ENSG00000150048 | HGLibA_09956 | 202 | 0.861507601 | CLEC1A | C-type lectin domain family 1 member A [Source:HGNC Symbol;Acc:HGNC:24355] |
| ENSG00000152939 | HGLibA_28382 | 202 | 0.861507601 | MARVELD2 | MARVEL domain containing 2 [Source:HGNC Symbol;Acc:HGNC:26401] |
| ENSG00000154305 | HGLibA_29188 | 202 | 0.861507601 | MIA3 | MIA SH3 domain ER export factor 3 [Source:HGNC Symbol;Acc:HGNC:24008] |
| ENSG00000155592 | HGLibA_55348 | 202 | 0.861507601 | ZKSCAN2 | zinc finger with KRAB and SCAN domains 2 [Source:HGNC Symbol;Acc:HGNC:25677] |
| ENSG00000160791 | HGLibA_08158 | 202 | 0.861507601 | CCR5 | C-C motif chemokine receptor 5 (gene/pseudogene) [Source:HGNC Symbol;Acc:HGNC:1606] |
| ENSG00000161681 | HGLibA_43987 | 202 | 0.861507601 | SHANK1 | SH3 and multiple ankyrin repeat domains 1 [Source:HGNC Symbol;Acc:HGNC:15474] |
| ENSG00000162188 | HGLibA_19522 | 202 | 0.861507601 | GNG3 | G protein subunit gamma 3 [Source:HGNC Symbol;Acc:HGNC:4405] |
| ENSG00000164520 | HGLibA_40119 | 202 | 0.861507601 | RAET1E | retinoic acid early transcript 1E [Source:HGNC Symbol;Acc:HGNC:16793] |
| ENSG00000165799 | HGLibA_41414 | 202 | 0.861507601 | RNASE7 | ribonuclease A family member 7 [Source:HGNC Symbol;Acc:HGNC:19278] |
| ENSG00000167702 | HGLibA_24973 | 202 | 0.861507601 | KIFC2 | kinesin family member C2 [Source:HGNC Symbol;Acc:HGNC:29530] |
| ENSG00000168818 | HGLibA_47631 | 202 | 0.861507601 | STX18 | syntaxin 18 [Source:HGNC Symbol;Acc:HGNC:15942] |
| ENSG00000169306 | HGLibA_23055 | 202 | 0.861507601 | IL1RAPL1 | interleukin 1 receptor accessory protein like 1 [Source:HGNC Symbol;Acc:HGNC:5996] |
| ENSG00000169435 | HGLibA_40388 | 202 | 0.861507601 | RASSF6 | Ras association domain family member 6 [Source:HGNC Symbol;Acc:HGNC:20796] |
| ENSG00000170500 | HGLibA_27024 | 202 | 0.861507601 | LONRF2 | LON peptidase N-terminal domain and ring finger 2 [Source:HGNC Symbol;Acc:HGNC:24788] |
| ENSG00000171551 | HGLibA_14387 | 202 | 0.861507601 | ECEL1 | endothelin converting enzyme like 1 [Source:HGNC Symbol;Acc:HGNC:3147] |
| ENSG00000172789 | HGLibA_21971 | 202 | 0.861507601 | HOXC5 | homeobox C5 [Source:HGNC Symbol;Acc:HGNC:5127] |
| ENSG00000173852 | HGLibA_13905 | 202 | 0.861507601 | DPY19L1 | dpy-19 like C-mannosyltransferase 1 [Source:HGNC Symbol;Acc:HGNC:22205] |
| ENSG00000176771 | HGLibA_31316 | 202 | 0.861507601 | NCKAP5 | NCK associated protein 5 [Source:HGNC Symbol;Acc:HGNC:29847] |
| ENSG00000178177 | HGLibA_26195 | 202 | 0.861507601 | LCORL | ligand dependent nuclear receptor corepressor like [Source:HGNC Symbol;Acc:HGNC:30776] |
| ENSG00000179021 | HGLibA_06243 | 202 | 0.861507601 | C3orf38 | chromosome 3 open reading frame 38 [Source:HGNC Symbol;Acc:HGNC:28384] |
| ENSG00000180104 | HGLibA_15737 | 202 | 0.861507601 | EXOC3 | exocyst complex component 3 [Source:HGNC Symbol;Acc:HGNC:30378] |
| ENSG00000182197 | HGLibA_15802 | 202 | 0.861507601 | EXT1 | exostosin glycosyltransferase 1 [Source:HGNC Symbol;Acc:HGNC:3512] |
| ENSG00000182334 | HGLibA_34350 | 202 | 0.861507601 | OR5P3 | olfactory receptor family 5 subfamily P member 3 [Source:HGNC Symbol;Acc:HGNC:14784] |
| ENSG00000184206 | HGLibA_19626 | 202 | 0.861507601 | GOLGA6L4 | golgin A6 family-like 4 [Source:HGNC Symbol;Acc:HGNC:27256] |
| ENSG00000184207 | HGLibA_36277 | 202 | 0.861507601 | PGP | phosphoglycolate phosphatase [Source:HGNC Symbol;Acc:HGNC:8909] |
| ENSG00000185774 | HGLibA_24242 | 202 | 0.861507601 | KCNIP4 | potassium voltage-gated channel interacting protein 4 [Source:HGNC Symbol;Acc:HGNC:30083] |
| ENSG00000186063 | HGLibA_01329 | 202 | 0.861507601 | AIDA | dorsalization associated [Source:HGNC Symbol;Acc:HGNC:25761]" |
| ENSG00000188895 | HGLibA_30210 | 202 | 0.861507601 | MSL1 | male specific lethal 1 homolog [Source:HGNC Symbol;Acc:HGNC:27905] |
| ENSG00000196470 | HGLibA_44106 | 202 | 0.861507601 | SIAH1 | siah E3 ubiquitin protein ligase 1 [Source:HGNC Symbol;Acc:HGNC:10857] |
| ENSG00000197006 | HGLibA_29023 | 202 | 0.861507601 | METTL9 | methyltransferase like 9 [Source:HGNC Symbol;Acc:HGNC:24586] |
| ENSG00000203772 | HGLibA_46799 | 202 | 0.861507601 | SPRN | shadow of prion protein [Source:HGNC Symbol;Acc:HGNC:16871] |
| ENSG00000203896 | HGLibA_26537 | 202 | 0.861507601 | LIME1 | Lck interacting transmembrane adaptor 1 [Source:HGNC Symbol;Acc:HGNC:26016] |
| ENSG00000204703 | HGLibA_33771 | 202 | 0.861507601 | OR2B3 | olfactory receptor family 2 subfamily B member 3 [Source:HGNC Symbol;Acc:HGNC:8238] |
| ENSG00000206560 | HGLibA_02054 | 202 | 0.861507601 | ANKRD28 | ankyrin repeat domain 28 [Source:HGNC Symbol;Acc:HGNC:29024] |
| ENSG00000221826 | HGLibA_39044 | 202 | 0.861507601 | PSG3 | pregnancy specific beta-1-glycoprotein 3 [Source:HGNC Symbol;Acc:HGNC:9520] |
| ENSG00000221970 | HGLibA_33719 | 202 | 0.861507601 | OR2A1 | olfactory receptor family 2 subfamily A member 1 [Source:HGNC Symbol;Acc:HGNC:8229] |
| ENSG00000231861 | HGLibA_34312 | 202 | 0.861507601 | OR5K2 | olfactory receptor family 5 subfamily K member 2 [Source:HGNC Symbol;Acc:HGNC:14774] |
| ENSG00000236104 | HGLibA_54844 | 202 | 0.861507601 | ZBTB22 | zinc finger and BTB domain containing 22 [Source:HGNC Symbol;Acc:HGNC:13085] |
| ENSG00000243543 | HGLibA_54272 | 202 | 0.861507601 | WFDC6 | WAP four-disulfide core domain 6 [Source:HGNC Symbol;Acc:HGNC:16164] |
| ENSG00000248483 | HGLibA_37793 | 202 | 0.861507601 | POU5F2 | transcription factor 2 [Source:HGNC Symbol;Acc:HGNC:26367]" |
| ENSG00000250741 | HGLibA_32808 | 202 | 0.861507601 | NT5C1B-RDH14 | NT5C1B-RDH14 readthrough [Source:HGNC Symbol;Acc:HGNC:38831] |
| ENSG00000255423 | HGLibA_14367 | 202 | 0.861507601 | EBLN2 | endogenous Bornavirus like nucleoprotein 2 [Source:HGNC Symbol;Acc:HGNC:25493] |
| hsa-mir-1228 | HGLibA_57311 | 202 | 0.861507601 | hsa-mir-1228 | - |
| hsa-mir-1249 | HGLibA_57396 | 202 | 0.861507601 | hsa-mir-1249 | - |
| hsa-mir-4292 | HGLibA_59879 | 202 | 0.861507601 | hsa-mir-4292 | - |
| hsa-mir-4321 | HGLibA_60007 | 202 | 0.861507601 | hsa-mir-4321 | - |
| hsa-mir-4662b | HGLibA_60763 | 202 | 0.861507601 | hsa-mir-4662b | - |
| hsa-mir-4727 | HGLibA_61006 | 202 | 0.861507601 | hsa-mir-4727 | - |
| hsa-mir-488 | HGLibA_61354 | 202 | 0.861507601 | hsa-mir-488 | - |
| hsa-mir-548ab | HGLibA_61858 | 202 | 0.861507601 | hsa-mir-548ab | - |
| hsa-mir-6503 | HGLibA_62863 | 202 | 0.861507601 | hsa-mir-6503 | - |
| hsa-mir-6715b | HGLibA_63020 | 202 | 0.861507601 | hsa-mir-6715b | - |
| hsa-mir-6837 | HGLibA_63525 | 202 | 0.861507601 | hsa-mir-6837 | - |
| hsa-mir-8089 | HGLibA_64200 | 202 | 0.861507601 | hsa-mir-8089 | - |
| LOC100507462 | HGLibA_26870 | 202 | 0.861507601 | LOC100507462 | - |
| PPAPDC1A | HGLibA_37826 | 202 | 0.861507601 | PPAPDC1A | - |
| PVRL1 | HGLibA_39623 | 202 | 0.861507601 | PVRL1 | - |
| CCDC147 | HGLibA_07632 | 201 | 0.851556564 | CCDC147 | - |
| CXorf40A | HGLibA_11924 | 201 | 0.851556564 | CXorf40A | - |
| ENSG00000007541 | HGLibA_36579 | 201 | 0.851556564 | PIGQ | phosphatidylinositol glycan anchor biosynthesis class Q [Source:HGNC Symbol;Acc:HGNC:14135] |
| ENSG00000022556 | HGLibA_32115 | 201 | 0.851556564 | NLRP2 | NLR family pyrin domain containing 2 [Source:HGNC Symbol;Acc:HGNC:22948] |
| ENSG00000033327 | HGLibA_18324 | 201 | 0.851556564 | GAB2 | GRB2 associated binding protein 2 [Source:HGNC Symbol;Acc:HGNC:14458] |
| ENSG00000061273 | HGLibA_21020 | 201 | 0.851556564 | HDAC7 | histone deacetylase 7 [Source:HGNC Symbol;Acc:HGNC:14067] |
| ENSG00000066185 | HGLibA_55418 | 201 | 0.851556564 | ZMYND12 | zinc finger MYND-type containing 12 [Source:HGNC Symbol;Acc:HGNC:21192] |
| ENSG00000089057 | HGLibA_44610 | 201 | 0.851556564 | SLC23A2 | solute carrier family 23 member 2 [Source:HGNC Symbol;Acc:HGNC:10973] |
| ENSG00000104723 | HGLibA_52348 | 201 | 0.851556564 | TUSC3 | tumor suppressor candidate 3 [Source:HGNC Symbol;Acc:HGNC:30242] |
| ENSG00000106686 | HGLibA_46525 | 201 | 0.851556564 | SPATA6L | spermatogenesis associated 6 like [Source:HGNC Symbol;Acc:HGNC:25472] |
| ENSG00000111181 | HGLibA_45296 | 201 | 0.851556564 | SLC6A12 | solute carrier family 6 member 12 [Source:HGNC Symbol;Acc:HGNC:11045] |
| ENSG00000111885 | HGLibA_28058 | 201 | 0.851556564 | MAN1A1 | mannosidase alpha class 1A member 1 [Source:HGNC Symbol;Acc:HGNC:6821] |
| ENSG00000119547 | HGLibA_33395 | 201 | 0.851556564 | ONECUT2 | one cut homeobox 2 [Source:HGNC Symbol;Acc:HGNC:8139] |
| ENSG00000120322 | HGLibA_35511 | 201 | 0.851556564 | PCDHB8 | protocadherin beta 8 [Source:HGNC Symbol;Acc:HGNC:8693] |
| ENSG00000122592 | HGLibA_21919 | 201 | 0.851556564 | HOXA7 | homeobox A7 [Source:HGNC Symbol;Acc:HGNC:5108] |
| ENSG00000123095 | HGLibA_04482 | 201 | 0.851556564 | BHLHE41 | basic helix-loop-helix family member e41 [Source:HGNC Symbol;Acc:HGNC:16617] |
| ENSG00000123700 | HGLibA_24272 | 201 | 0.851556564 | KCNJ2 | potassium voltage-gated channel subfamily J member 2 [Source:HGNC Symbol;Acc:HGNC:6263] |
| ENSG00000124092 | HGLibA_11599 | 201 | 0.851556564 | CTCFL | CCCTC-binding factor like [Source:HGNC Symbol;Acc:HGNC:16234] |
| ENSG00000125910 | HGLibA_42602 | 201 | 0.851556564 | S1PR4 | sphingosine-1-phosphate receptor 4 [Source:HGNC Symbol;Acc:HGNC:3170] |
| ENSG00000132321 | HGLibA_23487 | 201 | 0.851556564 | IQCA1 | IQ motif containing with AAA domain 1 [Source:HGNC Symbol;Acc:HGNC:26195] |
| ENSG00000133056 | HGLibA_36625 | 201 | 0.851556564 | PIK3C2B | phosphatidylinositol-4-phosphate 3-kinase catalytic subunit type 2 beta [Source:HGNC Symbol;Acc:HGNC:8972] |
| ENSG00000133488 | HGLibA_43200 | 201 | 0.851556564 | SEC14L4 | SEC14 like lipid binding 4 [Source:HGNC Symbol;Acc:HGNC:20627] |
| ENSG00000135324 | HGLibA_29810 | 201 | 0.851556564 | MRAP2 | melanocortin 2 receptor accessory protein 2 [Source:HGNC Symbol;Acc:HGNC:21232] |
| ENSG00000135373 | HGLibA_14678 | 201 | 0.851556564 | EHF | ETS homologous factor [Source:HGNC Symbol;Acc:HGNC:3246] |
| ENSG00000135916 | HGLibA_23836 | 201 | 0.851556564 | ITM2C | integral membrane protein 2C [Source:HGNC Symbol;Acc:HGNC:6175] |
| ENSG00000141429 | HGLibA_18526 | 201 | 0.851556564 | GALNT1 | polypeptide N-acetylgalactosaminyltransferase 1 [Source:HGNC Symbol;Acc:HGNC:4123] |
| ENSG00000141668 | HGLibA_07410 | 201 | 0.851556564 | CBLN2 | cerebellin 2 precursor [Source:HGNC Symbol;Acc:HGNC:1544] |
| ENSG00000144452 | HGLibA_00101 | 201 | 0.851556564 | ABCA12 | ATP binding cassette subfamily A member 12 [Source:HGNC Symbol;Acc:HGNC:14637] |
| ENSG00000146955 | HGLibA_39827 | 201 | 0.851556564 | RAB19 | member RAS oncogene family [Source:HGNC Symbol;Acc:HGNC:19982]" |
| ENSG00000150977 | HGLibA_41255 | 201 | 0.851556564 | RILPL2 | Rab interacting lysosomal protein like 2 [Source:HGNC Symbol;Acc:HGNC:28787] |
| ENSG00000152102 | HGLibA_16249 | 201 | 0.851556564 | FAM168B | family with sequence similarity 168 member B [Source:HGNC Symbol;Acc:HGNC:27016] |
| ENSG00000156150 | HGLibA_01737 | 201 | 0.851556564 | ALX3 | ALX homeobox 3 [Source:HGNC Symbol;Acc:HGNC:449] |
| ENSG00000157240 | HGLibA_18261 | 201 | 0.851556564 | FZD1 | frizzled class receptor 1 [Source:HGNC Symbol;Acc:HGNC:4038] |
| ENSG00000160051 | HGLibA_23493 | 201 | 0.851556564 | IQCC | IQ motif containing C [Source:HGNC Symbol;Acc:HGNC:25545] |
| ENSG00000160285 | HGLibA_27570 | 201 | 0.851556564 | LSS | lanosterol synthase [Source:HGNC Symbol;Acc:HGNC:6708] |
| ENSG00000163564 | HGLibA_39700 | 201 | 0.851556564 | PYHIN1 | pyrin and HIN domain family member 1 [Source:HGNC Symbol;Acc:HGNC:28894] |
| ENSG00000163746 | HGLibA_37235 | 201 | 0.851556564 | PLSCR2 | phospholipid scramblase 2 [Source:HGNC Symbol;Acc:HGNC:16494] |
| ENSG00000163823 | HGLibA_08144 | 201 | 0.851556564 | CCR1 | C-C motif chemokine receptor 1 [Source:HGNC Symbol;Acc:HGNC:1602] |
| ENSG00000164303 | HGLibA_15183 | 201 | 0.851556564 | ENPP6 | ectonucleotide pyrophosphatase/phosphodiesterase 6 [Source:HGNC Symbol;Acc:HGNC:23409] |
| ENSG00000165643 | HGLibA_46163 | 201 | 0.851556564 | SOHLH1 | spermatogenesis and oogenesis specific basic helix-loop-helix 1 [Source:HGNC Symbol;Acc:HGNC:27845] |
| ENSG00000165801 | HGLibA_02868 | 201 | 0.851556564 | ARHGEF40 | Rho guanine nucleotide exchange factor 40 [Source:HGNC Symbol;Acc:HGNC:25516] |
| ENSG00000166266 | HGLibA_11788 | 201 | 0.851556564 | CUL5 | cullin 5 [Source:HGNC Symbol;Acc:HGNC:2556] |
| ENSG00000166415 | HGLibA_54165 | 201 | 0.851556564 | WDR72 | WD repeat domain 72 [Source:HGNC Symbol;Acc:HGNC:26790] |
| ENSG00000169116 | HGLibA_35205 | 201 | 0.851556564 | PARM1 | prostate androgen-regulated mucin-like protein 1 [Source:HGNC Symbol;Acc:HGNC:24536] |
| ENSG00000172955 | HGLibA_00963 | 201 | 0.851556564 | ADH6 | alcohol dehydrogenase 6 (class V) [Source:HGNC Symbol;Acc:HGNC:255] |
| ENSG00000173334 | HGLibA_51391 | 201 | 0.851556564 | TRIB1 | tribbles pseudokinase 1 [Source:HGNC Symbol;Acc:HGNC:16891] |
| ENSG00000179583 | HGLibA_09705 | 201 | 0.851556564 | CIITA | class II major histocompatibility complex transactivator [Source:HGNC Symbol;Acc:HGNC:7067] |
| ENSG00000183034 | HGLibA_34738 | 201 | 0.851556564 | OTOP2 | otopetrin 2 [Source:HGNC Symbol;Acc:HGNC:19657] |
| ENSG00000183597 | HGLibA_48297 | 201 | 0.851556564 | TANGO2 | transport and golgi organization 2 homolog [Source:HGNC Symbol;Acc:HGNC:25439] |
| ENSG00000184347 | HGLibA_45509 | 201 | 0.851556564 | SLIT3 | slit guidance ligand 3 [Source:HGNC Symbol;Acc:HGNC:11087] |
| ENSG00000184574 | HGLibA_27064 | 201 | 0.851556564 | LPAR5 | lysophosphatidic acid receptor 5 [Source:HGNC Symbol;Acc:HGNC:13307] |
| ENSG00000185730 | HGLibA_56559 | 201 | 0.851556564 | ZNF696 | zinc finger protein 696 [Source:HGNC Symbol;Acc:HGNC:25872] |
| ENSG00000185821 | HGLibA_34412 | 201 | 0.851556564 | OR6C76 | olfactory receptor family 6 subfamily C member 76 [Source:HGNC Symbol;Acc:HGNC:31305] |
| ENSG00000186188 | HGLibA_17333 | 201 | 0.851556564 | FFAR4 | free fatty acid receptor 4 [Source:HGNC Symbol;Acc:HGNC:19061] |
| ENSG00000187664 | HGLibA_20849 | 201 | 0.851556564 | HAPLN4 | hyaluronan and proteoglycan link protein 4 [Source:HGNC Symbol;Acc:HGNC:31357] |
| ENSG00000196456 | HGLibA_56719 | 201 | 0.851556564 | ZNF775 | zinc finger protein 775 [Source:HGNC Symbol;Acc:HGNC:28501] |
| ENSG00000255223 | HGLibA_34334 | 201 | 0.851556564 | OR5M11 | olfactory receptor family 5 subfamily M member 11 [Source:HGNC Symbol;Acc:HGNC:15291] |
| ENSG00000278540 | HGLibA_00348 | 201 | 0.851556564 | ACACA | acetyl-CoA carboxylase alpha [Source:HGNC Symbol;Acc:HGNC:84] |
| ENSG00000288642 | HGLibA_08862 | 201 | 0.851556564 | CDR1 | - |
| FAM21B | HGLibA_16506 | 201 | 0.851556564 | FAM21B | - |
| hsa-mir-125a | HGLibA_57445 | 201 | 0.851556564 | hsa-mir-125a | - |
| hsa-mir-181b-2 | HGLibA_57971 | 201 | 0.851556564 | hsa-mir-181b-2 | - |
| hsa-mir-183 | HGLibA_57994 | 201 | 0.851556564 | hsa-mir-183 | - |
| hsa-mir-3130-1 | HGLibA_58571 | 201 | 0.851556564 | hsa-mir-3130-1 | - |
| hsa-mir-4505 | HGLibA_60446 | 201 | 0.851556564 | hsa-mir-4505 | - |
| hsa-mir-548p | HGLibA_62055 | 201 | 0.851556564 | hsa-mir-548p | - |
| hsa-mir-6509 | HGLibA_62887 | 201 | 0.851556564 | hsa-mir-6509 | - |
| hsa-mir-8054 | HGLibA_64053 | 201 | 0.851556564 | hsa-mir-8054 | - |
| hsa-mir-888 | HGLibA_64230 | 201 | 0.851556564 | hsa-mir-888 | - |
| KIAA0101 | HGLibA_24591 | 201 | 0.851556564 | KIAA0101 | - |
| NIM1 | HGLibA_31932 | 201 | 0.851556564 | NIM1 | - |
| ENSG00000004399 | HGLibA_37282 | 200 | 0.841605526 | PLXND1 | plexin D1 [Source:HGNC Symbol;Acc:HGNC:9107] |
| ENSG00000004848 | HGLibA_03179 | 200 | 0.841605526 | ARX | aristaless related homeobox [Source:HGNC Symbol;Acc:HGNC:18060] |

| | | | | | |
|---|---|---|---|---|---|
| ENSG00000006555 | HGLibA_52074 | 200 | 0.841605526 | TTC22 | tetratricopeptide repeat domain 22 [Source:HGNC Symbol;Acc:HGNC:26067] |
| ENSG00000011523 | HGLibA_09155 | 200 | 0.841605526 | CEP68 | centrosomal protein 68 [Source:HGNC Symbol;Acc:HGNC:29076] |
| ENSG00000069667 | HGLibA_41774 | 200 | 0.841605526 | RORA | RAR related orphan receptor A [Source:HGNC Symbol;Acc:HGNC:10258] |
| ENSG00000077935 | HGLibA_45644 | 200 | 0.841605526 | SMC1B | structural maintenance of chromosomes 1B [Source:HGNC Symbol;Acc:HGNC:11112] |
| ENSG00000085377 | HGLibA_38436 | 200 | 0.841605526 | PREP | prolyl endopeptidase [Source:HGNC Symbol;Acc:HGNC:9358] |
| ENSG00000096654 | HGLibA_55547 | 200 | 0.841605526 | ZNF184 | zinc finger protein 184 [Source:HGNC Symbol;Acc:HGNC:12975] |
| ENSG00000100038 | HGLibA_51003 | 200 | 0.841605526 | TOP3B | DNA topoisomerase III beta [Source:HGNC Symbol;Acc:HGNC:11993] |
| ENSG00000100196 | HGLibA_24485 | 200 | 0.841605526 | KDELR3 | KDEL endoplasmic reticulum protein retention receptor 3 [Source:HGNC Symbol;Acc:HGNC:6306] |
| ENSG00000100207 | HGLibA_48769 | 200 | 0.841605526 | TCF20 | transcription factor 20 [Source:HGNC Symbol;Acc:HGNC:11631] |
| ENSG00000105141 | HGLibA_07305 | 200 | 0.841605526 | CASP14 | caspase 14 [Source:HGNC Symbol;Acc:HGNC:1502] |
| ENSG00000105737 | HGLibA_20294 | 200 | 0.841605526 | GRIK5 | glutamate ionotropic receptor kainate type subunit 5 [Source:HGNC Symbol;Acc:HGNC:4583] |
| ENSG00000106541 | HGLibA_01243 | 200 | 0.841605526 | AGR2 | protein disulphide isomerase family member [Source:HGNC Symbol;Acc:HGNC:328]" |
| ENSG00000109436 | HGLibA_48571 | 200 | 0.841605526 | TBC1D9 | TBC1 domain family member 9 [Source:HGNC Symbol;Acc:HGNC:21710] |
| ENSG00000109684 | HGLibA_10086 | 200 | 0.841605526 | CLNK | cytokine dependent hematopoietic cell linker [Source:HGNC Symbol;Acc:HGNC:17438] |
| ENSG00000111305 | HGLibA_20418 | 200 | 0.841605526 | GSG1 | germ cell associated 1 [Source:HGNC Symbol;Acc:HGNC:19716] |
| ENSG00000112584 | HGLibA_16061 | 200 | 0.841605526 | FAM120B | family with sequence similarity 120B [Source:HGNC Symbol;Acc:HGNC:21109] |
| ENSG00000115109 | HGLibA_15256 | 200 | 0.841605526 | EPB41L5 | erythrocyte membrane protein band 4.1 like 5 [Source:HGNC Symbol;Acc:HGNC:19819] |
| ENSG00000116191 | HGLibA_40172 | 200 | 0.841605526 | RALGPS2 | Ral GEF with PH domain and SH3 binding motif 2 [Source:HGNC Symbol;Acc:HGNC:30279] |
| ENSG00000119397 | HGLibA_10437 | 200 | 0.841605526 | CNTRL | centriolin [Source:HGNC Symbol;Acc:HGNC:1858] |
| ENSG00000121742 | HGLibA_19172 | 200 | 0.841605526 | GJB6 | gap junction protein beta 6 [Source:HGNC Symbol;Acc:HGNC:4288] |
| ENSG00000124615 | HGLibA_29611 | 200 | 0.841605526 | MOCS1 | molybdenum cofactor synthesis 1 [Source:HGNC Symbol;Acc:HGNC:7190] |
| ENSG00000125967 | HGLibA_31590 | 200 | 0.841605526 | NECAB3 | N-terminal EF-hand calcium binding protein 3 [Source:HGNC Symbol;Acc:HGNC:15851] |
| ENSG00000128000 | HGLibA_56734 | 200 | 0.841605526 | ZNF780B | zinc finger protein 780B [Source:HGNC Symbol;Acc:HGNC:33109] |
| ENSG00000129455 | HGLibA_25269 | 200 | 0.841605526 | KLK8 | kallikrein related peptidase 8 [Source:HGNC Symbol;Acc:HGNC:6369] |
| ENSG00000130045 | HGLibA_33146 | 200 | 0.841605526 | NXNL2 | nucleoredoxin like 2 [Source:HGNC Symbol;Acc:HGNC:30482] |
| ENSG00000130538 | HGLibA_33569 | 200 | 0.841605526 | OR11H1 | olfactory receptor family 11 subfamily H member 1 [Source:HGNC Symbol;Acc:HGNC:15404] |
| ENSG00000130811 | HGLibA_14783 | 200 | 0.841605526 | EIF3G | eukaryotic translation initiation factor 3 subunit G [Source:HGNC Symbol;Acc:HGNC:3274] |
| ENSG00000135346 | HGLibA_09295 | 200 | 0.841605526 | CGA | alpha polypeptide [Source:HGNC Symbol;Acc:HGNC:1885]" |
| ENSG00000136014 | HGLibA_53371 | 200 | 0.841605526 | USP44 | ubiquitin specific peptidase 44 [Source:HGNC Symbol;Acc:HGNC:20064] |
| ENSG00000136783 | HGLibA_31974 | 200 | 0.841605526 | NIPSNAP3A | nipsnap homolog 3A [Source:HGNC Symbol;Acc:HGNC:23619] |
| ENSG00000142303 | HGLibA_00793 | 200 | 0.841605526 | ADAMTS10 | ADAM metallopeptidase with thrombospondin type 1 motif 10 [Source:HGNC Symbol;Acc:HGNC:13201] |
| ENSG00000143942 | HGLibA_09338 | 200 | 0.841605526 | CHAC2 | ChaC cation transport regulator homolog 2 [Source:HGNC Symbol;Acc:HGNC:32363] |
| ENSG00000145390 | HGLibA_53397 | 200 | 0.841605526 | USP53 | ubiquitin specific peptidase 53 [Source:HGNC Symbol;Acc:HGNC:29255] |
| ENSG00000146378 | HGLibA_48126 | 200 | 0.841605526 | TAAR2 | trace amine associated receptor 2 (gene/pseudogene) [Source:HGNC Symbol;Acc:HGNC:4514] |
| ENSG00000146674 | HGLibA_22821 | 200 | 0.841605526 | IGFBP3 | insulin like growth factor binding protein 3 [Source:HGNC Symbol;Acc:HGNC:5472] |
| ENSG00000147439 | HGLibA_04511 | 200 | 0.841605526 | BIN3 | bridging integrator 3 [Source:HGNC Symbol;Acc:HGNC:1054] |
| ENSG00000147853 | HGLibA_01382 | 200 | 0.841605526 | AK3 | adenylate kinase 3 [Source:HGNC Symbol;Acc:HGNC:17376] |
| ENSG00000151151 | HGLibA_23458 | 200 | 0.841605526 | IPMK | inositol polyphosphate multikinase [Source:HGNC Symbol;Acc:HGNC:20739] |
| ENSG00000158423 | HGLibA_41229 | 200 | 0.841605526 | RIBC1 | RIB43A domain with coiled-coils 1 [Source:HGNC Symbol;Acc:HGNC:26537] |
| ENSG00000163558 | HGLibA_38553 | 200 | 0.841605526 | PRKCI | protein kinase C iota [Source:HGNC Symbol;Acc:HGNC:9404] |
| ENSG00000163803 | HGLibA_36969 | 200 | 0.841605526 | PLB1 | phospholipase B1 [Source:HGNC Symbol;Acc:HGNC:30041] |
| ENSG00000164107 | HGLibA_20830 | 200 | 0.841605526 | HAND2 | heart and neural crest derivatives expressed 2 [Source:HGNC Symbol;Acc:HGNC:4808] |
| ENSG00000164733 | HGLibA_11693 | 200 | 0.841605526 | CTSB | cathepsin B [Source:HGNC Symbol;Acc:HGNC:2527] |
| ENSG00000167244 | HGLibA_22796 | 200 | 0.841605526 | IGF2 | insulin like growth factor 2 [Source:HGNC Symbol;Acc:HGNC:5466] |
| ENSG00000167394 | HGLibA_56493 | 200 | 0.841605526 | ZNF668 | zinc finger protein 668 [Source:HGNC Symbol;Acc:HGNC:25821] |
| ENSG00000167634 | HGLibA_32130 | 200 | 0.841605526 | NLRP7 | NLR family pyrin domain containing 7 [Source:HGNC Symbol;Acc:HGNC:22947] |
| ENSG00000167992 | HGLibA_53930 | 200 | 0.841605526 | VWCE | von Willebrand factor C and EGF domains [Source:HGNC Symbol;Acc:HGNC:26487] |
| ENSG00000168528 | HGLibA_43488 | 200 | 0.841605526 | SERINC2 | serine incorporator 2 [Source:HGNC Symbol;Acc:HGNC:23231] |
| ENSG00000175768 | HGLibA_50974 | 200 | 0.841605526 | TOMM5 | translocase of outer mitochondrial membrane 5 [Source:HGNC Symbol;Acc:HGNC:31369] |
| ENSG00000176435 | HGLibA_09933 | 200 | 0.841605526 | CLEC14A | C-type lectin domain containing 14A [Source:HGNC Symbol;Acc:HGNC:19832] |
| ENSG00000177990 | HGLibA_13907 | 200 | 0.841605526 | DPY19L2 | dpy-19 like 2 [Source:HGNC Symbol;Acc:HGNC:19414] |
| ENSG00000183783 | HGLibA_24467 | 200 | 0.841605526 | KCTD8 | potassium channel tetramerization domain containing 8 [Source:HGNC Symbol;Acc:HGNC:22394] |
| ENSG00000184216 | HGLibA_23552 | 200 | 0.841605526 | IRAK1 | interleukin 1 receptor associated kinase 1 [Source:HGNC Symbol;Acc:HGNC:6112] |
| ENSG00000185519 | HGLibA_16112 | 200 | 0.841605526 | FAM131C | family with sequence similarity 131 member C [Source:HGNC Symbol;Acc:HGNC:26717] |
| ENSG00000185926 | HGLibA_33959 | 200 | 0.841605526 | OR4C46 | olfactory receptor family 4 subfamily C member 46 [Source:HGNC Symbol;Acc:HGNC:31271] |
| ENSG00000197790 | HGLibA_34190 | 200 | 0.841605526 | OR52M1 | olfactory receptor family 52 subfamily M member 1 [Source:HGNC Symbol;Acc:HGNC:15225] |
| ENSG00000198890 | HGLibA_38631 | 200 | 0.841605526 | PRMT6 | protein arginine methyltransferase 6 [Source:HGNC Symbol;Acc:HGNC:18241] |
| ENSG00000198931 | HGLibA_02573 | 200 | 0.841605526 | APRT | adenine phosphoribosyltransferase [Source:HGNC Symbol;Acc:HGNC:626] |
| ENSG00000204099 | HGLibA_31719 | 200 | 0.841605526 | NEU4 | neuraminidase 4 [Source:HGNC Symbol;Acc:HGNC:21328] |
| ENSG00000205899 | HGLibA_04471 | 200 | 0.841605526 | BHLHA9 | basic helix-loop-helix family member a9 [Source:HGNC Symbol;Acc:HGNC:35126] |
| ENSG00000215356 | HGLibA_56591 | 200 | 0.841605526 | ZNF705B | zinc finger protein 705B [Source:HGNC Symbol;Acc:HGNC:32284] |
| ENSG00000215695 | HGLibA_42307 | 200 | 0.841605526 | RSC1A1 | regulator of solute carriers 1 [Source:HGNC Symbol;Acc:HGNC:10458] |
| ENSG00000267534 | HGLibA_42595 | 200 | 0.841605526 | S1PR2 | sphingosine-1-phosphate receptor 2 [Source:HGNC Symbol;Acc:HGNC:3169] |
| ENSG00000271303 | HGLibA_47096 | 200 | 0.841605526 | SRXN1 | sulfiredoxin 1 [Source:HGNC Symbol;Acc:HGNC:16132] |
| ENSG00000278570 | HGLibA_32606 | 200 | 0.841605526 | NR2E3 | nuclear receptor subfamily 2 group E member 3 [Source:HGNC Symbol;Acc:HGNC:7974] |
| hsa-mir-3116-1 | HGLibA_58503 | 200 | 0.841605526 | hsa-mir-3116-1 | - |
| hsa-mir-3683 | HGLibA_59285 | 200 | 0.841605526 | hsa-mir-3683 | - |
| hsa-mir-3692 | HGLibA_59359 | 200 | 0.841605526 | hsa-mir-3692 | - |
| hsa-mir-6084 | HGLibA_62595 | 200 | 0.841605526 | hsa-mir-6084 | - |
| hsa-mir-7161 | HGLibA_63871 | 200 | 0.841605526 | hsa-mir-7161 | - |
| hsa-mir-7704 | HGLibA_63953 | 200 | 0.841605526 | hsa-mir-7704 | - |
| KIAA1715 | HGLibA_24808 | 200 | 0.841605526 | KIAA1715 | - |
| C12orf79 | HGLibA_05307 | 199 | 0.831654489 | C12orf79 | - |
| C2orf71 | HGLibA_06173 | 199 | 0.831654489 | C2orf71 | - |
| C3orf67 | HGLibA_06259 | 199 | 0.831654489 | C3orf67 | - |
| CCDC135 | HGLibA_07601 | 199 | 0.831654489 | CCDC135 | - |
| ENSG00000008513 | HGLibA_47238 | 199 | 0.831654489 | ST3GAL1 | 3-sialyltransferase 1 [Source:HGNC Symbol;Acc:HGNC:10862]" |
| ENSG00000009780 | HGLibA_16721 | 199 | 0.831654489 | FAM76A | family with sequence similarity 76 member A [Source:HGNC Symbol;Acc:HGNC:28530] |
| ENSG00000060656 | HGLibA_39541 | 199 | 0.831654489 | PTPRU | receptor type U [Source:HGNC Symbol;Acc:HGNC:9683]" |
| ENSG00000064726 | HGLibA_04893 | 199 | 0.831654489 | BTBD1 | BTB domain containing 1 [Source:HGNC Symbol;Acc:HGNC:1120] |
| ENSG00000064989 | HGLibA_07006 | 199 | 0.831654489 | CALCRL | calcitonin receptor like receptor [Source:HGNC Symbol;Acc:HGNC:16709] |
| ENSG00000072182 | HGLibA_03307 | 199 | 0.831654489 | ASIC4 | acid sensing ion channel subunit family member 4 [Source:HGNC Symbol;Acc:HGNC:21263] |
| ENSG00000083535 | HGLibA_36518 | 199 | 0.831654489 | PIBF1 | progesterone immunomodulatory binding factor 1 [Source:HGNC Symbol;Acc:HGNC:23352] |
| ENSG00000100228 | HGLibA_39892 | 199 | 0.831654489 | RAB36 | member RAS oncogene family [Source:HGNC Symbol;Acc:HGNC:9775]" |
| ENSG00000101542 | HGLibA_08662 | 199 | 0.831654489 | CDH20 | cadherin 20 [Source:HGNC Symbol;Acc:HGNC:1760] |
| ENSG00000104290 | HGLibA_18270 | 199 | 0.831654489 | FZD3 | frizzled class receptor 3 [Source:HGNC Symbol;Acc:HGNC:4041] |
| ENSG00000104899 | HGLibA_01785 | 199 | 0.831654489 | AMH | anti-Mullerian hormone [Source:HGNC Symbol;Acc:HGNC:464] |
| ENSG00000111540 | HGLibA_39965 | 199 | 0.831654489 | RAB5B | member RAS oncogene family [Source:HGNC Symbol;Acc:HGNC:9784]" |
| ENSG00000113100 | HGLibA_08694 | 199 | 0.831654489 | CDH9 | cadherin 9 [Source:HGNC Symbol;Acc:HGNC:1768] |
| ENSG00000115648 | HGLibA_29432 | 199 | 0.831654489 | MLPH | melanophilin [Source:HGNC Symbol;Acc:HGNC:29643] |
| ENSG00000116017 | HGLibA_02892 | 199 | 0.831654489 | ARID3A | AT-rich interaction domain 3A [Source:HGNC Symbol;Acc:HGNC:3031] |
| ENSG00000117519 | HGLibA_10312 | 199 | 0.831654489 | CNN3 | calponin 3 [Source:HGNC Symbol;Acc:HGNC:2157] |
| ENSG00000120658 | HGLibA_15159 | 199 | 0.831654489 | ENOX1 | ecto-NOX disulfide-thiol exchanger 1 [Source:HGNC Symbol;Acc:HGNC:25474] |
| ENSG00000121361 | HGLibA_24287 | 199 | 0.831654489 | KCNJ8 | potassium voltage-gated channel subfamily J member 8 [Source:HGNC Symbol;Acc:HGNC:6269] |
| ENSG00000125812 | HGLibA_20715 | 199 | 0.831654489 | GZF1 | GDNF inducible zinc finger protein 1 [Source:HGNC Symbol;Acc:HGNC:15808] |
| ENSG00000127364 | HGLibA_48398 | 199 | 0.831654489 | TAS2R4 | taste 2 receptor member 4 [Source:HGNC Symbol;Acc:HGNC:14911] |
| ENSG00000130653 | HGLibA_37423 | 199 | 0.831654489 | PNPLA7 | patatin like phospholipase domain containing 7 [Source:HGNC Symbol;Acc:HGNC:24768] |
| ENSG00000131725 | HGLibA_54094 | 199 | 0.831654489 | WDR44 | WD repeat domain 44 [Source:HGNC Symbol;Acc:HGNC:30512] |
| ENSG00000135373 | HGLibA_14677 | 199 | 0.831654489 | EHF | ETS homologous factor [Source:HGNC Symbol;Acc:HGNC:3246] |
| ENSG00000135519 | HGLibA_24216 | 199 | 0.831654489 | KCNH3 | potassium voltage-gated channel subfamily H member 3 [Source:HGNC Symbol;Acc:HGNC:6252] |
| ENSG00000138030 | HGLibA_24575 | 199 | 0.831654489 | KHK | ketohexokinase [Source:HGNC Symbol;Acc:HGNC:6315] |
| ENSG00000139168 | HGLibA_55053 | 199 | 0.831654489 | ZCRB1 | zinc finger CCHC-type and RNA binding motif containing 1 [Source:HGNC Symbol;Acc:HGNC:29620] |
| ENSG00000142409 | HGLibA_56756 | 199 | 0.831654489 | ZNF787 | zinc finger protein 787 [Source:HGNC Symbol;Acc:HGNC:26998] |
| ENSG00000143341 | HGLibA_21675 | 199 | 0.831654489 | HMCN1 | hemicentin 1 [Source:HGNC Symbol;Acc:HGNC:19194] |
| ENSG00000144057 | HGLibA_47260 | 199 | 0.831654489 | ST6GAL2 | 6-sialyltransferase 2 [Source:HGNC Symbol;Acc:HGNC:10861]" |
| ENSG00000145020 | HGLibA_01836 | 199 | 0.831654489 | AMT | aminomethyltransferase [Source:HGNC Symbol;Acc:HGNC:473] |
| ENSG00000151240 | HGLibA_13252 | 199 | 0.831654489 | DIP2C | disco interacting protein 2 homolog C [Source:HGNC Symbol;Acc:HGNC:29150] |
| ENSG00000151348 | HGLibA_15806 | 199 | 0.831654489 | EXT2 | exostosin glycosyltransferase 2 [Source:HGNC Symbol;Acc:HGNC:3513] |
| ENSG00000156395 | HGLibA_46188 | 199 | 0.831654489 | SORCS3 | sortilin related VPS10 domain containing receptor 3 [Source:HGNC Symbol;Acc:HGNC:16699] |
| ENSG00000159650 | HGLibA_53152 | 199 | 0.831654489 | UROC1 | urocanate hydratase 1 [Source:HGNC Symbol;Acc:HGNC:26444] |
| ENSG00000160007 | HGLibA_02764 | 199 | 0.831654489 | ARHGAP35 | Rho GTPase activating protein 35 [Source:HGNC Symbol;Acc:HGNC:4591] |
| ENSG00000161281 | HGLibA_10857 | 199 | 0.831654489 | COX7A1 | cytochrome c oxidase subunit 7A1 [Source:HGNC Symbol;Acc:HGNC:2287] |
| ENSG00000163534 | HGLibA_17225 | 199 | 0.831654489 | FCRL1 | Fc receptor like 1 [Source:HGNC Symbol;Acc:HGNC:18509] |
| ENSG00000164062 | HGLibA_02404 | 199 | 0.831654489 | APEH | acylaminoacyl-peptide hydrolase [Source:HGNC Symbol;Acc:HGNC:586] |
| ENSG00000164399 | HGLibA_23126 | 199 | 0.831654489 | IL3 | interleukin 3 [Source:HGNC Symbol;Acc:HGNC:6011] |
| ENSG00000164597 | HGLibA_10483 | 199 | 0.831654489 | COG5 | component of oligomeric golgi complex 5 [Source:HGNC Symbol;Acc:HGNC:14857] |
| ENSG00000165071 | HGLibA_50505 | 199 | 0.831654489 | TMEM71 | transmembrane protein 71 [Source:HGNC Symbol;Acc:HGNC:26572] |
| ENSG00000168772 | HGLibA_11972 | 199 | 0.831654489 | CXXC4 | CXXC finger protein 4 [Source:HGNC Symbol;Acc:HGNC:24593] |
| ENSG00000168843 | HGLibA_18110 | 199 | 0.831654489 | FSTL5 | follistatin like 5 [Source:HGNC Symbol;Acc:HGNC:21386] |
| ENSG00000169895 | HGLibA_47908 | 199 | 0.831654489 | SYAP1 | synapse associated protein 1 [Source:HGNC Symbol;Acc:HGNC:16273] |
| ENSG00000170382 | HGLibA_27474 | 199 | 0.831654489 | LRRN2 | leucine rich repeat neuronal 2 [Source:HGNC Symbol;Acc:HGNC:16914] |
| ENSG00000170634 | HGLibA_00713 | 199 | 0.831654489 | ACYP2 | acylphosphatase 2 [Source:HGNC Symbol;Acc:HGNC:180] |
| ENSG00000171051 | HGLibA_17962 | 199 | 0.831654489 | FPR1 | formyl peptide receptor 1 [Source:HGNC Symbol;Acc:HGNC:3826] |
| ENSG00000171552 | HGLibA_04301 | 199 | 0.831654489 | BCL2L1 | BCL2 like 1 [Source:HGNC Symbol;Acc:HGNC:992] |
| ENSG00000174038 | HGLibA_06678 | 199 | 0.831654489 | C9orf131 | chromosome 9 open reading frame 131 [Source:HGNC Symbol;Acc:HGNC:31418] |
| ENSG00000174944 | HGLibA_34881 | 199 | 0.831654489 | P2RY14 | purinergic receptor P2Y14 [Source:HGNC Symbol;Acc:HGNC:16442] |
| ENSG00000177943 | HGLibA_28040 | 199 | 0.831654489 | MAMDC4 | MAM domain containing 4 [Source:HGNC Symbol;Acc:HGNC:24083] |
| ENSG00000179083 | HGLibA_16118 | 199 | 0.831654489 | FAM133A | family with sequence similarity 133 member A [Source:HGNC Symbol;Acc:HGNC:26748] |
| ENSG00000179919 | HGLibA_33476 | 199 | 0.831654489 | OR10A7 | olfactory receptor family 10 subfamily A member 7 [Source:HGNC Symbol;Acc:HGNC:15329] |
| ENSG00000182931 | HGLibA_54249 | 199 | 0.831654489 | WFDC10B | WAP four-disulfide core domain 10B [Source:HGNC Symbol;Acc:HGNC:20479] |
| ENSG00000183378 | HGLibA_34795 | 199 | 0.831654489 | OVCH2 | ovochymase 2 (gene/pseudogene) [Source:HGNC Symbol;Acc:HGNC:29970] |
| ENSG00000183624 | HGLibA_21673 | 199 | 0.831654489 | HMCES | ES cell specific [Source:HGNC Symbol;Acc:HGNC:24446]" |
| ENSG00000185359 | HGLibA_21247 | 199 | 0.831654489 | HGS | hepatocyte growth factor-regulated tyrosine kinase substrate [Source:HGNC Symbol;Acc:HGNC:4897] |
| ENSG00000188581 | HGLibA_25580 | 199 | 0.831654489 | KRTAP1-1 | keratin associated protein 1-1 [Source:HGNC Symbol;Acc:HGNC:16772] |
| ENSG00000196932 | HGLibA_50347 | 199 | 0.831654489 | TMEM26 | transmembrane protein 26 [Source:HGNC Symbol;Acc:HGNC:28550] |

| | | | | | |
|---|---|---|---|---|---|
| ENSG00000197506 | HGLibA_44836 | 199 | 0.831654489 | SLC28A3 | solute carrier family 28 member 3 [Source:HGNC Symbol;Acc:HGNC:16484] |
| ENSG00000204571 | HGLibA_25789 | 199 | 0.831654489 | KRTAP5-11 | keratin associated protein 5-11 [Source:HGNC Symbol;Acc:HGNC:23606] |
| ENSG00000205352 | HGLibA_38776 | 199 | 0.831654489 | PRR13 | proline rich 13 [Source:HGNC Symbol;Acc:HGNC:24528] |
| ENSG00000214087 | HGLibA_02948 | 199 | 0.831654489 | ARL16 | ADP ribosylation factor like GTPase 16 [Source:HGNC Symbol;Acc:HGNC:27902] |
| ENSG00000214376 | HGLibA_53872 | 199 | 0.831654489 | VSTM5 | V-set and transmembrane domain containing 5 [Source:HGNC Symbol;Acc:HGNC:34443] |
| ENSG00000221910 | HGLibA_33790 | 199 | 0.831654489 | OR2F2 | olfactory receptor family 2 subfamily F member 2 [Source:HGNC Symbol;Acc:HGNC:8247] |
| ENSG00000227268 | HGLibA_25279 | 199 | 0.831654489 | KLLN | p53 regulated DNA replication inhibitor [Source:HGNC Symbol;Acc:HGNC:37212]" |
| ENSG00000269502 | HGLibA_13434 | 199 | 0.831654489 | DMRTC1 | DMRT like family C1 [Source:HGNC Symbol;Acc:HGNC:13910] |
| FLJ44313 | HGLibA_17631 | 199 | 0.831654489 | FLJ44313 | - |
| HIST1H3D | HGLibA_21466 | 199 | 0.831654489 | HIST1H3D | - |
| hsa-mir-1286 | HGLibA_57607 | 199 | 0.831654489 | hsa-mir-1286 | - |
| hsa-mir-4532 | HGLibA_60586 | 199 | 0.831654489 | hsa-mir-4532 | - |
| hsa-mir-4699 | HGLibA_60904 | 199 | 0.831654489 | hsa-mir-4699 | - |
| hsa-mir-4773-1 | HGLibA_61191 | 199 | 0.831654489 | hsa-mir-4773-1 | - |
| hsa-mir-5692a-1 | HGLibA_62289 | 199 | 0.831654489 | hsa-mir-5692a-1 | - |
| hsa-mir-6718 | HGLibA_63033 | 199 | 0.831654489 | hsa-mir-6718 | - |
| hsa-mir-6765 | HGLibA_63222 | 199 | 0.831654489 | hsa-mir-6765 | - |
| IL8 | HGLibA_23196 | 199 | 0.831654489 | IL8 | - |
| NAT6 | HGLibA_31180 | 199 | 0.831654489 | NAT6 | - |
| 5-Sep | HGLibA_43444 | 199 | 0.831654489 | 5-Sep | - |
| SMEK1 | HGLibA_45691 | 199 | 0.831654489 | SMEK1 | - |
| C11orf70 | HGLibA_05176 | 198 | 0.821703451 | C11orf70 | - |
| ENSG00000012817 | HGLibA_24531 | 198 | 0.821703451 | KDM5D | lysine demethylase 5D [Source:HGNC Symbol;Acc:HGNC:11115] |
| ENSG00000054803 | HGLibA_07415 | 198 | 0.821703451 | CBLN4 | cerebellin 4 precursor [Source:HGNC Symbol;Acc:HGNC:16231] |
| ENSG00000065882 | HGLibA_48460 | 198 | 0.821703451 | TBC1D1 | TBC1 domain family member 1 [Source:HGNC Symbol;Acc:HGNC:11578] |
| ENSG00000065989 | HGLibA_35787 | 198 | 0.821703451 | PDE4A | phosphodiesterase 4A [Source:HGNC Symbol;Acc:HGNC:8780] |
| ENSG00000076053 | HGLibA_40580 | 198 | 0.821703451 | RBM7 | RNA binding motif protein 7 [Source:HGNC Symbol;Acc:HGNC:9904] |
| ENSG00000077616 | HGLibA_31008 | 198 | 0.821703451 | NAALAD2 | N-acetylated alpha-linked acidic dipeptidase 2 [Source:HGNC Symbol;Acc:HGNC:14526] |
| ENSG00000078403 | HGLibA_29411 | 198 | 0.821703451 | MLLT10 | histone lysine methyltransferase DOT1L cofactor [Source:HGNC Symbol;Acc:HGNC:16063]" |
| ENSG00000081237 | HGLibA_39490 | 198 | 0.821703451 | PTPRC | receptor type C [Source:HGNC Symbol;Acc:HGNC:9666]" |
| ENSG00000082497 | HGLibA_43614 | 198 | 0.821703451 | SERTAD4 | SERTA domain containing 4 [Source:HGNC Symbol;Acc:HGNC:25236] |
| ENSG00000090372 | HGLibA_47600 | 198 | 0.821703451 | STRN4 | striatin 4 [Source:HGNC Symbol;Acc:HGNC:15721] |
| ENSG00000090674 | HGLibA_28636 | 198 | 0.821703451 | MCOLN1 | mucolipin 1 [Source:HGNC Symbol;Acc:HGNC:13356] |
| ENSG00000094804 | HGLibA_08592 | 198 | 0.821703451 | CDC6 | cell division cycle 6 [Source:HGNC Symbol;Acc:HGNC:1744] |
| ENSG00000096696 | HGLibA_14037 | 198 | 0.821703451 | DSP | desmoplakin [Source:HGNC Symbol;Acc:HGNC:3052] |
| ENSG00000100601 | HGLibA_01645 | 198 | 0.821703451 | ALKBH1 | histone H2A dioxygenase [Source:HGNC Symbol;Acc:HGNC:17911]" |
| ENSG00000100767 | HGLibA_35132 | 198 | 0.821703451 | PAPLN | proteoglycan like sulfated glycoprotein [Source:HGNC Symbol;Acc:HGNC:19262]" |
| ENSG00000101825 | HGLibA_30622 | 198 | 0.821703451 | MXRA5 | matrix remodeling associated 5 [Source:HGNC Symbol;Acc:HGNC:7539] |
| ENSG00000102098 | HGLibA_42961 | 198 | 0.821703451 | SCML2 | Scm polycomb group protein like 2 [Source:HGNC Symbol;Acc:HGNC:10581] |
| ENSG00000103196 | HGLibA_11178 | 198 | 0.821703451 | CRISPLD2 | cysteine rich secretory protein LCCL domain containing 2 [Source:HGNC Symbol;Acc:HGNC:25248] |
| ENSG00000105438 | HGLibA_24478 | 198 | 0.821703451 | KDELR1 | KDEL endoplasmic reticulum protein retention receptor 1 [Source:HGNC Symbol;Acc:HGNC:6304] |
| ENSG00000106436 | HGLibA_30748 | 198 | 0.821703451 | MYL10 | myosin light chain 10 [Source:HGNC Symbol;Acc:HGNC:29825] |
| ENSG00000111261 | HGLibA_28099 | 198 | 0.821703451 | MANSC1 | MANSC domain containing 1 [Source:HGNC Symbol;Acc:HGNC:25505] |
| ENSG00000113924 | HGLibA_21238 | 198 | 0.821703451 | HGD | 2-dioxygenase [Source:HGNC Symbol;Acc:HGNC:4892]" |
| ENSG00000116977 | HGLibA_26378 | 198 | 0.821703451 | LGALS8 | galectin 8 [Source:HGNC Symbol;Acc:HGNC:6569] |
| ENSG00000118096 | HGLibA_22761 | 198 | 0.821703451 | IFT46 | intraflagellar transport 46 [Source:HGNC Symbol;Acc:HGNC:26146] |
| ENSG00000120278 | HGLibA_37101 | 198 | 0.821703451 | PLEKHG1 | pleckstrin homology and RhoGEF domain containing G1 [Source:HGNC Symbol;Acc:HGNC:20884] |
| ENSG00000121318 | HGLibA_48366 | 198 | 0.821703451 | TAS2R10 | taste 2 receptor member 10 [Source:HGNC Symbol;Acc:HGNC:14918] |
| ENSG00000125845 | HGLibA_04600 | 198 | 0.821703451 | BMP2 | bone morphogenetic protein 2 [Source:HGNC Symbol;Acc:HGNC:1069] |
| ENSG00000128276 | HGLibA_40936 | 198 | 0.821703451 | RFPL3 | ret finger protein like 3 [Source:HGNC Symbol;Acc:HGNC:9980] |
| ENSG00000131196 | HGLibA_31774 | 198 | 0.821703451 | NFATC1 | nuclear factor of activated T cells 1 [Source:HGNC Symbol;Acc:HGNC:7775] |
| ENSG00000131873 | HGLibA_09656 | 198 | 0.821703451 | CHSY1 | chondroitin sulfate synthase 1 [Source:HGNC Symbol;Acc:HGNC:17198] |
| ENSG00000131899 | HGLibA_26647 | 198 | 0.821703451 | LLGL1 | scribble cell polarity complex component [Source:HGNC Symbol;Acc:HGNC:6628]" |
| ENSG00000132164 | HGLibA_45291 | 198 | 0.821703451 | SLC6A11 | solute carrier family 6 member 11 [Source:HGNC Symbol;Acc:HGNC:11044] |
| ENSG00000132965 | HGLibA_01690 | 198 | 0.821703451 | ALOX5AP | arachidonate 5-lipoxygenase activating protein [Source:HGNC Symbol;Acc:HGNC:436] |
| ENSG00000133805 | HGLibA_01829 | 198 | 0.821703451 | AMPD3 | adenosine monophosphate deaminase 3 [Source:HGNC Symbol;Acc:HGNC:470] |
| ENSG00000134668 | HGLibA_46744 | 198 | 0.821703451 | SPOCD1 | SPOC domain containing 1 [Source:HGNC Symbol;Acc:HGNC:26338] |
| ENSG00000135406 | HGLibA_38749 | 198 | 0.821703451 | PRPH | peripherin [Source:HGNC Symbol;Acc:HGNC:9461] |
| ENSG00000136099 | HGLibA_35417 | 198 | 0.821703451 | PCDH8 | protocadherin 8 [Source:HGNC Symbol;Acc:HGNC:8660] |
| ENSG00000139178 | HGLibA_05973 | 198 | 0.821703451 | C1RL | complement C1r subcomponent like [Source:HGNC Symbol;Acc:HGNC:21265] |
| ENSG00000139722 | HGLibA_53778 | 198 | 0.821703451 | VPS37B | ESCRT-I subunit [Source:HGNC Symbol;Acc:HGNC:25754]" |
| ENSG00000142677 | HGLibA_23091 | 198 | 0.821703451 | IL22RA1 | interleukin 22 receptor subunit alpha 1 [Source:HGNC Symbol;Acc:HGNC:13700] |
| ENSG00000143337 | HGLibA_51018 | 198 | 0.821703451 | TOR1AIP1 | torsin 1A interacting protein 1 [Source:HGNC Symbol;Acc:HGNC:29456] |
| ENSG00000144488 | HGLibA_15577 | 198 | 0.821703451 | ESPNL | espin like [Source:HGNC Symbol;Acc:HGNC:27937] |
| ENSG00000151033 | HGLibA_27788 | 198 | 0.821703451 | LYZL2 | lysozyme like 2 [Source:HGNC Symbol;Acc:HGNC:29613] |
| ENSG00000154240 | HGLibA_09097 | 198 | 0.821703451 | CEP112 | centrosomal protein 112 [Source:HGNC Symbol;Acc:HGNC:28514] |
| ENSG00000157657 | HGLibA_56397 | 198 | 0.821703451 | ZNF618 | zinc finger protein 618 [Source:HGNC Symbol;Acc:HGNC:29416] |
| ENSG00000162341 | HGLibA_51108 | 198 | 0.821703451 | TPCN2 | two pore segment channel 2 [Source:HGNC Symbol;Acc:HGNC:20820] |
| ENSG00000165496 | HGLibA_41858 | 198 | 0.821703451 | RPL10L | ribosomal protein L10 like [Source:HGNC Symbol;Acc:HGNC:17976] |
| ENSG00000166159 | HGLibA_27502 | 198 | 0.821703451 | LRTM2 | leucine rich repeats and transmembrane domains 2 [Source:HGNC Symbol;Acc:HGNC:32443] |
| ENSG00000166477 | HGLibA_26307 | 198 | 0.821703451 | LEO1 | Paf1/RNA polymerase II complex component [Source:HGNC Symbol;Acc:HGNC:30401]" |
| ENSG00000167619 | HGLibA_49972 | 198 | 0.821703451 | TMEM145 | transmembrane protein 145 [Source:HGNC Symbol;Acc:HGNC:26912] |
| ENSG00000168778 | HGLibA_48874 | 198 | 0.821703451 | TCTN2 | tectonic family member 2 [Source:HGNC Symbol;Acc:HGNC:25774] |
| ENSG00000168824 | HGLibA_32756 | 198 | 0.821703451 | NSG1 | neuronal vesicle trafficking associated 1 [Source:HGNC Symbol;Acc:HGNC:18790] |
| ENSG00000169006 | HGLibA_32890 | 198 | 0.821703451 | NTSR2 | neurotensin receptor 2 [Source:HGNC Symbol;Acc:HGNC:8040] |
| ENSG00000169925 | HGLibA_04777 | 198 | 0.821703451 | BRD3 | bromodomain containing 3 [Source:HGNC Symbol;Acc:HGNC:1104] |
| ENSG00000172782 | HGLibA_15942 | 198 | 0.821703451 | FADS6 | fatty acid desaturase 6 [Source:HGNC Symbol;Acc:HGNC:30459] |
| ENSG00000172977 | HGLibA_24056 | 198 | 0.821703451 | KAT5 | lysine acetyltransferase 5 [Source:HGNC Symbol;Acc:HGNC:5275] |
| ENSG00000172995 | HGLibA_03100 | 198 | 0.821703451 | ARPP21 | cAMP regulated phosphoprotein 21 [Source:HGNC Symbol;Acc:HGNC:16968] |
| ENSG00000177383 | HGLibA_27987 | 198 | 0.821703451 | MAGEF1 | MAGE family member F1 [Source:HGNC Symbol;Acc:HGNC:29639] |
| ENSG00000177542 | HGLibA_44675 | 198 | 0.821703451 | SLC25A22 | solute carrier family 25 member 22 [Source:HGNC Symbol;Acc:HGNC:19954] |
| ENSG00000180537 | HGLibA_41585 | 198 | 0.821703451 | RNF182 | ring finger protein 182 [Source:HGNC Symbol;Acc:HGNC:28522] |
| ENSG00000183624 | HGLibA_21671 | 198 | 0.821703451 | HMCES | ES cell specific [Source:HGNC Symbol;Acc:HGNC:24446]" |
| ENSG00000196431 | HGLibA_11258 | 198 | 0.821703451 | CRYBA4 | crystallin beta A4 [Source:HGNC Symbol;Acc:HGNC:2396] |
| ENSG00000196539 | HGLibA_33872 | 198 | 0.821703451 | OR2T3 | olfactory receptor family 2 subfamily T member 3 [Source:HGNC Symbol;Acc:HGNC:14727] |
| ENSG00000196586 | HGLibA_30859 | 198 | 0.821703451 | MYO6 | myosin VI [Source:HGNC Symbol;Acc:HGNC:7605] |
| ENSG00000198128 | HGLibA_33824 | 198 | 0.821703451 | OR2L3 | olfactory receptor family 2 subfamily L member 3 [Source:HGNC Symbol;Acc:HGNC:15009] |
| ENSG00000198276 | HGLibA_52816 | 198 | 0.821703451 | UCKL1 | uridine-cytidine kinase 1 like 1 [Source:HGNC Symbol;Acc:HGNC:15938] |
| ENSG00000198814 | HGLibA_19196 | 198 | 0.821703451 | GK | glycerol kinase [Source:HGNC Symbol;Acc:HGNC:4289] |
| ENSG00000198824 | HGLibA_09354 | 198 | 0.821703451 | CHAMP1 | chromosome alignment maintaining phosphoprotein 1 [Source:HGNC Symbol;Acc:HGNC:20311] |
| ENSG00000203985 | HGLibA_26242 | 198 | 0.821703451 | LDLRAD1 | low density lipoprotein receptor class A domain containing 1 [Source:HGNC Symbol;Acc:HGNC:32069] |
| ENSG00000205426 | HGLibA_25559 | 198 | 0.821703451 | KRT81 | keratin 81 [Source:HGNC Symbol;Acc:HGNC:6458] |
| ENSG00000220201 | HGLibA_55304 | 198 | 0.821703451 | ZGLP1 | GATA-like protein 1 [Source:HGNC Symbol;Acc:HGNC:37245]" |
| ENSG00000225190 | HGLibA_37138 | 198 | 0.821703451 | PLEKHM1 | pleckstrin homology and RUN domain containing M1 [Source:HGNC Symbol;Acc:HGNC:29017] |
| ENSG00000232258 | HGLibA_49879 | 198 | 0.821703451 | TMEM114 | transmembrane protein 114 [Source:HGNC Symbol;Acc:HGNC:33227] |
| ENSG00000233608 | HGLibA_52377 | 198 | 0.821703451 | TWIST2 | twist family bHLH transcription factor 2 [Source:HGNC Symbol;Acc:HGNC:20670] |
| ENSG00000241186 | HGLibA_48883 | 198 | 0.821703451 | TDGF1 | teratocarcinoma-derived growth factor 1 [Source:HGNC Symbol;Acc:HGNC:11701] |
| ENSG00000255398 | HGLibA_20953 | 198 | 0.821703451 | HCAR3 | hydroxycarboxylic acid receptor 3 [Source:HGNC Symbol;Acc:HGNC:16824] |
| hsa-mir-1302-8 | HGLibA_57719 | 198 | 0.821703451 | hsa-mir-1302-8 | - |
| hsa-mir-3138 | HGLibA_58610 | 198 | 0.821703451 | hsa-mir-3138 | - |
| hsa-mir-4770 | HGLibA_61175 | 198 | 0.821703451 | hsa-mir-4770 | - |
| hsa-mir-8070 | HGLibA_64120 | 198 | 0.821703451 | hsa-mir-8070 | - |
| NRD1 | HGLibA_32664 | 198 | 0.821703451 | NRD1 | - |
| RAB7L1 | HGLibA_39983 | 198 | 0.821703451 | RAB7L1 | - |
| C2orf54 | HGLibA_06150 | 197 | 0.811752414 | C2orf54 | - |
| ENSG00000005448 | HGLibA_54124 | 197 | 0.811752414 | WDR54 | WD repeat domain 54 [Source:HGNC Symbol;Acc:HGNC:25770] |
| ENSG00000005893 | HGLibA_25980 | 197 | 0.811752414 | LAMP2 | lysosomal associated membrane protein 2 [Source:HGNC Symbol;Acc:HGNC:6501] |
| ENSG00000006071 | HGLibA_00185 | 197 | 0.811752414 | ABCC8 | ATP binding cassette subfamily C member 8 [Source:HGNC Symbol;Acc:HGNC:59] |
| ENSG00000011021 | HGLibA_09835 | 197 | 0.811752414 | CLCN6 | chloride voltage-gated channel 6 [Source:HGNC Symbol;Acc:HGNC:2024] |
| ENSG00000021645 | HGLibA_32739 | 197 | 0.811752414 | NRXN3 | neurexin 3 [Source:HGNC Symbol;Acc:HGNC:8010] |
| ENSG00000035720 | HGLibA_47343 | 197 | 0.811752414 | STAP1 | signal transducing adaptor family member 1 [Source:HGNC Symbol;Acc:HGNC:24133] |
| ENSG00000035928 | HGLibA_40901 | 197 | 0.811752414 | RFC1 | replication factor C subunit 1 [Source:HGNC Symbol;Acc:HGNC:9969] |
| ENSG00000063245 | HGLibA_15340 | 197 | 0.811752414 | EPN1 | epsin 1 [Source:HGNC Symbol;Acc:HGNC:21604] |
| ENSG00000068079 | HGLibA_22607 | 197 | 0.811752414 | IFI35 | interferon induced protein 35 [Source:HGNC Symbol;Acc:HGNC:5399] |
| ENSG00000070081 | HGLibA_32914 | 197 | 0.811752414 | NUCB2 | nucleobindin 2 [Source:HGNC Symbol;Acc:HGNC:8044] |
| ENSG00000070159 | HGLibA_39466 | 197 | 0.811752414 | PTPN3 | non-receptor type 3 [Source:HGNC Symbol;Acc:HGNC:9655]" |
| ENSG00000074201 | HGLibA_10090 | 197 | 0.811752414 | CLNS1A | chloride nucleotide-sensitive channel 1A [Source:HGNC Symbol;Acc:HGNC:2080] |
| ENSG00000076604 | HGLibA_51257 | 197 | 0.811752414 | TRAF4 | TNF receptor associated factor 4 [Source:HGNC Symbol;Acc:HGNC:12034] |
| ENSG00000090975 | HGLibA_36753 | 197 | 0.811752414 | PITPNM2 | phosphatidylinositol transfer protein membrane associated 2 [Source:HGNC Symbol;Acc:HGNC:21044] |
| ENSG00000095321 | HGLibA_11067 | 197 | 0.811752414 | CRAT | carnitine O-acetyltransferase [Source:HGNC Symbol;Acc:HGNC:2342] |
| ENSG00000095321 | HGLibA_11066 | 197 | 0.811752414 | CRAT | carnitine O-acetyltransferase [Source:HGNC Symbol;Acc:HGNC:2342] |
| ENSG00000100526 | HGLibA_08848 | 197 | 0.811752414 | CDKN3 | cyclin dependent kinase inhibitor 3 [Source:HGNC Symbol;Acc:HGNC:1791] |
| ENSG00000107807 | HGLibA_49669 | 197 | 0.811752414 | TLX1 | T cell leukemia homeobox 1 [Source:HGNC Symbol;Acc:HGNC:5056] |
| ENSG00000108244 | HGLibA_25454 | 197 | 0.811752414 | KRT23 | keratin 23 [Source:HGNC Symbol;Acc:HGNC:6438] |
| ENSG00000111245 | HGLibA_30758 | 197 | 0.811752414 | MYL2 | myosin light chain 2 [Source:HGNC Symbol;Acc:HGNC:7583] |
| ENSG00000111424 | HGLibA_53608 | 197 | 0.811752414 | VDR | vitamin D receptor [Source:HGNC Symbol;Acc:HGNC:12679] |
| ENSG00000112936 | HGLibA_06517 | 197 | 0.811752414 | C7 | complement C7 [Source:HGNC Symbol;Acc:HGNC:1346] |
| ENSG00000114251 | HGLibA_54372 | 197 | 0.811752414 | WNT5A | Wnt family member 5A [Source:HGNC Symbol;Acc:HGNC:12784] |
| ENSG00000121957 | HGLibA_20158 | 197 | 0.811752414 | GPSM2 | G protein signaling modulator 2 [Source:HGNC Symbol;Acc:HGNC:29501] |
| ENSG00000129151 | HGLibA_04202 | 197 | 0.811752414 | BBOX1 | gamma-butyrobetaine hydroxylase 1 [Source:HGNC Symbol;Acc:HGNC:964] |
| ENSG00000129682 | HGLibA_17371 | 197 | 0.811752414 | FGF13 | fibroblast growth factor 13 [Source:HGNC Symbol;Acc:HGNC:3670] |
| ENSG00000131724 | HGLibA_22974 | 197 | 0.811752414 | IL13RA1 | interleukin 13 receptor subunit alpha 1 [Source:HGNC Symbol;Acc:HGNC:5974] |
| ENSG00000132330 | HGLibA_42954 | 197 | 0.811752414 | SCLY | selenocysteine lyase [Source:HGNC Symbol;Acc:HGNC:18161] |
| ENSG00000134780 | HGLibA_12295 | 197 | 0.811752414 | DAGLA | diacylglycerol lipase alpha [Source:HGNC Symbol;Acc:HGNC:1165] |
| ENSG00000135898 | HGLibA_20053 | 197 | 0.811752414 | GPR55 | G protein-coupled receptor 55 [Source:HGNC Symbol;Acc:HGNC:4511] |

| | | | | | |
|---|---|---|---|---|---|
| ENSG00000137673 | HGLibA_29536 | 197 | 0.811752414 | MMP7 | matrix metallopeptidase 7 [Source:HGNC Symbol;Acc:HGNC:7174] |
| ENSG00000137727 | HGLibA_02724 | 197 | 0.811752414 | ARHGAP20 | Rho GTPase activating protein 20 [Source:HGNC Symbol;Acc:HGNC:18357] |
| ENSG00000143933 | HGLibA_07026 | 197 | 0.811752414 | CALM2 | calmodulin 2 [Source:HGNC Symbol;Acc:HGNC:1445] |
| ENSG00000144355 | HGLibA_13373 | 197 | 0.811752414 | DLX1 | distal-less homeobox 1 [Source:HGNC Symbol;Acc:HGNC:2914] |
| ENSG00000146676 | HGLibA_39588 | 197 | 0.811752414 | PURB | purine rich element binding protein B [Source:HGNC Symbol;Acc:HGNC:9702] |
| ENSG00000148734 | HGLibA_32440 | 197 | 0.811752414 | NPFFR1 | neuropeptide FF receptor 1 [Source:HGNC Symbol;Acc:HGNC:17425] |
| ENSG00000149970 | HGLibA_10299 | 197 | 0.811752414 | CNKSR2 | connector enhancer of kinase suppressor of Ras 2 [Source:HGNC Symbol;Acc:HGNC:19701] |
| ENSG00000150455 | HGLibA_49564 | 197 | 0.811752414 | TIRAP | TIR domain containing adaptor protein [Source:HGNC Symbol;Acc:HGNC:17192] |
| ENSG00000150455 | HGLibA_49565 | 197 | 0.811752414 | TIRAP | TIR domain containing adaptor protein [Source:HGNC Symbol;Acc:HGNC:17192] |
| ENSG00000152382 | HGLibA_48184 | 197 | 0.811752414 | TADA1 | transcriptional adaptor 1 [Source:HGNC Symbol;Acc:HGNC:30631] |
| ENSG00000153246 | HGLibA_36928 | 197 | 0.811752414 | PLA2R1 | phospholipase A2 receptor 1 [Source:HGNC Symbol;Acc:HGNC:9042] |
| ENSG00000157087 | HGLibA_03617 | 197 | 0.811752414 | ATP2B2 | ATPase plasma membrane Ca2+ transporting 2 [Source:HGNC Symbol;Acc:HGNC:815] |
| ENSG00000158477 | HGLibA_08265 | 197 | 0.811752414 | CD1A | CD1a molecule [Source:HGNC Symbol;Acc:HGNC:1634] |
| ENSG00000159714 | HGLibA_55065 | 197 | 0.811752414 | ZDHHC1 | zinc finger DHHC-type containing 1 [Source:HGNC Symbol;Acc:HGNC:17916] |
| ENSG00000162337 | HGLibA_27223 | 197 | 0.811752414 | LRP5 | LDL receptor related protein 5 [Source:HGNC Symbol;Acc:HGNC:6697] |
| ENSG00000162877 | HGLibA_37286 | 197 | 0.811752414 | PM20D1 | peptidase M20 domain containing 1 [Source:HGNC Symbol;Acc:HGNC:26518] |
| ENSG00000164120 | HGLibA_22032 | 197 | 0.811752414 | HPGD | 15-hydroxyprostaglandin dehydrogenase [Source:HGNC Symbol;Acc:HGNC:5154] |
| ENSG00000164244 | HGLibA_38858 | 197 | 0.811752414 | PRRC1 | proline rich coiled-coil 1 [Source:HGNC Symbol;Acc:HGNC:28164] |
| ENSG00000165458 | HGLibA_23365 | 197 | 0.811752414 | INPPL1 | inositol polyphosphate phosphatase like 1 [Source:HGNC Symbol;Acc:HGNC:6080] |
| ENSG00000166448 | HGLibA_49924 | 197 | 0.811752414 | TMEM130 | transmembrane protein 130 [Source:HGNC Symbol;Acc:HGNC:25429] |
| ENSG00000166848 | HGLibA_49016 | 197 | 0.811752414 | TERF2IP | TERF2 interacting protein [Source:HGNC Symbol;Acc:HGNC:19246] |
| ENSG00000168887 | HGLibA_06165 | 197 | 0.811752414 | C2orf68 | chromosome 2 open reading frame 68 [Source:HGNC Symbol;Acc:HGNC:34353] |
| ENSG00000171798 | HGLibA_25338 | 197 | 0.811752414 | KNDC1 | kinase non-catalytic C-lobe domain containing 1 [Source:HGNC Symbol;Acc:HGNC:29374] |
| ENSG00000175482 | HGLibA_37502 | 197 | 0.811752414 | POLD4 | accessory subunit [Source:HGNC Symbol;Acc:HGNC:14106]" |
| ENSG00000177189 | HGLibA_42165 | 197 | 0.811752414 | RPS6KA3 | ribosomal protein S6 kinase A3 [Source:HGNC Symbol;Acc:HGNC:10432] |
| ENSG00000177994 | HGLibA_06178 | 197 | 0.811752414 | C2orf73 | chromosome 2 open reading frame 73 [Source:HGNC Symbol;Acc:HGNC:26861] |
| ENSG00000179598 | HGLibA_37051 | 197 | 0.811752414 | PLD6 | phospholipase D family member 6 [Source:HGNC Symbol;Acc:HGNC:30447] |
| ENSG00000182405 | HGLibA_36226 | 197 | 0.811752414 | PGBD4 | piggyBac transposable element derived 4 [Source:HGNC Symbol;Acc:HGNC:19401] |
| ENSG00000184261 | HGLibA_24299 | 197 | 0.811752414 | KCNK12 | potassium two pore domain channel subfamily K member 12 [Source:HGNC Symbol;Acc:HGNC:6274] |
| ENSG00000184647 | HGLibA_38961 | 197 | 0.811752414 | PRSS55 | serine protease 55 [Source:HGNC Symbol;Acc:HGNC:30824] |
| ENSG00000187510 | HGLibA_37121 | 197 | 0.811752414 | PLEKHG7 | pleckstrin homology and RhoGEF domain containing G7 [Source:HGNC Symbol;Acc:HGNC:33829] |
| ENSG00000196177 | HGLibA_00374 | 197 | 0.811752414 | ACADSB | acyl-CoA dehydrogenase short/branched chain [Source:HGNC Symbol;Acc:HGNC:91] |
| ENSG00000196182 | HGLibA_47512 | 197 | 0.811752414 | STK40 | serine/threonine kinase 40 [Source:HGNC Symbol;Acc:HGNC:21373] |
| ENSG00000197245 | HGLibA_16021 | 197 | 0.811752414 | FAM110D | family with sequence similarity 110 member D [Source:HGNC Symbol;Acc:HGNC:25860] |
| ENSG00000213397 | HGLibA_20893 | 197 | 0.811752414 | HAUS7 | HAUS augmin like complex subunit 7 [Source:HGNC Symbol;Acc:HGNC:32979] |
| ENSG00000214814 | HGLibA_17289 | 197 | 0.811752414 | FER1L6 | fer-1 like family member 6 [Source:HGNC Symbol;Acc:HGNC:28065] |
| ENSG00000246922 | HGLibA_52550 | 197 | 0.811752414 | UBAP1L | ubiquitin associated protein 1 like [Source:HGNC Symbol;Acc:HGNC:40028] |
| ENSG00000255587 | HGLibA_39952 | 197 | 0.811752414 | RAB44 | member RAS oncogene family [Source:HGNC Symbol;Acc:HGNC:21068]" |
| ENSG00000261052 | HGLibA_47738 | 197 | 0.811752414 | SULT1A3 | sulfotransferase family 1A member 3 [Source:HGNC Symbol;Acc:HGNC:11455] |
| ENSG00000267534 | HGLibA_42596 | 197 | 0.811752414 | S1PR2 | sphingosine-1-phosphate receptor 2 [Source:HGNC Symbol;Acc:HGNC:3169] |
| ENSG00000273841 | HGLibA_48262 | 197 | 0.811752414 | TAF9 | TATA-box binding protein associated factor 9 [Source:HGNC Symbol;Acc:HGNC:11542] |
| ENSG00000282608 | HGLibA_01018 | 197 | 0.811752414 | ADORA3 | adenosine A3 receptor [Source:HGNC Symbol;Acc:HGNC:268] |
| GPR115 | HGLibA_19862 | 197 | 0.811752414 | GPR115 | - |
| hsa-mir-3154 | HGLibA_58674 | 197 | 0.811752414 | hsa-mir-3154 | - |
| hsa-mir-3913-1 | HGLibA_59520 | 197 | 0.811752414 | hsa-mir-3913-1 | - |
| hsa-mir-509-2 | HGLibA_61512 | 197 | 0.811752414 | hsa-mir-509-2 | - |
| hsa-mir-6855 | HGLibA_63594 | 197 | 0.811752414 | hsa-mir-6855 | - |
| hsa-mir-7854 | HGLibA_64004 | 197 | 0.811752414 | hsa-mir-7854 | - |
| C1orf111 | HGLibA_05746 | 196 | 0.801801376 | C1orf111 | - |
| CCBL1 | HGLibA_07496 | 196 | 0.801801376 | CCBL1 | - |
| CPSF3L | HGLibA_11003 | 196 | 0.801801376 | CPSF3L | - |
| ENSG00000007038 | HGLibA_38911 | 196 | 0.801801376 | PRSS21 | serine protease 21 [Source:HGNC Symbol;Acc:HGNC:9485] |
| ENSG00000011243 | HGLibA_01448 | 196 | 0.801801376 | AKAP8L | A-kinase anchoring protein 8 like [Source:HGNC Symbol;Acc:HGNC:29857] |
| ENSG00000018236 | HGLibA_10400 | 196 | 0.801801376 | CNTN1 | contactin 1 [Source:HGNC Symbol;Acc:HGNC:2171] |
| ENSG00000037637 | HGLibA_17100 | 196 | 0.801801376 | FBXO42 | F-box protein 42 [Source:HGNC Symbol;Acc:HGNC:29249] |
| ENSG00000050344 | HGLibA_31796 | 196 | 0.801801376 | NFE2L3 | erythroid 2 like 3 [Source:HGNC Symbol;Acc:HGNC:7783]" |
| ENSG00000070087 | HGLibA_36176 | 196 | 0.801801376 | PFN2 | profilin 2 [Source:HGNC Symbol;Acc:HGNC:8882] |
| ENSG00000087903 | HGLibA_40964 | 196 | 0.801801376 | RFX2 | regulatory factor X2 [Source:HGNC Symbol;Acc:HGNC:9983] |
| ENSG00000091181 | HGLibA_23177 | 196 | 0.801801376 | IL5RA | interleukin 5 receptor subunit alpha [Source:HGNC Symbol;Acc:HGNC:6017] |
| ENSG00000091592 | HGLibA_32098 | 196 | 0.801801376 | NLRP1 | NLR family pyrin domain containing 1 [Source:HGNC Symbol;Acc:HGNC:14374] |
| ENSG00000091972 | HGLibA_08283 | 196 | 0.801801376 | CD200 | CD200 molecule [Source:HGNC Symbol;Acc:HGNC:7203] |
| ENSG00000095383 | HGLibA_48493 | 196 | 0.801801376 | TBC1D2 | TBC1 domain family member 2 [Source:HGNC Symbol;Acc:HGNC:18026] |
| ENSG00000099985 | HGLibA_34692 | 196 | 0.801801376 | OSM | oncostatin M [Source:HGNC Symbol;Acc:HGNC:8506] |
| ENSG00000100290 | HGLibA_04504 | 196 | 0.801801376 | BIK | BCL2 interacting killer [Source:HGNC Symbol;Acc:HGNC:1051] |
| ENSG00000103490 | HGLibA_39669 | 196 | 0.801801376 | PYCARD | PYD and CARD domain containing [Source:HGNC Symbol;Acc:HGNC:16608] |
| ENSG00000104133 | HGLibA_46631 | 196 | 0.801801376 | SPG11 | spatacsin vesicle trafficking associated [Source:HGNC Symbol;Acc:HGNC:11226]" |
| ENSG00000105223 | HGLibA_37040 | 196 | 0.801801376 | PLD3 | phospholipase D family member 3 [Source:HGNC Symbol;Acc:HGNC:17158] |
| ENSG00000105576 | HGLibA_50902 | 196 | 0.801801376 | TNPO2 | transportin 2 [Source:HGNC Symbol;Acc:HGNC:19998] |
| ENSG00000106436 | HGLibA_30747 | 196 | 0.801801376 | MYL10 | myosin light chain 10 [Source:HGNC Symbol;Acc:HGNC:29825] |
| ENSG00000108423 | HGLibA_52296 | 196 | 0.801801376 | TUBD1 | tubulin delta 1 [Source:HGNC Symbol;Acc:HGNC:16811] |
| ENSG00000112812 | HGLibA_38904 | 196 | 0.801801376 | PRSS16 | serine protease 16 [Source:HGNC Symbol;Acc:HGNC:9480] |
| ENSG00000116791 | HGLibA_11294 | 196 | 0.801801376 | CRYZ | crystallin zeta [Source:HGNC Symbol;Acc:HGNC:2419] |
| ENSG00000124429 | HGLibA_37466 | 196 | 0.801801376 | POF1B | actin binding protein [Source:HGNC Symbol;Acc:HGNC:13711]" |
| ENSG00000125977 | HGLibA_14757 | 196 | 0.801801376 | EIF2S2 | eukaryotic translation initiation factor 2 subunit beta [Source:HGNC Symbol;Acc:HGNC:3266] |
| ENSG00000127743 | HGLibA_22991 | 196 | 0.801801376 | IL17B | interleukin 17B [Source:HGNC Symbol;Acc:HGNC:5982] |
| ENSG00000128253 | HGLibA_40931 | 196 | 0.801801376 | RFPL2 | ret finger protein like 2 [Source:HGNC Symbol;Acc:HGNC:9979] |
| ENSG00000128872 | HGLibA_50596 | 196 | 0.801801376 | TMOD2 | tropomodulin 2 [Source:HGNC Symbol;Acc:HGNC:11872] |
| ENSG00000131374 | HGLibA_48561 | 196 | 0.801801376 | TBC1D5 | TBC1 domain family member 5 [Source:HGNC Symbol;Acc:HGNC:19166] |
| ENSG00000135241 | HGLibA_37425 | 196 | 0.801801376 | PNPLA8 | patatin like phospholipase domain containing 8 [Source:HGNC Symbol;Acc:HGNC:28900] |
| ENSG00000136750 | HGLibA_18412 | 196 | 0.801801376 | GAD2 | glutamate decarboxylase 2 [Source:HGNC Symbol;Acc:HGNC:4093] |
| ENSG00000137563 | HGLibA_18998 | 196 | 0.801801376 | GGH | gamma-glutamyl hydrolase [Source:HGNC Symbol;Acc:HGNC:4248] |
| ENSG00000139914 | HGLibA_17540 | 196 | 0.801801376 | FITM1 | fat storage inducing transmembrane protein 1 [Source:HGNC Symbol;Acc:HGNC:33714] |
| ENSG00000140945 | HGLibA_08641 | 196 | 0.801801376 | CDH13 | cadherin 13 [Source:HGNC Symbol;Acc:HGNC:1753] |
| ENSG00000142549 | HGLibA_22872 | 196 | 0.801801376 | IGLON5 | IgLON family member 5 [Source:HGNC Symbol;Acc:HGNC:34550] |
| ENSG00000143514 | HGLibA_51056 | 196 | 0.801801376 | TP53BP2 | tumor protein p53 binding protein 2 [Source:HGNC Symbol;Acc:HGNC:12000] |
| ENSG00000143627 | HGLibA_36829 | 196 | 0.801801376 | PKLR | pyruvate kinase L/R [Source:HGNC Symbol;Acc:HGNC:9020] |
| ENSG00000146555 | HGLibA_43150 | 196 | 0.801801376 | SDK1 | sidekick cell adhesion molecule 1 [Source:HGNC Symbol;Acc:HGNC:19307] |
| ENSG00000152700 | HGLibA_42739 | 196 | 0.801801376 | SAR1B | secretion associated Ras related GTPase 1B [Source:HGNC Symbol;Acc:HGNC:10535] |
| ENSG00000154133 | HGLibA_41742 | 196 | 0.801801376 | ROBO4 | roundabout guidance receptor 4 [Source:HGNC Symbol;Acc:HGNC:17985] |
| ENSG00000155659 | HGLibA_53849 | 196 | 0.801801376 | VSIG4 | V-set and immunoglobulin domain containing 4 [Source:HGNC Symbol;Acc:HGNC:17032] |
| ENSG00000160712 | HGLibA_23185 | 196 | 0.801801376 | IL6R | interleukin 6 receptor [Source:HGNC Symbol;Acc:HGNC:6019] |
| ENSG00000160741 | HGLibA_11231 | 196 | 0.801801376 | CRTC2 | CREB regulated transcription coactivator 2 [Source:HGNC Symbol;Acc:HGNC:27301] |
| ENSG00000161013 | HGLibA_29145 | 196 | 0.801801376 | MGAT4B | 3-mannosyl-glycoprotein 4-beta-N-acetylglucosaminyltransferase B [Source:HGNC Symbol;Acc:HGNC:7048]" |
| ENSG00000162415 | HGLibA_57048 | 196 | 0.801801376 | ZSWIM5 | zinc finger SWIM-type containing 5 [Source:HGNC Symbol;Acc:HGNC:29299] |
| ENSG00000163083 | HGLibA_23304 | 196 | 0.801801376 | INHBB | inhibin subunit beta B [Source:HGNC Symbol;Acc:HGNC:6067] |
| ENSG00000163508 | HGLibA_15228 | 196 | 0.801801376 | EOMES | eomesodermin [Source:HGNC Symbol;Acc:HGNC:3372] |
| ENSG00000163827 | HGLibA_27278 | 196 | 0.801801376 | LRRC2 | leucine rich repeat containing 2 [Source:HGNC Symbol;Acc:HGNC:14676] |
| ENSG00000164056 | HGLibA_46835 | 196 | 0.801801376 | SPRY1 | sprouty RTK signaling antagonist 1 [Source:HGNC Symbol;Acc:HGNC:11269] |
| ENSG00000166821 | HGLibA_36083 | 196 | 0.801801376 | PEX11A | peroxisomal biogenesis factor 11 alpha [Source:HGNC Symbol;Acc:HGNC:8852] |
| ENSG00000166926 | HGLibA_30160 | 196 | 0.801801376 | MS4A6E | membrane spanning 4-domains A6E [Source:HGNC Symbol;Acc:HGNC:14285] |
| ENSG00000167548 | HGLibA_25329 | 196 | 0.801801376 | KMT2D | lysine methyltransferase 2D [Source:HGNC Symbol;Acc:HGNC:7133] |
| ENSG00000173679 | HGLibA_33686 | 196 | 0.801801376 | OR1L1 | olfactory receptor family 1 subfamily L member 1 [Source:HGNC Symbol;Acc:HGNC:8213] |
| ENSG00000174225 | HGLibA_02930 | 196 | 0.801801376 | ARL13A | ADP ribosylation factor like GTPase 13A [Source:HGNC Symbol;Acc:HGNC:31709] |
| ENSG00000174950 | HGLibA_08252 | 196 | 0.801801376 | CD164L2 | CD164 molecule like 2 [Source:HGNC Symbol;Acc:HGNC:32043] |
| ENSG00000175130 | HGLibA_28359 | 196 | 0.801801376 | MARCKSL1 | MARCKS like 1 [Source:HGNC Symbol;Acc:HGNC:7142] |
| ENSG00000176971 | HGLibA_17502 | 196 | 0.801801376 | FIBIN | fin bud initiation factor homolog [Source:HGNC Symbol;Acc:HGNC:33747] |
| ENSG00000178502 | HGLibA_25125 | 196 | 0.801801376 | KLHL11 | kelch like family member 11 [Source:HGNC Symbol;Acc:HGNC:19008] |
| ENSG00000183309 | HGLibA_56413 | 196 | 0.801801376 | ZNF623 | zinc finger protein 623 [Source:HGNC Symbol;Acc:HGNC:29084] |
| ENSG00000183317 | HGLibA_15286 | 196 | 0.801801376 | EPHA10 | EPH receptor A10 [Source:HGNC Symbol;Acc:HGNC:19987] |
| ENSG00000183747 | HGLibA_00573 | 196 | 0.801801376 | ACSM2A | acyl-CoA synthetase medium chain family member 2A [Source:HGNC Symbol;Acc:HGNC:32017] |
| ENSG00000184697 | HGLibA_09903 | 196 | 0.801801376 | CLDN6 | claudin 6 [Source:HGNC Symbol;Acc:HGNC:2048] |
| ENSG00000184911 | HGLibA_13438 | 196 | 0.801801376 | DMRTC1B | DMRT like family C1B [Source:HGNC Symbol;Acc:HGNC:31686] |
| ENSG00000184984 | HGLibA_09560 | 196 | 0.801801376 | CHRM5 | cholinergic receptor muscarinic 5 [Source:HGNC Symbol;Acc:HGNC:1954] |
| ENSG00000186231 | HGLibA_25185 | 196 | 0.801801376 | KLHL32 | kelch like family member 32 [Source:HGNC Symbol;Acc:HGNC:21221] |
| ENSG00000186567 | HGLibA_08928 | 196 | 0.801801376 | CEACAM19 | carcinoembryonic antigen related cell adhesion molecule 19 [Source:HGNC Symbol;Acc:HGNC:31951] |
| ENSG00000186925 | HGLibA_25679 | 196 | 0.801801376 | KRTAP19-6 | keratin associated protein 19-6 [Source:HGNC Symbol;Acc:HGNC:18941] |
| ENSG00000188529 | HGLibA_47063 | 196 | 0.801801376 | SRSF10 | serine and arginine rich splicing factor 10 [Source:HGNC Symbol;Acc:HGNC:16713] |
| ENSG00000189127 | HGLibA_02078 | 196 | 0.801801376 | ANKRD34B | ankyrin repeat domain 34B [Source:HGNC Symbol;Acc:HGNC:33736] |
| ENSG00000196152 | HGLibA_56760 | 196 | 0.801801376 | ZNF79 | zinc finger protein 79 [Source:HGNC Symbol;Acc:HGNC:13153] |
| ENSG00000197471 | HGLibA_46729 | 196 | 0.801801376 | SPN | sialophorin [Source:HGNC Symbol;Acc:HGNC:11249] |
| ENSG00000198746 | HGLibA_19744 | 196 | 0.801801376 | GPATCH3 | G-patch domain containing 3 [Source:HGNC Symbol;Acc:HGNC:25720] |
| ENSG00000204887 | HGLibA_25585 | 196 | 0.801801376 | KRTAP1-4 | keratin associated protein 1-4 [Source:HGNC Symbol;Acc:HGNC:18904] |
| ENSG00000205339 | HGLibA_23471 | 196 | 0.801801376 | IPO7 | importin 7 [Source:HGNC Symbol;Acc:HGNC:9852] |
| ENSG00000214681 | HGLibA_23511 | 196 | 0.801801376 | IQCF5 | IQ motif containing F5 [Source:HGNC Symbol;Acc:HGNC:35159] |
| ENSG00000263001 | HGLibA_20564 | 196 | 0.801801376 | GTF2I | general transcription factor IIi [Source:HGNC Symbol;Acc:HGNC:4659] |
| hsa-mir-106b | HGLibA_57191 | 196 | 0.801801376 | hsa-mir-106b | - |
| hsa-mir-1260b | HGLibA_57465 | 196 | 0.801801376 | hsa-mir-1260b | - |
| hsa-mir-302d | HGLibA_58449 | 196 | 0.801801376 | hsa-mir-302d | - |
| hsa-mir-3613 | HGLibA_59080 | 196 | 0.801801376 | hsa-mir-3613 | - |
| hsa-mir-4276 | HGLibA_59808 | 196 | 0.801801376 | hsa-mir-4276 | - |
| hsa-mir-4485 | HGLibA_60359 | 196 | 0.801801376 | hsa-mir-4485 | - |
| hsa-mir-4524b | HGLibA_60557 | 196 | 0.801801376 | hsa-mir-4524b | - |
| hsa-mir-4533 | HGLibA_60593 | 196 | 0.801801376 | hsa-mir-4533 | - |
| hsa-mir-4645 | HGLibA_60682 | 196 | 0.801801376 | hsa-mir-4645 | - |
| hsa-mir-4681 | HGLibA_60833 | 196 | 0.801801376 | hsa-mir-4681 | - |

| | | | | | |
|---|---|---|---|---|---|
| hsa-mir-548v | HGLibA_62073 | 196 | 0.801801376 | hsa-mir-548v | - |
| hsa-mir-5696 | HGLibA_62316 | 196 | 0.801801376 | hsa-mir-5696 | - |
| hsa-mir-5698 | HGLibA_62326 | 196 | 0.801801376 | hsa-mir-5698 | - |
| hsa-mir-8062 | HGLibA_64088 | 196 | 0.801801376 | hsa-mir-8062 | - |
| PQLC1 | HGLibA_38252 | 196 | 0.801801376 | PQLC1 | - |
| 3-Sep | HGLibA_43438 | 196 | 0.801801376 | 3-Sep | - |
| TBC1D29 | HGLibA_48523 | 196 | 0.801801376 | TBC1D29 | - |
| C14orf142 | HGLibA_05327 | 195 | 0.791850339 | C14orf142 | - |
| C9orf16 | HGLibA_06699 | 195 | 0.791850339 | C9orf16 | - |
| CCBL1 | HGLibA_07495 | 195 | 0.791850339 | CCBL1 | - |
| ENSG00000007520 | HGLibA_52001 | 195 | 0.791850339 | TSR3 | acp transferase ribosome maturation factor [Source:HGNC Symbol;Acc:HGNC:14175]" |
| ENSG00000053254 | HGLibA_17916 | 195 | 0.791850339 | FOXN3 | forkhead box N3 [Source:HGNC Symbol;Acc:HGNC:1928] |
| ENSG00000066629 | HGLibA_15057 | 195 | 0.791850339 | EML1 | echinoderm microtubule associated protein like 1 [Source:HGNC Symbol;Acc:HGNC:3330] |
| ENSG00000067798 | HGLibA_31199 | 195 | 0.791850339 | NAV3 | neuron navigator 3 [Source:HGNC Symbol;Acc:HGNC:15998] |
| ENSG00000069018 | HGLibA_51748 | 195 | 0.791850339 | TRPC7 | transient receptor potential cation channel subfamily C member 7 [Source:HGNC Symbol;Acc:HGNC:20754] |
| ENSG00000080573 | HGLibA_10592 | 195 | 0.791850339 | COL5A3 | collagen type V alpha 3 chain [Source:HGNC Symbol;Acc:HGNC:14864] |
| ENSG00000084207 | HGLibA_20494 | 195 | 0.791850339 | GSTP1 | glutathione S-transferase pi 1 [Source:HGNC Symbol;Acc:HGNC:4638] |
| ENSG00000102245 | HGLibA_08387 | 195 | 0.791850339 | CD40LG | CD40 ligand [Source:HGNC Symbol;Acc:HGNC:11935] |
| ENSG00000106397 | HGLibA_37214 | 195 | 0.791850339 | PLOD3 | 2-oxoglutarate 5-dioxygenase 3 [Source:HGNC Symbol;Acc:HGNC:9083]" |
| ENSG00000106536 | HGLibA_37797 | 195 | 0.791850339 | POU6F2 | POU class 6 homeobox 2 [Source:HGNC Symbol;Acc:HGNC:21694] |
| ENSG00000110665 | HGLibA_05121 | 195 | 0.791850339 | C11orf21 | chromosome 11 open reading frame 21 [Source:HGNC Symbol;Acc:HGNC:13231] |
| ENSG00000113396 | HGLibA_44828 | 195 | 0.791850339 | SLC27A6 | solute carrier family 27 member 6 [Source:HGNC Symbol;Acc:HGNC:11000] |
| ENSG00000120885 | HGLibA_10156 | 195 | 0.791850339 | CLU | clusterin [Source:HGNC Symbol;Acc:HGNC:2095] |
| ENSG00000123342 | HGLibA_29504 | 195 | 0.791850339 | MMP19 | matrix metallopeptidase 19 [Source:HGNC Symbol;Acc:HGNC:7165] |
| ENSG00000124196 | HGLibA_20631 | 195 | 0.791850339 | GTSF1L | gametocyte specific factor 1 like [Source:HGNC Symbol;Acc:HGNC:16198] |
| ENSG00000124766 | HGLibA_46263 | 195 | 0.791850339 | SOX4 | SRY-box 4 [Source:HGNC Symbol;Acc:HGNC:11200] |
| ENSG00000124882 | HGLibA_15459 | 195 | 0.791850339 | EREG | epiregulin [Source:HGNC Symbol;Acc:HGNC:3443] |
| ENSG00000129514 | HGLibA_17806 | 195 | 0.791850339 | FOXA1 | forkhead box A1 [Source:HGNC Symbol;Acc:HGNC:5021] |
| ENSG00000129988 | HGLibA_26079 | 195 | 0.791850339 | LBP | lipopolysaccharide binding protein [Source:HGNC Symbol;Acc:HGNC:6517] |
| ENSG00000131400 | HGLibA_31139 | 195 | 0.791850339 | NAPSA | napsin A aspartic peptidase [Source:HGNC Symbol;Acc:HGNC:13395] |
| ENSG00000133665 | HGLibA_14222 | 195 | 0.791850339 | DYDC2 | DPY30 domain containing 2 [Source:HGNC Symbol;Acc:HGNC:23468] |
| ENSG00000134287 | HGLibA_02650 | 195 | 0.791850339 | ARF3 | ADP ribosylation factor 3 [Source:HGNC Symbol;Acc:HGNC:654] |
| ENSG00000134851 | HGLibA_50036 | 195 | 0.791850339 | TMEM165 | transmembrane protein 165 [Source:HGNC Symbol;Acc:HGNC:30760] |
| ENSG00000135502 | HGLibA_44783 | 195 | 0.791850339 | SLC26A10 | solute carrier family 26 member 10 [Source:HGNC Symbol;Acc:HGNC:14470] |
| ENSG00000136068 | HGLibA_17641 | 195 | 0.791850339 | FLNB | filamin B [Source:HGNC Symbol;Acc:HGNC:3755] |
| ENSG00000136986 | HGLibA_12994 | 195 | 0.791850339 | DERL1 | derlin 1 [Source:HGNC Symbol;Acc:HGNC:28454] |
| ENSG00000140254 | HGLibA_14102 | 195 | 0.791850339 | DUOXA1 | dual oxidase maturation factor 1 [Source:HGNC Symbol;Acc:HGNC:26507] |
| ENSG00000140832 | HGLibA_28386 | 195 | 0.791850339 | MARVELD3 | MARVEL domain containing 3 [Source:HGNC Symbol;Acc:HGNC:30525] |
| ENSG00000140853 | HGLibA_32093 | 195 | 0.791850339 | NLRC5 | NLR family CARD domain containing 5 [Source:HGNC Symbol;Acc:HGNC:29933] |
| ENSG00000141013 | HGLibA_18659 | 195 | 0.791850339 | GAS8 | growth arrest specific 8 [Source:HGNC Symbol;Acc:HGNC:4166] |
| ENSG00000141219 | HGLibA_05581 | 195 | 0.791850339 | C17orf80 | chromosome 17 open reading frame 80 [Source:HGNC Symbol;Acc:HGNC:29601] |
| ENSG00000141985 | HGLibA_43945 | 195 | 0.791850339 | SH3GL1 | endophilin A2 [Source:HGNC Symbol;Acc:HGNC:10830]" |
| ENSG00000142694 | HGLibA_15675 | 195 | 0.791850339 | EVA1B | eva-1 homolog B [Source:HGNC Symbol;Acc:HGNC:25558] |
| ENSG00000143520 | HGLibA_17611 | 195 | 0.791850339 | FLG2 | filaggrin family member 2 [Source:HGNC Symbol;Acc:HGNC:33276] |
| ENSG00000145861 | HGLibA_05938 | 195 | 0.791850339 | C1QTNF2 | C1q and TNF related 2 [Source:HGNC Symbol;Acc:HGNC:14325] |
| ENSG00000152969 | HGLibA_23923 | 195 | 0.791850339 | JAKMIP1 | janus kinase and microtubule interacting protein 1 [Source:HGNC Symbol;Acc:HGNC:26460] |
| ENSG00000153822 | HGLibA_24268 | 195 | 0.791850339 | KCNJ16 | potassium voltage-gated channel subfamily J member 16 [Source:HGNC Symbol;Acc:HGNC:6262] |
| ENSG00000154479 | HGLibA_07699 | 195 | 0.791850339 | CCDC173 | coiled-coil domain containing 173 [Source:HGNC Symbol;Acc:HGNC:25064] |
| ENSG00000155008 | HGLibA_02553 | 195 | 0.791850339 | APOOL | apolipoprotein O like [Source:HGNC Symbol;Acc:HGNC:24009] |
| ENSG00000155729 | HGLibA_24437 | 195 | 0.791850339 | KCTD18 | potassium channel tetramerization domain containing 18 [Source:HGNC Symbol;Acc:HGNC:26446] |
| ENSG00000158458 | HGLibA_32680 | 195 | 0.791850339 | NRG2 | neuregulin 2 [Source:HGNC Symbol;Acc:HGNC:7998] |
| ENSG00000163098 | HGLibA_04529 | 195 | 0.791850339 | BIRC8 | baculoviral IAP repeat containing 8 [Source:HGNC Symbol;Acc:HGNC:14878] |
| ENSG00000163660 | HGLibA_08117 | 195 | 0.791850339 | CCNL1 | cyclin L1 [Source:HGNC Symbol;Acc:HGNC:20569] |
| ENSG00000164107 | HGLibA_20828 | 195 | 0.791850339 | HAND2 | heart and neural crest derivatives expressed 2 [Source:HGNC Symbol;Acc:HGNC:4808] |
| ENSG00000164176 | HGLibA_14465 | 195 | 0.791850339 | EDIL3 | EGF like repeats and discoidin domains 3 [Source:HGNC Symbol;Acc:HGNC:3173] |
| ENSG00000164871 | HGLibA_46347 | 195 | 0.791850339 | SPAG11B | sperm associated antigen 11B [Source:HGNC Symbol;Acc:HGNC:14534] |
| ENSG00000165915 | HGLibA_45099 | 195 | 0.791850339 | SLC39A13 | solute carrier family 39 member 13 [Source:HGNC Symbol;Acc:HGNC:20859] |
| ENSG00000169347 | HGLibA_19704 | 195 | 0.791850339 | GP2 | glycoprotein 2 [Source:HGNC Symbol;Acc:HGNC:4441] |
| ENSG00000169955 | HGLibA_56668 | 195 | 0.791850339 | ZNF747 | zinc finger protein 747 [Source:HGNC Symbol;Acc:HGNC:28350] |
| ENSG00000169992 | HGLibA_32071 | 195 | 0.791850339 | NLGN2 | neuroligin 2 [Source:HGNC Symbol;Acc:HGNC:14290] |
| ENSG00000170683 | HGLibA_33463 | 195 | 0.791850339 | OR10A3 | olfactory receptor family 10 subfamily A member 3 [Source:HGNC Symbol;Acc:HGNC:8162] |
| ENSG00000175489 | HGLibA_27290 | 195 | 0.791850339 | LRRC25 | leucine rich repeat containing 25 [Source:HGNC Symbol;Acc:HGNC:29806] |
| ENSG00000176371 | HGLibA_56995 | 195 | 0.791850339 | ZSCAN2 | zinc finger and SCAN domain containing 2 [Source:HGNC Symbol;Acc:HGNC:20994] |
| ENSG00000177519 | HGLibA_42052 | 195 | 0.791850339 | RPRM | TP53 dependent G2 arrest mediator homolog [Source:HGNC Symbol;Acc:HGNC:24201]" |
| ENSG00000178279 | HGLibA_50895 | 195 | 0.791850339 | TNP2 | transition protein 2 [Source:HGNC Symbol;Acc:HGNC:11952] |
| ENSG00000179639 | HGLibA_17169 | 195 | 0.791850339 | FCER1A | Fc fragment of IgE receptor Ia [Source:HGNC Symbol;Acc:HGNC:3609] |
| ENSG00000179751 | HGLibA_47924 | 195 | 0.791850339 | SYCN | syncollin [Source:HGNC Symbol;Acc:HGNC:18442] |
| ENSG00000181939 | HGLibA_33948 | 195 | 0.791850339 | OR4C15 | olfactory receptor family 4 subfamily C member 15 [Source:HGNC Symbol;Acc:HGNC:15171] |
| ENSG00000182223 | HGLibA_54778 | 195 | 0.791850339 | ZAR1 | zygote arrest 1 [Source:HGNC Symbol;Acc:HGNC:20436] |
| ENSG00000182931 | HGLibA_54251 | 195 | 0.791850339 | WFDC10B | WAP four-disulfide core domain 10B [Source:HGNC Symbol;Acc:HGNC:20479] |
| ENSG00000188338 | HGLibA_45066 | 195 | 0.791850339 | SLC38A3 | solute carrier family 38 member 3 [Source:HGNC Symbol;Acc:HGNC:18044] |
| ENSG00000196440 | HGLibA_03046 | 195 | 0.791850339 | ARMCX4 | armadillo repeat containing X-linked 4 [Source:HGNC Symbol;Acc:HGNC:28615] |
| ENSG00000196670 | HGLibA_55224 | 195 | 0.791850339 | ZFP62 | ZFP62 zinc finger protein [Source:HGNC Symbol;Acc:HGNC:23241] |
| ENSG00000196700 | HGLibA_56133 | 195 | 0.791850339 | ZNF512B | zinc finger protein 512B [Source:HGNC Symbol;Acc:HGNC:29212] |
| ENSG00000198089 | HGLibA_43719 | 195 | 0.791850339 | SFI1 | SFI1 centrin binding protein [Source:HGNC Symbol;Acc:HGNC:29064] |
| ENSG00000198633 | HGLibA_56182 | 195 | 0.791850339 | ZNF534 | zinc finger protein 534 [Source:HGNC Symbol;Acc:HGNC:26337] |
| ENSG00000203618 | HGLibA_19703 | 195 | 0.791850339 | GP1BB | glycoprotein Ib platelet subunit beta [Source:HGNC Symbol;Acc:HGNC:4440] |
| ENSG00000204193 | HGLibA_52426 | 195 | 0.791850339 | TXNDC8 | thioredoxin domain containing 8 [Source:HGNC Symbol;Acc:HGNC:31454] |
| ENSG00000204524 | HGLibA_56794 | 195 | 0.791850339 | ZNF805 | zinc finger protein 805 [Source:HGNC Symbol;Acc:HGNC:23272] |
| ENSG00000204538 | HGLibA_39221 | 195 | 0.791850339 | PSORS1C2 | psoriasis susceptibility 1 candidate 2 [Source:HGNC Symbol;Acc:HGNC:17199] |
| ENSG00000204590 | HGLibA_19545 | 195 | 0.791850339 | GNL1 | G protein nucleolar 1 (putative) [Source:HGNC Symbol;Acc:HGNC:4413] |
| ENSG00000239900 | HGLibA_01074 | 195 | 0.791850339 | ADSL | adenylosuccinate lyase [Source:HGNC Symbol;Acc:HGNC:291] |
| ENSG00000244355 | HGLibA_27667 | 195 | 0.791850339 | LY6G6D | lymphocyte antigen 6 family member G6D [Source:HGNC Symbol;Acc:HGNC:13935] |
| ENSG00000249860 | HGLibA_30483 | 195 | 0.791850339 | MTRNR2L5 | MT-RNR2 like 5 [Source:HGNC Symbol;Acc:HGNC:37162] |
| FAM160B2 | HGLibA_16214 | 195 | 0.791850339 | FAM160B2 | - |
| GATSL1 | HGLibA_18700 | 195 | 0.791850339 | GATSL1 | - |
| GTDC2 | HGLibA_20517 | 195 | 0.791850339 | GTDC2 | - |
| HMP19 | HGLibA_21752 | 195 | 0.791850339 | HMP19 | - |
| hsa-mir-1183 | HGLibA_57225 | 195 | 0.791850339 | hsa-mir-1183 | - |
| hsa-mir-137 | HGLibA_57807 | 195 | 0.791850339 | hsa-mir-137 | - |
| hsa-mir-1538 | HGLibA_57918 | 195 | 0.791850339 | hsa-mir-1538 | - |
| hsa-mir-181a-1 | HGLibA_57958 | 195 | 0.791850339 | hsa-mir-181a-1 | - |
| hsa-mir-2277 | HGLibA_58313 | 195 | 0.791850339 | hsa-mir-2277 | - |
| hsa-mir-4516 | HGLibA_60507 | 195 | 0.791850339 | hsa-mir-4516 | - |
| hsa-mir-4707 | HGLibA_60930 | 195 | 0.791850339 | hsa-mir-4707 | - |
| hsa-mir-5009 | HGLibA_61441 | 195 | 0.791850339 | hsa-mir-5009 | - |
| hsa-mir-5197 | HGLibA_61705 | 195 | 0.791850339 | hsa-mir-5197 | - |
| hsa-mir-574 | HGLibA_62391 | 195 | 0.791850339 | hsa-mir-574 | - |
| hsa-mir-6802 | HGLibA_63386 | 195 | 0.791850339 | hsa-mir-6802 | - |
| hsa-mir-7706 | HGLibA_63958 | 195 | 0.791850339 | hsa-mir-7706 | - |
| hsa-mir-7975 | HGLibA_64029 | 195 | 0.791850339 | hsa-mir-7975 | - |
| WAPAL | HGLibA_53941 | 195 | 0.791850339 | WAPAL | - |
| 7-Mar | HGLibA_28347 | 194 | 0.781899302 | 7-Mar | - |
| ADSS | HGLibA_01077 | 194 | 0.781899302 | ADSS | - |
| C15orf38-AP3S2 | HGLibA_05391 | 194 | 0.781899302 | C15orf38-AP3S2 | - |
| C6orf195 | HGLibA_06463 | 194 | 0.781899302 | C6orf195 | - |
| ENSG00000005187 | HGLibA_00577 | 194 | 0.781899302 | ACSM3 | acyl-CoA synthetase medium chain family member 3 [Source:HGNC Symbol;Acc:HGNC:10522] |
| ENSG00000040633 | HGLibA_36376 | 194 | 0.781899302 | PHF23 | PHD finger protein 23 [Source:HGNC Symbol;Acc:HGNC:28428] |
| ENSG00000044115 | HGLibA_11639 | 194 | 0.781899302 | CTNNA1 | catenin alpha 1 [Source:HGNC Symbol;Acc:HGNC:2509] |
| ENSG00000049245 | HGLibA_53495 | 194 | 0.781899302 | VAMP3 | vesicle associated membrane protein 3 [Source:HGNC Symbol;Acc:HGNC:12644] |
| ENSG00000057593 | HGLibA_15881 | 194 | 0.781899302 | F7 | coagulation factor VII [Source:HGNC Symbol;Acc:HGNC:3544] |
| ENSG00000058335 | HGLibA_40338 | 194 | 0.781899302 | RASGRF1 | Ras protein specific guanine nucleotide releasing factor 1 [Source:HGNC Symbol;Acc:HGNC:9875] |
| ENSG00000076003 | HGLibA_28619 | 194 | 0.781899302 | MCM6 | minichromosome maintenance complex component 6 [Source:HGNC Symbol;Acc:HGNC:6949] |
| ENSG00000076650 | HGLibA_19735 | 194 | 0.781899302 | GPATCH1 | G-patch domain containing 1 [Source:HGNC Symbol;Acc:HGNC:24658] |
| ENSG00000086015 | HGLibA_28402 | 194 | 0.781899302 | MAST2 | microtubule associated serine/threonine kinase 2 [Source:HGNC Symbol;Acc:HGNC:19035] |
| ENSG00000089682 | HGLibA_40549 | 194 | 0.781899302 | RBM41 | RNA binding motif protein 41 [Source:HGNC Symbol;Acc:HGNC:25617] |
| ENSG00000091128 | HGLibA_25965 | 194 | 0.781899302 | LAMB4 | laminin subunit beta 4 [Source:HGNC Symbol;Acc:HGNC:6491] |
| ENSG00000101916 | HGLibA_49663 | 194 | 0.781899302 | TLR8 | toll like receptor 8 [Source:HGNC Symbol;Acc:HGNC:15632] |
| ENSG00000102104 | HGLibA_42294 | 194 | 0.781899302 | RS1 | retinoschisin 1 [Source:HGNC Symbol;Acc:HGNC:10457] |
| ENSG00000102109 | HGLibA_35669 | 194 | 0.781899302 | PCSK1N | proprotein convertase subtilisin/kexin type 1 inhibitor [Source:HGNC Symbol;Acc:HGNC:17301] |
| ENSG00000103174 | HGLibA_31072 | 194 | 0.781899302 | NAGPA | N-acetylglucosamine-1-phosphodiester alpha-N-acetylglucosaminidase [Source:HGNC Symbol;Acc:HGNC:17378] |
| ENSG00000107551 | HGLibA_40384 | 194 | 0.781899302 | RASSF4 | Ras association domain family member 4 [Source:HGNC Symbol;Acc:HGNC:20793] |
| ENSG00000108848 | HGLibA_27627 | 194 | 0.781899302 | LUC7L3 | LUC7 like 3 pre-mRNA splicing factor [Source:HGNC Symbol;Acc:HGNC:24309] |
| ENSG00000113108 | HGLibA_02386 | 194 | 0.781899302 | APBB3 | amyloid beta precursor protein binding family B member 3 [Source:HGNC Symbol;Acc:HGNC:20708] |
| ENSG00000114841 | HGLibA_13467 | 194 | 0.781899302 | DNAH1 | dynein axonemal heavy chain 1 [Source:HGNC Symbol;Acc:HGNC:2940] |
| ENSG00000116786 | HGLibA_37140 | 194 | 0.781899302 | PLEKHM2 | pleckstrin homology and RUN domain containing M2 [Source:HGNC Symbol;Acc:HGNC:29131] |
| ENSG00000120451 | HGLibA_46053 | 194 | 0.781899302 | SNX19 | sorting nexin 19 [Source:HGNC Symbol;Acc:HGNC:21532] |
| ENSG00000122122 | HGLibA_42763 | 194 | 0.781899302 | SASH3 | SAM and SH3 domain containing 3 [Source:HGNC Symbol;Acc:HGNC:15975] |
| ENSG00000124664 | HGLibA_46567 | 194 | 0.781899302 | SPDEF | SAM pointed domain containing ETS transcription factor [Source:HGNC Symbol;Acc:HGNC:17257] |
| ENSG00000125810 | HGLibA_08481 | 194 | 0.781899302 | CD93 | CD93 molecule [Source:HGNC Symbol;Acc:HGNC:15855] |
| ENSG00000127588 | HGLibA_19518 | 194 | 0.781899302 | GNG13 | G protein subunit gamma 13 [Source:HGNC Symbol;Acc:HGNC:14131] |
| ENSG00000130592 | HGLibA_27564 | 194 | 0.781899302 | LSP1 | lymphocyte specific protein 1 [Source:HGNC Symbol;Acc:HGNC:6707] |
| ENSG00000130717 | HGLibA_52809 | 194 | 0.781899302 | UCK1 | uridine-cytidine kinase 1 [Source:HGNC Symbol;Acc:HGNC:14859] |
| ENSG00000136213 | HGLibA_09620 | 194 | 0.781899302 | CHST12 | carbohydrate sulfotransferase 12 [Source:HGNC Symbol;Acc:HGNC:17423] |
| ENSG00000138688 | HGLibA_24695 | 194 | 0.781899302 | KIAA1109 | KIAA1109 [Source:HGNC Symbol;Acc:HGNC:26953] |

| | | | | | |
|---|---|---|---|---|---|
| ENSG00000139269 | HGLibA_23311 | 194 | 0.781899302 | INHBE | inhibin subunit beta E [Source:HGNC Symbol;Acc:HGNC:24029] |
| ENSG00000140092 | HGLibA_16948 | 194 | 0.781899302 | FBLN5 | fibulin 5 [Source:HGNC Symbol;Acc:HGNC:3602] |
| ENSG00000140254 | HGLibA_14103 | 194 | 0.781899302 | DUOXA1 | dual oxidase maturation factor 1 [Source:HGNC Symbol;Acc:HGNC:26507] |
| ENSG00000142185 | HGLibA_51756 | 194 | 0.781899302 | TRPM2 | transient receptor potential cation channel subfamily M member 2 [Source:HGNC Symbol;Acc:HGNC:12339] |
| ENSG00000142405 | HGLibA_32107 | 194 | 0.781899302 | NLRP12 | NLR family pyrin domain containing 12 [Source:HGNC Symbol;Acc:HGNC:22938] |
| ENSG00000145192 | HGLibA_01319 | 194 | 0.781899302 | AHSG | alpha 2-HS glycoprotein [Source:HGNC Symbol;Acc:HGNC:349] |
| ENSG00000145996 | HGLibA_08802 | 194 | 0.781899302 | CDKAL1 | CDK5 regulatory subunit associated protein 1 like 1 [Source:HGNC Symbol;Acc:HGNC:21050] |
| ENSG00000146233 | HGLibA_12152 | 194 | 0.781899302 | CYP39A1 | cytochrome P450 family 39 subfamily A member 1 [Source:HGNC Symbol;Acc:HGNC:17449] |
| ENSG00000146378 | HGLibA_48127 | 194 | 0.781899302 | TAAR2 | trace amine associated receptor 2 (gene/pseudogene) [Source:HGNC Symbol;Acc:HGNC:4514] |
| ENSG00000148377 | HGLibA_22556 | 194 | 0.781899302 | IDI2 | isopentenyl-diphosphate delta isomerase 2 [Source:HGNC Symbol;Acc:HGNC:23487] |
| ENSG00000153066 | HGLibA_52406 | 194 | 0.781899302 | TXNDC11 | thioredoxin domain containing 11 [Source:HGNC Symbol;Acc:HGNC:28030] |
| ENSG00000155657 | HGLibA_52220 | 194 | 0.781899302 | TTN | titin [Source:HGNC Symbol;Acc:HGNC:12403] |
| ENSG00000157766 | HGLibA_00379 | 194 | 0.781899302 | ACAN | aggrecan [Source:HGNC Symbol;Acc:HGNC:319] |
| ENSG00000160862 | HGLibA_03930 | 194 | 0.781899302 | AZGP1 | zinc-binding [Source:HGNC Symbol;Acc:HGNC:910]" |
| ENSG00000164182 | HGLibA_31485 | 194 | 0.781899302 | NDUFAF2 | NADH:ubiquinone oxidoreductase complex assembly factor 2 [Source:HGNC Symbol;Acc:HGNC:28086] |
| ENSG00000165417 | HGLibA_20519 | 194 | 0.781899302 | GTF2A1 | general transcription factor IIA subunit 1 [Source:HGNC Symbol;Acc:HGNC:4646] |
| ENSG00000166501 | HGLibA_38537 | 194 | 0.781899302 | PRKCB | protein kinase C beta [Source:HGNC Symbol;Acc:HGNC:9395] |
| ENSG00000169131 | HGLibA_55860 | 194 | 0.781899302 | ZNF354A | zinc finger protein 354A [Source:HGNC Symbol;Acc:HGNC:11628] |
| ENSG00000172179 | HGLibA_38595 | 194 | 0.781899302 | PRL | prolactin [Source:HGNC Symbol;Acc:HGNC:9445] |
| ENSG00000172482 | HGLibA_01270 | 194 | 0.781899302 | AGXT | alanine--glyoxylate and serine--pyruvate aminotransferase [Source:HGNC Symbol;Acc:HGNC:341] |
| ENSG00000172987 | HGLibA_22065 | 194 | 0.781899302 | HPSE2 | heparanase 2 (inactive) [Source:HGNC Symbol;Acc:HGNC:18374] |
| ENSG00000174327 | HGLibA_44430 | 194 | 0.781899302 | SLC16A13 | solute carrier family 16 member 13 [Source:HGNC Symbol;Acc:HGNC:31037] |
| ENSG00000176230 | HGLibA_34029 | 194 | 0.781899302 | OR4K17 | olfactory receptor family 4 subfamily K member 17 [Source:HGNC Symbol;Acc:HGNC:15355] |
| ENSG00000179912 | HGLibA_39775 | 194 | 0.781899302 | R3HDM2 | R3H domain containing 2 [Source:HGNC Symbol;Acc:HGNC:29167] |
| ENSG00000180245 | HGLibA_42247 | 194 | 0.781899302 | RRH | retinal pigment epithelium-derived rhodopsin homolog [Source:HGNC Symbol;Acc:HGNC:10450] |
| ENSG00000181023 | HGLibA_34222 | 194 | 0.781899302 | OR56B1 | olfactory receptor family 56 subfamily B member 1 [Source:HGNC Symbol;Acc:HGNC:15245] |
| ENSG00000181616 | HGLibA_34170 | 194 | 0.781899302 | OR52H1 | olfactory receptor family 52 subfamily H member 1 [Source:HGNC Symbol;Acc:HGNC:15218] |
| ENSG00000181638 | HGLibA_55216 | 194 | 0.781899302 | ZFP41 | ZFP41 zinc finger protein [Source:HGNC Symbol;Acc:HGNC:26786] |
| ENSG00000184564 | HGLibA_45527 | 194 | 0.781899302 | SLITRK6 | SLIT and NTRK like family member 6 [Source:HGNC Symbol;Acc:HGNC:23503] |
| ENSG00000184697 | HGLibA_09905 | 194 | 0.781899302 | CLDN6 | claudin 6 [Source:HGNC Symbol;Acc:HGNC:2048] |
| ENSG00000187860 | HGLibA_07661 | 194 | 0.781899302 | CCDC157 | coiled-coil domain containing 157 [Source:HGNC Symbol;Acc:HGNC:33854] |
| ENSG00000188368 | HGLibA_38797 | 194 | 0.781899302 | PRR19 | proline rich 19 [Source:HGNC Symbol;Acc:HGNC:33728] |
| ENSG00000188582 | HGLibA_35175 | 194 | 0.781899302 | PAQR9 | progestin and adipoQ receptor family member 9 [Source:HGNC Symbol;Acc:HGNC:30131] |
| ENSG00000189132 | HGLibA_16611 | 194 | 0.781899302 | FAM47B | family with sequence similarity 47 member B [Source:HGNC Symbol;Acc:HGNC:26659] |
| ENSG00000196188 | HGLibA_11700 | 194 | 0.781899302 | CTSE | cathepsin E [Source:HGNC Symbol;Acc:HGNC:2530] |
| ENSG00000198464 | HGLibA_56072 | 194 | 0.781899302 | ZNF480 | zinc finger protein 480 [Source:HGNC Symbol;Acc:HGNC:23305] |
| ENSG00000223611 | HGLibA_47810 | 194 | 0.781899302 | SUPT20HL2 | SUPT20H like 2 [Source:HGNC Symbol;Acc:HGNC:31797] |
| ENSG00000235109 | HGLibA_57019 | 194 | 0.781899302 | ZSCAN31 | zinc finger and SCAN domain containing 31 [Source:HGNC Symbol;Acc:HGNC:14097] |
| ENSG00000258818 | HGLibA_41409 | 194 | 0.781899302 | RNASE4 | ribonuclease A family member 4 [Source:HGNC Symbol;Acc:HGNC:10047] |
| ENSG00000261787 | HGLibA_48780 | 194 | 0.781899302 | TCF24 | transcription factor 24 [Source:HGNC Symbol;Acc:HGNC:32275] |
| ENSG00000265190 | HGLibA_02251 | 194 | 0.781899302 | ANXA8 | annexin A8 [Source:HGNC Symbol;Acc:HGNC:546] |
| FAM84B | HGLibA_16768 | 194 | 0.781899302 | FAM84B | - |
| HIST1H4D | HGLibA_21496 | 194 | 0.781899302 | HIST1H4D | - |
| hsa-mir-3170 | HGLibA_58761 | 194 | 0.781899302 | hsa-mir-3170 | - |
| hsa-mir-4302 | HGLibA_59920 | 194 | 0.781899302 | hsa-mir-4302 | - |
| hsa-mir-4674 | HGLibA_60812 | 194 | 0.781899302 | hsa-mir-4674 | - |
| hsa-mir-4704 | HGLibA_60918 | 194 | 0.781899302 | hsa-mir-4704 | - |
| hsa-mir-568 | HGLibA_62232 | 194 | 0.781899302 | hsa-mir-568 | - |
| hsa-mir-628 | HGLibA_62751 | 194 | 0.781899302 | hsa-mir-628 | - |
| SGK110 | HGLibA_43821 | 194 | 0.781899302 | SGK110 | - |
| TMEM66 | HGLibA_50490 | 194 | 0.781899302 | TMEM66 | - |
| C20orf197 | HGLibA_06007 | 193 | 0.771948264 | C20orf197 | - |
| ENSG00000008283 | HGLibA_11977 | 193 | 0.771948264 | CYB561 | cytochrome b561 [Source:HGNC Symbol;Acc:HGNC:2571] |
| ENSG00000019186 | HGLibA_12082 | 193 | 0.771948264 | CYP24A1 | cytochrome P450 family 24 subfamily A member 1 [Source:HGNC Symbol;Acc:HGNC:2602] |
| ENSG00000025800 | HGLibA_25368 | 193 | 0.771948264 | KPNA6 | karyopherin subunit alpha 6 [Source:HGNC Symbol;Acc:HGNC:6399] |
| ENSG00000039068 | HGLibA_08629 | 193 | 0.771948264 | CDH1 | cadherin 1 [Source:HGNC Symbol;Acc:HGNC:1748] |
| ENSG00000049759 | HGLibA_31605 | 193 | 0.771948264 | NEDD4L | E3 ubiquitin protein ligase [Source:HGNC Symbol;Acc:HGNC:7728]" |
| ENSG00000054148 | HGLibA_36455 | 193 | 0.771948264 | PHPT1 | phosphohistidine phosphatase 1 [Source:HGNC Symbol;Acc:HGNC:30033] |
| ENSG00000058085 | HGLibA_25970 | 193 | 0.771948264 | LAMC2 | laminin subunit gamma 2 [Source:HGNC Symbol;Acc:HGNC:6493] |
| ENSG00000074527 | HGLibA_32859 | 193 | 0.771948264 | NTN4 | netrin 4 [Source:HGNC Symbol;Acc:HGNC:13658] |
| ENSG00000080493 | HGLibA_45219 | 193 | 0.771948264 | SLC4A4 | solute carrier family 4 member 4 [Source:HGNC Symbol;Acc:HGNC:11030] |
| ENSG00000080561 | HGLibA_29236 | 193 | 0.771948264 | MID2 | midline 2 [Source:HGNC Symbol;Acc:HGNC:7096] |
| ENSG00000084652 | HGLibA_52391 | 193 | 0.771948264 | TXLNA | taxilin alpha [Source:HGNC Symbol;Acc:HGNC:30685] |
| ENSG00000088035 | HGLibA_01635 | 193 | 0.771948264 | ALG6 | 3-glucosyltransferase [Source:HGNC Symbol;Acc:HGNC:23157]" |
| ENSG00000091664 | HGLibA_44473 | 193 | 0.771948264 | SLC17A6 | solute carrier family 17 member 6 [Source:HGNC Symbol;Acc:HGNC:16703] |
| ENSG00000100024 | HGLibA_53054 | 193 | 0.771948264 | UPB1 | beta-ureidopropionase 1 [Source:HGNC Symbol;Acc:HGNC:16297] |
| ENSG00000101367 | HGLibA_28312 | 193 | 0.771948264 | MAPRE1 | microtubule associated protein RP/EB family member 1 [Source:HGNC Symbol;Acc:HGNC:6890] |
| ENSG00000102539 | HGLibA_29430 | 193 | 0.771948264 | MLNR | motilin receptor [Source:HGNC Symbol;Acc:HGNC:4495] |
| ENSG00000105976 | HGLibA_28924 | 193 | 0.771948264 | MET | receptor tyrosine kinase [Source:HGNC Symbol;Acc:HGNC:7029]" |
| ENSG00000109472 | HGLibA_10915 | 193 | 0.771948264 | CPE | carboxypeptidase E [Source:HGNC Symbol;Acc:HGNC:2303] |
| ENSG00000111671 | HGLibA_46863 | 193 | 0.771948264 | SPSB2 | splA/ryanodine receptor domain and SOCS box containing 2 [Source:HGNC Symbol;Acc:HGNC:29522] |
| ENSG00000113966 | HGLibA_02985 | 193 | 0.771948264 | ARL6 | ADP ribosylation factor like GTPase 6 [Source:HGNC Symbol;Acc:HGNC:13210] |
| ENSG00000114698 | HGLibA_37241 | 193 | 0.771948264 | PLSCR4 | phospholipid scramblase 4 [Source:HGNC Symbol;Acc:HGNC:16497] |
| ENSG00000116793 | HGLibA_36463 | 193 | 0.771948264 | PHTF1 | putative homeodomain transcription factor 1 [Source:HGNC Symbol;Acc:HGNC:8939] |
| ENSG00000120549 | HGLibA_24722 | 193 | 0.771948264 | KIAA1217 | KIAA1217 [Source:HGNC Symbol;Acc:HGNC:25428] |
| ENSG00000124743 | HGLibA_25182 | 193 | 0.771948264 | KLHL31 | kelch like family member 31 [Source:HGNC Symbol;Acc:HGNC:21353] |
| ENSG00000125740 | HGLibA_17798 | 193 | 0.771948264 | FOSB | AP-1 transcription factor subunit [Source:HGNC Symbol;Acc:HGNC:3797]" |
| ENSG00000130733 | HGLibA_54673 | 193 | 0.771948264 | YIPF2 | Yip1 domain family member 2 [Source:HGNC Symbol;Acc:HGNC:28476] |
| ENSG00000134109 | HGLibA_14454 | 193 | 0.771948264 | EDEM1 | ER degradation enhancing alpha-mannosidase like protein 1 [Source:HGNC Symbol;Acc:HGNC:18967] |
| ENSG00000134443 | HGLibA_20368 | 193 | 0.771948264 | GRP | gastrin releasing peptide [Source:HGNC Symbol;Acc:HGNC:4605] |
| ENSG00000135452 | HGLibA_51929 | 193 | 0.771948264 | TSPAN31 | tetraspanin 31 [Source:HGNC Symbol;Acc:HGNC:10539] |
| ENSG00000141194 | HGLibA_33966 | 193 | 0.771948264 | OR4D1 | olfactory receptor family 4 subfamily D member 1 [Source:HGNC Symbol;Acc:HGNC:8293] |
| ENSG00000145687 | HGLibA_47117 | 193 | 0.771948264 | SSBP2 | single stranded DNA binding protein 2 [Source:HGNC Symbol;Acc:HGNC:15831] |
| ENSG00000146416 | HGLibA_01346 | 193 | 0.771948264 | AIG1 | androgen induced 1 [Source:HGNC Symbol;Acc:HGNC:21607] |
| ENSG00000146530 | HGLibA_53932 | 193 | 0.771948264 | VWDE | von Willebrand factor D and EGF domains [Source:HGNC Symbol;Acc:HGNC:21897] |
| ENSG00000151834 | HGLibA_18358 | 193 | 0.771948264 | GABRA2 | gamma-aminobutyric acid type A receptor alpha2 subunit [Source:HGNC Symbol;Acc:HGNC:4076] |
| ENSG00000154040 | HGLibA_06866 | 193 | 0.771948264 | CABYR | calcium binding tyrosine phosphorylation regulated [Source:HGNC Symbol;Acc:HGNC:15569] |
| ENSG00000160999 | HGLibA_43877 | 193 | 0.771948264 | SH2B2 | SH2B adaptor protein 2 [Source:HGNC Symbol;Acc:HGNC:17381] |
| ENSG00000162702 | HGLibA_55734 | 193 | 0.771948264 | ZNF281 | zinc finger protein 281 [Source:HGNC Symbol;Acc:HGNC:13075] |
| ENSG00000163218 | HGLibA_36258 | 193 | 0.771948264 | PGLYRP4 | peptidoglycan recognition protein 4 [Source:HGNC Symbol;Acc:HGNC:30015] |
| ENSG00000163431 | HGLibA_26716 | 193 | 0.771948264 | LMOD1 | leiomodin 1 [Source:HGNC Symbol;Acc:HGNC:6647] |
| ENSG00000163497 | HGLibA_17309 | 193 | 0.771948264 | FEV | ETS transcription factor [Source:HGNC Symbol;Acc:HGNC:18562]" |
| ENSG00000163512 | HGLibA_03937 | 193 | 0.771948264 | AZI2 | 5-azacytidine induced 2 [Source:HGNC Symbol;Acc:HGNC:24002] |
| ENSG00000163814 | HGLibA_08624 | 193 | 0.771948264 | CDCP1 | CUB domain containing protein 1 [Source:HGNC Symbol;Acc:HGNC:24357] |
| ENSG00000166002 | HGLibA_45671 | 193 | 0.771948264 | SMCO4 | single-pass membrane protein with coiled-coil domains 4 [Source:HGNC Symbol;Acc:HGNC:24810] |
| ENSG00000167094 | HGLibA_52057 | 193 | 0.771948264 | TTC16 | tetratricopeptide repeat domain 16 [Source:HGNC Symbol;Acc:HGNC:26536] |
| ENSG00000167916 | HGLibA_25456 | 193 | 0.771948264 | KRT24 | keratin 24 [Source:HGNC Symbol;Acc:HGNC:18527] |
| ENSG00000168818 | HGLibA_47630 | 193 | 0.771948264 | STX18 | syntaxin 18 [Source:HGNC Symbol;Acc:HGNC:15942] |
| ENSG00000173692 | HGLibA_39148 | 193 | 0.771948264 | PSMD1 | non-ATPase 1 [Source:HGNC Symbol;Acc:HGNC:9554]" |
| ENSG00000175879 | HGLibA_22003 | 193 | 0.771948264 | HOXD8 | homeobox D8 [Source:HGNC Symbol;Acc:HGNC:5139] |
| ENSG00000176219 | HGLibA_33581 | 193 | 0.771948264 | OR11H6 | olfactory receptor family 11 subfamily H member 6 [Source:HGNC Symbol;Acc:HGNC:15349] |
| ENSG00000177694 | HGLibA_31014 | 193 | 0.771948264 | NAALADL2 | N-acetylated alpha-linked acidic dipeptidase like 2 [Source:HGNC Symbol;Acc:HGNC:23219] |
| ENSG00000180481 | HGLibA_19264 | 193 | 0.771948264 | GLIPR1L2 | GLIPR1 like 2 [Source:HGNC Symbol;Acc:HGNC:28592] |
| ENSG00000181761 | HGLibA_34533 | 193 | 0.771948264 | OR8H3 | olfactory receptor family 8 subfamily H member 3 [Source:HGNC Symbol;Acc:HGNC:15309] |
| ENSG00000183208 | HGLibA_18905 | 193 | 0.771948264 | GDPGP1 | GDP-D-glucose phosphorylase 1 [Source:HGNC Symbol;Acc:HGNC:34360] |
| ENSG00000184716 | HGLibA_43494 | 193 | 0.771948264 | SERINC4 | serine incorporator 4 [Source:HGNC Symbol;Acc:HGNC:32237] |
| ENSG00000186827 | HGLibA_50783 | 193 | 0.771948264 | TNFRSF4 | TNF receptor superfamily member 4 [Source:HGNC Symbol;Acc:HGNC:11918] |
| ENSG00000187097 | HGLibA_15210 | 193 | 0.771948264 | ENTPD5 | ectonucleoside triphosphate diphosphohydrolase 5 [Source:HGNC Symbol;Acc:HGNC:3367] |
| ENSG00000187987 | HGLibA_57007 | 193 | 0.771948264 | ZSCAN23 | zinc finger and SCAN domain containing 23 [Source:HGNC Symbol;Acc:HGNC:21193] |
| ENSG00000188807 | HGLibA_50180 | 193 | 0.771948264 | TMEM201 | transmembrane protein 201 [Source:HGNC Symbol;Acc:HGNC:33719] |
| ENSG00000188997 | HGLibA_24449 | 193 | 0.771948264 | KCTD21 | potassium channel tetramerization domain containing 21 [Source:HGNC Symbol;Acc:HGNC:27452] |
| ENSG00000196860 | HGLibA_50957 | 193 | 0.771948264 | TOMM20L | translocase of outer mitochondrial membrane 20 like [Source:HGNC Symbol;Acc:HGNC:33752] |
| ENSG00000197380 | HGLibA_12287 | 193 | 0.771948264 | DACT3 | dishevelled binding antagonist of beta catenin 3 [Source:HGNC Symbol;Acc:HGNC:30745] |
| ENSG00000197417 | HGLibA_44078 | 193 | 0.771948264 | SHPK | sedoheptulokinase [Source:HGNC Symbol;Acc:HGNC:1492] |
| ENSG00000197776 | HGLibA_25090 | 193 | 0.771948264 | KLHDC1 | kelch domain containing 1 [Source:HGNC Symbol;Acc:HGNC:19836] |
| ENSG00000214216 | HGLibA_23524 | 193 | 0.771948264 | IQCJ | IQ motif containing J [Source:HGNC Symbol;Acc:HGNC:32406] |
| ENSG00000214435 | HGLibA_03181 | 193 | 0.771948264 | AS3MT | arsenite methyltransferase [Source:HGNC Symbol;Acc:HGNC:17452] |
| ENSG00000215262 | HGLibA_24399 | 193 | 0.771948264 | KCNU1 | potassium calcium-activated channel subfamily U member 1 [Source:HGNC Symbol;Acc:HGNC:18867] |
| ENSG00000268089 | HGLibA_18402 | 193 | 0.771948264 | GABRQ | gamma-aminobutyric acid type A receptor theta subunit [Source:HGNC Symbol;Acc:HGNC:14454] |
| ENSG00000268629 | HGLibA_49058 | 193 | 0.771948264 | TEX13A | testis expressed 13A [Source:HGNC Symbol;Acc:HGNC:11735] |
| ENSG00000274588 | HGLibA_13078 | 193 | 0.771948264 | DGKK | diacylglycerol kinase kappa [Source:HGNC Symbol;Acc:HGNC:32395] |
| ENSG00000287151 | HGLibA_06116 | 193 | 0.771948264 | C2orf27A | - |
| HIST3H2A | HGLibA_21555 | 193 | 0.771948264 | HIST3H2A | - |
| hsa-mir-1185-1 | HGLibA_57243 | 193 | 0.771948264 | hsa-mir-1185-1 | - |
| hsa-mir-183 | HGLibA_57993 | 193 | 0.771948264 | hsa-mir-183 | - |
| hsa-mir-18a | HGLibA_58019 | 193 | 0.771948264 | hsa-mir-18a | - |
| hsa-mir-302b | HGLibA_58439 | 193 | 0.771948264 | hsa-mir-302b | - |
| hsa-mir-3184 | HGLibA_58835 | 193 | 0.771948264 | hsa-mir-3184 | - |
| hsa-mir-337 | HGLibA_58999 | 193 | 0.771948264 | hsa-mir-337 | - |
| hsa-mir-4271 | HGLibA_59790 | 193 | 0.771948264 | hsa-mir-4271 | - |
| hsa-mir-4276 | HGLibA_59807 | 193 | 0.771948264 | hsa-mir-4276 | - |
| hsa-mir-4793 | HGLibA_61277 | 193 | 0.771948264 | hsa-mir-4793 | - |
| hsa-mir-509-1 | HGLibA_61508 | 193 | 0.771948264 | hsa-mir-509-1 | - |
| hsa-mir-6792 | HGLibA_63346 | 193 | 0.771948264 | hsa-mir-6792 | - |
| hsa-mir-6838 | HGLibA_63529 | 193 | 0.771948264 | hsa-mir-6838 | - |

| | | | | | |
|---|---|---|---|---|---|
| hsa-mir-6841 | HGLibA_63538 | 193 | 0.771948264 | hsa-mir-6841 | - |
| hsa-mir-708 | HGLibA_63779 | 193 | 0.771948264 | hsa-mir-708 | - |
| KIAA0368 | HGLibA_24630 | 193 | 0.771948264 | KIAA0368 | - |
| PTPLAD1 | HGLibA_39419 | 193 | 0.771948264 | PTPLAD1 | - |
| RRNAD1 | HGLibA_42263 | 193 | 0.771948264 | RRNAD1 | - |
| AKAP2 | HGLibA_01427 | 192 | 0.761997227 | AKAP2 | - |
| C16orf71 | HGLibA_05474 | 192 | 0.761997227 | C16orf71 | - |
| ENSG00000001626 | HGLibA_09293 | 192 | 0.761997227 | CFTR | cystic fibrosis transmembrane conductance regulator [Source:HGNC Symbol;Acc:HGNC:1884] |
| ENSG00000021461 | HGLibA_12157 | 192 | 0.761997227 | CYP3A43 | cytochrome P450 family 3 subfamily A member 43 [Source:HGNC Symbol;Acc:HGNC:17450] |
| ENSG00000021776 | HGLibA_02616 | 192 | 0.761997227 | AQR | aquarius intron-binding spliceosomal factor [Source:HGNC Symbol;Acc:HGNC:29513] |
| ENSG00000049541 | HGLibA_40906 | 192 | 0.761997227 | RFC2 | replication factor C subunit 2 [Source:HGNC Symbol;Acc:HGNC:9970] |
| ENSG00000064607 | HGLibA_47720 | 192 | 0.761997227 | SUGP2 | SURP and G-patch domain containing 2 [Source:HGNC Symbol;Acc:HGNC:18641] |
| ENSG00000064961 | HGLibA_21681 | 192 | 0.761997227 | HMG20B | high mobility group 20B [Source:HGNC Symbol;Acc:HGNC:5002] |
| ENSG00000077458 | HGLibA_16724 | 192 | 0.761997227 | FAM76B | family with sequence similarity 76 member B [Source:HGNC Symbol;Acc:HGNC:28492] |
| ENSG00000079308 | HGLibA_50923 | 192 | 0.761997227 | TNS1 | tensin 1 [Source:HGNC Symbol;Acc:HGNC:11973] |
| ENSG00000086205 | HGLibA_17780 | 192 | 0.761997227 | FOLH1 | folate hydrolase 1 [Source:HGNC Symbol;Acc:HGNC:3788] |
| ENSG00000087303 | HGLibA_31925 | 192 | 0.761997227 | NID2 | nidogen 2 [Source:HGNC Symbol;Acc:HGNC:13389] |
| ENSG00000088882 | HGLibA_11035 | 192 | 0.761997227 | CPXM1 | M14 family member 1 [Source:HGNC Symbol;Acc:HGNC:15771]" |
| ENSG00000089250 | HGLibA_32322 | 192 | 0.761997227 | NOS1 | nitric oxide synthase 1 [Source:HGNC Symbol;Acc:HGNC:7872] |
| ENSG00000099864 | HGLibA_35066 | 192 | 0.761997227 | PALM | paralemmin [Source:HGNC Symbol;Acc:HGNC:8594] |
| ENSG00000100036 | HGLibA_44997 | 192 | 0.761997227 | SLC35E4 | solute carrier family 35 member E4 [Source:HGNC Symbol;Acc:HGNC:17058] |
| ENSG00000100241 | HGLibA_42794 | 192 | 0.761997227 | SBF1 | SET binding factor 1 [Source:HGNC Symbol;Acc:HGNC:10542] |
| ENSG00000102316 | HGLibA_27971 | 192 | 0.761997227 | MAGED2 | MAGE family member D2 [Source:HGNC Symbol;Acc:HGNC:16353] |
| ENSG00000105954 | HGLibA_32540 | 192 | 0.761997227 | NPVF | neuropeptide VF precursor [Source:HGNC Symbol;Acc:HGNC:13782] |
| ENSG00000108946 | HGLibA_38520 | 192 | 0.761997227 | PRKAR1A | protein kinase cAMP-dependent type I regulatory subunit alpha [Source:HGNC Symbol;Acc:HGNC:9388] |
| ENSG00000111110 | HGLibA_37980 | 192 | 0.761997227 | PPM1H | Mg2+/Mn2+ dependent 1H [Source:HGNC Symbol;Acc:HGNC:18583]" |
| ENSG00000115295 | HGLibA_10050 | 192 | 0.761997227 | CLIP4 | CAP-Gly domain containing linker protein family member 4 [Source:HGNC Symbol;Acc:HGNC:26108] |
| ENSG00000117533 | HGLibA_53498 | 192 | 0.761997227 | VAMP4 | vesicle associated membrane protein 4 [Source:HGNC Symbol;Acc:HGNC:12645] |
| ENSG00000121864 | HGLibA_56441 | 192 | 0.761997227 | ZNF639 | zinc finger protein 639 [Source:HGNC Symbol;Acc:HGNC:30950] |
| ENSG00000122417 | HGLibA_33281 | 192 | 0.761997227 | ODF2L | outer dense fiber of sperm tails 2 like [Source:HGNC Symbol;Acc:HGNC:29225] |
| ENSG00000126549 | HGLibA_47401 | 192 | 0.761997227 | STATH | statherin [Source:HGNC Symbol;Acc:HGNC:11369] |
| ENSG00000127903 | HGLibA_56839 | 192 | 0.761997227 | ZNF835 | zinc finger protein 835 [Source:HGNC Symbol;Acc:HGNC:34332] |
| ENSG00000129680 | HGLibA_28240 | 192 | 0.761997227 | MAP7D3 | MAP7 domain containing 3 [Source:HGNC Symbol;Acc:HGNC:25742] |
| ENSG00000130204 | HGLibA_50967 | 192 | 0.761997227 | TOMM40 | translocase of outer mitochondrial membrane 40 [Source:HGNC Symbol;Acc:HGNC:18001] |
| ENSG00000130962 | HGLibA_38868 | 192 | 0.761997227 | PRRG1 | proline rich and Gla domain 1 [Source:HGNC Symbol;Acc:HGNC:9469] |
| ENSG00000131263 | HGLibA_41349 | 192 | 0.761997227 | RLIM | LIM domain interacting [Source:HGNC Symbol;Acc:HGNC:13429]" |
| ENSG00000131375 | HGLibA_07176 | 192 | 0.761997227 | CAPN7 | calpain 7 [Source:HGNC Symbol;Acc:HGNC:1484] |
| ENSG00000132254 | HGLibA_02680 | 192 | 0.761997227 | ARFIP2 | ADP ribosylation factor interacting protein 2 [Source:HGNC Symbol;Acc:HGNC:17160] |
| ENSG00000133243 | HGLibA_04911 | 192 | 0.761997227 | BTBD2 | BTB domain containing 2 [Source:HGNC Symbol;Acc:HGNC:15504] |
| ENSG00000133246 | HGLibA_38273 | 192 | 0.761997227 | PRAM1 | PML-RARA regulated adaptor molecule 1 [Source:HGNC Symbol;Acc:HGNC:30091] |
| ENSG00000140009 | HGLibA_15583 | 192 | 0.761997227 | ESR2 | estrogen receptor 2 [Source:HGNC Symbol;Acc:HGNC:3468] |
| ENSG00000143494 | HGLibA_53533 | 192 | 0.761997227 | VASH2 | vasohibin 2 [Source:HGNC Symbol;Acc:HGNC:25723] |
| ENSG00000146147 | HGLibA_29387 | 192 | 0.761997227 | MLIP | muscular LMNA interacting protein [Source:HGNC Symbol;Acc:HGNC:21355] |
| ENSG00000146701 | HGLibA_28688 | 192 | 0.761997227 | MDH2 | malate dehydrogenase 2 [Source:HGNC Symbol;Acc:HGNC:6971] |
| ENSG00000147596 | HGLibA_38378 | 192 | 0.761997227 | PRDM14 | PR/SET domain 14 [Source:HGNC Symbol;Acc:HGNC:14001] |
| ENSG00000147869 | HGLibA_09187 | 192 | 0.761997227 | CER1 | DAN family BMP antagonist [Source:HGNC Symbol;Acc:HGNC:1862]" |
| ENSG00000149090 | HGLibA_35089 | 192 | 0.761997227 | PAMR1 | peptidase domain containing associated with muscle regeneration 1 [Source:HGNC Symbol;Acc:HGNC:24554] |
| ENSG00000149260 | HGLibA_07168 | 192 | 0.761997227 | CAPN5 | calpain 5 [Source:HGNC Symbol;Acc:HGNC:1482] |
| ENSG00000151474 | HGLibA_18014 | 192 | 0.761997227 | FRMD4A | FERM domain containing 4A [Source:HGNC Symbol;Acc:HGNC:25491] |
| ENSG00000152380 | HGLibA_16166 | 192 | 0.761997227 | FAM151B | family with sequence similarity 151 member B [Source:HGNC Symbol;Acc:HGNC:33716] |
| ENSG00000153531 | HGLibA_01028 | 192 | 0.761997227 | ADPRHL1 | ADP-ribosylhydrolase like 1 [Source:HGNC Symbol;Acc:HGNC:21303] |
| ENSG00000153896 | HGLibA_56358 | 192 | 0.761997227 | ZNF599 | zinc finger protein 599 [Source:HGNC Symbol;Acc:HGNC:26408] |
| ENSG00000156256 | HGLibA_53213 | 192 | 0.761997227 | USP16 | ubiquitin specific peptidase 16 [Source:HGNC Symbol;Acc:HGNC:12614] |
| ENSG00000159527 | HGLibA_36256 | 192 | 0.761997227 | PGLYRP3 | peptidoglycan recognition protein 3 [Source:HGNC Symbol;Acc:HGNC:30014] |
| ENSG00000162877 | HGLibA_37284 | 192 | 0.761997227 | PM20D1 | peptidase M20 domain containing 1 [Source:HGNC Symbol;Acc:HGNC:26518] |
| ENSG00000163393 | HGLibA_44552 | 192 | 0.761997227 | SLC22A15 | solute carrier family 22 member 15 [Source:HGNC Symbol;Acc:HGNC:20301] |
| ENSG00000164291 | HGLibA_03156 | 192 | 0.761997227 | ARSK | arylsulfatase family member K [Source:HGNC Symbol;Acc:HGNC:25239] |
| ENSG00000164675 | HGLibA_23550 | 192 | 0.761997227 | IQUB | IQ motif and ubiquitin domain containing [Source:HGNC Symbol;Acc:HGNC:21995] |
| ENSG00000164694 | HGLibA_17745 | 192 | 0.761997227 | FNDC1 | fibronectin type III domain containing 1 [Source:HGNC Symbol;Acc:HGNC:21184] |
| ENSG00000166407 | HGLibA_26701 | 192 | 0.761997227 | LMO1 | LIM domain only 1 [Source:HGNC Symbol;Acc:HGNC:6641] |
| ENSG00000166527 | HGLibA_09986 | 192 | 0.761997227 | CLEC4D | C-type lectin domain family 4 member D [Source:HGNC Symbol;Acc:HGNC:14554] |
| ENSG00000167202 | HGLibA_48526 | 192 | 0.761997227 | TBC1D2B | TBC1 domain family member 2B [Source:HGNC Symbol;Acc:HGNC:29183] |
| ENSG00000167748 | HGLibA_25231 | 192 | 0.761997227 | KLK1 | kallikrein 1 [Source:HGNC Symbol;Acc:HGNC:6357] |
| ENSG00000168395 | HGLibA_23294 | 192 | 0.761997227 | ING5 | inhibitor of growth family member 5 [Source:HGNC Symbol;Acc:HGNC:19421] |
| ENSG00000168439 | HGLibA_47446 | 192 | 0.761997227 | STIP1 | stress induced phosphoprotein 1 [Source:HGNC Symbol;Acc:HGNC:11387] |
| ENSG00000177143 | HGLibA_09232 | 192 | 0.761997227 | CETN1 | centrin 1 [Source:HGNC Symbol;Acc:HGNC:1866] |
| ENSG00000177710 | HGLibA_45026 | 192 | 0.761997227 | SLC35G5 | solute carrier family 35 member G5 [Source:HGNC Symbol;Acc:HGNC:15546] |
| ENSG00000179922 | HGLibA_56745 | 192 | 0.761997227 | ZNF784 | zinc finger protein 784 [Source:HGNC Symbol;Acc:HGNC:33111] |
| ENSG00000182208 | HGLibA_29591 | 192 | 0.761997227 | MOB2 | MOB kinase activator 2 [Source:HGNC Symbol;Acc:HGNC:24904] |
| ENSG00000183576 | HGLibA_43647 | 192 | 0.761997227 | SETD3 | SET domain containing 3 [Source:HGNC Symbol;Acc:HGNC:20493] |
| ENSG00000184140 | HGLibA_34013 | 192 | 0.761997227 | OR4F6 | olfactory receptor family 4 subfamily F member 6 [Source:HGNC Symbol;Acc:HGNC:15372] |
| ENSG00000185220 | HGLibA_36220 | 192 | 0.761997227 | PGBD2 | piggyBac transposable element derived 2 [Source:HGNC Symbol;Acc:HGNC:19399] |
| ENSG00000187758 | HGLibA_00951 | 192 | 0.761997227 | ADH1A | alpha polypeptide [Source:HGNC Symbol;Acc:HGNC:249]" |
| ENSG00000187848 | HGLibA_34850 | 192 | 0.761997227 | P2RX2 | purinergic receptor P2X 2 [Source:HGNC Symbol;Acc:HGNC:15459] |
| ENSG00000188167 | HGLibA_50609 | 192 | 0.761997227 | TMPPE | transmembrane protein with metallophosphoesterase domain [Source:HGNC Symbol;Acc:HGNC:33865] |
| ENSG00000197381 | HGLibA_00871 | 192 | 0.761997227 | ADARB1 | RNA specific B1 [Source:HGNC Symbol;Acc:HGNC:226]" |
| ENSG00000197961 | HGLibA_55453 | 192 | 0.761997227 | ZNF121 | zinc finger protein 121 [Source:HGNC Symbol;Acc:HGNC:12904] |
| ENSG00000198092 | HGLibA_50624 | 192 | 0.761997227 | TMPRSS11F | transmembrane serine protease 11F [Source:HGNC Symbol;Acc:HGNC:29994] |
| ENSG00000204438 | HGLibA_19728 | 192 | 0.761997227 | GPANK1 | G-patch domain and ankyrin repeats 1 [Source:HGNC Symbol;Acc:HGNC:13920] |
| ENSG00000221938 | HGLibA_33726 | 192 | 0.761997227 | OR2A14 | olfactory receptor family 2 subfamily A member 14 [Source:HGNC Symbol;Acc:HGNC:15084] |
| ENSG00000241852 | HGLibA_06638 | 192 | 0.761997227 | C8orf58 | chromosome 8 open reading frame 58 [Source:HGNC Symbol;Acc:HGNC:32233] |
| ENSG00000285723 | HGLibA_29330 | 192 | 0.761997227 | MKKS | novel protein |
| GSTT1 | HGLibA_20497 | 192 | 0.761997227 | GSTT1 | - |
| hsa-mir-1587 | HGLibA_57935 | 192 | 0.761997227 | hsa-mir-1587 | - |
| hsa-mir-3193 | HGLibA_58870 | 192 | 0.761997227 | hsa-mir-3193 | - |
| hsa-mir-323b | HGLibA_58960 | 192 | 0.761997227 | hsa-mir-323b | - |
| hsa-mir-5008 | HGLibA_61435 | 192 | 0.761997227 | hsa-mir-5008 | - |
| hsa-mir-501 | HGLibA_61450 | 192 | 0.761997227 | hsa-mir-501 | - |
| hsa-mir-509-1 | HGLibA_61507 | 192 | 0.761997227 | hsa-mir-509-1 | - |
| hsa-mir-5197 | HGLibA_61706 | 192 | 0.761997227 | hsa-mir-5197 | - |
| hsa-mir-8077 | HGLibA_64152 | 192 | 0.761997227 | hsa-mir-8077 | - |
| KIAA1045 | HGLibA_24687 | 192 | 0.761997227 | KIAA1045 | - |
| KIAA1324L | HGLibA_24738 | 192 | 0.761997227 | KIAA1324L | - |
| LOC728819 | HGLibA_26994 | 192 | 0.761997227 | LOC728819 | - |
| PHOSPHO2-KLHL23 | HGLibA_36448 | 192 | 0.761997227 | PHOSPHO2-KLHL23 | - |
| PRAMEF16 | HGLibA_38295 | 192 | 0.761997227 | PRAMEF16 | - |
| PRR25 | HGLibA_38832 | 192 | 0.761997227 | PRR25 | - |
| QTRTD1 | HGLibA_39764 | 192 | 0.761997227 | QTRTD1 | - |
| TLDC1 | HGLibA_49601 | 192 | 0.761997227 | TLDC1 | - |
| BCMO1 | HGLibA_04355 | 191 | 0.752046189 | BCMO1 | - |
| C15orf26 | HGLibA_05377 | 191 | 0.752046189 | C15orf26 | - |
| C6orf211 | HGLibA_06473 | 191 | 0.752046189 | C6orf211 | - |
| ENSG00000003393 | HGLibA_01718 | 191 | 0.752046189 | ALS2 | alsin Rho guanine nucleotide exchange factor [Source:HGNC Symbol;Acc:HGNC:443]" |
| ENSG00000007402 | HGLibA_06909 | 191 | 0.752046189 | CACNA2D2 | calcium voltage-gated channel auxiliary subunit alpha2delta 2 [Source:HGNC Symbol;Acc:HGNC:1400] |
| ENSG00000008282 | HGLibA_48037 | 191 | 0.752046189 | SYPL1 | synaptophysin like 1 [Source:HGNC Symbol;Acc:HGNC:11507] |
| ENSG00000008323 | HGLibA_37119 | 191 | 0.752046189 | PLEKHG6 | pleckstrin homology and RhoGEF domain containing G6 [Source:HGNC Symbol;Acc:HGNC:25562] |
| ENSG00000008438 | HGLibA_36250 | 191 | 0.752046189 | PGLYRP1 | peptidoglycan recognition protein 1 [Source:HGNC Symbol;Acc:HGNC:8904] |
| ENSG00000009765 | HGLibA_23888 | 191 | 0.752046189 | IYD | iodotyrosine deiodinase [Source:HGNC Symbol;Acc:HGNC:21071] |
| ENSG00000065613 | HGLibA_45532 | 191 | 0.752046189 | SLK | STE20 like kinase [Source:HGNC Symbol;Acc:HGNC:11088] |
| ENSG00000080823 | HGLibA_29635 | 191 | 0.752046189 | MOK | MOK protein kinase [Source:HGNC Symbol;Acc:HGNC:9833] |
| ENSG00000085871 | HGLibA_29178 | 191 | 0.752046189 | MGST2 | microsomal glutathione S-transferase 2 [Source:HGNC Symbol;Acc:HGNC:7063] |
| ENSG00000100311 | HGLibA_35845 | 191 | 0.752046189 | PDGFB | platelet derived growth factor subunit B [Source:HGNC Symbol;Acc:HGNC:8800] |
| ENSG00000100344 | HGLibA_37411 | 191 | 0.752046189 | PNPLA3 | patatin like phospholipase domain containing 3 [Source:HGNC Symbol;Acc:HGNC:18590] |
| ENSG00000100351 | HGLibA_20215 | 191 | 0.752046189 | GRAP2 | GRB2 related adaptor protein 2 [Source:HGNC Symbol;Acc:HGNC:4563] |
| ENSG00000102309 | HGLibA_36688 | 191 | 0.752046189 | PIN4 | NIMA-interacting 4 [Source:HGNC Symbol;Acc:HGNC:8992]" |
| ENSG00000102359 | HGLibA_47037 | 191 | 0.752046189 | SRPX2 | sushi repeat containing protein X-linked 2 [Source:HGNC Symbol;Acc:HGNC:30668] |
| ENSG00000105607 | HGLibA_18769 | 191 | 0.752046189 | GCDH | glutaryl-CoA dehydrogenase [Source:HGNC Symbol;Acc:HGNC:4189] |
| ENSG00000105607 | HGLibA_18771 | 191 | 0.752046189 | GCDH | glutaryl-CoA dehydrogenase [Source:HGNC Symbol;Acc:HGNC:4189] |
| ENSG00000113073 | HGLibA_45231 | 191 | 0.752046189 | SLC4A9 | solute carrier family 4 member 9 [Source:HGNC Symbol;Acc:HGNC:11035] |
| ENSG00000113597 | HGLibA_51310 | 191 | 0.752046189 | TRAPPC13 | trafficking protein particle complex 13 [Source:HGNC Symbol;Acc:HGNC:25828] |
| ENSG00000115306 | HGLibA_46880 | 191 | 0.752046189 | SPTBN1 | non-erythrocytic 1 [Source:HGNC Symbol;Acc:HGNC:11275]" |
| ENSG00000116574 | HGLibA_41208 | 191 | 0.752046189 | RHOU | ras homolog family member U [Source:HGNC Symbol;Acc:HGNC:17794] |
| ENSG00000118496 | HGLibA_17067 | 191 | 0.752046189 | FBXO30 | F-box protein 30 [Source:HGNC Symbol;Acc:HGNC:15600] |
| ENSG00000121594 | HGLibA_08454 | 191 | 0.752046189 | CD80 | CD80 molecule [Source:HGNC Symbol;Acc:HGNC:1700] |
| ENSG00000124490 | HGLibA_11167 | 191 | 0.752046189 | CRISP2 | cysteine rich secretory protein 2 [Source:HGNC Symbol;Acc:HGNC:12024] |
| ENSG00000126804 | HGLibA_54812 | 191 | 0.752046189 | ZBTB1 | zinc finger and BTB domain containing 1 [Source:HGNC Symbol;Acc:HGNC:20259] |
| ENSG00000128311 | HGLibA_52023 | 191 | 0.752046189 | TST | thiosulfate sulfurtransferase [Source:HGNC Symbol;Acc:HGNC:12388] |
| ENSG00000130182 | HGLibA_56985 | 191 | 0.752046189 | ZSCAN10 | zinc finger and SCAN domain containing 10 [Source:HGNC Symbol;Acc:HGNC:12997] |
| ENSG00000130764 | HGLibA_27370 | 191 | 0.752046189 | LRRC47 | leucine rich repeat containing 47 [Source:HGNC Symbol;Acc:HGNC:29207] |
| ENSG00000133661 | HGLibA_43777 | 191 | 0.752046189 | SFTPD | surfactant protein D [Source:HGNC Symbol;Acc:HGNC:10803] |
| ENSG00000135686 | HGLibA_25197 | 191 | 0.752046189 | KLHL36 | kelch like family member 36 [Source:HGNC Symbol;Acc:HGNC:17844] |
| ENSG00000136048 | HGLibA_13943 | 191 | 0.752046189 | DRAM1 | DNA damage regulated autophagy modulator 1 [Source:HGNC Symbol;Acc:HGNC:25645] |
| ENSG00000136694 | HGLibA_23146 | 191 | 0.752046189 | IL36A | interleukin 36 alpha [Source:HGNC Symbol;Acc:HGNC:15562] |
| ENSG00000136867 | HGLibA_44929 | 191 | 0.752046189 | SLC31A2 | solute carrier family 31 member 2 [Source:HGNC Symbol;Acc:HGNC:11017] |
| ENSG00000137872 | HGLibA_43373 | 191 | 0.752046189 | SEMA6D | semaphorin 6D [Source:HGNC Symbol;Acc:HGNC:16770] |

| | | | | | |
|---|---|---|---|---|---|
| ENSG00000139835 | HGLibA_20382 | 191 | 0.752046189 | GRTP1 | growth hormone regulated TBC protein 1 [Source:HGNC Symbol;Acc:HGNC:20310] |
| ENSG00000140323 | HGLibA_13284 | 191 | 0.752046189 | DISP2 | dispatched RND transporter family member 2 [Source:HGNC Symbol;Acc:HGNC:19712] |
| ENSG00000141367 | HGLibA_10150 | 191 | 0.752046189 | CLTC | clathrin heavy chain [Source:HGNC Symbol;Acc:HGNC:2092] |
| ENSG00000143067 | HGLibA_56562 | 191 | 0.752046189 | ZNF697 | zinc finger protein 697 [Source:HGNC Symbol;Acc:HGNC:32034] |
| ENSG00000143162 | HGLibA_11120 | 191 | 0.752046189 | CREG1 | cellular repressor of E1A stimulated genes 1 [Source:HGNC Symbol;Acc:HGNC:2351] |
| ENSG00000143740 | HGLibA_45884 | 191 | 0.752046189 | SNAP47 | synaptosome associated protein 47 [Source:HGNC Symbol;Acc:HGNC:30669] |
| ENSG00000145864 | HGLibA_18377 | 191 | 0.752046189 | GABRB2 | gamma-aminobutyric acid type A receptor beta2 subunit [Source:HGNC Symbol;Acc:HGNC:4082] |
| ENSG00000150907 | HGLibA_17922 | 191 | 0.752046189 | FOXO1 | forkhead box O1 [Source:HGNC Symbol;Acc:HGNC:3819] |
| ENSG00000152944 | HGLibA_28776 | 191 | 0.752046189 | MED21 | mediator complex subunit 21 [Source:HGNC Symbol;Acc:HGNC:11473] |
| ENSG00000156052 | HGLibA_19467 | 191 | 0.752046189 | GNAQ | G protein subunit alpha q [Source:HGNC Symbol;Acc:HGNC:4390] |
| ENSG00000157426 | HGLibA_00076 | 191 | 0.752046189 | AASDH | aminoadipate-semialdehyde dehydrogenase [Source:HGNC Symbol;Acc:HGNC:23993] |
| ENSG00000157429 | HGLibA_55554 | 191 | 0.752046189 | ZNF19 | zinc finger protein 19 [Source:HGNC Symbol;Acc:HGNC:12981] |
| ENSG00000159398 | HGLibA_09229 | 191 | 0.752046189 | CES5A | carboxylesterase 5A [Source:HGNC Symbol;Acc:HGNC:26459] |
| ENSG00000160050 | HGLibA_07748 | 191 | 0.752046189 | CCDC28B | coiled-coil domain containing 28B [Source:HGNC Symbol;Acc:HGNC:28163] |
| ENSG00000161692 | HGLibA_12379 | 191 | 0.752046189 | DBF4B | DBF4 zinc finger B [Source:HGNC Symbol;Acc:HGNC:17883] |
| ENSG00000162222 | HGLibA_52165 | 191 | 0.752046189 | TTC9C | tetratricopeptide repeat domain 9C [Source:HGNC Symbol;Acc:HGNC:28432] |
| ENSG00000165832 | HGLibA_51802 | 191 | 0.752046189 | TRUB1 | TruB pseudouridine synthase family member 1 [Source:HGNC Symbol;Acc:HGNC:16060] |
| ENSG00000166863 | HGLibA_48153 | 191 | 0.752046189 | TAC3 | tachykinin 3 [Source:HGNC Symbol;Acc:HGNC:11521] |
| ENSG00000166888 | HGLibA_47398 | 191 | 0.752046189 | STAT6 | signal transducer and activator of transcription 6 [Source:HGNC Symbol;Acc:HGNC:11368] |
| ENSG00000167004 | HGLibA_35878 | 191 | 0.752046189 | PDIA3 | protein disulfide isomerase family A member 3 [Source:HGNC Symbol;Acc:HGNC:4606] |
| ENSG00000167515 | HGLibA_51314 | 191 | 0.752046189 | TRAPPC2L | trafficking protein particle complex 2 like [Source:HGNC Symbol;Acc:HGNC:30887] |
| ENSG00000168067 | HGLibA_28215 | 191 | 0.752046189 | MAP4K2 | mitogen-activated protein kinase kinase kinase kinase 2 [Source:HGNC Symbol;Acc:HGNC:6864] |
| ENSG00000169344 | HGLibA_52979 | 191 | 0.752046189 | UMOD | uromodulin [Source:HGNC Symbol;Acc:HGNC:12559] |
| ENSG00000170743 | HGLibA_48092 | 191 | 0.752046189 | SYT9 | synaptotagmin 9 [Source:HGNC Symbol;Acc:HGNC:19265] |
| ENSG00000171735 | HGLibA_07115 | 191 | 0.752046189 | CAMTA1 | calmodulin binding transcription activator 1 [Source:HGNC Symbol;Acc:HGNC:18806] |
| ENSG00000172403 | HGLibA_48022 | 191 | 0.752046189 | SYNPO2 | synaptopodin 2 [Source:HGNC Symbol;Acc:HGNC:17732] |
| ENSG00000173013 | HGLibA_07937 | 191 | 0.752046189 | CCDC96 | coiled-coil domain containing 96 [Source:HGNC Symbol;Acc:HGNC:26900] |
| ENSG00000174500 | HGLibA_18828 | 191 | 0.752046189 | GCSAM | germinal center associated signaling and motility [Source:HGNC Symbol;Acc:HGNC:20253] |
| ENSG00000175018 | HGLibA_49103 | 191 | 0.752046189 | TEX36 | testis expressed 36 [Source:HGNC Symbol;Acc:HGNC:31653] |
| ENSG00000179331 | HGLibA_39902 | 191 | 0.752046189 | RAB39A | member RAS oncogene family [Source:HGNC Symbol;Acc:HGNC:16521]" |
| ENSG00000180914 | HGLibA_34843 | 191 | 0.752046189 | OXTR | oxytocin receptor [Source:HGNC Symbol;Acc:HGNC:8529] |
| ENSG00000180938 | HGLibA_56281 | 191 | 0.752046189 | ZNF572 | zinc finger protein 572 [Source:HGNC Symbol;Acc:HGNC:26758] |
| ENSG00000181458 | HGLibA_50409 | 191 | 0.752046189 | TMEM45A | transmembrane protein 45A [Source:HGNC Symbol;Acc:HGNC:25480] |
| ENSG00000181718 | HGLibA_34357 | 191 | 0.752046189 | OR5T2 | olfactory receptor family 5 subfamily T member 2 [Source:HGNC Symbol;Acc:HGNC:15296] |
| ENSG00000181852 | HGLibA_41681 | 191 | 0.752046189 | RNF41 | ring finger protein 41 [Source:HGNC Symbol;Acc:HGNC:18401] |
| ENSG00000182271 | HGLibA_50584 | 191 | 0.752046189 | TMIGD1 | transmembrane and immunoglobulin domain containing 1 [Source:HGNC Symbol;Acc:HGNC:32431] |
| ENSG00000183049 | HGLibA_07060 | 191 | 0.752046189 | CAMK1D | calcium/calmodulin dependent protein kinase ID [Source:HGNC Symbol;Acc:HGNC:19341] |
| ENSG00000184924 | HGLibA_39557 | 191 | 0.752046189 | PTRHD1 | peptidyl-tRNA hydrolase domain containing 1 [Source:HGNC Symbol;Acc:HGNC:33782] |
| ENSG00000185352 | HGLibA_22148 | 191 | 0.752046189 | HS6ST3 | heparan sulfate 6-O-sulfotransferase 3 [Source:HGNC Symbol;Acc:HGNC:19134] |
| ENSG00000186723 | HGLibA_33506 | 191 | 0.752046189 | OR10H1 | olfactory receptor family 10 subfamily H member 1 [Source:HGNC Symbol;Acc:HGNC:8172] |
| ENSG00000187612 | HGLibA_34366 | 191 | 0.752046189 | OR5W2 | olfactory receptor family 5 subfamily W member 2 [Source:HGNC Symbol;Acc:HGNC:15299] |
| ENSG00000187862 | HGLibA_52084 | 191 | 0.752046189 | TTC24 | tetratricopeptide repeat domain 24 [Source:HGNC Symbol;Acc:HGNC:32348] |
| ENSG00000188089 | HGLibA_36912 | 191 | 0.752046189 | PLA2G4E | phospholipase A2 group IVE [Source:HGNC Symbol;Acc:HGNC:24791] |
| ENSG00000189067 | HGLibA_26639 | 191 | 0.752046189 | LITAF | lipopolysaccharide induced TNF factor [Source:HGNC Symbol;Acc:HGNC:16841] |
| ENSG00000197430 | HGLibA_33410 | 191 | 0.752046189 | OPALIN | oligodendrocytic myelin paranodal and inner loop protein [Source:HGNC Symbol;Acc:HGNC:20707] |
| ENSG00000198780 | HGLibA_16251 | 191 | 0.752046189 | FAM169A | family with sequence similarity 169 member A [Source:HGNC Symbol;Acc:HGNC:29138] |
| ENSG00000198951 | HGLibA_31063 | 191 | 0.752046189 | NAGA | alpha-N-acetylgalactosaminidase [Source:HGNC Symbol;Acc:HGNC:7631] |
| ENSG00000203756 | HGLibA_50296 | 191 | 0.752046189 | TMEM244 | transmembrane protein 244 [Source:HGNC Symbol;Acc:HGNC:21571] |
| ENSG00000205328 | HGLibA_34398 | 191 | 0.752046189 | OR6C65 | olfactory receptor family 6 subfamily C member 65 [Source:HGNC Symbol;Acc:HGNC:31295] |
| ENSG00000205593 | HGLibA_12968 | 191 | 0.752046189 | DENND6B | DENN domain containing 6B [Source:HGNC Symbol;Acc:HGNC:32690] |
| ENSG00000212900 | HGLibA_25744 | 191 | 0.752046189 | KRTAP3-2 | keratin associated protein 3-2 [Source:HGNC Symbol;Acc:HGNC:16779] |
| ENSG00000215375 | HGLibA_30765 | 191 | 0.752046189 | MYL5 | myosin light chain 5 [Source:HGNC Symbol;Acc:HGNC:7586] |
| ENSG00000215545 | HGLibA_12862 | 191 | 0.752046189 | DEFB116 | defensin beta 116 [Source:HGNC Symbol;Acc:HGNC:18097] |
| ENSG00000220008 | HGLibA_26590 | 191 | 0.752046189 | LINGO3 | leucine rich repeat and Ig domain containing 3 [Source:HGNC Symbol;Acc:HGNC:21206] |
| ENSG00000255423 | HGLibA_14368 | 191 | 0.752046189 | EBLN2 | endogenous Bornavirus like nucleoprotein 2 [Source:HGNC Symbol;Acc:HGNC:25493] |
| ENSG00000273749 | HGLibA_12031 | 191 | 0.752046189 | CYFIP1 | cytoplasmic FMR1 interacting protein 1 [Source:HGNC Symbol;Acc:HGNC:13759] |
| FAM73A | HGLibA_16717 | 191 | 0.752046189 | FAM73A | - |
| hsa-mir-1273c | HGLibA_57536 | 191 | 0.752046189 | hsa-mir-1273c | - |
| hsa-mir-1539 | HGLibA_57924 | 191 | 0.752046189 | hsa-mir-1539 | - |
| hsa-mir-3120 | HGLibA_58534 | 191 | 0.752046189 | hsa-mir-3120 | - |
| hsa-mir-3147 | HGLibA_58646 | 191 | 0.752046189 | hsa-mir-3147 | - |
| hsa-mir-3150a | HGLibA_58654 | 191 | 0.752046189 | hsa-mir-3150a | - |
| hsa-mir-3617 | HGLibA_59099 | 191 | 0.752046189 | hsa-mir-3617 | - |
| hsa-mir-3622b | HGLibA_59126 | 191 | 0.752046189 | hsa-mir-3622b | - |
| hsa-mir-363 | HGLibA_59131 | 191 | 0.752046189 | hsa-mir-363 | - |
| hsa-mir-4307 | HGLibA_59941 | 191 | 0.752046189 | hsa-mir-4307 | - |
| hsa-mir-4636 | HGLibA_60651 | 191 | 0.752046189 | hsa-mir-4636 | - |
| hsa-mir-4662a | HGLibA_60758 | 191 | 0.752046189 | hsa-mir-4662a | - |
| hsa-mir-4698 | HGLibA_60900 | 191 | 0.752046189 | hsa-mir-4698 | - |
| hsa-mir-5187 | HGLibA_61640 | 191 | 0.752046189 | hsa-mir-5187 | - |
| hsa-mir-611 | HGLibA_62635 | 191 | 0.752046189 | hsa-mir-611 | - |
| hsa-mir-6872 | HGLibA_63674 | 191 | 0.752046189 | hsa-mir-6872 | - |
| MKL1 | HGLibA_29333 | 191 | 0.752046189 | MKL1 | - |
| C17orf62 | HGLibA_05556 | 190 | 0.742095152 | C17orf62 | - |
| C3orf72 | HGLibA_06267 | 190 | 0.742095152 | C3orf72 | - |
| C6orf195 | HGLibA_06465 | 190 | 0.742095152 | C6orf195 | - |
| CCL4L1 | HGLibA_08030 | 190 | 0.742095152 | CCL4L1 | - |
| ENSG00000007080 | HGLibA_07575 | 190 | 0.742095152 | CCDC124 | coiled-coil domain containing 124 [Source:HGNC Symbol;Acc:HGNC:25171] |
| ENSG00000042781 | HGLibA_53178 | 190 | 0.742095152 | USH2A | usherin [Source:HGNC Symbol;Acc:HGNC:12601] |
| ENSG00000064932 | HGLibA_42810 | 190 | 0.742095152 | SBNO2 | strawberry notch homolog 2 [Source:HGNC Symbol;Acc:HGNC:29158] |
| ENSG00000087842 | HGLibA_36730 | 190 | 0.742095152 | PIR | pirin [Source:HGNC Symbol;Acc:HGNC:30048] |
| ENSG00000089820 | HGLibA_02773 | 190 | 0.742095152 | ARHGAP4 | Rho GTPase activating protein 4 [Source:HGNC Symbol;Acc:HGNC:674] |
| ENSG00000091656 | HGLibA_55176 | 190 | 0.742095152 | ZFHX4 | zinc finger homeobox 4 [Source:HGNC Symbol;Acc:HGNC:30939] |
| ENSG00000100276 | HGLibA_40359 | 190 | 0.742095152 | RASL10A | RAS like family 10 member A [Source:HGNC Symbol;Acc:HGNC:16954] |
| ENSG00000106688 | HGLibA_44506 | 190 | 0.742095152 | SLC1A1 | solute carrier family 1 member 1 [Source:HGNC Symbol;Acc:HGNC:10939] |
| ENSG00000107771 | HGLibA_08191 | 190 | 0.742095152 | CCSER2 | coiled-coil serine rich protein 2 [Source:HGNC Symbol;Acc:HGNC:29197] |
| ENSG00000108846 | HGLibA_00173 | 190 | 0.742095152 | ABCC3 | ATP binding cassette subfamily C member 3 [Source:HGNC Symbol;Acc:HGNC:54] |
| ENSG00000109861 | HGLibA_11695 | 190 | 0.742095152 | CTSC | cathepsin C [Source:HGNC Symbol;Acc:HGNC:2528] |
| ENSG00000110080 | HGLibA_47247 | 190 | 0.742095152 | ST3GAL4 | 3-sialyltransferase 4 [Source:HGNC Symbol;Acc:HGNC:10864]" |
| ENSG00000115170 | HGLibA_00685 | 190 | 0.742095152 | ACVR1 | activin A receptor type 1 [Source:HGNC Symbol;Acc:HGNC:171] |
| ENSG00000123975 | HGLibA_09789 | 190 | 0.742095152 | CKS2 | CDC28 protein kinase regulatory subunit 2 [Source:HGNC Symbol;Acc:HGNC:2000] |
| ENSG00000125498 | HGLibA_24981 | 190 | 0.742095152 | KIR2DL1 | two Ig domains and long cytoplasmic tail 1 [Source:HGNC Symbol;Acc:HGNC:6329]" |
| ENSG00000126746 | HGLibA_55895 | 190 | 0.742095152 | ZNF384 | zinc finger protein 384 [Source:HGNC Symbol;Acc:HGNC:11955] |
| ENSG00000128285 | HGLibA_28590 | 190 | 0.742095152 | MCHR1 | melanin concentrating hormone receptor 1 [Source:HGNC Symbol;Acc:HGNC:4479] |
| ENSG00000129911 | HGLibA_25059 | 190 | 0.742095152 | KLF16 | Kruppel like factor 16 [Source:HGNC Symbol;Acc:HGNC:16857] |
| ENSG00000131558 | HGLibA_15750 | 190 | 0.742095152 | EXOC4 | exocyst complex component 4 [Source:HGNC Symbol;Acc:HGNC:30389] |
| ENSG00000132970 | HGLibA_53960 | 190 | 0.742095152 | WASF3 | WAS protein family member 3 [Source:HGNC Symbol;Acc:HGNC:12734] |
| ENSG00000135643 | HGLibA_24350 | 190 | 0.742095152 | KCNMB4 | potassium calcium-activated channel subfamily M regulatory beta subunit 4 [Source:HGNC Symbol;Acc:HGNC:6289] |
| ENSG00000135821 | HGLibA_19368 | 190 | 0.742095152 | GLUL | glutamate-ammonia ligase [Source:HGNC Symbol;Acc:HGNC:4341] |
| ENSG00000137574 | HGLibA_49276 | 190 | 0.742095152 | TGS1 | trimethylguanosine synthase 1 [Source:HGNC Symbol;Acc:HGNC:17843] |
| ENSG00000138135 | HGLibA_09332 | 190 | 0.742095152 | CH25H | cholesterol 25-hydroxylase [Source:HGNC Symbol;Acc:HGNC:1907] |
| ENSG00000138443 | HGLibA_00293 | 190 | 0.742095152 | ABI2 | abl interactor 2 [Source:HGNC Symbol;Acc:HGNC:24011] |
| ENSG00000139547 | HGLibA_40746 | 190 | 0.742095152 | RDH16 | retinol dehydrogenase 16 [Source:HGNC Symbol;Acc:HGNC:29674] |
| ENSG00000140968 | HGLibA_23601 | 190 | 0.742095152 | IRF8 | interferon regulatory factor 8 [Source:HGNC Symbol;Acc:HGNC:5358] |
| ENSG00000142959 | HGLibA_04427 | 190 | 0.742095152 | BEST4 | bestrophin 4 [Source:HGNC Symbol;Acc:HGNC:17106] |
| ENSG00000144848 | HGLibA_03485 | 190 | 0.742095152 | ATG3 | autophagy related 3 [Source:HGNC Symbol;Acc:HGNC:20962] |
| ENSG00000149968 | HGLibA_29535 | 190 | 0.742095152 | MMP3 | matrix metallopeptidase 3 [Source:HGNC Symbol;Acc:HGNC:7173] |
| ENSG00000152082 | HGLibA_30946 | 190 | 0.742095152 | MZT2B | mitotic spindle organizing protein 2B [Source:HGNC Symbol;Acc:HGNC:25886] |
| ENSG00000156486 | HGLibA_24387 | 190 | 0.742095152 | KCNS2 | potassium voltage-gated channel modifier subfamily S member 2 [Source:HGNC Symbol;Acc:HGNC:6301] |
| ENSG00000157107 | HGLibA_17207 | 190 | 0.742095152 | FCHO2 | FCH domain only 2 [Source:HGNC Symbol;Acc:HGNC:25180] |
| ENSG00000158270 | HGLibA_10637 | 190 | 0.742095152 | COLEC12 | collectin subfamily member 12 [Source:HGNC Symbol;Acc:HGNC:16016] |
| ENSG00000158901 | HGLibA_54275 | 190 | 0.742095152 | WFDC8 | WAP four-disulfide core domain 8 [Source:HGNC Symbol;Acc:HGNC:16163] |
| ENSG00000160307 | HGLibA_42578 | 190 | 0.742095152 | S100B | S100 calcium binding protein B [Source:HGNC Symbol;Acc:HGNC:10500] |
| ENSG00000162390 | HGLibA_00471 | 190 | 0.742095152 | ACOT11 | acyl-CoA thioesterase 11 [Source:HGNC Symbol;Acc:HGNC:18156] |
| ENSG00000162931 | HGLibA_51424 | 190 | 0.742095152 | TRIM17 | tripartite motif containing 17 [Source:HGNC Symbol;Acc:HGNC:13430] |
| ENSG00000163867 | HGLibA_55405 | 190 | 0.742095152 | ZMYM6 | zinc finger MYM-type containing 6 [Source:HGNC Symbol;Acc:HGNC:13050] |
| ENSG00000164062 | HGLibA_02403 | 190 | 0.742095152 | APEH | acylaminoacyl-peptide hydrolase [Source:HGNC Symbol;Acc:HGNC:586] |
| ENSG00000165682 | HGLibA_09959 | 190 | 0.742095152 | CLEC1B | C-type lectin domain family 1 member B [Source:HGNC Symbol;Acc:HGNC:24356] |
| ENSG00000166682 | HGLibA_50645 | 190 | 0.742095152 | TMPRSS5 | transmembrane serine protease 5 [Source:HGNC Symbol;Acc:HGNC:14908] |
| ENSG00000167644 | HGLibA_05648 | 190 | 0.742095152 | C19orf33 | chromosome 19 open reading frame 33 [Source:HGNC Symbol;Acc:HGNC:16668] |
| ENSG00000167723 | HGLibA_51788 | 190 | 0.742095152 | TRPV3 | transient receptor potential cation channel subfamily V member 3 [Source:HGNC Symbol;Acc:HGNC:18084] |
| ENSG00000167815 | HGLibA_38409 | 190 | 0.742095152 | PRDX2 | peroxiredoxin 2 [Source:HGNC Symbol;Acc:HGNC:9353] |
| ENSG00000168913 | HGLibA_15132 | 190 | 0.742095152 | ENHO | energy homeostasis associated [Source:HGNC Symbol;Acc:HGNC:24838] |
| ENSG00000168961 | HGLibA_26382 | 190 | 0.742095152 | LGALS9 | galectin 9 [Source:HGNC Symbol;Acc:HGNC:6570] |
| ENSG00000170340 | HGLibA_03983 | 190 | 0.742095152 | B3GNT2 | 3-N-acetylglucosaminyltransferase 2 [Source:HGNC Symbol;Acc:HGNC:15629]" |
| ENSG00000171497 | HGLibA_37923 | 190 | 0.742095152 | PPID | peptidylprolyl isomerase D [Source:HGNC Symbol;Acc:HGNC:9257] |
| ENSG00000175325 | HGLibA_38685 | 190 | 0.742095152 | PROP1 | PROP paired-like homeobox 1 [Source:HGNC Symbol;Acc:HGNC:9455] |
| ENSG00000175820 | HGLibA_07679 | 190 | 0.742095152 | CCDC168 | coiled-coil domain containing 168 [Source:HGNC Symbol;Acc:HGNC:26851] |
| ENSG00000176401 | HGLibA_14700 | 190 | 0.742095152 | EID2B | EP300 interacting inhibitor of differentiation 2B [Source:HGNC Symbol;Acc:HGNC:26796] |
| ENSG00000178172 | HGLibA_46699 | 190 | 0.742095152 | SPINK6 | Kazal type 6 [Source:HGNC Symbol;Acc:HGNC:29486]" |
| ENSG00000178971 | HGLibA_11594 | 190 | 0.742095152 | CTC1 | CST telomere replication complex component 1 [Source:HGNC Symbol;Acc:HGNC:26169] |
| ENSG00000178997 | HGLibA_15715 | 190 | 0.742095152 | EXD1 | exonuclease 3'-5' domain containing 1 [Source:HGNC Symbol;Acc:HGNC:28507] |
| ENSG00000182379 | HGLibA_33170 | 190 | 0.742095152 | NXPH4 | neurexophilin 4 [Source:HGNC Symbol;Acc:HGNC:8078] |
| ENSG00000183828 | HGLibA_32946 | 190 | 0.742095152 | NUDT14 | nudix hydrolase 14 [Source:HGNC Symbol;Acc:HGNC:20141] |
| ENSG00000185966 | HGLibA_26143 | 190 | 0.742095152 | LCE3E | late cornified envelope 3E [Source:HGNC Symbol;Acc:HGNC:29463] |

| | | | | | |
|---|---|---|---|---|---|
| ENSG00000186891 | HGLibA_50766 | 190 | 0.742095152 | TNFRSF18 | TNF receptor superfamily member 18 [Source:HGNC Symbol;Acc:HGNC:11914] |
| ENSG00000187634 | HGLibA_42666 | 190 | 0.742095152 | SAMD11 | sterile alpha motif domain containing 11 [Source:HGNC Symbol;Acc:HGNC:28706] |
| ENSG00000189181 | HGLibA_33636 | 190 | 0.742095152 | OR14I1 | olfactory receptor family 14 subfamily I member 1 [Source:HGNC Symbol;Acc:HGNC:19575] |
| ENSG00000189186 | HGLibA_12462 | 190 | 0.742095152 | DCAF8L2 | DDB1 and CUL4 associated factor 8 like 2 [Source:HGNC Symbol;Acc:HGNC:31811] |
| ENSG00000196376 | HGLibA_45000 | 190 | 0.742095152 | SLC35F1 | solute carrier family 35 member F1 [Source:HGNC Symbol;Acc:HGNC:21483] |
| ENSG00000196814 | HGLibA_30593 | 190 | 0.742095152 | MVB12B | multivesicular body subunit 12B [Source:HGNC Symbol;Acc:HGNC:23368] |
| ENSG00000205279 | HGLibA_11762 | 190 | 0.742095152 | CTXN3 | cortexin 3 [Source:HGNC Symbol;Acc:HGNC:31110] |
| ENSG00000234560 | HGLibA_33500 | 190 | 0.742095152 | OR10G8 | olfactory receptor family 10 subfamily G member 8 [Source:HGNC Symbol;Acc:HGNC:14845] |
| ENSG00000243244 | HGLibA_47543 | 190 | 0.742095152 | STON1 | stonin 1 [Source:HGNC Symbol;Acc:HGNC:17003] |
| ENSG00000248905 | HGLibA_17688 | 190 | 0.742095152 | FMN1 | formin 1 [Source:HGNC Symbol;Acc:HGNC:3768] |
| ENSG00000257923 | HGLibA_11803 | 190 | 0.742095152 | CUX1 | cut like homeobox 1 [Source:HGNC Symbol;Acc:HGNC:2557] |
| ENSG00000258724 | HGLibA_36697 | 190 | 0.742095152 | PINX1 | PIN2 (TERF1) interacting telomerase inhibitor 1 [Source:HGNC Symbol;Acc:HGNC:30046] |
| hsa-mir-1205 | HGLibA_57277 | 190 | 0.742095152 | hsa-mir-1205 | - |
| hsa-mir-124-3 | HGLibA_57354 | 190 | 0.742095152 | hsa-mir-124-3 | - |
| hsa-mir-1248 | HGLibA_57391 | 190 | 0.742095152 | hsa-mir-1248 | - |
| hsa-mir-146a | HGLibA_57856 | 190 | 0.742095152 | hsa-mir-146a | - |
| hsa-mir-4277 | HGLibA_59811 | 190 | 0.742095152 | hsa-mir-4277 | - |
| hsa-mir-4447 | HGLibA_60193 | 190 | 0.742095152 | hsa-mir-4447 | - |
| hsa-mir-4684 | HGLibA_60846 | 190 | 0.742095152 | hsa-mir-4684 | - |
| hsa-mir-498 | HGLibA_61394 | 190 | 0.742095152 | hsa-mir-498 | - |
| hsa-mir-6784 | HGLibA_63313 | 190 | 0.742095152 | hsa-mir-6784 | - |
| hsa-mir-6791 | HGLibA_63340 | 190 | 0.742095152 | hsa-mir-6791 | - |
| hsa-mir-6796 | HGLibA_63361 | 190 | 0.742095152 | hsa-mir-6796 | - |
| PHF17 | HGLibA_36353 | 190 | 0.742095152 | PHF17 | - |
| PQLC3 | HGLibA_38256 | 190 | 0.742095152 | PQLC3 | - |
| PRAC | HGLibA_38260 | 190 | 0.742095152 | PRAC | - |
| C11orf57 | HGLibA_05161 | 189 | 0.732144115 | C11orf57 | - |
| C8orf42 | HGLibA_06620 | 189 | 0.732144115 | C8orf42 | - |
| CXorf27 | HGLibA_11911 | 189 | 0.732144115 | CXorf27 | - |
| ENSG00000004799 | HGLibA_35904 | 189 | 0.732144115 | PDK4 | pyruvate dehydrogenase kinase 4 [Source:HGNC Symbol;Acc:HGNC:8812] |
| ENSG00000004809 | HGLibA_44555 | 189 | 0.732144115 | SLC22A16 | solute carrier family 22 member 16 [Source:HGNC Symbol;Acc:HGNC:20302] |
| ENSG00000008130 | HGLibA_31046 | 189 | 0.732144115 | NADK | NAD kinase [Source:HGNC Symbol;Acc:HGNC:29831] |
| ENSG00000011422 | HGLibA_36966 | 189 | 0.732144115 | PLAUR | urokinase receptor [Source:HGNC Symbol;Acc:HGNC:9053]" |
| ENSG00000014641 | HGLibA_28683 | 189 | 0.732144115 | MDH1 | malate dehydrogenase 1 [Source:HGNC Symbol;Acc:HGNC:6970] |
| ENSG00000060971 | HGLibA_00340 | 189 | 0.732144115 | ACAA1 | acetyl-CoA acyltransferase 1 [Source:HGNC Symbol;Acc:HGNC:82] |
| ENSG00000064652 | HGLibA_46068 | 189 | 0.732144115 | SNX24 | sorting nexin 24 [Source:HGNC Symbol;Acc:HGNC:21533] |
| ENSG00000069248 | HGLibA_33021 | 189 | 0.732144115 | NUP133 | nucleoporin 133 [Source:HGNC Symbol;Acc:HGNC:18016] |
| ENSG00000070047 | HGLibA_36459 | 189 | 0.732144115 | PHRF1 | PHD and ring finger domains 1 [Source:HGNC Symbol;Acc:HGNC:24351] |
| ENSG00000082269 | HGLibA_16131 | 189 | 0.732144115 | FAM135A | family with sequence similarity 135 member A [Source:HGNC Symbol;Acc:HGNC:21084] |
| ENSG00000082512 | HGLibA_51260 | 189 | 0.732144115 | TRAF5 | TNF receptor associated factor 5 [Source:HGNC Symbol;Acc:HGNC:12035] |
| ENSG00000089280 | HGLibA_18176 | 189 | 0.732144115 | FUS | FUS RNA binding protein [Source:HGNC Symbol;Acc:HGNC:4010] |
| ENSG00000089356 | HGLibA_18231 | 189 | 0.732144115 | FXYD3 | FXYD domain containing ion transport regulator 3 [Source:HGNC Symbol;Acc:HGNC:4027] |
| ENSG00000100034 | HGLibA_37975 | 189 | 0.732144115 | PPM1F | Mg2+/Mn2+ dependent 1F [Source:HGNC Symbol;Acc:HGNC:19388]" |
| ENSG00000103021 | HGLibA_07546 | 189 | 0.732144115 | CCDC113 | coiled-coil domain containing 113 [Source:HGNC Symbol;Acc:HGNC:25002] |
| ENSG00000105198 | HGLibA_26351 | 189 | 0.732144115 | LGALS13 | galectin 13 [Source:HGNC Symbol;Acc:HGNC:15449] |
| ENSG00000105483 | HGLibA_07238 | 189 | 0.732144115 | CARD8 | caspase recruitment domain family member 8 [Source:HGNC Symbol;Acc:HGNC:17057] |
| ENSG00000107521 | HGLibA_22047 | 189 | 0.732144115 | HPS1 | biogenesis of lysosomal organelles complex 3 subunit 1 [Source:HGNC Symbol;Acc:HGNC:5163]" |
| ENSG00000112406 | HGLibA_21090 | 189 | 0.732144115 | HECA | cell cycle regulator [Source:HGNC Symbol;Acc:HGNC:21041]" |
| ENSG00000113555 | HGLibA_35398 | 189 | 0.732144115 | PCDH12 | protocadherin 12 [Source:HGNC Symbol;Acc:HGNC:8657] |
| ENSG00000114446 | HGLibA_22766 | 189 | 0.732144115 | IFT57 | intraflagellar transport 57 [Source:HGNC Symbol;Acc:HGNC:17367] |
| ENSG00000114529 | HGLibA_06247 | 189 | 0.732144115 | C3orf52 | chromosome 3 open reading frame 52 [Source:HGNC Symbol;Acc:HGNC:26255] |
| ENSG00000115486 | HGLibA_18994 | 189 | 0.732144115 | GGCX | gamma-glutamyl carboxylase [Source:HGNC Symbol;Acc:HGNC:4247] |
| ENSG00000116885 | HGLibA_34676 | 189 | 0.732144115 | OSCP1 | organic solute carrier partner 1 [Source:HGNC Symbol;Acc:HGNC:29971] |
| ENSG00000118246 | HGLibA_16903 | 189 | 0.732144115 | FASTKD2 | FAST kinase domains 2 [Source:HGNC Symbol;Acc:HGNC:29160] |
| ENSG00000119844 | HGLibA_01131 | 189 | 0.732144115 | AFTPH | aftiphilin [Source:HGNC Symbol;Acc:HGNC:25951] |
| ENSG00000122550 | HGLibA_25222 | 189 | 0.732144115 | KLHL7 | kelch like family member 7 [Source:HGNC Symbol;Acc:HGNC:15646] |
| ENSG00000123240 | HGLibA_33458 | 189 | 0.732144115 | OPTN | optineurin [Source:HGNC Symbol;Acc:HGNC:17142] |
| ENSG00000123453 | HGLibA_42742 | 189 | 0.732144115 | SARDH | sarcosine dehydrogenase [Source:HGNC Symbol;Acc:HGNC:10536] |
| ENSG00000123496 | HGLibA_22978 | 189 | 0.732144115 | IL13RA2 | interleukin 13 receptor subunit alpha 2 [Source:HGNC Symbol;Acc:HGNC:5975] |
| ENSG00000124104 | HGLibA_46064 | 189 | 0.732144115 | SNX21 | sorting nexin family member 21 [Source:HGNC Symbol;Acc:HGNC:16154] |
| ENSG00000125651 | HGLibA_20537 | 189 | 0.732144115 | GTF2F1 | general transcription factor IIF subunit 1 [Source:HGNC Symbol;Acc:HGNC:4652] |
| ENSG00000129255 | HGLibA_29712 | 189 | 0.732144115 | MPDU1 | mannose-P-dolichol utilization defect 1 [Source:HGNC Symbol;Acc:HGNC:7207] |
| ENSG00000133985 | HGLibA_52160 | 189 | 0.732144115 | TTC9 | tetratricopeptide repeat domain 9 [Source:HGNC Symbol;Acc:HGNC:20267] |
| ENSG00000138172 | HGLibA_07017 | 189 | 0.732144115 | CALHM2 | calcium homeostasis modulator family member 2 [Source:HGNC Symbol;Acc:HGNC:23493] |
| ENSG00000140157 | HGLibA_31954 | 189 | 0.732144115 | NIPA2 | NIPA magnesium transporter 2 [Source:HGNC Symbol;Acc:HGNC:17044] |
| ENSG00000141219 | HGLibA_05582 | 189 | 0.732144115 | C17orf80 | chromosome 17 open reading frame 80 [Source:HGNC Symbol;Acc:HGNC:29601] |
| ENSG00000149651 | HGLibA_46721 | 189 | 0.732144115 | SPINT4 | Kunitz type 4 [Source:HGNC Symbol;Acc:HGNC:16130]" |
| ENSG00000149968 | HGLibA_29534 | 189 | 0.732144115 | MMP3 | matrix metallopeptidase 3 [Source:HGNC Symbol;Acc:HGNC:7173] |
| ENSG00000151655 | HGLibA_23807 | 189 | 0.732144115 | ITIH2 | inter-alpha-trypsin inhibitor heavy chain 2 [Source:HGNC Symbol;Acc:HGNC:6167] |
| ENSG00000152465 | HGLibA_32207 | 189 | 0.732144115 | NMT2 | N-myristoyltransferase 2 [Source:HGNC Symbol;Acc:HGNC:7858] |
| ENSG00000157388 | HGLibA_06885 | 189 | 0.732144115 | CACNA1D | calcium voltage-gated channel subunit alpha1 D [Source:HGNC Symbol;Acc:HGNC:1391] |
| ENSG00000157992 | HGLibA_25856 | 189 | 0.732144115 | KRTCAP3 | keratinocyte associated protein 3 [Source:HGNC Symbol;Acc:HGNC:28943] |
| ENSG00000158050 | HGLibA_14156 | 189 | 0.732144115 | DUSP2 | dual specificity phosphatase 2 [Source:HGNC Symbol;Acc:HGNC:3068] |
| ENSG00000158156 | HGLibA_54532 | 189 | 0.732144115 | XKR8 | XK related 8 [Source:HGNC Symbol;Acc:HGNC:25508] |
| ENSG00000159069 | HGLibA_17150 | 189 | 0.732144115 | FBXW5 | F-box and WD repeat domain containing 5 [Source:HGNC Symbol;Acc:HGNC:13613] |
| ENSG00000162878 | HGLibA_36807 | 189 | 0.732144115 | PKDCC | cytoplasmic [Source:HGNC Symbol;Acc:HGNC:25123]" |
| ENSG00000163104 | HGLibA_45614 | 189 | 0.732144115 | SMARCAD1 | containing DEAD/H box 1 [Source:HGNC Symbol;Acc:HGNC:18398]" |
| ENSG00000163263 | HGLibA_05810 | 189 | 0.732144115 | C1orf189 | chromosome 1 open reading frame 189 [Source:HGNC Symbol;Acc:HGNC:32305] |
| ENSG00000165646 | HGLibA_44490 | 189 | 0.732144115 | SLC18A2 | solute carrier family 18 member A2 [Source:HGNC Symbol;Acc:HGNC:10935] |
| ENSG00000167110 | HGLibA_19596 | 189 | 0.732144115 | GOLGA2 | golgin A2 [Source:HGNC Symbol;Acc:HGNC:4425] |
| ENSG00000167208 | HGLibA_46060 | 189 | 0.732144115 | SNX20 | sorting nexin 20 [Source:HGNC Symbol;Acc:HGNC:30390] |
| ENSG00000167850 | HGLibA_08336 | 189 | 0.732144115 | CD300C | CD300c molecule [Source:HGNC Symbol;Acc:HGNC:19320] |
| ENSG00000168634 | HGLibA_54258 | 189 | 0.732144115 | WFDC13 | WAP four-disulfide core domain 13 [Source:HGNC Symbol;Acc:HGNC:16131] |
| ENSG00000170624 | HGLibA_43801 | 189 | 0.732144115 | SGCD | sarcoglycan delta [Source:HGNC Symbol;Acc:HGNC:10807] |
| ENSG00000171161 | HGLibA_56507 | 189 | 0.732144115 | ZNF672 | zinc finger protein 672 [Source:HGNC Symbol;Acc:HGNC:26179] |
| ENSG00000172061 | HGLibA_27260 | 189 | 0.732144115 | LRRC15 | leucine rich repeat containing 15 [Source:HGNC Symbol;Acc:HGNC:20818] |
| ENSG00000173757 | HGLibA_47397 | 189 | 0.732144115 | STAT5B | signal transducer and activator of transcription 5B [Source:HGNC Symbol;Acc:HGNC:11367] |
| ENSG00000173821 | HGLibA_41622 | 189 | 0.732144115 | RNF213 | ring finger protein 213 [Source:HGNC Symbol;Acc:HGNC:14539] |
| ENSG00000174990 | HGLibA_06813 | 189 | 0.732144115 | CA5A | carbonic anhydrase 5A [Source:HGNC Symbol;Acc:HGNC:1377] |
| ENSG00000176533 | HGLibA_19531 | 189 | 0.732144115 | GNG7 | G protein subunit gamma 7 [Source:HGNC Symbol;Acc:HGNC:4410] |
| ENSG00000177301 | HGLibA_24128 | 189 | 0.732144115 | KCNA2 | potassium voltage-gated channel subfamily A member 2 [Source:HGNC Symbol;Acc:HGNC:6220] |
| ENSG00000179218 | HGLibA_07045 | 189 | 0.732144115 | CALR | calreticulin [Source:HGNC Symbol;Acc:HGNC:1455] |
| ENSG00000179407 | HGLibA_13555 | 189 | 0.732144115 | DNAJB8 | DnaJ heat shock protein family (Hsp40) member B8 [Source:HGNC Symbol;Acc:HGNC:23699] |
| ENSG00000180383 | HGLibA_12876 | 189 | 0.732144115 | DEFB124 | defensin beta 124 [Source:HGNC Symbol;Acc:HGNC:18104] |
| ENSG00000182866 | HGLibA_26155 | 189 | 0.732144115 | LCK | Src family tyrosine kinase [Source:HGNC Symbol;Acc:HGNC:6524]" |
| ENSG00000184182 | HGLibA_52603 | 189 | 0.732144115 | UBE2F | ubiquitin conjugating enzyme E2 F (putative) [Source:HGNC Symbol;Acc:HGNC:12480] |
| ENSG00000185467 | HGLibA_25372 | 189 | 0.732144115 | KPNA7 | karyopherin subunit alpha 7 [Source:HGNC Symbol;Acc:HGNC:21839] |
| ENSG00000185737 | HGLibA_32683 | 189 | 0.732144115 | NRG3 | neuregulin 3 [Source:HGNC Symbol;Acc:HGNC:7999] |
| ENSG00000186642 | HGLibA_35777 | 189 | 0.732144115 | PDE2A | phosphodiesterase 2A [Source:HGNC Symbol;Acc:HGNC:8777] |
| ENSG00000187689 | HGLibA_01837 | 189 | 0.732144115 | AMTN | amelotin [Source:HGNC Symbol;Acc:HGNC:33188] |
| ENSG00000187994 | HGLibA_41301 | 189 | 0.732144115 | RINL | Ras and Rab interactor like [Source:HGNC Symbol;Acc:HGNC:24795] |
| ENSG00000188368 | HGLibA_38798 | 189 | 0.732144115 | PRR19 | proline rich 19 [Source:HGNC Symbol;Acc:HGNC:33728] |
| ENSG00000188559 | HGLibA_40160 | 189 | 0.732144115 | RALGAPA2 | Ral GTPase activating protein catalytic alpha subunit 2 [Source:HGNC Symbol;Acc:HGNC:16207] |
| ENSG00000196653 | HGLibA_56114 | 189 | 0.732144115 | ZNF502 | zinc finger protein 502 [Source:HGNC Symbol;Acc:HGNC:23718] |
| ENSG00000198598 | HGLibA_29502 | 189 | 0.732144115 | MMP17 | matrix metallopeptidase 17 [Source:HGNC Symbol;Acc:HGNC:7163] |
| ENSG00000198805 | HGLibA_37403 | 189 | 0.732144115 | PNP | purine nucleoside phosphorylase [Source:HGNC Symbol;Acc:HGNC:7892] |
| ENSG00000204227 | HGLibA_41299 | 189 | 0.732144115 | RING1 | ring finger protein 1 [Source:HGNC Symbol;Acc:HGNC:10018] |
| ENSG00000204310 | HGLibA_01216 | 189 | 0.732144115 | AGPAT1 | 1-acylglycerol-3-phosphate O-acyltransferase 1 [Source:HGNC Symbol;Acc:HGNC:324] |
| ENSG00000205882 | HGLibA_12906 | 189 | 0.732144115 | DEFB134 | defensin beta 134 [Source:HGNC Symbol;Acc:HGNC:32399] |
| ENSG00000211445 | HGLibA_20175 | 189 | 0.732144115 | GPX3 | glutathione peroxidase 3 [Source:HGNC Symbol;Acc:HGNC:4555] |
| ENSG00000213186 | HGLibA_51554 | 189 | 0.732144115 | TRIM59 | tripartite motif containing 59 [Source:HGNC Symbol;Acc:HGNC:30834] |
| ENSG00000213588 | HGLibA_54935 | 189 | 0.732144115 | ZBTB9 | zinc finger and BTB domain containing 9 [Source:HGNC Symbol;Acc:HGNC:28323] |
| ENSG00000233276 | HGLibA_20170 | 189 | 0.732144115 | GPX1 | glutathione peroxidase 1 [Source:HGNC Symbol;Acc:HGNC:4553] |
| ENSG00000243955 | HGLibA_20451 | 189 | 0.732144115 | GSTA1 | glutathione S-transferase alpha 1 [Source:HGNC Symbol;Acc:HGNC:4626] |
| ENSG00000248099 | HGLibA_23383 | 189 | 0.732144115 | INSL3 | insulin like 3 [Source:HGNC Symbol;Acc:HGNC:6086] |
| ENSG00000269699 | HGLibA_55338 | 189 | 0.732144115 | ZIM2 | zinc finger imprinted 2 [Source:HGNC Symbol;Acc:HGNC:12875] |
| ENSG00000270647 | HGLibA_48210 | 189 | 0.732144115 | TAF15 | TATA-box binding protein associated factor 15 [Source:HGNC Symbol;Acc:HGNC:11547] |
| hsa-mir-1470 | HGLibA_57866 | 189 | 0.732144115 | hsa-mir-1470 | - |
| hsa-mir-3190 | HGLibA_58858 | 189 | 0.732144115 | hsa-mir-3190 | - |
| hsa-mir-3664 | HGLibA_59208 | 189 | 0.732144115 | hsa-mir-3664 | - |
| hsa-mir-4453 | HGLibA_60219 | 189 | 0.732144115 | hsa-mir-4453 | - |
| hsa-mir-4540 | HGLibA_60627 | 189 | 0.732144115 | hsa-mir-4540 | - |
| hsa-mir-4700 | HGLibA_60907 | 189 | 0.732144115 | hsa-mir-4700 | - |
| hsa-mir-4760 | HGLibA_61137 | 189 | 0.732144115 | hsa-mir-4760 | - |
| hsa-mir-509-2 | HGLibA_61511 | 189 | 0.732144115 | hsa-mir-509-2 | - |
| hsa-mir-571 | HGLibA_62374 | 189 | 0.732144115 | hsa-mir-571 | - |
| hsa-mir-575 | HGLibA_62395 | 189 | 0.732144115 | hsa-mir-575 | - |
| hsa-mir-6083 | HGLibA_62594 | 189 | 0.732144115 | hsa-mir-6083 | - |
| hsa-mir-6083 | HGLibA_62593 | 189 | 0.732144115 | hsa-mir-6083 | - |
| hsa-mir-7705 | HGLibA_63955 | 189 | 0.732144115 | hsa-mir-7705 | - |
| OR5R1 | HGLibA_34353 | 189 | 0.732144115 | OR5R1 | - |
| C2orf48 | HGLibA_06138 | 188 | 0.722193077 | C2orf48 | - |
| ENSG00000006047 | HGLibA_54646 | 188 | 0.722193077 | YBX2 | Y-box binding protein 2 [Source:HGNC Symbol;Acc:HGNC:17948] |
| ENSG00000006432 | HGLibA_28205 | 188 | 0.722193077 | MAP3K9 | mitogen-activated protein kinase kinase kinase 9 [Source:HGNC Symbol;Acc:HGNC:6861] |
| ENSG00000009724 | HGLibA_28396 | 188 | 0.722193077 | MASP2 | mannan binding lectin serine peptidase 2 [Source:HGNC Symbol;Acc:HGNC:6902] |

| | | | | | |
|---|---|---|---|---|---|
| ENSG00000011143 | HGLibA_29357 | 188 | 0.722193077 | MKS1 | type 1 [Source:HGNC Symbol;Acc:HGNC:7121]" |
| ENSG00000065325 | HGLibA_19295 | 188 | 0.722193077 | GLP2R | glucagon like peptide 2 receptor [Source:HGNC Symbol;Acc:HGNC:4325] |
| ENSG00000077984 | HGLibA_11464 | 188 | 0.722193077 | CST7 | cystatin F [Source:HGNC Symbol;Acc:HGNC:2479] |
| ENSG00000080546 | HGLibA_43618 | 188 | 0.722193077 | SESN1 | sestrin 1 [Source:HGNC Symbol;Acc:HGNC:21595] |
| ENSG00000088256 | HGLibA_19436 | 188 | 0.722193077 | GNA11 | G protein subunit alpha 11 [Source:HGNC Symbol;Acc:HGNC:4379] |
| ENSG00000089048 | HGLibA_15567 | 188 | 0.722193077 | ESF1 | ESF1 nucleolar pre-rRNA processing protein homolog [Source:HGNC Symbol;Acc:HGNC:15898] |
| ENSG00000102349 | HGLibA_25084 | 188 | 0.722193077 | KLF8 | Kruppel like factor 8 [Source:HGNC Symbol;Acc:HGNC:6351] |
| ENSG00000102910 | HGLibA_27018 | 188 | 0.722193077 | LONP2 | peroxisomal [Source:HGNC Symbol;Acc:HGNC:20598]" |
| ENSG00000107833 | HGLibA_32494 | 188 | 0.722193077 | NPM3 | nucleophosmin/nucleoplasmin 3 [Source:HGNC Symbol;Acc:HGNC:7931] |
| ENSG00000109674 | HGLibA_31636 | 188 | 0.722193077 | NEIL3 | nei like DNA glycosylase 3 [Source:HGNC Symbol;Acc:HGNC:24573] |
| ENSG00000112706 | HGLibA_23263 | 188 | 0.722193077 | IMPG1 | interphotoreceptor matrix proteoglycan 1 [Source:HGNC Symbol;Acc:HGNC:6055] |
| ENSG00000113262 | HGLibA_20356 | 188 | 0.722193077 | GRM6 | glutamate metabotropic receptor 6 [Source:HGNC Symbol;Acc:HGNC:4598] |
| ENSG00000115257 | HGLibA_35677 | 188 | 0.722193077 | PCSK4 | proprotein convertase subtilisin/kexin type 4 [Source:HGNC Symbol;Acc:HGNC:8746] |
| ENSG00000115282 | HGLibA_52112 | 188 | 0.722193077 | TTC31 | tetratricopeptide repeat domain 31 [Source:HGNC Symbol;Acc:HGNC:25759] |
| ENSG00000115839 | HGLibA_39918 | 188 | 0.722193077 | RAB3GAP1 | RAB3 GTPase activating protein catalytic subunit 1 [Source:HGNC Symbol;Acc:HGNC:17063] |
| ENSG00000115896 | HGLibA_37014 | 188 | 0.722193077 | PLCL1 | phospholipase C like 1 (inactive) [Source:HGNC Symbol;Acc:HGNC:9063] |
| ENSG00000124194 | HGLibA_18843 | 188 | 0.722193077 | GDAP1L1 | ganglioside induced differentiation associated protein 1 like 1 [Source:HGNC Symbol;Acc:HGNC:4213] |
| ENSG00000124782 | HGLibA_42245 | 188 | 0.722193077 | RREB1 | ras responsive element binding protein 1 [Source:HGNC Symbol;Acc:HGNC:10449] |
| ENSG00000125931 | HGLibA_09743 | 188 | 0.722193077 | CITED1 | Cbp/p300 interacting transactivator with Glu/Asp rich carboxy-terminal domain 1 [Source:HGNC Symbol;Acc:HGNC:1986] |
| ENSG00000127152 | HGLibA_04294 | 188 | 0.722193077 | BCL11B | B cell CLL/lymphoma 11B [Source:HGNC Symbol;Acc:HGNC:13222] |
| ENSG00000128655 | HGLibA_35764 | 188 | 0.722193077 | PDE11A | phosphodiesterase 11A [Source:HGNC Symbol;Acc:HGNC:8773] |
| ENSG00000130511 | HGLibA_47124 | 188 | 0.722193077 | SSBP4 | single stranded DNA binding protein 4 [Source:HGNC Symbol;Acc:HGNC:15676] |
| ENSG00000134690 | HGLibA_08621 | 188 | 0.722193077 | CDCA8 | cell division cycle associated 8 [Source:HGNC Symbol;Acc:HGNC:14629] |
| ENSG00000134760 | HGLibA_14022 | 188 | 0.722193077 | DSG1 | desmoglein 1 [Source:HGNC Symbol;Acc:HGNC:3048] |
| ENSG00000136045 | HGLibA_39633 | 188 | 0.722193077 | PWP1 | endonuclein [Source:HGNC Symbol;Acc:HGNC:17015]" |
| ENSG00000137364 | HGLibA_51152 | 188 | 0.722193077 | TPMT | thiopurine S-methyltransferase [Source:HGNC Symbol;Acc:HGNC:12014] |
| ENSG00000137764 | HGLibA_28150 | 188 | 0.722193077 | MAP2K5 | mitogen-activated protein kinase kinase 5 [Source:HGNC Symbol;Acc:HGNC:6845] |
| ENSG00000139318 | HGLibA_14188 | 188 | 0.722193077 | DUSP6 | dual specificity phosphatase 6 [Source:HGNC Symbol;Acc:HGNC:3072] |
| ENSG00000140284 | HGLibA_44815 | 188 | 0.722193077 | SLC27A2 | solute carrier family 27 member 2 [Source:HGNC Symbol;Acc:HGNC:10996] |
| ENSG00000143816 | HGLibA_54394 | 188 | 0.722193077 | WNT9A | Wnt family member 9A [Source:HGNC Symbol;Acc:HGNC:12778] |
| ENSG00000145936 | HGLibA_24343 | 188 | 0.722193077 | KCNMB1 | potassium calcium-activated channel subfamily M regulatory beta subunit 1 [Source:HGNC Symbol;Acc:HGNC:6285] |
| ENSG00000147853 | HGLibA_01381 | 188 | 0.722193077 | AK3 | adenylate kinase 3 [Source:HGNC Symbol;Acc:HGNC:17376] |
| ENSG00000149136 | HGLibA_47164 | 188 | 0.722193077 | SSRP1 | structure specific recognition protein 1 [Source:HGNC Symbol;Acc:HGNC:11327] |
| ENSG00000150637 | HGLibA_08300 | 188 | 0.722193077 | CD226 | CD226 molecule [Source:HGNC Symbol;Acc:HGNC:16961] |
| ENSG00000153786 | HGLibA_55127 | 188 | 0.722193077 | ZDHHC7 | zinc finger DHHC-type containing 7 [Source:HGNC Symbol;Acc:HGNC:18459] |
| ENSG00000154359 | HGLibA_27019 | 188 | 0.722193077 | LONRF1 | LON peptidase N-terminal domain and ring finger 1 [Source:HGNC Symbol;Acc:HGNC:26302] |
| ENSG00000154415 | HGLibA_38097 | 188 | 0.722193077 | PPP1R3A | protein phosphatase 1 regulatory subunit 3A [Source:HGNC Symbol;Acc:HGNC:9291] |
| ENSG00000156515 | HGLibA_21578 | 188 | 0.722193077 | HK1 | hexokinase 1 [Source:HGNC Symbol;Acc:HGNC:4922] |
| ENSG00000160883 | HGLibA_21585 | 188 | 0.722193077 | HK3 | hexokinase 3 [Source:HGNC Symbol;Acc:HGNC:4925] |
| ENSG00000165309 | HGLibA_03018 | 188 | 0.722193077 | ARMC3 | armadillo repeat containing 3 [Source:HGNC Symbol;Acc:HGNC:30964] |
| ENSG00000166532 | HGLibA_41275 | 188 | 0.722193077 | RIMKLB | ribosomal modification protein rimK like family member B [Source:HGNC Symbol;Acc:HGNC:29228] |
| ENSG00000168032 | HGLibA_15202 | 188 | 0.722193077 | ENTPD3 | ectonucleoside triphosphate diphosphohydrolase 3 [Source:HGNC Symbol;Acc:HGNC:3365] |
| ENSG00000168621 | HGLibA_18887 | 188 | 0.722193077 | GDNF | glial cell derived neurotrophic factor [Source:HGNC Symbol;Acc:HGNC:4232] |
| ENSG00000170606 | HGLibA_22282 | 188 | 0.722193077 | HSPA4 | heat shock protein family A (Hsp70) member 4 [Source:HGNC Symbol;Acc:HGNC:5237] |
| ENSG00000171116 | HGLibA_22236 | 188 | 0.722193077 | HSFX1 | X-linked 1 [Source:HGNC Symbol;Acc:HGNC:29603]" |
| ENSG00000171757 | HGLibA_27319 | 188 | 0.722193077 | LRRC34 | leucine rich repeat containing 34 [Source:HGNC Symbol;Acc:HGNC:28408] |
| ENSG00000174652 | HGLibA_55700 | 188 | 0.722193077 | ZNF266 | zinc finger protein 266 [Source:HGNC Symbol;Acc:HGNC:13059] |
| ENSG00000175040 | HGLibA_09630 | 188 | 0.722193077 | CHST2 | carbohydrate sulfotransferase 2 [Source:HGNC Symbol;Acc:HGNC:1970] |
| ENSG00000175497 | HGLibA_13865 | 188 | 0.722193077 | DPP10 | dipeptidyl peptidase like 10 [Source:HGNC Symbol;Acc:HGNC:20823] |
| ENSG00000176273 | HGLibA_45017 | 188 | 0.722193077 | SLC35G1 | solute carrier family 35 member G1 [Source:HGNC Symbol;Acc:HGNC:26607] |
| ENSG00000176956 | HGLibA_27674 | 188 | 0.722193077 | LY6H | lymphocyte antigen 6 family member H [Source:HGNC Symbol;Acc:HGNC:6728] |
| ENSG00000178338 | HGLibA_55864 | 188 | 0.722193077 | ZNF354B | zinc finger protein 354B [Source:HGNC Symbol;Acc:HGNC:17197] |
| ENSG00000179399 | HGLibA_19773 | 188 | 0.722193077 | GPC5 | glypican 5 [Source:HGNC Symbol;Acc:HGNC:4453] |
| ENSG00000181026 | HGLibA_01090 | 188 | 0.722193077 | AEN | apoptosis enhancing nuclease [Source:HGNC Symbol;Acc:HGNC:25722] |
| ENSG00000181220 | HGLibA_56666 | 188 | 0.722193077 | ZNF746 | zinc finger protein 746 [Source:HGNC Symbol;Acc:HGNC:21948] |
| ENSG00000181518 | HGLibA_34514 | 188 | 0.722193077 | OR8D4 | olfactory receptor family 8 subfamily D member 4 [Source:HGNC Symbol;Acc:HGNC:14840] |
| ENSG00000186792 | HGLibA_22442 | 188 | 0.722193077 | HYAL3 | hyaluronidase 3 [Source:HGNC Symbol;Acc:HGNC:5322] |
| ENSG00000187513 | HGLibA_19145 | 188 | 0.722193077 | GJA4 | gap junction protein alpha 4 [Source:HGNC Symbol;Acc:HGNC:4278] |
| ENSG00000188095 | HGLibA_28919 | 188 | 0.722193077 | MESP2 | mesoderm posterior bHLH transcription factor 2 [Source:HGNC Symbol;Acc:HGNC:29659] |
| ENSG00000189420 | HGLibA_55247 | 188 | 0.722193077 | ZFP92 | ZFP92 zinc finger protein [Source:HGNC Symbol;Acc:HGNC:12865] |
| ENSG00000196151 | HGLibA_54230 | 188 | 0.722193077 | WDSUB1 | sterile alpha motif and U-box domain containing 1 [Source:HGNC Symbol;Acc:HGNC:26697]" |
| ENSG00000198354 | HGLibA_12425 | 188 | 0.722193077 | DCAF12L2 | DDB1 and CUL4 associated factor 12 like 2 [Source:HGNC Symbol;Acc:HGNC:32950] |
| ENSG00000205268 | HGLibA_35824 | 188 | 0.722193077 | PDE7A | phosphodiesterase 7A [Source:HGNC Symbol;Acc:HGNC:8791] |
| ENSG00000205838 | HGLibA_52080 | 188 | 0.722193077 | TTC23L | tetratricopeptide repeat domain 23 like [Source:HGNC Symbol;Acc:HGNC:26355] |
| ENSG00000213366 | HGLibA_20474 | 188 | 0.722193077 | GSTM2 | glutathione S-transferase mu 2 [Source:HGNC Symbol;Acc:HGNC:4634] |
| ENSG00000215187 | HGLibA_16238 | 188 | 0.722193077 | FAM166B | family with sequence similarity 166 member B [Source:HGNC Symbol;Acc:HGNC:34242] |
| ENSG00000242550 | HGLibA_43539 | 188 | 0.722193077 | SERPINB10 | serpin family B member 10 [Source:HGNC Symbol;Acc:HGNC:8942] |
| ENSG00000243364 | HGLibA_14590 | 188 | 0.722193077 | EFNA4 | ephrin A4 [Source:HGNC Symbol;Acc:HGNC:3224] |
| ENSG00000254737 | HGLibA_33495 | 188 | 0.722193077 | OR10G4 | olfactory receptor family 10 subfamily G member 4 [Source:HGNC Symbol;Acc:HGNC:14809] |
| ENSG00000280789 | HGLibA_35020 | 188 | 0.722193077 | PAGR1 | PAXIP1 associated glutamate rich protein 1 [Source:HGNC Symbol;Acc:HGNC:28707] |
| FAM57B | HGLibA_16648 | 188 | 0.722193077 | FAM57B | - |
| FAM65A | HGLibA_16668 | 188 | 0.722193077 | FAM65A | - |
| HIST1H4C | HGLibA_21492 | 188 | 0.722193077 | HIST1H4C | - |
| hsa-mir-1204 | HGLibA_57272 | 188 | 0.722193077 | hsa-mir-1204 | - |
| hsa-mir-205 | HGLibA_58180 | 188 | 0.722193077 | hsa-mir-205 | - |
| hsa-mir-4286 | HGLibA_59851 | 188 | 0.722193077 | hsa-mir-4286 | - |
| hsa-mir-451b | HGLibA_60528 | 188 | 0.722193077 | hsa-mir-451b | - |
| hsa-mir-5003 | HGLibA_61418 | 188 | 0.722193077 | hsa-mir-5003 | - |
| hsa-mir-5011 | HGLibA_61458 | 188 | 0.722193077 | hsa-mir-5011 | - |
| hsa-mir-5584 | HGLibA_62176 | 188 | 0.722193077 | hsa-mir-5584 | - |
| hsa-mir-939 | HGLibA_64329 | 188 | 0.722193077 | hsa-mir-939 | - |
| MYEOV2 | HGLibA_30693 | 188 | 0.722193077 | MYEOV2 | - |
| PQLC1 | HGLibA_38250 | 188 | 0.722193077 | PQLC1 | - |
| 8-Sep | HGLibA_43453 | 188 | 0.722193077 | 8-Sep | - |
| ATP5G1 | HGLibA_03655 | 187 | 0.71224204 | ATP5G1 | - |
| ENSG00000010932 | HGLibA_17702 | 187 | 0.71224204 | FMO1 | flavin containing monooxygenase 1 [Source:HGNC Symbol;Acc:HGNC:3769] |
| ENSG00000039600 | HGLibA_46262 | 187 | 0.71224204 | SOX30 | SRY-box 30 [Source:HGNC Symbol;Acc:HGNC:30635] |
| ENSG00000065371 | HGLibA_41761 | 187 | 0.71224204 | ROPN1 | rhophilin associated tail protein 1 [Source:HGNC Symbol;Acc:HGNC:17692] |
| ENSG00000065802 | HGLibA_03203 | 187 | 0.71224204 | ASB1 | ankyrin repeat and SOCS box containing 1 [Source:HGNC Symbol;Acc:HGNC:16011] |
| ENSG00000070214 | HGLibA_45157 | 187 | 0.71224204 | SLC44A1 | solute carrier family 44 member 1 [Source:HGNC Symbol;Acc:HGNC:18798] |
| ENSG00000071564 | HGLibA_48785 | 187 | 0.71224204 | TCF3 | transcription factor 3 [Source:HGNC Symbol;Acc:HGNC:11633] |
| ENSG00000088543 | HGLibA_06214 | 187 | 0.71224204 | C3orf18 | chromosome 3 open reading frame 18 [Source:HGNC Symbol;Acc:HGNC:24837] |
| ENSG00000088827 | HGLibA_44123 | 187 | 0.71224204 | SIGLEC1 | sialic acid binding Ig like lectin 1 [Source:HGNC Symbol;Acc:HGNC:11127] |
| ENSG00000092847 | HGLibA_01205 | 187 | 0.71224204 | AGO1 | RISC catalytic component [Source:HGNC Symbol;Acc:HGNC:3262]" |
| ENSG00000096654 | HGLibA_55545 | 187 | 0.71224204 | ZNF184 | zinc finger protein 184 [Source:HGNC Symbol;Acc:HGNC:12975] |
| ENSG00000096872 | HGLibA_22771 | 187 | 0.71224204 | IFT74 | intraflagellar transport 74 [Source:HGNC Symbol;Acc:HGNC:21424] |
| ENSG00000103494 | HGLibA_41842 | 187 | 0.71224204 | RPGRIP1L | RPGRIP1 like [Source:HGNC Symbol;Acc:HGNC:29168] |
| ENSG00000108759 | HGLibA_25476 | 187 | 0.71224204 | KRT32 | keratin 32 [Source:HGNC Symbol;Acc:HGNC:6449] |
| ENSG00000110107 | HGLibA_38714 | 187 | 0.71224204 | PRPF19 | pre-mRNA processing factor 19 [Source:HGNC Symbol;Acc:HGNC:17896] |
| ENSG00000111291 | HGLibA_20135 | 187 | 0.71224204 | GPRC5D | G protein-coupled receptor class C group 5 member D [Source:HGNC Symbol;Acc:HGNC:13310] |
| ENSG00000116991 | HGLibA_44195 | 187 | 0.71224204 | SIPA1L2 | signal induced proliferation associated 1 like 2 [Source:HGNC Symbol;Acc:HGNC:23800] |
| ENSG00000117395 | HGLibA_14369 | 187 | 0.71224204 | EBNA1BP2 | EBNA1 binding protein 2 [Source:HGNC Symbol;Acc:HGNC:15531] |
| ENSG00000118231 | HGLibA_11279 | 187 | 0.71224204 | CRYGD | crystallin gamma D [Source:HGNC Symbol;Acc:HGNC:2411] |
| ENSG00000127334 | HGLibA_14277 | 187 | 0.71224204 | DYRK2 | dual specificity tyrosine phosphorylation regulated kinase 2 [Source:HGNC Symbol;Acc:HGNC:3093] |
| ENSG00000130224 | HGLibA_27141 | 187 | 0.71224204 | LRCH2 | leucine rich repeats and calponin homology domain containing 2 [Source:HGNC Symbol;Acc:HGNC:29292] |
| ENSG00000133742 | HGLibA_06786 | 187 | 0.71224204 | CA1 | carbonic anhydrase 1 [Source:HGNC Symbol;Acc:HGNC:1368] |
| ENSG00000136327 | HGLibA_32046 | 187 | 0.71224204 | NKX2-8 | NK2 homeobox 8 [Source:HGNC Symbol;Acc:HGNC:16364] |
| ENSG00000136943 | HGLibA_11732 | 187 | 0.71224204 | CTSV | cathepsin V [Source:HGNC Symbol;Acc:HGNC:2538] |
| ENSG00000137252 | HGLibA_20991 | 187 | 0.71224204 | HCRTR2 | hypocretin receptor 2 [Source:HGNC Symbol;Acc:HGNC:4849] |
| ENSG00000137634 | HGLibA_33159 | 187 | 0.71224204 | NXPE4 | neurexophilin and PC-esterase domain family member 4 [Source:HGNC Symbol;Acc:HGNC:23117] |
| ENSG00000138100 | HGLibA_51543 | 187 | 0.71224204 | TRIM54 | tripartite motif containing 54 [Source:HGNC Symbol;Acc:HGNC:16008] |
| ENSG00000140557 | HGLibA_47292 | 187 | 0.71224204 | ST8SIA2 | 8-sialyltransferase 2 [Source:HGNC Symbol;Acc:HGNC:10870]" |
| ENSG00000141564 | HGLibA_42202 | 187 | 0.71224204 | RPTOR | regulatory associated protein of MTOR complex 1 [Source:HGNC Symbol;Acc:HGNC:30287] |
| ENSG00000142552 | HGLibA_40707 | 187 | 0.71224204 | RCN3 | reticulocalbin 3 [Source:HGNC Symbol;Acc:HGNC:21145] |
| ENSG00000149573 | HGLibA_29799 | 187 | 0.71224204 | MPZL2 | myelin protein zero like 2 [Source:HGNC Symbol;Acc:HGNC:3496] |
| ENSG00000155792 | HGLibA_12988 | 187 | 0.71224204 | DEPTOR | DEP domain containing MTOR interacting protein [Source:HGNC Symbol;Acc:HGNC:22953] |
| ENSG00000160007 | HGLibA_02766 | 187 | 0.71224204 | ARHGAP35 | Rho GTPase activating protein 35 [Source:HGNC Symbol;Acc:HGNC:4591] |
| ENSG00000161681 | HGLibA_43989 | 187 | 0.71224204 | SHANK1 | SH3 and multiple ankyrin repeat domains 1 [Source:HGNC Symbol;Acc:HGNC:15474] |
| ENSG00000162600 | HGLibA_33381 | 187 | 0.71224204 | OMA1 | OMA1 zinc metallopeptidase [Source:HGNC Symbol;Acc:HGNC:29661] |
| ENSG00000162910 | HGLibA_30023 | 187 | 0.71224204 | MRPL55 | mitochondrial ribosomal protein L55 [Source:HGNC Symbol;Acc:HGNC:16686] |
| ENSG00000163162 | HGLibA_41541 | 187 | 0.71224204 | RNF149 | ring finger protein 149 [Source:HGNC Symbol;Acc:HGNC:23137] |
| ENSG00000163357 | HGLibA_12529 | 187 | 0.71224204 | DCST1 | DC-STAMP domain containing 1 [Source:HGNC Symbol;Acc:HGNC:26539] |
| ENSG00000163558 | HGLibA_38555 | 187 | 0.71224204 | PRKCI | protein kinase C iota [Source:HGNC Symbol;Acc:HGNC:9404] |
| ENSG00000163624 | HGLibA_08881 | 187 | 0.71224204 | CDS1 | CDP-diacylglycerol synthase 1 [Source:HGNC Symbol;Acc:HGNC:1800] |
| ENSG00000163697 | HGLibA_02383 | 187 | 0.71224204 | APBB2 | amyloid beta precursor protein binding family B member 2 [Source:HGNC Symbol;Acc:HGNC:582] |
| ENSG00000165655 | HGLibA_56115 | 187 | 0.71224204 | ZNF503 | zinc finger protein 503 [Source:HGNC Symbol;Acc:HGNC:23589] |
| ENSG00000168246 | HGLibA_52768 | 187 | 0.71224204 | UBTD2 | ubiquitin domain containing 2 [Source:HGNC Symbol;Acc:HGNC:24463] |
| ENSG00000170075 | HGLibA_20034 | 187 | 0.71224204 | GPR37L1 | G protein-coupled receptor 37 like 1 [Source:HGNC Symbol;Acc:HGNC:14923] |
| ENSG00000170264 | HGLibA_16217 | 187 | 0.71224204 | FAM161A | centrosomal protein [Source:HGNC Symbol;Acc:HGNC:25808]" |
| ENSG00000170876 | HGLibA_50402 | 187 | 0.71224204 | TMEM43 | transmembrane protein 43 [Source:HGNC Symbol;Acc:HGNC:28472] |
| ENSG00000170927 | HGLibA_36814 | 187 | 0.71224204 | PKHD1 | fibrocystin/polyductin [Source:HGNC Symbol;Acc:HGNC:9016]" |
| ENSG00000172497 | HGLibA_00474 | 187 | 0.71224204 | ACOT12 | acyl-CoA thioesterase 12 [Source:HGNC Symbol;Acc:HGNC:24436] |
| ENSG00000174500 | HGLibA_18827 | 187 | 0.71224204 | GCSAM | germinal center associated signaling and motility [Source:HGNC Symbol;Acc:HGNC:20253] |
| ENSG00000177932 | HGLibA_55866 | 187 | 0.71224204 | ZNF354C | zinc finger protein 354C [Source:HGNC Symbol;Acc:HGNC:16736] |
| ENSG00000178802 | HGLibA_29733 | 187 | 0.71224204 | MPI | mannose phosphate isomerase [Source:HGNC Symbol;Acc:HGNC:7216] |

| | | | | | |
|---|---|---|---|---|---|
| ENSG00000179091 | HGLibA_12026 | 187 | 0.71224204 | CYC1 | cytochrome c1 [Source:HGNC Symbol;Acc:HGNC:2579] |
| ENSG00000179172 | HGLibA_21809 | 187 | 0.71224204 | HNRNPCL1 | heterogeneous nuclear ribonucleoprotein C-like 1 [Source:HGNC Symbol;Acc:HGNC:29295] |
| ENSG00000182247 | HGLibA_52597 | 187 | 0.71224204 | UBE2E2 | ubiquitin conjugating enzyme E2 E2 [Source:HGNC Symbol;Acc:HGNC:12478] |
| ENSG00000183979 | HGLibA_32410 | 187 | 0.71224204 | NPB | neuropeptide B [Source:HGNC Symbol;Acc:HGNC:30099] |
| ENSG00000184155 | HGLibA_33528 | 187 | 0.71224204 | OR10J5 | olfactory receptor family 10 subfamily J member 5 [Source:HGNC Symbol;Acc:HGNC:14993] |
| ENSG00000186790 | HGLibA_17860 | 187 | 0.71224204 | FOXE3 | forkhead box E3 [Source:HGNC Symbol;Acc:HGNC:3808] |
| ENSG00000187244 | HGLibA_04234 | 187 | 0.71224204 | BCAM | basal cell adhesion molecule (Lutheran blood group) [Source:HGNC Symbol;Acc:HGNC:6722] |
| ENSG00000188263 | HGLibA_23020 | 187 | 0.71224204 | IL17REL | interleukin 17 receptor E like [Source:HGNC Symbol;Acc:HGNC:33808] |
| ENSG00000189068 | HGLibA_53857 | 187 | 0.71224204 | VSTM1 | V-set and transmembrane domain containing 1 [Source:HGNC Symbol;Acc:HGNC:29455] |
| ENSG00000196724 | HGLibA_55951 | 187 | 0.71224204 | ZNF418 | zinc finger protein 418 [Source:HGNC Symbol;Acc:HGNC:20647] |
| ENSG00000196811 | HGLibA_09608 | 187 | 0.71224204 | CHRNG | cholinergic receptor nicotinic gamma subunit [Source:HGNC Symbol;Acc:HGNC:1967] |
| ENSG00000198535 | HGLibA_06094 | 187 | 0.71224204 | C2CD4A | C2 calcium dependent domain containing 4A [Source:HGNC Symbol;Acc:HGNC:33627] |
| ENSG00000198624 | HGLibA_07845 | 187 | 0.71224204 | CCDC69 | coiled-coil domain containing 69 [Source:HGNC Symbol;Acc:HGNC:24487] |
| ENSG00000198730 | HGLibA_11673 | 187 | 0.71224204 | CTR9 | Paf1/RNA polymerase II complex component [Source:HGNC Symbol;Acc:HGNC:16850]" |
| ENSG00000198854 | HGLibA_05892 | 187 | 0.71224204 | C1orf68 | chromosome 1 open reading frame 68 [Source:HGNC Symbol;Acc:HGNC:29468] |
| ENSG00000204382 | HGLibA_54470 | 187 | 0.71224204 | XAGE1B | X antigen family member 1B [Source:HGNC Symbol;Acc:HGNC:25400] |
| ENSG00000204872 | HGLibA_31186 | 187 | 0.71224204 | NAT8B | gene/pseudogene) [Source:HGNC Symbol;Acc:HGNC:30235]" |
| ENSG00000206503 | HGLibA_21594 | 187 | 0.71224204 | HLA-A | A [Source:HGNC Symbol;Acc:HGNC:4931]" |
| ENSG00000213221 | HGLibA_13679 | 187 | 0.71224204 | DNLZ | DNL-type zinc finger [Source:HGNC Symbol;Acc:HGNC:33879] |
| ENSG00000213563 | HGLibA_06649 | 187 | 0.71224204 | C8orf82 | chromosome 8 open reading frame 82 [Source:HGNC Symbol;Acc:HGNC:33826] |
| ENSG00000214402 | HGLibA_26191 | 187 | 0.71224204 | LCNL1 | lipocalin like 1 [Source:HGNC Symbol;Acc:HGNC:34436] |
| ENSG00000239713 | HGLibA_02491 | 187 | 0.71224204 | APOBEC3G | apolipoprotein B mRNA editing enzyme catalytic subunit 3G [Source:HGNC Symbol;Acc:HGNC:17357] |
| ENSG00000240694 | HGLibA_37369 | 187 | 0.71224204 | PNMA2 | PNMA family member 2 [Source:HGNC Symbol;Acc:HGNC:9159] |
| ENSG00000253846 | HGLibA_35520 | 187 | 0.71224204 | PCDHGA10 | 10 [Source:HGNC Symbol;Acc:HGNC:8697]" |
| ENSG00000254087 | HGLibA_27707 | 187 | 0.71224204 | LYN | Src family tyrosine kinase [Source:HGNC Symbol;Acc:HGNC:6735]" |
| ENSG00000255408 | HGLibA_35441 | 187 | 0.71224204 | PCDHA3 | protocadherin alpha 3 [Source:HGNC Symbol;Acc:HGNC:8669] |
| FAM46B | HGLibA_16600 | 187 | 0.71224204 | FAM46B | - |
| hsa-mir-1224 | HGLibA_57295 | 187 | 0.71224204 | hsa-mir-1224 | - |
| hsa-mir-138-2 | HGLibA_57817 | 187 | 0.71224204 | hsa-mir-138-2 | - |
| hsa-mir-1827 | HGLibA_57992 | 187 | 0.71224204 | hsa-mir-1827 | - |
| hsa-mir-30b | HGLibA_58475 | 187 | 0.71224204 | hsa-mir-30b | - |
| hsa-mir-4770 | HGLibA_61178 | 187 | 0.71224204 | hsa-mir-4770 | - |
| hsa-mir-758 | HGLibA_63892 | 187 | 0.71224204 | hsa-mir-758 | - |
| hsa-mir-762 | HGLibA_63906 | 187 | 0.71224204 | hsa-mir-762 | - |
| LOC401052 | HGLibA_26937 | 187 | 0.71224204 | LOC401052 | - |
| TMEM35 | HGLibA_50373 | 187 | 0.71224204 | TMEM35 | - |
| C15orf27 | HGLibA_05381 | 186 | 0.702291002 | C15orf27 | - |
| CSF2RA_Y | HGLibA_11334 | 186 | 0.702291002 | CSF2RA_Y | - |
| CXCR7 | HGLibA_11899 | 186 | 0.702291002 | CXCR7 | - |
| ENSG00000025770 | HGLibA_31277 | 186 | 0.702291002 | NCAPH2 | non-SMC condensin II complex subunit H2 [Source:HGNC Symbol;Acc:HGNC:25071] |
| ENSG00000035499 | HGLibA_12977 | 186 | 0.702291002 | DEPDC1B | DEP domain containing 1B [Source:HGNC Symbol;Acc:HGNC:24902] |
| ENSG00000035664 | HGLibA_12326 | 186 | 0.702291002 | DAPK2 | death associated protein kinase 2 [Source:HGNC Symbol;Acc:HGNC:2675] |
| ENSG00000047457 | HGLibA_10881 | 186 | 0.702291002 | CP | ceruloplasmin [Source:HGNC Symbol;Acc:HGNC:2295] |
| ENSG00000066294 | HGLibA_08464 | 186 | 0.702291002 | CD84 | CD84 molecule [Source:HGNC Symbol;Acc:HGNC:1704] |
| ENSG00000067221 | HGLibA_47535 | 186 | 0.702291002 | STOML1 | stomatin like 1 [Source:HGNC Symbol;Acc:HGNC:14560] |
| ENSG00000073060 | HGLibA_42870 | 186 | 0.702291002 | SCARB1 | scavenger receptor class B member 1 [Source:HGNC Symbol;Acc:HGNC:1664] |
| ENSG00000081320 | HGLibA_47464 | 186 | 0.702291002 | STK17B | serine/threonine kinase 17b [Source:HGNC Symbol;Acc:HGNC:11396] |
| ENSG00000085433 | HGLibA_54106 | 186 | 0.702291002 | WDR47 | WD repeat domain 47 [Source:HGNC Symbol;Acc:HGNC:29141] |
| ENSG00000091181 | HGLibA_23179 | 186 | 0.702291002 | IL5RA | interleukin 5 receptor subunit alpha [Source:HGNC Symbol;Acc:HGNC:6017] |
| ENSG00000092964 | HGLibA_13926 | 186 | 0.702291002 | DPYSL2 | dihydropyrimidinase like 2 [Source:HGNC Symbol;Acc:HGNC:3014] |
| ENSG00000099937 | HGLibA_43573 | 186 | 0.702291002 | SERPIND1 | serpin family D member 1 [Source:HGNC Symbol;Acc:HGNC:4838] |
| ENSG00000101856 | HGLibA_36289 | 186 | 0.702291002 | PGRMC1 | progesterone receptor membrane component 1 [Source:HGNC Symbol;Acc:HGNC:16090] |
| ENSG00000102181 | HGLibA_08495 | 186 | 0.702291002 | CD99L2 | CD99 molecule like 2 [Source:HGNC Symbol;Acc:HGNC:18237] |
| ENSG00000103196 | HGLibA_11177 | 186 | 0.702291002 | CRISPLD2 | cysteine rich secretory protein LCCL domain containing 2 [Source:HGNC Symbol;Acc:HGNC:25248] |
| ENSG00000103995 | HGLibA_09111 | 186 | 0.702291002 | CEP152 | centrosomal protein 152 [Source:HGNC Symbol;Acc:HGNC:29298] |
| ENSG00000104549 | HGLibA_46914 | 186 | 0.702291002 | SQLE | squalene epoxidase [Source:HGNC Symbol;Acc:HGNC:11279] |
| ENSG00000105889 | HGLibA_47423 | 186 | 0.702291002 | STEAP1B | STEAP family member 1B [Source:HGNC Symbol;Acc:HGNC:41907] |
| ENSG00000106400 | HGLibA_56923 | 186 | 0.702291002 | ZNHIT1 | zinc finger HIT-type containing 1 [Source:HGNC Symbol;Acc:HGNC:21688] |
| ENSG00000108582 | HGLibA_10912 | 186 | 0.702291002 | CPD | carboxypeptidase D [Source:HGNC Symbol;Acc:HGNC:2301] |
| ENSG00000111911 | HGLibA_21329 | 186 | 0.702291002 | HINT3 | histidine triad nucleotide binding protein 3 [Source:HGNC Symbol;Acc:HGNC:18468] |
| ENSG00000112246 | HGLibA_44173 | 186 | 0.702291002 | SIM1 | SIM bHLH transcription factor 1 [Source:HGNC Symbol;Acc:HGNC:10882] |
| ENSG00000112782 | HGLibA_10032 | 186 | 0.702291002 | CLIC5 | chloride intracellular channel 5 [Source:HGNC Symbol;Acc:HGNC:13517] |
| ENSG00000113194 | HGLibA_15950 | 186 | 0.702291002 | FAF2 | Fas associated factor family member 2 [Source:HGNC Symbol;Acc:HGNC:24666] |
| ENSG00000114933 | HGLibA_23327 | 186 | 0.702291002 | INO80D | INO80 complex subunit D [Source:HGNC Symbol;Acc:HGNC:25997] |
| ENSG00000119938 | HGLibA_38105 | 186 | 0.702291002 | PPP1R3C | protein phosphatase 1 regulatory subunit 3C [Source:HGNC Symbol;Acc:HGNC:9293] |
| ENSG00000120215 | HGLibA_29363 | 186 | 0.702291002 | MLANA | melan-A [Source:HGNC Symbol;Acc:HGNC:7124] |
| ENSG00000120500 | HGLibA_03104 | 186 | 0.702291002 | ARR3 | arrestin 3 [Source:HGNC Symbol;Acc:HGNC:710] |
| ENSG00000123570 | HGLibA_39994 | 186 | 0.702291002 | RAB9B | member RAS oncogene family [Source:HGNC Symbol;Acc:HGNC:14090]" |
| ENSG00000127081 | HGLibA_56076 | 186 | 0.702291002 | ZNF484 | zinc finger protein 484 [Source:HGNC Symbol;Acc:HGNC:23385] |
| ENSG00000129170 | HGLibA_11440 | 186 | 0.702291002 | CSRP3 | cysteine and glycine rich protein 3 [Source:HGNC Symbol;Acc:HGNC:2472] |
| ENSG00000130313 | HGLibA_36247 | 186 | 0.702291002 | PGLS | 6-phosphogluconolactonase [Source:HGNC Symbol;Acc:HGNC:8903] |
| ENSG00000131759 | HGLibA_40273 | 186 | 0.702291002 | RARA | retinoic acid receptor alpha [Source:HGNC Symbol;Acc:HGNC:9864] |
| ENSG00000134193 | HGLibA_40807 | 186 | 0.702291002 | REG4 | regenerating family member 4 [Source:HGNC Symbol;Acc:HGNC:22977] |
| ENSG00000134323 | HGLibA_30680 | 186 | 0.702291002 | MYCN | bHLH transcription factor [Source:HGNC Symbol;Acc:HGNC:7559]" |
| ENSG00000135111 | HGLibA_48667 | 186 | 0.702291002 | TBX3 | T-box 3 [Source:HGNC Symbol;Acc:HGNC:11602] |
| ENSG00000135441 | HGLibA_04557 | 186 | 0.702291002 | BLOC1S1 | biogenesis of lysosomal organelles complex 1 subunit 1 [Source:HGNC Symbol;Acc:HGNC:4200] |
| ENSG00000135472 | HGLibA_15966 | 186 | 0.702291002 | FAIM2 | Fas apoptotic inhibitory molecule 2 [Source:HGNC Symbol;Acc:HGNC:17067] |
| ENSG00000137078 | HGLibA_44237 | 186 | 0.702291002 | SIT1 | signaling threshold regulating transmembrane adaptor 1 [Source:HGNC Symbol;Acc:HGNC:17710] |
| ENSG00000138669 | HGLibA_38582 | 186 | 0.702291002 | PRKG2 | protein kinase cGMP-dependent 2 [Source:HGNC Symbol;Acc:HGNC:9416] |
| ENSG00000141968 | HGLibA_53549 | 186 | 0.702291002 | VAV1 | vav guanine nucleotide exchange factor 1 [Source:HGNC Symbol;Acc:HGNC:12657] |
| ENSG00000144935 | HGLibA_51729 | 186 | 0.702291002 | TRPC1 | transient receptor potential cation channel subfamily C member 1 [Source:HGNC Symbol;Acc:HGNC:12333] |
| ENSG00000156076 | HGLibA_54302 | 186 | 0.702291002 | WIF1 | WNT inhibitory factor 1 [Source:HGNC Symbol;Acc:HGNC:18081] |
| ENSG00000157193 | HGLibA_27234 | 186 | 0.702291002 | LRP8 | LDL receptor related protein 8 [Source:HGNC Symbol;Acc:HGNC:6700] |
| ENSG00000159314 | HGLibA_02743 | 186 | 0.702291002 | ARHGAP27 | Rho GTPase activating protein 27 [Source:HGNC Symbol;Acc:HGNC:31813] |
| ENSG00000162241 | HGLibA_44749 | 186 | 0.702291002 | SLC25A45 | solute carrier family 25 member 45 [Source:HGNC Symbol;Acc:HGNC:27442] |
| ENSG00000162763 | HGLibA_27387 | 186 | 0.702291002 | LRRC52 | leucine rich repeat containing 52 [Source:HGNC Symbol;Acc:HGNC:32156] |
| ENSG00000163159 | HGLibA_53814 | 186 | 0.702291002 | VPS72 | vacuolar protein sorting 72 homolog [Source:HGNC Symbol;Acc:HGNC:11644] |
| ENSG00000163295 | HGLibA_01698 | 186 | 0.702291002 | ALPI | intestinal [Source:HGNC Symbol;Acc:HGNC:437]" |
| ENSG00000163803 | HGLibA_36970 | 186 | 0.702291002 | PLB1 | phospholipase B1 [Source:HGNC Symbol;Acc:HGNC:30041] |
| ENSG00000163832 | HGLibA_14990 | 186 | 0.702291002 | ELP6 | elongator acetyltransferase complex subunit 6 [Source:HGNC Symbol;Acc:HGNC:25976] |
| ENSG00000166801 | HGLibA_16025 | 186 | 0.702291002 | FAM111A | family with sequence similarity 111 member A [Source:HGNC Symbol;Acc:HGNC:24725] |
| ENSG00000168137 | HGLibA_43653 | 186 | 0.702291002 | SETD5 | SET domain containing 5 [Source:HGNC Symbol;Acc:HGNC:25566] |
| ENSG00000172465 | HGLibA_48703 | 186 | 0.702291002 | TCEAL1 | transcription elongation factor A like 1 [Source:HGNC Symbol;Acc:HGNC:11616] |
| ENSG00000174038 | HGLibA_06676 | 186 | 0.702291002 | C9orf131 | chromosome 9 open reading frame 131 [Source:HGNC Symbol;Acc:HGNC:31418] |
| ENSG00000174130 | HGLibA_49657 | 186 | 0.702291002 | TLR6 | toll like receptor 6 [Source:HGNC Symbol;Acc:HGNC:16711] |
| ENSG00000176925 | HGLibA_34107 | 186 | 0.702291002 | OR51F2 | olfactory receptor family 51 subfamily F member 2 [Source:HGNC Symbol;Acc:HGNC:15197] |
| ENSG00000177202 | HGLibA_46330 | 186 | 0.702291002 | SPACA4 | sperm acrosome associated 4 [Source:HGNC Symbol;Acc:HGNC:16441] |
| ENSG00000177981 | HGLibA_03252 | 186 | 0.702291002 | ASB8 | ankyrin repeat and SOCS box containing 8 [Source:HGNC Symbol;Acc:HGNC:17183] |
| ENSG00000179021 | HGLibA_06242 | 186 | 0.702291002 | C3orf38 | chromosome 3 open reading frame 38 [Source:HGNC Symbol;Acc:HGNC:28384] |
| ENSG00000182983 | HGLibA_56480 | 186 | 0.702291002 | ZNF662 | zinc finger protein 662 [Source:HGNC Symbol;Acc:HGNC:31930] |
| ENSG00000183044 | HGLibA_00093 | 186 | 0.702291002 | ABAT | 4-aminobutyrate aminotransferase [Source:HGNC Symbol;Acc:HGNC:23] |
| ENSG00000184005 | HGLibA_47270 | 186 | 0.702291002 | ST6GALNAC3 | 6-sialyltransferase 3 [Source:HGNC Symbol;Acc:HGNC:19343]" |
| ENSG00000184831 | HGLibA_02551 | 186 | 0.702291002 | APOO | apolipoprotein O [Source:HGNC Symbol;Acc:HGNC:28727] |
| ENSG00000184933 | HGLibA_34370 | 186 | 0.702291002 | OR6A2 | olfactory receptor family 6 subfamily A member 2 [Source:HGNC Symbol;Acc:HGNC:15301] |
| ENSG00000185010 | HGLibA_15882 | 186 | 0.702291002 | F8 | coagulation factor VIII [Source:HGNC Symbol;Acc:HGNC:3546] |
| ENSG00000185338 | HGLibA_46124 | 186 | 0.702291002 | SOCS1 | suppressor of cytokine signaling 1 [Source:HGNC Symbol;Acc:HGNC:19383] |
| ENSG00000185982 | HGLibA_12887 | 186 | 0.702291002 | DEFB128 | defensin beta 128 [Source:HGNC Symbol;Acc:HGNC:18106] |
| ENSG00000187054 | HGLibA_50613 | 186 | 0.702291002 | TMPRSS11A | transmembrane serine protease 11A [Source:HGNC Symbol;Acc:HGNC:27954] |
| ENSG00000187486 | HGLibA_24252 | 186 | 0.702291002 | KCNJ11 | potassium voltage-gated channel subfamily J member 11 [Source:HGNC Symbol;Acc:HGNC:6257] |
| ENSG00000198178 | HGLibA_09981 | 186 | 0.702291002 | CLEC4C | C-type lectin domain family 4 member C [Source:HGNC Symbol;Acc:HGNC:13258] |
| ENSG00000198551 | HGLibA_56425 | 186 | 0.702291002 | ZNF627 | zinc finger protein 627 [Source:HGNC Symbol;Acc:HGNC:30570] |
| ENSG00000198851 | HGLibA_08372 | 186 | 0.702291002 | CD3E | CD3e molecule [Source:HGNC Symbol;Acc:HGNC:1674] |
| ENSG00000205138 | HGLibA_43135 | 186 | 0.702291002 | SDHAF1 | succinate dehydrogenase complex assembly factor 1 [Source:HGNC Symbol;Acc:HGNC:33867] |
| ENSG00000205277 | HGLibA_30524 | 186 | 0.702291002 | MUC12 | cell surface associated [Source:HGNC Symbol;Acc:HGNC:7510]" |
| ENSG00000235718 | HGLibA_29077 | 186 | 0.702291002 | MFRP | membrane frizzled-related protein [Source:HGNC Symbol;Acc:HGNC:18121] |
| ENSG00000243284 | HGLibA_53850 | 186 | 0.702291002 | VSIG8 | V-set and immunoglobulin domain containing 8 [Source:HGNC Symbol;Acc:HGNC:32063] |
| ENSG00000250565 | HGLibA_03750 | 186 | 0.702291002 | ATP6V1E2 | ATPase H+ transporting V1 subunit E2 [Source:HGNC Symbol;Acc:HGNC:18125] |
| ENSG00000254402 | HGLibA_27287 | 186 | 0.702291002 | LRRC24 | leucine rich repeat containing 24 [Source:HGNC Symbol;Acc:HGNC:28947] |
| ENSG00000258890 | HGLibA_09179 | 186 | 0.702291002 | CEP95 | centrosomal protein 95 [Source:HGNC Symbol;Acc:HGNC:25141] |
| HRASLS2 | HGLibA_22078 | 186 | 0.702291002 | HRASLS2 | - |
| hsa-mir-124-3 | HGLibA_57356 | 186 | 0.702291002 | hsa-mir-124-3 | - |
| hsa-mir-1283-1 | HGLibA_57588 | 186 | 0.702291002 | hsa-mir-1283-1 | - |
| hsa-mir-181b-1 | HGLibA_57966 | 186 | 0.702291002 | hsa-mir-181b-1 | - |
| hsa-mir-188 | HGLibA_58016 | 186 | 0.702291002 | hsa-mir-188 | - |
| hsa-mir-2113 | HGLibA_58224 | 186 | 0.702291002 | hsa-mir-2113 | - |
| hsa-mir-4428 | HGLibA_60098 | 186 | 0.702291002 | hsa-mir-4428 | - |
| hsa-mir-4730 | HGLibA_61018 | 186 | 0.702291002 | hsa-mir-4730 | - |
| hsa-mir-4740 | HGLibA_61057 | 186 | 0.702291002 | hsa-mir-4740 | - |
| hsa-mir-502 | HGLibA_61463 | 186 | 0.702291002 | hsa-mir-502 | - |
| hsa-mir-559 | HGLibA_62201 | 186 | 0.702291002 | hsa-mir-559 | - |
| hsa-mir-6500 | HGLibA_62852 | 186 | 0.702291002 | hsa-mir-6500 | - |
| hsa-mir-761 | HGLibA_63903 | 186 | 0.702291002 | hsa-mir-761 | - |
| KIAA0907 | HGLibA_24665 | 186 | 0.702291002 | KIAA0907 | - |
| KIAA1045 | HGLibA_24688 | 186 | 0.702291002 | KIAA1045 | - |
| LOC158434 | HGLibA_26901 | 186 | 0.702291002 | LOC158434 | - |

| | | | | | |
|---|---|---|---|---|---|
| PRSS46 | HGLibA_38948 | 186 | 0.702291002 | PRSS46 | - |
| SELT | HGLibA_43314 | 186 | 0.702291002 | SELT | - |
| C19orf80 | HGLibA_05711 | 185 | 0.692339965 | C19orf80 | - |
| CECR1 | HGLibA_08975 | 185 | 0.692339965 | CECR1 | - |
| DEFB130 | HGLibA_12895 | 185 | 0.692339965 | DEFB130 | - |
| ENSG00000002933 | HGLibA_50073 | 185 | 0.692339965 | TMEM176A | transmembrane protein 176A [Source:HGNC Symbol;Acc:HGNC:24930] |
| ENSG00000003987 | HGLibA_30435 | 185 | 0.692339965 | MTMR7 | myotubularin related protein 7 [Source:HGNC Symbol;Acc:HGNC:7454] |
| ENSG00000015520 | HGLibA_32422 | 185 | 0.692339965 | NPC1L1 | NPC1 like intracellular cholesterol transporter 1 [Source:HGNC Symbol;Acc:HGNC:7898] |
| ENSG00000019549 | HGLibA_45869 | 185 | 0.692339965 | SNAI2 | snail family transcriptional repressor 2 [Source:HGNC Symbol;Acc:HGNC:11094] |
| ENSG00000028277 | HGLibA_37758 | 185 | 0.692339965 | POU2F2 | POU class 2 homeobox 2 [Source:HGNC Symbol;Acc:HGNC:9213] |
| ENSG00000040933 | HGLibA_23338 | 185 | 0.692339965 | INPP4A | inositol polyphosphate-4-phosphatase type I A [Source:HGNC Symbol;Acc:HGNC:6074] |
| ENSG00000050344 | HGLibA_31797 | 185 | 0.692339965 | NFE2L3 | erythroid 2 like 3 [Source:HGNC Symbol;Acc:HGNC:7783]" |
| ENSG00000071677 | HGLibA_38600 | 185 | 0.692339965 | PRLH | prolactin releasing hormone [Source:HGNC Symbol;Acc:HGNC:17945] |
| ENSG00000075826 | HGLibA_43245 | 185 | 0.692339965 | SEC31B | COPII coat complex component [Source:HGNC Symbol;Acc:HGNC:23197]" |
| ENSG00000081985 | HGLibA_22968 | 185 | 0.692339965 | IL12RB2 | interleukin 12 receptor subunit beta 2 [Source:HGNC Symbol;Acc:HGNC:5972] |
| ENSG00000089060 | HGLibA_45396 | 185 | 0.692339965 | SLC8B1 | solute carrier family 8 member B1 [Source:HGNC Symbol;Acc:HGNC:26175] |
| ENSG00000092820 | HGLibA_15837 | 185 | 0.692339965 | EZR | ezrin [Source:HGNC Symbol;Acc:HGNC:12691] |
| ENSG00000101670 | HGLibA_26612 | 185 | 0.692339965 | LIPG | endothelial type [Source:HGNC Symbol;Acc:HGNC:6623]" |
| ENSG00000102970 | HGLibA_07980 | 185 | 0.692339965 | CCL17 | C-C motif chemokine ligand 17 [Source:HGNC Symbol;Acc:HGNC:10615] |
| ENSG00000104218 | HGLibA_11419 | 185 | 0.692339965 | CSPP1 | centrosome and spindle pole associated protein 1 [Source:HGNC Symbol;Acc:HGNC:26193] |
| ENSG00000105379 | HGLibA_15624 | 185 | 0.692339965 | ETFB | electron transfer flavoprotein subunit beta [Source:HGNC Symbol;Acc:HGNC:3482] |
| ENSG00000107651 | HGLibA_43226 | 185 | 0.692339965 | SEC23IP | SEC23 interacting protein [Source:HGNC Symbol;Acc:HGNC:17018] |
| ENSG00000108405 | HGLibA_34847 | 185 | 0.692339965 | P2RX1 | purinergic receptor P2X 1 [Source:HGNC Symbol;Acc:HGNC:8533] |
| ENSG00000110880 | HGLibA_10778 | 185 | 0.692339965 | CORO1C | coronin 1C [Source:HGNC Symbol;Acc:HGNC:2254] |
| ENSG00000116016 | HGLibA_15236 | 185 | 0.692339965 | EPAS1 | endothelial PAS domain protein 1 [Source:HGNC Symbol;Acc:HGNC:3374] |
| ENSG00000116218 | HGLibA_32456 | 185 | 0.692339965 | NPHS2 | podocin [Source:HGNC Symbol;Acc:HGNC:13394]" |
| ENSG00000116254 | HGLibA_09399 | 185 | 0.692339965 | CHD5 | chromodomain helicase DNA binding protein 5 [Source:HGNC Symbol;Acc:HGNC:16816] |
| ENSG00000116668 | HGLibA_47903 | 185 | 0.692339965 | SWT1 | RNA endoribonuclease homolog [Source:HGNC Symbol;Acc:HGNC:16785]" |
| ENSG00000119401 | HGLibA_51461 | 185 | 0.692339965 | TRIM32 | tripartite motif containing 32 [Source:HGNC Symbol;Acc:HGNC:16380] |
| ENSG00000119640 | HGLibA_00709 | 185 | 0.692339965 | ACYP1 | acylphosphatase 1 [Source:HGNC Symbol;Acc:HGNC:179] |
| ENSG00000120756 | HGLibA_37226 | 185 | 0.692339965 | PLS1 | plastin 1 [Source:HGNC Symbol;Acc:HGNC:9090] |
| ENSG00000120833 | HGLibA_46127 | 185 | 0.692339965 | SOCS2 | suppressor of cytokine signaling 2 [Source:HGNC Symbol;Acc:HGNC:19382] |
| ENSG00000123106 | HGLibA_07926 | 185 | 0.692339965 | CCDC91 | coiled-coil domain containing 91 [Source:HGNC Symbol;Acc:HGNC:24855] |
| ENSG00000123444 | HGLibA_24109 | 185 | 0.692339965 | KBTBD4 | kelch repeat and BTB domain containing 4 [Source:HGNC Symbol;Acc:HGNC:23761] |
| ENSG00000125503 | HGLibA_38027 | 185 | 0.692339965 | PPP1R12C | protein phosphatase 1 regulatory subunit 12C [Source:HGNC Symbol;Acc:HGNC:14947] |
| ENSG00000126233 | HGLibA_45553 | 185 | 0.692339965 | SLURP1 | secreted LY6/PLAUR domain containing 1 [Source:HGNC Symbol;Acc:HGNC:18746] |
| ENSG00000126353 | HGLibA_08164 | 185 | 0.692339965 | CCR7 | C-C motif chemokine receptor 7 [Source:HGNC Symbol;Acc:HGNC:1608] |
| ENSG00000127824 | HGLibA_52262 | 185 | 0.692339965 | TUBA4A | tubulin alpha 4a [Source:HGNC Symbol;Acc:HGNC:12407] |
| ENSG00000132481 | HGLibA_51515 | 185 | 0.692339965 | TRIM47 | tripartite motif containing 47 [Source:HGNC Symbol;Acc:HGNC:19020] |
| ENSG00000134222 | HGLibA_39232 | 185 | 0.692339965 | PSRC1 | proline and serine rich coiled-coil 1 [Source:HGNC Symbol;Acc:HGNC:24472] |
| ENSG00000134489 | HGLibA_22102 | 185 | 0.692339965 | HRH4 | histamine receptor H4 [Source:HGNC Symbol;Acc:HGNC:17383] |
| ENSG00000135636 | HGLibA_14287 | 185 | 0.692339965 | DYSF | dysferlin [Source:HGNC Symbol;Acc:HGNC:3097] |
| ENSG00000136002 | HGLibA_02863 | 185 | 0.692339965 | ARHGEF4 | Rho guanine nucleotide exchange factor 4 [Source:HGNC Symbol;Acc:HGNC:684] |
| ENSG00000136878 | HGLibA_53299 | 185 | 0.692339965 | USP20 | ubiquitin specific peptidase 20 [Source:HGNC Symbol;Acc:HGNC:12619] |
| ENSG00000137561 | HGLibA_52222 | 185 | 0.692339965 | TTPA | alpha tocopherol transfer protein [Source:HGNC Symbol;Acc:HGNC:12404] |
| ENSG00000143032 | HGLibA_04162 | 185 | 0.692339965 | BARHL2 | BarH like homeobox 2 [Source:HGNC Symbol;Acc:HGNC:954] |
| ENSG00000143632 | HGLibA_00595 | 185 | 0.692339965 | ACTA1 | skeletal muscle [Source:HGNC Symbol;Acc:HGNC:129]" |
| ENSG00000143653 | HGLibA_42882 | 185 | 0.692339965 | SCCPDH | saccharopine dehydrogenase (putative) [Source:HGNC Symbol;Acc:HGNC:24275] |
| ENSG00000145217 | HGLibA_44779 | 185 | 0.692339965 | SLC26A1 | solute carrier family 26 member 1 [Source:HGNC Symbol;Acc:HGNC:10993] |
| ENSG00000145335 | HGLibA_45907 | 185 | 0.692339965 | SNCA | synuclein alpha [Source:HGNC Symbol;Acc:HGNC:11138] |
| ENSG00000147894 | HGLibA_06757 | 185 | 0.692339965 | C9orf72 | chromosome 9 open reading frame 72 [Source:HGNC Symbol;Acc:HGNC:28337] |
| ENSG00000148840 | HGLibA_38229 | 185 | 0.692339965 | PPRC1 | coactivator-related 1 [Source:HGNC Symbol;Acc:HGNC:30025]" |
| ENSG00000154358 | HGLibA_33231 | 185 | 0.692339965 | OBSCN | cytoskeletal calmodulin and titin-interacting RhoGEF [Source:HGNC Symbol;Acc:HGNC:15719]" |
| ENSG00000155393 | HGLibA_21072 | 185 | 0.692339965 | HEATR3 | HEAT repeat containing 3 [Source:HGNC Symbol;Acc:HGNC:26087] |
| ENSG00000155530 | HGLibA_27170 | 185 | 0.692339965 | LRGUK | leucine rich repeats and guanylate kinase domain containing [Source:HGNC Symbol;Acc:HGNC:21964] |
| ENSG00000160271 | HGLibA_40166 | 185 | 0.692339965 | RALGDS | ral guanine nucleotide dissociation stimulator [Source:HGNC Symbol;Acc:HGNC:9842] |
| ENSG00000164305 | HGLibA_07309 | 185 | 0.692339965 | CASP3 | caspase 3 [Source:HGNC Symbol;Acc:HGNC:1504] |
| ENSG00000166024 | HGLibA_39768 | 185 | 0.692339965 | R3HCC1L | R3H domain and coiled-coil containing 1 like [Source:HGNC Symbol;Acc:HGNC:23512] |
| ENSG00000166086 | HGLibA_23934 | 185 | 0.692339965 | JAM3 | junctional adhesion molecule 3 [Source:HGNC Symbol;Acc:HGNC:15532] |
| ENSG00000166889 | HGLibA_35284 | 185 | 0.692339965 | PATL1 | processing body mRNA decay factor [Source:HGNC Symbol;Acc:HGNC:26721]" |
| ENSG00000168172 | HGLibA_21881 | 185 | 0.692339965 | HOOK3 | hook microtubule tethering protein 3 [Source:HGNC Symbol;Acc:HGNC:23576] |
| ENSG00000170779 | HGLibA_08607 | 185 | 0.692339965 | CDCA4 | cell division cycle associated 4 [Source:HGNC Symbol;Acc:HGNC:14625] |
| ENSG00000170953 | HGLibA_34493 | 185 | 0.692339965 | OR8B12 | olfactory receptor family 8 subfamily B member 12 [Source:HGNC Symbol;Acc:HGNC:15307] |
| ENSG00000173744 | HGLibA_01184 | 185 | 0.692339965 | AGFG1 | ArfGAP with FG repeats 1 [Source:HGNC Symbol;Acc:HGNC:5175] |
| ENSG00000174225 | HGLibA_02929 | 185 | 0.692339965 | ARL13A | ADP ribosylation factor like GTPase 13A [Source:HGNC Symbol;Acc:HGNC:31709] |
| ENSG00000175077 | HGLibA_42423 | 185 | 0.692339965 | RTP1 | receptor transporter protein 1 [Source:HGNC Symbol;Acc:HGNC:28580] |
| ENSG00000175390 | HGLibA_14779 | 185 | 0.692339965 | EIF3F | eukaryotic translation initiation factor 3 subunit F [Source:HGNC Symbol;Acc:HGNC:3275] |
| ENSG00000178209 | HGLibA_37053 | 185 | 0.692339965 | PLEC | plectin [Source:HGNC Symbol;Acc:HGNC:9069] |
| ENSG00000179826 | HGLibA_29853 | 185 | 0.692339965 | MRGPRX3 | MAS related GPR family member X3 [Source:HGNC Symbol;Acc:HGNC:17980] |
| ENSG00000181718 | HGLibA_34358 | 185 | 0.692339965 | OR5T2 | olfactory receptor family 5 subfamily T member 2 [Source:HGNC Symbol;Acc:HGNC:15296] |
| ENSG00000182177 | HGLibA_03231 | 185 | 0.692339965 | ASB18 | ankyrin repeat and SOCS box containing 18 [Source:HGNC Symbol;Acc:HGNC:19770] |
| ENSG00000182685 | HGLibA_04808 | 185 | 0.692339965 | BRICD5 | BRICHOS domain containing 5 [Source:HGNC Symbol;Acc:HGNC:28309] |
| ENSG00000185775 | HGLibA_46483 | 185 | 0.692339965 | SPATA31A6 | SPATA31 subfamily A member 6 [Source:HGNC Symbol;Acc:HGNC:32006] |
| ENSG00000186513 | HGLibA_34587 | 185 | 0.692339965 | OR9Q2 | olfactory receptor family 9 subfamily Q member 2 [Source:HGNC Symbol;Acc:HGNC:15328] |
| ENSG00000186790 | HGLibA_17861 | 185 | 0.692339965 | FOXE3 | forkhead box E3 [Source:HGNC Symbol;Acc:HGNC:3808] |
| ENSG00000187808 | HGLibA_46223 | 185 | 0.692339965 | SOWAHD | sosondowah ankyrin repeat domain family member D [Source:HGNC Symbol;Acc:HGNC:32960] |
| ENSG00000189037 | HGLibA_14161 | 185 | 0.692339965 | DUSP21 | dual specificity phosphatase 21 [Source:HGNC Symbol;Acc:HGNC:20476] |
| ENSG00000189056 | HGLibA_40824 | 185 | 0.692339965 | RELN | reelin [Source:HGNC Symbol;Acc:HGNC:9957] |
| ENSG00000197125 | HGLibA_34505 | 185 | 0.692339965 | OR8B8 | olfactory receptor family 8 subfamily B member 8 [Source:HGNC Symbol;Acc:HGNC:8477] |
| ENSG00000198948 | HGLibA_29047 | 185 | 0.692339965 | MFAP3L | microfibril associated protein 3 like [Source:HGNC Symbol;Acc:HGNC:29083] |
| ENSG00000198959 | HGLibA_49256 | 185 | 0.692339965 | TGM2 | transglutaminase 2 [Source:HGNC Symbol;Acc:HGNC:11778] |
| ENSG00000211452 | HGLibA_13238 | 185 | 0.692339965 | DIO1 | iodothyronine deiodinase 1 [Source:HGNC Symbol;Acc:HGNC:2883] |
| ENSG00000213160 | HGLibA_25160 | 185 | 0.692339965 | KLHL23 | kelch like family member 23 [Source:HGNC Symbol;Acc:HGNC:27506] |
| ENSG00000221933 | HGLibA_33730 | 185 | 0.692339965 | OR2A25 | olfactory receptor family 2 subfamily A member 25 [Source:HGNC Symbol;Acc:HGNC:19562] |
| ENSG00000243489 | HGLibA_25597 | 185 | 0.692339965 | KRTAP10-11 | keratin associated protein 10-11 [Source:HGNC Symbol;Acc:HGNC:20528] |
| hsa-mir-1199 | HGLibA_57257 | 185 | 0.692339965 | hsa-mir-1199 | - |
| hsa-mir-146b | HGLibA_57860 | 185 | 0.692339965 | hsa-mir-146b | - |
| hsa-mir-3191 | HGLibA_58862 | 185 | 0.692339965 | hsa-mir-3191 | - |
| hsa-mir-362 | HGLibA_59108 | 185 | 0.692339965 | hsa-mir-362 | - |
| hsa-mir-372 | HGLibA_59382 | 185 | 0.692339965 | hsa-mir-372 | - |
| hsa-mir-500a | HGLibA_61443 | 185 | 0.692339965 | hsa-mir-500a | - |
| hsa-mir-556 | HGLibA_62140 | 185 | 0.692339965 | hsa-mir-556 | - |
| hsa-mir-5579 | HGLibA_62156 | 185 | 0.692339965 | hsa-mir-5579 | - |
| hsa-mir-6715b | HGLibA_63021 | 185 | 0.692339965 | hsa-mir-6715b | - |
| hsa-mir-8089 | HGLibA_64197 | 185 | 0.692339965 | hsa-mir-8089 | - |
| KIAA1324 | HGLibA_24737 | 185 | 0.692339965 | KIAA1324 | - |
| PQLC1 | HGLibA_38251 | 185 | 0.692339965 | PQLC1 | - |
| TCEB3C | HGLibA_48745 | 185 | 0.692339965 | TCEB3C | - |
| C1orf234 | HGLibA_05849 | 184 | 0.682388927 | C1orf234 | - |
| ENSG00000008197 | HGLibA_49131 | 184 | 0.682388927 | TFAP2D | transcription factor AP-2 delta [Source:HGNC Symbol;Acc:HGNC:15581] |
| ENSG00000009307 | HGLibA_11314 | 184 | 0.682388927 | CSDE1 | cold shock domain containing E1 [Source:HGNC Symbol;Acc:HGNC:29905] |
| ENSG00000011132 | HGLibA_02373 | 184 | 0.682388927 | APBA3 | amyloid beta precursor protein binding family A member 3 [Source:HGNC Symbol;Acc:HGNC:580] |
| ENSG00000019582 | HGLibA_08443 | 184 | 0.682388927 | CD74 | CD74 molecule [Source:HGNC Symbol;Acc:HGNC:1697] |
| ENSG00000038210 | HGLibA_36494 | 184 | 0.682388927 | PI4K2B | phosphatidylinositol 4-kinase type 2 beta [Source:HGNC Symbol;Acc:HGNC:18215] |
| ENSG00000056736 | HGLibA_23006 | 184 | 0.682388927 | IL17RB | interleukin 17 receptor B [Source:HGNC Symbol;Acc:HGNC:18015] |
| ENSG00000069966 | HGLibA_19502 | 184 | 0.682388927 | GNB5 | G protein subunit beta 5 [Source:HGNC Symbol;Acc:HGNC:4401] |
| ENSG00000070601 | HGLibA_18031 | 184 | 0.682388927 | FRMPD1 | FERM and PDZ domain containing 1 [Source:HGNC Symbol;Acc:HGNC:29159] |
| ENSG00000077157 | HGLibA_38023 | 184 | 0.682388927 | PPP1R12B | protein phosphatase 1 regulatory subunit 12B [Source:HGNC Symbol;Acc:HGNC:7619] |
| ENSG00000077782 | HGLibA_17436 | 184 | 0.682388927 | FGFR1 | fibroblast growth factor receptor 1 [Source:HGNC Symbol;Acc:HGNC:3688] |
| ENSG00000078687 | HGLibA_50918 | 184 | 0.682388927 | TNRC6C | trinucleotide repeat containing 6C [Source:HGNC Symbol;Acc:HGNC:29318] |
| ENSG00000079557 | HGLibA_01121 | 184 | 0.682388927 | AFM | afamin [Source:HGNC Symbol;Acc:HGNC:316] |
| ENSG00000079950 | HGLibA_47657 | 184 | 0.682388927 | STX7 | syntaxin 7 [Source:HGNC Symbol;Acc:HGNC:11442] |
| ENSG00000082996 | HGLibA_41502 | 184 | 0.682388927 | RNF13 | ring finger protein 13 [Source:HGNC Symbol;Acc:HGNC:10057] |
| ENSG00000085365 | HGLibA_42837 | 184 | 0.682388927 | SCAMP1 | secretory carrier membrane protein 1 [Source:HGNC Symbol;Acc:HGNC:10563] |
| ENSG00000087274 | HGLibA_00941 | 184 | 0.682388927 | ADD1 | adducin 1 [Source:HGNC Symbol;Acc:HGNC:243] |
| ENSG00000088992 | HGLibA_49025 | 184 | 0.682388927 | TESC | tescalcin [Source:HGNC Symbol;Acc:HGNC:26065] |
| ENSG00000099715 | HGLibA_35394 | 184 | 0.682388927 | PCDH11Y | protocadherin 11 Y-linked [Source:HGNC Symbol;Acc:HGNC:15813] |
| ENSG00000100121 | HGLibA_19026 | 184 | 0.682388927 | GGTLC2 | gamma-glutamyltransferase light chain 2 [Source:HGNC Symbol;Acc:HGNC:18596] |
| ENSG00000100285 | HGLibA_31616 | 184 | 0.682388927 | NEFH | neurofilament heavy [Source:HGNC Symbol;Acc:HGNC:7737] |
| ENSG00000100968 | HGLibA_31786 | 184 | 0.682388927 | NFATC4 | nuclear factor of activated T cells 4 [Source:HGNC Symbol;Acc:HGNC:7778] |
| ENSG00000102096 | HGLibA_36678 | 184 | 0.682388927 | PIM2 | serine/threonine kinase [Source:HGNC Symbol;Acc:HGNC:8987]" |
| ENSG00000104537 | HGLibA_02229 | 184 | 0.682388927 | ANXA13 | annexin A13 [Source:HGNC Symbol;Acc:HGNC:536] |
| ENSG00000105204 | HGLibA_14274 | 184 | 0.682388927 | DYRK1B | dual specificity tyrosine phosphorylation regulated kinase 1B [Source:HGNC Symbol;Acc:HGNC:3092] |
| ENSG00000106052 | HGLibA_48449 | 184 | 0.682388927 | TAX1BP1 | Tax1 binding protein 1 [Source:HGNC Symbol;Acc:HGNC:11575] |
| ENSG00000106665 | HGLibA_10044 | 184 | 0.682388927 | CLIP2 | CAP-Gly domain containing linker protein 2 [Source:HGNC Symbol;Acc:HGNC:2586] |
| ENSG00000108883 | HGLibA_14618 | 184 | 0.682388927 | EFTUD2 | elongation factor Tu GTP binding domain containing 2 [Source:HGNC Symbol;Acc:HGNC:30858] |
| ENSG00000110987 | HGLibA_04339 | 184 | 0.682388927 | BCL7A | BCL tumor suppressor 7A [Source:HGNC Symbol;Acc:HGNC:1004] |
| ENSG00000116329 | HGLibA_33442 | 184 | 0.682388927 | OPRD1 | opioid receptor delta 1 [Source:HGNC Symbol;Acc:HGNC:8153] |
| ENSG00000117640 | HGLibA_30363 | 184 | 0.682388927 | MTFR1L | mitochondrial fission regulator 1 like [Source:HGNC Symbol;Acc:HGNC:28836] |
| ENSG00000117971 | HGLibA_09597 | 184 | 0.682388927 | CHRNB4 | cholinergic receptor nicotinic beta 4 subunit [Source:HGNC Symbol;Acc:HGNC:1964] |
| ENSG00000121964 | HGLibA_20513 | 184 | 0.682388927 | GTDC1 | glycosyltransferase like domain containing 1 [Source:HGNC Symbol;Acc:HGNC:20887] |
| ENSG00000124159 | HGLibA_28435 | 184 | 0.682388927 | MATN4 | matrilin 4 [Source:HGNC Symbol;Acc:HGNC:6910] |
| ENSG00000124491 | HGLibA_15853 | 184 | 0.682388927 | F13A1 | coagulation factor XIII A chain [Source:HGNC Symbol;Acc:HGNC:3531] |
| ENSG00000125746 | HGLibA_15059 | 184 | 0.682388927 | EML2 | echinoderm microtubule associated protein like 2 [Source:HGNC Symbol;Acc:HGNC:18035] |
| ENSG00000133392 | HGLibA_30710 | 184 | 0.682388927 | MYH11 | myosin heavy chain 11 [Source:HGNC Symbol;Acc:HGNC:7569] |

| | | | | | |
|---|---|---|---|---|---|
| ENSG00000135540 | HGLibA_31917 | 184 | 0.682388927 | NHSL1 | NHS like 1 [Source:HGNC Symbol;Acc:HGNC:21021] |
| ENSG00000138308 | HGLibA_36874 | 184 | 0.682388927 | PLA2G12B | phospholipase A2 group XIIB [Source:HGNC Symbol;Acc:HGNC:18555] |
| ENSG00000138363 | HGLibA_03511 | 184 | 0.682388927 | ATIC | 5-aminoimidazole-4-carboxamide ribonucleotide formyltransferase/IMP cyclohydrolase [Source:HGNC Symbol;Acc:HGNC:794] |
| ENSG00000143185 | HGLibA_54495 | 184 | 0.682388927 | XCL2 | X-C motif chemokine ligand 2 [Source:HGNC Symbol;Acc:HGNC:10646] |
| ENSG00000143845 | HGLibA_15636 | 184 | 0.682388927 | ETNK2 | ethanolamine kinase 2 [Source:HGNC Symbol;Acc:HGNC:25575] |
| ENSG00000144827 | HGLibA_00233 | 184 | 0.682388927 | ABHD10 | abhydrolase domain containing 10 [Source:HGNC Symbol;Acc:HGNC:25656] |
| ENSG00000149609 | HGLibA_05989 | 184 | 0.682388927 | C20orf144 | chromosome 20 open reading frame 144 [Source:HGNC Symbol;Acc:HGNC:16137] |
| ENSG00000151233 | HGLibA_20681 | 184 | 0.682388927 | GXYLT1 | glucoside xylosyltransferase 1 [Source:HGNC Symbol;Acc:HGNC:27482] |
| ENSG00000155961 | HGLibA_39903 | 184 | 0.682388927 | RAB39B | member RAS oncogene family [Source:HGNC Symbol;Acc:HGNC:16499]" |
| ENSG00000156959 | HGLibA_26437 | 184 | 0.682388927 | LHFPL4 | LHFPL tetraspan subfamily member 4 [Source:HGNC Symbol;Acc:HGNC:29568] |
| ENSG00000158525 | HGLibA_10895 | 184 | 0.682388927 | CPA5 | carboxypeptidase A5 [Source:HGNC Symbol;Acc:HGNC:15722] |
| ENSG00000161249 | HGLibA_13408 | 184 | 0.682388927 | DMKN | dermokine [Source:HGNC Symbol;Acc:HGNC:25063] |
| ENSG00000162040 | HGLibA_22139 | 184 | 0.682388927 | HS3ST6 | heparan sulfate-glucosamine 3-sulfotransferase 6 [Source:HGNC Symbol;Acc:HGNC:14178] |
| ENSG00000163746 | HGLibA_37234 | 184 | 0.682388927 | PLSCR2 | phospholipid scramblase 2 [Source:HGNC Symbol;Acc:HGNC:16494] |
| ENSG00000163817 | HGLibA_45322 | 184 | 0.682388927 | SLC6A20 | solute carrier family 6 member 20 [Source:HGNC Symbol;Acc:HGNC:30927] |
| ENSG00000164211 | HGLibA_47364 | 184 | 0.682388927 | STARD4 | StAR related lipid transfer domain containing 4 [Source:HGNC Symbol;Acc:HGNC:18058] |
| ENSG00000164576 | HGLibA_42727 | 184 | 0.682388927 | SAP30L | SAP30 like [Source:HGNC Symbol;Acc:HGNC:25663] |
| ENSG00000167580 | HGLibA_02590 | 184 | 0.682388927 | AQP2 | aquaporin 2 [Source:HGNC Symbol;Acc:HGNC:634] |
| ENSG00000168779 | HGLibA_44075 | 184 | 0.682388927 | SHOX2 | short stature homeobox 2 [Source:HGNC Symbol;Acc:HGNC:10854] |
| ENSG00000168828 | HGLibA_33625 | 184 | 0.682388927 | OR13J1 | olfactory receptor family 13 subfamily J member 1 [Source:HGNC Symbol;Acc:HGNC:15108] |
| ENSG00000168918 | HGLibA_23350 | 184 | 0.682388927 | INPP5D | inositol polyphosphate-5-phosphatase D [Source:HGNC Symbol;Acc:HGNC:6079] |
| ENSG00000169218 | HGLibA_42345 | 184 | 0.682388927 | RSPO1 | R-spondin 1 [Source:HGNC Symbol;Acc:HGNC:21679] |
| ENSG00000169744 | HGLibA_26213 | 184 | 0.682388927 | LDB2 | LIM domain binding 2 [Source:HGNC Symbol;Acc:HGNC:6533] |
| ENSG00000170482 | HGLibA_44606 | 184 | 0.682388927 | SLC23A1 | solute carrier family 23 member 1 [Source:HGNC Symbol;Acc:HGNC:10974] |
| ENSG00000171189 | HGLibA_20282 | 184 | 0.682388927 | GRIK1 | glutamate ionotropic receptor kainate type subunit 1 [Source:HGNC Symbol;Acc:HGNC:4579] |
| ENSG00000171388 | HGLibA_02435 | 184 | 0.682388927 | APLN | apelin [Source:HGNC Symbol;Acc:HGNC:16665] |
| ENSG00000171747 | HGLibA_26370 | 184 | 0.682388927 | LGALS4 | galectin 4 [Source:HGNC Symbol;Acc:HGNC:6565] |
| ENSG00000180185 | HGLibA_15954 | 184 | 0.682388927 | FAHD1 | fumarylacetoacetate hydrolase domain containing 1 [Source:HGNC Symbol;Acc:HGNC:14169] |
| ENSG00000181693 | HGLibA_34526 | 184 | 0.682388927 | OR8H1 | olfactory receptor family 8 subfamily H member 1 [Source:HGNC Symbol;Acc:HGNC:14824] |
| ENSG00000182035 | HGLibA_00973 | 184 | 0.682388927 | ADIG | adipogenin [Source:HGNC Symbol;Acc:HGNC:28606] |
| ENSG00000185479 | HGLibA_25516 | 184 | 0.682388927 | KRT6B | keratin 6B [Source:HGNC Symbol;Acc:HGNC:6444] |
| ENSG00000185761 | HGLibA_00860 | 184 | 0.682388927 | ADAMTSL5 | ADAMTS like 5 [Source:HGNC Symbol;Acc:HGNC:27912] |
| ENSG00000185862 | HGLibA_15688 | 184 | 0.682388927 | EVI2B | ecotropic viral integration site 2B [Source:HGNC Symbol;Acc:HGNC:3500] |
| ENSG00000185920 | HGLibA_39269 | 184 | 0.682388927 | PTCH1 | patched 1 [Source:HGNC Symbol;Acc:HGNC:9585] |
| ENSG00000186452 | HGLibA_50626 | 184 | 0.682388927 | TMPRSS12 | transmembrane serine protease 12 [Source:HGNC Symbol;Acc:HGNC:28779] |
| ENSG00000186818 | HGLibA_26515 | 184 | 0.682388927 | LILRB4 | leukocyte immunoglobulin like receptor B4 [Source:HGNC Symbol;Acc:HGNC:6608] |
| ENSG00000187140 | HGLibA_17834 | 184 | 0.682388927 | FOXD3 | forkhead box D3 [Source:HGNC Symbol;Acc:HGNC:3804] |
| ENSG00000187175 | HGLibA_25631 | 184 | 0.682388927 | KRTAP12-1 | keratin associated protein 12-1 [Source:HGNC Symbol;Acc:HGNC:20529] |
| ENSG00000189051 | HGLibA_41643 | 184 | 0.682388927 | RNF222 | ring finger protein 222 [Source:HGNC Symbol;Acc:HGNC:34517] |
| ENSG00000189409 | HGLibA_29515 | 184 | 0.682388927 | MMP23B | matrix metallopeptidase 23B [Source:HGNC Symbol;Acc:HGNC:7171] |
| ENSG00000196411 | HGLibA_15319 | 184 | 0.682388927 | EPHB4 | EPH receptor B4 [Source:HGNC Symbol;Acc:HGNC:3395] |
| ENSG00000197241 | HGLibA_44887 | 184 | 0.682388927 | SLC2A7 | solute carrier family 2 member 7 [Source:HGNC Symbol;Acc:HGNC:13445] |
| ENSG00000198142 | HGLibA_46220 | 184 | 0.682388927 | SOWAHC | sosondowah ankyrin repeat domain family member C [Source:HGNC Symbol;Acc:HGNC:26149] |
| ENSG00000198771 | HGLibA_40719 | 184 | 0.682388927 | RCSD1 | RCSD domain containing 1 [Source:HGNC Symbol;Acc:HGNC:28310] |
| ENSG00000213413 | HGLibA_39618 | 184 | 0.682388927 | PVRIG | PVR related immunoglobulin domain containing [Source:HGNC Symbol;Acc:HGNC:32190] |
| ENSG00000214694 | HGLibA_02849 | 184 | 0.682388927 | ARHGEF33 | Rho guanine nucleotide exchange factor 33 [Source:HGNC Symbol;Acc:HGNC:37252] |
| ENSG00000216937 | HGLibA_07847 | 184 | 0.682388927 | CCDC7 | coiled-coil domain containing 7 [Source:HGNC Symbol;Acc:HGNC:26533] |
| ENSG00000241635 | HGLibA_52877 | 184 | 0.682388927 | UGT1A1 | UDP glucuronosyltransferase family 1 member A1 [Source:HGNC Symbol;Acc:HGNC:12530] |
| ENSG00000248099 | HGLibA_23382 | 184 | 0.682388927 | INSL3 | insulin like 3 [Source:HGNC Symbol;Acc:HGNC:6086] |
| FAM65B | HGLibA_16671 | 184 | 0.682388927 | FAM65B | - |
| hsa-mir-3141 | HGLibA_58622 | 184 | 0.682388927 | hsa-mir-3141 | - |
| hsa-mir-3155a | HGLibA_58678 | 184 | 0.682388927 | hsa-mir-3155a | - |
| hsa-mir-3689e | HGLibA_59334 | 184 | 0.682388927 | hsa-mir-3689e | - |
| hsa-mir-4288 | HGLibA_59858 | 184 | 0.682388927 | hsa-mir-4288 | - |
| hsa-mir-4730 | HGLibA_61017 | 184 | 0.682388927 | hsa-mir-4730 | - |
| hsa-mir-499a | HGLibA_61400 | 184 | 0.682388927 | hsa-mir-499a | - |
| hsa-mir-6134 | HGLibA_62686 | 184 | 0.682388927 | hsa-mir-6134 | - |
| hsa-mir-631 | HGLibA_62762 | 184 | 0.682388927 | hsa-mir-631 | - |
| hsa-mir-6778 | HGLibA_63287 | 184 | 0.682388927 | hsa-mir-6778 | - |
| hsa-mir-887 | HGLibA_64227 | 184 | 0.682388927 | hsa-mir-887 | - |
| KIAA1147 | HGLibA_24700 | 184 | 0.682388927 | KIAA1147 | - |
| LOC100287036 | HGLibA_26817 | 184 | 0.682388927 | LOC100287036 | - |
| LOC100505841 | HGLibA_26854 | 184 | 0.682388927 | LOC100505841 | - |
| LOC100506422 | HGLibA_26861 | 184 | 0.682388927 | LOC100506422 | - |
| SGK196 | HGLibA_43824 | 184 | 0.682388927 | SGK196 | - |
| TMEM183B | HGLibA_50109 | 184 | 0.682388927 | TMEM183B | - |
| C2orf70 | HGLibA_06170 | 183 | 0.67243789 | C2orf70 | - |
| C4orf26 | HGLibA_06309 | 183 | 0.67243789 | C4orf26 | - |
| CCDC64 | HGLibA_07825 | 183 | 0.67243789 | CCDC64 | - |
| ENSG00000036530 | HGLibA_12170 | 183 | 0.67243789 | CYP46A1 | cytochrome P450 family 46 subfamily A member 1 [Source:HGNC Symbol;Acc:HGNC:2641] |
| ENSG00000064042 | HGLibA_26528 | 183 | 0.67243789 | LIMCH1 | LIM and calponin homology domains 1 [Source:HGNC Symbol;Acc:HGNC:29191] |
| ENSG00000074964 | HGLibA_02810 | 183 | 0.67243789 | ARHGEF10L | Rho guanine nucleotide exchange factor 10 like [Source:HGNC Symbol;Acc:HGNC:25540] |
| ENSG00000085514 | HGLibA_36670 | 183 | 0.67243789 | PILRA | paired immunoglobin like type 2 receptor alpha [Source:HGNC Symbol;Acc:HGNC:20396] |
| ENSG00000086061 | HGLibA_13510 | 183 | 0.67243789 | DNAJA1 | DnaJ heat shock protein family (Hsp40) member A1 [Source:HGNC Symbol;Acc:HGNC:5229] |
| ENSG00000099260 | HGLibA_35078 | 183 | 0.67243789 | PALMD | palmdelphin [Source:HGNC Symbol;Acc:HGNC:15846] |
| ENSG00000101004 | HGLibA_31943 | 183 | 0.67243789 | NINL | ninein like [Source:HGNC Symbol;Acc:HGNC:29163] |
| ENSG00000101489 | HGLibA_09012 | 183 | 0.67243789 | CELF4 | CUGBP Elav-like family member 4 [Source:HGNC Symbol;Acc:HGNC:14015] |
| ENSG00000103460 | HGLibA_51043 | 183 | 0.67243789 | TOX3 | TOX high mobility group box family member 3 [Source:HGNC Symbol;Acc:HGNC:11972] |
| ENSG00000104892 | HGLibA_25032 | 183 | 0.67243789 | KLC3 | kinesin light chain 3 [Source:HGNC Symbol;Acc:HGNC:20717] |
| ENSG00000105664 | HGLibA_10683 | 183 | 0.67243789 | COMP | cartilage oligomeric matrix protein [Source:HGNC Symbol;Acc:HGNC:2227] |
| ENSG00000106341 | HGLibA_38060 | 183 | 0.67243789 | PPP1R17 | protein phosphatase 1 regulatory subunit 17 [Source:HGNC Symbol;Acc:HGNC:16973] |
| ENSG00000109738 | HGLibA_19311 | 183 | 0.67243789 | GLRB | glycine receptor beta [Source:HGNC Symbol;Acc:HGNC:4329] |
| ENSG00000112293 | HGLibA_19816 | 183 | 0.67243789 | GPLD1 | glycosylphosphatidylinositol specific phospholipase D1 [Source:HGNC Symbol;Acc:HGNC:4459] |
| ENSG00000112852 | HGLibA_35494 | 183 | 0.67243789 | PCDHB2 | protocadherin beta 2 [Source:HGNC Symbol;Acc:HGNC:8687] |
| ENSG00000114646 | HGLibA_11418 | 183 | 0.67243789 | CSPG5 | chondroitin sulfate proteoglycan 5 [Source:HGNC Symbol;Acc:HGNC:2467] |
| ENSG00000116833 | HGLibA_32636 | 183 | 0.67243789 | NR5A2 | nuclear receptor subfamily 5 group A member 2 [Source:HGNC Symbol;Acc:HGNC:7984] |
| ENSG00000120549 | HGLibA_24721 | 183 | 0.67243789 | KIAA1217 | KIAA1217 [Source:HGNC Symbol;Acc:HGNC:25428] |
| ENSG00000122728 | HGLibA_48225 | 183 | 0.67243789 | TAF1L | TATA-box binding protein associated factor 1 like [Source:HGNC Symbol;Acc:HGNC:18056] |
| ENSG00000125841 | HGLibA_32727 | 183 | 0.67243789 | NRSN2 | neurensin 2 [Source:HGNC Symbol;Acc:HGNC:16229] |
| ENSG00000130653 | HGLibA_37424 | 183 | 0.67243789 | PNPLA7 | patatin like phospholipase domain containing 7 [Source:HGNC Symbol;Acc:HGNC:24768] |
| ENSG00000131737 | HGLibA_25485 | 183 | 0.67243789 | KRT34 | keratin 34 [Source:HGNC Symbol;Acc:HGNC:6452] |
| ENSG00000132122 | HGLibA_46522 | 183 | 0.67243789 | SPATA6 | spermatogenesis associated 6 [Source:HGNC Symbol;Acc:HGNC:18309] |
| ENSG00000132182 | HGLibA_33039 | 183 | 0.67243789 | NUP210 | nucleoporin 210 [Source:HGNC Symbol;Acc:HGNC:30052] |
| ENSG00000134243 | HGLibA_46198 | 183 | 0.67243789 | SORT1 | sortilin 1 [Source:HGNC Symbol;Acc:HGNC:11186] |
| ENSG00000134504 | HGLibA_24412 | 183 | 0.67243789 | KCTD1 | potassium channel tetramerization domain containing 1 [Source:HGNC Symbol;Acc:HGNC:18249] |
| ENSG00000135829 | HGLibA_13211 | 183 | 0.67243789 | DHX9 | DExH-box helicase 9 [Source:HGNC Symbol;Acc:HGNC:2750] |
| ENSG00000136478 | HGLibA_49072 | 183 | 0.67243789 | TEX2 | testis expressed 2 [Source:HGNC Symbol;Acc:HGNC:30884] |
| ENSG00000137757 | HGLibA_07316 | 183 | 0.67243789 | CASP5 | caspase 5 [Source:HGNC Symbol;Acc:HGNC:1506] |
| ENSG00000138152 | HGLibA_04903 | 183 | 0.67243789 | BTBD16 | BTB domain containing 16 [Source:HGNC Symbol;Acc:HGNC:26340] |
| ENSG00000142937 | HGLibA_42191 | 183 | 0.67243789 | RPS8 | ribosomal protein S8 [Source:HGNC Symbol;Acc:HGNC:10441] |
| ENSG00000145012 | HGLibA_27111 | 183 | 0.67243789 | LPP | LIM domain containing preferred translocation partner in lipoma [Source:HGNC Symbol;Acc:HGNC:6679] |
| ENSG00000148219 | HGLibA_03383 | 183 | 0.67243789 | ASTN2 | astrotactin 2 [Source:HGNC Symbol;Acc:HGNC:17021] |
| ENSG00000149823 | HGLibA_53803 | 183 | 0.67243789 | VPS51 | GARP complex subunit [Source:HGNC Symbol;Acc:HGNC:1172]" |
| ENSG00000150457 | HGLibA_26068 | 183 | 0.67243789 | LATS2 | large tumor suppressor kinase 2 [Source:HGNC Symbol;Acc:HGNC:6515] |
| ENSG00000152270 | HGLibA_35784 | 183 | 0.67243789 | PDE3B | phosphodiesterase 3B [Source:HGNC Symbol;Acc:HGNC:8779] |
| ENSG00000156925 | HGLibA_55328 | 183 | 0.67243789 | ZIC3 | Zic family member 3 [Source:HGNC Symbol;Acc:HGNC:12874] |
| ENSG00000158186 | HGLibA_29814 | 183 | 0.67243789 | MRAS | muscle RAS oncogene homolog [Source:HGNC Symbol;Acc:HGNC:7227] |
| ENSG00000160339 | HGLibA_17219 | 183 | 0.67243789 | FCN2 | ficolin 2 [Source:HGNC Symbol;Acc:HGNC:3624] |
| ENSG00000161960 | HGLibA_14803 | 183 | 0.67243789 | EIF4A1 | eukaryotic translation initiation factor 4A1 [Source:HGNC Symbol;Acc:HGNC:3282] |
| ENSG00000162600 | HGLibA_33380 | 183 | 0.67243789 | OMA1 | OMA1 zinc metallopeptidase [Source:HGNC Symbol;Acc:HGNC:29661] |
| ENSG00000164291 | HGLibA_03154 | 183 | 0.67243789 | ARSK | arylsulfatase family member K [Source:HGNC Symbol;Acc:HGNC:25239] |
| ENSG00000164332 | HGLibA_52717 | 183 | 0.67243789 | UBLCP1 | ubiquitin like domain containing CTD phosphatase 1 [Source:HGNC Symbol;Acc:HGNC:28110] |
| ENSG00000164707 | HGLibA_44392 | 183 | 0.67243789 | SLC13A4 | solute carrier family 13 member 4 [Source:HGNC Symbol;Acc:HGNC:15827] |
| ENSG00000164978 | HGLibA_32967 | 183 | 0.67243789 | NUDT2 | nudix hydrolase 2 [Source:HGNC Symbol;Acc:HGNC:8049] |
| ENSG00000165282 | HGLibA_36572 | 183 | 0.67243789 | PIGO | phosphatidylinositol glycan anchor biosynthesis class O [Source:HGNC Symbol;Acc:HGNC:23215] |
| ENSG00000165495 | HGLibA_36849 | 183 | 0.67243789 | PKNOX2 | PBX/knotted 1 homeobox 2 [Source:HGNC Symbol;Acc:HGNC:16714] |
| ENSG00000166323 | HGLibA_05170 | 183 | 0.67243789 | C11orf65 | chromosome 11 open reading frame 65 [Source:HGNC Symbol;Acc:HGNC:28519] |
| ENSG00000168397 | HGLibA_03491 | 183 | 0.67243789 | ATG4B | autophagy related 4B cysteine peptidase [Source:HGNC Symbol;Acc:HGNC:20790] |
| ENSG00000168412 | HGLibA_30445 | 183 | 0.67243789 | MTNR1A | melatonin receptor 1A [Source:HGNC Symbol;Acc:HGNC:7463] |
| ENSG00000170779 | HGLibA_08608 | 183 | 0.67243789 | CDCA4 | cell division cycle associated 4 [Source:HGNC Symbol;Acc:HGNC:14625] |
| ENSG00000172318 | HGLibA_03954 | 183 | 0.67243789 | B3GALT1 | 3-galactosyltransferase 1 [Source:HGNC Symbol;Acc:HGNC:916]" |
| ENSG00000173267 | HGLibA_45916 | 183 | 0.67243789 | SNCG | synuclein gamma [Source:HGNC Symbol;Acc:HGNC:11141] |
| ENSG00000173335 | HGLibA_11472 | 183 | 0.67243789 | CST9 | cystatin 9 [Source:HGNC Symbol;Acc:HGNC:13261] |
| ENSG00000173418 | HGLibA_30982 | 183 | 0.67243789 | NAA20 | NatB catalytic subunit [Source:HGNC Symbol;Acc:HGNC:15908]" |
| ENSG00000173868 | HGLibA_36442 | 183 | 0.67243789 | PHOSPHO1 | phosphoethanolamine/phosphocholine phosphatase [Source:HGNC Symbol;Acc:HGNC:16815] |
| ENSG00000174970 | HGLibA_33482 | 183 | 0.67243789 | OR10AG1 | olfactory receptor family 10 subfamily AG member 1 [Source:HGNC Symbol;Acc:HGNC:19607] |
| ENSG00000176390 | HGLibA_11194 | 183 | 0.67243789 | CRLF3 | cytokine receptor like factor 3 [Source:HGNC Symbol;Acc:HGNC:17177] |
| ENSG00000178175 | HGLibA_55878 | 183 | 0.67243789 | ZNF366 | zinc finger protein 366 [Source:HGNC Symbol;Acc:HGNC:18316] |
| ENSG00000180176 | HGLibA_49280 | 183 | 0.67243789 | TH | tyrosine hydroxylase [Source:HGNC Symbol;Acc:HGNC:11782] |
| ENSG00000180592 | HGLibA_44278 | 183 | 0.67243789 | SKIDA1 | SKI/DACH domain containing 1 [Source:HGNC Symbol;Acc:HGNC:32697] |
| ENSG00000182545 | HGLibA_41390 | 183 | 0.67243789 | RNASE10 | ribonuclease A family member 10 (inactive) [Source:HGNC Symbol;Acc:HGNC:19275] |
| ENSG00000182791 | HGLibA_07903 | 183 | 0.67243789 | CCDC87 | coiled-coil domain containing 87 [Source:HGNC Symbol;Acc:HGNC:25579] |
| ENSG00000183310 | HGLibA_33878 | 183 | 0.67243789 | OR2T34 | olfactory receptor family 2 subfamily T member 34 [Source:HGNC Symbol;Acc:HGNC:31256] |
| ENSG00000185640 | HGLibA_25549 | 183 | 0.67243789 | KRT79 | keratin 79 [Source:HGNC Symbol;Acc:HGNC:28930] |
| ENSG00000187068 | HGLibA_06263 | 183 | 0.67243789 | C3orf70 | chromosome 3 open reading frame 70 [Source:HGNC Symbol;Acc:HGNC:33731] |

| | | | | | |
|---|---|---|---|---|---|
| ENSG00000196131 | HGLibA_53701 | 183 | 0.67243789 | VN1R2 | vomeronasal 1 receptor 2 [Source:HGNC Symbol;Acc:HGNC:19872] |
| ENSG00000196228 | HGLibA_47750 | 183 | 0.67243789 | SULT1C3 | sulfotransferase family 1C member 3 [Source:HGNC Symbol;Acc:HGNC:33543] |
| ENSG00000196507 | HGLibA_48710 | 183 | 0.67243789 | TCEAL3 | transcription elongation factor A like 3 [Source:HGNC Symbol;Acc:HGNC:28247] |
| ENSG00000196793 | HGLibA_55653 | 183 | 0.67243789 | ZNF239 | zinc finger protein 239 [Source:HGNC Symbol;Acc:HGNC:13031] |
| ENSG00000196917 | HGLibA_20947 | 183 | 0.67243789 | HCAR1 | hydroxycarboxylic acid receptor 1 [Source:HGNC Symbol;Acc:HGNC:4532] |
| ENSG00000198719 | HGLibA_13359 | 183 | 0.67243789 | DLL1 | delta like canonical Notch ligand 1 [Source:HGNC Symbol;Acc:HGNC:2908] |
| ENSG00000204619 | HGLibA_38018 | 183 | 0.67243789 | PPP1R11 | protein phosphatase 1 regulatory inhibitor subunit 11 [Source:HGNC Symbol;Acc:HGNC:9285] |
| ENSG00000205978 | HGLibA_33185 | 183 | 0.67243789 | NYNRIN | NYN domain and retroviral integrase containing [Source:HGNC Symbol;Acc:HGNC:20165] |
| ENSG00000215029 | HGLibA_48850 | 183 | 0.67243789 | TCP11X2 | X-linked 2 [Source:HGNC Symbol;Acc:HGNC:48335]" |
| ENSG00000225899 | HGLibA_17999 | 183 | 0.67243789 | FRG2B | FSHD region gene 2 family member B [Source:HGNC Symbol;Acc:HGNC:33518] |
| ENSG00000227234 | HGLibA_46385 | 183 | 0.67243789 | SPANXB1 | SPANX family member B1 [Source:HGNC Symbol;Acc:HGNC:14329] |
| ENSG00000233670 | HGLibA_36733 | 183 | 0.67243789 | PIRT | phosphoinositide interacting regulator of transient receptor potential channels [Source:HGNC Symbol;Acc:HGNC:37239] |
| ENSG00000236609 | HGLibA_56876 | 183 | 0.67243789 | ZNF853 | zinc finger protein 853 [Source:HGNC Symbol;Acc:HGNC:21767] |
| ENSG00000239779 | HGLibA_53969 | 183 | 0.67243789 | WBP1 | WW domain binding protein 1 [Source:HGNC Symbol;Acc:HGNC:12737] |
| ENSG00000239961 | HGLibA_26498 | 183 | 0.67243789 | LILRA4 | leukocyte immunoglobulin like receptor A4 [Source:HGNC Symbol;Acc:HGNC:15503] |
| ENSG00000244414 | HGLibA_09262 | 183 | 0.67243789 | CFHR1 | complement factor H related 1 [Source:HGNC Symbol;Acc:HGNC:4888] |
| ENSG00000244694 | HGLibA_39282 | 183 | 0.67243789 | PTCHD4 | patched domain containing 4 [Source:HGNC Symbol;Acc:HGNC:21345] |
| ENSG00000265763 | HGLibA_56086 | 183 | 0.67243789 | ZNF488 | zinc finger protein 488 [Source:HGNC Symbol;Acc:HGNC:23535] |
| ENSG00000277462 | HGLibA_56499 | 183 | 0.67243789 | ZNF670 | zinc finger protein 670 [Source:HGNC Symbol;Acc:HGNC:28167] |
| FAM101A | HGLibA_15972 | 183 | 0.67243789 | FAM101A | - |
| FAM178A | HGLibA_16300 | 183 | 0.67243789 | FAM178A | - |
| FLJ45513 | HGLibA_17637 | 183 | 0.67243789 | FLJ45513 | - |
| hsa-let-7g | HGLibA_57133 | 183 | 0.67243789 | hsa-let-7g | - |
| hsa-mir-1343 | HGLibA_57787 | 183 | 0.67243789 | hsa-mir-1343 | - |
| hsa-mir-1470 | HGLibA_57863 | 183 | 0.67243789 | hsa-mir-1470 | - |
| hsa-mir-150 | HGLibA_57891 | 183 | 0.67243789 | hsa-mir-150 | - |
| hsa-mir-3126 | HGLibA_58556 | 183 | 0.67243789 | hsa-mir-3126 | - |
| hsa-mir-3130-2 | HGLibA_58575 | 183 | 0.67243789 | hsa-mir-3130-2 | - |
| hsa-mir-3689d-2 | HGLibA_59328 | 183 | 0.67243789 | hsa-mir-3689d-2 | - |
| hsa-mir-3690-2 | HGLibA_59351 | 183 | 0.67243789 | hsa-mir-3690-2 | - |
| hsa-mir-377 | HGLibA_59422 | 183 | 0.67243789 | hsa-mir-377 | - |
| hsa-mir-3939 | HGLibA_59616 | 183 | 0.67243789 | hsa-mir-3939 | - |
| hsa-mir-4309 | HGLibA_59947 | 183 | 0.67243789 | hsa-mir-4309 | - |
| hsa-mir-4476 | HGLibA_60316 | 183 | 0.67243789 | hsa-mir-4476 | - |
| hsa-mir-4496 | HGLibA_60401 | 183 | 0.67243789 | hsa-mir-4496 | - |
| hsa-mir-519c | HGLibA_61723 | 183 | 0.67243789 | hsa-mir-519c | - |
| hsa-mir-539 | HGLibA_61812 | 183 | 0.67243789 | hsa-mir-539 | - |
| hsa-mir-5706 | HGLibA_62363 | 183 | 0.67243789 | hsa-mir-5706 | - |
| hsa-mir-708 | HGLibA_63780 | 183 | 0.67243789 | hsa-mir-708 | - |
| hsa-mir-936 | HGLibA_64318 | 183 | 0.67243789 | hsa-mir-936 | - |
| MFSD4 | HGLibA_29101 | 183 | 0.67243789 | MFSD4 | - |
| TMEM2 | HGLibA_50168 | 183 | 0.67243789 | TMEM2 | - |
| C3orf79 | HGLibA_06268 | 182 | 0.662486853 | C3orf79 | - |
| CCDC155 | HGLibA_07657 | 182 | 0.662486853 | CCDC155 | - |
| ENSG00000012822 | HGLibA_06997 | 182 | 0.662486853 | CALCOCO1 | calcium binding and coiled-coil domain 1 [Source:HGNC Symbol;Acc:HGNC:29306] |
| ENSG00000044524 | HGLibA_15293 | 182 | 0.662486853 | EPHA3 | EPH receptor A3 [Source:HGNC Symbol;Acc:HGNC:3387] |
| ENSG00000055163 | HGLibA_12034 | 182 | 0.662486853 | CYFIP2 | cytoplasmic FMR1 interacting protein 2 [Source:HGNC Symbol;Acc:HGNC:13760] |
| ENSG00000069399 | HGLibA_04329 | 182 | 0.662486853 | BCL3 | B cell CLL/lymphoma 3 [Source:HGNC Symbol;Acc:HGNC:998] |
| ENSG00000072121 | HGLibA_55285 | 182 | 0.662486853 | ZFYVE26 | zinc finger FYVE-type containing 26 [Source:HGNC Symbol;Acc:HGNC:20761] |
| ENSG00000073146 | HGLibA_29695 | 182 | 0.662486853 | MOV10L1 | Mov10 like RISC complex RNA helicase 1 [Source:HGNC Symbol;Acc:HGNC:7201] |
| ENSG00000081913 | HGLibA_36435 | 182 | 0.662486853 | PHLPP1 | PH domain and leucine rich repeat protein phosphatase 1 [Source:HGNC Symbol;Acc:HGNC:20610] |
| ENSG00000087074 | HGLibA_38048 | 182 | 0.662486853 | PPP1R15A | protein phosphatase 1 regulatory subunit 15A [Source:HGNC Symbol;Acc:HGNC:14375] |
| ENSG00000101353 | HGLibA_29880 | 182 | 0.662486853 | MROH8 | maestro heat like repeat family member 8 [Source:HGNC Symbol;Acc:HGNC:16125] |
| ENSG00000102781 | HGLibA_24076 | 182 | 0.662486853 | KATNAL1 | katanin catalytic subunit A1 like 1 [Source:HGNC Symbol;Acc:HGNC:28361] |
| ENSG00000104327 | HGLibA_06986 | 182 | 0.662486853 | CALB1 | calbindin 1 [Source:HGNC Symbol;Acc:HGNC:1434] |
| ENSG00000104918 | HGLibA_40874 | 182 | 0.662486853 | RETN | resistin [Source:HGNC Symbol;Acc:HGNC:20389] |
| ENSG00000107147 | HGLibA_24393 | 182 | 0.662486853 | KCNT1 | potassium sodium-activated channel subfamily T member 1 [Source:HGNC Symbol;Acc:HGNC:18865] |
| ENSG00000109846 | HGLibA_11248 | 182 | 0.662486853 | CRYAB | crystallin alpha B [Source:HGNC Symbol;Acc:HGNC:2389] |
| ENSG00000111261 | HGLibA_28100 | 182 | 0.662486853 | MANSC1 | MANSC domain containing 1 [Source:HGNC Symbol;Acc:HGNC:25505] |
| ENSG00000111404 | HGLibA_40863 | 182 | 0.662486853 | RERGL | RERG like [Source:HGNC Symbol;Acc:HGNC:26213] |
| ENSG00000112116 | HGLibA_23002 | 182 | 0.662486853 | IL17F | interleukin 17F [Source:HGNC Symbol;Acc:HGNC:16404] |
| ENSG00000113391 | HGLibA_16272 | 182 | 0.662486853 | FAM172A | family with sequence similarity 172 member A [Source:HGNC Symbol;Acc:HGNC:25365] |
| ENSG00000114315 | HGLibA_21177 | 182 | 0.662486853 | HES1 | hes family bHLH transcription factor 1 [Source:HGNC Symbol;Acc:HGNC:5192] |
| ENSG00000118564 | HGLibA_17012 | 182 | 0.662486853 | FBXL5 | F-box and leucine rich repeat protein 5 [Source:HGNC Symbol;Acc:HGNC:13602] |
| ENSG00000125815 | HGLibA_11468 | 182 | 0.662486853 | CST8 | cystatin 8 [Source:HGNC Symbol;Acc:HGNC:2480] |
| ENSG00000127666 | HGLibA_49446 | 182 | 0.662486853 | TICAM1 | toll like receptor adaptor molecule 1 [Source:HGNC Symbol;Acc:HGNC:18348] |
| ENSG00000130032 | HGLibA_38876 | 182 | 0.662486853 | PRRG3 | proline rich and Gla domain 3 [Source:HGNC Symbol;Acc:HGNC:30798] |
| ENSG00000133030 | HGLibA_29776 | 182 | 0.662486853 | MPRIP | myosin phosphatase Rho interacting protein [Source:HGNC Symbol;Acc:HGNC:30321] |
| ENSG00000133195 | HGLibA_45094 | 182 | 0.662486853 | SLC39A11 | solute carrier family 39 member 11 [Source:HGNC Symbol;Acc:HGNC:14463] |
| ENSG00000133641 | HGLibA_05228 | 182 | 0.662486853 | C12orf29 | chromosome 12 open reading frame 29 [Source:HGNC Symbol;Acc:HGNC:25322] |
| ENSG00000134250 | HGLibA_32342 | 182 | 0.662486853 | NOTCH2 | notch 2 [Source:HGNC Symbol;Acc:HGNC:7882] |
| ENSG00000135083 | HGLibA_08110 | 182 | 0.662486853 | CCNJL | cyclin J like [Source:HGNC Symbol;Acc:HGNC:25876] |
| ENSG00000136250 | HGLibA_02263 | 182 | 0.662486853 | AOAH | acyloxyacyl hydrolase [Source:HGNC Symbol;Acc:HGNC:548] |
| ENSG00000137310 | HGLibA_48767 | 182 | 0.662486853 | TCF19 | transcription factor 19 [Source:HGNC Symbol;Acc:HGNC:11629] |
| ENSG00000137941 | HGLibA_52213 | 182 | 0.662486853 | TTLL7 | tubulin tyrosine ligase like 7 [Source:HGNC Symbol;Acc:HGNC:26242] |
| ENSG00000139436 | HGLibA_19134 | 182 | 0.662486853 | GIT2 | GIT ArfGAP 2 [Source:HGNC Symbol;Acc:HGNC:4273] |
| ENSG00000140090 | HGLibA_44624 | 182 | 0.662486853 | SLC24A4 | solute carrier family 24 member 4 [Source:HGNC Symbol;Acc:HGNC:10978] |
| ENSG00000141002 | HGLibA_48783 | 182 | 0.662486853 | TCF25 | transcription factor 25 [Source:HGNC Symbol;Acc:HGNC:29181] |
| ENSG00000144134 | HGLibA_40026 | 182 | 0.662486853 | RABL2A | member of RAS oncogene family like 2A [Source:HGNC Symbol;Acc:HGNC:9799]" |
| ENSG00000145824 | HGLibA_11856 | 182 | 0.662486853 | CXCL14 | C-X-C motif chemokine ligand 14 [Source:HGNC Symbol;Acc:HGNC:10640] |
| ENSG00000146426 | HGLibA_49442 | 182 | 0.662486853 | TIAM2 | T cell lymphoma invasion and metastasis 2 [Source:HGNC Symbol;Acc:HGNC:11806] |
| ENSG00000154274 | HGLibA_06299 | 182 | 0.662486853 | C4orf19 | chromosome 4 open reading frame 19 [Source:HGNC Symbol;Acc:HGNC:25618] |
| ENSG00000156194 | HGLibA_37870 | 182 | 0.662486853 | PPEF2 | protein phosphatase with EF-hand domain 2 [Source:HGNC Symbol;Acc:HGNC:9244] |
| ENSG00000160049 | HGLibA_13018 | 182 | 0.662486853 | DFFA | DNA fragmentation factor subunit alpha [Source:HGNC Symbol;Acc:HGNC:2772] |
| ENSG00000160233 | HGLibA_27306 | 182 | 0.662486853 | LRRC3 | leucine rich repeat containing 3 [Source:HGNC Symbol;Acc:HGNC:14965] |
| ENSG00000162702 | HGLibA_55736 | 182 | 0.662486853 | ZNF281 | zinc finger protein 281 [Source:HGNC Symbol;Acc:HGNC:13075] |
| ENSG00000162739 | HGLibA_44316 | 182 | 0.662486853 | SLAMF6 | SLAM family member 6 [Source:HGNC Symbol;Acc:HGNC:21392] |
| ENSG00000163257 | HGLibA_12432 | 182 | 0.662486853 | DCAF16 | DDB1 and CUL4 associated factor 16 [Source:HGNC Symbol;Acc:HGNC:25987] |
| ENSG00000164040 | HGLibA_36291 | 182 | 0.662486853 | PGRMC2 | progesterone receptor membrane component 2 [Source:HGNC Symbol;Acc:HGNC:16089] |
| ENSG00000164663 | HGLibA_53387 | 182 | 0.662486853 | USP49 | ubiquitin specific peptidase 49 [Source:HGNC Symbol;Acc:HGNC:20078] |
| ENSG00000165259 | HGLibA_21064 | 182 | 0.662486853 | HDX | highly divergent homeobox [Source:HGNC Symbol;Acc:HGNC:26411] |
| ENSG00000166819 | HGLibA_37170 | 182 | 0.662486853 | PLIN1 | perilipin 1 [Source:HGNC Symbol;Acc:HGNC:9076] |
| ENSG00000166900 | HGLibA_47644 | 182 | 0.662486853 | STX3 | syntaxin 3 [Source:HGNC Symbol;Acc:HGNC:11438] |
| ENSG00000167359 | HGLibA_34115 | 182 | 0.662486853 | OR51I1 | olfactory receptor family 51 subfamily I member 1 [Source:HGNC Symbol;Acc:HGNC:15200] |
| ENSG00000167525 | HGLibA_38654 | 182 | 0.662486853 | PROCA1 | protein interacting with cyclin A1 [Source:HGNC Symbol;Acc:HGNC:28600] |
| ENSG00000167842 | HGLibA_29302 | 182 | 0.662486853 | MIS12 | kinetochore complex component [Source:HGNC Symbol;Acc:HGNC:24967]" |
| ENSG00000168159 | HGLibA_41597 | 182 | 0.662486853 | RNF187 | ring finger protein 187 [Source:HGNC Symbol;Acc:HGNC:27146] |
| ENSG00000169184 | HGLibA_29559 | 182 | 0.662486853 | MN1 | transcriptional regulator [Source:HGNC Symbol;Acc:HGNC:7180]" |
| ENSG00000169903 | HGLibA_49704 | 182 | 0.662486853 | TM4SF4 | transmembrane 4 L six family member 4 [Source:HGNC Symbol;Acc:HGNC:11856] |
| ENSG00000171396 | HGLibA_25764 | 182 | 0.662486853 | KRTAP4-4 | keratin associated protein 4-4 [Source:HGNC Symbol;Acc:HGNC:16928] |
| ENSG00000172379 | HGLibA_03066 | 182 | 0.662486853 | ARNT2 | aryl hydrocarbon receptor nuclear translocator 2 [Source:HGNC Symbol;Acc:HGNC:16876] |
| ENSG00000176095 | HGLibA_23446 | 182 | 0.662486853 | IP6K1 | inositol hexakisphosphate kinase 1 [Source:HGNC Symbol;Acc:HGNC:18360] |
| ENSG00000176204 | HGLibA_27494 | 182 | 0.662486853 | LRRTM4 | leucine rich repeat transmembrane neuronal 4 [Source:HGNC Symbol;Acc:HGNC:19411] |
| ENSG00000177694 | HGLibA_31015 | 182 | 0.662486853 | NAALADL2 | N-acetylated alpha-linked acidic dipeptidase like 2 [Source:HGNC Symbol;Acc:HGNC:23219] |
| ENSG00000177706 | HGLibA_16451 | 182 | 0.662486853 | FAM20C | golgi associated secretory pathway kinase [Source:HGNC Symbol;Acc:HGNC:22140]" |
| ENSG00000183695 | HGLibA_29850 | 182 | 0.662486853 | MRGPRX2 | MAS related GPR family member X2 [Source:HGNC Symbol;Acc:HGNC:17983] |
| ENSG00000184995 | HGLibA_22704 | 182 | 0.662486853 | IFNE | interferon epsilon [Source:HGNC Symbol;Acc:HGNC:18163] |
| ENSG00000185737 | HGLibA_32681 | 182 | 0.662486853 | NRG3 | neuregulin 3 [Source:HGNC Symbol;Acc:HGNC:7999] |
| ENSG00000185800 | HGLibA_13450 | 182 | 0.662486853 | DMWD | WD repeat containing [Source:HGNC Symbol;Acc:HGNC:2936]" |
| ENSG00000187135 | HGLibA_53863 | 182 | 0.662486853 | VSTM2B | V-set and transmembrane domain containing 2B [Source:HGNC Symbol;Acc:HGNC:33595] |
| ENSG00000187243 | HGLibA_27978 | 182 | 0.662486853 | MAGED4B | MAGE family member D4B [Source:HGNC Symbol;Acc:HGNC:22880] |
| ENSG00000188379 | HGLibA_22673 | 182 | 0.662486853 | IFNA2 | interferon alpha 2 [Source:HGNC Symbol;Acc:HGNC:5423] |
| ENSG00000188404 | HGLibA_43295 | 182 | 0.662486853 | SELL | selectin L [Source:HGNC Symbol;Acc:HGNC:10720] |
| ENSG00000188624 | HGLibA_22846 | 182 | 0.662486853 | IGFL3 | IGF like family member 3 [Source:HGNC Symbol;Acc:HGNC:32930] |
| ENSG00000188785 | HGLibA_56207 | 182 | 0.662486853 | ZNF548 | zinc finger protein 548 [Source:HGNC Symbol;Acc:HGNC:26561] |
| ENSG00000196689 | HGLibA_51783 | 182 | 0.662486853 | TRPV1 | transient receptor potential cation channel subfamily V member 1 [Source:HGNC Symbol;Acc:HGNC:12716] |
| ENSG00000198088 | HGLibA_33067 | 182 | 0.662486853 | NUP62CL | nucleoporin 62 C-terminal like [Source:HGNC Symbol;Acc:HGNC:25960] |
| ENSG00000203910 | HGLibA_05775 | 182 | 0.662486853 | C1orf146 | chromosome 1 open reading frame 146 [Source:HGNC Symbol;Acc:HGNC:24032] |
| ENSG00000213638 | HGLibA_00885 | 182 | 0.662486853 | ADAT3 | tRNA specific 3 [Source:HGNC Symbol;Acc:HGNC:25151]" |
| ENSG00000223865 | HGLibA_21617 | 182 | 0.662486853 | HLA-DPB1 | DP beta 1 [Source:HGNC Symbol;Acc:HGNC:4940]" |
| ENSG00000239264 | HGLibA_52424 | 182 | 0.662486853 | TXNDC5 | thioredoxin domain containing 5 [Source:HGNC Symbol;Acc:HGNC:21073] |
| ENSG00000253304 | HGLibA_50174 | 182 | 0.662486853 | TMEM200B | transmembrane protein 200B [Source:HGNC Symbol;Acc:HGNC:33785] |
| ENSG00000256771 | HGLibA_55671 | 182 | 0.662486853 | ZNF253 | zinc finger protein 253 [Source:HGNC Symbol;Acc:HGNC:13497] |
| ENSG00000257446 | HGLibA_56886 | 182 | 0.662486853 | ZNF878 | zinc finger protein 878 [Source:HGNC Symbol;Acc:HGNC:37246] |
| ENSG00000270806 | HGLibA_05539 | 182 | 0.662486853 | C17orf50 | chromosome 17 open reading frame 50 [Source:HGNC Symbol;Acc:HGNC:29581] |
| ENSG00000280987 | HGLibA_28440 | 182 | 0.662486853 | MATR3 | matrin 3 [Source:NCBI gene;Acc:9782] |
| FAM154A | HGLibA_16175 | 182 | 0.662486853 | FAM154A | - |
| FAM160A2 | HGLibA_16208 | 182 | 0.662486853 | FAM160A2 | - |
| hsa-mir-1246 | HGLibA_57384 | 182 | 0.662486853 | hsa-mir-1246 | - |
| hsa-mir-328 | HGLibA_58976 | 182 | 0.662486853 | hsa-mir-328 | - |
| hsa-mir-3619 | HGLibA_59105 | 182 | 0.662486853 | hsa-mir-3619 | - |
| hsa-mir-4435-1 | HGLibA_60130 | 182 | 0.662486853 | hsa-mir-4435-1 | - |
| hsa-mir-543 | HGLibA_61822 | 182 | 0.662486853 | hsa-mir-543 | - |
| hsa-mir-548v | HGLibA_62076 | 182 | 0.662486853 | hsa-mir-548v | - |
| hsa-mir-585 | HGLibA_62440 | 182 | 0.662486853 | hsa-mir-585 | - |

| | | | | | |
|---|---|---|---|---|---|
| hsa-mir-6841 | HGLibA_63541 | 182 | 0.662486853 | hsa-mir-6841 | - |
| KIAA1432 | HGLibA_24758 | 182 | 0.662486853 | KIAA1432 | - |
| NAT6 | HGLibA_31179 | 182 | 0.662486853 | NAT6 | - |
| TMEM246 | HGLibA_50303 | 182 | 0.662486853 | TMEM246 | - |
| ZCCHC16 | HGLibA_55019 | 182 | 0.662486853 | ZCCHC16 | - |
| C3orf37 | HGLibA_06238 | 181 | 0.652535815 | C3orf37 | - |
| CTSL1 | HGLibA_11719 | 181 | 0.652535815 | CTSL1 | - |
| ENSG00000004948 | HGLibA_07004 | 181 | 0.652535815 | CALCR | calcitonin receptor [Source:HGNC Symbol;Acc:HGNC:1440] |
| ENSG00000005339 | HGLibA_11109 | 181 | 0.652535815 | CREBBP | CREB binding protein [Source:HGNC Symbol;Acc:HGNC:2348] |
| ENSG00000010310 | HGLibA_19126 | 181 | 0.652535815 | GIPR | gastric inhibitory polypeptide receptor [Source:HGNC Symbol;Acc:HGNC:4271] |
| ENSG00000036672 | HGLibA_53295 | 181 | 0.652535815 | USP2 | ubiquitin specific peptidase 2 [Source:HGNC Symbol;Acc:HGNC:12618] |
| ENSG00000044574 | HGLibA_22288 | 181 | 0.652535815 | HSPA5 | heat shock protein family A (Hsp70) member 5 [Source:HGNC Symbol;Acc:HGNC:5238] |
| ENSG00000052850 | HGLibA_01738 | 181 | 0.652535815 | ALX4 | ALX homeobox 4 [Source:HGNC Symbol;Acc:HGNC:450] |
| ENSG00000053747 | HGLibA_25946 | 181 | 0.652535815 | LAMA3 | laminin subunit alpha 3 [Source:HGNC Symbol;Acc:HGNC:6483] |
| ENSG00000059145 | HGLibA_53049 | 181 | 0.652535815 | UNKL | unkempt family like zinc finger [Source:HGNC Symbol;Acc:HGNC:14184] |
| ENSG00000064270 | HGLibA_03629 | 181 | 0.652535815 | ATP2C2 | ATPase secretory pathway Ca2+ transporting 2 [Source:HGNC Symbol;Acc:HGNC:29103] |
| ENSG00000075884 | HGLibA_02712 | 181 | 0.652535815 | ARHGAP15 | Rho GTPase activating protein 15 [Source:HGNC Symbol;Acc:HGNC:21030] |
| ENSG00000078304 | HGLibA_38177 | 181 | 0.652535815 | PPP2R5C | protein phosphatase 2 regulatory subunit B'gamma [Source:HGNC Symbol;Acc:HGNC:9311] |
| ENSG00000081853 | HGLibA_35528 | 181 | 0.652535815 | PCDHGA2 | 2 [Source:HGNC Symbol;Acc:HGNC:8700]" |
| ENSG00000090581 | HGLibA_19576 | 181 | 0.652535815 | GNPTG | N-acetylglucosamine-1-phosphate transferase subunit gamma [Source:HGNC Symbol;Acc:HGNC:23026] |
| ENSG00000090659 | HGLibA_08295 | 181 | 0.652535815 | CD209 | CD209 molecule [Source:HGNC Symbol;Acc:HGNC:1641] |
| ENSG00000091317 | HGLibA_10223 | 181 | 0.652535815 | CMTM6 | CKLF like MARVEL transmembrane domain containing 6 [Source:HGNC Symbol;Acc:HGNC:19177] |
| ENSG00000092931 | HGLibA_29086 | 181 | 0.652535815 | MFSD11 | major facilitator superfamily domain containing 11 [Source:HGNC Symbol;Acc:HGNC:25458] |
| ENSG00000101435 | HGLibA_11473 | 181 | 0.652535815 | CST9L | cystatin 9 like [Source:HGNC Symbol;Acc:HGNC:16233] |
| ENSG00000102287 | HGLibA_18387 | 181 | 0.652535815 | GABRE | gamma-aminobutyric acid type A receptor epsilon subunit [Source:HGNC Symbol;Acc:HGNC:4085] |
| ENSG00000103316 | HGLibA_11291 | 181 | 0.652535815 | CRYM | crystallin mu [Source:HGNC Symbol;Acc:HGNC:2418] |
| ENSG00000104055 | HGLibA_49266 | 181 | 0.652535815 | TGM5 | transglutaminase 5 [Source:HGNC Symbol;Acc:HGNC:11781] |
| ENSG00000104213 | HGLibA_35858 | 181 | 0.652535815 | PDGFRL | platelet derived growth factor receptor like [Source:HGNC Symbol;Acc:HGNC:8805] |
| ENSG00000104774 | HGLibA_28077 | 181 | 0.652535815 | MAN2B1 | mannosidase alpha class 2B member 1 [Source:HGNC Symbol;Acc:HGNC:6826] |
| ENSG00000108443 | HGLibA_42176 | 181 | 0.652535815 | RPS6KB1 | ribosomal protein S6 kinase B1 [Source:HGNC Symbol;Acc:HGNC:10436] |
| ENSG00000109320 | HGLibA_31815 | 181 | 0.652535815 | NFKB1 | nuclear factor kappa B subunit 1 [Source:HGNC Symbol;Acc:HGNC:7794] |
| ENSG00000111203 | HGLibA_23708 | 181 | 0.652535815 | ITFG2 | integrin alpha FG-GAP repeat containing 2 [Source:HGNC Symbol;Acc:HGNC:30879] |
| ENSG00000111912 | HGLibA_31353 | 181 | 0.652535815 | NCOA7 | nuclear receptor coactivator 7 [Source:HGNC Symbol;Acc:HGNC:21081] |
| ENSG00000113971 | HGLibA_32449 | 181 | 0.652535815 | NPHP3 | nephrocystin 3 [Source:HGNC Symbol;Acc:HGNC:7907] |
| ENSG00000119899 | HGLibA_44470 | 181 | 0.652535815 | SLC17A5 | solute carrier family 17 member 5 [Source:HGNC Symbol;Acc:HGNC:10933] |
| ENSG00000119965 | HGLibA_05100 | 181 | 0.652535815 | C10orf88 | chromosome 10 open reading frame 88 [Source:HGNC Symbol;Acc:HGNC:25822] |
| ENSG00000120992 | HGLibA_27738 | 181 | 0.652535815 | LYPLA1 | lysophospholipase I [Source:HGNC Symbol;Acc:HGNC:6737] |
| ENSG00000121769 | HGLibA_15916 | 181 | 0.652535815 | FABP3 | fatty acid binding protein 3 [Source:HGNC Symbol;Acc:HGNC:3557] |
| ENSG00000122678 | HGLibA_37541 | 181 | 0.652535815 | POLM | DNA polymerase mu [Source:HGNC Symbol;Acc:HGNC:9185] |
| ENSG00000123892 | HGLibA_39898 | 181 | 0.652535815 | RAB38 | member RAS oncogene family [Source:HGNC Symbol;Acc:HGNC:9776]" |
| ENSG00000126266 | HGLibA_17324 | 181 | 0.652535815 | FFAR1 | free fatty acid receptor 1 [Source:HGNC Symbol;Acc:HGNC:4498] |
| ENSG00000126500 | HGLibA_17655 | 181 | 0.652535815 | FLRT1 | fibronectin leucine rich transmembrane protein 1 [Source:HGNC Symbol;Acc:HGNC:3760] |
| ENSG00000129219 | HGLibA_37039 | 181 | 0.652535815 | PLD2 | phospholipase D2 [Source:HGNC Symbol;Acc:HGNC:9068] |
| ENSG00000131233 | HGLibA_19155 | 181 | 0.652535815 | GJA9 | gap junction protein alpha 9 [Source:HGNC Symbol;Acc:HGNC:19155] |
| ENSG00000133134 | HGLibA_04442 | 181 | 0.652535815 | BEX2 | brain expressed X-linked 2 [Source:HGNC Symbol;Acc:HGNC:30933] |
| ENSG00000133195 | HGLibA_45095 | 181 | 0.652535815 | SLC39A11 | solute carrier family 39 member 11 [Source:HGNC Symbol;Acc:HGNC:14463] |
| ENSG00000135116 | HGLibA_22107 | 181 | 0.652535815 | HRK | BCL2 interacting protein [Source:HGNC Symbol;Acc:HGNC:5185]" |
| ENSG00000135966 | HGLibA_49236 | 181 | 0.652535815 | TGFBRAP1 | transforming growth factor beta receptor associated protein 1 [Source:HGNC Symbol;Acc:HGNC:16836] |
| ENSG00000136859 | HGLibA_01913 | 181 | 0.652535815 | ANGPTL2 | angiopoietin like 2 [Source:HGNC Symbol;Acc:HGNC:490] |
| ENSG00000139304 | HGLibA_39528 | 181 | 0.652535815 | PTPRQ | receptor type Q [Source:HGNC Symbol;Acc:HGNC:9679]" |
| ENSG00000139437 | HGLibA_48809 | 181 | 0.652535815 | TCHP | trichoplein keratin filament binding [Source:HGNC Symbol;Acc:HGNC:28135] |
| ENSG00000140945 | HGLibA_08642 | 181 | 0.652535815 | CDH13 | cadherin 13 [Source:HGNC Symbol;Acc:HGNC:1753] |
| ENSG00000144451 | HGLibA_46350 | 181 | 0.652535815 | SPAG16 | sperm associated antigen 16 [Source:HGNC Symbol;Acc:HGNC:23225] |
| ENSG00000146276 | HGLibA_18405 | 181 | 0.652535815 | GABRR1 | gamma-aminobutyric acid type A receptor rho1 subunit [Source:HGNC Symbol;Acc:HGNC:4090] |
| ENSG00000146755 | HGLibA_51538 | 181 | 0.652535815 | TRIM50 | tripartite motif containing 50 [Source:HGNC Symbol;Acc:HGNC:19017] |
| ENSG00000150712 | HGLibA_30417 | 181 | 0.652535815 | MTMR12 | myotubularin related protein 12 [Source:HGNC Symbol;Acc:HGNC:18191] |
| ENSG00000151067 | HGLibA_06881 | 181 | 0.652535815 | CACNA1C | calcium voltage-gated channel subunit alpha1 C [Source:HGNC Symbol;Acc:HGNC:1390] |
| ENSG00000151632 | HGLibA_01484 | 181 | 0.652535815 | AKR1C2 | aldo-keto reductase family 1 member C2 [Source:HGNC Symbol;Acc:HGNC:385] |
| ENSG00000152253 | HGLibA_46555 | 181 | 0.652535815 | SPC25 | NDC80 kinetochore complex component [Source:HGNC Symbol;Acc:HGNC:24031]" |
| ENSG00000153179 | HGLibA_40379 | 181 | 0.652535815 | RASSF3 | Ras association domain family member 3 [Source:HGNC Symbol;Acc:HGNC:14271] |
| ENSG00000153443 | HGLibA_52540 | 181 | 0.652535815 | UBALD1 | UBA like domain containing 1 [Source:HGNC Symbol;Acc:HGNC:29576] |
| ENSG00000154258 | HGLibA_00129 | 181 | 0.652535815 | ABCA9 | ATP binding cassette subfamily A member 9 [Source:HGNC Symbol;Acc:HGNC:39] |
| ENSG00000154556 | HGLibA_46176 | 181 | 0.652535815 | SORBS2 | sorbin and SH3 domain containing 2 [Source:HGNC Symbol;Acc:HGNC:24098] |
| ENSG00000155760 | HGLibA_18282 | 181 | 0.652535815 | FZD7 | frizzled class receptor 7 [Source:HGNC Symbol;Acc:HGNC:4045] |
| ENSG00000158467 | HGLibA_01292 | 181 | 0.652535815 | AHCYL2 | adenosylhomocysteinase like 2 [Source:HGNC Symbol;Acc:HGNC:22204] |
| ENSG00000158882 | HGLibA_50969 | 181 | 0.652535815 | TOMM40L | translocase of outer mitochondrial membrane 40 like [Source:HGNC Symbol;Acc:HGNC:25756] |
| ENSG00000159658 | HGLibA_14532 | 181 | 0.652535815 | EFCAB14 | EF-hand calcium binding domain 14 [Source:HGNC Symbol;Acc:HGNC:29051] |
| ENSG00000160460 | HGLibA_46887 | 181 | 0.652535815 | SPTBN4 | non-erythrocytic 4 [Source:HGNC Symbol;Acc:HGNC:14896]" |
| ENSG00000160972 | HGLibA_38053 | 181 | 0.652535815 | PPP1R16A | protein phosphatase 1 regulatory subunit 16A [Source:HGNC Symbol;Acc:HGNC:14941] |
| ENSG00000162383 | HGLibA_44525 | 181 | 0.652535815 | SLC1A7 | solute carrier family 1 member 7 [Source:HGNC Symbol;Acc:HGNC:10945] |
| ENSG00000163138 | HGLibA_34950 | 181 | 0.652535815 | PACRGL | parkin coregulated like [Source:HGNC Symbol;Acc:HGNC:28442] |
| ENSG00000164081 | HGLibA_49086 | 181 | 0.652535815 | TEX264 | testis expressed 264 [Source:HGNC Symbol;Acc:HGNC:30247] |
| ENSG00000165724 | HGLibA_55423 | 181 | 0.652535815 | ZMYND19 | zinc finger MYND-type containing 19 [Source:HGNC Symbol;Acc:HGNC:21146] |
| ENSG00000167258 | HGLibA_08732 | 181 | 0.652535815 | CDK12 | cyclin dependent kinase 12 [Source:HGNC Symbol;Acc:HGNC:24224] |
| ENSG00000167526 | HGLibA_41868 | 181 | 0.652535815 | RPL13 | ribosomal protein L13 [Source:HGNC Symbol;Acc:HGNC:10303] |
| ENSG00000167614 | HGLibA_52231 | 181 | 0.652535815 | TTYH1 | tweety family member 1 [Source:HGNC Symbol;Acc:HGNC:13476] |
| ENSG00000167904 | HGLibA_50495 | 181 | 0.652535815 | TMEM68 | transmembrane protein 68 [Source:HGNC Symbol;Acc:HGNC:26510] |
| ENSG00000168594 | HGLibA_00768 | 181 | 0.652535815 | ADAM29 | ADAM metallopeptidase domain 29 [Source:HGNC Symbol;Acc:HGNC:207] |
| ENSG00000168610 | HGLibA_47388 | 181 | 0.652535815 | STAT3 | signal transducer and activator of transcription 3 [Source:HGNC Symbol;Acc:HGNC:11364] |
| ENSG00000168830 | HGLibA_22366 | 181 | 0.652535815 | HTR1E | 5-hydroxytryptamine receptor 1E [Source:HGNC Symbol;Acc:HGNC:5291] |
| ENSG00000168884 | HGLibA_50838 | 181 | 0.652535815 | TNIP2 | TNFAIP3 interacting protein 2 [Source:HGNC Symbol;Acc:HGNC:19118] |
| ENSG00000170364 | HGLibA_43674 | 181 | 0.652535815 | SETMAR | SET domain and mariner transposase fusion gene [Source:HGNC Symbol;Acc:HGNC:10762] |
| ENSG00000171446 | HGLibA_25466 | 181 | 0.652535815 | KRT27 | keratin 27 [Source:HGNC Symbol;Acc:HGNC:30841] |
| ENSG00000172243 | HGLibA_10007 | 181 | 0.652535815 | CLEC7A | C-type lectin domain containing 7A [Source:HGNC Symbol;Acc:HGNC:14558] |
| ENSG00000175003 | HGLibA_44533 | 181 | 0.652535815 | SLC22A1 | solute carrier family 22 member 1 [Source:HGNC Symbol;Acc:HGNC:10963] |
| ENSG00000176909 | HGLibA_28055 | 181 | 0.652535815 | MAMSTR | MEF2 activating motif and SAP domain containing transcriptional regulator [Source:HGNC Symbol;Acc:HGNC:26689] |
| ENSG00000178177 | HGLibA_26196 | 181 | 0.652535815 | LCORL | ligand dependent nuclear receptor corepressor like [Source:HGNC Symbol;Acc:HGNC:30776] |
| ENSG00000179630 | HGLibA_25909 | 181 | 0.652535815 | LACC1 | laccase domain containing 1 [Source:HGNC Symbol;Acc:HGNC:26789] |
| ENSG00000180008 | HGLibA_46131 | 181 | 0.652535815 | SOCS4 | suppressor of cytokine signaling 4 [Source:HGNC Symbol;Acc:HGNC:19392] |
| ENSG00000180777 | HGLibA_02062 | 181 | 0.652535815 | ANKRD30B | ankyrin repeat domain 30B [Source:HGNC Symbol;Acc:HGNC:24165] |
| ENSG00000182492 | HGLibA_04465 | 181 | 0.652535815 | BGN | biglycan [Source:HGNC Symbol;Acc:HGNC:1044] |
| ENSG00000182541 | HGLibA_26543 | 181 | 0.652535815 | LIMK2 | LIM domain kinase 2 [Source:HGNC Symbol;Acc:HGNC:6614] |
| ENSG00000182968 | HGLibA_46226 | 181 | 0.652535815 | SOX1 | SRY-box 1 [Source:HGNC Symbol;Acc:HGNC:11189] |
| ENSG00000184613 | HGLibA_31685 | 181 | 0.652535815 | NELL2 | neural EGFL like 2 [Source:HGNC Symbol;Acc:HGNC:7751] |
| ENSG00000186074 | HGLibA_08341 | 181 | 0.652535815 | CD300LF | CD300 molecule like family member f [Source:HGNC Symbol;Acc:HGNC:29883] |
| ENSG00000188153 | HGLibA_10580 | 181 | 0.652535815 | COL4A5 | collagen type IV alpha 5 chain [Source:HGNC Symbol;Acc:HGNC:2207] |
| ENSG00000188643 | HGLibA_42548 | 181 | 0.652535815 | S100A16 | S100 calcium binding protein A16 [Source:HGNC Symbol;Acc:HGNC:20441] |
| ENSG00000196366 | HGLibA_06701 | 181 | 0.652535815 | C9orf163 | chromosome 9 open reading frame 163 [Source:HGNC Symbol;Acc:HGNC:26718] |
| ENSG00000196876 | HGLibA_43003 | 181 | 0.652535815 | SCN8A | sodium voltage-gated channel alpha subunit 8 [Source:HGNC Symbol;Acc:HGNC:10596] |
| ENSG00000204577 | HGLibA_26512 | 181 | 0.652535815 | LILRB3 | leukocyte immunoglobulin like receptor B3 [Source:HGNC Symbol;Acc:HGNC:6607] |
| ENSG00000215041 | HGLibA_31731 | 181 | 0.652535815 | NEURL4 | neuralized E3 ubiquitin protein ligase 4 [Source:HGNC Symbol;Acc:HGNC:34410] |
| ENSG00000221963 | HGLibA_02542 | 181 | 0.652535815 | APOL6 | apolipoprotein L6 [Source:HGNC Symbol;Acc:HGNC:14870] |
| ENSG00000236981 | HGLibA_33504 | 181 | 0.652535815 | OR10G9 | olfactory receptor family 10 subfamily G member 9 [Source:HGNC Symbol;Acc:HGNC:15129] |
| HIST1H2BF | HGLibA_21427 | 181 | 0.652535815 | HIST1H2BF | - |
| hsa-mir-324 | HGLibA_58962 | 181 | 0.652535815 | hsa-mir-324 | - |
| hsa-mir-362 | HGLibA_59109 | 181 | 0.652535815 | hsa-mir-362 | - |
| hsa-mir-4319 | HGLibA_59995 | 181 | 0.652535815 | hsa-mir-4319 | - |
| hsa-mir-4496 | HGLibA_60402 | 181 | 0.652535815 | hsa-mir-4496 | - |
| hsa-mir-4689 | HGLibA_60867 | 181 | 0.652535815 | hsa-mir-4689 | - |
| hsa-mir-6715a | HGLibA_63016 | 181 | 0.652535815 | hsa-mir-6715a | - |
| hsa-mir-6769a | HGLibA_63239 | 181 | 0.652535815 | hsa-mir-6769a | - |
| hsa-mir-6810 | HGLibA_63416 | 181 | 0.652535815 | hsa-mir-6810 | - |
| hsa-mir-6811 | HGLibA_63422 | 181 | 0.652535815 | hsa-mir-6811 | - |
| LOC100129636 | HGLibA_26769 | 181 | 0.652535815 | LOC100129636 | - |
| TCEB3 | HGLibA_48741 | 181 | 0.652535815 | TCEB3 | - |
| TMEM206 | HGLibA_50194 | 181 | 0.652535815 | TMEM206 | - |
| ENSG00000005844 | HGLibA_23756 | 180 | 0.642584778 | ITGAL | integrin subunit alpha L [Source:HGNC Symbol;Acc:HGNC:6148] |
| ENSG00000006611 | HGLibA_53174 | 180 | 0.642584778 | USH1C | USH1 protein network component harmonin [Source:HGNC Symbol;Acc:HGNC:12597] |
| ENSG00000011465 | HGLibA_12517 | 180 | 0.642584778 | DCN | decorin [Source:HGNC Symbol;Acc:HGNC:2705] |
| ENSG00000058453 | HGLibA_11210 | 180 | 0.642584778 | CROCC | rootletin [Source:HGNC Symbol;Acc:HGNC:21299]" |
| ENSG00000062096 | HGLibA_03139 | 180 | 0.642584778 | ARSF | arylsulfatase F [Source:HGNC Symbol;Acc:HGNC:721] |
| ENSG00000072182 | HGLibA_03308 | 180 | 0.642584778 | ASIC4 | acid sensing ion channel subunit family member 4 [Source:HGNC Symbol;Acc:HGNC:21263] |
| ENSG00000072195 | HGLibA_46614 | 180 | 0.642584778 | SPEG | SPEG complex locus [Source:HGNC Symbol;Acc:HGNC:16901] |
| ENSG00000072422 | HGLibA_41171 | 180 | 0.642584778 | RHOBTB1 | Rho related BTB domain containing 1 [Source:HGNC Symbol;Acc:HGNC:18738] |
| ENSG00000079931 | HGLibA_29698 | 180 | 0.642584778 | MOXD1 | monooxygenase DBH like 1 [Source:HGNC Symbol;Acc:HGNC:21063] |
| ENSG00000090989 | HGLibA_15731 | 180 | 0.642584778 | EXOC1 | exocyst complex component 1 [Source:HGNC Symbol;Acc:HGNC:30380] |
| ENSG00000099338 | HGLibA_07372 | 180 | 0.642584778 | CATSPERG | cation channel sperm associated auxiliary subunit gamma [Source:HGNC Symbol;Acc:HGNC:25243] |
| ENSG00000100314 | HGLibA_06860 | 180 | 0.642584778 | CABP7 | calcium binding protein 7 [Source:HGNC Symbol;Acc:HGNC:20834] |
| ENSG00000100554 | HGLibA_03744 | 180 | 0.642584778 | ATP6V1D | ATPase H+ transporting V1 subunit D [Source:HGNC Symbol;Acc:HGNC:13527] |
| ENSG00000101441 | HGLibA_11455 | 180 | 0.642584778 | CST4 | cystatin S [Source:HGNC Symbol;Acc:HGNC:2476] |
| ENSG00000101977 | HGLibA_28576 | 180 | 0.642584778 | MCF2 | MCF.2 cell line derived transforming sequence [Source:HGNC Symbol;Acc:HGNC:6940] |
| ENSG00000104219 | HGLibA_55097 | 180 | 0.642584778 | ZDHHC2 | zinc finger DHHC-type containing 2 [Source:HGNC Symbol;Acc:HGNC:18469] |
| ENSG00000104814 | HGLibA_28210 | 180 | 0.642584778 | MAP4K1 | mitogen-activated protein kinase kinase kinase kinase 1 [Source:HGNC Symbol;Acc:HGNC:6863] |
| ENSG00000105287 | HGLibA_38570 | 180 | 0.642584778 | PRKD2 | protein kinase D2 [Source:HGNC Symbol;Acc:HGNC:17293] |
| ENSG00000105464 | HGLibA_20309 | 180 | 0.642584778 | GRIN2D | glutamate ionotropic receptor NMDA type subunit 2D [Source:HGNC Symbol;Acc:HGNC:4588] |

| | | | | | |
|---|---|---|---|---|---|
| ENSG00000107562 | HGLibA_11852 | 180 | 0.642584778 | CXCL12 | C-X-C motif chemokine ligand 12 [Source:HGNC Symbol;Acc:HGNC:10672] |
| ENSG00000111725 | HGLibA_38498 | 180 | 0.642584778 | PRKAB1 | protein kinase AMP-activated non-catalytic subunit beta 1 [Source:HGNC Symbol;Acc:HGNC:9378] |
| ENSG00000113569 | HGLibA_33026 | 180 | 0.642584778 | NUP155 | nucleoporin 155 [Source:HGNC Symbol;Acc:HGNC:8063] |
| ENSG00000113716 | HGLibA_21731 | 180 | 0.642584778 | HMGXB3 | HMG-box containing 3 [Source:HGNC Symbol;Acc:HGNC:28982] |
| ENSG00000114902 | HGLibA_46559 | 180 | 0.642584778 | SPCS1 | signal peptidase complex subunit 1 [Source:HGNC Symbol;Acc:HGNC:23401] |
| ENSG00000118557 | HGLibA_37310 | 180 | 0.642584778 | PMFBP1 | polyamine modulated factor 1 binding protein 1 [Source:HGNC Symbol;Acc:HGNC:17728] |
| ENSG00000119514 | HGLibA_18537 | 180 | 0.642584778 | GALNT12 | polypeptide N-acetylgalactosaminyltransferase 12 [Source:HGNC Symbol;Acc:HGNC:19877] |
| ENSG00000120242 | HGLibA_22693 | 180 | 0.642584778 | IFNA8 | interferon alpha 8 [Source:HGNC Symbol;Acc:HGNC:5429] |
| ENSG00000123505 | HGLibA_01756 | 180 | 0.642584778 | AMD1 | adenosylmethionine decarboxylase 1 [Source:HGNC Symbol;Acc:HGNC:457] |
| ENSG00000125084 | HGLibA_54342 | 180 | 0.642584778 | WNT1 | Wnt family member 1 [Source:HGNC Symbol;Acc:HGNC:12774] |
| ENSG00000125170 | HGLibA_13775 | 180 | 0.642584778 | DOK4 | docking protein 4 [Source:HGNC Symbol;Acc:HGNC:19868] |
| ENSG00000127526 | HGLibA_44987 | 180 | 0.642584778 | SLC35E1 | solute carrier family 35 member E1 [Source:HGNC Symbol;Acc:HGNC:20803] |
| ENSG00000129071 | HGLibA_28486 | 180 | 0.642584778 | MBD4 | DNA glycosylase [Source:HGNC Symbol;Acc:HGNC:6919]" |
| ENSG00000130589 | HGLibA_21126 | 180 | 0.642584778 | HELZ2 | helicase with zinc finger 2 [Source:HGNC Symbol;Acc:HGNC:30021] |
| ENSG00000131686 | HGLibA_06817 | 180 | 0.642584778 | CA6 | carbonic anhydrase 6 [Source:HGNC Symbol;Acc:HGNC:1380] |
| ENSG00000131721 | HGLibA_41218 | 180 | 0.642584778 | RHOXF2 | Rhox homeobox family member 2 [Source:HGNC Symbol;Acc:HGNC:30011] |
| ENSG00000135953 | HGLibA_29121 | 180 | 0.642584778 | MFSD9 | major facilitator superfamily domain containing 9 [Source:HGNC Symbol;Acc:HGNC:28158] |
| ENSG00000137496 | HGLibA_23025 | 180 | 0.642584778 | IL18BP | interleukin 18 binding protein [Source:HGNC Symbol;Acc:HGNC:5987] |
| ENSG00000139908 | HGLibA_52017 | 180 | 0.642584778 | TSSK4 | testis specific serine kinase 4 [Source:HGNC Symbol;Acc:HGNC:19825] |
| ENSG00000143499 | HGLibA_45854 | 180 | 0.642584778 | SMYD2 | SET and MYND domain containing 2 [Source:HGNC Symbol;Acc:HGNC:20982] |
| ENSG00000145040 | HGLibA_52824 | 180 | 0.642584778 | UCN2 | urocortin 2 [Source:HGNC Symbol;Acc:HGNC:18414] |
| ENSG00000145214 | HGLibA_13083 | 180 | 0.642584778 | DGKQ | diacylglycerol kinase theta [Source:HGNC Symbol;Acc:HGNC:2856] |
| ENSG00000149256 | HGLibA_49003 | 180 | 0.642584778 | TENM4 | teneurin transmembrane protein 4 [Source:HGNC Symbol;Acc:HGNC:29945] |
| ENSG00000149651 | HGLibA_46722 | 180 | 0.642584778 | SPINT4 | Kunitz type 4 [Source:HGNC Symbol;Acc:HGNC:16130]" |
| ENSG00000151962 | HGLibA_40564 | 180 | 0.642584778 | RBM46 | RNA binding motif protein 46 [Source:HGNC Symbol;Acc:HGNC:28401] |
| ENSG00000152076 | HGLibA_07865 | 180 | 0.642584778 | CCDC74B | coiled-coil domain containing 74B [Source:HGNC Symbol;Acc:HGNC:25267] |
| ENSG00000152595 | HGLibA_28904 | 180 | 0.642584778 | MEPE | matrix extracellular phosphoglycoprotein [Source:HGNC Symbol;Acc:HGNC:13361] |
| ENSG00000152904 | HGLibA_19008 | 180 | 0.642584778 | GGPS1 | geranylgeranyl diphosphate synthase 1 [Source:HGNC Symbol;Acc:HGNC:4249] |
| ENSG00000153002 | HGLibA_10905 | 180 | 0.642584778 | CPB1 | carboxypeptidase B1 [Source:HGNC Symbol;Acc:HGNC:2299] |
| ENSG00000158164 | HGLibA_50661 | 180 | 0.642584778 | TMSB15A | thymosin beta 15a [Source:HGNC Symbol;Acc:HGNC:30744] |
| ENSG00000159110 | HGLibA_22698 | 180 | 0.642584778 | IFNAR2 | interferon alpha and beta receptor subunit 2 [Source:HGNC Symbol;Acc:HGNC:5433] |
| ENSG00000162924 | HGLibA_40810 | 180 | 0.642584778 | REL | NF-kB subunit [Source:HGNC Symbol;Acc:HGNC:9954]" |
| ENSG00000163646 | HGLibA_10121 | 180 | 0.642584778 | CLRN1 | clarin 1 [Source:HGNC Symbol;Acc:HGNC:12605] |
| ENSG00000164400 | HGLibA_11328 | 180 | 0.642584778 | CSF2 | colony stimulating factor 2 [Source:HGNC Symbol;Acc:HGNC:2434] |
| ENSG00000164823 | HGLibA_34690 | 180 | 0.642584778 | OSGIN2 | oxidative stress induced growth inhibitor family member 2 [Source:HGNC Symbol;Acc:HGNC:1355] |
| ENSG00000165643 | HGLibA_46162 | 180 | 0.642584778 | SOHLH1 | spermatogenesis and oogenesis specific basic helix-loop-helix 1 [Source:HGNC Symbol;Acc:HGNC:27845] |
| ENSG00000166351 | HGLibA_37727 | 180 | 0.642584778 | POTED | POTE ankyrin domain family member D [Source:HGNC Symbol;Acc:HGNC:23822] |
| ENSG00000166971 | HGLibA_01516 | 180 | 0.642584778 | AKTIP | AKT interacting protein [Source:HGNC Symbol;Acc:HGNC:16710] |
| ENSG00000169223 | HGLibA_26663 | 180 | 0.642584778 | LMAN2 | mannose binding 2 [Source:HGNC Symbol;Acc:HGNC:16986]" |
| ENSG00000169856 | HGLibA_33393 | 180 | 0.642584778 | ONECUT1 | one cut homeobox 1 [Source:HGNC Symbol;Acc:HGNC:8138] |
| ENSG00000171102 | HGLibA_33227 | 180 | 0.642584778 | OBP2B | odorant binding protein 2B [Source:HGNC Symbol;Acc:HGNC:23381] |
| ENSG00000174807 | HGLibA_08312 | 180 | 0.642584778 | CD248 | CD248 molecule [Source:HGNC Symbol;Acc:HGNC:18219] |
| ENSG00000175073 | HGLibA_53578 | 180 | 0.642584778 | VCPIP1 | valosin containing protein interacting protein 1 [Source:HGNC Symbol;Acc:HGNC:30897] |
| ENSG00000175336 | HGLibA_02520 | 180 | 0.642584778 | APOF | apolipoprotein F [Source:HGNC Symbol;Acc:HGNC:615] |
| ENSG00000175573 | HGLibA_05172 | 180 | 0.642584778 | C11orf68 | chromosome 11 open reading frame 68 [Source:HGNC Symbol;Acc:HGNC:28801] |
| ENSG00000178928 | HGLibA_51192 | 180 | 0.642584778 | TPRX1 | tetrapeptide repeat homeobox 1 [Source:HGNC Symbol;Acc:HGNC:32174] |
| ENSG00000178974 | HGLibA_17078 | 180 | 0.642584778 | FBXO34 | F-box protein 34 [Source:HGNC Symbol;Acc:HGNC:20201] |
| ENSG00000185730 | HGLibA_56560 | 180 | 0.642584778 | ZNF696 | zinc finger protein 696 [Source:HGNC Symbol;Acc:HGNC:25872] |
| ENSG00000186732 | HGLibA_29772 | 180 | 0.642584778 | MPPED1 | metallophosphoesterase domain containing 1 [Source:HGNC Symbol;Acc:HGNC:1306] |
| ENSG00000187650 | HGLibA_53690 | 180 | 0.642584778 | VMAC | vimentin type intermediate filament associated coiled-coil protein [Source:HGNC Symbol;Acc:HGNC:33803] |
| ENSG00000196152 | HGLibA_56762 | 180 | 0.642584778 | ZNF79 | zinc finger protein 79 [Source:HGNC Symbol;Acc:HGNC:13153] |
| ENSG00000197134 | HGLibA_55680 | 180 | 0.642584778 | ZNF257 | zinc finger protein 257 [Source:HGNC Symbol;Acc:HGNC:13498] |
| ENSG00000198569 | HGLibA_44945 | 180 | 0.642584778 | SLC34A3 | solute carrier family 34 member 3 [Source:HGNC Symbol;Acc:HGNC:20305] |
| ENSG00000198816 | HGLibA_55869 | 180 | 0.642584778 | ZNF358 | zinc finger protein 358 [Source:HGNC Symbol;Acc:HGNC:16838] |
| ENSG00000204520 | HGLibA_29199 | 180 | 0.642584778 | MICA | MHC class I polypeptide-related sequence A [Source:HGNC Symbol;Acc:HGNC:7090] |
| ENSG00000204979 | HGLibA_30135 | 180 | 0.642584778 | MS4A13 | membrane spanning 4-domains A13 [Source:HGNC Symbol;Acc:HGNC:16674] |
| ENSG00000213186 | HGLibA_51556 | 180 | 0.642584778 | TRIM59 | tripartite motif containing 59 [Source:HGNC Symbol;Acc:HGNC:30834] |
| ENSG00000215883 | HGLibA_12014 | 180 | 0.642584778 | CYB5RL | cytochrome b5 reductase like [Source:HGNC Symbol;Acc:HGNC:32220] |
| ENSG00000241935 | HGLibA_21862 | 180 | 0.642584778 | HOGA1 | 4-hydroxy-2-oxoglutarate aldolase 1 [Source:HGNC Symbol;Acc:HGNC:25155] |
| ENSG00000251369 | HGLibA_56211 | 180 | 0.642584778 | ZNF550 | zinc finger protein 550 [Source:HGNC Symbol;Acc:HGNC:28643] |
| ENSG00000253626 | HGLibA_14860 | 180 | 0.642584778 | EIF5AL1 | eukaryotic translation initiation factor 5A-like 1 [Source:HGNC Symbol;Acc:HGNC:17419] |
| ENSG00000256683 | HGLibA_55857 | 180 | 0.642584778 | ZNF350 | zinc finger protein 350 [Source:HGNC Symbol;Acc:HGNC:16656] |
| ENSG00000257594 | HGLibA_18560 | 180 | 0.642584778 | GALNT4 | polypeptide N-acetylgalactosaminyltransferase 4 [Source:HGNC Symbol;Acc:HGNC:4126] |
| ENSG00000259120 | HGLibA_45765 | 180 | 0.642584778 | SMIM6 | small integral membrane protein 6 [Source:HGNC Symbol;Acc:HGNC:40032] |
| HFE2 | HGLibA_21227 | 180 | 0.642584778 | HFE2 | - |
| hsa-mir-1226 | HGLibA_57302 | 180 | 0.642584778 | hsa-mir-1226 | - |
| hsa-mir-196a-2 | HGLibA_58097 | 180 | 0.642584778 | hsa-mir-196a-2 | - |
| hsa-mir-3622a | HGLibA_59121 | 180 | 0.642584778 | hsa-mir-3622a | - |
| hsa-mir-4472-1 | HGLibA_60294 | 180 | 0.642584778 | hsa-mir-4472-1 | - |
| hsa-mir-4640 | HGLibA_60666 | 180 | 0.642584778 | hsa-mir-4640 | - |
| hsa-mir-4664 | HGLibA_60768 | 180 | 0.642584778 | hsa-mir-4664 | - |
| hsa-mir-4767 | HGLibA_61164 | 180 | 0.642584778 | hsa-mir-4767 | - |
| hsa-mir-507 | HGLibA_61487 | 180 | 0.642584778 | hsa-mir-507 | - |
| hsa-mir-7106 | HGLibA_63785 | 180 | 0.642584778 | hsa-mir-7106 | - |
| hsa-mir-8055 | HGLibA_64060 | 180 | 0.642584778 | hsa-mir-8055 | - |
| LOC100652824 | HGLibA_26872 | 180 | 0.642584778 | LOC100652824 | - |
| LOC286238 | HGLibA_26918 | 180 | 0.642584778 | LOC286238 | - |
| LRRC6 | HGLibA_27403 | 180 | 0.642584778 | LRRC6 | - |
| AES | HGLibA_01093 | 179 | 0.63263374 | AES | - |
| C19orf45 | HGLibA_05666 | 179 | 0.63263374 | C19orf45 | - |
| DUSP27 | HGLibA_14171 | 179 | 0.63263374 | DUSP27 | - |
| ENSG00000071889 | HGLibA_16575 | 179 | 0.63263374 | FAM3A | family with sequence similarity 3 member A [Source:HGNC Symbol;Acc:HGNC:13749] |
| ENSG00000076513 | HGLibA_01999 | 179 | 0.63263374 | ANKRD13A | ankyrin repeat domain 13A [Source:HGNC Symbol;Acc:HGNC:21268] |
| ENSG00000079102 | HGLibA_42457 | 179 | 0.63263374 | RUNX1T1 | RUNX1 translocation partner 1 [Source:HGNC Symbol;Acc:HGNC:1535] |
| ENSG00000079616 | HGLibA_24903 | 179 | 0.63263374 | KIF22 | kinesin family member 22 [Source:HGNC Symbol;Acc:HGNC:6391] |
| ENSG00000081277 | HGLibA_36851 | 179 | 0.63263374 | PKP1 | plakophilin 1 [Source:HGNC Symbol;Acc:HGNC:9023] |
| ENSG00000086827 | HGLibA_57064 | 179 | 0.63263374 | ZW10 | zw10 kinetochore protein [Source:HGNC Symbol;Acc:HGNC:13194] |
| ENSG00000087266 | HGLibA_43928 | 179 | 0.63263374 | SH3BP2 | SH3 domain binding protein 2 [Source:HGNC Symbol;Acc:HGNC:10825] |
| ENSG00000107796 | HGLibA_00598 | 179 | 0.63263374 | ACTA2 | aorta [Source:HGNC Symbol;Acc:HGNC:130]" |
| ENSG00000107831 | HGLibA_17420 | 179 | 0.63263374 | FGF8 | fibroblast growth factor 8 [Source:HGNC Symbol;Acc:HGNC:3686] |
| ENSG00000108561 | HGLibA_05918 | 179 | 0.63263374 | C1QBP | complement C1q binding protein [Source:HGNC Symbol;Acc:HGNC:1243] |
| ENSG00000108591 | HGLibA_13978 | 179 | 0.63263374 | DRG2 | developmentally regulated GTP binding protein 2 [Source:HGNC Symbol;Acc:HGNC:3030] |
| ENSG00000110171 | HGLibA_51457 | 179 | 0.63263374 | TRIM3 | tripartite motif containing 3 [Source:HGNC Symbol;Acc:HGNC:10064] |
| ENSG00000111863 | HGLibA_01083 | 179 | 0.63263374 | ADTRP | androgen dependent TFPI regulating protein [Source:HGNC Symbol;Acc:HGNC:21214] |
| ENSG00000112077 | HGLibA_41122 | 179 | 0.63263374 | RHAG | Rh associated glycoprotein [Source:HGNC Symbol;Acc:HGNC:10006] |
| ENSG00000113300 | HGLibA_10345 | 179 | 0.63263374 | CNOT6 | CCR4-NOT transcription complex subunit 6 [Source:HGNC Symbol;Acc:HGNC:14099] |
| ENSG00000114030 | HGLibA_25353 | 179 | 0.63263374 | KPNA1 | karyopherin subunit alpha 1 [Source:HGNC Symbol;Acc:HGNC:6394] |
| ENSG00000116198 | HGLibA_09094 | 179 | 0.63263374 | CEP104 | centrosomal protein 104 [Source:HGNC Symbol;Acc:HGNC:24866] |
| ENSG00000119685 | HGLibA_52207 | 179 | 0.63263374 | TTLL5 | tubulin tyrosine ligase like 5 [Source:HGNC Symbol;Acc:HGNC:19963] |
| ENSG00000121067 | HGLibA_46764 | 179 | 0.63263374 | SPOP | speckle type BTB/POZ protein [Source:HGNC Symbol;Acc:HGNC:11254] |
| ENSG00000123104 | HGLibA_23857 | 179 | 0.63263374 | ITPR2 | 5-trisphosphate receptor type 2 [Source:HGNC Symbol;Acc:HGNC:6181]" |
| ENSG00000124780 | HGLibA_24313 | 179 | 0.63263374 | KCNK17 | potassium two pore domain channel subfamily K member 17 [Source:HGNC Symbol;Acc:HGNC:14465] |
| ENSG00000125648 | HGLibA_44677 | 179 | 0.63263374 | SLC25A23 | solute carrier family 25 member 23 [Source:HGNC Symbol;Acc:HGNC:19375] |
| ENSG00000132376 | HGLibA_23361 | 179 | 0.63263374 | INPP5K | inositol polyphosphate-5-phosphatase K [Source:HGNC Symbol;Acc:HGNC:33882] |
| ENSG00000133477 | HGLibA_16754 | 179 | 0.63263374 | FAM83F | family with sequence similarity 83 member F [Source:HGNC Symbol;Acc:HGNC:25148] |
| ENSG00000135436 | HGLibA_16339 | 179 | 0.63263374 | FAM186B | family with sequence similarity 186 member B [Source:HGNC Symbol;Acc:HGNC:25296] |
| ENSG00000136152 | HGLibA_10476 | 179 | 0.63263374 | COG3 | component of oligomeric golgi complex 3 [Source:HGNC Symbol;Acc:HGNC:18619] |
| ENSG00000141540 | HGLibA_52235 | 179 | 0.63263374 | TTYH2 | tweety family member 2 [Source:HGNC Symbol;Acc:HGNC:13877] |
| ENSG00000141994 | HGLibA_14122 | 179 | 0.63263374 | DUS3L | dihydrouridine synthase 3 like [Source:HGNC Symbol;Acc:HGNC:26920] |
| ENSG00000143569 | HGLibA_52555 | 179 | 0.63263374 | UBAP2L | ubiquitin associated protein 2 like [Source:HGNC Symbol;Acc:HGNC:29877] |
| ENSG00000147573 | HGLibA_51547 | 179 | 0.63263374 | TRIM55 | tripartite motif containing 55 [Source:HGNC Symbol;Acc:HGNC:14215] |
| ENSG00000148218 | HGLibA_01521 | 179 | 0.63263374 | ALAD | aminolevulinate dehydratase [Source:HGNC Symbol;Acc:HGNC:395] |
| ENSG00000151952 | HGLibA_49936 | 179 | 0.63263374 | TMEM132D | transmembrane protein 132D [Source:HGNC Symbol;Acc:HGNC:29411] |
| ENSG00000153071 | HGLibA_12268 | 179 | 0.63263374 | DAB2 | clathrin adaptor protein [Source:HGNC Symbol;Acc:HGNC:2662]" |
| ENSG00000155849 | HGLibA_14929 | 179 | 0.63263374 | ELMO1 | engulfment and cell motility 1 [Source:HGNC Symbol;Acc:HGNC:16286] |
| ENSG00000157782 | HGLibA_06849 | 179 | 0.63263374 | CABP1 | calcium binding protein 1 [Source:HGNC Symbol;Acc:HGNC:1384] |
| ENSG00000163218 | HGLibA_36257 | 179 | 0.63263374 | PGLYRP4 | peptidoglycan recognition protein 4 [Source:HGNC Symbol;Acc:HGNC:30015] |
| ENSG00000163536 | HGLibA_43597 | 179 | 0.63263374 | SERPINI1 | serpin family I member 1 [Source:HGNC Symbol;Acc:HGNC:8943] |
| ENSG00000164056 | HGLibA_46834 | 179 | 0.63263374 | SPRY1 | sprouty RTK signaling antagonist 1 [Source:HGNC Symbol;Acc:HGNC:11269] |
| ENSG00000164073 | HGLibA_29118 | 179 | 0.63263374 | MFSD8 | major facilitator superfamily domain containing 8 [Source:HGNC Symbol;Acc:HGNC:28486] |
| ENSG00000164113 | HGLibA_00718 | 179 | 0.63263374 | ADAD1 | adenosine deaminase domain containing 1 [Source:HGNC Symbol;Acc:HGNC:30713] |
| ENSG00000164821 | HGLibA_12801 | 179 | 0.63263374 | DEFA4 | defensin alpha 4 [Source:HGNC Symbol;Acc:HGNC:2763] |
| ENSG00000165071 | HGLibA_50504 | 179 | 0.63263374 | TMEM71 | transmembrane protein 71 [Source:HGNC Symbol;Acc:HGNC:26572] |
| ENSG00000165650 | HGLibA_35986 | 179 | 0.63263374 | PDZD8 | PDZ domain containing 8 [Source:HGNC Symbol;Acc:HGNC:26974] |
| ENSG00000166183 | HGLibA_03347 | 179 | 0.63263374 | ASPG | asparaginase [Source:HGNC Symbol;Acc:HGNC:20123] |
| ENSG00000166326 | HGLibA_51508 | 179 | 0.63263374 | TRIM44 | tripartite motif containing 44 [Source:HGNC Symbol;Acc:HGNC:19016] |
| ENSG00000167531 | HGLibA_25939 | 179 | 0.63263374 | LALBA | lactalbumin alpha [Source:HGNC Symbol;Acc:HGNC:6480] |
| ENSG00000167768 | HGLibA_25413 | 179 | 0.63263374 | KRT1 | keratin 1 [Source:HGNC Symbol;Acc:HGNC:6412] |
| ENSG00000169777 | HGLibA_48364 | 179 | 0.63263374 | TAS2R1 | taste 2 receptor member 1 [Source:HGNC Symbol;Acc:HGNC:14909] |
| ENSG00000171495 | HGLibA_29869 | 179 | 0.63263374 | MROH2B | maestro heat like repeat family member 2B [Source:HGNC Symbol;Acc:HGNC:26857] |
| ENSG00000174669 | HGLibA_44842 | 179 | 0.63263374 | SLC29A2 | solute carrier family 29 member 2 [Source:HGNC Symbol;Acc:HGNC:11004] |
| ENSG00000175183 | HGLibA_11434 | 179 | 0.63263374 | CSRP2 | cysteine and glycine rich protein 2 [Source:HGNC Symbol;Acc:HGNC:2470] |
| ENSG00000177054 | HGLibA_55074 | 179 | 0.63263374 | ZDHHC13 | zinc finger DHHC-type containing 13 [Source:HGNC Symbol;Acc:HGNC:18413] |
| ENSG00000177426 | HGLibA_49237 | 179 | 0.63263374 | TGIF1 | TGFB induced factor homeobox 1 [Source:HGNC Symbol;Acc:HGNC:11776] |
| ENSG00000178381 | HGLibA_55146 | 179 | 0.63263374 | ZFAND2A | zinc finger AN1-type containing 2A [Source:HGNC Symbol;Acc:HGNC:28073] |

| | | | | | |
|---|---|---|---|---|---|
| ENSG00000180138 | HGLibA_11384 | 179 | 0.63263374 | CSNK1A1L | casein kinase 1 alpha 1 like [Source:HGNC Symbol;Acc:HGNC:20289] |
| ENSG00000180776 | HGLibA_55098 | 179 | 0.63263374 | ZDHHC20 | zinc finger DHHC-type containing 20 [Source:HGNC Symbol;Acc:HGNC:20749] |
| ENSG00000182518 | HGLibA_15990 | 179 | 0.63263374 | FAM104B | family with sequence similarity 104 member B [Source:HGNC Symbol;Acc:HGNC:25085] |
| ENSG00000184719 | HGLibA_41715 | 179 | 0.63263374 | RNLS | FAD dependent amine oxidase [Source:HGNC Symbol;Acc:HGNC:25641]" |
| ENSG00000185818 | HGLibA_31187 | 179 | 0.63263374 | NAT8L | N-acetyltransferase 8 like [Source:HGNC Symbol;Acc:HGNC:26742] |
| ENSG00000186732 | HGLibA_29771 | 179 | 0.63263374 | MPPED1 | metallophosphoesterase domain containing 1 [Source:HGNC Symbol;Acc:HGNC:1306] |
| ENSG00000188227 | HGLibA_56773 | 179 | 0.63263374 | ZNF793 | zinc finger protein 793 [Source:HGNC Symbol;Acc:HGNC:33115] |
| ENSG00000188818 | HGLibA_55069 | 179 | 0.63263374 | ZDHHC11 | zinc finger DHHC-type containing 11 [Source:HGNC Symbol;Acc:HGNC:19158] |
| ENSG00000189114 | HGLibA_04562 | 179 | 0.63263374 | BLOC1S3 | biogenesis of lysosomal organelles complex 1 subunit 3 [Source:HGNC Symbol;Acc:HGNC:20914] |
| ENSG00000189169 | HGLibA_25599 | 179 | 0.63263374 | KRTAP10-12 | keratin associated protein 10-12 [Source:HGNC Symbol;Acc:HGNC:20533] |
| ENSG00000196131 | HGLibA_53702 | 179 | 0.63263374 | VN1R2 | vomeronasal 1 receptor 2 [Source:HGNC Symbol;Acc:HGNC:19872] |
| ENSG00000196419 | HGLibA_54597 | 179 | 0.63263374 | XRCC6 | X-ray repair cross complementing 6 [Source:HGNC Symbol;Acc:HGNC:4055] |
| ENSG00000196531 | HGLibA_31030 | 179 | 0.63263374 | NACA | nascent polypeptide associated complex subunit alpha [Source:HGNC Symbol;Acc:HGNC:7629] |
| ENSG00000196576 | HGLibA_37274 | 179 | 0.63263374 | PLXNB2 | plexin B2 [Source:HGNC Symbol;Acc:HGNC:9104] |
| ENSG00000197959 | HGLibA_13691 | 179 | 0.63263374 | DNM3 | dynamin 3 [Source:HGNC Symbol;Acc:HGNC:29125] |
| ENSG00000198346 | HGLibA_56805 | 179 | 0.63263374 | ZNF813 | zinc finger protein 813 [Source:HGNC Symbol;Acc:HGNC:33257] |
| ENSG00000198680 | HGLibA_52342 | 179 | 0.63263374 | TUSC1 | tumor suppressor candidate 1 [Source:HGNC Symbol;Acc:HGNC:31010] |
| ENSG00000198720 | HGLibA_02004 | 179 | 0.63263374 | ANKRD13B | ankyrin repeat domain 13B [Source:HGNC Symbol;Acc:HGNC:26363] |
| ENSG00000198812 | HGLibA_27247 | 179 | 0.63263374 | LRRC10 | leucine rich repeat containing 10 [Source:HGNC Symbol;Acc:HGNC:20264] |
| ENSG00000198948 | HGLibA_29049 | 179 | 0.63263374 | MFAP3L | microfibril associated protein 3 like [Source:HGNC Symbol;Acc:HGNC:29083] |
| ENSG00000203666 | HGLibA_14535 | 179 | 0.63263374 | EFCAB2 | EF-hand calcium binding domain 2 [Source:HGNC Symbol;Acc:HGNC:28166] |
| ENSG00000213906 | HGLibA_27586 | 179 | 0.63263374 | LTB4R2 | leukotriene B4 receptor 2 [Source:HGNC Symbol;Acc:HGNC:19260] |
| ENSG00000225766 | HGLibA_13138 | 179 | 0.63263374 | DHRS4L1 | dehydrogenase/reductase 4 like 1 [Source:HGNC Symbol;Acc:HGNC:19732] |
| ENSG00000243896 | HGLibA_33743 | 179 | 0.63263374 | OR2A7 | olfactory receptor family 2 subfamily A member 7 [Source:HGNC Symbol;Acc:HGNC:8234] |
| ENSG00000262628 | HGLibA_33657 | 179 | 0.63263374 | OR1D5 | olfactory receptor family 1 subfamily D member 5 [Source:HGNC Symbol;Acc:HGNC:8186] |
| ENSG00000272920 | HGLibA_57409 | 179 | 0.63263374 | hsa-mir-1253 | hsa-mir-1253 [Source:miRBase;Acc:MI0006387] |
| ENSG00000277481 | HGLibA_36796 | 179 | 0.63263374 | PKD1L3 | transient receptor potential channel interacting [Source:HGNC Symbol;Acc:HGNC:21716]" |
| FAM46C | HGLibA_16601 | 179 | 0.63263374 | FAM46C | - |
| hsa-mir-1225 | HGLibA_57298 | 179 | 0.63263374 | hsa-mir-1225 | - |
| hsa-mir-1229 | HGLibA_57315 | 179 | 0.63263374 | hsa-mir-1229 | - |
| hsa-mir-21 | HGLibA_58207 | 179 | 0.63263374 | hsa-mir-21 | - |
| hsa-mir-3666 | HGLibA_59217 | 179 | 0.63263374 | hsa-mir-3666 | - |
| hsa-mir-4296 | HGLibA_59896 | 179 | 0.63263374 | hsa-mir-4296 | - |
| hsa-mir-4308 | HGLibA_59942 | 179 | 0.63263374 | hsa-mir-4308 | - |
| hsa-mir-4436b-1 | HGLibA_60143 | 179 | 0.63263374 | hsa-mir-4436b-1 | - |
| hsa-mir-4515 | HGLibA_60502 | 179 | 0.63263374 | hsa-mir-4515 | - |
| hsa-mir-4632 | HGLibA_60634 | 179 | 0.63263374 | hsa-mir-4632 | - |
| hsa-mir-4643 | HGLibA_60676 | 179 | 0.63263374 | hsa-mir-4643 | - |
| hsa-mir-4660 | HGLibA_60748 | 179 | 0.63263374 | hsa-mir-4660 | - |
| hsa-mir-4772 | HGLibA_61187 | 179 | 0.63263374 | hsa-mir-4772 | - |
| hsa-mir-568 | HGLibA_62231 | 179 | 0.63263374 | hsa-mir-568 | - |
| hsa-mir-5702 | HGLibA_62349 | 179 | 0.63263374 | hsa-mir-5702 | - |
| hsa-mir-571 | HGLibA_62375 | 179 | 0.63263374 | hsa-mir-571 | - |
| hsa-mir-6073 | HGLibA_62546 | 179 | 0.63263374 | hsa-mir-6073 | - |
| hsa-mir-612 | HGLibA_62638 | 179 | 0.63263374 | hsa-mir-612 | - |
| hsa-mir-6733 | HGLibA_63085 | 179 | 0.63263374 | hsa-mir-6733 | - |
| hsa-mir-6841 | HGLibA_63539 | 179 | 0.63263374 | hsa-mir-6841 | - |
| hsa-mir-874 | HGLibA_64206 | 179 | 0.63263374 | hsa-mir-874 | - |
| LRMP | HGLibA_27193 | 179 | 0.63263374 | LRMP | - |
| C10orf137 | HGLibA_05059 | 178 | 0.622682703 | C10orf137 | - |
| C2orf54 | HGLibA_06149 | 178 | 0.622682703 | C2orf54 | - |
| C9orf142 | HGLibA_06685 | 178 | 0.622682703 | C9orf142 | - |
| DYX1C1 | HGLibA_14291 | 178 | 0.622682703 | DYX1C1 | - |
| ENSG00000007129 | HGLibA_08936 | 178 | 0.622682703 | CEACAM21 | carcinoembryonic antigen related cell adhesion molecule 21 [Source:HGNC Symbol;Acc:HGNC:28834] |
| ENSG00000031003 | HGLibA_16143 | 178 | 0.622682703 | FAM13B | family with sequence similarity 13 member B [Source:HGNC Symbol;Acc:HGNC:1335] |
| ENSG00000033050 | HGLibA_00208 | 178 | 0.622682703 | ABCF2 | ATP binding cassette subfamily F member 2 [Source:HGNC Symbol;Acc:HGNC:71] |
| ENSG00000033627 | HGLibA_03702 | 178 | 0.622682703 | ATP6V0A1 | ATPase H+ transporting V0 subunit a1 [Source:HGNC Symbol;Acc:HGNC:865] |
| ENSG00000067167 | HGLibA_51282 | 178 | 0.622682703 | TRAM1 | translocation associated membrane protein 1 [Source:HGNC Symbol;Acc:HGNC:20568] |
| ENSG00000068400 | HGLibA_20322 | 178 | 0.622682703 | GRIPAP1 | GRIP1 associated protein 1 [Source:HGNC Symbol;Acc:HGNC:18706] |
| ENSG00000070081 | HGLibA_32912 | 178 | 0.622682703 | NUCB2 | nucleobindin 2 [Source:HGNC Symbol;Acc:HGNC:8044] |
| ENSG00000077522 | HGLibA_00642 | 178 | 0.622682703 | ACTN2 | actinin alpha 2 [Source:HGNC Symbol;Acc:HGNC:164] |
| ENSG00000078081 | HGLibA_25982 | 178 | 0.622682703 | LAMP3 | lysosomal associated membrane protein 3 [Source:HGNC Symbol;Acc:HGNC:14582] |
| ENSG00000078328 | HGLibA_40455 | 178 | 0.622682703 | RBFOX1 | RNA binding fox-1 homolog 1 [Source:HGNC Symbol;Acc:HGNC:18222] |
| ENSG00000080503 | HGLibA_45604 | 178 | 0.622682703 | SMARCA2 | member 2 [Source:HGNC Symbol;Acc:HGNC:11098]" |
| ENSG00000085224 | HGLibA_03825 | 178 | 0.622682703 | ATRX | chromatin remodeler [Source:HGNC Symbol;Acc:HGNC:886]" |
| ENSG00000089723 | HGLibA_34756 | 178 | 0.622682703 | OTUB2 | ubiquitin aldehyde binding 2 [Source:HGNC Symbol;Acc:HGNC:20351]" |
| ENSG00000095015 | HGLibA_28159 | 178 | 0.622682703 | MAP3K1 | mitogen-activated protein kinase kinase kinase 1 [Source:HGNC Symbol;Acc:HGNC:6848] |
| ENSG00000095794 | HGLibA_11133 | 178 | 0.622682703 | CREM | cAMP responsive element modulator [Source:HGNC Symbol;Acc:HGNC:2352] |
| ENSG00000100350 | HGLibA_17953 | 178 | 0.622682703 | FOXRED2 | FAD dependent oxidoreductase domain containing 2 [Source:HGNC Symbol;Acc:HGNC:26264] |
| ENSG00000101076 | HGLibA_21780 | 178 | 0.622682703 | HNF4A | hepatocyte nuclear factor 4 alpha [Source:HGNC Symbol;Acc:HGNC:5024] |
| ENSG00000101292 | HGLibA_38675 | 178 | 0.622682703 | PROKR2 | prokineticin receptor 2 [Source:HGNC Symbol;Acc:HGNC:15836] |
| ENSG00000101440 | HGLibA_03314 | 178 | 0.622682703 | ASIP | agouti signaling protein [Source:HGNC Symbol;Acc:HGNC:745] |
| ENSG00000101558 | HGLibA_53518 | 178 | 0.622682703 | VAPA | VAMP associated protein A [Source:HGNC Symbol;Acc:HGNC:12648] |
| ENSG00000102081 | HGLibA_17720 | 178 | 0.622682703 | FMR1 | fragile X mental retardation 1 [Source:HGNC Symbol;Acc:HGNC:3775] |
| ENSG00000103550 | HGLibA_25346 | 178 | 0.622682703 | KNOP1 | lysine rich nucleolar protein 1 [Source:HGNC Symbol;Acc:HGNC:34404] |
| ENSG00000104894 | HGLibA_08364 | 178 | 0.622682703 | CD37 | CD37 molecule [Source:HGNC Symbol;Acc:HGNC:1666] |
| ENSG00000105058 | HGLibA_16571 | 178 | 0.622682703 | FAM32A | family with sequence similarity 32 member A [Source:HGNC Symbol;Acc:HGNC:24563] |
| ENSG00000106034 | HGLibA_10928 | 178 | 0.622682703 | CPED1 | cadherin like and PC-esterase domain containing 1 [Source:HGNC Symbol;Acc:HGNC:26159] |
| ENSG00000106348 | HGLibA_23257 | 178 | 0.622682703 | IMPDH1 | inosine monophosphate dehydrogenase 1 [Source:HGNC Symbol;Acc:HGNC:6052] |
| ENSG00000107290 | HGLibA_43675 | 178 | 0.622682703 | SETX | senataxin [Source:HGNC Symbol;Acc:HGNC:445] |
| ENSG00000108590 | HGLibA_28806 | 178 | 0.622682703 | MED31 | mediator complex subunit 31 [Source:HGNC Symbol;Acc:HGNC:24260] |
| ENSG00000111701 | HGLibA_02468 | 178 | 0.622682703 | APOBEC1 | apolipoprotein B mRNA editing enzyme catalytic subunit 1 [Source:HGNC Symbol;Acc:HGNC:604] |
| ENSG00000112972 | HGLibA_21710 | 178 | 0.622682703 | HMGCS1 | 3-hydroxy-3-methylglutaryl-CoA synthase 1 [Source:HGNC Symbol;Acc:HGNC:5007] |
| ENSG00000113240 | HGLibA_10064 | 178 | 0.622682703 | CLK4 | CDC like kinase 4 [Source:HGNC Symbol;Acc:HGNC:13659] |
| ENSG00000113615 | HGLibA_43230 | 178 | 0.622682703 | SEC24A | COPII coat complex component [Source:HGNC Symbol;Acc:HGNC:10703]" |
| ENSG00000114648 | HGLibA_25145 | 178 | 0.622682703 | KLHL18 | kelch like family member 18 [Source:HGNC Symbol;Acc:HGNC:29120] |
| ENSG00000114654 | HGLibA_14558 | 178 | 0.622682703 | EFCC1 | EF-hand and coiled-coil domain containing 1 [Source:HGNC Symbol;Acc:HGNC:25692] |
| ENSG00000115255 | HGLibA_40790 | 178 | 0.622682703 | REEP6 | receptor accessory protein 6 [Source:HGNC Symbol;Acc:HGNC:30078] |
| ENSG00000117543 | HGLibA_13847 | 178 | 0.622682703 | DPH5 | diphthamide biosynthesis 5 [Source:HGNC Symbol;Acc:HGNC:24270] |
| ENSG00000118496 | HGLibA_17065 | 178 | 0.622682703 | FBXO30 | F-box protein 30 [Source:HGNC Symbol;Acc:HGNC:15600] |
| ENSG00000119862 | HGLibA_26389 | 178 | 0.622682703 | LGALSL | galectin like [Source:HGNC Symbol;Acc:HGNC:25012] |
| ENSG00000126368 | HGLibA_32574 | 178 | 0.622682703 | NR1D1 | nuclear receptor subfamily 1 group D member 1 [Source:HGNC Symbol;Acc:HGNC:7962] |
| ENSG00000126768 | HGLibA_49506 | 178 | 0.622682703 | TIMM17B | translocase of inner mitochondrial membrane 17B [Source:HGNC Symbol;Acc:HGNC:17310] |
| ENSG00000128272 | HGLibA_03441 | 178 | 0.622682703 | ATF4 | activating transcription factor 4 [Source:HGNC Symbol;Acc:HGNC:786] |
| ENSG00000128383 | HGLibA_02474 | 178 | 0.622682703 | APOBEC3A | apolipoprotein B mRNA editing enzyme catalytic subunit 3A [Source:HGNC Symbol;Acc:HGNC:17343] |
| ENSG00000134954 | HGLibA_15642 | 178 | 0.622682703 | ETS1 | transcription factor [Source:HGNC Symbol;Acc:HGNC:3488]" |
| ENSG00000135940 | HGLibA_10840 | 178 | 0.622682703 | COX5B | cytochrome c oxidase subunit 5B [Source:HGNC Symbol;Acc:HGNC:2269] |
| ENSG00000136918 | HGLibA_54083 | 178 | 0.622682703 | WDR38 | WD repeat domain 38 [Source:HGNC Symbol;Acc:HGNC:23745] |
| ENSG00000137142 | HGLibA_22836 | 178 | 0.622682703 | IGFBPL1 | insulin like growth factor binding protein like 1 [Source:HGNC Symbol;Acc:HGNC:20081] |
| ENSG00000137474 | HGLibA_30862 | 178 | 0.622682703 | MYO7A | myosin VIIA [Source:HGNC Symbol;Acc:HGNC:7606] |
| ENSG00000138615 | HGLibA_09708 | 178 | 0.622682703 | CILP | cartilage intermediate layer protein [Source:HGNC Symbol;Acc:HGNC:1980] |
| ENSG00000138741 | HGLibA_51732 | 178 | 0.622682703 | TRPC3 | transient receptor potential cation channel subfamily C member 3 [Source:HGNC Symbol;Acc:HGNC:12335] |
| ENSG00000138741 | HGLibA_51731 | 178 | 0.622682703 | TRPC3 | transient receptor potential cation channel subfamily C member 3 [Source:HGNC Symbol;Acc:HGNC:12335] |
| ENSG00000138821 | HGLibA_45124 | 178 | 0.622682703 | SLC39A8 | solute carrier family 39 member 8 [Source:HGNC Symbol;Acc:HGNC:20862] |
| ENSG00000140403 | HGLibA_13520 | 178 | 0.622682703 | DNAJA4 | DnaJ heat shock protein family (Hsp40) member A4 [Source:HGNC Symbol;Acc:HGNC:14885] |
| ENSG00000142627 | HGLibA_15290 | 178 | 0.622682703 | EPHA2 | EPH receptor A2 [Source:HGNC Symbol;Acc:HGNC:3386] |
| ENSG00000142945 | HGLibA_24931 | 178 | 0.622682703 | KIF2C | kinesin family member 2C [Source:HGNC Symbol;Acc:HGNC:6393] |
| ENSG00000143502 | HGLibA_47852 | 178 | 0.622682703 | SUSD4 | sushi domain containing 4 [Source:HGNC Symbol;Acc:HGNC:25470] |
| ENSG00000146776 | HGLibA_03851 | 178 | 0.622682703 | ATXN7L1 | ataxin 7 like 1 [Source:HGNC Symbol;Acc:HGNC:22210] |
| ENSG00000149761 | HGLibA_32973 | 178 | 0.622682703 | NUDT22 | nudix hydrolase 22 [Source:HGNC Symbol;Acc:HGNC:28189] |
| ENSG00000150527 | HGLibA_29186 | 178 | 0.622682703 | MIA2 | MIA SH3 domain ER export factor 2 [Source:HGNC Symbol;Acc:HGNC:18432] |
| ENSG00000150527 | HGLibA_29187 | 178 | 0.622682703 | MIA2 | MIA SH3 domain ER export factor 2 [Source:HGNC Symbol;Acc:HGNC:18432] |
| ENSG00000151657 | HGLibA_24979 | 178 | 0.622682703 | KIN | Kin17 DNA and RNA binding protein [Source:HGNC Symbol;Acc:HGNC:6327] |
| ENSG00000159374 | HGLibA_27812 | 178 | 0.622682703 | M1AP | meiosis 1 associated protein [Source:HGNC Symbol;Acc:HGNC:25183] |
| ENSG00000160072 | HGLibA_03414 | 178 | 0.622682703 | ATAD3B | AAA domain containing 3B [Source:HGNC Symbol;Acc:HGNC:24007]" |
| ENSG00000164398 | HGLibA_00567 | 178 | 0.622682703 | ACSL6 | acyl-CoA synthetase long chain family member 6 [Source:HGNC Symbol;Acc:HGNC:16496] |
| ENSG00000166510 | HGLibA_07840 | 178 | 0.622682703 | CCDC68 | coiled-coil domain containing 68 [Source:HGNC Symbol;Acc:HGNC:24350] |
| ENSG00000169836 | HGLibA_48178 | 178 | 0.622682703 | TACR3 | tachykinin receptor 3 [Source:HGNC Symbol;Acc:HGNC:11528] |
| ENSG00000170381 | HGLibA_43330 | 178 | 0.622682703 | SEMA3E | semaphorin 3E [Source:HGNC Symbol;Acc:HGNC:10727] |
| ENSG00000170852 | HGLibA_24103 | 178 | 0.622682703 | KBTBD2 | kelch repeat and BTB domain containing 2 [Source:HGNC Symbol;Acc:HGNC:21751] |
| ENSG00000173950 | HGLibA_54613 | 178 | 0.622682703 | XXYLT1 | xyloside xylosyltransferase 1 [Source:HGNC Symbol;Acc:HGNC:26639] |
| ENSG00000174791 | HGLibA_41288 | 178 | 0.622682703 | RIN1 | Ras and Rab interactor 1 [Source:HGNC Symbol;Acc:HGNC:18749] |
| ENSG00000177352 | HGLibA_07854 | 178 | 0.622682703 | CCDC71 | coiled-coil domain containing 71 [Source:HGNC Symbol;Acc:HGNC:25760] |
| ENSG00000177627 | HGLibA_05264 | 178 | 0.622682703 | C12orf54 | chromosome 12 open reading frame 54 [Source:HGNC Symbol;Acc:HGNC:28553] |
| ENSG00000178802 | HGLibA_29731 | 178 | 0.622682703 | MPI | mannose phosphate isomerase [Source:HGNC Symbol;Acc:HGNC:7216] |
| ENSG00000179304 | HGLibA_16188 | 178 | 0.622682703 | FAM156B | family with sequence similarity 156 member B [Source:HGNC Symbol;Acc:HGNC:31962] |
| ENSG00000180483 | HGLibA_12868 | 178 | 0.622682703 | DEFB119 | defensin beta 119 [Source:HGNC Symbol;Acc:HGNC:18099] |
| ENSG00000181982 | HGLibA_07638 | 178 | 0.622682703 | CCDC149 | coiled-coil domain containing 149 [Source:HGNC Symbol;Acc:HGNC:25405] |
| ENSG00000183397 | HGLibA_05702 | 178 | 0.622682703 | C19orf71 | chromosome 19 open reading frame 71 [Source:HGNC Symbol;Acc:HGNC:34496] |
| ENSG00000183801 | HGLibA_33357 | 178 | 0.622682703 | OLFML1 | olfactomedin like 1 [Source:HGNC Symbol;Acc:HGNC:24473] |
| ENSG00000185940 | HGLibA_25799 | 178 | 0.622682703 | KRTAP5-5 | keratin associated protein 5-5 [Source:HGNC Symbol;Acc:HGNC:23601] |
| ENSG00000187994 | HGLibA_41302 | 178 | 0.622682703 | RINL | Ras and Rab interactor like [Source:HGNC Symbol;Acc:HGNC:24795] |
| ENSG00000188158 | HGLibA_31913 | 178 | 0.622682703 | NHS | NHS actin remodeling regulator [Source:HGNC Symbol;Acc:HGNC:7820] |
| ENSG00000189129 | HGLibA_36949 | 178 | 0.622682703 | PLAC9 | placenta specific 9 [Source:HGNC Symbol;Acc:HGNC:19255] |
| ENSG00000197858 | HGLibA_19721 | 178 | 0.622682703 | GPAA1 | glycosylphosphatidylinositol anchor attachment 1 [Source:HGNC Symbol;Acc:HGNC:4446] |

| | | | | | |
|---|---|---|---|---|---|
| ENSG00000203970 | HGLibA_12846 | 178 | 0.622682703 | DEFB110 | defensin beta 110 [Source:HGNC Symbol;Acc:HGNC:18091] |
| ENSG00000204010 | HGLibA_22627 | 178 | 0.622682703 | IFIT1B | interferon induced protein with tetratricopeptide repeats 1B [Source:HGNC Symbol;Acc:HGNC:23442] |
| ENSG00000204449 | HGLibA_51528 | 178 | 0.622682703 | TRIM49C | tripartite motif containing 49C [Source:HGNC Symbol;Acc:HGNC:38877] |
| ENSG00000204923 | HGLibA_17117 | 178 | 0.622682703 | FBXO48 | F-box protein 48 [Source:HGNC Symbol;Acc:HGNC:33857] |
| ENSG00000212935 | HGLibA_25607 | 178 | 0.622682703 | KRTAP10-3 | keratin associated protein 10-3 [Source:HGNC Symbol;Acc:HGNC:22968] |
| ENSG00000213413 | HGLibA_39620 | 178 | 0.622682703 | PVRIG | PVR related immunoglobulin domain containing [Source:HGNC Symbol;Acc:HGNC:32190] |
| ENSG00000223802 | HGLibA_09200 | 178 | 0.622682703 | CERS1 | ceramide synthase 1 [Source:HGNC Symbol;Acc:HGNC:14253] |
| ENSG00000225828 | HGLibA_16541 | 178 | 0.622682703 | FAM229A | family with sequence similarity 229 member A [Source:HGNC Symbol;Acc:HGNC:44652] |
| ENSG00000244486 | HGLibA_42879 | 178 | 0.622682703 | SCARF2 | scavenger receptor class F member 2 [Source:HGNC Symbol;Acc:HGNC:19869] |
| ENSG00000253305 | HGLibA_35565 | 178 | 0.622682703 | PCDHGB6 | 6 [Source:HGNC Symbol;Acc:HGNC:8713]" |
| ENSG00000268350 | HGLibA_16186 | 178 | 0.622682703 | FAM156A | family with sequence similarity 156 member A [Source:HGNC Symbol;Acc:HGNC:30114] |
| ENSG00000268606 | HGLibA_27905 | 178 | 0.622682703 | MAGEA2 | MAGE family member A2 [Source:HGNC Symbol;Acc:HGNC:6800] |
| ENSG00000268738 | HGLibA_22237 | 178 | 0.622682703 | HSFX2 | X-linked 2 [Source:HGNC Symbol;Acc:HGNC:32701]" |
| GTPBP5 | HGLibA_20613 | 178 | 0.622682703 | GTPBP5 | - |
| H3F3B | HGLibA_20780 | 178 | 0.622682703 | H3F3B | - |
| hsa-mir-18b | HGLibA_58022 | 178 | 0.622682703 | hsa-mir-18b | - |
| hsa-mir-6081 | HGLibA_62586 | 178 | 0.622682703 | hsa-mir-6081 | - |
| hsa-mir-6738 | HGLibA_63105 | 178 | 0.622682703 | hsa-mir-6738 | - |
| hsa-mir-6863 | HGLibA_63638 | 178 | 0.622682703 | hsa-mir-6863 | - |
| hsa-mir-6892 | HGLibA_63753 | 178 | 0.622682703 | hsa-mir-6892 | - |
| hsa-mir-9-2 | HGLibA_64265 | 178 | 0.622682703 | hsa-mir-9-2 | - |
| KIAA1211 | HGLibA_24715 | 178 | 0.622682703 | KIAA1211 | - |
| LOC441155 | HGLibA_26952 | 178 | 0.622682703 | LOC441155 | - |
| LOC729020 | HGLibA_26996 | 178 | 0.622682703 | LOC729020 | - |
| NEURL | HGLibA_31722 | 178 | 0.622682703 | NEURL | - |
| SHOX_Y | HGLibA_44071 | 178 | 0.622682703 | SHOX_Y | - |
| ADSSL1 | HGLibA_01079 | 177 | 0.612731665 | ADSSL1 | - |
| AGPAT9 | HGLibA_01235 | 177 | 0.612731665 | AGPAT9 | - |
| C19orf10 | HGLibA_05628 | 177 | 0.612731665 | C19orf10 | - |
| DOPEY2 | HGLibA_13803 | 177 | 0.612731665 | DOPEY2 | - |
| ENSG00000029364 | HGLibA_45126 | 177 | 0.612731665 | SLC39A9 | solute carrier family 39 member 9 [Source:HGNC Symbol;Acc:HGNC:20182] |
| ENSG00000030110 | HGLibA_04135 | 177 | 0.612731665 | BAK1 | BCL2 antagonist/killer 1 [Source:HGNC Symbol;Acc:HGNC:949] |
| ENSG00000051128 | HGLibA_21869 | 177 | 0.612731665 | HOMER3 | homer scaffold protein 3 [Source:HGNC Symbol;Acc:HGNC:17514] |
| ENSG00000065320 | HGLibA_32852 | 177 | 0.612731665 | NTN1 | netrin 1 [Source:HGNC Symbol;Acc:HGNC:8029] |
| ENSG00000077080 | HGLibA_00623 | 177 | 0.612731665 | ACTL6B | actin like 6B [Source:HGNC Symbol;Acc:HGNC:160] |
| ENSG00000078061 | HGLibA_02621 | 177 | 0.612731665 | ARAF | serine/threonine kinase [Source:HGNC Symbol;Acc:HGNC:646]" |
| ENSG00000083307 | HGLibA_20252 | 177 | 0.612731665 | GRHL2 | grainyhead like transcription factor 2 [Source:HGNC Symbol;Acc:HGNC:2799] |
| ENSG00000083828 | HGLibA_56325 | 177 | 0.612731665 | ZNF586 | zinc finger protein 586 [Source:HGNC Symbol;Acc:HGNC:25949] |
| ENSG00000088280 | HGLibA_03201 | 177 | 0.612731665 | ASAP3 | ankyrin repeat and PH domain 3 [Source:HGNC Symbol;Acc:HGNC:14987]" |
| ENSG00000088882 | HGLibA_11037 | 177 | 0.612731665 | CPXM1 | M14 family member 1 [Source:HGNC Symbol;Acc:HGNC:15771]" |
| ENSG00000090382 | HGLibA_27782 | 177 | 0.612731665 | LYZ | lysozyme [Source:HGNC Symbol;Acc:HGNC:6740] |
| ENSG00000091106 | HGLibA_32091 | 177 | 0.612731665 | NLRC4 | NLR family CARD domain containing 4 [Source:HGNC Symbol;Acc:HGNC:16412] |
| ENSG00000095951 | HGLibA_21566 | 177 | 0.612731665 | HIVEP1 | human immunodeficiency virus type I enhancer binding protein 1 [Source:HGNC Symbol;Acc:HGNC:4920] |
| ENSG00000097021 | HGLibA_00488 | 177 | 0.612731665 | ACOT7 | acyl-CoA thioesterase 7 [Source:HGNC Symbol;Acc:HGNC:24157] |
| ENSG00000100320 | HGLibA_40457 | 177 | 0.612731665 | RBFOX2 | RNA binding fox-1 homolog 2 [Source:HGNC Symbol;Acc:HGNC:9906] |
| ENSG00000100612 | HGLibA_13144 | 177 | 0.612731665 | DHRS7 | dehydrogenase/reductase 7 [Source:HGNC Symbol;Acc:HGNC:21524] |
| ENSG00000100852 | HGLibA_02783 | 177 | 0.612731665 | ARHGAP5 | Rho GTPase activating protein 5 [Source:HGNC Symbol;Acc:HGNC:675] |
| ENSG00000101544 | HGLibA_01003 | 177 | 0.612731665 | ADNP2 | ADNP homeobox 2 [Source:HGNC Symbol;Acc:HGNC:23803] |
| ENSG00000103222 | HGLibA_00159 | 177 | 0.612731665 | ABCC1 | ATP binding cassette subfamily C member 1 [Source:HGNC Symbol;Acc:HGNC:51] |
| ENSG00000104763 | HGLibA_03184 | 177 | 0.612731665 | ASAH1 | N-acylsphingosine amidohydrolase 1 [Source:HGNC Symbol;Acc:HGNC:735] |
| ENSG00000106609 | HGLibA_50308 | 177 | 0.612731665 | TMEM248 | transmembrane protein 248 [Source:HGNC Symbol;Acc:HGNC:25476] |
| ENSG00000106633 | HGLibA_18789 | 177 | 0.612731665 | GCK | glucokinase [Source:HGNC Symbol;Acc:HGNC:4195] |
| ENSG00000107159 | HGLibA_06827 | 177 | 0.612731665 | CA9 | carbonic anhydrase 9 [Source:HGNC Symbol;Acc:HGNC:1383] |
| ENSG00000108759 | HGLibA_25477 | 177 | 0.612731665 | KRT32 | keratin 32 [Source:HGNC Symbol;Acc:HGNC:6449] |
| ENSG00000110801 | HGLibA_39188 | 177 | 0.612731665 | PSMD9 | non-ATPase 9 [Source:HGNC Symbol;Acc:HGNC:9567]" |
| ENSG00000115318 | HGLibA_27044 | 177 | 0.612731665 | LOXL3 | lysyl oxidase like 3 [Source:HGNC Symbol;Acc:HGNC:13869] |
| ENSG00000115694 | HGLibA_47473 | 177 | 0.612731665 | STK25 | serine/threonine kinase 25 [Source:HGNC Symbol;Acc:HGNC:11404] |
| ENSG00000115956 | HGLibA_37057 | 177 | 0.612731665 | PLEK | pleckstrin [Source:HGNC Symbol;Acc:HGNC:9070] |
| ENSG00000117859 | HGLibA_34670 | 177 | 0.612731665 | OSBPL9 | oxysterol binding protein like 9 [Source:HGNC Symbol;Acc:HGNC:16386] |
| ENSG00000122786 | HGLibA_07009 | 177 | 0.612731665 | CALD1 | caldesmon 1 [Source:HGNC Symbol;Acc:HGNC:1441] |
| ENSG00000123154 | HGLibA_54193 | 177 | 0.612731665 | WDR83 | WD repeat domain 83 [Source:HGNC Symbol;Acc:HGNC:32672] |
| ENSG00000124191 | HGLibA_51038 | 177 | 0.612731665 | TOX2 | TOX high mobility group box family member 2 [Source:HGNC Symbol;Acc:HGNC:16095] |
| ENSG00000127415 | HGLibA_22568 | 177 | 0.612731665 | IDUA | alpha-L- [Source:HGNC Symbol;Acc:HGNC:5391]" |
| ENSG00000127603 | HGLibA_27833 | 177 | 0.612731665 | MACF1 | microtubule-actin crosslinking factor 1 [Source:HGNC Symbol;Acc:HGNC:13664] |
| ENSG00000129990 | HGLibA_48081 | 177 | 0.612731665 | SYT5 | synaptotagmin 5 [Source:HGNC Symbol;Acc:HGNC:11513] |
| ENSG00000133488 | HGLibA_43199 | 177 | 0.612731665 | SEC14L4 | SEC14 like lipid binding 4 [Source:HGNC Symbol;Acc:HGNC:20627] |
| ENSG00000135074 | HGLibA_00746 | 177 | 0.612731665 | ADAM19 | ADAM metallopeptidase domain 19 [Source:HGNC Symbol;Acc:HGNC:197] |
| ENSG00000136457 | HGLibA_09341 | 177 | 0.612731665 | CHAD | chondroadherin [Source:HGNC Symbol;Acc:HGNC:1909] |
| ENSG00000136541 | HGLibA_15505 | 177 | 0.612731665 | ERMN | ermin [Source:HGNC Symbol;Acc:HGNC:29208] |
| ENSG00000136944 | HGLibA_26736 | 177 | 0.612731665 | LMX1B | LIM homeobox transcription factor 1 beta [Source:HGNC Symbol;Acc:HGNC:6654] |
| ENSG00000137764 | HGLibA_28151 | 177 | 0.612731665 | MAP2K5 | mitogen-activated protein kinase kinase 5 [Source:HGNC Symbol;Acc:HGNC:6845] |
| ENSG00000137869 | HGLibA_12064 | 177 | 0.612731665 | CYP19A1 | cytochrome P450 family 19 subfamily A member 1 [Source:HGNC Symbol;Acc:HGNC:2594] |
| ENSG00000138347 | HGLibA_30911 | 177 | 0.612731665 | MYPN | myopalladin [Source:HGNC Symbol;Acc:HGNC:23246] |
| ENSG00000139438 | HGLibA_16521 | 177 | 0.612731665 | FAM222A | family with sequence similarity 222 member A [Source:HGNC Symbol;Acc:HGNC:25915] |
| ENSG00000140416 | HGLibA_51142 | 177 | 0.612731665 | TPM1 | tropomyosin 1 [Source:HGNC Symbol;Acc:HGNC:12010] |
| ENSG00000140488 | HGLibA_09019 | 177 | 0.612731665 | CELF6 | CUGBP Elav-like family member 6 [Source:HGNC Symbol;Acc:HGNC:14059] |
| ENSG00000140836 | HGLibA_55174 | 177 | 0.612731665 | ZFHX3 | zinc finger homeobox 3 [Source:HGNC Symbol;Acc:HGNC:777] |
| ENSG00000141279 | HGLibA_32434 | 177 | 0.612731665 | NPEPPS | aminopeptidase puromycin sensitive [Source:HGNC Symbol;Acc:HGNC:7900] |
| ENSG00000144331 | HGLibA_55899 | 177 | 0.612731665 | ZNF385B | zinc finger protein 385B [Source:HGNC Symbol;Acc:HGNC:26332] |
| ENSG00000144460 | HGLibA_33183 | 177 | 0.612731665 | NYAP2 | neuronal tyrosine-phosphorylated phosphoinositide-3-kinase adaptor 2 [Source:HGNC Symbol;Acc:HGNC:29291] |
| ENSG00000145362 | HGLibA_01938 | 177 | 0.612731665 | ANK2 | ankyrin 2 [Source:HGNC Symbol;Acc:HGNC:493] |
| ENSG00000147457 | HGLibA_09503 | 177 | 0.612731665 | CHMP7 | charged multivesicular body protein 7 [Source:HGNC Symbol;Acc:HGNC:28439] |
| ENSG00000147883 | HGLibA_08840 | 177 | 0.612731665 | CDKN2B | cyclin dependent kinase inhibitor 2B [Source:HGNC Symbol;Acc:HGNC:1788] |
| ENSG00000151882 | HGLibA_08016 | 177 | 0.612731665 | CCL28 | C-C motif chemokine ligand 28 [Source:HGNC Symbol;Acc:HGNC:17700] |
| ENSG00000152683 | HGLibA_44915 | 177 | 0.612731665 | SLC30A6 | solute carrier family 30 member 6 [Source:HGNC Symbol;Acc:HGNC:19305] |
| ENSG00000155275 | HGLibA_51688 | 177 | 0.612731665 | TRMT44 | tRNA methyltransferase 44 homolog [Source:HGNC Symbol;Acc:HGNC:26653] |
| ENSG00000160613 | HGLibA_35684 | 177 | 0.612731665 | PCSK7 | proprotein convertase subtilisin/kexin type 7 [Source:HGNC Symbol;Acc:HGNC:8748] |
| ENSG00000162366 | HGLibA_35994 | 177 | 0.612731665 | PDZK1IP1 | PDZK1 interacting protein 1 [Source:HGNC Symbol;Acc:HGNC:16887] |
| ENSG00000162572 | HGLibA_43020 | 177 | 0.612731665 | SCNN1D | sodium channel epithelial 1 delta subunit [Source:HGNC Symbol;Acc:HGNC:10601] |
| ENSG00000163755 | HGLibA_22048 | 177 | 0.612731665 | HPS3 | biogenesis of lysosomal organelles complex 2 subunit 1 [Source:HGNC Symbol;Acc:HGNC:15597]" |
| ENSG00000164330 | HGLibA_14350 | 177 | 0.612731665 | EBF1 | EBF transcription factor 1 [Source:HGNC Symbol;Acc:HGNC:3126] |
| ENSG00000165887 | HGLibA_02023 | 177 | 0.612731665 | ANKRD2 | ankyrin repeat domain 2 [Source:HGNC Symbol;Acc:HGNC:495] |
| ENSG00000166211 | HGLibA_46657 | 177 | 0.612731665 | SPIC | Spi-C transcription factor [Source:HGNC Symbol;Acc:HGNC:29549] |
| ENSG00000166979 | HGLibA_15678 | 177 | 0.612731665 | EVA1C | eva-1 homolog C [Source:HGNC Symbol;Acc:HGNC:13239] |
| ENSG00000167371 | HGLibA_38885 | 177 | 0.612731665 | PRRT2 | proline rich transmembrane protein 2 [Source:HGNC Symbol;Acc:HGNC:30500] |
| ENSG00000168060 | HGLibA_31013 | 177 | 0.612731665 | NAALADL1 | N-acetylated alpha-linked acidic dipeptidase like 1 [Source:HGNC Symbol;Acc:HGNC:23536] |
| ENSG00000168229 | HGLibA_39304 | 177 | 0.612731665 | PTGDR | prostaglandin D2 receptor [Source:HGNC Symbol;Acc:HGNC:9591] |
| ENSG00000168803 | HGLibA_00725 | 177 | 0.612731665 | ADAL | adenosine deaminase like [Source:HGNC Symbol;Acc:HGNC:31853] |
| ENSG00000168830 | HGLibA_22364 | 177 | 0.612731665 | HTR1E | 5-hydroxytryptamine receptor 1E [Source:HGNC Symbol;Acc:HGNC:5291] |
| ENSG00000168913 | HGLibA_15131 | 177 | 0.612731665 | ENHO | energy homeostasis associated [Source:HGNC Symbol;Acc:HGNC:24838] |
| ENSG00000168993 | HGLibA_10931 | 177 | 0.612731665 | CPLX1 | complexin 1 [Source:HGNC Symbol;Acc:HGNC:2309] |
| ENSG00000169609 | HGLibA_05397 | 177 | 0.612731665 | C15orf40 | chromosome 15 open reading frame 40 [Source:HGNC Symbol;Acc:HGNC:28443] |
| ENSG00000170909 | HGLibA_34672 | 177 | 0.612731665 | OSCAR | immunoglobulin-like receptor [Source:HGNC Symbol;Acc:HGNC:29960]" |
| ENSG00000171204 | HGLibA_49914 | 177 | 0.612731665 | TMEM126B | transmembrane protein 126B [Source:HGNC Symbol;Acc:HGNC:30883] |
| ENSG00000171396 | HGLibA_25763 | 177 | 0.612731665 | KRTAP4-4 | keratin associated protein 4-4 [Source:HGNC Symbol;Acc:HGNC:16928] |
| ENSG00000173401 | HGLibA_19262 | 177 | 0.612731665 | GLIPR1L1 | GLIPR1 like 1 [Source:HGNC Symbol;Acc:HGNC:28392] |
| ENSG00000174358 | HGLibA_45317 | 177 | 0.612731665 | SLC6A19 | solute carrier family 6 member 19 [Source:HGNC Symbol;Acc:HGNC:27960] |
| ENSG00000174871 | HGLibA_10286 | 177 | 0.612731665 | CNIH2 | cornichon family AMPA receptor auxiliary protein 2 [Source:HGNC Symbol;Acc:HGNC:28744] |
| ENSG00000174989 | HGLibA_17155 | 177 | 0.612731665 | FBXW8 | F-box and WD repeat domain containing 8 [Source:HGNC Symbol;Acc:HGNC:13597] |
| ENSG00000175920 | HGLibA_13786 | 177 | 0.612731665 | DOK7 | docking protein 7 [Source:HGNC Symbol;Acc:HGNC:26594] |
| ENSG00000178222 | HGLibA_41618 | 177 | 0.612731665 | RNF212 | ring finger protein 212 [Source:HGNC Symbol;Acc:HGNC:27729] |
| ENSG00000179604 | HGLibA_08575 | 177 | 0.612731665 | CDC42EP4 | CDC42 effector protein 4 [Source:HGNC Symbol;Acc:HGNC:17147] |
| ENSG00000179943 | HGLibA_17545 | 177 | 0.612731665 | FIZ1 | FLT3 interacting zinc finger 1 [Source:HGNC Symbol;Acc:HGNC:25917] |
| ENSG00000181023 | HGLibA_34224 | 177 | 0.612731665 | OR56B1 | olfactory receptor family 56 subfamily B member 1 [Source:HGNC Symbol;Acc:HGNC:15245] |
| ENSG00000181045 | HGLibA_44787 | 177 | 0.612731665 | SLC26A11 | solute carrier family 26 member 11 [Source:HGNC Symbol;Acc:HGNC:14471] |
| ENSG00000182175 | HGLibA_41014 | 177 | 0.612731665 | RGMA | repulsive guidance molecule BMP co-receptor a [Source:HGNC Symbol;Acc:HGNC:30308] |
| ENSG00000183479 | HGLibA_51376 | 177 | 0.612731665 | TREX2 | three prime repair exonuclease 2 [Source:HGNC Symbol;Acc:HGNC:12270] |
| ENSG00000184545 | HGLibA_14193 | 177 | 0.612731665 | DUSP8 | dual specificity phosphatase 8 [Source:HGNC Symbol;Acc:HGNC:3074] |
| ENSG00000184986 | HGLibA_49900 | 177 | 0.612731665 | TMEM121 | transmembrane protein 121 [Source:HGNC Symbol;Acc:HGNC:20511] |
| ENSG00000185972 | HGLibA_07949 | 177 | 0.612731665 | CCIN | calicin [Source:HGNC Symbol;Acc:HGNC:1568] |
| ENSG00000186300 | HGLibA_56225 | 177 | 0.612731665 | ZNF555 | zinc finger protein 555 [Source:HGNC Symbol;Acc:HGNC:28382] |
| ENSG00000186723 | HGLibA_33507 | 177 | 0.612731665 | OR10H1 | olfactory receptor family 10 subfamily H member 1 [Source:HGNC Symbol;Acc:HGNC:8172] |
| ENSG00000187187 | HGLibA_56201 | 177 | 0.612731665 | ZNF546 | zinc finger protein 546 [Source:HGNC Symbol;Acc:HGNC:28671] |
| ENSG00000187840 | HGLibA_14828 | 177 | 0.612731665 | EIF4EBP1 | eukaryotic translation initiation factor 4E binding protein 1 [Source:HGNC Symbol;Acc:HGNC:3288] |
| ENSG00000188522 | HGLibA_16759 | 177 | 0.612731665 | FAM83G | family with sequence similarity 83 member G [Source:HGNC Symbol;Acc:HGNC:32554] |
| ENSG00000189023 | HGLibA_27934 | 177 | 0.612731665 | MAGEB16 | MAGE family member B16 [Source:HGNC Symbol;Acc:HGNC:21188] |
| ENSG00000198429 | HGLibA_56549 | 177 | 0.612731665 | ZNF69 | zinc finger protein 69 [Source:HGNC Symbol;Acc:HGNC:13138] |
| ENSG00000198692 | HGLibA_14715 | 177 | 0.612731665 | EIF1AY | eukaryotic translation initiation factor 1A Y-linked [Source:HGNC Symbol;Acc:HGNC:3252] |
| ENSG00000204614 | HGLibA_51491 | 177 | 0.612731665 | TRIM40 | tripartite motif containing 40 [Source:HGNC Symbol;Acc:HGNC:18736] |
| ENSG00000204632 | HGLibA_21648 | 177 | 0.612731665 | HLA-G | G [Source:HGNC Symbol;Acc:HGNC:4964]" |
| ENSG00000214102 | HGLibA_54241 | 177 | 0.612731665 | WEE2 | WEE1 homolog 2 [Source:HGNC Symbol;Acc:HGNC:19684] |
| ENSG00000214510 | HGLibA_46686 | 177 | 0.612731665 | SPINK13 | Kazal type 13 (putative) [Source:HGNC Symbol;Acc:HGNC:27200]" |
| ENSG00000221866 | HGLibA_37268 | 177 | 0.612731665 | PLXNA4 | plexin A4 [Source:HGNC Symbol;Acc:HGNC:9102] |
| ENSG00000221994 | HGLibA_56434 | 177 | 0.612731665 | ZNF630 | zinc finger protein 630 [Source:HGNC Symbol;Acc:HGNC:28855] |
| ENSG00000227802 | HGLibA_13540 | 177 | 0.612731665 | DNAJB3 | DnaJ heat shock protein family (Hsp40) member B3 [Source:HGNC Symbol;Acc:HGNC:32397] |

| | | | | | |
|---|---|---|---|---|---|
| ENSG00000244624 | HGLibA_25699 | 177 | 0.612731665 | KRTAP20-1 | keratin associated protein 20-1 [Source:HGNC Symbol;Acc:HGNC:18943] |
| ENSG00000254122 | HGLibA_35569 | 177 | 0.612731665 | PCDHGB7 | 7 [Source:HGNC Symbol;Acc:HGNC:8714]" |
| ENSG00000256806 | HGLibA_05517 | 177 | 0.612731665 | C17orf100 | chromosome 17 open reading frame 100 [Source:HGNC Symbol;Acc:HGNC:34494] |
| ENSG00000274102 | HGLibA_34042 | 177 | 0.612731665 | OR4M2 | olfactory receptor family 4 subfamily M member 2 [Source:HGNC Symbol;Acc:HGNC:15373] |
| FAM64A | HGLibA_16665 | 177 | 0.612731665 | FAM64A | - |
| hsa-mir-1205 | HGLibA_57278 | 177 | 0.612731665 | hsa-mir-1205 | - |
| hsa-mir-2116 | HGLibA_58235 | 177 | 0.612731665 | hsa-mir-2116 | - |
| hsa-mir-4268 | HGLibA_59781 | 177 | 0.612731665 | hsa-mir-4268 | - |
| hsa-mir-552 | HGLibA_62128 | 177 | 0.612731665 | hsa-mir-552 | - |
| hsa-mir-586 | HGLibA_62442 | 177 | 0.612731665 | hsa-mir-586 | - |
| hsa-mir-6894 | HGLibA_63760 | 177 | 0.612731665 | hsa-mir-6894 | - |
| hsa-mir-7106 | HGLibA_63784 | 177 | 0.612731665 | hsa-mir-7106 | - |
| hsa-mir-8074 | HGLibA_64139 | 177 | 0.612731665 | hsa-mir-8074 | - |
| KIAA1644 | HGLibA_24795 | 177 | 0.612731665 | KIAA1644 | - |
| LOC100130357 | HGLibA_26780 | 177 | 0.612731665 | LOC100130357 | - |
| LOC729059 | HGLibA_26998 | 177 | 0.612731665 | LOC729059 | - |
| MUM1L1 | HGLibA_30568 | 177 | 0.612731665 | MUM1L1 | - |
| OCLM | HGLibA_33252 | 177 | 0.612731665 | OCLM | - |
| PAPD7 | HGLibA_35128 | 177 | 0.612731665 | PAPD7 | - |
| AES | HGLibA_01095 | 176 | 0.602780628 | AES | - |
| AKAP17A_X | HGLibA_01421 | 176 | 0.602780628 | AKAP17A_X | - |
| C17orf47 | HGLibA_05535 | 176 | 0.602780628 | C17orf47 | - |
| CCBP2 | HGLibA_07501 | 176 | 0.602780628 | CCBP2 | - |
| CXorf21 | HGLibA_11902 | 176 | 0.602780628 | CXorf21 | - |
| ENSG00000010361 | HGLibA_18213 | 176 | 0.602780628 | FUZ | fuzzy planar cell polarity protein [Source:HGNC Symbol;Acc:HGNC:26219] |
| ENSG00000013573 | HGLibA_12653 | 176 | 0.602780628 | DDX11 | DEAD/H-box helicase 11 [Source:HGNC Symbol;Acc:HGNC:2736] |
| ENSG00000035141 | HGLibA_16139 | 176 | 0.602780628 | FAM136A | family with sequence similarity 136 member A [Source:HGNC Symbol;Acc:HGNC:25911] |
| ENSG00000065618 | HGLibA_10522 | 176 | 0.602780628 | COL17A1 | collagen type XVII alpha 1 chain [Source:HGNC Symbol;Acc:HGNC:2194] |
| ENSG00000073861 | HGLibA_48662 | 176 | 0.602780628 | TBX21 | T-box 21 [Source:HGNC Symbol;Acc:HGNC:11599] |
| ENSG00000077080 | HGLibA_00624 | 176 | 0.602780628 | ACTL6B | actin like 6B [Source:HGNC Symbol;Acc:HGNC:160] |
| ENSG00000083807 | HGLibA_44824 | 176 | 0.602780628 | SLC27A5 | solute carrier family 27 member 5 [Source:HGNC Symbol;Acc:HGNC:10999] |
| ENSG00000091181 | HGLibA_23178 | 176 | 0.602780628 | IL5RA | interleukin 5 receptor subunit alpha [Source:HGNC Symbol;Acc:HGNC:6017] |
| ENSG00000100941 | HGLibA_37392 | 176 | 0.602780628 | PNN | desmosome associated protein [Source:HGNC Symbol;Acc:HGNC:9162]" |
| ENSG00000101213 | HGLibA_39385 | 176 | 0.602780628 | PTK6 | protein tyrosine kinase 6 [Source:HGNC Symbol;Acc:HGNC:9617] |
| ENSG00000101464 | HGLibA_36590 | 176 | 0.602780628 | PIGU | phosphatidylinositol glycan anchor biosynthesis class U [Source:HGNC Symbol;Acc:HGNC:15791] |
| ENSG00000102030 | HGLibA_30971 | 176 | 0.602780628 | NAA10 | NatA catalytic subunit [Source:HGNC Symbol;Acc:HGNC:18704]" |
| ENSG00000104883 | HGLibA_36089 | 176 | 0.602780628 | PEX11G | peroxisomal biogenesis factor 11 gamma [Source:HGNC Symbol;Acc:HGNC:20208] |
| ENSG00000105398 | HGLibA_47759 | 176 | 0.602780628 | SULT2A1 | sulfotransferase family 2A member 1 [Source:HGNC Symbol;Acc:HGNC:11458] |
| ENSG00000105409 | HGLibA_03587 | 176 | 0.602780628 | ATP1A3 | ATPase Na+/K+ transporting subunit alpha 3 [Source:HGNC Symbol;Acc:HGNC:801] |
| ENSG00000105605 | HGLibA_06948 | 176 | 0.602780628 | CACNG7 | calcium voltage-gated channel auxiliary subunit gamma 7 [Source:HGNC Symbol;Acc:HGNC:13626] |
| ENSG00000105698 | HGLibA_53170 | 176 | 0.602780628 | USF2 | c-fos interacting [Source:HGNC Symbol;Acc:HGNC:12594]" |
| ENSG00000105767 | HGLibA_06974 | 176 | 0.602780628 | CADM4 | cell adhesion molecule 4 [Source:HGNC Symbol;Acc:HGNC:30825] |
| ENSG00000105887 | HGLibA_30459 | 176 | 0.602780628 | MTPN | myotrophin [Source:HGNC Symbol;Acc:HGNC:15667] |
| ENSG00000106211 | HGLibA_22300 | 176 | 0.602780628 | HSPB1 | heat shock protein family B (small) member 1 [Source:HGNC Symbol;Acc:HGNC:5246] |
| ENSG00000108849 | HGLibA_38245 | 176 | 0.602780628 | PPY | pancreatic polypeptide [Source:HGNC Symbol;Acc:HGNC:9327] |
| ENSG00000116198 | HGLibA_09095 | 176 | 0.602780628 | CEP104 | centrosomal protein 104 [Source:HGNC Symbol;Acc:HGNC:24866] |
| ENSG00000120262 | HGLibA_07691 | 176 | 0.602780628 | CCDC170 | coiled-coil domain containing 170 [Source:HGNC Symbol;Acc:HGNC:21177] |
| ENSG00000121316 | HGLibA_36973 | 176 | 0.602780628 | PLBD1 | phospholipase B domain containing 1 [Source:HGNC Symbol;Acc:HGNC:26215] |
| ENSG00000121381 | HGLibA_48433 | 176 | 0.602780628 | TAS2R9 | taste 2 receptor member 9 [Source:HGNC Symbol;Acc:HGNC:14917] |
| ENSG00000122033 | HGLibA_30400 | 176 | 0.602780628 | MTIF3 | mitochondrial translational initiation factor 3 [Source:HGNC Symbol;Acc:HGNC:29788] |
| ENSG00000124490 | HGLibA_11169 | 176 | 0.602780628 | CRISP2 | cysteine rich secretory protein 2 [Source:HGNC Symbol;Acc:HGNC:12024] |
| ENSG00000124942 | HGLibA_01302 | 176 | 0.602780628 | AHNAK | AHNAK nucleoprotein [Source:HGNC Symbol;Acc:HGNC:347] |
| ENSG00000126243 | HGLibA_27158 | 176 | 0.602780628 | LRFN3 | leucine rich repeat and fibronectin type III domain containing 3 [Source:HGNC Symbol;Acc:HGNC:28370] |
| ENSG00000130023 | HGLibA_15503 | 176 | 0.602780628 | ERMARD | ER membrane associated RNA degradation [Source:HGNC Symbol;Acc:HGNC:21056] |
| ENSG00000130779 | HGLibA_10043 | 176 | 0.602780628 | CLIP1 | CAP-Gly domain containing linker protein 1 [Source:HGNC Symbol;Acc:HGNC:10461] |
| ENSG00000131127 | HGLibA_55486 | 176 | 0.602780628 | ZNF141 | zinc finger protein 141 [Source:HGNC Symbol;Acc:HGNC:12926] |
| ENSG00000132915 | HGLibA_35806 | 176 | 0.602780628 | PDE6A | phosphodiesterase 6A [Source:HGNC Symbol;Acc:HGNC:8785] |
| ENSG00000132972 | HGLibA_41571 | 176 | 0.602780628 | RNF17 | ring finger protein 17 [Source:HGNC Symbol;Acc:HGNC:10060] |
| ENSG00000133048 | HGLibA_09437 | 176 | 0.602780628 | CHI3L1 | chitinase 3 like 1 [Source:HGNC Symbol;Acc:HGNC:1932] |
| ENSG00000133884 | HGLibA_13831 | 176 | 0.602780628 | DPF2 | double PHD fingers 2 [Source:HGNC Symbol;Acc:HGNC:9964] |
| ENSG00000134375 | HGLibA_49502 | 176 | 0.602780628 | TIMM17A | translocase of inner mitochondrial membrane 17A [Source:HGNC Symbol;Acc:HGNC:17315] |
| ENSG00000135324 | HGLibA_29811 | 176 | 0.602780628 | MRAP2 | melanocortin 2 receptor accessory protein 2 [Source:HGNC Symbol;Acc:HGNC:21232] |
| ENSG00000138594 | HGLibA_50600 | 176 | 0.602780628 | TMOD3 | tropomodulin 3 [Source:HGNC Symbol;Acc:HGNC:11873] |
| ENSG00000139514 | HGLibA_45347 | 176 | 0.602780628 | SLC7A1 | solute carrier family 7 member 1 [Source:HGNC Symbol;Acc:HGNC:11057] |
| ENSG00000140526 | HGLibA_00271 | 176 | 0.602780628 | ABHD2 | abhydrolase domain containing 2 [Source:HGNC Symbol;Acc:HGNC:18717] |
| ENSG00000141682 | HGLibA_37291 | 176 | 0.602780628 | PMAIP1 | phorbol-12-myristate-13-acetate-induced protein 1 [Source:HGNC Symbol;Acc:HGNC:9108] |
| ENSG00000142453 | HGLibA_07253 | 176 | 0.602780628 | CARM1 | coactivator associated arginine methyltransferase 1 [Source:HGNC Symbol;Acc:HGNC:23393] |
| ENSG00000143171 | HGLibA_42512 | 176 | 0.602780628 | RXRG | retinoid X receptor gamma [Source:HGNC Symbol;Acc:HGNC:10479] |
| ENSG00000144821 | HGLibA_30719 | 176 | 0.602780628 | MYH15 | myosin heavy chain 15 [Source:HGNC Symbol;Acc:HGNC:31073] |
| ENSG00000145864 | HGLibA_18378 | 176 | 0.602780628 | GABRB2 | gamma-aminobutyric acid type A receptor beta2 subunit [Source:HGNC Symbol;Acc:HGNC:4082] |
| ENSG00000151062 | HGLibA_06913 | 176 | 0.602780628 | CACNA2D4 | calcium voltage-gated channel auxiliary subunit alpha2delta 4 [Source:HGNC Symbol;Acc:HGNC:20202] |
| ENSG00000151806 | HGLibA_20670 | 176 | 0.602780628 | GUF1 | GTPase [Source:HGNC Symbol;Acc:HGNC:25799]" |
| ENSG00000158201 | HGLibA_00275 | 176 | 0.602780628 | ABHD3 | abhydrolase domain containing 3 [Source:HGNC Symbol;Acc:HGNC:18718] |
| ENSG00000159128 | HGLibA_22712 | 176 | 0.602780628 | IFNGR2 | interferon gamma receptor 2 [Source:HGNC Symbol;Acc:HGNC:5440] |
| ENSG00000159197 | HGLibA_24190 | 176 | 0.602780628 | KCNE2 | potassium voltage-gated channel subfamily E regulatory subunit 2 [Source:HGNC Symbol;Acc:HGNC:6242] |
| ENSG00000162711 | HGLibA_32117 | 176 | 0.602780628 | NLRP3 | NLR family pyrin domain containing 3 [Source:HGNC Symbol;Acc:HGNC:16400] |
| ENSG00000163312 | HGLibA_21116 | 176 | 0.602780628 | HELQ | POLQ like [Source:HGNC Symbol;Acc:HGNC:18536]" |
| ENSG00000163374 | HGLibA_54759 | 176 | 0.602780628 | YY1AP1 | YY1 associated protein 1 [Source:HGNC Symbol;Acc:HGNC:30935] |
| ENSG00000164978 | HGLibA_32968 | 176 | 0.602780628 | NUDT2 | nudix hydrolase 2 [Source:HGNC Symbol;Acc:HGNC:8049] |
| ENSG00000165091 | HGLibA_49748 | 176 | 0.602780628 | TMC1 | transmembrane channel like 1 [Source:HGNC Symbol;Acc:HGNC:16513] |
| ENSG00000165246 | HGLibA_32079 | 176 | 0.602780628 | NLGN4Y | neuroligin 4 Y-linked [Source:HGNC Symbol;Acc:HGNC:15529] |
| ENSG00000166106 | HGLibA_00807 | 176 | 0.602780628 | ADAMTS15 | ADAM metallopeptidase with thrombospondin type 1 motif 15 [Source:HGNC Symbol;Acc:HGNC:16305] |
| ENSG00000168291 | HGLibA_35868 | 176 | 0.602780628 | PDHB | pyruvate dehydrogenase E1 beta subunit [Source:HGNC Symbol;Acc:HGNC:8808] |
| ENSG00000169918 | HGLibA_34776 | 176 | 0.602780628 | OTUD7A | OTU deubiquitinase 7A [Source:HGNC Symbol;Acc:HGNC:20718] |
| ENSG00000169972 | HGLibA_39609 | 176 | 0.602780628 | PUSL1 | pseudouridine synthase like 1 [Source:HGNC Symbol;Acc:HGNC:26914] |
| ENSG00000170275 | HGLibA_11224 | 176 | 0.602780628 | CRTAP | cartilage associated protein [Source:HGNC Symbol;Acc:HGNC:2379] |
| ENSG00000171174 | HGLibA_40463 | 176 | 0.602780628 | RBKS | ribokinase [Source:HGNC Symbol;Acc:HGNC:30325] |
| ENSG00000171206 | HGLibA_51615 | 176 | 0.602780628 | TRIM8 | tripartite motif containing 8 [Source:HGNC Symbol;Acc:HGNC:15579] |
| ENSG00000171453 | HGLibA_37556 | 176 | 0.602780628 | POLR1C | RNA polymerase I and III subunit C [Source:HGNC Symbol;Acc:HGNC:20194] |
| ENSG00000171819 | HGLibA_01928 | 176 | 0.602780628 | ANGPTL7 | angiopoietin like 7 [Source:HGNC Symbol;Acc:HGNC:24078] |
| ENSG00000172188 | HGLibA_33937 | 176 | 0.602780628 | OR4C11 | olfactory receptor family 4 subfamily C member 11 [Source:HGNC Symbol;Acc:HGNC:15167] |
| ENSG00000173409 | HGLibA_03174 | 176 | 0.602780628 | ARV1 | fatty acid homeostasis modulator [Source:HGNC Symbol;Acc:HGNC:29561]" |
| ENSG00000174775 | HGLibA_22073 | 176 | 0.602780628 | HRAS | GTPase [Source:HGNC Symbol;Acc:HGNC:5173]" |
| ENSG00000175115 | HGLibA_34954 | 176 | 0.602780628 | PACS1 | phosphofurin acidic cluster sorting protein 1 [Source:HGNC Symbol;Acc:HGNC:30032] |
| ENSG00000175646 | HGLibA_38608 | 176 | 0.602780628 | PRM1 | protamine 1 [Source:HGNC Symbol;Acc:HGNC:9447] |
| ENSG00000176381 | HGLibA_38794 | 176 | 0.602780628 | PRR18 | proline rich 18 [Source:HGNC Symbol;Acc:HGNC:28574] |
| ENSG00000177963 | HGLibA_41236 | 176 | 0.602780628 | RIC8A | RIC8 guanine nucleotide exchange factor A [Source:HGNC Symbol;Acc:HGNC:29550] |
| ENSG00000178184 | HGLibA_35190 | 176 | 0.602780628 | PARD6G | par-6 family cell polarity regulator gamma [Source:HGNC Symbol;Acc:HGNC:16076] |
| ENSG00000178188 | HGLibA_43873 | 176 | 0.602780628 | SH2B1 | SH2B adaptor protein 1 [Source:HGNC Symbol;Acc:HGNC:30417] |
| ENSG00000178445 | HGLibA_19235 | 176 | 0.602780628 | GLDC | glycine decarboxylase [Source:HGNC Symbol;Acc:HGNC:4313] |
| ENSG00000179833 | HGLibA_43607 | 176 | 0.602780628 | SERTAD2 | SERTA domain containing 2 [Source:HGNC Symbol;Acc:HGNC:30784] |
| ENSG00000180398 | HGLibA_28585 | 176 | 0.602780628 | MCFD2 | multiple coagulation factor deficiency 2 [Source:HGNC Symbol;Acc:HGNC:18451] |
| ENSG00000181222 | HGLibA_37565 | 176 | 0.602780628 | POLR2A | RNA polymerase II subunit A [Source:HGNC Symbol;Acc:HGNC:9187] |
| ENSG00000182809 | HGLibA_11153 | 176 | 0.602780628 | CRIP2 | cysteine rich protein 2 [Source:HGNC Symbol;Acc:HGNC:2361] |
| ENSG00000184838 | HGLibA_38792 | 176 | 0.602780628 | PRR16 | proline rich 16 [Source:HGNC Symbol;Acc:HGNC:29654] |
| ENSG00000185982 | HGLibA_12888 | 176 | 0.602780628 | DEFB128 | defensin beta 128 [Source:HGNC Symbol;Acc:HGNC:18106] |
| ENSG00000186106 | HGLibA_02107 | 176 | 0.602780628 | ANKRD46 | ankyrin repeat domain 46 [Source:HGNC Symbol;Acc:HGNC:27229] |
| ENSG00000186160 | HGLibA_12205 | 176 | 0.602780628 | CYP4Z1 | cytochrome P450 family 4 subfamily Z member 1 [Source:HGNC Symbol;Acc:HGNC:20583] |
| ENSG00000186193 | HGLibA_42733 | 176 | 0.602780628 | SAPCD2 | suppressor APC domain containing 2 [Source:HGNC Symbol;Acc:HGNC:28055] |
| ENSG00000196712 | HGLibA_31758 | 176 | 0.602780628 | NF1 | neurofibromin 1 [Source:HGNC Symbol;Acc:HGNC:7765] |
| ENSG00000197579 | HGLibA_51013 | 176 | 0.602780628 | TOPORS | TOP1 binding arginine/serine rich protein [Source:HGNC Symbol;Acc:HGNC:21653] |
| ENSG00000197879 | HGLibA_30827 | 176 | 0.602780628 | MYO1C | myosin IC [Source:HGNC Symbol;Acc:HGNC:7597] |
| ENSG00000198742 | HGLibA_45845 | 176 | 0.602780628 | SMURF1 | SMAD specific E3 ubiquitin protein ligase 1 [Source:HGNC Symbol;Acc:HGNC:16807] |
| ENSG00000198912 | HGLibA_05798 | 176 | 0.602780628 | C1orf174 | chromosome 1 open reading frame 174 [Source:HGNC Symbol;Acc:HGNC:27915] |
| ENSG00000205328 | HGLibA_34396 | 176 | 0.602780628 | OR6C65 | olfactory receptor family 6 subfamily C member 65 [Source:HGNC Symbol;Acc:HGNC:31295] |
| ENSG00000205423 | HGLibA_10256 | 176 | 0.602780628 | CNEP1R1 | CTD nuclear envelope phosphatase 1 regulatory subunit 1 [Source:HGNC Symbol;Acc:HGNC:26759] |
| ENSG00000213246 | HGLibA_47817 | 176 | 0.602780628 | SUPT4H1 | DSIF elongation factor subunit [Source:HGNC Symbol;Acc:HGNC:11467]" |
| ENSG00000226650 | HGLibA_24945 | 176 | 0.602780628 | KIF4B | kinesin family member 4B [Source:HGNC Symbol;Acc:HGNC:6322] |
| ENSG00000228083 | HGLibA_22664 | 176 | 0.602780628 | IFNA14 | interferon alpha 14 [Source:HGNC Symbol;Acc:HGNC:5420] |
| ENSG00000232268 | HGLibA_34172 | 176 | 0.602780628 | OR52I1 | olfactory receptor family 52 subfamily I member 1 [Source:HGNC Symbol;Acc:HGNC:15220] |
| ENSG00000242441 | HGLibA_20524 | 176 | 0.602780628 | GTF2A1L | general transcription factor IIA subunit 1 like [Source:HGNC Symbol;Acc:HGNC:30727] |
| ENSG00000248167 | HGLibA_51485 | 176 | 0.602780628 | TRIM39-RPP21 | TRIM39-RPP21 readthrough [Source:HGNC Symbol;Acc:HGNC:38845] |
| ENSG00000254598 | HGLibA_11407 | 176 | 0.602780628 | CSNK2A3 | casein kinase 2 alpha 3 [Source:HGNC Symbol;Acc:HGNC:2458] |
| ENSG00000256045 | HGLibA_30473 | 176 | 0.602780628 | MTRNR2L10 | MT-RNR2 like 10 [Source:HGNC Symbol;Acc:HGNC:37167] |
| ENSG00000270806 | HGLibA_05540 | 176 | 0.602780628 | C17orf50 | chromosome 17 open reading frame 50 [Source:HGNC Symbol;Acc:HGNC:29581] |
| ENSG00000277149 | HGLibA_52483 | 176 | 0.602780628 | TYW1B | tRNA-yW synthesizing protein 1 homolog B [Source:HGNC Symbol;Acc:HGNC:33908] |
| FAM160A2 | HGLibA_16206 | 176 | 0.602780628 | FAM160A2 | - |
| FAM194B | HGLibA_16372 | 176 | 0.602780628 | FAM194B | - |
| hsa-mir-1294 | HGLibA_57651 | 176 | 0.602780628 | hsa-mir-1294 | - |
| hsa-mir-2053 | HGLibA_58185 | 176 | 0.602780628 | hsa-mir-2053 | - |
| hsa-mir-4283-1 | HGLibA_59837 | 176 | 0.602780628 | hsa-mir-4283-1 | - |
| hsa-mir-4757 | HGLibA_61127 | 176 | 0.602780628 | hsa-mir-4757 | - |
| hsa-mir-6818 | HGLibA_63449 | 176 | 0.602780628 | hsa-mir-6818 | - |
| hsa-mir-7155 | HGLibA_63848 | 176 | 0.602780628 | hsa-mir-7155 | - |
| C8orf46 | HGLibA_06630 | 175 | 0.592829591 | C8orf46 | - |

| | | | | | |
|---|---|---|---|---|---|
| C9orf170 | HGLibA_06707 | 175 | 0.592829591 | C9orf170 | - |
| ENSG00000006210 | HGLibA_11832 | 175 | 0.592829591 | CX3CL1 | C-X3-C motif chemokine ligand 1 [Source:HGNC Symbol;Acc:HGNC:10647] |
| ENSG00000007952 | HGLibA_32370 | 175 | 0.592829591 | NOX1 | NADPH oxidase 1 [Source:HGNC Symbol;Acc:HGNC:7889] |
| ENSG00000008394 | HGLibA_29174 | 175 | 0.592829591 | MGST1 | microsomal glutathione S-transferase 1 [Source:HGNC Symbol;Acc:HGNC:7061] |
| ENSG00000011083 | HGLibA_45336 | 175 | 0.592829591 | SLC6A7 | solute carrier family 6 member 7 [Source:HGNC Symbol;Acc:HGNC:11054] |
| ENSG00000026559 | HGLibA_24197 | 175 | 0.592829591 | KCNG1 | potassium voltage-gated channel modifier subfamily G member 1 [Source:HGNC Symbol;Acc:HGNC:6248] |
| ENSG00000065150 | HGLibA_23470 | 175 | 0.592829591 | IPO5 | importin 5 [Source:HGNC Symbol;Acc:HGNC:6402] |
| ENSG00000066923 | HGLibA_47327 | 175 | 0.592829591 | STAG3 | stromal antigen 3 [Source:HGNC Symbol;Acc:HGNC:11356] |
| ENSG00000072736 | HGLibA_31783 | 175 | 0.592829591 | NFATC3 | nuclear factor of activated T cells 3 [Source:HGNC Symbol;Acc:HGNC:7777] |
| ENSG00000075043 | HGLibA_24370 | 175 | 0.592829591 | KCNQ2 | potassium voltage-gated channel subfamily Q member 2 [Source:HGNC Symbol;Acc:HGNC:6296] |
| ENSG00000078814 | HGLibA_30736 | 175 | 0.592829591 | MYH7B | myosin heavy chain 7B [Source:HGNC Symbol;Acc:HGNC:15906] |
| ENSG00000080910 | HGLibA_09265 | 175 | 0.592829591 | CFHR2 | complement factor H related 2 [Source:HGNC Symbol;Acc:HGNC:4890] |
| ENSG00000085511 | HGLibA_28186 | 175 | 0.592829591 | MAP3K4 | mitogen-activated protein kinase kinase kinase 4 [Source:HGNC Symbol;Acc:HGNC:6856] |
| ENSG00000100784 | HGLibA_42170 | 175 | 0.592829591 | RPS6KA5 | ribosomal protein S6 kinase A5 [Source:HGNC Symbol;Acc:HGNC:10434] |
| ENSG00000100911 | HGLibA_39193 | 175 | 0.592829591 | PSME2 | proteasome activator subunit 2 [Source:HGNC Symbol;Acc:HGNC:9569] |
| ENSG00000101280 | HGLibA_01906 | 175 | 0.592829591 | ANGPT4 | angiopoietin 4 [Source:HGNC Symbol;Acc:HGNC:487] |
| ENSG00000105261 | HGLibA_34806 | 175 | 0.592829591 | OVOL3 | ovo like zinc finger 3 [Source:HGNC Symbol;Acc:HGNC:14186] |
| ENSG00000107736 | HGLibA_08669 | 175 | 0.592829591 | CDH23 | cadherin related 23 [Source:HGNC Symbol;Acc:HGNC:13733] |
| ENSG00000109103 | HGLibA_52986 | 175 | 0.592829591 | UNC119 | unc-119 lipid binding chaperone [Source:HGNC Symbol;Acc:HGNC:12565] |
| ENSG00000110944 | HGLibA_23096 | 175 | 0.592829591 | IL23A | interleukin 23 subunit alpha [Source:HGNC Symbol;Acc:HGNC:15488] |
| ENSG00000111012 | HGLibA_12096 | 175 | 0.592829591 | CYP27B1 | cytochrome P450 family 27 subfamily B member 1 [Source:HGNC Symbol;Acc:HGNC:2606] |
| ENSG00000111275 | HGLibA_01559 | 175 | 0.592829591 | ALDH2 | aldehyde dehydrogenase 2 family member [Source:HGNC Symbol;Acc:HGNC:404] |
| ENSG00000111664 | HGLibA_19495 | 175 | 0.592829591 | GNB3 | G protein subunit beta 3 [Source:HGNC Symbol;Acc:HGNC:4400] |
| ENSG00000113356 | HGLibA_37626 | 175 | 0.592829591 | POLR3G | RNA polymerase III subunit G [Source:HGNC Symbol;Acc:HGNC:30075] |
| ENSG00000115875 | HGLibA_47088 | 175 | 0.592829591 | SRSF7 | serine and arginine rich splicing factor 7 [Source:HGNC Symbol;Acc:HGNC:10789] |
| ENSG00000116489 | HGLibA_07206 | 175 | 0.592829591 | CAPZA1 | capping actin protein of muscle Z-line subunit alpha 1 [Source:HGNC Symbol;Acc:HGNC:1488] |
| ENSG00000116514 | HGLibA_41605 | 175 | 0.592829591 | RNF19B | ring finger protein 19B [Source:HGNC Symbol;Acc:HGNC:26886] |
| ENSG00000123384 | HGLibA_27198 | 175 | 0.592829591 | LRP1 | LDL receptor related protein 1 [Source:HGNC Symbol;Acc:HGNC:6692] |
| ENSG00000124812 | HGLibA_11164 | 175 | 0.592829591 | CRISP1 | cysteine rich secretory protein 1 [Source:HGNC Symbol;Acc:HGNC:304] |
| ENSG00000124920 | HGLibA_30915 | 175 | 0.592829591 | MYRF | myelin regulatory factor [Source:HGNC Symbol;Acc:HGNC:1181] |
| ENSG00000125846 | HGLibA_55466 | 175 | 0.592829591 | ZNF133 | zinc finger protein 133 [Source:HGNC Symbol;Acc:HGNC:12917] |
| ENSG00000126001 | HGLibA_09127 | 175 | 0.592829591 | CEP250 | centrosomal protein 250 [Source:HGNC Symbol;Acc:HGNC:1859] |
| ENSG00000126233 | HGLibA_45554 | 175 | 0.592829591 | SLURP1 | secreted LY6/PLAUR domain containing 1 [Source:HGNC Symbol;Acc:HGNC:18746] |
| ENSG00000126952 | HGLibA_33138 | 175 | 0.592829591 | NXF5 | nuclear RNA export factor 5 [Source:HGNC Symbol;Acc:HGNC:8075] |
| ENSG00000128185 | HGLibA_13051 | 175 | 0.592829591 | DGCR6L | DiGeorge syndrome critical region gene 6 like [Source:HGNC Symbol;Acc:HGNC:18551] |
| ENSG00000129535 | HGLibA_32702 | 175 | 0.592829591 | NRL | neural retina leucine zipper [Source:HGNC Symbol;Acc:HGNC:8002] |
| ENSG00000130948 | HGLibA_22190 | 175 | 0.592829591 | HSD17B3 | hydroxysteroid 17-beta dehydrogenase 3 [Source:HGNC Symbol;Acc:HGNC:5212] |
| ENSG00000132185 | HGLibA_17243 | 175 | 0.592829591 | FCRLA | Fc receptor like A [Source:HGNC Symbol;Acc:HGNC:18504] |
| ENSG00000136881 | HGLibA_04054 | 175 | 0.592829591 | BAAT | bile acid-CoA:amino acid N-acyltransferase [Source:HGNC Symbol;Acc:HGNC:932] |
| ENSG00000137218 | HGLibA_18049 | 175 | 0.592829591 | FRS3 | fibroblast growth factor receptor substrate 3 [Source:HGNC Symbol;Acc:HGNC:16970] |
| ENSG00000138668 | HGLibA_21813 | 175 | 0.592829591 | HNRNPD | heterogeneous nuclear ribonucleoprotein D [Source:HGNC Symbol;Acc:HGNC:5036] |
| ENSG00000138798 | HGLibA_14622 | 175 | 0.592829591 | EGF | epidermal growth factor [Source:HGNC Symbol;Acc:HGNC:3229] |
| ENSG00000140274 | HGLibA_14107 | 175 | 0.592829591 | DUOXA2 | dual oxidase maturation factor 2 [Source:HGNC Symbol;Acc:HGNC:32698] |
| ENSG00000141579 | HGLibA_56675 | 175 | 0.592829591 | ZNF750 | zinc finger protein 750 [Source:HGNC Symbol;Acc:HGNC:25843] |
| ENSG00000141753 | HGLibA_22825 | 175 | 0.592829591 | IGFBP4 | insulin like growth factor binding protein 4 [Source:HGNC Symbol;Acc:HGNC:5473] |
| ENSG00000144029 | HGLibA_30103 | 175 | 0.592829591 | MRPS5 | mitochondrial ribosomal protein S5 [Source:HGNC Symbol;Acc:HGNC:14498] |
| ENSG00000144233 | HGLibA_01805 | 175 | 0.592829591 | AMMECR1L | AMMECR1 like [Source:HGNC Symbol;Acc:HGNC:28658] |
| ENSG00000144655 | HGLibA_11422 | 175 | 0.592829591 | CSRNP1 | cysteine and serine rich nuclear protein 1 [Source:HGNC Symbol;Acc:HGNC:14300] |
| ENSG00000147381 | HGLibA_27914 | 175 | 0.592829591 | MAGEA4 | MAGE family member A4 [Source:HGNC Symbol;Acc:HGNC:6802] |
| ENSG00000148459 | HGLibA_35948 | 175 | 0.592829591 | PDSS1 | decaprenyl diphosphate synthase subunit 1 [Source:HGNC Symbol;Acc:HGNC:17759] |
| ENSG00000150867 | HGLibA_36705 | 175 | 0.592829591 | PIP4K2A | phosphatidylinositol-5-phosphate 4-kinase type 2 alpha [Source:HGNC Symbol;Acc:HGNC:8997] |
| ENSG00000151348 | HGLibA_15804 | 175 | 0.592829591 | EXT2 | exostosin glycosyltransferase 2 [Source:HGNC Symbol;Acc:HGNC:3513] |
| ENSG00000153253 | HGLibA_42986 | 175 | 0.592829591 | SCN3A | sodium voltage-gated channel alpha subunit 3 [Source:HGNC Symbol;Acc:HGNC:10590] |
| ENSG00000156150 | HGLibA_01736 | 175 | 0.592829591 | ALX3 | ALX homeobox 3 [Source:HGNC Symbol;Acc:HGNC:449] |
| ENSG00000160445 | HGLibA_55142 | 175 | 0.592829591 | ZER1 | zyg-11 related cell cycle regulator [Source:HGNC Symbol;Acc:HGNC:30960] |
| ENSG00000162076 | HGLibA_17684 | 175 | 0.592829591 | FLYWCH2 | FLYWCH family member 2 [Source:HGNC Symbol;Acc:HGNC:25178] |
| ENSG00000162302 | HGLibA_42168 | 175 | 0.592829591 | RPS6KA4 | ribosomal protein S6 kinase A4 [Source:HGNC Symbol;Acc:HGNC:10433] |
| ENSG00000162949 | HGLibA_07155 | 175 | 0.592829591 | CAPN13 | calpain 13 [Source:HGNC Symbol;Acc:HGNC:16663] |
| ENSG00000165030 | HGLibA_31808 | 175 | 0.592829591 | NFIL3 | interleukin 3 regulated [Source:HGNC Symbol;Acc:HGNC:7787]" |
| ENSG00000165424 | HGLibA_55031 | 175 | 0.592829591 | ZCCHC24 | zinc finger CCHC-type containing 24 [Source:HGNC Symbol;Acc:HGNC:26911] |
| ENSG00000166262 | HGLibA_16531 | 175 | 0.592829591 | FAM227B | family with sequence similarity 227 member B [Source:HGNC Symbol;Acc:HGNC:26543] |
| ENSG00000167964 | HGLibA_39854 | 175 | 0.592829591 | RAB26 | member RAS oncogene family [Source:HGNC Symbol;Acc:HGNC:14259]" |
| ENSG00000169813 | HGLibA_21819 | 175 | 0.592829591 | HNRNPF | heterogeneous nuclear ribonucleoprotein F [Source:HGNC Symbol;Acc:HGNC:5039] |
| ENSG00000171467 | HGLibA_55787 | 175 | 0.592829591 | ZNF318 | zinc finger protein 318 [Source:HGNC Symbol;Acc:HGNC:13578] |
| ENSG00000172320 | HGLibA_34228 | 175 | 0.592829591 | OR5A1 | olfactory receptor family 5 subfamily A member 1 [Source:HGNC Symbol;Acc:HGNC:8319] |
| ENSG00000172497 | HGLibA_00473 | 175 | 0.592829591 | ACOT12 | acyl-CoA thioesterase 12 [Source:HGNC Symbol;Acc:HGNC:24436] |
| ENSG00000173083 | HGLibA_22060 | 175 | 0.592829591 | HPSE | heparanase [Source:HGNC Symbol;Acc:HGNC:5164] |
| ENSG00000173153 | HGLibA_15593 | 175 | 0.592829591 | ESRRA | estrogen related receptor alpha [Source:HGNC Symbol;Acc:HGNC:3471] |
| ENSG00000174099 | HGLibA_30246 | 175 | 0.592829591 | MSRB3 | methionine sulfoxide reductase B3 [Source:HGNC Symbol;Acc:HGNC:27375] |
| ENSG00000178795 | HGLibA_18900 | 175 | 0.592829591 | GDPD4 | glycerophosphodiester phosphodiesterase domain containing 4 [Source:HGNC Symbol;Acc:HGNC:24849] |
| ENSG00000185122 | HGLibA_22221 | 175 | 0.592829591 | HSF1 | heat shock transcription factor 1 [Source:HGNC Symbol;Acc:HGNC:5224] |
| ENSG00000185344 | HGLibA_03707 | 175 | 0.592829591 | ATP6V0A2 | ATPase H+ transporting V0 subunit a2 [Source:HGNC Symbol;Acc:HGNC:18481] |
| ENSG00000186191 | HGLibA_04728 | 175 | 0.592829591 | BPIFB4 | BPI fold containing family B member 4 [Source:HGNC Symbol;Acc:HGNC:16179] |
| ENSG00000187258 | HGLibA_32527 | 175 | 0.592829591 | NPSR1 | neuropeptide S receptor 1 [Source:HGNC Symbol;Acc:HGNC:23631] |
| ENSG00000188747 | HGLibA_32381 | 175 | 0.592829591 | NOXA1 | NADPH oxidase activator 1 [Source:HGNC Symbol;Acc:HGNC:10668] |
| ENSG00000196460 | HGLibA_40983 | 175 | 0.592829591 | RFX8 | lacking RFX DNA binding domain [Source:HGNC Symbol;Acc:HGNC:37253]" |
| ENSG00000197045 | HGLibA_19404 | 175 | 0.592829591 | GMFB | glia maturation factor beta [Source:HGNC Symbol;Acc:HGNC:4373] |
| ENSG00000198246 | HGLibA_44846 | 175 | 0.592829591 | SLC29A3 | solute carrier family 29 member 3 [Source:HGNC Symbol;Acc:HGNC:23096] |
| ENSG00000198252 | HGLibA_47688 | 175 | 0.592829591 | STYX | serine/threonine/tyrosine interacting protein [Source:HGNC Symbol;Acc:HGNC:11447] |
| ENSG00000198704 | HGLibA_20186 | 175 | 0.592829591 | GPX6 | glutathione peroxidase 6 [Source:HGNC Symbol;Acc:HGNC:4558] |
| ENSG00000198846 | HGLibA_51035 | 175 | 0.592829591 | TOX | thymocyte selection associated high mobility group box [Source:HGNC Symbol;Acc:HGNC:18988] |
| ENSG00000205358 | HGLibA_30291 | 175 | 0.592829591 | MT1H | metallothionein 1H [Source:HGNC Symbol;Acc:HGNC:7400] |
| ENSG00000232810 | HGLibA_50702 | 175 | 0.592829591 | TNF | tumor necrosis factor [Source:HGNC Symbol;Acc:HGNC:11892] |
| ENSG00000239998 | HGLibA_26493 | 175 | 0.592829591 | LILRA2 | leukocyte immunoglobulin like receptor A2 [Source:HGNC Symbol;Acc:HGNC:6603] |
| ENSG00000243725 | HGLibA_52141 | 175 | 0.592829591 | TTC4 | tetratricopeptide repeat domain 4 [Source:HGNC Symbol;Acc:HGNC:12394] |
| ENSG00000250254 | HGLibA_39567 | 175 | 0.592829591 | PTTG2 | pituitary tumor-transforming 2 [Source:HGNC Symbol;Acc:HGNC:9691] |
| ENSG00000264364 | HGLibA_14256 | 175 | 0.592829591 | DYNLL2 | dynein light chain LC8-type 2 [Source:HGNC Symbol;Acc:HGNC:24596] |
| ENSG00000268182 | HGLibA_45737 | 175 | 0.592829591 | SMIM17 | small integral membrane protein 17 [Source:HGNC Symbol;Acc:HGNC:27114] |
| ENSG00000273820 | HGLibA_53318 | 175 | 0.592829591 | USP27X | ubiquitin specific peptidase 27 X-linked [Source:HGNC Symbol;Acc:HGNC:13486] |
| ENSG00000277399 | HGLibA_19979 | 175 | 0.592829591 | GPR179 | G protein-coupled receptor 179 [Source:HGNC Symbol;Acc:HGNC:31371] |
| FAM122C | HGLibA_16073 | 175 | 0.592829591 | FAM122C | - |
| hsa-mir-1254-1 | HGLibA_57413 | 175 | 0.592829591 | hsa-mir-1254-1 | - |
| hsa-mir-1287 | HGLibA_57612 | 175 | 0.592829591 | hsa-mir-1287 | - |
| hsa-mir-4314 | HGLibA_59973 | 175 | 0.592829591 | hsa-mir-4314 | - |
| hsa-mir-4529 | HGLibA_60577 | 175 | 0.592829591 | hsa-mir-4529 | - |
| hsa-mir-486-2 | HGLibA_61339 | 175 | 0.592829591 | hsa-mir-486-2 | - |
| hsa-mir-579 | HGLibA_62414 | 175 | 0.592829591 | hsa-mir-579 | - |
| hsa-mir-6124 | HGLibA_62644 | 175 | 0.592829591 | hsa-mir-6124 | - |
| hsa-mir-6877 | HGLibA_63692 | 175 | 0.592829591 | hsa-mir-6877 | - |
| hsa-mir-758 | HGLibA_63891 | 175 | 0.592829591 | hsa-mir-758 | - |
| LOC100287177 | HGLibA_26818 | 175 | 0.592829591 | LOC100287177 | - |
| NEURL | HGLibA_31721 | 175 | 0.592829591 | NEURL | - |
| PPAP2B | HGLibA_37818 | 175 | 0.592829591 | PPAP2B | - |
| PRSS42 | HGLibA_38940 | 175 | 0.592829591 | PRSS42 | - |
| WTH3DI | HGLibA_54434 | 175 | 0.592829591 | WTH3DI | - |
| ALPPL2 | HGLibA_01714 | 174 | 0.582878553 | ALPPL2 | - |
| ALS2CR12 | HGLibA_01728 | 174 | 0.582878553 | ALS2CR12 | - |
| C19orf24 | HGLibA_05638 | 174 | 0.582878553 | C19orf24 | - |
| C1orf168 | HGLibA_05790 | 174 | 0.582878553 | C1orf168 | - |
| C20orf78 | HGLibA_06027 | 174 | 0.582878553 | C20orf78 | - |
| CCDC111 | HGLibA_07542 | 174 | 0.582878553 | CCDC111 | - |
| ENSG00000005206 | HGLibA_46777 | 174 | 0.582878553 | SPPL2B | signal peptide peptidase like 2B [Source:HGNC Symbol;Acc:HGNC:30627] |
| ENSG00000034713 | HGLibA_18339 | 174 | 0.582878553 | GABARAPL2 | GABA type A receptor associated protein like 2 [Source:HGNC Symbol;Acc:HGNC:13291] |
| ENSG00000066933 | HGLibA_30869 | 174 | 0.582878553 | MYO9A | myosin IXA [Source:HGNC Symbol;Acc:HGNC:7608] |
| ENSG00000072133 | HGLibA_42173 | 174 | 0.582878553 | RPS6KA6 | ribosomal protein S6 kinase A6 [Source:HGNC Symbol;Acc:HGNC:10435] |
| ENSG00000072858 | HGLibA_44115 | 174 | 0.582878553 | SIDT1 | SID1 transmembrane family member 1 [Source:HGNC Symbol;Acc:HGNC:25967] |
| ENSG00000073331 | HGLibA_01699 | 174 | 0.582878553 | ALPK1 | alpha kinase 1 [Source:HGNC Symbol;Acc:HGNC:20917] |
| ENSG00000073921 | HGLibA_36521 | 174 | 0.582878553 | PICALM | phosphatidylinositol binding clathrin assembly protein [Source:HGNC Symbol;Acc:HGNC:15514] |
| ENSG00000089248 | HGLibA_15528 | 174 | 0.582878553 | ERP29 | endoplasmic reticulum protein 29 [Source:HGNC Symbol;Acc:HGNC:13799] |
| ENSG00000092421 | HGLibA_43365 | 174 | 0.582878553 | SEMA6A | semaphorin 6A [Source:HGNC Symbol;Acc:HGNC:10738] |
| ENSG00000100503 | HGLibA_31934 | 174 | 0.582878553 | NIN | ninein [Source:HGNC Symbol;Acc:HGNC:14906] |
| ENSG00000100591 | HGLibA_01312 | 174 | 0.582878553 | AHSA1 | activator of HSP90 ATPase activity 1 [Source:HGNC Symbol;Acc:HGNC:1189] |
| ENSG00000101162 | HGLibA_52272 | 174 | 0.582878553 | TUBB1 | tubulin beta 1 class VI [Source:HGNC Symbol;Acc:HGNC:16257] |
| ENSG00000102554 | HGLibA_25075 | 174 | 0.582878553 | KLF5 | Kruppel like factor 5 [Source:HGNC Symbol;Acc:HGNC:6349] |
| ENSG00000102996 | HGLibA_29494 | 174 | 0.582878553 | MMP15 | matrix metallopeptidase 15 [Source:HGNC Symbol;Acc:HGNC:7161] |
| ENSG00000103355 | HGLibA_38926 | 174 | 0.582878553 | PRSS33 | serine protease 33 [Source:HGNC Symbol;Acc:HGNC:30405] |
| ENSG00000106682 | HGLibA_14848 | 174 | 0.582878553 | EIF4H | eukaryotic translation initiation factor 4H [Source:HGNC Symbol;Acc:HGNC:12741] |
| ENSG00000106689 | HGLibA_26450 | 174 | 0.582878553 | LHX2 | LIM homeobox 2 [Source:HGNC Symbol;Acc:HGNC:6594] |
| ENSG00000107742 | HGLibA_46751 | 174 | 0.582878553 | SPOCK2 | cwcv and kazal like domains proteoglycan 2 [Source:HGNC Symbol;Acc:HGNC:13564]" |
| ENSG00000107902 | HGLibA_26445 | 174 | 0.582878553 | LHPP | phospholysine phosphohistidine inorganic pyrophosphate phosphatase [Source:HGNC Symbol;Acc:HGNC:30042] |
| ENSG00000108448 | HGLibA_51420 | 174 | 0.582878553 | TRIM16L | tripartite motif containing 16 like [Source:HGNC Symbol;Acc:HGNC:32670] |
| ENSG00000111046 | HGLibA_30700 | 174 | 0.582878553 | MYF6 | myogenic factor 6 [Source:HGNC Symbol;Acc:HGNC:7566] |
| ENSG00000116396 | HGLibA_24172 | 174 | 0.582878553 | KCNC4 | potassium voltage-gated channel subfamily C member 4 [Source:HGNC Symbol;Acc:HGNC:6236] |
| ENSG00000119402 | HGLibA_17144 | 174 | 0.582878553 | FBXW2 | F-box and WD repeat domain containing 2 [Source:HGNC Symbol;Acc:HGNC:13608] |
| ENSG00000120708 | HGLibA_49220 | 174 | 0.582878553 | TGFBI | transforming growth factor beta induced [Source:HGNC Symbol;Acc:HGNC:11771] |

| | | | | | |
|---|---|---|---|---|---|
| ENSG00000123472 | HGLibA_03798 | 174 | 0.582878553 | ATPAF1 | ATP synthase mitochondrial F1 complex assembly factor 1 [Source:HGNC Symbol;Acc:HGNC:18803] |
| ENSG00000123737 | HGLibA_15795 | 174 | 0.582878553 | EXOSC9 | exosome component 9 [Source:HGNC Symbol;Acc:HGNC:9137] |
| ENSG00000124074 | HGLibA_15134 | 174 | 0.582878553 | ENKD1 | enkurin domain containing 1 [Source:HGNC Symbol;Acc:HGNC:25246] |
| ENSG00000126247 | HGLibA_07184 | 174 | 0.582878553 | CAPNS1 | calpain small subunit 1 [Source:HGNC Symbol;Acc:HGNC:1481] |
| ENSG00000130513 | HGLibA_18861 | 174 | 0.582878553 | GDF15 | growth differentiation factor 15 [Source:HGNC Symbol;Acc:HGNC:30142] |
| ENSG00000132170 | HGLibA_37844 | 174 | 0.582878553 | PPARG | peroxisome proliferator activated receptor gamma [Source:HGNC Symbol;Acc:HGNC:9236] |
| ENSG00000133105 | HGLibA_42497 | 174 | 0.582878553 | RXFP2 | relaxin family peptide receptor 2 [Source:HGNC Symbol;Acc:HGNC:17318] |
| ENSG00000134207 | HGLibA_48085 | 174 | 0.582878553 | SYT6 | synaptotagmin 6 [Source:HGNC Symbol;Acc:HGNC:18638] |
| ENSG00000134248 | HGLibA_26001 | 174 | 0.582878553 | LAMTOR5 | MAPK and MTOR activator 5 [Source:HGNC Symbol;Acc:HGNC:17955]" |
| ENSG00000135045 | HGLibA_06728 | 174 | 0.582878553 | C9orf40 | chromosome 9 open reading frame 40 [Source:HGNC Symbol;Acc:HGNC:23433] |
| ENSG00000135838 | HGLibA_32481 | 174 | 0.582878553 | NPL | N-acetylneuraminate pyruvate lyase [Source:HGNC Symbol;Acc:HGNC:16781] |
| ENSG00000136643 | HGLibA_42183 | 174 | 0.582878553 | RPS6KC1 | ribosomal protein S6 kinase C1 [Source:HGNC Symbol;Acc:HGNC:10439] |
| ENSG00000141349 | HGLibA_18312 | 174 | 0.582878553 | G6PC3 | glucose-6-phosphatase catalytic subunit 3 [Source:HGNC Symbol;Acc:HGNC:24861] |
| ENSG00000141425 | HGLibA_42045 | 174 | 0.582878553 | RPRD1A | regulation of nuclear pre-mRNA domain containing 1A [Source:HGNC Symbol;Acc:HGNC:25560] |
| ENSG00000143344 | HGLibA_41002 | 174 | 0.582878553 | RGL1 | ral guanine nucleotide dissociation stimulator like 1 [Source:HGNC Symbol;Acc:HGNC:30281] |
| ENSG00000143387 | HGLibA_11713 | 174 | 0.582878553 | CTSK | cathepsin K [Source:HGNC Symbol;Acc:HGNC:2536] |
| ENSG00000144031 | HGLibA_02119 | 174 | 0.582878553 | ANKRD53 | ankyrin repeat domain 53 [Source:HGNC Symbol;Acc:HGNC:25691] |
| ENSG00000144233 | HGLibA_01806 | 174 | 0.582878553 | AMMECR1L | AMMECR1 like [Source:HGNC Symbol;Acc:HGNC:28658] |
| ENSG00000144674 | HGLibA_19602 | 174 | 0.582878553 | GOLGA4 | golgin A4 [Source:HGNC Symbol;Acc:HGNC:4427] |
| ENSG00000144891 | HGLibA_01262 | 174 | 0.582878553 | AGTR1 | angiotensin II receptor type 1 [Source:HGNC Symbol;Acc:HGNC:336] |
| ENSG00000146674 | HGLibA_22822 | 174 | 0.582878553 | IGFBP3 | insulin like growth factor binding protein 3 [Source:HGNC Symbol;Acc:HGNC:5472] |
| ENSG00000147140 | HGLibA_32298 | 174 | 0.582878553 | NONO | non-POU domain containing octamer binding [Source:HGNC Symbol;Acc:HGNC:7871] |
| ENSG00000148225 | HGLibA_54064 | 174 | 0.582878553 | WDR31 | WD repeat domain 31 [Source:HGNC Symbol;Acc:HGNC:21421] |
| ENSG00000149256 | HGLibA_49004 | 174 | 0.582878553 | TENM4 | teneurin transmembrane protein 4 [Source:HGNC Symbol;Acc:HGNC:29945] |
| ENSG00000153208 | HGLibA_28906 | 174 | 0.582878553 | MERTK | tyrosine kinase [Source:HGNC Symbol;Acc:HGNC:7027]" |
| ENSG00000154162 | HGLibA_08638 | 174 | 0.582878553 | CDH12 | cadherin 12 [Source:HGNC Symbol;Acc:HGNC:1751] |
| ENSG00000158955 | HGLibA_54398 | 174 | 0.582878553 | WNT9B | Wnt family member 9B [Source:HGNC Symbol;Acc:HGNC:12779] |
| ENSG00000160789 | HGLibA_26694 | 174 | 0.582878553 | LMNA | lamin A/C [Source:HGNC Symbol;Acc:HGNC:6636] |
| ENSG00000163106 | HGLibA_22035 | 174 | 0.582878553 | HPGDS | hematopoietic prostaglandin D synthase [Source:HGNC Symbol;Acc:HGNC:17890] |
| ENSG00000164038 | HGLibA_45436 | 174 | 0.582878553 | SLC9B2 | solute carrier family 9 member B2 [Source:HGNC Symbol;Acc:HGNC:25143] |
| ENSG00000167588 | HGLibA_19782 | 174 | 0.582878553 | GPD1 | glycerol-3-phosphate dehydrogenase 1 [Source:HGNC Symbol;Acc:HGNC:4455] |
| ENSG00000167799 | HGLibA_32992 | 174 | 0.582878553 | NUDT8 | nudix hydrolase 8 [Source:HGNC Symbol;Acc:HGNC:8055] |
| ENSG00000168237 | HGLibA_19381 | 174 | 0.582878553 | GLYCTK | glycerate kinase [Source:HGNC Symbol;Acc:HGNC:24247] |
| ENSG00000168807 | HGLibA_46002 | 174 | 0.582878553 | SNTB2 | syntrophin beta 2 [Source:HGNC Symbol;Acc:HGNC:11169] |
| ENSG00000172456 | HGLibA_17459 | 174 | 0.582878553 | FGGY | FGGY carbohydrate kinase domain containing [Source:HGNC Symbol;Acc:HGNC:25610] |
| ENSG00000172728 | HGLibA_18181 | 174 | 0.582878553 | FUT10 | fucosyltransferase 10 [Source:HGNC Symbol;Acc:HGNC:19234] |
| ENSG00000172954 | HGLibA_26157 | 174 | 0.582878553 | LCLAT1 | lysocardiolipin acyltransferase 1 [Source:HGNC Symbol;Acc:HGNC:26756] |
| ENSG00000174325 | HGLibA_13265 | 174 | 0.582878553 | DIRC1 | disrupted in renal carcinoma 1 [Source:HGNC Symbol;Acc:HGNC:15760] |
| ENSG00000178821 | HGLibA_50433 | 174 | 0.582878553 | TMEM52 | transmembrane protein 52 [Source:HGNC Symbol;Acc:HGNC:27916] |
| ENSG00000180611 | HGLibA_28459 | 174 | 0.582878553 | MB21D2 | Mab-21 domain containing 2 [Source:HGNC Symbol;Acc:HGNC:30438] |
| ENSG00000180730 | HGLibA_44032 | 174 | 0.582878553 | SHISA2 | shisa family member 2 [Source:HGNC Symbol;Acc:HGNC:20366] |
| ENSG00000180818 | HGLibA_21955 | 174 | 0.582878553 | HOXC10 | homeobox C10 [Source:HGNC Symbol;Acc:HGNC:5122] |
| ENSG00000181408 | HGLibA_53468 | 174 | 0.582878553 | UTS2R | urotensin 2 receptor [Source:HGNC Symbol;Acc:HGNC:4468] |
| ENSG00000183309 | HGLibA_56412 | 174 | 0.582878553 | ZNF623 | zinc finger protein 623 [Source:HGNC Symbol;Acc:HGNC:29084] |
| ENSG00000184110 | HGLibA_14767 | 174 | 0.582878553 | EIF3C | eukaryotic translation initiation factor 3 subunit C [Source:HGNC Symbol;Acc:HGNC:3279] |
| ENSG00000185294 | HGLibA_46780 | 174 | 0.582878553 | SPPL2C | signal peptide peptidase like 2C [Source:HGNC Symbol;Acc:HGNC:28902] |
| ENSG00000186090 | HGLibA_22389 | 174 | 0.582878553 | HTR3D | 5-hydroxytryptamine receptor 3D [Source:HGNC Symbol;Acc:HGNC:24004] |
| ENSG00000196652 | HGLibA_55356 | 174 | 0.582878553 | ZKSCAN5 | zinc finger with KRAB and SCAN domains 5 [Source:HGNC Symbol;Acc:HGNC:12867] |
| ENSG00000196811 | HGLibA_09606 | 174 | 0.582878553 | CHRNG | cholinergic receptor nicotinic gamma subunit [Source:HGNC Symbol;Acc:HGNC:1967] |
| ENSG00000198728 | HGLibA_26210 | 174 | 0.582878553 | LDB1 | LIM domain binding 1 [Source:HGNC Symbol;Acc:HGNC:6532] |
| ENSG00000198889 | HGLibA_12422 | 174 | 0.582878553 | DCAF12L1 | DDB1 and CUL4 associated factor 12 like 1 [Source:HGNC Symbol;Acc:HGNC:29395] |
| ENSG00000204414 | HGLibA_11357 | 174 | 0.582878553 | CSHL1 | chorionic somatomammotropin hormone like 1 [Source:HGNC Symbol;Acc:HGNC:2442] |
| ENSG00000204531 | HGLibA_37785 | 174 | 0.582878553 | POU5F1 | POU class 5 homeobox 1 [Source:HGNC Symbol;Acc:HGNC:9221] |
| ENSG00000204548 | HGLibA_12869 | 174 | 0.582878553 | DEFB121 | defensin beta 121 [Source:HGNC Symbol;Acc:HGNC:18101] |
| ENSG00000205155 | HGLibA_39031 | 174 | 0.582878553 | PSENEN | gamma-secretase subunit [Source:HGNC Symbol;Acc:HGNC:30100]" |
| ENSG00000221988 | HGLibA_38236 | 174 | 0.582878553 | PPT2 | palmitoyl-protein thioesterase 2 [Source:HGNC Symbol;Acc:HGNC:9326] |
| ENSG00000223731 | HGLibA_47807 | 174 | 0.582878553 | SUPT20HL1 | SUPT20H like 1 [Source:HGNC Symbol;Acc:HGNC:30773] |
| ENSG00000229676 | HGLibA_56095 | 174 | 0.582878553 | ZNF492 | zinc finger protein 492 [Source:HGNC Symbol;Acc:HGNC:23707] |
| ENSG00000230246 | HGLibA_46489 | 174 | 0.582878553 | SPATA31C1 | SPATA31 subfamily C member 1 [Source:HGNC Symbol;Acc:HGNC:27846] |
| ENSG00000285723 | HGLibA_29331 | 174 | 0.582878553 | MKKS | novel protein |
| HDHD1 | HGLibA_21050 | 174 | 0.582878553 | HDHD1 | - |
| HIST1H2AB | HGLibA_21378 | 174 | 0.582878553 | HIST1H2AB | - |
| hsa-mir-3150b | HGLibA_58658 | 174 | 0.582878553 | hsa-mir-3150b | - |
| hsa-mir-320b-1 | HGLibA_58929 | 174 | 0.582878553 | hsa-mir-320b-1 | - |
| hsa-mir-3677 | HGLibA_59258 | 174 | 0.582878553 | hsa-mir-3677 | - |
| hsa-mir-4493 | HGLibA_60391 | 174 | 0.582878553 | hsa-mir-4493 | - |
| hsa-mir-4701 | HGLibA_60913 | 174 | 0.582878553 | hsa-mir-4701 | - |
| hsa-mir-532 | HGLibA_61805 | 174 | 0.582878553 | hsa-mir-532 | - |
| hsa-mir-5581 | HGLibA_62165 | 174 | 0.582878553 | hsa-mir-5581 | - |
| hsa-mir-6785 | HGLibA_63317 | 174 | 0.582878553 | hsa-mir-6785 | - |
| hsa-mir-6875 | HGLibA_63683 | 174 | 0.582878553 | hsa-mir-6875 | - |
| hsa-mir-6880 | HGLibA_63706 | 174 | 0.582878553 | hsa-mir-6880 | - |
| LPPR1 | HGLibA_27113 | 174 | 0.582878553 | LPPR1 | - |
| NDNL2 | HGLibA_31405 | 174 | 0.582878553 | NDNL2 | - |
| C1orf234 | HGLibA_05848 | 173 | 0.572927516 | C1orf234 | - |
| ENSG00000003147 | HGLibA_22487 | 173 | 0.572927516 | ICA1 | islet cell autoantigen 1 [Source:HGNC Symbol;Acc:HGNC:5343] |
| ENSG00000067066 | HGLibA_46286 | 173 | 0.572927516 | SP100 | SP100 nuclear antigen [Source:HGNC Symbol;Acc:HGNC:11206] |
| ENSG00000068985 | HGLibA_35005 | 173 | 0.572927516 | PAGE1 | PAGE family member 1 [Source:HGNC Symbol;Acc:HGNC:4107] |
| ENSG00000072182 | HGLibA_03309 | 173 | 0.572927516 | ASIC4 | acid sensing ion channel subunit family member 4 [Source:HGNC Symbol;Acc:HGNC:21263] |
| ENSG00000075429 | HGLibA_06940 | 173 | 0.572927516 | CACNG5 | calcium voltage-gated channel auxiliary subunit gamma 5 [Source:HGNC Symbol;Acc:HGNC:1409] |
| ENSG00000080503 | HGLibA_45605 | 173 | 0.572927516 | SMARCA2 | member 2 [Source:HGNC Symbol;Acc:HGNC:11098]" |
| ENSG00000081041 | HGLibA_11867 | 173 | 0.572927516 | CXCL2 | C-X-C motif chemokine ligand 2 [Source:HGNC Symbol;Acc:HGNC:4603] |
| ENSG00000090447 | HGLibA_49136 | 173 | 0.572927516 | TFAP4 | transcription factor AP-4 [Source:HGNC Symbol;Acc:HGNC:11745] |
| ENSG00000090470 | HGLibA_35744 | 173 | 0.572927516 | PDCD7 | programmed cell death 7 [Source:HGNC Symbol;Acc:HGNC:8767] |
| ENSG00000099337 | HGLibA_24329 | 173 | 0.572927516 | KCNK6 | potassium two pore domain channel subfamily K member 6 [Source:HGNC Symbol;Acc:HGNC:6281] |
| ENSG00000101224 | HGLibA_08531 | 173 | 0.572927516 | CDC25B | cell division cycle 25B [Source:HGNC Symbol;Acc:HGNC:1726] |
| ENSG00000101447 | HGLibA_16750 | 173 | 0.572927516 | FAM83D | family with sequence similarity 83 member D [Source:HGNC Symbol;Acc:HGNC:16122] |
| ENSG00000101596 | HGLibA_45661 | 173 | 0.572927516 | SMCHD1 | structural maintenance of chromosomes flexible hinge domain containing 1 [Source:HGNC Symbol;Acc:HGNC:29090] |
| ENSG00000101911 | HGLibA_38760 | 173 | 0.572927516 | PRPS2 | phosphoribosyl pyrophosphate synthetase 2 [Source:HGNC Symbol;Acc:HGNC:9465] |
| ENSG00000101938 | HGLibA_09539 | 173 | 0.572927516 | CHRDL1 | chordin like 1 [Source:HGNC Symbol;Acc:HGNC:29861] |
| ENSG00000105711 | HGLibA_42976 | 173 | 0.572927516 | SCN1B | sodium voltage-gated channel beta subunit 1 [Source:HGNC Symbol;Acc:HGNC:10586] |
| ENSG00000110436 | HGLibA_44509 | 173 | 0.572927516 | SLC1A2 | solute carrier family 1 member 2 [Source:HGNC Symbol;Acc:HGNC:10940] |
| ENSG00000111725 | HGLibA_38496 | 173 | 0.572927516 | PRKAB1 | protein kinase AMP-activated non-catalytic subunit beta 1 [Source:HGNC Symbol;Acc:HGNC:9378] |
| ENSG00000111729 | HGLibA_09978 | 173 | 0.572927516 | CLEC4A | C-type lectin domain family 4 member A [Source:HGNC Symbol;Acc:HGNC:13257] |
| ENSG00000111785 | HGLibA_41239 | 173 | 0.572927516 | RIC8B | RIC8 guanine nucleotide exchange factor B [Source:HGNC Symbol;Acc:HGNC:25555] |
| ENSG00000111801 | HGLibA_04973 | 173 | 0.572927516 | BTN3A3 | butyrophilin subfamily 3 member A3 [Source:HGNC Symbol;Acc:HGNC:1140] |
| ENSG00000112305 | HGLibA_45595 | 173 | 0.572927516 | SMAP1 | small ArfGAP 1 [Source:HGNC Symbol;Acc:HGNC:19651] |
| ENSG00000119457 | HGLibA_45186 | 173 | 0.572927516 | SLC46A2 | solute carrier family 46 member 2 [Source:HGNC Symbol;Acc:HGNC:16055] |
| ENSG00000122299 | HGLibA_54983 | 173 | 0.572927516 | ZC3H7A | zinc finger CCCH-type containing 7A [Source:HGNC Symbol;Acc:HGNC:30959] |
| ENSG00000123200 | HGLibA_54960 | 173 | 0.572927516 | ZC3H13 | zinc finger CCCH-type containing 13 [Source:HGNC Symbol;Acc:HGNC:20368] |
| ENSG00000124212 | HGLibA_39350 | 173 | 0.572927516 | PTGIS | prostaglandin I2 synthase [Source:HGNC Symbol;Acc:HGNC:9603] |
| ENSG00000124449 | HGLibA_23609 | 173 | 0.572927516 | IRGC | immunity related GTPase cinema [Source:HGNC Symbol;Acc:HGNC:28835] |
| ENSG00000125457 | HGLibA_29263 | 173 | 0.572927516 | MIF4GD | MIF4G domain containing [Source:HGNC Symbol;Acc:HGNC:24030] |
| ENSG00000125869 | HGLibA_25986 | 173 | 0.572927516 | LAMP5 | lysosomal associated membrane protein family member 5 [Source:HGNC Symbol;Acc:HGNC:16097] |
| ENSG00000127527 | HGLibA_15372 | 173 | 0.572927516 | EPS15L1 | epidermal growth factor receptor pathway substrate 15 like 1 [Source:HGNC Symbol;Acc:HGNC:24634] |
| ENSG00000127528 | HGLibA_25067 | 173 | 0.572927516 | KLF2 | Kruppel like factor 2 [Source:HGNC Symbol;Acc:HGNC:6347] |
| ENSG00000127980 | HGLibA_36078 | 173 | 0.572927516 | PEX1 | peroxisomal biogenesis factor 1 [Source:HGNC Symbol;Acc:HGNC:8850] |
| ENSG00000128165 | HGLibA_00995 | 173 | 0.572927516 | ADM2 | adrenomedullin 2 [Source:HGNC Symbol;Acc:HGNC:28898] |
| ENSG00000130950 | HGLibA_33108 | 173 | 0.572927516 | NUTM2F | NUT family member 2F [Source:HGNC Symbol;Acc:HGNC:23450] |
| ENSG00000132305 | HGLibA_23236 | 173 | 0.572927516 | IMMT | inner membrane mitochondrial protein [Source:HGNC Symbol;Acc:HGNC:6047] |
| ENSG00000133874 | HGLibA_41487 | 173 | 0.572927516 | RNF122 | ring finger protein 122 [Source:HGNC Symbol;Acc:HGNC:21147] |
| ENSG00000136834 | HGLibA_33675 | 173 | 0.572927516 | OR1J1 | olfactory receptor family 1 subfamily J member 1 [Source:HGNC Symbol;Acc:HGNC:8208] |
| ENSG00000137845 | HGLibA_00727 | 173 | 0.572927516 | ADAM10 | ADAM metallopeptidase domain 10 [Source:HGNC Symbol;Acc:HGNC:188] |
| ENSG00000138669 | HGLibA_38580 | 173 | 0.572927516 | PRKG2 | protein kinase cGMP-dependent 2 [Source:HGNC Symbol;Acc:HGNC:9416] |
| ENSG00000138821 | HGLibA_45125 | 173 | 0.572927516 | SLC39A8 | solute carrier family 39 member 8 [Source:HGNC Symbol;Acc:HGNC:20862] |
| ENSG00000142227 | HGLibA_15080 | 173 | 0.572927516 | EMP3 | epithelial membrane protein 3 [Source:HGNC Symbol;Acc:HGNC:3335] |
| ENSG00000142546 | HGLibA_32334 | 173 | 0.572927516 | NOSIP | nitric oxide synthase interacting protein [Source:HGNC Symbol;Acc:HGNC:17946] |
| ENSG00000143093 | HGLibA_47589 | 173 | 0.572927516 | STRIP1 | striatin interacting protein 1 [Source:HGNC Symbol;Acc:HGNC:25916] |
| ENSG00000144535 | HGLibA_13276 | 173 | 0.572927516 | DIS3L2 | DIS3 like 3'-5' exoribonuclease 2 [Source:HGNC Symbol;Acc:HGNC:28648] |
| ENSG00000148795 | HGLibA_12061 | 173 | 0.572927516 | CYP17A1 | cytochrome P450 family 17 subfamily A member 1 [Source:HGNC Symbol;Acc:HGNC:2593] |
| ENSG00000152591 | HGLibA_14041 | 173 | 0.572927516 | DSPP | dentin sialophosphoprotein [Source:HGNC Symbol;Acc:HGNC:3054] |
| ENSG00000156313 | HGLibA_41834 | 173 | 0.572927516 | RPGR | retinitis pigmentosa GTPase regulator [Source:HGNC Symbol;Acc:HGNC:10295] |
| ENSG00000158711 | HGLibA_14917 | 173 | 0.572927516 | ELK4 | ETS transcription factor [Source:HGNC Symbol;Acc:HGNC:3326]" |
| ENSG00000160404 | HGLibA_51028 | 173 | 0.572927516 | TOR2A | torsin family 2 member A [Source:HGNC Symbol;Acc:HGNC:11996] |
| ENSG00000163520 | HGLibA_16947 | 173 | 0.572927516 | FBLN2 | fibulin 2 [Source:HGNC Symbol;Acc:HGNC:3601] |
| ENSG00000165555 | HGLibA_32387 | 173 | 0.572927516 | NOXRED1 | NADP dependent oxidoreductase domain containing 1 [Source:HGNC Symbol;Acc:HGNC:20487] |
| ENSG00000166169 | HGLibA_37537 | 173 | 0.572927516 | POLL | DNA polymerase lambda [Source:HGNC Symbol;Acc:HGNC:9184] |
| ENSG00000166961 | HGLibA_30143 | 173 | 0.572927516 | MS4A15 | membrane spanning 4-domains A15 [Source:HGNC Symbol;Acc:HGNC:28573] |
| ENSG00000168993 | HGLibA_10932 | 173 | 0.572927516 | CPLX1 | complexin 1 [Source:HGNC Symbol;Acc:HGNC:2309] |
| ENSG00000169047 | HGLibA_23620 | 173 | 0.572927516 | IRS1 | insulin receptor substrate 1 [Source:HGNC Symbol;Acc:HGNC:6125] |
| ENSG00000169469 | HGLibA_46806 | 173 | 0.572927516 | SPRR1B | small proline rich protein 1B [Source:HGNC Symbol;Acc:HGNC:11260] |
| ENSG00000170011 | HGLibA_30918 | 173 | 0.572927516 | MYRIP | myosin VIIA and Rab interacting protein [Source:HGNC Symbol;Acc:HGNC:19156] |
| ENSG00000170231 | HGLibA_15926 | 173 | 0.572927516 | FABP6 | fatty acid binding protein 6 [Source:HGNC Symbol;Acc:HGNC:3561] |
| ENSG00000170426 | HGLibA_43169 | 173 | 0.572927516 | SDR9C7 | short chain dehydrogenase/reductase family 9C member 7 [Source:HGNC Symbol;Acc:HGNC:29958] |
| ENSG00000170959 | HGLibA_12480 | 173 | 0.572927516 | DCDC1 | doublecortin domain containing 1 [Source:HGNC Symbol;Acc:HGNC:20625] |

| | | | | | |
|---|---|---|---|---|---|
| ENSG00000171109 | HGLibA_29069 | 173 | 0.572927516 | MFN1 | mitofusin 1 [Source:HGNC Symbol;Acc:HGNC:18262] |
| ENSG00000171310 | HGLibA_09615 | 173 | 0.572927516 | CHST11 | carbohydrate sulfotransferase 11 [Source:HGNC Symbol;Acc:HGNC:17422] |
| ENSG00000171522 | HGLibA_39322 | 173 | 0.572927516 | PTGER4 | prostaglandin E receptor 4 [Source:HGNC Symbol;Acc:HGNC:9596] |
| ENSG00000172006 | HGLibA_56222 | 173 | 0.572927516 | ZNF554 | zinc finger protein 554 [Source:HGNC Symbol;Acc:HGNC:26629] |
| ENSG00000175274 | HGLibA_51060 | 173 | 0.572927516 | TP53I11 | tumor protein p53 inducible protein 11 [Source:HGNC Symbol;Acc:HGNC:16842] |
| ENSG00000176422 | HGLibA_46853 | 173 | 0.572927516 | SPRYD4 | SPRY domain containing 4 [Source:HGNC Symbol;Acc:HGNC:27468] |
| ENSG00000176909 | HGLibA_28056 | 173 | 0.572927516 | MAMSTR | MEF2 activating motif and SAP domain containing transcriptional regulator [Source:HGNC Symbol;Acc:HGNC:26689] |
| ENSG00000177047 | HGLibA_22733 | 173 | 0.572927516 | IFNW1 | interferon omega 1 [Source:HGNC Symbol;Acc:HGNC:5448] |
| ENSG00000179088 | HGLibA_05240 | 173 | 0.572927516 | C12orf42 | chromosome 12 open reading frame 42 [Source:HGNC Symbol;Acc:HGNC:24729] |
| ENSG00000179832 | HGLibA_29866 | 173 | 0.572927516 | MROH1 | maestro heat like repeat family member 1 [Source:HGNC Symbol;Acc:HGNC:26958] |
| ENSG00000180155 | HGLibA_27709 | 173 | 0.572927516 | LYNX1 | Ly6/neurotoxin 1 [Source:HGNC Symbol;Acc:HGNC:29604] |
| ENSG00000181031 | HGLibA_41848 | 173 | 0.572927516 | RPH3AL | rabphilin 3A like (without C2 domains) [Source:HGNC Symbol;Acc:HGNC:10296] |
| ENSG00000181826 | HGLibA_40817 | 173 | 0.572927516 | RELL1 | RELT like 1 [Source:HGNC Symbol;Acc:HGNC:27379] |
| ENSG00000183454 | HGLibA_20300 | 173 | 0.572927516 | GRIN2A | glutamate ionotropic receptor NMDA type subunit 2A [Source:HGNC Symbol;Acc:HGNC:4585] |
| ENSG00000184924 | HGLibA_39556 | 173 | 0.572927516 | PTRHD1 | peptidyl-tRNA hydrolase domain containing 1 [Source:HGNC Symbol;Acc:HGNC:33782] |
| ENSG00000185652 | HGLibA_32837 | 173 | 0.572927516 | NTF3 | neurotrophin 3 [Source:HGNC Symbol;Acc:HGNC:8023] |
| ENSG00000186318 | HGLibA_04058 | 173 | 0.572927516 | BACE1 | beta-secretase 1 [Source:HGNC Symbol;Acc:HGNC:933] |
| ENSG00000187545 | HGLibA_38281 | 173 | 0.572927516 | PRAMEF10 | PRAME family member 10 [Source:HGNC Symbol;Acc:HGNC:27997] |
| ENSG00000188389 | HGLibA_35714 | 173 | 0.572927516 | PDCD1 | programmed cell death 1 [Source:HGNC Symbol;Acc:HGNC:8760] |
| ENSG00000188816 | HGLibA_21762 | 173 | 0.572927516 | HMX2 | H6 family homeobox 2 [Source:HGNC Symbol;Acc:HGNC:5018] |
| ENSG00000188959 | HGLibA_06688 | 173 | 0.572927516 | C9orf152 | chromosome 9 open reading frame 152 [Source:HGNC Symbol;Acc:HGNC:31455] |
| ENSG00000189079 | HGLibA_02887 | 173 | 0.572927516 | ARID2 | AT-rich interaction domain 2 [Source:HGNC Symbol;Acc:HGNC:18037] |
| ENSG00000189157 | HGLibA_16617 | 173 | 0.572927516 | FAM47E | family with sequence similarity 47 member E [Source:HGNC Symbol;Acc:HGNC:34343] |
| ENSG00000196917 | HGLibA_20945 | 173 | 0.572927516 | HCAR1 | hydroxycarboxylic acid receptor 1 [Source:HGNC Symbol;Acc:HGNC:4532] |
| ENSG00000197532 | HGLibA_34454 | 173 | 0.572927516 | OR6Y1 | olfactory receptor family 6 subfamily Y member 1 [Source:HGNC Symbol;Acc:HGNC:14823] |
| ENSG00000197696 | HGLibA_32142 | 173 | 0.572927516 | NMB | neuromedin B [Source:HGNC Symbol;Acc:HGNC:7842] |
| ENSG00000198681 | HGLibA_27890 | 173 | 0.572927516 | MAGEA1 | MAGE family member A1 [Source:HGNC Symbol;Acc:HGNC:6796] |
| ENSG00000198719 | HGLibA_13360 | 173 | 0.572927516 | DLL1 | delta like canonical Notch ligand 1 [Source:HGNC Symbol;Acc:HGNC:2908] |
| ENSG00000198797 | HGLibA_04815 | 173 | 0.572927516 | BRINP2 | BMP/retinoic acid inducible neural specific 2 [Source:HGNC Symbol;Acc:HGNC:13746] |
| ENSG00000198883 | HGLibA_37376 | 173 | 0.572927516 | PNMA5 | PNMA family member 5 [Source:HGNC Symbol;Acc:HGNC:18743] |
| ENSG00000204516 | HGLibA_29218 | 173 | 0.572927516 | MICB | MHC class I polypeptide-related sequence B [Source:HGNC Symbol;Acc:HGNC:7091] |
| ENSG00000213901 | HGLibA_44611 | 173 | 0.572927516 | SLC23A3 | solute carrier family 23 member 3 [Source:HGNC Symbol;Acc:HGNC:20601] |
| ENSG00000214413 | HGLibA_04199 | 173 | 0.572927516 | BBIP1 | BBSome interacting protein 1 [Source:HGNC Symbol;Acc:HGNC:28093] |
| ENSG00000214686 | HGLibA_23513 | 173 | 0.572927516 | IQCF6 | IQ motif containing F6 [Source:HGNC Symbol;Acc:HGNC:35158] |
| ENSG00000245680 | HGLibA_56324 | 173 | 0.572927516 | ZNF585B | zinc finger protein 585B [Source:HGNC Symbol;Acc:HGNC:30948] |
| ENSG00000255833 | HGLibA_49459 | 173 | 0.572927516 | TIFAB | TIFA inhibitor [Source:HGNC Symbol;Acc:HGNC:34024] |
| ENSG00000275835 | HGLibA_52317 | 173 | 0.572927516 | TUBGCP5 | tubulin gamma complex associated protein 5 [Source:HGNC Symbol;Acc:HGNC:18600] |
| FAM155B | HGLibA_16182 | 173 | 0.572927516 | FAM155B | - |
| FAM159A | HGLibA_16199 | 173 | 0.572927516 | FAM159A | - |
| hsa-mir-1306 | HGLibA_57740 | 173 | 0.572927516 | hsa-mir-1306 | - |
| hsa-mir-3121 | HGLibA_58538 | 173 | 0.572927516 | hsa-mir-3121 | - |
| hsa-mir-3155b | HGLibA_58682 | 173 | 0.572927516 | hsa-mir-3155b | - |
| hsa-mir-3165 | HGLibA_58741 | 173 | 0.572927516 | hsa-mir-3165 | - |
| hsa-mir-3182 | HGLibA_58827 | 173 | 0.572927516 | hsa-mir-3182 | - |
| hsa-mir-4277 | HGLibA_59812 | 173 | 0.572927516 | hsa-mir-4277 | - |
| hsa-mir-5004 | HGLibA_61425 | 173 | 0.572927516 | hsa-mir-5004 | - |
| hsa-mir-548ai | HGLibA_61884 | 173 | 0.572927516 | hsa-mir-548ai | - |
| hsa-mir-6726 | HGLibA_63059 | 173 | 0.572927516 | hsa-mir-6726 | - |
| MTERF | HGLibA_30337 | 173 | 0.572927516 | MTERF | - |
| TCEB3 | HGLibA_48739 | 173 | 0.572927516 | TCEB3 | - |
| TMEM194B | HGLibA_50155 | 173 | 0.572927516 | TMEM194B | - |
| C7orf49 | HGLibA_06549 | 172 | 0.562976478 | C7orf49 | - |
| ENSG00000007384 | HGLibA_41134 | 172 | 0.562976478 | RHBDF1 | rhomboid 5 homolog 1 [Source:HGNC Symbol;Acc:HGNC:20561] |
| ENSG00000013297 | HGLibA_09854 | 172 | 0.562976478 | CLDN11 | claudin 11 [Source:HGNC Symbol;Acc:HGNC:8514] |
| ENSG00000029639 | HGLibA_49138 | 172 | 0.562976478 | TFB1M | mitochondrial [Source:HGNC Symbol;Acc:HGNC:17037]" |
| ENSG00000054654 | HGLibA_47979 | 172 | 0.562976478 | SYNE2 | spectrin repeat containing nuclear envelope protein 2 [Source:HGNC Symbol;Acc:HGNC:17084] |
| ENSG00000066827 | HGLibA_55165 | 172 | 0.562976478 | ZFAT | zinc finger and AT-hook domain containing [Source:HGNC Symbol;Acc:HGNC:19899] |
| ENSG00000069011 | HGLibA_36761 | 172 | 0.562976478 | PITX1 | paired like homeodomain 1 [Source:HGNC Symbol;Acc:HGNC:9004] |
| ENSG00000073050 | HGLibA_54582 | 172 | 0.562976478 | XRCC1 | X-ray repair cross complementing 1 [Source:HGNC Symbol;Acc:HGNC:12828] |
| ENSG00000075568 | HGLibA_49929 | 172 | 0.562976478 | TMEM131 | transmembrane protein 131 [Source:HGNC Symbol;Acc:HGNC:30366] |
| ENSG00000078053 | HGLibA_01832 | 172 | 0.562976478 | AMPH | amphiphysin [Source:HGNC Symbol;Acc:HGNC:471] |
| ENSG00000081248 | HGLibA_06901 | 172 | 0.562976478 | CACNA1S | calcium voltage-gated channel subunit alpha1 S [Source:HGNC Symbol;Acc:HGNC:1397] |
| ENSG00000095794 | HGLibA_11131 | 172 | 0.562976478 | CREM | cAMP responsive element modulator [Source:HGNC Symbol;Acc:HGNC:2352] |
| ENSG00000100156 | HGLibA_44454 | 172 | 0.562976478 | SLC16A8 | solute carrier family 16 member 8 [Source:HGNC Symbol;Acc:HGNC:16270] |
| ENSG00000100505 | HGLibA_51619 | 172 | 0.562976478 | TRIM9 | tripartite motif containing 9 [Source:HGNC Symbol;Acc:HGNC:16288] |
| ENSG00000100522 | HGLibA_19570 | 172 | 0.562976478 | GNPNAT1 | glucosamine-phosphate N-acetyltransferase 1 [Source:HGNC Symbol;Acc:HGNC:19980] |
| ENSG00000101298 | HGLibA_45936 | 172 | 0.562976478 | SNPH | syntaphilin [Source:HGNC Symbol;Acc:HGNC:15931] |
| ENSG00000102057 | HGLibA_24173 | 172 | 0.562976478 | KCND1 | potassium voltage-gated channel subfamily D member 1 [Source:HGNC Symbol;Acc:HGNC:6237] |
| ENSG00000103381 | HGLibA_10984 | 172 | 0.562976478 | CPPED1 | calcineurin like phosphoesterase domain containing 1 [Source:HGNC Symbol;Acc:HGNC:25632] |
| ENSG00000104946 | HGLibA_48487 | 172 | 0.562976478 | TBC1D17 | TBC1 domain family member 17 [Source:HGNC Symbol;Acc:HGNC:25699] |
| ENSG00000106829 | HGLibA_49617 | 172 | 0.562976478 | TLE4 | transducin like enhancer of split 4 [Source:HGNC Symbol;Acc:HGNC:11840] |
| ENSG00000110195 | HGLibA_17783 | 172 | 0.562976478 | FOLR1 | folate receptor 1 [Source:HGNC Symbol;Acc:HGNC:3791] |
| ENSG00000111215 | HGLibA_38840 | 172 | 0.562976478 | PRR4 | proline rich 4 [Source:HGNC Symbol;Acc:HGNC:18020] |
| ENSG00000111801 | HGLibA_04974 | 172 | 0.562976478 | BTN3A3 | butyrophilin subfamily 3 member A3 [Source:HGNC Symbol;Acc:HGNC:1140] |
| ENSG00000112038 | HGLibA_33453 | 172 | 0.562976478 | OPRM1 | opioid receptor mu 1 [Source:HGNC Symbol;Acc:HGNC:8156] |
| ENSG00000114999 | HGLibA_52183 | 172 | 0.562976478 | TTL | tubulin tyrosine ligase [Source:HGNC Symbol;Acc:HGNC:21586] |
| ENSG00000115993 | HGLibA_51279 | 172 | 0.562976478 | TRAK2 | trafficking kinesin protein 2 [Source:HGNC Symbol;Acc:HGNC:13206] |
| ENSG00000119318 | HGLibA_40071 | 172 | 0.562976478 | RAD23B | nucleotide excision repair protein [Source:HGNC Symbol;Acc:HGNC:9813]" |
| ENSG00000119938 | HGLibA_38103 | 172 | 0.562976478 | PPP1R3C | protein phosphatase 1 regulatory subunit 3C [Source:HGNC Symbol;Acc:HGNC:9293] |
| ENSG00000120616 | HGLibA_15267 | 172 | 0.562976478 | EPC1 | enhancer of polycomb homolog 1 [Source:HGNC Symbol;Acc:HGNC:19876] |
| ENSG00000121552 | HGLibA_11478 | 172 | 0.562976478 | CSTA | cystatin A [Source:HGNC Symbol;Acc:HGNC:2481] |
| ENSG00000122367 | HGLibA_26217 | 172 | 0.562976478 | LDB3 | LIM domain binding 3 [Source:HGNC Symbol;Acc:HGNC:15710] |
| ENSG00000122873 | HGLibA_09726 | 172 | 0.562976478 | CISD1 | CDGSH iron sulfur domain 1 [Source:HGNC Symbol;Acc:HGNC:30880] |
| ENSG00000125492 | HGLibA_04158 | 172 | 0.562976478 | BARHL1 | BarH like homeobox 1 [Source:HGNC Symbol;Acc:HGNC:953] |
| ENSG00000125998 | HGLibA_16746 | 172 | 0.562976478 | FAM83C | family with sequence similarity 83 member C [Source:HGNC Symbol;Acc:HGNC:16121] |
| ENSG00000128340 | HGLibA_40046 | 172 | 0.562976478 | RAC2 | Rac family small GTPase 2 [Source:HGNC Symbol;Acc:HGNC:9802] |
| ENSG00000128652 | HGLibA_21997 | 172 | 0.562976478 | HOXD3 | homeobox D3 [Source:HGNC Symbol;Acc:HGNC:5137] |
| ENSG00000130656 | HGLibA_20942 | 172 | 0.562976478 | HBZ | hemoglobin subunit zeta [Source:HGNC Symbol;Acc:HGNC:4835] |
| ENSG00000131018 | HGLibA_47975 | 172 | 0.562976478 | SYNE1 | spectrin repeat containing nuclear envelope protein 1 [Source:HGNC Symbol;Acc:HGNC:17089] |
| ENSG00000134193 | HGLibA_40805 | 172 | 0.562976478 | REG4 | regenerating family member 4 [Source:HGNC Symbol;Acc:HGNC:22977] |
| ENSG00000135709 | HGLibA_24640 | 172 | 0.562976478 | KIAA0513 | KIAA0513 [Source:HGNC Symbol;Acc:HGNC:29058] |
| ENSG00000137198 | HGLibA_19427 | 172 | 0.562976478 | GMPR | guanosine monophosphate reductase [Source:HGNC Symbol;Acc:HGNC:4376] |
| ENSG00000137501 | HGLibA_48098 | 172 | 0.562976478 | SYTL2 | synaptotagmin like 2 [Source:HGNC Symbol;Acc:HGNC:15585] |
| ENSG00000139651 | HGLibA_56663 | 172 | 0.562976478 | ZNF740 | zinc finger protein 740 [Source:HGNC Symbol;Acc:HGNC:27465] |
| ENSG00000140443 | HGLibA_22795 | 172 | 0.562976478 | IGF1R | insulin like growth factor 1 receptor [Source:HGNC Symbol;Acc:HGNC:5465] |
| ENSG00000140932 | HGLibA_10213 | 172 | 0.562976478 | CMTM2 | CKLF like MARVEL transmembrane domain containing 2 [Source:HGNC Symbol;Acc:HGNC:19173] |
| ENSG00000140948 | HGLibA_55014 | 172 | 0.562976478 | ZCCHC14 | zinc finger CCHC-type containing 14 [Source:HGNC Symbol;Acc:HGNC:24134] |
| ENSG00000141295 | HGLibA_43051 | 172 | 0.562976478 | SCRN2 | secernin 2 [Source:HGNC Symbol;Acc:HGNC:30381] |
| ENSG00000143067 | HGLibA_56564 | 172 | 0.562976478 | ZNF697 | zinc finger protein 697 [Source:HGNC Symbol;Acc:HGNC:32034] |
| ENSG00000151093 | HGLibA_34835 | 172 | 0.562976478 | OXSM | mitochondrial [Source:HGNC Symbol;Acc:HGNC:26063]" |
| ENSG00000152256 | HGLibA_35895 | 172 | 0.562976478 | PDK1 | pyruvate dehydrogenase kinase 1 [Source:HGNC Symbol;Acc:HGNC:8809] |
| ENSG00000154478 | HGLibA_20007 | 172 | 0.562976478 | GPR26 | G protein-coupled receptor 26 [Source:HGNC Symbol;Acc:HGNC:4481] |
| ENSG00000154589 | HGLibA_27693 | 172 | 0.562976478 | LY96 | lymphocyte antigen 96 [Source:HGNC Symbol;Acc:HGNC:17156] |
| ENSG00000156313 | HGLibA_41835 | 172 | 0.562976478 | RPGR | retinitis pigmentosa GTPase regulator [Source:HGNC Symbol;Acc:HGNC:10295] |
| ENSG00000158747 | HGLibA_31215 | 172 | 0.562976478 | NBL1 | DAN family BMP antagonist [Source:HGNC Symbol;Acc:HGNC:7650]" |
| ENSG00000161509 | HGLibA_20305 | 172 | 0.562976478 | GRIN2C | glutamate ionotropic receptor NMDA type subunit 2C [Source:HGNC Symbol;Acc:HGNC:4587] |
| ENSG00000161544 | HGLibA_12036 | 172 | 0.562976478 | CYGB | cytoglobin [Source:HGNC Symbol;Acc:HGNC:16505] |
| ENSG00000162144 | HGLibA_11980 | 172 | 0.562976478 | CYB561A3 | cytochrome b561 family member A3 [Source:HGNC Symbol;Acc:HGNC:23014] |
| ENSG00000162595 | HGLibA_13262 | 172 | 0.562976478 | DIRAS3 | DIRAS family GTPase 3 [Source:HGNC Symbol;Acc:HGNC:687] |
| ENSG00000163216 | HGLibA_46814 | 172 | 0.562976478 | SPRR2D | small proline rich protein 2D [Source:HGNC Symbol;Acc:HGNC:11264] |
| ENSG00000163576 | HGLibA_14567 | 172 | 0.562976478 | EFHB | EF-hand domain family member B [Source:HGNC Symbol;Acc:HGNC:26330] |
| ENSG00000163811 | HGLibA_54090 | 172 | 0.562976478 | WDR43 | WD repeat domain 43 [Source:HGNC Symbol;Acc:HGNC:28945] |
| ENSG00000164330 | HGLibA_14349 | 172 | 0.562976478 | EBF1 | EBF transcription factor 1 [Source:HGNC Symbol;Acc:HGNC:3126] |
| ENSG00000164647 | HGLibA_47421 | 172 | 0.562976478 | STEAP1 | STEAP family member 1 [Source:HGNC Symbol;Acc:HGNC:11378] |
| ENSG00000164830 | HGLibA_34832 | 172 | 0.562976478 | OXR1 | oxidation resistance 1 [Source:HGNC Symbol;Acc:HGNC:15822] |
| ENSG00000165059 | HGLibA_38509 | 172 | 0.562976478 | PRKACG | protein kinase cAMP-activated catalytic subunit gamma [Source:HGNC Symbol;Acc:HGNC:9382] |
| ENSG00000165643 | HGLibA_46161 | 172 | 0.562976478 | SOHLH1 | spermatogenesis and oogenesis specific basic helix-loop-helix 1 [Source:HGNC Symbol;Acc:HGNC:27845] |
| ENSG00000166260 | HGLibA_10806 | 172 | 0.562976478 | COX11 | cytochrome c oxidase copper chaperone COX11 [Source:HGNC Symbol;Acc:HGNC:2261] |
| ENSG00000167554 | HGLibA_56381 | 172 | 0.562976478 | ZNF610 | zinc finger protein 610 [Source:HGNC Symbol;Acc:HGNC:26687] |
| ENSG00000168356 | HGLibA_42971 | 172 | 0.562976478 | SCN11A | sodium voltage-gated channel alpha subunit 11 [Source:HGNC Symbol;Acc:HGNC:10583] |
| ENSG00000169727 | HGLibA_20149 | 172 | 0.562976478 | GPS1 | G protein pathway suppressor 1 [Source:HGNC Symbol;Acc:HGNC:4549] |
| ENSG00000169914 | HGLibA_34763 | 172 | 0.562976478 | OTUD3 | OTU deubiquitinase 3 [Source:HGNC Symbol;Acc:HGNC:29038] |
| ENSG00000171501 | HGLibA_33708 | 172 | 0.562976478 | OR1N2 | olfactory receptor family 1 subfamily N member 2 [Source:HGNC Symbol;Acc:HGNC:15111] |
| ENSG00000171723 | HGLibA_19803 | 172 | 0.562976478 | GPHN | gephyrin [Source:HGNC Symbol;Acc:HGNC:15465] |
| ENSG00000172586 | HGLibA_09359 | 172 | 0.562976478 | CHCHD1 | coiled-coil-helix-coiled-coil-helix domain containing 1 [Source:HGNC Symbol;Acc:HGNC:23518] |
| ENSG00000174371 | HGLibA_15725 | 172 | 0.562976478 | EXO1 | exonuclease 1 [Source:HGNC Symbol;Acc:HGNC:3511] |
| ENSG00000175305 | HGLibA_08088 | 172 | 0.562976478 | CCNE2 | cyclin E2 [Source:HGNC Symbol;Acc:HGNC:1590] |
| ENSG00000175485 | HGLibA_34209 | 172 | 0.562976478 | OR52W1 | olfactory receptor family 52 subfamily W member 1 [Source:HGNC Symbol;Acc:HGNC:15239] |
| ENSG00000175535 | HGLibA_37355 | 172 | 0.562976478 | PNLIP | pancreatic lipase [Source:HGNC Symbol;Acc:HGNC:9155] |
| ENSG00000177548 | HGLibA_40003 | 172 | 0.562976478 | RABEP2 | RAB GTPase binding effector protein 2 [Source:HGNC Symbol;Acc:HGNC:24817]" |
| ENSG00000177674 | HGLibA_01269 | 172 | 0.562976478 | AGTRAP | angiotensin II receptor associated protein [Source:HGNC Symbol;Acc:HGNC:13539] |
| ENSG00000178038 | HGLibA_01721 | 172 | 0.562976478 | ALS2CL | ALS2 C-terminal like [Source:HGNC Symbol;Acc:HGNC:20605] |
| ENSG00000180089 | HGLibA_50529 | 172 | 0.562976478 | TMEM86B | transmembrane protein 86B [Source:HGNC Symbol;Acc:HGNC:28448] |
| ENSG00000182255 | HGLibA_24136 | 172 | 0.562976478 | KCNA4 | potassium voltage-gated channel subfamily A member 4 [Source:HGNC Symbol;Acc:HGNC:6222] |
| ENSG00000183206 | HGLibA_37723 | 172 | 0.562976478 | POTEC | POTE ankyrin domain family member C [Source:HGNC Symbol;Acc:HGNC:33894] |

| | | | | | |
|---|---|---|---|---|---|
| ENSG00000183287 | HGLibA_07494 | 172 | 0.562976478 | CCBE1 | collagen and calcium binding EGF domains 1 [Source:HGNC Symbol;Acc:HGNC:29426] |
| ENSG00000183780 | HGLibA_45007 | 172 | 0.562976478 | SLC35F3 | solute carrier family 35 member F3 [Source:HGNC Symbol;Acc:HGNC:23616] |
| ENSG00000185900 | HGLibA_37664 | 172 | 0.562976478 | POMK | protein-O-mannose kinase [Source:HGNC Symbol;Acc:HGNC:26267] |
| ENSG00000186908 | HGLibA_55088 | 172 | 0.562976478 | ZDHHC17 | zinc finger DHHC-type containing 17 [Source:HGNC Symbol;Acc:HGNC:18412] |
| ENSG00000188283 | HGLibA_55892 | 172 | 0.562976478 | ZNF383 | zinc finger protein 383 [Source:HGNC Symbol;Acc:HGNC:18609] |
| ENSG00000189227 | HGLibA_05428 | 172 | 0.562976478 | C15orf61 | chromosome 15 open reading frame 61 [Source:HGNC Symbol;Acc:HGNC:34453] |
| ENSG00000196782 | HGLibA_28048 | 172 | 0.562976478 | MAML3 | mastermind like transcriptional coactivator 3 [Source:HGNC Symbol;Acc:HGNC:16272] |
| ENSG00000196998 | HGLibA_54096 | 172 | 0.562976478 | WDR45 | WD repeat domain 45 [Source:HGNC Symbol;Acc:HGNC:28912] |
| ENSG00000197566 | HGLibA_56416 | 172 | 0.562976478 | ZNF624 | zinc finger protein 624 [Source:HGNC Symbol;Acc:HGNC:29254] |
| ENSG00000198018 | HGLibA_15215 | 172 | 0.562976478 | ENTPD7 | ectonucleoside triphosphate diphosphohydrolase 7 [Source:HGNC Symbol;Acc:HGNC:19745] |
| ENSG00000198173 | HGLibA_16615 | 172 | 0.562976478 | FAM47C | family with sequence similarity 47 member C [Source:HGNC Symbol;Acc:HGNC:25301] |
| ENSG00000203705 | HGLibA_48446 | 172 | 0.562976478 | TATDN3 | TatD DNase domain containing 3 [Source:HGNC Symbol;Acc:HGNC:27010] |
| ENSG00000205413 | HGLibA_42699 | 172 | 0.562976478 | SAMD9 | sterile alpha motif domain containing 9 [Source:HGNC Symbol;Acc:HGNC:1348] |
| ENSG00000213762 | HGLibA_55467 | 172 | 0.562976478 | ZNF134 | zinc finger protein 134 [Source:HGNC Symbol;Acc:HGNC:12918] |
| ENSG00000215695 | HGLibA_42308 | 172 | 0.562976478 | RSC1A1 | regulator of solute carriers 1 [Source:HGNC Symbol;Acc:HGNC:10458] |
| ENSG00000222047 | HGLibA_05079 | 172 | 0.562976478 | C10orf55 | chromosome 10 open reading frame 55 [Source:HGNC Symbol;Acc:HGNC:31008] |
| ENSG00000227051 | HGLibA_05324 | 172 | 0.562976478 | C14orf132 | chromosome 14 open reading frame 132 [Source:HGNC Symbol;Acc:HGNC:20346] |
| hsa-mir-1249 | HGLibA_57395 | 172 | 0.562976478 | hsa-mir-1249 | - |
| hsa-mir-2117 | HGLibA_58240 | 172 | 0.562976478 | hsa-mir-2117 | - |
| hsa-mir-3129 | HGLibA_58567 | 172 | 0.562976478 | hsa-mir-3129 | - |
| hsa-mir-4531 | HGLibA_60582 | 172 | 0.562976478 | hsa-mir-4531 | - |
| hsa-mir-6824 | HGLibA_63473 | 172 | 0.562976478 | hsa-mir-6824 | - |
| hsa-mir-7159 | HGLibA_63863 | 172 | 0.562976478 | hsa-mir-7159 | - |
| hsa-mir-7849 | HGLibA_63985 | 172 | 0.562976478 | hsa-mir-7849 | - |
| hsa-mir-7849 | HGLibA_63987 | 172 | 0.562976478 | hsa-mir-7849 | - |
| hsa-mir-8074 | HGLibA_64137 | 172 | 0.562976478 | hsa-mir-8074 | - |
| PQLC2 | HGLibA_38253 | 172 | 0.562976478 | PQLC2 | - |
| ATP5J2-PTCD1 | HGLibA_03676 | 171 | 0.553025441 | ATP5J2-PTCD1 | - |
| ENSG00000007255 | HGLibA_51330 | 171 | 0.553025441 | TRAPPC6A | trafficking protein particle complex 6A [Source:HGNC Symbol;Acc:HGNC:23069] |
| ENSG00000023892 | HGLibA_12786 | 171 | 0.553025441 | DEF6 | guanine nucleotide exchange factor [Source:HGNC Symbol;Acc:HGNC:2760]" |
| ENSG00000027697 | HGLibA_22709 | 171 | 0.553025441 | IFNGR1 | interferon gamma receptor 1 [Source:HGNC Symbol;Acc:HGNC:5439] |
| ENSG00000057757 | HGLibA_36737 | 171 | 0.553025441 | PITHD1 | PITH domain containing 1 [Source:HGNC Symbol;Acc:HGNC:25022] |
| ENSG00000067840 | HGLibA_35980 | 171 | 0.553025441 | PDZD4 | PDZ domain containing 4 [Source:HGNC Symbol;Acc:HGNC:21167] |
| ENSG00000068489 | HGLibA_38769 | 171 | 0.553025441 | PRR11 | proline rich 11 [Source:HGNC Symbol;Acc:HGNC:25619] |
| ENSG00000073670 | HGLibA_00730 | 171 | 0.553025441 | ADAM11 | ADAM metallopeptidase domain 11 [Source:HGNC Symbol;Acc:HGNC:189] |
| ENSG00000078295 | HGLibA_00912 | 171 | 0.553025441 | ADCY2 | adenylate cyclase 2 [Source:HGNC Symbol;Acc:HGNC:233] |
| ENSG00000082258 | HGLibA_08129 | 171 | 0.553025441 | CCNT2 | cyclin T2 [Source:HGNC Symbol;Acc:HGNC:1600] |
| ENSG00000083720 | HGLibA_34813 | 171 | 0.553025441 | OXCT1 | 3-oxoacid CoA-transferase 1 [Source:HGNC Symbol;Acc:HGNC:8527] |
| ENSG00000084710 | HGLibA_14610 | 171 | 0.553025441 | EFR3B | EFR3 homolog B [Source:HGNC Symbol;Acc:HGNC:29155] |
| ENSG00000086504 | HGLibA_29941 | 171 | 0.553025441 | MRPL28 | mitochondrial ribosomal protein L28 [Source:HGNC Symbol;Acc:HGNC:14484] |
| ENSG00000088280 | HGLibA_03199 | 171 | 0.553025441 | ASAP3 | ankyrin repeat and PH domain 3 [Source:HGNC Symbol;Acc:HGNC:14987]" |
| ENSG00000094661 | HGLibA_33670 | 171 | 0.553025441 | OR1I1 | olfactory receptor family 1 subfamily I member 1 [Source:HGNC Symbol;Acc:HGNC:8207] |
| ENSG00000100336 | HGLibA_02535 | 171 | 0.553025441 | APOL4 | apolipoprotein L4 [Source:HGNC Symbol;Acc:HGNC:14867] |
| ENSG00000100427 | HGLibA_29365 | 171 | 0.553025441 | MLC1 | megalencephalic leukoencephalopathy with subcortical cysts 1 [Source:HGNC Symbol;Acc:HGNC:17082] |
| ENSG00000100764 | HGLibA_39127 | 171 | 0.553025441 | PSMC1 | ATPase 1 [Source:HGNC Symbol;Acc:HGNC:9547]" |
| ENSG00000100865 | HGLibA_09714 | 171 | 0.553025441 | CINP | cyclin dependent kinase 2 interacting protein [Source:HGNC Symbol;Acc:HGNC:23789] |
| ENSG00000101439 | HGLibA_11453 | 171 | 0.553025441 | CST3 | cystatin C [Source:HGNC Symbol;Acc:HGNC:2475] |
| ENSG00000107829 | HGLibA_17146 | 171 | 0.553025441 | FBXW4 | F-box and WD repeat domain containing 4 [Source:HGNC Symbol;Acc:HGNC:10847] |
| ENSG00000111790 | HGLibA_17440 | 171 | 0.553025441 | FGFR1OP2 | FGFR1 oncogene partner 2 [Source:HGNC Symbol;Acc:HGNC:23098] |
| ENSG00000112039 | HGLibA_16845 | 171 | 0.553025441 | FANCE | FA complementation group E [Source:HGNC Symbol;Acc:HGNC:3586] |
| ENSG00000115325 | HGLibA_13766 | 171 | 0.553025441 | DOK1 | docking protein 1 [Source:HGNC Symbol;Acc:HGNC:2990] |
| ENSG00000118007 | HGLibA_47320 | 171 | 0.553025441 | STAG1 | stromal antigen 1 [Source:HGNC Symbol;Acc:HGNC:11354] |
| ENSG00000121691 | HGLibA_07351 | 171 | 0.553025441 | CAT | catalase [Source:HGNC Symbol;Acc:HGNC:1516] |
| ENSG00000122550 | HGLibA_25221 | 171 | 0.553025441 | KLHL7 | kelch like family member 7 [Source:HGNC Symbol;Acc:HGNC:15646] |
| ENSG00000126752 | HGLibA_47194 | 171 | 0.553025441 | SSX1 | SSX family member 1 [Source:HGNC Symbol;Acc:HGNC:11335] |
| ENSG00000128482 | HGLibA_41470 | 171 | 0.553025441 | RNF112 | ring finger protein 112 [Source:HGNC Symbol;Acc:HGNC:12968] |
| ENSG00000128609 | HGLibA_31463 | 171 | 0.553025441 | NDUFA5 | NADH:ubiquinone oxidoreductase subunit A5 [Source:HGNC Symbol;Acc:HGNC:7688] |
| ENSG00000129038 | HGLibA_27039 | 171 | 0.553025441 | LOXL1 | lysyl oxidase like 1 [Source:HGNC Symbol;Acc:HGNC:6665] |
| ENSG00000129472 | HGLibA_39869 | 171 | 0.553025441 | RAB2B | member RAS oncogene family [Source:HGNC Symbol;Acc:HGNC:20246]" |
| ENSG00000129968 | HGLibA_00262 | 171 | 0.553025441 | ABHD17A | abhydrolase domain containing 17A [Source:HGNC Symbol;Acc:HGNC:28756] |
| ENSG00000131626 | HGLibA_37873 | 171 | 0.553025441 | PPFIA1 | PTPRF interacting protein alpha 1 [Source:HGNC Symbol;Acc:HGNC:9245] |
| ENSG00000133250 | HGLibA_55938 | 171 | 0.553025441 | ZNF414 | zinc finger protein 414 [Source:HGNC Symbol;Acc:HGNC:20630] |
| ENSG00000136861 | HGLibA_08784 | 171 | 0.553025441 | CDK5RAP2 | CDK5 regulatory subunit associated protein 2 [Source:HGNC Symbol;Acc:HGNC:18672] |
| ENSG00000137198 | HGLibA_19428 | 171 | 0.553025441 | GMPR | guanosine monophosphate reductase [Source:HGNC Symbol;Acc:HGNC:4376] |
| ENSG00000138031 | HGLibA_00913 | 171 | 0.553025441 | ADCY3 | adenylate cyclase 3 [Source:HGNC Symbol;Acc:HGNC:234] |
| ENSG00000138032 | HGLibA_37967 | 171 | 0.553025441 | PPM1B | Mg2+/Mn2+ dependent 1B [Source:HGNC Symbol;Acc:HGNC:9276]" |
| ENSG00000138182 | HGLibA_24894 | 171 | 0.553025441 | KIF20B | kinesin family member 20B [Source:HGNC Symbol;Acc:HGNC:7212] |
| ENSG00000139880 | HGLibA_08670 | 171 | 0.553025441 | CDH24 | cadherin 24 [Source:HGNC Symbol;Acc:HGNC:14265] |
| ENSG00000140983 | HGLibA_41204 | 171 | 0.553025441 | RHOT2 | ras homolog family member T2 [Source:HGNC Symbol;Acc:HGNC:21169] |
| ENSG00000141576 | HGLibA_41554 | 171 | 0.553025441 | RNF157 | ring finger protein 157 [Source:HGNC Symbol;Acc:HGNC:29402] |
| ENSG00000142188 | HGLibA_50425 | 171 | 0.553025441 | TMEM50B | transmembrane protein 50B [Source:HGNC Symbol;Acc:HGNC:1280] |
| ENSG00000144233 | HGLibA_01804 | 171 | 0.553025441 | AMMECR1L | AMMECR1 like [Source:HGNC Symbol;Acc:HGNC:28658] |
| ENSG00000145687 | HGLibA_47116 | 171 | 0.553025441 | SSBP2 | single stranded DNA binding protein 2 [Source:HGNC Symbol;Acc:HGNC:15831] |
| ENSG00000146094 | HGLibA_13772 | 171 | 0.553025441 | DOK3 | docking protein 3 [Source:HGNC Symbol;Acc:HGNC:24583] |
| ENSG00000146830 | HGLibA_19064 | 171 | 0.553025441 | GIGYF1 | GRB10 interacting GYF protein 1 [Source:HGNC Symbol;Acc:HGNC:9126] |
| ENSG00000147050 | HGLibA_24532 | 171 | 0.553025441 | KDM6A | lysine demethylase 6A [Source:HGNC Symbol;Acc:HGNC:12637] |
| ENSG00000147081 | HGLibA_01434 | 171 | 0.553025441 | AKAP4 | A-kinase anchoring protein 4 [Source:HGNC Symbol;Acc:HGNC:374] |
| ENSG00000151892 | HGLibA_18965 | 171 | 0.553025441 | GFRA1 | GDNF family receptor alpha 1 [Source:HGNC Symbol;Acc:HGNC:4243] |
| ENSG00000154545 | HGLibA_27974 | 171 | 0.553025441 | MAGED4 | MAGE family member D4 [Source:HGNC Symbol;Acc:HGNC:23793] |
| ENSG00000157191 | HGLibA_31597 | 171 | 0.553025441 | NECAP2 | NECAP endocytosis associated 2 [Source:HGNC Symbol;Acc:HGNC:25528] |
| ENSG00000160233 | HGLibA_27304 | 171 | 0.553025441 | LRRC3 | leucine rich repeat containing 3 [Source:HGNC Symbol;Acc:HGNC:14965] |
| ENSG00000161610 | HGLibA_20986 | 171 | 0.553025441 | HCRT | hypocretin neuropeptide precursor [Source:HGNC Symbol;Acc:HGNC:4847] |
| ENSG00000163170 | HGLibA_04688 | 171 | 0.553025441 | BOLA3 | bolA family member 3 [Source:HGNC Symbol;Acc:HGNC:24415] |
| ENSG00000163527 | HGLibA_47609 | 171 | 0.553025441 | STT3B | catalytic subunit of the oligosaccharyltransferase complex [Source:HGNC Symbol;Acc:HGNC:30611]" |
| ENSG00000166086 | HGLibA_23935 | 171 | 0.553025441 | JAM3 | junctional adhesion molecule 3 [Source:HGNC Symbol;Acc:HGNC:15532] |
| ENSG00000166340 | HGLibA_51160 | 171 | 0.553025441 | TPP1 | tripeptidyl peptidase 1 [Source:HGNC Symbol;Acc:HGNC:2073] |
| ENSG00000167118 | HGLibA_53148 | 171 | 0.553025441 | URM1 | ubiquitin related modifier 1 [Source:HGNC Symbol;Acc:HGNC:28378] |
| ENSG00000167323 | HGLibA_47442 | 171 | 0.553025441 | STIM1 | stromal interaction molecule 1 [Source:HGNC Symbol;Acc:HGNC:11386] |
| ENSG00000167608 | HGLibA_49756 | 171 | 0.553025441 | TMC4 | transmembrane channel like 4 [Source:HGNC Symbol;Acc:HGNC:22998] |
| ENSG00000168765 | HGLibA_20482 | 171 | 0.553025441 | GSTM4 | glutathione S-transferase mu 4 [Source:HGNC Symbol;Acc:HGNC:4636] |
| ENSG00000169474 | HGLibA_46802 | 171 | 0.553025441 | SPRR1A | small proline rich protein 1A [Source:HGNC Symbol;Acc:HGNC:11259] |
| ENSG00000172780 | HGLibA_39948 | 171 | 0.553025441 | RAB43 | member RAS oncogene family [Source:HGNC Symbol;Acc:HGNC:19983]" |
| ENSG00000173572 | HGLibA_32110 | 171 | 0.553025441 | NLRP13 | NLR family pyrin domain containing 13 [Source:HGNC Symbol;Acc:HGNC:22937] |
| ENSG00000175224 | HGLibA_03468 | 171 | 0.553025441 | ATG13 | autophagy related 13 [Source:HGNC Symbol;Acc:HGNC:29091] |
| ENSG00000176055 | HGLibA_28504 | 171 | 0.553025441 | MBLAC2 | metallo-beta-lactamase domain containing 2 [Source:HGNC Symbol;Acc:HGNC:33711] |
| ENSG00000176547 | HGLibA_33953 | 171 | 0.553025441 | OR4C3 | olfactory receptor family 4 subfamily C member 3 [Source:HGNC Symbol;Acc:HGNC:14697] |
| ENSG00000178409 | HGLibA_04403 | 171 | 0.553025441 | BEND3 | BEN domain containing 3 [Source:HGNC Symbol;Acc:HGNC:23040] |
| ENSG00000178425 | HGLibA_32820 | 171 | 0.553025441 | NT5DC1 | 5'-nucleotidase domain containing 1 [Source:HGNC Symbol;Acc:HGNC:21556] |
| ENSG00000179588 | HGLibA_55252 | 171 | 0.553025441 | ZFPM1 | FOG family member 1 [Source:HGNC Symbol;Acc:HGNC:19762]" |
| ENSG00000181322 | HGLibA_32177 | 171 | 0.553025441 | NME9 | NME/NM23 family member 9 [Source:HGNC Symbol;Acc:HGNC:21343] |
| ENSG00000181562 | HGLibA_14448 | 171 | 0.553025441 | EDDM3A | epididymal protein 3A [Source:HGNC Symbol;Acc:HGNC:16978] |
| ENSG00000184828 | HGLibA_54922 | 171 | 0.553025441 | ZBTB7C | zinc finger and BTB domain containing 7C [Source:HGNC Symbol;Acc:HGNC:31700] |
| ENSG00000185739 | HGLibA_46990 | 171 | 0.553025441 | SRL | sarcalumenin [Source:HGNC Symbol;Acc:HGNC:11295] |
| ENSG00000188691 | HGLibA_34221 | 171 | 0.553025441 | OR56A5 | olfactory receptor family 56 subfamily A member 5 [Source:HGNC Symbol;Acc:HGNC:14792] |
| ENSG00000189221 | HGLibA_28106 | 171 | 0.553025441 | MAOA | monoamine oxidase A [Source:HGNC Symbol;Acc:HGNC:6833] |
| ENSG00000196363 | HGLibA_54115 | 171 | 0.553025441 | WDR5 | WD repeat domain 5 [Source:HGNC Symbol;Acc:HGNC:12757] |
| ENSG00000196526 | HGLibA_01096 | 171 | 0.553025441 | AFAP1 | actin filament associated protein 1 [Source:HGNC Symbol;Acc:HGNC:24017] |
| ENSG00000196646 | HGLibA_55473 | 171 | 0.553025441 | ZNF136 | zinc finger protein 136 [Source:HGNC Symbol;Acc:HGNC:12920] |
| ENSG00000197457 | HGLibA_47523 | 171 | 0.553025441 | STMN3 | stathmin 3 [Source:HGNC Symbol;Acc:HGNC:15926] |
| ENSG00000198185 | HGLibA_55823 | 171 | 0.553025441 | ZNF334 | zinc finger protein 334 [Source:HGNC Symbol;Acc:HGNC:15806] |
| ENSG00000198798 | HGLibA_27946 | 171 | 0.553025441 | MAGEB3 | MAGE family member B3 [Source:HGNC Symbol;Acc:HGNC:6810] |
| ENSG00000204001 | HGLibA_26183 | 171 | 0.553025441 | LCN8 | lipocalin 8 [Source:HGNC Symbol;Acc:HGNC:27038] |
| ENSG00000205683 | HGLibA_13833 | 171 | 0.553025441 | DPF3 | double PHD fingers 3 [Source:HGNC Symbol;Acc:HGNC:17427] |
| ENSG00000205784 | HGLibA_03124 | 171 | 0.553025441 | ARRDC5 | arrestin domain containing 5 [Source:HGNC Symbol;Acc:HGNC:31407] |
| ENSG00000206562 | HGLibA_29016 | 171 | 0.553025441 | METTL6 | methyltransferase like 6 [Source:HGNC Symbol;Acc:HGNC:28343] |
| ENSG00000213741 | HGLibA_42134 | 171 | 0.553025441 | RPS29 | ribosomal protein S29 [Source:HGNC Symbol;Acc:HGNC:10419] |
| ENSG00000213931 | HGLibA_20918 | 171 | 0.553025441 | HBE1 | hemoglobin subunit epsilon 1 [Source:HGNC Symbol;Acc:HGNC:4830] |
| ENSG00000214309 | HGLibA_28501 | 171 | 0.553025441 | MBLAC1 | metallo-beta-lactamase domain containing 1 [Source:HGNC Symbol;Acc:HGNC:22180] |
| ENSG00000214338 | HGLibA_46158 | 171 | 0.553025441 | SOGA3 | SOGA family member 3 [Source:HGNC Symbol;Acc:HGNC:21494] |
| ENSG00000214814 | HGLibA_17288 | 171 | 0.553025441 | FER1L6 | fer-1 like family member 6 [Source:HGNC Symbol;Acc:HGNC:28065] |
| ENSG00000221970 | HGLibA_33718 | 171 | 0.553025441 | OR2A1 | olfactory receptor family 2 subfamily A member 1 [Source:HGNC Symbol;Acc:HGNC:8229] |
| ENSG00000224440 | HGLibA_11939 | 171 | 0.553025441 | CXorf51A | chromosome X open reading frame 51A [Source:HGNC Symbol;Acc:HGNC:30533] |
| ENSG00000271043 | HGLibA_30477 | 171 | 0.553025441 | MTRNR2L2 | MT-RNR2 like 2 [Source:HGNC Symbol;Acc:HGNC:37156] |
| ENSG00000278195 | HGLibA_47181 | 171 | 0.553025441 | SSTR3 | somatostatin receptor 3 [Source:HGNC Symbol;Acc:HGNC:11332] |
| ENSG00000286237 | HGLibA_03054 | 171 | 0.553025441 | ARMCX5-GPRASP2 | - |
| GAREML | HGLibA_18629 | 171 | 0.553025441 | GAREML | - |
| HNRPLL | HGLibA_21858 | 171 | 0.553025441 | HNRPLL | - |
| hsa-mir-1193 | HGLibA_57250 | 171 | 0.553025441 | hsa-mir-1193 | - |
| hsa-mir-1469 | HGLibA_57851 | 171 | 0.553025441 | hsa-mir-1469 | - |
| hsa-mir-23c | HGLibA_58337 | 171 | 0.553025441 | hsa-mir-23c | - |
| hsa-mir-3617 | HGLibA_59097 | 171 | 0.553025441 | hsa-mir-3617 | - |
| hsa-mir-378e | HGLibA_59445 | 171 | 0.553025441 | hsa-mir-378e | - |
| hsa-mir-4293 | HGLibA_59884 | 171 | 0.553025441 | hsa-mir-4293 | - |
| hsa-mir-4639 | HGLibA_60659 | 171 | 0.553025441 | hsa-mir-4639 | - |
| hsa-mir-4700 | HGLibA_60909 | 171 | 0.553025441 | hsa-mir-4700 | - |

| | | | | | |
|---|---|---|---|---|---|
| hsa-mir-4768 | HGLibA_61170 | 171 | 0.553025441 | hsa-mir-4768 | - |
| hsa-mir-548az | HGLibA_61946 | 171 | 0.553025441 | hsa-mir-548az | - |
| hsa-mir-611 | HGLibA_62637 | 171 | 0.553025441 | hsa-mir-611 | - |
| hsa-mir-6715a | HGLibA_63017 | 171 | 0.553025441 | hsa-mir-6715a | - |
| hsa-mir-6738 | HGLibA_63106 | 171 | 0.553025441 | hsa-mir-6738 | - |
| KIAA1683 | HGLibA_24803 | 171 | 0.553025441 | KIAA1683 | - |
| LOC100130451 | HGLibA_26786 | 171 | 0.553025441 | LOC100130451 | - |
| LOC440563 | HGLibA_26949 | 171 | 0.553025441 | LOC440563 | - |
| NGFRAP1 | HGLibA_31877 | 171 | 0.553025441 | NGFRAP1 | - |
| C17orf112 | HGLibA_05531 | 170 | 0.543074403 | C17orf112 | - |
| C20orf24 | HGLibA_06018 | 170 | 0.543074403 | C20orf24 | - |
| ENSG00000002330 | HGLibA_04072 | 170 | 0.543074403 | BAD | BCL2 associated agonist of cell death [Source:HGNC Symbol;Acc:HGNC:936] |
| ENSG00000002587 | HGLibA_22122 | 170 | 0.543074403 | HS3ST1 | heparan sulfate-glucosamine 3-sulfotransferase 1 [Source:HGNC Symbol;Acc:HGNC:5194] |
| ENSG00000002746 | HGLibA_21101 | 170 | 0.543074403 | HECW1 | C2 and WW domain containing E3 ubiquitin protein ligase 1 [Source:HGNC Symbol;Acc:HGNC:22195]" |
| ENSG00000010072 | HGLibA_46832 | 170 | 0.543074403 | SPRTN | SprT-like N-terminal domain [Source:HGNC Symbol;Acc:HGNC:25356] |
| ENSG00000028839 | HGLibA_48627 | 170 | 0.543074403 | TBPL1 | TATA-box binding protein like 1 [Source:HGNC Symbol;Acc:HGNC:11589] |
| ENSG00000041802 | HGLibA_27515 | 170 | 0.543074403 | LSG1 | large 60S subunit nuclear export GTPase 1 [Source:HGNC Symbol;Acc:HGNC:25652] |
| ENSG00000043093 | HGLibA_12564 | 170 | 0.543074403 | DCUN1D1 | defective in cullin neddylation 1 domain containing 1 [Source:HGNC Symbol;Acc:HGNC:18184] |
| ENSG00000056097 | HGLibA_55259 | 170 | 0.543074403 | ZFR | zinc finger RNA binding protein [Source:HGNC Symbol;Acc:HGNC:17277] |
| ENSG00000067057 | HGLibA_36170 | 170 | 0.543074403 | PFKP | platelet [Source:HGNC Symbol;Acc:HGNC:8878]" |
| ENSG00000068781 | HGLibA_47547 | 170 | 0.543074403 | STON1-GTF2A1L | STON1-GTF2A1L readthrough [Source:HGNC Symbol;Acc:HGNC:30651] |
| ENSG00000076928 | HGLibA_02805 | 170 | 0.543074403 | ARHGEF1 | Rho guanine nucleotide exchange factor 1 [Source:HGNC Symbol;Acc:HGNC:681] |
| ENSG00000077097 | HGLibA_50997 | 170 | 0.543074403 | TOP2B | DNA topoisomerase II beta [Source:HGNC Symbol;Acc:HGNC:11990] |
| ENSG00000080644 | HGLibA_09570 | 170 | 0.543074403 | CHRNA3 | cholinergic receptor nicotinic alpha 3 subunit [Source:HGNC Symbol;Acc:HGNC:1957] |
| ENSG00000084110 | HGLibA_20820 | 170 | 0.543074403 | HAL | histidine ammonia-lyase [Source:HGNC Symbol;Acc:HGNC:4806] |
| ENSG00000087299 | HGLibA_25891 | 170 | 0.543074403 | L2HGDH | L-2-hydroxyglutarate dehydrogenase [Source:HGNC Symbol;Acc:HGNC:20499] |
| ENSG00000099256 | HGLibA_38976 | 170 | 0.543074403 | PRTFDC1 | phosphoribosyl transferase domain containing 1 [Source:HGNC Symbol;Acc:HGNC:23333] |
| ENSG00000101190 | HGLibA_48800 | 170 | 0.543074403 | TCFL5 | transcription factor like 5 [Source:HGNC Symbol;Acc:HGNC:11646] |
| ENSG00000101353 | HGLibA_29878 | 170 | 0.543074403 | MROH8 | maestro heat like repeat family member 8 [Source:HGNC Symbol;Acc:HGNC:16125] |
| ENSG00000105393 | HGLibA_04056 | 170 | 0.543074403 | BABAM1 | BRISC and BRCA1 A complex member 1 [Source:HGNC Symbol;Acc:HGNC:25008] |
| ENSG00000105576 | HGLibA_50901 | 170 | 0.543074403 | TNPO2 | transportin 2 [Source:HGNC Symbol;Acc:HGNC:19998] |
| ENSG00000105650 | HGLibA_35792 | 170 | 0.543074403 | PDE4C | phosphodiesterase 4C [Source:HGNC Symbol;Acc:HGNC:8782] |
| ENSG00000106460 | HGLibA_49856 | 170 | 0.543074403 | TMEM106B | transmembrane protein 106B [Source:HGNC Symbol;Acc:HGNC:22407] |
| ENSG00000107077 | HGLibA_24511 | 170 | 0.543074403 | KDM4C | lysine demethylase 4C [Source:HGNC Symbol;Acc:HGNC:17071] |
| ENSG00000109189 | HGLibA_53378 | 170 | 0.543074403 | USP46 | ubiquitin specific peptidase 46 [Source:HGNC Symbol;Acc:HGNC:20075] |
| ENSG00000111321 | HGLibA_27603 | 170 | 0.543074403 | LTBR | lymphotoxin beta receptor [Source:HGNC Symbol;Acc:HGNC:6718] |
| ENSG00000112246 | HGLibA_44175 | 170 | 0.543074403 | SIM1 | SIM bHLH transcription factor 1 [Source:HGNC Symbol;Acc:HGNC:10882] |
| ENSG00000112561 | HGLibA_49163 | 170 | 0.543074403 | TFEB | transcription factor EB [Source:HGNC Symbol;Acc:HGNC:11753] |
| ENSG00000113719 | HGLibA_15468 | 170 | 0.543074403 | ERGIC1 | endoplasmic reticulum-golgi intermediate compartment 1 [Source:HGNC Symbol;Acc:HGNC:29205] |
| ENSG00000114737 | HGLibA_09737 | 170 | 0.543074403 | CISH | cytokine inducible SH2 containing protein [Source:HGNC Symbol;Acc:HGNC:1984] |
| ENSG00000116514 | HGLibA_41603 | 170 | 0.543074403 | RNF19B | ring finger protein 19B [Source:HGNC Symbol;Acc:HGNC:26886] |
| ENSG00000117281 | HGLibA_08239 | 170 | 0.543074403 | CD160 | CD160 molecule [Source:HGNC Symbol;Acc:HGNC:17013] |
| ENSG00000118690 | HGLibA_03014 | 170 | 0.543074403 | ARMC2 | armadillo repeat containing 2 [Source:HGNC Symbol;Acc:HGNC:23045] |
| ENSG00000119711 | HGLibA_01579 | 170 | 0.543074403 | ALDH6A1 | aldehyde dehydrogenase 6 family member A1 [Source:HGNC Symbol;Acc:HGNC:7179] |
| ENSG00000121940 | HGLibA_09815 | 170 | 0.543074403 | CLCC1 | chloride channel CLIC like 1 [Source:HGNC Symbol;Acc:HGNC:29675] |
| ENSG00000124789 | HGLibA_33023 | 170 | 0.543074403 | NUP153 | nucleoporin 153 [Source:HGNC Symbol;Acc:HGNC:8062] |
| ENSG00000125841 | HGLibA_32726 | 170 | 0.543074403 | NRSN2 | neurensin 2 [Source:HGNC Symbol;Acc:HGNC:16229] |
| ENSG00000127423 | HGLibA_03868 | 170 | 0.543074403 | AUNIP | aurora kinase A and ninein interacting protein [Source:HGNC Symbol;Acc:HGNC:28363] |
| ENSG00000128266 | HGLibA_19482 | 170 | 0.543074403 | GNAZ | G protein subunit alpha z [Source:HGNC Symbol;Acc:HGNC:4395] |
| ENSG00000129007 | HGLibA_07033 | 170 | 0.543074403 | CALML4 | calmodulin like 4 [Source:HGNC Symbol;Acc:HGNC:18445] |
| ENSG00000131068 | HGLibA_12864 | 170 | 0.543074403 | DEFB118 | defensin beta 118 [Source:HGNC Symbol;Acc:HGNC:16196] |
| ENSG00000132406 | HGLibA_49919 | 170 | 0.543074403 | TMEM128 | transmembrane protein 128 [Source:HGNC Symbol;Acc:HGNC:28201] |
| ENSG00000134668 | HGLibA_46746 | 170 | 0.543074403 | SPOCD1 | SPOC domain containing 1 [Source:HGNC Symbol;Acc:HGNC:26338] |
| ENSG00000134817 | HGLibA_02437 | 170 | 0.543074403 | APLNR | apelin receptor [Source:HGNC Symbol;Acc:HGNC:339] |
| ENSG00000135870 | HGLibA_40663 | 170 | 0.543074403 | RC3H1 | ring finger and CCCH-type domains 1 [Source:HGNC Symbol;Acc:HGNC:29434] |
| ENSG00000136167 | HGLibA_26200 | 170 | 0.543074403 | LCP1 | lymphocyte cytosolic protein 1 [Source:HGNC Symbol;Acc:HGNC:6528] |
| ENSG00000139187 | HGLibA_25309 | 170 | 0.543074403 | KLRG1 | killer cell lectin like receptor G1 [Source:HGNC Symbol;Acc:HGNC:6380] |
| ENSG00000139574 | HGLibA_32436 | 170 | 0.543074403 | NPFF | neuropeptide FF-amide peptide precursor [Source:HGNC Symbol;Acc:HGNC:7901] |
| ENSG00000144136 | HGLibA_44527 | 170 | 0.543074403 | SLC20A1 | solute carrier family 20 member 1 [Source:HGNC Symbol;Acc:HGNC:10946] |
| ENSG00000144648 | HGLibA_00442 | 170 | 0.543074403 | ACKR2 | atypical chemokine receptor 2 [Source:HGNC Symbol;Acc:HGNC:1565] |
| ENSG00000146707 | HGLibA_37676 | 170 | 0.543074403 | POMZP3 | POM121 and ZP3 fusion [Source:HGNC Symbol;Acc:HGNC:9203] |
| ENSG00000148219 | HGLibA_03384 | 170 | 0.543074403 | ASTN2 | astrotactin 2 [Source:HGNC Symbol;Acc:HGNC:17021] |
| ENSG00000152465 | HGLibA_32209 | 170 | 0.543074403 | NMT2 | N-myristoyltransferase 2 [Source:HGNC Symbol;Acc:HGNC:7858] |
| ENSG00000153574 | HGLibA_41851 | 170 | 0.543074403 | RPIA | ribose 5-phosphate isomerase A [Source:HGNC Symbol;Acc:HGNC:10297] |
| ENSG00000154743 | HGLibA_51832 | 170 | 0.543074403 | TSEN2 | tRNA splicing endonuclease subunit 2 [Source:HGNC Symbol;Acc:HGNC:28422] |
| ENSG00000156521 | HGLibA_52476 | 170 | 0.543074403 | TYSND1 | trypsin domain containing 1 [Source:HGNC Symbol;Acc:HGNC:28531] |
| ENSG00000157680 | HGLibA_13076 | 170 | 0.543074403 | DGKI | diacylglycerol kinase iota [Source:HGNC Symbol;Acc:HGNC:2855] |
| ENSG00000157999 | HGLibA_02131 | 170 | 0.543074403 | ANKRD61 | ankyrin repeat domain 61 [Source:HGNC Symbol;Acc:HGNC:22467] |
| ENSG00000158079 | HGLibA_39413 | 170 | 0.543074403 | PTPDC1 | protein tyrosine phosphatase domain containing 1 [Source:HGNC Symbol;Acc:HGNC:30184] |
| ENSG00000158747 | HGLibA_31216 | 170 | 0.543074403 | NBL1 | DAN family BMP antagonist [Source:HGNC Symbol;Acc:HGNC:7650]" |
| ENSG00000161381 | HGLibA_37253 | 170 | 0.543074403 | PLXDC1 | plexin domain containing 1 [Source:HGNC Symbol;Acc:HGNC:20945] |
| ENSG00000162456 | HGLibA_25336 | 170 | 0.543074403 | KNCN | kinocilin [Source:HGNC Symbol;Acc:HGNC:26488] |
| ENSG00000164850 | HGLibA_19796 | 170 | 0.543074403 | GPER1 | G protein-coupled estrogen receptor 1 [Source:HGNC Symbol;Acc:HGNC:4485] |
| ENSG00000165443 | HGLibA_36477 | 170 | 0.543074403 | PHYHIPL | phytanoyl-CoA 2-hydroxylase interacting protein like [Source:HGNC Symbol;Acc:HGNC:29378] |
| ENSG00000166405 | HGLibA_41232 | 170 | 0.543074403 | RIC3 | RIC3 acetylcholine receptor chaperone [Source:HGNC Symbol;Acc:HGNC:30338] |
| ENSG00000167173 | HGLibA_05393 | 170 | 0.543074403 | C15orf39 | chromosome 15 open reading frame 39 [Source:HGNC Symbol;Acc:HGNC:24497] |
| ENSG00000167346 | HGLibA_29526 | 170 | 0.543074403 | MMP26 | matrix metallopeptidase 26 [Source:HGNC Symbol;Acc:HGNC:14249] |
| ENSG00000167515 | HGLibA_51316 | 170 | 0.543074403 | TRAPPC2L | trafficking protein particle complex 2 like [Source:HGNC Symbol;Acc:HGNC:30887] |
| ENSG00000168280 | HGLibA_24954 | 170 | 0.543074403 | KIF5C | kinesin family member 5C [Source:HGNC Symbol;Acc:HGNC:6325] |
| ENSG00000172568 | HGLibA_17762 | 170 | 0.543074403 | FNDC9 | fibronectin type III domain containing 9 [Source:HGNC Symbol;Acc:HGNC:33547] |
| ENSG00000172927 | HGLibA_30691 | 170 | 0.543074403 | MYEOV | myeloma overexpressed [Source:HGNC Symbol;Acc:HGNC:7563] |
| ENSG00000173431 | HGLibA_41417 | 170 | 0.543074403 | RNASE8 | ribonuclease A family member 8 [Source:HGNC Symbol;Acc:HGNC:19277] |
| ENSG00000173575 | HGLibA_09388 | 170 | 0.543074403 | CHD2 | chromodomain helicase DNA binding protein 2 [Source:HGNC Symbol;Acc:HGNC:1917] |
| ENSG00000175198 | HGLibA_35378 | 170 | 0.543074403 | PCCA | propionyl-CoA carboxylase subunit alpha [Source:HGNC Symbol;Acc:HGNC:8653] |
| ENSG00000176294 | HGLibA_34045 | 170 | 0.543074403 | OR4N2 | olfactory receptor family 4 subfamily N member 2 [Source:HGNC Symbol;Acc:HGNC:14742] |
| ENSG00000176624 | HGLibA_29032 | 170 | 0.543074403 | MEX3C | mex-3 RNA binding family member C [Source:HGNC Symbol;Acc:HGNC:28040] |
| ENSG00000176692 | HGLibA_17825 | 170 | 0.543074403 | FOXC2 | forkhead box C2 [Source:HGNC Symbol;Acc:HGNC:3801] |
| ENSG00000180720 | HGLibA_09557 | 170 | 0.543074403 | CHRM4 | cholinergic receptor muscarinic 4 [Source:HGNC Symbol;Acc:HGNC:1953] |
| ENSG00000181274 | HGLibA_17982 | 170 | 0.543074403 | FRAT2 | WNT signaling pathway regulator [Source:HGNC Symbol;Acc:HGNC:16048]" |
| ENSG00000182986 | HGLibA_55794 | 170 | 0.543074403 | ZNF320 | zinc finger protein 320 [Source:HGNC Symbol;Acc:HGNC:13842] |
| ENSG00000185129 | HGLibA_39585 | 170 | 0.543074403 | PURA | purine rich element binding protein A [Source:HGNC Symbol;Acc:HGNC:9701] |
| ENSG00000185262 | HGLibA_52544 | 170 | 0.543074403 | UBALD2 | UBA like domain containing 2 [Source:HGNC Symbol;Acc:HGNC:28438] |
| ENSG00000185432 | HGLibA_29019 | 170 | 0.543074403 | METTL7A | methyltransferase like 7A [Source:HGNC Symbol;Acc:HGNC:24550] |
| ENSG00000187231 | HGLibA_43628 | 170 | 0.543074403 | SESTD1 | SEC14 and spectrin domain containing 1 [Source:HGNC Symbol;Acc:HGNC:18379] |
| ENSG00000187764 | HGLibA_43349 | 170 | 0.543074403 | SEMA4D | semaphorin 4D [Source:HGNC Symbol;Acc:HGNC:10732] |
| ENSG00000188785 | HGLibA_56206 | 170 | 0.543074403 | ZNF548 | zinc finger protein 548 [Source:HGNC Symbol;Acc:HGNC:26561] |
| ENSG00000197016 | HGLibA_56053 | 170 | 0.543074403 | ZNF470 | zinc finger protein 470 [Source:HGNC Symbol;Acc:HGNC:22220] |
| ENSG00000198551 | HGLibA_56426 | 170 | 0.543074403 | ZNF627 | zinc finger protein 627 [Source:HGNC Symbol;Acc:HGNC:30570] |
| ENSG00000203778 | HGLibA_16542 | 170 | 0.543074403 | FAM229B | family with sequence similarity 229 member B [Source:HGNC Symbol;Acc:HGNC:33858] |
| ENSG00000203896 | HGLibA_26538 | 170 | 0.543074403 | LIME1 | Lck interacting transmembrane adaptor 1 [Source:HGNC Symbol;Acc:HGNC:26016] |
| ENSG00000204947 | HGLibA_55962 | 170 | 0.543074403 | ZNF425 | zinc finger protein 425 [Source:HGNC Symbol;Acc:HGNC:20690] |
| ENSG00000243130 | HGLibA_39037 | 170 | 0.543074403 | PSG11 | pregnancy specific beta-1-glycoprotein 11 [Source:HGNC Symbol;Acc:HGNC:9516] |
| ENSG00000261701 | HGLibA_22040 | 170 | 0.543074403 | HPR | haptoglobin-related protein [Source:HGNC Symbol;Acc:HGNC:5156] |
| ENSG00000274286 | HGLibA_01048 | 170 | 0.543074403 | ADRA2B | adrenoceptor alpha 2B [Source:HGNC Symbol;Acc:HGNC:282] |
| ENSG00000278535 | HGLibA_13121 | 170 | 0.543074403 | DHRS11 | dehydrogenase/reductase 11 [Source:HGNC Symbol;Acc:HGNC:28639] |
| ENSG00000278570 | HGLibA_32607 | 170 | 0.543074403 | NR2E3 | nuclear receptor subfamily 2 group E member 3 [Source:HGNC Symbol;Acc:HGNC:7974] |
| FAM49A | HGLibA_16623 | 170 | 0.543074403 | FAM49A | - |
| FYB | HGLibA_18247 | 170 | 0.543074403 | FYB | - |
| GAGE12I | HGLibA_18457 | 170 | 0.543074403 | GAGE12I | - |
| hsa-mir-320a | HGLibA_58925 | 170 | 0.543074403 | hsa-mir-320a | - |
| hsa-mir-3689e | HGLibA_59332 | 170 | 0.543074403 | hsa-mir-3689e | - |
| hsa-mir-4289 | HGLibA_59865 | 170 | 0.543074403 | hsa-mir-4289 | - |
| hsa-mir-431 | HGLibA_59952 | 170 | 0.543074403 | hsa-mir-431 | - |
| hsa-mir-4482 | HGLibA_60346 | 170 | 0.543074403 | hsa-mir-4482 | - |
| hsa-mir-4531 | HGLibA_60584 | 170 | 0.543074403 | hsa-mir-4531 | - |
| hsa-mir-4721 | HGLibA_60981 | 170 | 0.543074403 | hsa-mir-4721 | - |
| hsa-mir-4762 | HGLibA_61143 | 170 | 0.543074403 | hsa-mir-4762 | - |
| hsa-mir-548a-2 | HGLibA_61840 | 170 | 0.543074403 | hsa-mir-548a-2 | - |
| hsa-mir-625 | HGLibA_62738 | 170 | 0.543074403 | hsa-mir-625 | - |
| hsa-mir-651 | HGLibA_62889 | 170 | 0.543074403 | hsa-mir-651 | - |
| hsa-mir-6806 | HGLibA_63402 | 170 | 0.543074403 | hsa-mir-6806 | - |
| hsa-mir-6865 | HGLibA_63644 | 170 | 0.543074403 | hsa-mir-6865 | - |
| hsa-mir-7150 | HGLibA_63827 | 170 | 0.543074403 | hsa-mir-7150 | - |
| LRRC6 | HGLibA_27404 | 170 | 0.543074403 | LRRC6 | - |
| SMCR7L | HGLibA_45681 | 170 | 0.543074403 | SMCR7L | - |
| SUV420H1 | HGLibA_47864 | 170 | 0.543074403 | SUV420H1 | - |
| WBSCR27 | HGLibA_53997 | 170 | 0.543074403 | WBSCR27 | - |
| CCDC114 | HGLibA_07551 | 169 | 0.533123366 | CCDC114 | - |
| DUPD1 | HGLibA_14110 | 169 | 0.533123366 | DUPD1 | - |
| ENSG00000011009 | HGLibA_27741 | 169 | 0.533123366 | LYPLA2 | lysophospholipase II [Source:HGNC Symbol;Acc:HGNC:6738] |
| ENSG00000049130 | HGLibA_25019 | 169 | 0.533123366 | KITLG | KIT ligand [Source:HGNC Symbol;Acc:HGNC:6343] |
| ENSG00000054965 | HGLibA_16245 | 169 | 0.533123366 | FAM168A | family with sequence similarity 168 member A [Source:HGNC Symbol;Acc:HGNC:28999] |
| ENSG00000055211 | HGLibA_19099 | 169 | 0.533123366 | GINM1 | glycoprotein integral membrane 1 [Source:HGNC Symbol;Acc:HGNC:21074] |
| ENSG00000073584 | HGLibA_45638 | 169 | 0.533123366 | SMARCE1 | member 1 [Source:HGNC Symbol;Acc:HGNC:11109]" |
| ENSG00000077312 | HGLibA_45963 | 169 | 0.533123366 | SNRPA | small nuclear ribonucleoprotein polypeptide A [Source:HGNC Symbol;Acc:HGNC:11151] |

| | | | | | |
|---|---|---|---|---|---|
| ENSG00000100147 | HGLibA_07598 | 169 | 0.533123366 | CCDC134 | coiled-coil domain containing 134 [Source:HGNC Symbol;Acc:HGNC:26185] |
| ENSG00000106348 | HGLibA_23255 | 169 | 0.533123366 | IMPDH1 | inosine monophosphate dehydrogenase 1 [Source:HGNC Symbol;Acc:HGNC:6052] |
| ENSG00000106536 | HGLibA_37799 | 169 | 0.533123366 | POU6F2 | POU class 6 homeobox 2 [Source:HGNC Symbol;Acc:HGNC:21694] |
| ENSG00000108826 | HGLibA_29940 | 169 | 0.533123366 | MRPL27 | mitochondrial ribosomal protein L27 [Source:HGNC Symbol;Acc:HGNC:14483] |
| ENSG00000109323 | HGLibA_28085 | 169 | 0.533123366 | MANBA | mannosidase beta [Source:HGNC Symbol;Acc:HGNC:6831] |
| ENSG00000109625 | HGLibA_11043 | 169 | 0.533123366 | CPZ | carboxypeptidase Z [Source:HGNC Symbol;Acc:HGNC:2333] |
| ENSG00000110697 | HGLibA_36751 | 169 | 0.533123366 | PITPNM1 | phosphatidylinositol transfer protein membrane associated 1 [Source:HGNC Symbol;Acc:HGNC:9003] |
| ENSG00000113100 | HGLibA_08696 | 169 | 0.533123366 | CDH9 | cadherin 9 [Source:HGNC Symbol;Acc:HGNC:1768] |
| ENSG00000114796 | HGLibA_25161 | 169 | 0.533123366 | KLHL24 | kelch like family member 24 [Source:HGNC Symbol;Acc:HGNC:25947] |
| ENSG00000115239 | HGLibA_03235 | 169 | 0.533123366 | ASB3 | ankyrin repeat and SOCS box containing 3 [Source:HGNC Symbol;Acc:HGNC:16013] |
| ENSG00000116885 | HGLibA_34674 | 169 | 0.533123366 | OSCP1 | organic solute carrier partner 1 [Source:HGNC Symbol;Acc:HGNC:29971] |
| ENSG00000118900 | HGLibA_52721 | 169 | 0.533123366 | UBN1 | ubinuclein 1 [Source:HGNC Symbol;Acc:HGNC:12506] |
| ENSG00000118960 | HGLibA_22115 | 169 | 0.533123366 | HS1BP3 | HCLS1 binding protein 3 [Source:HGNC Symbol;Acc:HGNC:24979] |
| ENSG00000122035 | HGLibA_40365 | 169 | 0.533123366 | RASL11A | RAS like family 11 member A [Source:HGNC Symbol;Acc:HGNC:23802] |
| ENSG00000122882 | HGLibA_14380 | 169 | 0.533123366 | ECD | ecdysoneless cell cycle regulator [Source:HGNC Symbol;Acc:HGNC:17029] |
| ENSG00000123159 | HGLibA_19119 | 169 | 0.533123366 | GIPC1 | GIPC PDZ domain containing family member 1 [Source:HGNC Symbol;Acc:HGNC:1226] |
| ENSG00000124226 | HGLibA_41479 | 169 | 0.533123366 | RNF114 | ring finger protein 114 [Source:HGNC Symbol;Acc:HGNC:13094] |
| ENSG00000124532 | HGLibA_30119 | 169 | 0.533123366 | MRS2 | magnesium transporter MRS2 [Source:HGNC Symbol;Acc:HGNC:13785] |
| ENSG00000126216 | HGLibA_52311 | 169 | 0.533123366 | TUBGCP3 | tubulin gamma complex associated protein 3 [Source:HGNC Symbol;Acc:HGNC:18598] |
| ENSG00000127083 | HGLibA_33384 | 169 | 0.533123366 | OMD | osteomodulin [Source:HGNC Symbol;Acc:HGNC:8134] |
| ENSG00000127364 | HGLibA_48399 | 169 | 0.533123366 | TAS2R4 | taste 2 receptor member 4 [Source:HGNC Symbol;Acc:HGNC:14911] |
| ENSG00000130164 | HGLibA_26239 | 169 | 0.533123366 | LDLR | low density lipoprotein receptor [Source:HGNC Symbol;Acc:HGNC:6547] |
| ENSG00000130699 | HGLibA_48234 | 169 | 0.533123366 | TAF4 | TATA-box binding protein associated factor 4 [Source:HGNC Symbol;Acc:HGNC:11537] |
| ENSG00000130826 | HGLibA_13290 | 169 | 0.533123366 | DKC1 | dyskerin pseudouridine synthase 1 [Source:HGNC Symbol;Acc:HGNC:2890] |
| ENSG00000131669 | HGLibA_31937 | 169 | 0.533123366 | NINJ1 | ninjurin 1 [Source:HGNC Symbol;Acc:HGNC:7824] |
| ENSG00000132661 | HGLibA_33173 | 169 | 0.533123366 | NXT1 | nuclear transport factor 2 like export factor 1 [Source:HGNC Symbol;Acc:HGNC:15913] |
| ENSG00000132763 | HGLibA_29455 | 169 | 0.533123366 | MMACHC | metabolism of cobalamin associated C [Source:HGNC Symbol;Acc:HGNC:24525] |
| ENSG00000133657 | HGLibA_03573 | 169 | 0.533123366 | ATP13A3 | ATPase 13A3 [Source:HGNC Symbol;Acc:HGNC:24113] |
| ENSG00000134817 | HGLibA_02436 | 169 | 0.533123366 | APLNR | apelin receptor [Source:HGNC Symbol;Acc:HGNC:339] |
| ENSG00000136834 | HGLibA_33673 | 169 | 0.533123366 | OR1J1 | olfactory receptor family 1 subfamily J member 1 [Source:HGNC Symbol;Acc:HGNC:8208] |
| ENSG00000138650 | HGLibA_35388 | 169 | 0.533123366 | PCDH10 | protocadherin 10 [Source:HGNC Symbol;Acc:HGNC:13404] |
| ENSG00000139083 | HGLibA_15665 | 169 | 0.533123366 | ETV6 | ETS variant 6 [Source:HGNC Symbol;Acc:HGNC:3495] |
| ENSG00000143164 | HGLibA_12452 | 169 | 0.533123366 | DCAF6 | DDB1 and CUL4 associated factor 6 [Source:HGNC Symbol;Acc:HGNC:30002] |
| ENSG00000143185 | HGLibA_54496 | 169 | 0.533123366 | XCL2 | X-C motif chemokine ligand 2 [Source:HGNC Symbol;Acc:HGNC:10646] |
| ENSG00000143373 | HGLibA_56538 | 169 | 0.533123366 | ZNF687 | zinc finger protein 687 [Source:HGNC Symbol;Acc:HGNC:29277] |
| ENSG00000144843 | HGLibA_01025 | 169 | 0.533123366 | ADPRH | ADP-ribosylarginine hydrolase [Source:HGNC Symbol;Acc:HGNC:269] |
| ENSG00000144857 | HGLibA_04665 | 169 | 0.533123366 | BOC | oncogene regulated [Source:HGNC Symbol;Acc:HGNC:17173]" |
| ENSG00000145945 | HGLibA_16633 | 169 | 0.533123366 | FAM50B | family with sequence similarity 50 member B [Source:HGNC Symbol;Acc:HGNC:18789] |
| ENSG00000146755 | HGLibA_51537 | 169 | 0.533123366 | TRIM50 | tripartite motif containing 50 [Source:HGNC Symbol;Acc:HGNC:19017] |
| ENSG00000148572 | HGLibA_32652 | 169 | 0.533123366 | NRBF2 | nuclear receptor binding factor 2 [Source:HGNC Symbol;Acc:HGNC:19692] |
| ENSG00000154262 | HGLibA_00118 | 169 | 0.533123366 | ABCA6 | ATP binding cassette subfamily A member 6 [Source:HGNC Symbol;Acc:HGNC:36] |
| ENSG00000157211 | HGLibA_08626 | 169 | 0.533123366 | CDCP2 | CUB domain containing protein 2 [Source:HGNC Symbol;Acc:HGNC:27297] |
| ENSG00000158639 | HGLibA_35015 | 169 | 0.533123366 | PAGE5 | PAGE family member 5 [Source:HGNC Symbol;Acc:HGNC:29992] |
| ENSG00000158715 | HGLibA_45177 | 169 | 0.533123366 | SLC45A3 | solute carrier family 45 member 3 [Source:HGNC Symbol;Acc:HGNC:8642] |
| ENSG00000159263 | HGLibA_44178 | 169 | 0.533123366 | SIM2 | SIM bHLH transcription factor 2 [Source:HGNC Symbol;Acc:HGNC:10883] |
| ENSG00000160094 | HGLibA_55874 | 169 | 0.533123366 | ZNF362 | zinc finger protein 362 [Source:HGNC Symbol;Acc:HGNC:18079] |
| ENSG00000160688 | HGLibA_17604 | 169 | 0.533123366 | FLAD1 | flavin adenine dinucleotide synthetase 1 [Source:HGNC Symbol;Acc:HGNC:24671] |
| ENSG00000161642 | HGLibA_55896 | 169 | 0.533123366 | ZNF385A | zinc finger protein 385A [Source:HGNC Symbol;Acc:HGNC:17521] |
| ENSG00000163530 | HGLibA_13888 | 169 | 0.533123366 | DPPA2 | developmental pluripotency associated 2 [Source:HGNC Symbol;Acc:HGNC:19197] |
| ENSG00000163735 | HGLibA_11871 | 169 | 0.533123366 | CXCL5 | C-X-C motif chemokine ligand 5 [Source:HGNC Symbol;Acc:HGNC:10642] |
| ENSG00000163814 | HGLibA_08622 | 169 | 0.533123366 | CDCP1 | CUB domain containing protein 1 [Source:HGNC Symbol;Acc:HGNC:24357] |
| ENSG00000164047 | HGLibA_07104 | 169 | 0.533123366 | CAMP | cathelicidin antimicrobial peptide [Source:HGNC Symbol;Acc:HGNC:1472] |
| ENSG00000165288 | HGLibA_04857 | 169 | 0.533123366 | BRWD3 | bromodomain and WD repeat domain containing 3 [Source:HGNC Symbol;Acc:HGNC:17342] |
| ENSG00000168546 | HGLibA_18969 | 169 | 0.533123366 | GFRA2 | GDNF family receptor alpha 2 [Source:HGNC Symbol;Acc:HGNC:4244] |
| ENSG00000168872 | HGLibA_12662 | 169 | 0.533123366 | DDX19A | DEAD-box helicase 19A [Source:HGNC Symbol;Acc:HGNC:25628] |
| ENSG00000168884 | HGLibA_50839 | 169 | 0.533123366 | TNIP2 | TNFAIP3 interacting protein 2 [Source:HGNC Symbol;Acc:HGNC:19118] |
| ENSG00000169021 | HGLibA_53118 | 169 | 0.533123366 | UQCRFS1 | Rieske iron-sulfur polypeptide 1 [Source:HGNC Symbol;Acc:HGNC:12587]" |
| ENSG00000170613 | HGLibA_16689 | 169 | 0.533123366 | FAM71B | family with sequence similarity 71 member B [Source:HGNC Symbol;Acc:HGNC:28397] |
| ENSG00000171017 | HGLibA_27440 | 169 | 0.533123366 | LRRC8E | leucine rich repeat containing 8 VRAC subunit E [Source:HGNC Symbol;Acc:HGNC:26272] |
| ENSG00000173114 | HGLibA_27476 | 169 | 0.533123366 | LRRN3 | leucine rich repeat neuronal 3 [Source:HGNC Symbol;Acc:HGNC:17200] |
| ENSG00000173705 | HGLibA_47855 | 169 | 0.533123366 | SUSD5 | sushi domain containing 5 [Source:HGNC Symbol;Acc:HGNC:29061] |
| ENSG00000176040 | HGLibA_50651 | 169 | 0.533123366 | TMPRSS7 | transmembrane serine protease 7 [Source:HGNC Symbol;Acc:HGNC:30846] |
| ENSG00000181019 | HGLibA_32563 | 169 | 0.533123366 | NQO1 | NAD(P)H quinone dehydrogenase 1 [Source:HGNC Symbol;Acc:HGNC:2874] |
| ENSG00000181761 | HGLibA_34531 | 169 | 0.533123366 | OR8H3 | olfactory receptor family 8 subfamily H member 3 [Source:HGNC Symbol;Acc:HGNC:15309] |
| ENSG00000182676 | HGLibA_38082 | 169 | 0.533123366 | PPP1R27 | protein phosphatase 1 regulatory subunit 27 [Source:HGNC Symbol;Acc:HGNC:16813] |
| ENSG00000182771 | HGLibA_20271 | 169 | 0.533123366 | GRID1 | glutamate ionotropic receptor delta type subunit 1 [Source:HGNC Symbol;Acc:HGNC:4575] |
| ENSG00000183305 | HGLibA_27909 | 169 | 0.533123366 | MAGEA2B | MAGE family member A2B [Source:HGNC Symbol;Acc:HGNC:19340] |
| ENSG00000183742 | HGLibA_27829 | 169 | 0.533123366 | MACC1 | MET transcriptional regulator MACC1 [Source:HGNC Symbol;Acc:HGNC:30215] |
| ENSG00000183850 | HGLibA_56645 | 169 | 0.533123366 | ZNF730 | zinc finger protein 730 [Source:HGNC Symbol;Acc:HGNC:32470] |
| ENSG00000185156 | HGLibA_29112 | 169 | 0.533123366 | MFSD6L | major facilitator superfamily domain containing 6 like [Source:HGNC Symbol;Acc:HGNC:26656] |
| ENSG00000186448 | HGLibA_55561 | 169 | 0.533123366 | ZNF197 | zinc finger protein 197 [Source:HGNC Symbol;Acc:HGNC:12988] |
| ENSG00000189181 | HGLibA_33634 | 169 | 0.533123366 | OR14I1 | olfactory receptor family 14 subfamily I member 1 [Source:HGNC Symbol;Acc:HGNC:19575] |
| ENSG00000189326 | HGLibA_46411 | 169 | 0.533123366 | SPANXN4 | SPANX family member N4 [Source:HGNC Symbol;Acc:HGNC:33177] |
| ENSG00000196839 | HGLibA_00715 | 169 | 0.533123366 | ADA | adenosine deaminase [Source:HGNC Symbol;Acc:HGNC:186] |
| ENSG00000197406 | HGLibA_13243 | 169 | 0.533123366 | DIO3 | iodothyronine deiodinase 3 [Source:HGNC Symbol;Acc:HGNC:2885] |
| ENSG00000197769 | HGLibA_28131 | 169 | 0.533123366 | MAP1LC3C | microtubule associated protein 1 light chain 3 gamma [Source:HGNC Symbol;Acc:HGNC:13353] |
| ENSG00000203785 | HGLibA_46816 | 169 | 0.533123366 | SPRR2E | small proline rich protein 2E [Source:HGNC Symbol;Acc:HGNC:11265] |
| ENSG00000203970 | HGLibA_12845 | 169 | 0.533123366 | DEFB110 | defensin beta 110 [Source:HGNC Symbol;Acc:HGNC:18091] |
| ENSG00000204172 | HGLibA_01164 | 169 | 0.533123366 | AGAP9 | ankyrin repeat and PH domain 9 [Source:HGNC Symbol;Acc:HGNC:23463]" |
| ENSG00000205269 | HGLibA_50058 | 169 | 0.533123366 | TMEM170B | transmembrane protein 170B [Source:HGNC Symbol;Acc:HGNC:34244] |
| ENSG00000213923 | HGLibA_11391 | 169 | 0.533123366 | CSNK1E | casein kinase 1 epsilon [Source:HGNC Symbol;Acc:HGNC:2453] |
| ENSG00000215262 | HGLibA_24400 | 169 | 0.533123366 | KCNU1 | potassium calcium-activated channel subfamily U member 1 [Source:HGNC Symbol;Acc:HGNC:18867] |
| ENSG00000231068 | HGLibA_25713 | 169 | 0.533123366 | KRTAP21-3 | keratin associated protein 21-3 [Source:HGNC Symbol;Acc:HGNC:34216] |
| ENSG00000235194 | HGLibA_38111 | 169 | 0.533123366 | PPP1R3E | protein phosphatase 1 regulatory subunit 3E [Source:HGNC Symbol;Acc:HGNC:14943] |
| ENSG00000241685 | HGLibA_03074 | 169 | 0.533123366 | ARPC1A | actin related protein 2/3 complex subunit 1A [Source:HGNC Symbol;Acc:HGNC:703] |
| ENSG00000244486 | HGLibA_42878 | 169 | 0.533123366 | SCARF2 | scavenger receptor class F member 2 [Source:HGNC Symbol;Acc:HGNC:19869] |
| ENSG00000284922 | HGLibA_27506 | 169 | 0.533123366 | LRTOMT | novel protein |
| EPRS | HGLibA_15364 | 169 | 0.533123366 | EPRS | - |
| FAM19A3 | HGLibA_16403 | 169 | 0.533123366 | FAM19A3 | - |
| FIGF | HGLibA_17512 | 169 | 0.533123366 | FIGF | - |
| hsa-mir-339 | HGLibA_59006 | 169 | 0.533123366 | hsa-mir-339 | - |
| hsa-mir-3681 | HGLibA_59277 | 169 | 0.533123366 | hsa-mir-3681 | - |
| hsa-mir-3691 | HGLibA_59354 | 169 | 0.533123366 | hsa-mir-3691 | - |
| hsa-mir-3714 | HGLibA_59368 | 169 | 0.533123366 | hsa-mir-3714 | - |
| hsa-mir-425 | HGLibA_59708 | 169 | 0.533123366 | hsa-mir-425 | - |
| hsa-mir-4297 | HGLibA_59899 | 169 | 0.533123366 | hsa-mir-4297 | - |
| hsa-mir-451a | HGLibA_60524 | 169 | 0.533123366 | hsa-mir-451a | - |
| hsa-mir-4527 | HGLibA_60571 | 169 | 0.533123366 | hsa-mir-4527 | - |
| hsa-mir-601 | HGLibA_62496 | 169 | 0.533123366 | hsa-mir-601 | - |
| hsa-mir-6129 | HGLibA_62665 | 169 | 0.533123366 | hsa-mir-6129 | - |
| hsa-mir-7156 | HGLibA_63852 | 169 | 0.533123366 | hsa-mir-7156 | - |
| WDR66 | HGLibA_54155 | 169 | 0.533123366 | WDR66 | - |
| C15orf57 | HGLibA_05420 | 168 | 0.523172329 | C15orf57 | - |
| ENSG00000008735 | HGLibA_28289 | 168 | 0.523172329 | MAPK8IP2 | mitogen-activated protein kinase 8 interacting protein 2 [Source:HGNC Symbol;Acc:HGNC:6883] |
| ENSG00000015520 | HGLibA_32420 | 168 | 0.523172329 | NPC1L1 | NPC1 like intracellular cholesterol transporter 1 [Source:HGNC Symbol;Acc:HGNC:7898] |
| ENSG00000047648 | HGLibA_02785 | 168 | 0.523172329 | ARHGAP6 | Rho GTPase activating protein 6 [Source:HGNC Symbol;Acc:HGNC:676] |
| ENSG00000049449 | HGLibA_40700 | 168 | 0.523172329 | RCN1 | reticulocalbin 1 [Source:HGNC Symbol;Acc:HGNC:9934] |
| ENSG00000050628 | HGLibA_39318 | 168 | 0.523172329 | PTGER3 | prostaglandin E receptor 3 [Source:HGNC Symbol;Acc:HGNC:9595] |
| ENSG00000076344 | HGLibA_41056 | 168 | 0.523172329 | RGS11 | regulator of G protein signaling 11 [Source:HGNC Symbol;Acc:HGNC:9993] |
| ENSG00000078668 | HGLibA_53605 | 168 | 0.523172329 | VDAC3 | voltage dependent anion channel 3 [Source:HGNC Symbol;Acc:HGNC:12674] |
| ENSG00000082516 | HGLibA_18918 | 168 | 0.523172329 | GEMIN5 | gem nuclear organelle associated protein 5 [Source:HGNC Symbol;Acc:HGNC:20043] |
| ENSG00000083312 | HGLibA_50899 | 168 | 0.523172329 | TNPO1 | transportin 1 [Source:HGNC Symbol;Acc:HGNC:6401] |
| ENSG00000100078 | HGLibA_36896 | 168 | 0.523172329 | PLA2G3 | phospholipase A2 group III [Source:HGNC Symbol;Acc:HGNC:17934] |
| ENSG00000100426 | HGLibA_54800 | 168 | 0.523172329 | ZBED4 | zinc finger BED-type containing 4 [Source:HGNC Symbol;Acc:HGNC:20721] |
| ENSG00000100814 | HGLibA_08059 | 168 | 0.523172329 | CCNB1IP1 | cyclin B1 interacting protein 1 [Source:HGNC Symbol;Acc:HGNC:19437] |
| ENSG00000102543 | HGLibA_08500 | 168 | 0.523172329 | CDADC1 | cytidine and dCMP deaminase domain containing 1 [Source:HGNC Symbol;Acc:HGNC:20299] |
| ENSG00000102804 | HGLibA_51816 | 168 | 0.523172329 | TSC22D1 | TSC22 domain family member 1 [Source:HGNC Symbol;Acc:HGNC:16826] |
| ENSG00000104883 | HGLibA_36091 | 168 | 0.523172329 | PEX11G | peroxisomal biogenesis factor 11 gamma [Source:HGNC Symbol;Acc:HGNC:20208] |
| ENSG00000104915 | HGLibA_47614 | 168 | 0.523172329 | STX10 | syntaxin 10 [Source:HGNC Symbol;Acc:HGNC:11428] |
| ENSG00000108639 | HGLibA_47993 | 168 | 0.523172329 | SYNGR2 | synaptogyrin 2 [Source:HGNC Symbol;Acc:HGNC:11499] |
| ENSG00000109163 | HGLibA_19585 | 168 | 0.523172329 | GNRHR | gonadotropin releasing hormone receptor [Source:HGNC Symbol;Acc:HGNC:4421] |
| ENSG00000112511 | HGLibA_36329 | 168 | 0.523172329 | PHF1 | PHD finger protein 1 [Source:HGNC Symbol;Acc:HGNC:8919] |
| ENSG00000114771 | HGLibA_00028 | 168 | 0.523172329 | AADAC | arylacetamide deacetylase [Source:HGNC Symbol;Acc:HGNC:17] |
| ENSG00000115266 | HGLibA_02391 | 168 | 0.523172329 | APC2 | WNT signaling pathway regulator [Source:HGNC Symbol;Acc:HGNC:24036]" |
| ENSG00000115365 | HGLibA_26004 | 168 | 0.523172329 | LANCL1 | LanC like 1 [Source:HGNC Symbol;Acc:HGNC:6508] |
| ENSG00000116062 | HGLibA_30201 | 168 | 0.523172329 | MSH6 | mutS homolog 6 [Source:HGNC Symbol;Acc:HGNC:7329] |
| ENSG00000122574 | HGLibA_54309 | 168 | 0.523172329 | WIPF3 | WAS/WASL interacting protein family member 3 [Source:HGNC Symbol;Acc:HGNC:22004] |
| ENSG00000123977 | HGLibA_12347 | 168 | 0.523172329 | DAW1 | dynein assembly factor with WD repeats 1 [Source:HGNC Symbol;Acc:HGNC:26383] |
| ENSG00000124357 | HGLibA_31067 | 168 | 0.523172329 | NAGK | N-acetylglucosamine kinase [Source:HGNC Symbol;Acc:HGNC:17174] |
| ENSG00000124657 | HGLibA_33772 | 168 | 0.523172329 | OR2B6 | olfactory receptor family 2 subfamily B member 6 [Source:HGNC Symbol;Acc:HGNC:8241] |
| ENSG00000124780 | HGLibA_24312 | 168 | 0.523172329 | KCNK17 | potassium two pore domain channel subfamily K member 17 [Source:HGNC Symbol;Acc:HGNC:14465] |
| ENSG00000126970 | HGLibA_55000 | 168 | 0.523172329 | ZC4H2 | zinc finger C4H2-type containing [Source:HGNC Symbol;Acc:HGNC:24931] |
| ENSG00000127083 | HGLibA_33382 | 168 | 0.523172329 | OMD | osteomodulin [Source:HGNC Symbol;Acc:HGNC:8134] |
| ENSG00000127585 | HGLibA_16984 | 168 | 0.523172329 | FBXL16 | F-box and leucine rich repeat protein 16 [Source:HGNC Symbol;Acc:HGNC:14150] |
| ENSG00000129993 | HGLibA_07389 | 168 | 0.523172329 | CBFA2T3 | CBFA2/RUNX1 translocation partner 3 [Source:HGNC Symbol;Acc:HGNC:1537] |

| | | | | | |
|---|---|---|---|---|---|
| ENSG00000132429 | HGLibA_37702 | 168 | 0.523172329 | POPDC3 | popeye domain containing 3 [Source:HGNC Symbol;Acc:HGNC:17649] |
| ENSG00000132855 | HGLibA_01915 | 168 | 0.523172329 | ANGPTL3 | angiopoietin like 3 [Source:HGNC Symbol;Acc:HGNC:491] |
| ENSG00000135517 | HGLibA_29290 | 168 | 0.523172329 | MIP | major intrinsic protein of lens fiber [Source:HGNC Symbol;Acc:HGNC:7103] |
| ENSG00000135686 | HGLibA_25198 | 168 | 0.523172329 | KLHL36 | kelch like family member 36 [Source:HGNC Symbol;Acc:HGNC:17844] |
| ENSG00000136352 | HGLibA_32032 | 168 | 0.523172329 | NKX2-1 | NK2 homeobox 1 [Source:HGNC Symbol;Acc:HGNC:11825] |
| ENSG00000137825 | HGLibA_23844 | 168 | 0.523172329 | ITPKA | inositol-trisphosphate 3-kinase A [Source:HGNC Symbol;Acc:HGNC:6178] |
| ENSG00000138081 | HGLibA_17028 | 168 | 0.523172329 | FBXO11 | F-box protein 11 [Source:HGNC Symbol;Acc:HGNC:13590] |
| ENSG00000139350 | HGLibA_31600 | 168 | 0.523172329 | NEDD1 | developmentally down-regulated 1 [Source:HGNC Symbol;Acc:HGNC:7723]" |
| ENSG00000141198 | HGLibA_50949 | 168 | 0.523172329 | TOM1L1 | target of myb1 like 1 membrane trafficking protein [Source:HGNC Symbol;Acc:HGNC:11983] |
| ENSG00000141295 | HGLibA_43053 | 168 | 0.523172329 | SCRN2 | secernin 2 [Source:HGNC Symbol;Acc:HGNC:30381] |
| ENSG00000143452 | HGLibA_21888 | 168 | 0.523172329 | HORMAD1 | HORMA domain containing 1 [Source:HGNC Symbol;Acc:HGNC:25245] |
| ENSG00000145779 | HGLibA_50717 | 168 | 0.523172329 | TNFAIP8 | TNF alpha induced protein 8 [Source:HGNC Symbol;Acc:HGNC:17260] |
| ENSG00000148926 | HGLibA_00993 | 168 | 0.523172329 | ADM | adrenomedullin [Source:HGNC Symbol;Acc:HGNC:259] |
| ENSG00000158639 | HGLibA_35017 | 168 | 0.523172329 | PAGE5 | PAGE family member 5 [Source:HGNC Symbol;Acc:HGNC:29992] |
| ENSG00000160613 | HGLibA_35685 | 168 | 0.523172329 | PCSK7 | proprotein convertase subtilisin/kexin type 7 [Source:HGNC Symbol;Acc:HGNC:8748] |
| ENSG00000163519 | HGLibA_51343 | 168 | 0.523172329 | TRAT1 | T cell receptor associated transmembrane adaptor 1 [Source:HGNC Symbol;Acc:HGNC:30698] |
| ENSG00000163607 | HGLibA_20622 | 168 | 0.523172329 | GTPBP8 | GTP binding protein 8 (putative) [Source:HGNC Symbol;Acc:HGNC:25007] |
| ENSG00000165025 | HGLibA_47950 | 168 | 0.523172329 | SYK | spleen associated tyrosine kinase [Source:HGNC Symbol;Acc:HGNC:11491] |
| ENSG00000165475 | HGLibA_11287 | 168 | 0.523172329 | CRYL1 | crystallin lambda 1 [Source:HGNC Symbol;Acc:HGNC:18246] |
| ENSG00000169247 | HGLibA_43983 | 168 | 0.523172329 | SH3TC2 | SH3 domain and tetratricopeptide repeats 2 [Source:HGNC Symbol;Acc:HGNC:29427] |
| ENSG00000169570 | HGLibA_14077 | 168 | 0.523172329 | DTWD2 | DTW domain containing 2 [Source:HGNC Symbol;Acc:HGNC:19334] |
| ENSG00000169696 | HGLibA_03364 | 168 | 0.523172329 | ASPSCR1 | UBX domain containing tether for SLC2A4 [Source:HGNC Symbol;Acc:HGNC:13825]" |
| ENSG00000170370 | HGLibA_15096 | 168 | 0.523172329 | EMX2 | empty spiracles homeobox 2 [Source:HGNC Symbol;Acc:HGNC:3341] |
| ENSG00000171148 | HGLibA_48193 | 168 | 0.523172329 | TADA3 | transcriptional adaptor 3 [Source:HGNC Symbol;Acc:HGNC:19422] |
| ENSG00000172365 | HGLibA_34262 | 168 | 0.523172329 | OR5B2 | olfactory receptor family 5 subfamily B member 2 [Source:HGNC Symbol;Acc:HGNC:8323] |
| ENSG00000172548 | HGLibA_31964 | 168 | 0.523172329 | NIPAL4 | NIPA like domain containing 4 [Source:HGNC Symbol;Acc:HGNC:28018] |
| ENSG00000172551 | HGLibA_30559 | 168 | 0.523172329 | MUCL1 | mucin like 1 [Source:HGNC Symbol;Acc:HGNC:30588] |
| ENSG00000173950 | HGLibA_54612 | 168 | 0.523172329 | XXYLT1 | xyloside xylosyltransferase 1 [Source:HGNC Symbol;Acc:HGNC:26639] |
| ENSG00000174460 | HGLibA_55009 | 168 | 0.523172329 | ZCCHC12 | zinc finger CCHC-type containing 12 [Source:HGNC Symbol;Acc:HGNC:27273] |
| ENSG00000174957 | HGLibA_34307 | 168 | 0.523172329 | OR5J2 | olfactory receptor family 5 subfamily J member 2 [Source:HGNC Symbol;Acc:HGNC:19612] |
| ENSG00000176402 | HGLibA_19184 | 168 | 0.523172329 | GJC3 | gap junction protein gamma 3 [Source:HGNC Symbol;Acc:HGNC:17495] |
| ENSG00000177150 | HGLibA_16452 | 168 | 0.523172329 | FAM210A | family with sequence similarity 210 member A [Source:HGNC Symbol;Acc:HGNC:28346] |
| ENSG00000177706 | HGLibA_16449 | 168 | 0.523172329 | FAM20C | golgi associated secretory pathway kinase [Source:HGNC Symbol;Acc:HGNC:22140]" |
| ENSG00000179071 | HGLibA_07915 | 168 | 0.523172329 | CCDC89 | coiled-coil domain containing 89 [Source:HGNC Symbol;Acc:HGNC:26762] |
| ENSG00000180720 | HGLibA_09555 | 168 | 0.523172329 | CHRM4 | cholinergic receptor muscarinic 4 [Source:HGNC Symbol;Acc:HGNC:1953] |
| ENSG00000181035 | HGLibA_44741 | 168 | 0.523172329 | SLC25A42 | solute carrier family 25 member 42 [Source:HGNC Symbol;Acc:HGNC:28380] |
| ENSG00000181903 | HGLibA_33963 | 168 | 0.523172329 | OR4C6 | olfactory receptor family 4 subfamily C member 6 [Source:HGNC Symbol;Acc:HGNC:14743] |
| ENSG00000182793 | HGLibA_20463 | 168 | 0.523172329 | GSTA5 | glutathione S-transferase alpha 5 [Source:HGNC Symbol;Acc:HGNC:19662] |
| ENSG00000184351 | HGLibA_25662 | 168 | 0.523172329 | KRTAP19-1 | keratin associated protein 19-1 [Source:HGNC Symbol;Acc:HGNC:18936] |
| ENSG00000184860 | HGLibA_43166 | 168 | 0.523172329 | SDR42E1 | member 1 [Source:HGNC Symbol;Acc:HGNC:29834]" |
| ENSG00000185177 | HGLibA_56064 | 168 | 0.523172329 | ZNF479 | zinc finger protein 479 [Source:HGNC Symbol;Acc:HGNC:23258] |
| ENSG00000186198 | HGLibA_45241 | 168 | 0.523172329 | SLC51B | solute carrier family 51 beta subunit [Source:HGNC Symbol;Acc:HGNC:29956] |
| ENSG00000186575 | HGLibA_31760 | 168 | 0.523172329 | NF2 | neurofibromin 2 [Source:HGNC Symbol;Acc:HGNC:7773] |
| ENSG00000187033 | HGLibA_42692 | 168 | 0.523172329 | SAMD7 | sterile alpha motif domain containing 7 [Source:HGNC Symbol;Acc:HGNC:25394] |
| ENSG00000187605 | HGLibA_49044 | 168 | 0.523172329 | TET3 | tet methylcytosine dioxygenase 3 [Source:HGNC Symbol;Acc:HGNC:28313] |
| ENSG00000187753 | HGLibA_06691 | 168 | 0.523172329 | C9orf153 | chromosome 9 open reading frame 153 [Source:HGNC Symbol;Acc:HGNC:31456] |
| ENSG00000187824 | HGLibA_50234 | 168 | 0.523172329 | TMEM220 | transmembrane protein 220 [Source:HGNC Symbol;Acc:HGNC:33757] |
| ENSG00000187860 | HGLibA_07662 | 168 | 0.523172329 | CCDC157 | coiled-coil domain containing 157 [Source:HGNC Symbol;Acc:HGNC:33854] |
| ENSG00000188338 | HGLibA_45068 | 168 | 0.523172329 | SLC38A3 | solute carrier family 38 member 3 [Source:HGNC Symbol;Acc:HGNC:18044] |
| ENSG00000188394 | HGLibA_19998 | 168 | 0.523172329 | GPR21 | G protein-coupled receptor 21 [Source:HGNC Symbol;Acc:HGNC:4476] |
| ENSG00000196196 | HGLibA_22088 | 168 | 0.523172329 | HRCT1 | histidine rich carboxyl terminus 1 [Source:HGNC Symbol;Acc:HGNC:33872] |
| ENSG00000196208 | HGLibA_20235 | 168 | 0.523172329 | GREB1 | growth regulating estrogen receptor binding 1 [Source:HGNC Symbol;Acc:HGNC:24885] |
| ENSG00000196338 | HGLibA_32074 | 168 | 0.523172329 | NLGN3 | neuroligin 3 [Source:HGNC Symbol;Acc:HGNC:14289] |
| ENSG00000196611 | HGLibA_29478 | 168 | 0.523172329 | MMP1 | matrix metallopeptidase 1 [Source:HGNC Symbol;Acc:HGNC:7155] |
| ENSG00000196946 | HGLibA_56588 | 168 | 0.523172329 | ZNF705A | zinc finger protein 705A [Source:HGNC Symbol;Acc:HGNC:32281] |
| ENSG00000197734 | HGLibA_05344 | 168 | 0.523172329 | C14orf178 | chromosome 14 open reading frame 178 [Source:HGNC Symbol;Acc:HGNC:26385] |
| ENSG00000197892 | HGLibA_24860 | 168 | 0.523172329 | KIF13B | kinesin family member 13B [Source:HGNC Symbol;Acc:HGNC:14405] |
| ENSG00000198570 | HGLibA_40724 | 168 | 0.523172329 | RD3 | GUCY2D regulator [Source:HGNC Symbol;Acc:HGNC:19689]" |
| ENSG00000198573 | HGLibA_46391 | 168 | 0.523172329 | SPANXC | SPANX family member C [Source:HGNC Symbol;Acc:HGNC:14331] |
| ENSG00000205409 | HGLibA_34163 | 168 | 0.523172329 | OR52E6 | olfactory receptor family 52 subfamily E member 6 [Source:HGNC Symbol;Acc:HGNC:15215] |
| ENSG00000206107 | HGLibA_25734 | 168 | 0.523172329 | KRTAP27-1 | keratin associated protein 27-1 [Source:HGNC Symbol;Acc:HGNC:33864] |
| ENSG00000225614 | HGLibA_56049 | 168 | 0.523172329 | ZNF469 | zinc finger protein 469 [Source:HGNC Symbol;Acc:HGNC:23216] |
| ENSG00000230062 | HGLibA_02142 | 168 | 0.523172329 | ANKRD66 | ankyrin repeat domain 66 [Source:HGNC Symbol;Acc:HGNC:44669] |
| ENSG00000238269 | HGLibA_35009 | 168 | 0.523172329 | PAGE2B | PAGE family member 2B [Source:HGNC Symbol;Acc:HGNC:31805] |
| ENSG00000261594 | HGLibA_51101 | 168 | 0.523172329 | TPBGL | trophoblast glycoprotein like [Source:HGNC Symbol;Acc:HGNC:44159] |
| ENSG00000265491 | HGLibA_41482 | 168 | 0.523172329 | RNF115 | ring finger protein 115 [Source:HGNC Symbol;Acc:HGNC:18154] |
| ENSG00000266524 | HGLibA_18855 | 168 | 0.523172329 | GDF10 | growth differentiation factor 10 [Source:HGNC Symbol;Acc:HGNC:4215] |
| ENSG00000280165 | HGLibA_35412 | 168 | 0.523172329 | PCDH20 | protocadherin 20 [Source:HGNC Symbol;Acc:HGNC:14257] |
| GPR116 | HGLibA_19863 | 168 | 0.523172329 | GPR116 | - |
| hsa-mir-222 | HGLibA_58295 | 168 | 0.523172329 | hsa-mir-222 | - |
| hsa-mir-3167 | HGLibA_58746 | 168 | 0.523172329 | hsa-mir-3167 | - |
| hsa-mir-3199-2 | HGLibA_58900 | 168 | 0.523172329 | hsa-mir-3199-2 | - |
| hsa-mir-4456 | HGLibA_60232 | 168 | 0.523172329 | hsa-mir-4456 | - |
| hsa-mir-4464 | HGLibA_60263 | 168 | 0.523172329 | hsa-mir-4464 | - |
| hsa-mir-449a | HGLibA_60418 | 168 | 0.523172329 | hsa-mir-449a | - |
| hsa-mir-4690 | HGLibA_60870 | 168 | 0.523172329 | hsa-mir-4690 | - |
| hsa-mir-548aj-2 | HGLibA_61893 | 168 | 0.523172329 | hsa-mir-548aj-2 | - |
| hsa-mir-5583-1 | HGLibA_62170 | 168 | 0.523172329 | hsa-mir-5583-1 | - |
| hsa-mir-760 | HGLibA_63899 | 168 | 0.523172329 | hsa-mir-760 | - |
| hsa-mir-8062 | HGLibA_64087 | 168 | 0.523172329 | hsa-mir-8062 | - |
| hsa-mir-8065 | HGLibA_64098 | 168 | 0.523172329 | hsa-mir-8065 | - |
| METTL11B | HGLibA_28949 | 168 | 0.523172329 | METTL11B | - |
| MTSS1L | HGLibA_30498 | 168 | 0.523172329 | MTSS1L | - |
| NUTMF | HGLibA_33114 | 168 | 0.523172329 | NUTMF | - |
| C3orf58 | HGLibA_06254 | 167 | 0.513221291 | C3orf58 | - |
| ENSG00000007080 | HGLibA_07573 | 167 | 0.513221291 | CCDC124 | coiled-coil domain containing 124 [Source:HGNC Symbol;Acc:HGNC:25171] |
| ENSG00000023287 | HGLibA_40415 | 167 | 0.513221291 | RB1CC1 | RB1 inducible coiled-coil 1 [Source:HGNC Symbol;Acc:HGNC:15574] |
| ENSG00000031081 | HGLibA_02756 | 167 | 0.513221291 | ARHGAP31 | Rho GTPase activating protein 31 [Source:HGNC Symbol;Acc:HGNC:29216] |
| ENSG00000042317 | HGLibA_46529 | 167 | 0.513221291 | SPATA7 | spermatogenesis associated 7 [Source:HGNC Symbol;Acc:HGNC:20423] |
| ENSG00000047621 | HGLibA_05236 | 167 | 0.513221291 | C12orf4 | chromosome 12 open reading frame 4 [Source:HGNC Symbol;Acc:HGNC:1184] |
| ENSG00000067836 | HGLibA_41751 | 167 | 0.513221291 | ROGDI | rogdi homolog [Source:HGNC Symbol;Acc:HGNC:29478] |
| ENSG00000068878 | HGLibA_39199 | 167 | 0.513221291 | PSME4 | proteasome activator subunit 4 [Source:HGNC Symbol;Acc:HGNC:20635] |
| ENSG00000071991 | HGLibA_08656 | 167 | 0.513221291 | CDH19 | cadherin 19 [Source:HGNC Symbol;Acc:HGNC:1758] |
| ENSG00000085276 | HGLibA_28725 | 167 | 0.513221291 | MECOM | MDS1 and EVI1 complex locus [Source:HGNC Symbol;Acc:HGNC:3498] |
| ENSG00000087116 | HGLibA_00822 | 167 | 0.513221291 | ADAMTS2 | ADAM metallopeptidase with thrombospondin type 1 motif 2 [Source:HGNC Symbol;Acc:HGNC:218] |
| ENSG00000089006 | HGLibA_46098 | 167 | 0.513221291 | SNX5 | sorting nexin 5 [Source:HGNC Symbol;Acc:HGNC:14969] |
| ENSG00000091129 | HGLibA_32660 | 167 | 0.513221291 | NRCAM | neuronal cell adhesion molecule [Source:HGNC Symbol;Acc:HGNC:7994] |
| ENSG00000095303 | HGLibA_39358 | 167 | 0.513221291 | PTGS1 | prostaglandin-endoperoxide synthase 1 [Source:HGNC Symbol;Acc:HGNC:9604] |
| ENSG00000101470 | HGLibA_50868 | 167 | 0.513221291 | TNNC2 | fast skeletal type [Source:HGNC Symbol;Acc:HGNC:11944]" |
| ENSG00000102054 | HGLibA_40436 | 167 | 0.513221291 | RBBP7 | chromatin remodeling factor [Source:HGNC Symbol;Acc:HGNC:9890]" |
| ENSG00000102891 | HGLibA_30306 | 167 | 0.513221291 | MT4 | metallothionein 4 [Source:HGNC Symbol;Acc:HGNC:18705] |
| ENSG00000103310 | HGLibA_56952 | 167 | 0.513221291 | ZP2 | zona pellucida glycoprotein 2 [Source:HGNC Symbol;Acc:HGNC:13188] |
| ENSG00000105642 | HGLibA_24354 | 167 | 0.513221291 | KCNN1 | potassium calcium-activated channel subfamily N member 1 [Source:HGNC Symbol;Acc:HGNC:6290] |
| ENSG00000108349 | HGLibA_07274 | 167 | 0.513221291 | CASC3 | exon junction complex subunit [Source:HGNC Symbol;Acc:HGNC:17040]" |
| ENSG00000111859 | HGLibA_31615 | 167 | 0.513221291 | NEDD9 | developmentally down-regulated 9 [Source:HGNC Symbol;Acc:HGNC:7733]" |
| ENSG00000112182 | HGLibA_04069 | 167 | 0.513221291 | BACH2 | BTB domain and CNC homolog 2 [Source:HGNC Symbol;Acc:HGNC:14078] |
| ENSG00000113369 | HGLibA_03120 | 167 | 0.513221291 | ARRDC3 | arrestin domain containing 3 [Source:HGNC Symbol;Acc:HGNC:29263] |
| ENSG00000114779 | HGLibA_00252 | 167 | 0.513221291 | ABHD14B | abhydrolase domain containing 14B [Source:HGNC Symbol;Acc:HGNC:28235] |
| ENSG00000116726 | HGLibA_38288 | 167 | 0.513221291 | PRAMEF12 | PRAME family member 12 [Source:HGNC Symbol;Acc:HGNC:22125] |
| ENSG00000117834 | HGLibA_45285 | 167 | 0.513221291 | SLC5A9 | solute carrier family 5 member 9 [Source:HGNC Symbol;Acc:HGNC:22146] |
| ENSG00000121742 | HGLibA_19173 | 167 | 0.513221291 | GJB6 | gap junction protein beta 6 [Source:HGNC Symbol;Acc:HGNC:4288] |
| ENSG00000124089 | HGLibA_28547 | 167 | 0.513221291 | MC3R | melanocortin 3 receptor [Source:HGNC Symbol;Acc:HGNC:6931] |
| ENSG00000124256 | HGLibA_54808 | 167 | 0.513221291 | ZBP1 | Z-DNA binding protein 1 [Source:HGNC Symbol;Acc:HGNC:16176] |
| ENSG00000125522 | HGLibA_32416 | 167 | 0.513221291 | NPBWR2 | neuropeptides B and W receptor 2 [Source:HGNC Symbol;Acc:HGNC:4530] |
| ENSG00000125551 | HGLibA_37164 | 167 | 0.513221291 | PLGLB2 | plasminogen-like B2 [Source:HGNC Symbol;Acc:HGNC:9073] |
| ENSG00000134830 | HGLibA_06357 | 167 | 0.513221291 | C5AR2 | complement component 5a receptor 2 [Source:HGNC Symbol;Acc:HGNC:4527] |
| ENSG00000137210 | HGLibA_49983 | 167 | 0.513221291 | TMEM14B | transmembrane protein 14B [Source:HGNC Symbol;Acc:HGNC:21384] |
| ENSG00000139624 | HGLibA_09213 | 167 | 0.513221291 | CERS5 | ceramide synthase 5 [Source:HGNC Symbol;Acc:HGNC:23749] |
| ENSG00000140067 | HGLibA_16319 | 167 | 0.513221291 | FAM181A | family with sequence similarity 181 member A [Source:HGNC Symbol;Acc:HGNC:20491] |
| ENSG00000145975 | HGLibA_16488 | 167 | 0.513221291 | FAM217A | family with sequence similarity 217 member A [Source:HGNC Symbol;Acc:HGNC:21362] |
| ENSG00000149243 | HGLibA_25194 | 167 | 0.513221291 | KLHL35 | kelch like family member 35 [Source:HGNC Symbol;Acc:HGNC:26597] |
| ENSG00000150347 | HGLibA_02909 | 167 | 0.513221291 | ARID5B | AT-rich interaction domain 5B [Source:HGNC Symbol;Acc:HGNC:17362] |
| ENSG00000152284 | HGLibA_48795 | 167 | 0.513221291 | TCF7L1 | transcription factor 7 like 1 [Source:HGNC Symbol;Acc:HGNC:11640] |
| ENSG00000154114 | HGLibA_48597 | 167 | 0.513221291 | TBCEL | tubulin folding cofactor E like [Source:HGNC Symbol;Acc:HGNC:28115] |
| ENSG00000155506 | HGLibA_26028 | 167 | 0.513221291 | LARP1 | La ribonucleoprotein domain family member 1 [Source:HGNC Symbol;Acc:HGNC:29531] |
| ENSG00000155545 | HGLibA_29258 | 167 | 0.513221291 | MIER3 | MIER family member 3 [Source:HGNC Symbol;Acc:HGNC:26678] |
| ENSG00000157873 | HGLibA_50761 | 167 | 0.513221291 | TNFRSF14 | TNF receptor superfamily member 14 [Source:HGNC Symbol;Acc:HGNC:11912] |
| ENSG00000159556 | HGLibA_23668 | 167 | 0.513221291 | ISL2 | ISL LIM homeobox 2 [Source:HGNC Symbol;Acc:HGNC:18524] |
| ENSG00000162729 | HGLibA_22902 | 167 | 0.513221291 | IGSF8 | immunoglobulin superfamily member 8 [Source:HGNC Symbol;Acc:HGNC:17813] |
| ENSG00000163515 | HGLibA_40879 | 167 | 0.513221291 | RETNLB | resistin like beta [Source:HGNC Symbol;Acc:HGNC:20388] |
| ENSG00000163545 | HGLibA_32896 | 167 | 0.513221291 | NUAK2 | NUAK family kinase 2 [Source:HGNC Symbol;Acc:HGNC:29558] |
| ENSG00000163565 | HGLibA_22594 | 167 | 0.513221291 | IFI16 | interferon gamma inducible protein 16 [Source:HGNC Symbol;Acc:HGNC:5395] |
| ENSG00000164038 | HGLibA_45435 | 167 | 0.513221291 | SLC9B2 | solute carrier family 9 member B2 [Source:HGNC Symbol;Acc:HGNC:25143] |
| ENSG00000165948 | HGLibA_22600 | 167 | 0.513221291 | IFI27L1 | interferon alpha inducible protein 27 like 1 [Source:HGNC Symbol;Acc:HGNC:19754] |
| ENSG00000168135 | HGLibA_24279 | 167 | 0.513221291 | KCNJ4 | potassium voltage-gated channel subfamily J member 4 [Source:HGNC Symbol;Acc:HGNC:6265] |

| | | | | | |
|---|---|---|---|---|---|
| ENSG00000168398 | HGLibA_04383 | 167 | 0.513221291 | BDKRB2 | bradykinin receptor B2 [Source:HGNC Symbol;Acc:HGNC:1030] |
| ENSG00000170619 | HGLibA_10669 | 167 | 0.513221291 | COMMD5 | COMM domain containing 5 [Source:HGNC Symbol;Acc:HGNC:17902] |
| ENSG00000172458 | HGLibA_22998 | 167 | 0.513221291 | IL17D | interleukin 17D [Source:HGNC Symbol;Acc:HGNC:5984] |
| ENSG00000175093 | HGLibA_46867 | 167 | 0.513221291 | SPSB4 | splA/ryanodine receptor domain and SOCS box containing 4 [Source:HGNC Symbol;Acc:HGNC:30630] |
| ENSG00000175175 | HGLibA_37971 | 167 | 0.513221291 | PPM1E | Mg2+/Mn2+ dependent 1E [Source:HGNC Symbol;Acc:HGNC:19322]" |
| ENSG00000175782 | HGLibA_44994 | 167 | 0.513221291 | SLC35E3 | solute carrier family 35 member E3 [Source:HGNC Symbol;Acc:HGNC:20864] |
| ENSG00000179242 | HGLibA_08680 | 167 | 0.513221291 | CDH4 | cadherin 4 [Source:HGNC Symbol;Acc:HGNC:1763] |
| ENSG00000179841 | HGLibA_01435 | 167 | 0.513221291 | AKAP5 | A-kinase anchoring protein 5 [Source:HGNC Symbol;Acc:HGNC:375] |
| ENSG00000180386 | HGLibA_25845 | 167 | 0.513221291 | KRTAP9-7 | keratin associated protein 9-7 [Source:HGNC Symbol;Acc:HGNC:18915] |
| ENSG00000181927 | HGLibA_34056 | 167 | 0.513221291 | OR4P4 | olfactory receptor family 4 subfamily P member 4 [Source:HGNC Symbol;Acc:HGNC:15180] |
| ENSG00000182053 | HGLibA_51525 | 167 | 0.513221291 | TRIM49B | tripartite motif containing 49B [Source:HGNC Symbol;Acc:HGNC:42955] |
| ENSG00000183576 | HGLibA_43645 | 167 | 0.513221291 | SETD3 | SET domain containing 3 [Source:HGNC Symbol;Acc:HGNC:20493] |
| ENSG00000183726 | HGLibA_50422 | 167 | 0.513221291 | TMEM50A | transmembrane protein 50A [Source:HGNC Symbol;Acc:HGNC:30590] |
| ENSG00000185467 | HGLibA_25373 | 167 | 0.513221291 | KPNA7 | karyopherin subunit alpha 7 [Source:HGNC Symbol;Acc:HGNC:21839] |
| ENSG00000185897 | HGLibA_17331 | 167 | 0.513221291 | FFAR3 | free fatty acid receptor 3 [Source:HGNC Symbol;Acc:HGNC:4499] |
| ENSG00000186792 | HGLibA_22443 | 167 | 0.513221291 | HYAL3 | hyaluronidase 3 [Source:HGNC Symbol;Acc:HGNC:5322] |
| ENSG00000187003 | HGLibA_00627 | 167 | 0.513221291 | ACTL7A | actin like 7A [Source:HGNC Symbol;Acc:HGNC:161] |
| ENSG00000188848 | HGLibA_04406 | 167 | 0.513221291 | BEND4 | BEN domain containing 4 [Source:HGNC Symbol;Acc:HGNC:23815] |
| ENSG00000196196 | HGLibA_22087 | 167 | 0.513221291 | HRCT1 | histidine rich carboxyl terminus 1 [Source:HGNC Symbol;Acc:HGNC:33872] |
| ENSG00000196196 | HGLibA_22089 | 167 | 0.513221291 | HRCT1 | histidine rich carboxyl terminus 1 [Source:HGNC Symbol;Acc:HGNC:33872] |
| ENSG00000198646 | HGLibA_31351 | 167 | 0.513221291 | NCOA6 | nuclear receptor coactivator 6 [Source:HGNC Symbol;Acc:HGNC:15936] |
| ENSG00000204653 | HGLibA_03345 | 167 | 0.513221291 | ASPDH | aspartate dehydrogenase domain containing [Source:HGNC Symbol;Acc:HGNC:33856] |
| ENSG00000205923 | HGLibA_09030 | 167 | 0.513221291 | CEMP1 | cementum protein 1 [Source:HGNC Symbol;Acc:HGNC:32553] |
| ENSG00000214842 | HGLibA_40084 | 167 | 0.513221291 | RAD51AP2 | RAD51 associated protein 2 [Source:HGNC Symbol;Acc:HGNC:34417] |
| ENSG00000224940 | HGLibA_38890 | 167 | 0.513221291 | PRRT4 | proline rich transmembrane protein 4 [Source:HGNC Symbol;Acc:HGNC:37280] |
| ENSG00000236699 | HGLibA_02858 | 167 | 0.513221291 | ARHGEF38 | Rho guanine nucleotide exchange factor 38 [Source:HGNC Symbol;Acc:HGNC:25968] |
| ENSG00000258724 | HGLibA_36695 | 167 | 0.513221291 | PINX1 | PIN2 (TERF1) interacting telomerase inhibitor 1 [Source:HGNC Symbol;Acc:HGNC:30046] |
| ENSG00000274391 | HGLibA_51212 | 167 | 0.513221291 | TPTE | transmembrane phosphatase with tensin homology [Source:HGNC Symbol;Acc:HGNC:12023] |
| ENSG00000280236 | HGLibA_33586 | 167 | 0.513221291 | OR12D2 | olfactory receptor family 12 subfamily D member 2 (gene/pseudogene) [Source:HGNC Symbol;Acc:HGNC:8178] |
| ENSG00000283706 | HGLibA_38954 | 167 | 0.513221291 | PRSS50 | serine protease 50 [Source:NCBI gene;Acc:29122] |
| FAM175B | HGLibA_16291 | 167 | 0.513221291 | FAM175B | - |
| FAM35A | HGLibA_16574 | 167 | 0.513221291 | FAM35A | - |
| FAM46B | HGLibA_16599 | 167 | 0.513221291 | FAM46B | - |
| hsa-mir-2115 | HGLibA_58234 | 167 | 0.513221291 | hsa-mir-2115 | - |
| hsa-mir-4522 | HGLibA_60546 | 167 | 0.513221291 | hsa-mir-4522 | - |
| hsa-mir-500b | HGLibA_61447 | 167 | 0.513221291 | hsa-mir-500b | - |
| hsa-mir-548ax | HGLibA_61939 | 167 | 0.513221291 | hsa-mir-548ax | - |
| hsa-mir-6079 | HGLibA_62572 | 167 | 0.513221291 | hsa-mir-6079 | - |
| hsa-mir-668 | HGLibA_63004 | 167 | 0.513221291 | hsa-mir-668 | - |
| IMPAD1 | HGLibA_23253 | 167 | 0.513221291 | IMPAD1 | - |
| KIAA1715 | HGLibA_24807 | 167 | 0.513221291 | KIAA1715 | - |
| LOC100653515 | HGLibA_26876 | 167 | 0.513221291 | LOC100653515 | - |
| SLC24A6 | HGLibA_44631 | 167 | 0.513221291 | SLC24A6 | - |
| C2orf54 | HGLibA_06148 | 166 | 0.503270254 | C2orf54 | - |
| ENSG00000004776 | HGLibA_22314 | 166 | 0.503270254 | HSPB6 | heat shock protein family B (small) member 6 [Source:HGNC Symbol;Acc:HGNC:26511] |
| ENSG00000006756 | HGLibA_03135 | 166 | 0.503270254 | ARSD | arylsulfatase D [Source:HGNC Symbol;Acc:HGNC:717] |
| ENSG00000007237 | HGLibA_18655 | 166 | 0.503270254 | GAS7 | growth arrest specific 7 [Source:HGNC Symbol;Acc:HGNC:4169] |
| ENSG00000025423 | HGLibA_22195 | 166 | 0.503270254 | HSD17B6 | hydroxysteroid 17-beta dehydrogenase 6 [Source:HGNC Symbol;Acc:HGNC:23316] |
| ENSG00000033170 | HGLibA_18207 | 166 | 0.503270254 | FUT8 | fucosyltransferase 8 [Source:HGNC Symbol;Acc:HGNC:4019] |
| ENSG00000060491 | HGLibA_33316 | 166 | 0.503270254 | OGFR | opioid growth factor receptor [Source:HGNC Symbol;Acc:HGNC:15768] |
| ENSG00000065802 | HGLibA_03202 | 166 | 0.503270254 | ASB1 | ankyrin repeat and SOCS box containing 1 [Source:HGNC Symbol;Acc:HGNC:16011] |
| ENSG00000066651 | HGLibA_51668 | 166 | 0.503270254 | TRMT11 | tRNA methyltransferase 11 homolog [Source:HGNC Symbol;Acc:HGNC:21080] |
| ENSG00000068028 | HGLibA_40375 | 166 | 0.503270254 | RASSF1 | Ras association domain family member 1 [Source:HGNC Symbol;Acc:HGNC:9882] |
| ENSG00000088727 | HGLibA_24965 | 166 | 0.503270254 | KIF9 | kinesin family member 9 [Source:HGNC Symbol;Acc:HGNC:16666] |
| ENSG00000100234 | HGLibA_49540 | 166 | 0.503270254 | TIMP3 | TIMP metallopeptidase inhibitor 3 [Source:HGNC Symbol;Acc:HGNC:11822] |
| ENSG00000100342 | HGLibA_02526 | 166 | 0.503270254 | APOL1 | apolipoprotein L1 [Source:HGNC Symbol;Acc:HGNC:618] |
| ENSG00000100373 | HGLibA_53076 | 166 | 0.503270254 | UPK3A | uroplakin 3A [Source:HGNC Symbol;Acc:HGNC:12580] |
| ENSG00000100711 | HGLibA_55282 | 166 | 0.503270254 | ZFYVE21 | zinc finger FYVE-type containing 21 [Source:HGNC Symbol;Acc:HGNC:20760] |
| ENSG00000100979 | HGLibA_37246 | 166 | 0.503270254 | PLTP | phospholipid transfer protein [Source:HGNC Symbol;Acc:HGNC:9093] |
| ENSG00000102034 | HGLibA_14901 | 166 | 0.503270254 | ELF4 | E74 like ETS transcription factor 4 [Source:HGNC Symbol;Acc:HGNC:3319] |
| ENSG00000102683 | HGLibA_43809 | 166 | 0.503270254 | SGCG | sarcoglycan gamma [Source:HGNC Symbol;Acc:HGNC:10809] |
| ENSG00000106591 | HGLibA_29950 | 166 | 0.503270254 | MRPL32 | mitochondrial ribosomal protein L32 [Source:HGNC Symbol;Acc:HGNC:14035] |
| ENSG00000107864 | HGLibA_10924 | 166 | 0.503270254 | CPEB3 | cytoplasmic polyadenylation element binding protein 3 [Source:HGNC Symbol;Acc:HGNC:21746] |
| ENSG00000108924 | HGLibA_21655 | 166 | 0.503270254 | HLF | PAR bZIP transcription factor [Source:HGNC Symbol;Acc:HGNC:4977]" |
| ENSG00000111252 | HGLibA_43881 | 166 | 0.503270254 | SH2B3 | SH2B adaptor protein 3 [Source:HGNC Symbol;Acc:HGNC:29605] |
| ENSG00000113732 | HGLibA_03725 | 166 | 0.503270254 | ATP6V0E1 | ATPase H+ transporting V0 subunit e1 [Source:HGNC Symbol;Acc:HGNC:863] |
| ENSG00000114026 | HGLibA_33323 | 166 | 0.503270254 | OGG1 | 8-oxoguanine DNA glycosylase [Source:HGNC Symbol;Acc:HGNC:8125] |
| ENSG00000114757 | HGLibA_36121 | 166 | 0.503270254 | PEX5L | peroxisomal biogenesis factor 5 like [Source:HGNC Symbol;Acc:HGNC:30024] |
| ENSG00000115590 | HGLibA_23049 | 166 | 0.503270254 | IL1R2 | interleukin 1 receptor type 2 [Source:HGNC Symbol;Acc:HGNC:5994] |
| ENSG00000116337 | HGLibA_01827 | 166 | 0.503270254 | AMPD2 | adenosine monophosphate deaminase 2 [Source:HGNC Symbol;Acc:HGNC:469] |
| ENSG00000116761 | HGLibA_11625 | 166 | 0.503270254 | CTH | cystathionine gamma-lyase [Source:HGNC Symbol;Acc:HGNC:2501] |
| ENSG00000117009 | HGLibA_25317 | 166 | 0.503270254 | KMO | kynurenine 3-monooxygenase [Source:HGNC Symbol;Acc:HGNC:6381] |
| ENSG00000119231 | HGLibA_43393 | 166 | 0.503270254 | SENP5 | SUMO specific peptidase 5 [Source:HGNC Symbol;Acc:HGNC:28407] |
| ENSG00000120068 | HGLibA_21950 | 166 | 0.503270254 | HOXB8 | homeobox B8 [Source:HGNC Symbol;Acc:HGNC:5119] |
| ENSG00000123064 | HGLibA_12744 | 166 | 0.503270254 | DDX54 | DEAD-box helicase 54 [Source:HGNC Symbol;Acc:HGNC:20084] |
| ENSG00000124568 | HGLibA_44460 | 166 | 0.503270254 | SLC17A1 | solute carrier family 17 member 1 [Source:HGNC Symbol;Acc:HGNC:10929] |
| ENSG00000127914 | HGLibA_01451 | 166 | 0.503270254 | AKAP9 | A-kinase anchoring protein 9 [Source:HGNC Symbol;Acc:HGNC:379] |
| ENSG00000128463 | HGLibA_15014 | 166 | 0.503270254 | EMC4 | ER membrane protein complex subunit 4 [Source:HGNC Symbol;Acc:HGNC:28032] |
| ENSG00000128482 | HGLibA_41468 | 166 | 0.503270254 | RNF112 | ring finger protein 112 [Source:HGNC Symbol;Acc:HGNC:12968] |
| ENSG00000128536 | HGLibA_08704 | 166 | 0.503270254 | CDHR3 | cadherin related family member 3 [Source:HGNC Symbol;Acc:HGNC:26308] |
| ENSG00000129151 | HGLibA_04204 | 166 | 0.503270254 | BBOX1 | gamma-butyrobetaine hydroxylase 1 [Source:HGNC Symbol;Acc:HGNC:964] |
| ENSG00000129255 | HGLibA_29711 | 166 | 0.503270254 | MPDU1 | mannose-P-dolichol utilization defect 1 [Source:HGNC Symbol;Acc:HGNC:7207] |
| ENSG00000130812 | HGLibA_01925 | 166 | 0.503270254 | ANGPTL6 | angiopoietin like 6 [Source:HGNC Symbol;Acc:HGNC:23140] |
| ENSG00000131943 | HGLibA_05631 | 166 | 0.503270254 | C19orf12 | chromosome 19 open reading frame 12 [Source:HGNC Symbol;Acc:HGNC:25443] |
| ENSG00000132464 | HGLibA_15106 | 166 | 0.503270254 | ENAM | enamelin [Source:HGNC Symbol;Acc:HGNC:3344] |
| ENSG00000132763 | HGLibA_29457 | 166 | 0.503270254 | MMACHC | metabolism of cobalamin associated C [Source:HGNC Symbol;Acc:HGNC:24525] |
| ENSG00000132793 | HGLibA_27100 | 166 | 0.503270254 | LPIN3 | lipin 3 [Source:HGNC Symbol;Acc:HGNC:14451] |
| ENSG00000135914 | HGLibA_22374 | 166 | 0.503270254 | HTR2B | 5-hydroxytryptamine receptor 2B [Source:HGNC Symbol;Acc:HGNC:5294] |
| ENSG00000136492 | HGLibA_04821 | 166 | 0.503270254 | BRIP1 | BRCA1 interacting protein C-terminal helicase 1 [Source:HGNC Symbol;Acc:HGNC:20473] |
| ENSG00000136541 | HGLibA_15506 | 166 | 0.503270254 | ERMN | ermin [Source:HGNC Symbol;Acc:HGNC:29208] |
| ENSG00000136936 | HGLibA_54546 | 166 | 0.503270254 | XPA | DNA damage recognition and repair factor [Source:HGNC Symbol;Acc:HGNC:12814]" |
| ENSG00000137872 | HGLibA_43374 | 166 | 0.503270254 | SEMA6D | semaphorin 6D [Source:HGNC Symbol;Acc:HGNC:16770] |
| ENSG00000137975 | HGLibA_09809 | 166 | 0.503270254 | CLCA2 | chloride channel accessory 2 [Source:HGNC Symbol;Acc:HGNC:2016] |
| ENSG00000139725 | HGLibA_41186 | 166 | 0.503270254 | RHOF | filopodia associated [Source:HGNC Symbol;Acc:HGNC:15703]" |
| ENSG00000140395 | HGLibA_54142 | 166 | 0.503270254 | WDR61 | WD repeat domain 61 [Source:HGNC Symbol;Acc:HGNC:30300] |
| ENSG00000141338 | HGLibA_00126 | 166 | 0.503270254 | ABCA8 | ATP binding cassette subfamily A member 8 [Source:HGNC Symbol;Acc:HGNC:38] |
| ENSG00000142627 | HGLibA_15291 | 166 | 0.503270254 | EPHA2 | EPH receptor A2 [Source:HGNC Symbol;Acc:HGNC:3386] |
| ENSG00000143314 | HGLibA_29935 | 166 | 0.503270254 | MRPL24 | mitochondrial ribosomal protein L24 [Source:HGNC Symbol;Acc:HGNC:14037] |
| ENSG00000143333 | HGLibA_41066 | 166 | 0.503270254 | RGS16 | regulator of G protein signaling 16 [Source:HGNC Symbol;Acc:HGNC:9997] |
| ENSG00000144035 | HGLibA_31183 | 166 | 0.503270254 | NAT8 | N-acetyltransferase 8 (putative) [Source:HGNC Symbol;Acc:HGNC:18069] |
| ENSG00000145996 | HGLibA_08804 | 166 | 0.503270254 | CDKAL1 | CDK5 regulatory subunit associated protein 1 like 1 [Source:HGNC Symbol;Acc:HGNC:21050] |
| ENSG00000149635 | HGLibA_33266 | 166 | 0.503270254 | OCSTAMP | osteoclast stimulatory transmembrane protein [Source:HGNC Symbol;Acc:HGNC:16116] |
| ENSG00000152076 | HGLibA_07864 | 166 | 0.503270254 | CCDC74B | coiled-coil domain containing 74B [Source:HGNC Symbol;Acc:HGNC:25267] |
| ENSG00000152240 | HGLibA_20873 | 166 | 0.503270254 | HAUS1 | HAUS augmin like complex subunit 1 [Source:HGNC Symbol;Acc:HGNC:25174] |
| ENSG00000154856 | HGLibA_02394 | 166 | 0.503270254 | APCDD1 | APC down-regulated 1 [Source:HGNC Symbol;Acc:HGNC:15718] |
| ENSG00000155629 | HGLibA_36619 | 166 | 0.503270254 | PIK3AP1 | phosphoinositide-3-kinase adaptor protein 1 [Source:HGNC Symbol;Acc:HGNC:30034] |
| ENSG00000156269 | HGLibA_30972 | 166 | 0.503270254 | NAA11 | NatA catalytic subunit [Source:HGNC Symbol;Acc:HGNC:28125]" |
| ENSG00000156298 | HGLibA_51945 | 166 | 0.503270254 | TSPAN7 | tetraspanin 7 [Source:HGNC Symbol;Acc:HGNC:11854] |
| ENSG00000156885 | HGLibA_10846 | 166 | 0.503270254 | COX6A2 | cytochrome c oxidase subunit 6A2 [Source:HGNC Symbol;Acc:HGNC:2279] |
| ENSG00000160013 | HGLibA_39345 | 166 | 0.503270254 | PTGIR | prostaglandin I2 receptor [Source:HGNC Symbol;Acc:HGNC:9602] |
| ENSG00000163584 | HGLibA_41900 | 166 | 0.503270254 | RPL22L1 | ribosomal protein L22 like 1 [Source:HGNC Symbol;Acc:HGNC:27610] |
| ENSG00000164930 | HGLibA_18277 | 166 | 0.503270254 | FZD6 | frizzled class receptor 6 [Source:HGNC Symbol;Acc:HGNC:4044] |
| ENSG00000165078 | HGLibA_10899 | 166 | 0.503270254 | CPA6 | carboxypeptidase A6 [Source:HGNC Symbol;Acc:HGNC:17245] |
| ENSG00000165097 | HGLibA_24490 | 166 | 0.503270254 | KDM1B | lysine demethylase 1B [Source:HGNC Symbol;Acc:HGNC:21577] |
| ENSG00000166090 | HGLibA_23106 | 166 | 0.503270254 | IL25 | interleukin 25 [Source:HGNC Symbol;Acc:HGNC:13765] |
| ENSG00000166326 | HGLibA_51506 | 166 | 0.503270254 | TRIM44 | tripartite motif containing 44 [Source:HGNC Symbol;Acc:HGNC:19016] |
| ENSG00000167941 | HGLibA_46207 | 166 | 0.503270254 | SOST | sclerostin [Source:HGNC Symbol;Acc:HGNC:13771] |
| ENSG00000168101 | HGLibA_32956 | 166 | 0.503270254 | NUDT16L1 | nudix hydrolase 16 like 1 [Source:HGNC Symbol;Acc:HGNC:28154] |
| ENSG00000168269 | HGLibA_17875 | 166 | 0.503270254 | FOXI1 | forkhead box I1 [Source:HGNC Symbol;Acc:HGNC:3815] |
| ENSG00000168955 | HGLibA_49700 | 166 | 0.503270254 | TM4SF20 | transmembrane 4 L six family member 20 [Source:HGNC Symbol;Acc:HGNC:26230] |
| ENSG00000169184 | HGLibA_29557 | 166 | 0.503270254 | MN1 | transcriptional regulator [Source:HGNC Symbol;Acc:HGNC:7180]" |
| ENSG00000171954 | HGLibA_12189 | 166 | 0.503270254 | CYP4F22 | cytochrome P450 family 4 subfamily F member 22 [Source:HGNC Symbol;Acc:HGNC:26820] |
| ENSG00000172336 | HGLibA_37696 | 166 | 0.503270254 | POP7 | ribonuclease P/MRP subunit [Source:HGNC Symbol;Acc:HGNC:19949]" |
| ENSG00000172459 | HGLibA_34246 | 166 | 0.503270254 | OR5AR1 | olfactory receptor family 5 subfamily AR member 1 (gene/pseudogene) [Source:HGNC Symbol;Acc:HGNC:15260] |
| ENSG00000172771 | HGLibA_14527 | 166 | 0.503270254 | EFCAB12 | EF-hand calcium binding domain 12 [Source:HGNC Symbol;Acc:HGNC:28061] |
| ENSG00000174327 | HGLibA_44429 | 166 | 0.503270254 | SLC16A13 | solute carrier family 16 member 13 [Source:HGNC Symbol;Acc:HGNC:31037] |
| ENSG00000174428 | HGLibA_20573 | 166 | 0.503270254 | GTF2IRD2B | GTF2I repeat domain containing 2B [Source:HGNC Symbol;Acc:HGNC:33125] |
| ENSG00000178401 | HGLibA_13601 | 166 | 0.503270254 | DNAJC22 | DnaJ heat shock protein family (Hsp40) member C22 [Source:HGNC Symbol;Acc:HGNC:25802] |
| ENSG00000178828 | HGLibA_41595 | 166 | 0.503270254 | RNF186 | ring finger protein 186 [Source:HGNC Symbol;Acc:HGNC:25978] |
| ENSG00000184022 | HGLibA_33853 | 166 | 0.503270254 | OR2T10 | olfactory receptor family 2 subfamily T member 10 [Source:HGNC Symbol;Acc:HGNC:19573] |
| ENSG00000184492 | HGLibA_17841 | 166 | 0.503270254 | FOXD4L1 | forkhead box D4 like 1 [Source:HGNC Symbol;Acc:HGNC:18521] |
| ENSG00000185019 | HGLibA_52726 | 166 | 0.503270254 | UBOX5 | U-box domain containing 5 [Source:HGNC Symbol;Acc:HGNC:17777] |
| ENSG00000185198 | HGLibA_38967 | 166 | 0.503270254 | PRSS57 | serine protease 57 [Source:HGNC Symbol;Acc:HGNC:31397] |
| ENSG00000187672 | HGLibA_15428 | 166 | 0.503270254 | ERC2 | ELKS/RAB6-interacting/CAST family member 2 [Source:HGNC Symbol;Acc:HGNC:31922] |
| ENSG00000188760 | HGLibA_50163 | 166 | 0.503270254 | TMEM198 | transmembrane protein 198 [Source:HGNC Symbol;Acc:HGNC:33704] |

| | | | | | |
|---|---|---|---|---|---|
| ENSG00000189144 | HGLibA_56283 | 166 | 0.503270254 | ZNF573 | zinc finger protein 573 [Source:HGNC Symbol;Acc:HGNC:26420] |
| ENSG00000189241 | HGLibA_51978 | 166 | 0.503270254 | TSPYL1 | TSPY like 1 [Source:HGNC Symbol;Acc:HGNC:12382] |
| ENSG00000204952 | HGLibA_17113 | 166 | 0.503270254 | FBXO47 | F-box protein 47 [Source:HGNC Symbol;Acc:HGNC:31969] |
| ENSG00000213614 | HGLibA_21200 | 166 | 0.503270254 | HEXA | hexosaminidase subunit alpha [Source:HGNC Symbol;Acc:HGNC:4878] |
| ENSG00000213853 | HGLibA_15078 | 166 | 0.503270254 | EMP2 | epithelial membrane protein 2 [Source:HGNC Symbol;Acc:HGNC:3334] |
| ENSG00000215009 | HGLibA_00582 | 166 | 0.503270254 | ACSM4 | acyl-CoA synthetase medium chain family member 4 [Source:HGNC Symbol;Acc:HGNC:32016] |
| ENSG00000230797 | HGLibA_54762 | 166 | 0.503270254 | YY2 | YY2 transcription factor [Source:HGNC Symbol;Acc:HGNC:31684] |
| ENSG00000232268 | HGLibA_34171 | 166 | 0.503270254 | OR52I1 | olfactory receptor family 52 subfamily I member 1 [Source:HGNC Symbol;Acc:HGNC:15220] |
| ENSG00000232423 | HGLibA_38323 | 166 | 0.503270254 | PRAMEF6 | PRAME family member 6 [Source:HGNC Symbol;Acc:HGNC:30583] |
| ENSG00000232629 | HGLibA_21631 | 166 | 0.503270254 | HLA-DQB2 | DQ beta 2 [Source:HGNC Symbol;Acc:HGNC:4945]" |
| ENSG00000234745 | HGLibA_21597 | 166 | 0.503270254 | HLA-B | B [Source:HGNC Symbol;Acc:HGNC:4932]" |
| ENSG00000242419 | HGLibA_35575 | 166 | 0.503270254 | PCDHGC4 | 4 [Source:HGNC Symbol;Acc:HGNC:8717]" |
| ENSG00000243566 | HGLibA_53080 | 166 | 0.503270254 | UPK3B | uroplakin 3B [Source:HGNC Symbol;Acc:HGNC:21444] |
| ENSG00000257727 | HGLibA_10366 | 166 | 0.503270254 | CNPY2 | canopy FGF signaling regulator 2 [Source:HGNC Symbol;Acc:HGNC:13529] |
| ENSG00000270757 | HGLibA_22338 | 166 | 0.503270254 | HSPE1-MOB4 | HSPE1-MOB4 readthrough [Source:HGNC Symbol;Acc:HGNC:49184] |
| ENSG00000276293 | HGLibA_36709 | 166 | 0.503270254 | PIP4K2B | phosphatidylinositol-5-phosphate 4-kinase type 2 beta [Source:HGNC Symbol;Acc:HGNC:8998] |
| hsa-mir-141 | HGLibA_57827 | 166 | 0.503270254 | hsa-mir-141 | - |
| hsa-mir-16-1 | HGLibA_57945 | 166 | 0.503270254 | hsa-mir-16-1 | - |
| hsa-mir-187 | HGLibA_58011 | 166 | 0.503270254 | hsa-mir-187 | - |
| hsa-mir-3650 | HGLibA_59147 | 166 | 0.503270254 | hsa-mir-3650 | - |
| hsa-mir-3679 | HGLibA_59265 | 166 | 0.503270254 | hsa-mir-3679 | - |
| hsa-mir-4690 | HGLibA_60869 | 166 | 0.503270254 | hsa-mir-4690 | - |
| hsa-mir-5692a-2 | HGLibA_62293 | 166 | 0.503270254 | hsa-mir-5692a-2 | - |
| hsa-mir-7854 | HGLibA_64007 | 166 | 0.503270254 | hsa-mir-7854 | - |
| hsa-mir-98 | HGLibA_64375 | 166 | 0.503270254 | hsa-mir-98 | - |
| KIAA1462 | HGLibA_24762 | 166 | 0.503270254 | KIAA1462 | - |
| SLC25A6_Y | HGLibA_44778 | 166 | 0.503270254 | SLC25A6_Y | - |
| SPERT | HGLibA_46625 | 166 | 0.503270254 | SPERT | - |
| WDYHV1 | HGLibA_54235 | 166 | 0.503270254 | WDYHV1 | - |
| C16orf13 | HGLibA_05440 | 165 | 0.493319216 | C16orf13 | - |
| C2orf27B | HGLibA_06120 | 165 | 0.493319216 | C2orf27B | - |
| C5orf55 | HGLibA_06411 | 165 | 0.493319216 | C5orf55 | - |
| CCDC58 | HGLibA_07806 | 165 | 0.493319216 | CCDC58 | - |
| DARC | HGLibA_12338 | 165 | 0.493319216 | DARC | - |
| ENSG00000022976 | HGLibA_56849 | 165 | 0.493319216 | ZNF839 | zinc finger protein 839 [Source:HGNC Symbol;Acc:HGNC:20345] |
| ENSG00000026559 | HGLibA_24198 | 165 | 0.493319216 | KCNG1 | potassium voltage-gated channel modifier subfamily G member 1 [Source:HGNC Symbol;Acc:HGNC:6248] |
| ENSG00000051382 | HGLibA_36635 | 165 | 0.493319216 | PIK3CB | 5-bisphosphate 3-kinase catalytic subunit beta [Source:HGNC Symbol;Acc:HGNC:8976]" |
| ENSG00000055070 | HGLibA_48115 | 165 | 0.493319216 | SZRD1 | SUZ RNA binding domain containing 1 [Source:HGNC Symbol;Acc:HGNC:30232] |
| ENSG00000063854 | HGLibA_20814 | 165 | 0.493319216 | HAGH | hydroxyacylglutathione hydrolase [Source:HGNC Symbol;Acc:HGNC:4805] |
| ENSG00000092108 | HGLibA_42892 | 165 | 0.493319216 | SCFD1 | sec1 family domain containing 1 [Source:HGNC Symbol;Acc:HGNC:20726] |
| ENSG00000092758 | HGLibA_10626 | 165 | 0.493319216 | COL9A3 | collagen type IX alpha 3 chain [Source:HGNC Symbol;Acc:HGNC:2219] |
| ENSG00000100949 | HGLibA_40017 | 165 | 0.493319216 | RABGGTA | Rab geranylgeranyltransferase subunit alpha [Source:HGNC Symbol;Acc:HGNC:9795] |
| ENSG00000101997 | HGLibA_07731 | 165 | 0.493319216 | CCDC22 | coiled-coil domain containing 22 [Source:HGNC Symbol;Acc:HGNC:28909] |
| ENSG00000103657 | HGLibA_21153 | 165 | 0.493319216 | HERC1 | HECT and RLD domain containing E3 ubiquitin protein ligase family member 1 [Source:HGNC Symbol;Acc:HGNC:4867] |
| ENSG00000103932 | HGLibA_41815 | 165 | 0.493319216 | RPAP1 | RNA polymerase II associated protein 1 [Source:HGNC Symbol;Acc:HGNC:24567] |
| ENSG00000104267 | HGLibA_06802 | 165 | 0.493319216 | CA2 | carbonic anhydrase 2 [Source:HGNC Symbol;Acc:HGNC:1373] |
| ENSG00000105467 | HGLibA_47999 | 165 | 0.493319216 | SYNGR4 | synaptogyrin 4 [Source:HGNC Symbol;Acc:HGNC:11502] |
| ENSG00000106683 | HGLibA_26539 | 165 | 0.493319216 | LIMK1 | LIM domain kinase 1 [Source:HGNC Symbol;Acc:HGNC:6613] |
| ENSG00000108602 | HGLibA_01561 | 165 | 0.493319216 | ALDH3A1 | aldehyde dehydrogenase 3 family member A1 [Source:HGNC Symbol;Acc:HGNC:405] |
| ENSG00000109381 | HGLibA_14894 | 165 | 0.493319216 | ELF2 | E74 like ETS transcription factor 2 [Source:HGNC Symbol;Acc:HGNC:3317] |
| ENSG00000109466 | HGLibA_25147 | 165 | 0.493319216 | KLHL2 | kelch like family member 2 [Source:HGNC Symbol;Acc:HGNC:6353] |
| ENSG00000110060 | HGLibA_39600 | 165 | 0.493319216 | PUS3 | pseudouridine synthase 3 [Source:HGNC Symbol;Acc:HGNC:25461] |
| ENSG00000110801 | HGLibA_39186 | 165 | 0.493319216 | PSMD9 | non-ATPase 9 [Source:HGNC Symbol;Acc:HGNC:9567]" |
| ENSG00000113369 | HGLibA_03118 | 165 | 0.493319216 | ARRDC3 | arrestin domain containing 3 [Source:HGNC Symbol;Acc:HGNC:29263] |
| ENSG00000115041 | HGLibA_24241 | 165 | 0.493319216 | KCNIP3 | potassium voltage-gated channel interacting protein 3 [Source:HGNC Symbol;Acc:HGNC:15523] |
| ENSG00000116729 | HGLibA_54330 | 165 | 0.493319216 | WLS | wntless Wnt ligand secretion mediator [Source:HGNC Symbol;Acc:HGNC:30238] |
| ENSG00000117245 | HGLibA_24872 | 165 | 0.493319216 | KIF17 | kinesin family member 17 [Source:HGNC Symbol;Acc:HGNC:19167] |
| ENSG00000117472 | HGLibA_51893 | 165 | 0.493319216 | TSPAN1 | tetraspanin 1 [Source:HGNC Symbol;Acc:HGNC:20657] |
| ENSG00000117595 | HGLibA_23594 | 165 | 0.493319216 | IRF6 | interferon regulatory factor 6 [Source:HGNC Symbol;Acc:HGNC:6121] |
| ENSG00000118017 | HGLibA_00020 | 165 | 0.493319216 | A4GNT | 4-N-acetylglucosaminyltransferase [Source:HGNC Symbol;Acc:HGNC:17968]" |
| ENSG00000124249 | HGLibA_24307 | 165 | 0.493319216 | KCNK15 | potassium two pore domain channel subfamily K member 15 [Source:HGNC Symbol;Acc:HGNC:13814] |
| ENSG00000125652 | HGLibA_01665 | 165 | 0.493319216 | ALKBH7 | alkB homolog 7 [Source:HGNC Symbol;Acc:HGNC:21306] |
| ENSG00000126756 | HGLibA_53481 | 165 | 0.493319216 | UXT | ubiquitously expressed prefoldin like chaperone [Source:HGNC Symbol;Acc:HGNC:12641] |
| ENSG00000128578 | HGLibA_47592 | 165 | 0.493319216 | STRIP2 | striatin interacting protein 2 [Source:HGNC Symbol;Acc:HGNC:22209] |
| ENSG00000128928 | HGLibA_23877 | 165 | 0.493319216 | IVD | isovaleryl-CoA dehydrogenase [Source:HGNC Symbol;Acc:HGNC:6186] |
| ENSG00000129354 | HGLibA_02294 | 165 | 0.493319216 | AP1M2 | adaptor related protein complex 1 subunit mu 2 [Source:HGNC Symbol;Acc:HGNC:558] |
| ENSG00000132703 | HGLibA_02400 | 165 | 0.493319216 | APCS | serum [Source:HGNC Symbol;Acc:HGNC:584]" |
| ENSG00000133106 | HGLibA_15387 | 165 | 0.493319216 | EPSTI1 | epithelial stromal interaction 1 [Source:HGNC Symbol;Acc:HGNC:16465] |
| ENSG00000135052 | HGLibA_19653 | 165 | 0.493319216 | GOLM1 | golgi membrane protein 1 [Source:HGNC Symbol;Acc:HGNC:15451] |
| ENSG00000137674 | HGLibA_29511 | 165 | 0.493319216 | MMP20 | matrix metallopeptidase 20 [Source:HGNC Symbol;Acc:HGNC:7167] |
| ENSG00000137869 | HGLibA_12063 | 165 | 0.493319216 | CYP19A1 | cytochrome P450 family 19 subfamily A member 1 [Source:HGNC Symbol;Acc:HGNC:2594] |
| ENSG00000138674 | HGLibA_43240 | 165 | 0.493319216 | SEC31A | COPII coat complex component [Source:HGNC Symbol;Acc:HGNC:17052]" |
| ENSG00000138792 | HGLibA_15165 | 165 | 0.493319216 | ENPEP | glutamyl aminopeptidase [Source:HGNC Symbol;Acc:HGNC:3355] |
| ENSG00000138834 | HGLibA_28293 | 165 | 0.493319216 | MAPK8IP3 | mitogen-activated protein kinase 8 interacting protein 3 [Source:HGNC Symbol;Acc:HGNC:6884] |
| ENSG00000141522 | HGLibA_02795 | 165 | 0.493319216 | ARHGDIA | Rho GDP dissociation inhibitor alpha [Source:HGNC Symbol;Acc:HGNC:678] |
| ENSG00000142669 | HGLibA_43922 | 165 | 0.493319216 | SH3BGRL3 | SH3 domain binding glutamate rich protein like 3 [Source:HGNC Symbol;Acc:HGNC:15568] |
| ENSG00000147168 | HGLibA_23125 | 165 | 0.493319216 | IL2RG | interleukin 2 receptor subunit gamma [Source:HGNC Symbol;Acc:HGNC:6010] |
| ENSG00000148835 | HGLibA_48241 | 165 | 0.493319216 | TAF5 | TATA-box binding protein associated factor 5 [Source:HGNC Symbol;Acc:HGNC:11539] |
| ENSG00000154646 | HGLibA_50634 | 165 | 0.493319216 | TMPRSS15 | transmembrane serine protease 15 [Source:HGNC Symbol;Acc:HGNC:9490] |
| ENSG00000156398 | HGLibA_43783 | 165 | 0.493319216 | SFXN2 | sideroflexin 2 [Source:HGNC Symbol;Acc:HGNC:16086] |
| ENSG00000157429 | HGLibA_55556 | 165 | 0.493319216 | ZNF19 | zinc finger protein 19 [Source:HGNC Symbol;Acc:HGNC:12981] |
| ENSG00000157617 | HGLibA_06087 | 165 | 0.493319216 | C2CD2 | C2 calcium dependent domain containing 2 [Source:HGNC Symbol;Acc:HGNC:1266] |
| ENSG00000158258 | HGLibA_10136 | 165 | 0.493319216 | CLSTN2 | calsyntenin 2 [Source:HGNC Symbol;Acc:HGNC:17448] |
| ENSG00000158887 | HGLibA_29792 | 165 | 0.493319216 | MPZ | myelin protein zero [Source:HGNC Symbol;Acc:HGNC:7225] |
| ENSG00000161847 | HGLibA_40402 | 165 | 0.493319216 | RAVER1 | PTB binding 1 [Source:HGNC Symbol;Acc:HGNC:30296]" |
| ENSG00000164122 | HGLibA_03241 | 165 | 0.493319216 | ASB5 | ankyrin repeat and SOCS box containing 5 [Source:HGNC Symbol;Acc:HGNC:17180] |
| ENSG00000164418 | HGLibA_20285 | 165 | 0.493319216 | GRIK2 | glutamate ionotropic receptor kainate type subunit 2 [Source:HGNC Symbol;Acc:HGNC:4580] |
| ENSG00000165568 | HGLibA_01496 | 165 | 0.493319216 | AKR1E2 | aldo-keto reductase family 1 member E2 [Source:HGNC Symbol;Acc:HGNC:23437] |
| ENSG00000165795 | HGLibA_31417 | 165 | 0.493319216 | NDRG2 | NDRG family member 2 [Source:HGNC Symbol;Acc:HGNC:14460] |
| ENSG00000166685 | HGLibA_10471 | 165 | 0.493319216 | COG1 | component of oligomeric golgi complex 1 [Source:HGNC Symbol;Acc:HGNC:6545] |
| ENSG00000167987 | HGLibA_53781 | 165 | 0.493319216 | VPS37C | ESCRT-I subunit [Source:HGNC Symbol;Acc:HGNC:26097]" |
| ENSG00000167995 | HGLibA_04420 | 165 | 0.493319216 | BEST1 | bestrophin 1 [Source:HGNC Symbol;Acc:HGNC:12703] |
| ENSG00000168878 | HGLibA_43773 | 165 | 0.493319216 | SFTPB | surfactant protein B [Source:HGNC Symbol;Acc:HGNC:10801] |
| ENSG00000169344 | HGLibA_52978 | 165 | 0.493319216 | UMOD | uromodulin [Source:HGNC Symbol;Acc:HGNC:12559] |
| ENSG00000169609 | HGLibA_05396 | 165 | 0.493319216 | C15orf40 | chromosome 15 open reading frame 40 [Source:HGNC Symbol;Acc:HGNC:28443] |
| ENSG00000169895 | HGLibA_47906 | 165 | 0.493319216 | SYAP1 | synapse associated protein 1 [Source:HGNC Symbol;Acc:HGNC:16273] |
| ENSG00000170190 | HGLibA_44443 | 165 | 0.493319216 | SLC16A5 | solute carrier family 16 member 5 [Source:HGNC Symbol;Acc:HGNC:10926] |
| ENSG00000170893 | HGLibA_51379 | 165 | 0.493319216 | TRH | thyrotropin releasing hormone [Source:HGNC Symbol;Acc:HGNC:12298] |
| ENSG00000170965 | HGLibA_36932 | 165 | 0.493319216 | PLAC1 | placenta specific 1 [Source:HGNC Symbol;Acc:HGNC:9044] |
| ENSG00000172738 | HGLibA_50226 | 165 | 0.493319216 | TMEM217 | transmembrane protein 217 [Source:HGNC Symbol;Acc:HGNC:21238] |
| ENSG00000174586 | HGLibA_56104 | 165 | 0.493319216 | ZNF497 | zinc finger protein 497 [Source:HGNC Symbol;Acc:HGNC:23714] |
| ENSG00000175048 | HGLibA_55079 | 165 | 0.493319216 | ZDHHC14 | zinc finger DHHC-type containing 14 [Source:HGNC Symbol;Acc:HGNC:20341] |
| ENSG00000175711 | HGLibA_04007 | 165 | 0.493319216 | B3GNTL1 | 3-N-acetylglucosaminyltransferase like 1 [Source:HGNC Symbol;Acc:HGNC:21727]" |
| ENSG00000177683 | HGLibA_49303 | 165 | 0.493319216 | THAP5 | THAP domain containing 5 [Source:HGNC Symbol;Acc:HGNC:23188] |
| ENSG00000178695 | HGLibA_24421 | 165 | 0.493319216 | KCTD12 | potassium channel tetramerization domain containing 12 [Source:HGNC Symbol;Acc:HGNC:14678] |
| ENSG00000179284 | HGLibA_12306 | 165 | 0.493319216 | DAND5 | DAN domain BMP antagonist family member 5 [Source:HGNC Symbol;Acc:HGNC:26780] |
| ENSG00000179695 | HGLibA_34384 | 165 | 0.493319216 | OR6C2 | olfactory receptor family 6 subfamily C member 2 [Source:HGNC Symbol;Acc:HGNC:15436] |
| ENSG00000185245 | HGLibA_19700 | 165 | 0.493319216 | GP1BA | glycoprotein Ib platelet subunit alpha [Source:HGNC Symbol;Acc:HGNC:4439] |
| ENSG00000187239 | HGLibA_17736 | 165 | 0.493319216 | FNBP1 | formin binding protein 1 [Source:HGNC Symbol;Acc:HGNC:17069] |
| ENSG00000187796 | HGLibA_07240 | 165 | 0.493319216 | CARD9 | caspase recruitment domain family member 9 [Source:HGNC Symbol;Acc:HGNC:16391] |
| ENSG00000188107 | HGLibA_15829 | 165 | 0.493319216 | EYS | eyes shut homolog [Source:HGNC Symbol;Acc:HGNC:21555] |
| ENSG00000188167 | HGLibA_50608 | 165 | 0.493319216 | TMPPE | transmembrane protein with metallophosphoesterase domain [Source:HGNC Symbol;Acc:HGNC:33865] |
| ENSG00000189001 | HGLibA_42811 | 165 | 0.493319216 | SBSN | suprabasin [Source:HGNC Symbol;Acc:HGNC:24950] |
| ENSG00000198771 | HGLibA_40718 | 165 | 0.493319216 | RCSD1 | RCSD domain containing 1 [Source:HGNC Symbol;Acc:HGNC:28310] |
| ENSG00000198844 | HGLibA_02818 | 165 | 0.493319216 | ARHGEF15 | Rho guanine nucleotide exchange factor 15 [Source:HGNC Symbol;Acc:HGNC:15590] |
| ENSG00000204428 | HGLibA_27661 | 165 | 0.493319216 | LY6G5C | lymphocyte antigen 6 family member G5C [Source:HGNC Symbol;Acc:HGNC:13932] |
| ENSG00000214285 | HGLibA_32524 | 165 | 0.493319216 | NPS | neuropeptide S [Source:HGNC Symbol;Acc:HGNC:33940] |
| ENSG00000215009 | HGLibA_00581 | 165 | 0.493319216 | ACSM4 | acyl-CoA synthetase medium chain family member 4 [Source:HGNC Symbol;Acc:HGNC:32016] |
| ENSG00000215187 | HGLibA_16236 | 165 | 0.493319216 | FAM166B | family with sequence similarity 166 member B [Source:HGNC Symbol;Acc:HGNC:34242] |
| ENSG00000236320 | HGLibA_45494 | 165 | 0.493319216 | SLFN14 | schlafen family member 14 [Source:HGNC Symbol;Acc:HGNC:32689] |
| ENSG00000240720 | HGLibA_27446 | 165 | 0.493319216 | LRRD1 | leucine rich repeats and death domain containing 1 [Source:HGNC Symbol;Acc:HGNC:34300] |
| ENSG00000243480 | HGLibA_01851 | 165 | 0.493319216 | AMY2A | alpha 2A (pancreatic) [Source:HGNC Symbol;Acc:HGNC:477]" |
| ENSG00000248098 | HGLibA_04278 | 165 | 0.493319216 | BCKDHA | alpha polypeptide [Source:HGNC Symbol;Acc:HGNC:986]" |
| ENSG00000250254 | HGLibA_39569 | 165 | 0.493319216 | PTTG2 | pituitary tumor-transforming 2 [Source:HGNC Symbol;Acc:HGNC:9691] |
| ENSG00000280236 | HGLibA_33588 | 165 | 0.493319216 | OR12D2 | olfactory receptor family 12 subfamily D member 2 (gene/pseudogene) [Source:HGNC Symbol;Acc:HGNC:8178] |
| FAM84A | HGLibA_16765 | 165 | 0.493319216 | FAM84A | - |
| GSTT1 | HGLibA_20496 | 165 | 0.493319216 | GSTT1 | - |
| HDGFRP3 | HGLibA_21049 | 165 | 0.493319216 | HDGFRP3 | - |
| hsa-mir-1281 | HGLibA_57579 | 165 | 0.493319216 | hsa-mir-1281 | - |
| hsa-mir-3180-2 | HGLibA_58807 | 165 | 0.493319216 | hsa-mir-3180-2 | - |
| hsa-mir-323a | HGLibA_58955 | 165 | 0.493319216 | hsa-mir-323a | - |
| hsa-mir-3689b | HGLibA_59319 | 165 | 0.493319216 | hsa-mir-3689b | - |
| hsa-mir-410 | HGLibA_59679 | 165 | 0.493319216 | hsa-mir-410 | - |
| hsa-mir-4658 | HGLibA_60741 | 165 | 0.493319216 | hsa-mir-4658 | - |
| hsa-mir-4674 | HGLibA_60811 | 165 | 0.493319216 | hsa-mir-4674 | - |

| | | | | | |
|---|---|---|---|---|---|
| hsa-mir-4687 | HGLibA_60858 | 165 | 0.493319216 | hsa-mir-4687 | - |
| hsa-mir-5687 | HGLibA_62264 | 165 | 0.493319216 | hsa-mir-5687 | - |
| hsa-mir-590 | HGLibA_62458 | 165 | 0.493319216 | hsa-mir-590 | - |
| hsa-mir-7111 | HGLibA_63810 | 165 | 0.493319216 | hsa-mir-7111 | - |
| PALM2-AKAP2 | HGLibA_35072 | 165 | 0.493319216 | PALM2-AKAP2 | - |
| RGAG4 | HGLibA_40995 | 165 | 0.493319216 | RGAG4 | - |
| TMEM133 | HGLibA_49942 | 165 | 0.493319216 | TMEM133 | - |
| TSSC1 | HGLibA_52004 | 165 | 0.493319216 | TSSC1 | - |
| AES | HGLibA_01094 | 164 | 0.483368179 | AES | - |
| C11orf74 | HGLibA_05185 | 164 | 0.483368179 | C11orf74 | - |
| C1orf65 | HGLibA_05887 | 164 | 0.483368179 | C1orf65 | - |
| ENSG00000019485 | HGLibA_38368 | 164 | 0.483368179 | PRDM11 | PR/SET domain 11 [Source:HGNC Symbol;Acc:HGNC:13996] |
| ENSG00000060558 | HGLibA_19449 | 164 | 0.483368179 | GNA15 | G protein subunit alpha 15 [Source:HGNC Symbol;Acc:HGNC:4383] |
| ENSG00000068001 | HGLibA_22440 | 164 | 0.483368179 | HYAL2 | hyaluronidase 2 [Source:HGNC Symbol;Acc:HGNC:5321] |
| ENSG00000076944 | HGLibA_47666 | 164 | 0.483368179 | STXBP2 | syntaxin binding protein 2 [Source:HGNC Symbol;Acc:HGNC:11445] |
| ENSG00000090863 | HGLibA_19245 | 164 | 0.483368179 | GLG1 | golgi glycoprotein 1 [Source:HGNC Symbol;Acc:HGNC:4316] |
| ENSG00000099246 | HGLibA_39824 | 164 | 0.483368179 | RAB18 | member RAS oncogene family [Source:HGNC Symbol;Acc:HGNC:14244]" |
| ENSG00000099622 | HGLibA_09720 | 164 | 0.483368179 | CIRBP | cold inducible RNA binding protein [Source:HGNC Symbol;Acc:HGNC:1982] |
| ENSG00000099999 | HGLibA_41628 | 164 | 0.483368179 | RNF215 | ring finger protein 215 [Source:HGNC Symbol;Acc:HGNC:33434] |
| ENSG00000100065 | HGLibA_07217 | 164 | 0.483368179 | CARD10 | caspase recruitment domain family member 10 [Source:HGNC Symbol;Acc:HGNC:16422] |
| ENSG00000101850 | HGLibA_19914 | 164 | 0.483368179 | GPR143 | G protein-coupled receptor 143 [Source:HGNC Symbol;Acc:HGNC:20145] |
| ENSG00000101916 | HGLibA_49664 | 164 | 0.483368179 | TLR8 | toll like receptor 8 [Source:HGNC Symbol;Acc:HGNC:15632] |
| ENSG00000102390 | HGLibA_35329 | 164 | 0.483368179 | PBDC1 | polysaccharide biosynthesis domain containing 1 [Source:HGNC Symbol;Acc:HGNC:28790] |
| ENSG00000105697 | HGLibA_20823 | 164 | 0.483368179 | HAMP | hepcidin antimicrobial peptide [Source:HGNC Symbol;Acc:HGNC:15598] |
| ENSG00000106804 | HGLibA_06349 | 164 | 0.483368179 | C5 | complement C5 [Source:HGNC Symbol;Acc:HGNC:1331] |
| ENSG00000106927 | HGLibA_01750 | 164 | 0.483368179 | AMBP | alpha-1-microglobulin/bikunin precursor [Source:HGNC Symbol;Acc:HGNC:453] |
| ENSG00000108515 | HGLibA_15145 | 164 | 0.483368179 | ENO3 | enolase 3 [Source:HGNC Symbol;Acc:HGNC:3354] |
| ENSG00000109906 | HGLibA_54826 | 164 | 0.483368179 | ZBTB16 | zinc finger and BTB domain containing 16 [Source:HGNC Symbol;Acc:HGNC:12930] |
| ENSG00000110455 | HGLibA_00413 | 164 | 0.483368179 | ACCS | 1-aminocyclopropane-1-carboxylate synthase homolog (inactive) [Source:HGNC Symbol;Acc:HGNC:23989] |
| ENSG00000112658 | HGLibA_46960 | 164 | 0.483368179 | SRF | serum response factor [Source:HGNC Symbol;Acc:HGNC:11291] |
| ENSG00000112877 | HGLibA_09161 | 164 | 0.483368179 | CEP72 | centrosomal protein 72 [Source:HGNC Symbol;Acc:HGNC:25547] |
| ENSG00000114030 | HGLibA_25355 | 164 | 0.483368179 | KPNA1 | karyopherin subunit alpha 1 [Source:HGNC Symbol;Acc:HGNC:6394] |
| ENSG00000120093 | HGLibA_21935 | 164 | 0.483368179 | HOXB3 | homeobox B3 [Source:HGNC Symbol;Acc:HGNC:5114] |
| ENSG00000120662 | HGLibA_30465 | 164 | 0.483368179 | MTRF1 | mitochondrial translation release factor 1 [Source:HGNC Symbol;Acc:HGNC:7469] |
| ENSG00000120937 | HGLibA_32501 | 164 | 0.483368179 | NPPB | natriuretic peptide B [Source:HGNC Symbol;Acc:HGNC:7940] |
| ENSG00000122711 | HGLibA_46695 | 164 | 0.483368179 | SPINK4 | Kazal type 4 [Source:HGNC Symbol;Acc:HGNC:16646]" |
| ENSG00000122787 | HGLibA_01494 | 164 | 0.483368179 | AKR1D1 | aldo-keto reductase family 1 member D1 [Source:HGNC Symbol;Acc:HGNC:388] |
| ENSG00000124155 | HGLibA_36589 | 164 | 0.483368179 | PIGT | phosphatidylinositol glycan anchor biosynthesis class T [Source:HGNC Symbol;Acc:HGNC:14938] |
| ENSG00000125386 | HGLibA_16362 | 164 | 0.483368179 | FAM193A | family with sequence similarity 193 member A [Source:HGNC Symbol;Acc:HGNC:16822] |
| ENSG00000125888 | HGLibA_04143 | 164 | 0.483368179 | BANF2 | barrier to autointegration factor 2 [Source:HGNC Symbol;Acc:HGNC:16172] |
| ENSG00000128294 | HGLibA_51207 | 164 | 0.483368179 | TPST2 | tyrosylprotein sulfotransferase 2 [Source:HGNC Symbol;Acc:HGNC:12021] |
| ENSG00000130829 | HGLibA_14196 | 164 | 0.483368179 | DUSP9 | dual specificity phosphatase 9 [Source:HGNC Symbol;Acc:HGNC:3076] |
| ENSG00000131791 | HGLibA_38499 | 164 | 0.483368179 | PRKAB2 | protein kinase AMP-activated non-catalytic subunit beta 2 [Source:HGNC Symbol;Acc:HGNC:9379] |
| ENSG00000133657 | HGLibA_03571 | 164 | 0.483368179 | ATP13A3 | ATPase 13A3 [Source:HGNC Symbol;Acc:HGNC:24113] |
| ENSG00000137996 | HGLibA_42372 | 164 | 0.483368179 | RTCA | RNA 3'-terminal phosphate cyclase [Source:HGNC Symbol;Acc:HGNC:17981] |
| ENSG00000138623 | HGLibA_43375 | 164 | 0.483368179 | SEMA7A | semaphorin 7A (John Milton Hagen blood group) [Source:HGNC Symbol;Acc:HGNC:10741] |
| ENSG00000139433 | HGLibA_19343 | 164 | 0.483368179 | GLTP | glycolipid transfer protein [Source:HGNC Symbol;Acc:HGNC:24867] |
| ENSG00000139546 | HGLibA_48333 | 164 | 0.483368179 | TARBP2 | RISC loading complex RNA binding subunit [Source:HGNC Symbol;Acc:HGNC:11569]" |
| ENSG00000140450 | HGLibA_03121 | 164 | 0.483368179 | ARRDC4 | arrestin domain containing 4 [Source:HGNC Symbol;Acc:HGNC:28087] |
| ENSG00000140557 | HGLibA_47290 | 164 | 0.483368179 | ST8SIA2 | 8-sialyltransferase 2 [Source:HGNC Symbol;Acc:HGNC:10870]" |
| ENSG00000140612 | HGLibA_43180 | 164 | 0.483368179 | SEC11A | signal peptidase complex subunit [Source:HGNC Symbol;Acc:HGNC:17718]" |
| ENSG00000141086 | HGLibA_11686 | 164 | 0.483368179 | CTRL | chymotrypsin like [Source:HGNC Symbol;Acc:HGNC:2524] |
| ENSG00000142149 | HGLibA_22421 | 164 | 0.483368179 | HUNK | hormonally up-regulated Neu-associated kinase [Source:HGNC Symbol;Acc:HGNC:13326] |
| ENSG00000143442 | HGLibA_37481 | 164 | 0.483368179 | POGZ | pogo transposable element derived with ZNF domain [Source:HGNC Symbol;Acc:HGNC:18801] |
| ENSG00000144635 | HGLibA_14241 | 164 | 0.483368179 | DYNC1LI1 | dynein cytoplasmic 1 light intermediate chain 1 [Source:HGNC Symbol;Acc:HGNC:18745] |
| ENSG00000145103 | HGLibA_23207 | 164 | 0.483368179 | ILDR1 | immunoglobulin like domain containing receptor 1 [Source:HGNC Symbol;Acc:HGNC:28741] |
| ENSG00000146859 | HGLibA_49960 | 164 | 0.483368179 | TMEM140 | transmembrane protein 140 [Source:HGNC Symbol;Acc:HGNC:21870] |
| ENSG00000147027 | HGLibA_50413 | 164 | 0.483368179 | TMEM47 | transmembrane protein 47 [Source:HGNC Symbol;Acc:HGNC:18515] |
| ENSG00000147121 | HGLibA_25397 | 164 | 0.483368179 | KRBOX4 | KRAB box domain containing 4 [Source:HGNC Symbol;Acc:HGNC:26007] |
| ENSG00000147485 | HGLibA_39652 | 164 | 0.483368179 | PXDNL | peroxidasin like [Source:HGNC Symbol;Acc:HGNC:26359] |
| ENSG00000149100 | HGLibA_14802 | 164 | 0.483368179 | EIF3M | eukaryotic translation initiation factor 3 subunit M [Source:HGNC Symbol;Acc:HGNC:24460] |
| ENSG00000151465 | HGLibA_08506 | 164 | 0.483368179 | CDC123 | cell division cycle 123 [Source:HGNC Symbol;Acc:HGNC:16827] |
| ENSG00000152127 | HGLibA_29149 | 164 | 0.483368179 | MGAT5 | 6-mannosylglycoprotein 6-beta-N-acetylglucosaminyltransferase [Source:HGNC Symbol;Acc:HGNC:7049]" |
| ENSG00000152128 | HGLibA_50029 | 164 | 0.483368179 | TMEM163 | transmembrane protein 163 [Source:HGNC Symbol;Acc:HGNC:25380] |
| ENSG00000154016 | HGLibA_20212 | 164 | 0.483368179 | GRAP | GRB2 related adaptor protein [Source:HGNC Symbol;Acc:HGNC:4562] |
| ENSG00000159423 | HGLibA_01574 | 164 | 0.483368179 | ALDH4A1 | aldehyde dehydrogenase 4 family member A1 [Source:HGNC Symbol;Acc:HGNC:406] |
| ENSG00000161807 | HGLibA_34480 | 164 | 0.483368179 | OR7G1 | olfactory receptor family 7 subfamily G member 1 [Source:HGNC Symbol;Acc:HGNC:8465] |
| ENSG00000162510 | HGLibA_28427 | 164 | 0.483368179 | MATN1 | matrilin 1 [Source:HGNC Symbol;Acc:HGNC:6907] |
| ENSG00000163464 | HGLibA_11881 | 164 | 0.483368179 | CXCR1 | C-X-C motif chemokine receptor 1 [Source:HGNC Symbol;Acc:HGNC:6026] |
| ENSG00000163485 | HGLibA_01010 | 164 | 0.483368179 | ADORA1 | adenosine A1 receptor [Source:HGNC Symbol;Acc:HGNC:262] |
| ENSG00000163611 | HGLibA_46662 | 164 | 0.483368179 | SPICE1 | spindle and centriole associated protein 1 [Source:HGNC Symbol;Acc:HGNC:25083] |
| ENSG00000165204 | HGLibA_33682 | 164 | 0.483368179 | OR1K1 | olfactory receptor family 1 subfamily K member 1 [Source:HGNC Symbol;Acc:HGNC:8212] |
| ENSG00000166278 | HGLibA_05979 | 164 | 0.483368179 | C2 | complement C2 [Source:HGNC Symbol;Acc:HGNC:1248] |
| ENSG00000166928 | HGLibA_30138 | 164 | 0.483368179 | MS4A14 | membrane spanning 4-domains A14 [Source:HGNC Symbol;Acc:HGNC:30706] |
| ENSG00000167749 | HGLibA_25257 | 164 | 0.483368179 | KLK4 | kallikrein related peptidase 4 [Source:HGNC Symbol;Acc:HGNC:6365] |
| ENSG00000167881 | HGLibA_47009 | 164 | 0.483368179 | SRP68 | signal recognition particle 68 [Source:HGNC Symbol;Acc:HGNC:11302] |
| ENSG00000168477 | HGLibA_50930 | 164 | 0.483368179 | TNXB | tenascin XB [Source:HGNC Symbol;Acc:HGNC:11976] |
| ENSG00000168612 | HGLibA_57038 | 164 | 0.483368179 | ZSWIM1 | zinc finger SWIM-type containing 1 [Source:HGNC Symbol;Acc:HGNC:16155] |
| ENSG00000169220 | HGLibA_41063 | 164 | 0.483368179 | RGS14 | regulator of G protein signaling 14 [Source:HGNC Symbol;Acc:HGNC:9996] |
| ENSG00000169432 | HGLibA_43008 | 164 | 0.483368179 | SCN9A | sodium voltage-gated channel alpha subunit 9 [Source:HGNC Symbol;Acc:HGNC:10597] |
| ENSG00000171115 | HGLibA_19092 | 164 | 0.483368179 | GIMAP8 | IMAP family member 8 [Source:HGNC Symbol;Acc:HGNC:21792]" |
| ENSG00000171360 | HGLibA_25497 | 164 | 0.483368179 | KRT38 | keratin 38 [Source:HGNC Symbol;Acc:HGNC:6456] |
| ENSG00000171617 | HGLibA_15111 | 164 | 0.483368179 | ENC1 | ectodermal-neural cortex 1 [Source:HGNC Symbol;Acc:HGNC:3345] |
| ENSG00000172845 | HGLibA_46300 | 164 | 0.483368179 | SP3 | Sp3 transcription factor [Source:HGNC Symbol;Acc:HGNC:11208] |
| ENSG00000174370 | HGLibA_05142 | 164 | 0.483368179 | C11orf45 | chromosome 11 open reading frame 45 [Source:HGNC Symbol;Acc:HGNC:28584] |
| ENSG00000178694 | HGLibA_32784 | 164 | 0.483368179 | NSUN3 | NOP2/Sun RNA methyltransferase family member 3 [Source:HGNC Symbol;Acc:HGNC:26208] |
| ENSG00000178878 | HGLibA_02545 | 164 | 0.483368179 | APOLD1 | apolipoprotein L domain containing 1 [Source:HGNC Symbol;Acc:HGNC:25268] |
| ENSG00000180044 | HGLibA_06271 | 164 | 0.483368179 | C3orf80 | chromosome 3 open reading frame 80 [Source:HGNC Symbol;Acc:HGNC:40048] |
| ENSG00000181773 | HGLibA_20015 | 164 | 0.483368179 | GPR3 | G protein-coupled receptor 3 [Source:HGNC Symbol;Acc:HGNC:4484] |
| ENSG00000182070 | HGLibA_34139 | 164 | 0.483368179 | OR52A1 | olfactory receptor family 52 subfamily A member 1 [Source:HGNC Symbol;Acc:HGNC:8318] |
| ENSG00000183092 | HGLibA_04399 | 164 | 0.483368179 | BEGAIN | brain enriched guanylate kinase associated [Source:HGNC Symbol;Acc:HGNC:24163] |
| ENSG00000183831 | HGLibA_02106 | 164 | 0.483368179 | ANKRD45 | ankyrin repeat domain 45 [Source:HGNC Symbol;Acc:HGNC:24786] |
| ENSG00000184227 | HGLibA_00467 | 164 | 0.483368179 | ACOT1 | acyl-CoA thioesterase 1 [Source:HGNC Symbol;Acc:HGNC:33128] |
| ENSG00000184502 | HGLibA_18663 | 164 | 0.483368179 | GAST | gastrin [Source:HGNC Symbol;Acc:HGNC:4164] |
| ENSG00000188176 | HGLibA_45835 | 164 | 0.483368179 | SMTNL2 | smoothelin like 2 [Source:HGNC Symbol;Acc:HGNC:24764] |
| ENSG00000188848 | HGLibA_04408 | 164 | 0.483368179 | BEND4 | BEN domain containing 4 [Source:HGNC Symbol;Acc:HGNC:23815] |
| ENSG00000189401 | HGLibA_34772 | 164 | 0.483368179 | OTUD6A | OTU deubiquitinase 6A [Source:HGNC Symbol;Acc:HGNC:32312] |
| ENSG00000197461 | HGLibA_35841 | 164 | 0.483368179 | PDGFA | platelet derived growth factor subunit A [Source:HGNC Symbol;Acc:HGNC:8799] |
| ENSG00000197992 | HGLibA_10009 | 164 | 0.483368179 | CLEC9A | C-type lectin domain containing 9A [Source:HGNC Symbol;Acc:HGNC:26705] |
| ENSG00000198270 | HGLibA_49886 | 164 | 0.483368179 | TMEM116 | transmembrane protein 116 [Source:HGNC Symbol;Acc:HGNC:25084] |
| ENSG00000198759 | HGLibA_14625 | 164 | 0.483368179 | EGFL6 | EGF like domain multiple 6 [Source:HGNC Symbol;Acc:HGNC:3235] |
| ENSG00000203877 | HGLibA_41330 | 164 | 0.483368179 | RIPPLY2 | ripply transcriptional repressor 2 [Source:HGNC Symbol;Acc:HGNC:21390] |
| ENSG00000204576 | HGLibA_38835 | 164 | 0.483368179 | PRR3 | proline rich 3 [Source:HGNC Symbol;Acc:HGNC:21149] |
| ENSG00000204909 | HGLibA_46710 | 164 | 0.483368179 | SPINK9 | Kazal type 9 [Source:HGNC Symbol;Acc:HGNC:32951]" |
| ENSG00000205327 | HGLibA_34399 | 164 | 0.483368179 | OR6C68 | olfactory receptor family 6 subfamily C member 68 [Source:HGNC Symbol;Acc:HGNC:31297] |
| ENSG00000205445 | HGLibA_25602 | 164 | 0.483368179 | KRTAP10-2 | keratin associated protein 10-2 [Source:HGNC Symbol;Acc:HGNC:22967] |
| ENSG00000224531 | HGLibA_45727 | 164 | 0.483368179 | SMIM13 | small integral membrane protein 13 [Source:HGNC Symbol;Acc:HGNC:27356] |
| ENSG00000242950 | HGLibA_15551 | 164 | 0.483368179 | ERVW-1 | envelope [Source:HGNC Symbol;Acc:HGNC:13525]" |
| ENSG00000255690 | HGLibA_51398 | 164 | 0.483368179 | TRIL | TLR4 interactor with leucine rich repeats [Source:HGNC Symbol;Acc:HGNC:22200] |
| ENSG00000263001 | HGLibA_20566 | 164 | 0.483368179 | GTF2I | general transcription factor IIi [Source:HGNC Symbol;Acc:HGNC:4659] |
| ENSG00000270672 | HGLibA_30485 | 164 | 0.483368179 | MTRNR2L6 | MT-RNR2 like 6 [Source:HGNC Symbol;Acc:HGNC:37163] |
| FAM49A | HGLibA_16624 | 164 | 0.483368179 | FAM49A | - |
| hsa-mir-218-2 | HGLibA_58273 | 164 | 0.483368179 | hsa-mir-218-2 | - |
| hsa-mir-3913-2 | HGLibA_59524 | 164 | 0.483368179 | hsa-mir-3913-2 | - |
| hsa-mir-431 | HGLibA_59950 | 164 | 0.483368179 | hsa-mir-431 | - |
| hsa-mir-4489 | HGLibA_60376 | 164 | 0.483368179 | hsa-mir-4489 | - |
| hsa-mir-4642 | HGLibA_60671 | 164 | 0.483368179 | hsa-mir-4642 | - |
| hsa-mir-548ay | HGLibA_61945 | 164 | 0.483368179 | hsa-mir-548ay | - |
| hsa-mir-588 | HGLibA_62450 | 164 | 0.483368179 | hsa-mir-588 | - |
| hsa-mir-635 | HGLibA_62779 | 164 | 0.483368179 | hsa-mir-635 | - |
| hsa-mir-6731 | HGLibA_63078 | 164 | 0.483368179 | hsa-mir-6731 | - |
| hsa-mir-6788 | HGLibA_63331 | 164 | 0.483368179 | hsa-mir-6788 | - |
| hsa-mir-8083 | HGLibA_64173 | 164 | 0.483368179 | hsa-mir-8083 | - |
| JHDM1D | HGLibA_23947 | 164 | 0.483368179 | JHDM1D | - |
| KDELC2 | HGLibA_24477 | 164 | 0.483368179 | KDELC2 | - |
| ACN9 | HGLibA_00457 | 163 | 0.473417141 | ACN9 | - |
| AGXT2L1 | HGLibA_01276 | 163 | 0.473417141 | AGXT2L1 | - |
| BAI1 | HGLibA_04114 | 163 | 0.473417141 | BAI1 | - |
| DSCR3 | HGLibA_14008 | 163 | 0.473417141 | DSCR3 | - |
| ENSG00000022567 | HGLibA_45180 | 163 | 0.473417141 | SLC45A4 | solute carrier family 45 member 4 [Source:HGNC Symbol;Acc:HGNC:29196] |
| ENSG00000049449 | HGLibA_40702 | 163 | 0.473417141 | RCN1 | reticulocalbin 1 [Source:HGNC Symbol;Acc:HGNC:9934] |
| ENSG00000069764 | HGLibA_36867 | 163 | 0.473417141 | PLA2G10 | phospholipase A2 group X [Source:HGNC Symbol;Acc:HGNC:9029] |
| ENSG00000070476 | HGLibA_57078 | 163 | 0.473417141 | ZXDC | ZXD family zinc finger C [Source:HGNC Symbol;Acc:HGNC:28160] |
| ENSG00000076716 | HGLibA_19770 | 163 | 0.473417141 | GPC4 | glypican 4 [Source:HGNC Symbol;Acc:HGNC:4452] |
| ENSG00000082269 | HGLibA_16133 | 163 | 0.473417141 | FAM135A | family with sequence similarity 135 member A [Source:HGNC Symbol;Acc:HGNC:21084] |

| | | | | | |
|---|---|---|---|---|---|
| ENSG00000087245 | HGLibA_29506 | 163 | 0.473417141 | MMP2 | matrix metallopeptidase 2 [Source:HGNC Symbol;Acc:HGNC:7166] |
| ENSG00000099953 | HGLibA_29484 | 163 | 0.473417141 | MMP11 | matrix metallopeptidase 11 [Source:HGNC Symbol;Acc:HGNC:7157] |
| ENSG00000101134 | HGLibA_13780 | 163 | 0.473417141 | DOK5 | docking protein 5 [Source:HGNC Symbol;Acc:HGNC:16173] |
| ENSG00000102076 | HGLibA_33422 | 163 | 0.473417141 | OPN1LW | long wave sensitive [Source:HGNC Symbol;Acc:HGNC:9936]" |
| ENSG00000103034 | HGLibA_31423 | 163 | 0.473417141 | NDRG4 | NDRG family member 4 [Source:HGNC Symbol;Acc:HGNC:14466] |
| ENSG00000103034 | HGLibA_31421 | 163 | 0.473417141 | NDRG4 | NDRG family member 4 [Source:HGNC Symbol;Acc:HGNC:14466] |
| ENSG00000103876 | HGLibA_15952 | 163 | 0.473417141 | FAH | fumarylacetoacetate hydrolase [Source:HGNC Symbol;Acc:HGNC:3579] |
| ENSG00000105639 | HGLibA_23920 | 163 | 0.473417141 | JAK3 | Janus kinase 3 [Source:HGNC Symbol;Acc:HGNC:6193] |
| ENSG00000105755 | HGLibA_15630 | 163 | 0.473417141 | ETHE1 | persulfide dioxygenase [Source:HGNC Symbol;Acc:HGNC:23287]" |
| ENSG00000105851 | HGLibA_36641 | 163 | 0.473417141 | PIK3CG | 5-bisphosphate 3-kinase catalytic subunit gamma [Source:HGNC Symbol;Acc:HGNC:8978]" |
| ENSG00000106927 | HGLibA_01751 | 163 | 0.473417141 | AMBP | alpha-1-microglobulin/bikunin precursor [Source:HGNC Symbol;Acc:HGNC:453] |
| ENSG00000108352 | HGLibA_40263 | 163 | 0.473417141 | RAPGEFL1 | Rap guanine nucleotide exchange factor like 1 [Source:HGNC Symbol;Acc:HGNC:17428] |
| ENSG00000108786 | HGLibA_22169 | 163 | 0.473417141 | HSD17B1 | hydroxysteroid 17-beta dehydrogenase 1 [Source:HGNC Symbol;Acc:HGNC:5210] |
| ENSG00000109956 | HGLibA_03973 | 163 | 0.473417141 | B3GAT1 | 3-glucuronyltransferase 1 [Source:HGNC Symbol;Acc:HGNC:921]" |
| ENSG00000111269 | HGLibA_11110 | 163 | 0.473417141 | CREBL2 | cAMP responsive element binding protein like 2 [Source:HGNC Symbol;Acc:HGNC:2350] |
| ENSG00000112137 | HGLibA_36298 | 163 | 0.473417141 | PHACTR1 | phosphatase and actin regulator 1 [Source:HGNC Symbol;Acc:HGNC:20990] |
| ENSG00000112232 | HGLibA_24569 | 163 | 0.473417141 | KHDRBS2 | signal transduction associated 2 [Source:HGNC Symbol;Acc:HGNC:18114]" |
| ENSG00000112697 | HGLibA_50359 | 163 | 0.473417141 | TMEM30A | transmembrane protein 30A [Source:HGNC Symbol;Acc:HGNC:16667] |
| ENSG00000115446 | HGLibA_53011 | 163 | 0.473417141 | UNC50 | unc-50 inner nuclear membrane RNA binding protein [Source:HGNC Symbol;Acc:HGNC:16046] |
| ENSG00000116649 | HGLibA_46993 | 163 | 0.473417141 | SRM | spermidine synthase [Source:HGNC Symbol;Acc:HGNC:11296] |
| ENSG00000117620 | HGLibA_44952 | 163 | 0.473417141 | SLC35A3 | solute carrier family 35 member A3 [Source:HGNC Symbol;Acc:HGNC:11023] |
| ENSG00000119699 | HGLibA_49218 | 163 | 0.473417141 | TGFB3 | transforming growth factor beta 3 [Source:HGNC Symbol;Acc:HGNC:11769] |
| ENSG00000119965 | HGLibA_05101 | 163 | 0.473417141 | C10orf88 | chromosome 10 open reading frame 88 [Source:HGNC Symbol;Acc:HGNC:25822] |
| ENSG00000120690 | HGLibA_14892 | 163 | 0.473417141 | ELF1 | E74 like ETS transcription factor 1 [Source:HGNC Symbol;Acc:HGNC:3316] |
| ENSG00000124256 | HGLibA_54807 | 163 | 0.473417141 | ZBP1 | Z-DNA binding protein 1 [Source:HGNC Symbol;Acc:HGNC:16176] |
| ENSG00000124564 | HGLibA_44464 | 163 | 0.473417141 | SLC17A3 | solute carrier family 17 member 3 [Source:HGNC Symbol;Acc:HGNC:10931] |
| ENSG00000124702 | HGLibA_25100 | 163 | 0.473417141 | KLHDC3 | kelch domain containing 3 [Source:HGNC Symbol;Acc:HGNC:20704] |
| ENSG00000125844 | HGLibA_42242 | 163 | 0.473417141 | RRBP1 | ribosome binding protein 1 [Source:HGNC Symbol;Acc:HGNC:10448] |
| ENSG00000127129 | HGLibA_14471 | 163 | 0.473417141 | EDN2 | endothelin 2 [Source:HGNC Symbol;Acc:HGNC:3177] |
| ENSG00000127415 | HGLibA_22570 | 163 | 0.473417141 | IDUA | alpha-L- [Source:HGNC Symbol;Acc:HGNC:5391]" |
| ENSG00000128242 | HGLibA_18497 | 163 | 0.473417141 | GAL3ST1 | galactose-3-O-sulfotransferase 1 [Source:HGNC Symbol;Acc:HGNC:24240] |
| ENSG00000128335 | HGLibA_02529 | 163 | 0.473417141 | APOL2 | apolipoprotein L2 [Source:HGNC Symbol;Acc:HGNC:619] |
| ENSG00000130203 | HGLibA_02517 | 163 | 0.473417141 | APOE | apolipoprotein E [Source:HGNC Symbol;Acc:HGNC:613] |
| ENSG00000130244 | HGLibA_16816 | 163 | 0.473417141 | FAM98C | family with sequence similarity 98 member C [Source:HGNC Symbol;Acc:HGNC:27119] |
| ENSG00000134398 | HGLibA_15514 | 163 | 0.473417141 | ERN2 | endoplasmic reticulum to nucleus signaling 2 [Source:HGNC Symbol;Acc:HGNC:16942] |
| ENSG00000134852 | HGLibA_10093 | 163 | 0.473417141 | CLOCK | clock circadian regulator [Source:HGNC Symbol;Acc:HGNC:2082] |
| ENSG00000134940 | HGLibA_00538 | 163 | 0.473417141 | ACRV1 | acrosomal vesicle protein 1 [Source:HGNC Symbol;Acc:HGNC:127] |
| ENSG00000139209 | HGLibA_45069 | 163 | 0.473417141 | SLC38A4 | solute carrier family 38 member 4 [Source:HGNC Symbol;Acc:HGNC:14679] |
| ENSG00000139613 | HGLibA_45626 | 163 | 0.473417141 | SMARCC2 | actin dependent regulator of chromatin subfamily c member 2 [Source:HGNC Symbol;Acc:HGNC:11105]" |
| ENSG00000140092 | HGLibA_16950 | 163 | 0.473417141 | FBLN5 | fibulin 5 [Source:HGNC Symbol;Acc:HGNC:3602] |
| ENSG00000141497 | HGLibA_55420 | 163 | 0.473417141 | ZMYND15 | zinc finger MYND-type containing 15 [Source:HGNC Symbol;Acc:HGNC:20997] |
| ENSG00000141527 | HGLibA_07224 | 163 | 0.473417141 | CARD14 | caspase recruitment domain family member 14 [Source:HGNC Symbol;Acc:HGNC:16446] |
| ENSG00000142512 | HGLibA_44127 | 163 | 0.473417141 | SIGLEC10 | sialic acid binding Ig like lectin 10 [Source:HGNC Symbol;Acc:HGNC:15620] |
| ENSG00000142798 | HGLibA_22341 | 163 | 0.473417141 | HSPG2 | heparan sulfate proteoglycan 2 [Source:HGNC Symbol;Acc:HGNC:5273] |
| ENSG00000144426 | HGLibA_31209 | 163 | 0.473417141 | NBEAL1 | neurobeachin like 1 [Source:HGNC Symbol;Acc:HGNC:20681] |
| ENSG00000144749 | HGLibA_27175 | 163 | 0.473417141 | LRIG1 | leucine rich repeats and immunoglobulin like domains 1 [Source:HGNC Symbol;Acc:HGNC:17360] |
| ENSG00000145826 | HGLibA_26268 | 163 | 0.473417141 | LECT2 | leukocyte cell derived chemotaxin 2 [Source:HGNC Symbol;Acc:HGNC:6550] |
| ENSG00000147454 | HGLibA_44722 | 163 | 0.473417141 | SLC25A37 | solute carrier family 25 member 37 [Source:HGNC Symbol;Acc:HGNC:29786] |
| ENSG00000148840 | HGLibA_38230 | 163 | 0.473417141 | PPRC1 | coactivator-related 1 [Source:HGNC Symbol;Acc:HGNC:30025]" |
| ENSG00000149403 | HGLibA_20291 | 163 | 0.473417141 | GRIK4 | glutamate ionotropic receptor kainate type subunit 4 [Source:HGNC Symbol;Acc:HGNC:4582] |
| ENSG00000149596 | HGLibA_23983 | 163 | 0.473417141 | JPH2 | junctophilin 2 [Source:HGNC Symbol;Acc:HGNC:14202] |
| ENSG00000150316 | HGLibA_11812 | 163 | 0.473417141 | CWC15 | CWC15 spliceosome associated protein homolog [Source:HGNC Symbol;Acc:HGNC:26939] |
| ENSG00000151687 | HGLibA_01943 | 163 | 0.473417141 | ANKAR | ankyrin and armadillo repeat containing [Source:HGNC Symbol;Acc:HGNC:26350] |
| ENSG00000153066 | HGLibA_52405 | 163 | 0.473417141 | TXNDC11 | thioredoxin domain containing 11 [Source:HGNC Symbol;Acc:HGNC:28030] |
| ENSG00000153774 | HGLibA_09258 | 163 | 0.473417141 | CFDP1 | craniofacial development protein 1 [Source:HGNC Symbol;Acc:HGNC:1873] |
| ENSG00000155897 | HGLibA_00928 | 163 | 0.473417141 | ADCY8 | adenylate cyclase 8 [Source:HGNC Symbol;Acc:HGNC:239] |
| ENSG00000160551 | HGLibA_48305 | 163 | 0.473417141 | TAOK1 | TAO kinase 1 [Source:HGNC Symbol;Acc:HGNC:29259] |
| ENSG00000163520 | HGLibA_16946 | 163 | 0.473417141 | FBLN2 | fibulin 2 [Source:HGNC Symbol;Acc:HGNC:3601] |
| ENSG00000166847 | HGLibA_12555 | 163 | 0.473417141 | DCTN5 | dynactin subunit 5 [Source:HGNC Symbol;Acc:HGNC:24594] |
| ENSG00000167080 | HGLibA_04013 | 163 | 0.473417141 | B4GALNT2 | 4-N-acetyl-galactosaminyltransferase 2 [Source:HGNC Symbol;Acc:HGNC:24136]" |
| ENSG00000168273 | HGLibA_45759 | 163 | 0.473417141 | SMIM4 | small integral membrane protein 4 [Source:HGNC Symbol;Acc:HGNC:37257] |
| ENSG00000168505 | HGLibA_18753 | 163 | 0.473417141 | GBX2 | gastrulation brain homeobox 2 [Source:HGNC Symbol;Acc:HGNC:4186] |
| ENSG00000168619 | HGLibA_00742 | 163 | 0.473417141 | ADAM18 | ADAM metallopeptidase domain 18 [Source:HGNC Symbol;Acc:HGNC:196] |
| ENSG00000168676 | HGLibA_24442 | 163 | 0.473417141 | KCTD19 | potassium channel tetramerization domain containing 19 [Source:HGNC Symbol;Acc:HGNC:24753] |
| ENSG00000168758 | HGLibA_43347 | 163 | 0.473417141 | SEMA4C | semaphorin 4C [Source:HGNC Symbol;Acc:HGNC:10731] |
| ENSG00000169727 | HGLibA_20150 | 163 | 0.473417141 | GPS1 | G protein pathway suppressor 1 [Source:HGNC Symbol;Acc:HGNC:4549] |
| ENSG00000169851 | HGLibA_35414 | 163 | 0.473417141 | PCDH7 | protocadherin 7 [Source:HGNC Symbol;Acc:HGNC:8659] |
| ENSG00000171136 | HGLibA_41359 | 163 | 0.473417141 | RLN3 | relaxin 3 [Source:HGNC Symbol;Acc:HGNC:17135] |
| ENSG00000175104 | HGLibA_51265 | 163 | 0.473417141 | TRAF6 | TNF receptor associated factor 6 [Source:HGNC Symbol;Acc:HGNC:12036] |
| ENSG00000176095 | HGLibA_23444 | 163 | 0.473417141 | IP6K1 | inositol hexakisphosphate kinase 1 [Source:HGNC Symbol;Acc:HGNC:18360] |
| ENSG00000177291 | HGLibA_19192 | 163 | 0.473417141 | GJD4 | gap junction protein delta 4 [Source:HGNC Symbol;Acc:HGNC:23296] |
| ENSG00000178075 | HGLibA_20201 | 163 | 0.473417141 | GRAMD1C | GRAM domain containing 1C [Source:HGNC Symbol;Acc:HGNC:25252] |
| ENSG00000181016 | HGLibA_27557 | 163 | 0.473417141 | LSMEM1 | leucine rich single-pass membrane protein 1 [Source:HGNC Symbol;Acc:HGNC:22036] |
| ENSG00000181323 | HGLibA_46618 | 163 | 0.473417141 | SPEM1 | spermatid maturation 1 [Source:HGNC Symbol;Acc:HGNC:32429] |
| ENSG00000181396 | HGLibA_33315 | 163 | 0.473417141 | OGFOD3 | 2-oxoglutarate and iron dependent oxygenase domain containing 3 [Source:HGNC Symbol;Acc:HGNC:26174] |
| ENSG00000182489 | HGLibA_54538 | 163 | 0.473417141 | XKRX | XK related X-linked [Source:HGNC Symbol;Acc:HGNC:29845] |
| ENSG00000182712 | HGLibA_10192 | 163 | 0.473417141 | CMC4 | C-X9-C motif containing 4 [Source:HGNC Symbol;Acc:HGNC:35428] |
| ENSG00000182890 | HGLibA_19363 | 163 | 0.473417141 | GLUD2 | glutamate dehydrogenase 2 [Source:HGNC Symbol;Acc:HGNC:4336] |
| ENSG00000185518 | HGLibA_47877 | 163 | 0.473417141 | SV2B | synaptic vesicle glycoprotein 2B [Source:HGNC Symbol;Acc:HGNC:16874] |
| ENSG00000186136 | HGLibA_48407 | 163 | 0.473417141 | TAS2R42 | taste 2 receptor member 42 [Source:HGNC Symbol;Acc:HGNC:18888] |
| ENSG00000187556 | HGLibA_31103 | 163 | 0.473417141 | NANOS3 | nanos C2HC-type zinc finger 3 [Source:HGNC Symbol;Acc:HGNC:22048] |
| ENSG00000187634 | HGLibA_42664 | 163 | 0.473417141 | SAMD11 | sterile alpha motif domain containing 11 [Source:HGNC Symbol;Acc:HGNC:28706] |
| ENSG00000187783 | HGLibA_50507 | 163 | 0.473417141 | TMEM72 | transmembrane protein 72 [Source:HGNC Symbol;Acc:HGNC:31658] |
| ENSG00000188010 | HGLibA_29669 | 163 | 0.473417141 | MORN2 | MORN repeat containing 2 [Source:HGNC Symbol;Acc:HGNC:30166] |
| ENSG00000188508 | HGLibA_25861 | 163 | 0.473417141 | KRTDAP | keratinocyte differentiation associated protein [Source:HGNC Symbol;Acc:HGNC:16313] |
| ENSG00000188937 | HGLibA_33189 | 163 | 0.473417141 | NYX | nyctalopin [Source:HGNC Symbol;Acc:HGNC:8082] |
| ENSG00000196218 | HGLibA_42520 | 163 | 0.473417141 | RYR1 | ryanodine receptor 1 [Source:HGNC Symbol;Acc:HGNC:10483] |
| ENSG00000197361 | HGLibA_17004 | 163 | 0.473417141 | FBXL22 | F-box and leucine rich repeat protein 22 [Source:HGNC Symbol;Acc:HGNC:27537] |
| ENSG00000197540 | HGLibA_20731 | 163 | 0.473417141 | GZMM | granzyme M [Source:HGNC Symbol;Acc:HGNC:4712] |
| ENSG00000197978 | HGLibA_19632 | 163 | 0.473417141 | GOLGA6L9 | golgin A6 family-like 9 [Source:HGNC Symbol;Acc:HGNC:37229] |
| ENSG00000198028 | HGLibA_56242 | 163 | 0.473417141 | ZNF560 | zinc finger protein 560 [Source:HGNC Symbol;Acc:HGNC:26484] |
| ENSG00000204713 | HGLibA_51447 | 163 | 0.473417141 | TRIM27 | tripartite motif containing 27 [Source:HGNC Symbol;Acc:HGNC:9975] |
| ENSG00000205864 | HGLibA_25803 | 163 | 0.473417141 | KRTAP5-6 | keratin associated protein 5-6 [Source:HGNC Symbol;Acc:HGNC:23600] |
| ENSG00000212127 | HGLibA_48373 | 163 | 0.473417141 | TAS2R14 | taste 2 receptor member 14 [Source:HGNC Symbol;Acc:HGNC:14920] |
| ENSG00000214063 | HGLibA_51938 | 163 | 0.473417141 | TSPAN4 | tetraspanin 4 [Source:HGNC Symbol;Acc:HGNC:11859] |
| ENSG00000228474 | HGLibA_34706 | 163 | 0.473417141 | OST4 | non-catalytic [Source:HGNC Symbol;Acc:HGNC:32483]" |
| ENSG00000236362 | HGLibA_18448 | 163 | 0.473417141 | GAGE12F | G antigen 12F [Source:HGNC Symbol;Acc:HGNC:31906] |
| ENSG00000242802 | HGLibA_02363 | 163 | 0.473417141 | AP5Z1 | adaptor related protein complex 5 subunit zeta 1 [Source:HGNC Symbol;Acc:HGNC:22197] |
| GPR75-ASB3 | HGLibA_20083 | 163 | 0.473417141 | GPR75-ASB3 | - |
| hsa-mir-3170 | HGLibA_58759 | 163 | 0.473417141 | hsa-mir-3170 | - |
| hsa-mir-3199-1 | HGLibA_58896 | 163 | 0.473417141 | hsa-mir-3199-1 | - |
| hsa-mir-34c | HGLibA_59045 | 163 | 0.473417141 | hsa-mir-34c | - |
| hsa-mir-380 | HGLibA_59473 | 163 | 0.473417141 | hsa-mir-380 | - |
| hsa-mir-4492 | HGLibA_60386 | 163 | 0.473417141 | hsa-mir-4492 | - |
| hsa-mir-4746 | HGLibA_61081 | 163 | 0.473417141 | hsa-mir-4746 | - |
| hsa-mir-668 | HGLibA_63005 | 163 | 0.473417141 | hsa-mir-668 | - |
| hsa-mir-6717 | HGLibA_63029 | 163 | 0.473417141 | hsa-mir-6717 | - |
| hsa-mir-6814 | HGLibA_63434 | 163 | 0.473417141 | hsa-mir-6814 | - |
| LOC100133267 | HGLibA_26811 | 163 | 0.473417141 | LOC100133267 | - |
| LPPR1 | HGLibA_27112 | 163 | 0.473417141 | LPPR1 | - |
| SLC25A6_X | HGLibA_44775 | 163 | 0.473417141 | SLC25A6_X | - |
| SSSCA1 | HGLibA_47167 | 163 | 0.473417141 | SSSCA1 | - |
| C10orf114 | HGLibA_05037 | 162 | 0.463466104 | C10orf114 | - |
| CXCR7 | HGLibA_11898 | 162 | 0.463466104 | CXCR7 | - |
| ENSG00000002745 | HGLibA_54355 | 162 | 0.463466104 | WNT16 | Wnt family member 16 [Source:HGNC Symbol;Acc:HGNC:16267] |
| ENSG00000041880 | HGLibA_35236 | 162 | 0.463466104 | PARP3 | poly(ADP-ribose) polymerase family member 3 [Source:HGNC Symbol;Acc:HGNC:273] |
| ENSG00000057294 | HGLibA_36855 | 162 | 0.463466104 | PKP2 | plakophilin 2 [Source:HGNC Symbol;Acc:HGNC:9024] |
| ENSG00000066697 | HGLibA_30176 | 162 | 0.463466104 | MSANTD3 | Myb/SANT DNA binding domain containing 3 [Source:HGNC Symbol;Acc:HGNC:23370] |
| ENSG00000070367 | HGLibA_15751 | 162 | 0.463466104 | EXOC5 | exocyst complex component 5 [Source:HGNC Symbol;Acc:HGNC:10696] |
| ENSG00000074276 | HGLibA_08702 | 162 | 0.463466104 | CDHR2 | cadherin related family member 2 [Source:HGNC Symbol;Acc:HGNC:18231] |
| ENSG00000075035 | HGLibA_54424 | 162 | 0.463466104 | WSCD2 | WSC domain containing 2 [Source:HGNC Symbol;Acc:HGNC:29117] |
| ENSG00000076864 | HGLibA_40228 | 162 | 0.463466104 | RAP1GAP | RAP1 GTPase activating protein [Source:HGNC Symbol;Acc:HGNC:9858] |
| ENSG00000077279 | HGLibA_12580 | 162 | 0.463466104 | DCX | doublecortin [Source:HGNC Symbol;Acc:HGNC:2714] |
| ENSG00000080824 | HGLibA_22249 | 162 | 0.463466104 | HSP90AA1 | heat shock protein 90 alpha family class A member 1 [Source:HGNC Symbol;Acc:HGNC:5253] |
| ENSG00000094661 | HGLibA_33671 | 162 | 0.463466104 | OR1I1 | olfactory receptor family 1 subfamily I member 1 [Source:HGNC Symbol;Acc:HGNC:8207] |
| ENSG00000099203 | HGLibA_49798 | 162 | 0.463466104 | TMED1 | transmembrane p24 trafficking protein 1 [Source:HGNC Symbol;Acc:HGNC:17291] |
| ENSG00000099960 | HGLibA_45366 | 162 | 0.463466104 | SLC7A4 | solute carrier family 7 member 4 [Source:HGNC Symbol;Acc:HGNC:11062] |
| ENSG00000100151 | HGLibA_36525 | 162 | 0.463466104 | PICK1 | protein interacting with PRKCA 1 [Source:HGNC Symbol;Acc:HGNC:9394] |
| ENSG00000102935 | HGLibA_55959 | 162 | 0.463466104 | ZNF423 | zinc finger protein 423 [Source:HGNC Symbol;Acc:HGNC:16762] |
| ENSG00000104381 | HGLibA_18838 | 162 | 0.463466104 | GDAP1 | ganglioside induced differentiation associated protein 1 [Source:HGNC Symbol;Acc:HGNC:15968] |
| ENSG00000105613 | HGLibA_28399 | 162 | 0.463466104 | MAST1 | microtubule associated serine/threonine kinase 1 [Source:HGNC Symbol;Acc:HGNC:19034] |
| ENSG00000105701 | HGLibA_17587 | 162 | 0.463466104 | FKBP8 | FK506 binding protein 8 [Source:HGNC Symbol;Acc:HGNC:3724] |
| ENSG00000106785 | HGLibA_51411 | 162 | 0.463466104 | TRIM14 | tripartite motif containing 14 [Source:HGNC Symbol;Acc:HGNC:16283] |
| ENSG00000111058 | HGLibA_00594 | 162 | 0.463466104 | ACSS3 | acyl-CoA synthetase short chain family member 3 [Source:HGNC Symbol;Acc:HGNC:24723] |
| ENSG00000113269 | HGLibA_41505 | 162 | 0.463466104 | RNF130 | ring finger protein 130 [Source:HGNC Symbol;Acc:HGNC:18280] |

| | | | | | |
|---|---|---|---|---|---|
| ENSG00000116819 | HGLibA_49132 | 162 | 0.463466104 | TFAP2E | transcription factor AP-2 epsilon [Source:HGNC Symbol;Acc:HGNC:30774] |
| ENSG00000117505 | HGLibA_13940 | 162 | 0.463466104 | DR1 | down-regulator of transcription 1 [Source:HGNC Symbol;Acc:HGNC:3017] |
| ENSG00000119729 | HGLibA_41198 | 162 | 0.463466104 | RHOQ | ras homolog family member Q [Source:HGNC Symbol;Acc:HGNC:17736] |
| ENSG00000121068 | HGLibA_48656 | 162 | 0.463466104 | TBX2 | T-box 2 [Source:HGNC Symbol;Acc:HGNC:11597] |
| ENSG00000121743 | HGLibA_19141 | 162 | 0.463466104 | GJA3 | gap junction protein alpha 3 [Source:HGNC Symbol;Acc:HGNC:4277] |
| ENSG00000124383 | HGLibA_29723 | 162 | 0.463466104 | MPHOSPH10 | M-phase phosphoprotein 10 [Source:HGNC Symbol;Acc:HGNC:7213] |
| ENSG00000126759 | HGLibA_09289 | 162 | 0.463466104 | CFP | complement factor properdin [Source:HGNC Symbol;Acc:HGNC:8864] |
| ENSG00000126947 | HGLibA_03039 | 162 | 0.463466104 | ARMCX1 | armadillo repeat containing X-linked 1 [Source:HGNC Symbol;Acc:HGNC:18073] |
| ENSG00000127529 | HGLibA_34468 | 162 | 0.463466104 | OR7C2 | olfactory receptor family 7 subfamily C member 2 [Source:HGNC Symbol;Acc:HGNC:8374] |
| ENSG00000128713 | HGLibA_21990 | 162 | 0.463466104 | HOXD11 | homeobox D11 [Source:HGNC Symbol;Acc:HGNC:5134] |
| ENSG00000130560 | HGLibA_52533 | 162 | 0.463466104 | UBAC1 | UBA domain containing 1 [Source:HGNC Symbol;Acc:HGNC:30221] |
| ENSG00000132394 | HGLibA_14515 | 162 | 0.463466104 | EEFSEC | selenocysteine-tRNA specific [Source:HGNC Symbol;Acc:HGNC:24614]" |
| ENSG00000132622 | HGLibA_22263 | 162 | 0.463466104 | HSPA12B | heat shock protein family A (Hsp70) member 12B [Source:HGNC Symbol;Acc:HGNC:16193] |
| ENSG00000133063 | HGLibA_09454 | 162 | 0.463466104 | CHIT1 | chitinase 1 [Source:HGNC Symbol;Acc:HGNC:1936] |
| ENSG00000134780 | HGLibA_12296 | 162 | 0.463466104 | DAGLA | diacylglycerol lipase alpha [Source:HGNC Symbol;Acc:HGNC:1165] |
| ENSG00000135272 | HGLibA_28674 | 162 | 0.463466104 | MDFIC | MyoD family inhibitor domain containing [Source:HGNC Symbol;Acc:HGNC:28870] |
| ENSG00000135365 | HGLibA_36369 | 162 | 0.463466104 | PHF21A | PHD finger protein 21A [Source:HGNC Symbol;Acc:HGNC:24156] |
| ENSG00000135835 | HGLibA_24794 | 162 | 0.463466104 | KIAA1614 | KIAA1614 [Source:HGNC Symbol;Acc:HGNC:29327] |
| ENSG00000135899 | HGLibA_46288 | 162 | 0.463466104 | SP110 | SP110 nuclear body protein [Source:HGNC Symbol;Acc:HGNC:5401] |
| ENSG00000136240 | HGLibA_24483 | 162 | 0.463466104 | KDELR2 | KDEL endoplasmic reticulum protein retention receptor 2 [Source:HGNC Symbol;Acc:HGNC:6305] |
| ENSG00000136689 | HGLibA_23066 | 162 | 0.463466104 | IL1RN | interleukin 1 receptor antagonist [Source:HGNC Symbol;Acc:HGNC:6000] |
| ENSG00000136732 | HGLibA_20702 | 162 | 0.463466104 | GYPC | glycophorin C (Gerbich blood group) [Source:HGNC Symbol;Acc:HGNC:4704] |
| ENSG00000139985 | HGLibA_00755 | 162 | 0.463466104 | ADAM21 | ADAM metallopeptidase domain 21 [Source:HGNC Symbol;Acc:HGNC:200] |
| ENSG00000140006 | HGLibA_54211 | 162 | 0.463466104 | WDR89 | WD repeat domain 89 [Source:HGNC Symbol;Acc:HGNC:20489] |
| ENSG00000143179 | HGLibA_52812 | 162 | 0.463466104 | UCK2 | uridine-cytidine kinase 2 [Source:HGNC Symbol;Acc:HGNC:12562] |
| ENSG00000144026 | HGLibA_56139 | 162 | 0.463466104 | ZNF514 | zinc finger protein 514 [Source:HGNC Symbol;Acc:HGNC:25894] |
| ENSG00000149050 | HGLibA_55593 | 162 | 0.463466104 | ZNF214 | zinc finger protein 214 [Source:HGNC Symbol;Acc:HGNC:13006] |
| ENSG00000150337 | HGLibA_17183 | 162 | 0.463466104 | FCGR1A | Fc fragment of IgG receptor Ia [Source:HGNC Symbol;Acc:HGNC:3613] |
| ENSG00000150773 | HGLibA_36611 | 162 | 0.463466104 | PIH1D2 | PIH1 domain containing 2 [Source:HGNC Symbol;Acc:HGNC:25210] |
| ENSG00000151690 | HGLibA_29108 | 162 | 0.463466104 | MFSD6 | major facilitator superfamily domain containing 6 [Source:HGNC Symbol;Acc:HGNC:24711] |
| ENSG00000153291 | HGLibA_44689 | 162 | 0.463466104 | SLC25A27 | solute carrier family 25 member 27 [Source:HGNC Symbol;Acc:HGNC:21065] |
| ENSG00000154930 | HGLibA_00586 | 162 | 0.463466104 | ACSS1 | acyl-CoA synthetase short chain family member 1 [Source:HGNC Symbol;Acc:HGNC:16091] |
| ENSG00000155011 | HGLibA_13298 | 162 | 0.463466104 | DKK2 | dickkopf WNT signaling pathway inhibitor 2 [Source:HGNC Symbol;Acc:HGNC:2892] |
| ENSG00000155100 | HGLibA_34775 | 162 | 0.463466104 | OTUD6B | OTU domain containing 6B [Source:HGNC Symbol;Acc:HGNC:24281] |
| ENSG00000155833 | HGLibA_12045 | 162 | 0.463466104 | CYLC2 | cylicin 2 [Source:HGNC Symbol;Acc:HGNC:2583] |
| ENSG00000157131 | HGLibA_06592 | 162 | 0.463466104 | C8A | complement C8 alpha chain [Source:HGNC Symbol;Acc:HGNC:1352] |
| ENSG00000158786 | HGLibA_36894 | 162 | 0.463466104 | PLA2G2F | phospholipase A2 group IIF [Source:HGNC Symbol;Acc:HGNC:30040] |
| ENSG00000159023 | HGLibA_15238 | 162 | 0.463466104 | EPB41 | erythrocyte membrane protein band 4.1 [Source:HGNC Symbol;Acc:HGNC:3377] |
| ENSG00000159579 | HGLibA_42356 | 162 | 0.463466104 | RSPRY1 | ring finger and SPRY domain containing 1 [Source:HGNC Symbol;Acc:HGNC:29420] |
| ENSG00000160058 | HGLibA_04862 | 162 | 0.463466104 | BSDC1 | BSD domain containing 1 [Source:HGNC Symbol;Acc:HGNC:25501] |
| ENSG00000160654 | HGLibA_08379 | 162 | 0.463466104 | CD3G | CD3g molecule [Source:HGNC Symbol;Acc:HGNC:1675] |
| ENSG00000160767 | HGLibA_16357 | 162 | 0.463466104 | FAM189B | family with sequence similarity 189 member B [Source:HGNC Symbol;Acc:HGNC:1233] |
| ENSG00000161970 | HGLibA_41913 | 162 | 0.463466104 | RPL26 | ribosomal protein L26 [Source:HGNC Symbol;Acc:HGNC:10327] |
| ENSG00000163209 | HGLibA_46827 | 162 | 0.463466104 | SPRR3 | small proline rich protein 3 [Source:HGNC Symbol;Acc:HGNC:11268] |
| ENSG00000165383 | HGLibA_27272 | 162 | 0.463466104 | LRRC18 | leucine rich repeat containing 18 [Source:HGNC Symbol;Acc:HGNC:23199] |
| ENSG00000166046 | HGLibA_48849 | 162 | 0.463466104 | TCP11L2 | t-complex 11 like 2 [Source:HGNC Symbol;Acc:HGNC:28627] |
| ENSG00000166446 | HGLibA_08921 | 162 | 0.463466104 | CDYL2 | chromodomain Y like 2 [Source:HGNC Symbol;Acc:HGNC:23030] |
| ENSG00000167257 | HGLibA_41624 | 162 | 0.463466104 | RNF214 | ring finger protein 214 [Source:HGNC Symbol;Acc:HGNC:25335] |
| ENSG00000167525 | HGLibA_38652 | 162 | 0.463466104 | PROCA1 | protein interacting with cyclin A1 [Source:HGNC Symbol;Acc:HGNC:28600] |
| ENSG00000168453 | HGLibA_22070 | 162 | 0.463466104 | HR | lysine demethylase and nuclear receptor corepressor [Source:HGNC Symbol;Acc:HGNC:5172]" |
| ENSG00000169016 | HGLibA_14322 | 162 | 0.463466104 | E2F6 | E2F transcription factor 6 [Source:HGNC Symbol;Acc:HGNC:3120] |
| ENSG00000171496 | HGLibA_33699 | 162 | 0.463466104 | OR1L8 | olfactory receptor family 1 subfamily L member 8 [Source:HGNC Symbol;Acc:HGNC:15110] |
| ENSG00000173464 | HGLibA_41393 | 162 | 0.463466104 | RNASE11 | ribonuclease A family member 11 (inactive) [Source:HGNC Symbol;Acc:HGNC:19269] |
| ENSG00000174010 | HGLibA_25139 | 162 | 0.463466104 | KLHL15 | kelch like family member 15 [Source:HGNC Symbol;Acc:HGNC:29347] |
| ENSG00000174233 | HGLibA_00923 | 162 | 0.463466104 | ADCY6 | adenylate cyclase 6 [Source:HGNC Symbol;Acc:HGNC:237] |
| ENSG00000175785 | HGLibA_38487 | 162 | 0.463466104 | PRIMA1 | proline rich membrane anchor 1 [Source:HGNC Symbol;Acc:HGNC:18319] |
| ENSG00000176018 | HGLibA_27769 | 162 | 0.463466104 | LYSMD3 | LysM domain containing 3 [Source:HGNC Symbol;Acc:HGNC:26969] |
| ENSG00000177989 | HGLibA_33286 | 162 | 0.463466104 | ODF3B | outer dense fiber of sperm tails 3B [Source:HGNC Symbol;Acc:HGNC:34388] |
| ENSG00000180818 | HGLibA_21957 | 162 | 0.463466104 | HOXC10 | homeobox C10 [Source:HGNC Symbol;Acc:HGNC:5122] |
| ENSG00000181961 | HGLibA_33926 | 162 | 0.463466104 | OR4A16 | olfactory receptor family 4 subfamily A member 16 [Source:HGNC Symbol;Acc:HGNC:15153] |
| ENSG00000182459 | HGLibA_49070 | 162 | 0.463466104 | TEX19 | testis expressed 19 [Source:HGNC Symbol;Acc:HGNC:33802] |
| ENSG00000182463 | HGLibA_51866 | 162 | 0.463466104 | TSHZ2 | teashirt zinc finger homeobox 2 [Source:HGNC Symbol;Acc:HGNC:13010] |
| ENSG00000185448 | HGLibA_16608 | 162 | 0.463466104 | FAM47A | family with sequence similarity 47 member A [Source:HGNC Symbol;Acc:HGNC:29962] |
| ENSG00000185787 | HGLibA_29661 | 162 | 0.463466104 | MORF4L1 | mortality factor 4 like 1 [Source:HGNC Symbol;Acc:HGNC:16989] |
| ENSG00000185885 | HGLibA_22639 | 162 | 0.463466104 | IFITM1 | interferon induced transmembrane protein 1 [Source:HGNC Symbol;Acc:HGNC:5412] |
| ENSG00000185933 | HGLibA_07012 | 162 | 0.463466104 | CALHM1 | calcium homeostasis modulator 1 [Source:HGNC Symbol;Acc:HGNC:23494] |
| ENSG00000187969 | HGLibA_55012 | 162 | 0.463466104 | ZCCHC13 | zinc finger CCHC-type containing 13 [Source:HGNC Symbol;Acc:HGNC:31749] |
| ENSG00000188295 | HGLibA_56496 | 162 | 0.463466104 | ZNF669 | zinc finger protein 669 [Source:HGNC Symbol;Acc:HGNC:25736] |
| ENSG00000188807 | HGLibA_50179 | 162 | 0.463466104 | TMEM201 | transmembrane protein 201 [Source:HGNC Symbol;Acc:HGNC:33719] |
| ENSG00000189319 | HGLibA_16637 | 162 | 0.463466104 | FAM53B | family with sequence similarity 53 member B [Source:HGNC Symbol;Acc:HGNC:28968] |
| ENSG00000196405 | HGLibA_15699 | 162 | 0.463466104 | EVL | Enah/Vasp-like [Source:HGNC Symbol;Acc:HGNC:20234] |
| ENSG00000196914 | HGLibA_02816 | 162 | 0.463466104 | ARHGEF12 | Rho guanine nucleotide exchange factor 12 [Source:HGNC Symbol;Acc:HGNC:14193] |
| ENSG00000196944 | HGLibA_33883 | 162 | 0.463466104 | OR2T4 | olfactory receptor family 2 subfamily T member 4 [Source:HGNC Symbol;Acc:HGNC:15016] |
| ENSG00000197363 | HGLibA_56145 | 162 | 0.463466104 | ZNF517 | zinc finger protein 517 [Source:HGNC Symbol;Acc:HGNC:27984] |
| ENSG00000197982 | HGLibA_05758 | 162 | 0.463466104 | C1orf122 | chromosome 1 open reading frame 122 [Source:HGNC Symbol;Acc:HGNC:24789] |
| ENSG00000198885 | HGLibA_23864 | 162 | 0.463466104 | ITPRIPL1 | ITPRIP like 1 [Source:HGNC Symbol;Acc:HGNC:29371] |
| ENSG00000198911 | HGLibA_46953 | 162 | 0.463466104 | SREBF2 | sterol regulatory element binding transcription factor 2 [Source:HGNC Symbol;Acc:HGNC:11290] |
| ENSG00000204472 | HGLibA_01331 | 162 | 0.463466104 | AIF1 | allograft inflammatory factor 1 [Source:HGNC Symbol;Acc:HGNC:352] |
| ENSG00000212864 | HGLibA_41616 | 162 | 0.463466104 | RNF208 | ring finger protein 208 [Source:HGNC Symbol;Acc:HGNC:25420] |
| ENSG00000213931 | HGLibA_20920 | 162 | 0.463466104 | HBE1 | hemoglobin subunit epsilon 1 [Source:HGNC Symbol;Acc:HGNC:4830] |
| ENSG00000215455 | HGLibA_25591 | 162 | 0.463466104 | KRTAP10-1 | keratin associated protein 10-1 [Source:HGNC Symbol;Acc:HGNC:22966] |
| ENSG00000240857 | HGLibA_40744 | 162 | 0.463466104 | RDH14 | retinol dehydrogenase 14 [Source:HGNC Symbol;Acc:HGNC:19979] |
| ENSG00000244122 | HGLibA_52893 | 162 | 0.463466104 | UGT1A7 | UDP glucuronosyltransferase family 1 member A7 [Source:HGNC Symbol;Acc:HGNC:12539] |
| ENSG00000269343 | HGLibA_56332 | 162 | 0.463466104 | ZNF587B | zinc finger protein 587B [Source:HGNC Symbol;Acc:HGNC:37142] |
| ENSG00000274274 | HGLibA_18463 | 162 | 0.463466104 | GAGE13 | G antigen 13 [Source:HGNC Symbol;Acc:HGNC:29081] |
| ENSG00000275113 | HGLibA_18478 | 162 | 0.463466104 | GAGE2E | G antigen 2E [Source:HGNC Symbol;Acc:HGNC:31960] |
| ENSG00000279000 | HGLibA_33472 | 162 | 0.463466104 | OR10A6 | olfactory receptor family 10 subfamily A member 6 (gene/pseudogene) [Source:HGNC Symbol;Acc:HGNC:15132] |
| ENSG00000279116 | HGLibA_34512 | 162 | 0.463466104 | OR8D2 | olfactory receptor family 8 subfamily D member 2 (gene/pseudogene) [Source:HGNC Symbol;Acc:HGNC:8482] |
| FAM122C | HGLibA_16072 | 162 | 0.463466104 | FAM122C | - |
| FAM155B | HGLibA_16184 | 162 | 0.463466104 | FAM155B | - |
| HIST1H2AC | HGLibA_21381 | 162 | 0.463466104 | HIST1H2AC | - |
| HIST1H3I | HGLibA_21480 | 162 | 0.463466104 | HIST1H3I | - |
| hsa-mir-129-1 | HGLibA_57629 | 162 | 0.463466104 | hsa-mir-129-1 | - |
| hsa-mir-1915 | HGLibA_58065 | 162 | 0.463466104 | hsa-mir-1915 | - |
| hsa-mir-2682 | HGLibA_58359 | 162 | 0.463466104 | hsa-mir-2682 | - |
| hsa-mir-3916 | HGLibA_59540 | 162 | 0.463466104 | hsa-mir-3916 | - |
| hsa-mir-548e | HGLibA_61967 | 162 | 0.463466104 | hsa-mir-548e | - |
| hsa-mir-6786 | HGLibA_63321 | 162 | 0.463466104 | hsa-mir-6786 | - |
| hsa-mir-7158 | HGLibA_63860 | 162 | 0.463466104 | hsa-mir-7158 | - |
| hsa-mir-7160 | HGLibA_63870 | 162 | 0.463466104 | hsa-mir-7160 | - |
| LOC149373 | HGLibA_26890 | 162 | 0.463466104 | LOC149373 | - |
| LOC388849 | HGLibA_26928 | 162 | 0.463466104 | LOC388849 | - |
| ATHL1 | HGLibA_03510 | 161 | 0.453515067 | ATHL1 | - |
| ENSG00000004534 | HGLibA_40579 | 161 | 0.453515067 | RBM6 | RNA binding motif protein 6 [Source:HGNC Symbol;Acc:HGNC:9903] |
| ENSG00000004975 | HGLibA_14210 | 161 | 0.453515067 | DVL2 | dishevelled segment polarity protein 2 [Source:HGNC Symbol;Acc:HGNC:3086] |
| ENSG00000007129 | HGLibA_08935 | 161 | 0.453515067 | CEACAM21 | carcinoembryonic antigen related cell adhesion molecule 21 [Source:HGNC Symbol;Acc:HGNC:28834] |
| ENSG00000048545 | HGLibA_20633 | 161 | 0.453515067 | GUCA1A | guanylate cyclase activator 1A [Source:HGNC Symbol;Acc:HGNC:4678] |
| ENSG00000058091 | HGLibA_08737 | 161 | 0.453515067 | CDK14 | cyclin dependent kinase 14 [Source:HGNC Symbol;Acc:HGNC:8883] |
| ENSG00000065485 | HGLibA_35884 | 161 | 0.453515067 | PDIA5 | protein disulfide isomerase family A member 5 [Source:HGNC Symbol;Acc:HGNC:24811] |
| ENSG00000067365 | HGLibA_28989 | 161 | 0.453515067 | METTL22 | methyltransferase like 22 [Source:HGNC Symbol;Acc:HGNC:28368] |
| ENSG00000077092 | HGLibA_40274 | 161 | 0.453515067 | RARB | retinoic acid receptor beta [Source:HGNC Symbol;Acc:HGNC:9865] |
| ENSG00000082397 | HGLibA_15247 | 161 | 0.453515067 | EPB41L3 | erythrocyte membrane protein band 4.1 like 3 [Source:HGNC Symbol;Acc:HGNC:3380] |
| ENSG00000086062 | HGLibA_04024 | 161 | 0.453515067 | B4GALT1 | 4-galactosyltransferase 1 [Source:HGNC Symbol;Acc:HGNC:924]" |
| ENSG00000099256 | HGLibA_38977 | 161 | 0.453515067 | PRTFDC1 | phosphoribosyl transferase domain containing 1 [Source:HGNC Symbol;Acc:HGNC:23333] |
| ENSG00000100600 | HGLibA_26404 | 161 | 0.453515067 | LGMN | legumain [Source:HGNC Symbol;Acc:HGNC:9472] |
| ENSG00000103260 | HGLibA_28938 | 161 | 0.453515067 | METRN | glial cell differentiation regulator [Source:HGNC Symbol;Acc:HGNC:14151]" |
| ENSG00000105352 | HGLibA_08940 | 161 | 0.453515067 | CEACAM4 | carcinoembryonic antigen related cell adhesion molecule 4 [Source:HGNC Symbol;Acc:HGNC:1816] |
| ENSG00000105559 | HGLibA_37072 | 161 | 0.453515067 | PLEKHA4 | pleckstrin homology domain containing A4 [Source:HGNC Symbol;Acc:HGNC:14339] |
| ENSG00000105948 | HGLibA_52091 | 161 | 0.453515067 | TTC26 | tetratricopeptide repeat domain 26 [Source:HGNC Symbol;Acc:HGNC:21882] |
| ENSG00000106246 | HGLibA_39260 | 161 | 0.453515067 | PTCD1 | pentatricopeptide repeat domain 1 [Source:HGNC Symbol;Acc:HGNC:22198] |
| ENSG00000106266 | HGLibA_46107 | 161 | 0.453515067 | SNX8 | sorting nexin 8 [Source:HGNC Symbol;Acc:HGNC:14972] |
| ENSG00000109381 | HGLibA_14893 | 161 | 0.453515067 | ELF2 | E74 like ETS transcription factor 2 [Source:HGNC Symbol;Acc:HGNC:3317] |
| ENSG00000109654 | HGLibA_51425 | 161 | 0.453515067 | TRIM2 | tripartite motif containing 2 [Source:HGNC Symbol;Acc:HGNC:15974] |
| ENSG00000110077 | HGLibA_30156 | 161 | 0.453515067 | MS4A6A | membrane spanning 4-domains A6A [Source:HGNC Symbol;Acc:HGNC:13375] |
| ENSG00000111266 | HGLibA_14148 | 161 | 0.453515067 | DUSP16 | dual specificity phosphatase 16 [Source:HGNC Symbol;Acc:HGNC:17909] |
| ENSG00000113303 | HGLibA_04979 | 161 | 0.453515067 | BTNL8 | butyrophilin like 8 [Source:HGNC Symbol;Acc:HGNC:26131] |
| ENSG00000115841 | HGLibA_41366 | 161 | 0.453515067 | RMDN2 | regulator of microtubule dynamics 2 [Source:HGNC Symbol;Acc:HGNC:26567] |
| ENSG00000116786 | HGLibA_37139 | 161 | 0.453515067 | PLEKHM2 | pleckstrin homology and RUN domain containing M2 [Source:HGNC Symbol;Acc:HGNC:29131] |
| ENSG00000117620 | HGLibA_44953 | 161 | 0.453515067 | SLC35A3 | solute carrier family 35 member A3 [Source:HGNC Symbol;Acc:HGNC:11023] |
| ENSG00000118965 | HGLibA_54073 | 161 | 0.453515067 | WDR35 | WD repeat domain 35 [Source:HGNC Symbol;Acc:HGNC:29250] |
| ENSG00000119777 | HGLibA_50216 | 161 | 0.453515067 | TMEM214 | transmembrane protein 214 [Source:HGNC Symbol;Acc:HGNC:25983] |
| ENSG00000120057 | HGLibA_43746 | 161 | 0.453515067 | SFRP5 | secreted frizzled related protein 5 [Source:HGNC Symbol;Acc:HGNC:10779] |
| ENSG00000122971 | HGLibA_00370 | 161 | 0.453515067 | ACADS | acyl-CoA dehydrogenase short chain [Source:HGNC Symbol;Acc:HGNC:90] |
| ENSG00000126254 | HGLibA_40551 | 161 | 0.453515067 | RBM42 | RNA binding motif protein 42 [Source:HGNC Symbol;Acc:HGNC:28117] |
| ENSG00000127928 | HGLibA_19537 | 161 | 0.453515067 | GNGT1 | G protein subunit gamma transducin 1 [Source:HGNC Symbol;Acc:HGNC:4411] |

| | | | | | |
|---|---|---|---|---|---|
| ENSG00000128268 | HGLibA_29137 | 161 | 0.453515067 | MGAT3 | 4-N-acetylglucosaminyltransferase [Source:HGNC Symbol;Acc:HGNC:7046]" |
| ENSG00000130312 | HGLibA_29957 | 161 | 0.453515067 | MRPL34 | mitochondrial ribosomal protein L34 [Source:HGNC Symbol;Acc:HGNC:14488] |
| ENSG00000132823 | HGLibA_34678 | 161 | 0.453515067 | OSER1 | oxidative stress responsive serine rich 1 [Source:HGNC Symbol;Acc:HGNC:16105] |
| ENSG00000135549 | HGLibA_36823 | 161 | 0.453515067 | PKIB | cAMP-dependent protein kinase inhibitor beta [Source:HGNC Symbol;Acc:HGNC:9018] |
| ENSG00000135773 | HGLibA_07182 | 161 | 0.453515067 | CAPN9 | calpain 9 [Source:HGNC Symbol;Acc:HGNC:1486] |
| ENSG00000136240 | HGLibA_24481 | 161 | 0.453515067 | KDELR2 | KDEL endoplasmic reticulum protein retention receptor 2 [Source:HGNC Symbol;Acc:HGNC:6305] |
| ENSG00000137076 | HGLibA_49634 | 161 | 0.453515067 | TLN1 | talin 1 [Source:HGNC Symbol;Acc:HGNC:11845] |
| ENSG00000137177 | HGLibA_24857 | 161 | 0.453515067 | KIF13A | kinesin family member 13A [Source:HGNC Symbol;Acc:HGNC:14566] |
| ENSG00000138435 | HGLibA_09563 | 161 | 0.453515067 | CHRNA1 | cholinergic receptor nicotinic alpha 1 subunit [Source:HGNC Symbol;Acc:HGNC:1955] |
| ENSG00000139194 | HGLibA_40640 | 161 | 0.453515067 | RBP5 | retinol binding protein 5 [Source:HGNC Symbol;Acc:HGNC:15847] |
| ENSG00000139436 | HGLibA_19133 | 161 | 0.453515067 | GIT2 | GIT ArfGAP 2 [Source:HGNC Symbol;Acc:HGNC:4273] |
| ENSG00000139613 | HGLibA_45625 | 161 | 0.453515067 | SMARCC2 | actin dependent regulator of chromatin subfamily c member 2 [Source:HGNC Symbol;Acc:HGNC:11105]" |
| ENSG00000140403 | HGLibA_13519 | 161 | 0.453515067 | DNAJA4 | DnaJ heat shock protein family (Hsp40) member A4 [Source:HGNC Symbol;Acc:HGNC:14885] |
| ENSG00000141576 | HGLibA_41553 | 161 | 0.453515067 | RNF157 | ring finger protein 157 [Source:HGNC Symbol;Acc:HGNC:29402] |
| ENSG00000142186 | HGLibA_43084 | 161 | 0.453515067 | SCYL1 | SCY1 like pseudokinase 1 [Source:HGNC Symbol;Acc:HGNC:14372] |
| ENSG00000142632 | HGLibA_02832 | 161 | 0.453515067 | ARHGEF19 | Rho guanine nucleotide exchange factor 19 [Source:HGNC Symbol;Acc:HGNC:26604] |
| ENSG00000142798 | HGLibA_22339 | 161 | 0.453515067 | HSPG2 | heparan sulfate proteoglycan 2 [Source:HGNC Symbol;Acc:HGNC:5273] |
| ENSG00000144962 | HGLibA_46432 | 161 | 0.453515067 | SPATA16 | spermatogenesis associated 16 [Source:HGNC Symbol;Acc:HGNC:29935] |
| ENSG00000147262 | HGLibA_19868 | 161 | 0.453515067 | GPR119 | G protein-coupled receptor 119 [Source:HGNC Symbol;Acc:HGNC:19060] |
| ENSG00000147526 | HGLibA_48160 | 161 | 0.453515067 | TACC1 | transforming acidic coiled-coil containing protein 1 [Source:HGNC Symbol;Acc:HGNC:11522] |
| ENSG00000149231 | HGLibA_07882 | 161 | 0.453515067 | CCDC82 | coiled-coil domain containing 82 [Source:HGNC Symbol;Acc:HGNC:26282] |
| ENSG00000149657 | HGLibA_27532 | 161 | 0.453515067 | LSM14B | LSM family member 14B [Source:HGNC Symbol;Acc:HGNC:15887] |
| ENSG00000151640 | HGLibA_13933 | 161 | 0.453515067 | DPYSL4 | dihydropyrimidinase like 4 [Source:HGNC Symbol;Acc:HGNC:3016] |
| ENSG00000163793 | HGLibA_13629 | 161 | 0.453515067 | DNAJC5G | DnaJ heat shock protein family (Hsp40) member C5 gamma [Source:HGNC Symbol;Acc:HGNC:24844] |
| ENSG00000164253 | HGLibA_54089 | 161 | 0.453515067 | WDR41 | WD repeat domain 41 [Source:HGNC Symbol;Acc:HGNC:25601] |
| ENSG00000164900 | HGLibA_18749 | 161 | 0.453515067 | GBX1 | gastrulation brain homeobox 1 [Source:HGNC Symbol;Acc:HGNC:4185] |
| ENSG00000165509 | HGLibA_27966 | 161 | 0.453515067 | MAGEC3 | MAGE family member C3 [Source:HGNC Symbol;Acc:HGNC:23798] |
| ENSG00000165525 | HGLibA_31689 | 161 | 0.453515067 | NEMF | nuclear export mediator factor [Source:HGNC Symbol;Acc:HGNC:10663] |
| ENSG00000166046 | HGLibA_48848 | 161 | 0.453515067 | TCP11L2 | t-complex 11 like 2 [Source:HGNC Symbol;Acc:HGNC:28627] |
| ENSG00000166148 | HGLibA_03903 | 161 | 0.453515067 | AVPR1A | arginine vasopressin receptor 1A [Source:HGNC Symbol;Acc:HGNC:895] |
| ENSG00000168930 | HGLibA_51521 | 161 | 0.453515067 | TRIM49 | tripartite motif containing 49 [Source:HGNC Symbol;Acc:HGNC:13431] |
| ENSG00000169018 | HGLibA_17276 | 161 | 0.453515067 | FEM1B | fem-1 homolog B [Source:HGNC Symbol;Acc:HGNC:3649] |
| ENSG00000169972 | HGLibA_39611 | 161 | 0.453515067 | PUSL1 | pseudouridine synthase like 1 [Source:HGNC Symbol;Acc:HGNC:26914] |
| ENSG00000170448 | HGLibA_31850 | 161 | 0.453515067 | NFXL1 | X-box binding like 1 [Source:HGNC Symbol;Acc:HGNC:18726]" |
| ENSG00000170927 | HGLibA_36812 | 161 | 0.453515067 | PKHD1 | fibrocystin/polyductin [Source:HGNC Symbol;Acc:HGNC:9016]" |
| ENSG00000171053 | HGLibA_35272 | 161 | 0.453515067 | PATE1 | prostate and testis expressed 1 [Source:HGNC Symbol;Acc:HGNC:24664] |
| ENSG00000171115 | HGLibA_19090 | 161 | 0.453515067 | GIMAP8 | IMAP family member 8 [Source:HGNC Symbol;Acc:HGNC:21792]" |
| ENSG00000171126 | HGLibA_24203 | 161 | 0.453515067 | KCNG3 | potassium voltage-gated channel modifier subfamily G member 3 [Source:HGNC Symbol;Acc:HGNC:18306] |
| ENSG00000171174 | HGLibA_40464 | 161 | 0.453515067 | RBKS | ribokinase [Source:HGNC Symbol;Acc:HGNC:30325] |
| ENSG00000172936 | HGLibA_30685 | 161 | 0.453515067 | MYD88 | innate immune signal transduction adaptor [Source:HGNC Symbol;Acc:HGNC:7562]" |
| ENSG00000173546 | HGLibA_11413 | 161 | 0.453515067 | CSPG4 | chondroitin sulfate proteoglycan 4 [Source:HGNC Symbol;Acc:HGNC:2466] |
| ENSG00000175216 | HGLibA_09764 | 161 | 0.453515067 | CKAP5 | cytoskeleton associated protein 5 [Source:HGNC Symbol;Acc:HGNC:28959] |
| ENSG00000175445 | HGLibA_27103 | 161 | 0.453515067 | LPL | lipoprotein lipase [Source:HGNC Symbol;Acc:HGNC:6677] |
| ENSG00000177374 | HGLibA_21287 | 161 | 0.453515067 | HIC1 | HIC ZBTB transcriptional repressor 1 [Source:HGNC Symbol;Acc:HGNC:4909] |
| ENSG00000177606 | HGLibA_24004 | 161 | 0.453515067 | JUN | AP-1 transcription factor subunit [Source:HGNC Symbol;Acc:HGNC:6204]" |
| ENSG00000178718 | HGLibA_42028 | 161 | 0.453515067 | RPP25 | ribonuclease P and MRP subunit p25 [Source:HGNC Symbol;Acc:HGNC:30361] |
| ENSG00000178904 | HGLibA_13911 | 161 | 0.453515067 | DPY19L3 | dpy-19 like C-mannosyltransferase 3 [Source:HGNC Symbol;Acc:HGNC:27120] |
| ENSG00000180138 | HGLibA_11383 | 161 | 0.453515067 | CSNK1A1L | casein kinase 1 alpha 1 like [Source:HGNC Symbol;Acc:HGNC:20289] |
| ENSG00000180209 | HGLibA_30796 | 161 | 0.453515067 | MYLPF | fast skeletal muscle [Source:HGNC Symbol;Acc:HGNC:29824]" |
| ENSG00000180914 | HGLibA_34844 | 161 | 0.453515067 | OXTR | oxytocin receptor [Source:HGNC Symbol;Acc:HGNC:8529] |
| ENSG00000183310 | HGLibA_33879 | 161 | 0.453515067 | OR2T34 | olfactory receptor family 2 subfamily T member 34 [Source:HGNC Symbol;Acc:HGNC:31256] |
| ENSG00000185386 | HGLibA_28252 | 161 | 0.453515067 | MAPK11 | mitogen-activated protein kinase 11 [Source:HGNC Symbol;Acc:HGNC:6873] |
| ENSG00000186038 | HGLibA_22392 | 161 | 0.453515067 | HTR3E | 5-hydroxytryptamine receptor 3E [Source:HGNC Symbol;Acc:HGNC:24005] |
| ENSG00000186049 | HGLibA_25532 | 161 | 0.453515067 | KRT73 | keratin 73 [Source:HGNC Symbol;Acc:HGNC:28928] |
| ENSG00000186815 | HGLibA_51105 | 161 | 0.453515067 | TPCN1 | two pore segment channel 1 [Source:HGNC Symbol;Acc:HGNC:18182] |
| ENSG00000188133 | HGLibA_50219 | 161 | 0.453515067 | TMEM215 | transmembrane protein 215 [Source:HGNC Symbol;Acc:HGNC:33816] |
| ENSG00000196329 | HGLibA_19082 | 161 | 0.453515067 | GIMAP5 | IMAP family member 5 [Source:HGNC Symbol;Acc:HGNC:18005]" |
| ENSG00000197496 | HGLibA_44856 | 161 | 0.453515067 | SLC2A10 | solute carrier family 2 member 10 [Source:HGNC Symbol;Acc:HGNC:13444] |
| ENSG00000197579 | HGLibA_51011 | 161 | 0.453515067 | TOPORS | TOP1 binding arginine/serine rich protein [Source:HGNC Symbol;Acc:HGNC:21653] |
| ENSG00000214042 | HGLibA_22689 | 161 | 0.453515067 | IFNA7 | interferon alpha 7 [Source:HGNC Symbol;Acc:HGNC:5428] |
| ENSG00000214753 | HGLibA_21851 | 161 | 0.453515067 | HNRNPUL2 | heterogeneous nuclear ribonucleoprotein U like 2 [Source:HGNC Symbol;Acc:HGNC:25451] |
| ENSG00000214943 | HGLibA_20024 | 161 | 0.453515067 | GPR33 | G protein-coupled receptor 33 (gene/pseudogene) [Source:HGNC Symbol;Acc:HGNC:4489] |
| ENSG00000249915 | HGLibA_35738 | 161 | 0.453515067 | PDCD6 | programmed cell death 6 [Source:HGNC Symbol;Acc:HGNC:8765] |
| ENSG00000288702 | HGLibA_52881 | 161 | 0.453515067 | UGT1A3 | - |
| H2AFV | HGLibA_20756 | 161 | 0.453515067 | H2AFV | - |
| hsa-mir-138-1 | HGLibA_57812 | 161 | 0.453515067 | hsa-mir-138-1 | - |
| hsa-mir-202 | HGLibA_58162 | 161 | 0.453515067 | hsa-mir-202 | - |
| hsa-mir-3183 | HGLibA_58828 | 161 | 0.453515067 | hsa-mir-3183 | - |
| hsa-mir-3610 | HGLibA_59074 | 161 | 0.453515067 | hsa-mir-3610 | - |
| hsa-mir-3684 | HGLibA_59291 | 161 | 0.453515067 | hsa-mir-3684 | - |
| hsa-mir-3919 | HGLibA_59554 | 161 | 0.453515067 | hsa-mir-3919 | - |
| hsa-mir-4431 | HGLibA_60111 | 161 | 0.453515067 | hsa-mir-4431 | - |
| hsa-mir-4786 | HGLibA_61253 | 161 | 0.453515067 | hsa-mir-4786 | - |
| hsa-mir-6754 | HGLibA_63175 | 161 | 0.453515067 | hsa-mir-6754 | - |
| hsa-mir-6762 | HGLibA_63210 | 161 | 0.453515067 | hsa-mir-6762 | - |
| hsa-mir-921 | HGLibA_64278 | 161 | 0.453515067 | hsa-mir-921 | - |
| hsa-mir-92a-1 | HGLibA_64291 | 161 | 0.453515067 | hsa-mir-92a-1 | - |
| KIAA1024L | HGLibA_24681 | 161 | 0.453515067 | KIAA1024L | - |
| LOC200726 | HGLibA_26903 | 161 | 0.453515067 | LOC200726 | - |
| 6-Sep | HGLibA_43447 | 161 | 0.453515067 | 6-Sep | - |
| C17orf102 | HGLibA_05518 | 160 | 0.443564029 | C17orf102 | - |
| C17orf51 | HGLibA_05544 | 160 | 0.443564029 | C17orf51 | - |
| CSF2RA_X | HGLibA_11331 | 160 | 0.443564029 | CSF2RA_X | - |
| ENSG00000005073 | HGLibA_21898 | 160 | 0.443564029 | HOXA11 | homeobox A11 [Source:HGNC Symbol;Acc:HGNC:5101] |
| ENSG00000005243 | HGLibA_10739 | 160 | 0.443564029 | COPZ2 | coatomer protein complex subunit zeta 2 [Source:HGNC Symbol;Acc:HGNC:19356] |
| ENSG00000055147 | HGLibA_16032 | 160 | 0.443564029 | FAM114A2 | family with sequence similarity 114 member A2 [Source:HGNC Symbol;Acc:HGNC:1333] |
| ENSG00000073849 | HGLibA_47257 | 160 | 0.443564029 | ST6GAL1 | 6-sialyltransferase 1 [Source:HGNC Symbol;Acc:HGNC:10860]" |
| ENSG00000074319 | HGLibA_51844 | 160 | 0.443564029 | TSG101 | tumor susceptibility 101 [Source:HGNC Symbol;Acc:HGNC:15971] |
| ENSG00000074706 | HGLibA_23453 | 160 | 0.443564029 | IPCEF1 | interaction protein for cytohesin exchange factors 1 [Source:HGNC Symbol;Acc:HGNC:21204] |
| ENSG00000076382 | HGLibA_46359 | 160 | 0.443564029 | SPAG5 | sperm associated antigen 5 [Source:HGNC Symbol;Acc:HGNC:13452] |
| ENSG00000078018 | HGLibA_28135 | 160 | 0.443564029 | MAP2 | microtubule associated protein 2 [Source:HGNC Symbol;Acc:HGNC:6839] |
| ENSG00000091129 | HGLibA_32661 | 160 | 0.443564029 | NRCAM | neuronal cell adhesion molecule [Source:HGNC Symbol;Acc:HGNC:7994] |
| ENSG00000091947 | HGLibA_49841 | 160 | 0.443564029 | TMEM101 | transmembrane protein 101 [Source:HGNC Symbol;Acc:HGNC:28653] |
| ENSG00000100034 | HGLibA_37976 | 160 | 0.443564029 | PPM1F | Mg2+/Mn2+ dependent 1F [Source:HGNC Symbol;Acc:HGNC:19388]" |
| ENSG00000102606 | HGLibA_02876 | 160 | 0.443564029 | ARHGEF7 | Rho guanine nucleotide exchange factor 7 [Source:HGNC Symbol;Acc:HGNC:15607] |
| ENSG00000103966 | HGLibA_14674 | 160 | 0.443564029 | EHD4 | EH domain containing 4 [Source:HGNC Symbol;Acc:HGNC:3245] |
| ENSG00000107404 | HGLibA_14207 | 160 | 0.443564029 | DVL1 | dishevelled segment polarity protein 1 [Source:HGNC Symbol;Acc:HGNC:3084] |
| ENSG00000107593 | HGLibA_36800 | 160 | 0.443564029 | PKD2L1 | transient receptor potential cation channel [Source:HGNC Symbol;Acc:HGNC:9011]" |
| ENSG00000107819 | HGLibA_43788 | 160 | 0.443564029 | SFXN3 | sideroflexin 3 [Source:HGNC Symbol;Acc:HGNC:16087] |
| ENSG00000113262 | HGLibA_20355 | 160 | 0.443564029 | GRM6 | glutamate metabotropic receptor 6 [Source:HGNC Symbol;Acc:HGNC:4598] |
| ENSG00000113361 | HGLibA_08687 | 160 | 0.443564029 | CDH6 | cadherin 6 [Source:HGNC Symbol;Acc:HGNC:1765] |
| ENSG00000114455 | HGLibA_21270 | 160 | 0.443564029 | HHLA2 | HERV-H LTR-associating 2 [Source:HGNC Symbol;Acc:HGNC:4905] |
| ENSG00000115380 | HGLibA_14562 | 160 | 0.443564029 | EFEMP1 | EGF containing fibulin extracellular matrix protein 1 [Source:HGNC Symbol;Acc:HGNC:3218] |
| ENSG00000118194 | HGLibA_50887 | 160 | 0.443564029 | TNNT2 | cardiac type [Source:HGNC Symbol;Acc:HGNC:11949]" |
| ENSG00000120129 | HGLibA_14127 | 160 | 0.443564029 | DUSP1 | dual specificity phosphatase 1 [Source:HGNC Symbol;Acc:HGNC:3064] |
| ENSG00000120889 | HGLibA_50736 | 160 | 0.443564029 | TNFRSF10B | TNF receptor superfamily member 10b [Source:HGNC Symbol;Acc:HGNC:11905] |
| ENSG00000121680 | HGLibA_36102 | 160 | 0.443564029 | PEX16 | peroxisomal biogenesis factor 16 [Source:HGNC Symbol;Acc:HGNC:8857] |
| ENSG00000124574 | HGLibA_00162 | 160 | 0.443564029 | ABCC10 | ATP binding cassette subfamily C member 10 [Source:HGNC Symbol;Acc:HGNC:52] |
| ENSG00000125650 | HGLibA_39228 | 160 | 0.443564029 | PSPN | persephin [Source:HGNC Symbol;Acc:HGNC:9579] |
| ENSG00000125753 | HGLibA_53538 | 160 | 0.443564029 | VASP | vasodilator stimulated phosphoprotein [Source:HGNC Symbol;Acc:HGNC:12652] |
| ENSG00000125846 | HGLibA_55465 | 160 | 0.443564029 | ZNF133 | zinc finger protein 133 [Source:HGNC Symbol;Acc:HGNC:12917] |
| ENSG00000125991 | HGLibA_15474 | 160 | 0.443564029 | ERGIC3 | ERGIC and golgi 3 [Source:HGNC Symbol;Acc:HGNC:15927] |
| ENSG00000131503 | HGLibA_01963 | 160 | 0.443564029 | ANKHD1 | ankyrin repeat and KH domain containing 1 [Source:HGNC Symbol;Acc:HGNC:24714] |
| ENSG00000131746 | HGLibA_50929 | 160 | 0.443564029 | TNS4 | tensin 4 [Source:HGNC Symbol;Acc:HGNC:24352] |
| ENSG00000132026 | HGLibA_42369 | 160 | 0.443564029 | RTBDN | retbindin [Source:HGNC Symbol;Acc:HGNC:30310] |
| ENSG00000132330 | HGLibA_42952 | 160 | 0.443564029 | SCLY | selenocysteine lyase [Source:HGNC Symbol;Acc:HGNC:18161] |
| ENSG00000133083 | HGLibA_12500 | 160 | 0.443564029 | DCLK1 | doublecortin like kinase 1 [Source:HGNC Symbol;Acc:HGNC:2700] |
| ENSG00000134871 | HGLibA_10568 | 160 | 0.443564029 | COL4A2 | collagen type IV alpha 2 chain [Source:HGNC Symbol;Acc:HGNC:2203] |
| ENSG00000134982 | HGLibA_02388 | 160 | 0.443564029 | APC | WNT signaling pathway regulator [Source:HGNC Symbol;Acc:HGNC:583]" |
| ENSG00000135312 | HGLibA_22360 | 160 | 0.443564029 | HTR1B | 5-hydroxytryptamine receptor 1B [Source:HGNC Symbol;Acc:HGNC:5287] |
| ENSG00000135519 | HGLibA_24215 | 160 | 0.443564029 | KCNH3 | potassium voltage-gated channel subfamily H member 3 [Source:HGNC Symbol;Acc:HGNC:6252] |
| ENSG00000137154 | HGLibA_42155 | 160 | 0.443564029 | RPS6 | ribosomal protein S6 [Source:HGNC Symbol;Acc:HGNC:10429] |
| ENSG00000137166 | HGLibA_17940 | 160 | 0.443564029 | FOXP4 | forkhead box P4 [Source:HGNC Symbol;Acc:HGNC:20842] |
| ENSG00000138085 | HGLibA_03812 | 160 | 0.443564029 | ATRAID | all-trans retinoic acid induced differentiation factor [Source:HGNC Symbol;Acc:HGNC:24090] |
| ENSG00000139737 | HGLibA_44306 | 160 | 0.443564029 | SLAIN1 | SLAIN motif family member 1 [Source:HGNC Symbol;Acc:HGNC:26387] |
| ENSG00000139926 | HGLibA_18023 | 160 | 0.443564029 | FRMD6 | FERM domain containing 6 [Source:HGNC Symbol;Acc:HGNC:19839] |
| ENSG00000141682 | HGLibA_37290 | 160 | 0.443564029 | PMAIP1 | phorbol-12-myristate-13-acetate-induced protein 1 [Source:HGNC Symbol;Acc:HGNC:9108] |
| ENSG00000142185 | HGLibA_51754 | 160 | 0.443564029 | TRPM2 | transient receptor potential cation channel subfamily M member 2 [Source:HGNC Symbol;Acc:HGNC:12339] |
| ENSG00000145348 | HGLibA_48598 | 160 | 0.443564029 | TBCK | TBC1 domain containing kinase [Source:HGNC Symbol;Acc:HGNC:28261] |
| ENSG00000145506 | HGLibA_32009 | 160 | 0.443564029 | NKD2 | naked cuticle homolog 2 [Source:HGNC Symbol;Acc:HGNC:17046] |
| ENSG00000147383 | HGLibA_32747 | 160 | 0.443564029 | NSDHL | NAD(P) dependent steroid dehydrogenase-like [Source:HGNC Symbol;Acc:HGNC:13398] |
| ENSG00000149218 | HGLibA_15112 | 160 | 0.443564029 | ENDOD1 | endonuclease domain containing 1 [Source:HGNC Symbol;Acc:HGNC:29129] |
| ENSG00000151470 | HGLibA_06324 | 160 | 0.443564029 | C4orf33 | chromosome 4 open reading frame 33 [Source:HGNC Symbol;Acc:HGNC:27025] |
| ENSG00000151612 | HGLibA_56824 | 160 | 0.443564029 | ZNF827 | zinc finger protein 827 [Source:HGNC Symbol;Acc:HGNC:27193] |
| ENSG00000151693 | HGLibA_03196 | 160 | 0.443564029 | ASAP2 | ankyrin repeat and PH domain 2 [Source:HGNC Symbol;Acc:HGNC:2721]" |

| | | | | | |
|---|---|---|---|---|---|
| ENSG00000152492 | HGLibA_07794 | 160 | 0.443564029 | CCDC50 | coiled-coil domain containing 50 [Source:HGNC Symbol;Acc:HGNC:18111] |
| ENSG00000153094 | HGLibA_04307 | 160 | 0.443564029 | BCL2L11 | BCL2 like 11 [Source:HGNC Symbol;Acc:HGNC:994] |
| ENSG00000153140 | HGLibA_09239 | 160 | 0.443564029 | CETN3 | centrin 3 [Source:HGNC Symbol;Acc:HGNC:1868] |
| ENSG00000154162 | HGLibA_08637 | 160 | 0.443564029 | CDH12 | cadherin 12 [Source:HGNC Symbol;Acc:HGNC:1751] |
| ENSG00000154429 | HGLibA_08186 | 160 | 0.443564029 | CCSAP | cilia and spindle associated protein [Source:HGNC Symbol;Acc:HGNC:29578]" |
| ENSG00000161649 | HGLibA_08346 | 160 | 0.443564029 | CD300LG | CD300 molecule like family member g [Source:HGNC Symbol;Acc:HGNC:30455] |
| ENSG00000164600 | HGLibA_31742 | 160 | 0.443564029 | NEUROD6 | neuronal differentiation 6 [Source:HGNC Symbol;Acc:HGNC:13804] |
| ENSG00000165805 | HGLibA_05258 | 160 | 0.443564029 | C12orf50 | chromosome 12 open reading frame 50 [Source:HGNC Symbol;Acc:HGNC:26665] |
| ENSG00000166359 | HGLibA_54209 | 160 | 0.443564029 | WDR88 | WD repeat domain 88 [Source:HGNC Symbol;Acc:HGNC:26999] |
| ENSG00000167601 | HGLibA_03926 | 160 | 0.443564029 | AXL | AXL receptor tyrosine kinase [Source:HGNC Symbol;Acc:HGNC:905] |
| ENSG00000167971 | HGLibA_07288 | 160 | 0.443564029 | CASKIN1 | CASK interacting protein 1 [Source:HGNC Symbol;Acc:HGNC:20879] |
| ENSG00000168646 | HGLibA_03924 | 160 | 0.443564029 | AXIN2 | axin 2 [Source:HGNC Symbol;Acc:HGNC:904] |
| ENSG00000171206 | HGLibA_51616 | 160 | 0.443564029 | TRIM8 | tripartite motif containing 8 [Source:HGNC Symbol;Acc:HGNC:15579] |
| ENSG00000171456 | HGLibA_03390 | 160 | 0.443564029 | ASXL1 | ASXL transcriptional regulator 1 [Source:HGNC Symbol;Acc:HGNC:18318] |
| ENSG00000171812 | HGLibA_10616 | 160 | 0.443564029 | COL8A2 | collagen type VIII alpha 2 chain [Source:HGNC Symbol;Acc:HGNC:2216] |
| ENSG00000172689 | HGLibA_30129 | 160 | 0.443564029 | MS4A10 | membrane spanning 4-domains A10 [Source:HGNC Symbol;Acc:HGNC:13368] |
| ENSG00000174405 | HGLibA_26485 | 160 | 0.443564029 | LIG4 | DNA ligase 4 [Source:HGNC Symbol;Acc:HGNC:6601] |
| ENSG00000174705 | HGLibA_43966 | 160 | 0.443564029 | SH3PXD2B | SH3 and PX domains 2B [Source:HGNC Symbol;Acc:HGNC:29242] |
| ENSG00000175287 | HGLibA_36470 | 160 | 0.443564029 | PHYHD1 | phytanoyl-CoA dioxygenase domain containing 1 [Source:HGNC Symbol;Acc:HGNC:23396] |
| ENSG00000175866 | HGLibA_04123 | 160 | 0.443564029 | BAIAP2 | BAI1 associated protein 2 [Source:HGNC Symbol;Acc:HGNC:947] |
| ENSG00000176204 | HGLibA_27493 | 160 | 0.443564029 | LRRTM4 | leucine rich repeat transmembrane neuronal 4 [Source:HGNC Symbol;Acc:HGNC:19411] |
| ENSG00000176884 | HGLibA_20295 | 160 | 0.443564029 | GRIN1 | glutamate ionotropic receptor NMDA type subunit 1 [Source:HGNC Symbol;Acc:HGNC:4584] |
| ENSG00000179344 | HGLibA_21628 | 160 | 0.443564029 | HLA-DQB1 | DQ beta 1 [Source:HGNC Symbol;Acc:HGNC:4944]" |
| ENSG00000179409 | HGLibA_18913 | 160 | 0.443564029 | GEMIN4 | gem nuclear organelle associated protein 4 [Source:HGNC Symbol;Acc:HGNC:15717] |
| ENSG00000179615 | HGLibA_33757 | 160 | 0.443564029 | OR2AP1 | olfactory receptor family 2 subfamily AP member 1 [Source:HGNC Symbol;Acc:HGNC:15335] |
| ENSG00000181001 | HGLibA_34192 | 160 | 0.443564029 | OR52N1 | olfactory receptor family 52 subfamily N member 1 [Source:HGNC Symbol;Acc:HGNC:14853] |
| ENSG00000181135 | HGLibA_56599 | 160 | 0.443564029 | ZNF707 | zinc finger protein 707 [Source:HGNC Symbol;Acc:HGNC:27815] |
| ENSG00000183775 | HGLibA_24431 | 160 | 0.443564029 | KCTD16 | potassium channel tetramerization domain containing 16 [Source:HGNC Symbol;Acc:HGNC:29244] |
| ENSG00000184166 | HGLibA_33653 | 160 | 0.443564029 | OR1D2 | olfactory receptor family 1 subfamily D member 2 [Source:HGNC Symbol;Acc:HGNC:8183] |
| ENSG00000185475 | HGLibA_50089 | 160 | 0.443564029 | TMEM179B | transmembrane protein 179B [Source:HGNC Symbol;Acc:HGNC:33744] |
| ENSG00000185760 | HGLibA_24379 | 160 | 0.443564029 | KCNQ5 | potassium voltage-gated channel subfamily Q member 5 [Source:HGNC Symbol;Acc:HGNC:6299] |
| ENSG00000186575 | HGLibA_31762 | 160 | 0.443564029 | NF2 | neurofibromin 2 [Source:HGNC Symbol;Acc:HGNC:7773] |
| ENSG00000187037 | HGLibA_19908 | 160 | 0.443564029 | GPR141 | G protein-coupled receptor 141 [Source:HGNC Symbol;Acc:HGNC:19997] |
| ENSG00000187288 | HGLibA_09702 | 160 | 0.443564029 | CIDEC | cell death inducing DFFA like effector c [Source:HGNC Symbol;Acc:HGNC:24229] |
| ENSG00000187942 | HGLibA_26243 | 160 | 0.443564029 | LDLRAD2 | low density lipoprotein receptor class A domain containing 2 [Source:HGNC Symbol;Acc:HGNC:32071] |
| ENSG00000196139 | HGLibA_01488 | 160 | 0.443564029 | AKR1C3 | aldo-keto reductase family 1 member C3 [Source:HGNC Symbol;Acc:HGNC:386] |
| ENSG00000196224 | HGLibA_25793 | 160 | 0.443564029 | KRTAP5-3 | keratin associated protein 5-3 [Source:HGNC Symbol;Acc:HGNC:23598] |
| ENSG00000196335 | HGLibA_47481 | 160 | 0.443564029 | STK31 | serine/threonine kinase 31 [Source:HGNC Symbol;Acc:HGNC:11407] |
| ENSG00000196565 | HGLibA_20928 | 160 | 0.443564029 | HBG2 | hemoglobin subunit gamma 2 [Source:HGNC Symbol;Acc:HGNC:4832] |
| ENSG00000196570 | HGLibA_36179 | 160 | 0.443564029 | PFN3 | profilin 3 [Source:HGNC Symbol;Acc:HGNC:18627] |
| ENSG00000197885 | HGLibA_32016 | 160 | 0.443564029 | NKIRAS1 | NFKB inhibitor interacting Ras like 1 [Source:HGNC Symbol;Acc:HGNC:17899] |
| ENSG00000198466 | HGLibA_56329 | 160 | 0.443564029 | ZNF587 | zinc finger protein 587 [Source:HGNC Symbol;Acc:HGNC:30955] |
| ENSG00000198812 | HGLibA_27249 | 160 | 0.443564029 | LRRC10 | leucine rich repeat containing 10 [Source:HGNC Symbol;Acc:HGNC:20264] |
| ENSG00000204954 | HGLibA_05290 | 160 | 0.443564029 | C12orf73 | chromosome 12 open reading frame 73 [Source:HGNC Symbol;Acc:HGNC:34450] |
| ENSG00000205456 | HGLibA_51086 | 160 | 0.443564029 | TP53TG3D | TP53 target 3D [Source:HGNC Symbol;Acc:HGNC:44657] |
| ENSG00000205846 | HGLibA_10003 | 160 | 0.443564029 | CLEC6A | C-type lectin domain containing 6A [Source:HGNC Symbol;Acc:HGNC:14556] |
| ENSG00000213901 | HGLibA_44612 | 160 | 0.443564029 | SLC23A3 | solute carrier family 23 member 3 [Source:HGNC Symbol;Acc:HGNC:20601] |
| ENSG00000214013 | HGLibA_18609 | 160 | 0.443564029 | GANC | neutral C [Source:HGNC Symbol;Acc:HGNC:4139]" |
| ENSG00000242366 | HGLibA_52896 | 160 | 0.443564029 | UGT1A8 | UDP glucuronosyltransferase family 1 member A8 [Source:HGNC Symbol;Acc:HGNC:12540] |
| ENSG00000266265 | HGLibA_25055 | 160 | 0.443564029 | KLF14 | Kruppel like factor 14 [Source:HGNC Symbol;Acc:HGNC:23025] |
| ENSG00000274226 | HGLibA_48553 | 160 | 0.443564029 | TBC1D3H | TBC1 domain family member 3H [Source:HGNC Symbol;Acc:HGNC:30708] |
| FAM150A | HGLibA_16157 | 160 | 0.443564029 | FAM150A | - |
| hsa-mir-107 | HGLibA_57194 | 160 | 0.443564029 | hsa-mir-107 | - |
| hsa-mir-1226 | HGLibA_57301 | 160 | 0.443564029 | hsa-mir-1226 | - |
| hsa-mir-144 | HGLibA_57840 | 160 | 0.443564029 | hsa-mir-144 | - |
| hsa-mir-337 | HGLibA_58998 | 160 | 0.443564029 | hsa-mir-337 | - |
| hsa-mir-4299 | HGLibA_59907 | 160 | 0.443564029 | hsa-mir-4299 | - |
| hsa-mir-4325 | HGLibA_60023 | 160 | 0.443564029 | hsa-mir-4325 | - |
| hsa-mir-4688 | HGLibA_60863 | 160 | 0.443564029 | hsa-mir-4688 | - |
| hsa-mir-4748 | HGLibA_61091 | 160 | 0.443564029 | hsa-mir-4748 | - |
| hsa-mir-4773-2 | HGLibA_61195 | 160 | 0.443564029 | hsa-mir-4773-2 | - |
| hsa-mir-8080 | HGLibA_64162 | 160 | 0.443564029 | hsa-mir-8080 | - |
| KIAA1009 | HGLibA_24675 | 160 | 0.443564029 | KIAA1009 | - |
| KIAA1024L | HGLibA_24683 | 160 | 0.443564029 | KIAA1024L | - |
| LPPR3 | HGLibA_27120 | 160 | 0.443564029 | LPPR3 | - |
| MRVI1 | HGLibA_30123 | 160 | 0.443564029 | MRVI1 | - |
| PALM2 | HGLibA_35069 | 160 | 0.443564029 | PALM2 | - |
| ATP5J | HGLibA_03671 | 159 | 0.433612992 | ATP5J | - |
| ENSG00000005206 | HGLibA_46778 | 159 | 0.433612992 | SPPL2B | signal peptide peptidase like 2B [Source:HGNC Symbol;Acc:HGNC:30627] |
| ENSG00000050426 | HGLibA_26339 | 159 | 0.433612992 | LETMD1 | LETM1 domain containing 1 [Source:HGNC Symbol;Acc:HGNC:24241] |
| ENSG00000067064 | HGLibA_22553 | 159 | 0.433612992 | IDI1 | isopentenyl-diphosphate delta isomerase 1 [Source:HGNC Symbol;Acc:HGNC:5387] |
| ENSG00000078269 | HGLibA_48006 | 159 | 0.433612992 | SYNJ2 | synaptojanin 2 [Source:HGNC Symbol;Acc:HGNC:11504] |
| ENSG00000078725 | HGLibA_04811 | 159 | 0.433612992 | BRINP1 | BMP/retinoic acid inducible neural specific 1 [Source:HGNC Symbol;Acc:HGNC:2687] |
| ENSG00000085117 | HGLibA_08459 | 159 | 0.433612992 | CD82 | CD82 molecule [Source:HGNC Symbol;Acc:HGNC:6210] |
| ENSG00000087076 | HGLibA_22184 | 159 | 0.433612992 | HSD17B14 | hydroxysteroid 17-beta dehydrogenase 14 [Source:HGNC Symbol;Acc:HGNC:23238] |
| ENSG00000092850 | HGLibA_48973 | 159 | 0.433612992 | TEKT2 | tektin 2 [Source:HGNC Symbol;Acc:HGNC:11725] |
| ENSG00000095951 | HGLibA_21568 | 159 | 0.433612992 | HIVEP1 | human immunodeficiency virus type I enhancer binding protein 1 [Source:HGNC Symbol;Acc:HGNC:4920] |
| ENSG00000095970 | HGLibA_51358 | 159 | 0.433612992 | TREM2 | triggering receptor expressed on myeloid cells 2 [Source:HGNC Symbol;Acc:HGNC:17761] |
| ENSG00000100307 | HGLibA_07463 | 159 | 0.433612992 | CBX7 | chromobox 7 [Source:HGNC Symbol;Acc:HGNC:1557] |
| ENSG00000100593 | HGLibA_23679 | 159 | 0.433612992 | ISM2 | isthmin 2 [Source:HGNC Symbol;Acc:HGNC:23176] |
| ENSG00000101871 | HGLibA_29230 | 159 | 0.433612992 | MID1 | midline 1 [Source:HGNC Symbol;Acc:HGNC:7095] |
| ENSG00000102539 | HGLibA_29428 | 159 | 0.433612992 | MLNR | motilin receptor [Source:HGNC Symbol;Acc:HGNC:4495] |
| ENSG00000102890 | HGLibA_14937 | 159 | 0.433612992 | ELMO3 | engulfment and cell motility 3 [Source:HGNC Symbol;Acc:HGNC:17289] |
| ENSG00000106355 | HGLibA_27546 | 159 | 0.433612992 | LSM5 | U6 small nuclear RNA and mRNA degradation associated [Source:HGNC Symbol;Acc:HGNC:17162]" |
| ENSG00000106948 | HGLibA_01464 | 159 | 0.433612992 | AKNA | AT-hook transcription factor [Source:HGNC Symbol;Acc:HGNC:24108] |
| ENSG00000107317 | HGLibA_39309 | 159 | 0.433612992 | PTGDS | prostaglandin D2 synthase [Source:HGNC Symbol;Acc:HGNC:9592] |
| ENSG00000108932 | HGLibA_44446 | 159 | 0.433612992 | SLC16A6 | solute carrier family 16 member 6 [Source:HGNC Symbol;Acc:HGNC:10927] |
| ENSG00000109943 | HGLibA_11221 | 159 | 0.433612992 | CRTAM | cytotoxic and regulatory T cell molecule [Source:HGNC Symbol;Acc:HGNC:24313] |
| ENSG00000110090 | HGLibA_11019 | 159 | 0.433612992 | CPT1A | carnitine palmitoyltransferase 1A [Source:HGNC Symbol;Acc:HGNC:2328] |
| ENSG00000110888 | HGLibA_07193 | 159 | 0.433612992 | CAPRIN2 | caprin family member 2 [Source:HGNC Symbol;Acc:HGNC:21259] |
| ENSG00000116882 | HGLibA_20835 | 159 | 0.433612992 | HAO2 | hydroxyacid oxidase 2 [Source:HGNC Symbol;Acc:HGNC:4810] |
| ENSG00000117009 | HGLibA_25318 | 159 | 0.433612992 | KMO | kynurenine 3-monooxygenase [Source:HGNC Symbol;Acc:HGNC:6381] |
| ENSG00000118058 | HGLibA_25320 | 159 | 0.433612992 | KMT2A | lysine methyltransferase 2A [Source:HGNC Symbol;Acc:HGNC:7132] |
| ENSG00000118705 | HGLibA_42020 | 159 | 0.433612992 | RPN2 | ribophorin II [Source:HGNC Symbol;Acc:HGNC:10382] |
| ENSG00000119414 | HGLibA_38219 | 159 | 0.433612992 | PPP6C | protein phosphatase 6 catalytic subunit [Source:HGNC Symbol;Acc:HGNC:9323] |
| ENSG00000119638 | HGLibA_31669 | 159 | 0.433612992 | NEK9 | NIMA related kinase 9 [Source:HGNC Symbol;Acc:HGNC:18591] |
| ENSG00000121380 | HGLibA_04318 | 159 | 0.433612992 | BCL2L14 | BCL2 like 14 [Source:HGNC Symbol;Acc:HGNC:16657] |
| ENSG00000126561 | HGLibA_47392 | 159 | 0.433612992 | STAT5A | signal transducer and activator of transcription 5A [Source:HGNC Symbol;Acc:HGNC:11366] |
| ENSG00000129566 | HGLibA_49008 | 159 | 0.433612992 | TEP1 | telomerase associated protein 1 [Source:HGNC Symbol;Acc:HGNC:11726] |
| ENSG00000131115 | HGLibA_55627 | 159 | 0.433612992 | ZNF227 | zinc finger protein 227 [Source:HGNC Symbol;Acc:HGNC:13020] |
| ENSG00000132781 | HGLibA_30587 | 159 | 0.433612992 | MUTYH | mutY DNA glycosylase [Source:HGNC Symbol;Acc:HGNC:7527] |
| ENSG00000133020 | HGLibA_30738 | 159 | 0.433612992 | MYH8 | myosin heavy chain 8 [Source:HGNC Symbol;Acc:HGNC:7578] |
| ENSG00000133067 | HGLibA_26415 | 159 | 0.433612992 | LGR6 | leucine rich repeat containing G protein-coupled receptor 6 [Source:HGNC Symbol;Acc:HGNC:19719] |
| ENSG00000135272 | HGLibA_28672 | 159 | 0.433612992 | MDFIC | MyoD family inhibitor domain containing [Source:HGNC Symbol;Acc:HGNC:28870] |
| ENSG00000135424 | HGLibA_23741 | 159 | 0.433612992 | ITGA7 | integrin subunit alpha 7 [Source:HGNC Symbol;Acc:HGNC:6143] |
| ENSG00000135776 | HGLibA_00135 | 159 | 0.433612992 | ABCB10 | ATP binding cassette subfamily B member 10 [Source:HGNC Symbol;Acc:HGNC:41] |
| ENSG00000137270 | HGLibA_18799 | 159 | 0.433612992 | GCM1 | glial cells missing homolog 1 [Source:HGNC Symbol;Acc:HGNC:4197] |
| ENSG00000138347 | HGLibA_30910 | 159 | 0.433612992 | MYPN | myopalladin [Source:HGNC Symbol;Acc:HGNC:23246] |
| ENSG00000138606 | HGLibA_44023 | 159 | 0.433612992 | SHF | Src homology 2 domain containing F [Source:HGNC Symbol;Acc:HGNC:25116] |
| ENSG00000138629 | HGLibA_52713 | 159 | 0.433612992 | UBL7 | ubiquitin like 7 [Source:HGNC Symbol;Acc:HGNC:28221] |
| ENSG00000139531 | HGLibA_47800 | 159 | 0.433612992 | SUOX | sulfite oxidase [Source:HGNC Symbol;Acc:HGNC:11460] |
| ENSG00000139842 | HGLibA_11781 | 159 | 0.433612992 | CUL4A | cullin 4A [Source:HGNC Symbol;Acc:HGNC:2554] |
| ENSG00000139973 | HGLibA_48066 | 159 | 0.433612992 | SYT16 | synaptotagmin 16 [Source:HGNC Symbol;Acc:HGNC:23142] |
| ENSG00000142599 | HGLibA_40857 | 159 | 0.433612992 | RERE | arginine-glutamic acid dipeptide repeats [Source:HGNC Symbol;Acc:HGNC:9965] |
| ENSG00000142619 | HGLibA_34975 | 159 | 0.433612992 | PADI3 | peptidyl arginine deiminase 3 [Source:HGNC Symbol;Acc:HGNC:18337] |
| ENSG00000142632 | HGLibA_02830 | 159 | 0.433612992 | ARHGEF19 | Rho guanine nucleotide exchange factor 19 [Source:HGNC Symbol;Acc:HGNC:26604] |
| ENSG00000143382 | HGLibA_00856 | 159 | 0.433612992 | ADAMTSL4 | ADAMTS like 4 [Source:HGNC Symbol;Acc:HGNC:19706] |
| ENSG00000143631 | HGLibA_17608 | 159 | 0.433612992 | FLG | filaggrin [Source:HGNC Symbol;Acc:HGNC:3748] |
| ENSG00000143870 | HGLibA_35887 | 159 | 0.433612992 | PDIA6 | protein disulfide isomerase family A member 6 [Source:HGNC Symbol;Acc:HGNC:30168] |
| ENSG00000143977 | HGLibA_45990 | 159 | 0.433612992 | SNRPG | small nuclear ribonucleoprotein polypeptide G [Source:HGNC Symbol;Acc:HGNC:11163] |
| ENSG00000143995 | HGLibA_28872 | 159 | 0.433612992 | MEIS1 | Meis homeobox 1 [Source:HGNC Symbol;Acc:HGNC:7000] |
| ENSG00000144369 | HGLibA_16269 | 159 | 0.433612992 | FAM171B | family with sequence similarity 171 member B [Source:HGNC Symbol;Acc:HGNC:29412] |
| ENSG00000148082 | HGLibA_44005 | 159 | 0.433612992 | SHC3 | SHC adaptor protein 3 [Source:HGNC Symbol;Acc:HGNC:18181] |
| ENSG00000149742 | HGLibA_44602 | 159 | 0.433612992 | SLC22A9 | solute carrier family 22 member 9 [Source:HGNC Symbol;Acc:HGNC:16261] |
| ENSG00000151292 | HGLibA_11399 | 159 | 0.433612992 | CSNK1G3 | casein kinase 1 gamma 3 [Source:HGNC Symbol;Acc:HGNC:2456] |
| ENSG00000152822 | HGLibA_20342 | 159 | 0.433612992 | GRM1 | glutamate metabotropic receptor 1 [Source:HGNC Symbol;Acc:HGNC:4593] |
| ENSG00000153822 | HGLibA_24267 | 159 | 0.433612992 | KCNJ16 | potassium voltage-gated channel subfamily J member 16 [Source:HGNC Symbol;Acc:HGNC:6262] |
| ENSG00000157557 | HGLibA_15643 | 159 | 0.433612992 | ETS2 | transcription factor [Source:HGNC Symbol;Acc:HGNC:3489]" |
| ENSG00000158411 | HGLibA_29316 | 159 | 0.433612992 | MITD1 | microtubule interacting and trafficking domain containing 1 [Source:HGNC Symbol;Acc:HGNC:25207] |
| ENSG00000158796 | HGLibA_12779 | 159 | 0.433612992 | DEDD | death effector domain containing [Source:HGNC Symbol;Acc:HGNC:2755] |
| ENSG00000160993 | HGLibA_01654 | 159 | 0.433612992 | ALKBH4 | lysine demethylase [Source:HGNC Symbol;Acc:HGNC:21900]" |
| ENSG00000162302 | HGLibA_42167 | 159 | 0.433612992 | RPS6KA4 | ribosomal protein S6 kinase A4 [Source:HGNC Symbol;Acc:HGNC:10433] |
| ENSG00000163430 | HGLibA_18100 | 159 | 0.433612992 | FSTL1 | follistatin like 1 [Source:HGNC Symbol;Acc:HGNC:3972] |
| ENSG00000164841 | HGLibA_50511 | 159 | 0.433612992 | TMEM74 | transmembrane protein 74 [Source:HGNC Symbol;Acc:HGNC:26409] |
| ENSG00000165120 | HGLibA_47140 | 159 | 0.433612992 | SSMEM1 | serine rich single-pass membrane protein 1 [Source:HGNC Symbol;Acc:HGNC:29580] |

| | | | | | |
|---|---|---|---|---|---|
| ENSG00000166006 | HGLibA_24166 | 159 | 0.433612992 | KCNC2 | potassium voltage-gated channel subfamily C member 2 [Source:HGNC Symbol;Acc:HGNC:6234] |
| ENSG00000166387 | HGLibA_37889 | 159 | 0.433612992 | PPFIBP2 | PPFIA binding protein 2 [Source:HGNC Symbol;Acc:HGNC:9250] |
| ENSG00000167588 | HGLibA_19783 | 159 | 0.433612992 | GPD1 | glycerol-3-phosphate dehydrogenase 1 [Source:HGNC Symbol;Acc:HGNC:4455] |
| ENSG00000168256 | HGLibA_32020 | 159 | 0.433612992 | NKIRAS2 | NFKB inhibitor interacting Ras like 2 [Source:HGNC Symbol;Acc:HGNC:17898] |
| ENSG00000169933 | HGLibA_18038 | 159 | 0.433612992 | FRMPD4 | FERM and PDZ domain containing 4 [Source:HGNC Symbol;Acc:HGNC:29007] |
| ENSG00000170178 | HGLibA_21991 | 159 | 0.433612992 | HOXD12 | homeobox D12 [Source:HGNC Symbol;Acc:HGNC:5135] |
| ENSG00000170271 | HGLibA_16932 | 159 | 0.433612992 | FAXDC2 | fatty acid hydroxylase domain containing 2 [Source:HGNC Symbol;Acc:HGNC:1334] |
| ENSG00000171189 | HGLibA_20281 | 159 | 0.433612992 | GRIK1 | glutamate ionotropic receptor kainate type subunit 1 [Source:HGNC Symbol;Acc:HGNC:4579] |
| ENSG00000171552 | HGLibA_04302 | 159 | 0.433612992 | BCL2L1 | BCL2 like 1 [Source:HGNC Symbol;Acc:HGNC:992] |
| ENSG00000171747 | HGLibA_26369 | 159 | 0.433612992 | LGALS4 | galectin 4 [Source:HGNC Symbol;Acc:HGNC:6565] |
| ENSG00000172260 | HGLibA_31627 | 159 | 0.433612992 | NEGR1 | neuronal growth regulator 1 [Source:HGNC Symbol;Acc:HGNC:17302] |
| ENSG00000174562 | HGLibA_25249 | 159 | 0.433612992 | KLK15 | kallikrein related peptidase 15 [Source:HGNC Symbol;Acc:HGNC:20453] |
| ENSG00000176428 | HGLibA_53786 | 159 | 0.433612992 | VPS37D | ESCRT-I subunit [Source:HGNC Symbol;Acc:HGNC:18287]" |
| ENSG00000182534 | HGLibA_30626 | 159 | 0.433612992 | MXRA7 | matrix remodeling associated 7 [Source:HGNC Symbol;Acc:HGNC:7541] |
| ENSG00000182545 | HGLibA_41391 | 159 | 0.433612992 | RNASE10 | ribonuclease A family member 10 (inactive) [Source:HGNC Symbol;Acc:HGNC:19275] |
| ENSG00000183742 | HGLibA_27831 | 159 | 0.433612992 | MACC1 | MET transcriptional regulator MACC1 [Source:HGNC Symbol;Acc:HGNC:30215] |
| ENSG00000186625 | HGLibA_24072 | 159 | 0.433612992 | KATNA1 | katanin catalytic subunit A1 [Source:HGNC Symbol;Acc:HGNC:6216] |
| ENSG00000187173 | HGLibA_26119 | 159 | 0.433612992 | LCE2A | late cornified envelope 2A [Source:HGNC Symbol;Acc:HGNC:29469] |
| ENSG00000196812 | HGLibA_56989 | 159 | 0.433612992 | ZSCAN16 | zinc finger and SCAN domain containing 16 [Source:HGNC Symbol;Acc:HGNC:20813] |
| ENSG00000198929 | HGLibA_32324 | 159 | 0.433612992 | NOS1AP | nitric oxide synthase 1 adaptor protein [Source:HGNC Symbol;Acc:HGNC:16859] |
| ENSG00000198929 | HGLibA_32326 | 159 | 0.433612992 | NOS1AP | nitric oxide synthase 1 adaptor protein [Source:HGNC Symbol;Acc:HGNC:16859] |
| ENSG00000198933 | HGLibA_48604 | 159 | 0.433612992 | TBKBP1 | TBK1 binding protein 1 [Source:HGNC Symbol;Acc:HGNC:30140] |
| ENSG00000198948 | HGLibA_29048 | 159 | 0.433612992 | MFAP3L | microfibril associated protein 3 like [Source:HGNC Symbol;Acc:HGNC:29083] |
| ENSG00000205084 | HGLibA_50259 | 159 | 0.433612992 | TMEM231 | transmembrane protein 231 [Source:HGNC Symbol;Acc:HGNC:37234] |
| ENSG00000205838 | HGLibA_52081 | 159 | 0.433612992 | TTC23L | tetratricopeptide repeat domain 23 like [Source:HGNC Symbol;Acc:HGNC:26355] |
| ENSG00000213402 | HGLibA_39492 | 159 | 0.433612992 | PTPRCAP | receptor type C associated protein [Source:HGNC Symbol;Acc:HGNC:9667]" |
| ENSG00000213672 | HGLibA_31323 | 159 | 0.433612992 | NCKIPSD | NCK interacting protein with SH3 domain [Source:HGNC Symbol;Acc:HGNC:15486] |
| ENSG00000214946 | HGLibA_48519 | 159 | 0.433612992 | TBC1D26 | TBC1 domain family member 26 [Source:HGNC Symbol;Acc:HGNC:28745] |
| ENSG00000227500 | HGLibA_42844 | 159 | 0.433612992 | SCAMP4 | secretory carrier membrane protein 4 [Source:HGNC Symbol;Acc:HGNC:30385] |
| ENSG00000233701 | HGLibA_38827 | 159 | 0.433612992 | PRR23C | proline rich 23C [Source:HGNC Symbol;Acc:HGNC:37173] |
| ENSG00000239732 | HGLibA_49667 | 159 | 0.433612992 | TLR9 | toll like receptor 9 [Source:HGNC Symbol;Acc:HGNC:15633] |
| ENSG00000242110 | HGLibA_01745 | 159 | 0.433612992 | AMACR | alpha-methylacyl-CoA racemase [Source:HGNC Symbol;Acc:HGNC:451] |
| ENSG00000279270 | HGLibA_34204 | 159 | 0.433612992 | OR52R1 | olfactory receptor family 52 subfamily R member 1 (gene/pseudogene) [Source:HGNC Symbol;Acc:HGNC:15235] |
| ENSG00000288649 | HGLibA_00618 | 159 | 0.433612992 | ACTL10 | - |
| hsa-let-7a-1 | HGLibA_57096 | 159 | 0.433612992 | hsa-let-7a-1 | - |
| hsa-mir-19b-2 | HGLibA_58146 | 159 | 0.433612992 | hsa-mir-19b-2 | - |
| hsa-mir-3649 | HGLibA_59143 | 159 | 0.433612992 | hsa-mir-3649 | - |
| hsa-mir-4320 | HGLibA_60003 | 159 | 0.433612992 | hsa-mir-4320 | - |
| hsa-mir-4474 | HGLibA_60306 | 159 | 0.433612992 | hsa-mir-4474 | - |
| hsa-mir-4684 | HGLibA_60848 | 159 | 0.433612992 | hsa-mir-4684 | - |
| hsa-mir-519b | HGLibA_61720 | 159 | 0.433612992 | hsa-mir-519b | - |
| hsa-mir-6514 | HGLibA_62928 | 159 | 0.433612992 | hsa-mir-6514 | - |
| KIAA0141 | HGLibA_24594 | 159 | 0.433612992 | KIAA0141 | - |
| KIAA0556 | HGLibA_24644 | 159 | 0.433612992 | KIAA0556 | - |
| LPPR4 | HGLibA_27123 | 159 | 0.433612992 | LPPR4 | - |
| PTCHD2 | HGLibA_39277 | 159 | 0.433612992 | PTCHD2 | - |
| SMEK1 | HGLibA_45693 | 159 | 0.433612992 | SMEK1 | - |
| SUV420H1 | HGLibA_47865 | 159 | 0.433612992 | SUV420H1 | - |
| TCTE3 | HGLibA_48859 | 159 | 0.433612992 | TCTE3 | - |
| XAGE1E | HGLibA_54479 | 159 | 0.433612992 | XAGE1E | - |
| C14orf182 | HGLibA_05352 | 158 | 0.423661954 | C14orf182 | - |
| C4orf29 | HGLibA_06314 | 158 | 0.423661954 | C4orf29 | - |
| CECR5 | HGLibA_08979 | 158 | 0.423661954 | CECR5 | - |
| DGCR14 | HGLibA_13043 | 158 | 0.423661954 | DGCR14 | - |
| EMR1 | HGLibA_15084 | 158 | 0.423661954 | EMR1 | - |
| ENSG00000002834 | HGLibA_26056 | 158 | 0.423661954 | LASP1 | LIM and SH3 protein 1 [Source:HGNC Symbol;Acc:HGNC:6513] |
| ENSG00000003393 | HGLibA_01719 | 158 | 0.423661954 | ALS2 | alsin Rho guanine nucleotide exchange factor [Source:HGNC Symbol;Acc:HGNC:443]" |
| ENSG00000013297 | HGLibA_09852 | 158 | 0.423661954 | CLDN11 | claudin 11 [Source:HGNC Symbol;Acc:HGNC:8514] |
| ENSG00000022267 | HGLibA_17481 | 158 | 0.423661954 | FHL1 | four and a half LIM domains 1 [Source:HGNC Symbol;Acc:HGNC:3702] |
| ENSG00000048828 | HGLibA_16055 | 158 | 0.423661954 | FAM120A | family with sequence similarity 120A [Source:HGNC Symbol;Acc:HGNC:13247] |
| ENSG00000049130 | HGLibA_25017 | 158 | 0.423661954 | KITLG | KIT ligand [Source:HGNC Symbol;Acc:HGNC:6343] |
| ENSG00000054796 | HGLibA_46741 | 158 | 0.423661954 | SPO11 | initiator of meiotic double stranded breaks [Source:HGNC Symbol;Acc:HGNC:11250]" |
| ENSG00000065675 | HGLibA_38557 | 158 | 0.423661954 | PRKCQ | protein kinase C theta [Source:HGNC Symbol;Acc:HGNC:9410] |
| ENSG00000066248 | HGLibA_31868 | 158 | 0.423661954 | NGEF | neuronal guanine nucleotide exchange factor [Source:HGNC Symbol;Acc:HGNC:7807] |
| ENSG00000075239 | HGLibA_00391 | 158 | 0.423661954 | ACAT1 | acetyl-CoA acetyltransferase 1 [Source:HGNC Symbol;Acc:HGNC:93] |
| ENSG00000075673 | HGLibA_03564 | 158 | 0.423661954 | ATP12A | ATPase H+/K+ transporting non-gastric alpha2 subunit [Source:HGNC Symbol;Acc:HGNC:13816] |
| ENSG00000081138 | HGLibA_08690 | 158 | 0.423661954 | CDH7 | cadherin 7 [Source:HGNC Symbol;Acc:HGNC:1766] |
| ENSG00000099998 | HGLibA_19012 | 158 | 0.423661954 | GGT5 | gamma-glutamyltransferase 5 [Source:HGNC Symbol;Acc:HGNC:4260] |
| ENSG00000100385 | HGLibA_23122 | 158 | 0.423661954 | IL2RB | interleukin 2 receptor subunit beta [Source:HGNC Symbol;Acc:HGNC:6009] |
| ENSG00000103024 | HGLibA_32159 | 158 | 0.423661954 | NME3 | NME/NM23 nucleoside diphosphate kinase 3 [Source:HGNC Symbol;Acc:HGNC:7851] |
| ENSG00000103196 | HGLibA_11176 | 158 | 0.423661954 | CRISPLD2 | cysteine rich secretory protein LCCL domain containing 2 [Source:HGNC Symbol;Acc:HGNC:25248] |
| ENSG00000104976 | HGLibA_45892 | 158 | 0.423661954 | SNAPC2 | small nuclear RNA activating complex polypeptide 2 [Source:HGNC Symbol;Acc:HGNC:11135] |
| ENSG00000105880 | HGLibA_13385 | 158 | 0.423661954 | DLX5 | distal-less homeobox 5 [Source:HGNC Symbol;Acc:HGNC:2918] |
| ENSG00000105939 | HGLibA_54990 | 158 | 0.423661954 | ZC3HAV1 | antiviral 1 [Source:HGNC Symbol;Acc:HGNC:23721]" |
| ENSG00000108639 | HGLibA_47994 | 158 | 0.423661954 | SYNGR2 | synaptogyrin 2 [Source:HGNC Symbol;Acc:HGNC:11499] |
| ENSG00000110075 | HGLibA_38226 | 158 | 0.423661954 | PPP6R3 | protein phosphatase 6 regulatory subunit 3 [Source:HGNC Symbol;Acc:HGNC:1173] |
| ENSG00000111305 | HGLibA_20419 | 158 | 0.423661954 | GSG1 | germ cell associated 1 [Source:HGNC Symbol;Acc:HGNC:19716] |
| ENSG00000115159 | HGLibA_19790 | 158 | 0.423661954 | GPD2 | glycerol-3-phosphate dehydrogenase 2 [Source:HGNC Symbol;Acc:HGNC:4456] |
| ENSG00000116604 | HGLibA_28840 | 158 | 0.423661954 | MEF2D | myocyte enhancer factor 2D [Source:HGNC Symbol;Acc:HGNC:6997] |
| ENSG00000118733 | HGLibA_33351 | 158 | 0.423661954 | OLFM3 | olfactomedin 3 [Source:HGNC Symbol;Acc:HGNC:17990] |
| ENSG00000122359 | HGLibA_02226 | 158 | 0.423661954 | ANXA11 | annexin A11 [Source:HGNC Symbol;Acc:HGNC:535] |
| ENSG00000122585 | HGLibA_32546 | 158 | 0.423661954 | NPY | neuropeptide Y [Source:HGNC Symbol;Acc:HGNC:7955] |
| ENSG00000123307 | HGLibA_31739 | 158 | 0.423661954 | NEUROD4 | neuronal differentiation 4 [Source:HGNC Symbol;Acc:HGNC:13802] |
| ENSG00000123562 | HGLibA_29664 | 158 | 0.423661954 | MORF4L2 | mortality factor 4 like 2 [Source:HGNC Symbol;Acc:HGNC:16849] |
| ENSG00000123810 | HGLibA_04046 | 158 | 0.423661954 | B9D2 | B9 domain containing 2 [Source:HGNC Symbol;Acc:HGNC:28636] |
| ENSG00000124839 | HGLibA_39821 | 158 | 0.423661954 | RAB17 | member RAS oncogene family [Source:HGNC Symbol;Acc:HGNC:16523]" |
| ENSG00000125726 | HGLibA_08438 | 158 | 0.423661954 | CD70 | CD70 molecule [Source:HGNC Symbol;Acc:HGNC:11937] |
| ENSG00000125945 | HGLibA_55989 | 158 | 0.423661954 | ZNF436 | zinc finger protein 436 [Source:HGNC Symbol;Acc:HGNC:20814] |
| ENSG00000126067 | HGLibA_39103 | 158 | 0.423661954 | PSMB2 | proteasome subunit beta 2 [Source:HGNC Symbol;Acc:HGNC:9539] |
| ENSG00000128656 | HGLibA_09504 | 158 | 0.423661954 | CHN1 | chimerin 1 [Source:HGNC Symbol;Acc:HGNC:1943] |
| ENSG00000128714 | HGLibA_21994 | 158 | 0.423661954 | HOXD13 | homeobox D13 [Source:HGNC Symbol;Acc:HGNC:5136] |
| ENSG00000129932 | HGLibA_13765 | 158 | 0.423661954 | DOHH | deoxyhypusine hydroxylase [Source:HGNC Symbol;Acc:HGNC:28662] |
| ENSG00000129993 | HGLibA_07387 | 158 | 0.423661954 | CBFA2T3 | CBFA2/RUNX1 translocation partner 3 [Source:HGNC Symbol;Acc:HGNC:1537] |
| ENSG00000130203 | HGLibA_02518 | 158 | 0.423661954 | APOE | apolipoprotein E [Source:HGNC Symbol;Acc:HGNC:613] |
| ENSG00000130402 | HGLibA_00644 | 158 | 0.423661954 | ACTN4 | actinin alpha 4 [Source:HGNC Symbol;Acc:HGNC:166] |
| ENSG00000131055 | HGLibA_10833 | 158 | 0.423661954 | COX4I2 | cytochrome c oxidase subunit 4I2 [Source:HGNC Symbol;Acc:HGNC:16232] |
| ENSG00000132424 | HGLibA_37345 | 158 | 0.423661954 | PNISR | PNN interacting serine and arginine rich protein [Source:HGNC Symbol;Acc:HGNC:21222] |
| ENSG00000133114 | HGLibA_19723 | 158 | 0.423661954 | GPALPP1 | GPALPP motifs containing 1 [Source:HGNC Symbol;Acc:HGNC:20298] |
| ENSG00000134897 | HGLibA_04534 | 158 | 0.423661954 | BIVM | immunoglobulin-like variable motif containing [Source:HGNC Symbol;Acc:HGNC:16034]" |
| ENSG00000135443 | HGLibA_25569 | 158 | 0.423661954 | KRT85 | keratin 85 [Source:HGNC Symbol;Acc:HGNC:6462] |
| ENSG00000135677 | HGLibA_19590 | 158 | 0.423661954 | GNS | glucosamine (N-acetyl)-6-sulfatase [Source:HGNC Symbol;Acc:HGNC:4422] |
| ENSG00000137634 | HGLibA_33160 | 158 | 0.423661954 | NXPE4 | neurexophilin and PC-esterase domain family member 4 [Source:HGNC Symbol;Acc:HGNC:23117] |
| ENSG00000137942 | HGLibA_17737 | 158 | 0.423661954 | FNBP1L | formin binding protein 1 like [Source:HGNC Symbol;Acc:HGNC:20851] |
| ENSG00000138080 | HGLibA_15046 | 158 | 0.423661954 | EMILIN1 | elastin microfibril interfacer 1 [Source:HGNC Symbol;Acc:HGNC:19880] |
| ENSG00000138639 | HGLibA_02735 | 158 | 0.423661954 | ARHGAP24 | Rho GTPase activating protein 24 [Source:HGNC Symbol;Acc:HGNC:25361] |
| ENSG00000140548 | HGLibA_56612 | 158 | 0.423661954 | ZNF710 | zinc finger protein 710 [Source:HGNC Symbol;Acc:HGNC:25352] |
| ENSG00000141052 | HGLibA_30878 | 158 | 0.423661954 | MYOCD | myocardin [Source:HGNC Symbol;Acc:HGNC:16067] |
| ENSG00000142235 | HGLibA_26729 | 158 | 0.423661954 | LMTK3 | lemur tyrosine kinase 3 [Source:HGNC Symbol;Acc:HGNC:19295] |
| ENSG00000142910 | HGLibA_49549 | 158 | 0.423661954 | TINAGL1 | tubulointerstitial nephritis antigen like 1 [Source:HGNC Symbol;Acc:HGNC:19168] |
| ENSG00000143320 | HGLibA_11056 | 158 | 0.423661954 | CRABP2 | cellular retinoic acid binding protein 2 [Source:HGNC Symbol;Acc:HGNC:2339] |
| ENSG00000143869 | HGLibA_18874 | 158 | 0.423661954 | GDF7 | growth differentiation factor 7 [Source:HGNC Symbol;Acc:HGNC:4222] |
| ENSG00000147394 | HGLibA_55548 | 158 | 0.423661954 | ZNF185 | zinc finger protein 185 with LIM domain [Source:HGNC Symbol;Acc:HGNC:12976] |
| ENSG00000149654 | HGLibA_08664 | 158 | 0.423661954 | CDH22 | cadherin 22 [Source:HGNC Symbol;Acc:HGNC:13251] |
| ENSG00000154252 | HGLibA_18499 | 158 | 0.423661954 | GAL3ST2 | galactose-3-O-sulfotransferase 2 [Source:HGNC Symbol;Acc:HGNC:24869] |
| ENSG00000156515 | HGLibA_21579 | 158 | 0.423661954 | HK1 | hexokinase 1 [Source:HGNC Symbol;Acc:HGNC:4922] |
| ENSG00000158301 | HGLibA_20121 | 158 | 0.423661954 | GPRASP2 | G protein-coupled receptor associated sorting protein 2 [Source:HGNC Symbol;Acc:HGNC:25169] |
| ENSG00000158555 | HGLibA_18902 | 158 | 0.423661954 | GDPD5 | glycerophosphodiester phosphodiesterase domain containing 5 [Source:HGNC Symbol;Acc:HGNC:28804] |
| ENSG00000160207 | HGLibA_22225 | 158 | 0.423661954 | HSF2BP | heat shock transcription factor 2 binding protein [Source:HGNC Symbol;Acc:HGNC:5226] |
| ENSG00000163013 | HGLibA_17095 | 158 | 0.423661954 | FBXO41 | F-box protein 41 [Source:HGNC Symbol;Acc:HGNC:29409] |
| ENSG00000163257 | HGLibA_12434 | 158 | 0.423661954 | DCAF16 | DDB1 and CUL4 associated factor 16 [Source:HGNC Symbol;Acc:HGNC:25987] |
| ENSG00000163406 | HGLibA_44408 | 158 | 0.423661954 | SLC15A2 | solute carrier family 15 member 2 [Source:HGNC Symbol;Acc:HGNC:10921] |
| ENSG00000163421 | HGLibA_38668 | 158 | 0.423661954 | PROK2 | prokineticin 2 [Source:HGNC Symbol;Acc:HGNC:18455] |
| ENSG00000164089 | HGLibA_15637 | 158 | 0.423661954 | ETNPPL | ethanolamine-phosphate phospho-lyase [Source:HGNC Symbol;Acc:HGNC:14404] |
| ENSG00000166337 | HGLibA_48197 | 158 | 0.423661954 | TAF10 | TATA-box binding protein associated factor 10 [Source:HGNC Symbol;Acc:HGNC:11543] |
| ENSG00000166391 | HGLibA_29628 | 158 | 0.423661954 | MOGAT2 | monoacylglycerol O-acyltransferase 2 [Source:HGNC Symbol;Acc:HGNC:23248] |
| ENSG00000167800 | HGLibA_48644 | 158 | 0.423661954 | TBX10 | T-box 10 [Source:HGNC Symbol;Acc:HGNC:11593] |
| ENSG00000168158 | HGLibA_33776 | 158 | 0.423661954 | OR2C1 | olfactory receptor family 2 subfamily C member 1 [Source:HGNC Symbol;Acc:HGNC:8242] |
| ENSG00000169567 | HGLibA_21325 | 158 | 0.423661954 | HINT1 | histidine triad nucleotide binding protein 1 [Source:HGNC Symbol;Acc:HGNC:4912] |
| ENSG00000172508 | HGLibA_07255 | 158 | 0.423661954 | CARNS1 | carnosine synthase 1 [Source:HGNC Symbol;Acc:HGNC:29268] |
| ENSG00000172831 | HGLibA_09221 | 158 | 0.423661954 | CES2 | carboxylesterase 2 [Source:HGNC Symbol;Acc:HGNC:1864] |
| ENSG00000173207 | HGLibA_09787 | 158 | 0.423661954 | CKS1B | CDC28 protein kinase regulatory subunit 1B [Source:HGNC Symbol;Acc:HGNC:19083] |
| ENSG00000173212 | HGLibA_27827 | 158 | 0.423661954 | MAB21L3 | mab-21 like 3 [Source:HGNC Symbol;Acc:HGNC:26787] |
| ENSG00000173227 | HGLibA_48054 | 158 | 0.423661954 | SYT12 | synaptotagmin 12 [Source:HGNC Symbol;Acc:HGNC:18381] |
| ENSG00000173327 | HGLibA_28165 | 158 | 0.423661954 | MAP3K11 | mitogen-activated protein kinase kinase kinase 11 [Source:HGNC Symbol;Acc:HGNC:6850] |
| ENSG00000174469 | HGLibA_10423 | 158 | 0.423661954 | CNTNAP2 | contactin associated protein like 2 [Source:HGNC Symbol;Acc:HGNC:13830] |

| | | | | | |
|---|---|---|---|---|---|
| ENSG00000176160 | HGLibA_22233 | 158 | 0.423661954 | HSF5 | heat shock transcription factor 5 [Source:HGNC Symbol;Acc:HGNC:26862] |
| ENSG00000177169 | HGLibA_52968 | 158 | 0.423661954 | ULK1 | unc-51 like autophagy activating kinase 1 [Source:HGNC Symbol;Acc:HGNC:12558] |
| ENSG00000177576 | HGLibA_05612 | 158 | 0.423661954 | C18orf32 | chromosome 18 open reading frame 32 [Source:HGNC Symbol;Acc:HGNC:31690] |
| ENSG00000178031 | HGLibA_00848 | 158 | 0.423661954 | ADAMTSL1 | ADAMTS like 1 [Source:HGNC Symbol;Acc:HGNC:14632] |
| ENSG00000179813 | HGLibA_16486 | 158 | 0.423661954 | FAM216B | family with sequence similarity 216 member B [Source:HGNC Symbol;Acc:HGNC:26883] |
| ENSG00000180638 | HGLibA_45197 | 158 | 0.423661954 | SLC47A2 | solute carrier family 47 member 2 [Source:HGNC Symbol;Acc:HGNC:26439] |
| ENSG00000181752 | HGLibA_34551 | 158 | 0.423661954 | OR8K5 | olfactory receptor family 8 subfamily K member 5 [Source:HGNC Symbol;Acc:HGNC:15315] |
| ENSG00000182087 | HGLibA_50344 | 158 | 0.423661954 | TMEM259 | transmembrane protein 259 [Source:HGNC Symbol;Acc:HGNC:17039] |
| ENSG00000182645 | HGLibA_07696 | 158 | 0.423661954 | CCDC172 | coiled-coil domain containing 172 [Source:HGNC Symbol;Acc:HGNC:30524] |
| ENSG00000183018 | HGLibA_46736 | 158 | 0.423661954 | SPNS2 | sphingolipid transporter 2 [Source:HGNC Symbol;Acc:HGNC:26992] |
| ENSG00000183077 | HGLibA_01123 | 158 | 0.423661954 | AFMID | arylformamidase [Source:HGNC Symbol;Acc:HGNC:20910] |
| ENSG00000183305 | HGLibA_27907 | 158 | 0.423661954 | MAGEA2B | MAGE family member A2B [Source:HGNC Symbol;Acc:HGNC:19340] |
| ENSG00000183389 | HGLibA_34218 | 158 | 0.423661954 | OR56A4 | olfactory receptor family 56 subfamily A member 4 [Source:HGNC Symbol;Acc:HGNC:14791] |
| ENSG00000184647 | HGLibA_38963 | 158 | 0.423661954 | PRSS55 | serine protease 55 [Source:HGNC Symbol;Acc:HGNC:30824] |
| ENSG00000185958 | HGLibA_16337 | 158 | 0.423661954 | FAM186A | family with sequence similarity 186 member A [Source:HGNC Symbol;Acc:HGNC:26980] |
| ENSG00000187912 | HGLibA_09940 | 158 | 0.423661954 | CLEC17A | C-type lectin domain containing 17A [Source:HGNC Symbol;Acc:HGNC:34520] |
| ENSG00000188994 | HGLibA_55760 | 158 | 0.423661954 | ZNF292 | zinc finger protein 292 [Source:HGNC Symbol;Acc:HGNC:18410] |
| ENSG00000189056 | HGLibA_40823 | 158 | 0.423661954 | RELN | reelin [Source:HGNC Symbol;Acc:HGNC:9957] |
| ENSG00000196224 | HGLibA_25795 | 158 | 0.423661954 | KRTAP5-3 | keratin associated protein 5-3 [Source:HGNC Symbol;Acc:HGNC:23598] |
| ENSG00000196715 | HGLibA_53679 | 158 | 0.423661954 | VKORC1L1 | vitamin K epoxide reductase complex subunit 1 like 1 [Source:HGNC Symbol;Acc:HGNC:21492] |
| ENSG00000196792 | HGLibA_47596 | 158 | 0.423661954 | STRN3 | striatin 3 [Source:HGNC Symbol;Acc:HGNC:15720] |
| ENSG00000198131 | HGLibA_56197 | 158 | 0.423661954 | ZNF544 | zinc finger protein 544 [Source:HGNC Symbol;Acc:HGNC:16759] |
| ENSG00000198417 | HGLibA_30287 | 158 | 0.423661954 | MT1F | metallothionein 1F [Source:HGNC Symbol;Acc:HGNC:7398] |
| ENSG00000198435 | HGLibA_32645 | 158 | 0.423661954 | NRARP | NOTCH regulated ankyrin repeat protein [Source:HGNC Symbol;Acc:HGNC:33843] |
| ENSG00000198863 | HGLibA_42447 | 158 | 0.423661954 | RUNDC1 | RUN domain containing 1 [Source:HGNC Symbol;Acc:HGNC:25418] |
| ENSG00000204952 | HGLibA_17114 | 158 | 0.423661954 | FBXO47 | F-box protein 47 [Source:HGNC Symbol;Acc:HGNC:31969] |
| ENSG00000204961 | HGLibA_35461 | 158 | 0.423661954 | PCDHA9 | protocadherin alpha 9 [Source:HGNC Symbol;Acc:HGNC:8675] |
| ENSG00000211452 | HGLibA_13239 | 158 | 0.423661954 | DIO1 | iodothyronine deiodinase 1 [Source:HGNC Symbol;Acc:HGNC:2883] |
| ENSG00000213760 | HGLibA_03760 | 158 | 0.423661954 | ATP6V1G2 | ATPase H+ transporting V1 subunit G2 [Source:HGNC Symbol;Acc:HGNC:862] |
| ENSG00000214700 | HGLibA_05287 | 158 | 0.423661954 | C12orf71 | chromosome 12 open reading frame 71 [Source:HGNC Symbol;Acc:HGNC:34452] |
| ENSG00000217236 | HGLibA_46317 | 158 | 0.423661954 | SP9 | Sp9 transcription factor [Source:HGNC Symbol;Acc:HGNC:30690] |
| ENSG00000221859 | HGLibA_25593 | 158 | 0.423661954 | KRTAP10-10 | keratin associated protein 10-10 [Source:HGNC Symbol;Acc:HGNC:22972] |
| ENSG00000243989 | HGLibA_00704 | 158 | 0.423661954 | ACY1 | aminoacylase 1 [Source:HGNC Symbol;Acc:HGNC:177] |
| ENSG00000277322 | HGLibA_19627 | 158 | 0.423661954 | GOLGA6L6 | golgin A6 family-like 6 [Source:HGNC Symbol;Acc:HGNC:37225] |
| FTH1P18 | HGLibA_18120 | 158 | 0.423661954 | FTH1P18 | - |
| FTSJD1 | HGLibA_18142 | 158 | 0.423661954 | FTSJD1 | - |
| hsa-mir-10b | HGLibA_57202 | 158 | 0.423661954 | hsa-mir-10b | - |
| hsa-mir-26b | HGLibA_58373 | 158 | 0.423661954 | hsa-mir-26b | - |
| hsa-mir-3158-2 | HGLibA_58709 | 158 | 0.423661954 | hsa-mir-3158-2 | - |
| hsa-mir-3916 | HGLibA_59541 | 158 | 0.423661954 | hsa-mir-3916 | - |
| hsa-mir-4317 | HGLibA_59988 | 158 | 0.423661954 | hsa-mir-4317 | - |
| hsa-mir-4505 | HGLibA_60448 | 158 | 0.423661954 | hsa-mir-4505 | - |
| hsa-mir-4792 | HGLibA_61273 | 158 | 0.423661954 | hsa-mir-4792 | - |
| hsa-mir-519d | HGLibA_61726 | 158 | 0.423661954 | hsa-mir-519d | - |
| hsa-mir-643 | HGLibA_62817 | 158 | 0.423661954 | hsa-mir-643 | - |
| hsa-mir-644a | HGLibA_62818 | 158 | 0.423661954 | hsa-mir-644a | - |
| hsa-mir-6757 | HGLibA_63187 | 158 | 0.423661954 | hsa-mir-6757 | - |
| hsa-mir-6794 | HGLibA_63353 | 158 | 0.423661954 | hsa-mir-6794 | - |
| hsa-mir-6865 | HGLibA_63643 | 158 | 0.423661954 | hsa-mir-6865 | - |
| IGJ | HGLibA_22862 | 158 | 0.423661954 | IGJ | - |
| KIAA1033 | HGLibA_24685 | 158 | 0.423661954 | KIAA1033 | - |
| MURC | HGLibA_30572 | 158 | 0.423661954 | MURC | - |
| TMEM183B | HGLibA_50108 | 158 | 0.423661954 | TMEM183B | - |
| TMEM5 | HGLibA_50421 | 158 | 0.423661954 | TMEM5 | - |
| TSSC1 | HGLibA_52002 | 158 | 0.423661954 | TSSC1 | - |
| ATP5E | HGLibA_03649 | 157 | 0.413710917 | ATP5E | - |
| BAGE | HGLibA_04091 | 157 | 0.413710917 | BAGE | - |
| C15orf59 | HGLibA_05424 | 157 | 0.413710917 | C15orf59 | - |
| C16orf80 | HGLibA_05485 | 157 | 0.413710917 | C16orf80 | - |
| C1orf168 | HGLibA_05788 | 157 | 0.413710917 | C1orf168 | - |
| C1orf177 | HGLibA_05801 | 157 | 0.413710917 | C1orf177 | - |
| CTGF | HGLibA_11624 | 157 | 0.413710917 | CTGF | - |
| DOPEY1 | HGLibA_13801 | 157 | 0.413710917 | DOPEY1 | - |
| ENSG00000058453 | HGLibA_11209 | 157 | 0.413710917 | CROCC | rootletin [Source:HGNC Symbol;Acc:HGNC:21299]" |
| ENSG00000062716 | HGLibA_53695 | 157 | 0.413710917 | VMP1 | vacuole membrane protein 1 [Source:HGNC Symbol;Acc:HGNC:29559] |
| ENSG00000077420 | HGLibA_02380 | 157 | 0.413710917 | APBB1IP | amyloid beta precursor protein binding family B member 1 interacting protein [Source:HGNC Symbol;Acc:HGNC:17379] |
| ENSG00000084112 | HGLibA_47133 | 157 | 0.413710917 | SSH1 | slingshot protein phosphatase 1 [Source:HGNC Symbol;Acc:HGNC:30579] |
| ENSG00000092421 | HGLibA_43364 | 157 | 0.413710917 | SEMA6A | semaphorin 6A [Source:HGNC Symbol;Acc:HGNC:10738] |
| ENSG00000100266 | HGLibA_34964 | 157 | 0.413710917 | PACSIN2 | protein kinase C and casein kinase substrate in neurons 2 [Source:HGNC Symbol;Acc:HGNC:8571] |
| ENSG00000101489 | HGLibA_09014 | 157 | 0.413710917 | CELF4 | CUGBP Elav-like family member 4 [Source:HGNC Symbol;Acc:HGNC:14015] |
| ENSG00000102230 | HGLibA_35702 | 157 | 0.413710917 | PCYT1B | beta [Source:HGNC Symbol;Acc:HGNC:8755]" |
| ENSG00000102383 | HGLibA_55080 | 157 | 0.413710917 | ZDHHC15 | zinc finger DHHC-type containing 15 [Source:HGNC Symbol;Acc:HGNC:20342] |
| ENSG00000103710 | HGLibA_40371 | 157 | 0.413710917 | RASL12 | RAS like family 12 [Source:HGNC Symbol;Acc:HGNC:30289] |
| ENSG00000104823 | HGLibA_14390 | 157 | 0.413710917 | ECH1 | enoyl-CoA hydratase 1 [Source:HGNC Symbol;Acc:HGNC:3149] |
| ENSG00000104881 | HGLibA_38031 | 157 | 0.413710917 | PPP1R13L | protein phosphatase 1 regulatory subunit 13 like [Source:HGNC Symbol;Acc:HGNC:18838] |
| ENSG00000105329 | HGLibA_49209 | 157 | 0.413710917 | TGFB1 | transforming growth factor beta 1 [Source:HGNC Symbol;Acc:HGNC:11766] |
| ENSG00000105929 | HGLibA_03709 | 157 | 0.413710917 | ATP6V0A4 | ATPase H+ transporting V0 subunit a4 [Source:HGNC Symbol;Acc:HGNC:866] |
| ENSG00000107859 | HGLibA_36768 | 157 | 0.413710917 | PITX3 | paired like homeodomain 3 [Source:HGNC Symbol;Acc:HGNC:9006] |
| ENSG00000110395 | HGLibA_07393 | 157 | 0.413710917 | CBL | Cbl proto-oncogene [Source:HGNC Symbol;Acc:HGNC:1541] |
| ENSG00000111554 | HGLibA_28694 | 157 | 0.413710917 | MDM1 | Mdm1 nuclear protein [Source:HGNC Symbol;Acc:HGNC:29917] |
| ENSG00000112039 | HGLibA_16844 | 157 | 0.413710917 | FANCE | FA complementation group E [Source:HGNC Symbol;Acc:HGNC:3586] |
| ENSG00000112365 | HGLibA_54848 | 157 | 0.413710917 | ZBTB24 | zinc finger and BTB domain containing 24 [Source:HGNC Symbol;Acc:HGNC:21143] |
| ENSG00000113083 | HGLibA_27033 | 157 | 0.413710917 | LOX | lysyl oxidase [Source:HGNC Symbol;Acc:HGNC:6664] |
| ENSG00000113108 | HGLibA_02387 | 157 | 0.413710917 | APBB3 | amyloid beta precursor protein binding family B member 3 [Source:HGNC Symbol;Acc:HGNC:20708] |
| ENSG00000113790 | HGLibA_14682 | 157 | 0.413710917 | EHHADH | enoyl-CoA hydratase and 3-hydroxyacyl CoA dehydrogenase [Source:HGNC Symbol;Acc:HGNC:3247] |
| ENSG00000114126 | HGLibA_49155 | 157 | 0.413710917 | TFDP2 | transcription factor Dp-2 [Source:HGNC Symbol;Acc:HGNC:11751] |
| ENSG00000115183 | HGLibA_48290 | 157 | 0.413710917 | TANC1 | ankyrin repeat and coiled-coil containing 1 [Source:HGNC Symbol;Acc:HGNC:29364]" |
| ENSG00000115194 | HGLibA_44905 | 157 | 0.413710917 | SLC30A3 | solute carrier family 30 member 3 [Source:HGNC Symbol;Acc:HGNC:11014] |
| ENSG00000117707 | HGLibA_38701 | 157 | 0.413710917 | PROX1 | prospero homeobox 1 [Source:HGNC Symbol;Acc:HGNC:9459] |
| ENSG00000119004 | HGLibA_12075 | 157 | 0.413710917 | CYP20A1 | cytochrome P450 family 20 subfamily A member 1 [Source:HGNC Symbol;Acc:HGNC:20576] |
| ENSG00000121446 | HGLibA_41119 | 157 | 0.413710917 | RGSL1 | regulator of G protein signaling like 1 [Source:HGNC Symbol;Acc:HGNC:18636] |
| ENSG00000124795 | HGLibA_12927 | 157 | 0.413710917 | DEK | DEK proto-oncogene [Source:HGNC Symbol;Acc:HGNC:2768] |
| ENSG00000125650 | HGLibA_39229 | 157 | 0.413710917 | PSPN | persephin [Source:HGNC Symbol;Acc:HGNC:9579] |
| ENSG00000127954 | HGLibA_47433 | 157 | 0.413710917 | STEAP4 | STEAP4 metalloreductase [Source:HGNC Symbol;Acc:HGNC:21923] |
| ENSG00000128228 | HGLibA_43127 | 157 | 0.413710917 | SDF2L1 | stromal cell derived factor 2 like 1 [Source:HGNC Symbol;Acc:HGNC:10676] |
| ENSG00000128606 | HGLibA_27269 | 157 | 0.413710917 | LRRC17 | leucine rich repeat containing 17 [Source:HGNC Symbol;Acc:HGNC:16895] |
| ENSG00000129317 | HGLibA_39608 | 157 | 0.413710917 | PUS7L | pseudouridine synthase 7 like [Source:HGNC Symbol;Acc:HGNC:25276] |
| ENSG00000131171 | HGLibA_43917 | 157 | 0.413710917 | SH3BGRL | SH3 domain binding glutamate rich protein like [Source:HGNC Symbol;Acc:HGNC:10823] |
| ENSG00000134955 | HGLibA_45046 | 157 | 0.413710917 | SLC37A2 | solute carrier family 37 member 2 [Source:HGNC Symbol;Acc:HGNC:20644] |
| ENSG00000135845 | HGLibA_36550 | 157 | 0.413710917 | PIGC | phosphatidylinositol glycan anchor biosynthesis class C [Source:HGNC Symbol;Acc:HGNC:8960] |
| ENSG00000135929 | HGLibA_12093 | 157 | 0.413710917 | CYP27A1 | cytochrome P450 family 27 subfamily A member 1 [Source:HGNC Symbol;Acc:HGNC:2605] |
| ENSG00000136929 | HGLibA_21130 | 157 | 0.413710917 | HEMGN | hemogen [Source:HGNC Symbol;Acc:HGNC:17509] |
| ENSG00000137834 | HGLibA_45584 | 157 | 0.413710917 | SMAD6 | SMAD family member 6 [Source:HGNC Symbol;Acc:HGNC:6772] |
| ENSG00000139233 | HGLibA_26654 | 157 | 0.413710917 | LLPH | long-term synaptic facilitation factor [Source:HGNC Symbol;Acc:HGNC:28229]" |
| ENSG00000143776 | HGLibA_08557 | 157 | 0.413710917 | CDC42BPA | CDC42 binding protein kinase alpha [Source:HGNC Symbol;Acc:HGNC:1737] |
| ENSG00000143786 | HGLibA_10290 | 157 | 0.413710917 | CNIH3 | cornichon family AMPA receptor auxiliary protein 3 [Source:HGNC Symbol;Acc:HGNC:26802] |
| ENSG00000144647 | HGLibA_37659 | 157 | 0.413710917 | POMGNT2 | 4-) [Source:HGNC Symbol;Acc:HGNC:25902]" |
| ENSG00000145087 | HGLibA_47678 | 157 | 0.413710917 | STXBP5L | syntaxin binding protein 5 like [Source:HGNC Symbol;Acc:HGNC:30757] |
| ENSG00000147160 | HGLibA_03914 | 157 | 0.413710917 | AWAT2 | acyl-CoA wax alcohol acyltransferase 2 [Source:HGNC Symbol;Acc:HGNC:23251] |
| ENSG00000148399 | HGLibA_13854 | 157 | 0.413710917 | DPH7 | diphthamide biosynthesis 7 [Source:HGNC Symbol;Acc:HGNC:25199] |
| ENSG00000148730 | HGLibA_14832 | 157 | 0.413710917 | EIF4EBP2 | eukaryotic translation initiation factor 4E binding protein 2 [Source:HGNC Symbol;Acc:HGNC:3289] |
| ENSG00000149054 | HGLibA_55598 | 157 | 0.413710917 | ZNF215 | zinc finger protein 215 [Source:HGNC Symbol;Acc:HGNC:13007] |
| ENSG00000149090 | HGLibA_35088 | 157 | 0.413710917 | PAMR1 | peptidase domain containing associated with muscle regeneration 1 [Source:HGNC Symbol;Acc:HGNC:24554] |
| ENSG00000149596 | HGLibA_23982 | 157 | 0.413710917 | JPH2 | junctophilin 2 [Source:HGNC Symbol;Acc:HGNC:14202] |
| ENSG00000150967 | HGLibA_00155 | 157 | 0.413710917 | ABCB9 | ATP binding cassette subfamily B member 9 [Source:HGNC Symbol;Acc:HGNC:50] |
| ENSG00000151065 | HGLibA_12520 | 157 | 0.413710917 | DCP1B | decapping mRNA 1B [Source:HGNC Symbol;Acc:HGNC:24451] |
| ENSG00000152670 | HGLibA_12707 | 157 | 0.413710917 | DDX4 | DEAD-box helicase 4 [Source:HGNC Symbol;Acc:HGNC:18700] |
| ENSG00000152749 | HGLibA_19984 | 157 | 0.413710917 | GPR180 | G protein-coupled receptor 180 [Source:HGNC Symbol;Acc:HGNC:28899] |
| ENSG00000152910 | HGLibA_10432 | 157 | 0.413710917 | CNTNAP4 | contactin associated protein like 4 [Source:HGNC Symbol;Acc:HGNC:18747] |
| ENSG00000153902 | HGLibA_26402 | 157 | 0.413710917 | LGI4 | leucine rich repeat LGI family member 4 [Source:HGNC Symbol;Acc:HGNC:18712] |
| ENSG00000156097 | HGLibA_20061 | 157 | 0.413710917 | GPR61 | G protein-coupled receptor 61 [Source:HGNC Symbol;Acc:HGNC:13300] |
| ENSG00000157873 | HGLibA_50760 | 157 | 0.413710917 | TNFRSF14 | TNF receptor superfamily member 14 [Source:HGNC Symbol;Acc:HGNC:11912] |
| ENSG00000158125 | HGLibA_54503 | 157 | 0.413710917 | XDH | xanthine dehydrogenase [Source:HGNC Symbol;Acc:HGNC:12805] |
| ENSG00000159339 | HGLibA_34977 | 157 | 0.413710917 | PADI4 | peptidyl arginine deiminase 4 [Source:HGNC Symbol;Acc:HGNC:18368] |
| ENSG00000161031 | HGLibA_36252 | 157 | 0.413710917 | PGLYRP2 | peptidoglycan recognition protein 2 [Source:HGNC Symbol;Acc:HGNC:30013] |
| ENSG00000162600 | HGLibA_33379 | 157 | 0.413710917 | OMA1 | OMA1 zinc metallopeptidase [Source:HGNC Symbol;Acc:HGNC:29661] |
| ENSG00000165195 | HGLibA_36544 | 157 | 0.413710917 | PIGA | phosphatidylinositol glycan anchor biosynthesis class A [Source:HGNC Symbol;Acc:HGNC:8957] |
| ENSG00000165548 | HGLibA_50481 | 157 | 0.413710917 | TMEM63C | transmembrane protein 63C [Source:HGNC Symbol;Acc:HGNC:23787] |
| ENSG00000166535 | HGLibA_00012 | 157 | 0.413710917 | A2ML1 | alpha-2-macroglobulin like 1 [Source:HGNC Symbol;Acc:HGNC:23336] |
| ENSG00000168067 | HGLibA_28214 | 157 | 0.413710917 | MAP4K2 | mitogen-activated protein kinase kinase kinase kinase 2 [Source:HGNC Symbol;Acc:HGNC:6864] |
| ENSG00000168209 | HGLibA_12617 | 157 | 0.413710917 | DDIT4 | DNA damage inducible transcript 4 [Source:HGNC Symbol;Acc:HGNC:24944] |
| ENSG00000168386 | HGLibA_17530 | 157 | 0.413710917 | FILIP1L | filamin A interacting protein 1 like [Source:HGNC Symbol;Acc:HGNC:24589] |
| ENSG00000168913 | HGLibA_15130 | 157 | 0.413710917 | ENHO | energy homeostasis associated [Source:HGNC Symbol;Acc:HGNC:24838] |
| ENSG00000168930 | HGLibA_51522 | 157 | 0.413710917 | TRIM49 | tripartite motif containing 49 [Source:HGNC Symbol;Acc:HGNC:13431] |
| ENSG00000170385 | HGLibA_44897 | 157 | 0.413710917 | SLC30A1 | solute carrier family 30 member 1 [Source:HGNC Symbol;Acc:HGNC:11012] |

| | | | | | |
|---|---|---|---|---|---|
| ENSG00000170545 | HGLibA_45594 | 157 | 0.413710917 | SMAGP | small cell adhesion glycoprotein [Source:HGNC Symbol;Acc:HGNC:26918] |
| ENSG00000170615 | HGLibA_44799 | 157 | 0.413710917 | SLC26A5 | solute carrier family 26 member 5 [Source:HGNC Symbol;Acc:HGNC:9359] |
| ENSG00000171161 | HGLibA_56505 | 157 | 0.413710917 | ZNF672 | zinc finger protein 672 [Source:HGNC Symbol;Acc:HGNC:26179] |
| ENSG00000174106 | HGLibA_26292 | 157 | 0.413710917 | LEMD3 | LEM domain containing 3 [Source:HGNC Symbol;Acc:HGNC:28887] |
| ENSG00000174628 | HGLibA_23530 | 157 | 0.413710917 | IQCK | IQ motif containing K [Source:HGNC Symbol;Acc:HGNC:28556] |
| ENSG00000175065 | HGLibA_14031 | 157 | 0.413710917 | DSG4 | desmoglein 4 [Source:HGNC Symbol;Acc:HGNC:21307] |
| ENSG00000175646 | HGLibA_38609 | 157 | 0.413710917 | PRM1 | protamine 1 [Source:HGNC Symbol;Acc:HGNC:9447] |
| ENSG00000178502 | HGLibA_25126 | 157 | 0.413710917 | KLHL11 | kelch like family member 11 [Source:HGNC Symbol;Acc:HGNC:19008] |
| ENSG00000178919 | HGLibA_17859 | 157 | 0.413710917 | FOXE1 | forkhead box E1 [Source:HGNC Symbol;Acc:HGNC:3806] |
| ENSG00000179111 | HGLibA_21194 | 157 | 0.413710917 | HES7 | hes family bHLH transcription factor 7 [Source:HGNC Symbol;Acc:HGNC:15977] |
| ENSG00000179630 | HGLibA_25907 | 157 | 0.413710917 | LACC1 | laccase domain containing 1 [Source:HGNC Symbol;Acc:HGNC:26789] |
| ENSG00000180061 | HGLibA_49994 | 157 | 0.413710917 | TMEM150B | transmembrane protein 150B [Source:HGNC Symbol;Acc:HGNC:34415] |
| ENSG00000180532 | HGLibA_57024 | 157 | 0.413710917 | ZSCAN4 | zinc finger and SCAN domain containing 4 [Source:HGNC Symbol;Acc:HGNC:23709] |
| ENSG00000180644 | HGLibA_38449 | 157 | 0.413710917 | PRF1 | perforin 1 [Source:HGNC Symbol;Acc:HGNC:9360] |
| ENSG00000182652 | HGLibA_34057 | 157 | 0.413710917 | OR4Q3 | olfactory receptor family 4 subfamily Q member 3 [Source:HGNC Symbol;Acc:HGNC:15426] |
| ENSG00000182783 | HGLibA_33869 | 157 | 0.413710917 | OR2T29 | olfactory receptor family 2 subfamily T member 29 [Source:HGNC Symbol;Acc:HGNC:31253] |
| ENSG00000183044 | HGLibA_00092 | 157 | 0.413710917 | ABAT | 4-aminobutyrate aminotransferase [Source:HGNC Symbol;Acc:HGNC:23] |
| ENSG00000183090 | HGLibA_17989 | 157 | 0.413710917 | FREM3 | FRAS1 related extracellular matrix 3 [Source:HGNC Symbol;Acc:HGNC:25172] |
| ENSG00000183607 | HGLibA_19210 | 157 | 0.413710917 | GKN2 | gastrokine 2 [Source:HGNC Symbol;Acc:HGNC:24588] |
| ENSG00000185420 | HGLibA_45857 | 157 | 0.413710917 | SMYD3 | SET and MYND domain containing 3 [Source:HGNC Symbol;Acc:HGNC:15513] |
| ENSG00000185686 | HGLibA_38276 | 157 | 0.413710917 | PRAME | preferentially expressed antigen in melanoma [Source:HGNC Symbol;Acc:HGNC:9336] |
| ENSG00000185888 | HGLibA_38938 | 157 | 0.413710917 | PRSS38 | serine protease 38 [Source:HGNC Symbol;Acc:HGNC:29625] |
| ENSG00000187049 | HGLibA_50223 | 157 | 0.413710917 | TMEM216 | transmembrane protein 216 [Source:HGNC Symbol;Acc:HGNC:25018] |
| ENSG00000187257 | HGLibA_42304 | 157 | 0.413710917 | RSBN1L | round spermatid basic protein 1 like [Source:HGNC Symbol;Acc:HGNC:24765] |
| ENSG00000187266 | HGLibA_15353 | 157 | 0.413710917 | EPOR | erythropoietin receptor [Source:HGNC Symbol;Acc:HGNC:3416] |
| ENSG00000196098 | HGLibA_34320 | 157 | 0.413710917 | OR5K4 | olfactory receptor family 5 subfamily K member 4 [Source:HGNC Symbol;Acc:HGNC:31291] |
| ENSG00000196341 | HGLibA_34509 | 157 | 0.413710917 | OR8D1 | olfactory receptor family 8 subfamily D member 1 [Source:HGNC Symbol;Acc:HGNC:8481] |
| ENSG00000198015 | HGLibA_29985 | 157 | 0.413710917 | MRPL42 | mitochondrial ribosomal protein L42 [Source:HGNC Symbol;Acc:HGNC:14493] |
| ENSG00000198046 | HGLibA_56491 | 157 | 0.413710917 | ZNF667 | zinc finger protein 667 [Source:HGNC Symbol;Acc:HGNC:28854] |
| ENSG00000198087 | HGLibA_08327 | 157 | 0.413710917 | CD2AP | CD2 associated protein [Source:HGNC Symbol;Acc:HGNC:14258] |
| ENSG00000204186 | HGLibA_55062 | 157 | 0.413710917 | ZDBF2 | zinc finger DBF-type containing 2 [Source:HGNC Symbol;Acc:HGNC:29313] |
| ENSG00000204262 | HGLibA_10591 | 157 | 0.413710917 | COL5A2 | collagen type V alpha 2 chain [Source:HGNC Symbol;Acc:HGNC:2210] |
| ENSG00000205177 | HGLibA_05211 | 157 | 0.413710917 | C11orf91 | chromosome 11 open reading frame 91 [Source:HGNC Symbol;Acc:HGNC:34444] |
| ENSG00000205667 | HGLibA_03145 | 157 | 0.413710917 | ARSH | arylsulfatase family member H [Source:HGNC Symbol;Acc:HGNC:32488] |
| ENSG00000213658 | HGLibA_26059 | 157 | 0.413710917 | LAT | linker for activation of T cells [Source:HGNC Symbol;Acc:HGNC:18874] |
| ENSG00000215375 | HGLibA_30767 | 157 | 0.413710917 | MYL5 | myosin light chain 5 [Source:HGNC Symbol;Acc:HGNC:7586] |
| ENSG00000215397 | HGLibA_43061 | 157 | 0.413710917 | SCRT2 | scratch family transcriptional repressor 2 [Source:HGNC Symbol;Acc:HGNC:15952] |
| ENSG00000215595 | HGLibA_06014 | 157 | 0.413710917 | C20orf202 | chromosome 20 open reading frame 202 [Source:HGNC Symbol;Acc:HGNC:37254] |
| ENSG00000226174 | HGLibA_49075 | 157 | 0.413710917 | TEX22 | testis expressed 22 [Source:HGNC Symbol;Acc:HGNC:40026] |
| ENSG00000229937 | HGLibA_38757 | 157 | 0.413710917 | PRPS1L1 | phosphoribosyl pyrophosphate synthetase 1-like 1 [Source:HGNC Symbol;Acc:HGNC:9463] |
| ENSG00000233013 | HGLibA_16195 | 157 | 0.413710917 | FAM157B | family with sequence similarity 157 member B [Source:HGNC Symbol;Acc:HGNC:34080] |
| ENSG00000239886 | HGLibA_25832 | 157 | 0.413710917 | KRTAP9-2 | keratin associated protein 9-2 [Source:HGNC Symbol;Acc:HGNC:16926] |
| ENSG00000243364 | HGLibA_14591 | 157 | 0.413710917 | EFNA4 | ephrin A4 [Source:HGNC Symbol;Acc:HGNC:3224] |
| ENSG00000244624 | HGLibA_25700 | 157 | 0.413710917 | KRTAP20-1 | keratin associated protein 20-1 [Source:HGNC Symbol;Acc:HGNC:18943] |
| ENSG00000254788 | HGLibA_09771 | 157 | 0.413710917 | CKLF-CMTM1 | CKLF-CMTM1 readthrough [Source:HGNC Symbol;Acc:HGNC:39977] |
| ENSG00000277149 | HGLibA_52482 | 157 | 0.413710917 | TYW1B | tRNA-yW synthesizing protein 1 homolog B [Source:HGNC Symbol;Acc:HGNC:33908] |
| FAM101B | HGLibA_15976 | 157 | 0.413710917 | FAM101B | - |
| GPR133 | HGLibA_19891 | 157 | 0.413710917 | GPR133 | - |
| hsa-mir-127 | HGLibA_57512 | 157 | 0.413710917 | hsa-mir-127 | - |
| hsa-mir-15a | HGLibA_57939 | 157 | 0.413710917 | hsa-mir-15a | - |
| hsa-mir-18b | HGLibA_58021 | 157 | 0.413710917 | hsa-mir-18b | - |
| hsa-mir-200b | HGLibA_58153 | 157 | 0.413710917 | hsa-mir-200b | - |
| hsa-mir-28 | HGLibA_58383 | 157 | 0.413710917 | hsa-mir-28 | - |
| hsa-mir-3200 | HGLibA_58907 | 157 | 0.413710917 | hsa-mir-3200 | - |
| hsa-mir-328 | HGLibA_58975 | 157 | 0.413710917 | hsa-mir-328 | - |
| hsa-mir-378b | HGLibA_59431 | 157 | 0.413710917 | hsa-mir-378b | - |
| hsa-mir-3919 | HGLibA_59553 | 157 | 0.413710917 | hsa-mir-3919 | - |
| hsa-mir-4436a | HGLibA_60138 | 157 | 0.413710917 | hsa-mir-4436a | - |
| hsa-mir-4461 | HGLibA_60250 | 157 | 0.413710917 | hsa-mir-4461 | - |
| hsa-mir-4483 | HGLibA_60351 | 157 | 0.413710917 | hsa-mir-4483 | - |
| hsa-mir-4633 | HGLibA_60639 | 157 | 0.413710917 | hsa-mir-4633 | - |
| hsa-mir-548ba | HGLibA_61954 | 157 | 0.413710917 | hsa-mir-548ba | - |
| hsa-mir-559 | HGLibA_62200 | 157 | 0.413710917 | hsa-mir-559 | - |
| hsa-mir-602 | HGLibA_62499 | 157 | 0.413710917 | hsa-mir-602 | - |
| hsa-mir-6752 | HGLibA_63164 | 157 | 0.413710917 | hsa-mir-6752 | - |
| NUTMF | HGLibA_33113 | 157 | 0.413710917 | NUTMF | - |
| SLMO1 | HGLibA_45538 | 157 | 0.413710917 | SLMO1 | - |
| C7orf10 | HGLibA_06521 | 156 | 0.40375988 | C7orf10 | - |
| ENSG00000001036 | HGLibA_18158 | 156 | 0.40375988 | FUCA2 | alpha-L-fucosidase 2 [Source:HGNC Symbol;Acc:HGNC:4008] |
| ENSG00000006210 | HGLibA_11834 | 156 | 0.40375988 | CX3CL1 | C-X3-C motif chemokine ligand 1 [Source:HGNC Symbol;Acc:HGNC:10647] |
| ENSG00000007129 | HGLibA_08934 | 156 | 0.40375988 | CEACAM21 | carcinoembryonic antigen related cell adhesion molecule 21 [Source:HGNC Symbol;Acc:HGNC:28834] |
| ENSG00000012779 | HGLibA_01688 | 156 | 0.40375988 | ALOX5 | arachidonate 5-lipoxygenase [Source:HGNC Symbol;Acc:HGNC:435] |
| ENSG00000041515 | HGLibA_30807 | 156 | 0.40375988 | MYO16 | myosin XVI [Source:HGNC Symbol;Acc:HGNC:29822] |
| ENSG00000048162 | HGLibA_32306 | 156 | 0.40375988 | NOP16 | NOP16 nucleolar protein [Source:HGNC Symbol;Acc:HGNC:26934] |
| ENSG00000050405 | HGLibA_26525 | 156 | 0.40375988 | LIMA1 | LIM domain and actin binding 1 [Source:HGNC Symbol;Acc:HGNC:24636] |
| ENSG00000053524 | HGLibA_28582 | 156 | 0.40375988 | MCF2L2 | MCF.2 cell line derived transforming sequence-like 2 [Source:HGNC Symbol;Acc:HGNC:30319] |
| ENSG00000053702 | HGLibA_32695 | 156 | 0.40375988 | NRIP2 | nuclear receptor interacting protein 2 [Source:HGNC Symbol;Acc:HGNC:23078] |
| ENSG00000058063 | HGLibA_03556 | 156 | 0.40375988 | ATP11B | ATPase phospholipid transporting 11B (putative) [Source:HGNC Symbol;Acc:HGNC:13553] |
| ENSG00000064655 | HGLibA_15820 | 156 | 0.40375988 | EYA2 | EYA transcriptional coactivator and phosphatase 2 [Source:HGNC Symbol;Acc:HGNC:3520] |
| ENSG00000065361 | HGLibA_15418 | 156 | 0.40375988 | ERBB3 | erb-b2 receptor tyrosine kinase 3 [Source:HGNC Symbol;Acc:HGNC:3431] |
| ENSG00000075391 | HGLibA_40315 | 156 | 0.40375988 | RASAL2 | RAS protein activator like 2 [Source:HGNC Symbol;Acc:HGNC:9874] |
| ENSG00000075826 | HGLibA_43244 | 156 | 0.40375988 | SEC31B | COPII coat complex component [Source:HGNC Symbol;Acc:HGNC:23197]" |
| ENSG00000087250 | HGLibA_30304 | 156 | 0.40375988 | MT3 | metallothionein 3 [Source:HGNC Symbol;Acc:HGNC:7408] |
| ENSG00000102886 | HGLibA_18896 | 156 | 0.40375988 | GDPD3 | glycerophosphodiester phosphodiesterase domain containing 3 [Source:HGNC Symbol;Acc:HGNC:28638] |
| ENSG00000103274 | HGLibA_32902 | 156 | 0.40375988 | NUBP1 | nucleotide binding protein 1 [Source:HGNC Symbol;Acc:HGNC:8041] |
| ENSG00000104312 | HGLibA_41320 | 156 | 0.40375988 | RIPK2 | receptor interacting serine/threonine kinase 2 [Source:HGNC Symbol;Acc:HGNC:10020] |
| ENSG00000107807 | HGLibA_49671 | 156 | 0.40375988 | TLX1 | T cell leukemia homeobox 1 [Source:HGNC Symbol;Acc:HGNC:5056] |
| ENSG00000111880 | HGLibA_41709 | 156 | 0.40375988 | RNGTT | RNA guanylyltransferase and 5'-phosphatase [Source:HGNC Symbol;Acc:HGNC:10073] |
| ENSG00000112715 | HGLibA_53611 | 156 | 0.40375988 | VEGFA | vascular endothelial growth factor A [Source:HGNC Symbol;Acc:HGNC:12680] |
| ENSG00000113100 | HGLibA_08695 | 156 | 0.40375988 | CDH9 | cadherin 9 [Source:HGNC Symbol;Acc:HGNC:1768] |
| ENSG00000113196 | HGLibA_20825 | 156 | 0.40375988 | HAND1 | heart and neural crest derivatives expressed 1 [Source:HGNC Symbol;Acc:HGNC:4807] |
| ENSG00000113504 | HGLibA_44375 | 156 | 0.40375988 | SLC12A7 | solute carrier family 12 member 7 [Source:HGNC Symbol;Acc:HGNC:10915] |
| ENSG00000113946 | HGLibA_09866 | 156 | 0.40375988 | CLDN16 | claudin 16 [Source:HGNC Symbol;Acc:HGNC:2037] |
| ENSG00000115234 | HGLibA_46048 | 156 | 0.40375988 | SNX17 | sorting nexin 17 [Source:HGNC Symbol;Acc:HGNC:14979] |
| ENSG00000116809 | HGLibA_54830 | 156 | 0.40375988 | ZBTB17 | zinc finger and BTB domain containing 17 [Source:HGNC Symbol;Acc:HGNC:12936] |
| ENSG00000120279 | HGLibA_30683 | 156 | 0.40375988 | MYCT1 | MYC target 1 [Source:HGNC Symbol;Acc:HGNC:23172] |
| ENSG00000123908 | HGLibA_01208 | 156 | 0.40375988 | AGO2 | RISC catalytic component [Source:HGNC Symbol;Acc:HGNC:3263]" |
| ENSG00000124440 | HGLibA_21302 | 156 | 0.40375988 | HIF3A | hypoxia inducible factor 3 subunit alpha [Source:HGNC Symbol;Acc:HGNC:15825] |
| ENSG00000126247 | HGLibA_07183 | 156 | 0.40375988 | CAPNS1 | calpain small subunit 1 [Source:HGNC Symbol;Acc:HGNC:1481] |
| ENSG00000130182 | HGLibA_56987 | 156 | 0.40375988 | ZSCAN10 | zinc finger and SCAN domain containing 10 [Source:HGNC Symbol;Acc:HGNC:12997] |
| ENSG00000130856 | HGLibA_55651 | 156 | 0.40375988 | ZNF236 | zinc finger protein 236 [Source:HGNC Symbol;Acc:HGNC:13028] |
| ENSG00000132386 | HGLibA_43587 | 156 | 0.40375988 | SERPINF1 | serpin family F member 1 [Source:HGNC Symbol;Acc:HGNC:8824] |
| ENSG00000133816 | HGLibA_29203 | 156 | 0.40375988 | MICAL2 | calponin and LIM domain containing 2 [Source:HGNC Symbol;Acc:HGNC:24693]" |
| ENSG00000133863 | HGLibA_49067 | 156 | 0.40375988 | TEX15 | meiosis and synapsis associated [Source:HGNC Symbol;Acc:HGNC:11738]" |
| ENSG00000134900 | HGLibA_51162 | 156 | 0.40375988 | TPP2 | tripeptidyl peptidase 2 [Source:HGNC Symbol;Acc:HGNC:12016] |
| ENSG00000136367 | HGLibA_55172 | 156 | 0.40375988 | ZFHX2 | zinc finger homeobox 2 [Source:HGNC Symbol;Acc:HGNC:20152] |
| ENSG00000139547 | HGLibA_40745 | 156 | 0.40375988 | RDH16 | retinol dehydrogenase 16 [Source:HGNC Symbol;Acc:HGNC:29674] |
| ENSG00000139714 | HGLibA_29672 | 156 | 0.40375988 | MORN3 | MORN repeat containing 3 [Source:HGNC Symbol;Acc:HGNC:29807] |
| ENSG00000139832 | HGLibA_39836 | 156 | 0.40375988 | RAB20 | member RAS oncogene family [Source:HGNC Symbol;Acc:HGNC:18260]" |
| ENSG00000139973 | HGLibA_48067 | 156 | 0.40375988 | SYT16 | synaptotagmin 16 [Source:HGNC Symbol;Acc:HGNC:23142] |
| ENSG00000141965 | HGLibA_17272 | 156 | 0.40375988 | FEM1A | fem-1 homolog A [Source:HGNC Symbol;Acc:HGNC:16934] |
| ENSG00000144119 | HGLibA_05928 | 156 | 0.40375988 | C1QL2 | complement C1q like 2 [Source:HGNC Symbol;Acc:HGNC:24181] |
| ENSG00000144597 | HGLibA_14333 | 156 | 0.40375988 | EAF1 | ELL associated factor 1 [Source:HGNC Symbol;Acc:HGNC:20907] |
| ENSG00000146966 | HGLibA_12938 | 156 | 0.40375988 | DENND2A | DENN domain containing 2A [Source:HGNC Symbol;Acc:HGNC:22212] |
| ENSG00000147481 | HGLibA_46005 | 156 | 0.40375988 | SNTG1 | syntrophin gamma 1 [Source:HGNC Symbol;Acc:HGNC:13740] |
| ENSG00000147873 | HGLibA_22684 | 156 | 0.40375988 | IFNA5 | interferon alpha 5 [Source:HGNC Symbol;Acc:HGNC:5426] |
| ENSG00000148175 | HGLibA_47531 | 156 | 0.40375988 | STOM | stomatin [Source:HGNC Symbol;Acc:HGNC:3383] |
| ENSG00000149452 | HGLibA_44600 | 156 | 0.40375988 | SLC22A8 | solute carrier family 22 member 8 [Source:HGNC Symbol;Acc:HGNC:10972] |
| ENSG00000149633 | HGLibA_24818 | 156 | 0.40375988 | KIAA1755 | KIAA1755 [Source:HGNC Symbol;Acc:HGNC:29372] |
| ENSG00000153060 | HGLibA_48983 | 156 | 0.40375988 | TEKT5 | tektin 5 [Source:HGNC Symbol;Acc:HGNC:26554] |
| ENSG00000153064 | HGLibA_04147 | 156 | 0.40375988 | BANK1 | B cell scaffold protein with ankyrin repeats 1 [Source:HGNC Symbol;Acc:HGNC:18233] |
| ENSG00000156531 | HGLibA_36384 | 156 | 0.40375988 | PHF6 | PHD finger protein 6 [Source:HGNC Symbol;Acc:HGNC:18145] |
| ENSG00000157064 | HGLibA_32187 | 156 | 0.40375988 | NMNAT2 | nicotinamide nucleotide adenylyltransferase 2 [Source:HGNC Symbol;Acc:HGNC:16789] |
| ENSG00000159840 | HGLibA_57088 | 156 | 0.40375988 | ZYX | zyxin [Source:HGNC Symbol;Acc:HGNC:13200] |
| ENSG00000159884 | HGLibA_07525 | 156 | 0.40375988 | CCDC107 | coiled-coil domain containing 107 [Source:HGNC Symbol;Acc:HGNC:28465] |
| ENSG00000161179 | HGLibA_54653 | 156 | 0.40375988 | YDJC | YdjC chitooligosaccharide deacetylase homolog [Source:HGNC Symbol;Acc:HGNC:27158] |
| ENSG00000162517 | HGLibA_36025 | 156 | 0.40375988 | PEF1 | penta-EF-hand domain containing 1 [Source:HGNC Symbol;Acc:HGNC:30009] |
| ENSG00000162613 | HGLibA_18149 | 156 | 0.40375988 | FUBP1 | far upstream element binding protein 1 [Source:HGNC Symbol;Acc:HGNC:4004] |
| ENSG00000162849 | HGLibA_24920 | 156 | 0.40375988 | KIF26B | kinesin family member 26B [Source:HGNC Symbol;Acc:HGNC:25484] |
| ENSG00000163131 | HGLibA_11729 | 156 | 0.40375988 | CTSS | cathepsin S [Source:HGNC Symbol;Acc:HGNC:2545] |
| ENSG00000163219 | HGLibA_02737 | 156 | 0.40375988 | ARHGAP25 | Rho GTPase activating protein 25 [Source:HGNC Symbol;Acc:HGNC:28951] |
| ENSG00000163357 | HGLibA_12530 | 156 | 0.40375988 | DCST1 | DC-STAMP domain containing 1 [Source:HGNC Symbol;Acc:HGNC:26539] |
| ENSG00000163453 | HGLibA_22834 | 156 | 0.40375988 | IGFBP7 | insulin like growth factor binding protein 7 [Source:HGNC Symbol;Acc:HGNC:5476] |
| ENSG00000163599 | HGLibA_11635 | 156 | 0.40375988 | CTLA4 | cytotoxic T-lymphocyte associated protein 4 [Source:HGNC Symbol;Acc:HGNC:2505] |
| ENSG00000163823 | HGLibA_08143 | 156 | 0.40375988 | CCR1 | C-C motif chemokine receptor 1 [Source:HGNC Symbol;Acc:HGNC:1602] |

| | | | | | |
|---|---|---|---|---|---|
| ENSG00000165972 | HGLibA_07768 | 156 | 0.40375988 | CCDC38 | coiled-coil domain containing 38 [Source:HGNC Symbol;Acc:HGNC:26843] |
| ENSG00000166265 | HGLibA_12254 | 156 | 0.40375988 | CYYR1 | cysteine and tyrosine rich 1 [Source:HGNC Symbol;Acc:HGNC:16274] |
| ENSG00000166317 | HGLibA_48025 | 156 | 0.40375988 | SYNPO2L | synaptopodin 2 like [Source:HGNC Symbol;Acc:HGNC:23532] |
| ENSG00000166823 | HGLibA_28917 | 156 | 0.40375988 | MESP1 | mesoderm posterior bHLH transcription factor 1 [Source:HGNC Symbol;Acc:HGNC:29658] |
| ENSG00000168137 | HGLibA_43651 | 156 | 0.40375988 | SETD5 | SET domain containing 5 [Source:HGNC Symbol;Acc:HGNC:25566] |
| ENSG00000170315 | HGLibA_52564 | 156 | 0.40375988 | UBB | ubiquitin B [Source:HGNC Symbol;Acc:HGNC:12463] |
| ENSG00000171223 | HGLibA_24006 | 156 | 0.40375988 | JUNB | AP-1 transcription factor subunit [Source:HGNC Symbol;Acc:HGNC:6205]" |
| ENSG00000171700 | HGLibA_41077 | 156 | 0.40375988 | RGS19 | regulator of G protein signaling 19 [Source:HGNC Symbol;Acc:HGNC:13735] |
| ENSG00000173264 | HGLibA_19896 | 156 | 0.40375988 | GPR137 | G protein-coupled receptor 137 [Source:HGNC Symbol;Acc:HGNC:24300] |
| ENSG00000175143 | HGLibA_33852 | 156 | 0.40375988 | OR2T1 | olfactory receptor family 2 subfamily T member 1 [Source:HGNC Symbol;Acc:HGNC:8277] |
| ENSG00000175336 | HGLibA_02522 | 156 | 0.40375988 | APOF | apolipoprotein F [Source:HGNC Symbol;Acc:HGNC:615] |
| ENSG00000177519 | HGLibA_42054 | 156 | 0.40375988 | RPRM | TP53 dependent G2 arrest mediator homolog [Source:HGNC Symbol;Acc:HGNC:24201]" |
| ENSG00000178338 | HGLibA_55863 | 156 | 0.40375988 | ZNF354B | zinc finger protein 354B [Source:HGNC Symbol;Acc:HGNC:17197] |
| ENSG00000178952 | HGLibA_52323 | 156 | 0.40375988 | TUFM | mitochondrial [Source:HGNC Symbol;Acc:HGNC:12420]" |
| ENSG00000182053 | HGLibA_51524 | 156 | 0.40375988 | TRIM49B | tripartite motif containing 49B [Source:HGNC Symbol;Acc:HGNC:42955] |
| ENSG00000182108 | HGLibA_13016 | 156 | 0.40375988 | DEXI | Dexi homolog [Source:HGNC Symbol;Acc:HGNC:13267] |
| ENSG00000184144 | HGLibA_10404 | 156 | 0.40375988 | CNTN2 | contactin 2 [Source:HGNC Symbol;Acc:HGNC:2172] |
| ENSG00000184162 | HGLibA_32601 | 156 | 0.40375988 | NR2C2AP | nuclear receptor 2C2 associated protein [Source:HGNC Symbol;Acc:HGNC:30763] |
| ENSG00000186416 | HGLibA_32021 | 156 | 0.40375988 | NKRF | NFKB repressing factor [Source:HGNC Symbol;Acc:HGNC:19374] |
| ENSG00000186599 | HGLibA_12828 | 156 | 0.40375988 | DEFB105B | defensin beta 105B [Source:HGNC Symbol;Acc:HGNC:29930] |
| ENSG00000187492 | HGLibA_08708 | 156 | 0.40375988 | CDHR4 | cadherin related family member 4 [Source:HGNC Symbol;Acc:HGNC:34527] |
| ENSG00000187624 | HGLibA_05596 | 156 | 0.40375988 | C17orf97 | chromosome 17 open reading frame 97 [Source:HGNC Symbol;Acc:HGNC:33800] |
| ENSG00000187942 | HGLibA_26244 | 156 | 0.40375988 | LDLRAD2 | low density lipoprotein receptor class A domain containing 2 [Source:HGNC Symbol;Acc:HGNC:32071] |
| ENSG00000196242 | HGLibA_33780 | 156 | 0.40375988 | OR2C3 | olfactory receptor family 2 subfamily C member 3 [Source:HGNC Symbol;Acc:HGNC:15005] |
| ENSG00000196990 | HGLibA_16232 | 156 | 0.40375988 | FAM163B | family with sequence similarity 163 member B [Source:HGNC Symbol;Acc:HGNC:33277] |
| ENSG00000197705 | HGLibA_25136 | 156 | 0.40375988 | KLHL14 | kelch like family member 14 [Source:HGNC Symbol;Acc:HGNC:29266] |
| ENSG00000197745 | HGLibA_42920 | 156 | 0.40375988 | SCGB1D4 | secretoglobin family 1D member 4 [Source:HGNC Symbol;Acc:HGNC:31748] |
| ENSG00000197938 | HGLibA_34298 | 156 | 0.40375988 | OR5H2 | olfactory receptor family 5 subfamily H member 2 [Source:HGNC Symbol;Acc:HGNC:14752] |
| ENSG00000198561 | HGLibA_11660 | 156 | 0.40375988 | CTNND1 | catenin delta 1 [Source:HGNC Symbol;Acc:HGNC:2515] |
| ENSG00000204104 | HGLibA_51250 | 156 | 0.40375988 | TRAF3IP1 | TRAF3 interacting protein 1 [Source:HGNC Symbol;Acc:HGNC:17861] |
| ENSG00000204209 | HGLibA_12349 | 156 | 0.40375988 | DAXX | death domain associated protein [Source:HGNC Symbol;Acc:HGNC:2681] |
| ENSG00000204478 | HGLibA_38308 | 156 | 0.40375988 | PRAMEF20 | PRAME family member 20 [Source:HGNC Symbol;Acc:HGNC:25224] |
| ENSG00000205076 | HGLibA_26371 | 156 | 0.40375988 | LGALS7 | galectin 7 [Source:HGNC Symbol;Acc:HGNC:6568] |
| ENSG00000205669 | HGLibA_00484 | 156 | 0.40375988 | ACOT6 | acyl-CoA thioesterase 6 [Source:HGNC Symbol;Acc:HGNC:33159] |
| ENSG00000205777 | HGLibA_18430 | 156 | 0.40375988 | GAGE1 | G antigen 1 [Source:HGNC Symbol;Acc:HGNC:4098] |
| ENSG00000213722 | HGLibA_12590 | 156 | 0.40375988 | DDAH2 | dimethylarginine dimethylaminohydrolase 2 [Source:HGNC Symbol;Acc:HGNC:2716] |
| ENSG00000223609 | HGLibA_20916 | 156 | 0.40375988 | HBD | hemoglobin subunit delta [Source:HGNC Symbol;Acc:HGNC:4829] |
| ENSG00000228623 | HGLibA_56896 | 156 | 0.40375988 | ZNF883 | zinc finger protein 883 [Source:HGNC Symbol;Acc:HGNC:27271] |
| ENSG00000234127 | HGLibA_51443 | 156 | 0.40375988 | TRIM26 | tripartite motif containing 26 [Source:HGNC Symbol;Acc:HGNC:12962] |
| ENSG00000243232 | HGLibA_35465 | 156 | 0.40375988 | PCDHAC2 | 2 [Source:HGNC Symbol;Acc:HGNC:8677]" |
| ENSG00000259207 | HGLibA_23782 | 156 | 0.40375988 | ITGB3 | integrin subunit beta 3 [Source:HGNC Symbol;Acc:HGNC:6156] |
| ENSG00000272920 | HGLibA_57410 | 156 | 0.40375988 | hsa-mir-1253 | hsa-mir-1253 [Source:miRBase;Acc:MI0006387] |
| GLTPD1 | HGLibA_19347 | 156 | 0.40375988 | GLTPD1 | - |
| GPR128 | HGLibA_19884 | 156 | 0.40375988 | GPR128 | - |
| hsa-mir-1178 | HGLibA_57204 | 156 | 0.40375988 | hsa-mir-1178 | - |
| hsa-mir-1276 | HGLibA_57558 | 156 | 0.40375988 | hsa-mir-1276 | - |
| hsa-mir-1972-1 | HGLibA_58107 | 156 | 0.40375988 | hsa-mir-1972-1 | - |
| hsa-mir-3194 | HGLibA_58872 | 156 | 0.40375988 | hsa-mir-3194 | - |
| hsa-mir-3200 | HGLibA_58908 | 156 | 0.40375988 | hsa-mir-3200 | - |
| hsa-mir-337 | HGLibA_59001 | 156 | 0.40375988 | hsa-mir-337 | - |
| hsa-mir-4314 | HGLibA_59972 | 156 | 0.40375988 | hsa-mir-4314 | - |
| hsa-mir-4740 | HGLibA_61059 | 156 | 0.40375988 | hsa-mir-4740 | - |
| hsa-mir-518a-2 | HGLibA_61655 | 156 | 0.40375988 | hsa-mir-518a-2 | - |
| hsa-mir-6780b | HGLibA_63298 | 156 | 0.40375988 | hsa-mir-6780b | - |
| hsa-mir-6854 | HGLibA_63587 | 156 | 0.40375988 | hsa-mir-6854 | - |
| hsa-mir-9-3 | HGLibA_64270 | 156 | 0.40375988 | hsa-mir-9-3 | - |
| LPPR5 | HGLibA_27125 | 156 | 0.40375988 | LPPR5 | - |
| TCTE3 | HGLibA_48861 | 156 | 0.40375988 | TCTE3 | - |
| 6-Mar | HGLibA_28344 | 155 | 0.393808842 | 6-Mar | - |
| ATP5G1 | HGLibA_03654 | 155 | 0.393808842 | ATP5G1 | - |
| ATP5S | HGLibA_03688 | 155 | 0.393808842 | ATP5S | - |
| BRE | HGLibA_04794 | 155 | 0.393808842 | BRE | - |
| C1orf101 | HGLibA_05731 | 155 | 0.393808842 | C1orf101 | - |
| C4orf32 | HGLibA_06319 | 155 | 0.393808842 | C4orf32 | - |
| C6orf1 | HGLibA_06429 | 155 | 0.393808842 | C6orf1 | - |
| ENSG00000005108 | HGLibA_49407 | 155 | 0.393808842 | THSD7A | thrombospondin type 1 domain containing 7A [Source:HGNC Symbol;Acc:HGNC:22207] |
| ENSG00000005187 | HGLibA_00579 | 155 | 0.393808842 | ACSM3 | acyl-CoA synthetase medium chain family member 3 [Source:HGNC Symbol;Acc:HGNC:10522] |
| ENSG00000008441 | HGLibA_31811 | 155 | 0.393808842 | NFIX | nuclear factor I X [Source:HGNC Symbol;Acc:HGNC:7788] |
| ENSG00000010030 | HGLibA_15667 | 155 | 0.393808842 | ETV7 | ETS variant 7 [Source:HGNC Symbol;Acc:HGNC:18160] |
| ENSG00000025039 | HGLibA_42232 | 155 | 0.393808842 | RRAGD | Ras related GTP binding D [Source:HGNC Symbol;Acc:HGNC:19903] |
| ENSG00000030304 | HGLibA_30578 | 155 | 0.393808842 | MUSK | muscle associated receptor tyrosine kinase [Source:HGNC Symbol;Acc:HGNC:7525] |
| ENSG00000043462 | HGLibA_26201 | 155 | 0.393808842 | LCP2 | lymphocyte cytosolic protein 2 [Source:HGNC Symbol;Acc:HGNC:6529] |
| ENSG00000053918 | HGLibA_24365 | 155 | 0.393808842 | KCNQ1 | potassium voltage-gated channel subfamily Q member 1 [Source:HGNC Symbol;Acc:HGNC:6294] |
| ENSG00000056291 | HGLibA_32441 | 155 | 0.393808842 | NPFFR2 | neuropeptide FF receptor 2 [Source:HGNC Symbol;Acc:HGNC:4525] |
| ENSG00000064652 | HGLibA_46070 | 155 | 0.393808842 | SNX24 | sorting nexin 24 [Source:HGNC Symbol;Acc:HGNC:21533] |
| ENSG00000066294 | HGLibA_08466 | 155 | 0.393808842 | CD84 | CD84 molecule [Source:HGNC Symbol;Acc:HGNC:1704] |
| ENSG00000072062 | HGLibA_38502 | 155 | 0.393808842 | PRKACA | protein kinase cAMP-activated catalytic subunit alpha [Source:HGNC Symbol;Acc:HGNC:9380] |
| ENSG00000074582 | HGLibA_04370 | 155 | 0.393808842 | BCS1L | ubiquinol-cytochrome c reductase complex chaperone [Source:HGNC Symbol;Acc:HGNC:1020]" |
| ENSG00000078596 | HGLibA_23830 | 155 | 0.393808842 | ITM2A | integral membrane protein 2A [Source:HGNC Symbol;Acc:HGNC:6173] |
| ENSG00000100258 | HGLibA_26687 | 155 | 0.393808842 | LMF2 | lipase maturation factor 2 [Source:HGNC Symbol;Acc:HGNC:25096] |
| ENSG00000100297 | HGLibA_28617 | 155 | 0.393808842 | MCM5 | minichromosome maintenance complex component 5 [Source:HGNC Symbol;Acc:HGNC:6948] |
| ENSG00000100749 | HGLibA_53824 | 155 | 0.393808842 | VRK1 | VRK serine/threonine kinase 1 [Source:HGNC Symbol;Acc:HGNC:12718] |
| ENSG00000100897 | HGLibA_12416 | 155 | 0.393808842 | DCAF11 | DDB1 and CUL4 associated factor 11 [Source:HGNC Symbol;Acc:HGNC:20258] |
| ENSG00000101194 | HGLibA_44482 | 155 | 0.393808842 | SLC17A9 | solute carrier family 17 member 9 [Source:HGNC Symbol;Acc:HGNC:16192] |
| ENSG00000102174 | HGLibA_36327 | 155 | 0.393808842 | PHEX | phosphate regulating endopeptidase homolog X-linked [Source:HGNC Symbol;Acc:HGNC:8918] |
| ENSG00000104447 | HGLibA_51775 | 155 | 0.393808842 | TRPS1 | transcriptional repressor GATA binding 1 [Source:HGNC Symbol;Acc:HGNC:12340] |
| ENSG00000104755 | HGLibA_00749 | 155 | 0.393808842 | ADAM2 | ADAM metallopeptidase domain 2 [Source:HGNC Symbol;Acc:HGNC:198] |
| ENSG00000104889 | HGLibA_41428 | 155 | 0.393808842 | RNASEH2A | ribonuclease H2 subunit A [Source:HGNC Symbol;Acc:HGNC:18518] |
| ENSG00000105122 | HGLibA_40318 | 155 | 0.393808842 | RASAL3 | RAS protein activator like 3 [Source:HGNC Symbol;Acc:HGNC:26129] |
| ENSG00000107518 | HGLibA_03823 | 155 | 0.393808842 | ATRNL1 | attractin like 1 [Source:HGNC Symbol;Acc:HGNC:29063] |
| ENSG00000111796 | HGLibA_25281 | 155 | 0.393808842 | KLRB1 | killer cell lectin like receptor B1 [Source:HGNC Symbol;Acc:HGNC:6373] |
| ENSG00000112214 | HGLibA_17490 | 155 | 0.393808842 | FHL5 | four and a half LIM domains 5 [Source:HGNC Symbol;Acc:HGNC:17371] |
| ENSG00000114019 | HGLibA_01821 | 155 | 0.393808842 | AMOTL2 | angiomotin like 2 [Source:HGNC Symbol;Acc:HGNC:17812] |
| ENSG00000115844 | HGLibA_13374 | 155 | 0.393808842 | DLX2 | distal-less homeobox 2 [Source:HGNC Symbol;Acc:HGNC:2915] |
| ENSG00000116882 | HGLibA_20836 | 155 | 0.393808842 | HAO2 | hydroxyacid oxidase 2 [Source:HGNC Symbol;Acc:HGNC:4810] |
| ENSG00000123352 | HGLibA_46545 | 155 | 0.393808842 | SPATS2 | spermatogenesis associated serine rich 2 [Source:HGNC Symbol;Acc:HGNC:18650] |
| ENSG00000124749 | HGLibA_10538 | 155 | 0.393808842 | COL21A1 | collagen type XXI alpha 1 chain [Source:HGNC Symbol;Acc:HGNC:17025] |
| ENSG00000126351 | HGLibA_49389 | 155 | 0.393808842 | THRA | thyroid hormone receptor alpha [Source:HGNC Symbol;Acc:HGNC:11796] |
| ENSG00000126752 | HGLibA_47195 | 155 | 0.393808842 | SSX1 | SSX family member 1 [Source:HGNC Symbol;Acc:HGNC:11335] |
| ENSG00000129167 | HGLibA_51129 | 155 | 0.393808842 | TPH1 | tryptophan hydroxylase 1 [Source:HGNC Symbol;Acc:HGNC:12008] |
| ENSG00000129255 | HGLibA_29710 | 155 | 0.393808842 | MPDU1 | mannose-P-dolichol utilization defect 1 [Source:HGNC Symbol;Acc:HGNC:7207] |
| ENSG00000130234 | HGLibA_00426 | 155 | 0.393808842 | ACE2 | angiotensin I converting enzyme 2 [Source:HGNC Symbol;Acc:HGNC:13557] |
| ENSG00000131238 | HGLibA_38232 | 155 | 0.393808842 | PPT1 | palmitoyl-protein thioesterase 1 [Source:HGNC Symbol;Acc:HGNC:9325] |
| ENSG00000132170 | HGLibA_37843 | 155 | 0.393808842 | PPARG | peroxisome proliferator activated receptor gamma [Source:HGNC Symbol;Acc:HGNC:9236] |
| ENSG00000134769 | HGLibA_14064 | 155 | 0.393808842 | DTNA | dystrobrevin alpha [Source:HGNC Symbol;Acc:HGNC:3057] |
| ENSG00000136271 | HGLibA_12750 | 155 | 0.393808842 | DDX56 | DEAD-box helicase 56 [Source:HGNC Symbol;Acc:HGNC:18193] |
| ENSG00000137077 | HGLibA_07993 | 155 | 0.393808842 | CCL21 | C-C motif chemokine ligand 21 [Source:HGNC Symbol;Acc:HGNC:10620] |
| ENSG00000137486 | HGLibA_03107 | 155 | 0.393808842 | ARRB1 | arrestin beta 1 [Source:HGNC Symbol;Acc:HGNC:711] |
| ENSG00000137501 | HGLibA_48100 | 155 | 0.393808842 | SYTL2 | synaptotagmin like 2 [Source:HGNC Symbol;Acc:HGNC:15585] |
| ENSG00000139722 | HGLibA_53779 | 155 | 0.393808842 | VPS37B | ESCRT-I subunit [Source:HGNC Symbol;Acc:HGNC:25754]" |
| ENSG00000141971 | HGLibA_30589 | 155 | 0.393808842 | MVB12A | multivesicular body subunit 12A [Source:HGNC Symbol;Acc:HGNC:25153] |
| ENSG00000143590 | HGLibA_14588 | 155 | 0.393808842 | EFNA3 | ephrin A3 [Source:HGNC Symbol;Acc:HGNC:3223] |
| ENSG00000143811 | HGLibA_39675 | 155 | 0.393808842 | PYCR2 | pyrroline-5-carboxylate reductase 2 [Source:HGNC Symbol;Acc:HGNC:30262] |
| ENSG00000145040 | HGLibA_52825 | 155 | 0.393808842 | UCN2 | urocortin 2 [Source:HGNC Symbol;Acc:HGNC:18414] |
| ENSG00000145113 | HGLibA_30547 | 155 | 0.393808842 | MUC4 | cell surface associated [Source:HGNC Symbol;Acc:HGNC:7514]" |
| ENSG00000146374 | HGLibA_42349 | 155 | 0.393808842 | RSPO3 | R-spondin 3 [Source:HGNC Symbol;Acc:HGNC:20866] |
| ENSG00000146555 | HGLibA_43151 | 155 | 0.393808842 | SDK1 | sidekick cell adhesion molecule 1 [Source:HGNC Symbol;Acc:HGNC:19307] |
| ENSG00000146802 | HGLibA_50044 | 155 | 0.393808842 | TMEM168 | transmembrane protein 168 [Source:HGNC Symbol;Acc:HGNC:25826] |
| ENSG00000148604 | HGLibA_41047 | 155 | 0.393808842 | RGR | retinal G protein coupled receptor [Source:HGNC Symbol;Acc:HGNC:9990] |
| ENSG00000148702 | HGLibA_20793 | 155 | 0.393808842 | HABP2 | hyaluronan binding protein 2 [Source:HGNC Symbol;Acc:HGNC:4798] |
| ENSG00000149735 | HGLibA_19798 | 155 | 0.393808842 | GPHA2 | glycoprotein hormone alpha 2 [Source:HGNC Symbol;Acc:HGNC:18054] |
| ENSG00000151500 | HGLibA_49428 | 155 | 0.393808842 | THYN1 | thymocyte nuclear protein 1 [Source:HGNC Symbol;Acc:HGNC:29560] |
| ENSG00000152503 | HGLibA_51475 | 155 | 0.393808842 | TRIM36 | tripartite motif containing 36 [Source:HGNC Symbol;Acc:HGNC:16280] |
| ENSG00000153339 | HGLibA_51336 | 155 | 0.393808842 | TRAPPC8 | trafficking protein particle complex 8 [Source:HGNC Symbol;Acc:HGNC:29169] |
| ENSG00000155719 | HGLibA_34724 | 155 | 0.393808842 | OTOA | otoancorin [Source:HGNC Symbol;Acc:HGNC:16378] |
| ENSG00000157625 | HGLibA_48147 | 155 | 0.393808842 | TAB3 | TGF-beta activated kinase 1 (MAP3K7) binding protein 3 [Source:HGNC Symbol;Acc:HGNC:30681] |
| ENSG00000158773 | HGLibA_53167 | 155 | 0.393808842 | USF1 | upstream transcription factor 1 [Source:HGNC Symbol;Acc:HGNC:12593] |
| ENSG00000162078 | HGLibA_55301 | 155 | 0.393808842 | ZG16B | zymogen granule protein 16B [Source:HGNC Symbol;Acc:HGNC:30456] |
| ENSG00000163251 | HGLibA_18275 | 155 | 0.393808842 | FZD5 | frizzled class receptor 5 [Source:HGNC Symbol;Acc:HGNC:4043] |
| ENSG00000163737 | HGLibA_36129 | 155 | 0.393808842 | PF4 | platelet factor 4 [Source:HGNC Symbol;Acc:HGNC:8861] |
| ENSG00000164574 | HGLibA_18530 | 155 | 0.393808842 | GALNT10 | polypeptide N-acetylgalactosaminyltransferase 10 [Source:HGNC Symbol;Acc:HGNC:19873] |
| ENSG00000165355 | HGLibA_17074 | 155 | 0.393808842 | FBXO33 | F-box protein 33 [Source:HGNC Symbol;Acc:HGNC:19833] |
| ENSG00000165376 | HGLibA_09877 | 155 | 0.393808842 | CLDN2 | claudin 2 [Source:HGNC Symbol;Acc:HGNC:2041] |
| ENSG00000166822 | HGLibA_50054 | 155 | 0.393808842 | TMEM170A | transmembrane protein 170A [Source:HGNC Symbol;Acc:HGNC:29577] |
| ENSG00000167165 | HGLibA_52890 | 155 | 0.393808842 | UGT1A6 | UDP glucuronosyltransferase family 1 member A6 [Source:HGNC Symbol;Acc:HGNC:12538] |

| | | | | | |
|---|---|---|---|---|---|
| ENSG00000167641 | HGLibA_38035 | 155 | 0.393808842 | PPP1R14A | protein phosphatase 1 regulatory inhibitor subunit 14A [Source:HGNC Symbol;Acc:HGNC:14871] |
| ENSG00000167880 | HGLibA_15700 | 155 | 0.393808842 | EVPL | envoplakin [Source:HGNC Symbol;Acc:HGNC:3503] |
| ENSG00000171603 | HGLibA_10135 | 155 | 0.393808842 | CLSTN1 | calsyntenin 1 [Source:HGNC Symbol;Acc:HGNC:17447] |
| ENSG00000172057 | HGLibA_34631 | 155 | 0.393808842 | ORMDL3 | ORMDL sphingolipid biosynthesis regulator 3 [Source:HGNC Symbol;Acc:HGNC:16038] |
| ENSG00000173302 | HGLibA_19922 | 155 | 0.393808842 | GPR148 | G protein-coupled receptor 148 [Source:HGNC Symbol;Acc:HGNC:23623] |
| ENSG00000174851 | HGLibA_54665 | 155 | 0.393808842 | YIF1A | membrane trafficking protein [Source:HGNC Symbol;Acc:HGNC:16688]" |
| ENSG00000177000 | HGLibA_30388 | 155 | 0.393808842 | MTHFR | methylenetetrahydrofolate reductase [Source:HGNC Symbol;Acc:HGNC:7436] |
| ENSG00000179021 | HGLibA_06241 | 155 | 0.393808842 | C3orf38 | chromosome 3 open reading frame 38 [Source:HGNC Symbol;Acc:HGNC:28384] |
| ENSG00000179299 | HGLibA_32797 | 155 | 0.393808842 | NSUN7 | NOP2/Sun RNA methyltransferase family member 7 [Source:HGNC Symbol;Acc:HGNC:25857] |
| ENSG00000179981 | HGLibA_51861 | 155 | 0.393808842 | TSHZ1 | teashirt zinc finger homeobox 1 [Source:HGNC Symbol;Acc:HGNC:10669] |
| ENSG00000180901 | HGLibA_24443 | 155 | 0.393808842 | KCTD2 | potassium channel tetramerization domain containing 2 [Source:HGNC Symbol;Acc:HGNC:21294] |
| ENSG00000181467 | HGLibA_40239 | 155 | 0.393808842 | RAP2B | member of RAS oncogene family [Source:HGNC Symbol;Acc:HGNC:9862]" |
| ENSG00000181585 | HGLibA_50581 | 155 | 0.393808842 | TMIE | transmembrane inner ear [Source:HGNC Symbol;Acc:HGNC:30800] |
| ENSG00000184276 | HGLibA_12843 | 155 | 0.393808842 | DEFB108B | defensin beta 108B [Source:HGNC Symbol;Acc:HGNC:29966] |
| ENSG00000184634 | HGLibA_28741 | 155 | 0.393808842 | MED12 | mediator complex subunit 12 [Source:HGNC Symbol;Acc:HGNC:11957] |
| ENSG00000185808 | HGLibA_36577 | 155 | 0.393808842 | PIGP | phosphatidylinositol glycan anchor biosynthesis class P [Source:HGNC Symbol;Acc:HGNC:3046] |
| ENSG00000185825 | HGLibA_04243 | 155 | 0.393808842 | BCAP31 | B cell receptor associated protein 31 [Source:HGNC Symbol;Acc:HGNC:16695] |
| ENSG00000185899 | HGLibA_48422 | 155 | 0.393808842 | TAS2R60 | taste 2 receptor member 60 [Source:HGNC Symbol;Acc:HGNC:20639] |
| ENSG00000186517 | HGLibA_02753 | 155 | 0.393808842 | ARHGAP30 | Rho GTPase activating protein 30 [Source:HGNC Symbol;Acc:HGNC:27414] |
| ENSG00000187210 | HGLibA_18808 | 155 | 0.393808842 | GCNT1 | core 2 [Source:HGNC Symbol;Acc:HGNC:4203]" |
| ENSG00000189091 | HGLibA_43708 | 155 | 0.393808842 | SF3B3 | splicing factor 3b subunit 3 [Source:HGNC Symbol;Acc:HGNC:10770] |
| ENSG00000196071 | HGLibA_33819 | 155 | 0.393808842 | OR2L13 | olfactory receptor family 2 subfamily L member 13 [Source:HGNC Symbol;Acc:HGNC:19578] |
| ENSG00000196387 | HGLibA_55482 | 155 | 0.393808842 | ZNF140 | zinc finger protein 140 [Source:HGNC Symbol;Acc:HGNC:12925] |
| ENSG00000196420 | HGLibA_42561 | 155 | 0.393808842 | S100A5 | S100 calcium binding protein A5 [Source:HGNC Symbol;Acc:HGNC:10495] |
| ENSG00000196754 | HGLibA_42551 | 155 | 0.393808842 | S100A2 | S100 calcium binding protein A2 [Source:HGNC Symbol;Acc:HGNC:10492] |
| ENSG00000197714 | HGLibA_56035 | 155 | 0.393808842 | ZNF460 | zinc finger protein 460 [Source:HGNC Symbol;Acc:HGNC:21628] |
| ENSG00000204165 | HGLibA_11958 | 155 | 0.393808842 | CXorf65 | chromosome X open reading frame 65 [Source:HGNC Symbol;Acc:HGNC:33713] |
| ENSG00000205116 | HGLibA_50540 | 155 | 0.393808842 | TMEM88B | transmembrane protein 88B [Source:HGNC Symbol;Acc:HGNC:37099] |
| ENSG00000205403 | HGLibA_09278 | 155 | 0.393808842 | CFI | complement factor I [Source:HGNC Symbol;Acc:HGNC:5394] |
| ENSG00000214435 | HGLibA_03183 | 155 | 0.393808842 | AS3MT | arsenite methyltransferase [Source:HGNC Symbol;Acc:HGNC:17452] |
| ENSG00000224659 | HGLibA_18461 | 155 | 0.393808842 | GAGE12J | G antigen 12J [Source:HGNC Symbol;Acc:HGNC:17778] |
| ENSG00000225828 | HGLibA_16540 | 155 | 0.393808842 | FAM229A | family with sequence similarity 229 member A [Source:HGNC Symbol;Acc:HGNC:44652] |
| ENSG00000226479 | HGLibA_50124 | 155 | 0.393808842 | TMEM185B | transmembrane protein 185B [Source:HGNC Symbol;Acc:HGNC:18896] |
| ENSG00000228083 | HGLibA_22666 | 155 | 0.393808842 | IFNA14 | interferon alpha 14 [Source:HGNC Symbol;Acc:HGNC:5420] |
| ENSG00000237693 | HGLibA_23614 | 155 | 0.393808842 | IRGM | immunity related GTPase M [Source:HGNC Symbol;Acc:HGNC:29597] |
| ENSG00000240021 | HGLibA_49100 | 155 | 0.393808842 | TEX35 | testis expressed 35 [Source:HGNC Symbol;Acc:HGNC:25366] |
| ENSG00000249459 | HGLibA_55754 | 155 | 0.393808842 | ZNF286B | zinc finger protein 286B [Source:HGNC Symbol;Acc:HGNC:33241] |
| ENSG00000255713 | HGLibA_33973 | 155 | 0.393808842 | OR4D2 | olfactory receptor family 4 subfamily D member 2 [Source:HGNC Symbol;Acc:HGNC:8294] |
| ENSG00000256980 | HGLibA_24559 | 155 | 0.393808842 | KHDC1L | KH domain containing 1 like [Source:HGNC Symbol;Acc:HGNC:37274] |
| GAGE7 | HGLibA_18487 | 155 | 0.393808842 | GAGE7 | - |
| GUCY1A3 | HGLibA_20654 | 155 | 0.393808842 | GUCY1A3 | - |
| hsa-mir-3196 | HGLibA_58881 | 155 | 0.393808842 | hsa-mir-3196 | - |
| hsa-mir-3689d-2 | HGLibA_59331 | 155 | 0.393808842 | hsa-mir-3689d-2 | - |
| hsa-mir-4491 | HGLibA_60381 | 155 | 0.393808842 | hsa-mir-4491 | - |
| hsa-mir-4782 | HGLibA_61234 | 155 | 0.393808842 | hsa-mir-4782 | - |
| hsa-mir-518d | HGLibA_61669 | 155 | 0.393808842 | hsa-mir-518d | - |
| hsa-mir-532 | HGLibA_61806 | 155 | 0.393808842 | hsa-mir-532 | - |
| hsa-mir-5700 | HGLibA_62337 | 155 | 0.393808842 | hsa-mir-5700 | - |
| hsa-mir-614 | HGLibA_62690 | 155 | 0.393808842 | hsa-mir-614 | - |
| hsa-mir-657 | HGLibA_62962 | 155 | 0.393808842 | hsa-mir-657 | - |
| hsa-mir-676 | HGLibA_63198 | 155 | 0.393808842 | hsa-mir-676 | - |
| hsa-mir-7160 | HGLibA_63868 | 155 | 0.393808842 | hsa-mir-7160 | - |
| hsa-mir-7974 | HGLibA_64025 | 155 | 0.393808842 | hsa-mir-7974 | - |
| LOC100507462 | HGLibA_26871 | 155 | 0.393808842 | LOC100507462 | - |
| SMCR9 | HGLibA_45686 | 155 | 0.393808842 | SMCR9 | - |
| ZFP112 | HGLibA_55185 | 155 | 0.393808842 | ZFP112 | - |
| ADPRHL2 | HGLibA_01032 | 154 | 0.383857805 | ADPRHL2 | - |
| ATP5O | HGLibA_03686 | 154 | 0.383857805 | ATP5O | - |
| ENSG00000008735 | HGLibA_28290 | 154 | 0.383857805 | MAPK8IP2 | mitogen-activated protein kinase 8 interacting protein 2 [Source:HGNC Symbol;Acc:HGNC:6883] |
| ENSG00000013275 | HGLibA_39140 | 154 | 0.383857805 | PSMC4 | ATPase 4 [Source:HGNC Symbol;Acc:HGNC:9551]" |
| ENSG00000023516 | HGLibA_01409 | 154 | 0.383857805 | AKAP11 | A-kinase anchoring protein 11 [Source:HGNC Symbol;Acc:HGNC:369] |
| ENSG00000025039 | HGLibA_42233 | 154 | 0.383857805 | RRAGD | Ras related GTP binding D [Source:HGNC Symbol;Acc:HGNC:19903] |
| ENSG00000031081 | HGLibA_02755 | 154 | 0.383857805 | ARHGAP31 | Rho GTPase activating protein 31 [Source:HGNC Symbol;Acc:HGNC:29216] |
| ENSG00000060982 | HGLibA_04263 | 154 | 0.383857805 | BCAT1 | branched chain amino acid transaminase 1 [Source:HGNC Symbol;Acc:HGNC:976] |
| ENSG00000061936 | HGLibA_43747 | 154 | 0.383857805 | SFSWAP | splicing factor SWAP [Source:HGNC Symbol;Acc:HGNC:10790] |
| ENSG00000064545 | HGLibA_50025 | 154 | 0.383857805 | TMEM161A | transmembrane protein 161A [Source:HGNC Symbol;Acc:HGNC:26020] |
| ENSG00000065154 | HGLibA_33211 | 154 | 0.383857805 | OAT | ornithine aminotransferase [Source:HGNC Symbol;Acc:HGNC:8091] |
| ENSG00000070371 | HGLibA_10152 | 154 | 0.383857805 | CLTCL1 | clathrin heavy chain like 1 [Source:HGNC Symbol;Acc:HGNC:2093] |
| ENSG00000076321 | HGLibA_25150 | 154 | 0.383857805 | KLHL20 | kelch like family member 20 [Source:HGNC Symbol;Acc:HGNC:25056] |
| ENSG00000079263 | HGLibA_46291 | 154 | 0.383857805 | SP140 | SP140 nuclear body protein [Source:HGNC Symbol;Acc:HGNC:17133] |
| ENSG00000090020 | HGLibA_45400 | 154 | 0.383857805 | SLC9A1 | solute carrier family 9 member A1 [Source:HGNC Symbol;Acc:HGNC:11071] |
| ENSG00000092009 | HGLibA_10177 | 154 | 0.383857805 | CMA1 | chymase 1 [Source:HGNC Symbol;Acc:HGNC:2097] |
| ENSG00000100266 | HGLibA_34963 | 154 | 0.383857805 | PACSIN2 | protein kinase C and casein kinase substrate in neurons 2 [Source:HGNC Symbol;Acc:HGNC:8571] |
| ENSG00000100568 | HGLibA_53889 | 154 | 0.383857805 | VTI1B | vesicle transport through interaction with t-SNAREs 1B [Source:HGNC Symbol;Acc:HGNC:17793] |
| ENSG00000100813 | HGLibA_00439 | 154 | 0.383857805 | ACIN1 | apoptotic chromatin condensation inducer 1 [Source:HGNC Symbol;Acc:HGNC:17066] |
| ENSG00000101049 | HGLibA_43826 | 154 | 0.383857805 | SGK2 | serine/threonine kinase 2 [Source:HGNC Symbol;Acc:HGNC:13900]" |
| ENSG00000101445 | HGLibA_38055 | 154 | 0.383857805 | PPP1R16B | protein phosphatase 1 regulatory subunit 16B [Source:HGNC Symbol;Acc:HGNC:15850] |
| ENSG00000102384 | HGLibA_09059 | 154 | 0.383857805 | CENPI | centromere protein I [Source:HGNC Symbol;Acc:HGNC:3968] |
| ENSG00000102539 | HGLibA_29429 | 154 | 0.383857805 | MLNR | motilin receptor [Source:HGNC Symbol;Acc:HGNC:4495] |
| ENSG00000102543 | HGLibA_08502 | 154 | 0.383857805 | CDADC1 | cytidine and dCMP deaminase domain containing 1 [Source:HGNC Symbol;Acc:HGNC:20299] |
| ENSG00000103647 | HGLibA_10784 | 154 | 0.383857805 | CORO2B | coronin 2B [Source:HGNC Symbol;Acc:HGNC:2256] |
| ENSG00000104313 | HGLibA_15817 | 154 | 0.383857805 | EYA1 | EYA transcriptional coactivator and phosphatase 1 [Source:HGNC Symbol;Acc:HGNC:3519] |
| ENSG00000104881 | HGLibA_38032 | 154 | 0.383857805 | PPP1R13L | protein phosphatase 1 regulatory subunit 13 like [Source:HGNC Symbol;Acc:HGNC:18838] |
| ENSG00000105255 | HGLibA_18076 | 154 | 0.383857805 | FSD1 | fibronectin type III and SPRY domain containing 1 [Source:HGNC Symbol;Acc:HGNC:13745] |
| ENSG00000105698 | HGLibA_53171 | 154 | 0.383857805 | USF2 | c-fos interacting [Source:HGNC Symbol;Acc:HGNC:12594]" |
| ENSG00000110169 | HGLibA_22067 | 154 | 0.383857805 | HPX | hemopexin [Source:HGNC Symbol;Acc:HGNC:5171] |
| ENSG00000110244 | HGLibA_02458 | 154 | 0.383857805 | APOA4 | apolipoprotein A4 [Source:HGNC Symbol;Acc:HGNC:602] |
| ENSG00000110777 | HGLibA_37753 | 154 | 0.383857805 | POU2AF1 | POU class 2 associating factor 1 [Source:HGNC Symbol;Acc:HGNC:9211] |
| ENSG00000111801 | HGLibA_04975 | 154 | 0.383857805 | BTN3A3 | butyrophilin subfamily 3 member A3 [Source:HGNC Symbol;Acc:HGNC:1140] |
| ENSG00000112541 | HGLibA_35759 | 154 | 0.383857805 | PDE10A | phosphodiesterase 10A [Source:HGNC Symbol;Acc:HGNC:8772] |
| ENSG00000115415 | HGLibA_47381 | 154 | 0.383857805 | STAT1 | signal transducer and activator of transcription 1 [Source:HGNC Symbol;Acc:HGNC:11362] |
| ENSG00000115641 | HGLibA_17482 | 154 | 0.383857805 | FHL2 | four and a half LIM domains 2 [Source:HGNC Symbol;Acc:HGNC:3703] |
| ENSG00000117228 | HGLibA_18729 | 154 | 0.383857805 | GBP1 | guanylate binding protein 1 [Source:HGNC Symbol;Acc:HGNC:4182] |
| ENSG00000117501 | HGLibA_29883 | 154 | 0.383857805 | MROH9 | maestro heat like repeat family member 9 [Source:HGNC Symbol;Acc:HGNC:26287] |
| ENSG00000118454 | HGLibA_02005 | 154 | 0.383857805 | ANKRD13C | ankyrin repeat domain 13C [Source:HGNC Symbol;Acc:HGNC:25374] |
| ENSG00000118900 | HGLibA_52720 | 154 | 0.383857805 | UBN1 | ubinuclein 1 [Source:HGNC Symbol;Acc:HGNC:12506] |
| ENSG00000120055 | HGLibA_05108 | 154 | 0.383857805 | C10orf95 | chromosome 10 open reading frame 95 [Source:HGNC Symbol;Acc:HGNC:25880] |
| ENSG00000121297 | HGLibA_51868 | 154 | 0.383857805 | TSHZ3 | teashirt zinc finger homeobox 3 [Source:HGNC Symbol;Acc:HGNC:30700] |
| ENSG00000122482 | HGLibA_56447 | 154 | 0.383857805 | ZNF644 | zinc finger protein 644 [Source:HGNC Symbol;Acc:HGNC:29222] |
| ENSG00000122705 | HGLibA_10143 | 154 | 0.383857805 | CLTA | clathrin light chain A [Source:HGNC Symbol;Acc:HGNC:2090] |
| ENSG00000124198 | HGLibA_02673 | 154 | 0.383857805 | ARFGEF2 | ADP ribosylation factor guanine nucleotide exchange factor 2 [Source:HGNC Symbol;Acc:HGNC:15853] |
| ENSG00000130706 | HGLibA_01070 | 154 | 0.383857805 | ADRM1 | adhesion regulating molecule 1 [Source:HGNC Symbol;Acc:HGNC:15759] |
| ENSG00000132768 | HGLibA_13838 | 154 | 0.383857805 | DPH2 | DPH2 homolog [Source:HGNC Symbol;Acc:HGNC:3004] |
| ENSG00000134245 | HGLibA_54361 | 154 | 0.383857805 | WNT2B | Wnt family member 2B [Source:HGNC Symbol;Acc:HGNC:12781] |
| ENSG00000135124 | HGLibA_34856 | 154 | 0.383857805 | P2RX4 | purinergic receptor P2X 4 [Source:HGNC Symbol;Acc:HGNC:8535] |
| ENSG00000136872 | HGLibA_01594 | 154 | 0.383857805 | ALDOB | fructose-bisphosphate B [Source:HGNC Symbol;Acc:HGNC:417]" |
| ENSG00000136908 | HGLibA_13860 | 154 | 0.383857805 | DPM2 | regulatory [Source:HGNC Symbol;Acc:HGNC:3006]" |
| ENSG00000137070 | HGLibA_22957 | 154 | 0.383857805 | IL11RA | interleukin 11 receptor subunit alpha [Source:HGNC Symbol;Acc:HGNC:5967] |
| ENSG00000139428 | HGLibA_29452 | 154 | 0.383857805 | MMAB | metabolism of cobalamin associated B [Source:HGNC Symbol;Acc:HGNC:19331] |
| ENSG00000139625 | HGLibA_28170 | 154 | 0.383857805 | MAP3K12 | mitogen-activated protein kinase kinase kinase 12 [Source:HGNC Symbol;Acc:HGNC:6851] |
| ENSG00000140396 | HGLibA_31338 | 154 | 0.383857805 | NCOA2 | nuclear receptor coactivator 2 [Source:HGNC Symbol;Acc:HGNC:7669] |
| ENSG00000143867 | HGLibA_34698 | 154 | 0.383857805 | OSR1 | odd-skipped related transciption factor 1 [Source:HGNC Symbol;Acc:HGNC:8111] |
| ENSG00000147383 | HGLibA_32748 | 154 | 0.383857805 | NSDHL | NAD(P) dependent steroid dehydrogenase-like [Source:HGNC Symbol;Acc:HGNC:13398] |
| ENSG00000149948 | HGLibA_21687 | 154 | 0.383857805 | HMGA2 | high mobility group AT-hook 2 [Source:HGNC Symbol;Acc:HGNC:5009] |
| ENSG00000149968 | HGLibA_29533 | 154 | 0.383857805 | MMP3 | matrix metallopeptidase 3 [Source:HGNC Symbol;Acc:HGNC:7173] |
| ENSG00000153395 | HGLibA_27070 | 154 | 0.383857805 | LPCAT1 | lysophosphatidylcholine acyltransferase 1 [Source:HGNC Symbol;Acc:HGNC:25718] |
| ENSG00000157193 | HGLibA_27232 | 154 | 0.383857805 | LRP8 | LDL receptor related protein 8 [Source:HGNC Symbol;Acc:HGNC:6700] |
| ENSG00000158711 | HGLibA_14919 | 154 | 0.383857805 | ELK4 | ETS transcription factor [Source:HGNC Symbol;Acc:HGNC:3326]" |
| ENSG00000159455 | HGLibA_26121 | 154 | 0.383857805 | LCE2B | late cornified envelope 2B [Source:HGNC Symbol;Acc:HGNC:16610] |
| ENSG00000160678 | HGLibA_42529 | 154 | 0.383857805 | S100A1 | S100 calcium binding protein A1 [Source:HGNC Symbol;Acc:HGNC:10486] |
| ENSG00000160801 | HGLibA_39367 | 154 | 0.383857805 | PTH1R | parathyroid hormone 1 receptor [Source:HGNC Symbol;Acc:HGNC:9608] |
| ENSG00000161395 | HGLibA_36214 | 154 | 0.383857805 | PGAP3 | post-GPI attachment to proteins 3 [Source:HGNC Symbol;Acc:HGNC:23719] |
| ENSG00000162344 | HGLibA_17387 | 154 | 0.383857805 | FGF19 | fibroblast growth factor 19 [Source:HGNC Symbol;Acc:HGNC:3675] |
| ENSG00000162927 | HGLibA_39598 | 154 | 0.383857805 | PUS10 | pseudouridine synthase 10 [Source:HGNC Symbol;Acc:HGNC:26505] |
| ENSG00000163485 | HGLibA_01011 | 154 | 0.383857805 | ADORA1 | adenosine A1 receptor [Source:HGNC Symbol;Acc:HGNC:262] |
| ENSG00000163606 | HGLibA_08285 | 154 | 0.383857805 | CD200R1 | CD200 receptor 1 [Source:HGNC Symbol;Acc:HGNC:24235] |
| ENSG00000163820 | HGLibA_18251 | 154 | 0.383857805 | FYCO1 | FYVE and coiled-coil domain containing 1 [Source:HGNC Symbol;Acc:HGNC:14673] |
| ENSG00000163960 | HGLibA_52795 | 154 | 0.383857805 | UBXN7 | UBX domain protein 7 [Source:HGNC Symbol;Acc:HGNC:29119] |
| ENSG00000166153 | HGLibA_12981 | 154 | 0.383857805 | DEPDC4 | DEP domain containing 4 [Source:HGNC Symbol;Acc:HGNC:22952] |
| ENSG00000166828 | HGLibA_43022 | 154 | 0.383857805 | SCNN1G | sodium channel epithelial 1 gamma subunit [Source:HGNC Symbol;Acc:HGNC:10602] |
| ENSG00000167196 | HGLibA_17048 | 154 | 0.383857805 | FBXO22 | F-box protein 22 [Source:HGNC Symbol;Acc:HGNC:13593] |
| ENSG00000168398 | HGLibA_04382 | 154 | 0.383857805 | BDKRB2 | bradykinin receptor B2 [Source:HGNC Symbol;Acc:HGNC:1030] |
| ENSG00000168438 | HGLibA_08552 | 154 | 0.383857805 | CDC40 | cell division cycle 40 [Source:HGNC Symbol;Acc:HGNC:17350] |
| ENSG00000170128 | HGLibA_20004 | 154 | 0.383857805 | GPR25 | G protein-coupled receptor 25 [Source:HGNC Symbol;Acc:HGNC:4480] |
| ENSG00000170426 | HGLibA_43168 | 154 | 0.383857805 | SDR9C7 | short chain dehydrogenase/reductase family 9C member 7 [Source:HGNC Symbol;Acc:HGNC:29958] |

| | | | | | |
|---|---|---|---|---|---|
| ENSG00000171435 | HGLibA_25866 | 154 | 0.383857805 | KSR2 | kinase suppressor of ras 2 [Source:HGNC Symbol;Acc:HGNC:18610] |
| ENSG00000171561 | HGLibA_33762 | 154 | 0.383857805 | OR2AT4 | olfactory receptor family 2 subfamily AT member 4 [Source:HGNC Symbol;Acc:HGNC:19620] |
| ENSG00000174721 | HGLibA_17431 | 154 | 0.383857805 | FGFBP3 | fibroblast growth factor binding protein 3 [Source:HGNC Symbol;Acc:HGNC:23428] |
| ENSG00000176697 | HGLibA_04387 | 154 | 0.383857805 | BDNF | brain derived neurotrophic factor [Source:HGNC Symbol;Acc:HGNC:1033] |
| ENSG00000177189 | HGLibA_42164 | 154 | 0.383857805 | RPS6KA3 | ribosomal protein S6 kinase A3 [Source:HGNC Symbol;Acc:HGNC:10432] |
| ENSG00000180917 | HGLibA_10237 | 154 | 0.383857805 | CMTR2 | cap methyltransferase 2 [Source:HGNC Symbol;Acc:HGNC:25635] |
| ENSG00000183647 | HGLibA_56175 | 154 | 0.383857805 | ZNF530 | zinc finger protein 530 [Source:HGNC Symbol;Acc:HGNC:29297] |
| ENSG00000184144 | HGLibA_10403 | 154 | 0.383857805 | CNTN2 | contactin 2 [Source:HGNC Symbol;Acc:HGNC:2172] |
| ENSG00000184408 | HGLibA_24178 | 154 | 0.383857805 | KCND2 | potassium voltage-gated channel subfamily D member 2 [Source:HGNC Symbol;Acc:HGNC:6238] |
| ENSG00000184995 | HGLibA_22705 | 154 | 0.383857805 | IFNE | interferon epsilon [Source:HGNC Symbol;Acc:HGNC:18163] |
| ENSG00000185000 | HGLibA_13033 | 154 | 0.383857805 | DGAT1 | diacylglycerol O-acyltransferase 1 [Source:HGNC Symbol;Acc:HGNC:2843] |
| ENSG00000186334 | HGLibA_45036 | 154 | 0.383857805 | SLC36A3 | solute carrier family 36 member 3 [Source:HGNC Symbol;Acc:HGNC:19659] |
| ENSG00000186526 | HGLibA_12195 | 154 | 0.383857805 | CYP4F8 | cytochrome P450 family 4 subfamily F member 8 [Source:HGNC Symbol;Acc:HGNC:2648] |
| ENSG00000187792 | HGLibA_56571 | 154 | 0.383857805 | ZNF70 | zinc finger protein 70 [Source:HGNC Symbol;Acc:HGNC:13140] |
| ENSG00000188257 | HGLibA_36886 | 154 | 0.383857805 | PLA2G2A | phospholipase A2 group IIA [Source:HGNC Symbol;Acc:HGNC:9031] |
| ENSG00000196345 | HGLibA_55359 | 154 | 0.383857805 | ZKSCAN7 | zinc finger with KRAB and SCAN domains 7 [Source:HGNC Symbol;Acc:HGNC:12955] |
| ENSG00000196844 | HGLibA_35275 | 154 | 0.383857805 | PATE2 | prostate and testis expressed 2 [Source:HGNC Symbol;Acc:HGNC:32249] |
| ENSG00000197213 | HGLibA_57030 | 154 | 0.383857805 | ZSCAN5B | zinc finger and SCAN domain containing 5B [Source:HGNC Symbol;Acc:HGNC:34246] |
| ENSG00000198093 | HGLibA_56458 | 154 | 0.383857805 | ZNF649 | zinc finger protein 649 [Source:HGNC Symbol;Acc:HGNC:25741] |
| ENSG00000198517 | HGLibA_27884 | 154 | 0.383857805 | MAFK | MAF bZIP transcription factor K [Source:HGNC Symbol;Acc:HGNC:6782] |
| ENSG00000198846 | HGLibA_51036 | 154 | 0.383857805 | TOX | thymocyte selection associated high mobility group box [Source:HGNC Symbol;Acc:HGNC:18988] |
| ENSG00000198934 | HGLibA_27980 | 154 | 0.383857805 | MAGEE1 | MAGE family member E1 [Source:HGNC Symbol;Acc:HGNC:24934] |
| ENSG00000203870 | HGLibA_45772 | 154 | 0.383857805 | SMIM9 | small integral membrane protein 9 [Source:HGNC Symbol;Acc:HGNC:41915] |
| ENSG00000204388 | HGLibA_22273 | 154 | 0.383857805 | HSPA1B | heat shock protein family A (Hsp70) member 1B [Source:HGNC Symbol;Acc:HGNC:5233] |
| ENSG00000205420 | HGLibA_25512 | 154 | 0.383857805 | KRT6A | keratin 6A [Source:HGNC Symbol;Acc:HGNC:6443] |
| ENSG00000205549 | HGLibA_06779 | 154 | 0.383857805 | C9orf92 | chromosome 9 open reading frame 92 [Source:HGNC Symbol;Acc:HGNC:19054] |
| ENSG00000205810 | HGLibA_25290 | 154 | 0.383857805 | KLRC3 | killer cell lectin like receptor C3 [Source:HGNC Symbol;Acc:HGNC:6376] |
| ENSG00000206432 | HGLibA_50178 | 154 | 0.383857805 | TMEM200C | transmembrane protein 200C [Source:HGNC Symbol;Acc:HGNC:37208] |
| ENSG00000213139 | HGLibA_11284 | 154 | 0.383857805 | CRYGS | crystallin gamma S [Source:HGNC Symbol;Acc:HGNC:2417] |
| ENSG00000213139 | HGLibA_11285 | 154 | 0.383857805 | CRYGS | crystallin gamma S [Source:HGNC Symbol;Acc:HGNC:2417] |
| ENSG00000236311 | HGLibA_49674 | 154 | 0.383857805 | TLX1NB | TLX1 neighbor [Source:HGNC Symbol;Acc:HGNC:37183] |
| ENSG00000255012 | HGLibA_34328 | 154 | 0.383857805 | OR5M1 | olfactory receptor family 5 subfamily M member 1 [Source:HGNC Symbol;Acc:HGNC:8352] |
| ENSG00000255181 | HGLibA_07673 | 154 | 0.383857805 | CCDC166 | coiled-coil domain containing 166 [Source:HGNC Symbol;Acc:HGNC:41910] |
| ENSG00000256463 | HGLibA_42654 | 154 | 0.383857805 | SALL3 | spalt like transcription factor 3 [Source:HGNC Symbol;Acc:HGNC:10527] |
| ENSG00000264424 | HGLibA_30726 | 154 | 0.383857805 | MYH4 | myosin heavy chain 4 [Source:HGNC Symbol;Acc:HGNC:7574] |
| ENSG00000278129 | HGLibA_56778 | 154 | 0.383857805 | ZNF8 | zinc finger protein 8 [Source:HGNC Symbol;Acc:HGNC:13154] |
| ENSG00000288611 | HGLibA_32413 | 154 | 0.383857805 | NPBWR1 | - |
| GLTSCR2 | HGLibA_19358 | 154 | 0.383857805 | GLTSCR2 | - |
| GRASP | HGLibA_20220 | 154 | 0.383857805 | GRASP | - |
| hsa-mir-1228 | HGLibA_57312 | 154 | 0.383857805 | hsa-mir-1228 | - |
| hsa-mir-1302-4 | HGLibA_57703 | 154 | 0.383857805 | hsa-mir-1302-4 | - |
| hsa-mir-1302-4 | HGLibA_57702 | 154 | 0.383857805 | hsa-mir-1302-4 | - |
| hsa-mir-151a | HGLibA_57894 | 154 | 0.383857805 | hsa-mir-151a | - |
| hsa-mir-30d | HGLibA_58486 | 154 | 0.383857805 | hsa-mir-30d | - |
| hsa-mir-4317 | HGLibA_59987 | 154 | 0.383857805 | hsa-mir-4317 | - |
| hsa-mir-502 | HGLibA_61465 | 154 | 0.383857805 | hsa-mir-502 | - |
| hsa-mir-5571 | HGLibA_62149 | 154 | 0.383857805 | hsa-mir-5571 | - |
| hsa-mir-575 | HGLibA_62393 | 154 | 0.383857805 | hsa-mir-575 | - |
| hsa-mir-7156 | HGLibA_63851 | 154 | 0.383857805 | hsa-mir-7156 | - |
| hsa-mir-8060 | HGLibA_64077 | 154 | 0.383857805 | hsa-mir-8060 | - |
| hsa-mir-8084 | HGLibA_64177 | 154 | 0.383857805 | hsa-mir-8084 | - |
| WBSCR22 | HGLibA_53996 | 154 | 0.383857805 | WBSCR22 | - |
| BAGE | HGLibA_04092 | 153 | 0.373906767 | BAGE | - |
| ENSG00000004866 | HGLibA_47281 | 153 | 0.373906767 | ST7 | suppression of tumorigenicity 7 [Source:HGNC Symbol;Acc:HGNC:11351] |
| ENSG00000007216 | HGLibA_44388 | 153 | 0.373906767 | SLC13A2 | solute carrier family 13 member 2 [Source:HGNC Symbol;Acc:HGNC:10917] |
| ENSG00000064655 | HGLibA_15821 | 153 | 0.373906767 | EYA2 | EYA transcriptional coactivator and phosphatase 2 [Source:HGNC Symbol;Acc:HGNC:3520] |
| ENSG00000072042 | HGLibA_40734 | 153 | 0.373906767 | RDH11 | retinol dehydrogenase 11 [Source:HGNC Symbol;Acc:HGNC:17964] |
| ENSG00000072201 | HGLibA_26743 | 153 | 0.373906767 | LNX1 | ligand of numb-protein X 1 [Source:HGNC Symbol;Acc:HGNC:6657] |
| ENSG00000075429 | HGLibA_06941 | 153 | 0.373906767 | CACNG5 | calcium voltage-gated channel auxiliary subunit gamma 5 [Source:HGNC Symbol;Acc:HGNC:1409] |
| ENSG00000095110 | HGLibA_33149 | 153 | 0.373906767 | NXPE1 | neurexophilin and PC-esterase domain family member 1 [Source:HGNC Symbol;Acc:HGNC:28527] |
| ENSG00000100284 | HGLibA_50945 | 153 | 0.373906767 | TOM1 | target of myb1 membrane trafficking protein [Source:HGNC Symbol;Acc:HGNC:11982] |
| ENSG00000100418 | HGLibA_13008 | 153 | 0.373906767 | DESI1 | desumoylating isopeptidase 1 [Source:HGNC Symbol;Acc:HGNC:24577] |
| ENSG00000100604 | HGLibA_09431 | 153 | 0.373906767 | CHGA | chromogranin A [Source:HGNC Symbol;Acc:HGNC:1929] |
| ENSG00000100911 | HGLibA_39192 | 153 | 0.373906767 | PSME2 | proteasome activator subunit 2 [Source:HGNC Symbol;Acc:HGNC:9569] |
| ENSG00000102317 | HGLibA_40529 | 153 | 0.373906767 | RBM3 | RNA binding motif protein 3 [Source:HGNC Symbol;Acc:HGNC:9900] |
| ENSG00000103257 | HGLibA_45369 | 153 | 0.373906767 | SLC7A5 | solute carrier family 7 member 5 [Source:HGNC Symbol;Acc:HGNC:11063] |
| ENSG00000104723 | HGLibA_52347 | 153 | 0.373906767 | TUSC3 | tumor suppressor candidate 3 [Source:HGNC Symbol;Acc:HGNC:30242] |
| ENSG00000105383 | HGLibA_08353 | 153 | 0.373906767 | CD33 | CD33 molecule [Source:HGNC Symbol;Acc:HGNC:1659] |
| ENSG00000105438 | HGLibA_24480 | 153 | 0.373906767 | KDELR1 | KDEL endoplasmic reticulum protein retention receptor 1 [Source:HGNC Symbol;Acc:HGNC:6304] |
| ENSG00000105722 | HGLibA_15461 | 153 | 0.373906767 | ERF | ETS2 repressor factor [Source:HGNC Symbol;Acc:HGNC:3444] |
| ENSG00000106443 | HGLibA_36345 | 153 | 0.373906767 | PHF14 | PHD finger protein 14 [Source:HGNC Symbol;Acc:HGNC:22203] |
| ENSG00000106565 | HGLibA_50076 | 153 | 0.373906767 | TMEM176B | transmembrane protein 176B [Source:HGNC Symbol;Acc:HGNC:29596] |
| ENSG00000108784 | HGLibA_31068 | 153 | 0.373906767 | NAGLU | N-acetyl-alpha-glucosaminidase [Source:HGNC Symbol;Acc:HGNC:7632] |
| ENSG00000109819 | HGLibA_37845 | 153 | 0.373906767 | PPARGC1A | PPARG coactivator 1 alpha [Source:HGNC Symbol;Acc:HGNC:9237] |
| ENSG00000111796 | HGLibA_25282 | 153 | 0.373906767 | KLRB1 | killer cell lectin like receptor B1 [Source:HGNC Symbol;Acc:HGNC:6373] |
| ENSG00000115361 | HGLibA_00366 | 153 | 0.373906767 | ACADL | acyl-CoA dehydrogenase long chain [Source:HGNC Symbol;Acc:HGNC:88] |
| ENSG00000116288 | HGLibA_35199 | 153 | 0.373906767 | PARK7 | Parkinsonism associated deglycase [Source:HGNC Symbol;Acc:HGNC:16369] |
| ENSG00000117480 | HGLibA_15900 | 153 | 0.373906767 | FAAH | fatty acid amide hydrolase [Source:HGNC Symbol;Acc:HGNC:3553] |
| ENSG00000118620 | HGLibA_55979 | 153 | 0.373906767 | ZNF430 | zinc finger protein 430 [Source:HGNC Symbol;Acc:HGNC:20808] |
| ENSG00000118849 | HGLibA_40281 | 153 | 0.373906767 | RARRES1 | retinoic acid receptor responder 1 [Source:HGNC Symbol;Acc:HGNC:9867] |
| ENSG00000120265 | HGLibA_35624 | 153 | 0.373906767 | PCMT1 | protein-L-isoaspartate (D-aspartate) O-methyltransferase [Source:HGNC Symbol;Acc:HGNC:8728] |
| ENSG00000121390 | HGLibA_39222 | 153 | 0.373906767 | PSPC1 | paraspeckle component 1 [Source:HGNC Symbol;Acc:HGNC:20320] |
| ENSG00000123178 | HGLibA_46857 | 153 | 0.373906767 | SPRYD7 | SPRY domain containing 7 [Source:HGNC Symbol;Acc:HGNC:14297] |
| ENSG00000124019 | HGLibA_16079 | 153 | 0.373906767 | FAM124B | family with sequence similarity 124 member B [Source:HGNC Symbol;Acc:HGNC:26224] |
| ENSG00000124151 | HGLibA_31341 | 153 | 0.373906767 | NCOA3 | nuclear receptor coactivator 3 [Source:HGNC Symbol;Acc:HGNC:7670] |
| ENSG00000124222 | HGLibA_47623 | 153 | 0.373906767 | STX16 | syntaxin 16 [Source:HGNC Symbol;Acc:HGNC:11431] |
| ENSG00000125968 | HGLibA_22525 | 153 | 0.373906767 | ID1 | HLH protein [Source:HGNC Symbol;Acc:HGNC:5360]" |
| ENSG00000126070 | HGLibA_01210 | 153 | 0.373906767 | AGO3 | RISC catalytic component [Source:HGNC Symbol;Acc:HGNC:18421]" |
| ENSG00000128602 | HGLibA_45790 | 153 | 0.373906767 | SMO | frizzled class receptor [Source:HGNC Symbol;Acc:HGNC:11119]" |
| ENSG00000129437 | HGLibA_25245 | 153 | 0.373906767 | KLK14 | kallikrein related peptidase 14 [Source:HGNC Symbol;Acc:HGNC:6362] |
| ENSG00000130950 | HGLibA_33107 | 153 | 0.373906767 | NUTM2F | NUT family member 2F [Source:HGNC Symbol;Acc:HGNC:23450] |
| ENSG00000131398 | HGLibA_24169 | 153 | 0.373906767 | KCNC3 | potassium voltage-gated channel subfamily C member 3 [Source:HGNC Symbol;Acc:HGNC:6235] |
| ENSG00000131626 | HGLibA_37872 | 153 | 0.373906767 | PPFIA1 | PTPRF interacting protein alpha 1 [Source:HGNC Symbol;Acc:HGNC:9245] |
| ENSG00000132294 | HGLibA_14606 | 153 | 0.373906767 | EFR3A | EFR3 homolog A [Source:HGNC Symbol;Acc:HGNC:28970] |
| ENSG00000134864 | HGLibA_18988 | 153 | 0.373906767 | GGACT | gamma-glutamylamine cyclotransferase [Source:HGNC Symbol;Acc:HGNC:25100] |
| ENSG00000135655 | HGLibA_53208 | 153 | 0.373906767 | USP15 | ubiquitin specific peptidase 15 [Source:HGNC Symbol;Acc:HGNC:12613] |
| ENSG00000136542 | HGLibA_18563 | 153 | 0.373906767 | GALNT5 | polypeptide N-acetylgalactosaminyltransferase 5 [Source:HGNC Symbol;Acc:HGNC:4127] |
| ENSG00000136867 | HGLibA_44930 | 153 | 0.373906767 | SLC31A2 | solute carrier family 31 member 2 [Source:HGNC Symbol;Acc:HGNC:11017] |
| ENSG00000137575 | HGLibA_43109 | 153 | 0.373906767 | SDCBP | syndecan binding protein [Source:HGNC Symbol;Acc:HGNC:10662] |
| ENSG00000138780 | HGLibA_20466 | 153 | 0.373906767 | GSTCD | glutathione S-transferase C-terminal domain containing [Source:HGNC Symbol;Acc:HGNC:25806] |
| ENSG00000140259 | HGLibA_29040 | 153 | 0.373906767 | MFAP1 | microfibril associated protein 1 [Source:HGNC Symbol;Acc:HGNC:7032] |
| ENSG00000143341 | HGLibA_21676 | 153 | 0.373906767 | HMCN1 | hemicentin 1 [Source:HGNC Symbol;Acc:HGNC:19194] |
| ENSG00000143450 | HGLibA_33219 | 153 | 0.373906767 | OAZ3 | ornithine decarboxylase antizyme 3 [Source:HGNC Symbol;Acc:HGNC:8097] |
| ENSG00000143450 | HGLibA_33218 | 153 | 0.373906767 | OAZ3 | ornithine decarboxylase antizyme 3 [Source:HGNC Symbol;Acc:HGNC:8097] |
| ENSG00000143479 | HGLibA_14280 | 153 | 0.373906767 | DYRK3 | dual specificity tyrosine phosphorylation regulated kinase 3 [Source:HGNC Symbol;Acc:HGNC:3094] |
| ENSG00000144191 | HGLibA_10269 | 153 | 0.373906767 | CNGA3 | cyclic nucleotide gated channel alpha 3 [Source:HGNC Symbol;Acc:HGNC:2150] |
| ENSG00000148296 | HGLibA_47841 | 153 | 0.373906767 | SURF6 | surfeit 6 [Source:HGNC Symbol;Acc:HGNC:11478] |
| ENSG00000148335 | HGLibA_32850 | 153 | 0.373906767 | NTMT1 | N-terminal Xaa-Pro-Lys N-methyltransferase 1 [Source:HGNC Symbol;Acc:HGNC:23373] |
| ENSG00000148482 | HGLibA_45098 | 153 | 0.373906767 | SLC39A12 | solute carrier family 39 member 12 [Source:HGNC Symbol;Acc:HGNC:20860] |
| ENSG00000149922 | HGLibA_48678 | 153 | 0.373906767 | TBX6 | T-box 6 [Source:HGNC Symbol;Acc:HGNC:11605] |
| ENSG00000151743 | HGLibA_01810 | 153 | 0.373906767 | AMN1 | antagonist of mitotic exit network 1 homolog [Source:HGNC Symbol;Acc:HGNC:27281] |
| ENSG00000154217 | HGLibA_36748 | 153 | 0.373906767 | PITPNC1 | phosphatidylinositol transfer protein cytoplasmic 1 [Source:HGNC Symbol;Acc:HGNC:21045] |
| ENSG00000154493 | HGLibA_05102 | 153 | 0.373906767 | C10orf90 | chromosome 10 open reading frame 90 [Source:HGNC Symbol;Acc:HGNC:26563] |
| ENSG00000155850 | HGLibA_44790 | 153 | 0.373906767 | SLC26A2 | solute carrier family 26 member 2 [Source:HGNC Symbol;Acc:HGNC:10994] |
| ENSG00000156282 | HGLibA_09869 | 153 | 0.373906767 | CLDN17 | claudin 17 [Source:HGNC Symbol;Acc:HGNC:2038] |
| ENSG00000162104 | HGLibA_00931 | 153 | 0.373906767 | ADCY9 | adenylate cyclase 9 [Source:HGNC Symbol;Acc:HGNC:240] |
| ENSG00000162623 | HGLibA_52485 | 153 | 0.373906767 | TYW3 | tRNA-yW synthesizing protein 3 homolog [Source:HGNC Symbol;Acc:HGNC:24757] |
| ENSG00000162753 | HGLibA_45441 | 153 | 0.373906767 | SLC9C2 | solute carrier family 9 member C2 (putative) [Source:HGNC Symbol;Acc:HGNC:28664] |
| ENSG00000163444 | HGLibA_50106 | 153 | 0.373906767 | TMEM183A | transmembrane protein 183A [Source:HGNC Symbol;Acc:HGNC:20173] |
| ENSG00000164967 | HGLibA_42032 | 153 | 0.373906767 | RPP25L | ribonuclease P/MRP subunit p25 like [Source:HGNC Symbol;Acc:HGNC:19909] |
| ENSG00000165695 | HGLibA_01398 | 153 | 0.373906767 | AK8 | adenylate kinase 8 [Source:HGNC Symbol;Acc:HGNC:26526] |
| ENSG00000166012 | HGLibA_48221 | 153 | 0.373906767 | TAF1D | RNA polymerase I subunit D [Source:HGNC Symbol;Acc:HGNC:28759]" |
| ENSG00000166263 | HGLibA_47671 | 153 | 0.373906767 | STXBP4 | syntaxin binding protein 4 [Source:HGNC Symbol;Acc:HGNC:19694] |
| ENSG00000168418 | HGLibA_24207 | 153 | 0.373906767 | KCNG4 | potassium voltage-gated channel modifier subfamily G member 4 [Source:HGNC Symbol;Acc:HGNC:19697] |
| ENSG00000168447 | HGLibA_43016 | 153 | 0.373906767 | SCNN1B | sodium channel epithelial 1 beta subunit [Source:HGNC Symbol;Acc:HGNC:10600] |
| ENSG00000169562 | HGLibA_19156 | 153 | 0.373906767 | GJB1 | gap junction protein beta 1 [Source:HGNC Symbol;Acc:HGNC:4283] |
| ENSG00000169598 | HGLibA_13023 | 153 | 0.373906767 | DFFB | DNA fragmentation factor subunit beta [Source:HGNC Symbol;Acc:HGNC:2773] |
| ENSG00000170540 | HGLibA_02988 | 153 | 0.373906767 | ARL6IP1 | ADP ribosylation factor like GTPase 6 interacting protein 1 [Source:HGNC Symbol;Acc:HGNC:697] |
| ENSG00000171303 | HGLibA_24322 | 153 | 0.373906767 | KCNK3 | potassium two pore domain channel subfamily K member 3 [Source:HGNC Symbol;Acc:HGNC:6278] |
| ENSG00000171557 | HGLibA_17457 | 153 | 0.373906767 | FGG | fibrinogen gamma chain [Source:HGNC Symbol;Acc:HGNC:3694] |
| ENSG00000172456 | HGLibA_17460 | 153 | 0.373906767 | FGGY | FGGY carbohydrate kinase domain containing [Source:HGNC Symbol;Acc:HGNC:25610] |
| ENSG00000173535 | HGLibA_50740 | 153 | 0.373906767 | TNFRSF10C | TNF receptor superfamily member 10c [Source:HGNC Symbol;Acc:HGNC:11906] |
| ENSG00000174136 | HGLibA_41015 | 153 | 0.373906767 | RGMB | repulsive guidance molecule BMP co-receptor b [Source:HGNC Symbol;Acc:HGNC:26896] |
| ENSG00000176601 | HGLibA_28178 | 153 | 0.373906767 | MAP3K19 | mitogen-activated protein kinase kinase kinase 19 [Source:HGNC Symbol;Acc:HGNC:26249] |
| ENSG00000177494 | HGLibA_54794 | 153 | 0.373906767 | ZBED2 | zinc finger BED-type containing 2 [Source:HGNC Symbol;Acc:HGNC:20710] |

| | | | | | |
|---|---|---|---|---|---|
| ENSG00000178093 | HGLibA_52020 | 153 | 0.373906767 | TSSK6 | testis specific serine kinase 6 [Source:HGNC Symbol;Acc:HGNC:30410] |
| ENSG00000178234 | HGLibA_18532 | 153 | 0.373906767 | GALNT11 | polypeptide N-acetylgalactosaminyltransferase 11 [Source:HGNC Symbol;Acc:HGNC:19875] |
| ENSG00000179772 | HGLibA_17958 | 153 | 0.373906767 | FOXS1 | forkhead box S1 [Source:HGNC Symbol;Acc:HGNC:3735] |
| ENSG00000182533 | HGLibA_07382 | 153 | 0.373906767 | CAV3 | caveolin 3 [Source:HGNC Symbol;Acc:HGNC:1529] |
| ENSG00000182983 | HGLibA_56479 | 153 | 0.373906767 | ZNF662 | zinc finger protein 662 [Source:HGNC Symbol;Acc:HGNC:31930] |
| ENSG00000188687 | HGLibA_45224 | 153 | 0.373906767 | SLC4A5 | solute carrier family 4 member 5 [Source:HGNC Symbol;Acc:HGNC:18168] |
| ENSG00000196345 | HGLibA_55361 | 153 | 0.373906767 | ZKSCAN7 | zinc finger with KRAB and SCAN domains 7 [Source:HGNC Symbol;Acc:HGNC:12955] |
| ENSG00000196767 | HGLibA_37775 | 153 | 0.373906767 | POU3F4 | POU class 3 homeobox 4 [Source:HGNC Symbol;Acc:HGNC:9217] |
| ENSG00000197019 | HGLibA_43603 | 153 | 0.373906767 | SERTAD1 | SERTA domain containing 1 [Source:HGNC Symbol;Acc:HGNC:17932] |
| ENSG00000197044 | HGLibA_56005 | 153 | 0.373906767 | ZNF441 | zinc finger protein 441 [Source:HGNC Symbol;Acc:HGNC:20875] |
| ENSG00000197084 | HGLibA_26105 | 153 | 0.373906767 | LCE1C | late cornified envelope 1C [Source:HGNC Symbol;Acc:HGNC:29464] |
| ENSG00000197587 | HGLibA_13396 | 153 | 0.373906767 | DMBX1 | diencephalon/mesencephalon homeobox 1 [Source:HGNC Symbol;Acc:HGNC:19026] |
| ENSG00000197746 | HGLibA_39002 | 153 | 0.373906767 | PSAP | prosaposin [Source:HGNC Symbol;Acc:HGNC:9498] |
| ENSG00000197818 | HGLibA_45427 | 153 | 0.373906767 | SLC9A8 | solute carrier family 9 member A8 [Source:HGNC Symbol;Acc:HGNC:20728] |
| ENSG00000198283 | HGLibA_34264 | 153 | 0.373906767 | OR5B21 | olfactory receptor family 5 subfamily B member 21 [Source:HGNC Symbol;Acc:HGNC:19616] |
| ENSG00000198553 | HGLibA_24381 | 153 | 0.373906767 | KCNRG | potassium channel regulator [Source:HGNC Symbol;Acc:HGNC:18893] |
| ENSG00000204569 | HGLibA_38013 | 153 | 0.373906767 | PPP1R10 | protein phosphatase 1 regulatory subunit 10 [Source:HGNC Symbol;Acc:HGNC:9284] |
| ENSG00000212659 | HGLibA_25843 | 153 | 0.373906767 | KRTAP9-6 | keratin associated protein 9-6 [Source:HGNC Symbol;Acc:HGNC:18914] |
| ENSG00000256683 | HGLibA_55858 | 153 | 0.373906767 | ZNF350 | zinc finger protein 350 [Source:HGNC Symbol;Acc:HGNC:16656] |
| ENSG00000263761 | HGLibA_18862 | 153 | 0.373906767 | GDF2 | growth differentiation factor 2 [Source:HGNC Symbol;Acc:HGNC:4217] |
| GAREM | HGLibA_18626 | 153 | 0.373906767 | GAREM | - |
| hsa-mir-1254-2 | HGLibA_57417 | 153 | 0.373906767 | hsa-mir-1254-2 | - |
| hsa-mir-1269b | HGLibA_57507 | 153 | 0.373906767 | hsa-mir-1269b | - |
| hsa-mir-127 | HGLibA_57510 | 153 | 0.373906767 | hsa-mir-127 | - |
| hsa-mir-1275 | HGLibA_57556 | 153 | 0.373906767 | hsa-mir-1275 | - |
| hsa-mir-145 | HGLibA_57843 | 153 | 0.373906767 | hsa-mir-145 | - |
| hsa-mir-20b | HGLibA_58203 | 153 | 0.373906767 | hsa-mir-20b | - |
| hsa-mir-3158-1 | HGLibA_58705 | 153 | 0.373906767 | hsa-mir-3158-1 | - |
| hsa-mir-3168 | HGLibA_58750 | 153 | 0.373906767 | hsa-mir-3168 | - |
| hsa-mir-4529 | HGLibA_60575 | 153 | 0.373906767 | hsa-mir-4529 | - |
| hsa-mir-4664 | HGLibA_60769 | 153 | 0.373906767 | hsa-mir-4664 | - |
| hsa-mir-4752 | HGLibA_61106 | 153 | 0.373906767 | hsa-mir-4752 | - |
| hsa-mir-492 | HGLibA_61367 | 153 | 0.373906767 | hsa-mir-492 | - |
| hsa-mir-6794 | HGLibA_63352 | 153 | 0.373906767 | hsa-mir-6794 | - |
| hsa-mir-8066 | HGLibA_64102 | 153 | 0.373906767 | hsa-mir-8066 | - |
| LPPR5 | HGLibA_27126 | 153 | 0.373906767 | LPPR5 | - |
| APOPT1 | HGLibA_02558 | 152 | 0.36395573 | APOPT1 | - |
| C1orf51 | HGLibA_05865 | 152 | 0.36395573 | C1orf51 | - |
| C3orf79 | HGLibA_06270 | 152 | 0.36395573 | C3orf79 | - |
| C6orf7 | HGLibA_06505 | 152 | 0.36395573 | C6orf7 | - |
| DDX26B | HGLibA_12685 | 152 | 0.36395573 | DDX26B | - |
| ENSG00000012124 | HGLibA_08297 | 152 | 0.36395573 | CD22 | CD22 molecule [Source:HGNC Symbol;Acc:HGNC:1643] |
| ENSG00000020577 | HGLibA_42684 | 152 | 0.36395573 | SAMD4A | sterile alpha motif domain containing 4A [Source:HGNC Symbol;Acc:HGNC:23023] |
| ENSG00000028116 | HGLibA_53826 | 152 | 0.36395573 | VRK2 | VRK serine/threonine kinase 2 [Source:HGNC Symbol;Acc:HGNC:12719] |
| ENSG00000055609 | HGLibA_25328 | 152 | 0.36395573 | KMT2C | lysine methyltransferase 2C [Source:HGNC Symbol;Acc:HGNC:13726] |
| ENSG00000063176 | HGLibA_46645 | 152 | 0.36395573 | SPHK2 | sphingosine kinase 2 [Source:HGNC Symbol;Acc:HGNC:18859] |
| ENSG00000066032 | HGLibA_11641 | 152 | 0.36395573 | CTNNA2 | catenin alpha 2 [Source:HGNC Symbol;Acc:HGNC:2510] |
| ENSG00000066056 | HGLibA_49455 | 152 | 0.36395573 | TIE1 | tyrosine kinase with immunoglobulin like and EGF like domains 1 [Source:HGNC Symbol;Acc:HGNC:11809] |
| ENSG00000066135 | HGLibA_24507 | 152 | 0.36395573 | KDM4A | lysine demethylase 4A [Source:HGNC Symbol;Acc:HGNC:22978] |
| ENSG00000067445 | HGLibA_51717 | 152 | 0.36395573 | TRO | trophinin [Source:HGNC Symbol;Acc:HGNC:12326] |
| ENSG00000068781 | HGLibA_47546 | 152 | 0.36395573 | STON1-GTF2A1L | STON1-GTF2A1L readthrough [Source:HGNC Symbol;Acc:HGNC:30651] |
| ENSG00000074416 | HGLibA_29158 | 152 | 0.36395573 | MGLL | monoglyceride lipase [Source:HGNC Symbol;Acc:HGNC:17038] |
| ENSG00000078399 | HGLibA_21923 | 152 | 0.36395573 | HOXA9 | homeobox A9 [Source:HGNC Symbol;Acc:HGNC:5109] |
| ENSG00000082126 | HGLibA_29757 | 152 | 0.36395573 | MPP4 | membrane palmitoylated protein 4 [Source:HGNC Symbol;Acc:HGNC:13680] |
| ENSG00000089163 | HGLibA_44224 | 152 | 0.36395573 | SIRT4 | sirtuin 4 [Source:HGNC Symbol;Acc:HGNC:14932] |
| ENSG00000091513 | HGLibA_49116 | 152 | 0.36395573 | TF | transferrin [Source:HGNC Symbol;Acc:HGNC:11740] |
| ENSG00000100439 | HGLibA_00277 | 152 | 0.36395573 | ABHD4 | abhydrolase domain containing 4 [Source:HGNC Symbol;Acc:HGNC:20154] |
| ENSG00000101198 | HGLibA_31997 | 152 | 0.36395573 | NKAIN4 | sodium/potassium transporting ATPase interacting 4 [Source:HGNC Symbol;Acc:HGNC:16191] |
| ENSG00000101605 | HGLibA_30889 | 152 | 0.36395573 | MYOM1 | myomesin 1 [Source:HGNC Symbol;Acc:HGNC:7613] |
| ENSG00000101680 | HGLibA_25941 | 152 | 0.36395573 | LAMA1 | laminin subunit alpha 1 [Source:HGNC Symbol;Acc:HGNC:6481] |
| ENSG00000104921 | HGLibA_17175 | 152 | 0.36395573 | FCER2 | Fc fragment of IgE receptor II [Source:HGNC Symbol;Acc:HGNC:3612] |
| ENSG00000105327 | HGLibA_04198 | 152 | 0.36395573 | BBC3 | BCL2 binding component 3 [Source:HGNC Symbol;Acc:HGNC:17868] |
| ENSG00000105409 | HGLibA_03588 | 152 | 0.36395573 | ATP1A3 | ATPase Na+/K+ transporting subunit alpha 3 [Source:HGNC Symbol;Acc:HGNC:801] |
| ENSG00000105982 | HGLibA_41665 | 152 | 0.36395573 | RNF32 | ring finger protein 32 [Source:HGNC Symbol;Acc:HGNC:17118] |
| ENSG00000108306 | HGLibA_17000 | 152 | 0.36395573 | FBXL20 | F-box and leucine rich repeat protein 20 [Source:HGNC Symbol;Acc:HGNC:24679] |
| ENSG00000108961 | HGLibA_40218 | 152 | 0.36395573 | RANGRF | RAN guanine nucleotide release factor [Source:HGNC Symbol;Acc:HGNC:17679] |
| ENSG00000110077 | HGLibA_30158 | 152 | 0.36395573 | MS4A6A | membrane spanning 4-domains A6A [Source:HGNC Symbol;Acc:HGNC:13375] |
| ENSG00000110243 | HGLibA_02461 | 152 | 0.36395573 | APOA5 | apolipoprotein A5 [Source:HGNC Symbol;Acc:HGNC:17288] |
| ENSG00000112276 | HGLibA_05005 | 152 | 0.36395573 | BVES | blood vessel epicardial substance [Source:HGNC Symbol;Acc:HGNC:1152] |
| ENSG00000112619 | HGLibA_38751 | 152 | 0.36395573 | PRPH2 | peripherin 2 [Source:HGNC Symbol;Acc:HGNC:9942] |
| ENSG00000113532 | HGLibA_47296 | 152 | 0.36395573 | ST8SIA4 | 8-sialyltransferase 4 [Source:HGNC Symbol;Acc:HGNC:10871]" |
| ENSG00000113721 | HGLibA_35855 | 152 | 0.36395573 | PDGFRB | platelet derived growth factor receptor beta [Source:HGNC Symbol;Acc:HGNC:8804] |
| ENSG00000114013 | HGLibA_08468 | 152 | 0.36395573 | CD86 | CD86 molecule [Source:HGNC Symbol;Acc:HGNC:1705] |
| ENSG00000115350 | HGLibA_37520 | 152 | 0.36395573 | POLE4 | accessory subunit [Source:HGNC Symbol;Acc:HGNC:18755]" |
| ENSG00000116406 | HGLibA_14461 | 152 | 0.36395573 | EDEM3 | ER degradation enhancing alpha-mannosidase like protein 3 [Source:HGNC Symbol;Acc:HGNC:16787] |
| ENSG00000119280 | HGLibA_05818 | 152 | 0.36395573 | C1orf198 | chromosome 1 open reading frame 198 [Source:HGNC Symbol;Acc:HGNC:25900] |
| ENSG00000123130 | HGLibA_00495 | 152 | 0.36395573 | ACOT9 | acyl-CoA thioesterase 9 [Source:HGNC Symbol;Acc:HGNC:17152] |
| ENSG00000123612 | HGLibA_00693 | 152 | 0.36395573 | ACVR1C | activin A receptor type 1C [Source:HGNC Symbol;Acc:HGNC:18123] |
| ENSG00000126003 | HGLibA_36957 | 152 | 0.36395573 | PLAGL2 | PLAG1 like zinc finger 2 [Source:HGNC Symbol;Acc:HGNC:9047] |
| ENSG00000127529 | HGLibA_34470 | 152 | 0.36395573 | OR7C2 | olfactory receptor family 7 subfamily C member 2 [Source:HGNC Symbol;Acc:HGNC:8374] |
| ENSG00000127529 | HGLibA_34469 | 152 | 0.36395573 | OR7C2 | olfactory receptor family 7 subfamily C member 2 [Source:HGNC Symbol;Acc:HGNC:8374] |
| ENSG00000128654 | HGLibA_30514 | 152 | 0.36395573 | MTX2 | metaxin 2 [Source:HGNC Symbol;Acc:HGNC:7506] |
| ENSG00000130702 | HGLibA_25953 | 152 | 0.36395573 | LAMA5 | laminin subunit alpha 5 [Source:HGNC Symbol;Acc:HGNC:6485] |
| ENSG00000130762 | HGLibA_02821 | 152 | 0.36395573 | ARHGEF16 | Rho guanine nucleotide exchange factor 16 [Source:HGNC Symbol;Acc:HGNC:15515] |
| ENSG00000130881 | HGLibA_27218 | 152 | 0.36395573 | LRP3 | LDL receptor related protein 3 [Source:HGNC Symbol;Acc:HGNC:6695] |
| ENSG00000132259 | HGLibA_10271 | 152 | 0.36395573 | CNGA4 | cyclic nucleotide gated channel alpha 4 [Source:HGNC Symbol;Acc:HGNC:2152] |
| ENSG00000136160 | HGLibA_14482 | 152 | 0.36395573 | EDNRB | endothelin receptor type B [Source:HGNC Symbol;Acc:HGNC:3180] |
| ENSG00000137133 | HGLibA_21326 | 152 | 0.36395573 | HINT2 | histidine triad nucleotide binding protein 2 [Source:HGNC Symbol;Acc:HGNC:18344] |
| ENSG00000137802 | HGLibA_28310 | 152 | 0.36395573 | MAPKBP1 | mitogen-activated protein kinase binding protein 1 [Source:HGNC Symbol;Acc:HGNC:29536] |
| ENSG00000138604 | HGLibA_19231 | 152 | 0.36395573 | GLCE | glucuronic acid epimerase [Source:HGNC Symbol;Acc:HGNC:17855] |
| ENSG00000140497 | HGLibA_42840 | 152 | 0.36395573 | SCAMP2 | secretory carrier membrane protein 2 [Source:HGNC Symbol;Acc:HGNC:10564] |
| ENSG00000143387 | HGLibA_11712 | 152 | 0.36395573 | CTSK | cathepsin K [Source:HGNC Symbol;Acc:HGNC:2536] |
| ENSG00000144671 | HGLibA_44550 | 152 | 0.36395573 | SLC22A14 | solute carrier family 22 member 14 [Source:HGNC Symbol;Acc:HGNC:8495] |
| ENSG00000144671 | HGLibA_44549 | 152 | 0.36395573 | SLC22A14 | solute carrier family 22 member 14 [Source:HGNC Symbol;Acc:HGNC:8495] |
| ENSG00000144847 | HGLibA_22881 | 152 | 0.36395573 | IGSF11 | immunoglobulin superfamily member 11 [Source:HGNC Symbol;Acc:HGNC:16669] |
| ENSG00000145919 | HGLibA_04667 | 152 | 0.36395573 | BOD1 | biorientation of chromosomes in cell division 1 [Source:HGNC Symbol;Acc:HGNC:25114] |
| ENSG00000147206 | HGLibA_33136 | 152 | 0.36395573 | NXF3 | nuclear RNA export factor 3 [Source:HGNC Symbol;Acc:HGNC:8073] |
| ENSG00000148411 | HGLibA_31042 | 152 | 0.36395573 | NACC2 | NACC family member 2 [Source:HGNC Symbol;Acc:HGNC:23846] |
| ENSG00000148680 | HGLibA_22403 | 152 | 0.36395573 | HTR7 | 5-hydroxytryptamine receptor 7 [Source:HGNC Symbol;Acc:HGNC:5302] |
| ENSG00000149115 | HGLibA_50852 | 152 | 0.36395573 | TNKS1BP1 | tankyrase 1 binding protein 1 [Source:HGNC Symbol;Acc:HGNC:19081] |
| ENSG00000152214 | HGLibA_41339 | 152 | 0.36395573 | RIT2 | Ras like without CAAX 2 [Source:HGNC Symbol;Acc:HGNC:10017] |
| ENSG00000153283 | HGLibA_08484 | 152 | 0.36395573 | CD96 | CD96 molecule [Source:HGNC Symbol;Acc:HGNC:16892] |
| ENSG00000154478 | HGLibA_20009 | 152 | 0.36395573 | GPR26 | G protein-coupled receptor 26 [Source:HGNC Symbol;Acc:HGNC:4481] |
| ENSG00000154640 | HGLibA_04946 | 152 | 0.36395573 | BTG3 | BTG anti-proliferation factor 3 [Source:HGNC Symbol;Acc:HGNC:1132] |
| ENSG00000155755 | HGLibA_50275 | 152 | 0.36395573 | TMEM237 | transmembrane protein 237 [Source:HGNC Symbol;Acc:HGNC:14432] |
| ENSG00000161036 | HGLibA_27508 | 152 | 0.36395573 | LRWD1 | leucine rich repeats and WD repeat domain containing 1 [Source:HGNC Symbol;Acc:HGNC:21769] |
| ENSG00000161798 | HGLibA_02598 | 152 | 0.36395573 | AQP5 | aquaporin 5 [Source:HGNC Symbol;Acc:HGNC:638] |
| ENSG00000162383 | HGLibA_44526 | 152 | 0.36395573 | SLC1A7 | solute carrier family 1 member 7 [Source:HGNC Symbol;Acc:HGNC:10945] |
| ENSG00000162520 | HGLibA_47963 | 152 | 0.36395573 | SYNC | intermediate filament protein [Source:HGNC Symbol;Acc:HGNC:28897]" |
| ENSG00000164346 | HGLibA_32743 | 152 | 0.36395573 | NSA2 | ribosome biogenesis homolog [Source:HGNC Symbol;Acc:HGNC:30728]" |
| ENSG00000166068 | HGLibA_46789 | 152 | 0.36395573 | SPRED1 | sprouty related EVH1 domain containing 1 [Source:HGNC Symbol;Acc:HGNC:20249] |
| ENSG00000167720 | HGLibA_47040 | 152 | 0.36395573 | SRR | serine racemase [Source:HGNC Symbol;Acc:HGNC:14398] |
| ENSG00000168421 | HGLibA_41193 | 152 | 0.36395573 | RHOH | ras homolog family member H [Source:HGNC Symbol;Acc:HGNC:686] |
| ENSG00000170390 | HGLibA_12503 | 152 | 0.36395573 | DCLK2 | doublecortin like kinase 2 [Source:HGNC Symbol;Acc:HGNC:19002] |
| ENSG00000172156 | HGLibA_07965 | 152 | 0.36395573 | CCL11 | C-C motif chemokine ligand 11 [Source:HGNC Symbol;Acc:HGNC:10610] |
| ENSG00000172500 | HGLibA_17504 | 152 | 0.36395573 | FIBP | FGF1 intracellular binding protein [Source:HGNC Symbol;Acc:HGNC:3705] |
| ENSG00000172939 | HGLibA_34836 | 152 | 0.36395573 | OXSR1 | oxidative stress responsive 1 [Source:HGNC Symbol;Acc:HGNC:8508] |
| ENSG00000173239 | HGLibA_26628 | 152 | 0.36395573 | LIPM | lipase family member M [Source:HGNC Symbol;Acc:HGNC:23455] |
| ENSG00000173372 | HGLibA_05913 | 152 | 0.36395573 | C1QA | complement C1q A chain [Source:HGNC Symbol;Acc:HGNC:1241] |
| ENSG00000175183 | HGLibA_11436 | 152 | 0.36395573 | CSRP2 | cysteine and glycine rich protein 2 [Source:HGNC Symbol;Acc:HGNC:2470] |
| ENSG00000175497 | HGLibA_13866 | 152 | 0.36395573 | DPP10 | dipeptidyl peptidase like 10 [Source:HGNC Symbol;Acc:HGNC:20823] |
| ENSG00000176222 | HGLibA_55923 | 152 | 0.36395573 | ZNF404 | zinc finger protein 404 [Source:HGNC Symbol;Acc:HGNC:19417] |
| ENSG00000178209 | HGLibA_37052 | 152 | 0.36395573 | PLEC | plectin [Source:HGNC Symbol;Acc:HGNC:9069] |
| ENSG00000179583 | HGLibA_09707 | 152 | 0.36395573 | CIITA | class II major histocompatibility complex transactivator [Source:HGNC Symbol;Acc:HGNC:7067] |
| ENSG00000179627 | HGLibA_54886 | 152 | 0.36395573 | ZBTB42 | zinc finger and BTB domain containing 42 [Source:HGNC Symbol;Acc:HGNC:32550] |
| ENSG00000181240 | HGLibA_44737 | 152 | 0.36395573 | SLC25A41 | solute carrier family 25 member 41 [Source:HGNC Symbol;Acc:HGNC:28533] |
| ENSG00000181523 | HGLibA_43857 | 152 | 0.36395573 | SGSH | N-sulfoglucosamine sulfohydrolase [Source:HGNC Symbol;Acc:HGNC:10818] |
| ENSG00000182993 | HGLibA_05272 | 152 | 0.36395573 | C12orf60 | chromosome 12 open reading frame 60 [Source:HGNC Symbol;Acc:HGNC:28726] |
| ENSG00000184602 | HGLibA_45933 | 152 | 0.36395573 | SNN | stannin [Source:HGNC Symbol;Acc:HGNC:11149] |
| ENSG00000185247 | HGLibA_27899 | 152 | 0.36395573 | MAGEA11 | MAGE family member A11 [Source:HGNC Symbol;Acc:HGNC:6798] |
| ENSG00000185989 | HGLibA_40303 | 152 | 0.36395573 | RASA3 | RAS p21 protein activator 3 [Source:HGNC Symbol;Acc:HGNC:20331] |
| ENSG00000188611 | HGLibA_03188 | 152 | 0.36395573 | ASAH2 | N-acylsphingosine amidohydrolase 2 [Source:HGNC Symbol;Acc:HGNC:18860] |
| ENSG00000188906 | HGLibA_27466 | 152 | 0.36395573 | LRRK2 | leucine rich repeat kinase 2 [Source:HGNC Symbol;Acc:HGNC:18618] |
| ENSG00000189410 | HGLibA_43903 | 152 | 0.36395573 | SH2D5 | SH2 domain containing 5 [Source:HGNC Symbol;Acc:HGNC:28819] |
| ENSG00000198062 | HGLibA_37738 | 152 | 0.36395573 | POTEH | POTE ankyrin domain family member H [Source:HGNC Symbol;Acc:HGNC:133] |

| | | | | | |
|---|---|---|---|---|---|
| ENSG00000203883 | HGLibA_46248 | 152 | 0.36395573 | SOX18 | SRY-box 18 [Source:HGNC Symbol;Acc:HGNC:11194] |
| ENSG00000213231 | HGLibA_48817 | 152 | 0.36395573 | TCL1B | T cell leukemia/lymphoma 1B [Source:HGNC Symbol;Acc:HGNC:11649] |
| ENSG00000221840 | HGLibA_33933 | 152 | 0.36395573 | OR4A5 | olfactory receptor family 4 subfamily A member 5 [Source:HGNC Symbol;Acc:HGNC:15162] |
| ENSG00000221937 | HGLibA_48403 | 152 | 0.36395573 | TAS2R40 | taste 2 receptor member 40 [Source:HGNC Symbol;Acc:HGNC:18885] |
| ENSG00000225556 | HGLibA_06104 | 152 | 0.36395573 | C2CD4D | C2 calcium dependent domain containing 4D [Source:HGNC Symbol;Acc:HGNC:37210] |
| ENSG00000237515 | HGLibA_44054 | 152 | 0.36395573 | SHISA9 | shisa family member 9 [Source:HGNC Symbol;Acc:HGNC:37231] |
| ENSG00000244414 | HGLibA_09263 | 152 | 0.36395573 | CFHR1 | complement factor H related 1 [Source:HGNC Symbol;Acc:HGNC:4888] |
| ENSG00000249459 | HGLibA_55753 | 152 | 0.36395573 | ZNF286B | zinc finger protein 286B [Source:HGNC Symbol;Acc:HGNC:33241] |
| ENSG00000249693 | HGLibA_49337 | 152 | 0.36395573 | THEGL | theg spermatid protein like [Source:HGNC Symbol;Acc:HGNC:43771] |
| ENSG00000259075 | HGLibA_37448 | 152 | 0.36395573 | POC1B-GALNT4 | POC1B-GALNT4 readthrough [Source:HGNC Symbol;Acc:HGNC:42957] |
| GAGE2D | HGLibA_18475 | 152 | 0.36395573 | GAGE2D | - |
| HIST1H4C | HGLibA_21493 | 152 | 0.36395573 | HIST1H4C | - |
| hsa-mir-1267 | HGLibA_57492 | 152 | 0.36395573 | hsa-mir-1267 | - |
| hsa-mir-1321 | HGLibA_57760 | 152 | 0.36395573 | hsa-mir-1321 | - |
| hsa-mir-1324 | HGLibA_57768 | 152 | 0.36395573 | hsa-mir-1324 | - |
| hsa-mir-3126 | HGLibA_58558 | 152 | 0.36395573 | hsa-mir-3126 | - |
| hsa-mir-323a | HGLibA_58956 | 152 | 0.36395573 | hsa-mir-323a | - |
| hsa-mir-4316 | HGLibA_59984 | 152 | 0.36395573 | hsa-mir-4316 | - |
| hsa-mir-4429 | HGLibA_60101 | 152 | 0.36395573 | hsa-mir-4429 | - |
| hsa-mir-4510 | HGLibA_60485 | 152 | 0.36395573 | hsa-mir-4510 | - |
| hsa-mir-4713 | HGLibA_60953 | 152 | 0.36395573 | hsa-mir-4713 | - |
| hsa-mir-4731 | HGLibA_61023 | 152 | 0.36395573 | hsa-mir-4731 | - |
| hsa-mir-4752 | HGLibA_61104 | 152 | 0.36395573 | hsa-mir-4752 | - |
| hsa-mir-5100 | HGLibA_61550 | 152 | 0.36395573 | hsa-mir-5100 | - |
| hsa-mir-627 | HGLibA_62747 | 152 | 0.36395573 | hsa-mir-627 | - |
| hsa-mir-647 | HGLibA_62831 | 152 | 0.36395573 | hsa-mir-647 | - |
| hsa-mir-6774 | HGLibA_63268 | 152 | 0.36395573 | hsa-mir-6774 | - |
| LOC401052 | HGLibA_26936 | 152 | 0.36395573 | LOC401052 | - |
| LPPR3 | HGLibA_27119 | 152 | 0.36395573 | LPPR3 | - |
| ELTD1 | HGLibA_14996 | 151 | 0.354004692 | ELTD1 | - |
| ELTD1 | HGLibA_14997 | 151 | 0.354004692 | ELTD1 | - |
| ENSG00000000971 | HGLibA_09261 | 151 | 0.354004692 | CFH | complement factor H [Source:HGNC Symbol;Acc:HGNC:4883] |
| ENSG00000005889 | HGLibA_55263 | 151 | 0.354004692 | ZFX | zinc finger protein X-linked [Source:HGNC Symbol;Acc:HGNC:12869] |
| ENSG00000041982 | HGLibA_50700 | 151 | 0.354004692 | TNC | tenascin C [Source:HGNC Symbol;Acc:HGNC:5318] |
| ENSG00000058668 | HGLibA_03623 | 151 | 0.354004692 | ATP2B4 | ATPase plasma membrane Ca2+ transporting 4 [Source:HGNC Symbol;Acc:HGNC:817] |
| ENSG00000059378 | HGLibA_35221 | 151 | 0.354004692 | PARP12 | poly(ADP-ribose) polymerase family member 12 [Source:HGNC Symbol;Acc:HGNC:21919] |
| ENSG00000070961 | HGLibA_03613 | 151 | 0.354004692 | ATP2B1 | ATPase plasma membrane Ca2+ transporting 1 [Source:HGNC Symbol;Acc:HGNC:814] |
| ENSG00000075303 | HGLibA_44736 | 151 | 0.354004692 | SLC25A40 | solute carrier family 25 member 40 [Source:HGNC Symbol;Acc:HGNC:29680] |
| ENSG00000077279 | HGLibA_12581 | 151 | 0.354004692 | DCX | doublecortin [Source:HGNC Symbol;Acc:HGNC:2714] |
| ENSG00000081803 | HGLibA_06981 | 151 | 0.354004692 | CADPS2 | calcium dependent secretion activator 2 [Source:HGNC Symbol;Acc:HGNC:16018] |
| ENSG00000082684 | HGLibA_43362 | 151 | 0.354004692 | SEMA5B | semaphorin 5B [Source:HGNC Symbol;Acc:HGNC:10737] |
| ENSG00000090776 | HGLibA_14598 | 151 | 0.354004692 | EFNB1 | ephrin B1 [Source:HGNC Symbol;Acc:HGNC:3226] |
| ENSG00000091592 | HGLibA_32096 | 151 | 0.354004692 | NLRP1 | NLR family pyrin domain containing 1 [Source:HGNC Symbol;Acc:HGNC:14374] |
| ENSG00000102898 | HGLibA_33098 | 151 | 0.354004692 | NUTF2 | nuclear transport factor 2 [Source:HGNC Symbol;Acc:HGNC:13722] |
| ENSG00000103024 | HGLibA_32161 | 151 | 0.354004692 | NME3 | NME/NM23 nucleoside diphosphate kinase 3 [Source:HGNC Symbol;Acc:HGNC:7851] |
| ENSG00000104951 | HGLibA_23170 | 151 | 0.354004692 | IL4I1 | interleukin 4 induced 1 [Source:HGNC Symbol;Acc:HGNC:19094] |
| ENSG00000105393 | HGLibA_04057 | 151 | 0.354004692 | BABAM1 | BRISC and BRCA1 A complex member 1 [Source:HGNC Symbol;Acc:HGNC:25008] |
| ENSG00000106392 | HGLibA_05722 | 151 | 0.354004692 | C1GALT1 | glycoprotein-N-acetylgalactosamine 3-beta-galactosyltransferase 1 [Source:HGNC Symbol;Acc:HGNC:24337]" |
| ENSG00000108406 | HGLibA_13200 | 151 | 0.354004692 | DHX40 | DEAH-box helicase 40 [Source:HGNC Symbol;Acc:HGNC:18018] |
| ENSG00000109424 | HGLibA_52832 | 151 | 0.354004692 | UCP1 | uncoupling protein 1 [Source:HGNC Symbol;Acc:HGNC:12517] |
| ENSG00000111249 | HGLibA_11805 | 151 | 0.354004692 | CUX2 | cut like homeobox 2 [Source:HGNC Symbol;Acc:HGNC:19347] |
| ENSG00000114166 | HGLibA_24054 | 151 | 0.354004692 | KAT2B | lysine acetyltransferase 2B [Source:HGNC Symbol;Acc:HGNC:8638] |
| ENSG00000116212 | HGLibA_27358 | 151 | 0.354004692 | LRRC42 | leucine rich repeat containing 42 [Source:HGNC Symbol;Acc:HGNC:28792] |
| ENSG00000122735 | HGLibA_13503 | 151 | 0.354004692 | DNAI1 | dynein axonemal intermediate chain 1 [Source:HGNC Symbol;Acc:HGNC:2954] |
| ENSG00000123472 | HGLibA_03797 | 151 | 0.354004692 | ATPAF1 | ATP synthase mitochondrial F1 complex assembly factor 1 [Source:HGNC Symbol;Acc:HGNC:18803] |
| ENSG00000123552 | HGLibA_53373 | 151 | 0.354004692 | USP45 | ubiquitin specific peptidase 45 [Source:HGNC Symbol;Acc:HGNC:20080] |
| ENSG00000125629 | HGLibA_23378 | 151 | 0.354004692 | INSIG2 | insulin induced gene 2 [Source:HGNC Symbol;Acc:HGNC:20452] |
| ENSG00000126653 | HGLibA_32778 | 151 | 0.354004692 | NSRP1 | nuclear speckle splicing regulatory protein 1 [Source:HGNC Symbol;Acc:HGNC:25305] |
| ENSG00000127377 | HGLibA_11281 | 151 | 0.354004692 | CRYGN | crystallin gamma N [Source:HGNC Symbol;Acc:HGNC:20458] |
| ENSG00000127561 | HGLibA_47996 | 151 | 0.354004692 | SYNGR3 | synaptogyrin 3 [Source:HGNC Symbol;Acc:HGNC:11501] |
| ENSG00000128284 | HGLibA_02534 | 151 | 0.354004692 | APOL3 | apolipoprotein L3 [Source:HGNC Symbol;Acc:HGNC:14868] |
| ENSG00000128917 | HGLibA_13366 | 151 | 0.354004692 | DLL4 | delta like canonical Notch ligand 4 [Source:HGNC Symbol;Acc:HGNC:2910] |
| ENSG00000131080 | HGLibA_14434 | 151 | 0.354004692 | EDA2R | ectodysplasin A2 receptor [Source:HGNC Symbol;Acc:HGNC:17756] |
| ENSG00000131966 | HGLibA_00647 | 151 | 0.354004692 | ACTR10 | actin related protein 10 homolog [Source:HGNC Symbol;Acc:HGNC:17372] |
| ENSG00000131981 | HGLibA_26364 | 151 | 0.354004692 | LGALS3 | galectin 3 [Source:HGNC Symbol;Acc:HGNC:6563] |
| ENSG00000133020 | HGLibA_30740 | 151 | 0.354004692 | MYH8 | myosin heavy chain 8 [Source:HGNC Symbol;Acc:HGNC:7578] |
| ENSG00000133265 | HGLibA_22328 | 151 | 0.354004692 | HSPBP1 | HSPA (Hsp70) binding protein 1 [Source:HGNC Symbol;Acc:HGNC:24989] |
| ENSG00000134294 | HGLibA_45063 | 151 | 0.354004692 | SLC38A2 | solute carrier family 38 member 2 [Source:HGNC Symbol;Acc:HGNC:13448] |
| ENSG00000134762 | HGLibA_13995 | 151 | 0.354004692 | DSC3 | desmocollin 3 [Source:HGNC Symbol;Acc:HGNC:3037] |
| ENSG00000135472 | HGLibA_15968 | 151 | 0.354004692 | FAIM2 | Fas apoptotic inhibitory molecule 2 [Source:HGNC Symbol;Acc:HGNC:17067] |
| ENSG00000135637 | HGLibA_07618 | 151 | 0.354004692 | CCDC142 | coiled-coil domain containing 142 [Source:HGNC Symbol;Acc:HGNC:25889] |
| ENSG00000136541 | HGLibA_15507 | 151 | 0.354004692 | ERMN | ermin [Source:HGNC Symbol;Acc:HGNC:29208] |
| ENSG00000137992 | HGLibA_12405 | 151 | 0.354004692 | DBT | dihydrolipoamide branched chain transacylase E2 [Source:HGNC Symbol;Acc:HGNC:2698] |
| ENSG00000138964 | HGLibA_35263 | 151 | 0.354004692 | PARVG | parvin gamma [Source:HGNC Symbol;Acc:HGNC:14654] |
| ENSG00000139540 | HGLibA_45115 | 151 | 0.354004692 | SLC39A5 | solute carrier family 39 member 5 [Source:HGNC Symbol;Acc:HGNC:20502] |
| ENSG00000141568 | HGLibA_17896 | 151 | 0.354004692 | FOXK2 | forkhead box K2 [Source:HGNC Symbol;Acc:HGNC:6036] |
| ENSG00000144036 | HGLibA_15757 | 151 | 0.354004692 | EXOC6B | exocyst complex component 6B [Source:HGNC Symbol;Acc:HGNC:17085] |
| ENSG00000145321 | HGLibA_18755 | 151 | 0.354004692 | GC | vitamin D binding protein [Source:HGNC Symbol;Acc:HGNC:4187]" |
| ENSG00000145936 | HGLibA_24342 | 151 | 0.354004692 | KCNMB1 | potassium calcium-activated channel subfamily M regulatory beta subunit 1 [Source:HGNC Symbol;Acc:HGNC:6285] |
| ENSG00000149761 | HGLibA_32974 | 151 | 0.354004692 | NUDT22 | nudix hydrolase 22 [Source:HGNC Symbol;Acc:HGNC:28189] |
| ENSG00000151704 | HGLibA_24245 | 151 | 0.354004692 | KCNJ1 | potassium voltage-gated channel subfamily J member 1 [Source:HGNC Symbol;Acc:HGNC:6255] |
| ENSG00000152213 | HGLibA_02928 | 151 | 0.354004692 | ARL11 | ADP ribosylation factor like GTPase 11 [Source:HGNC Symbol;Acc:HGNC:24046] |
| ENSG00000153214 | HGLibA_50533 | 151 | 0.354004692 | TMEM87B | transmembrane protein 87B [Source:HGNC Symbol;Acc:HGNC:25913] |
| ENSG00000155275 | HGLibA_51690 | 151 | 0.354004692 | TRMT44 | tRNA methyltransferase 44 homolog [Source:HGNC Symbol;Acc:HGNC:26653] |
| ENSG00000158457 | HGLibA_51935 | 151 | 0.354004692 | TSPAN33 | tetraspanin 33 [Source:HGNC Symbol;Acc:HGNC:28743] |
| ENSG00000158850 | HGLibA_04028 | 151 | 0.354004692 | B4GALT3 | 4-galactosyltransferase 3 [Source:HGNC Symbol;Acc:HGNC:926]" |
| ENSG00000162572 | HGLibA_43019 | 151 | 0.354004692 | SCNN1D | sodium channel epithelial 1 delta subunit [Source:HGNC Symbol;Acc:HGNC:10601] |
| ENSG00000162627 | HGLibA_46106 | 151 | 0.354004692 | SNX7 | sorting nexin 7 [Source:HGNC Symbol;Acc:HGNC:14971] |
| ENSG00000163032 | HGLibA_53853 | 151 | 0.354004692 | VSNL1 | visinin like 1 [Source:HGNC Symbol;Acc:HGNC:12722] |
| ENSG00000163064 | HGLibA_15098 | 151 | 0.354004692 | EN1 | engrailed homeobox 1 [Source:HGNC Symbol;Acc:HGNC:3342] |
| ENSG00000163444 | HGLibA_50105 | 151 | 0.354004692 | TMEM183A | transmembrane protein 183A [Source:HGNC Symbol;Acc:HGNC:20173] |
| ENSG00000163513 | HGLibA_49226 | 151 | 0.354004692 | TGFBR2 | transforming growth factor beta receptor 2 [Source:HGNC Symbol;Acc:HGNC:11773] |
| ENSG00000164073 | HGLibA_29117 | 151 | 0.354004692 | MFSD8 | major facilitator superfamily domain containing 8 [Source:HGNC Symbol;Acc:HGNC:28486] |
| ENSG00000164100 | HGLibA_31432 | 151 | 0.354004692 | NDST3 | N-deacetylase and N-sulfotransferase 3 [Source:HGNC Symbol;Acc:HGNC:7682] |
| ENSG00000164236 | HGLibA_02072 | 151 | 0.354004692 | ANKRD33B | ankyrin repeat domain 33B [Source:HGNC Symbol;Acc:HGNC:35240] |
| ENSG00000164615 | HGLibA_07099 | 151 | 0.354004692 | CAMLG | calcium modulating ligand [Source:HGNC Symbol;Acc:HGNC:1471] |
| ENSG00000165097 | HGLibA_24492 | 151 | 0.354004692 | KDM1B | lysine demethylase 1B [Source:HGNC Symbol;Acc:HGNC:21577] |
| ENSG00000165762 | HGLibA_34030 | 151 | 0.354004692 | OR4K2 | olfactory receptor family 4 subfamily K member 2 [Source:HGNC Symbol;Acc:HGNC:14728] |
| ENSG00000165959 | HGLibA_10070 | 151 | 0.354004692 | CLMN | calmin [Source:HGNC Symbol;Acc:HGNC:19972] |
| ENSG00000167098 | HGLibA_47796 | 151 | 0.354004692 | SUN5 | Sad1 and UNC84 domain containing 5 [Source:HGNC Symbol;Acc:HGNC:16252] |
| ENSG00000167378 | HGLibA_23617 | 151 | 0.354004692 | IRGQ | immunity related GTPase Q [Source:HGNC Symbol;Acc:HGNC:24868] |
| ENSG00000167994 | HGLibA_39926 | 151 | 0.354004692 | RAB3IL1 | RAB3A interacting protein like 1 [Source:HGNC Symbol;Acc:HGNC:9780] |
| ENSG00000168496 | HGLibA_17282 | 151 | 0.354004692 | FEN1 | flap structure-specific endonuclease 1 [Source:HGNC Symbol;Acc:HGNC:3650] |
| ENSG00000168763 | HGLibA_10320 | 151 | 0.354004692 | CNNM3 | cyclin and CBS domain divalent metal cation transport mediator 3 [Source:HGNC Symbol;Acc:HGNC:104] |
| ENSG00000168894 | HGLibA_41582 | 151 | 0.354004692 | RNF181 | ring finger protein 181 [Source:HGNC Symbol;Acc:HGNC:28037] |
| ENSG00000169385 | HGLibA_41404 | 151 | 0.354004692 | RNASE2 | ribonuclease A family member 2 [Source:HGNC Symbol;Acc:HGNC:10045] |
| ENSG00000169436 | HGLibA_10542 | 151 | 0.354004692 | COL22A1 | collagen type XXII alpha 1 chain [Source:HGNC Symbol;Acc:HGNC:22989] |
| ENSG00000169676 | HGLibA_13972 | 151 | 0.354004692 | DRD5 | dopamine receptor D5 [Source:HGNC Symbol;Acc:HGNC:3026] |
| ENSG00000171295 | HGLibA_56003 | 151 | 0.354004692 | ZNF440 | zinc finger protein 440 [Source:HGNC Symbol;Acc:HGNC:20874] |
| ENSG00000171540 | HGLibA_34751 | 151 | 0.354004692 | OTP | orthopedia homeobox [Source:HGNC Symbol;Acc:HGNC:8518] |
| ENSG00000172322 | HGLibA_09929 | 151 | 0.354004692 | CLEC12A | C-type lectin domain family 12 member A [Source:HGNC Symbol;Acc:HGNC:31713] |
| ENSG00000172538 | HGLibA_16261 | 151 | 0.354004692 | FAM170B | family with sequence similarity 170 member B [Source:HGNC Symbol;Acc:HGNC:19736] |
| ENSG00000173372 | HGLibA_05911 | 151 | 0.354004692 | C1QA | complement C1q A chain [Source:HGNC Symbol;Acc:HGNC:1241] |
| ENSG00000173821 | HGLibA_41623 | 151 | 0.354004692 | RNF213 | ring finger protein 213 [Source:HGNC Symbol;Acc:HGNC:14539] |
| ENSG00000174236 | HGLibA_40842 | 151 | 0.354004692 | REP15 | RAB15 effector protein [Source:HGNC Symbol;Acc:HGNC:33748] |
| ENSG00000174970 | HGLibA_33483 | 151 | 0.354004692 | OR10AG1 | olfactory receptor family 10 subfamily AG member 1 [Source:HGNC Symbol;Acc:HGNC:19607] |
| ENSG00000176884 | HGLibA_20297 | 151 | 0.354004692 | GRIN1 | glutamate ionotropic receptor NMDA type subunit 1 [Source:HGNC Symbol;Acc:HGNC:4584] |
| ENSG00000177302 | HGLibA_51000 | 151 | 0.354004692 | TOP3A | DNA topoisomerase III alpha [Source:HGNC Symbol;Acc:HGNC:11992] |
| ENSG00000178597 | HGLibA_39004 | 151 | 0.354004692 | PSAPL1 | prosaposin like 1 (gene/pseudogene) [Source:HGNC Symbol;Acc:HGNC:33131] |
| ENSG00000180592 | HGLibA_44280 | 151 | 0.354004692 | SKIDA1 | SKI/DACH domain containing 1 [Source:HGNC Symbol;Acc:HGNC:32697] |
| ENSG00000182308 | HGLibA_12442 | 151 | 0.354004692 | DCAF4L1 | DDB1 and CUL4 associated factor 4 like 1 [Source:HGNC Symbol;Acc:HGNC:27723] |
| ENSG00000182916 | HGLibA_48721 | 151 | 0.354004692 | TCEAL7 | transcription elongation factor A like 7 [Source:HGNC Symbol;Acc:HGNC:28336] |
| ENSG00000183072 | HGLibA_32043 | 151 | 0.354004692 | NKX2-5 | NK2 homeobox 5 [Source:HGNC Symbol;Acc:HGNC:2488] |
| ENSG00000183971 | HGLibA_32545 | 151 | 0.354004692 | NPW | neuropeptide W [Source:HGNC Symbol;Acc:HGNC:30509] |
| ENSG00000184860 | HGLibA_43165 | 151 | 0.354004692 | SDR42E1 | member 1 [Source:HGNC Symbol;Acc:HGNC:29834]" |
| ENSG00000184903 | HGLibA_23233 | 151 | 0.354004692 | IMMP2L | inner mitochondrial membrane peptidase subunit 2 [Source:HGNC Symbol;Acc:HGNC:14598] |
| ENSG00000185198 | HGLibA_38969 | 151 | 0.354004692 | PRSS57 | serine protease 57 [Source:HGNC Symbol;Acc:HGNC:31397] |
| ENSG00000185324 | HGLibA_08723 | 151 | 0.354004692 | CDK10 | cyclin dependent kinase 10 [Source:HGNC Symbol;Acc:HGNC:1770] |
| ENSG00000186212 | HGLibA_46216 | 151 | 0.354004692 | SOWAHB | sosondowah ankyrin repeat domain family member B [Source:HGNC Symbol;Acc:HGNC:32958] |
| ENSG00000186675 | HGLibA_27982 | 151 | 0.354004692 | MAGEE2 | MAGE family member E2 [Source:HGNC Symbol;Acc:HGNC:24935] |
| ENSG00000187824 | HGLibA_50233 | 151 | 0.354004692 | TMEM220 | transmembrane protein 220 [Source:HGNC Symbol;Acc:HGNC:33757] |
| ENSG00000188290 | HGLibA_21186 | 151 | 0.354004692 | HES4 | hes family bHLH transcription factor 4 [Source:HGNC Symbol;Acc:HGNC:24149] |
| ENSG00000197870 | HGLibA_38345 | 151 | 0.354004692 | PRB3 | proline rich protein BstNI subfamily 3 [Source:HGNC Symbol;Acc:HGNC:9339] |
| ENSG00000198218 | HGLibA_39743 | 151 | 0.354004692 | QRICH1 | glutamine rich 1 [Source:HGNC Symbol;Acc:HGNC:24713] |
| ENSG00000198471 | HGLibA_42425 | 151 | 0.354004692 | RTP2 | receptor transporter protein 2 [Source:HGNC Symbol;Acc:HGNC:32486] |

| | | | | | |
|---|---|---|---|---|---|
| ENSG00000203710 | HGLibA_11044 | 151 | 0.354004692 | CR1 | complement C3b/C4b receptor 1 (Knops blood group) [Source:HGNC Symbol;Acc:HGNC:2334] |
| ENSG00000204694 | HGLibA_33563 | 151 | 0.354004692 | OR11A1 | olfactory receptor family 11 subfamily A member 1 [Source:HGNC Symbol;Acc:HGNC:8176] |
| ENSG00000205362 | HGLibA_30276 | 151 | 0.354004692 | MT1A | metallothionein 1A [Source:HGNC Symbol;Acc:HGNC:7393] |
| ENSG00000214063 | HGLibA_51937 | 151 | 0.354004692 | TSPAN4 | tetraspanin 4 [Source:HGNC Symbol;Acc:HGNC:11859] |
| ENSG00000224902 | HGLibA_18456 | 151 | 0.354004692 | GAGE12H | G antigen 12H [Source:HGNC Symbol;Acc:HGNC:31908] |
| ENSG00000226761 | HGLibA_48415 | 151 | 0.354004692 | TAS2R46 | taste 2 receptor member 46 [Source:HGNC Symbol;Acc:HGNC:18877] |
| ENSG00000232838 | HGLibA_36076 | 151 | 0.354004692 | PET117 | PET117 homolog [Source:HGNC Symbol;Acc:HGNC:40045] |
| ENSG00000232995 | HGLibA_41097 | 151 | 0.354004692 | RGS5 | regulator of G protein signaling 5 [Source:NCBI gene;Acc:8490] |
| ENSG00000241119 | HGLibA_52899 | 151 | 0.354004692 | UGT1A9 | UDP glucuronosyltransferase family 1 member A9 [Source:HGNC Symbol;Acc:HGNC:12541] |
| ENSG00000267467 | HGLibA_02511 | 151 | 0.354004692 | APOC4 | apolipoprotein C4 [Source:HGNC Symbol;Acc:HGNC:611] |
| ENSG00000270757 | HGLibA_22336 | 151 | 0.354004692 | HSPE1-MOB4 | HSPE1-MOB4 readthrough [Source:HGNC Symbol;Acc:HGNC:49184] |
| FAM127B | HGLibA_16091 | 151 | 0.354004692 | FAM127B | - |
| FAM195A | HGLibA_16374 | 151 | 0.354004692 | FAM195A | - |
| hsa-mir-206 | HGLibA_58192 | 151 | 0.354004692 | hsa-mir-206 | - |
| hsa-mir-4479 | HGLibA_60331 | 151 | 0.354004692 | hsa-mir-4479 | - |
| hsa-mir-4534 | HGLibA_60596 | 151 | 0.354004692 | hsa-mir-4534 | - |
| hsa-mir-4675 | HGLibA_60815 | 151 | 0.354004692 | hsa-mir-4675 | - |
| hsa-mir-4763 | HGLibA_61149 | 151 | 0.354004692 | hsa-mir-4763 | - |
| hsa-mir-501 | HGLibA_61453 | 151 | 0.354004692 | hsa-mir-501 | - |
| hsa-mir-6510 | HGLibA_62892 | 151 | 0.354004692 | hsa-mir-6510 | - |
| hsa-mir-6738 | HGLibA_63107 | 151 | 0.354004692 | hsa-mir-6738 | - |
| hsa-mir-6746 | HGLibA_63139 | 151 | 0.354004692 | hsa-mir-6746 | - |
| METTL21B | HGLibA_28980 | 151 | 0.354004692 | METTL21B | - |
| TLDC1 | HGLibA_49600 | 151 | 0.354004692 | TLDC1 | - |
| TMEM133 | HGLibA_49944 | 151 | 0.354004692 | TMEM133 | - |
| C10orf107 | HGLibA_05024 | 150 | 0.344053655 | C10orf107 | - |
| C6orf165 | HGLibA_06459 | 150 | 0.344053655 | C6orf165 | - |
| ENSG00000003137 | HGLibA_12087 | 150 | 0.344053655 | CYP26B1 | cytochrome P450 family 26 subfamily B member 1 [Source:HGNC Symbol;Acc:HGNC:20581] |
| ENSG00000004478 | HGLibA_17575 | 150 | 0.344053655 | FKBP4 | FK506 binding protein 4 [Source:HGNC Symbol;Acc:HGNC:3720] |
| ENSG00000006025 | HGLibA_34663 | 150 | 0.344053655 | OSBPL7 | oxysterol binding protein like 7 [Source:HGNC Symbol;Acc:HGNC:16387] |
| ENSG00000006377 | HGLibA_13386 | 150 | 0.344053655 | DLX6 | distal-less homeobox 6 [Source:HGNC Symbol;Acc:HGNC:2919] |
| ENSG00000006740 | HGLibA_02779 | 150 | 0.344053655 | ARHGAP44 | Rho GTPase activating protein 44 [Source:HGNC Symbol;Acc:HGNC:29096] |
| ENSG00000008283 | HGLibA_11978 | 150 | 0.344053655 | CYB561 | cytochrome b561 [Source:HGNC Symbol;Acc:HGNC:2571] |
| ENSG00000057608 | HGLibA_18885 | 150 | 0.344053655 | GDI2 | GDP dissociation inhibitor 2 [Source:HGNC Symbol;Acc:HGNC:4227] |
| ENSG00000066455 | HGLibA_19604 | 150 | 0.344053655 | GOLGA5 | golgin A5 [Source:HGNC Symbol;Acc:HGNC:4428] |
| ENSG00000069812 | HGLibA_21180 | 150 | 0.344053655 | HES2 | hes family bHLH transcription factor 2 [Source:HGNC Symbol;Acc:HGNC:16005] |
| ENSG00000073756 | HGLibA_39362 | 150 | 0.344053655 | PTGS2 | prostaglandin-endoperoxide synthase 2 [Source:HGNC Symbol;Acc:HGNC:9605] |
| ENSG00000082258 | HGLibA_08128 | 150 | 0.344053655 | CCNT2 | cyclin T2 [Source:HGNC Symbol;Acc:HGNC:1600] |
| ENSG00000088826 | HGLibA_45801 | 150 | 0.344053655 | SMOX | spermine oxidase [Source:HGNC Symbol;Acc:HGNC:15862] |
| ENSG00000089063 | HGLibA_50255 | 150 | 0.344053655 | TMEM230 | transmembrane protein 230 [Source:HGNC Symbol;Acc:HGNC:15876] |
| ENSG00000089486 | HGLibA_08714 | 150 | 0.344053655 | CDIP1 | cell death inducing p53 target 1 [Source:HGNC Symbol;Acc:HGNC:13234] |
| ENSG00000091831 | HGLibA_15580 | 150 | 0.344053655 | ESR1 | estrogen receptor 1 [Source:HGNC Symbol;Acc:HGNC:3467] |
| ENSG00000096092 | HGLibA_49980 | 150 | 0.344053655 | TMEM14A | transmembrane protein 14A [Source:HGNC Symbol;Acc:HGNC:21076] |
| ENSG00000100024 | HGLibA_53053 | 150 | 0.344053655 | UPB1 | beta-ureidopropionase 1 [Source:HGNC Symbol;Acc:HGNC:16297] |
| ENSG00000100242 | HGLibA_47791 | 150 | 0.344053655 | SUN2 | Sad1 and UNC84 domain containing 2 [Source:HGNC Symbol;Acc:HGNC:14210] |
| ENSG00000100485 | HGLibA_46205 | 150 | 0.344053655 | SOS2 | SOS Ras/Rho guanine nucleotide exchange factor 2 [Source:HGNC Symbol;Acc:HGNC:11188] |
| ENSG00000102312 | HGLibA_37708 | 150 | 0.344053655 | PORCN | porcupine O-acyltransferase [Source:HGNC Symbol;Acc:HGNC:17652] |
| ENSG00000102984 | HGLibA_56819 | 150 | 0.344053655 | ZNF821 | zinc finger protein 821 [Source:HGNC Symbol;Acc:HGNC:28043] |
| ENSG00000104972 | HGLibA_26507 | 150 | 0.344053655 | LILRB1 | leukocyte immunoglobulin like receptor B1 [Source:HGNC Symbol;Acc:HGNC:6605] |
| ENSG00000105388 | HGLibA_08945 | 150 | 0.344053655 | CEACAM5 | carcinoembryonic antigen related cell adhesion molecule 5 [Source:HGNC Symbol;Acc:HGNC:1817] |
| ENSG00000105639 | HGLibA_23918 | 150 | 0.344053655 | JAK3 | Janus kinase 3 [Source:HGNC Symbol;Acc:HGNC:6193] |
| ENSG00000105996 | HGLibA_21906 | 150 | 0.344053655 | HOXA2 | homeobox A2 [Source:HGNC Symbol;Acc:HGNC:5103] |
| ENSG00000107249 | HGLibA_19277 | 150 | 0.344053655 | GLIS3 | GLIS family zinc finger 3 [Source:HGNC Symbol;Acc:HGNC:28510] |
| ENSG00000107438 | HGLibA_35906 | 150 | 0.344053655 | PDLIM1 | PDZ and LIM domain 1 [Source:HGNC Symbol;Acc:HGNC:2067] |
| ENSG00000107864 | HGLibA_10922 | 150 | 0.344053655 | CPEB3 | cytoplasmic polyadenylation element binding protein 3 [Source:HGNC Symbol;Acc:HGNC:21746] |
| ENSG00000107957 | HGLibA_43963 | 150 | 0.344053655 | SH3PXD2A | SH3 and PX domains 2A [Source:HGNC Symbol;Acc:HGNC:23664] |
| ENSG00000109471 | HGLibA_23071 | 150 | 0.344053655 | IL2 | interleukin 2 [Source:HGNC Symbol;Acc:HGNC:6001] |
| ENSG00000110244 | HGLibA_02457 | 150 | 0.344053655 | APOA4 | apolipoprotein A4 [Source:HGNC Symbol;Acc:HGNC:602] |
| ENSG00000110446 | HGLibA_44410 | 150 | 0.344053655 | SLC15A3 | solute carrier family 15 member 3 [Source:HGNC Symbol;Acc:HGNC:18068] |
| ENSG00000111684 | HGLibA_27076 | 150 | 0.344053655 | LPCAT3 | lysophosphatidylcholine acyltransferase 3 [Source:HGNC Symbol;Acc:HGNC:30244] |
| ENSG00000112294 | HGLibA_01578 | 150 | 0.344053655 | ALDH5A1 | aldehyde dehydrogenase 5 family member A1 [Source:HGNC Symbol;Acc:HGNC:408] |
| ENSG00000115226 | HGLibA_17754 | 150 | 0.344053655 | FNDC4 | fibronectin type III domain containing 4 [Source:HGNC Symbol;Acc:HGNC:20239] |
| ENSG00000115828 | HGLibA_39728 | 150 | 0.344053655 | QPCT | glutaminyl-peptide cyclotransferase [Source:HGNC Symbol;Acc:HGNC:9753] |
| ENSG00000115993 | HGLibA_51280 | 150 | 0.344053655 | TRAK2 | trafficking kinesin protein 2 [Source:HGNC Symbol;Acc:HGNC:13206] |
| ENSG00000116209 | HGLibA_50460 | 150 | 0.344053655 | TMEM59 | transmembrane protein 59 [Source:HGNC Symbol;Acc:HGNC:1239] |
| ENSG00000116218 | HGLibA_32457 | 150 | 0.344053655 | NPHS2 | podocin [Source:HGNC Symbol;Acc:HGNC:13394]" |
| ENSG00000116273 | HGLibA_36343 | 150 | 0.344053655 | PHF13 | PHD finger protein 13 [Source:HGNC Symbol;Acc:HGNC:22983] |
| ENSG00000116962 | HGLibA_31922 | 150 | 0.344053655 | NID1 | nidogen 1 [Source:HGNC Symbol;Acc:HGNC:7821] |
| ENSG00000117586 | HGLibA_50823 | 150 | 0.344053655 | TNFSF4 | TNF superfamily member 4 [Source:HGNC Symbol;Acc:HGNC:11934] |
| ENSG00000119698 | HGLibA_38208 | 150 | 0.344053655 | PPP4R4 | protein phosphatase 4 regulatory subunit 4 [Source:HGNC Symbol;Acc:HGNC:23788] |
| ENSG00000119820 | HGLibA_54679 | 150 | 0.344053655 | YIPF4 | Yip1 domain family member 4 [Source:HGNC Symbol;Acc:HGNC:28145] |
| ENSG00000120318 | HGLibA_02632 | 150 | 0.344053655 | ARAP3 | ankyrin repeat and PH domain 3 [Source:HGNC Symbol;Acc:HGNC:24097]" |
| ENSG00000120963 | HGLibA_56597 | 150 | 0.344053655 | ZNF706 | zinc finger protein 706 [Source:HGNC Symbol;Acc:HGNC:24992] |
| ENSG00000122136 | HGLibA_33225 | 150 | 0.344053655 | OBP2A | odorant binding protein 2A [Source:HGNC Symbol;Acc:HGNC:23380] |
| ENSG00000124449 | HGLibA_23611 | 150 | 0.344053655 | IRGC | immunity related GTPase cinema [Source:HGNC Symbol;Acc:HGNC:28835] |
| ENSG00000125434 | HGLibA_44718 | 150 | 0.344053655 | SLC25A35 | solute carrier family 25 member 35 [Source:HGNC Symbol;Acc:HGNC:31921] |
| ENSG00000125817 | HGLibA_09041 | 150 | 0.344053655 | CENPB | centromere protein B [Source:HGNC Symbol;Acc:HGNC:1852] |
| ENSG00000125878 | HGLibA_48763 | 150 | 0.344053655 | TCF15 | transcription factor 15 [Source:HGNC Symbol;Acc:HGNC:11627] |
| ENSG00000126562 | HGLibA_54341 | 150 | 0.344053655 | WNK4 | WNK lysine deficient protein kinase 4 [Source:HGNC Symbol;Acc:HGNC:14544] |
| ENSG00000127022 | HGLibA_07129 | 150 | 0.344053655 | CANX | calnexin [Source:HGNC Symbol;Acc:HGNC:1473] |
| ENSG00000131015 | HGLibA_52963 | 150 | 0.344053655 | ULBP2 | UL16 binding protein 2 [Source:HGNC Symbol;Acc:HGNC:14894] |
| ENSG00000131183 | HGLibA_44937 | 150 | 0.344053655 | SLC34A1 | solute carrier family 34 member 1 [Source:HGNC Symbol;Acc:HGNC:11019] |
| ENSG00000131373 | HGLibA_20803 | 150 | 0.344053655 | HACL1 | 2-hydroxyacyl-CoA lyase 1 [Source:HGNC Symbol;Acc:HGNC:17856] |
| ENSG00000132471 | HGLibA_53978 | 150 | 0.344053655 | WBP2 | WW domain binding protein 2 [Source:HGNC Symbol;Acc:HGNC:12738] |
| ENSG00000133101 | HGLibA_08050 | 150 | 0.344053655 | CCNA1 | cyclin A1 [Source:HGNC Symbol;Acc:HGNC:1577] |
| ENSG00000133665 | HGLibA_14224 | 150 | 0.344053655 | DYDC2 | DPY30 domain containing 2 [Source:HGNC Symbol;Acc:HGNC:23468] |
| ENSG00000134461 | HGLibA_02011 | 150 | 0.344053655 | ANKRD16 | ankyrin repeat domain 16 [Source:HGNC Symbol;Acc:HGNC:23471] |
| ENSG00000136717 | HGLibA_04505 | 150 | 0.344053655 | BIN1 | bridging integrator 1 [Source:HGNC Symbol;Acc:HGNC:1052] |
| ENSG00000137672 | HGLibA_51747 | 150 | 0.344053655 | TRPC6 | transient receptor potential cation channel subfamily C member 6 [Source:HGNC Symbol;Acc:HGNC:12338] |
| ENSG00000137675 | HGLibA_29528 | 150 | 0.344053655 | MMP27 | matrix metallopeptidase 27 [Source:HGNC Symbol;Acc:HGNC:14250] |
| ENSG00000138166 | HGLibA_14185 | 150 | 0.344053655 | DUSP5 | dual specificity phosphatase 5 [Source:HGNC Symbol;Acc:HGNC:3071] |
| ENSG00000138867 | HGLibA_20648 | 150 | 0.344053655 | GUCD1 | guanylyl cyclase domain containing 1 [Source:HGNC Symbol;Acc:HGNC:14237] |
| ENSG00000139117 | HGLibA_10974 | 150 | 0.344053655 | CPNE8 | copine 8 [Source:HGNC Symbol;Acc:HGNC:23498] |
| ENSG00000139636 | HGLibA_26672 | 150 | 0.344053655 | LMBR1L | limb development membrane protein 1 like [Source:HGNC Symbol;Acc:HGNC:18268] |
| ENSG00000143443 | HGLibA_05877 | 150 | 0.344053655 | C1orf56 | chromosome 1 open reading frame 56 [Source:HGNC Symbol;Acc:HGNC:26045] |
| ENSG00000145220 | HGLibA_27694 | 150 | 0.344053655 | LYAR | Ly1 antibody reactive [Source:HGNC Symbol;Acc:HGNC:26021] |
| ENSG00000147160 | HGLibA_03915 | 150 | 0.344053655 | AWAT2 | acyl-CoA wax alcohol acyltransferase 2 [Source:HGNC Symbol;Acc:HGNC:23251] |
| ENSG00000147873 | HGLibA_22682 | 150 | 0.344053655 | IFNA5 | interferon alpha 5 [Source:HGNC Symbol;Acc:HGNC:5426] |
| ENSG00000148411 | HGLibA_31041 | 150 | 0.344053655 | NACC2 | NACC family member 2 [Source:HGNC Symbol;Acc:HGNC:23846] |
| ENSG00000151929 | HGLibA_04081 | 150 | 0.344053655 | BAG3 | BCL2 associated athanogene 3 [Source:HGNC Symbol;Acc:HGNC:939] |
| ENSG00000153558 | HGLibA_16997 | 150 | 0.344053655 | FBXL2 | F-box and leucine rich repeat protein 2 [Source:HGNC Symbol;Acc:HGNC:13598] |
| ENSG00000153563 | HGLibA_08470 | 150 | 0.344053655 | CD8A | CD8a molecule [Source:HGNC Symbol;Acc:HGNC:1706] |
| ENSG00000154277 | HGLibA_52802 | 150 | 0.344053655 | UCHL1 | ubiquitin C-terminal hydrolase L1 [Source:HGNC Symbol;Acc:HGNC:12513] |
| ENSG00000156299 | HGLibA_49439 | 150 | 0.344053655 | TIAM1 | T cell lymphoma invasion and metastasis 1 [Source:HGNC Symbol;Acc:HGNC:11805] |
| ENSG00000158077 | HGLibA_32111 | 150 | 0.344053655 | NLRP14 | NLR family pyrin domain containing 14 [Source:HGNC Symbol;Acc:HGNC:22939] |
| ENSG00000158286 | HGLibA_41612 | 150 | 0.344053655 | RNF207 | ring finger protein 207 [Source:HGNC Symbol;Acc:HGNC:32947] |
| ENSG00000160349 | HGLibA_26165 | 150 | 0.344053655 | LCN1 | lipocalin 1 [Source:HGNC Symbol;Acc:HGNC:6525] |
| ENSG00000163618 | HGLibA_06978 | 150 | 0.344053655 | CADPS | calcium dependent secretion activator [Source:HGNC Symbol;Acc:HGNC:1426] |
| ENSG00000164172 | HGLibA_29616 | 150 | 0.344053655 | MOCS2 | molybdenum cofactor synthesis 2 [Source:HGNC Symbol;Acc:HGNC:7193] |
| ENSG00000165632 | HGLibA_48230 | 150 | 0.344053655 | TAF3 | TATA-box binding protein associated factor 3 [Source:HGNC Symbol;Acc:HGNC:17303] |
| ENSG00000166272 | HGLibA_53973 | 150 | 0.344053655 | WBP1L | WW domain binding protein 1 like [Source:HGNC Symbol;Acc:HGNC:23510] |
| ENSG00000166526 | HGLibA_55766 | 150 | 0.344053655 | ZNF3 | zinc finger protein 3 [Source:HGNC Symbol;Acc:HGNC:13089] |
| ENSG00000167077 | HGLibA_28862 | 150 | 0.344053655 | MEI1 | meiotic double-stranded break formation protein 1 [Source:HGNC Symbol;Acc:HGNC:28613] |
| ENSG00000167080 | HGLibA_04015 | 150 | 0.344053655 | B4GALNT2 | 4-N-acetyl-galactosaminyltransferase 2 [Source:HGNC Symbol;Acc:HGNC:24136]" |
| ENSG00000167625 | HGLibA_56165 | 150 | 0.344053655 | ZNF526 | zinc finger protein 526 [Source:HGNC Symbol;Acc:HGNC:29415] |
| ENSG00000168394 | HGLibA_48314 | 150 | 0.344053655 | TAP1 | ATP binding cassette subfamily B member [Source:HGNC Symbol;Acc:HGNC:43]" |
| ENSG00000169131 | HGLibA_55862 | 150 | 0.344053655 | ZNF354A | zinc finger protein 354A [Source:HGNC Symbol;Acc:HGNC:11628] |
| ENSG00000170604 | HGLibA_23578 | 150 | 0.344053655 | IRF2BP1 | interferon regulatory factor 2 binding protein 1 [Source:HGNC Symbol;Acc:HGNC:21728] |
| ENSG00000172005 | HGLibA_28022 | 150 | 0.344053655 | MAL | T cell differentiation protein [Source:HGNC Symbol;Acc:HGNC:6817]" |
| ENSG00000172071 | HGLibA_14729 | 150 | 0.344053655 | EIF2AK3 | eukaryotic translation initiation factor 2 alpha kinase 3 [Source:HGNC Symbol;Acc:HGNC:3255] |
| ENSG00000172724 | HGLibA_07984 | 150 | 0.344053655 | CCL19 | C-C motif chemokine ligand 19 [Source:HGNC Symbol;Acc:HGNC:10617] |
| ENSG00000173250 | HGLibA_19932 | 150 | 0.344053655 | GPR151 | G protein-coupled receptor 151 [Source:HGNC Symbol;Acc:HGNC:23624] |
| ENSG00000173699 | HGLibA_46464 | 150 | 0.344053655 | SPATA3 | spermatogenesis associated 3 [Source:HGNC Symbol;Acc:HGNC:17884] |
| ENSG00000173889 | HGLibA_36323 | 150 | 0.344053655 | PHC3 | polyhomeotic homolog 3 [Source:HGNC Symbol;Acc:HGNC:15682] |
| ENSG00000173928 | HGLibA_47901 | 150 | 0.344053655 | SWSAP1 | SWIM-type zinc finger 7 associated protein 1 [Source:HGNC Symbol;Acc:HGNC:26638] |
| ENSG00000174021 | HGLibA_19530 | 150 | 0.344053655 | GNG5 | G protein subunit gamma 5 [Source:HGNC Symbol;Acc:HGNC:4408] |
| ENSG00000176798 | HGLibA_34122 | 150 | 0.344053655 | OR51L1 | olfactory receptor family 51 subfamily L member 1 [Source:HGNC Symbol;Acc:HGNC:14759] |
| ENSG00000177300 | HGLibA_09884 | 150 | 0.344053655 | CLDN22 | claudin 22 [Source:HGNC Symbol;Acc:HGNC:2044] |
| ENSG00000177674 | HGLibA_01268 | 150 | 0.344053655 | AGTRAP | angiotensin II receptor associated protein [Source:HGNC Symbol;Acc:HGNC:13539] |
| ENSG00000178385 | HGLibA_37144 | 150 | 0.344053655 | PLEKHM3 | pleckstrin homology domain containing M3 [Source:HGNC Symbol;Acc:HGNC:34006] |
| ENSG00000179041 | HGLibA_42289 | 150 | 0.344053655 | RRS1 | ribosome biogenesis regulator homolog [Source:HGNC Symbol;Acc:HGNC:17083] |
| ENSG00000180035 | HGLibA_56067 | 150 | 0.344053655 | ZNF48 | zinc finger protein 48 [Source:HGNC Symbol;Acc:HGNC:13114] |
| ENSG00000184500 | HGLibA_38688 | 150 | 0.344053655 | PROS1 | protein S [Source:HGNC Symbol;Acc:HGNC:9456] |
| ENSG00000186153 | HGLibA_54449 | 150 | 0.344053655 | WWOX | WW domain containing oxidoreductase [Source:HGNC Symbol;Acc:HGNC:12799] |
| ENSG00000187223 | HGLibA_26126 | 150 | 0.344053655 | LCE2D | late cornified envelope 2D [Source:HGNC Symbol;Acc:HGNC:16518] |

| | | | | | |
|---|---|---|---|---|---|
| ENSG00000188554 | HGLibA_31248 | 150 | 0.344053655 | NBR1 | autophagy cargo receptor [Source:HGNC Symbol;Acc:HGNC:6746]" |
| ENSG00000189143 | HGLibA_09898 | 150 | 0.344053655 | CLDN4 | claudin 4 [Source:HGNC Symbol;Acc:HGNC:2046] |
| ENSG00000196263 | HGLibA_56055 | 150 | 0.344053655 | ZNF471 | zinc finger protein 471 [Source:HGNC Symbol;Acc:HGNC:23226] |
| ENSG00000196547 | HGLibA_28072 | 150 | 0.344053655 | MAN2A2 | mannosidase alpha class 2A member 2 [Source:HGNC Symbol;Acc:HGNC:6825] |
| ENSG00000196814 | HGLibA_30591 | 150 | 0.344053655 | MVB12B | multivesicular body subunit 12B [Source:HGNC Symbol;Acc:HGNC:23368] |
| ENSG00000197992 | HGLibA_10010 | 150 | 0.344053655 | CLEC9A | C-type lectin domain containing 9A [Source:HGNC Symbol;Acc:HGNC:26705] |
| ENSG00000198856 | HGLibA_34709 | 150 | 0.344053655 | OSTC | oligosaccharyltransferase complex non-catalytic subunit [Source:HGNC Symbol;Acc:HGNC:24448] |
| ENSG00000204700 | HGLibA_33808 | 150 | 0.344053655 | OR2J2 | olfactory receptor family 2 subfamily J member 2 [Source:HGNC Symbol;Acc:HGNC:8260] |
| ENSG00000205423 | HGLibA_10258 | 150 | 0.344053655 | CNEP1R1 | CTD nuclear envelope phosphatase 1 regulatory subunit 1 [Source:HGNC Symbol;Acc:HGNC:26759] |
| ENSG00000205784 | HGLibA_03125 | 150 | 0.344053655 | ARRDC5 | arrestin domain containing 5 [Source:HGNC Symbol;Acc:HGNC:31407] |
| ENSG00000205835 | HGLibA_19414 | 150 | 0.344053655 | GMNC | geminin coiled-coil domain containing [Source:HGNC Symbol;Acc:HGNC:40049] |
| ENSG00000213927 | HGLibA_08011 | 150 | 0.344053655 | CCL27 | C-C motif chemokine ligand 27 [Source:HGNC Symbol;Acc:HGNC:10626] |
| ENSG00000215912 | HGLibA_52119 | 150 | 0.344053655 | TTC34 | tetratricopeptide repeat domain 34 [Source:HGNC Symbol;Acc:HGNC:34297] |
| ENSG00000221890 | HGLibA_32537 | 150 | 0.344053655 | NPTXR | neuronal pentraxin receptor [Source:HGNC Symbol;Acc:HGNC:7954] |
| ENSG00000223638 | HGLibA_40939 | 150 | 0.344053655 | RFPL4A | ret finger protein like 4A [Source:HGNC Symbol;Acc:HGNC:16449] |
| ENSG00000229549 | HGLibA_51976 | 150 | 0.344053655 | TSPY8 | testis specific protein Y-linked 8 [Source:HGNC Symbol;Acc:HGNC:37471] |
| ENSG00000244474 | HGLibA_52884 | 150 | 0.344053655 | UGT1A4 | UDP glucuronosyltransferase family 1 member A4 [Source:HGNC Symbol;Acc:HGNC:12536] |
| ENSG00000270601 | HGLibA_38320 | 150 | 0.344053655 | PRAMEF5 | PRAME family member 5 [Source:HGNC Symbol;Acc:HGNC:27995] |
| ENSG00000275183 | HGLibA_26304 | 150 | 0.344053655 | LENG9 | leukocyte receptor cluster member 9 [Source:HGNC Symbol;Acc:HGNC:16306] |
| ENSG00000279169 | HGLibA_38291 | 150 | 0.344053655 | PRAMEF13 | PRAME family member 13 [Source:HGNC Symbol;Acc:HGNC:13262] |
| H2AFY2 | HGLibA_20767 | 150 | 0.344053655 | H2AFY2 | - |
| HNRPDL | HGLibA_21854 | 150 | 0.344053655 | HNRPDL | - |
| hsa-mir-103b-1 | HGLibA_57171 | 150 | 0.344053655 | hsa-mir-103b-1 | - |
| hsa-mir-17 | HGLibA_57955 | 150 | 0.344053655 | hsa-mir-17 | - |
| hsa-mir-299 | HGLibA_58407 | 150 | 0.344053655 | hsa-mir-299 | - |
| hsa-mir-3191 | HGLibA_58863 | 150 | 0.344053655 | hsa-mir-3191 | - |
| hsa-mir-378d-1 | HGLibA_59438 | 150 | 0.344053655 | hsa-mir-378d-1 | - |
| hsa-mir-4681 | HGLibA_60836 | 150 | 0.344053655 | hsa-mir-4681 | - |
| hsa-mir-520b | HGLibA_61737 | 150 | 0.344053655 | hsa-mir-520b | - |
| hsa-mir-6772 | HGLibA_63261 | 150 | 0.344053655 | hsa-mir-6772 | - |
| hsa-mir-6824 | HGLibA_63475 | 150 | 0.344053655 | hsa-mir-6824 | - |
| LOC100129083 | HGLibA_26754 | 150 | 0.344053655 | LOC100129083 | - |
| LOC643669 | HGLibA_26970 | 150 | 0.344053655 | LOC643669 | - |
| LRRC33 | HGLibA_27316 | 150 | 0.344053655 | LRRC33 | - |
| ST5 | HGLibA_47254 | 150 | 0.344053655 | ST5 | - |
| TMEM189 | HGLibA_50132 | 150 | 0.344053655 | TMEM189 | - |
| ZFP106 | HGLibA_55183 | 150 | 0.344053655 | ZFP106 | - |
| C4orf32 | HGLibA_06320 | 149 | 0.334102618 | C4orf32 | - |
| C6orf195 | HGLibA_06464 | 149 | 0.334102618 | C6orf195 | - |
| ENSG00000007402 | HGLibA_06907 | 149 | 0.334102618 | CACNA2D2 | calcium voltage-gated channel auxiliary subunit alpha2delta 2 [Source:HGNC Symbol;Acc:HGNC:1400] |
| ENSG00000008869 | HGLibA_21079 | 149 | 0.334102618 | HEATR5B | HEAT repeat containing 5B [Source:HGNC Symbol;Acc:HGNC:29273] |
| ENSG00000010318 | HGLibA_36386 | 149 | 0.334102618 | PHF7 | PHD finger protein 7 [Source:HGNC Symbol;Acc:HGNC:18458] |
| ENSG00000020256 | HGLibA_55227 | 149 | 0.334102618 | ZFP64 | ZFP64 zinc finger protein [Source:HGNC Symbol;Acc:HGNC:15940] |
| ENSG00000021355 | HGLibA_43534 | 149 | 0.334102618 | SERPINB1 | serpin family B member 1 [Source:HGNC Symbol;Acc:HGNC:3311] |
| ENSG00000023287 | HGLibA_40417 | 149 | 0.334102618 | RB1CC1 | RB1 inducible coiled-coil 1 [Source:HGNC Symbol;Acc:HGNC:15574] |
| ENSG00000052802 | HGLibA_30227 | 149 | 0.334102618 | MSMO1 | methylsterol monooxygenase 1 [Source:HGNC Symbol;Acc:HGNC:10545] |
| ENSG00000058866 | HGLibA_13071 | 149 | 0.334102618 | DGKG | diacylglycerol kinase gamma [Source:HGNC Symbol;Acc:HGNC:2853] |
| ENSG00000065054 | HGLibA_45411 | 149 | 0.334102618 | SLC9A3R2 | SLC9A3 regulator 2 [Source:HGNC Symbol;Acc:HGNC:11076] |
| ENSG00000079102 | HGLibA_42459 | 149 | 0.334102618 | RUNX1T1 | RUNX1 translocation partner 1 [Source:HGNC Symbol;Acc:HGNC:1535] |
| ENSG00000086730 | HGLibA_26062 | 149 | 0.334102618 | LAT2 | linker for activation of T cells family member 2 [Source:HGNC Symbol;Acc:HGNC:12749] |
| ENSG00000089335 | HGLibA_55773 | 149 | 0.334102618 | ZNF302 | zinc finger protein 302 [Source:HGNC Symbol;Acc:HGNC:13848] |
| ENSG00000090857 | HGLibA_35936 | 149 | 0.334102618 | PDPR | pyruvate dehydrogenase phosphatase regulatory subunit [Source:HGNC Symbol;Acc:HGNC:30264] |
| ENSG00000091542 | HGLibA_01658 | 149 | 0.334102618 | ALKBH5 | RNA demethylase [Source:HGNC Symbol;Acc:HGNC:25996]" |
| ENSG00000092470 | HGLibA_54179 | 149 | 0.334102618 | WDR76 | WD repeat domain 76 [Source:HGNC Symbol;Acc:HGNC:25773] |
| ENSG00000100739 | HGLibA_04380 | 149 | 0.334102618 | BDKRB1 | bradykinin receptor B1 [Source:HGNC Symbol;Acc:HGNC:1029] |
| ENSG00000101751 | HGLibA_37531 | 149 | 0.334102618 | POLI | DNA polymerase iota [Source:HGNC Symbol;Acc:HGNC:9182] |
| ENSG00000104412 | HGLibA_15007 | 149 | 0.334102618 | EMC2 | ER membrane protein complex subunit 2 [Source:HGNC Symbol;Acc:HGNC:28963] |
| ENSG00000105401 | HGLibA_08546 | 149 | 0.334102618 | CDC37 | cell division cycle 37 [Source:HGNC Symbol;Acc:HGNC:1735] |
| ENSG00000105607 | HGLibA_18770 | 149 | 0.334102618 | GCDH | glutaryl-CoA dehydrogenase [Source:HGNC Symbol;Acc:HGNC:4189] |
| ENSG00000105983 | HGLibA_26669 | 149 | 0.334102618 | LMBR1 | limb development membrane protein 1 [Source:HGNC Symbol;Acc:HGNC:13243] |
| ENSG00000106009 | HGLibA_04758 | 149 | 0.334102618 | BRAT1 | BRCA1 associated ATM activator 1 [Source:HGNC Symbol;Acc:HGNC:21701] |
| ENSG00000106066 | HGLibA_11030 | 149 | 0.334102618 | CPVL | vitellogenic like [Source:HGNC Symbol;Acc:HGNC:14399]" |
| ENSG00000112425 | HGLibA_15336 | 149 | 0.334102618 | EPM2A | laforin glucan phosphatase [Source:HGNC Symbol;Acc:HGNC:3413]" |
| ENSG00000113240 | HGLibA_10062 | 149 | 0.334102618 | CLK4 | CDC like kinase 4 [Source:HGNC Symbol;Acc:HGNC:13659] |
| ENSG00000115109 | HGLibA_15258 | 149 | 0.334102618 | EPB41L5 | erythrocyte membrane protein band 4.1 like 5 [Source:HGNC Symbol;Acc:HGNC:19819] |
| ENSG00000115607 | HGLibA_23030 | 149 | 0.334102618 | IL18RAP | interleukin 18 receptor accessory protein [Source:HGNC Symbol;Acc:HGNC:5989] |
| ENSG00000119927 | HGLibA_19725 | 149 | 0.334102618 | GPAM | mitochondrial [Source:HGNC Symbol;Acc:HGNC:24865]" |
| ENSG00000121314 | HGLibA_48428 | 149 | 0.334102618 | TAS2R8 | taste 2 receptor member 8 [Source:HGNC Symbol;Acc:HGNC:14915] |
| ENSG00000122679 | HGLibA_40187 | 149 | 0.334102618 | RAMP3 | receptor activity modifying protein 3 [Source:HGNC Symbol;Acc:HGNC:9845] |
| ENSG00000124006 | HGLibA_33235 | 149 | 0.334102618 | OBSL1 | obscurin like 1 [Source:HGNC Symbol;Acc:HGNC:29092] |
| ENSG00000124214 | HGLibA_47406 | 149 | 0.334102618 | STAU1 | staufen double-stranded RNA binding protein 1 [Source:HGNC Symbol;Acc:HGNC:11370] |
| ENSG00000124731 | HGLibA_51355 | 149 | 0.334102618 | TREM1 | triggering receptor expressed on myeloid cells 1 [Source:HGNC Symbol;Acc:HGNC:17760] |
| ENSG00000124743 | HGLibA_25183 | 149 | 0.334102618 | KLHL31 | kelch like family member 31 [Source:HGNC Symbol;Acc:HGNC:21353] |
| ENSG00000125834 | HGLibA_47495 | 149 | 0.334102618 | STK35 | serine/threonine kinase 35 [Source:HGNC Symbol;Acc:HGNC:16254] |
| ENSG00000129003 | HGLibA_53738 | 149 | 0.334102618 | VPS13C | vacuolar protein sorting 13 homolog C [Source:HGNC Symbol;Acc:HGNC:23594] |
| ENSG00000129221 | HGLibA_01363 | 149 | 0.334102618 | AIPL1 | aryl hydrocarbon receptor interacting protein like 1 [Source:HGNC Symbol;Acc:HGNC:359] |
| ENSG00000133114 | HGLibA_19722 | 149 | 0.334102618 | GPALPP1 | GPALPP motifs containing 1 [Source:HGNC Symbol;Acc:HGNC:20298] |
| ENSG00000134640 | HGLibA_30448 | 149 | 0.334102618 | MTNR1B | melatonin receptor 1B [Source:HGNC Symbol;Acc:HGNC:7464] |
| ENSG00000135211 | HGLibA_50466 | 149 | 0.334102618 | TMEM60 | transmembrane protein 60 [Source:HGNC Symbol;Acc:HGNC:21754] |
| ENSG00000136238 | HGLibA_40043 | 149 | 0.334102618 | RAC1 | Rac family small GTPase 1 [Source:HGNC Symbol;Acc:HGNC:9801] |
| ENSG00000137309 | HGLibA_21684 | 149 | 0.334102618 | HMGA1 | high mobility group AT-hook 1 [Source:HGNC Symbol;Acc:HGNC:5010] |
| ENSG00000137393 | HGLibA_41530 | 149 | 0.334102618 | RNF144B | ring finger protein 144B [Source:HGNC Symbol;Acc:HGNC:21578] |
| ENSG00000137814 | HGLibA_20878 | 149 | 0.334102618 | HAUS2 | HAUS augmin like complex subunit 2 [Source:HGNC Symbol;Acc:HGNC:25530] |
| ENSG00000137825 | HGLibA_23843 | 149 | 0.334102618 | ITPKA | inositol-trisphosphate 3-kinase A [Source:HGNC Symbol;Acc:HGNC:6178] |
| ENSG00000138182 | HGLibA_24895 | 149 | 0.334102618 | KIF20B | kinesin family member 20B [Source:HGNC Symbol;Acc:HGNC:7212] |
| ENSG00000138382 | HGLibA_29013 | 149 | 0.334102618 | METTL5 | methyltransferase like 5 [Source:HGNC Symbol;Acc:HGNC:25006] |
| ENSG00000139287 | HGLibA_51133 | 149 | 0.334102618 | TPH2 | tryptophan hydroxylase 2 [Source:HGNC Symbol;Acc:HGNC:20692] |
| ENSG00000139826 | HGLibA_00244 | 149 | 0.334102618 | ABHD13 | abhydrolase domain containing 13 [Source:HGNC Symbol;Acc:HGNC:20293] |
| ENSG00000139910 | HGLibA_32364 | 149 | 0.334102618 | NOVA1 | NOVA alternative splicing regulator 1 [Source:HGNC Symbol;Acc:HGNC:7886] |
| ENSG00000140398 | HGLibA_31630 | 149 | 0.334102618 | NEIL1 | nei like DNA glycosylase 1 [Source:HGNC Symbol;Acc:HGNC:18448] |
| ENSG00000141968 | HGLibA_53547 | 149 | 0.334102618 | VAV1 | vav guanine nucleotide exchange factor 1 [Source:HGNC Symbol;Acc:HGNC:12657] |
| ENSG00000142687 | HGLibA_24624 | 149 | 0.334102618 | KIAA0319L | KIAA0319 like [Source:HGNC Symbol;Acc:HGNC:30071] |
| ENSG00000143761 | HGLibA_02646 | 149 | 0.334102618 | ARF1 | ADP ribosylation factor 1 [Source:HGNC Symbol;Acc:HGNC:652] |
| ENSG00000144191 | HGLibA_10268 | 149 | 0.334102618 | CNGA3 | cyclic nucleotide gated channel alpha 3 [Source:HGNC Symbol;Acc:HGNC:2150] |
| ENSG00000144451 | HGLibA_46351 | 149 | 0.334102618 | SPAG16 | sperm associated antigen 16 [Source:HGNC Symbol;Acc:HGNC:23225] |
| ENSG00000145283 | HGLibA_44346 | 149 | 0.334102618 | SLC10A6 | solute carrier family 10 member 6 [Source:HGNC Symbol;Acc:HGNC:30603] |
| ENSG00000146416 | HGLibA_01347 | 149 | 0.334102618 | AIG1 | androgen induced 1 [Source:HGNC Symbol;Acc:HGNC:21607] |
| ENSG00000147606 | HGLibA_44805 | 149 | 0.334102618 | SLC26A7 | solute carrier family 26 member 7 [Source:HGNC Symbol;Acc:HGNC:14467] |
| ENSG00000148082 | HGLibA_44006 | 149 | 0.334102618 | SHC3 | SHC adaptor protein 3 [Source:HGNC Symbol;Acc:HGNC:18181] |
| ENSG00000152092 | HGLibA_03380 | 149 | 0.334102618 | ASTN1 | astrotactin 1 [Source:HGNC Symbol;Acc:HGNC:773] |
| ENSG00000152778 | HGLibA_22634 | 149 | 0.334102618 | IFIT5 | interferon induced protein with tetratricopeptide repeats 5 [Source:HGNC Symbol;Acc:HGNC:13328] |
| ENSG00000153140 | HGLibA_09240 | 149 | 0.334102618 | CETN3 | centrin 3 [Source:HGNC Symbol;Acc:HGNC:1868] |
| ENSG00000153822 | HGLibA_24266 | 149 | 0.334102618 | KCNJ16 | potassium voltage-gated channel subfamily J member 16 [Source:HGNC Symbol;Acc:HGNC:6262] |
| ENSG00000155287 | HGLibA_44694 | 149 | 0.334102618 | SLC25A28 | solute carrier family 25 member 28 [Source:HGNC Symbol;Acc:HGNC:23472] |
| ENSG00000156486 | HGLibA_24386 | 149 | 0.334102618 | KCNS2 | potassium voltage-gated channel modifier subfamily S member 2 [Source:HGNC Symbol;Acc:HGNC:6301] |
| ENSG00000156787 | HGLibA_48536 | 149 | 0.334102618 | TBC1D31 | TBC1 domain family member 31 [Source:HGNC Symbol;Acc:HGNC:30888] |
| ENSG00000157064 | HGLibA_32186 | 149 | 0.334102618 | NMNAT2 | nicotinamide nucleotide adenylyltransferase 2 [Source:HGNC Symbol;Acc:HGNC:16789] |
| ENSG00000161813 | HGLibA_26035 | 149 | 0.334102618 | LARP4 | La ribonucleoprotein domain family member 4 [Source:HGNC Symbol;Acc:HGNC:24320] |
| ENSG00000162494 | HGLibA_27337 | 149 | 0.334102618 | LRRC38 | leucine rich repeat containing 38 [Source:HGNC Symbol;Acc:HGNC:27005] |
| ENSG00000163347 | HGLibA_09847 | 149 | 0.334102618 | CLDN1 | claudin 1 [Source:HGNC Symbol;Acc:HGNC:2032] |
| ENSG00000163517 | HGLibA_21004 | 149 | 0.334102618 | HDAC11 | histone deacetylase 11 [Source:HGNC Symbol;Acc:HGNC:19086] |
| ENSG00000163687 | HGLibA_13662 | 149 | 0.334102618 | DNASE1L3 | deoxyribonuclease 1 like 3 [Source:HGNC Symbol;Acc:HGNC:2959] |
| ENSG00000164182 | HGLibA_31486 | 149 | 0.334102618 | NDUFAF2 | NADH:ubiquinone oxidoreductase complex assembly factor 2 [Source:HGNC Symbol;Acc:HGNC:28086] |
| ENSG00000164308 | HGLibA_15408 | 149 | 0.334102618 | ERAP2 | endoplasmic reticulum aminopeptidase 2 [Source:HGNC Symbol;Acc:HGNC:29499] |
| ENSG00000165192 | HGLibA_03209 | 149 | 0.334102618 | ASB11 | ankyrin repeat and SOCS box containing 11 [Source:HGNC Symbol;Acc:HGNC:17186] |
| ENSG00000165527 | HGLibA_02657 | 149 | 0.334102618 | ARF6 | ADP ribosylation factor 6 [Source:HGNC Symbol;Acc:HGNC:659] |
| ENSG00000167397 | HGLibA_53678 | 149 | 0.334102618 | VKORC1 | vitamin K epoxide reductase complex subunit 1 [Source:HGNC Symbol;Acc:HGNC:23663] |
| ENSG00000168542 | HGLibA_10563 | 149 | 0.334102618 | COL3A1 | collagen type III alpha 1 chain [Source:HGNC Symbol;Acc:HGNC:2201] |
| ENSG00000169067 | HGLibA_00606 | 149 | 0.334102618 | ACTBL2 | beta like 2 [Source:HGNC Symbol;Acc:HGNC:17780]" |
| ENSG00000169213 | HGLibA_39909 | 149 | 0.334102618 | RAB3B | member RAS oncogene family [Source:HGNC Symbol;Acc:HGNC:9778]" |
| ENSG00000169427 | HGLibA_24335 | 149 | 0.334102618 | KCNK9 | potassium two pore domain channel subfamily K member 9 [Source:HGNC Symbol;Acc:HGNC:6283] |
| ENSG00000170113 | HGLibA_31951 | 149 | 0.334102618 | NIPA1 | NIPA magnesium transporter 1 [Source:HGNC Symbol;Acc:HGNC:17043] |
| ENSG00000171004 | HGLibA_22144 | 149 | 0.334102618 | HS6ST2 | heparan sulfate 6-O-sulfotransferase 2 [Source:HGNC Symbol;Acc:HGNC:19133] |
| ENSG00000171847 | HGLibA_16790 | 149 | 0.334102618 | FAM90A1 | family with sequence similarity 90 member A1 [Source:HGNC Symbol;Acc:HGNC:25526] |
| ENSG00000173349 | HGLibA_43756 | 149 | 0.334102618 | SFT2D3 | SFT2 domain containing 3 [Source:HGNC Symbol;Acc:HGNC:28767] |
| ENSG00000178150 | HGLibA_55445 | 149 | 0.334102618 | ZNF114 | zinc finger protein 114 [Source:HGNC Symbol;Acc:HGNC:12894] |
| ENSG00000179168 | HGLibA_19002 | 149 | 0.334102618 | GGN | gametogenetin [Source:HGNC Symbol;Acc:HGNC:18869] |
| ENSG00000179284 | HGLibA_12307 | 149 | 0.334102618 | DAND5 | DAN domain BMP antagonist family member 5 [Source:HGNC Symbol;Acc:HGNC:26780] |
| ENSG00000180340 | HGLibA_18266 | 149 | 0.334102618 | FZD2 | frizzled class receptor 2 [Source:HGNC Symbol;Acc:HGNC:4040] |
| ENSG00000181631 | HGLibA_34878 | 149 | 0.334102618 | P2RY13 | purinergic receptor P2Y13 [Source:HGNC Symbol;Acc:HGNC:4537] |
| ENSG00000181761 | HGLibA_34532 | 149 | 0.334102618 | OR8H3 | olfactory receptor family 8 subfamily H member 3 [Source:HGNC Symbol;Acc:HGNC:15309] |
| ENSG00000182698 | HGLibA_40866 | 149 | 0.334102618 | RESP18 | regulated endocrine specific protein 18 [Source:HGNC Symbol;Acc:HGNC:33762] |
| ENSG00000183172 | HGLibA_45688 | 149 | 0.334102618 | SMDT1 | single-pass membrane protein with aspartate rich tail 1 [Source:HGNC Symbol;Acc:HGNC:25055] |
| ENSG00000183862 | HGLibA_10267 | 149 | 0.334102618 | CNGA2 | cyclic nucleotide gated channel alpha 2 [Source:HGNC Symbol;Acc:HGNC:2149] |
| ENSG00000185917 | HGLibA_43650 | 149 | 0.334102618 | SETD4 | SET domain containing 4 [Source:HGNC Symbol;Acc:HGNC:1258] |

| | | | | | |
|---|---|---|---|---|---|
| ENSG00000186132 | HGLibA_06182 | 149 | 0.334102618 | C2orf76 | chromosome 2 open reading frame 76 [Source:HGNC Symbol;Acc:HGNC:27017] |
| ENSG00000186364 | HGLibA_32957 | 149 | 0.334102618 | NUDT17 | nudix hydrolase 17 [Source:HGNC Symbol;Acc:HGNC:26618] |
| ENSG00000188984 | HGLibA_00035 | 149 | 0.334102618 | AADACL3 | arylacetamide deacetylase like 3 [Source:HGNC Symbol;Acc:HGNC:32037] |
| ENSG00000188996 | HGLibA_22427 | 149 | 0.334102618 | HUS1B | HUS1 checkpoint clamp component B [Source:HGNC Symbol;Acc:HGNC:16485] |
| ENSG00000198624 | HGLibA_07844 | 149 | 0.334102618 | CCDC69 | coiled-coil domain containing 69 [Source:HGNC Symbol;Acc:HGNC:24487] |
| ENSG00000198721 | HGLibA_14410 | 149 | 0.334102618 | ECI2 | enoyl-CoA delta isomerase 2 [Source:HGNC Symbol;Acc:HGNC:14601] |
| ENSG00000203965 | HGLibA_14551 | 149 | 0.334102618 | EFCAB7 | EF-hand calcium binding domain 7 [Source:HGNC Symbol;Acc:HGNC:29379] |
| ENSG00000204536 | HGLibA_07946 | 149 | 0.334102618 | CCHCR1 | coiled-coil alpha-helical rod protein 1 [Source:HGNC Symbol;Acc:HGNC:13930] |
| ENSG00000204618 | HGLibA_41674 | 149 | 0.334102618 | RNF39 | ring finger protein 39 [Source:HGNC Symbol;Acc:HGNC:18064] |
| ENSG00000229937 | HGLibA_38759 | 149 | 0.334102618 | PRPS1L1 | phosphoribosyl pyrophosphate synthetase 1-like 1 [Source:HGNC Symbol;Acc:HGNC:9463] |
| ENSG00000230657 | HGLibA_38347 | 149 | 0.334102618 | PRB4 | proline rich protein BstNI subfamily 4 [Source:HGNC Symbol;Acc:HGNC:9340] |
| ENSG00000234068 | HGLibA_35006 | 149 | 0.334102618 | PAGE2 | PAGE family member 2 [Source:HGNC Symbol;Acc:HGNC:31804] |
| ENSG00000239388 | HGLibA_03217 | 149 | 0.334102618 | ASB14 | ankyrin repeat and SOCS box containing 14 [Source:HGNC Symbol;Acc:HGNC:19766] |
| ENSG00000258227 | HGLibA_09999 | 149 | 0.334102618 | CLEC5A | C-type lectin domain containing 5A [Source:HGNC Symbol;Acc:HGNC:2054] |
| ENSG00000277556 | HGLibA_33605 | 149 | 0.334102618 | OR13C5 | olfactory receptor family 13 subfamily C member 5 [Source:HGNC Symbol;Acc:HGNC:15100] |
| hsa-mir-1197 | HGLibA_57255 | 149 | 0.334102618 | hsa-mir-1197 | - |
| hsa-mir-1252 | HGLibA_57406 | 149 | 0.334102618 | hsa-mir-1252 | - |
| hsa-mir-1265 | HGLibA_57483 | 149 | 0.334102618 | hsa-mir-1265 | - |
| hsa-mir-1324 | HGLibA_57770 | 149 | 0.334102618 | hsa-mir-1324 | - |
| hsa-mir-15a | HGLibA_57937 | 149 | 0.334102618 | hsa-mir-15a | - |
| hsa-mir-184 | HGLibA_57997 | 149 | 0.334102618 | hsa-mir-184 | - |
| hsa-mir-3198-1 | HGLibA_58888 | 149 | 0.334102618 | hsa-mir-3198-1 | - |
| hsa-mir-4425 | HGLibA_60086 | 149 | 0.334102618 | hsa-mir-4425 | - |
| hsa-mir-4714 | HGLibA_60958 | 149 | 0.334102618 | hsa-mir-4714 | - |
| hsa-mir-4742 | HGLibA_61066 | 149 | 0.334102618 | hsa-mir-4742 | - |
| hsa-mir-506 | HGLibA_61482 | 149 | 0.334102618 | hsa-mir-506 | - |
| hsa-mir-516a-2 | HGLibA_61614 | 149 | 0.334102618 | hsa-mir-516a-2 | - |
| hsa-mir-611 | HGLibA_62634 | 149 | 0.334102618 | hsa-mir-611 | - |
| hsa-mir-639 | HGLibA_62795 | 149 | 0.334102618 | hsa-mir-639 | - |
| hsa-mir-8084 | HGLibA_64179 | 149 | 0.334102618 | hsa-mir-8084 | - |
| hsa-mir-939 | HGLibA_64331 | 149 | 0.334102618 | hsa-mir-939 | - |
| KIAA1462 | HGLibA_24764 | 149 | 0.334102618 | KIAA1462 | - |
| PPAPDC2 | HGLibA_37832 | 149 | 0.334102618 | PPAPDC2 | - |
| RTFDC1 | HGLibA_42386 | 149 | 0.334102618 | RTFDC1 | - |
| TMEM99 | HGLibA_50572 | 149 | 0.334102618 | TMEM99 | - |
| XAGE1C | HGLibA_54473 | 149 | 0.334102618 | XAGE1C | - |
| 6-Mar | HGLibA_28343 | 148 | 0.32415158 | 6-Mar | - |
| BAI1 | HGLibA_04112 | 148 | 0.32415158 | BAI1 | - |
| C10orf128 | HGLibA_05050 | 148 | 0.32415158 | C10orf128 | - |
| C15orf38-AP3S2 | HGLibA_05390 | 148 | 0.32415158 | C15orf38-AP3S2 | - |
| C1orf151-NBL1 | HGLibA_05778 | 148 | 0.32415158 | C1orf151-NBL1 | - |
| ENSG00000010818 | HGLibA_21569 | 148 | 0.32415158 | HIVEP2 | human immunodeficiency virus type I enhancer binding protein 2 [Source:HGNC Symbol;Acc:HGNC:4921] |
| ENSG00000012660 | HGLibA_14968 | 148 | 0.32415158 | ELOVL5 | ELOVL fatty acid elongase 5 [Source:HGNC Symbol;Acc:HGNC:21308] |
| ENSG00000012822 | HGLibA_06998 | 148 | 0.32415158 | CALCOCO1 | calcium binding and coiled-coil domain 1 [Source:HGNC Symbol;Acc:HGNC:29306] |
| ENSG00000013725 | HGLibA_08424 | 148 | 0.32415158 | CD6 | CD6 molecule [Source:HGNC Symbol;Acc:HGNC:1691] |
| ENSG00000026559 | HGLibA_24199 | 148 | 0.32415158 | KCNG1 | potassium voltage-gated channel modifier subfamily G member 1 [Source:HGNC Symbol;Acc:HGNC:6248] |
| ENSG00000049239 | HGLibA_20787 | 148 | 0.32415158 | H6PD | hexose-6-phosphate dehydrogenase/glucose 1-dehydrogenase [Source:HGNC Symbol;Acc:HGNC:4795] |
| ENSG00000053372 | HGLibA_30121 | 148 | 0.32415158 | MRTO4 | ribosome maturation factor [Source:HGNC Symbol;Acc:HGNC:18477]" |
| ENSG00000064995 | HGLibA_48202 | 148 | 0.32415158 | TAF11 | TATA-box binding protein associated factor 11 [Source:HGNC Symbol;Acc:HGNC:11544] |
| ENSG00000065665 | HGLibA_43250 | 148 | 0.32415158 | SEC61A2 | Sec61 translocon alpha 2 subunit [Source:HGNC Symbol;Acc:HGNC:17702] |
| ENSG00000065833 | HGLibA_28709 | 148 | 0.32415158 | ME1 | malic enzyme 1 [Source:HGNC Symbol;Acc:HGNC:6983] |
| ENSG00000066735 | HGLibA_24916 | 148 | 0.32415158 | KIF26A | kinesin family member 26A [Source:HGNC Symbol;Acc:HGNC:20226] |
| ENSG00000068354 | HGLibA_48516 | 148 | 0.32415158 | TBC1D25 | TBC1 domain family member 25 [Source:HGNC Symbol;Acc:HGNC:8092] |
| ENSG00000069020 | HGLibA_28409 | 148 | 0.32415158 | MAST4 | microtubule associated serine/threonine kinase family member 4 [Source:HGNC Symbol;Acc:HGNC:19037] |
| ENSG00000072832 | HGLibA_11201 | 148 | 0.32415158 | CRMP1 | collapsin response mediator protein 1 [Source:HGNC Symbol;Acc:HGNC:2365] |
| ENSG00000074527 | HGLibA_32858 | 148 | 0.32415158 | NTN4 | netrin 4 [Source:HGNC Symbol;Acc:HGNC:13658] |
| ENSG00000080224 | HGLibA_15303 | 148 | 0.32415158 | EPHA6 | EPH receptor A6 [Source:HGNC Symbol;Acc:HGNC:19296] |
| ENSG00000081479 | HGLibA_27212 | 148 | 0.32415158 | LRP2 | LDL receptor related protein 2 [Source:HGNC Symbol;Acc:HGNC:6694] |
| ENSG00000085185 | HGLibA_04364 | 148 | 0.32415158 | BCORL1 | BCL6 corepressor like 1 [Source:HGNC Symbol;Acc:HGNC:25657] |
| ENSG00000090932 | HGLibA_13364 | 148 | 0.32415158 | DLL3 | delta like canonical Notch ligand 3 [Source:HGNC Symbol;Acc:HGNC:2909] |
| ENSG00000095059 | HGLibA_13115 | 148 | 0.32415158 | DHPS | deoxyhypusine synthase [Source:HGNC Symbol;Acc:HGNC:2869] |
| ENSG00000100034 | HGLibA_37974 | 148 | 0.32415158 | PPM1F | Mg2+/Mn2+ dependent 1F [Source:HGNC Symbol;Acc:HGNC:19388]" |
| ENSG00000101441 | HGLibA_11456 | 148 | 0.32415158 | CST4 | cystatin S [Source:HGNC Symbol;Acc:HGNC:2476] |
| ENSG00000102554 | HGLibA_25074 | 148 | 0.32415158 | KLF5 | Kruppel like factor 5 [Source:HGNC Symbol;Acc:HGNC:6349] |
| ENSG00000104497 | HGLibA_46044 | 148 | 0.32415158 | SNX16 | sorting nexin 16 [Source:HGNC Symbol;Acc:HGNC:14980] |
| ENSG00000106052 | HGLibA_48448 | 148 | 0.32415158 | TAX1BP1 | Tax1 binding protein 1 [Source:HGNC Symbol;Acc:HGNC:11575] |
| ENSG00000107679 | HGLibA_37063 | 148 | 0.32415158 | PLEKHA1 | pleckstrin homology domain containing A1 [Source:HGNC Symbol;Acc:HGNC:14335] |
| ENSG00000107745 | HGLibA_29223 | 148 | 0.32415158 | MICU1 | mitochondrial calcium uptake 1 [Source:HGNC Symbol;Acc:HGNC:1530] |
| ENSG00000108242 | HGLibA_12114 | 148 | 0.32415158 | CYP2C18 | cytochrome P450 family 2 subfamily C member 18 [Source:HGNC Symbol;Acc:HGNC:2620] |
| ENSG00000111832 | HGLibA_42478 | 148 | 0.32415158 | RWDD1 | RWD domain containing 1 [Source:HGNC Symbol;Acc:HGNC:20993] |
| ENSG00000114554 | HGLibA_37259 | 148 | 0.32415158 | PLXNA1 | plexin A1 [Source:HGNC Symbol;Acc:HGNC:9099] |
| ENSG00000115488 | HGLibA_31713 | 148 | 0.32415158 | NEU2 | neuraminidase 2 [Source:HGNC Symbol;Acc:HGNC:7759] |
| ENSG00000116106 | HGLibA_15296 | 148 | 0.32415158 | EPHA4 | EPH receptor A4 [Source:HGNC Symbol;Acc:HGNC:3388] |
| ENSG00000117472 | HGLibA_51892 | 148 | 0.32415158 | TSPAN1 | tetraspanin 1 [Source:HGNC Symbol;Acc:HGNC:20657] |
| ENSG00000117859 | HGLibA_34669 | 148 | 0.32415158 | OSBPL9 | oxysterol binding protein like 9 [Source:HGNC Symbol;Acc:HGNC:16386] |
| ENSG00000118242 | HGLibA_29825 | 148 | 0.32415158 | MREG | melanoregulin [Source:HGNC Symbol;Acc:HGNC:25478] |
| ENSG00000118971 | HGLibA_08075 | 148 | 0.32415158 | CCND2 | cyclin D2 [Source:HGNC Symbol;Acc:HGNC:1583] |
| ENSG00000119411 | HGLibA_04877 | 148 | 0.32415158 | BSPRY | B-box and SPRY domain containing [Source:HGNC Symbol;Acc:HGNC:18232] |
| ENSG00000119714 | HGLibA_20076 | 148 | 0.32415158 | GPR68 | G protein-coupled receptor 68 [Source:HGNC Symbol;Acc:HGNC:4519] |
| ENSG00000120306 | HGLibA_12233 | 148 | 0.32415158 | CYSTM1 | cysteine rich transmembrane module containing 1 [Source:HGNC Symbol;Acc:HGNC:30239] |
| ENSG00000120659 | HGLibA_50800 | 148 | 0.32415158 | TNFSF11 | TNF superfamily member 11 [Source:HGNC Symbol;Acc:HGNC:11926] |
| ENSG00000121486 | HGLibA_51680 | 148 | 0.32415158 | TRMT1L | tRNA methyltransferase 1 like [Source:HGNC Symbol;Acc:HGNC:16782] |
| ENSG00000124217 | HGLibA_29617 | 148 | 0.32415158 | MOCS3 | molybdenum cofactor synthesis 3 [Source:HGNC Symbol;Acc:HGNC:15765] |
| ENSG00000126890 | HGLibA_11563 | 148 | 0.32415158 | CTAG2 | cancer/testis antigen 2 [Source:HGNC Symbol;Acc:HGNC:2492] |
| ENSG00000128524 | HGLibA_03754 | 148 | 0.32415158 | ATP6V1F | ATPase H+ transporting V1 subunit F [Source:HGNC Symbol;Acc:HGNC:16832] |
| ENSG00000128928 | HGLibA_23878 | 148 | 0.32415158 | IVD | isovaleryl-CoA dehydrogenase [Source:HGNC Symbol;Acc:HGNC:6186] |
| ENSG00000130714 | HGLibA_37669 | 148 | 0.32415158 | POMT1 | protein O-mannosyltransferase 1 [Source:HGNC Symbol;Acc:HGNC:9202] |
| ENSG00000131471 | HGLibA_02274 | 148 | 0.32415158 | AOC3 | copper containing 3 [Source:HGNC Symbol;Acc:HGNC:550]" |
| ENSG00000131773 | HGLibA_24573 | 148 | 0.32415158 | KHDRBS3 | signal transduction associated 3 [Source:HGNC Symbol;Acc:HGNC:18117]" |
| ENSG00000132510 | HGLibA_24537 | 148 | 0.32415158 | KDM6B | lysine demethylase 6B [Source:HGNC Symbol;Acc:HGNC:29012] |
| ENSG00000132514 | HGLibA_09922 | 148 | 0.32415158 | CLEC10A | C-type lectin domain containing 10A [Source:HGNC Symbol;Acc:HGNC:16916] |
| ENSG00000132823 | HGLibA_34677 | 148 | 0.32415158 | OSER1 | oxidative stress responsive serine rich 1 [Source:HGNC Symbol;Acc:HGNC:16105] |
| ENSG00000133315 | HGLibA_27836 | 148 | 0.32415158 | MACROD1 | MACRO domain containing 1 [Source:HGNC Symbol;Acc:HGNC:29598] |
| ENSG00000135090 | HGLibA_48311 | 148 | 0.32415158 | TAOK3 | TAO kinase 3 [Source:HGNC Symbol;Acc:HGNC:18133] |
| ENSG00000135218 | HGLibA_08360 | 148 | 0.32415158 | CD36 | CD36 molecule [Source:HGNC Symbol;Acc:HGNC:1663] |
| ENSG00000135222 | HGLibA_11375 | 148 | 0.32415158 | CSN2 | casein beta [Source:HGNC Symbol;Acc:HGNC:2447] |
| ENSG00000137878 | HGLibA_18824 | 148 | 0.32415158 | GCOM1 | GRINL1A complex locus 1 [Source:HGNC Symbol;Acc:HGNC:26424] |
| ENSG00000138336 | HGLibA_49038 | 148 | 0.32415158 | TET1 | tet methylcytosine dioxygenase 1 [Source:HGNC Symbol;Acc:HGNC:29484] |
| ENSG00000138738 | HGLibA_38391 | 148 | 0.32415158 | PRDM5 | PR/SET domain 5 [Source:HGNC Symbol;Acc:HGNC:9349] |
| ENSG00000139437 | HGLibA_48808 | 148 | 0.32415158 | TCHP | trichoplein keratin filament binding [Source:HGNC Symbol;Acc:HGNC:28135] |
| ENSG00000140299 | HGLibA_04653 | 148 | 0.32415158 | BNIP2 | BCL2 interacting protein 2 [Source:HGNC Symbol;Acc:HGNC:1083] |
| ENSG00000140519 | HGLibA_41148 | 148 | 0.32415158 | RHCG | Rh family C glycoprotein [Source:HGNC Symbol;Acc:HGNC:18140] |
| ENSG00000142227 | HGLibA_15081 | 148 | 0.32415158 | EMP3 | epithelial membrane protein 3 [Source:HGNC Symbol;Acc:HGNC:3335] |
| ENSG00000145494 | HGLibA_31560 | 148 | 0.32415158 | NDUFS6 | NADH:ubiquinone oxidoreductase subunit S6 [Source:HGNC Symbol;Acc:HGNC:7713] |
| ENSG00000147224 | HGLibA_38756 | 148 | 0.32415158 | PRPS1 | phosphoribosyl pyrophosphate synthetase 1 [Source:HGNC Symbol;Acc:HGNC:9462] |
| ENSG00000147606 | HGLibA_44803 | 148 | 0.32415158 | SLC26A7 | solute carrier family 26 member 7 [Source:HGNC Symbol;Acc:HGNC:14467] |
| ENSG00000148158 | HGLibA_46085 | 148 | 0.32415158 | SNX30 | sorting nexin family member 30 [Source:HGNC Symbol;Acc:HGNC:23685] |
| ENSG00000148671 | HGLibA_00986 | 148 | 0.32415158 | ADIRF | adipogenesis regulatory factor [Source:HGNC Symbol;Acc:HGNC:24043] |
| ENSG00000149201 | HGLibA_07879 | 148 | 0.32415158 | CCDC81 | coiled-coil domain containing 81 [Source:HGNC Symbol;Acc:HGNC:26281] |
| ENSG00000149651 | HGLibA_46720 | 148 | 0.32415158 | SPINT4 | Kunitz type 4 [Source:HGNC Symbol;Acc:HGNC:16130]" |
| ENSG00000150627 | HGLibA_54036 | 148 | 0.32415158 | WDR17 | WD repeat domain 17 [Source:HGNC Symbol;Acc:HGNC:16661] |
| ENSG00000152292 | HGLibA_43906 | 148 | 0.32415158 | SH2D6 | SH2 domain containing 6 [Source:HGNC Symbol;Acc:HGNC:30439] |
| ENSG00000153266 | HGLibA_17322 | 148 | 0.32415158 | FEZF2 | FEZ family zinc finger 2 [Source:HGNC Symbol;Acc:HGNC:13506] |
| ENSG00000153944 | HGLibA_30209 | 148 | 0.32415158 | MSI2 | musashi RNA binding protein 2 [Source:HGNC Symbol;Acc:HGNC:18585] |
| ENSG00000154845 | HGLibA_38202 | 148 | 0.32415158 | PPP4R1 | protein phosphatase 4 regulatory subunit 1 [Source:HGNC Symbol;Acc:HGNC:9320] |
| ENSG00000155592 | HGLibA_55349 | 148 | 0.32415158 | ZKSCAN2 | zinc finger with KRAB and SCAN domains 2 [Source:HGNC Symbol;Acc:HGNC:25677] |
| ENSG00000155974 | HGLibA_20319 | 148 | 0.32415158 | GRIP1 | glutamate receptor interacting protein 1 [Source:HGNC Symbol;Acc:HGNC:18708] |
| ENSG00000156509 | HGLibA_17101 | 148 | 0.32415158 | FBXO43 | F-box protein 43 [Source:HGNC Symbol;Acc:HGNC:28521] |
| ENSG00000159173 | HGLibA_50872 | 148 | 0.32415158 | TNNI1 | slow skeletal type [Source:HGNC Symbol;Acc:HGNC:11945]" |
| ENSG00000159958 | HGLibA_50757 | 148 | 0.32415158 | TNFRSF13C | TNF receptor superfamily member 13C [Source:HGNC Symbol;Acc:HGNC:17755] |
| ENSG00000160999 | HGLibA_43876 | 148 | 0.32415158 | SH2B2 | SH2B adaptor protein 2 [Source:HGNC Symbol;Acc:HGNC:17381] |
| ENSG00000161813 | HGLibA_26033 | 148 | 0.32415158 | LARP4 | La ribonucleoprotein domain family member 4 [Source:HGNC Symbol;Acc:HGNC:24320] |
| ENSG00000162692 | HGLibA_53567 | 148 | 0.32415158 | VCAM1 | vascular cell adhesion molecule 1 [Source:HGNC Symbol;Acc:HGNC:12663] |
| ENSG00000162761 | HGLibA_26732 | 148 | 0.32415158 | LMX1A | LIM homeobox transcription factor 1 alpha [Source:HGNC Symbol;Acc:HGNC:6653] |
| ENSG00000162814 | HGLibA_46435 | 148 | 0.32415158 | SPATA17 | spermatogenesis associated 17 [Source:HGNC Symbol;Acc:HGNC:25184] |
| ENSG00000163449 | HGLibA_50048 | 148 | 0.32415158 | TMEM169 | transmembrane protein 169 [Source:HGNC Symbol;Acc:HGNC:25130] |
| ENSG00000163629 | HGLibA_39438 | 148 | 0.32415158 | PTPN13 | non-receptor type 13 [Source:HGNC Symbol;Acc:HGNC:9646]" |
| ENSG00000163870 | HGLibA_51178 | 148 | 0.32415158 | TPRA1 | transmembrane protein adipocyte associated 1 [Source:HGNC Symbol;Acc:HGNC:30413] |
| ENSG00000164900 | HGLibA_18748 | 148 | 0.32415158 | GBX1 | gastrulation brain homeobox 1 [Source:HGNC Symbol;Acc:HGNC:4185] |
| ENSG00000164916 | HGLibA_17893 | 148 | 0.32415158 | FOXK1 | forkhead box K1 [Source:HGNC Symbol;Acc:HGNC:23480] |
| ENSG00000165626 | HGLibA_04416 | 148 | 0.32415158 | BEND7 | BEN domain containing 7 [Source:HGNC Symbol;Acc:HGNC:23514] |
| ENSG00000167700 | HGLibA_29100 | 148 | 0.32415158 | MFSD3 | major facilitator superfamily domain containing 3 [Source:HGNC Symbol;Acc:HGNC:25157] |
| ENSG00000170396 | HGLibA_56789 | 148 | 0.32415158 | ZNF804A | zinc finger protein 804A [Source:HGNC Symbol;Acc:HGNC:21711] |
| ENSG00000170633 | HGLibA_41667 | 148 | 0.32415158 | RNF34 | ring finger protein 34 [Source:HGNC Symbol;Acc:HGNC:17297] |
| ENSG00000171540 | HGLibA_34749 | 148 | 0.32415158 | OTP | orthopedia homeobox [Source:HGNC Symbol;Acc:HGNC:8518] |

| | | | | | |
|---|---|---|---|---|---|
| ENSG00000172209 | HGLibA_20003 | 148 | 0.32415158 | GPR22 | G protein-coupled receptor 22 [Source:HGNC Symbol;Acc:HGNC:4477] |
| ENSG00000174010 | HGLibA_25137 | 148 | 0.32415158 | KLHL15 | kelch like family member 15 [Source:HGNC Symbol;Acc:HGNC:29347] |
| ENSG00000175787 | HGLibA_55519 | 148 | 0.32415158 | ZNF169 | zinc finger protein 169 [Source:HGNC Symbol;Acc:HGNC:12957] |
| ENSG00000176095 | HGLibA_23445 | 148 | 0.32415158 | IP6K1 | inositol hexakisphosphate kinase 1 [Source:HGNC Symbol;Acc:HGNC:18360] |
| ENSG00000179593 | HGLibA_01685 | 148 | 0.32415158 | ALOX15B | type B [Source:HGNC Symbol;Acc:HGNC:434]" |
| ENSG00000181588 | HGLibA_29035 | 148 | 0.32415158 | MEX3D | mex-3 RNA binding family member D [Source:HGNC Symbol;Acc:HGNC:16734] |
| ENSG00000182220 | HGLibA_03700 | 148 | 0.32415158 | ATP6AP2 | ATPase H+ transporting accessory protein 2 [Source:HGNC Symbol;Acc:HGNC:18305] |
| ENSG00000182459 | HGLibA_49069 | 148 | 0.32415158 | TEX19 | testis expressed 19 [Source:HGNC Symbol;Acc:HGNC:33802] |
| ENSG00000183862 | HGLibA_10266 | 148 | 0.32415158 | CNGA2 | cyclic nucleotide gated channel alpha 2 [Source:HGNC Symbol;Acc:HGNC:2149] |
| ENSG00000185332 | HGLibA_49850 | 148 | 0.32415158 | TMEM105 | TMEM105 long non-coding RNA [Source:HGNC Symbol;Acc:HGNC:26794] |
| ENSG00000185480 | HGLibA_35251 | 148 | 0.32415158 | PARPBP | PARP1 binding protein [Source:HGNC Symbol;Acc:HGNC:26074] |
| ENSG00000185915 | HGLibA_25193 | 148 | 0.32415158 | KLHL34 | kelch like family member 34 [Source:HGNC Symbol;Acc:HGNC:26634] |
| ENSG00000187555 | HGLibA_53410 | 148 | 0.32415158 | USP7 | ubiquitin specific peptidase 7 [Source:HGNC Symbol;Acc:HGNC:12630] |
| ENSG00000188488 | HGLibA_43522 | 148 | 0.32415158 | SERPINA5 | serpin family A member 5 [Source:HGNC Symbol;Acc:HGNC:8723] |
| ENSG00000188783 | HGLibA_38433 | 148 | 0.32415158 | PRELP | proline and arginine rich end leucine rich repeat protein [Source:HGNC Symbol;Acc:HGNC:9357] |
| ENSG00000196189 | HGLibA_43339 | 148 | 0.32415158 | SEMA4A | semaphorin 4A [Source:HGNC Symbol;Acc:HGNC:10729] |
| ENSG00000196227 | HGLibA_16491 | 148 | 0.32415158 | FAM217B | family with sequence similarity 217 member B [Source:HGNC Symbol;Acc:HGNC:16170] |
| ENSG00000196743 | HGLibA_19387 | 148 | 0.32415158 | GM2A | GM2 ganglioside activator [Source:HGNC Symbol;Acc:HGNC:4367] |
| ENSG00000197272 | HGLibA_23113 | 148 | 0.32415158 | IL27 | interleukin 27 [Source:HGNC Symbol;Acc:HGNC:19157] |
| ENSG00000197406 | HGLibA_13245 | 148 | 0.32415158 | DIO3 | iodothyronine deiodinase 3 [Source:HGNC Symbol;Acc:HGNC:2885] |
| ENSG00000197841 | HGLibA_55539 | 148 | 0.32415158 | ZNF181 | zinc finger protein 181 [Source:HGNC Symbol;Acc:HGNC:12971] |
| ENSG00000197943 | HGLibA_37004 | 148 | 0.32415158 | PLCG2 | phospholipase C gamma 2 [Source:HGNC Symbol;Acc:HGNC:9066] |
| ENSG00000198585 | HGLibA_32953 | 148 | 0.32415158 | NUDT16 | nudix hydrolase 16 [Source:HGNC Symbol;Acc:HGNC:26442] |
| ENSG00000204033 | HGLibA_27188 | 148 | 0.32415158 | LRIT2 | Ig-like and transmembrane domains 2 [Source:HGNC Symbol;Acc:HGNC:23443]" |
| ENSG00000204217 | HGLibA_04636 | 148 | 0.32415158 | BMPR2 | bone morphogenetic protein receptor type 2 [Source:HGNC Symbol;Acc:HGNC:1078] |
| ENSG00000204449 | HGLibA_51527 | 148 | 0.32415158 | TRIM49C | tripartite motif containing 49C [Source:HGNC Symbol;Acc:HGNC:38877] |
| ENSG00000212658 | HGLibA_25737 | 148 | 0.32415158 | KRTAP29-1 | keratin associated protein 29-1 [Source:HGNC Symbol;Acc:HGNC:34211] |
| ENSG00000213424 | HGLibA_25449 | 148 | 0.32415158 | KRT222 | keratin 222 [Source:HGNC Symbol;Acc:HGNC:28695] |
| ENSG00000213465 | HGLibA_02958 | 148 | 0.32415158 | ARL2 | ADP ribosylation factor like GTPase 2 [Source:HGNC Symbol;Acc:HGNC:693] |
| ENSG00000213672 | HGLibA_31324 | 148 | 0.32415158 | NCKIPSD | NCK interacting protein with SH3 domain [Source:HGNC Symbol;Acc:HGNC:15486] |
| ENSG00000213934 | HGLibA_20925 | 148 | 0.32415158 | HBG1 | hemoglobin subunit gamma 1 [Source:HGNC Symbol;Acc:HGNC:4831] |
| ENSG00000213967 | HGLibA_56633 | 148 | 0.32415158 | ZNF726 | zinc finger protein 726 [Source:HGNC Symbol;Acc:HGNC:32462] |
| ENSG00000214891 | HGLibA_51581 | 148 | 0.32415158 | TRIM64C | tripartite motif containing 64C [Source:HGNC Symbol;Acc:HGNC:37148] |
| ENSG00000225830 | HGLibA_15445 | 148 | 0.32415158 | ERCC6 | chromatin remodeling factor [Source:HGNC Symbol;Acc:HGNC:3438]" |
| ENSG00000231924 | HGLibA_39033 | 148 | 0.32415158 | PSG1 | pregnancy specific beta-1-glycoprotein 1 [Source:HGNC Symbol;Acc:HGNC:9514] |
| ENSG00000235863 | HGLibA_03961 | 148 | 0.32415158 | B3GALT4 | 3-galactosyltransferase 4 [Source:HGNC Symbol;Acc:HGNC:919]" |
| ENSG00000254440 | HGLibA_35338 | 148 | 0.32415158 | PBOV1 | prostate and breast cancer overexpressed 1 [Source:HGNC Symbol;Acc:HGNC:21079] |
| ENSG00000255150 | HGLibA_14701 | 148 | 0.32415158 | EID3 | EP300 interacting inhibitor of differentiation 3 [Source:HGNC Symbol;Acc:HGNC:32961] |
| ENSG00000261678 | HGLibA_43057 | 148 | 0.32415158 | SCRT1 | scratch family transcriptional repressor 1 [Source:HGNC Symbol;Acc:HGNC:15950] |
| ENSG00000273540 | HGLibA_01166 | 148 | 0.32415158 | AGBL1 | ATP/GTP binding protein like 1 [Source:HGNC Symbol;Acc:HGNC:26504] |
| ENSG00000284723 | HGLibA_34552 | 148 | 0.32415158 | OR8S1 | olfactory receptor family 8 subfamily S member 1 [Source:HGNC Symbol;Acc:HGNC:19628] |
| FBXO18 | HGLibA_17040 | 148 | 0.32415158 | FBXO18 | - |
| GATSL2 | HGLibA_18704 | 148 | 0.32415158 | GATSL2 | - |
| GPR114 | HGLibA_19858 | 148 | 0.32415158 | GPR114 | - |
| GTDC2 | HGLibA_20518 | 148 | 0.32415158 | GTDC2 | - |
| hsa-let-7a-2 | HGLibA_57101 | 148 | 0.32415158 | hsa-let-7a-2 | - |
| hsa-mir-10b | HGLibA_57201 | 148 | 0.32415158 | hsa-mir-10b | - |
| hsa-mir-1263 | HGLibA_57477 | 148 | 0.32415158 | hsa-mir-1263 | - |
| hsa-mir-19b-1 | HGLibA_58142 | 148 | 0.32415158 | hsa-mir-19b-1 | - |
| hsa-mir-4263 | HGLibA_59758 | 148 | 0.32415158 | hsa-mir-4263 | - |
| hsa-mir-4682 | HGLibA_60838 | 148 | 0.32415158 | hsa-mir-4682 | - |
| hsa-mir-4730 | HGLibA_61016 | 148 | 0.32415158 | hsa-mir-4730 | - |
| hsa-mir-4747 | HGLibA_61086 | 148 | 0.32415158 | hsa-mir-4747 | - |
| hsa-mir-5000 | HGLibA_61406 | 148 | 0.32415158 | hsa-mir-5000 | - |
| hsa-mir-571 | HGLibA_62376 | 148 | 0.32415158 | hsa-mir-571 | - |
| hsa-mir-6823 | HGLibA_63470 | 148 | 0.32415158 | hsa-mir-6823 | - |
| hsa-mir-6838 | HGLibA_63528 | 148 | 0.32415158 | hsa-mir-6838 | - |
| hsa-mir-6853 | HGLibA_63585 | 148 | 0.32415158 | hsa-mir-6853 | - |
| hsa-mir-885 | HGLibA_64223 | 148 | 0.32415158 | hsa-mir-885 | - |
| LOC147646 | HGLibA_26889 | 148 | 0.32415158 | LOC147646 | - |
| LRRC16A | HGLibA_27262 | 148 | 0.32415158 | LRRC16A | - |
| SHOX_Y | HGLibA_44072 | 148 | 0.32415158 | SHOX_Y | - |
| YAE1D1 | HGLibA_54626 | 148 | 0.32415158 | YAE1D1 | - |
| C19orf35 | HGLibA_05651 | 147 | 0.314200543 | C19orf35 | - |
| ENSG00000001630 | HGLibA_12209 | 147 | 0.314200543 | CYP51A1 | cytochrome P450 family 51 subfamily A member 1 [Source:HGNC Symbol;Acc:HGNC:2649] |
| ENSG00000002822 | HGLibA_27842 | 147 | 0.314200543 | MAD1L1 | mitotic arrest deficient 1 like 1 [Source:HGNC Symbol;Acc:HGNC:6762] |
| ENSG00000003056 | HGLibA_27815 | 147 | 0.314200543 | M6PR | cation dependent [Source:HGNC Symbol;Acc:HGNC:6752]" |
| ENSG00000011600 | HGLibA_52472 | 147 | 0.314200543 | TYROBP | TYRO protein tyrosine kinase binding protein [Source:HGNC Symbol;Acc:HGNC:12449] |
| ENSG00000033050 | HGLibA_00210 | 147 | 0.314200543 | ABCF2 | ATP binding cassette subfamily F member 2 [Source:HGNC Symbol;Acc:HGNC:71] |
| ENSG00000052126 | HGLibA_37074 | 147 | 0.314200543 | PLEKHA5 | pleckstrin homology domain containing A5 [Source:HGNC Symbol;Acc:HGNC:30036] |
| ENSG00000070808 | HGLibA_07068 | 147 | 0.314200543 | CAMK2A | calcium/calmodulin dependent protein kinase II alpha [Source:HGNC Symbol;Acc:HGNC:1460] |
| ENSG00000075223 | HGLibA_43324 | 147 | 0.314200543 | SEMA3C | semaphorin 3C [Source:HGNC Symbol;Acc:HGNC:10725] |
| ENSG00000079257 | HGLibA_27649 | 147 | 0.314200543 | LXN | latexin [Source:HGNC Symbol;Acc:HGNC:13347] |
| ENSG00000079805 | HGLibA_13689 | 147 | 0.314200543 | DNM2 | dynamin 2 [Source:HGNC Symbol;Acc:HGNC:2974] |
| ENSG00000087086 | HGLibA_18124 | 147 | 0.314200543 | FTL | ferritin light chain [Source:HGNC Symbol;Acc:HGNC:3999] |
| ENSG00000089639 | HGLibA_19409 | 147 | 0.314200543 | GMIP | GEM interacting protein [Source:HGNC Symbol;Acc:HGNC:24852] |
| ENSG00000091972 | HGLibA_08282 | 147 | 0.314200543 | CD200 | CD200 molecule [Source:HGNC Symbol;Acc:HGNC:7203] |
| ENSG00000097021 | HGLibA_00487 | 147 | 0.314200543 | ACOT7 | acyl-CoA thioesterase 7 [Source:HGNC Symbol;Acc:HGNC:24157] |
| ENSG00000100429 | HGLibA_21001 | 147 | 0.314200543 | HDAC10 | histone deacetylase 10 [Source:HGNC Symbol;Acc:HGNC:18128] |
| ENSG00000100908 | HGLibA_15026 | 147 | 0.314200543 | EMC9 | ER membrane protein complex subunit 9 [Source:HGNC Symbol;Acc:HGNC:20273] |
| ENSG00000101966 | HGLibA_54508 | 147 | 0.314200543 | XIAP | X-linked inhibitor of apoptosis [Source:HGNC Symbol;Acc:HGNC:592] |
| ENSG00000103148 | HGLibA_32519 | 147 | 0.314200543 | NPRL3 | GATOR1 complex subunit [Source:HGNC Symbol;Acc:HGNC:14124]" |
| ENSG00000103522 | HGLibA_23086 | 147 | 0.314200543 | IL21R | interleukin 21 receptor [Source:HGNC Symbol;Acc:HGNC:6006] |
| ENSG00000106009 | HGLibA_04757 | 147 | 0.314200543 | BRAT1 | BRCA1 associated ATM activator 1 [Source:HGNC Symbol;Acc:HGNC:21701] |
| ENSG00000108878 | HGLibA_06930 | 147 | 0.314200543 | CACNG1 | calcium voltage-gated channel auxiliary subunit gamma 1 [Source:HGNC Symbol;Acc:HGNC:1405] |
| ENSG00000109065 | HGLibA_31191 | 147 | 0.314200543 | NAT9 | N-acetyltransferase 9 (putative) [Source:HGNC Symbol;Acc:HGNC:23133] |
| ENSG00000111732 | HGLibA_01326 | 147 | 0.314200543 | AICDA | activation induced cytidine deaminase [Source:HGNC Symbol;Acc:HGNC:13203] |
| ENSG00000112033 | HGLibA_37839 | 147 | 0.314200543 | PPARD | peroxisome proliferator activated receptor delta [Source:HGNC Symbol;Acc:HGNC:9235] |
| ENSG00000112984 | HGLibA_24892 | 147 | 0.314200543 | KIF20A | kinesin family member 20A [Source:HGNC Symbol;Acc:HGNC:9787] |
| ENSG00000115942 | HGLibA_34603 | 147 | 0.314200543 | ORC2 | origin recognition complex subunit 2 [Source:HGNC Symbol;Acc:HGNC:8488] |
| ENSG00000118113 | HGLibA_29540 | 147 | 0.314200543 | MMP8 | matrix metallopeptidase 8 [Source:HGNC Symbol;Acc:HGNC:7175] |
| ENSG00000118263 | HGLibA_25081 | 147 | 0.314200543 | KLF7 | Kruppel like factor 7 [Source:HGNC Symbol;Acc:HGNC:6350] |
| ENSG00000119771 | HGLibA_25173 | 147 | 0.314200543 | KLHL29 | kelch like family member 29 [Source:HGNC Symbol;Acc:HGNC:29404] |
| ENSG00000121413 | HGLibA_56991 | 147 | 0.314200543 | ZSCAN18 | zinc finger and SCAN domain containing 18 [Source:HGNC Symbol;Acc:HGNC:21037] |
| ENSG00000123908 | HGLibA_01207 | 147 | 0.314200543 | AGO2 | RISC catalytic component [Source:HGNC Symbol;Acc:HGNC:3263]" |
| ENSG00000125730 | HGLibA_06202 | 147 | 0.314200543 | C3 | complement C3 [Source:HGNC Symbol;Acc:HGNC:1318] |
| ENSG00000126895 | HGLibA_03908 | 147 | 0.314200543 | AVPR2 | arginine vasopressin receptor 2 [Source:HGNC Symbol;Acc:HGNC:897] |
| ENSG00000129625 | HGLibA_40788 | 147 | 0.314200543 | REEP5 | receptor accessory protein 5 [Source:HGNC Symbol;Acc:HGNC:30077] |
| ENSG00000130193 | HGLibA_49346 | 147 | 0.314200543 | THEM6 | thioesterase superfamily member 6 [Source:HGNC Symbol;Acc:HGNC:29656] |
| ENSG00000130943 | HGLibA_36810 | 147 | 0.314200543 | PKDREJ | polycystin family receptor for egg jelly [Source:HGNC Symbol;Acc:HGNC:9015] |
| ENSG00000130958 | HGLibA_44981 | 147 | 0.314200543 | SLC35D2 | solute carrier family 35 member D2 [Source:HGNC Symbol;Acc:HGNC:20799] |
| ENSG00000131059 | HGLibA_04715 | 147 | 0.314200543 | BPIFA3 | BPI fold containing family A member 3 [Source:HGNC Symbol;Acc:HGNC:16204] |
| ENSG00000131668 | HGLibA_04164 | 147 | 0.314200543 | BARX1 | BARX homeobox 1 [Source:HGNC Symbol;Acc:HGNC:955] |
| ENSG00000133392 | HGLibA_30708 | 147 | 0.314200543 | MYH11 | myosin heavy chain 11 [Source:HGNC Symbol;Acc:HGNC:7569] |
| ENSG00000134313 | HGLibA_24848 | 147 | 0.314200543 | KIDINS220 | kinase D interacting substrate 220 [Source:HGNC Symbol;Acc:HGNC:29508] |
| ENSG00000134686 | HGLibA_36322 | 147 | 0.314200543 | PHC2 | polyhomeotic homolog 2 [Source:HGNC Symbol;Acc:HGNC:3183] |
| ENSG00000135766 | HGLibA_14640 | 147 | 0.314200543 | EGLN1 | egl-9 family hypoxia inducible factor 1 [Source:HGNC Symbol;Acc:HGNC:1232] |
| ENSG00000137692 | HGLibA_12578 | 147 | 0.314200543 | DCUN1D5 | defective in cullin neddylation 1 domain containing 5 [Source:HGNC Symbol;Acc:HGNC:28409] |
| ENSG00000139287 | HGLibA_51132 | 147 | 0.314200543 | TPH2 | tryptophan hydroxylase 2 [Source:HGNC Symbol;Acc:HGNC:20692] |
| ENSG00000140274 | HGLibA_14105 | 147 | 0.314200543 | DUOXA2 | dual oxidase maturation factor 2 [Source:HGNC Symbol;Acc:HGNC:32698] |
| ENSG00000140365 | HGLibA_10664 | 147 | 0.314200543 | COMMD4 | COMM domain containing 4 [Source:HGNC Symbol;Acc:HGNC:26027] |
| ENSG00000141219 | HGLibA_05583 | 147 | 0.314200543 | C17orf80 | chromosome 17 open reading frame 80 [Source:HGNC Symbol;Acc:HGNC:29601] |
| ENSG00000141505 | HGLibA_03286 | 147 | 0.314200543 | ASGR1 | asialoglycoprotein receptor 1 [Source:HGNC Symbol;Acc:HGNC:742] |
| ENSG00000141664 | HGLibA_55026 | 147 | 0.314200543 | ZCCHC2 | zinc finger CCHC-type containing 2 [Source:HGNC Symbol;Acc:HGNC:22916] |
| ENSG00000143442 | HGLibA_37480 | 147 | 0.314200543 | POGZ | pogo transposable element derived with ZNF domain [Source:HGNC Symbol;Acc:HGNC:18801] |
| ENSG00000147475 | HGLibA_15497 | 147 | 0.314200543 | ERLIN2 | ER lipid raft associated 2 [Source:HGNC Symbol;Acc:HGNC:1356] |
| ENSG00000149257 | HGLibA_43596 | 147 | 0.314200543 | SERPINH1 | serpin family H member 1 [Source:HGNC Symbol;Acc:HGNC:1546] |
| ENSG00000150656 | HGLibA_10250 | 147 | 0.314200543 | CNDP1 | carnosine dipeptidase 1 [Source:HGNC Symbol;Acc:HGNC:20675] |
| ENSG00000152061 | HGLibA_40012 | 147 | 0.314200543 | RABGAP1L | RAB GTPase activating protein 1 like [Source:HGNC Symbol;Acc:HGNC:24663] |
| ENSG00000154415 | HGLibA_38099 | 147 | 0.314200543 | PPP1R3A | protein phosphatase 1 regulatory subunit 3A [Source:HGNC Symbol;Acc:HGNC:9291] |
| ENSG00000156873 | HGLibA_36414 | 147 | 0.314200543 | PHKG2 | phosphorylase kinase catalytic subunit gamma 2 [Source:HGNC Symbol;Acc:HGNC:8931] |
| ENSG00000156970 | HGLibA_04992 | 147 | 0.314200543 | BUB1B | BUB1 mitotic checkpoint serine/threonine kinase B [Source:HGNC Symbol;Acc:HGNC:1149] |
| ENSG00000162065 | HGLibA_48513 | 147 | 0.314200543 | TBC1D24 | TBC1 domain family member 24 [Source:HGNC Symbol;Acc:HGNC:29203] |
| ENSG00000162231 | HGLibA_33126 | 147 | 0.314200543 | NXF1 | nuclear RNA export factor 1 [Source:HGNC Symbol;Acc:HGNC:8071] |
| ENSG00000163110 | HGLibA_35919 | 147 | 0.314200543 | PDLIM5 | PDZ and LIM domain 5 [Source:HGNC Symbol;Acc:HGNC:17468] |
| ENSG00000163815 | HGLibA_09977 | 147 | 0.314200543 | CLEC3B | C-type lectin domain family 3 member B [Source:HGNC Symbol;Acc:HGNC:11891] |
| ENSG00000164070 | HGLibA_22285 | 147 | 0.314200543 | HSPA4L | heat shock protein family A (Hsp70) member 4 like [Source:HGNC Symbol;Acc:HGNC:17041] |
| ENSG00000165029 | HGLibA_00095 | 147 | 0.314200543 | ABCA1 | ATP binding cassette subfamily A member 1 [Source:HGNC Symbol;Acc:HGNC:29] |
| ENSG00000166046 | HGLibA_48847 | 147 | 0.314200543 | TCP11L2 | t-complex 11 like 2 [Source:HGNC Symbol;Acc:HGNC:28627] |
| ENSG00000167654 | HGLibA_03426 | 147 | 0.314200543 | ATCAY | caytaxin [Source:HGNC Symbol;Acc:HGNC:779]" |
| ENSG00000168209 | HGLibA_12618 | 147 | 0.314200543 | DDIT4 | DNA damage inducible transcript 4 [Source:HGNC Symbol;Acc:HGNC:24944] |
| ENSG00000168758 | HGLibA_43345 | 147 | 0.314200543 | SEMA4C | semaphorin 4C [Source:HGNC Symbol;Acc:HGNC:10731] |
| ENSG00000168928 | HGLibA_11680 | 147 | 0.314200543 | CTRB2 | chymotrypsinogen B2 [Source:HGNC Symbol;Acc:HGNC:2522] |
| ENSG00000169047 | HGLibA_23618 | 147 | 0.314200543 | IRS1 | insulin receptor substrate 1 [Source:HGNC Symbol;Acc:HGNC:6125] |
| ENSG00000169249 | HGLibA_56980 | 147 | 0.314200543 | ZRSR2 | RNA binding motif and serine/arginine rich 2 [Source:HGNC Symbol;Acc:HGNC:23019]" |

| | | | | | |
|---|---|---|---|---|---|
| ENSG00000169594 | HGLibA_04645 | 147 | 0.314200543 | BNC1 | basonuclin 1 [Source:HGNC Symbol;Acc:HGNC:1081] |
| ENSG00000170633 | HGLibA_41668 | 147 | 0.314200543 | RNF34 | ring finger protein 34 [Source:HGNC Symbol;Acc:HGNC:17297] |
| ENSG00000171223 | HGLibA_24005 | 147 | 0.314200543 | JUNB | AP-1 transcription factor subunit [Source:HGNC Symbol;Acc:HGNC:6205]" |
| ENSG00000171246 | HGLibA_32533 | 147 | 0.314200543 | NPTX1 | neuronal pentraxin 1 [Source:HGNC Symbol;Acc:HGNC:7952] |
| ENSG00000171865 | HGLibA_41425 | 147 | 0.314200543 | RNASEH1 | ribonuclease H1 [Source:HGNC Symbol;Acc:HGNC:18466] |
| ENSG00000172673 | HGLibA_49348 | 147 | 0.314200543 | THEMIS | thymocyte selection associated [Source:HGNC Symbol;Acc:HGNC:21569] |
| ENSG00000173627 | HGLibA_02498 | 147 | 0.314200543 | APOBEC4 | apolipoprotein B mRNA editing enzyme catalytic polypeptide like 4 [Source:HGNC Symbol;Acc:HGNC:32152] |
| ENSG00000174599 | HGLibA_51285 | 147 | 0.314200543 | TRAM1L1 | translocation associated membrane protein 1 like 1 [Source:HGNC Symbol;Acc:HGNC:28371] |
| ENSG00000175387 | HGLibA_45571 | 147 | 0.314200543 | SMAD2 | SMAD family member 2 [Source:HGNC Symbol;Acc:HGNC:6768] |
| ENSG00000175489 | HGLibA_27291 | 147 | 0.314200543 | LRRC25 | leucine rich repeat containing 25 [Source:HGNC Symbol;Acc:HGNC:29806] |
| ENSG00000177106 | HGLibA_15380 | 147 | 0.314200543 | EPS8L2 | EPS8 like 2 [Source:HGNC Symbol;Acc:HGNC:21296] |
| ENSG00000178919 | HGLibA_17857 | 147 | 0.314200543 | FOXE1 | forkhead box E1 [Source:HGNC Symbol;Acc:HGNC:3806] |
| ENSG00000181754 | HGLibA_01793 | 147 | 0.314200543 | AMIGO1 | adhesion molecule with Ig like domain 1 [Source:HGNC Symbol;Acc:HGNC:20824] |
| ENSG00000181938 | HGLibA_19108 | 147 | 0.314200543 | GINS3 | GINS complex subunit 3 [Source:HGNC Symbol;Acc:HGNC:25851] |
| ENSG00000182348 | HGLibA_56791 | 147 | 0.314200543 | ZNF804B | zinc finger protein 804B [Source:HGNC Symbol;Acc:HGNC:21958] |
| ENSG00000183172 | HGLibA_45689 | 147 | 0.314200543 | SMDT1 | single-pass membrane protein with aspartate rich tail 1 [Source:HGNC Symbol;Acc:HGNC:25055] |
| ENSG00000183571 | HGLibA_36282 | 147 | 0.314200543 | PGPEP1L | pyroglutamyl-peptidase I like [Source:HGNC Symbol;Acc:HGNC:27080] |
| ENSG00000183914 | HGLibA_13484 | 147 | 0.314200543 | DNAH2 | dynein axonemal heavy chain 2 [Source:HGNC Symbol;Acc:HGNC:2948] |
| ENSG00000184831 | HGLibA_02552 | 147 | 0.314200543 | APOO | apolipoprotein O [Source:HGNC Symbol;Acc:HGNC:28727] |
| ENSG00000187244 | HGLibA_04232 | 147 | 0.314200543 | BCAM | basal cell adhesion molecule (Lutheran blood group) [Source:HGNC Symbol;Acc:HGNC:6722] |
| ENSG00000188321 | HGLibA_56235 | 147 | 0.314200543 | ZNF559 | zinc finger protein 559 [Source:HGNC Symbol;Acc:HGNC:28197] |
| ENSG00000188613 | HGLibA_31096 | 147 | 0.314200543 | NANOS1 | nanos C2HC-type zinc finger 1 [Source:HGNC Symbol;Acc:HGNC:23044] |
| ENSG00000189007 | HGLibA_00880 | 147 | 0.314200543 | ADAT2 | tRNA specific 2 [Source:HGNC Symbol;Acc:HGNC:21172]" |
| ENSG00000196628 | HGLibA_48788 | 147 | 0.314200543 | TCF4 | transcription factor 4 [Source:HGNC Symbol;Acc:HGNC:11634] |
| ENSG00000197165 | HGLibA_47735 | 147 | 0.314200543 | SULT1A2 | sulfotransferase family 1A member 2 [Source:HGNC Symbol;Acc:HGNC:11454] |
| ENSG00000197213 | HGLibA_57031 | 147 | 0.314200543 | ZSCAN5B | zinc finger and SCAN domain containing 5B [Source:HGNC Symbol;Acc:HGNC:34246] |
| ENSG00000198680 | HGLibA_52343 | 147 | 0.314200543 | TUSC1 | tumor suppressor candidate 1 [Source:HGNC Symbol;Acc:HGNC:31010] |
| ENSG00000203727 | HGLibA_42688 | 147 | 0.314200543 | SAMD5 | sterile alpha motif domain containing 5 [Source:HGNC Symbol;Acc:HGNC:21180] |
| ENSG00000204688 | HGLibA_33802 | 147 | 0.314200543 | OR2H1 | olfactory receptor family 2 subfamily H member 1 [Source:HGNC Symbol;Acc:HGNC:8252] |
| ENSG00000213983 | HGLibA_02288 | 147 | 0.314200543 | AP1G2 | adaptor related protein complex 1 subunit gamma 2 [Source:HGNC Symbol;Acc:HGNC:556] |
| ENSG00000214415 | HGLibA_19479 | 147 | 0.314200543 | GNAT3 | G protein subunit alpha transducin 3 [Source:HGNC Symbol;Acc:HGNC:22800] |
| ENSG00000224982 | HGLibA_50263 | 147 | 0.314200543 | TMEM233 | transmembrane protein 233 [Source:HGNC Symbol;Acc:HGNC:37219] |
| ENSG00000231213 | HGLibA_37243 | 147 | 0.314200543 | PLSCR5 | phospholipid scramblase family member 5 [Source:HGNC Symbol;Acc:HGNC:19952] |
| ENSG00000239810 | HGLibA_38284 | 147 | 0.314200543 | PRAMEF11 | PRAME family member 11 [Source:HGNC Symbol;Acc:HGNC:14086] |
| ENSG00000242221 | HGLibA_39039 | 147 | 0.314200543 | PSG2 | pregnancy specific beta-1-glycoprotein 2 [Source:HGNC Symbol;Acc:HGNC:9519] |
| ENSG00000248905 | HGLibA_17686 | 147 | 0.314200543 | FMN1 | formin 1 [Source:HGNC Symbol;Acc:HGNC:3768] |
| ENSG00000254470 | HGLibA_02352 | 147 | 0.314200543 | AP5B1 | adaptor related protein complex 5 subunit beta 1 [Source:HGNC Symbol;Acc:HGNC:25104] |
| ENSG00000257727 | HGLibA_10364 | 147 | 0.314200543 | CNPY2 | canopy FGF signaling regulator 2 [Source:HGNC Symbol;Acc:HGNC:13529] |
| ENSG00000267467 | HGLibA_02512 | 147 | 0.314200543 | APOC4 | apolipoprotein C4 [Source:HGNC Symbol;Acc:HGNC:611] |
| ENSG00000273820 | HGLibA_53316 | 147 | 0.314200543 | USP27X | ubiquitin specific peptidase 27 X-linked [Source:HGNC Symbol;Acc:HGNC:13486] |
| ENSG00000280969 | HGLibA_42149 | 147 | 0.314200543 | RPS4Y2 | ribosomal protein S4 Y-linked 2 [Source:HGNC Symbol;Acc:HGNC:18501] |
| FAM159B | HGLibA_16202 | 147 | 0.314200543 | FAM159B | - |
| GAGE2B | HGLibA_18469 | 147 | 0.314200543 | GAGE2B | - |
| GLTPD1 | HGLibA_19345 | 147 | 0.314200543 | GLTPD1 | - |
| hsa-mir-1469 | HGLibA_57854 | 147 | 0.314200543 | hsa-mir-1469 | - |
| hsa-mir-3615 | HGLibA_59088 | 147 | 0.314200543 | hsa-mir-3615 | - |
| hsa-mir-3677 | HGLibA_59256 | 147 | 0.314200543 | hsa-mir-3677 | - |
| hsa-mir-375 | HGLibA_59403 | 147 | 0.314200543 | hsa-mir-375 | - |
| hsa-mir-4293 | HGLibA_59883 | 147 | 0.314200543 | hsa-mir-4293 | - |
| hsa-mir-4682 | HGLibA_60837 | 147 | 0.314200543 | hsa-mir-4682 | - |
| hsa-mir-4789 | HGLibA_61263 | 147 | 0.314200543 | hsa-mir-4789 | - |
| hsa-mir-4799 | HGLibA_61301 | 147 | 0.314200543 | hsa-mir-4799 | - |
| hsa-mir-516b-2 | HGLibA_61620 | 147 | 0.314200543 | hsa-mir-516b-2 | - |
| hsa-mir-6132 | HGLibA_62680 | 147 | 0.314200543 | hsa-mir-6132 | - |
| hsa-mir-630 | HGLibA_62759 | 147 | 0.314200543 | hsa-mir-630 | - |
| hsa-mir-649 | HGLibA_62839 | 147 | 0.314200543 | hsa-mir-649 | - |
| hsa-mir-660 | HGLibA_62973 | 147 | 0.314200543 | hsa-mir-660 | - |
| hsa-mir-7158 | HGLibA_63862 | 147 | 0.314200543 | hsa-mir-7158 | - |
| hsa-mir-8082 | HGLibA_64170 | 147 | 0.314200543 | hsa-mir-8082 | - |
| KIAA0556 | HGLibA_24643 | 147 | 0.314200543 | KIAA0556 | - |
| LOC100129216 | HGLibA_26756 | 147 | 0.314200543 | LOC100129216 | - |
| MNF1 | HGLibA_29570 | 147 | 0.314200543 | MNF1 | - |
| SOGA2 | HGLibA_46155 | 147 | 0.314200543 | SOGA2 | - |
| SOGA2 | HGLibA_46157 | 147 | 0.314200543 | SOGA2 | - |
| SPANXB2 | HGLibA_46387 | 147 | 0.314200543 | SPANXB2 | - |
| SPANXB2 | HGLibA_46388 | 147 | 0.314200543 | SPANXB2 | - |
| SQRDL | HGLibA_46917 | 147 | 0.314200543 | SQRDL | - |
| C19orf66 | HGLibA_05693 | 146 | 0.304249505 | C19orf66 | - |
| C6orf222 | HGLibA_06477 | 146 | 0.304249505 | C6orf222 | - |
| CCDC132 | HGLibA_07596 | 146 | 0.304249505 | CCDC132 | - |
| ENSG00000004700 | HGLibA_40768 | 146 | 0.304249505 | RECQL | RecQ like helicase [Source:HGNC Symbol;Acc:HGNC:9948] |
| ENSG00000005302 | HGLibA_30217 | 146 | 0.304249505 | MSL3 | MSL complex subunit 3 [Source:HGNC Symbol;Acc:HGNC:7370] |
| ENSG00000057657 | HGLibA_38361 | 146 | 0.304249505 | PRDM1 | PR/SET domain 1 [Source:HGNC Symbol;Acc:HGNC:9346] |
| ENSG00000065135 | HGLibA_19458 | 146 | 0.304249505 | GNAI3 | G protein subunit alpha i3 [Source:HGNC Symbol;Acc:HGNC:4387] |
| ENSG00000065308 | HGLibA_51288 | 146 | 0.304249505 | TRAM2 | translocation associated membrane protein 2 [Source:HGNC Symbol;Acc:HGNC:16855] |
| ENSG00000068137 | HGLibA_37130 | 146 | 0.304249505 | PLEKHH3 | MyTH4 and FERM domain containing H3 [Source:HGNC Symbol;Acc:HGNC:26105]" |
| ENSG00000071082 | HGLibA_41936 | 146 | 0.304249505 | RPL31 | ribosomal protein L31 [Source:HGNC Symbol;Acc:HGNC:10334] |
| ENSG00000073737 | HGLibA_13153 | 146 | 0.304249505 | DHRS9 | dehydrogenase/reductase 9 [Source:HGNC Symbol;Acc:HGNC:16888] |
| ENSG00000091317 | HGLibA_10224 | 146 | 0.304249505 | CMTM6 | CKLF like MARVEL transmembrane domain containing 6 [Source:HGNC Symbol;Acc:HGNC:19177] |
| ENSG00000099337 | HGLibA_24331 | 146 | 0.304249505 | KCNK6 | potassium two pore domain channel subfamily K member 6 [Source:HGNC Symbol;Acc:HGNC:6281] |
| ENSG00000100226 | HGLibA_20599 | 146 | 0.304249505 | GTPBP1 | GTP binding protein 1 [Source:HGNC Symbol;Acc:HGNC:4669] |
| ENSG00000100290 | HGLibA_04502 | 146 | 0.304249505 | BIK | BCL2 interacting killer [Source:HGNC Symbol;Acc:HGNC:1051] |
| ENSG00000100867 | HGLibA_13131 | 146 | 0.304249505 | DHRS2 | dehydrogenase/reductase 2 [Source:HGNC Symbol;Acc:HGNC:18349] |
| ENSG00000102468 | HGLibA_22372 | 146 | 0.304249505 | HTR2A | 5-hydroxytryptamine receptor 2A [Source:HGNC Symbol;Acc:HGNC:5293] |
| ENSG00000102710 | HGLibA_47806 | 146 | 0.304249505 | SUPT20H | SAGA complex component [Source:HGNC Symbol;Acc:HGNC:20596]" |
| ENSG00000105649 | HGLibA_39906 | 146 | 0.304249505 | RAB3A | member RAS oncogene family [Source:HGNC Symbol;Acc:HGNC:9777]" |
| ENSG00000105989 | HGLibA_54357 | 146 | 0.304249505 | WNT2 | Wnt family member 2 [Source:HGNC Symbol;Acc:HGNC:12780] |
| ENSG00000106701 | HGLibA_18079 | 146 | 0.304249505 | FSD1L | fibronectin type III and SPRY domain containing 1 like [Source:HGNC Symbol;Acc:HGNC:13753] |
| ENSG00000107551 | HGLibA_40383 | 146 | 0.304249505 | RASSF4 | Ras association domain family member 4 [Source:HGNC Symbol;Acc:HGNC:20793] |
| ENSG00000108001 | HGLibA_14356 | 146 | 0.304249505 | EBF3 | EBF transcription factor 3 [Source:HGNC Symbol;Acc:HGNC:19087] |
| ENSG00000108349 | HGLibA_07275 | 146 | 0.304249505 | CASC3 | exon junction complex subunit [Source:HGNC Symbol;Acc:HGNC:17040]" |
| ENSG00000108506 | HGLibA_23415 | 146 | 0.304249505 | INTS2 | integrator complex subunit 2 [Source:HGNC Symbol;Acc:HGNC:29241] |
| ENSG00000112200 | HGLibA_56028 | 146 | 0.304249505 | ZNF451 | zinc finger protein 451 [Source:HGNC Symbol;Acc:HGNC:21091] |
| ENSG00000112981 | HGLibA_32167 | 146 | 0.304249505 | NME5 | NME/NM23 family member 5 [Source:HGNC Symbol;Acc:HGNC:7853] |
| ENSG00000115163 | HGLibA_09038 | 146 | 0.304249505 | CENPA | centromere protein A [Source:HGNC Symbol;Acc:HGNC:1851] |
| ENSG00000115380 | HGLibA_14560 | 146 | 0.304249505 | EFEMP1 | EGF containing fibulin extracellular matrix protein 1 [Source:HGNC Symbol;Acc:HGNC:3218] |
| ENSG00000115540 | HGLibA_29603 | 146 | 0.304249505 | MOB4 | phocein [Source:HGNC Symbol;Acc:HGNC:17261]" |
| ENSG00000117475 | HGLibA_04581 | 146 | 0.304249505 | BLZF1 | basic leucine zipper nuclear factor 1 [Source:HGNC Symbol;Acc:HGNC:1065] |
| ENSG00000120669 | HGLibA_46166 | 146 | 0.304249505 | SOHLH2 | spermatogenesis and oogenesis specific basic helix-loop-helix 2 [Source:HGNC Symbol;Acc:HGNC:26026] |
| ENSG00000120805 | HGLibA_02920 | 146 | 0.304249505 | ARL1 | ADP ribosylation factor like GTPase 1 [Source:HGNC Symbol;Acc:HGNC:692] |
| ENSG00000121318 | HGLibA_48367 | 146 | 0.304249505 | TAS2R10 | taste 2 receptor member 10 [Source:HGNC Symbol;Acc:HGNC:14918] |
| ENSG00000121716 | HGLibA_36671 | 146 | 0.304249505 | PILRB | paired immunoglobin-like type 2 receptor beta [Source:HGNC Symbol;Acc:HGNC:18297] |
| ENSG00000123473 | HGLibA_47438 | 146 | 0.304249505 | STIL | centriolar assembly protein [Source:HGNC Symbol;Acc:HGNC:10879]" |
| ENSG00000123989 | HGLibA_09522 | 146 | 0.304249505 | CHPF | chondroitin polymerizing factor [Source:HGNC Symbol;Acc:HGNC:24291] |
| ENSG00000124429 | HGLibA_37464 | 146 | 0.304249505 | POF1B | actin binding protein [Source:HGNC Symbol;Acc:HGNC:13711]" |
| ENSG00000124688 | HGLibA_27849 | 146 | 0.304249505 | MAD2L1BP | MAD2L1 binding protein [Source:HGNC Symbol;Acc:HGNC:21059] |
| ENSG00000125611 | HGLibA_09373 | 146 | 0.304249505 | CHCHD5 | coiled-coil-helix-coiled-coil-helix domain containing 5 [Source:HGNC Symbol;Acc:HGNC:17840] |
| ENSG00000125798 | HGLibA_17810 | 146 | 0.304249505 | FOXA2 | forkhead box A2 [Source:HGNC Symbol;Acc:HGNC:5022] |
| ENSG00000133392 | HGLibA_30709 | 146 | 0.304249505 | MYH11 | myosin heavy chain 11 [Source:HGNC Symbol;Acc:HGNC:7569] |
| ENSG00000134249 | HGLibA_00769 | 146 | 0.304249505 | ADAM30 | ADAM metallopeptidase domain 30 [Source:HGNC Symbol;Acc:HGNC:208] |
| ENSG00000134438 | HGLibA_40408 | 146 | 0.304249505 | RAX | retina and anterior neural fold homeobox [Source:HGNC Symbol;Acc:HGNC:18662] |
| ENSG00000136153 | HGLibA_26714 | 146 | 0.304249505 | LMO7 | LIM domain 7 [Source:HGNC Symbol;Acc:HGNC:6646] |
| ENSG00000138767 | HGLibA_10348 | 146 | 0.304249505 | CNOT6L | CCR4-NOT transcription complex subunit 6 like [Source:HGNC Symbol;Acc:HGNC:18042] |
| ENSG00000138835 | HGLibA_41091 | 146 | 0.304249505 | RGS3 | regulator of G protein signaling 3 [Source:HGNC Symbol;Acc:HGNC:9999] |
| ENSG00000141258 | HGLibA_43861 | 146 | 0.304249505 | SGSM2 | small G protein signaling modulator 2 [Source:HGNC Symbol;Acc:HGNC:29026] |
| ENSG00000141956 | HGLibA_38379 | 146 | 0.304249505 | PRDM15 | PR/SET domain 15 [Source:HGNC Symbol;Acc:HGNC:13999] |
| ENSG00000143858 | HGLibA_48071 | 146 | 0.304249505 | SYT2 | synaptotagmin 2 [Source:HGNC Symbol;Acc:HGNC:11510] |
| ENSG00000143858 | HGLibA_48073 | 146 | 0.304249505 | SYT2 | synaptotagmin 2 [Source:HGNC Symbol;Acc:HGNC:11510] |
| ENSG00000144476 | HGLibA_00445 | 146 | 0.304249505 | ACKR3 | atypical chemokine receptor 3 [Source:HGNC Symbol;Acc:HGNC:23692] |
| ENSG00000144504 | HGLibA_01979 | 146 | 0.304249505 | ANKMY1 | ankyrin repeat and MYND domain containing 1 [Source:HGNC Symbol;Acc:HGNC:20987] |
| ENSG00000144724 | HGLibA_39506 | 146 | 0.304249505 | PTPRG | receptor type G [Source:HGNC Symbol;Acc:HGNC:9671]" |
| ENSG00000144868 | HGLibA_49866 | 146 | 0.304249505 | TMEM108 | transmembrane protein 108 [Source:HGNC Symbol;Acc:HGNC:28451] |
| ENSG00000144909 | HGLibA_34645 | 146 | 0.304249505 | OSBPL11 | oxysterol binding protein like 11 [Source:HGNC Symbol;Acc:HGNC:16397] |
| ENSG00000149451 | HGLibA_00775 | 146 | 0.304249505 | ADAM33 | ADAM metallopeptidase domain 33 [Source:HGNC Symbol;Acc:HGNC:15478] |
| ENSG00000149599 | HGLibA_14145 | 146 | 0.304249505 | DUSP15 | dual specificity phosphatase 15 [Source:HGNC Symbol;Acc:HGNC:16236] |
| ENSG00000151240 | HGLibA_13253 | 146 | 0.304249505 | DIP2C | disco interacting protein 2 homolog C [Source:HGNC Symbol;Acc:HGNC:29150] |
| ENSG00000151376 | HGLibA_28715 | 146 | 0.304249505 | ME3 | malic enzyme 3 [Source:HGNC Symbol;Acc:HGNC:6985] |
| ENSG00000151502 | HGLibA_53756 | 146 | 0.304249505 | VPS26B | retromer complex component B [Source:HGNC Symbol;Acc:HGNC:28119]" |
| ENSG00000152229 | HGLibA_39240 | 146 | 0.304249505 | PSTPIP2 | proline-serine-threonine phosphatase interacting protein 2 [Source:HGNC Symbol;Acc:HGNC:9581] |
| ENSG00000152377 | HGLibA_46749 | 146 | 0.304249505 | SPOCK1 | cwcv and kazal like domains proteoglycan 1 [Source:HGNC Symbol;Acc:HGNC:11251]" |
| ENSG00000154451 | HGLibA_18741 | 146 | 0.304249505 | GBP5 | guanylate binding protein 5 [Source:HGNC Symbol;Acc:HGNC:19895] |
| ENSG00000155511 | HGLibA_20260 | 146 | 0.304249505 | GRIA1 | glutamate ionotropic receptor AMPA type subunit 1 [Source:HGNC Symbol;Acc:HGNC:4571] |
| ENSG00000157350 | HGLibA_47240 | 146 | 0.304249505 | ST3GAL2 | 3-sialyltransferase 2 [Source:HGNC Symbol;Acc:HGNC:10863]" |
| ENSG00000159167 | HGLibA_47413 | 146 | 0.304249505 | STC1 | stanniocalcin 1 [Source:HGNC Symbol;Acc:HGNC:11373] |
| ENSG00000159176 | HGLibA_11432 | 146 | 0.304249505 | CSRP1 | cysteine and glycine rich protein 1 [Source:HGNC Symbol;Acc:HGNC:2469] |

| | | | | | |
|---|---|---|---|---|---|
| ENSG00000159516 | HGLibA_46824 | 146 | 0.304249505 | SPRR2G | small proline rich protein 2G [Source:HGNC Symbol;Acc:HGNC:11267] |
| ENSG00000162892 | HGLibA_23102 | 146 | 0.304249505 | IL24 | interleukin 24 [Source:HGNC Symbol;Acc:HGNC:11346] |
| ENSG00000163638 | HGLibA_00846 | 146 | 0.304249505 | ADAMTS9 | ADAM metallopeptidase with thrombospondin type 1 motif 9 [Source:HGNC Symbol;Acc:HGNC:13202] |
| ENSG00000165169 | HGLibA_14268 | 146 | 0.304249505 | DYNLT3 | dynein light chain Tctex-type 3 [Source:HGNC Symbol;Acc:HGNC:11694] |
| ENSG00000165970 | HGLibA_45330 | 146 | 0.304249505 | SLC6A5 | solute carrier family 6 member 5 [Source:HGNC Symbol;Acc:HGNC:11051] |
| ENSG00000166450 | HGLibA_38979 | 146 | 0.304249505 | PRTG | protogenin [Source:HGNC Symbol;Acc:HGNC:26373] |
| ENSG00000167085 | HGLibA_36312 | 146 | 0.304249505 | PHB | prohibitin [Source:HGNC Symbol;Acc:HGNC:8912] |
| ENSG00000167771 | HGLibA_40714 | 146 | 0.304249505 | RCOR2 | REST corepressor 2 [Source:HGNC Symbol;Acc:HGNC:27455] |
| ENSG00000167900 | HGLibA_49580 | 146 | 0.304249505 | TK1 | thymidine kinase 1 [Source:HGNC Symbol;Acc:HGNC:11830] |
| ENSG00000168453 | HGLibA_22071 | 146 | 0.304249505 | HR | lysine demethylase and nuclear receptor corepressor [Source:HGNC Symbol;Acc:HGNC:5172]" |
| ENSG00000169359 | HGLibA_44934 | 146 | 0.304249505 | SLC33A1 | solute carrier family 33 member 1 [Source:HGNC Symbol;Acc:HGNC:95] |
| ENSG00000170577 | HGLibA_44247 | 146 | 0.304249505 | SIX2 | SIX homeobox 2 [Source:HGNC Symbol;Acc:HGNC:10888] |
| ENSG00000171234 | HGLibA_52931 | 146 | 0.304249505 | UGT2B7 | UDP glucuronosyltransferase family 2 member B7 [Source:HGNC Symbol;Acc:HGNC:12554] |
| ENSG00000171649 | HGLibA_55336 | 146 | 0.304249505 | ZIK1 | zinc finger protein interacting with K protein 1 [Source:HGNC Symbol;Acc:HGNC:33104] |
| ENSG00000171724 | HGLibA_53545 | 146 | 0.304249505 | VAT1L | vesicle amine transport 1 like [Source:HGNC Symbol;Acc:HGNC:29315] |
| ENSG00000171864 | HGLibA_38640 | 146 | 0.304249505 | PRND | prion like protein doppel [Source:HGNC Symbol;Acc:HGNC:15748] |
| ENSG00000172208 | HGLibA_34069 | 146 | 0.304249505 | OR4X2 | olfactory receptor family 4 subfamily X member 2 (gene/pseudogene) [Source:HGNC Symbol;Acc:HGNC:15184] |
| ENSG00000173638 | HGLibA_44499 | 146 | 0.304249505 | SLC19A1 | solute carrier family 19 member 1 [Source:HGNC Symbol;Acc:HGNC:10937] |
| ENSG00000173809 | HGLibA_48902 | 146 | 0.304249505 | TDRD12 | tudor domain containing 12 [Source:HGNC Symbol;Acc:HGNC:25044] |
| ENSG00000175066 | HGLibA_19201 | 146 | 0.304249505 | GK5 | glycerol kinase 5 [Source:HGNC Symbol;Acc:HGNC:28635] |
| ENSG00000177025 | HGLibA_05632 | 146 | 0.304249505 | C19orf18 | chromosome 19 open reading frame 18 [Source:HGNC Symbol;Acc:HGNC:28642] |
| ENSG00000177479 | HGLibA_02916 | 146 | 0.304249505 | ARIH2 | ariadne RBR E3 ubiquitin protein ligase 2 [Source:HGNC Symbol;Acc:HGNC:690] |
| ENSG00000177807 | HGLibA_24250 | 146 | 0.304249505 | KCNJ10 | potassium voltage-gated channel subfamily J member 10 [Source:HGNC Symbol;Acc:HGNC:6256] |
| ENSG00000178750 | HGLibA_47634 | 146 | 0.304249505 | STX19 | syntaxin 19 [Source:HGNC Symbol;Acc:HGNC:19300] |
| ENSG00000178764 | HGLibA_55314 | 146 | 0.304249505 | ZHX2 | zinc fingers and homeoboxes 2 [Source:HGNC Symbol;Acc:HGNC:18513] |
| ENSG00000179133 | HGLibA_05084 | 146 | 0.304249505 | C10orf67 | chromosome 10 open reading frame 67 [Source:HGNC Symbol;Acc:HGNC:28716] |
| ENSG00000180509 | HGLibA_24183 | 146 | 0.304249505 | KCNE1 | potassium voltage-gated channel subfamily E regulatory subunit 1 [Source:HGNC Symbol;Acc:HGNC:6240] |
| ENSG00000180891 | HGLibA_11766 | 146 | 0.304249505 | CUEDC1 | CUE domain containing 1 [Source:HGNC Symbol;Acc:HGNC:31350] |
| ENSG00000181904 | HGLibA_06369 | 146 | 0.304249505 | C5orf24 | chromosome 5 open reading frame 24 [Source:HGNC Symbol;Acc:HGNC:26746] |
| ENSG00000181929 | HGLibA_38513 | 146 | 0.304249505 | PRKAG1 | protein kinase AMP-activated non-catalytic subunit gamma 1 [Source:HGNC Symbol;Acc:HGNC:9385] |
| ENSG00000182197 | HGLibA_15803 | 146 | 0.304249505 | EXT1 | exostosin glycosyltransferase 1 [Source:HGNC Symbol;Acc:HGNC:3512] |
| ENSG00000183423 | HGLibA_27190 | 146 | 0.304249505 | LRIT3 | Ig-like and transmembrane domains 3 [Source:HGNC Symbol;Acc:HGNC:24783]" |
| ENSG00000183496 | HGLibA_29030 | 146 | 0.304249505 | MEX3B | mex-3 RNA binding family member B [Source:HGNC Symbol;Acc:HGNC:25297] |
| ENSG00000183850 | HGLibA_56643 | 146 | 0.304249505 | ZNF730 | zinc finger protein 730 [Source:HGNC Symbol;Acc:HGNC:32470] |
| ENSG00000184735 | HGLibA_12742 | 146 | 0.304249505 | DDX53 | DEAD-box helicase 53 [Source:HGNC Symbol;Acc:HGNC:20083] |
| ENSG00000185955 | HGLibA_06565 | 146 | 0.304249505 | C7orf61 | chromosome 7 open reading frame 61 [Source:HGNC Symbol;Acc:HGNC:22135] |
| ENSG00000187566 | HGLibA_31895 | 146 | 0.304249505 | NHLRC1 | NHL repeat containing E3 ubiquitin protein ligase 1 [Source:HGNC Symbol;Acc:HGNC:21576] |
| ENSG00000188227 | HGLibA_56774 | 146 | 0.304249505 | ZNF793 | zinc finger protein 793 [Source:HGNC Symbol;Acc:HGNC:33115] |
| ENSG00000188263 | HGLibA_23019 | 146 | 0.304249505 | IL17REL | interleukin 17 receptor E like [Source:HGNC Symbol;Acc:HGNC:33808] |
| ENSG00000188322 | HGLibA_42800 | 146 | 0.304249505 | SBK1 | SH3 domain binding kinase 1 [Source:HGNC Symbol;Acc:HGNC:17699] |
| ENSG00000189007 | HGLibA_00881 | 146 | 0.304249505 | ADAT2 | tRNA specific 2 [Source:HGNC Symbol;Acc:HGNC:21172]" |
| ENSG00000197885 | HGLibA_32017 | 146 | 0.304249505 | NKIRAS1 | NFKB inhibitor interacting Ras like 1 [Source:HGNC Symbol;Acc:HGNC:17899] |
| ENSG00000198785 | HGLibA_20312 | 146 | 0.304249505 | GRIN3A | glutamate ionotropic receptor NMDA type subunit 3A [Source:HGNC Symbol;Acc:HGNC:16767] |
| ENSG00000205423 | HGLibA_10257 | 146 | 0.304249505 | CNEP1R1 | CTD nuclear envelope phosphatase 1 regulatory subunit 1 [Source:HGNC Symbol;Acc:HGNC:26759] |
| ENSG00000213316 | HGLibA_27606 | 146 | 0.304249505 | LTC4S | leukotriene C4 synthase [Source:HGNC Symbol;Acc:HGNC:6719] |
| ENSG00000214046 | HGLibA_45767 | 146 | 0.304249505 | SMIM7 | small integral membrane protein 7 [Source:HGNC Symbol;Acc:HGNC:28419] |
| ENSG00000218336 | HGLibA_49002 | 146 | 0.304249505 | TENM3 | teneurin transmembrane protein 3 [Source:HGNC Symbol;Acc:HGNC:29944] |
| ENSG00000237521 | HGLibA_34478 | 146 | 0.304249505 | OR7E24 | olfactory receptor family 7 subfamily E member 24 [Source:HGNC Symbol;Acc:HGNC:8396] |
| ENSG00000237671 | HGLibA_18439 | 146 | 0.304249505 | GAGE12C | G antigen 12C [Source:HGNC Symbol;Acc:HGNC:28402] |
| ENSG00000241123 | HGLibA_25611 | 146 | 0.304249505 | KRTAP10-5 | keratin associated protein 10-5 [Source:HGNC Symbol;Acc:HGNC:22969] |
| ENSG00000242515 | HGLibA_52878 | 146 | 0.304249505 | UGT1A10 | UDP glucuronosyltransferase family 1 member A10 [Source:HGNC Symbol;Acc:HGNC:12531] |
| ENSG00000244486 | HGLibA_42877 | 146 | 0.304249505 | SCARF2 | scavenger receptor class F member 2 [Source:HGNC Symbol;Acc:HGNC:19869] |
| ENSG00000249459 | HGLibA_55752 | 146 | 0.304249505 | ZNF286B | zinc finger protein 286B [Source:HGNC Symbol;Acc:HGNC:33241] |
| ENSG00000255690 | HGLibA_51400 | 146 | 0.304249505 | TRIL | TLR4 interactor with leucine rich repeats [Source:HGNC Symbol;Acc:HGNC:22200] |
| ENSG00000257218 | HGLibA_18692 | 146 | 0.304249505 | GATC | glutamyl-tRNA amidotransferase subunit C [Source:HGNC Symbol;Acc:HGNC:25068] |
| ENSG00000258873 | HGLibA_14205 | 146 | 0.304249505 | DUXA | double homeobox A [Source:HGNC Symbol;Acc:HGNC:32179] |
| ENSG00000262246 | HGLibA_10792 | 146 | 0.304249505 | CORO7 | coronin 7 [Source:HGNC Symbol;Acc:HGNC:26161] |
| ENSG00000269113 | HGLibA_51235 | 146 | 0.304249505 | TRABD2B | TraB domain containing 2B [Source:HGNC Symbol;Acc:HGNC:44200] |
| ENSG00000276293 | HGLibA_36708 | 146 | 0.304249505 | PIP4K2B | phosphatidylinositol-5-phosphate 4-kinase type 2 beta [Source:HGNC Symbol;Acc:HGNC:8998] |
| FIGF | HGLibA_17513 | 146 | 0.304249505 | FIGF | - |
| H2AFJ | HGLibA_20753 | 146 | 0.304249505 | H2AFJ | - |
| hsa-mir-147a | HGLibA_57872 | 146 | 0.304249505 | hsa-mir-147a | - |
| hsa-mir-331 | HGLibA_58993 | 146 | 0.304249505 | hsa-mir-331 | - |
| hsa-mir-4269 | HGLibA_59784 | 146 | 0.304249505 | hsa-mir-4269 | - |
| hsa-mir-4277 | HGLibA_59813 | 146 | 0.304249505 | hsa-mir-4277 | - |
| hsa-mir-4306 | HGLibA_59934 | 146 | 0.304249505 | hsa-mir-4306 | - |
| hsa-mir-4454 | HGLibA_60223 | 146 | 0.304249505 | hsa-mir-4454 | - |
| hsa-mir-4530 | HGLibA_60579 | 146 | 0.304249505 | hsa-mir-4530 | - |
| hsa-mir-4737 | HGLibA_61047 | 146 | 0.304249505 | hsa-mir-4737 | - |
| hsa-mir-4779 | HGLibA_61223 | 146 | 0.304249505 | hsa-mir-4779 | - |
| hsa-mir-5187 | HGLibA_61639 | 146 | 0.304249505 | hsa-mir-5187 | - |
| hsa-mir-599 | HGLibA_62487 | 146 | 0.304249505 | hsa-mir-599 | - |
| hsa-mir-601 | HGLibA_62495 | 146 | 0.304249505 | hsa-mir-601 | - |
| hsa-mir-651 | HGLibA_62888 | 146 | 0.304249505 | hsa-mir-651 | - |
| hsa-mir-6790 | HGLibA_63336 | 146 | 0.304249505 | hsa-mir-6790 | - |
| hsa-mir-6816 | HGLibA_63442 | 146 | 0.304249505 | hsa-mir-6816 | - |
| hsa-mir-6846 | HGLibA_63556 | 146 | 0.304249505 | hsa-mir-6846 | - |
| hsa-mir-7161 | HGLibA_63872 | 146 | 0.304249505 | hsa-mir-7161 | - |
| hsa-mir-7847 | HGLibA_63979 | 146 | 0.304249505 | hsa-mir-7847 | - |
| hsa-mir-941-1 | HGLibA_64339 | 146 | 0.304249505 | hsa-mir-941-1 | - |
| KIAA1432 | HGLibA_24757 | 146 | 0.304249505 | KIAA1432 | - |
| SHOX_X | HGLibA_44068 | 146 | 0.304249505 | SHOX_X | - |
| ADSSL1 | HGLibA_01078 | 145 | 0.294298468 | ADSSL1 | - |
| ANXA8L2 | HGLibA_02257 | 145 | 0.294298468 | ANXA8L2 | - |
| C11orf84 | HGLibA_05196 | 145 | 0.294298468 | C11orf84 | - |
| C12orf49 | HGLibA_05253 | 145 | 0.294298468 | C12orf49 | - |
| C12orf74 | HGLibA_05293 | 145 | 0.294298468 | C12orf74 | - |
| ENSG00000003436 | HGLibA_49185 | 145 | 0.294298468 | TFPI | tissue factor pathway inhibitor [Source:HGNC Symbol;Acc:HGNC:11760] |
| ENSG00000004838 | HGLibA_55411 | 145 | 0.294298468 | ZMYND10 | zinc finger MYND-type containing 10 [Source:HGNC Symbol;Acc:HGNC:19412] |
| ENSG00000006625 | HGLibA_18992 | 145 | 0.294298468 | GGCT | gamma-glutamylcyclotransferase [Source:HGNC Symbol;Acc:HGNC:21705] |
| ENSG00000007372 | HGLibA_35309 | 145 | 0.294298468 | PAX6 | paired box 6 [Source:HGNC Symbol;Acc:HGNC:8620] |
| ENSG00000010610 | HGLibA_08380 | 145 | 0.294298468 | CD4 | CD4 molecule [Source:HGNC Symbol;Acc:HGNC:1678] |
| ENSG00000011347 | HGLibA_48086 | 145 | 0.294298468 | SYT7 | synaptotagmin 7 [Source:HGNC Symbol;Acc:HGNC:11514] |
| ENSG00000013810 | HGLibA_48164 | 145 | 0.294298468 | TACC3 | transforming acidic coiled-coil containing protein 3 [Source:HGNC Symbol;Acc:HGNC:11524] |
| ENSG00000035403 | HGLibA_53572 | 145 | 0.294298468 | VCL | vinculin [Source:HGNC Symbol;Acc:HGNC:12665] |
| ENSG00000057149 | HGLibA_43551 | 145 | 0.294298468 | SERPINB3 | serpin family B member 3 [Source:HGNC Symbol;Acc:HGNC:10569] |
| ENSG00000060138 | HGLibA_54648 | 145 | 0.294298468 | YBX3 | Y-box binding protein 3 [Source:HGNC Symbol;Acc:HGNC:2428] |
| ENSG00000061676 | HGLibA_31312 | 145 | 0.294298468 | NCKAP1 | NCK associated protein 1 [Source:HGNC Symbol;Acc:HGNC:7666] |
| ENSG00000066185 | HGLibA_55416 | 145 | 0.294298468 | ZMYND12 | zinc finger MYND-type containing 12 [Source:HGNC Symbol;Acc:HGNC:21192] |
| ENSG00000066735 | HGLibA_24917 | 145 | 0.294298468 | KIF26A | kinesin family member 26A [Source:HGNC Symbol;Acc:HGNC:20226] |
| ENSG00000068654 | HGLibA_37550 | 145 | 0.294298468 | POLR1A | RNA polymerase I subunit A [Source:HGNC Symbol;Acc:HGNC:17264] |
| ENSG00000070882 | HGLibA_34654 | 145 | 0.294298468 | OSBPL3 | oxysterol binding protein like 3 [Source:HGNC Symbol;Acc:HGNC:16370] |
| ENSG00000078589 | HGLibA_34871 | 145 | 0.294298468 | P2RY10 | P2Y receptor family member 10 [Source:HGNC Symbol;Acc:HGNC:19906] |
| ENSG00000081853 | HGLibA_35530 | 145 | 0.294298468 | PCDHGA2 | 2 [Source:HGNC Symbol;Acc:HGNC:8700]" |
| ENSG00000083812 | HGLibA_55802 | 145 | 0.294298468 | ZNF324 | zinc finger protein 324 [Source:HGNC Symbol;Acc:HGNC:14096] |
| ENSG00000092054 | HGLibA_30734 | 145 | 0.294298468 | MYH7 | myosin heavy chain 7 [Source:HGNC Symbol;Acc:HGNC:7577] |
| ENSG00000092964 | HGLibA_13927 | 145 | 0.294298468 | DPYSL2 | dihydropyrimidinase like 2 [Source:HGNC Symbol;Acc:HGNC:3014] |
| ENSG00000094880 | HGLibA_08525 | 145 | 0.294298468 | CDC23 | cell division cycle 23 [Source:HGNC Symbol;Acc:HGNC:1724] |
| ENSG00000094963 | HGLibA_17706 | 145 | 0.294298468 | FMO2 | flavin containing monooxygenase 2 [Source:HGNC Symbol;Acc:HGNC:3770] |
| ENSG00000100292 | HGLibA_21746 | 145 | 0.294298468 | HMOX1 | heme oxygenase 1 [Source:HGNC Symbol;Acc:HGNC:5013] |
| ENSG00000102904 | HGLibA_51890 | 145 | 0.294298468 | TSNAXIP1 | translin associated factor X interacting protein 1 [Source:HGNC Symbol;Acc:HGNC:18586] |
| ENSG00000105559 | HGLibA_37070 | 145 | 0.294298468 | PLEKHA4 | pleckstrin homology domain containing A4 [Source:HGNC Symbol;Acc:HGNC:14339] |
| ENSG00000105672 | HGLibA_15649 | 145 | 0.294298468 | ETV2 | ETS variant 2 [Source:HGNC Symbol;Acc:HGNC:3491] |
| ENSG00000108465 | HGLibA_08787 | 145 | 0.294298468 | CDK5RAP3 | CDK5 regulatory subunit associated protein 3 [Source:HGNC Symbol;Acc:HGNC:18673] |
| ENSG00000108479 | HGLibA_18514 | 145 | 0.294298468 | GALK1 | galactokinase 1 [Source:HGNC Symbol;Acc:HGNC:4118] |
| ENSG00000110046 | HGLibA_03480 | 145 | 0.294298468 | ATG2A | autophagy related 2A [Source:HGNC Symbol;Acc:HGNC:29028] |
| ENSG00000111652 | HGLibA_10728 | 145 | 0.294298468 | COPS7A | COP9 signalosome subunit 7A [Source:HGNC Symbol;Acc:HGNC:16758] |
| ENSG00000111886 | HGLibA_18406 | 145 | 0.294298468 | GABRR2 | gamma-aminobutyric acid type A receptor rho2 subunit [Source:HGNC Symbol;Acc:HGNC:4091] |
| ENSG00000112218 | HGLibA_20068 | 145 | 0.294298468 | GPR63 | G protein-coupled receptor 63 [Source:HGNC Symbol;Acc:HGNC:13302] |
| ENSG00000115183 | HGLibA_48291 | 145 | 0.294298468 | TANC1 | ankyrin repeat and coiled-coil containing 1 [Source:HGNC Symbol;Acc:HGNC:29364]" |
| ENSG00000116741 | HGLibA_41079 | 145 | 0.294298468 | RGS2 | regulator of G protein signaling 2 [Source:HGNC Symbol;Acc:HGNC:9998] |
| ENSG00000117245 | HGLibA_24870 | 145 | 0.294298468 | KIF17 | kinesin family member 17 [Source:HGNC Symbol;Acc:HGNC:19167] |
| ENSG00000122557 | HGLibA_21173 | 145 | 0.294298468 | HERPUD2 | HERPUD family member 2 [Source:HGNC Symbol;Acc:HGNC:21915] |
| ENSG00000123178 | HGLibA_46855 | 145 | 0.294298468 | SPRYD7 | SPRY domain containing 7 [Source:HGNC Symbol;Acc:HGNC:14297] |
| ENSG00000124181 | HGLibA_37002 | 145 | 0.294298468 | PLCG1 | phospholipase C gamma 1 [Source:HGNC Symbol;Acc:HGNC:9065] |
| ENSG00000125249 | HGLibA_40236 | 145 | 0.294298468 | RAP2A | member of RAS oncogene family [Source:HGNC Symbol;Acc:HGNC:9861]" |
| ENSG00000126767 | HGLibA_14911 | 145 | 0.294298468 | ELK1 | ETS transcription factor [Source:HGNC Symbol;Acc:HGNC:3321]" |
| ENSG00000128311 | HGLibA_52024 | 145 | 0.294298468 | TST | thiosulfate sulfurtransferase [Source:HGNC Symbol;Acc:HGNC:12388] |
| ENSG00000129472 | HGLibA_39868 | 145 | 0.294298468 | RAB2B | member RAS oncogene family [Source:HGNC Symbol;Acc:HGNC:20246]" |
| ENSG00000130150 | HGLibA_29688 | 145 | 0.294298468 | MOSPD2 | motile sperm domain containing 2 [Source:HGNC Symbol;Acc:HGNC:28381] |
| ENSG00000130766 | HGLibA_43621 | 145 | 0.294298468 | SESN2 | sestrin 2 [Source:HGNC Symbol;Acc:HGNC:20746] |
| ENSG00000131368 | HGLibA_30074 | 145 | 0.294298468 | MRPS25 | mitochondrial ribosomal protein S25 [Source:HGNC Symbol;Acc:HGNC:14511] |
| ENSG00000132671 | HGLibA_47183 | 145 | 0.294298468 | SSTR4 | somatostatin receptor 4 [Source:HGNC Symbol;Acc:HGNC:11333] |
| ENSG00000133878 | HGLibA_14168 | 145 | 0.294298468 | DUSP26 | dual specificity phosphatase 26 [Source:HGNC Symbol;Acc:HGNC:28161] |
| ENSG00000135374 | HGLibA_14903 | 145 | 0.294298468 | ELF5 | E74 like ETS transcription factor 5 [Source:HGNC Symbol;Acc:HGNC:3320] |

| | | | | | |
|---|---|---|---|---|---|
| ENSG00000135902 | HGLibA_09600 | 145 | 0.294298468 | CHRND | cholinergic receptor nicotinic delta subunit [Source:HGNC Symbol;Acc:HGNC:1965] |
| ENSG00000136881 | HGLibA_04053 | 145 | 0.294298468 | BAAT | bile acid-CoA:amino acid N-acyltransferase [Source:HGNC Symbol;Acc:HGNC:932] |
| ENSG00000138134 | HGLibA_47338 | 145 | 0.294298468 | STAMBPL1 | STAM binding protein like 1 [Source:HGNC Symbol;Acc:HGNC:24105] |
| ENSG00000138641 | HGLibA_21158 | 145 | 0.294298468 | HERC3 | HECT and RLD domain containing E3 ubiquitin protein ligase 3 [Source:HGNC Symbol;Acc:HGNC:4876] |
| ENSG00000142046 | HGLibA_50557 | 145 | 0.294298468 | TMEM91 | transmembrane protein 91 [Source:HGNC Symbol;Acc:HGNC:32393] |
| ENSG00000142677 | HGLibA_23092 | 145 | 0.294298468 | IL22RA1 | interleukin 22 receptor subunit alpha 1 [Source:HGNC Symbol;Acc:HGNC:13700] |
| ENSG00000143486 | HGLibA_14749 | 145 | 0.294298468 | EIF2D | eukaryotic translation initiation factor 2D [Source:HGNC Symbol;Acc:HGNC:6583] |
| ENSG00000146039 | HGLibA_44468 | 145 | 0.294298468 | SLC17A4 | solute carrier family 17 member 4 [Source:HGNC Symbol;Acc:HGNC:10932] |
| ENSG00000147852 | HGLibA_53684 | 145 | 0.294298468 | VLDLR | very low density lipoprotein receptor [Source:HGNC Symbol;Acc:HGNC:12698] |
| ENSG00000149781 | HGLibA_17301 | 145 | 0.294298468 | FERMT3 | fermitin family member 3 [Source:HGNC Symbol;Acc:HGNC:23151] |
| ENSG00000155052 | HGLibA_10435 | 145 | 0.294298468 | CNTNAP5 | contactin associated protein like 5 [Source:HGNC Symbol;Acc:HGNC:18748] |
| ENSG00000155265 | HGLibA_19638 | 145 | 0.294298468 | GOLGA7B | golgin A7 family member B [Source:HGNC Symbol;Acc:HGNC:31668] |
| ENSG00000156531 | HGLibA_36383 | 145 | 0.294298468 | PHF6 | PHD finger protein 6 [Source:HGNC Symbol;Acc:HGNC:18145] |
| ENSG00000157510 | HGLibA_01099 | 145 | 0.294298468 | AFAP1L1 | actin filament associated protein 1 like 1 [Source:HGNC Symbol;Acc:HGNC:26714] |
| ENSG00000158156 | HGLibA_54533 | 145 | 0.294298468 | XKR8 | XK related 8 [Source:HGNC Symbol;Acc:HGNC:25508] |
| ENSG00000158158 | HGLibA_10323 | 145 | 0.294298468 | CNNM4 | cyclin and CBS domain divalent metal cation transport mediator 4 [Source:HGNC Symbol;Acc:HGNC:105] |
| ENSG00000158796 | HGLibA_12781 | 145 | 0.294298468 | DEDD | death effector domain containing [Source:HGNC Symbol;Acc:HGNC:2755] |
| ENSG00000158828 | HGLibA_36691 | 145 | 0.294298468 | PINK1 | PTEN induced kinase 1 [Source:HGNC Symbol;Acc:HGNC:14581] |
| ENSG00000159840 | HGLibA_57087 | 145 | 0.294298468 | ZYX | zyxin [Source:HGNC Symbol;Acc:HGNC:13200] |
| ENSG00000161914 | HGLibA_56465 | 145 | 0.294298468 | ZNF653 | zinc finger protein 653 [Source:HGNC Symbol;Acc:HGNC:25196] |
| ENSG00000162378 | HGLibA_57084 | 145 | 0.294298468 | ZYG11B | cell cycle regulator [Source:HGNC Symbol;Acc:HGNC:25820]" |
| ENSG00000162511 | HGLibA_26022 | 145 | 0.294298468 | LAPTM5 | lysosomal protein transmembrane 5 [Source:HGNC Symbol;Acc:HGNC:29612] |
| ENSG00000162520 | HGLibA_47965 | 145 | 0.294298468 | SYNC | intermediate filament protein [Source:HGNC Symbol;Acc:HGNC:28897]" |
| ENSG00000162545 | HGLibA_07078 | 145 | 0.294298468 | CAMK2N1 | calcium/calmodulin dependent protein kinase II inhibitor 1 [Source:HGNC Symbol;Acc:HGNC:24190] |
| ENSG00000162804 | HGLibA_45924 | 145 | 0.294298468 | SNED1 | nidogen and EGF like domains 1 [Source:HGNC Symbol;Acc:HGNC:24696]" |
| ENSG00000163017 | HGLibA_00614 | 145 | 0.294298468 | ACTG2 | enteric [Source:HGNC Symbol;Acc:HGNC:145]" |
| ENSG00000163288 | HGLibA_18375 | 145 | 0.294298468 | GABRB1 | gamma-aminobutyric acid type A receptor beta1 subunit [Source:HGNC Symbol;Acc:HGNC:4081] |
| ENSG00000163823 | HGLibA_08145 | 145 | 0.294298468 | CCR1 | C-C motif chemokine receptor 1 [Source:HGNC Symbol;Acc:HGNC:1602] |
| ENSG00000165084 | HGLibA_06611 | 145 | 0.294298468 | C8orf34 | chromosome 8 open reading frame 34 [Source:HGNC Symbol;Acc:HGNC:30905] |
| ENSG00000166167 | HGLibA_04987 | 145 | 0.294298468 | BTRC | beta-transducin repeat containing E3 ubiquitin protein ligase [Source:HGNC Symbol;Acc:HGNC:1144] |
| ENSG00000169488 | HGLibA_34026 | 145 | 0.294298468 | OR4K15 | olfactory receptor family 4 subfamily K member 15 [Source:HGNC Symbol;Acc:HGNC:15353] |
| ENSG00000169955 | HGLibA_56667 | 145 | 0.294298468 | ZNF747 | zinc finger protein 747 [Source:HGNC Symbol;Acc:HGNC:28350] |
| ENSG00000170381 | HGLibA_43332 | 145 | 0.294298468 | SEMA3E | semaphorin 3E [Source:HGNC Symbol;Acc:HGNC:10727] |
| ENSG00000171130 | HGLibA_03728 | 145 | 0.294298468 | ATP6V0E2 | ATPase H+ transporting V0 subunit e2 [Source:HGNC Symbol;Acc:HGNC:21723] |
| ENSG00000171561 | HGLibA_33761 | 145 | 0.294298468 | OR2AT4 | olfactory receptor family 2 subfamily AT member 4 [Source:HGNC Symbol;Acc:HGNC:19620] |
| ENSG00000172464 | HGLibA_34245 | 145 | 0.294298468 | OR5AP2 | olfactory receptor family 5 subfamily AP member 2 [Source:HGNC Symbol;Acc:HGNC:15258] |
| ENSG00000172831 | HGLibA_09222 | 145 | 0.294298468 | CES2 | carboxylesterase 2 [Source:HGNC Symbol;Acc:HGNC:1864] |
| ENSG00000173157 | HGLibA_00823 | 145 | 0.294298468 | ADAMTS20 | ADAM metallopeptidase with thrombospondin type 1 motif 20 [Source:HGNC Symbol;Acc:HGNC:17178] |
| ENSG00000174325 | HGLibA_13266 | 145 | 0.294298468 | DIRC1 | disrupted in renal carcinoma 1 [Source:HGNC Symbol;Acc:HGNC:15760] |
| ENSG00000175087 | HGLibA_35888 | 145 | 0.294298468 | PDIK1L | PDLIM1 interacting kinase 1 like [Source:HGNC Symbol;Acc:HGNC:18981] |
| ENSG00000175267 | HGLibA_53901 | 145 | 0.294298468 | VWA3A | von Willebrand factor A domain containing 3A [Source:HGNC Symbol;Acc:HGNC:27088] |
| ENSG00000175356 | HGLibA_43074 | 145 | 0.294298468 | SCUBE2 | CUB domain and EGF like domain containing 2 [Source:HGNC Symbol;Acc:HGNC:30425]" |
| ENSG00000176024 | HGLibA_56385 | 145 | 0.294298468 | ZNF613 | zinc finger protein 613 [Source:HGNC Symbol;Acc:HGNC:25827] |
| ENSG00000178772 | HGLibA_10950 | 145 | 0.294298468 | CPN2 | carboxypeptidase N subunit 2 [Source:HGNC Symbol;Acc:HGNC:2313] |
| ENSG00000179562 | HGLibA_18764 | 145 | 0.294298468 | GCC1 | GRIP and coiled-coil domain containing 1 [Source:HGNC Symbol;Acc:HGNC:19095] |
| ENSG00000180447 | HGLibA_18641 | 145 | 0.294298468 | GAS1 | growth arrest specific 1 [Source:HGNC Symbol;Acc:HGNC:4165] |
| ENSG00000180785 | HGLibA_34097 | 145 | 0.294298468 | OR51E1 | olfactory receptor family 51 subfamily E member 1 [Source:HGNC Symbol;Acc:HGNC:15194] |
| ENSG00000181896 | HGLibA_55436 | 145 | 0.294298468 | ZNF101 | zinc finger protein 101 [Source:HGNC Symbol;Acc:HGNC:12881] |
| ENSG00000182575 | HGLibA_33169 | 145 | 0.294298468 | NXPH3 | neurexophilin 3 [Source:HGNC Symbol;Acc:HGNC:8077] |
| ENSG00000183530 | HGLibA_38782 | 145 | 0.294298468 | PRR14L | proline rich 14 like [Source:HGNC Symbol;Acc:HGNC:28738] |
| ENSG00000183960 | HGLibA_24232 | 145 | 0.294298468 | KCNH8 | potassium voltage-gated channel subfamily H member 8 [Source:HGNC Symbol;Acc:HGNC:18864] |
| ENSG00000184719 | HGLibA_41716 | 145 | 0.294298468 | RNLS | FAD dependent amine oxidase [Source:HGNC Symbol;Acc:HGNC:25641]" |
| ENSG00000186635 | HGLibA_02626 | 145 | 0.294298468 | ARAP1 | ankyrin repeat and PH domain 1 [Source:HGNC Symbol;Acc:HGNC:16925]" |
| ENSG00000187742 | HGLibA_43264 | 145 | 0.294298468 | SECISBP2 | SECIS binding protein 2 [Source:HGNC Symbol;Acc:HGNC:30972] |
| ENSG00000189037 | HGLibA_14159 | 145 | 0.294298468 | DUSP21 | dual specificity phosphatase 21 [Source:HGNC Symbol;Acc:HGNC:20476] |
| ENSG00000197535 | HGLibA_30850 | 145 | 0.294298468 | MYO5A | myosin VA [Source:HGNC Symbol;Acc:HGNC:7602] |
| ENSG00000197647 | HGLibA_55986 | 145 | 0.294298468 | ZNF433 | zinc finger protein 433 [Source:HGNC Symbol;Acc:HGNC:20811] |
| ENSG00000198681 | HGLibA_27891 | 145 | 0.294298468 | MAGEA1 | MAGE family member A1 [Source:HGNC Symbol;Acc:HGNC:6796] |
| ENSG00000198734 | HGLibA_15876 | 145 | 0.294298468 | F5 | coagulation factor V [Source:HGNC Symbol;Acc:HGNC:3542] |
| ENSG00000204099 | HGLibA_31718 | 145 | 0.294298468 | NEU4 | neuraminidase 4 [Source:HGNC Symbol;Acc:HGNC:21328] |
| ENSG00000204174 | HGLibA_32556 | 145 | 0.294298468 | NPY4R | neuropeptide Y receptor Y4 [Source:HGNC Symbol;Acc:HGNC:9329] |
| ENSG00000204501 | HGLibA_38294 | 145 | 0.294298468 | PRAMEF15 | PRAME family member 15 [Source:HGNC Symbol;Acc:HGNC:26764] |
| ENSG00000213341 | HGLibA_09669 | 145 | 0.294298468 | CHUK | conserved helix-loop-helix ubiquitous kinase [Source:HGNC Symbol;Acc:HGNC:1974] |
| ENSG00000213892 | HGLibA_08926 | 145 | 0.294298468 | CEACAM16 | carcinoembryonic antigen related cell adhesion molecule 16 [Source:HGNC Symbol;Acc:HGNC:31948] |
| ENSG00000214842 | HGLibA_40083 | 145 | 0.294298468 | RAD51AP2 | RAD51 associated protein 2 [Source:HGNC Symbol;Acc:HGNC:34417] |
| ENSG00000221938 | HGLibA_33725 | 145 | 0.294298468 | OR2A14 | olfactory receptor family 2 subfamily A member 14 [Source:HGNC Symbol;Acc:HGNC:15084] |
| ENSG00000227488 | HGLibA_18442 | 145 | 0.294298468 | GAGE12D | G antigen 12D [Source:HGNC Symbol;Acc:HGNC:31904] |
| ENSG00000229292 | HGLibA_40940 | 145 | 0.294298468 | RFPL4AL1 | ret finger protein like 4A like 1 [Source:HGNC Symbol;Acc:HGNC:45147] |
| ENSG00000232859 | HGLibA_27761 | 145 | 0.294298468 | LYRM9 | LYR motif containing 9 [Source:HGNC Symbol;Acc:HGNC:27314] |
| ENSG00000233198 | HGLibA_41650 | 145 | 0.294298468 | RNF224 | ring finger protein 224 [Source:HGNC Symbol;Acc:HGNC:41912] |
| ENSG00000236609 | HGLibA_56875 | 145 | 0.294298468 | ZNF853 | zinc finger protein 853 [Source:HGNC Symbol;Acc:HGNC:21767] |
| ENSG00000237541 | HGLibA_21623 | 145 | 0.294298468 | HLA-DQA2 | DQ alpha 2 [Source:HGNC Symbol;Acc:HGNC:4943]" |
| ENSG00000243709 | HGLibA_26273 | 145 | 0.294298468 | LEFTY1 | left-right determination factor 1 [Source:HGNC Symbol;Acc:HGNC:6552] |
| ENSG00000244005 | HGLibA_31843 | 145 | 0.294298468 | NFS1 | cysteine desulfurase [Source:HGNC Symbol;Acc:HGNC:15910]" |
| ENSG00000254521 | HGLibA_44132 | 145 | 0.294298468 | SIGLEC12 | sialic acid binding Ig like lectin 12 (gene/pseudogene) [Source:HGNC Symbol;Acc:HGNC:15482] |
| ENSG00000256463 | HGLibA_42653 | 145 | 0.294298468 | SALL3 | spalt like transcription factor 3 [Source:HGNC Symbol;Acc:HGNC:10527] |
| ENSG00000261236 | HGLibA_04696 | 145 | 0.294298468 | BOP1 | block of proliferation 1 [Source:HGNC Symbol;Acc:HGNC:15519] |
| ENSG00000265203 | HGLibA_40636 | 145 | 0.294298468 | RBP3 | retinol binding protein 3 [Source:HGNC Symbol;Acc:HGNC:9921] |
| GAGE2C | HGLibA_18472 | 145 | 0.294298468 | GAGE2C | - |
| GPR115 | HGLibA_19861 | 145 | 0.294298468 | GPR115 | - |
| GTDC2 | HGLibA_20516 | 145 | 0.294298468 | GTDC2 | - |
| HIST1H2BL | HGLibA_21445 | 145 | 0.294298468 | HIST1H2BL | - |
| hsa-mir-1200 | HGLibA_57261 | 145 | 0.294298468 | hsa-mir-1200 | - |
| hsa-mir-4306 | HGLibA_59935 | 145 | 0.294298468 | hsa-mir-4306 | - |
| hsa-mir-4672 | HGLibA_60804 | 145 | 0.294298468 | hsa-mir-4672 | - |
| hsa-mir-558 | HGLibA_62161 | 145 | 0.294298468 | hsa-mir-558 | - |
| hsa-mir-5697 | HGLibA_62320 | 145 | 0.294298468 | hsa-mir-5697 | - |
| hsa-mir-608 | HGLibA_62576 | 145 | 0.294298468 | hsa-mir-608 | - |
| hsa-mir-6133 | HGLibA_62682 | 145 | 0.294298468 | hsa-mir-6133 | - |
| hsa-mir-6804 | HGLibA_63393 | 145 | 0.294298468 | hsa-mir-6804 | - |
| hsa-mir-891b | HGLibA_64248 | 145 | 0.294298468 | hsa-mir-891b | - |
| METTL11B | HGLibA_28950 | 145 | 0.294298468 | METTL11B | - |
| TMEM110-MUSTN1 | HGLibA_49876 | 145 | 0.294298468 | TMEM110-MUSTN1 | - |
| ARMC4 | HGLibA_03020 | 144 | 0.28434743 | ARMC4 | - |
| C12orf65 | HGLibA_05280 | 144 | 0.28434743 | C12orf65 | - |
| C17orf59 | HGLibA_05551 | 144 | 0.28434743 | C17orf59 | - |
| C4orf40 | HGLibA_06330 | 144 | 0.28434743 | C4orf40 | - |
| ENSG00000016864 | HGLibA_19338 | 144 | 0.28434743 | GLT8D1 | glycosyltransferase 8 domain containing 1 [Source:HGNC Symbol;Acc:HGNC:24870] |
| ENSG00000049249 | HGLibA_50793 | 144 | 0.28434743 | TNFRSF9 | TNF receptor superfamily member 9 [Source:HGNC Symbol;Acc:HGNC:11924] |
| ENSG00000050767 | HGLibA_10546 | 144 | 0.28434743 | COL23A1 | collagen type XXIII alpha 1 chain [Source:HGNC Symbol;Acc:HGNC:22990] |
| ENSG00000053501 | HGLibA_53164 | 144 | 0.28434743 | USE1 | unconventional SNARE in the ER 1 [Source:HGNC Symbol;Acc:HGNC:30882] |
| ENSG00000055070 | HGLibA_48113 | 144 | 0.28434743 | SZRD1 | SUZ RNA binding domain containing 1 [Source:HGNC Symbol;Acc:HGNC:30232] |
| ENSG00000068305 | HGLibA_28825 | 144 | 0.28434743 | MEF2A | myocyte enhancer factor 2A [Source:HGNC Symbol;Acc:HGNC:6993] |
| ENSG00000077943 | HGLibA_23746 | 144 | 0.28434743 | ITGA8 | integrin subunit alpha 8 [Source:HGNC Symbol;Acc:HGNC:6144] |
| ENSG00000079739 | HGLibA_36260 | 144 | 0.28434743 | PGM1 | phosphoglucomutase 1 [Source:HGNC Symbol;Acc:HGNC:8905] |
| ENSG00000088832 | HGLibA_17565 | 144 | 0.28434743 | FKBP1A | FK506 binding protein 1A [Source:HGNC Symbol;Acc:HGNC:3711] |
| ENSG00000090013 | HGLibA_04579 | 144 | 0.28434743 | BLVRB | biliverdin reductase B [Source:HGNC Symbol;Acc:HGNC:1063] |
| ENSG00000100385 | HGLibA_23120 | 144 | 0.28434743 | IL2RB | interleukin 2 receptor subunit beta [Source:HGNC Symbol;Acc:HGNC:6009] |
| ENSG00000100422 | HGLibA_09194 | 144 | 0.28434743 | CERK | ceramide kinase [Source:HGNC Symbol;Acc:HGNC:19256] |
| ENSG00000100804 | HGLibA_39112 | 144 | 0.28434743 | PSMB5 | proteasome subunit beta 5 [Source:HGNC Symbol;Acc:HGNC:9542] |
| ENSG00000101115 | HGLibA_42655 | 144 | 0.28434743 | SALL4 | spalt like transcription factor 4 [Source:HGNC Symbol;Acc:HGNC:15924] |
| ENSG00000101160 | HGLibA_11737 | 144 | 0.28434743 | CTSZ | cathepsin Z [Source:HGNC Symbol;Acc:HGNC:2547] |
| ENSG00000102531 | HGLibA_17746 | 144 | 0.28434743 | FNDC3A | fibronectin type III domain containing 3A [Source:HGNC Symbol;Acc:HGNC:20296] |
| ENSG00000102699 | HGLibA_35239 | 144 | 0.28434743 | PARP4 | poly(ADP-ribose) polymerase family member 4 [Source:HGNC Symbol;Acc:HGNC:271] |
| ENSG00000104447 | HGLibA_51776 | 144 | 0.28434743 | TRPS1 | transcriptional repressor GATA binding 1 [Source:HGNC Symbol;Acc:HGNC:12340] |
| ENSG00000104969 | HGLibA_43867 | 144 | 0.28434743 | SGTA | small glutamine rich tetratricopeptide repeat containing alpha [Source:HGNC Symbol;Acc:HGNC:10819] |
| ENSG00000106479 | HGLibA_56881 | 144 | 0.28434743 | ZNF862 | zinc finger protein 862 [Source:HGNC Symbol;Acc:HGNC:34519] |
| ENSG00000106609 | HGLibA_50310 | 144 | 0.28434743 | TMEM248 | transmembrane protein 248 [Source:HGNC Symbol;Acc:HGNC:25476] |
| ENSG00000107537 | HGLibA_36468 | 144 | 0.28434743 | PHYH | phytanoyl-CoA 2-hydroxylase [Source:HGNC Symbol;Acc:HGNC:8940] |
| ENSG00000108830 | HGLibA_41449 | 144 | 0.28434743 | RND2 | Rho family GTPase 2 [Source:HGNC Symbol;Acc:HGNC:18315] |
| ENSG00000110442 | HGLibA_10681 | 144 | 0.28434743 | COMMD9 | COMM domain containing 9 [Source:HGNC Symbol;Acc:HGNC:25014] |
| ENSG00000111424 | HGLibA_53607 | 144 | 0.28434743 | VDR | vitamin D receptor [Source:HGNC Symbol;Acc:HGNC:12679] |
| ENSG00000115840 | HGLibA_44641 | 144 | 0.28434743 | SLC25A12 | solute carrier family 25 member 12 [Source:HGNC Symbol;Acc:HGNC:10982] |
| ENSG00000118898 | HGLibA_37959 | 144 | 0.28434743 | PPL | periplakin [Source:HGNC Symbol;Acc:HGNC:9273] |
| ENSG00000120656 | HGLibA_48203 | 144 | 0.28434743 | TAF12 | TATA-box binding protein associated factor 12 [Source:HGNC Symbol;Acc:HGNC:11545] |
| ENSG00000122641 | HGLibA_23302 | 144 | 0.28434743 | INHBA | inhibin subunit beta A [Source:HGNC Symbol;Acc:HGNC:6066] |
| ENSG00000124217 | HGLibA_29619 | 144 | 0.28434743 | MOCS3 | molybdenum cofactor synthesis 3 [Source:HGNC Symbol;Acc:HGNC:15765] |
| ENSG00000124812 | HGLibA_11166 | 144 | 0.28434743 | CRISP1 | cysteine rich secretory protein 1 [Source:HGNC Symbol;Acc:HGNC:304] |
| ENSG00000125398 | HGLibA_46279 | 144 | 0.28434743 | SOX9 | SRY-box 9 [Source:HGNC Symbol;Acc:HGNC:11204] |
| ENSG00000125648 | HGLibA_44679 | 144 | 0.28434743 | SLC25A23 | solute carrier family 25 member 23 [Source:HGNC Symbol;Acc:HGNC:19375] |
| ENSG00000127318 | HGLibA_23089 | 144 | 0.28434743 | IL22 | interleukin 22 [Source:HGNC Symbol;Acc:HGNC:14900] |
| ENSG00000129559 | HGLibA_31609 | 144 | 0.28434743 | NEDD8 | developmentally down-regulated 8 [Source:HGNC Symbol;Acc:HGNC:7732]" |
| ENSG00000131746 | HGLibA_50927 | 144 | 0.28434743 | TNS4 | tensin 4 [Source:HGNC Symbol;Acc:HGNC:24352] |
| ENSG00000132004 | HGLibA_17158 | 144 | 0.28434743 | FBXW9 | F-box and WD repeat domain containing 9 [Source:HGNC Symbol;Acc:HGNC:28136] |
| ENSG00000132182 | HGLibA_33040 | 144 | 0.28434743 | NUP210 | nucleoporin 210 [Source:HGNC Symbol;Acc:HGNC:30052] |

| | | | | | |
|---|---|---|---|---|---|
| ENSG00000132382 | HGLibA_30640 | 144 | 0.28434743 | MYBBP1A | MYB binding protein 1a [Source:HGNC Symbol;Acc:HGNC:7546] |
| ENSG00000132688 | HGLibA_31699 | 144 | 0.28434743 | NES | nestin [Source:HGNC Symbol;Acc:HGNC:7756] |
| ENSG00000132781 | HGLibA_30585 | 144 | 0.28434743 | MUTYH | mutY DNA glycosylase [Source:HGNC Symbol;Acc:HGNC:7527] |
| ENSG00000132824 | HGLibA_43491 | 144 | 0.28434743 | SERINC3 | serine incorporator 3 [Source:HGNC Symbol;Acc:HGNC:11699] |
| ENSG00000133030 | HGLibA_29777 | 144 | 0.28434743 | MPRIP | myosin phosphatase Rho interacting protein [Source:HGNC Symbol;Acc:HGNC:30321] |
| ENSG00000133063 | HGLibA_09455 | 144 | 0.28434743 | CHIT1 | chitinase 1 [Source:HGNC Symbol;Acc:HGNC:1936] |
| ENSG00000134594 | HGLibA_39881 | 144 | 0.28434743 | RAB33A | member RAS oncogene family [Source:HGNC Symbol;Acc:HGNC:9773]" |
| ENSG00000134970 | HGLibA_49819 | 144 | 0.28434743 | TMED7 | transmembrane p24 trafficking protein 7 [Source:HGNC Symbol;Acc:HGNC:24253] |
| ENSG00000136044 | HGLibA_02568 | 144 | 0.28434743 | APPL2 | phosphotyrosine interacting with PH domain and leucine zipper 2 [Source:HGNC Symbol;Acc:HGNC:18242]" |
| ENSG00000137171 | HGLibA_25036 | 144 | 0.28434743 | KLC4 | kinesin light chain 4 [Source:HGNC Symbol;Acc:HGNC:21624] |
| ENSG00000138036 | HGLibA_14250 | 144 | 0.28434743 | DYNC2LI1 | dynein cytoplasmic 2 light intermediate chain 1 [Source:HGNC Symbol;Acc:HGNC:24595] |
| ENSG00000140090 | HGLibA_44623 | 144 | 0.28434743 | SLC24A4 | solute carrier family 24 member 4 [Source:HGNC Symbol;Acc:HGNC:10978] |
| ENSG00000140379 | HGLibA_04300 | 144 | 0.28434743 | BCL2A1 | BCL2 related protein A1 [Source:HGNC Symbol;Acc:HGNC:991] |
| ENSG00000141627 | HGLibA_14227 | 144 | 0.28434743 | DYM | dymeclin [Source:HGNC Symbol;Acc:HGNC:21317] |
| ENSG00000143771 | HGLibA_10292 | 144 | 0.28434743 | CNIH4 | cornichon family AMPA receptor auxiliary protein 4 [Source:HGNC Symbol;Acc:HGNC:25013] |
| ENSG00000143776 | HGLibA_08558 | 144 | 0.28434743 | CDC42BPA | CDC42 binding protein kinase alpha [Source:HGNC Symbol;Acc:HGNC:1737] |
| ENSG00000143889 | HGLibA_21836 | 144 | 0.28434743 | HNRNPLL | heterogeneous nuclear ribonucleoprotein L like [Source:HGNC Symbol;Acc:HGNC:25127] |
| ENSG00000144959 | HGLibA_31294 | 144 | 0.28434743 | NCEH1 | neutral cholesterol ester hydrolase 1 [Source:HGNC Symbol;Acc:HGNC:29260] |
| ENSG00000145781 | HGLibA_10654 | 144 | 0.28434743 | COMMD10 | COMM domain containing 10 [Source:HGNC Symbol;Acc:HGNC:30201] |
| ENSG00000146950 | HGLibA_44089 | 144 | 0.28434743 | SHROOM2 | shroom family member 2 [Source:HGNC Symbol;Acc:HGNC:630] |
| ENSG00000148143 | HGLibA_56040 | 144 | 0.28434743 | ZNF462 | zinc finger protein 462 [Source:HGNC Symbol;Acc:HGNC:21684] |
| ENSG00000150510 | HGLibA_16075 | 144 | 0.28434743 | FAM124A | family with sequence similarity 124 member A [Source:HGNC Symbol;Acc:HGNC:26413] |
| ENSG00000151247 | HGLibA_14815 | 144 | 0.28434743 | EIF4E | eukaryotic translation initiation factor 4E [Source:HGNC Symbol;Acc:HGNC:3287] |
| ENSG00000152894 | HGLibA_39513 | 144 | 0.28434743 | PTPRK | receptor type K [Source:HGNC Symbol;Acc:HGNC:9674]" |
| ENSG00000155090 | HGLibA_25043 | 144 | 0.28434743 | KLF10 | Kruppel like factor 10 [Source:HGNC Symbol;Acc:HGNC:11810] |
| ENSG00000158856 | HGLibA_13447 | 144 | 0.28434743 | DMTN | dematin actin binding protein [Source:HGNC Symbol;Acc:HGNC:3382] |
| ENSG00000160055 | HGLibA_50268 | 144 | 0.28434743 | TMEM234 | transmembrane protein 234 [Source:HGNC Symbol;Acc:HGNC:28837] |
| ENSG00000160447 | HGLibA_36843 | 144 | 0.28434743 | PKN3 | protein kinase N3 [Source:HGNC Symbol;Acc:HGNC:17999] |
| ENSG00000162267 | HGLibA_23812 | 144 | 0.28434743 | ITIH3 | inter-alpha-trypsin inhibitor heavy chain 3 [Source:HGNC Symbol;Acc:HGNC:6168] |
| ENSG00000162999 | HGLibA_14154 | 144 | 0.28434743 | DUSP19 | dual specificity phosphatase 19 [Source:HGNC Symbol;Acc:HGNC:18894] |
| ENSG00000163145 | HGLibA_05954 | 144 | 0.28434743 | C1QTNF7 | C1q and TNF related 7 [Source:HGNC Symbol;Acc:HGNC:14342] |
| ENSG00000164675 | HGLibA_23549 | 144 | 0.28434743 | IQUB | IQ motif and ubiquitin domain containing [Source:HGNC Symbol;Acc:HGNC:21995] |
| ENSG00000165195 | HGLibA_36542 | 144 | 0.28434743 | PIGA | phosphatidylinositol glycan anchor biosynthesis class A [Source:HGNC Symbol;Acc:HGNC:8957] |
| ENSG00000165934 | HGLibA_10998 | 144 | 0.28434743 | CPSF2 | cleavage and polyadenylation specific factor 2 [Source:HGNC Symbol;Acc:HGNC:2325] |
| ENSG00000166363 | HGLibA_33470 | 144 | 0.28434743 | OR10A5 | olfactory receptor family 10 subfamily A member 5 [Source:HGNC Symbol;Acc:HGNC:15131] |
| ENSG00000167202 | HGLibA_48528 | 144 | 0.28434743 | TBC1D2B | TBC1 domain family member 2B [Source:HGNC Symbol;Acc:HGNC:29183] |
| ENSG00000167699 | HGLibA_19285 | 144 | 0.28434743 | GLOD4 | glyoxalase domain containing 4 [Source:HGNC Symbol;Acc:HGNC:14111] |
| ENSG00000167703 | HGLibA_45151 | 144 | 0.28434743 | SLC43A2 | solute carrier family 43 member 2 [Source:HGNC Symbol;Acc:HGNC:23087] |
| ENSG00000168904 | HGLibA_27298 | 144 | 0.28434743 | LRRC28 | leucine rich repeat containing 28 [Source:HGNC Symbol;Acc:HGNC:28355] |
| ENSG00000169760 | HGLibA_32066 | 144 | 0.28434743 | NLGN1 | neuroligin 1 [Source:HGNC Symbol;Acc:HGNC:14291] |
| ENSG00000170571 | HGLibA_14999 | 144 | 0.28434743 | EMB | embigin [Source:HGNC Symbol;Acc:HGNC:30465] |
| ENSG00000171595 | HGLibA_13508 | 144 | 0.28434743 | DNAI2 | dynein axonemal intermediate chain 2 [Source:HGNC Symbol;Acc:HGNC:18744] |
| ENSG00000171813 | HGLibA_39644 | 144 | 0.28434743 | PWWP2B | PWWP domain containing 2B [Source:HGNC Symbol;Acc:HGNC:25150] |
| ENSG00000172548 | HGLibA_31966 | 144 | 0.28434743 | NIPAL4 | NIPA like domain containing 4 [Source:HGNC Symbol;Acc:HGNC:28018] |
| ENSG00000173432 | HGLibA_42609 | 144 | 0.28434743 | SAA1 | serum amyloid A1 [Source:HGNC Symbol;Acc:HGNC:10513] |
| ENSG00000175664 | HGLibA_49080 | 144 | 0.28434743 | TEX26 | testis expressed 26 [Source:HGNC Symbol;Acc:HGNC:28622] |
| ENSG00000176055 | HGLibA_28506 | 144 | 0.28434743 | MBLAC2 | metallo-beta-lactamase domain containing 2 [Source:HGNC Symbol;Acc:HGNC:33711] |
| ENSG00000178074 | HGLibA_06167 | 144 | 0.28434743 | C2orf69 | chromosome 2 open reading frame 69 [Source:HGNC Symbol;Acc:HGNC:26799] |
| ENSG00000178913 | HGLibA_48252 | 144 | 0.28434743 | TAF7 | TATA-box binding protein associated factor 7 [Source:HGNC Symbol;Acc:HGNC:11541] |
| ENSG00000179841 | HGLibA_01437 | 144 | 0.28434743 | AKAP5 | A-kinase anchoring protein 5 [Source:HGNC Symbol;Acc:HGNC:375] |
| ENSG00000179921 | HGLibA_19753 | 144 | 0.28434743 | GPBAR1 | G protein-coupled bile acid receptor 1 [Source:HGNC Symbol;Acc:HGNC:19680] |
| ENSG00000180938 | HGLibA_56280 | 144 | 0.28434743 | ZNF572 | zinc finger protein 572 [Source:HGNC Symbol;Acc:HGNC:26758] |
| ENSG00000181444 | HGLibA_56045 | 144 | 0.28434743 | ZNF467 | zinc finger protein 467 [Source:HGNC Symbol;Acc:HGNC:23154] |
| ENSG00000181961 | HGLibA_33925 | 144 | 0.28434743 | OR4A16 | olfactory receptor family 4 subfamily A member 16 [Source:HGNC Symbol;Acc:HGNC:15153] |
| ENSG00000183067 | HGLibA_22895 | 144 | 0.28434743 | IGSF5 | immunoglobulin superfamily member 5 [Source:HGNC Symbol;Acc:HGNC:5952] |
| ENSG00000183648 | HGLibA_31503 | 144 | 0.28434743 | NDUFB1 | NADH:ubiquinone oxidoreductase subunit B1 [Source:HGNC Symbol;Acc:HGNC:7695] |
| ENSG00000184486 | HGLibA_37767 | 144 | 0.28434743 | POU3F2 | POU class 3 homeobox 2 [Source:HGNC Symbol;Acc:HGNC:9215] |
| ENSG00000185149 | HGLibA_32553 | 144 | 0.28434743 | NPY2R | neuropeptide Y receptor Y2 [Source:HGNC Symbol;Acc:HGNC:7957] |
| ENSG00000187783 | HGLibA_50508 | 144 | 0.28434743 | TMEM72 | transmembrane protein 72 [Source:HGNC Symbol;Acc:HGNC:31658] |
| ENSG00000187902 | HGLibA_44049 | 144 | 0.28434743 | SHISA7 | shisa family member 7 [Source:HGNC Symbol;Acc:HGNC:35409] |
| ENSG00000188186 | HGLibA_25997 | 144 | 0.28434743 | LAMTOR4 | MAPK and MTOR activator 4 [Source:HGNC Symbol;Acc:HGNC:33772]" |
| ENSG00000188452 | HGLibA_09196 | 144 | 0.28434743 | CERKL | ceramide kinase like [Source:HGNC Symbol;Acc:HGNC:21699] |
| ENSG00000189369 | HGLibA_20442 | 144 | 0.28434743 | GSPT2 | G1 to S phase transition 2 [Source:HGNC Symbol;Acc:HGNC:4622] |
| ENSG00000196372 | HGLibA_03215 | 144 | 0.28434743 | ASB13 | ankyrin repeat and SOCS box containing 13 [Source:HGNC Symbol;Acc:HGNC:19765] |
| ENSG00000197584 | HGLibA_24345 | 144 | 0.28434743 | KCNMB2 | potassium calcium-activated channel subfamily M regulatory beta subunit 2 [Source:HGNC Symbol;Acc:HGNC:6286] |
| ENSG00000197959 | HGLibA_13692 | 144 | 0.28434743 | DNM3 | dynamin 3 [Source:HGNC Symbol;Acc:HGNC:29125] |
| ENSG00000198010 | HGLibA_13343 | 144 | 0.28434743 | DLGAP2 | DLG associated protein 2 [Source:HGNC Symbol;Acc:HGNC:2906] |
| ENSG00000198160 | HGLibA_29252 | 144 | 0.28434743 | MIER1 | MIER1 transcriptional regulator [Source:HGNC Symbol;Acc:HGNC:29657] |
| ENSG00000198736 | HGLibA_30240 | 144 | 0.28434743 | MSRB1 | methionine sulfoxide reductase B1 [Source:HGNC Symbol;Acc:HGNC:14133] |
| ENSG00000198876 | HGLibA_12417 | 144 | 0.28434743 | DCAF12 | DDB1 and CUL4 associated factor 12 [Source:HGNC Symbol;Acc:HGNC:19911] |
| ENSG00000204379 | HGLibA_54467 | 144 | 0.28434743 | XAGE1A | X antigen family member 1A [Source:HGNC Symbol;Acc:HGNC:4111] |
| ENSG00000205856 | HGLibA_06076 | 144 | 0.28434743 | C22orf42 | chromosome 22 open reading frame 42 [Source:HGNC Symbol;Acc:HGNC:27160] |
| ENSG00000206106 | HGLibA_25718 | 144 | 0.28434743 | KRTAP22-2 | keratin associated protein 22-2 [Source:HGNC Symbol;Acc:HGNC:37091] |
| ENSG00000214078 | HGLibA_10953 | 144 | 0.28434743 | CPNE1 | copine 1 [Source:HGNC Symbol;Acc:HGNC:2314] |
| ENSG00000222047 | HGLibA_05078 | 144 | 0.28434743 | C10orf55 | chromosome 10 open reading frame 55 [Source:HGNC Symbol;Acc:HGNC:31008] |
| ENSG00000224586 | HGLibA_20181 | 144 | 0.28434743 | GPX5 | glutathione peroxidase 5 [Source:HGNC Symbol;Acc:HGNC:4557] |
| ENSG00000229972 | HGLibA_23507 | 144 | 0.28434743 | IQCF3 | IQ motif containing F3 [Source:HGNC Symbol;Acc:HGNC:31816] |
| ENSG00000235169 | HGLibA_45716 | 144 | 0.28434743 | SMIM1 | small integral membrane protein 1 (Vel blood group) [Source:HGNC Symbol;Acc:HGNC:44204] |
| ENSG00000244362 | HGLibA_25680 | 144 | 0.28434743 | KRTAP19-7 | keratin associated protein 19-7 [Source:HGNC Symbol;Acc:HGNC:18942] |
| ENSG00000276234 | HGLibA_48187 | 144 | 0.28434743 | TADA2A | transcriptional adaptor 2A [Source:HGNC Symbol;Acc:HGNC:11531] |
| ENSG00000277494 | HGLibA_19810 | 144 | 0.28434743 | GPIHBP1 | glycosylphosphatidylinositol anchored high density lipoprotein binding protein 1 [Source:HGNC Symbol;Acc:HGNC:24945] |
| ENSG00000278195 | HGLibA_47179 | 144 | 0.28434743 | SSTR3 | somatostatin receptor 3 [Source:HGNC Symbol;Acc:HGNC:11332] |
| FAM195A | HGLibA_16376 | 144 | 0.28434743 | FAM195A | - |
| FTH1P18 | HGLibA_18118 | 144 | 0.28434743 | FTH1P18 | - |
| hsa-mir-214 | HGLibA_58250 | 144 | 0.28434743 | hsa-mir-214 | - |
| hsa-mir-376a-1 | HGLibA_59405 | 144 | 0.28434743 | hsa-mir-376a-1 | - |
| hsa-mir-3935 | HGLibA_59600 | 144 | 0.28434743 | hsa-mir-3935 | - |
| hsa-mir-3944 | HGLibA_59636 | 144 | 0.28434743 | hsa-mir-3944 | - |
| hsa-mir-4325 | HGLibA_60025 | 144 | 0.28434743 | hsa-mir-4325 | - |
| hsa-mir-4676 | HGLibA_60820 | 144 | 0.28434743 | hsa-mir-4676 | - |
| hsa-mir-4724 | HGLibA_60994 | 144 | 0.28434743 | hsa-mir-4724 | - |
| hsa-mir-519e | HGLibA_61732 | 144 | 0.28434743 | hsa-mir-519e | - |
| hsa-mir-665 | HGLibA_63001 | 144 | 0.28434743 | hsa-mir-665 | - |
| hsa-mir-6875 | HGLibA_63686 | 144 | 0.28434743 | hsa-mir-6875 | - |
| hsa-mir-6884 | HGLibA_63721 | 144 | 0.28434743 | hsa-mir-6884 | - |
| hsa-mir-7976 | HGLibA_64035 | 144 | 0.28434743 | hsa-mir-7976 | - |
| hsa-mir-8058 | HGLibA_64069 | 144 | 0.28434743 | hsa-mir-8058 | - |
| KIAA1430 | HGLibA_24755 | 144 | 0.28434743 | KIAA1430 | - |
| TCTEX1D1 | HGLibA_48864 | 144 | 0.28434743 | TCTEX1D1 | - |
| TMEM194A | HGLibA_50152 | 144 | 0.28434743 | TMEM194A | - |
| USP17L1P | HGLibA_53239 | 144 | 0.28434743 | USP17L1P | - |
| CASC10 | HGLibA_07271 | 143 | 0.274396393 | CASC10 | - |
| ENSG00000006607 | HGLibA_16876 | 143 | 0.274396393 | FARP2 | ARH/RhoGEF and pleckstrin domain protein 2 [Source:HGNC Symbol;Acc:HGNC:16460]" |
| ENSG00000013375 | HGLibA_36271 | 143 | 0.274396393 | PGM3 | phosphoglucomutase 3 [Source:HGNC Symbol;Acc:HGNC:8907] |
| ENSG00000042445 | HGLibA_40882 | 143 | 0.274396393 | RETSAT | retinol saturase [Source:HGNC Symbol;Acc:HGNC:25991] |
| ENSG00000069399 | HGLibA_04330 | 143 | 0.274396393 | BCL3 | B cell CLL/lymphoma 3 [Source:HGNC Symbol;Acc:HGNC:998] |
| ENSG00000078747 | HGLibA_23703 | 143 | 0.274396393 | ITCH | itchy E3 ubiquitin protein ligase [Source:HGNC Symbol;Acc:HGNC:13890] |
| ENSG00000081665 | HGLibA_56118 | 143 | 0.274396393 | ZNF506 | zinc finger protein 506 [Source:HGNC Symbol;Acc:HGNC:23780] |
| ENSG00000083535 | HGLibA_36520 | 143 | 0.274396393 | PIBF1 | progesterone immunomodulatory binding factor 1 [Source:HGNC Symbol;Acc:HGNC:23352] |
| ENSG00000091490 | HGLibA_43282 | 143 | 0.274396393 | SEL1L3 | SEL1L family member 3 [Source:HGNC Symbol;Acc:HGNC:29108] |
| ENSG00000100982 | HGLibA_35611 | 143 | 0.274396393 | PCIF1 | PDX1 C-terminal inhibiting factor 1 [Source:HGNC Symbol;Acc:HGNC:16200] |
| ENSG00000101336 | HGLibA_20966 | 143 | 0.274396393 | HCK | Src family tyrosine kinase [Source:HGNC Symbol;Acc:HGNC:4840]" |
| ENSG00000102081 | HGLibA_17719 | 143 | 0.274396393 | FMR1 | fragile X mental retardation 1 [Source:HGNC Symbol;Acc:HGNC:3775] |
| ENSG00000105443 | HGLibA_12238 | 143 | 0.274396393 | CYTH2 | cytohesin 2 [Source:HGNC Symbol;Acc:HGNC:9502] |
| ENSG00000105677 | HGLibA_49975 | 143 | 0.274396393 | TMEM147 | transmembrane protein 147 [Source:HGNC Symbol;Acc:HGNC:30414] |
| ENSG00000106537 | HGLibA_51900 | 143 | 0.274396393 | TSPAN13 | tetraspanin 13 [Source:HGNC Symbol;Acc:HGNC:21643] |
| ENSG00000106633 | HGLibA_18788 | 143 | 0.274396393 | GCK | glucokinase [Source:HGNC Symbol;Acc:HGNC:4195] |
| ENSG00000107282 | HGLibA_02367 | 143 | 0.274396393 | APBA1 | amyloid beta precursor protein binding family A member 1 [Source:HGNC Symbol;Acc:HGNC:578] |
| ENSG00000107798 | HGLibA_26600 | 143 | 0.274396393 | LIPA | lysosomal acid type [Source:HGNC Symbol;Acc:HGNC:6617]" |
| ENSG00000108439 | HGLibA_37429 | 143 | 0.274396393 | PNPO | pyridoxamine 5'-phosphate oxidase [Source:HGNC Symbol;Acc:HGNC:30260] |
| ENSG00000111704 | HGLibA_31089 | 143 | 0.274396393 | NANOG | Nanog homeobox [Source:HGNC Symbol;Acc:HGNC:20857] |
| ENSG00000112116 | HGLibA_23001 | 143 | 0.274396393 | IL17F | interleukin 17F [Source:HGNC Symbol;Acc:HGNC:16404] |
| ENSG00000112559 | HGLibA_28670 | 143 | 0.274396393 | MDFI | MyoD family inhibitor [Source:HGNC Symbol;Acc:HGNC:6967] |
| ENSG00000113763 | HGLibA_53013 | 143 | 0.274396393 | UNC5A | unc-5 netrin receptor A [Source:HGNC Symbol;Acc:HGNC:12567] |
| ENSG00000117593 | HGLibA_12344 | 143 | 0.274396393 | DARS2 | mitochondrial [Source:HGNC Symbol;Acc:HGNC:25538]" |
| ENSG00000120436 | HGLibA_20017 | 143 | 0.274396393 | GPR31 | G protein-coupled receptor 31 [Source:HGNC Symbol;Acc:HGNC:4486] |
| ENSG00000120725 | HGLibA_44172 | 143 | 0.274396393 | SIL1 | SIL1 nucleotide exchange factor [Source:HGNC Symbol;Acc:HGNC:24624] |
| ENSG00000123353 | HGLibA_34629 | 143 | 0.274396393 | ORMDL2 | ORMDL sphingolipid biosynthesis regulator 2 [Source:HGNC Symbol;Acc:HGNC:16037] |
| ENSG00000123636 | HGLibA_04193 | 143 | 0.274396393 | BAZ2B | bromodomain adjacent to zinc finger domain 2B [Source:HGNC Symbol;Acc:HGNC:963] |
| ENSG00000123700 | HGLibA_24274 | 143 | 0.274396393 | KCNJ2 | potassium voltage-gated channel subfamily J member 2 [Source:HGNC Symbol;Acc:HGNC:6263] |
| ENSG00000124089 | HGLibA_28546 | 143 | 0.274396393 | MC3R | melanocortin 3 receptor [Source:HGNC Symbol;Acc:HGNC:6931] |
| ENSG00000124469 | HGLibA_08952 | 143 | 0.274396393 | CEACAM8 | carcinoembryonic antigen related cell adhesion molecule 8 [Source:HGNC Symbol;Acc:HGNC:1820] |
| ENSG00000124733 | HGLibA_28717 | 143 | 0.274396393 | MEA1 | male-enhanced antigen 1 [Source:HGNC Symbol;Acc:HGNC:6986] |
| ENSG00000124831 | HGLibA_27449 | 143 | 0.274396393 | LRRFIP1 | LRR binding FLII interacting protein 1 [Source:HGNC Symbol;Acc:HGNC:6702] |
| ENSG00000125788 | HGLibA_12882 | 143 | 0.274396393 | DEFB126 | defensin beta 126 [Source:HGNC Symbol;Acc:HGNC:15900] |

| | | | | | |
|---|---|---|---|---|---|
| ENSG00000125850 | HGLibA_34805 | 143 | 0.274396393 | OVOL2 | ovo like zinc finger 2 [Source:HGNC Symbol;Acc:HGNC:15804] |
| ENSG00000126838 | HGLibA_39716 | 143 | 0.274396393 | PZP | alpha-2-macroglobulin like [Source:HGNC Symbol;Acc:HGNC:9750]" |
| ENSG00000128610 | HGLibA_17318 | 143 | 0.274396393 | FEZF1 | FEZ family zinc finger 1 [Source:HGNC Symbol;Acc:HGNC:22788] |
| ENSG00000130643 | HGLibA_07055 | 143 | 0.274396393 | CALY | calcyon neuron specific vesicular protein [Source:HGNC Symbol;Acc:HGNC:17938] |
| ENSG00000134765 | HGLibA_13989 | 143 | 0.274396393 | DSC1 | desmocollin 1 [Source:HGNC Symbol;Acc:HGNC:3035] |
| ENSG00000134884 | HGLibA_02693 | 143 | 0.274396393 | ARGLU1 | arginine and glutamate rich 1 [Source:HGNC Symbol;Acc:HGNC:25482] |
| ENSG00000135392 | HGLibA_13576 | 143 | 0.274396393 | DNAJC14 | DnaJ heat shock protein family (Hsp40) member C14 [Source:HGNC Symbol;Acc:HGNC:24581] |
| ENSG00000136942 | HGLibA_41947 | 143 | 0.274396393 | RPL35 | ribosomal protein L35 [Source:HGNC Symbol;Acc:HGNC:10344] |
| ENSG00000137500 | HGLibA_07923 | 143 | 0.274396393 | CCDC90B | coiled-coil domain containing 90B [Source:HGNC Symbol;Acc:HGNC:28108] |
| ENSG00000138193 | HGLibA_37000 | 143 | 0.274396393 | PLCE1 | phospholipase C epsilon 1 [Source:HGNC Symbol;Acc:HGNC:17175] |
| ENSG00000138735 | HGLibA_35803 | 143 | 0.274396393 | PDE5A | phosphodiesterase 5A [Source:HGNC Symbol;Acc:HGNC:8784] |
| ENSG00000142186 | HGLibA_43085 | 143 | 0.274396393 | SCYL1 | SCY1 like pseudokinase 1 [Source:HGNC Symbol;Acc:HGNC:14372] |
| ENSG00000142694 | HGLibA_15674 | 143 | 0.274396393 | EVA1B | eva-1 homolog B [Source:HGNC Symbol;Acc:HGNC:25558] |
| ENSG00000143119 | HGLibA_08408 | 143 | 0.274396393 | CD53 | CD53 molecule [Source:HGNC Symbol;Acc:HGNC:1686] |
| ENSG00000143867 | HGLibA_34699 | 143 | 0.274396393 | OSR1 | odd-skipped related transciption factor 1 [Source:HGNC Symbol;Acc:HGNC:8111] |
| ENSG00000144224 | HGLibA_52788 | 143 | 0.274396393 | UBXN4 | UBX domain protein 4 [Source:HGNC Symbol;Acc:HGNC:14860] |
| ENSG00000144227 | HGLibA_33165 | 143 | 0.274396393 | NXPH2 | neurexophilin 2 [Source:HGNC Symbol;Acc:HGNC:8076] |
| ENSG00000144589 | HGLibA_47457 | 143 | 0.274396393 | STK11IP | serine/threonine kinase 11 interacting protein [Source:HGNC Symbol;Acc:HGNC:19184] |
| ENSG00000145088 | HGLibA_14337 | 143 | 0.274396393 | EAF2 | ELL associated factor 2 [Source:HGNC Symbol;Acc:HGNC:23115] |
| ENSG00000146232 | HGLibA_31831 | 143 | 0.274396393 | NFKBIE | NFKB inhibitor epsilon [Source:HGNC Symbol;Acc:HGNC:7799] |
| ENSG00000146540 | HGLibA_06551 | 143 | 0.274396393 | C7orf50 | chromosome 7 open reading frame 50 [Source:HGNC Symbol;Acc:HGNC:22421] |
| ENSG00000147526 | HGLibA_48159 | 143 | 0.274396393 | TACC1 | transforming acidic coiled-coil containing protein 1 [Source:HGNC Symbol;Acc:HGNC:11522] |
| ENSG00000150201 | HGLibA_18232 | 143 | 0.274396393 | FXYD4 | FXYD domain containing ion transport regulator 4 [Source:HGNC Symbol;Acc:HGNC:4028] |
| ENSG00000151502 | HGLibA_53755 | 143 | 0.274396393 | VPS26B | retromer complex component B [Source:HGNC Symbol;Acc:HGNC:28119]" |
| ENSG00000152270 | HGLibA_35785 | 143 | 0.274396393 | PDE3B | phosphodiesterase 3B [Source:HGNC Symbol;Acc:HGNC:8779] |
| ENSG00000154710 | HGLibA_40015 | 143 | 0.274396393 | RABGEF1 | RAB guanine nucleotide exchange factor 1 [Source:HGNC Symbol;Acc:HGNC:17676] |
| ENSG00000156587 | HGLibA_52631 | 143 | 0.274396393 | UBE2L6 | ubiquitin conjugating enzyme E2 L6 [Source:HGNC Symbol;Acc:HGNC:12490] |
| ENSG00000157890 | HGLibA_28851 | 143 | 0.274396393 | MEGF11 | multiple EGF like domains 11 [Source:HGNC Symbol;Acc:HGNC:29635] |
| ENSG00000158106 | HGLibA_41224 | 143 | 0.274396393 | RHPN1 | rhophilin Rho GTPase binding protein 1 [Source:HGNC Symbol;Acc:HGNC:19973] |
| ENSG00000158186 | HGLibA_29812 | 143 | 0.274396393 | MRAS | muscle RAS oncogene homolog [Source:HGNC Symbol;Acc:HGNC:7227] |
| ENSG00000159596 | HGLibA_50498 | 143 | 0.274396393 | TMEM69 | transmembrane protein 69 [Source:HGNC Symbol;Acc:HGNC:28035] |
| ENSG00000160746 | HGLibA_02173 | 143 | 0.274396393 | ANO10 | anoctamin 10 [Source:HGNC Symbol;Acc:HGNC:25519] |
| ENSG00000162365 | HGLibA_12174 | 143 | 0.274396393 | CYP4A22 | cytochrome P450 family 4 subfamily A member 22 [Source:HGNC Symbol;Acc:HGNC:20575] |
| ENSG00000162441 | HGLibA_27797 | 143 | 0.274396393 | LZIC | leucine zipper and CTNNBIP1 domain containing [Source:HGNC Symbol;Acc:HGNC:17497] |
| ENSG00000162461 | HGLibA_44713 | 143 | 0.274396393 | SLC25A34 | solute carrier family 25 member 34 [Source:HGNC Symbol;Acc:HGNC:27653] |
| ENSG00000162650 | HGLibA_03855 | 143 | 0.274396393 | ATXN7L2 | ataxin 7 like 2 [Source:HGNC Symbol;Acc:HGNC:28713] |
| ENSG00000164114 | HGLibA_28245 | 143 | 0.274396393 | MAP9 | microtubule associated protein 9 [Source:HGNC Symbol;Acc:HGNC:26118] |
| ENSG00000166548 | HGLibA_49582 | 143 | 0.274396393 | TK2 | mitochondrial [Source:HGNC Symbol;Acc:HGNC:11831]" |
| ENSG00000167384 | HGLibA_55538 | 143 | 0.274396393 | ZNF180 | zinc finger protein 180 [Source:HGNC Symbol;Acc:HGNC:12970] |
| ENSG00000167895 | HGLibA_49768 | 143 | 0.274396393 | TMC8 | transmembrane channel like 8 [Source:HGNC Symbol;Acc:HGNC:20474] |
| ENSG00000168916 | HGLibA_56374 | 143 | 0.274396393 | ZNF608 | zinc finger protein 608 [Source:HGNC Symbol;Acc:HGNC:29238] |
| ENSG00000169184 | HGLibA_29558 | 143 | 0.274396393 | MN1 | transcriptional regulator [Source:HGNC Symbol;Acc:HGNC:7180]" |
| ENSG00000169756 | HGLibA_26547 | 143 | 0.274396393 | LIMS1 | LIM zinc finger domain containing 1 [Source:HGNC Symbol;Acc:HGNC:6616] |
| ENSG00000170954 | HGLibA_55942 | 143 | 0.274396393 | ZNF415 | zinc finger protein 415 [Source:HGNC Symbol;Acc:HGNC:20636] |
| ENSG00000171316 | HGLibA_09405 | 143 | 0.274396393 | CHD7 | chromodomain helicase DNA binding protein 7 [Source:HGNC Symbol;Acc:HGNC:20626] |
| ENSG00000171872 | HGLibA_25064 | 143 | 0.274396393 | KLF17 | Kruppel like factor 17 [Source:HGNC Symbol;Acc:HGNC:18830] |
| ENSG00000171951 | HGLibA_42900 | 143 | 0.274396393 | SCG2 | secretogranin II [Source:HGNC Symbol;Acc:HGNC:10575] |
| ENSG00000172350 | HGLibA_00222 | 143 | 0.274396393 | ABCG4 | ATP binding cassette subfamily G member 4 [Source:HGNC Symbol;Acc:HGNC:13884] |
| ENSG00000174255 | HGLibA_56781 | 143 | 0.274396393 | ZNF80 | zinc finger protein 80 [Source:HGNC Symbol;Acc:HGNC:13155] |
| ENSG00000174358 | HGLibA_45316 | 143 | 0.274396393 | SLC6A19 | solute carrier family 6 member 19 [Source:HGNC Symbol;Acc:HGNC:27960] |
| ENSG00000175518 | HGLibA_52743 | 143 | 0.274396393 | UBQLNL | ubiquilin like [Source:HGNC Symbol;Acc:HGNC:28294] |
| ENSG00000175573 | HGLibA_05171 | 143 | 0.274396393 | C11orf68 | chromosome 11 open reading frame 68 [Source:HGNC Symbol;Acc:HGNC:28801] |
| ENSG00000178163 | HGLibA_56151 | 143 | 0.274396393 | ZNF518B | zinc finger protein 518B [Source:HGNC Symbol;Acc:HGNC:29365] |
| ENSG00000179776 | HGLibA_08682 | 143 | 0.274396393 | CDH5 | cadherin 5 [Source:HGNC Symbol;Acc:HGNC:1764] |
| ENSG00000181929 | HGLibA_38511 | 143 | 0.274396393 | PRKAG1 | protein kinase AMP-activated non-catalytic subunit gamma 1 [Source:HGNC Symbol;Acc:HGNC:9385] |
| ENSG00000182704 | HGLibA_51875 | 143 | 0.274396393 | TSKU | small leucine rich proteoglycan [Source:HGNC Symbol;Acc:HGNC:28850]" |
| ENSG00000183010 | HGLibA_39672 | 143 | 0.274396393 | PYCR1 | pyrroline-5-carboxylate reductase 1 [Source:HGNC Symbol;Acc:HGNC:9721] |
| ENSG00000185624 | HGLibA_34908 | 143 | 0.274396393 | P4HB | prolyl 4-hydroxylase subunit beta [Source:HGNC Symbol;Acc:HGNC:8548] |
| ENSG00000185869 | HGLibA_56827 | 143 | 0.274396393 | ZNF829 | zinc finger protein 829 [Source:HGNC Symbol;Acc:HGNC:34032] |
| ENSG00000186090 | HGLibA_22388 | 143 | 0.274396393 | HTR3D | 5-hydroxytryptamine receptor 3D [Source:HGNC Symbol;Acc:HGNC:24004] |
| ENSG00000186439 | HGLibA_51347 | 143 | 0.274396393 | TRDN | triadin [Source:HGNC Symbol;Acc:HGNC:12261] |
| ENSG00000186562 | HGLibA_12824 | 143 | 0.274396393 | DEFB105A | defensin beta 105A [Source:HGNC Symbol;Acc:HGNC:18087] |
| ENSG00000187583 | HGLibA_37146 | 143 | 0.274396393 | PLEKHN1 | pleckstrin homology domain containing N1 [Source:HGNC Symbol;Acc:HGNC:25284] |
| ENSG00000188393 | HGLibA_09960 | 143 | 0.274396393 | CLEC2A | C-type lectin domain family 2 member A [Source:HGNC Symbol;Acc:HGNC:24191] |
| ENSG00000189409 | HGLibA_29516 | 143 | 0.274396393 | MMP23B | matrix metallopeptidase 23B [Source:HGNC Symbol;Acc:HGNC:7171] |
| ENSG00000196368 | HGLibA_32937 | 143 | 0.274396393 | NUDT11 | nudix hydrolase 11 [Source:HGNC Symbol;Acc:HGNC:18011] |
| ENSG00000197124 | HGLibA_56529 | 143 | 0.274396393 | ZNF682 | zinc finger protein 682 [Source:HGNC Symbol;Acc:HGNC:28857] |
| ENSG00000197647 | HGLibA_55987 | 143 | 0.274396393 | ZNF433 | zinc finger protein 433 [Source:HGNC Symbol;Acc:HGNC:20811] |
| ENSG00000197818 | HGLibA_45426 | 143 | 0.274396393 | SLC9A8 | solute carrier family 9 member A8 [Source:HGNC Symbol;Acc:HGNC:20728] |
| ENSG00000197928 | HGLibA_56518 | 143 | 0.274396393 | ZNF677 | zinc finger protein 677 [Source:HGNC Symbol;Acc:HGNC:28730] |
| ENSG00000197933 | HGLibA_56822 | 143 | 0.274396393 | ZNF823 | zinc finger protein 823 [Source:HGNC Symbol;Acc:HGNC:30936] |
| ENSG00000203943 | HGLibA_42672 | 143 | 0.274396393 | SAMD13 | sterile alpha motif domain containing 13 [Source:HGNC Symbol;Acc:HGNC:24582] |
| ENSG00000204410 | HGLibA_30200 | 143 | 0.274396393 | MSH5 | mutS homolog 5 [Source:HGNC Symbol;Acc:HGNC:7328] |
| ENSG00000205609 | HGLibA_14772 | 143 | 0.274396393 | EIF3CL | eukaryotic translation initiation factor 3 subunit C like [Source:HGNC Symbol;Acc:HGNC:26347] |
| ENSG00000213578 | HGLibA_10937 | 143 | 0.274396393 | CPLX3 | complexin 3 [Source:HGNC Symbol;Acc:HGNC:27652] |
| ENSG00000213886 | HGLibA_52570 | 143 | 0.274396393 | UBD | ubiquitin D [Source:HGNC Symbol;Acc:HGNC:18795] |
| ENSG00000215269 | HGLibA_18451 | 143 | 0.274396393 | GAGE12G | G antigen 12G [Source:HGNC Symbol;Acc:HGNC:31907] |
| ENSG00000221843 | HGLibA_06113 | 143 | 0.274396393 | C2orf16 | chromosome 2 open reading frame 16 [Source:HGNC Symbol;Acc:HGNC:25275] |
| ENSG00000231389 | HGLibA_21614 | 143 | 0.274396393 | HLA-DPA1 | DP alpha 1 [Source:HGNC Symbol;Acc:HGNC:4938]" |
| ENSG00000235034 | HGLibA_05714 | 143 | 0.274396393 | C19orf81 | chromosome 19 open reading frame 81 [Source:HGNC Symbol;Acc:HGNC:40041] |
| ENSG00000236032 | HGLibA_34291 | 143 | 0.274396393 | OR5H14 | olfactory receptor family 5 subfamily H member 14 [Source:HGNC Symbol;Acc:HGNC:31286] |
| ENSG00000241484 | HGLibA_02788 | 143 | 0.274396393 | ARHGAP8 | Rho GTPase activating protein 8 [Source:HGNC Symbol;Acc:HGNC:677] |
| ENSG00000251201 | HGLibA_49823 | 143 | 0.274396393 | TMED7-TICAM2 | TMED7-TICAM2 readthrough [Source:HGNC Symbol;Acc:HGNC:33945] |
| ENSG00000251664 | HGLibA_35434 | 143 | 0.274396393 | PCDHA12 | protocadherin alpha 12 [Source:HGNC Symbol;Acc:HGNC:8666] |
| ENSG00000273274 | HGLibA_54927 | 143 | 0.274396393 | ZBTB8B | zinc finger and BTB domain containing 8B [Source:HGNC Symbol;Acc:HGNC:37057] |
| FAM109A | HGLibA_16007 | 143 | 0.274396393 | FAM109A | - |
| GAGE6 | HGLibA_18484 | 143 | 0.274396393 | GAGE6 | - |
| hsa-mir-1236 | HGLibA_57336 | 143 | 0.274396393 | hsa-mir-1236 | - |
| hsa-mir-1273f | HGLibA_57545 | 143 | 0.274396393 | hsa-mir-1273f | - |
| hsa-mir-1296 | HGLibA_57663 | 143 | 0.274396393 | hsa-mir-1296 | - |
| hsa-mir-204 | HGLibA_58175 | 143 | 0.274396393 | hsa-mir-204 | - |
| hsa-mir-2117 | HGLibA_58239 | 143 | 0.274396393 | hsa-mir-2117 | - |
| hsa-mir-23b | HGLibA_58333 | 143 | 0.274396393 | hsa-mir-23b | - |
| hsa-mir-378e | HGLibA_59444 | 143 | 0.274396393 | hsa-mir-378e | - |
| hsa-mir-4524b | HGLibA_60559 | 143 | 0.274396393 | hsa-mir-4524b | - |
| hsa-mir-548ae-1 | HGLibA_61870 | 143 | 0.274396393 | hsa-mir-548ae-1 | - |
| hsa-mir-548o-2 | HGLibA_62053 | 143 | 0.274396393 | hsa-mir-548o-2 | - |
| hsa-mir-590 | HGLibA_62457 | 143 | 0.274396393 | hsa-mir-590 | - |
| hsa-mir-591 | HGLibA_62461 | 143 | 0.274396393 | hsa-mir-591 | - |
| hsa-mir-6739 | HGLibA_63109 | 143 | 0.274396393 | hsa-mir-6739 | - |
| hsa-mir-6847 | HGLibA_63561 | 143 | 0.274396393 | hsa-mir-6847 | - |
| hsa-mir-8066 | HGLibA_64103 | 143 | 0.274396393 | hsa-mir-8066 | - |
| hsa-mir-936 | HGLibA_64317 | 143 | 0.274396393 | hsa-mir-936 | - |
| KAL1 | HGLibA_24019 | 143 | 0.274396393 | KAL1 | - |
| MFI2 | HGLibA_29066 | 143 | 0.274396393 | MFI2 | - |
| WHSC1 | HGLibA_54293 | 143 | 0.274396393 | WHSC1 | - |
| XAGE1D | HGLibA_54476 | 143 | 0.274396393 | XAGE1D | - |
| ZCCHC6 | HGLibA_55043 | 143 | 0.274396393 | ZCCHC6 | - |
| AGPAT6 | HGLibA_01231 | 142 | 0.264445356 | AGPAT6 | - |
| C10orf2 | HGLibA_05062 | 142 | 0.264445356 | C10orf2 | - |
| C2orf61 | HGLibA_06156 | 142 | 0.264445356 | C2orf61 | - |
| C2orf91 | HGLibA_06201 | 142 | 0.264445356 | C2orf91 | - |
| ELMSAN1 | HGLibA_14948 | 142 | 0.264445356 | ELMSAN1 | - |
| ENSG00000013588 | HGLibA_20126 | 142 | 0.264445356 | GPRC5A | G protein-coupled receptor class C group 5 member A [Source:HGNC Symbol;Acc:HGNC:9836] |
| ENSG00000023608 | HGLibA_45890 | 142 | 0.264445356 | SNAPC1 | small nuclear RNA activating complex polypeptide 1 [Source:HGNC Symbol;Acc:HGNC:11134] |
| ENSG00000033100 | HGLibA_09525 | 142 | 0.264445356 | CHPF2 | chondroitin polymerizing factor 2 [Source:HGNC Symbol;Acc:HGNC:29270] |
| ENSG00000049245 | HGLibA_53494 | 142 | 0.264445356 | VAMP3 | vesicle associated membrane protein 3 [Source:HGNC Symbol;Acc:HGNC:12644] |
| ENSG00000052344 | HGLibA_38973 | 142 | 0.264445356 | PRSS8 | serine protease 8 [Source:HGNC Symbol;Acc:HGNC:9491] |
| ENSG00000054796 | HGLibA_46742 | 142 | 0.264445356 | SPO11 | initiator of meiotic double stranded breaks [Source:HGNC Symbol;Acc:HGNC:11250]" |
| ENSG00000057294 | HGLibA_36856 | 142 | 0.264445356 | PKP2 | plakophilin 2 [Source:HGNC Symbol;Acc:HGNC:9024] |
| ENSG00000065060 | HGLibA_52948 | 142 | 0.264445356 | UHRF1BP1 | UHRF1 binding protein 1 [Source:HGNC Symbol;Acc:HGNC:21216] |
| ENSG00000072756 | HGLibA_51713 | 142 | 0.264445356 | TRNT1 | tRNA nucleotidyl transferase 1 [Source:HGNC Symbol;Acc:HGNC:17341] |
| ENSG00000077264 | HGLibA_35045 | 142 | 0.264445356 | PAK3 | p21 (RAC1) activated kinase 3 [Source:HGNC Symbol;Acc:HGNC:8592] |
| ENSG00000078177 | HGLibA_30951 | 142 | 0.264445356 | N4BP2 | NEDD4 binding protein 2 [Source:HGNC Symbol;Acc:HGNC:29851] |
| ENSG00000081026 | HGLibA_28000 | 142 | 0.264445356 | MAGI3 | WW and PDZ domain containing 3 [Source:HGNC Symbol;Acc:HGNC:29647]" |
| ENSG00000087076 | HGLibA_22185 | 142 | 0.264445356 | HSD17B14 | hydroxysteroid 17-beta dehydrogenase 14 [Source:HGNC Symbol;Acc:HGNC:23238] |
| ENSG00000088876 | HGLibA_55842 | 142 | 0.264445356 | ZNF343 | zinc finger protein 343 [Source:HGNC Symbol;Acc:HGNC:16017] |
| ENSG00000101146 | HGLibA_40117 | 142 | 0.264445356 | RAE1 | ribonucleic acid export 1 [Source:HGNC Symbol;Acc:HGNC:9828] |
| ENSG00000101188 | HGLibA_32886 | 142 | 0.264445356 | NTSR1 | neurotensin receptor 1 [Source:HGNC Symbol;Acc:HGNC:8039] |
| ENSG00000101363 | HGLibA_28088 | 142 | 0.264445356 | MANBAL | mannosidase beta like [Source:HGNC Symbol;Acc:HGNC:15799] |
| ENSG00000103150 | HGLibA_29448 | 142 | 0.264445356 | MLYCD | malonyl-CoA decarboxylase [Source:HGNC Symbol;Acc:HGNC:7150] |
| ENSG00000103264 | HGLibA_17069 | 142 | 0.264445356 | FBXO31 | F-box protein 31 [Source:HGNC Symbol;Acc:HGNC:16510] |
| ENSG00000104312 | HGLibA_41318 | 142 | 0.264445356 | RIPK2 | receptor interacting serine/threonine kinase 2 [Source:HGNC Symbol;Acc:HGNC:10020] |
| ENSG00000105185 | HGLibA_35737 | 142 | 0.264445356 | PDCD5 | programmed cell death 5 [Source:HGNC Symbol;Acc:HGNC:8764] |
| ENSG00000105327 | HGLibA_04196 | 142 | 0.264445356 | BBC3 | BCL2 binding component 3 [Source:HGNC Symbol;Acc:HGNC:17868] |

| | | | | | |
|---|---|---|---|---|---|
| ENSG00000106290 | HGLibA_48245 | 142 | 0.264445356 | TAF6 | TATA-box binding protein associated factor 6 [Source:HGNC Symbol;Acc:HGNC:11540] |
| ENSG00000109956 | HGLibA_03971 | 142 | 0.264445356 | B3GAT1 | 3-glucuronyltransferase 1 [Source:HGNC Symbol;Acc:HGNC:921]" |
| ENSG00000111897 | HGLibA_43484 | 142 | 0.264445356 | SERINC1 | serine incorporator 1 [Source:HGNC Symbol;Acc:HGNC:13464] |
| ENSG00000112367 | HGLibA_17509 | 142 | 0.264445356 | FIG4 | FIG4 phosphoinositide 5-phosphatase [Source:HGNC Symbol;Acc:HGNC:16873] |
| ENSG00000113282 | HGLibA_10040 | 142 | 0.264445356 | CLINT1 | clathrin interactor 1 [Source:HGNC Symbol;Acc:HGNC:23186] |
| ENSG00000114416 | HGLibA_18217 | 142 | 0.264445356 | FXR1 | FMR1 autosomal homolog 1 [Source:HGNC Symbol;Acc:HGNC:4023] |
| ENSG00000115758 | HGLibA_33273 | 142 | 0.264445356 | ODC1 | ornithine decarboxylase 1 [Source:HGNC Symbol;Acc:HGNC:8109] |
| ENSG00000117394 | HGLibA_44853 | 142 | 0.264445356 | SLC2A1 | solute carrier family 2 member 1 [Source:HGNC Symbol;Acc:HGNC:11005] |
| ENSG00000117560 | HGLibA_16893 | 142 | 0.264445356 | FASLG | Fas ligand [Source:HGNC Symbol;Acc:HGNC:11936] |
| ENSG00000117601 | HGLibA_43572 | 142 | 0.264445356 | SERPINC1 | serpin family C member 1 [Source:HGNC Symbol;Acc:HGNC:775] |
| ENSG00000118217 | HGLibA_03447 | 142 | 0.264445356 | ATF6 | activating transcription factor 6 [Source:HGNC Symbol;Acc:HGNC:791] |
| ENSG00000119280 | HGLibA_05820 | 142 | 0.264445356 | C1orf198 | chromosome 1 open reading frame 198 [Source:HGNC Symbol;Acc:HGNC:25900] |
| ENSG00000119878 | HGLibA_11161 | 142 | 0.264445356 | CRIPT | CXXC repeat containing interactor of PDZ3 domain [Source:HGNC Symbol;Acc:HGNC:14312] |
| ENSG00000120075 | HGLibA_21941 | 142 | 0.264445356 | HOXB5 | homeobox B5 [Source:HGNC Symbol;Acc:HGNC:5116] |
| ENSG00000123496 | HGLibA_22976 | 142 | 0.264445356 | IL13RA2 | interleukin 13 receptor subunit alpha 2 [Source:HGNC Symbol;Acc:HGNC:5975] |
| ENSG00000125703 | HGLibA_03494 | 142 | 0.264445356 | ATG4C | autophagy related 4C cysteine peptidase [Source:HGNC Symbol;Acc:HGNC:16040] |
| ENSG00000128268 | HGLibA_29138 | 142 | 0.264445356 | MGAT3 | 4-N-acetylglucosaminyltransferase [Source:HGNC Symbol;Acc:HGNC:7046]" |
| ENSG00000129744 | HGLibA_03159 | 142 | 0.264445356 | ART1 | ADP-ribosyltransferase 1 [Source:HGNC Symbol;Acc:HGNC:723] |
| ENSG00000131864 | HGLibA_53323 | 142 | 0.264445356 | USP29 | ubiquitin specific peptidase 29 [Source:HGNC Symbol;Acc:HGNC:18563] |
| ENSG00000134955 | HGLibA_45047 | 142 | 0.264445356 | SLC37A2 | solute carrier family 37 member 2 [Source:HGNC Symbol;Acc:HGNC:20644] |
| ENSG00000136231 | HGLibA_22806 | 142 | 0.264445356 | IGF2BP3 | insulin like growth factor 2 mRNA binding protein 3 [Source:HGNC Symbol;Acc:HGNC:28868] |
| ENSG00000140835 | HGLibA_09636 | 142 | 0.264445356 | CHST4 | carbohydrate sulfotransferase 4 [Source:HGNC Symbol;Acc:HGNC:1972] |
| ENSG00000141873 | HGLibA_45108 | 142 | 0.264445356 | SLC39A3 | solute carrier family 39 member 3 [Source:HGNC Symbol;Acc:HGNC:17128] |
| ENSG00000142599 | HGLibA_40856 | 142 | 0.264445356 | RERE | arginine-glutamic acid dipeptide repeats [Source:HGNC Symbol;Acc:HGNC:9965] |
| ENSG00000142623 | HGLibA_34969 | 142 | 0.264445356 | PADI1 | peptidyl arginine deiminase 1 [Source:HGNC Symbol;Acc:HGNC:18367] |
| ENSG00000144290 | HGLibA_45205 | 142 | 0.264445356 | SLC4A10 | solute carrier family 4 member 10 [Source:HGNC Symbol;Acc:HGNC:13811] |
| ENSG00000144550 | HGLibA_10976 | 142 | 0.264445356 | CPNE9 | copine family member 9 [Source:HGNC Symbol;Acc:HGNC:24336] |
| ENSG00000144681 | HGLibA_47311 | 142 | 0.264445356 | STAC | SH3 and cysteine rich domain [Source:HGNC Symbol;Acc:HGNC:11353] |
| ENSG00000146122 | HGLibA_12261 | 142 | 0.264445356 | DAAM2 | dishevelled associated activator of morphogenesis 2 [Source:HGNC Symbol;Acc:HGNC:18143] |
| ENSG00000146232 | HGLibA_31830 | 142 | 0.264445356 | NFKBIE | NFKB inhibitor epsilon [Source:HGNC Symbol;Acc:HGNC:7799] |
| ENSG00000146802 | HGLibA_50046 | 142 | 0.264445356 | TMEM168 | transmembrane protein 168 [Source:HGNC Symbol;Acc:HGNC:25826] |
| ENSG00000146856 | HGLibA_01173 | 142 | 0.264445356 | AGBL3 | ATP/GTP binding protein like 3 [Source:HGNC Symbol;Acc:HGNC:27981] |
| ENSG00000147655 | HGLibA_42347 | 142 | 0.264445356 | RSPO2 | R-spondin 2 [Source:HGNC Symbol;Acc:HGNC:28583] |
| ENSG00000149289 | HGLibA_54958 | 142 | 0.264445356 | ZC3H12C | zinc finger CCCH-type containing 12C [Source:HGNC Symbol;Acc:HGNC:29362] |
| ENSG00000150656 | HGLibA_10251 | 142 | 0.264445356 | CNDP1 | carnosine dipeptidase 1 [Source:HGNC Symbol;Acc:HGNC:20675] |
| ENSG00000151006 | HGLibA_38956 | 142 | 0.264445356 | PRSS53 | serine protease 53 [Source:HGNC Symbol;Acc:HGNC:34407] |
| ENSG00000154122 | HGLibA_01962 | 142 | 0.264445356 | ANKH | ANKH inorganic pyrophosphate transport regulator [Source:HGNC Symbol;Acc:HGNC:15492] |
| ENSG00000154262 | HGLibA_00120 | 142 | 0.264445356 | ABCA6 | ATP binding cassette subfamily A member 6 [Source:HGNC Symbol;Acc:HGNC:36] |
| ENSG00000155330 | HGLibA_05492 | 142 | 0.264445356 | C16orf87 | chromosome 16 open reading frame 87 [Source:HGNC Symbol;Acc:HGNC:33754] |
| ENSG00000157927 | HGLibA_40115 | 142 | 0.264445356 | RADIL | Rap associating with DIL domain [Source:HGNC Symbol;Acc:HGNC:22226] |
| ENSG00000159082 | HGLibA_48004 | 142 | 0.264445356 | SYNJ1 | synaptojanin 1 [Source:HGNC Symbol;Acc:HGNC:11503] |
| ENSG00000161905 | HGLibA_01682 | 142 | 0.264445356 | ALOX15 | arachidonate 15-lipoxygenase [Source:HGNC Symbol;Acc:HGNC:433] |
| ENSG00000162607 | HGLibA_53192 | 142 | 0.264445356 | USP1 | ubiquitin specific peptidase 1 [Source:HGNC Symbol;Acc:HGNC:12607] |
| ENSG00000163132 | HGLibA_30270 | 142 | 0.264445356 | MSX1 | msh homeobox 1 [Source:HGNC Symbol;Acc:HGNC:7391] |
| ENSG00000164296 | HGLibA_49477 | 142 | 0.264445356 | TIGD6 | tigger transposable element derived 6 [Source:HGNC Symbol;Acc:HGNC:18332] |
| ENSG00000166068 | HGLibA_46791 | 142 | 0.264445356 | SPRED1 | sprouty related EVH1 domain containing 1 [Source:HGNC Symbol;Acc:HGNC:20249] |
| ENSG00000166140 | HGLibA_55276 | 142 | 0.264445356 | ZFYVE19 | zinc finger FYVE-type containing 19 [Source:HGNC Symbol;Acc:HGNC:20758] |
| ENSG00000166192 | HGLibA_43402 | 142 | 0.264445356 | SENP8 | NEDD8 specific [Source:HGNC Symbol;Acc:HGNC:22992]" |
| ENSG00000166546 | HGLibA_04393 | 142 | 0.264445356 | BEAN1 | brain expressed associated with NEDD4 1 [Source:HGNC Symbol;Acc:HGNC:24160] |
| ENSG00000166689 | HGLibA_37079 | 142 | 0.264445356 | PLEKHA7 | pleckstrin homology domain containing A7 [Source:HGNC Symbol;Acc:HGNC:27049] |
| ENSG00000167346 | HGLibA_29525 | 142 | 0.264445356 | MMP26 | matrix metallopeptidase 26 [Source:HGNC Symbol;Acc:HGNC:14249] |
| ENSG00000167380 | HGLibA_55624 | 142 | 0.264445356 | ZNF226 | zinc finger protein 226 [Source:HGNC Symbol;Acc:HGNC:13019] |
| ENSG00000167751 | HGLibA_25251 | 142 | 0.264445356 | KLK2 | kallikrein related peptidase 2 [Source:HGNC Symbol;Acc:HGNC:6363] |
| ENSG00000168546 | HGLibA_18968 | 142 | 0.264445356 | GFRA2 | GDNF family receptor alpha 2 [Source:HGNC Symbol;Acc:HGNC:4244] |
| ENSG00000168944 | HGLibA_09100 | 142 | 0.264445356 | CEP120 | centrosomal protein 120 [Source:HGNC Symbol;Acc:HGNC:26690] |
| ENSG00000171044 | HGLibA_54525 | 142 | 0.264445356 | XKR6 | XK related 6 [Source:HGNC Symbol;Acc:HGNC:27806] |
| ENSG00000171109 | HGLibA_29068 | 142 | 0.264445356 | MFN1 | mitofusin 1 [Source:HGNC Symbol;Acc:HGNC:18262] |
| ENSG00000171431 | HGLibA_25447 | 142 | 0.264445356 | KRT20 | keratin 20 [Source:HGNC Symbol;Acc:HGNC:20412] |
| ENSG00000171462 | HGLibA_13358 | 142 | 0.264445356 | DLK2 | delta like non-canonical Notch ligand 2 [Source:HGNC Symbol;Acc:HGNC:21113] |
| ENSG00000173171 | HGLibA_30510 | 142 | 0.264445356 | MTX1 | metaxin 1 [Source:HGNC Symbol;Acc:HGNC:7504] |
| ENSG00000173389 | HGLibA_23501 | 142 | 0.264445356 | IQCF1 | IQ motif containing F1 [Source:HGNC Symbol;Acc:HGNC:28607] |
| ENSG00000173546 | HGLibA_11414 | 142 | 0.264445356 | CSPG4 | chondroitin sulfate proteoglycan 4 [Source:HGNC Symbol;Acc:HGNC:2466] |
| ENSG00000174898 | HGLibA_07371 | 142 | 0.264445356 | CATSPERD | cation channel sperm associated auxiliary subunit delta [Source:HGNC Symbol;Acc:HGNC:28598] |
| ENSG00000175324 | HGLibA_27518 | 142 | 0.264445356 | LSM1 | mRNA degradation associated [Source:HGNC Symbol;Acc:HGNC:20472]" |
| ENSG00000175463 | HGLibA_48471 | 142 | 0.264445356 | TBC1D10C | TBC1 domain family member 10C [Source:HGNC Symbol;Acc:HGNC:24702] |
| ENSG00000175535 | HGLibA_37353 | 142 | 0.264445356 | PNLIP | pancreatic lipase [Source:HGNC Symbol;Acc:HGNC:9155] |
| ENSG00000175575 | HGLibA_34919 | 142 | 0.264445356 | PAAF1 | proteasomal ATPase associated factor 1 [Source:HGNC Symbol;Acc:HGNC:25687] |
| ENSG00000175582 | HGLibA_39970 | 142 | 0.264445356 | RAB6A | member RAS oncogene family [Source:HGNC Symbol;Acc:HGNC:9786]" |
| ENSG00000176742 | HGLibA_34135 | 142 | 0.264445356 | OR51V1 | olfactory receptor family 51 subfamily V member 1 [Source:HGNC Symbol;Acc:HGNC:19597] |
| ENSG00000177842 | HGLibA_56405 | 142 | 0.264445356 | ZNF620 | zinc finger protein 620 [Source:HGNC Symbol;Acc:HGNC:28742] |
| ENSG00000178177 | HGLibA_26197 | 142 | 0.264445356 | LCORL | ligand dependent nuclear receptor corepressor like [Source:HGNC Symbol;Acc:HGNC:30776] |
| ENSG00000178449 | HGLibA_10809 | 142 | 0.264445356 | COX14 | cytochrome c oxidase assembly factor COX14 [Source:HGNC Symbol;Acc:HGNC:28216] |
| ENSG00000178814 | HGLibA_33419 | 142 | 0.264445356 | OPLAH | ATP-hydrolysing [Source:HGNC Symbol;Acc:HGNC:8149]" |
| ENSG00000178826 | HGLibA_49957 | 142 | 0.264445356 | TMEM139 | transmembrane protein 139 [Source:HGNC Symbol;Acc:HGNC:22058] |
| ENSG00000179750 | HGLibA_02478 | 142 | 0.264445356 | APOBEC3B | apolipoprotein B mRNA editing enzyme catalytic subunit 3B [Source:HGNC Symbol;Acc:HGNC:17352] |
| ENSG00000179761 | HGLibA_36725 | 142 | 0.264445356 | PIPOX | pipecolic acid and sarcosine oxidase [Source:HGNC Symbol;Acc:HGNC:17804] |
| ENSG00000179820 | HGLibA_30632 | 142 | 0.264445356 | MYADM | myeloid associated differentiation marker [Source:HGNC Symbol;Acc:HGNC:7544] |
| ENSG00000180016 | HGLibA_33660 | 142 | 0.264445356 | OR1E1 | olfactory receptor family 1 subfamily E member 1 [Source:HGNC Symbol;Acc:HGNC:8189] |
| ENSG00000182179 | HGLibA_52532 | 142 | 0.264445356 | UBA7 | ubiquitin like modifier activating enzyme 7 [Source:HGNC Symbol;Acc:HGNC:12471] |
| ENSG00000182218 | HGLibA_21265 | 142 | 0.264445356 | HHIPL1 | HHIP like 1 [Source:HGNC Symbol;Acc:HGNC:19710] |
| ENSG00000182674 | HGLibA_24160 | 142 | 0.264445356 | KCNB2 | potassium voltage-gated channel subfamily B member 2 [Source:HGNC Symbol;Acc:HGNC:6232] |
| ENSG00000183971 | HGLibA_32543 | 142 | 0.264445356 | NPW | neuropeptide W [Source:HGNC Symbol;Acc:HGNC:30509] |
| ENSG00000184900 | HGLibA_47783 | 142 | 0.264445356 | SUMO3 | small ubiquitin-like modifier 3 [Source:HGNC Symbol;Acc:HGNC:11124] |
| ENSG00000185745 | HGLibA_22622 | 142 | 0.264445356 | IFIT1 | interferon induced protein with tetratricopeptide repeats 1 [Source:HGNC Symbol;Acc:HGNC:5407] |
| ENSG00000185899 | HGLibA_48423 | 142 | 0.264445356 | TAS2R60 | taste 2 receptor member 60 [Source:HGNC Symbol;Acc:HGNC:20639] |
| ENSG00000185947 | HGLibA_55701 | 142 | 0.264445356 | ZNF267 | zinc finger protein 267 [Source:HGNC Symbol;Acc:HGNC:13060] |
| ENSG00000186326 | HGLibA_41114 | 142 | 0.264445356 | RGS9BP | regulator of G protein signaling 9 binding protein [Source:HGNC Symbol;Acc:HGNC:30304] |
| ENSG00000186714 | HGLibA_07859 | 142 | 0.264445356 | CCDC73 | coiled-coil domain containing 73 [Source:HGNC Symbol;Acc:HGNC:23261] |
| ENSG00000186897 | HGLibA_05933 | 142 | 0.264445356 | C1QL4 | complement C1q like 4 [Source:HGNC Symbol;Acc:HGNC:31416] |
| ENSG00000188394 | HGLibA_19999 | 142 | 0.264445356 | GPR21 | G protein-coupled receptor 21 [Source:HGNC Symbol;Acc:HGNC:4476] |
| ENSG00000188558 | HGLibA_33799 | 142 | 0.264445356 | OR2G6 | olfactory receptor family 2 subfamily G member 6 [Source:HGNC Symbol;Acc:HGNC:27019] |
| ENSG00000188976 | HGLibA_32235 | 142 | 0.264445356 | NOC2L | NOC2 like nucleolar associated transcriptional repressor [Source:HGNC Symbol;Acc:HGNC:24517] |
| ENSG00000189143 | HGLibA_09899 | 142 | 0.264445356 | CLDN4 | claudin 4 [Source:HGNC Symbol;Acc:HGNC:2046] |
| ENSG00000189227 | HGLibA_05430 | 142 | 0.264445356 | C15orf61 | chromosome 15 open reading frame 61 [Source:HGNC Symbol;Acc:HGNC:34453] |
| ENSG00000197273 | HGLibA_20642 | 142 | 0.264445356 | GUCA2A | guanylate cyclase activator 2A [Source:HGNC Symbol;Acc:HGNC:4682] |
| ENSG00000197487 | HGLibA_18584 | 142 | 0.264445356 | GALP | galanin like peptide [Source:HGNC Symbol;Acc:HGNC:24840] |
| ENSG00000204882 | HGLibA_19997 | 142 | 0.264445356 | GPR20 | G protein-coupled receptor 20 [Source:HGNC Symbol;Acc:HGNC:4475] |
| ENSG00000205220 | HGLibA_39097 | 142 | 0.264445356 | PSMB10 | proteasome subunit beta 10 [Source:HGNC Symbol;Acc:HGNC:9538] |
| ENSG00000205923 | HGLibA_09032 | 142 | 0.264445356 | CEMP1 | cementum protein 1 [Source:HGNC Symbol;Acc:HGNC:32553] |
| ENSG00000206172 | HGLibA_20907 | 142 | 0.264445356 | HBA1 | hemoglobin subunit alpha 1 [Source:HGNC Symbol;Acc:HGNC:4823] |
| ENSG00000213722 | HGLibA_12591 | 142 | 0.264445356 | DDAH2 | dimethylarginine dimethylaminohydrolase 2 [Source:HGNC Symbol;Acc:HGNC:2716] |
| ENSG00000214686 | HGLibA_23515 | 142 | 0.264445356 | IQCF6 | IQ motif containing F6 [Source:HGNC Symbol;Acc:HGNC:35158] |
| ENSG00000215203 | HGLibA_20389 | 142 | 0.264445356 | GRXCR1 | glutaredoxin and cysteine rich domain containing 1 [Source:HGNC Symbol;Acc:HGNC:31673] |
| ENSG00000215712 | HGLibA_50291 | 142 | 0.264445356 | TMEM242 | transmembrane protein 242 [Source:HGNC Symbol;Acc:HGNC:17206] |
| ENSG00000240871 | HGLibA_25773 | 142 | 0.264445356 | KRTAP4-7 | keratin associated protein 4-7 [Source:HGNC Symbol;Acc:HGNC:18898] |
| ENSG00000254656 | HGLibA_42395 | 142 | 0.264445356 | RTL1 | retrotransposon Gag like 1 [Source:HGNC Symbol;Acc:HGNC:14665] |
| ENSG00000255359 | HGLibA_07719 | 142 | 0.264445356 | CCDC179 | coiled-coil domain containing 179 [Source:HGNC Symbol;Acc:HGNC:44653] |
| ENSG00000269113 | HGLibA_51234 | 142 | 0.264445356 | TRABD2B | TraB domain containing 2B [Source:HGNC Symbol;Acc:HGNC:44200] |
| ENSG00000278889 | HGLibA_33849 | 142 | 0.264445356 | OR2S2 | olfactory receptor family 2 subfamily S member 2 (gene/pseudogene) [Source:HGNC Symbol;Acc:HGNC:8276] |
| FAM179B | HGLibA_16308 | 142 | 0.264445356 | FAM179B | - |
| hsa-mir-1265 | HGLibA_57485 | 142 | 0.264445356 | hsa-mir-1265 | - |
| hsa-mir-1304 | HGLibA_57733 | 142 | 0.264445356 | hsa-mir-1304 | - |
| hsa-mir-147b | HGLibA_57877 | 142 | 0.264445356 | hsa-mir-147b | - |
| hsa-mir-2467 | HGLibA_58349 | 142 | 0.264445356 | hsa-mir-2467 | - |
| hsa-mir-30a | HGLibA_58472 | 142 | 0.264445356 | hsa-mir-30a | - |
| hsa-mir-3188 | HGLibA_58850 | 142 | 0.264445356 | hsa-mir-3188 | - |
| hsa-mir-4649 | HGLibA_60699 | 142 | 0.264445356 | hsa-mir-4649 | - |
| hsa-mir-4694 | HGLibA_60886 | 142 | 0.264445356 | hsa-mir-4694 | - |
| hsa-mir-5047 | HGLibA_61474 | 142 | 0.264445356 | hsa-mir-5047 | - |
| hsa-mir-508 | HGLibA_61492 | 142 | 0.264445356 | hsa-mir-508 | - |
| hsa-mir-5088 | HGLibA_61500 | 142 | 0.264445356 | hsa-mir-5088 | - |
| hsa-mir-6895 | HGLibA_63764 | 142 | 0.264445356 | hsa-mir-6895 | - |
| hsa-mir-7150 | HGLibA_63828 | 142 | 0.264445356 | hsa-mir-7150 | - |
| LOC100130880 | HGLibA_26799 | 142 | 0.264445356 | LOC100130880 | - |
| 14-Sep | HGLibA_43407 | 142 | 0.264445356 | 14-Sep | - |
| ZCCHC11 | HGLibA_55006 | 142 | 0.264445356 | ZCCHC11 | - |
| C7orf66 | HGLibA_06579 | 141 | 0.254494318 | C7orf66 | - |
| CARKD | HGLibA_07250 | 141 | 0.254494318 | CARKD | - |
| DFNB59 | HGLibA_13030 | 141 | 0.254494318 | DFNB59 | - |
| DGCR14 | HGLibA_13042 | 141 | 0.254494318 | DGCR14 | - |
| ENSG00000007038 | HGLibA_38910 | 141 | 0.254494318 | PRSS21 | serine protease 21 [Source:HGNC Symbol;Acc:HGNC:9485] |
| ENSG00000011465 | HGLibA_12518 | 141 | 0.254494318 | DCN | decorin [Source:HGNC Symbol;Acc:HGNC:2705] |
| ENSG00000041988 | HGLibA_49298 | 141 | 0.254494318 | THAP3 | THAP domain containing 3 [Source:HGNC Symbol;Acc:HGNC:20855] |
| ENSG00000047457 | HGLibA_10880 | 141 | 0.254494318 | CP | ceruloplasmin [Source:HGNC Symbol;Acc:HGNC:2295] |
| ENSG00000070087 | HGLibA_36178 | 141 | 0.254494318 | PFN2 | profilin 2 [Source:HGNC Symbol;Acc:HGNC:8882] |

| | | | | | |
|---|---|---|---|---|---|
| ENSG00000071967 | HGLibA_12022 | 141 | 0.254494318 | CYBRD1 | cytochrome b reductase 1 [Source:HGNC Symbol;Acc:HGNC:20797] |
| ENSG00000086544 | HGLibA_23850 | 141 | 0.254494318 | ITPKC | inositol-trisphosphate 3-kinase C [Source:HGNC Symbol;Acc:HGNC:14897] |
| ENSG00000089335 | HGLibA_55774 | 141 | 0.254494318 | ZNF302 | zinc finger protein 302 [Source:HGNC Symbol;Acc:HGNC:13848] |
| ENSG00000092068 | HGLibA_45382 | 141 | 0.254494318 | SLC7A8 | solute carrier family 7 member 8 [Source:HGNC Symbol;Acc:HGNC:11066] |
| ENSG00000100665 | HGLibA_43521 | 141 | 0.254494318 | SERPINA4 | serpin family A member 4 [Source:HGNC Symbol;Acc:HGNC:8948] |
| ENSG00000102904 | HGLibA_51888 | 141 | 0.254494318 | TSNAXIP1 | translin associated factor X interacting protein 1 [Source:HGNC Symbol;Acc:HGNC:18586] |
| ENSG00000104059 | HGLibA_16350 | 141 | 0.254494318 | FAM189A1 | family with sequence similarity 189 member A1 [Source:HGNC Symbol;Acc:HGNC:29075] |
| ENSG00000104365 | HGLibA_22919 | 141 | 0.254494318 | IKBKB | inhibitor of nuclear factor kappa B kinase subunit beta [Source:HGNC Symbol;Acc:HGNC:5960] |
| ENSG00000104691 | HGLibA_52798 | 141 | 0.254494318 | UBXN8 | UBX domain protein 8 [Source:HGNC Symbol;Acc:HGNC:30307] |
| ENSG00000105379 | HGLibA_15623 | 141 | 0.254494318 | ETFB | electron transfer flavoprotein subunit beta [Source:HGNC Symbol;Acc:HGNC:3482] |
| ENSG00000105426 | HGLibA_39535 | 141 | 0.254494318 | PTPRS | receptor type S [Source:HGNC Symbol;Acc:HGNC:9681]" |
| ENSG00000106397 | HGLibA_37212 | 141 | 0.254494318 | PLOD3 | 2-oxoglutarate 5-dioxygenase 3 [Source:HGNC Symbol;Acc:HGNC:9083]" |
| ENSG00000107679 | HGLibA_37062 | 141 | 0.254494318 | PLEKHA1 | pleckstrin homology domain containing A1 [Source:HGNC Symbol;Acc:HGNC:14335] |
| ENSG00000108100 | HGLibA_08132 | 141 | 0.254494318 | CCNY | cyclin Y [Source:HGNC Symbol;Acc:HGNC:23354] |
| ENSG00000108231 | HGLibA_26393 | 141 | 0.254494318 | LGI1 | leucine rich glioma inactivated 1 [Source:HGNC Symbol;Acc:HGNC:6572] |
| ENSG00000115593 | HGLibA_45851 | 141 | 0.254494318 | SMYD1 | SET and MYND domain containing 1 [Source:HGNC Symbol;Acc:HGNC:20986] |
| ENSG00000115594 | HGLibA_23045 | 141 | 0.254494318 | IL1R1 | interleukin 1 receptor type 1 [Source:HGNC Symbol;Acc:HGNC:5993] |
| ENSG00000115756 | HGLibA_22020 | 141 | 0.254494318 | HPCAL1 | hippocalcin like 1 [Source:HGNC Symbol;Acc:HGNC:5145] |
| ENSG00000118985 | HGLibA_14925 | 141 | 0.254494318 | ELL2 | elongation factor for RNA polymerase II 2 [Source:HGNC Symbol;Acc:HGNC:17064] |
| ENSG00000120254 | HGLibA_30379 | 141 | 0.254494318 | MTHFD1L | methylenetetrahydrofolate dehydrogenase (NADP+ dependent) 1 like [Source:HGNC Symbol;Acc:HGNC:21055] |
| ENSG00000125520 | HGLibA_44879 | 141 | 0.254494318 | SLC2A4RG | SLC2A4 regulator [Source:HGNC Symbol;Acc:HGNC:15930] |
| ENSG00000126062 | HGLibA_49883 | 141 | 0.254494318 | TMEM115 | transmembrane protein 115 [Source:HGNC Symbol;Acc:HGNC:30055] |
| ENSG00000126803 | HGLibA_22281 | 141 | 0.254494318 | HSPA2 | heat shock protein family A (Hsp70) member 2 [Source:HGNC Symbol;Acc:HGNC:5235] |
| ENSG00000126814 | HGLibA_51691 | 141 | 0.254494318 | TRMT5 | tRNA methyltransferase 5 [Source:HGNC Symbol;Acc:HGNC:23141] |
| ENSG00000129988 | HGLibA_26080 | 141 | 0.254494318 | LBP | lipopolysaccharide binding protein [Source:HGNC Symbol;Acc:HGNC:6517] |
| ENSG00000130475 | HGLibA_17204 | 141 | 0.254494318 | FCHO1 | FCH domain only 1 [Source:HGNC Symbol;Acc:HGNC:29002] |
| ENSG00000130720 | HGLibA_17498 | 141 | 0.254494318 | FIBCD1 | fibrinogen C domain containing 1 [Source:HGNC Symbol;Acc:HGNC:25922] |
| ENSG00000131470 | HGLibA_39135 | 141 | 0.254494318 | PSMC3IP | PSMC3 interacting protein [Source:HGNC Symbol;Acc:HGNC:17928] |
| ENSG00000132199 | HGLibA_15154 | 141 | 0.254494318 | ENOSF1 | enolase superfamily member 1 [Source:HGNC Symbol;Acc:HGNC:30365] |
| ENSG00000132434 | HGLibA_26007 | 141 | 0.254494318 | LANCL2 | LanC like 2 [Source:HGNC Symbol;Acc:HGNC:6509] |
| ENSG00000132938 | HGLibA_30508 | 141 | 0.254494318 | MTUS2 | microtubule associated scaffold protein 2 [Source:HGNC Symbol;Acc:HGNC:20595] |
| ENSG00000133121 | HGLibA_47355 | 141 | 0.254494318 | STARD13 | StAR related lipid transfer domain containing 13 [Source:HGNC Symbol;Acc:HGNC:19164] |
| ENSG00000134056 | HGLibA_30102 | 141 | 0.254494318 | MRPS36 | mitochondrial ribosomal protein S36 [Source:HGNC Symbol;Acc:HGNC:16631] |
| ENSG00000137270 | HGLibA_18800 | 141 | 0.254494318 | GCM1 | glial cells missing homolog 1 [Source:HGNC Symbol;Acc:HGNC:4197] |
| ENSG00000137463 | HGLibA_29129 | 141 | 0.254494318 | MGARP | mitochondria localized glutamic acid rich protein [Source:HGNC Symbol;Acc:HGNC:29969] |
| ENSG00000138604 | HGLibA_19232 | 141 | 0.254494318 | GLCE | glucuronic acid epimerase [Source:HGNC Symbol;Acc:HGNC:17855] |
| ENSG00000138813 | HGLibA_06297 | 141 | 0.254494318 | C4orf17 | chromosome 4 open reading frame 17 [Source:HGNC Symbol;Acc:HGNC:25274] |
| ENSG00000140521 | HGLibA_37523 | 141 | 0.254494318 | POLG | catalytic subunit [Source:HGNC Symbol;Acc:HGNC:9179]" |
| ENSG00000142867 | HGLibA_04287 | 141 | 0.254494318 | BCL10 | B cell CLL/lymphoma 10 [Source:HGNC Symbol;Acc:HGNC:989] |
| ENSG00000143153 | HGLibA_03594 | 141 | 0.254494318 | ATP1B1 | ATPase Na+/K+ transporting subunit beta 1 [Source:HGNC Symbol;Acc:HGNC:804] |
| ENSG00000143631 | HGLibA_17610 | 141 | 0.254494318 | FLG | filaggrin [Source:HGNC Symbol;Acc:HGNC:3748] |
| ENSG00000150625 | HGLibA_19820 | 141 | 0.254494318 | GPM6A | glycoprotein M6A [Source:HGNC Symbol;Acc:HGNC:4460] |
| ENSG00000151778 | HGLibA_43503 | 141 | 0.254494318 | SERP2 | stress associated endoplasmic reticulum protein family member 2 [Source:HGNC Symbol;Acc:HGNC:20607] |
| ENSG00000151789 | HGLibA_55903 | 141 | 0.254494318 | ZNF385D | zinc finger protein 385D [Source:HGNC Symbol;Acc:HGNC:26191] |
| ENSG00000154813 | HGLibA_13841 | 141 | 0.254494318 | DPH3 | diphthamide biosynthesis 3 [Source:HGNC Symbol;Acc:HGNC:27717] |
| ENSG00000156273 | HGLibA_04064 | 141 | 0.254494318 | BACH1 | BTB domain and CNC homolog 1 [Source:HGNC Symbol;Acc:HGNC:935] |
| ENSG00000158865 | HGLibA_45260 | 141 | 0.254494318 | SLC5A11 | solute carrier family 5 member 11 [Source:HGNC Symbol;Acc:HGNC:23091] |
| ENSG00000159905 | HGLibA_55609 | 141 | 0.254494318 | ZNF221 | zinc finger protein 221 [Source:HGNC Symbol;Acc:HGNC:13014] |
| ENSG00000161011 | HGLibA_46918 | 141 | 0.254494318 | SQSTM1 | sequestosome 1 [Source:HGNC Symbol;Acc:HGNC:11280] |
| ENSG00000161973 | HGLibA_07782 | 141 | 0.254494318 | CCDC42 | coiled-coil domain containing 42 [Source:HGNC Symbol;Acc:HGNC:26528] |
| ENSG00000162419 | HGLibA_19397 | 141 | 0.254494318 | GMEB1 | glucocorticoid modulatory element binding protein 1 [Source:HGNC Symbol;Acc:HGNC:4370] |
| ENSG00000163349 | HGLibA_21339 | 141 | 0.254494318 | HIPK1 | homeodomain interacting protein kinase 1 [Source:HGNC Symbol;Acc:HGNC:19006] |
| ENSG00000163701 | HGLibA_23017 | 141 | 0.254494318 | IL17RE | interleukin 17 receptor E [Source:HGNC Symbol;Acc:HGNC:18439] |
| ENSG00000164253 | HGLibA_54088 | 141 | 0.254494318 | WDR41 | WD repeat domain 41 [Source:HGNC Symbol;Acc:HGNC:25601] |
| ENSG00000165072 | HGLibA_28038 | 141 | 0.254494318 | MAMDC2 | MAM domain containing 2 [Source:HGNC Symbol;Acc:HGNC:23673] |
| ENSG00000165661 | HGLibA_39758 | 141 | 0.254494318 | QSOX2 | quiescin sulfhydryl oxidase 2 [Source:HGNC Symbol;Acc:HGNC:30249] |
| ENSG00000165684 | HGLibA_45898 | 141 | 0.254494318 | SNAPC4 | small nuclear RNA activating complex polypeptide 4 [Source:HGNC Symbol;Acc:HGNC:11137] |
| ENSG00000165821 | HGLibA_42650 | 141 | 0.254494318 | SALL2 | spalt like transcription factor 2 [Source:HGNC Symbol;Acc:HGNC:10526] |
| ENSG00000167257 | HGLibA_41625 | 141 | 0.254494318 | RNF214 | ring finger protein 214 [Source:HGNC Symbol;Acc:HGNC:25335] |
| ENSG00000168283 | HGLibA_04586 | 141 | 0.254494318 | BMI1 | polycomb ring finger [Source:HGNC Symbol;Acc:HGNC:1066]" |
| ENSG00000168824 | HGLibA_32758 | 141 | 0.254494318 | NSG1 | neuronal vesicle trafficking associated 1 [Source:HGNC Symbol;Acc:HGNC:18790] |
| ENSG00000168955 | HGLibA_49701 | 141 | 0.254494318 | TM4SF20 | transmembrane 4 L six family member 20 [Source:HGNC Symbol;Acc:HGNC:26230] |
| ENSG00000169688 | HGLibA_30281 | 141 | 0.254494318 | MT1B | metallothionein 1B [Source:HGNC Symbol;Acc:HGNC:7394] |
| ENSG00000170222 | HGLibA_01033 | 141 | 0.254494318 | ADPRM | manganese dependent [Source:HGNC Symbol;Acc:HGNC:30925]" |
| ENSG00000171307 | HGLibA_55083 | 141 | 0.254494318 | ZDHHC16 | zinc finger DHHC-type containing 16 [Source:HGNC Symbol;Acc:HGNC:20714] |
| ENSG00000171385 | HGLibA_24181 | 141 | 0.254494318 | KCND3 | potassium voltage-gated channel subfamily D member 3 [Source:HGNC Symbol;Acc:HGNC:6239] |
| ENSG00000171700 | HGLibA_41075 | 141 | 0.254494318 | RGS19 | regulator of G protein signaling 19 [Source:HGNC Symbol;Acc:HGNC:13735] |
| ENSG00000171806 | HGLibA_28971 | 141 | 0.254494318 | METTL18 | methyltransferase like 18 [Source:HGNC Symbol;Acc:HGNC:28793] |
| ENSG00000172137 | HGLibA_06990 | 141 | 0.254494318 | CALB2 | calbindin 2 [Source:HGNC Symbol;Acc:HGNC:1435] |
| ENSG00000172154 | HGLibA_34536 | 141 | 0.254494318 | OR8I2 | olfactory receptor family 8 subfamily I member 2 [Source:HGNC Symbol;Acc:HGNC:15310] |
| ENSG00000172331 | HGLibA_04700 | 141 | 0.254494318 | BPGM | bisphosphoglycerate mutase [Source:HGNC Symbol;Acc:HGNC:1093] |
| ENSG00000174485 | HGLibA_12949 | 141 | 0.254494318 | DENND4A | DENN domain containing 4A [Source:HGNC Symbol;Acc:HGNC:24321] |
| ENSG00000175575 | HGLibA_34918 | 141 | 0.254494318 | PAAF1 | proteasomal ATPase associated factor 1 [Source:HGNC Symbol;Acc:HGNC:25687] |
| ENSG00000176842 | HGLibA_23640 | 141 | 0.254494318 | IRX5 | iroquois homeobox 5 [Source:HGNC Symbol;Acc:HGNC:14361] |
| ENSG00000178445 | HGLibA_19234 | 141 | 0.254494318 | GLDC | glycine decarboxylase [Source:HGNC Symbol;Acc:HGNC:4313] |
| ENSG00000178467 | HGLibA_34912 | 141 | 0.254494318 | P4HTM | transmembrane [Source:HGNC Symbol;Acc:HGNC:28858]" |
| ENSG00000179889 | HGLibA_35959 | 141 | 0.254494318 | PDXDC1 | pyridoxal dependent decarboxylase domain containing 1 [Source:HGNC Symbol;Acc:HGNC:28995] |
| ENSG00000180697 | HGLibA_06222 | 141 | 0.254494318 | C3orf22 | chromosome 3 open reading frame 22 [Source:HGNC Symbol;Acc:HGNC:28534] |
| ENSG00000180884 | HGLibA_56769 | 141 | 0.254494318 | ZNF792 | zinc finger protein 792 [Source:HGNC Symbol;Acc:HGNC:24751] |
| ENSG00000181827 | HGLibA_40980 | 141 | 0.254494318 | RFX7 | regulatory factor X7 [Source:HGNC Symbol;Acc:HGNC:25777] |
| ENSG00000182938 | HGLibA_34740 | 141 | 0.254494318 | OTOP3 | otopetrin 3 [Source:HGNC Symbol;Acc:HGNC:19658] |
| ENSG00000184166 | HGLibA_33652 | 141 | 0.254494318 | OR1D2 | olfactory receptor family 1 subfamily D member 2 [Source:HGNC Symbol;Acc:HGNC:8183] |
| ENSG00000185813 | HGLibA_35706 | 141 | 0.254494318 | PCYT2 | ethanolamine [Source:HGNC Symbol;Acc:HGNC:8756]" |
| ENSG00000186409 | HGLibA_07754 | 141 | 0.254494318 | CCDC30 | coiled-coil domain containing 30 [Source:HGNC Symbol;Acc:HGNC:26103] |
| ENSG00000186810 | HGLibA_11886 | 141 | 0.254494318 | CXCR3 | C-X-C motif chemokine receptor 3 [Source:HGNC Symbol;Acc:HGNC:4540] |
| ENSG00000187323 | HGLibA_12476 | 141 | 0.254494318 | DCC | DCC netrin 1 receptor [Source:HGNC Symbol;Acc:HGNC:2701] |
| ENSG00000187514 | HGLibA_39390 | 141 | 0.254494318 | PTMA | prothymosin alpha [Source:HGNC Symbol;Acc:HGNC:9623] |
| ENSG00000187758 | HGLibA_00950 | 141 | 0.254494318 | ADH1A | alpha polypeptide [Source:HGNC Symbol;Acc:HGNC:249]" |
| ENSG00000187764 | HGLibA_43350 | 141 | 0.254494318 | SEMA4D | semaphorin 4D [Source:HGNC Symbol;Acc:HGNC:10732] |
| ENSG00000196240 | HGLibA_33862 | 141 | 0.254494318 | OR2T2 | olfactory receptor family 2 subfamily T member 2 [Source:HGNC Symbol;Acc:HGNC:14725] |
| ENSG00000197635 | HGLibA_13873 | 141 | 0.254494318 | DPP4 | dipeptidyl peptidase 4 [Source:HGNC Symbol;Acc:HGNC:3009] |
| ENSG00000198021 | HGLibA_46379 | 141 | 0.254494318 | SPANXA1 | family member A1 [Source:HGNC Symbol;Acc:HGNC:11218]" |
| ENSG00000205279 | HGLibA_11761 | 141 | 0.254494318 | CTXN3 | cortexin 3 [Source:HGNC Symbol;Acc:HGNC:31110] |
| ENSG00000205726 | HGLibA_23872 | 141 | 0.254494318 | ITSN1 | intersectin 1 [Source:HGNC Symbol;Acc:HGNC:6183] |
| ENSG00000212938 | HGLibA_25820 | 141 | 0.254494318 | KRTAP6-3 | keratin associated protein 6-3 [Source:HGNC Symbol;Acc:HGNC:18933] |
| ENSG00000213722 | HGLibA_12592 | 141 | 0.254494318 | DDAH2 | dimethylarginine dimethylaminohydrolase 2 [Source:HGNC Symbol;Acc:HGNC:2716] |
| ENSG00000215183 | HGLibA_30229 | 141 | 0.254494318 | MSMP | prostate associated [Source:HGNC Symbol;Acc:HGNC:29663]" |
| ENSG00000215187 | HGLibA_16237 | 141 | 0.254494318 | FAM166B | family with sequence similarity 166 member B [Source:HGNC Symbol;Acc:HGNC:34242] |
| ENSG00000221931 | HGLibA_34451 | 141 | 0.254494318 | OR6X1 | olfactory receptor family 6 subfamily X member 1 [Source:HGNC Symbol;Acc:HGNC:14737] |
| ENSG00000229292 | HGLibA_40941 | 141 | 0.254494318 | RFPL4AL1 | ret finger protein like 4A like 1 [Source:HGNC Symbol;Acc:HGNC:45147] |
| ENSG00000278023 | HGLibA_40755 | 141 | 0.254494318 | RDM1 | RAD52 motif containing 1 [Source:HGNC Symbol;Acc:HGNC:19950] |
| ENSG00000284609 | HGLibA_34498 | 141 | 0.254494318 | OR8B3 | olfactory receptor family 8 subfamily B member 3 [Source:HGNC Symbol;Acc:HGNC:8472] |
| FAM26D | HGLibA_16560 | 141 | 0.254494318 | FAM26D | - |
| hsa-mir-1237 | HGLibA_57337 | 141 | 0.254494318 | hsa-mir-1237 | - |
| hsa-mir-1265 | HGLibA_57484 | 141 | 0.254494318 | hsa-mir-1265 | - |
| hsa-mir-1913 | HGLibA_58055 | 141 | 0.254494318 | hsa-mir-1913 | - |
| hsa-mir-3163 | HGLibA_58732 | 141 | 0.254494318 | hsa-mir-3163 | - |
| hsa-mir-3202-1 | HGLibA_58917 | 141 | 0.254494318 | hsa-mir-3202-1 | - |
| hsa-mir-339 | HGLibA_59008 | 141 | 0.254494318 | hsa-mir-339 | - |
| hsa-mir-3660 | HGLibA_59192 | 141 | 0.254494318 | hsa-mir-3660 | - |
| hsa-mir-3666 | HGLibA_59214 | 141 | 0.254494318 | hsa-mir-3666 | - |
| hsa-mir-4260 | HGLibA_59748 | 141 | 0.254494318 | hsa-mir-4260 | - |
| hsa-mir-431 | HGLibA_59951 | 141 | 0.254494318 | hsa-mir-431 | - |
| hsa-mir-4449 | HGLibA_60201 | 141 | 0.254494318 | hsa-mir-4449 | - |
| hsa-mir-597 | HGLibA_62482 | 141 | 0.254494318 | hsa-mir-597 | - |
| hsa-mir-6732 | HGLibA_63083 | 141 | 0.254494318 | hsa-mir-6732 | - |
| hsa-mir-6733 | HGLibA_63084 | 141 | 0.254494318 | hsa-mir-6733 | - |
| hsa-mir-6802 | HGLibA_63385 | 141 | 0.254494318 | hsa-mir-6802 | - |
| KIAA0196 | HGLibA_24601 | 141 | 0.254494318 | KIAA0196 | - |
| KIAA0947 | HGLibA_24673 | 141 | 0.254494318 | KIAA0947 | - |
| PPP2R3B_Y | HGLibA_38161 | 141 | 0.254494318 | PPP2R3B_Y | - |
| UTP11L | HGLibA_53430 | 141 | 0.254494318 | UTP11L | - |
| 7-Mar | HGLibA_28346 | 140 | 0.244543281 | 7-Mar | - |
| C3orf27 | HGLibA_06225 | 140 | 0.244543281 | C3orf27 | - |
| C5orf45 | HGLibA_06387 | 140 | 0.244543281 | C5orf45 | - |
| CRLF2_Y | HGLibA_11192 | 140 | 0.244543281 | CRLF2_Y | - |
| CXXC11 | HGLibA_11967 | 140 | 0.244543281 | CXXC11 | - |
| ENSG00000002834 | HGLibA_26055 | 140 | 0.244543281 | LASP1 | LIM and SH3 protein 1 [Source:HGNC Symbol;Acc:HGNC:6513] |
| ENSG00000004468 | HGLibA_08366 | 140 | 0.244543281 | CD38 | CD38 molecule [Source:HGNC Symbol;Acc:HGNC:1667] |
| ENSG00000007923 | HGLibA_13567 | 140 | 0.244543281 | DNAJC11 | DnaJ heat shock protein family (Hsp40) member C11 [Source:HGNC Symbol;Acc:HGNC:25570] |
| ENSG00000007968 | HGLibA_14311 | 140 | 0.244543281 | E2F2 | E2F transcription factor 2 [Source:HGNC Symbol;Acc:HGNC:3114] |
| ENSG00000011198 | HGLibA_00280 | 140 | 0.244543281 | ABHD5 | abhydrolase domain containing 5 [Source:HGNC Symbol;Acc:HGNC:21396] |
| ENSG00000021355 | HGLibA_43535 | 140 | 0.244543281 | SERPINB1 | serpin family B member 1 [Source:HGNC Symbol;Acc:HGNC:3311] |
| ENSG00000021776 | HGLibA_02617 | 140 | 0.244543281 | AQR | aquarius intron-binding spliceosomal factor [Source:HGNC Symbol;Acc:HGNC:29513] |
| ENSG00000048649 | HGLibA_42311 | 140 | 0.244543281 | RSF1 | remodeling and spacing factor 1 [Source:HGNC Symbol;Acc:HGNC:18118] |

| | | | | | |
|---|---|---|---|---|---|
| ENSG00000051341 | HGLibA_37545 | 140 | 0.244543281 | POLQ | DNA polymerase theta [Source:HGNC Symbol;Acc:HGNC:9186] |
| ENSG00000054690 | HGLibA_37126 | 140 | 0.244543281 | PLEKHH1 | MyTH4 and FERM domain containing H1 [Source:HGNC Symbol;Acc:HGNC:17733]" |
| ENSG00000064012 | HGLibA_07324 | 140 | 0.244543281 | CASP8 | caspase 8 [Source:HGNC Symbol;Acc:HGNC:1509] |
| ENSG00000067560 | HGLibA_41167 | 140 | 0.244543281 | RHOA | ras homolog family member A [Source:HGNC Symbol;Acc:HGNC:667] |
| ENSG00000068697 | HGLibA_26017 | 140 | 0.244543281 | LAPTM4A | lysosomal protein transmembrane 4 alpha [Source:HGNC Symbol;Acc:HGNC:6924] |
| ENSG00000072958 | HGLibA_02291 | 140 | 0.244543281 | AP1M1 | adaptor related protein complex 1 subunit mu 1 [Source:HGNC Symbol;Acc:HGNC:13667] |
| ENSG00000073910 | HGLibA_18053 | 140 | 0.244543281 | FRY | FRY microtubule binding protein [Source:HGNC Symbol;Acc:HGNC:20367] |
| ENSG00000084734 | HGLibA_18791 | 140 | 0.244543281 | GCKR | glucokinase regulator [Source:HGNC Symbol;Acc:HGNC:4196] |
| ENSG00000085662 | HGLibA_01473 | 140 | 0.244543281 | AKR1B1 | aldo-keto reductase family 1 member B [Source:HGNC Symbol;Acc:HGNC:381] |
| ENSG00000087008 | HGLibA_00502 | 140 | 0.244543281 | ACOX3 | pristanoyl [Source:HGNC Symbol;Acc:HGNC:121]" |
| ENSG00000090857 | HGLibA_35938 | 140 | 0.244543281 | PDPR | pyruvate dehydrogenase phosphatase regulatory subunit [Source:HGNC Symbol;Acc:HGNC:30264] |
| ENSG00000094631 | HGLibA_21017 | 140 | 0.244543281 | HDAC6 | histone deacetylase 6 [Source:HGNC Symbol;Acc:HGNC:14064] |
| ENSG00000095564 | HGLibA_04891 | 140 | 0.244543281 | BTAF1 | B-TFIID TATA-box binding protein associated factor 1 [Source:HGNC Symbol;Acc:HGNC:17307] |
| ENSG00000099377 | HGLibA_22210 | 140 | 0.244543281 | HSD3B7 | 3 beta- and steroid delta-isomerase 7 [Source:HGNC Symbol;Acc:HGNC:18324]" |
| ENSG00000100294 | HGLibA_28558 | 140 | 0.244543281 | MCAT | malonyl-CoA-acyl carrier protein transacylase [Source:HGNC Symbol;Acc:HGNC:29622] |
| ENSG00000100473 | HGLibA_10466 | 140 | 0.244543281 | COCH | cochlin [Source:HGNC Symbol;Acc:HGNC:2180] |
| ENSG00000101441 | HGLibA_11457 | 140 | 0.244543281 | CST4 | cystatin S [Source:HGNC Symbol;Acc:HGNC:2476] |
| ENSG00000102038 | HGLibA_45602 | 140 | 0.244543281 | SMARCA1 | member 1 [Source:HGNC Symbol;Acc:HGNC:11097]" |
| ENSG00000102230 | HGLibA_35703 | 140 | 0.244543281 | PCYT1B | beta [Source:HGNC Symbol;Acc:HGNC:8755]" |
| ENSG00000103310 | HGLibA_56953 | 140 | 0.244543281 | ZP2 | zona pellucida glycoprotein 2 [Source:HGNC Symbol;Acc:HGNC:13188] |
| ENSG00000104043 | HGLibA_03790 | 140 | 0.244543281 | ATP8B4 | ATPase phospholipid transporting 8B4 (putative) [Source:HGNC Symbol;Acc:HGNC:13536] |
| ENSG00000104983 | HGLibA_07818 | 140 | 0.244543281 | CCDC61 | coiled-coil domain containing 61 [Source:HGNC Symbol;Acc:HGNC:33629] |
| ENSG00000105298 | HGLibA_06952 | 140 | 0.244543281 | CACTIN | spliceosome C complex subunit [Source:HGNC Symbol;Acc:HGNC:29938]" |
| ENSG00000105357 | HGLibA_30715 | 140 | 0.244543281 | MYH14 | myosin heavy chain 14 [Source:HGNC Symbol;Acc:HGNC:23212] |
| ENSG00000106013 | HGLibA_02144 | 140 | 0.244543281 | ANKRD7 | ankyrin repeat domain 7 [Source:HGNC Symbol;Acc:HGNC:18588] |
| ENSG00000109654 | HGLibA_51426 | 140 | 0.244543281 | TRIM2 | tripartite motif containing 2 [Source:HGNC Symbol;Acc:HGNC:15974] |
| ENSG00000112599 | HGLibA_20637 | 140 | 0.244543281 | GUCA1B | guanylate cyclase activator 1B [Source:HGNC Symbol;Acc:HGNC:4679] |
| ENSG00000115145 | HGLibA_47332 | 140 | 0.244543281 | STAM2 | signal transducing adaptor molecule 2 [Source:HGNC Symbol;Acc:HGNC:11358] |
| ENSG00000117226 | HGLibA_18733 | 140 | 0.244543281 | GBP3 | guanylate binding protein 3 [Source:HGNC Symbol;Acc:HGNC:4184] |
| ENSG00000120458 | HGLibA_30173 | 140 | 0.244543281 | MSANTD2 | Myb/SANT DNA binding domain containing 2 [Source:HGNC Symbol;Acc:HGNC:26266] |
| ENSG00000120992 | HGLibA_27737 | 140 | 0.244543281 | LYPLA1 | lysophospholipase I [Source:HGNC Symbol;Acc:HGNC:6737] |
| ENSG00000122136 | HGLibA_33224 | 140 | 0.244543281 | OBP2A | odorant binding protein 2A [Source:HGNC Symbol;Acc:HGNC:23380] |
| ENSG00000123358 | HGLibA_32624 | 140 | 0.244543281 | NR4A1 | nuclear receptor subfamily 4 group A member 1 [Source:HGNC Symbol;Acc:HGNC:7980] |
| ENSG00000124383 | HGLibA_29724 | 140 | 0.244543281 | MPHOSPH10 | M-phase phosphoprotein 10 [Source:HGNC Symbol;Acc:HGNC:7213] |
| ENSG00000127838 | HGLibA_37347 | 140 | 0.244543281 | PNKD | MBL domain containing [Source:HGNC Symbol;Acc:HGNC:9153]" |
| ENSG00000128011 | HGLibA_27151 | 140 | 0.244543281 | LRFN1 | leucine rich repeat and fibronectin type III domain containing 1 [Source:HGNC Symbol;Acc:HGNC:29290] |
| ENSG00000128512 | HGLibA_13747 | 140 | 0.244543281 | DOCK4 | dedicator of cytokinesis 4 [Source:HGNC Symbol;Acc:HGNC:19192] |
| ENSG00000129437 | HGLibA_25247 | 140 | 0.244543281 | KLK14 | kallikrein related peptidase 14 [Source:HGNC Symbol;Acc:HGNC:6362] |
| ENSG00000132436 | HGLibA_17521 | 140 | 0.244543281 | FIGNL1 | fidgetin like 1 [Source:HGNC Symbol;Acc:HGNC:13286] |
| ENSG00000132446 | HGLibA_18121 | 140 | 0.244543281 | FTHL17 | ferritin heavy chain like 17 [Source:HGNC Symbol;Acc:HGNC:3987] |
| ENSG00000135956 | HGLibA_49915 | 140 | 0.244543281 | TMEM127 | transmembrane protein 127 [Source:HGNC Symbol;Acc:HGNC:26038] |
| ENSG00000141384 | HGLibA_48237 | 140 | 0.244543281 | TAF4B | TATA-box binding protein associated factor 4b [Source:HGNC Symbol;Acc:HGNC:11538] |
| ENSG00000142634 | HGLibA_14580 | 140 | 0.244543281 | EFHD2 | EF-hand domain family member D2 [Source:HGNC Symbol;Acc:HGNC:28670] |
| ENSG00000145284 | HGLibA_42888 | 140 | 0.244543281 | SCD5 | stearoyl-CoA desaturase 5 [Source:HGNC Symbol;Acc:HGNC:21088] |
| ENSG00000145908 | HGLibA_55771 | 140 | 0.244543281 | ZNF300 | zinc finger protein 300 [Source:HGNC Symbol;Acc:HGNC:13091] |
| ENSG00000146070 | HGLibA_36923 | 140 | 0.244543281 | PLA2G7 | phospholipase A2 group VII [Source:HGNC Symbol;Acc:HGNC:9040] |
| ENSG00000147010 | HGLibA_43960 | 140 | 0.244543281 | SH3KBP1 | SH3 domain containing kinase binding protein 1 [Source:HGNC Symbol;Acc:HGNC:13867] |
| ENSG00000151418 | HGLibA_03764 | 140 | 0.244543281 | ATP6V1G3 | ATPase H+ transporting V1 subunit G3 [Source:HGNC Symbol;Acc:HGNC:18265] |
| ENSG00000155629 | HGLibA_36617 | 140 | 0.244543281 | PIK3AP1 | phosphoinositide-3-kinase adaptor protein 1 [Source:HGNC Symbol;Acc:HGNC:30034] |
| ENSG00000157470 | HGLibA_16733 | 140 | 0.244543281 | FAM81A | family with sequence similarity 81 member A [Source:HGNC Symbol;Acc:HGNC:28379] |
| ENSG00000157557 | HGLibA_15644 | 140 | 0.244543281 | ETS2 | transcription factor [Source:HGNC Symbol;Acc:HGNC:3489]" |
| ENSG00000158528 | HGLibA_38128 | 140 | 0.244543281 | PPP1R9A | protein phosphatase 1 regulatory subunit 9A [Source:HGNC Symbol;Acc:HGNC:14946] |
| ENSG00000158874 | HGLibA_02456 | 140 | 0.244543281 | APOA2 | apolipoprotein A2 [Source:HGNC Symbol;Acc:HGNC:601] |
| ENSG00000161265 | HGLibA_52494 | 140 | 0.244543281 | U2AF1L4 | U2 small nuclear RNA auxiliary factor 1 like 4 [Source:HGNC Symbol;Acc:HGNC:23020] |
| ENSG00000161265 | HGLibA_52496 | 140 | 0.244543281 | U2AF1L4 | U2 small nuclear RNA auxiliary factor 1 like 4 [Source:HGNC Symbol;Acc:HGNC:23020] |
| ENSG00000162139 | HGLibA_31715 | 140 | 0.244543281 | NEU3 | neuraminidase 3 [Source:HGNC Symbol;Acc:HGNC:7760] |
| ENSG00000162753 | HGLibA_45442 | 140 | 0.244543281 | SLC9C2 | solute carrier family 9 member C2 (putative) [Source:HGNC Symbol;Acc:HGNC:28664] |
| ENSG00000163520 | HGLibA_16945 | 140 | 0.244543281 | FBLN2 | fibulin 2 [Source:HGNC Symbol;Acc:HGNC:3601] |
| ENSG00000163875 | HGLibA_28722 | 140 | 0.244543281 | MEAF6 | MYST/Esa1 associated factor 6 [Source:HGNC Symbol;Acc:HGNC:25674] |
| ENSG00000164040 | HGLibA_36290 | 140 | 0.244543281 | PGRMC2 | progesterone receptor membrane component 2 [Source:HGNC Symbol;Acc:HGNC:16089] |
| ENSG00000166260 | HGLibA_10805 | 140 | 0.244543281 | COX11 | cytochrome c oxidase copper chaperone COX11 [Source:HGNC Symbol;Acc:HGNC:2261] |
| ENSG00000166311 | HGLibA_45802 | 140 | 0.244543281 | SMPD1 | sphingomyelin phosphodiesterase 1 [Source:HGNC Symbol;Acc:HGNC:11120] |
| ENSG00000168374 | HGLibA_02652 | 140 | 0.244543281 | ARF4 | ADP ribosylation factor 4 [Source:HGNC Symbol;Acc:HGNC:655] |
| ENSG00000170004 | HGLibA_09392 | 140 | 0.244543281 | CHD3 | chromodomain helicase DNA binding protein 3 [Source:HGNC Symbol;Acc:HGNC:1918] |
| ENSG00000170683 | HGLibA_33465 | 140 | 0.244543281 | OR10A3 | olfactory receptor family 10 subfamily A member 3 [Source:HGNC Symbol;Acc:HGNC:8162] |
| ENSG00000171487 | HGLibA_32124 | 140 | 0.244543281 | NLRP5 | NLR family pyrin domain containing 5 [Source:HGNC Symbol;Acc:HGNC:21269] |
| ENSG00000171505 | HGLibA_33704 | 140 | 0.244543281 | OR1N1 | olfactory receptor family 1 subfamily N member 1 [Source:HGNC Symbol;Acc:HGNC:8221] |
| ENSG00000172716 | HGLibA_45481 | 140 | 0.244543281 | SLFN11 | schlafen family member 11 [Source:HGNC Symbol;Acc:HGNC:26633] |
| ENSG00000173531 | HGLibA_30253 | 140 | 0.244543281 | MST1 | macrophage stimulating 1 [Source:HGNC Symbol;Acc:HGNC:7380] |
| ENSG00000175294 | HGLibA_07355 | 140 | 0.244543281 | CATSPER1 | cation channel sperm associated 1 [Source:HGNC Symbol;Acc:HGNC:17116] |
| ENSG00000176956 | HGLibA_27673 | 140 | 0.244543281 | LY6H | lymphocyte antigen 6 family member H [Source:HGNC Symbol;Acc:HGNC:6728] |
| ENSG00000177103 | HGLibA_14000 | 140 | 0.244543281 | DSCAML1 | DS cell adhesion molecule like 1 [Source:HGNC Symbol;Acc:HGNC:14656] |
| ENSG00000177156 | HGLibA_48285 | 140 | 0.244543281 | TALDO1 | transaldolase 1 [Source:HGNC Symbol;Acc:HGNC:11559] |
| ENSG00000178685 | HGLibA_35214 | 140 | 0.244543281 | PARP10 | poly(ADP-ribose) polymerase family member 10 [Source:HGNC Symbol;Acc:HGNC:25895] |
| ENSG00000179869 | HGLibA_00105 | 140 | 0.244543281 | ABCA13 | ATP binding cassette subfamily A member 13 [Source:HGNC Symbol;Acc:HGNC:14638] |
| ENSG00000179914 | HGLibA_23824 | 140 | 0.244543281 | ITLN1 | intelectin 1 [Source:HGNC Symbol;Acc:HGNC:18259] |
| ENSG00000180667 | HGLibA_54704 | 140 | 0.244543281 | YOD1 | YOD1 deubiquitinase [Source:HGNC Symbol;Acc:HGNC:25035] |
| ENSG00000181418 | HGLibA_12623 | 140 | 0.244543281 | DDN | dendrin [Source:HGNC Symbol;Acc:HGNC:24458] |
| ENSG00000182158 | HGLibA_11095 | 140 | 0.244543281 | CREB3L2 | cAMP responsive element binding protein 3 like 2 [Source:HGNC Symbol;Acc:HGNC:23720] |
| ENSG00000182308 | HGLibA_12441 | 140 | 0.244543281 | DCAF4L1 | DDB1 and CUL4 associated factor 4 like 1 [Source:HGNC Symbol;Acc:HGNC:27723] |
| ENSG00000182330 | HGLibA_38330 | 140 | 0.244543281 | PRAMEF8 | PRAME family member 8 [Source:HGNC Symbol;Acc:HGNC:24074] |
| ENSG00000183090 | HGLibA_17990 | 140 | 0.244543281 | FREM3 | FRAS1 related extracellular matrix 3 [Source:HGNC Symbol;Acc:HGNC:25172] |
| ENSG00000183281 | HGLibA_37162 | 140 | 0.244543281 | PLGLB1 | plasminogen-like B1 [Source:HGNC Symbol;Acc:HGNC:9072] |
| ENSG00000184005 | HGLibA_47271 | 140 | 0.244543281 | ST6GALNAC3 | 6-sialyltransferase 3 [Source:HGNC Symbol;Acc:HGNC:19343]" |
| ENSG00000184232 | HGLibA_33192 | 140 | 0.244543281 | OAF | out at first homolog [Source:HGNC Symbol;Acc:HGNC:28752] |
| ENSG00000185236 | HGLibA_39789 | 140 | 0.244543281 | RAB11B | member RAS oncogene family [Source:HGNC Symbol;Acc:HGNC:9761]" |
| ENSG00000186767 | HGLibA_46679 | 140 | 0.244543281 | SPIN4 | spindlin family member 4 [Source:HGNC Symbol;Acc:HGNC:27040] |
| ENSG00000186930 | HGLibA_25819 | 140 | 0.244543281 | KRTAP6-2 | keratin associated protein 6-2 [Source:HGNC Symbol;Acc:HGNC:18932] |
| ENSG00000187239 | HGLibA_17735 | 140 | 0.244543281 | FNBP1 | formin binding protein 1 [Source:HGNC Symbol;Acc:HGNC:17069] |
| ENSG00000187498 | HGLibA_10565 | 140 | 0.244543281 | COL4A1 | collagen type IV alpha 1 chain [Source:HGNC Symbol;Acc:HGNC:2202] |
| ENSG00000188629 | HGLibA_55531 | 140 | 0.244543281 | ZNF177 | zinc finger protein 177 [Source:HGNC Symbol;Acc:HGNC:12966] |
| ENSG00000188886 | HGLibA_03377 | 140 | 0.244543281 | ASTL | astacin like metalloendopeptidase [Source:HGNC Symbol;Acc:HGNC:31704] |
| ENSG00000189068 | HGLibA_53858 | 140 | 0.244543281 | VSTM1 | V-set and transmembrane domain containing 1 [Source:HGNC Symbol;Acc:HGNC:29455] |
| ENSG00000196415 | HGLibA_38983 | 140 | 0.244543281 | PRTN3 | proteinase 3 [Source:HGNC Symbol;Acc:HGNC:9495] |
| ENSG00000196663 | HGLibA_48945 | 140 | 0.244543281 | TECPR2 | tectonin beta-propeller repeat containing 2 [Source:HGNC Symbol;Acc:HGNC:19957] |
| ENSG00000197102 | HGLibA_14231 | 140 | 0.244543281 | DYNC1H1 | dynein cytoplasmic 1 heavy chain 1 [Source:HGNC Symbol;Acc:HGNC:2961] |
| ENSG00000197465 | HGLibA_20707 | 140 | 0.244543281 | GYPE | glycophorin E (MNS blood group) [Source:HGNC Symbol;Acc:HGNC:4705] |
| ENSG00000198732 | HGLibA_45793 | 140 | 0.244543281 | SMOC1 | SPARC related modular calcium binding 1 [Source:HGNC Symbol;Acc:HGNC:20318] |
| ENSG00000205517 | HGLibA_41007 | 140 | 0.244543281 | RGL3 | ral guanine nucleotide dissociation stimulator like 3 [Source:HGNC Symbol;Acc:HGNC:30282] |
| ENSG00000214652 | HGLibA_56636 | 140 | 0.244543281 | ZNF727 | zinc finger protein 727 [Source:HGNC Symbol;Acc:HGNC:22785] |
| ENSG00000215113 | HGLibA_11936 | 140 | 0.244543281 | CXorf49B | chromosome X open reading frame 49B [Source:HGNC Symbol;Acc:HGNC:34229] |
| ENSG00000230522 | HGLibA_28475 | 140 | 0.244543281 | MBD3L2 | methyl-CpG binding domain protein 3 like 2 [Source:HGNC Symbol;Acc:HGNC:18532] |
| ENSG00000231925 | HGLibA_48320 | 140 | 0.244543281 | TAPBP | TAP binding protein [Source:HGNC Symbol;Acc:HGNC:11566] |
| ENSG00000237765 | HGLibA_16413 | 140 | 0.244543281 | FAM200B | family with sequence similarity 200 member B [Source:HGNC Symbol;Acc:HGNC:27740] |
| ENSG00000241644 | HGLibA_23317 | 140 | 0.244543281 | INMT | indolethylamine N-methyltransferase [Source:HGNC Symbol;Acc:HGNC:6069] |
| ENSG00000242110 | HGLibA_01746 | 140 | 0.244543281 | AMACR | alpha-methylacyl-CoA racemase [Source:HGNC Symbol;Acc:HGNC:451] |
| ENSG00000256977 | HGLibA_26553 | 140 | 0.244543281 | LIMS3 | LIM zinc finger domain containing 3 [Source:HGNC Symbol;Acc:HGNC:30047] |
| ENSG00000269897 | HGLibA_10661 | 140 | 0.244543281 | COMMD3-BMI1 | COMMD3-BMI1 readthrough [Source:HGNC Symbol;Acc:HGNC:48326] |
| ENSG00000272031 | HGLibA_02076 | 140 | 0.244543281 | ANKRD34A | ankyrin repeat domain 34A [Source:HGNC Symbol;Acc:HGNC:27639] |
| FAM179A | HGLibA_16307 | 140 | 0.244543281 | FAM179A | - |
| HIST1H3A | HGLibA_21457 | 140 | 0.244543281 | HIST1H3A | - |
| hsa-mir-1269b | HGLibA_57506 | 140 | 0.244543281 | hsa-mir-1269b | - |
| hsa-mir-127 | HGLibA_57513 | 140 | 0.244543281 | hsa-mir-127 | - |
| hsa-mir-151b | HGLibA_57901 | 140 | 0.244543281 | hsa-mir-151b | - |
| hsa-mir-181c | HGLibA_57976 | 140 | 0.244543281 | hsa-mir-181c | - |
| hsa-mir-183 | HGLibA_57995 | 140 | 0.244543281 | hsa-mir-183 | - |
| hsa-mir-3115 | HGLibA_58498 | 140 | 0.244543281 | hsa-mir-3115 | - |
| hsa-mir-378i | HGLibA_59462 | 140 | 0.244543281 | hsa-mir-378i | - |
| hsa-mir-429 | HGLibA_59867 | 140 | 0.244543281 | hsa-mir-429 | - |
| hsa-mir-4422 | HGLibA_60076 | 140 | 0.244543281 | hsa-mir-4422 | - |
| hsa-mir-449a | HGLibA_60416 | 140 | 0.244543281 | hsa-mir-449a | - |
| hsa-mir-504 | HGLibA_61471 | 140 | 0.244543281 | hsa-mir-504 | - |
| hsa-mir-516a-1 | HGLibA_61610 | 140 | 0.244543281 | hsa-mir-516a-1 | - |
| hsa-mir-5193 | HGLibA_61689 | 140 | 0.244543281 | hsa-mir-5193 | - |
| hsa-mir-5194 | HGLibA_61693 | 140 | 0.244543281 | hsa-mir-5194 | - |
| hsa-mir-548w | HGLibA_62079 | 140 | 0.244543281 | hsa-mir-548w | - |
| hsa-mir-621 | HGLibA_62725 | 140 | 0.244543281 | hsa-mir-621 | - |
| hsa-mir-6825 | HGLibA_63477 | 140 | 0.244543281 | hsa-mir-6825 | - |
| OR8G2 | HGLibA_34521 | 140 | 0.244543281 | OR8G2 | - |
| RBAK-LOC389458 | HGLibA_40423 | 140 | 0.244543281 | RBAK-LOC389458 | - |
| REXO1L1 | HGLibA_40892 | 140 | 0.244543281 | REXO1L1 | - |
| SPATA31A2 | HGLibA_46471 | 140 | 0.244543281 | SPATA31A2 | - |
| TMEM206 | HGLibA_50195 | 140 | 0.244543281 | TMEM206 | - |
| C19orf66 | HGLibA_05694 | 139 | 0.234592243 | C19orf66 | - |
| C5orf54 | HGLibA_06408 | 139 | 0.234592243 | C5orf54 | - |

| | | | | | |
|---|---|---|---|---|---|
| C9orf37 | HGLibA_06725 | 139 | 0.234592243 | C9orf37 | - |
| CXorf22 | HGLibA_11904 | 139 | 0.234592243 | CXorf22 | - |
| CXorf61 | HGLibA_11953 | 139 | 0.234592243 | CXorf61 | - |
| EMR3 | HGLibA_15090 | 139 | 0.234592243 | EMR3 | - |
| ENSG00000005893 | HGLibA_25981 | 139 | 0.234592243 | LAMP2 | lysosomal associated membrane protein 2 [Source:HGNC Symbol;Acc:HGNC:6501] |
| ENSG00000007062 | HGLibA_38681 | 139 | 0.234592243 | PROM1 | prominin 1 [Source:HGNC Symbol;Acc:HGNC:9454] |
| ENSG00000008382 | HGLibA_29742 | 139 | 0.234592243 | MPND | MPN domain containing [Source:HGNC Symbol;Acc:HGNC:25934] |
| ENSG00000019102 | HGLibA_53844 | 139 | 0.234592243 | VSIG2 | V-set and immunoglobulin domain containing 2 [Source:HGNC Symbol;Acc:HGNC:17149] |
| ENSG00000040531 | HGLibA_11665 | 139 | 0.234592243 | CTNS | lysosomal cystine transporter [Source:HGNC Symbol;Acc:HGNC:2518]" |
| ENSG00000044446 | HGLibA_36404 | 139 | 0.234592243 | PHKA2 | phosphorylase kinase regulatory subunit alpha 2 [Source:HGNC Symbol;Acc:HGNC:8926] |
| ENSG00000051596 | HGLibA_49369 | 139 | 0.234592243 | THOC3 | THO complex 3 [Source:HGNC Symbol;Acc:HGNC:19072] |
| ENSG00000069966 | HGLibA_19503 | 139 | 0.234592243 | GNB5 | G protein subunit beta 5 [Source:HGNC Symbol;Acc:HGNC:4401] |
| ENSG00000073146 | HGLibA_29697 | 139 | 0.234592243 | MOV10L1 | Mov10 like RISC complex RNA helicase 1 [Source:HGNC Symbol;Acc:HGNC:7201] |
| ENSG00000081248 | HGLibA_06902 | 139 | 0.234592243 | CACNA1S | calcium voltage-gated channel subunit alpha1 S [Source:HGNC Symbol;Acc:HGNC:1397] |
| ENSG00000085872 | HGLibA_09426 | 139 | 0.234592243 | CHERP | calcium homeostasis endoplasmic reticulum protein [Source:HGNC Symbol;Acc:HGNC:16930] |
| ENSG00000091137 | HGLibA_44794 | 139 | 0.234592243 | SLC26A4 | solute carrier family 26 member 4 [Source:HGNC Symbol;Acc:HGNC:8818] |
| ENSG00000099385 | HGLibA_04343 | 139 | 0.234592243 | BCL7C | BCL tumor suppressor 7C [Source:HGNC Symbol;Acc:HGNC:1006] |
| ENSG00000101310 | HGLibA_43223 | 139 | 0.234592243 | SEC23B | coat complex II component [Source:HGNC Symbol;Acc:HGNC:10702]" |
| ENSG00000103175 | HGLibA_54243 | 139 | 0.234592243 | WFDC1 | WAP four-disulfide core domain 1 [Source:HGNC Symbol;Acc:HGNC:15466] |
| ENSG00000104093 | HGLibA_13457 | 139 | 0.234592243 | DMXL2 | Dmx like 2 [Source:HGNC Symbol;Acc:HGNC:2938] |
| ENSG00000105428 | HGLibA_56947 | 139 | 0.234592243 | ZNRF4 | zinc and ring finger 4 [Source:HGNC Symbol;Acc:HGNC:17726] |
| ENSG00000106012 | HGLibA_23499 | 139 | 0.234592243 | IQCE | IQ motif containing E [Source:HGNC Symbol;Acc:HGNC:29171] |
| ENSG00000106028 | HGLibA_47113 | 139 | 0.234592243 | SSBP1 | single stranded DNA binding protein 1 [Source:HGNC Symbol;Acc:HGNC:11317] |
| ENSG00000107295 | HGLibA_43948 | 139 | 0.234592243 | SH3GL2 | endophilin A1 [Source:HGNC Symbol;Acc:HGNC:10831]" |
| ENSG00000107581 | HGLibA_14763 | 139 | 0.234592243 | EIF3A | eukaryotic translation initiation factor 3 subunit A [Source:HGNC Symbol;Acc:HGNC:3271] |
| ENSG00000109072 | HGLibA_53894 | 139 | 0.234592243 | VTN | vitronectin [Source:HGNC Symbol;Acc:HGNC:12724] |
| ENSG00000109133 | HGLibA_50368 | 139 | 0.234592243 | TMEM33 | transmembrane protein 33 [Source:HGNC Symbol;Acc:HGNC:25541] |
| ENSG00000110321 | HGLibA_14844 | 139 | 0.234592243 | EIF4G2 | eukaryotic translation initiation factor 4 gamma 2 [Source:HGNC Symbol;Acc:HGNC:3297] |
| ENSG00000110442 | HGLibA_10679 | 139 | 0.234592243 | COMMD9 | COMM domain containing 9 [Source:HGNC Symbol;Acc:HGNC:25014] |
| ENSG00000111331 | HGLibA_33204 | 139 | 0.234592243 | OAS3 | 2'-5'-oligoadenylate synthetase 3 [Source:HGNC Symbol;Acc:HGNC:8088] |
| ENSG00000112079 | HGLibA_47502 | 139 | 0.234592243 | STK38 | serine/threonine kinase 38 [Source:HGNC Symbol;Acc:HGNC:17847] |
| ENSG00000112494 | HGLibA_53034 | 139 | 0.234592243 | UNC93A | unc-93 homolog A [Source:HGNC Symbol;Acc:HGNC:12570] |
| ENSG00000114854 | HGLibA_50866 | 139 | 0.234592243 | TNNC1 | slow skeletal and cardiac type [Source:HGNC Symbol;Acc:HGNC:11943]" |
| ENSG00000115507 | HGLibA_34782 | 139 | 0.234592243 | OTX1 | orthodenticle homeobox 1 [Source:HGNC Symbol;Acc:HGNC:8521] |
| ENSG00000115652 | HGLibA_53480 | 139 | 0.234592243 | UXS1 | UDP-glucuronate decarboxylase 1 [Source:HGNC Symbol;Acc:HGNC:17729] |
| ENSG00000116157 | HGLibA_20189 | 139 | 0.234592243 | GPX7 | glutathione peroxidase 7 [Source:HGNC Symbol;Acc:HGNC:4559] |
| ENSG00000118418 | HGLibA_21724 | 139 | 0.234592243 | HMGN3 | high mobility group nucleosomal binding domain 3 [Source:HGNC Symbol;Acc:HGNC:12312] |
| ENSG00000118972 | HGLibA_17401 | 139 | 0.234592243 | FGF23 | fibroblast growth factor 23 [Source:HGNC Symbol;Acc:HGNC:3680] |
| ENSG00000119401 | HGLibA_51463 | 139 | 0.234592243 | TRIM32 | tripartite motif containing 32 [Source:HGNC Symbol;Acc:HGNC:16380] |
| ENSG00000120925 | HGLibA_41574 | 139 | 0.234592243 | RNF170 | ring finger protein 170 [Source:HGNC Symbol;Acc:HGNC:25358] |
| ENSG00000122592 | HGLibA_21921 | 139 | 0.234592243 | HOXA7 | homeobox A7 [Source:HGNC Symbol;Acc:HGNC:5108] |
| ENSG00000125823 | HGLibA_11496 | 139 | 0.234592243 | CSTL1 | cystatin like 1 [Source:HGNC Symbol;Acc:HGNC:15958] |
| ENSG00000129250 | HGLibA_24890 | 139 | 0.234592243 | KIF1C | kinesin family member 1C [Source:HGNC Symbol;Acc:HGNC:6317] |
| ENSG00000129534 | HGLibA_29310 | 139 | 0.234592243 | MIS18BP1 | MIS18 binding protein 1 [Source:HGNC Symbol;Acc:HGNC:20190] |
| ENSG00000130827 | HGLibA_37265 | 139 | 0.234592243 | PLXNA3 | plexin A3 [Source:HGNC Symbol;Acc:HGNC:9101] |
| ENSG00000131171 | HGLibA_43915 | 139 | 0.234592243 | SH3BGRL | SH3 domain binding glutamate rich protein like [Source:HGNC Symbol;Acc:HGNC:10823] |
| ENSG00000131203 | HGLibA_22564 | 139 | 0.234592243 | IDO1 | 3-dioxygenase 1 [Source:HGNC Symbol;Acc:HGNC:6059]" |
| ENSG00000134222 | HGLibA_39231 | 139 | 0.234592243 | PSRC1 | proline and serine rich coiled-coil 1 [Source:HGNC Symbol;Acc:HGNC:24472] |
| ENSG00000135622 | HGLibA_43352 | 139 | 0.234592243 | SEMA4F | ssemaphorin 4F [Source:HGNC Symbol;Acc:HGNC:10734] |
| ENSG00000136717 | HGLibA_04507 | 139 | 0.234592243 | BIN1 | bridging integrator 1 [Source:HGNC Symbol;Acc:HGNC:1052] |
| ENSG00000138068 | HGLibA_47768 | 139 | 0.234592243 | SULT6B1 | sulfotransferase family 6B member 1 [Source:HGNC Symbol;Acc:HGNC:33433] |
| ENSG00000138119 | HGLibA_30882 | 139 | 0.234592243 | MYOF | myoferlin [Source:HGNC Symbol;Acc:HGNC:3656] |
| ENSG00000138399 | HGLibA_16902 | 139 | 0.234592243 | FASTKD1 | FAST kinase domains 1 [Source:HGNC Symbol;Acc:HGNC:26150] |
| ENSG00000138778 | HGLibA_09051 | 139 | 0.234592243 | CENPE | centromere protein E [Source:HGNC Symbol;Acc:HGNC:1856] |
| ENSG00000141084 | HGLibA_40196 | 139 | 0.234592243 | RANBP10 | RAN binding protein 10 [Source:HGNC Symbol;Acc:HGNC:29285] |
| ENSG00000141337 | HGLibA_03144 | 139 | 0.234592243 | ARSG | arylsulfatase G [Source:HGNC Symbol;Acc:HGNC:24102] |
| ENSG00000142511 | HGLibA_20020 | 139 | 0.234592243 | GPR32 | G protein-coupled receptor 32 [Source:HGNC Symbol;Acc:HGNC:4487] |
| ENSG00000143851 | HGLibA_39479 | 139 | 0.234592243 | PTPN7 | non-receptor type 7 [Source:HGNC Symbol;Acc:HGNC:9659]" |
| ENSG00000143947 | HGLibA_42125 | 139 | 0.234592243 | RPS27A | ribosomal protein S27a [Source:HGNC Symbol;Acc:HGNC:10417] |
| ENSG00000144229 | HGLibA_49410 | 139 | 0.234592243 | THSD7B | thrombospondin type 1 domain containing 7B [Source:HGNC Symbol;Acc:HGNC:29348] |
| ENSG00000145194 | HGLibA_14385 | 139 | 0.234592243 | ECE2 | endothelin converting enzyme 2 [Source:HGNC Symbol;Acc:HGNC:13275] |
| ENSG00000145757 | HGLibA_46533 | 139 | 0.234592243 | SPATA9 | spermatogenesis associated 9 [Source:HGNC Symbol;Acc:HGNC:22988] |
| ENSG00000148153 | HGLibA_23312 | 139 | 0.234592243 | INIP | INTS3 and NABP interacting protein [Source:HGNC Symbol;Acc:HGNC:24994] |
| ENSG00000148154 | HGLibA_52861 | 139 | 0.234592243 | UGCG | UDP-glucose ceramide glucosyltransferase [Source:HGNC Symbol;Acc:HGNC:12524] |
| ENSG00000148734 | HGLibA_32439 | 139 | 0.234592243 | NPFFR1 | neuropeptide FF receptor 1 [Source:HGNC Symbol;Acc:HGNC:17425] |
| ENSG00000149591 | HGLibA_48269 | 139 | 0.234592243 | TAGLN | transgelin [Source:HGNC Symbol;Acc:HGNC:11553] |
| ENSG00000150281 | HGLibA_11621 | 139 | 0.234592243 | CTF1 | cardiotrophin 1 [Source:HGNC Symbol;Acc:HGNC:2499] |
| ENSG00000150681 | HGLibA_41072 | 139 | 0.234592243 | RGS18 | regulator of G protein signaling 18 [Source:HGNC Symbol;Acc:HGNC:14261] |
| ENSG00000157191 | HGLibA_31596 | 139 | 0.234592243 | NECAP2 | NECAP endocytosis associated 2 [Source:HGNC Symbol;Acc:HGNC:25528] |
| ENSG00000161267 | HGLibA_04374 | 139 | 0.234592243 | BDH1 | 3-hydroxybutyrate dehydrogenase 1 [Source:HGNC Symbol;Acc:HGNC:1027] |
| ENSG00000163293 | HGLibA_31955 | 139 | 0.234592243 | NIPAL1 | NIPA like domain containing 1 [Source:HGNC Symbol;Acc:HGNC:27194] |
| ENSG00000163879 | HGLibA_13651 | 139 | 0.234592243 | DNALI1 | dynein axonemal light intermediate chain 1 [Source:HGNC Symbol;Acc:HGNC:14353] |
| ENSG00000167130 | HGLibA_13791 | 139 | 0.234592243 | DOLPP1 | dolichyldiphosphatase 1 [Source:HGNC Symbol;Acc:HGNC:29565] |
| ENSG00000168421 | HGLibA_41194 | 139 | 0.234592243 | RHOH | ras homolog family member H [Source:HGNC Symbol;Acc:HGNC:686] |
| ENSG00000170835 | HGLibA_08985 | 139 | 0.234592243 | CEL | carboxyl ester lipase [Source:HGNC Symbol;Acc:HGNC:1848] |
| ENSG00000171049 | HGLibA_17965 | 139 | 0.234592243 | FPR2 | formyl peptide receptor 2 [Source:HGNC Symbol;Acc:HGNC:3827] |
| ENSG00000171121 | HGLibA_24347 | 139 | 0.234592243 | KCNMB3 | potassium calcium-activated channel subfamily M regulatory beta subunit 3 [Source:HGNC Symbol;Acc:HGNC:6287] |
| ENSG00000171161 | HGLibA_56506 | 139 | 0.234592243 | ZNF672 | zinc finger protein 672 [Source:HGNC Symbol;Acc:HGNC:26179] |
| ENSG00000172671 | HGLibA_55155 | 139 | 0.234592243 | ZFAND4 | zinc finger AN1-type containing 4 [Source:HGNC Symbol;Acc:HGNC:23504] |
| ENSG00000176720 | HGLibA_04678 | 139 | 0.234592243 | BOK | BCL2 family apoptosis regulator BOK [Source:HGNC Symbol;Acc:HGNC:1087] |
| ENSG00000177551 | HGLibA_31894 | 139 | 0.234592243 | NHLH2 | nescient helix-loop-helix 2 [Source:HGNC Symbol;Acc:HGNC:7818] |
| ENSG00000178287 | HGLibA_46344 | 139 | 0.234592243 | SPAG11A | sperm associated antigen 11A [Source:HGNC Symbol;Acc:HGNC:33342] |
| ENSG00000179361 | HGLibA_02895 | 139 | 0.234592243 | ARID3B | AT-rich interaction domain 3B [Source:HGNC Symbol;Acc:HGNC:14350] |
| ENSG00000179889 | HGLibA_35958 | 139 | 0.234592243 | PDXDC1 | pyridoxal dependent decarboxylase domain containing 1 [Source:HGNC Symbol;Acc:HGNC:28995] |
| ENSG00000180257 | HGLibA_56812 | 139 | 0.234592243 | ZNF816 | zinc finger protein 816 [Source:HGNC Symbol;Acc:HGNC:26995] |
| ENSG00000180479 | HGLibA_56278 | 139 | 0.234592243 | ZNF571 | zinc finger protein 571 [Source:HGNC Symbol;Acc:HGNC:25000] |
| ENSG00000182621 | HGLibA_36979 | 139 | 0.234592243 | PLCB1 | phospholipase C beta 1 [Source:HGNC Symbol;Acc:HGNC:15917] |
| ENSG00000182979 | HGLibA_30310 | 139 | 0.234592243 | MTA1 | metastasis associated 1 [Source:HGNC Symbol;Acc:HGNC:7410] |
| ENSG00000183530 | HGLibA_38781 | 139 | 0.234592243 | PRR14L | proline rich 14 like [Source:HGNC Symbol;Acc:HGNC:28738] |
| ENSG00000185361 | HGLibA_50720 | 139 | 0.234592243 | TNFAIP8L1 | TNF alpha induced protein 8 like 1 [Source:HGNC Symbol;Acc:HGNC:28279] |
| ENSG00000185900 | HGLibA_37663 | 139 | 0.234592243 | POMK | protein-O-mannose kinase [Source:HGNC Symbol;Acc:HGNC:26267] |
| ENSG00000185926 | HGLibA_33960 | 139 | 0.234592243 | OR4C46 | olfactory receptor family 4 subfamily C member 46 [Source:HGNC Symbol;Acc:HGNC:31271] |
| ENSG00000186191 | HGLibA_04729 | 139 | 0.234592243 | BPIFB4 | BPI fold containing family B member 4 [Source:HGNC Symbol;Acc:HGNC:16179] |
| ENSG00000186409 | HGLibA_07753 | 139 | 0.234592243 | CCDC30 | coiled-coil domain containing 30 [Source:HGNC Symbol;Acc:HGNC:26103] |
| ENSG00000187754 | HGLibA_47218 | 139 | 0.234592243 | SSX7 | SSX family member 7 [Source:HGNC Symbol;Acc:HGNC:19653] |
| ENSG00000188277 | HGLibA_05432 | 139 | 0.234592243 | C15orf62 | chromosome 15 open reading frame 62 [Source:HGNC Symbol;Acc:HGNC:34489] |
| ENSG00000188906 | HGLibA_27467 | 139 | 0.234592243 | LRRK2 | leucine rich repeat kinase 2 [Source:HGNC Symbol;Acc:HGNC:18618] |
| ENSG00000196363 | HGLibA_54114 | 139 | 0.234592243 | WDR5 | WD repeat domain 5 [Source:HGNC Symbol;Acc:HGNC:12757] |
| ENSG00000196605 | HGLibA_56866 | 139 | 0.234592243 | ZNF846 | zinc finger protein 846 [Source:HGNC Symbol;Acc:HGNC:27260] |
| ENSG00000196867 | HGLibA_55194 | 139 | 0.234592243 | ZFP28 | ZFP28 zinc finger protein [Source:HGNC Symbol;Acc:HGNC:17801] |
| ENSG00000197323 | HGLibA_51465 | 139 | 0.234592243 | TRIM33 | tripartite motif containing 33 [Source:HGNC Symbol;Acc:HGNC:16290] |
| ENSG00000197385 | HGLibA_56878 | 139 | 0.234592243 | ZNF860 | zinc finger protein 860 [Source:HGNC Symbol;Acc:HGNC:34513] |
| ENSG00000198121 | HGLibA_27053 | 139 | 0.234592243 | LPAR1 | lysophosphatidic acid receptor 1 [Source:HGNC Symbol;Acc:HGNC:3166] |
| ENSG00000198208 | HGLibA_42184 | 139 | 0.234592243 | RPS6KL1 | ribosomal protein S6 kinase like 1 [Source:HGNC Symbol;Acc:HGNC:20222] |
| ENSG00000198677 | HGLibA_52125 | 139 | 0.234592243 | TTC37 | tetratricopeptide repeat domain 37 [Source:HGNC Symbol;Acc:HGNC:23639] |
| ENSG00000198729 | HGLibA_38042 | 139 | 0.234592243 | PPP1R14C | protein phosphatase 1 regulatory inhibitor subunit 14C [Source:HGNC Symbol;Acc:HGNC:14952] |
| ENSG00000198874 | HGLibA_52479 | 139 | 0.234592243 | TYW1 | tRNA-yW synthesizing protein 1 homolog [Source:HGNC Symbol;Acc:HGNC:25598] |
| ENSG00000203909 | HGLibA_13897 | 139 | 0.234592243 | DPPA5 | developmental pluripotency associated 5 [Source:HGNC Symbol;Acc:HGNC:19201] |
| ENSG00000204583 | HGLibA_27150 | 139 | 0.234592243 | LRCOL1 | leucine rich colipase like 1 [Source:HGNC Symbol;Acc:HGNC:44160] |
| ENSG00000204704 | HGLibA_33901 | 139 | 0.234592243 | OR2W1 | olfactory receptor family 2 subfamily W member 1 [Source:HGNC Symbol;Acc:HGNC:8281] |
| ENSG00000205726 | HGLibA_23871 | 139 | 0.234592243 | ITSN1 | intersectin 1 [Source:HGNC Symbol;Acc:HGNC:6183] |
| ENSG00000213366 | HGLibA_20476 | 139 | 0.234592243 | GSTM2 | glutathione S-transferase mu 2 [Source:HGNC Symbol;Acc:HGNC:4634] |
| ENSG00000226979 | HGLibA_27575 | 139 | 0.234592243 | LTA | lymphotoxin alpha [Source:HGNC Symbol;Acc:HGNC:6709] |
| ENSG00000230937 | HGLibA_29301 | 139 | 0.234592243 | MIR205HG | MIR205 host gene [Source:HGNC Symbol;Acc:HGNC:43562] |
| ENSG00000242715 | HGLibA_07682 | 139 | 0.234592243 | CCDC169 | coiled-coil domain containing 169 [Source:HGNC Symbol;Acc:HGNC:34361] |
| ENSG00000255245 | HGLibA_18242 | 139 | 0.234592243 | FXYD6-FXYD2 | FXYD6-FXYD2 readthrough [Source:HGNC Symbol;Acc:HGNC:39978] |
| ENSG00000275111 | HGLibA_55563 | 139 | 0.234592243 | ZNF2 | zinc finger protein 2 [Source:HGNC Symbol;Acc:HGNC:12991] |
| ENSG00000286237 | HGLibA_03053 | 139 | 0.234592243 | ARMCX5-GPRASP2 | - |
| FAM196A | HGLibA_16382 | 139 | 0.234592243 | FAM196A | - |
| FAM208B | HGLibA_16436 | 139 | 0.234592243 | FAM208B | - |
| hsa-mir-1302-6 | HGLibA_57710 | 139 | 0.234592243 | hsa-mir-1302-6 | - |
| hsa-mir-378f | HGLibA_59450 | 139 | 0.234592243 | hsa-mir-378f | - |
| hsa-mir-425 | HGLibA_59706 | 139 | 0.234592243 | hsa-mir-425 | - |
| hsa-mir-4301 | HGLibA_59915 | 139 | 0.234592243 | hsa-mir-4301 | - |
| hsa-mir-4450 | HGLibA_60208 | 139 | 0.234592243 | hsa-mir-4450 | - |
| hsa-mir-448 | HGLibA_60334 | 139 | 0.234592243 | hsa-mir-448 | - |
| hsa-mir-4529 | HGLibA_60576 | 139 | 0.234592243 | hsa-mir-4529 | - |
| hsa-mir-4651 | HGLibA_60712 | 139 | 0.234592243 | hsa-mir-4651 | - |
| hsa-mir-4737 | HGLibA_61046 | 139 | 0.234592243 | hsa-mir-4737 | - |
| hsa-mir-521-1 | HGLibA_61768 | 139 | 0.234592243 | hsa-mir-521-1 | - |
| hsa-mir-5583-2 | HGLibA_62174 | 139 | 0.234592243 | hsa-mir-5583-2 | - |
| hsa-mir-6744 | HGLibA_63130 | 139 | 0.234592243 | hsa-mir-6744 | - |
| hsa-mir-6785 | HGLibA_63316 | 139 | 0.234592243 | hsa-mir-6785 | - |
| hsa-mir-6876 | HGLibA_63689 | 139 | 0.234592243 | hsa-mir-6876 | - |
| hsa-mir-8061 | HGLibA_64082 | 139 | 0.234592243 | hsa-mir-8061 | - |

| | | | | | |
|---|---|---|---|---|---|
| KIAA1468 | HGLibA_24768 | 139 | 0.234592243 | KIAA1468 | - |
| LOC149373 | HGLibA_26892 | 139 | 0.234592243 | LOC149373 | - |
| MNF1 | HGLibA_29569 | 139 | 0.234592243 | MNF1 | - |
| AKAP17A_Y | HGLibA_01424 | 138 | 0.224641206 | AKAP17A_Y | - |
| C4orf22 | HGLibA_06305 | 138 | 0.224641206 | C4orf22 | - |
| C8orf42 | HGLibA_06621 | 138 | 0.224641206 | C8orf42 | - |
| CCDC130 | HGLibA_07592 | 138 | 0.224641206 | CCDC130 | - |
| DUS2L | HGLibA_14119 | 138 | 0.224641206 | DUS2L | - |
| ENSG00000002933 | HGLibA_50071 | 138 | 0.224641206 | TMEM176A | transmembrane protein 176A [Source:HGNC Symbol;Acc:HGNC:24930] |
| ENSG00000004142 | HGLibA_37504 | 138 | 0.224641206 | POLDIP2 | DNA polymerase delta interacting protein 2 [Source:HGNC Symbol;Acc:HGNC:23781] |
| ENSG00000013016 | HGLibA_14672 | 138 | 0.224641206 | EHD3 | EH domain containing 3 [Source:HGNC Symbol;Acc:HGNC:3244] |
| ENSG00000024048 | HGLibA_52750 | 138 | 0.224641206 | UBR2 | ubiquitin protein ligase E3 component n-recognin 2 [Source:HGNC Symbol;Acc:HGNC:21289] |
| ENSG00000033178 | HGLibA_52527 | 138 | 0.224641206 | UBA6 | ubiquitin like modifier activating enzyme 6 [Source:HGNC Symbol;Acc:HGNC:25581] |
| ENSG00000047579 | HGLibA_14070 | 138 | 0.224641206 | DTNBP1 | dystrobrevin binding protein 1 [Source:HGNC Symbol;Acc:HGNC:17328] |
| ENSG00000054796 | HGLibA_46743 | 138 | 0.224641206 | SPO11 | initiator of meiotic double stranded breaks [Source:HGNC Symbol;Acc:HGNC:11250]" |
| ENSG00000055332 | HGLibA_14727 | 138 | 0.224641206 | EIF2AK2 | eukaryotic translation initiation factor 2 alpha kinase 2 [Source:HGNC Symbol;Acc:HGNC:9437] |
| ENSG00000064300 | HGLibA_31876 | 138 | 0.224641206 | NGFR | nerve growth factor receptor [Source:HGNC Symbol;Acc:HGNC:7809] |
| ENSG00000066583 | HGLibA_23682 | 138 | 0.224641206 | ISOC1 | isochorismatase domain containing 1 [Source:HGNC Symbol;Acc:HGNC:24254] |
| ENSG00000071203 | HGLibA_30134 | 138 | 0.224641206 | MS4A12 | membrane spanning 4-domains A12 [Source:HGNC Symbol;Acc:HGNC:13370] |
| ENSG00000072858 | HGLibA_44114 | 138 | 0.224641206 | SIDT1 | SID1 transmembrane family member 1 [Source:HGNC Symbol;Acc:HGNC:25967] |
| ENSG00000074047 | HGLibA_19249 | 138 | 0.224641206 | GLI2 | GLI family zinc finger 2 [Source:HGNC Symbol;Acc:HGNC:4318] |
| ENSG00000074317 | HGLibA_45913 | 138 | 0.224641206 | SNCB | synuclein beta [Source:HGNC Symbol;Acc:HGNC:11140] |
| ENSG00000075035 | HGLibA_54425 | 138 | 0.224641206 | WSCD2 | WSC domain containing 2 [Source:HGNC Symbol;Acc:HGNC:29117] |
| ENSG00000075043 | HGLibA_24368 | 138 | 0.224641206 | KCNQ2 | potassium voltage-gated channel subfamily Q member 2 [Source:HGNC Symbol;Acc:HGNC:6296] |
| ENSG00000075151 | HGLibA_14847 | 138 | 0.224641206 | EIF4G3 | eukaryotic translation initiation factor 4 gamma 3 [Source:HGNC Symbol;Acc:HGNC:3298] |
| ENSG00000079337 | HGLibA_40252 | 138 | 0.224641206 | RAPGEF3 | Rap guanine nucleotide exchange factor 3 [Source:HGNC Symbol;Acc:HGNC:16629] |
| ENSG00000084693 | HGLibA_01178 | 138 | 0.224641206 | AGBL5 | ATP/GTP binding protein like 5 [Source:HGNC Symbol;Acc:HGNC:26147] |
| ENSG00000086991 | HGLibA_32375 | 138 | 0.224641206 | NOX4 | NADPH oxidase 4 [Source:HGNC Symbol;Acc:HGNC:7891] |
| ENSG00000097033 | HGLibA_43956 | 138 | 0.224641206 | SH3GLB1 | endophilin B1 [Source:HGNC Symbol;Acc:HGNC:10833]" |
| ENSG00000100271 | HGLibA_52187 | 138 | 0.224641206 | TTLL1 | tubulin tyrosine ligase like 1 [Source:HGNC Symbol;Acc:HGNC:1312] |
| ENSG00000100902 | HGLibA_39086 | 138 | 0.224641206 | PSMA6 | proteasome subunit alpha 6 [Source:HGNC Symbol;Acc:HGNC:9535] |
| ENSG00000100908 | HGLibA_15025 | 138 | 0.224641206 | EMC9 | ER membrane protein complex subunit 9 [Source:HGNC Symbol;Acc:HGNC:20273] |
| ENSG00000101115 | HGLibA_42657 | 138 | 0.224641206 | SALL4 | spalt like transcription factor 4 [Source:HGNC Symbol;Acc:HGNC:15924] |
| ENSG00000101222 | HGLibA_46609 | 138 | 0.224641206 | SPEF1 | sperm flagellar 1 [Source:HGNC Symbol;Acc:HGNC:15874] |
| ENSG00000102104 | HGLibA_42292 | 138 | 0.224641206 | RS1 | retinoschisin 1 [Source:HGNC Symbol;Acc:HGNC:10457] |
| ENSG00000102189 | HGLibA_14484 | 138 | 0.224641206 | EEA1 | early endosome antigen 1 [Source:HGNC Symbol;Acc:HGNC:3185] |
| ENSG00000105996 | HGLibA_21905 | 138 | 0.224641206 | HOXA2 | homeobox A2 [Source:HGNC Symbol;Acc:HGNC:5103] |
| ENSG00000106327 | HGLibA_49193 | 138 | 0.224641206 | TFR2 | transferrin receptor 2 [Source:HGNC Symbol;Acc:HGNC:11762] |
| ENSG00000109066 | HGLibA_49846 | 138 | 0.224641206 | TMEM104 | transmembrane protein 104 [Source:HGNC Symbol;Acc:HGNC:25984] |
| ENSG00000109099 | HGLibA_37324 | 138 | 0.224641206 | PMP22 | peripheral myelin protein 22 [Source:HGNC Symbol;Acc:HGNC:9118] |
| ENSG00000110321 | HGLibA_14842 | 138 | 0.224641206 | EIF4G2 | eukaryotic translation initiation factor 4 gamma 2 [Source:HGNC Symbol;Acc:HGNC:3297] |
| ENSG00000110628 | HGLibA_44561 | 138 | 0.224641206 | SLC22A18 | solute carrier family 22 member 18 [Source:HGNC Symbol;Acc:HGNC:10964] |
| ENSG00000110911 | HGLibA_44354 | 138 | 0.224641206 | SLC11A2 | solute carrier family 11 member 2 [Source:HGNC Symbol;Acc:HGNC:10908] |
| ENSG00000112378 | HGLibA_36063 | 138 | 0.224641206 | PERP | TP53 apoptosis effector [Source:HGNC Symbol;Acc:HGNC:17637]" |
| ENSG00000113494 | HGLibA_38605 | 138 | 0.224641206 | PRLR | prolactin receptor [Source:HGNC Symbol;Acc:HGNC:9446] |
| ENSG00000114805 | HGLibA_37009 | 138 | 0.224641206 | PLCH1 | phospholipase C eta 1 [Source:HGNC Symbol;Acc:HGNC:29185] |
| ENSG00000116141 | HGLibA_28364 | 138 | 0.224641206 | MARK1 | microtubule affinity regulating kinase 1 [Source:HGNC Symbol;Acc:HGNC:6896] |
| ENSG00000119688 | HGLibA_00199 | 138 | 0.224641206 | ABCD4 | ATP binding cassette subfamily D member 4 [Source:HGNC Symbol;Acc:HGNC:68] |
| ENSG00000121690 | HGLibA_12987 | 138 | 0.224641206 | DEPDC7 | DEP domain containing 7 [Source:HGNC Symbol;Acc:HGNC:29899] |
| ENSG00000121903 | HGLibA_56997 | 138 | 0.224641206 | ZSCAN20 | zinc finger and SCAN domain containing 20 [Source:HGNC Symbol;Acc:HGNC:13093] |
| ENSG00000122543 | HGLibA_33256 | 138 | 0.224641206 | OCM | oncomodulin [Source:HGNC Symbol;Acc:HGNC:8105] |
| ENSG00000125877 | HGLibA_23838 | 138 | 0.224641206 | ITPA | inosine triphosphatase [Source:HGNC Symbol;Acc:HGNC:6176] |
| ENSG00000125970 | HGLibA_40174 | 138 | 0.224641206 | RALY | RALY heterogeneous nuclear ribonucleoprotein [Source:HGNC Symbol;Acc:HGNC:15921] |
| ENSG00000127329 | HGLibA_39486 | 138 | 0.224641206 | PTPRB | receptor type B [Source:HGNC Symbol;Acc:HGNC:9665]" |
| ENSG00000128383 | HGLibA_02472 | 138 | 0.224641206 | APOBEC3A | apolipoprotein B mRNA editing enzyme catalytic subunit 3A [Source:HGNC Symbol;Acc:HGNC:17343] |
| ENSG00000129084 | HGLibA_39070 | 138 | 0.224641206 | PSMA1 | proteasome subunit alpha 1 [Source:HGNC Symbol;Acc:HGNC:9530] |
| ENSG00000129595 | HGLibA_15250 | 138 | 0.224641206 | EPB41L4A | erythrocyte membrane protein band 4.1 like 4A [Source:HGNC Symbol;Acc:HGNC:13278] |
| ENSG00000132874 | HGLibA_44401 | 138 | 0.224641206 | SLC14A2 | solute carrier family 14 member 2 [Source:HGNC Symbol;Acc:HGNC:10919] |
| ENSG00000133574 | HGLibA_19080 | 138 | 0.224641206 | GIMAP4 | IMAP family member 4 [Source:HGNC Symbol;Acc:HGNC:21872]" |
| ENSG00000134339 | HGLibA_42611 | 138 | 0.224641206 | SAA2 | serum amyloid A2 [Source:HGNC Symbol;Acc:HGNC:10514] |
| ENSG00000135374 | HGLibA_14902 | 138 | 0.224641206 | ELF5 | E74 like ETS transcription factor 5 [Source:HGNC Symbol;Acc:HGNC:3320] |
| ENSG00000137693 | HGLibA_54631 | 138 | 0.224641206 | YAP1 | Yes associated protein 1 [Source:HGNC Symbol;Acc:HGNC:16262] |
| ENSG00000137841 | HGLibA_36980 | 138 | 0.224641206 | PLCB2 | phospholipase C beta 2 [Source:HGNC Symbol;Acc:HGNC:9055] |
| ENSG00000138002 | HGLibA_22749 | 138 | 0.224641206 | IFT172 | intraflagellar transport 172 [Source:HGNC Symbol;Acc:HGNC:30391] |
| ENSG00000143322 | HGLibA_00305 | 138 | 0.224641206 | ABL2 | non-receptor tyrosine kinase [Source:HGNC Symbol;Acc:HGNC:77]" |
| ENSG00000143412 | HGLibA_02262 | 138 | 0.224641206 | ANXA9 | annexin A9 [Source:HGNC Symbol;Acc:HGNC:547] |
| ENSG00000143772 | HGLibA_23847 | 138 | 0.224641206 | ITPKB | inositol-trisphosphate 3-kinase B [Source:HGNC Symbol;Acc:HGNC:6179] |
| ENSG00000146215 | HGLibA_11157 | 138 | 0.224641206 | CRIP3 | cysteine rich protein 3 [Source:HGNC Symbol;Acc:HGNC:17751] |
| ENSG00000146383 | HGLibA_48132 | 138 | 0.224641206 | TAAR6 | trace amine associated receptor 6 [Source:HGNC Symbol;Acc:HGNC:20978] |
| ENSG00000146834 | HGLibA_28900 | 138 | 0.224641206 | MEPCE | methylphosphate capping enzyme [Source:HGNC Symbol;Acc:HGNC:20247] |
| ENSG00000148053 | HGLibA_32877 | 138 | 0.224641206 | NTRK2 | neurotrophic receptor tyrosine kinase 2 [Source:HGNC Symbol;Acc:HGNC:8032] |
| ENSG00000150753 | HGLibA_08203 | 138 | 0.224641206 | CCT5 | chaperonin containing TCP1 subunit 5 [Source:HGNC Symbol;Acc:HGNC:1618] |
| ENSG00000151067 | HGLibA_06880 | 138 | 0.224641206 | CACNA1C | calcium voltage-gated channel subunit alpha1 C [Source:HGNC Symbol;Acc:HGNC:1390] |
| ENSG00000151790 | HGLibA_48887 | 138 | 0.224641206 | TDO2 | 3-dioxygenase [Source:HGNC Symbol;Acc:HGNC:11708]" |
| ENSG00000153820 | HGLibA_46648 | 138 | 0.224641206 | SPHKAP | AKAP domain containing [Source:HGNC Symbol;Acc:HGNC:30619]" |
| ENSG00000153976 | HGLibA_22128 | 138 | 0.224641206 | HS3ST3A1 | heparan sulfate-glucosamine 3-sulfotransferase 3A1 [Source:HGNC Symbol;Acc:HGNC:5196] |
| ENSG00000154803 | HGLibA_17605 | 138 | 0.224641206 | FLCN | folliculin [Source:HGNC Symbol;Acc:HGNC:27310] |
| ENSG00000155508 | HGLibA_10352 | 138 | 0.224641206 | CNOT8 | CCR4-NOT transcription complex subunit 8 [Source:HGNC Symbol;Acc:HGNC:9207] |
| ENSG00000156219 | HGLibA_03160 | 138 | 0.224641206 | ART3 | ADP-ribosyltransferase 3 [Source:HGNC Symbol;Acc:HGNC:725] |
| ENSG00000156508 | HGLibA_14490 | 138 | 0.224641206 | EEF1A1 | eukaryotic translation elongation factor 1 alpha 1 [Source:HGNC Symbol;Acc:HGNC:3189] |
| ENSG00000158014 | HGLibA_44903 | 138 | 0.224641206 | SLC30A2 | solute carrier family 30 member 2 [Source:HGNC Symbol;Acc:HGNC:11013] |
| ENSG00000159182 | HGLibA_38264 | 138 | 0.224641206 | PRAC1 | PRAC1 small nuclear protein [Source:HGNC Symbol;Acc:HGNC:30591] |
| ENSG00000160214 | HGLibA_42265 | 138 | 0.224641206 | RRP1 | ribosomal RNA processing 1 [Source:HGNC Symbol;Acc:HGNC:18785] |
| ENSG00000160961 | HGLibA_55818 | 138 | 0.224641206 | ZNF333 | zinc finger protein 333 [Source:HGNC Symbol;Acc:HGNC:15624] |
| ENSG00000163354 | HGLibA_12531 | 138 | 0.224641206 | DCST2 | DC-STAMP domain containing 2 [Source:HGNC Symbol;Acc:HGNC:26562] |
| ENSG00000163660 | HGLibA_08118 | 138 | 0.224641206 | CCNL1 | cyclin L1 [Source:HGNC Symbol;Acc:HGNC:20569] |
| ENSG00000163794 | HGLibA_52823 | 138 | 0.224641206 | UCN | urocortin [Source:HGNC Symbol;Acc:HGNC:12516] |
| ENSG00000163814 | HGLibA_08623 | 138 | 0.224641206 | CDCP1 | CUB domain containing protein 1 [Source:HGNC Symbol;Acc:HGNC:24357] |
| ENSG00000164087 | HGLibA_37441 | 138 | 0.224641206 | POC1A | POC1 centriolar protein A [Source:HGNC Symbol;Acc:HGNC:24488] |
| ENSG00000164893 | HGLibA_45356 | 138 | 0.224641206 | SLC7A13 | solute carrier family 7 member 13 [Source:HGNC Symbol;Acc:HGNC:23092] |
| ENSG00000166847 | HGLibA_12557 | 138 | 0.224641206 | DCTN5 | dynactin subunit 5 [Source:HGNC Symbol;Acc:HGNC:24594] |
| ENSG00000167693 | HGLibA_33142 | 138 | 0.224641206 | NXN | nucleoredoxin [Source:HGNC Symbol;Acc:HGNC:18008] |
| ENSG00000168096 | HGLibA_02157 | 138 | 0.224641206 | ANKS3 | ankyrin repeat and sterile alpha motif domain containing 3 [Source:HGNC Symbol;Acc:HGNC:29422] |
| ENSG00000168268 | HGLibA_32824 | 138 | 0.224641206 | NT5DC2 | 5'-nucleotidase domain containing 2 [Source:HGNC Symbol;Acc:HGNC:25717] |
| ENSG00000168811 | HGLibA_22958 | 138 | 0.224641206 | IL12A | interleukin 12A [Source:HGNC Symbol;Acc:HGNC:5969] |
| ENSG00000168952 | HGLibA_47680 | 138 | 0.224641206 | STXBP6 | syntaxin binding protein 6 [Source:HGNC Symbol;Acc:HGNC:19666] |
| ENSG00000169252 | HGLibA_01058 | 138 | 0.224641206 | ADRB2 | adrenoceptor beta 2 [Source:HGNC Symbol;Acc:HGNC:286] |
| ENSG00000169327 | HGLibA_34253 | 138 | 0.224641206 | OR5AU1 | olfactory receptor family 5 subfamily AU member 1 [Source:HGNC Symbol;Acc:HGNC:15362] |
| ENSG00000170027 | HGLibA_54745 | 138 | 0.224641206 | YWHAG | tyrosine 3-monooxygenase/tryptophan 5-monooxygenase activation protein gamma [Source:HGNC Symbol;Acc:HGNC:12852] |
| ENSG00000170421 | HGLibA_25552 | 138 | 0.224641206 | KRT8 | keratin 8 [Source:HGNC Symbol;Acc:HGNC:6446] |
| ENSG00000171987 | HGLibA_05135 | 138 | 0.224641206 | C11orf40 | chromosome 11 open reading frame 40 [Source:HGNC Symbol;Acc:HGNC:23986] |
| ENSG00000172828 | HGLibA_09225 | 138 | 0.224641206 | CES3 | carboxylesterase 3 [Source:HGNC Symbol;Acc:HGNC:1865] |
| ENSG00000172986 | HGLibA_20684 | 138 | 0.224641206 | GXYLT2 | glucoside xylosyltransferase 2 [Source:HGNC Symbol;Acc:HGNC:33383] |
| ENSG00000173276 | HGLibA_54842 | 138 | 0.224641206 | ZBTB21 | zinc finger and BTB domain containing 21 [Source:HGNC Symbol;Acc:HGNC:13083] |
| ENSG00000173714 | HGLibA_54284 | 138 | 0.224641206 | WFIKKN2 | kunitz and netrin domain containing 2 [Source:HGNC Symbol;Acc:HGNC:30916]" |
| ENSG00000173801 | HGLibA_24013 | 138 | 0.224641206 | JUP | junction plakoglobin [Source:HGNC Symbol;Acc:HGNC:6207] |
| ENSG00000176495 | HGLibA_34242 | 138 | 0.224641206 | OR5AN1 | olfactory receptor family 5 subfamily AN member 1 [Source:HGNC Symbol;Acc:HGNC:15255] |
| ENSG00000179002 | HGLibA_48358 | 138 | 0.224641206 | TAS1R2 | taste 1 receptor member 2 [Source:HGNC Symbol;Acc:HGNC:14905] |
| ENSG00000179930 | HGLibA_56456 | 138 | 0.224641206 | ZNF648 | zinc finger protein 648 [Source:HGNC Symbol;Acc:HGNC:18190] |
| ENSG00000181856 | HGLibA_44876 | 138 | 0.224641206 | SLC2A4 | solute carrier family 2 member 4 [Source:HGNC Symbol;Acc:HGNC:11009] |
| ENSG00000182631 | HGLibA_42501 | 138 | 0.224641206 | RXFP3 | relaxin family peptide receptor 3 [Source:HGNC Symbol;Acc:HGNC:24883] |
| ENSG00000183134 | HGLibA_39307 | 138 | 0.224641206 | PTGDR2 | prostaglandin D2 receptor 2 [Source:HGNC Symbol;Acc:HGNC:4502] |
| ENSG00000184347 | HGLibA_45510 | 138 | 0.224641206 | SLIT3 | slit guidance ligand 3 [Source:HGNC Symbol;Acc:HGNC:11087] |
| ENSG00000185105 | HGLibA_30633 | 138 | 0.224641206 | MYADML2 | myeloid associated differentiation marker like 2 [Source:HGNC Symbol;Acc:HGNC:34548] |
| ENSG00000185155 | HGLibA_29320 | 138 | 0.224641206 | MIXL1 | Mix paired-like homeobox [Source:HGNC Symbol;Acc:HGNC:13363] |
| ENSG00000186026 | HGLibA_55744 | 138 | 0.224641206 | ZNF284 | zinc finger protein 284 [Source:HGNC Symbol;Acc:HGNC:13078] |
| ENSG00000186526 | HGLibA_12197 | 138 | 0.224641206 | CYP4F8 | cytochrome P450 family 4 subfamily F member 8 [Source:HGNC Symbol;Acc:HGNC:2648] |
| ENSG00000186881 | HGLibA_33618 | 138 | 0.224641206 | OR13F1 | olfactory receptor family 13 subfamily F member 1 [Source:HGNC Symbol;Acc:HGNC:14723] |
| ENSG00000187609 | HGLibA_15722 | 138 | 0.224641206 | EXD3 | exonuclease 3'-5' domain containing 3 [Source:HGNC Symbol;Acc:HGNC:26023] |
| ENSG00000187950 | HGLibA_34793 | 138 | 0.224641206 | OVCH1 | ovochymase 1 [Source:HGNC Symbol;Acc:HGNC:23080] |
| ENSG00000188603 | HGLibA_10076 | 138 | 0.224641206 | CLN3 | battenin [Source:HGNC Symbol;Acc:HGNC:2074]" |
| ENSG00000189023 | HGLibA_27936 | 138 | 0.224641206 | MAGEB16 | MAGE family member B16 [Source:HGNC Symbol;Acc:HGNC:21188] |
| ENSG00000196396 | HGLibA_39431 | 138 | 0.224641206 | PTPN1 | non-receptor type 1 [Source:HGNC Symbol;Acc:HGNC:9642]" |
| ENSG00000196782 | HGLibA_28049 | 138 | 0.224641206 | MAML3 | mastermind like transcriptional coactivator 3 [Source:HGNC Symbol;Acc:HGNC:16272] |
| ENSG00000197353 | HGLibA_27717 | 138 | 0.224641206 | LYPD2 | LY6/PLAUR domain containing 2 [Source:HGNC Symbol;Acc:HGNC:25215] |
| ENSG00000197601 | HGLibA_16867 | 138 | 0.224641206 | FAR1 | fatty acyl-CoA reductase 1 [Source:HGNC Symbol;Acc:HGNC:26222] |
| ENSG00000212122 | HGLibA_52008 | 138 | 0.224641206 | TSSK1B | testis specific serine kinase 1B [Source:HGNC Symbol;Acc:HGNC:14968] |
| ENSG00000213316 | HGLibA_27605 | 138 | 0.224641206 | LTC4S | leukotriene C4 synthase [Source:HGNC Symbol;Acc:HGNC:6719] |
| ENSG00000213983 | HGLibA_02287 | 138 | 0.224641206 | AP1G2 | adaptor related protein complex 1 subunit gamma 2 [Source:HGNC Symbol;Acc:HGNC:556] |
| ENSG00000219200 | HGLibA_41437 | 138 | 0.224641206 | RNASEK | ribonuclease K [Source:HGNC Symbol;Acc:HGNC:33911] |
| ENSG00000222038 | HGLibA_37743 | 138 | 0.224641206 | POTEJ | POTE ankyrin domain family member J [Source:HGNC Symbol;Acc:HGNC:37094] |
| ENSG00000228075 | HGLibA_04674 | 138 | 0.224641206 | BOD1L2 | biorientation of chromosomes in cell division 1 like 2 [Source:HGNC Symbol;Acc:HGNC:28505] |
| ENSG00000240972 | HGLibA_29261 | 138 | 0.224641206 | MIF | macrophage migration inhibitory factor [Source:HGNC Symbol;Acc:HGNC:7097] |
| ENSG00000241563 | HGLibA_10797 | 138 | 0.224641206 | CORT | cortistatin [Source:HGNC Symbol;Acc:HGNC:2257] |
| ENSG00000247746 | HGLibA_53396 | 138 | 0.224641206 | USP51 | ubiquitin specific peptidase 51 [Source:HGNC Symbol;Acc:HGNC:23086] |

| | | | | | |
|---|---|---|---|---|---|
| ENSG00000254709 | HGLibA_22870 | 138 | 0.224641206 | IGLL5 | immunoglobulin lambda like polypeptide 5 [Source:HGNC Symbol;Acc:HGNC:38476] |
| ENSG00000279761 | HGLibA_34275 | 138 | 0.224641206 | OR5D13 | olfactory receptor family 5 subfamily D member 13 (gene/pseudogene) [Source:HGNC Symbol;Acc:HGNC:15280] |
| GTF2H2D | HGLibA_20554 | 138 | 0.224641206 | GTF2H2D | - |
| hsa-mir-153-2 | HGLibA_57912 | 138 | 0.224641206 | hsa-mir-153-2 | - |
| hsa-mir-3191 | HGLibA_58861 | 138 | 0.224641206 | hsa-mir-3191 | - |
| hsa-mir-3192 | HGLibA_58866 | 138 | 0.224641206 | hsa-mir-3192 | - |
| hsa-mir-3945 | HGLibA_59639 | 138 | 0.224641206 | hsa-mir-3945 | - |
| hsa-mir-4418 | HGLibA_60057 | 138 | 0.224641206 | hsa-mir-4418 | - |
| hsa-mir-4497 | HGLibA_60405 | 138 | 0.224641206 | hsa-mir-4497 | - |
| hsa-mir-4655 | HGLibA_60728 | 138 | 0.224641206 | hsa-mir-4655 | - |
| hsa-mir-4661 | HGLibA_60753 | 138 | 0.224641206 | hsa-mir-4661 | - |
| hsa-mir-532 | HGLibA_61808 | 138 | 0.224641206 | hsa-mir-532 | - |
| hsa-mir-548ag-1 | HGLibA_61876 | 138 | 0.224641206 | hsa-mir-548ag-1 | - |
| hsa-mir-548h-4 | HGLibA_62005 | 138 | 0.224641206 | hsa-mir-548h-4 | - |
| hsa-mir-5680 | HGLibA_62234 | 138 | 0.224641206 | hsa-mir-5680 | - |
| hsa-mir-6765 | HGLibA_63223 | 138 | 0.224641206 | hsa-mir-6765 | - |
| hsa-mir-6767 | HGLibA_63229 | 138 | 0.224641206 | hsa-mir-6767 | - |
| hsa-mir-6786 | HGLibA_63322 | 138 | 0.224641206 | hsa-mir-6786 | - |
| hsa-mir-6862-2 | HGLibA_63633 | 138 | 0.224641206 | hsa-mir-6862-2 | - |
| hsa-mir-8079 | HGLibA_64160 | 138 | 0.224641206 | hsa-mir-8079 | - |
| KIAA1211L | HGLibA_24719 | 138 | 0.224641206 | KIAA1211L | - |
| LRRC16A | HGLibA_27264 | 138 | 0.224641206 | LRRC16A | - |
| MLLT4 | HGLibA_29420 | 138 | 0.224641206 | MLLT4 | - |
| SHOX_X | HGLibA_44069 | 138 | 0.224641206 | SHOX_X | - |
| C10orf12 | HGLibA_05043 | 137 | 0.214690168 | C10orf12 | - |
| C12orf66 | HGLibA_05282 | 137 | 0.214690168 | C12orf66 | - |
| C5orf20 | HGLibA_06363 | 137 | 0.214690168 | C5orf20 | - |
| CCDC37 | HGLibA_07766 | 137 | 0.214690168 | CCDC37 | - |
| ENSG00000012779 | HGLibA_01687 | 137 | 0.214690168 | ALOX5 | arachidonate 5-lipoxygenase [Source:HGNC Symbol;Acc:HGNC:435] |
| ENSG00000044459 | HGLibA_10399 | 137 | 0.214690168 | CNTLN | centlein [Source:HGNC Symbol;Acc:HGNC:23432] |
| ENSG00000059758 | HGLibA_08746 | 137 | 0.214690168 | CDK17 | cyclin dependent kinase 17 [Source:HGNC Symbol;Acc:HGNC:8750] |
| ENSG00000063046 | HGLibA_14813 | 137 | 0.214690168 | EIF4B | eukaryotic translation initiation factor 4B [Source:HGNC Symbol;Acc:HGNC:3285] |
| ENSG00000065413 | HGLibA_02102 | 137 | 0.214690168 | ANKRD44 | ankyrin repeat domain 44 [Source:HGNC Symbol;Acc:HGNC:25259] |
| ENSG00000070193 | HGLibA_17362 | 137 | 0.214690168 | FGF10 | fibroblast growth factor 10 [Source:HGNC Symbol;Acc:HGNC:3666] |
| ENSG00000071909 | HGLibA_30847 | 137 | 0.214690168 | MYO3B | myosin IIIB [Source:HGNC Symbol;Acc:HGNC:15576] |
| ENSG00000072657 | HGLibA_51380 | 137 | 0.214690168 | TRHDE | thyrotropin releasing hormone degrading enzyme [Source:HGNC Symbol;Acc:HGNC:30748] |
| ENSG00000077585 | HGLibA_19899 | 137 | 0.214690168 | GPR137B | G protein-coupled receptor 137B [Source:HGNC Symbol;Acc:HGNC:11862] |
| ENSG00000078902 | HGLibA_50942 | 137 | 0.214690168 | TOLLIP | toll interacting protein [Source:HGNC Symbol;Acc:HGNC:16476] |
| ENSG00000082458 | HGLibA_13331 | 137 | 0.214690168 | DLG3 | discs large MAGUK scaffold protein 3 [Source:HGNC Symbol;Acc:HGNC:2902] |
| ENSG00000086758 | HGLibA_22430 | 137 | 0.214690168 | HUWE1 | E3 ubiquitin protein ligase [Source:HGNC Symbol;Acc:HGNC:30892]" |
| ENSG00000090612 | HGLibA_55705 | 137 | 0.214690168 | ZNF268 | zinc finger protein 268 [Source:HGNC Symbol;Acc:HGNC:13061] |
| ENSG00000102531 | HGLibA_17748 | 137 | 0.214690168 | FNDC3A | fibronectin type III domain containing 3A [Source:HGNC Symbol;Acc:HGNC:20296] |
| ENSG00000103042 | HGLibA_45080 | 137 | 0.214690168 | SLC38A7 | solute carrier family 38 member 7 [Source:HGNC Symbol;Acc:HGNC:25582] |
| ENSG00000103647 | HGLibA_10785 | 137 | 0.214690168 | CORO2B | coronin 2B [Source:HGNC Symbol;Acc:HGNC:2256] |
| ENSG00000105519 | HGLibA_07196 | 137 | 0.214690168 | CAPS | calcyphosine [Source:HGNC Symbol;Acc:HGNC:1487] |
| ENSG00000107099 | HGLibA_13758 | 137 | 0.214690168 | DOCK8 | dedicator of cytokinesis 8 [Source:HGNC Symbol;Acc:HGNC:19191] |
| ENSG00000109065 | HGLibA_31190 | 137 | 0.214690168 | NAT9 | N-acetyltransferase 9 (putative) [Source:HGNC Symbol;Acc:HGNC:23133] |
| ENSG00000109686 | HGLibA_43940 | 137 | 0.214690168 | SH3D19 | SH3 domain containing 19 [Source:HGNC Symbol;Acc:HGNC:30418] |
| ENSG00000110092 | HGLibA_08071 | 137 | 0.214690168 | CCND1 | cyclin D1 [Source:HGNC Symbol;Acc:HGNC:1582] |
| ENSG00000110218 | HGLibA_35110 | 137 | 0.214690168 | PANX1 | pannexin 1 [Source:HGNC Symbol;Acc:HGNC:8599] |
| ENSG00000111328 | HGLibA_08762 | 137 | 0.214690168 | CDK2AP1 | cyclin dependent kinase 2 associated protein 1 [Source:HGNC Symbol;Acc:HGNC:14002] |
| ENSG00000112486 | HGLibA_08163 | 137 | 0.214690168 | CCR6 | C-C motif chemokine receptor 6 [Source:HGNC Symbol;Acc:HGNC:1607] |
| ENSG00000112576 | HGLibA_08078 | 137 | 0.214690168 | CCND3 | cyclin D3 [Source:HGNC Symbol;Acc:HGNC:1585] |
| ENSG00000116213 | HGLibA_54404 | 137 | 0.214690168 | WRAP73 | antisense to TP73 [Source:HGNC Symbol;Acc:HGNC:12759]" |
| ENSG00000116906 | HGLibA_19561 | 137 | 0.214690168 | GNPAT | glyceronephosphate O-acyltransferase [Source:HGNC Symbol;Acc:HGNC:4416] |
| ENSG00000118557 | HGLibA_37309 | 137 | 0.214690168 | PMFBP1 | polyamine modulated factor 1 binding protein 1 [Source:HGNC Symbol;Acc:HGNC:17728] |
| ENSG00000119547 | HGLibA_33394 | 137 | 0.214690168 | ONECUT2 | one cut homeobox 2 [Source:HGNC Symbol;Acc:HGNC:8139] |
| ENSG00000120332 | HGLibA_50863 | 137 | 0.214690168 | TNN | tenascin N [Source:HGNC Symbol;Acc:HGNC:22942] |
| ENSG00000120526 | HGLibA_32923 | 137 | 0.214690168 | NUDCD1 | NudC domain containing 1 [Source:HGNC Symbol;Acc:HGNC:24306] |
| ENSG00000122257 | HGLibA_40433 | 137 | 0.214690168 | RBBP6 | ubiquitin ligase [Source:HGNC Symbol;Acc:HGNC:9889]" |
| ENSG00000122335 | HGLibA_43463 | 137 | 0.214690168 | SERAC1 | serine active site containing 1 [Source:HGNC Symbol;Acc:HGNC:21061] |
| ENSG00000123358 | HGLibA_32625 | 137 | 0.214690168 | NR4A1 | nuclear receptor subfamily 4 group A member 1 [Source:HGNC Symbol;Acc:HGNC:7980] |
| ENSG00000124786 | HGLibA_44964 | 137 | 0.214690168 | SLC35B3 | solute carrier family 35 member B3 [Source:HGNC Symbol;Acc:HGNC:21601] |
| ENSG00000125910 | HGLibA_42603 | 137 | 0.214690168 | S1PR4 | sphingosine-1-phosphate receptor 4 [Source:HGNC Symbol;Acc:HGNC:3170] |
| ENSG00000127241 | HGLibA_28393 | 137 | 0.214690168 | MASP1 | mannan binding lectin serine peptidase 1 [Source:HGNC Symbol;Acc:HGNC:6901] |
| ENSG00000127314 | HGLibA_40224 | 137 | 0.214690168 | RAP1B | member of RAS oncogene family [Source:HGNC Symbol;Acc:HGNC:9857]" |
| ENSG00000129152 | HGLibA_30879 | 137 | 0.214690168 | MYOD1 | myogenic differentiation 1 [Source:HGNC Symbol;Acc:HGNC:7611] |
| ENSG00000131408 | HGLibA_32580 | 137 | 0.214690168 | NR1H2 | nuclear receptor subfamily 1 group H member 2 [Source:HGNC Symbol;Acc:HGNC:7965] |
| ENSG00000131724 | HGLibA_22975 | 137 | 0.214690168 | IL13RA1 | interleukin 13 receptor subunit alpha 1 [Source:HGNC Symbol;Acc:HGNC:5974] |
| ENSG00000132155 | HGLibA_40130 | 137 | 0.214690168 | RAF1 | serine/threonine kinase [Source:HGNC Symbol;Acc:HGNC:9829]" |
| ENSG00000133119 | HGLibA_40907 | 137 | 0.214690168 | RFC3 | replication factor C subunit 3 [Source:HGNC Symbol;Acc:HGNC:9971] |
| ENSG00000136425 | HGLibA_09684 | 137 | 0.214690168 | CIB2 | calcium and integrin binding family member 2 [Source:HGNC Symbol;Acc:HGNC:24579] |
| ENSG00000137251 | HGLibA_49547 | 137 | 0.214690168 | TINAG | tubulointerstitial nephritis antigen [Source:HGNC Symbol;Acc:HGNC:14599] |
| ENSG00000137948 | HGLibA_04792 | 137 | 0.214690168 | BRDT | bromodomain testis associated [Source:HGNC Symbol;Acc:HGNC:1105] |
| ENSG00000138375 | HGLibA_45616 | 137 | 0.214690168 | SMARCAL1 | subfamily a like 1 [Source:HGNC Symbol;Acc:HGNC:11102]" |
| ENSG00000138398 | HGLibA_37934 | 137 | 0.214690168 | PPIG | peptidylprolyl isomerase G [Source:HGNC Symbol;Acc:HGNC:14650] |
| ENSG00000138780 | HGLibA_20467 | 137 | 0.214690168 | GSTCD | glutathione S-transferase C-terminal domain containing [Source:HGNC Symbol;Acc:HGNC:25806] |
| ENSG00000139174 | HGLibA_38469 | 137 | 0.214690168 | PRICKLE1 | prickle planar cell polarity protein 1 [Source:HGNC Symbol;Acc:HGNC:17019] |
| ENSG00000139714 | HGLibA_29673 | 137 | 0.214690168 | MORN3 | MORN repeat containing 3 [Source:HGNC Symbol;Acc:HGNC:29807] |
| ENSG00000140876 | HGLibA_32988 | 137 | 0.214690168 | NUDT7 | nudix hydrolase 7 [Source:HGNC Symbol;Acc:HGNC:8054] |
| ENSG00000142528 | HGLibA_56059 | 137 | 0.214690168 | ZNF473 | zinc finger protein 473 [Source:HGNC Symbol;Acc:HGNC:23239] |
| ENSG00000144824 | HGLibA_36428 | 137 | 0.214690168 | PHLDB2 | pleckstrin homology like domain family B member 2 [Source:HGNC Symbol;Acc:HGNC:29573] |
| ENSG00000144834 | HGLibA_48276 | 137 | 0.214690168 | TAGLN3 | transgelin 3 [Source:HGNC Symbol;Acc:HGNC:29868] |
| ENSG00000145217 | HGLibA_44781 | 137 | 0.214690168 | SLC26A1 | solute carrier family 26 member 1 [Source:HGNC Symbol;Acc:HGNC:10993] |
| ENSG00000145384 | HGLibA_15912 | 137 | 0.214690168 | FABP2 | fatty acid binding protein 2 [Source:HGNC Symbol;Acc:HGNC:3556] |
| ENSG00000148572 | HGLibA_32651 | 137 | 0.214690168 | NRBF2 | nuclear receptor binding factor 2 [Source:HGNC Symbol;Acc:HGNC:19692] |
| ENSG00000148798 | HGLibA_23269 | 137 | 0.214690168 | INA | internexin neuronal intermediate filament protein alpha [Source:HGNC Symbol;Acc:HGNC:6057] |
| ENSG00000148908 | HGLibA_41051 | 137 | 0.214690168 | RGS10 | regulator of G protein signaling 10 [Source:HGNC Symbol;Acc:HGNC:9992] |
| ENSG00000149428 | HGLibA_22460 | 137 | 0.214690168 | HYOU1 | hypoxia up-regulated 1 [Source:HGNC Symbol;Acc:HGNC:16931] |
| ENSG00000149452 | HGLibA_44599 | 137 | 0.214690168 | SLC22A8 | solute carrier family 22 member 8 [Source:HGNC Symbol;Acc:HGNC:10972] |
| ENSG00000149532 | HGLibA_11016 | 137 | 0.214690168 | CPSF7 | cleavage and polyadenylation specific factor 7 [Source:HGNC Symbol;Acc:HGNC:30098] |
| ENSG00000149633 | HGLibA_24817 | 137 | 0.214690168 | KIAA1755 | KIAA1755 [Source:HGNC Symbol;Acc:HGNC:29372] |
| ENSG00000149970 | HGLibA_10300 | 137 | 0.214690168 | CNKSR2 | connector enhancer of kinase suppressor of Ras 2 [Source:HGNC Symbol;Acc:HGNC:19701] |
| ENSG00000150054 | HGLibA_29766 | 137 | 0.214690168 | MPP7 | membrane palmitoylated protein 7 [Source:HGNC Symbol;Acc:HGNC:26542] |
| ENSG00000150667 | HGLibA_18091 | 137 | 0.214690168 | FSIP1 | fibrous sheath interacting protein 1 [Source:HGNC Symbol;Acc:HGNC:21674] |
| ENSG00000151893 | HGLibA_06957 | 137 | 0.214690168 | CACUL1 | CDK2 associated cullin domain 1 [Source:HGNC Symbol;Acc:HGNC:23727] |
| ENSG00000153721 | HGLibA_10303 | 137 | 0.214690168 | CNKSR3 | CNKSR family member 3 [Source:HGNC Symbol;Acc:HGNC:23034] |
| ENSG00000154122 | HGLibA_01961 | 137 | 0.214690168 | ANKH | ANKH inorganic pyrophosphate transport regulator [Source:HGNC Symbol;Acc:HGNC:15492] |
| ENSG00000154127 | HGLibA_52561 | 137 | 0.214690168 | UBASH3B | ubiquitin associated and SH3 domain containing B [Source:HGNC Symbol;Acc:HGNC:29884] |
| ENSG00000155849 | HGLibA_14931 | 137 | 0.214690168 | ELMO1 | engulfment and cell motility 1 [Source:HGNC Symbol;Acc:HGNC:16286] |
| ENSG00000156222 | HGLibA_44831 | 137 | 0.214690168 | SLC28A1 | solute carrier family 28 member 1 [Source:HGNC Symbol;Acc:HGNC:11001] |
| ENSG00000158966 | HGLibA_06871 | 137 | 0.214690168 | CACHD1 | cache domain containing 1 [Source:HGNC Symbol;Acc:HGNC:29314] |
| ENSG00000160216 | HGLibA_01222 | 137 | 0.214690168 | AGPAT3 | 1-acylglycerol-3-phosphate O-acyltransferase 3 [Source:HGNC Symbol;Acc:HGNC:326] |
| ENSG00000163171 | HGLibA_08572 | 137 | 0.214690168 | CDC42EP3 | CDC42 effector protein 3 [Source:HGNC Symbol;Acc:HGNC:16943] |
| ENSG00000163491 | HGLibA_31642 | 137 | 0.214690168 | NEK10 | NIMA related kinase 10 [Source:HGNC Symbol;Acc:HGNC:18592] |
| ENSG00000163510 | HGLibA_11815 | 137 | 0.214690168 | CWC22 | CWC22 spliceosome associated protein homolog [Source:HGNC Symbol;Acc:HGNC:29322] |
| ENSG00000165501 | HGLibA_27242 | 137 | 0.214690168 | LRR1 | leucine rich repeat protein 1 [Source:HGNC Symbol;Acc:HGNC:19742] |
| ENSG00000166035 | HGLibA_26602 | 137 | 0.214690168 | LIPC | hepatic type [Source:HGNC Symbol;Acc:HGNC:6619]" |
| ENSG00000166317 | HGLibA_48023 | 137 | 0.214690168 | SYNPO2L | synaptopodin 2 like [Source:HGNC Symbol;Acc:HGNC:23532] |
| ENSG00000167193 | HGLibA_11179 | 137 | 0.214690168 | CRK | adaptor protein [Source:HGNC Symbol;Acc:HGNC:2362]" |
| ENSG00000167644 | HGLibA_05647 | 137 | 0.214690168 | C19orf33 | chromosome 19 open reading frame 33 [Source:HGNC Symbol;Acc:HGNC:16668] |
| ENSG00000168925 | HGLibA_11678 | 137 | 0.214690168 | CTRB1 | chymotrypsinogen B1 [Source:HGNC Symbol;Acc:HGNC:2521] |
| ENSG00000169288 | HGLibA_29887 | 137 | 0.214690168 | MRPL1 | mitochondrial ribosomal protein L1 [Source:HGNC Symbol;Acc:HGNC:14275] |
| ENSG00000169756 | HGLibA_26545 | 137 | 0.214690168 | LIMS1 | LIM zinc finger domain containing 1 [Source:HGNC Symbol;Acc:HGNC:6616] |
| ENSG00000171557 | HGLibA_17455 | 137 | 0.214690168 | FGG | fibrinogen gamma chain [Source:HGNC Symbol;Acc:HGNC:3694] |
| ENSG00000172288 | HGLibA_08905 | 137 | 0.214690168 | CDY1 | chromodomain Y-linked 1 [Source:HGNC Symbol;Acc:HGNC:1809] |
| ENSG00000172345 | HGLibA_47365 | 137 | 0.214690168 | STARD5 | StAR related lipid transfer domain containing 5 [Source:HGNC Symbol;Acc:HGNC:18065] |
| ENSG00000173171 | HGLibA_30512 | 137 | 0.214690168 | MTX1 | metaxin 1 [Source:HGNC Symbol;Acc:HGNC:7504] |
| ENSG00000173239 | HGLibA_26626 | 137 | 0.214690168 | LIPM | lipase family member M [Source:HGNC Symbol;Acc:HGNC:23455] |
| ENSG00000173786 | HGLibA_10357 | 137 | 0.214690168 | CNP | 3'-cyclic nucleotide 3' phosphodiesterase [Source:HGNC Symbol;Acc:HGNC:2158]" |
| ENSG00000175220 | HGLibA_02697 | 137 | 0.214690168 | ARHGAP1 | Rho GTPase activating protein 1 [Source:HGNC Symbol;Acc:HGNC:673] |
| ENSG00000175550 | HGLibA_13951 | 137 | 0.214690168 | DRAP1 | DR1 associated protein 1 [Source:HGNC Symbol;Acc:HGNC:3019] |
| ENSG00000175787 | HGLibA_55520 | 137 | 0.214690168 | ZNF169 | zinc finger protein 169 [Source:HGNC Symbol;Acc:HGNC:12957] |
| ENSG00000176125 | HGLibA_52856 | 137 | 0.214690168 | UFSP1 | UFM1 specific peptidase 1 (inactive) [Source:HGNC Symbol;Acc:HGNC:33821] |
| ENSG00000179588 | HGLibA_55253 | 137 | 0.214690168 | ZFPM1 | FOG family member 1 [Source:HGNC Symbol;Acc:HGNC:19762]" |
| ENSG00000179636 | HGLibA_51169 | 137 | 0.214690168 | TPPP2 | tubulin polymerization promoting protein family member 2 [Source:HGNC Symbol;Acc:HGNC:19293] |
| ENSG00000180386 | HGLibA_25844 | 137 | 0.214690168 | KRTAP9-7 | keratin associated protein 9-7 [Source:HGNC Symbol;Acc:HGNC:18915] |
| ENSG00000180537 | HGLibA_41586 | 137 | 0.214690168 | RNF182 | ring finger protein 182 [Source:HGNC Symbol;Acc:HGNC:28522] |
| ENSG00000181852 | HGLibA_41682 | 137 | 0.214690168 | RNF41 | ring finger protein 41 [Source:HGNC Symbol;Acc:HGNC:18401] |
| ENSG00000182326 | HGLibA_05974 | 137 | 0.214690168 | C1S | complement C1s [Source:HGNC Symbol;Acc:HGNC:1247] |
| ENSG00000183605 | HGLibA_43789 | 137 | 0.214690168 | SFXN4 | sideroflexin 4 [Source:HGNC Symbol;Acc:HGNC:16088] |
| ENSG00000183856 | HGLibA_23539 | 137 | 0.214690168 | IQGAP3 | IQ motif containing GTPase activating protein 3 [Source:HGNC Symbol;Acc:HGNC:20669] |
| ENSG00000184113 | HGLibA_09901 | 137 | 0.214690168 | CLDN5 | claudin 5 [Source:HGNC Symbol;Acc:HGNC:2047] |
| ENSG00000184156 | HGLibA_24372 | 137 | 0.214690168 | KCNQ3 | potassium voltage-gated channel subfamily Q member 3 [Source:HGNC Symbol;Acc:HGNC:6297] |
| ENSG00000184205 | HGLibA_51982 | 137 | 0.214690168 | TSPYL2 | TSPY like 2 [Source:HGNC Symbol;Acc:HGNC:24358] |

| | | | | | |
|---|---|---|---|---|---|
| ENSG00000184724 | HGLibA_25816 | 137 | 0.214690168 | KRTAP6-1 | keratin associated protein 6-1 [Source:HGNC Symbol;Acc:HGNC:18931] |
| ENSG00000186111 | HGLibA_36720 | 137 | 0.214690168 | PIP5K1C | phosphatidylinositol-4-phosphate 5-kinase type 1 gamma [Source:HGNC Symbol;Acc:HGNC:8996] |
| ENSG00000186190 | HGLibA_04724 | 137 | 0.214690168 | BPIFB3 | BPI fold containing family B member 3 [Source:HGNC Symbol;Acc:HGNC:16178] |
| ENSG00000186283 | HGLibA_51030 | 137 | 0.214690168 | TOR3A | torsin family 3 member A [Source:HGNC Symbol;Acc:HGNC:11997] |
| ENSG00000196218 | HGLibA_42521 | 137 | 0.214690168 | RYR1 | ryanodine receptor 1 [Source:HGNC Symbol;Acc:HGNC:10483] |
| ENSG00000203783 | HGLibA_38853 | 137 | 0.214690168 | PRR9 | proline rich 9 [Source:HGNC Symbol;Acc:HGNC:32057] |
| ENSG00000204147 | HGLibA_03192 | 137 | 0.214690168 | ASAH2B | N-acylsphingosine amidohydrolase 2B [Source:HGNC Symbol;Acc:HGNC:23456] |
| ENSG00000204439 | HGLibA_06489 | 137 | 0.214690168 | C6orf47 | chromosome 6 open reading frame 47 [Source:HGNC Symbol;Acc:HGNC:19076] |
| ENSG00000213694 | HGLibA_42598 | 137 | 0.214690168 | S1PR3 | sphingosine-1-phosphate receptor 3 [Source:HGNC Symbol;Acc:HGNC:3167] |
| ENSG00000221914 | HGLibA_38146 | 137 | 0.214690168 | PPP2R2A | protein phosphatase 2 regulatory subunit Balpha [Source:HGNC Symbol;Acc:HGNC:9304] |
| ENSG00000228741 | HGLibA_46428 | 137 | 0.214690168 | SPATA13 | spermatogenesis associated 13 [Source:NCBI gene;Acc:221178] |
| ENSG00000241635 | HGLibA_52875 | 137 | 0.214690168 | UGT1A1 | UDP glucuronosyltransferase family 1 member A1 [Source:HGNC Symbol;Acc:HGNC:12530] |
| ENSG00000256463 | HGLibA_42652 | 137 | 0.214690168 | SALL3 | spalt like transcription factor 3 [Source:HGNC Symbol;Acc:HGNC:10527] |
| ENSG00000257335 | HGLibA_29125 | 137 | 0.214690168 | MGAM | maltase-glucoamylase [Source:HGNC Symbol;Acc:HGNC:7043] |
| ENSG00000263155 | HGLibA_30931 | 137 | 0.214690168 | MYZAP | myocardial zonula adherens protein [Source:HGNC Symbol;Acc:HGNC:43444] |
| ENSG00000270231 | HGLibA_31241 | 137 | 0.214690168 | NBPF8 | NBPF member 8 [Source:HGNC Symbol;Acc:HGNC:31990] |
| ENSG00000273079 | HGLibA_20301 | 137 | 0.214690168 | GRIN2B | glutamate ionotropic receptor NMDA type subunit 2B [Source:HGNC Symbol;Acc:HGNC:4586] |
| ENSG00000275395 | HGLibA_17179 | 137 | 0.214690168 | FCGBP | Fc fragment of IgG binding protein [Source:HGNC Symbol;Acc:HGNC:13572] |
| ENSG00000288705 | HGLibA_52887 | 137 | 0.214690168 | UGT1A5 | - |
| FAM211B | HGLibA_16462 | 137 | 0.214690168 | FAM211B | - |
| GAGE5 | HGLibA_18481 | 137 | 0.214690168 | GAGE5 | - |
| hsa-mir-124-3 | HGLibA_57353 | 137 | 0.214690168 | hsa-mir-124-3 | - |
| hsa-mir-1254-2 | HGLibA_57416 | 137 | 0.214690168 | hsa-mir-1254-2 | - |
| hsa-mir-1302-1 | HGLibA_57683 | 137 | 0.214690168 | hsa-mir-1302-1 | - |
| hsa-mir-194-1 | HGLibA_58082 | 137 | 0.214690168 | hsa-mir-194-1 | - |
| hsa-mir-2276 | HGLibA_58310 | 137 | 0.214690168 | hsa-mir-2276 | - |
| hsa-mir-3152 | HGLibA_58667 | 137 | 0.214690168 | hsa-mir-3152 | - |
| hsa-mir-3912 | HGLibA_59515 | 137 | 0.214690168 | hsa-mir-3912 | - |
| hsa-mir-3916 | HGLibA_59539 | 137 | 0.214690168 | hsa-mir-3916 | - |
| hsa-mir-410 | HGLibA_59680 | 137 | 0.214690168 | hsa-mir-410 | - |
| hsa-mir-4643 | HGLibA_60677 | 137 | 0.214690168 | hsa-mir-4643 | - |
| hsa-mir-4781 | HGLibA_61231 | 137 | 0.214690168 | hsa-mir-4781 | - |
| hsa-mir-4786 | HGLibA_61252 | 137 | 0.214690168 | hsa-mir-4786 | - |
| hsa-mir-5093 | HGLibA_61531 | 137 | 0.214690168 | hsa-mir-5093 | - |
| hsa-mir-548az | HGLibA_61947 | 137 | 0.214690168 | hsa-mir-548az | - |
| hsa-mir-668 | HGLibA_63007 | 137 | 0.214690168 | hsa-mir-668 | - |
| hsa-mir-6804 | HGLibA_63395 | 137 | 0.214690168 | hsa-mir-6804 | - |
| hsa-mir-6824 | HGLibA_63474 | 137 | 0.214690168 | hsa-mir-6824 | - |
| hsa-mir-6850 | HGLibA_63573 | 137 | 0.214690168 | hsa-mir-6850 | - |
| hsa-mir-6869 | HGLibA_63661 | 137 | 0.214690168 | hsa-mir-6869 | - |
| hsa-mir-6887 | HGLibA_63734 | 137 | 0.214690168 | hsa-mir-6887 | - |
| hsa-mir-7107 | HGLibA_63788 | 137 | 0.214690168 | hsa-mir-7107 | - |
| hsa-mir-7151 | HGLibA_63831 | 137 | 0.214690168 | hsa-mir-7151 | - |
| hsa-mir-8066 | HGLibA_64104 | 137 | 0.214690168 | hsa-mir-8066 | - |
| KIAA2022 | HGLibA_24844 | 137 | 0.214690168 | KIAA2022 | - |
| WDR65 | HGLibA_54150 | 137 | 0.214690168 | WDR65 | - |
| C2orf40 | HGLibA_06121 | 136 | 0.204739131 | C2orf40 | - |
| C3orf67 | HGLibA_06261 | 136 | 0.204739131 | C3orf67 | - |
| C7orf69 | HGLibA_06580 | 136 | 0.204739131 | C7orf69 | - |
| C9orf91 | HGLibA_06776 | 136 | 0.204739131 | C9orf91 | - |
| ENSG00000005483 | HGLibA_25333 | 136 | 0.204739131 | KMT2E | lysine methyltransferase 2E [Source:HGNC Symbol;Acc:HGNC:18541] |
| ENSG00000006638 | HGLibA_48681 | 136 | 0.204739131 | TBXA2R | thromboxane A2 receptor [Source:HGNC Symbol;Acc:HGNC:11608] |
| ENSG00000009844 | HGLibA_53881 | 136 | 0.204739131 | VTA1 | vesicle trafficking 1 [Source:HGNC Symbol;Acc:HGNC:20954] |
| ENSG00000042286 | HGLibA_01339 | 136 | 0.204739131 | AIFM2 | mitochondria associated 2 [Source:HGNC Symbol;Acc:HGNC:21411]" |
| ENSG00000056277 | HGLibA_55728 | 136 | 0.204739131 | ZNF280C | zinc finger protein 280C [Source:HGNC Symbol;Acc:HGNC:25955] |
| ENSG00000060656 | HGLibA_39542 | 136 | 0.204739131 | PTPRU | receptor type U [Source:HGNC Symbol;Acc:HGNC:9683]" |
| ENSG00000070182 | HGLibA_46876 | 136 | 0.204739131 | SPTB | erythrocytic [Source:HGNC Symbol;Acc:HGNC:11274]" |
| ENSG00000082701 | HGLibA_20430 | 136 | 0.204739131 | GSK3B | glycogen synthase kinase 3 beta [Source:HGNC Symbol;Acc:HGNC:4617] |
| ENSG00000101493 | HGLibA_56143 | 136 | 0.204739131 | ZNF516 | zinc finger protein 516 [Source:HGNC Symbol;Acc:HGNC:28990] |
| ENSG00000101605 | HGLibA_30888 | 136 | 0.204739131 | MYOM1 | myomesin 1 [Source:HGNC Symbol;Acc:HGNC:7613] |
| ENSG00000102878 | HGLibA_22228 | 136 | 0.204739131 | HSF4 | heat shock transcription factor 4 [Source:HGNC Symbol;Acc:HGNC:5227] |
| ENSG00000105576 | HGLibA_50900 | 136 | 0.204739131 | TNPO2 | transportin 2 [Source:HGNC Symbol;Acc:HGNC:19998] |
| ENSG00000106299 | HGLibA_53965 | 136 | 0.204739131 | WASL | Wiskott-Aldrich syndrome like [Source:HGNC Symbol;Acc:HGNC:12735] |
| ENSG00000106415 | HGLibA_19230 | 136 | 0.204739131 | GLCCI1 | glucocorticoid induced 1 [Source:HGNC Symbol;Acc:HGNC:18713] |
| ENSG00000107863 | HGLibA_02727 | 136 | 0.204739131 | ARHGAP21 | Rho GTPase activating protein 21 [Source:HGNC Symbol;Acc:HGNC:23725] |
| ENSG00000108829 | HGLibA_27402 | 136 | 0.204739131 | LRRC59 | leucine rich repeat containing 59 [Source:HGNC Symbol;Acc:HGNC:28817] |
| ENSG00000111266 | HGLibA_14149 | 136 | 0.204739131 | DUSP16 | dual specificity phosphatase 16 [Source:HGNC Symbol;Acc:HGNC:17909] |
| ENSG00000112210 | HGLibA_39844 | 136 | 0.204739131 | RAB23 | member RAS oncogene family [Source:HGNC Symbol;Acc:HGNC:14263]" |
| ENSG00000112599 | HGLibA_20636 | 136 | 0.204739131 | GUCA1B | guanylate cyclase activator 1B [Source:HGNC Symbol;Acc:HGNC:4679] |
| ENSG00000116473 | HGLibA_40221 | 136 | 0.204739131 | RAP1A | member of RAS oncogene family [Source:HGNC Symbol;Acc:HGNC:9855]" |
| ENSG00000117682 | HGLibA_13096 | 136 | 0.204739131 | DHDDS | dehydrodolichyl diphosphate synthase subunit [Source:HGNC Symbol;Acc:HGNC:20603] |
| ENSG00000118200 | HGLibA_07109 | 136 | 0.204739131 | CAMSAP2 | calmodulin regulated spectrin associated protein family member 2 [Source:HGNC Symbol;Acc:HGNC:29188] |
| ENSG00000118402 | HGLibA_14965 | 136 | 0.204739131 | ELOVL4 | ELOVL fatty acid elongase 4 [Source:HGNC Symbol;Acc:HGNC:14415] |
| ENSG00000120949 | HGLibA_50789 | 136 | 0.204739131 | TNFRSF8 | TNF receptor superfamily member 8 [Source:HGNC Symbol;Acc:HGNC:11923] |
| ENSG00000121104 | HGLibA_16043 | 136 | 0.204739131 | FAM117A | family with sequence similarity 117 member A [Source:HGNC Symbol;Acc:HGNC:24179] |
| ENSG00000121486 | HGLibA_51679 | 136 | 0.204739131 | TRMT1L | tRNA methyltransferase 1 like [Source:HGNC Symbol;Acc:HGNC:16782] |
| ENSG00000121931 | HGLibA_27174 | 136 | 0.204739131 | LRIF1 | ligand dependent nuclear receptor interacting factor 1 [Source:HGNC Symbol;Acc:HGNC:30299] |
| ENSG00000122375 | HGLibA_33436 | 136 | 0.204739131 | OPN4 | opsin 4 [Source:HGNC Symbol;Acc:HGNC:14449] |
| ENSG00000123908 | HGLibA_01209 | 136 | 0.204739131 | AGO2 | RISC catalytic component [Source:HGNC Symbol;Acc:HGNC:3263]" |
| ENSG00000124091 | HGLibA_18822 | 136 | 0.204739131 | GCNT7 | glucosaminyl (N-acetyl) transferase family member 7 [Source:HGNC Symbol;Acc:HGNC:16099] |
| ENSG00000126814 | HGLibA_51693 | 136 | 0.204739131 | TRMT5 | tRNA methyltransferase 5 [Source:HGNC Symbol;Acc:HGNC:23141] |
| ENSG00000128918 | HGLibA_01543 | 136 | 0.204739131 | ALDH1A2 | aldehyde dehydrogenase 1 family member A2 [Source:HGNC Symbol;Acc:HGNC:15472] |
| ENSG00000130528 | HGLibA_22084 | 136 | 0.204739131 | HRC | histidine rich calcium binding protein [Source:HGNC Symbol;Acc:HGNC:5178] |
| ENSG00000130812 | HGLibA_01924 | 136 | 0.204739131 | ANGPTL6 | angiopoietin like 6 [Source:HGNC Symbol;Acc:HGNC:23140] |
| ENSG00000131471 | HGLibA_02273 | 136 | 0.204739131 | AOC3 | copper containing 3 [Source:HGNC Symbol;Acc:HGNC:550]" |
| ENSG00000135519 | HGLibA_24217 | 136 | 0.204739131 | KCNH3 | potassium voltage-gated channel subfamily H member 3 [Source:HGNC Symbol;Acc:HGNC:6252] |
| ENSG00000135862 | HGLibA_25968 | 136 | 0.204739131 | LAMC1 | laminin subunit gamma 1 [Source:HGNC Symbol;Acc:HGNC:6492] |
| ENSG00000136425 | HGLibA_09685 | 136 | 0.204739131 | CIB2 | calcium and integrin binding family member 2 [Source:HGNC Symbol;Acc:HGNC:24579] |
| ENSG00000137648 | HGLibA_50641 | 136 | 0.204739131 | TMPRSS4 | transmembrane serine protease 4 [Source:HGNC Symbol;Acc:HGNC:11878] |
| ENSG00000139289 | HGLibA_36418 | 136 | 0.204739131 | PHLDA1 | pleckstrin homology like domain family A member 1 [Source:HGNC Symbol;Acc:HGNC:8933] |
| ENSG00000139734 | HGLibA_13223 | 136 | 0.204739131 | DIAPH3 | diaphanous related formin 3 [Source:HGNC Symbol;Acc:HGNC:15480] |
| ENSG00000139832 | HGLibA_39834 | 136 | 0.204739131 | RAB20 | member RAS oncogene family [Source:HGNC Symbol;Acc:HGNC:18260]" |
| ENSG00000142408 | HGLibA_06951 | 136 | 0.204739131 | CACNG8 | calcium voltage-gated channel auxiliary subunit gamma 8 [Source:HGNC Symbol;Acc:HGNC:13628] |
| ENSG00000143126 | HGLibA_09026 | 136 | 0.204739131 | CELSR2 | cadherin EGF LAG seven-pass G-type receptor 2 [Source:HGNC Symbol;Acc:HGNC:3231] |
| ENSG00000144868 | HGLibA_49865 | 136 | 0.204739131 | TMEM108 | transmembrane protein 108 [Source:HGNC Symbol;Acc:HGNC:28451] |
| ENSG00000145882 | HGLibA_35697 | 136 | 0.204739131 | PCYOX1L | prenylcysteine oxidase 1 like [Source:HGNC Symbol;Acc:HGNC:28477] |
| ENSG00000146411 | HGLibA_44860 | 136 | 0.204739131 | SLC2A12 | solute carrier family 2 member 12 [Source:HGNC Symbol;Acc:HGNC:18067] |
| ENSG00000147138 | HGLibA_19971 | 136 | 0.204739131 | GPR174 | G protein-coupled receptor 174 [Source:HGNC Symbol;Acc:HGNC:30245] |
| ENSG00000147166 | HGLibA_23776 | 136 | 0.204739131 | ITGB1BP2 | integrin subunit beta 1 binding protein 2 [Source:HGNC Symbol;Acc:HGNC:6154] |
| ENSG00000147576 | HGLibA_00967 | 136 | 0.204739131 | ADHFE1 | iron containing 1 [Source:HGNC Symbol;Acc:HGNC:16354]" |
| ENSG00000148215 | HGLibA_34272 | 136 | 0.204739131 | OR5C1 | olfactory receptor family 5 subfamily C member 1 [Source:HGNC Symbol;Acc:HGNC:8331] |
| ENSG00000150261 | HGLibA_34544 | 136 | 0.204739131 | OR8K1 | olfactory receptor family 8 subfamily K member 1 [Source:HGNC Symbol;Acc:HGNC:14831] |
| ENSG00000150403 | HGLibA_49787 | 136 | 0.204739131 | TMCO3 | transmembrane and coiled-coil domains 3 [Source:HGNC Symbol;Acc:HGNC:20329] |
| ENSG00000151458 | HGLibA_02115 | 136 | 0.204739131 | ANKRD50 | ankyrin repeat domain 50 [Source:HGNC Symbol;Acc:HGNC:29223] |
| ENSG00000156510 | HGLibA_21587 | 136 | 0.204739131 | HKDC1 | hexokinase domain containing 1 [Source:HGNC Symbol;Acc:HGNC:23302] |
| ENSG00000157800 | HGLibA_45048 | 136 | 0.204739131 | SLC37A3 | solute carrier family 37 member 3 [Source:HGNC Symbol;Acc:HGNC:20651] |
| ENSG00000157884 | HGLibA_09692 | 136 | 0.204739131 | CIB4 | calcium and integrin binding family member 4 [Source:HGNC Symbol;Acc:HGNC:33703] |
| ENSG00000160877 | HGLibA_31038 | 136 | 0.204739131 | NACC1 | nucleus accumbens associated 1 [Source:HGNC Symbol;Acc:HGNC:20967] |
| ENSG00000162551 | HGLibA_01709 | 136 | 0.204739131 | ALPL | biomineralization associated [Source:HGNC Symbol;Acc:HGNC:438]" |
| ENSG00000163357 | HGLibA_12528 | 136 | 0.204739131 | DCST1 | DC-STAMP domain containing 1 [Source:HGNC Symbol;Acc:HGNC:26539] |
| ENSG00000163655 | HGLibA_19433 | 136 | 0.204739131 | GMPS | guanine monophosphate synthase [Source:HGNC Symbol;Acc:HGNC:4378] |
| ENSG00000163935 | HGLibA_43722 | 136 | 0.204739131 | SFMBT1 | Scm like with four mbt domains 1 [Source:HGNC Symbol;Acc:HGNC:20255] |
| ENSG00000164066 | HGLibA_23438 | 136 | 0.204739131 | INTU | inturned planar cell polarity protein [Source:HGNC Symbol;Acc:HGNC:29239] |
| ENSG00000164086 | HGLibA_14190 | 136 | 0.204739131 | DUSP7 | dual specificity phosphatase 7 [Source:HGNC Symbol;Acc:HGNC:3073] |
| ENSG00000164209 | HGLibA_44753 | 136 | 0.204739131 | SLC25A46 | solute carrier family 25 member 46 [Source:HGNC Symbol;Acc:HGNC:25198] |
| ENSG00000164970 | HGLibA_16499 | 136 | 0.204739131 | FAM219A | family with sequence similarity 219 member A [Source:HGNC Symbol;Acc:HGNC:19920] |
| ENSG00000165568 | HGLibA_01497 | 136 | 0.204739131 | AKR1E2 | aldo-keto reductase family 1 member E2 [Source:HGNC Symbol;Acc:HGNC:23437] |
| ENSG00000165732 | HGLibA_12673 | 136 | 0.204739131 | DDX21 | DExD-box helicase 21 [Source:HGNC Symbol;Acc:HGNC:2744] |
| ENSG00000166670 | HGLibA_29480 | 136 | 0.204739131 | MMP10 | matrix metallopeptidase 10 [Source:HGNC Symbol;Acc:HGNC:7156] |
| ENSG00000169635 | HGLibA_21291 | 136 | 0.204739131 | HIC2 | HIC ZBTB transcriptional repressor 2 [Source:HGNC Symbol;Acc:HGNC:18595] |
| ENSG00000169750 | HGLibA_40047 | 136 | 0.204739131 | RAC3 | Rac family small GTPase 3 [Source:HGNC Symbol;Acc:HGNC:9803] |
| ENSG00000171587 | HGLibA_13998 | 136 | 0.204739131 | DSCAM | DS cell adhesion molecule [Source:HGNC Symbol;Acc:HGNC:3039] |
| ENSG00000172382 | HGLibA_38919 | 136 | 0.204739131 | PRSS27 | serine protease 27 [Source:HGNC Symbol;Acc:HGNC:15475] |
| ENSG00000172878 | HGLibA_28931 | 136 | 0.204739131 | METAP1D | mitochondrial [Source:HGNC Symbol;Acc:HGNC:32583]" |
| ENSG00000173166 | HGLibA_40266 | 136 | 0.204739131 | RAPH1 | Ras association (RalGDS/AF-6) and pleckstrin homology domains 1 [Source:HGNC Symbol;Acc:HGNC:14436] |
| ENSG00000173391 | HGLibA_33378 | 136 | 0.204739131 | OLR1 | oxidized low density lipoprotein receptor 1 [Source:HGNC Symbol;Acc:HGNC:8133] |
| ENSG00000173452 | HGLibA_50158 | 136 | 0.204739131 | TMEM196 | transmembrane protein 196 [Source:HGNC Symbol;Acc:HGNC:22431] |
| ENSG00000174004 | HGLibA_32720 | 136 | 0.204739131 | NRROS | negative regulator of reactive oxygen species [Source:HGNC Symbol;Acc:HGNC:24613] |
| ENSG00000174939 | HGLibA_03353 | 136 | 0.204739131 | ASPHD1 | aspartate beta-hydroxylase domain containing 1 [Source:HGNC Symbol;Acc:HGNC:27380] |
| ENSG00000175121 | HGLibA_54268 | 136 | 0.204739131 | WFDC5 | WAP four-disulfide core domain 5 [Source:HGNC Symbol;Acc:HGNC:20477] |
| ENSG00000175329 | HGLibA_23691 | 136 | 0.204739131 | ISX | intestine specific homeobox [Source:HGNC Symbol;Acc:HGNC:28084] |
| ENSG00000177989 | HGLibA_33287 | 136 | 0.204739131 | ODF3B | outer dense fiber of sperm tails 3B [Source:HGNC Symbol;Acc:HGNC:34388] |
| ENSG00000180044 | HGLibA_06272 | 136 | 0.204739131 | C3orf80 | chromosome 3 open reading frame 80 [Source:HGNC Symbol;Acc:HGNC:40048] |
| ENSG00000182415 | HGLibA_08910 | 136 | 0.204739131 | CDY2A | chromodomain Y-linked 2A [Source:HGNC Symbol;Acc:HGNC:1810] |
| ENSG00000182816 | HGLibA_25645 | 136 | 0.204739131 | KRTAP13-2 | keratin associated protein 13-2 [Source:HGNC Symbol;Acc:HGNC:18923] |

| | | | | | |
|---|---|---|---|---|---|
| ENSG00000184009 | HGLibA_00610 | 136 | 0.204739131 | ACTG1 | actin gamma 1 [Source:HGNC Symbol;Acc:HGNC:144] |
| ENSG00000185219 | HGLibA_56018 | 136 | 0.204739131 | ZNF445 | zinc finger protein 445 [Source:HGNC Symbol;Acc:HGNC:21018] |
| ENSG00000185252 | HGLibA_56658 | 136 | 0.204739131 | ZNF74 | zinc finger protein 74 [Source:HGNC Symbol;Acc:HGNC:13144] |
| ENSG00000186081 | HGLibA_25510 | 136 | 0.204739131 | KRT5 | keratin 5 [Source:HGNC Symbol;Acc:HGNC:6442] |
| ENSG00000186980 | HGLibA_25722 | 136 | 0.204739131 | KRTAP23-1 | keratin associated protein 23-1 [Source:HGNC Symbol;Acc:HGNC:18928] |
| ENSG00000188163 | HGLibA_16233 | 136 | 0.204739131 | FAM166A | family with sequence similarity 166 member A [Source:HGNC Symbol;Acc:HGNC:33818] |
| ENSG00000188295 | HGLibA_56498 | 136 | 0.204739131 | ZNF669 | zinc finger protein 669 [Source:HGNC Symbol;Acc:HGNC:25736] |
| ENSG00000188822 | HGLibA_10376 | 136 | 0.204739131 | CNR2 | cannabinoid receptor 2 [Source:HGNC Symbol;Acc:HGNC:2160] |
| ENSG00000188937 | HGLibA_33188 | 136 | 0.204739131 | NYX | nyctalopin [Source:HGNC Symbol;Acc:HGNC:8082] |
| ENSG00000188984 | HGLibA_00036 | 136 | 0.204739131 | AADACL3 | arylacetamide deacetylase like 3 [Source:HGNC Symbol;Acc:HGNC:32037] |
| ENSG00000189064 | HGLibA_18468 | 136 | 0.204739131 | GAGE2A | G antigen 2A [Source:HGNC Symbol;Acc:HGNC:4099] |
| ENSG00000189171 | HGLibA_42541 | 136 | 0.204739131 | S100A13 | S100 calcium binding protein A13 [Source:HGNC Symbol;Acc:HGNC:10490] |
| ENSG00000197121 | HGLibA_36208 | 136 | 0.204739131 | PGAP1 | post-GPI attachment to proteins 1 [Source:HGNC Symbol;Acc:HGNC:25712] |
| ENSG00000197142 | HGLibA_00562 | 136 | 0.204739131 | ACSL5 | acyl-CoA synthetase long chain family member 5 [Source:HGNC Symbol;Acc:HGNC:16526] |
| ENSG00000197548 | HGLibA_03502 | 136 | 0.204739131 | ATG7 | autophagy related 7 [Source:HGNC Symbol;Acc:HGNC:16935] |
| ENSG00000197763 | HGLibA_52451 | 136 | 0.204739131 | TXNRD3 | thioredoxin reductase 3 [Source:HGNC Symbol;Acc:HGNC:20667] |
| ENSG00000197982 | HGLibA_05760 | 136 | 0.204739131 | C1orf122 | chromosome 1 open reading frame 122 [Source:HGNC Symbol;Acc:HGNC:24789] |
| ENSG00000198399 | HGLibA_23873 | 136 | 0.204739131 | ITSN2 | intersectin 2 [Source:HGNC Symbol;Acc:HGNC:6184] |
| ENSG00000198445 | HGLibA_08219 | 136 | 0.204739131 | CCT8L2 | chaperonin containing TCP1 subunit 8 like 2 [Source:HGNC Symbol;Acc:HGNC:15553] |
| ENSG00000198885 | HGLibA_23865 | 136 | 0.204739131 | ITPRIPL1 | ITPRIP like 1 [Source:HGNC Symbol;Acc:HGNC:29371] |
| ENSG00000203697 | HGLibA_07178 | 136 | 0.204739131 | CAPN8 | calpain 8 [Source:HGNC Symbol;Acc:HGNC:1485] |
| ENSG00000204099 | HGLibA_31720 | 136 | 0.204739131 | NEU4 | neuraminidase 4 [Source:HGNC Symbol;Acc:HGNC:21328] |
| ENSG00000204271 | HGLibA_46675 | 136 | 0.204739131 | SPIN3 | spindlin family member 3 [Source:HGNC Symbol;Acc:HGNC:27272] |
| ENSG00000204634 | HGLibA_48567 | 136 | 0.204739131 | TBC1D8 | TBC1 domain family member 8 [Source:HGNC Symbol;Acc:HGNC:17791] |
| ENSG00000205456 | HGLibA_51087 | 136 | 0.204739131 | TP53TG3D | TP53 target 3D [Source:HGNC Symbol;Acc:HGNC:44657] |
| ENSG00000213171 | HGLibA_26593 | 136 | 0.204739131 | LINGO4 | leucine rich repeat and Ig domain containing 4 [Source:HGNC Symbol;Acc:HGNC:31814] |
| ENSG00000214642 | HGLibA_12853 | 136 | 0.204739131 | DEFB113 | defensin beta 113 [Source:HGNC Symbol;Acc:HGNC:18094] |
| ENSG00000221888 | HGLibA_33650 | 136 | 0.204739131 | OR1C1 | olfactory receptor family 1 subfamily C member 1 [Source:HGNC Symbol;Acc:HGNC:8182] |
| ENSG00000226288 | HGLibA_34176 | 136 | 0.204739131 | OR52I2 | olfactory receptor family 52 subfamily I member 2 [Source:HGNC Symbol;Acc:HGNC:15221] |
| ENSG00000257727 | HGLibA_10365 | 136 | 0.204739131 | CNPY2 | canopy FGF signaling regulator 2 [Source:HGNC Symbol;Acc:HGNC:13529] |
| ENSG00000261456 | HGLibA_52294 | 136 | 0.204739131 | TUBB8 | tubulin beta 8 class VIII [Source:HGNC Symbol;Acc:HGNC:20773] |
| ENSG00000267631 | HGLibA_09302 | 136 | 0.204739131 | CGB1 | chorionic gonadotropin subunit beta 1 [Source:HGNC Symbol;Acc:HGNC:16721] |
| ENSG00000268182 | HGLibA_45736 | 136 | 0.204739131 | SMIM17 | small integral membrane protein 17 [Source:HGNC Symbol;Acc:HGNC:27114] |
| ENSG00000269858 | HGLibA_14643 | 136 | 0.204739131 | EGLN2 | egl-9 family hypoxia inducible factor 2 [Source:HGNC Symbol;Acc:HGNC:14660] |
| FAM26F | HGLibA_16567 | 136 | 0.204739131 | FAM26F | - |
| GATS | HGLibA_18698 | 136 | 0.204739131 | GATS | - |
| GPR64 | HGLibA_20071 | 136 | 0.204739131 | GPR64 | - |
| hsa-mir-1283-1 | HGLibA_57587 | 136 | 0.204739131 | hsa-mir-1283-1 | - |
| hsa-mir-1539 | HGLibA_57921 | 136 | 0.204739131 | hsa-mir-1539 | - |
| hsa-mir-3181 | HGLibA_58820 | 136 | 0.204739131 | hsa-mir-3181 | - |
| hsa-mir-3198-1 | HGLibA_58890 | 136 | 0.204739131 | hsa-mir-3198-1 | - |
| hsa-mir-34b | HGLibA_59038 | 136 | 0.204739131 | hsa-mir-34b | - |
| hsa-mir-4323 | HGLibA_60017 | 136 | 0.204739131 | hsa-mir-4323 | - |
| hsa-mir-4667 | HGLibA_60785 | 136 | 0.204739131 | hsa-mir-4667 | - |
| hsa-mir-4711 | HGLibA_60943 | 136 | 0.204739131 | hsa-mir-4711 | - |
| hsa-mir-4792 | HGLibA_61275 | 136 | 0.204739131 | hsa-mir-4792 | - |
| hsa-mir-5001 | HGLibA_61410 | 136 | 0.204739131 | hsa-mir-5001 | - |
| hsa-mir-5010 | HGLibA_61457 | 136 | 0.204739131 | hsa-mir-5010 | - |
| hsa-mir-5091 | HGLibA_61521 | 136 | 0.204739131 | hsa-mir-5091 | - |
| hsa-mir-5100 | HGLibA_61549 | 136 | 0.204739131 | hsa-mir-5100 | - |
| hsa-mir-5190 | HGLibA_61678 | 136 | 0.204739131 | hsa-mir-5190 | - |
| hsa-mir-573 | HGLibA_62382 | 136 | 0.204739131 | hsa-mir-573 | - |
| hsa-mir-6079 | HGLibA_62573 | 136 | 0.204739131 | hsa-mir-6079 | - |
| hsa-mir-6080 | HGLibA_62579 | 136 | 0.204739131 | hsa-mir-6080 | - |
| hsa-mir-941-2 | HGLibA_64343 | 136 | 0.204739131 | hsa-mir-941-2 | - |
| LOC100132146 | HGLibA_26804 | 136 | 0.204739131 | LOC100132146 | - |
| PTRF | HGLibA_39547 | 136 | 0.204739131 | PTRF | - |
| AMICA1 | HGLibA_01791 | 135 | 0.194788094 | AMICA1 | - |
| CCDC104 | HGLibA_07517 | 135 | 0.194788094 | CCDC104 | - |
| ENSG00000008952 | HGLibA_43260 | 135 | 0.194788094 | SEC62 | preprotein translocation factor [Source:HGNC Symbol;Acc:HGNC:11846]" |
| ENSG00000011677 | HGLibA_18362 | 135 | 0.194788094 | GABRA3 | gamma-aminobutyric acid type A receptor alpha3 subunit [Source:HGNC Symbol;Acc:HGNC:4077] |
| ENSG00000019505 | HGLibA_48057 | 135 | 0.194788094 | SYT13 | synaptotagmin 13 [Source:HGNC Symbol;Acc:HGNC:14962] |
| ENSG00000047849 | HGLibA_28209 | 135 | 0.194788094 | MAP4 | microtubule associated protein 4 [Source:HGNC Symbol;Acc:HGNC:6862] |
| ENSG00000054116 | HGLibA_51318 | 135 | 0.194788094 | TRAPPC3 | trafficking protein particle complex 3 [Source:HGNC Symbol;Acc:HGNC:19942] |
| ENSG00000057019 | HGLibA_12472 | 135 | 0.194788094 | DCBLD2 | CUB and LCCL domain containing 2 [Source:HGNC Symbol;Acc:HGNC:24627]" |
| ENSG00000068784 | HGLibA_46924 | 135 | 0.194788094 | SRBD1 | S1 RNA binding domain 1 [Source:HGNC Symbol;Acc:HGNC:25521] |
| ENSG00000078549 | HGLibA_00937 | 135 | 0.194788094 | ADCYAP1R1 | ADCYAP receptor type I [Source:HGNC Symbol;Acc:HGNC:242] |
| ENSG00000088448 | HGLibA_01992 | 135 | 0.194788094 | ANKRD10 | ankyrin repeat domain 10 [Source:HGNC Symbol;Acc:HGNC:20265] |
| ENSG00000091317 | HGLibA_10225 | 135 | 0.194788094 | CMTM6 | CKLF like MARVEL transmembrane domain containing 6 [Source:HGNC Symbol;Acc:HGNC:19177] |
| ENSG00000095637 | HGLibA_46173 | 135 | 0.194788094 | SORBS1 | sorbin and SH3 domain containing 1 [Source:HGNC Symbol;Acc:HGNC:14565] |
| ENSG00000096088 | HGLibA_36228 | 135 | 0.194788094 | PGC | progastricsin [Source:HGNC Symbol;Acc:HGNC:8890] |
| ENSG00000099795 | HGLibA_31527 | 135 | 0.194788094 | NDUFB7 | NADH:ubiquinone oxidoreductase subunit B7 [Source:HGNC Symbol;Acc:HGNC:7702] |
| ENSG00000100221 | HGLibA_23973 | 135 | 0.194788094 | JOSD1 | Josephin domain containing 1 [Source:HGNC Symbol;Acc:HGNC:28953] |
| ENSG00000100418 | HGLibA_13007 | 135 | 0.194788094 | DESI1 | desumoylating isopeptidase 1 [Source:HGNC Symbol;Acc:HGNC:24577] |
| ENSG00000100593 | HGLibA_23678 | 135 | 0.194788094 | ISM2 | isthmin 2 [Source:HGNC Symbol;Acc:HGNC:23176] |
| ENSG00000100744 | HGLibA_20435 | 135 | 0.194788094 | GSKIP | GSK3B interacting protein [Source:HGNC Symbol;Acc:HGNC:20343] |
| ENSG00000101425 | HGLibA_04707 | 135 | 0.194788094 | BPI | bactericidal permeability increasing protein [Source:HGNC Symbol;Acc:HGNC:1095] |
| ENSG00000101463 | HGLibA_47970 | 135 | 0.194788094 | SYNDIG1 | synapse differentiation inducing 1 [Source:HGNC Symbol;Acc:HGNC:15885] |
| ENSG00000102103 | HGLibA_38248 | 135 | 0.194788094 | PQBP1 | polyglutamine binding protein 1 [Source:HGNC Symbol;Acc:HGNC:9330] |
| ENSG00000103152 | HGLibA_29719 | 135 | 0.194788094 | MPG | N-methylpurine DNA glycosylase [Source:HGNC Symbol;Acc:HGNC:7211] |
| ENSG00000104112 | HGLibA_42903 | 135 | 0.194788094 | SCG3 | secretogranin III [Source:HGNC Symbol;Acc:HGNC:13707] |
| ENSG00000104325 | HGLibA_12773 | 135 | 0.194788094 | DECR1 | 4-dienoyl-CoA reductase 1 [Source:HGNC Symbol;Acc:HGNC:2753]" |
| ENSG00000104756 | HGLibA_24470 | 135 | 0.194788094 | KCTD9 | potassium channel tetramerization domain containing 9 [Source:HGNC Symbol;Acc:HGNC:22401] |
| ENSG00000104899 | HGLibA_01783 | 135 | 0.194788094 | AMH | anti-Mullerian hormone [Source:HGNC Symbol;Acc:HGNC:464] |
| ENSG00000105366 | HGLibA_44151 | 135 | 0.194788094 | SIGLEC8 | sialic acid binding Ig like lectin 8 [Source:HGNC Symbol;Acc:HGNC:10877] |
| ENSG00000106852 | HGLibA_26463 | 135 | 0.194788094 | LHX6 | LIM homeobox 6 [Source:HGNC Symbol;Acc:HGNC:21735] |
| ENSG00000107929 | HGLibA_26036 | 135 | 0.194788094 | LARP4B | La ribonucleoprotein domain family member 4B [Source:HGNC Symbol;Acc:HGNC:28987] |
| ENSG00000108702 | HGLibA_07962 | 135 | 0.194788094 | CCL1 | C-C motif chemokine ligand 1 [Source:HGNC Symbol;Acc:HGNC:10609] |
| ENSG00000114948 | HGLibA_00761 | 135 | 0.194788094 | ADAM23 | ADAM metallopeptidase domain 23 [Source:HGNC Symbol;Acc:HGNC:202] |
| ENSG00000115966 | HGLibA_03435 | 135 | 0.194788094 | ATF2 | activating transcription factor 2 [Source:HGNC Symbol;Acc:HGNC:784] |
| ENSG00000118785 | HGLibA_46768 | 135 | 0.194788094 | SPP1 | secreted phosphoprotein 1 [Source:HGNC Symbol;Acc:HGNC:11255] |
| ENSG00000120696 | HGLibA_24114 | 135 | 0.194788094 | KBTBD7 | kelch repeat and BTB domain containing 7 [Source:HGNC Symbol;Acc:HGNC:25266] |
| ENSG00000124215 | HGLibA_08673 | 135 | 0.194788094 | CDH26 | cadherin 26 [Source:HGNC Symbol;Acc:HGNC:15902] |
| ENSG00000126003 | HGLibA_36956 | 135 | 0.194788094 | PLAGL2 | PLAG1 like zinc finger 2 [Source:HGNC Symbol;Acc:HGNC:9047] |
| ENSG00000126895 | HGLibA_03910 | 135 | 0.194788094 | AVPR2 | arginine vasopressin receptor 2 [Source:HGNC Symbol;Acc:HGNC:897] |
| ENSG00000129595 | HGLibA_15252 | 135 | 0.194788094 | EPB41L4A | erythrocyte membrane protein band 4.1 like 4A [Source:HGNC Symbol;Acc:HGNC:13278] |
| ENSG00000130943 | HGLibA_36811 | 135 | 0.194788094 | PKDREJ | polycystin family receptor for egg jelly [Source:HGNC Symbol;Acc:HGNC:9015] |
| ENSG00000131069 | HGLibA_00589 | 135 | 0.194788094 | ACSS2 | acyl-CoA synthetase short chain family member 2 [Source:HGNC Symbol;Acc:HGNC:15814] |
| ENSG00000133816 | HGLibA_29204 | 135 | 0.194788094 | MICAL2 | calponin and LIM domain containing 2 [Source:HGNC Symbol;Acc:HGNC:24693]" |
| ENSG00000134086 | HGLibA_53648 | 135 | 0.194788094 | VHL | von Hippel-Lindau tumor suppressor [Source:HGNC Symbol;Acc:HGNC:12687] |
| ENSG00000134308 | HGLibA_54752 | 135 | 0.194788094 | YWHAQ | tyrosine 3-monooxygenase/tryptophan 5-monooxygenase activation protein theta [Source:HGNC Symbol;Acc:HGNC:12854] |
| ENSG00000135373 | HGLibA_14679 | 135 | 0.194788094 | EHF | ETS homologous factor [Source:HGNC Symbol;Acc:HGNC:3246] |
| ENSG00000136143 | HGLibA_47696 | 135 | 0.194788094 | SUCLA2 | succinate-CoA ligase ADP-forming beta subunit [Source:HGNC Symbol;Acc:HGNC:11448] |
| ENSG00000136546 | HGLibA_43001 | 135 | 0.194788094 | SCN7A | sodium voltage-gated channel alpha subunit 7 [Source:HGNC Symbol;Acc:HGNC:10594] |
| ENSG00000137714 | HGLibA_17257 | 135 | 0.194788094 | FDX1 | ferredoxin 1 [Source:HGNC Symbol;Acc:HGNC:3638] |
| ENSG00000138829 | HGLibA_16958 | 135 | 0.194788094 | FBN2 | fibrillin 2 [Source:HGNC Symbol;Acc:HGNC:3604] |
| ENSG00000140993 | HGLibA_49480 | 135 | 0.194788094 | TIGD7 | tigger transposable element derived 7 [Source:HGNC Symbol;Acc:HGNC:18331] |
| ENSG00000141437 | HGLibA_44767 | 135 | 0.194788094 | SLC25A52 | solute carrier family 25 member 52 [Source:HGNC Symbol;Acc:HGNC:23324] |
| ENSG00000146221 | HGLibA_48856 | 135 | 0.194788094 | TCTE1 | t-complex-associated-testis-expressed 1 [Source:HGNC Symbol;Acc:HGNC:11693] |
| ENSG00000149054 | HGLibA_55597 | 135 | 0.194788094 | ZNF215 | zinc finger protein 215 [Source:HGNC Symbol;Acc:HGNC:13007] |
| ENSG00000150961 | HGLibA_43237 | 135 | 0.194788094 | SEC24D | COPII coat complex component [Source:HGNC Symbol;Acc:HGNC:10706]" |
| ENSG00000151090 | HGLibA_49393 | 135 | 0.194788094 | THRB | thyroid hormone receptor beta [Source:HGNC Symbol;Acc:HGNC:11799] |
| ENSG00000153237 | HGLibA_07633 | 135 | 0.194788094 | CCDC148 | coiled-coil domain containing 148 [Source:HGNC Symbol;Acc:HGNC:25191] |
| ENSG00000154102 | HGLibA_05480 | 135 | 0.194788094 | C16orf74 | chromosome 16 open reading frame 74 [Source:HGNC Symbol;Acc:HGNC:23362] |
| ENSG00000155034 | HGLibA_16990 | 135 | 0.194788094 | FBXL18 | F-box and leucine rich repeat protein 18 [Source:HGNC Symbol;Acc:HGNC:21874] |
| ENSG00000155367 | HGLibA_37983 | 135 | 0.194788094 | PPM1J | Mg2+/Mn2+ dependent 1J [Source:HGNC Symbol;Acc:HGNC:20785]" |
| ENSG00000159423 | HGLibA_01575 | 135 | 0.194788094 | ALDH4A1 | aldehyde dehydrogenase 4 family member A1 [Source:HGNC Symbol;Acc:HGNC:406] |
| ENSG00000159496 | HGLibA_41010 | 135 | 0.194788094 | RGL4 | ral guanine nucleotide dissociation stimulator like 4 [Source:HGNC Symbol;Acc:HGNC:31911] |
| ENSG00000160199 | HGLibA_36846 | 135 | 0.194788094 | PKNOX1 | PBX/knotted 1 homeobox 1 [Source:HGNC Symbol;Acc:HGNC:9022] |
| ENSG00000160408 | HGLibA_47279 | 135 | 0.194788094 | ST6GALNAC6 | 6-sialyltransferase 6 [Source:HGNC Symbol;Acc:HGNC:23364]" |
| ENSG00000161850 | HGLibA_25562 | 135 | 0.194788094 | KRT82 | keratin 82 [Source:HGNC Symbol;Acc:HGNC:6459] |
| ENSG00000163218 | HGLibA_36259 | 135 | 0.194788094 | PGLYRP4 | peptidoglycan recognition protein 4 [Source:HGNC Symbol;Acc:HGNC:30015] |
| ENSG00000163331 | HGLibA_12332 | 135 | 0.194788094 | DAPL1 | death associated protein like 1 [Source:HGNC Symbol;Acc:HGNC:21490] |
| ENSG00000164136 | HGLibA_22980 | 135 | 0.194788094 | IL15 | interleukin 15 [Source:HGNC Symbol;Acc:HGNC:5977] |
| ENSG00000165794 | HGLibA_45107 | 135 | 0.194788094 | SLC39A2 | solute carrier family 39 member 2 [Source:HGNC Symbol;Acc:HGNC:17127] |
| ENSG00000166188 | HGLibA_55789 | 135 | 0.194788094 | ZNF319 | zinc finger protein 319 [Source:HGNC Symbol;Acc:HGNC:13644] |
| ENSG00000168079 | HGLibA_42865 | 135 | 0.194788094 | SCARA5 | scavenger receptor class A member 5 [Source:HGNC Symbol;Acc:HGNC:28701] |
| ENSG00000168938 | HGLibA_37921 | 135 | 0.194788094 | PPIC | peptidylprolyl isomerase C [Source:HGNC Symbol;Acc:HGNC:9256] |
| ENSG00000170417 | HGLibA_50102 | 135 | 0.194788094 | TMEM182 | transmembrane protein 182 [Source:HGNC Symbol;Acc:HGNC:26391] |
| ENSG00000170458 | HGLibA_08233 | 135 | 0.194788094 | CD14 | CD14 molecule [Source:HGNC Symbol;Acc:HGNC:1628] |
| ENSG00000171291 | HGLibA_55996 | 135 | 0.194788094 | ZNF439 | zinc finger protein 439 [Source:HGNC Symbol;Acc:HGNC:20873] |
| ENSG00000171502 | HGLibA_10547 | 135 | 0.194788094 | COL24A1 | collagen type XXIV alpha 1 chain [Source:HGNC Symbol;Acc:HGNC:20821] |
| ENSG00000171794 | HGLibA_53428 | 135 | 0.194788094 | UTF1 | undifferentiated embryonic cell transcription factor 1 [Source:HGNC Symbol;Acc:HGNC:12634] |
| ENSG00000173482 | HGLibA_39518 | 135 | 0.194788094 | PTPRM | receptor type M [Source:HGNC Symbol;Acc:HGNC:9675]" |
| ENSG00000173890 | HGLibA_19954 | 135 | 0.194788094 | GPR160 | G protein-coupled receptor 160 [Source:HGNC Symbol;Acc:HGNC:23693] |

| | | | | | |
|---|---|---|---|---|---|
| ENSG00000175718 | HGLibA_40607 | 135 | 0.194788094 | RBMXL3 | RBMX like 3 [Source:HGNC Symbol;Acc:HGNC:26859] |
| ENSG00000180190 | HGLibA_48922 | 135 | 0.194788094 | TDRP | testis development related protein [Source:HGNC Symbol;Acc:HGNC:26951] |
| ENSG00000181786 | HGLibA_00634 | 135 | 0.194788094 | ACTL9 | actin like 9 [Source:HGNC Symbol;Acc:HGNC:28494] |
| ENSG00000182272 | HGLibA_04021 | 135 | 0.194788094 | B4GALNT4 | 4-N-acetyl-galactosaminyltransferase 4 [Source:HGNC Symbol;Acc:HGNC:26315]" |
| ENSG00000183682 | HGLibA_04620 | 135 | 0.194788094 | BMP8A | bone morphogenetic protein 8a [Source:HGNC Symbol;Acc:HGNC:21650] |
| ENSG00000185634 | HGLibA_44009 | 135 | 0.194788094 | SHC4 | SHC adaptor protein 4 [Source:HGNC Symbol;Acc:HGNC:16743] |
| ENSG00000185669 | HGLibA_45872 | 135 | 0.194788094 | SNAI3 | snail family transcriptional repressor 3 [Source:HGNC Symbol;Acc:HGNC:18411] |
| ENSG00000186417 | HGLibA_19238 | 135 | 0.194788094 | GLDN | gliomedin [Source:HGNC Symbol;Acc:HGNC:29514] |
| ENSG00000186642 | HGLibA_35779 | 135 | 0.194788094 | PDE2A | phosphodiesterase 2A [Source:HGNC Symbol;Acc:HGNC:8777] |
| ENSG00000187240 | HGLibA_14248 | 135 | 0.194788094 | DYNC2H1 | dynein cytoplasmic 2 heavy chain 1 [Source:HGNC Symbol;Acc:HGNC:2962] |
| ENSG00000188037 | HGLibA_09819 | 135 | 0.194788094 | CLCN1 | chloride voltage-gated channel 1 [Source:HGNC Symbol;Acc:HGNC:2019] |
| ENSG00000189051 | HGLibA_41642 | 135 | 0.194788094 | RNF222 | ring finger protein 222 [Source:HGNC Symbol;Acc:HGNC:34517] |
| ENSG00000196350 | HGLibA_56641 | 135 | 0.194788094 | ZNF729 | zinc finger protein 729 [Source:HGNC Symbol;Acc:HGNC:32464] |
| ENSG00000196378 | HGLibA_55838 | 135 | 0.194788094 | ZNF34 | zinc finger protein 34 [Source:HGNC Symbol;Acc:HGNC:13098] |
| ENSG00000197457 | HGLibA_47521 | 135 | 0.194788094 | STMN3 | stathmin 3 [Source:HGNC Symbol;Acc:HGNC:15926] |
| ENSG00000198704 | HGLibA_20185 | 135 | 0.194788094 | GPX6 | glutathione peroxidase 6 [Source:HGNC Symbol;Acc:HGNC:4558] |
| ENSG00000198794 | HGLibA_42849 | 135 | 0.194788094 | SCAMP5 | secretory carrier membrane protein 5 [Source:HGNC Symbol;Acc:HGNC:30386] |
| ENSG00000198856 | HGLibA_34708 | 135 | 0.194788094 | OSTC | oligosaccharyltransferase complex non-catalytic subunit [Source:HGNC Symbol;Acc:HGNC:24448] |
| ENSG00000203485 | HGLibA_23280 | 135 | 0.194788094 | INF2 | FH2 and WH2 domain containing [Source:HGNC Symbol;Acc:HGNC:23791]" |
| ENSG00000203909 | HGLibA_13895 | 135 | 0.194788094 | DPPA5 | developmental pluripotency associated 5 [Source:HGNC Symbol;Acc:HGNC:19201] |
| ENSG00000214013 | HGLibA_18607 | 135 | 0.194788094 | GANC | neutral C [Source:HGNC Symbol;Acc:HGNC:4139]" |
| ENSG00000214655 | HGLibA_57058 | 135 | 0.194788094 | ZSWIM8 | zinc finger SWIM-type containing 8 [Source:HGNC Symbol;Acc:HGNC:23528] |
| ENSG00000215784 | HGLibA_16714 | 135 | 0.194788094 | FAM72D | family with sequence similarity 72 member D [Source:HGNC Symbol;Acc:HGNC:33593] |
| ENSG00000222038 | HGLibA_37744 | 135 | 0.194788094 | POTEJ | POTE ankyrin domain family member J [Source:HGNC Symbol;Acc:HGNC:37094] |
| ENSG00000231192 | HGLibA_34290 | 135 | 0.194788094 | OR5H1 | olfactory receptor family 5 subfamily H member 1 [Source:HGNC Symbol;Acc:HGNC:8346] |
| ENSG00000236027 | HGLibA_35276 | 135 | 0.194788094 | PATE3 | prostate and testis expressed 3 [Source:HGNC Symbol;Acc:HGNC:35426] |
| ENSG00000239789 | HGLibA_30046 | 135 | 0.194788094 | MRPS17 | mitochondrial ribosomal protein S17 [Source:HGNC Symbol;Acc:HGNC:14047] |
| ENSG00000255302 | HGLibA_14693 | 135 | 0.194788094 | EID1 | EP300 interacting inhibitor of differentiation 1 [Source:HGNC Symbol;Acc:HGNC:1191] |
| ENSG00000259384 | HGLibA_19029 | 135 | 0.194788094 | GH1 | growth hormone 1 [Source:HGNC Symbol;Acc:HGNC:4261] |
| FAIM3 | HGLibA_15969 | 135 | 0.194788094 | FAIM3 | - |
| FAM46C | HGLibA_16602 | 135 | 0.194788094 | FAM46C | - |
| hsa-mir-132 | HGLibA_57755 | 135 | 0.194788094 | hsa-mir-132 | - |
| hsa-mir-217 | HGLibA_58265 | 135 | 0.194788094 | hsa-mir-217 | - |
| hsa-mir-4290 | HGLibA_59871 | 135 | 0.194788094 | hsa-mir-4290 | - |
| hsa-mir-4434 | HGLibA_60127 | 135 | 0.194788094 | hsa-mir-4434 | - |
| hsa-mir-573 | HGLibA_62384 | 135 | 0.194788094 | hsa-mir-573 | - |
| hsa-mir-6767 | HGLibA_63230 | 135 | 0.194788094 | hsa-mir-6767 | - |
| hsa-mir-6775 | HGLibA_63274 | 135 | 0.194788094 | hsa-mir-6775 | - |
| hsa-mir-6784 | HGLibA_63312 | 135 | 0.194788094 | hsa-mir-6784 | - |
| hsa-mir-6787 | HGLibA_63324 | 135 | 0.194788094 | hsa-mir-6787 | - |
| hsa-mir-6894 | HGLibA_63761 | 135 | 0.194788094 | hsa-mir-6894 | - |
| PHF16 | HGLibA_36351 | 135 | 0.194788094 | PHF16 | - |
| TMEM14E | HGLibA_49989 | 135 | 0.194788094 | TMEM14E | - |
| ADRBK1 | HGLibA_01065 | 134 | 0.184837056 | ADRBK1 | - |
| C15orf26 | HGLibA_05379 | 134 | 0.184837056 | C15orf26 | - |
| C17orf53 | HGLibA_05546 | 134 | 0.184837056 | C17orf53 | - |
| C20orf111 | HGLibA_05982 | 134 | 0.184837056 | C20orf111 | - |
| C9orf173 | HGLibA_06717 | 134 | 0.184837056 | C9orf173 | - |
| DSCR6 | HGLibA_14012 | 134 | 0.184837056 | DSCR6 | - |
| ENSG00000008283 | HGLibA_11976 | 134 | 0.184837056 | CYB561 | cytochrome b561 [Source:HGNC Symbol;Acc:HGNC:2571] |
| ENSG00000010322 | HGLibA_31979 | 134 | 0.184837056 | NISCH | nischarin [Source:HGNC Symbol;Acc:HGNC:18006] |
| ENSG00000026025 | HGLibA_53657 | 134 | 0.184837056 | VIM | vimentin [Source:HGNC Symbol;Acc:HGNC:12692] |
| ENSG00000052841 | HGLibA_52059 | 134 | 0.184837056 | TTC17 | tetratricopeptide repeat domain 17 [Source:HGNC Symbol;Acc:HGNC:25596] |
| ENSG00000064933 | HGLibA_37332 | 134 | 0.184837056 | PMS1 | mismatch repair system component [Source:HGNC Symbol;Acc:HGNC:9121]" |
| ENSG00000068912 | HGLibA_15492 | 134 | 0.184837056 | ERLEC1 | endoplasmic reticulum lectin 1 [Source:HGNC Symbol;Acc:HGNC:25222] |
| ENSG00000070388 | HGLibA_17400 | 134 | 0.184837056 | FGF22 | fibroblast growth factor 22 [Source:HGNC Symbol;Acc:HGNC:3679] |
| ENSG00000071054 | HGLibA_28221 | 134 | 0.184837056 | MAP4K4 | mitogen-activated protein kinase kinase kinase kinase 4 [Source:HGNC Symbol;Acc:HGNC:6866] |
| ENSG00000077092 | HGLibA_40276 | 134 | 0.184837056 | RARB | retinoic acid receptor beta [Source:HGNC Symbol;Acc:HGNC:9865] |
| ENSG00000081189 | HGLibA_28837 | 134 | 0.184837056 | MEF2C | myocyte enhancer factor 2C [Source:HGNC Symbol;Acc:HGNC:6996] |
| ENSG00000085265 | HGLibA_17215 | 134 | 0.184837056 | FCN1 | ficolin 1 [Source:HGNC Symbol;Acc:HGNC:3623] |
| ENSG00000085741 | HGLibA_54353 | 134 | 0.184837056 | WNT11 | Wnt family member 11 [Source:HGNC Symbol;Acc:HGNC:12776] |
| ENSG00000089199 | HGLibA_09433 | 134 | 0.184837056 | CHGB | chromogranin B [Source:HGNC Symbol;Acc:HGNC:1930] |
| ENSG00000099617 | HGLibA_14585 | 134 | 0.184837056 | EFNA2 | ephrin A2 [Source:HGNC Symbol;Acc:HGNC:3222] |
| ENSG00000100196 | HGLibA_24486 | 134 | 0.184837056 | KDELR3 | KDEL endoplasmic reticulum protein retention receptor 3 [Source:HGNC Symbol;Acc:HGNC:6306] |
| ENSG00000100979 | HGLibA_37245 | 134 | 0.184837056 | PLTP | phospholipid transfer protein [Source:HGNC Symbol;Acc:HGNC:9093] |
| ENSG00000101928 | HGLibA_29683 | 134 | 0.184837056 | MOSPD1 | motile sperm domain containing 1 [Source:HGNC Symbol;Acc:HGNC:25235] |
| ENSG00000101935 | HGLibA_01801 | 134 | 0.184837056 | AMMECR1 | midface hypoplasia and elliptocytosis chromosomal region gene 1 [Source:HGNC Symbol;Acc:HGNC:467]" |
| ENSG00000102891 | HGLibA_30308 | 134 | 0.184837056 | MT4 | metallothionein 4 [Source:HGNC Symbol;Acc:HGNC:18705] |
| ENSG00000103066 | HGLibA_36877 | 134 | 0.184837056 | PLA2G15 | phospholipase A2 group XV [Source:HGNC Symbol;Acc:HGNC:17163] |
| ENSG00000103202 | HGLibA_32164 | 134 | 0.184837056 | NME4 | NME/NM23 nucleoside diphosphate kinase 4 [Source:HGNC Symbol;Acc:HGNC:7852] |
| ENSG00000104783 | HGLibA_24363 | 134 | 0.184837056 | KCNN4 | potassium calcium-activated channel subfamily N member 4 [Source:HGNC Symbol;Acc:HGNC:6293] |
| ENSG00000104894 | HGLibA_08363 | 134 | 0.184837056 | CD37 | CD37 molecule [Source:HGNC Symbol;Acc:HGNC:1666] |
| ENSG00000105281 | HGLibA_44518 | 134 | 0.184837056 | SLC1A5 | solute carrier family 1 member 5 [Source:HGNC Symbol;Acc:HGNC:10943] |
| ENSG00000105329 | HGLibA_49207 | 134 | 0.184837056 | TGFB1 | transforming growth factor beta 1 [Source:HGNC Symbol;Acc:HGNC:11766] |
| ENSG00000105483 | HGLibA_07239 | 134 | 0.184837056 | CARD8 | caspase recruitment domain family member 8 [Source:HGNC Symbol;Acc:HGNC:17057] |
| ENSG00000105821 | HGLibA_13594 | 134 | 0.184837056 | DNAJC2 | DnaJ heat shock protein family (Hsp40) member C2 [Source:HGNC Symbol;Acc:HGNC:13192] |
| ENSG00000107669 | HGLibA_03427 | 134 | 0.184837056 | ATE1 | arginyltransferase 1 [Source:HGNC Symbol;Acc:HGNC:782] |
| ENSG00000108515 | HGLibA_15146 | 134 | 0.184837056 | ENO3 | enolase 3 [Source:HGNC Symbol;Acc:HGNC:3354] |
| ENSG00000109089 | HGLibA_08870 | 134 | 0.184837056 | CDR2L | cerebellar degeneration related protein 2 like [Source:HGNC Symbol;Acc:HGNC:29999] |
| ENSG00000111254 | HGLibA_01431 | 134 | 0.184837056 | AKAP3 | A-kinase anchoring protein 3 [Source:HGNC Symbol;Acc:HGNC:373] |
| ENSG00000111679 | HGLibA_39476 | 134 | 0.184837056 | PTPN6 | non-receptor type 6 [Source:HGNC Symbol;Acc:HGNC:9658]" |
| ENSG00000112715 | HGLibA_53612 | 134 | 0.184837056 | VEGFA | vascular endothelial growth factor A [Source:HGNC Symbol;Acc:HGNC:12680] |
| ENSG00000112769 | HGLibA_25951 | 134 | 0.184837056 | LAMA4 | laminin subunit alpha 4 [Source:HGNC Symbol;Acc:HGNC:6484] |
| ENSG00000112983 | HGLibA_04786 | 134 | 0.184837056 | BRD8 | bromodomain containing 8 [Source:HGNC Symbol;Acc:HGNC:19874] |
| ENSG00000113327 | HGLibA_18392 | 134 | 0.184837056 | GABRG2 | gamma-aminobutyric acid type A receptor gamma2 subunit [Source:HGNC Symbol;Acc:HGNC:4087] |
| ENSG00000115423 | HGLibA_13492 | 134 | 0.184837056 | DNAH6 | dynein axonemal heavy chain 6 [Source:HGNC Symbol;Acc:HGNC:2951] |
| ENSG00000115616 | HGLibA_45404 | 134 | 0.184837056 | SLC9A2 | solute carrier family 9 member A2 [Source:HGNC Symbol;Acc:HGNC:11072] |
| ENSG00000116882 | HGLibA_20834 | 134 | 0.184837056 | HAO2 | hydroxyacid oxidase 2 [Source:HGNC Symbol;Acc:HGNC:4810] |
| ENSG00000117281 | HGLibA_08241 | 134 | 0.184837056 | CD160 | CD160 molecule [Source:HGNC Symbol;Acc:HGNC:17013] |
| ENSG00000119705 | HGLibA_45501 | 134 | 0.184837056 | SLIRP | SRA stem-loop interacting RNA binding protein [Source:HGNC Symbol;Acc:HGNC:20495] |
| ENSG00000119912 | HGLibA_22537 | 134 | 0.184837056 | IDE | insulin degrading enzyme [Source:HGNC Symbol;Acc:HGNC:5381] |
| ENSG00000121989 | HGLibA_00696 | 134 | 0.184837056 | ACVR2A | activin A receptor type 2A [Source:HGNC Symbol;Acc:HGNC:173] |
| ENSG00000123992 | HGLibA_13710 | 134 | 0.184837056 | DNPEP | aspartyl aminopeptidase [Source:HGNC Symbol;Acc:HGNC:2981] |
| ENSG00000124003 | HGLibA_29624 | 134 | 0.184837056 | MOGAT1 | monoacylglycerol O-acyltransferase 1 [Source:HGNC Symbol;Acc:HGNC:18210] |
| ENSG00000124140 | HGLibA_44368 | 134 | 0.184837056 | SLC12A5 | solute carrier family 12 member 5 [Source:HGNC Symbol;Acc:HGNC:13818] |
| ENSG00000126562 | HGLibA_54339 | 134 | 0.184837056 | WNK4 | WNK lysine deficient protein kinase 4 [Source:HGNC Symbol;Acc:HGNC:14544] |
| ENSG00000126709 | HGLibA_22617 | 134 | 0.184837056 | IFI6 | interferon alpha inducible protein 6 [Source:HGNC Symbol;Acc:HGNC:4054] |
| ENSG00000126947 | HGLibA_03037 | 134 | 0.184837056 | ARMCX1 | armadillo repeat containing X-linked 1 [Source:HGNC Symbol;Acc:HGNC:18073] |
| ENSG00000130159 | HGLibA_14421 | 134 | 0.184837056 | ECSIT | ECSIT signalling integrator [Source:HGNC Symbol;Acc:HGNC:29548] |
| ENSG00000130702 | HGLibA_25952 | 134 | 0.184837056 | LAMA5 | laminin subunit alpha 5 [Source:HGNC Symbol;Acc:HGNC:6485] |
| ENSG00000131446 | HGLibA_29131 | 134 | 0.184837056 | MGAT1 | 2-N-acetylglucosaminyltransferase [Source:HGNC Symbol;Acc:HGNC:7044]" |
| ENSG00000131725 | HGLibA_54093 | 134 | 0.184837056 | WDR44 | WD repeat domain 44 [Source:HGNC Symbol;Acc:HGNC:30512] |
| ENSG00000132361 | HGLibA_10162 | 134 | 0.184837056 | CLUH | clustered mitochondria homolog [Source:HGNC Symbol;Acc:HGNC:29094] |
| ENSG00000132612 | HGLibA_53798 | 134 | 0.184837056 | VPS4A | vacuolar protein sorting 4 homolog A [Source:HGNC Symbol;Acc:HGNC:13488] |
| ENSG00000134056 | HGLibA_30101 | 134 | 0.184837056 | MRPS36 | mitochondrial ribosomal protein S36 [Source:HGNC Symbol;Acc:HGNC:16631] |
| ENSG00000134324 | HGLibA_27095 | 134 | 0.184837056 | LPIN1 | lipin 1 [Source:HGNC Symbol;Acc:HGNC:13345] |
| ENSG00000135454 | HGLibA_04011 | 134 | 0.184837056 | B4GALNT1 | 4-N-acetyl-galactosaminyltransferase 1 [Source:HGNC Symbol;Acc:HGNC:4117]" |
| ENSG00000136104 | HGLibA_41429 | 134 | 0.184837056 | RNASEH2B | ribonuclease H2 subunit B [Source:HGNC Symbol;Acc:HGNC:25671] |
| ENSG00000137185 | HGLibA_57035 | 134 | 0.184837056 | ZSCAN9 | zinc finger and SCAN domain containing 9 [Source:HGNC Symbol;Acc:HGNC:12984] |
| ENSG00000138303 | HGLibA_03258 | 134 | 0.184837056 | ASCC1 | activating signal cointegrator 1 complex subunit 1 [Source:HGNC Symbol;Acc:HGNC:24268] |
| ENSG00000139151 | HGLibA_37033 | 134 | 0.184837056 | PLCZ1 | phospholipase C zeta 1 [Source:HGNC Symbol;Acc:HGNC:19218] |
| ENSG00000142227 | HGLibA_15079 | 134 | 0.184837056 | EMP3 | epithelial membrane protein 3 [Source:HGNC Symbol;Acc:HGNC:3335] |
| ENSG00000142347 | HGLibA_30834 | 134 | 0.184837056 | MYO1F | myosin IF [Source:HGNC Symbol;Acc:HGNC:7600] |
| ENSG00000143398 | HGLibA_36713 | 134 | 0.184837056 | PIP5K1A | phosphatidylinositol-4-phosphate 5-kinase type 1 alpha [Source:HGNC Symbol;Acc:HGNC:8994] |
| ENSG00000143727 | HGLibA_00508 | 134 | 0.184837056 | ACP1 | acid phosphatase 1 [Source:HGNC Symbol;Acc:HGNC:122] |
| ENSG00000144647 | HGLibA_37660 | 134 | 0.184837056 | POMGNT2 | 4-) [Source:HGNC Symbol;Acc:HGNC:25902]" |
| ENSG00000145391 | HGLibA_43658 | 134 | 0.184837056 | SETD7 | SET domain containing lysine methyltransferase 7 [Source:HGNC Symbol;Acc:HGNC:30412] |
| ENSG00000146197 | HGLibA_43076 | 134 | 0.184837056 | SCUBE3 | CUB domain and EGF like domain containing 3 [Source:HGNC Symbol;Acc:HGNC:13655]" |
| ENSG00000149781 | HGLibA_17299 | 134 | 0.184837056 | FERMT3 | fermitin family member 3 [Source:HGNC Symbol;Acc:HGNC:23151] |
| ENSG00000152558 | HGLibA_49904 | 134 | 0.184837056 | TMEM123 | transmembrane protein 123 [Source:HGNC Symbol;Acc:HGNC:30138] |
| ENSG00000153956 | HGLibA_06904 | 134 | 0.184837056 | CACNA2D1 | calcium voltage-gated channel auxiliary subunit alpha2delta 1 [Source:HGNC Symbol;Acc:HGNC:1399] |
| ENSG00000156127 | HGLibA_04172 | 134 | 0.184837056 | BATF | basic leucine zipper ATF-like transcription factor [Source:HGNC Symbol;Acc:HGNC:958] |
| ENSG00000157613 | HGLibA_11094 | 134 | 0.184837056 | CREB3L1 | cAMP responsive element binding protein 3 like 1 [Source:HGNC Symbol;Acc:HGNC:18856] |
| ENSG00000158485 | HGLibA_08268 | 134 | 0.184837056 | CD1B | CD1b molecule [Source:HGNC Symbol;Acc:HGNC:1635] |
| ENSG00000159708 | HGLibA_27323 | 134 | 0.184837056 | LRRC36 | leucine rich repeat containing 36 [Source:HGNC Symbol;Acc:HGNC:25615] |
| ENSG00000162739 | HGLibA_44315 | 134 | 0.184837056 | SLAMF6 | SLAM family member 6 [Source:HGNC Symbol;Acc:HGNC:21392] |
| ENSG00000164190 | HGLibA_31968 | 134 | 0.184837056 | NIPBL | cohesin loading factor [Source:HGNC Symbol;Acc:HGNC:28862]" |
| ENSG00000164741 | HGLibA_13311 | 134 | 0.184837056 | DLC1 | DLC1 Rho GTPase activating protein [Source:HGNC Symbol;Acc:HGNC:2897] |
| ENSG00000165030 | HGLibA_31810 | 134 | 0.184837056 | NFIL3 | interleukin 3 regulated [Source:HGNC Symbol;Acc:HGNC:7787]" |
| ENSG00000166527 | HGLibA_09984 | 134 | 0.184837056 | CLEC4D | C-type lectin domain family 4 member D [Source:HGNC Symbol;Acc:HGNC:14554] |
| ENSG00000167962 | HGLibA_56355 | 134 | 0.184837056 | ZNF598 | zinc finger protein 598 [Source:HGNC Symbol;Acc:HGNC:28079] |
| ENSG00000168159 | HGLibA_41598 | 134 | 0.184837056 | RNF187 | ring finger protein 187 [Source:HGNC Symbol;Acc:HGNC:27146] |
| ENSG00000168175 | HGLibA_28268 | 134 | 0.184837056 | MAPK1IP1L | mitogen-activated protein kinase 1 interacting protein 1 like [Source:HGNC Symbol;Acc:HGNC:19840] |
| ENSG00000168269 | HGLibA_17876 | 134 | 0.184837056 | FOXI1 | forkhead box I1 [Source:HGNC Symbol;Acc:HGNC:3815] |
| ENSG00000168495 | HGLibA_37619 | 134 | 0.184837056 | POLR3D | RNA polymerase III subunit D [Source:HGNC Symbol;Acc:HGNC:1080] |

| | | | | | |
|---|---|---|---|---|---|
| ENSG00000168748 | HGLibA_06821 | 134 | 0.184837056 | CA7 | carbonic anhydrase 7 [Source:HGNC Symbol;Acc:HGNC:1381] |
| ENSG00000168795 | HGLibA_54909 | 134 | 0.184837056 | ZBTB5 | zinc finger and BTB domain containing 5 [Source:HGNC Symbol;Acc:HGNC:23836] |
| ENSG00000169062 | HGLibA_53061 | 134 | 0.184837056 | UPF3A | regulator of nonsense mediated mRNA decay [Source:HGNC Symbol;Acc:HGNC:20332]" |
| ENSG00000169379 | HGLibA_02932 | 134 | 0.184837056 | ARL13B | ADP ribosylation factor like GTPase 13B [Source:HGNC Symbol;Acc:HGNC:25419] |
| ENSG00000169499 | HGLibA_37064 | 134 | 0.184837056 | PLEKHA2 | pleckstrin homology domain containing A2 [Source:HGNC Symbol;Acc:HGNC:14336] |
| ENSG00000169682 | HGLibA_46734 | 134 | 0.184837056 | SPNS1 | sphingolipid transporter 1 (putative) [Source:HGNC Symbol;Acc:HGNC:30621] |
| ENSG00000169856 | HGLibA_33391 | 134 | 0.184837056 | ONECUT1 | one cut homeobox 1 [Source:HGNC Symbol;Acc:HGNC:8138] |
| ENSG00000171475 | HGLibA_54308 | 134 | 0.184837056 | WIPF2 | WAS/WASL interacting protein family member 2 [Source:HGNC Symbol;Acc:HGNC:30923] |
| ENSG00000173638 | HGLibA_44497 | 134 | 0.184837056 | SLC19A1 | solute carrier family 19 member 1 [Source:HGNC Symbol;Acc:HGNC:10937] |
| ENSG00000175857 | HGLibA_18621 | 134 | 0.184837056 | GAPT | transmembrane [Source:HGNC Symbol;Acc:HGNC:26588]" |
| ENSG00000176692 | HGLibA_17826 | 134 | 0.184837056 | FOXC2 | forkhead box C2 [Source:HGNC Symbol;Acc:HGNC:3801] |
| ENSG00000177103 | HGLibA_14002 | 134 | 0.184837056 | DSCAML1 | DS cell adhesion molecule like 1 [Source:HGNC Symbol;Acc:HGNC:14656] |
| ENSG00000178187 | HGLibA_56032 | 134 | 0.184837056 | ZNF454 | zinc finger protein 454 [Source:HGNC Symbol;Acc:HGNC:21200] |
| ENSG00000180767 | HGLibA_09621 | 134 | 0.184837056 | CHST13 | carbohydrate sulfotransferase 13 [Source:HGNC Symbol;Acc:HGNC:21755] |
| ENSG00000181885 | HGLibA_09906 | 134 | 0.184837056 | CLDN7 | claudin 7 [Source:HGNC Symbol;Acc:HGNC:2049] |
| ENSG00000183908 | HGLibA_27390 | 134 | 0.184837056 | LRRC55 | leucine rich repeat containing 55 [Source:HGNC Symbol;Acc:HGNC:32324] |
| ENSG00000185664 | HGLibA_37298 | 134 | 0.184837056 | PMEL | premelanosome protein [Source:HGNC Symbol;Acc:HGNC:10880] |
| ENSG00000186115 | HGLibA_12186 | 134 | 0.184837056 | CYP4F2 | cytochrome P450 family 4 subfamily F member 2 [Source:HGNC Symbol;Acc:HGNC:2645] |
| ENSG00000186487 | HGLibA_30928 | 134 | 0.184837056 | MYT1L | myelin transcription factor 1 like [Source:HGNC Symbol;Acc:HGNC:7623] |
| ENSG00000187609 | HGLibA_15721 | 134 | 0.184837056 | EXD3 | exonuclease 3'-5' domain containing 3 [Source:HGNC Symbol;Acc:HGNC:26023] |
| ENSG00000187783 | HGLibA_50506 | 134 | 0.184837056 | TMEM72 | transmembrane protein 72 [Source:HGNC Symbol;Acc:HGNC:31658] |
| ENSG00000188687 | HGLibA_45223 | 134 | 0.184837056 | SLC4A5 | solute carrier family 4 member 5 [Source:HGNC Symbol;Acc:HGNC:18168] |
| ENSG00000197008 | HGLibA_55476 | 134 | 0.184837056 | ZNF138 | zinc finger protein 138 [Source:HGNC Symbol;Acc:HGNC:12922] |
| ENSG00000198090 | HGLibA_25770 | 134 | 0.184837056 | KRTAP4-6 | keratin associated protein 4-6 [Source:HGNC Symbol;Acc:HGNC:18909] |
| ENSG00000198883 | HGLibA_37375 | 134 | 0.184837056 | PNMA5 | PNMA family member 5 [Source:HGNC Symbol;Acc:HGNC:18743] |
| ENSG00000204219 | HGLibA_48702 | 134 | 0.184837056 | TCEA3 | transcription elongation factor A3 [Source:HGNC Symbol;Acc:HGNC:11615] |
| ENSG00000215115 | HGLibA_11933 | 134 | 0.184837056 | CXorf49 | chromosome X open reading frame 49 [Source:HGNC Symbol;Acc:HGNC:30891] |
| ENSG00000223802 | HGLibA_09199 | 134 | 0.184837056 | CERS1 | ceramide synthase 1 [Source:HGNC Symbol;Acc:HGNC:14253] |
| ENSG00000235034 | HGLibA_05715 | 134 | 0.184837056 | C19orf81 | chromosome 19 open reading frame 81 [Source:HGNC Symbol;Acc:HGNC:40041] |
| ENSG00000255112 | HGLibA_09475 | 134 | 0.184837056 | CHMP1B | charged multivesicular body protein 1B [Source:HGNC Symbol;Acc:HGNC:24287] |
| ENSG00000269343 | HGLibA_56331 | 134 | 0.184837056 | ZNF587B | zinc finger protein 587B [Source:HGNC Symbol;Acc:HGNC:37142] |
| ENSG00000278259 | HGLibA_30817 | 134 | 0.184837056 | MYO19 | myosin XIX [Source:HGNC Symbol;Acc:HGNC:26234] |
| GBAS | HGLibA_18717 | 134 | 0.184837056 | GBAS | - |
| HIST1H4G | HGLibA_21503 | 134 | 0.184837056 | HIST1H4G | - |
| hsa-mir-136 | HGLibA_57805 | 134 | 0.184837056 | hsa-mir-136 | - |
| hsa-mir-3197 | HGLibA_58887 | 134 | 0.184837056 | hsa-mir-3197 | - |
| hsa-mir-3616 | HGLibA_59095 | 134 | 0.184837056 | hsa-mir-3616 | - |
| hsa-mir-4421 | HGLibA_60070 | 134 | 0.184837056 | hsa-mir-4421 | - |
| hsa-mir-621 | HGLibA_62722 | 134 | 0.184837056 | hsa-mir-621 | - |
| hsa-mir-627 | HGLibA_62746 | 134 | 0.184837056 | hsa-mir-627 | - |
| hsa-mir-6842 | HGLibA_63544 | 134 | 0.184837056 | hsa-mir-6842 | - |
| hsa-mir-7108 | HGLibA_63792 | 134 | 0.184837056 | hsa-mir-7108 | - |
| hsa-mir-8069 | HGLibA_64113 | 134 | 0.184837056 | hsa-mir-8069 | - |
| hsa-mir-92a-2 | HGLibA_64295 | 134 | 0.184837056 | hsa-mir-92a-2 | - |
| LACE1 | HGLibA_25910 | 134 | 0.184837056 | LACE1 | - |
| LOC729020 | HGLibA_26995 | 134 | 0.184837056 | LOC729020 | - |
| LRRC16B | HGLibA_27266 | 134 | 0.184837056 | LRRC16B | - |
| 10-Mar | HGLibA_28328 | 133 | 0.174886019 | 10-Mar | - |
| ENSG00000003147 | HGLibA_22489 | 133 | 0.174886019 | ICA1 | islet cell autoantigen 1 [Source:HGNC Symbol;Acc:HGNC:5343] |
| ENSG00000006025 | HGLibA_34664 | 133 | 0.174886019 | OSBPL7 | oxysterol binding protein like 7 [Source:HGNC Symbol;Acc:HGNC:16387] |
| ENSG00000011332 | HGLibA_13826 | 133 | 0.174886019 | DPF1 | double PHD fingers 1 [Source:HGNC Symbol;Acc:HGNC:20225] |
| ENSG00000016602 | HGLibA_09810 | 133 | 0.174886019 | CLCA4 | chloride channel accessory 4 [Source:HGNC Symbol;Acc:HGNC:2018] |
| ENSG00000029993 | HGLibA_21697 | 133 | 0.174886019 | HMGB3 | high mobility group box 3 [Source:HGNC Symbol;Acc:HGNC:5004] |
| ENSG00000037241 | HGLibA_41915 | 133 | 0.174886019 | RPL26L1 | ribosomal protein L26 like 1 [Source:HGNC Symbol;Acc:HGNC:17050] |
| ENSG00000037280 | HGLibA_17671 | 133 | 0.174886019 | FLT4 | fms related tyrosine kinase 4 [Source:HGNC Symbol;Acc:HGNC:3767] |
| ENSG00000039537 | HGLibA_06424 | 133 | 0.174886019 | C6 | complement C6 [Source:HGNC Symbol;Acc:HGNC:1339] |
| ENSG00000057608 | HGLibA_18884 | 133 | 0.174886019 | GDI2 | GDP dissociation inhibitor 2 [Source:HGNC Symbol;Acc:HGNC:4227] |
| ENSG00000063015 | HGLibA_43680 | 133 | 0.174886019 | SEZ6 | seizure related 6 homolog [Source:HGNC Symbol;Acc:HGNC:15955] |
| ENSG00000068885 | HGLibA_22773 | 133 | 0.174886019 | IFT80 | intraflagellar transport 80 [Source:HGNC Symbol;Acc:HGNC:29262] |
| ENSG00000071994 | HGLibA_35728 | 133 | 0.174886019 | PDCD2 | programmed cell death 2 [Source:HGNC Symbol;Acc:HGNC:8762] |
| ENSG00000076258 | HGLibA_17712 | 133 | 0.174886019 | FMO4 | flavin containing monooxygenase 4 [Source:HGNC Symbol;Acc:HGNC:3772] |
| ENSG00000088756 | HGLibA_02748 | 133 | 0.174886019 | ARHGAP28 | Rho GTPase activating protein 28 [Source:HGNC Symbol;Acc:HGNC:25509] |
| ENSG00000092850 | HGLibA_48975 | 133 | 0.174886019 | TEKT2 | tektin 2 [Source:HGNC Symbol;Acc:HGNC:11725] |
| ENSG00000099812 | HGLibA_29312 | 133 | 0.174886019 | MISP | mitotic spindle positioning [Source:HGNC Symbol;Acc:HGNC:27000] |
| ENSG00000100105 | HGLibA_35288 | 133 | 0.174886019 | PATZ1 | POZ/BTB and AT hook containing zinc finger 1 [Source:HGNC Symbol;Acc:HGNC:13071] |
| ENSG00000101298 | HGLibA_45934 | 133 | 0.174886019 | SNPH | syntaphilin [Source:HGNC Symbol;Acc:HGNC:15931] |
| ENSG00000102290 | HGLibA_35392 | 133 | 0.174886019 | PCDH11X | protocadherin 11 X-linked [Source:HGNC Symbol;Acc:HGNC:8656] |
| ENSG00000102572 | HGLibA_47470 | 133 | 0.174886019 | STK24 | serine/threonine kinase 24 [Source:HGNC Symbol;Acc:HGNC:11403] |
| ENSG00000104368 | HGLibA_36959 | 133 | 0.174886019 | PLAT | tissue type [Source:HGNC Symbol;Acc:HGNC:9051]" |
| ENSG00000105810 | HGLibA_08791 | 133 | 0.174886019 | CDK6 | cyclin dependent kinase 6 [Source:HGNC Symbol;Acc:HGNC:1777] |
| ENSG00000109472 | HGLibA_10914 | 133 | 0.174886019 | CPE | carboxypeptidase E [Source:HGNC Symbol;Acc:HGNC:2303] |
| ENSG00000110011 | HGLibA_13621 | 133 | 0.174886019 | DNAJC4 | DnaJ heat shock protein family (Hsp40) member C4 [Source:HGNC Symbol;Acc:HGNC:5271] |
| ENSG00000115216 | HGLibA_32654 | 133 | 0.174886019 | NRBP1 | nuclear receptor binding protein 1 [Source:HGNC Symbol;Acc:HGNC:7993] |
| ENSG00000116266 | HGLibA_47670 | 133 | 0.174886019 | STXBP3 | syntaxin binding protein 3 [Source:HGNC Symbol;Acc:HGNC:11446] |
| ENSG00000119283 | HGLibA_51588 | 133 | 0.174886019 | TRIM67 | tripartite motif containing 67 [Source:HGNC Symbol;Acc:HGNC:31859] |
| ENSG00000119943 | HGLibA_39705 | 133 | 0.174886019 | PYROXD2 | pyridine nucleotide-disulphide oxidoreductase domain 2 [Source:HGNC Symbol;Acc:HGNC:23517] |
| ENSG00000120278 | HGLibA_37102 | 133 | 0.174886019 | PLEKHG1 | pleckstrin homology and RhoGEF domain containing G1 [Source:HGNC Symbol;Acc:HGNC:20884] |
| ENSG00000122122 | HGLibA_42764 | 133 | 0.174886019 | SASH3 | SAM and SH3 domain containing 3 [Source:HGNC Symbol;Acc:HGNC:15975] |
| ENSG00000124466 | HGLibA_27720 | 133 | 0.174886019 | LYPD3 | LY6/PLAUR domain containing 3 [Source:HGNC Symbol;Acc:HGNC:24880] |
| ENSG00000125675 | HGLibA_20265 | 133 | 0.174886019 | GRIA3 | glutamate ionotropic receptor AMPA type subunit 3 [Source:HGNC Symbol;Acc:HGNC:4573] |
| ENSG00000126231 | HGLibA_38707 | 133 | 0.174886019 | PROZ | vitamin K dependent plasma glycoprotein [Source:HGNC Symbol;Acc:HGNC:9460]" |
| ENSG00000129355 | HGLibA_08845 | 133 | 0.174886019 | CDKN2D | cyclin dependent kinase inhibitor 2D [Source:HGNC Symbol;Acc:HGNC:1790] |
| ENSG00000130640 | HGLibA_52310 | 133 | 0.174886019 | TUBGCP2 | tubulin gamma complex associated protein 2 [Source:HGNC Symbol;Acc:HGNC:18599] |
| ENSG00000131044 | HGLibA_52217 | 133 | 0.174886019 | TTLL9 | tubulin tyrosine ligase like 9 [Source:HGNC Symbol;Acc:HGNC:16118] |
| ENSG00000131435 | HGLibA_35917 | 133 | 0.174886019 | PDLIM4 | PDZ and LIM domain 4 [Source:HGNC Symbol;Acc:HGNC:16501] |
| ENSG00000132470 | HGLibA_23786 | 133 | 0.174886019 | ITGB4 | integrin subunit beta 4 [Source:HGNC Symbol;Acc:HGNC:6158] |
| ENSG00000133106 | HGLibA_15386 | 133 | 0.174886019 | EPSTI1 | epithelial stromal interaction 1 [Source:HGNC Symbol;Acc:HGNC:16465] |
| ENSG00000135314 | HGLibA_24557 | 133 | 0.174886019 | KHDC1 | KH domain containing 1 [Source:HGNC Symbol;Acc:HGNC:21366] |
| ENSG00000135837 | HGLibA_09134 | 133 | 0.174886019 | CEP350 | centrosomal protein 350 [Source:HGNC Symbol;Acc:HGNC:24238] |
| ENSG00000136827 | HGLibA_51015 | 133 | 0.174886019 | TOR1A | torsin family 1 member A [Source:HGNC Symbol;Acc:HGNC:3098] |
| ENSG00000136848 | HGLibA_12272 | 133 | 0.174886019 | DAB2IP | DAB2 interacting protein [Source:HGNC Symbol;Acc:HGNC:17294] |
| ENSG00000136859 | HGLibA_01914 | 133 | 0.174886019 | ANGPTL2 | angiopoietin like 2 [Source:HGNC Symbol;Acc:HGNC:490] |
| ENSG00000137203 | HGLibA_49122 | 133 | 0.174886019 | TFAP2A | transcription factor AP-2 alpha [Source:HGNC Symbol;Acc:HGNC:11742] |
| ENSG00000138835 | HGLibA_41090 | 133 | 0.174886019 | RGS3 | regulator of G protein signaling 3 [Source:HGNC Symbol;Acc:HGNC:9999] |
| ENSG00000139572 | HGLibA_20096 | 133 | 0.174886019 | GPR84 | G protein-coupled receptor 84 [Source:HGNC Symbol;Acc:HGNC:4535] |
| ENSG00000139767 | HGLibA_47050 | 133 | 0.174886019 | SRRM4 | serine/arginine repetitive matrix 4 [Source:HGNC Symbol;Acc:HGNC:29389] |
| ENSG00000140332 | HGLibA_49612 | 133 | 0.174886019 | TLE3 | transducin like enhancer of split 3 [Source:HGNC Symbol;Acc:HGNC:11839] |
| ENSG00000140403 | HGLibA_13518 | 133 | 0.174886019 | DNAJA4 | DnaJ heat shock protein family (Hsp40) member A4 [Source:HGNC Symbol;Acc:HGNC:14885] |
| ENSG00000141384 | HGLibA_48238 | 133 | 0.174886019 | TAF4B | TATA-box binding protein associated factor 4b [Source:HGNC Symbol;Acc:HGNC:11538] |
| ENSG00000143033 | HGLibA_30351 | 133 | 0.174886019 | MTF2 | metal response element binding transcription factor 2 [Source:HGNC Symbol;Acc:HGNC:29535] |
| ENSG00000143226 | HGLibA_17188 | 133 | 0.174886019 | FCGR2A | Fc fragment of IgG receptor IIa [Source:HGNC Symbol;Acc:HGNC:3616] |
| ENSG00000143630 | HGLibA_20980 | 133 | 0.174886019 | HCN3 | hyperpolarization activated cyclic nucleotide gated potassium channel 3 [Source:HGNC Symbol;Acc:HGNC:19183] |
| ENSG00000144381 | HGLibA_22330 | 133 | 0.174886019 | HSPD1 | heat shock protein family D (Hsp60) member 1 [Source:HGNC Symbol;Acc:HGNC:5261] |
| ENSG00000145545 | HGLibA_46939 | 133 | 0.174886019 | SRD5A1 | steroid 5 alpha-reductase 1 [Source:HGNC Symbol;Acc:HGNC:11284] |
| ENSG00000146857 | HGLibA_47570 | 133 | 0.174886019 | STRA8 | stimulated by retinoic acid 8 [Source:HGNC Symbol;Acc:HGNC:30653] |
| ENSG00000147081 | HGLibA_01433 | 133 | 0.174886019 | AKAP4 | A-kinase anchoring protein 4 [Source:HGNC Symbol;Acc:HGNC:374] |
| ENSG00000147894 | HGLibA_06758 | 133 | 0.174886019 | C9orf72 | chromosome 9 open reading frame 72 [Source:HGNC Symbol;Acc:HGNC:28337] |
| ENSG00000151952 | HGLibA_49938 | 133 | 0.174886019 | TMEM132D | transmembrane protein 132D [Source:HGNC Symbol;Acc:HGNC:29411] |
| ENSG00000152463 | HGLibA_33341 | 133 | 0.174886019 | OLAH | oleoyl-ACP hydrolase [Source:HGNC Symbol;Acc:HGNC:25625] |
| ENSG00000156136 | HGLibA_12495 | 133 | 0.174886019 | DCK | deoxycytidine kinase [Source:HGNC Symbol;Acc:HGNC:2704] |
| ENSG00000157005 | HGLibA_47172 | 133 | 0.174886019 | SST | somatostatin [Source:HGNC Symbol;Acc:HGNC:11329] |
| ENSG00000157103 | HGLibA_45290 | 133 | 0.174886019 | SLC6A1 | solute carrier family 6 member 1 [Source:HGNC Symbol;Acc:HGNC:11042] |
| ENSG00000158553 | HGLibA_37651 | 133 | 0.174886019 | POM121L2 | POM121 transmembrane nucleoporin like 2 [Source:HGNC Symbol;Acc:HGNC:13973] |
| ENSG00000159082 | HGLibA_48002 | 133 | 0.174886019 | SYNJ1 | synaptojanin 1 [Source:HGNC Symbol;Acc:HGNC:11503] |
| ENSG00000160877 | HGLibA_31039 | 133 | 0.174886019 | NACC1 | nucleus accumbens associated 1 [Source:HGNC Symbol;Acc:HGNC:20967] |
| ENSG00000162599 | HGLibA_31800 | 133 | 0.174886019 | NFIA | nuclear factor I A [Source:HGNC Symbol;Acc:HGNC:7784] |
| ENSG00000163069 | HGLibA_43799 | 133 | 0.174886019 | SGCB | sarcoglycan beta [Source:HGNC Symbol;Acc:HGNC:10806] |
| ENSG00000163220 | HGLibA_42575 | 133 | 0.174886019 | S100A9 | S100 calcium binding protein A9 [Source:HGNC Symbol;Acc:HGNC:10499] |
| ENSG00000163687 | HGLibA_13663 | 133 | 0.174886019 | DNASE1L3 | deoxyribonuclease 1 like 3 [Source:HGNC Symbol;Acc:HGNC:2959] |
| ENSG00000164070 | HGLibA_22286 | 133 | 0.174886019 | HSPA4L | heat shock protein family A (Hsp70) member 4 like [Source:HGNC Symbol;Acc:HGNC:17041] |
| ENSG00000164756 | HGLibA_44920 | 133 | 0.174886019 | SLC30A8 | solute carrier family 30 member 8 [Source:HGNC Symbol;Acc:HGNC:20303] |
| ENSG00000164761 | HGLibA_50747 | 133 | 0.174886019 | TNFRSF11B | TNF receptor superfamily member 11b [Source:HGNC Symbol;Acc:HGNC:11909] |
| ENSG00000166147 | HGLibA_16954 | 133 | 0.174886019 | FBN1 | fibrillin 1 [Source:HGNC Symbol;Acc:HGNC:3603] |
| ENSG00000166257 | HGLibA_42989 | 133 | 0.174886019 | SCN3B | sodium voltage-gated channel beta subunit 3 [Source:HGNC Symbol;Acc:HGNC:20665] |
| ENSG00000166801 | HGLibA_16023 | 133 | 0.174886019 | FAM111A | family with sequence similarity 111 member A [Source:HGNC Symbol;Acc:HGNC:24725] |
| ENSG00000166886 | HGLibA_31020 | 133 | 0.174886019 | NAB2 | NGFI-A binding protein 2 [Source:HGNC Symbol;Acc:HGNC:7627] |
| ENSG00000167635 | HGLibA_55495 | 133 | 0.174886019 | ZNF146 | zinc finger protein 146 [Source:HGNC Symbol;Acc:HGNC:12931] |
| ENSG00000168394 | HGLibA_48316 | 133 | 0.174886019 | TAP1 | ATP binding cassette subfamily B member [Source:HGNC Symbol;Acc:HGNC:43]" |
| ENSG00000169193 | HGLibA_07581 | 133 | 0.174886019 | CCDC126 | coiled-coil domain containing 126 [Source:HGNC Symbol;Acc:HGNC:22398] |
| ENSG00000169679 | HGLibA_04989 | 133 | 0.174886019 | BUB1 | BUB1 mitotic checkpoint serine/threonine kinase [Source:HGNC Symbol;Acc:HGNC:1148] |
| ENSG00000170162 | HGLibA_53637 | 133 | 0.174886019 | VGLL2 | vestigial like family member 2 [Source:HGNC Symbol;Acc:HGNC:20232] |
| ENSG00000171469 | HGLibA_56244 | 133 | 0.174886019 | ZNF561 | zinc finger protein 561 [Source:HGNC Symbol;Acc:HGNC:28684] |
| ENSG00000171496 | HGLibA_33698 | 133 | 0.174886019 | OR1L8 | olfactory receptor family 1 subfamily L member 8 [Source:HGNC Symbol;Acc:HGNC:15110] |
| ENSG00000174516 | HGLibA_36038 | 133 | 0.174886019 | PELI3 | pellino E3 ubiquitin protein ligase family member 3 [Source:HGNC Symbol;Acc:HGNC:30010] |
| ENSG00000174903 | HGLibA_39833 | 133 | 0.174886019 | RAB1B | member RAS oncogene family [Source:HGNC Symbol;Acc:HGNC:18370]" |

| | | | | | |
|---|---|---|---|---|---|
| ENSG00000176410 | HGLibA_13617 | 133 | 0.174886019 | DNAJC30 | DnaJ heat shock protein family (Hsp40) member C30 [Source:HGNC Symbol;Acc:HGNC:16410] |
| ENSG00000176809 | HGLibA_27332 | 133 | 0.174886019 | LRRC37A3 | leucine rich repeat containing 37 member A3 [Source:HGNC Symbol;Acc:HGNC:32427] |
| ENSG00000176973 | HGLibA_16785 | 133 | 0.174886019 | FAM89B | family with sequence similarity 89 member B [Source:HGNC Symbol;Acc:HGNC:16708] |
| ENSG00000176986 | HGLibA_43234 | 133 | 0.174886019 | SEC24C | COPII coat complex component [Source:HGNC Symbol;Acc:HGNC:10705]" |
| ENSG00000177363 | HGLibA_27481 | 133 | 0.174886019 | LRRN4CL | LRRN4 C-terminal like [Source:HGNC Symbol;Acc:HGNC:33724] |
| ENSG00000177669 | HGLibA_28524 | 133 | 0.174886019 | MBOAT4 | membrane bound O-acyltransferase domain containing 4 [Source:HGNC Symbol;Acc:HGNC:32311] |
| ENSG00000178538 | HGLibA_06823 | 133 | 0.174886019 | CA8 | carbonic anhydrase 8 [Source:HGNC Symbol;Acc:HGNC:1382] |
| ENSG00000179083 | HGLibA_16116 | 133 | 0.174886019 | FAM133A | family with sequence similarity 133 member A [Source:HGNC Symbol;Acc:HGNC:26748] |
| ENSG00000180638 | HGLibA_45195 | 133 | 0.174886019 | SLC47A2 | solute carrier family 47 member 2 [Source:HGNC Symbol;Acc:HGNC:26439] |
| ENSG00000180957 | HGLibA_36744 | 133 | 0.174886019 | PITPNB | phosphatidylinositol transfer protein beta [Source:HGNC Symbol;Acc:HGNC:9002] |
| ENSG00000182022 | HGLibA_09627 | 133 | 0.174886019 | CHST15 | carbohydrate sulfotransferase 15 [Source:HGNC Symbol;Acc:HGNC:18137] |
| ENSG00000183484 | HGLibA_19889 | 133 | 0.174886019 | GPR132 | G protein-coupled receptor 132 [Source:HGNC Symbol;Acc:HGNC:17482] |
| ENSG00000184857 | HGLibA_50125 | 133 | 0.174886019 | TMEM186 | transmembrane protein 186 [Source:HGNC Symbol;Acc:HGNC:24530] |
| ENSG00000184986 | HGLibA_49901 | 133 | 0.174886019 | TMEM121 | transmembrane protein 121 [Source:HGNC Symbol;Acc:HGNC:20511] |
| ENSG00000184988 | HGLibA_49853 | 133 | 0.174886019 | TMEM106A | transmembrane protein 106A [Source:HGNC Symbol;Acc:HGNC:28288] |
| ENSG00000186132 | HGLibA_06183 | 133 | 0.174886019 | C2orf76 | chromosome 2 open reading frame 76 [Source:HGNC Symbol;Acc:HGNC:27017] |
| ENSG00000186994 | HGLibA_24030 | 133 | 0.174886019 | KANK3 | KN motif and ankyrin repeat domains 3 [Source:HGNC Symbol;Acc:HGNC:24796] |
| ENSG00000187556 | HGLibA_31102 | 133 | 0.174886019 | NANOS3 | nanos C2HC-type zinc finger 3 [Source:HGNC Symbol;Acc:HGNC:22048] |
| ENSG00000188379 | HGLibA_22674 | 133 | 0.174886019 | IFNA2 | interferon alpha 2 [Source:HGNC Symbol;Acc:HGNC:5423] |
| ENSG00000188993 | HGLibA_27409 | 133 | 0.174886019 | LRRC66 | leucine rich repeat containing 66 [Source:HGNC Symbol;Acc:HGNC:34299] |
| ENSG00000197261 | HGLibA_06448 | 133 | 0.174886019 | C6orf141 | chromosome 6 open reading frame 141 [Source:HGNC Symbol;Acc:HGNC:21351] |
| ENSG00000197566 | HGLibA_56415 | 133 | 0.174886019 | ZNF624 | zinc finger protein 624 [Source:HGNC Symbol;Acc:HGNC:29254] |
| ENSG00000197763 | HGLibA_52449 | 133 | 0.174886019 | TXNRD3 | thioredoxin reductase 3 [Source:HGNC Symbol;Acc:HGNC:20667] |
| ENSG00000197766 | HGLibA_09253 | 133 | 0.174886019 | CFD | complement factor D [Source:HGNC Symbol;Acc:HGNC:2771] |
| ENSG00000197774 | HGLibA_15039 | 133 | 0.174886019 | EME2 | essential meiotic structure-specific endonuclease subunit 2 [Source:HGNC Symbol;Acc:HGNC:27289] |
| ENSG00000198682 | HGLibA_35154 | 133 | 0.174886019 | PAPSS2 | 3'-phosphoadenosine 5'-phosphosulfate synthase 2 [Source:HGNC Symbol;Acc:HGNC:8604] |
| ENSG00000203780 | HGLibA_16863 | 133 | 0.174886019 | FANK1 | fibronectin type III and ankyrin repeat domains 1 [Source:HGNC Symbol;Acc:HGNC:23527] |
| ENSG00000203867 | HGLibA_40507 | 133 | 0.174886019 | RBM20 | RNA binding motif protein 20 [Source:HGNC Symbol;Acc:HGNC:27424] |
| ENSG00000204271 | HGLibA_46676 | 133 | 0.174886019 | SPIN3 | spindlin family member 3 [Source:HGNC Symbol;Acc:HGNC:27272] |
| ENSG00000204923 | HGLibA_17116 | 133 | 0.174886019 | FBXO48 | F-box protein 48 [Source:HGNC Symbol;Acc:HGNC:33857] |
| ENSG00000205269 | HGLibA_50056 | 133 | 0.174886019 | TMEM170B | transmembrane protein 170B [Source:HGNC Symbol;Acc:HGNC:34244] |
| ENSG00000205581 | HGLibA_21717 | 133 | 0.174886019 | HMGN1 | high mobility group nucleosome binding domain 1 [Source:HGNC Symbol;Acc:HGNC:4984] |
| ENSG00000205922 | HGLibA_33399 | 133 | 0.174886019 | ONECUT3 | one cut homeobox 3 [Source:HGNC Symbol;Acc:HGNC:13399] |
| ENSG00000212126 | HGLibA_48419 | 133 | 0.174886019 | TAS2R50 | taste 2 receptor member 50 [Source:HGNC Symbol;Acc:HGNC:18882] |
| ENSG00000213337 | HGLibA_02092 | 133 | 0.174886019 | ANKRD39 | ankyrin repeat domain 39 [Source:HGNC Symbol;Acc:HGNC:28640] |
| ENSG00000215454 | HGLibA_25609 | 133 | 0.174886019 | KRTAP10-4 | keratin associated protein 10-4 [Source:HGNC Symbol;Acc:HGNC:20521] |
| ENSG00000227057 | HGLibA_54103 | 133 | 0.174886019 | WDR46 | WD repeat domain 46 [Source:HGNC Symbol;Acc:HGNC:13923] |
| ENSG00000233493 | HGLibA_50278 | 133 | 0.174886019 | TMEM238 | transmembrane protein 238 [Source:HGNC Symbol;Acc:HGNC:40042] |
| ENSG00000236737 | HGLibA_18436 | 133 | 0.174886019 | GAGE12B | G antigen 12B [Source:HGNC Symbol;Acc:HGNC:26779] |
| ENSG00000248385 | HGLibA_48338 | 133 | 0.174886019 | TARM1 | activating receptor on myeloid cells 1 [Source:HGNC Symbol;Acc:HGNC:37250]" |
| ENSG00000248672 | HGLibA_27682 | 133 | 0.174886019 | LY75-CD302 | LY75-CD302 readthrough [Source:HGNC Symbol;Acc:HGNC:38828] |
| ENSG00000255307 | HGLibA_34144 | 133 | 0.174886019 | OR52B2 | olfactory receptor family 52 subfamily B member 2 [Source:HGNC Symbol;Acc:HGNC:15207] |
| ENSG00000273540 | HGLibA_01165 | 133 | 0.174886019 | AGBL1 | ATP/GTP binding protein like 1 [Source:HGNC Symbol;Acc:HGNC:26504] |
| ENSG00000280090 | HGLibA_34501 | 133 | 0.174886019 | OR8B4 | olfactory receptor family 8 subfamily B member 4 (gene/pseudogene) [Source:HGNC Symbol;Acc:HGNC:8473] |
| FAM194A | HGLibA_16369 | 133 | 0.174886019 | FAM194A | - |
| FOPNL | HGLibA_17792 | 133 | 0.174886019 | FOPNL | - |
| HIST1H2AB | HGLibA_21377 | 133 | 0.174886019 | HIST1H2AB | - |
| hsa-mir-1251 | HGLibA_57403 | 133 | 0.174886019 | hsa-mir-1251 | - |
| hsa-mir-1291 | HGLibA_57641 | 133 | 0.174886019 | hsa-mir-1291 | - |
| hsa-mir-1293 | HGLibA_57648 | 133 | 0.174886019 | hsa-mir-1293 | - |
| hsa-mir-193a | HGLibA_58074 | 133 | 0.174886019 | hsa-mir-193a | - |
| hsa-mir-3196 | HGLibA_58880 | 133 | 0.174886019 | hsa-mir-3196 | - |
| hsa-mir-4282 | HGLibA_59830 | 133 | 0.174886019 | hsa-mir-4282 | - |
| hsa-mir-4473 | HGLibA_60304 | 133 | 0.174886019 | hsa-mir-4473 | - |
| hsa-mir-5007 | HGLibA_61433 | 133 | 0.174886019 | hsa-mir-5007 | - |
| hsa-mir-5692b | HGLibA_62295 | 133 | 0.174886019 | hsa-mir-5692b | - |
| hsa-mir-617 | HGLibA_62709 | 133 | 0.174886019 | hsa-mir-617 | - |
| hsa-mir-6782 | HGLibA_63307 | 133 | 0.174886019 | hsa-mir-6782 | - |
| hsa-mir-6805 | HGLibA_63399 | 133 | 0.174886019 | hsa-mir-6805 | - |
| hsa-mir-6846 | HGLibA_63557 | 133 | 0.174886019 | hsa-mir-6846 | - |
| hsa-mir-6866 | HGLibA_63650 | 133 | 0.174886019 | hsa-mir-6866 | - |
| hsa-mir-8052 | HGLibA_64047 | 133 | 0.174886019 | hsa-mir-8052 | - |
| LOC643037 | HGLibA_26962 | 133 | 0.174886019 | LOC643037 | - |
| APOBEC3A_B | HGLibA_02475 | 132 | 0.164934981 | APOBEC3A_B | - |
| C22orf43 | HGLibA_06080 | 132 | 0.164934981 | C22orf43 | - |
| C5orf45 | HGLibA_06386 | 132 | 0.164934981 | C5orf45 | - |
| C9orf173 | HGLibA_06715 | 132 | 0.164934981 | C9orf173 | - |
| ENSG00000040531 | HGLibA_11664 | 132 | 0.164934981 | CTNS | lysosomal cystine transporter [Source:HGNC Symbol;Acc:HGNC:2518]" |
| ENSG00000050344 | HGLibA_31798 | 132 | 0.164934981 | NFE2L3 | erythroid 2 like 3 [Source:HGNC Symbol;Acc:HGNC:7783]" |
| ENSG00000055332 | HGLibA_14726 | 132 | 0.164934981 | EIF2AK2 | eukaryotic translation initiation factor 2 alpha kinase 2 [Source:HGNC Symbol;Acc:HGNC:9437] |
| ENSG00000061987 | HGLibA_29646 | 132 | 0.164934981 | MON2 | regulator of endosome-to-Golgi trafficking [Source:HGNC Symbol;Acc:HGNC:29177]" |
| ENSG00000063015 | HGLibA_43678 | 132 | 0.164934981 | SEZ6 | seizure related 6 homolog [Source:HGNC Symbol;Acc:HGNC:15955] |
| ENSG00000065548 | HGLibA_54967 | 132 | 0.164934981 | ZC3H15 | zinc finger CCCH-type containing 15 [Source:HGNC Symbol;Acc:HGNC:29528] |
| ENSG00000071889 | HGLibA_16576 | 132 | 0.164934981 | FAM3A | family with sequence similarity 3 member A [Source:HGNC Symbol;Acc:HGNC:13749] |
| ENSG00000081051 | HGLibA_01127 | 132 | 0.164934981 | AFP | alpha fetoprotein [Source:HGNC Symbol;Acc:HGNC:317] |
| ENSG00000088833 | HGLibA_32754 | 132 | 0.164934981 | NSFL1C | NSFL1 cofactor [Source:HGNC Symbol;Acc:HGNC:15912] |
| ENSG00000090060 | HGLibA_35135 | 132 | 0.164934981 | PAPOLA | poly(A) polymerase alpha [Source:HGNC Symbol;Acc:HGNC:14981] |
| ENSG00000099250 | HGLibA_32715 | 132 | 0.164934981 | NRP1 | neuropilin 1 [Source:HGNC Symbol;Acc:HGNC:8004] |
| ENSG00000100068 | HGLibA_27226 | 132 | 0.164934981 | LRP5L | LDL receptor related protein 5 like [Source:HGNC Symbol;Acc:HGNC:25323] |
| ENSG00000100206 | HGLibA_13400 | 132 | 0.164934981 | DMC1 | DNA meiotic recombinase 1 [Source:HGNC Symbol;Acc:HGNC:2927] |
| ENSG00000100376 | HGLibA_16048 | 132 | 0.164934981 | FAM118A | family with sequence similarity 118 member A [Source:HGNC Symbol;Acc:HGNC:1313] |
| ENSG00000100380 | HGLibA_47222 | 132 | 0.164934981 | ST13 | Hsp70 interacting protein [Source:HGNC Symbol;Acc:HGNC:11343]" |
| ENSG00000101144 | HGLibA_04618 | 132 | 0.164934981 | BMP7 | bone morphogenetic protein 7 [Source:HGNC Symbol;Acc:HGNC:1074] |
| ENSG00000102967 | HGLibA_13113 | 132 | 0.164934981 | DHODH | dihydroorotate dehydrogenase (quinone) [Source:HGNC Symbol;Acc:HGNC:2867] |
| ENSG00000104325 | HGLibA_12775 | 132 | 0.164934981 | DECR1 | 4-dienoyl-CoA reductase 1 [Source:HGNC Symbol;Acc:HGNC:2753]" |
| ENSG00000105392 | HGLibA_11238 | 132 | 0.164934981 | CRX | cone-rod homeobox [Source:HGNC Symbol;Acc:HGNC:2383] |
| ENSG00000105723 | HGLibA_20427 | 132 | 0.164934981 | GSK3A | glycogen synthase kinase 3 alpha [Source:HGNC Symbol;Acc:HGNC:4616] |
| ENSG00000107669 | HGLibA_03429 | 132 | 0.164934981 | ATE1 | arginyltransferase 1 [Source:HGNC Symbol;Acc:HGNC:782] |
| ENSG00000107829 | HGLibA_17147 | 132 | 0.164934981 | FBXW4 | F-box and WD repeat domain containing 4 [Source:HGNC Symbol;Acc:HGNC:10847] |
| ENSG00000108813 | HGLibA_13380 | 132 | 0.164934981 | DLX4 | distal-less homeobox 4 [Source:HGNC Symbol;Acc:HGNC:2917] |
| ENSG00000109861 | HGLibA_11694 | 132 | 0.164934981 | CTSC | cathepsin C [Source:HGNC Symbol;Acc:HGNC:2528] |
| ENSG00000112041 | HGLibA_52330 | 132 | 0.164934981 | TULP1 | tubby like protein 1 [Source:HGNC Symbol;Acc:HGNC:12423] |
| ENSG00000117395 | HGLibA_14371 | 132 | 0.164934981 | EBNA1BP2 | EBNA1 binding protein 2 [Source:HGNC Symbol;Acc:HGNC:15531] |
| ENSG00000117461 | HGLibA_36654 | 132 | 0.164934981 | PIK3R3 | phosphoinositide-3-kinase regulatory subunit 3 [Source:HGNC Symbol;Acc:HGNC:8981] |
| ENSG00000122035 | HGLibA_40366 | 132 | 0.164934981 | RASL11A | RAS like family 11 member A [Source:HGNC Symbol;Acc:HGNC:23802] |
| ENSG00000125454 | HGLibA_44662 | 132 | 0.164934981 | SLC25A19 | solute carrier family 25 member 19 [Source:HGNC Symbol;Acc:HGNC:14409] |
| ENSG00000126903 | HGLibA_44335 | 132 | 0.164934981 | SLC10A3 | solute carrier family 10 member 3 [Source:HGNC Symbol;Acc:HGNC:22979] |
| ENSG00000129048 | HGLibA_00448 | 132 | 0.164934981 | ACKR4 | atypical chemokine receptor 4 [Source:HGNC Symbol;Acc:HGNC:1611] |
| ENSG00000129460 | HGLibA_31865 | 132 | 0.164934981 | NGDN | neuroguidin [Source:HGNC Symbol;Acc:HGNC:20271] |
| ENSG00000129625 | HGLibA_40787 | 132 | 0.164934981 | REEP5 | receptor accessory protein 5 [Source:HGNC Symbol;Acc:HGNC:30077] |
| ENSG00000129673 | HGLibA_00060 | 132 | 0.164934981 | AANAT | aralkylamine N-acetyltransferase [Source:HGNC Symbol;Acc:HGNC:19] |
| ENSG00000130270 | HGLibA_03786 | 132 | 0.164934981 | ATP8B3 | ATPase phospholipid transporting 8B3 [Source:HGNC Symbol;Acc:HGNC:13535] |
| ENSG00000134853 | HGLibA_35852 | 132 | 0.164934981 | PDGFRA | platelet derived growth factor receptor alpha [Source:HGNC Symbol;Acc:HGNC:8803] |
| ENSG00000135525 | HGLibA_28233 | 132 | 0.164934981 | MAP7 | microtubule associated protein 7 [Source:HGNC Symbol;Acc:HGNC:6869] |
| ENSG00000135905 | HGLibA_13733 | 132 | 0.164934981 | DOCK10 | dedicator of cytokinesis 10 [Source:HGNC Symbol;Acc:HGNC:23479] |
| ENSG00000135960 | HGLibA_14437 | 132 | 0.164934981 | EDAR | ectodysplasin A receptor [Source:HGNC Symbol;Acc:HGNC:2895] |
| ENSG00000136286 | HGLibA_30837 | 132 | 0.164934981 | MYO1G | myosin IG [Source:HGNC Symbol;Acc:HGNC:13880] |
| ENSG00000137154 | HGLibA_42154 | 132 | 0.164934981 | RPS6 | ribosomal protein S6 [Source:HGNC Symbol;Acc:HGNC:10429] |
| ENSG00000138750 | HGLibA_33060 | 132 | 0.164934981 | NUP54 | nucleoporin 54 [Source:HGNC Symbol;Acc:HGNC:17359] |
| ENSG00000140285 | HGLibA_17418 | 132 | 0.164934981 | FGF7 | fibroblast growth factor 7 [Source:HGNC Symbol;Acc:HGNC:3685] |
| ENSG00000141447 | HGLibA_34650 | 132 | 0.164934981 | OSBPL1A | oxysterol binding protein like 1A [Source:HGNC Symbol;Acc:HGNC:16398] |
| ENSG00000141622 | HGLibA_41557 | 132 | 0.164934981 | RNF165 | ring finger protein 165 [Source:HGNC Symbol;Acc:HGNC:31696] |
| ENSG00000142583 | HGLibA_44883 | 132 | 0.164934981 | SLC2A5 | solute carrier family 2 member 5 [Source:HGNC Symbol;Acc:HGNC:11010] |
| ENSG00000143322 | HGLibA_00306 | 132 | 0.164934981 | ABL2 | non-receptor tyrosine kinase [Source:HGNC Symbol;Acc:HGNC:77]" |
| ENSG00000145741 | HGLibA_04934 | 132 | 0.164934981 | BTF3 | basic transcription factor 3 [Source:HGNC Symbol;Acc:HGNC:1125] |
| ENSG00000147421 | HGLibA_21666 | 132 | 0.164934981 | HMBOX1 | homeobox containing 1 [Source:HGNC Symbol;Acc:HGNC:26137] |
| ENSG00000151617 | HGLibA_14478 | 132 | 0.164934981 | EDNRA | endothelin receptor type A [Source:HGNC Symbol;Acc:HGNC:3179] |
| ENSG00000159214 | HGLibA_07736 | 132 | 0.164934981 | CCDC24 | coiled-coil domain containing 24 [Source:HGNC Symbol;Acc:HGNC:28688] |
| ENSG00000159217 | HGLibA_22800 | 132 | 0.164934981 | IGF2BP1 | insulin like growth factor 2 mRNA binding protein 1 [Source:HGNC Symbol;Acc:HGNC:28866] |
| ENSG00000159873 | HGLibA_07559 | 132 | 0.164934981 | CCDC117 | coiled-coil domain containing 117 [Source:HGNC Symbol;Acc:HGNC:26599] |
| ENSG00000160182 | HGLibA_49170 | 132 | 0.164934981 | TFF1 | trefoil factor 1 [Source:HGNC Symbol;Acc:HGNC:11755] |
| ENSG00000160190 | HGLibA_45042 | 132 | 0.164934981 | SLC37A1 | solute carrier family 37 member 1 [Source:HGNC Symbol;Acc:HGNC:11024] |
| ENSG00000160392 | HGLibA_05668 | 132 | 0.164934981 | C19orf47 | chromosome 19 open reading frame 47 [Source:HGNC Symbol;Acc:HGNC:26723] |
| ENSG00000160999 | HGLibA_43878 | 132 | 0.164934981 | SH2B2 | SH2B adaptor protein 2 [Source:HGNC Symbol;Acc:HGNC:17381] |
| ENSG00000162783 | HGLibA_22581 | 132 | 0.164934981 | IER5 | immediate early response 5 [Source:HGNC Symbol;Acc:HGNC:5393] |
| ENSG00000162852 | HGLibA_10384 | 132 | 0.164934981 | CNST | connexin sorting protein [Source:HGNC Symbol;Acc:HGNC:26486]" |
| ENSG00000163352 | HGLibA_26293 | 132 | 0.164934981 | LENEP | lens epithelial protein [Source:HGNC Symbol;Acc:HGNC:14429] |
| ENSG00000164684 | HGLibA_56583 | 132 | 0.164934981 | ZNF704 | zinc finger protein 704 [Source:HGNC Symbol;Acc:HGNC:32291] |
| ENSG00000166152 | HGLibA_05482 | 132 | 0.164934981 | C16orf78 | chromosome 16 open reading frame 78 [Source:HGNC Symbol;Acc:HGNC:28479] |
| ENSG00000166439 | HGLibA_41568 | 132 | 0.164934981 | RNF169 | ring finger protein 169 [Source:HGNC Symbol;Acc:HGNC:26961] |
| ENSG00000166598 | HGLibA_22257 | 132 | 0.164934981 | HSP90B1 | heat shock protein 90 beta family member 1 [Source:HGNC Symbol;Acc:HGNC:12028] |
| ENSG00000167311 | HGLibA_03167 | 132 | 0.164934981 | ART5 | ADP-ribosyltransferase 5 [Source:HGNC Symbol;Acc:HGNC:24049] |
| ENSG00000167914 | HGLibA_20403 | 132 | 0.164934981 | GSDMA | gasdermin A [Source:HGNC Symbol;Acc:HGNC:13311] |
| ENSG00000168214 | HGLibA_40647 | 132 | 0.164934981 | RBPJ | recombination signal binding protein for immunoglobulin kappa J region [Source:HGNC Symbol;Acc:HGNC:5724] |
| ENSG00000170848 | HGLibA_39052 | 132 | 0.164934981 | PSG6 | pregnancy specific beta-1-glycoprotein 6 [Source:HGNC Symbol;Acc:HGNC:9523] |

| | | | | | |
|---|---|---|---|---|---|
| ENSG00000170871 | HGLibA_24611 | 132 | 0.164934981 | KIAA0232 | KIAA0232 [Source:HGNC Symbol;Acc:HGNC:28992] |
| ENSG00000171056 | HGLibA_46272 | 132 | 0.164934981 | SOX7 | SRY-box 7 [Source:HGNC Symbol;Acc:HGNC:18196] |
| ENSG00000171116 | HGLibA_22235 | 132 | 0.164934981 | HSFX1 | X-linked 1 [Source:HGNC Symbol;Acc:HGNC:29603]" |
| ENSG00000171501 | HGLibA_33706 | 132 | 0.164934981 | OR1N2 | olfactory receptor family 1 subfamily N member 2 [Source:HGNC Symbol;Acc:HGNC:15111] |
| ENSG00000171798 | HGLibA_25340 | 132 | 0.164934981 | KNDC1 | kinase non-catalytic C-lobe domain containing 1 [Source:HGNC Symbol;Acc:HGNC:29374] |
| ENSG00000171817 | HGLibA_56189 | 132 | 0.164934981 | ZNF540 | zinc finger protein 540 [Source:HGNC Symbol;Acc:HGNC:25331] |
| ENSG00000172367 | HGLibA_35977 | 132 | 0.164934981 | PDZD3 | PDZ domain containing 3 [Source:HGNC Symbol;Acc:HGNC:19891] |
| ENSG00000172725 | HGLibA_10777 | 132 | 0.164934981 | CORO1B | coronin 1B [Source:HGNC Symbol;Acc:HGNC:2253] |
| ENSG00000173611 | HGLibA_42832 | 132 | 0.164934981 | SCAI | suppressor of cancer cell invasion [Source:HGNC Symbol;Acc:HGNC:26709] |
| ENSG00000173889 | HGLibA_36324 | 132 | 0.164934981 | PHC3 | polyhomeotic homolog 3 [Source:HGNC Symbol;Acc:HGNC:15682] |
| ENSG00000173928 | HGLibA_47902 | 132 | 0.164934981 | SWSAP1 | SWIM-type zinc finger 7 associated protein 1 [Source:HGNC Symbol;Acc:HGNC:26638] |
| ENSG00000173976 | HGLibA_40411 | 132 | 0.164934981 | RAX2 | retina and anterior neural fold homeobox 2 [Source:HGNC Symbol;Acc:HGNC:18286] |
| ENSG00000174165 | HGLibA_55110 | 132 | 0.164934981 | ZDHHC24 | zinc finger DHHC-type containing 24 [Source:HGNC Symbol;Acc:HGNC:27387] |
| ENSG00000174485 | HGLibA_12951 | 132 | 0.164934981 | DENND4A | DENN domain containing 4A [Source:HGNC Symbol;Acc:HGNC:24321] |
| ENSG00000174804 | HGLibA_18272 | 132 | 0.164934981 | FZD4 | frizzled class receptor 4 [Source:HGNC Symbol;Acc:HGNC:4042] |
| ENSG00000174842 | HGLibA_19281 | 132 | 0.164934981 | GLMN | FKBP associated protein [Source:HGNC Symbol;Acc:HGNC:14373]" |
| ENSG00000175832 | HGLibA_15660 | 132 | 0.164934981 | ETV4 | ETS variant 4 [Source:HGNC Symbol;Acc:HGNC:3493] |
| ENSG00000176679 | HGLibA_49250 | 132 | 0.164934981 | TGIF2LY | TGFB induced factor homeobox 2 like Y-linked [Source:HGNC Symbol;Acc:HGNC:18569] |
| ENSG00000178171 | HGLibA_01779 | 132 | 0.164934981 | AMER3 | APC membrane recruitment protein 3 [Source:HGNC Symbol;Acc:HGNC:26771] |
| ENSG00000178623 | HGLibA_20028 | 132 | 0.164934981 | GPR35 | G protein-coupled receptor 35 [Source:HGNC Symbol;Acc:HGNC:4492] |
| ENSG00000179292 | HGLibA_50000 | 132 | 0.164934981 | TMEM151A | transmembrane protein 151A [Source:HGNC Symbol;Acc:HGNC:28497] |
| ENSG00000180801 | HGLibA_03153 | 132 | 0.164934981 | ARSJ | arylsulfatase family member J [Source:HGNC Symbol;Acc:HGNC:26286] |
| ENSG00000180871 | HGLibA_11884 | 132 | 0.164934981 | CXCR2 | C-X-C motif chemokine receptor 2 [Source:HGNC Symbol;Acc:HGNC:6027] |
| ENSG00000181830 | HGLibA_44972 | 132 | 0.164934981 | SLC35C1 | solute carrier family 35 member C1 [Source:HGNC Symbol;Acc:HGNC:20197] |
| ENSG00000182552 | HGLibA_42490 | 132 | 0.164934981 | RWDD4 | RWD domain containing 4 [Source:HGNC Symbol;Acc:HGNC:23750] |
| ENSG00000182931 | HGLibA_54250 | 132 | 0.164934981 | WFDC10B | WAP four-disulfide core domain 10B [Source:HGNC Symbol;Acc:HGNC:20479] |
| ENSG00000183813 | HGLibA_08156 | 132 | 0.164934981 | CCR4 | C-C motif chemokine receptor 4 [Source:HGNC Symbol;Acc:HGNC:1605] |
| ENSG00000184792 | HGLibA_34639 | 132 | 0.164934981 | OSBP2 | oxysterol binding protein 2 [Source:HGNC Symbol;Acc:HGNC:8504] |
| ENSG00000185527 | HGLibA_35816 | 132 | 0.164934981 | PDE6G | phosphodiesterase 6G [Source:HGNC Symbol;Acc:HGNC:8789] |
| ENSG00000186130 | HGLibA_54912 | 132 | 0.164934981 | ZBTB6 | zinc finger and BTB domain containing 6 [Source:HGNC Symbol;Acc:HGNC:16764] |
| ENSG00000188373 | HGLibA_05112 | 132 | 0.164934981 | C10orf99 | chromosome 10 open reading frame 99 [Source:HGNC Symbol;Acc:HGNC:31428] |
| ENSG00000189233 | HGLibA_33007 | 132 | 0.164934981 | NUGGC | germinal center associated [Source:HGNC Symbol;Acc:HGNC:33550]" |
| ENSG00000196268 | HGLibA_56099 | 132 | 0.164934981 | ZNF493 | zinc finger protein 493 [Source:HGNC Symbol;Acc:HGNC:23708] |
| ENSG00000196511 | HGLibA_51138 | 132 | 0.164934981 | TPK1 | thiamin pyrophosphokinase 1 [Source:HGNC Symbol;Acc:HGNC:17358] |
| ENSG00000196584 | HGLibA_54587 | 132 | 0.164934981 | XRCC2 | X-ray repair cross complementing 2 [Source:HGNC Symbol;Acc:HGNC:12829] |
| ENSG00000196865 | HGLibA_31899 | 132 | 0.164934981 | NHLRC2 | NHL repeat containing 2 [Source:HGNC Symbol;Acc:HGNC:24731] |
| ENSG00000197506 | HGLibA_44838 | 132 | 0.164934981 | SLC28A3 | solute carrier family 28 member 3 [Source:HGNC Symbol;Acc:HGNC:16484] |
| ENSG00000198160 | HGLibA_29251 | 132 | 0.164934981 | MIER1 | MIER1 transcriptional regulator [Source:HGNC Symbol;Acc:HGNC:29657] |
| ENSG00000198502 | HGLibA_21640 | 132 | 0.164934981 | HLA-DRB5 | DR beta 5 [Source:HGNC Symbol;Acc:HGNC:4953]" |
| ENSG00000204390 | HGLibA_22276 | 132 | 0.164934981 | HSPA1L | heat shock protein family A (Hsp70) member 1 like [Source:HGNC Symbol;Acc:HGNC:5234] |
| ENSG00000204889 | HGLibA_25508 | 132 | 0.164934981 | KRT40 | keratin 40 [Source:HGNC Symbol;Acc:HGNC:26707] |
| ENSG00000205090 | HGLibA_50284 | 132 | 0.164934981 | TMEM240 | transmembrane protein 240 [Source:HGNC Symbol;Acc:HGNC:25186] |
| ENSG00000213145 | HGLibA_11150 | 132 | 0.164934981 | CRIP1 | cysteine rich protein 1 [Source:HGNC Symbol;Acc:HGNC:2360] |
| ENSG00000215252 | HGLibA_19643 | 132 | 0.164934981 | GOLGA8B | golgin A8 family member B [Source:HGNC Symbol;Acc:HGNC:31973] |
| ENSG00000225556 | HGLibA_06103 | 132 | 0.164934981 | C2CD4D | C2 calcium dependent domain containing 4D [Source:HGNC Symbol;Acc:HGNC:37210] |
| ENSG00000244274 | HGLibA_12393 | 132 | 0.164934981 | DBNDD2 | dysbindin domain containing 2 [Source:HGNC Symbol;Acc:HGNC:15881] |
| ENSG00000256222 | HGLibA_30478 | 132 | 0.164934981 | MTRNR2L3 | MT-RNR2 like 3 [Source:HGNC Symbol;Acc:HGNC:37157] |
| ENSG00000263002 | HGLibA_55644 | 132 | 0.164934981 | ZNF234 | zinc finger protein 234 [Source:HGNC Symbol;Acc:HGNC:13027] |
| ENSG00000268988 | HGLibA_46405 | 132 | 0.164934981 | SPANXN2 | SPANX family member N2 [Source:HGNC Symbol;Acc:HGNC:33175] |
| ENSG00000269096 | HGLibA_11504 | 132 | 0.164934981 | CT45A3 | cancer/testis antigen family 45 member A3 [Source:HGNC Symbol;Acc:HGNC:33268] |
| ENSG00000276380 | HGLibA_52639 | 132 | 0.164934981 | UBE2NL | ubiquitin conjugating enzyme E2 N like (gene/pseudogene) [Source:HGNC Symbol;Acc:HGNC:31710] |
| ENSG00000279111 | HGLibA_33558 | 132 | 0.164934981 | OR10X1 | olfactory receptor family 10 subfamily X member 1 (gene/pseudogene) [Source:HGNC Symbol;Acc:HGNC:14995] |
| FAM188B | HGLibA_16349 | 132 | 0.164934981 | FAM188B | - |
| FAM63A | HGLibA_16660 | 132 | 0.164934981 | FAM63A | - |
| GCN1L1 | HGLibA_18805 | 132 | 0.164934981 | GCN1L1 | - |
| HIST1H4L | HGLibA_21520 | 132 | 0.164934981 | HIST1H4L | - |
| hsa-let-7a-2 | HGLibA_57102 | 132 | 0.164934981 | hsa-let-7a-2 | - |
| hsa-mir-1200 | HGLibA_57262 | 132 | 0.164934981 | hsa-mir-1200 | - |
| hsa-mir-125b-2 | HGLibA_57451 | 132 | 0.164934981 | hsa-mir-125b-2 | - |
| hsa-mir-1273f | HGLibA_57542 | 132 | 0.164934981 | hsa-mir-1273f | - |
| hsa-mir-3115 | HGLibA_58501 | 132 | 0.164934981 | hsa-mir-3115 | - |
| hsa-mir-3619 | HGLibA_59104 | 132 | 0.164934981 | hsa-mir-3619 | - |
| hsa-mir-3945 | HGLibA_59641 | 132 | 0.164934981 | hsa-mir-3945 | - |
| hsa-mir-424 | HGLibA_59704 | 132 | 0.164934981 | hsa-mir-424 | - |
| hsa-mir-450a-2 | HGLibA_60478 | 132 | 0.164934981 | hsa-mir-450a-2 | - |
| hsa-mir-4522 | HGLibA_60544 | 132 | 0.164934981 | hsa-mir-4522 | - |
| hsa-mir-612 | HGLibA_62639 | 132 | 0.164934981 | hsa-mir-612 | - |
| hsa-mir-6793 | HGLibA_63350 | 132 | 0.164934981 | hsa-mir-6793 | - |
| hsa-mir-6876 | HGLibA_63687 | 132 | 0.164934981 | hsa-mir-6876 | - |
| NUTMG | HGLibA_33116 | 132 | 0.164934981 | NUTMG | - |
| PIDD | HGLibA_36531 | 132 | 0.164934981 | PIDD | - |
| TROVE2 | HGLibA_51723 | 132 | 0.164934981 | TROVE2 | - |
| C20orf26 | HGLibA_06020 | 131 | 0.154983944 | C20orf26 | - |
| C5orf48 | HGLibA_06394 | 131 | 0.154983944 | C5orf48 | - |
| C9orf66 | HGLibA_06752 | 131 | 0.154983944 | C9orf66 | - |
| ENSG00000003756 | HGLibA_40575 | 131 | 0.154983944 | RBM5 | RNA binding motif protein 5 [Source:HGNC Symbol;Acc:HGNC:9902] |
| ENSG00000022840 | HGLibA_41455 | 131 | 0.154983944 | RNF10 | ring finger protein 10 [Source:HGNC Symbol;Acc:HGNC:10055] |
| ENSG00000025156 | HGLibA_22223 | 131 | 0.154983944 | HSF2 | heat shock transcription factor 2 [Source:HGNC Symbol;Acc:HGNC:5225] |
| ENSG00000037757 | HGLibA_29857 | 131 | 0.154983944 | MRI1 | methylthioribose-1-phosphate isomerase 1 [Source:HGNC Symbol;Acc:HGNC:28469] |
| ENSG00000058063 | HGLibA_03557 | 131 | 0.154983944 | ATP11B | ATPase phospholipid transporting 11B (putative) [Source:HGNC Symbol;Acc:HGNC:13553] |
| ENSG00000061492 | HGLibA_54388 | 131 | 0.154983944 | WNT8A | Wnt family member 8A [Source:HGNC Symbol;Acc:HGNC:12788] |
| ENSG00000070985 | HGLibA_51763 | 131 | 0.154983944 | TRPM5 | transient receptor potential cation channel subfamily M member 5 [Source:HGNC Symbol;Acc:HGNC:14323] |
| ENSG00000074276 | HGLibA_08701 | 131 | 0.154983944 | CDHR2 | cadherin related family member 2 [Source:HGNC Symbol;Acc:HGNC:18231] |
| ENSG00000085760 | HGLibA_30397 | 131 | 0.154983944 | MTIF2 | mitochondrial translational initiation factor 2 [Source:HGNC Symbol;Acc:HGNC:7441] |
| ENSG00000087338 | HGLibA_19391 | 131 | 0.154983944 | GMCL1 | spermatogenesis associated 1 [Source:HGNC Symbol;Acc:HGNC:23843]" |
| ENSG00000089327 | HGLibA_18235 | 131 | 0.154983944 | FXYD5 | FXYD domain containing ion transport regulator 5 [Source:HGNC Symbol;Acc:HGNC:4029] |
| ENSG00000095002 | HGLibA_30189 | 131 | 0.154983944 | MSH2 | mutS homolog 2 [Source:HGNC Symbol;Acc:HGNC:7325] |
| ENSG00000099977 | HGLibA_12641 | 131 | 0.154983944 | DDT | D-dopachrome tautomerase [Source:HGNC Symbol;Acc:HGNC:2732] |
| ENSG00000100299 | HGLibA_03128 | 131 | 0.154983944 | ARSA | arylsulfatase A [Source:HGNC Symbol;Acc:HGNC:713] |
| ENSG00000100714 | HGLibA_30376 | 131 | 0.154983944 | MTHFD1 | cyclohydrolase and formyltetrahydrofolate synthetase 1 [Source:HGNC Symbol;Acc:HGNC:7432]" |
| ENSG00000101311 | HGLibA_17294 | 131 | 0.154983944 | FERMT1 | fermitin family member 1 [Source:HGNC Symbol;Acc:HGNC:15889] |
| ENSG00000102195 | HGLibA_20046 | 131 | 0.154983944 | GPR50 | G protein-coupled receptor 50 [Source:HGNC Symbol;Acc:HGNC:4506] |
| ENSG00000102962 | HGLibA_07998 | 131 | 0.154983944 | CCL22 | C-C motif chemokine ligand 22 [Source:HGNC Symbol;Acc:HGNC:10621] |
| ENSG00000105550 | HGLibA_17396 | 131 | 0.154983944 | FGF21 | fibroblast growth factor 21 [Source:HGNC Symbol;Acc:HGNC:3678] |
| ENSG00000105695 | HGLibA_27887 | 131 | 0.154983944 | MAG | myelin associated glycoprotein [Source:HGNC Symbol;Acc:HGNC:6783] |
| ENSG00000105974 | HGLibA_07376 | 131 | 0.154983944 | CAV1 | caveolin 1 [Source:HGNC Symbol;Acc:HGNC:1527] |
| ENSG00000107338 | HGLibA_43995 | 131 | 0.154983944 | SHB | SH2 domain containing adaptor protein B [Source:HGNC Symbol;Acc:HGNC:10838] |
| ENSG00000108797 | HGLibA_10418 | 131 | 0.154983944 | CNTNAP1 | contactin associated protein 1 [Source:HGNC Symbol;Acc:HGNC:8011] |
| ENSG00000110237 | HGLibA_02826 | 131 | 0.154983944 | ARHGEF17 | Rho guanine nucleotide exchange factor 17 [Source:HGNC Symbol;Acc:HGNC:21726] |
| ENSG00000110455 | HGLibA_00414 | 131 | 0.154983944 | ACCS | 1-aminocyclopropane-1-carboxylate synthase homolog (inactive) [Source:HGNC Symbol;Acc:HGNC:23989] |
| ENSG00000110911 | HGLibA_44355 | 131 | 0.154983944 | SLC11A2 | solute carrier family 11 member 2 [Source:HGNC Symbol;Acc:HGNC:10908] |
| ENSG00000111536 | HGLibA_23109 | 131 | 0.154983944 | IL26 | interleukin 26 [Source:HGNC Symbol;Acc:HGNC:17119] |
| ENSG00000111860 | HGLibA_09174 | 131 | 0.154983944 | CEP85L | centrosomal protein 85 like [Source:HGNC Symbol;Acc:HGNC:21638] |
| ENSG00000112530 | HGLibA_34949 | 131 | 0.154983944 | PACRG | parkin coregulated [Source:HGNC Symbol;Acc:HGNC:19152] |
| ENSG00000117266 | HGLibA_08750 | 131 | 0.154983944 | CDK18 | cyclin dependent kinase 18 [Source:HGNC Symbol;Acc:HGNC:8751] |
| ENSG00000118137 | HGLibA_02450 | 131 | 0.154983944 | APOA1 | apolipoprotein A1 [Source:HGNC Symbol;Acc:HGNC:600] |
| ENSG00000118922 | HGLibA_25047 | 131 | 0.154983944 | KLF12 | Kruppel like factor 12 [Source:HGNC Symbol;Acc:HGNC:6346] |
| ENSG00000119321 | HGLibA_17561 | 131 | 0.154983944 | FKBP15 | FK506 binding protein 15 [Source:HGNC Symbol;Acc:HGNC:23397] |
| ENSG00000119508 | HGLibA_32631 | 131 | 0.154983944 | NR4A3 | nuclear receptor subfamily 4 group A member 3 [Source:HGNC Symbol;Acc:HGNC:7982] |
| ENSG00000119787 | HGLibA_03519 | 131 | 0.154983944 | ATL2 | atlastin GTPase 2 [Source:HGNC Symbol;Acc:HGNC:24047] |
| ENSG00000121454 | HGLibA_26457 | 131 | 0.154983944 | LHX4 | LIM homeobox 4 [Source:HGNC Symbol;Acc:HGNC:21734] |
| ENSG00000123130 | HGLibA_00493 | 131 | 0.154983944 | ACOT9 | acyl-CoA thioesterase 9 [Source:HGNC Symbol;Acc:HGNC:17152] |
| ENSG00000124459 | HGLibA_56025 | 131 | 0.154983944 | ZNF45 | zinc finger protein 45 [Source:HGNC Symbol;Acc:HGNC:13111] |
| ENSG00000124713 | HGLibA_19558 | 131 | 0.154983944 | GNMT | glycine N-methyltransferase [Source:HGNC Symbol;Acc:HGNC:4415] |
| ENSG00000124783 | HGLibA_47153 | 131 | 0.154983944 | SSR1 | signal sequence receptor subunit 1 [Source:HGNC Symbol;Acc:HGNC:11323] |
| ENSG00000125810 | HGLibA_08479 | 131 | 0.154983944 | CD93 | CD93 molecule [Source:HGNC Symbol;Acc:HGNC:15855] |
| ENSG00000128713 | HGLibA_21989 | 131 | 0.154983944 | HOXD11 | homeobox D11 [Source:HGNC Symbol;Acc:HGNC:5134] |
| ENSG00000130234 | HGLibA_00424 | 131 | 0.154983944 | ACE2 | angiotensin I converting enzyme 2 [Source:HGNC Symbol;Acc:HGNC:13557] |
| ENSG00000132207 | HGLibA_45558 | 131 | 0.154983944 | SLX1A | structure-specific endonuclease subunit [Source:HGNC Symbol;Acc:HGNC:20922]" |
| ENSG00000132819 | HGLibA_40539 | 131 | 0.154983944 | RBM38 | RNA binding motif protein 38 [Source:HGNC Symbol;Acc:HGNC:15818] |
| ENSG00000134250 | HGLibA_32344 | 131 | 0.154983944 | NOTCH2 | notch 2 [Source:HGNC Symbol;Acc:HGNC:7882] |
| ENSG00000134986 | HGLibA_32671 | 131 | 0.154983944 | NREP | neuronal regeneration related protein [Source:HGNC Symbol;Acc:HGNC:16834] |
| ENSG00000137193 | HGLibA_36675 | 131 | 0.154983944 | PIM1 | serine/threonine kinase [Source:HGNC Symbol;Acc:HGNC:8986]" |
| ENSG00000137204 | HGLibA_44597 | 131 | 0.154983944 | SLC22A7 | solute carrier family 22 member 7 [Source:HGNC Symbol;Acc:HGNC:10971] |
| ENSG00000138696 | HGLibA_04631 | 131 | 0.154983944 | BMPR1B | bone morphogenetic protein receptor type 1B [Source:HGNC Symbol;Acc:HGNC:1077] |
| ENSG00000138798 | HGLibA_14620 | 131 | 0.154983944 | EGF | epidermal growth factor [Source:HGNC Symbol;Acc:HGNC:3229] |
| ENSG00000139344 | HGLibA_01760 | 131 | 0.154983944 | AMDHD1 | amidohydrolase domain containing 1 [Source:HGNC Symbol;Acc:HGNC:28577] |
| ENSG00000140006 | HGLibA_54210 | 131 | 0.154983944 | WDR89 | WD repeat domain 89 [Source:HGNC Symbol;Acc:HGNC:20489] |
| ENSG00000140836 | HGLibA_55175 | 131 | 0.154983944 | ZFHX3 | zinc finger homeobox 3 [Source:HGNC Symbol;Acc:HGNC:777] |
| ENSG00000140848 | HGLibA_10956 | 131 | 0.154983944 | CPNE2 | copine 2 [Source:HGNC Symbol;Acc:HGNC:2315] |
| ENSG00000141750 | HGLibA_47316 | 131 | 0.154983944 | STAC2 | SH3 and cysteine rich domain 2 [Source:HGNC Symbol;Acc:HGNC:23990] |
| ENSG00000142794 | HGLibA_31233 | 131 | 0.154983944 | NBPF3 | NBPF member 3 [Source:HGNC Symbol;Acc:HGNC:25076] |
| ENSG00000143162 | HGLibA_11119 | 131 | 0.154983944 | CREG1 | cellular repressor of E1A stimulated genes 1 [Source:HGNC Symbol;Acc:HGNC:2351] |
| ENSG00000143258 | HGLibA_53301 | 131 | 0.154983944 | USP21 | ubiquitin specific peptidase 21 [Source:HGNC Symbol;Acc:HGNC:12620] |
| ENSG00000143612 | HGLibA_05857 | 131 | 0.154983944 | C1orf43 | chromosome 1 open reading frame 43 [Source:HGNC Symbol;Acc:HGNC:29876] |

| | | | | | |
|---|---|---|---|---|---|
| ENSG00000143622 | HGLibA_41338 | 131 | 0.154983944 | RIT1 | Ras like without CAAX 1 [Source:HGNC Symbol;Acc:HGNC:10023] |
| ENSG00000143811 | HGLibA_39677 | 131 | 0.154983944 | PYCR2 | pyrroline-5-carboxylate reductase 2 [Source:HGNC Symbol;Acc:HGNC:30262] |
| ENSG00000145214 | HGLibA_13082 | 131 | 0.154983944 | DGKQ | diacylglycerol kinase theta [Source:HGNC Symbol;Acc:HGNC:2856] |
| ENSG00000147439 | HGLibA_04513 | 131 | 0.154983944 | BIN3 | bridging integrator 3 [Source:HGNC Symbol;Acc:HGNC:1054] |
| ENSG00000147724 | HGLibA_16134 | 131 | 0.154983944 | FAM135B | family with sequence similarity 135 member B [Source:HGNC Symbol;Acc:HGNC:28029] |
| ENSG00000147883 | HGLibA_08839 | 131 | 0.154983944 | CDKN2B | cyclin dependent kinase inhibitor 2B [Source:HGNC Symbol;Acc:HGNC:1788] |
| ENSG00000148484 | HGLibA_42366 | 131 | 0.154983944 | RSU1 | Ras suppressor protein 1 [Source:HGNC Symbol;Acc:HGNC:10464] |
| ENSG00000149084 | HGLibA_22178 | 131 | 0.154983944 | HSD17B12 | hydroxysteroid 17-beta dehydrogenase 12 [Source:HGNC Symbol;Acc:HGNC:18646] |
| ENSG00000149435 | HGLibA_19021 | 131 | 0.154983944 | GGTLC1 | gamma-glutamyltransferase light chain 1 [Source:HGNC Symbol;Acc:HGNC:16437] |
| ENSG00000150938 | HGLibA_11148 | 131 | 0.154983944 | CRIM1 | cysteine rich transmembrane BMP regulator 1 [Source:HGNC Symbol;Acc:HGNC:2359] |
| ENSG00000151694 | HGLibA_00741 | 131 | 0.154983944 | ADAM17 | ADAM metallopeptidase domain 17 [Source:HGNC Symbol;Acc:HGNC:195] |
| ENSG00000151923 | HGLibA_49435 | 131 | 0.154983944 | TIAL1 | TIA1 cytotoxic granule associated RNA binding protein like 1 [Source:HGNC Symbol;Acc:HGNC:11804] |
| ENSG00000151967 | HGLibA_42941 | 131 | 0.154983944 | SCHIP1 | schwannomin interacting protein 1 [Source:HGNC Symbol;Acc:HGNC:15678] |
| ENSG00000152433 | HGLibA_56204 | 131 | 0.154983944 | ZNF547 | zinc finger protein 547 [Source:HGNC Symbol;Acc:HGNC:26432] |
| ENSG00000152779 | HGLibA_44427 | 131 | 0.154983944 | SLC16A12 | solute carrier family 16 member 12 [Source:HGNC Symbol;Acc:HGNC:23094] |
| ENSG00000153406 | HGLibA_32194 | 131 | 0.154983944 | NMRAL1 | NmrA like redox sensor 1 [Source:HGNC Symbol;Acc:HGNC:24987] |
| ENSG00000153802 | HGLibA_50617 | 131 | 0.154983944 | TMPRSS11D | transmembrane serine protease 11D [Source:HGNC Symbol;Acc:HGNC:24059] |
| ENSG00000154493 | HGLibA_05104 | 131 | 0.154983944 | C10orf90 | chromosome 10 open reading frame 90 [Source:HGNC Symbol;Acc:HGNC:26563] |
| ENSG00000154645 | HGLibA_09511 | 131 | 0.154983944 | CHODL | chondrolectin [Source:HGNC Symbol;Acc:HGNC:17807] |
| ENSG00000155868 | HGLibA_28814 | 131 | 0.154983944 | MED7 | mediator complex subunit 7 [Source:HGNC Symbol;Acc:HGNC:2378] |
| ENSG00000159322 | HGLibA_01023 | 131 | 0.154983944 | ADPGK | ADP dependent glucokinase [Source:HGNC Symbol;Acc:HGNC:25250] |
| ENSG00000160781 | HGLibA_35165 | 131 | 0.154983944 | PAQR6 | progestin and adipoQ receptor family member 6 [Source:HGNC Symbol;Acc:HGNC:30132] |
| ENSG00000161277 | HGLibA_49312 | 131 | 0.154983944 | THAP8 | THAP domain containing 8 [Source:HGNC Symbol;Acc:HGNC:23191] |
| ENSG00000163516 | HGLibA_02166 | 131 | 0.154983944 | ANKZF1 | ankyrin repeat and zinc finger domain containing 1 [Source:HGNC Symbol;Acc:HGNC:25527] |
| ENSG00000163596 | HGLibA_22490 | 131 | 0.154983944 | ICA1L | islet cell autoantigen 1 like [Source:HGNC Symbol;Acc:HGNC:14442] |
| ENSG00000163704 | HGLibA_38887 | 131 | 0.154983944 | PRRT3 | proline rich transmembrane protein 3 [Source:HGNC Symbol;Acc:HGNC:26591] |
| ENSG00000164082 | HGLibA_20343 | 131 | 0.154983944 | GRM2 | glutamate metabotropic receptor 2 [Source:HGNC Symbol;Acc:HGNC:4594] |
| ENSG00000164294 | HGLibA_20191 | 131 | 0.154983944 | GPX8 | glutathione peroxidase 8 (putative) [Source:HGNC Symbol;Acc:HGNC:33100] |
| ENSG00000164591 | HGLibA_30908 | 131 | 0.154983944 | MYOZ3 | myozenin 3 [Source:HGNC Symbol;Acc:HGNC:18565] |
| ENSG00000164742 | HGLibA_00906 | 131 | 0.154983944 | ADCY1 | adenylate cyclase 1 [Source:HGNC Symbol;Acc:HGNC:232] |
| ENSG00000165025 | HGLibA_47948 | 131 | 0.154983944 | SYK | spleen associated tyrosine kinase [Source:HGNC Symbol;Acc:HGNC:11491] |
| ENSG00000165443 | HGLibA_36478 | 131 | 0.154983944 | PHYHIPL | phytanoyl-CoA 2-hydroxylase interacting protein like [Source:HGNC Symbol;Acc:HGNC:29378] |
| ENSG00000166167 | HGLibA_04985 | 131 | 0.154983944 | BTRC | beta-transducin repeat containing E3 ubiquitin protein ligase [Source:HGNC Symbol;Acc:HGNC:1144] |
| ENSG00000166333 | HGLibA_23219 | 131 | 0.154983944 | ILK | integrin linked kinase [Source:HGNC Symbol;Acc:HGNC:6040] |
| ENSG00000166743 | HGLibA_00568 | 131 | 0.154983944 | ACSM1 | acyl-CoA synthetase medium chain family member 1 [Source:HGNC Symbol;Acc:HGNC:18049] |
| ENSG00000167081 | HGLibA_35348 | 131 | 0.154983944 | PBX3 | PBX homeobox 3 [Source:HGNC Symbol;Acc:HGNC:8634] |
| ENSG00000167191 | HGLibA_20128 | 131 | 0.154983944 | GPRC5B | G protein-coupled receptor class C group 5 member B [Source:HGNC Symbol;Acc:HGNC:13308] |
| ENSG00000168461 | HGLibA_39875 | 131 | 0.154983944 | RAB31 | member RAS oncogene family [Source:HGNC Symbol;Acc:HGNC:9771]" |
| ENSG00000168807 | HGLibA_46003 | 131 | 0.154983944 | SNTB2 | syntrophin beta 2 [Source:HGNC Symbol;Acc:HGNC:11169] |
| ENSG00000170236 | HGLibA_53392 | 131 | 0.154983944 | USP50 | ubiquitin specific peptidase 50 [Source:HGNC Symbol;Acc:HGNC:20079] |
| ENSG00000170476 | HGLibA_30934 | 131 | 0.154983944 | MZB1 | marginal zone B and B1 cell specific protein [Source:HGNC Symbol;Acc:HGNC:30125] |
| ENSG00000171345 | HGLibA_25442 | 131 | 0.154983944 | KRT19 | keratin 19 [Source:HGNC Symbol;Acc:HGNC:6436] |
| ENSG00000172247 | HGLibA_05944 | 131 | 0.154983944 | C1QTNF4 | C1q and TNF related 4 [Source:HGNC Symbol;Acc:HGNC:14346] |
| ENSG00000173273 | HGLibA_50849 | 131 | 0.154983944 | TNKS | tankyrase [Source:HGNC Symbol;Acc:HGNC:11941] |
| ENSG00000173418 | HGLibA_30983 | 131 | 0.154983944 | NAA20 | NatB catalytic subunit [Source:HGNC Symbol;Acc:HGNC:15908]" |
| ENSG00000174652 | HGLibA_55699 | 131 | 0.154983944 | ZNF266 | zinc finger protein 266 [Source:HGNC Symbol;Acc:HGNC:13059] |
| ENSG00000174903 | HGLibA_39831 | 131 | 0.154983944 | RAB1B | member RAS oncogene family [Source:HGNC Symbol;Acc:HGNC:18370]" |
| ENSG00000180806 | HGLibA_21980 | 131 | 0.154983944 | HOXC9 | homeobox C9 [Source:HGNC Symbol;Acc:HGNC:5130] |
| ENSG00000180992 | HGLibA_29902 | 131 | 0.154983944 | MRPL14 | mitochondrial ribosomal protein L14 [Source:HGNC Symbol;Acc:HGNC:14279] |
| ENSG00000181004 | HGLibA_04213 | 131 | 0.154983944 | BBS12 | Bardet-Biedl syndrome 12 [Source:HGNC Symbol;Acc:HGNC:26648] |
| ENSG00000181803 | HGLibA_34443 | 131 | 0.154983944 | OR6S1 | olfactory receptor family 6 subfamily S member 1 [Source:HGNC Symbol;Acc:HGNC:15363] |
| ENSG00000182132 | HGLibA_24234 | 131 | 0.154983944 | KCNIP1 | potassium voltage-gated channel interacting protein 1 [Source:HGNC Symbol;Acc:HGNC:15521] |
| ENSG00000182676 | HGLibA_38083 | 131 | 0.154983944 | PPP1R27 | protein phosphatase 1 regulatory subunit 27 [Source:HGNC Symbol;Acc:HGNC:16813] |
| ENSG00000183632 | HGLibA_51079 | 131 | 0.154983944 | TP53TG3 | TP53 target 3 [Source:HGNC Symbol;Acc:HGNC:30759] |
| ENSG00000184208 | HGLibA_06082 | 131 | 0.154983944 | C22orf46 | chromosome 22 open reading frame 46 [Source:HGNC Symbol;Acc:HGNC:26294] |
| ENSG00000184611 | HGLibA_24229 | 131 | 0.154983944 | KCNH7 | potassium voltage-gated channel subfamily H member 7 [Source:HGNC Symbol;Acc:HGNC:18863] |
| ENSG00000185069 | HGLibA_25541 | 131 | 0.154983944 | KRT76 | keratin 76 [Source:HGNC Symbol;Acc:HGNC:24430] |
| ENSG00000185112 | HGLibA_16586 | 131 | 0.154983944 | FAM43A | family with sequence similarity 43 member A [Source:HGNC Symbol;Acc:HGNC:26888] |
| ENSG00000185630 | HGLibA_35343 | 131 | 0.154983944 | PBX1 | PBX homeobox 1 [Source:HGNC Symbol;Acc:HGNC:8632] |
| ENSG00000186526 | HGLibA_12196 | 131 | 0.154983944 | CYP4F8 | cytochrome P450 family 4 subfamily F member 8 [Source:HGNC Symbol;Acc:HGNC:2648] |
| ENSG00000187094 | HGLibA_07951 | 131 | 0.154983944 | CCK | cholecystokinin [Source:HGNC Symbol;Acc:HGNC:1569] |
| ENSG00000187950 | HGLibA_34791 | 131 | 0.154983944 | OVCH1 | ovochymase 1 [Source:HGNC Symbol;Acc:HGNC:23080] |
| ENSG00000196166 | HGLibA_06653 | 131 | 0.154983944 | C8orf86 | chromosome 8 open reading frame 86 [Source:HGNC Symbol;Acc:HGNC:33774] |
| ENSG00000196420 | HGLibA_42560 | 131 | 0.154983944 | S100A5 | S100 calcium binding protein A5 [Source:HGNC Symbol;Acc:HGNC:10495] |
| ENSG00000197487 | HGLibA_18583 | 131 | 0.154983944 | GALP | galanin like peptide [Source:HGNC Symbol;Acc:HGNC:24840] |
| ENSG00000197561 | HGLibA_14876 | 131 | 0.154983944 | ELANE | neutrophil expressed [Source:HGNC Symbol;Acc:HGNC:3309]" |
| ENSG00000197938 | HGLibA_34297 | 131 | 0.154983944 | OR5H2 | olfactory receptor family 5 subfamily H member 2 [Source:HGNC Symbol;Acc:HGNC:14752] |
| ENSG00000198081 | HGLibA_54823 | 131 | 0.154983944 | ZBTB14 | zinc finger and BTB domain containing 14 [Source:HGNC Symbol;Acc:HGNC:12860] |
| ENSG00000198393 | HGLibA_55688 | 131 | 0.154983944 | ZNF26 | zinc finger protein 26 [Source:HGNC Symbol;Acc:HGNC:13053] |
| ENSG00000198597 | HGLibA_56186 | 131 | 0.154983944 | ZNF536 | zinc finger protein 536 [Source:HGNC Symbol;Acc:HGNC:29025] |
| ENSG00000203663 | HGLibA_33821 | 131 | 0.154983944 | OR2L2 | olfactory receptor family 2 subfamily L member 2 [Source:HGNC Symbol;Acc:HGNC:8266] |
| ENSG00000204209 | HGLibA_12350 | 131 | 0.154983944 | DAXX | death domain associated protein [Source:HGNC Symbol;Acc:HGNC:2681] |
| ENSG00000204421 | HGLibA_27666 | 131 | 0.154983944 | LY6G6C | lymphocyte antigen 6 family member G6C [Source:HGNC Symbol;Acc:HGNC:13936] |
| ENSG00000204576 | HGLibA_38836 | 131 | 0.154983944 | PRR3 | proline rich 3 [Source:HGNC Symbol;Acc:HGNC:21149] |
| ENSG00000204930 | HGLibA_16518 | 131 | 0.154983944 | FAM221B | family with sequence similarity 221 member B [Source:HGNC Symbol;Acc:HGNC:30762] |
| ENSG00000205302 | HGLibA_46058 | 131 | 0.154983944 | SNX2 | sorting nexin 2 [Source:HGNC Symbol;Acc:HGNC:11173] |
| ENSG00000205927 | HGLibA_33371 | 131 | 0.154983944 | OLIG2 | oligodendrocyte transcription factor 2 [Source:HGNC Symbol;Acc:HGNC:9398] |
| ENSG00000214338 | HGLibA_46159 | 131 | 0.154983944 | SOGA3 | SOGA family member 3 [Source:HGNC Symbol;Acc:HGNC:21494] |
| ENSG00000214413 | HGLibA_04200 | 131 | 0.154983944 | BBIP1 | BBSome interacting protein 1 [Source:HGNC Symbol;Acc:HGNC:28093] |
| ENSG00000215356 | HGLibA_56590 | 131 | 0.154983944 | ZNF705B | zinc finger protein 705B [Source:HGNC Symbol;Acc:HGNC:32284] |
| ENSG00000215915 | HGLibA_03415 | 131 | 0.154983944 | ATAD3C | AAA domain containing 3C [Source:HGNC Symbol;Acc:HGNC:32151]" |
| ENSG00000221855 | HGLibA_48405 | 131 | 0.154983944 | TAS2R41 | taste 2 receptor member 41 [Source:HGNC Symbol;Acc:HGNC:18883] |
| ENSG00000221963 | HGLibA_02541 | 131 | 0.154983944 | APOL6 | apolipoprotein L6 [Source:HGNC Symbol;Acc:HGNC:14870] |
| ENSG00000221988 | HGLibA_38235 | 131 | 0.154983944 | PPT2 | palmitoyl-protein thioesterase 2 [Source:HGNC Symbol;Acc:HGNC:9326] |
| ENSG00000221989 | HGLibA_33727 | 131 | 0.154983944 | OR2A2 | olfactory receptor family 2 subfamily A member 2 [Source:HGNC Symbol;Acc:HGNC:8230] |
| ENSG00000226941 | HGLibA_40626 | 131 | 0.154983944 | RBMY1J | member J [Source:HGNC Symbol;Acc:HGNC:23917]" |
| ENSG00000227234 | HGLibA_46384 | 131 | 0.154983944 | SPANXB1 | SPANX family member B1 [Source:HGNC Symbol;Acc:HGNC:14329] |
| ENSG00000233670 | HGLibA_36732 | 131 | 0.154983944 | PIRT | phosphoinositide interacting regulator of transient receptor potential channels [Source:HGNC Symbol;Acc:HGNC:37239] |
| ENSG00000237440 | HGLibA_56655 | 131 | 0.154983944 | ZNF737 | zinc finger protein 737 [Source:HGNC Symbol;Acc:HGNC:32468] |
| ENSG00000244411 | HGLibA_25806 | 131 | 0.154983944 | KRTAP5-7 | keratin associated protein 5-7 [Source:HGNC Symbol;Acc:HGNC:23602] |
| ENSG00000266200 | HGLibA_37361 | 131 | 0.154983944 | PNLIPRP2 | pancreatic lipase related protein 2 (gene/pseudogene) [Source:HGNC Symbol;Acc:HGNC:9157] |
| FUK | HGLibA_18160 | 131 | 0.154983944 | FUK | - |
| GIF | HGLibA_19062 | 131 | 0.154983944 | GIF | - |
| hsa-mir-1199 | HGLibA_57259 | 131 | 0.154983944 | hsa-mir-1199 | - |
| hsa-mir-214 | HGLibA_58249 | 131 | 0.154983944 | hsa-mir-214 | - |
| hsa-mir-29c | HGLibA_58420 | 131 | 0.154983944 | hsa-mir-29c | - |
| hsa-mir-3150a | HGLibA_58657 | 131 | 0.154983944 | hsa-mir-3150a | - |
| hsa-mir-376c | HGLibA_59418 | 131 | 0.154983944 | hsa-mir-376c | - |
| hsa-mir-4255 | HGLibA_59728 | 131 | 0.154983944 | hsa-mir-4255 | - |
| hsa-mir-4430 | HGLibA_60106 | 131 | 0.154983944 | hsa-mir-4430 | - |
| hsa-mir-4454 | HGLibA_60222 | 131 | 0.154983944 | hsa-mir-4454 | - |
| hsa-mir-4704 | HGLibA_60916 | 131 | 0.154983944 | hsa-mir-4704 | - |
| hsa-mir-4768 | HGLibA_61167 | 131 | 0.154983944 | hsa-mir-4768 | - |
| hsa-mir-512-2 | HGLibA_61566 | 131 | 0.154983944 | hsa-mir-512-2 | - |
| hsa-mir-520f | HGLibA_61756 | 131 | 0.154983944 | hsa-mir-520f | - |
| hsa-mir-5707 | HGLibA_62368 | 131 | 0.154983944 | hsa-mir-5707 | - |
| hsa-mir-573 | HGLibA_62383 | 131 | 0.154983944 | hsa-mir-573 | - |
| hsa-mir-578 | HGLibA_62408 | 131 | 0.154983944 | hsa-mir-578 | - |
| hsa-mir-647 | HGLibA_62832 | 131 | 0.154983944 | hsa-mir-647 | - |
| hsa-mir-670 | HGLibA_63008 | 131 | 0.154983944 | hsa-mir-670 | - |
| hsa-mir-8070 | HGLibA_64119 | 131 | 0.154983944 | hsa-mir-8070 | - |
| hsa-mir-8077 | HGLibA_64149 | 131 | 0.154983944 | hsa-mir-8077 | - |
| IL3RA_Y | HGLibA_23163 | 131 | 0.154983944 | IL3RA_Y | - |
| LOC401052 | HGLibA_26935 | 131 | 0.154983944 | LOC401052 | - |
| C10orf129 | HGLibA_05053 | 130 | 0.145032906 | C10orf129 | - |
| C13orf35 | HGLibA_05309 | 130 | 0.145032906 | C13orf35 | - |
| C1orf204 | HGLibA_05823 | 130 | 0.145032906 | C1orf204 | - |
| C20orf197 | HGLibA_06009 | 130 | 0.145032906 | C20orf197 | - |
| CCDC64 | HGLibA_07827 | 130 | 0.145032906 | CCDC64 | - |
| ENSG00000005001 | HGLibA_38913 | 130 | 0.145032906 | PRSS22 | serine protease 22 [Source:HGNC Symbol;Acc:HGNC:14368] |
| ENSG00000005381 | HGLibA_29743 | 130 | 0.145032906 | MPO | myeloperoxidase [Source:HGNC Symbol;Acc:HGNC:7218] |
| ENSG00000008516 | HGLibA_29522 | 130 | 0.145032906 | MMP25 | matrix metallopeptidase 25 [Source:HGNC Symbol;Acc:HGNC:14246] |
| ENSG00000011009 | HGLibA_27740 | 130 | 0.145032906 | LYPLA2 | lysophospholipase II [Source:HGNC Symbol;Acc:HGNC:6738] |
| ENSG00000015171 | HGLibA_55414 | 130 | 0.145032906 | ZMYND11 | zinc finger MYND-type containing 11 [Source:HGNC Symbol;Acc:HGNC:16966] |
| ENSG00000029725 | HGLibA_40000 | 130 | 0.145032906 | RABEP1 | RAB GTPase binding effector protein 1 [Source:HGNC Symbol;Acc:HGNC:17677]" |
| ENSG00000035681 | HGLibA_32763 | 130 | 0.145032906 | NSMAF | neutral sphingomyelinase activation associated factor [Source:HGNC Symbol;Acc:HGNC:8017] |
| ENSG00000054611 | HGLibA_48503 | 130 | 0.145032906 | TBC1D22A | TBC1 domain family member 22A [Source:HGNC Symbol;Acc:HGNC:1309] |
| ENSG00000065615 | HGLibA_12011 | 130 | 0.145032906 | CYB5R4 | cytochrome b5 reductase 4 [Source:HGNC Symbol;Acc:HGNC:20147] |
| ENSG00000067057 | HGLibA_36171 | 130 | 0.145032906 | PFKP | platelet [Source:HGNC Symbol;Acc:HGNC:8878]" |
| ENSG00000069329 | HGLibA_53769 | 130 | 0.145032906 | VPS35 | retromer complex component [Source:HGNC Symbol;Acc:HGNC:13487]" |
| ENSG00000070778 | HGLibA_39457 | 130 | 0.145032906 | PTPN21 | non-receptor type 21 [Source:HGNC Symbol;Acc:HGNC:9651]" |
| ENSG00000071282 | HGLibA_26682 | 130 | 0.145032906 | LMCD1 | LIM and cysteine rich domains 1 [Source:HGNC Symbol;Acc:HGNC:6633] |
| ENSG00000077458 | HGLibA_16726 | 130 | 0.145032906 | FAM76B | family with sequence similarity 76 member B [Source:HGNC Symbol;Acc:HGNC:28492] |
| ENSG00000079432 | HGLibA_09693 | 130 | 0.145032906 | CIC | capicua transcriptional repressor [Source:HGNC Symbol;Acc:HGNC:14214] |
| ENSG00000082684 | HGLibA_43360 | 130 | 0.145032906 | SEMA5B | semaphorin 5B [Source:HGNC Symbol;Acc:HGNC:10737] |

| | | | | | |
|---|---|---|---|---|---|
| ENSG00000082805 | HGLibA_15424 | 130 | 0.145032906 | ERC1 | ELKS/RAB6-interacting/CAST family member 1 [Source:HGNC Symbol;Acc:HGNC:17072] |
| ENSG00000088205 | HGLibA_12661 | 130 | 0.145032906 | DDX18 | DEAD-box helicase 18 [Source:HGNC Symbol;Acc:HGNC:2741] |
| ENSG00000090487 | HGLibA_46636 | 130 | 0.145032906 | SPG21 | maspardin [Source:HGNC Symbol;Acc:HGNC:20373]" |
| ENSG00000090612 | HGLibA_55704 | 130 | 0.145032906 | ZNF268 | zinc finger protein 268 [Source:HGNC Symbol;Acc:HGNC:13061] |
| ENSG00000091732 | HGLibA_54997 | 130 | 0.145032906 | ZC3HC1 | zinc finger C3HC-type containing 1 [Source:HGNC Symbol;Acc:HGNC:29913] |
| ENSG00000094975 | HGLibA_47709 | 130 | 0.145032906 | SUCO | SUN domain containing ossification factor [Source:HGNC Symbol;Acc:HGNC:1240] |
| ENSG00000100100 | HGLibA_36646 | 130 | 0.145032906 | PIK3IP1 | phosphoinositide-3-kinase interacting protein 1 [Source:HGNC Symbol;Acc:HGNC:24942] |
| ENSG00000101574 | HGLibA_29010 | 130 | 0.145032906 | METTL4 | methyltransferase like 4 [Source:HGNC Symbol;Acc:HGNC:24726] |
| ENSG00000101938 | HGLibA_09538 | 130 | 0.145032906 | CHRDL1 | chordin like 1 [Source:HGNC Symbol;Acc:HGNC:29861] |
| ENSG00000102032 | HGLibA_40838 | 130 | 0.145032906 | RENBP | renin binding protein [Source:HGNC Symbol;Acc:HGNC:9959] |
| ENSG00000102393 | HGLibA_19214 | 130 | 0.145032906 | GLA | galactosidase alpha [Source:HGNC Symbol;Acc:HGNC:4296] |
| ENSG00000102996 | HGLibA_29495 | 130 | 0.145032906 | MMP15 | matrix metallopeptidase 15 [Source:HGNC Symbol;Acc:HGNC:7161] |
| ENSG00000105808 | HGLibA_40306 | 130 | 0.145032906 | RASA4 | RAS p21 protein activator 4 [Source:HGNC Symbol;Acc:HGNC:23181] |
| ENSG00000107185 | HGLibA_41022 | 130 | 0.145032906 | RGP1 | RAB6A GEF complex partner 1 [Source:HGNC Symbol;Acc:HGNC:21965]" |
| ENSG00000108064 | HGLibA_49119 | 130 | 0.145032906 | TFAM | mitochondrial [Source:HGNC Symbol;Acc:HGNC:11741]" |
| ENSG00000108819 | HGLibA_38131 | 130 | 0.145032906 | PPP1R9B | protein phosphatase 1 regulatory subunit 9B [Source:HGNC Symbol;Acc:HGNC:9298] |
| ENSG00000111707 | HGLibA_47710 | 130 | 0.145032906 | SUDS3 | SIN3A corepressor complex component [Source:HGNC Symbol;Acc:HGNC:29545]" |
| ENSG00000111799 | HGLibA_10505 | 130 | 0.145032906 | COL12A1 | collagen type XII alpha 1 chain [Source:HGNC Symbol;Acc:HGNC:2188] |
| ENSG00000112343 | HGLibA_51479 | 130 | 0.145032906 | TRIM38 | tripartite motif containing 38 [Source:HGNC Symbol;Acc:HGNC:10059] |
| ENSG00000112462 | HGLibA_33591 | 130 | 0.145032906 | OR12D3 | olfactory receptor family 12 subfamily D member 3 [Source:HGNC Symbol;Acc:HGNC:13963] |
| ENSG00000113083 | HGLibA_27031 | 130 | 0.145032906 | LOX | lysyl oxidase [Source:HGNC Symbol;Acc:HGNC:6664] |
| ENSG00000113448 | HGLibA_35795 | 130 | 0.145032906 | PDE4D | phosphodiesterase 4D [Source:HGNC Symbol;Acc:HGNC:8783] |
| ENSG00000114353 | HGLibA_19453 | 130 | 0.145032906 | GNAI2 | G protein subunit alpha i2 [Source:HGNC Symbol;Acc:HGNC:4385] |
| ENSG00000115159 | HGLibA_19788 | 130 | 0.145032906 | GPD2 | glycerol-3-phosphate dehydrogenase 2 [Source:HGNC Symbol;Acc:HGNC:4456] |
| ENSG00000115295 | HGLibA_10052 | 130 | 0.145032906 | CLIP4 | CAP-Gly domain containing linker protein family member 4 [Source:HGNC Symbol;Acc:HGNC:26108] |
| ENSG00000116062 | HGLibA_30203 | 130 | 0.145032906 | MSH6 | mutS homolog 6 [Source:HGNC Symbol;Acc:HGNC:7329] |
| ENSG00000116473 | HGLibA_40222 | 130 | 0.145032906 | RAP1A | member of RAS oncogene family [Source:HGNC Symbol;Acc:HGNC:9855]" |
| ENSG00000116903 | HGLibA_15762 | 130 | 0.145032906 | EXOC8 | exocyst complex component 8 [Source:HGNC Symbol;Acc:HGNC:24659] |
| ENSG00000117335 | HGLibA_08392 | 130 | 0.145032906 | CD46 | CD46 molecule [Source:HGNC Symbol;Acc:HGNC:6953] |
| ENSG00000117419 | HGLibA_15485 | 130 | 0.145032906 | ERI3 | ERI1 exoribonuclease family member 3 [Source:HGNC Symbol;Acc:HGNC:17276] |
| ENSG00000118156 | HGLibA_56192 | 130 | 0.145032906 | ZNF541 | zinc finger protein 541 [Source:HGNC Symbol;Acc:HGNC:25294] |
| ENSG00000119689 | HGLibA_13370 | 130 | 0.145032906 | DLST | dihydrolipoamide S-succinyltransferase [Source:HGNC Symbol;Acc:HGNC:2911] |
| ENSG00000119703 | HGLibA_54943 | 130 | 0.145032906 | ZC2HC1C | zinc finger C2HC-type containing 1C [Source:HGNC Symbol;Acc:HGNC:20354] |
| ENSG00000120254 | HGLibA_30380 | 130 | 0.145032906 | MTHFD1L | methylenetetrahydrofolate dehydrogenase (NADP+ dependent) 1 like [Source:HGNC Symbol;Acc:HGNC:21055] |
| ENSG00000120693 | HGLibA_45591 | 130 | 0.145032906 | SMAD9 | SMAD family member 9 [Source:HGNC Symbol;Acc:HGNC:6774] |
| ENSG00000121310 | HGLibA_14397 | 130 | 0.145032906 | ECHDC2 | enoyl-CoA hydratase domain containing 2 [Source:HGNC Symbol;Acc:HGNC:23408] |
| ENSG00000121766 | HGLibA_55021 | 130 | 0.145032906 | ZCCHC17 | zinc finger CCHC-type containing 17 [Source:HGNC Symbol;Acc:HGNC:30246] |
| ENSG00000122482 | HGLibA_56446 | 130 | 0.145032906 | ZNF644 | zinc finger protein 644 [Source:HGNC Symbol;Acc:HGNC:29222] |
| ENSG00000123685 | HGLibA_04179 | 130 | 0.145032906 | BATF3 | basic leucine zipper ATF-like transcription factor 3 [Source:HGNC Symbol;Acc:HGNC:28915] |
| ENSG00000124570 | HGLibA_43560 | 130 | 0.145032906 | SERPINB6 | serpin family B member 6 [Source:HGNC Symbol;Acc:HGNC:8950] |
| ENSG00000125652 | HGLibA_01664 | 130 | 0.145032906 | ALKBH7 | alkB homolog 7 [Source:HGNC Symbol;Acc:HGNC:21306] |
| ENSG00000128645 | HGLibA_21984 | 130 | 0.145032906 | HOXD1 | homeobox D1 [Source:HGNC Symbol;Acc:HGNC:5132] |
| ENSG00000130147 | HGLibA_43932 | 130 | 0.145032906 | SH3BP4 | SH3 domain binding protein 4 [Source:HGNC Symbol;Acc:HGNC:10826] |
| ENSG00000130699 | HGLibA_48233 | 130 | 0.145032906 | TAF4 | TATA-box binding protein associated factor 4 [Source:HGNC Symbol;Acc:HGNC:11537] |
| ENSG00000131061 | HGLibA_55839 | 130 | 0.145032906 | ZNF341 | zinc finger protein 341 [Source:HGNC Symbol;Acc:HGNC:15992] |
| ENSG00000131504 | HGLibA_13218 | 130 | 0.145032906 | DIAPH1 | diaphanous related formin 1 [Source:HGNC Symbol;Acc:HGNC:2876] |
| ENSG00000132185 | HGLibA_17244 | 130 | 0.145032906 | FCRLA | Fc receptor like A [Source:HGNC Symbol;Acc:HGNC:18504] |
| ENSG00000132464 | HGLibA_15108 | 130 | 0.145032906 | ENAM | enamelin [Source:HGNC Symbol;Acc:HGNC:3344] |
| ENSG00000134910 | HGLibA_47605 | 130 | 0.145032906 | STT3A | catalytic subunit of the oligosaccharyltransferase complex [Source:HGNC Symbol;Acc:HGNC:6172]" |
| ENSG00000135108 | HGLibA_17045 | 130 | 0.145032906 | FBXO21 | F-box protein 21 [Source:HGNC Symbol;Acc:HGNC:13592] |
| ENSG00000136261 | HGLibA_05015 | 130 | 0.145032906 | BZW2 | basic leucine zipper and W2 domains 2 [Source:HGNC Symbol;Acc:HGNC:18808] |
| ENSG00000136699 | HGLibA_45812 | 130 | 0.145032906 | SMPD4 | sphingomyelin phosphodiesterase 4 [Source:HGNC Symbol;Acc:HGNC:32949] |
| ENSG00000136897 | HGLibA_30009 | 130 | 0.145032906 | MRPL50 | mitochondrial ribosomal protein L50 [Source:HGNC Symbol;Acc:HGNC:16654] |
| ENSG00000137275 | HGLibA_41317 | 130 | 0.145032906 | RIPK1 | receptor interacting serine/threonine kinase 1 [Source:HGNC Symbol;Acc:HGNC:10019] |
| ENSG00000137364 | HGLibA_51153 | 130 | 0.145032906 | TPMT | thiopurine S-methyltransferase [Source:HGNC Symbol;Acc:HGNC:12014] |
| ENSG00000138136 | HGLibA_26086 | 130 | 0.145032906 | LBX1 | ladybird homeobox 1 [Source:HGNC Symbol;Acc:HGNC:16960] |
| ENSG00000141977 | HGLibA_09689 | 130 | 0.145032906 | CIB3 | calcium and integrin binding family member 3 [Source:HGNC Symbol;Acc:HGNC:24580] |
| ENSG00000142173 | HGLibA_10598 | 130 | 0.145032906 | COL6A2 | collagen type VI alpha 2 chain [Source:HGNC Symbol;Acc:HGNC:2212] |
| ENSG00000142973 | HGLibA_12179 | 130 | 0.145032906 | CYP4B1 | cytochrome P450 family 4 subfamily B member 1 [Source:HGNC Symbol;Acc:HGNC:2644] |
| ENSG00000143153 | HGLibA_03592 | 130 | 0.145032906 | ATP1B1 | ATPase Na+/K+ transporting subunit beta 1 [Source:HGNC Symbol;Acc:HGNC:804] |
| ENSG00000143434 | HGLibA_43370 | 130 | 0.145032906 | SEMA6C | semaphorin 6C [Source:HGNC Symbol;Acc:HGNC:10740] |
| ENSG00000143740 | HGLibA_45883 | 130 | 0.145032906 | SNAP47 | synaptosome associated protein 47 [Source:HGNC Symbol;Acc:HGNC:30669] |
| ENSG00000143772 | HGLibA_23848 | 130 | 0.145032906 | ITPKB | inositol-trisphosphate 3-kinase B [Source:HGNC Symbol;Acc:HGNC:6179] |
| ENSG00000145309 | HGLibA_06862 | 130 | 0.145032906 | CABS1 | spermatid associated 1 [Source:HGNC Symbol;Acc:HGNC:30710]" |
| ENSG00000146205 | HGLibA_02190 | 130 | 0.145032906 | ANO7 | anoctamin 7 [Source:HGNC Symbol;Acc:HGNC:31677] |
| ENSG00000147570 | HGLibA_13627 | 130 | 0.145032906 | DNAJC5B | DnaJ heat shock protein family (Hsp40) member C5 beta [Source:HGNC Symbol;Acc:HGNC:24138] |
| ENSG00000147613 | HGLibA_39067 | 130 | 0.145032906 | PSKH2 | protein serine kinase H2 [Source:HGNC Symbol;Acc:HGNC:18997] |
| ENSG00000150782 | HGLibA_23023 | 130 | 0.145032906 | IL18 | interleukin 18 [Source:HGNC Symbol;Acc:HGNC:5986] |
| ENSG00000152672 | HGLibA_09991 | 130 | 0.145032906 | CLEC4F | C-type lectin domain family 4 member F [Source:HGNC Symbol;Acc:HGNC:25357] |
| ENSG00000154734 | HGLibA_00791 | 130 | 0.145032906 | ADAMTS1 | ADAM metallopeptidase with thrombospondin type 1 motif 1 [Source:HGNC Symbol;Acc:HGNC:217] |
| ENSG00000155330 | HGLibA_05491 | 130 | 0.145032906 | C16orf87 | chromosome 16 open reading frame 87 [Source:HGNC Symbol;Acc:HGNC:33754] |
| ENSG00000155744 | HGLibA_16085 | 130 | 0.145032906 | FAM126B | family with sequence similarity 126 member B [Source:HGNC Symbol;Acc:HGNC:28593] |
| ENSG00000156049 | HGLibA_19444 | 130 | 0.145032906 | GNA14 | G protein subunit alpha 14 [Source:HGNC Symbol;Acc:HGNC:4382] |
| ENSG00000158545 | HGLibA_54970 | 130 | 0.145032906 | ZC3H18 | zinc finger CCCH-type containing 18 [Source:HGNC Symbol;Acc:HGNC:25091] |
| ENSG00000159423 | HGLibA_01573 | 130 | 0.145032906 | ALDH4A1 | aldehyde dehydrogenase 4 family member A1 [Source:HGNC Symbol;Acc:HGNC:406] |
| ENSG00000160216 | HGLibA_01223 | 130 | 0.145032906 | AGPAT3 | 1-acylglycerol-3-phosphate O-acyltransferase 3 [Source:HGNC Symbol;Acc:HGNC:326] |
| ENSG00000161652 | HGLibA_23895 | 130 | 0.145032906 | IZUMO2 | IZUMO family member 2 [Source:HGNC Symbol;Acc:HGNC:28518] |
| ENSG00000162687 | HGLibA_24397 | 130 | 0.145032906 | KCNT2 | potassium sodium-activated channel subfamily T member 2 [Source:HGNC Symbol;Acc:HGNC:18866] |
| ENSG00000162971 | HGLibA_52488 | 130 | 0.145032906 | TYW5 | tRNA-yW synthesizing protein 5 [Source:HGNC Symbol;Acc:HGNC:26754] |
| ENSG00000163156 | HGLibA_43011 | 130 | 0.145032906 | SCNM1 | sodium channel modifier 1 [Source:HGNC Symbol;Acc:HGNC:23136] |
| ENSG00000163743 | HGLibA_40695 | 130 | 0.145032906 | RCHY1 | ring finger and CHY zinc finger domain containing 1 [Source:HGNC Symbol;Acc:HGNC:17479] |
| ENSG00000164292 | HGLibA_41177 | 130 | 0.145032906 | RHOBTB3 | Rho related BTB domain containing 3 [Source:HGNC Symbol;Acc:HGNC:18757] |
| ENSG00000166394 | HGLibA_12003 | 130 | 0.145032906 | CYB5R2 | cytochrome b5 reductase 2 [Source:HGNC Symbol;Acc:HGNC:24376] |
| ENSG00000166532 | HGLibA_41273 | 130 | 0.145032906 | RIMKLB | ribosomal modification protein rimK like family member B [Source:HGNC Symbol;Acc:HGNC:29228] |
| ENSG00000166960 | HGLibA_07716 | 130 | 0.145032906 | CCDC178 | coiled-coil domain containing 178 [Source:HGNC Symbol;Acc:HGNC:29588] |
| ENSG00000166984 | HGLibA_48840 | 130 | 0.145032906 | TCP10L2 | t-complex 10 like 2 [Source:HGNC Symbol;Acc:HGNC:21254] |
| ENSG00000167671 | HGLibA_52792 | 130 | 0.145032906 | UBXN6 | UBX domain protein 6 [Source:HGNC Symbol;Acc:HGNC:14928] |
| ENSG00000168748 | HGLibA_06822 | 130 | 0.145032906 | CA7 | carbonic anhydrase 7 [Source:HGNC Symbol;Acc:HGNC:1381] |
| ENSG00000168936 | HGLibA_49923 | 130 | 0.145032906 | TMEM129 | transmembrane protein 129 [Source:HGNC Symbol;Acc:HGNC:25137] |
| ENSG00000169035 | HGLibA_25266 | 130 | 0.145032906 | KLK7 | kallikrein related peptidase 7 [Source:HGNC Symbol;Acc:HGNC:6368] |
| ENSG00000170523 | HGLibA_25564 | 130 | 0.145032906 | KRT83 | keratin 83 [Source:HGNC Symbol;Acc:HGNC:6460] |
| ENSG00000170523 | HGLibA_25565 | 130 | 0.145032906 | KRT83 | keratin 83 [Source:HGNC Symbol;Acc:HGNC:6460] |
| ENSG00000171492 | HGLibA_27437 | 130 | 0.145032906 | LRRC8D | leucine rich repeat containing 8 VRAC subunit D [Source:HGNC Symbol;Acc:HGNC:16992] |
| ENSG00000172216 | HGLibA_08958 | 130 | 0.145032906 | CEBPB | CCAAT enhancer binding protein beta [Source:HGNC Symbol;Acc:HGNC:1834] |
| ENSG00000172840 | HGLibA_35927 | 130 | 0.145032906 | PDP2 | pyruvate dehyrogenase phosphatase catalytic subunit 2 [Source:HGNC Symbol;Acc:HGNC:30263] |
| ENSG00000172915 | HGLibA_31206 | 130 | 0.145032906 | NBEA | neurobeachin [Source:HGNC Symbol;Acc:HGNC:7648] |
| ENSG00000172995 | HGLibA_03102 | 130 | 0.145032906 | ARPP21 | cAMP regulated phosphoprotein 21 [Source:HGNC Symbol;Acc:HGNC:16968] |
| ENSG00000174640 | HGLibA_45461 | 130 | 0.145032906 | SLCO2A1 | solute carrier organic anion transporter family member 2A1 [Source:HGNC Symbol;Acc:HGNC:10955] |
| ENSG00000175073 | HGLibA_53577 | 130 | 0.145032906 | VCPIP1 | valosin containing protein interacting protein 1 [Source:HGNC Symbol;Acc:HGNC:30897] |
| ENSG00000176231 | HGLibA_33514 | 130 | 0.145032906 | OR10H4 | olfactory receptor family 10 subfamily H member 4 [Source:HGNC Symbol;Acc:HGNC:15388] |
| ENSG00000176695 | HGLibA_33995 | 130 | 0.145032906 | OR4F17 | olfactory receptor family 4 subfamily F member 17 [Source:HGNC Symbol;Acc:HGNC:15381] |
| ENSG00000177311 | HGLibA_54871 | 130 | 0.145032906 | ZBTB38 | zinc finger and BTB domain containing 38 [Source:HGNC Symbol;Acc:HGNC:26636] |
| ENSG00000179151 | HGLibA_14442 | 130 | 0.145032906 | EDC3 | enhancer of mRNA decapping 3 [Source:HGNC Symbol;Acc:HGNC:26114] |
| ENSG00000179912 | HGLibA_39776 | 130 | 0.145032906 | R3HDM2 | R3H domain containing 2 [Source:HGNC Symbol;Acc:HGNC:29167] |
| ENSG00000180772 | HGLibA_01266 | 130 | 0.145032906 | AGTR2 | angiotensin II receptor type 2 [Source:HGNC Symbol;Acc:HGNC:338] |
| ENSG00000181027 | HGLibA_17598 | 130 | 0.145032906 | FKRP | fukutin related protein [Source:HGNC Symbol;Acc:HGNC:17997] |
| ENSG00000181704 | HGLibA_54685 | 130 | 0.145032906 | YIPF6 | Yip1 domain family member 6 [Source:HGNC Symbol;Acc:HGNC:28304] |
| ENSG00000181817 | HGLibA_27522 | 130 | 0.145032906 | LSM10 | U7 small nuclear RNA associated [Source:HGNC Symbol;Acc:HGNC:17562]" |
| ENSG00000184588 | HGLibA_35790 | 130 | 0.145032906 | PDE4B | phosphodiesterase 4B [Source:HGNC Symbol;Acc:HGNC:8781] |
| ENSG00000185787 | HGLibA_29659 | 130 | 0.145032906 | MORF4L1 | mortality factor 4 like 1 [Source:HGNC Symbol;Acc:HGNC:16989] |
| ENSG00000186136 | HGLibA_48408 | 130 | 0.145032906 | TAS2R42 | taste 2 receptor member 42 [Source:HGNC Symbol;Acc:HGNC:18888] |
| ENSG00000188277 | HGLibA_05433 | 130 | 0.145032906 | C15orf62 | chromosome 15 open reading frame 62 [Source:HGNC Symbol;Acc:HGNC:34489] |
| ENSG00000188452 | HGLibA_09198 | 130 | 0.145032906 | CERKL | ceramide kinase like [Source:HGNC Symbol;Acc:HGNC:21699] |
| ENSG00000189357 | HGLibA_46500 | 130 | 0.145032906 | SPATA31D4 | SPATA31 subfamily D member 4 [Source:HGNC Symbol;Acc:HGNC:38601] |
| ENSG00000196700 | HGLibA_56135 | 130 | 0.145032906 | ZNF512B | zinc finger protein 512B [Source:HGNC Symbol;Acc:HGNC:29212] |
| ENSG00000198369 | HGLibA_46792 | 130 | 0.145032906 | SPRED2 | sprouty related EVH1 domain containing 2 [Source:HGNC Symbol;Acc:HGNC:17722] |
| ENSG00000204348 | HGLibA_14218 | 130 | 0.145032906 | DXO | decapping exoribonuclease [Source:HGNC Symbol;Acc:HGNC:2992] |
| ENSG00000204352 | HGLibA_06675 | 130 | 0.145032906 | C9orf129 | chromosome 9 open reading frame 129 [Source:HGNC Symbol;Acc:HGNC:31116] |
| ENSG00000204842 | HGLibA_03838 | 130 | 0.145032906 | ATXN2 | ataxin 2 [Source:HGNC Symbol;Acc:HGNC:10555] |
| ENSG00000205744 | HGLibA_12935 | 130 | 0.145032906 | DENND1C | DENN domain containing 1C [Source:HGNC Symbol;Acc:HGNC:26225] |
| ENSG00000212916 | HGLibA_28111 | 130 | 0.145032906 | MAP10 | microtubule associated protein 10 [Source:HGNC Symbol;Acc:HGNC:29265] |
| ENSG00000213047 | HGLibA_12933 | 130 | 0.145032906 | DENND1B | DENN domain containing 1B [Source:HGNC Symbol;Acc:HGNC:28404] |
| ENSG00000214946 | HGLibA_48518 | 130 | 0.145032906 | TBC1D26 | TBC1 domain family member 26 [Source:HGNC Symbol;Acc:HGNC:28745] |
| ENSG00000215271 | HGLibA_21873 | 130 | 0.145032906 | HOMEZ | homeobox and leucine zipper encoding [Source:HGNC Symbol;Acc:HGNC:20164] |
| ENSG00000217128 | HGLibA_17765 | 130 | 0.145032906 | FNIP1 | folliculin interacting protein 1 [Source:HGNC Symbol;Acc:HGNC:29418] |
| ENSG00000221946 | HGLibA_18244 | 130 | 0.145032906 | FXYD7 | FXYD domain containing ion transport regulator 7 [Source:HGNC Symbol;Acc:HGNC:4034] |
| ENSG00000230989 | HGLibA_22151 | 130 | 0.145032906 | HSBP1 | heat shock factor binding protein 1 [Source:HGNC Symbol;Acc:HGNC:5203] |
| ENSG00000231924 | HGLibA_39035 | 130 | 0.145032906 | PSG1 | pregnancy specific beta-1-glycoprotein 1 [Source:HGNC Symbol;Acc:HGNC:9514] |
| ENSG00000242550 | HGLibA_43538 | 130 | 0.145032906 | SERPINB10 | serpin family B member 10 [Source:HGNC Symbol;Acc:HGNC:8942] |
| ENSG00000249853 | HGLibA_22137 | 130 | 0.145032906 | HS3ST5 | heparan sulfate-glucosamine 3-sulfotransferase 5 [Source:HGNC Symbol;Acc:HGNC:19419] |
| ENSG00000256229 | HGLibA_56084 | 130 | 0.145032906 | ZNF486 | zinc finger protein 486 [Source:HGNC Symbol;Acc:HGNC:20807] |
| ENSG00000275152 | HGLibA_07976 | 130 | 0.145032906 | CCL16 | C-C motif chemokine ligand 16 [Source:HGNC Symbol;Acc:HGNC:10614] |
| ENSG00000278505 | HGLibA_05579 | 130 | 0.145032906 | C17orf78 | chromosome 17 open reading frame 78 [Source:HGNC Symbol;Acc:HGNC:26831] |
| FAM160B1 | HGLibA_16211 | 130 | 0.145032906 | FAM160B1 | - |
| FAM213A | HGLibA_16470 | 130 | 0.145032906 | FAM213A | - |

| | | | | | |
|---|---|---|---|---|---|
| hsa-mir-1183 | HGLibA_57227 | 130 | 0.145032906 | hsa-mir-1183 | - |
| hsa-mir-1208 | HGLibA_57286 | 130 | 0.145032906 | hsa-mir-1208 | - |
| hsa-mir-146a | HGLibA_57858 | 130 | 0.145032906 | hsa-mir-146a | - |
| hsa-mir-184 | HGLibA_57999 | 130 | 0.145032906 | hsa-mir-184 | - |
| hsa-mir-34a | HGLibA_59036 | 130 | 0.145032906 | hsa-mir-34a | - |
| hsa-mir-34c | HGLibA_59043 | 130 | 0.145032906 | hsa-mir-34c | - |
| hsa-mir-4461 | HGLibA_60252 | 130 | 0.145032906 | hsa-mir-4461 | - |
| hsa-mir-516b-1 | HGLibA_61616 | 130 | 0.145032906 | hsa-mir-516b-1 | - |
| hsa-mir-521-1 | HGLibA_61766 | 130 | 0.145032906 | hsa-mir-521-1 | - |
| hsa-mir-548a-3 | HGLibA_61845 | 130 | 0.145032906 | hsa-mir-548a-3 | - |
| hsa-mir-551b | HGLibA_62123 | 130 | 0.145032906 | hsa-mir-551b | - |
| hsa-mir-6756 | HGLibA_63183 | 130 | 0.145032906 | hsa-mir-6756 | - |
| LEPREL2 | HGLibA_26322 | 130 | 0.145032906 | LEPREL2 | - |
| TGIF2-C20orf24 | HGLibA_49244 | 130 | 0.145032906 | TGIF2-C20orf24 | - |
| TMEM136 | HGLibA_49953 | 130 | 0.145032906 | TMEM136 | - |
| C19orf82 | HGLibA_05716 | 129 | 0.135081869 | C19orf82 | - |
| C20orf112 | HGLibA_05985 | 129 | 0.135081869 | C20orf112 | - |
| CECR5 | HGLibA_08981 | 129 | 0.135081869 | CECR5 | - |
| ENSG00000000457 | HGLibA_43092 | 129 | 0.135081869 | SCYL3 | SCY1 like pseudokinase 3 [Source:HGNC Symbol;Acc:HGNC:19285] |
| ENSG00000001167 | HGLibA_31853 | 129 | 0.135081869 | NFYA | nuclear transcription factor Y subunit alpha [Source:HGNC Symbol;Acc:HGNC:7804] |
| ENSG00000010803 | HGLibA_42955 | 129 | 0.135081869 | SCMH1 | Scm polycomb group protein homolog 1 [Source:HGNC Symbol;Acc:HGNC:19003] |
| ENSG00000011132 | HGLibA_02374 | 129 | 0.135081869 | APBA3 | amyloid beta precursor protein binding family A member 3 [Source:HGNC Symbol;Acc:HGNC:580] |
| ENSG00000011677 | HGLibA_18361 | 129 | 0.135081869 | GABRA3 | gamma-aminobutyric acid type A receptor alpha3 subunit [Source:HGNC Symbol;Acc:HGNC:4077] |
| ENSG00000016490 | HGLibA_09805 | 129 | 0.135081869 | CLCA1 | chloride channel accessory 1 [Source:HGNC Symbol;Acc:HGNC:2015] |
| ENSG00000030419 | HGLibA_22933 | 129 | 0.135081869 | IKZF2 | IKAROS family zinc finger 2 [Source:HGNC Symbol;Acc:HGNC:13177] |
| ENSG00000036054 | HGLibA_48508 | 129 | 0.135081869 | TBC1D23 | TBC1 domain family member 23 [Source:HGNC Symbol;Acc:HGNC:25622] |
| ENSG00000055957 | HGLibA_23806 | 129 | 0.135081869 | ITIH1 | inter-alpha-trypsin inhibitor heavy chain 1 [Source:HGNC Symbol;Acc:HGNC:6166] |
| ENSG00000075413 | HGLibA_28371 | 129 | 0.135081869 | MARK3 | microtubule affinity regulating kinase 3 [Source:HGNC Symbol;Acc:HGNC:6897] |
| ENSG00000078687 | HGLibA_50919 | 129 | 0.135081869 | TNRC6C | trinucleotide repeat containing 6C [Source:HGNC Symbol;Acc:HGNC:29318] |
| ENSG00000081760 | HGLibA_00026 | 129 | 0.135081869 | AACS | acetoacetyl-CoA synthetase [Source:HGNC Symbol;Acc:HGNC:21298] |
| ENSG00000084453 | HGLibA_45445 | 129 | 0.135081869 | SLCO1A2 | solute carrier organic anion transporter family member 1A2 [Source:HGNC Symbol;Acc:HGNC:10956] |
| ENSG00000088053 | HGLibA_19711 | 129 | 0.135081869 | GP6 | glycoprotein VI platelet [Source:HGNC Symbol;Acc:HGNC:14388] |
| ENSG00000088247 | HGLibA_24581 | 129 | 0.135081869 | KHSRP | KH-type splicing regulatory protein [Source:HGNC Symbol;Acc:HGNC:6316] |
| ENSG00000094975 | HGLibA_47708 | 129 | 0.135081869 | SUCO | SUN domain containing ossification factor [Source:HGNC Symbol;Acc:HGNC:1240] |
| ENSG00000099977 | HGLibA_12642 | 129 | 0.135081869 | DDT | D-dopachrome tautomerase [Source:HGNC Symbol;Acc:HGNC:2732] |
| ENSG00000100401 | HGLibA_40214 | 129 | 0.135081869 | RANGAP1 | Ran GTPase activating protein 1 [Source:HGNC Symbol;Acc:HGNC:9854] |
| ENSG00000100453 | HGLibA_20721 | 129 | 0.135081869 | GZMB | granzyme B [Source:HGNC Symbol;Acc:HGNC:4709] |
| ENSG00000101443 | HGLibA_54263 | 129 | 0.135081869 | WFDC2 | WAP four-disulfide core domain 2 [Source:HGNC Symbol;Acc:HGNC:15939] |
| ENSG00000104998 | HGLibA_23116 | 129 | 0.135081869 | IL27RA | interleukin 27 receptor subunit alpha [Source:HGNC Symbol;Acc:HGNC:17290] |
| ENSG00000105205 | HGLibA_09802 | 129 | 0.135081869 | CLC | Charcot-Leyden crystal galectin [Source:HGNC Symbol;Acc:HGNC:2014] |
| ENSG00000105997 | HGLibA_21908 | 129 | 0.135081869 | HOXA3 | homeobox A3 [Source:HGNC Symbol;Acc:HGNC:5104] |
| ENSG00000107798 | HGLibA_26599 | 129 | 0.135081869 | LIPA | lysosomal acid type [Source:HGNC Symbol;Acc:HGNC:6617]" |
| ENSG00000108179 | HGLibA_37929 | 129 | 0.135081869 | PPIF | peptidylprolyl isomerase F [Source:HGNC Symbol;Acc:HGNC:9259] |
| ENSG00000108774 | HGLibA_39966 | 129 | 0.135081869 | RAB5C | member RAS oncogene family [Source:HGNC Symbol;Acc:HGNC:9785]" |
| ENSG00000110422 | HGLibA_21345 | 129 | 0.135081869 | HIPK3 | homeodomain interacting protein kinase 3 [Source:HGNC Symbol;Acc:HGNC:4915] |
| ENSG00000111110 | HGLibA_37981 | 129 | 0.135081869 | PPM1H | Mg2+/Mn2+ dependent 1H [Source:HGNC Symbol;Acc:HGNC:18583]" |
| ENSG00000112039 | HGLibA_16846 | 129 | 0.135081869 | FANCE | FA complementation group E [Source:HGNC Symbol;Acc:HGNC:3586] |
| ENSG00000112186 | HGLibA_07135 | 129 | 0.135081869 | CAP2 | cyclase associated actin cytoskeleton regulatory protein 2 [Source:HGNC Symbol;Acc:HGNC:20039] |
| ENSG00000113790 | HGLibA_14681 | 129 | 0.135081869 | EHHADH | enoyl-CoA hydratase and 3-hydroxyacyl CoA dehydrogenase [Source:HGNC Symbol;Acc:HGNC:3247] |
| ENSG00000120160 | HGLibA_15398 | 129 | 0.135081869 | EQTN | equatorin [Source:HGNC Symbol;Acc:HGNC:1359] |
| ENSG00000121904 | HGLibA_11365 | 129 | 0.135081869 | CSMD2 | CUB and Sushi multiple domains 2 [Source:HGNC Symbol;Acc:HGNC:19290] |
| ENSG00000125398 | HGLibA_46280 | 129 | 0.135081869 | SOX9 | SRY-box 9 [Source:HGNC Symbol;Acc:HGNC:11204] |
| ENSG00000126777 | HGLibA_25871 | 129 | 0.135081869 | KTN1 | kinectin 1 [Source:HGNC Symbol;Acc:HGNC:6467] |
| ENSG00000128683 | HGLibA_18410 | 129 | 0.135081869 | GAD1 | glutamate decarboxylase 1 [Source:HGNC Symbol;Acc:HGNC:4092] |
| ENSG00000130368 | HGLibA_28389 | 129 | 0.135081869 | MAS1 | G protein-coupled receptor [Source:HGNC Symbol;Acc:HGNC:6899]" |
| ENSG00000130429 | HGLibA_03076 | 129 | 0.135081869 | ARPC1B | actin related protein 2/3 complex subunit 1B [Source:HGNC Symbol;Acc:HGNC:704] |
| ENSG00000130856 | HGLibA_55650 | 129 | 0.135081869 | ZNF236 | zinc finger protein 236 [Source:HGNC Symbol;Acc:HGNC:13028] |
| ENSG00000131015 | HGLibA_52962 | 129 | 0.135081869 | ULBP2 | UL16 binding protein 2 [Source:HGNC Symbol;Acc:HGNC:14894] |
| ENSG00000131584 | HGLibA_00389 | 129 | 0.135081869 | ACAP3 | ankyrin repeat and PH domains 3 [Source:HGNC Symbol;Acc:HGNC:16754]" |
| ENSG00000132170 | HGLibA_37842 | 129 | 0.135081869 | PPARG | peroxisome proliferator activated receptor gamma [Source:HGNC Symbol;Acc:HGNC:9236] |
| ENSG00000132958 | HGLibA_51217 | 129 | 0.135081869 | TPTE2 | transmembrane phosphoinositide 3-phosphatase and tensin homolog 2 [Source:HGNC Symbol;Acc:HGNC:17299] |
| ENSG00000133640 | HGLibA_27455 | 129 | 0.135081869 | LRRIQ1 | leucine rich repeats and IQ motif containing 1 [Source:HGNC Symbol;Acc:HGNC:25708] |
| ENSG00000135250 | HGLibA_47022 | 129 | 0.135081869 | SRPK2 | SRSF protein kinase 2 [Source:HGNC Symbol;Acc:HGNC:11306] |
| ENSG00000135362 | HGLibA_38847 | 129 | 0.135081869 | PRR5L | proline rich 5 like [Source:HGNC Symbol;Acc:HGNC:25878] |
| ENSG00000135968 | HGLibA_18766 | 129 | 0.135081869 | GCC2 | GRIP and coiled-coil domain containing 2 [Source:HGNC Symbol;Acc:HGNC:23218] |
| ENSG00000136943 | HGLibA_11730 | 129 | 0.135081869 | CTSV | cathepsin V [Source:HGNC Symbol;Acc:HGNC:2538] |
| ENSG00000137077 | HGLibA_07994 | 129 | 0.135081869 | CCL21 | C-C motif chemokine ligand 21 [Source:HGNC Symbol;Acc:HGNC:10620] |
| ENSG00000137185 | HGLibA_57034 | 129 | 0.135081869 | ZSCAN9 | zinc finger and SCAN domain containing 9 [Source:HGNC Symbol;Acc:HGNC:12984] |
| ENSG00000137571 | HGLibA_45476 | 129 | 0.135081869 | SLCO5A1 | solute carrier organic anion transporter family member 5A1 [Source:HGNC Symbol;Acc:HGNC:19046] |
| ENSG00000138109 | HGLibA_12125 | 129 | 0.135081869 | CYP2C9 | cytochrome P450 family 2 subfamily C member 9 [Source:HGNC Symbol;Acc:HGNC:2623] |
| ENSG00000138311 | HGLibA_55877 | 129 | 0.135081869 | ZNF365 | zinc finger protein 365 [Source:HGNC Symbol;Acc:HGNC:18194] |
| ENSG00000139620 | HGLibA_24041 | 129 | 0.135081869 | KANSL2 | KAT8 regulatory NSL complex subunit 2 [Source:HGNC Symbol;Acc:HGNC:26024] |
| ENSG00000140678 | HGLibA_23765 | 129 | 0.135081869 | ITGAX | integrin subunit alpha X [Source:HGNC Symbol;Acc:HGNC:6152] |
| ENSG00000142615 | HGLibA_08993 | 129 | 0.135081869 | CELA2A | chymotrypsin like elastase family member 2A [Source:HGNC Symbol;Acc:HGNC:24609] |
| ENSG00000144460 | HGLibA_33182 | 129 | 0.135081869 | NYAP2 | neuronal tyrosine-phosphorylated phosphoinositide-3-kinase adaptor 2 [Source:HGNC Symbol;Acc:HGNC:29291] |
| ENSG00000144655 | HGLibA_11423 | 129 | 0.135081869 | CSRNP1 | cysteine and serine rich nuclear protein 1 [Source:HGNC Symbol;Acc:HGNC:14300] |
| ENSG00000145391 | HGLibA_43659 | 129 | 0.135081869 | SETD7 | SET domain containing lysine methyltransferase 7 [Source:HGNC Symbol;Acc:HGNC:30412] |
| ENSG00000145888 | HGLibA_19299 | 129 | 0.135081869 | GLRA1 | glycine receptor alpha 1 [Source:HGNC Symbol;Acc:HGNC:4326] |
| ENSG00000146587 | HGLibA_40420 | 129 | 0.135081869 | RBAK | RB associated KRAB zinc finger [Source:HGNC Symbol;Acc:HGNC:17680] |
| ENSG00000152595 | HGLibA_28903 | 129 | 0.135081869 | MEPE | matrix extracellular phosphoglycoprotein [Source:HGNC Symbol;Acc:HGNC:13361] |
| ENSG00000154611 | HGLibA_39091 | 129 | 0.135081869 | PSMA8 | proteasome subunit alpha 8 [Source:HGNC Symbol;Acc:HGNC:22985] |
| ENSG00000154975 | HGLibA_06787 | 129 | 0.135081869 | CA10 | carbonic anhydrase 10 [Source:HGNC Symbol;Acc:HGNC:1369] |
| ENSG00000155975 | HGLibA_53776 | 129 | 0.135081869 | VPS37A | ESCRT-I subunit [Source:HGNC Symbol;Acc:HGNC:24928]" |
| ENSG00000159110 | HGLibA_22699 | 129 | 0.135081869 | IFNAR2 | interferon alpha and beta receptor subunit 2 [Source:HGNC Symbol;Acc:HGNC:5433] |
| ENSG00000160695 | HGLibA_53727 | 129 | 0.135081869 | VPS11 | CORVET/HOPS core subunit [Source:HGNC Symbol;Acc:HGNC:14583]" |
| ENSG00000163359 | HGLibA_10603 | 129 | 0.135081869 | COL6A3 | collagen type VI alpha 3 chain [Source:HGNC Symbol;Acc:HGNC:2213] |
| ENSG00000163840 | HGLibA_14088 | 129 | 0.135081869 | DTX3L | deltex E3 ubiquitin ligase 3L [Source:HGNC Symbol;Acc:HGNC:30323] |
| ENSG00000164304 | HGLibA_06983 | 129 | 0.135081869 | CAGE1 | cancer antigen 1 [Source:HGNC Symbol;Acc:HGNC:21622] |
| ENSG00000164989 | HGLibA_07695 | 129 | 0.135081869 | CCDC171 | coiled-coil domain containing 171 [Source:HGNC Symbol;Acc:HGNC:29828] |
| ENSG00000165102 | HGLibA_21249 | 129 | 0.135081869 | HGSNAT | heparan-alpha-glucosaminide N-acetyltransferase [Source:HGNC Symbol;Acc:HGNC:26527] |
| ENSG00000166451 | HGLibA_09075 | 129 | 0.135081869 | CENPN | centromere protein N [Source:HGNC Symbol;Acc:HGNC:30873] |
| ENSG00000166997 | HGLibA_10370 | 129 | 0.135081869 | CNPY4 | canopy FGF signaling regulator 4 [Source:HGNC Symbol;Acc:HGNC:28631] |
| ENSG00000167395 | HGLibA_56451 | 129 | 0.135081869 | ZNF646 | zinc finger protein 646 [Source:HGNC Symbol;Acc:HGNC:29004] |
| ENSG00000168026 | HGLibA_52070 | 129 | 0.135081869 | TTC21A | tetratricopeptide repeat domain 21A [Source:HGNC Symbol;Acc:HGNC:30761] |
| ENSG00000168306 | HGLibA_00499 | 129 | 0.135081869 | ACOX2 | acyl-CoA oxidase 2 [Source:HGNC Symbol;Acc:HGNC:120] |
| ENSG00000169018 | HGLibA_17275 | 129 | 0.135081869 | FEM1B | fem-1 homolog B [Source:HGNC Symbol;Acc:HGNC:3649] |
| ENSG00000169436 | HGLibA_10541 | 129 | 0.135081869 | COL22A1 | collagen type XXII alpha 1 chain [Source:HGNC Symbol;Acc:HGNC:22989] |
| ENSG00000170425 | HGLibA_01015 | 129 | 0.135081869 | ADORA2B | adenosine A2b receptor [Source:HGNC Symbol;Acc:HGNC:264] |
| ENSG00000170667 | HGLibA_40307 | 129 | 0.135081869 | RASA4B | RAS p21 protein activator 4B [Source:HGNC Symbol;Acc:HGNC:35202] |
| ENSG00000171530 | HGLibA_48579 | 129 | 0.135081869 | TBCA | tubulin folding cofactor A [Source:HGNC Symbol;Acc:HGNC:11579] |
| ENSG00000172188 | HGLibA_33938 | 129 | 0.135081869 | OR4C11 | olfactory receptor family 4 subfamily C member 11 [Source:HGNC Symbol;Acc:HGNC:15167] |
| ENSG00000172421 | HGLibA_14538 | 129 | 0.135081869 | EFCAB3 | EF-hand calcium binding domain 3 [Source:HGNC Symbol;Acc:HGNC:26379] |
| ENSG00000175520 | HGLibA_52738 | 129 | 0.135081869 | UBQLN3 | ubiquilin 3 [Source:HGNC Symbol;Acc:HGNC:12510] |
| ENSG00000175643 | HGLibA_41376 | 129 | 0.135081869 | RMI2 | RecQ mediated genome instability 2 [Source:HGNC Symbol;Acc:HGNC:28349] |
| ENSG00000176171 | HGLibA_04657 | 129 | 0.135081869 | BNIP3 | BCL2 interacting protein 3 [Source:HGNC Symbol;Acc:HGNC:1084] |
| ENSG00000176302 | HGLibA_17944 | 129 | 0.135081869 | FOXR1 | forkhead box R1 [Source:HGNC Symbol;Acc:HGNC:29980] |
| ENSG00000176635 | HGLibA_21890 | 129 | 0.135081869 | HORMAD2 | HORMA domain containing 2 [Source:HGNC Symbol;Acc:HGNC:28383] |
| ENSG00000178585 | HGLibA_11653 | 129 | 0.135081869 | CTNNBIP1 | catenin beta interacting protein 1 [Source:HGNC Symbol;Acc:HGNC:16913] |
| ENSG00000179456 | HGLibA_54833 | 129 | 0.135081869 | ZBTB18 | zinc finger and BTB domain containing 18 [Source:HGNC Symbol;Acc:HGNC:13030] |
| ENSG00000179873 | HGLibA_32102 | 129 | 0.135081869 | NLRP11 | NLR family pyrin domain containing 11 [Source:HGNC Symbol;Acc:HGNC:22945] |
| ENSG00000179919 | HGLibA_33477 | 129 | 0.135081869 | OR10A7 | olfactory receptor family 10 subfamily A member 7 [Source:HGNC Symbol;Acc:HGNC:15329] |
| ENSG00000180658 | HGLibA_33734 | 129 | 0.135081869 | OR2A4 | olfactory receptor family 2 subfamily A member 4 [Source:HGNC Symbol;Acc:HGNC:14729] |
| ENSG00000180758 | HGLibA_19949 | 129 | 0.135081869 | GPR157 | G protein-coupled receptor 157 [Source:HGNC Symbol;Acc:HGNC:23687] |
| ENSG00000182372 | HGLibA_10085 | 129 | 0.135081869 | CLN8 | transmembrane ER and ERGIC protein [Source:HGNC Symbol;Acc:HGNC:2079]" |
| ENSG00000183020 | HGLibA_02309 | 129 | 0.135081869 | AP2A2 | adaptor related protein complex 2 subunit alpha 2 [Source:HGNC Symbol;Acc:HGNC:562] |
| ENSG00000183150 | HGLibA_19992 | 129 | 0.135081869 | GPR19 | G protein-coupled receptor 19 [Source:HGNC Symbol;Acc:HGNC:4473] |
| ENSG00000185231 | HGLibA_28545 | 129 | 0.135081869 | MC2R | melanocortin 2 receptor [Source:HGNC Symbol;Acc:HGNC:6930] |
| ENSG00000185344 | HGLibA_03705 | 129 | 0.135081869 | ATP6V0A2 | ATPase H+ transporting V0 subunit a2 [Source:HGNC Symbol;Acc:HGNC:18481] |
| ENSG00000187323 | HGLibA_12474 | 129 | 0.135081869 | DCC | DCC netrin 1 receptor [Source:HGNC Symbol;Acc:HGNC:2701] |
| ENSG00000188467 | HGLibA_44628 | 129 | 0.135081869 | SLC24A5 | solute carrier family 24 member 5 [Source:HGNC Symbol;Acc:HGNC:20611] |
| ENSG00000189319 | HGLibA_16638 | 129 | 0.135081869 | FAM53B | family with sequence similarity 53 member B [Source:HGNC Symbol;Acc:HGNC:28968] |
| ENSG00000189375 | HGLibA_48522 | 129 | 0.135081869 | TBC1D28 | TBC1 domain family member 28 [Source:HGNC Symbol;Acc:HGNC:26858] |
| ENSG00000196510 | HGLibA_01888 | 129 | 0.135081869 | ANAPC7 | anaphase promoting complex subunit 7 [Source:HGNC Symbol;Acc:HGNC:17380] |
| ENSG00000196569 | HGLibA_25945 | 129 | 0.135081869 | LAMA2 | laminin subunit alpha 2 [Source:HGNC Symbol;Acc:HGNC:6482] |
| ENSG00000197757 | HGLibA_21973 | 129 | 0.135081869 | HOXC6 | homeobox C6 [Source:HGNC Symbol;Acc:HGNC:5128] |
| ENSG00000198597 | HGLibA_56185 | 129 | 0.135081869 | ZNF536 | zinc finger protein 536 [Source:HGNC Symbol;Acc:HGNC:29025] |
| ENSG00000198598 | HGLibA_29501 | 129 | 0.135081869 | MMP17 | matrix metallopeptidase 17 [Source:HGNC Symbol;Acc:HGNC:7163] |
| ENSG00000198909 | HGLibA_28185 | 129 | 0.135081869 | MAP3K3 | mitogen-activated protein kinase kinase kinase 3 [Source:HGNC Symbol;Acc:HGNC:6855] |
| ENSG00000204577 | HGLibA_26513 | 129 | 0.135081869 | LILRB3 | leukocyte immunoglobulin like receptor B3 [Source:HGNC Symbol;Acc:HGNC:6607] |
| ENSG00000213523 | HGLibA_46921 | 129 | 0.135081869 | SRA1 | steroid receptor RNA activator 1 [Source:HGNC Symbol;Acc:HGNC:11281] |
| ENSG00000213973 | HGLibA_56916 | 129 | 0.135081869 | ZNF99 | zinc finger protein 99 [Source:HGNC Symbol;Acc:HGNC:13175] |
| ENSG00000235961 | HGLibA_37379 | 129 | 0.135081869 | PNMA6A | PNMA family member 6A [Source:HGNC Symbol;Acc:HGNC:28248] |
| ENSG00000240386 | HGLibA_26115 | 129 | 0.135081869 | LCE1F | late cornified envelope 1F [Source:HGNC Symbol;Acc:HGNC:29467] |
| ENSG00000241399 | HGLibA_08347 | 129 | 0.135081869 | CD302 | CD302 molecule [Source:HGNC Symbol;Acc:HGNC:30843] |
| ENSG00000243709 | HGLibA_26275 | 129 | 0.135081869 | LEFTY1 | left-right determination factor 1 [Source:HGNC Symbol;Acc:HGNC:6552] |

| | | | | | |
|---|---|---|---|---|---|
| ENSG00000244094 | HGLibA_46820 | 129 | 0.135081869 | SPRR2F | small proline rich protein 2F [Source:HGNC Symbol;Acc:HGNC:11266] |
| ENSG00000244165 | HGLibA_34873 | 129 | 0.135081869 | P2RY11 | purinergic receptor P2Y11 [Source:HGNC Symbol;Acc:HGNC:8540] |
| ENSG00000256574 | HGLibA_33594 | 129 | 0.135081869 | OR13A1 | olfactory receptor family 13 subfamily A member 1 [Source:HGNC Symbol;Acc:HGNC:14772] |
| ENSG00000268606 | HGLibA_27906 | 129 | 0.135081869 | MAGEA2 | MAGE family member A2 [Source:HGNC Symbol;Acc:HGNC:6800] |
| ENSG00000277443 | HGLibA_28355 | 129 | 0.135081869 | MARCKS | myristoylated alanine rich protein kinase C substrate [Source:HGNC Symbol;Acc:HGNC:6759] |
| FAM49A | HGLibA_16622 | 129 | 0.135081869 | FAM49A | - |
| GPR115 | HGLibA_19860 | 129 | 0.135081869 | GPR115 | - |
| hsa-mir-1228 | HGLibA_57309 | 129 | 0.135081869 | hsa-mir-1228 | - |
| hsa-mir-153-2 | HGLibA_57910 | 129 | 0.135081869 | hsa-mir-153-2 | - |
| hsa-mir-3617 | HGLibA_59096 | 129 | 0.135081869 | hsa-mir-3617 | - |
| hsa-mir-373 | HGLibA_59385 | 129 | 0.135081869 | hsa-mir-373 | - |
| hsa-mir-450a-2 | HGLibA_60477 | 129 | 0.135081869 | hsa-mir-450a-2 | - |
| hsa-mir-4704 | HGLibA_60917 | 129 | 0.135081869 | hsa-mir-4704 | - |
| hsa-mir-4705 | HGLibA_60919 | 129 | 0.135081869 | hsa-mir-4705 | - |
| hsa-mir-4777 | HGLibA_61217 | 129 | 0.135081869 | hsa-mir-4777 | - |
| hsa-mir-491 | HGLibA_61364 | 129 | 0.135081869 | hsa-mir-491 | - |
| hsa-mir-496 | HGLibA_61385 | 129 | 0.135081869 | hsa-mir-496 | - |
| hsa-mir-507 | HGLibA_61488 | 129 | 0.135081869 | hsa-mir-507 | - |
| hsa-mir-517c | HGLibA_61630 | 129 | 0.135081869 | hsa-mir-517c | - |
| hsa-mir-544b | HGLibA_61827 | 129 | 0.135081869 | hsa-mir-544b | - |
| hsa-mir-548ae-1 | HGLibA_61869 | 129 | 0.135081869 | hsa-mir-548ae-1 | - |
| hsa-mir-6822 | HGLibA_63467 | 129 | 0.135081869 | hsa-mir-6822 | - |
| hsa-mir-6829 | HGLibA_63493 | 129 | 0.135081869 | hsa-mir-6829 | - |
| hsa-mir-6862-1 | HGLibA_63627 | 129 | 0.135081869 | hsa-mir-6862-1 | - |
| hsa-mir-6892 | HGLibA_63752 | 129 | 0.135081869 | hsa-mir-6892 | - |
| hsa-mir-921 | HGLibA_64280 | 129 | 0.135081869 | hsa-mir-921 | - |
| hsa-mir-941-4 | HGLibA_64350 | 129 | 0.135081869 | hsa-mir-941-4 | - |
| KIAA2022 | HGLibA_24845 | 129 | 0.135081869 | KIAA2022 | - |
| LOC100132146 | HGLibA_26805 | 129 | 0.135081869 | LOC100132146 | - |
| LOC441155 | HGLibA_26950 | 129 | 0.135081869 | LOC441155 | - |
| RAB7L1 | HGLibA_39982 | 129 | 0.135081869 | RAB7L1 | - |
| 9-Sep | HGLibA_43456 | 129 | 0.135081869 | 9-Sep | - |
| SPANXB2 | HGLibA_46386 | 129 | 0.135081869 | SPANXB2 | - |
| WISP2 | HGLibA_54321 | 129 | 0.135081869 | WISP2 | - |
| 1-Dec | HGLibA_12770 | 128 | 0.125130832 | 1-Dec | - |
| AIM1 | HGLibA_01348 | 128 | 0.125130832 | AIM1 | - |
| ARSE | HGLibA_03136 | 128 | 0.125130832 | ARSE | - |
| ATP5G3 | HGLibA_03661 | 128 | 0.125130832 | ATP5G3 | - |
| C10orf2 | HGLibA_05061 | 128 | 0.125130832 | C10orf2 | - |
| C7orf73 | HGLibA_06590 | 128 | 0.125130832 | C7orf73 | - |
| CCDC155 | HGLibA_07659 | 128 | 0.125130832 | CCDC155 | - |
| CNIH | HGLibA_10280 | 128 | 0.125130832 | CNIH | - |
| ENSG00000004776 | HGLibA_22312 | 128 | 0.125130832 | HSPB6 | heat shock protein family B (small) member 6 [Source:HGNC Symbol;Acc:HGNC:26511] |
| ENSG00000005187 | HGLibA_00578 | 128 | 0.125130832 | ACSM3 | acyl-CoA synthetase medium chain family member 3 [Source:HGNC Symbol;Acc:HGNC:10522] |
| ENSG00000018408 | HGLibA_54456 | 128 | 0.125130832 | WWTR1 | WW domain containing transcription regulator 1 [Source:HGNC Symbol;Acc:HGNC:24042] |
| ENSG00000023191 | HGLibA_41712 | 128 | 0.125130832 | RNH1 | ribonuclease/angiogenin inhibitor 1 [Source:HGNC Symbol;Acc:HGNC:10074] |
| ENSG00000025039 | HGLibA_42234 | 128 | 0.125130832 | RRAGD | Ras related GTP binding D [Source:HGNC Symbol;Acc:HGNC:19903] |
| ENSG00000035499 | HGLibA_12976 | 128 | 0.125130832 | DEPDC1B | DEP domain containing 1B [Source:HGNC Symbol;Acc:HGNC:24902] |
| ENSG00000047936 | HGLibA_41784 | 128 | 0.125130832 | ROS1 | receptor tyrosine kinase [Source:HGNC Symbol;Acc:HGNC:10261]" |
| ENSG00000050130 | HGLibA_23950 | 128 | 0.125130832 | JKAMP | JNK1/MAPK8 associated membrane protein [Source:HGNC Symbol;Acc:HGNC:20184] |
| ENSG00000054219 | HGLibA_27680 | 128 | 0.125130832 | LY75 | lymphocyte antigen 75 [Source:HGNC Symbol;Acc:HGNC:6729] |
| ENSG00000064687 | HGLibA_00121 | 128 | 0.125130832 | ABCA7 | ATP binding cassette subfamily A member 7 [Source:HGNC Symbol;Acc:HGNC:37] |
| ENSG00000065150 | HGLibA_23469 | 128 | 0.125130832 | IPO5 | importin 5 [Source:HGNC Symbol;Acc:HGNC:6402] |
| ENSG00000065833 | HGLibA_28710 | 128 | 0.125130832 | ME1 | malic enzyme 1 [Source:HGNC Symbol;Acc:HGNC:6983] |
| ENSG00000073111 | HGLibA_28605 | 128 | 0.125130832 | MCM2 | minichromosome maintenance complex component 2 [Source:HGNC Symbol;Acc:HGNC:6944] |
| ENSG00000078070 | HGLibA_28565 | 128 | 0.125130832 | MCCC1 | methylcrotonoyl-CoA carboxylase 1 [Source:HGNC Symbol;Acc:HGNC:6936] |
| ENSG00000079739 | HGLibA_36262 | 128 | 0.125130832 | PGM1 | phosphoglucomutase 1 [Source:HGNC Symbol;Acc:HGNC:8905] |
| ENSG00000080298 | HGLibA_40969 | 128 | 0.125130832 | RFX3 | regulatory factor X3 [Source:HGNC Symbol;Acc:HGNC:9984] |
| ENSG00000090372 | HGLibA_47599 | 128 | 0.125130832 | STRN4 | striatin 4 [Source:HGNC Symbol;Acc:HGNC:15721] |
| ENSG00000097096 | HGLibA_47944 | 128 | 0.125130832 | SYDE2 | synapse defective Rho GTPase homolog 2 [Source:HGNC Symbol;Acc:HGNC:25841] |
| ENSG00000100345 | HGLibA_30741 | 128 | 0.125130832 | MYH9 | myosin heavy chain 9 [Source:HGNC Symbol;Acc:HGNC:7579] |
| ENSG00000100600 | HGLibA_26405 | 128 | 0.125130832 | LGMN | legumain [Source:HGNC Symbol;Acc:HGNC:9472] |
| ENSG00000100982 | HGLibA_35610 | 128 | 0.125130832 | PCIF1 | PDX1 C-terminal inhibiting factor 1 [Source:HGNC Symbol;Acc:HGNC:16200] |
| ENSG00000101126 | HGLibA_01000 | 128 | 0.125130832 | ADNP | activity dependent neuroprotector homeobox [Source:HGNC Symbol;Acc:HGNC:15766] |
| ENSG00000102786 | HGLibA_23426 | 128 | 0.125130832 | INTS6 | integrator complex subunit 6 [Source:HGNC Symbol;Acc:HGNC:14879] |
| ENSG00000103168 | HGLibA_48218 | 128 | 0.125130832 | TAF1C | RNA polymerase I subunit C [Source:HGNC Symbol;Acc:HGNC:11534]" |
| ENSG00000105610 | HGLibA_25038 | 128 | 0.125130832 | KLF1 | Kruppel like factor 1 [Source:HGNC Symbol;Acc:HGNC:6345] |
| ENSG00000108773 | HGLibA_24051 | 128 | 0.125130832 | KAT2A | lysine acetyltransferase 2A [Source:HGNC Symbol;Acc:HGNC:4201] |
| ENSG00000108946 | HGLibA_38522 | 128 | 0.125130832 | PRKAR1A | protein kinase cAMP-dependent type I regulatory subunit alpha [Source:HGNC Symbol;Acc:HGNC:9388] |
| ENSG00000109062 | HGLibA_45409 | 128 | 0.125130832 | SLC9A3R1 | SLC9A3 regulator 1 [Source:HGNC Symbol;Acc:HGNC:11075] |
| ENSG00000112494 | HGLibA_53035 | 128 | 0.125130832 | UNC93A | unc-93 homolog A [Source:HGNC Symbol;Acc:HGNC:12570] |
| ENSG00000114270 | HGLibA_10610 | 128 | 0.125130832 | COL7A1 | collagen type VII alpha 1 chain [Source:HGNC Symbol;Acc:HGNC:2214] |
| ENSG00000114544 | HGLibA_45145 | 128 | 0.125130832 | SLC41A3 | solute carrier family 41 member 3 [Source:HGNC Symbol;Acc:HGNC:31046] |
| ENSG00000114742 | HGLibA_54110 | 128 | 0.125130832 | WDR48 | WD repeat domain 48 [Source:HGNC Symbol;Acc:HGNC:30914] |
| ENSG00000116198 | HGLibA_09096 | 128 | 0.125130832 | CEP104 | centrosomal protein 104 [Source:HGNC Symbol;Acc:HGNC:24866] |
| ENSG00000116721 | HGLibA_38279 | 128 | 0.125130832 | PRAMEF1 | PRAME family member 1 [Source:HGNC Symbol;Acc:HGNC:28840] |
| ENSG00000116922 | HGLibA_05740 | 128 | 0.125130832 | C1orf109 | chromosome 1 open reading frame 109 [Source:HGNC Symbol;Acc:HGNC:26039] |
| ENSG00000118046 | HGLibA_47454 | 128 | 0.125130832 | STK11 | serine/threonine kinase 11 [Source:HGNC Symbol;Acc:HGNC:11389] |
| ENSG00000124574 | HGLibA_00161 | 128 | 0.125130832 | ABCC10 | ATP binding cassette subfamily C member 10 [Source:HGNC Symbol;Acc:HGNC:52] |
| ENSG00000126550 | HGLibA_22351 | 128 | 0.125130832 | HTN1 | histatin 1 [Source:HGNC Symbol;Acc:HGNC:5283] |
| ENSG00000127863 | HGLibA_50769 | 128 | 0.125130832 | TNFRSF19 | TNF receptor superfamily member 19 [Source:HGNC Symbol;Acc:HGNC:11915] |
| ENSG00000128989 | HGLibA_03099 | 128 | 0.125130832 | ARPP19 | cAMP regulated phosphoprotein 19 [Source:HGNC Symbol;Acc:HGNC:16967] |
| ENSG00000129103 | HGLibA_47772 | 128 | 0.125130832 | SUMF2 | sulfatase modifying factor 2 [Source:HGNC Symbol;Acc:HGNC:20415] |
| ENSG00000129204 | HGLibA_53404 | 128 | 0.125130832 | USP6 | ubiquitin specific peptidase 6 [Source:HGNC Symbol;Acc:HGNC:12629] |
| ENSG00000130303 | HGLibA_04883 | 128 | 0.125130832 | BST2 | bone marrow stromal cell antigen 2 [Source:HGNC Symbol;Acc:HGNC:1119] |
| ENSG00000130377 | HGLibA_00544 | 128 | 0.125130832 | ACSBG2 | acyl-CoA synthetase bubblegum family member 2 [Source:HGNC Symbol;Acc:HGNC:24174] |
| ENSG00000131374 | HGLibA_48560 | 128 | 0.125130832 | TBC1D5 | TBC1 domain family member 5 [Source:HGNC Symbol;Acc:HGNC:19166] |
| ENSG00000131748 | HGLibA_47356 | 128 | 0.125130832 | STARD3 | StAR related lipid transfer domain containing 3 [Source:HGNC Symbol;Acc:HGNC:17579] |
| ENSG00000131864 | HGLibA_53324 | 128 | 0.125130832 | USP29 | ubiquitin specific peptidase 29 [Source:HGNC Symbol;Acc:HGNC:18563] |
| ENSG00000132953 | HGLibA_54566 | 128 | 0.125130832 | XPO4 | exportin 4 [Source:HGNC Symbol;Acc:HGNC:17796] |
| ENSG00000134070 | HGLibA_23560 | 128 | 0.125130832 | IRAK2 | interleukin 1 receptor associated kinase 2 [Source:HGNC Symbol;Acc:HGNC:6113] |
| ENSG00000134369 | HGLibA_31195 | 128 | 0.125130832 | NAV1 | neuron navigator 1 [Source:HGNC Symbol;Acc:HGNC:15989] |
| ENSG00000134627 | HGLibA_36780 | 128 | 0.125130832 | PIWIL4 | piwi like RNA-mediated gene silencing 4 [Source:HGNC Symbol;Acc:HGNC:18444] |
| ENSG00000135968 | HGLibA_18767 | 128 | 0.125130832 | GCC2 | GRIP and coiled-coil domain containing 2 [Source:HGNC Symbol;Acc:HGNC:23218] |
| ENSG00000136270 | HGLibA_48639 | 128 | 0.125130832 | TBRG4 | transforming growth factor beta regulator 4 [Source:HGNC Symbol;Acc:HGNC:17443] |
| ENSG00000137094 | HGLibA_13546 | 128 | 0.125130832 | DNAJB5 | DnaJ heat shock protein family (Hsp40) member B5 [Source:HGNC Symbol;Acc:HGNC:14887] |
| ENSG00000137207 | HGLibA_54675 | 128 | 0.125130832 | YIPF3 | Yip1 domain family member 3 [Source:HGNC Symbol;Acc:HGNC:21023] |
| ENSG00000137673 | HGLibA_29538 | 128 | 0.125130832 | MMP7 | matrix metallopeptidase 7 [Source:HGNC Symbol;Acc:HGNC:7174] |
| ENSG00000138399 | HGLibA_16901 | 128 | 0.125130832 | FASTKD1 | FAST kinase domains 1 [Source:HGNC Symbol;Acc:HGNC:26150] |
| ENSG00000139151 | HGLibA_37032 | 128 | 0.125130832 | PLCZ1 | phospholipase C zeta 1 [Source:HGNC Symbol;Acc:HGNC:19218] |
| ENSG00000140848 | HGLibA_10957 | 128 | 0.125130832 | CPNE2 | copine 2 [Source:HGNC Symbol;Acc:HGNC:2315] |
| ENSG00000140992 | HGLibA_35930 | 128 | 0.125130832 | PDPK1 | 3-phosphoinositide dependent protein kinase 1 [Source:HGNC Symbol;Acc:HGNC:8816] |
| ENSG00000142484 | HGLibA_49706 | 128 | 0.125130832 | TM4SF5 | transmembrane 4 L six family member 5 [Source:HGNC Symbol;Acc:HGNC:11857] |
| ENSG00000142507 | HGLibA_39115 | 128 | 0.125130832 | PSMB6 | proteasome subunit beta 6 [Source:HGNC Symbol;Acc:HGNC:9543] |
| ENSG00000143297 | HGLibA_17236 | 128 | 0.125130832 | FCRL5 | Fc receptor like 5 [Source:HGNC Symbol;Acc:HGNC:18508] |
| ENSG00000143374 | HGLibA_48348 | 128 | 0.125130832 | TARS2 | mitochondrial [Source:HGNC Symbol;Acc:HGNC:30740]" |
| ENSG00000145911 | HGLibA_30962 | 128 | 0.125130832 | N4BP3 | NEDD4 binding protein 3 [Source:HGNC Symbol;Acc:HGNC:29852] |
| ENSG00000146054 | HGLibA_51597 | 128 | 0.125130832 | TRIM7 | tripartite motif containing 7 [Source:HGNC Symbol;Acc:HGNC:16278] |
| ENSG00000147475 | HGLibA_15498 | 128 | 0.125130832 | ERLIN2 | ER lipid raft associated 2 [Source:HGNC Symbol;Acc:HGNC:1356] |
| ENSG00000149380 | HGLibA_34907 | 128 | 0.125130832 | P4HA3 | prolyl 4-hydroxylase subunit alpha 3 [Source:HGNC Symbol;Acc:HGNC:30135] |
| ENSG00000149557 | HGLibA_17313 | 128 | 0.125130832 | FEZ1 | fasciculation and elongation protein zeta 1 [Source:HGNC Symbol;Acc:HGNC:3659] |
| ENSG00000151892 | HGLibA_18964 | 128 | 0.125130832 | GFRA1 | GDNF family receptor alpha 1 [Source:HGNC Symbol;Acc:HGNC:4243] |
| ENSG00000152208 | HGLibA_20274 | 128 | 0.125130832 | GRID2 | glutamate ionotropic receptor delta type subunit 2 [Source:HGNC Symbol;Acc:HGNC:4576] |
| ENSG00000152217 | HGLibA_43635 | 128 | 0.125130832 | SETBP1 | SET binding protein 1 [Source:HGNC Symbol;Acc:HGNC:15573] |
| ENSG00000152463 | HGLibA_33342 | 128 | 0.125130832 | OLAH | oleoyl-ACP hydrolase [Source:HGNC Symbol;Acc:HGNC:25625] |
| ENSG00000157036 | HGLibA_15767 | 128 | 0.125130832 | EXOG | exo/endonuclease G [Source:HGNC Symbol;Acc:HGNC:3347] |
| ENSG00000160392 | HGLibA_05669 | 128 | 0.125130832 | C19orf47 | chromosome 19 open reading frame 47 [Source:HGNC Symbol;Acc:HGNC:26723] |
| ENSG00000163380 | HGLibA_26723 | 128 | 0.125130832 | LMOD3 | leiomodin 3 [Source:HGNC Symbol;Acc:HGNC:6649] |
| ENSG00000164037 | HGLibA_45432 | 128 | 0.125130832 | SLC9B1 | solute carrier family 9 member B1 [Source:HGNC Symbol;Acc:HGNC:24244] |
| ENSG00000164674 | HGLibA_48102 | 128 | 0.125130832 | SYTL3 | synaptotagmin like 3 [Source:HGNC Symbol;Acc:HGNC:15587] |
| ENSG00000165124 | HGLibA_47883 | 128 | 0.125130832 | SVEP1 | EGF and pentraxin domain containing 1 [Source:HGNC Symbol;Acc:HGNC:15985]" |
| ENSG00000165617 | HGLibA_12279 | 128 | 0.125130832 | DACT1 | dishevelled binding antagonist of beta catenin 1 [Source:HGNC Symbol;Acc:HGNC:17748] |
| ENSG00000166002 | HGLibA_45672 | 128 | 0.125130832 | SMCO4 | single-pass membrane protein with coiled-coil domains 4 [Source:HGNC Symbol;Acc:HGNC:24810] |
| ENSG00000166199 | HGLibA_01653 | 128 | 0.125130832 | ALKBH3 | alpha-ketoglutaratedependent dioxygenase [Source:HGNC Symbol;Acc:HGNC:30141]" |
| ENSG00000167323 | HGLibA_47441 | 128 | 0.125130832 | STIM1 | stromal interaction molecule 1 [Source:HGNC Symbol;Acc:HGNC:11386] |
| ENSG00000167491 | HGLibA_18685 | 128 | 0.125130832 | GATAD2A | GATA zinc finger domain containing 2A [Source:HGNC Symbol;Acc:HGNC:29989] |
| ENSG00000167791 | HGLibA_06851 | 128 | 0.125130832 | CABP2 | calcium binding protein 2 [Source:HGNC Symbol;Acc:HGNC:1385] |
| ENSG00000167797 | HGLibA_08764 | 128 | 0.125130832 | CDK2AP2 | cyclin dependent kinase 2 associated protein 2 [Source:HGNC Symbol;Acc:HGNC:30833] |
| ENSG00000170613 | HGLibA_16688 | 128 | 0.125130832 | FAM71B | family with sequence similarity 71 member B [Source:HGNC Symbol;Acc:HGNC:28397] |
| ENSG00000170631 | HGLibA_55509 | 128 | 0.125130832 | ZNF16 | zinc finger protein 16 [Source:HGNC Symbol;Acc:HGNC:12947] |
| ENSG00000170632 | HGLibA_03009 | 128 | 0.125130832 | ARMC10 | armadillo repeat containing 10 [Source:HGNC Symbol;Acc:HGNC:21706] |
| ENSG00000170653 | HGLibA_03452 | 128 | 0.125130832 | ATF7 | activating transcription factor 7 [Source:HGNC Symbol;Acc:HGNC:792] |
| ENSG00000170917 | HGLibA_32985 | 128 | 0.125130832 | NUDT6 | nudix hydrolase 6 [Source:HGNC Symbol;Acc:HGNC:8053] |
| ENSG00000171603 | HGLibA_10133 | 128 | 0.125130832 | CLSTN1 | calsyntenin 1 [Source:HGNC Symbol;Acc:HGNC:17447] |
| ENSG00000172543 | HGLibA_11734 | 128 | 0.125130832 | CTSW | cathepsin W [Source:HGNC Symbol;Acc:HGNC:2546] |

| | | | | | |
|---|---|---|---|---|---|
| ENSG00000172869 | HGLibA_13452 | 128 | 0.125130832 | DMXL1 | Dmx like 1 [Source:HGNC Symbol;Acc:HGNC:2937] |
| ENSG00000173662 | HGLibA_48354 | 128 | 0.125130832 | TAS1R1 | taste 1 receptor member 1 [Source:HGNC Symbol;Acc:HGNC:14448] |
| ENSG00000174236 | HGLibA_40843 | 128 | 0.125130832 | REP15 | RAB15 effector protein [Source:HGNC Symbol;Acc:HGNC:33748] |
| ENSG00000174564 | HGLibA_23080 | 128 | 0.125130832 | IL20RB | interleukin 20 receptor subunit beta [Source:HGNC Symbol;Acc:HGNC:6004] |
| ENSG00000175756 | HGLibA_03877 | 128 | 0.125130832 | AURKAIP1 | aurora kinase A interacting protein 1 [Source:HGNC Symbol;Acc:HGNC:24114] |
| ENSG00000176087 | HGLibA_44955 | 128 | 0.125130832 | SLC35A4 | solute carrier family 35 member A4 [Source:HGNC Symbol;Acc:HGNC:20753] |
| ENSG00000176256 | HGLibA_21699 | 128 | 0.125130832 | HMGB4 | high mobility group box 4 [Source:HGNC Symbol;Acc:HGNC:24954] |
| ENSG00000179873 | HGLibA_32103 | 128 | 0.125130832 | NLRP11 | NLR family pyrin domain containing 11 [Source:HGNC Symbol;Acc:HGNC:22945] |
| ENSG00000180481 | HGLibA_19266 | 128 | 0.125130832 | GLIPR1L2 | GLIPR1 like 2 [Source:HGNC Symbol;Acc:HGNC:28592] |
| ENSG00000180616 | HGLibA_47177 | 128 | 0.125130832 | SSTR2 | somatostatin receptor 2 [Source:HGNC Symbol;Acc:HGNC:11331] |
| ENSG00000180953 | HGLibA_47231 | 128 | 0.125130832 | ST20 | suppressor of tumorigenicity 20 [Source:HGNC Symbol;Acc:HGNC:33520] |
| ENSG00000181803 | HGLibA_34442 | 128 | 0.125130832 | OR6S1 | olfactory receptor family 6 subfamily S member 1 [Source:HGNC Symbol;Acc:HGNC:15363] |
| ENSG00000182324 | HGLibA_24262 | 128 | 0.125130832 | KCNJ14 | potassium voltage-gated channel subfamily J member 14 [Source:HGNC Symbol;Acc:HGNC:6260] |
| ENSG00000182938 | HGLibA_34741 | 128 | 0.125130832 | OTOP3 | otopetrin 3 [Source:HGNC Symbol;Acc:HGNC:19658] |
| ENSG00000183145 | HGLibA_41335 | 128 | 0.125130832 | RIPPLY3 | ripply transcriptional repressor 3 [Source:HGNC Symbol;Acc:HGNC:3047] |
| ENSG00000183303 | HGLibA_34346 | 128 | 0.125130832 | OR5P2 | olfactory receptor family 5 subfamily P member 2 [Source:HGNC Symbol;Acc:HGNC:14783] |
| ENSG00000184110 | HGLibA_14769 | 128 | 0.125130832 | EIF3C | eukaryotic translation initiation factor 3 subunit C [Source:HGNC Symbol;Acc:HGNC:3279] |
| ENSG00000185010 | HGLibA_15883 | 128 | 0.125130832 | F8 | coagulation factor VIII [Source:HGNC Symbol;Acc:HGNC:3546] |
| ENSG00000185897 | HGLibA_17330 | 128 | 0.125130832 | FFAR3 | free fatty acid receptor 3 [Source:HGNC Symbol;Acc:HGNC:4499] |
| ENSG00000186998 | HGLibA_15044 | 128 | 0.125130832 | EMID1 | EMI domain containing 1 [Source:HGNC Symbol;Acc:HGNC:18036] |
| ENSG00000187733 | HGLibA_01847 | 128 | 0.125130832 | AMY1C | alpha 1C (salivary) [Source:HGNC Symbol;Acc:HGNC:476]" |
| ENSG00000188089 | HGLibA_36911 | 128 | 0.125130832 | PLA2G4E | phospholipase A2 group IVE [Source:HGNC Symbol;Acc:HGNC:24791] |
| ENSG00000188269 | HGLibA_34464 | 128 | 0.125130832 | OR7A5 | olfactory receptor family 7 subfamily A member 5 [Source:HGNC Symbol;Acc:HGNC:8368] |
| ENSG00000188324 | HGLibA_34393 | 128 | 0.125130832 | OR6C6 | olfactory receptor family 6 subfamily C member 6 [Source:HGNC Symbol;Acc:HGNC:31293] |
| ENSG00000188487 | HGLibA_23372 | 128 | 0.125130832 | INSC | spindle orientation adaptor protein [Source:HGNC Symbol;Acc:HGNC:33116]" |
| ENSG00000188582 | HGLibA_35176 | 128 | 0.125130832 | PAQR9 | progestin and adipoQ receptor family member 9 [Source:HGNC Symbol;Acc:HGNC:30131] |
| ENSG00000189046 | HGLibA_01649 | 128 | 0.125130832 | ALKBH2 | alpha-ketoglutarate dependent dioxygenase [Source:HGNC Symbol;Acc:HGNC:32487]" |
| ENSG00000196189 | HGLibA_43340 | 128 | 0.125130832 | SEMA4A | semaphorin 4A [Source:HGNC Symbol;Acc:HGNC:10729] |
| ENSG00000196428 | HGLibA_51820 | 128 | 0.125130832 | TSC22D2 | TSC22 domain family member 2 [Source:HGNC Symbol;Acc:HGNC:29095] |
| ENSG00000196547 | HGLibA_28073 | 128 | 0.125130832 | MAN2A2 | mannosidase alpha class 2A member 2 [Source:HGNC Symbol;Acc:HGNC:6825] |
| ENSG00000197296 | HGLibA_17544 | 128 | 0.125130832 | FITM2 | fat storage inducing transmembrane protein 2 [Source:HGNC Symbol;Acc:HGNC:16135] |
| ENSG00000198860 | HGLibA_51830 | 128 | 0.125130832 | TSEN15 | tRNA splicing endonuclease subunit 15 [Source:HGNC Symbol;Acc:HGNC:16791] |
| ENSG00000204444 | HGLibA_02547 | 128 | 0.125130832 | APOM | apolipoprotein M [Source:HGNC Symbol;Acc:HGNC:13916] |
| ENSG00000204498 | HGLibA_31833 | 128 | 0.125130832 | NFKBIL1 | NFKB inhibitor like 1 [Source:HGNC Symbol;Acc:HGNC:7800] |
| ENSG00000214414 | HGLibA_51613 | 128 | 0.125130832 | TRIM77 | tripartite motif containing 77 [Source:HGNC Symbol;Acc:HGNC:34228] |
| ENSG00000215704 | HGLibA_08996 | 128 | 0.125130832 | CELA2B | chymotrypsin like elastase family member 2B [Source:HGNC Symbol;Acc:HGNC:29995] |
| ENSG00000244509 | HGLibA_02481 | 128 | 0.125130832 | APOBEC3C | apolipoprotein B mRNA editing enzyme catalytic subunit 3C [Source:HGNC Symbol;Acc:HGNC:17353] |
| ENSG00000249471 | HGLibA_55805 | 128 | 0.125130832 | ZNF324B | zinc finger protein 324B [Source:HGNC Symbol;Acc:HGNC:33107] |
| ENSG00000255526 | HGLibA_31612 | 128 | 0.125130832 | NEDD8-MDP1 | NEDD8-MDP1 readthrough [Source:HGNC Symbol;Acc:HGNC:39551] |
| ENSG00000261272 | HGLibA_30543 | 128 | 0.125130832 | MUC22 | mucin 22 [Source:HGNC Symbol;Acc:HGNC:39755] |
| ENSG00000264424 | HGLibA_30728 | 128 | 0.125130832 | MYH4 | myosin heavy chain 4 [Source:HGNC Symbol;Acc:HGNC:7574] |
| ENSG00000269190 | HGLibA_17037 | 128 | 0.125130832 | FBXO17 | F-box protein 17 [Source:HGNC Symbol;Acc:HGNC:18754] |
| ENSG00000275793 | HGLibA_41262 | 128 | 0.125130832 | RIMBP3 | RIMS binding protein 3 [Source:HGNC Symbol;Acc:HGNC:29344] |
| ENSG00000277556 | HGLibA_33606 | 128 | 0.125130832 | OR13C5 | olfactory receptor family 13 subfamily C member 5 [Source:HGNC Symbol;Acc:HGNC:15100] |
| FAM211A | HGLibA_16460 | 128 | 0.125130832 | FAM211A | - |
| HIST1H3G | HGLibA_21475 | 128 | 0.125130832 | HIST1H3G | - |
| hsa-mir-129-1 | HGLibA_57628 | 128 | 0.125130832 | hsa-mir-129-1 | - |
| hsa-mir-153-1 | HGLibA_57906 | 128 | 0.125130832 | hsa-mir-153-1 | - |
| hsa-mir-296 | HGLibA_58398 | 128 | 0.125130832 | hsa-mir-296 | - |
| hsa-mir-298 | HGLibA_58402 | 128 | 0.125130832 | hsa-mir-298 | - |
| hsa-mir-3132 | HGLibA_58582 | 128 | 0.125130832 | hsa-mir-3132 | - |
| hsa-mir-3138 | HGLibA_58612 | 128 | 0.125130832 | hsa-mir-3138 | - |
| hsa-mir-3148 | HGLibA_58651 | 128 | 0.125130832 | hsa-mir-3148 | - |
| hsa-mir-3692 | HGLibA_59356 | 128 | 0.125130832 | hsa-mir-3692 | - |
| hsa-mir-378g | HGLibA_59452 | 128 | 0.125130832 | hsa-mir-378g | - |
| hsa-mir-3911 | HGLibA_59511 | 128 | 0.125130832 | hsa-mir-3911 | - |
| hsa-mir-421 | HGLibA_59691 | 128 | 0.125130832 | hsa-mir-421 | - |
| hsa-mir-4446 | HGLibA_60191 | 128 | 0.125130832 | hsa-mir-4446 | - |
| hsa-mir-4505 | HGLibA_60447 | 128 | 0.125130832 | hsa-mir-4505 | - |
| hsa-mir-4540 | HGLibA_60626 | 128 | 0.125130832 | hsa-mir-4540 | - |
| hsa-mir-4737 | HGLibA_61044 | 128 | 0.125130832 | hsa-mir-4737 | - |
| hsa-mir-4780 | HGLibA_61228 | 128 | 0.125130832 | hsa-mir-4780 | - |
| hsa-mir-488 | HGLibA_61351 | 128 | 0.125130832 | hsa-mir-488 | - |
| hsa-mir-518a-1 | HGLibA_61652 | 128 | 0.125130832 | hsa-mir-518a-1 | - |
| hsa-mir-5192 | HGLibA_61686 | 128 | 0.125130832 | hsa-mir-5192 | - |
| hsa-mir-520h | HGLibA_61761 | 128 | 0.125130832 | hsa-mir-520h | - |
| hsa-mir-6085 | HGLibA_62602 | 128 | 0.125130832 | hsa-mir-6085 | - |
| hsa-mir-6770-3 | HGLibA_63253 | 128 | 0.125130832 | hsa-mir-6770-3 | - |
| hsa-mir-6809 | HGLibA_63415 | 128 | 0.125130832 | hsa-mir-6809 | - |
| LOC646498 | HGLibA_26975 | 128 | 0.125130832 | LOC646498 | - |
| MLL3 | HGLibA_29399 | 128 | 0.125130832 | MLL3 | - |
| PNMA6C | HGLibA_37382 | 128 | 0.125130832 | PNMA6C | - |
| QTRTD1 | HGLibA_39763 | 128 | 0.125130832 | QTRTD1 | - |
| 6-Sep | HGLibA_43449 | 128 | 0.125130832 | 6-Sep | - |
| SPANXF1 | HGLibA_46400 | 128 | 0.125130832 | SPANXF1 | - |
| TSTA3 | HGLibA_52028 | 128 | 0.125130832 | TSTA3 | - |
| 11-Mar | HGLibA_28332 | 127 | 0.115179794 | 11-Mar | - |
| C14orf80 | HGLibA_05373 | 127 | 0.115179794 | C14orf80 | - |
| C9orf9 | HGLibA_06773 | 127 | 0.115179794 | C9orf9 | - |
| ENSG00000005486 | HGLibA_41127 | 127 | 0.115179794 | RHBDD2 | rhomboid domain containing 2 [Source:HGNC Symbol;Acc:HGNC:23082] |
| ENSG00000009413 | HGLibA_40887 | 127 | 0.115179794 | REV3L | DNA directed polymerase zeta catalytic subunit [Source:HGNC Symbol;Acc:HGNC:9968]" |
| ENSG00000024422 | HGLibA_14669 | 127 | 0.115179794 | EHD2 | EH domain containing 2 [Source:HGNC Symbol;Acc:HGNC:3243] |
| ENSG00000027869 | HGLibA_43890 | 127 | 0.115179794 | SH2D2A | SH2 domain containing 2A [Source:HGNC Symbol;Acc:HGNC:10821] |
| ENSG00000049769 | HGLibA_38113 | 127 | 0.115179794 | PPP1R3F | protein phosphatase 1 regulatory subunit 3F [Source:HGNC Symbol;Acc:HGNC:14944] |
| ENSG00000050165 | HGLibA_13300 | 127 | 0.115179794 | DKK3 | dickkopf WNT signaling pathway inhibitor 3 [Source:HGNC Symbol;Acc:HGNC:2893] |
| ENSG00000064199 | HGLibA_46320 | 127 | 0.115179794 | SPA17 | sperm autoantigenic protein 17 [Source:HGNC Symbol;Acc:HGNC:11210] |
| ENSG00000064835 | HGLibA_37750 | 127 | 0.115179794 | POU1F1 | POU class 1 homeobox 1 [Source:HGNC Symbol;Acc:HGNC:9210] |
| ENSG00000068078 | HGLibA_17446 | 127 | 0.115179794 | FGFR3 | fibroblast growth factor receptor 3 [Source:HGNC Symbol;Acc:HGNC:3690] |
| ENSG00000071203 | HGLibA_30133 | 127 | 0.115179794 | MS4A12 | membrane spanning 4-domains A12 [Source:HGNC Symbol;Acc:HGNC:13370] |
| ENSG00000084764 | HGLibA_28318 | 127 | 0.115179794 | MAPRE3 | microtubule associated protein RP/EB family member 3 [Source:HGNC Symbol;Acc:HGNC:6892] |
| ENSG00000088538 | HGLibA_13742 | 127 | 0.115179794 | DOCK3 | dedicator of cytokinesis 3 [Source:HGNC Symbol;Acc:HGNC:2989] |
| ENSG00000100100 | HGLibA_36644 | 127 | 0.115179794 | PIK3IP1 | phosphoinositide-3-kinase interacting protein 1 [Source:HGNC Symbol;Acc:HGNC:24942] |
| ENSG00000100191 | HGLibA_45272 | 127 | 0.115179794 | SLC5A4 | solute carrier family 5 member 4 [Source:HGNC Symbol;Acc:HGNC:11039] |
| ENSG00000100302 | HGLibA_40323 | 127 | 0.115179794 | RASD2 | RASD family member 2 [Source:HGNC Symbol;Acc:HGNC:18229] |
| ENSG00000100312 | HGLibA_00529 | 127 | 0.115179794 | ACR | acrosin [Source:HGNC Symbol;Acc:HGNC:126] |
| ENSG00000102096 | HGLibA_36677 | 127 | 0.115179794 | PIM2 | serine/threonine kinase [Source:HGNC Symbol;Acc:HGNC:8987]" |
| ENSG00000102225 | HGLibA_08742 | 127 | 0.115179794 | CDK16 | cyclin dependent kinase 16 [Source:HGNC Symbol;Acc:HGNC:8749] |
| ENSG00000102245 | HGLibA_08386 | 127 | 0.115179794 | CD40LG | CD40 ligand [Source:HGNC Symbol;Acc:HGNC:11935] |
| ENSG00000104695 | HGLibA_38138 | 127 | 0.115179794 | PPP2CB | protein phosphatase 2 catalytic subunit beta [Source:HGNC Symbol;Acc:HGNC:9300] |
| ENSG00000105185 | HGLibA_35735 | 127 | 0.115179794 | PDCD5 | programmed cell death 5 [Source:HGNC Symbol;Acc:HGNC:8764] |
| ENSG00000105865 | HGLibA_14123 | 127 | 0.115179794 | DUS4L | dihydrouridine synthase 4 like [Source:HGNC Symbol;Acc:HGNC:21517] |
| ENSG00000106078 | HGLibA_10462 | 127 | 0.115179794 | COBL | cordon-bleu WH2 repeat protein [Source:HGNC Symbol;Acc:HGNC:22199] |
| ENSG00000109047 | HGLibA_40721 | 127 | 0.115179794 | RCVRN | recoverin [Source:HGNC Symbol;Acc:HGNC:9937] |
| ENSG00000112139 | HGLibA_28676 | 127 | 0.115179794 | MDGA1 | MAM domain containing glycosylphosphatidylinositol anchor 1 [Source:HGNC Symbol;Acc:HGNC:19267] |
| ENSG00000112303 | HGLibA_53714 | 127 | 0.115179794 | VNN2 | vanin 2 [Source:HGNC Symbol;Acc:HGNC:12706] |
| ENSG00000112335 | HGLibA_46081 | 127 | 0.115179794 | SNX3 | sorting nexin 3 [Source:HGNC Symbol;Acc:HGNC:11174] |
| ENSG00000112699 | HGLibA_19395 | 127 | 0.115179794 | GMDS | 6-dehydratase [Source:HGNC Symbol;Acc:HGNC:4369]" |
| ENSG00000114316 | HGLibA_53360 | 127 | 0.115179794 | USP4 | ubiquitin specific peptidase 4 [Source:HGNC Symbol;Acc:HGNC:12627] |
| ENSG00000114331 | HGLibA_00386 | 127 | 0.115179794 | ACAP2 | ankyrin repeat and PH domains 2 [Source:HGNC Symbol;Acc:HGNC:16469]" |
| ENSG00000114942 | HGLibA_14495 | 127 | 0.115179794 | EEF1B2 | eukaryotic translation elongation factor 1 beta 2 [Source:HGNC Symbol;Acc:HGNC:3208] |
| ENSG00000116701 | HGLibA_31299 | 127 | 0.115179794 | NCF2 | neutrophil cytosolic factor 2 [Source:HGNC Symbol;Acc:HGNC:7661] |
| ENSG00000118762 | HGLibA_36799 | 127 | 0.115179794 | PKD2 | transient receptor potential cation channel [Source:HGNC Symbol;Acc:HGNC:9009]" |
| ENSG00000119041 | HGLibA_20587 | 127 | 0.115179794 | GTF3C3 | general transcription factor IIIC subunit 3 [Source:HGNC Symbol;Acc:HGNC:4666] |
| ENSG00000119608 | HGLibA_38704 | 127 | 0.115179794 | PROX2 | prospero homeobox 2 [Source:HGNC Symbol;Acc:HGNC:26715] |
| ENSG00000125363 | HGLibA_01767 | 127 | 0.115179794 | AMELX | amelogenin X-linked [Source:HGNC Symbol;Acc:HGNC:461] |
| ENSG00000125878 | HGLibA_48764 | 127 | 0.115179794 | TCF15 | transcription factor 15 [Source:HGNC Symbol;Acc:HGNC:11627] |
| ENSG00000128519 | HGLibA_48376 | 127 | 0.115179794 | TAS2R16 | taste 2 receptor member 16 [Source:HGNC Symbol;Acc:HGNC:14921] |
| ENSG00000130045 | HGLibA_33147 | 127 | 0.115179794 | NXNL2 | nucleoredoxin like 2 [Source:HGNC Symbol;Acc:HGNC:30482] |
| ENSG00000130544 | HGLibA_56229 | 127 | 0.115179794 | ZNF557 | zinc finger protein 557 [Source:HGNC Symbol;Acc:HGNC:28632] |
| ENSG00000130643 | HGLibA_07056 | 127 | 0.115179794 | CALY | calcyon neuron specific vesicular protein [Source:HGNC Symbol;Acc:HGNC:17938] |
| ENSG00000131044 | HGLibA_52215 | 127 | 0.115179794 | TTLL9 | tubulin tyrosine ligase like 9 [Source:HGNC Symbol;Acc:HGNC:16118] |
| ENSG00000131116 | HGLibA_55968 | 127 | 0.115179794 | ZNF428 | zinc finger protein 428 [Source:HGNC Symbol;Acc:HGNC:20804] |
| ENSG00000131650 | HGLibA_25406 | 127 | 0.115179794 | KREMEN2 | kringle containing transmembrane protein 2 [Source:HGNC Symbol;Acc:HGNC:18797] |
| ENSG00000131848 | HGLibA_57027 | 127 | 0.115179794 | ZSCAN5A | zinc finger and SCAN domain containing 5A [Source:HGNC Symbol;Acc:HGNC:23710] |
| ENSG00000132002 | HGLibA_13523 | 127 | 0.115179794 | DNAJB1 | DnaJ heat shock protein family (Hsp40) member B1 [Source:HGNC Symbol;Acc:HGNC:5270] |
| ENSG00000136492 | HGLibA_04820 | 127 | 0.115179794 | BRIP1 | BRCA1 interacting protein C-terminal helicase 1 [Source:HGNC Symbol;Acc:HGNC:20473] |
| ENSG00000137269 | HGLibA_27246 | 127 | 0.115179794 | LRRC1 | leucine rich repeat containing 1 [Source:HGNC Symbol;Acc:HGNC:14307] |
| ENSG00000137752 | HGLibA_07294 | 127 | 0.115179794 | CASP1 | caspase 1 [Source:HGNC Symbol;Acc:HGNC:1499] |
| ENSG00000139133 | HGLibA_01604 | 127 | 0.115179794 | ALG10 | 2-glucosyltransferase [Source:HGNC Symbol;Acc:HGNC:23162]" |
| ENSG00000139793 | HGLibA_28511 | 127 | 0.115179794 | MBNL2 | muscleblind like splicing regulator 2 [Source:HGNC Symbol;Acc:HGNC:16746] |
| ENSG00000141086 | HGLibA_11687 | 127 | 0.115179794 | CTRL | chymotrypsin like [Source:HGNC Symbol;Acc:HGNC:2524] |
| ENSG00000141560 | HGLibA_17733 | 127 | 0.115179794 | FN3KRP | fructosamine 3 kinase related protein [Source:HGNC Symbol;Acc:HGNC:25700] |
| ENSG00000142864 | HGLibA_43465 | 127 | 0.115179794 | SERBP1 | SERPINE1 mRNA binding protein 1 [Source:HGNC Symbol;Acc:HGNC:17860] |
| ENSG00000143153 | HGLibA_03593 | 127 | 0.115179794 | ATP1B1 | ATPase Na+/K+ transporting subunit beta 1 [Source:HGNC Symbol;Acc:HGNC:804] |
| ENSG00000144161 | HGLibA_54987 | 127 | 0.115179794 | ZC3H8 | zinc finger CCCH-type containing 8 [Source:HGNC Symbol;Acc:HGNC:30941] |

| | | | | | |
|---|---|---|---|---|---|
| ENSG00000147896 | HGLibA_22715 | 127 | 0.115179794 | IFNK | interferon kappa [Source:HGNC Symbol;Acc:HGNC:21714] |
| ENSG00000148773 | HGLibA_29324 | 127 | 0.115179794 | MKI67 | marker of proliferation Ki-67 [Source:HGNC Symbol;Acc:HGNC:7107] |
| ENSG00000150995 | HGLibA_23854 | 127 | 0.115179794 | ITPR1 | 5-trisphosphate receptor type 1 [Source:HGNC Symbol;Acc:HGNC:6180]" |
| ENSG00000151490 | HGLibA_39527 | 127 | 0.115179794 | PTPRO | receptor type O [Source:HGNC Symbol;Acc:HGNC:9678]" |
| ENSG00000152127 | HGLibA_29150 | 127 | 0.115179794 | MGAT5 | 6-mannosylglycoprotein 6-beta-N-acetylglucosaminyltransferase [Source:HGNC Symbol;Acc:HGNC:7049]" |
| ENSG00000153879 | HGLibA_08969 | 127 | 0.115179794 | CEBPG | CCAAT enhancer binding protein gamma [Source:HGNC Symbol;Acc:HGNC:1837] |
| ENSG00000154080 | HGLibA_09652 | 127 | 0.115179794 | CHST9 | carbohydrate sulfotransferase 9 [Source:HGNC Symbol;Acc:HGNC:19898] |
| ENSG00000154240 | HGLibA_09099 | 127 | 0.115179794 | CEP112 | centrosomal protein 112 [Source:HGNC Symbol;Acc:HGNC:28514] |
| ENSG00000158955 | HGLibA_54397 | 127 | 0.115179794 | WNT9B | Wnt family member 9B [Source:HGNC Symbol;Acc:HGNC:12779] |
| ENSG00000159212 | HGLibA_10037 | 127 | 0.115179794 | CLIC6 | chloride intracellular channel 6 [Source:HGNC Symbol;Acc:HGNC:2065] |
| ENSG00000160182 | HGLibA_49168 | 127 | 0.115179794 | TFF1 | trefoil factor 1 [Source:HGNC Symbol;Acc:HGNC:11755] |
| ENSG00000160298 | HGLibA_06042 | 127 | 0.115179794 | C21orf58 | chromosome 21 open reading frame 58 [Source:HGNC Symbol;Acc:HGNC:1300] |
| ENSG00000162438 | HGLibA_11684 | 127 | 0.115179794 | CTRC | chymotrypsin C [Source:HGNC Symbol;Acc:HGNC:2523] |
| ENSG00000165671 | HGLibA_32744 | 127 | 0.115179794 | NSD1 | nuclear receptor binding SET domain protein 1 [Source:HGNC Symbol;Acc:HGNC:14234] |
| ENSG00000165685 | HGLibA_50434 | 127 | 0.115179794 | TMEM52B | transmembrane protein 52B [Source:HGNC Symbol;Acc:HGNC:26438] |
| ENSG00000166002 | HGLibA_45670 | 127 | 0.115179794 | SMCO4 | single-pass membrane protein with coiled-coil domains 4 [Source:HGNC Symbol;Acc:HGNC:24810] |
| ENSG00000166341 | HGLibA_12489 | 127 | 0.115179794 | DCHS1 | dachsous cadherin-related 1 [Source:HGNC Symbol;Acc:HGNC:13681] |
| ENSG00000167193 | HGLibA_11181 | 127 | 0.115179794 | CRK | adaptor protein [Source:HGNC Symbol;Acc:HGNC:2362]" |
| ENSG00000167395 | HGLibA_56453 | 127 | 0.115179794 | ZNF646 | zinc finger protein 646 [Source:HGNC Symbol;Acc:HGNC:29004] |
| ENSG00000167723 | HGLibA_51789 | 127 | 0.115179794 | TRPV3 | transient receptor potential cation channel subfamily V member 3 [Source:HGNC Symbol;Acc:HGNC:18084] |
| ENSG00000167779 | HGLibA_22831 | 127 | 0.115179794 | IGFBP6 | insulin like growth factor binding protein 6 [Source:HGNC Symbol;Acc:HGNC:5475] |
| ENSG00000168028 | HGLibA_42197 | 127 | 0.115179794 | RPSA | ribosomal protein SA [Source:HGNC Symbol;Acc:HGNC:6502] |
| ENSG00000168158 | HGLibA_33777 | 127 | 0.115179794 | OR2C1 | olfactory receptor family 2 subfamily C member 1 [Source:HGNC Symbol;Acc:HGNC:8242] |
| ENSG00000168743 | HGLibA_32495 | 127 | 0.115179794 | NPNT | nephronectin [Source:HGNC Symbol;Acc:HGNC:27405] |
| ENSG00000170145 | HGLibA_44166 | 127 | 0.115179794 | SIK2 | salt inducible kinase 2 [Source:HGNC Symbol;Acc:HGNC:21680] |
| ENSG00000171459 | HGLibA_33694 | 127 | 0.115179794 | OR1L6 | olfactory receptor family 1 subfamily L member 6 [Source:HGNC Symbol;Acc:HGNC:8218] |
| ENSG00000172081 | HGLibA_29594 | 127 | 0.115179794 | MOB3A | MOB kinase activator 3A [Source:HGNC Symbol;Acc:HGNC:29802] |
| ENSG00000173226 | HGLibA_23490 | 127 | 0.115179794 | IQCB1 | IQ motif containing B1 [Source:HGNC Symbol;Acc:HGNC:28949] |
| ENSG00000175414 | HGLibA_02925 | 127 | 0.115179794 | ARL10 | ADP ribosylation factor like GTPase 10 [Source:HGNC Symbol;Acc:HGNC:22042] |
| ENSG00000177169 | HGLibA_52970 | 127 | 0.115179794 | ULK1 | unc-51 like autophagy activating kinase 1 [Source:HGNC Symbol;Acc:HGNC:12558] |
| ENSG00000180988 | HGLibA_34197 | 127 | 0.115179794 | OR52N2 | olfactory receptor family 52 subfamily N member 2 [Source:HGNC Symbol;Acc:HGNC:15228] |
| ENSG00000181019 | HGLibA_32562 | 127 | 0.115179794 | NQO1 | NAD(P)H quinone dehydrogenase 1 [Source:HGNC Symbol;Acc:HGNC:2874] |
| ENSG00000181634 | HGLibA_50816 | 127 | 0.115179794 | TNFSF15 | TNF superfamily member 15 [Source:HGNC Symbol;Acc:HGNC:11931] |
| ENSG00000181847 | HGLibA_49484 | 127 | 0.115179794 | TIGIT | T cell immunoreceptor with Ig and ITIM domains [Source:HGNC Symbol;Acc:HGNC:26838] |
| ENSG00000183718 | HGLibA_51540 | 127 | 0.115179794 | TRIM52 | tripartite motif containing 52 [Source:HGNC Symbol;Acc:HGNC:19024] |
| ENSG00000184047 | HGLibA_13215 | 127 | 0.115179794 | DIABLO | diablo IAP-binding mitochondrial protein [Source:HGNC Symbol;Acc:HGNC:21528] |
| ENSG00000184530 | HGLibA_06500 | 127 | 0.115179794 | C6orf58 | chromosome 6 open reading frame 58 [Source:HGNC Symbol;Acc:HGNC:20960] |
| ENSG00000186020 | HGLibA_56173 | 127 | 0.115179794 | ZNF529 | zinc finger protein 529 [Source:HGNC Symbol;Acc:HGNC:29328] |
| ENSG00000186462 | HGLibA_31115 | 127 | 0.115179794 | NAP1L2 | nucleosome assembly protein 1 like 2 [Source:HGNC Symbol;Acc:HGNC:7638] |
| ENSG00000187553 | HGLibA_12091 | 127 | 0.115179794 | CYP26C1 | cytochrome P450 family 26 subfamily C member 1 [Source:HGNC Symbol;Acc:HGNC:20577] |
| ENSG00000187626 | HGLibA_55355 | 127 | 0.115179794 | ZKSCAN4 | zinc finger with KRAB and SCAN domains 4 [Source:HGNC Symbol;Acc:HGNC:13854] |
| ENSG00000187753 | HGLibA_06692 | 127 | 0.115179794 | C9orf153 | chromosome 9 open reading frame 153 [Source:HGNC Symbol;Acc:HGNC:31456] |
| ENSG00000188352 | HGLibA_17777 | 127 | 0.115179794 | FOCAD | focadhesin [Source:HGNC Symbol;Acc:HGNC:23377] |
| ENSG00000188687 | HGLibA_45222 | 127 | 0.115179794 | SLC4A5 | solute carrier family 4 member 5 [Source:HGNC Symbol;Acc:HGNC:18168] |
| ENSG00000196169 | HGLibA_24881 | 127 | 0.115179794 | KIF19 | kinesin family member 19 [Source:HGNC Symbol;Acc:HGNC:26735] |
| ENSG00000196209 | HGLibA_44207 | 127 | 0.115179794 | SIRPB2 | signal regulatory protein beta 2 [Source:HGNC Symbol;Acc:HGNC:16247] |
| ENSG00000196214 | HGLibA_56696 | 127 | 0.115179794 | ZNF766 | zinc finger protein 766 [Source:HGNC Symbol;Acc:HGNC:28063] |
| ENSG00000196422 | HGLibA_38079 | 127 | 0.115179794 | PPP1R26 | protein phosphatase 1 regulatory subunit 26 [Source:HGNC Symbol;Acc:HGNC:29089] |
| ENSG00000197044 | HGLibA_56004 | 127 | 0.115179794 | ZNF441 | zinc finger protein 441 [Source:HGNC Symbol;Acc:HGNC:20875] |
| ENSG00000197256 | HGLibA_24027 | 127 | 0.115179794 | KANK2 | KN motif and ankyrin repeat domains 2 [Source:HGNC Symbol;Acc:HGNC:29300] |
| ENSG00000197296 | HGLibA_17543 | 127 | 0.115179794 | FITM2 | fat storage inducing transmembrane protein 2 [Source:HGNC Symbol;Acc:HGNC:16135] |
| ENSG00000197429 | HGLibA_23482 | 127 | 0.115179794 | IPP | intracisternal A particle-promoted polypeptide [Source:HGNC Symbol;Acc:HGNC:6108] |
| ENSG00000198019 | HGLibA_17185 | 127 | 0.115179794 | FCGR1B | Fc fragment of IgG receptor Ib [Source:HGNC Symbol;Acc:HGNC:3614] |
| ENSG00000203663 | HGLibA_33822 | 127 | 0.115179794 | OR2L2 | olfactory receptor family 2 subfamily L member 2 [Source:HGNC Symbol;Acc:HGNC:8266] |
| ENSG00000204381 | HGLibA_26072 | 127 | 0.115179794 | LAYN | layilin [Source:HGNC Symbol;Acc:HGNC:29471] |
| ENSG00000205084 | HGLibA_50258 | 127 | 0.115179794 | TMEM231 | transmembrane protein 231 [Source:HGNC Symbol;Acc:HGNC:37234] |
| ENSG00000205097 | HGLibA_17995 | 127 | 0.115179794 | FRG2 | FSHD region gene 2 [Source:HGNC Symbol;Acc:HGNC:19136] |
| ENSG00000212126 | HGLibA_48421 | 127 | 0.115179794 | TAS2R50 | taste 2 receptor member 50 [Source:HGNC Symbol;Acc:HGNC:18882] |
| ENSG00000212900 | HGLibA_25742 | 127 | 0.115179794 | KRTAP3-2 | keratin associated protein 3-2 [Source:HGNC Symbol;Acc:HGNC:16779] |
| ENSG00000213096 | HGLibA_55674 | 127 | 0.115179794 | ZNF254 | zinc finger protein 254 [Source:HGNC Symbol;Acc:HGNC:13047] |
| ENSG00000213339 | HGLibA_39760 | 127 | 0.115179794 | QTRT1 | queuine tRNA-ribosyltransferase catalytic subunit 1 [Source:HGNC Symbol;Acc:HGNC:23797] |
| ENSG00000214360 | HGLibA_14554 | 127 | 0.115179794 | EFCAB9 | EF-hand calcium binding domain 9 [Source:HGNC Symbol;Acc:HGNC:34530] |
| ENSG00000214688 | HGLibA_05022 | 127 | 0.115179794 | C10orf105 | chromosome 10 open reading frame 105 [Source:HGNC Symbol;Acc:HGNC:20304] |
| ENSG00000215454 | HGLibA_25608 | 127 | 0.115179794 | KRTAP10-4 | keratin associated protein 10-4 [Source:HGNC Symbol;Acc:HGNC:20521] |
| ENSG00000221954 | HGLibA_33941 | 127 | 0.115179794 | OR4C12 | olfactory receptor family 4 subfamily C member 12 [Source:HGNC Symbol;Acc:HGNC:15168] |
| ENSG00000222036 | HGLibA_37746 | 127 | 0.115179794 | POTEM | POTE ankyrin domain family member M [Source:HGNC Symbol;Acc:HGNC:37096] |
| ENSG00000231192 | HGLibA_34289 | 127 | 0.115179794 | OR5H1 | olfactory receptor family 5 subfamily H member 1 [Source:HGNC Symbol;Acc:HGNC:8346] |
| ENSG00000241595 | HGLibA_25839 | 127 | 0.115179794 | KRTAP9-4 | keratin associated protein 9-4 [Source:HGNC Symbol;Acc:HGNC:18902] |
| ENSG00000251349 | HGLibA_30177 | 127 | 0.115179794 | MSANTD3-TMEFF1 | MSANTD3-TMEFF1 readthrough [Source:HGNC Symbol;Acc:HGNC:38838] |
| ENSG00000255501 | HGLibA_07233 | 127 | 0.115179794 | CARD18 | caspase recruitment domain family member 18 [Source:HGNC Symbol;Acc:HGNC:28861] |
| ENSG00000255690 | HGLibA_51399 | 127 | 0.115179794 | TRIL | TLR4 interactor with leucine rich repeats [Source:HGNC Symbol;Acc:HGNC:22200] |
| ENSG00000268223 | HGLibA_02942 | 127 | 0.115179794 | ARL14EPL | ADP ribosylation factor like GTPase 14 effector protein like [Source:HGNC Symbol;Acc:HGNC:44201] |
| ENSG00000271449 | HGLibA_11501 | 127 | 0.115179794 | CT45A2 | cancer/testis antigen family 45 member A2 [Source:HGNC Symbol;Acc:HGNC:28400] |
| ENSG00000280021 | HGLibA_34103 | 127 | 0.115179794 | OR51F1 | olfactory receptor family 51 subfamily F member 1 (gene/pseudogene) [Source:HGNC Symbol;Acc:HGNC:15196] |
| ENSG00000283632 | HGLibA_15742 | 127 | 0.115179794 | EXOC3L2 | exocyst complex component 3 like 2 [Source:HGNC Symbol;Acc:HGNC:30162] |
| FAM115A | HGLibA_16036 | 127 | 0.115179794 | FAM115A | - |
| HIST1H2BJ | HGLibA_21439 | 127 | 0.115179794 | HIST1H2BJ | - |
| hsa-mir-1266 | HGLibA_57486 | 127 | 0.115179794 | hsa-mir-1266 | - |
| hsa-mir-1285-1 | HGLibA_57600 | 127 | 0.115179794 | hsa-mir-1285-1 | - |
| hsa-mir-1825 | HGLibA_57985 | 127 | 0.115179794 | hsa-mir-1825 | - |
| hsa-mir-3681 | HGLibA_59279 | 127 | 0.115179794 | hsa-mir-3681 | - |
| hsa-mir-4290 | HGLibA_59870 | 127 | 0.115179794 | hsa-mir-4290 | - |
| hsa-mir-4441 | HGLibA_60168 | 127 | 0.115179794 | hsa-mir-4441 | - |
| hsa-mir-4470 | HGLibA_60288 | 127 | 0.115179794 | hsa-mir-4470 | - |
| hsa-mir-4510 | HGLibA_60484 | 127 | 0.115179794 | hsa-mir-4510 | - |
| hsa-mir-4640 | HGLibA_60663 | 127 | 0.115179794 | hsa-mir-4640 | - |
| hsa-mir-4708 | HGLibA_60931 | 127 | 0.115179794 | hsa-mir-4708 | - |
| hsa-mir-520g | HGLibA_61758 | 127 | 0.115179794 | hsa-mir-520g | - |
| hsa-mir-524 | HGLibA_61784 | 127 | 0.115179794 | hsa-mir-524 | - |
| hsa-mir-647 | HGLibA_62830 | 127 | 0.115179794 | hsa-mir-647 | - |
| hsa-mir-660 | HGLibA_62974 | 127 | 0.115179794 | hsa-mir-660 | - |
| hsa-mir-7856 | HGLibA_64012 | 127 | 0.115179794 | hsa-mir-7856 | - |
| hsa-mir-891a | HGLibA_64243 | 127 | 0.115179794 | hsa-mir-891a | - |
| hsa-mir-941-3 | HGLibA_64346 | 127 | 0.115179794 | hsa-mir-941-3 | - |
| KIAA0754 | HGLibA_24651 | 127 | 0.115179794 | KIAA0754 | - |
| KIAA1524 | HGLibA_24774 | 127 | 0.115179794 | KIAA1524 | - |
| MB21D1 | HGLibA_28456 | 127 | 0.115179794 | MB21D1 | - |
| MICALCL | HGLibA_29209 | 127 | 0.115179794 | MICALCL | - |
| MLL5 | HGLibA_29406 | 127 | 0.115179794 | MLL5 | - |
| PAPD5 | HGLibA_35125 | 127 | 0.115179794 | PAPD5 | - |
| TNFAIP8L2-SCNM1 | HGLibA_50728 | 127 | 0.115179794 | TNFAIP8L2-SCNM1 | - |
| TROVE2 | HGLibA_51722 | 127 | 0.115179794 | TROVE2 | - |
| ENSG00000005486 | HGLibA_41126 | 126 | 0.105228757 | RHBDD2 | rhomboid domain containing 2 [Source:HGNC Symbol;Acc:HGNC:23082] |
| ENSG00000010810 | HGLibA_18254 | 126 | 0.105228757 | FYN | Src family tyrosine kinase [Source:HGNC Symbol;Acc:HGNC:4037]" |
| ENSG00000014138 | HGLibA_37486 | 126 | 0.105228757 | POLA2 | accessory subunit [Source:HGNC Symbol;Acc:HGNC:30073]" |
| ENSG00000017427 | HGLibA_22792 | 126 | 0.105228757 | IGF1 | insulin like growth factor 1 [Source:HGNC Symbol;Acc:HGNC:5464] |
| ENSG00000034713 | HGLibA_18338 | 126 | 0.105228757 | GABARAPL2 | GABA type A receptor associated protein like 2 [Source:HGNC Symbol;Acc:HGNC:13291] |
| ENSG00000035928 | HGLibA_40903 | 126 | 0.105228757 | RFC1 | replication factor C subunit 1 [Source:HGNC Symbol;Acc:HGNC:9969] |
| ENSG00000038274 | HGLibA_28420 | 126 | 0.105228757 | MAT2B | methionine adenosyltransferase 2B [Source:HGNC Symbol;Acc:HGNC:6905] |
| ENSG00000054392 | HGLibA_21252 | 126 | 0.105228757 | HHAT | hedgehog acyltransferase [Source:HGNC Symbol;Acc:HGNC:18270] |
| ENSG00000056998 | HGLibA_20691 | 126 | 0.105228757 | GYG2 | glycogenin 2 [Source:HGNC Symbol;Acc:HGNC:4700] |
| ENSG00000067533 | HGLibA_42273 | 126 | 0.105228757 | RRP15 | ribosomal RNA processing 15 homolog [Source:HGNC Symbol;Acc:HGNC:24255] |
| ENSG00000067715 | HGLibA_48045 | 126 | 0.105228757 | SYT1 | synaptotagmin 1 [Source:HGNC Symbol;Acc:HGNC:11509] |
| ENSG00000069869 | HGLibA_31603 | 126 | 0.105228757 | NEDD4 | E3 ubiquitin protein ligase [Source:HGNC Symbol;Acc:HGNC:7727]" |
| ENSG00000071054 | HGLibA_28220 | 126 | 0.105228757 | MAP4K4 | mitogen-activated protein kinase kinase kinase kinase 4 [Source:HGNC Symbol;Acc:HGNC:6866] |
| ENSG00000075886 | HGLibA_52255 | 126 | 0.105228757 | TUBA3D | tubulin alpha 3d [Source:HGNC Symbol;Acc:HGNC:24071] |
| ENSG00000081181 | HGLibA_02689 | 126 | 0.105228757 | ARG2 | arginase 2 [Source:HGNC Symbol;Acc:HGNC:664] |
| ENSG00000086712 | HGLibA_52396 | 126 | 0.105228757 | TXLNG | taxilin gamma [Source:HGNC Symbol;Acc:HGNC:18578] |
| ENSG00000090266 | HGLibA_31511 | 126 | 0.105228757 | NDUFB2 | NADH:ubiquinone oxidoreductase subunit B2 [Source:HGNC Symbol;Acc:HGNC:7697] |
| ENSG00000095261 | HGLibA_39176 | 126 | 0.105228757 | PSMD5 | non-ATPase 5 [Source:HGNC Symbol;Acc:HGNC:9563]" |
| ENSG00000100243 | HGLibA_12008 | 126 | 0.105228757 | CYB5R3 | cytochrome b5 reductase 3 [Source:HGNC Symbol;Acc:HGNC:2873] |
| ENSG00000100604 | HGLibA_09430 | 126 | 0.105228757 | CHGA | chromogranin A [Source:HGNC Symbol;Acc:HGNC:1929] |
| ENSG00000100883 | HGLibA_47006 | 126 | 0.105228757 | SRP54 | signal recognition particle 54 [Source:HGNC Symbol;Acc:HGNC:11301] |
| ENSG00000100938 | HGLibA_19429 | 126 | 0.105228757 | GMPR2 | guanosine monophosphate reductase 2 [Source:HGNC Symbol;Acc:HGNC:4377] |
| ENSG00000101294 | HGLibA_21664 | 126 | 0.105228757 | HM13 | histocompatibility minor 13 [Source:HGNC Symbol;Acc:HGNC:16435] |
| ENSG00000101842 | HGLibA_53837 | 126 | 0.105228757 | VSIG1 | V-set and immunoglobulin domain containing 1 [Source:HGNC Symbol;Acc:HGNC:28675] |
| ENSG00000101928 | HGLibA_29685 | 126 | 0.105228757 | MOSPD1 | motile sperm domain containing 1 [Source:HGNC Symbol;Acc:HGNC:25235] |
| ENSG00000102760 | HGLibA_40998 | 126 | 0.105228757 | RGCC | regulator of cell cycle [Source:HGNC Symbol;Acc:HGNC:20369] |
| ENSG00000103227 | HGLibA_26683 | 126 | 0.105228757 | LMF1 | lipase maturation factor 1 [Source:HGNC Symbol;Acc:HGNC:14154] |
| ENSG00000103852 | HGLibA_52078 | 126 | 0.105228757 | TTC23 | tetratricopeptide repeat domain 23 [Source:HGNC Symbol;Acc:HGNC:25730] |
| ENSG00000105197 | HGLibA_49520 | 126 | 0.105228757 | TIMM50 | translocase of inner mitochondrial membrane 50 [Source:HGNC Symbol;Acc:HGNC:23656] |
| ENSG00000105339 | HGLibA_12948 | 126 | 0.105228757 | DENND3 | DENN domain containing 3 [Source:HGNC Symbol;Acc:HGNC:29134] |
| ENSG00000105810 | HGLibA_08792 | 126 | 0.105228757 | CDK6 | cyclin dependent kinase 6 [Source:HGNC Symbol;Acc:HGNC:1777] |
| ENSG00000106302 | HGLibA_22445 | 126 | 0.105228757 | HYAL4 | hyaluronidase 4 [Source:HGNC Symbol;Acc:HGNC:5323] |
| ENSG00000106635 | HGLibA_04341 | 126 | 0.105228757 | BCL7B | BCL tumor suppressor 7B [Source:HGNC Symbol;Acc:HGNC:1005] |

| | | | | | |
|---|---|---|---|---|---|
| ENSG00000107262 | HGLibA_04074 | 126 | 0.105228757 | BAG1 | BCL2 associated athanogene 1 [Source:HGNC Symbol;Acc:HGNC:937] |
| ENSG00000107796 | HGLibA_00599 | 126 | 0.105228757 | ACTA2 | aorta [Source:HGNC Symbol;Acc:HGNC:130]" |
| ENSG00000107984 | HGLibA_13294 | 126 | 0.105228757 | DKK1 | dickkopf WNT signaling pathway inhibitor 1 [Source:HGNC Symbol;Acc:HGNC:2891] |
| ENSG00000108846 | HGLibA_00172 | 126 | 0.105228757 | ABCC3 | ATP binding cassette subfamily C member 3 [Source:HGNC Symbol;Acc:HGNC:54] |
| ENSG00000108984 | HGLibA_28154 | 126 | 0.105228757 | MAP2K6 | mitogen-activated protein kinase kinase 6 [Source:HGNC Symbol;Acc:HGNC:6846] |
| ENSG00000109576 | HGLibA_00042 | 126 | 0.105228757 | AADAT | aminoadipate aminotransferase [Source:HGNC Symbol;Acc:HGNC:17929] |
| ENSG00000113070 | HGLibA_20921 | 126 | 0.105228757 | HBEGF | heparin binding EGF like growth factor [Source:HGNC Symbol;Acc:HGNC:3059] |
| ENSG00000115325 | HGLibA_13767 | 126 | 0.105228757 | DOK1 | docking protein 1 [Source:HGNC Symbol;Acc:HGNC:2990] |
| ENSG00000115355 | HGLibA_07906 | 126 | 0.105228757 | CCDC88A | coiled-coil domain containing 88A [Source:HGNC Symbol;Acc:HGNC:25523] |
| ENSG00000116473 | HGLibA_40220 | 126 | 0.105228757 | RAP1A | member of RAS oncogene family [Source:HGNC Symbol;Acc:HGNC:9855]" |
| ENSG00000118804 | HGLibA_47411 | 126 | 0.105228757 | STBD1 | starch binding domain 1 [Source:HGNC Symbol;Acc:HGNC:24854] |
| ENSG00000119514 | HGLibA_18536 | 126 | 0.105228757 | GALNT12 | polypeptide N-acetylgalactosaminyltransferase 12 [Source:HGNC Symbol;Acc:HGNC:19877] |
| ENSG00000119655 | HGLibA_32425 | 126 | 0.105228757 | NPC2 | NPC intracellular cholesterol transporter 2 [Source:HGNC Symbol;Acc:HGNC:14537] |
| ENSG00000119673 | HGLibA_00479 | 126 | 0.105228757 | ACOT2 | acyl-CoA thioesterase 2 [Source:HGNC Symbol;Acc:HGNC:18431] |
| ENSG00000121410 | HGLibA_00001 | 126 | 0.105228757 | A1BG | alpha-1-B glycoprotein [Source:HGNC Symbol;Acc:HGNC:5] |
| ENSG00000122484 | HGLibA_41817 | 126 | 0.105228757 | RPAP2 | RNA polymerase II associated protein 2 [Source:HGNC Symbol;Acc:HGNC:25791] |
| ENSG00000126883 | HGLibA_33046 | 126 | 0.105228757 | NUP214 | nucleoporin 214 [Source:HGNC Symbol;Acc:HGNC:8064] |
| ENSG00000128594 | HGLibA_27351 | 126 | 0.105228757 | LRRC4 | leucine rich repeat containing 4 [Source:HGNC Symbol;Acc:HGNC:15586] |
| ENSG00000128881 | HGLibA_52043 | 126 | 0.105228757 | TTBK2 | tau tubulin kinase 2 [Source:HGNC Symbol;Acc:HGNC:19141] |
| ENSG00000129682 | HGLibA_17373 | 126 | 0.105228757 | FGF13 | fibroblast growth factor 13 [Source:HGNC Symbol;Acc:HGNC:3670] |
| ENSG00000130037 | HGLibA_24137 | 126 | 0.105228757 | KCNA5 | potassium voltage-gated channel subfamily A member 5 [Source:HGNC Symbol;Acc:HGNC:6224] |
| ENSG00000131732 | HGLibA_55051 | 126 | 0.105228757 | ZCCHC9 | zinc finger CCHC-type containing 9 [Source:HGNC Symbol;Acc:HGNC:25424] |
| ENSG00000132329 | HGLibA_40180 | 126 | 0.105228757 | RAMP1 | receptor activity modifying protein 1 [Source:HGNC Symbol;Acc:HGNC:9843] |
| ENSG00000133812 | HGLibA_42797 | 126 | 0.105228757 | SBF2 | SET binding factor 2 [Source:HGNC Symbol;Acc:HGNC:2135] |
| ENSG00000134874 | HGLibA_14298 | 126 | 0.105228757 | DZIP1 | DAZ interacting zinc finger protein 1 [Source:HGNC Symbol;Acc:HGNC:20908] |
| ENSG00000135175 | HGLibA_33259 | 126 | 0.105228757 | OCM2 | oncomodulin 2 [Source:HGNC Symbol;Acc:HGNC:34396] |
| ENSG00000135605 | HGLibA_48938 | 126 | 0.105228757 | TEC | tec protein tyrosine kinase [Source:HGNC Symbol;Acc:HGNC:11719] |
| ENSG00000135824 | HGLibA_41109 | 126 | 0.105228757 | RGS8 | regulator of G protein signaling 8 [Source:HGNC Symbol;Acc:HGNC:16810] |
| ENSG00000136144 | HGLibA_40676 | 126 | 0.105228757 | RCBTB1 | RCC1 and BTB domain containing protein 1 [Source:HGNC Symbol;Acc:HGNC:18243] |
| ENSG00000136295 | HGLibA_52237 | 126 | 0.105228757 | TTYH3 | tweety family member 3 [Source:HGNC Symbol;Acc:HGNC:22222] |
| ENSG00000136404 | HGLibA_49710 | 126 | 0.105228757 | TM6SF1 | transmembrane 6 superfamily member 1 [Source:HGNC Symbol;Acc:HGNC:11860] |
| ENSG00000137392 | HGLibA_10105 | 126 | 0.105228757 | CLPS | colipase [Source:HGNC Symbol;Acc:HGNC:2085] |
| ENSG00000138639 | HGLibA_02736 | 126 | 0.105228757 | ARHGAP24 | Rho GTPase activating protein 24 [Source:HGNC Symbol;Acc:HGNC:25361] |
| ENSG00000140284 | HGLibA_44817 | 126 | 0.105228757 | SLC27A2 | solute carrier family 27 member 2 [Source:HGNC Symbol;Acc:HGNC:10996] |
| ENSG00000140859 | HGLibA_24977 | 126 | 0.105228757 | KIFC3 | kinesin family member C3 [Source:HGNC Symbol;Acc:HGNC:6326] |
| ENSG00000142065 | HGLibA_55190 | 126 | 0.105228757 | ZFP14 | ZFP14 zinc finger protein [Source:HGNC Symbol;Acc:HGNC:29312] |
| ENSG00000142700 | HGLibA_13430 | 126 | 0.105228757 | DMRTA2 | DMRT like family A2 [Source:HGNC Symbol;Acc:HGNC:13908] |
| ENSG00000143416 | HGLibA_43288 | 126 | 0.105228757 | SELENBP1 | selenium binding protein 1 [Source:HGNC Symbol;Acc:HGNC:10719] |
| ENSG00000144061 | HGLibA_32446 | 126 | 0.105228757 | NPHP1 | nephrocystin 1 [Source:HGNC Symbol;Acc:HGNC:7905] |
| ENSG00000144935 | HGLibA_51728 | 126 | 0.105228757 | TRPC1 | transient receptor potential cation channel subfamily C member 1 [Source:HGNC Symbol;Acc:HGNC:12333] |
| ENSG00000145879 | HGLibA_46703 | 126 | 0.105228757 | SPINK7 | Kazal type 7 (putative) [Source:HGNC Symbol;Acc:HGNC:24643]" |
| ENSG00000146376 | HGLibA_02717 | 126 | 0.105228757 | ARHGAP18 | Rho GTPase activating protein 18 [Source:HGNC Symbol;Acc:HGNC:21035] |
| ENSG00000146842 | HGLibA_50205 | 126 | 0.105228757 | TMEM209 | transmembrane protein 209 [Source:HGNC Symbol;Acc:HGNC:21898] |
| ENSG00000148297 | HGLibA_28777 | 126 | 0.105228757 | MED22 | mediator complex subunit 22 [Source:HGNC Symbol;Acc:HGNC:11477] |
| ENSG00000148297 | HGLibA_28779 | 126 | 0.105228757 | MED22 | mediator complex subunit 22 [Source:HGNC Symbol;Acc:HGNC:11477] |
| ENSG00000148483 | HGLibA_50274 | 126 | 0.105228757 | TMEM236 | transmembrane protein 236 [Source:HGNC Symbol;Acc:HGNC:23473] |
| ENSG00000150556 | HGLibA_27731 | 126 | 0.105228757 | LYPD6B | LY6/PLAUR domain containing 6B [Source:HGNC Symbol;Acc:HGNC:27018] |
| ENSG00000151692 | HGLibA_41526 | 126 | 0.105228757 | RNF144A | ring finger protein 144A [Source:HGNC Symbol;Acc:HGNC:20457] |
| ENSG00000152457 | HGLibA_12514 | 126 | 0.105228757 | DCLRE1C | DNA cross-link repair 1C [Source:HGNC Symbol;Acc:HGNC:17642] |
| ENSG00000156103 | HGLibA_29499 | 126 | 0.105228757 | MMP16 | matrix metallopeptidase 16 [Source:HGNC Symbol;Acc:HGNC:7162] |
| ENSG00000158458 | HGLibA_32679 | 126 | 0.105228757 | NRG2 | neuregulin 2 [Source:HGNC Symbol;Acc:HGNC:7998] |
| ENSG00000160808 | HGLibA_30759 | 126 | 0.105228757 | MYL3 | myosin light chain 3 [Source:HGNC Symbol;Acc:HGNC:7584] |
| ENSG00000162188 | HGLibA_19523 | 126 | 0.105228757 | GNG3 | G protein subunit gamma 3 [Source:HGNC Symbol;Acc:HGNC:4405] |
| ENSG00000162458 | HGLibA_16940 | 126 | 0.105228757 | FBLIM1 | filamin binding LIM protein 1 [Source:HGNC Symbol;Acc:HGNC:24686] |
| ENSG00000162496 | HGLibA_13132 | 126 | 0.105228757 | DHRS3 | dehydrogenase/reductase 3 [Source:HGNC Symbol;Acc:HGNC:17693] |
| ENSG00000162642 | HGLibA_05866 | 126 | 0.105228757 | C1orf52 | chromosome 1 open reading frame 52 [Source:HGNC Symbol;Acc:HGNC:24871] |
| ENSG00000163630 | HGLibA_48027 | 126 | 0.105228757 | SYNPR | synaptoporin [Source:HGNC Symbol;Acc:HGNC:16507] |
| ENSG00000164893 | HGLibA_45354 | 126 | 0.105228757 | SLC7A13 | solute carrier family 7 member 13 [Source:HGNC Symbol;Acc:HGNC:23092] |
| ENSG00000165621 | HGLibA_34823 | 126 | 0.105228757 | OXGR1 | oxoglutarate receptor 1 [Source:HGNC Symbol;Acc:HGNC:4531] |
| ENSG00000166394 | HGLibA_12005 | 126 | 0.105228757 | CYB5R2 | cytochrome b5 reductase 2 [Source:HGNC Symbol;Acc:HGNC:24376] |
| ENSG00000166526 | HGLibA_55764 | 126 | 0.105228757 | ZNF3 | zinc finger protein 3 [Source:HGNC Symbol;Acc:HGNC:13089] |
| ENSG00000167476 | HGLibA_23996 | 126 | 0.105228757 | JSRP1 | junctional sarcoplasmic reticulum protein 1 [Source:HGNC Symbol;Acc:HGNC:24963] |
| ENSG00000167822 | HGLibA_34542 | 126 | 0.105228757 | OR8J3 | olfactory receptor family 8 subfamily J member 3 [Source:HGNC Symbol;Acc:HGNC:15312] |
| ENSG00000168314 | HGLibA_29607 | 126 | 0.105228757 | MOBP | myelin-associated oligodendrocyte basic protein [Source:HGNC Symbol;Acc:HGNC:7189] |
| ENSG00000168779 | HGLibA_44076 | 126 | 0.105228757 | SHOX2 | short stature homeobox 2 [Source:HGNC Symbol;Acc:HGNC:10854] |
| ENSG00000169180 | HGLibA_54571 | 126 | 0.105228757 | XPO6 | exportin 6 [Source:HGNC Symbol;Acc:HGNC:19733] |
| ENSG00000169379 | HGLibA_02933 | 126 | 0.105228757 | ARL13B | ADP ribosylation factor like GTPase 13B [Source:HGNC Symbol;Acc:HGNC:25419] |
| ENSG00000169856 | HGLibA_33392 | 126 | 0.105228757 | ONECUT1 | one cut homeobox 1 [Source:HGNC Symbol;Acc:HGNC:8138] |
| ENSG00000170142 | HGLibA_52593 | 126 | 0.105228757 | UBE2E1 | ubiquitin conjugating enzyme E2 E1 [Source:HGNC Symbol;Acc:HGNC:12477] |
| ENSG00000170345 | HGLibA_17795 | 126 | 0.105228757 | FOS | AP-1 transcription factor subunit [Source:HGNC Symbol;Acc:HGNC:3796]" |
| ENSG00000171954 | HGLibA_12190 | 126 | 0.105228757 | CYP4F22 | cytochrome P450 family 4 subfamily F member 22 [Source:HGNC Symbol;Acc:HGNC:26820] |
| ENSG00000172399 | HGLibA_30905 | 126 | 0.105228757 | MYOZ2 | myozenin 2 [Source:HGNC Symbol;Acc:HGNC:1330] |
| ENSG00000173163 | HGLibA_10650 | 126 | 0.105228757 | COMMD1 | copper metabolism domain containing 1 [Source:HGNC Symbol;Acc:HGNC:23024] |
| ENSG00000173267 | HGLibA_45918 | 126 | 0.105228757 | SNCG | synuclein gamma [Source:HGNC Symbol;Acc:HGNC:11141] |
| ENSG00000174796 | HGLibA_49308 | 126 | 0.105228757 | THAP6 | THAP domain containing 6 [Source:HGNC Symbol;Acc:HGNC:23189] |
| ENSG00000175106 | HGLibA_52364 | 126 | 0.105228757 | TVP23C | trans-golgi network vesicle protein 23 homolog C [Source:HGNC Symbol;Acc:HGNC:30453] |
| ENSG00000176024 | HGLibA_56386 | 126 | 0.105228757 | ZNF613 | zinc finger protein 613 [Source:HGNC Symbol;Acc:HGNC:25827] |
| ENSG00000176567 | HGLibA_34068 | 126 | 0.105228757 | OR4X1 | olfactory receptor family 4 subfamily X member 1 (gene/pseudogene) [Source:HGNC Symbol;Acc:HGNC:14854] |
| ENSG00000176681 | HGLibA_27326 | 126 | 0.105228757 | LRRC37A | leucine rich repeat containing 37A [Source:HGNC Symbol;Acc:HGNC:29069] |
| ENSG00000177693 | HGLibA_34008 | 126 | 0.105228757 | OR4F4 | olfactory receptor family 4 subfamily F member 4 [Source:HGNC Symbol;Acc:HGNC:8301] |
| ENSG00000177697 | HGLibA_08238 | 126 | 0.105228757 | CD151 | CD151 molecule (Raph blood group) [Source:HGNC Symbol;Acc:HGNC:1630] |
| ENSG00000177853 | HGLibA_56150 | 126 | 0.105228757 | ZNF518A | zinc finger protein 518A [Source:HGNC Symbol;Acc:HGNC:29009] |
| ENSG00000178163 | HGLibA_56152 | 126 | 0.105228757 | ZNF518B | zinc finger protein 518B [Source:HGNC Symbol;Acc:HGNC:29365] |
| ENSG00000178184 | HGLibA_35191 | 126 | 0.105228757 | PARD6G | par-6 family cell polarity regulator gamma [Source:HGNC Symbol;Acc:HGNC:16076] |
| ENSG00000179632 | HGLibA_27869 | 126 | 0.105228757 | MAF1 | negative regulator of RNA polymerase III [Source:HGNC Symbol;Acc:HGNC:24966]" |
| ENSG00000179632 | HGLibA_27870 | 126 | 0.105228757 | MAF1 | negative regulator of RNA polymerase III [Source:HGNC Symbol;Acc:HGNC:24966]" |
| ENSG00000184389 | HGLibA_00015 | 126 | 0.105228757 | A3GALT2 | 3-galactosyltransferase 2 [Source:HGNC Symbol;Acc:HGNC:30005]" |
| ENSG00000184545 | HGLibA_14194 | 126 | 0.105228757 | DUSP8 | dual specificity phosphatase 8 [Source:HGNC Symbol;Acc:HGNC:3074] |
| ENSG00000185477 | HGLibA_20147 | 126 | 0.105228757 | GPRIN3 | GPRIN family member 3 [Source:HGNC Symbol;Acc:HGNC:27733] |
| ENSG00000185499 | HGLibA_30519 | 126 | 0.105228757 | MUC1 | cell surface associated [Source:HGNC Symbol;Acc:HGNC:7508]" |
| ENSG00000185915 | HGLibA_25192 | 126 | 0.105228757 | KLHL34 | kelch like family member 34 [Source:HGNC Symbol;Acc:HGNC:26634] |
| ENSG00000186092 | HGLibA_34011 | 126 | 0.105228757 | OR4F5 | olfactory receptor family 4 subfamily F member 5 [Source:HGNC Symbol;Acc:HGNC:14825] |
| ENSG00000186184 | HGLibA_37558 | 126 | 0.105228757 | POLR1D | RNA polymerase I and III subunit D [Source:HGNC Symbol;Acc:HGNC:20422] |
| ENSG00000186335 | HGLibA_45035 | 126 | 0.105228757 | SLC36A2 | solute carrier family 36 member 2 [Source:HGNC Symbol;Acc:HGNC:18762] |
| ENSG00000188056 | HGLibA_51366 | 126 | 0.105228757 | TREML4 | triggering receptor expressed on myeloid cells like 4 [Source:HGNC Symbol;Acc:HGNC:30807] |
| ENSG00000196344 | HGLibA_00965 | 126 | 0.105228757 | ADH7 | mu or sigma polypeptide [Source:HGNC Symbol;Acc:HGNC:256]" |
| ENSG00000196428 | HGLibA_51821 | 126 | 0.105228757 | TSC22D2 | TSC22 domain family member 2 [Source:HGNC Symbol;Acc:HGNC:29095] |
| ENSG00000196517 | HGLibA_45344 | 126 | 0.105228757 | SLC6A9 | solute carrier family 6 member 9 [Source:HGNC Symbol;Acc:HGNC:11056] |
| ENSG00000196569 | HGLibA_25944 | 126 | 0.105228757 | LAMA2 | laminin subunit alpha 2 [Source:HGNC Symbol;Acc:HGNC:6482] |
| ENSG00000196604 | HGLibA_37733 | 126 | 0.105228757 | POTEF | POTE ankyrin domain family member F [Source:HGNC Symbol;Acc:HGNC:33905] |
| ENSG00000197540 | HGLibA_20729 | 126 | 0.105228757 | GZMM | granzyme M [Source:HGNC Symbol;Acc:HGNC:4712] |
| ENSG00000198689 | HGLibA_45420 | 126 | 0.105228757 | SLC9A6 | solute carrier family 9 member A6 [Source:HGNC Symbol;Acc:HGNC:11079] |
| ENSG00000198924 | HGLibA_12508 | 126 | 0.105228757 | DCLRE1A | DNA cross-link repair 1A [Source:HGNC Symbol;Acc:HGNC:17660] |
| ENSG00000204525 | HGLibA_21601 | 126 | 0.105228757 | HLA-C | C [Source:HGNC Symbol;Acc:HGNC:4933]" |
| ENSG00000205544 | HGLibA_50336 | 126 | 0.105228757 | TMEM256 | transmembrane protein 256 [Source:HGNC Symbol;Acc:HGNC:28618] |
| ENSG00000205981 | HGLibA_13592 | 126 | 0.105228757 | DNAJC19 | DnaJ heat shock protein family (Hsp40) member C19 [Source:HGNC Symbol;Acc:HGNC:30528] |
| ENSG00000206535 | HGLibA_26739 | 126 | 0.105228757 | LNP1 | leukemia NUP98 fusion partner 1 [Source:HGNC Symbol;Acc:HGNC:28014] |
| ENSG00000206557 | HGLibA_51600 | 126 | 0.105228757 | TRIM71 | tripartite motif containing 71 [Source:HGNC Symbol;Acc:HGNC:32669] |
| ENSG00000212710 | HGLibA_11568 | 126 | 0.105228757 | CTAGE1 | cutaneous T cell lymphoma-associated antigen 1 [Source:HGNC Symbol;Acc:HGNC:24346] |
| ENSG00000221931 | HGLibA_34450 | 126 | 0.105228757 | OR6X1 | olfactory receptor family 6 subfamily X member 1 [Source:HGNC Symbol;Acc:HGNC:14737] |
| ENSG00000222028 | HGLibA_39099 | 126 | 0.105228757 | PSMB11 | proteasome subunit beta 11 [Source:HGNC Symbol;Acc:HGNC:31963] |
| ENSG00000239697 | HGLibA_50802 | 126 | 0.105228757 | TNFSF12 | TNF superfamily member 12 [Source:HGNC Symbol;Acc:HGNC:11927] |
| ENSG00000242173 | HGLibA_02801 | 126 | 0.105228757 | ARHGDIG | Rho GDP dissociation inhibitor gamma [Source:HGNC Symbol;Acc:HGNC:680] |
| ENSG00000256436 | HGLibA_48390 | 126 | 0.105228757 | TAS2R31 | taste 2 receptor member 31 [Source:HGNC Symbol;Acc:HGNC:19113] |
| FAM203A | HGLibA_16418 | 126 | 0.105228757 | FAM203A | - |
| HIST1H4I | HGLibA_21509 | 126 | 0.105228757 | HIST1H4I | - |
| hsa-mir-1272 | HGLibA_57526 | 126 | 0.105228757 | hsa-mir-1272 | - |
| hsa-mir-181c | HGLibA_57975 | 126 | 0.105228757 | hsa-mir-181c | - |
| hsa-mir-376b | HGLibA_59413 | 126 | 0.105228757 | hsa-mir-376b | - |
| hsa-mir-379 | HGLibA_59471 | 126 | 0.105228757 | hsa-mir-379 | - |
| hsa-mir-424 | HGLibA_59703 | 126 | 0.105228757 | hsa-mir-424 | - |
| hsa-mir-515-2 | HGLibA_61605 | 126 | 0.105228757 | hsa-mir-515-2 | - |
| hsa-mir-5193 | HGLibA_61691 | 126 | 0.105228757 | hsa-mir-5193 | - |
| hsa-mir-521-2 | HGLibA_61772 | 126 | 0.105228757 | hsa-mir-521-2 | - |
| hsa-mir-569 | HGLibA_62274 | 126 | 0.105228757 | hsa-mir-569 | - |
| hsa-mir-6131 | HGLibA_62675 | 126 | 0.105228757 | hsa-mir-6131 | - |
| hsa-mir-6817 | HGLibA_63447 | 126 | 0.105228757 | hsa-mir-6817 | - |
| hsa-mir-6833 | HGLibA_63511 | 126 | 0.105228757 | hsa-mir-6833 | - |
| hsa-mir-6839 | HGLibA_63531 | 126 | 0.105228757 | hsa-mir-6839 | - |
| hsa-mir-7-2 | HGLibA_63773 | 126 | 0.105228757 | hsa-mir-7-2 | - |
| KIAA0391 | HGLibA_24633 | 126 | 0.105228757 | KIAA0391 | - |
| 2-Mar | HGLibA_28326 | 126 | 0.105228757 | 2-Mar | - |
| MLL3 | HGLibA_29398 | 126 | 0.105228757 | MLL3 | - |
| OR8U8 | HGLibA_34560 | 126 | 0.105228757 | OR8U8 | - |
| PPAPDC1A | HGLibA_37825 | 126 | 0.105228757 | PPAPDC1A | - |

| | | | | | |
|---|---|---|---|---|---|
| RGAG1 | HGLibA_40992 | 126 | 0.105228757 | RGAG1 | - |
| SGK110 | HGLibA_43820 | 126 | 0.105228757 | SGK110 | - |
| SMIM11 | HGLibA_45722 | 126 | 0.105228757 | SMIM11 | - |
| 11-Mar | HGLibA_28331 | 125 | 0.095277719 | 11-Mar | - |
| ENSG00000001617 | HGLibA_43334 | 125 | 0.095277719 | SEMA3F | semaphorin 3F [Source:HGNC Symbol;Acc:HGNC:10728] |
| ENSG00000020129 | HGLibA_31291 | 125 | 0.095277719 | NCDN | neurochondrin [Source:HGNC Symbol;Acc:HGNC:17597] |
| ENSG00000038427 | HGLibA_53568 | 125 | 0.095277719 | VCAN | versican [Source:HGNC Symbol;Acc:HGNC:2464] |
| ENSG00000040633 | HGLibA_36374 | 125 | 0.095277719 | PHF23 | PHD finger protein 23 [Source:HGNC Symbol;Acc:HGNC:28428] |
| ENSG00000043462 | HGLibA_26203 | 125 | 0.095277719 | LCP2 | lymphocyte cytosolic protein 2 [Source:HGNC Symbol;Acc:HGNC:6529] |
| ENSG00000056487 | HGLibA_36373 | 125 | 0.095277719 | PHF21B | PHD finger protein 21B [Source:HGNC Symbol;Acc:HGNC:25161] |
| ENSG00000071564 | HGLibA_48784 | 125 | 0.095277719 | TCF3 | transcription factor 3 [Source:HGNC Symbol;Acc:HGNC:11633] |
| ENSG00000075340 | HGLibA_00943 | 125 | 0.095277719 | ADD2 | adducin 2 [Source:HGNC Symbol;Acc:HGNC:244] |
| ENSG00000076248 | HGLibA_53043 | 125 | 0.095277719 | UNG | uracil DNA glycosylase [Source:HGNC Symbol;Acc:HGNC:12572] |
| ENSG00000079112 | HGLibA_08649 | 125 | 0.095277719 | CDH17 | cadherin 17 [Source:HGNC Symbol;Acc:HGNC:1756] |
| ENSG00000084110 | HGLibA_20821 | 125 | 0.095277719 | HAL | histidine ammonia-lyase [Source:HGNC Symbol;Acc:HGNC:4806] |
| ENSG00000085274 | HGLibA_30800 | 125 | 0.095277719 | MYNN | myoneurin [Source:HGNC Symbol;Acc:HGNC:14955] |
| ENSG00000088386 | HGLibA_44405 | 125 | 0.095277719 | SLC15A1 | solute carrier family 15 member 1 [Source:HGNC Symbol;Acc:HGNC:10920] |
| ENSG00000094661 | HGLibA_33672 | 125 | 0.095277719 | OR1I1 | olfactory receptor family 1 subfamily I member 1 [Source:HGNC Symbol;Acc:HGNC:8207] |
| ENSG00000095539 | HGLibA_43356 | 125 | 0.095277719 | SEMA4G | semaphorin 4G [Source:HGNC Symbol;Acc:HGNC:10735] |
| ENSG00000101323 | HGLibA_20833 | 125 | 0.095277719 | HAO1 | hydroxyacid oxidase 1 [Source:HGNC Symbol;Acc:HGNC:4809] |
| ENSG00000101405 | HGLibA_34841 | 125 | 0.095277719 | OXT | oxytocin/neurophysin I prepropeptide [Source:HGNC Symbol;Acc:HGNC:8528] |
| ENSG00000102879 | HGLibA_10774 | 125 | 0.095277719 | CORO1A | coronin 1A [Source:HGNC Symbol;Acc:HGNC:2252] |
| ENSG00000104870 | HGLibA_17201 | 125 | 0.095277719 | FCGRT | Fc fragment of IgG receptor and transporter [Source:HGNC Symbol;Acc:HGNC:3621] |
| ENSG00000105549 | HGLibA_49333 | 125 | 0.095277719 | THEG | theg spermatid protein [Source:HGNC Symbol;Acc:HGNC:13706] |
| ENSG00000106100 | HGLibA_32244 | 125 | 0.095277719 | NOD1 | nucleotide binding oligomerization domain containing 1 [Source:HGNC Symbol;Acc:HGNC:16390] |
| ENSG00000106348 | HGLibA_23256 | 125 | 0.095277719 | IMPDH1 | inosine monophosphate dehydrogenase 1 [Source:HGNC Symbol;Acc:HGNC:6052] |
| ENSG00000107165 | HGLibA_52474 | 125 | 0.095277719 | TYRP1 | tyrosinase related protein 1 [Source:HGNC Symbol;Acc:HGNC:12450] |
| ENSG00000107789 | HGLibA_29281 | 125 | 0.095277719 | MINPP1 | multiple inositol-polyphosphate phosphatase 1 [Source:HGNC Symbol;Acc:HGNC:7102] |
| ENSG00000108370 | HGLibA_41113 | 125 | 0.095277719 | RGS9 | regulator of G protein signaling 9 [Source:HGNC Symbol;Acc:HGNC:10004] |
| ENSG00000109814 | HGLibA_52865 | 125 | 0.095277719 | UGDH | UDP-glucose 6-dehydrogenase [Source:HGNC Symbol;Acc:HGNC:12525] |
| ENSG00000110448 | HGLibA_08403 | 125 | 0.095277719 | CD5 | CD5 molecule [Source:HGNC Symbol;Acc:HGNC:1685] |
| ENSG00000111335 | HGLibA_33200 | 125 | 0.095277719 | OAS2 | 2'-5'-oligoadenylate synthetase 2 [Source:HGNC Symbol;Acc:HGNC:8087] |
| ENSG00000111642 | HGLibA_09395 | 125 | 0.095277719 | CHD4 | chromodomain helicase DNA binding protein 4 [Source:HGNC Symbol;Acc:HGNC:1919] |
| ENSG00000112539 | HGLibA_06437 | 125 | 0.095277719 | C6orf118 | chromosome 6 open reading frame 118 [Source:HGNC Symbol;Acc:HGNC:21233] |
| ENSG00000113851 | HGLibA_11079 | 125 | 0.095277719 | CRBN | cereblon [Source:HGNC Symbol;Acc:HGNC:30185] |
| ENSG00000115523 | HGLibA_19555 | 125 | 0.095277719 | GNLY | granulysin [Source:HGNC Symbol;Acc:HGNC:4414] |
| ENSG00000117528 | HGLibA_00198 | 125 | 0.095277719 | ABCD3 | ATP binding cassette subfamily D member 3 [Source:HGNC Symbol;Acc:HGNC:67] |
| ENSG00000119280 | HGLibA_05819 | 125 | 0.095277719 | C1orf198 | chromosome 1 open reading frame 198 [Source:HGNC Symbol;Acc:HGNC:25900] |
| ENSG00000119487 | HGLibA_28298 | 125 | 0.095277719 | MAPKAP1 | mitogen-activated protein kinase associated protein 1 [Source:HGNC Symbol;Acc:HGNC:18752] |
| ENSG00000120253 | HGLibA_33053 | 125 | 0.095277719 | NUP43 | nucleoporin 43 [Source:HGNC Symbol;Acc:HGNC:21182] |
| ENSG00000120915 | HGLibA_15327 | 125 | 0.095277719 | EPHX2 | epoxide hydrolase 2 [Source:HGNC Symbol;Acc:HGNC:3402] |
| ENSG00000124120 | HGLibA_52225 | 125 | 0.095277719 | TTPAL | alpha tocopherol transfer protein like [Source:HGNC Symbol;Acc:HGNC:16114] |
| ENSG00000124145 | HGLibA_43107 | 125 | 0.095277719 | SDC4 | syndecan 4 [Source:HGNC Symbol;Acc:HGNC:10661] |
| ENSG00000124164 | HGLibA_53521 | 125 | 0.095277719 | VAPB | VAMP associated protein B and C [Source:HGNC Symbol;Acc:HGNC:12649] |
| ENSG00000124733 | HGLibA_28718 | 125 | 0.095277719 | MEA1 | male-enhanced antigen 1 [Source:HGNC Symbol;Acc:HGNC:6986] |
| ENSG00000124762 | HGLibA_08821 | 125 | 0.095277719 | CDKN1A | cyclin dependent kinase inhibitor 1A [Source:HGNC Symbol;Acc:HGNC:1784] |
| ENSG00000124772 | HGLibA_10964 | 125 | 0.095277719 | CPNE5 | copine 5 [Source:HGNC Symbol;Acc:HGNC:2318] |
| ENSG00000125124 | HGLibA_04214 | 125 | 0.095277719 | BBS2 | Bardet-Biedl syndrome 2 [Source:HGNC Symbol;Acc:HGNC:967] |
| ENSG00000125498 | HGLibA_24983 | 125 | 0.095277719 | KIR2DL1 | two Ig domains and long cytoplasmic tail 1 [Source:HGNC Symbol;Acc:HGNC:6329]" |
| ENSG00000129351 | HGLibA_23216 | 125 | 0.095277719 | ILF3 | interleukin enhancer binding factor 3 [Source:HGNC Symbol;Acc:HGNC:6038] |
| ENSG00000130175 | HGLibA_38559 | 125 | 0.095277719 | PRKCSH | protein kinase C substrate 80K-H [Source:HGNC Symbol;Acc:HGNC:9411] |
| ENSG00000131042 | HGLibA_26510 | 125 | 0.095277719 | LILRB2 | leukocyte immunoglobulin like receptor B2 [Source:HGNC Symbol;Acc:HGNC:6606] |
| ENSG00000131148 | HGLibA_15022 | 125 | 0.095277719 | EMC8 | ER membrane protein complex subunit 8 [Source:HGNC Symbol;Acc:HGNC:7864] |
| ENSG00000131398 | HGLibA_24168 | 125 | 0.095277719 | KCNC3 | potassium voltage-gated channel subfamily C member 3 [Source:HGNC Symbol;Acc:HGNC:6235] |
| ENSG00000131943 | HGLibA_05629 | 125 | 0.095277719 | C19orf12 | chromosome 19 open reading frame 12 [Source:HGNC Symbol;Acc:HGNC:25443] |
| ENSG00000134365 | HGLibA_09271 | 125 | 0.095277719 | CFHR4 | complement factor H related 4 [Source:HGNC Symbol;Acc:HGNC:16979] |
| ENSG00000134597 | HGLibA_40600 | 125 | 0.095277719 | RBMX2 | RNA binding motif protein X-linked 2 [Source:HGNC Symbol;Acc:HGNC:24282] |
| ENSG00000135333 | HGLibA_15306 | 125 | 0.095277719 | EPHA7 | EPH receptor A7 [Source:HGNC Symbol;Acc:HGNC:3390] |
| ENSG00000138395 | HGLibA_08740 | 125 | 0.095277719 | CDK15 | cyclin dependent kinase 15 [Source:HGNC Symbol;Acc:HGNC:14434] |
| ENSG00000140464 | HGLibA_37311 | 125 | 0.095277719 | PML | promyelocytic leukemia [Source:HGNC Symbol;Acc:HGNC:9113] |
| ENSG00000141469 | HGLibA_44398 | 125 | 0.095277719 | SLC14A1 | solute carrier family 14 member 1 (Kidd blood group) [Source:HGNC Symbol;Acc:HGNC:10918] |
| ENSG00000141750 | HGLibA_47314 | 125 | 0.095277719 | STAC2 | SH3 and cysteine rich domain 2 [Source:HGNC Symbol;Acc:HGNC:23990] |
| ENSG00000142546 | HGLibA_32333 | 125 | 0.095277719 | NOSIP | nitric oxide synthase interacting protein [Source:HGNC Symbol;Acc:HGNC:17946] |
| ENSG00000144136 | HGLibA_44529 | 125 | 0.095277719 | SLC20A1 | solute carrier family 20 member 1 [Source:HGNC Symbol;Acc:HGNC:10946] |
| ENSG00000144357 | HGLibA_52754 | 125 | 0.095277719 | UBR3 | ubiquitin protein ligase E3 component n-recognin 3 [Source:HGNC Symbol;Acc:HGNC:30467] |
| ENSG00000144381 | HGLibA_22332 | 125 | 0.095277719 | HSPD1 | heat shock protein family D (Hsp60) member 1 [Source:HGNC Symbol;Acc:HGNC:5261] |
| ENSG00000144724 | HGLibA_39504 | 125 | 0.095277719 | PTPRG | receptor type G [Source:HGNC Symbol;Acc:HGNC:9671]" |
| ENSG00000146013 | HGLibA_18972 | 125 | 0.095277719 | GFRA3 | GDNF family receptor alpha 3 [Source:HGNC Symbol;Acc:HGNC:4245] |
| ENSG00000146215 | HGLibA_11156 | 125 | 0.095277719 | CRIP3 | cysteine rich protein 3 [Source:HGNC Symbol;Acc:HGNC:17751] |
| ENSG00000147144 | HGLibA_07564 | 125 | 0.095277719 | CCDC120 | coiled-coil domain containing 120 [Source:HGNC Symbol;Acc:HGNC:28910] |
| ENSG00000147677 | HGLibA_14785 | 125 | 0.095277719 | EIF3H | eukaryotic translation initiation factor 3 subunit H [Source:HGNC Symbol;Acc:HGNC:3273] |
| ENSG00000148158 | HGLibA_46083 | 125 | 0.095277719 | SNX30 | sorting nexin family member 30 [Source:HGNC Symbol;Acc:HGNC:23685] |
| ENSG00000148175 | HGLibA_47532 | 125 | 0.095277719 | STOM | stomatin [Source:HGNC Symbol;Acc:HGNC:3383] |
| ENSG00000148832 | HGLibA_35119 | 125 | 0.095277719 | PAOX | polyamine oxidase [Source:HGNC Symbol;Acc:HGNC:20837] |
| ENSG00000149357 | HGLibA_25990 | 125 | 0.095277719 | LAMTOR1 | MAPK and MTOR activator 1 [Source:HGNC Symbol;Acc:HGNC:26068]" |
| ENSG00000152467 | HGLibA_56983 | 125 | 0.095277719 | ZSCAN1 | zinc finger and SCAN domain containing 1 [Source:HGNC Symbol;Acc:HGNC:23712] |
| ENSG00000152782 | HGLibA_35096 | 125 | 0.095277719 | PANK1 | pantothenate kinase 1 [Source:HGNC Symbol;Acc:HGNC:8598] |
| ENSG00000153162 | HGLibA_04615 | 125 | 0.095277719 | BMP6 | bone morphogenetic protein 6 [Source:HGNC Symbol;Acc:HGNC:1073] |
| ENSG00000153976 | HGLibA_22126 | 125 | 0.095277719 | HS3ST3A1 | heparan sulfate-glucosamine 3-sulfotransferase 3A1 [Source:HGNC Symbol;Acc:HGNC:5196] |
| ENSG00000156282 | HGLibA_09867 | 125 | 0.095277719 | CLDN17 | claudin 17 [Source:HGNC Symbol;Acc:HGNC:2038] |
| ENSG00000157796 | HGLibA_54042 | 125 | 0.095277719 | WDR19 | WD repeat domain 19 [Source:HGNC Symbol;Acc:HGNC:18340] |
| ENSG00000157827 | HGLibA_17696 | 125 | 0.095277719 | FMNL2 | formin like 2 [Source:HGNC Symbol;Acc:HGNC:18267] |
| ENSG00000158806 | HGLibA_32490 | 125 | 0.095277719 | NPM2 | nucleophosmin/nucleoplasmin 2 [Source:HGNC Symbol;Acc:HGNC:7930] |
| ENSG00000160072 | HGLibA_03412 | 125 | 0.095277719 | ATAD3B | AAA domain containing 3B [Source:HGNC Symbol;Acc:HGNC:24007]" |
| ENSG00000160182 | HGLibA_49169 | 125 | 0.095277719 | TFF1 | trefoil factor 1 [Source:HGNC Symbol;Acc:HGNC:11755] |
| ENSG00000161920 | HGLibA_28739 | 125 | 0.095277719 | MED11 | mediator complex subunit 11 [Source:HGNC Symbol;Acc:HGNC:32687] |
| ENSG00000162620 | HGLibA_27458 | 125 | 0.095277719 | LRRIQ3 | leucine rich repeats and IQ motif containing 3 [Source:HGNC Symbol;Acc:HGNC:28318] |
| ENSG00000162849 | HGLibA_24918 | 125 | 0.095277719 | KIF26B | kinesin family member 26B [Source:HGNC Symbol;Acc:HGNC:25484] |
| ENSG00000163348 | HGLibA_39697 | 125 | 0.095277719 | PYGO2 | pygopus family PHD finger 2 [Source:HGNC Symbol;Acc:HGNC:30257] |
| ENSG00000165434 | HGLibA_36268 | 125 | 0.095277719 | PGM2L1 | phosphoglucomutase 2 like 1 [Source:HGNC Symbol;Acc:HGNC:20898] |
| ENSG00000166321 | HGLibA_32942 | 125 | 0.095277719 | NUDT13 | nudix hydrolase 13 [Source:HGNC Symbol;Acc:HGNC:18827] |
| ENSG00000166411 | HGLibA_22544 | 125 | 0.095277719 | IDH3A | isocitrate dehydrogenase 3 (NAD(+)) alpha [Source:HGNC Symbol;Acc:HGNC:5384] |
| ENSG00000166736 | HGLibA_22381 | 125 | 0.095277719 | HTR3A | 5-hydroxytryptamine receptor 3A [Source:HGNC Symbol;Acc:HGNC:5297] |
| ENSG00000166926 | HGLibA_30159 | 125 | 0.095277719 | MS4A6E | membrane spanning 4-domains A6E [Source:HGNC Symbol;Acc:HGNC:14285] |
| ENSG00000167136 | HGLibA_15115 | 125 | 0.095277719 | ENDOG | endonuclease G [Source:HGNC Symbol;Acc:HGNC:3346] |
| ENSG00000167554 | HGLibA_56380 | 125 | 0.095277719 | ZNF610 | zinc finger protein 610 [Source:HGNC Symbol;Acc:HGNC:26687] |
| ENSG00000168439 | HGLibA_47447 | 125 | 0.095277719 | STIP1 | stress induced phosphoprotein 1 [Source:HGNC Symbol;Acc:HGNC:11387] |
| ENSG00000170289 | HGLibA_10279 | 125 | 0.095277719 | CNGB3 | cyclic nucleotide gated channel beta 3 [Source:HGNC Symbol;Acc:HGNC:2153] |
| ENSG00000170442 | HGLibA_25573 | 125 | 0.095277719 | KRT86 | keratin 86 [Source:HGNC Symbol;Acc:HGNC:6463] |
| ENSG00000171448 | HGLibA_54852 | 125 | 0.095277719 | ZBTB26 | zinc finger and BTB domain containing 26 [Source:HGNC Symbol;Acc:HGNC:23383] |
| ENSG00000171860 | HGLibA_06207 | 125 | 0.095277719 | C3AR1 | complement C3a receptor 1 [Source:HGNC Symbol;Acc:HGNC:1319] |
| ENSG00000173852 | HGLibA_13906 | 125 | 0.095277719 | DPY19L1 | dpy-19 like C-mannosyltransferase 1 [Source:HGNC Symbol;Acc:HGNC:22205] |
| ENSG00000173918 | HGLibA_05937 | 125 | 0.095277719 | C1QTNF1 | C1q and TNF related 1 [Source:HGNC Symbol;Acc:HGNC:14324] |
| ENSG00000177374 | HGLibA_21289 | 125 | 0.095277719 | HIC1 | HIC ZBTB transcriptional repressor 1 [Source:HGNC Symbol;Acc:HGNC:4909] |
| ENSG00000177414 | HGLibA_52664 | 125 | 0.095277719 | UBE2U | ubiquitin conjugating enzyme E2 U [Source:HGNC Symbol;Acc:HGNC:28559] |
| ENSG00000177947 | HGLibA_33285 | 125 | 0.095277719 | ODF3 | outer dense fiber of sperm tails 3 [Source:HGNC Symbol;Acc:HGNC:19905] |
| ENSG00000182628 | HGLibA_44263 | 125 | 0.095277719 | SKA2 | spindle and kinetochore associated complex subunit 2 [Source:HGNC Symbol;Acc:HGNC:28006] |
| ENSG00000184524 | HGLibA_09035 | 125 | 0.095277719 | CEND1 | cell cycle exit and neuronal differentiation 1 [Source:HGNC Symbol;Acc:HGNC:24153] |
| ENSG00000186468 | HGLibA_42109 | 125 | 0.095277719 | RPS23 | ribosomal protein S23 [Source:HGNC Symbol;Acc:HGNC:10410] |
| ENSG00000187531 | HGLibA_44235 | 125 | 0.095277719 | SIRT7 | sirtuin 7 [Source:HGNC Symbol;Acc:HGNC:14935] |
| ENSG00000187758 | HGLibA_00949 | 125 | 0.095277719 | ADH1A | alpha polypeptide [Source:HGNC Symbol;Acc:HGNC:249]" |
| ENSG00000196507 | HGLibA_48711 | 125 | 0.095277719 | TCEAL3 | transcription elongation factor A like 3 [Source:HGNC Symbol;Acc:HGNC:28247] |
| ENSG00000196932 | HGLibA_50348 | 125 | 0.095277719 | TMEM26 | transmembrane protein 26 [Source:HGNC Symbol;Acc:HGNC:28550] |
| ENSG00000197119 | HGLibA_44695 | 125 | 0.095277719 | SLC25A29 | solute carrier family 25 member 29 [Source:HGNC Symbol;Acc:HGNC:20116] |
| ENSG00000198556 | HGLibA_56757 | 125 | 0.095277719 | ZNF789 | zinc finger protein 789 [Source:HGNC Symbol;Acc:HGNC:27801] |
| ENSG00000198670 | HGLibA_27050 | 125 | 0.095277719 | LPA | lipoprotein(a) [Source:HGNC Symbol;Acc:HGNC:6667] |
| ENSG00000204511 | HGLibA_28572 | 125 | 0.095277719 | MCCD1 | mitochondrial coiled-coil domain 1 [Source:HGNC Symbol;Acc:HGNC:20668] |
| ENSG00000213465 | HGLibA_02956 | 125 | 0.095277719 | ARL2 | ADP ribosylation factor like GTPase 2 [Source:HGNC Symbol;Acc:HGNC:693] |
| ENSG00000229292 | HGLibA_40942 | 125 | 0.095277719 | RFPL4AL1 | ret finger protein like 4A like 1 [Source:HGNC Symbol;Acc:HGNC:45147] |
| ENSG00000229314 | HGLibA_34619 | 125 | 0.095277719 | ORM1 | orosomucoid 1 [Source:HGNC Symbol;Acc:HGNC:8498] |
| ENSG00000229809 | HGLibA_56543 | 125 | 0.095277719 | ZNF688 | zinc finger protein 688 [Source:HGNC Symbol;Acc:HGNC:30489] |
| ENSG00000253797 | HGLibA_53437 | 125 | 0.095277719 | UTP14C | small subunit processome component [Source:HGNC Symbol;Acc:HGNC:20321]" |
| ENSG00000274211 | HGLibA_46142 | 125 | 0.095277719 | SOCS7 | suppressor of cytokine signaling 7 [Source:HGNC Symbol;Acc:HGNC:29846] |
| GPR114 | HGLibA_19859 | 125 | 0.095277719 | GPR114 | - |
| HIST3H3 | HGLibA_21560 | 125 | 0.095277719 | HIST3H3 | - |
| hsa-mir-124-1 | HGLibA_57348 | 125 | 0.095277719 | hsa-mir-124-1 | - |
| hsa-mir-191 | HGLibA_58043 | 125 | 0.095277719 | hsa-mir-191 | - |
| hsa-mir-1911 | HGLibA_58049 | 125 | 0.095277719 | hsa-mir-1911 | - |
| hsa-mir-194-1 | HGLibA_58081 | 125 | 0.095277719 | hsa-mir-194-1 | - |
| hsa-mir-3164 | HGLibA_58734 | 125 | 0.095277719 | hsa-mir-3164 | - |
| hsa-mir-4280 | HGLibA_59822 | 125 | 0.095277719 | hsa-mir-4280 | - |
| hsa-mir-4462 | HGLibA_60255 | 125 | 0.095277719 | hsa-mir-4462 | - |
| hsa-mir-5091 | HGLibA_61522 | 125 | 0.095277719 | hsa-mir-5091 | - |
| hsa-mir-512-1 | HGLibA_61562 | 125 | 0.095277719 | hsa-mir-512-1 | - |
| hsa-mir-526b | HGLibA_61797 | 125 | 0.095277719 | hsa-mir-526b | - |

| | | | | | |
|---|---|---|---|---|---|
| hsa-mir-587 | HGLibA_62448 | 125 | 0.095277719 | hsa-mir-587 | - |
| hsa-mir-6086 | HGLibA_62604 | 125 | 0.095277719 | hsa-mir-6086 | - |
| hsa-mir-9-1 | HGLibA_64261 | 125 | 0.095277719 | hsa-mir-9-1 | - |
| hsa-mir-941-4 | HGLibA_64351 | 125 | 0.095277719 | hsa-mir-941-4 | - |
| MFSD7 | HGLibA_29114 | 125 | 0.095277719 | MFSD7 | - |
| NARR | HGLibA_31152 | 125 | 0.095277719 | NARR | - |
| TTC25 | HGLibA_52088 | 125 | 0.095277719 | TTC25 | - |
| ZFP106 | HGLibA_55184 | 125 | 0.095277719 | ZFP106 | - |
| C14orf166 | HGLibA_05332 | 124 | 0.085326682 | C14orf166 | - |
| C2orf47 | HGLibA_06133 | 124 | 0.085326682 | C2orf47 | - |
| CCDC19 | HGLibA_07728 | 124 | 0.085326682 | CCDC19 | - |
| DBC1 | HGLibA_12374 | 124 | 0.085326682 | DBC1 | - |
| ENSG00000002746 | HGLibA_21102 | 124 | 0.085326682 | HECW1 | C2 and WW domain containing E3 ubiquitin protein ligase 1 [Source:HGNC Symbol;Acc:HGNC:22195]" |
| ENSG00000007908 | HGLibA_43286 | 124 | 0.085326682 | SELE | selectin E [Source:HGNC Symbol;Acc:HGNC:10718] |
| ENSG00000008086 | HGLibA_08817 | 124 | 0.085326682 | CDKL5 | cyclin dependent kinase like 5 [Source:HGNC Symbol;Acc:HGNC:11411] |
| ENSG00000009954 | HGLibA_04187 | 124 | 0.085326682 | BAZ1B | bromodomain adjacent to zinc finger domain 1B [Source:HGNC Symbol;Acc:HGNC:961] |
| ENSG00000010361 | HGLibA_18211 | 124 | 0.085326682 | FUZ | fuzzy planar cell polarity protein [Source:HGNC Symbol;Acc:HGNC:26219] |
| ENSG00000021852 | HGLibA_06595 | 124 | 0.085326682 | C8B | complement C8 beta chain [Source:HGNC Symbol;Acc:HGNC:1353] |
| ENSG00000037241 | HGLibA_41916 | 124 | 0.085326682 | RPL26L1 | ribosomal protein L26 like 1 [Source:HGNC Symbol;Acc:HGNC:17050] |
| ENSG00000046604 | HGLibA_14026 | 124 | 0.085326682 | DSG2 | desmoglein 2 [Source:HGNC Symbol;Acc:HGNC:3049] |
| ENSG00000048740 | HGLibA_09006 | 124 | 0.085326682 | CELF2 | CUGBP Elav-like family member 2 [Source:HGNC Symbol;Acc:HGNC:2550] |
| ENSG00000065000 | HGLibA_02326 | 124 | 0.085326682 | AP3D1 | adaptor related protein complex 3 subunit delta 1 [Source:HGNC Symbol;Acc:HGNC:568] |
| ENSG00000070759 | HGLibA_49032 | 124 | 0.085326682 | TESK2 | testis associated actin remodelling kinase 2 [Source:HGNC Symbol;Acc:HGNC:11732] |
| ENSG00000072315 | HGLibA_51740 | 124 | 0.085326682 | TRPC5 | transient receptor potential cation channel subfamily C member 5 [Source:HGNC Symbol;Acc:HGNC:12337] |
| ENSG00000073803 | HGLibA_28173 | 124 | 0.085326682 | MAP3K13 | mitogen-activated protein kinase kinase kinase 13 [Source:HGNC Symbol;Acc:HGNC:6852] |
| ENSG00000073910 | HGLibA_18052 | 124 | 0.085326682 | FRY | FRY microtubule binding protein [Source:HGNC Symbol;Acc:HGNC:20367] |
| ENSG00000075151 | HGLibA_14845 | 124 | 0.085326682 | EIF4G3 | eukaryotic translation initiation factor 4 gamma 3 [Source:HGNC Symbol;Acc:HGNC:3298] |
| ENSG00000076248 | HGLibA_53045 | 124 | 0.085326682 | UNG | uracil DNA glycosylase [Source:HGNC Symbol;Acc:HGNC:12572] |
| ENSG00000077463 | HGLibA_44230 | 124 | 0.085326682 | SIRT6 | sirtuin 6 [Source:HGNC Symbol;Acc:HGNC:14934] |
| ENSG00000083750 | HGLibA_42228 | 124 | 0.085326682 | RRAGB | Ras related GTP binding B [Source:HGNC Symbol;Acc:HGNC:19901] |
| ENSG00000083857 | HGLibA_16913 | 124 | 0.085326682 | FAT1 | FAT atypical cadherin 1 [Source:HGNC Symbol;Acc:HGNC:3595] |
| ENSG00000085840 | HGLibA_34601 | 124 | 0.085326682 | ORC1 | origin recognition complex subunit 1 [Source:HGNC Symbol;Acc:HGNC:8487] |
| ENSG00000087053 | HGLibA_30425 | 124 | 0.085326682 | MTMR2 | myotubularin related protein 2 [Source:HGNC Symbol;Acc:HGNC:7450] |
| ENSG00000087253 | HGLibA_27074 | 124 | 0.085326682 | LPCAT2 | lysophosphatidylcholine acyltransferase 2 [Source:HGNC Symbol;Acc:HGNC:26032] |
| ENSG00000087842 | HGLibA_36729 | 124 | 0.085326682 | PIR | pirin [Source:HGNC Symbol;Acc:HGNC:30048] |
| ENSG00000092377 | HGLibA_48613 | 124 | 0.085326682 | TBL1Y | transducin beta like 1 Y-linked [Source:HGNC Symbol;Acc:HGNC:18502] |
| ENSG00000100142 | HGLibA_37578 | 124 | 0.085326682 | POLR2F | RNA polymerase II subunit F [Source:HGNC Symbol;Acc:HGNC:9193] |
| ENSG00000100721 | HGLibA_48815 | 124 | 0.085326682 | TCL1A | T cell leukemia/lymphoma 1A [Source:HGNC Symbol;Acc:HGNC:11648] |
| ENSG00000101074 | HGLibA_39781 | 124 | 0.085326682 | R3HDML | R3H domain containing like [Source:HGNC Symbol;Acc:HGNC:16249] |
| ENSG00000102385 | HGLibA_13987 | 124 | 0.085326682 | DRP2 | dystrophin related protein 2 [Source:HGNC Symbol;Acc:HGNC:3032] |
| ENSG00000102471 | HGLibA_31396 | 124 | 0.085326682 | NDFIP2 | Nedd4 family interacting protein 2 [Source:HGNC Symbol;Acc:HGNC:18537] |
| ENSG00000103932 | HGLibA_41814 | 124 | 0.085326682 | RPAP1 | RNA polymerase II associated protein 1 [Source:HGNC Symbol;Acc:HGNC:24567] |
| ENSG00000103994 | HGLibA_55438 | 124 | 0.085326682 | ZNF106 | zinc finger protein 106 [Source:HGNC Symbol;Acc:HGNC:12886] |
| ENSG00000104432 | HGLibA_23189 | 124 | 0.085326682 | IL7 | interleukin 7 [Source:HGNC Symbol;Acc:HGNC:6023] |
| ENSG00000104825 | HGLibA_31824 | 124 | 0.085326682 | NFKBIB | NFKB inhibitor beta [Source:HGNC Symbol;Acc:HGNC:7798] |
| ENSG00000107185 | HGLibA_41021 | 124 | 0.085326682 | RGP1 | RAB6A GEF complex partner 1 [Source:HGNC Symbol;Acc:HGNC:21965]" |
| ENSG00000107789 | HGLibA_29283 | 124 | 0.085326682 | MINPP1 | multiple inositol-polyphosphate phosphatase 1 [Source:HGNC Symbol;Acc:HGNC:7102] |
| ENSG00000108187 | HGLibA_35333 | 124 | 0.085326682 | PBLD | phenazine biosynthesis like protein domain containing [Source:HGNC Symbol;Acc:HGNC:23301] |
| ENSG00000109193 | HGLibA_47756 | 124 | 0.085326682 | SULT1E1 | sulfotransferase family 1E member 1 [Source:HGNC Symbol;Acc:HGNC:11377] |
| ENSG00000111057 | HGLibA_25439 | 124 | 0.085326682 | KRT18 | keratin 18 [Source:HGNC Symbol;Acc:HGNC:6430] |
| ENSG00000112062 | HGLibA_28261 | 124 | 0.085326682 | MAPK14 | mitogen-activated protein kinase 14 [Source:HGNC Symbol;Acc:HGNC:6876] |
| ENSG00000112333 | HGLibA_32605 | 124 | 0.085326682 | NR2E1 | nuclear receptor subfamily 2 group E member 1 [Source:HGNC Symbol;Acc:HGNC:7973] |
| ENSG00000114923 | HGLibA_45218 | 124 | 0.085326682 | SLC4A3 | solute carrier family 4 member 3 [Source:HGNC Symbol;Acc:HGNC:11029] |
| ENSG00000115604 | HGLibA_23027 | 124 | 0.085326682 | IL18R1 | interleukin 18 receptor 1 [Source:HGNC Symbol;Acc:HGNC:5988] |
| ENSG00000116786 | HGLibA_37141 | 124 | 0.085326682 | PLEKHM2 | pleckstrin homology and RUN domain containing M2 [Source:HGNC Symbol;Acc:HGNC:29131] |
| ENSG00000117983 | HGLibA_30551 | 124 | 0.085326682 | MUC5B | oligomeric mucus/gel-forming [Source:HGNC Symbol;Acc:HGNC:7516]" |
| ENSG00000118939 | HGLibA_52805 | 124 | 0.085326682 | UCHL3 | ubiquitin C-terminal hydrolase L3 [Source:HGNC Symbol;Acc:HGNC:12515] |
| ENSG00000120318 | HGLibA_02630 | 124 | 0.085326682 | ARAP3 | ankyrin repeat and PH domain 3 [Source:HGNC Symbol;Acc:HGNC:24097]" |
| ENSG00000120669 | HGLibA_46164 | 124 | 0.085326682 | SOHLH2 | spermatogenesis and oogenesis specific basic helix-loop-helix 2 [Source:HGNC Symbol;Acc:HGNC:26026] |
| ENSG00000121053 | HGLibA_15391 | 124 | 0.085326682 | EPX | eosinophil peroxidase [Source:HGNC Symbol;Acc:HGNC:3423] |
| ENSG00000121454 | HGLibA_26456 | 124 | 0.085326682 | LHX4 | LIM homeobox 4 [Source:HGNC Symbol;Acc:HGNC:21734] |
| ENSG00000123307 | HGLibA_31741 | 124 | 0.085326682 | NEUROD4 | neuronal differentiation 4 [Source:HGNC Symbol;Acc:HGNC:13802] |
| ENSG00000124249 | HGLibA_24305 | 124 | 0.085326682 | KCNK15 | potassium two pore domain channel subfamily K member 15 [Source:HGNC Symbol;Acc:HGNC:13814] |
| ENSG00000125166 | HGLibA_19697 | 124 | 0.085326682 | GOT2 | glutamic-oxaloacetic transaminase 2 [Source:HGNC Symbol;Acc:HGNC:4433] |
| ENSG00000125780 | HGLibA_49259 | 124 | 0.085326682 | TGM3 | transglutaminase 3 [Source:HGNC Symbol;Acc:HGNC:11779] |
| ENSG00000125965 | HGLibA_18869 | 124 | 0.085326682 | GDF5 | growth differentiation factor 5 [Source:HGNC Symbol;Acc:HGNC:4220] |
| ENSG00000128714 | HGLibA_21996 | 124 | 0.085326682 | HOXD13 | homeobox D13 [Source:HGNC Symbol;Acc:HGNC:5136] |
| ENSG00000129667 | HGLibA_41136 | 124 | 0.085326682 | RHBDF2 | rhomboid 5 homolog 2 [Source:HGNC Symbol;Acc:HGNC:20788] |
| ENSG00000134291 | HGLibA_49860 | 124 | 0.085326682 | TMEM106C | transmembrane protein 106C [Source:HGNC Symbol;Acc:HGNC:28775] |
| ENSG00000135392 | HGLibA_13575 | 124 | 0.085326682 | DNAJC14 | DnaJ heat shock protein family (Hsp40) member C14 [Source:HGNC Symbol;Acc:HGNC:24581] |
| ENSG00000135587 | HGLibA_45807 | 124 | 0.085326682 | SMPD2 | sphingomyelin phosphodiesterase 2 [Source:HGNC Symbol;Acc:HGNC:11121] |
| ENSG00000135821 | HGLibA_19367 | 124 | 0.085326682 | GLUL | glutamate-ammonia ligase [Source:HGNC Symbol;Acc:HGNC:4341] |
| ENSG00000135905 | HGLibA_13734 | 124 | 0.085326682 | DOCK10 | dedicator of cytokinesis 10 [Source:HGNC Symbol;Acc:HGNC:23479] |
| ENSG00000136014 | HGLibA_53370 | 124 | 0.085326682 | USP44 | ubiquitin specific peptidase 44 [Source:HGNC Symbol;Acc:HGNC:20064] |
| ENSG00000136840 | HGLibA_47274 | 124 | 0.085326682 | ST6GALNAC4 | 6-sialyltransferase 4 [Source:HGNC Symbol;Acc:HGNC:17846]" |
| ENSG00000136940 | HGLibA_35748 | 124 | 0.085326682 | PDCL | phosducin like [Source:HGNC Symbol;Acc:HGNC:8770] |
| ENSG00000137055 | HGLibA_36929 | 124 | 0.085326682 | PLAA | phospholipase A2 activating protein [Source:HGNC Symbol;Acc:HGNC:9043] |
| ENSG00000137331 | HGLibA_22576 | 124 | 0.085326682 | IER3 | immediate early response 3 [Source:HGNC Symbol;Acc:HGNC:5392] |
| ENSG00000137338 | HGLibA_36217 | 124 | 0.085326682 | PGBD1 | piggyBac transposable element derived 1 [Source:HGNC Symbol;Acc:HGNC:19398] |
| ENSG00000138271 | HGLibA_20100 | 124 | 0.085326682 | GPR87 | G protein-coupled receptor 87 [Source:HGNC Symbol;Acc:HGNC:4538] |
| ENSG00000138674 | HGLibA_43241 | 124 | 0.085326682 | SEC31A | COPII coat complex component [Source:HGNC Symbol;Acc:HGNC:17052]" |
| ENSG00000138760 | HGLibA_42871 | 124 | 0.085326682 | SCARB2 | scavenger receptor class B member 2 [Source:HGNC Symbol;Acc:HGNC:1665] |
| ENSG00000138814 | HGLibA_38184 | 124 | 0.085326682 | PPP3CA | protein phosphatase 3 catalytic subunit alpha [Source:HGNC Symbol;Acc:HGNC:9314] |
| ENSG00000139197 | HGLibA_36117 | 124 | 0.085326682 | PEX5 | peroxisomal biogenesis factor 5 [Source:HGNC Symbol;Acc:HGNC:9719] |
| ENSG00000139915 | HGLibA_28678 | 124 | 0.085326682 | MDGA2 | MAM domain containing glycosylphosphatidylinositol anchor 2 [Source:HGNC Symbol;Acc:HGNC:19835] |
| ENSG00000140632 | HGLibA_19386 | 124 | 0.085326682 | GLYR1 | glyoxylate reductase 1 homolog [Source:HGNC Symbol;Acc:HGNC:24434] |
| ENSG00000143549 | HGLibA_51148 | 124 | 0.085326682 | TPM3 | tropomyosin 3 [Source:HGNC Symbol;Acc:HGNC:12012] |
| ENSG00000143669 | HGLibA_27776 | 124 | 0.085326682 | LYST | lysosomal trafficking regulator [Source:HGNC Symbol;Acc:HGNC:1968] |
| ENSG00000144815 | HGLibA_33155 | 124 | 0.085326682 | NXPE3 | neurexophilin and PC-esterase domain family member 3 [Source:HGNC Symbol;Acc:HGNC:28238] |
| ENSG00000145451 | HGLibA_19305 | 124 | 0.085326682 | GLRA3 | glycine receptor alpha 3 [Source:HGNC Symbol;Acc:HGNC:4328] |
| ENSG00000145975 | HGLibA_16490 | 124 | 0.085326682 | FAM217A | family with sequence similarity 217 member A [Source:HGNC Symbol;Acc:HGNC:21362] |
| ENSG00000146858 | HGLibA_54993 | 124 | 0.085326682 | ZC3HAV1L | antiviral 1 like [Source:HGNC Symbol;Acc:HGNC:22423]" |
| ENSG00000150722 | HGLibA_38071 | 124 | 0.085326682 | PPP1R1C | protein phosphatase 1 regulatory inhibitor subunit 1C [Source:HGNC Symbol;Acc:HGNC:14940] |
| ENSG00000151090 | HGLibA_49395 | 124 | 0.085326682 | THRB | thyroid hormone receptor beta [Source:HGNC Symbol;Acc:HGNC:11799] |
| ENSG00000152402 | HGLibA_20653 | 124 | 0.085326682 | GUCY1A2 | guanylate cyclase 1 soluble subunit alpha 2 [Source:HGNC Symbol;Acc:HGNC:4684] |
| ENSG00000155463 | HGLibA_34809 | 124 | 0.085326682 | OXA1L | mitochondrial inner membrane protein [Source:HGNC Symbol;Acc:HGNC:8526]" |
| ENSG00000157315 | HGLibA_49818 | 124 | 0.085326682 | TMED6 | transmembrane p24 trafficking protein 6 [Source:HGNC Symbol;Acc:HGNC:28331] |
| ENSG00000158164 | HGLibA_50660 | 124 | 0.085326682 | TMSB15A | thymosin beta 15a [Source:HGNC Symbol;Acc:HGNC:30744] |
| ENSG00000160321 | HGLibA_55581 | 124 | 0.085326682 | ZNF208 | zinc finger protein 208 [Source:HGNC Symbol;Acc:HGNC:12999] |
| ENSG00000162813 | HGLibA_04736 | 124 | 0.085326682 | BPNT1 | 5'-bisphosphate nucleotidase 1 [Source:HGNC Symbol;Acc:HGNC:1096]" |
| ENSG00000163453 | HGLibA_22833 | 124 | 0.085326682 | IGFBP7 | insulin like growth factor binding protein 7 [Source:HGNC Symbol;Acc:HGNC:5476] |
| ENSG00000163888 | HGLibA_07082 | 124 | 0.085326682 | CAMK2N2 | calcium/calmodulin dependent protein kinase II inhibitor 2 [Source:HGNC Symbol;Acc:HGNC:24197] |
| ENSG00000164325 | HGLibA_50066 | 124 | 0.085326682 | TMEM174 | transmembrane protein 174 [Source:HGNC Symbol;Acc:HGNC:28187] |
| ENSG00000164506 | HGLibA_47675 | 124 | 0.085326682 | STXBP5 | syntaxin binding protein 5 [Source:HGNC Symbol;Acc:HGNC:19665] |
| ENSG00000165078 | HGLibA_10900 | 124 | 0.085326682 | CPA6 | carboxypeptidase A6 [Source:HGNC Symbol;Acc:HGNC:17245] |
| ENSG00000166845 | HGLibA_05619 | 124 | 0.085326682 | C18orf54 | chromosome 18 open reading frame 54 [Source:HGNC Symbol;Acc:HGNC:13796] |
| ENSG00000168491 | HGLibA_07538 | 124 | 0.085326682 | CCDC110 | coiled-coil domain containing 110 [Source:HGNC Symbol;Acc:HGNC:28504] |
| ENSG00000169372 | HGLibA_11060 | 124 | 0.085326682 | CRADD | CASP2 and RIPK1 domain containing adaptor with death domain [Source:HGNC Symbol;Acc:HGNC:2340] |
| ENSG00000170423 | HGLibA_25545 | 124 | 0.085326682 | KRT78 | keratin 78 [Source:HGNC Symbol;Acc:HGNC:28926] |
| ENSG00000170500 | HGLibA_27023 | 124 | 0.085326682 | LONRF2 | LON peptidase N-terminal domain and ring finger 2 [Source:HGNC Symbol;Acc:HGNC:24788] |
| ENSG00000170790 | HGLibA_33462 | 124 | 0.085326682 | OR10A2 | olfactory receptor family 10 subfamily A member 2 [Source:HGNC Symbol;Acc:HGNC:8161] |
| ENSG00000172146 | HGLibA_33640 | 124 | 0.085326682 | OR1A1 | olfactory receptor family 1 subfamily A member 1 [Source:HGNC Symbol;Acc:HGNC:8179] |
| ENSG00000172183 | HGLibA_23658 | 124 | 0.085326682 | ISG20 | interferon stimulated exonuclease gene 20 [Source:HGNC Symbol;Acc:HGNC:6130] |
| ENSG00000173917 | HGLibA_21933 | 124 | 0.085326682 | HOXB2 | homeobox B2 [Source:HGNC Symbol;Acc:HGNC:5113] |
| ENSG00000173992 | HGLibA_08182 | 124 | 0.085326682 | CCS | copper chaperone for superoxide dismutase [Source:HGNC Symbol;Acc:HGNC:1613] |
| ENSG00000174013 | HGLibA_17109 | 124 | 0.085326682 | FBXO45 | F-box protein 45 [Source:HGNC Symbol;Acc:HGNC:29148] |
| ENSG00000174473 | HGLibA_18580 | 124 | 0.085326682 | GALNTL6 | polypeptide N-acetylgalactosaminyltransferase like 6 [Source:HGNC Symbol;Acc:HGNC:33844] |
| ENSG00000174640 | HGLibA_45460 | 124 | 0.085326682 | SLCO2A1 | solute carrier organic anion transporter family member 2A1 [Source:HGNC Symbol;Acc:HGNC:10955] |
| ENSG00000175137 | HGLibA_43938 | 124 | 0.085326682 | SH3BP5L | SH3 binding domain protein 5 like [Source:HGNC Symbol;Acc:HGNC:29360] |
| ENSG00000175520 | HGLibA_52737 | 124 | 0.085326682 | UBQLN3 | ubiquilin 3 [Source:HGNC Symbol;Acc:HGNC:12510] |
| ENSG00000175550 | HGLibA_13949 | 124 | 0.085326682 | DRAP1 | DR1 associated protein 1 [Source:HGNC Symbol;Acc:HGNC:3019] |
| ENSG00000175745 | HGLibA_32611 | 124 | 0.085326682 | NR2F1 | nuclear receptor subfamily 2 group F member 1 [Source:HGNC Symbol;Acc:HGNC:7975] |
| ENSG00000178591 | HGLibA_12880 | 124 | 0.085326682 | DEFB125 | defensin beta 125 [Source:HGNC Symbol;Acc:HGNC:18105] |
| ENSG00000180934 | HGLibA_34210 | 124 | 0.085326682 | OR56A1 | olfactory receptor family 56 subfamily A member 1 [Source:HGNC Symbol;Acc:HGNC:14781] |
| ENSG00000182040 | HGLibA_53175 | 124 | 0.085326682 | USH1G | USH1 protein network component sans [Source:HGNC Symbol;Acc:HGNC:16356] |
| ENSG00000182175 | HGLibA_41012 | 124 | 0.085326682 | RGMA | repulsive guidance molecule BMP co-receptor a [Source:HGNC Symbol;Acc:HGNC:30308] |
| ENSG00000182472 | HGLibA_07151 | 124 | 0.085326682 | CAPN12 | calpain 12 [Source:HGNC Symbol;Acc:HGNC:13249] |
| ENSG00000184009 | HGLibA_00611 | 124 | 0.085326682 | ACTG1 | actin gamma 1 [Source:HGNC Symbol;Acc:HGNC:144] |
| ENSG00000187912 | HGLibA_09939 | 124 | 0.085326682 | CLEC17A | C-type lectin domain containing 17A [Source:HGNC Symbol;Acc:HGNC:34520] |
| ENSG00000196116 | HGLibA_48913 | 124 | 0.085326682 | TDRD7 | tudor domain containing 7 [Source:HGNC Symbol;Acc:HGNC:30831] |
| ENSG00000196834 | HGLibA_37740 | 124 | 0.085326682 | POTEI | POTE ankyrin domain family member I [Source:HGNC Symbol;Acc:HGNC:37093] |
| ENSG00000197279 | HGLibA_55516 | 124 | 0.085326682 | ZNF165 | zinc finger protein 165 [Source:HGNC Symbol;Acc:HGNC:12953] |
| ENSG00000197912 | HGLibA_46639 | 124 | 0.085326682 | SPG7 | paraplegin matrix AAA peptidase subunit [Source:HGNC Symbol;Acc:HGNC:11237]" |
| ENSG00000204450 | HGLibA_51577 | 124 | 0.085326682 | TRIM64 | tripartite motif containing 64 [Source:HGNC Symbol;Acc:HGNC:14663] |
| ENSG00000214517 | HGLibA_37998 | 124 | 0.085326682 | PPME1 | protein phosphatase methylesterase 1 [Source:HGNC Symbol;Acc:HGNC:30178] |

| | | | | | |
|---|---|---|---|---|---|
| ENSG00000215218 | HGLibA_52650 | 124 | 0.085326682 | UBE2QL1 | ubiquitin conjugating enzyme E2 Q family like 1 [Source:HGNC Symbol;Acc:HGNC:37269] |
| ENSG00000228474 | HGLibA_34704 | 124 | 0.085326682 | OST4 | non-catalytic [Source:HGNC Symbol;Acc:HGNC:32483]" |
| ENSG00000231887 | HGLibA_38461 | 124 | 0.085326682 | PRH1 | proline rich protein HaeIII subfamily 1 [Source:HGNC Symbol;Acc:HGNC:9366] |
| ENSG00000242265 | HGLibA_36027 | 124 | 0.085326682 | PEG10 | paternally expressed 10 [Source:HGNC Symbol;Acc:HGNC:14005] |
| ENSG00000245848 | HGLibA_08957 | 124 | 0.085326682 | CEBPA | CCAAT enhancer binding protein alpha [Source:HGNC Symbol;Acc:HGNC:1833] |
| ENSG00000261011 | HGLibA_01140 | 124 | 0.085326682 | AGAP11 | ankyrin repeat and PH domain family (AGAP) pseudogene" |
| ENSG00000267796 | HGLibA_26564 | 124 | 0.085326682 | LIN37 | lin-37 DREAM MuvB core complex component [Source:HGNC Symbol;Acc:HGNC:33234] |
| hsa-mir-1208 | HGLibA_57288 | 124 | 0.085326682 | hsa-mir-1208 | - |
| hsa-mir-2276 | HGLibA_58307 | 124 | 0.085326682 | hsa-mir-2276 | - |
| hsa-mir-24-2 | HGLibA_58343 | 124 | 0.085326682 | hsa-mir-24-2 | - |
| hsa-mir-520g | HGLibA_61760 | 124 | 0.085326682 | hsa-mir-520g | - |
| hsa-mir-577 | HGLibA_62403 | 124 | 0.085326682 | hsa-mir-577 | - |
| hsa-mir-6798 | HGLibA_63371 | 124 | 0.085326682 | hsa-mir-6798 | - |
| hsa-mir-6891 | HGLibA_63750 | 124 | 0.085326682 | hsa-mir-6891 | - |
| hsa-mir-7112-2 | HGLibA_63816 | 124 | 0.085326682 | hsa-mir-7112-2 | - |
| hsa-mir-7846 | HGLibA_63975 | 124 | 0.085326682 | hsa-mir-7846 | - |
| hsa-mir-8072 | HGLibA_64130 | 124 | 0.085326682 | hsa-mir-8072 | - |
| hsa-mir-885 | HGLibA_64224 | 124 | 0.085326682 | hsa-mir-885 | - |
| TMEM189-UBE2V1 | HGLibA_50135 | 124 | 0.085326682 | TMEM189-UBE2V1 | - |
| WDR34 | HGLibA_54071 | 124 | 0.085326682 | WDR34 | - |
| WDR63 | HGLibA_54148 | 124 | 0.085326682 | WDR63 | - |
| C2orf61 | HGLibA_06155 | 123 | 0.075375644 | C2orf61 | - |
| ENSG00000004468 | HGLibA_08365 | 123 | 0.075375644 | CD38 | CD38 molecule [Source:HGNC Symbol;Acc:HGNC:1667] |
| ENSG00000005469 | HGLibA_11212 | 123 | 0.075375644 | CROT | carnitine O-octanoyltransferase [Source:HGNC Symbol;Acc:HGNC:2366] |
| ENSG00000018625 | HGLibA_03583 | 123 | 0.075375644 | ATP1A2 | ATPase Na+/K+ transporting subunit alpha 2 [Source:HGNC Symbol;Acc:HGNC:800] |
| ENSG00000063761 | HGLibA_00891 | 123 | 0.075375644 | ADCK1 | aarF domain containing kinase 1 [Source:HGNC Symbol;Acc:HGNC:19038] |
| ENSG00000065057 | HGLibA_32845 | 123 | 0.075375644 | NTHL1 | nth like DNA glycosylase 1 [Source:HGNC Symbol;Acc:HGNC:8028] |
| ENSG00000065621 | HGLibA_20490 | 123 | 0.075375644 | GSTO2 | glutathione S-transferase omega 2 [Source:HGNC Symbol;Acc:HGNC:23064] |
| ENSG00000075290 | HGLibA_54392 | 123 | 0.075375644 | WNT8B | Wnt family member 8B [Source:HGNC Symbol;Acc:HGNC:12789] |
| ENSG00000079841 | HGLibA_41276 | 123 | 0.075375644 | RIMS1 | regulating synaptic membrane exocytosis 1 [Source:HGNC Symbol;Acc:HGNC:17282] |
| ENSG00000084072 | HGLibA_37927 | 123 | 0.075375644 | PPIE | peptidylprolyl isomerase E [Source:HGNC Symbol;Acc:HGNC:9258] |
| ENSG00000088387 | HGLibA_13762 | 123 | 0.075375644 | DOCK9 | dedicator of cytokinesis 9 [Source:HGNC Symbol;Acc:HGNC:14132] |
| ENSG00000090060 | HGLibA_35136 | 123 | 0.075375644 | PAPOLA | poly(A) polymerase alpha [Source:HGNC Symbol;Acc:HGNC:14981] |
| ENSG00000091009 | HGLibA_40524 | 123 | 0.075375644 | RBM27 | RNA binding motif protein 27 [Source:HGNC Symbol;Acc:HGNC:29243] |
| ENSG00000092853 | HGLibA_10131 | 123 | 0.075375644 | CLSPN | claspin [Source:HGNC Symbol;Acc:HGNC:19715] |
| ENSG00000095002 | HGLibA_30191 | 123 | 0.075375644 | MSH2 | mutS homolog 2 [Source:HGNC Symbol;Acc:HGNC:7325] |
| ENSG00000096996 | HGLibA_22964 | 123 | 0.075375644 | IL12RB1 | interleukin 12 receptor subunit beta 1 [Source:HGNC Symbol;Acc:HGNC:5971] |
| ENSG00000100147 | HGLibA_07597 | 123 | 0.075375644 | CCDC134 | coiled-coil domain containing 134 [Source:HGNC Symbol;Acc:HGNC:26185] |
| ENSG00000100162 | HGLibA_09070 | 123 | 0.075375644 | CENPM | centromere protein M [Source:HGNC Symbol;Acc:HGNC:18352] |
| ENSG00000100897 | HGLibA_12415 | 123 | 0.075375644 | DCAF11 | DDB1 and CUL4 associated factor 11 [Source:HGNC Symbol;Acc:HGNC:20258] |
| ENSG00000101665 | HGLibA_45587 | 123 | 0.075375644 | SMAD7 | SMAD family member 7 [Source:HGNC Symbol;Acc:HGNC:6773] |
| ENSG00000101680 | HGLibA_25942 | 123 | 0.075375644 | LAMA1 | laminin subunit alpha 1 [Source:HGNC Symbol;Acc:HGNC:6481] |
| ENSG00000101882 | HGLibA_32000 | 123 | 0.075375644 | NKAP | NFKB activating protein [Source:HGNC Symbol;Acc:HGNC:29873] |
| ENSG00000102001 | HGLibA_06890 | 123 | 0.075375644 | CACNA1F | calcium voltage-gated channel subunit alpha1 F [Source:HGNC Symbol;Acc:HGNC:1393] |
| ENSG00000103047 | HGLibA_48301 | 123 | 0.075375644 | TANGO6 | transport and golgi organization 6 homolog [Source:HGNC Symbol;Acc:HGNC:25749] |
| ENSG00000104154 | HGLibA_44909 | 123 | 0.075375644 | SLC30A4 | solute carrier family 30 member 4 [Source:HGNC Symbol;Acc:HGNC:11015] |
| ENSG00000104490 | HGLibA_31252 | 123 | 0.075375644 | NCALD | neurocalcin delta [Source:HGNC Symbol;Acc:HGNC:7655] |
| ENSG00000105323 | HGLibA_21850 | 123 | 0.075375644 | HNRNPUL1 | heterogeneous nuclear ribonucleoprotein U like 1 [Source:HGNC Symbol;Acc:HGNC:17011] |
| ENSG00000105438 | HGLibA_24479 | 123 | 0.075375644 | KDELR1 | KDEL endoplasmic reticulum protein retention receptor 1 [Source:HGNC Symbol;Acc:HGNC:6304] |
| ENSG00000105641 | HGLibA_45274 | 123 | 0.075375644 | SLC5A5 | solute carrier family 5 member 5 [Source:HGNC Symbol;Acc:HGNC:11040] |
| ENSG00000107779 | HGLibA_04630 | 123 | 0.075375644 | BMPR1A | bone morphogenetic protein receptor type 1A [Source:HGNC Symbol;Acc:HGNC:1076] |
| ENSG00000109452 | HGLibA_23339 | 123 | 0.075375644 | INPP4B | inositol polyphosphate-4-phosphatase type II B [Source:HGNC Symbol;Acc:HGNC:6075] |
| ENSG00000109452 | HGLibA_23341 | 123 | 0.075375644 | INPP4B | inositol polyphosphate-4-phosphatase type II B [Source:HGNC Symbol;Acc:HGNC:6075] |
| ENSG00000111832 | HGLibA_42479 | 123 | 0.075375644 | RWDD1 | RWD domain containing 1 [Source:HGNC Symbol;Acc:HGNC:20993] |
| ENSG00000111897 | HGLibA_43485 | 123 | 0.075375644 | SERINC1 | serine incorporator 1 [Source:HGNC Symbol;Acc:HGNC:13464] |
| ENSG00000112337 | HGLibA_44461 | 123 | 0.075375644 | SLC17A2 | solute carrier family 17 member 2 [Source:HGNC Symbol;Acc:HGNC:10930] |
| ENSG00000113248 | HGLibA_35486 | 123 | 0.075375644 | PCDHB15 | protocadherin beta 15 [Source:HGNC Symbol;Acc:HGNC:8686] |
| ENSG00000114423 | HGLibA_07397 | 123 | 0.075375644 | CBLB | Cbl proto-oncogene B [Source:HGNC Symbol;Acc:HGNC:1542] |
| ENSG00000117151 | HGLibA_11589 | 123 | 0.075375644 | CTBS | chitobiase [Source:HGNC Symbol;Acc:HGNC:2496] |
| ENSG00000117318 | HGLibA_22529 | 123 | 0.075375644 | ID3 | HLH protein [Source:HGNC Symbol;Acc:HGNC:5362]" |
| ENSG00000119283 | HGLibA_51587 | 123 | 0.075375644 | TRIM67 | tripartite motif containing 67 [Source:HGNC Symbol;Acc:HGNC:31859] |
| ENSG00000119661 | HGLibA_13645 | 123 | 0.075375644 | DNAL1 | dynein axonemal light chain 1 [Source:HGNC Symbol;Acc:HGNC:23247] |
| ENSG00000120896 | HGLibA_46179 | 123 | 0.075375644 | SORBS3 | sorbin and SH3 domain containing 3 [Source:HGNC Symbol;Acc:HGNC:30907] |
| ENSG00000123739 | HGLibA_36869 | 123 | 0.075375644 | PLA2G12A | phospholipase A2 group XIIA [Source:HGNC Symbol;Acc:HGNC:18554] |
| ENSG00000124205 | HGLibA_14475 | 123 | 0.075375644 | EDN3 | endothelin 3 [Source:HGNC Symbol;Acc:HGNC:3178] |
| ENSG00000124207 | HGLibA_11319 | 123 | 0.075375644 | CSE1L | chromosome segregation 1 like [Source:HGNC Symbol;Acc:HGNC:2431] |
| ENSG00000124767 | HGLibA_19282 | 123 | 0.075375644 | GLO1 | glyoxalase I [Source:HGNC Symbol;Acc:HGNC:4323] |
| ENSG00000125629 | HGLibA_23379 | 123 | 0.075375644 | INSIG2 | insulin induced gene 2 [Source:HGNC Symbol;Acc:HGNC:20452] |
| ENSG00000125780 | HGLibA_49258 | 123 | 0.075375644 | TGM3 | transglutaminase 3 [Source:HGNC Symbol;Acc:HGNC:11779] |
| ENSG00000126709 | HGLibA_22616 | 123 | 0.075375644 | IFI6 | interferon alpha inducible protein 6 [Source:HGNC Symbol;Acc:HGNC:4054] |
| ENSG00000126903 | HGLibA_44337 | 123 | 0.075375644 | SLC10A3 | solute carrier family 10 member 3 [Source:HGNC Symbol;Acc:HGNC:22979] |
| ENSG00000127081 | HGLibA_56078 | 123 | 0.075375644 | ZNF484 | zinc finger protein 484 [Source:HGNC Symbol;Acc:HGNC:23385] |
| ENSG00000127452 | HGLibA_16974 | 123 | 0.075375644 | FBXL12 | F-box and leucine rich repeat protein 12 [Source:HGNC Symbol;Acc:HGNC:13611] |
| ENSG00000128310 | HGLibA_18594 | 123 | 0.075375644 | GALR3 | galanin receptor 3 [Source:HGNC Symbol;Acc:HGNC:4134] |
| ENSG00000130638 | HGLibA_03832 | 123 | 0.075375644 | ATXN10 | ataxin 10 [Source:HGNC Symbol;Acc:HGNC:10549] |
| ENSG00000132256 | HGLibA_51533 | 123 | 0.075375644 | TRIM5 | tripartite motif containing 5 [Source:HGNC Symbol;Acc:HGNC:16276] |
| ENSG00000132670 | HGLibA_39484 | 123 | 0.075375644 | PTPRA | receptor type A [Source:HGNC Symbol;Acc:HGNC:9664]" |
| ENSG00000134287 | HGLibA_02648 | 123 | 0.075375644 | ARF3 | ADP ribosylation factor 3 [Source:HGNC Symbol;Acc:HGNC:654] |
| ENSG00000134775 | HGLibA_17496 | 123 | 0.075375644 | FHOD3 | formin homology 2 domain containing 3 [Source:HGNC Symbol;Acc:HGNC:26178] |
| ENSG00000134986 | HGLibA_32670 | 123 | 0.075375644 | NREP | neuronal regeneration related protein [Source:HGNC Symbol;Acc:HGNC:16834] |
| ENSG00000135413 | HGLibA_25915 | 123 | 0.075375644 | LACRT | lacritin [Source:HGNC Symbol;Acc:HGNC:16430] |
| ENSG00000135443 | HGLibA_25570 | 123 | 0.075375644 | KRT85 | keratin 85 [Source:HGNC Symbol;Acc:HGNC:6462] |
| ENSG00000136436 | HGLibA_07000 | 123 | 0.075375644 | CALCOCO2 | calcium binding and coiled-coil domain 2 [Source:HGNC Symbol;Acc:HGNC:29912] |
| ENSG00000136935 | HGLibA_19592 | 123 | 0.075375644 | GOLGA1 | golgin A1 [Source:HGNC Symbol;Acc:HGNC:4424] |
| ENSG00000137699 | HGLibA_51453 | 123 | 0.075375644 | TRIM29 | tripartite motif containing 29 [Source:HGNC Symbol;Acc:HGNC:17274] |
| ENSG00000137713 | HGLibA_38144 | 123 | 0.075375644 | PPP2R1B | protein phosphatase 2 scaffold subunit Abeta [Source:HGNC Symbol;Acc:HGNC:9303] |
| ENSG00000138449 | HGLibA_45135 | 123 | 0.075375644 | SLC40A1 | solute carrier family 40 member 1 [Source:HGNC Symbol;Acc:HGNC:10909] |
| ENSG00000139055 | HGLibA_15523 | 123 | 0.075375644 | ERP27 | endoplasmic reticulum protein 27 [Source:HGNC Symbol;Acc:HGNC:26495] |
| ENSG00000139192 | HGLibA_48323 | 123 | 0.075375644 | TAPBPL | TAP binding protein like [Source:HGNC Symbol;Acc:HGNC:30683] |
| ENSG00000140386 | HGLibA_42859 | 123 | 0.075375644 | SCAPER | S-phase cyclin A associated protein in the ER [Source:HGNC Symbol;Acc:HGNC:13081] |
| ENSG00000141642 | HGLibA_14870 | 123 | 0.075375644 | ELAC1 | elaC ribonuclease Z 1 [Source:HGNC Symbol;Acc:HGNC:14197] |
| ENSG00000141668 | HGLibA_07409 | 123 | 0.075375644 | CBLN2 | cerebellin 2 precursor [Source:HGNC Symbol;Acc:HGNC:1544] |
| ENSG00000144369 | HGLibA_16271 | 123 | 0.075375644 | FAM171B | family with sequence similarity 171 member B [Source:HGNC Symbol;Acc:HGNC:29412] |
| ENSG00000145284 | HGLibA_42886 | 123 | 0.075375644 | SCD5 | stearoyl-CoA desaturase 5 [Source:HGNC Symbol;Acc:HGNC:21088] |
| ENSG00000145850 | HGLibA_49486 | 123 | 0.075375644 | TIMD4 | T cell immunoglobulin and mucin domain containing 4 [Source:HGNC Symbol;Acc:HGNC:25132] |
| ENSG00000146755 | HGLibA_51536 | 123 | 0.075375644 | TRIM50 | tripartite motif containing 50 [Source:HGNC Symbol;Acc:HGNC:19017] |
| ENSG00000147119 | HGLibA_09645 | 123 | 0.075375644 | CHST7 | carbohydrate sulfotransferase 7 [Source:HGNC Symbol;Acc:HGNC:13817] |
| ENSG00000147576 | HGLibA_00969 | 123 | 0.075375644 | ADHFE1 | iron containing 1 [Source:HGNC Symbol;Acc:HGNC:16354]" |
| ENSG00000148677 | HGLibA_01988 | 123 | 0.075375644 | ANKRD1 | ankyrin repeat domain 1 [Source:HGNC Symbol;Acc:HGNC:15819] |
| ENSG00000152683 | HGLibA_44913 | 123 | 0.075375644 | SLC30A6 | solute carrier family 30 member 6 [Source:HGNC Symbol;Acc:HGNC:19305] |
| ENSG00000154358 | HGLibA_33232 | 123 | 0.075375644 | OBSCN | cytoskeletal calmodulin and titin-interacting RhoGEF [Source:HGNC Symbol;Acc:HGNC:15719]" |
| ENSG00000154655 | HGLibA_25904 | 123 | 0.075375644 | L3MBTL4 | histone methyl-lysine binding protein [Source:HGNC Symbol;Acc:HGNC:26677]" |
| ENSG00000155090 | HGLibA_25042 | 123 | 0.075375644 | KLF10 | Kruppel like factor 10 [Source:HGNC Symbol;Acc:HGNC:11810] |
| ENSG00000156049 | HGLibA_19445 | 123 | 0.075375644 | GNA14 | G protein subunit alpha 14 [Source:HGNC Symbol;Acc:HGNC:4382] |
| ENSG00000156413 | HGLibA_18201 | 123 | 0.075375644 | FUT6 | fucosyltransferase 6 [Source:HGNC Symbol;Acc:HGNC:4017] |
| ENSG00000157514 | HGLibA_51823 | 123 | 0.075375644 | TSC22D3 | TSC22 domain family member 3 [Source:HGNC Symbol;Acc:HGNC:3051] |
| ENSG00000158578 | HGLibA_01526 | 123 | 0.075375644 | ALAS2 | 5'-aminolevulinate synthase 2 [Source:HGNC Symbol;Acc:HGNC:397] |
| ENSG00000160691 | HGLibA_43999 | 123 | 0.075375644 | SHC1 | SHC adaptor protein 1 [Source:HGNC Symbol;Acc:HGNC:10840] |
| ENSG00000160710 | HGLibA_00869 | 123 | 0.075375644 | ADAR | RNA specific [Source:HGNC Symbol;Acc:HGNC:225]" |
| ENSG00000161048 | HGLibA_31131 | 123 | 0.075375644 | NAPEPLD | N-acyl phosphatidylethanolamine phospholipase D [Source:HGNC Symbol;Acc:HGNC:21683] |
| ENSG00000163568 | HGLibA_01353 | 123 | 0.075375644 | AIM2 | absent in melanoma 2 [Source:HGNC Symbol;Acc:HGNC:357] |
| ENSG00000163864 | HGLibA_32189 | 123 | 0.075375644 | NMNAT3 | nicotinamide nucleotide adenylyltransferase 3 [Source:HGNC Symbol;Acc:HGNC:20989] |
| ENSG00000163945 | HGLibA_53477 | 123 | 0.075375644 | UVSSA | UV stimulated scaffold protein A [Source:HGNC Symbol;Acc:HGNC:29304] |
| ENSG00000164334 | HGLibA_16259 | 123 | 0.075375644 | FAM170A | family with sequence similarity 170 member A [Source:HGNC Symbol;Acc:HGNC:27963] |
| ENSG00000164530 | HGLibA_36485 | 123 | 0.075375644 | PI16 | peptidase inhibitor 16 [Source:HGNC Symbol;Acc:HGNC:21245] |
| ENSG00000165868 | HGLibA_22260 | 123 | 0.075375644 | HSPA12A | heat shock protein family A (Hsp70) member 12A [Source:HGNC Symbol;Acc:HGNC:19022] |
| ENSG00000166037 | HGLibA_09147 | 123 | 0.075375644 | CEP57 | centrosomal protein 57 [Source:HGNC Symbol;Acc:HGNC:30794] |
| ENSG00000166199 | HGLibA_01651 | 123 | 0.075375644 | ALKBH3 | alpha-ketoglutaratedependent dioxygenase [Source:HGNC Symbol;Acc:HGNC:30141]" |
| ENSG00000166225 | HGLibA_18046 | 123 | 0.075375644 | FRS2 | fibroblast growth factor receptor substrate 2 [Source:HGNC Symbol;Acc:HGNC:16971] |
| ENSG00000166963 | HGLibA_28115 | 123 | 0.075375644 | MAP1A | microtubule associated protein 1A [Source:HGNC Symbol;Acc:HGNC:6835] |
| ENSG00000167785 | HGLibA_56232 | 123 | 0.075375644 | ZNF558 | zinc finger protein 558 [Source:HGNC Symbol;Acc:HGNC:26422] |
| ENSG00000169059 | HGLibA_53586 | 123 | 0.075375644 | VCX3A | variable charge X-linked 3A [Source:HGNC Symbol;Acc:HGNC:18159] |
| ENSG00000169905 | HGLibA_51021 | 123 | 0.075375644 | TOR1AIP2 | torsin 1A interacting protein 2 [Source:HGNC Symbol;Acc:HGNC:24055] |
| ENSG00000169964 | HGLibA_50399 | 123 | 0.075375644 | TMEM42 | transmembrane protein 42 [Source:HGNC Symbol;Acc:HGNC:28444] |
| ENSG00000170421 | HGLibA_25551 | 123 | 0.075375644 | KRT8 | keratin 8 [Source:HGNC Symbol;Acc:HGNC:6446] |
| ENSG00000171759 | HGLibA_35022 | 123 | 0.075375644 | PAH | phenylalanine hydroxylase [Source:HGNC Symbol;Acc:HGNC:8582] |
| ENSG00000171956 | HGLibA_17816 | 123 | 0.075375644 | FOXB1 | forkhead box B1 [Source:HGNC Symbol;Acc:HGNC:3799] |
| ENSG00000172352 | HGLibA_08907 | 123 | 0.075375644 | CDY1B | chromodomain Y-linked 1B [Source:HGNC Symbol;Acc:HGNC:23920] |
| ENSG00000172531 | HGLibA_38005 | 123 | 0.075375644 | PPP1CA | protein phosphatase 1 catalytic subunit alpha [Source:HGNC Symbol;Acc:HGNC:9281] |
| ENSG00000172867 | HGLibA_25443 | 123 | 0.075375644 | KRT2 | keratin 2 [Source:HGNC Symbol;Acc:HGNC:6439] |
| ENSG00000173250 | HGLibA_19933 | 123 | 0.075375644 | GPR151 | G protein-coupled receptor 151 [Source:HGNC Symbol;Acc:HGNC:23624] |
| ENSG00000174437 | HGLibA_03609 | 123 | 0.075375644 | ATP2A2 | ATPase sarcoplasmic/endoplasmic reticulum Ca2+ transporting 2 [Source:HGNC Symbol;Acc:HGNC:812] |
| ENSG00000175893 | HGLibA_55101 | 123 | 0.075375644 | ZDHHC21 | zinc finger DHHC-type containing 21 [Source:HGNC Symbol;Acc:HGNC:20750] |
| ENSG00000176014 | HGLibA_52291 | 123 | 0.075375644 | TUBB6 | tubulin beta 6 class V [Source:HGNC Symbol;Acc:HGNC:20776] |
| ENSG00000176566 | HGLibA_12445 | 123 | 0.075375644 | DCAF4L2 | DDB1 and CUL4 associated factor 4 like 2 [Source:HGNC Symbol;Acc:HGNC:26657] |

| | | | | | |
|---|---|---|---|---|---|
| ENSG00000177888 | HGLibA_54884 | 123 | 0.075375644 | ZBTB41 | zinc finger and BTB domain containing 41 [Source:HGNC Symbol;Acc:HGNC:24819] |
| ENSG00000178607 | HGLibA_15511 | 123 | 0.075375644 | ERN1 | endoplasmic reticulum to nucleus signaling 1 [Source:HGNC Symbol;Acc:HGNC:3449] |
| ENSG00000180089 | HGLibA_50528 | 123 | 0.075375644 | TMEM86B | transmembrane protein 86B [Source:HGNC Symbol;Acc:HGNC:28448] |
| ENSG00000181803 | HGLibA_34441 | 123 | 0.075375644 | OR6S1 | olfactory receptor family 6 subfamily S member 1 [Source:HGNC Symbol;Acc:HGNC:15363] |
| ENSG00000181867 | HGLibA_18129 | 123 | 0.075375644 | FTMT | ferritin mitochondrial [Source:HGNC Symbol;Acc:HGNC:17345] |
| ENSG00000181885 | HGLibA_09907 | 123 | 0.075375644 | CLDN7 | claudin 7 [Source:HGNC Symbol;Acc:HGNC:2049] |
| ENSG00000182318 | HGLibA_57003 | 123 | 0.075375644 | ZSCAN22 | zinc finger and SCAN domain containing 22 [Source:HGNC Symbol;Acc:HGNC:4929] |
| ENSG00000182325 | HGLibA_17015 | 123 | 0.075375644 | FBXL6 | F-box and leucine rich repeat protein 6 [Source:HGNC Symbol;Acc:HGNC:13603] |
| ENSG00000182326 | HGLibA_05975 | 123 | 0.075375644 | C1S | complement C1s [Source:HGNC Symbol;Acc:HGNC:1247] |
| ENSG00000182782 | HGLibA_20949 | 123 | 0.075375644 | HCAR2 | hydroxycarboxylic acid receptor 2 [Source:HGNC Symbol;Acc:HGNC:24827] |
| ENSG00000183542 | HGLibA_25293 | 123 | 0.075375644 | KLRC4 | killer cell lectin like receptor C4 [Source:HGNC Symbol;Acc:HGNC:6377] |
| ENSG00000184164 | HGLibA_11129 | 123 | 0.075375644 | CRELD2 | cysteine rich with EGF like domains 2 [Source:HGNC Symbol;Acc:HGNC:28150] |
| ENSG00000186075 | HGLibA_56965 | 123 | 0.075375644 | ZPBP2 | zona pellucida binding protein 2 [Source:HGNC Symbol;Acc:HGNC:20678] |
| ENSG00000187608 | HGLibA_23656 | 123 | 0.075375644 | ISG15 | ISG15 ubiquitin-like modifier [Source:HGNC Symbol;Acc:HGNC:4053] |
| ENSG00000188778 | HGLibA_01062 | 123 | 0.075375644 | ADRB3 | adrenoceptor beta 3 [Source:HGNC Symbol;Acc:HGNC:288] |
| ENSG00000189299 | HGLibA_17948 | 123 | 0.075375644 | FOXR2 | forkhead box R2 [Source:HGNC Symbol;Acc:HGNC:30469] |
| ENSG00000196220 | HGLibA_46982 | 123 | 0.075375644 | SRGAP3 | SLIT-ROBO Rho GTPase activating protein 3 [Source:HGNC Symbol;Acc:HGNC:19744] |
| ENSG00000196407 | HGLibA_49342 | 123 | 0.075375644 | THEM5 | thioesterase superfamily member 5 [Source:HGNC Symbol;Acc:HGNC:26755] |
| ENSG00000196498 | HGLibA_31358 | 123 | 0.075375644 | NCOR2 | nuclear receptor corepressor 2 [Source:HGNC Symbol;Acc:HGNC:7673] |
| ENSG00000196683 | HGLibA_50979 | 123 | 0.075375644 | TOMM7 | translocase of outer mitochondrial membrane 7 [Source:HGNC Symbol;Acc:HGNC:21648] |
| ENSG00000197170 | HGLibA_39156 | 123 | 0.075375644 | PSMD12 | non-ATPase 12 [Source:HGNC Symbol;Acc:HGNC:9557]" |
| ENSG00000198203 | HGLibA_47745 | 123 | 0.075375644 | SULT1C2 | sulfotransferase family 1C member 2 [Source:HGNC Symbol;Acc:HGNC:11456] |
| ENSG00000198354 | HGLibA_12424 | 123 | 0.075375644 | DCAF12L2 | DDB1 and CUL4 associated factor 12 like 2 [Source:HGNC Symbol;Acc:HGNC:32950] |
| ENSG00000204020 | HGLibA_26631 | 123 | 0.075375644 | LIPN | lipase family member N [Source:HGNC Symbol;Acc:HGNC:23452] |
| ENSG00000204103 | HGLibA_27876 | 123 | 0.075375644 | MAFB | MAF bZIP transcription factor B [Source:HGNC Symbol;Acc:HGNC:6408] |
| ENSG00000204406 | HGLibA_28490 | 123 | 0.075375644 | MBD5 | methyl-CpG binding domain protein 5 [Source:HGNC Symbol;Acc:HGNC:20444] |
| ENSG00000204410 | HGLibA_30198 | 123 | 0.075375644 | MSH5 | mutS homolog 5 [Source:HGNC Symbol;Acc:HGNC:7328] |
| ENSG00000204444 | HGLibA_02548 | 123 | 0.075375644 | APOM | apolipoprotein M [Source:HGNC Symbol;Acc:HGNC:13916] |
| ENSG00000204611 | HGLibA_56394 | 123 | 0.075375644 | ZNF616 | zinc finger protein 616 [Source:HGNC Symbol;Acc:HGNC:28062] |
| ENSG00000204887 | HGLibA_25584 | 123 | 0.075375644 | KRTAP1-4 | keratin associated protein 1-4 [Source:HGNC Symbol;Acc:HGNC:18904] |
| ENSG00000205758 | HGLibA_11298 | 123 | 0.075375644 | CRYZL1 | crystallin zeta like 1 [Source:HGNC Symbol;Acc:HGNC:2420] |
| ENSG00000213699 | HGLibA_45012 | 123 | 0.075375644 | SLC35F6 | solute carrier family 35 member F6 [Source:HGNC Symbol;Acc:HGNC:26055] |
| ENSG00000233954 | HGLibA_53125 | 123 | 0.075375644 | UQCRHL | ubiquinol-cytochrome c reductase hinge protein like [Source:HGNC Symbol;Acc:HGNC:51714] |
| ENSG00000243137 | HGLibA_39047 | 123 | 0.075375644 | PSG4 | pregnancy specific beta-1-glycoprotein 4 [Source:HGNC Symbol;Acc:HGNC:9521] |
| ENSG00000244509 | HGLibA_02482 | 123 | 0.075375644 | APOBEC3C | apolipoprotein B mRNA editing enzyme catalytic subunit 3C [Source:HGNC Symbol;Acc:HGNC:17353] |
| ENSG00000258986 | HGLibA_50087 | 123 | 0.075375644 | TMEM179 | transmembrane protein 179 [Source:HGNC Symbol;Acc:HGNC:20137] |
| ENSG00000266560 | HGLibA_27895 | 123 | 0.075375644 | MAGEA10-MAGEA5 | MAGEA10-MAGEA5 readthrough [Source:NCBI gene;Acc:100533997] |
| ENSG00000268651 | HGLibA_11559 | 123 | 0.075375644 | CTAG1A | cancer/testis antigen 1A [Source:HGNC Symbol;Acc:HGNC:24198] |
| ENSG00000269699 | HGLibA_55340 | 123 | 0.075375644 | ZIM2 | zinc finger imprinted 2 [Source:HGNC Symbol;Acc:HGNC:12875] |
| ENSG00000272573 | HGLibA_30580 | 123 | 0.075375644 | MUSTN1 | embryonic nuclear protein 1 [Source:HGNC Symbol;Acc:HGNC:22144]" |
| GAREML | HGLibA_18628 | 123 | 0.075375644 | GAREML | - |
| GPR112 | HGLibA_19852 | 123 | 0.075375644 | GPR112 | - |
| hsa-mir-1290 | HGLibA_57635 | 123 | 0.075375644 | hsa-mir-1290 | - |
| hsa-mir-302f | HGLibA_58455 | 123 | 0.075375644 | hsa-mir-302f | - |
| hsa-mir-326 | HGLibA_58971 | 123 | 0.075375644 | hsa-mir-326 | - |
| hsa-mir-4288 | HGLibA_59860 | 123 | 0.075375644 | hsa-mir-4288 | - |
| hsa-mir-4474 | HGLibA_60308 | 123 | 0.075375644 | hsa-mir-4474 | - |
| hsa-mir-4512 | HGLibA_60490 | 123 | 0.075375644 | hsa-mir-4512 | - |
| hsa-mir-4645 | HGLibA_60685 | 123 | 0.075375644 | hsa-mir-4645 | - |
| hsa-mir-4650-1 | HGLibA_60702 | 123 | 0.075375644 | hsa-mir-4650-1 | - |
| hsa-mir-7843 | HGLibA_63961 | 123 | 0.075375644 | hsa-mir-7843 | - |
| hsa-mir-941-2 | HGLibA_64342 | 123 | 0.075375644 | hsa-mir-941-2 | - |
| KIAA1377 | HGLibA_24746 | 123 | 0.075375644 | KIAA1377 | - |
| NARFL | HGLibA_31147 | 123 | 0.075375644 | NARFL | - |
| PDDC1 | HGLibA_35758 | 123 | 0.075375644 | PDDC1 | - |
| C10orf32 | HGLibA_05067 | 122 | 0.065424607 | C10orf32 | - |
| C14orf182 | HGLibA_05351 | 122 | 0.065424607 | C14orf182 | - |
| C17orf70 | HGLibA_05564 | 122 | 0.065424607 | C17orf70 | - |
| C22orf29 | HGLibA_06069 | 122 | 0.065424607 | C22orf29 | - |
| CCDC67 | HGLibA_07837 | 122 | 0.065424607 | CCDC67 | - |
| DFNA5 | HGLibA_13024 | 122 | 0.065424607 | DFNA5 | - |
| ENSG00000003402 | HGLibA_09288 | 122 | 0.065424607 | CFLAR | CASP8 and FADD like apoptosis regulator [Source:HGNC Symbol;Acc:HGNC:1876] |
| ENSG00000003436 | HGLibA_49183 | 122 | 0.065424607 | TFPI | tissue factor pathway inhibitor [Source:HGNC Symbol;Acc:HGNC:11760] |
| ENSG00000007237 | HGLibA_18656 | 122 | 0.065424607 | GAS7 | growth arrest specific 7 [Source:HGNC Symbol;Acc:HGNC:4169] |
| ENSG00000010810 | HGLibA_18253 | 122 | 0.065424607 | FYN | Src family tyrosine kinase [Source:HGNC Symbol;Acc:HGNC:4037]" |
| ENSG00000018236 | HGLibA_10401 | 122 | 0.065424607 | CNTN1 | contactin 1 [Source:HGNC Symbol;Acc:HGNC:2171] |
| ENSG00000026103 | HGLibA_16889 | 122 | 0.065424607 | FAS | Fas cell surface death receptor [Source:HGNC Symbol;Acc:HGNC:11920] |
| ENSG00000028528 | HGLibA_46023 | 122 | 0.065424607 | SNX1 | sorting nexin 1 [Source:HGNC Symbol;Acc:HGNC:11172] |
| ENSG00000029725 | HGLibA_40001 | 122 | 0.065424607 | RABEP1 | RAB GTPase binding effector protein 1 [Source:HGNC Symbol;Acc:HGNC:17677]" |
| ENSG00000030110 | HGLibA_04133 | 122 | 0.065424607 | BAK1 | BCL2 antagonist/killer 1 [Source:HGNC Symbol;Acc:HGNC:949] |
| ENSG00000064687 | HGLibA_00122 | 122 | 0.065424607 | ABCA7 | ATP binding cassette subfamily A member 7 [Source:HGNC Symbol;Acc:HGNC:37] |
| ENSG00000065268 | HGLibA_54040 | 122 | 0.065424607 | WDR18 | WD repeat domain 18 [Source:HGNC Symbol;Acc:HGNC:17956] |
| ENSG00000071243 | HGLibA_23290 | 122 | 0.065424607 | ING3 | inhibitor of growth family member 3 [Source:HGNC Symbol;Acc:HGNC:14587] |
| ENSG00000081052 | HGLibA_10578 | 122 | 0.065424607 | COL4A4 | collagen type IV alpha 4 chain [Source:HGNC Symbol;Acc:HGNC:2206] |
| ENSG00000083937 | HGLibA_09482 | 122 | 0.065424607 | CHMP2B | charged multivesicular body protein 2B [Source:HGNC Symbol;Acc:HGNC:24537] |
| ENSG00000086102 | HGLibA_31848 | 122 | 0.065424607 | NFX1 | X-box binding 1 [Source:HGNC Symbol;Acc:HGNC:7803]" |
| ENSG00000095203 | HGLibA_15254 | 122 | 0.065424607 | EPB41L4B | erythrocyte membrane protein band 4.1 like 4B [Source:HGNC Symbol;Acc:HGNC:19818] |
| ENSG00000095752 | HGLibA_22953 | 122 | 0.065424607 | IL11 | interleukin 11 [Source:HGNC Symbol;Acc:HGNC:5966] |
| ENSG00000095917 | HGLibA_51197 | 122 | 0.065424607 | TPSD1 | tryptase delta 1 [Source:HGNC Symbol;Acc:HGNC:14118] |
| ENSG00000099814 | HGLibA_09118 | 122 | 0.065424607 | CEP170B | centrosomal protein 170B [Source:HGNC Symbol;Acc:HGNC:20362] |
| ENSG00000100350 | HGLibA_17955 | 122 | 0.065424607 | FOXRED2 | FAD dependent oxidoreductase domain containing 2 [Source:HGNC Symbol;Acc:HGNC:26264] |
| ENSG00000101443 | HGLibA_54262 | 122 | 0.065424607 | WFDC2 | WAP four-disulfide core domain 2 [Source:HGNC Symbol;Acc:HGNC:15939] |
| ENSG00000104321 | HGLibA_51725 | 122 | 0.065424607 | TRPA1 | transient receptor potential cation channel subfamily A member 1 [Source:HGNC Symbol;Acc:HGNC:497] |
| ENSG00000105364 | HGLibA_29974 | 122 | 0.065424607 | MRPL4 | mitochondrial ribosomal protein L4 [Source:HGNC Symbol;Acc:HGNC:14276] |
| ENSG00000105550 | HGLibA_17395 | 122 | 0.065424607 | FGF21 | fibroblast growth factor 21 [Source:HGNC Symbol;Acc:HGNC:3678] |
| ENSG00000105642 | HGLibA_24353 | 122 | 0.065424607 | KCNN1 | potassium calcium-activated channel subfamily N member 1 [Source:HGNC Symbol;Acc:HGNC:6290] |
| ENSG00000106305 | HGLibA_01357 | 122 | 0.065424607 | AIMP2 | aminoacyl tRNA synthetase complex interacting multifunctional protein 2 [Source:HGNC Symbol;Acc:HGNC:20609] |
| ENSG00000106344 | HGLibA_40526 | 122 | 0.065424607 | RBM28 | RNA binding motif protein 28 [Source:HGNC Symbol;Acc:HGNC:21863] |
| ENSG00000108641 | HGLibA_04043 | 122 | 0.065424607 | B9D1 | B9 domain containing 1 [Source:HGNC Symbol;Acc:HGNC:24123] |
| ENSG00000109881 | HGLibA_07761 | 122 | 0.065424607 | CCDC34 | coiled-coil domain containing 34 [Source:HGNC Symbol;Acc:HGNC:25079] |
| ENSG00000112038 | HGLibA_33452 | 122 | 0.065424607 | OPRM1 | opioid receptor mu 1 [Source:HGNC Symbol;Acc:HGNC:8156] |
| ENSG00000115263 | HGLibA_18777 | 122 | 0.065424607 | GCG | glucagon [Source:HGNC Symbol;Acc:HGNC:4191] |
| ENSG00000117676 | HGLibA_42159 | 122 | 0.065424607 | RPS6KA1 | ribosomal protein S6 kinase A1 [Source:HGNC Symbol;Acc:HGNC:10430] |
| ENSG00000124491 | HGLibA_15854 | 122 | 0.065424607 | F13A1 | coagulation factor XIII A chain [Source:HGNC Symbol;Acc:HGNC:3531] |
| ENSG00000124942 | HGLibA_01300 | 122 | 0.065424607 | AHNAK | AHNAK nucleoprotein [Source:HGNC Symbol;Acc:HGNC:347] |
| ENSG00000127540 | HGLibA_53106 | 122 | 0.065424607 | UQCR11 | complex III subunit XI [Source:HGNC Symbol;Acc:HGNC:30862]" |
| ENSG00000130313 | HGLibA_36245 | 122 | 0.065424607 | PGLS | 6-phosphogluconolactonase [Source:HGNC Symbol;Acc:HGNC:8903] |
| ENSG00000130768 | HGLibA_45819 | 122 | 0.065424607 | SMPDL3B | sphingomyelin phosphodiesterase acid like 3B [Source:HGNC Symbol;Acc:HGNC:21416] |
| ENSG00000130856 | HGLibA_55652 | 122 | 0.065424607 | ZNF236 | zinc finger protein 236 [Source:HGNC Symbol;Acc:HGNC:13028] |
| ENSG00000131773 | HGLibA_24571 | 122 | 0.065424607 | KHDRBS3 | signal transduction associated 3 [Source:HGNC Symbol;Acc:HGNC:18117]" |
| ENSG00000131831 | HGLibA_40144 | 122 | 0.065424607 | RAI2 | retinoic acid induced 2 [Source:HGNC Symbol;Acc:HGNC:9835] |
| ENSG00000132694 | HGLibA_02814 | 122 | 0.065424607 | ARHGEF11 | Rho guanine nucleotide exchange factor 11 [Source:HGNC Symbol;Acc:HGNC:14580] |
| ENSG00000132840 | HGLibA_04488 | 122 | 0.065424607 | BHMT2 | betaine--homocysteine S-methyltransferase 2 [Source:HGNC Symbol;Acc:HGNC:1048] |
| ENSG00000133063 | HGLibA_09453 | 122 | 0.065424607 | CHIT1 | chitinase 1 [Source:HGNC Symbol;Acc:HGNC:1936] |
| ENSG00000134324 | HGLibA_27096 | 122 | 0.065424607 | LPIN1 | lipin 1 [Source:HGNC Symbol;Acc:HGNC:13345] |
| ENSG00000135111 | HGLibA_48668 | 122 | 0.065424607 | TBX3 | T-box 3 [Source:HGNC Symbol;Acc:HGNC:11602] |
| ENSG00000135362 | HGLibA_38849 | 122 | 0.065424607 | PRR5L | proline rich 5 like [Source:HGNC Symbol;Acc:HGNC:25878] |
| ENSG00000137463 | HGLibA_29130 | 122 | 0.065424607 | MGARP | mitochondria localized glutamic acid rich protein [Source:HGNC Symbol;Acc:HGNC:29969] |
| ENSG00000138308 | HGLibA_36873 | 122 | 0.065424607 | PLA2G12B | phospholipase A2 group XIIB [Source:HGNC Symbol;Acc:HGNC:18555] |
| ENSG00000138778 | HGLibA_09050 | 122 | 0.065424607 | CENPE | centromere protein E [Source:HGNC Symbol;Acc:HGNC:1856] |
| ENSG00000139055 | HGLibA_15525 | 122 | 0.065424607 | ERP27 | endoplasmic reticulum protein 27 [Source:HGNC Symbol;Acc:HGNC:26495] |
| ENSG00000139734 | HGLibA_13222 | 122 | 0.065424607 | DIAPH3 | diaphanous related formin 3 [Source:HGNC Symbol;Acc:HGNC:15480] |
| ENSG00000143401 | HGLibA_02208 | 122 | 0.065424607 | ANP32E | acidic nuclear phosphoprotein 32 family member E [Source:HGNC Symbol;Acc:HGNC:16673] |
| ENSG00000143850 | HGLibA_37078 | 122 | 0.065424607 | PLEKHA6 | pleckstrin homology domain containing A6 [Source:HGNC Symbol;Acc:HGNC:17053] |
| ENSG00000144290 | HGLibA_45204 | 122 | 0.065424607 | SLC4A10 | solute carrier family 4 member 10 [Source:HGNC Symbol;Acc:HGNC:13811] |
| ENSG00000144339 | HGLibA_49834 | 122 | 0.065424607 | TMEFF2 | transmembrane protein with EGF like and two follistatin like domains 2 [Source:HGNC Symbol;Acc:HGNC:11867] |
| ENSG00000144746 | HGLibA_02990 | 122 | 0.065424607 | ARL6IP5 | ADP ribosylation factor like GTPase 6 interacting protein 5 [Source:HGNC Symbol;Acc:HGNC:16937] |
| ENSG00000145819 | HGLibA_02741 | 122 | 0.065424607 | ARHGAP26 | Rho GTPase activating protein 26 [Source:HGNC Symbol;Acc:HGNC:17073] |
| ENSG00000146021 | HGLibA_25177 | 122 | 0.065424607 | KLHL3 | kelch like family member 3 [Source:HGNC Symbol;Acc:HGNC:6354] |
| ENSG00000148358 | HGLibA_19844 | 122 | 0.065424607 | GPR107 | G protein-coupled receptor 107 [Source:HGNC Symbol;Acc:HGNC:17830] |
| ENSG00000151611 | HGLibA_29449 | 122 | 0.065424607 | MMAA | metabolism of cobalamin associated A [Source:HGNC Symbol;Acc:HGNC:18871] |
| ENSG00000155115 | HGLibA_20595 | 122 | 0.065424607 | GTF3C6 | general transcription factor IIIC subunit 6 [Source:HGNC Symbol;Acc:HGNC:20872] |
| ENSG00000155393 | HGLibA_21071 | 122 | 0.065424607 | HEATR3 | HEAT repeat containing 3 [Source:HGNC Symbol;Acc:HGNC:26087] |
| ENSG00000157240 | HGLibA_18259 | 122 | 0.065424607 | FZD1 | frizzled class receptor 1 [Source:HGNC Symbol;Acc:HGNC:4038] |
| ENSG00000159692 | HGLibA_11584 | 122 | 0.065424607 | CTBP1 | C-terminal binding protein 1 [Source:HGNC Symbol;Acc:HGNC:2494] |
| ENSG00000160299 | HGLibA_35640 | 122 | 0.065424607 | PCNT | pericentrin [Source:HGNC Symbol;Acc:HGNC:16068] |
| ENSG00000161996 | HGLibA_54215 | 122 | 0.065424607 | WDR90 | WD repeat domain 90 [Source:HGNC Symbol;Acc:HGNC:26960] |
| ENSG00000162552 | HGLibA_54370 | 122 | 0.065424607 | WNT4 | Wnt family member 4 [Source:HGNC Symbol;Acc:HGNC:12783] |
| ENSG00000163098 | HGLibA_04531 | 122 | 0.065424607 | BIRC8 | baculoviral IAP repeat containing 8 [Source:HGNC Symbol;Acc:HGNC:14878] |
| ENSG00000163746 | HGLibA_37233 | 122 | 0.065424607 | PLSCR2 | phospholipid scramblase 2 [Source:HGNC Symbol;Acc:HGNC:16494] |
| ENSG00000164023 | HGLibA_43838 | 122 | 0.065424607 | SGMS2 | sphingomyelin synthase 2 [Source:HGNC Symbol;Acc:HGNC:28395] |
| ENSG00000164742 | HGLibA_00904 | 122 | 0.065424607 | ADCY1 | adenylate cyclase 1 [Source:HGNC Symbol;Acc:HGNC:232] |
| ENSG00000165917 | HGLibA_40268 | 122 | 0.065424607 | RAPSN | receptor associated protein of the synapse [Source:HGNC Symbol;Acc:HGNC:9863] |
| ENSG00000166105 | HGLibA_19225 | 122 | 0.065424607 | GLB1L3 | galactosidase beta 1 like 3 [Source:HGNC Symbol;Acc:HGNC:25147] |
| ENSG00000166278 | HGLibA_05977 | 122 | 0.065424607 | C2 | complement C2 [Source:HGNC Symbol;Acc:HGNC:1248] |

| | | | | | |
|---|---|---|---|---|---|
| ENSG00000166743 | HGLibA_00569 | 122 | 0.065424607 | ACSM1 | acyl-CoA synthetase medium chain family member 1 [Source:HGNC Symbol;Acc:HGNC:18049] |
| ENSG00000167232 | HGLibA_56905 | 122 | 0.065424607 | ZNF91 | zinc finger protein 91 [Source:HGNC Symbol;Acc:HGNC:13166] |
| ENSG00000168256 | HGLibA_32018 | 122 | 0.065424607 | NKIRAS2 | NFKB inhibitor interacting Ras like 2 [Source:HGNC Symbol;Acc:HGNC:17898] |
| ENSG00000168477 | HGLibA_50932 | 122 | 0.065424607 | TNXB | tenascin XB [Source:HGNC Symbol;Acc:HGNC:11976] |
| ENSG00000168676 | HGLibA_24441 | 122 | 0.065424607 | KCTD19 | potassium channel tetramerization domain containing 19 [Source:HGNC Symbol;Acc:HGNC:24753] |
| ENSG00000169418 | HGLibA_32508 | 122 | 0.065424607 | NPR1 | natriuretic peptide receptor 1 [Source:HGNC Symbol;Acc:HGNC:7943] |
| ENSG00000169418 | HGLibA_32507 | 122 | 0.065424607 | NPR1 | natriuretic peptide receptor 1 [Source:HGNC Symbol;Acc:HGNC:7943] |
| ENSG00000169946 | HGLibA_55256 | 122 | 0.065424607 | ZFPM2 | FOG family member 2 [Source:HGNC Symbol;Acc:HGNC:16700]" |
| ENSG00000170011 | HGLibA_30919 | 122 | 0.065424607 | MYRIP | myosin VIIA and Rab interacting protein [Source:HGNC Symbol;Acc:HGNC:19156] |
| ENSG00000170054 | HGLibA_43532 | 122 | 0.065424607 | SERPINA9 | serpin family A member 9 [Source:HGNC Symbol;Acc:HGNC:15995] |
| ENSG00000171388 | HGLibA_02434 | 122 | 0.065424607 | APLN | apelin [Source:HGNC Symbol;Acc:HGNC:16665] |
| ENSG00000171502 | HGLibA_10548 | 122 | 0.065424607 | COL24A1 | collagen type XXIV alpha 1 chain [Source:HGNC Symbol;Acc:HGNC:20821] |
| ENSG00000172116 | HGLibA_08474 | 122 | 0.065424607 | CD8B | CD8b molecule [Source:HGNC Symbol;Acc:HGNC:1707] |
| ENSG00000172243 | HGLibA_10005 | 122 | 0.065424607 | CLEC7A | C-type lectin domain containing 7A [Source:HGNC Symbol;Acc:HGNC:14558] |
| ENSG00000173905 | HGLibA_19652 | 122 | 0.065424607 | GOLIM4 | golgi integral membrane protein 4 [Source:HGNC Symbol;Acc:HGNC:15448] |
| ENSG00000175591 | HGLibA_34884 | 122 | 0.065424607 | P2RY2 | purinergic receptor P2Y2 [Source:HGNC Symbol;Acc:HGNC:8541] |
| ENSG00000176022 | HGLibA_03965 | 122 | 0.065424607 | B3GALT6 | 3-galactosyltransferase 6 [Source:HGNC Symbol;Acc:HGNC:17978]" |
| ENSG00000176444 | HGLibA_10057 | 122 | 0.065424607 | CLK2 | CDC like kinase 2 [Source:HGNC Symbol;Acc:HGNC:2069] |
| ENSG00000177663 | HGLibA_23003 | 122 | 0.065424607 | IL17RA | interleukin 17 receptor A [Source:HGNC Symbol;Acc:HGNC:5985] |
| ENSG00000178764 | HGLibA_55315 | 122 | 0.065424607 | ZHX2 | zinc fingers and homeoboxes 2 [Source:HGNC Symbol;Acc:HGNC:18513] |
| ENSG00000179008 | HGLibA_05365 | 122 | 0.065424607 | C14orf39 | chromosome 14 open reading frame 39 [Source:HGNC Symbol;Acc:HGNC:19849] |
| ENSG00000179148 | HGLibA_01694 | 122 | 0.065424607 | ALOXE3 | arachidonate lipoxygenase 3 [Source:HGNC Symbol;Acc:HGNC:13743] |
| ENSG00000180011 | HGLibA_54770 | 122 | 0.065424607 | ZADH2 | zinc binding alcohol dehydrogenase domain containing 2 [Source:HGNC Symbol;Acc:HGNC:28697] |
| ENSG00000181085 | HGLibA_28265 | 122 | 0.065424607 | MAPK15 | mitogen-activated protein kinase 15 [Source:HGNC Symbol;Acc:HGNC:24667] |
| ENSG00000182827 | HGLibA_00398 | 122 | 0.065424607 | ACBD3 | acyl-CoA binding domain containing 3 [Source:HGNC Symbol;Acc:HGNC:15453] |
| ENSG00000182854 | HGLibA_33989 | 122 | 0.065424607 | OR4F15 | olfactory receptor family 4 subfamily F member 15 [Source:HGNC Symbol;Acc:HGNC:15078] |
| ENSG00000182890 | HGLibA_19365 | 122 | 0.065424607 | GLUD2 | glutamate dehydrogenase 2 [Source:HGNC Symbol;Acc:HGNC:4336] |
| ENSG00000183317 | HGLibA_15288 | 122 | 0.065424607 | EPHA10 | EPH receptor A10 [Source:HGNC Symbol;Acc:HGNC:19987] |
| ENSG00000183960 | HGLibA_24231 | 122 | 0.065424607 | KCNH8 | potassium voltage-gated channel subfamily H member 8 [Source:HGNC Symbol;Acc:HGNC:18864] |
| ENSG00000184140 | HGLibA_34014 | 122 | 0.065424607 | OR4F6 | olfactory receptor family 4 subfamily F member 6 [Source:HGNC Symbol;Acc:HGNC:15372] |
| ENSG00000184481 | HGLibA_17927 | 122 | 0.065424607 | FOXO4 | forkhead box O4 [Source:HGNC Symbol;Acc:HGNC:7139] |
| ENSG00000185559 | HGLibA_13353 | 122 | 0.065424607 | DLK1 | delta like non-canonical Notch ligand 1 [Source:HGNC Symbol;Acc:HGNC:2907] |
| ENSG00000186212 | HGLibA_46215 | 122 | 0.065424607 | SOWAHB | sosondowah ankyrin repeat domain family member B [Source:HGNC Symbol;Acc:HGNC:32958] |
| ENSG00000186376 | HGLibA_56681 | 122 | 0.065424607 | ZNF75D | zinc finger protein 75D [Source:HGNC Symbol;Acc:HGNC:13145] |
| ENSG00000186951 | HGLibA_37838 | 122 | 0.065424607 | PPARA | peroxisome proliferator activated receptor alpha [Source:HGNC Symbol;Acc:HGNC:9232] |
| ENSG00000187546 | HGLibA_01202 | 122 | 0.065424607 | AGMO | alkylglycerol monooxygenase [Source:HGNC Symbol;Acc:HGNC:33784] |
| ENSG00000187581 | HGLibA_10879 | 122 | 0.065424607 | COX8C | cytochrome c oxidase subunit 8C [Source:HGNC Symbol;Acc:HGNC:24382] |
| ENSG00000187715 | HGLibA_24096 | 122 | 0.065424607 | KBTBD12 | kelch repeat and BTB domain containing 12 [Source:HGNC Symbol;Acc:HGNC:25731] |
| ENSG00000196141 | HGLibA_46548 | 122 | 0.065424607 | SPATS2L | spermatogenesis associated serine rich 2 like [Source:HGNC Symbol;Acc:HGNC:24574] |
| ENSG00000196152 | HGLibA_56761 | 122 | 0.065424607 | ZNF79 | zinc finger protein 79 [Source:HGNC Symbol;Acc:HGNC:13153] |
| ENSG00000196376 | HGLibA_45002 | 122 | 0.065424607 | SLC35F1 | solute carrier family 35 member F1 [Source:HGNC Symbol;Acc:HGNC:21483] |
| ENSG00000197057 | HGLibA_14058 | 122 | 0.065424607 | DTHD1 | death domain containing 1 [Source:HGNC Symbol;Acc:HGNC:37261] |
| ENSG00000197566 | HGLibA_56417 | 122 | 0.065424607 | ZNF624 | zinc finger protein 624 [Source:HGNC Symbol;Acc:HGNC:29254] |
| ENSG00000198626 | HGLibA_42524 | 122 | 0.065424607 | RYR2 | ryanodine receptor 2 [Source:HGNC Symbol;Acc:HGNC:10484] |
| ENSG00000198682 | HGLibA_35155 | 122 | 0.065424607 | PAPSS2 | 3'-phosphoadenosine 5'-phosphosulfate synthase 2 [Source:HGNC Symbol;Acc:HGNC:8604] |
| ENSG00000198752 | HGLibA_08560 | 122 | 0.065424607 | CDC42BPB | CDC42 binding protein kinase beta [Source:HGNC Symbol;Acc:HGNC:1738] |
| ENSG00000198807 | HGLibA_35318 | 122 | 0.065424607 | PAX9 | paired box 9 [Source:HGNC Symbol;Acc:HGNC:8623] |
| ENSG00000203734 | HGLibA_14426 | 122 | 0.065424607 | ECT2L | epithelial cell transforming 2 like [Source:HGNC Symbol;Acc:HGNC:21118] |
| ENSG00000204227 | HGLibA_41298 | 122 | 0.065424607 | RING1 | ring finger protein 1 [Source:HGNC Symbol;Acc:HGNC:10018] |
| ENSG00000204397 | HGLibA_07227 | 122 | 0.065424607 | CARD16 | caspase recruitment domain family member 16 [Source:HGNC Symbol;Acc:HGNC:33701] |
| ENSG00000204657 | HGLibA_33805 | 122 | 0.065424607 | OR2H2 | olfactory receptor family 2 subfamily H member 2 [Source:HGNC Symbol;Acc:HGNC:8253] |
| ENSG00000213551 | HGLibA_13642 | 122 | 0.065424607 | DNAJC9 | DnaJ heat shock protein family (Hsp40) member C9 [Source:HGNC Symbol;Acc:HGNC:19123] |
| ENSG00000223953 | HGLibA_05947 | 122 | 0.065424607 | C1QTNF5 | C1q and TNF related 5 [Source:HGNC Symbol;Acc:HGNC:14344] |
| ENSG00000238205 | HGLibA_29704 | 122 | 0.065424607 | MPC1L | mitochondrial pyruvate carrier 1 like [Source:HGNC Symbol;Acc:HGNC:44205] |
| ENSG00000250423 | HGLibA_24711 | 122 | 0.065424607 | KIAA1210 | KIAA1210 [Source:HGNC Symbol;Acc:HGNC:29218] |
| ENSG00000255408 | HGLibA_35442 | 122 | 0.065424607 | PCDHA3 | protocadherin alpha 3 [Source:HGNC Symbol;Acc:HGNC:8669] |
| ENSG00000257138 | HGLibA_48393 | 122 | 0.065424607 | TAS2R38 | taste 2 receptor member 38 [Source:HGNC Symbol;Acc:HGNC:9584] |
| ENSG00000270181 | HGLibA_04536 | 122 | 0.065424607 | BIVM-ERCC5 | BIVM-ERCC5 readthrough [Source:HGNC Symbol;Acc:HGNC:43690] |
| ENSG00000270231 | HGLibA_31242 | 122 | 0.065424607 | NBPF8 | NBPF member 8 [Source:HGNC Symbol;Acc:HGNC:31990] |
| ENSG00000275074 | HGLibA_32961 | 122 | 0.065424607 | NUDT18 | nudix hydrolase 18 [Source:HGNC Symbol;Acc:HGNC:26194] |
| FAM196B | HGLibA_16383 | 122 | 0.065424607 | FAM196B | - |
| FAM26E | HGLibA_16563 | 122 | 0.065424607 | FAM26E | - |
| FUK | HGLibA_18162 | 122 | 0.065424607 | FUK | - |
| H2AFB2 | HGLibA_20747 | 122 | 0.065424607 | H2AFB2 | - |
| hsa-mir-1180 | HGLibA_57213 | 122 | 0.065424607 | hsa-mir-1180 | - |
| hsa-mir-181b-2 | HGLibA_57972 | 122 | 0.065424607 | hsa-mir-181b-2 | - |
| hsa-mir-196b | HGLibA_58100 | 122 | 0.065424607 | hsa-mir-196b | - |
| hsa-mir-1972-2 | HGLibA_58113 | 122 | 0.065424607 | hsa-mir-1972-2 | - |
| hsa-mir-3130-2 | HGLibA_58576 | 122 | 0.065424607 | hsa-mir-3130-2 | - |
| hsa-mir-3146 | HGLibA_58642 | 122 | 0.065424607 | hsa-mir-3146 | - |
| hsa-mir-3529 | HGLibA_59047 | 122 | 0.065424607 | hsa-mir-3529 | - |
| hsa-mir-3680-2 | HGLibA_59275 | 122 | 0.065424607 | hsa-mir-3680-2 | - |
| hsa-mir-4422 | HGLibA_60077 | 122 | 0.065424607 | hsa-mir-4422 | - |
| hsa-mir-4499 | HGLibA_60412 | 122 | 0.065424607 | hsa-mir-4499 | - |
| hsa-mir-4653 | HGLibA_60719 | 122 | 0.065424607 | hsa-mir-4653 | - |
| hsa-mir-5006 | HGLibA_61429 | 122 | 0.065424607 | hsa-mir-5006 | - |
| hsa-mir-5193 | HGLibA_61692 | 122 | 0.065424607 | hsa-mir-5193 | - |
| hsa-mir-581 | HGLibA_62424 | 122 | 0.065424607 | hsa-mir-581 | - |
| hsa-mir-586 | HGLibA_62444 | 122 | 0.065424607 | hsa-mir-586 | - |
| hsa-mir-6513 | HGLibA_62924 | 122 | 0.065424607 | hsa-mir-6513 | - |
| hsa-mir-6802 | HGLibA_63387 | 122 | 0.065424607 | hsa-mir-6802 | - |
| hsa-mir-7161 | HGLibA_63873 | 122 | 0.065424607 | hsa-mir-7161 | - |
| hsa-mir-8077 | HGLibA_64150 | 122 | 0.065424607 | hsa-mir-8077 | - |
| KIAA0247 | HGLibA_24613 | 122 | 0.065424607 | KIAA0247 | - |
| KIAA1239 | HGLibA_24724 | 122 | 0.065424607 | KIAA1239 | - |
| KIAA1704 | HGLibA_24804 | 122 | 0.065424607 | KIAA1704 | - |
| PET112 | HGLibA_36072 | 122 | 0.065424607 | PET112 | - |
| SPATA31A2 | HGLibA_46472 | 122 | 0.065424607 | SPATA31A2 | - |
| 9-Mar | HGLibA_28353 | 121 | 0.05547357 | 9-Mar | - |
| ACRC | HGLibA_00537 | 121 | 0.05547357 | ACRC | - |
| C20orf201 | HGLibA_06010 | 121 | 0.05547357 | C20orf201 | - |
| ENSG00000006062 | HGLibA_28176 | 121 | 0.05547357 | MAP3K14 | mitogen-activated protein kinase kinase kinase 14 [Source:HGNC Symbol;Acc:HGNC:6853] |
| ENSG00000006757 | HGLibA_37414 | 121 | 0.05547357 | PNPLA4 | patatin like phospholipase domain containing 4 [Source:HGNC Symbol;Acc:HGNC:24887] |
| ENSG00000010219 | HGLibA_14282 | 121 | 0.05547357 | DYRK4 | dual specificity tyrosine phosphorylation regulated kinase 4 [Source:HGNC Symbol;Acc:HGNC:3095] |
| ENSG00000020633 | HGLibA_42465 | 121 | 0.05547357 | RUNX3 | runt related transcription factor 3 [Source:HGNC Symbol;Acc:HGNC:10473] |
| ENSG00000065135 | HGLibA_19456 | 121 | 0.05547357 | GNAI3 | G protein subunit alpha i3 [Source:HGNC Symbol;Acc:HGNC:4387] |
| ENSG00000065534 | HGLibA_30783 | 121 | 0.05547357 | MYLK | myosin light chain kinase [Source:HGNC Symbol;Acc:HGNC:7590] |
| ENSG00000067365 | HGLibA_28987 | 121 | 0.05547357 | METTL22 | methyltransferase like 22 [Source:HGNC Symbol;Acc:HGNC:28368] |
| ENSG00000070190 | HGLibA_12335 | 121 | 0.05547357 | DAPP1 | dual adaptor of phosphotyrosine and 3-phosphoinositides 1 [Source:HGNC Symbol;Acc:HGNC:16500] |
| ENSG00000070748 | HGLibA_09355 | 121 | 0.05547357 | CHAT | choline O-acetyltransferase [Source:HGNC Symbol;Acc:HGNC:1912] |
| ENSG00000071189 | HGLibA_46035 | 121 | 0.05547357 | SNX13 | sorting nexin 13 [Source:HGNC Symbol;Acc:HGNC:21335] |
| ENSG00000071575 | HGLibA_51392 | 121 | 0.05547357 | TRIB2 | tribbles pseudokinase 2 [Source:HGNC Symbol;Acc:HGNC:30809] |
| ENSG00000073282 | HGLibA_51094 | 121 | 0.05547357 | TP63 | tumor protein p63 [Source:HGNC Symbol;Acc:HGNC:15979] |
| ENSG00000075336 | HGLibA_49508 | 121 | 0.05547357 | TIMM21 | translocase of inner mitochondrial membrane 21 [Source:HGNC Symbol;Acc:HGNC:25010] |
| ENSG00000079101 | HGLibA_10163 | 121 | 0.05547357 | CLUL1 | clusterin like 1 [Source:HGNC Symbol;Acc:HGNC:2096] |
| ENSG00000092098 | HGLibA_41660 | 121 | 0.05547357 | RNF31 | ring finger protein 31 [Source:HGNC Symbol;Acc:HGNC:16031] |
| ENSG00000100124 | HGLibA_02122 | 121 | 0.05547357 | ANKRD54 | ankyrin repeat domain 54 [Source:HGNC Symbol;Acc:HGNC:25185] |
| ENSG00000100246 | HGLibA_13648 | 121 | 0.05547357 | DNAL4 | dynein axonemal light chain 4 [Source:HGNC Symbol;Acc:HGNC:2955] |
| ENSG00000101438 | HGLibA_44933 | 121 | 0.05547357 | SLC32A1 | solute carrier family 32 member 1 [Source:HGNC Symbol;Acc:HGNC:11018] |
| ENSG00000102100 | HGLibA_44951 | 121 | 0.05547357 | SLC35A2 | solute carrier family 35 member A2 [Source:HGNC Symbol;Acc:HGNC:11022] |
| ENSG00000102678 | HGLibA_17424 | 121 | 0.05547357 | FGF9 | fibroblast growth factor 9 [Source:HGNC Symbol;Acc:HGNC:3687] |
| ENSG00000102897 | HGLibA_27746 | 121 | 0.05547357 | LYRM1 | LYR motif containing 1 [Source:HGNC Symbol;Acc:HGNC:25074] |
| ENSG00000103932 | HGLibA_41813 | 121 | 0.05547357 | RPAP1 | RNA polymerase II associated protein 1 [Source:HGNC Symbol;Acc:HGNC:24567] |
| ENSG00000104804 | HGLibA_52333 | 121 | 0.05547357 | TULP2 | tubby like protein 2 [Source:HGNC Symbol;Acc:HGNC:12424] |
| ENSG00000105989 | HGLibA_54359 | 121 | 0.05547357 | WNT2 | Wnt family member 2 [Source:HGNC Symbol;Acc:HGNC:12780] |
| ENSG00000106537 | HGLibA_51901 | 121 | 0.05547357 | TSPAN13 | tetraspanin 13 [Source:HGNC Symbol;Acc:HGNC:21643] |
| ENSG00000107819 | HGLibA_43786 | 121 | 0.05547357 | SFXN3 | sideroflexin 3 [Source:HGNC Symbol;Acc:HGNC:16087] |
| ENSG00000108828 | HGLibA_53542 | 121 | 0.05547357 | VAT1 | vesicle amine transport 1 [Source:HGNC Symbol;Acc:HGNC:16919] |
| ENSG00000109208 | HGLibA_45824 | 121 | 0.05547357 | SMR3A | submaxillary gland androgen regulated protein 3A [Source:HGNC Symbol;Acc:HGNC:19216] |
| ENSG00000110243 | HGLibA_02462 | 121 | 0.05547357 | APOA5 | apolipoprotein A5 [Source:HGNC Symbol;Acc:HGNC:17288] |
| ENSG00000110680 | HGLibA_06991 | 121 | 0.05547357 | CALCA | calcitonin related polypeptide alpha [Source:HGNC Symbol;Acc:HGNC:1437] |
| ENSG00000111678 | HGLibA_05269 | 121 | 0.05547357 | C12orf57 | chromosome 12 open reading frame 57 [Source:HGNC Symbol;Acc:HGNC:29521] |
| ENSG00000111877 | HGLibA_28628 | 121 | 0.05547357 | MCM9 | minichromosome maintenance 9 homologous recombination repair factor [Source:HGNC Symbol;Acc:HGNC:21484] |
| ENSG00000113211 | HGLibA_35504 | 121 | 0.05547357 | PCDHB6 | protocadherin beta 6 [Source:HGNC Symbol;Acc:HGNC:8691] |
| ENSG00000116030 | HGLibA_47776 | 121 | 0.05547357 | SUMO1 | small ubiquitin-like modifier 1 [Source:HGNC Symbol;Acc:HGNC:12502] |
| ENSG00000116539 | HGLibA_03293 | 121 | 0.05547357 | ASH1L | ASH1 like histone lysine methyltransferase [Source:HGNC Symbol;Acc:HGNC:19088] |
| ENSG00000116539 | HGLibA_03292 | 121 | 0.05547357 | ASH1L | ASH1 like histone lysine methyltransferase [Source:HGNC Symbol;Acc:HGNC:19088] |
| ENSG00000116791 | HGLibA_11293 | 121 | 0.05547357 | CRYZ | crystallin zeta [Source:HGNC Symbol;Acc:HGNC:2419] |
| ENSG00000117620 | HGLibA_44954 | 121 | 0.05547357 | SLC35A3 | solute carrier family 35 member A3 [Source:HGNC Symbol;Acc:HGNC:11023] |
| ENSG00000117707 | HGLibA_38702 | 121 | 0.05547357 | PROX1 | prospero homeobox 1 [Source:HGNC Symbol;Acc:HGNC:9459] |
| ENSG00000118733 | HGLibA_33349 | 121 | 0.05547357 | OLFM3 | olfactomedin 3 [Source:HGNC Symbol;Acc:HGNC:17990] |
| ENSG00000119715 | HGLibA_15595 | 121 | 0.05547357 | ESRRB | estrogen related receptor beta [Source:HGNC Symbol;Acc:HGNC:3473] |
| ENSG00000124279 | HGLibA_16906 | 121 | 0.05547357 | FASTKD3 | FAST kinase domains 3 [Source:HGNC Symbol;Acc:HGNC:28758] |
| ENSG00000126856 | HGLibA_38397 | 121 | 0.05547357 | PRDM7 | PR/SET domain 7 [Source:HGNC Symbol;Acc:HGNC:9351] |
| ENSG00000127663 | HGLibA_24508 | 121 | 0.05547357 | KDM4B | lysine demethylase 4B [Source:HGNC Symbol;Acc:HGNC:29136] |

| | | | | | |
|---|---|---|---|---|---|
| ENSG00000130592 | HGLibA_27563 | 121 | 0.05547357 | LSP1 | lymphocyte specific protein 1 [Source:HGNC Symbol;Acc:HGNC:6707] |
| ENSG00000131095 | HGLibA_18935 | 121 | 0.05547357 | GFAP | glial fibrillary acidic protein [Source:HGNC Symbol;Acc:HGNC:4235] |
| ENSG00000131504 | HGLibA_13216 | 121 | 0.05547357 | DIAPH1 | diaphanous related formin 1 [Source:HGNC Symbol;Acc:HGNC:2876] |
| ENSG00000131634 | HGLibA_50189 | 121 | 0.05547357 | TMEM204 | transmembrane protein 204 [Source:HGNC Symbol;Acc:HGNC:14158] |
| ENSG00000131781 | HGLibA_17714 | 121 | 0.05547357 | FMO5 | flavin containing monooxygenase 5 [Source:HGNC Symbol;Acc:HGNC:3773] |
| ENSG00000132423 | HGLibA_10753 | 121 | 0.05547357 | COQ3 | methyltransferase [Source:HGNC Symbol;Acc:HGNC:18175]" |
| ENSG00000134183 | HGLibA_19475 | 121 | 0.05547357 | GNAT2 | G protein subunit alpha transducin 2 [Source:HGNC Symbol;Acc:HGNC:4394] |
| ENSG00000135144 | HGLibA_14080 | 121 | 0.05547357 | DTX1 | deltex E3 ubiquitin ligase 1 [Source:HGNC Symbol;Acc:HGNC:3060] |
| ENSG00000135245 | HGLibA_21319 | 121 | 0.05547357 | HILPDA | hypoxia inducible lipid droplet associated [Source:HGNC Symbol;Acc:HGNC:28859] |
| ENSG00000136143 | HGLibA_47697 | 121 | 0.05547357 | SUCLA2 | succinate-CoA ligase ADP-forming beta subunit [Source:HGNC Symbol;Acc:HGNC:11448] |
| ENSG00000136715 | HGLibA_42713 | 121 | 0.05547357 | SAP130 | Sin3A associated protein 130 [Source:HGNC Symbol;Acc:HGNC:29813] |
| ENSG00000136931 | HGLibA_32634 | 121 | 0.05547357 | NR5A1 | nuclear receptor subfamily 5 group A member 1 [Source:HGNC Symbol;Acc:HGNC:7983] |
| ENSG00000137133 | HGLibA_21327 | 121 | 0.05547357 | HINT2 | histidine triad nucleotide binding protein 2 [Source:HGNC Symbol;Acc:HGNC:18344] |
| ENSG00000137504 | HGLibA_11118 | 121 | 0.05547357 | CREBZF | CREB/ATF bZIP transcription factor [Source:HGNC Symbol;Acc:HGNC:24905] |
| ENSG00000137628 | HGLibA_12762 | 121 | 0.05547357 | DDX60 | DExD/H-box helicase 60 [Source:HGNC Symbol;Acc:HGNC:25942] |
| ENSG00000138069 | HGLibA_39830 | 121 | 0.05547357 | RAB1A | member RAS oncogene family [Source:HGNC Symbol;Acc:HGNC:9758]" |
| ENSG00000138100 | HGLibA_51542 | 121 | 0.05547357 | TRIM54 | tripartite motif containing 54 [Source:HGNC Symbol;Acc:HGNC:16008] |
| ENSG00000138617 | HGLibA_35229 | 121 | 0.05547357 | PARP16 | poly(ADP-ribose) polymerase family member 16 [Source:HGNC Symbol;Acc:HGNC:26040] |
| ENSG00000138802 | HGLibA_43231 | 121 | 0.05547357 | SEC24B | COPII coat complex component [Source:HGNC Symbol;Acc:HGNC:10704]" |
| ENSG00000139899 | HGLibA_07411 | 121 | 0.05547357 | CBLN3 | cerebellin 3 precursor [Source:HGNC Symbol;Acc:HGNC:20146] |
| ENSG00000139914 | HGLibA_17541 | 121 | 0.05547357 | FITM1 | fat storage inducing transmembrane protein 1 [Source:HGNC Symbol;Acc:HGNC:33714] |
| ENSG00000142507 | HGLibA_39114 | 121 | 0.05547357 | PSMB6 | proteasome subunit beta 6 [Source:HGNC Symbol;Acc:HGNC:9543] |
| ENSG00000142583 | HGLibA_44881 | 121 | 0.05547357 | SLC2A5 | solute carrier family 2 member 5 [Source:HGNC Symbol;Acc:HGNC:11010] |
| ENSG00000142945 | HGLibA_24932 | 121 | 0.05547357 | KIF2C | kinesin family member 2C [Source:HGNC Symbol;Acc:HGNC:6393] |
| ENSG00000143226 | HGLibA_17190 | 121 | 0.05547357 | FCGR2A | Fc fragment of IgG receptor IIa [Source:HGNC Symbol;Acc:HGNC:3616] |
| ENSG00000143624 | HGLibA_23417 | 121 | 0.05547357 | INTS3 | integrator complex subunit 3 [Source:HGNC Symbol;Acc:HGNC:26153] |
| ENSG00000143801 | HGLibA_39027 | 121 | 0.05547357 | PSEN2 | presenilin 2 [Source:HGNC Symbol;Acc:HGNC:9509] |
| ENSG00000146757 | HGLibA_56908 | 121 | 0.05547357 | ZNF92 | zinc finger protein 92 [Source:HGNC Symbol;Acc:HGNC:13168] |
| ENSG00000148288 | HGLibA_18725 | 121 | 0.05547357 | GBGT1 | 3-N-acetylgalactosaminyltransferase 1 (FORS blood group) [Source:HGNC Symbol;Acc:HGNC:20460]" |
| ENSG00000148400 | HGLibA_32339 | 121 | 0.05547357 | NOTCH1 | notch 1 [Source:HGNC Symbol;Acc:HGNC:7881] |
| ENSG00000151657 | HGLibA_24980 | 121 | 0.05547357 | KIN | Kin17 DNA and RNA binding protein [Source:HGNC Symbol;Acc:HGNC:6327] |
| ENSG00000152409 | HGLibA_23969 | 121 | 0.05547357 | JMY | p53 cofactor [Source:HGNC Symbol;Acc:HGNC:28916]" |
| ENSG00000153560 | HGLibA_52729 | 121 | 0.05547357 | UBP1 | upstream binding protein 1 [Source:HGNC Symbol;Acc:HGNC:12507] |
| ENSG00000155621 | HGLibA_06768 | 121 | 0.05547357 | C9orf85 | chromosome 9 open reading frame 85 [Source:HGNC Symbol;Acc:HGNC:28784] |
| ENSG00000156218 | HGLibA_00853 | 121 | 0.05547357 | ADAMTSL3 | ADAMTS like 3 [Source:HGNC Symbol;Acc:HGNC:14633] |
| ENSG00000158710 | HGLibA_48273 | 121 | 0.05547357 | TAGLN2 | transgelin 2 [Source:HGNC Symbol;Acc:HGNC:11554] |
| ENSG00000162723 | HGLibA_44324 | 121 | 0.05547357 | SLAMF9 | SLAM family member 9 [Source:HGNC Symbol;Acc:HGNC:18430] |
| ENSG00000163104 | HGLibA_45613 | 121 | 0.05547357 | SMARCAD1 | containing DEAD/H box 1 [Source:HGNC Symbol;Acc:HGNC:18398]" |
| ENSG00000164045 | HGLibA_08526 | 121 | 0.05547357 | CDC25A | cell division cycle 25A [Source:HGNC Symbol;Acc:HGNC:1725] |
| ENSG00000164296 | HGLibA_49479 | 121 | 0.05547357 | TIGD6 | tigger transposable element derived 6 [Source:HGNC Symbol;Acc:HGNC:18332] |
| ENSG00000164411 | HGLibA_19174 | 121 | 0.05547357 | GJB7 | gap junction protein beta 7 [Source:HGNC Symbol;Acc:HGNC:16690] |
| ENSG00000165506 | HGLibA_13463 | 121 | 0.05547357 | DNAAF2 | dynein axonemal assembly factor 2 [Source:HGNC Symbol;Acc:HGNC:20188] |
| ENSG00000166405 | HGLibA_41233 | 121 | 0.05547357 | RIC3 | RIC3 acetylcholine receptor chaperone [Source:HGNC Symbol;Acc:HGNC:30338] |
| ENSG00000166484 | HGLibA_28279 | 121 | 0.05547357 | MAPK7 | mitogen-activated protein kinase 7 [Source:HGNC Symbol;Acc:HGNC:6880] |
| ENSG00000166928 | HGLibA_30139 | 121 | 0.05547357 | MS4A14 | membrane spanning 4-domains A14 [Source:HGNC Symbol;Acc:HGNC:30706] |
| ENSG00000167554 | HGLibA_56379 | 121 | 0.05547357 | ZNF610 | zinc finger protein 610 [Source:HGNC Symbol;Acc:HGNC:26687] |
| ENSG00000167895 | HGLibA_49770 | 121 | 0.05547357 | TMC8 | transmembrane channel like 8 [Source:HGNC Symbol;Acc:HGNC:20474] |
| ENSG00000168528 | HGLibA_43486 | 121 | 0.05547357 | SERINC2 | serine incorporator 2 [Source:HGNC Symbol;Acc:HGNC:23231] |
| ENSG00000168685 | HGLibA_23194 | 121 | 0.05547357 | IL7R | interleukin 7 receptor [Source:HGNC Symbol;Acc:HGNC:6024] |
| ENSG00000168925 | HGLibA_11677 | 121 | 0.05547357 | CTRB1 | chymotrypsinogen B1 [Source:HGNC Symbol;Acc:HGNC:2521] |
| ENSG00000169083 | HGLibA_02618 | 121 | 0.05547357 | AR | androgen receptor [Source:HGNC Symbol;Acc:HGNC:644] |
| ENSG00000169136 | HGLibA_03442 | 121 | 0.05547357 | ATF5 | activating transcription factor 5 [Source:HGNC Symbol;Acc:HGNC:790] |
| ENSG00000170962 | HGLibA_35849 | 121 | 0.05547357 | PDGFD | platelet derived growth factor D [Source:HGNC Symbol;Acc:HGNC:30620] |
| ENSG00000171234 | HGLibA_52930 | 121 | 0.05547357 | UGT2B7 | UDP glucuronosyltransferase family 2 member B7 [Source:HGNC Symbol;Acc:HGNC:12554] |
| ENSG00000171533 | HGLibA_28226 | 121 | 0.05547357 | MAP6 | microtubule associated protein 6 [Source:HGNC Symbol;Acc:HGNC:6868] |
| ENSG00000171786 | HGLibA_31891 | 121 | 0.05547357 | NHLH1 | nescient helix-loop-helix 1 [Source:HGNC Symbol;Acc:HGNC:7817] |
| ENSG00000172594 | HGLibA_45815 | 121 | 0.05547357 | SMPDL3A | sphingomyelin phosphodiesterase acid like 3A [Source:HGNC Symbol;Acc:HGNC:17389] |
| ENSG00000173542 | HGLibA_29588 | 121 | 0.05547357 | MOB1B | MOB kinase activator 1B [Source:HGNC Symbol;Acc:HGNC:29801] |
| ENSG00000173548 | HGLibA_46093 | 121 | 0.05547357 | SNX33 | sorting nexin 33 [Source:HGNC Symbol;Acc:HGNC:28468] |
| ENSG00000174453 | HGLibA_53926 | 121 | 0.05547357 | VWC2L | von Willebrand factor C domain containing 2 like [Source:HGNC Symbol;Acc:HGNC:37203] |
| ENSG00000175573 | HGLibA_05173 | 121 | 0.05547357 | C11orf68 | chromosome 11 open reading frame 68 [Source:HGNC Symbol;Acc:HGNC:28801] |
| ENSG00000176194 | HGLibA_09696 | 121 | 0.05547357 | CIDEA | cell death-inducing DFFA-like effector a [Source:HGNC Symbol;Acc:HGNC:1976] |
| ENSG00000176619 | HGLibA_26699 | 121 | 0.05547357 | LMNB2 | lamin B2 [Source:HGNC Symbol;Acc:HGNC:6638] |
| ENSG00000177613 | HGLibA_11490 | 121 | 0.05547357 | CSTF2T | cleavage stimulation factor subunit 2 tau variant [Source:HGNC Symbol;Acc:HGNC:17086] |
| ENSG00000177694 | HGLibA_31016 | 121 | 0.05547357 | NAALADL2 | N-acetylated alpha-linked acidic dipeptidase like 2 [Source:HGNC Symbol;Acc:HGNC:23219] |
| ENSG00000178187 | HGLibA_56033 | 121 | 0.05547357 | ZNF454 | zinc finger protein 454 [Source:HGNC Symbol;Acc:HGNC:21200] |
| ENSG00000179044 | HGLibA_15739 | 121 | 0.05547357 | EXOC3L1 | exocyst complex component 3 like 1 [Source:HGNC Symbol;Acc:HGNC:27540] |
| ENSG00000180305 | HGLibA_54246 | 121 | 0.05547357 | WFDC10A | WAP four-disulfide core domain 10A [Source:HGNC Symbol;Acc:HGNC:16139] |
| ENSG00000183389 | HGLibA_34217 | 121 | 0.05547357 | OR56A4 | olfactory receptor family 56 subfamily A member 4 [Source:HGNC Symbol;Acc:HGNC:14791] |
| ENSG00000183733 | HGLibA_17517 | 121 | 0.05547357 | FIGLA | folliculogenesis specific bHLH transcription factor [Source:HGNC Symbol;Acc:HGNC:24669] |
| ENSG00000185615 | HGLibA_35874 | 121 | 0.05547357 | PDIA2 | protein disulfide isomerase family A member 2 [Source:HGNC Symbol;Acc:HGNC:14180] |
| ENSG00000187714 | HGLibA_44491 | 121 | 0.05547357 | SLC18A3 | solute carrier family 18 member A3 [Source:HGNC Symbol;Acc:HGNC:10936] |
| ENSG00000188784 | HGLibA_36890 | 121 | 0.05547357 | PLA2G2E | phospholipase A2 group IIE [Source:HGNC Symbol;Acc:HGNC:13414] |
| ENSG00000188910 | HGLibA_19164 | 121 | 0.05547357 | GJB3 | gap junction protein beta 3 [Source:HGNC Symbol;Acc:HGNC:4285] |
| ENSG00000196224 | HGLibA_25794 | 121 | 0.05547357 | KRTAP5-3 | keratin associated protein 5-3 [Source:HGNC Symbol;Acc:HGNC:23598] |
| ENSG00000196248 | HGLibA_33545 | 121 | 0.05547357 | OR10S1 | olfactory receptor family 10 subfamily S member 1 [Source:HGNC Symbol;Acc:HGNC:14807] |
| ENSG00000197261 | HGLibA_06449 | 121 | 0.05547357 | C6orf141 | chromosome 6 open reading frame 141 [Source:HGNC Symbol;Acc:HGNC:21351] |
| ENSG00000197724 | HGLibA_36360 | 121 | 0.05547357 | PHF2 | PHD finger protein 2 [Source:HGNC Symbol;Acc:HGNC:8920] |
| ENSG00000197891 | HGLibA_44542 | 121 | 0.05547357 | SLC22A12 | solute carrier family 22 member 12 [Source:HGNC Symbol;Acc:HGNC:17989] |
| ENSG00000198400 | HGLibA_32873 | 121 | 0.05547357 | NTRK1 | neurotrophic receptor tyrosine kinase 1 [Source:HGNC Symbol;Acc:HGNC:8031] |
| ENSG00000198924 | HGLibA_12509 | 121 | 0.05547357 | DCLRE1A | DNA cross-link repair 1A [Source:HGNC Symbol;Acc:HGNC:17660] |
| ENSG00000204381 | HGLibA_26073 | 121 | 0.05547357 | LAYN | layilin [Source:HGNC Symbol;Acc:HGNC:29471] |
| ENSG00000204564 | HGLibA_06447 | 121 | 0.05547357 | C6orf136 | chromosome 6 open reading frame 136 [Source:HGNC Symbol;Acc:HGNC:21301] |
| ENSG00000204592 | HGLibA_21643 | 121 | 0.05547357 | HLA-E | E [Source:HGNC Symbol;Acc:HGNC:4962]" |
| ENSG00000213413 | HGLibA_39619 | 121 | 0.05547357 | PVRIG | PVR related immunoglobulin domain containing [Source:HGNC Symbol;Acc:HGNC:32190] |
| ENSG00000215475 | HGLibA_44111 | 121 | 0.05547357 | SIAH3 | siah E3 ubiquitin protein ligase family member 3 [Source:HGNC Symbol;Acc:HGNC:30553] |
| ENSG00000221836 | HGLibA_33741 | 121 | 0.05547357 | OR2A5 | olfactory receptor family 2 subfamily A member 5 [Source:HGNC Symbol;Acc:HGNC:8232] |
| ENSG00000221955 | HGLibA_44379 | 121 | 0.05547357 | SLC12A8 | solute carrier family 12 member 8 [Source:HGNC Symbol;Acc:HGNC:15595] |
| ENSG00000231924 | HGLibA_39034 | 121 | 0.05547357 | PSG1 | pregnancy specific beta-1-glycoprotein 1 [Source:HGNC Symbol;Acc:HGNC:9514] |
| ENSG00000233838 | HGLibA_13845 | 121 | 0.05547357 | DPH3P1 | diphthamide biosynthesis 3 pseudogene 1 [Source:HGNC Symbol;Acc:HGNC:16136] |
| ENSG00000243902 | HGLibA_14910 | 121 | 0.05547357 | ELFN2 | extracellular leucine rich repeat and fibronectin type III domain containing 2 [Source:NCBI gene;Acc:114794] |
| ENSG00000246922 | HGLibA_52549 | 121 | 0.05547357 | UBAP1L | ubiquitin associated protein 1 like [Source:HGNC Symbol;Acc:HGNC:40028] |
| ENSG00000257008 | HGLibA_19913 | 121 | 0.05547357 | GPR142 | G protein-coupled receptor 142 [Source:HGNC Symbol;Acc:HGNC:20088] |
| ENSG00000257520 | HGLibA_43764 | 121 | 0.05547357 | SFTA3 | surfactant associated 3 [Source:NCBI gene;Acc:253970] |
| ENSG00000260314 | HGLibA_29815 | 121 | 0.05547357 | MRC1 | mannose receptor C-type 1 [Source:HGNC Symbol;Acc:HGNC:7228] |
| ENSG00000260903 | HGLibA_54529 | 121 | 0.05547357 | XKR7 | XK related 7 [Source:HGNC Symbol;Acc:HGNC:23062] |
| ENSG00000270617 | HGLibA_53142 | 121 | 0.05547357 | URGCP-MRPS24 | URGCP-MRPS24 readthrough [Source:HGNC Symbol;Acc:HGNC:49188] |
| ENSG00000279761 | HGLibA_34274 | 121 | 0.05547357 | OR5D13 | olfactory receptor family 5 subfamily D member 13 (gene/pseudogene) [Source:HGNC Symbol;Acc:HGNC:15280] |
| ENSG00000280314 | HGLibA_34547 | 121 | 0.05547357 | OR8K3 | olfactory receptor family 8 subfamily K member 3 (gene/pseudogene) [Source:HGNC Symbol;Acc:HGNC:15313] |
| hsa-mir-1285-1 | HGLibA_57601 | 121 | 0.05547357 | hsa-mir-1285-1 | - |
| hsa-mir-149 | HGLibA_57886 | 121 | 0.05547357 | hsa-mir-149 | - |
| hsa-mir-205 | HGLibA_58179 | 121 | 0.05547357 | hsa-mir-205 | - |
| hsa-mir-214 | HGLibA_58247 | 121 | 0.05547357 | hsa-mir-214 | - |
| hsa-mir-3132 | HGLibA_58583 | 121 | 0.05547357 | hsa-mir-3132 | - |
| hsa-mir-3662 | HGLibA_59201 | 121 | 0.05547357 | hsa-mir-3662 | - |
| hsa-mir-3680-1 | HGLibA_59268 | 121 | 0.05547357 | hsa-mir-3680-1 | - |
| hsa-mir-376a-2 | HGLibA_59409 | 121 | 0.05547357 | hsa-mir-376a-2 | - |
| hsa-mir-4306 | HGLibA_59936 | 121 | 0.05547357 | hsa-mir-4306 | - |
| hsa-mir-4501 | HGLibA_60431 | 121 | 0.05547357 | hsa-mir-4501 | - |
| hsa-mir-4688 | HGLibA_60861 | 121 | 0.05547357 | hsa-mir-4688 | - |
| hsa-mir-4695 | HGLibA_60890 | 121 | 0.05547357 | hsa-mir-4695 | - |
| hsa-mir-4748 | HGLibA_61089 | 121 | 0.05547357 | hsa-mir-4748 | - |
| hsa-mir-499a | HGLibA_61401 | 121 | 0.05547357 | hsa-mir-499a | - |
| hsa-mir-512-2 | HGLibA_61565 | 121 | 0.05547357 | hsa-mir-512-2 | - |
| hsa-mir-520a | HGLibA_61733 | 121 | 0.05547357 | hsa-mir-520a | - |
| hsa-mir-526b | HGLibA_61800 | 121 | 0.05547357 | hsa-mir-526b | - |
| hsa-mir-548ag-2 | HGLibA_61879 | 121 | 0.05547357 | hsa-mir-548ag-2 | - |
| hsa-mir-6815 | HGLibA_63437 | 121 | 0.05547357 | hsa-mir-6815 | - |
| hsa-mir-6815 | HGLibA_63438 | 121 | 0.05547357 | hsa-mir-6815 | - |
| hsa-mir-891b | HGLibA_64247 | 121 | 0.05547357 | hsa-mir-891b | - |
| INADL | HGLibA_23272 | 121 | 0.05547357 | INADL | - |
| PLA2G16 | HGLibA_36879 | 121 | 0.05547357 | PLA2G16 | - |
| SELO | HGLibA_43301 | 121 | 0.05547357 | SELO | - |
| C6orf25 | HGLibA_06486 | 120 | 0.045522532 | C6orf25 | - |
| DFNB31 | HGLibA_13029 | 120 | 0.045522532 | DFNB31 | - |
| ENSG00000004939 | HGLibA_45202 | 120 | 0.045522532 | SLC4A1 | solute carrier family 4 member 1 (Diego blood group) [Source:HGNC Symbol;Acc:HGNC:11027] |
| ENSG00000005436 | HGLibA_18772 | 120 | 0.045522532 | GCFC2 | GC-rich sequence DNA-binding factor 2 [Source:HGNC Symbol;Acc:HGNC:1317] |
| ENSG00000009694 | HGLibA_48995 | 120 | 0.045522532 | TENM1 | teneurin transmembrane protein 1 [Source:HGNC Symbol;Acc:HGNC:8117] |
| ENSG00000012504 | HGLibA_32587 | 120 | 0.045522532 | NR1H4 | nuclear receptor subfamily 1 group H member 4 [Source:HGNC Symbol;Acc:HGNC:7967] |
| ENSG00000039319 | HGLibA_55272 | 120 | 0.045522532 | ZFYVE16 | zinc finger FYVE-type containing 16 [Source:HGNC Symbol;Acc:HGNC:20756] |
| ENSG00000044459 | HGLibA_10397 | 120 | 0.045522532 | CNTLN | centlein [Source:HGNC Symbol;Acc:HGNC:23432] |
| ENSG00000063854 | HGLibA_20815 | 120 | 0.045522532 | HAGH | hydroxyacylglutathione hydrolase [Source:HGNC Symbol;Acc:HGNC:4805] |
| ENSG00000065548 | HGLibA_54966 | 120 | 0.045522532 | ZC3H15 | zinc finger CCCH-type containing 15 [Source:HGNC Symbol;Acc:HGNC:29528] |
| ENSG00000066084 | HGLibA_13251 | 120 | 0.045522532 | DIP2B | disco interacting protein 2 homolog B [Source:HGNC Symbol;Acc:HGNC:29284] |
| ENSG00000069493 | HGLibA_09966 | 120 | 0.045522532 | CLEC2D | C-type lectin domain family 2 member D [Source:HGNC Symbol;Acc:HGNC:14351] |

| | | | | | |
|---|---|---|---|---|---|
| ENSG00000069696 | HGLibA_13969 | 120 | 0.045522532 | DRD4 | dopamine receptor D4 [Source:HGNC Symbol;Acc:HGNC:3025] |
| ENSG00000069943 | HGLibA_36547 | 120 | 0.045522532 | PIGB | phosphatidylinositol glycan anchor biosynthesis class B [Source:HGNC Symbol;Acc:HGNC:8959] |
| ENSG00000077327 | HGLibA_46364 | 120 | 0.045522532 | SPAG6 | sperm associated antigen 6 [Source:HGNC Symbol;Acc:HGNC:11215] |
| ENSG00000077935 | HGLibA_45645 | 120 | 0.045522532 | SMC1B | structural maintenance of chromosomes 1B [Source:HGNC Symbol;Acc:HGNC:11112] |
| ENSG00000079931 | HGLibA_29699 | 120 | 0.045522532 | MOXD1 | monooxygenase DBH like 1 [Source:HGNC Symbol;Acc:HGNC:21063] |
| ENSG00000085733 | HGLibA_11739 | 120 | 0.045522532 | CTTN | cortactin [Source:HGNC Symbol;Acc:HGNC:3338] |
| ENSG00000088386 | HGLibA_44404 | 120 | 0.045522532 | SLC15A1 | solute carrier family 15 member 1 [Source:HGNC Symbol;Acc:HGNC:10920] |
| ENSG00000089159 | HGLibA_39664 | 120 | 0.045522532 | PXN | paxillin [Source:HGNC Symbol;Acc:HGNC:9718] |
| ENSG00000090054 | HGLibA_46891 | 120 | 0.045522532 | SPTLC1 | serine palmitoyltransferase long chain base subunit 1 [Source:HGNC Symbol;Acc:HGNC:11277] |
| ENSG00000095981 | HGLibA_24308 | 120 | 0.045522532 | KCNK16 | potassium two pore domain channel subfamily K member 16 [Source:HGNC Symbol;Acc:HGNC:14464] |
| ENSG00000100325 | HGLibA_03259 | 120 | 0.045522532 | ASCC2 | activating signal cointegrator 1 complex subunit 2 [Source:HGNC Symbol;Acc:HGNC:24103] |
| ENSG00000101000 | HGLibA_38656 | 120 | 0.045522532 | PROCR | protein C receptor [Source:HGNC Symbol;Acc:HGNC:9452] |
| ENSG00000101187 | HGLibA_45470 | 120 | 0.045522532 | SLCO4A1 | solute carrier organic anion transporter family member 4A1 [Source:HGNC Symbol;Acc:HGNC:10953] |
| ENSG00000101331 | HGLibA_08047 | 120 | 0.045522532 | CCM2L | CCM2 like scaffold protein [Source:HGNC Symbol;Acc:HGNC:16153] |
| ENSG00000102934 | HGLibA_37202 | 120 | 0.045522532 | PLLP | plasmolipin [Source:HGNC Symbol;Acc:HGNC:18553] |
| ENSG00000102970 | HGLibA_07979 | 120 | 0.045522532 | CCL17 | C-C motif chemokine ligand 17 [Source:HGNC Symbol;Acc:HGNC:10615] |
| ENSG00000103043 | HGLibA_53485 | 120 | 0.045522532 | VAC14 | PIKFYVE complex component [Source:HGNC Symbol;Acc:HGNC:25507]" |
| ENSG00000103343 | HGLibA_55526 | 120 | 0.045522532 | ZNF174 | zinc finger protein 174 [Source:HGNC Symbol;Acc:HGNC:12963] |
| ENSG00000104375 | HGLibA_47476 | 120 | 0.045522532 | STK3 | serine/threonine kinase 3 [Source:HGNC Symbol;Acc:HGNC:11406] |
| ENSG00000105514 | HGLibA_39915 | 120 | 0.045522532 | RAB3D | member RAS oncogene family [Source:HGNC Symbol;Acc:HGNC:9779]" |
| ENSG00000105991 | HGLibA_21892 | 120 | 0.045522532 | HOXA1 | homeobox A1 [Source:HGNC Symbol;Acc:HGNC:5099] |
| ENSG00000108825 | HGLibA_39336 | 120 | 0.045522532 | PTGES3L-AARSD1 | PTGES3L-AARSD1 readthrough [Source:HGNC Symbol;Acc:HGNC:43946] |
| ENSG00000112280 | HGLibA_10620 | 120 | 0.045522532 | COL9A1 | collagen type IX alpha 1 chain [Source:HGNC Symbol;Acc:HGNC:2217] |
| ENSG00000115091 | HGLibA_00659 | 120 | 0.045522532 | ACTR3 | ARP3 actin related protein 3 homolog [Source:HGNC Symbol;Acc:HGNC:170] |
| ENSG00000115275 | HGLibA_29634 | 120 | 0.045522532 | MOGS | mannosyl-oligosaccharide glucosidase [Source:HGNC Symbol;Acc:HGNC:24862] |
| ENSG00000118515 | HGLibA_43817 | 120 | 0.045522532 | SGK1 | serum/glucocorticoid regulated kinase 1 [Source:HGNC Symbol;Acc:HGNC:10810] |
| ENSG00000120563 | HGLibA_27785 | 120 | 0.045522532 | LYZL1 | lysozyme like 1 [Source:HGNC Symbol;Acc:HGNC:30502] |
| ENSG00000122254 | HGLibA_22125 | 120 | 0.045522532 | HS3ST2 | heparan sulfate-glucosamine 3-sulfotransferase 2 [Source:HGNC Symbol;Acc:HGNC:5195] |
| ENSG00000122691 | HGLibA_52376 | 120 | 0.045522532 | TWIST1 | twist family bHLH transcription factor 1 [Source:HGNC Symbol;Acc:HGNC:12428] |
| ENSG00000122729 | HGLibA_00460 | 120 | 0.045522532 | ACO1 | aconitase 1 [Source:HGNC Symbol;Acc:HGNC:117] |
| ENSG00000123999 | HGLibA_23298 | 120 | 0.045522532 | INHA | inhibin subunit alpha [Source:HGNC Symbol;Acc:HGNC:6065] |
| ENSG00000125813 | HGLibA_35296 | 120 | 0.045522532 | PAX1 | paired box 1 [Source:HGNC Symbol;Acc:HGNC:8615] |
| ENSG00000125843 | HGLibA_02358 | 120 | 0.045522532 | AP5S1 | adaptor related protein complex 5 subunit sigma 1 [Source:HGNC Symbol;Acc:HGNC:15875] |
| ENSG00000126001 | HGLibA_09129 | 120 | 0.045522532 | CEP250 | centrosomal protein 250 [Source:HGNC Symbol;Acc:HGNC:1859] |
| ENSG00000126821 | HGLibA_43849 | 120 | 0.045522532 | SGPP1 | sphingosine-1-phosphate phosphatase 1 [Source:HGNC Symbol;Acc:HGNC:17720] |
| ENSG00000127540 | HGLibA_53107 | 120 | 0.045522532 | UQCR11 | complex III subunit XI [Source:HGNC Symbol;Acc:HGNC:30862]" |
| ENSG00000127780 | HGLibA_33663 | 120 | 0.045522532 | OR1E2 | olfactory receptor family 1 subfamily E member 2 [Source:HGNC Symbol;Acc:HGNC:8190] |
| ENSG00000127914 | HGLibA_01450 | 120 | 0.045522532 | AKAP9 | A-kinase anchoring protein 9 [Source:HGNC Symbol;Acc:HGNC:379] |
| ENSG00000127993 | HGLibA_40570 | 120 | 0.045522532 | RBM48 | RNA binding motif protein 48 [Source:HGNC Symbol;Acc:HGNC:21785] |
| ENSG00000128310 | HGLibA_18592 | 120 | 0.045522532 | GALR3 | galanin receptor 3 [Source:HGNC Symbol;Acc:HGNC:4134] |
| ENSG00000128607 | HGLibA_25093 | 120 | 0.045522532 | KLHDC10 | kelch domain containing 10 [Source:HGNC Symbol;Acc:HGNC:22194] |
| ENSG00000128944 | HGLibA_25347 | 120 | 0.045522532 | KNSTRN | kinetochore localized astrin (SPAG5) binding protein [Source:HGNC Symbol;Acc:HGNC:30767] |
| ENSG00000129467 | HGLibA_00917 | 120 | 0.045522532 | ADCY4 | adenylate cyclase 4 [Source:HGNC Symbol;Acc:HGNC:235] |
| ENSG00000130720 | HGLibA_17499 | 120 | 0.045522532 | FIBCD1 | fibrinogen C domain containing 1 [Source:HGNC Symbol;Acc:HGNC:25922] |
| ENSG00000131061 | HGLibA_55840 | 120 | 0.045522532 | ZNF341 | zinc finger protein 341 [Source:HGNC Symbol;Acc:HGNC:15992] |
| ENSG00000131127 | HGLibA_55485 | 120 | 0.045522532 | ZNF141 | zinc finger protein 141 [Source:HGNC Symbol;Acc:HGNC:12926] |
| ENSG00000132819 | HGLibA_40538 | 120 | 0.045522532 | RBM38 | RNA binding motif protein 38 [Source:HGNC Symbol;Acc:HGNC:15818] |
| ENSG00000135045 | HGLibA_06727 | 120 | 0.045522532 | C9orf40 | chromosome 9 open reading frame 40 [Source:HGNC Symbol;Acc:HGNC:23433] |
| ENSG00000135457 | HGLibA_49144 | 120 | 0.045522532 | TFCP2 | transcription factor CP2 [Source:HGNC Symbol;Acc:HGNC:11748] |
| ENSG00000135736 | HGLibA_07509 | 120 | 0.045522532 | CCDC102A | coiled-coil domain containing 102A [Source:HGNC Symbol;Acc:HGNC:28097] |
| ENSG00000135951 | HGLibA_51847 | 120 | 0.045522532 | TSGA10 | testis specific 10 [Source:HGNC Symbol;Acc:HGNC:14927] |
| ENSG00000136695 | HGLibA_23155 | 120 | 0.045522532 | IL36RN | interleukin 36 receptor antagonist [Source:HGNC Symbol;Acc:HGNC:15561] |
| ENSG00000136918 | HGLibA_54082 | 120 | 0.045522532 | WDR38 | WD repeat domain 38 [Source:HGNC Symbol;Acc:HGNC:23745] |
| ENSG00000137225 | HGLibA_07149 | 120 | 0.045522532 | CAPN11 | calpain 11 [Source:HGNC Symbol;Acc:HGNC:1478] |
| ENSG00000137699 | HGLibA_51452 | 120 | 0.045522532 | TRIM29 | tripartite motif containing 29 [Source:HGNC Symbol;Acc:HGNC:17274] |
| ENSG00000138735 | HGLibA_35802 | 120 | 0.045522532 | PDE5A | phosphodiesterase 5A [Source:HGNC Symbol;Acc:HGNC:8784] |
| ENSG00000139144 | HGLibA_36628 | 120 | 0.045522532 | PIK3C2G | phosphatidylinositol-4-phosphate 3-kinase catalytic subunit type 2 gamma [Source:HGNC Symbol;Acc:HGNC:8973] |
| ENSG00000139679 | HGLibA_27067 | 120 | 0.045522532 | LPAR6 | lysophosphatidic acid receptor 6 [Source:HGNC Symbol;Acc:HGNC:15520] |
| ENSG00000139998 | HGLibA_39818 | 120 | 0.045522532 | RAB15 | member RAS oncogene family [Source:HGNC Symbol;Acc:HGNC:20150]" |
| ENSG00000140443 | HGLibA_22794 | 120 | 0.045522532 | IGF1R | insulin like growth factor 1 receptor [Source:HGNC Symbol;Acc:HGNC:5465] |
| ENSG00000143546 | HGLibA_42572 | 120 | 0.045522532 | S100A8 | S100 calcium binding protein A8 [Source:HGNC Symbol;Acc:HGNC:10498] |
| ENSG00000145246 | HGLibA_03551 | 120 | 0.045522532 | ATP10D | ATPase phospholipid transporting 10D (putative) [Source:HGNC Symbol;Acc:HGNC:13549] |
| ENSG00000145743 | HGLibA_16988 | 120 | 0.045522532 | FBXL17 | F-box and leucine rich repeat protein 17 [Source:HGNC Symbol;Acc:HGNC:13615] |
| ENSG00000147202 | HGLibA_13219 | 120 | 0.045522532 | DIAPH2 | diaphanous related formin 2 [Source:HGNC Symbol;Acc:HGNC:2877] |
| ENSG00000150093 | HGLibA_23768 | 120 | 0.045522532 | ITGB1 | integrin subunit beta 1 [Source:HGNC Symbol;Acc:HGNC:6153] |
| ENSG00000152795 | HGLibA_21815 | 120 | 0.045522532 | HNRNPDL | heterogeneous nuclear ribonucleoprotein D like [Source:HGNC Symbol;Acc:HGNC:5037] |
| ENSG00000153093 | HGLibA_00506 | 120 | 0.045522532 | ACOXL | acyl-CoA oxidase like [Source:HGNC Symbol;Acc:HGNC:25621] |
| ENSG00000153767 | HGLibA_20533 | 120 | 0.045522532 | GTF2E1 | general transcription factor IIE subunit 1 [Source:HGNC Symbol;Acc:HGNC:4650] |
| ENSG00000154188 | HGLibA_01902 | 120 | 0.045522532 | ANGPT1 | angiopoietin 1 [Source:HGNC Symbol;Acc:HGNC:484] |
| ENSG00000154305 | HGLibA_29190 | 120 | 0.045522532 | MIA3 | MIA SH3 domain ER export factor 3 [Source:HGNC Symbol;Acc:HGNC:24008] |
| ENSG00000156689 | HGLibA_19375 | 120 | 0.045522532 | GLYATL2 | glycine-N-acyltransferase like 2 [Source:HGNC Symbol;Acc:HGNC:24178] |
| ENSG00000157093 | HGLibA_27791 | 120 | 0.045522532 | LYZL4 | lysozyme like 4 [Source:HGNC Symbol;Acc:HGNC:28387] |
| ENSG00000157315 | HGLibA_49816 | 120 | 0.045522532 | TMED6 | transmembrane p24 trafficking protein 6 [Source:HGNC Symbol;Acc:HGNC:28331] |
| ENSG00000157423 | HGLibA_22450 | 120 | 0.045522532 | HYDIN | axonemal central pair apparatus protein [Source:HGNC Symbol;Acc:HGNC:19368]" |
| ENSG00000161956 | HGLibA_43392 | 120 | 0.045522532 | SENP3 | SUMO specific peptidase 3 [Source:HGNC Symbol;Acc:HGNC:17862] |
| ENSG00000162399 | HGLibA_04872 | 120 | 0.045522532 | BSND | barttin CLCNK type accessory beta subunit [Source:HGNC Symbol;Acc:HGNC:16512] |
| ENSG00000162409 | HGLibA_38493 | 120 | 0.045522532 | PRKAA2 | protein kinase AMP-activated catalytic subunit alpha 2 [Source:HGNC Symbol;Acc:HGNC:9377] |
| ENSG00000163098 | HGLibA_04530 | 120 | 0.045522532 | BIRC8 | baculoviral IAP repeat containing 8 [Source:HGNC Symbol;Acc:HGNC:14878] |
| ENSG00000163581 | HGLibA_44871 | 120 | 0.045522532 | SLC2A2 | solute carrier family 2 member 2 [Source:HGNC Symbol;Acc:HGNC:11006] |
| ENSG00000163611 | HGLibA_46661 | 120 | 0.045522532 | SPICE1 | spindle and centriole associated protein 1 [Source:HGNC Symbol;Acc:HGNC:25083] |
| ENSG00000163644 | HGLibA_37986 | 120 | 0.045522532 | PPM1K | Mg2+/Mn2+ dependent 1K [Source:HGNC Symbol;Acc:HGNC:25415]" |
| ENSG00000163810 | HGLibA_49261 | 120 | 0.045522532 | TGM4 | transglutaminase 4 [Source:HGNC Symbol;Acc:HGNC:11780] |
| ENSG00000164284 | HGLibA_20373 | 120 | 0.045522532 | GRPEL2 | mitochondrial [Source:HGNC Symbol;Acc:HGNC:21060]" |
| ENSG00000164331 | HGLibA_01984 | 120 | 0.045522532 | ANKRA2 | ankyrin repeat family A member 2 [Source:HGNC Symbol;Acc:HGNC:13208] |
| ENSG00000164548 | HGLibA_51221 | 120 | 0.045522532 | TRA2A | transformer 2 alpha homolog [Source:HGNC Symbol;Acc:HGNC:16645] |
| ENSG00000165304 | HGLibA_28880 | 120 | 0.045522532 | MELK | maternal embryonic leucine zipper kinase [Source:HGNC Symbol;Acc:HGNC:16870] |
| ENSG00000165392 | HGLibA_54410 | 120 | 0.045522532 | WRN | Werner syndrome RecQ like helicase [Source:HGNC Symbol;Acc:HGNC:12791] |
| ENSG00000165935 | HGLibA_45665 | 120 | 0.045522532 | SMCO2 | single-pass membrane protein with coiled-coil domains 2 [Source:HGNC Symbol;Acc:HGNC:34448] |
| ENSG00000166173 | HGLibA_26040 | 120 | 0.045522532 | LARP6 | La ribonucleoprotein domain family member 6 [Source:HGNC Symbol;Acc:HGNC:24012] |
| ENSG00000167183 | HGLibA_38788 | 120 | 0.045522532 | PRR15L | proline rich 15 like [Source:HGNC Symbol;Acc:HGNC:28149] |
| ENSG00000167978 | HGLibA_47049 | 120 | 0.045522532 | SRRM2 | serine/arginine repetitive matrix 2 [Source:HGNC Symbol;Acc:HGNC:16639] |
| ENSG00000168071 | HGLibA_07911 | 120 | 0.045522532 | CCDC88B | coiled-coil domain containing 88B [Source:HGNC Symbol;Acc:HGNC:26757] |
| ENSG00000168411 | HGLibA_40958 | 120 | 0.045522532 | RFWD3 | ring finger and WD repeat domain 3 [Source:HGNC Symbol;Acc:HGNC:25539] |
| ENSG00000169154 | HGLibA_19694 | 120 | 0.045522532 | GOT1L1 | glutamic-oxaloacetic transaminase 1 like 1 [Source:HGNC Symbol;Acc:HGNC:28487] |
| ENSG00000169181 | HGLibA_20421 | 120 | 0.045522532 | GSG1L | GSG1 like [Source:HGNC Symbol;Acc:HGNC:28283] |
| ENSG00000169188 | HGLibA_02410 | 120 | 0.045522532 | APEX2 | apurinic/apyrimidinic endodeoxyribonuclease 2 [Source:HGNC Symbol;Acc:HGNC:17889] |
| ENSG00000169717 | HGLibA_00679 | 120 | 0.045522532 | ACTRT2 | actin related protein T2 [Source:HGNC Symbol;Acc:HGNC:24026] |
| ENSG00000169962 | HGLibA_48361 | 120 | 0.045522532 | TAS1R3 | taste 1 receptor member 3 [Source:HGNC Symbol;Acc:HGNC:15661] |
| ENSG00000169994 | HGLibA_30865 | 120 | 0.045522532 | MYO7B | myosin VIIB [Source:HGNC Symbol;Acc:HGNC:7607] |
| ENSG00000171163 | HGLibA_56554 | 120 | 0.045522532 | ZNF692 | zinc finger protein 692 [Source:HGNC Symbol;Acc:HGNC:26049] |
| ENSG00000171316 | HGLibA_09403 | 120 | 0.045522532 | CHD7 | chromodomain helicase DNA binding protein 7 [Source:HGNC Symbol;Acc:HGNC:20626] |
| ENSG00000172671 | HGLibA_55156 | 120 | 0.045522532 | ZFAND4 | zinc finger AN1-type containing 4 [Source:HGNC Symbol;Acc:HGNC:23504] |
| ENSG00000172869 | HGLibA_13454 | 120 | 0.045522532 | DMXL1 | Dmx like 1 [Source:HGNC Symbol;Acc:HGNC:2937] |
| ENSG00000173875 | HGLibA_56767 | 120 | 0.045522532 | ZNF791 | zinc finger protein 791 [Source:HGNC Symbol;Acc:HGNC:26895] |
| ENSG00000175084 | HGLibA_13004 | 120 | 0.045522532 | DES | desmin [Source:HGNC Symbol;Acc:HGNC:2770] |
| ENSG00000175414 | HGLibA_02923 | 120 | 0.045522532 | ARL10 | ADP ribosylation factor like GTPase 10 [Source:HGNC Symbol;Acc:HGNC:22042] |
| ENSG00000175567 | HGLibA_52833 | 120 | 0.045522532 | UCP2 | uncoupling protein 2 [Source:HGNC Symbol;Acc:HGNC:12518] |
| ENSG00000177103 | HGLibA_14001 | 120 | 0.045522532 | DSCAML1 | DS cell adhesion molecule like 1 [Source:HGNC Symbol;Acc:HGNC:14656] |
| ENSG00000178028 | HGLibA_13390 | 120 | 0.045522532 | DMAP1 | DNA methyltransferase 1 associated protein 1 [Source:HGNC Symbol;Acc:HGNC:18291] |
| ENSG00000178125 | HGLibA_38118 | 120 | 0.045522532 | PPP1R42 | protein phosphatase 1 regulatory subunit 42 [Source:HGNC Symbol;Acc:HGNC:33732] |
| ENSG00000179335 | HGLibA_10061 | 120 | 0.045522532 | CLK3 | CDC like kinase 3 [Source:HGNC Symbol;Acc:HGNC:2071] |
| ENSG00000180263 | HGLibA_17357 | 120 | 0.045522532 | FGD6 | RhoGEF and PH domain containing 6 [Source:HGNC Symbol;Acc:HGNC:21740]" |
| ENSG00000180628 | HGLibA_35601 | 120 | 0.045522532 | PCGF5 | polycomb group ring finger 5 [Source:HGNC Symbol;Acc:HGNC:28264] |
| ENSG00000180878 | HGLibA_05139 | 120 | 0.045522532 | C11orf42 | chromosome 11 open reading frame 42 [Source:HGNC Symbol;Acc:HGNC:28541] |
| ENSG00000184979 | HGLibA_53290 | 120 | 0.045522532 | USP18 | ubiquitin specific peptidase 18 [Source:HGNC Symbol;Acc:HGNC:12616] |
| ENSG00000186458 | HGLibA_12899 | 120 | 0.045522532 | DEFB132 | defensin beta 132 [Source:HGNC Symbol;Acc:HGNC:33806] |
| ENSG00000187537 | HGLibA_37734 | 120 | 0.045522532 | POTEG | POTE ankyrin domain family member G [Source:HGNC Symbol;Acc:HGNC:33896] |
| ENSG00000187715 | HGLibA_24097 | 120 | 0.045522532 | KBTBD12 | kelch repeat and BTB domain containing 12 [Source:HGNC Symbol;Acc:HGNC:25731] |
| ENSG00000188419 | HGLibA_09466 | 120 | 0.045522532 | CHM | Rab escort protein 1 [Source:HGNC Symbol;Acc:HGNC:1940]" |
| ENSG00000188958 | HGLibA_53465 | 120 | 0.045522532 | UTS2B | urotensin 2B [Source:HGNC Symbol;Acc:HGNC:30894] |
| ENSG00000196663 | HGLibA_48943 | 120 | 0.045522532 | TECPR2 | tectonin beta-propeller repeat containing 2 [Source:HGNC Symbol;Acc:HGNC:19957] |
| ENSG00000196975 | HGLibA_02239 | 120 | 0.045522532 | ANXA4 | annexin A4 [Source:HGNC Symbol;Acc:HGNC:542] |
| ENSG00000197217 | HGLibA_15207 | 120 | 0.045522532 | ENTPD4 | ectonucleoside triphosphate diphosphohydrolase 4 [Source:HGNC Symbol;Acc:HGNC:14573] |
| ENSG00000197724 | HGLibA_36359 | 120 | 0.045522532 | PHF2 | PHD finger protein 2 [Source:HGNC Symbol;Acc:HGNC:8920] |
| ENSG00000198055 | HGLibA_20335 | 120 | 0.045522532 | GRK6 | G protein-coupled receptor kinase 6 [Source:HGNC Symbol;Acc:HGNC:4545] |
| ENSG00000203883 | HGLibA_46250 | 120 | 0.045522532 | SOX18 | SRY-box 18 [Source:HGNC Symbol;Acc:HGNC:11194] |
| ENSG00000204052 | HGLibA_27426 | 120 | 0.045522532 | LRRC73 | leucine rich repeat containing 73 [Source:HGNC Symbol;Acc:HGNC:21375] |
| ENSG00000205212 | HGLibA_07626 | 120 | 0.045522532 | CCDC144NL | N-terminal like [Source:HGNC Symbol;Acc:HGNC:33735]" |
| ENSG00000205221 | HGLibA_53673 | 120 | 0.045522532 | VIT | vitrin [Source:HGNC Symbol;Acc:HGNC:12697] |
| ENSG00000206140 | HGLibA_50146 | 120 | 0.045522532 | TMEM191C | transmembrane protein 191C [Source:HGNC Symbol;Acc:HGNC:33601] |
| ENSG00000214113 | HGLibA_27753 | 120 | 0.045522532 | LYRM4 | LYR motif containing 4 [Source:HGNC Symbol;Acc:HGNC:21365] |
| ENSG00000214518 | HGLibA_25690 | 120 | 0.045522532 | KRTAP2-2 | keratin associated protein 2-2 [Source:HGNC Symbol;Acc:HGNC:18905] |
| ENSG00000226979 | HGLibA_27576 | 120 | 0.045522532 | LTA | lymphotoxin alpha [Source:HGNC Symbol;Acc:HGNC:6709] |
| ENSG00000231192 | HGLibA_34288 | 120 | 0.045522532 | OR5H1 | olfactory receptor family 5 subfamily H member 1 [Source:HGNC Symbol;Acc:HGNC:8346] |
| ENSG00000231738 | HGLibA_51919 | 120 | 0.045522532 | TSPAN19 | tetraspanin 19 [Source:HGNC Symbol;Acc:HGNC:31886] |

| | | | | | |
|---|---|---|---|---|---|
| ENSG00000234906 | HGLibA_02505 | 120 | 0.045522532 | APOC2 | apolipoprotein C2 [Source:HGNC Symbol;Acc:HGNC:609] |
| ENSG00000236980 | HGLibA_06278 | 120 | 0.045522532 | C3orf84 | chromosome 3 open reading frame 84 [Source:HGNC Symbol;Acc:HGNC:44666] |
| ENSG00000237172 | HGLibA_04006 | 120 | 0.045522532 | B3GNT9 | 3-N-acetylglucosaminyltransferase 9 [Source:HGNC Symbol;Acc:HGNC:28714]" |
| ENSG00000254470 | HGLibA_02354 | 120 | 0.045522532 | AP5B1 | adaptor related protein complex 5 subunit beta 1 [Source:HGNC Symbol;Acc:HGNC:25104] |
| ENSG00000257315 | HGLibA_54803 | 120 | 0.045522532 | ZBED6 | zinc finger BED-type containing 6 [Source:HGNC Symbol;Acc:HGNC:33273] |
| ENSG00000261052 | HGLibA_47736 | 120 | 0.045522532 | SULT1A3 | sulfotransferase family 1A member 3 [Source:HGNC Symbol;Acc:HGNC:11455] |
| ENSG00000279514 | HGLibA_33949 | 120 | 0.045522532 | OR4C16 | olfactory receptor family 4 subfamily C member 16 (gene/pseudogene) [Source:HGNC Symbol;Acc:HGNC:15172] |
| hsa-mir-103b-2 | HGLibA_57175 | 120 | 0.045522532 | hsa-mir-103b-2 | - |
| hsa-mir-1205 | HGLibA_57279 | 120 | 0.045522532 | hsa-mir-1205 | - |
| hsa-mir-129-2 | HGLibA_57632 | 120 | 0.045522532 | hsa-mir-129-2 | - |
| hsa-mir-19b-2 | HGLibA_58147 | 120 | 0.045522532 | hsa-mir-19b-2 | - |
| hsa-mir-301a | HGLibA_58430 | 120 | 0.045522532 | hsa-mir-301a | - |
| hsa-mir-3713 | HGLibA_59366 | 120 | 0.045522532 | hsa-mir-3713 | - |
| hsa-mir-3925 | HGLibA_59573 | 120 | 0.045522532 | hsa-mir-3925 | - |
| hsa-mir-4442 | HGLibA_60169 | 120 | 0.045522532 | hsa-mir-4442 | - |
| hsa-mir-4694 | HGLibA_60884 | 120 | 0.045522532 | hsa-mir-4694 | - |
| hsa-mir-4750 | HGLibA_61099 | 120 | 0.045522532 | hsa-mir-4750 | - |
| hsa-mir-592 | HGLibA_62466 | 120 | 0.045522532 | hsa-mir-592 | - |
| hsa-mir-6510 | HGLibA_62895 | 120 | 0.045522532 | hsa-mir-6510 | - |
| hsa-mir-6805 | HGLibA_63398 | 120 | 0.045522532 | hsa-mir-6805 | - |
| hsa-mir-6885 | HGLibA_63725 | 120 | 0.045522532 | hsa-mir-6885 | - |
| hsa-mir-7159 | HGLibA_63865 | 120 | 0.045522532 | hsa-mir-7159 | - |
| LEPREL2 | HGLibA_26320 | 120 | 0.045522532 | LEPREL2 | - |
| TMEM189-UBE2V1 | HGLibA_50136 | 120 | 0.045522532 | TMEM189-UBE2V1 | - |
| ZFP112 | HGLibA_55186 | 120 | 0.045522532 | ZFP112 | - |
| C6orf222 | HGLibA_06475 | 119 | 0.035571495 | C6orf222 | - |
| C8orf31 | HGLibA_06604 | 119 | 0.035571495 | C8orf31 | - |
| C9orf62 | HGLibA_06745 | 119 | 0.035571495 | C9orf62 | - |
| ENSG00000003400 | HGLibA_07298 | 119 | 0.035571495 | CASP10 | caspase 10 [Source:HGNC Symbol;Acc:HGNC:1500] |
| ENSG00000006607 | HGLibA_16877 | 119 | 0.035571495 | FARP2 | ARH/RhoGEF and pleckstrin domain protein 2 [Source:HGNC Symbol;Acc:HGNC:16460]" |
| ENSG00000013288 | HGLibA_28080 | 119 | 0.035571495 | MAN2B2 | mannosidase alpha class 2B member 2 [Source:HGNC Symbol;Acc:HGNC:29623] |
| ENSG00000054938 | HGLibA_09541 | 119 | 0.035571495 | CHRDL2 | chordin like 2 [Source:HGNC Symbol;Acc:HGNC:24168] |
| ENSG00000057704 | HGLibA_49777 | 119 | 0.035571495 | TMCC3 | transmembrane and coiled-coil domain family 3 [Source:HGNC Symbol;Acc:HGNC:29199] |
| ENSG00000062524 | HGLibA_27611 | 119 | 0.035571495 | LTK | leukocyte receptor tyrosine kinase [Source:HGNC Symbol;Acc:HGNC:6721] |
| ENSG00000063515 | HGLibA_20402 | 119 | 0.035571495 | GSC2 | goosecoid homeobox 2 [Source:HGNC Symbol;Acc:HGNC:4613] |
| ENSG00000065717 | HGLibA_49609 | 119 | 0.035571495 | TLE2 | transducin like enhancer of split 2 [Source:HGNC Symbol;Acc:HGNC:11838] |
| ENSG00000065923 | HGLibA_45424 | 119 | 0.035571495 | SLC9A7 | solute carrier family 9 member A7 [Source:HGNC Symbol;Acc:HGNC:17123] |
| ENSG00000066027 | HGLibA_38171 | 119 | 0.035571495 | PPP2R5A | protein phosphatase 2 regulatory subunit B'alpha [Source:HGNC Symbol;Acc:HGNC:9309] |
| ENSG00000073150 | HGLibA_35111 | 119 | 0.035571495 | PANX2 | pannexin 2 [Source:HGNC Symbol;Acc:HGNC:8600] |
| ENSG00000085831 | HGLibA_52133 | 119 | 0.035571495 | TTC39A | tetratricopeptide repeat domain 39A [Source:HGNC Symbol;Acc:HGNC:18657] |
| ENSG00000088305 | HGLibA_13704 | 119 | 0.035571495 | DNMT3B | DNA methyltransferase 3 beta [Source:HGNC Symbol;Acc:HGNC:2979] |
| ENSG00000099875 | HGLibA_29346 | 119 | 0.035571495 | MKNK2 | MAP kinase interacting serine/threonine kinase 2 [Source:HGNC Symbol;Acc:HGNC:7111] |
| ENSG00000100360 | HGLibA_22756 | 119 | 0.035571495 | IFT27 | intraflagellar transport 27 [Source:HGNC Symbol;Acc:HGNC:18626] |
| ENSG00000100505 | HGLibA_51618 | 119 | 0.035571495 | TRIM9 | tripartite motif containing 9 [Source:HGNC Symbol;Acc:HGNC:16288] |
| ENSG00000101158 | HGLibA_31678 | 119 | 0.035571495 | NELFCD | negative elongation factor complex member C/D [Source:HGNC Symbol;Acc:HGNC:15934] |
| ENSG00000101448 | HGLibA_15357 | 119 | 0.035571495 | EPPIN | epididymal peptidase inhibitor [Source:HGNC Symbol;Acc:HGNC:15932] |
| ENSG00000101680 | HGLibA_25940 | 119 | 0.035571495 | LAMA1 | laminin subunit alpha 1 [Source:HGNC Symbol;Acc:HGNC:6481] |
| ENSG00000101888 | HGLibA_33177 | 119 | 0.035571495 | NXT2 | nuclear transport factor 2 like export factor 2 [Source:HGNC Symbol;Acc:HGNC:18151] |
| ENSG00000102781 | HGLibA_24075 | 119 | 0.035571495 | KATNAL1 | katanin catalytic subunit A1 like 1 [Source:HGNC Symbol;Acc:HGNC:28361] |
| ENSG00000103375 | HGLibA_02608 | 119 | 0.035571495 | AQP8 | aquaporin 8 [Source:HGNC Symbol;Acc:HGNC:642] |
| ENSG00000103710 | HGLibA_40370 | 119 | 0.035571495 | RASL12 | RAS like family 12 [Source:HGNC Symbol;Acc:HGNC:30289] |
| ENSG00000104671 | HGLibA_12560 | 119 | 0.035571495 | DCTN6 | dynactin subunit 6 [Source:HGNC Symbol;Acc:HGNC:16964] |
| ENSG00000106100 | HGLibA_32245 | 119 | 0.035571495 | NOD1 | nucleotide binding oligomerization domain containing 1 [Source:HGNC Symbol;Acc:HGNC:16390] |
| ENSG00000106258 | HGLibA_12161 | 119 | 0.035571495 | CYP3A5 | cytochrome P450 family 3 subfamily A member 5 [Source:HGNC Symbol;Acc:HGNC:2638] |
| ENSG00000107566 | HGLibA_15495 | 119 | 0.035571495 | ERLIN1 | ER lipid raft associated 1 [Source:HGNC Symbol;Acc:HGNC:16947] |
| ENSG00000108091 | HGLibA_07811 | 119 | 0.035571495 | CCDC6 | coiled-coil domain containing 6 [Source:HGNC Symbol;Acc:HGNC:18782] |
| ENSG00000111536 | HGLibA_23108 | 119 | 0.035571495 | IL26 | interleukin 26 [Source:HGNC Symbol;Acc:HGNC:17119] |
| ENSG00000111799 | HGLibA_10507 | 119 | 0.035571495 | COL12A1 | collagen type XII alpha 1 chain [Source:HGNC Symbol;Acc:HGNC:2188] |
| ENSG00000113296 | HGLibA_49331 | 119 | 0.035571495 | THBS4 | thrombospondin 4 [Source:HGNC Symbol;Acc:HGNC:11788] |
| ENSG00000115008 | HGLibA_23036 | 119 | 0.035571495 | IL1A | interleukin 1 alpha [Source:HGNC Symbol;Acc:HGNC:5991] |
| ENSG00000115602 | HGLibA_23062 | 119 | 0.035571495 | IL1RL1 | interleukin 1 receptor like 1 [Source:HGNC Symbol;Acc:HGNC:5998] |
| ENSG00000115947 | HGLibA_34610 | 119 | 0.035571495 | ORC4 | origin recognition complex subunit 4 [Source:HGNC Symbol;Acc:HGNC:8490] |
| ENSG00000116396 | HGLibA_24170 | 119 | 0.035571495 | KCNC4 | potassium voltage-gated channel subfamily C member 4 [Source:HGNC Symbol;Acc:HGNC:6236] |
| ENSG00000116406 | HGLibA_14459 | 119 | 0.035571495 | EDEM3 | ER degradation enhancing alpha-mannosidase like protein 3 [Source:HGNC Symbol;Acc:HGNC:16787] |
| ENSG00000117475 | HGLibA_04582 | 119 | 0.035571495 | BLZF1 | basic leucine zipper nuclear factor 1 [Source:HGNC Symbol;Acc:HGNC:1065] |
| ENSG00000118503 | HGLibA_50713 | 119 | 0.035571495 | TNFAIP3 | TNF alpha induced protein 3 [Source:HGNC Symbol;Acc:HGNC:11896] |
| ENSG00000119402 | HGLibA_17145 | 119 | 0.035571495 | FBXW2 | F-box and WD repeat domain containing 2 [Source:HGNC Symbol;Acc:HGNC:13608] |
| ENSG00000120217 | HGLibA_08317 | 119 | 0.035571495 | CD274 | CD274 molecule [Source:HGNC Symbol;Acc:HGNC:17635] |
| ENSG00000120896 | HGLibA_46181 | 119 | 0.035571495 | SORBS3 | sorbin and SH3 domain containing 3 [Source:HGNC Symbol;Acc:HGNC:30907] |
| ENSG00000122550 | HGLibA_25223 | 119 | 0.035571495 | KLHL7 | kelch like family member 7 [Source:HGNC Symbol;Acc:HGNC:15646] |
| ENSG00000122644 | HGLibA_02966 | 119 | 0.035571495 | ARL4A | ADP ribosylation factor like GTPase 4A [Source:HGNC Symbol;Acc:HGNC:695] |
| ENSG00000124232 | HGLibA_40649 | 119 | 0.035571495 | RBPJL | recombination signal binding protein for immunoglobulin kappa J region like [Source:HGNC Symbol;Acc:HGNC:13761] |
| ENSG00000124356 | HGLibA_47337 | 119 | 0.035571495 | STAMBP | STAM binding protein [Source:HGNC Symbol;Acc:HGNC:16950] |
| ENSG00000125966 | HGLibA_29520 | 119 | 0.035571495 | MMP24 | matrix metallopeptidase 24 [Source:HGNC Symbol;Acc:HGNC:7172] |
| ENSG00000128908 | HGLibA_23318 | 119 | 0.035571495 | INO80 | INO80 complex subunit [Source:HGNC Symbol;Acc:HGNC:26956] |
| ENSG00000128944 | HGLibA_25349 | 119 | 0.035571495 | KNSTRN | kinetochore localized astrin (SPAG5) binding protein [Source:HGNC Symbol;Acc:HGNC:30767] |
| ENSG00000129873 | HGLibA_08913 | 119 | 0.035571495 | CDY2B | chromodomain Y-linked 2B [Source:HGNC Symbol;Acc:HGNC:23921] |
| ENSG00000130347 | HGLibA_42409 | 119 | 0.035571495 | RTN4IP1 | reticulon 4 interacting protein 1 [Source:HGNC Symbol;Acc:HGNC:18647] |
| ENSG00000130558 | HGLibA_33344 | 119 | 0.035571495 | OLFM1 | olfactomedin 1 [Source:HGNC Symbol;Acc:HGNC:17187] |
| ENSG00000131016 | HGLibA_01411 | 119 | 0.035571495 | AKAP12 | A-kinase anchoring protein 12 [Source:HGNC Symbol;Acc:HGNC:370] |
| ENSG00000133069 | HGLibA_49776 | 119 | 0.035571495 | TMCC2 | transmembrane and coiled-coil domain family 2 [Source:HGNC Symbol;Acc:HGNC:24239] |
| ENSG00000133131 | HGLibA_29656 | 119 | 0.035571495 | MORC4 | MORC family CW-type zinc finger 4 [Source:HGNC Symbol;Acc:HGNC:23485] |
| ENSG00000134899 | HGLibA_15442 | 119 | 0.035571495 | ERCC5 | endonuclease [Source:HGNC Symbol;Acc:HGNC:3437]" |
| ENSG00000135063 | HGLibA_16353 | 119 | 0.035571495 | FAM189A2 | family with sequence similarity 189 member A2 [Source:HGNC Symbol;Acc:HGNC:24820] |
| ENSG00000135077 | HGLibA_20901 | 119 | 0.035571495 | HAVCR2 | hepatitis A virus cellular receptor 2 [Source:HGNC Symbol;Acc:HGNC:18437] |
| ENSG00000135269 | HGLibA_49022 | 119 | 0.035571495 | TES | testin LIM domain protein [Source:HGNC Symbol;Acc:HGNC:14620] |
| ENSG00000135426 | HGLibA_49033 | 119 | 0.035571495 | TESPA1 | positive selection associated 1 [Source:HGNC Symbol;Acc:HGNC:29109]" |
| ENSG00000135437 | HGLibA_40750 | 119 | 0.035571495 | RDH5 | retinol dehydrogenase 5 [Source:HGNC Symbol;Acc:HGNC:9940] |
| ENSG00000135929 | HGLibA_12095 | 119 | 0.035571495 | CYP27A1 | cytochrome P450 family 27 subfamily A member 1 [Source:HGNC Symbol;Acc:HGNC:2605] |
| ENSG00000136261 | HGLibA_05017 | 119 | 0.035571495 | BZW2 | basic leucine zipper and W2 domains 2 [Source:HGNC Symbol;Acc:HGNC:18808] |
| ENSG00000136866 | HGLibA_55212 | 119 | 0.035571495 | ZFP37 | ZFP37 zinc finger protein [Source:HGNC Symbol;Acc:HGNC:12863] |
| ENSG00000137875 | HGLibA_04306 | 119 | 0.035571495 | BCL2L10 | BCL2 like 10 [Source:HGNC Symbol;Acc:HGNC:993] |
| ENSG00000138160 | HGLibA_24851 | 119 | 0.035571495 | KIF11 | kinesin family member 11 [Source:HGNC Symbol;Acc:HGNC:6388] |
| ENSG00000138495 | HGLibA_10818 | 119 | 0.035571495 | COX17 | cytochrome c oxidase copper chaperone COX17 [Source:HGNC Symbol;Acc:HGNC:2264] |
| ENSG00000139209 | HGLibA_45071 | 119 | 0.035571495 | SLC38A4 | solute carrier family 38 member 4 [Source:HGNC Symbol;Acc:HGNC:14679] |
| ENSG00000139351 | HGLibA_47937 | 119 | 0.035571495 | SYCP3 | synaptonemal complex protein 3 [Source:HGNC Symbol;Acc:HGNC:18130] |
| ENSG00000140043 | HGLibA_39355 | 119 | 0.035571495 | PTGR2 | prostaglandin reductase 2 [Source:HGNC Symbol;Acc:HGNC:20149] |
| ENSG00000140488 | HGLibA_09018 | 119 | 0.035571495 | CELF6 | CUGBP Elav-like family member 6 [Source:HGNC Symbol;Acc:HGNC:14059] |
| ENSG00000140859 | HGLibA_24975 | 119 | 0.035571495 | KIFC3 | kinesin family member C3 [Source:HGNC Symbol;Acc:HGNC:6326] |
| ENSG00000141194 | HGLibA_33964 | 119 | 0.035571495 | OR4D1 | olfactory receptor family 4 subfamily D member 1 [Source:HGNC Symbol;Acc:HGNC:8293] |
| ENSG00000141401 | HGLibA_23246 | 119 | 0.035571495 | IMPA2 | inositol monophosphatase 2 [Source:HGNC Symbol;Acc:HGNC:6051] |
| ENSG00000141431 | HGLibA_03394 | 119 | 0.035571495 | ASXL3 | ASXL transcriptional regulator 3 [Source:HGNC Symbol;Acc:HGNC:29357] |
| ENSG00000141905 | HGLibA_31806 | 119 | 0.035571495 | NFIC | nuclear factor I C [Source:HGNC Symbol;Acc:HGNC:7786] |
| ENSG00000142541 | HGLibA_41870 | 119 | 0.035571495 | RPL13A | ribosomal protein L13a [Source:HGNC Symbol;Acc:HGNC:10304] |
| ENSG00000143147 | HGLibA_19958 | 119 | 0.035571495 | GPR161 | G protein-coupled receptor 161 [Source:HGNC Symbol;Acc:HGNC:23694] |
| ENSG00000143228 | HGLibA_32997 | 119 | 0.035571495 | NUF2 | NDC80 kinetochore complex component NUF2 [Source:HGNC Symbol;Acc:HGNC:14621] |
| ENSG00000143622 | HGLibA_41336 | 119 | 0.035571495 | RIT1 | Ras like without CAAX 1 [Source:HGNC Symbol;Acc:HGNC:10023] |
| ENSG00000143756 | HGLibA_17059 | 119 | 0.035571495 | FBXO28 | F-box protein 28 [Source:HGNC Symbol;Acc:HGNC:29046] |
| ENSG00000144591 | HGLibA_19420 | 119 | 0.035571495 | GMPPA | GDP-mannose pyrophosphorylase A [Source:HGNC Symbol;Acc:HGNC:22923] |
| ENSG00000145808 | HGLibA_00818 | 119 | 0.035571495 | ADAMTS19 | ADAM metallopeptidase with thrombospondin type 1 motif 19 [Source:HGNC Symbol;Acc:HGNC:17111] |
| ENSG00000148341 | HGLibA_43958 | 119 | 0.035571495 | SH3GLB2 | endophilin B2 [Source:HGNC Symbol;Acc:HGNC:10834]" |
| ENSG00000150403 | HGLibA_49786 | 119 | 0.035571495 | TMCO3 | transmembrane and coiled-coil domains 3 [Source:HGNC Symbol;Acc:HGNC:20329] |
| ENSG00000152467 | HGLibA_56982 | 119 | 0.035571495 | ZSCAN1 | zinc finger and SCAN domain containing 1 [Source:HGNC Symbol;Acc:HGNC:23712] |
| ENSG00000152766 | HGLibA_02039 | 119 | 0.035571495 | ANKRD22 | ankyrin repeat domain 22 [Source:HGNC Symbol;Acc:HGNC:28321] |
| ENSG00000153130 | HGLibA_43032 | 119 | 0.035571495 | SCOC | short coiled-coil protein [Source:HGNC Symbol;Acc:HGNC:20335] |
| ENSG00000154451 | HGLibA_18739 | 119 | 0.035571495 | GBP5 | guanylate binding protein 5 [Source:HGNC Symbol;Acc:HGNC:19895] |
| ENSG00000154639 | HGLibA_11838 | 119 | 0.035571495 | CXADR | Ig-like cell adhesion molecule [Source:HGNC Symbol;Acc:HGNC:2559]" |
| ENSG00000156873 | HGLibA_36413 | 119 | 0.035571495 | PHKG2 | phosphorylase kinase catalytic subunit gamma 2 [Source:HGNC Symbol;Acc:HGNC:8931] |
| ENSG00000156973 | HGLibA_35815 | 119 | 0.035571495 | PDE6D | phosphodiesterase 6D [Source:HGNC Symbol;Acc:HGNC:8788] |
| ENSG00000157823 | HGLibA_02339 | 119 | 0.035571495 | AP3S2 | adaptor related protein complex 3 subunit sigma 2 [Source:HGNC Symbol;Acc:HGNC:571] |
| ENSG00000158516 | HGLibA_10886 | 119 | 0.035571495 | CPA2 | carboxypeptidase A2 [Source:HGNC Symbol;Acc:HGNC:2297] |
| ENSG00000158806 | HGLibA_32489 | 119 | 0.035571495 | NPM2 | nucleophosmin/nucleoplasmin 2 [Source:HGNC Symbol;Acc:HGNC:7930] |
| ENSG00000160796 | HGLibA_31213 | 119 | 0.035571495 | NBEAL2 | neurobeachin like 2 [Source:HGNC Symbol;Acc:HGNC:31928] |
| ENSG00000161664 | HGLibA_03224 | 119 | 0.035571495 | ASB16 | ankyrin repeat and SOCS box containing 16 [Source:HGNC Symbol;Acc:HGNC:19768] |
| ENSG00000161791 | HGLibA_17699 | 119 | 0.035571495 | FMNL3 | formin like 3 [Source:HGNC Symbol;Acc:HGNC:23698] |
| ENSG00000162636 | HGLibA_15981 | 119 | 0.035571495 | FAM102B | family with sequence similarity 102 member B [Source:HGNC Symbol;Acc:HGNC:27637] |
| ENSG00000162670 | HGLibA_04818 | 119 | 0.035571495 | BRINP3 | BMP/retinoic acid inducible neural specific 3 [Source:HGNC Symbol;Acc:HGNC:22393] |
| ENSG00000162881 | HGLibA_34820 | 119 | 0.035571495 | OXER1 | oxoeicosanoid receptor 1 [Source:HGNC Symbol;Acc:HGNC:24884] |
| ENSG00000163281 | HGLibA_19567 | 119 | 0.035571495 | GNPDA2 | glucosamine-6-phosphate deaminase 2 [Source:HGNC Symbol;Acc:HGNC:21526] |
| ENSG00000163793 | HGLibA_13631 | 119 | 0.035571495 | DNAJC5G | DnaJ heat shock protein family (Hsp40) member C5 gamma [Source:HGNC Symbol;Acc:HGNC:24844] |
| ENSG00000164144 | HGLibA_02677 | 119 | 0.035571495 | ARFIP1 | ADP ribosylation factor interacting protein 1 [Source:HGNC Symbol;Acc:HGNC:21496] |
| ENSG00000164270 | HGLibA_22394 | 119 | 0.035571495 | HTR4 | 5-hydroxytryptamine receptor 4 [Source:HGNC Symbol;Acc:HGNC:5299] |
| ENSG00000164708 | HGLibA_36199 | 119 | 0.035571495 | PGAM2 | phosphoglycerate mutase 2 [Source:HGNC Symbol;Acc:HGNC:8889] |
| ENSG00000164761 | HGLibA_50749 | 119 | 0.035571495 | TNFRSF11B | TNF receptor superfamily member 11b [Source:HGNC Symbol;Acc:HGNC:11909] |
| ENSG00000164975 | HGLibA_45895 | 119 | 0.035571495 | SNAPC3 | small nuclear RNA activating complex polypeptide 3 [Source:HGNC Symbol;Acc:HGNC:11136] |

| | | | | | |
|---|---|---|---|---|---|
| ENSG00000165061 | HGLibA_55374 | 119 | 0.035571495 | ZMAT4 | zinc finger matrin-type 4 [Source:HGNC Symbol;Acc:HGNC:25844] |
| ENSG00000165434 | HGLibA_36267 | 119 | 0.035571495 | PGM2L1 | phosphoglucomutase 2 like 1 [Source:HGNC Symbol;Acc:HGNC:20898] |
| ENSG00000165985 | HGLibA_05930 | 119 | 0.035571495 | C1QL3 | complement C1q like 3 [Source:HGNC Symbol;Acc:HGNC:19359] |
| ENSG00000166313 | HGLibA_02377 | 119 | 0.035571495 | APBB1 | amyloid beta precursor protein binding family B member 1 [Source:HGNC Symbol;Acc:HGNC:581] |
| ENSG00000167476 | HGLibA_23997 | 119 | 0.035571495 | JSRP1 | junctional sarcoplasmic reticulum protein 1 [Source:HGNC Symbol;Acc:HGNC:24963] |
| ENSG00000167528 | HGLibA_56443 | 119 | 0.035571495 | ZNF641 | zinc finger protein 641 [Source:HGNC Symbol;Acc:HGNC:31834] |
| ENSG00000167799 | HGLibA_32990 | 119 | 0.035571495 | NUDT8 | nudix hydrolase 8 [Source:HGNC Symbol;Acc:HGNC:8055] |
| ENSG00000167971 | HGLibA_07289 | 119 | 0.035571495 | CASKIN1 | CASK interacting protein 1 [Source:HGNC Symbol;Acc:HGNC:20879] |
| ENSG00000169174 | HGLibA_35687 | 119 | 0.035571495 | PCSK9 | proprotein convertase subtilisin/kexin type 9 [Source:HGNC Symbol;Acc:HGNC:20001] |
| ENSG00000169583 | HGLibA_10028 | 119 | 0.035571495 | CLIC3 | chloride intracellular channel 3 [Source:HGNC Symbol;Acc:HGNC:2064] |
| ENSG00000170525 | HGLibA_36160 | 119 | 0.035571495 | PFKFB3 | 6-biphosphatase 3 [Source:HGNC Symbol;Acc:HGNC:8874]" |
| ENSG00000170909 | HGLibA_34673 | 119 | 0.035571495 | OSCAR | immunoglobulin-like receptor [Source:HGNC Symbol;Acc:HGNC:29960]" |
| ENSG00000171456 | HGLibA_03389 | 119 | 0.035571495 | ASXL1 | ASXL transcriptional regulator 1 [Source:HGNC Symbol;Acc:HGNC:18318] |
| ENSG00000172059 | HGLibA_25045 | 119 | 0.035571495 | KLF11 | Kruppel like factor 11 [Source:HGNC Symbol;Acc:HGNC:11811] |
| ENSG00000172296 | HGLibA_46898 | 119 | 0.035571495 | SPTLC3 | serine palmitoyltransferase long chain base subunit 3 [Source:HGNC Symbol;Acc:HGNC:16253] |
| ENSG00000174990 | HGLibA_06812 | 119 | 0.035571495 | CA5A | carbonic anhydrase 5A [Source:HGNC Symbol;Acc:HGNC:1377] |
| ENSG00000175175 | HGLibA_37972 | 119 | 0.035571495 | PPM1E | Mg2+/Mn2+ dependent 1E [Source:HGNC Symbol;Acc:HGNC:19322]" |
| ENSG00000177058 | HGLibA_45085 | 119 | 0.035571495 | SLC38A9 | solute carrier family 38 member 9 [Source:HGNC Symbol;Acc:HGNC:26907] |
| ENSG00000177504 | HGLibA_53584 | 119 | 0.035571495 | VCX2 | variable charge X-linked 2 [Source:HGNC Symbol;Acc:HGNC:18158] |
| ENSG00000179363 | HGLibA_50367 | 119 | 0.035571495 | TMEM31 | transmembrane protein 31 [Source:HGNC Symbol;Acc:HGNC:28601] |
| ENSG00000179564 | HGLibA_27559 | 119 | 0.035571495 | LSMEM2 | leucine rich single-pass membrane protein 2 [Source:HGNC Symbol;Acc:HGNC:26781] |
| ENSG00000182621 | HGLibA_36978 | 119 | 0.035571495 | PLCB1 | phospholipase C beta 1 [Source:HGNC Symbol;Acc:HGNC:15917] |
| ENSG00000182700 | HGLibA_22859 | 119 | 0.035571495 | IGIP | IgA inducing protein [Source:HGNC Symbol;Acc:HGNC:33847] |
| ENSG00000182968 | HGLibA_46225 | 119 | 0.035571495 | SOX1 | SRY-box 1 [Source:HGNC Symbol;Acc:HGNC:11189] |
| ENSG00000183778 | HGLibA_03962 | 119 | 0.035571495 | B3GALT5 | 3-galactosyltransferase 5 [Source:HGNC Symbol;Acc:HGNC:920]" |
| ENSG00000184381 | HGLibA_36921 | 119 | 0.035571495 | PLA2G6 | phospholipase A2 group VI [Source:HGNC Symbol;Acc:HGNC:9039] |
| ENSG00000184900 | HGLibA_47782 | 119 | 0.035571495 | SUMO3 | small ubiquitin-like modifier 3 [Source:HGNC Symbol;Acc:HGNC:11124] |
| ENSG00000185155 | HGLibA_29321 | 119 | 0.035571495 | MIXL1 | Mix paired-like homeobox [Source:HGNC Symbol;Acc:HGNC:13363] |
| ENSG00000186847 | HGLibA_25425 | 119 | 0.035571495 | KRT14 | keratin 14 [Source:HGNC Symbol;Acc:HGNC:6416] |
| ENSG00000187033 | HGLibA_42691 | 119 | 0.035571495 | SAMD7 | sterile alpha motif domain containing 7 [Source:HGNC Symbol;Acc:HGNC:25394] |
| ENSG00000187054 | HGLibA_50612 | 119 | 0.035571495 | TMPRSS11A | transmembrane serine protease 11A [Source:HGNC Symbol;Acc:HGNC:27954] |
| ENSG00000187537 | HGLibA_37736 | 119 | 0.035571495 | POTEG | POTE ankyrin domain family member G [Source:HGNC Symbol;Acc:HGNC:33896] |
| ENSG00000188152 | HGLibA_33110 | 119 | 0.035571495 | NUTM2G | NUT family member 2G [Source:HGNC Symbol;Acc:HGNC:23449] |
| ENSG00000188559 | HGLibA_40158 | 119 | 0.035571495 | RALGAPA2 | Ral GTPase activating protein catalytic alpha subunit 2 [Source:HGNC Symbol;Acc:HGNC:16207] |
| ENSG00000189139 | HGLibA_18066 | 119 | 0.035571495 | FSCB | fibrous sheath CABYR binding protein [Source:HGNC Symbol;Acc:HGNC:20494] |
| ENSG00000196357 | HGLibA_56257 | 119 | 0.035571495 | ZNF565 | zinc finger protein 565 [Source:HGNC Symbol;Acc:HGNC:26726] |
| ENSG00000197128 | HGLibA_56709 | 119 | 0.035571495 | ZNF772 | zinc finger protein 772 [Source:HGNC Symbol;Acc:HGNC:33106] |
| ENSG00000197838 | HGLibA_12102 | 119 | 0.035571495 | CYP2A13 | cytochrome P450 family 2 subfamily A member 13 [Source:HGNC Symbol;Acc:HGNC:2608] |
| ENSG00000198854 | HGLibA_05890 | 119 | 0.035571495 | C1orf68 | chromosome 1 open reading frame 68 [Source:HGNC Symbol;Acc:HGNC:29468] |
| ENSG00000205111 | HGLibA_08814 | 119 | 0.035571495 | CDKL4 | cyclin dependent kinase like 4 [Source:HGNC Symbol;Acc:HGNC:19287] |
| ENSG00000205436 | HGLibA_15746 | 119 | 0.035571495 | EXOC3L4 | exocyst complex component 3 like 4 [Source:HGNC Symbol;Acc:HGNC:20120] |
| ENSG00000205464 | HGLibA_03697 | 119 | 0.035571495 | ATP6AP1L | ATPase H+ transporting accessory protein 1 like [Source:HGNC Symbol;Acc:HGNC:28091] |
| ENSG00000214216 | HGLibA_23522 | 119 | 0.035571495 | IQCJ | IQ motif containing J [Source:HGNC Symbol;Acc:HGNC:32406] |
| ENSG00000221883 | HGLibA_02919 | 119 | 0.035571495 | ARIH2OS | ariadne RBR E3 ubiquitin protein ligase 2 opposite strand [Source:HGNC Symbol;Acc:HGNC:34425] |
| ENSG00000228927 | HGLibA_51970 | 119 | 0.035571495 | TSPY3 | testis specific protein Y-linked 3 [Source:HGNC Symbol;Acc:HGNC:33876] |
| ENSG00000259332 | HGLibA_47233 | 119 | 0.035571495 | ST20-MTHFS | ST20-MTHFS readthrough [Source:HGNC Symbol;Acc:HGNC:44655] |
| ENSG00000283154 | HGLibA_23526 | 119 | 0.035571495 | IQCJ-SCHIP1 | IQCJ-SCHIP1 readthrough [Source:HGNC Symbol;Acc:HGNC:38842] |
| ENSG00000288596 | HGLibA_06623 | 119 | 0.035571495 | C8orf44 | - |
| HIST1H2AH | HGLibA_21392 | 119 | 0.035571495 | HIST1H2AH | - |
| HIST1H2BK | HGLibA_21441 | 119 | 0.035571495 | HIST1H2BK | - |
| hsa-let-7c | HGLibA_57115 | 119 | 0.035571495 | hsa-let-7c | - |
| hsa-mir-128-1 | HGLibA_57571 | 119 | 0.035571495 | hsa-mir-128-1 | - |
| hsa-mir-155 | HGLibA_57929 | 119 | 0.035571495 | hsa-mir-155 | - |
| hsa-mir-198 | HGLibA_58124 | 119 | 0.035571495 | hsa-mir-198 | - |
| hsa-mir-3198-2 | HGLibA_58894 | 119 | 0.035571495 | hsa-mir-3198-2 | - |
| hsa-mir-3910-2 | HGLibA_59507 | 119 | 0.035571495 | hsa-mir-3910-2 | - |
| hsa-mir-4304 | HGLibA_59926 | 119 | 0.035571495 | hsa-mir-4304 | - |
| hsa-mir-4490 | HGLibA_60380 | 119 | 0.035571495 | hsa-mir-4490 | - |
| hsa-mir-450a-1 | HGLibA_60473 | 119 | 0.035571495 | hsa-mir-450a-1 | - |
| hsa-mir-4632 | HGLibA_60633 | 119 | 0.035571495 | hsa-mir-4632 | - |
| hsa-mir-4633 | HGLibA_60636 | 119 | 0.035571495 | hsa-mir-4633 | - |
| hsa-mir-4769 | HGLibA_61174 | 119 | 0.035571495 | hsa-mir-4769 | - |
| hsa-mir-504 | HGLibA_61473 | 119 | 0.035571495 | hsa-mir-504 | - |
| hsa-mir-514a-2 | HGLibA_61592 | 119 | 0.035571495 | hsa-mir-514a-2 | - |
| hsa-mir-548i-3 | HGLibA_62021 | 119 | 0.035571495 | hsa-mir-548i-3 | - |
| hsa-mir-5581 | HGLibA_62162 | 119 | 0.035571495 | hsa-mir-5581 | - |
| hsa-mir-576 | HGLibA_62397 | 119 | 0.035571495 | hsa-mir-576 | - |
| hsa-mir-586 | HGLibA_62441 | 119 | 0.035571495 | hsa-mir-586 | - |
| hsa-mir-661 | HGLibA_62976 | 119 | 0.035571495 | hsa-mir-661 | - |
| hsa-mir-6840 | HGLibA_63534 | 119 | 0.035571495 | hsa-mir-6840 | - |
| hsa-mir-892a | HGLibA_64249 | 119 | 0.035571495 | hsa-mir-892a | - |
| hsa-mir-936 | HGLibA_64319 | 119 | 0.035571495 | hsa-mir-936 | - |
| KIAA0317 | HGLibA_24618 | 119 | 0.035571495 | KIAA0317 | - |
| KIAA1279 | HGLibA_24734 | 119 | 0.035571495 | KIAA1279 | - |
| KIAA1324L | HGLibA_24740 | 119 | 0.035571495 | KIAA1324L | - |
| KIAA1462 | HGLibA_24763 | 119 | 0.035571495 | KIAA1462 | - |
| PDDC1 | HGLibA_35756 | 119 | 0.035571495 | PDDC1 | - |
| SGK110 | HGLibA_43819 | 119 | 0.035571495 | SGK110 | - |
| SLC24A6 | HGLibA_44629 | 119 | 0.035571495 | SLC24A6 | - |
| TMEM56-RWDD3 | HGLibA_50453 | 119 | 0.035571495 | TMEM56-RWDD3 | - |
| WBSCR28 | HGLibA_54000 | 119 | 0.035571495 | WBSCR28 | - |
| ZNRD1 | HGLibA_56936 | 119 | 0.035571495 | ZNRD1 | - |
| C10orf11 | HGLibA_05027 | 118 | 0.025620457 | C10orf11 | - |
| C7orf43 | HGLibA_06546 | 118 | 0.025620457 | C7orf43 | - |
| CLLU1OS | HGLibA_10066 | 118 | 0.025620457 | CLLU1OS | - |
| CLLU1OS | HGLibA_10067 | 118 | 0.025620457 | CLLU1OS | - |
| CSB-PGBD3 | HGLibA_11308 | 118 | 0.025620457 | CSB-PGBD3 | - |
| CXorf21 | HGLibA_11903 | 118 | 0.025620457 | CXorf21 | - |
| ENSG00000006116 | HGLibA_06935 | 118 | 0.025620457 | CACNG3 | calcium voltage-gated channel auxiliary subunit gamma 3 [Source:HGNC Symbol;Acc:HGNC:1407] |
| ENSG00000016490 | HGLibA_09806 | 118 | 0.025620457 | CLCA1 | chloride channel accessory 1 [Source:HGNC Symbol;Acc:HGNC:2015] |
| ENSG00000035862 | HGLibA_49539 | 118 | 0.025620457 | TIMP2 | TIMP metallopeptidase inhibitor 2 [Source:HGNC Symbol;Acc:HGNC:11821] |
| ENSG00000039537 | HGLibA_06426 | 118 | 0.025620457 | C6 | complement C6 [Source:HGNC Symbol;Acc:HGNC:1339] |
| ENSG00000043355 | HGLibA_55323 | 118 | 0.025620457 | ZIC2 | Zic family member 2 [Source:HGNC Symbol;Acc:HGNC:12873] |
| ENSG00000046651 | HGLibA_33298 | 118 | 0.025620457 | OFD1 | centriole and centriolar satellite protein [Source:HGNC Symbol;Acc:HGNC:2567]" |
| ENSG00000061455 | HGLibA_38394 | 118 | 0.025620457 | PRDM6 | PR/SET domain 6 [Source:HGNC Symbol;Acc:HGNC:9350] |
| ENSG00000065029 | HGLibA_56683 | 118 | 0.025620457 | ZNF76 | zinc finger protein 76 [Source:HGNC Symbol;Acc:HGNC:13149] |
| ENSG00000067829 | HGLibA_22550 | 118 | 0.025620457 | IDH3G | isocitrate dehydrogenase 3 (NAD(+)) gamma [Source:HGNC Symbol;Acc:HGNC:5386] |
| ENSG00000072062 | HGLibA_38504 | 118 | 0.025620457 | PRKACA | protein kinase cAMP-activated catalytic subunit alpha [Source:HGNC Symbol;Acc:HGNC:9380] |
| ENSG00000072518 | HGLibA_28366 | 118 | 0.025620457 | MARK2 | microtubule affinity regulating kinase 2 [Source:HGNC Symbol;Acc:HGNC:3332] |
| ENSG00000075413 | HGLibA_28370 | 118 | 0.025620457 | MARK3 | microtubule affinity regulating kinase 3 [Source:HGNC Symbol;Acc:HGNC:6897] |
| ENSG00000085733 | HGLibA_11741 | 118 | 0.025620457 | CTTN | cortactin [Source:HGNC Symbol;Acc:HGNC:3338] |
| ENSG00000085978 | HGLibA_03473 | 118 | 0.025620457 | ATG16L1 | autophagy related 16 like 1 [Source:HGNC Symbol;Acc:HGNC:21498] |
| ENSG00000089050 | HGLibA_40446 | 118 | 0.025620457 | RBBP9 | serine hydrolase [Source:HGNC Symbol;Acc:HGNC:9892]" |
| ENSG00000101204 | HGLibA_09575 | 118 | 0.025620457 | CHRNA4 | cholinergic receptor nicotinic alpha 4 subunit [Source:HGNC Symbol;Acc:HGNC:1958] |
| ENSG00000102302 | HGLibA_17342 | 118 | 0.025620457 | FGD1 | RhoGEF and PH domain containing 1 [Source:HGNC Symbol;Acc:HGNC:3663]" |
| ENSG00000102796 | HGLibA_13125 | 118 | 0.025620457 | DHRS12 | dehydrogenase/reductase 12 [Source:HGNC Symbol;Acc:HGNC:25832] |
| ENSG00000103145 | HGLibA_20960 | 118 | 0.025620457 | HCFC1R1 | host cell factor C1 regulator 1 [Source:HGNC Symbol;Acc:HGNC:21198] |
| ENSG00000104375 | HGLibA_47477 | 118 | 0.025620457 | STK3 | serine/threonine kinase 3 [Source:HGNC Symbol;Acc:HGNC:11406] |
| ENSG00000105325 | HGLibA_18290 | 118 | 0.025620457 | FZR1 | fizzy and cell division cycle 20 related 1 [Source:HGNC Symbol;Acc:HGNC:24824] |
| ENSG00000106025 | HGLibA_51899 | 118 | 0.025620457 | TSPAN12 | tetraspanin 12 [Source:HGNC Symbol;Acc:HGNC:21641] |
| ENSG00000106080 | HGLibA_17557 | 118 | 0.025620457 | FKBP14 | FK506 binding protein 14 [Source:HGNC Symbol;Acc:HGNC:18625] |
| ENSG00000106638 | HGLibA_48617 | 118 | 0.025620457 | TBL2 | transducin beta like 2 [Source:HGNC Symbol;Acc:HGNC:11586] |
| ENSG00000106701 | HGLibA_18080 | 118 | 0.025620457 | FSD1L | fibronectin type III and SPRY domain containing 1 like [Source:HGNC Symbol;Acc:HGNC:13753] |
| ENSG00000108510 | HGLibA_28748 | 118 | 0.025620457 | MED13 | mediator complex subunit 13 [Source:HGNC Symbol;Acc:HGNC:22474] |
| ENSG00000110048 | HGLibA_34637 | 118 | 0.025620457 | OSBP | oxysterol binding protein [Source:HGNC Symbol;Acc:HGNC:8503] |
| ENSG00000110344 | HGLibA_52690 | 118 | 0.025620457 | UBE4A | ubiquitination factor E4A [Source:HGNC Symbol;Acc:HGNC:12499] |
| ENSG00000112494 | HGLibA_53036 | 118 | 0.025620457 | UNC93A | unc-93 homolog A [Source:HGNC Symbol;Acc:HGNC:12570] |
| ENSG00000112977 | HGLibA_12316 | 118 | 0.025620457 | DAP | death associated protein [Source:HGNC Symbol;Acc:HGNC:2672] |
| ENSG00000113088 | HGLibA_20728 | 118 | 0.025620457 | GZMK | granzyme K [Source:HGNC Symbol;Acc:HGNC:4711] |
| ENSG00000116133 | HGLibA_13090 | 118 | 0.025620457 | DHCR24 | 24-dehydrocholesterol reductase [Source:HGNC Symbol;Acc:HGNC:2859] |
| ENSG00000116237 | HGLibA_22513 | 118 | 0.025620457 | ICMT | isoprenylcysteine carboxyl methyltransferase [Source:HGNC Symbol;Acc:HGNC:5350] |
| ENSG00000117322 | HGLibA_11050 | 118 | 0.025620457 | CR2 | complement C3d receptor 2 [Source:HGNC Symbol;Acc:HGNC:2336] |
| ENSG00000120211 | HGLibA_23385 | 118 | 0.025620457 | INSL4 | insulin like 4 [Source:HGNC Symbol;Acc:HGNC:6087] |
| ENSG00000120457 | HGLibA_24281 | 118 | 0.025620457 | KCNJ5 | potassium voltage-gated channel subfamily J member 5 [Source:HGNC Symbol;Acc:HGNC:6266] |
| ENSG00000121671 | HGLibA_11244 | 118 | 0.025620457 | CRY2 | cryptochrome circadian regulator 2 [Source:HGNC Symbol;Acc:HGNC:2385] |
| ENSG00000123268 | HGLibA_03431 | 118 | 0.025620457 | ATF1 | activating transcription factor 1 [Source:HGNC Symbol;Acc:HGNC:783] |
| ENSG00000126803 | HGLibA_22280 | 118 | 0.025620457 | HSPA2 | heat shock protein family A (Hsp70) member 2 [Source:HGNC Symbol;Acc:HGNC:5235] |
| ENSG00000127184 | HGLibA_10873 | 118 | 0.025620457 | COX7C | cytochrome c oxidase subunit 7C [Source:HGNC Symbol;Acc:HGNC:2292] |
| ENSG00000127334 | HGLibA_14278 | 118 | 0.025620457 | DYRK2 | dual specificity tyrosine phosphorylation regulated kinase 2 [Source:HGNC Symbol;Acc:HGNC:3093] |
| ENSG00000127588 | HGLibA_19517 | 118 | 0.025620457 | GNG13 | G protein subunit gamma 13 [Source:HGNC Symbol;Acc:HGNC:14131] |
| ENSG00000129244 | HGLibA_03595 | 118 | 0.025620457 | ATP1B2 | ATPase Na+/K+ transporting subunit beta 2 [Source:HGNC Symbol;Acc:HGNC:805] |
| ENSG00000129484 | HGLibA_35233 | 118 | 0.025620457 | PARP2 | poly(ADP-ribose) polymerase 2 [Source:HGNC Symbol;Acc:HGNC:272] |
| ENSG00000131023 | HGLibA_26065 | 118 | 0.025620457 | LATS1 | large tumor suppressor kinase 1 [Source:HGNC Symbol;Acc:HGNC:6514] |
| ENSG00000131115 | HGLibA_55626 | 118 | 0.025620457 | ZNF227 | zinc finger protein 227 [Source:HGNC Symbol;Acc:HGNC:13020] |
| ENSG00000132561 | HGLibA_28430 | 118 | 0.025620457 | MATN2 | matrilin 2 [Source:HGNC Symbol;Acc:HGNC:6908] |

| | | | | | |
|---|---|---|---|---|---|
| ENSG00000133789 | HGLibA_47899 | 118 | 0.025620457 | SWAP70 | switching B cell complex subunit SWAP70 [Source:HGNC Symbol;Acc:HGNC:17070] |
| ENSG00000134317 | HGLibA_20247 | 118 | 0.025620457 | GRHL1 | grainyhead like transcription factor 1 [Source:HGNC Symbol;Acc:HGNC:17923] |
| ENSG00000134717 | HGLibA_04937 | 118 | 0.025620457 | BTF3L4 | basic transcription factor 3 like 4 [Source:HGNC Symbol;Acc:HGNC:30547] |
| ENSG00000134825 | HGLibA_50341 | 118 | 0.025620457 | TMEM258 | transmembrane protein 258 [Source:HGNC Symbol;Acc:HGNC:1164] |
| ENSG00000135506 | HGLibA_34634 | 118 | 0.025620457 | OS9 | endoplasmic reticulum lectin [Source:HGNC Symbol;Acc:HGNC:16994]" |
| ENSG00000135966 | HGLibA_49234 | 118 | 0.025620457 | TGFBRAP1 | transforming growth factor beta receptor associated protein 1 [Source:HGNC Symbol;Acc:HGNC:16836] |
| ENSG00000136828 | HGLibA_40169 | 118 | 0.025620457 | RALGPS1 | Ral GEF with PH domain and SH3 binding motif 1 [Source:HGNC Symbol;Acc:HGNC:16851] |
| ENSG00000137675 | HGLibA_29529 | 118 | 0.025620457 | MMP27 | matrix metallopeptidase 27 [Source:HGNC Symbol;Acc:HGNC:14250] |
| ENSG00000138092 | HGLibA_09078 | 118 | 0.025620457 | CENPO | centromere protein O [Source:HGNC Symbol;Acc:HGNC:28152] |
| ENSG00000138496 | HGLibA_35247 | 118 | 0.025620457 | PARP9 | poly(ADP-ribose) polymerase family member 9 [Source:HGNC Symbol;Acc:HGNC:24118] |
| ENSG00000139178 | HGLibA_05972 | 118 | 0.025620457 | C1RL | complement C1r subcomponent like [Source:HGNC Symbol;Acc:HGNC:21265] |
| ENSG00000140279 | HGLibA_14099 | 118 | 0.025620457 | DUOX2 | dual oxidase 2 [Source:HGNC Symbol;Acc:HGNC:13273] |
| ENSG00000143842 | HGLibA_46236 | 118 | 0.025620457 | SOX13 | SRY-box 13 [Source:HGNC Symbol;Acc:HGNC:11192] |
| ENSG00000145103 | HGLibA_23209 | 118 | 0.025620457 | ILDR1 | immunoglobulin like domain containing receptor 1 [Source:HGNC Symbol;Acc:HGNC:28741] |
| ENSG00000146414 | HGLibA_44081 | 118 | 0.025620457 | SHPRH | SNF2 histone linker PHD RING helicase [Source:HGNC Symbol;Acc:HGNC:19336] |
| ENSG00000147166 | HGLibA_23774 | 118 | 0.025620457 | ITGB1BP2 | integrin subunit beta 1 binding protein 2 [Source:HGNC Symbol;Acc:HGNC:6154] |
| ENSG00000147588 | HGLibA_37321 | 118 | 0.025620457 | PMP2 | peripheral myelin protein 2 [Source:HGNC Symbol;Acc:HGNC:9117] |
| ENSG00000149577 | HGLibA_44116 | 118 | 0.025620457 | SIDT2 | SID1 transmembrane family member 2 [Source:HGNC Symbol;Acc:HGNC:24272] |
| ENSG00000151093 | HGLibA_34833 | 118 | 0.025620457 | OXSM | mitochondrial [Source:HGNC Symbol;Acc:HGNC:26063]" |
| ENSG00000151458 | HGLibA_02114 | 118 | 0.025620457 | ANKRD50 | ankyrin repeat domain 50 [Source:HGNC Symbol;Acc:HGNC:29223] |
| ENSG00000151615 | HGLibA_37780 | 118 | 0.025620457 | POU4F2 | POU class 4 homeobox 2 [Source:HGNC Symbol;Acc:HGNC:9219] |
| ENSG00000153157 | HGLibA_47934 | 118 | 0.025620457 | SYCP2L | synaptonemal complex protein 2 like [Source:HGNC Symbol;Acc:HGNC:21537] |
| ENSG00000154889 | HGLibA_29769 | 118 | 0.025620457 | MPPE1 | metallophosphoesterase 1 [Source:HGNC Symbol;Acc:HGNC:15988] |
| ENSG00000156265 | HGLibA_28198 | 118 | 0.025620457 | MAP3K7CL | MAP3K7 C-terminal like [Source:HGNC Symbol;Acc:HGNC:16457] |
| ENSG00000158296 | HGLibA_44391 | 118 | 0.025620457 | SLC13A3 | solute carrier family 13 member 3 [Source:HGNC Symbol;Acc:HGNC:14430] |
| ENSG00000160439 | HGLibA_40739 | 118 | 0.025620457 | RDH13 | retinol dehydrogenase 13 [Source:HGNC Symbol;Acc:HGNC:19978] |
| ENSG00000160932 | HGLibA_27655 | 118 | 0.025620457 | LY6E | lymphocyte antigen 6 family member E [Source:HGNC Symbol;Acc:HGNC:6727] |
| ENSG00000161547 | HGLibA_47072 | 118 | 0.025620457 | SRSF2 | serine and arginine rich splicing factor 2 [Source:HGNC Symbol;Acc:HGNC:10783] |
| ENSG00000161610 | HGLibA_20985 | 118 | 0.025620457 | HCRT | hypocretin neuropeptide precursor [Source:HGNC Symbol;Acc:HGNC:4847] |
| ENSG00000162772 | HGLibA_03436 | 118 | 0.025620457 | ATF3 | activating transcription factor 3 [Source:HGNC Symbol;Acc:HGNC:785] |
| ENSG00000163114 | HGLibA_35864 | 118 | 0.025620457 | PDHA2 | pyruvate dehydrogenase E1 alpha 2 subunit [Source:HGNC Symbol;Acc:HGNC:8807] |
| ENSG00000163512 | HGLibA_03935 | 118 | 0.025620457 | AZI2 | 5-azacytidine induced 2 [Source:HGNC Symbol;Acc:HGNC:24002] |
| ENSG00000165724 | HGLibA_55424 | 118 | 0.025620457 | ZMYND19 | zinc finger MYND-type containing 19 [Source:HGNC Symbol;Acc:HGNC:21146] |
| ENSG00000165731 | HGLibA_40873 | 118 | 0.025620457 | RET | ret proto-oncogene [Source:HGNC Symbol;Acc:HGNC:9967] |
| ENSG00000166292 | HGLibA_49838 | 118 | 0.025620457 | TMEM100 | transmembrane protein 100 [Source:HGNC Symbol;Acc:HGNC:25607] |
| ENSG00000166634 | HGLibA_43542 | 118 | 0.025620457 | SERPINB12 | serpin family B member 12 [Source:HGNC Symbol;Acc:HGNC:14220] |
| ENSG00000167083 | HGLibA_19540 | 118 | 0.025620457 | GNGT2 | G protein subunit gamma transducin 2 [Source:HGNC Symbol;Acc:HGNC:4412] |
| ENSG00000167705 | HGLibA_41249 | 118 | 0.025620457 | RILP | Rab interacting lysosomal protein [Source:HGNC Symbol;Acc:HGNC:30266] |
| ENSG00000168490 | HGLibA_36475 | 118 | 0.025620457 | PHYHIP | phytanoyl-CoA 2-hydroxylase interacting protein [Source:HGNC Symbol;Acc:HGNC:16865] |
| ENSG00000168894 | HGLibA_41583 | 118 | 0.025620457 | RNF181 | ring finger protein 181 [Source:HGNC Symbol;Acc:HGNC:28037] |
| ENSG00000168925 | HGLibA_11676 | 118 | 0.025620457 | CTRB1 | chymotrypsinogen B1 [Source:HGNC Symbol;Acc:HGNC:2521] |
| ENSG00000169228 | HGLibA_39848 | 118 | 0.025620457 | RAB24 | member RAS oncogene family [Source:HGNC Symbol;Acc:HGNC:9765]" |
| ENSG00000169598 | HGLibA_13022 | 118 | 0.025620457 | DFFB | DNA fragmentation factor subunit beta [Source:HGNC Symbol;Acc:HGNC:2773] |
| ENSG00000169756 | HGLibA_26546 | 118 | 0.025620457 | LIMS1 | LIM zinc finger domain containing 1 [Source:HGNC Symbol;Acc:HGNC:6616] |
| ENSG00000169989 | HGLibA_49471 | 118 | 0.025620457 | TIGD4 | tigger transposable element derived 4 [Source:HGNC Symbol;Acc:HGNC:18335] |
| ENSG00000171773 | HGLibA_33145 | 118 | 0.025620457 | NXNL1 | nucleoredoxin like 1 [Source:HGNC Symbol;Acc:HGNC:25179] |
| ENSG00000172888 | HGLibA_56406 | 118 | 0.025620457 | ZNF621 | zinc finger protein 621 [Source:HGNC Symbol;Acc:HGNC:24787] |
| ENSG00000173137 | HGLibA_00901 | 118 | 0.025620457 | ADCK5 | aarF domain containing kinase 5 [Source:HGNC Symbol;Acc:HGNC:21738] |
| ENSG00000173621 | HGLibA_27160 | 118 | 0.025620457 | LRFN4 | leucine rich repeat and fibronectin type III domain containing 4 [Source:HGNC Symbol;Acc:HGNC:28456] |
| ENSG00000173930 | HGLibA_45473 | 118 | 0.025620457 | SLCO4C1 | solute carrier organic anion transporter family member 4C1 [Source:HGNC Symbol;Acc:HGNC:23612] |
| ENSG00000176209 | HGLibA_45742 | 118 | 0.025620457 | SMIM19 | small integral membrane protein 19 [Source:HGNC Symbol;Acc:HGNC:25166] |
| ENSG00000176834 | HGLibA_53838 | 118 | 0.025620457 | VSIG10 | V-set and immunoglobulin domain containing 10 [Source:HGNC Symbol;Acc:HGNC:26078] |
| ENSG00000176928 | HGLibA_18817 | 118 | 0.025620457 | GCNT4 | core 2 [Source:HGNC Symbol;Acc:HGNC:17973]" |
| ENSG00000176988 | HGLibA_17723 | 118 | 0.025620457 | FMR1NB | FMR1 neighbor [Source:HGNC Symbol;Acc:HGNC:26372] |
| ENSG00000177191 | HGLibA_04003 | 118 | 0.025620457 | B3GNT8 | 3-N-acetylglucosaminyltransferase 8 [Source:HGNC Symbol;Acc:HGNC:24139]" |
| ENSG00000177494 | HGLibA_54792 | 118 | 0.025620457 | ZBED2 | zinc finger BED-type containing 2 [Source:HGNC Symbol;Acc:HGNC:20710] |
| ENSG00000178257 | HGLibA_38615 | 118 | 0.025620457 | PRM3 | protamine 3 [Source:HGNC Symbol;Acc:HGNC:13732] |
| ENSG00000179256 | HGLibA_45668 | 118 | 0.025620457 | SMCO3 | single-pass membrane protein with coiled-coil domains 3 [Source:HGNC Symbol;Acc:HGNC:34401] |
| ENSG00000179922 | HGLibA_56747 | 118 | 0.025620457 | ZNF784 | zinc finger protein 784 [Source:HGNC Symbol;Acc:HGNC:33111] |
| ENSG00000181007 | HGLibA_55236 | 118 | 0.025620457 | ZFP82 | ZFP82 zinc finger protein [Source:HGNC Symbol;Acc:HGNC:28682] |
| ENSG00000181704 | HGLibA_54684 | 118 | 0.025620457 | YIPF6 | Yip1 domain family member 6 [Source:HGNC Symbol;Acc:HGNC:28304] |
| ENSG00000182798 | HGLibA_27937 | 118 | 0.025620457 | MAGEB17 | MAGE family member B17 [Source:HGNC Symbol;Acc:HGNC:17418] |
| ENSG00000184698 | HGLibA_34124 | 118 | 0.025620457 | OR51M1 | olfactory receptor family 51 subfamily M member 1 [Source:HGNC Symbol;Acc:HGNC:14847] |
| ENSG00000184743 | HGLibA_03521 | 118 | 0.025620457 | ATL3 | atlastin GTPase 3 [Source:HGNC Symbol;Acc:HGNC:24526] |
| ENSG00000184937 | HGLibA_54428 | 118 | 0.025620457 | WT1 | Wilms tumor 1 [Source:HGNC Symbol;Acc:HGNC:12796] |
| ENSG00000185002 | HGLibA_40977 | 118 | 0.025620457 | RFX6 | regulatory factor X6 [Source:HGNC Symbol;Acc:HGNC:21478] |
| ENSG00000185432 | HGLibA_29017 | 118 | 0.025620457 | METTL7A | methyltransferase like 7A [Source:HGNC Symbol;Acc:HGNC:24550] |
| ENSG00000186925 | HGLibA_25677 | 118 | 0.025620457 | KRTAP19-6 | keratin associated protein 19-6 [Source:HGNC Symbol;Acc:HGNC:18941] |
| ENSG00000187080 | HGLibA_33754 | 118 | 0.025620457 | OR2AK2 | olfactory receptor family 2 subfamily AK member 2 [Source:HGNC Symbol;Acc:HGNC:19569] |
| ENSG00000188735 | HGLibA_49897 | 118 | 0.025620457 | TMEM120B | transmembrane protein 120B [Source:HGNC Symbol;Acc:HGNC:32008] |
| ENSG00000196381 | HGLibA_56738 | 118 | 0.025620457 | ZNF781 | zinc finger protein 781 [Source:HGNC Symbol;Acc:HGNC:26745] |
| ENSG00000196834 | HGLibA_37741 | 118 | 0.025620457 | POTEI | POTE ankyrin domain family member I [Source:HGNC Symbol;Acc:HGNC:37093] |
| ENSG00000197150 | HGLibA_00152 | 118 | 0.025620457 | ABCB8 | ATP binding cassette subfamily B member 8 [Source:HGNC Symbol;Acc:HGNC:49] |
| ENSG00000197520 | HGLibA_16297 | 118 | 0.025620457 | FAM177B | family with sequence similarity 177 member B [Source:HGNC Symbol;Acc:HGNC:34395] |
| ENSG00000197992 | HGLibA_10008 | 118 | 0.025620457 | CLEC9A | C-type lectin domain containing 9A [Source:HGNC Symbol;Acc:HGNC:26705] |
| ENSG00000198062 | HGLibA_37739 | 118 | 0.025620457 | POTEH | POTE ankyrin domain family member H [Source:HGNC Symbol;Acc:HGNC:133] |
| ENSG00000204410 | HGLibA_30199 | 118 | 0.025620457 | MSH5 | mutS homolog 5 [Source:HGNC Symbol;Acc:HGNC:7328] |
| ENSG00000204889 | HGLibA_25507 | 118 | 0.025620457 | KRT40 | keratin 40 [Source:HGNC Symbol;Acc:HGNC:26707] |
| ENSG00000204897 | HGLibA_25459 | 118 | 0.025620457 | KRT25 | keratin 25 [Source:HGNC Symbol;Acc:HGNC:30839] |
| ENSG00000204923 | HGLibA_17118 | 118 | 0.025620457 | FBXO48 | F-box protein 48 [Source:HGNC Symbol;Acc:HGNC:33857] |
| ENSG00000204983 | HGLibA_38900 | 118 | 0.025620457 | PRSS1 | serine protease 1 [Source:HGNC Symbol;Acc:HGNC:9475] |
| ENSG00000205208 | HGLibA_06335 | 118 | 0.025620457 | C4orf46 | chromosome 4 open reading frame 46 [Source:HGNC Symbol;Acc:HGNC:27320] |
| ENSG00000213096 | HGLibA_55676 | 118 | 0.025620457 | ZNF254 | zinc finger protein 254 [Source:HGNC Symbol;Acc:HGNC:13047] |
| ENSG00000213145 | HGLibA_11149 | 118 | 0.025620457 | CRIP1 | cysteine rich protein 1 [Source:HGNC Symbol;Acc:HGNC:2360] |
| ENSG00000213676 | HGLibA_03450 | 118 | 0.025620457 | ATF6B | activating transcription factor 6 beta [Source:HGNC Symbol;Acc:HGNC:2349] |
| ENSG00000215915 | HGLibA_03417 | 118 | 0.025620457 | ATAD3C | AAA domain containing 3C [Source:HGNC Symbol;Acc:HGNC:32151]" |
| ENSG00000223601 | HGLibA_14363 | 118 | 0.025620457 | EBLN1 | endogenous Bornavirus like nucleoprotein 1 [Source:HGNC Symbol;Acc:HGNC:39430] |
| ENSG00000226430 | HGLibA_53284 | 118 | 0.025620457 | USP17L7 | ubiquitin specific peptidase 17-like family member 7 [Source:HGNC Symbol;Acc:HGNC:37180] |
| ENSG00000227507 | HGLibA_27582 | 118 | 0.025620457 | LTB | lymphotoxin beta [Source:HGNC Symbol;Acc:HGNC:6711] |
| ENSG00000231068 | HGLibA_25714 | 118 | 0.025620457 | KRTAP21-3 | keratin associated protein 21-3 [Source:HGNC Symbol;Acc:HGNC:34216] |
| ENSG00000243649 | HGLibA_09245 | 118 | 0.025620457 | CFB | complement factor B [Source:HGNC Symbol;Acc:HGNC:1037] |
| ENSG00000243772 | HGLibA_24986 | 118 | 0.025620457 | KIR2DL3 | two Ig domains and long cytoplasmic tail 3 [Source:HGNC Symbol;Acc:HGNC:6331]" |
| ENSG00000256223 | HGLibA_55430 | 118 | 0.025620457 | ZNF10 | zinc finger protein 10 [Source:HGNC Symbol;Acc:HGNC:12879] |
| ENSG00000258992 | HGLibA_51963 | 118 | 0.025620457 | TSPY1 | testis specific protein Y-linked 1 [Source:HGNC Symbol;Acc:HGNC:12381] |
| ENSG00000261272 | HGLibA_30545 | 118 | 0.025620457 | MUC22 | mucin 22 [Source:HGNC Symbol;Acc:HGNC:39755] |
| ENSG00000261509 | HGLibA_51081 | 118 | 0.025620457 | TP53TG3B | TP53 target 3B [Source:HGNC Symbol;Acc:HGNC:37202] |
| ENSG00000272636 | HGLibA_13729 | 118 | 0.025620457 | DOC2B | double C2 domain beta [Source:HGNC Symbol;Acc:HGNC:2986] |
| ENSG00000273111 | HGLibA_27722 | 118 | 0.025620457 | LYPD4 | LY6/PLAUR domain containing 4 [Source:HGNC Symbol;Acc:HGNC:28659] |
| FAM155A | HGLibA_16181 | 118 | 0.025620457 | FAM155A | - |
| GPR124 | HGLibA_19875 | 118 | 0.025620457 | GPR124 | - |
| GSG2 | HGLibA_20424 | 118 | 0.025620457 | GSG2 | - |
| hsa-mir-1203 | HGLibA_57271 | 118 | 0.025620457 | hsa-mir-1203 | - |
| hsa-mir-1302-8 | HGLibA_57718 | 118 | 0.025620457 | hsa-mir-1302-8 | - |
| hsa-mir-199a-1 | HGLibA_58128 | 118 | 0.025620457 | hsa-mir-199a-1 | - |
| hsa-mir-301b | HGLibA_58434 | 118 | 0.025620457 | hsa-mir-301b | - |
| hsa-mir-3202-2 | HGLibA_58921 | 118 | 0.025620457 | hsa-mir-3202-2 | - |
| hsa-mir-3691 | HGLibA_59355 | 118 | 0.025620457 | hsa-mir-3691 | - |
| hsa-mir-371b | HGLibA_59377 | 118 | 0.025620457 | hsa-mir-371b | - |
| hsa-mir-378h | HGLibA_59457 | 118 | 0.025620457 | hsa-mir-378h | - |
| hsa-mir-4289 | HGLibA_59863 | 118 | 0.025620457 | hsa-mir-4289 | - |
| hsa-mir-4430 | HGLibA_60108 | 118 | 0.025620457 | hsa-mir-4430 | - |
| hsa-mir-4498 | HGLibA_60410 | 118 | 0.025620457 | hsa-mir-4498 | - |
| hsa-mir-4660 | HGLibA_60750 | 118 | 0.025620457 | hsa-mir-4660 | - |
| hsa-mir-4691 | HGLibA_60873 | 118 | 0.025620457 | hsa-mir-4691 | - |
| hsa-mir-4700 | HGLibA_60910 | 118 | 0.025620457 | hsa-mir-4700 | - |
| hsa-mir-4783 | HGLibA_61240 | 118 | 0.025620457 | hsa-mir-4783 | - |
| hsa-mir-5002 | HGLibA_61417 | 118 | 0.025620457 | hsa-mir-5002 | - |
| hsa-mir-581 | HGLibA_62421 | 118 | 0.025620457 | hsa-mir-581 | - |
| hsa-mir-8056 | HGLibA_64063 | 118 | 0.025620457 | hsa-mir-8056 | - |
| KIAA0355 | HGLibA_24626 | 118 | 0.025620457 | KIAA0355 | - |
| KIAA0907 | HGLibA_24663 | 118 | 0.025620457 | KIAA0907 | - |
| LOC100130357 | HGLibA_26779 | 118 | 0.025620457 | LOC100130357 | - |
| LOC286238 | HGLibA_26917 | 118 | 0.025620457 | LOC286238 | - |
| LOC339862 | HGLibA_26922 | 118 | 0.025620457 | LOC339862 | - |
| MPP5 | HGLibA_29758 | 118 | 0.025620457 | MPP5 | - |
| MTSS1L | HGLibA_30499 | 118 | 0.025620457 | MTSS1L | - |
| ATP5H | HGLibA_03663 | 117 | 0.01566942 | ATP5H | - |
| C14orf166B | HGLibA_05337 | 117 | 0.01566942 | C14orf166B | - |
| C20orf112 | HGLibA_05984 | 117 | 0.01566942 | C20orf112 | - |
| C5orf54 | HGLibA_06407 | 117 | 0.01566942 | C5orf54 | - |
| CCDC147 | HGLibA_07631 | 117 | 0.01566942 | CCDC147 | - |
| DPCR1 | HGLibA_13814 | 117 | 0.01566942 | DPCR1 | - |
| ENSG00000002822 | HGLibA_27841 | 117 | 0.01566942 | MAD1L1 | mitotic arrest deficient 1 like 1 [Source:HGNC Symbol;Acc:HGNC:6762] |
| ENSG00000007171 | HGLibA_32328 | 117 | 0.01566942 | NOS2 | nitric oxide synthase 2 [Source:HGNC Symbol;Acc:HGNC:7873] |
| ENSG00000007264 | HGLibA_28424 | 117 | 0.01566942 | MATK | megakaryocyte-associated tyrosine kinase [Source:HGNC Symbol;Acc:HGNC:6906] |

| | | | | | |
|---|---|---|---|---|---|
| ENSG00000007541 | HGLibA_36578 | 117 | 0.01566942 | PIGQ | phosphatidylinositol glycan anchor biosynthesis class Q [Source:HGNC Symbol;Acc:HGNC:14135] |
| ENSG00000008869 | HGLibA_21077 | 117 | 0.01566942 | HEATR5B | HEAT repeat containing 5B [Source:HGNC Symbol;Acc:HGNC:29273] |
| ENSG00000010319 | HGLibA_43336 | 117 | 0.01566942 | SEMA3G | semaphorin 3G [Source:HGNC Symbol;Acc:HGNC:30400] |
| ENSG00000013573 | HGLibA_12654 | 117 | 0.01566942 | DDX11 | DEAD/H-box helicase 11 [Source:HGNC Symbol;Acc:HGNC:2736] |
| ENSG00000047346 | HGLibA_16478 | 117 | 0.01566942 | FAM214A | family with sequence similarity 214 member A [Source:HGNC Symbol;Acc:HGNC:25609] |
| ENSG00000048471 | HGLibA_46078 | 117 | 0.01566942 | SNX29 | sorting nexin 29 [Source:HGNC Symbol;Acc:HGNC:30542] |
| ENSG00000059758 | HGLibA_08747 | 117 | 0.01566942 | CDK17 | cyclin dependent kinase 17 [Source:HGNC Symbol;Acc:HGNC:8750] |
| ENSG00000069702 | HGLibA_49229 | 117 | 0.01566942 | TGFBR3 | transforming growth factor beta receptor 3 [Source:HGNC Symbol;Acc:HGNC:11774] |
| ENSG00000088782 | HGLibA_12884 | 117 | 0.01566942 | DEFB127 | defensin beta 127 [Source:HGNC Symbol;Acc:HGNC:16206] |
| ENSG00000088833 | HGLibA_32755 | 117 | 0.01566942 | NSFL1C | NSFL1 cofactor [Source:HGNC Symbol;Acc:HGNC:15912] |
| ENSG00000091128 | HGLibA_25966 | 117 | 0.01566942 | LAMB4 | laminin subunit beta 4 [Source:HGNC Symbol;Acc:HGNC:6491] |
| ENSG00000092203 | HGLibA_51044 | 117 | 0.01566942 | TOX4 | TOX high mobility group box family member 4 [Source:HGNC Symbol;Acc:HGNC:20161] |
| ENSG00000094841 | HGLibA_53091 | 117 | 0.01566942 | UPRT | uracil phosphoribosyltransferase homolog [Source:HGNC Symbol;Acc:HGNC:28334] |
| ENSG00000099960 | HGLibA_45368 | 117 | 0.01566942 | SLC7A4 | solute carrier family 7 member 4 [Source:HGNC Symbol;Acc:HGNC:11062] |
| ENSG00000100564 | HGLibA_36558 | 117 | 0.01566942 | PIGH | phosphatidylinositol glycan anchor biosynthesis class H [Source:HGNC Symbol;Acc:HGNC:8964] |
| ENSG00000100596 | HGLibA_46895 | 117 | 0.01566942 | SPTLC2 | serine palmitoyltransferase long chain base subunit 2 [Source:HGNC Symbol;Acc:HGNC:11278] |
| ENSG00000100908 | HGLibA_15027 | 117 | 0.01566942 | EMC9 | ER membrane protein complex subunit 9 [Source:HGNC Symbol;Acc:HGNC:20273] |
| ENSG00000102043 | HGLibA_30439 | 117 | 0.01566942 | MTMR8 | myotubularin related protein 8 [Source:HGNC Symbol;Acc:HGNC:16825] |
| ENSG00000103160 | HGLibA_22215 | 117 | 0.01566942 | HSDL1 | hydroxysteroid dehydrogenase like 1 [Source:HGNC Symbol;Acc:HGNC:16475] |
| ENSG00000103353 | HGLibA_52695 | 117 | 0.01566942 | UBFD1 | ubiquitin family domain containing 1 [Source:HGNC Symbol;Acc:HGNC:30565] |
| ENSG00000103549 | HGLibA_41680 | 117 | 0.01566942 | RNF40 | ring finger protein 40 [Source:HGNC Symbol;Acc:HGNC:16867] |
| ENSG00000104332 | HGLibA_43737 | 117 | 0.01566942 | SFRP1 | secreted frizzled related protein 1 [Source:HGNC Symbol;Acc:HGNC:10776] |
| ENSG00000105202 | HGLibA_16938 | 117 | 0.01566942 | FBL | fibrillarin [Source:HGNC Symbol;Acc:HGNC:3599] |
| ENSG00000105610 | HGLibA_25039 | 117 | 0.01566942 | KLF1 | Kruppel like factor 1 [Source:HGNC Symbol;Acc:HGNC:6345] |
| ENSG00000106246 | HGLibA_39259 | 117 | 0.01566942 | PTCD1 | pentatricopeptide repeat domain 1 [Source:HGNC Symbol;Acc:HGNC:22198] |
| ENSG00000106299 | HGLibA_53964 | 117 | 0.01566942 | WASL | Wiskott-Aldrich syndrome like [Source:HGNC Symbol;Acc:HGNC:12735] |
| ENSG00000106927 | HGLibA_01752 | 117 | 0.01566942 | AMBP | alpha-1-microglobulin/bikunin precursor [Source:HGNC Symbol;Acc:HGNC:453] |
| ENSG00000108823 | HGLibA_43797 | 117 | 0.01566942 | SGCA | sarcoglycan alpha [Source:HGNC Symbol;Acc:HGNC:10805] |
| ENSG00000109101 | HGLibA_17910 | 117 | 0.01566942 | FOXN1 | forkhead box N1 [Source:HGNC Symbol;Acc:HGNC:12765] |
| ENSG00000110900 | HGLibA_51895 | 117 | 0.01566942 | TSPAN11 | tetraspanin 11 [Source:HGNC Symbol;Acc:HGNC:30795] |
| ENSG00000111142 | HGLibA_28934 | 117 | 0.01566942 | METAP2 | methionyl aminopeptidase 2 [Source:HGNC Symbol;Acc:HGNC:16672] |
| ENSG00000111325 | HGLibA_33312 | 117 | 0.01566942 | OGFOD2 | 2-oxoglutarate and iron dependent oxygenase domain containing 2 [Source:HGNC Symbol;Acc:HGNC:25823] |
| ENSG00000111554 | HGLibA_28693 | 117 | 0.01566942 | MDM1 | Mdm1 nuclear protein [Source:HGNC Symbol;Acc:HGNC:29917] |
| ENSG00000111837 | HGLibA_28017 | 117 | 0.01566942 | MAK | male germ cell associated kinase [Source:HGNC Symbol;Acc:HGNC:6816] |
| ENSG00000112290 | HGLibA_53952 | 117 | 0.01566942 | WASF1 | WAS protein family member 1 [Source:HGNC Symbol;Acc:HGNC:12732] |
| ENSG00000114853 | HGLibA_54900 | 117 | 0.01566942 | ZBTB47 | zinc finger and BTB domain containing 47 [Source:HGNC Symbol;Acc:HGNC:26955] |
| ENSG00000115419 | HGLibA_19324 | 117 | 0.01566942 | GLS | glutaminase [Source:HGNC Symbol;Acc:HGNC:4331] |
| ENSG00000116005 | HGLibA_35695 | 117 | 0.01566942 | PCYOX1 | prenylcysteine oxidase 1 [Source:HGNC Symbol;Acc:HGNC:20588] |
| ENSG00000117519 | HGLibA_10311 | 117 | 0.01566942 | CNN3 | calponin 3 [Source:HGNC Symbol;Acc:HGNC:2157] |
| ENSG00000118257 | HGLibA_32717 | 117 | 0.01566942 | NRP2 | neuropilin 2 [Source:HGNC Symbol;Acc:HGNC:8005] |
| ENSG00000119522 | HGLibA_12930 | 117 | 0.01566942 | DENND1A | DENN domain containing 1A [Source:HGNC Symbol;Acc:HGNC:29324] |
| ENSG00000120306 | HGLibA_12231 | 117 | 0.01566942 | CYSTM1 | cysteine rich transmembrane module containing 1 [Source:HGNC Symbol;Acc:HGNC:30239] |
| ENSG00000120725 | HGLibA_44171 | 117 | 0.01566942 | SIL1 | SIL1 nucleotide exchange factor [Source:HGNC Symbol;Acc:HGNC:24624] |
| ENSG00000123415 | HGLibA_45841 | 117 | 0.01566942 | SMUG1 | single-strand-selective monofunctional uracil-DNA glycosylase 1 [Source:HGNC Symbol;Acc:HGNC:17148] |
| ENSG00000124116 | HGLibA_54265 | 117 | 0.01566942 | WFDC3 | WAP four-disulfide core domain 3 [Source:HGNC Symbol;Acc:HGNC:15957] |
| ENSG00000124486 | HGLibA_53415 | 117 | 0.01566942 | USP9X | ubiquitin specific peptidase 9 X-linked [Source:HGNC Symbol;Acc:HGNC:12632] |
| ENSG00000125503 | HGLibA_38025 | 117 | 0.01566942 | PPP1R12C | protein phosphatase 1 regulatory subunit 12C [Source:HGNC Symbol;Acc:HGNC:14947] |
| ENSG00000128699 | HGLibA_34624 | 117 | 0.01566942 | ORMDL1 | ORMDL sphingolipid biosynthesis regulator 1 [Source:HGNC Symbol;Acc:HGNC:16036] |
| ENSG00000129518 | HGLibA_14340 | 117 | 0.01566942 | EAPP | E2F associated phosphoprotein [Source:HGNC Symbol;Acc:HGNC:19312] |
| ENSG00000130590 | HGLibA_42663 | 117 | 0.01566942 | SAMD10 | sterile alpha motif domain containing 10 [Source:HGNC Symbol;Acc:HGNC:16129] |
| ENSG00000130711 | HGLibA_38372 | 117 | 0.01566942 | PRDM12 | PR/SET domain 12 [Source:HGNC Symbol;Acc:HGNC:13997] |
| ENSG00000132478 | HGLibA_53046 | 117 | 0.01566942 | UNK | unkempt family zinc finger [Source:HGNC Symbol;Acc:HGNC:29369] |
| ENSG00000132855 | HGLibA_01917 | 117 | 0.01566942 | ANGPTL3 | angiopoietin like 3 [Source:HGNC Symbol;Acc:HGNC:491] |
| ENSG00000133874 | HGLibA_41486 | 117 | 0.01566942 | RNF122 | ring finger protein 122 [Source:HGNC Symbol;Acc:HGNC:21147] |
| ENSG00000134716 | HGLibA_12137 | 117 | 0.01566942 | CYP2J2 | cytochrome P450 family 2 subfamily J member 2 [Source:HGNC Symbol;Acc:HGNC:2634] |
| ENSG00000135211 | HGLibA_50464 | 117 | 0.01566942 | TMEM60 | transmembrane protein 60 [Source:HGNC Symbol;Acc:HGNC:21754] |
| ENSG00000135336 | HGLibA_34607 | 117 | 0.01566942 | ORC3 | origin recognition complex subunit 3 [Source:HGNC Symbol;Acc:HGNC:8489] |
| ENSG00000135604 | HGLibA_47619 | 117 | 0.01566942 | STX11 | syntaxin 11 [Source:HGNC Symbol;Acc:HGNC:11429] |
| ENSG00000136045 | HGLibA_39634 | 117 | 0.01566942 | PWP1 | endonuclein [Source:HGNC Symbol;Acc:HGNC:17015]" |
| ENSG00000136114 | HGLibA_49401 | 117 | 0.01566942 | THSD1 | thrombospondin type 1 domain containing 1 [Source:HGNC Symbol;Acc:HGNC:17754] |
| ENSG00000136696 | HGLibA_23148 | 117 | 0.01566942 | IL36B | interleukin 36 beta [Source:HGNC Symbol;Acc:HGNC:15564] |
| ENSG00000136938 | HGLibA_02202 | 117 | 0.01566942 | ANP32B | acidic nuclear phosphoprotein 32 family member B [Source:HGNC Symbol;Acc:HGNC:16677] |
| ENSG00000137166 | HGLibA_17938 | 117 | 0.01566942 | FOXP4 | forkhead box P4 [Source:HGNC Symbol;Acc:HGNC:20842] |
| ENSG00000138600 | HGLibA_46775 | 117 | 0.01566942 | SPPL2A | signal peptide peptidase like 2A [Source:HGNC Symbol;Acc:HGNC:30227] |
| ENSG00000139304 | HGLibA_39530 | 117 | 0.01566942 | PTPRQ | receptor type Q [Source:HGNC Symbol;Acc:HGNC:9679]" |
| ENSG00000140022 | HGLibA_47549 | 117 | 0.01566942 | STON2 | stonin 2 [Source:HGNC Symbol;Acc:HGNC:30652] |
| ENSG00000142910 | HGLibA_49551 | 117 | 0.01566942 | TINAGL1 | tubulointerstitial nephritis antigen like 1 [Source:HGNC Symbol;Acc:HGNC:19168] |
| ENSG00000143155 | HGLibA_49563 | 117 | 0.01566942 | TIPRL | TOR signaling pathway regulator [Source:HGNC Symbol;Acc:HGNC:30231] |
| ENSG00000143786 | HGLibA_10291 | 117 | 0.01566942 | CNIH3 | cornichon family AMPA receptor auxiliary protein 3 [Source:HGNC Symbol;Acc:HGNC:26802] |
| ENSG00000145191 | HGLibA_14746 | 117 | 0.01566942 | EIF2B5 | eukaryotic translation initiation factor 2B subunit epsilon [Source:HGNC Symbol;Acc:HGNC:3261] |
| ENSG00000145244 | HGLibA_10770 | 117 | 0.01566942 | CORIN | serine peptidase [Source:HGNC Symbol;Acc:HGNC:19012]" |
| ENSG00000145826 | HGLibA_26267 | 117 | 0.01566942 | LECT2 | leukocyte cell derived chemotaxin 2 [Source:HGNC Symbol;Acc:HGNC:6550] |
| ENSG00000146005 | HGLibA_39016 | 117 | 0.01566942 | PSD2 | pleckstrin and Sec7 domain containing 2 [Source:HGNC Symbol;Acc:HGNC:19092] |
| ENSG00000147526 | HGLibA_48158 | 117 | 0.01566942 | TACC1 | transforming acidic coiled-coil containing protein 1 [Source:HGNC Symbol;Acc:HGNC:11522] |
| ENSG00000148426 | HGLibA_38699 | 117 | 0.01566942 | PROSER2 | proline and serine rich 2 [Source:HGNC Symbol;Acc:HGNC:23728] |
| ENSG00000151360 | HGLibA_01670 | 117 | 0.01566942 | ALLC | allantoicase [Source:HGNC Symbol;Acc:HGNC:17377] |
| ENSG00000154165 | HGLibA_19927 | 117 | 0.01566942 | GPR15 | G protein-coupled receptor 15 [Source:HGNC Symbol;Acc:HGNC:4469] |
| ENSG00000155366 | HGLibA_41182 | 117 | 0.01566942 | RHOC | ras homolog family member C [Source:HGNC Symbol;Acc:HGNC:669] |
| ENSG00000158435 | HGLibA_10333 | 117 | 0.01566942 | CNOT11 | CCR4-NOT transcription complex subunit 11 [Source:HGNC Symbol;Acc:HGNC:25217] |
| ENSG00000158869 | HGLibA_17170 | 117 | 0.01566942 | FCER1G | Fc fragment of IgE receptor Ig [Source:HGNC Symbol;Acc:HGNC:3611] |
| ENSG00000161940 | HGLibA_04335 | 117 | 0.01566942 | BCL6B | B cell CLL/lymphoma 6B [Source:HGNC Symbol;Acc:HGNC:1002] |
| ENSG00000161999 | HGLibA_23968 | 117 | 0.01566942 | JMJD8 | jumonji domain containing 8 [Source:HGNC Symbol;Acc:HGNC:14148] |
| ENSG00000162591 | HGLibA_28852 | 117 | 0.01566942 | MEGF6 | multiple EGF like domains 6 [Source:HGNC Symbol;Acc:HGNC:3232] |
| ENSG00000163312 | HGLibA_21118 | 117 | 0.01566942 | HELQ | POLQ like [Source:HGNC Symbol;Acc:HGNC:18536]" |
| ENSG00000163530 | HGLibA_13886 | 117 | 0.01566942 | DPPA2 | developmental pluripotency associated 2 [Source:HGNC Symbol;Acc:HGNC:19197] |
| ENSG00000163531 | HGLibA_31767 | 117 | 0.01566942 | NFASC | neurofascin [Source:HGNC Symbol;Acc:HGNC:29866] |
| ENSG00000164117 | HGLibA_17128 | 117 | 0.01566942 | FBXO8 | F-box protein 8 [Source:HGNC Symbol;Acc:HGNC:13587] |
| ENSG00000164118 | HGLibA_09141 | 117 | 0.01566942 | CEP44 | centrosomal protein 44 [Source:HGNC Symbol;Acc:HGNC:29356] |
| ENSG00000164442 | HGLibA_09745 | 117 | 0.01566942 | CITED2 | Cbp/p300 interacting transactivator with Glu/Asp rich carboxy-terminal domain 2 [Source:HGNC Symbol;Acc:HGNC:1987] |
| ENSG00000164796 | HGLibA_11369 | 117 | 0.01566942 | CSMD3 | CUB and Sushi multiple domains 3 [Source:HGNC Symbol;Acc:HGNC:19291] |
| ENSG00000164933 | HGLibA_44708 | 117 | 0.01566942 | SLC25A32 | solute carrier family 25 member 32 [Source:HGNC Symbol;Acc:HGNC:29683] |
| ENSG00000165269 | HGLibA_02605 | 117 | 0.01566942 | AQP7 | aquaporin 7 [Source:HGNC Symbol;Acc:HGNC:640] |
| ENSG00000165410 | HGLibA_09284 | 117 | 0.01566942 | CFL2 | cofilin 2 [Source:HGNC Symbol;Acc:HGNC:1875] |
| ENSG00000166333 | HGLibA_23221 | 117 | 0.01566942 | ILK | integrin linked kinase [Source:HGNC Symbol;Acc:HGNC:6040] |
| ENSG00000167785 | HGLibA_56233 | 117 | 0.01566942 | ZNF558 | zinc finger protein 558 [Source:HGNC Symbol;Acc:HGNC:26422] |
| ENSG00000168282 | HGLibA_29134 | 117 | 0.01566942 | MGAT2 | 2-N-acetylglucosaminyltransferase [Source:HGNC Symbol;Acc:HGNC:7045]" |
| ENSG00000168724 | HGLibA_13596 | 117 | 0.01566942 | DNAJC21 | DnaJ heat shock protein family (Hsp40) member C21 [Source:HGNC Symbol;Acc:HGNC:27030] |
| ENSG00000169813 | HGLibA_21820 | 117 | 0.01566942 | HNRNPF | heterogeneous nuclear ribonucleoprotein F [Source:HGNC Symbol;Acc:HGNC:5039] |
| ENSG00000170128 | HGLibA_20006 | 117 | 0.01566942 | GPR25 | G protein-coupled receptor 25 [Source:HGNC Symbol;Acc:HGNC:4480] |
| ENSG00000170627 | HGLibA_20627 | 117 | 0.01566942 | GTSF1 | gametocyte specific factor 1 [Source:HGNC Symbol;Acc:HGNC:26565] |
| ENSG00000170775 | HGLibA_20033 | 117 | 0.01566942 | GPR37 | G protein-coupled receptor 37 [Source:HGNC Symbol;Acc:HGNC:4494] |
| ENSG00000171611 | HGLibA_39286 | 117 | 0.01566942 | PTCRA | pre T cell antigen receptor alpha [Source:HGNC Symbol;Acc:HGNC:21290] |
| ENSG00000172269 | HGLibA_13810 | 117 | 0.01566942 | DPAGT1 | dolichyl-phosphate N-acetylglucosaminephosphotransferase 1 [Source:HGNC Symbol;Acc:HGNC:2995] |
| ENSG00000172380 | HGLibA_19514 | 117 | 0.01566942 | GNG12 | G protein subunit gamma 12 [Source:HGNC Symbol;Acc:HGNC:19663] |
| ENSG00000172464 | HGLibA_34244 | 117 | 0.01566942 | OR5AP2 | olfactory receptor family 5 subfamily AP member 2 [Source:HGNC Symbol;Acc:HGNC:15258] |
| ENSG00000173207 | HGLibA_09788 | 117 | 0.01566942 | CKS1B | CDC28 protein kinase regulatory subunit 1B [Source:HGNC Symbol;Acc:HGNC:19083] |
| ENSG00000173210 | HGLibA_00315 | 117 | 0.01566942 | ABLIM3 | actin binding LIM protein family member 3 [Source:HGNC Symbol;Acc:HGNC:29132] |
| ENSG00000173786 | HGLibA_10355 | 117 | 0.01566942 | CNP | 3'-cyclic nucleotide 3' phosphodiesterase [Source:HGNC Symbol;Acc:HGNC:2158]" |
| ENSG00000173992 | HGLibA_08184 | 117 | 0.01566942 | CCS | copper chaperone for superoxide dismutase [Source:HGNC Symbol;Acc:HGNC:1613] |
| ENSG00000174276 | HGLibA_56927 | 117 | 0.01566942 | ZNHIT2 | zinc finger HIT-type containing 2 [Source:HGNC Symbol;Acc:HGNC:1177] |
| ENSG00000174521 | HGLibA_52161 | 117 | 0.01566942 | TTC9B | tetratricopeptide repeat domain 9B [Source:HGNC Symbol;Acc:HGNC:26395] |
| ENSG00000174527 | HGLibA_30842 | 117 | 0.01566942 | MYO1H | myosin IH [Source:HGNC Symbol;Acc:HGNC:13879] |
| ENSG00000174844 | HGLibA_13478 | 117 | 0.01566942 | DNAH12 | dynein axonemal heavy chain 12 [Source:HGNC Symbol;Acc:HGNC:2943] |
| ENSG00000177938 | HGLibA_07211 | 117 | 0.01566942 | CAPZA3 | capping actin protein of muscle Z-line subunit alpha 3 [Source:HGNC Symbol;Acc:HGNC:24205] |
| ENSG00000177954 | HGLibA_42121 | 117 | 0.01566942 | RPS27 | ribosomal protein S27 [Source:HGNC Symbol;Acc:HGNC:10416] |
| ENSG00000178015 | HGLibA_19930 | 117 | 0.01566942 | GPR150 | G protein-coupled receptor 150 [Source:HGNC Symbol;Acc:HGNC:23628] |
| ENSG00000179761 | HGLibA_36726 | 117 | 0.01566942 | PIPOX | pipecolic acid and sarcosine oxidase [Source:HGNC Symbol;Acc:HGNC:17804] |
| ENSG00000179919 | HGLibA_33475 | 117 | 0.01566942 | OR10A7 | olfactory receptor family 10 subfamily A member 7 [Source:HGNC Symbol;Acc:HGNC:15329] |
| ENSG00000180739 | HGLibA_42605 | 117 | 0.01566942 | S1PR5 | sphingosine-1-phosphate receptor 5 [Source:HGNC Symbol;Acc:HGNC:14299] |
| ENSG00000180773 | HGLibA_45040 | 117 | 0.01566942 | SLC36A4 | solute carrier family 36 member 4 [Source:HGNC Symbol;Acc:HGNC:19660] |
| ENSG00000181631 | HGLibA_34880 | 117 | 0.01566942 | P2RY13 | purinergic receptor P2Y13 [Source:HGNC Symbol;Acc:HGNC:4537] |
| ENSG00000181781 | HGLibA_33293 | 117 | 0.01566942 | ODF3L2 | outer dense fiber of sperm tails 3 like 2 [Source:HGNC Symbol;Acc:HGNC:26841] |
| ENSG00000182054 | HGLibA_22541 | 117 | 0.01566942 | IDH2 | mitochondrial [Source:HGNC Symbol;Acc:HGNC:5383]" |
| ENSG00000182083 | HGLibA_34377 | 117 | 0.01566942 | OR6B2 | olfactory receptor family 6 subfamily B member 2 [Source:HGNC Symbol;Acc:HGNC:15041] |
| ENSG00000182261 | HGLibA_32101 | 117 | 0.01566942 | NLRP10 | NLR family pyrin domain containing 10 [Source:HGNC Symbol;Acc:HGNC:21464] |
| ENSG00000183092 | HGLibA_04398 | 117 | 0.01566942 | BEGAIN | brain enriched guanylate kinase associated [Source:HGNC Symbol;Acc:HGNC:24163] |
| ENSG00000183876 | HGLibA_03150 | 117 | 0.01566942 | ARSI | arylsulfatase family member I [Source:HGNC Symbol;Acc:HGNC:32521] |
| ENSG00000186965 | HGLibA_25667 | 117 | 0.01566942 | KRTAP19-2 | keratin associated protein 19-2 [Source:HGNC Symbol;Acc:HGNC:18937] |
| ENSG00000187268 | HGLibA_16823 | 117 | 0.01566942 | FAM9C | family with sequence similarity 9 member C [Source:HGNC Symbol;Acc:HGNC:18405] |
| ENSG00000187569 | HGLibA_13889 | 117 | 0.01566942 | DPPA3 | developmental pluripotency associated 3 [Source:HGNC Symbol;Acc:HGNC:19199] |
| ENSG00000188816 | HGLibA_21761 | 117 | 0.01566942 | HMX2 | H6 family homeobox 2 [Source:HGNC Symbol;Acc:HGNC:5018] |
| ENSG00000188817 | HGLibA_46013 | 117 | 0.01566942 | SNTN | cilia apical structure protein [Source:HGNC Symbol;Acc:HGNC:33706]" |
| ENSG00000196329 | HGLibA_19081 | 117 | 0.01566942 | GIMAP5 | IMAP family member 5 [Source:HGNC Symbol;Acc:HGNC:18005]" |
| ENSG00000197616 | HGLibA_30729 | 117 | 0.01566942 | MYH6 | myosin heavy chain 6 [Source:HGNC Symbol;Acc:HGNC:7576] |
| ENSG00000198342 | HGLibA_56009 | 117 | 0.01566942 | ZNF442 | zinc finger protein 442 [Source:HGNC Symbol;Acc:HGNC:20877] |

| | | | | | |
|---|---|---|---|---|---|
| ENSG00000198342 | HGLibA_56007 | 117 | 0.01566942 | ZNF442 | zinc finger protein 442 [Source:HGNC Symbol;Acc:HGNC:20877] |
| ENSG00000198515 | HGLibA_10262 | 117 | 0.01566942 | CNGA1 | cyclic nucleotide gated channel alpha 1 [Source:HGNC Symbol;Acc:HGNC:2148] |
| ENSG00000203783 | HGLibA_38854 | 117 | 0.01566942 | PRR9 | proline rich 9 [Source:HGNC Symbol;Acc:HGNC:32057] |
| ENSG00000203989 | HGLibA_41221 | 117 | 0.01566942 | RHOXF2B | Rhox homeobox family member 2B [Source:HGNC Symbol;Acc:HGNC:33519] |
| ENSG00000205213 | HGLibA_26409 | 117 | 0.01566942 | LGR4 | leucine rich repeat containing G protein-coupled receptor 4 [Source:HGNC Symbol;Acc:HGNC:13299] |
| ENSG00000205810 | HGLibA_25292 | 117 | 0.01566942 | KLRC3 | killer cell lectin like receptor C3 [Source:HGNC Symbol;Acc:HGNC:6376] |
| ENSG00000205981 | HGLibA_13591 | 117 | 0.01566942 | DNAJC19 | DnaJ heat shock protein family (Hsp40) member C19 [Source:HGNC Symbol;Acc:HGNC:30528] |
| ENSG00000213023 | HGLibA_48076 | 117 | 0.01566942 | SYT3 | synaptotagmin 3 [Source:HGNC Symbol;Acc:HGNC:11511] |
| ENSG00000213648 | HGLibA_47739 | 117 | 0.01566942 | SULT1A4 | sulfotransferase family 1A member 4 [Source:HGNC Symbol;Acc:HGNC:30004] |
| ENSG00000214022 | HGLibA_40844 | 117 | 0.01566942 | REPIN1 | replication initiator 1 [Source:HGNC Symbol;Acc:HGNC:17922] |
| ENSG00000234776 | HGLibA_05217 | 117 | 0.01566942 | C11orf94 | chromosome 11 open reading frame 94 [Source:HGNC Symbol;Acc:HGNC:37213] |
| ENSG00000242950 | HGLibA_15552 | 117 | 0.01566942 | ERVW-1 | envelope [Source:HGNC Symbol;Acc:HGNC:13525]" |
| ENSG00000248643 | HGLibA_40489 | 117 | 0.01566942 | RBM14-RBM4 | RBM14-RBM4 readthrough [Source:HGNC Symbol;Acc:HGNC:38840] |
| ENSG00000275969 | HGLibA_46475 | 117 | 0.01566942 | SPATA31A3 | SPATA31 subfamily A member 3 [Source:HGNC Symbol;Acc:HGNC:32003] |
| ENSG00000278224 | HGLibA_38479 | 117 | 0.01566942 | PRICKLE4 | prickle planar cell polarity protein 4 [Source:HGNC Symbol;Acc:HGNC:16805] |
| hsa-mir-1273h | HGLibA_57553 | 117 | 0.01566942 | hsa-mir-1273h | - |
| hsa-mir-1914 | HGLibA_58059 | 117 | 0.01566942 | hsa-mir-1914 | - |
| hsa-mir-194-2 | HGLibA_58085 | 117 | 0.01566942 | hsa-mir-194-2 | - |
| hsa-mir-197 | HGLibA_58106 | 117 | 0.01566942 | hsa-mir-197 | - |
| hsa-mir-216a | HGLibA_58257 | 117 | 0.01566942 | hsa-mir-216a | - |
| hsa-mir-3918 | HGLibA_59548 | 117 | 0.01566942 | hsa-mir-3918 | - |
| hsa-mir-4650-2 | HGLibA_60707 | 117 | 0.01566942 | hsa-mir-4650-2 | - |
| hsa-mir-5586 | HGLibA_62187 | 117 | 0.01566942 | hsa-mir-5586 | - |
| hsa-mir-5702 | HGLibA_62347 | 117 | 0.01566942 | hsa-mir-5702 | - |
| hsa-mir-6748 | HGLibA_63145 | 117 | 0.01566942 | hsa-mir-6748 | - |
| hsa-mir-6812 | HGLibA_63426 | 117 | 0.01566942 | hsa-mir-6812 | - |
| hsa-mir-6874 | HGLibA_63680 | 117 | 0.01566942 | hsa-mir-6874 | - |
| hsa-mir-7851 | HGLibA_63993 | 117 | 0.01566942 | hsa-mir-7851 | - |
| hsa-mir-8082 | HGLibA_64169 | 117 | 0.01566942 | hsa-mir-8082 | - |
| hsa-mir-8088 | HGLibA_64195 | 117 | 0.01566942 | hsa-mir-8088 | - |
| hsa-mir-941-3 | HGLibA_64347 | 117 | 0.01566942 | hsa-mir-941-3 | - |
| KARS | HGLibA_24047 | 117 | 0.01566942 | KARS | - |
| LOC643037 | HGLibA_26964 | 117 | 0.01566942 | LOC643037 | - |
| LOC643355 | HGLibA_26965 | 117 | 0.01566942 | LOC643355 | - |
| PRAC | HGLibA_38261 | 117 | 0.01566942 | PRAC | - |
| TCEB3B | HGLibA_48744 | 117 | 0.01566942 | TCEB3B | - |
| AMICA1 | HGLibA_01789 | 116 | 0.005718383 | AMICA1 | - |
| ATP5A1 | HGLibA_03639 | 116 | 0.005718383 | ATP5A1 | - |
| C11orf82 | HGLibA_05189 | 116 | 0.005718383 | C11orf82 | - |
| C6orf10 | HGLibA_06432 | 116 | 0.005718383 | C6orf10 | - |
| C6orf170 | HGLibA_06460 | 116 | 0.005718383 | C6orf170 | - |
| C6orf99 | HGLibA_06514 | 116 | 0.005718383 | C6orf99 | - |
| C9orf47 | HGLibA_06738 | 116 | 0.005718383 | C9orf47 | - |
| ENSG00000000419 | HGLibA_13858 | 116 | 0.005718383 | DPM1 | catalytic [Source:HGNC Symbol;Acc:HGNC:3005]" |
| ENSG00000026751 | HGLibA_44318 | 116 | 0.005718383 | SLAMF7 | SLAM family member 7 [Source:HGNC Symbol;Acc:HGNC:21394] |
| ENSG00000028137 | HGLibA_50775 | 116 | 0.005718383 | TNFRSF1B | TNF receptor superfamily member 1B [Source:HGNC Symbol;Acc:HGNC:11917] |
| ENSG00000042445 | HGLibA_40880 | 116 | 0.005718383 | RETSAT | retinol saturase [Source:HGNC Symbol;Acc:HGNC:25991] |
| ENSG00000047932 | HGLibA_19672 | 116 | 0.005718383 | GOPC | golgi associated PDZ and coiled-coil motif containing [Source:HGNC Symbol;Acc:HGNC:17643] |
| ENSG00000048140 | HGLibA_51914 | 116 | 0.005718383 | TSPAN17 | tetraspanin 17 [Source:HGNC Symbol;Acc:HGNC:13594] |
| ENSG00000069275 | HGLibA_32915 | 116 | 0.005718383 | NUCKS1 | nuclear casein kinase and cyclin dependent kinase substrate 1 [Source:HGNC Symbol;Acc:HGNC:29923] |
| ENSG00000075234 | HGLibA_52129 | 116 | 0.005718383 | TTC38 | tetratricopeptide repeat domain 38 [Source:HGNC Symbol;Acc:HGNC:26082] |
| ENSG00000077585 | HGLibA_19901 | 116 | 0.005718383 | GPR137B | G protein-coupled receptor 137B [Source:HGNC Symbol;Acc:HGNC:11862] |
| ENSG00000085982 | HGLibA_53361 | 116 | 0.005718383 | USP40 | ubiquitin specific peptidase 40 [Source:HGNC Symbol;Acc:HGNC:20069] |
| ENSG00000087266 | HGLibA_43927 | 116 | 0.005718383 | SH3BP2 | SH3 domain binding protein 2 [Source:HGNC Symbol;Acc:HGNC:10825] |
| ENSG00000096433 | HGLibA_23858 | 116 | 0.005718383 | ITPR3 | 5-trisphosphate receptor type 3 [Source:HGNC Symbol;Acc:HGNC:6182]" |
| ENSG00000100012 | HGLibA_43195 | 116 | 0.005718383 | SEC14L3 | SEC14 like lipid binding 3 [Source:HGNC Symbol;Acc:HGNC:18655] |
| ENSG00000100453 | HGLibA_20722 | 116 | 0.005718383 | GZMB | granzyme B [Source:HGNC Symbol;Acc:HGNC:4709] |
| ENSG00000102024 | HGLibA_37229 | 116 | 0.005718383 | PLS3 | plastin 3 [Source:HGNC Symbol;Acc:HGNC:9091] |
| ENSG00000103599 | HGLibA_23519 | 116 | 0.005718383 | IQCH | IQ motif containing H [Source:HGNC Symbol;Acc:HGNC:25721] |
| ENSG00000104375 | HGLibA_47478 | 116 | 0.005718383 | STK3 | serine/threonine kinase 3 [Source:HGNC Symbol;Acc:HGNC:11406] |
| ENSG00000105352 | HGLibA_08941 | 116 | 0.005718383 | CEACAM4 | carcinoembryonic antigen related cell adhesion molecule 4 [Source:HGNC Symbol;Acc:HGNC:1816] |
| ENSG00000105404 | HGLibA_39996 | 116 | 0.005718383 | RABAC1 | Rab acceptor 1 [Source:HGNC Symbol;Acc:HGNC:9794] |
| ENSG00000105697 | HGLibA_20822 | 116 | 0.005718383 | HAMP | hepcidin antimicrobial peptide [Source:HGNC Symbol;Acc:HGNC:15598] |
| ENSG00000106331 | HGLibA_35303 | 116 | 0.005718383 | PAX4 | paired box 4 [Source:HGNC Symbol;Acc:HGNC:8618] |
| ENSG00000106701 | HGLibA_18081 | 116 | 0.005718383 | FSD1L | fibronectin type III and SPRY domain containing 1 like [Source:HGNC Symbol;Acc:HGNC:13753] |
| ENSG00000107819 | HGLibA_43787 | 116 | 0.005718383 | SFXN3 | sideroflexin 3 [Source:HGNC Symbol;Acc:HGNC:16087] |
| ENSG00000107949 | HGLibA_04269 | 116 | 0.005718383 | BCCIP | BRCA2 and CDKN1A interacting protein [Source:HGNC Symbol;Acc:HGNC:978] |
| ENSG00000109103 | HGLibA_52988 | 116 | 0.005718383 | UNC119 | unc-119 lipid binding chaperone [Source:HGNC Symbol;Acc:HGNC:12565] |
| ENSG00000109158 | HGLibA_18365 | 116 | 0.005718383 | GABRA4 | gamma-aminobutyric acid type A receptor alpha4 subunit [Source:HGNC Symbol;Acc:HGNC:4078] |
| ENSG00000110011 | HGLibA_13622 | 116 | 0.005718383 | DNAJC4 | DnaJ heat shock protein family (Hsp40) member C4 [Source:HGNC Symbol;Acc:HGNC:5271] |
| ENSG00000112378 | HGLibA_36064 | 116 | 0.005718383 | PERP | TP53 apoptosis effector [Source:HGNC Symbol;Acc:HGNC:17637]" |
| ENSG00000112539 | HGLibA_06438 | 116 | 0.005718383 | C6orf118 | chromosome 6 open reading frame 118 [Source:HGNC Symbol;Acc:HGNC:21233] |
| ENSG00000113328 | HGLibA_08093 | 116 | 0.005718383 | CCNG1 | cyclin G1 [Source:HGNC Symbol;Acc:HGNC:1592] |
| ENSG00000114098 | HGLibA_03032 | 116 | 0.005718383 | ARMC8 | armadillo repeat containing 8 [Source:HGNC Symbol;Acc:HGNC:24999] |
| ENSG00000114520 | HGLibA_46095 | 116 | 0.005718383 | SNX4 | sorting nexin 4 [Source:HGNC Symbol;Acc:HGNC:11175] |
| ENSG00000115598 | HGLibA_23065 | 116 | 0.005718383 | IL1RL2 | interleukin 1 receptor like 2 [Source:HGNC Symbol;Acc:HGNC:5999] |
| ENSG00000116745 | HGLibA_41827 | 116 | 0.005718383 | RPE65 | retinoid isomerohydrolase [Source:HGNC Symbol;Acc:HGNC:10294]" |
| ENSG00000117602 | HGLibA_40673 | 116 | 0.005718383 | RCAN3 | RCAN family member 3 [Source:HGNC Symbol;Acc:HGNC:3042] |
| ENSG00000118707 | HGLibA_49242 | 116 | 0.005718383 | TGIF2 | TGFB induced factor homeobox 2 [Source:HGNC Symbol;Acc:HGNC:15764] |
| ENSG00000119778 | HGLibA_03407 | 116 | 0.005718383 | ATAD2B | AAA domain containing 2B [Source:HGNC Symbol;Acc:HGNC:29230]" |
| ENSG00000121057 | HGLibA_01402 | 116 | 0.005718383 | AKAP1 | A-kinase anchoring protein 1 [Source:HGNC Symbol;Acc:HGNC:367] |
| ENSG00000121807 | HGLibA_08151 | 116 | 0.005718383 | CCR2 | C-C motif chemokine receptor 2 [Source:HGNC Symbol;Acc:HGNC:1603] |
| ENSG00000121964 | HGLibA_20514 | 116 | 0.005718383 | GTDC1 | glycosyltransferase like domain containing 1 [Source:HGNC Symbol;Acc:HGNC:20887] |
| ENSG00000122359 | HGLibA_02224 | 116 | 0.005718383 | ANXA11 | annexin A11 [Source:HGNC Symbol;Acc:HGNC:535] |
| ENSG00000123106 | HGLibA_07925 | 116 | 0.005718383 | CCDC91 | coiled-coil domain containing 91 [Source:HGNC Symbol;Acc:HGNC:24855] |
| ENSG00000123810 | HGLibA_04048 | 116 | 0.005718383 | B9D2 | B9 domain containing 2 [Source:HGNC Symbol;Acc:HGNC:28636] |
| ENSG00000124164 | HGLibA_53520 | 116 | 0.005718383 | VAPB | VAMP associated protein B and C [Source:HGNC Symbol;Acc:HGNC:12649] |
| ENSG00000125457 | HGLibA_29264 | 116 | 0.005718383 | MIF4GD | MIF4G domain containing [Source:HGNC Symbol;Acc:HGNC:24030] |
| ENSG00000126214 | HGLibA_25026 | 116 | 0.005718383 | KLC1 | kinesin light chain 1 [Source:HGNC Symbol;Acc:HGNC:6387] |
| ENSG00000127419 | HGLibA_50070 | 116 | 0.005718383 | TMEM175 | transmembrane protein 175 [Source:HGNC Symbol;Acc:HGNC:28709] |
| ENSG00000127616 | HGLibA_45607 | 116 | 0.005718383 | SMARCA4 | member 4 [Source:HGNC Symbol;Acc:HGNC:11100]" |
| ENSG00000130508 | HGLibA_39649 | 116 | 0.005718383 | PXDN | peroxidasin [Source:HGNC Symbol;Acc:HGNC:14966] |
| ENSG00000130726 | HGLibA_51450 | 116 | 0.005718383 | TRIM28 | tripartite motif containing 28 [Source:HGNC Symbol;Acc:HGNC:16384] |
| ENSG00000130775 | HGLibA_49352 | 116 | 0.005718383 | THEMIS2 | thymocyte selection associated family member 2 [Source:HGNC Symbol;Acc:HGNC:16839] |
| ENSG00000131016 | HGLibA_01413 | 116 | 0.005718383 | AKAP12 | A-kinase anchoring protein 12 [Source:HGNC Symbol;Acc:HGNC:370] |
| ENSG00000132334 | HGLibA_39498 | 116 | 0.005718383 | PTPRE | receptor type E [Source:HGNC Symbol;Acc:HGNC:9669]" |
| ENSG00000132669 | HGLibA_41291 | 116 | 0.005718383 | RIN2 | Ras and Rab interactor 2 [Source:HGNC Symbol;Acc:HGNC:18750] |
| ENSG00000133678 | HGLibA_50328 | 116 | 0.005718383 | TMEM254 | transmembrane protein 254 [Source:HGNC Symbol;Acc:HGNC:25804] |
| ENSG00000135241 | HGLibA_37426 | 116 | 0.005718383 | PNPLA8 | patatin like phospholipase domain containing 8 [Source:HGNC Symbol;Acc:HGNC:28900] |
| ENSG00000135823 | HGLibA_47655 | 116 | 0.005718383 | STX6 | syntaxin 6 [Source:HGNC Symbol;Acc:HGNC:11441] |
| ENSG00000135912 | HGLibA_52203 | 116 | 0.005718383 | TTLL4 | tubulin tyrosine ligase like 4 [Source:HGNC Symbol;Acc:HGNC:28976] |
| ENSG00000136011 | HGLibA_47310 | 116 | 0.005718383 | STAB2 | stabilin 2 [Source:HGNC Symbol;Acc:HGNC:18629] |
| ENSG00000136197 | HGLibA_06525 | 116 | 0.005718383 | C7orf25 | chromosome 7 open reading frame 25 [Source:HGNC Symbol;Acc:HGNC:21703] |
| ENSG00000136235 | HGLibA_19833 | 116 | 0.005718383 | GPNMB | glycoprotein nmb [Source:HGNC Symbol;Acc:HGNC:4462] |
| ENSG00000137413 | HGLibA_48259 | 116 | 0.005718383 | TAF8 | TATA-box binding protein associated factor 8 [Source:HGNC Symbol;Acc:HGNC:17300] |
| ENSG00000137634 | HGLibA_33158 | 116 | 0.005718383 | NXPE4 | neurexophilin and PC-esterase domain family member 4 [Source:HGNC Symbol;Acc:HGNC:23117] |
| ENSG00000138640 | HGLibA_16140 | 116 | 0.005718383 | FAM13A | family with sequence similarity 13 member A [Source:HGNC Symbol;Acc:HGNC:19367] |
| ENSG00000139133 | HGLibA_01605 | 116 | 0.005718383 | ALG10 | 2-glucosyltransferase [Source:HGNC Symbol;Acc:HGNC:23162]" |
| ENSG00000139278 | HGLibA_19258 | 116 | 0.005718383 | GLIPR1 | GLI pathogenesis related 1 [Source:HGNC Symbol;Acc:HGNC:17001] |
| ENSG00000139517 | HGLibA_26746 | 116 | 0.005718383 | LNX2 | ligand of numb-protein X 2 [Source:HGNC Symbol;Acc:HGNC:20421] |
| ENSG00000139597 | HGLibA_30956 | 116 | 0.005718383 | N4BP2L1 | NEDD4 binding protein 2 like 1 [Source:HGNC Symbol;Acc:HGNC:25037] |
| ENSG00000141279 | HGLibA_32433 | 116 | 0.005718383 | NPEPPS | aminopeptidase puromycin sensitive [Source:HGNC Symbol;Acc:HGNC:7900] |
| ENSG00000141959 | HGLibA_36166 | 116 | 0.005718383 | PFKL | liver type [Source:HGNC Symbol;Acc:HGNC:8876]" |
| ENSG00000142046 | HGLibA_50559 | 116 | 0.005718383 | TMEM91 | transmembrane protein 91 [Source:HGNC Symbol;Acc:HGNC:32393] |
| ENSG00000143183 | HGLibA_49782 | 116 | 0.005718383 | TMCO1 | transmembrane and coiled-coil domains 1 [Source:HGNC Symbol;Acc:HGNC:18188] |
| ENSG00000143278 | HGLibA_15856 | 116 | 0.005718383 | F13B | coagulation factor XIII B chain [Source:HGNC Symbol;Acc:HGNC:3534] |
| ENSG00000143514 | HGLibA_51058 | 116 | 0.005718383 | TP53BP2 | tumor protein p53 binding protein 2 [Source:HGNC Symbol;Acc:HGNC:12000] |
| ENSG00000144339 | HGLibA_49835 | 116 | 0.005718383 | TMEFF2 | transmembrane protein with EGF like and two follistatin like domains 2 [Source:HGNC Symbol;Acc:HGNC:11867] |
| ENSG00000145283 | HGLibA_44344 | 116 | 0.005718383 | SLC10A6 | solute carrier family 10 member 6 [Source:HGNC Symbol;Acc:HGNC:30603] |
| ENSG00000148334 | HGLibA_39329 | 116 | 0.005718383 | PTGES2 | prostaglandin E synthase 2 [Source:HGNC Symbol;Acc:HGNC:17822] |
| ENSG00000148680 | HGLibA_22405 | 116 | 0.005718383 | HTR7 | 5-hydroxytryptamine receptor 7 [Source:HGNC Symbol;Acc:HGNC:5302] |
| ENSG00000149257 | HGLibA_43595 | 116 | 0.005718383 | SERPINH1 | serpin family H member 1 [Source:HGNC Symbol;Acc:HGNC:1546] |
| ENSG00000149927 | HGLibA_13726 | 116 | 0.005718383 | DOC2A | double C2 domain alpha [Source:HGNC Symbol;Acc:HGNC:2985] |
| ENSG00000150627 | HGLibA_54037 | 116 | 0.005718383 | WDR17 | WD repeat domain 17 [Source:HGNC Symbol;Acc:HGNC:16661] |
| ENSG00000151532 | HGLibA_53887 | 116 | 0.005718383 | VTI1A | vesicle transport through interaction with t-SNAREs 1A [Source:HGNC Symbol;Acc:HGNC:17792] |
| ENSG00000155085 | HGLibA_01400 | 116 | 0.005718383 | AK9 | adenylate kinase 9 [Source:HGNC Symbol;Acc:HGNC:33814] |
| ENSG00000155926 | HGLibA_44300 | 116 | 0.005718383 | SLA | Src like adaptor [Source:HGNC Symbol;Acc:HGNC:10902] |
| ENSG00000157766 | HGLibA_00380 | 116 | 0.005718383 | ACAN | aggrecan [Source:HGNC Symbol;Acc:HGNC:319] |
| ENSG00000158014 | HGLibA_44902 | 116 | 0.005718383 | SLC30A2 | solute carrier family 30 member 2 [Source:HGNC Symbol;Acc:HGNC:11013] |
| ENSG00000158578 | HGLibA_01525 | 116 | 0.005718383 | ALAS2 | 5'-aminolevulinate synthase 2 [Source:HGNC Symbol;Acc:HGNC:397] |
| ENSG00000159289 | HGLibA_19607 | 116 | 0.005718383 | GOLGA6A | golgin A6 family member A [Source:HGNC Symbol;Acc:HGNC:13567] |
| ENSG00000159640 | HGLibA_00423 | 116 | 0.005718383 | ACE | angiotensin I converting enzyme [Source:HGNC Symbol;Acc:HGNC:2707] |
| ENSG00000162402 | HGLibA_53308 | 116 | 0.005718383 | USP24 | ubiquitin specific peptidase 24 [Source:HGNC Symbol;Acc:HGNC:12623] |
| ENSG00000162757 | HGLibA_05895 | 116 | 0.005718383 | C1orf74 | chromosome 1 open reading frame 74 [Source:HGNC Symbol;Acc:HGNC:26319] |
| ENSG00000163435 | HGLibA_14898 | 116 | 0.005718383 | ELF3 | E74 like ETS transcription factor 3 [Source:HGNC Symbol;Acc:HGNC:3318] |

| | | | | | |
|---|---|---|---|---|---|
| ENSG00000163586 | HGLibA_15908 | 116 | 0.005718383 | FABP1 | fatty acid binding protein 1 [Source:HGNC Symbol;Acc:HGNC:3555] |
| ENSG00000163719 | HGLibA_30422 | 116 | 0.005718383 | MTMR14 | myotubularin related protein 14 [Source:HGNC Symbol;Acc:HGNC:26190] |
| ENSG00000163807 | HGLibA_24697 | 116 | 0.005718383 | KIAA1143 | KIAA1143 [Source:HGNC Symbol;Acc:HGNC:29198] |
| ENSG00000164197 | HGLibA_41580 | 116 | 0.005718383 | RNF180 | ring finger protein 180 [Source:HGNC Symbol;Acc:HGNC:27752] |
| ENSG00000164398 | HGLibA_00566 | 116 | 0.005718383 | ACSL6 | acyl-CoA synthetase long chain family member 6 [Source:HGNC Symbol;Acc:HGNC:16496] |
| ENSG00000164404 | HGLibA_18879 | 116 | 0.005718383 | GDF9 | growth differentiation factor 9 [Source:HGNC Symbol;Acc:HGNC:4224] |
| ENSG00000166118 | HGLibA_46441 | 116 | 0.005718383 | SPATA19 | spermatogenesis associated 19 [Source:HGNC Symbol;Acc:HGNC:30614] |
| ENSG00000167333 | HGLibA_51592 | 116 | 0.005718383 | TRIM68 | tripartite motif containing 68 [Source:HGNC Symbol;Acc:HGNC:21161] |
| ENSG00000168216 | HGLibA_26675 | 116 | 0.005718383 | LMBRD1 | LMBR1 domain containing 1 [Source:HGNC Symbol;Acc:HGNC:23038] |
| ENSG00000170248 | HGLibA_35742 | 116 | 0.005718383 | PDCD6IP | programmed cell death 6 interacting protein [Source:HGNC Symbol;Acc:HGNC:8766] |
| ENSG00000170477 | HGLibA_25505 | 116 | 0.005718383 | KRT4 | keratin 4 [Source:HGNC Symbol;Acc:HGNC:6441] |
| ENSG00000170537 | HGLibA_49767 | 116 | 0.005718383 | TMC7 | transmembrane channel like 7 [Source:HGNC Symbol;Acc:HGNC:23000] |
| ENSG00000171241 | HGLibA_44011 | 116 | 0.005718383 | SHCBP1 | SHC binding and spindle associated 1 [Source:HGNC Symbol;Acc:HGNC:29547] |
| ENSG00000171451 | HGLibA_14019 | 116 | 0.005718383 | DSEL | dermatan sulfate epimerase like [Source:HGNC Symbol;Acc:HGNC:18144] |
| ENSG00000171680 | HGLibA_37115 | 116 | 0.005718383 | PLEKHG5 | pleckstrin homology and RhoGEF domain containing G5 [Source:HGNC Symbol;Acc:HGNC:29105] |
| ENSG00000171735 | HGLibA_07116 | 116 | 0.005718383 | CAMTA1 | calmodulin binding transcription activator 1 [Source:HGNC Symbol;Acc:HGNC:18806] |
| ENSG00000172179 | HGLibA_38597 | 116 | 0.005718383 | PRL | prolactin [Source:HGNC Symbol;Acc:HGNC:9445] |
| ENSG00000172640 | HGLibA_33480 | 116 | 0.005718383 | OR10AD1 | olfactory receptor family 10 subfamily AD member 1 [Source:HGNC Symbol;Acc:HGNC:14819] |
| ENSG00000173153 | HGLibA_15592 | 116 | 0.005718383 | ESRRA | estrogen related receptor alpha [Source:HGNC Symbol;Acc:HGNC:3471] |
| ENSG00000173334 | HGLibA_51390 | 116 | 0.005718383 | TRIB1 | tribbles pseudokinase 1 [Source:HGNC Symbol;Acc:HGNC:16891] |
| ENSG00000173389 | HGLibA_23502 | 116 | 0.005718383 | IQCF1 | IQ motif containing F1 [Source:HGNC Symbol;Acc:HGNC:28607] |
| ENSG00000174576 | HGLibA_32402 | 116 | 0.005718383 | NPAS4 | neuronal PAS domain protein 4 [Source:HGNC Symbol;Acc:HGNC:18983] |
| ENSG00000175564 | HGLibA_52838 | 116 | 0.005718383 | UCP3 | uncoupling protein 3 [Source:HGNC Symbol;Acc:HGNC:12519] |
| ENSG00000176749 | HGLibA_08776 | 116 | 0.005718383 | CDK5R1 | cyclin dependent kinase 5 regulatory subunit 1 [Source:HGNC Symbol;Acc:HGNC:1775] |
| ENSG00000177257 | HGLibA_12916 | 116 | 0.005718383 | DEFB4B | defensin beta 4B [Source:HGNC Symbol;Acc:HGNC:30193] |
| ENSG00000177570 | HGLibA_42669 | 116 | 0.005718383 | SAMD12 | sterile alpha motif domain containing 12 [Source:HGNC Symbol;Acc:HGNC:31750] |
| ENSG00000177684 | HGLibA_12856 | 116 | 0.005718383 | DEFB114 | defensin beta 114 [Source:HGNC Symbol;Acc:HGNC:18095] |
| ENSG00000178568 | HGLibA_15423 | 116 | 0.005718383 | ERBB4 | erb-b2 receptor tyrosine kinase 4 [Source:HGNC Symbol;Acc:HGNC:3432] |
| ENSG00000178718 | HGLibA_42029 | 116 | 0.005718383 | RPP25 | ribonuclease P and MRP subunit p25 [Source:HGNC Symbol;Acc:HGNC:30361] |
| ENSG00000179222 | HGLibA_27968 | 116 | 0.005718383 | MAGED1 | MAGE family member D1 [Source:HGNC Symbol;Acc:HGNC:6813] |
| ENSG00000179902 | HGLibA_05815 | 116 | 0.005718383 | C1orf194 | chromosome 1 open reading frame 194 [Source:HGNC Symbol;Acc:HGNC:32331] |
| ENSG00000180011 | HGLibA_54769 | 116 | 0.005718383 | ZADH2 | zinc binding alcohol dehydrogenase domain containing 2 [Source:HGNC Symbol;Acc:HGNC:28697] |
| ENSG00000180871 | HGLibA_11885 | 116 | 0.005718383 | CXCR2 | C-X-C motif chemokine receptor 2 [Source:HGNC Symbol;Acc:HGNC:6027] |
| ENSG00000182544 | HGLibA_29104 | 116 | 0.005718383 | MFSD5 | major facilitator superfamily domain containing 5 [Source:HGNC Symbol;Acc:HGNC:28156] |
| ENSG00000182585 | HGLibA_15282 | 116 | 0.005718383 | EPGN | epithelial mitogen [Source:HGNC Symbol;Acc:HGNC:17470] |
| ENSG00000182606 | HGLibA_51276 | 116 | 0.005718383 | TRAK1 | trafficking kinesin protein 1 [Source:HGNC Symbol;Acc:HGNC:29947] |
| ENSG00000183208 | HGLibA_18904 | 116 | 0.005718383 | GDPGP1 | GDP-D-glucose phosphorylase 1 [Source:HGNC Symbol;Acc:HGNC:34360] |
| ENSG00000184140 | HGLibA_34012 | 116 | 0.005718383 | OR4F6 | olfactory receptor family 4 subfamily F member 6 [Source:HGNC Symbol;Acc:HGNC:15372] |
| ENSG00000184220 | HGLibA_10206 | 116 | 0.005718383 | CMSS1 | cms1 ribosomal small subunit homolog [Source:HGNC Symbol;Acc:HGNC:28666] |
| ENSG00000185630 | HGLibA_35342 | 116 | 0.005718383 | PBX1 | PBX homeobox 1 [Source:HGNC Symbol;Acc:HGNC:8632] |
| ENSG00000185803 | HGLibA_45246 | 116 | 0.005718383 | SLC52A2 | solute carrier family 52 member 2 [Source:HGNC Symbol;Acc:HGNC:30224] |
| ENSG00000185962 | HGLibA_26130 | 116 | 0.005718383 | LCE3A | late cornified envelope 3A [Source:HGNC Symbol;Acc:HGNC:29461] |
| ENSG00000186113 | HGLibA_34277 | 116 | 0.005718383 | OR5D14 | olfactory receptor family 5 subfamily D member 14 [Source:HGNC Symbol;Acc:HGNC:15281] |
| ENSG00000186207 | HGLibA_26148 | 116 | 0.005718383 | LCE5A | late cornified envelope 5A [Source:HGNC Symbol;Acc:HGNC:16614] |
| ENSG00000187091 | HGLibA_36991 | 116 | 0.005718383 | PLCD1 | phospholipase C delta 1 [Source:HGNC Symbol;Acc:HGNC:9060] |
| ENSG00000187105 | HGLibA_21075 | 116 | 0.005718383 | HEATR4 | HEAT repeat containing 4 [Source:HGNC Symbol;Acc:HGNC:16761] |
| ENSG00000187474 | HGLibA_17970 | 116 | 0.005718383 | FPR3 | formyl peptide receptor 3 [Source:HGNC Symbol;Acc:HGNC:3828] |
| ENSG00000196155 | HGLibA_37110 | 116 | 0.005718383 | PLEKHG4 | pleckstrin homology and RhoGEF domain containing G4 [Source:HGNC Symbol;Acc:HGNC:24501] |
| ENSG00000197233 | HGLibA_33676 | 116 | 0.005718383 | OR1J2 | olfactory receptor family 1 subfamily J member 2 [Source:HGNC Symbol;Acc:HGNC:8209] |
| ENSG00000197563 | HGLibA_36570 | 116 | 0.005718383 | PIGN | phosphatidylinositol glycan anchor biosynthesis class N [Source:HGNC Symbol;Acc:HGNC:8967] |
| ENSG00000203705 | HGLibA_48447 | 116 | 0.005718383 | TATDN3 | TatD DNase domain containing 3 [Source:HGNC Symbol;Acc:HGNC:27010] |
| ENSG00000204020 | HGLibA_26630 | 116 | 0.005718383 | LIPN | lipase family member N [Source:HGNC Symbol;Acc:HGNC:23452] |
| ENSG00000205085 | HGLibA_16704 | 116 | 0.005718383 | FAM71F2 | family with sequence similarity 71 member F2 [Source:HGNC Symbol;Acc:HGNC:27998] |
| ENSG00000213658 | HGLibA_26057 | 116 | 0.005718383 | LAT | linker for activation of T cells [Source:HGNC Symbol;Acc:HGNC:18874] |
| ENSG00000213930 | HGLibA_18597 | 116 | 0.005718383 | GALT | galactose-1-phosphate uridylyltransferase [Source:HGNC Symbol;Acc:HGNC:4135] |
| ENSG00000216649 | HGLibA_18445 | 116 | 0.005718383 | GAGE12E | G antigen 12E [Source:HGNC Symbol;Acc:HGNC:31905] |
| ENSG00000228474 | HGLibA_34705 | 116 | 0.005718383 | OST4 | non-catalytic [Source:HGNC Symbol;Acc:HGNC:32483]" |
| ENSG00000243414 | HGLibA_49448 | 116 | 0.005718383 | TICAM2 | toll like receptor adaptor molecule 2 [Source:HGNC Symbol;Acc:HGNC:21354] |
| ENSG00000253304 | HGLibA_50173 | 116 | 0.005718383 | TMEM200B | transmembrane protein 200B [Source:HGNC Symbol;Acc:HGNC:33785] |
| ENSG00000255398 | HGLibA_20952 | 116 | 0.005718383 | HCAR3 | hydroxycarboxylic acid receptor 3 [Source:HGNC Symbol;Acc:HGNC:16824] |
| ENSG00000257115 | HGLibA_33572 | 116 | 0.005718383 | OR11H12 | olfactory receptor family 11 subfamily H member 12 [Source:HGNC Symbol;Acc:HGNC:30738] |
| ENSG00000257591 | HGLibA_56418 | 116 | 0.005718383 | ZNF625 | zinc finger protein 625 [Source:HGNC Symbol;Acc:HGNC:30571] |
| ENSG00000258659 | HGLibA_51469 | 116 | 0.005718383 | TRIM34 | tripartite motif containing 34 [Source:HGNC Symbol;Acc:HGNC:10063] |
| ENSG00000258713 | HGLibA_05986 | 116 | 0.005718383 | C20orf141 | chromosome 20 open reading frame 141 [Source:HGNC Symbol;Acc:HGNC:16134] |
| ENSG00000275152 | HGLibA_07975 | 116 | 0.005718383 | CCL16 | C-C motif chemokine ligand 16 [Source:HGNC Symbol;Acc:HGNC:10614] |
| ERO1LB | HGLibA_15522 | 116 | 0.005718383 | ERO1LB | - |
| FAM192A | HGLibA_16361 | 116 | 0.005718383 | FAM192A | - |
| HIST2H3D | HGLibA_21547 | 116 | 0.005718383 | HIST2H3D | - |
| hsa-mir-1281 | HGLibA_57580 | 116 | 0.005718383 | hsa-mir-1281 | - |
| hsa-mir-3169 | HGLibA_58754 | 116 | 0.005718383 | hsa-mir-3169 | - |
| hsa-mir-328 | HGLibA_58977 | 116 | 0.005718383 | hsa-mir-328 | - |
| hsa-mir-369 | HGLibA_59341 | 116 | 0.005718383 | hsa-mir-369 | - |
| hsa-mir-422a | HGLibA_59697 | 116 | 0.005718383 | hsa-mir-422a | - |
| hsa-mir-4313 | HGLibA_59968 | 116 | 0.005718383 | hsa-mir-4313 | - |
| hsa-mir-4687 | HGLibA_60857 | 116 | 0.005718383 | hsa-mir-4687 | - |
| hsa-mir-4717 | HGLibA_60968 | 116 | 0.005718383 | hsa-mir-4717 | - |
| hsa-mir-497 | HGLibA_61390 | 116 | 0.005718383 | hsa-mir-497 | - |
| hsa-mir-523 | HGLibA_61780 | 116 | 0.005718383 | hsa-mir-523 | - |
| hsa-mir-548aa-1 | HGLibA_61850 | 116 | 0.005718383 | hsa-mir-548aa-1 | - |
| hsa-mir-552 | HGLibA_62127 | 116 | 0.005718383 | hsa-mir-552 | - |
| hsa-mir-610 | HGLibA_62632 | 116 | 0.005718383 | hsa-mir-610 | - |
| hsa-mir-6132 | HGLibA_62678 | 116 | 0.005718383 | hsa-mir-6132 | - |
| hsa-mir-6502 | HGLibA_62860 | 116 | 0.005718383 | hsa-mir-6502 | - |
| hsa-mir-6722 | HGLibA_63049 | 116 | 0.005718383 | hsa-mir-6722 | - |
| hsa-mir-6837 | HGLibA_63522 | 116 | 0.005718383 | hsa-mir-6837 | - |
| hsa-mir-6862-1 | HGLibA_63630 | 116 | 0.005718383 | hsa-mir-6862-1 | - |
| hsa-mir-7112-1 | HGLibA_63812 | 116 | 0.005718383 | hsa-mir-7112-1 | - |
| hsa-mir-767 | HGLibA_63933 | 116 | 0.005718383 | hsa-mir-767 | - |
| hsa-mir-7850 | HGLibA_63990 | 116 | 0.005718383 | hsa-mir-7850 | - |
| hsa-mir-8085 | HGLibA_64183 | 116 | 0.005718383 | hsa-mir-8085 | - |
| KIAA1407 | HGLibA_24748 | 116 | 0.005718383 | KIAA1407 | - |
| LOC284385 | HGLibA_26914 | 116 | 0.005718383 | LOC284385 | - |
| 10-Sep | HGLibA_43427 | 116 | 0.005718383 | 10-Sep | - |
| ALS2CR8 | HGLibA_01731 | 115 | -0.004232655 | ALS2CR8 | - |
| C17orf74 | HGLibA_05570 | 115 | -0.004232655 | C17orf74 | - |
| C1orf192 | HGLibA_05812 | 115 | -0.004232655 | C1orf192 | - |
| C1orf51 | HGLibA_05864 | 115 | -0.004232655 | C1orf51 | - |
| CXorf40B | HGLibA_11926 | 115 | -0.004232655 | CXorf40B | - |
| ENSG00000006453 | HGLibA_04124 | 115 | -0.004232655 | BAIAP2L1 | BAI1 associated protein 2 like 1 [Source:HGNC Symbol;Acc:HGNC:21649] |
| ENSG00000008952 | HGLibA_43258 | 115 | -0.004232655 | SEC62 | preprotein translocation factor [Source:HGNC Symbol;Acc:HGNC:11846]" |
| ENSG00000029153 | HGLibA_03071 | 115 | -0.004232655 | ARNTL2 | aryl hydrocarbon receptor nuclear translocator like 2 [Source:HGNC Symbol;Acc:HGNC:18984] |
| ENSG00000037474 | HGLibA_32782 | 115 | -0.004232655 | NSUN2 | NOP2/Sun RNA methyltransferase family member 2 [Source:HGNC Symbol;Acc:HGNC:25994] |
| ENSG00000041880 | HGLibA_35235 | 115 | -0.004232655 | PARP3 | poly(ADP-ribose) polymerase family member 3 [Source:HGNC Symbol;Acc:HGNC:273] |
| ENSG00000049245 | HGLibA_53493 | 115 | -0.004232655 | VAMP3 | vesicle associated membrane protein 3 [Source:HGNC Symbol;Acc:HGNC:12644] |
| ENSG00000058262 | HGLibA_43247 | 115 | -0.004232655 | SEC61A1 | Sec61 translocon alpha 1 subunit [Source:HGNC Symbol;Acc:HGNC:18276] |
| ENSG00000073150 | HGLibA_35113 | 115 | -0.004232655 | PANX2 | pannexin 2 [Source:HGNC Symbol;Acc:HGNC:8600] |
| ENSG00000074319 | HGLibA_51843 | 115 | -0.004232655 | TSG101 | tumor susceptibility 101 [Source:HGNC Symbol;Acc:HGNC:15971] |
| ENSG00000076662 | HGLibA_22500 | 115 | -0.004232655 | ICAM3 | intercellular adhesion molecule 3 [Source:HGNC Symbol;Acc:HGNC:5346] |
| ENSG00000077942 | HGLibA_16944 | 115 | -0.004232655 | FBLN1 | fibulin 1 [Source:HGNC Symbol;Acc:HGNC:3600] |
| ENSG00000089157 | HGLibA_42008 | 115 | -0.004232655 | RPLP0 | ribosomal protein lateral stalk subunit P0 [Source:HGNC Symbol;Acc:HGNC:10371] |
| ENSG00000089558 | HGLibA_24219 | 115 | -0.004232655 | KCNH4 | potassium voltage-gated channel subfamily H member 4 [Source:HGNC Symbol;Acc:HGNC:6253] |
| ENSG00000090924 | HGLibA_37104 | 115 | -0.004232655 | PLEKHG2 | pleckstrin homology and RhoGEF domain containing G2 [Source:HGNC Symbol;Acc:HGNC:29515] |
| ENSG00000099282 | HGLibA_51906 | 115 | -0.004232655 | TSPAN15 | tetraspanin 15 [Source:HGNC Symbol;Acc:HGNC:23298] |
| ENSG00000100532 | HGLibA_09328 | 115 | -0.004232655 | CGRRF1 | cell growth regulator with ring finger domain 1 [Source:HGNC Symbol;Acc:HGNC:15528] |
| ENSG00000102547 | HGLibA_06837 | 115 | -0.004232655 | CAB39L | calcium binding protein 39 like [Source:HGNC Symbol;Acc:HGNC:20290] |
| ENSG00000103005 | HGLibA_53160 | 115 | -0.004232655 | USB1 | U6 snRNA biogenesis phosphodiesterase 1 [Source:HGNC Symbol;Acc:HGNC:25792] |
| ENSG00000103067 | HGLibA_15589 | 115 | -0.004232655 | ESRP2 | epithelial splicing regulatory protein 2 [Source:HGNC Symbol;Acc:HGNC:26152] |
| ENSG00000103126 | HGLibA_03922 | 115 | -0.004232655 | AXIN1 | axin 1 [Source:HGNC Symbol;Acc:HGNC:903] |
| ENSG00000104368 | HGLibA_36961 | 115 | -0.004232655 | PLAT | tissue type [Source:HGNC Symbol;Acc:HGNC:9051]" |
| ENSG00000104679 | HGLibA_39767 | 115 | -0.004232655 | R3HCC1 | R3H domain and coiled-coil containing 1 [Source:HGNC Symbol;Acc:HGNC:27329] |
| ENSG00000104687 | HGLibA_20445 | 115 | -0.004232655 | GSR | glutathione-disulfide reductase [Source:HGNC Symbol;Acc:HGNC:4623] |
| ENSG00000105171 | HGLibA_37689 | 115 | -0.004232655 | POP4 | ribonuclease P/MRP subunit [Source:HGNC Symbol;Acc:HGNC:30081]" |
| ENSG00000105967 | HGLibA_49166 | 115 | -0.004232655 | TFEC | transcription factor EC [Source:HGNC Symbol;Acc:HGNC:11754] |
| ENSG00000106524 | HGLibA_01983 | 115 | -0.004232655 | ANKMY2 | ankyrin repeat and MYND domain containing 2 [Source:HGNC Symbol;Acc:HGNC:25370] |
| ENSG00000106541 | HGLibA_01244 | 115 | -0.004232655 | AGR2 | protein disulphide isomerase family member [Source:HGNC Symbol;Acc:HGNC:328]" |
| ENSG00000107957 | HGLibA_43965 | 115 | -0.004232655 | SH3PXD2A | SH3 and PX domains 2A [Source:HGNC Symbol;Acc:HGNC:23664] |
| ENSG00000110911 | HGLibA_44353 | 115 | -0.004232655 | SLC11A2 | solute carrier family 11 member 2 [Source:HGNC Symbol;Acc:HGNC:10908] |
| ENSG00000111405 | HGLibA_15118 | 115 | -0.004232655 | ENDOU | poly(U) specific [Source:HGNC Symbol;Acc:HGNC:14369]" |
| ENSG00000113361 | HGLibA_08686 | 115 | -0.004232655 | CDH6 | cadherin 6 [Source:HGNC Symbol;Acc:HGNC:1765] |
| ENSG00000113758 | HGLibA_12389 | 115 | -0.004232655 | DBN1 | drebrin 1 [Source:HGNC Symbol;Acc:HGNC:2695] |
| ENSG00000114126 | HGLibA_49153 | 115 | -0.004232655 | TFDP2 | transcription factor Dp-2 [Source:HGNC Symbol;Acc:HGNC:11751] |
| ENSG00000114248 | HGLibA_27311 | 115 | -0.004232655 | LRRC31 | leucine rich repeat containing 31 [Source:HGNC Symbol;Acc:HGNC:26261] |
| ENSG00000114544 | HGLibA_45144 | 115 | -0.004232655 | SLC41A3 | solute carrier family 41 member 3 [Source:HGNC Symbol;Acc:HGNC:31046] |
| ENSG00000114654 | HGLibA_14559 | 115 | -0.004232655 | EFCC1 | EF-hand and coiled-coil domain containing 1 [Source:HGNC Symbol;Acc:HGNC:25692] |
| ENSG00000115042 | HGLibA_15959 | 115 | -0.004232655 | FAHD2A | fumarylacetoacetate hydrolase domain containing 2A [Source:HGNC Symbol;Acc:HGNC:24252] |

| | | | | | |
|---|---|---|---|---|---|
| ENSG00000115290 | HGLibA_20226 | 115 | -0.004232655 | GRB14 | growth factor receptor bound protein 14 [Source:HGNC Symbol;Acc:HGNC:4565] |
| ENSG00000116586 | HGLibA_25993 | 115 | -0.004232655 | LAMTOR2 | MAPK and MTOR activator 2 [Source:HGNC Symbol;Acc:HGNC:29796]" |
| ENSG00000117569 | HGLibA_39252 | 115 | -0.004232655 | PTBP2 | polypyrimidine tract binding protein 2 [Source:HGNC Symbol;Acc:HGNC:17662] |
| ENSG00000117691 | HGLibA_31692 | 115 | -0.004232655 | NENF | neudesin neurotrophic factor [Source:HGNC Symbol;Acc:HGNC:30384] |
| ENSG00000118094 | HGLibA_51350 | 115 | -0.004232655 | TREH | trehalase [Source:HGNC Symbol;Acc:HGNC:12266] |
| ENSG00000120242 | HGLibA_22691 | 115 | -0.004232655 | IFNA8 | interferon alpha 8 [Source:HGNC Symbol;Acc:HGNC:5429] |
| ENSG00000123545 | HGLibA_31490 | 115 | -0.004232655 | NDUFAF4 | NADH:ubiquinone oxidoreductase complex assembly factor 4 [Source:HGNC Symbol;Acc:HGNC:21034] |
| ENSG00000123610 | HGLibA_50714 | 115 | -0.004232655 | TNFAIP6 | TNF alpha induced protein 6 [Source:HGNC Symbol;Acc:HGNC:11898] |
| ENSG00000124279 | HGLibA_16908 | 115 | -0.004232655 | FASTKD3 | FAST kinase domains 3 [Source:HGNC Symbol;Acc:HGNC:28758] |
| ENSG00000124467 | HGLibA_39056 | 115 | -0.004232655 | PSG8 | pregnancy specific beta-1-glycoprotein 8 [Source:HGNC Symbol;Acc:HGNC:9525] |
| ENSG00000125430 | HGLibA_22131 | 115 | -0.004232655 | HS3ST3B1 | heparan sulfate-glucosamine 3-sulfotransferase 3B1 [Source:HGNC Symbol;Acc:HGNC:5198] |
| ENSG00000127990 | HGLibA_43804 | 115 | -0.004232655 | SGCE | sarcoglycan epsilon [Source:HGNC Symbol;Acc:HGNC:10808] |
| ENSG00000130844 | HGLibA_55817 | 115 | -0.004232655 | ZNF331 | zinc finger protein 331 [Source:HGNC Symbol;Acc:HGNC:15489] |
| ENSG00000131408 | HGLibA_32581 | 115 | -0.004232655 | NR1H2 | nuclear receptor subfamily 1 group H member 2 [Source:HGNC Symbol;Acc:HGNC:7965] |
| ENSG00000132640 | HGLibA_04914 | 115 | -0.004232655 | BTBD3 | BTB domain containing 3 [Source:HGNC Symbol;Acc:HGNC:15854] |
| ENSG00000132693 | HGLibA_11216 | 115 | -0.004232655 | CRP | C-reactive protein [Source:HGNC Symbol;Acc:HGNC:2367] |
| ENSG00000132965 | HGLibA_01692 | 115 | -0.004232655 | ALOX5AP | arachidonate 5-lipoxygenase activating protein [Source:HGNC Symbol;Acc:HGNC:436] |
| ENSG00000134285 | HGLibA_17554 | 115 | -0.004232655 | FKBP11 | FK506 binding protein 11 [Source:HGNC Symbol;Acc:HGNC:18624] |
| ENSG00000134709 | HGLibA_21877 | 115 | -0.004232655 | HOOK1 | hook microtubule tethering protein 1 [Source:HGNC Symbol;Acc:HGNC:19884] |
| ENSG00000135643 | HGLibA_24352 | 115 | -0.004232655 | KCNMB4 | potassium calcium-activated channel subfamily M regulatory beta subunit 4 [Source:HGNC Symbol;Acc:HGNC:6289] |
| ENSG00000136631 | HGLibA_53795 | 115 | -0.004232655 | VPS45 | vacuolar protein sorting 45 homolog [Source:HGNC Symbol;Acc:HGNC:14579] |
| ENSG00000136944 | HGLibA_26735 | 115 | -0.004232655 | LMX1B | LIM homeobox transcription factor 1 beta [Source:HGNC Symbol;Acc:HGNC:6654] |
| ENSG00000137055 | HGLibA_36931 | 115 | -0.004232655 | PLAA | phospholipase A2 activating protein [Source:HGNC Symbol;Acc:HGNC:9043] |
| ENSG00000137074 | HGLibA_02574 | 115 | -0.004232655 | APTX | aprataxin [Source:HGNC Symbol;Acc:HGNC:15984] |
| ENSG00000137575 | HGLibA_43108 | 115 | -0.004232655 | SDCBP | syndecan binding protein [Source:HGNC Symbol;Acc:HGNC:10662] |
| ENSG00000138135 | HGLibA_09331 | 115 | -0.004232655 | CH25H | cholesterol 25-hydroxylase [Source:HGNC Symbol;Acc:HGNC:1907] |
| ENSG00000139908 | HGLibA_52019 | 115 | -0.004232655 | TSSK4 | testis specific serine kinase 4 [Source:HGNC Symbol;Acc:HGNC:19825] |
| ENSG00000140332 | HGLibA_49613 | 115 | -0.004232655 | TLE3 | transducin like enhancer of split 3 [Source:HGNC Symbol;Acc:HGNC:11839] |
| ENSG00000140983 | HGLibA_41205 | 115 | -0.004232655 | RHOT2 | ras homolog family member T2 [Source:HGNC Symbol;Acc:HGNC:21169] |
| ENSG00000141434 | HGLibA_28897 | 115 | -0.004232655 | MEP1B | meprin A subunit beta [Source:HGNC Symbol;Acc:HGNC:7020] |
| ENSG00000142619 | HGLibA_34974 | 115 | -0.004232655 | PADI3 | peptidyl arginine deiminase 3 [Source:HGNC Symbol;Acc:HGNC:18337] |
| ENSG00000142677 | HGLibA_23090 | 115 | -0.004232655 | IL22RA1 | interleukin 22 receptor subunit alpha 1 [Source:HGNC Symbol;Acc:HGNC:13700] |
| ENSG00000142864 | HGLibA_43467 | 115 | -0.004232655 | SERBP1 | SERPINE1 mRNA binding protein 1 [Source:HGNC Symbol;Acc:HGNC:17860] |
| ENSG00000143862 | HGLibA_02997 | 115 | -0.004232655 | ARL8A | ADP ribosylation factor like GTPase 8A [Source:HGNC Symbol;Acc:HGNC:25192] |
| ENSG00000144290 | HGLibA_45206 | 115 | -0.004232655 | SLC4A10 | solute carrier family 4 member 10 [Source:HGNC Symbol;Acc:HGNC:13811] |
| ENSG00000145321 | HGLibA_18754 | 115 | -0.004232655 | GC | vitamin D binding protein [Source:HGNC Symbol;Acc:HGNC:4187]" |
| ENSG00000145384 | HGLibA_15914 | 115 | -0.004232655 | FABP2 | fatty acid binding protein 2 [Source:HGNC Symbol;Acc:HGNC:3556] |
| ENSG00000146242 | HGLibA_51099 | 115 | -0.004232655 | TPBG | trophoblast glycoprotein [Source:HGNC Symbol;Acc:HGNC:12004] |
| ENSG00000147677 | HGLibA_14787 | 115 | -0.004232655 | EIF3H | eukaryotic translation initiation factor 3 subunit H [Source:HGNC Symbol;Acc:HGNC:3273] |
| ENSG00000148057 | HGLibA_22559 | 115 | -0.004232655 | IDNK | gluconokinase [Source:HGNC Symbol;Acc:HGNC:31367]" |
| ENSG00000148835 | HGLibA_48239 | 115 | -0.004232655 | TAF5 | TATA-box binding protein associated factor 5 [Source:HGNC Symbol;Acc:HGNC:11539] |
| ENSG00000149179 | HGLibA_05149 | 115 | -0.004232655 | C11orf49 | chromosome 11 open reading frame 49 [Source:HGNC Symbol;Acc:HGNC:28720] |
| ENSG00000150667 | HGLibA_18093 | 115 | -0.004232655 | FSIP1 | fibrous sheath interacting protein 1 [Source:HGNC Symbol;Acc:HGNC:21674] |
| ENSG00000150681 | HGLibA_41073 | 115 | -0.004232655 | RGS18 | regulator of G protein signaling 18 [Source:HGNC Symbol;Acc:HGNC:14261] |
| ENSG00000150961 | HGLibA_43239 | 115 | -0.004232655 | SEC24D | COPII coat complex component [Source:HGNC Symbol;Acc:HGNC:10706]" |
| ENSG00000151694 | HGLibA_00740 | 115 | -0.004232655 | ADAM17 | ADAM metallopeptidase domain 17 [Source:HGNC Symbol;Acc:HGNC:195] |
| ENSG00000153130 | HGLibA_43031 | 115 | -0.004232655 | SCOC | short coiled-coil protein [Source:HGNC Symbol;Acc:HGNC:20335] |
| ENSG00000154065 | HGLibA_02056 | 115 | -0.004232655 | ANKRD29 | ankyrin repeat domain 29 [Source:HGNC Symbol;Acc:HGNC:27110] |
| ENSG00000154319 | HGLibA_16241 | 115 | -0.004232655 | FAM167A | family with sequence similarity 167 member A [Source:HGNC Symbol;Acc:HGNC:15549] |
| ENSG00000154945 | HGLibA_02097 | 115 | -0.004232655 | ANKRD40 | ankyrin repeat domain 40 [Source:HGNC Symbol;Acc:HGNC:28233] |
| ENSG00000156006 | HGLibA_31176 | 115 | -0.004232655 | NAT2 | N-acetyltransferase 2 [Source:HGNC Symbol;Acc:HGNC:7646] |
| ENSG00000157796 | HGLibA_54044 | 115 | -0.004232655 | WDR19 | WD repeat domain 19 [Source:HGNC Symbol;Acc:HGNC:18340] |
| ENSG00000158480 | HGLibA_46443 | 115 | -0.004232655 | SPATA2 | spermatogenesis associated 2 [Source:HGNC Symbol;Acc:HGNC:14681] |
| ENSG00000160050 | HGLibA_07747 | 115 | -0.004232655 | CCDC28B | coiled-coil domain containing 28B [Source:HGNC Symbol;Acc:HGNC:28163] |
| ENSG00000160208 | HGLibA_42274 | 115 | -0.004232655 | RRP1B | ribosomal RNA processing 1B [Source:HGNC Symbol;Acc:HGNC:23818] |
| ENSG00000160471 | HGLibA_10850 | 115 | -0.004232655 | COX6B2 | cytochrome c oxidase subunit 6B2 [Source:HGNC Symbol;Acc:HGNC:24380] |
| ENSG00000160679 | HGLibA_09667 | 115 | -0.004232655 | CHTOP | chromatin target of PRMT1 [Source:HGNC Symbol;Acc:HGNC:24511] |
| ENSG00000162733 | HGLibA_12637 | 115 | -0.004232655 | DDR2 | discoidin domain receptor tyrosine kinase 2 [Source:HGNC Symbol;Acc:HGNC:2731] |
| ENSG00000163220 | HGLibA_42574 | 115 | -0.004232655 | S100A9 | S100 calcium binding protein A9 [Source:HGNC Symbol;Acc:HGNC:10499] |
| ENSG00000163872 | HGLibA_54655 | 115 | -0.004232655 | YEATS2 | YEATS domain containing 2 [Source:HGNC Symbol;Acc:HGNC:25489] |
| ENSG00000164106 | HGLibA_43042 | 115 | -0.004232655 | SCRG1 | stimulator of chondrogenesis 1 [Source:HGNC Symbol;Acc:HGNC:17036] |
| ENSG00000164168 | HGLibA_50116 | 115 | -0.004232655 | TMEM184C | transmembrane protein 184C [Source:HGNC Symbol;Acc:HGNC:25587] |
| ENSG00000165244 | HGLibA_55881 | 115 | -0.004232655 | ZNF367 | zinc finger protein 367 [Source:HGNC Symbol;Acc:HGNC:18320] |
| ENSG00000165379 | HGLibA_27164 | 115 | -0.004232655 | LRFN5 | leucine rich repeat and fibronectin type III domain containing 5 [Source:HGNC Symbol;Acc:HGNC:20360] |
| ENSG00000166165 | HGLibA_09766 | 115 | -0.004232655 | CKB | creatine kinase B [Source:HGNC Symbol;Acc:HGNC:1991] |
| ENSG00000166343 | HGLibA_30250 | 115 | -0.004232655 | MSS51 | MSS51 mitochondrial translational activator [Source:HGNC Symbol;Acc:HGNC:21000] |
| ENSG00000166484 | HGLibA_28281 | 115 | -0.004232655 | MAPK7 | mitogen-activated protein kinase 7 [Source:HGNC Symbol;Acc:HGNC:6880] |
| ENSG00000166908 | HGLibA_36712 | 115 | -0.004232655 | PIP4K2C | phosphatidylinositol-5-phosphate 4-kinase type 2 gamma [Source:HGNC Symbol;Acc:HGNC:23786] |
| ENSG00000166946 | HGLibA_08081 | 115 | -0.004232655 | CCNDBP1 | cyclin D1 binding protein 1 [Source:HGNC Symbol;Acc:HGNC:1587] |
| ENSG00000166971 | HGLibA_01518 | 115 | -0.004232655 | AKTIP | AKT interacting protein [Source:HGNC Symbol;Acc:HGNC:16710] |
| ENSG00000167549 | HGLibA_10788 | 115 | -0.004232655 | CORO6 | coronin 6 [Source:HGNC Symbol;Acc:HGNC:21356] |
| ENSG00000167618 | HGLibA_25935 | 115 | -0.004232655 | LAIR2 | leukocyte associated immunoglobulin like receptor 2 [Source:HGNC Symbol;Acc:HGNC:6478] |
| ENSG00000167986 | HGLibA_12593 | 115 | -0.004232655 | DDB1 | damage specific DNA binding protein 1 [Source:HGNC Symbol;Acc:HGNC:2717] |
| ENSG00000168062 | HGLibA_04177 | 115 | -0.004232655 | BATF2 | basic leucine zipper ATF-like transcription factor 2 [Source:HGNC Symbol;Acc:HGNC:25163] |
| ENSG00000168310 | HGLibA_23573 | 115 | -0.004232655 | IRF2 | interferon regulatory factor 2 [Source:HGNC Symbol;Acc:HGNC:6117] |
| ENSG00000168329 | HGLibA_11835 | 115 | -0.004232655 | CX3CR1 | C-X3-C motif chemokine receptor 1 [Source:HGNC Symbol;Acc:HGNC:2558] |
| ENSG00000168564 | HGLibA_08833 | 115 | -0.004232655 | CDKN2AIP | CDKN2A interacting protein [Source:HGNC Symbol;Acc:HGNC:24325] |
| ENSG00000168646 | HGLibA_03925 | 115 | -0.004232655 | AXIN2 | axin 2 [Source:HGNC Symbol;Acc:HGNC:904] |
| ENSG00000168785 | HGLibA_51940 | 115 | -0.004232655 | TSPAN5 | tetraspanin 5 [Source:HGNC Symbol;Acc:HGNC:17753] |
| ENSG00000168993 | HGLibA_10933 | 115 | -0.004232655 | CPLX1 | complexin 1 [Source:HGNC Symbol;Acc:HGNC:2309] |
| ENSG00000169385 | HGLibA_41403 | 115 | -0.004232655 | RNASE2 | ribonuclease A family member 2 [Source:HGNC Symbol;Acc:HGNC:10045] |
| ENSG00000170122 | HGLibA_17836 | 115 | -0.004232655 | FOXD4 | forkhead box D4 [Source:HGNC Symbol;Acc:HGNC:3805] |
| ENSG00000171223 | HGLibA_24007 | 115 | -0.004232655 | JUNB | AP-1 transcription factor subunit [Source:HGNC Symbol;Acc:HGNC:6205]" |
| ENSG00000171489 | HGLibA_46333 | 115 | -0.004232655 | SPACA5 | sperm acrosome associated 5 [Source:HGNC Symbol;Acc:HGNC:31353] |
| ENSG00000171657 | HGLibA_20089 | 115 | -0.004232655 | GPR82 | G protein-coupled receptor 82 [Source:HGNC Symbol;Acc:HGNC:4533] |
| ENSG00000172667 | HGLibA_55373 | 115 | -0.004232655 | ZMAT3 | zinc finger matrin-type 3 [Source:HGNC Symbol;Acc:HGNC:29983] |
| ENSG00000174564 | HGLibA_23078 | 115 | -0.004232655 | IL20RB | interleukin 20 receptor subunit beta [Source:HGNC Symbol;Acc:HGNC:6004] |
| ENSG00000176029 | HGLibA_05117 | 115 | -0.004232655 | C11orf16 | chromosome 11 open reading frame 16 [Source:HGNC Symbol;Acc:HGNC:1169] |
| ENSG00000177669 | HGLibA_28523 | 115 | -0.004232655 | MBOAT4 | membrane bound O-acyltransferase domain containing 4 [Source:HGNC Symbol;Acc:HGNC:32311] |
| ENSG00000178233 | HGLibA_50003 | 115 | -0.004232655 | TMEM151B | transmembrane protein 151B [Source:HGNC Symbol;Acc:HGNC:21315] |
| ENSG00000179213 | HGLibA_44157 | 115 | -0.004232655 | SIGLECL1 | SIGLEC family like 1 [Source:HGNC Symbol;Acc:HGNC:26856] |
| ENSG00000179598 | HGLibA_37050 | 115 | -0.004232655 | PLD6 | phospholipase D family member 6 [Source:HGNC Symbol;Acc:HGNC:30447] |
| ENSG00000181192 | HGLibA_13163 | 115 | -0.004232655 | DHTKD1 | dehydrogenase E1 and transketolase domain containing 1 [Source:HGNC Symbol;Acc:HGNC:23537] |
| ENSG00000181625 | HGLibA_45561 | 115 | -0.004232655 | SLX1B | structure-specific endonuclease subunit [Source:HGNC Symbol;Acc:HGNC:28748]" |
| ENSG00000183128 | HGLibA_07020 | 115 | -0.004232655 | CALHM3 | calcium homeostasis modulator 3 [Source:HGNC Symbol;Acc:HGNC:23458] |
| ENSG00000183323 | HGLibA_07577 | 115 | -0.004232655 | CCDC125 | coiled-coil domain containing 125 [Source:HGNC Symbol;Acc:HGNC:28924] |
| ENSG00000183439 | HGLibA_51567 | 115 | -0.004232655 | TRIM61 | tripartite motif containing 61 [Source:HGNC Symbol;Acc:HGNC:24339] |
| ENSG00000184012 | HGLibA_50635 | 115 | -0.004232655 | TMPRSS2 | transmembrane serine protease 2 [Source:HGNC Symbol;Acc:HGNC:11876] |
| ENSG00000185002 | HGLibA_40978 | 115 | -0.004232655 | RFX6 | regulatory factor X6 [Source:HGNC Symbol;Acc:HGNC:21478] |
| ENSG00000185385 | HGLibA_34459 | 115 | -0.004232655 | OR7A17 | olfactory receptor family 7 subfamily A member 17 [Source:HGNC Symbol;Acc:HGNC:8363] |
| ENSG00000186393 | HGLibA_25463 | 115 | -0.004232655 | KRT26 | keratin 26 [Source:HGNC Symbol;Acc:HGNC:30840] |
| ENSG00000186642 | HGLibA_35778 | 115 | -0.004232655 | PDE2A | phosphodiesterase 2A [Source:HGNC Symbol;Acc:HGNC:8777] |
| ENSG00000186803 | HGLibA_22659 | 115 | -0.004232655 | IFNA10 | interferon alpha 10 [Source:HGNC Symbol;Acc:HGNC:5418] |
| ENSG00000187017 | HGLibA_15574 | 115 | -0.004232655 | ESPN | espin [Source:HGNC Symbol;Acc:HGNC:13281] |
| ENSG00000196344 | HGLibA_00966 | 115 | -0.004232655 | ADH7 | mu or sigma polypeptide [Source:HGNC Symbol;Acc:HGNC:256]" |
| ENSG00000196549 | HGLibA_29469 | 115 | -0.004232655 | MME | membrane metalloendopeptidase [Source:HGNC Symbol;Acc:HGNC:7154] |
| ENSG00000196715 | HGLibA_53681 | 115 | -0.004232655 | VKORC1L1 | vitamin K epoxide reductase complex subunit 1 like 1 [Source:HGNC Symbol;Acc:HGNC:21492] |
| ENSG00000196878 | HGLibA_25961 | 115 | -0.004232655 | LAMB3 | laminin subunit beta 3 [Source:HGNC Symbol;Acc:HGNC:6490] |
| ENSG00000197403 | HGLibA_34429 | 115 | -0.004232655 | OR6N1 | olfactory receptor family 6 subfamily N member 1 [Source:HGNC Symbol;Acc:HGNC:15034] |
| ENSG00000198919 | HGLibA_14304 | 115 | -0.004232655 | DZIP3 | DAZ interacting zinc finger protein 3 [Source:HGNC Symbol;Acc:HGNC:30938] |
| ENSG00000203880 | HGLibA_35631 | 115 | -0.004232655 | PCMTD2 | protein-L-isoaspartate (D-aspartate) O-methyltransferase domain containing 2 [Source:HGNC Symbol;Acc:HGNC:15882] |
| ENSG00000204673 | HGLibA_01509 | 115 | -0.004232655 | AKT1S1 | AKT1 substrate 1 [Source:HGNC Symbol;Acc:HGNC:28426] |
| ENSG00000206172 | HGLibA_20906 | 115 | -0.004232655 | HBA1 | hemoglobin subunit alpha 1 [Source:HGNC Symbol;Acc:HGNC:4823] |
| ENSG00000212659 | HGLibA_25841 | 115 | -0.004232655 | KRTAP9-6 | keratin associated protein 9-6 [Source:HGNC Symbol;Acc:HGNC:18914] |
| ENSG00000214686 | HGLibA_23514 | 115 | -0.004232655 | IQCF6 | IQ motif containing F6 [Source:HGNC Symbol;Acc:HGNC:35158] |
| ENSG00000214943 | HGLibA_20022 | 115 | -0.004232655 | GPR33 | G protein-coupled receptor 33 (gene/pseudogene) [Source:HGNC Symbol;Acc:HGNC:4489] |
| ENSG00000215018 | HGLibA_10556 | 115 | -0.004232655 | COL28A1 | collagen type XXVIII alpha 1 chain [Source:HGNC Symbol;Acc:HGNC:22442] |
| ENSG00000242419 | HGLibA_35574 | 115 | -0.004232655 | PCDHGC4 | 4 [Source:HGNC Symbol;Acc:HGNC:8717]" |
| ENSG00000243073 | HGLibA_38316 | 115 | -0.004232655 | PRAMEF4 | PRAME family member 4 [Source:HGNC Symbol;Acc:HGNC:31971] |
| ENSG00000244752 | HGLibA_11263 | 115 | -0.004232655 | CRYBB2 | crystallin beta B2 [Source:HGNC Symbol;Acc:HGNC:2398] |
| ENSG00000261594 | HGLibA_51102 | 115 | -0.004232655 | TPBGL | trophoblast glycoprotein like [Source:HGNC Symbol;Acc:HGNC:44159] |
| ENSG00000268350 | HGLibA_16185 | 115 | -0.004232655 | FAM156A | family with sequence similarity 156 member A [Source:HGNC Symbol;Acc:HGNC:30114] |
| ENSG00000270885 | HGLibA_40363 | 115 | -0.004232655 | RASL10B | RAS like family 10 member B [Source:HGNC Symbol;Acc:HGNC:30295] |
| ENSG00000272968 | HGLibA_40426 | 115 | -0.004232655 | RBAK-RBAKDN | RBAK-RBAKDN readthrough [Source:HGNC Symbol;Acc:HGNC:42971] |
| ENSG00000289549 | HGLibA_37907 | 115 | -0.004232655 | PPIAL4D | - |
| GPR128 | HGLibA_19885 | 115 | -0.004232655 | GPR128 | - |
| H2AFB1 | HGLibA_20745 | 115 | -0.004232655 | H2AFB1 | - |
| hsa-mir-105-1 | HGLibA_57179 | 115 | -0.004232655 | hsa-mir-105-1 | - |
| hsa-mir-215 | HGLibA_58254 | 115 | -0.004232655 | hsa-mir-215 | - |
| hsa-mir-3175 | HGLibA_58774 | 115 | -0.004232655 | hsa-mir-3175 | - |
| hsa-mir-326 | HGLibA_58972 | 115 | -0.004232655 | hsa-mir-326 | - |
| hsa-mir-3973 | HGLibA_59651 | 115 | -0.004232655 | hsa-mir-3973 | - |
| hsa-mir-4317 | HGLibA_59986 | 115 | -0.004232655 | hsa-mir-4317 | - |
| hsa-mir-4441 | HGLibA_60167 | 115 | -0.004232655 | hsa-mir-4441 | - |
| hsa-mir-4499 | HGLibA_60414 | 115 | -0.004232655 | hsa-mir-4499 | - |

| | | | | | |
|---|---|---|---|---|---|
| hsa-mir-450a-2 | HGLibA_60475 | 115 | -0.004232655 | hsa-mir-450a-2 | - |
| hsa-mir-4685 | HGLibA_60849 | 115 | -0.004232655 | hsa-mir-4685 | - |
| hsa-mir-4705 | HGLibA_60922 | 115 | -0.004232655 | hsa-mir-4705 | - |
| hsa-mir-4740 | HGLibA_61056 | 115 | -0.004232655 | hsa-mir-4740 | - |
| hsa-mir-4764 | HGLibA_61154 | 115 | -0.004232655 | hsa-mir-4764 | - |
| hsa-mir-492 | HGLibA_61368 | 115 | -0.004232655 | hsa-mir-492 | - |
| hsa-mir-5087 | HGLibA_61496 | 115 | -0.004232655 | hsa-mir-5087 | - |
| hsa-mir-6128 | HGLibA_62659 | 115 | -0.004232655 | hsa-mir-6128 | - |
| hsa-mir-6129 | HGLibA_62664 | 115 | -0.004232655 | hsa-mir-6129 | - |
| hsa-mir-614 | HGLibA_62691 | 115 | -0.004232655 | hsa-mir-614 | - |
| hsa-mir-6739 | HGLibA_63110 | 115 | -0.004232655 | hsa-mir-6739 | - |
| hsa-mir-6823 | HGLibA_63469 | 115 | -0.004232655 | hsa-mir-6823 | - |
| hsa-mir-6851 | HGLibA_63575 | 115 | -0.004232655 | hsa-mir-6851 | - |
| hsa-mir-8059 | HGLibA_64075 | 115 | -0.004232655 | hsa-mir-8059 | - |
| hsa-mir-939 | HGLibA_64330 | 115 | -0.004232655 | hsa-mir-939 | - |
| LIMS3L | HGLibA_26554 | 115 | -0.004232655 | LIMS3L | - |
| MGEA5 | HGLibA_29156 | 115 | -0.004232655 | MGEA5 | - |
| SHOX_X | HGLibA_44070 | 115 | -0.004232655 | SHOX_X | - |
| SMCR7 | HGLibA_45677 | 115 | -0.004232655 | SMCR7 | - |
| 7-Mar | HGLibA_28345 | 114 | -0.014183692 | 7-Mar | - |
| ENSG00000006468 | HGLibA_15647 | 114 | -0.014183692 | ETV1 | ETS variant 1 [Source:HGNC Symbol;Acc:HGNC:3490] |
| ENSG00000008277 | HGLibA_00759 | 114 | -0.014183692 | ADAM22 | ADAM metallopeptidase domain 22 [Source:HGNC Symbol;Acc:HGNC:201] |
| ENSG00000011332 | HGLibA_13827 | 114 | -0.014183692 | DPF1 | double PHD fingers 1 [Source:HGNC Symbol;Acc:HGNC:20225] |
| ENSG00000024048 | HGLibA_52751 | 114 | -0.014183692 | UBR2 | ubiquitin protein ligase E3 component n-recognin 2 [Source:HGNC Symbol;Acc:HGNC:21289] |
| ENSG00000049323 | HGLibA_27591 | 114 | -0.014183692 | LTBP1 | latent transforming growth factor beta binding protein 1 [Source:HGNC Symbol;Acc:HGNC:6714] |
| ENSG00000062524 | HGLibA_27610 | 114 | -0.014183692 | LTK | leukocyte receptor tyrosine kinase [Source:HGNC Symbol;Acc:HGNC:6721] |
| ENSG00000064201 | HGLibA_51930 | 114 | -0.014183692 | TSPAN32 | tetraspanin 32 [Source:HGNC Symbol;Acc:HGNC:13410] |
| ENSG00000070886 | HGLibA_15309 | 114 | -0.014183692 | EPHA8 | EPH receptor A8 [Source:HGNC Symbol;Acc:HGNC:3391] |
| ENSG00000074370 | HGLibA_03611 | 114 | -0.014183692 | ATP2A3 | ATPase sarcoplasmic/endoplasmic reticulum Ca2+ transporting 3 [Source:HGNC Symbol;Acc:HGNC:813] |
| ENSG00000074603 | HGLibA_13882 | 114 | -0.014183692 | DPP8 | dipeptidyl peptidase 8 [Source:HGNC Symbol;Acc:HGNC:16490] |
| ENSG00000074771 | HGLibA_32374 | 114 | -0.014183692 | NOX3 | NADPH oxidase 3 [Source:HGNC Symbol;Acc:HGNC:7890] |
| ENSG00000080839 | HGLibA_40466 | 114 | -0.014183692 | RBL1 | RB transcriptional corepressor like 1 [Source:HGNC Symbol;Acc:HGNC:9893] |
| ENSG00000087085 | HGLibA_00438 | 114 | -0.014183692 | ACHE | acetylcholinesterase (Cartwright blood group) [Source:HGNC Symbol;Acc:HGNC:108] |
| ENSG00000089847 | HGLibA_02044 | 114 | -0.014183692 | ANKRD24 | ankyrin repeat domain 24 [Source:HGNC Symbol;Acc:HGNC:29424] |
| ENSG00000091138 | HGLibA_44793 | 114 | -0.014183692 | SLC26A3 | solute carrier family 26 member 3 [Source:HGNC Symbol;Acc:HGNC:3018] |
| ENSG00000092068 | HGLibA_45383 | 114 | -0.014183692 | SLC7A8 | solute carrier family 7 member 8 [Source:HGNC Symbol;Acc:HGNC:11066] |
| ENSG00000093183 | HGLibA_43217 | 114 | -0.014183692 | SEC22C | vesicle trafficking protein [Source:HGNC Symbol;Acc:HGNC:16828]" |
| ENSG00000100354 | HGLibA_50916 | 114 | -0.014183692 | TNRC6B | trinucleotide repeat containing 6B [Source:HGNC Symbol;Acc:HGNC:29190] |
| ENSG00000100911 | HGLibA_39194 | 114 | -0.014183692 | PSME2 | proteasome activator subunit 2 [Source:HGNC Symbol;Acc:HGNC:9569] |
| ENSG00000101180 | HGLibA_22099 | 114 | -0.014183692 | HRH3 | histamine receptor H3 [Source:HGNC Symbol;Acc:HGNC:5184] |
| ENSG00000103642 | HGLibA_25918 | 114 | -0.014183692 | LACTB | lactamase beta [Source:HGNC Symbol;Acc:HGNC:16468] |
| ENSG00000105048 | HGLibA_50884 | 114 | -0.014183692 | TNNT1 | slow skeletal type [Source:HGNC Symbol;Acc:HGNC:11948]" |
| ENSG00000106829 | HGLibA_49615 | 114 | -0.014183692 | TLE4 | transducin like enhancer of split 4 [Source:HGNC Symbol;Acc:HGNC:11840] |
| ENSG00000107175 | HGLibA_11090 | 114 | -0.014183692 | CREB3 | cAMP responsive element binding protein 3 [Source:HGNC Symbol;Acc:HGNC:2347] |
| ENSG00000107736 | HGLibA_08667 | 114 | -0.014183692 | CDH23 | cadherin related 23 [Source:HGNC Symbol;Acc:HGNC:13733] |
| ENSG00000109794 | HGLibA_16149 | 114 | -0.014183692 | FAM149A | family with sequence similarity 149 member A [Source:HGNC Symbol;Acc:HGNC:24527] |
| ENSG00000110497 | HGLibA_01754 | 114 | -0.014183692 | AMBRA1 | autophagy and beclin 1 regulator 1 [Source:HGNC Symbol;Acc:HGNC:25990] |
| ENSG00000110931 | HGLibA_07092 | 114 | -0.014183692 | CAMKK2 | calcium/calmodulin dependent protein kinase kinase 2 [Source:HGNC Symbol;Acc:HGNC:1470] |
| ENSG00000111344 | HGLibA_40310 | 114 | -0.014183692 | RASAL1 | RAS protein activator like 1 [Source:HGNC Symbol;Acc:HGNC:9873] |
| ENSG00000112659 | HGLibA_11794 | 114 | -0.014183692 | CUL9 | cullin 9 [Source:HGNC Symbol;Acc:HGNC:15982] |
| ENSG00000112799 | HGLibA_27686 | 114 | -0.014183692 | LY86 | lymphocyte antigen 86 [Source:HGNC Symbol;Acc:HGNC:16837] |
| ENSG00000114115 | HGLibA_40628 | 114 | -0.014183692 | RBP1 | retinol binding protein 1 [Source:HGNC Symbol;Acc:HGNC:9919] |
| ENSG00000116885 | HGLibA_34675 | 114 | -0.014183692 | OSCP1 | organic solute carrier partner 1 [Source:HGNC Symbol;Acc:HGNC:29971] |
| ENSG00000117501 | HGLibA_29882 | 114 | -0.014183692 | MROH9 | maestro heat like repeat family member 9 [Source:HGNC Symbol;Acc:HGNC:26287] |
| ENSG00000120235 | HGLibA_22685 | 114 | -0.014183692 | IFNA6 | interferon alpha 6 [Source:HGNC Symbol;Acc:HGNC:5427] |
| ENSG00000123329 | HGLibA_02791 | 114 | -0.014183692 | ARHGAP9 | Rho GTPase activating protein 9 [Source:HGNC Symbol;Acc:HGNC:14130] |
| ENSG00000126453 | HGLibA_04310 | 114 | -0.014183692 | BCL2L12 | BCL2 like 12 [Source:HGNC Symbol;Acc:HGNC:13787] |
| ENSG00000126746 | HGLibA_55893 | 114 | -0.014183692 | ZNF384 | zinc finger protein 384 [Source:HGNC Symbol;Acc:HGNC:11955] |
| ENSG00000127586 | HGLibA_09661 | 114 | -0.014183692 | CHTF18 | chromosome transmission fidelity factor 18 [Source:HGNC Symbol;Acc:HGNC:18435] |
| ENSG00000129595 | HGLibA_15251 | 114 | -0.014183692 | EPB41L4A | erythrocyte membrane protein band 4.1 like 4A [Source:HGNC Symbol;Acc:HGNC:13278] |
| ENSG00000131116 | HGLibA_55969 | 114 | -0.014183692 | ZNF428 | zinc finger protein 428 [Source:HGNC Symbol;Acc:HGNC:20804] |
| ENSG00000132207 | HGLibA_45557 | 114 | -0.014183692 | SLX1A | structure-specific endonuclease subunit [Source:HGNC Symbol;Acc:HGNC:20922]" |
| ENSG00000132635 | HGLibA_35583 | 114 | -0.014183692 | PCED1A | PC-esterase domain containing 1A [Source:HGNC Symbol;Acc:HGNC:16212] |
| ENSG00000132872 | HGLibA_48079 | 114 | -0.014183692 | SYT4 | synaptotagmin 4 [Source:HGNC Symbol;Acc:HGNC:11512] |
| ENSG00000133111 | HGLibA_40990 | 114 | -0.014183692 | RFXAP | regulatory factor X associated protein [Source:HGNC Symbol;Acc:HGNC:9988] |
| ENSG00000134207 | HGLibA_48083 | 114 | -0.014183692 | SYT6 | synaptotagmin 6 [Source:HGNC Symbol;Acc:HGNC:18638] |
| ENSG00000134532 | HGLibA_46267 | 114 | -0.014183692 | SOX5 | SRY-box 5 [Source:HGNC Symbol;Acc:HGNC:11201] |
| ENSG00000135249 | HGLibA_41305 | 114 | -0.014183692 | RINT1 | RAD50 interactor 1 [Source:HGNC Symbol;Acc:HGNC:21876] |
| ENSG00000135472 | HGLibA_15967 | 114 | -0.014183692 | FAIM2 | Fas apoptotic inhibitory molecule 2 [Source:HGNC Symbol;Acc:HGNC:17067] |
| ENSG00000137074 | HGLibA_02575 | 114 | -0.014183692 | APTX | aprataxin [Source:HGNC Symbol;Acc:HGNC:15984] |
| ENSG00000137168 | HGLibA_37939 | 114 | -0.014183692 | PPIL1 | peptidylprolyl isomerase like 1 [Source:HGNC Symbol;Acc:HGNC:9260] |
| ENSG00000137509 | HGLibA_38359 | 114 | -0.014183692 | PRCP | prolylcarboxypeptidase [Source:HGNC Symbol;Acc:HGNC:9344] |
| ENSG00000137558 | HGLibA_36484 | 114 | -0.014183692 | PI15 | peptidase inhibitor 15 [Source:HGNC Symbol;Acc:HGNC:8946] |
| ENSG00000137700 | HGLibA_45051 | 114 | -0.014183692 | SLC37A4 | solute carrier family 37 member 4 [Source:HGNC Symbol;Acc:HGNC:4061] |
| ENSG00000137745 | HGLibA_29488 | 114 | -0.014183692 | MMP13 | matrix metallopeptidase 13 [Source:HGNC Symbol;Acc:HGNC:7159] |
| ENSG00000137857 | HGLibA_14096 | 114 | -0.014183692 | DUOX1 | dual oxidase 1 [Source:HGNC Symbol;Acc:HGNC:3062] |
| ENSG00000138675 | HGLibA_17412 | 114 | -0.014183692 | FGF5 | fibroblast growth factor 5 [Source:HGNC Symbol;Acc:HGNC:3683] |
| ENSG00000139219 | HGLibA_10561 | 114 | -0.014183692 | COL2A1 | collagen type II alpha 1 chain [Source:HGNC Symbol;Acc:HGNC:2200] |
| ENSG00000139537 | HGLibA_07833 | 114 | -0.014183692 | CCDC65 | coiled-coil domain containing 65 [Source:HGNC Symbol;Acc:HGNC:29937] |
| ENSG00000140391 | HGLibA_51924 | 114 | -0.014183692 | TSPAN3 | tetraspanin 3 [Source:HGNC Symbol;Acc:HGNC:17752] |
| ENSG00000140506 | HGLibA_26660 | 114 | -0.014183692 | LMAN1L | mannose binding 1 like [Source:HGNC Symbol;Acc:HGNC:6632]" |
| ENSG00000140526 | HGLibA_00273 | 114 | -0.014183692 | ABHD2 | abhydrolase domain containing 2 [Source:HGNC Symbol;Acc:HGNC:18717] |
| ENSG00000140848 | HGLibA_10955 | 114 | -0.014183692 | CPNE2 | copine 2 [Source:HGNC Symbol;Acc:HGNC:2315] |
| ENSG00000141968 | HGLibA_53548 | 114 | -0.014183692 | VAV1 | vav guanine nucleotide exchange factor 1 [Source:HGNC Symbol;Acc:HGNC:12657] |
| ENSG00000142973 | HGLibA_12177 | 114 | -0.014183692 | CYP4B1 | cytochrome P450 family 4 subfamily B member 1 [Source:HGNC Symbol;Acc:HGNC:2644] |
| ENSG00000143067 | HGLibA_56563 | 114 | -0.014183692 | ZNF697 | zinc finger protein 697 [Source:HGNC Symbol;Acc:HGNC:32034] |
| ENSG00000143641 | HGLibA_18553 | 114 | -0.014183692 | GALNT2 | polypeptide N-acetylgalactosaminyltransferase 2 [Source:HGNC Symbol;Acc:HGNC:4124] |
| ENSG00000144063 | HGLibA_28029 | 114 | -0.014183692 | MALL | T cell differentiation protein like [Source:HGNC Symbol;Acc:HGNC:6818]" |
| ENSG00000144591 | HGLibA_19421 | 114 | -0.014183692 | GMPPA | GDP-mannose pyrophosphorylase A [Source:HGNC Symbol;Acc:HGNC:22923] |
| ENSG00000144645 | HGLibA_34644 | 114 | -0.014183692 | OSBPL10 | oxysterol binding protein like 10 [Source:HGNC Symbol;Acc:HGNC:16395] |
| ENSG00000146007 | HGLibA_55368 | 114 | -0.014183692 | ZMAT2 | zinc finger matrin-type 2 [Source:HGNC Symbol;Acc:HGNC:26433] |
| ENSG00000146070 | HGLibA_36924 | 114 | -0.014183692 | PLA2G7 | phospholipase A2 group VII [Source:HGNC Symbol;Acc:HGNC:9040] |
| ENSG00000146830 | HGLibA_19063 | 114 | -0.014183692 | GIGYF1 | GRB10 interacting GYF protein 1 [Source:HGNC Symbol;Acc:HGNC:9126] |
| ENSG00000146858 | HGLibA_54995 | 114 | -0.014183692 | ZC3HAV1L | antiviral 1 like [Source:HGNC Symbol;Acc:HGNC:22423]" |
| ENSG00000151276 | HGLibA_27994 | 114 | -0.014183692 | MAGI1 | WW and PDZ domain containing 1 [Source:HGNC Symbol;Acc:HGNC:946]" |
| ENSG00000151500 | HGLibA_49426 | 114 | -0.014183692 | THYN1 | thymocyte nuclear protein 1 [Source:HGNC Symbol;Acc:HGNC:29560] |
| ENSG00000151729 | HGLibA_44731 | 114 | -0.014183692 | SLC25A4 | solute carrier family 25 member 4 [Source:HGNC Symbol;Acc:HGNC:10990] |
| ENSG00000153162 | HGLibA_04614 | 114 | -0.014183692 | BMP6 | bone morphogenetic protein 6 [Source:HGNC Symbol;Acc:HGNC:1073] |
| ENSG00000153779 | HGLibA_49248 | 114 | -0.014183692 | TGIF2LX | TGFB induced factor homeobox 2 like X-linked [Source:HGNC Symbol;Acc:HGNC:18570] |
| ENSG00000155313 | HGLibA_53311 | 114 | -0.014183692 | USP25 | ubiquitin specific peptidase 25 [Source:HGNC Symbol;Acc:HGNC:12624] |
| ENSG00000155962 | HGLibA_10023 | 114 | -0.014183692 | CLIC2 | chloride intracellular channel 2 [Source:HGNC Symbol;Acc:HGNC:2063] |
| ENSG00000158161 | HGLibA_15824 | 114 | -0.014183692 | EYA3 | EYA transcriptional coactivator and phosphatase 3 [Source:HGNC Symbol;Acc:HGNC:3521] |
| ENSG00000162222 | HGLibA_52164 | 114 | -0.014183692 | TTC9C | tetratricopeptide repeat domain 9C [Source:HGNC Symbol;Acc:HGNC:28432] |
| ENSG00000162373 | HGLibA_04410 | 114 | -0.014183692 | BEND5 | BEN domain containing 5 [Source:HGNC Symbol;Acc:HGNC:25668] |
| ENSG00000162630 | HGLibA_03958 | 114 | -0.014183692 | B3GALT2 | 3-galactosyltransferase 2 [Source:HGNC Symbol;Acc:HGNC:917]" |
| ENSG00000164024 | HGLibA_28928 | 114 | -0.014183692 | METAP1 | methionyl aminopeptidase 1 [Source:HGNC Symbol;Acc:HGNC:15789] |
| ENSG00000164024 | HGLibA_28927 | 114 | -0.014183692 | METAP1 | methionyl aminopeptidase 1 [Source:HGNC Symbol;Acc:HGNC:15789] |
| ENSG00000164031 | HGLibA_13533 | 114 | -0.014183692 | DNAJB14 | DnaJ heat shock protein family (Hsp40) member B14 [Source:HGNC Symbol;Acc:HGNC:25881] |
| ENSG00000164049 | HGLibA_17142 | 114 | -0.014183692 | FBXW12 | F-box and WD repeat domain containing 12 [Source:HGNC Symbol;Acc:HGNC:20729] |
| ENSG00000164338 | HGLibA_53440 | 114 | -0.014183692 | UTP15 | small subunit processome component [Source:HGNC Symbol;Acc:HGNC:25758]" |
| ENSG00000164972 | HGLibA_06720 | 114 | -0.014183692 | C9orf24 | chromosome 9 open reading frame 24 [Source:HGNC Symbol;Acc:HGNC:19919] |
| ENSG00000165076 | HGLibA_38935 | 114 | -0.014183692 | PRSS37 | serine protease 37 [Source:HGNC Symbol;Acc:HGNC:29211] |
| ENSG00000166342 | HGLibA_31705 | 114 | -0.014183692 | NETO1 | neuropilin and tolloid like 1 [Source:HGNC Symbol;Acc:HGNC:13823] |
| ENSG00000166396 | HGLibA_43561 | 114 | -0.014183692 | SERPINB7 | serpin family B member 7 [Source:HGNC Symbol;Acc:HGNC:13902] |
| ENSG00000167034 | HGLibA_32048 | 114 | -0.014183692 | NKX3-1 | NK3 homeobox 1 [Source:HGNC Symbol;Acc:HGNC:7838] |
| ENSG00000167654 | HGLibA_03424 | 114 | -0.014183692 | ATCAY | caytaxin [Source:HGNC Symbol;Acc:HGNC:779]" |
| ENSG00000169122 | HGLibA_16015 | 114 | -0.014183692 | FAM110B | family with sequence similarity 110 member B [Source:HGNC Symbol;Acc:HGNC:28587] |
| ENSG00000169302 | HGLibA_47483 | 114 | -0.014183692 | STK32A | serine/threonine kinase 32A [Source:HGNC Symbol;Acc:HGNC:28317] |
| ENSG00000169599 | HGLibA_31846 | 114 | -0.014183692 | NFU1 | NFU1 iron-sulfur cluster scaffold [Source:HGNC Symbol;Acc:HGNC:16287] |
| ENSG00000169635 | HGLibA_21290 | 114 | -0.014183692 | HIC2 | HIC ZBTB transcriptional repressor 2 [Source:HGNC Symbol;Acc:HGNC:18595] |
| ENSG00000170043 | HGLibA_51296 | 114 | -0.014183692 | TRAPPC1 | trafficking protein particle complex 1 [Source:HGNC Symbol;Acc:HGNC:19894] |
| ENSG00000170276 | HGLibA_22307 | 114 | -0.014183692 | HSPB2 | heat shock protein family B (small) member 2 [Source:HGNC Symbol;Acc:HGNC:5247] |
| ENSG00000171314 | HGLibA_36194 | 114 | -0.014183692 | PGAM1 | phosphoglycerate mutase 1 [Source:HGNC Symbol;Acc:HGNC:8888] |
| ENSG00000171365 | HGLibA_09831 | 114 | -0.014183692 | CLCN5 | chloride voltage-gated channel 5 [Source:HGNC Symbol;Acc:HGNC:2023] |
| ENSG00000171813 | HGLibA_39642 | 114 | -0.014183692 | PWWP2B | PWWP domain containing 2B [Source:HGNC Symbol;Acc:HGNC:25150] |
| ENSG00000172590 | HGLibA_30013 | 114 | -0.014183692 | MRPL52 | mitochondrial ribosomal protein L52 [Source:HGNC Symbol;Acc:HGNC:16655] |
| ENSG00000174123 | HGLibA_49644 | 114 | -0.014183692 | TLR10 | toll like receptor 10 [Source:HGNC Symbol;Acc:HGNC:15634] |
| ENSG00000174358 | HGLibA_45315 | 114 | -0.014183692 | SLC6A19 | solute carrier family 6 member 19 [Source:HGNC Symbol;Acc:HGNC:27960] |
| ENSG00000174738 | HGLibA_32578 | 114 | -0.014183692 | NR1D2 | nuclear receptor subfamily 1 group D member 2 [Source:HGNC Symbol;Acc:HGNC:7963] |
| ENSG00000175029 | HGLibA_11586 | 114 | -0.014183692 | CTBP2 | C-terminal binding protein 2 [Source:HGNC Symbol;Acc:HGNC:2495] |
| ENSG00000175224 | HGLibA_03466 | 114 | -0.014183692 | ATG13 | autophagy related 13 [Source:HGNC Symbol;Acc:HGNC:29091] |
| ENSG00000175376 | HGLibA_14708 | 114 | -0.014183692 | EIF1AD | eukaryotic translation initiation factor 1A domain containing [Source:HGNC Symbol;Acc:HGNC:28147] |
| ENSG00000176531 | HGLibA_36433 | 114 | -0.014183692 | PHLDB3 | pleckstrin homology like domain family B member 3 [Source:HGNC Symbol;Acc:HGNC:30499] |
| ENSG00000176597 | HGLibA_03994 | 114 | -0.014183692 | B3GNT5 | 3-N-acetylglucosaminyltransferase 5 [Source:HGNC Symbol;Acc:HGNC:15684]" |
| ENSG00000177212 | HGLibA_33876 | 114 | -0.014183692 | OR2T33 | olfactory receptor family 2 subfamily T member 33 [Source:HGNC Symbol;Acc:HGNC:31255] |

| | | | | | |
|---|---|---|---|---|---|
| ENSG00000177483 | HGLibA_40558 | 114 | -0.014183692 | RBM44 | RNA binding motif protein 44 [Source:HGNC Symbol;Acc:HGNC:24756] |
| ENSG00000179029 | HGLibA_49862 | 114 | -0.014183692 | TMEM107 | transmembrane protein 107 [Source:HGNC Symbol;Acc:HGNC:28128] |
| ENSG00000179119 | HGLibA_46906 | 114 | -0.014183692 | SPTY2D1 | SPT2 chromatin protein domain containing 1 [Source:HGNC Symbol;Acc:HGNC:26818] |
| ENSG00000180370 | HGLibA_35044 | 114 | -0.014183692 | PAK2 | p21 (RAC1) activated kinase 2 [Source:HGNC Symbol;Acc:HGNC:8591] |
| ENSG00000180708 | HGLibA_33532 | 114 | -0.014183692 | OR10K2 | olfactory receptor family 10 subfamily K member 2 [Source:HGNC Symbol;Acc:HGNC:14826] |
| ENSG00000181773 | HGLibA_20014 | 114 | -0.014183692 | GPR3 | G protein-coupled receptor 3 [Source:HGNC Symbol;Acc:HGNC:4484] |
| ENSG00000182199 | HGLibA_44064 | 114 | -0.014183692 | SHMT2 | serine hydroxymethyltransferase 2 [Source:HGNC Symbol;Acc:HGNC:10852] |
| ENSG00000183010 | HGLibA_39674 | 114 | -0.014183692 | PYCR1 | pyrroline-5-carboxylate reductase 1 [Source:HGNC Symbol;Acc:HGNC:9721] |
| ENSG00000183067 | HGLibA_22897 | 114 | -0.014183692 | IGSF5 | immunoglobulin superfamily member 5 [Source:HGNC Symbol;Acc:HGNC:5952] |
| ENSG00000183578 | HGLibA_50729 | 114 | -0.014183692 | TNFAIP8L3 | TNF alpha induced protein 8 like 3 [Source:HGNC Symbol;Acc:HGNC:20620] |
| ENSG00000183876 | HGLibA_03148 | 114 | -0.014183692 | ARSI | arylsulfatase family member I [Source:HGNC Symbol;Acc:HGNC:32521] |
| ENSG00000186815 | HGLibA_51106 | 114 | -0.014183692 | TPCN1 | two pore segment channel 1 [Source:HGNC Symbol;Acc:HGNC:18182] |
| ENSG00000187147 | HGLibA_41639 | 114 | -0.014183692 | RNF220 | ring finger protein 220 [Source:HGNC Symbol;Acc:HGNC:25552] |
| ENSG00000188219 | HGLibA_37729 | 114 | -0.014183692 | POTEE | POTE ankyrin domain family member E [Source:HGNC Symbol;Acc:HGNC:33895] |
| ENSG00000188603 | HGLibA_10074 | 114 | -0.014183692 | CLN3 | battenin [Source:HGNC Symbol;Acc:HGNC:2074]" |
| ENSG00000188868 | HGLibA_56250 | 114 | -0.014183692 | ZNF563 | zinc finger protein 563 [Source:HGNC Symbol;Acc:HGNC:30498] |
| ENSG00000196209 | HGLibA_44206 | 114 | -0.014183692 | SIRPB2 | signal regulatory protein beta 2 [Source:HGNC Symbol;Acc:HGNC:16247] |
| ENSG00000196267 | HGLibA_56843 | 114 | -0.014183692 | ZNF836 | zinc finger protein 836 [Source:HGNC Symbol;Acc:HGNC:34333] |
| ENSG00000196684 | HGLibA_22248 | 114 | -0.014183692 | HSH2D | hematopoietic SH2 domain containing [Source:HGNC Symbol;Acc:HGNC:24920] |
| ENSG00000196968 | HGLibA_18186 | 114 | -0.014183692 | FUT11 | fucosyltransferase 11 [Source:HGNC Symbol;Acc:HGNC:19233] |
| ENSG00000197616 | HGLibA_30730 | 114 | -0.014183692 | MYH6 | myosin heavy chain 6 [Source:HGNC Symbol;Acc:HGNC:7576] |
| ENSG00000198453 | HGLibA_56267 | 114 | -0.014183692 | ZNF568 | zinc finger protein 568 [Source:HGNC Symbol;Acc:HGNC:25392] |
| ENSG00000205126 | HGLibA_00416 | 114 | -0.014183692 | ACCSL | 1-aminocyclopropane-1-carboxylate synthase homolog (inactive) like [Source:HGNC Symbol;Acc:HGNC:34391] |
| ENSG00000205744 | HGLibA_12934 | 114 | -0.014183692 | DENND1C | DENN domain containing 1C [Source:HGNC Symbol;Acc:HGNC:26225] |
| ENSG00000213648 | HGLibA_47741 | 114 | -0.014183692 | SULT1A4 | sulfotransferase family 1A member 4 [Source:HGNC Symbol;Acc:HGNC:30004] |
| ENSG00000215455 | HGLibA_25592 | 114 | -0.014183692 | KRTAP10-1 | keratin associated protein 10-1 [Source:HGNC Symbol;Acc:HGNC:22966] |
| ENSG00000215845 | HGLibA_52030 | 114 | -0.014183692 | TSTD1 | thiosulfate sulfurtransferase like domain containing 1 [Source:HGNC Symbol;Acc:HGNC:35410] |
| ENSG00000221843 | HGLibA_06112 | 114 | -0.014183692 | C2orf16 | chromosome 2 open reading frame 16 [Source:HGNC Symbol;Acc:HGNC:25275] |
| ENSG00000231852 | HGLibA_12078 | 114 | -0.014183692 | CYP21A2 | cytochrome P450 family 21 subfamily A member 2 [Source:HGNC Symbol;Acc:HGNC:2600] |
| ENSG00000242389 | HGLibA_40620 | 114 | -0.014183692 | RBMY1E | member E [Source:HGNC Symbol;Acc:HGNC:23916]" |
| ENSG00000250709 | HGLibA_07686 | 114 | -0.014183692 | CCDC169-SOHLH2 | CCDC169-SOHLH2 readthrough [Source:HGNC Symbol;Acc:HGNC:38866] |
| ENSG00000255819 | HGLibA_25297 | 114 | -0.014183692 | KLRC4-KLRK1 | KLRC4-KLRK1 readthrough [Source:HGNC Symbol;Acc:HGNC:48357] |
| ENSG00000267041 | HGLibA_56872 | 114 | -0.014183692 | ZNF850 | zinc finger protein 850 [Source:HGNC Symbol;Acc:HGNC:27994] |
| ENSG00000276231 | HGLibA_36663 | 114 | -0.014183692 | PIK3R6 | phosphoinositide-3-kinase regulatory subunit 6 [Source:HGNC Symbol;Acc:HGNC:27101] |
| ENSG00000278870 | HGLibA_34108 | 114 | -0.014183692 | OR51G1 | olfactory receptor family 51 subfamily G member 1 (gene/pseudogene) [Source:HGNC Symbol;Acc:HGNC:14738] |
| GPR56 | HGLibA_20055 | 114 | -0.014183692 | GPR56 | - |
| hsa-mir-1-1 | HGLibA_57142 | 114 | -0.014183692 | hsa-mir-1-1 | - |
| hsa-mir-1197 | HGLibA_57254 | 114 | -0.014183692 | hsa-mir-1197 | - |
| hsa-mir-1207 | HGLibA_57281 | 114 | -0.014183692 | hsa-mir-1207 | - |
| hsa-mir-1238 | HGLibA_57341 | 114 | -0.014183692 | hsa-mir-1238 | - |
| hsa-mir-1295a | HGLibA_57656 | 114 | -0.014183692 | hsa-mir-1295a | - |
| hsa-mir-145 | HGLibA_57845 | 114 | -0.014183692 | hsa-mir-145 | - |
| hsa-mir-196b | HGLibA_58099 | 114 | -0.014183692 | hsa-mir-196b | - |
| hsa-mir-29c | HGLibA_58422 | 114 | -0.014183692 | hsa-mir-29c | - |
| hsa-mir-3190 | HGLibA_58859 | 114 | -0.014183692 | hsa-mir-3190 | - |
| hsa-mir-3926-1 | HGLibA_59578 | 114 | -0.014183692 | hsa-mir-3926-1 | - |
| hsa-mir-4256 | HGLibA_59733 | 114 | -0.014183692 | hsa-mir-4256 | - |
| hsa-mir-4488 | HGLibA_60372 | 114 | -0.014183692 | hsa-mir-4488 | - |
| hsa-mir-4506 | HGLibA_60450 | 114 | -0.014183692 | hsa-mir-4506 | - |
| hsa-mir-4666a | HGLibA_60777 | 114 | -0.014183692 | hsa-mir-4666a | - |
| hsa-mir-4683 | HGLibA_60843 | 114 | -0.014183692 | hsa-mir-4683 | - |
| hsa-mir-4751 | HGLibA_61103 | 114 | -0.014183692 | hsa-mir-4751 | - |
| hsa-mir-498 | HGLibA_61391 | 114 | -0.014183692 | hsa-mir-498 | - |
| hsa-mir-514a-1 | HGLibA_61586 | 114 | -0.014183692 | hsa-mir-514a-1 | - |
| hsa-mir-515-1 | HGLibA_61602 | 114 | -0.014183692 | hsa-mir-515-1 | - |
| hsa-mir-636 | HGLibA_62782 | 114 | -0.014183692 | hsa-mir-636 | - |
| hsa-mir-8061 | HGLibA_64083 | 114 | -0.014183692 | hsa-mir-8061 | - |
| IFLTD1 | HGLibA_22654 | 114 | -0.014183692 | IFLTD1 | - |
| LARGE | HGLibA_26024 | 114 | -0.014183692 | LARGE | - |
| LINS | HGLibA_26597 | 114 | -0.014183692 | LINS | - |
| MKL1 | HGLibA_29334 | 114 | -0.014183692 | MKL1 | - |
| TCEB2 | HGLibA_48736 | 114 | -0.014183692 | TCEB2 | - |
| C19orf82 | HGLibA_05718 | 113 | -0.02413473 | C19orf82 | - |
| ENSG00000006837 | HGLibA_08811 | 113 | -0.02413473 | CDKL3 | cyclin dependent kinase like 3 [Source:HGNC Symbol;Acc:HGNC:15483] |
| ENSG00000007952 | HGLibA_32371 | 113 | -0.02413473 | NOX1 | NADPH oxidase 1 [Source:HGNC Symbol;Acc:HGNC:7889] |
| ENSG00000028116 | HGLibA_53828 | 113 | -0.02413473 | VRK2 | VRK serine/threonine kinase 2 [Source:HGNC Symbol;Acc:HGNC:12719] |
| ENSG00000038945 | HGLibA_30235 | 113 | -0.02413473 | MSR1 | macrophage scavenger receptor 1 [Source:HGNC Symbol;Acc:HGNC:7376] |
| ENSG00000050438 | HGLibA_45229 | 113 | -0.02413473 | SLC4A8 | solute carrier family 4 member 8 [Source:HGNC Symbol;Acc:HGNC:11034] |
| ENSG00000056586 | HGLibA_40664 | 113 | -0.02413473 | RC3H2 | ring finger and CCCH-type domains 2 [Source:HGNC Symbol;Acc:HGNC:21461] |
| ENSG00000060971 | HGLibA_00341 | 113 | -0.02413473 | ACAA1 | acetyl-CoA acyltransferase 1 [Source:HGNC Symbol;Acc:HGNC:82] |
| ENSG00000063244 | HGLibA_52497 | 113 | -0.02413473 | U2AF2 | U2 small nuclear RNA auxiliary factor 2 [Source:HGNC Symbol;Acc:HGNC:23156] |
| ENSG00000064961 | HGLibA_21682 | 113 | -0.02413473 | HMG20B | high mobility group 20B [Source:HGNC Symbol;Acc:HGNC:5002] |
| ENSG00000068697 | HGLibA_26015 | 113 | -0.02413473 | LAPTM4A | lysosomal protein transmembrane 4 alpha [Source:HGNC Symbol;Acc:HGNC:6924] |
| ENSG00000082126 | HGLibA_29755 | 113 | -0.02413473 | MPP4 | membrane palmitoylated protein 4 [Source:HGNC Symbol;Acc:HGNC:13680] |
| ENSG00000087074 | HGLibA_38046 | 113 | -0.02413473 | PPP1R15A | protein phosphatase 1 regulatory subunit 15A [Source:HGNC Symbol;Acc:HGNC:14375] |
| ENSG00000092621 | HGLibA_36392 | 113 | -0.02413473 | PHGDH | phosphoglycerate dehydrogenase [Source:HGNC Symbol;Acc:HGNC:8923] |
| ENSG00000094755 | HGLibA_18397 | 113 | -0.02413473 | GABRP | gamma-aminobutyric acid type A receptor pi subunit [Source:HGNC Symbol;Acc:HGNC:4089] |
| ENSG00000099260 | HGLibA_35079 | 113 | -0.02413473 | PALMD | palmdelphin [Source:HGNC Symbol;Acc:HGNC:15846] |
| ENSG00000099364 | HGLibA_16995 | 113 | -0.02413473 | FBXL19 | F-box and leucine rich repeat protein 19 [Source:HGNC Symbol;Acc:HGNC:25300] |
| ENSG00000100253 | HGLibA_29289 | 113 | -0.02413473 | MIOX | myo-inositol oxygenase [Source:HGNC Symbol;Acc:HGNC:14522] |
| ENSG00000100372 | HGLibA_44657 | 113 | -0.02413473 | SLC25A17 | solute carrier family 25 member 17 [Source:HGNC Symbol;Acc:HGNC:10987] |
| ENSG00000101457 | HGLibA_13718 | 113 | -0.02413473 | DNTTIP1 | deoxynucleotidyltransferase terminal interacting protein 1 [Source:HGNC Symbol;Acc:HGNC:16160] |
| ENSG00000102738 | HGLibA_30088 | 113 | -0.02413473 | MRPS31 | mitochondrial ribosomal protein S31 [Source:HGNC Symbol;Acc:HGNC:16632] |
| ENSG00000103249 | HGLibA_09839 | 113 | -0.02413473 | CLCN7 | chloride voltage-gated channel 7 [Source:HGNC Symbol;Acc:HGNC:2025] |
| ENSG00000103365 | HGLibA_18984 | 113 | -0.02413473 | GGA2 | ARF binding protein 2 [Source:HGNC Symbol;Acc:HGNC:16064]" |
| ENSG00000104047 | HGLibA_14073 | 113 | -0.02413473 | DTWD1 | DTW domain containing 1 [Source:HGNC Symbol;Acc:HGNC:30926] |
| ENSG00000105136 | HGLibA_55953 | 113 | -0.02413473 | ZNF419 | zinc finger protein 419 [Source:HGNC Symbol;Acc:HGNC:20648] |
| ENSG00000105523 | HGLibA_16753 | 113 | -0.02413473 | FAM83E | family with sequence similarity 83 member E [Source:HGNC Symbol;Acc:HGNC:25972] |
| ENSG00000108106 | HGLibA_52658 | 113 | -0.02413473 | UBE2S | ubiquitin conjugating enzyme E2 S [Source:HGNC Symbol;Acc:HGNC:17895] |
| ENSG00000108588 | HGLibA_07791 | 113 | -0.02413473 | CCDC47 | coiled-coil domain containing 47 [Source:HGNC Symbol;Acc:HGNC:24856] |
| ENSG00000109689 | HGLibA_47443 | 113 | -0.02413473 | STIM2 | stromal interaction molecule 2 [Source:HGNC Symbol;Acc:HGNC:19205] |
| ENSG00000110079 | HGLibA_30150 | 113 | -0.02413473 | MS4A4A | membrane spanning 4-domains A4A [Source:HGNC Symbol;Acc:HGNC:13371] |
| ENSG00000112164 | HGLibA_19292 | 113 | -0.02413473 | GLP1R | glucagon like peptide 1 receptor [Source:HGNC Symbol;Acc:HGNC:4324] |
| ENSG00000112273 | HGLibA_21042 | 113 | -0.02413473 | HDGFL1 | HDGF like 1 [Source:HGNC Symbol;Acc:HGNC:21095] |
| ENSG00000113161 | HGLibA_21708 | 113 | -0.02413473 | HMGCR | 3-hydroxy-3-methylglutaryl-CoA reductase [Source:HGNC Symbol;Acc:HGNC:5006] |
| ENSG00000113742 | HGLibA_10926 | 113 | -0.02413473 | CPEB4 | cytoplasmic polyadenylation element binding protein 4 [Source:HGNC Symbol;Acc:HGNC:21747] |
| ENSG00000114805 | HGLibA_37008 | 113 | -0.02413473 | PLCH1 | phospholipase C eta 1 [Source:HGNC Symbol;Acc:HGNC:29185] |
| ENSG00000116288 | HGLibA_35200 | 113 | -0.02413473 | PARK7 | Parkinsonism associated deglycase [Source:HGNC Symbol;Acc:HGNC:16369] |
| ENSG00000116661 | HGLibA_17042 | 113 | -0.02413473 | FBXO2 | F-box protein 2 [Source:HGNC Symbol;Acc:HGNC:13581] |
| ENSG00000117148 | HGLibA_00631 | 113 | -0.02413473 | ACTL8 | actin like 8 [Source:HGNC Symbol;Acc:HGNC:24018] |
| ENSG00000118156 | HGLibA_56190 | 113 | -0.02413473 | ZNF541 | zinc finger protein 541 [Source:HGNC Symbol;Acc:HGNC:25294] |
| ENSG00000118434 | HGLibA_46324 | 113 | -0.02413473 | SPACA1 | sperm acrosome associated 1 [Source:HGNC Symbol;Acc:HGNC:14967] |
| ENSG00000119121 | HGLibA_51766 | 113 | -0.02413473 | TRPM6 | transient receptor potential cation channel subfamily M member 6 [Source:HGNC Symbol;Acc:HGNC:17995] |
| ENSG00000121764 | HGLibA_20989 | 113 | -0.02413473 | HCRTR1 | hypocretin receptor 1 [Source:HGNC Symbol;Acc:HGNC:4848] |
| ENSG00000122188 | HGLibA_26069 | 113 | -0.02413473 | LAX1 | lymphocyte transmembrane adaptor 1 [Source:HGNC Symbol;Acc:HGNC:26005] |
| ENSG00000123595 | HGLibA_39990 | 113 | -0.02413473 | RAB9A | member RAS oncogene family [Source:HGNC Symbol;Acc:HGNC:9792]" |
| ENSG00000124466 | HGLibA_27718 | 113 | -0.02413473 | LYPD3 | LY6/PLAUR domain containing 3 [Source:HGNC Symbol;Acc:HGNC:24880] |
| ENSG00000125505 | HGLibA_28526 | 113 | -0.02413473 | MBOAT7 | membrane bound O-acyltransferase domain containing 7 [Source:HGNC Symbol;Acc:HGNC:15505] |
| ENSG00000125971 | HGLibA_14259 | 113 | -0.02413473 | DYNLRB1 | dynein light chain roadblock-type 1 [Source:HGNC Symbol;Acc:HGNC:15468] |
| ENSG00000126581 | HGLibA_04396 | 113 | -0.02413473 | BECN1 | beclin 1 [Source:HGNC Symbol;Acc:HGNC:1034] |
| ENSG00000128016 | HGLibA_55205 | 113 | -0.02413473 | ZFP36 | ZFP36 ring finger protein [Source:HGNC Symbol;Acc:HGNC:12862] |
| ENSG00000129170 | HGLibA_11441 | 113 | -0.02413473 | CSRP3 | cysteine and glycine rich protein 3 [Source:HGNC Symbol;Acc:HGNC:2472] |
| ENSG00000131507 | HGLibA_31393 | 113 | -0.02413473 | NDFIP1 | Nedd4 family interacting protein 1 [Source:HGNC Symbol;Acc:HGNC:17592] |
| ENSG00000131748 | HGLibA_47358 | 113 | -0.02413473 | STARD3 | StAR related lipid transfer domain containing 3 [Source:HGNC Symbol;Acc:HGNC:17579] |
| ENSG00000132423 | HGLibA_10751 | 113 | -0.02413473 | COQ3 | methyltransferase [Source:HGNC Symbol;Acc:HGNC:18175]" |
| ENSG00000135046 | HGLibA_02219 | 113 | -0.02413473 | ANXA1 | annexin A1 [Source:HGNC Symbol;Acc:HGNC:533] |
| ENSG00000135052 | HGLibA_19655 | 113 | -0.02413473 | GOLM1 | golgi membrane protein 1 [Source:HGNC Symbol;Acc:HGNC:15451] |
| ENSG00000137142 | HGLibA_22837 | 113 | -0.02413473 | IGFBPL1 | insulin like growth factor binding protein like 1 [Source:HGNC Symbol;Acc:HGNC:20081] |
| ENSG00000138653 | HGLibA_31433 | 113 | -0.02413473 | NDST4 | N-deacetylase and N-sulfotransferase 4 [Source:HGNC Symbol;Acc:HGNC:20779] |
| ENSG00000138686 | HGLibA_04225 | 113 | -0.02413473 | BBS7 | Bardet-Biedl syndrome 7 [Source:HGNC Symbol;Acc:HGNC:18758] |
| ENSG00000139287 | HGLibA_51131 | 113 | -0.02413473 | TPH2 | tryptophan hydroxylase 2 [Source:HGNC Symbol;Acc:HGNC:20692] |
| ENSG00000139797 | HGLibA_41475 | 113 | -0.02413473 | RNF113B | ring finger protein 113B [Source:HGNC Symbol;Acc:HGNC:17267] |
| ENSG00000139899 | HGLibA_07413 | 113 | -0.02413473 | CBLN3 | cerebellin 3 precursor [Source:HGNC Symbol;Acc:HGNC:20146] |
| ENSG00000140382 | HGLibA_21679 | 113 | -0.02413473 | HMG20A | high mobility group 20A [Source:HGNC Symbol;Acc:HGNC:5001] |
| ENSG00000140398 | HGLibA_31628 | 113 | -0.02413473 | NEIL1 | nei like DNA glycosylase 1 [Source:HGNC Symbol;Acc:HGNC:18448] |
| ENSG00000141738 | HGLibA_20234 | 113 | -0.02413473 | GRB7 | growth factor receptor bound protein 7 [Source:HGNC Symbol;Acc:HGNC:4567] |
| ENSG00000143702 | HGLibA_09117 | 113 | -0.02413473 | CEP170 | centrosomal protein 170 [Source:HGNC Symbol;Acc:HGNC:28920] |
| ENSG00000145287 | HGLibA_36943 | 113 | -0.02413473 | PLAC8 | placenta specific 8 [Source:HGNC Symbol;Acc:HGNC:19254] |
| ENSG00000146109 | HGLibA_00331 | 113 | -0.02413473 | ABT1 | activator of basal transcription 1 [Source:HGNC Symbol;Acc:HGNC:17369] |
| ENSG00000146373 | HGLibA_41635 | 113 | -0.02413473 | RNF217 | ring finger protein 217 [Source:HGNC Symbol;Acc:HGNC:21487] |
| ENSG00000147571 | HGLibA_11136 | 113 | -0.02413473 | CRH | corticotropin releasing hormone [Source:HGNC Symbol;Acc:HGNC:2355] |
| ENSG00000147650 | HGLibA_27207 | 113 | -0.02413473 | LRP12 | LDL receptor related protein 12 [Source:HGNC Symbol;Acc:HGNC:31708] |
| ENSG00000147724 | HGLibA_16135 | 113 | -0.02413473 | FAM135B | family with sequence similarity 135 member B [Source:HGNC Symbol;Acc:HGNC:28029] |
| ENSG00000148840 | HGLibA_38231 | 113 | -0.02413473 | PPRC1 | coactivator-related 1 [Source:HGNC Symbol;Acc:HGNC:30025]" |
| ENSG00000151388 | HGLibA_00797 | 113 | -0.02413473 | ADAMTS12 | ADAM metallopeptidase with thrombospondin type 1 motif 12 [Source:HGNC Symbol;Acc:HGNC:14605] |

| | | | | | |
|---|---|---|---|---|---|
| ENSG00000151715 | HGLibA_50411 | 113 | -0.02413473 | TMEM45B | transmembrane protein 45B [Source:HGNC Symbol;Acc:HGNC:25194] |
| ENSG00000152315 | HGLibA_24304 | 113 | -0.02413473 | KCNK13 | potassium two pore domain channel subfamily K member 13 [Source:HGNC Symbol;Acc:HGNC:6275] |
| ENSG00000153802 | HGLibA_50619 | 113 | -0.02413473 | TMPRSS11D | transmembrane serine protease 11D [Source:HGNC Symbol;Acc:HGNC:24059] |
| ENSG00000154832 | HGLibA_11964 | 113 | -0.02413473 | CXXC1 | CXXC finger protein 1 [Source:HGNC Symbol;Acc:HGNC:24343] |
| ENSG00000155465 | HGLibA_45379 | 113 | -0.02413473 | SLC7A7 | solute carrier family 7 member 7 [Source:HGNC Symbol;Acc:HGNC:11065] |
| ENSG00000156218 | HGLibA_00854 | 113 | -0.02413473 | ADAMTSL3 | ADAMTS like 3 [Source:HGNC Symbol;Acc:HGNC:14633] |
| ENSG00000156234 | HGLibA_11854 | 113 | -0.02413473 | CXCL13 | C-X-C motif chemokine ligand 13 [Source:HGNC Symbol;Acc:HGNC:10639] |
| ENSG00000158220 | HGLibA_15611 | 113 | -0.02413473 | ESYT3 | extended synaptotagmin 3 [Source:HGNC Symbol;Acc:HGNC:24295] |
| ENSG00000158604 | HGLibA_49810 | 113 | -0.02413473 | TMED4 | transmembrane p24 trafficking protein 4 [Source:HGNC Symbol;Acc:HGNC:22301] |
| ENSG00000158987 | HGLibA_40259 | 113 | -0.02413473 | RAPGEF6 | Rap guanine nucleotide exchange factor 6 [Source:HGNC Symbol;Acc:HGNC:20655] |
| ENSG00000159184 | HGLibA_21928 | 113 | -0.02413473 | HOXB13 | homeobox B13 [Source:HGNC Symbol;Acc:HGNC:5112] |
| ENSG00000159445 | HGLibA_49341 | 113 | -0.02413473 | THEM4 | thioesterase superfamily member 4 [Source:HGNC Symbol;Acc:HGNC:17947] |
| ENSG00000161634 | HGLibA_12477 | 113 | -0.02413473 | DCD | dermcidin [Source:HGNC Symbol;Acc:HGNC:14669] |
| ENSG00000161653 | HGLibA_31075 | 113 | -0.02413473 | NAGS | N-acetylglutamate synthase [Source:HGNC Symbol;Acc:HGNC:17996] |
| ENSG00000162413 | HGLibA_25152 | 113 | -0.02413473 | KLHL21 | kelch like family member 21 [Source:HGNC Symbol;Acc:HGNC:29041] |
| ENSG00000162631 | HGLibA_32864 | 113 | -0.02413473 | NTNG1 | netrin G1 [Source:HGNC Symbol;Acc:HGNC:23319] |
| ENSG00000162688 | HGLibA_01195 | 113 | -0.02413473 | AGL | 4-alpha-glucanotransferase [Source:HGNC Symbol;Acc:HGNC:321]" |
| ENSG00000166359 | HGLibA_54207 | 113 | -0.02413473 | WDR88 | WD repeat domain 88 [Source:HGNC Symbol;Acc:HGNC:26999] |
| ENSG00000166455 | HGLibA_05450 | 113 | -0.02413473 | C16orf46 | chromosome 16 open reading frame 46 [Source:HGNC Symbol;Acc:HGNC:26525] |
| ENSG00000167487 | HGLibA_25167 | 113 | -0.02413473 | KLHL26 | kelch like family member 26 [Source:HGNC Symbol;Acc:HGNC:25623] |
| ENSG00000168481 | HGLibA_26398 | 113 | -0.02413473 | LGI3 | leucine rich repeat LGI family member 3 [Source:HGNC Symbol;Acc:HGNC:18711] |
| ENSG00000168734 | HGLibA_36825 | 113 | -0.02413473 | PKIG | cAMP-dependent protein kinase inhibitor gamma [Source:HGNC Symbol;Acc:HGNC:9019] |
| ENSG00000168754 | HGLibA_16303 | 113 | -0.02413473 | FAM178B | family with sequence similarity 178 member B [Source:HGNC Symbol;Acc:HGNC:28036] |
| ENSG00000168757 | HGLibA_51968 | 113 | -0.02413473 | TSPY2 | testis specific protein Y-linked 2 [Source:HGNC Symbol;Acc:HGNC:23924] |
| ENSG00000169347 | HGLibA_19705 | 113 | -0.02413473 | GP2 | glycoprotein 2 [Source:HGNC Symbol;Acc:HGNC:4441] |
| ENSG00000170323 | HGLibA_15920 | 113 | -0.02413473 | FABP4 | fatty acid binding protein 4 [Source:HGNC Symbol;Acc:HGNC:3559] |
| ENSG00000170776 | HGLibA_01414 | 113 | -0.02413473 | AKAP13 | A-kinase anchoring protein 13 [Source:HGNC Symbol;Acc:HGNC:371] |
| ENSG00000170837 | HGLibA_20011 | 113 | -0.02413473 | GPR27 | G protein-coupled receptor 27 [Source:HGNC Symbol;Acc:HGNC:4482] |
| ENSG00000171405 | HGLibA_54488 | 113 | -0.02413473 | XAGE5 | X antigen family member 5 [Source:HGNC Symbol;Acc:HGNC:30930] |
| ENSG00000171885 | HGLibA_02597 | 113 | -0.02413473 | AQP4 | aquaporin 4 [Source:HGNC Symbol;Acc:HGNC:637] |
| ENSG00000172150 | HGLibA_33645 | 113 | -0.02413473 | OR1A2 | olfactory receptor family 1 subfamily A member 2 [Source:HGNC Symbol;Acc:HGNC:8180] |
| ENSG00000172742 | HGLibA_33982 | 113 | -0.02413473 | OR4D9 | olfactory receptor family 4 subfamily D member 9 [Source:HGNC Symbol;Acc:HGNC:15178] |
| ENSG00000173599 | HGLibA_35359 | 113 | -0.02413473 | PC | pyruvate carboxylase [Source:HGNC Symbol;Acc:HGNC:8636] |
| ENSG00000174807 | HGLibA_08313 | 113 | -0.02413473 | CD248 | CD248 molecule [Source:HGNC Symbol;Acc:HGNC:18219] |
| ENSG00000174989 | HGLibA_17156 | 113 | -0.02413473 | FBXW8 | F-box and WD repeat domain containing 8 [Source:HGNC Symbol;Acc:HGNC:13597] |
| ENSG00000175137 | HGLibA_43936 | 113 | -0.02413473 | SH3BP5L | SH3 binding domain protein 5 like [Source:HGNC Symbol;Acc:HGNC:29360] |
| ENSG00000176058 | HGLibA_51189 | 113 | -0.02413473 | TPRN | taperin [Source:HGNC Symbol;Acc:HGNC:26894] |
| ENSG00000176842 | HGLibA_23641 | 113 | -0.02413473 | IRX5 | iroquois homeobox 5 [Source:HGNC Symbol;Acc:HGNC:14361] |
| ENSG00000177324 | HGLibA_04402 | 113 | -0.02413473 | BEND2 | BEN domain containing 2 [Source:HGNC Symbol;Acc:HGNC:28509] |
| ENSG00000177854 | HGLibA_50130 | 113 | -0.02413473 | TMEM187 | transmembrane protein 187 [Source:HGNC Symbol;Acc:HGNC:13705] |
| ENSG00000178537 | HGLibA_44668 | 113 | -0.02413473 | SLC25A20 | solute carrier family 25 member 20 [Source:HGNC Symbol;Acc:HGNC:1421] |
| ENSG00000180479 | HGLibA_56277 | 113 | -0.02413473 | ZNF571 | zinc finger protein 571 [Source:HGNC Symbol;Acc:HGNC:25000] |
| ENSG00000180730 | HGLibA_44034 | 113 | -0.02413473 | SHISA2 | shisa family member 2 [Source:HGNC Symbol;Acc:HGNC:20366] |
| ENSG00000181513 | HGLibA_00401 | 113 | -0.02413473 | ACBD4 | acyl-CoA binding domain containing 4 [Source:HGNC Symbol;Acc:HGNC:23337] |
| ENSG00000181585 | HGLibA_50583 | 113 | -0.02413473 | TMIE | transmembrane inner ear [Source:HGNC Symbol;Acc:HGNC:30800] |
| ENSG00000181616 | HGLibA_34169 | 113 | -0.02413473 | OR52H1 | olfactory receptor family 52 subfamily H member 1 [Source:HGNC Symbol;Acc:HGNC:15218] |
| ENSG00000182759 | HGLibA_27871 | 113 | -0.02413473 | MAFA | MAF bZIP transcription factor A [Source:HGNC Symbol;Acc:HGNC:23145] |
| ENSG00000183090 | HGLibA_17991 | 113 | -0.02413473 | FREM3 | FRAS1 related extracellular matrix 3 [Source:HGNC Symbol;Acc:HGNC:25172] |
| ENSG00000183246 | HGLibA_41267 | 113 | -0.02413473 | RIMBP3C | RIMS binding protein 3C [Source:HGNC Symbol;Acc:HGNC:33892] |
| ENSG00000183479 | HGLibA_51375 | 113 | -0.02413473 | TREX2 | three prime repair exonuclease 2 [Source:HGNC Symbol;Acc:HGNC:12270] |
| ENSG00000183530 | HGLibA_38783 | 113 | -0.02413473 | PRR14L | proline rich 14 like [Source:HGNC Symbol;Acc:HGNC:28738] |
| ENSG00000183655 | HGLibA_25166 | 113 | -0.02413473 | KLHL25 | kelch like family member 25 [Source:HGNC Symbol;Acc:HGNC:25732] |
| ENSG00000183844 | HGLibA_16578 | 113 | -0.02413473 | FAM3B | family with sequence similarity 3 member B [Source:HGNC Symbol;Acc:HGNC:1253] |
| ENSG00000184451 | HGLibA_08147 | 113 | -0.02413473 | CCR10 | C-C motif chemokine receptor 10 [Source:HGNC Symbol;Acc:HGNC:4474] |
| ENSG00000185176 | HGLibA_02588 | 113 | -0.02413473 | AQP12B | aquaporin 12B [Source:HGNC Symbol;Acc:HGNC:6096] |
| ENSG00000187678 | HGLibA_46848 | 113 | -0.02413473 | SPRY4 | sprouty RTK signaling antagonist 4 [Source:HGNC Symbol;Acc:HGNC:15533] |
| ENSG00000187747 | HGLibA_34152 | 113 | -0.02413473 | OR52B6 | olfactory receptor family 52 subfamily B member 6 [Source:HGNC Symbol;Acc:HGNC:15211] |
| ENSG00000188283 | HGLibA_55891 | 113 | -0.02413473 | ZNF383 | zinc finger protein 383 [Source:HGNC Symbol;Acc:HGNC:18609] |
| ENSG00000196616 | HGLibA_00953 | 113 | -0.02413473 | ADH1B | beta polypeptide [Source:HGNC Symbol;Acc:HGNC:250]" |
| ENSG00000196620 | HGLibA_52917 | 113 | -0.02413473 | UGT2B15 | UDP glucuronosyltransferase family 2 member B15 [Source:HGNC Symbol;Acc:HGNC:12546] |
| ENSG00000197953 | HGLibA_00033 | 113 | -0.02413473 | AADACL2 | arylacetamide deacetylase like 2 [Source:HGNC Symbol;Acc:HGNC:24427] |
| ENSG00000198315 | HGLibA_55362 | 113 | -0.02413473 | ZKSCAN8 | zinc finger with KRAB and SCAN domains 8 [Source:HGNC Symbol;Acc:HGNC:12983] |
| ENSG00000203667 | HGLibA_10826 | 113 | -0.02413473 | COX20 | cytochrome c oxidase assembly factor COX20 [Source:HGNC Symbol;Acc:HGNC:26970] |
| ENSG00000204539 | HGLibA_08887 | 113 | -0.02413473 | CDSN | corneodesmosin [Source:HGNC Symbol;Acc:HGNC:1802] |
| ENSG00000206104 | HGLibA_25706 | 113 | -0.02413473 | KRTAP20-3 | keratin associated protein 20-3 [Source:HGNC Symbol;Acc:HGNC:34001] |
| ENSG00000221923 | HGLibA_56892 | 113 | -0.02413473 | ZNF880 | zinc finger protein 880 [Source:HGNC Symbol;Acc:HGNC:37249] |
| ENSG00000226372 | HGLibA_12459 | 113 | -0.02413473 | DCAF8L1 | DDB1 and CUL4 associated factor 8 like 1 [Source:HGNC Symbol;Acc:HGNC:31810] |
| ENSG00000233803 | HGLibA_51972 | 113 | -0.02413473 | TSPY4 | testis specific protein Y-linked 4 [Source:HGNC Symbol;Acc:HGNC:37287] |
| ENSG00000236371 | HGLibA_11515 | 113 | -0.02413473 | CT47A1 | member A1 [Source:HGNC Symbol;Acc:HGNC:33282]" |
| ENSG00000244395 | HGLibA_40617 | 113 | -0.02413473 | RBMY1D | member D [Source:HGNC Symbol;Acc:HGNC:23915]" |
| ENSG00000253148 | HGLibA_41085 | 113 | -0.02413473 | RGS21 | regulator of G protein signaling 21 [Source:HGNC Symbol;Acc:HGNC:26839] |
| ENSG00000259024 | HGLibA_52367 | 113 | -0.02413473 | TVP23C-CDRT4 | TVP23C-CDRT4 readthrough [Source:HGNC Symbol;Acc:HGNC:42961] |
| ENSG00000275302 | HGLibA_08027 | 113 | -0.02413473 | CCL4 | C-C motif chemokine ligand 4 [Source:HGNC Symbol;Acc:HGNC:10630] |
| HDHD1 | HGLibA_21052 | 113 | -0.02413473 | HDHD1 | - |
| hsa-mir-1224 | HGLibA_57293 | 113 | -0.02413473 | hsa-mir-1224 | - |
| hsa-mir-1302-1 | HGLibA_57685 | 113 | -0.02413473 | hsa-mir-1302-1 | - |
| hsa-mir-1302-1 | HGLibA_57682 | 113 | -0.02413473 | hsa-mir-1302-1 | - |
| hsa-mir-1324 | HGLibA_57769 | 113 | -0.02413473 | hsa-mir-1324 | - |
| hsa-mir-3123 | HGLibA_58545 | 113 | -0.02413473 | hsa-mir-3123 | - |
| hsa-mir-3131 | HGLibA_58580 | 113 | -0.02413473 | hsa-mir-3131 | - |
| hsa-mir-3136 | HGLibA_58601 | 113 | -0.02413473 | hsa-mir-3136 | - |
| hsa-mir-3681 | HGLibA_59278 | 113 | -0.02413473 | hsa-mir-3681 | - |
| hsa-mir-3935 | HGLibA_59599 | 113 | -0.02413473 | hsa-mir-3935 | - |
| hsa-mir-4514 | HGLibA_60500 | 113 | -0.02413473 | hsa-mir-4514 | - |
| hsa-mir-4650-1 | HGLibA_60705 | 113 | -0.02413473 | hsa-mir-4650-1 | - |
| hsa-mir-4734 | HGLibA_61035 | 113 | -0.02413473 | hsa-mir-4734 | - |
| hsa-mir-526b | HGLibA_61799 | 113 | -0.02413473 | hsa-mir-526b | - |
| hsa-mir-5697 | HGLibA_62319 | 113 | -0.02413473 | hsa-mir-5697 | - |
| hsa-mir-7856 | HGLibA_64014 | 113 | -0.02413473 | hsa-mir-7856 | - |
| hsa-mir-8069 | HGLibA_64114 | 113 | -0.02413473 | hsa-mir-8069 | - |
| KCNE1L | HGLibA_24186 | 113 | -0.02413473 | KCNE1L | - |
| LOC100996485 | HGLibA_26885 | 113 | -0.02413473 | LOC100996485 | - |
| PRKCDBP | HGLibA_38541 | 113 | -0.02413473 | PRKCDBP | - |
| SSFA2 | HGLibA_47129 | 113 | -0.02413473 | SSFA2 | - |
| C10orf10 | HGLibA_05020 | 112 | -0.034085767 | C10orf10 | - |
| C1QTNF9B-AS1 | HGLibA_05967 | 112 | -0.034085767 | C1QTNF9B-AS1 | - |
| C7orf55-LUC7L2 | HGLibA_06557 | 112 | -0.034085767 | C7orf55-LUC7L2 | - |
| ENSG00000007952 | HGLibA_32369 | 112 | -0.034085767 | NOX1 | NADPH oxidase 1 [Source:HGNC Symbol;Acc:HGNC:7889] |
| ENSG00000019186 | HGLibA_12083 | 112 | -0.034085767 | CYP24A1 | cytochrome P450 family 24 subfamily A member 1 [Source:HGNC Symbol;Acc:HGNC:2602] |
| ENSG00000025772 | HGLibA_50964 | 112 | -0.034085767 | TOMM34 | translocase of outer mitochondrial membrane 34 [Source:HGNC Symbol;Acc:HGNC:15746] |
| ENSG00000034510 | HGLibA_50658 | 112 | -0.034085767 | TMSB10 | thymosin beta 10 [Source:HGNC Symbol;Acc:HGNC:11879] |
| ENSG00000048405 | HGLibA_56784 | 112 | -0.034085767 | ZNF800 | zinc finger protein 800 [Source:HGNC Symbol;Acc:HGNC:27267] |
| ENSG00000054523 | HGLibA_24885 | 112 | -0.034085767 | KIF1B | kinesin family member 1B [Source:HGNC Symbol;Acc:HGNC:16636] |
| ENSG00000068438 | HGLibA_18134 | 112 | -0.034085767 | FTSJ1 | FtsJ RNA methyltransferase homolog 1 [Source:HGNC Symbol;Acc:HGNC:13254] |
| ENSG00000072042 | HGLibA_40733 | 112 | -0.034085767 | RDH11 | retinol dehydrogenase 11 [Source:HGNC Symbol;Acc:HGNC:17964] |
| ENSG00000077943 | HGLibA_23744 | 112 | -0.034085767 | ITGA8 | integrin subunit alpha 8 [Source:HGNC Symbol;Acc:HGNC:6144] |
| ENSG00000078070 | HGLibA_28566 | 112 | -0.034085767 | MCCC1 | methylcrotonoyl-CoA carboxylase 1 [Source:HGNC Symbol;Acc:HGNC:6936] |
| ENSG00000080815 | HGLibA_39026 | 112 | -0.034085767 | PSEN1 | presenilin 1 [Source:HGNC Symbol;Acc:HGNC:9508] |
| ENSG00000087589 | HGLibA_07343 | 112 | -0.034085767 | CASS4 | Cas scaffold protein family member 4 [Source:HGNC Symbol;Acc:HGNC:15878] |
| ENSG00000089123 | HGLibA_48434 | 112 | -0.034085767 | TASP1 | taspase 1 [Source:HGNC Symbol;Acc:HGNC:15859] |
| ENSG00000089248 | HGLibA_15526 | 112 | -0.034085767 | ERP29 | endoplasmic reticulum protein 29 [Source:HGNC Symbol;Acc:HGNC:13799] |
| ENSG00000097033 | HGLibA_43955 | 112 | -0.034085767 | SH3GLB1 | endophilin B1 [Source:HGNC Symbol;Acc:HGNC:10833]" |
| ENSG00000100368 | HGLibA_11337 | 112 | -0.034085767 | CSF2RB | colony stimulating factor 2 receptor beta common subunit [Source:HGNC Symbol;Acc:HGNC:2436] |
| ENSG00000100528 | HGLibA_10283 | 112 | -0.034085767 | CNIH1 | cornichon family AMPA receptor auxiliary protein 1 [Source:HGNC Symbol;Acc:HGNC:19431] |
| ENSG00000100678 | HGLibA_45395 | 112 | -0.034085767 | SLC8A3 | solute carrier family 8 member A3 [Source:HGNC Symbol;Acc:HGNC:11070] |
| ENSG00000100916 | HGLibA_04833 | 112 | -0.034085767 | BRMS1L | BRMS1 like transcriptional repressor [Source:HGNC Symbol;Acc:HGNC:20512] |
| ENSG00000102316 | HGLibA_27970 | 112 | -0.034085767 | MAGED2 | MAGE family member D2 [Source:HGNC Symbol;Acc:HGNC:16353] |
| ENSG00000103064 | HGLibA_45373 | 112 | -0.034085767 | SLC7A6 | solute carrier family 7 member 6 [Source:HGNC Symbol;Acc:HGNC:11064] |
| ENSG00000105136 | HGLibA_55954 | 112 | -0.034085767 | ZNF419 | zinc finger protein 419 [Source:HGNC Symbol;Acc:HGNC:20648] |
| ENSG00000105251 | HGLibA_44018 | 112 | -0.034085767 | SHD | Src homology 2 domain containing transforming protein D [Source:HGNC Symbol;Acc:HGNC:30633] |
| ENSG00000106483 | HGLibA_43741 | 112 | -0.034085767 | SFRP4 | secreted frizzled related protein 4 [Source:HGNC Symbol;Acc:HGNC:10778] |
| ENSG00000106638 | HGLibA_48616 | 112 | -0.034085767 | TBL2 | transducin beta like 2 [Source:HGNC Symbol;Acc:HGNC:11586] |
| ENSG00000108312 | HGLibA_52771 | 112 | -0.034085767 | UBTF | upstream binding transcription factor [Source:HGNC Symbol;Acc:HGNC:12511] |
| ENSG00000108960 | HGLibA_29463 | 112 | -0.034085767 | MMD | monocyte to macrophage differentiation associated [Source:HGNC Symbol;Acc:HGNC:7153] |
| ENSG00000109501 | HGLibA_54286 | 112 | -0.034085767 | WFS1 | wolframin ER transmembrane glycoprotein [Source:HGNC Symbol;Acc:HGNC:12762] |
| ENSG00000110756 | HGLibA_22054 | 112 | -0.034085767 | HPS5 | biogenesis of lysosomal organelles complex 2 subunit 2 [Source:HGNC Symbol;Acc:HGNC:17022]" |
| ENSG00000111729 | HGLibA_09979 | 112 | -0.034085767 | CLEC4A | C-type lectin domain family 4 member A [Source:HGNC Symbol;Acc:HGNC:13257] |
| ENSG00000112245 | HGLibA_39403 | 112 | -0.034085767 | PTP4A1 | member 1 [Source:HGNC Symbol;Acc:HGNC:9634]" |
| ENSG00000113494 | HGLibA_38606 | 112 | -0.034085767 | PRLR | prolactin receptor [Source:HGNC Symbol;Acc:HGNC:9446] |
| ENSG00000115840 | HGLibA_44642 | 112 | -0.034085767 | SLC25A12 | solute carrier family 25 member 12 [Source:HGNC Symbol;Acc:HGNC:10982] |
| ENSG00000115966 | HGLibA_03433 | 112 | -0.034085767 | ATF2 | activating transcription factor 2 [Source:HGNC Symbol;Acc:HGNC:784] |
| ENSG00000117481 | HGLibA_32786 | 112 | -0.034085767 | NSUN4 | NOP2/Sun RNA methyltransferase family member 4 [Source:HGNC Symbol;Acc:HGNC:31802] |
| ENSG00000117569 | HGLibA_39253 | 112 | -0.034085767 | PTBP2 | polypyrimidine tract binding protein 2 [Source:HGNC Symbol;Acc:HGNC:17662] |
| ENSG00000119487 | HGLibA_28299 | 112 | -0.034085767 | MAPKAP1 | mitogen-activated protein kinase associated protein 1 [Source:HGNC Symbol;Acc:HGNC:18752] |
| ENSG00000119820 | HGLibA_54678 | 112 | -0.034085767 | YIPF4 | Yip1 domain family member 4 [Source:HGNC Symbol;Acc:HGNC:28145] |

| | | | | | |
|---|---|---|---|---|---|
| ENSG00000122591 | HGLibA_16082 | 112 | -0.034085767 | FAM126A | family with sequence similarity 126 member A [Source:HGNC Symbol;Acc:HGNC:24587] |
| ENSG00000124574 | HGLibA_00160 | 112 | -0.034085767 | ABCC10 | ATP binding cassette subfamily C member 10 [Source:HGNC Symbol;Acc:HGNC:52] |
| ENSG00000126456 | HGLibA_23587 | 112 | -0.034085767 | IRF3 | interferon regulatory factor 3 [Source:HGNC Symbol;Acc:HGNC:6118] |
| ENSG00000127022 | HGLibA_07130 | 112 | -0.034085767 | CANX | calnexin [Source:HGNC Symbol;Acc:HGNC:1473] |
| ENSG00000128340 | HGLibA_40044 | 112 | -0.034085767 | RAC2 | Rac family small GTPase 2 [Source:HGNC Symbol;Acc:HGNC:9802] |
| ENSG00000128590 | HGLibA_13557 | 112 | -0.034085767 | DNAJB9 | DnaJ heat shock protein family (Hsp40) member B9 [Source:HGNC Symbol;Acc:HGNC:6968] |
| ENSG00000130957 | HGLibA_16967 | 112 | -0.034085767 | FBP2 | fructose-bisphosphatase 2 [Source:HGNC Symbol;Acc:HGNC:3607] |
| ENSG00000132109 | HGLibA_51428 | 112 | -0.034085767 | TRIM21 | tripartite motif containing 21 [Source:HGNC Symbol;Acc:HGNC:11312] |
| ENSG00000133985 | HGLibA_52158 | 112 | -0.034085767 | TTC9 | tetratricopeptide repeat domain 9 [Source:HGNC Symbol;Acc:HGNC:20267] |
| ENSG00000135114 | HGLibA_33206 | 112 | -0.034085767 | OASL | 2'-5'-oligoadenylate synthetase like [Source:HGNC Symbol;Acc:HGNC:8090] |
| ENSG00000136193 | HGLibA_43048 | 112 | -0.034085767 | SCRN1 | secernin 1 [Source:HGNC Symbol;Acc:HGNC:22192] |
| ENSG00000136783 | HGLibA_31973 | 112 | -0.034085767 | NIPSNAP3A | nipsnap homolog 3A [Source:HGNC Symbol;Acc:HGNC:23619] |
| ENSG00000136982 | HGLibA_14004 | 112 | -0.034085767 | DSCC1 | DNA replication and sister chromatid cohesion 1 [Source:HGNC Symbol;Acc:HGNC:24453] |
| ENSG00000137807 | HGLibA_24906 | 112 | -0.034085767 | KIF23 | kinesin family member 23 [Source:HGNC Symbol;Acc:HGNC:6392] |
| ENSG00000138802 | HGLibA_43232 | 112 | -0.034085767 | SEC24B | COPII coat complex component [Source:HGNC Symbol;Acc:HGNC:10704]" |
| ENSG00000138823 | HGLibA_30501 | 112 | -0.034085767 | MTTP | microsomal triglyceride transfer protein [Source:HGNC Symbol;Acc:HGNC:7467] |
| ENSG00000139668 | HGLibA_54007 | 112 | -0.034085767 | WDFY2 | WD repeat and FYVE domain containing 2 [Source:HGNC Symbol;Acc:HGNC:20482] |
| ENSG00000140030 | HGLibA_20075 | 112 | -0.034085767 | GPR65 | G protein-coupled receptor 65 [Source:HGNC Symbol;Acc:HGNC:4517] |
| ENSG00000141349 | HGLibA_18310 | 112 | -0.034085767 | G6PC3 | glucose-6-phosphatase catalytic subunit 3 [Source:HGNC Symbol;Acc:HGNC:24861] |
| ENSG00000141437 | HGLibA_44769 | 112 | -0.034085767 | SLC25A52 | solute carrier family 25 member 52 [Source:HGNC Symbol;Acc:HGNC:23324] |
| ENSG00000141946 | HGLibA_55342 | 112 | -0.034085767 | ZIM3 | zinc finger imprinted 3 [Source:HGNC Symbol;Acc:HGNC:16366] |
| ENSG00000143110 | HGLibA_05787 | 112 | -0.034085767 | C1orf162 | chromosome 1 open reading frame 162 [Source:HGNC Symbol;Acc:HGNC:28344] |
| ENSG00000144036 | HGLibA_15758 | 112 | -0.034085767 | EXOC6B | exocyst complex component 6B [Source:HGNC Symbol;Acc:HGNC:17085] |
| ENSG00000145362 | HGLibA_01937 | 112 | -0.034085767 | ANK2 | ankyrin 2 [Source:HGNC Symbol;Acc:HGNC:493] |
| ENSG00000145868 | HGLibA_17084 | 112 | -0.034085767 | FBXO38 | F-box protein 38 [Source:HGNC Symbol;Acc:HGNC:28844] |
| ENSG00000146872 | HGLibA_49626 | 112 | -0.034085767 | TLK2 | tousled like kinase 2 [Source:HGNC Symbol;Acc:HGNC:11842] |
| ENSG00000147155 | HGLibA_14373 | 112 | -0.034085767 | EBP | cholestenol delta-isomerase [Source:HGNC Symbol;Acc:HGNC:3133]" |
| ENSG00000147400 | HGLibA_09237 | 112 | -0.034085767 | CETN2 | centrin 2 [Source:HGNC Symbol;Acc:HGNC:1867] |
| ENSG00000147679 | HGLibA_53448 | 112 | -0.034085767 | UTP23 | small subunit processome component [Source:HGNC Symbol;Acc:HGNC:28224]" |
| ENSG00000148158 | HGLibA_46084 | 112 | -0.034085767 | SNX30 | sorting nexin family member 30 [Source:HGNC Symbol;Acc:HGNC:23685] |
| ENSG00000148444 | HGLibA_10658 | 112 | -0.034085767 | COMMD3 | COMM domain containing 3 [Source:HGNC Symbol;Acc:HGNC:23332] |
| ENSG00000148824 | HGLibA_30369 | 112 | -0.034085767 | MTG1 | mitochondrial ribosome associated GTPase 1 [Source:HGNC Symbol;Acc:HGNC:32159] |
| ENSG00000155096 | HGLibA_03938 | 112 | -0.034085767 | AZIN1 | antizyme inhibitor 1 [Source:HGNC Symbol;Acc:HGNC:16432] |
| ENSG00000155729 | HGLibA_24439 | 112 | -0.034085767 | KCTD18 | potassium channel tetramerization domain containing 18 [Source:HGNC Symbol;Acc:HGNC:26446] |
| ENSG00000157060 | HGLibA_44015 | 112 | -0.034085767 | SHCBP1L | SHC binding and spindle associated 1 like [Source:HGNC Symbol;Acc:HGNC:16788] |
| ENSG00000157107 | HGLibA_17208 | 112 | -0.034085767 | FCHO2 | FCH domain only 2 [Source:HGNC Symbol;Acc:HGNC:25180] |
| ENSG00000157881 | HGLibA_35107 | 112 | -0.034085767 | PANK4 | pantothenate kinase 4 [Source:HGNC Symbol;Acc:HGNC:19366] |
| ENSG00000158321 | HGLibA_03885 | 112 | -0.034085767 | AUTS2 | activator of transcription and developmental regulator [Source:HGNC Symbol;Acc:HGNC:14262]" |
| ENSG00000158486 | HGLibA_13486 | 112 | -0.034085767 | DNAH3 | dynein axonemal heavy chain 3 [Source:HGNC Symbol;Acc:HGNC:2949] |
| ENSG00000160094 | HGLibA_55873 | 112 | -0.034085767 | ZNF362 | zinc finger protein 362 [Source:HGNC Symbol;Acc:HGNC:18079] |
| ENSG00000160781 | HGLibA_35166 | 112 | -0.034085767 | PAQR6 | progestin and adipoQ receptor family member 6 [Source:HGNC Symbol;Acc:HGNC:30132] |
| ENSG00000161798 | HGLibA_02600 | 112 | -0.034085767 | AQP5 | aquaporin 5 [Source:HGNC Symbol;Acc:HGNC:638] |
| ENSG00000162374 | HGLibA_14888 | 112 | -0.034085767 | ELAVL4 | ELAV like RNA binding protein 4 [Source:HGNC Symbol;Acc:HGNC:3315] |
| ENSG00000162714 | HGLibA_56100 | 112 | -0.034085767 | ZNF496 | zinc finger protein 496 [Source:HGNC Symbol;Acc:HGNC:23713] |
| ENSG00000163395 | HGLibA_22854 | 112 | -0.034085767 | IGFN1 | immunoglobulin-like and fibronectin type III domain containing 1 [Source:HGNC Symbol;Acc:HGNC:24607] |
| ENSG00000163406 | HGLibA_44407 | 112 | -0.034085767 | SLC15A2 | solute carrier family 15 member 2 [Source:HGNC Symbol;Acc:HGNC:10921] |
| ENSG00000163482 | HGLibA_47498 | 112 | -0.034085767 | STK36 | serine/threonine kinase 36 [Source:HGNC Symbol;Acc:HGNC:17209] |
| ENSG00000163602 | HGLibA_42515 | 112 | -0.034085767 | RYBP | RING1 and YY1 binding protein [Source:HGNC Symbol;Acc:HGNC:10480] |
| ENSG00000163995 | HGLibA_00312 | 112 | -0.034085767 | ABLIM2 | actin binding LIM protein family member 2 [Source:HGNC Symbol;Acc:HGNC:19195] |
| ENSG00000164093 | HGLibA_36765 | 112 | -0.034085767 | PITX2 | paired like homeodomain 2 [Source:HGNC Symbol;Acc:HGNC:9005] |
| ENSG00000164237 | HGLibA_10181 | 112 | -0.034085767 | CMBL | carboxymethylenebutenolidase homolog [Source:HGNC Symbol;Acc:HGNC:25090] |
| ENSG00000164294 | HGLibA_20192 | 112 | -0.034085767 | GPX8 | glutathione peroxidase 8 (putative) [Source:HGNC Symbol;Acc:HGNC:33100] |
| ENSG00000164627 | HGLibA_24959 | 112 | -0.034085767 | KIF6 | kinesin family member 6 [Source:HGNC Symbol;Acc:HGNC:21202] |
| ENSG00000165156 | HGLibA_55310 | 112 | -0.034085767 | ZHX1 | zinc fingers and homeoboxes 1 [Source:HGNC Symbol;Acc:HGNC:12871] |
| ENSG00000165383 | HGLibA_27273 | 112 | -0.034085767 | LRRC18 | leucine rich repeat containing 18 [Source:HGNC Symbol;Acc:HGNC:23199] |
| ENSG00000166105 | HGLibA_19227 | 112 | -0.034085767 | GLB1L3 | galactosidase beta 1 like 3 [Source:HGNC Symbol;Acc:HGNC:25147] |
| ENSG00000167617 | HGLibA_08578 | 112 | -0.034085767 | CDC42EP5 | CDC42 effector protein 5 [Source:HGNC Symbol;Acc:HGNC:17408] |
| ENSG00000167850 | HGLibA_08337 | 112 | -0.034085767 | CD300C | CD300c molecule [Source:HGNC Symbol;Acc:HGNC:19320] |
| ENSG00000168124 | HGLibA_33666 | 112 | -0.034085767 | OR1F1 | olfactory receptor family 1 subfamily F member 1 [Source:HGNC Symbol;Acc:HGNC:8194] |
| ENSG00000168263 | HGLibA_24404 | 112 | -0.034085767 | KCNV2 | potassium voltage-gated channel modifier subfamily V member 2 [Source:HGNC Symbol;Acc:HGNC:19698] |
| ENSG00000168671 | HGLibA_52935 | 112 | -0.034085767 | UGT3A2 | UDP glycosyltransferase family 3 member A2 [Source:HGNC Symbol;Acc:HGNC:27266] |
| ENSG00000168928 | HGLibA_11679 | 112 | -0.034085767 | CTRB2 | chymotrypsinogen B2 [Source:HGNC Symbol;Acc:HGNC:2522] |
| ENSG00000169220 | HGLibA_41065 | 112 | -0.034085767 | RGS14 | regulator of G protein signaling 14 [Source:HGNC Symbol;Acc:HGNC:9996] |
| ENSG00000169562 | HGLibA_19158 | 112 | -0.034085767 | GJB1 | gap junction protein beta 1 [Source:HGNC Symbol;Acc:HGNC:4283] |
| ENSG00000169807 | HGLibA_38998 | 112 | -0.034085767 | PRY2 | PTPN13 like Y-linked 2 [Source:HGNC Symbol;Acc:HGNC:21504] |
| ENSG00000170145 | HGLibA_44164 | 112 | -0.034085767 | SIK2 | salt inducible kinase 2 [Source:HGNC Symbol;Acc:HGNC:21680] |
| ENSG00000170381 | HGLibA_43331 | 112 | -0.034085767 | SEMA3E | semaphorin 3E [Source:HGNC Symbol;Acc:HGNC:10727] |
| ENSG00000170703 | HGLibA_52209 | 112 | -0.034085767 | TTLL6 | tubulin tyrosine ligase like 6 [Source:HGNC Symbol;Acc:HGNC:26664] |
| ENSG00000171204 | HGLibA_49912 | 112 | -0.034085767 | TMEM126B | transmembrane protein 126B [Source:HGNC Symbol;Acc:HGNC:30883] |
| ENSG00000174013 | HGLibA_17107 | 112 | -0.034085767 | FBXO45 | F-box protein 45 [Source:HGNC Symbol;Acc:HGNC:29148] |
| ENSG00000174326 | HGLibA_44424 | 112 | -0.034085767 | SLC16A11 | solute carrier family 16 member 11 [Source:HGNC Symbol;Acc:HGNC:23093] |
| ENSG00000177409 | HGLibA_42700 | 112 | -0.034085767 | SAMD9L | sterile alpha motif domain containing 9 like [Source:HGNC Symbol;Acc:HGNC:1349] |
| ENSG00000177917 | HGLibA_02994 | 112 | -0.034085767 | ARL6IP6 | ADP ribosylation factor like GTPase 6 interacting protein 6 [Source:HGNC Symbol;Acc:HGNC:24048] |
| ENSG00000181074 | HGLibA_34199 | 112 | -0.034085767 | OR52N4 | olfactory receptor family 52 subfamily N member 4 (gene/pseudogene) [Source:HGNC Symbol;Acc:HGNC:15230] |
| ENSG00000183091 | HGLibA_31579 | 112 | -0.034085767 | NEB | nebulin [Source:HGNC Symbol;Acc:HGNC:7720] |
| ENSG00000183434 | HGLibA_49156 | 112 | -0.034085767 | TFDP3 | transcription factor Dp family member 3 [Source:HGNC Symbol;Acc:HGNC:24603] |
| ENSG00000183773 | HGLibA_01343 | 112 | -0.034085767 | AIFM3 | mitochondria associated 3 [Source:HGNC Symbol;Acc:HGNC:26398]" |
| ENSG00000183778 | HGLibA_03964 | 112 | -0.034085767 | B3GALT5 | 3-galactosyltransferase 5 [Source:HGNC Symbol;Acc:HGNC:920]" |
| ENSG00000184210 | HGLibA_13041 | 112 | -0.034085767 | DGAT2L6 | diacylglycerol O-acyltransferase 2 like 6 [Source:HGNC Symbol;Acc:HGNC:23250] |
| ENSG00000184344 | HGLibA_18867 | 112 | -0.034085767 | GDF3 | growth differentiation factor 3 [Source:HGNC Symbol;Acc:HGNC:4218] |
| ENSG00000185009 | HGLibA_02328 | 112 | -0.034085767 | AP3M1 | adaptor related protein complex 3 subunit mu 1 [Source:HGNC Symbol;Acc:HGNC:569] |
| ENSG00000185272 | HGLibA_40476 | 112 | -0.034085767 | RBM11 | RNA binding motif protein 11 [Source:HGNC Symbol;Acc:HGNC:9897] |
| ENSG00000186970 | HGLibA_25654 | 112 | -0.034085767 | KRTAP15-1 | keratin associated protein 15-1 [Source:HGNC Symbol;Acc:HGNC:18927] |
| ENSG00000188334 | HGLibA_04874 | 112 | -0.034085767 | BSPH1 | binder of sperm protein homolog 1 [Source:HGNC Symbol;Acc:HGNC:33906] |
| ENSG00000196284 | HGLibA_47813 | 112 | -0.034085767 | SUPT3H | SAGA and STAGA complex component [Source:HGNC Symbol;Acc:HGNC:11466]" |
| ENSG00000196597 | HGLibA_56741 | 112 | -0.034085767 | ZNF782 | zinc finger protein 782 [Source:HGNC Symbol;Acc:HGNC:33110] |
| ENSG00000197213 | HGLibA_57032 | 112 | -0.034085767 | ZSCAN5B | zinc finger and SCAN domain containing 5B [Source:HGNC Symbol;Acc:HGNC:34246] |
| ENSG00000197233 | HGLibA_33677 | 112 | -0.034085767 | OR1J2 | olfactory receptor family 1 subfamily J member 2 [Source:HGNC Symbol;Acc:HGNC:8209] |
| ENSG00000205209 | HGLibA_42928 | 112 | -0.034085767 | SCGB2B2 | secretoglobin family 2B member 2 [Source:HGNC Symbol;Acc:HGNC:27616] |
| ENSG00000205884 | HGLibA_12910 | 112 | -0.034085767 | DEFB136 | defensin beta 136 [Source:HGNC Symbol;Acc:HGNC:34433] |
| ENSG00000212657 | HGLibA_25656 | 112 | -0.034085767 | KRTAP16-1 | keratin associated protein 16-1 [Source:HGNC Symbol;Acc:HGNC:18916] |
| ENSG00000213965 | HGLibA_32963 | 112 | -0.034085767 | NUDT19 | nudix hydrolase 19 [Source:HGNC Symbol;Acc:HGNC:32036] |
| ENSG00000214212 | HGLibA_05654 | 112 | -0.034085767 | C19orf38 | chromosome 19 open reading frame 38 [Source:HGNC Symbol;Acc:HGNC:34073] |
| ENSG00000214510 | HGLibA_46685 | 112 | -0.034085767 | SPINK13 | Kazal type 13 (putative) [Source:HGNC Symbol;Acc:HGNC:27200]" |
| ENSG00000225899 | HGLibA_17998 | 112 | -0.034085767 | FRG2B | FSHD region gene 2 family member B [Source:HGNC Symbol;Acc:HGNC:33518] |
| ENSG00000234829 | HGLibA_22671 | 112 | -0.034085767 | IFNA17 | interferon alpha 17 [Source:HGNC Symbol;Acc:HGNC:5422] |
| ENSG00000235568 | HGLibA_31763 | 112 | -0.034085767 | NFAM1 | NFAT activating protein with ITAM motif 1 [Source:HGNC Symbol;Acc:HGNC:29872] |
| ENSG00000236981 | HGLibA_33503 | 112 | -0.034085767 | OR10G9 | olfactory receptor family 10 subfamily G member 9 [Source:HGNC Symbol;Acc:HGNC:15129] |
| ENSG00000238269 | HGLibA_35010 | 112 | -0.034085767 | PAGE2B | PAGE family member 2B [Source:HGNC Symbol;Acc:HGNC:31805] |
| ENSG00000241553 | HGLibA_03085 | 112 | -0.034085767 | ARPC4 | actin related protein 2/3 complex subunit 4 [Source:HGNC Symbol;Acc:HGNC:707] |
| ENSG00000249471 | HGLibA_55803 | 112 | -0.034085767 | ZNF324B | zinc finger protein 324B [Source:HGNC Symbol;Acc:HGNC:33107] |
| ENSG00000260027 | HGLibA_21946 | 112 | -0.034085767 | HOXB7 | homeobox B7 [Source:HGNC Symbol;Acc:HGNC:5118] |
| ENSG00000263155 | HGLibA_30932 | 112 | -0.034085767 | MYZAP | myocardial zonula adherens protein [Source:HGNC Symbol;Acc:HGNC:43444] |
| ENSG00000271079 | HGLibA_11571 | 112 | -0.034085767 | CTAGE15 | CTAGE family member 15 [Source:HGNC Symbol;Acc:HGNC:37295] |
| ENSG00000272414 | HGLibA_16619 | 112 | -0.034085767 | FAM47E-STBD1 | FAM47E-STBD1 readthrough [Source:HGNC Symbol;Acc:HGNC:44667] |
| ENSG00000276581 | HGLibA_46480 | 112 | -0.034085767 | SPATA31A5 | SPATA31 subfamily A member 5 [Source:HGNC Symbol;Acc:HGNC:32005] |
| HDGFRP2 | HGLibA_21046 | 112 | -0.034085767 | HDGFRP2 | - |
| HIST1H2AH | HGLibA_21394 | 112 | -0.034085767 | HIST1H2AH | - |
| HNRPDL | HGLibA_21856 | 112 | -0.034085767 | HNRPDL | - |
| hsa-mir-101-1 | HGLibA_57155 | 112 | -0.034085767 | hsa-mir-101-1 | - |
| hsa-mir-1272 | HGLibA_57527 | 112 | -0.034085767 | hsa-mir-1272 | - |
| hsa-mir-1283-1 | HGLibA_57589 | 112 | -0.034085767 | hsa-mir-1283-1 | - |
| hsa-mir-1302-7 | HGLibA_57715 | 112 | -0.034085767 | hsa-mir-1302-7 | - |
| hsa-mir-3144 | HGLibA_58636 | 112 | -0.034085767 | hsa-mir-3144 | - |
| hsa-mir-3146 | HGLibA_58643 | 112 | -0.034085767 | hsa-mir-3146 | - |
| hsa-mir-3178 | HGLibA_58787 | 112 | -0.034085767 | hsa-mir-3178 | - |
| hsa-mir-3180-1 | HGLibA_58800 | 112 | -0.034085767 | hsa-mir-3180-1 | - |
| hsa-mir-345 | HGLibA_59026 | 112 | -0.034085767 | hsa-mir-345 | - |
| hsa-mir-378b | HGLibA_59428 | 112 | -0.034085767 | hsa-mir-378b | - |
| hsa-mir-4278 | HGLibA_59816 | 112 | -0.034085767 | hsa-mir-4278 | - |
| hsa-mir-4535 | HGLibA_60600 | 112 | -0.034085767 | hsa-mir-4535 | - |
| hsa-mir-4716 | HGLibA_60966 | 112 | -0.034085767 | hsa-mir-4716 | - |
| hsa-mir-4756 | HGLibA_61122 | 112 | -0.034085767 | hsa-mir-4756 | - |
| hsa-mir-4769 | HGLibA_61172 | 112 | -0.034085767 | hsa-mir-4769 | - |
| hsa-mir-511 | HGLibA_61554 | 112 | -0.034085767 | hsa-mir-511 | - |
| hsa-mir-549a | HGLibA_62094 | 112 | -0.034085767 | hsa-mir-549a | - |
| LEPREL4 | HGLibA_26323 | 112 | -0.034085767 | LEPREL4 | - |
| LOC100130301 | HGLibA_26773 | 112 | -0.034085767 | LOC100130301 | - |
| LOC100130451 | HGLibA_26787 | 112 | -0.034085767 | LOC100130451 | - |
| LOC730183 | HGLibA_27004 | 112 | -0.034085767 | LOC730183 | - |
| TMEM189 | HGLibA_50133 | 112 | -0.034085767 | TMEM189 | - |
| WDR67 | HGLibA_54158 | 112 | -0.034085767 | WDR67 | - |
| C12orf10 | HGLibA_05222 | 111 | -0.044036805 | C12orf10 | - |
| ENSG00000004948 | HGLibA_07003 | 111 | -0.044036805 | CALCR | calcitonin receptor [Source:HGNC Symbol;Acc:HGNC:1440] |
| ENSG00000055147 | HGLibA_16034 | 111 | -0.044036805 | FAM114A2 | family with sequence similarity 114 member A2 [Source:HGNC Symbol;Acc:HGNC:1333] |

| | | | | | |
|---|---|---|---|---|---|
| ENSG00000064763 | HGLibA_16872 | 111 | -0.044036805 | FAR2 | fatty acyl-CoA reductase 2 [Source:HGNC Symbol;Acc:HGNC:25531] |
| ENSG00000066084 | HGLibA_13249 | 111 | -0.044036805 | DIP2B | disco interacting protein 2 homolog B [Source:HGNC Symbol;Acc:HGNC:29284] |
| ENSG00000068489 | HGLibA_38771 | 111 | -0.044036805 | PRR11 | proline rich 11 [Source:HGNC Symbol;Acc:HGNC:25619] |
| ENSG00000069431 | HGLibA_00189 | 111 | -0.044036805 | ABCC9 | ATP binding cassette subfamily C member 9 [Source:HGNC Symbol;Acc:HGNC:60] |
| ENSG00000073464 | HGLibA_09829 | 111 | -0.044036805 | CLCN4 | chloride voltage-gated channel 4 [Source:HGNC Symbol;Acc:HGNC:2022] |
| ENSG00000076108 | HGLibA_04190 | 111 | -0.044036805 | BAZ2A | bromodomain adjacent to zinc finger domain 2A [Source:HGNC Symbol;Acc:HGNC:962] |
| ENSG00000078902 | HGLibA_50943 | 111 | -0.044036805 | TOLLIP | toll interacting protein [Source:HGNC Symbol;Acc:HGNC:16476] |
| ENSG00000085382 | HGLibA_20799 | 111 | -0.044036805 | HACE1 | HECT domain and ankyrin repeat containing E3 ubiquitin protein ligase 1 [Source:HGNC Symbol;Acc:HGNC:21033] |
| ENSG00000089248 | HGLibA_15527 | 111 | -0.044036805 | ERP29 | endoplasmic reticulum protein 29 [Source:HGNC Symbol;Acc:HGNC:13799] |
| ENSG00000089558 | HGLibA_24220 | 111 | -0.044036805 | KCNH4 | potassium voltage-gated channel subfamily H member 4 [Source:HGNC Symbol;Acc:HGNC:6253] |
| ENSG00000089876 | HGLibA_13177 | 111 | -0.044036805 | DHX32 | DEAH-box helicase 32 (putative) [Source:HGNC Symbol;Acc:HGNC:16717] |
| ENSG00000092841 | HGLibA_30768 | 111 | -0.044036805 | MYL6 | myosin light chain 6 [Source:HGNC Symbol;Acc:HGNC:7587] |
| ENSG00000094755 | HGLibA_18398 | 111 | -0.044036805 | GABRP | gamma-aminobutyric acid type A receptor pi subunit [Source:HGNC Symbol;Acc:HGNC:4089] |
| ENSG00000099866 | HGLibA_27855 | 111 | -0.044036805 | MADCAM1 | mucosal vascular addressin cell adhesion molecule 1 [Source:HGNC Symbol;Acc:HGNC:6765] |
| ENSG00000100473 | HGLibA_10468 | 111 | -0.044036805 | COCH | cochlin [Source:HGNC Symbol;Acc:HGNC:2180] |
| ENSG00000100526 | HGLibA_08847 | 111 | -0.044036805 | CDKN3 | cyclin dependent kinase inhibitor 3 [Source:HGNC Symbol;Acc:HGNC:1791] |
| ENSG00000103249 | HGLibA_09837 | 111 | -0.044036805 | CLCN7 | chloride voltage-gated channel 7 [Source:HGNC Symbol;Acc:HGNC:2025] |
| ENSG00000104412 | HGLibA_15008 | 111 | -0.044036805 | EMC2 | ER membrane protein complex subunit 2 [Source:HGNC Symbol;Acc:HGNC:28963] |
| ENSG00000104643 | HGLibA_30442 | 111 | -0.044036805 | MTMR9 | myotubularin related protein 9 [Source:HGNC Symbol;Acc:HGNC:14596] |
| ENSG00000105173 | HGLibA_08085 | 111 | -0.044036805 | CCNE1 | cyclin E1 [Source:HGNC Symbol;Acc:HGNC:1589] |
| ENSG00000105258 | HGLibA_37589 | 111 | -0.044036805 | POLR2I | RNA polymerase II subunit I [Source:HGNC Symbol;Acc:HGNC:9196] |
| ENSG00000106526 | HGLibA_00665 | 111 | -0.044036805 | ACTR3C | ARP3 actin related protein 3 homolog C [Source:HGNC Symbol;Acc:HGNC:37282] |
| ENSG00000111325 | HGLibA_33310 | 111 | -0.044036805 | OGFOD2 | 2-oxoglutarate and iron dependent oxygenase domain containing 2 [Source:HGNC Symbol;Acc:HGNC:25823] |
| ENSG00000111785 | HGLibA_41238 | 111 | -0.044036805 | RIC8B | RIC8 guanine nucleotide exchange factor B [Source:HGNC Symbol;Acc:HGNC:25555] |
| ENSG00000112164 | HGLibA_19291 | 111 | -0.044036805 | GLP1R | glucagon like peptide 1 receptor [Source:HGNC Symbol;Acc:HGNC:4324] |
| ENSG00000113273 | HGLibA_03131 | 111 | -0.044036805 | ARSB | arylsulfatase B [Source:HGNC Symbol;Acc:HGNC:714] |
| ENSG00000113615 | HGLibA_43228 | 111 | -0.044036805 | SEC24A | COPII coat complex component [Source:HGNC Symbol;Acc:HGNC:10703]" |
| ENSG00000114331 | HGLibA_00387 | 111 | -0.044036805 | ACAP2 | ankyrin repeat and PH domains 2 [Source:HGNC Symbol;Acc:HGNC:16469]" |
| ENSG00000114738 | HGLibA_28305 | 111 | -0.044036805 | MAPKAPK3 | mitogen-activated protein kinase-activated protein kinase 3 [Source:HGNC Symbol;Acc:HGNC:6888] |
| ENSG00000115255 | HGLibA_40792 | 111 | -0.044036805 | REEP6 | receptor accessory protein 6 [Source:HGNC Symbol;Acc:HGNC:30078] |
| ENSG00000116679 | HGLibA_23883 | 111 | -0.044036805 | IVNS1ABP | influenza virus NS1A binding protein [Source:HGNC Symbol;Acc:HGNC:16951] |
| ENSG00000116704 | HGLibA_44976 | 111 | -0.044036805 | SLC35D1 | solute carrier family 35 member D1 [Source:HGNC Symbol;Acc:HGNC:20800] |
| ENSG00000117215 | HGLibA_36888 | 111 | -0.044036805 | PLA2G2D | phospholipase A2 group IID [Source:HGNC Symbol;Acc:HGNC:9033] |
| ENSG00000120647 | HGLibA_07867 | 111 | -0.044036805 | CCDC77 | coiled-coil domain containing 77 [Source:HGNC Symbol;Acc:HGNC:28203] |
| ENSG00000120659 | HGLibA_50799 | 111 | -0.044036805 | TNFSF11 | TNF superfamily member 11 [Source:HGNC Symbol;Acc:HGNC:11926] |
| ENSG00000121381 | HGLibA_48431 | 111 | -0.044036805 | TAS2R9 | taste 2 receptor member 9 [Source:HGNC Symbol;Acc:HGNC:14917] |
| ENSG00000122203 | HGLibA_24705 | 111 | -0.044036805 | KIAA1191 | KIAA1191 [Source:HGNC Symbol;Acc:HGNC:29209] |
| ENSG00000123689 | HGLibA_18294 | 111 | -0.044036805 | G0S2 | G0/G1 switch 2 [Source:HGNC Symbol;Acc:HGNC:30229] |
| ENSG00000124788 | HGLibA_03829 | 111 | -0.044036805 | ATXN1 | ataxin 1 [Source:HGNC Symbol;Acc:HGNC:10548] |
| ENSG00000125522 | HGLibA_32415 | 111 | -0.044036805 | NPBWR2 | neuropeptides B and W receptor 2 [Source:HGNC Symbol;Acc:HGNC:4530] |
| ENSG00000125831 | HGLibA_11447 | 111 | -0.044036805 | CST11 | cystatin 11 [Source:HGNC Symbol;Acc:HGNC:15959] |
| ENSG00000125875 | HGLibA_48497 | 111 | -0.044036805 | TBC1D20 | TBC1 domain family member 20 [Source:HGNC Symbol;Acc:HGNC:16133] |
| ENSG00000125967 | HGLibA_31591 | 111 | -0.044036805 | NECAB3 | N-terminal EF-hand calcium binding protein 3 [Source:HGNC Symbol;Acc:HGNC:15851] |
| ENSG00000128313 | HGLibA_02540 | 111 | -0.044036805 | APOL5 | apolipoprotein L5 [Source:HGNC Symbol;Acc:HGNC:14869] |
| ENSG00000128512 | HGLibA_13746 | 111 | -0.044036805 | DOCK4 | dedicator of cytokinesis 4 [Source:HGNC Symbol;Acc:HGNC:19192] |
| ENSG00000128594 | HGLibA_27350 | 111 | -0.044036805 | LRRC4 | leucine rich repeat containing 4 [Source:HGNC Symbol;Acc:HGNC:15586] |
| ENSG00000129757 | HGLibA_08826 | 111 | -0.044036805 | CDKN1C | cyclin dependent kinase inhibitor 1C [Source:HGNC Symbol;Acc:HGNC:1786] |
| ENSG00000131050 | HGLibA_04714 | 111 | -0.044036805 | BPIFA2 | BPI fold containing family A member 2 [Source:HGNC Symbol;Acc:HGNC:16203] |
| ENSG00000131730 | HGLibA_09783 | 111 | -0.044036805 | CKMT2 | mitochondrial 2 [Source:HGNC Symbol;Acc:HGNC:1996]" |
| ENSG00000132434 | HGLibA_26008 | 111 | -0.044036805 | LANCL2 | LanC like 2 [Source:HGNC Symbol;Acc:HGNC:6509] |
| ENSG00000132522 | HGLibA_20152 | 111 | -0.044036805 | GPS2 | G protein pathway suppressor 2 [Source:HGNC Symbol;Acc:HGNC:4550] |
| ENSG00000133131 | HGLibA_29657 | 111 | -0.044036805 | MORC4 | MORC family CW-type zinc finger 4 [Source:HGNC Symbol;Acc:HGNC:23485] |
| ENSG00000133134 | HGLibA_04443 | 111 | -0.044036805 | BEX2 | brain expressed X-linked 2 [Source:HGNC Symbol;Acc:HGNC:30933] |
| ENSG00000133401 | HGLibA_35973 | 111 | -0.044036805 | PDZD2 | PDZ domain containing 2 [Source:HGNC Symbol;Acc:HGNC:18486] |
| ENSG00000133597 | HGLibA_00892 | 111 | -0.044036805 | ADCK2 | aarF domain containing kinase 2 [Source:HGNC Symbol;Acc:HGNC:19039] |
| ENSG00000134193 | HGLibA_40806 | 111 | -0.044036805 | REG4 | regenerating family member 4 [Source:HGNC Symbol;Acc:HGNC:22977] |
| ENSG00000134259 | HGLibA_31873 | 111 | -0.044036805 | NGF | nerve growth factor [Source:HGNC Symbol;Acc:HGNC:7808] |
| ENSG00000135074 | HGLibA_00747 | 111 | -0.044036805 | ADAM19 | ADAM metallopeptidase domain 19 [Source:HGNC Symbol;Acc:HGNC:197] |
| ENSG00000135423 | HGLibA_19327 | 111 | -0.044036805 | GLS2 | glutaminase 2 [Source:HGNC Symbol;Acc:HGNC:29570] |
| ENSG00000136002 | HGLibA_02865 | 111 | -0.044036805 | ARHGEF4 | Rho guanine nucleotide exchange factor 4 [Source:HGNC Symbol;Acc:HGNC:684] |
| ENSG00000136052 | HGLibA_45142 | 111 | -0.044036805 | SLC41A2 | solute carrier family 41 member 2 [Source:HGNC Symbol;Acc:HGNC:31045] |
| ENSG00000137207 | HGLibA_54677 | 111 | -0.044036805 | YIPF3 | Yip1 domain family member 3 [Source:HGNC Symbol;Acc:HGNC:21023] |
| ENSG00000137558 | HGLibA_36482 | 111 | -0.044036805 | PI15 | peptidase inhibitor 15 [Source:HGNC Symbol;Acc:HGNC:8946] |
| ENSG00000137845 | HGLibA_00729 | 111 | -0.044036805 | ADAM10 | ADAM metallopeptidase domain 10 [Source:HGNC Symbol;Acc:HGNC:188] |
| ENSG00000138316 | HGLibA_00804 | 111 | -0.044036805 | ADAMTS14 | ADAM metallopeptidase with thrombospondin type 1 motif 14 [Source:HGNC Symbol;Acc:HGNC:14899] |
| ENSG00000138439 | HGLibA_16046 | 111 | -0.044036805 | FAM117B | family with sequence similarity 117 member B [Source:HGNC Symbol;Acc:HGNC:14440] |
| ENSG00000138814 | HGLibA_38185 | 111 | -0.044036805 | PPP3CA | protein phosphatase 3 catalytic subunit alpha [Source:HGNC Symbol;Acc:HGNC:9314] |
| ENSG00000139182 | HGLibA_10140 | 111 | -0.044036805 | CLSTN3 | calsyntenin 3 [Source:HGNC Symbol;Acc:HGNC:18371] |
| ENSG00000139220 | HGLibA_37876 | 111 | -0.044036805 | PPFIA2 | PTPRF interacting protein alpha 2 [Source:HGNC Symbol;Acc:HGNC:9246] |
| ENSG00000139990 | HGLibA_12449 | 111 | -0.044036805 | DCAF5 | DDB1 and CUL4 associated factor 5 [Source:HGNC Symbol;Acc:HGNC:20224] |
| ENSG00000140534 | HGLibA_49452 | 111 | -0.044036805 | TICRR | TOPBP1 interacting checkpoint and replication regulator [Source:HGNC Symbol;Acc:HGNC:28704] |
| ENSG00000143228 | HGLibA_32998 | 111 | -0.044036805 | NUF2 | NDC80 kinetochore complex component NUF2 [Source:HGNC Symbol;Acc:HGNC:14621] |
| ENSG00000145014 | HGLibA_50405 | 111 | -0.044036805 | TMEM44 | transmembrane protein 44 [Source:HGNC Symbol;Acc:HGNC:25120] |
| ENSG00000145050 | HGLibA_28098 | 111 | -0.044036805 | MANF | mesencephalic astrocyte derived neurotrophic factor [Source:HGNC Symbol;Acc:HGNC:15461] |
| ENSG00000145819 | HGLibA_02742 | 111 | -0.044036805 | ARHGAP26 | Rho GTPase activating protein 26 [Source:HGNC Symbol;Acc:HGNC:17073] |
| ENSG00000147408 | HGLibA_11344 | 111 | -0.044036805 | CSGALNACT1 | chondroitin sulfate N-acetylgalactosaminyltransferase 1 [Source:HGNC Symbol;Acc:HGNC:24290] |
| ENSG00000148384 | HGLibA_23351 | 111 | -0.044036805 | INPP5E | inositol polyphosphate-5-phosphatase E [Source:HGNC Symbol;Acc:HGNC:21474] |
| ENSG00000151033 | HGLibA_27787 | 111 | -0.044036805 | LYZL2 | lysozyme like 2 [Source:HGNC Symbol;Acc:HGNC:29613] |
| ENSG00000153885 | HGLibA_24428 | 111 | -0.044036805 | KCTD15 | potassium channel tetramerization domain containing 15 [Source:HGNC Symbol;Acc:HGNC:23297] |
| ENSG00000153896 | HGLibA_56359 | 111 | -0.044036805 | ZNF599 | zinc finger protein 599 [Source:HGNC Symbol;Acc:HGNC:26408] |
| ENSG00000156603 | HGLibA_28769 | 111 | -0.044036805 | MED19 | mediator complex subunit 19 [Source:HGNC Symbol;Acc:HGNC:29600] |
| ENSG00000158497 | HGLibA_21740 | 111 | -0.044036805 | HMHB1 | histocompatibility minor HB-1 [Source:HGNC Symbol;Acc:HGNC:29677] |
| ENSG00000158941 | HGLibA_07490 | 111 | -0.044036805 | CCAR2 | cell cycle and apoptosis regulator 2 [Source:HGNC Symbol;Acc:HGNC:23360] |
| ENSG00000160007 | HGLibA_02765 | 111 | -0.044036805 | ARHGAP35 | Rho GTPase activating protein 35 [Source:HGNC Symbol;Acc:HGNC:4591] |
| ENSG00000161533 | HGLibA_00497 | 111 | -0.044036805 | ACOX1 | acyl-CoA oxidase 1 [Source:HGNC Symbol;Acc:HGNC:119] |
| ENSG00000161940 | HGLibA_04336 | 111 | -0.044036805 | BCL6B | B cell CLL/lymphoma 6B [Source:HGNC Symbol;Acc:HGNC:1002] |
| ENSG00000162039 | HGLibA_28869 | 111 | -0.044036805 | MEIOB | meiosis specific with OB domains [Source:HGNC Symbol;Acc:HGNC:28569] |
| ENSG00000162337 | HGLibA_27225 | 111 | -0.044036805 | LRP5 | LDL receptor related protein 5 [Source:HGNC Symbol;Acc:HGNC:6697] |
| ENSG00000162391 | HGLibA_16161 | 111 | -0.044036805 | FAM151A | family with sequence similarity 151 member A [Source:HGNC Symbol;Acc:HGNC:25032] |
| ENSG00000163710 | HGLibA_35654 | 111 | -0.044036805 | PCOLCE2 | procollagen C-endopeptidase enhancer 2 [Source:HGNC Symbol;Acc:HGNC:8739] |
| ENSG00000164220 | HGLibA_15869 | 111 | -0.044036805 | F2RL2 | coagulation factor II thrombin receptor like 2 [Source:HGNC Symbol;Acc:HGNC:3539] |
| ENSG00000164924 | HGLibA_54753 | 111 | -0.044036805 | YWHAZ | tyrosine 3-monooxygenase/tryptophan 5-monooxygenase activation protein zeta [Source:HGNC Symbol;Acc:HGNC:12855] |
| ENSG00000164938 | HGLibA_51070 | 111 | -0.044036805 | TP53INP1 | tumor protein p53 inducible nuclear protein 1 [Source:HGNC Symbol;Acc:HGNC:18022] |
| ENSG00000165066 | HGLibA_32061 | 111 | -0.044036805 | NKX6-3 | NK6 homeobox 3 [Source:HGNC Symbol;Acc:HGNC:26328] |
| ENSG00000165322 | HGLibA_02708 | 111 | -0.044036805 | ARHGAP12 | Rho GTPase activating protein 12 [Source:HGNC Symbol;Acc:HGNC:16348] |
| ENSG00000165449 | HGLibA_44457 | 111 | -0.044036805 | SLC16A9 | solute carrier family 16 member 9 [Source:HGNC Symbol;Acc:HGNC:23520] |
| ENSG00000165566 | HGLibA_01776 | 111 | -0.044036805 | AMER2 | APC membrane recruitment protein 2 [Source:HGNC Symbol;Acc:HGNC:26360] |
| ENSG00000167637 | HGLibA_55741 | 111 | -0.044036805 | ZNF283 | zinc finger protein 283 [Source:HGNC Symbol;Acc:HGNC:13077] |
| ENSG00000168237 | HGLibA_19382 | 111 | -0.044036805 | GLYCTK | glycerate kinase [Source:HGNC Symbol;Acc:HGNC:24247] |
| ENSG00000168334 | HGLibA_54512 | 111 | -0.044036805 | XIRP1 | xin actin binding repeat containing 1 [Source:HGNC Symbol;Acc:HGNC:14301] |
| ENSG00000169413 | HGLibA_41413 | 111 | -0.044036805 | RNASE6 | ribonuclease A family member k6 [Source:HGNC Symbol;Acc:HGNC:10048] |
| ENSG00000170689 | HGLibA_21953 | 111 | -0.044036805 | HOXB9 | homeobox B9 [Source:HGNC Symbol;Acc:HGNC:5120] |
| ENSG00000171574 | HGLibA_56317 | 111 | -0.044036805 | ZNF584 | zinc finger protein 584 [Source:HGNC Symbol;Acc:HGNC:27318] |
| ENSG00000171853 | HGLibA_51307 | 111 | -0.044036805 | TRAPPC12 | trafficking protein particle complex 12 [Source:HGNC Symbol;Acc:HGNC:24284] |
| ENSG00000172493 | HGLibA_01107 | 111 | -0.044036805 | AFF1 | AF4/FMR2 family member 1 [Source:HGNC Symbol;Acc:HGNC:7135] |
| ENSG00000176009 | HGLibA_03272 | 111 | -0.044036805 | ASCL3 | achaete-scute family bHLH transcription factor 3 [Source:HGNC Symbol;Acc:HGNC:740] |
| ENSG00000176230 | HGLibA_34028 | 111 | -0.044036805 | OR4K17 | olfactory receptor family 4 subfamily K member 17 [Source:HGNC Symbol;Acc:HGNC:15355] |
| ENSG00000177058 | HGLibA_45086 | 111 | -0.044036805 | SLC38A9 | solute carrier family 38 member 9 [Source:HGNC Symbol;Acc:HGNC:26907] |
| ENSG00000177646 | HGLibA_00361 | 111 | -0.044036805 | ACAD9 | acyl-CoA dehydrogenase family member 9 [Source:HGNC Symbol;Acc:HGNC:21497] |
| ENSG00000177732 | HGLibA_46233 | 111 | -0.044036805 | SOX12 | SRY-box 12 [Source:HGNC Symbol;Acc:HGNC:11198] |
| ENSG00000178573 | HGLibA_27866 | 111 | -0.044036805 | MAF | MAF bZIP transcription factor [Source:HGNC Symbol;Acc:HGNC:6776] |
| ENSG00000179242 | HGLibA_08679 | 111 | -0.044036805 | CDH4 | cadherin 4 [Source:HGNC Symbol;Acc:HGNC:1763] |
| ENSG00000179431 | HGLibA_17550 | 111 | -0.044036805 | FJX1 | four-jointed box kinase 1 [Source:HGNC Symbol;Acc:HGNC:17166] |
| ENSG00000180210 | HGLibA_15860 | 111 | -0.044036805 | F2 | thrombin [Source:HGNC Symbol;Acc:HGNC:3535]" |
| ENSG00000180891 | HGLibA_11768 | 111 | -0.044036805 | CUEDC1 | CUE domain containing 1 [Source:HGNC Symbol;Acc:HGNC:31350] |
| ENSG00000181019 | HGLibA_32561 | 111 | -0.044036805 | NQO1 | NAD(P)H quinone dehydrogenase 1 [Source:HGNC Symbol;Acc:HGNC:2874] |
| ENSG00000181894 | HGLibA_55810 | 111 | -0.044036805 | ZNF329 | zinc finger protein 329 [Source:HGNC Symbol;Acc:HGNC:14209] |
| ENSG00000182687 | HGLibA_18589 | 111 | -0.044036805 | GALR2 | galanin receptor 2 [Source:HGNC Symbol;Acc:HGNC:4133] |
| ENSG00000183049 | HGLibA_07062 | 111 | -0.044036805 | CAMK1D | calcium/calmodulin dependent protein kinase ID [Source:HGNC Symbol;Acc:HGNC:19341] |
| ENSG00000183161 | HGLibA_16849 | 111 | -0.044036805 | FANCF | FA complementation group F [Source:HGNC Symbol;Acc:HGNC:3587] |
| ENSG00000183401 | HGLibA_07668 | 111 | -0.044036805 | CCDC159 | coiled-coil domain containing 159 [Source:HGNC Symbol;Acc:HGNC:26996] |
| ENSG00000184434 | HGLibA_27276 | 111 | -0.044036805 | LRRC19 | leucine rich repeat containing 19 [Source:HGNC Symbol;Acc:HGNC:23379] |
| ENSG00000185085 | HGLibA_23423 | 111 | -0.044036805 | INTS5 | integrator complex subunit 5 [Source:HGNC Symbol;Acc:HGNC:29352] |
| ENSG00000185432 | HGLibA_29018 | 111 | -0.044036805 | METTL7A | methyltransferase like 7A [Source:HGNC Symbol;Acc:HGNC:24550] |
| ENSG00000186001 | HGLibA_27144 | 111 | -0.044036805 | LRCH3 | leucine rich repeats and calponin homology domain containing 3 [Source:HGNC Symbol;Acc:HGNC:28637] |
| ENSG00000186104 | HGLibA_12138 | 111 | -0.044036805 | CYP2R1 | cytochrome P450 family 2 subfamily R member 1 [Source:HGNC Symbol;Acc:HGNC:20580] |
| ENSG00000186860 | HGLibA_25661 | 111 | -0.044036805 | KRTAP17-1 | keratin associated protein 17-1 [Source:HGNC Symbol;Acc:HGNC:18917] |
| ENSG00000187079 | HGLibA_48926 | 111 | -0.044036805 | TEAD1 | TEA domain transcription factor 1 [Source:HGNC Symbol;Acc:HGNC:11714] |
| ENSG00000188785 | HGLibA_56205 | 111 | -0.044036805 | ZNF548 | zinc finger protein 548 [Source:HGNC Symbol;Acc:HGNC:26561] |
| ENSG00000188992 | HGLibA_26619 | 111 | -0.044036805 | LIPI | lipase I [Source:HGNC Symbol;Acc:HGNC:18821] |
| ENSG00000196172 | HGLibA_56527 | 111 | -0.044036805 | ZNF681 | zinc finger protein 681 [Source:HGNC Symbol;Acc:HGNC:26457] |
| ENSG00000196335 | HGLibA_47480 | 111 | -0.044036805 | STK31 | serine/threonine kinase 31 [Source:HGNC Symbol;Acc:HGNC:11407] |
| ENSG00000196366 | HGLibA_06700 | 111 | -0.044036805 | C9orf163 | chromosome 9 open reading frame 163 [Source:HGNC Symbol;Acc:HGNC:26718] |
| ENSG00000198759 | HGLibA_14624 | 111 | -0.044036805 | EGFL6 | EGF like domain multiple 6 [Source:HGNC Symbol;Acc:HGNC:3235] |
| ENSG00000198793 | HGLibA_30454 | 111 | -0.044036805 | MTOR | mechanistic target of rapamycin kinase [Source:HGNC Symbol;Acc:HGNC:3942] |

| | | | | | |
|---|---|---|---|---|---|
| ENSG00000203907 | HGLibA_33400 | 111 | -0.044036805 | OOEP | oocyte expressed protein [Source:HGNC Symbol;Acc:HGNC:21382] |
| ENSG00000204463 | HGLibA_04090 | 111 | -0.044036805 | BAG6 | BCL2 associated athanogene 6 [Source:HGNC Symbol;Acc:HGNC:13919] |
| ENSG00000204644 | HGLibA_55222 | 111 | -0.044036805 | ZFP57 | ZFP57 zinc finger protein [Source:HGNC Symbol;Acc:HGNC:18791] |
| ENSG00000204882 | HGLibA_19996 | 111 | -0.044036805 | GPR20 | G protein-coupled receptor 20 [Source:HGNC Symbol;Acc:HGNC:4475] |
| ENSG00000205667 | HGLibA_03146 | 111 | -0.044036805 | ARSH | arylsulfatase family member H [Source:HGNC Symbol;Acc:HGNC:32488] |
| ENSG00000205867 | HGLibA_25792 | 111 | -0.044036805 | KRTAP5-2 | keratin associated protein 5-2 [Source:HGNC Symbol;Acc:HGNC:23597] |
| ENSG00000206026 | HGLibA_45748 | 111 | -0.044036805 | SMIM21 | small integral membrane protein 21 [Source:HGNC Symbol;Acc:HGNC:27598] |
| ENSG00000206422 | HGLibA_27308 | 111 | -0.044036805 | LRRC30 | leucine rich repeat containing 30 [Source:HGNC Symbol;Acc:HGNC:30219] |
| ENSG00000213462 | HGLibA_15535 | 111 | -0.044036805 | ERV3-1 | envelope [Source:HGNC Symbol;Acc:HGNC:3454]" |
| ENSG00000215021 | HGLibA_36315 | 111 | -0.044036805 | PHB2 | prohibitin 2 [Source:HGNC Symbol;Acc:HGNC:30306] |
| ENSG00000224420 | HGLibA_00998 | 111 | -0.044036805 | ADM5 | adrenomedullin 5 (putative) [Source:HGNC Symbol;Acc:HGNC:27293] |
| ENSG00000225697 | HGLibA_44801 | 111 | -0.044036805 | SLC26A6 | solute carrier family 26 member 6 [Source:HGNC Symbol;Acc:HGNC:14472] |
| ENSG00000226979 | HGLibA_27574 | 111 | -0.044036805 | LTA | lymphotoxin alpha [Source:HGNC Symbol;Acc:HGNC:6709] |
| ENSG00000227729 | HGLibA_40728 | 111 | -0.044036805 | RD3L | retinal degeneration 3 like [Source:HGNC Symbol;Acc:HGNC:40912] |
| ENSG00000240857 | HGLibA_40743 | 111 | -0.044036805 | RDH14 | retinol dehydrogenase 14 [Source:HGNC Symbol;Acc:HGNC:19979] |
| ENSG00000241878 | HGLibA_36735 | 111 | -0.044036805 | PISD | phosphatidylserine decarboxylase [Source:HGNC Symbol;Acc:HGNC:8999] |
| ENSG00000242247 | HGLibA_02667 | 111 | -0.044036805 | ARFGAP3 | ADP ribosylation factor GTPase activating protein 3 [Source:HGNC Symbol;Acc:HGNC:661] |
| ENSG00000243414 | HGLibA_49449 | 111 | -0.044036805 | TICAM2 | toll like receptor adaptor molecule 2 [Source:HGNC Symbol;Acc:HGNC:21354] |
| ENSG00000248672 | HGLibA_27683 | 111 | -0.044036805 | LY75-CD302 | LY75-CD302 readthrough [Source:HGNC Symbol;Acc:HGNC:38828] |
| ENSG00000250868 | HGLibA_54542 | 111 | -0.044036805 | XKRY | Y-linked [Source:HGNC Symbol;Acc:HGNC:18571]" |
| ENSG00000258817 | HGLibA_33944 | 111 | -0.044036805 | OR4C13 | olfactory receptor family 4 subfamily C member 13 [Source:HGNC Symbol;Acc:HGNC:15169] |
| ENSG00000272804 | HGLibA_25617 | 111 | -0.044036805 | KRTAP10-7 | keratin associated protein 10-7 [Source:HGNC Symbol;Acc:HGNC:22970] |
| ENSG00000276040 | HGLibA_46487 | 111 | -0.044036805 | SPATA31A7 | SPATA31 subfamily A member 7 [Source:HGNC Symbol;Acc:HGNC:32007] |
| FAM122B | HGLibA_16069 | 111 | -0.044036805 | FAM122B | - |
| FAM160B2 | HGLibA_16212 | 111 | -0.044036805 | FAM160B2 | - |
| FAM208B | HGLibA_16434 | 111 | -0.044036805 | FAM208B | - |
| FAM46A | HGLibA_16597 | 111 | -0.044036805 | FAM46A | - |
| hsa-mir-1231 | HGLibA_57319 | 111 | -0.044036805 | hsa-mir-1231 | - |
| hsa-mir-147a | HGLibA_57874 | 111 | -0.044036805 | hsa-mir-147a | - |
| hsa-mir-1909 | HGLibA_58031 | 111 | -0.044036805 | hsa-mir-1909 | - |
| hsa-mir-190a | HGLibA_58036 | 111 | -0.044036805 | hsa-mir-190a | - |
| hsa-mir-195 | HGLibA_58089 | 111 | -0.044036805 | hsa-mir-195 | - |
| hsa-mir-196a-1 | HGLibA_58093 | 111 | -0.044036805 | hsa-mir-196a-1 | - |
| hsa-mir-2278 | HGLibA_58317 | 111 | -0.044036805 | hsa-mir-2278 | - |
| hsa-mir-24-1 | HGLibA_58340 | 111 | -0.044036805 | hsa-mir-24-1 | - |
| hsa-mir-3189 | HGLibA_58853 | 111 | -0.044036805 | hsa-mir-3189 | - |
| hsa-mir-3680-2 | HGLibA_59272 | 111 | -0.044036805 | hsa-mir-3680-2 | - |
| hsa-mir-3686 | HGLibA_59297 | 111 | -0.044036805 | hsa-mir-3686 | - |
| hsa-mir-378d-1 | HGLibA_59439 | 111 | -0.044036805 | hsa-mir-378d-1 | - |
| hsa-mir-3910-1 | HGLibA_59503 | 111 | -0.044036805 | hsa-mir-3910-1 | - |
| hsa-mir-3912 | HGLibA_59517 | 111 | -0.044036805 | hsa-mir-3912 | - |
| hsa-mir-4267 | HGLibA_59774 | 111 | -0.044036805 | hsa-mir-4267 | - |
| hsa-mir-4643 | HGLibA_60675 | 111 | -0.044036805 | hsa-mir-4643 | - |
| hsa-mir-4725 | HGLibA_60998 | 111 | -0.044036805 | hsa-mir-4725 | - |
| hsa-mir-513a-1 | HGLibA_61571 | 111 | -0.044036805 | hsa-mir-513a-1 | - |
| hsa-mir-587 | HGLibA_62445 | 111 | -0.044036805 | hsa-mir-587 | - |
| hsa-mir-598 | HGLibA_62486 | 111 | -0.044036805 | hsa-mir-598 | - |
| hsa-mir-6075 | HGLibA_62551 | 111 | -0.044036805 | hsa-mir-6075 | - |
| hsa-mir-633 | HGLibA_62772 | 111 | -0.044036805 | hsa-mir-633 | - |
| hsa-mir-6792 | HGLibA_63345 | 111 | -0.044036805 | hsa-mir-6792 | - |
| hsa-mir-6815 | HGLibA_63439 | 111 | -0.044036805 | hsa-mir-6815 | - |
| hsa-mir-6852 | HGLibA_63579 | 111 | -0.044036805 | hsa-mir-6852 | - |
| hsa-mir-6871 | HGLibA_63669 | 111 | -0.044036805 | hsa-mir-6871 | - |
| hsa-mir-7157 | HGLibA_63856 | 111 | -0.044036805 | hsa-mir-7157 | - |
| hsa-mir-8065 | HGLibA_64100 | 111 | -0.044036805 | hsa-mir-8065 | - |
| hsa-mir-935 | HGLibA_64315 | 111 | -0.044036805 | hsa-mir-935 | - |
| hsa-mir-95 | HGLibA_64365 | 111 | -0.044036805 | hsa-mir-95 | - |
| TMEM56 | HGLibA_50450 | 111 | -0.044036805 | TMEM56 | - |
| WAPAL | HGLibA_53942 | 111 | -0.044036805 | WAPAL | - |
| ZMYM6NB | HGLibA_55408 | 111 | -0.044036805 | ZMYM6NB | - |
| C14orf2 | HGLibA_05358 | 110 | -0.053987842 | C14orf2 | - |
| C17orf66 | HGLibA_05560 | 110 | -0.053987842 | C17orf66 | - |
| C17orf85 | HGLibA_05588 | 110 | -0.053987842 | C17orf85 | - |
| C7orf49 | HGLibA_06548 | 110 | -0.053987842 | C7orf49 | - |
| ENSG00000006047 | HGLibA_54647 | 110 | -0.053987842 | YBX2 | Y-box binding protein 2 [Source:HGNC Symbol;Acc:HGNC:17948] |
| ENSG00000011485 | HGLibA_38211 | 110 | -0.053987842 | PPP5C | protein phosphatase 5 catalytic subunit [Source:HGNC Symbol;Acc:HGNC:9322] |
| ENSG00000021826 | HGLibA_10990 | 110 | -0.053987842 | CPS1 | carbamoyl-phosphate synthase 1 [Source:HGNC Symbol;Acc:HGNC:2323] |
| ENSG00000027869 | HGLibA_43888 | 110 | -0.053987842 | SH2D2A | SH2 domain containing 2A [Source:HGNC Symbol;Acc:HGNC:10821] |
| ENSG00000048405 | HGLibA_56786 | 110 | -0.053987842 | ZNF800 | zinc finger protein 800 [Source:HGNC Symbol;Acc:HGNC:27267] |
| ENSG00000058668 | HGLibA_03622 | 110 | -0.053987842 | ATP2B4 | ATPase plasma membrane Ca2+ transporting 4 [Source:HGNC Symbol;Acc:HGNC:817] |
| ENSG00000064225 | HGLibA_47252 | 110 | -0.053987842 | ST3GAL6 | 3-sialyltransferase 6 [Source:HGNC Symbol;Acc:HGNC:18080]" |
| ENSG00000069482 | HGLibA_18493 | 110 | -0.053987842 | GAL | galanin and GMAP prepropeptide [Source:HGNC Symbol;Acc:HGNC:4114] |
| ENSG00000069812 | HGLibA_21179 | 110 | -0.053987842 | HES2 | hes family bHLH transcription factor 2 [Source:HGNC Symbol;Acc:HGNC:16005] |
| ENSG00000070413 | HGLibA_13046 | 110 | -0.053987842 | DGCR2 | DiGeorge syndrome critical region gene 2 [Source:HGNC Symbol;Acc:HGNC:2845] |
| ENSG00000073969 | HGLibA_32750 | 110 | -0.053987842 | NSF | vesicle fusing ATPase [Source:HGNC Symbol;Acc:HGNC:8016]" |
| ENSG00000077327 | HGLibA_46363 | 110 | -0.053987842 | SPAG6 | sperm associated antigen 6 [Source:HGNC Symbol;Acc:HGNC:11215] |
| ENSG00000083642 | HGLibA_35945 | 110 | -0.053987842 | PDS5B | PDS5 cohesin associated factor B [Source:HGNC Symbol;Acc:HGNC:20418] |
| ENSG00000083896 | HGLibA_54724 | 110 | -0.053987842 | YTHDC1 | YTH domain containing 1 [Source:HGNC Symbol;Acc:HGNC:30626] |
| ENSG00000084092 | HGLibA_32228 | 110 | -0.053987842 | NOA1 | nitric oxide associated 1 [Source:HGNC Symbol;Acc:HGNC:28473] |
| ENSG00000099341 | HGLibA_39183 | 110 | -0.053987842 | PSMD8 | non-ATPase 8 [Source:HGNC Symbol;Acc:HGNC:9566]" |
| ENSG00000099834 | HGLibA_08710 | 110 | -0.053987842 | CDHR5 | cadherin related family member 5 [Source:HGNC Symbol;Acc:HGNC:7521] |
| ENSG00000100097 | HGLibA_26344 | 110 | -0.053987842 | LGALS1 | galectin 1 [Source:HGNC Symbol;Acc:HGNC:6561] |
| ENSG00000100302 | HGLibA_40324 | 110 | -0.053987842 | RASD2 | RASD family member 2 [Source:HGNC Symbol;Acc:HGNC:18229] |
| ENSG00000100416 | HGLibA_51703 | 110 | -0.053987842 | TRMU | tRNA 5-methylaminomethyl-2-thiouridylate methyltransferase [Source:HGNC Symbol;Acc:HGNC:25481] |
| ENSG00000100982 | HGLibA_35609 | 110 | -0.053987842 | PCIF1 | PDX1 C-terminal inhibiting factor 1 [Source:HGNC Symbol;Acc:HGNC:16200] |
| ENSG00000100994 | HGLibA_39685 | 110 | -0.053987842 | PYGB | glycogen phosphorylase B [Source:HGNC Symbol;Acc:HGNC:9723] |
| ENSG00000101162 | HGLibA_52274 | 110 | -0.053987842 | TUBB1 | tubulin beta 1 class VI [Source:HGNC Symbol;Acc:HGNC:16257] |
| ENSG00000101255 | HGLibA_51397 | 110 | -0.053987842 | TRIB3 | tribbles pseudokinase 3 [Source:HGNC Symbol;Acc:HGNC:16228] |
| ENSG00000104833 | HGLibA_52285 | 110 | -0.053987842 | TUBB4A | tubulin beta 4A class IVa [Source:HGNC Symbol;Acc:HGNC:20774] |
| ENSG00000105549 | HGLibA_49335 | 110 | -0.053987842 | THEG | theg spermatid protein [Source:HGNC Symbol;Acc:HGNC:13706] |
| ENSG00000105647 | HGLibA_36652 | 110 | -0.053987842 | PIK3R2 | phosphoinositide-3-kinase regulatory subunit 2 [Source:HGNC Symbol;Acc:HGNC:8980] |
| ENSG00000106799 | HGLibA_49224 | 110 | -0.053987842 | TGFBR1 | transforming growth factor beta receptor 1 [Source:HGNC Symbol;Acc:HGNC:11772] |
| ENSG00000107854 | HGLibA_50856 | 110 | -0.053987842 | TNKS2 | tankyrase 2 [Source:HGNC Symbol;Acc:HGNC:15677] |
| ENSG00000108061 | HGLibA_44067 | 110 | -0.053987842 | SHOC2 | leucine rich repeat scaffold protein [Source:HGNC Symbol;Acc:HGNC:15454]" |
| ENSG00000108379 | HGLibA_54364 | 110 | -0.053987842 | WNT3 | Wnt family member 3 [Source:HGNC Symbol;Acc:HGNC:12782] |
| ENSG00000108561 | HGLibA_05917 | 110 | -0.053987842 | C1QBP | complement C1q binding protein [Source:HGNC Symbol;Acc:HGNC:1243] |
| ENSG00000108773 | HGLibA_24050 | 110 | -0.053987842 | KAT2A | lysine acetyltransferase 2A [Source:HGNC Symbol;Acc:HGNC:4201] |
| ENSG00000108861 | HGLibA_14178 | 110 | -0.053987842 | DUSP3 | dual specificity phosphatase 3 [Source:HGNC Symbol;Acc:HGNC:3069] |
| ENSG00000109063 | HGLibA_30723 | 110 | -0.053987842 | MYH3 | myosin heavy chain 3 [Source:HGNC Symbol;Acc:HGNC:7573] |
| ENSG00000109445 | HGLibA_55812 | 110 | -0.053987842 | ZNF330 | zinc finger protein 330 [Source:HGNC Symbol;Acc:HGNC:15462] |
| ENSG00000110713 | HGLibA_33077 | 110 | -0.053987842 | NUP98 | nucleoporin 98 [Source:HGNC Symbol;Acc:HGNC:8068] |
| ENSG00000111540 | HGLibA_39963 | 110 | -0.053987842 | RAB5B | member RAS oncogene family [Source:HGNC Symbol;Acc:HGNC:9784]" |
| ENSG00000113492 | HGLibA_01275 | 110 | -0.053987842 | AGXT2 | alanine--glyoxylate aminotransferase 2 [Source:HGNC Symbol;Acc:HGNC:14412] |
| ENSG00000116260 | HGLibA_39755 | 110 | -0.053987842 | QSOX1 | quiescin sulfhydryl oxidase 1 [Source:HGNC Symbol;Acc:HGNC:9756] |
| ENSG00000116983 | HGLibA_22021 | 110 | -0.053987842 | HPCAL4 | hippocalcin like 4 [Source:HGNC Symbol;Acc:HGNC:18212] |
| ENSG00000119922 | HGLibA_22629 | 110 | -0.053987842 | IFIT2 | interferon induced protein with tetratricopeptide repeats 2 [Source:HGNC Symbol;Acc:HGNC:5409] |
| ENSG00000120053 | HGLibA_19691 | 110 | -0.053987842 | GOT1 | glutamic-oxaloacetic transaminase 1 [Source:HGNC Symbol;Acc:HGNC:4432] |
| ENSG00000120837 | HGLibA_31856 | 110 | -0.053987842 | NFYB | nuclear transcription factor Y subunit beta [Source:HGNC Symbol;Acc:HGNC:7805] |
| ENSG00000122133 | HGLibA_34983 | 110 | -0.053987842 | PAEP | progestagen associated endometrial protein [Source:HGNC Symbol;Acc:HGNC:8573] |
| ENSG00000122417 | HGLibA_33282 | 110 | -0.053987842 | ODF2L | outer dense fiber of sperm tails 2 like [Source:HGNC Symbol;Acc:HGNC:29225] |
| ENSG00000123106 | HGLibA_07924 | 110 | -0.053987842 | CCDC91 | coiled-coil domain containing 91 [Source:HGNC Symbol;Acc:HGNC:24855] |
| ENSG00000126464 | HGLibA_38774 | 110 | -0.053987842 | PRR12 | proline rich 12 [Source:HGNC Symbol;Acc:HGNC:29217] |
| ENSG00000127329 | HGLibA_39487 | 110 | -0.053987842 | PTPRB | receptor type B [Source:HGNC Symbol;Acc:HGNC:9665]" |
| ENSG00000130402 | HGLibA_00643 | 110 | -0.053987842 | ACTN4 | actinin alpha 4 [Source:HGNC Symbol;Acc:HGNC:166] |
| ENSG00000130561 | HGLibA_42641 | 110 | -0.053987842 | SAG | S-antigen visual arrestin [Source:HGNC Symbol;Acc:HGNC:10521] |
| ENSG00000130997 | HGLibA_37543 | 110 | -0.053987842 | POLN | DNA polymerase nu [Source:HGNC Symbol;Acc:HGNC:18870] |
| ENSG00000131788 | HGLibA_36513 | 110 | -0.053987842 | PIAS3 | protein inhibitor of activated STAT 3 [Source:HGNC Symbol;Acc:HGNC:16861] |
| ENSG00000134571 | HGLibA_30654 | 110 | -0.053987842 | MYBPC3 | cardiac [Source:HGNC Symbol;Acc:HGNC:7551]" |
| ENSG00000134757 | HGLibA_14027 | 110 | -0.053987842 | DSG3 | desmoglein 3 [Source:HGNC Symbol;Acc:HGNC:3050] |
| ENSG00000136490 | HGLibA_26535 | 110 | -0.053987842 | LIMD2 | LIM domain containing 2 [Source:HGNC Symbol;Acc:HGNC:28142] |
| ENSG00000138073 | HGLibA_38426 | 110 | -0.053987842 | PREB | prolactin regulatory element binding [Source:HGNC Symbol;Acc:HGNC:9356] |
| ENSG00000139223 | HGLibA_02204 | 110 | -0.053987842 | ANP32D | acidic nuclear phosphoprotein 32 family member D [Source:HGNC Symbol;Acc:HGNC:16676] |
| ENSG00000139318 | HGLibA_14186 | 110 | -0.053987842 | DUSP6 | dual specificity phosphatase 6 [Source:HGNC Symbol;Acc:HGNC:3072] |
| ENSG00000140543 | HGLibA_13012 | 110 | -0.053987842 | DET1 | COP1 ubiquitin ligase partner [Source:HGNC Symbol;Acc:HGNC:25477]" |
| ENSG00000140577 | HGLibA_11233 | 110 | -0.053987842 | CRTC3 | CREB regulated transcription coactivator 3 [Source:HGNC Symbol;Acc:HGNC:26148] |
| ENSG00000141337 | HGLibA_03143 | 110 | -0.053987842 | ARSG | arylsulfatase G [Source:HGNC Symbol;Acc:HGNC:24102] |
| ENSG00000142347 | HGLibA_30836 | 110 | -0.053987842 | MYO1F | myosin IF [Source:HGNC Symbol;Acc:HGNC:7600] |
| ENSG00000142409 | HGLibA_56755 | 110 | -0.053987842 | ZNF787 | zinc finger protein 787 [Source:HGNC Symbol;Acc:HGNC:26998] |
| ENSG00000146223 | HGLibA_42000 | 110 | -0.053987842 | RPL7L1 | ribosomal protein L7 like 1 [Source:HGNC Symbol;Acc:HGNC:21370] |
| ENSG00000147724 | HGLibA_16136 | 110 | -0.053987842 | FAM135B | family with sequence similarity 135 member B [Source:HGNC Symbol;Acc:HGNC:28029] |
| ENSG00000149115 | HGLibA_50853 | 110 | -0.053987842 | TNKS1BP1 | tankyrase 1 binding protein 1 [Source:HGNC Symbol;Acc:HGNC:19081] |
| ENSG00000151575 | HGLibA_49112 | 110 | -0.053987842 | TEX9 | testis expressed 9 [Source:HGNC Symbol;Acc:HGNC:29585] |
| ENSG00000151726 | HGLibA_00555 | 110 | -0.053987842 | ACSL1 | acyl-CoA synthetase long chain family member 1 [Source:HGNC Symbol;Acc:HGNC:3569] |
| ENSG00000154162 | HGLibA_08639 | 110 | -0.053987842 | CDH12 | cadherin 12 [Source:HGNC Symbol;Acc:HGNC:1751] |
| ENSG00000155962 | HGLibA_10024 | 110 | -0.053987842 | CLIC2 | chloride intracellular channel 2 [Source:HGNC Symbol;Acc:HGNC:2063] |

| | | | | | |
|---|---|---|---|---|---|
| ENSG00000155975 | HGLibA_53777 | 110 | -0.053987842 | VPS37A | ESCRT-I subunit [Source:HGNC Symbol;Acc:HGNC:24928]" |
| ENSG00000157349 | HGLibA_12665 | 110 | -0.053987842 | DDX19B | DEAD-box helicase 19B [Source:HGNC Symbol;Acc:HGNC:2742] |
| ENSG00000157978 | HGLibA_26254 | 110 | -0.053987842 | LDLRAP1 | low density lipoprotein receptor adaptor protein 1 [Source:HGNC Symbol;Acc:HGNC:18640] |
| ENSG00000158290 | HGLibA_11785 | 110 | -0.053987842 | CUL4B | cullin 4B [Source:HGNC Symbol;Acc:HGNC:2555] |
| ENSG00000159214 | HGLibA_07735 | 110 | -0.053987842 | CCDC24 | coiled-coil domain containing 24 [Source:HGNC Symbol;Acc:HGNC:28688] |
| ENSG00000160685 | HGLibA_54919 | 110 | -0.053987842 | ZBTB7B | zinc finger and BTB domain containing 7B [Source:HGNC Symbol;Acc:HGNC:18668] |
| ENSG00000160883 | HGLibA_21584 | 110 | -0.053987842 | HK3 | hexokinase 3 [Source:HGNC Symbol;Acc:HGNC:4925] |
| ENSG00000161055 | HGLibA_42932 | 110 | -0.053987842 | SCGB3A1 | secretoglobin family 3A member 1 [Source:HGNC Symbol;Acc:HGNC:18384] |
| ENSG00000161526 | HGLibA_42725 | 110 | -0.053987842 | SAP30BP | SAP30 binding protein [Source:HGNC Symbol;Acc:HGNC:30785] |
| ENSG00000162482 | HGLibA_01502 | 110 | -0.053987842 | AKR7A3 | aldo-keto reductase family 7 member A3 [Source:HGNC Symbol;Acc:HGNC:390] |
| ENSG00000162994 | HGLibA_10018 | 110 | -0.053987842 | CLHC1 | clathrin heavy chain linker domain containing 1 [Source:HGNC Symbol;Acc:HGNC:26453] |
| ENSG00000164690 | HGLibA_44031 | 110 | -0.053987842 | SHH | sonic hedgehog [Source:HGNC Symbol;Acc:HGNC:10848] |
| ENSG00000164825 | HGLibA_12809 | 110 | -0.053987842 | DEFB1 | defensin beta 1 [Source:HGNC Symbol;Acc:HGNC:2766] |
| ENSG00000165675 | HGLibA_15162 | 110 | -0.053987842 | ENOX2 | ecto-NOX disulfide-thiol exchanger 2 [Source:HGNC Symbol;Acc:HGNC:2259] |
| ENSG00000167671 | HGLibA_52791 | 110 | -0.053987842 | UBXN6 | UBX domain protein 6 [Source:HGNC Symbol;Acc:HGNC:14928] |
| ENSG00000167977 | HGLibA_24457 | 110 | -0.053987842 | KCTD5 | potassium channel tetramerization domain containing 5 [Source:HGNC Symbol;Acc:HGNC:21423] |
| ENSG00000168310 | HGLibA_23575 | 110 | -0.053987842 | IRF2 | interferon regulatory factor 2 [Source:HGNC Symbol;Acc:HGNC:6117] |
| ENSG00000169299 | HGLibA_36264 | 110 | -0.053987842 | PGM2 | phosphoglucomutase 2 [Source:HGNC Symbol;Acc:HGNC:8906] |
| ENSG00000169314 | HGLibA_06054 | 110 | -0.053987842 | C22orf15 | chromosome 22 open reading frame 15 [Source:HGNC Symbol;Acc:HGNC:15558] |
| ENSG00000171102 | HGLibA_33229 | 110 | -0.053987842 | OBP2B | odorant binding protein 2B [Source:HGNC Symbol;Acc:HGNC:23381] |
| ENSG00000171155 | HGLibA_05727 | 110 | -0.053987842 | C1GALT1C1 | C1GALT1 specific chaperone 1 [Source:HGNC Symbol;Acc:HGNC:24338] |
| ENSG00000171236 | HGLibA_27167 | 110 | -0.053987842 | LRG1 | leucine rich alpha-2-glycoprotein 1 [Source:HGNC Symbol;Acc:HGNC:29480] |
| ENSG00000171298 | HGLibA_18318 | 110 | -0.053987842 | GAA | acid [Source:HGNC Symbol;Acc:HGNC:4065]" |
| ENSG00000171357 | HGLibA_27632 | 110 | -0.053987842 | LURAP1 | leucine rich adaptor protein 1 [Source:HGNC Symbol;Acc:HGNC:32327] |
| ENSG00000171843 | HGLibA_29417 | 110 | -0.053987842 | MLLT3 | super elongation complex subunit [Source:HGNC Symbol;Acc:HGNC:7136]" |
| ENSG00000172215 | HGLibA_11895 | 110 | -0.053987842 | CXCR6 | C-X-C motif chemokine receptor 6 [Source:HGNC Symbol;Acc:HGNC:16647] |
| ENSG00000172247 | HGLibA_05945 | 110 | -0.053987842 | C1QTNF4 | C1q and TNF related 4 [Source:HGNC Symbol;Acc:HGNC:14346] |
| ENSG00000173272 | HGLibA_30942 | 110 | -0.053987842 | MZT2A | mitotic spindle organizing protein 2A [Source:HGNC Symbol;Acc:HGNC:33187] |
| ENSG00000173818 | HGLibA_15123 | 110 | -0.053987842 | ENDOV | endonuclease V [Source:HGNC Symbol;Acc:HGNC:26640] |
| ENSG00000178104 | HGLibA_35800 | 110 | -0.053987842 | PDE4DIP | phosphodiesterase 4D interacting protein [Source:HGNC Symbol;Acc:HGNC:15580] |
| ENSG00000178796 | HGLibA_41248 | 110 | -0.053987842 | RIIAD1 | regulatory subunit of type II PKA R-subunit (RIIa) domain containing 1 [Source:HGNC Symbol;Acc:HGNC:26686] |
| ENSG00000179580 | HGLibA_41548 | 110 | -0.053987842 | RNF151 | ring finger protein 151 [Source:HGNC Symbol;Acc:HGNC:23235] |
| ENSG00000180228 | HGLibA_38583 | 110 | -0.053987842 | PRKRA | protein activator of interferon induced protein kinase EIF2AK2 [Source:HGNC Symbol;Acc:HGNC:9438] |
| ENSG00000180881 | HGLibA_07200 | 110 | -0.053987842 | CAPS2 | calcyphosine 2 [Source:HGNC Symbol;Acc:HGNC:16471] |
| ENSG00000182810 | HGLibA_12691 | 110 | -0.053987842 | DDX28 | DEAD-box helicase 28 [Source:HGNC Symbol;Acc:HGNC:17330] |
| ENSG00000183665 | HGLibA_51675 | 110 | -0.053987842 | TRMT12 | tRNA methyltransferase 12 homolog [Source:HGNC Symbol;Acc:HGNC:26091] |
| ENSG00000183808 | HGLibA_40481 | 110 | -0.053987842 | RBM12B | RNA binding motif protein 12B [Source:HGNC Symbol;Acc:HGNC:32310] |
| ENSG00000184922 | HGLibA_17694 | 110 | -0.053987842 | FMNL1 | formin like 1 [Source:HGNC Symbol;Acc:HGNC:1212] |
| ENSG00000185880 | HGLibA_51594 | 110 | -0.053987842 | TRIM69 | tripartite motif containing 69 [Source:HGNC Symbol;Acc:HGNC:17857] |
| ENSG00000186510 | HGLibA_09842 | 110 | -0.053987842 | CLCNKA | chloride voltage-gated channel Ka [Source:HGNC Symbol;Acc:HGNC:2026] |
| ENSG00000187838 | HGLibA_37237 | 110 | -0.053987842 | PLSCR3 | phospholipid scramblase 3 [Source:HGNC Symbol;Acc:HGNC:16495] |
| ENSG00000188219 | HGLibA_37728 | 110 | -0.053987842 | POTEE | POTE ankyrin domain family member E [Source:HGNC Symbol;Acc:HGNC:33895] |
| ENSG00000188419 | HGLibA_09465 | 110 | -0.053987842 | CHM | Rab escort protein 1 [Source:HGNC Symbol;Acc:HGNC:1940]" |
| ENSG00000188542 | HGLibA_14176 | 110 | -0.053987842 | DUSP28 | dual specificity phosphatase 28 [Source:HGNC Symbol;Acc:HGNC:33237] |
| ENSG00000188613 | HGLibA_31095 | 110 | -0.053987842 | NANOS1 | nanos C2HC-type zinc finger 1 [Source:HGNC Symbol;Acc:HGNC:23044] |
| ENSG00000188833 | HGLibA_15218 | 110 | -0.053987842 | ENTPD8 | ectonucleoside triphosphate diphosphohydrolase 8 [Source:HGNC Symbol;Acc:HGNC:24860] |
| ENSG00000196743 | HGLibA_19388 | 110 | -0.053987842 | GM2A | GM2 ganglioside activator [Source:HGNC Symbol;Acc:HGNC:4367] |
| ENSG00000196757 | HGLibA_56574 | 110 | -0.053987842 | ZNF700 | zinc finger protein 700 [Source:HGNC Symbol;Acc:HGNC:25292] |
| ENSG00000197093 | HGLibA_18505 | 110 | -0.053987842 | GAL3ST4 | galactose-3-O-sulfotransferase 4 [Source:HGNC Symbol;Acc:HGNC:24145] |
| ENSG00000197870 | HGLibA_38344 | 110 | -0.053987842 | PRB3 | proline rich protein BstNI subfamily 3 [Source:HGNC Symbol;Acc:HGNC:9339] |
| ENSG00000197905 | HGLibA_48935 | 110 | -0.053987842 | TEAD4 | TEA domain transcription factor 4 [Source:HGNC Symbol;Acc:HGNC:11717] |
| ENSG00000198049 | HGLibA_03907 | 110 | -0.053987842 | AVPR1B | arginine vasopressin receptor 1B [Source:HGNC Symbol;Acc:HGNC:896] |
| ENSG00000198105 | HGLibA_55660 | 110 | -0.053987842 | ZNF248 | zinc finger protein 248 [Source:HGNC Symbol;Acc:HGNC:13041] |
| ENSG00000198185 | HGLibA_55821 | 110 | -0.053987842 | ZNF334 | zinc finger protein 334 [Source:HGNC Symbol;Acc:HGNC:15806] |
| ENSG00000203883 | HGLibA_46249 | 110 | -0.053987842 | SOX18 | SRY-box 18 [Source:HGNC Symbol;Acc:HGNC:11194] |
| ENSG00000204524 | HGLibA_56795 | 110 | -0.053987842 | ZNF805 | zinc finger protein 805 [Source:HGNC Symbol;Acc:HGNC:23272] |
| ENSG00000205221 | HGLibA_53674 | 110 | -0.053987842 | VIT | vitrin [Source:HGNC Symbol;Acc:HGNC:12697] |
| ENSG00000213186 | HGLibA_51555 | 110 | -0.053987842 | TRIM59 | tripartite motif containing 59 [Source:HGNC Symbol;Acc:HGNC:30834] |
| ENSG00000213996 | HGLibA_49712 | 110 | -0.053987842 | TM6SF2 | transmembrane 6 superfamily member 2 [Source:HGNC Symbol;Acc:HGNC:11861] |
| ENSG00000214513 | HGLibA_32356 | 110 | -0.053987842 | NOTO | notochord homeobox [Source:HGNC Symbol;Acc:HGNC:31839] |
| ENSG00000240230 | HGLibA_10823 | 110 | -0.053987842 | COX19 | cytochrome c oxidase assembly factor COX19 [Source:HGNC Symbol;Acc:HGNC:28074] |
| ENSG00000248167 | HGLibA_51486 | 110 | -0.053987842 | TRIM39-RPP21 | TRIM39-RPP21 readthrough [Source:HGNC Symbol;Acc:HGNC:38845] |
| ENSG00000257017 | HGLibA_22009 | 110 | -0.053987842 | HP | haptoglobin [Source:HGNC Symbol;Acc:HGNC:5141] |
| ENSG00000258436 | HGLibA_41397 | 110 | -0.053987842 | RNASE12 | ribonuclease A family member 12 (inactive) [Source:HGNC Symbol;Acc:HGNC:24211] |
| ENSG00000264522 | HGLibA_34781 | 110 | -0.053987842 | OTUD7B | OTU deubiquitinase 7B [Source:HGNC Symbol;Acc:HGNC:16683] |
| ENSG00000265241 | HGLibA_40585 | 110 | -0.053987842 | RBM8A | RNA binding motif protein 8A [Source:HGNC Symbol;Acc:HGNC:9905] |
| ENSG00000283597 | HGLibA_16256 | 110 | -0.053987842 | FAM169B | family with sequence similarity 169 member B [Source:HGNC Symbol;Acc:HGNC:26835] |
| ENSG00000288649 | HGLibA_00617 | 110 | -0.053987842 | ACTL10 | - |
| FAM203A | HGLibA_16417 | 110 | -0.053987842 | FAM203A | - |
| FTH1P18 | HGLibA_18119 | 110 | -0.053987842 | FTH1P18 | - |
| HIST2H4A | HGLibA_21549 | 110 | -0.053987842 | HIST2H4A | - |
| hsa-mir-1183 | HGLibA_57226 | 110 | -0.053987842 | hsa-mir-1183 | - |
| hsa-mir-1271 | HGLibA_57525 | 110 | -0.053987842 | hsa-mir-1271 | - |
| hsa-mir-1294 | HGLibA_57653 | 110 | -0.053987842 | hsa-mir-1294 | - |
| hsa-mir-194-1 | HGLibA_58080 | 110 | -0.053987842 | hsa-mir-194-1 | - |
| hsa-mir-2115 | HGLibA_58231 | 110 | -0.053987842 | hsa-mir-2115 | - |
| hsa-mir-2467 | HGLibA_58348 | 110 | -0.053987842 | hsa-mir-2467 | - |
| hsa-mir-3074 | HGLibA_58467 | 110 | -0.053987842 | hsa-mir-3074 | - |
| hsa-mir-378j | HGLibA_59465 | 110 | -0.053987842 | hsa-mir-378j | - |
| hsa-mir-3927 | HGLibA_59584 | 110 | -0.053987842 | hsa-mir-3927 | - |
| hsa-mir-424 | HGLibA_59705 | 110 | -0.053987842 | hsa-mir-424 | - |
| hsa-mir-4326 | HGLibA_60026 | 110 | -0.053987842 | hsa-mir-4326 | - |
| hsa-mir-4420 | HGLibA_60068 | 110 | -0.053987842 | hsa-mir-4420 | - |
| hsa-mir-4426 | HGLibA_60092 | 110 | -0.053987842 | hsa-mir-4426 | - |
| hsa-mir-448 | HGLibA_60336 | 110 | -0.053987842 | hsa-mir-448 | - |
| hsa-mir-4494 | HGLibA_60395 | 110 | -0.053987842 | hsa-mir-4494 | - |
| hsa-mir-4511 | HGLibA_60488 | 110 | -0.053987842 | hsa-mir-4511 | - |
| hsa-mir-4523 | HGLibA_60551 | 110 | -0.053987842 | hsa-mir-4523 | - |
| hsa-mir-4682 | HGLibA_60839 | 110 | -0.053987842 | hsa-mir-4682 | - |
| hsa-mir-4797 | HGLibA_61293 | 110 | -0.053987842 | hsa-mir-4797 | - |
| hsa-mir-5001 | HGLibA_61411 | 110 | -0.053987842 | hsa-mir-5001 | - |
| hsa-mir-511 | HGLibA_61558 | 110 | -0.053987842 | hsa-mir-511 | - |
| hsa-mir-548n | HGLibA_62046 | 110 | -0.053987842 | hsa-mir-548n | - |
| hsa-mir-5704 | HGLibA_62357 | 110 | -0.053987842 | hsa-mir-5704 | - |
| hsa-mir-5707 | HGLibA_62367 | 110 | -0.053987842 | hsa-mir-5707 | - |
| hsa-mir-6804 | HGLibA_63392 | 110 | -0.053987842 | hsa-mir-6804 | - |
| hsa-mir-6817 | HGLibA_63445 | 110 | -0.053987842 | hsa-mir-6817 | - |
| hsa-mir-6821 | HGLibA_63460 | 110 | -0.053987842 | hsa-mir-6821 | - |
| hsa-mir-7113 | HGLibA_63819 | 110 | -0.053987842 | hsa-mir-7113 | - |
| hsa-mir-889 | HGLibA_64233 | 110 | -0.053987842 | hsa-mir-889 | - |
| LPHN1 | HGLibA_27085 | 110 | -0.053987842 | LPHN1 | - |
| NADKD1 | HGLibA_31052 | 110 | -0.053987842 | NADKD1 | - |
| PVRL2 | HGLibA_39624 | 110 | -0.053987842 | PVRL2 | - |
| 2-Sep | HGLibA_43436 | 110 | -0.053987842 | 2-Sep | - |
| SLC22A20 | HGLibA_44570 | 110 | -0.053987842 | SLC22A20 | - |
| TCP10 | HGLibA_48832 | 110 | -0.053987842 | TCP10 | - |
| WBSCR16 | HGLibA_53990 | 110 | -0.053987842 | WBSCR16 | - |
| WDR85 | HGLibA_54200 | 110 | -0.053987842 | WDR85 | - |
| ATPIF1 | HGLibA_03808 | 109 | -0.063938879 | ATPIF1 | - |
| BAI3 | HGLibA_04119 | 109 | -0.063938879 | BAI3 | - |
| BET3L | HGLibA_04438 | 109 | -0.063938879 | BET3L | - |
| C1orf85 | HGLibA_05898 | 109 | -0.063938879 | C1orf85 | - |
| C3orf37 | HGLibA_06239 | 109 | -0.063938879 | C3orf37 | - |
| C9orf69 | HGLibA_06756 | 109 | -0.063938879 | C9orf69 | - |
| CCDC130 | HGLibA_07593 | 109 | -0.063938879 | CCDC130 | - |
| ENSG00000004864 | HGLibA_44645 | 109 | -0.063938879 | SLC25A13 | solute carrier family 25 member 13 [Source:HGNC Symbol;Acc:HGNC:10983] |
| ENSG00000005381 | HGLibA_29745 | 109 | -0.063938879 | MPO | myeloperoxidase [Source:HGNC Symbol;Acc:HGNC:7218] |
| ENSG00000008853 | HGLibA_41176 | 109 | -0.063938879 | RHOBTB2 | Rho related BTB domain containing 2 [Source:HGNC Symbol;Acc:HGNC:18756] |
| ENSG00000011114 | HGLibA_04920 | 109 | -0.063938879 | BTBD7 | BTB domain containing 7 [Source:HGNC Symbol;Acc:HGNC:18269] |
| ENSG00000011478 | HGLibA_39731 | 109 | -0.063938879 | QPCTL | glutaminyl-peptide cyclotransferase like [Source:HGNC Symbol;Acc:HGNC:25952] |
| ENSG00000013306 | HGLibA_44730 | 109 | -0.063938879 | SLC25A39 | solute carrier family 25 member 39 [Source:HGNC Symbol;Acc:HGNC:24279] |
| ENSG00000023902 | HGLibA_37149 | 109 | -0.063938879 | PLEKHO1 | pleckstrin homology domain containing O1 [Source:HGNC Symbol;Acc:HGNC:24310] |
| ENSG00000047621 | HGLibA_05234 | 109 | -0.063938879 | C12orf4 | chromosome 12 open reading frame 4 [Source:HGNC Symbol;Acc:HGNC:1184] |
| ENSG00000058056 | HGLibA_53202 | 109 | -0.063938879 | USP13 | ubiquitin specific peptidase 13 [Source:HGNC Symbol;Acc:HGNC:12611] |
| ENSG00000059122 | HGLibA_17681 | 109 | -0.063938879 | FLYWCH1 | FLYWCH-type zinc finger 1 [Source:HGNC Symbol;Acc:HGNC:25404] |
| ENSG00000067596 | HGLibA_13208 | 109 | -0.063938879 | DHX8 | DEAH-box helicase 8 [Source:HGNC Symbol;Acc:HGNC:2749] |
| ENSG00000070526 | HGLibA_47265 | 109 | -0.063938879 | ST6GALNAC1 | 6-sialyltransferase 1 [Source:HGNC Symbol;Acc:HGNC:23614]" |
| ENSG00000072134 | HGLibA_15343 | 109 | -0.063938879 | EPN2 | epsin 2 [Source:HGNC Symbol;Acc:HGNC:18639] |
| ENSG00000082293 | HGLibA_10527 | 109 | -0.063938879 | COL19A1 | collagen type XIX alpha 1 chain [Source:HGNC Symbol;Acc:HGNC:2196] |
| ENSG00000085982 | HGLibA_53363 | 109 | -0.063938879 | USP40 | ubiquitin specific peptidase 40 [Source:HGNC Symbol;Acc:HGNC:20069] |
| ENSG00000089050 | HGLibA_40445 | 109 | -0.063938879 | RBBP9 | serine hydrolase [Source:HGNC Symbol;Acc:HGNC:9892]" |
| ENSG00000100558 | HGLibA_37059 | 109 | -0.063938879 | PLEK2 | pleckstrin 2 [Source:HGNC Symbol;Acc:HGNC:19238] |
| ENSG00000100842 | HGLibA_14611 | 109 | -0.063938879 | EFS | embryonal Fyn-associated substrate [Source:HGNC Symbol;Acc:HGNC:16898] |
| ENSG00000101144 | HGLibA_04617 | 109 | -0.063938879 | BMP7 | bone morphogenetic protein 7 [Source:HGNC Symbol;Acc:HGNC:1074] |
| ENSG00000101265 | HGLibA_40377 | 109 | -0.063938879 | RASSF2 | Ras association domain family member 2 [Source:HGNC Symbol;Acc:HGNC:9883] |

| | | | | | |
|---|---|---|---|---|---|
| ENSG00000102076 | HGLibA_33423 | 109 | -0.063938879 | OPN1LW | long wave sensitive [Source:HGNC Symbol;Acc:HGNC:9936]" |
| ENSG00000102385 | HGLibA_13985 | 109 | -0.063938879 | DRP2 | dystrophin related protein 2 [Source:HGNC Symbol;Acc:HGNC:3032] |
| ENSG00000103356 | HGLibA_14342 | 109 | -0.063938879 | EARS2 | mitochondrial [Source:HGNC Symbol;Acc:HGNC:29419]" |
| ENSG00000103671 | HGLibA_51644 | 109 | -0.063938879 | TRIP4 | thyroid hormone receptor interactor 4 [Source:HGNC Symbol;Acc:HGNC:12310] |
| ENSG00000103994 | HGLibA_55437 | 109 | -0.063938879 | ZNF106 | zinc finger protein 106 [Source:HGNC Symbol;Acc:HGNC:12886] |
| ENSG00000105894 | HGLibA_39397 | 109 | -0.063938879 | PTN | pleiotrophin [Source:HGNC Symbol;Acc:HGNC:9630] |
| ENSG00000106546 | HGLibA_01307 | 109 | -0.063938879 | AHR | aryl hydrocarbon receptor [Source:HGNC Symbol;Acc:HGNC:348] |
| ENSG00000107554 | HGLibA_13694 | 109 | -0.063938879 | DNMBP | dynamin binding protein [Source:HGNC Symbol;Acc:HGNC:30373] |
| ENSG00000109929 | HGLibA_42818 | 109 | -0.063938879 | SC5D | sterol-C5-desaturase [Source:HGNC Symbol;Acc:HGNC:10547] |
| ENSG00000110497 | HGLibA_01755 | 109 | -0.063938879 | AMBRA1 | autophagy and beclin 1 regulator 1 [Source:HGNC Symbol;Acc:HGNC:25990] |
| ENSG00000111218 | HGLibA_38637 | 109 | -0.063938879 | PRMT8 | protein arginine methyltransferase 8 [Source:HGNC Symbol;Acc:HGNC:5188] |
| ENSG00000111241 | HGLibA_17413 | 109 | -0.063938879 | FGF6 | fibroblast growth factor 6 [Source:HGNC Symbol;Acc:HGNC:3684] |
| ENSG00000111817 | HGLibA_14017 | 109 | -0.063938879 | DSE | dermatan sulfate epimerase [Source:HGNC Symbol;Acc:HGNC:21144] |
| ENSG00000113196 | HGLibA_20826 | 109 | -0.063938879 | HAND1 | heart and neural crest derivatives expressed 1 [Source:HGNC Symbol;Acc:HGNC:4807] |
| ENSG00000113761 | HGLibA_55850 | 109 | -0.063938879 | ZNF346 | zinc finger protein 346 [Source:HGNC Symbol;Acc:HGNC:16403] |
| ENSG00000113812 | HGLibA_00673 | 109 | -0.063938879 | ACTR8 | ARP8 actin related protein 8 homolog [Source:HGNC Symbol;Acc:HGNC:14672] |
| ENSG00000114738 | HGLibA_28303 | 109 | -0.063938879 | MAPKAPK3 | mitogen-activated protein kinase-activated protein kinase 3 [Source:HGNC Symbol;Acc:HGNC:6888] |
| ENSG00000114988 | HGLibA_26666 | 109 | -0.063938879 | LMAN2L | mannose binding 2 like [Source:HGNC Symbol;Acc:HGNC:19263]" |
| ENSG00000117477 | HGLibA_07724 | 109 | -0.063938879 | CCDC181 | coiled-coil domain containing 181 [Source:HGNC Symbol;Acc:HGNC:28051] |
| ENSG00000120727 | HGLibA_35032 | 109 | -0.063938879 | PAIP2 | poly(A) binding protein interacting protein 2 [Source:HGNC Symbol;Acc:HGNC:17970] |
| ENSG00000121270 | HGLibA_00163 | 109 | -0.063938879 | ABCC11 | ATP binding cassette subfamily C member 11 [Source:HGNC Symbol;Acc:HGNC:14639] |
| ENSG00000121895 | HGLibA_50013 | 109 | -0.063938879 | TMEM156 | transmembrane protein 156 [Source:HGNC Symbol;Acc:HGNC:26260] |
| ENSG00000123171 | HGLibA_07850 | 109 | -0.063938879 | CCDC70 | coiled-coil domain containing 70 [Source:HGNC Symbol;Acc:HGNC:25303] |
| ENSG00000123405 | HGLibA_31787 | 109 | -0.063938879 | NFE2 | erythroid 2 [Source:HGNC Symbol;Acc:HGNC:7780]" |
| ENSG00000126391 | HGLibA_18030 | 109 | -0.063938879 | FRMD8 | FERM domain containing 8 [Source:HGNC Symbol;Acc:HGNC:25462] |
| ENSG00000126603 | HGLibA_19275 | 109 | -0.063938879 | GLIS2 | GLIS family zinc finger 2 [Source:HGNC Symbol;Acc:HGNC:29450] |
| ENSG00000127920 | HGLibA_19511 | 109 | -0.063938879 | GNG11 | G protein subunit gamma 11 [Source:HGNC Symbol;Acc:HGNC:4403] |
| ENSG00000128989 | HGLibA_03098 | 109 | -0.063938879 | ARPP19 | cAMP regulated phosphoprotein 19 [Source:HGNC Symbol;Acc:HGNC:16967] |
| ENSG00000129455 | HGLibA_25270 | 109 | -0.063938879 | KLK8 | kallikrein related peptidase 8 [Source:HGNC Symbol;Acc:HGNC:6369] |
| ENSG00000129625 | HGLibA_40789 | 109 | -0.063938879 | REEP5 | receptor accessory protein 5 [Source:HGNC Symbol;Acc:HGNC:30077] |
| ENSG00000130517 | HGLibA_36280 | 109 | -0.063938879 | PGPEP1 | pyroglutamyl-peptidase I [Source:HGNC Symbol;Acc:HGNC:13568] |
| ENSG00000133169 | HGLibA_04441 | 109 | -0.063938879 | BEX1 | brain expressed X-linked 1 [Source:HGNC Symbol;Acc:HGNC:1036] |
| ENSG00000133422 | HGLibA_29652 | 109 | -0.063938879 | MORC2 | MORC family CW-type zinc finger 2 [Source:HGNC Symbol;Acc:HGNC:23573] |
| ENSG00000133612 | HGLibA_01145 | 109 | -0.063938879 | AGAP3 | ankyrin repeat and PH domain 3 [Source:HGNC Symbol;Acc:HGNC:16923]" |
| ENSG00000134240 | HGLibA_21713 | 109 | -0.063938879 | HMGCS2 | 3-hydroxy-3-methylglutaryl-CoA synthase 2 [Source:HGNC Symbol;Acc:HGNC:5008] |
| ENSG00000134369 | HGLibA_31194 | 109 | -0.063938879 | NAV1 | neuron navigator 1 [Source:HGNC Symbol;Acc:HGNC:15989] |
| ENSG00000134463 | HGLibA_14401 | 109 | -0.063938879 | ECHDC3 | enoyl-CoA hydratase domain containing 3 [Source:HGNC Symbol;Acc:HGNC:23489] |
| ENSG00000134490 | HGLibA_50287 | 109 | -0.063938879 | TMEM241 | transmembrane protein 241 [Source:HGNC Symbol;Acc:HGNC:31723] |
| ENSG00000136869 | HGLibA_49653 | 109 | -0.063938879 | TLR4 | toll like receptor 4 [Source:HGNC Symbol;Acc:HGNC:11850] |
| ENSG00000137878 | HGLibA_18825 | 109 | -0.063938879 | GCOM1 | GRINL1A complex locus 1 [Source:HGNC Symbol;Acc:HGNC:26424] |
| ENSG00000138109 | HGLibA_12123 | 109 | -0.063938879 | CYP2C9 | cytochrome P450 family 2 subfamily C member 9 [Source:HGNC Symbol;Acc:HGNC:2623] |
| ENSG00000139233 | HGLibA_26653 | 109 | -0.063938879 | LLPH | long-term synaptic facilitation factor [Source:HGNC Symbol;Acc:HGNC:28229]" |
| ENSG00000139410 | HGLibA_43175 | 109 | -0.063938879 | SDSL | serine dehydratase like [Source:HGNC Symbol;Acc:HGNC:30404] |
| ENSG00000142252 | HGLibA_18922 | 109 | -0.063938879 | GEMIN7 | gem nuclear organelle associated protein 7 [Source:HGNC Symbol;Acc:HGNC:20045] |
| ENSG00000142748 | HGLibA_17223 | 109 | -0.063938879 | FCN3 | ficolin 3 [Source:HGNC Symbol;Acc:HGNC:3625] |
| ENSG00000143149 | HGLibA_01588 | 109 | -0.063938879 | ALDH9A1 | aldehyde dehydrogenase 9 family member A1 [Source:HGNC Symbol;Acc:HGNC:412] |
| ENSG00000144792 | HGLibA_56475 | 109 | -0.063938879 | ZNF660 | zinc finger protein 660 [Source:HGNC Symbol;Acc:HGNC:26720] |
| ENSG00000144962 | HGLibA_46433 | 109 | -0.063938879 | SPATA16 | spermatogenesis associated 16 [Source:HGNC Symbol;Acc:HGNC:29935] |
| ENSG00000145439 | HGLibA_07425 | 109 | -0.063938879 | CBR4 | carbonyl reductase 4 [Source:HGNC Symbol;Acc:HGNC:25891] |
| ENSG00000147140 | HGLibA_32297 | 109 | -0.063938879 | NONO | non-POU domain containing octamer binding [Source:HGNC Symbol;Acc:HGNC:7871] |
| ENSG00000148926 | HGLibA_00991 | 109 | -0.063938879 | ADM | adrenomedullin [Source:HGNC Symbol;Acc:HGNC:259] |
| ENSG00000153012 | HGLibA_26395 | 109 | -0.063938879 | LGI2 | leucine rich repeat LGI family member 2 [Source:HGNC Symbol;Acc:HGNC:18710] |
| ENSG00000153495 | HGLibA_49090 | 109 | -0.063938879 | TEX29 | testis expressed 29 [Source:HGNC Symbol;Acc:HGNC:20370] |
| ENSG00000154328 | HGLibA_31633 | 109 | -0.063938879 | NEIL2 | nei like DNA glycosylase 2 [Source:HGNC Symbol;Acc:HGNC:18956] |
| ENSG00000155592 | HGLibA_55347 | 109 | -0.063938879 | ZKSCAN2 | zinc finger with KRAB and SCAN domains 2 [Source:HGNC Symbol;Acc:HGNC:25677] |
| ENSG00000157110 | HGLibA_40652 | 109 | -0.063938879 | RBPMS | mRNA processing factor [Source:HGNC Symbol;Acc:HGNC:19097]" |
| ENSG00000157259 | HGLibA_18683 | 109 | -0.063938879 | GATAD1 | GATA zinc finger domain containing 1 [Source:HGNC Symbol;Acc:HGNC:29941] |
| ENSG00000158079 | HGLibA_39411 | 109 | -0.063938879 | PTPDC1 | protein tyrosine phosphatase domain containing 1 [Source:HGNC Symbol;Acc:HGNC:30184] |
| ENSG00000158201 | HGLibA_00274 | 109 | -0.063938879 | ABHD3 | abhydrolase domain containing 3 [Source:HGNC Symbol;Acc:HGNC:18718] |
| ENSG00000160213 | HGLibA_11480 | 109 | -0.063938879 | CSTB | cystatin B [Source:HGNC Symbol;Acc:HGNC:2482] |
| ENSG00000161013 | HGLibA_29143 | 109 | -0.063938879 | MGAT4B | 3-mannosyl-glycoprotein 4-beta-N-acetylglucosaminyltransferase B [Source:HGNC Symbol;Acc:HGNC:7048]" |
| ENSG00000161973 | HGLibA_07781 | 109 | -0.063938879 | CCDC42 | coiled-coil domain containing 42 [Source:HGNC Symbol;Acc:HGNC:26528] |
| ENSG00000162545 | HGLibA_07080 | 109 | -0.063938879 | CAMK2N1 | calcium/calmodulin dependent protein kinase II inhibitor 1 [Source:HGNC Symbol;Acc:HGNC:24190] |
| ENSG00000162992 | HGLibA_31733 | 109 | -0.063938879 | NEUROD1 | neuronal differentiation 1 [Source:HGNC Symbol;Acc:HGNC:7762] |
| ENSG00000164171 | HGLibA_23725 | 109 | -0.063938879 | ITGA2 | integrin subunit alpha 2 [Source:HGNC Symbol;Acc:HGNC:6137] |
| ENSG00000164588 | HGLibA_20974 | 109 | -0.063938879 | HCN1 | hyperpolarization activated cyclic nucleotide gated potassium channel 1 [Source:HGNC Symbol;Acc:HGNC:4845] |
| ENSG00000165113 | HGLibA_19205 | 109 | -0.063938879 | GKAP1 | G kinase anchoring protein 1 [Source:HGNC Symbol;Acc:HGNC:17496] |
| ENSG00000165868 | HGLibA_22258 | 109 | -0.063938879 | HSPA12A | heat shock protein family A (Hsp70) member 12A [Source:HGNC Symbol;Acc:HGNC:19022] |
| ENSG00000165886 | HGLibA_52765 | 109 | -0.063938879 | UBTD1 | ubiquitin domain containing 1 [Source:HGNC Symbol;Acc:HGNC:25683] |
| ENSG00000165949 | HGLibA_22595 | 109 | -0.063938879 | IFI27 | interferon alpha inducible protein 27 [Source:HGNC Symbol;Acc:HGNC:5397] |
| ENSG00000166313 | HGLibA_02376 | 109 | -0.063938879 | APBB1 | amyloid beta precursor protein binding family B member 1 [Source:HGNC Symbol;Acc:HGNC:581] |
| ENSG00000166813 | HGLibA_24962 | 109 | -0.063938879 | KIF7 | kinesin family member 7 [Source:HGNC Symbol;Acc:HGNC:30497] |
| ENSG00000167900 | HGLibA_49579 | 109 | -0.063938879 | TK1 | thymidine kinase 1 [Source:HGNC Symbol;Acc:HGNC:11830] |
| ENSG00000167941 | HGLibA_46206 | 109 | -0.063938879 | SOST | sclerostin [Source:HGNC Symbol;Acc:HGNC:13771] |
| ENSG00000168958 | HGLibA_29056 | 109 | -0.063938879 | MFF | mitochondrial fission factor [Source:HGNC Symbol;Acc:HGNC:24858] |
| ENSG00000169807 | HGLibA_38997 | 109 | -0.063938879 | PRY2 | PTPN13 like Y-linked 2 [Source:HGNC Symbol;Acc:HGNC:21504] |
| ENSG00000169885 | HGLibA_07039 | 109 | -0.063938879 | CALML6 | calmodulin like 6 [Source:HGNC Symbol;Acc:HGNC:24193] |
| ENSG00000172020 | HGLibA_18611 | 109 | -0.063938879 | GAP43 | growth associated protein 43 [Source:HGNC Symbol;Acc:HGNC:4140] |
| ENSG00000172992 | HGLibA_12467 | 109 | -0.063938879 | DCAKD | dephospho-CoA kinase domain containing [Source:HGNC Symbol;Acc:HGNC:26238] |
| ENSG00000173253 | HGLibA_13421 | 109 | -0.063938879 | DMRT2 | doublesex and mab-3 related transcription factor 2 [Source:HGNC Symbol;Acc:HGNC:2935] |
| ENSG00000173531 | HGLibA_30252 | 109 | -0.063938879 | MST1 | macrophage stimulating 1 [Source:HGNC Symbol;Acc:HGNC:7380] |
| ENSG00000173889 | HGLibA_36325 | 109 | -0.063938879 | PHC3 | polyhomeotic homolog 3 [Source:HGNC Symbol;Acc:HGNC:15682] |
| ENSG00000174405 | HGLibA_26487 | 109 | -0.063938879 | LIG4 | DNA ligase 4 [Source:HGNC Symbol;Acc:HGNC:6601] |
| ENSG00000174442 | HGLibA_57068 | 109 | -0.063938879 | ZWILCH | zwilch kinetochore protein [Source:HGNC Symbol;Acc:HGNC:25468] |
| ENSG00000175556 | HGLibA_27025 | 109 | -0.063938879 | LONRF3 | LON peptidase N-terminal domain and ring finger 3 [Source:HGNC Symbol;Acc:HGNC:21152] |
| ENSG00000176782 | HGLibA_12818 | 109 | -0.063938879 | DEFB104A | defensin beta 104A [Source:HGNC Symbol;Acc:HGNC:18115] |
| ENSG00000178235 | HGLibA_45514 | 109 | -0.063938879 | SLITRK1 | SLIT and NTRK like family member 1 [Source:HGNC Symbol;Acc:HGNC:20297] |
| ENSG00000178295 | HGLibA_18928 | 109 | -0.063938879 | GEN1 | Holliday junction 5' flap endonuclease [Source:HGNC Symbol;Acc:HGNC:26881]" |
| ENSG00000179134 | HGLibA_42686 | 109 | -0.063938879 | SAMD4B | sterile alpha motif domain containing 4B [Source:HGNC Symbol;Acc:HGNC:25492] |
| ENSG00000181143 | HGLibA_30530 | 109 | -0.063938879 | MUC16 | cell surface associated [Source:HGNC Symbol;Acc:HGNC:15582]" |
| ENSG00000181191 | HGLibA_36783 | 109 | -0.063938879 | PJA1 | praja ring finger ubiquitin ligase 1 [Source:HGNC Symbol;Acc:HGNC:16648] |
| ENSG00000182070 | HGLibA_34140 | 109 | -0.063938879 | OR52A1 | olfactory receptor family 52 subfamily A member 1 [Source:HGNC Symbol;Acc:HGNC:8318] |
| ENSG00000182492 | HGLibA_04464 | 109 | -0.063938879 | BGN | biglycan [Source:HGNC Symbol;Acc:HGNC:1044] |
| ENSG00000183036 | HGLibA_35661 | 109 | -0.063938879 | PCP4 | Purkinje cell protein 4 [Source:HGNC Symbol;Acc:HGNC:8742] |
| ENSG00000183580 | HGLibA_17018 | 109 | -0.063938879 | FBXL7 | F-box and leucine rich repeat protein 7 [Source:HGNC Symbol;Acc:HGNC:13604] |
| ENSG00000183632 | HGLibA_51077 | 109 | -0.063938879 | TP53TG3 | TP53 target 3 [Source:HGNC Symbol;Acc:HGNC:30759] |
| ENSG00000183691 | HGLibA_32252 | 109 | -0.063938879 | NOG | noggin [Source:HGNC Symbol;Acc:HGNC:7866] |
| ENSG00000184108 | HGLibA_51621 | 109 | -0.063938879 | TRIML1 | tripartite motif family like 1 [Source:HGNC Symbol;Acc:HGNC:26698] |
| ENSG00000184155 | HGLibA_33527 | 109 | -0.063938879 | OR10J5 | olfactory receptor family 10 subfamily J member 5 [Source:HGNC Symbol;Acc:HGNC:14993] |
| ENSG00000185129 | HGLibA_39587 | 109 | -0.063938879 | PURA | purine rich element binding protein A [Source:HGNC Symbol;Acc:HGNC:9701] |
| ENSG00000186104 | HGLibA_12139 | 109 | -0.063938879 | CYP2R1 | cytochrome P450 family 2 subfamily R member 1 [Source:HGNC Symbol;Acc:HGNC:20580] |
| ENSG00000186318 | HGLibA_04060 | 109 | -0.063938879 | BACE1 | beta-secretase 1 [Source:HGNC Symbol;Acc:HGNC:933] |
| ENSG00000188386 | HGLibA_38196 | 109 | -0.063938879 | PPP3R2 | beta [Source:HGNC Symbol;Acc:HGNC:9318]" |
| ENSG00000188536 | HGLibA_20910 | 109 | -0.063938879 | HBA2 | hemoglobin subunit alpha 2 [Source:HGNC Symbol;Acc:HGNC:4824] |
| ENSG00000189037 | HGLibA_14160 | 109 | -0.063938879 | DUSP21 | dual specificity phosphatase 21 [Source:HGNC Symbol;Acc:HGNC:20476] |
| ENSG00000189167 | HGLibA_54781 | 109 | -0.063938879 | ZAR1L | zygote arrest 1 like [Source:HGNC Symbol;Acc:HGNC:37116] |
| ENSG00000196116 | HGLibA_48915 | 109 | -0.063938879 | TDRD7 | tudor domain containing 7 [Source:HGNC Symbol;Acc:HGNC:30831] |
| ENSG00000196455 | HGLibA_36657 | 109 | -0.063938879 | PIK3R4 | phosphoinositide-3-kinase regulatory subunit 4 [Source:HGNC Symbol;Acc:HGNC:8982] |
| ENSG00000196660 | HGLibA_44900 | 109 | -0.063938879 | SLC30A10 | solute carrier family 30 member 10 [Source:HGNC Symbol;Acc:HGNC:25355] |
| ENSG00000197181 | HGLibA_36775 | 109 | -0.063938879 | PIWIL2 | piwi like RNA-mediated gene silencing 2 [Source:HGNC Symbol;Acc:HGNC:17644] |
| ENSG00000197888 | HGLibA_52921 | 109 | -0.063938879 | UGT2B17 | UDP glucuronosyltransferase family 2 member B17 [Source:HGNC Symbol;Acc:HGNC:12547] |
| ENSG00000198556 | HGLibA_56758 | 109 | -0.063938879 | ZNF789 | zinc finger protein 789 [Source:HGNC Symbol;Acc:HGNC:27801] |
| ENSG00000198815 | HGLibA_17892 | 109 | -0.063938879 | FOXJ3 | forkhead box J3 [Source:HGNC Symbol;Acc:HGNC:29178] |
| ENSG00000204149 | HGLibA_01153 | 109 | -0.063938879 | AGAP6 | ankyrin repeat and PH domain 6 [Source:HGNC Symbol;Acc:HGNC:23466]" |
| ENSG00000204347 | HGLibA_04904 | 109 | -0.063938879 | BTBD17 | BTB domain containing 17 [Source:HGNC Symbol;Acc:HGNC:33758] |
| ENSG00000204475 | HGLibA_31367 | 109 | -0.063938879 | NCR3 | natural cytotoxicity triggering receptor 3 [Source:HGNC Symbol;Acc:HGNC:19077] |
| ENSG00000205445 | HGLibA_25603 | 109 | -0.063938879 | KRTAP10-2 | keratin associated protein 10-2 [Source:HGNC Symbol;Acc:HGNC:22967] |
| ENSG00000205669 | HGLibA_00486 | 109 | -0.063938879 | ACOT6 | acyl-CoA thioesterase 6 [Source:HGNC Symbol;Acc:HGNC:33159] |
| ENSG00000213246 | HGLibA_47816 | 109 | -0.063938879 | SUPT4H1 | DSIF elongation factor subunit [Source:HGNC Symbol;Acc:HGNC:11467]" |
| ENSG00000213799 | HGLibA_56864 | 109 | -0.063938879 | ZNF845 | zinc finger protein 845 [Source:HGNC Symbol;Acc:HGNC:25112] |
| ENSG00000215251 | HGLibA_16909 | 109 | -0.063938879 | FASTKD5 | FAST kinase domains 5 [Source:HGNC Symbol;Acc:HGNC:25790] |
| ENSG00000215343 | HGLibA_56592 | 109 | -0.063938879 | ZNF705D | zinc finger protein 705D [Source:HGNC Symbol;Acc:HGNC:33202] |
| ENSG00000217128 | HGLibA_17766 | 109 | -0.063938879 | FNIP1 | folliculin interacting protein 1 [Source:HGNC Symbol;Acc:HGNC:29418] |
| ENSG00000223443 | HGLibA_53242 | 109 | -0.063938879 | USP17L2 | ubiquitin specific peptidase 17-like family member 2 [Source:HGNC Symbol;Acc:HGNC:34434] |
| ENSG00000240747 | HGLibA_25392 | 109 | -0.063938879 | KRBOX1 | KRAB box domain containing 1 [Source:HGNC Symbol;Acc:HGNC:38708] |
| ENSG00000248383 | HGLibA_35464 | 109 | -0.063938879 | PCDHAC1 | 1 [Source:HGNC Symbol;Acc:HGNC:8676]" |
| ENSG00000253148 | HGLibA_41086 | 109 | -0.063938879 | RGS21 | regulator of G protein signaling 21 [Source:HGNC Symbol;Acc:HGNC:26839] |
| ENSG00000254415 | HGLibA_44136 | 109 | -0.063938879 | SIGLEC14 | sialic acid binding Ig like lectin 14 [Source:HGNC Symbol;Acc:HGNC:32926] |
| ENSG00000272920 | HGLibA_57411 | 109 | -0.063938879 | hsa-mir-1253 | hsa-mir-1253 [Source:miRBase;Acc:MI0006387] |
| ENSG00000277150 | HGLibA_15891 | 109 | -0.063938879 | F8A3 | coagulation factor VIII associated 3 [Source:HGNC Symbol;Acc:HGNC:31850] |
| ENSG00000278540 | HGLibA_00347 | 109 | -0.063938879 | ACACA | acetyl-CoA carboxylase alpha [Source:HGNC Symbol;Acc:HGNC:84] |
| FAM86C1 | HGLibA_16779 | 109 | -0.063938879 | FAM86C1 | - |
| GATSL2 | HGLibA_18705 | 109 | -0.063938879 | GATSL2 | - |
| GPR56 | HGLibA_20057 | 109 | -0.063938879 | GPR56 | - |
| HIST1H3G | HGLibA_21474 | 109 | -0.063938879 | HIST1H3G | - |

| | | | | | |
|---|---|---|---|---|---|
| hsa-mir-142 | HGLibA_57832 | 109 | -0.063938879 | hsa-mir-142 | - |
| hsa-mir-3926-2 | HGLibA_59581 | 109 | -0.063938879 | hsa-mir-3926-2 | - |
| hsa-mir-4436b-2 | HGLibA_60148 | 109 | -0.063938879 | hsa-mir-4436b-2 | - |
| hsa-mir-4474 | HGLibA_60307 | 109 | -0.063938879 | hsa-mir-4474 | - |
| hsa-mir-513a-2 | HGLibA_61574 | 109 | -0.063938879 | hsa-mir-513a-2 | - |
| hsa-mir-548u | HGLibA_62069 | 109 | -0.063938879 | hsa-mir-548u | - |
| hsa-mir-548z | HGLibA_62092 | 109 | -0.063938879 | hsa-mir-548z | - |
| hsa-mir-670 | HGLibA_63010 | 109 | -0.063938879 | hsa-mir-670 | - |
| hsa-mir-6771 | HGLibA_63258 | 109 | -0.063938879 | hsa-mir-6771 | - |
| hsa-mir-6787 | HGLibA_63325 | 109 | -0.063938879 | hsa-mir-6787 | - |
| hsa-mir-6789 | HGLibA_63335 | 109 | -0.063938879 | hsa-mir-6789 | - |
| hsa-mir-6872 | HGLibA_63672 | 109 | -0.063938879 | hsa-mir-6872 | - |
| hsa-mir-7-3 | HGLibA_63776 | 109 | -0.063938879 | hsa-mir-7-3 | - |
| hsa-mir-876 | HGLibA_64216 | 109 | -0.063938879 | hsa-mir-876 | - |
| KIAA0226L | HGLibA_24608 | 109 | -0.063938879 | KIAA0226L | - |
| KIAA1377 | HGLibA_24745 | 109 | -0.063938879 | KIAA1377 | - |
| LHFP | HGLibA_26426 | 109 | -0.063938879 | LHFP | - |
| PIH1D3 | HGLibA_36615 | 109 | -0.063938879 | PIH1D3 | - |
| 4-Sep | HGLibA_43443 | 109 | -0.063938879 | 4-Sep | - |
| WBSCR22 | HGLibA_53994 | 109 | -0.063938879 | WBSCR22 | - |
| ACPP | HGLibA_00523 | 108 | -0.073889917 | ACPP | - |
| ASUN | HGLibA_03387 | 108 | -0.073889917 | ASUN | - |
| ATP5SL | HGLibA_03691 | 108 | -0.073889917 | ATP5SL | - |
| C14orf177 | HGLibA_05341 | 108 | -0.073889917 | C14orf177 | - |
| C6orf70 | HGLibA_06508 | 108 | -0.073889917 | C6orf70 | - |
| CSRP2BP | HGLibA_11438 | 108 | -0.073889917 | CSRP2BP | - |
| DUPD1 | HGLibA_14108 | 108 | -0.073889917 | DUPD1 | - |
| ENSG00000008196 | HGLibA_49125 | 108 | -0.073889917 | TFAP2B | transcription factor AP-2 beta [Source:HGNC Symbol;Acc:HGNC:11743] |
| ENSG00000021461 | HGLibA_12158 | 108 | -0.073889917 | CYP3A43 | cytochrome P450 family 3 subfamily A member 43 [Source:HGNC Symbol;Acc:HGNC:17450] |
| ENSG00000044090 | HGLibA_11790 | 108 | -0.073889917 | CUL7 | cullin 7 [Source:HGNC Symbol;Acc:HGNC:21024] |
| ENSG00000049246 | HGLibA_36061 | 108 | -0.073889917 | PER3 | period circadian regulator 3 [Source:HGNC Symbol;Acc:HGNC:8847] |
| ENSG00000049618 | HGLibA_02884 | 108 | -0.073889917 | ARID1B | AT-rich interaction domain 1B [Source:HGNC Symbol;Acc:HGNC:18040] |
| ENSG00000051180 | HGLibA_40079 | 108 | -0.073889917 | RAD51 | RAD51 recombinase [Source:HGNC Symbol;Acc:HGNC:9817] |
| ENSG00000055955 | HGLibA_23815 | 108 | -0.073889917 | ITIH4 | inter-alpha-trypsin inhibitor heavy chain family member 4 [Source:HGNC Symbol;Acc:HGNC:6169] |
| ENSG00000065665 | HGLibA_43251 | 108 | -0.073889917 | SEC61A2 | Sec61 translocon alpha 2 subunit [Source:HGNC Symbol;Acc:HGNC:17702] |
| ENSG00000074660 | HGLibA_42876 | 108 | -0.073889917 | SCARF1 | scavenger receptor class F member 1 [Source:HGNC Symbol;Acc:HGNC:16820] |
| ENSG00000075292 | HGLibA_56436 | 108 | -0.073889917 | ZNF638 | zinc finger protein 638 [Source:HGNC Symbol;Acc:HGNC:17894] |
| ENSG00000075884 | HGLibA_02710 | 108 | -0.073889917 | ARHGAP15 | Rho GTPase activating protein 15 [Source:HGNC Symbol;Acc:HGNC:21030] |
| ENSG00000076864 | HGLibA_40226 | 108 | -0.073889917 | RAP1GAP | RAP1 GTPase activating protein [Source:HGNC Symbol;Acc:HGNC:9858] |
| ENSG00000078246 | HGLibA_52337 | 108 | -0.073889917 | TULP3 | tubby like protein 3 [Source:HGNC Symbol;Acc:HGNC:12425] |
| ENSG00000081307 | HGLibA_52523 | 108 | -0.073889917 | UBA5 | ubiquitin like modifier activating enzyme 5 [Source:HGNC Symbol;Acc:HGNC:23230] |
| ENSG00000082175 | HGLibA_36284 | 108 | -0.073889917 | PGR | progesterone receptor [Source:HGNC Symbol;Acc:HGNC:8910] |
| ENSG00000084754 | HGLibA_20809 | 108 | -0.073889917 | HADHA | hydroxyacyl-CoA dehydrogenase trifunctional multienzyme complex subunit alpha [Source:HGNC Symbol;Acc:HGNC:4801] |
| ENSG00000091127 | HGLibA_39605 | 108 | -0.073889917 | PUS7 | pseudouridine synthase 7 [Source:HGNC Symbol;Acc:HGNC:26033] |
| ENSG00000091583 | HGLibA_02525 | 108 | -0.073889917 | APOH | apolipoprotein H [Source:HGNC Symbol;Acc:HGNC:616] |
| ENSG00000094631 | HGLibA_21019 | 108 | -0.073889917 | HDAC6 | histone deacetylase 6 [Source:HGNC Symbol;Acc:HGNC:14064] |
| ENSG00000099203 | HGLibA_49799 | 108 | -0.073889917 | TMED1 | transmembrane p24 trafficking protein 1 [Source:HGNC Symbol;Acc:HGNC:17291] |
| ENSG00000100219 | HGLibA_54489 | 108 | -0.073889917 | XBP1 | X-box binding protein 1 [Source:HGNC Symbol;Acc:HGNC:12801] |
| ENSG00000100722 | HGLibA_54964 | 108 | -0.073889917 | ZC3H14 | zinc finger CCCH-type containing 14 [Source:HGNC Symbol;Acc:HGNC:20509] |
| ENSG00000102804 | HGLibA_51817 | 108 | -0.073889917 | TSC22D1 | TSC22 domain family member 1 [Source:HGNC Symbol;Acc:HGNC:16826] |
| ENSG00000103064 | HGLibA_45374 | 108 | -0.073889917 | SLC7A6 | solute carrier family 7 member 6 [Source:HGNC Symbol;Acc:HGNC:11064] |
| ENSG00000103275 | HGLibA_52614 | 108 | -0.073889917 | UBE2I | ubiquitin conjugating enzyme E2 I [Source:HGNC Symbol;Acc:HGNC:12485] |
| ENSG00000105246 | HGLibA_14362 | 108 | -0.073889917 | EBI3 | Epstein-Barr virus induced 3 [Source:HGNC Symbol;Acc:HGNC:3129] |
| ENSG00000105523 | HGLibA_16751 | 108 | -0.073889917 | FAM83E | family with sequence similarity 83 member E [Source:HGNC Symbol;Acc:HGNC:25972] |
| ENSG00000106631 | HGLibA_30774 | 108 | -0.073889917 | MYL7 | myosin light chain 7 [Source:HGNC Symbol;Acc:HGNC:21719] |
| ENSG00000110987 | HGLibA_04337 | 108 | -0.073889917 | BCL7A | BCL tumor suppressor 7A [Source:HGNC Symbol;Acc:HGNC:1004] |
| ENSG00000112701 | HGLibA_43397 | 108 | -0.073889917 | SENP6 | SUMO specific peptidase 6 [Source:HGNC Symbol;Acc:HGNC:20944] |
| ENSG00000113319 | HGLibA_40341 | 108 | -0.073889917 | RASGRF2 | Ras protein specific guanine nucleotide releasing factor 2 [Source:HGNC Symbol;Acc:HGNC:9876] |
| ENSG00000113721 | HGLibA_35856 | 108 | -0.073889917 | PDGFRB | platelet derived growth factor receptor beta [Source:HGNC Symbol;Acc:HGNC:8804] |
| ENSG00000114745 | HGLibA_19677 | 108 | -0.073889917 | GORASP1 | golgi reassembly stacking protein 1 [Source:HGNC Symbol;Acc:HGNC:16769] |
| ENSG00000115232 | HGLibA_23734 | 108 | -0.073889917 | ITGA4 | integrin subunit alpha 4 [Source:HGNC Symbol;Acc:HGNC:6140] |
| ENSG00000115274 | HGLibA_23323 | 108 | -0.073889917 | INO80B | INO80 complex subunit B [Source:HGNC Symbol;Acc:HGNC:13324] |
| ENSG00000115457 | HGLibA_22818 | 108 | -0.073889917 | IGFBP2 | insulin like growth factor binding protein 2 [Source:HGNC Symbol;Acc:HGNC:5471] |
| ENSG00000115718 | HGLibA_38650 | 108 | -0.073889917 | PROC | inactivator of coagulation factors Va and VIIIa [Source:HGNC Symbol;Acc:HGNC:9451]" |
| ENSG00000117632 | HGLibA_47515 | 108 | -0.073889917 | STMN1 | stathmin 1 [Source:HGNC Symbol;Acc:HGNC:6510] |
| ENSG00000117758 | HGLibA_47622 | 108 | -0.073889917 | STX12 | syntaxin 12 [Source:HGNC Symbol;Acc:HGNC:11430] |
| ENSG00000118257 | HGLibA_32719 | 108 | -0.073889917 | NRP2 | neuropilin 2 [Source:HGNC Symbol;Acc:HGNC:8005] |
| ENSG00000119121 | HGLibA_51767 | 108 | -0.073889917 | TRPM6 | transient receptor potential cation channel subfamily M member 6 [Source:HGNC Symbol;Acc:HGNC:17995] |
| ENSG00000119915 | HGLibA_14962 | 108 | -0.073889917 | ELOVL3 | ELOVL fatty acid elongase 3 [Source:HGNC Symbol;Acc:HGNC:18047] |
| ENSG00000120329 | HGLibA_44667 | 108 | -0.073889917 | SLC25A2 | solute carrier family 25 member 2 [Source:HGNC Symbol;Acc:HGNC:22921] |
| ENSG00000120471 | HGLibA_51052 | 108 | -0.073889917 | TP53AIP1 | tumor protein p53 regulated apoptosis inducing protein 1 [Source:HGNC Symbol;Acc:HGNC:29984] |
| ENSG00000120756 | HGLibA_37224 | 108 | -0.073889917 | PLS1 | plastin 1 [Source:HGNC Symbol;Acc:HGNC:9090] |
| ENSG00000121966 | HGLibA_11889 | 108 | -0.073889917 | CXCR4 | C-X-C motif chemokine receptor 4 [Source:HGNC Symbol;Acc:HGNC:2561] |
| ENSG00000126368 | HGLibA_32575 | 108 | -0.073889917 | NR1D1 | nuclear receptor subfamily 1 group D member 1 [Source:HGNC Symbol;Acc:HGNC:7962] |
| ENSG00000128965 | HGLibA_09336 | 108 | -0.073889917 | CHAC1 | ChaC glutathione specific gamma-glutamylcyclotransferase 1 [Source:HGNC Symbol;Acc:HGNC:28680] |
| ENSG00000129437 | HGLibA_25246 | 108 | -0.073889917 | KLK14 | kallikrein related peptidase 14 [Source:HGNC Symbol;Acc:HGNC:6362] |
| ENSG00000131389 | HGLibA_45335 | 108 | -0.073889917 | SLC6A6 | solute carrier family 6 member 6 [Source:HGNC Symbol;Acc:HGNC:11052] |
| ENSG00000131779 | HGLibA_36086 | 108 | -0.073889917 | PEX11B | peroxisomal biogenesis factor 11 beta [Source:HGNC Symbol;Acc:HGNC:8853] |
| ENSG00000132254 | HGLibA_02678 | 108 | -0.073889917 | ARFIP2 | ADP ribosylation factor interacting protein 2 [Source:HGNC Symbol;Acc:HGNC:17160] |
| ENSG00000132356 | HGLibA_38491 | 108 | -0.073889917 | PRKAA1 | protein kinase AMP-activated catalytic subunit alpha 1 [Source:HGNC Symbol;Acc:HGNC:9376] |
| ENSG00000132581 | HGLibA_43123 | 108 | -0.073889917 | SDF2 | stromal cell derived factor 2 [Source:HGNC Symbol;Acc:HGNC:10675] |
| ENSG00000132671 | HGLibA_47184 | 108 | -0.073889917 | SSTR4 | somatostatin receptor 4 [Source:HGNC Symbol;Acc:HGNC:11333] |
| ENSG00000134287 | HGLibA_02649 | 108 | -0.073889917 | ARF3 | ADP ribosylation factor 3 [Source:HGNC Symbol;Acc:HGNC:654] |
| ENSG00000134389 | HGLibA_09275 | 108 | -0.073889917 | CFHR5 | complement factor H related 5 [Source:HGNC Symbol;Acc:HGNC:24668] |
| ENSG00000134827 | HGLibA_48821 | 108 | -0.073889917 | TCN1 | transcobalamin 1 [Source:HGNC Symbol;Acc:HGNC:11652] |
| ENSG00000134873 | HGLibA_09849 | 108 | -0.073889917 | CLDN10 | claudin 10 [Source:HGNC Symbol;Acc:HGNC:2033] |
| ENSG00000135338 | HGLibA_26092 | 108 | -0.073889917 | LCA5 | lebercilin [Source:HGNC Symbol;Acc:HGNC:31923]" |
| ENSG00000135778 | HGLibA_32872 | 108 | -0.073889917 | NTPCR | cancer-related [Source:HGNC Symbol;Acc:HGNC:28204]" |
| ENSG00000136247 | HGLibA_55116 | 108 | -0.073889917 | ZDHHC4 | zinc finger DHHC-type containing 4 [Source:HGNC Symbol;Acc:HGNC:18471] |
| ENSG00000137726 | HGLibA_18239 | 108 | -0.073889917 | FXYD6 | FXYD domain containing ion transport regulator 6 [Source:HGNC Symbol;Acc:HGNC:4030] |
| ENSG00000138646 | HGLibA_21165 | 108 | -0.073889917 | HERC5 | HECT and RLD domain containing E3 ubiquitin protein ligase 5 [Source:HGNC Symbol;Acc:HGNC:24368] |
| ENSG00000139155 | HGLibA_45457 | 108 | -0.073889917 | SLCO1C1 | solute carrier organic anion transporter family member 1C1 [Source:HGNC Symbol;Acc:HGNC:13819] |
| ENSG00000139351 | HGLibA_47938 | 108 | -0.073889917 | SYCP3 | synaptonemal complex protein 3 [Source:HGNC Symbol;Acc:HGNC:18130] |
| ENSG00000143575 | HGLibA_20904 | 108 | -0.073889917 | HAX1 | HCLS1 associated protein X-1 [Source:HGNC Symbol;Acc:HGNC:16915] |
| ENSG00000143595 | HGLibA_02582 | 108 | -0.073889917 | AQP10 | aquaporin 10 [Source:HGNC Symbol;Acc:HGNC:16029] |
| ENSG00000143799 | HGLibA_35211 | 108 | -0.073889917 | PARP1 | poly(ADP-ribose) polymerase 1 [Source:HGNC Symbol;Acc:HGNC:270] |
| ENSG00000143919 | HGLibA_07093 | 108 | -0.073889917 | CAMKMT | calmodulin-lysine N-methyltransferase [Source:HGNC Symbol;Acc:HGNC:26276] |
| ENSG00000145358 | HGLibA_12620 | 108 | -0.073889917 | DDIT4L | DNA damage inducible transcript 4 like [Source:HGNC Symbol;Acc:HGNC:30555] |
| ENSG00000145703 | HGLibA_23535 | 108 | -0.073889917 | IQGAP2 | IQ motif containing GTPase activating protein 2 [Source:HGNC Symbol;Acc:HGNC:6111] |
| ENSG00000146147 | HGLibA_29388 | 108 | -0.073889917 | MLIP | muscular LMNA interacting protein [Source:HGNC Symbol;Acc:HGNC:21355] |
| ENSG00000148602 | HGLibA_27186 | 108 | -0.073889917 | LRIT1 | Ig-like and transmembrane domains 1 [Source:HGNC Symbol;Acc:HGNC:23404]" |
| ENSG00000150990 | HGLibA_13192 | 108 | -0.073889917 | DHX37 | DEAH-box helicase 37 [Source:HGNC Symbol;Acc:HGNC:17210] |
| ENSG00000151418 | HGLibA_03763 | 108 | -0.073889917 | ATP6V1G3 | ATPase H+ transporting V1 subunit G3 [Source:HGNC Symbol;Acc:HGNC:18265] |
| ENSG00000152291 | HGLibA_49274 | 108 | -0.073889917 | TGOLN2 | trans-golgi network protein 2 [Source:HGNC Symbol;Acc:HGNC:15450] |
| ENSG00000153982 | HGLibA_18889 | 108 | -0.073889917 | GDPD1 | glycerophosphodiester phosphodiesterase domain containing 1 [Source:HGNC Symbol;Acc:HGNC:20883] |
| ENSG00000155011 | HGLibA_13296 | 108 | -0.073889917 | DKK2 | dickkopf WNT signaling pathway inhibitor 2 [Source:HGNC Symbol;Acc:HGNC:2892] |
| ENSG00000155026 | HGLibA_42325 | 108 | -0.073889917 | RSPH10B | radial spoke head 10 homolog B [Source:HGNC Symbol;Acc:HGNC:27362] |
| ENSG00000158987 | HGLibA_40261 | 108 | -0.073889917 | RAPGEF6 | Rap guanine nucleotide exchange factor 6 [Source:HGNC Symbol;Acc:HGNC:20655] |
| ENSG00000159251 | HGLibA_00608 | 108 | -0.073889917 | ACTC1 | cardiac muscle 1 [Source:HGNC Symbol;Acc:HGNC:143]" |
| ENSG00000160321 | HGLibA_55583 | 108 | -0.073889917 | ZNF208 | zinc finger protein 208 [Source:HGNC Symbol;Acc:HGNC:12999] |
| ENSG00000161920 | HGLibA_28738 | 108 | -0.073889917 | MED11 | mediator complex subunit 11 [Source:HGNC Symbol;Acc:HGNC:32687] |
| ENSG00000164054 | HGLibA_44042 | 108 | -0.073889917 | SHISA5 | shisa family member 5 [Source:HGNC Symbol;Acc:HGNC:30376] |
| ENSG00000164104 | HGLibA_21693 | 108 | -0.073889917 | HMGB2 | high mobility group box 2 [Source:HGNC Symbol;Acc:HGNC:5000] |
| ENSG00000164619 | HGLibA_04627 | 108 | -0.073889917 | BMPER | BMP binding endothelial regulator [Source:HGNC Symbol;Acc:HGNC:24154] |
| ENSG00000164631 | HGLibA_55449 | 108 | -0.073889917 | ZNF12 | zinc finger protein 12 [Source:HGNC Symbol;Acc:HGNC:12902] |
| ENSG00000165914 | HGLibA_52154 | 108 | -0.073889917 | TTC7B | tetratricopeptide repeat domain 7B [Source:HGNC Symbol;Acc:HGNC:19858] |
| ENSG00000167515 | HGLibA_51315 | 108 | -0.073889917 | TRAPPC2L | trafficking protein particle complex 2 like [Source:HGNC Symbol;Acc:HGNC:30887] |
| ENSG00000167632 | HGLibA_51340 | 108 | -0.073889917 | TRAPPC9 | trafficking protein particle complex 9 [Source:HGNC Symbol;Acc:HGNC:30832] |
| ENSG00000168268 | HGLibA_32823 | 108 | -0.073889917 | NT5DC2 | 5'-nucleotidase domain containing 2 [Source:HGNC Symbol;Acc:HGNC:25717] |
| ENSG00000169594 | HGLibA_04644 | 108 | -0.073889917 | BNC1 | basonuclin 1 [Source:HGNC Symbol;Acc:HGNC:1081] |
| ENSG00000169896 | HGLibA_23761 | 108 | -0.073889917 | ITGAM | integrin subunit alpha M [Source:HGNC Symbol;Acc:HGNC:6149] |
| ENSG00000170476 | HGLibA_30935 | 108 | -0.073889917 | MZB1 | marginal zone B and B1 cell specific protein [Source:HGNC Symbol;Acc:HGNC:30125] |
| ENSG00000170515 | HGLibA_34915 | 108 | -0.073889917 | PA2G4 | proliferation-associated 2G4 [Source:HGNC Symbol;Acc:HGNC:8550] |
| ENSG00000171033 | HGLibA_36819 | 108 | -0.073889917 | PKIA | cAMP-dependent protein kinase inhibitor alpha [Source:HGNC Symbol;Acc:HGNC:9017] |
| ENSG00000171431 | HGLibA_25446 | 108 | -0.073889917 | KRT20 | keratin 20 [Source:HGNC Symbol;Acc:HGNC:20412] |
| ENSG00000173480 | HGLibA_55949 | 108 | -0.073889917 | ZNF417 | zinc finger protein 417 [Source:HGNC Symbol;Acc:HGNC:20646] |
| ENSG00000173610 | HGLibA_52903 | 108 | -0.073889917 | UGT2A1 | UDP glucuronosyltransferase family 2 member A1 complex locus [Source:HGNC Symbol;Acc:HGNC:12542] |
| ENSG00000174498 | HGLibA_22785 | 108 | -0.073889917 | IGDCC3 | immunoglobulin superfamily DCC subclass member 3 [Source:HGNC Symbol;Acc:HGNC:9700] |
| ENSG00000174992 | HGLibA_55298 | 108 | -0.073889917 | ZG16 | zymogen granule protein 16 [Source:HGNC Symbol;Acc:HGNC:30961] |
| ENSG00000175395 | HGLibA_55664 | 108 | -0.073889917 | ZNF25 | zinc finger protein 25 [Source:HGNC Symbol;Acc:HGNC:13043] |
| ENSG00000176244 | HGLibA_00411 | 108 | -0.073889917 | ACBD7 | acyl-CoA binding domain containing 7 [Source:HGNC Symbol;Acc:HGNC:17715] |
| ENSG00000176248 | HGLibA_01879 | 108 | -0.073889917 | ANAPC2 | anaphase promoting complex subunit 2 [Source:HGNC Symbol;Acc:HGNC:19989] |
| ENSG00000176444 | HGLibA_10058 | 108 | -0.073889917 | CLK2 | CDC like kinase 2 [Source:HGNC Symbol;Acc:HGNC:2069] |
| ENSG00000178035 | HGLibA_23259 | 108 | -0.073889917 | IMPDH2 | inosine monophosphate dehydrogenase 2 [Source:HGNC Symbol;Acc:HGNC:6053] |
| ENSG00000179886 | HGLibA_49476 | 108 | -0.073889917 | TIGD5 | tigger transposable element derived 5 [Source:HGNC Symbol;Acc:HGNC:18336] |

| | | | | | |
|---|---|---|---|---|---|
| ENSG00000180376 | HGLibA_07835 | 108 | -0.073889917 | CCDC66 | coiled-coil domain containing 66 [Source:HGNC Symbol;Acc:HGNC:27709] |
| ENSG00000180423 | HGLibA_20854 | 108 | -0.073889917 | HARBI1 | harbinger transposase derived 1 [Source:HGNC Symbol;Acc:HGNC:26522] |
| ENSG00000180881 | HGLibA_07198 | 108 | -0.073889917 | CAPS2 | calcyphosine 2 [Source:HGNC Symbol;Acc:HGNC:16471] |
| ENSG00000183684 | HGLibA_01742 | 108 | -0.073889917 | ALYREF | Aly/REF export factor [Source:HGNC Symbol;Acc:HGNC:19071] |
| ENSG00000184619 | HGLibA_25389 | 108 | -0.073889917 | KRBA2 | KRAB-A domain containing 2 [Source:HGNC Symbol;Acc:HGNC:26989] |
| ENSG00000187260 | HGLibA_54201 | 108 | -0.073889917 | WDR86 | WD repeat domain 86 [Source:HGNC Symbol;Acc:HGNC:28020] |
| ENSG00000188624 | HGLibA_22845 | 108 | -0.073889917 | IGFL3 | IGF like family member 3 [Source:HGNC Symbol;Acc:HGNC:32930] |
| ENSG00000189253 | HGLibA_51580 | 108 | -0.073889917 | TRIM64B | tripartite motif containing 64B [Source:HGNC Symbol;Acc:HGNC:37147] |
| ENSG00000189326 | HGLibA_46410 | 108 | -0.073889917 | SPANXN4 | SPANX family member N4 [Source:HGNC Symbol;Acc:HGNC:33177] |
| ENSG00000196074 | HGLibA_47931 | 108 | -0.073889917 | SYCP2 | synaptonemal complex protein 2 [Source:HGNC Symbol;Acc:HGNC:11490] |
| ENSG00000197417 | HGLibA_44077 | 108 | -0.073889917 | SHPK | sedoheptulokinase [Source:HGNC Symbol;Acc:HGNC:1492] |
| ENSG00000197442 | HGLibA_28190 | 108 | -0.073889917 | MAP3K5 | mitogen-activated protein kinase kinase kinase 5 [Source:HGNC Symbol;Acc:HGNC:6857] |
| ENSG00000197956 | HGLibA_42562 | 108 | -0.073889917 | S100A6 | S100 calcium binding protein A6 [Source:HGNC Symbol;Acc:HGNC:10496] |
| ENSG00000198300 | HGLibA_36031 | 108 | -0.073889917 | PEG3 | paternally expressed 3 [Source:HGNC Symbol;Acc:HGNC:8826] |
| ENSG00000198522 | HGLibA_19824 | 108 | -0.073889917 | GPN1 | GPN-loop GTPase 1 [Source:HGNC Symbol;Acc:HGNC:17030] |
| ENSG00000204396 | HGLibA_53915 | 108 | -0.073889917 | VWA7 | von Willebrand factor A domain containing 7 [Source:HGNC Symbol;Acc:HGNC:13939] |
| ENSG00000204479 | HGLibA_38299 | 108 | -0.073889917 | PRAMEF17 | PRAME family member 17 [Source:HGNC Symbol;Acc:HGNC:29485] |
| ENSG00000205413 | HGLibA_42698 | 108 | -0.073889917 | SAMD9 | sterile alpha motif domain containing 9 [Source:HGNC Symbol;Acc:HGNC:1348] |
| ENSG00000206052 | HGLibA_13782 | 108 | -0.073889917 | DOK6 | docking protein 6 [Source:HGNC Symbol;Acc:HGNC:28301] |
| ENSG00000211460 | HGLibA_51880 | 108 | -0.073889917 | TSN | translin [Source:HGNC Symbol;Acc:HGNC:12379] |
| ENSG00000213024 | HGLibA_33063 | 108 | -0.073889917 | NUP62 | nucleoporin 62 [Source:HGNC Symbol;Acc:HGNC:8066] |
| ENSG00000213416 | HGLibA_25755 | 108 | -0.073889917 | KRTAP4-12 | keratin associated protein 4-12 [Source:HGNC Symbol;Acc:HGNC:16776] |
| ENSG00000215131 | HGLibA_05498 | 108 | -0.073889917 | C16orf90 | chromosome 16 open reading frame 90 [Source:HGNC Symbol;Acc:HGNC:34455] |
| ENSG00000218891 | HGLibA_56302 | 108 | -0.073889917 | ZNF579 | zinc finger protein 579 [Source:HGNC Symbol;Acc:HGNC:26646] |
| ENSG00000230937 | HGLibA_29299 | 108 | -0.073889917 | MIR205HG | MIR205 host gene [Source:HGNC Symbol;Acc:HGNC:43562] |
| ENSG00000236398 | HGLibA_48395 | 108 | -0.073889917 | TAS2R39 | taste 2 receptor member 39 [Source:HGNC Symbol;Acc:HGNC:18886] |
| ENSG00000251349 | HGLibA_30178 | 108 | -0.073889917 | MSANTD3-TMEFF1 | MSANTD3-TMEFF1 readthrough [Source:HGNC Symbol;Acc:HGNC:38838] |
| ENSG00000254737 | HGLibA_33493 | 108 | -0.073889917 | OR10G4 | olfactory receptor family 10 subfamily G member 4 [Source:HGNC Symbol;Acc:HGNC:14809] |
| ENSG00000277322 | HGLibA_19629 | 108 | -0.073889917 | GOLGA6L6 | golgin A6 family-like 6 [Source:HGNC Symbol;Acc:HGNC:37225] |
| ENSG00000280789 | HGLibA_35019 | 108 | -0.073889917 | PAGR1 | PAXIP1 associated glutamate rich protein 1 [Source:HGNC Symbol;Acc:HGNC:28707] |
| FAM103A1 | HGLibA_15984 | 108 | -0.073889917 | FAM103A1 | - |
| FAM134B | HGLibA_16126 | 108 | -0.073889917 | FAM134B | - |
| H2AFB3 | HGLibA_20752 | 108 | -0.073889917 | H2AFB3 | - |
| H2AFJ | HGLibA_20754 | 108 | -0.073889917 | H2AFJ | - |
| hsa-mir-1-1 | HGLibA_57141 | 108 | -0.073889917 | hsa-mir-1-1 | - |
| hsa-mir-1284 | HGLibA_57598 | 108 | -0.073889917 | hsa-mir-1284 | - |
| hsa-mir-138-1 | HGLibA_57811 | 108 | -0.073889917 | hsa-mir-138-1 | - |
| hsa-mir-141 | HGLibA_57828 | 108 | -0.073889917 | hsa-mir-141 | - |
| hsa-mir-1468 | HGLibA_57847 | 108 | -0.073889917 | hsa-mir-1468 | - |
| hsa-mir-147b | HGLibA_57876 | 108 | -0.073889917 | hsa-mir-147b | - |
| hsa-mir-23c | HGLibA_58338 | 108 | -0.073889917 | hsa-mir-23c | - |
| hsa-mir-26a-1 | HGLibA_58364 | 108 | -0.073889917 | hsa-mir-26a-1 | - |
| hsa-mir-26a-2 | HGLibA_58367 | 108 | -0.073889917 | hsa-mir-26a-2 | - |
| hsa-mir-3605 | HGLibA_59054 | 108 | -0.073889917 | hsa-mir-3605 | - |
| hsa-mir-4452 | HGLibA_60216 | 108 | -0.073889917 | hsa-mir-4452 | - |
| hsa-mir-4511 | HGLibA_60487 | 108 | -0.073889917 | hsa-mir-4511 | - |
| hsa-mir-4659a | HGLibA_60744 | 108 | -0.073889917 | hsa-mir-4659a | - |
| hsa-mir-4659b | HGLibA_60747 | 108 | -0.073889917 | hsa-mir-4659b | - |
| hsa-mir-513c | HGLibA_61581 | 108 | -0.073889917 | hsa-mir-513c | - |
| hsa-mir-519b | HGLibA_61718 | 108 | -0.073889917 | hsa-mir-519b | - |
| hsa-mir-548d-1 | HGLibA_61962 | 108 | -0.073889917 | hsa-mir-548d-1 | - |
| hsa-mir-659 | HGLibA_62971 | 108 | -0.073889917 | hsa-mir-659 | - |
| hsa-mir-6821 | HGLibA_63462 | 108 | -0.073889917 | hsa-mir-6821 | - |
| hsa-mir-7110 | HGLibA_63806 | 108 | -0.073889917 | hsa-mir-7110 | - |
| hsa-mir-7702 | HGLibA_63943 | 108 | -0.073889917 | hsa-mir-7702 | - |
| hsa-mir-802 | HGLibA_64044 | 108 | -0.073889917 | hsa-mir-802 | - |
| hsa-mir-92b | HGLibA_64300 | 108 | -0.073889917 | hsa-mir-92b | - |
| hsa-mir-944 | HGLibA_64361 | 108 | -0.073889917 | hsa-mir-944 | - |
| LOC100652824 | HGLibA_26873 | 108 | -0.073889917 | LOC100652824 | - |
| NUPR1L | HGLibA_33091 | 108 | -0.073889917 | NUPR1L | - |
| PRSS45 | HGLibA_38945 | 108 | -0.073889917 | PRSS45 | - |
| TCTEX1D1 | HGLibA_48862 | 108 | -0.073889917 | TCTEX1D1 | - |
| C8orf22 | HGLibA_06603 | 107 | -0.083840954 | C8orf22 | - |
| CCDC94 | HGLibA_07933 | 107 | -0.083840954 | CCDC94 | - |
| CTGF | HGLibA_11622 | 107 | -0.083840954 | CTGF | - |
| ENSG00000002016 | HGLibA_40097 | 107 | -0.083840954 | RAD52 | DNA repair protein [Source:HGNC Symbol;Acc:HGNC:9824]" |
| ENSG00000006659 | HGLibA_26354 | 107 | -0.083840954 | LGALS14 | galectin 14 [Source:HGNC Symbol;Acc:HGNC:30054] |
| ENSG00000013441 | HGLibA_10054 | 107 | -0.083840954 | CLK1 | CDC like kinase 1 [Source:HGNC Symbol;Acc:HGNC:2068] |
| ENSG00000015475 | HGLibA_04499 | 107 | -0.083840954 | BID | BH3 interacting domain death agonist [Source:HGNC Symbol;Acc:HGNC:1050] |
| ENSG00000038219 | HGLibA_04672 | 107 | -0.083840954 | BOD1L1 | biorientation of chromosomes in cell division 1 like 1 [Source:HGNC Symbol;Acc:HGNC:31792] |
| ENSG00000044115 | HGLibA_11637 | 107 | -0.083840954 | CTNNA1 | catenin alpha 1 [Source:HGNC Symbol;Acc:HGNC:2509] |
| ENSG00000044446 | HGLibA_36406 | 107 | -0.083840954 | PHKA2 | phosphorylase kinase regulatory subunit alpha 2 [Source:HGNC Symbol;Acc:HGNC:8926] |
| ENSG00000047621 | HGLibA_05235 | 107 | -0.083840954 | C12orf4 | chromosome 12 open reading frame 4 [Source:HGNC Symbol;Acc:HGNC:1184] |
| ENSG00000062725 | HGLibA_02564 | 107 | -0.083840954 | APPBP2 | amyloid beta precursor protein binding protein 2 [Source:HGNC Symbol;Acc:HGNC:622] |
| ENSG00000065911 | HGLibA_30381 | 107 | -0.083840954 | MTHFD2 | methenyltetrahydrofolate cyclohydrolase [Source:HGNC Symbol;Acc:HGNC:7434]" |
| ENSG00000066583 | HGLibA_23683 | 107 | -0.083840954 | ISOC1 | isochorismatase domain containing 1 [Source:HGNC Symbol;Acc:HGNC:24254] |
| ENSG00000071189 | HGLibA_46037 | 107 | -0.083840954 | SNX13 | sorting nexin 13 [Source:HGNC Symbol;Acc:HGNC:21335] |
| ENSG00000072786 | HGLibA_47449 | 107 | -0.083840954 | STK10 | serine/threonine kinase 10 [Source:HGNC Symbol;Acc:HGNC:11388] |
| ENSG00000077147 | HGLibA_49728 | 107 | -0.083840954 | TM9SF3 | transmembrane 9 superfamily member 3 [Source:HGNC Symbol;Acc:HGNC:21529] |
| ENSG00000078900 | HGLibA_51097 | 107 | -0.083840954 | TP73 | tumor protein p73 [Source:HGNC Symbol;Acc:HGNC:12003] |
| ENSG00000094914 | HGLibA_00022 | 107 | -0.083840954 | AAAS | aladin WD repeat nucleoporin [Source:HGNC Symbol;Acc:HGNC:13666] |
| ENSG00000099974 | HGLibA_12646 | 107 | -0.083840954 | DDTL | D-dopachrome tautomerase like [Source:HGNC Symbol;Acc:HGNC:33446] |
| ENSG00000100311 | HGLibA_35844 | 107 | -0.083840954 | PDGFB | platelet derived growth factor subunit B [Source:HGNC Symbol;Acc:HGNC:8800] |
| ENSG00000100425 | HGLibA_04771 | 107 | -0.083840954 | BRD1 | bromodomain containing 1 [Source:HGNC Symbol;Acc:HGNC:1102] |
| ENSG00000100614 | HGLibA_37962 | 107 | -0.083840954 | PPM1A | Mg2+/Mn2+ dependent 1A [Source:HGNC Symbol;Acc:HGNC:9275]" |
| ENSG00000100644 | HGLibA_21297 | 107 | -0.083840954 | HIF1A | hypoxia inducible factor 1 subunit alpha [Source:HGNC Symbol;Acc:HGNC:4910] |
| ENSG00000102226 | HGLibA_53198 | 107 | -0.083840954 | USP11 | ubiquitin specific peptidase 11 [Source:HGNC Symbol;Acc:HGNC:12609] |
| ENSG00000102349 | HGLibA_25083 | 107 | -0.083840954 | KLF8 | Kruppel like factor 8 [Source:HGNC Symbol;Acc:HGNC:6351] |
| ENSG00000102802 | HGLibA_28822 | 107 | -0.083840954 | MEDAG | mesenteric estrogen dependent adipogenesis [Source:HGNC Symbol;Acc:HGNC:25926] |
| ENSG00000102962 | HGLibA_07996 | 107 | -0.083840954 | CCL22 | C-C motif chemokine ligand 22 [Source:HGNC Symbol;Acc:HGNC:10621] |
| ENSG00000102984 | HGLibA_56817 | 107 | -0.083840954 | ZNF821 | zinc finger protein 821 [Source:HGNC Symbol;Acc:HGNC:28043] |
| ENSG00000103187 | HGLibA_10800 | 107 | -0.083840954 | COTL1 | coactosin like F-actin binding protein 1 [Source:HGNC Symbol;Acc:HGNC:18304] |
| ENSG00000103995 | HGLibA_09109 | 107 | -0.083840954 | CEP152 | centrosomal protein 152 [Source:HGNC Symbol;Acc:HGNC:29298] |
| ENSG00000104907 | HGLibA_51657 | 107 | -0.083840954 | TRMT1 | tRNA methyltransferase 1 [Source:HGNC Symbol;Acc:HGNC:25980] |
| ENSG00000105254 | HGLibA_48580 | 107 | -0.083840954 | TBCB | tubulin folding cofactor B [Source:HGNC Symbol;Acc:HGNC:1989] |
| ENSG00000105989 | HGLibA_54358 | 107 | -0.083840954 | WNT2 | Wnt family member 2 [Source:HGNC Symbol;Acc:HGNC:12780] |
| ENSG00000106018 | HGLibA_53671 | 107 | -0.083840954 | VIPR2 | vasoactive intestinal peptide receptor 2 [Source:HGNC Symbol;Acc:HGNC:12695] |
| ENSG00000106328 | HGLibA_18073 | 107 | -0.083840954 | FSCN3 | fascin actin-bundling protein 3 [Source:HGNC Symbol;Acc:HGNC:3961] |
| ENSG00000109321 | HGLibA_02639 | 107 | -0.083840954 | AREG | amphiregulin [Source:HGNC Symbol;Acc:HGNC:651] |
| ENSG00000110756 | HGLibA_22056 | 107 | -0.083840954 | HPS5 | biogenesis of lysosomal organelles complex 2 subunit 2 [Source:HGNC Symbol;Acc:HGNC:17022]" |
| ENSG00000111144 | HGLibA_27578 | 107 | -0.083840954 | LTA4H | leukotriene A4 hydrolase [Source:HGNC Symbol;Acc:HGNC:6710] |
| ENSG00000111481 | HGLibA_10736 | 107 | -0.083840954 | COPZ1 | coatomer protein complex subunit zeta 1 [Source:HGNC Symbol;Acc:HGNC:2243] |
| ENSG00000111981 | HGLibA_52961 | 107 | -0.083840954 | ULBP1 | UL16 binding protein 1 [Source:HGNC Symbol;Acc:HGNC:14893] |
| ENSG00000112697 | HGLibA_50360 | 107 | -0.083840954 | TMEM30A | transmembrane protein 30A [Source:HGNC Symbol;Acc:HGNC:16667] |
| ENSG00000113013 | HGLibA_22298 | 107 | -0.083840954 | HSPA9 | heat shock protein family A (Hsp70) member 9 [Source:HGNC Symbol;Acc:HGNC:5244] |
| ENSG00000113389 | HGLibA_32513 | 107 | -0.083840954 | NPR3 | natriuretic peptide receptor 3 [Source:HGNC Symbol;Acc:HGNC:7945] |
| ENSG00000113721 | HGLibA_35857 | 107 | -0.083840954 | PDGFRB | platelet derived growth factor receptor beta [Source:HGNC Symbol;Acc:HGNC:8804] |
| ENSG00000114354 | HGLibA_49178 | 107 | -0.083840954 | TFG | TRK-fused gene [Source:HGNC Symbol;Acc:HGNC:11758] |
| ENSG00000115165 | HGLibA_12248 | 107 | -0.083840954 | CYTIP | cytohesin 1 interacting protein [Source:HGNC Symbol;Acc:HGNC:9506] |
| ENSG00000115207 | HGLibA_20582 | 107 | -0.083840954 | GTF3C2 | general transcription factor IIIC subunit 2 [Source:HGNC Symbol;Acc:HGNC:4665] |
| ENSG00000115474 | HGLibA_24258 | 107 | -0.083840954 | KCNJ13 | potassium voltage-gated channel subfamily J member 13 [Source:HGNC Symbol;Acc:HGNC:6259] |
| ENSG00000116039 | HGLibA_03734 | 107 | -0.083840954 | ATP6V1B1 | ATPase H+ transporting V1 subunit B1 [Source:HGNC Symbol;Acc:HGNC:853] |
| ENSG00000116044 | HGLibA_31795 | 107 | -0.083840954 | NFE2L2 | erythroid 2 like 2 [Source:HGNC Symbol;Acc:HGNC:7782]" |
| ENSG00000116138 | HGLibA_13582 | 107 | -0.083840954 | DNAJC16 | DnaJ heat shock protein family (Hsp40) member C16 [Source:HGNC Symbol;Acc:HGNC:29157] |
| ENSG00000117408 | HGLibA_23463 | 107 | -0.083840954 | IPO13 | importin 13 [Source:HGNC Symbol;Acc:HGNC:16853] |
| ENSG00000117862 | HGLibA_52409 | 107 | -0.083840954 | TXNDC12 | thioredoxin domain containing 12 [Source:HGNC Symbol;Acc:HGNC:24626] |
| ENSG00000119509 | HGLibA_23443 | 107 | -0.083840954 | INVS | inversin [Source:HGNC Symbol;Acc:HGNC:17870] |
| ENSG00000119632 | HGLibA_22603 | 107 | -0.083840954 | IFI27L2 | interferon alpha inducible protein 27 like 2 [Source:HGNC Symbol;Acc:HGNC:19753] |
| ENSG00000120526 | HGLibA_32922 | 107 | -0.083840954 | NUDCD1 | NudC domain containing 1 [Source:HGNC Symbol;Acc:HGNC:24306] |
| ENSG00000121542 | HGLibA_43215 | 107 | -0.083840954 | SEC22A | vesicle trafficking protein [Source:HGNC Symbol;Acc:HGNC:20260]" |
| ENSG00000121634 | HGLibA_19150 | 107 | -0.083840954 | GJA8 | gap junction protein alpha 8 [Source:HGNC Symbol;Acc:HGNC:4281] |
| ENSG00000122547 | HGLibA_14518 | 107 | -0.083840954 | EEPD1 | endonuclease/exonuclease/phosphatase family domain containing 1 [Source:HGNC Symbol;Acc:HGNC:22223] |
| ENSG00000126790 | HGLibA_25894 | 107 | -0.083840954 | L3HYPDH | trans-L-3-hydroxyproline dehydratase [Source:HGNC Symbol;Acc:HGNC:20488] |
| ENSG00000128016 | HGLibA_55204 | 107 | -0.083840954 | ZFP36 | ZFP36 ring finger protein [Source:HGNC Symbol;Acc:HGNC:12862] |
| ENSG00000128564 | HGLibA_53631 | 107 | -0.083840954 | VGF | VGF nerve growth factor inducible [Source:HGNC Symbol;Acc:HGNC:12684] |
| ENSG00000128973 | HGLibA_10080 | 107 | -0.083840954 | CLN6 | transmembrane ER protein [Source:HGNC Symbol;Acc:HGNC:2077]" |
| ENSG00000129515 | HGLibA_46101 | 107 | -0.083840954 | SNX6 | sorting nexin 6 [Source:HGNC Symbol;Acc:HGNC:14970] |
| ENSG00000129824 | HGLibA_42145 | 107 | -0.083840954 | RPS4Y1 | ribosomal protein S4 Y-linked 1 [Source:HGNC Symbol;Acc:HGNC:10425] |
| ENSG00000130300 | HGLibA_37249 | 107 | -0.083840954 | PLVAP | plasmalemma vesicle associated protein [Source:HGNC Symbol;Acc:HGNC:13635] |
| ENSG00000130338 | HGLibA_52338 | 107 | -0.083840954 | TULP4 | tubby like protein 4 [Source:HGNC Symbol;Acc:HGNC:15530] |
| ENSG00000131979 | HGLibA_18782 | 107 | -0.083840954 | GCH1 | GTP cyclohydrolase 1 [Source:HGNC Symbol;Acc:HGNC:4193] |
| ENSG00000134313 | HGLibA_24847 | 107 | -0.083840954 | KIDINS220 | kinase D interacting substrate 220 [Source:HGNC Symbol;Acc:HGNC:29508] |
| ENSG00000135175 | HGLibA_33260 | 107 | -0.083840954 | OCM2 | oncomodulin 2 [Source:HGNC Symbol;Acc:HGNC:34396] |
| ENSG00000135185 | HGLibA_50293 | 107 | -0.083840954 | TMEM243 | transmembrane protein 243 [Source:HGNC Symbol;Acc:HGNC:21707] |
| ENSG00000135723 | HGLibA_17491 | 107 | -0.083840954 | FHOD1 | formin homology 2 domain containing 1 [Source:HGNC Symbol;Acc:HGNC:17905] |

| | | | | | |
|---|---|---|---|---|---|
| ENSG00000136449 | HGLibA_30669 | 107 | -0.083840954 | MYCBPAP | MYCBP associated protein [Source:HGNC Symbol;Acc:HGNC:19677] |
| ENSG00000137404 | HGLibA_32706 | 107 | -0.083840954 | NRM | nurim [Source:HGNC Symbol;Acc:HGNC:8003] |
| ENSG00000137877 | HGLibA_46890 | 107 | -0.083840954 | SPTBN5 | non-erythrocytic 5 [Source:HGNC Symbol;Acc:HGNC:15680]" |
| ENSG00000139737 | HGLibA_44305 | 107 | -0.083840954 | SLAIN1 | SLAIN motif family member 1 [Source:HGNC Symbol;Acc:HGNC:26387] |
| ENSG00000140497 | HGLibA_42838 | 107 | -0.083840954 | SCAMP2 | secretory carrier membrane protein 2 [Source:HGNC Symbol;Acc:HGNC:10564] |
| ENSG00000140943 | HGLibA_28536 | 107 | -0.083840954 | MBTPS1 | site 1 [Source:HGNC Symbol;Acc:HGNC:15456]" |
| ENSG00000142556 | HGLibA_56388 | 107 | -0.083840954 | ZNF614 | zinc finger protein 614 [Source:HGNC Symbol;Acc:HGNC:24722] |
| ENSG00000142765 | HGLibA_48097 | 107 | -0.083840954 | SYTL1 | synaptotagmin like 1 [Source:HGNC Symbol;Acc:HGNC:15584] |
| ENSG00000143621 | HGLibA_23215 | 107 | -0.083840954 | ILF2 | interleukin enhancer binding factor 2 [Source:HGNC Symbol;Acc:HGNC:6037] |
| ENSG00000144229 | HGLibA_49408 | 107 | -0.083840954 | THSD7B | thrombospondin type 1 domain containing 7B [Source:HGNC Symbol;Acc:HGNC:29348] |
| ENSG00000144810 | HGLibA_10614 | 107 | -0.083840954 | COL8A1 | collagen type VIII alpha 1 chain [Source:HGNC Symbol;Acc:HGNC:2215] |
| ENSG00000145022 | HGLibA_48853 | 107 | -0.083840954 | TCTA | T cell leukemia translocation altered [Source:HGNC Symbol;Acc:HGNC:11692] |
| ENSG00000145779 | HGLibA_50719 | 107 | -0.083840954 | TNFAIP8 | TNF alpha induced protein 8 [Source:HGNC Symbol;Acc:HGNC:17260] |
| ENSG00000149782 | HGLibA_36984 | 107 | -0.083840954 | PLCB3 | phospholipase C beta 3 [Source:HGNC Symbol;Acc:HGNC:9056] |
| ENSG00000150636 | HGLibA_07512 | 107 | -0.083840954 | CCDC102B | coiled-coil domain containing 102B [Source:HGNC Symbol;Acc:HGNC:26295] |
| ENSG00000152086 | HGLibA_52259 | 107 | -0.083840954 | TUBA3E | tubulin alpha 3e [Source:HGNC Symbol;Acc:HGNC:20765] |
| ENSG00000153391 | HGLibA_23325 | 107 | -0.083840954 | INO80C | INO80 complex subunit C [Source:HGNC Symbol;Acc:HGNC:26994] |
| ENSG00000155329 | HGLibA_55002 | 107 | -0.083840954 | ZCCHC10 | zinc finger CCHC-type containing 10 [Source:HGNC Symbol;Acc:HGNC:25954] |
| ENSG00000157766 | HGLibA_00381 | 107 | -0.083840954 | ACAN | aggrecan [Source:HGNC Symbol;Acc:HGNC:319] |
| ENSG00000158258 | HGLibA_10137 | 107 | -0.083840954 | CLSTN2 | calsyntenin 2 [Source:HGNC Symbol;Acc:HGNC:17448] |
| ENSG00000159788 | HGLibA_41057 | 107 | -0.083840954 | RGS12 | regulator of G protein signaling 12 [Source:HGNC Symbol;Acc:HGNC:9994] |
| ENSG00000160298 | HGLibA_06040 | 107 | -0.083840954 | C21orf58 | chromosome 21 open reading frame 58 [Source:HGNC Symbol;Acc:HGNC:1300] |
| ENSG00000162989 | HGLibA_24275 | 107 | -0.083840954 | KCNJ3 | potassium voltage-gated channel subfamily J member 3 [Source:HGNC Symbol;Acc:HGNC:6264] |
| ENSG00000163421 | HGLibA_38669 | 107 | -0.083840954 | PROK2 | prokineticin 2 [Source:HGNC Symbol;Acc:HGNC:18455] |
| ENSG00000163623 | HGLibA_32054 | 107 | -0.083840954 | NKX6-1 | NK6 homeobox 1 [Source:HGNC Symbol;Acc:HGNC:7839] |
| ENSG00000163704 | HGLibA_38888 | 107 | -0.083840954 | PRRT3 | proline rich transmembrane protein 3 [Source:HGNC Symbol;Acc:HGNC:26591] |
| ENSG00000164086 | HGLibA_14189 | 107 | -0.083840954 | DUSP7 | dual specificity phosphatase 7 [Source:HGNC Symbol;Acc:HGNC:3073] |
| ENSG00000166971 | HGLibA_01517 | 107 | -0.083840954 | AKTIP | AKT interacting protein [Source:HGNC Symbol;Acc:HGNC:16710] |
| ENSG00000167360 | HGLibA_34127 | 107 | -0.083840954 | OR51Q1 | olfactory receptor family 51 subfamily Q member 1 (gene/pseudogene) [Source:HGNC Symbol;Acc:HGNC:14851] |
| ENSG00000167555 | HGLibA_56171 | 107 | -0.083840954 | ZNF528 | zinc finger protein 528 [Source:HGNC Symbol;Acc:HGNC:29384] |
| ENSG00000167580 | HGLibA_02589 | 107 | -0.083840954 | AQP2 | aquaporin 2 [Source:HGNC Symbol;Acc:HGNC:634] |
| ENSG00000167822 | HGLibA_34541 | 107 | -0.083840954 | OR8J3 | olfactory receptor family 8 subfamily J member 3 [Source:HGNC Symbol;Acc:HGNC:15312] |
| ENSG00000168092 | HGLibA_34993 | 107 | -0.083840954 | PAFAH1B2 | platelet activating factor acetylhydrolase 1b catalytic subunit 2 [Source:HGNC Symbol;Acc:HGNC:8575] |
| ENSG00000168209 | HGLibA_12619 | 107 | -0.083840954 | DDIT4 | DNA damage inducible transcript 4 [Source:HGNC Symbol;Acc:HGNC:24944] |
| ENSG00000168594 | HGLibA_00767 | 107 | -0.083840954 | ADAM29 | ADAM metallopeptidase domain 29 [Source:HGNC Symbol;Acc:HGNC:207] |
| ENSG00000168890 | HGLibA_49990 | 107 | -0.083840954 | TMEM150A | transmembrane protein 150A [Source:HGNC Symbol;Acc:HGNC:24677] |
| ENSG00000170364 | HGLibA_43673 | 107 | -0.083840954 | SETMAR | SET domain and mariner transposase fusion gene [Source:HGNC Symbol;Acc:HGNC:10762] |
| ENSG00000170373 | HGLibA_11445 | 107 | -0.083840954 | CST1 | cystatin SN [Source:HGNC Symbol;Acc:HGNC:2473] |
| ENSG00000170571 | HGLibA_14998 | 107 | -0.083840954 | EMB | embigin [Source:HGNC Symbol;Acc:HGNC:30465] |
| ENSG00000171051 | HGLibA_17964 | 107 | -0.083840954 | FPR1 | formyl peptide receptor 1 [Source:HGNC Symbol;Acc:HGNC:3826] |
| ENSG00000171532 | HGLibA_31738 | 107 | -0.083840954 | NEUROD2 | neuronal differentiation 2 [Source:HGNC Symbol;Acc:HGNC:7763] |
| ENSG00000171970 | HGLibA_56272 | 107 | -0.083840954 | ZNF57 | zinc finger protein 57 [Source:HGNC Symbol;Acc:HGNC:13125] |
| ENSG00000172468 | HGLibA_22242 | 107 | -0.083840954 | HSFY1 | heat shock transcription factor Y-linked 1 [Source:HGNC Symbol;Acc:HGNC:18568] |
| ENSG00000172771 | HGLibA_14525 | 107 | -0.083840954 | EFCAB12 | EF-hand calcium binding domain 12 [Source:HGNC Symbol;Acc:HGNC:28061] |
| ENSG00000173208 | HGLibA_00193 | 107 | -0.083840954 | ABCD2 | ATP binding cassette subfamily D member 2 [Source:HGNC Symbol;Acc:HGNC:66] |
| ENSG00000173728 | HGLibA_05730 | 107 | -0.083840954 | C1orf100 | chromosome 1 open reading frame 100 [Source:HGNC Symbol;Acc:HGNC:30435] |
| ENSG00000175544 | HGLibA_06853 | 107 | -0.083840954 | CABP4 | calcium binding protein 4 [Source:HGNC Symbol;Acc:HGNC:1386] |
| ENSG00000175595 | HGLibA_15440 | 107 | -0.083840954 | ERCC4 | endonuclease catalytic subunit [Source:HGNC Symbol;Acc:HGNC:3436]" |
| ENSG00000177182 | HGLibA_10168 | 107 | -0.083840954 | CLVS1 | clavesin 1 [Source:HGNC Symbol;Acc:HGNC:23139] |
| ENSG00000177646 | HGLibA_00363 | 107 | -0.083840954 | ACAD9 | acyl-CoA dehydrogenase family member 9 [Source:HGNC Symbol;Acc:HGNC:21497] |
| ENSG00000177688 | HGLibA_47784 | 107 | -0.083840954 | SUMO4 | small ubiquitin-like modifier 4 [Source:HGNC Symbol;Acc:HGNC:21181] |
| ENSG00000179387 | HGLibA_14942 | 107 | -0.083840954 | ELMOD2 | ELMO domain containing 2 [Source:HGNC Symbol;Acc:HGNC:28111] |
| ENSG00000181004 | HGLibA_04212 | 107 | -0.083840954 | BBS12 | Bardet-Biedl syndrome 12 [Source:HGNC Symbol;Acc:HGNC:26648] |
| ENSG00000182330 | HGLibA_38329 | 107 | -0.083840954 | PRAMEF8 | PRAME family member 8 [Source:HGNC Symbol;Acc:HGNC:24074] |
| ENSG00000183347 | HGLibA_18744 | 107 | -0.083840954 | GBP6 | guanylate binding protein family member 6 [Source:HGNC Symbol;Acc:HGNC:25395] |
| ENSG00000183978 | HGLibA_10447 | 107 | -0.083840954 | COA3 | cytochrome c oxidase assembly factor 3 [Source:HGNC Symbol;Acc:HGNC:24990] |
| ENSG00000184162 | HGLibA_32600 | 107 | -0.083840954 | NR2C2AP | nuclear receptor 2C2 associated protein [Source:HGNC Symbol;Acc:HGNC:30763] |
| ENSG00000188322 | HGLibA_42801 | 107 | -0.083840954 | SBK1 | SH3 domain binding kinase 1 [Source:HGNC Symbol;Acc:HGNC:17699] |
| ENSG00000188338 | HGLibA_45067 | 107 | -0.083840954 | SLC38A3 | solute carrier family 38 member 3 [Source:HGNC Symbol;Acc:HGNC:18044] |
| ENSG00000188501 | HGLibA_26207 | 107 | -0.083840954 | LCTL | lactase like [Source:HGNC Symbol;Acc:HGNC:15583] |
| ENSG00000196132 | HGLibA_30925 | 107 | -0.083840954 | MYT1 | myelin transcription factor 1 [Source:HGNC Symbol;Acc:HGNC:7622] |
| ENSG00000196353 | HGLibA_10961 | 107 | -0.083840954 | CPNE4 | copine 4 [Source:HGNC Symbol;Acc:HGNC:2317] |
| ENSG00000196616 | HGLibA_00954 | 107 | -0.083840954 | ADH1B | beta polypeptide [Source:HGNC Symbol;Acc:HGNC:250]" |
| ENSG00000196715 | HGLibA_53680 | 107 | -0.083840954 | VKORC1L1 | vitamin K epoxide reductase complex subunit 1 like 1 [Source:HGNC Symbol;Acc:HGNC:21492] |
| ENSG00000197714 | HGLibA_56034 | 107 | -0.083840954 | ZNF460 | zinc finger protein 460 [Source:HGNC Symbol;Acc:HGNC:21628] |
| ENSG00000198298 | HGLibA_56079 | 107 | -0.083840954 | ZNF485 | zinc finger protein 485 [Source:HGNC Symbol;Acc:HGNC:23440] |
| ENSG00000198483 | HGLibA_02084 | 107 | -0.083840954 | ANKRD35 | ankyrin repeat domain 35 [Source:HGNC Symbol;Acc:HGNC:26323] |
| ENSG00000198792 | HGLibA_50114 | 107 | -0.083840954 | TMEM184B | transmembrane protein 184B [Source:HGNC Symbol;Acc:HGNC:1310] |
| ENSG00000203783 | HGLibA_38855 | 107 | -0.083840954 | PRR9 | proline rich 9 [Source:HGNC Symbol;Acc:HGNC:32057] |
| ENSG00000205403 | HGLibA_09277 | 107 | -0.083840954 | CFI | complement factor I [Source:HGNC Symbol;Acc:HGNC:5394] |
| ENSG00000205629 | HGLibA_26161 | 107 | -0.083840954 | LCMT1 | leucine carboxyl methyltransferase 1 [Source:HGNC Symbol;Acc:HGNC:17557] |
| ENSG00000206052 | HGLibA_13783 | 107 | -0.083840954 | DOK6 | docking protein 6 [Source:HGNC Symbol;Acc:HGNC:28301] |
| ENSG00000212657 | HGLibA_25658 | 107 | -0.083840954 | KRTAP16-1 | keratin associated protein 16-1 [Source:HGNC Symbol;Acc:HGNC:18916] |
| ENSG00000213199 | HGLibA_03305 | 107 | -0.083840954 | ASIC3 | acid sensing ion channel subunit 3 [Source:HGNC Symbol;Acc:HGNC:101] |
| ENSG00000213463 | HGLibA_48008 | 107 | -0.083840954 | SYNJ2BP | synaptojanin 2 binding protein [Source:HGNC Symbol;Acc:HGNC:18955] |
| ENSG00000214300 | HGLibA_46590 | 107 | -0.083840954 | SPDYE3 | speedy/RINGO cell cycle regulator family member E3 [Source:HGNC Symbol;Acc:HGNC:35462] |
| ENSG00000223501 | HGLibA_53807 | 107 | -0.083840954 | VPS52 | GARP complex subunit [Source:HGNC Symbol;Acc:HGNC:10518]" |
| ENSG00000240053 | HGLibA_27658 | 107 | -0.083840954 | LY6G5B | lymphocyte antigen 6 family member G5B [Source:HGNC Symbol;Acc:HGNC:13931] |
| ENSG00000243543 | HGLibA_54270 | 107 | -0.083840954 | WFDC6 | WAP four-disulfide core domain 6 [Source:HGNC Symbol;Acc:HGNC:16164] |
| ENSG00000250067 | HGLibA_54690 | 107 | -0.083840954 | YJEFN3 | YjeF N-terminal domain containing 3 [Source:HGNC Symbol;Acc:HGNC:24785] |
| ENSG00000254598 | HGLibA_11408 | 107 | -0.083840954 | CSNK2A3 | casein kinase 2 alpha 3 [Source:HGNC Symbol;Acc:HGNC:2458] |
| ENSG00000257315 | HGLibA_54801 | 107 | -0.083840954 | ZBED6 | zinc finger BED-type containing 6 [Source:HGNC Symbol;Acc:HGNC:33273] |
| ENSG00000257446 | HGLibA_56887 | 107 | -0.083840954 | ZNF878 | zinc finger protein 878 [Source:HGNC Symbol;Acc:HGNC:37246] |
| ENSG00000275722 | HGLibA_27794 | 107 | -0.083840954 | LYZL6 | lysozyme like 6 [Source:HGNC Symbol;Acc:HGNC:29614] |
| ENSG00000280314 | HGLibA_34548 | 107 | -0.083840954 | OR8K3 | olfactory receptor family 8 subfamily K member 3 (gene/pseudogene) [Source:HGNC Symbol;Acc:HGNC:15313] |
| FAM57A | HGLibA_16645 | 107 | -0.083840954 | FAM57A | - |
| hsa-mir-106a | HGLibA_57184 | 107 | -0.083840954 | hsa-mir-106a | - |
| hsa-mir-124-2 | HGLibA_57352 | 107 | -0.083840954 | hsa-mir-124-2 | - |
| hsa-mir-1282 | HGLibA_57585 | 107 | -0.083840954 | hsa-mir-1282 | - |
| hsa-mir-1306 | HGLibA_57741 | 107 | -0.083840954 | hsa-mir-1306 | - |
| hsa-mir-196a-1 | HGLibA_58092 | 107 | -0.083840954 | hsa-mir-196a-1 | - |
| hsa-mir-197 | HGLibA_58105 | 107 | -0.083840954 | hsa-mir-197 | - |
| hsa-mir-2110 | HGLibA_58222 | 107 | -0.083840954 | hsa-mir-2110 | - |
| hsa-mir-302b | HGLibA_58441 | 107 | -0.083840954 | hsa-mir-302b | - |
| hsa-mir-3150b | HGLibA_58661 | 107 | -0.083840954 | hsa-mir-3150b | - |
| hsa-mir-330 | HGLibA_58987 | 107 | -0.083840954 | hsa-mir-330 | - |
| hsa-mir-4295 | HGLibA_59890 | 107 | -0.083840954 | hsa-mir-4295 | - |
| hsa-mir-432 | HGLibA_60001 | 107 | -0.083840954 | hsa-mir-432 | - |
| hsa-mir-4320 | HGLibA_60004 | 107 | -0.083840954 | hsa-mir-4320 | - |
| hsa-mir-4682 | HGLibA_60840 | 107 | -0.083840954 | hsa-mir-4682 | - |
| hsa-mir-4722 | HGLibA_60987 | 107 | -0.083840954 | hsa-mir-4722 | - |
| hsa-mir-4793 | HGLibA_61280 | 107 | -0.083840954 | hsa-mir-4793 | - |
| hsa-mir-514a-3 | HGLibA_61596 | 107 | -0.083840954 | hsa-mir-514a-3 | - |
| hsa-mir-548a-3 | HGLibA_61844 | 107 | -0.083840954 | hsa-mir-548a-3 | - |
| hsa-mir-632 | HGLibA_62768 | 107 | -0.083840954 | hsa-mir-632 | - |
| hsa-mir-6516 | HGLibA_62937 | 107 | -0.083840954 | hsa-mir-6516 | - |
| hsa-mir-658 | HGLibA_62965 | 107 | -0.083840954 | hsa-mir-658 | - |
| hsa-mir-6806 | HGLibA_63401 | 107 | -0.083840954 | hsa-mir-6806 | - |
| hsa-mir-6814 | HGLibA_63433 | 107 | -0.083840954 | hsa-mir-6814 | - |
| hsa-mir-6866 | HGLibA_63648 | 107 | -0.083840954 | hsa-mir-6866 | - |
| hsa-mir-7843 | HGLibA_63960 | 107 | -0.083840954 | hsa-mir-7843 | - |
| hsa-mir-8063 | HGLibA_64092 | 107 | -0.083840954 | hsa-mir-8063 | - |
| hsa-mir-876 | HGLibA_64214 | 107 | -0.083840954 | hsa-mir-876 | - |
| hsa-mir-938 | HGLibA_64327 | 107 | -0.083840954 | hsa-mir-938 | - |
| KIAA1324 | HGLibA_24736 | 107 | -0.083840954 | KIAA1324 | - |
| KIAA1737 | HGLibA_24815 | 107 | -0.083840954 | KIAA1737 | - |
| NARS | HGLibA_31154 | 107 | -0.083840954 | NARS | - |
| PPP2R3B_X | HGLibA_38158 | 107 | -0.083840954 | PPP2R3B_X | - |
| PRR21 | HGLibA_38816 | 107 | -0.083840954 | PRR21 | - |
| VIMP | HGLibA_53660 | 107 | -0.083840954 | VIMP | - |
| ZCCHC16 | HGLibA_55017 | 107 | -0.083840954 | ZCCHC16 | - |
| CRLF2_X | HGLibA_11189 | 106 | -0.093791992 | CRLF2_X | - |
| DFNB31 | HGLibA_13028 | 106 | -0.093791992 | DFNB31 | - |
| ENSG00000000003 | HGLibA_51944 | 106 | -0.093791992 | TSPAN6 | tetraspanin 6 [Source:HGNC Symbol;Acc:HGNC:11858] |
| ENSG00000008056 | HGLibA_47955 | 106 | -0.093791992 | SYN1 | synapsin I [Source:HGNC Symbol;Acc:HGNC:11494] |
| ENSG00000010438 | HGLibA_38922 | 106 | -0.093791992 | PRSS3 | serine protease 3 [Source:HGNC Symbol;Acc:HGNC:9486] |
| ENSG00000065717 | HGLibA_49611 | 106 | -0.093791992 | TLE2 | transducin like enhancer of split 2 [Source:HGNC Symbol;Acc:HGNC:11838] |
| ENSG00000066279 | HGLibA_03359 | 106 | -0.093791992 | ASPM | abnormal spindle microtubule assembly [Source:HGNC Symbol;Acc:HGNC:19048] |
| ENSG00000067064 | HGLibA_22554 | 106 | -0.093791992 | IDI1 | isopentenyl-diphosphate delta isomerase 1 [Source:HGNC Symbol;Acc:HGNC:5387] |
| ENSG00000071553 | HGLibA_03695 | 106 | -0.093791992 | ATP6AP1 | ATPase H+ transporting accessory protein 1 [Source:HGNC Symbol;Acc:HGNC:868] |
| ENSG00000071655 | HGLibA_28470 | 106 | -0.093791992 | MBD3 | methyl-CpG binding domain protein 3 [Source:HGNC Symbol;Acc:HGNC:6918] |
| ENSG00000073578 | HGLibA_43134 | 106 | -0.093791992 | SDHA | succinate dehydrogenase complex flavoprotein subunit A [Source:HGNC Symbol;Acc:HGNC:10680] |
| ENSG00000081692 | HGLibA_23956 | 106 | -0.093791992 | JMJD4 | jumonji domain containing 4 [Source:HGNC Symbol;Acc:HGNC:25724] |
| ENSG00000082397 | HGLibA_15248 | 106 | -0.093791992 | EPB41L3 | erythrocyte membrane protein band 4.1 like 3 [Source:HGNC Symbol;Acc:HGNC:3380] |
| ENSG00000093217 | HGLibA_54616 | 106 | -0.093791992 | XYLB | xylulokinase [Source:HGNC Symbol;Acc:HGNC:12839] |

| | | | | | |
|---|---|---|---|---|---|
| ENSG00000100784 | HGLibA_42169 | 106 | -0.093791992 | RPS6KA5 | ribosomal protein S6 kinase A5 [Source:HGNC Symbol;Acc:HGNC:10434] |
| ENSG00000101216 | HGLibA_19399 | 106 | -0.093791992 | GMEB2 | glucocorticoid modulatory element binding protein 2 [Source:HGNC Symbol;Acc:HGNC:4371] |
| ENSG00000101251 | HGLibA_43280 | 106 | -0.093791992 | SEL1L2 | ERAD E3 ligase adaptor subunit [Source:HGNC Symbol;Acc:HGNC:15897]" |
| ENSG00000103375 | HGLibA_02609 | 106 | -0.093791992 | AQP8 | aquaporin 8 [Source:HGNC Symbol;Acc:HGNC:642] |
| ENSG00000104921 | HGLibA_17173 | 106 | -0.093791992 | FCER2 | Fc fragment of IgE receptor II [Source:HGNC Symbol;Acc:HGNC:3612] |
| ENSG00000105220 | HGLibA_19806 | 106 | -0.093791992 | GPI | glucose-6-phosphate isomerase [Source:HGNC Symbol;Acc:HGNC:4458] |
| ENSG00000105538 | HGLibA_40355 | 106 | -0.093791992 | RASIP1 | Ras interacting protein 1 [Source:HGNC Symbol;Acc:HGNC:24716] |
| ENSG00000106078 | HGLibA_10460 | 106 | -0.093791992 | COBL | cordon-bleu WH2 repeat protein [Source:HGNC Symbol;Acc:HGNC:22199] |
| ENSG00000106086 | HGLibA_37082 | 106 | -0.093791992 | PLEKHA8 | pleckstrin homology domain containing A8 [Source:HGNC Symbol;Acc:HGNC:30037] |
| ENSG00000107130 | HGLibA_31375 | 106 | -0.093791992 | NCS1 | neuronal calcium sensor 1 [Source:HGNC Symbol;Acc:HGNC:3953] |
| ENSG00000108947 | HGLibA_14604 | 106 | -0.093791992 | EFNB3 | ephrin B3 [Source:HGNC Symbol;Acc:HGNC:3228] |
| ENSG00000109586 | HGLibA_18569 | 106 | -0.093791992 | GALNT7 | polypeptide N-acetylgalactosaminyltransferase 7 [Source:HGNC Symbol;Acc:HGNC:4129] |
| ENSG00000114200 | HGLibA_04274 | 106 | -0.093791992 | BCHE | butyrylcholinesterase [Source:HGNC Symbol;Acc:HGNC:983] |
| ENSG00000114353 | HGLibA_19455 | 106 | -0.093791992 | GNAI2 | G protein subunit alpha i2 [Source:HGNC Symbol;Acc:HGNC:4385] |
| ENSG00000114631 | HGLibA_37461 | 106 | -0.093791992 | PODXL2 | podocalyxin like 2 [Source:HGNC Symbol;Acc:HGNC:17936] |
| ENSG00000115661 | HGLibA_47458 | 106 | -0.093791992 | STK16 | serine/threonine kinase 16 [Source:HGNC Symbol;Acc:HGNC:11394] |
| ENSG00000116039 | HGLibA_03733 | 106 | -0.093791992 | ATP6V1B1 | ATPase H+ transporting V1 subunit B1 [Source:HGNC Symbol;Acc:HGNC:853] |
| ENSG00000116497 | HGLibA_42587 | 106 | -0.093791992 | S100PBP | S100P binding protein [Source:HGNC Symbol;Acc:HGNC:25768] |
| ENSG00000116717 | HGLibA_18417 | 106 | -0.093791992 | GADD45A | growth arrest and DNA damage inducible alpha [Source:HGNC Symbol;Acc:HGNC:4095] |
| ENSG00000117640 | HGLibA_30364 | 106 | -0.093791992 | MTFR1L | mitochondrial fission regulator 1 like [Source:HGNC Symbol;Acc:HGNC:28836] |
| ENSG00000119899 | HGLibA_44471 | 106 | -0.093791992 | SLC17A5 | solute carrier family 17 member 5 [Source:HGNC Symbol;Acc:HGNC:10933] |
| ENSG00000120063 | HGLibA_19441 | 106 | -0.093791992 | GNA13 | G protein subunit alpha 13 [Source:HGNC Symbol;Acc:HGNC:4381] |
| ENSG00000120324 | HGLibA_35471 | 106 | -0.093791992 | PCDHB10 | protocadherin beta 10 [Source:HGNC Symbol;Acc:HGNC:8681] |
| ENSG00000120738 | HGLibA_14648 | 106 | -0.093791992 | EGR1 | early growth response 1 [Source:HGNC Symbol;Acc:HGNC:3238] |
| ENSG00000121064 | HGLibA_43040 | 106 | -0.093791992 | SCPEP1 | serine carboxypeptidase 1 [Source:HGNC Symbol;Acc:HGNC:29507] |
| ENSG00000122180 | HGLibA_30886 | 106 | -0.093791992 | MYOG | myogenin [Source:HGNC Symbol;Acc:HGNC:7612] |
| ENSG00000122367 | HGLibA_26216 | 106 | -0.093791992 | LDB3 | LIM domain binding 3 [Source:HGNC Symbol;Acc:HGNC:15710] |
| ENSG00000122643 | HGLibA_32815 | 106 | -0.093791992 | NT5C3A | cytosolic IIIA [Source:HGNC Symbol;Acc:HGNC:17820]" |
| ENSG00000122756 | HGLibA_10394 | 106 | -0.093791992 | CNTFR | ciliary neurotrophic factor receptor [Source:HGNC Symbol;Acc:HGNC:2170] |
| ENSG00000123358 | HGLibA_32626 | 106 | -0.093791992 | NR4A1 | nuclear receptor subfamily 4 group A member 1 [Source:HGNC Symbol;Acc:HGNC:7980] |
| ENSG00000124787 | HGLibA_42042 | 106 | -0.093791992 | RPP40 | ribonuclease P/MRP subunit p40 [Source:HGNC Symbol;Acc:HGNC:20992] |
| ENSG00000126264 | HGLibA_20993 | 106 | -0.093791992 | HCST | hematopoietic cell signal transducer [Source:HGNC Symbol;Acc:HGNC:16977] |
| ENSG00000127074 | HGLibA_41062 | 106 | -0.093791992 | RGS13 | regulator of G protein signaling 13 [Source:HGNC Symbol;Acc:HGNC:9995] |
| ENSG00000127948 | HGLibA_37704 | 106 | -0.093791992 | POR | cytochrome p450 oxidoreductase [Source:HGNC Symbol;Acc:HGNC:9208] |
| ENSG00000128626 | HGLibA_30035 | 106 | -0.093791992 | MRPS12 | mitochondrial ribosomal protein S12 [Source:HGNC Symbol;Acc:HGNC:10380] |
| ENSG00000130119 | HGLibA_19553 | 106 | -0.093791992 | GNL3L | G protein nucleolar 3 like [Source:HGNC Symbol;Acc:HGNC:25553] |
| ENSG00000130821 | HGLibA_45341 | 106 | -0.093791992 | SLC6A8 | solute carrier family 6 member 8 [Source:HGNC Symbol;Acc:HGNC:11055] |
| ENSG00000132466 | HGLibA_02015 | 106 | -0.093791992 | ANKRD17 | ankyrin repeat domain 17 [Source:HGNC Symbol;Acc:HGNC:23575] |
| ENSG00000133731 | HGLibA_23243 | 106 | -0.093791992 | IMPA1 | inositol monophosphatase 1 [Source:HGNC Symbol;Acc:HGNC:6050] |
| ENSG00000134200 | HGLibA_51857 | 106 | -0.093791992 | TSHB | thyroid stimulating hormone beta [Source:HGNC Symbol;Acc:HGNC:12372] |
| ENSG00000134882 | HGLibA_52538 | 106 | -0.093791992 | UBAC2 | UBA domain containing 2 [Source:HGNC Symbol;Acc:HGNC:20486] |
| ENSG00000135114 | HGLibA_33207 | 106 | -0.093791992 | OASL | 2'-5'-oligoadenylate synthetase like [Source:HGNC Symbol;Acc:HGNC:8090] |
| ENSG00000135436 | HGLibA_16338 | 106 | -0.093791992 | FAM186B | family with sequence similarity 186 member B [Source:HGNC Symbol;Acc:HGNC:25296] |
| ENSG00000135577 | HGLibA_32144 | 106 | -0.093791992 | NMBR | neuromedin B receptor [Source:HGNC Symbol;Acc:HGNC:7843] |
| ENSG00000137269 | HGLibA_27244 | 106 | -0.093791992 | LRRC1 | leucine rich repeat containing 1 [Source:HGNC Symbol;Acc:HGNC:14307] |
| ENSG00000137404 | HGLibA_32707 | 106 | -0.093791992 | NRM | nurim [Source:HGNC Symbol;Acc:HGNC:8003] |
| ENSG00000138162 | HGLibA_48162 | 106 | -0.093791992 | TACC2 | transforming acidic coiled-coil containing protein 2 [Source:HGNC Symbol;Acc:HGNC:11523] |
| ENSG00000139428 | HGLibA_29453 | 106 | -0.093791992 | MMAB | metabolism of cobalamin associated B [Source:HGNC Symbol;Acc:HGNC:19331] |
| ENSG00000139631 | HGLibA_11302 | 106 | -0.093791992 | CSAD | cysteine sulfinic acid decarboxylase [Source:HGNC Symbol;Acc:HGNC:18966] |
| ENSG00000140505 | HGLibA_12069 | 106 | -0.093791992 | CYP1A2 | cytochrome P450 family 1 subfamily A member 2 [Source:HGNC Symbol;Acc:HGNC:2596] |
| ENSG00000140983 | HGLibA_41206 | 106 | -0.093791992 | RHOT2 | ras homolog family member T2 [Source:HGNC Symbol;Acc:HGNC:21169] |
| ENSG00000141040 | HGLibA_55756 | 106 | -0.093791992 | ZNF287 | zinc finger protein 287 [Source:HGNC Symbol;Acc:HGNC:13502] |
| ENSG00000141748 | HGLibA_02981 | 106 | -0.093791992 | ARL5C | ADP ribosylation factor like GTPase 5C [Source:HGNC Symbol;Acc:HGNC:31111] |
| ENSG00000142039 | HGLibA_07941 | 106 | -0.093791992 | CCDC97 | coiled-coil domain containing 97 [Source:HGNC Symbol;Acc:HGNC:28289] |
| ENSG00000143954 | HGLibA_40802 | 106 | -0.093791992 | REG3G | regenerating family member 3 gamma [Source:HGNC Symbol;Acc:HGNC:29595] |
| ENSG00000145088 | HGLibA_14338 | 106 | -0.093791992 | EAF2 | ELL associated factor 2 [Source:HGNC Symbol;Acc:HGNC:23115] |
| ENSG00000145354 | HGLibA_09731 | 106 | -0.093791992 | CISD2 | CDGSH iron sulfur domain 2 [Source:HGNC Symbol;Acc:HGNC:24212] |
| ENSG00000145794 | HGLibA_28847 | 106 | -0.093791992 | MEGF10 | multiple EGF like domains 10 [Source:HGNC Symbol;Acc:HGNC:29634] |
| ENSG00000146910 | HGLibA_10363 | 106 | -0.093791992 | CNPY1 | canopy FGF signaling regulator 1 [Source:HGNC Symbol;Acc:HGNC:27786] |
| ENSG00000148153 | HGLibA_23313 | 106 | -0.093791992 | INIP | INTS3 and NABP interacting protein [Source:HGNC Symbol;Acc:HGNC:24994] |
| ENSG00000148943 | HGLibA_26579 | 106 | -0.093791992 | LIN7C | crumbs cell polarity complex component [Source:HGNC Symbol;Acc:HGNC:17789]" |
| ENSG00000151468 | HGLibA_07750 | 106 | -0.093791992 | CCDC3 | coiled-coil domain containing 3 [Source:HGNC Symbol;Acc:HGNC:23813] |
| ENSG00000151612 | HGLibA_56825 | 106 | -0.093791992 | ZNF827 | zinc finger protein 827 [Source:HGNC Symbol;Acc:HGNC:27193] |
| ENSG00000151773 | HGLibA_07570 | 106 | -0.093791992 | CCDC122 | coiled-coil domain containing 122 [Source:HGNC Symbol;Acc:HGNC:26478] |
| ENSG00000151835 | HGLibA_42628 | 106 | -0.093791992 | SACS | sacsin molecular chaperone [Source:HGNC Symbol;Acc:HGNC:10519] |
| ENSG00000153266 | HGLibA_17321 | 106 | -0.093791992 | FEZF2 | FEZ family zinc finger 2 [Source:HGNC Symbol;Acc:HGNC:13506] |
| ENSG00000155087 | HGLibA_33275 | 106 | -0.093791992 | ODF1 | outer dense fiber of sperm tails 1 [Source:HGNC Symbol;Acc:HGNC:8113] |
| ENSG00000156299 | HGLibA_49440 | 106 | -0.093791992 | TIAM1 | T cell lymphoma invasion and metastasis 1 [Source:HGNC Symbol;Acc:HGNC:11805] |
| ENSG00000157326 | HGLibA_13135 | 106 | -0.093791992 | DHRS4 | dehydrogenase/reductase 4 [Source:HGNC Symbol;Acc:HGNC:16985] |
| ENSG00000158158 | HGLibA_10324 | 106 | -0.093791992 | CNNM4 | cyclin and CBS domain divalent metal cation transport mediator 4 [Source:HGNC Symbol;Acc:HGNC:105] |
| ENSG00000159248 | HGLibA_19188 | 106 | -0.093791992 | GJD2 | gap junction protein delta 2 [Source:HGNC Symbol;Acc:HGNC:19154] |
| ENSG00000159648 | HGLibA_49010 | 106 | -0.093791992 | TEPP | prostate and placenta expressed [Source:HGNC Symbol;Acc:HGNC:33745]" |
| ENSG00000159958 | HGLibA_50758 | 106 | -0.093791992 | TNFRSF13C | TNF receptor superfamily member 13C [Source:HGNC Symbol;Acc:HGNC:17755] |
| ENSG00000160087 | HGLibA_52620 | 106 | -0.093791992 | UBE2J2 | ubiquitin conjugating enzyme E2 J2 [Source:HGNC Symbol;Acc:HGNC:19268] |
| ENSG00000162783 | HGLibA_22580 | 106 | -0.093791992 | IER5 | immediate early response 5 [Source:HGNC Symbol;Acc:HGNC:5393] |
| ENSG00000162946 | HGLibA_13280 | 106 | -0.093791992 | DISC1 | DISC1 scaffold protein [Source:HGNC Symbol;Acc:HGNC:2888] |
| ENSG00000163002 | HGLibA_33049 | 106 | -0.093791992 | NUP35 | nucleoporin 35 [Source:HGNC Symbol;Acc:HGNC:29797] |
| ENSG00000164066 | HGLibA_23440 | 106 | -0.093791992 | INTU | inturned planar cell polarity protein [Source:HGNC Symbol;Acc:HGNC:29239] |
| ENSG00000164305 | HGLibA_07311 | 106 | -0.093791992 | CASP3 | caspase 3 [Source:HGNC Symbol;Acc:HGNC:1504] |
| ENSG00000164485 | HGLibA_23093 | 106 | -0.093791992 | IL22RA2 | interleukin 22 receptor subunit alpha 2 [Source:HGNC Symbol;Acc:HGNC:14901] |
| ENSG00000164509 | HGLibA_23134 | 106 | -0.093791992 | IL31RA | interleukin 31 receptor A [Source:HGNC Symbol;Acc:HGNC:18969] |
| ENSG00000165055 | HGLibA_29004 | 106 | -0.093791992 | METTL2B | methyltransferase like 2B [Source:HGNC Symbol;Acc:HGNC:18272] |
| ENSG00000165272 | HGLibA_02594 | 106 | -0.093791992 | AQP3 | aquaporin 3 (Gill blood group) [Source:HGNC Symbol;Acc:HGNC:636] |
| ENSG00000165389 | HGLibA_46900 | 106 | -0.093791992 | SPTSSA | serine palmitoyltransferase small subunit A [Source:HGNC Symbol;Acc:HGNC:20361] |
| ENSG00000165943 | HGLibA_29582 | 106 | -0.093791992 | MOAP1 | modulator of apoptosis 1 [Source:HGNC Symbol;Acc:HGNC:16658] |
| ENSG00000166135 | HGLibA_21299 | 106 | -0.093791992 | HIF1AN | hypoxia inducible factor 1 subunit alpha inhibitor [Source:HGNC Symbol;Acc:HGNC:17113] |
| ENSG00000166347 | HGLibA_11989 | 106 | -0.093791992 | CYB5A | cytochrome b5 type A [Source:HGNC Symbol;Acc:HGNC:2570] |
| ENSG00000166819 | HGLibA_37169 | 106 | -0.093791992 | PLIN1 | perilipin 1 [Source:HGNC Symbol;Acc:HGNC:9076] |
| ENSG00000167114 | HGLibA_44822 | 106 | -0.093791992 | SLC27A4 | solute carrier family 27 member 4 [Source:HGNC Symbol;Acc:HGNC:10998] |
| ENSG00000167513 | HGLibA_08889 | 106 | -0.093791992 | CDT1 | chromatin licensing and DNA replication factor 1 [Source:HGNC Symbol;Acc:HGNC:24576] |
| ENSG00000167613 | HGLibA_25933 | 106 | -0.093791992 | LAIR1 | leukocyte associated immunoglobulin like receptor 1 [Source:HGNC Symbol;Acc:HGNC:6477] |
| ENSG00000167815 | HGLibA_38411 | 106 | -0.093791992 | PRDX2 | peroxiredoxin 2 [Source:HGNC Symbol;Acc:HGNC:9353] |
| ENSG00000167880 | HGLibA_15702 | 106 | -0.093791992 | EVPL | envoplakin [Source:HGNC Symbol;Acc:HGNC:3503] |
| ENSG00000168056 | HGLibA_27596 | 106 | -0.093791992 | LTBP3 | latent transforming growth factor beta binding protein 3 [Source:HGNC Symbol;Acc:HGNC:6716] |
| ENSG00000169564 | HGLibA_35368 | 106 | -0.093791992 | PCBP1 | poly(rC) binding protein 1 [Source:HGNC Symbol;Acc:HGNC:8647] |
| ENSG00000170615 | HGLibA_44797 | 106 | -0.093791992 | SLC26A5 | solute carrier family 26 member 5 [Source:HGNC Symbol;Acc:HGNC:9359] |
| ENSG00000170965 | HGLibA_36933 | 106 | -0.093791992 | PLAC1 | placenta specific 1 [Source:HGNC Symbol;Acc:HGNC:9044] |
| ENSG00000171478 | HGLibA_46336 | 106 | -0.093791992 | SPACA5B | sperm acrosome associated 5B [Source:HGNC Symbol;Acc:HGNC:19142] |
| ENSG00000172115 | HGLibA_12027 | 106 | -0.093791992 | CYCS | somatic [Source:HGNC Symbol;Acc:HGNC:19986]" |
| ENSG00000172890 | HGLibA_31053 | 106 | -0.093791992 | NADSYN1 | NAD synthetase 1 [Source:HGNC Symbol;Acc:HGNC:29832] |
| ENSG00000173457 | HGLibA_38038 | 106 | -0.093791992 | PPP1R14B | protein phosphatase 1 regulatory inhibitor subunit 14B [Source:HGNC Symbol;Acc:HGNC:9057] |
| ENSG00000173641 | HGLibA_22316 | 106 | -0.093791992 | HSPB7 | heat shock protein family B (small) member 7 [Source:HGNC Symbol;Acc:HGNC:5249] |
| ENSG00000173890 | HGLibA_19953 | 106 | -0.093791992 | GPR160 | G protein-coupled receptor 160 [Source:HGNC Symbol;Acc:HGNC:23693] |
| ENSG00000174898 | HGLibA_07369 | 106 | -0.093791992 | CATSPERD | cation channel sperm associated auxiliary subunit delta [Source:HGNC Symbol;Acc:HGNC:28598] |
| ENSG00000176769 | HGLibA_48759 | 106 | -0.093791992 | TCERG1L | transcription elongation regulator 1 like [Source:HGNC Symbol;Acc:HGNC:23533] |
| ENSG00000178409 | HGLibA_04405 | 106 | -0.093791992 | BEND3 | BEN domain containing 3 [Source:HGNC Symbol;Acc:HGNC:23040] |
| ENSG00000179023 | HGLibA_25105 | 106 | -0.093791992 | KLHDC7A | kelch domain containing 7A [Source:HGNC Symbol;Acc:HGNC:26791] |
| ENSG00000179165 | HGLibA_39666 | 106 | -0.093791992 | PXT1 | peroxisomal testis enriched protein 1 [Source:HGNC Symbol;Acc:HGNC:18312] |
| ENSG00000180269 | HGLibA_19906 | 106 | -0.093791992 | GPR139 | G protein-coupled receptor 139 [Source:HGNC Symbol;Acc:HGNC:19995] |
| ENSG00000180481 | HGLibA_19265 | 106 | -0.093791992 | GLIPR1L2 | GLIPR1 like 2 [Source:HGNC Symbol;Acc:HGNC:28592] |
| ENSG00000180694 | HGLibA_50484 | 106 | -0.093791992 | TMEM64 | transmembrane protein 64 [Source:HGNC Symbol;Acc:HGNC:25441] |
| ENSG00000181045 | HGLibA_44786 | 106 | -0.093791992 | SLC26A11 | solute carrier family 26 member 11 [Source:HGNC Symbol;Acc:HGNC:14471] |
| ENSG00000183615 | HGLibA_16244 | 106 | -0.093791992 | FAM167B | family with sequence similarity 167 member B [Source:HGNC Symbol;Acc:HGNC:28133] |
| ENSG00000184182 | HGLibA_52604 | 106 | -0.093791992 | UBE2F | ubiquitin conjugating enzyme E2 F (putative) [Source:HGNC Symbol;Acc:HGNC:12480] |
| ENSG00000184611 | HGLibA_24228 | 106 | -0.093791992 | KCNH7 | potassium voltage-gated channel subfamily H member 7 [Source:HGNC Symbol;Acc:HGNC:18863] |
| ENSG00000185681 | HGLibA_29678 | 106 | -0.093791992 | MORN5 | MORN repeat containing 5 [Source:HGNC Symbol;Acc:HGNC:17841] |
| ENSG00000186501 | HGLibA_50241 | 106 | -0.093791992 | TMEM222 | transmembrane protein 222 [Source:HGNC Symbol;Acc:HGNC:25363] |
| ENSG00000186788 | HGLibA_46498 | 106 | -0.093791992 | SPATA31D3 | SPATA31 subfamily D member 3 [Source:HGNC Symbol;Acc:HGNC:38603] |
| ENSG00000196187 | HGLibA_50473 | 106 | -0.093791992 | TMEM63A | transmembrane protein 63A [Source:HGNC Symbol;Acc:HGNC:29118] |
| ENSG00000196367 | HGLibA_51799 | 106 | -0.093791992 | TRRAP | transformation/transcription domain associated protein [Source:HGNC Symbol;Acc:HGNC:12347] |
| ENSG00000196767 | HGLibA_37773 | 106 | -0.093791992 | POU3F4 | POU class 3 homeobox 4 [Source:HGNC Symbol;Acc:HGNC:9217] |
| ENSG00000196812 | HGLibA_56988 | 106 | -0.093791992 | ZSCAN16 | zinc finger and SCAN domain containing 16 [Source:HGNC Symbol;Acc:HGNC:20813] |
| ENSG00000198535 | HGLibA_06095 | 106 | -0.093791992 | C2CD4A | C2 calcium dependent domain containing 4A [Source:HGNC Symbol;Acc:HGNC:33627] |
| ENSG00000198822 | HGLibA_20346 | 106 | -0.093791992 | GRM3 | glutamate metabotropic receptor 3 [Source:HGNC Symbol;Acc:HGNC:4595] |
| ENSG00000204351 | HGLibA_44286 | 106 | -0.093791992 | SKIV2L | Ski2 like RNA helicase [Source:HGNC Symbol;Acc:HGNC:10898] |
| ENSG00000204659 | HGLibA_07473 | 106 | -0.093791992 | CBY3 | chibby family member 3 [Source:HGNC Symbol;Acc:HGNC:33278] |
| ENSG00000204897 | HGLibA_25458 | 106 | -0.093791992 | KRT25 | keratin 25 [Source:HGNC Symbol;Acc:HGNC:30839] |
| ENSG00000213920 | HGLibA_28705 | 106 | -0.093791992 | MDP1 | magnesium dependent phosphatase 1 [Source:HGNC Symbol;Acc:HGNC:28781] |
| ENSG00000223510 | HGLibA_08876 | 106 | -0.093791992 | CDRT15 | CMT1A duplicated region transcript 15 [Source:HGNC Symbol;Acc:HGNC:14395] |
| ENSG00000237038 | HGLibA_53287 | 106 | -0.093791992 | USP17L8 | ubiquitin specific peptidase 17-like family member 8 [Source:HGNC Symbol;Acc:HGNC:37181] |
| ENSG00000239961 | HGLibA_26497 | 106 | -0.093791992 | LILRA4 | leukocyte immunoglobulin like receptor A4 [Source:HGNC Symbol;Acc:HGNC:15503] |
| ENSG00000240065 | HGLibA_39123 | 106 | -0.093791992 | PSMB9 | proteasome subunit beta 9 [Source:HGNC Symbol;Acc:HGNC:9546] |
| ENSG00000243244 | HGLibA_47542 | 106 | -0.093791992 | STON1 | stonin 1 [Source:HGNC Symbol;Acc:HGNC:17003] |
| ENSG00000256162 | HGLibA_45780 | 106 | -0.093791992 | SMLR1 | small leucine rich protein 1 [Source:HGNC Symbol;Acc:HGNC:44670] |
| ENSG00000257138 | HGLibA_48394 | 106 | -0.093791992 | TAS2R38 | taste 2 receptor member 38 [Source:HGNC Symbol;Acc:HGNC:9584] |

| | | | | | |
|---|---|---|---|---|---|
| ENSG00000259803 | HGLibA_44586 | 106 | -0.093791992 | SLC22A31 | solute carrier family 22 member 31 [Source:HGNC Symbol;Acc:HGNC:27091] |
| ENSG00000272395 | HGLibA_22727 | 106 | -0.093791992 | IFNL4 | interferon lambda 4 (gene/pseudogene) [Source:HGNC Symbol;Acc:HGNC:44480] |
| ENSG00000273274 | HGLibA_54928 | 106 | -0.093791992 | ZBTB8B | zinc finger and BTB domain containing 8B [Source:HGNC Symbol;Acc:HGNC:37057] |
| ENSG00000275004 | HGLibA_55725 | 106 | -0.093791992 | ZNF280B | zinc finger protein 280B [Source:HGNC Symbol;Acc:HGNC:23022] |
| ENSG00000278224 | HGLibA_38478 | 106 | -0.093791992 | PRICKLE4 | prickle planar cell polarity protein 4 [Source:HGNC Symbol;Acc:HGNC:16805] |
| FAM188A | HGLibA_16344 | 106 | -0.093791992 | FAM188A | - |
| GPR97 | HGLibA_20112 | 106 | -0.093791992 | GPR97 | - |
| hsa-mir-1469 | HGLibA_57852 | 106 | -0.093791992 | hsa-mir-1469 | - |
| hsa-mir-199a-2 | HGLibA_58131 | 106 | -0.093791992 | hsa-mir-199a-2 | - |
| hsa-mir-202 | HGLibA_58161 | 106 | -0.093791992 | hsa-mir-202 | - |
| hsa-mir-30e | HGLibA_58490 | 106 | -0.093791992 | hsa-mir-30e | - |
| hsa-mir-3128 | HGLibA_58565 | 106 | -0.093791992 | hsa-mir-3128 | - |
| hsa-mir-3161 | HGLibA_58724 | 106 | -0.093791992 | hsa-mir-3161 | - |
| hsa-mir-3927 | HGLibA_59586 | 106 | -0.093791992 | hsa-mir-3927 | - |
| hsa-mir-3929 | HGLibA_59592 | 106 | -0.093791992 | hsa-mir-3929 | - |
| hsa-mir-3940 | HGLibA_59620 | 106 | -0.093791992 | hsa-mir-3940 | - |
| hsa-mir-4261 | HGLibA_59752 | 106 | -0.093791992 | hsa-mir-4261 | - |
| hsa-mir-4305 | HGLibA_59931 | 106 | -0.093791992 | hsa-mir-4305 | - |
| hsa-mir-4453 | HGLibA_60218 | 106 | -0.093791992 | hsa-mir-4453 | - |
| hsa-mir-4474 | HGLibA_60305 | 106 | -0.093791992 | hsa-mir-4474 | - |
| hsa-mir-4686 | HGLibA_60855 | 106 | -0.093791992 | hsa-mir-4686 | - |
| hsa-mir-4777 | HGLibA_61215 | 106 | -0.093791992 | hsa-mir-4777 | - |
| hsa-mir-6759 | HGLibA_63193 | 106 | -0.093791992 | hsa-mir-6759 | - |
| hsa-mir-6778 | HGLibA_63286 | 106 | -0.093791992 | hsa-mir-6778 | - |
| hsa-mir-6856 | HGLibA_63595 | 106 | -0.093791992 | hsa-mir-6856 | - |
| LOC650293 | HGLibA_26984 | 106 | -0.093791992 | LOC650293 | - |
| TCTEX1D4 | HGLibA_48868 | 106 | -0.093791992 | TCTEX1D4 | - |
| VARS | HGLibA_53525 | 106 | -0.093791992 | VARS | - |
| WIBG | HGLibA_54297 | 106 | -0.093791992 | WIBG | - |
| C17orf104 | HGLibA_05523 | 105 | -0.103743029 | C17orf104 | - |
| C18orf8 | HGLibA_05624 | 105 | -0.103743029 | C18orf8 | - |
| CRIPAK | HGLibA_11160 | 105 | -0.103743029 | CRIPAK | - |
| CXorf56 | HGLibA_11945 | 105 | -0.103743029 | CXorf56 | - |
| ENSG00000005469 | HGLibA_11213 | 105 | -0.103743029 | CROT | carnitine O-octanoyltransferase [Source:HGNC Symbol;Acc:HGNC:2366] |
| ENSG00000011677 | HGLibA_18363 | 105 | -0.103743029 | GABRA3 | gamma-aminobutyric acid type A receptor alpha3 subunit [Source:HGNC Symbol;Acc:HGNC:4077] |
| ENSG00000023909 | HGLibA_18796 | 105 | -0.103743029 | GCLM | glutamate-cysteine ligase modifier subunit [Source:HGNC Symbol;Acc:HGNC:4312] |
| ENSG00000031691 | HGLibA_09084 | 105 | -0.103743029 | CENPQ | centromere protein Q [Source:HGNC Symbol;Acc:HGNC:21347] |
| ENSG00000063515 | HGLibA_20401 | 105 | -0.103743029 | GSC2 | goosecoid homeobox 2 [Source:HGNC Symbol;Acc:HGNC:4613] |
| ENSG00000067191 | HGLibA_06918 | 105 | -0.103743029 | CACNB1 | calcium voltage-gated channel auxiliary subunit beta 1 [Source:HGNC Symbol;Acc:HGNC:1401] |
| ENSG00000067992 | HGLibA_35902 | 105 | -0.103743029 | PDK3 | pyruvate dehydrogenase kinase 3 [Source:HGNC Symbol;Acc:HGNC:8811] |
| ENSG00000068781 | HGLibA_47545 | 105 | -0.103743029 | STON1-GTF2A1L | STON1-GTF2A1L readthrough [Source:HGNC Symbol;Acc:HGNC:30651] |
| ENSG00000070019 | HGLibA_20660 | 105 | -0.103743029 | GUCY2C | guanylate cyclase 2C [Source:HGNC Symbol;Acc:HGNC:4688] |
| ENSG00000077232 | HGLibA_13564 | 105 | -0.103743029 | DNAJC10 | DnaJ heat shock protein family (Hsp40) member C10 [Source:HGNC Symbol;Acc:HGNC:24637] |
| ENSG00000078177 | HGLibA_30952 | 105 | -0.103743029 | N4BP2 | NEDD4 binding protein 2 [Source:HGNC Symbol;Acc:HGNC:29851] |
| ENSG00000085117 | HGLibA_08458 | 105 | -0.103743029 | CD82 | CD82 molecule [Source:HGNC Symbol;Acc:HGNC:6210] |
| ENSG00000085741 | HGLibA_54352 | 105 | -0.103743029 | WNT11 | Wnt family member 11 [Source:HGNC Symbol;Acc:HGNC:12776] |
| ENSG00000089505 | HGLibA_10210 | 105 | -0.103743029 | CMTM1 | CKLF like MARVEL transmembrane domain containing 1 [Source:HGNC Symbol;Acc:HGNC:19172] |
| ENSG00000089818 | HGLibA_31592 | 105 | -0.103743029 | NECAP1 | NECAP endocytosis associated 1 [Source:HGNC Symbol;Acc:HGNC:24539] |
| ENSG00000100105 | HGLibA_35289 | 105 | -0.103743029 | PATZ1 | POZ/BTB and AT hook containing zinc finger 1 [Source:HGNC Symbol;Acc:HGNC:13071] |
| ENSG00000101190 | HGLibA_48799 | 105 | -0.103743029 | TCFL5 | transcription factor like 5 [Source:HGNC Symbol;Acc:HGNC:11646] |
| ENSG00000101350 | HGLibA_24937 | 105 | -0.103743029 | KIF3B | kinesin family member 3B [Source:HGNC Symbol;Acc:HGNC:6320] |
| ENSG00000102053 | HGLibA_54955 | 105 | -0.103743029 | ZC3H12B | zinc finger CCCH-type containing 12B [Source:HGNC Symbol;Acc:HGNC:17407] |
| ENSG00000102575 | HGLibA_00516 | 105 | -0.103743029 | ACP5 | tartrate resistant [Source:HGNC Symbol;Acc:HGNC:124]" |
| ENSG00000103978 | HGLibA_50531 | 105 | -0.103743029 | TMEM87A | transmembrane protein 87A [Source:HGNC Symbol;Acc:HGNC:24522] |
| ENSG00000104825 | HGLibA_31823 | 105 | -0.103743029 | NFKBIB | NFKB inhibitor beta [Source:HGNC Symbol;Acc:HGNC:7798] |
| ENSG00000104904 | HGLibA_33214 | 105 | -0.103743029 | OAZ1 | ornithine decarboxylase antizyme 1 [Source:HGNC Symbol;Acc:HGNC:8095] |
| ENSG00000105053 | HGLibA_53830 | 105 | -0.103743029 | VRK3 | VRK serine/threonine kinase 3 [Source:HGNC Symbol;Acc:HGNC:18996] |
| ENSG00000106153 | HGLibA_09365 | 105 | -0.103743029 | CHCHD2 | coiled-coil-helix-coiled-coil-helix domain containing 2 [Source:HGNC Symbol;Acc:HGNC:21645] |
| ENSG00000106268 | HGLibA_32931 | 105 | -0.103743029 | NUDT1 | nudix hydrolase 1 [Source:HGNC Symbol;Acc:HGNC:8048] |
| ENSG00000106333 | HGLibA_35652 | 105 | -0.103743029 | PCOLCE | procollagen C-endopeptidase enhancer [Source:HGNC Symbol;Acc:HGNC:8738] |
| ENSG00000106591 | HGLibA_29951 | 105 | -0.103743029 | MRPL32 | mitochondrial ribosomal protein L32 [Source:HGNC Symbol;Acc:HGNC:14035] |
| ENSG00000108395 | HGLibA_51477 | 105 | -0.103743029 | TRIM37 | tripartite motif containing 37 [Source:HGNC Symbol;Acc:HGNC:7523] |
| ENSG00000108679 | HGLibA_26366 | 105 | -0.103743029 | LGALS3BP | galectin 3 binding protein [Source:HGNC Symbol;Acc:HGNC:6564] |
| ENSG00000110047 | HGLibA_14667 | 105 | -0.103743029 | EHD1 | EH domain containing 1 [Source:HGNC Symbol;Acc:HGNC:3242] |
| ENSG00000111142 | HGLibA_28935 | 105 | -0.103743029 | METAP2 | methionyl aminopeptidase 2 [Source:HGNC Symbol;Acc:HGNC:16672] |
| ENSG00000111262 | HGLibA_24123 | 105 | -0.103743029 | KCNA1 | potassium voltage-gated channel subfamily A member 1 [Source:HGNC Symbol;Acc:HGNC:6218] |
| ENSG00000111271 | HGLibA_00352 | 105 | -0.103743029 | ACAD10 | acyl-CoA dehydrogenase family member 10 [Source:HGNC Symbol;Acc:HGNC:21597] |
| ENSG00000111907 | HGLibA_51114 | 105 | -0.103743029 | TPD52L1 | TPD52 like 1 [Source:HGNC Symbol;Acc:HGNC:12006] |
| ENSG00000113312 | HGLibA_52046 | 105 | -0.103743029 | TTC1 | tetratricopeptide repeat domain 1 [Source:HGNC Symbol;Acc:HGNC:12391] |
| ENSG00000114529 | HGLibA_06249 | 105 | -0.103743029 | C3orf52 | chromosome 3 open reading frame 52 [Source:HGNC Symbol;Acc:HGNC:26255] |
| ENSG00000115561 | HGLibA_09485 | 105 | -0.103743029 | CHMP3 | charged multivesicular body protein 3 [Source:HGNC Symbol;Acc:HGNC:29865] |
| ENSG00000116703 | HGLibA_35713 | 105 | -0.103743029 | PDC | phosducin [Source:HGNC Symbol;Acc:HGNC:8759] |
| ENSG00000117153 | HGLibA_25128 | 105 | -0.103743029 | KLHL12 | kelch like family member 12 [Source:HGNC Symbol;Acc:HGNC:19360] |
| ENSG00000118246 | HGLibA_16905 | 105 | -0.103743029 | FASTKD2 | FAST kinase domains 2 [Source:HGNC Symbol;Acc:HGNC:29160] |
| ENSG00000118690 | HGLibA_03015 | 105 | -0.103743029 | ARMC2 | armadillo repeat containing 2 [Source:HGNC Symbol;Acc:HGNC:23045] |
| ENSG00000118960 | HGLibA_22114 | 105 | -0.103743029 | HS1BP3 | HCLS1 binding protein 3 [Source:HGNC Symbol;Acc:HGNC:24979] |
| ENSG00000119574 | HGLibA_54894 | 105 | -0.103743029 | ZBTB45 | zinc finger and BTB domain containing 45 [Source:HGNC Symbol;Acc:HGNC:23715] |
| ENSG00000120907 | HGLibA_01038 | 105 | -0.103743029 | ADRA1A | adrenoceptor alpha 1A [Source:HGNC Symbol;Acc:HGNC:277] |
| ENSG00000122863 | HGLibA_09634 | 105 | -0.103743029 | CHST3 | carbohydrate sulfotransferase 3 [Source:HGNC Symbol;Acc:HGNC:1971] |
| ENSG00000122986 | HGLibA_22433 | 105 | -0.103743029 | HVCN1 | hydrogen voltage gated channel 1 [Source:HGNC Symbol;Acc:HGNC:28240] |
| ENSG00000125538 | HGLibA_23041 | 105 | -0.103743029 | IL1B | interleukin 1 beta [Source:HGNC Symbol;Acc:HGNC:5992] |
| ENSG00000129235 | HGLibA_52416 | 105 | -0.103743029 | TXNDC17 | thioredoxin domain containing 17 [Source:HGNC Symbol;Acc:HGNC:28218] |
| ENSG00000131044 | HGLibA_52216 | 105 | -0.103743029 | TTLL9 | tubulin tyrosine ligase like 9 [Source:HGNC Symbol;Acc:HGNC:16118] |
| ENSG00000133193 | HGLibA_15987 | 105 | -0.103743029 | FAM104A | family with sequence similarity 104 member A [Source:HGNC Symbol;Acc:HGNC:25918] |
| ENSG00000133477 | HGLibA_16755 | 105 | -0.103743029 | FAM83F | family with sequence similarity 83 member F [Source:HGNC Symbol;Acc:HGNC:25148] |
| ENSG00000134256 | HGLibA_08229 | 105 | -0.103743029 | CD101 | CD101 molecule [Source:HGNC Symbol;Acc:HGNC:5949] |
| ENSG00000134398 | HGLibA_15516 | 105 | -0.103743029 | ERN2 | endoplasmic reticulum to nucleus signaling 2 [Source:HGNC Symbol;Acc:HGNC:16942] |
| ENSG00000135111 | HGLibA_48669 | 105 | -0.103743029 | TBX3 | T-box 3 [Source:HGNC Symbol;Acc:HGNC:11602] |
| ENSG00000135423 | HGLibA_19329 | 105 | -0.103743029 | GLS2 | glutaminase 2 [Source:HGNC Symbol;Acc:HGNC:29570] |
| ENSG00000137161 | HGLibA_10367 | 105 | -0.103743029 | CNPY3 | canopy FGF signaling regulator 3 [Source:HGNC Symbol;Acc:HGNC:11968] |
| ENSG00000140575 | HGLibA_23533 | 105 | -0.103743029 | IQGAP1 | IQ motif containing GTPase activating protein 1 [Source:HGNC Symbol;Acc:HGNC:6110] |
| ENSG00000140830 | HGLibA_52441 | 105 | -0.103743029 | TXNL4B | thioredoxin like 4B [Source:HGNC Symbol;Acc:HGNC:26041] |
| ENSG00000141543 | HGLibA_14809 | 105 | -0.103743029 | EIF4A3 | eukaryotic translation initiation factor 4A3 [Source:HGNC Symbol;Acc:HGNC:18683] |
| ENSG00000142235 | HGLibA_26728 | 105 | -0.103743029 | LMTK3 | lemur tyrosine kinase 3 [Source:HGNC Symbol;Acc:HGNC:19295] |
| ENSG00000143971 | HGLibA_15615 | 105 | -0.103743029 | ETAA1 | ATR kinase activator [Source:HGNC Symbol;Acc:HGNC:24648]" |
| ENSG00000144824 | HGLibA_36429 | 105 | -0.103743029 | PHLDB2 | pleckstrin homology like domain family B member 2 [Source:HGNC Symbol;Acc:HGNC:29573] |
| ENSG00000148965 | HGLibA_42616 | 105 | -0.103743029 | SAA4 | constitutive [Source:HGNC Symbol;Acc:HGNC:10516]" |
| ENSG00000149043 | HGLibA_48091 | 105 | -0.103743029 | SYT8 | synaptotagmin 8 [Source:HGNC Symbol;Acc:HGNC:19264] |
| ENSG00000149575 | HGLibA_42983 | 105 | -0.103743029 | SCN2B | sodium voltage-gated channel beta subunit 2 [Source:HGNC Symbol;Acc:HGNC:10589] |
| ENSG00000151005 | HGLibA_49592 | 105 | -0.103743029 | TKTL2 | transketolase like 2 [Source:HGNC Symbol;Acc:HGNC:25313] |
| ENSG00000151475 | HGLibA_44704 | 105 | -0.103743029 | SLC25A31 | solute carrier family 25 member 31 [Source:HGNC Symbol;Acc:HGNC:25319] |
| ENSG00000151498 | HGLibA_00358 | 105 | -0.103743029 | ACAD8 | acyl-CoA dehydrogenase family member 8 [Source:HGNC Symbol;Acc:HGNC:87] |
| ENSG00000152484 | HGLibA_53199 | 105 | -0.103743029 | USP12 | ubiquitin specific peptidase 12 [Source:HGNC Symbol;Acc:HGNC:20485] |
| ENSG00000153767 | HGLibA_20531 | 105 | -0.103743029 | GTF2E1 | general transcription factor IIE subunit 1 [Source:HGNC Symbol;Acc:HGNC:4650] |
| ENSG00000153898 | HGLibA_28641 | 105 | -0.103743029 | MCOLN2 | mucolipin 2 [Source:HGNC Symbol;Acc:HGNC:13357] |
| ENSG00000155890 | HGLibA_51498 | 105 | -0.103743029 | TRIM42 | tripartite motif containing 42 [Source:HGNC Symbol;Acc:HGNC:19014] |
| ENSG00000155974 | HGLibA_20320 | 105 | -0.103743029 | GRIP1 | glutamate receptor interacting protein 1 [Source:HGNC Symbol;Acc:HGNC:18708] |
| ENSG00000156603 | HGLibA_28768 | 105 | -0.103743029 | MED19 | mediator complex subunit 19 [Source:HGNC Symbol;Acc:HGNC:29600] |
| ENSG00000158352 | HGLibA_44095 | 105 | -0.103743029 | SHROOM4 | shroom family member 4 [Source:HGNC Symbol;Acc:HGNC:29215] |
| ENSG00000158985 | HGLibA_08585 | 105 | -0.103743029 | CDC42SE2 | CDC42 small effector 2 [Source:HGNC Symbol;Acc:HGNC:18547] |
| ENSG00000159658 | HGLibA_14533 | 105 | -0.103743029 | EFCAB14 | EF-hand calcium binding domain 14 [Source:HGNC Symbol;Acc:HGNC:29051] |
| ENSG00000159720 | HGLibA_03719 | 105 | -0.103743029 | ATP6V0D1 | ATPase H+ transporting V0 subunit d1 [Source:HGNC Symbol;Acc:HGNC:13724] |
| ENSG00000160194 | HGLibA_31575 | 105 | -0.103743029 | NDUFV3 | NADH:ubiquinone oxidoreductase subunit V3 [Source:HGNC Symbol;Acc:HGNC:7719] |
| ENSG00000160957 | HGLibA_40771 | 105 | -0.103743029 | RECQL4 | RecQ like helicase 4 [Source:HGNC Symbol;Acc:HGNC:9949] |
| ENSG00000161281 | HGLibA_10858 | 105 | -0.103743029 | COX7A1 | cytochrome c oxidase subunit 7A1 [Source:HGNC Symbol;Acc:HGNC:2287] |
| ENSG00000161551 | HGLibA_56295 | 105 | -0.103743029 | ZNF577 | zinc finger protein 577 [Source:HGNC Symbol;Acc:HGNC:28673] |
| ENSG00000162433 | HGLibA_01385 | 105 | -0.103743029 | AK4 | adenylate kinase 4 [Source:HGNC Symbol;Acc:HGNC:363] |
| ENSG00000162782 | HGLibA_48907 | 105 | -0.103743029 | TDRD5 | tudor domain containing 5 [Source:HGNC Symbol;Acc:HGNC:20614] |
| ENSG00000163012 | HGLibA_57040 | 105 | -0.103743029 | ZSWIM2 | zinc finger SWIM-type containing 2 [Source:HGNC Symbol;Acc:HGNC:30990] |
| ENSG00000163808 | HGLibA_24866 | 105 | -0.103743029 | KIF15 | kinesin family member 15 [Source:HGNC Symbol;Acc:HGNC:17273] |
| ENSG00000164007 | HGLibA_09875 | 105 | -0.103743029 | CLDN19 | claudin 19 [Source:HGNC Symbol;Acc:HGNC:2040] |
| ENSG00000164164 | HGLibA_34765 | 105 | -0.103743029 | OTUD4 | OTU deubiquitinase 4 [Source:HGNC Symbol;Acc:HGNC:24949] |
| ENSG00000164463 | HGLibA_11115 | 105 | -0.103743029 | CREBRF | CREB3 regulatory factor [Source:HGNC Symbol;Acc:HGNC:24050] |
| ENSG00000165973 | HGLibA_31684 | 105 | -0.103743029 | NELL1 | neural EGFL like 1 [Source:HGNC Symbol;Acc:HGNC:7750] |
| ENSG00000166869 | HGLibA_09519 | 105 | -0.103743029 | CHP2 | calcineurin like EF-hand protein 2 [Source:HGNC Symbol;Acc:HGNC:24927] |
| ENSG00000166922 | HGLibA_42906 | 105 | -0.103743029 | SCG5 | secretogranin V [Source:HGNC Symbol;Acc:HGNC:10816] |
| ENSG00000168140 | HGLibA_53535 | 105 | -0.103743029 | VASN | vasorin [Source:HGNC Symbol;Acc:HGNC:18517] |
| ENSG00000168754 | HGLibA_16304 | 105 | -0.103743029 | FAM178B | family with sequence similarity 178 member B [Source:HGNC Symbol;Acc:HGNC:28036] |
| ENSG00000168781 | HGLibA_37954 | 105 | -0.103743029 | PPIP5K1 | diphosphoinositol pentakisphosphate kinase 1 [Source:HGNC Symbol;Acc:HGNC:29023] |
| ENSG00000170439 | HGLibA_29022 | 105 | -0.103743029 | METTL7B | methyltransferase like 7B [Source:HGNC Symbol;Acc:HGNC:28276] |
| ENSG00000171587 | HGLibA_13997 | 105 | -0.103743029 | DSCAM | DS cell adhesion molecule [Source:HGNC Symbol;Acc:HGNC:3039] |
| ENSG00000172264 | HGLibA_27840 | 105 | -0.103743029 | MACROD2 | MACRO domain containing 2 [Source:HGNC Symbol;Acc:HGNC:16126] |
| ENSG00000172640 | HGLibA_33479 | 105 | -0.103743029 | OR10AD1 | olfactory receptor family 10 subfamily AD member 1 [Source:HGNC Symbol;Acc:HGNC:14819] |
| ENSG00000173198 | HGLibA_12225 | 105 | -0.103743029 | CYSLTR1 | cysteinyl leukotriene receptor 1 [Source:HGNC Symbol;Acc:HGNC:17451] |
| ENSG00000174007 | HGLibA_09123 | 105 | -0.103743029 | CEP19 | centrosomal protein 19 [Source:HGNC Symbol;Acc:HGNC:28209] |

| | | | | | |
|---|---|---|---|---|---|
| ENSG00000174156 | HGLibA_20456 | 105 | -0.103743029 | GSTA3 | glutathione S-transferase alpha 3 [Source:HGNC Symbol;Acc:HGNC:4628] |
| ENSG00000175806 | HGLibA_30238 | 105 | -0.103743029 | MSRA | methionine sulfoxide reductase A [Source:HGNC Symbol;Acc:HGNC:7377] |
| ENSG00000175868 | HGLibA_06995 | 105 | -0.103743029 | CALCB | calcitonin related polypeptide beta [Source:HGNC Symbol;Acc:HGNC:1438] |
| ENSG00000176732 | HGLibA_36184 | 105 | -0.103743029 | PFN4 | profilin family member 4 [Source:HGNC Symbol;Acc:HGNC:31103] |
| ENSG00000176946 | HGLibA_49301 | 105 | -0.103743029 | THAP4 | THAP domain containing 4 [Source:HGNC Symbol;Acc:HGNC:23187] |
| ENSG00000177363 | HGLibA_27483 | 105 | -0.103743029 | LRRN4CL | LRRN4 C-terminal like [Source:HGNC Symbol;Acc:HGNC:33724] |
| ENSG00000177689 | HGLibA_27932 | 105 | -0.103743029 | MAGEB10 | MAGE family member B10 [Source:HGNC Symbol;Acc:HGNC:25377] |
| ENSG00000177791 | HGLibA_30900 | 105 | -0.103743029 | MYOZ1 | myozenin 1 [Source:HGNC Symbol;Acc:HGNC:13752] |
| ENSG00000177839 | HGLibA_35513 | 105 | -0.103743029 | PCDHB9 | protocadherin beta 9 [Source:HGNC Symbol;Acc:HGNC:8694] |
| ENSG00000178690 | HGLibA_14229 | 105 | -0.103743029 | DYNAP | dynactin associated protein [Source:HGNC Symbol;Acc:HGNC:26808] |
| ENSG00000178982 | HGLibA_14795 | 105 | -0.103743029 | EIF3K | eukaryotic translation initiation factor 3 subunit K [Source:HGNC Symbol;Acc:HGNC:24656] |
| ENSG00000179071 | HGLibA_07916 | 105 | -0.103743029 | CCDC89 | coiled-coil domain containing 89 [Source:HGNC Symbol;Acc:HGNC:26762] |
| ENSG00000179520 | HGLibA_44480 | 105 | -0.103743029 | SLC17A8 | solute carrier family 17 member 8 [Source:HGNC Symbol;Acc:HGNC:20151] |
| ENSG00000179943 | HGLibA_17547 | 105 | -0.103743029 | FIZ1 | FLT3 interacting zinc finger 1 [Source:HGNC Symbol;Acc:HGNC:25917] |
| ENSG00000182872 | HGLibA_40474 | 105 | -0.103743029 | RBM10 | RNA binding motif protein 10 [Source:HGNC Symbol;Acc:HGNC:9896] |
| ENSG00000183580 | HGLibA_17017 | 105 | -0.103743029 | FBXL7 | F-box and leucine rich repeat protein 7 [Source:HGNC Symbol;Acc:HGNC:13604] |
| ENSG00000184156 | HGLibA_24371 | 105 | -0.103743029 | KCNQ3 | potassium voltage-gated channel subfamily Q member 3 [Source:HGNC Symbol;Acc:HGNC:6297] |
| ENSG00000184345 | HGLibA_23506 | 105 | -0.103743029 | IQCF2 | IQ motif containing F2 [Source:HGNC Symbol;Acc:HGNC:31815] |
| ENSG00000186376 | HGLibA_56679 | 105 | -0.103743029 | ZNF75D | zinc finger protein 75D [Source:HGNC Symbol;Acc:HGNC:13145] |
| ENSG00000186919 | HGLibA_54767 | 105 | -0.103743029 | ZACN | zinc activated ion channel [Source:HGNC Symbol;Acc:HGNC:29504] |
| ENSG00000187049 | HGLibA_50222 | 105 | -0.103743029 | TMEM216 | transmembrane protein 216 [Source:HGNC Symbol;Acc:HGNC:25018] |
| ENSG00000188786 | HGLibA_30349 | 105 | -0.103743029 | MTF1 | metal regulatory transcription factor 1 [Source:HGNC Symbol;Acc:HGNC:7428] |
| ENSG00000189357 | HGLibA_46502 | 105 | -0.103743029 | SPATA31D4 | SPATA31 subfamily D member 4 [Source:HGNC Symbol;Acc:HGNC:38601] |
| ENSG00000189376 | HGLibA_06646 | 105 | -0.103743029 | C8orf76 | chromosome 8 open reading frame 76 [Source:HGNC Symbol;Acc:HGNC:25924] |
| ENSG00000196482 | HGLibA_15599 | 105 | -0.103743029 | ESRRG | estrogen related receptor gamma [Source:HGNC Symbol;Acc:HGNC:3474] |
| ENSG00000196503 | HGLibA_03001 | 105 | -0.103743029 | ARL9 | ADP ribosylation factor like GTPase 9 [Source:HGNC Symbol;Acc:HGNC:23592] |
| ENSG00000196653 | HGLibA_56112 | 105 | -0.103743029 | ZNF502 | zinc finger protein 502 [Source:HGNC Symbol;Acc:HGNC:23718] |
| ENSG00000198586 | HGLibA_49621 | 105 | -0.103743029 | TLK1 | tousled like kinase 1 [Source:HGNC Symbol;Acc:HGNC:11841] |
| ENSG00000198589 | HGLibA_27133 | 105 | -0.103743029 | LRBA | LPS responsive beige-like anchor protein [Source:HGNC Symbol;Acc:HGNC:1742] |
| ENSG00000205097 | HGLibA_17996 | 105 | -0.103743029 | FRG2 | FSHD region gene 2 [Source:HGNC Symbol;Acc:HGNC:19136] |
| ENSG00000212126 | HGLibA_48420 | 105 | -0.103743029 | TAS2R50 | taste 2 receptor member 50 [Source:HGNC Symbol;Acc:HGNC:18882] |
| ENSG00000212128 | HGLibA_48370 | 105 | -0.103743029 | TAS2R13 | taste 2 receptor member 13 [Source:HGNC Symbol;Acc:HGNC:14919] |
| ENSG00000213064 | HGLibA_43755 | 105 | -0.103743029 | SFT2D2 | SFT2 domain containing 2 [Source:HGNC Symbol;Acc:HGNC:25140] |
| ENSG00000213199 | HGLibA_03304 | 105 | -0.103743029 | ASIC3 | acid sensing ion channel subunit 3 [Source:HGNC Symbol;Acc:HGNC:101] |
| ENSG00000214285 | HGLibA_32523 | 105 | -0.103743029 | NPS | neuropeptide S [Source:HGNC Symbol;Acc:HGNC:33940] |
| ENSG00000214643 | HGLibA_12903 | 105 | -0.103743029 | DEFB133 | defensin beta 133 [Source:HGNC Symbol;Acc:HGNC:31331] |
| ENSG00000223569 | HGLibA_53227 | 105 | -0.103743029 | USP17L15 | ubiquitin specific peptidase 17-like family member 15 [Source:HGNC Symbol;Acc:HGNC:44443] |
| ENSG00000227051 | HGLibA_05323 | 105 | -0.103743029 | C14orf132 | chromosome 14 open reading frame 132 [Source:HGNC Symbol;Acc:HGNC:20346] |
| ENSG00000233412 | HGLibA_34294 | 105 | -0.103743029 | OR5H15 | olfactory receptor family 5 subfamily H member 15 [Source:HGNC Symbol;Acc:HGNC:31287] |
| ENSG00000236027 | HGLibA_35278 | 105 | -0.103743029 | PATE3 | prostate and testis expressed 3 [Source:HGNC Symbol;Acc:HGNC:35426] |
| ENSG00000241123 | HGLibA_25613 | 105 | -0.103743029 | KRTAP10-5 | keratin associated protein 10-5 [Source:HGNC Symbol;Acc:HGNC:22969] |
| ENSG00000242715 | HGLibA_07681 | 105 | -0.103743029 | CCDC169 | coiled-coil domain containing 169 [Source:HGNC Symbol;Acc:HGNC:34361] |
| ENSG00000243279 | HGLibA_38269 | 105 | -0.103743029 | PRAF2 | PRA1 domain family member 2 [Source:HGNC Symbol;Acc:HGNC:28911] |
| ENSG00000267978 | HGLibA_27926 | 105 | -0.103743029 | MAGEA9B | MAGE family member A9B [Source:HGNC Symbol;Acc:HGNC:31909] |
| HIST1H4F | HGLibA_21501 | 105 | -0.103743029 | HIST1H4F | - |
| HNRPLL | HGLibA_21857 | 105 | -0.103743029 | HNRPLL | - |
| hsa-mir-1247 | HGLibA_57385 | 105 | -0.103743029 | hsa-mir-1247 | - |
| hsa-mir-1289-1 | HGLibA_57620 | 105 | -0.103743029 | hsa-mir-1289-1 | - |
| hsa-mir-1298 | HGLibA_57672 | 105 | -0.103743029 | hsa-mir-1298 | - |
| hsa-mir-182 | HGLibA_57982 | 105 | -0.103743029 | hsa-mir-182 | - |
| hsa-mir-1909 | HGLibA_58030 | 105 | -0.103743029 | hsa-mir-1909 | - |
| hsa-mir-3198-2 | HGLibA_58892 | 105 | -0.103743029 | hsa-mir-3198-2 | - |
| hsa-mir-3664 | HGLibA_59207 | 105 | -0.103743029 | hsa-mir-3664 | - |
| hsa-mir-411 | HGLibA_59683 | 105 | -0.103743029 | hsa-mir-411 | - |
| hsa-mir-4309 | HGLibA_59949 | 105 | -0.103743029 | hsa-mir-4309 | - |
| hsa-mir-4462 | HGLibA_60253 | 105 | -0.103743029 | hsa-mir-4462 | - |
| hsa-mir-4473 | HGLibA_60302 | 105 | -0.103743029 | hsa-mir-4473 | - |
| hsa-mir-548a-2 | HGLibA_61842 | 105 | -0.103743029 | hsa-mir-548a-2 | - |
| hsa-mir-613 | HGLibA_62666 | 105 | -0.103743029 | hsa-mir-613 | - |
| hsa-mir-660 | HGLibA_62975 | 105 | -0.103743029 | hsa-mir-660 | - |
| hsa-mir-662 | HGLibA_62983 | 105 | -0.103743029 | hsa-mir-662 | - |
| hsa-mir-6716 | HGLibA_63024 | 105 | -0.103743029 | hsa-mir-6716 | - |
| hsa-mir-6814 | HGLibA_63435 | 105 | -0.103743029 | hsa-mir-6814 | - |
| hsa-mir-6851 | HGLibA_63576 | 105 | -0.103743029 | hsa-mir-6851 | - |
| hsa-mir-8085 | HGLibA_64182 | 105 | -0.103743029 | hsa-mir-8085 | - |
| hsa-mir-92a-2 | HGLibA_64293 | 105 | -0.103743029 | hsa-mir-92a-2 | - |
| LRRC33 | HGLibA_27318 | 105 | -0.103743029 | LRRC33 | - |
| PPIAL4B | HGLibA_37901 | 105 | -0.103743029 | PPIAL4B | - |
| SHOX_Y | HGLibA_44073 | 105 | -0.103743029 | SHOX_Y | - |
| TMEM56 | HGLibA_50449 | 105 | -0.103743029 | TMEM56 | - |
| TSTA3 | HGLibA_52026 | 105 | -0.103743029 | TSTA3 | - |
| WISP1 | HGLibA_54320 | 105 | -0.103743029 | WISP1 | - |
| C19orf45 | HGLibA_05665 | 104 | -0.113694067 | C19orf45 | - |
| C1orf63 | HGLibA_05882 | 104 | -0.113694067 | C1orf63 | - |
| CD97 | HGLibA_08486 | 104 | -0.113694067 | CD97 | - |
| ENSG00000000003 | HGLibA_51943 | 104 | -0.113694067 | TSPAN6 | tetraspanin 6 [Source:HGNC Symbol;Acc:HGNC:11858] |
| ENSG00000007312 | HGLibA_08450 | 104 | -0.113694067 | CD79B | CD79b molecule [Source:HGNC Symbol;Acc:HGNC:1699] |
| ENSG00000017427 | HGLibA_22791 | 104 | -0.113694067 | IGF1 | insulin like growth factor 1 [Source:HGNC Symbol;Acc:HGNC:5464] |
| ENSG00000036257 | HGLibA_11780 | 104 | -0.113694067 | CUL3 | cullin 3 [Source:HGNC Symbol;Acc:HGNC:2553] |
| ENSG00000037042 | HGLibA_52306 | 104 | -0.113694067 | TUBG2 | tubulin gamma 2 [Source:HGNC Symbol;Acc:HGNC:12419] |
| ENSG00000062038 | HGLibA_08676 | 104 | -0.113694067 | CDH3 | cadherin 3 [Source:HGNC Symbol;Acc:HGNC:1762] |
| ENSG00000068912 | HGLibA_15490 | 104 | -0.113694067 | ERLEC1 | endoplasmic reticulum lectin 1 [Source:HGNC Symbol;Acc:HGNC:25222] |
| ENSG00000069188 | HGLibA_43154 | 104 | -0.113694067 | SDK2 | sidekick cell adhesion molecule 2 [Source:HGNC Symbol;Acc:HGNC:19308] |
| ENSG00000077150 | HGLibA_31819 | 104 | -0.113694067 | NFKB2 | nuclear factor kappa B subunit 2 [Source:HGNC Symbol;Acc:HGNC:7795] |
| ENSG00000083457 | HGLibA_23753 | 104 | -0.113694067 | ITGAE | integrin subunit alpha E [Source:HGNC Symbol;Acc:HGNC:6147] |
| ENSG00000085719 | HGLibA_10958 | 104 | -0.113694067 | CPNE3 | copine 3 [Source:HGNC Symbol;Acc:HGNC:2316] |
| ENSG00000095370 | HGLibA_43895 | 104 | -0.113694067 | SH2D3C | SH2 domain containing 3C [Source:HGNC Symbol;Acc:HGNC:16884] |
| ENSG00000099940 | HGLibA_45880 | 104 | -0.113694067 | SNAP29 | synaptosome associated protein 29 [Source:HGNC Symbol;Acc:HGNC:11133] |
| ENSG00000102043 | HGLibA_30438 | 104 | -0.113694067 | MTMR8 | myotubularin related protein 8 [Source:HGNC Symbol;Acc:HGNC:16825] |
| ENSG00000104221 | HGLibA_04800 | 104 | -0.113694067 | BRF2 | RNA polymerase III transcription initiation factor subunit [Source:HGNC Symbol;Acc:HGNC:17298]" |
| ENSG00000104812 | HGLibA_20710 | 104 | -0.113694067 | GYS1 | glycogen synthase 1 [Source:HGNC Symbol;Acc:HGNC:4706] |
| ENSG00000105732 | HGLibA_56286 | 104 | -0.113694067 | ZNF574 | zinc finger protein 574 [Source:HGNC Symbol;Acc:HGNC:26166] |
| ENSG00000106078 | HGLibA_10461 | 104 | -0.113694067 | COBL | cordon-bleu WH2 repeat protein [Source:HGNC Symbol;Acc:HGNC:22199] |
| ENSG00000107331 | HGLibA_00107 | 104 | -0.113694067 | ABCA2 | ATP binding cassette subfamily A member 2 [Source:HGNC Symbol;Acc:HGNC:32] |
| ENSG00000107341 | HGLibA_52653 | 104 | -0.113694067 | UBE2R2 | ubiquitin conjugating enzyme E2 R2 [Source:HGNC Symbol;Acc:HGNC:19907] |
| ENSG00000108587 | HGLibA_19684 | 104 | -0.113694067 | GOSR1 | golgi SNAP receptor complex member 1 [Source:HGNC Symbol;Acc:HGNC:4430] |
| ENSG00000109971 | HGLibA_22296 | 104 | -0.113694067 | HSPA8 | heat shock protein family A (Hsp70) member 8 [Source:HGNC Symbol;Acc:HGNC:5241] |
| ENSG00000110675 | HGLibA_14940 | 104 | -0.113694067 | ELMOD1 | ELMO domain containing 1 [Source:HGNC Symbol;Acc:HGNC:25334] |
| ENSG00000111218 | HGLibA_38638 | 104 | -0.113694067 | PRMT8 | protein arginine methyltransferase 8 [Source:HGNC Symbol;Acc:HGNC:5188] |
| ENSG00000111653 | HGLibA_23291 | 104 | -0.113694067 | ING4 | inhibitor of growth family member 4 [Source:HGNC Symbol;Acc:HGNC:19423] |
| ENSG00000112053 | HGLibA_44807 | 104 | -0.113694067 | SLC26A8 | solute carrier family 26 member 8 [Source:HGNC Symbol;Acc:HGNC:14468] |
| ENSG00000112234 | HGLibA_17009 | 104 | -0.113694067 | FBXL4 | F-box and leucine rich repeat protein 4 [Source:HGNC Symbol;Acc:HGNC:13601] |
| ENSG00000117226 | HGLibA_18734 | 104 | -0.113694067 | GBP3 | guanylate binding protein 3 [Source:HGNC Symbol;Acc:HGNC:4184] |
| ENSG00000118495 | HGLibA_36953 | 104 | -0.113694067 | PLAGL1 | PLAG1 like zinc finger 1 [Source:HGNC Symbol;Acc:HGNC:9046] |
| ENSG00000120235 | HGLibA_22687 | 104 | -0.113694067 | IFNA6 | interferon alpha 6 [Source:HGNC Symbol;Acc:HGNC:5427] |
| ENSG00000121691 | HGLibA_07352 | 104 | -0.113694067 | CAT | catalase [Source:HGNC Symbol;Acc:HGNC:1516] |
| ENSG00000121858 | HGLibA_50796 | 104 | -0.113694067 | TNFSF10 | TNF superfamily member 10 [Source:HGNC Symbol;Acc:HGNC:11925] |
| ENSG00000122435 | HGLibA_51677 | 104 | -0.113694067 | TRMT13 | tRNA methyltransferase 13 homolog [Source:HGNC Symbol;Acc:HGNC:25502] |
| ENSG00000122574 | HGLibA_54310 | 104 | -0.113694067 | WIPF3 | WAS/WASL interacting protein family member 3 [Source:HGNC Symbol;Acc:HGNC:22004] |
| ENSG00000123338 | HGLibA_31313 | 104 | -0.113694067 | NCKAP1L | NCK associated protein 1 like [Source:HGNC Symbol;Acc:HGNC:4862] |
| ENSG00000123505 | HGLibA_01757 | 104 | -0.113694067 | AMD1 | adenosylmethionine decarboxylase 1 [Source:HGNC Symbol;Acc:HGNC:457] |
| ENSG00000123607 | HGLibA_52072 | 104 | -0.113694067 | TTC21B | tetratricopeptide repeat domain 21B [Source:HGNC Symbol;Acc:HGNC:25660] |
| ENSG00000124767 | HGLibA_19284 | 104 | -0.113694067 | GLO1 | glyoxalase I [Source:HGNC Symbol;Acc:HGNC:4323] |
| ENSG00000126522 | HGLibA_03317 | 104 | -0.113694067 | ASL | argininosuccinate lyase [Source:HGNC Symbol;Acc:HGNC:746] |
| ENSG00000128422 | HGLibA_25434 | 104 | -0.113694067 | KRT17 | keratin 17 [Source:HGNC Symbol;Acc:HGNC:6427] |
| ENSG00000129422 | HGLibA_30504 | 104 | -0.113694067 | MTUS1 | microtubule associated scaffold protein 1 [Source:HGNC Symbol;Acc:HGNC:29789] |
| ENSG00000130222 | HGLibA_18422 | 104 | -0.113694067 | GADD45G | growth arrest and DNA damage inducible gamma [Source:HGNC Symbol;Acc:HGNC:4097] |
| ENSG00000130508 | HGLibA_39650 | 104 | -0.113694067 | PXDN | peroxidasin [Source:HGNC Symbol;Acc:HGNC:14966] |
| ENSG00000131378 | HGLibA_40949 | 104 | -0.113694067 | RFTN1 | lipid raft linker 1 [Source:HGNC Symbol;Acc:HGNC:30278]" |
| ENSG00000132128 | HGLibA_27355 | 104 | -0.113694067 | LRRC41 | leucine rich repeat containing 41 [Source:HGNC Symbol;Acc:HGNC:16917] |
| ENSG00000133134 | HGLibA_04444 | 104 | -0.113694067 | BEX2 | brain expressed X-linked 2 [Source:HGNC Symbol;Acc:HGNC:30933] |
| ENSG00000134595 | HGLibA_46257 | 104 | -0.113694067 | SOX3 | SRY-box 3 [Source:HGNC Symbol;Acc:HGNC:11199] |
| ENSG00000135763 | HGLibA_53138 | 104 | -0.113694067 | URB2 | URB2 ribosome biogenesis homolog [Source:HGNC Symbol;Acc:HGNC:28967] |
| ENSG00000137090 | HGLibA_13416 | 104 | -0.113694067 | DMRT1 | doublesex and mab-3 related transcription factor 1 [Source:HGNC Symbol;Acc:HGNC:2934] |
| ENSG00000137154 | HGLibA_42156 | 104 | -0.113694067 | RPS6 | ribosomal protein S6 [Source:HGNC Symbol;Acc:HGNC:10429] |
| ENSG00000137504 | HGLibA_11116 | 104 | -0.113694067 | CREBZF | CREB/ATF bZIP transcription factor [Source:HGNC Symbol;Acc:HGNC:24905] |
| ENSG00000138829 | HGLibA_16959 | 104 | -0.113694067 | FBN2 | fibrillin 2 [Source:HGNC Symbol;Acc:HGNC:3604] |
| ENSG00000139269 | HGLibA_23310 | 104 | -0.113694067 | INHBE | inhibin subunit beta E [Source:HGNC Symbol;Acc:HGNC:24029] |
| ENSG00000139410 | HGLibA_43174 | 104 | -0.113694067 | SDSL | serine dehydratase like [Source:HGNC Symbol;Acc:HGNC:30404] |
| ENSG00000140025 | HGLibA_14523 | 104 | -0.113694067 | EFCAB11 | EF-hand calcium binding domain 11 [Source:HGNC Symbol;Acc:HGNC:20357] |
| ENSG00000140093 | HGLibA_43507 | 104 | -0.113694067 | SERPINA10 | serpin family A member 10 [Source:HGNC Symbol;Acc:HGNC:15996] |
| ENSG00000140450 | HGLibA_03122 | 104 | -0.113694067 | ARRDC4 | arrestin domain containing 4 [Source:HGNC Symbol;Acc:HGNC:28087] |
| ENSG00000140538 | HGLibA_32879 | 104 | -0.113694067 | NTRK3 | neurotrophic receptor tyrosine kinase 3 [Source:HGNC Symbol;Acc:HGNC:8033] |
| ENSG00000141446 | HGLibA_15557 | 104 | -0.113694067 | ESCO1 | establishment of sister chromatid cohesion N-acetyltransferase 1 [Source:HGNC Symbol;Acc:HGNC:24645] |
| ENSG00000141449 | HGLibA_20238 | 104 | -0.113694067 | GREB1L | GREB1 like retinoic acid receptor coactivator [Source:HGNC Symbol;Acc:HGNC:31042] |

| | | | | | |
|---|---|---|---|---|---|
| ENSG00000141837 | HGLibA_06874 | 104 | -0.113694067 | CACNA1A | calcium voltage-gated channel subunit alpha1 A [Source:HGNC Symbol;Acc:HGNC:1388] |
| ENSG00000142089 | HGLibA_22647 | 104 | -0.113694067 | IFITM3 | interferon induced transmembrane protein 3 [Source:HGNC Symbol;Acc:HGNC:5414] |
| ENSG00000143125 | HGLibA_38665 | 104 | -0.113694067 | PROK1 | prokineticin 1 [Source:HGNC Symbol;Acc:HGNC:18454] |
| ENSG00000143196 | HGLibA_13903 | 104 | -0.113694067 | DPT | dermatopontin [Source:HGNC Symbol;Acc:HGNC:3011] |
| ENSG00000143603 | HGLibA_24359 | 104 | -0.113694067 | KCNN3 | potassium calcium-activated channel subfamily N member 3 [Source:HGNC Symbol;Acc:HGNC:6292] |
| ENSG00000146410 | HGLibA_30368 | 104 | -0.113694067 | MTFR2 | mitochondrial fission regulator 2 [Source:HGNC Symbol;Acc:HGNC:21115] |
| ENSG00000147256 | HGLibA_02769 | 104 | -0.113694067 | ARHGAP36 | Rho GTPase activating protein 36 [Source:HGNC Symbol;Acc:HGNC:26388] |
| ENSG00000152910 | HGLibA_10431 | 104 | -0.113694067 | CNTNAP4 | contactin associated protein like 4 [Source:HGNC Symbol;Acc:HGNC:18747] |
| ENSG00000152926 | HGLibA_55446 | 104 | -0.113694067 | ZNF117 | zinc finger protein 117 [Source:HGNC Symbol;Acc:HGNC:12897] |
| ENSG00000154227 | HGLibA_09206 | 104 | -0.113694067 | CERS3 | ceramide synthase 3 [Source:HGNC Symbol;Acc:HGNC:23752] |
| ENSG00000154262 | HGLibA_00119 | 104 | -0.113694067 | ABCA6 | ATP binding cassette subfamily A member 6 [Source:HGNC Symbol;Acc:HGNC:36] |
| ENSG00000157445 | HGLibA_06910 | 104 | -0.113694067 | CACNA2D3 | calcium voltage-gated channel auxiliary subunit alpha2delta 3 [Source:HGNC Symbol;Acc:HGNC:15460] |
| ENSG00000158470 | HGLibA_04034 | 104 | -0.113694067 | B4GALT5 | 4-galactosyltransferase 5 [Source:HGNC Symbol;Acc:HGNC:928]" |
| ENSG00000162551 | HGLibA_01708 | 104 | -0.113694067 | ALPL | biomineralization associated [Source:HGNC Symbol;Acc:HGNC:438]" |
| ENSG00000163235 | HGLibA_49205 | 104 | -0.113694067 | TGFA | transforming growth factor alpha [Source:HGNC Symbol;Acc:HGNC:11765] |
| ENSG00000163993 | HGLibA_42585 | 104 | -0.113694067 | S100P | S100 calcium binding protein P [Source:HGNC Symbol;Acc:HGNC:10504] |
| ENSG00000164362 | HGLibA_49019 | 104 | -0.113694067 | TERT | telomerase reverse transcriptase [Source:HGNC Symbol;Acc:HGNC:11730] |
| ENSG00000164442 | HGLibA_09744 | 104 | -0.113694067 | CITED2 | Cbp/p300 interacting transactivator with Glu/Asp rich carboxy-terminal domain 2 [Source:HGNC Symbol;Acc:HGNC:1987] |
| ENSG00000164651 | HGLibA_46315 | 104 | -0.113694067 | SP8 | Sp8 transcription factor [Source:HGNC Symbol;Acc:HGNC:19196] |
| ENSG00000165168 | HGLibA_12019 | 104 | -0.113694067 | CYBB | cytochrome b-245 beta chain [Source:HGNC Symbol;Acc:HGNC:2578] |
| ENSG00000165204 | HGLibA_33683 | 104 | -0.113694067 | OR1K1 | olfactory receptor family 1 subfamily K member 1 [Source:HGNC Symbol;Acc:HGNC:8212] |
| ENSG00000167656 | HGLibA_27653 | 104 | -0.113694067 | LY6D | lymphocyte antigen 6 family member D [Source:HGNC Symbol;Acc:HGNC:13348] |
| ENSG00000168477 | HGLibA_50931 | 104 | -0.113694067 | TNXB | tenascin XB [Source:HGNC Symbol;Acc:HGNC:11976] |
| ENSG00000169154 | HGLibA_19692 | 104 | -0.113694067 | GOT1L1 | glutamic-oxaloacetic transaminase 1 like 1 [Source:HGNC Symbol;Acc:HGNC:28487] |
| ENSG00000169155 | HGLibA_54889 | 104 | -0.113694067 | ZBTB43 | zinc finger and BTB domain containing 43 [Source:HGNC Symbol;Acc:HGNC:17908] |
| ENSG00000170122 | HGLibA_17838 | 104 | -0.113694067 | FOXD4 | forkhead box D4 [Source:HGNC Symbol;Acc:HGNC:3805] |
| ENSG00000170310 | HGLibA_47659 | 104 | -0.113694067 | STX8 | syntaxin 8 [Source:HGNC Symbol;Acc:HGNC:11443] |
| ENSG00000171100 | HGLibA_30407 | 104 | -0.113694067 | MTM1 | myotubularin 1 [Source:HGNC Symbol;Acc:HGNC:7448] |
| ENSG00000171497 | HGLibA_37924 | 104 | -0.113694067 | PPID | peptidylprolyl isomerase D [Source:HGNC Symbol;Acc:HGNC:9257] |
| ENSG00000173083 | HGLibA_22062 | 104 | -0.113694067 | HPSE | heparanase [Source:HGNC Symbol;Acc:HGNC:5164] |
| ENSG00000173230 | HGLibA_19649 | 104 | -0.113694067 | GOLGB1 | golgin B1 [Source:HGNC Symbol;Acc:HGNC:4429] |
| ENSG00000173517 | HGLibA_36007 | 104 | -0.113694067 | PEAK1 | pseudopodium enriched atypical kinase 1 [Source:HGNC Symbol;Acc:HGNC:29431] |
| ENSG00000173898 | HGLibA_46884 | 104 | -0.113694067 | SPTBN2 | non-erythrocytic 2 [Source:HGNC Symbol;Acc:HGNC:11276]" |
| ENSG00000174136 | HGLibA_41016 | 104 | -0.113694067 | RGMB | repulsive guidance molecule BMP co-receptor b [Source:HGNC Symbol;Acc:HGNC:26896] |
| ENSG00000175445 | HGLibA_27104 | 104 | -0.113694067 | LPL | lipoprotein lipase [Source:HGNC Symbol;Acc:HGNC:6677] |
| ENSG00000175756 | HGLibA_03876 | 104 | -0.113694067 | AURKAIP1 | aurora kinase A interacting protein 1 [Source:HGNC Symbol;Acc:HGNC:24114] |
| ENSG00000175899 | HGLibA_00008 | 104 | -0.113694067 | A2M | alpha-2-macroglobulin [Source:HGNC Symbol;Acc:HGNC:7] |
| ENSG00000176401 | HGLibA_14699 | 104 | -0.113694067 | EID2B | EP300 interacting inhibitor of differentiation 2B [Source:HGNC Symbol;Acc:HGNC:26796] |
| ENSG00000176473 | HGLibA_54052 | 104 | -0.113694067 | WDR25 | WD repeat domain 25 [Source:HGNC Symbol;Acc:HGNC:21064] |
| ENSG00000176723 | HGLibA_56857 | 104 | -0.113694067 | ZNF843 | zinc finger protein 843 [Source:HGNC Symbol;Acc:HGNC:28710] |
| ENSG00000176903 | HGLibA_37367 | 104 | -0.113694067 | PNMA1 | PNMA family member 1 [Source:HGNC Symbol;Acc:HGNC:9158] |
| ENSG00000177283 | HGLibA_18283 | 104 | -0.113694067 | FZD8 | frizzled class receptor 8 [Source:HGNC Symbol;Acc:HGNC:4046] |
| ENSG00000178928 | HGLibA_51193 | 104 | -0.113694067 | TPRX1 | tetrapeptide repeat homeobox 1 [Source:HGNC Symbol;Acc:HGNC:32174] |
| ENSG00000181982 | HGLibA_07636 | 104 | -0.113694067 | CCDC149 | coiled-coil domain containing 149 [Source:HGNC Symbol;Acc:HGNC:25405] |
| ENSG00000182263 | HGLibA_17518 | 104 | -0.113694067 | FIGN | microtubule severing factor [Source:HGNC Symbol;Acc:HGNC:13285]" |
| ENSG00000182810 | HGLibA_12689 | 104 | -0.113694067 | DDX28 | DEAD-box helicase 28 [Source:HGNC Symbol;Acc:HGNC:17330] |
| ENSG00000184083 | HGLibA_16062 | 104 | -0.113694067 | FAM120C | family with sequence similarity 120C [Source:HGNC Symbol;Acc:HGNC:16949] |
| ENSG00000184451 | HGLibA_08146 | 104 | -0.113694067 | CCR10 | C-C motif chemokine receptor 10 [Source:HGNC Symbol;Acc:HGNC:4474] |
| ENSG00000184908 | HGLibA_09845 | 104 | -0.113694067 | CLCNKB | chloride voltage-gated channel Kb [Source:HGNC Symbol;Acc:HGNC:2027] |
| ENSG00000185019 | HGLibA_52727 | 104 | -0.113694067 | UBOX5 | U-box domain containing 5 [Source:HGNC Symbol;Acc:HGNC:17777] |
| ENSG00000185101 | HGLibA_02194 | 104 | -0.113694067 | ANO9 | anoctamin 9 [Source:HGNC Symbol;Acc:HGNC:20679] |
| ENSG00000186364 | HGLibA_32959 | 104 | -0.113694067 | NUDT17 | nudix hydrolase 17 [Source:HGNC Symbol;Acc:HGNC:26618] |
| ENSG00000186715 | HGLibA_30256 | 104 | -0.113694067 | MST1L | macrophage stimulating 1 like [Source:HGNC Symbol;Acc:HGNC:7390] |
| ENSG00000186918 | HGLibA_55912 | 104 | -0.113694067 | ZNF395 | zinc finger protein 395 [Source:HGNC Symbol;Acc:HGNC:18737] |
| ENSG00000187372 | HGLibA_35482 | 104 | -0.113694067 | PCDHB13 | protocadherin beta 13 [Source:HGNC Symbol;Acc:HGNC:8684] |
| ENSG00000187527 | HGLibA_03579 | 104 | -0.113694067 | ATP13A5 | ATPase 13A5 [Source:HGNC Symbol;Acc:HGNC:31789] |
| ENSG00000187736 | HGLibA_31886 | 104 | -0.113694067 | NHEJ1 | non-homologous end joining factor 1 [Source:HGNC Symbol;Acc:HGNC:25737] |
| ENSG00000187792 | HGLibA_56573 | 104 | -0.113694067 | ZNF70 | zinc finger protein 70 [Source:HGNC Symbol;Acc:HGNC:13140] |
| ENSG00000188211 | HGLibA_31371 | 104 | -0.113694067 | NCR3LG1 | natural killer cell cytotoxicity receptor 3 ligand 1 [Source:HGNC Symbol;Acc:HGNC:42400] |
| ENSG00000188878 | HGLibA_16935 | 104 | -0.113694067 | FBF1 | Fas binding factor 1 [Source:HGNC Symbol;Acc:HGNC:24674] |
| ENSG00000197119 | HGLibA_44696 | 104 | -0.113694067 | SLC25A29 | solute carrier family 25 member 29 [Source:HGNC Symbol;Acc:HGNC:20116] |
| ENSG00000198010 | HGLibA_13342 | 104 | -0.113694067 | DLGAP2 | DLG associated protein 2 [Source:HGNC Symbol;Acc:HGNC:2906] |
| ENSG00000198855 | HGLibA_17507 | 104 | -0.113694067 | FICD | FIC domain containing [Source:HGNC Symbol;Acc:HGNC:18416] |
| ENSG00000198910 | HGLibA_25884 | 104 | -0.113694067 | L1CAM | L1 cell adhesion molecule [Source:HGNC Symbol;Acc:HGNC:6470] |
| ENSG00000204505 | HGLibA_38331 | 104 | -0.113694067 | PRAMEF9 | PRAME family member 9 [Source:HGNC Symbol;Acc:HGNC:27996] |
| ENSG00000204842 | HGLibA_03837 | 104 | -0.113694067 | ATXN2 | ataxin 2 [Source:HGNC Symbol;Acc:HGNC:10555] |
| ENSG00000205327 | HGLibA_34400 | 104 | -0.113694067 | OR6C68 | olfactory receptor family 6 subfamily C member 68 [Source:HGNC Symbol;Acc:HGNC:31297] |
| ENSG00000211448 | HGLibA_13242 | 104 | -0.113694067 | DIO2 | iodothyronine deiodinase 2 [Source:HGNC Symbol;Acc:HGNC:2884] |
| ENSG00000213516 | HGLibA_40601 | 104 | -0.113694067 | RBMXL1 | RBMX like 1 [Source:HGNC Symbol;Acc:HGNC:25073] |
| ENSG00000213967 | HGLibA_56632 | 104 | -0.113694067 | ZNF726 | zinc finger protein 726 [Source:HGNC Symbol;Acc:HGNC:32462] |
| ENSG00000243646 | HGLibA_22950 | 104 | -0.113694067 | IL10RB | interleukin 10 receptor subunit beta [Source:HGNC Symbol;Acc:HGNC:5965] |
| ENSG00000255819 | HGLibA_25296 | 104 | -0.113694067 | KLRC4-KLRK1 | KLRC4-KLRK1 readthrough [Source:HGNC Symbol;Acc:HGNC:48357] |
| ENSG00000258405 | HGLibA_56299 | 104 | -0.113694067 | ZNF578 | zinc finger protein 578 [Source:HGNC Symbol;Acc:HGNC:26449] |
| ENSG00000261210 | HGLibA_09952 | 104 | -0.113694067 | CLEC19A | C-type lectin domain containing 19A [Source:HGNC Symbol;Acc:HGNC:34522] |
| ENSG00000263353 | HGLibA_37898 | 104 | -0.113694067 | PPIAL4A | peptidylprolyl isomerase A like 4A [Source:HGNC Symbol;Acc:HGNC:24369] |
| ENSG00000269743 | HGLibA_44770 | 104 | -0.113694067 | SLC25A53 | solute carrier family 25 member 53 [Source:HGNC Symbol;Acc:HGNC:31894] |
| ENSG00000277632 | HGLibA_08017 | 104 | -0.113694067 | CCL3 | C-C motif chemokine ligand 3 [Source:HGNC Symbol;Acc:HGNC:10627] |
| ENSG00000277972 | HGLibA_09732 | 104 | -0.113694067 | CISD3 | CDGSH iron sulfur domain 3 [Source:HGNC Symbol;Acc:HGNC:27578] |
| FAM46A | HGLibA_16596 | 104 | -0.113694067 | FAM46A | - |
| hsa-mir-3130-1 | HGLibA_58572 | 104 | -0.113694067 | hsa-mir-3130-1 | - |
| hsa-mir-3615 | HGLibA_59089 | 104 | -0.113694067 | hsa-mir-3615 | - |
| hsa-mir-3657 | HGLibA_59172 | 104 | -0.113694067 | hsa-mir-3657 | - |
| hsa-mir-3690-1 | HGLibA_59346 | 104 | -0.113694067 | hsa-mir-3690-1 | - |
| hsa-mir-378h | HGLibA_59459 | 104 | -0.113694067 | hsa-mir-378h | - |
| hsa-mir-4297 | HGLibA_59898 | 104 | -0.113694067 | hsa-mir-4297 | - |
| hsa-mir-4303 | HGLibA_59925 | 104 | -0.113694067 | hsa-mir-4303 | - |
| hsa-mir-4418 | HGLibA_60055 | 104 | -0.113694067 | hsa-mir-4418 | - |
| hsa-mir-4651 | HGLibA_60710 | 104 | -0.113694067 | hsa-mir-4651 | - |
| hsa-mir-4752 | HGLibA_61105 | 104 | -0.113694067 | hsa-mir-4752 | - |
| hsa-mir-4804 | HGLibA_61322 | 104 | -0.113694067 | hsa-mir-4804 | - |
| hsa-mir-498 | HGLibA_61393 | 104 | -0.113694067 | hsa-mir-498 | - |
| hsa-mir-5197 | HGLibA_61707 | 104 | -0.113694067 | hsa-mir-5197 | - |
| hsa-mir-526a-1 | HGLibA_61790 | 104 | -0.113694067 | hsa-mir-526a-1 | - |
| hsa-mir-548f-3 | HGLibA_61979 | 104 | -0.113694067 | hsa-mir-548f-3 | - |
| hsa-mir-551b | HGLibA_62122 | 104 | -0.113694067 | hsa-mir-551b | - |
| hsa-mir-6069 | HGLibA_62526 | 104 | -0.113694067 | hsa-mir-6069 | - |
| hsa-mir-609 | HGLibA_62623 | 104 | -0.113694067 | hsa-mir-609 | - |
| hsa-mir-6888 | HGLibA_63736 | 104 | -0.113694067 | hsa-mir-6888 | - |
| hsa-mir-924 | HGLibA_64288 | 104 | -0.113694067 | hsa-mir-924 | - |
| MTERFD1 | HGLibA_30341 | 104 | -0.113694067 | MTERFD1 | - |
| RLTPR | HGLibA_41362 | 104 | -0.113694067 | RLTPR | - |
| TENC1 | HGLibA_48992 | 104 | -0.113694067 | TENC1 | - |
| TMEM55A | HGLibA_50444 | 104 | -0.113694067 | TMEM55A | - |
| ACN9 | HGLibA_00459 | 103 | -0.123645104 | ACN9 | - |
| C12orf39 | HGLibA_05232 | 103 | -0.123645104 | C12orf39 | - |
| C13orf35 | HGLibA_05310 | 103 | -0.123645104 | C13orf35 | - |
| C17orf59 | HGLibA_05553 | 103 | -0.123645104 | C17orf59 | - |
| C19orf21 | HGLibA_05635 | 103 | -0.123645104 | C19orf21 | - |
| CASC10 | HGLibA_07270 | 103 | -0.123645104 | CASC10 | - |
| ENSG00000006042 | HGLibA_50569 | 103 | -0.123645104 | TMEM98 | transmembrane protein 98 [Source:HGNC Symbol;Acc:HGNC:24529] |
| ENSG00000006059 | HGLibA_25481 | 103 | -0.123645104 | KRT33A | keratin 33A [Source:HGNC Symbol;Acc:HGNC:6450] |
| ENSG00000006740 | HGLibA_02780 | 103 | -0.123645104 | ARHGAP44 | Rho GTPase activating protein 44 [Source:HGNC Symbol;Acc:HGNC:29096] |
| ENSG00000025708 | HGLibA_52458 | 103 | -0.123645104 | TYMP | thymidine phosphorylase [Source:HGNC Symbol;Acc:HGNC:3148] |
| ENSG00000026297 | HGLibA_41441 | 103 | -0.123645104 | RNASET2 | ribonuclease T2 [Source:HGNC Symbol;Acc:HGNC:21686] |
| ENSG00000036448 | HGLibA_30893 | 103 | -0.123645104 | MYOM2 | myomesin 2 [Source:HGNC Symbol;Acc:HGNC:7614] |
| ENSG00000039068 | HGLibA_08630 | 103 | -0.123645104 | CDH1 | cadherin 1 [Source:HGNC Symbol;Acc:HGNC:1748] |
| ENSG00000049167 | HGLibA_15454 | 103 | -0.123645104 | ERCC8 | CSA ubiquitin ligase complex subunit [Source:HGNC Symbol;Acc:HGNC:3439]" |
| ENSG00000049323 | HGLibA_27589 | 103 | -0.123645104 | LTBP1 | latent transforming growth factor beta binding protein 1 [Source:HGNC Symbol;Acc:HGNC:6714] |
| ENSG00000062194 | HGLibA_19755 | 103 | -0.123645104 | GPBP1 | GC-rich promoter binding protein 1 [Source:HGNC Symbol;Acc:HGNC:29520] |
| ENSG00000065883 | HGLibA_08733 | 103 | -0.123645104 | CDK13 | cyclin dependent kinase 13 [Source:HGNC Symbol;Acc:HGNC:1733] |
| ENSG00000070413 | HGLibA_13045 | 103 | -0.123645104 | DGCR2 | DiGeorge syndrome critical region gene 2 [Source:HGNC Symbol;Acc:HGNC:2845] |
| ENSG00000074966 | HGLibA_52388 | 103 | -0.123645104 | TXK | TXK tyrosine kinase [Source:HGNC Symbol;Acc:HGNC:12434] |
| ENSG00000076258 | HGLibA_17711 | 103 | -0.123645104 | FMO4 | flavin containing monooxygenase 4 [Source:HGNC Symbol;Acc:HGNC:3772] |
| ENSG00000077800 | HGLibA_17582 | 103 | -0.123645104 | FKBP6 | FK506 binding protein 6 [Source:HGNC Symbol;Acc:HGNC:3722] |
| ENSG00000086288 | HGLibA_32176 | 103 | -0.123645104 | NME8 | NME/NM23 family member 8 [Source:HGNC Symbol;Acc:HGNC:16473] |
| ENSG00000089022 | HGLibA_28306 | 103 | -0.123645104 | MAPKAPK5 | mitogen-activated protein kinase-activated protein kinase 5 [Source:HGNC Symbol;Acc:HGNC:6889] |
| ENSG00000099385 | HGLibA_04345 | 103 | -0.123645104 | BCL7C | BCL tumor suppressor 7C [Source:HGNC Symbol;Acc:HGNC:1006] |
| ENSG00000100276 | HGLibA_40358 | 103 | -0.123645104 | RASL10A | RAS like family 10 member A [Source:HGNC Symbol;Acc:HGNC:16954] |
| ENSG00000100288 | HGLibA_09461 | 103 | -0.123645104 | CHKB | choline kinase beta [Source:HGNC Symbol;Acc:HGNC:1938] |
| ENSG00000100575 | HGLibA_49529 | 103 | -0.123645104 | TIMM9 | translocase of inner mitochondrial membrane 9 [Source:HGNC Symbol;Acc:HGNC:11819] |
| ENSG00000100604 | HGLibA_09432 | 103 | -0.123645104 | CHGA | chromogranin A [Source:HGNC Symbol;Acc:HGNC:1929] |
| ENSG00000103145 | HGLibA_20962 | 103 | -0.123645104 | HCFC1R1 | host cell factor C1 regulator 1 [Source:HGNC Symbol;Acc:HGNC:21198] |
| ENSG00000104660 | HGLibA_26329 | 103 | -0.123645104 | LEPROTL1 | leptin receptor overlapping transcript like 1 [Source:HGNC Symbol;Acc:HGNC:6555] |
| ENSG00000105821 | HGLibA_13595 | 103 | -0.123645104 | DNAJC2 | DnaJ heat shock protein family (Hsp40) member C2 [Source:HGNC Symbol;Acc:HGNC:13192] |
| ENSG00000106336 | HGLibA_17051 | 103 | -0.123645104 | FBXO24 | F-box protein 24 [Source:HGNC Symbol;Acc:HGNC:13595] |

| | | | | | |
|---|---|---|---|---|---|
| ENSG00000106608 | HGLibA_53141 | 103 | -0.123645104 | URGCP | upregulator of cell proliferation [Source:HGNC Symbol;Acc:HGNC:30890] |
| ENSG00000108702 | HGLibA_07961 | 103 | -0.123645104 | CCL1 | C-C motif chemokine ligand 1 [Source:HGNC Symbol;Acc:HGNC:10609] |
| ENSG00000109790 | HGLibA_25215 | 103 | -0.123645104 | KLHL5 | kelch like family member 5 [Source:HGNC Symbol;Acc:HGNC:6356] |
| ENSG00000110851 | HGLibA_38388 | 103 | -0.123645104 | PRDM4 | PR/SET domain 4 [Source:HGNC Symbol;Acc:HGNC:9348] |
| ENSG00000111269 | HGLibA_11111 | 103 | -0.123645104 | CREBL2 | cAMP responsive element binding protein like 2 [Source:HGNC Symbol;Acc:HGNC:2350] |
| ENSG00000111713 | HGLibA_20712 | 103 | -0.123645104 | GYS2 | glycogen synthase 2 [Source:HGNC Symbol;Acc:HGNC:4707] |
| ENSG00000112280 | HGLibA_10619 | 103 | -0.123645104 | COL9A1 | collagen type IX alpha 1 chain [Source:HGNC Symbol;Acc:HGNC:2217] |
| ENSG00000114988 | HGLibA_26665 | 103 | -0.123645104 | LMAN2L | mannose binding 2 like [Source:HGNC Symbol;Acc:HGNC:19263]" |
| ENSG00000116761 | HGLibA_11626 | 103 | -0.123645104 | CTH | cystathionine gamma-lyase [Source:HGNC Symbol;Acc:HGNC:2501] |
| ENSG00000117748 | HGLibA_41802 | 103 | -0.123645104 | RPA2 | replication protein A2 [Source:HGNC Symbol;Acc:HGNC:10290] |
| ENSG00000118156 | HGLibA_56191 | 103 | -0.123645104 | ZNF541 | zinc finger protein 541 [Source:HGNC Symbol;Acc:HGNC:25294] |
| ENSG00000119986 | HGLibA_03900 | 103 | -0.123645104 | AVPI1 | arginine vasopressin induced 1 [Source:HGNC Symbol;Acc:HGNC:30898] |
| ENSG00000122547 | HGLibA_14517 | 103 | -0.123645104 | EEPD1 | endonuclease/exonuclease/phosphatase family domain containing 1 [Source:HGNC Symbol;Acc:HGNC:22223] |
| ENSG00000123407 | HGLibA_21962 | 103 | -0.123645104 | HOXC12 | homeobox C12 [Source:HGNC Symbol;Acc:HGNC:5124] |
| ENSG00000123570 | HGLibA_39995 | 103 | -0.123645104 | RAB9B | member RAS oncogene family [Source:HGNC Symbol;Acc:HGNC:14090]" |
| ENSG00000123584 | HGLibA_27922 | 103 | -0.123645104 | MAGEA9 | MAGE family member A9 [Source:HGNC Symbol;Acc:HGNC:6807] |
| ENSG00000124209 | HGLibA_39842 | 103 | -0.123645104 | RAB22A | member RAS oncogene family [Source:HGNC Symbol;Acc:HGNC:9764]" |
| ENSG00000125355 | HGLibA_50331 | 103 | -0.123645104 | TMEM255A | transmembrane protein 255A [Source:HGNC Symbol;Acc:HGNC:26086] |
| ENSG00000125999 | HGLibA_04719 | 103 | -0.123645104 | BPIFB1 | BPI fold containing family B member 1 [Source:HGNC Symbol;Acc:HGNC:16108] |
| ENSG00000126249 | HGLibA_35729 | 103 | -0.123645104 | PDCD2L | programmed cell death 2 like [Source:HGNC Symbol;Acc:HGNC:28194] |
| ENSG00000127412 | HGLibA_51794 | 103 | -0.123645104 | TRPV5 | transient receptor potential cation channel subfamily V member 5 [Source:HGNC Symbol;Acc:HGNC:3145] |
| ENSG00000128271 | HGLibA_01014 | 103 | -0.123645104 | ADORA2A | adenosine A2a receptor [Source:HGNC Symbol;Acc:HGNC:263] |
| ENSG00000130303 | HGLibA_04885 | 103 | -0.123645104 | BST2 | bone marrow stromal cell antigen 2 [Source:HGNC Symbol;Acc:HGNC:1119] |
| ENSG00000130522 | HGLibA_24008 | 103 | -0.123645104 | JUND | AP-1 transcription factor subunit [Source:HGNC Symbol;Acc:HGNC:6206]" |
| ENSG00000130656 | HGLibA_20944 | 103 | -0.123645104 | HBZ | hemoglobin subunit zeta [Source:HGNC Symbol;Acc:HGNC:4835] |
| ENSG00000130751 | HGLibA_32393 | 103 | -0.123645104 | NPAS1 | neuronal PAS domain protein 1 [Source:HGNC Symbol;Acc:HGNC:7894] |
| ENSG00000130935 | HGLibA_32259 | 103 | -0.123645104 | NOL11 | nucleolar protein 11 [Source:HGNC Symbol;Acc:HGNC:24557] |
| ENSG00000130956 | HGLibA_20795 | 103 | -0.123645104 | HABP4 | hyaluronan binding protein 4 [Source:HGNC Symbol;Acc:HGNC:17062] |
| ENSG00000131437 | HGLibA_24934 | 103 | -0.123645104 | KIF3A | kinesin family member 3A [Source:HGNC Symbol;Acc:HGNC:6319] |
| ENSG00000131941 | HGLibA_41225 | 103 | -0.123645104 | RHPN2 | rhophilin Rho GTPase binding protein 2 [Source:HGNC Symbol;Acc:HGNC:19974] |
| ENSG00000133983 | HGLibA_10814 | 103 | -0.123645104 | COX16 | cytochrome c oxidase assembly factor COX16 [Source:HGNC Symbol;Acc:HGNC:20213] |
| ENSG00000134765 | HGLibA_13990 | 103 | -0.123645104 | DSC1 | desmocollin 1 [Source:HGNC Symbol;Acc:HGNC:3035] |
| ENSG00000134970 | HGLibA_49821 | 103 | -0.123645104 | TMED7 | transmembrane p24 trafficking protein 7 [Source:HGNC Symbol;Acc:HGNC:24253] |
| ENSG00000135631 | HGLibA_39806 | 103 | -0.123645104 | RAB11FIP5 | RAB11 family interacting protein 5 [Source:HGNC Symbol;Acc:HGNC:24845] |
| ENSG00000136098 | HGLibA_31651 | 103 | -0.123645104 | NEK3 | NIMA related kinase 3 [Source:HGNC Symbol;Acc:HGNC:7746] |
| ENSG00000136378 | HGLibA_00838 | 103 | -0.123645104 | ADAMTS7 | ADAM metallopeptidase with thrombospondin type 1 motif 7 [Source:HGNC Symbol;Acc:HGNC:223] |
| ENSG00000136514 | HGLibA_42431 | 103 | -0.123645104 | RTP4 | receptor transporter protein 4 [Source:HGNC Symbol;Acc:HGNC:23992] |
| ENSG00000137275 | HGLibA_41315 | 103 | -0.123645104 | RIPK1 | receptor interacting serine/threonine kinase 1 [Source:HGNC Symbol;Acc:HGNC:10019] |
| ENSG00000137815 | HGLibA_42384 | 103 | -0.123645104 | RTF1 | Paf1/RNA polymerase II complex component [Source:HGNC Symbol;Acc:HGNC:28996]" |
| ENSG00000138050 | HGLibA_49419 | 103 | -0.123645104 | THUMPD2 | THUMP domain containing 2 [Source:HGNC Symbol;Acc:HGNC:14890] |
| ENSG00000138175 | HGLibA_02964 | 103 | -0.123645104 | ARL3 | ADP ribosylation factor like GTPase 3 [Source:HGNC Symbol;Acc:HGNC:694] |
| ENSG00000139131 | HGLibA_54637 | 103 | -0.123645104 | YARS2 | tyrosyl-tRNA synthetase 2 [Source:HGNC Symbol;Acc:HGNC:24249] |
| ENSG00000139291 | HGLibA_50138 | 103 | -0.123645104 | TMEM19 | transmembrane protein 19 [Source:HGNC Symbol;Acc:HGNC:25605] |
| ENSG00000139684 | HGLibA_15563 | 103 | -0.123645104 | ESD | esterase D [Source:HGNC Symbol;Acc:HGNC:3465] |
| ENSG00000140093 | HGLibA_43509 | 103 | -0.123645104 | SERPINA10 | serpin family A member 10 [Source:HGNC Symbol;Acc:HGNC:15996] |
| ENSG00000144134 | HGLibA_40027 | 103 | -0.123645104 | RABL2A | member of RAS oncogene family like 2A [Source:HGNC Symbol;Acc:HGNC:9799]" |
| ENSG00000144369 | HGLibA_16270 | 103 | -0.123645104 | FAM171B | family with sequence similarity 171 member B [Source:HGNC Symbol;Acc:HGNC:29412] |
| ENSG00000145241 | HGLibA_09045 | 103 | -0.123645104 | CENPC | centromere protein C [Source:HGNC Symbol;Acc:HGNC:1854] |
| ENSG00000146411 | HGLibA_44861 | 103 | -0.123645104 | SLC2A12 | solute carrier family 2 member 12 [Source:HGNC Symbol;Acc:HGNC:18067] |
| ENSG00000148798 | HGLibA_23268 | 103 | -0.123645104 | INA | internexin neuronal intermediate filament protein alpha [Source:HGNC Symbol;Acc:HGNC:6057] |
| ENSG00000150261 | HGLibA_34545 | 103 | -0.123645104 | OR8K1 | olfactory receptor family 8 subfamily K member 1 [Source:HGNC Symbol;Acc:HGNC:14831] |
| ENSG00000151320 | HGLibA_01439 | 103 | -0.123645104 | AKAP6 | A-kinase anchoring protein 6 [Source:HGNC Symbol;Acc:HGNC:376] |
| ENSG00000154165 | HGLibA_19928 | 103 | -0.123645104 | GPR15 | G protein-coupled receptor 15 [Source:HGNC Symbol;Acc:HGNC:4469] |
| ENSG00000154822 | HGLibA_37018 | 103 | -0.123645104 | PLCL2 | phospholipase C like 2 [Source:HGNC Symbol;Acc:HGNC:9064] |
| ENSG00000154845 | HGLibA_38204 | 103 | -0.123645104 | PPP4R1 | protein phosphatase 4 regulatory subunit 1 [Source:HGNC Symbol;Acc:HGNC:9320] |
| ENSG00000156298 | HGLibA_51946 | 103 | -0.123645104 | TSPAN7 | tetraspanin 7 [Source:HGNC Symbol;Acc:HGNC:11854] |
| ENSG00000158815 | HGLibA_17381 | 103 | -0.123645104 | FGF17 | fibroblast growth factor 17 [Source:HGNC Symbol;Acc:HGNC:3673] |
| ENSG00000158865 | HGLibA_45258 | 103 | -0.123645104 | SLC5A11 | solute carrier family 5 member 11 [Source:HGNC Symbol;Acc:HGNC:23091] |
| ENSG00000159723 | HGLibA_01253 | 103 | -0.123645104 | AGRP | agouti related neuropeptide [Source:HGNC Symbol;Acc:HGNC:330] |
| ENSG00000160323 | HGLibA_00800 | 103 | -0.123645104 | ADAMTS13 | ADAM metallopeptidase with thrombospondin type 1 motif 13 [Source:HGNC Symbol;Acc:HGNC:1366] |
| ENSG00000161929 | HGLibA_42943 | 103 | -0.123645104 | SCIMP | SLP adaptor and CSK interacting membrane protein [Source:HGNC Symbol;Acc:HGNC:33504] |
| ENSG00000162366 | HGLibA_35995 | 103 | -0.123645104 | PDZK1IP1 | PDZK1 interacting protein 1 [Source:HGNC Symbol;Acc:HGNC:16887] |
| ENSG00000162694 | HGLibA_15811 | 103 | -0.123645104 | EXTL2 | exostosin like glycosyltransferase 2 [Source:HGNC Symbol;Acc:HGNC:3516] |
| ENSG00000163093 | HGLibA_04221 | 103 | -0.123645104 | BBS5 | Bardet-Biedl syndrome 5 [Source:HGNC Symbol;Acc:HGNC:970] |
| ENSG00000163497 | HGLibA_17310 | 103 | -0.123645104 | FEV | ETS transcription factor [Source:HGNC Symbol;Acc:HGNC:18562]" |
| ENSG00000164136 | HGLibA_22981 | 103 | -0.123645104 | IL15 | interleukin 15 [Source:HGNC Symbol;Acc:HGNC:5977] |
| ENSG00000164434 | HGLibA_15927 | 103 | -0.123645104 | FABP7 | fatty acid binding protein 7 [Source:HGNC Symbol;Acc:HGNC:3562] |
| ENSG00000164822 | HGLibA_12808 | 103 | -0.123645104 | DEFA6 | defensin alpha 6 [Source:HGNC Symbol;Acc:HGNC:2765] |
| ENSG00000165192 | HGLibA_03208 | 103 | -0.123645104 | ASB11 | ankyrin repeat and SOCS box containing 11 [Source:HGNC Symbol;Acc:HGNC:17186] |
| ENSG00000165288 | HGLibA_04856 | 103 | -0.123645104 | BRWD3 | bromodomain and WD repeat domain containing 3 [Source:HGNC Symbol;Acc:HGNC:17342] |
| ENSG00000166509 | HGLibA_09972 | 103 | -0.123645104 | CLEC3A | C-type lectin domain family 3 member A [Source:HGNC Symbol;Acc:HGNC:2052] |
| ENSG00000167037 | HGLibA_43858 | 103 | -0.123645104 | SGSM1 | small G protein signaling modulator 1 [Source:HGNC Symbol;Acc:HGNC:29410] |
| ENSG00000167178 | HGLibA_23672 | 103 | -0.123645104 | ISLR2 | immunoglobulin superfamily containing leucine rich repeat 2 [Source:HGNC Symbol;Acc:HGNC:29286] |
| ENSG00000167525 | HGLibA_38653 | 103 | -0.123645104 | PROCA1 | protein interacting with cyclin A1 [Source:HGNC Symbol;Acc:HGNC:28600] |
| ENSG00000167657 | HGLibA_12328 | 103 | -0.123645104 | DAPK3 | death associated protein kinase 3 [Source:HGNC Symbol;Acc:HGNC:2676] |
| ENSG00000167800 | HGLibA_48643 | 103 | -0.123645104 | TBX10 | T-box 10 [Source:HGNC Symbol;Acc:HGNC:11593] |
| ENSG00000168065 | HGLibA_44539 | 103 | -0.123645104 | SLC22A11 | solute carrier family 22 member 11 [Source:HGNC Symbol;Acc:HGNC:18120] |
| ENSG00000169504 | HGLibA_10031 | 103 | -0.123645104 | CLIC4 | chloride intracellular channel 4 [Source:HGNC Symbol;Acc:HGNC:13518] |
| ENSG00000170836 | HGLibA_37970 | 103 | -0.123645104 | PPM1D | Mg2+/Mn2+ dependent 1D [Source:HGNC Symbol;Acc:HGNC:9277]" |
| ENSG00000170921 | HGLibA_48294 | 103 | -0.123645104 | TANC2 | ankyrin repeat and coiled-coil containing 2 [Source:HGNC Symbol;Acc:HGNC:30212]" |
| ENSG00000171729 | HGLibA_50430 | 103 | -0.123645104 | TMEM51 | transmembrane protein 51 [Source:HGNC Symbol;Acc:HGNC:25488] |
| ENSG00000171766 | HGLibA_18695 | 103 | -0.123645104 | GATM | glycine amidinotransferase [Source:HGNC Symbol;Acc:HGNC:4175] |
| ENSG00000172399 | HGLibA_30904 | 103 | -0.123645104 | MYOZ2 | myozenin 2 [Source:HGNC Symbol;Acc:HGNC:1330] |
| ENSG00000173660 | HGLibA_53123 | 103 | -0.123645104 | UQCRH | ubiquinol-cytochrome c reductase hinge protein [Source:HGNC Symbol;Acc:HGNC:12590] |
| ENSG00000174307 | HGLibA_36424 | 103 | -0.123645104 | PHLDA3 | pleckstrin homology like domain family A member 3 [Source:HGNC Symbol;Acc:HGNC:8934] |
| ENSG00000174521 | HGLibA_52163 | 103 | -0.123645104 | TTC9B | tetratricopeptide repeat domain 9B [Source:HGNC Symbol;Acc:HGNC:26395] |
| ENSG00000174951 | HGLibA_18179 | 103 | -0.123645104 | FUT1 | fucosyltransferase 1 (H blood group) [Source:HGNC Symbol;Acc:HGNC:4012] |
| ENSG00000174996 | HGLibA_25031 | 103 | -0.123645104 | KLC2 | kinesin light chain 2 [Source:HGNC Symbol;Acc:HGNC:20716] |
| ENSG00000175931 | HGLibA_52641 | 103 | -0.123645104 | UBE2O | ubiquitin conjugating enzyme E2 O [Source:HGNC Symbol;Acc:HGNC:29554] |
| ENSG00000176142 | HGLibA_50383 | 103 | -0.123645104 | TMEM39A | transmembrane protein 39A [Source:HGNC Symbol;Acc:HGNC:25600] |
| ENSG00000176171 | HGLibA_04656 | 103 | -0.123645104 | BNIP3 | BCL2 interacting protein 3 [Source:HGNC Symbol;Acc:HGNC:1084] |
| ENSG00000176358 | HGLibA_48156 | 103 | -0.123645104 | TAC4 | tachykinin 4 [Source:HGNC Symbol;Acc:HGNC:16641] |
| ENSG00000176797 | HGLibA_12814 | 103 | -0.123645104 | DEFB103A | defensin beta 103A [Source:HGNC Symbol;Acc:HGNC:15967] |
| ENSG00000178502 | HGLibA_25127 | 103 | -0.123645104 | KLHL11 | kelch like family member 11 [Source:HGNC Symbol;Acc:HGNC:19008] |
| ENSG00000178662 | HGLibA_11430 | 103 | -0.123645104 | CSRNP3 | cysteine and serine rich nuclear protein 3 [Source:HGNC Symbol;Acc:HGNC:30729] |
| ENSG00000181418 | HGLibA_12625 | 103 | -0.123645104 | DDN | dendrin [Source:HGNC Symbol;Acc:HGNC:24458] |
| ENSG00000181939 | HGLibA_33947 | 103 | -0.123645104 | OR4C15 | olfactory receptor family 4 subfamily C member 15 [Source:HGNC Symbol;Acc:HGNC:15171] |
| ENSG00000182168 | HGLibA_53019 | 103 | -0.123645104 | UNC5C | unc-5 netrin receptor C [Source:HGNC Symbol;Acc:HGNC:12569] |
| ENSG00000183060 | HGLibA_27772 | 103 | -0.123645104 | LYSMD4 | LysM domain containing 4 [Source:HGNC Symbol;Acc:HGNC:26571] |
| ENSG00000183060 | HGLibA_27773 | 103 | -0.123645104 | LYSMD4 | LysM domain containing 4 [Source:HGNC Symbol;Acc:HGNC:26571] |
| ENSG00000183862 | HGLibA_10265 | 103 | -0.123645104 | CNGA2 | cyclic nucleotide gated channel alpha 2 [Source:HGNC Symbol;Acc:HGNC:2149] |
| ENSG00000184033 | HGLibA_11562 | 103 | -0.123645104 | CTAG1B | cancer/testis antigen 1B [Source:HGNC Symbol;Acc:HGNC:2491] |
| ENSG00000184887 | HGLibA_04918 | 103 | -0.123645104 | BTBD6 | BTB domain containing 6 [Source:HGNC Symbol;Acc:HGNC:19897] |
| ENSG00000184898 | HGLibA_40555 | 103 | -0.123645104 | RBM43 | RNA binding motif protein 43 [Source:HGNC Symbol;Acc:HGNC:24790] |
| ENSG00000185252 | HGLibA_56660 | 103 | -0.123645104 | ZNF74 | zinc finger protein 74 [Source:HGNC Symbol;Acc:HGNC:13144] |
| ENSG00000185479 | HGLibA_25517 | 103 | -0.123645104 | KRT6B | keratin 6B [Source:HGNC Symbol;Acc:HGNC:6444] |
| ENSG00000185513 | HGLibA_25897 | 103 | -0.123645104 | L3MBTL1 | histone methyl-lysine binding protein [Source:HGNC Symbol;Acc:HGNC:15905]" |
| ENSG00000186777 | HGLibA_56648 | 103 | -0.123645104 | ZNF732 | zinc finger protein 732 [Source:HGNC Symbol;Acc:HGNC:37138] |
| ENSG00000187080 | HGLibA_33755 | 103 | -0.123645104 | OR2AK2 | olfactory receptor family 2 subfamily AK member 2 [Source:HGNC Symbol;Acc:HGNC:19569] |
| ENSG00000187091 | HGLibA_36990 | 103 | -0.123645104 | PLCD1 | phospholipase C delta 1 [Source:HGNC Symbol;Acc:HGNC:9060] |
| ENSG00000187969 | HGLibA_55013 | 103 | -0.123645104 | ZCCHC13 | zinc finger CCHC-type containing 13 [Source:HGNC Symbol;Acc:HGNC:31749] |
| ENSG00000188039 | HGLibA_33123 | 103 | -0.123645104 | NWD1 | NACHT and WD repeat domain containing 1 [Source:HGNC Symbol;Acc:HGNC:27619] |
| ENSG00000188386 | HGLibA_38197 | 103 | -0.123645104 | PPP3R2 | beta [Source:HGNC Symbol;Acc:HGNC:9318]" |
| ENSG00000188828 | HGLibA_19306 | 103 | -0.123645104 | GLRA4 | glycine receptor alpha 4 [Source:HGNC Symbol;Acc:HGNC:31715] |
| ENSG00000196914 | HGLibA_02815 | 103 | -0.123645104 | ARHGEF12 | Rho guanine nucleotide exchange factor 12 [Source:HGNC Symbol;Acc:HGNC:14193] |
| ENSG00000197448 | HGLibA_20469 | 103 | -0.123645104 | GSTK1 | glutathione S-transferase kappa 1 [Source:HGNC Symbol;Acc:HGNC:16906] |
| ENSG00000197753 | HGLibA_26441 | 103 | -0.123645104 | LHFPL5 | LHFPL tetraspan subfamily member 5 [Source:HGNC Symbol;Acc:HGNC:21253] |
| ENSG00000197816 | HGLibA_07721 | 103 | -0.123645104 | CCDC180 | coiled-coil domain containing 180 [Source:HGNC Symbol;Acc:HGNC:29303] |
| ENSG00000198336 | HGLibA_30762 | 103 | -0.123645104 | MYL4 | myosin light chain 4 [Source:HGNC Symbol;Acc:HGNC:7585] |
| ENSG00000198642 | HGLibA_25227 | 103 | -0.123645104 | KLHL9 | kelch like family member 9 [Source:HGNC Symbol;Acc:HGNC:18732] |
| ENSG00000198963 | HGLibA_41779 | 103 | -0.123645104 | RORB | RAR related orphan receptor B [Source:HGNC Symbol;Acc:HGNC:10259] |
| ENSG00000203618 | HGLibA_19702 | 103 | -0.123645104 | GP1BB | glycoprotein Ib platelet subunit beta [Source:HGNC Symbol;Acc:HGNC:4440] |
| ENSG00000203722 | HGLibA_40123 | 103 | -0.123645104 | RAET1G | retinoic acid early transcript 1G [Source:HGNC Symbol;Acc:HGNC:16795] |
| ENSG00000204130 | HGLibA_42439 | 103 | -0.123645104 | RUFY2 | RUN and FYVE domain containing 2 [Source:HGNC Symbol;Acc:HGNC:19761] |
| ENSG00000204518 | HGLibA_00038 | 103 | -0.123645104 | AADACL4 | arylacetamide deacetylase like 4 [Source:HGNC Symbol;Acc:HGNC:32038] |
| ENSG00000204539 | HGLibA_08886 | 103 | -0.123645104 | CDSN | corneodesmosin [Source:HGNC Symbol;Acc:HGNC:1802] |
| ENSG00000205496 | HGLibA_34073 | 103 | -0.123645104 | OR51A2 | olfactory receptor family 51 subfamily A member 2 [Source:HGNC Symbol;Acc:HGNC:14764] |
| ENSG00000205593 | HGLibA_12969 | 103 | -0.123645104 | DENND6B | DENN domain containing 6B [Source:HGNC Symbol;Acc:HGNC:32690] |
| ENSG00000206203 | HGLibA_52011 | 103 | -0.123645104 | TSSK2 | testis specific serine kinase 2 [Source:HGNC Symbol;Acc:HGNC:11401] |
| ENSG00000212127 | HGLibA_48371 | 103 | -0.123645104 | TAS2R14 | taste 2 receptor member 14 [Source:HGNC Symbol;Acc:HGNC:14920] |
| ENSG00000215568 | HGLibA_18330 | 103 | -0.123645104 | GAB4 | GRB2 associated binding protein family member 4 [Source:HGNC Symbol;Acc:HGNC:18325] |
| ENSG00000229972 | HGLibA_23509 | 103 | -0.123645104 | IQCF3 | IQ motif containing F3 [Source:HGNC Symbol;Acc:HGNC:31816] |
| ENSG00000237172 | HGLibA_04005 | 103 | -0.123645104 | B3GNT9 | 3-N-acetylglucosaminyltransferase 9 [Source:HGNC Symbol;Acc:HGNC:28714]" |
| ENSG00000237515 | HGLibA_44053 | 103 | -0.123645104 | SHISA9 | shisa family member 9 [Source:HGNC Symbol;Acc:HGNC:37231] |
| ENSG00000241563 | HGLibA_10798 | 103 | -0.123645104 | CORT | cortistatin [Source:HGNC Symbol;Acc:HGNC:2257] |

| | | | | | |
|---|---|---|---|---|---|
| ENSG00000253831 | HGLibA_15656 | 103 | -0.123645104 | ETV3L | ETS variant 3 like [Source:HGNC Symbol;Acc:HGNC:33834] |
| ENSG00000258644 | HGLibA_48011 | 103 | -0.123645104 | SYNJ2BP-COX16 | SYNJ2BP-COX16 readthrough [Source:HGNC Symbol;Acc:HGNC:48350] |
| ENSG00000273976 | HGLibA_19618 | 103 | -0.123645104 | GOLGA6L1 | golgin A6 family-like 1 [Source:HGNC Symbol;Acc:HGNC:37444] |
| FLJ22184 | HGLibA_17621 | 103 | -0.123645104 | FLJ22184 | - |
| HKR1 | HGLibA_21592 | 103 | -0.123645104 | HKR1 | - |
| HRASLS5 | HGLibA_22082 | 103 | -0.123645104 | HRASLS5 | - |
| hsa-mir-1204 | HGLibA_57274 | 103 | -0.123645104 | hsa-mir-1204 | - |
| hsa-mir-1296 | HGLibA_57664 | 103 | -0.123645104 | hsa-mir-1296 | - |
| hsa-mir-1301 | HGLibA_57680 | 103 | -0.123645104 | hsa-mir-1301 | - |
| hsa-mir-181a-1 | HGLibA_57960 | 103 | -0.123645104 | hsa-mir-181a-1 | - |
| hsa-mir-1827 | HGLibA_57991 | 103 | -0.123645104 | hsa-mir-1827 | - |
| hsa-mir-186 | HGLibA_58006 | 103 | -0.123645104 | hsa-mir-186 | - |
| hsa-mir-196a-2 | HGLibA_58095 | 103 | -0.123645104 | hsa-mir-196a-2 | - |
| hsa-mir-26b | HGLibA_58371 | 103 | -0.123645104 | hsa-mir-26b | - |
| hsa-mir-32 | HGLibA_58904 | 103 | -0.123645104 | hsa-mir-32 | - |
| hsa-mir-325 | HGLibA_58968 | 103 | -0.123645104 | hsa-mir-325 | - |
| hsa-mir-4273 | HGLibA_59797 | 103 | -0.123645104 | hsa-mir-4273 | - |
| hsa-mir-4425 | HGLibA_60089 | 103 | -0.123645104 | hsa-mir-4425 | - |
| hsa-mir-4460 | HGLibA_60248 | 103 | -0.123645104 | hsa-mir-4460 | - |
| hsa-mir-4471 | HGLibA_60290 | 103 | -0.123645104 | hsa-mir-4471 | - |
| hsa-mir-4472-1 | HGLibA_60295 | 103 | -0.123645104 | hsa-mir-4472-1 | - |
| hsa-mir-4662a | HGLibA_60757 | 103 | -0.123645104 | hsa-mir-4662a | - |
| hsa-mir-504 | HGLibA_61470 | 103 | -0.123645104 | hsa-mir-504 | - |
| hsa-mir-5091 | HGLibA_61523 | 103 | -0.123645104 | hsa-mir-5091 | - |
| hsa-mir-604 | HGLibA_62508 | 103 | -0.123645104 | hsa-mir-604 | - |
| hsa-mir-628 | HGLibA_62750 | 103 | -0.123645104 | hsa-mir-628 | - |
| hsa-mir-640 | HGLibA_62799 | 103 | -0.123645104 | hsa-mir-640 | - |
| hsa-mir-6733 | HGLibA_63086 | 103 | -0.123645104 | hsa-mir-6733 | - |
| hsa-mir-6759 | HGLibA_63194 | 103 | -0.123645104 | hsa-mir-6759 | - |
| hsa-mir-6786 | HGLibA_63323 | 103 | -0.123645104 | hsa-mir-6786 | - |
| hsa-mir-6843 | HGLibA_63548 | 103 | -0.123645104 | hsa-mir-6843 | - |
| hsa-mir-892b | HGLibA_64253 | 103 | -0.123645104 | hsa-mir-892b | - |
| KIAA0355 | HGLibA_24627 | 103 | -0.123645104 | KIAA0355 | - |
| KIAA1731 | HGLibA_24811 | 103 | -0.123645104 | KIAA1731 | - |
| MFSD4 | HGLibA_29102 | 103 | -0.123645104 | MFSD4 | - |
| P2RY8_Y | HGLibA_34897 | 103 | -0.123645104 | P2RY8_Y | - |
| TMEM27 | HGLibA_50356 | 103 | -0.123645104 | TMEM27 | - |
| BET3L | HGLibA_04437 | 102 | -0.133596141 | BET3L | - |
| C15orf54 | HGLibA_05415 | 102 | -0.133596141 | C15orf54 | - |
| C2orf43 | HGLibA_06128 | 102 | -0.133596141 | C2orf43 | - |
| CXorf27 | HGLibA_11910 | 102 | -0.133596141 | CXorf27 | - |
| CYR61 | HGLibA_12220 | 102 | -0.133596141 | CYR61 | - |
| ENSG00000000003 | HGLibA_51942 | 102 | -0.133596141 | TSPAN6 | tetraspanin 6 [Source:HGNC Symbol;Acc:HGNC:11858] |
| ENSG00000015592 | HGLibA_47524 | 102 | -0.133596141 | STMN4 | stathmin 4 [Source:HGNC Symbol;Acc:HGNC:16078] |
| ENSG00000035141 | HGLibA_16138 | 102 | -0.133596141 | FAM136A | family with sequence similarity 136 member A [Source:HGNC Symbol;Acc:HGNC:25911] |
| ENSG00000049283 | HGLibA_15346 | 102 | -0.133596141 | EPN3 | epsin 3 [Source:HGNC Symbol;Acc:HGNC:18235] |
| ENSG00000056558 | HGLibA_51239 | 102 | -0.133596141 | TRAF1 | TNF receptor associated factor 1 [Source:HGNC Symbol;Acc:HGNC:12031] |
| ENSG00000058866 | HGLibA_13070 | 102 | -0.133596141 | DGKG | diacylglycerol kinase gamma [Source:HGNC Symbol;Acc:HGNC:2853] |
| ENSG00000066468 | HGLibA_17443 | 102 | -0.133596141 | FGFR2 | fibroblast growth factor receptor 2 [Source:HGNC Symbol;Acc:HGNC:3689] |
| ENSG00000068745 | HGLibA_23447 | 102 | -0.133596141 | IP6K2 | inositol hexakisphosphate kinase 2 [Source:HGNC Symbol;Acc:HGNC:17313] |
| ENSG00000071246 | HGLibA_53529 | 102 | -0.133596141 | VASH1 | vasohibin 1 [Source:HGNC Symbol;Acc:HGNC:19964] |
| ENSG00000071991 | HGLibA_08657 | 102 | -0.133596141 | CDH19 | cadherin 19 [Source:HGNC Symbol;Acc:HGNC:1758] |
| ENSG00000074219 | HGLibA_48929 | 102 | -0.133596141 | TEAD2 | TEA domain transcription factor 2 [Source:HGNC Symbol;Acc:HGNC:11715] |
| ENSG00000075336 | HGLibA_49509 | 102 | -0.133596141 | TIMM21 | translocase of inner mitochondrial membrane 21 [Source:HGNC Symbol;Acc:HGNC:25010] |
| ENSG00000078081 | HGLibA_25984 | 102 | -0.133596141 | LAMP3 | lysosomal associated membrane protein 3 [Source:HGNC Symbol;Acc:HGNC:14582] |
| ENSG00000078328 | HGLibA_40454 | 102 | -0.133596141 | RBFOX1 | RNA binding fox-1 homolog 1 [Source:HGNC Symbol;Acc:HGNC:18222] |
| ENSG00000081026 | HGLibA_28001 | 102 | -0.133596141 | MAGI3 | WW and PDZ domain containing 3 [Source:HGNC Symbol;Acc:HGNC:29647]" |
| ENSG00000083750 | HGLibA_42226 | 102 | -0.133596141 | RRAGB | Ras related GTP binding B [Source:HGNC Symbol;Acc:HGNC:19901] |
| ENSG00000086506 | HGLibA_20937 | 102 | -0.133596141 | HBQ1 | hemoglobin subunit theta 1 [Source:HGNC Symbol;Acc:HGNC:4833] |
| ENSG00000092758 | HGLibA_10625 | 102 | -0.133596141 | COL9A3 | collagen type IX alpha 3 chain [Source:HGNC Symbol;Acc:HGNC:2219] |
| ENSG00000094841 | HGLibA_53092 | 102 | -0.133596141 | UPRT | uracil phosphoribosyltransferase homolog [Source:HGNC Symbol;Acc:HGNC:28334] |
| ENSG00000099968 | HGLibA_04313 | 102 | -0.133596141 | BCL2L13 | BCL2 like 13 [Source:HGNC Symbol;Acc:HGNC:17164] |
| ENSG00000100350 | HGLibA_17954 | 102 | -0.133596141 | FOXRED2 | FAD dependent oxidoreductase domain containing 2 [Source:HGNC Symbol;Acc:HGNC:26264] |
| ENSG00000100888 | HGLibA_09408 | 102 | -0.133596141 | CHD8 | chromodomain helicase DNA binding protein 8 [Source:HGNC Symbol;Acc:HGNC:20153] |
| ENSG00000101188 | HGLibA_32885 | 102 | -0.133596141 | NTSR1 | neurotensin receptor 1 [Source:HGNC Symbol;Acc:HGNC:8039] |
| ENSG00000101307 | HGLibA_44204 | 102 | -0.133596141 | SIRPB1 | signal regulatory protein beta 1 [Source:HGNC Symbol;Acc:HGNC:15928] |
| ENSG00000101337 | HGLibA_49729 | 102 | -0.133596141 | TM9SF4 | transmembrane 9 superfamily member 4 [Source:HGNC Symbol;Acc:HGNC:30797] |
| ENSG00000103226 | HGLibA_32294 | 102 | -0.133596141 | NOMO3 | NODAL modulator 3 [Source:HGNC Symbol;Acc:HGNC:25242] |
| ENSG00000104140 | HGLibA_41212 | 102 | -0.133596141 | RHOV | ras homolog family member V [Source:HGNC Symbol;Acc:HGNC:18313] |
| ENSG00000104427 | HGLibA_54937 | 102 | -0.133596141 | ZC2HC1A | zinc finger C2HC-type containing 1A [Source:HGNC Symbol;Acc:HGNC:24277] |
| ENSG00000104611 | HGLibA_43897 | 102 | -0.133596141 | SH2D4A | SH2 domain containing 4A [Source:HGNC Symbol;Acc:HGNC:26102] |
| ENSG00000105568 | HGLibA_38141 | 102 | -0.133596141 | PPP2R1A | protein phosphatase 2 scaffold subunit Aalpha [Source:HGNC Symbol;Acc:HGNC:9302] |
| ENSG00000105662 | HGLibA_11228 | 102 | -0.133596141 | CRTC1 | CREB regulated transcription coactivator 1 [Source:HGNC Symbol;Acc:HGNC:16062] |
| ENSG00000106853 | HGLibA_39353 | 102 | -0.133596141 | PTGR1 | prostaglandin reductase 1 [Source:HGNC Symbol;Acc:HGNC:18429] |
| ENSG00000110080 | HGLibA_47246 | 102 | -0.133596141 | ST3GAL4 | 3-sialyltransferase 4 [Source:HGNC Symbol;Acc:HGNC:10864]" |
| ENSG00000111786 | HGLibA_47092 | 102 | -0.133596141 | SRSF9 | serine and arginine rich splicing factor 9 [Source:HGNC Symbol;Acc:HGNC:10791] |
| ENSG00000113595 | HGLibA_51434 | 102 | -0.133596141 | TRIM23 | tripartite motif containing 23 [Source:HGNC Symbol;Acc:HGNC:660] |
| ENSG00000114554 | HGLibA_37257 | 102 | -0.133596141 | PLXNA1 | plexin A1 [Source:HGNC Symbol;Acc:HGNC:9099] |
| ENSG00000114859 | HGLibA_09824 | 102 | -0.133596141 | CLCN2 | chloride voltage-gated channel 2 [Source:HGNC Symbol;Acc:HGNC:2020] |
| ENSG00000115129 | HGLibA_51067 | 102 | -0.133596141 | TP53I3 | tumor protein p53 inducible protein 3 [Source:HGNC Symbol;Acc:HGNC:19373] |
| ENSG00000115762 | HGLibA_37090 | 102 | -0.133596141 | PLEKHB2 | pleckstrin homology domain containing B2 [Source:HGNC Symbol;Acc:HGNC:19236] |
| ENSG00000116212 | HGLibA_27359 | 102 | -0.133596141 | LRRC42 | leucine rich repeat containing 42 [Source:HGNC Symbol;Acc:HGNC:28792] |
| ENSG00000116663 | HGLibA_17124 | 102 | -0.133596141 | FBXO6 | F-box protein 6 [Source:HGNC Symbol;Acc:HGNC:13585] |
| ENSG00000117758 | HGLibA_47621 | 102 | -0.133596141 | STX12 | syntaxin 12 [Source:HGNC Symbol;Acc:HGNC:11430] |
| ENSG00000119431 | HGLibA_21056 | 102 | -0.133596141 | HDHD3 | haloacid dehalogenase like hydrolase domain containing 3 [Source:HGNC Symbol;Acc:HGNC:28171] |
| ENSG00000121440 | HGLibA_35998 | 102 | -0.133596141 | PDZRN3 | PDZ domain containing ring finger 3 [Source:HGNC Symbol;Acc:HGNC:17704] |
| ENSG00000121964 | HGLibA_20515 | 102 | -0.133596141 | GTDC1 | glycosyltransferase like domain containing 1 [Source:HGNC Symbol;Acc:HGNC:20887] |
| ENSG00000122068 | HGLibA_18257 | 102 | -0.133596141 | FYTTD1 | forty-two-three domain containing 1 [Source:HGNC Symbol;Acc:HGNC:25407] |
| ENSG00000122257 | HGLibA_40434 | 102 | -0.133596141 | RBBP6 | ubiquitin ligase [Source:HGNC Symbol;Acc:HGNC:9889]" |
| ENSG00000123453 | HGLibA_42743 | 102 | -0.133596141 | SARDH | sarcosine dehydrogenase [Source:HGNC Symbol;Acc:HGNC:10536] |
| ENSG00000124203 | HGLibA_56836 | 102 | -0.133596141 | ZNF831 | zinc finger protein 831 [Source:HGNC Symbol;Acc:HGNC:16167] |
| ENSG00000124613 | HGLibA_55906 | 102 | -0.133596141 | ZNF391 | zinc finger protein 391 [Source:HGNC Symbol;Acc:HGNC:18779] |
| ENSG00000125675 | HGLibA_20266 | 102 | -0.133596141 | GRIA3 | glutamate ionotropic receptor AMPA type subunit 3 [Source:HGNC Symbol;Acc:HGNC:4573] |
| ENSG00000126778 | HGLibA_44243 | 102 | -0.133596141 | SIX1 | SIX homeobox 1 [Source:HGNC Symbol;Acc:HGNC:10887] |
| ENSG00000126821 | HGLibA_43851 | 102 | -0.133596141 | SGPP1 | sphingosine-1-phosphate phosphatase 1 [Source:HGNC Symbol;Acc:HGNC:17720] |
| ENSG00000127074 | HGLibA_41061 | 102 | -0.133596141 | RGS13 | regulator of G protein signaling 13 [Source:HGNC Symbol;Acc:HGNC:9995] |
| ENSG00000129028 | HGLibA_49288 | 102 | -0.133596141 | THAP10 | THAP domain containing 10 [Source:HGNC Symbol;Acc:HGNC:23193] |
| ENSG00000131591 | HGLibA_05782 | 102 | -0.133596141 | C1orf159 | chromosome 1 open reading frame 159 [Source:HGNC Symbol;Acc:HGNC:26062] |
| ENSG00000131634 | HGLibA_50190 | 102 | -0.133596141 | TMEM204 | transmembrane protein 204 [Source:HGNC Symbol;Acc:HGNC:14158] |
| ENSG00000133275 | HGLibA_11396 | 102 | -0.133596141 | CSNK1G2 | casein kinase 1 gamma 2 [Source:HGNC Symbol;Acc:HGNC:2455] |
| ENSG00000134443 | HGLibA_20367 | 102 | -0.133596141 | GRP | gastrin releasing peptide [Source:HGNC Symbol;Acc:HGNC:4605] |
| ENSG00000134533 | HGLibA_40860 | 102 | -0.133596141 | RERG | RAS like estrogen regulated growth inhibitor [Source:HGNC Symbol;Acc:HGNC:15980] |
| ENSG00000134686 | HGLibA_36321 | 102 | -0.133596141 | PHC2 | polyhomeotic homolog 2 [Source:HGNC Symbol;Acc:HGNC:3183] |
| ENSG00000135148 | HGLibA_51270 | 102 | -0.133596141 | TRAFD1 | TRAF-type zinc finger domain containing 1 [Source:HGNC Symbol;Acc:HGNC:24808] |
| ENSG00000135413 | HGLibA_25914 | 102 | -0.133596141 | LACRT | lacritin [Source:HGNC Symbol;Acc:HGNC:16430] |
| ENSG00000136839 | HGLibA_33612 | 102 | -0.133596141 | OR13C9 | olfactory receptor family 13 subfamily C member 9 [Source:HGNC Symbol;Acc:HGNC:15104] |
| ENSG00000137441 | HGLibA_17430 | 102 | -0.133596141 | FGFBP2 | fibroblast growth factor binding protein 2 [Source:HGNC Symbol;Acc:HGNC:29451] |
| ENSG00000138185 | HGLibA_15197 | 102 | -0.133596141 | ENTPD1 | ectonucleoside triphosphate diphosphohydrolase 1 [Source:HGNC Symbol;Acc:HGNC:3363] |
| ENSG00000138190 | HGLibA_15753 | 102 | -0.133596141 | EXOC6 | exocyst complex component 6 [Source:HGNC Symbol;Acc:HGNC:23196] |
| ENSG00000138400 | HGLibA_28686 | 102 | -0.133596141 | MDH1B | malate dehydrogenase 1B [Source:HGNC Symbol;Acc:HGNC:17836] |
| ENSG00000138760 | HGLibA_42873 | 102 | -0.133596141 | SCARB2 | scavenger receptor class B member 2 [Source:HGNC Symbol;Acc:HGNC:1665] |
| ENSG00000139278 | HGLibA_19259 | 102 | -0.133596141 | GLIPR1 | GLI pathogenesis related 1 [Source:HGNC Symbol;Acc:HGNC:17001] |
| ENSG00000139842 | HGLibA_11783 | 102 | -0.133596141 | CUL4A | cullin 4A [Source:HGNC Symbol;Acc:HGNC:2554] |
| ENSG00000139946 | HGLibA_36037 | 102 | -0.133596141 | PELI2 | pellino E3 ubiquitin protein ligase family member 2 [Source:HGNC Symbol;Acc:HGNC:8828] |
| ENSG00000140961 | HGLibA_34686 | 102 | -0.133596141 | OSGIN1 | oxidative stress induced growth inhibitor 1 [Source:HGNC Symbol;Acc:HGNC:30093] |
| ENSG00000140995 | HGLibA_12790 | 102 | -0.133596141 | DEF8 | differentially expressed in FDCP 8 homolog [Source:HGNC Symbol;Acc:HGNC:25969] |
| ENSG00000141068 | HGLibA_25862 | 102 | -0.133596141 | KSR1 | kinase suppressor of ras 1 [Source:HGNC Symbol;Acc:HGNC:6465] |
| ENSG00000141580 | HGLibA_54099 | 102 | -0.133596141 | WDR45B | WD repeat domain 45B [Source:HGNC Symbol;Acc:HGNC:25072] |
| ENSG00000142230 | HGLibA_42632 | 102 | -0.133596141 | SAE1 | SUMO1 activating enzyme subunit 1 [Source:HGNC Symbol;Acc:HGNC:30660] |
| ENSG00000143297 | HGLibA_17237 | 102 | -0.133596141 | FCRL5 | Fc receptor like 5 [Source:HGNC Symbol;Acc:HGNC:18508] |
| ENSG00000145029 | HGLibA_31920 | 102 | -0.133596141 | NICN1 | nicolin 1 [Source:HGNC Symbol;Acc:HGNC:18317] |
| ENSG00000145113 | HGLibA_30548 | 102 | -0.133596141 | MUC4 | cell surface associated [Source:HGNC Symbol;Acc:HGNC:7514]" |
| ENSG00000145730 | HGLibA_35083 | 102 | -0.133596141 | PAM | peptidylglycine alpha-amidating monooxygenase [Source:HGNC Symbol;Acc:HGNC:8596] |
| ENSG00000146094 | HGLibA_13773 | 102 | -0.133596141 | DOK3 | docking protein 3 [Source:HGNC Symbol;Acc:HGNC:24583] |
| ENSG00000146540 | HGLibA_06550 | 102 | -0.133596141 | C7orf50 | chromosome 7 open reading frame 50 [Source:HGNC Symbol;Acc:HGNC:22421] |
| ENSG00000147912 | HGLibA_17023 | 102 | -0.133596141 | FBXO10 | F-box protein 10 [Source:HGNC Symbol;Acc:HGNC:13589] |
| ENSG00000148700 | HGLibA_00948 | 102 | -0.133596141 | ADD3 | adducin 3 [Source:HGNC Symbol;Acc:HGNC:245] |
| ENSG00000150093 | HGLibA_23770 | 102 | -0.133596141 | ITGB1 | integrin subunit beta 1 [Source:HGNC Symbol;Acc:HGNC:6153] |
| ENSG00000150394 | HGLibA_08691 | 102 | -0.133596141 | CDH8 | cadherin 8 [Source:HGNC Symbol;Acc:HGNC:1767] |
| ENSG00000151466 | HGLibA_42949 | 102 | -0.133596141 | SCLT1 | sodium channel and clathrin linker 1 [Source:HGNC Symbol;Acc:HGNC:26406] |
| ENSG00000152464 | HGLibA_42037 | 102 | -0.133596141 | RPP38 | ribonuclease P/MRP subunit p38 [Source:HGNC Symbol;Acc:HGNC:30329] |
| ENSG00000153208 | HGLibA_28908 | 102 | -0.133596141 | MERTK | tyrosine kinase [Source:HGNC Symbol;Acc:HGNC:7027]" |
| ENSG00000153936 | HGLibA_22118 | 102 | -0.133596141 | HS2ST1 | heparan sulfate 2-O-sulfotransferase 1 [Source:HGNC Symbol;Acc:HGNC:5193] |
| ENSG00000155666 | HGLibA_24540 | 102 | -0.133596141 | KDM8 | lysine demethylase 8 [Source:HGNC Symbol;Acc:HGNC:25840] |
| ENSG00000156467 | HGLibA_53111 | 102 | -0.133596141 | UQCRB | ubiquinol-cytochrome c reductase binding protein [Source:HGNC Symbol;Acc:HGNC:12582] |

| | | | | | |
|---|---|---|---|---|---|
| ENSG00000158106 | HGLibA_41223 | 102 | -0.133596141 | RHPN1 | rhophilin Rho GTPase binding protein 1 [Source:HGNC Symbol;Acc:HGNC:19973] |
| ENSG00000158321 | HGLibA_03884 | 102 | -0.133596141 | AUTS2 | activator of transcription and developmental regulator [Source:HGNC Symbol;Acc:HGNC:14262]" |
| ENSG00000158545 | HGLibA_54969 | 102 | -0.133596141 | ZC3H18 | zinc finger CCCH-type containing 18 [Source:HGNC Symbol;Acc:HGNC:25091] |
| ENSG00000158639 | HGLibA_35016 | 102 | -0.133596141 | PAGE5 | PAGE family member 5 [Source:HGNC Symbol;Acc:HGNC:29992] |
| ENSG00000158825 | HGLibA_08497 | 102 | -0.133596141 | CDA | cytidine deaminase [Source:HGNC Symbol;Acc:HGNC:1712] |
| ENSG00000159216 | HGLibA_42455 | 102 | -0.133596141 | RUNX1 | runt related transcription factor 1 [Source:HGNC Symbol;Acc:HGNC:10471] |
| ENSG00000159261 | HGLibA_09858 | 102 | -0.133596141 | CLDN14 | claudin 14 [Source:HGNC Symbol;Acc:HGNC:2035] |
| ENSG00000159516 | HGLibA_46823 | 102 | -0.133596141 | SPRR2G | small proline rich protein 2G [Source:HGNC Symbol;Acc:HGNC:11267] |
| ENSG00000159905 | HGLibA_55608 | 102 | -0.133596141 | ZNF221 | zinc finger protein 221 [Source:HGNC Symbol;Acc:HGNC:13014] |
| ENSG00000159958 | HGLibA_50756 | 102 | -0.133596141 | TNFRSF13C | TNF receptor superfamily member 13C [Source:HGNC Symbol;Acc:HGNC:17755] |
| ENSG00000160087 | HGLibA_52622 | 102 | -0.133596141 | UBE2J2 | ubiquitin conjugating enzyme E2 J2 [Source:HGNC Symbol;Acc:HGNC:19268] |
| ENSG00000161618 | HGLibA_01534 | 102 | -0.133596141 | ALDH16A1 | aldehyde dehydrogenase 16 family member A1 [Source:HGNC Symbol;Acc:HGNC:28114] |
| ENSG00000161956 | HGLibA_43390 | 102 | -0.133596141 | SENP3 | SUMO specific peptidase 3 [Source:HGNC Symbol;Acc:HGNC:17862] |
| ENSG00000162771 | HGLibA_16686 | 102 | -0.133596141 | FAM71A | family with sequence similarity 71 member A [Source:HGNC Symbol;Acc:HGNC:26541] |
| ENSG00000163166 | HGLibA_23887 | 102 | -0.133596141 | IWS1 | SUPT6H interacting protein [Source:HGNC Symbol;Acc:HGNC:25467]" |
| ENSG00000163389 | HGLibA_37477 | 102 | -0.133596141 | POGLUT1 | protein O-glucosyltransferase 1 [Source:HGNC Symbol;Acc:HGNC:22954] |
| ENSG00000163734 | HGLibA_11868 | 102 | -0.133596141 | CXCL3 | C-X-C motif chemokine ligand 3 [Source:HGNC Symbol;Acc:HGNC:4604] |
| ENSG00000164144 | HGLibA_02676 | 102 | -0.133596141 | ARFIP1 | ADP ribosylation factor interacting protein 1 [Source:HGNC Symbol;Acc:HGNC:21496] |
| ENSG00000164305 | HGLibA_07310 | 102 | -0.133596141 | CASP3 | caspase 3 [Source:HGNC Symbol;Acc:HGNC:1504] |
| ENSG00000164532 | HGLibA_48658 | 102 | -0.133596141 | TBX20 | T-box 20 [Source:HGNC Symbol;Acc:HGNC:11598] |
| ENSG00000165471 | HGLibA_28499 | 102 | -0.133596141 | MBL2 | mannose binding lectin 2 [Source:HGNC Symbol;Acc:HGNC:6922] |
| ENSG00000166169 | HGLibA_37538 | 102 | -0.133596141 | POLL | DNA polymerase lambda [Source:HGNC Symbol;Acc:HGNC:9184] |
| ENSG00000166192 | HGLibA_43403 | 102 | -0.133596141 | SENP8 | NEDD8 specific [Source:HGNC Symbol;Acc:HGNC:22992]" |
| ENSG00000166716 | HGLibA_56337 | 102 | -0.133596141 | ZNF592 | zinc finger protein 592 [Source:HGNC Symbol;Acc:HGNC:28986] |
| ENSG00000166840 | HGLibA_19374 | 102 | -0.133596141 | GLYATL1 | glycine-N-acyltransferase like 1 [Source:HGNC Symbol;Acc:HGNC:30519] |
| ENSG00000167258 | HGLibA_08730 | 102 | -0.133596141 | CDK12 | cyclin dependent kinase 12 [Source:HGNC Symbol;Acc:HGNC:24224] |
| ENSG00000167468 | HGLibA_20179 | 102 | -0.133596141 | GPX4 | glutathione peroxidase 4 [Source:HGNC Symbol;Acc:HGNC:4556] |
| ENSG00000168826 | HGLibA_54907 | 102 | -0.133596141 | ZBTB49 | zinc finger and BTB domain containing 49 [Source:HGNC Symbol;Acc:HGNC:19883] |
| ENSG00000170748 | HGLibA_40604 | 102 | -0.133596141 | RBMXL2 | RBMX like 2 [Source:HGNC Symbol;Acc:HGNC:17886] |
| ENSG00000173894 | HGLibA_07447 | 102 | -0.133596141 | CBX2 | chromobox 2 [Source:HGNC Symbol;Acc:HGNC:1552] |
| ENSG00000174227 | HGLibA_36554 | 102 | -0.133596141 | PIGG | phosphatidylinositol glycan anchor biosynthesis class G [Source:HGNC Symbol;Acc:HGNC:25985] |
| ENSG00000175445 | HGLibA_27105 | 102 | -0.133596141 | LPL | lipoprotein lipase [Source:HGNC Symbol;Acc:HGNC:6677] |
| ENSG00000175866 | HGLibA_04122 | 102 | -0.133596141 | BAIAP2 | BAI1 associated protein 2 [Source:HGNC Symbol;Acc:HGNC:947] |
| ENSG00000175938 | HGLibA_34596 | 102 | -0.133596141 | ORAI3 | ORAI calcium release-activated calcium modulator 3 [Source:HGNC Symbol;Acc:HGNC:28185] |
| ENSG00000176472 | HGLibA_56291 | 102 | -0.133596141 | ZNF575 | zinc finger protein 575 [Source:HGNC Symbol;Acc:HGNC:27606] |
| ENSG00000176973 | HGLibA_16784 | 102 | -0.133596141 | FAM89B | family with sequence similarity 89 member B [Source:HGNC Symbol;Acc:HGNC:16708] |
| ENSG00000177427 | HGLibA_29246 | 102 | -0.133596141 | MIEF2 | mitochondrial elongation factor 2 [Source:HGNC Symbol;Acc:HGNC:17920] |
| ENSG00000177432 | HGLibA_31124 | 102 | -0.133596141 | NAP1L5 | nucleosome assembly protein 1 like 5 [Source:HGNC Symbol;Acc:HGNC:19968] |
| ENSG00000177606 | HGLibA_24002 | 102 | -0.133596141 | JUN | AP-1 transcription factor subunit [Source:HGNC Symbol;Acc:HGNC:6204]" |
| ENSG00000178602 | HGLibA_34747 | 102 | -0.133596141 | OTOS | otospiralin [Source:HGNC Symbol;Acc:HGNC:22644] |
| ENSG00000178732 | HGLibA_19707 | 102 | -0.133596141 | GP5 | glycoprotein V platelet [Source:HGNC Symbol;Acc:HGNC:4443] |
| ENSG00000179751 | HGLibA_47925 | 102 | -0.133596141 | SYCN | syncollin [Source:HGNC Symbol;Acc:HGNC:18442] |
| ENSG00000180209 | HGLibA_30795 | 102 | -0.133596141 | MYLPF | fast skeletal muscle [Source:HGNC Symbol;Acc:HGNC:29824]" |
| ENSG00000181433 | HGLibA_42643 | 102 | -0.133596141 | SAGE1 | sarcoma antigen 1 [Source:HGNC Symbol;Acc:HGNC:30369] |
| ENSG00000181625 | HGLibA_45560 | 102 | -0.133596141 | SLX1B | structure-specific endonuclease subunit [Source:HGNC Symbol;Acc:HGNC:28748]" |
| ENSG00000181690 | HGLibA_36950 | 102 | -0.133596141 | PLAG1 | PLAG1 zinc finger [Source:HGNC Symbol;Acc:HGNC:9045] |
| ENSG00000182022 | HGLibA_09628 | 102 | -0.133596141 | CHST15 | carbohydrate sulfotransferase 15 [Source:HGNC Symbol;Acc:HGNC:18137] |
| ENSG00000182141 | HGLibA_56601 | 102 | -0.133596141 | ZNF708 | zinc finger protein 708 [Source:HGNC Symbol;Acc:HGNC:12945] |
| ENSG00000182541 | HGLibA_26544 | 102 | -0.133596141 | LIMK2 | LIM domain kinase 2 [Source:HGNC Symbol;Acc:HGNC:6614] |
| ENSG00000183145 | HGLibA_41333 | 102 | -0.133596141 | RIPPLY3 | ripply transcriptional repressor 3 [Source:HGNC Symbol;Acc:HGNC:3047] |
| ENSG00000184347 | HGLibA_45511 | 102 | -0.133596141 | SLIT3 | slit guidance ligand 3 [Source:HGNC Symbol;Acc:HGNC:11087] |
| ENSG00000184378 | HGLibA_00684 | 102 | -0.133596141 | ACTRT3 | actin related protein T3 [Source:HGNC Symbol;Acc:HGNC:24022] |
| ENSG00000185963 | HGLibA_04498 | 102 | -0.133596141 | BICD2 | BICD cargo adaptor 2 [Source:HGNC Symbol;Acc:HGNC:17208] |
| ENSG00000186395 | HGLibA_25417 | 102 | -0.133596141 | KRT10 | keratin 10 [Source:HGNC Symbol;Acc:HGNC:6413] |
| ENSG00000188373 | HGLibA_05113 | 102 | -0.133596141 | C10orf99 | chromosome 10 open reading frame 99 [Source:HGNC Symbol;Acc:HGNC:31428] |
| ENSG00000188760 | HGLibA_50161 | 102 | -0.133596141 | TMEM198 | transmembrane protein 198 [Source:HGNC Symbol;Acc:HGNC:33704] |
| ENSG00000189043 | HGLibA_31457 | 102 | -0.133596141 | NDUFA4 | mitochondrial complex associated [Source:HGNC Symbol;Acc:HGNC:7687]" |
| ENSG00000196214 | HGLibA_56694 | 102 | -0.133596141 | ZNF766 | zinc finger protein 766 [Source:HGNC Symbol;Acc:HGNC:28063] |
| ENSG00000196242 | HGLibA_33778 | 102 | -0.133596141 | OR2C3 | olfactory receptor family 2 subfamily C member 3 [Source:HGNC Symbol;Acc:HGNC:15005] |
| ENSG00000196365 | HGLibA_27015 | 102 | -0.133596141 | LONP1 | mitochondrial [Source:HGNC Symbol;Acc:HGNC:9479]" |
| ENSG00000196502 | HGLibA_47730 | 102 | -0.133596141 | SULT1A1 | sulfotransferase family 1A member 1 [Source:HGNC Symbol;Acc:HGNC:11453] |
| ENSG00000196655 | HGLibA_51325 | 102 | -0.133596141 | TRAPPC4 | trafficking protein particle complex 4 [Source:HGNC Symbol;Acc:HGNC:19943] |
| ENSG00000196935 | HGLibA_46967 | 102 | -0.133596141 | SRGAP1 | SLIT-ROBO Rho GTPase activating protein 1 [Source:HGNC Symbol;Acc:HGNC:17382] |
| ENSG00000197360 | HGLibA_56915 | 102 | -0.133596141 | ZNF98 | zinc finger protein 98 [Source:HGNC Symbol;Acc:HGNC:13174] |
| ENSG00000197530 | HGLibA_29196 | 102 | -0.133596141 | MIB2 | mindbomb E3 ubiquitin protein ligase 2 [Source:HGNC Symbol;Acc:HGNC:30577] |
| ENSG00000198467 | HGLibA_51145 | 102 | -0.133596141 | TPM2 | tropomyosin 2 [Source:HGNC Symbol;Acc:HGNC:12011] |
| ENSG00000198728 | HGLibA_26212 | 102 | -0.133596141 | LDB1 | LIM domain binding 1 [Source:HGNC Symbol;Acc:HGNC:6532] |
| ENSG00000198730 | HGLibA_11674 | 102 | -0.133596141 | CTR9 | Paf1/RNA polymerase II complex component [Source:HGNC Symbol;Acc:HGNC:16850]" |
| ENSG00000204084 | HGLibA_23346 | 102 | -0.133596141 | INPP5B | inositol polyphosphate-5-phosphatase B [Source:HGNC Symbol;Acc:HGNC:6077] |
| ENSG00000204256 | HGLibA_04774 | 102 | -0.133596141 | BRD2 | bromodomain containing 2 [Source:HGNC Symbol;Acc:HGNC:1103] |
| ENSG00000204385 | HGLibA_45166 | 102 | -0.133596141 | SLC44A4 | solute carrier family 44 member 4 [Source:HGNC Symbol;Acc:HGNC:13941] |
| ENSG00000204501 | HGLibA_38292 | 102 | -0.133596141 | PRAMEF15 | PRAME family member 15 [Source:HGNC Symbol;Acc:HGNC:26764] |
| ENSG00000204540 | HGLibA_39217 | 102 | -0.133596141 | PSORS1C1 | psoriasis susceptibility 1 candidate 1 [Source:HGNC Symbol;Acc:HGNC:17202] |
| ENSG00000204866 | HGLibA_22841 | 102 | -0.133596141 | IGFL2 | IGF like family member 2 [Source:HGNC Symbol;Acc:HGNC:32929] |
| ENSG00000205189 | HGLibA_54815 | 102 | -0.133596141 | ZBTB10 | zinc finger and BTB domain containing 10 [Source:HGNC Symbol;Acc:HGNC:30953] |
| ENSG00000213988 | HGLibA_56903 | 102 | -0.133596141 | ZNF90 | zinc finger protein 90 [Source:HGNC Symbol;Acc:HGNC:13165] |
| ENSG00000223496 | HGLibA_15788 | 102 | -0.133596141 | EXOSC6 | exosome component 6 [Source:HGNC Symbol;Acc:HGNC:19055] |
| ENSG00000226742 | HGLibA_22154 | 102 | -0.133596141 | HSBP1L1 | heat shock factor binding protein 1 like 1 [Source:HGNC Symbol;Acc:HGNC:37243] |
| ENSG00000227234 | HGLibA_46383 | 102 | -0.133596141 | SPANXB1 | SPANX family member B1 [Source:HGNC Symbol;Acc:HGNC:14329] |
| ENSG00000229314 | HGLibA_34620 | 102 | -0.133596141 | ORM1 | orosomucoid 1 [Source:HGNC Symbol;Acc:HGNC:8498] |
| ENSG00000253159 | HGLibA_35525 | 102 | -0.133596141 | PCDHGA12 | 12 [Source:HGNC Symbol;Acc:HGNC:8699]" |
| ENSG00000255501 | HGLibA_07232 | 102 | -0.133596141 | CARD18 | caspase recruitment domain family member 18 [Source:HGNC Symbol;Acc:HGNC:28861] |
| ENSG00000256525 | HGLibA_37525 | 102 | -0.133596141 | POLG2 | accessory subunit [Source:HGNC Symbol;Acc:HGNC:9180]" |
| ENSG00000257335 | HGLibA_29126 | 102 | -0.133596141 | MGAM | maltase-glucoamylase [Source:HGNC Symbol;Acc:HGNC:7043] |
| ENSG00000257923 | HGLibA_11802 | 102 | -0.133596141 | CUX1 | cut like homeobox 1 [Source:HGNC Symbol;Acc:HGNC:2557] |
| ENSG00000268223 | HGLibA_02941 | 102 | -0.133596141 | ARL14EPL | ADP ribosylation factor like GTPase 14 effector protein like [Source:HGNC Symbol;Acc:HGNC:44201] |
| FAM212B | HGLibA_16467 | 102 | -0.133596141 | FAM212B | - |
| HIST1H2AE | HGLibA_21388 | 102 | -0.133596141 | HIST1H2AE | - |
| HIST1H4H | HGLibA_21506 | 102 | -0.133596141 | HIST1H4H | - |
| hsa-mir-1246 | HGLibA_57383 | 102 | -0.133596141 | hsa-mir-1246 | - |
| hsa-mir-139 | HGLibA_57821 | 102 | -0.133596141 | hsa-mir-139 | - |
| hsa-mir-223 | HGLibA_58302 | 102 | -0.133596141 | hsa-mir-223 | - |
| hsa-mir-3180-3 | HGLibA_58810 | 102 | -0.133596141 | hsa-mir-3180-3 | - |
| hsa-mir-3658 | HGLibA_59176 | 102 | -0.133596141 | hsa-mir-3658 | - |
| hsa-mir-371a | HGLibA_59374 | 102 | -0.133596141 | hsa-mir-371a | - |
| hsa-mir-4514 | HGLibA_60499 | 102 | -0.133596141 | hsa-mir-4514 | - |
| hsa-mir-4683 | HGLibA_60841 | 102 | -0.133596141 | hsa-mir-4683 | - |
| hsa-mir-4744 | HGLibA_61074 | 102 | -0.133596141 | hsa-mir-4744 | - |
| hsa-mir-4776-2 | HGLibA_61211 | 102 | -0.133596141 | hsa-mir-4776-2 | - |
| hsa-mir-548f-2 | HGLibA_61975 | 102 | -0.133596141 | hsa-mir-548f-2 | - |
| hsa-mir-5701-2 | HGLibA_62345 | 102 | -0.133596141 | hsa-mir-5701-2 | - |
| hsa-mir-6075 | HGLibA_62554 | 102 | -0.133596141 | hsa-mir-6075 | - |
| hsa-mir-6129 | HGLibA_62662 | 102 | -0.133596141 | hsa-mir-6129 | - |
| hsa-mir-638 | HGLibA_62791 | 102 | -0.133596141 | hsa-mir-638 | - |
| hsa-mir-6852 | HGLibA_63581 | 102 | -0.133596141 | hsa-mir-6852 | - |
| hsa-mir-6875 | HGLibA_63684 | 102 | -0.133596141 | hsa-mir-6875 | - |
| hsa-mir-8070 | HGLibA_64118 | 102 | -0.133596141 | hsa-mir-8070 | - |
| hsa-mir-99b | HGLibA_64383 | 102 | -0.133596141 | hsa-mir-99b | - |
| IL9R_X | HGLibA_23203 | 102 | -0.133596141 | IL9R_X | - |
| LOC100129636 | HGLibA_26768 | 102 | -0.133596141 | LOC100129636 | - |
| NAPRT1 | HGLibA_31137 | 102 | -0.133596141 | NAPRT1 | - |
| TMEM56-RWDD3 | HGLibA_50452 | 102 | -0.133596141 | TMEM56-RWDD3 | - |
| TMEM57 | HGLibA_50457 | 102 | -0.133596141 | TMEM57 | - |
| ADRBK2 | HGLibA_01068 | 101 | -0.143547179 | ADRBK2 | - |
| AKAP17A_Y | HGLibA_01423 | 101 | -0.143547179 | AKAP17A_Y | - |
| C1orf192 | HGLibA_05813 | 101 | -0.143547179 | C1orf192 | - |
| C22orf24 | HGLibA_06058 | 101 | -0.143547179 | C22orf24 | - |
| C22orf29 | HGLibA_06067 | 101 | -0.143547179 | C22orf29 | - |
| C4orf22 | HGLibA_06306 | 101 | -0.143547179 | C4orf22 | - |
| CCDC101 | HGLibA_07504 | 101 | -0.143547179 | CCDC101 | - |
| ENSG00000003096 | HGLibA_25131 | 101 | -0.143547179 | KLHL13 | kelch like family member 13 [Source:HGNC Symbol;Acc:HGNC:22931] |
| ENSG00000010438 | HGLibA_38924 | 101 | -0.143547179 | PRSS3 | serine protease 3 [Source:HGNC Symbol;Acc:HGNC:9486] |
| ENSG00000011021 | HGLibA_09836 | 101 | -0.143547179 | CLCN6 | chloride voltage-gated channel 6 [Source:HGNC Symbol;Acc:HGNC:2024] |
| ENSG00000011600 | HGLibA_52471 | 101 | -0.143547179 | TYROBP | TYRO protein tyrosine kinase binding protein [Source:HGNC Symbol;Acc:HGNC:12449] |
| ENSG00000014164 | HGLibA_54973 | 101 | -0.143547179 | ZC3H3 | zinc finger CCCH-type containing 3 [Source:HGNC Symbol;Acc:HGNC:28972] |
| ENSG00000019549 | HGLibA_45870 | 101 | -0.143547179 | SNAI2 | snail family transcriptional repressor 2 [Source:HGNC Symbol;Acc:HGNC:11094] |
| ENSG00000021826 | HGLibA_10991 | 101 | -0.143547179 | CPS1 | carbamoyl-phosphate synthase 1 [Source:HGNC Symbol;Acc:HGNC:2323] |
| ENSG00000025770 | HGLibA_31278 | 101 | -0.143547179 | NCAPH2 | non-SMC condensin II complex subunit H2 [Source:HGNC Symbol;Acc:HGNC:25071] |
| ENSG00000029559 | HGLibA_22481 | 101 | -0.143547179 | IBSP | integrin binding sialoprotein [Source:HGNC Symbol;Acc:HGNC:5341] |
| ENSG00000034510 | HGLibA_50656 | 101 | -0.143547179 | TMSB10 | thymosin beta 10 [Source:HGNC Symbol;Acc:HGNC:11879] |
| ENSG00000035681 | HGLibA_32762 | 101 | -0.143547179 | NSMAF | neutral sphingomyelinase activation associated factor [Source:HGNC Symbol;Acc:HGNC:8017] |
| ENSG00000042753 | HGLibA_02316 | 101 | -0.143547179 | AP2S1 | adaptor related protein complex 2 subunit sigma 1 [Source:HGNC Symbol;Acc:HGNC:565] |
| ENSG00000047346 | HGLibA_16476 | 101 | -0.143547179 | FAM214A | family with sequence similarity 214 member A [Source:HGNC Symbol;Acc:HGNC:25609] |
| ENSG00000048392 | HGLibA_42256 | 101 | -0.143547179 | RRM2B | ribonucleotide reductase regulatory TP53 inducible subunit M2B [Source:HGNC Symbol;Acc:HGNC:17296] |
| ENSG00000059573 | HGLibA_01539 | 101 | -0.143547179 | ALDH18A1 | aldehyde dehydrogenase 18 family member A1 [Source:HGNC Symbol;Acc:HGNC:9722] |

| | | | | | |
|---|---|---|---|---|---|
| ENSG00000062282 | HGLibA_13037 | 101 | -0.143547179 | DGAT2 | diacylglycerol O-acyltransferase 2 [Source:HGNC Symbol;Acc:HGNC:16940] |
| ENSG00000075673 | HGLibA_03563 | 101 | -0.143547179 | ATP12A | ATPase H+/K+ transporting non-gastric alpha2 subunit [Source:HGNC Symbol;Acc:HGNC:13816] |
| ENSG00000075884 | HGLibA_02711 | 101 | -0.143547179 | ARHGAP15 | Rho GTPase activating protein 15 [Source:HGNC Symbol;Acc:HGNC:21030] |
| ENSG00000082014 | HGLibA_45636 | 101 | -0.143547179 | SMARCD3 | member 3 [Source:HGNC Symbol;Acc:HGNC:11108]" |
| ENSG00000086544 | HGLibA_23851 | 101 | -0.143547179 | ITPKC | inositol-trisphosphate 3-kinase C [Source:HGNC Symbol;Acc:HGNC:14897] |
| ENSG00000088387 | HGLibA_13760 | 101 | -0.143547179 | DOCK9 | dedicator of cytokinesis 9 [Source:HGNC Symbol;Acc:HGNC:14132] |
| ENSG00000089737 | HGLibA_12679 | 101 | -0.143547179 | DDX24 | DEAD-box helicase 24 [Source:HGNC Symbol;Acc:HGNC:13266] |
| ENSG00000090054 | HGLibA_46892 | 101 | -0.143547179 | SPTLC1 | serine palmitoyltransferase long chain base subunit 1 [Source:HGNC Symbol;Acc:HGNC:11277] |
| ENSG00000099957 | HGLibA_34860 | 101 | -0.143547179 | P2RX6 | purinergic receptor P2X 6 [Source:HGNC Symbol;Acc:HGNC:8538] |
| ENSG00000100095 | HGLibA_43683 | 101 | -0.143547179 | SEZ6L | seizure related 6 homolog like [Source:HGNC Symbol;Acc:HGNC:10763] |
| ENSG00000100124 | HGLibA_02124 | 101 | -0.143547179 | ANKRD54 | ankyrin repeat domain 54 [Source:HGNC Symbol;Acc:HGNC:25185] |
| ENSG00000100209 | HGLibA_22156 | 101 | -0.143547179 | HSCB | HscB mitochondrial iron-sulfur cluster cochaperone [Source:HGNC Symbol;Acc:HGNC:28913] |
| ENSG00000100300 | HGLibA_51958 | 101 | -0.143547179 | TSPO | translocator protein [Source:HGNC Symbol;Acc:HGNC:1158] |
| ENSG00000100625 | HGLibA_44253 | 101 | -0.143547179 | SIX4 | SIX homeobox 4 [Source:HGNC Symbol;Acc:HGNC:10890] |
| ENSG00000101945 | HGLibA_47858 | 101 | -0.143547179 | SUV39H1 | suppressor of variegation 3-9 homolog 1 [Source:HGNC Symbol;Acc:HGNC:11479] |
| ENSG00000102313 | HGLibA_23818 | 101 | -0.143547179 | ITIH6 | inter-alpha-trypsin inhibitor heavy chain family member 6 [Source:HGNC Symbol;Acc:HGNC:28907] |
| ENSG00000102996 | HGLibA_29496 | 101 | -0.143547179 | MMP15 | matrix metallopeptidase 15 [Source:HGNC Symbol;Acc:HGNC:7161] |
| ENSG00000103528 | HGLibA_48069 | 101 | -0.143547179 | SYT17 | synaptotagmin 17 [Source:HGNC Symbol;Acc:HGNC:24119] |
| ENSG00000104626 | HGLibA_15480 | 101 | -0.143547179 | ERI1 | exoribonuclease 1 [Source:HGNC Symbol;Acc:HGNC:23994] |
| ENSG00000105676 | HGLibA_03025 | 101 | -0.143547179 | ARMC6 | armadillo repeat containing 6 [Source:HGNC Symbol;Acc:HGNC:25049] |
| ENSG00000105677 | HGLibA_49976 | 101 | -0.143547179 | TMEM147 | transmembrane protein 147 [Source:HGNC Symbol;Acc:HGNC:30414] |
| ENSG00000105997 | HGLibA_21909 | 101 | -0.143547179 | HOXA3 | homeobox A3 [Source:HGNC Symbol;Acc:HGNC:5104] |
| ENSG00000106635 | HGLibA_04342 | 101 | -0.143547179 | BCL7B | BCL tumor suppressor 7B [Source:HGNC Symbol;Acc:HGNC:1005] |
| ENSG00000107242 | HGLibA_36716 | 101 | -0.143547179 | PIP5K1B | phosphatidylinositol-4-phosphate 5-kinase type 1 beta [Source:HGNC Symbol;Acc:HGNC:8995] |
| ENSG00000107560 | HGLibA_39796 | 101 | -0.143547179 | RAB11FIP2 | RAB11 family interacting protein 2 [Source:HGNC Symbol;Acc:HGNC:29152] |
| ENSG00000109610 | HGLibA_46149 | 101 | -0.143547179 | SOD3 | superoxide dismutase 3 [Source:HGNC Symbol;Acc:HGNC:11181] |
| ENSG00000109790 | HGLibA_25217 | 101 | -0.143547179 | KLHL5 | kelch like family member 5 [Source:HGNC Symbol;Acc:HGNC:6356] |
| ENSG00000110108 | HGLibA_49869 | 101 | -0.143547179 | TMEM109 | transmembrane protein 109 [Source:HGNC Symbol;Acc:HGNC:28771] |
| ENSG00000110244 | HGLibA_02459 | 101 | -0.143547179 | APOA4 | apolipoprotein A4 [Source:HGNC Symbol;Acc:HGNC:602] |
| ENSG00000112511 | HGLibA_36331 | 101 | -0.143547179 | PHF1 | PHD finger protein 1 [Source:HGNC Symbol;Acc:HGNC:8919] |
| ENSG00000112514 | HGLibA_11798 | 101 | -0.143547179 | CUTA | cutA divalent cation tolerance homolog [Source:HGNC Symbol;Acc:HGNC:21101] |
| ENSG00000112697 | HGLibA_50361 | 101 | -0.143547179 | TMEM30A | transmembrane protein 30A [Source:HGNC Symbol;Acc:HGNC:16667] |
| ENSG00000112855 | HGLibA_20860 | 101 | -0.143547179 | HARS2 | mitochondrial [Source:HGNC Symbol;Acc:HGNC:4817]" |
| ENSG00000114867 | HGLibA_14840 | 101 | -0.143547179 | EIF4G1 | eukaryotic translation initiation factor 4 gamma 1 [Source:HGNC Symbol;Acc:HGNC:3296] |
| ENSG00000115548 | HGLibA_24501 | 101 | -0.143547179 | KDM3A | lysine demethylase 3A [Source:HGNC Symbol;Acc:HGNC:20815] |
| ENSG00000115850 | HGLibA_26205 | 101 | -0.143547179 | LCT | lactase [Source:HGNC Symbol;Acc:HGNC:6530] |
| ENSG00000116251 | HGLibA_41897 | 101 | -0.143547179 | RPL22 | ribosomal protein L22 [Source:HGNC Symbol;Acc:HGNC:10315] |
| ENSG00000118939 | HGLibA_52803 | 101 | -0.143547179 | UCHL3 | ubiquitin C-terminal hydrolase L3 [Source:HGNC Symbol;Acc:HGNC:12515] |
| ENSG00000121621 | HGLibA_24875 | 101 | -0.143547179 | KIF18A | kinesin family member 18A [Source:HGNC Symbol;Acc:HGNC:29441] |
| ENSG00000122012 | HGLibA_47880 | 101 | -0.143547179 | SV2C | synaptic vesicle glycoprotein 2C [Source:HGNC Symbol;Acc:HGNC:30670] |
| ENSG00000122304 | HGLibA_38611 | 101 | -0.143547179 | PRM2 | protamine 2 [Source:HGNC Symbol;Acc:HGNC:9448] |
| ENSG00000122477 | HGLibA_27340 | 101 | -0.143547179 | LRRC39 | leucine rich repeat containing 39 [Source:HGNC Symbol;Acc:HGNC:28228] |
| ENSG00000123561 | HGLibA_43530 | 101 | -0.143547179 | SERPINA7 | serpin family A member 7 [Source:HGNC Symbol;Acc:HGNC:11583] |
| ENSG00000125124 | HGLibA_04215 | 101 | -0.143547179 | BBS2 | Bardet-Biedl syndrome 2 [Source:HGNC Symbol;Acc:HGNC:967] |
| ENSG00000125449 | HGLibA_03029 | 101 | -0.143547179 | ARMC7 | armadillo repeat containing 7 [Source:HGNC Symbol;Acc:HGNC:26168] |
| ENSG00000125903 | HGLibA_12891 | 101 | -0.143547179 | DEFB129 | defensin beta 129 [Source:HGNC Symbol;Acc:HGNC:16218] |
| ENSG00000129675 | HGLibA_02872 | 101 | -0.143547179 | ARHGEF6 | Rac/Cdc42 guanine nucleotide exchange factor 6 [Source:HGNC Symbol;Acc:HGNC:685] |
| ENSG00000132010 | HGLibA_55567 | 101 | -0.143547179 | ZNF20 | zinc finger protein 20 [Source:HGNC Symbol;Acc:HGNC:12992] |
| ENSG00000132846 | HGLibA_54796 | 101 | -0.143547179 | ZBED3 | zinc finger BED-type containing 3 [Source:HGNC Symbol;Acc:HGNC:20711] |
| ENSG00000133937 | HGLibA_20399 | 101 | -0.143547179 | GSC | goosecoid homeobox [Source:HGNC Symbol;Acc:HGNC:4612] |
| ENSG00000134897 | HGLibA_04532 | 101 | -0.143547179 | BIVM | immunoglobulin-like variable motif containing [Source:HGNC Symbol;Acc:HGNC:16034]" |
| ENSG00000135094 | HGLibA_43171 | 101 | -0.143547179 | SDS | serine dehydratase [Source:HGNC Symbol;Acc:HGNC:10691] |
| ENSG00000135406 | HGLibA_38750 | 101 | -0.143547179 | PRPH | peripherin [Source:HGNC Symbol;Acc:HGNC:9461] |
| ENSG00000135587 | HGLibA_45805 | 101 | -0.143547179 | SMPD2 | sphingomyelin phosphodiesterase 2 [Source:HGNC Symbol;Acc:HGNC:11121] |
| ENSG00000136250 | HGLibA_02264 | 101 | -0.143547179 | AOAH | acyloxyacyl hydrolase [Source:HGNC Symbol;Acc:HGNC:548] |
| ENSG00000136720 | HGLibA_22141 | 101 | -0.143547179 | HS6ST1 | heparan sulfate 6-O-sulfotransferase 1 [Source:HGNC Symbol;Acc:HGNC:5201] |
| ENSG00000136811 | HGLibA_33277 | 101 | -0.143547179 | ODF2 | outer dense fiber of sperm tails 2 [Source:HGNC Symbol;Acc:HGNC:8114] |
| ENSG00000137460 | HGLibA_17473 | 101 | -0.143547179 | FHDC1 | FH2 domain containing 1 [Source:HGNC Symbol;Acc:HGNC:29363] |
| ENSG00000137707 | HGLibA_04949 | 101 | -0.143547179 | BTG4 | BTG anti-proliferation factor 4 [Source:HGNC Symbol;Acc:HGNC:13862] |
| ENSG00000137766 | HGLibA_53000 | 101 | -0.143547179 | UNC13C | unc-13 homolog C [Source:HGNC Symbol;Acc:HGNC:23149] |
| ENSG00000138002 | HGLibA_22750 | 101 | -0.143547179 | IFT172 | intraflagellar transport 172 [Source:HGNC Symbol;Acc:HGNC:30391] |
| ENSG00000139684 | HGLibA_15564 | 101 | -0.143547179 | ESD | esterase D [Source:HGNC Symbol;Acc:HGNC:3465] |
| ENSG00000139970 | HGLibA_42397 | 101 | -0.143547179 | RTN1 | reticulon 1 [Source:HGNC Symbol;Acc:HGNC:10467] |
| ENSG00000139988 | HGLibA_40736 | 101 | -0.143547179 | RDH12 | retinol dehydrogenase 12 [Source:HGNC Symbol;Acc:HGNC:19977] |
| ENSG00000141750 | HGLibA_47315 | 101 | -0.143547179 | STAC2 | SH3 and cysteine rich domain 2 [Source:HGNC Symbol;Acc:HGNC:23990] |
| ENSG00000142528 | HGLibA_56058 | 101 | -0.143547179 | ZNF473 | zinc finger protein 473 [Source:HGNC Symbol;Acc:HGNC:23239] |
| ENSG00000143294 | HGLibA_38352 | 101 | -0.143547179 | PRCC | proline rich mitotic checkpoint control factor [Source:HGNC Symbol;Acc:HGNC:9343] |
| ENSG00000143376 | HGLibA_46075 | 101 | -0.143547179 | SNX27 | sorting nexin family member 27 [Source:HGNC Symbol;Acc:HGNC:20073] |
| ENSG00000143545 | HGLibA_39811 | 101 | -0.143547179 | RAB13 | member RAS oncogene family [Source:HGNC Symbol;Acc:HGNC:9762]" |
| ENSG00000143921 | HGLibA_00227 | 101 | -0.143547179 | ABCG8 | ATP binding cassette subfamily G member 8 [Source:HGNC Symbol;Acc:HGNC:13887] |
| ENSG00000143951 | HGLibA_54018 | 101 | -0.143547179 | WDPCP | WD repeat containing planar cell polarity effector [Source:HGNC Symbol;Acc:HGNC:28027] |
| ENSG00000144840 | HGLibA_40034 | 101 | -0.143547179 | RABL3 | member of RAS oncogene family like 3 [Source:HGNC Symbol;Acc:HGNC:18072]" |
| ENSG00000145491 | HGLibA_41767 | 101 | -0.143547179 | ROPN1L | rhophilin associated tail protein 1 like [Source:HGNC Symbol;Acc:HGNC:24060] |
| ENSG00000146242 | HGLibA_51100 | 101 | -0.143547179 | TPBG | trophoblast glycoprotein [Source:HGNC Symbol;Acc:HGNC:12004] |
| ENSG00000146243 | HGLibA_23557 | 101 | -0.143547179 | IRAK1BP1 | interleukin 1 receptor associated kinase 1 binding protein 1 [Source:HGNC Symbol;Acc:HGNC:17368] |
| ENSG00000148719 | HGLibA_13528 | 101 | -0.143547179 | DNAJB12 | DnaJ heat shock protein family (Hsp40) member B12 [Source:HGNC Symbol;Acc:HGNC:14891] |
| ENSG00000148719 | HGLibA_13527 | 101 | -0.143547179 | DNAJB12 | DnaJ heat shock protein family (Hsp40) member B12 [Source:HGNC Symbol;Acc:HGNC:14891] |
| ENSG00000148834 | HGLibA_20487 | 101 | -0.143547179 | GSTO1 | glutathione S-transferase omega 1 [Source:HGNC Symbol;Acc:HGNC:13312] |
| ENSG00000149089 | HGLibA_02422 | 101 | -0.143547179 | APIP | APAF1 interacting protein [Source:HGNC Symbol;Acc:HGNC:17581] |
| ENSG00000151704 | HGLibA_24246 | 101 | -0.143547179 | KCNJ1 | potassium voltage-gated channel subfamily J member 1 [Source:HGNC Symbol;Acc:HGNC:6255] |
| ENSG00000151726 | HGLibA_00554 | 101 | -0.143547179 | ACSL1 | acyl-CoA synthetase long chain family member 1 [Source:HGNC Symbol;Acc:HGNC:3569] |
| ENSG00000154473 | HGLibA_04994 | 101 | -0.143547179 | BUB3 | mitotic checkpoint protein [Source:HGNC Symbol;Acc:HGNC:1151]" |
| ENSG00000156414 | HGLibA_48916 | 101 | -0.143547179 | TDRD9 | tudor domain containing 9 [Source:HGNC Symbol;Acc:HGNC:20122] |
| ENSG00000156508 | HGLibA_14491 | 101 | -0.143547179 | EEF1A1 | eukaryotic translation elongation factor 1 alpha 1 [Source:HGNC Symbol;Acc:HGNC:3189] |
| ENSG00000156858 | HGLibA_38779 | 101 | -0.143547179 | PRR14 | proline rich 14 [Source:HGNC Symbol;Acc:HGNC:28458] |
| ENSG00000157060 | HGLibA_44014 | 101 | -0.143547179 | SHCBP1L | SHC binding and spindle associated 1 like [Source:HGNC Symbol;Acc:HGNC:16788] |
| ENSG00000157212 | HGLibA_35326 | 101 | -0.143547179 | PAXIP1 | PAX interacting protein 1 [Source:HGNC Symbol;Acc:HGNC:8624] |
| ENSG00000157851 | HGLibA_13934 | 101 | -0.143547179 | DPYSL5 | dihydropyrimidinase like 5 [Source:HGNC Symbol;Acc:HGNC:20637] |
| ENSG00000158169 | HGLibA_16835 | 101 | -0.143547179 | FANCC | FA complementation group C [Source:HGNC Symbol;Acc:HGNC:3584] |
| ENSG00000159231 | HGLibA_07420 | 101 | -0.143547179 | CBR3 | carbonyl reductase 3 [Source:HGNC Symbol;Acc:HGNC:1549] |
| ENSG00000161513 | HGLibA_17268 | 101 | -0.143547179 | FDXR | ferredoxin reductase [Source:HGNC Symbol;Acc:HGNC:3642] |
| ENSG00000161526 | HGLibA_42724 | 101 | -0.143547179 | SAP30BP | SAP30 binding protein [Source:HGNC Symbol;Acc:HGNC:30785] |
| ENSG00000162434 | HGLibA_23914 | 101 | -0.143547179 | JAK1 | Janus kinase 1 [Source:HGNC Symbol;Acc:HGNC:6190] |
| ENSG00000162927 | HGLibA_39599 | 101 | -0.143547179 | PUS10 | pseudouridine synthase 10 [Source:HGNC Symbol;Acc:HGNC:26505] |
| ENSG00000163563 | HGLibA_29567 | 101 | -0.143547179 | MNDA | myeloid cell nuclear differentiation antigen [Source:HGNC Symbol;Acc:HGNC:7183] |
| ENSG00000164035 | HGLibA_15029 | 101 | -0.143547179 | EMCN | endomucin [Source:HGNC Symbol;Acc:HGNC:16041] |
| ENSG00000164266 | HGLibA_46682 | 101 | -0.143547179 | SPINK1 | Kazal type 1 [Source:HGNC Symbol;Acc:HGNC:11244]" |
| ENSG00000164347 | HGLibA_18951 | 101 | -0.143547179 | GFM2 | G elongation factor mitochondrial 2 [Source:HGNC Symbol;Acc:HGNC:29682] |
| ENSG00000164362 | HGLibA_49020 | 101 | -0.143547179 | TERT | telomerase reverse transcriptase [Source:HGNC Symbol;Acc:HGNC:11730] |
| ENSG00000164841 | HGLibA_50509 | 101 | -0.143547179 | TMEM74 | transmembrane protein 74 [Source:HGNC Symbol;Acc:HGNC:26409] |
| ENSG00000164938 | HGLibA_51069 | 101 | -0.143547179 | TP53INP1 | tumor protein p53 inducible nuclear protein 1 [Source:HGNC Symbol;Acc:HGNC:18022] |
| ENSG00000165801 | HGLibA_02867 | 101 | -0.143547179 | ARHGEF40 | Rho guanine nucleotide exchange factor 40 [Source:HGNC Symbol;Acc:HGNC:25516] |
| ENSG00000167614 | HGLibA_52230 | 101 | -0.143547179 | TTYH1 | tweety family member 1 [Source:HGNC Symbol;Acc:HGNC:13476] |
| ENSG00000167749 | HGLibA_25259 | 101 | -0.143547179 | KLK4 | kallikrein related peptidase 4 [Source:HGNC Symbol;Acc:HGNC:6365] |
| ENSG00000167858 | HGLibA_48972 | 101 | -0.143547179 | TEKT1 | tektin 1 [Source:HGNC Symbol;Acc:HGNC:15534] |
| ENSG00000168283 | HGLibA_04588 | 101 | -0.143547179 | BMI1 | polycomb ring finger [Source:HGNC Symbol;Acc:HGNC:1066]" |
| ENSG00000168309 | HGLibA_15999 | 101 | -0.143547179 | FAM107A | family with sequence similarity 107 member A [Source:HGNC Symbol;Acc:HGNC:30827] |
| ENSG00000169169 | HGLibA_11024 | 101 | -0.143547179 | CPT1C | carnitine palmitoyltransferase 1C [Source:HGNC Symbol;Acc:HGNC:18540] |
| ENSG00000169807 | HGLibA_38999 | 101 | -0.143547179 | PRY2 | PTPN13 like Y-linked 2 [Source:HGNC Symbol;Acc:HGNC:21504] |
| ENSG00000169967 | HGLibA_28180 | 101 | -0.143547179 | MAP3K2 | mitogen-activated protein kinase kinase kinase 2 [Source:HGNC Symbol;Acc:HGNC:6854] |
| ENSG00000170185 | HGLibA_53353 | 101 | -0.143547179 | USP38 | ubiquitin specific peptidase 38 [Source:HGNC Symbol;Acc:HGNC:20067] |
| ENSG00000170290 | HGLibA_45544 | 101 | -0.143547179 | SLN | sarcolipin [Source:HGNC Symbol;Acc:HGNC:11089] |
| ENSG00000170782 | HGLibA_33468 | 101 | -0.143547179 | OR10A4 | olfactory receptor family 10 subfamily A member 4 [Source:HGNC Symbol;Acc:HGNC:15130] |
| ENSG00000172878 | HGLibA_28930 | 101 | -0.143547179 | METAP1D | mitochondrial [Source:HGNC Symbol;Acc:HGNC:32583]" |
| ENSG00000173267 | HGLibA_45917 | 101 | -0.143547179 | SNCG | synuclein gamma [Source:HGNC Symbol;Acc:HGNC:11141] |
| ENSG00000173826 | HGLibA_24224 | 101 | -0.143547179 | KCNH6 | potassium voltage-gated channel subfamily H member 6 [Source:HGNC Symbol;Acc:HGNC:18862] |
| ENSG00000175193 | HGLibA_35201 | 101 | -0.143547179 | PARL | presenilin associated rhomboid like [Source:HGNC Symbol;Acc:HGNC:18253] |
| ENSG00000175348 | HGLibA_50575 | 101 | -0.143547179 | TMEM9B | TMEM9 domain family member B [Source:HGNC Symbol;Acc:HGNC:1168] |
| ENSG00000175497 | HGLibA_13867 | 101 | -0.143547179 | DPP10 | dipeptidyl peptidase like 10 [Source:HGNC Symbol;Acc:HGNC:20823] |
| ENSG00000177257 | HGLibA_12918 | 101 | -0.143547179 | DEFB4B | defensin beta 4B [Source:HGNC Symbol;Acc:HGNC:30193] |
| ENSG00000178607 | HGLibA_15512 | 101 | -0.143547179 | ERN1 | endoplasmic reticulum to nucleus signaling 1 [Source:HGNC Symbol;Acc:HGNC:3449] |
| ENSG00000179476 | HGLibA_05361 | 101 | -0.143547179 | C14orf28 | chromosome 14 open reading frame 28 [Source:HGNC Symbol;Acc:HGNC:19834] |
| ENSG00000179709 | HGLibA_32133 | 101 | -0.143547179 | NLRP8 | NLR family pyrin domain containing 8 [Source:HGNC Symbol;Acc:HGNC:22940] |
| ENSG00000181026 | HGLibA_01091 | 101 | -0.143547179 | AEN | apoptosis enhancing nuclease [Source:HGNC Symbol;Acc:HGNC:25722] |
| ENSG00000182218 | HGLibA_21263 | 101 | -0.143547179 | HHIPL1 | HHIP like 1 [Source:HGNC Symbol;Acc:HGNC:19710] |
| ENSG00000182670 | HGLibA_52101 | 101 | -0.143547179 | TTC3 | tetratricopeptide repeat domain 3 [Source:HGNC Symbol;Acc:HGNC:12393] |
| ENSG00000183023 | HGLibA_45388 | 101 | -0.143547179 | SLC8A1 | solute carrier family 8 member A1 [Source:HGNC Symbol;Acc:HGNC:11068] |
| ENSG00000183690 | HGLibA_14573 | 101 | -0.143547179 | EFHC2 | EF-hand domain containing 2 [Source:HGNC Symbol;Acc:HGNC:26233] |
| ENSG00000184544 | HGLibA_13151 | 101 | -0.143547179 | DHRS7C | dehydrogenase/reductase 7C [Source:HGNC Symbol;Acc:HGNC:32423] |
| ENSG00000185448 | HGLibA_16607 | 101 | -0.143547179 | FAM47A | family with sequence similarity 47 member A [Source:HGNC Symbol;Acc:HGNC:29962] |
| ENSG00000186009 | HGLibA_03634 | 101 | -0.143547179 | ATP4B | ATPase H+/K+ transporting subunit beta [Source:HGNC Symbol;Acc:HGNC:820] |
| ENSG00000186106 | HGLibA_02108 | 101 | -0.143547179 | ANKRD46 | ankyrin repeat domain 46 [Source:HGNC Symbol;Acc:HGNC:27229] |
| ENSG00000186866 | HGLibA_37471 | 101 | -0.143547179 | POFUT2 | protein O-fucosyltransferase 2 [Source:HGNC Symbol;Acc:HGNC:14683] |
| ENSG00000187037 | HGLibA_19909 | 101 | -0.143547179 | GPR141 | G protein-coupled receptor 141 [Source:HGNC Symbol;Acc:HGNC:19997] |
| ENSG00000187672 | HGLibA_15429 | 101 | -0.143547179 | ERC2 | ELKS/RAB6-interacting/CAST family member 2 [Source:HGNC Symbol;Acc:HGNC:31922] |

| | | | | | |
|---|---|---|---|---|---|
| ENSG00000188171 | HGLibA_56423 | 101 | -0.143547179 | ZNF626 | zinc finger protein 626 [Source:HGNC Symbol;Acc:HGNC:30461] |
| ENSG00000188730 | HGLibA_53924 | 101 | -0.143547179 | VWC2 | von Willebrand factor C domain containing 2 [Source:HGNC Symbol;Acc:HGNC:30200] |
| ENSG00000189433 | HGLibA_19167 | 101 | -0.143547179 | GJB4 | gap junction protein beta 4 [Source:HGNC Symbol;Acc:HGNC:4286] |
| ENSG00000196335 | HGLibA_47479 | 101 | -0.143547179 | STK31 | serine/threonine kinase 31 [Source:HGNC Symbol;Acc:HGNC:11407] |
| ENSG00000196407 | HGLibA_49344 | 101 | -0.143547179 | THEM5 | thioesterase superfamily member 5 [Source:HGNC Symbol;Acc:HGNC:26755] |
| ENSG00000196502 | HGLibA_47731 | 101 | -0.143547179 | SULT1A1 | sulfotransferase family 1A member 1 [Source:HGNC Symbol;Acc:HGNC:11453] |
| ENSG00000196611 | HGLibA_29476 | 101 | -0.143547179 | MMP1 | matrix metallopeptidase 1 [Source:HGNC Symbol;Acc:HGNC:7155] |
| ENSG00000197472 | HGLibA_56557 | 101 | -0.143547179 | ZNF695 | zinc finger protein 695 [Source:HGNC Symbol;Acc:HGNC:30954] |
| ENSG00000197535 | HGLibA_30849 | 101 | -0.143547179 | MYO5A | myosin VA [Source:HGNC Symbol;Acc:HGNC:7602] |
| ENSG00000197651 | HGLibA_07944 | 101 | -0.143547179 | CCER1 | coiled-coil glutamate rich protein 1 [Source:HGNC Symbol;Acc:HGNC:28373] |
| ENSG00000197860 | HGLibA_43872 | 101 | -0.143547179 | SGTB | small glutamine rich tetratricopeptide repeat containing beta [Source:HGNC Symbol;Acc:HGNC:23567] |
| ENSG00000198039 | HGLibA_55709 | 101 | -0.143547179 | ZNF273 | zinc finger protein 273 [Source:HGNC Symbol;Acc:HGNC:13067] |
| ENSG00000198855 | HGLibA_17508 | 101 | -0.143547179 | FICD | FIC domain containing [Source:HGNC Symbol;Acc:HGNC:18416] |
| ENSG00000203872 | HGLibA_06456 | 101 | -0.143547179 | C6orf163 | chromosome 6 open reading frame 163 [Source:HGNC Symbol;Acc:HGNC:21403] |
| ENSG00000204256 | HGLibA_04772 | 101 | -0.143547179 | BRD2 | bromodomain containing 2 [Source:HGNC Symbol;Acc:HGNC:1103] |
| ENSG00000204469 | HGLibA_38861 | 101 | -0.143547179 | PRRC2A | proline rich coiled-coil 2A [Source:HGNC Symbol;Acc:HGNC:13918] |
| ENSG00000212864 | HGLibA_41615 | 101 | -0.143547179 | RNF208 | ring finger protein 208 [Source:HGNC Symbol;Acc:HGNC:25420] |
| ENSG00000214681 | HGLibA_23510 | 101 | -0.143547179 | IQCF5 | IQ motif containing F5 [Source:HGNC Symbol;Acc:HGNC:35159] |
| ENSG00000237110 | HGLibA_48137 | 101 | -0.143547179 | TAAR9 | trace amine associated receptor 9 (gene/pseudogene) [Source:HGNC Symbol;Acc:HGNC:20977] |
| ENSG00000253731 | HGLibA_35537 | 101 | -0.143547179 | PCDHGA6 | 6 [Source:HGNC Symbol;Acc:HGNC:8704]" |
| ENSG00000257520 | HGLibA_43763 | 101 | -0.143547179 | SFTA3 | surfactant associated 3 [Source:NCBI gene;Acc:253970] |
| ENSG00000261115 | HGLibA_50083 | 101 | -0.143547179 | TMEM178B | transmembrane protein 178B [Source:HGNC Symbol;Acc:HGNC:44112] |
| ENSG00000261701 | HGLibA_22041 | 101 | -0.143547179 | HPR | haptoglobin-related protein [Source:HGNC Symbol;Acc:HGNC:5156] |
| ENSG00000268500 | HGLibA_44142 | 101 | -0.143547179 | SIGLEC5 | sense intronic to AC018755.18" |
| ENSG00000270181 | HGLibA_04535 | 101 | -0.143547179 | BIVM-ERCC5 | BIVM-ERCC5 readthrough [Source:HGNC Symbol;Acc:HGNC:43690] |
| ENSG00000275718 | HGLibA_07972 | 101 | -0.143547179 | CCL15 | C-C motif chemokine ligand 15 [Source:HGNC Symbol;Acc:HGNC:10613] |
| ENSG00000278540 | HGLibA_00346 | 101 | -0.143547179 | ACACA | acetyl-CoA carboxylase alpha [Source:HGNC Symbol;Acc:HGNC:84] |
| H2AFB3 | HGLibA_20751 | 101 | -0.143547179 | H2AFB3 | - |
| HIST2H2BF | HGLibA_21538 | 101 | -0.143547179 | HIST2H2BF | - |
| hsa-mir-128-2 | HGLibA_57578 | 101 | -0.143547179 | hsa-mir-128-2 | - |
| hsa-mir-133a-2 | HGLibA_57778 | 101 | -0.143547179 | hsa-mir-133a-2 | - |
| hsa-mir-16-1 | HGLibA_57947 | 101 | -0.143547179 | hsa-mir-16-1 | - |
| hsa-mir-2114 | HGLibA_58229 | 101 | -0.143547179 | hsa-mir-2114 | - |
| hsa-mir-3190 | HGLibA_58857 | 101 | -0.143547179 | hsa-mir-3190 | - |
| hsa-mir-3650 | HGLibA_59146 | 101 | -0.143547179 | hsa-mir-3650 | - |
| hsa-mir-4308 | HGLibA_59944 | 101 | -0.143547179 | hsa-mir-4308 | - |
| hsa-mir-4540 | HGLibA_60625 | 101 | -0.143547179 | hsa-mir-4540 | - |
| hsa-mir-4644 | HGLibA_60681 | 101 | -0.143547179 | hsa-mir-4644 | - |
| hsa-mir-4797 | HGLibA_61294 | 101 | -0.143547179 | hsa-mir-4797 | - |
| hsa-mir-5100 | HGLibA_61552 | 101 | -0.143547179 | hsa-mir-5100 | - |
| hsa-mir-588 | HGLibA_62451 | 101 | -0.143547179 | hsa-mir-588 | - |
| hsa-mir-6510 | HGLibA_62893 | 101 | -0.143547179 | hsa-mir-6510 | - |
| hsa-mir-7515 | HGLibA_63888 | 101 | -0.143547179 | hsa-mir-7515 | - |
| KIAA0355 | HGLibA_24625 | 101 | -0.143547179 | KIAA0355 | - |
| MTL5 | HGLibA_30404 | 101 | -0.143547179 | MTL5 | - |
| NRD1 | HGLibA_32663 | 101 | -0.143547179 | NRD1 | - |
| TMEM8A | HGLibA_50547 | 101 | -0.143547179 | TMEM8A | - |
| ANKRD20A2 | HGLibA_02031 | 100 | -0.153498216 | ANKRD20A2 | - |
| C10orf11 | HGLibA_05028 | 100 | -0.153498216 | C10orf11 | - |
| C11orf31 | HGLibA_05129 | 100 | -0.153498216 | C11orf31 | - |
| C16orf71 | HGLibA_05473 | 100 | -0.153498216 | C16orf71 | - |
| C1orf85 | HGLibA_05896 | 100 | -0.153498216 | C1orf85 | - |
| CARKD | HGLibA_07251 | 100 | -0.153498216 | CARKD | - |
| CTSL2 | HGLibA_11723 | 100 | -0.153498216 | CTSL2 | - |
| DSCR6 | HGLibA_14014 | 100 | -0.153498216 | DSCR6 | - |
| ENSG00000005812 | HGLibA_17006 | 100 | -0.153498216 | FBXL3 | F-box and leucine rich repeat protein 3 [Source:HGNC Symbol;Acc:HGNC:13599] |
| ENSG00000013293 | HGLibA_45359 | 100 | -0.153498216 | SLC7A14 | solute carrier family 7 member 14 [Source:HGNC Symbol;Acc:HGNC:29326] |
| ENSG00000014641 | HGLibA_28682 | 100 | -0.153498216 | MDH1 | malate dehydrogenase 1 [Source:HGNC Symbol;Acc:HGNC:6970] |
| ENSG00000026025 | HGLibA_53656 | 100 | -0.153498216 | VIM | vimentin [Source:HGNC Symbol;Acc:HGNC:12692] |
| ENSG00000033178 | HGLibA_52528 | 100 | -0.153498216 | UBA6 | ubiquitin like modifier activating enzyme 6 [Source:HGNC Symbol;Acc:HGNC:25581] |
| ENSG00000042832 | HGLibA_49199 | 100 | -0.153498216 | TG | thyroglobulin [Source:HGNC Symbol;Acc:HGNC:11764] |
| ENSG00000055609 | HGLibA_25327 | 100 | -0.153498216 | KMT2C | lysine methyltransferase 2C [Source:HGNC Symbol;Acc:HGNC:13726] |
| ENSG00000059728 | HGLibA_30611 | 100 | -0.153498216 | MXD1 | MAX dimerization protein 1 [Source:HGNC Symbol;Acc:HGNC:6761] |
| ENSG00000065526 | HGLibA_46620 | 100 | -0.153498216 | SPEN | spen family transcriptional repressor [Source:HGNC Symbol;Acc:HGNC:17575] |
| ENSG00000065613 | HGLibA_45531 | 100 | -0.153498216 | SLK | STE20 like kinase [Source:HGNC Symbol;Acc:HGNC:11088] |
| ENSG00000067064 | HGLibA_22555 | 100 | -0.153498216 | IDI1 | isopentenyl-diphosphate delta isomerase 1 [Source:HGNC Symbol;Acc:HGNC:5387] |
| ENSG00000069849 | HGLibA_03598 | 100 | -0.153498216 | ATP1B3 | ATPase Na+/K+ transporting subunit beta 3 [Source:HGNC Symbol;Acc:HGNC:806] |
| ENSG00000070814 | HGLibA_48827 | 100 | -0.153498216 | TCOF1 | treacle ribosome biogenesis factor 1 [Source:HGNC Symbol;Acc:HGNC:11654] |
| ENSG00000073849 | HGLibA_47259 | 100 | -0.153498216 | ST6GAL1 | 6-sialyltransferase 1 [Source:HGNC Symbol;Acc:HGNC:10860]" |
| ENSG00000082996 | HGLibA_41501 | 100 | -0.153498216 | RNF13 | ring finger protein 13 [Source:HGNC Symbol;Acc:HGNC:10057] |
| ENSG00000083535 | HGLibA_36519 | 100 | -0.153498216 | PIBF1 | progesterone immunomodulatory binding factor 1 [Source:HGNC Symbol;Acc:HGNC:23352] |
| ENSG00000089063 | HGLibA_50256 | 100 | -0.153498216 | TMEM230 | transmembrane protein 230 [Source:HGNC Symbol;Acc:HGNC:15876] |
| ENSG00000092096 | HGLibA_44557 | 100 | -0.153498216 | SLC22A17 | solute carrier family 22 member 17 [Source:HGNC Symbol;Acc:HGNC:23095] |
| ENSG00000092531 | HGLibA_45874 | 100 | -0.153498216 | SNAP23 | synaptosome associated protein 23 [Source:HGNC Symbol;Acc:HGNC:11131] |
| ENSG00000095066 | HGLibA_21880 | 100 | -0.153498216 | HOOK2 | hook microtubule tethering protein 2 [Source:HGNC Symbol;Acc:HGNC:19885] |
| ENSG00000101098 | HGLibA_41285 | 100 | -0.153498216 | RIMS4 | regulating synaptic membrane exocytosis 4 [Source:HGNC Symbol;Acc:HGNC:16183] |
| ENSG00000101542 | HGLibA_08663 | 100 | -0.153498216 | CDH20 | cadherin 20 [Source:HGNC Symbol;Acc:HGNC:1760] |
| ENSG00000102243 | HGLibA_53634 | 100 | -0.153498216 | VGLL1 | vestigial like family member 1 [Source:HGNC Symbol;Acc:HGNC:20985] |
| ENSG00000103148 | HGLibA_32521 | 100 | -0.153498216 | NPRL3 | GATOR1 complex subunit [Source:HGNC Symbol;Acc:HGNC:14124]" |
| ENSG00000103260 | HGLibA_28937 | 100 | -0.153498216 | METRN | glial cell differentiation regulator [Source:HGNC Symbol;Acc:HGNC:14151]" |
| ENSG00000104140 | HGLibA_41210 | 100 | -0.153498216 | RHOV | ras homolog family member V [Source:HGNC Symbol;Acc:HGNC:18313] |
| ENSG00000104231 | HGLibA_55144 | 100 | -0.153498216 | ZFAND1 | zinc finger AN1-type containing 1 [Source:HGNC Symbol;Acc:HGNC:25858] |
| ENSG00000104880 | HGLibA_02827 | 100 | -0.153498216 | ARHGEF18 | Rho/Rac guanine nucleotide exchange factor 18 [Source:HGNC Symbol;Acc:HGNC:17090] |
| ENSG00000105186 | HGLibA_02050 | 100 | -0.153498216 | ANKRD27 | ankyrin repeat domain 27 [Source:HGNC Symbol;Acc:HGNC:25310] |
| ENSG00000105464 | HGLibA_20307 | 100 | -0.153498216 | GRIN2D | glutamate ionotropic receptor NMDA type subunit 2D [Source:HGNC Symbol;Acc:HGNC:4588] |
| ENSG00000105613 | HGLibA_28400 | 100 | -0.153498216 | MAST1 | microtubule associated serine/threonine kinase 1 [Source:HGNC Symbol;Acc:HGNC:19034] |
| ENSG00000106003 | HGLibA_26342 | 100 | -0.153498216 | LFNG | LFNG O-fucosylpeptide 3-beta-N-acetylglucosaminyltransferase [Source:HGNC Symbol;Acc:HGNC:6560] |
| ENSG00000107014 | HGLibA_41354 | 100 | -0.153498216 | RLN2 | relaxin 2 [Source:HGNC Symbol;Acc:HGNC:10027] |
| ENSG00000107902 | HGLibA_26444 | 100 | -0.153498216 | LHPP | phospholysine phosphohistidine inorganic pyrophosphate phosphatase [Source:HGNC Symbol;Acc:HGNC:30042] |
| ENSG00000108849 | HGLibA_38246 | 100 | -0.153498216 | PPY | pancreatic polypeptide [Source:HGNC Symbol;Acc:HGNC:9327] |
| ENSG00000110060 | HGLibA_39601 | 100 | -0.153498216 | PUS3 | pseudouridine synthase 3 [Source:HGNC Symbol;Acc:HGNC:25461] |
| ENSG00000110092 | HGLibA_08072 | 100 | -0.153498216 | CCND1 | cyclin D1 [Source:HGNC Symbol;Acc:HGNC:1582] |
| ENSG00000111341 | HGLibA_29168 | 100 | -0.153498216 | MGP | matrix Gla protein [Source:HGNC Symbol;Acc:HGNC:7060] |
| ENSG00000112212 | HGLibA_51962 | 100 | -0.153498216 | TSPO2 | translocator protein 2 [Source:HGNC Symbol;Acc:HGNC:21256] |
| ENSG00000112763 | HGLibA_04961 | 100 | -0.153498216 | BTN2A1 | butyrophilin subfamily 2 member A1 [Source:HGNC Symbol;Acc:HGNC:1136] |
| ENSG00000113302 | HGLibA_22962 | 100 | -0.153498216 | IL12B | interleukin 12B [Source:HGNC Symbol;Acc:HGNC:5970] |
| ENSG00000113649 | HGLibA_48754 | 100 | -0.153498216 | TCERG1 | transcription elongation regulator 1 [Source:HGNC Symbol;Acc:HGNC:15630] |
| ENSG00000115380 | HGLibA_14561 | 100 | -0.153498216 | EFEMP1 | EGF containing fibulin extracellular matrix protein 1 [Source:HGNC Symbol;Acc:HGNC:3218] |
| ENSG00000115827 | HGLibA_12436 | 100 | -0.153498216 | DCAF17 | DDB1 and CUL4 associated factor 17 [Source:HGNC Symbol;Acc:HGNC:25784] |
| ENSG00000116147 | HGLibA_50907 | 100 | -0.153498216 | TNR | tenascin R [Source:HGNC Symbol;Acc:HGNC:11953] |
| ENSG00000117090 | HGLibA_44313 | 100 | -0.153498216 | SLAMF1 | signaling lymphocytic activation molecule family member 1 [Source:HGNC Symbol;Acc:HGNC:10903] |
| ENSG00000118705 | HGLibA_42019 | 100 | -0.153498216 | RPN2 | ribophorin II [Source:HGNC Symbol;Acc:HGNC:10382] |
| ENSG00000119673 | HGLibA_00480 | 100 | -0.153498216 | ACOT2 | acyl-CoA thioesterase 2 [Source:HGNC Symbol;Acc:HGNC:18431] |
| ENSG00000119919 | HGLibA_32038 | 100 | -0.153498216 | NKX2-3 | NK2 homeobox 3 [Source:HGNC Symbol;Acc:HGNC:7836] |
| ENSG00000123933 | HGLibA_30615 | 100 | -0.153498216 | MXD4 | MAX dimerization protein 4 [Source:HGNC Symbol;Acc:HGNC:13906] |
| ENSG00000124813 | HGLibA_42460 | 100 | -0.153498216 | RUNX2 | runt related transcription factor 2 [Source:HGNC Symbol;Acc:HGNC:10472] |
| ENSG00000125730 | HGLibA_06203 | 100 | -0.153498216 | C3 | complement C3 [Source:HGNC Symbol;Acc:HGNC:1318] |
| ENSG00000127946 | HGLibA_21333 | 100 | -0.153498216 | HIP1 | huntingtin interacting protein 1 [Source:HGNC Symbol;Acc:HGNC:4913] |
| ENSG00000128271 | HGLibA_01012 | 100 | -0.153498216 | ADORA2A | adenosine A2a receptor [Source:HGNC Symbol;Acc:HGNC:263] |
| ENSG00000128567 | HGLibA_37458 | 100 | -0.153498216 | PODXL | podocalyxin like [Source:HGNC Symbol;Acc:HGNC:9171] |
| ENSG00000129007 | HGLibA_07034 | 100 | -0.153498216 | CALML4 | calmodulin like 4 [Source:HGNC Symbol;Acc:HGNC:18445] |
| ENSG00000129824 | HGLibA_42146 | 100 | -0.153498216 | RPS4Y1 | ribosomal protein S4 Y-linked 1 [Source:HGNC Symbol;Acc:HGNC:10425] |
| ENSG00000130529 | HGLibA_51760 | 100 | -0.153498216 | TRPM4 | transient receptor potential cation channel subfamily M member 4 [Source:HGNC Symbol;Acc:HGNC:17993] |
| ENSG00000130545 | HGLibA_11075 | 100 | -0.153498216 | CRB3 | cell polarity complex component [Source:HGNC Symbol;Acc:HGNC:20237]" |
| ENSG00000131187 | HGLibA_15851 | 100 | -0.153498216 | F12 | coagulation factor XII [Source:HGNC Symbol;Acc:HGNC:3530] |
| ENSG00000131899 | HGLibA_26648 | 100 | -0.153498216 | LLGL1 | scribble cell polarity complex component [Source:HGNC Symbol;Acc:HGNC:6628]" |
| ENSG00000131931 | HGLibA_49285 | 100 | -0.153498216 | THAP1 | THAP domain containing 1 [Source:HGNC Symbol;Acc:HGNC:20856] |
| ENSG00000132002 | HGLibA_13522 | 100 | -0.153498216 | DNAJB1 | DnaJ heat shock protein family (Hsp40) member B1 [Source:HGNC Symbol;Acc:HGNC:5270] |
| ENSG00000132631 | HGLibA_43036 | 100 | -0.153498216 | SCP2D1 | SCP2 sterol binding domain containing 1 [Source:HGNC Symbol;Acc:HGNC:16211] |
| ENSG00000134138 | HGLibA_28873 | 100 | -0.153498216 | MEIS2 | Meis homeobox 2 [Source:HGNC Symbol;Acc:HGNC:7001] |
| ENSG00000134343 | HGLibA_02177 | 100 | -0.153498216 | ANO3 | anoctamin 3 [Source:HGNC Symbol;Acc:HGNC:14004] |
| ENSG00000135049 | HGLibA_01259 | 100 | -0.153498216 | AGTPBP1 | ATP/GTP binding protein 1 [Source:HGNC Symbol;Acc:HGNC:17258] |
| ENSG00000135249 | HGLibA_41304 | 100 | -0.153498216 | RINT1 | RAD50 interactor 1 [Source:HGNC Symbol;Acc:HGNC:21876] |
| ENSG00000135617 | HGLibA_38267 | 100 | -0.153498216 | PRADC1 | protease associated domain containing 1 [Source:HGNC Symbol;Acc:HGNC:16047] |
| ENSG00000135631 | HGLibA_39805 | 100 | -0.153498216 | RAB11FIP5 | RAB11 family interacting protein 5 [Source:HGNC Symbol;Acc:HGNC:24845] |
| ENSG00000137040 | HGLibA_40209 | 100 | -0.153498216 | RANBP6 | RAN binding protein 6 [Source:HGNC Symbol;Acc:HGNC:9851] |
| ENSG00000137642 | HGLibA_46196 | 100 | -0.153498216 | SORL1 | sortilin related receptor 1 [Source:HGNC Symbol;Acc:HGNC:11185] |
| ENSG00000137801 | HGLibA_49322 | 100 | -0.153498216 | THBS1 | thrombospondin 1 [Source:HGNC Symbol;Acc:HGNC:11785] |
| ENSG00000138675 | HGLibA_17410 | 100 | -0.153498216 | FGF5 | fibroblast growth factor 5 [Source:HGNC Symbol;Acc:HGNC:3683] |
| ENSG00000139269 | HGLibA_23309 | 100 | -0.153498216 | INHBE | inhibin subunit beta E [Source:HGNC Symbol;Acc:HGNC:24029] |
| ENSG00000140280 | HGLibA_27767 | 100 | -0.153498216 | LYSMD2 | LysM domain containing 2 [Source:HGNC Symbol;Acc:HGNC:28571] |
| ENSG00000140451 | HGLibA_36538 | 100 | -0.153498216 | PIF1 | PIF1 5'-to-3' DNA helicase [Source:HGNC Symbol;Acc:HGNC:26220] |
| ENSG00000140675 | HGLibA_45265 | 100 | -0.153498216 | SLC5A2 | solute carrier family 5 member 2 [Source:HGNC Symbol;Acc:HGNC:11037] |
| ENSG00000140873 | HGLibA_00815 | 100 | -0.153498216 | ADAMTS18 | ADAM metallopeptidase with thrombospondin type 1 motif 18 [Source:HGNC Symbol;Acc:HGNC:17110] |
| ENSG00000141424 | HGLibA_45118 | 100 | -0.153498216 | SLC39A6 | solute carrier family 39 member 6 [Source:HGNC Symbol;Acc:HGNC:18607] |

| | | | | | |
|---|---|---|---|---|---|
| ENSG00000142235 | HGLibA_26730 | 100 | -0.153498216 | LMTK3 | lemur tyrosine kinase 3 [Source:HGNC Symbol;Acc:HGNC:19295] |
| ENSG00000142949 | HGLibA_39503 | 100 | -0.153498216 | PTPRF | receptor type F [Source:HGNC Symbol;Acc:HGNC:9670]" |
| ENSG00000147642 | HGLibA_47911 | 100 | -0.153498216 | SYBU | syntabulin [Source:HGNC Symbol;Acc:HGNC:26011] |
| ENSG00000148156 | HGLibA_00628 | 100 | -0.153498216 | ACTL7B | actin like 7B [Source:HGNC Symbol;Acc:HGNC:162] |
| ENSG00000149182 | HGLibA_02664 | 100 | -0.153498216 | ARFGAP2 | ADP ribosylation factor GTPase activating protein 2 [Source:HGNC Symbol;Acc:HGNC:13504] |
| ENSG00000149516 | HGLibA_30148 | 100 | -0.153498216 | MS4A3 | membrane spanning 4-domains A3 [Source:HGNC Symbol;Acc:HGNC:7317] |
| ENSG00000151164 | HGLibA_40112 | 100 | -0.153498216 | RAD9B | RAD9 checkpoint clamp component B [Source:HGNC Symbol;Acc:HGNC:21700] |
| ENSG00000153029 | HGLibA_29804 | 100 | -0.153498216 | MR1 | class I-related [Source:HGNC Symbol;Acc:HGNC:4975]" |
| ENSG00000155087 | HGLibA_33276 | 100 | -0.153498216 | ODF1 | outer dense fiber of sperm tails 1 [Source:HGNC Symbol;Acc:HGNC:8113] |
| ENSG00000156273 | HGLibA_04065 | 100 | -0.153498216 | BACH1 | BTB domain and CNC homolog 1 [Source:HGNC Symbol;Acc:HGNC:935] |
| ENSG00000156689 | HGLibA_19377 | 100 | -0.153498216 | GLYATL2 | glycine-N-acyltransferase like 2 [Source:HGNC Symbol;Acc:HGNC:24178] |
| ENSG00000157216 | HGLibA_47121 | 100 | -0.153498216 | SSBP3 | single stranded DNA binding protein 3 [Source:HGNC Symbol;Acc:HGNC:15674] |
| ENSG00000158714 | HGLibA_44321 | 100 | -0.153498216 | SLAMF8 | SLAM family member 8 [Source:HGNC Symbol;Acc:HGNC:21391] |
| ENSG00000159131 | HGLibA_18637 | 100 | -0.153498216 | GART | phosphoribosylaminoimidazole synthetase [Source:HGNC Symbol;Acc:HGNC:4163]" |
| ENSG00000159263 | HGLibA_44176 | 100 | -0.153498216 | SIM2 | SIM bHLH transcription factor 2 [Source:HGNC Symbol;Acc:HGNC:10883] |
| ENSG00000159885 | HGLibA_55611 | 100 | -0.153498216 | ZNF222 | zinc finger protein 222 [Source:HGNC Symbol;Acc:HGNC:13015] |
| ENSG00000160282 | HGLibA_18112 | 100 | -0.153498216 | FTCD | formimidoyltransferase cyclodeaminase [Source:HGNC Symbol;Acc:HGNC:3974] |
| ENSG00000162039 | HGLibA_28868 | 100 | -0.153498216 | MEIOB | meiosis specific with OB domains [Source:HGNC Symbol;Acc:HGNC:28569] |
| ENSG00000162512 | HGLibA_43103 | 100 | -0.153498216 | SDC3 | syndecan 3 [Source:HGNC Symbol;Acc:HGNC:10660] |
| ENSG00000162592 | HGLibA_07742 | 100 | -0.153498216 | CCDC27 | coiled-coil domain containing 27 [Source:HGNC Symbol;Acc:HGNC:26546] |
| ENSG00000162873 | HGLibA_25111 | 100 | -0.153498216 | KLHDC8A | kelch domain containing 8A [Source:HGNC Symbol;Acc:HGNC:25573] |
| ENSG00000162897 | HGLibA_17162 | 100 | -0.153498216 | FCAMR | Fc fragment of IgA and IgM receptor [Source:HGNC Symbol;Acc:HGNC:24692] |
| ENSG00000163114 | HGLibA_35865 | 100 | -0.153498216 | PDHA2 | pyruvate dehydrogenase E1 alpha 2 subunit [Source:HGNC Symbol;Acc:HGNC:8807] |
| ENSG00000163513 | HGLibA_49225 | 100 | -0.153498216 | TGFBR2 | transforming growth factor beta receptor 2 [Source:HGNC Symbol;Acc:HGNC:11773] |
| ENSG00000163714 | HGLibA_52501 | 100 | -0.153498216 | U2SURP | U2 snRNP associated SURP domain containing [Source:HGNC Symbol;Acc:HGNC:30855] |
| ENSG00000164638 | HGLibA_44849 | 100 | -0.153498216 | SLC29A4 | solute carrier family 29 member 4 [Source:HGNC Symbol;Acc:HGNC:23097] |
| ENSG00000165025 | HGLibA_47949 | 100 | -0.153498216 | SYK | spleen associated tyrosine kinase [Source:HGNC Symbol;Acc:HGNC:11491] |
| ENSG00000166183 | HGLibA_03346 | 100 | -0.153498216 | ASPG | asparaginase [Source:HGNC Symbol;Acc:HGNC:20123] |
| ENSG00000166831 | HGLibA_40655 | 100 | -0.153498216 | RBPMS2 | mRNA processing factor 2 [Source:HGNC Symbol;Acc:HGNC:19098]" |
| ENSG00000166884 | HGLibA_33980 | 100 | -0.153498216 | OR4D6 | olfactory receptor family 4 subfamily D member 6 [Source:HGNC Symbol;Acc:HGNC:15175] |
| ENSG00000167104 | HGLibA_04731 | 100 | -0.153498216 | BPIFB6 | BPI fold containing family B member 6 [Source:HGNC Symbol;Acc:HGNC:16504] |
| ENSG00000167306 | HGLibA_30852 | 100 | -0.153498216 | MYO5B | myosin VB [Source:HGNC Symbol;Acc:HGNC:7603] |
| ENSG00000167552 | HGLibA_52243 | 100 | -0.153498216 | TUBA1A | tubulin alpha 1a [Source:HGNC Symbol;Acc:HGNC:20766] |
| ENSG00000167759 | HGLibA_25242 | 100 | -0.153498216 | KLK13 | kallikrein related peptidase 13 [Source:HGNC Symbol;Acc:HGNC:6361] |
| ENSG00000168830 | HGLibA_22365 | 100 | -0.153498216 | HTR1E | 5-hydroxytryptamine receptor 1E [Source:HGNC Symbol;Acc:HGNC:5291] |
| ENSG00000169508 | HGLibA_19989 | 100 | -0.153498216 | GPR183 | G protein-coupled receptor 183 [Source:HGNC Symbol;Acc:HGNC:3128] |
| ENSG00000169789 | HGLibA_38994 | 100 | -0.153498216 | PRY | PTPN13 like Y-linked [Source:HGNC Symbol;Acc:HGNC:14024] |
| ENSG00000170689 | HGLibA_21952 | 100 | -0.153498216 | HOXB9 | homeobox B9 [Source:HGNC Symbol;Acc:HGNC:5120] |
| ENSG00000170876 | HGLibA_50401 | 100 | -0.153498216 | TMEM43 | transmembrane protein 43 [Source:HGNC Symbol;Acc:HGNC:28472] |
| ENSG00000171241 | HGLibA_44013 | 100 | -0.153498216 | SHCBP1 | SHC binding and spindle associated 1 [Source:HGNC Symbol;Acc:HGNC:29547] |
| ENSG00000171992 | HGLibA_48018 | 100 | -0.153498216 | SYNPO | synaptopodin [Source:HGNC Symbol;Acc:HGNC:30672] |
| ENSG00000172780 | HGLibA_39949 | 100 | -0.153498216 | RAB43 | member RAS oncogene family [Source:HGNC Symbol;Acc:HGNC:19983]" |
| ENSG00000174599 | HGLibA_51284 | 100 | -0.153498216 | TRAM1L1 | translocation associated membrane protein 1 like 1 [Source:HGNC Symbol;Acc:HGNC:28371] |
| ENSG00000174951 | HGLibA_18180 | 100 | -0.153498216 | FUT1 | fucosyltransferase 1 (H blood group) [Source:HGNC Symbol;Acc:HGNC:4012] |
| ENSG00000175175 | HGLibA_37973 | 100 | -0.153498216 | PPM1E | Mg2+/Mn2+ dependent 1E [Source:HGNC Symbol;Acc:HGNC:19322]" |
| ENSG00000175215 | HGLibA_11611 | 100 | -0.153498216 | CTDSP2 | CTD small phosphatase 2 [Source:HGNC Symbol;Acc:HGNC:17077] |
| ENSG00000175662 | HGLibA_50951 | 100 | -0.153498216 | TOM1L2 | target of myb1 like 2 membrane trafficking protein [Source:HGNC Symbol;Acc:HGNC:11984] |
| ENSG00000176101 | HGLibA_47143 | 100 | -0.153498216 | SSNA1 | SS nuclear autoantigen 1 [Source:HGNC Symbol;Acc:HGNC:11321] |
| ENSG00000176358 | HGLibA_48157 | 100 | -0.153498216 | TAC4 | tachykinin 4 [Source:HGNC Symbol;Acc:HGNC:16641] |
| ENSG00000176834 | HGLibA_53839 | 100 | -0.153498216 | VSIG10 | V-set and immunoglobulin domain containing 10 [Source:HGNC Symbol;Acc:HGNC:26078] |
| ENSG00000177243 | HGLibA_12817 | 100 | -0.153498216 | DEFB103B | defensin beta 103B [Source:HGNC Symbol;Acc:HGNC:31702] |
| ENSG00000177842 | HGLibA_56403 | 100 | -0.153498216 | ZNF620 | zinc finger protein 620 [Source:HGNC Symbol;Acc:HGNC:28742] |
| ENSG00000178171 | HGLibA_01778 | 100 | -0.153498216 | AMER3 | APC membrane recruitment protein 3 [Source:HGNC Symbol;Acc:HGNC:26771] |
| ENSG00000179431 | HGLibA_17549 | 100 | -0.153498216 | FJX1 | four-jointed box kinase 1 [Source:HGNC Symbol;Acc:HGNC:17166] |
| ENSG00000179774 | HGLibA_03535 | 100 | -0.153498216 | ATOH7 | atonal bHLH transcription factor 7 [Source:HGNC Symbol;Acc:HGNC:13907] |
| ENSG00000180999 | HGLibA_05736 | 100 | -0.153498216 | C1orf105 | chromosome 1 open reading frame 105 [Source:HGNC Symbol;Acc:HGNC:29591] |
| ENSG00000181036 | HGLibA_17239 | 100 | -0.153498216 | FCRL6 | Fc receptor like 6 [Source:HGNC Symbol;Acc:HGNC:31910] |
| ENSG00000181315 | HGLibA_55799 | 100 | -0.153498216 | ZNF322 | zinc finger protein 322 [Source:HGNC Symbol;Acc:HGNC:23640] |
| ENSG00000181896 | HGLibA_55435 | 100 | -0.153498216 | ZNF101 | zinc finger protein 101 [Source:HGNC Symbol;Acc:HGNC:12881] |
| ENSG00000181963 | HGLibA_34184 | 100 | -0.153498216 | OR52K2 | olfactory receptor family 52 subfamily K member 2 [Source:HGNC Symbol;Acc:HGNC:15223] |
| ENSG00000183024 | HGLibA_33667 | 100 | -0.153498216 | OR1G1 | olfactory receptor family 1 subfamily G member 1 [Source:HGNC Symbol;Acc:HGNC:8204] |
| ENSG00000183801 | HGLibA_33356 | 100 | -0.153498216 | OLFML1 | olfactomedin like 1 [Source:HGNC Symbol;Acc:HGNC:24473] |
| ENSG00000185338 | HGLibA_46122 | 100 | -0.153498216 | SOCS1 | suppressor of cytokine signaling 1 [Source:HGNC Symbol;Acc:HGNC:19383] |
| ENSG00000187238 | HGLibA_26134 | 100 | -0.153498216 | LCE3B | late cornified envelope 3B [Source:HGNC Symbol;Acc:HGNC:29462] |
| ENSG00000187821 | HGLibA_21121 | 100 | -0.153498216 | HELT | helt bHLH transcription factor [Source:HGNC Symbol;Acc:HGNC:33783] |
| ENSG00000188076 | HGLibA_42912 | 100 | -0.153498216 | SCGB1C1 | secretoglobin family 1C member 1 [Source:HGNC Symbol;Acc:HGNC:18394] |
| ENSG00000196156 | HGLibA_25762 | 100 | -0.153498216 | KRTAP4-3 | keratin associated protein 4-3 [Source:HGNC Symbol;Acc:HGNC:18908] |
| ENSG00000196208 | HGLibA_20237 | 100 | -0.153498216 | GREB1 | growth regulating estrogen receptor binding 1 [Source:HGNC Symbol;Acc:HGNC:24885] |
| ENSG00000196248 | HGLibA_33544 | 100 | -0.153498216 | OR10S1 | olfactory receptor family 10 subfamily S member 1 [Source:HGNC Symbol;Acc:HGNC:14807] |
| ENSG00000196323 | HGLibA_54892 | 100 | -0.153498216 | ZBTB44 | zinc finger and BTB domain containing 44 [Source:HGNC Symbol;Acc:HGNC:25001] |
| ENSG00000196361 | HGLibA_14886 | 100 | -0.153498216 | ELAVL3 | ELAV like RNA binding protein 3 [Source:HGNC Symbol;Acc:HGNC:3314] |
| ENSG00000196724 | HGLibA_55952 | 100 | -0.153498216 | ZNF418 | zinc finger protein 418 [Source:HGNC Symbol;Acc:HGNC:20647] |
| ENSG00000197619 | HGLibA_56392 | 100 | -0.153498216 | ZNF615 | zinc finger protein 615 [Source:HGNC Symbol;Acc:HGNC:24740] |
| ENSG00000197915 | HGLibA_22108 | 100 | -0.153498216 | HRNR | hornerin [Source:HGNC Symbol;Acc:HGNC:20846] |
| ENSG00000197980 | HGLibA_26280 | 100 | -0.153498216 | LEKR1 | glutamate and lysine rich 1 [Source:HGNC Symbol;Acc:HGNC:33765]" |
| ENSG00000198087 | HGLibA_08328 | 100 | -0.153498216 | CD2AP | CD2 associated protein [Source:HGNC Symbol;Acc:HGNC:14258] |
| ENSG00000198792 | HGLibA_50113 | 100 | -0.153498216 | TMEM184B | transmembrane protein 184B [Source:HGNC Symbol;Acc:HGNC:1310] |
| ENSG00000203872 | HGLibA_06455 | 100 | -0.153498216 | C6orf163 | chromosome 6 open reading frame 163 [Source:HGNC Symbol;Acc:HGNC:21403] |
| ENSG00000204006 | HGLibA_05805 | 100 | -0.153498216 | C1orf185 | chromosome 1 open reading frame 185 [Source:HGNC Symbol;Acc:HGNC:28096] |
| ENSG00000204873 | HGLibA_25835 | 100 | -0.153498216 | KRTAP9-3 | keratin associated protein 9-3 [Source:HGNC Symbol;Acc:HGNC:16927] |
| ENSG00000205045 | HGLibA_45486 | 100 | -0.153498216 | SLFN12L | schlafen family member 12 like [Source:HGNC Symbol;Acc:HGNC:33920] |
| ENSG00000205420 | HGLibA_25513 | 100 | -0.153498216 | KRT6A | keratin 6A [Source:HGNC Symbol;Acc:HGNC:6443] |
| ENSG00000206422 | HGLibA_27307 | 100 | -0.153498216 | LRRC30 | leucine rich repeat containing 30 [Source:HGNC Symbol;Acc:HGNC:30219] |
| ENSG00000213977 | HGLibA_48452 | 100 | -0.153498216 | TAX1BP3 | Tax1 binding protein 3 [Source:HGNC Symbol;Acc:HGNC:30684] |
| ENSG00000214290 | HGLibA_10628 | 100 | -0.153498216 | COLCA2 | colorectal cancer associated 2 [Source:HGNC Symbol;Acc:HGNC:26978] |
| ENSG00000221867 | HGLibA_27910 | 100 | -0.153498216 | MAGEA3 | MAGE family member A3 [Source:HGNC Symbol;Acc:HGNC:6801] |
| ENSG00000225781 | HGLibA_34449 | 100 | -0.153498216 | OR6V1 | olfactory receptor family 6 subfamily V member 1 [Source:HGNC Symbol;Acc:HGNC:15090] |
| ENSG00000225968 | HGLibA_14906 | 100 | -0.153498216 | ELFN1 | extracellular leucine rich repeat and fibronectin type III domain containing 1 [Source:HGNC Symbol;Acc:HGNC:33154] |
| ENSG00000239887 | HGLibA_05834 | 100 | -0.153498216 | C1orf226 | chromosome 1 open reading frame 226 [Source:HGNC Symbol;Acc:HGNC:34351] |
| ENSG00000241697 | HGLibA_49833 | 100 | -0.153498216 | TMEFF1 | transmembrane protein with EGF like and two follistatin like domains 1 [Source:HGNC Symbol;Acc:HGNC:11866] |
| ENSG00000243955 | HGLibA_20450 | 100 | -0.153498216 | GSTA1 | glutathione S-transferase alpha 1 [Source:HGNC Symbol;Acc:HGNC:4626] |
| ENSG00000248167 | HGLibA_51487 | 100 | -0.153498216 | TRIM39-RPP21 | TRIM39-RPP21 readthrough [Source:HGNC Symbol;Acc:HGNC:38845] |
| ENSG00000250361 | HGLibA_20699 | 100 | -0.153498216 | GYPB | glycophorin B (MNS blood group) [Source:HGNC Symbol;Acc:HGNC:4703] |
| ENSG00000258839 | HGLibA_28540 | 100 | -0.153498216 | MC1R | melanocortin 1 receptor [Source:HGNC Symbol;Acc:HGNC:6929] |
| ENSG00000271601 | HGLibA_26646 | 100 | -0.153498216 | LIX1L | limb and CNS expressed 1 like [Source:HGNC Symbol;Acc:HGNC:28715] |
| ENSG00000276045 | HGLibA_34588 | 100 | -0.153498216 | ORAI1 | ORAI calcium release-activated calcium modulator 1 [Source:HGNC Symbol;Acc:HGNC:25896] |
| ENSG00000282608 | HGLibA_01020 | 100 | -0.153498216 | ADORA3 | adenosine A3 receptor [Source:HGNC Symbol;Acc:HGNC:268] |
| FAM73A | HGLibA_16716 | 100 | -0.153498216 | FAM73A | - |
| HIST1H2BC | HGLibA_21417 | 100 | -0.153498216 | HIST1H2BC | - |
| hsa-mir-1278 | HGLibA_57566 | 100 | -0.153498216 | hsa-mir-1278 | - |
| hsa-mir-1302-4 | HGLibA_57705 | 100 | -0.153498216 | hsa-mir-1302-4 | - |
| hsa-mir-2682 | HGLibA_58361 | 100 | -0.153498216 | hsa-mir-2682 | - |
| hsa-mir-302a | HGLibA_58438 | 100 | -0.153498216 | hsa-mir-302a | - |
| hsa-mir-3156-1 | HGLibA_58689 | 100 | -0.153498216 | hsa-mir-3156-1 | - |
| hsa-mir-3914-1 | HGLibA_59529 | 100 | -0.153498216 | hsa-mir-3914-1 | - |
| hsa-mir-3960 | HGLibA_59643 | 100 | -0.153498216 | hsa-mir-3960 | - |
| hsa-mir-409 | HGLibA_59676 | 100 | -0.153498216 | hsa-mir-409 | - |
| hsa-mir-4265 | HGLibA_59767 | 100 | -0.153498216 | hsa-mir-4265 | - |
| hsa-mir-4265 | HGLibA_59766 | 100 | -0.153498216 | hsa-mir-4265 | - |
| hsa-mir-4276 | HGLibA_59806 | 100 | -0.153498216 | hsa-mir-4276 | - |
| hsa-mir-4638 | HGLibA_60657 | 100 | -0.153498216 | hsa-mir-4638 | - |
| hsa-mir-4647 | HGLibA_60692 | 100 | -0.153498216 | hsa-mir-4647 | - |
| hsa-mir-4650-2 | HGLibA_60709 | 100 | -0.153498216 | hsa-mir-4650-2 | - |
| hsa-mir-496 | HGLibA_61386 | 100 | -0.153498216 | hsa-mir-496 | - |
| hsa-mir-500a | HGLibA_61442 | 100 | -0.153498216 | hsa-mir-500a | - |
| hsa-mir-520e | HGLibA_61751 | 100 | -0.153498216 | hsa-mir-520e | - |
| hsa-mir-548ax | HGLibA_61941 | 100 | -0.153498216 | hsa-mir-548ax | - |
| hsa-mir-550a-3 | HGLibA_62105 | 100 | -0.153498216 | hsa-mir-550a-3 | - |
| hsa-mir-5586 | HGLibA_62184 | 100 | -0.153498216 | hsa-mir-5586 | - |
| hsa-mir-5589 | HGLibA_62197 | 100 | -0.153498216 | hsa-mir-5589 | - |
| hsa-mir-6072 | HGLibA_62539 | 100 | -0.153498216 | hsa-mir-6072 | - |
| hsa-mir-6769a | HGLibA_63238 | 100 | -0.153498216 | hsa-mir-6769a | - |
| hsa-mir-6848 | HGLibA_63565 | 100 | -0.153498216 | hsa-mir-6848 | - |
| hsa-mir-7702 | HGLibA_63942 | 100 | -0.153498216 | hsa-mir-7702 | - |
| hsa-mir-8072 | HGLibA_64131 | 100 | -0.153498216 | hsa-mir-8072 | - |
| hsa-mir-8076 | HGLibA_64148 | 100 | -0.153498216 | hsa-mir-8076 | - |
| LOC100129216 | HGLibA_26755 | 100 | -0.153498216 | LOC100129216 | - |
| LRRC48 | HGLibA_27374 | 100 | -0.153498216 | LRRC48 | - |
| LUZP6 | HGLibA_27648 | 100 | -0.153498216 | LUZP6 | - |
| MPP6 | HGLibA_29763 | 100 | -0.153498216 | MPP6 | - |
| SLC35E2 | HGLibA_44988 | 100 | -0.153498216 | SLC35E2 | - |
| SUV420H2 | HGLibA_47869 | 100 | -0.153498216 | SUV420H2 | - |
| TCTEX1D1 | HGLibA_48863 | 100 | -0.153498216 | TCTEX1D1 | - |
| TMEM246 | HGLibA_50304 | 100 | -0.153498216 | TMEM246 | - |
| C12orf23 | HGLibA_05227 | 99 | -0.163449254 | C12orf23 | - |

| | | | | | |
|---|---|---|---|---|---|
| C16orf11 | HGLibA_05438 | 99 | -0.163449254 | C16orf11 | - |
| C4B_2 | HGLibA_06288 | 99 | -0.163449254 | C4B_2 | - |
| C5orf42 | HGLibA_06383 | 99 | -0.163449254 | C5orf42 | - |
| ENSG00000006634 | HGLibA_12376 | 99 | -0.163449254 | DBF4 | DBF4 zinc finger [Source:HGNC Symbol;Acc:HGNC:17364] |
| ENSG00000007062 | HGLibA_38680 | 99 | -0.163449254 | PROM1 | prominin 1 [Source:HGNC Symbol;Acc:HGNC:9454] |
| ENSG00000008394 | HGLibA_29173 | 99 | -0.163449254 | MGST1 | microsomal glutathione S-transferase 1 [Source:HGNC Symbol;Acc:HGNC:7061] |
| ENSG00000010404 | HGLibA_22566 | 99 | -0.163449254 | IDS | iduronate 2-sulfatase [Source:HGNC Symbol;Acc:HGNC:5389] |
| ENSG00000010704 | HGLibA_21225 | 99 | -0.163449254 | HFE | homeostatic iron regulator [Source:HGNC Symbol;Acc:HGNC:4886] |
| ENSG00000013503 | HGLibA_37613 | 99 | -0.163449254 | POLR3B | RNA polymerase III subunit B [Source:HGNC Symbol;Acc:HGNC:30348] |
| ENSG00000018625 | HGLibA_03585 | 99 | -0.163449254 | ATP1A2 | ATPase Na+/K+ transporting subunit alpha 2 [Source:HGNC Symbol;Acc:HGNC:800] |
| ENSG00000022976 | HGLibA_56847 | 99 | -0.163449254 | ZNF839 | zinc finger protein 839 [Source:HGNC Symbol;Acc:HGNC:20345] |
| ENSG00000039560 | HGLibA_40140 | 99 | -0.163449254 | RAI14 | retinoic acid induced 14 [Source:HGNC Symbol;Acc:HGNC:14873] |
| ENSG00000065029 | HGLibA_56682 | 99 | -0.163449254 | ZNF76 | zinc finger protein 76 [Source:HGNC Symbol;Acc:HGNC:13149] |
| ENSG00000071073 | HGLibA_29142 | 99 | -0.163449254 | MGAT4A | 3-mannosyl-glycoprotein 4-beta-N-acetylglucosaminyltransferase A [Source:HGNC Symbol;Acc:HGNC:7047]" |
| ENSG00000072401 | HGLibA_52581 | 99 | -0.163449254 | UBE2D1 | ubiquitin conjugating enzyme E2 D1 [Source:HGNC Symbol;Acc:HGNC:12474] |
| ENSG00000074219 | HGLibA_48930 | 99 | -0.163449254 | TEAD2 | TEA domain transcription factor 2 [Source:HGNC Symbol;Acc:HGNC:11715] |
| ENSG00000079332 | HGLibA_42738 | 99 | -0.163449254 | SAR1A | secretion associated Ras related GTPase 1A [Source:HGNC Symbol;Acc:HGNC:10534] |
| ENSG00000081277 | HGLibA_36852 | 99 | -0.163449254 | PKP1 | plakophilin 1 [Source:HGNC Symbol;Acc:HGNC:9023] |
| ENSG00000087085 | HGLibA_00437 | 99 | -0.163449254 | ACHE | acetylcholinesterase (Cartwright blood group) [Source:HGNC Symbol;Acc:HGNC:108] |
| ENSG00000091482 | HGLibA_45820 | 99 | -0.163449254 | SMPX | small muscle protein X-linked [Source:HGNC Symbol;Acc:HGNC:11122] |
| ENSG00000092020 | HGLibA_38164 | 99 | -0.163449254 | PPP2R3C | protein phosphatase 2 regulatory subunit B''gamma [Source:HGNC Symbol;Acc:HGNC:17485] |
| ENSG00000092067 | HGLibA_08966 | 99 | -0.163449254 | CEBPE | CCAAT enhancer binding protein epsilon [Source:HGNC Symbol;Acc:HGNC:1836] |
| ENSG00000095585 | HGLibA_04554 | 99 | -0.163449254 | BLNK | B cell linker [Source:HGNC Symbol;Acc:HGNC:14211] |
| ENSG00000096996 | HGLibA_22965 | 99 | -0.163449254 | IL12RB1 | interleukin 12 receptor subunit beta 1 [Source:HGNC Symbol;Acc:HGNC:5971] |
| ENSG00000100129 | HGLibA_14797 | 99 | -0.163449254 | EIF3L | eukaryotic translation initiation factor 3 subunit L [Source:HGNC Symbol;Acc:HGNC:18138] |
| ENSG00000100445 | HGLibA_43164 | 99 | -0.163449254 | SDR39U1 | short chain dehydrogenase/reductase family 39U member 1 [Source:HGNC Symbol;Acc:HGNC:20275] |
| ENSG00000100580 | HGLibA_49826 | 99 | -0.163449254 | TMED8 | transmembrane p24 trafficking protein family member 8 [Source:HGNC Symbol;Acc:HGNC:18633] |
| ENSG00000100726 | HGLibA_48987 | 99 | -0.163449254 | TELO2 | telomere maintenance 2 [Source:HGNC Symbol;Acc:HGNC:29099] |
| ENSG00000101400 | HGLibA_45997 | 99 | -0.163449254 | SNTA1 | syntrophin alpha 1 [Source:HGNC Symbol;Acc:HGNC:11167] |
| ENSG00000102362 | HGLibA_48104 | 99 | -0.163449254 | SYTL4 | synaptotagmin like 4 [Source:HGNC Symbol;Acc:HGNC:15588] |
| ENSG00000102886 | HGLibA_18895 | 99 | -0.163449254 | GDPD3 | glycerophosphodiester phosphodiesterase domain containing 3 [Source:HGNC Symbol;Acc:HGNC:28638] |
| ENSG00000103707 | HGLibA_30356 | 99 | -0.163449254 | MTFMT | mitochondrial methionyl-tRNA formyltransferase [Source:HGNC Symbol;Acc:HGNC:29666] |
| ENSG00000105612 | HGLibA_13665 | 99 | -0.163449254 | DNASE2 | lysosomal [Source:HGNC Symbol;Acc:HGNC:2960]" |
| ENSG00000105679 | HGLibA_18617 | 99 | -0.163449254 | GAPDHS | spermatogenic [Source:HGNC Symbol;Acc:HGNC:24864]" |
| ENSG00000106608 | HGLibA_53139 | 99 | -0.163449254 | URGCP | upregulator of cell proliferation [Source:HGNC Symbol;Acc:HGNC:30890] |
| ENSG00000109163 | HGLibA_19586 | 99 | -0.163449254 | GNRHR | gonadotropin releasing hormone receptor [Source:HGNC Symbol;Acc:HGNC:4421] |
| ENSG00000109171 | HGLibA_44308 | 99 | -0.163449254 | SLAIN2 | SLAIN motif family member 2 [Source:HGNC Symbol;Acc:HGNC:29282] |
| ENSG00000109684 | HGLibA_10087 | 99 | -0.163449254 | CLNK | cytokine dependent hematopoietic cell linker [Source:HGNC Symbol;Acc:HGNC:17438] |
| ENSG00000112818 | HGLibA_28895 | 99 | -0.163449254 | MEP1A | meprin A subunit alpha [Source:HGNC Symbol;Acc:HGNC:7015] |
| ENSG00000113749 | HGLibA_22098 | 99 | -0.163449254 | HRH2 | histamine receptor H2 [Source:HGNC Symbol;Acc:HGNC:5183] |
| ENSG00000113749 | HGLibA_22096 | 99 | -0.163449254 | HRH2 | histamine receptor H2 [Source:HGNC Symbol;Acc:HGNC:5183] |
| ENSG00000114349 | HGLibA_19473 | 99 | -0.163449254 | GNAT1 | G protein subunit alpha transducin 1 [Source:HGNC Symbol;Acc:HGNC:4393] |
| ENSG00000114626 | HGLibA_00336 | 99 | -0.163449254 | ABTB1 | ankyrin repeat and BTB domain containing 1 [Source:HGNC Symbol;Acc:HGNC:18275] |
| ENSG00000114978 | HGLibA_29586 | 99 | -0.163449254 | MOB1A | MOB kinase activator 1A [Source:HGNC Symbol;Acc:HGNC:16015] |
| ENSG00000115138 | HGLibA_37655 | 99 | -0.163449254 | POMC | proopiomelanocortin [Source:HGNC Symbol;Acc:HGNC:9201] |
| ENSG00000115159 | HGLibA_19789 | 99 | -0.163449254 | GPD2 | glycerol-3-phosphate dehydrogenase 2 [Source:HGNC Symbol;Acc:HGNC:4456] |
| ENSG00000115902 | HGLibA_44516 | 99 | -0.163449254 | SLC1A4 | solute carrier family 1 member 4 [Source:HGNC Symbol;Acc:HGNC:10942] |
| ENSG00000116096 | HGLibA_46788 | 99 | -0.163449254 | SPR | sepiapterin reductase [Source:HGNC Symbol;Acc:HGNC:11257] |
| ENSG00000116668 | HGLibA_47904 | 99 | -0.163449254 | SWT1 | RNA endoribonuclease homolog [Source:HGNC Symbol;Acc:HGNC:16785]" |
| ENSG00000117650 | HGLibA_31648 | 99 | -0.163449254 | NEK2 | NIMA related kinase 2 [Source:HGNC Symbol;Acc:HGNC:7745] |
| ENSG00000118985 | HGLibA_14924 | 99 | -0.163449254 | ELL2 | elongation factor for RNA polymerase II 2 [Source:HGNC Symbol;Acc:HGNC:17064] |
| ENSG00000119013 | HGLibA_31516 | 99 | -0.163449254 | NDUFB3 | NADH:ubiquinone oxidoreductase subunit B3 [Source:HGNC Symbol;Acc:HGNC:7698] |
| ENSG00000119326 | HGLibA_11648 | 99 | -0.163449254 | CTNNAL1 | catenin alpha like 1 [Source:HGNC Symbol;Acc:HGNC:2512] |
| ENSG00000119408 | HGLibA_31660 | 99 | -0.163449254 | NEK6 | NIMA related kinase 6 [Source:HGNC Symbol;Acc:HGNC:7749] |
| ENSG00000119522 | HGLibA_12928 | 99 | -0.163449254 | DENND1A | DENN domain containing 1A [Source:HGNC Symbol;Acc:HGNC:29324] |
| ENSG00000120539 | HGLibA_28413 | 99 | -0.163449254 | MASTL | microtubule associated serine/threonine kinase like [Source:HGNC Symbol;Acc:HGNC:19042] |
| ENSG00000120697 | HGLibA_01630 | 99 | -0.163449254 | ALG5 | dolichyl-phosphate beta-glucosyltransferase [Source:HGNC Symbol;Acc:HGNC:20266]" |
| ENSG00000120820 | HGLibA_19341 | 99 | -0.163449254 | GLT8D2 | glycosyltransferase 8 domain containing 2 [Source:HGNC Symbol;Acc:HGNC:24890] |
| ENSG00000121879 | HGLibA_36632 | 99 | -0.163449254 | PIK3CA | 5-bisphosphate 3-kinase catalytic subunit alpha [Source:HGNC Symbol;Acc:HGNC:8975]" |
| ENSG00000121989 | HGLibA_00694 | 99 | -0.163449254 | ACVR2A | activin A receptor type 2A [Source:HGNC Symbol;Acc:HGNC:173] |
| ENSG00000124275 | HGLibA_30493 | 99 | -0.163449254 | MTRR | 5-methyltetrahydrofolate-homocysteine methyltransferase reductase [Source:HGNC Symbol;Acc:HGNC:7473] |
| ENSG00000124356 | HGLibA_47336 | 99 | -0.163449254 | STAMBP | STAM binding protein [Source:HGNC Symbol;Acc:HGNC:16950] |
| ENSG00000124641 | HGLibA_28773 | 99 | -0.163449254 | MED20 | mediator complex subunit 20 [Source:HGNC Symbol;Acc:HGNC:16840] |
| ENSG00000124920 | HGLibA_30916 | 99 | -0.163449254 | MYRF | myelin regulatory factor [Source:HGNC Symbol;Acc:HGNC:1181] |
| ENSG00000125352 | HGLibA_41473 | 99 | -0.163449254 | RNF113A | ring finger protein 113A [Source:HGNC Symbol;Acc:HGNC:12974] |
| ENSG00000125414 | HGLibA_30722 | 99 | -0.163449254 | MYH2 | myosin heavy chain 2 [Source:HGNC Symbol;Acc:HGNC:7572] |
| ENSG00000125656 | HGLibA_10101 | 99 | -0.163449254 | CLPP | caseinolytic mitochondrial matrix peptidase proteolytic subunit [Source:HGNC Symbol;Acc:HGNC:2084] |
| ENSG00000125912 | HGLibA_31330 | 99 | -0.163449254 | NCLN | nicalin [Source:HGNC Symbol;Acc:HGNC:26923] |
| ENSG00000125962 | HGLibA_03051 | 99 | -0.163449254 | ARMCX5 | armadillo repeat containing X-linked 5 [Source:HGNC Symbol;Acc:HGNC:25772] |
| ENSG00000127603 | HGLibA_27834 | 99 | -0.163449254 | MACF1 | microtubule-actin crosslinking factor 1 [Source:HGNC Symbol;Acc:HGNC:13664] |
| ENSG00000129450 | HGLibA_44152 | 99 | -0.163449254 | SIGLEC9 | sialic acid binding Ig like lectin 9 [Source:HGNC Symbol;Acc:HGNC:10878] |
| ENSG00000130332 | HGLibA_27552 | 99 | -0.163449254 | LSM7 | U6 small nuclear RNA and mRNA degradation associated [Source:HGNC Symbol;Acc:HGNC:20470]" |
| ENSG00000130702 | HGLibA_25954 | 99 | -0.163449254 | LAMA5 | laminin subunit alpha 5 [Source:HGNC Symbol;Acc:HGNC:6485] |
| ENSG00000132196 | HGLibA_22199 | 99 | -0.163449254 | HSD17B7 | hydroxysteroid 17-beta dehydrogenase 7 [Source:HGNC Symbol;Acc:HGNC:5215] |
| ENSG00000132676 | HGLibA_12318 | 99 | -0.163449254 | DAP3 | death associated protein 3 [Source:HGNC Symbol;Acc:HGNC:2673] |
| ENSG00000133687 | HGLibA_50671 | 99 | -0.163449254 | TMTC1 | transmembrane and tetratricopeptide repeat containing 1 [Source:HGNC Symbol;Acc:HGNC:24099] |
| ENSG00000134049 | HGLibA_22577 | 99 | -0.163449254 | IER3IP1 | immediate early response 3 interacting protein 1 [Source:HGNC Symbol;Acc:HGNC:18550] |
| ENSG00000134815 | HGLibA_13185 | 99 | -0.163449254 | DHX34 | DExH-box helicase 34 [Source:HGNC Symbol;Acc:HGNC:16719] |
| ENSG00000134897 | HGLibA_04533 | 99 | -0.163449254 | BIVM | immunoglobulin-like variable motif containing [Source:HGNC Symbol;Acc:HGNC:16034]" |
| ENSG00000135047 | HGLibA_11717 | 99 | -0.163449254 | CTSL | cathepsin L [Source:HGNC Symbol;Acc:HGNC:2537] |
| ENSG00000135316 | HGLibA_47968 | 99 | -0.163449254 | SYNCRIP | synaptotagmin binding cytoplasmic RNA interacting protein [Source:HGNC Symbol;Acc:HGNC:16918] |
| ENSG00000136280 | HGLibA_08044 | 99 | -0.163449254 | CCM2 | CCM2 scaffold protein [Source:HGNC Symbol;Acc:HGNC:21708] |
| ENSG00000136731 | HGLibA_52868 | 99 | -0.163449254 | UGGT1 | UDP-glucose glycoprotein glucosyltransferase 1 [Source:HGNC Symbol;Acc:HGNC:15663] |
| ENSG00000136839 | HGLibA_33610 | 99 | -0.163449254 | OR13C9 | olfactory receptor family 13 subfamily C member 9 [Source:HGNC Symbol;Acc:HGNC:15104] |
| ENSG00000137161 | HGLibA_10368 | 99 | -0.163449254 | CNPY3 | canopy FGF signaling regulator 3 [Source:HGNC Symbol;Acc:HGNC:11968] |
| ENSG00000137216 | HGLibA_50477 | 99 | -0.163449254 | TMEM63B | transmembrane protein 63B [Source:HGNC Symbol;Acc:HGNC:17735] |
| ENSG00000137221 | HGLibA_49569 | 99 | -0.163449254 | TJAP1 | tight junction associated protein 1 [Source:HGNC Symbol;Acc:HGNC:17949] |
| ENSG00000139610 | HGLibA_08990 | 99 | -0.163449254 | CELA1 | chymotrypsin like elastase family member 1 [Source:HGNC Symbol;Acc:HGNC:3308] |
| ENSG00000141101 | HGLibA_32232 | 99 | -0.163449254 | NOB1 | NIN1 (RPN12) binding protein 1 homolog [Source:HGNC Symbol;Acc:HGNC:29540] |
| ENSG00000142856 | HGLibA_23785 | 99 | -0.163449254 | ITGB3BP | integrin subunit beta 3 binding protein [Source:HGNC Symbol;Acc:HGNC:6157] |
| ENSG00000143486 | HGLibA_14751 | 99 | -0.163449254 | EIF2D | eukaryotic translation initiation factor 2D [Source:HGNC Symbol;Acc:HGNC:6583] |
| ENSG00000143643 | HGLibA_52052 | 99 | -0.163449254 | TTC13 | tetratricopeptide repeat domain 13 [Source:HGNC Symbol;Acc:HGNC:26204] |
| ENSG00000143847 | HGLibA_37881 | 99 | -0.163449254 | PPFIA4 | PTPRF interacting protein alpha 4 [Source:HGNC Symbol;Acc:HGNC:9248] |
| ENSG00000144354 | HGLibA_08614 | 99 | -0.163449254 | CDCA7 | cell division cycle associated 7 [Source:HGNC Symbol;Acc:HGNC:14628] |
| ENSG00000144426 | HGLibA_31208 | 99 | -0.163449254 | NBEAL1 | neurobeachin like 1 [Source:HGNC Symbol;Acc:HGNC:20681] |
| ENSG00000144579 | HGLibA_11607 | 99 | -0.163449254 | CTDSP1 | CTD small phosphatase 1 [Source:HGNC Symbol;Acc:HGNC:21614] |
| ENSG00000144852 | HGLibA_32588 | 99 | -0.163449254 | NR1I2 | nuclear receptor subfamily 1 group I member 2 [Source:HGNC Symbol;Acc:HGNC:7968] |
| ENSG00000145335 | HGLibA_45909 | 99 | -0.163449254 | SNCA | synuclein alpha [Source:HGNC Symbol;Acc:HGNC:11138] |
| ENSG00000146285 | HGLibA_42966 | 99 | -0.163449254 | SCML4 | Scm polycomb group protein like 4 [Source:HGNC Symbol;Acc:HGNC:21397] |
| ENSG00000147223 | HGLibA_41327 | 99 | -0.163449254 | RIPPLY1 | ripply transcriptional repressor 1 [Source:HGNC Symbol;Acc:HGNC:25117] |
| ENSG00000147854 | HGLibA_52953 | 99 | -0.163449254 | UHRF2 | ubiquitin like with PHD and ring finger domains 2 [Source:HGNC Symbol;Acc:HGNC:12557] |
| ENSG00000148082 | HGLibA_44007 | 99 | -0.163449254 | SHC3 | SHC adaptor protein 3 [Source:HGNC Symbol;Acc:HGNC:18181] |
| ENSG00000148450 | HGLibA_30243 | 99 | -0.163449254 | MSRB2 | methionine sulfoxide reductase B2 [Source:HGNC Symbol;Acc:HGNC:17061] |
| ENSG00000148935 | HGLibA_18645 | 99 | -0.163449254 | GAS2 | growth arrest specific 2 [Source:HGNC Symbol;Acc:HGNC:4167] |
| ENSG00000149357 | HGLibA_25989 | 99 | -0.163449254 | LAMTOR1 | MAPK and MTOR activator 1 [Source:HGNC Symbol;Acc:HGNC:26068]" |
| ENSG00000150401 | HGLibA_12569 | 99 | -0.163449254 | DCUN1D2 | defective in cullin neddylation 1 domain containing 2 [Source:HGNC Symbol;Acc:HGNC:20328] |
| ENSG00000150594 | HGLibA_01047 | 99 | -0.163449254 | ADRA2A | adrenoceptor alpha 2A [Source:HGNC Symbol;Acc:HGNC:281] |
| ENSG00000151789 | HGLibA_55902 | 99 | -0.163449254 | ZNF385D | zinc finger protein 385D [Source:HGNC Symbol;Acc:HGNC:26191] |
| ENSG00000153130 | HGLibA_43030 | 99 | -0.163449254 | SCOC | short coiled-coil protein [Source:HGNC Symbol;Acc:HGNC:20335] |
| ENSG00000153914 | HGLibA_46954 | 99 | -0.163449254 | SREK1 | splicing regulatory glutamic acid and lysine rich protein 1 [Source:HGNC Symbol;Acc:HGNC:17882] |
| ENSG00000154822 | HGLibA_37017 | 99 | -0.163449254 | PLCL2 | phospholipase C like 2 [Source:HGNC Symbol;Acc:HGNC:9064] |
| ENSG00000155714 | HGLibA_35989 | 99 | -0.163449254 | PDZD9 | PDZ domain containing 9 [Source:HGNC Symbol;Acc:HGNC:28740] |
| ENSG00000156050 | HGLibA_16218 | 99 | -0.163449254 | FAM161B | centrosomal protein [Source:HGNC Symbol;Acc:HGNC:19854]" |
| ENSG00000157456 | HGLibA_08062 | 99 | -0.163449254 | CCNB2 | cyclin B2 [Source:HGNC Symbol;Acc:HGNC:1580] |
| ENSG00000157514 | HGLibA_51822 | 99 | -0.163449254 | TSC22D3 | TSC22 domain family member 3 [Source:HGNC Symbol;Acc:HGNC:3051] |
| ENSG00000157551 | HGLibA_24264 | 99 | -0.163449254 | KCNJ15 | potassium voltage-gated channel subfamily J member 15 [Source:HGNC Symbol;Acc:HGNC:6261] |
| ENSG00000158109 | HGLibA_51182 | 99 | -0.163449254 | TPRG1L | tumor protein p63 regulated 1 like [Source:HGNC Symbol;Acc:HGNC:27007] |
| ENSG00000158470 | HGLibA_04035 | 99 | -0.163449254 | B4GALT5 | 4-galactosyltransferase 5 [Source:HGNC Symbol;Acc:HGNC:928]" |
| ENSG00000160255 | HGLibA_23777 | 99 | -0.163449254 | ITGB2 | integrin subunit beta 2 [Source:HGNC Symbol;Acc:HGNC:6155] |
| ENSG00000162444 | HGLibA_40644 | 99 | -0.163449254 | RBP7 | retinol binding protein 7 [Source:HGNC Symbol;Acc:HGNC:30316] |
| ENSG00000163131 | HGLibA_11727 | 99 | -0.163449254 | CTSS | cathepsin S [Source:HGNC Symbol;Acc:HGNC:2545] |
| ENSG00000163440 | HGLibA_35752 | 99 | -0.163449254 | PDCL2 | phosducin like 2 [Source:HGNC Symbol;Acc:HGNC:29524] |
| ENSG00000163633 | HGLibA_06325 | 99 | -0.163449254 | C4orf36 | chromosome 4 open reading frame 36 [Source:HGNC Symbol;Acc:HGNC:28386] |
| ENSG00000163704 | HGLibA_38886 | 99 | -0.163449254 | PRRT3 | proline rich transmembrane protein 3 [Source:HGNC Symbol;Acc:HGNC:26591] |
| ENSG00000164188 | HGLibA_40207 | 99 | -0.163449254 | RANBP3L | RAN binding protein 3 like [Source:HGNC Symbol;Acc:HGNC:26353] |
| ENSG00000164344 | HGLibA_25277 | 99 | -0.163449254 | KLKB1 | kallikrein B1 [Source:HGNC Symbol;Acc:HGNC:6371] |
| ENSG00000164597 | HGLibA_10482 | 99 | -0.163449254 | COG5 | component of oligomeric golgi complex 5 [Source:HGNC Symbol;Acc:HGNC:14857] |
| ENSG00000164743 | HGLibA_06636 | 99 | -0.163449254 | C8orf48 | chromosome 8 open reading frame 48 [Source:HGNC Symbol;Acc:HGNC:26345] |
| ENSG00000165805 | HGLibA_05260 | 99 | -0.163449254 | C12orf50 | chromosome 12 open reading frame 50 [Source:HGNC Symbol;Acc:HGNC:26665] |
| ENSG00000165995 | HGLibA_06921 | 99 | -0.163449254 | CACNB2 | calcium voltage-gated channel auxiliary subunit beta 2 [Source:HGNC Symbol;Acc:HGNC:1402] |
| ENSG00000166426 | HGLibA_11055 | 99 | -0.163449254 | CRABP1 | cellular retinoic acid binding protein 1 [Source:HGNC Symbol;Acc:HGNC:2338] |
| ENSG00000167065 | HGLibA_14151 | 99 | -0.163449254 | DUSP18 | dual specificity phosphatase 18 [Source:HGNC Symbol;Acc:HGNC:18484] |
| ENSG00000167157 | HGLibA_38896 | 99 | -0.163449254 | PRRX2 | paired related homeobox 2 [Source:HGNC Symbol;Acc:HGNC:21338] |
| ENSG00000167491 | HGLibA_18687 | 99 | -0.163449254 | GATAD2A | GATA zinc finger domain containing 2A [Source:HGNC Symbol;Acc:HGNC:29989] |
| ENSG00000167755 | HGLibA_25264 | 99 | -0.163449254 | KLK6 | kallikrein related peptidase 6 [Source:HGNC Symbol;Acc:HGNC:6367] |

| | | | | | |
|---|---|---|---|---|---|
| ENSG00000168038 | HGLibA_52975 | 99 | -0.163449254 | ULK4 | unc-51 like kinase 4 [Source:HGNC Symbol;Acc:HGNC:15784] |
| ENSG00000168300 | HGLibA_35627 | 99 | -0.163449254 | PCMTD1 | protein-L-isoaspartate (D-aspartate) O-methyltransferase domain containing 1 [Source:HGNC Symbol;Acc:HGNC:30483] |
| ENSG00000168329 | HGLibA_11836 | 99 | -0.163449254 | CX3CR1 | C-X3-C motif chemokine receptor 1 [Source:HGNC Symbol;Acc:HGNC:2558] |
| ENSG00000169083 | HGLibA_02620 | 99 | -0.163449254 | AR | androgen receptor [Source:HGNC Symbol;Acc:HGNC:644] |
| ENSG00000169288 | HGLibA_29889 | 99 | -0.163449254 | MRPL1 | mitochondrial ribosomal protein L1 [Source:HGNC Symbol;Acc:HGNC:14275] |
| ENSG00000170430 | HGLibA_29164 | 99 | -0.163449254 | MGMT | O-6-methylguanine-DNA methyltransferase [Source:HGNC Symbol;Acc:HGNC:7059] |
| ENSG00000170786 | HGLibA_43161 | 99 | -0.163449254 | SDR16C5 | short chain dehydrogenase/reductase family 16C member 5 [Source:HGNC Symbol;Acc:HGNC:30311] |
| ENSG00000170891 | HGLibA_12249 | 99 | -0.163449254 | CYTL1 | cytokine like 1 [Source:HGNC Symbol;Acc:HGNC:24435] |
| ENSG00000171195 | HGLibA_30557 | 99 | -0.163449254 | MUC7 | secreted [Source:HGNC Symbol;Acc:HGNC:7518]" |
| ENSG00000172575 | HGLibA_40343 | 99 | -0.163449254 | RASGRP1 | RAS guanyl releasing protein 1 [Source:HGNC Symbol;Acc:HGNC:9878] |
| ENSG00000172671 | HGLibA_55157 | 99 | -0.163449254 | ZFAND4 | zinc finger AN1-type containing 4 [Source:HGNC Symbol;Acc:HGNC:23504] |
| ENSG00000172725 | HGLibA_10775 | 99 | -0.163449254 | CORO1B | coronin 1B [Source:HGNC Symbol;Acc:HGNC:2253] |
| ENSG00000173548 | HGLibA_46094 | 99 | -0.163449254 | SNX33 | sorting nexin 33 [Source:HGNC Symbol;Acc:HGNC:28468] |
| ENSG00000174851 | HGLibA_54663 | 99 | -0.163449254 | YIF1A | membrane trafficking protein [Source:HGNC Symbol;Acc:HGNC:16688]" |
| ENSG00000175183 | HGLibA_11435 | 99 | -0.163449254 | CSRP2 | cysteine and glycine rich protein 2 [Source:HGNC Symbol;Acc:HGNC:2470] |
| ENSG00000176623 | HGLibA_41365 | 99 | -0.163449254 | RMDN1 | regulator of microtubule dynamics 1 [Source:HGNC Symbol;Acc:HGNC:24285] |
| ENSG00000179152 | HGLibA_48689 | 99 | -0.163449254 | TCAIM | mitochondrial [Source:HGNC Symbol;Acc:HGNC:25241]" |
| ENSG00000179399 | HGLibA_19775 | 99 | -0.163449254 | GPC5 | glypican 5 [Source:HGNC Symbol;Acc:HGNC:4453] |
| ENSG00000180660 | HGLibA_27820 | 99 | -0.163449254 | MAB21L1 | mab-21 like 1 [Source:HGNC Symbol;Acc:HGNC:6757] |
| ENSG00000181085 | HGLibA_28266 | 99 | -0.163449254 | MAPK15 | mitogen-activated protein kinase 15 [Source:HGNC Symbol;Acc:HGNC:24667] |
| ENSG00000183310 | HGLibA_33877 | 99 | -0.163449254 | OR2T34 | olfactory receptor family 2 subfamily T member 34 [Source:HGNC Symbol;Acc:HGNC:31256] |
| ENSG00000185177 | HGLibA_56065 | 99 | -0.163449254 | ZNF479 | zinc finger protein 479 [Source:HGNC Symbol;Acc:HGNC:23258] |
| ENSG00000185761 | HGLibA_00859 | 99 | -0.163449254 | ADAMTSL5 | ADAMTS like 5 [Source:HGNC Symbol;Acc:HGNC:27912] |
| ENSG00000185899 | HGLibA_48424 | 99 | -0.163449254 | TAS2R60 | taste 2 receptor member 60 [Source:HGNC Symbol;Acc:HGNC:20639] |
| ENSG00000185920 | HGLibA_39267 | 99 | -0.163449254 | PTCH1 | patched 1 [Source:HGNC Symbol;Acc:HGNC:9585] |
| ENSG00000186007 | HGLibA_26285 | 99 | -0.163449254 | LEMD1 | LEM domain containing 1 [Source:HGNC Symbol;Acc:HGNC:18725] |
| ENSG00000186231 | HGLibA_25186 | 99 | -0.163449254 | KLHL32 | kelch like family member 32 [Source:HGNC Symbol;Acc:HGNC:21221] |
| ENSG00000187005 | HGLibA_25707 | 99 | -0.163449254 | KRTAP21-1 | keratin associated protein 21-1 [Source:HGNC Symbol;Acc:HGNC:18945] |
| ENSG00000187792 | HGLibA_56572 | 99 | -0.163449254 | ZNF70 | zinc finger protein 70 [Source:HGNC Symbol;Acc:HGNC:13140] |
| ENSG00000188064 | HGLibA_54386 | 99 | -0.163449254 | WNT7B | Wnt family member 7B [Source:HGNC Symbol;Acc:HGNC:12787] |
| ENSG00000189180 | HGLibA_55832 | 99 | -0.163449254 | ZNF33A | zinc finger protein 33A [Source:HGNC Symbol;Acc:HGNC:13096] |
| ENSG00000189221 | HGLibA_28105 | 99 | -0.163449254 | MAOA | monoamine oxidase A [Source:HGNC Symbol;Acc:HGNC:6833] |
| ENSG00000189401 | HGLibA_34770 | 99 | -0.163449254 | OTUD6A | OTU deubiquitinase 6A [Source:HGNC Symbol;Acc:HGNC:32312] |
| ENSG00000196227 | HGLibA_16492 | 99 | -0.163449254 | FAM217B | family with sequence similarity 217 member B [Source:HGNC Symbol;Acc:HGNC:16170] |
| ENSG00000196482 | HGLibA_15598 | 99 | -0.163449254 | ESRRG | estrogen related receptor gamma [Source:HGNC Symbol;Acc:HGNC:3474] |
| ENSG00000196998 | HGLibA_54097 | 99 | -0.163449254 | WDR45 | WD repeat domain 45 [Source:HGNC Symbol;Acc:HGNC:28912] |
| ENSG00000198131 | HGLibA_56196 | 99 | -0.163449254 | ZNF544 | zinc finger protein 544 [Source:HGNC Symbol;Acc:HGNC:16759] |
| ENSG00000198178 | HGLibA_09983 | 99 | -0.163449254 | CLEC4C | C-type lectin domain family 4 member C [Source:HGNC Symbol;Acc:HGNC:13258] |
| ENSG00000198513 | HGLibA_03516 | 99 | -0.163449254 | ATL1 | atlastin GTPase 1 [Source:HGNC Symbol;Acc:HGNC:11231] |
| ENSG00000198791 | HGLibA_10350 | 99 | -0.163449254 | CNOT7 | CCR4-NOT transcription complex subunit 7 [Source:HGNC Symbol;Acc:HGNC:14101] |
| ENSG00000198908 | HGLibA_04472 | 99 | -0.163449254 | BHLHB9 | basic helix-loop-helix family member b9 [Source:HGNC Symbol;Acc:HGNC:29353] |
| ENSG00000198961 | HGLibA_36787 | 99 | -0.163449254 | PJA2 | praja ring finger ubiquitin ligase 2 [Source:HGNC Symbol;Acc:HGNC:17481] |
| ENSG00000204314 | HGLibA_38882 | 99 | -0.163449254 | PRRT1 | proline rich transmembrane protein 1 [Source:HGNC Symbol;Acc:HGNC:13943] |
| ENSG00000204632 | HGLibA_21649 | 99 | -0.163449254 | HLA-G | G [Source:HGNC Symbol;Acc:HGNC:4964]" |
| ENSG00000205464 | HGLibA_03698 | 99 | -0.163449254 | ATP6AP1L | ATPase H+ transporting accessory protein 1 like [Source:HGNC Symbol;Acc:HGNC:28091] |
| ENSG00000213064 | HGLibA_43753 | 99 | -0.163449254 | SFT2D2 | SFT2 domain containing 2 [Source:HGNC Symbol;Acc:HGNC:25140] |
| ENSG00000213366 | HGLibA_20475 | 99 | -0.163449254 | GSTM2 | glutathione S-transferase mu 2 [Source:HGNC Symbol;Acc:HGNC:4634] |
| ENSG00000213390 | HGLibA_02719 | 99 | -0.163449254 | ARHGAP19 | Rho GTPase activating protein 19 [Source:HGNC Symbol;Acc:HGNC:23724] |
| ENSG00000215845 | HGLibA_52029 | 99 | -0.163449254 | TSTD1 | thiosulfate sulfurtransferase like domain containing 1 [Source:HGNC Symbol;Acc:HGNC:35410] |
| ENSG00000218336 | HGLibA_49001 | 99 | -0.163449254 | TENM3 | teneurin transmembrane protein 3 [Source:HGNC Symbol;Acc:HGNC:29944] |
| ENSG00000223547 | HGLibA_56859 | 99 | -0.163449254 | ZNF844 | zinc finger protein 844 [Source:HGNC Symbol;Acc:HGNC:25932] |
| ENSG00000224089 | HGLibA_11518 | 99 | -0.163449254 | CT47A10 | member A10 [Source:HGNC Symbol;Acc:HGNC:33291]" |
| ENSG00000229314 | HGLibA_34618 | 99 | -0.163449254 | ORM1 | orosomucoid 1 [Source:HGNC Symbol;Acc:HGNC:8498] |
| ENSG00000233608 | HGLibA_52379 | 99 | -0.163449254 | TWIST2 | twist family bHLH transcription factor 2 [Source:HGNC Symbol;Acc:HGNC:20670] |
| ENSG00000242366 | HGLibA_52898 | 99 | -0.163449254 | UGT1A8 | UDP glucuronosyltransferase family 1 member A8 [Source:HGNC Symbol;Acc:HGNC:12540] |
| ENSG00000242866 | HGLibA_47585 | 99 | -0.163449254 | STRC | stereocilin [Source:HGNC Symbol;Acc:HGNC:16035] |
| ENSG00000243130 | HGLibA_39036 | 99 | -0.163449254 | PSG11 | pregnancy specific beta-1-glycoprotein 11 [Source:HGNC Symbol;Acc:HGNC:9516] |
| ENSG00000244646 | HGLibA_54545 | 99 | -0.163449254 | XKRY2 | Y-linked 2 [Source:HGNC Symbol;Acc:HGNC:23922]" |
| ENSG00000253276 | HGLibA_07857 | 99 | -0.163449254 | CCDC71L | coiled-coil domain containing 71 like [Source:HGNC Symbol;Acc:HGNC:26685] |
| ENSG00000256977 | HGLibA_26552 | 99 | -0.163449254 | LIMS3 | LIM zinc finger domain containing 3 [Source:HGNC Symbol;Acc:HGNC:30047] |
| ENSG00000275835 | HGLibA_52318 | 99 | -0.163449254 | TUBGCP5 | tubulin gamma complex associated protein 5 [Source:HGNC Symbol;Acc:HGNC:18600] |
| FAM192A | HGLibA_16359 | 99 | -0.163449254 | FAM192A | - |
| FAM21C | HGLibA_16510 | 99 | -0.163449254 | FAM21C | - |
| HDGFRP2 | HGLibA_21044 | 99 | -0.163449254 | HDGFRP2 | - |
| hsa-let-7f-2 | HGLibA_57130 | 99 | -0.163449254 | hsa-let-7f-2 | - |
| hsa-mir-1254-1 | HGLibA_57415 | 99 | -0.163449254 | hsa-mir-1254-1 | - |
| hsa-mir-1910 | HGLibA_58047 | 99 | -0.163449254 | hsa-mir-1910 | - |
| hsa-mir-216b | HGLibA_58260 | 99 | -0.163449254 | hsa-mir-216b | - |
| hsa-mir-23c | HGLibA_58336 | 99 | -0.163449254 | hsa-mir-23c | - |
| hsa-mir-27b | HGLibA_58381 | 99 | -0.163449254 | hsa-mir-27b | - |
| hsa-mir-3129 | HGLibA_58569 | 99 | -0.163449254 | hsa-mir-3129 | - |
| hsa-mir-378a | HGLibA_59424 | 99 | -0.163449254 | hsa-mir-378a | - |
| hsa-mir-3909 | HGLibA_59502 | 99 | -0.163449254 | hsa-mir-3909 | - |
| hsa-mir-3934 | HGLibA_59598 | 99 | -0.163449254 | hsa-mir-3934 | - |
| hsa-mir-3944 | HGLibA_59634 | 99 | -0.163449254 | hsa-mir-3944 | - |
| hsa-mir-4271 | HGLibA_59791 | 99 | -0.163449254 | hsa-mir-4271 | - |
| hsa-mir-4419b | HGLibA_60062 | 99 | -0.163449254 | hsa-mir-4419b | - |
| hsa-mir-4639 | HGLibA_60662 | 99 | -0.163449254 | hsa-mir-4639 | - |
| hsa-mir-4685 | HGLibA_60851 | 99 | -0.163449254 | hsa-mir-4685 | - |
| hsa-mir-4745 | HGLibA_61079 | 99 | -0.163449254 | hsa-mir-4745 | - |
| hsa-mir-548h-3 | HGLibA_62001 | 99 | -0.163449254 | hsa-mir-548h-3 | - |
| hsa-mir-624 | HGLibA_62734 | 99 | -0.163449254 | hsa-mir-624 | - |
| hsa-mir-649 | HGLibA_62841 | 99 | -0.163449254 | hsa-mir-649 | - |
| hsa-mir-6848 | HGLibA_63566 | 99 | -0.163449254 | hsa-mir-6848 | - |
| hsa-mir-8071-2 | HGLibA_64126 | 99 | -0.163449254 | hsa-mir-8071-2 | - |
| hsa-mir-920 | HGLibA_64273 | 99 | -0.163449254 | hsa-mir-920 | - |
| hsa-mir-939 | HGLibA_64332 | 99 | -0.163449254 | hsa-mir-939 | - |
| KIAA1644 | HGLibA_24796 | 99 | -0.163449254 | KIAA1644 | - |
| KIAA2018 | HGLibA_24841 | 99 | -0.163449254 | KIAA2018 | - |
| LOC100288255 | HGLibA_26827 | 99 | -0.163449254 | LOC100288255 | - |
| MPP6 | HGLibA_29762 | 99 | -0.163449254 | MPP6 | - |
| PTPN20A | HGLibA_39452 | 99 | -0.163449254 | PTPN20A | - |
| PTPN20B | HGLibA_39455 | 99 | -0.163449254 | PTPN20B | - |
| SCXA | HGLibA_43080 | 99 | -0.163449254 | SCXA | - |
| WDR60 | HGLibA_54139 | 99 | -0.163449254 | WDR60 | - |
| ZHX1-C8ORF76 | HGLibA_55311 | 99 | -0.163449254 | ZHX1-C8ORF76 | - |
| ADCK3 | HGLibA_00897 | 98 | -0.173400291 | ADCK3 | - |
| C12orf74 | HGLibA_05295 | 98 | -0.173400291 | C12orf74 | - |
| C19orf43 | HGLibA_05660 | 98 | -0.173400291 | C19orf43 | - |
| C9orf96 | HGLibA_06781 | 98 | -0.173400291 | C9orf96 | - |
| ENSG00000006757 | HGLibA_37413 | 98 | -0.173400291 | PNPLA4 | patatin like phospholipase domain containing 4 [Source:HGNC Symbol;Acc:HGNC:24887] |
| ENSG00000008294 | HGLibA_46371 | 98 | -0.173400291 | SPAG9 | sperm associated antigen 9 [Source:HGNC Symbol;Acc:HGNC:14524] |
| ENSG00000046653 | HGLibA_19822 | 98 | -0.173400291 | GPM6B | glycoprotein M6B [Source:HGNC Symbol;Acc:HGNC:4461] |
| ENSG00000051596 | HGLibA_49371 | 98 | -0.173400291 | THOC3 | THO complex 3 [Source:HGNC Symbol;Acc:HGNC:19072] |
| ENSG00000053108 | HGLibA_18107 | 98 | -0.173400291 | FSTL4 | follistatin like 4 [Source:HGNC Symbol;Acc:HGNC:21389] |
| ENSG00000070019 | HGLibA_20662 | 98 | -0.173400291 | GUCY2C | guanylate cyclase 2C [Source:HGNC Symbol;Acc:HGNC:4688] |
| ENSG00000070371 | HGLibA_10153 | 98 | -0.173400291 | CLTCL1 | clathrin heavy chain like 1 [Source:HGNC Symbol;Acc:HGNC:2093] |
| ENSG00000079387 | HGLibA_43384 | 98 | -0.173400291 | SENP1 | SUMO specific peptidase 1 [Source:HGNC Symbol;Acc:HGNC:17927] |
| ENSG00000080839 | HGLibA_40467 | 98 | -0.173400291 | RBL1 | RB transcriptional corepressor like 1 [Source:HGNC Symbol;Acc:HGNC:9893] |
| ENSG00000082175 | HGLibA_36286 | 98 | -0.173400291 | PGR | progesterone receptor [Source:HGNC Symbol;Acc:HGNC:8910] |
| ENSG00000086589 | HGLibA_40510 | 98 | -0.173400291 | RBM22 | RNA binding motif protein 22 [Source:HGNC Symbol;Acc:HGNC:25503] |
| ENSG00000099364 | HGLibA_16993 | 98 | -0.173400291 | FBXL19 | F-box and leucine rich repeat protein 19 [Source:HGNC Symbol;Acc:HGNC:25300] |
| ENSG00000100116 | HGLibA_18762 | 98 | -0.173400291 | GCAT | glycine C-acetyltransferase [Source:HGNC Symbol;Acc:HGNC:4188] |
| ENSG00000100206 | HGLibA_13398 | 98 | -0.173400291 | DMC1 | DNA meiotic recombinase 1 [Source:HGNC Symbol;Acc:HGNC:2927] |
| ENSG00000100360 | HGLibA_22755 | 98 | -0.173400291 | IFT27 | intraflagellar transport 27 [Source:HGNC Symbol;Acc:HGNC:18626] |
| ENSG00000100395 | HGLibA_25900 | 98 | -0.173400291 | L3MBTL2 | polycomb repressive complex 1 subunit [Source:HGNC Symbol;Acc:HGNC:18594]" |
| ENSG00000100804 | HGLibA_39111 | 98 | -0.173400291 | PSMB5 | proteasome subunit beta 5 [Source:HGNC Symbol;Acc:HGNC:9542] |
| ENSG00000103522 | HGLibA_23084 | 98 | -0.173400291 | IL21R | interleukin 21 receptor [Source:HGNC Symbol;Acc:HGNC:6006] |
| ENSG00000104388 | HGLibA_39866 | 98 | -0.173400291 | RAB2A | member RAS oncogene family [Source:HGNC Symbol;Acc:HGNC:9763]" |
| ENSG00000104852 | HGLibA_45959 | 98 | -0.173400291 | SNRNP70 | small nuclear ribonucleoprotein U1 subunit 70 [Source:HGNC Symbol;Acc:HGNC:11150] |
| ENSG00000105290 | HGLibA_02440 | 98 | -0.173400291 | APLP1 | amyloid beta precursor like protein 1 [Source:HGNC Symbol;Acc:HGNC:597] |
| ENSG00000105364 | HGLibA_29976 | 98 | -0.173400291 | MRPL4 | mitochondrial ribosomal protein L4 [Source:HGNC Symbol;Acc:HGNC:14276] |
| ENSG00000105374 | HGLibA_32013 | 98 | -0.173400291 | NKG7 | natural killer cell granule protein 7 [Source:HGNC Symbol;Acc:HGNC:7830] |
| ENSG00000105700 | HGLibA_25875 | 98 | -0.173400291 | KXD1 | KxDL motif containing 1 [Source:HGNC Symbol;Acc:HGNC:28420] |
| ENSG00000106049 | HGLibA_21281 | 98 | -0.173400291 | HIBADH | 3-hydroxyisobutyrate dehydrogenase [Source:HGNC Symbol;Acc:HGNC:4907] |
| ENSG00000106648 | HGLibA_18578 | 98 | -0.173400291 | GALNTL5 | polypeptide N-acetylgalactosaminyltransferase like 5 [Source:HGNC Symbol;Acc:HGNC:21725] |
| ENSG00000107018 | HGLibA_41352 | 98 | -0.173400291 | RLN1 | relaxin 1 [Source:HGNC Symbol;Acc:HGNC:10026] |
| ENSG00000107362 | HGLibA_00267 | 98 | -0.173400291 | ABHD17B | abhydrolase domain containing 17B [Source:HGNC Symbol;Acc:HGNC:24278] |
| ENSG00000108389 | HGLibA_30431 | 98 | -0.173400291 | MTMR4 | myotubularin related protein 4 [Source:HGNC Symbol;Acc:HGNC:7452] |
| ENSG00000108469 | HGLibA_40773 | 98 | -0.173400291 | RECQL5 | RecQ like helicase 5 [Source:HGNC Symbol;Acc:HGNC:9950] |
| ENSG00000108771 | HGLibA_13205 | 98 | -0.173400291 | DHX58 | DExH-box helicase 58 [Source:HGNC Symbol;Acc:HGNC:29517] |
| ENSG00000109680 | HGLibA_48490 | 98 | -0.173400291 | TBC1D19 | TBC1 domain family member 19 [Source:HGNC Symbol;Acc:HGNC:25624] |
| ENSG00000110048 | HGLibA_34638 | 98 | -0.173400291 | OSBP | oxysterol binding protein [Source:HGNC Symbol;Acc:HGNC:8503] |
| ENSG00000110203 | HGLibA_17788 | 98 | -0.173400291 | FOLR3 | folate receptor 3 [Source:HGNC Symbol;Acc:HGNC:3795] |
| ENSG00000111057 | HGLibA_25437 | 98 | -0.173400291 | KRT18 | keratin 18 [Source:HGNC Symbol;Acc:HGNC:6430] |

| | | | | | |
|---|---|---|---|---|---|
| ENSG00000111602 | HGLibA_49489 | 98 | -0.173400291 | TIMELESS | timeless circadian regulator [Source:HGNC Symbol;Acc:HGNC:11813] |
| ENSG00000111728 | HGLibA_47288 | 98 | -0.173400291 | ST8SIA1 | 8-sialyltransferase 1 [Source:HGNC Symbol;Acc:HGNC:10869]" |
| ENSG00000112031 | HGLibA_30468 | 98 | -0.173400291 | MTRF1L | mitochondrial translational release factor 1 like [Source:HGNC Symbol;Acc:HGNC:21051] |
| ENSG00000112167 | HGLibA_42789 | 98 | -0.173400291 | SAYSD1 | SAYSVFN motif domain containing 1 [Source:HGNC Symbol;Acc:HGNC:21025] |
| ENSG00000114812 | HGLibA_53668 | 98 | -0.173400291 | VIPR1 | vasoactive intestinal peptide receptor 1 [Source:HGNC Symbol;Acc:HGNC:12694] |
| ENSG00000115216 | HGLibA_32656 | 98 | -0.173400291 | NRBP1 | nuclear receptor binding protein 1 [Source:HGNC Symbol;Acc:HGNC:7993] |
| ENSG00000115844 | HGLibA_13375 | 98 | -0.173400291 | DLX2 | distal-less homeobox 2 [Source:HGNC Symbol;Acc:HGNC:2915] |
| ENSG00000116981 | HGLibA_32802 | 98 | -0.173400291 | NT5C1A | cytosolic IA [Source:HGNC Symbol;Acc:HGNC:17819]" |
| ENSG00000117069 | HGLibA_47275 | 98 | -0.173400291 | ST6GALNAC5 | 6-sialyltransferase 5 [Source:HGNC Symbol;Acc:HGNC:19342]" |
| ENSG00000118640 | HGLibA_53510 | 98 | -0.173400291 | VAMP8 | vesicle associated membrane protein 8 [Source:HGNC Symbol;Acc:HGNC:12647] |
| ENSG00000118873 | HGLibA_39921 | 98 | -0.173400291 | RAB3GAP2 | RAB3 GTPase activating non-catalytic protein subunit 2 [Source:HGNC Symbol;Acc:HGNC:17168] |
| ENSG00000120057 | HGLibA_43744 | 98 | -0.173400291 | SFRP5 | secreted frizzled related protein 5 [Source:HGNC Symbol;Acc:HGNC:10779] |
| ENSG00000120071 | HGLibA_24036 | 98 | -0.173400291 | KANSL1 | KAT8 regulatory NSL complex subunit 1 [Source:HGNC Symbol;Acc:HGNC:24565] |
| ENSG00000121851 | HGLibA_37630 | 98 | -0.173400291 | POLR3GL | RNA polymerase III subunit G like [Source:HGNC Symbol;Acc:HGNC:28466] |
| ENSG00000122390 | HGLibA_31004 | 98 | -0.173400291 | NAA60 | NatF catalytic subunit [Source:HGNC Symbol;Acc:HGNC:25875]" |
| ENSG00000122678 | HGLibA_37540 | 98 | -0.173400291 | POLM | DNA polymerase mu [Source:HGNC Symbol;Acc:HGNC:9185] |
| ENSG00000122707 | HGLibA_40763 | 98 | -0.173400291 | RECK | reversion inducing cysteine rich protein with kazal motifs [Source:HGNC Symbol;Acc:HGNC:11345] |
| ENSG00000123131 | HGLibA_38415 | 98 | -0.173400291 | PRDX4 | peroxiredoxin 4 [Source:HGNC Symbol;Acc:HGNC:17169] |
| ENSG00000123364 | HGLibA_21966 | 98 | -0.173400291 | HOXC13 | homeobox C13 [Source:HGNC Symbol;Acc:HGNC:5125] |
| ENSG00000123983 | HGLibA_00558 | 98 | -0.173400291 | ACSL3 | acyl-CoA synthetase long chain family member 3 [Source:HGNC Symbol;Acc:HGNC:3570] |
| ENSG00000125356 | HGLibA_31438 | 98 | -0.173400291 | NDUFA1 | NADH:ubiquinone oxidoreductase subunit A1 [Source:HGNC Symbol;Acc:HGNC:7683] |
| ENSG00000126259 | HGLibA_25002 | 98 | -0.173400291 | KIRREL2 | kirre like nephrin family adhesion molecule 2 [Source:HGNC Symbol;Acc:HGNC:18816] |
| ENSG00000129292 | HGLibA_36365 | 98 | -0.173400291 | PHF20L1 | PHD finger protein 20 like 1 [Source:HGNC Symbol;Acc:HGNC:24280] |
| ENSG00000131943 | HGLibA_05630 | 98 | -0.173400291 | C19orf12 | chromosome 19 open reading frame 12 [Source:HGNC Symbol;Acc:HGNC:25443] |
| ENSG00000132326 | HGLibA_36056 | 98 | -0.173400291 | PER2 | period circadian regulator 2 [Source:HGNC Symbol;Acc:HGNC:8846] |
| ENSG00000133069 | HGLibA_49774 | 98 | -0.173400291 | TMCC2 | transmembrane and coiled-coil domain family 2 [Source:HGNC Symbol;Acc:HGNC:24239] |
| ENSG00000134200 | HGLibA_51856 | 98 | -0.173400291 | TSHB | thyroid stimulating hormone beta [Source:HGNC Symbol;Acc:HGNC:12372] |
| ENSG00000134352 | HGLibA_23188 | 98 | -0.173400291 | IL6ST | interleukin 6 signal transducer [Source:HGNC Symbol;Acc:HGNC:6021] |
| ENSG00000134545 | HGLibA_25286 | 98 | -0.173400291 | KLRC1 | killer cell lectin like receptor C1 [Source:HGNC Symbol;Acc:HGNC:6374] |
| ENSG00000134824 | HGLibA_15938 | 98 | -0.173400291 | FADS2 | fatty acid desaturase 2 [Source:HGNC Symbol;Acc:HGNC:3575] |
| ENSG00000135220 | HGLibA_52909 | 98 | -0.173400291 | UGT2A3 | UDP glucuronosyltransferase family 2 member A3 [Source:HGNC Symbol;Acc:HGNC:28528] |
| ENSG00000135974 | HGLibA_06140 | 98 | -0.173400291 | C2orf49 | chromosome 2 open reading frame 49 [Source:HGNC Symbol;Acc:HGNC:28772] |
| ENSG00000136143 | HGLibA_47695 | 98 | -0.173400291 | SUCLA2 | succinate-CoA ligase ADP-forming beta subunit [Source:HGNC Symbol;Acc:HGNC:11448] |
| ENSG00000136274 | HGLibA_31036 | 98 | -0.173400291 | NACAD | NAC alpha domain containing [Source:HGNC Symbol;Acc:HGNC:22196] |
| ENSG00000136720 | HGLibA_22142 | 98 | -0.173400291 | HS6ST1 | heparan sulfate 6-O-sulfotransferase 1 [Source:HGNC Symbol;Acc:HGNC:5201] |
| ENSG00000136895 | HGLibA_18631 | 98 | -0.173400291 | GARNL3 | GTPase activating Rap/RanGAP domain like 3 [Source:HGNC Symbol;Acc:HGNC:25425] |
| ENSG00000137101 | HGLibA_08441 | 98 | -0.173400291 | CD72 | CD72 molecule [Source:HGNC Symbol;Acc:HGNC:1696] |
| ENSG00000137497 | HGLibA_33008 | 98 | -0.173400291 | NUMA1 | nuclear mitotic apparatus protein 1 [Source:HGNC Symbol;Acc:HGNC:8059] |
| ENSG00000137936 | HGLibA_04249 | 98 | -0.173400291 | BCAR3 | NSP family adaptor protein [Source:HGNC Symbol;Acc:HGNC:973]" |
| ENSG00000138162 | HGLibA_48163 | 98 | -0.173400291 | TACC2 | transforming acidic coiled-coil containing protein 2 [Source:HGNC Symbol;Acc:HGNC:11523] |
| ENSG00000138735 | HGLibA_35801 | 98 | -0.173400291 | PDE5A | phosphodiesterase 5A [Source:HGNC Symbol;Acc:HGNC:8784] |
| ENSG00000140443 | HGLibA_22793 | 98 | -0.173400291 | IGF1R | insulin like growth factor 1 receptor [Source:HGNC Symbol;Acc:HGNC:5465] |
| ENSG00000140829 | HGLibA_13195 | 98 | -0.173400291 | DHX38 | DEAH-box helicase 38 [Source:HGNC Symbol;Acc:HGNC:17211] |
| ENSG00000143774 | HGLibA_20673 | 98 | -0.173400291 | GUK1 | guanylate kinase 1 [Source:HGNC Symbol;Acc:HGNC:4693] |
| ENSG00000144061 | HGLibA_32445 | 98 | -0.173400291 | NPHP1 | nephrocystin 1 [Source:HGNC Symbol;Acc:HGNC:7905] |
| ENSG00000144278 | HGLibA_18539 | 98 | -0.173400291 | GALNT13 | polypeptide N-acetylgalactosaminyltransferase 13 [Source:HGNC Symbol;Acc:HGNC:23242] |
| ENSG00000145220 | HGLibA_27696 | 98 | -0.173400291 | LYAR | Ly1 antibody reactive [Source:HGNC Symbol;Acc:HGNC:26021] |
| ENSG00000145287 | HGLibA_36942 | 98 | -0.173400291 | PLAC8 | placenta specific 8 [Source:HGNC Symbol;Acc:HGNC:19254] |
| ENSG00000146352 | HGLibA_10169 | 98 | -0.173400291 | CLVS2 | clavesin 2 [Source:HGNC Symbol;Acc:HGNC:23046] |
| ENSG00000147065 | HGLibA_30231 | 98 | -0.173400291 | MSN | moesin [Source:HGNC Symbol;Acc:HGNC:7373] |
| ENSG00000149489 | HGLibA_41754 | 98 | -0.173400291 | ROM1 | retinal outer segment membrane protein 1 [Source:HGNC Symbol;Acc:HGNC:10254] |
| ENSG00000149534 | HGLibA_30146 | 98 | -0.173400291 | MS4A2 | membrane spanning 4-domains A2 [Source:HGNC Symbol;Acc:HGNC:7316] |
| ENSG00000149658 | HGLibA_54731 | 98 | -0.173400291 | YTHDF1 | YTH N6-methyladenosine RNA binding protein 1 [Source:HGNC Symbol;Acc:HGNC:15867] |
| ENSG00000149922 | HGLibA_48676 | 98 | -0.173400291 | TBX6 | T-box 6 [Source:HGNC Symbol;Acc:HGNC:11605] |
| ENSG00000152086 | HGLibA_52258 | 98 | -0.173400291 | TUBA3E | tubulin alpha 3e [Source:HGNC Symbol;Acc:HGNC:20765] |
| ENSG00000152242 | HGLibA_05610 | 98 | -0.173400291 | C18orf25 | chromosome 18 open reading frame 25 [Source:HGNC Symbol;Acc:HGNC:28172] |
| ENSG00000152443 | HGLibA_56722 | 98 | -0.173400291 | ZNF776 | zinc finger protein 776 [Source:HGNC Symbol;Acc:HGNC:26765] |
| ENSG00000152518 | HGLibA_55209 | 98 | -0.173400291 | ZFP36L2 | ZFP36 ring finger protein like 2 [Source:HGNC Symbol;Acc:HGNC:1108] |
| ENSG00000153015 | HGLibA_11822 | 98 | -0.173400291 | CWC27 | CWC27 spliceosome associated protein homolog [Source:HGNC Symbol;Acc:HGNC:10664] |
| ENSG00000153902 | HGLibA_26401 | 98 | -0.173400291 | LGI4 | leucine rich repeat LGI family member 4 [Source:HGNC Symbol;Acc:HGNC:18712] |
| ENSG00000154258 | HGLibA_00128 | 98 | -0.173400291 | ABCA9 | ATP binding cassette subfamily A member 9 [Source:HGNC Symbol;Acc:HGNC:39] |
| ENSG00000154978 | HGLibA_53717 | 98 | -0.173400291 | VOPP1 | WBP1/VOPP1 family member [Source:HGNC Symbol;Acc:HGNC:34518]" |
| ENSG00000155918 | HGLibA_40127 | 98 | -0.173400291 | RAET1L | retinoic acid early transcript 1L [Source:HGNC Symbol;Acc:HGNC:16798] |
| ENSG00000157093 | HGLibA_27792 | 98 | -0.173400291 | LYZL4 | lysozyme like 4 [Source:HGNC Symbol;Acc:HGNC:28387] |
| ENSG00000157985 | HGLibA_01135 | 98 | -0.173400291 | AGAP1 | ankyrin repeat and PH domain 1 [Source:HGNC Symbol;Acc:HGNC:16922]" |
| ENSG00000158270 | HGLibA_10638 | 98 | -0.173400291 | COLEC12 | collectin subfamily member 12 [Source:HGNC Symbol;Acc:HGNC:16016] |
| ENSG00000158481 | HGLibA_08269 | 98 | -0.173400291 | CD1C | CD1c molecule [Source:HGNC Symbol;Acc:HGNC:1636] |
| ENSG00000159885 | HGLibA_55612 | 98 | -0.173400291 | ZNF222 | zinc finger protein 222 [Source:HGNC Symbol;Acc:HGNC:13015] |
| ENSG00000160179 | HGLibA_00215 | 98 | -0.173400291 | ABCG1 | ATP binding cassette subfamily G member 1 [Source:HGNC Symbol;Acc:HGNC:73] |
| ENSG00000161956 | HGLibA_43391 | 98 | -0.173400291 | SENP3 | SUMO specific peptidase 3 [Source:HGNC Symbol;Acc:HGNC:17862] |
| ENSG00000162144 | HGLibA_11981 | 98 | -0.173400291 | CYB561A3 | cytochrome b561 family member A3 [Source:HGNC Symbol;Acc:HGNC:23014] |
| ENSG00000162367 | HGLibA_48280 | 98 | -0.173400291 | TAL1 | erythroid differentiation factor [Source:HGNC Symbol;Acc:HGNC:11556]" |
| ENSG00000162607 | HGLibA_53191 | 98 | -0.173400291 | USP1 | ubiquitin specific peptidase 1 [Source:HGNC Symbol;Acc:HGNC:12607] |
| ENSG00000164530 | HGLibA_36487 | 98 | -0.173400291 | PI16 | peptidase inhibitor 16 [Source:HGNC Symbol;Acc:HGNC:21245] |
| ENSG00000164690 | HGLibA_44030 | 98 | -0.173400291 | SHH | sonic hedgehog [Source:HGNC Symbol;Acc:HGNC:10848] |
| ENSG00000164904 | HGLibA_01584 | 98 | -0.173400291 | ALDH7A1 | aldehyde dehydrogenase 7 family member A1 [Source:HGNC Symbol;Acc:HGNC:877] |
| ENSG00000165300 | HGLibA_45526 | 98 | -0.173400291 | SLITRK5 | SLIT and NTRK like family member 5 [Source:HGNC Symbol;Acc:HGNC:20295] |
| ENSG00000165675 | HGLibA_15161 | 98 | -0.173400291 | ENOX2 | ecto-NOX disulfide-thiol exchanger 2 [Source:HGNC Symbol;Acc:HGNC:2259] |
| ENSG00000165702 | HGLibA_18945 | 98 | -0.173400291 | GFI1B | growth factor independent 1B transcriptional repressor [Source:HGNC Symbol;Acc:HGNC:4238] |
| ENSG00000165898 | HGLibA_23650 | 98 | -0.173400291 | ISCA2 | iron-sulfur cluster assembly 2 [Source:HGNC Symbol;Acc:HGNC:19857] |
| ENSG00000166147 | HGLibA_16956 | 98 | -0.173400291 | FBN1 | fibrillin 1 [Source:HGNC Symbol;Acc:HGNC:3603] |
| ENSG00000166401 | HGLibA_43566 | 98 | -0.173400291 | SERPINB8 | serpin family B member 8 [Source:HGNC Symbol;Acc:HGNC:8952] |
| ENSG00000167081 | HGLibA_35349 | 98 | -0.173400291 | PBX3 | PBX homeobox 3 [Source:HGNC Symbol;Acc:HGNC:8634] |
| ENSG00000167193 | HGLibA_11180 | 98 | -0.173400291 | CRK | adaptor protein [Source:HGNC Symbol;Acc:HGNC:2362]" |
| ENSG00000169876 | HGLibA_30532 | 98 | -0.173400291 | MUC17 | cell surface associated [Source:HGNC Symbol;Acc:HGNC:16800]" |
| ENSG00000170624 | HGLibA_43802 | 98 | -0.173400291 | SGCD | sarcoglycan delta [Source:HGNC Symbol;Acc:HGNC:10807] |
| ENSG00000171130 | HGLibA_03727 | 98 | -0.173400291 | ATP6V0E2 | ATPase H+ transporting V0 subunit e2 [Source:HGNC Symbol;Acc:HGNC:21723] |
| ENSG00000171160 | HGLibA_29674 | 98 | -0.173400291 | MORN4 | MORN repeat containing 4 [Source:HGNC Symbol;Acc:HGNC:24001] |
| ENSG00000171595 | HGLibA_13507 | 98 | -0.173400291 | DNAI2 | dynein axonemal intermediate chain 2 [Source:HGNC Symbol;Acc:HGNC:18744] |
| ENSG00000171823 | HGLibA_16978 | 98 | -0.173400291 | FBXL14 | F-box and leucine rich repeat protein 14 [Source:HGNC Symbol;Acc:HGNC:28624] |
| ENSG00000173614 | HGLibA_32183 | 98 | -0.173400291 | NMNAT1 | nicotinamide nucleotide adenylyltransferase 1 [Source:HGNC Symbol;Acc:HGNC:17877] |
| ENSG00000173706 | HGLibA_21108 | 98 | -0.173400291 | HEG1 | heart development protein with EGF like domains 1 [Source:HGNC Symbol;Acc:HGNC:29227] |
| ENSG00000174453 | HGLibA_53927 | 98 | -0.173400291 | VWC2L | von Willebrand factor C domain containing 2 like [Source:HGNC Symbol;Acc:HGNC:37203] |
| ENSG00000176244 | HGLibA_00410 | 98 | -0.173400291 | ACBD7 | acyl-CoA binding domain containing 7 [Source:HGNC Symbol;Acc:HGNC:17715] |
| ENSG00000176988 | HGLibA_17724 | 98 | -0.173400291 | FMR1NB | FMR1 neighbor [Source:HGNC Symbol;Acc:HGNC:26372] |
| ENSG00000177731 | HGLibA_17619 | 98 | -0.173400291 | FLII | actin remodeling protein [Source:HGNC Symbol;Acc:HGNC:3750]" |
| ENSG00000178591 | HGLibA_12879 | 98 | -0.173400291 | DEFB125 | defensin beta 125 [Source:HGNC Symbol;Acc:HGNC:18105] |
| ENSG00000179218 | HGLibA_07047 | 98 | -0.173400291 | CALR | calreticulin [Source:HGNC Symbol;Acc:HGNC:1455] |
| ENSG00000179262 | HGLibA_40068 | 98 | -0.173400291 | RAD23A | nucleotide excision repair protein [Source:HGNC Symbol;Acc:HGNC:9812]" |
| ENSG00000180424 | HGLibA_12873 | 98 | -0.173400291 | DEFB123 | defensin beta 123 [Source:HGNC Symbol;Acc:HGNC:18103] |
| ENSG00000180767 | HGLibA_09623 | 98 | -0.173400291 | CHST13 | carbohydrate sulfotransferase 13 [Source:HGNC Symbol;Acc:HGNC:21755] |
| ENSG00000182240 | HGLibA_04061 | 98 | -0.173400291 | BACE2 | beta-secretase 2 [Source:HGNC Symbol;Acc:HGNC:934] |
| ENSG00000182963 | HGLibA_19178 | 98 | -0.173400291 | GJC1 | gap junction protein gamma 1 [Source:HGNC Symbol;Acc:HGNC:4280] |
| ENSG00000183496 | HGLibA_29029 | 98 | -0.173400291 | MEX3B | mex-3 RNA binding family member B [Source:HGNC Symbol;Acc:HGNC:25297] |
| ENSG00000185246 | HGLibA_38727 | 98 | -0.173400291 | PRPF39 | pre-mRNA processing factor 39 [Source:HGNC Symbol;Acc:HGNC:20314] |
| ENSG00000185386 | HGLibA_28253 | 98 | -0.173400291 | MAPK11 | mitogen-activated protein kinase 11 [Source:HGNC Symbol;Acc:HGNC:6873] |
| ENSG00000185674 | HGLibA_27700 | 98 | -0.173400291 | LYG2 | lysozyme g2 [Source:HGNC Symbol;Acc:HGNC:29615] |
| ENSG00000186094 | HGLibA_01174 | 98 | -0.173400291 | AGBL4 | ATP/GTP binding protein like 4 [Source:HGNC Symbol;Acc:HGNC:25892] |
| ENSG00000186687 | HGLibA_27759 | 98 | -0.173400291 | LYRM7 | LYR motif containing 7 [Source:HGNC Symbol;Acc:HGNC:28072] |
| ENSG00000187134 | HGLibA_01482 | 98 | -0.173400291 | AKR1C1 | aldo-keto reductase family 1 member C1 [Source:HGNC Symbol;Acc:HGNC:384] |
| ENSG00000187806 | HGLibA_50184 | 98 | -0.173400291 | TMEM202 | transmembrane protein 202 [Source:HGNC Symbol;Acc:HGNC:33733] |
| ENSG00000187955 | HGLibA_10511 | 98 | -0.173400291 | COL14A1 | collagen type XIV alpha 1 chain [Source:HGNC Symbol;Acc:HGNC:2191] |
| ENSG00000188243 | HGLibA_10670 | 98 | -0.173400291 | COMMD6 | COMM domain containing 6 [Source:HGNC Symbol;Acc:HGNC:24015] |
| ENSG00000188505 | HGLibA_31288 | 98 | -0.173400291 | NCCRP1 | non-specific cytotoxic cell receptor protein 1 homolog (zebrafish) [Source:HGNC Symbol;Acc:HGNC:33739] |
| ENSG00000189171 | HGLibA_42542 | 98 | -0.173400291 | S100A13 | S100 calcium binding protein A13 [Source:HGNC Symbol;Acc:HGNC:10490] |
| ENSG00000189298 | HGLibA_55350 | 98 | -0.173400291 | ZKSCAN3 | zinc finger with KRAB and SCAN domains 3 [Source:HGNC Symbol;Acc:HGNC:13853] |
| ENSG00000196911 | HGLibA_25366 | 98 | -0.173400291 | KPNA5 | karyopherin subunit alpha 5 [Source:HGNC Symbol;Acc:HGNC:6398] |
| ENSG00000197226 | HGLibA_48574 | 98 | -0.173400291 | TBC1D9B | TBC1 domain family member 9B [Source:HGNC Symbol;Acc:HGNC:29097] |
| ENSG00000197410 | HGLibA_12493 | 98 | -0.173400291 | DCHS2 | dachsous cadherin-related 2 [Source:HGNC Symbol;Acc:HGNC:23111] |
| ENSG00000197461 | HGLibA_35840 | 98 | -0.173400291 | PDGFA | platelet derived growth factor subunit A [Source:HGNC Symbol;Acc:HGNC:8799] |
| ENSG00000197953 | HGLibA_00032 | 98 | -0.173400291 | AADACL2 | arylacetamide deacetylase like 2 [Source:HGNC Symbol;Acc:HGNC:24427] |
| ENSG00000198049 | HGLibA_03905 | 98 | -0.173400291 | AVPR1B | arginine vasopressin receptor 1B [Source:HGNC Symbol;Acc:HGNC:896] |
| ENSG00000198121 | HGLibA_27054 | 98 | -0.173400291 | LPAR1 | lysophosphatidic acid receptor 1 [Source:HGNC Symbol;Acc:HGNC:3166] |
| ENSG00000198183 | HGLibA_04711 | 98 | -0.173400291 | BPIFA1 | BPI fold containing family A member 1 [Source:HGNC Symbol;Acc:HGNC:15749] |
| ENSG00000198965 | HGLibA_33541 | 98 | -0.173400291 | OR10R2 | olfactory receptor family 10 subfamily R member 2 [Source:HGNC Symbol;Acc:HGNC:14820] |
| ENSG00000198967 | HGLibA_33559 | 98 | -0.173400291 | OR10Z1 | olfactory receptor family 10 subfamily Z member 1 [Source:HGNC Symbol;Acc:HGNC:14996] |
| ENSG00000204315 | HGLibA_17593 | 98 | -0.173400291 | FKBPL | FK506 binding protein like [Source:HGNC Symbol;Acc:HGNC:13949] |
| ENSG00000204899 | HGLibA_30939 | 98 | -0.173400291 | MZT1 | mitotic spindle organizing protein 1 [Source:HGNC Symbol;Acc:HGNC:33830] |
| ENSG00000205457 | HGLibA_51084 | 98 | -0.173400291 | TP53TG3C | TP53 target 3C [Source:HGNC Symbol;Acc:HGNC:42962] |
| ENSG00000205609 | HGLibA_14770 | 98 | -0.173400291 | EIF3CL | eukaryotic translation initiation factor 3 subunit C like [Source:HGNC Symbol;Acc:HGNC:26347] |
| ENSG00000205659 | HGLibA_26567 | 98 | -0.173400291 | LIN52 | lin-52 DREAM MuvB core complex component [Source:HGNC Symbol;Acc:HGNC:19856] |
| ENSG00000215251 | HGLibA_16911 | 98 | -0.173400291 | FASTKD5 | FAST kinase domains 5 [Source:HGNC Symbol;Acc:HGNC:25790] |
| ENSG00000221933 | HGLibA_33731 | 98 | -0.173400291 | OR2A25 | olfactory receptor family 2 subfamily A member 25 [Source:HGNC Symbol;Acc:HGNC:19562] |
| ENSG00000221996 | HGLibA_34147 | 98 | -0.173400291 | OR52B4 | olfactory receptor family 52 subfamily B member 4 (gene/pseudogene) [Source:HGNC Symbol;Acc:HGNC:15209] |
| ENSG00000237190 | HGLibA_08837 | 98 | -0.173400291 | CDKN2AIPNL | CDKN2A interacting protein N-terminal like [Source:HGNC Symbol;Acc:HGNC:30545] |

| | | | | | |
|---|---|---|---|---|---|
| ENSG00000250151 | HGLibA_03088 | 98 | -0.173400291 | ARPC4-TTLL3 | ARPC4-TTLL3 readthrough [Source:HGNC Symbol;Acc:HGNC:38830] |
| ENSG00000254221 | HGLibA_35551 | 98 | -0.173400291 | PCDHGB1 | 1 [Source:HGNC Symbol;Acc:HGNC:8708]" |
| ENSG00000254788 | HGLibA_09773 | 98 | -0.173400291 | CKLF-CMTM1 | CKLF-CMTM1 readthrough [Source:HGNC Symbol;Acc:HGNC:39977] |
| ENSG00000256436 | HGLibA_48391 | 98 | -0.173400291 | TAS2R31 | taste 2 receptor member 31 [Source:HGNC Symbol;Acc:HGNC:19113] |
| ENSG00000269067 | HGLibA_56639 | 98 | -0.173400291 | ZNF728 | zinc finger protein 728 [Source:HGNC Symbol;Acc:HGNC:32463] |
| ENSG00000271601 | HGLibA_26645 | 98 | -0.173400291 | LIX1L | limb and CNS expressed 1 like [Source:HGNC Symbol;Acc:HGNC:28715] |
| ENSG00000272031 | HGLibA_02074 | 98 | -0.173400291 | ANKRD34A | ankyrin repeat domain 34A [Source:HGNC Symbol;Acc:HGNC:27639] |
| ENSG00000275896 | HGLibA_38909 | 98 | -0.173400291 | PRSS2 | serine protease 2 [Source:HGNC Symbol;Acc:HGNC:9483] |
| ENSG00000279804 | HGLibA_38303 | 98 | -0.173400291 | PRAMEF18 | PRAME family member 18 [Source:HGNC Symbol;Acc:HGNC:30693] |
| ENSG00000284662 | HGLibA_33993 | 98 | -0.173400291 | OR4F16 | olfactory receptor family 4 subfamily F member 16 [Source:HGNC Symbol;Acc:HGNC:15079] |
| FAM69C | HGLibA_16682 | 98 | -0.173400291 | FAM69C | - |
| FLJ45513 | HGLibA_17635 | 98 | -0.173400291 | FLJ45513 | - |
| GPR126 | HGLibA_19881 | 98 | -0.173400291 | GPR126 | - |
| HIST1H1E | HGLibA_21370 | 98 | -0.173400291 | HIST1H1E | - |
| hsa-mir-106a | HGLibA_57187 | 98 | -0.173400291 | hsa-mir-106a | - |
| hsa-mir-1343 | HGLibA_57790 | 98 | -0.173400291 | hsa-mir-1343 | - |
| hsa-mir-148b | HGLibA_57883 | 98 | -0.173400291 | hsa-mir-148b | - |
| hsa-mir-3120 | HGLibA_58531 | 98 | -0.173400291 | hsa-mir-3120 | - |
| hsa-mir-3154 | HGLibA_58675 | 98 | -0.173400291 | hsa-mir-3154 | - |
| hsa-mir-3682 | HGLibA_59280 | 98 | -0.173400291 | hsa-mir-3682 | - |
| hsa-mir-4498 | HGLibA_60408 | 98 | -0.173400291 | hsa-mir-4498 | - |
| hsa-mir-4669 | HGLibA_60794 | 98 | -0.173400291 | hsa-mir-4669 | - |
| hsa-mir-4761 | HGLibA_61141 | 98 | -0.173400291 | hsa-mir-4761 | - |
| hsa-mir-4767 | HGLibA_61166 | 98 | -0.173400291 | hsa-mir-4767 | - |
| hsa-mir-485 | HGLibA_61334 | 98 | -0.173400291 | hsa-mir-485 | - |
| hsa-mir-512-1 | HGLibA_61561 | 98 | -0.173400291 | hsa-mir-512-1 | - |
| hsa-mir-519b | HGLibA_61717 | 98 | -0.173400291 | hsa-mir-519b | - |
| hsa-mir-548as | HGLibA_61922 | 98 | -0.173400291 | hsa-mir-548as | - |
| hsa-mir-6744 | HGLibA_63129 | 98 | -0.173400291 | hsa-mir-6744 | - |
| hsa-mir-6750 | HGLibA_63159 | 98 | -0.173400291 | hsa-mir-6750 | - |
| hsa-mir-6887 | HGLibA_63731 | 98 | -0.173400291 | hsa-mir-6887 | - |
| hsa-mir-7-2 | HGLibA_63771 | 98 | -0.173400291 | hsa-mir-7-2 | - |
| hsa-mir-92b | HGLibA_64298 | 98 | -0.173400291 | hsa-mir-92b | - |
| LHFP | HGLibA_26425 | 98 | -0.173400291 | LHFP | - |
| LOC100289561 | HGLibA_26844 | 98 | -0.173400291 | LOC100289561 | - |
| NARFL | HGLibA_31146 | 98 | -0.173400291 | NARFL | - |
| PNMAL1 | HGLibA_37385 | 98 | -0.173400291 | PNMAL1 | - |
| SGK223 | HGLibA_43829 | 98 | -0.173400291 | SGK223 | - |
| SRCRB4D | HGLibA_46938 | 98 | -0.173400291 | SRCRB4D | - |
| T | HGLibA_48120 | 98 | -0.173400291 | T | - |
| TMEM136 | HGLibA_49952 | 98 | -0.173400291 | TMEM136 | - |
| ZUFSP | HGLibA_57062 | 98 | -0.173400291 | ZUFSP | - |
| AGPAT6 | HGLibA_01232 | 97 | -0.183351329 | AGPAT6 | - |
| C10orf114 | HGLibA_05036 | 97 | -0.183351329 | C10orf114 | - |
| C10orf128 | HGLibA_05049 | 97 | -0.183351329 | C10orf128 | - |
| C10orf54 | HGLibA_05075 | 97 | -0.183351329 | C10orf54 | - |
| C16orf97 | HGLibA_05512 | 97 | -0.183351329 | C16orf97 | - |
| C2orf62 | HGLibA_06158 | 97 | -0.183351329 | C2orf62 | - |
| C8orf4 | HGLibA_06616 | 97 | -0.183351329 | C8orf4 | - |
| CCDC104 | HGLibA_07518 | 97 | -0.183351329 | CCDC104 | - |
| ENSG00000006059 | HGLibA_25480 | 97 | -0.183351329 | KRT33A | keratin 33A [Source:HGNC Symbol;Acc:HGNC:6450] |
| ENSG00000006125 | HGLibA_02312 | 97 | -0.183351329 | AP2B1 | adaptor related protein complex 2 subunit beta 1 [Source:HGNC Symbol;Acc:HGNC:563] |
| ENSG00000006659 | HGLibA_26355 | 97 | -0.183351329 | LGALS14 | galectin 14 [Source:HGNC Symbol;Acc:HGNC:30054] |
| ENSG00000006715 | HGLibA_53791 | 97 | -0.183351329 | VPS41 | HOPS complex subunit [Source:HGNC Symbol;Acc:HGNC:12713]" |
| ENSG00000017483 | HGLibA_45073 | 97 | -0.183351329 | SLC38A5 | solute carrier family 38 member 5 [Source:HGNC Symbol;Acc:HGNC:18070] |
| ENSG00000018869 | HGLibA_56312 | 97 | -0.183351329 | ZNF582 | zinc finger protein 582 [Source:HGNC Symbol;Acc:HGNC:26421] |
| ENSG00000034693 | HGLibA_36113 | 97 | -0.183351329 | PEX3 | peroxisomal biogenesis factor 3 [Source:HGNC Symbol;Acc:HGNC:8858] |
| ENSG00000041357 | HGLibA_39079 | 97 | -0.183351329 | PSMA4 | proteasome subunit alpha 4 [Source:HGNC Symbol;Acc:HGNC:9533] |
| ENSG00000046889 | HGLibA_38445 | 97 | -0.183351329 | PREX2 | 5-trisphosphate dependent Rac exchange factor 2 [Source:HGNC Symbol;Acc:HGNC:22950]" |
| ENSG00000048052 | HGLibA_21027 | 97 | -0.183351329 | HDAC9 | histone deacetylase 9 [Source:HGNC Symbol;Acc:HGNC:14065] |
| ENSG00000055118 | HGLibA_24212 | 97 | -0.183351329 | KCNH2 | potassium voltage-gated channel subfamily H member 2 [Source:HGNC Symbol;Acc:HGNC:6251] |
| ENSG00000060140 | HGLibA_47685 | 97 | -0.183351329 | STYK1 | serine/threonine/tyrosine kinase 1 [Source:HGNC Symbol;Acc:HGNC:18889] |
| ENSG00000064999 | HGLibA_02149 | 97 | -0.183351329 | ANKS1A | ankyrin repeat and sterile alpha motif domain containing 1A [Source:HGNC Symbol;Acc:HGNC:20961] |
| ENSG00000067191 | HGLibA_06917 | 97 | -0.183351329 | CACNB1 | calcium voltage-gated channel auxiliary subunit beta 1 [Source:HGNC Symbol;Acc:HGNC:1401] |
| ENSG00000072803 | HGLibA_17138 | 97 | -0.183351329 | FBXW11 | F-box and WD repeat domain containing 11 [Source:HGNC Symbol;Acc:HGNC:13607] |
| ENSG00000074603 | HGLibA_13881 | 97 | -0.183351329 | DPP8 | dipeptidyl peptidase 8 [Source:HGNC Symbol;Acc:HGNC:16490] |
| ENSG00000076242 | HGLibA_29382 | 97 | -0.183351329 | MLH1 | mutL homolog 1 [Source:HGNC Symbol;Acc:HGNC:7127] |
| ENSG00000078549 | HGLibA_00939 | 97 | -0.183351329 | ADCYAP1R1 | ADCYAP receptor type I [Source:HGNC Symbol;Acc:HGNC:242] |
| ENSG00000080371 | HGLibA_39838 | 97 | -0.183351329 | RAB21 | member RAS oncogene family [Source:HGNC Symbol;Acc:HGNC:18263]" |
| ENSG00000083454 | HGLibA_34859 | 97 | -0.183351329 | P2RX5 | purinergic receptor P2X 5 [Source:HGNC Symbol;Acc:HGNC:8536] |
| ENSG00000086289 | HGLibA_15276 | 97 | -0.183351329 | EPDR1 | ependymin related 1 [Source:HGNC Symbol;Acc:HGNC:17572] |
| ENSG00000087245 | HGLibA_29508 | 97 | -0.183351329 | MMP2 | matrix metallopeptidase 2 [Source:HGNC Symbol;Acc:HGNC:7166] |
| ENSG00000090402 | HGLibA_44100 | 97 | -0.183351329 | SI | sucrase-isomaltase [Source:HGNC Symbol;Acc:HGNC:10856] |
| ENSG00000095970 | HGLibA_51356 | 97 | -0.183351329 | TREM2 | triggering receptor expressed on myeloid cells 2 [Source:HGNC Symbol;Acc:HGNC:17761] |
| ENSG00000099910 | HGLibA_25157 | 97 | -0.183351329 | KLHL22 | kelch like family member 22 [Source:HGNC Symbol;Acc:HGNC:25888] |
| ENSG00000100122 | HGLibA_11260 | 97 | -0.183351329 | CRYBB1 | crystallin beta B1 [Source:HGNC Symbol;Acc:HGNC:2397] |
| ENSG00000101843 | HGLibA_39151 | 97 | -0.183351329 | PSMD10 | non-ATPase 10 [Source:HGNC Symbol;Acc:HGNC:9555]" |
| ENSG00000102226 | HGLibA_53196 | 97 | -0.183351329 | USP11 | ubiquitin specific peptidase 11 [Source:HGNC Symbol;Acc:HGNC:12609] |
| ENSG00000103671 | HGLibA_51646 | 97 | -0.183351329 | TRIP4 | thyroid hormone receptor interactor 4 [Source:HGNC Symbol;Acc:HGNC:12310] |
| ENSG00000104969 | HGLibA_43868 | 97 | -0.183351329 | SGTA | small glutamine rich tetratricopeptide repeat containing alpha [Source:HGNC Symbol;Acc:HGNC:10819] |
| ENSG00000105427 | HGLibA_10259 | 97 | -0.183351329 | CNFN | cornifelin [Source:HGNC Symbol;Acc:HGNC:30183] |
| ENSG00000105643 | HGLibA_03116 | 97 | -0.183351329 | ARRDC2 | arrestin domain containing 2 [Source:HGNC Symbol;Acc:HGNC:25225] |
| ENSG00000107560 | HGLibA_39797 | 97 | -0.183351329 | RAB11FIP2 | RAB11 family interacting protein 2 [Source:HGNC Symbol;Acc:HGNC:29152] |
| ENSG00000111371 | HGLibA_45055 | 97 | -0.183351329 | SLC38A1 | solute carrier family 38 member 1 [Source:HGNC Symbol;Acc:HGNC:13447] |
| ENSG00000113504 | HGLibA_44374 | 97 | -0.183351329 | SLC12A7 | solute carrier family 12 member 7 [Source:HGNC Symbol;Acc:HGNC:10915] |
| ENSG00000113600 | HGLibA_06658 | 97 | -0.183351329 | C9 | complement C9 [Source:HGNC Symbol;Acc:HGNC:1358] |
| ENSG00000114942 | HGLibA_14497 | 97 | -0.183351329 | EEF1B2 | eukaryotic translation elongation factor 1 beta 2 [Source:HGNC Symbol;Acc:HGNC:3208] |
| ENSG00000115339 | HGLibA_18558 | 97 | -0.183351329 | GALNT3 | polypeptide N-acetylgalactosaminyltransferase 3 [Source:HGNC Symbol;Acc:HGNC:4125] |
| ENSG00000115425 | HGLibA_36020 | 97 | -0.183351329 | PECR | peroxisomal trans-2-enoyl-CoA reductase [Source:HGNC Symbol;Acc:HGNC:18281] |
| ENSG00000115687 | HGLibA_35268 | 97 | -0.183351329 | PASK | PAS domain containing serine/threonine kinase [Source:HGNC Symbol;Acc:HGNC:17270] |
| ENSG00000115756 | HGLibA_22019 | 97 | -0.183351329 | HPCAL1 | hippocalcin like 1 [Source:HGNC Symbol;Acc:HGNC:5145] |
| ENSG00000115963 | HGLibA_41451 | 97 | -0.183351329 | RND3 | Rho family GTPase 3 [Source:HGNC Symbol;Acc:HGNC:671] |
| ENSG00000120256 | HGLibA_27204 | 97 | -0.183351329 | LRP11 | LDL receptor related protein 11 [Source:HGNC Symbol;Acc:HGNC:16936] |
| ENSG00000120314 | HGLibA_54128 | 97 | -0.183351329 | WDR55 | WD repeat domain 55 [Source:HGNC Symbol;Acc:HGNC:25971] |
| ENSG00000120688 | HGLibA_53982 | 97 | -0.183351329 | WBP4 | WW domain binding protein 4 [Source:HGNC Symbol;Acc:HGNC:12739] |
| ENSG00000121053 | HGLibA_15392 | 97 | -0.183351329 | EPX | eosinophil peroxidase [Source:HGNC Symbol;Acc:HGNC:3423] |
| ENSG00000124479 | HGLibA_31410 | 97 | -0.183351329 | NDP | norrin cystine knot growth factor [Source:HGNC Symbol;Acc:HGNC:7678]" |
| ENSG00000125207 | HGLibA_36770 | 97 | -0.183351329 | PIWIL1 | piwi like RNA-mediated gene silencing 1 [Source:HGNC Symbol;Acc:HGNC:9007] |
| ENSG00000125266 | HGLibA_14601 | 97 | -0.183351329 | EFNB2 | ephrin B2 [Source:HGNC Symbol;Acc:HGNC:3227] |
| ENSG00000126106 | HGLibA_50437 | 97 | -0.183351329 | TMEM53 | transmembrane protein 53 [Source:HGNC Symbol;Acc:HGNC:26186] |
| ENSG00000126107 | HGLibA_21098 | 97 | -0.183351329 | HECTD3 | HECT domain E3 ubiquitin protein ligase 3 [Source:HGNC Symbol;Acc:HGNC:26117] |
| ENSG00000127418 | HGLibA_17452 | 97 | -0.183351329 | FGFRL1 | fibroblast growth factor receptor like 1 [Source:HGNC Symbol;Acc:HGNC:3693] |
| ENSG00000128298 | HGLibA_04129 | 97 | -0.183351329 | BAIAP2L2 | BAI1 associated protein 2 like 2 [Source:HGNC Symbol;Acc:HGNC:26203] |
| ENSG00000128510 | HGLibA_10892 | 97 | -0.183351329 | CPA4 | carboxypeptidase A4 [Source:HGNC Symbol;Acc:HGNC:15740] |
| ENSG00000129538 | HGLibA_41387 | 97 | -0.183351329 | RNASE1 | pancreatic [Source:HGNC Symbol;Acc:HGNC:10044]" |
| ENSG00000130244 | HGLibA_16814 | 97 | -0.183351329 | FAM98C | family with sequence similarity 98 member C [Source:HGNC Symbol;Acc:HGNC:27119] |
| ENSG00000130303 | HGLibA_04884 | 97 | -0.183351329 | BST2 | bone marrow stromal cell antigen 2 [Source:HGNC Symbol;Acc:HGNC:1119] |
| ENSG00000132702 | HGLibA_20844 | 97 | -0.183351329 | HAPLN2 | hyaluronan and proteoglycan link protein 2 [Source:HGNC Symbol;Acc:HGNC:17410] |
| ENSG00000132821 | HGLibA_53867 | 97 | -0.183351329 | VSTM2L | V-set and transmembrane domain containing 2 like [Source:HGNC Symbol;Acc:HGNC:16096] |
| ENSG00000133105 | HGLibA_42498 | 97 | -0.183351329 | RXFP2 | relaxin family peptide receptor 2 [Source:HGNC Symbol;Acc:HGNC:17318] |
| ENSG00000133574 | HGLibA_19078 | 97 | -0.183351329 | GIMAP4 | IMAP family member 4 [Source:HGNC Symbol;Acc:HGNC:21872]" |
| ENSG00000133597 | HGLibA_00893 | 97 | -0.183351329 | ADCK2 | aarF domain containing kinase 2 [Source:HGNC Symbol;Acc:HGNC:19039] |
| ENSG00000133962 | HGLibA_07366 | 97 | -0.183351329 | CATSPERB | cation channel sperm associated auxiliary subunit beta [Source:HGNC Symbol;Acc:HGNC:20500] |
| ENSG00000134222 | HGLibA_39233 | 97 | -0.183351329 | PSRC1 | proline and serine rich coiled-coil 1 [Source:HGNC Symbol;Acc:HGNC:24472] |
| ENSG00000134569 | HGLibA_27221 | 97 | -0.183351329 | LRP4 | LDL receptor related protein 4 [Source:HGNC Symbol;Acc:HGNC:6696] |
| ENSG00000135114 | HGLibA_33208 | 97 | -0.183351329 | OASL | 2'-5'-oligoadenylate synthetase like [Source:HGNC Symbol;Acc:HGNC:8090] |
| ENSG00000135269 | HGLibA_49023 | 97 | -0.183351329 | TES | testin LIM domain protein [Source:HGNC Symbol;Acc:HGNC:14620] |
| ENSG00000135452 | HGLibA_51928 | 97 | -0.183351329 | TSPAN31 | tetraspanin 31 [Source:HGNC Symbol;Acc:HGNC:10539] |
| ENSG00000135480 | HGLibA_25523 | 97 | -0.183351329 | KRT7 | keratin 7 [Source:HGNC Symbol;Acc:HGNC:6445] |
| ENSG00000135547 | HGLibA_21220 | 97 | -0.183351329 | HEY2 | hes related family bHLH transcription factor with YRPW motif 2 [Source:HGNC Symbol;Acc:HGNC:4881] |
| ENSG00000135577 | HGLibA_32145 | 97 | -0.183351329 | NMBR | neuromedin B receptor [Source:HGNC Symbol;Acc:HGNC:7843] |
| ENSG00000135655 | HGLibA_53209 | 97 | -0.183351329 | USP15 | ubiquitin specific peptidase 15 [Source:HGNC Symbol;Acc:HGNC:12613] |
| ENSG00000135723 | HGLibA_17492 | 97 | -0.183351329 | FHOD1 | formin homology 2 domain containing 1 [Source:HGNC Symbol;Acc:HGNC:17905] |
| ENSG00000135763 | HGLibA_53136 | 97 | -0.183351329 | URB2 | URB2 ribosome biogenesis homolog [Source:HGNC Symbol;Acc:HGNC:28967] |
| ENSG00000135778 | HGLibA_32870 | 97 | -0.183351329 | NTPCR | cancer-related [Source:HGNC Symbol;Acc:HGNC:28204]" |
| ENSG00000135902 | HGLibA_09602 | 97 | -0.183351329 | CHRND | cholinergic receptor nicotinic delta subunit [Source:HGNC Symbol;Acc:HGNC:1965] |
| ENSG00000135926 | HGLibA_49740 | 97 | -0.183351329 | TMBIM1 | transmembrane BAX inhibitor motif containing 1 [Source:HGNC Symbol;Acc:HGNC:23410] |
| ENSG00000136881 | HGLibA_04052 | 97 | -0.183351329 | BAAT | bile acid-CoA:amino acid N-acyltransferase [Source:HGNC Symbol;Acc:HGNC:932] |
| ENSG00000137288 | HGLibA_53102 | 97 | -0.183351329 | UQCC2 | ubiquinol-cytochrome c reductase complex assembly factor 2 [Source:HGNC Symbol;Acc:HGNC:21237] |
| ENSG00000137648 | HGLibA_50643 | 97 | -0.183351329 | TMPRSS4 | transmembrane serine protease 4 [Source:HGNC Symbol;Acc:HGNC:11878] |
| ENSG00000137959 | HGLibA_22615 | 97 | -0.183351329 | IFI44L | interferon induced protein 44 like [Source:HGNC Symbol;Acc:HGNC:17817] |
| ENSG00000138380 | HGLibA_07243 | 97 | -0.183351329 | CARF | calcium responsive transcription factor [Source:HGNC Symbol;Acc:HGNC:14435] |
| ENSG00000139726 | HGLibA_12970 | 97 | -0.183351329 | DENR | density regulated re-initiation and release factor [Source:HGNC Symbol;Acc:HGNC:2769] |
| ENSG00000140463 | HGLibA_04219 | 97 | -0.183351329 | BBS4 | Bardet-Biedl syndrome 4 [Source:HGNC Symbol;Acc:HGNC:969] |
| ENSG00000140694 | HGLibA_35207 | 97 | -0.183351329 | PARN | poly(A)-specific ribonuclease [Source:HGNC Symbol;Acc:HGNC:8609] |

| | | | | | |
|---|---|---|---|---|---|
| ENSG00000141258 | HGLibA_43863 | 97 | -0.183351329 | SGSM2 | small G protein signaling modulator 2 [Source:HGNC Symbol;Acc:HGNC:29026] |
| ENSG00000141338 | HGLibA_00124 | 97 | -0.183351329 | ABCA8 | ATP binding cassette subfamily A member 8 [Source:HGNC Symbol;Acc:HGNC:38] |
| ENSG00000143119 | HGLibA_08409 | 97 | -0.183351329 | CD53 | CD53 molecule [Source:HGNC Symbol;Acc:HGNC:1686] |
| ENSG00000143458 | HGLibA_18353 | 97 | -0.183351329 | GABPB2 | GA binding protein transcription factor subunit beta 2 [Source:HGNC Symbol;Acc:HGNC:28441] |
| ENSG00000143748 | HGLibA_33120 | 97 | -0.183351329 | NVL | nuclear VCP-like [Source:HGNC Symbol;Acc:HGNC:8070] |
| ENSG00000144115 | HGLibA_49360 | 97 | -0.183351329 | THNSL2 | threonine synthase like 2 [Source:HGNC Symbol;Acc:HGNC:25602] |
| ENSG00000144857 | HGLibA_04666 | 97 | -0.183351329 | BOC | oncogene regulated [Source:HGNC Symbol;Acc:HGNC:17173]" |
| ENSG00000145293 | HGLibA_15151 | 97 | -0.183351329 | ENOPH1 | enolase-phosphatase 1 [Source:HGNC Symbol;Acc:HGNC:24599] |
| ENSG00000147576 | HGLibA_00968 | 97 | -0.183351329 | ADHFE1 | iron containing 1 [Source:HGNC Symbol;Acc:HGNC:16354]" |
| ENSG00000150510 | HGLibA_16074 | 97 | -0.183351329 | FAM124A | family with sequence similarity 124 member A [Source:HGNC Symbol;Acc:HGNC:26413] |
| ENSG00000151572 | HGLibA_02181 | 97 | -0.183351329 | ANO4 | anoctamin 4 [Source:HGNC Symbol;Acc:HGNC:23837] |
| ENSG00000151623 | HGLibA_32621 | 97 | -0.183351329 | NR3C2 | nuclear receptor subfamily 3 group C member 2 [Source:HGNC Symbol;Acc:HGNC:7979] |
| ENSG00000152217 | HGLibA_43633 | 97 | -0.183351329 | SETBP1 | SET binding protein 1 [Source:HGNC Symbol;Acc:HGNC:15573] |
| ENSG00000152977 | HGLibA_55321 | 97 | -0.183351329 | ZIC1 | Zic family member 1 [Source:HGNC Symbol;Acc:HGNC:12872] |
| ENSG00000153064 | HGLibA_04145 | 97 | -0.183351329 | BANK1 | B cell scaffold protein with ankyrin repeats 1 [Source:HGNC Symbol;Acc:HGNC:18233] |
| ENSG00000153498 | HGLibA_46339 | 97 | -0.183351329 | SPACA7 | sperm acrosome associated 7 [Source:HGNC Symbol;Acc:HGNC:29575] |
| ENSG00000153827 | HGLibA_51638 | 97 | -0.183351329 | TRIP12 | thyroid hormone receptor interactor 12 [Source:HGNC Symbol;Acc:HGNC:12306] |
| ENSG00000154438 | HGLibA_03398 | 97 | -0.183351329 | ASZ1 | SAM and basic leucine zipper domain containing 1 [Source:HGNC Symbol;Acc:HGNC:1350]" |
| ENSG00000155158 | HGLibA_52136 | 97 | -0.183351329 | TTC39B | tetratricopeptide repeat domain 39B [Source:HGNC Symbol;Acc:HGNC:23704] |
| ENSG00000156535 | HGLibA_08231 | 97 | -0.183351329 | CD109 | CD109 molecule [Source:HGNC Symbol;Acc:HGNC:21685] |
| ENSG00000159164 | HGLibA_47875 | 97 | -0.183351329 | SV2A | synaptic vesicle glycoprotein 2A [Source:HGNC Symbol;Acc:HGNC:20566] |
| ENSG00000159459 | HGLibA_52747 | 97 | -0.183351329 | UBR1 | ubiquitin protein ligase E3 component n-recognin 1 [Source:HGNC Symbol;Acc:HGNC:16808] |
| ENSG00000160181 | HGLibA_49172 | 97 | -0.183351329 | TFF2 | trefoil factor 2 [Source:HGNC Symbol;Acc:HGNC:11756] |
| ENSG00000160710 | HGLibA_00870 | 97 | -0.183351329 | ADAR | RNA specific [Source:HGNC Symbol;Acc:HGNC:225]" |
| ENSG00000162627 | HGLibA_46104 | 97 | -0.183351329 | SNX7 | sorting nexin 7 [Source:HGNC Symbol;Acc:HGNC:14971] |
| ENSG00000162736 | HGLibA_31376 | 97 | -0.183351329 | NCSTN | nicastrin [Source:HGNC Symbol;Acc:HGNC:17091] |
| ENSG00000162992 | HGLibA_31735 | 97 | -0.183351329 | NEUROD1 | neuronal differentiation 1 [Source:HGNC Symbol;Acc:HGNC:7762] |
| ENSG00000163352 | HGLibA_26295 | 97 | -0.183351329 | LENEP | lens epithelial protein [Source:HGNC Symbol;Acc:HGNC:14429] |
| ENSG00000163606 | HGLibA_08286 | 97 | -0.183351329 | CD200R1 | CD200 receptor 1 [Source:HGNC Symbol;Acc:HGNC:24235] |
| ENSG00000163867 | HGLibA_55404 | 97 | -0.183351329 | ZMYM6 | zinc finger MYM-type containing 6 [Source:HGNC Symbol;Acc:HGNC:13050] |
| ENSG00000164045 | HGLibA_08527 | 97 | -0.183351329 | CDC25A | cell division cycle 25A [Source:HGNC Symbol;Acc:HGNC:1725] |
| ENSG00000164080 | HGLibA_40105 | 97 | -0.183351329 | RAD54L2 | RAD54 like 2 [Source:HGNC Symbol;Acc:HGNC:29123] |
| ENSG00000164692 | HGLibA_10533 | 97 | -0.183351329 | COL1A2 | collagen type I alpha 2 chain [Source:HGNC Symbol;Acc:HGNC:2198] |
| ENSG00000164970 | HGLibA_16498 | 97 | -0.183351329 | FAM219A | family with sequence similarity 219 member A [Source:HGNC Symbol;Acc:HGNC:19920] |
| ENSG00000165115 | HGLibA_24921 | 97 | -0.183351329 | KIF27 | kinesin family member 27 [Source:HGNC Symbol;Acc:HGNC:18632] |
| ENSG00000165527 | HGLibA_02658 | 97 | -0.183351329 | ARF6 | ADP ribosylation factor 6 [Source:HGNC Symbol;Acc:HGNC:659] |
| ENSG00000167165 | HGLibA_52892 | 97 | -0.183351329 | UGT1A6 | UDP glucuronosyltransferase family 1 member A6 [Source:HGNC Symbol;Acc:HGNC:12538] |
| ENSG00000167208 | HGLibA_46059 | 97 | -0.183351329 | SNX20 | sorting nexin 20 [Source:HGNC Symbol;Acc:HGNC:30390] |
| ENSG00000168036 | HGLibA_11650 | 97 | -0.183351329 | CTNNB1 | catenin beta 1 [Source:HGNC Symbol;Acc:HGNC:2514] |
| ENSG00000168491 | HGLibA_07539 | 97 | -0.183351329 | CCDC110 | coiled-coil domain containing 110 [Source:HGNC Symbol;Acc:HGNC:28504] |
| ENSG00000168679 | HGLibA_44442 | 97 | -0.183351329 | SLC16A4 | solute carrier family 16 member 4 [Source:HGNC Symbol;Acc:HGNC:10925] |
| ENSG00000170605 | HGLibA_34581 | 97 | -0.183351329 | OR9K2 | olfactory receptor family 9 subfamily K member 2 [Source:HGNC Symbol;Acc:HGNC:15339] |
| ENSG00000170807 | HGLibA_26720 | 97 | -0.183351329 | LMOD2 | leiomodin 2 [Source:HGNC Symbol;Acc:HGNC:6648] |
| ENSG00000170899 | HGLibA_20460 | 97 | -0.183351329 | GSTA4 | glutathione S-transferase alpha 4 [Source:HGNC Symbol;Acc:HGNC:4629] |
| ENSG00000171202 | HGLibA_49910 | 97 | -0.183351329 | TMEM126A | transmembrane protein 126A [Source:HGNC Symbol;Acc:HGNC:25382] |
| ENSG00000171944 | HGLibA_34143 | 97 | -0.183351329 | OR52A5 | olfactory receptor family 52 subfamily A member 5 [Source:HGNC Symbol;Acc:HGNC:19580] |
| ENSG00000172375 | HGLibA_06089 | 97 | -0.183351329 | C2CD2L | C2CD2 like [Source:HGNC Symbol;Acc:HGNC:29000] |
| ENSG00000172377 | HGLibA_34576 | 97 | -0.183351329 | OR9I1 | olfactory receptor family 9 subfamily I member 1 [Source:HGNC Symbol;Acc:HGNC:14718] |
| ENSG00000172410 | HGLibA_23389 | 97 | -0.183351329 | INSL5 | insulin like 5 [Source:HGNC Symbol;Acc:HGNC:6088] |
| ENSG00000172456 | HGLibA_17458 | 97 | -0.183351329 | FGGY | FGGY carbohydrate kinase domain containing [Source:HGNC Symbol;Acc:HGNC:25610] |
| ENSG00000172482 | HGLibA_01271 | 97 | -0.183351329 | AGXT | alanine--glyoxylate and serine--pyruvate aminotransferase [Source:HGNC Symbol;Acc:HGNC:341] |
| ENSG00000172940 | HGLibA_44545 | 97 | -0.183351329 | SLC22A13 | solute carrier family 22 member 13 [Source:HGNC Symbol;Acc:HGNC:8494] |
| ENSG00000174697 | HGLibA_26308 | 97 | -0.183351329 | LEP | leptin [Source:HGNC Symbol;Acc:HGNC:6553] |
| ENSG00000176018 | HGLibA_27770 | 97 | -0.183351329 | LYSMD3 | LysM domain containing 3 [Source:HGNC Symbol;Acc:HGNC:26969] |
| ENSG00000178252 | HGLibA_54135 | 97 | -0.183351329 | WDR6 | WD repeat domain 6 [Source:HGNC Symbol;Acc:HGNC:12758] |
| ENSG00000179008 | HGLibA_05366 | 97 | -0.183351329 | C14orf39 | chromosome 14 open reading frame 39 [Source:HGNC Symbol;Acc:HGNC:19849] |
| ENSG00000182816 | HGLibA_25646 | 97 | -0.183351329 | KRTAP13-2 | keratin associated protein 13-2 [Source:HGNC Symbol;Acc:HGNC:18923] |
| ENSG00000183421 | HGLibA_41326 | 97 | -0.183351329 | RIPK4 | receptor interacting serine/threonine kinase 4 [Source:HGNC Symbol;Acc:HGNC:496] |
| ENSG00000183778 | HGLibA_03963 | 97 | -0.183351329 | B3GALT5 | 3-galactosyltransferase 5 [Source:HGNC Symbol;Acc:HGNC:920]" |
| ENSG00000183943 | HGLibA_38592 | 97 | -0.183351329 | PRKX | protein kinase X-linked [Source:HGNC Symbol;Acc:HGNC:9441] |
| ENSG00000185049 | HGLibA_31671 | 97 | -0.183351329 | NELFA | negative elongation factor complex member A [Source:HGNC Symbol;Acc:HGNC:12768] |
| ENSG00000185674 | HGLibA_27701 | 97 | -0.183351329 | LYG2 | lysozyme g2 [Source:HGNC Symbol;Acc:HGNC:29615] |
| ENSG00000186063 | HGLibA_01327 | 97 | -0.183351329 | AIDA | dorsalization associated [Source:HGNC Symbol;Acc:HGNC:25761]" |
| ENSG00000186472 | HGLibA_35619 | 97 | -0.183351329 | PCLO | piccolo presynaptic cytomatrix protein [Source:HGNC Symbol;Acc:HGNC:13406] |
| ENSG00000186684 | HGLibA_12101 | 97 | -0.183351329 | CYP27C1 | cytochrome P450 family 27 subfamily C member 1 [Source:HGNC Symbol;Acc:HGNC:33480] |
| ENSG00000187140 | HGLibA_17833 | 97 | -0.183351329 | FOXD3 | forkhead box D3 [Source:HGNC Symbol;Acc:HGNC:3804] |
| ENSG00000187144 | HGLibA_46450 | 97 | -0.183351329 | SPATA21 | spermatogenesis associated 21 [Source:HGNC Symbol;Acc:HGNC:28026] |
| ENSG00000187741 | HGLibA_16829 | 97 | -0.183351329 | FANCA | FA complementation group A [Source:HGNC Symbol;Acc:HGNC:3582] |
| ENSG00000189046 | HGLibA_01650 | 97 | -0.183351329 | ALKBH2 | alpha-ketoglutarate dependent dioxygenase [Source:HGNC Symbol;Acc:HGNC:32487]" |
| ENSG00000189366 | HGLibA_01621 | 97 | -0.183351329 | ALG1L | chitobiosyldiphosphodolichol beta-mannosyltransferase like [Source:HGNC Symbol;Acc:HGNC:33721]" |
| ENSG00000196247 | HGLibA_55440 | 97 | -0.183351329 | ZNF107 | zinc finger protein 107 [Source:HGNC Symbol;Acc:HGNC:12887] |
| ENSG00000196581 | HGLibA_01371 | 97 | -0.183351329 | AJAP1 | adherens junctions associated protein 1 [Source:HGNC Symbol;Acc:HGNC:30801] |
| ENSG00000197008 | HGLibA_55478 | 97 | -0.183351329 | ZNF138 | zinc finger protein 138 [Source:HGNC Symbol;Acc:HGNC:12922] |
| ENSG00000197037 | HGLibA_57009 | 97 | -0.183351329 | ZSCAN25 | zinc finger and SCAN domain containing 25 [Source:HGNC Symbol;Acc:HGNC:21961] |
| ENSG00000198398 | HGLibA_50198 | 97 | -0.183351329 | TMEM207 | transmembrane protein 207 [Source:HGNC Symbol;Acc:HGNC:33705] |
| ENSG00000198833 | HGLibA_52619 | 97 | -0.183351329 | UBE2J1 | ubiquitin conjugating enzyme E2 J1 [Source:HGNC Symbol;Acc:HGNC:17598] |
| ENSG00000204386 | HGLibA_31711 | 97 | -0.183351329 | NEU1 | neuraminidase 1 [Source:HGNC Symbol;Acc:HGNC:7758] |
| ENSG00000204438 | HGLibA_19729 | 97 | -0.183351329 | GPANK1 | G-patch domain and ankyrin repeats 1 [Source:HGNC Symbol;Acc:HGNC:13920] |
| ENSG00000205108 | HGLibA_16424 | 97 | -0.183351329 | FAM205A | family with sequence similarity 205 member A [Source:HGNC Symbol;Acc:HGNC:41911] |
| ENSG00000205189 | HGLibA_54813 | 97 | -0.183351329 | ZBTB10 | zinc finger and BTB domain containing 10 [Source:HGNC Symbol;Acc:HGNC:30953] |
| ENSG00000205456 | HGLibA_51088 | 97 | -0.183351329 | TP53TG3D | TP53 target 3D [Source:HGNC Symbol;Acc:HGNC:44657] |
| ENSG00000206535 | HGLibA_26738 | 97 | -0.183351329 | LNP1 | leukemia NUP98 fusion partner 1 [Source:HGNC Symbol;Acc:HGNC:28014] |
| ENSG00000215356 | HGLibA_56589 | 97 | -0.183351329 | ZNF705B | zinc finger protein 705B [Source:HGNC Symbol;Acc:HGNC:32284] |
| ENSG00000226763 | HGLibA_47054 | 97 | -0.183351329 | SRRM5 | serine/arginine repetitive matrix 5 [Source:HGNC Symbol;Acc:HGNC:37248] |
| ENSG00000230246 | HGLibA_46490 | 97 | -0.183351329 | SPATA31C1 | SPATA31 subfamily C member 1 [Source:HGNC Symbol;Acc:HGNC:27846] |
| ENSG00000240204 | HGLibA_45776 | 97 | -0.183351329 | SMKR1 | small lysine rich protein 1 [Source:HGNC Symbol;Acc:HGNC:43561] |
| ENSG00000242389 | HGLibA_40621 | 97 | -0.183351329 | RBMY1E | member E [Source:HGNC Symbol;Acc:HGNC:23916]" |
| ENSG00000255071 | HGLibA_42615 | 97 | -0.183351329 | SAA2-SAA4 | SAA2-SAA4 readthrough [Source:HGNC Symbol;Acc:HGNC:39550] |
| ENSG00000265817 | HGLibA_18061 | 97 | -0.183351329 | FSBP | fibrinogen silencer binding protein [Source:HGNC Symbol;Acc:HGNC:43653] |
| ENSG00000268500 | HGLibA_44141 | 97 | -0.183351329 | SIGLEC5 | sense intronic to AC018755.18" |
| ENSG00000278057 | HGLibA_49089 | 97 | -0.183351329 | TEX28 | testis expressed 28 [Source:HGNC Symbol;Acc:HGNC:2563] |
| HIST2H4B | HGLibA_21551 | 97 | -0.183351329 | HIST2H4B | - |
| hsa-mir-21 | HGLibA_58209 | 97 | -0.183351329 | hsa-mir-21 | - |
| hsa-mir-23b | HGLibA_58334 | 97 | -0.183351329 | hsa-mir-23b | - |
| hsa-mir-27a | HGLibA_58378 | 97 | -0.183351329 | hsa-mir-27a | - |
| hsa-mir-29b-1 | HGLibA_58415 | 97 | -0.183351329 | hsa-mir-29b-1 | - |
| hsa-mir-3173 | HGLibA_58767 | 97 | -0.183351329 | hsa-mir-3173 | - |
| hsa-mir-330 | HGLibA_58988 | 97 | -0.183351329 | hsa-mir-330 | - |
| hsa-mir-365a | HGLibA_59186 | 97 | -0.183351329 | hsa-mir-365a | - |
| hsa-mir-4256 | HGLibA_59730 | 97 | -0.183351329 | hsa-mir-4256 | - |
| hsa-mir-4275 | HGLibA_59805 | 97 | -0.183351329 | hsa-mir-4275 | - |
| hsa-mir-4325 | HGLibA_60024 | 97 | -0.183351329 | hsa-mir-4325 | - |
| hsa-mir-451a | HGLibA_60525 | 97 | -0.183351329 | hsa-mir-451a | - |
| hsa-mir-4646 | HGLibA_60686 | 97 | -0.183351329 | hsa-mir-4646 | - |
| hsa-mir-4666b | HGLibA_60780 | 97 | -0.183351329 | hsa-mir-4666b | - |
| hsa-mir-5195 | HGLibA_61699 | 97 | -0.183351329 | hsa-mir-5195 | - |
| hsa-mir-519c | HGLibA_61721 | 97 | -0.183351329 | hsa-mir-519c | - |
| hsa-mir-548d-1 | HGLibA_61959 | 97 | -0.183351329 | hsa-mir-548d-1 | - |
| hsa-mir-548e | HGLibA_61969 | 97 | -0.183351329 | hsa-mir-548e | - |
| hsa-mir-5705 | HGLibA_62359 | 97 | -0.183351329 | hsa-mir-5705 | - |
| hsa-mir-7848 | HGLibA_63981 | 97 | -0.183351329 | hsa-mir-7848 | - |
| hsa-mir-891a | HGLibA_64241 | 97 | -0.183351329 | hsa-mir-891a | - |
| hsa-mir-96 | HGLibA_64369 | 97 | -0.183351329 | hsa-mir-96 | - |
| LOC100129924 | HGLibA_26772 | 97 | -0.183351329 | LOC100129924 | - |
| LOC391322 | HGLibA_26934 | 97 | -0.183351329 | LOC391322 | - |
| MST4 | HGLibA_30261 | 97 | -0.183351329 | MST4 | - |
| SDPR | HGLibA_43158 | 97 | -0.183351329 | SDPR | - |
| TMEM14E | HGLibA_49987 | 97 | -0.183351329 | TMEM14E | - |
| TMEM189-UBE2V1 | HGLibA_50134 | 97 | -0.183351329 | TMEM189-UBE2V1 | - |
| TMEM8A | HGLibA_50545 | 97 | -0.183351329 | TMEM8A | - |
| C2orf47 | HGLibA_06135 | 96 | -0.193302366 | C2orf47 | - |
| C9orf117 | HGLibA_06667 | 96 | -0.193302366 | C9orf117 | - |
| C9orf169 | HGLibA_06705 | 96 | -0.193302366 | C9orf169 | - |
| DIRC2 | HGLibA_13268 | 96 | -0.193302366 | DIRC2 | - |
| ENSG00000002726 | HGLibA_02268 | 96 | -0.193302366 | AOC1 | copper containing 1 [Source:HGNC Symbol;Acc:HGNC:80]" |
| ENSG00000002919 | HGLibA_46031 | 96 | -0.193302366 | SNX11 | sorting nexin 11 [Source:HGNC Symbol;Acc:HGNC:14975] |
| ENSG00000009790 | HGLibA_51254 | 96 | -0.193302366 | TRAF3IP3 | TRAF3 interacting protein 3 [Source:HGNC Symbol;Acc:HGNC:30766] |
| ENSG00000014138 | HGLibA_37487 | 96 | -0.193302366 | POLA2 | accessory subunit [Source:HGNC Symbol;Acc:HGNC:30073]" |
| ENSG00000019549 | HGLibA_45868 | 96 | -0.193302366 | SNAI2 | snail family transcriptional repressor 2 [Source:HGNC Symbol;Acc:HGNC:11094] |
| ENSG00000022840 | HGLibA_41454 | 96 | -0.193302366 | RNF10 | ring finger protein 10 [Source:HGNC Symbol;Acc:HGNC:10055] |
| ENSG00000038382 | HGLibA_51628 | 96 | -0.193302366 | TRIO | trio Rho guanine nucleotide exchange factor [Source:HGNC Symbol;Acc:HGNC:12303] |
| ENSG00000040731 | HGLibA_08632 | 96 | -0.193302366 | CDH10 | cadherin 10 [Source:HGNC Symbol;Acc:HGNC:1749] |
| ENSG00000051128 | HGLibA_21871 | 96 | -0.193302366 | HOMER3 | homer scaffold protein 3 [Source:HGNC Symbol;Acc:HGNC:17514] |
| ENSG00000054598 | HGLibA_17821 | 96 | -0.193302366 | FOXC1 | forkhead box C1 [Source:HGNC Symbol;Acc:HGNC:3800] |
| ENSG00000065060 | HGLibA_52947 | 96 | -0.193302366 | UHRF1BP1 | UHRF1 binding protein 1 [Source:HGNC Symbol;Acc:HGNC:21216] |

| | | | | | |
|---|---|---|---|---|---|
| ENSG00000066468 | HGLibA_17444 | 96 | -0.193302366 | FGFR2 | fibroblast growth factor receptor 2 [Source:HGNC Symbol;Acc:HGNC:3689] |
| ENSG00000067646 | HGLibA_55268 | 96 | -0.193302366 | ZFY | zinc finger protein Y-linked [Source:HGNC Symbol;Acc:HGNC:12870] |
| ENSG00000078246 | HGLibA_52336 | 96 | -0.193302366 | TULP3 | tubby like protein 3 [Source:HGNC Symbol;Acc:HGNC:12425] |
| ENSG00000084764 | HGLibA_28320 | 96 | -0.193302366 | MAPRE3 | microtubule associated protein RP/EB family member 3 [Source:HGNC Symbol;Acc:HGNC:6892] |
| ENSG00000085224 | HGLibA_03826 | 96 | -0.193302366 | ATRX | chromatin remodeler [Source:HGNC Symbol;Acc:HGNC:886]" |
| ENSG00000088325 | HGLibA_51220 | 96 | -0.193302366 | TPX2 | microtubule nucleation factor [Source:HGNC Symbol;Acc:HGNC:1249]" |
| ENSG00000088367 | HGLibA_15242 | 96 | -0.193302366 | EPB41L1 | erythrocyte membrane protein band 4.1 like 1 [Source:HGNC Symbol;Acc:HGNC:3378] |
| ENSG00000090104 | HGLibA_41050 | 96 | -0.193302366 | RGS1 | regulator of G protein signaling 1 [Source:HGNC Symbol;Acc:HGNC:9991] |
| ENSG00000091010 | HGLibA_37783 | 96 | -0.193302366 | POU4F3 | POU class 4 homeobox 3 [Source:HGNC Symbol;Acc:HGNC:9220] |
| ENSG00000091640 | HGLibA_46367 | 96 | -0.193302366 | SPAG7 | sperm associated antigen 7 [Source:HGNC Symbol;Acc:HGNC:11216] |
| ENSG00000099949 | HGLibA_27804 | 96 | -0.193302366 | LZTR1 | leucine zipper like transcription regulator 1 [Source:HGNC Symbol;Acc:HGNC:6742] |
| ENSG00000100280 | HGLibA_02280 | 96 | -0.193302366 | AP1B1 | adaptor related protein complex 1 subunit beta 1 [Source:HGNC Symbol;Acc:HGNC:554] |
| ENSG00000100522 | HGLibA_19571 | 96 | -0.193302366 | GNPNAT1 | glucosamine-phosphate N-acetyltransferase 1 [Source:HGNC Symbol;Acc:HGNC:19980] |
| ENSG00000100652 | HGLibA_44331 | 96 | -0.193302366 | SLC10A1 | solute carrier family 10 member 1 [Source:HGNC Symbol;Acc:HGNC:10905] |
| ENSG00000100721 | HGLibA_48816 | 96 | -0.193302366 | TCL1A | T cell leukemia/lymphoma 1A [Source:HGNC Symbol;Acc:HGNC:11648] |
| ENSG00000100784 | HGLibA_42171 | 96 | -0.193302366 | RPS6KA5 | ribosomal protein S6 kinase A5 [Source:HGNC Symbol;Acc:HGNC:10434] |
| ENSG00000101489 | HGLibA_09013 | 96 | -0.193302366 | CELF4 | CUGBP Elav-like family member 4 [Source:HGNC Symbol;Acc:HGNC:14015] |
| ENSG00000101557 | HGLibA_53206 | 96 | -0.193302366 | USP14 | ubiquitin specific peptidase 14 [Source:HGNC Symbol;Acc:HGNC:12612] |
| ENSG00000102760 | HGLibA_40997 | 96 | -0.193302366 | RGCC | regulator of cell cycle [Source:HGNC Symbol;Acc:HGNC:20369] |
| ENSG00000103319 | HGLibA_14511 | 96 | -0.193302366 | EEF2K | eukaryotic elongation factor 2 kinase [Source:HGNC Symbol;Acc:HGNC:24615] |
| ENSG00000103740 | HGLibA_00543 | 96 | -0.193302366 | ACSBG1 | acyl-CoA synthetase bubblegum family member 1 [Source:HGNC Symbol;Acc:HGNC:29567] |
| ENSG00000104059 | HGLibA_16352 | 96 | -0.193302366 | FAM189A1 | family with sequence similarity 189 member A1 [Source:HGNC Symbol;Acc:HGNC:29075] |
| ENSG00000104327 | HGLibA_06985 | 96 | -0.193302366 | CALB1 | calbindin 1 [Source:HGNC Symbol;Acc:HGNC:1434] |
| ENSG00000104894 | HGLibA_08362 | 96 | -0.193302366 | CD37 | CD37 molecule [Source:HGNC Symbol;Acc:HGNC:1666] |
| ENSG00000105664 | HGLibA_10682 | 96 | -0.193302366 | COMP | cartilage oligomeric matrix protein [Source:HGNC Symbol;Acc:HGNC:2227] |
| ENSG00000107187 | HGLibA_26452 | 96 | -0.193302366 | LHX3 | LIM homeobox 3 [Source:HGNC Symbol;Acc:HGNC:6595] |
| ENSG00000110011 | HGLibA_13620 | 96 | -0.193302366 | DNAJC4 | DnaJ heat shock protein family (Hsp40) member C4 [Source:HGNC Symbol;Acc:HGNC:5271] |
| ENSG00000110721 | HGLibA_09458 | 96 | -0.193302366 | CHKA | choline kinase alpha [Source:HGNC Symbol;Acc:HGNC:1937] |
| ENSG00000111052 | HGLibA_26574 | 96 | -0.193302366 | LIN7A | crumbs cell polarity complex component [Source:HGNC Symbol;Acc:HGNC:17787]" |
| ENSG00000111144 | HGLibA_27579 | 96 | -0.193302366 | LTA4H | leukotriene A4 hydrolase [Source:HGNC Symbol;Acc:HGNC:6710] |
| ENSG00000111536 | HGLibA_23110 | 96 | -0.193302366 | IL26 | interleukin 26 [Source:HGNC Symbol;Acc:HGNC:17119] |
| ENSG00000111886 | HGLibA_18407 | 96 | -0.193302366 | GABRR2 | gamma-aminobutyric acid type A receptor rho2 subunit [Source:HGNC Symbol;Acc:HGNC:4091] |
| ENSG00000112053 | HGLibA_44806 | 96 | -0.193302366 | SLC26A8 | solute carrier family 26 member 8 [Source:HGNC Symbol;Acc:HGNC:14468] |
| ENSG00000117425 | HGLibA_39272 | 96 | -0.193302366 | PTCH2 | patched 2 [Source:HGNC Symbol;Acc:HGNC:9586] |
| ENSG00000118007 | HGLibA_47321 | 96 | -0.193302366 | STAG1 | stromal antigen 1 [Source:HGNC Symbol;Acc:HGNC:11354] |
| ENSG00000118515 | HGLibA_43816 | 96 | -0.193302366 | SGK1 | serum/glucocorticoid regulated kinase 1 [Source:HGNC Symbol;Acc:HGNC:10810] |
| ENSG00000119729 | HGLibA_41199 | 96 | -0.193302366 | RHOQ | ras homolog family member Q [Source:HGNC Symbol;Acc:HGNC:17736] |
| ENSG00000120659 | HGLibA_50798 | 96 | -0.193302366 | TNFSF11 | TNF superfamily member 11 [Source:HGNC Symbol;Acc:HGNC:11926] |
| ENSG00000121716 | HGLibA_36672 | 96 | -0.193302366 | PILRB | paired immunoglobin-like type 2 receptor beta [Source:HGNC Symbol;Acc:HGNC:18297] |
| ENSG00000123700 | HGLibA_24273 | 96 | -0.193302366 | KCNJ2 | potassium voltage-gated channel subfamily J member 2 [Source:HGNC Symbol;Acc:HGNC:6263] |
| ENSG00000124140 | HGLibA_44370 | 96 | -0.193302366 | SLC12A5 | solute carrier family 12 member 5 [Source:HGNC Symbol;Acc:HGNC:13818] |
| ENSG00000125508 | HGLibA_46998 | 96 | -0.193302366 | SRMS | src-related kinase lacking C-terminal regulatory tyrosine and N-terminal myristylation sites [Source:HGNC Symbol;Acc:HGNC:11298] |
| ENSG00000125637 | HGLibA_39021 | 96 | -0.193302366 | PSD4 | pleckstrin and Sec7 domain containing 4 [Source:HGNC Symbol;Acc:HGNC:19096] |
| ENSG00000128536 | HGLibA_08705 | 96 | -0.193302366 | CDHR3 | cadherin related family member 3 [Source:HGNC Symbol;Acc:HGNC:26308] |
| ENSG00000128596 | HGLibA_07605 | 96 | -0.193302366 | CCDC136 | coiled-coil domain containing 136 [Source:HGNC Symbol;Acc:HGNC:22225] |
| ENSG00000130538 | HGLibA_33570 | 96 | -0.193302366 | OR11H1 | olfactory receptor family 11 subfamily H member 1 [Source:HGNC Symbol;Acc:HGNC:15404] |
| ENSG00000131061 | HGLibA_55841 | 96 | -0.193302366 | ZNF341 | zinc finger protein 341 [Source:HGNC Symbol;Acc:HGNC:15992] |
| ENSG00000132205 | HGLibA_15051 | 96 | -0.193302366 | EMILIN2 | elastin microfibril interfacer 2 [Source:HGNC Symbol;Acc:HGNC:19881] |
| ENSG00000132693 | HGLibA_11215 | 96 | -0.193302366 | CRP | C-reactive protein [Source:HGNC Symbol;Acc:HGNC:2367] |
| ENSG00000133138 | HGLibA_48570 | 96 | -0.193302366 | TBC1D8B | TBC1 domain family member 8B [Source:HGNC Symbol;Acc:HGNC:24715] |
| ENSG00000135018 | HGLibA_52731 | 96 | -0.193302366 | UBQLN1 | ubiquilin 1 [Source:HGNC Symbol;Acc:HGNC:12508] |
| ENSG00000135119 | HGLibA_41705 | 96 | -0.193302366 | RNFT2 | transmembrane 2 [Source:HGNC Symbol;Acc:HGNC:25905]" |
| ENSG00000135837 | HGLibA_09135 | 96 | -0.193302366 | CEP350 | centrosomal protein 350 [Source:HGNC Symbol;Acc:HGNC:24238] |
| ENSG00000136856 | HGLibA_44891 | 96 | -0.193302366 | SLC2A8 | solute carrier family 2 member 8 [Source:HGNC Symbol;Acc:HGNC:13812] |
| ENSG00000137818 | HGLibA_42012 | 96 | -0.193302366 | RPLP1 | ribosomal protein lateral stalk subunit P1 [Source:HGNC Symbol;Acc:HGNC:10372] |
| ENSG00000138439 | HGLibA_16045 | 96 | -0.193302366 | FAM117B | family with sequence similarity 117 member B [Source:HGNC Symbol;Acc:HGNC:14440] |
| ENSG00000138780 | HGLibA_20465 | 96 | -0.193302366 | GSTCD | glutathione S-transferase C-terminal domain containing [Source:HGNC Symbol;Acc:HGNC:25806] |
| ENSG00000139508 | HGLibA_45190 | 96 | -0.193302366 | SLC46A3 | solute carrier family 46 member 3 [Source:HGNC Symbol;Acc:HGNC:27501] |
| ENSG00000139977 | HGLibA_30987 | 96 | -0.193302366 | NAA30 | NatC catalytic subunit [Source:HGNC Symbol;Acc:HGNC:19844]" |
| ENSG00000140563 | HGLibA_28654 | 96 | -0.193302366 | MCTP2 | multiple C2 and transmembrane domain containing 2 [Source:HGNC Symbol;Acc:HGNC:25636] |
| ENSG00000140993 | HGLibA_49482 | 96 | -0.193302366 | TIGD7 | tigger transposable element derived 7 [Source:HGNC Symbol;Acc:HGNC:18331] |
| ENSG00000142149 | HGLibA_22422 | 96 | -0.193302366 | HUNK | hormonally up-regulated Neu-associated kinase [Source:HGNC Symbol;Acc:HGNC:13326] |
| ENSG00000142583 | HGLibA_44882 | 96 | -0.193302366 | SLC2A5 | solute carrier family 2 member 5 [Source:HGNC Symbol;Acc:HGNC:11010] |
| ENSG00000143376 | HGLibA_46076 | 96 | -0.193302366 | SNX27 | sorting nexin family member 27 [Source:HGNC Symbol;Acc:HGNC:20073] |
| ENSG00000144227 | HGLibA_33164 | 96 | -0.193302366 | NXPH2 | neurexophilin 2 [Source:HGNC Symbol;Acc:HGNC:8076] |
| ENSG00000144635 | HGLibA_14242 | 96 | -0.193302366 | DYNC1LI1 | dynein cytoplasmic 1 light intermediate chain 1 [Source:HGNC Symbol;Acc:HGNC:18745] |
| ENSG00000144837 | HGLibA_36865 | 96 | -0.193302366 | PLA1A | phospholipase A1 member A [Source:HGNC Symbol;Acc:HGNC:17661] |
| ENSG00000146776 | HGLibA_03852 | 96 | -0.193302366 | ATXN7L1 | ataxin 7 like 1 [Source:HGNC Symbol;Acc:HGNC:22210] |
| ENSG00000147065 | HGLibA_30233 | 96 | -0.193302366 | MSN | moesin [Source:HGNC Symbol;Acc:HGNC:7373] |
| ENSG00000149212 | HGLibA_43626 | 96 | -0.193302366 | SESN3 | sestrin 3 [Source:HGNC Symbol;Acc:HGNC:23060] |
| ENSG00000150676 | HGLibA_07885 | 96 | -0.193302366 | CCDC83 | coiled-coil domain containing 83 [Source:HGNC Symbol;Acc:HGNC:28535] |
| ENSG00000150768 | HGLibA_13310 | 96 | -0.193302366 | DLAT | dihydrolipoamide S-acetyltransferase [Source:HGNC Symbol;Acc:HGNC:2896] |
| ENSG00000151388 | HGLibA_00796 | 96 | -0.193302366 | ADAMTS12 | ADAM metallopeptidase with thrombospondin type 1 motif 12 [Source:HGNC Symbol;Acc:HGNC:14605] |
| ENSG00000152795 | HGLibA_21817 | 96 | -0.193302366 | HNRNPDL | heterogeneous nuclear ribonucleoprotein D like [Source:HGNC Symbol;Acc:HGNC:5037] |
| ENSG00000154027 | HGLibA_01388 | 96 | -0.193302366 | AK5 | adenylate kinase 5 [Source:HGNC Symbol;Acc:HGNC:365] |
| ENSG00000155052 | HGLibA_10433 | 96 | -0.193302366 | CNTNAP5 | contactin associated protein like 5 [Source:HGNC Symbol;Acc:HGNC:18748] |
| ENSG00000157211 | HGLibA_08627 | 96 | -0.193302366 | CDCP2 | CUB domain containing protein 2 [Source:HGNC Symbol;Acc:HGNC:27297] |
| ENSG00000157617 | HGLibA_06085 | 96 | -0.193302366 | C2CD2 | C2 calcium dependent domain containing 2 [Source:HGNC Symbol;Acc:HGNC:1266] |
| ENSG00000163159 | HGLibA_53815 | 96 | -0.193302366 | VPS72 | vacuolar protein sorting 72 homolog [Source:HGNC Symbol;Acc:HGNC:11644] |
| ENSG00000163568 | HGLibA_01351 | 96 | -0.193302366 | AIM2 | absent in melanoma 2 [Source:HGNC Symbol;Acc:HGNC:357] |
| ENSG00000163930 | HGLibA_04153 | 96 | -0.193302366 | BAP1 | BRCA1 associated protein 1 [Source:HGNC Symbol;Acc:HGNC:950] |
| ENSG00000163958 | HGLibA_55094 | 96 | -0.193302366 | ZDHHC19 | zinc finger DHHC-type containing 19 [Source:HGNC Symbol;Acc:HGNC:20713] |
| ENSG00000164022 | HGLibA_01355 | 96 | -0.193302366 | AIMP1 | aminoacyl tRNA synthetase complex interacting multifunctional protein 1 [Source:HGNC Symbol;Acc:HGNC:10648] |
| ENSG00000164663 | HGLibA_53385 | 96 | -0.193302366 | USP49 | ubiquitin specific peptidase 49 [Source:HGNC Symbol;Acc:HGNC:20078] |
| ENSG00000164715 | HGLibA_26726 | 96 | -0.193302366 | LMTK2 | lemur tyrosine kinase 2 [Source:HGNC Symbol;Acc:HGNC:17880] |
| ENSG00000164823 | HGLibA_34691 | 96 | -0.193302366 | OSGIN2 | oxidative stress induced growth inhibitor family member 2 [Source:HGNC Symbol;Acc:HGNC:1355] |
| ENSG00000165138 | HGLibA_02161 | 96 | -0.193302366 | ANKS6 | ankyrin repeat and sterile alpha motif domain containing 6 [Source:HGNC Symbol;Acc:HGNC:26724] |
| ENSG00000165202 | HGLibA_33709 | 96 | -0.193302366 | OR1Q1 | olfactory receptor family 1 subfamily Q member 1 [Source:HGNC Symbol;Acc:HGNC:8223] |
| ENSG00000165606 | HGLibA_13980 | 96 | -0.193302366 | DRGX | dorsal root ganglia homeobox [Source:HGNC Symbol;Acc:HGNC:21536] |
| ENSG00000165678 | HGLibA_19038 | 96 | -0.193302366 | GHITM | growth hormone inducible transmembrane protein [Source:HGNC Symbol;Acc:HGNC:17281] |
| ENSG00000165863 | HGLibA_05098 | 96 | -0.193302366 | C10orf82 | chromosome 10 open reading frame 82 [Source:HGNC Symbol;Acc:HGNC:28500] |
| ENSG00000166024 | HGLibA_39770 | 96 | -0.193302366 | R3HCC1L | R3H domain and coiled-coil containing 1 like [Source:HGNC Symbol;Acc:HGNC:23512] |
| ENSG00000166118 | HGLibA_46442 | 96 | -0.193302366 | SPATA19 | spermatogenesis associated 19 [Source:HGNC Symbol;Acc:HGNC:30614] |
| ENSG00000166278 | HGLibA_05978 | 96 | -0.193302366 | C2 | complement C2 [Source:HGNC Symbol;Acc:HGNC:1248] |
| ENSG00000166546 | HGLibA_04392 | 96 | -0.193302366 | BEAN1 | brain expressed associated with NEDD4 1 [Source:HGNC Symbol;Acc:HGNC:24160] |
| ENSG00000167461 | HGLibA_39984 | 96 | -0.193302366 | RAB8A | member RAS oncogene family [Source:HGNC Symbol;Acc:HGNC:7007]" |
| ENSG00000168096 | HGLibA_02155 | 96 | -0.193302366 | ANKS3 | ankyrin repeat and sterile alpha motif domain containing 3 [Source:HGNC Symbol;Acc:HGNC:29422] |
| ENSG00000168404 | HGLibA_29389 | 96 | -0.193302366 | MLKL | mixed lineage kinase domain like pseudokinase [Source:HGNC Symbol;Acc:HGNC:26617] |
| ENSG00000168621 | HGLibA_18888 | 96 | -0.193302366 | GDNF | glial cell derived neurotrophic factor [Source:HGNC Symbol;Acc:HGNC:4232] |
| ENSG00000168807 | HGLibA_46004 | 96 | -0.193302366 | SNTB2 | syntrophin beta 2 [Source:HGNC Symbol;Acc:HGNC:11169] |
| ENSG00000168894 | HGLibA_41584 | 96 | -0.193302366 | RNF181 | ring finger protein 181 [Source:HGNC Symbol;Acc:HGNC:28037] |
| ENSG00000168952 | HGLibA_47682 | 96 | -0.193302366 | STXBP6 | syntaxin binding protein 6 [Source:HGNC Symbol;Acc:HGNC:19666] |
| ENSG00000169604 | HGLibA_02212 | 96 | -0.193302366 | ANTXR1 | ANTXR cell adhesion molecule 1 [Source:HGNC Symbol;Acc:HGNC:21014] |
| ENSG00000170266 | HGLibA_19217 | 96 | -0.193302366 | GLB1 | galactosidase beta 1 [Source:HGNC Symbol;Acc:HGNC:4298] |
| ENSG00000170509 | HGLibA_22181 | 96 | -0.193302366 | HSD17B13 | hydroxysteroid 17-beta dehydrogenase 13 [Source:HGNC Symbol;Acc:HGNC:18685] |
| ENSG00000170775 | HGLibA_20032 | 96 | -0.193302366 | GPR37 | G protein-coupled receptor 37 [Source:HGNC Symbol;Acc:HGNC:4494] |
| ENSG00000170777 | HGLibA_51121 | 96 | -0.193302366 | TPD52L3 | TPD52 like 3 [Source:HGNC Symbol;Acc:HGNC:23382] |
| ENSG00000171444 | HGLibA_28562 | 96 | -0.193302366 | MCC | WNT signaling pathway regulator [Source:HGNC Symbol;Acc:HGNC:6935]" |
| ENSG00000171812 | HGLibA_10618 | 96 | -0.193302366 | COL8A2 | collagen type VIII alpha 2 chain [Source:HGNC Symbol;Acc:HGNC:2216] |
| ENSG00000171860 | HGLibA_06205 | 96 | -0.193302366 | C3AR1 | complement C3a receptor 1 [Source:HGNC Symbol;Acc:HGNC:1319] |
| ENSG00000171943 | HGLibA_46975 | 96 | -0.193302366 | SRGAP2C | SLIT-ROBO Rho GTPase activating protein 2C [Source:HGNC Symbol;Acc:HGNC:30584] |
| ENSG00000172179 | HGLibA_38596 | 96 | -0.193302366 | PRL | prolactin [Source:HGNC Symbol;Acc:HGNC:9445] |
| ENSG00000172915 | HGLibA_31205 | 96 | -0.193302366 | NBEA | neurobeachin [Source:HGNC Symbol;Acc:HGNC:7648] |
| ENSG00000175182 | HGLibA_16104 | 96 | -0.193302366 | FAM131A | family with sequence similarity 131 member A [Source:HGNC Symbol;Acc:HGNC:28308] |
| ENSG00000175356 | HGLibA_43073 | 96 | -0.193302366 | SCUBE2 | CUB domain and EGF like domain containing 2 [Source:HGNC Symbol;Acc:HGNC:30425]" |
| ENSG00000175793 | HGLibA_43726 | 96 | -0.193302366 | SFN | stratifin [Source:HGNC Symbol;Acc:HGNC:10773] |
| ENSG00000175931 | HGLibA_52643 | 96 | -0.193302366 | UBE2O | ubiquitin conjugating enzyme E2 O [Source:HGNC Symbol;Acc:HGNC:29554] |
| ENSG00000176463 | HGLibA_45465 | 96 | -0.193302366 | SLCO3A1 | solute carrier organic anion transporter family member 3A1 [Source:HGNC Symbol;Acc:HGNC:10952] |
| ENSG00000178295 | HGLibA_18930 | 96 | -0.193302366 | GEN1 | Holliday junction 5' flap endonuclease [Source:HGNC Symbol;Acc:HGNC:26881]" |
| ENSG00000178828 | HGLibA_41596 | 96 | -0.193302366 | RNF186 | ring finger protein 186 [Source:HGNC Symbol;Acc:HGNC:25978] |
| ENSG00000178904 | HGLibA_13910 | 96 | -0.193302366 | DPY19L3 | dpy-19 like C-mannosyltransferase 3 [Source:HGNC Symbol;Acc:HGNC:27120] |
| ENSG00000179468 | HGLibA_34562 | 96 | -0.193302366 | OR9A2 | olfactory receptor family 9 subfamily A member 2 [Source:HGNC Symbol;Acc:HGNC:15093] |
| ENSG00000179477 | HGLibA_01678 | 96 | -0.193302366 | ALOX12B | 12R type [Source:HGNC Symbol;Acc:HGNC:430]" |
| ENSG00000179750 | HGLibA_02479 | 96 | -0.193302366 | APOBEC3B | apolipoprotein B mRNA editing enzyme catalytic subunit 3B [Source:HGNC Symbol;Acc:HGNC:17352] |
| ENSG00000182197 | HGLibA_15801 | 96 | -0.193302366 | EXT1 | exostosin glycosyltransferase 1 [Source:HGNC Symbol;Acc:HGNC:3512] |
| ENSG00000183795 | HGLibA_04746 | 96 | -0.193302366 | BPY2B | basic charge Y-linked 2B [Source:HGNC Symbol;Acc:HGNC:25449] |
| ENSG00000184205 | HGLibA_51981 | 96 | -0.193302366 | TSPYL2 | TSPY like 2 [Source:HGNC Symbol;Acc:HGNC:24358] |
| ENSG00000185104 | HGLibA_15946 | 96 | -0.193302366 | FAF1 | Fas associated factor 1 [Source:HGNC Symbol;Acc:HGNC:3578] |
| ENSG00000185220 | HGLibA_36218 | 96 | -0.193302366 | PGBD2 | piggyBac transposable element derived 2 [Source:HGNC Symbol;Acc:HGNC:19399] |
| ENSG00000185261 | HGLibA_24656 | 96 | -0.193302366 | KIAA0825 | KIAA0825 [Source:HGNC Symbol;Acc:HGNC:28532] |
| ENSG00000185332 | HGLibA_49851 | 96 | -0.193302366 | TMEM105 | TMEM105 long non-coding RNA [Source:HGNC Symbol;Acc:HGNC:26794] |
| ENSG00000185339 | HGLibA_48824 | 96 | -0.193302366 | TCN2 | transcobalamin 2 [Source:HGNC Symbol;Acc:HGNC:11653] |
| ENSG00000185608 | HGLibA_29979 | 96 | -0.193302366 | MRPL40 | mitochondrial ribosomal protein L40 [Source:HGNC Symbol;Acc:HGNC:14491] |
| ENSG00000186049 | HGLibA_25530 | 96 | -0.193302366 | KRT73 | keratin 73 [Source:HGNC Symbol;Acc:HGNC:28928] |
| ENSG00000186092 | HGLibA_34009 | 96 | -0.193302366 | OR4F5 | olfactory receptor family 4 subfamily F member 5 [Source:HGNC Symbol;Acc:HGNC:14825] |

| | | | | | |
|---|---|---|---|---|---|
| ENSG00000186187 | HGLibA_56937 | 96 | -0.193302366 | ZNRF1 | zinc and ring finger 1 [Source:HGNC Symbol;Acc:HGNC:18452] |
| ENSG00000186529 | HGLibA_12194 | 96 | -0.193302366 | CYP4F3 | cytochrome P450 family 4 subfamily F member 3 [Source:HGNC Symbol;Acc:HGNC:2646] |
| ENSG00000186654 | HGLibA_38843 | 96 | -0.193302366 | PRR5 | proline rich 5 [Source:HGNC Symbol;Acc:HGNC:31682] |
| ENSG00000186891 | HGLibA_50767 | 96 | -0.193302366 | TNFRSF18 | TNF receptor superfamily member 18 [Source:HGNC Symbol;Acc:HGNC:11914] |
| ENSG00000187720 | HGLibA_49402 | 96 | -0.193302366 | THSD4 | thrombospondin type 1 domain containing 4 [Source:HGNC Symbol;Acc:HGNC:25835] |
| ENSG00000188452 | HGLibA_09197 | 96 | -0.193302366 | CERKL | ceramide kinase like [Source:HGNC Symbol;Acc:HGNC:21699] |
| ENSG00000188910 | HGLibA_19163 | 96 | -0.193302366 | GJB3 | gap junction protein beta 3 [Source:HGNC Symbol;Acc:HGNC:4285] |
| ENSG00000189377 | HGLibA_11862 | 96 | -0.193302366 | CXCL17 | C-X-C motif chemokine ligand 17 [Source:HGNC Symbol;Acc:HGNC:19232] |
| ENSG00000196184 | HGLibA_33521 | 96 | -0.193302366 | OR10J1 | olfactory receptor family 10 subfamily J member 1 [Source:HGNC Symbol;Acc:HGNC:8175] |
| ENSG00000196456 | HGLibA_56718 | 96 | -0.193302366 | ZNF775 | zinc finger protein 775 [Source:HGNC Symbol;Acc:HGNC:28501] |
| ENSG00000196591 | HGLibA_21006 | 96 | -0.193302366 | HDAC2 | histone deacetylase 2 [Source:HGNC Symbol;Acc:HGNC:4853] |
| ENSG00000197122 | HGLibA_46927 | 96 | -0.193302366 | SRC | non-receptor tyrosine kinase [Source:HGNC Symbol;Acc:HGNC:11283]" |
| ENSG00000197993 | HGLibA_24552 | 96 | -0.193302366 | KEL | Kell metallo-endopeptidase (Kell blood group) [Source:HGNC Symbol;Acc:HGNC:6308] |
| ENSG00000198791 | HGLibA_10349 | 96 | -0.193302366 | CNOT7 | CCR4-NOT transcription complex subunit 7 [Source:HGNC Symbol;Acc:HGNC:14101] |
| ENSG00000198798 | HGLibA_27947 | 96 | -0.193302366 | MAGEB3 | MAGE family member B3 [Source:HGNC Symbol;Acc:HGNC:6810] |
| ENSG00000204977 | HGLibA_51409 | 96 | -0.193302366 | TRIM13 | tripartite motif containing 13 [Source:HGNC Symbol;Acc:HGNC:9976] |
| ENSG00000204979 | HGLibA_30136 | 96 | -0.193302366 | MS4A13 | membrane spanning 4-domains A13 [Source:HGNC Symbol;Acc:HGNC:16674] |
| ENSG00000205754 | HGLibA_45454 | 96 | -0.193302366 | SLCO1B7 | solute carrier organic anion transporter family member 1B7 (putative) [Source:HGNC Symbol;Acc:HGNC:32934] |
| ENSG00000205810 | HGLibA_25291 | 96 | -0.193302366 | KLRC3 | killer cell lectin like receptor C3 [Source:HGNC Symbol;Acc:HGNC:6376] |
| ENSG00000212725 | HGLibA_25687 | 96 | -0.193302366 | KRTAP2-1 | keratin associated protein 2-1 [Source:HGNC Symbol;Acc:HGNC:16775] |
| ENSG00000213047 | HGLibA_12932 | 96 | -0.193302366 | DENND1B | DENN domain containing 1B [Source:HGNC Symbol;Acc:HGNC:28404] |
| ENSG00000215183 | HGLibA_30230 | 96 | -0.193302366 | MSMP | prostate associated [Source:HGNC Symbol;Acc:HGNC:29663]" |
| ENSG00000215784 | HGLibA_16712 | 96 | -0.193302366 | FAM72D | family with sequence similarity 72 member D [Source:HGNC Symbol;Acc:HGNC:33593] |
| ENSG00000221829 | HGLibA_16852 | 96 | -0.193302366 | FANCG | FA complementation group G [Source:HGNC Symbol;Acc:HGNC:3588] |
| ENSG00000240747 | HGLibA_25393 | 96 | -0.193302366 | KRBOX1 | KRAB box domain containing 1 [Source:HGNC Symbol;Acc:HGNC:38708] |
| ENSG00000241404 | HGLibA_14630 | 96 | -0.193302366 | EGFL8 | EGF like domain multiple 8 [Source:HGNC Symbol;Acc:HGNC:13944] |
| ENSG00000242259 | HGLibA_06073 | 96 | -0.193302366 | C22orf39 | chromosome 22 open reading frame 39 [Source:HGNC Symbol;Acc:HGNC:27012] |
| ENSG00000242515 | HGLibA_52880 | 96 | -0.193302366 | UGT1A10 | UDP glucuronosyltransferase family 1 member A10 [Source:HGNC Symbol;Acc:HGNC:12531] |
| ENSG00000248712 | HGLibA_07651 | 96 | -0.193302366 | CCDC153 | coiled-coil domain containing 153 [Source:HGNC Symbol;Acc:HGNC:27446] |
| ENSG00000249751 | HGLibA_14418 | 96 | -0.193302366 | ECSCR | endothelial cell surface expressed chemotaxis and apoptosis regulator [Source:HGNC Symbol;Acc:HGNC:35454] |
| ENSG00000254726 | HGLibA_29028 | 96 | -0.193302366 | MEX3A | mex-3 RNA binding family member A [Source:HGNC Symbol;Acc:HGNC:33482] |
| ENSG00000260691 | HGLibA_02028 | 96 | -0.193302366 | ANKRD20A1 | ankyrin repeat domain 20 family member A1 [Source:HGNC Symbol;Acc:HGNC:23665] |
| ENSG00000277363 | HGLibA_46935 | 96 | -0.193302366 | SRCIN1 | SRC kinase signaling inhibitor 1 [Source:HGNC Symbol;Acc:HGNC:29506] |
| ENSG00000278318 | HGLibA_55631 | 96 | -0.193302366 | ZNF229 | zinc finger protein 229 [Source:HGNC Symbol;Acc:HGNC:13022] |
| FAM155A | HGLibA_16180 | 96 | -0.193302366 | FAM155A | - |
| FAM21A | HGLibA_16504 | 96 | -0.193302366 | FAM21A | - |
| HIST1H3E | HGLibA_21467 | 96 | -0.193302366 | HIST1H3E | - |
| hsa-let-7g | HGLibA_57135 | 96 | -0.193302366 | hsa-let-7g | - |
| hsa-mir-1260a | HGLibA_57458 | 96 | -0.193302366 | hsa-mir-1260a | - |
| hsa-mir-1281 | HGLibA_57581 | 96 | -0.193302366 | hsa-mir-1281 | - |
| hsa-mir-182 | HGLibA_57984 | 96 | -0.193302366 | hsa-mir-182 | - |
| hsa-mir-202 | HGLibA_58164 | 96 | -0.193302366 | hsa-mir-202 | - |
| hsa-mir-205 | HGLibA_58178 | 96 | -0.193302366 | hsa-mir-205 | - |
| hsa-mir-3177 | HGLibA_58783 | 96 | -0.193302366 | hsa-mir-3177 | - |
| hsa-mir-3605 | HGLibA_59055 | 96 | -0.193302366 | hsa-mir-3605 | - |
| hsa-mir-3690-1 | HGLibA_59345 | 96 | -0.193302366 | hsa-mir-3690-1 | - |
| hsa-mir-382 | HGLibA_59482 | 96 | -0.193302366 | hsa-mir-382 | - |
| hsa-mir-3910-1 | HGLibA_59504 | 96 | -0.193302366 | hsa-mir-3910-1 | - |
| hsa-mir-4254 | HGLibA_59724 | 96 | -0.193302366 | hsa-mir-4254 | - |
| hsa-mir-4324 | HGLibA_60018 | 96 | -0.193302366 | hsa-mir-4324 | - |
| hsa-mir-4429 | HGLibA_60102 | 96 | -0.193302366 | hsa-mir-4429 | - |
| hsa-mir-4432 | HGLibA_60113 | 96 | -0.193302366 | hsa-mir-4432 | - |
| hsa-mir-4513 | HGLibA_60494 | 96 | -0.193302366 | hsa-mir-4513 | - |
| hsa-mir-4520a | HGLibA_60533 | 96 | -0.193302366 | hsa-mir-4520a | - |
| hsa-mir-455 | HGLibA_60628 | 96 | -0.193302366 | hsa-mir-455 | - |
| hsa-mir-4649 | HGLibA_60700 | 96 | -0.193302366 | hsa-mir-4649 | - |
| hsa-mir-4689 | HGLibA_60868 | 96 | -0.193302366 | hsa-mir-4689 | - |
| hsa-mir-483 | HGLibA_61324 | 96 | -0.193302366 | hsa-mir-483 | - |
| hsa-mir-544b | HGLibA_61830 | 96 | -0.193302366 | hsa-mir-544b | - |
| hsa-mir-548ab | HGLibA_61857 | 96 | -0.193302366 | hsa-mir-548ab | - |
| hsa-mir-6084 | HGLibA_62596 | 96 | -0.193302366 | hsa-mir-6084 | - |
| hsa-mir-659 | HGLibA_62968 | 96 | -0.193302366 | hsa-mir-659 | - |
| hsa-mir-6789 | HGLibA_63333 | 96 | -0.193302366 | hsa-mir-6789 | - |
| hsa-mir-6801 | HGLibA_63383 | 96 | -0.193302366 | hsa-mir-6801 | - |
| hsa-mir-761 | HGLibA_63904 | 96 | -0.193302366 | hsa-mir-761 | - |
| LILRA3 | HGLibA_26495 | 96 | -0.193302366 | LILRA3 | - |
| LOC256021 | HGLibA_26907 | 96 | -0.193302366 | LOC256021 | - |
| LOC643669 | HGLibA_26968 | 96 | -0.193302366 | LOC643669 | - |
| LRRC16B | HGLibA_27267 | 96 | -0.193302366 | LRRC16B | - |
| LYRM5 | HGLibA_27754 | 96 | -0.193302366 | LYRM5 | - |
| OR5R1 | HGLibA_34352 | 96 | -0.193302366 | OR5R1 | - |
| TGIF2-C20orf24 | HGLibA_49245 | 96 | -0.193302366 | TGIF2-C20orf24 | - |
| TMEM189 | HGLibA_50131 | 96 | -0.193302366 | TMEM189 | - |
| ATP5B | HGLibA_03642 | 95 | -0.203253403 | ATP5B | - |
| C22orf24 | HGLibA_06059 | 95 | -0.203253403 | C22orf24 | - |
| C3orf27 | HGLibA_06223 | 95 | -0.203253403 | C3orf27 | - |
| C6orf203 | HGLibA_06469 | 95 | -0.203253403 | C6orf203 | - |
| CCDC36 | HGLibA_07763 | 95 | -0.203253403 | CCDC36 | - |
| ENSG00000011295 | HGLibA_52065 | 95 | -0.203253403 | TTC19 | tetratricopeptide repeat domain 19 [Source:HGNC Symbol;Acc:HGNC:26006] |
| ENSG00000027001 | HGLibA_29293 | 95 | -0.203253403 | MIPEP | mitochondrial intermediate peptidase [Source:HGNC Symbol;Acc:HGNC:7104] |
| ENSG00000036257 | HGLibA_11778 | 95 | -0.203253403 | CUL3 | cullin 3 [Source:HGNC Symbol;Acc:HGNC:2553] |
| ENSG00000047230 | HGLibA_11672 | 95 | -0.203253403 | CTPS2 | CTP synthase 2 [Source:HGNC Symbol;Acc:HGNC:2520] |
| ENSG00000054282 | HGLibA_43117 | 95 | -0.203253403 | SDCCAG8 | serologically defined colon cancer antigen 8 [Source:HGNC Symbol;Acc:HGNC:10671] |
| ENSG00000058272 | HGLibA_38021 | 95 | -0.203253403 | PPP1R12A | protein phosphatase 1 regulatory subunit 12A [Source:HGNC Symbol;Acc:HGNC:7618] |
| ENSG00000064201 | HGLibA_51931 | 95 | -0.203253403 | TSPAN32 | tetraspanin 32 [Source:HGNC Symbol;Acc:HGNC:13410] |
| ENSG00000066926 | HGLibA_17271 | 95 | -0.203253403 | FECH | ferrochelatase [Source:HGNC Symbol;Acc:HGNC:3647] |
| ENSG00000067445 | HGLibA_51716 | 95 | -0.203253403 | TRO | trophinin [Source:HGNC Symbol;Acc:HGNC:12326] |
| ENSG00000068971 | HGLibA_38173 | 95 | -0.203253403 | PPP2R5B | protein phosphatase 2 regulatory subunit B'beta [Source:HGNC Symbol;Acc:HGNC:9310] |
| ENSG00000069667 | HGLibA_41775 | 95 | -0.203253403 | RORA | RAR related orphan receptor A [Source:HGNC Symbol;Acc:HGNC:10258] |
| ENSG00000070770 | HGLibA_11404 | 95 | -0.203253403 | CSNK2A2 | casein kinase 2 alpha 2 [Source:HGNC Symbol;Acc:HGNC:2459] |
| ENSG00000073050 | HGLibA_54583 | 95 | -0.203253403 | XRCC1 | X-ray repair cross complementing 1 [Source:HGNC Symbol;Acc:HGNC:12828] |
| ENSG00000083457 | HGLibA_23754 | 95 | -0.203253403 | ITGAE | integrin subunit alpha E [Source:HGNC Symbol;Acc:HGNC:6147] |
| ENSG00000085491 | HGLibA_44681 | 95 | -0.203253403 | SLC25A24 | solute carrier family 25 member 24 [Source:HGNC Symbol;Acc:HGNC:20662] |
| ENSG00000086848 | HGLibA_01639 | 95 | -0.203253403 | ALG9 | 2-mannosyltransferase [Source:HGNC Symbol;Acc:HGNC:15672]" |
| ENSG00000089127 | HGLibA_33199 | 95 | -0.203253403 | OAS1 | 2'-5'-oligoadenylate synthetase 1 [Source:HGNC Symbol;Acc:HGNC:8086] |
| ENSG00000095539 | HGLibA_43355 | 95 | -0.203253403 | SEMA4G | semaphorin 4G [Source:HGNC Symbol;Acc:HGNC:10735] |
| ENSG00000099204 | HGLibA_00309 | 95 | -0.203253403 | ABLIM1 | actin binding LIM protein 1 [Source:HGNC Symbol;Acc:HGNC:78] |
| ENSG00000099954 | HGLibA_08977 | 95 | -0.203253403 | CECR2 | histone acetyl-lysine reader [Source:HGNC Symbol;Acc:HGNC:1840]" |
| ENSG00000100038 | HGLibA_51002 | 95 | -0.203253403 | TOP3B | DNA topoisomerase III beta [Source:HGNC Symbol;Acc:HGNC:11993] |
| ENSG00000100150 | HGLibA_12984 | 95 | -0.203253403 | DEPDC5 | DEP domain containing 5 [Source:HGNC Symbol;Acc:HGNC:18423] |
| ENSG00000101577 | HGLibA_27098 | 95 | -0.203253403 | LPIN2 | lipin 2 [Source:HGNC Symbol;Acc:HGNC:14450] |
| ENSG00000102241 | HGLibA_22349 | 95 | -0.203253403 | HTATSF1 | HIV-1 Tat specific factor 1 [Source:HGNC Symbol;Acc:HGNC:5276] |
| ENSG00000104499 | HGLibA_19413 | 95 | -0.203253403 | GML | glycosylphosphatidylinositol anchored molecule like [Source:HGNC Symbol;Acc:HGNC:4375] |
| ENSG00000105447 | HGLibA_20386 | 95 | -0.203253403 | GRWD1 | glutamate rich WD repeat containing 1 [Source:HGNC Symbol;Acc:HGNC:21270] |
| ENSG00000105514 | HGLibA_39916 | 95 | -0.203253403 | RAB3D | member RAS oncogene family [Source:HGNC Symbol;Acc:HGNC:9779]" |
| ENSG00000105664 | HGLibA_10684 | 95 | -0.203253403 | COMP | cartilage oligomeric matrix protein [Source:HGNC Symbol;Acc:HGNC:2227] |
| ENSG00000105707 | HGLibA_22036 | 95 | -0.203253403 | HPN | hepsin [Source:HGNC Symbol;Acc:HGNC:5155] |
| ENSG00000105879 | HGLibA_07403 | 95 | -0.203253403 | CBLL1 | Cbl proto-oncogene like 1 [Source:HGNC Symbol;Acc:HGNC:21225] |
| ENSG00000106178 | HGLibA_08004 | 95 | -0.203253403 | CCL24 | C-C motif chemokine ligand 24 [Source:HGNC Symbol;Acc:HGNC:10623] |
| ENSG00000106236 | HGLibA_32535 | 95 | -0.203253403 | NPTX2 | neuronal pentraxin 2 [Source:HGNC Symbol;Acc:HGNC:7953] |
| ENSG00000106565 | HGLibA_50075 | 95 | -0.203253403 | TMEM176B | transmembrane protein 176B [Source:HGNC Symbol;Acc:HGNC:29596] |
| ENSG00000106714 | HGLibA_10424 | 95 | -0.203253403 | CNTNAP3 | contactin associated protein like 3 [Source:HGNC Symbol;Acc:HGNC:13834] |
| ENSG00000106823 | HGLibA_14414 | 95 | -0.203253403 | ECM2 | extracellular matrix protein 2 [Source:HGNC Symbol;Acc:HGNC:3154] |
| ENSG00000107372 | HGLibA_55158 | 95 | -0.203253403 | ZFAND5 | zinc finger AN1-type containing 5 [Source:HGNC Symbol;Acc:HGNC:13008] |
| ENSG00000108405 | HGLibA_34846 | 95 | -0.203253403 | P2RX1 | purinergic receptor P2X 1 [Source:HGNC Symbol;Acc:HGNC:8533] |
| ENSG00000109674 | HGLibA_31634 | 95 | -0.203253403 | NEIL3 | nei like DNA glycosylase 3 [Source:HGNC Symbol;Acc:HGNC:24573] |
| ENSG00000110871 | HGLibA_10759 | 95 | -0.203253403 | COQ5 | methyltransferase [Source:HGNC Symbol;Acc:HGNC:28722]" |
| ENSG00000111371 | HGLibA_45054 | 95 | -0.203253403 | SLC38A1 | solute carrier family 38 member 1 [Source:HGNC Symbol;Acc:HGNC:13447] |
| ENSG00000111537 | HGLibA_22708 | 95 | -0.203253403 | IFNG | interferon gamma [Source:HGNC Symbol;Acc:HGNC:5438] |
| ENSG00000112337 | HGLibA_44463 | 95 | -0.203253403 | SLC17A2 | solute carrier family 17 member 2 [Source:HGNC Symbol;Acc:HGNC:10930] |
| ENSG00000112419 | HGLibA_36300 | 95 | -0.203253403 | PHACTR2 | phosphatase and actin regulator 2 [Source:HGNC Symbol;Acc:HGNC:20956] |
| ENSG00000113068 | HGLibA_36138 | 95 | -0.203253403 | PFDN1 | prefoldin subunit 1 [Source:HGNC Symbol;Acc:HGNC:8866] |
| ENSG00000113734 | HGLibA_04650 | 95 | -0.203253403 | BNIP1 | BCL2 interacting protein 1 [Source:HGNC Symbol;Acc:HGNC:1082] |
| ENSG00000115761 | HGLibA_32257 | 95 | -0.203253403 | NOL10 | nucleolar protein 10 [Source:HGNC Symbol;Acc:HGNC:25862] |
| ENSG00000116521 | HGLibA_42841 | 95 | -0.203253403 | SCAMP3 | secretory carrier membrane protein 3 [Source:HGNC Symbol;Acc:HGNC:10565] |
| ENSG00000116761 | HGLibA_11627 | 95 | -0.203253403 | CTH | cystathionine gamma-lyase [Source:HGNC Symbol;Acc:HGNC:2501] |
| ENSG00000116819 | HGLibA_49134 | 95 | -0.203253403 | TFAP2E | transcription factor AP-2 epsilon [Source:HGNC Symbol;Acc:HGNC:30774] |
| ENSG00000116833 | HGLibA_32638 | 95 | -0.203253403 | NR5A2 | nuclear receptor subfamily 5 group A member 2 [Source:HGNC Symbol;Acc:HGNC:7984] |
| ENSG00000117305 | HGLibA_21702 | 95 | -0.203253403 | HMGCL | 3-hydroxy-3-methylglutaryl-CoA lyase [Source:HGNC Symbol;Acc:HGNC:5005] |
| ENSG00000117984 | HGLibA_11697 | 95 | -0.203253403 | CTSD | cathepsin D [Source:HGNC Symbol;Acc:HGNC:2529] |
| ENSG00000121075 | HGLibA_48670 | 95 | -0.203253403 | TBX4 | T-box 4 [Source:HGNC Symbol;Acc:HGNC:11603] |
| ENSG00000122390 | HGLibA_31003 | 95 | -0.203253403 | NAA60 | NatF catalytic subunit [Source:HGNC Symbol;Acc:HGNC:25875]" |
| ENSG00000122694 | HGLibA_19268 | 95 | -0.203253403 | GLIPR2 | GLI pathogenesis related 2 [Source:HGNC Symbol;Acc:HGNC:18007] |
| ENSG00000122756 | HGLibA_10395 | 95 | -0.203253403 | CNTFR | ciliary neurotrophic factor receptor [Source:HGNC Symbol;Acc:HGNC:2170] |
| ENSG00000125207 | HGLibA_36771 | 95 | -0.203253403 | PIWIL1 | piwi like RNA-mediated gene silencing 1 [Source:HGNC Symbol;Acc:HGNC:9007] |

| | | | | | |
|---|---|---|---|---|---|
| ENSG00000125868 | HGLibA_14046 | 95 | -0.203253403 | DSTN | actin depolymerizing factor [Source:HGNC Symbol;Acc:HGNC:15750]" |
| ENSG00000125901 | HGLibA_30077 | 95 | -0.203253403 | MRPS26 | mitochondrial ribosomal protein S26 [Source:HGNC Symbol;Acc:HGNC:14045] |
| ENSG00000127084 | HGLibA_17348 | 95 | -0.203253403 | FGD3 | RhoGEF and PH domain containing 3 [Source:HGNC Symbol;Acc:HGNC:16027]" |
| ENSG00000127124 | HGLibA_21574 | 95 | -0.203253403 | HIVEP3 | human immunodeficiency virus type I enhancer binding protein 3 [Source:HGNC Symbol;Acc:HGNC:13561] |
| ENSG00000127533 | HGLibA_15872 | 95 | -0.203253403 | F2RL3 | F2R like thrombin or trypsin receptor 3 [Source:HGNC Symbol;Acc:HGNC:3540] |
| ENSG00000128335 | HGLibA_02530 | 95 | -0.203253403 | APOL2 | apolipoprotein L2 [Source:HGNC Symbol;Acc:HGNC:619] |
| ENSG00000128833 | HGLibA_30857 | 95 | -0.203253403 | MYO5C | myosin VC [Source:HGNC Symbol;Acc:HGNC:7604] |
| ENSG00000131473 | HGLibA_00452 | 95 | -0.203253403 | ACLY | ATP citrate lyase [Source:HGNC Symbol;Acc:HGNC:115] |
| ENSG00000131507 | HGLibA_31392 | 95 | -0.203253403 | NDFIP1 | Nedd4 family interacting protein 1 [Source:HGNC Symbol;Acc:HGNC:17592] |
| ENSG00000132464 | HGLibA_15107 | 95 | -0.203253403 | ENAM | enamelin [Source:HGNC Symbol;Acc:HGNC:3344] |
| ENSG00000132471 | HGLibA_53977 | 95 | -0.203253403 | WBP2 | WW domain binding protein 2 [Source:HGNC Symbol;Acc:HGNC:12738] |
| ENSG00000132510 | HGLibA_24535 | 95 | -0.203253403 | KDM6B | lysine demethylase 6B [Source:HGNC Symbol;Acc:HGNC:29012] |
| ENSG00000133636 | HGLibA_32884 | 95 | -0.203253403 | NTS | neurotensin [Source:HGNC Symbol;Acc:HGNC:8038] |
| ENSG00000134152 | HGLibA_24083 | 95 | -0.203253403 | KATNBL1 | katanin regulatory subunit B1 like 1 [Source:HGNC Symbol;Acc:HGNC:26199] |
| ENSG00000134262 | HGLibA_02342 | 95 | -0.203253403 | AP4B1 | adaptor related protein complex 4 subunit beta 1 [Source:HGNC Symbol;Acc:HGNC:572] |
| ENSG00000134852 | HGLibA_10092 | 95 | -0.203253403 | CLOCK | clock circadian regulator [Source:HGNC Symbol;Acc:HGNC:2082] |
| ENSG00000135083 | HGLibA_08112 | 95 | -0.203253403 | CCNJL | cyclin J like [Source:HGNC Symbol;Acc:HGNC:25876] |
| ENSG00000135100 | HGLibA_21775 | 95 | -0.203253403 | HNF1A | HNF1 homeobox A [Source:HGNC Symbol;Acc:HGNC:11621] |
| ENSG00000136750 | HGLibA_18414 | 95 | -0.203253403 | GAD2 | glutamate decarboxylase 2 [Source:HGNC Symbol;Acc:HGNC:4093] |
| ENSG00000136931 | HGLibA_32633 | 95 | -0.203253403 | NR5A1 | nuclear receptor subfamily 5 group A member 1 [Source:HGNC Symbol;Acc:HGNC:7983] |
| ENSG00000137312 | HGLibA_17649 | 95 | -0.203253403 | FLOT1 | flotillin 1 [Source:HGNC Symbol;Acc:HGNC:3757] |
| ENSG00000139624 | HGLibA_09212 | 95 | -0.203253403 | CERS5 | ceramide synthase 5 [Source:HGNC Symbol;Acc:HGNC:23749] |
| ENSG00000140153 | HGLibA_54045 | 95 | -0.203253403 | WDR20 | WD repeat domain 20 [Source:HGNC Symbol;Acc:HGNC:19667] |
| ENSG00000141664 | HGLibA_55028 | 95 | -0.203253403 | ZCCHC2 | zinc finger CCHC-type containing 2 [Source:HGNC Symbol;Acc:HGNC:22916] |
| ENSG00000143179 | HGLibA_52814 | 95 | -0.203253403 | UCK2 | uridine-cytidine kinase 2 [Source:HGNC Symbol;Acc:HGNC:12562] |
| ENSG00000143811 | HGLibA_39676 | 95 | -0.203253403 | PYCR2 | pyrroline-5-carboxylate reductase 2 [Source:HGNC Symbol;Acc:HGNC:30262] |
| ENSG00000144010 | HGLibA_51503 | 95 | -0.203253403 | TRIM43B | tripartite motif containing 43B [Source:HGNC Symbol;Acc:HGNC:37146] |
| ENSG00000144711 | HGLibA_23542 | 95 | -0.203253403 | IQSEC1 | IQ motif and Sec7 domain 1 [Source:HGNC Symbol;Acc:HGNC:29112] |
| ENSG00000145817 | HGLibA_54681 | 95 | -0.203253403 | YIPF5 | Yip1 domain family member 5 [Source:HGNC Symbol;Acc:HGNC:24877] |
| ENSG00000145920 | HGLibA_10936 | 95 | -0.203253403 | CPLX2 | complexin 2 [Source:HGNC Symbol;Acc:HGNC:2310] |
| ENSG00000146090 | HGLibA_40334 | 95 | -0.203253403 | RASGEF1C | RasGEF domain family member 1C [Source:HGNC Symbol;Acc:HGNC:27400] |
| ENSG00000146267 | HGLibA_16929 | 95 | -0.203253403 | FAXC | failed axon connections homolog [Source:HGNC Symbol;Acc:HGNC:20742] |
| ENSG00000146276 | HGLibA_18404 | 95 | -0.203253403 | GABRR1 | gamma-aminobutyric acid type A receptor rho1 subunit [Source:HGNC Symbol;Acc:HGNC:4090] |
| ENSG00000148300 | HGLibA_40900 | 95 | -0.203253403 | REXO4 | 3'-5' exonuclease [Source:HGNC Symbol;Acc:HGNC:12820]" |
| ENSG00000148604 | HGLibA_41046 | 95 | -0.203253403 | RGR | retinal G protein coupled receptor [Source:HGNC Symbol;Acc:HGNC:9990] |
| ENSG00000149488 | HGLibA_49752 | 95 | -0.203253403 | TMC2 | transmembrane channel like 2 [Source:HGNC Symbol;Acc:HGNC:16527] |
| ENSG00000149600 | HGLibA_10674 | 95 | -0.203253403 | COMMD7 | COMM domain containing 7 [Source:HGNC Symbol;Acc:HGNC:16223] |
| ENSG00000151327 | HGLibA_16295 | 95 | -0.203253403 | FAM177A1 | family with sequence similarity 177 member A1 [Source:HGNC Symbol;Acc:HGNC:19829] |
| ENSG00000151552 | HGLibA_39721 | 95 | -0.203253403 | QDPR | quinoid dihydropteridine reductase [Source:HGNC Symbol;Acc:HGNC:9752] |
| ENSG00000152208 | HGLibA_20275 | 95 | -0.203253403 | GRID2 | glutamate ionotropic receptor delta type subunit 2 [Source:HGNC Symbol;Acc:HGNC:4576] |
| ENSG00000152556 | HGLibA_36168 | 95 | -0.203253403 | PFKM | muscle [Source:HGNC Symbol;Acc:HGNC:8877]" |
| ENSG00000153283 | HGLibA_08482 | 95 | -0.203253403 | CD96 | CD96 molecule [Source:HGNC Symbol;Acc:HGNC:16892] |
| ENSG00000154358 | HGLibA_33230 | 95 | -0.203253403 | OBSCN | cytoskeletal calmodulin and titin-interacting RhoGEF [Source:HGNC Symbol;Acc:HGNC:15719]" |
| ENSG00000154678 | HGLibA_35775 | 95 | -0.203253403 | PDE1C | phosphodiesterase 1C [Source:HGNC Symbol;Acc:HGNC:8776] |
| ENSG00000156011 | HGLibA_39018 | 95 | -0.203253403 | PSD3 | pleckstrin and Sec7 domain containing 3 [Source:HGNC Symbol;Acc:HGNC:19093] |
| ENSG00000157551 | HGLibA_24265 | 95 | -0.203253403 | KCNJ15 | potassium voltage-gated channel subfamily J member 15 [Source:HGNC Symbol;Acc:HGNC:6261] |
| ENSG00000158113 | HGLibA_27363 | 95 | -0.203253403 | LRRC43 | leucine rich repeat containing 43 [Source:HGNC Symbol;Acc:HGNC:28562] |
| ENSG00000160959 | HGLibA_27255 | 95 | -0.203253403 | LRRC14 | leucine rich repeat containing 14 [Source:HGNC Symbol;Acc:HGNC:20419] |
| ENSG00000161618 | HGLibA_01535 | 95 | -0.203253403 | ALDH16A1 | aldehyde dehydrogenase 16 family member A1 [Source:HGNC Symbol;Acc:HGNC:28114] |
| ENSG00000162598 | HGLibA_05904 | 95 | -0.203253403 | C1orf87 | chromosome 1 open reading frame 87 [Source:HGNC Symbol;Acc:HGNC:28547] |
| ENSG00000163596 | HGLibA_22491 | 95 | -0.203253403 | ICA1L | islet cell autoantigen 1 like [Source:HGNC Symbol;Acc:HGNC:14442] |
| ENSG00000164024 | HGLibA_28929 | 95 | -0.203253403 | METAP1 | methionyl aminopeptidase 1 [Source:HGNC Symbol;Acc:HGNC:15789] |
| ENSG00000164049 | HGLibA_17140 | 95 | -0.203253403 | FBXW12 | F-box and WD repeat domain containing 12 [Source:HGNC Symbol;Acc:HGNC:20729] |
| ENSG00000164099 | HGLibA_38901 | 95 | -0.203253403 | PRSS12 | serine protease 12 [Source:HGNC Symbol;Acc:HGNC:9477] |
| ENSG00000164332 | HGLibA_52716 | 95 | -0.203253403 | UBLCP1 | ubiquitin like domain containing CTD phosphatase 1 [Source:HGNC Symbol;Acc:HGNC:28110] |
| ENSG00000165621 | HGLibA_34822 | 95 | -0.203253403 | OXGR1 | oxoglutarate receptor 1 [Source:HGNC Symbol;Acc:HGNC:4531] |
| ENSG00000165886 | HGLibA_52764 | 95 | -0.203253403 | UBTD1 | ubiquitin domain containing 1 [Source:HGNC Symbol;Acc:HGNC:25683] |
| ENSG00000165983 | HGLibA_39297 | 95 | -0.203253403 | PTER | phosphotriesterase related [Source:HGNC Symbol;Acc:HGNC:9590] |
| ENSG00000166250 | HGLibA_10071 | 95 | -0.203253403 | CLMP | CXADR like membrane protein [Source:HGNC Symbol;Acc:HGNC:24039] |
| ENSG00000166831 | HGLibA_40657 | 95 | -0.203253403 | RBPMS2 | mRNA processing factor 2 [Source:HGNC Symbol;Acc:HGNC:19098]" |
| ENSG00000167751 | HGLibA_25252 | 95 | -0.203253403 | KLK2 | kallikrein related peptidase 2 [Source:HGNC Symbol;Acc:HGNC:6363] |
| ENSG00000168081 | HGLibA_37400 | 95 | -0.203253403 | PNOC | prepronociceptin [Source:HGNC Symbol;Acc:HGNC:9163] |
| ENSG00000169359 | HGLibA_44935 | 95 | -0.203253403 | SLC33A1 | solute carrier family 33 member 1 [Source:HGNC Symbol;Acc:HGNC:95] |
| ENSG00000169488 | HGLibA_34024 | 95 | -0.203253403 | OR4K15 | olfactory receptor family 4 subfamily K member 15 [Source:HGNC Symbol;Acc:HGNC:15353] |
| ENSG00000169550 | HGLibA_30526 | 95 | -0.203253403 | MUC15 | cell surface associated [Source:HGNC Symbol;Acc:HGNC:14956]" |
| ENSG00000169836 | HGLibA_48177 | 95 | -0.203253403 | TACR3 | tachykinin receptor 3 [Source:HGNC Symbol;Acc:HGNC:11528] |
| ENSG00000169855 | HGLibA_41733 | 95 | -0.203253403 | ROBO1 | roundabout guidance receptor 1 [Source:HGNC Symbol;Acc:HGNC:10249] |
| ENSG00000170128 | HGLibA_20005 | 95 | -0.203253403 | GPR25 | G protein-coupled receptor 25 [Source:HGNC Symbol;Acc:HGNC:4480] |
| ENSG00000170265 | HGLibA_55739 | 95 | -0.203253403 | ZNF282 | zinc finger protein 282 [Source:HGNC Symbol;Acc:HGNC:13076] |
| ENSG00000170925 | HGLibA_49062 | 95 | -0.203253403 | TEX13B | testis expressed 13B [Source:HGNC Symbol;Acc:HGNC:11736] |
| ENSG00000171222 | HGLibA_42851 | 95 | -0.203253403 | SCAND1 | SCAN domain containing 1 [Source:HGNC Symbol;Acc:HGNC:10566] |
| ENSG00000171560 | HGLibA_17337 | 95 | -0.203253403 | FGA | fibrinogen alpha chain [Source:HGNC Symbol;Acc:HGNC:3661] |
| ENSG00000172159 | HGLibA_18010 | 95 | -0.203253403 | FRMD3 | FERM domain containing 3 [Source:HGNC Symbol;Acc:HGNC:24125] |
| ENSG00000172459 | HGLibA_34248 | 95 | -0.203253403 | OR5AR1 | olfactory receptor family 5 subfamily AR member 1 (gene/pseudogene) [Source:HGNC Symbol;Acc:HGNC:15260] |
| ENSG00000173947 | HGLibA_36541 | 95 | -0.203253403 | PIFO | primary cilia formation [Source:HGNC Symbol;Acc:HGNC:27009] |
| ENSG00000174125 | HGLibA_49640 | 95 | -0.203253403 | TLR1 | toll like receptor 1 [Source:HGNC Symbol;Acc:HGNC:11847] |
| ENSG00000174197 | HGLibA_29123 | 95 | -0.203253403 | MGA | MAX dimerization protein [Source:HGNC Symbol;Acc:HGNC:14010]" |
| ENSG00000174339 | HGLibA_33909 | 95 | -0.203253403 | OR2Y1 | olfactory receptor family 2 subfamily Y member 1 [Source:HGNC Symbol;Acc:HGNC:14837] |
| ENSG00000175619 | HGLibA_33936 | 95 | -0.203253403 | OR4B1 | olfactory receptor family 4 subfamily B member 1 [Source:HGNC Symbol;Acc:HGNC:8290] |
| ENSG00000175906 | HGLibA_02971 | 95 | -0.203253403 | ARL4D | ADP ribosylation factor like GTPase 4D [Source:HGNC Symbol;Acc:HGNC:656] |
| ENSG00000176177 | HGLibA_15191 | 95 | -0.203253403 | ENTHD1 | ENTH domain containing 1 [Source:HGNC Symbol;Acc:HGNC:26352] |
| ENSG00000176209 | HGLibA_45744 | 95 | -0.203253403 | SMIM19 | small integral membrane protein 19 [Source:HGNC Symbol;Acc:HGNC:25166] |
| ENSG00000177000 | HGLibA_30389 | 95 | -0.203253403 | MTHFR | methylenetetrahydrofolate reductase [Source:HGNC Symbol;Acc:HGNC:7436] |
| ENSG00000177174 | HGLibA_33633 | 95 | -0.203253403 | OR14C36 | olfactory receptor family 14 subfamily C member 36 [Source:HGNC Symbol;Acc:HGNC:15026] |
| ENSG00000177354 | HGLibA_05090 | 95 | -0.203253403 | C10orf71 | chromosome 10 open reading frame 71 [Source:HGNC Symbol;Acc:HGNC:26973] |
| ENSG00000177946 | HGLibA_09044 | 95 | -0.203253403 | CENPBD1 | CENPB DNA-binding domain containing 1 [Source:HGNC Symbol;Acc:HGNC:28272] |
| ENSG00000178585 | HGLibA_11652 | 95 | -0.203253403 | CTNNBIP1 | catenin beta interacting protein 1 [Source:HGNC Symbol;Acc:HGNC:16913] |
| ENSG00000178974 | HGLibA_17079 | 95 | -0.203253403 | FBXO34 | F-box protein 34 [Source:HGNC Symbol;Acc:HGNC:20201] |
| ENSG00000179172 | HGLibA_21811 | 95 | -0.203253403 | HNRNPCL1 | heterogeneous nuclear ribonucleoprotein C-like 1 [Source:HGNC Symbol;Acc:HGNC:29295] |
| ENSG00000179593 | HGLibA_01686 | 95 | -0.203253403 | ALOX15B | type B [Source:HGNC Symbol;Acc:HGNC:434]" |
| ENSG00000179673 | HGLibA_42056 | 95 | -0.203253403 | RPRML | reprimo like [Source:HGNC Symbol;Acc:HGNC:32422] |
| ENSG00000180934 | HGLibA_34212 | 95 | -0.203253403 | OR56A1 | olfactory receptor family 56 subfamily A member 1 [Source:HGNC Symbol;Acc:HGNC:14781] |
| ENSG00000181577 | HGLibA_06480 | 95 | -0.203253403 | C6orf223 | chromosome 6 open reading frame 223 [Source:HGNC Symbol;Acc:HGNC:28692] |
| ENSG00000182103 | HGLibA_16322 | 95 | -0.203253403 | FAM181B | family with sequence similarity 181 member B [Source:HGNC Symbol;Acc:HGNC:28512] |
| ENSG00000183570 | HGLibA_35372 | 95 | -0.203253403 | PCBP3 | poly(rC) binding protein 3 [Source:HGNC Symbol;Acc:HGNC:8651] |
| ENSG00000183625 | HGLibA_08152 | 95 | -0.203253403 | CCR3 | C-C motif chemokine receptor 3 [Source:HGNC Symbol;Acc:HGNC:1604] |
| ENSG00000184459 | HGLibA_04735 | 95 | -0.203253403 | BPIFC | BPI fold containing family C [Source:HGNC Symbol;Acc:HGNC:16503] |
| ENSG00000184924 | HGLibA_39555 | 95 | -0.203253403 | PTRHD1 | peptidyl-tRNA hydrolase domain containing 1 [Source:HGNC Symbol;Acc:HGNC:33782] |
| ENSG00000185272 | HGLibA_40475 | 95 | -0.203253403 | RBM11 | RNA binding motif protein 11 [Source:HGNC Symbol;Acc:HGNC:9897] |
| ENSG00000186090 | HGLibA_22390 | 95 | -0.203253403 | HTR3D | 5-hydroxytryptamine receptor 3D [Source:HGNC Symbol;Acc:HGNC:24004] |
| ENSG00000187082 | HGLibA_12834 | 95 | -0.203253403 | DEFB106B | defensin beta 106B [Source:HGNC Symbol;Acc:HGNC:28879] |
| ENSG00000187189 | HGLibA_51985 | 95 | -0.203253403 | TSPYL4 | TSPY like 4 [Source:HGNC Symbol;Acc:HGNC:21559] |
| ENSG00000188394 | HGLibA_20000 | 95 | -0.203253403 | GPR21 | G protein-coupled receptor 21 [Source:HGNC Symbol;Acc:HGNC:4476] |
| ENSG00000188536 | HGLibA_20909 | 95 | -0.203253403 | HBA2 | hemoglobin subunit alpha 2 [Source:HGNC Symbol;Acc:HGNC:4824] |
| ENSG00000196832 | HGLibA_33566 | 95 | -0.203253403 | OR11G2 | olfactory receptor family 11 subfamily G member 2 [Source:HGNC Symbol;Acc:HGNC:15346] |
| ENSG00000204103 | HGLibA_27875 | 95 | -0.203253403 | MAFB | MAF bZIP transcription factor B [Source:HGNC Symbol;Acc:HGNC:6408] |
| ENSG00000204822 | HGLibA_30017 | 95 | -0.203253403 | MRPL53 | mitochondrial ribosomal protein L53 [Source:HGNC Symbol;Acc:HGNC:16684] |
| ENSG00000205639 | HGLibA_29095 | 95 | -0.203253403 | MFSD2B | major facilitator superfamily domain containing 2B [Source:HGNC Symbol;Acc:HGNC:37207] |
| ENSG00000212899 | HGLibA_25747 | 95 | -0.203253403 | KRTAP3-3 | keratin associated protein 3-3 [Source:HGNC Symbol;Acc:HGNC:18890] |
| ENSG00000214107 | HGLibA_27930 | 95 | -0.203253403 | MAGEB1 | MAGE family member B1 [Source:HGNC Symbol;Acc:HGNC:6808] |
| ENSG00000214510 | HGLibA_46684 | 95 | -0.203253403 | SPINK13 | Kazal type 13 (putative) [Source:HGNC Symbol;Acc:HGNC:27200]" |
| ENSG00000215045 | HGLibA_20279 | 95 | -0.203253403 | GRID2IP | Grid2 interacting protein [Source:HGNC Symbol;Acc:HGNC:18464] |
| ENSG00000215131 | HGLibA_05497 | 95 | -0.203253403 | C16orf90 | chromosome 16 open reading frame 90 [Source:HGNC Symbol;Acc:HGNC:34455] |
| ENSG00000215186 | HGLibA_19609 | 95 | -0.203253403 | GOLGA6B | golgin A6 family member B [Source:HGNC Symbol;Acc:HGNC:32205] |
| ENSG00000221910 | HGLibA_33791 | 95 | -0.203253403 | OR2F2 | olfactory receptor family 2 subfamily F member 2 [Source:HGNC Symbol;Acc:HGNC:8247] |
| ENSG00000224586 | HGLibA_20183 | 95 | -0.203253403 | GPX5 | glutathione peroxidase 5 [Source:HGNC Symbol;Acc:HGNC:4557] |
| ENSG00000239382 | HGLibA_01661 | 95 | -0.203253403 | ALKBH6 | alkB homolog 6 [Source:HGNC Symbol;Acc:HGNC:28243] |
| ENSG00000241476 | HGLibA_47199 | 95 | -0.203253403 | SSX2 | SSX family member 2 [Source:HGNC Symbol;Acc:HGNC:11336] |
| ENSG00000272398 | HGLibA_08303 | 95 | -0.203253403 | CD24 | CD24 molecule [Source:HGNC Symbol;Acc:HGNC:1645] |
| ENSG00000272674 | HGLibA_35490 | 95 | -0.203253403 | PCDHB16 | protocadherin beta 16 [Source:HGNC Symbol;Acc:HGNC:14546] |
| FAM211B | HGLibA_16463 | 95 | -0.203253403 | FAM211B | - |
| FAM46A | HGLibA_16595 | 95 | -0.203253403 | FAM46A | - |
| GAREM | HGLibA_18625 | 95 | -0.203253403 | GAREM | - |
| GRAMD2 | HGLibA_20204 | 95 | -0.203253403 | GRAMD2 | - |
| hsa-mir-1208 | HGLibA_57285 | 95 | -0.203253403 | hsa-mir-1208 | - |
| hsa-mir-185 | HGLibA_58003 | 95 | -0.203253403 | hsa-mir-185 | - |
| hsa-mir-3119-1 | HGLibA_58525 | 95 | -0.203253403 | hsa-mir-3119-1 | - |
| hsa-mir-3689f | HGLibA_59338 | 95 | -0.203253403 | hsa-mir-3689f | - |
| hsa-mir-4488 | HGLibA_60369 | 95 | -0.203253403 | hsa-mir-4488 | - |
| hsa-mir-4722 | HGLibA_60984 | 95 | -0.203253403 | hsa-mir-4722 | - |
| hsa-mir-5095 | HGLibA_61539 | 95 | -0.203253403 | hsa-mir-5095 | - |
| hsa-mir-548ah | HGLibA_61881 | 95 | -0.203253403 | hsa-mir-548ah | - |
| hsa-mir-548c | HGLibA_61958 | 95 | -0.203253403 | hsa-mir-548c | - |
| hsa-mir-613 | HGLibA_62667 | 95 | -0.203253403 | hsa-mir-613 | - |
| hsa-mir-7706 | HGLibA_63956 | 95 | -0.203253403 | hsa-mir-7706 | - |

| | | | | | |
|---|---|---|---|---|---|
| hsa-mir-874 | HGLibA_64207 | 95 | -0.203253403 | hsa-mir-874 | - |
| KIAA0922 | HGLibA_24668 | 95 | -0.203253403 | KIAA0922 | - |
| KIAA1024 | HGLibA_24680 | 95 | -0.203253403 | KIAA1024 | - |
| LOC154872 | HGLibA_26897 | 95 | -0.203253403 | LOC154872 | - |
| MINOS1 | HGLibA_29278 | 95 | -0.203253403 | MINOS1 | - |
| NAPRT1 | HGLibA_31138 | 95 | -0.203253403 | NAPRT1 | - |
| PLCXD1_X | HGLibA_37021 | 95 | -0.203253403 | PLCXD1_X | - |
| RSG1 | HGLibA_42313 | 95 | -0.203253403 | RSG1 | - |
| SQRDL | HGLibA_46916 | 95 | -0.203253403 | SQRDL | - |
| TMEM5 | HGLibA_50420 | 95 | -0.203253403 | TMEM5 | - |
| ADCK3 | HGLibA_00895 | 94 | -0.213204441 | ADCK3 | - |
| C16orf62 | HGLibA_05469 | 94 | -0.213204441 | C16orf62 | - |
| C17orf82 | HGLibA_05586 | 94 | -0.213204441 | C17orf82 | - |
| ENSG00000004846 | HGLibA_00143 | 94 | -0.213204441 | ABCB5 | ATP binding cassette subfamily B member 5 [Source:HGNC Symbol;Acc:HGNC:46] |
| ENSG00000007402 | HGLibA_06908 | 94 | -0.213204441 | CACNA2D2 | calcium voltage-gated channel auxiliary subunit alpha2delta 2 [Source:HGNC Symbol;Acc:HGNC:1400] |
| ENSG00000023228 | HGLibA_31544 | 94 | -0.213204441 | NDUFS1 | NADH:ubiquinone oxidoreductase core subunit S1 [Source:HGNC Symbol;Acc:HGNC:7707] |
| ENSG00000044459 | HGLibA_10398 | 94 | -0.213204441 | CNTLN | centlein [Source:HGNC Symbol;Acc:HGNC:23432] |
| ENSG00000055118 | HGLibA_24213 | 94 | -0.213204441 | KCNH2 | potassium voltage-gated channel subfamily H member 2 [Source:HGNC Symbol;Acc:HGNC:6251] |
| ENSG00000059588 | HGLibA_48329 | 94 | -0.213204441 | TARBP1 | TAR (HIV-1) RNA binding protein 1 [Source:HGNC Symbol;Acc:HGNC:11568] |
| ENSG00000060642 | HGLibA_36594 | 94 | -0.213204441 | PIGV | phosphatidylinositol glycan anchor biosynthesis class V [Source:HGNC Symbol;Acc:HGNC:26031] |
| ENSG00000062485 | HGLibA_11299 | 94 | -0.213204441 | CS | citrate synthase [Source:HGNC Symbol;Acc:HGNC:2422] |
| ENSG00000063244 | HGLibA_52499 | 94 | -0.213204441 | U2AF2 | U2 small nuclear RNA auxiliary factor 2 [Source:HGNC Symbol;Acc:HGNC:23156] |
| ENSG00000065413 | HGLibA_02101 | 94 | -0.213204441 | ANKRD44 | ankyrin repeat domain 44 [Source:HGNC Symbol;Acc:HGNC:25259] |
| ENSG00000069974 | HGLibA_39857 | 94 | -0.213204441 | RAB27A | member RAS oncogene family [Source:HGNC Symbol;Acc:HGNC:9766]" |
| ENSG00000077274 | HGLibA_07173 | 94 | -0.213204441 | CAPN6 | calpain 6 [Source:HGNC Symbol;Acc:HGNC:1483] |
| ENSG00000092871 | HGLibA_40921 | 94 | -0.213204441 | RFFL | ring finger and FYVE like domain containing E3 ubiquitin protein ligase [Source:HGNC Symbol;Acc:HGNC:24821] |
| ENSG00000099219 | HGLibA_15510 | 94 | -0.213204441 | ERMP1 | endoplasmic reticulum metallopeptidase 1 [Source:HGNC Symbol;Acc:HGNC:23703] |
| ENSG00000100211 | HGLibA_07468 | 94 | -0.213204441 | CBY1 | beta catenin antagonist [Source:HGNC Symbol;Acc:HGNC:1307]" |
| ENSG00000100221 | HGLibA_23974 | 94 | -0.213204441 | JOSD1 | Josephin domain containing 1 [Source:HGNC Symbol;Acc:HGNC:28953] |
| ENSG00000100395 | HGLibA_25899 | 94 | -0.213204441 | L3MBTL2 | polycomb repressive complex 1 subunit [Source:HGNC Symbol;Acc:HGNC:18594]" |
| ENSG00000100448 | HGLibA_11707 | 94 | -0.213204441 | CTSG | cathepsin G [Source:HGNC Symbol;Acc:HGNC:2532] |
| ENSG00000101346 | HGLibA_37467 | 94 | -0.213204441 | POFUT1 | protein O-fucosyltransferase 1 [Source:HGNC Symbol;Acc:HGNC:14988] |
| ENSG00000102387 | HGLibA_48256 | 94 | -0.213204441 | TAF7L | TATA-box binding protein associated factor 7 like [Source:HGNC Symbol;Acc:HGNC:11548] |
| ENSG00000102580 | HGLibA_13614 | 94 | -0.213204441 | DNAJC3 | DnaJ heat shock protein family (Hsp40) member C3 [Source:HGNC Symbol;Acc:HGNC:9439] |
| ENSG00000104808 | HGLibA_13101 | 94 | -0.213204441 | DHDH | dihydrodiol dehydrogenase [Source:HGNC Symbol;Acc:HGNC:17887] |
| ENSG00000105877 | HGLibA_13473 | 94 | -0.213204441 | DNAH11 | dynein axonemal heavy chain 11 [Source:HGNC Symbol;Acc:HGNC:2942] |
| ENSG00000108187 | HGLibA_35334 | 94 | -0.213204441 | PBLD | phenazine biosynthesis like protein domain containing [Source:HGNC Symbol;Acc:HGNC:23301] |
| ENSG00000110841 | HGLibA_37885 | 94 | -0.213204441 | PPFIBP1 | PPFIA binding protein 1 [Source:HGNC Symbol;Acc:HGNC:9249] |
| ENSG00000111252 | HGLibA_43880 | 94 | -0.213204441 | SH2B3 | SH2B adaptor protein 3 [Source:HGNC Symbol;Acc:HGNC:29605] |
| ENSG00000112367 | HGLibA_17510 | 94 | -0.213204441 | FIG4 | FIG4 phosphoinositide 5-phosphatase [Source:HGNC Symbol;Acc:HGNC:16873] |
| ENSG00000113525 | HGLibA_23174 | 94 | -0.213204441 | IL5 | interleukin 5 [Source:HGNC Symbol;Acc:HGNC:6016] |
| ENSG00000115363 | HGLibA_15672 | 94 | -0.213204441 | EVA1A | regulator of programmed cell death [Source:HGNC Symbol;Acc:HGNC:25816]" |
| ENSG00000116991 | HGLibA_44196 | 94 | -0.213204441 | SIPA1L2 | signal induced proliferation associated 1 like 2 [Source:HGNC Symbol;Acc:HGNC:23800] |
| ENSG00000117266 | HGLibA_08748 | 94 | -0.213204441 | CDK18 | cyclin dependent kinase 18 [Source:HGNC Symbol;Acc:HGNC:8751] |
| ENSG00000117500 | HGLibA_49815 | 94 | -0.213204441 | TMED5 | transmembrane p24 trafficking protein 5 [Source:HGNC Symbol;Acc:HGNC:24251] |
| ENSG00000121858 | HGLibA_50797 | 94 | -0.213204441 | TNFSF10 | TNF superfamily member 10 [Source:HGNC Symbol;Acc:HGNC:11925] |
| ENSG00000123136 | HGLibA_12695 | 94 | -0.213204441 | DDX39A | DExD-box helicase 39A [Source:HGNC Symbol;Acc:HGNC:17821] |
| ENSG00000124074 | HGLibA_15133 | 94 | -0.213204441 | ENKD1 | enkurin domain containing 1 [Source:HGNC Symbol;Acc:HGNC:25246] |
| ENSG00000124209 | HGLibA_39841 | 94 | -0.213204441 | RAB22A | member RAS oncogene family [Source:HGNC Symbol;Acc:HGNC:9764]" |
| ENSG00000124721 | HGLibA_13498 | 94 | -0.213204441 | DNAH8 | dynein axonemal heavy chain 8 [Source:HGNC Symbol;Acc:HGNC:2952] |
| ENSG00000126266 | HGLibA_17323 | 94 | -0.213204441 | FFAR1 | free fatty acid receptor 1 [Source:HGNC Symbol;Acc:HGNC:4498] |
| ENSG00000128641 | HGLibA_30823 | 94 | -0.213204441 | MYO1B | myosin IB [Source:HGNC Symbol;Acc:HGNC:7596] |
| ENSG00000130803 | HGLibA_55784 | 94 | -0.213204441 | ZNF317 | zinc finger protein 317 [Source:HGNC Symbol;Acc:HGNC:13507] |
| ENSG00000131475 | HGLibA_53748 | 94 | -0.213204441 | VPS25 | vacuolar protein sorting 25 homolog [Source:HGNC Symbol;Acc:HGNC:28122] |
| ENSG00000132694 | HGLibA_02813 | 94 | -0.213204441 | ARHGEF11 | Rho guanine nucleotide exchange factor 11 [Source:HGNC Symbol;Acc:HGNC:14580] |
| ENSG00000133028 | HGLibA_43026 | 94 | -0.213204441 | SCO1 | cytochrome c oxidase assembly protein [Source:HGNC Symbol;Acc:HGNC:10603]" |
| ENSG00000133398 | HGLibA_28736 | 94 | -0.213204441 | MED10 | mediator complex subunit 10 [Source:HGNC Symbol;Acc:HGNC:28760] |
| ENSG00000134057 | HGLibA_08056 | 94 | -0.213204441 | CCNB1 | cyclin B1 [Source:HGNC Symbol;Acc:HGNC:1579] |
| ENSG00000134389 | HGLibA_09276 | 94 | -0.213204441 | CFHR5 | complement factor H related 5 [Source:HGNC Symbol;Acc:HGNC:24668] |
| ENSG00000134463 | HGLibA_14400 | 94 | -0.213204441 | ECHDC3 | enoyl-CoA hydratase domain containing 3 [Source:HGNC Symbol;Acc:HGNC:23489] |
| ENSG00000135333 | HGLibA_15305 | 94 | -0.213204441 | EPHA7 | EPH receptor A7 [Source:HGNC Symbol;Acc:HGNC:3390] |
| ENSG00000135426 | HGLibA_49035 | 94 | -0.213204441 | TESPA1 | positive selection associated 1 [Source:HGNC Symbol;Acc:HGNC:29109]" |
| ENSG00000135698 | HGLibA_29725 | 94 | -0.213204441 | MPHOSPH6 | M-phase phosphoprotein 6 [Source:HGNC Symbol;Acc:HGNC:7214] |
| ENSG00000136152 | HGLibA_10475 | 94 | -0.213204441 | COG3 | component of oligomeric golgi complex 3 [Source:HGNC Symbol;Acc:HGNC:18619] |
| ENSG00000137500 | HGLibA_07922 | 94 | -0.213204441 | CCDC90B | coiled-coil domain containing 90B [Source:HGNC Symbol;Acc:HGNC:28108] |
| ENSG00000137502 | HGLibA_39871 | 94 | -0.213204441 | RAB30 | member RAS oncogene family [Source:HGNC Symbol;Acc:HGNC:9770]" |
| ENSG00000138801 | HGLibA_35151 | 94 | -0.213204441 | PAPSS1 | 3'-phosphoadenosine 5'-phosphosulfate synthase 1 [Source:HGNC Symbol;Acc:HGNC:8603] |
| ENSG00000139163 | HGLibA_15633 | 94 | -0.213204441 | ETNK1 | ethanolamine kinase 1 [Source:HGNC Symbol;Acc:HGNC:24649] |
| ENSG00000142632 | HGLibA_02831 | 94 | -0.213204441 | ARHGEF19 | Rho guanine nucleotide exchange factor 19 [Source:HGNC Symbol;Acc:HGNC:26604] |
| ENSG00000143799 | HGLibA_35210 | 94 | -0.213204441 | PARP1 | poly(ADP-ribose) polymerase 1 [Source:HGNC Symbol;Acc:HGNC:270] |
| ENSG00000144134 | HGLibA_40028 | 94 | -0.213204441 | RABL2A | member of RAS oncogene family like 2A [Source:HGNC Symbol;Acc:HGNC:9799]" |
| ENSG00000144730 | HGLibA_23012 | 94 | -0.213204441 | IL17RD | interleukin 17 receptor D [Source:HGNC Symbol;Acc:HGNC:17616] |
| ENSG00000144810 | HGLibA_10613 | 94 | -0.213204441 | COL8A1 | collagen type VIII alpha 1 chain [Source:HGNC Symbol;Acc:HGNC:2215] |
| ENSG00000145817 | HGLibA_54683 | 94 | -0.213204441 | YIPF5 | Yip1 domain family member 5 [Source:HGNC Symbol;Acc:HGNC:24877] |
| ENSG00000146350 | HGLibA_48538 | 94 | -0.213204441 | TBC1D32 | TBC1 domain family member 32 [Source:HGNC Symbol;Acc:HGNC:21485] |
| ENSG00000149485 | HGLibA_15933 | 94 | -0.213204441 | FADS1 | fatty acid desaturase 1 [Source:HGNC Symbol;Acc:HGNC:3574] |
| ENSG00000152056 | HGLibA_02301 | 94 | -0.213204441 | AP1S3 | adaptor related protein complex 1 subunit sigma 3 [Source:HGNC Symbol;Acc:HGNC:18971] |
| ENSG00000152795 | HGLibA_21816 | 94 | -0.213204441 | HNRNPDL | heterogeneous nuclear ribonucleoprotein D like [Source:HGNC Symbol;Acc:HGNC:5037] |
| ENSG00000153560 | HGLibA_52728 | 94 | -0.213204441 | UBP1 | upstream binding protein 1 [Source:HGNC Symbol;Acc:HGNC:12507] |
| ENSG00000153774 | HGLibA_09256 | 94 | -0.213204441 | CFDP1 | craniofacial development protein 1 [Source:HGNC Symbol;Acc:HGNC:1873] |
| ENSG00000154370 | HGLibA_51404 | 94 | -0.213204441 | TRIM11 | tripartite motif containing 11 [Source:HGNC Symbol;Acc:HGNC:16281] |
| ENSG00000154620 | HGLibA_50667 | 94 | -0.213204441 | TMSB4Y | thymosin beta 4 Y-linked [Source:HGNC Symbol;Acc:HGNC:11882] |
| ENSG00000157800 | HGLibA_45050 | 94 | -0.213204441 | SLC37A3 | solute carrier family 37 member 3 [Source:HGNC Symbol;Acc:HGNC:20651] |
| ENSG00000158717 | HGLibA_41560 | 94 | -0.213204441 | RNF166 | ring finger protein 166 [Source:HGNC Symbol;Acc:HGNC:28856] |
| ENSG00000160345 | HGLibA_06666 | 94 | -0.213204441 | C9orf116 | chromosome 9 open reading frame 116 [Source:HGNC Symbol;Acc:HGNC:28435] |
| ENSG00000162592 | HGLibA_07741 | 94 | -0.213204441 | CCDC27 | coiled-coil domain containing 27 [Source:HGNC Symbol;Acc:HGNC:26546] |
| ENSG00000162624 | HGLibA_26466 | 94 | -0.213204441 | LHX8 | LIM homeobox 8 [Source:HGNC Symbol;Acc:HGNC:28838] |
| ENSG00000163528 | HGLibA_09371 | 94 | -0.213204441 | CHCHD4 | coiled-coil-helix-coiled-coil-helix domain containing 4 [Source:HGNC Symbol;Acc:HGNC:26467] |
| ENSG00000164080 | HGLibA_40106 | 94 | -0.213204441 | RAD54L2 | RAD54 like 2 [Source:HGNC Symbol;Acc:HGNC:29123] |
| ENSG00000164105 | HGLibA_42723 | 94 | -0.213204441 | SAP30 | Sin3A associated protein 30 [Source:HGNC Symbol;Acc:HGNC:10532] |
| ENSG00000164182 | HGLibA_31484 | 94 | -0.213204441 | NDUFAF2 | NADH:ubiquinone oxidoreductase complex assembly factor 2 [Source:HGNC Symbol;Acc:HGNC:28086] |
| ENSG00000164438 | HGLibA_49678 | 94 | -0.213204441 | TLX3 | T cell leukemia homeobox 3 [Source:HGNC Symbol;Acc:HGNC:13532] |
| ENSG00000164512 | HGLibA_02125 | 94 | -0.213204441 | ANKRD55 | ankyrin repeat domain 55 [Source:HGNC Symbol;Acc:HGNC:25681] |
| ENSG00000166532 | HGLibA_41274 | 94 | -0.213204441 | RIMKLB | ribosomal modification protein rimK like family member B [Source:HGNC Symbol;Acc:HGNC:29228] |
| ENSG00000166582 | HGLibA_09089 | 94 | -0.213204441 | CENPV | centromere protein V [Source:HGNC Symbol;Acc:HGNC:29920] |
| ENSG00000167380 | HGLibA_55625 | 94 | -0.213204441 | ZNF226 | zinc finger protein 226 [Source:HGNC Symbol;Acc:HGNC:13019] |
| ENSG00000167711 | HGLibA_43588 | 94 | -0.213204441 | SERPINF2 | serpin family F member 2 [Source:HGNC Symbol;Acc:HGNC:9075] |
| ENSG00000168116 | HGLibA_24787 | 94 | -0.213204441 | KIAA1586 | KIAA1586 [Source:HGNC Symbol;Acc:HGNC:21360] |
| ENSG00000168792 | HGLibA_00253 | 94 | -0.213204441 | ABHD15 | abhydrolase domain containing 15 [Source:HGNC Symbol;Acc:HGNC:26971] |
| ENSG00000169297 | HGLibA_32568 | 94 | -0.213204441 | NR0B1 | nuclear receptor subfamily 0 group B member 1 [Source:HGNC Symbol;Acc:HGNC:7960] |
| ENSG00000169504 | HGLibA_10029 | 94 | -0.213204441 | CLIC4 | chloride intracellular channel 4 [Source:HGNC Symbol;Acc:HGNC:13518] |
| ENSG00000169684 | HGLibA_09577 | 94 | -0.213204441 | CHRNA5 | cholinergic receptor nicotinic alpha 5 subunit [Source:HGNC Symbol;Acc:HGNC:1959] |
| ENSG00000170892 | HGLibA_51836 | 94 | -0.213204441 | TSEN34 | tRNA splicing endonuclease subunit 34 [Source:HGNC Symbol;Acc:HGNC:15506] |
| ENSG00000171119 | HGLibA_32731 | 94 | -0.213204441 | NRTN | neurturin [Source:HGNC Symbol;Acc:HGNC:8007] |
| ENSG00000172322 | HGLibA_09928 | 94 | -0.213204441 | CLEC12A | C-type lectin domain family 12 member A [Source:HGNC Symbol;Acc:HGNC:31713] |
| ENSG00000172977 | HGLibA_24058 | 94 | -0.213204441 | KAT5 | lysine acetyltransferase 5 [Source:HGNC Symbol;Acc:HGNC:5275] |
| ENSG00000175065 | HGLibA_14030 | 94 | -0.213204441 | DSG4 | desmoglein 4 [Source:HGNC Symbol;Acc:HGNC:21307] |
| ENSG00000175985 | HGLibA_37091 | 94 | -0.213204441 | PLEKHD1 | pleckstrin homology and coiled-coil domain containing D1 [Source:HGNC Symbol;Acc:HGNC:20148] |
| ENSG00000176009 | HGLibA_03271 | 94 | -0.213204441 | ASCL3 | achaete-scute family bHLH transcription factor 3 [Source:HGNC Symbol;Acc:HGNC:740] |
| ENSG00000176953 | HGLibA_31778 | 94 | -0.213204441 | NFATC2IP | nuclear factor of activated T cells 2 interacting protein [Source:HGNC Symbol;Acc:HGNC:25906] |
| ENSG00000177192 | HGLibA_39596 | 94 | -0.213204441 | PUS1 | pseudouridine synthase 1 [Source:HGNC Symbol;Acc:HGNC:15508] |
| ENSG00000177462 | HGLibA_33894 | 94 | -0.213204441 | OR2T8 | olfactory receptor family 2 subfamily T member 8 [Source:HGNC Symbol;Acc:HGNC:15020] |
| ENSG00000178226 | HGLibA_38932 | 94 | -0.213204441 | PRSS36 | serine protease 36 [Source:HGNC Symbol;Acc:HGNC:26906] |
| ENSG00000178896 | HGLibA_15782 | 94 | -0.213204441 | EXOSC4 | exosome component 4 [Source:HGNC Symbol;Acc:HGNC:18189] |
| ENSG00000179242 | HGLibA_08681 | 94 | -0.213204441 | CDH4 | cadherin 4 [Source:HGNC Symbol;Acc:HGNC:1763] |
| ENSG00000179954 | HGLibA_47126 | 94 | -0.213204441 | SSC5D | scavenger receptor cysteine rich family member with 5 domains [Source:HGNC Symbol;Acc:HGNC:26641] |
| ENSG00000180035 | HGLibA_56068 | 94 | -0.213204441 | ZNF48 | zinc finger protein 48 [Source:HGNC Symbol;Acc:HGNC:13114] |
| ENSG00000180116 | HGLibA_05237 | 94 | -0.213204441 | C12orf40 | chromosome 12 open reading frame 40 [Source:HGNC Symbol;Acc:HGNC:26846] |
| ENSG00000180332 | HGLibA_24455 | 94 | -0.213204441 | KCTD4 | potassium channel tetramerization domain containing 4 [Source:HGNC Symbol;Acc:HGNC:23227] |
| ENSG00000181195 | HGLibA_36049 | 94 | -0.213204441 | PENK | proenkephalin [Source:HGNC Symbol;Acc:HGNC:8831] |
| ENSG00000181481 | HGLibA_41510 | 94 | -0.213204441 | RNF135 | ring finger protein 135 [Source:HGNC Symbol;Acc:HGNC:21158] |
| ENSG00000181552 | HGLibA_14452 | 94 | -0.213204441 | EDDM3B | epididymal protein 3B [Source:HGNC Symbol;Acc:HGNC:19223] |
| ENSG00000182168 | HGLibA_53020 | 94 | -0.213204441 | UNC5C | unc-5 netrin receptor C [Source:HGNC Symbol;Acc:HGNC:12569] |
| ENSG00000182218 | HGLibA_21264 | 94 | -0.213204441 | HHIPL1 | HHIP like 1 [Source:HGNC Symbol;Acc:HGNC:19710] |
| ENSG00000182613 | HGLibA_33899 | 94 | -0.213204441 | OR2V2 | olfactory receptor family 2 subfamily V member 2 [Source:HGNC Symbol;Acc:HGNC:15341] |
| ENSG00000183117 | HGLibA_11364 | 94 | -0.213204441 | CSMD1 | CUB and Sushi multiple domains 1 [Source:HGNC Symbol;Acc:HGNC:14026] |
| ENSG00000183807 | HGLibA_16224 | 94 | -0.213204441 | FAM162B | family with sequence similarity 162 member B [Source:HGNC Symbol;Acc:HGNC:21549] |
| ENSG00000184203 | HGLibA_38075 | 94 | -0.213204441 | PPP1R2 | protein phosphatase 1 regulatory inhibitor subunit 2 [Source:HGNC Symbol;Acc:HGNC:9288] |
| ENSG00000184271 | HGLibA_37795 | 94 | -0.213204441 | POU6F1 | POU class 6 homeobox 1 [Source:HGNC Symbol;Acc:HGNC:9224] |
| ENSG00000184304 | HGLibA_38567 | 94 | -0.213204441 | PRKD1 | protein kinase D1 [Source:HGNC Symbol;Acc:HGNC:9407] |
| ENSG00000184574 | HGLibA_27065 | 94 | -0.213204441 | LPAR5 | lysophosphatidic acid receptor 5 [Source:HGNC Symbol;Acc:HGNC:13307] |
| ENSG00000184988 | HGLibA_49854 | 94 | -0.213204441 | TMEM106A | transmembrane protein 106A [Source:HGNC Symbol;Acc:HGNC:28288] |
| ENSG00000185313 | HGLibA_42969 | 94 | -0.213204441 | SCN10A | sodium voltage-gated channel alpha subunit 10 [Source:HGNC Symbol;Acc:HGNC:10582] |
| ENSG00000185634 | HGLibA_44010 | 94 | -0.213204441 | SHC4 | SHC adaptor protein 4 [Source:HGNC Symbol;Acc:HGNC:16743] |
| ENSG00000186010 | HGLibA_31449 | 94 | -0.213204441 | NDUFA13 | NADH:ubiquinone oxidoreductase subunit A13 [Source:HGNC Symbol;Acc:HGNC:17194] |

| | | | | | |
|---|---|---|---|---|---|
| ENSG00000186714 | HGLibA_07858 | 94 | -0.213204441 | CCDC73 | coiled-coil domain containing 73 [Source:HGNC Symbol;Acc:HGNC:23261] |
| ENSG00000186723 | HGLibA_33505 | 94 | -0.213204441 | OR10H1 | olfactory receptor family 10 subfamily H member 1 [Source:HGNC Symbol;Acc:HGNC:8172] |
| ENSG00000187134 | HGLibA_01481 | 94 | -0.213204441 | AKR1C1 | aldo-keto reductase family 1 member C1 [Source:HGNC Symbol;Acc:HGNC:384] |
| ENSG00000187535 | HGLibA_22747 | 94 | -0.213204441 | IFT140 | intraflagellar transport 140 [Source:HGNC Symbol;Acc:HGNC:29077] |
| ENSG00000187627 | HGLibA_41026 | 94 | -0.213204441 | RGPD1 | RANBP2-like and GRIP domain containing 1 [Source:HGNC Symbol;Acc:HGNC:32414] |
| ENSG00000188385 | HGLibA_23929 | 94 | -0.213204441 | JAKMIP3 | Janus kinase and microtubule interacting protein 3 [Source:HGNC Symbol;Acc:HGNC:23523] |
| ENSG00000188613 | HGLibA_31097 | 94 | -0.213204441 | NANOS1 | nanos C2HC-type zinc finger 1 [Source:HGNC Symbol;Acc:HGNC:23044] |
| ENSG00000189433 | HGLibA_19165 | 94 | -0.213204441 | GJB4 | gap junction protein beta 4 [Source:HGNC Symbol;Acc:HGNC:4286] |
| ENSG00000196230 | HGLibA_52270 | 94 | -0.213204441 | TUBB | tubulin beta class I [Source:HGNC Symbol;Acc:HGNC:20778] |
| ENSG00000196511 | HGLibA_51137 | 94 | -0.213204441 | TPK1 | thiamin pyrophosphokinase 1 [Source:HGNC Symbol;Acc:HGNC:17358] |
| ENSG00000197343 | HGLibA_56471 | 94 | -0.213204441 | ZNF655 | zinc finger protein 655 [Source:HGNC Symbol;Acc:HGNC:30899] |
| ENSG00000197887 | HGLibA_33716 | 94 | -0.213204441 | OR1S2 | olfactory receptor family 1 subfamily S member 2 [Source:HGNC Symbol;Acc:HGNC:15141] |
| ENSG00000198089 | HGLibA_43717 | 94 | -0.213204441 | SFI1 | SFI1 centrin binding protein [Source:HGNC Symbol;Acc:HGNC:29064] |
| ENSG00000198176 | HGLibA_49151 | 94 | -0.213204441 | TFDP1 | transcription factor Dp-1 [Source:HGNC Symbol;Acc:HGNC:11749] |
| ENSG00000203722 | HGLibA_40124 | 94 | -0.213204441 | RAET1G | retinoic acid early transcript 1G [Source:HGNC Symbol;Acc:HGNC:16795] |
| ENSG00000204386 | HGLibA_31709 | 94 | -0.213204441 | NEU1 | neuraminidase 1 [Source:HGNC Symbol;Acc:HGNC:7758] |
| ENSG00000204531 | HGLibA_37787 | 94 | -0.213204441 | POU5F1 | POU class 5 homeobox 1 [Source:HGNC Symbol;Acc:HGNC:9221] |
| ENSG00000204544 | HGLibA_30542 | 94 | -0.213204441 | MUC21 | cell surface associated [Source:HGNC Symbol;Acc:HGNC:21661]" |
| ENSG00000204634 | HGLibA_48566 | 94 | -0.213204441 | TBC1D8 | TBC1 domain family member 8 [Source:HGNC Symbol;Acc:HGNC:17791] |
| ENSG00000213699 | HGLibA_45014 | 94 | -0.213204441 | SLC35F6 | solute carrier family 35 member F6 [Source:HGNC Symbol;Acc:HGNC:26055] |
| ENSG00000225614 | HGLibA_56050 | 94 | -0.213204441 | ZNF469 | zinc finger protein 469 [Source:HGNC Symbol;Acc:HGNC:23216] |
| ENSG00000226600 | HGLibA_11548 | 94 | -0.213204441 | CT47A9 | member A9 [Source:HGNC Symbol;Acc:HGNC:33290]" |
| ENSG00000241595 | HGLibA_25838 | 94 | -0.213204441 | KRTAP9-4 | keratin associated protein 9-4 [Source:HGNC Symbol;Acc:HGNC:18902] |
| ENSG00000242259 | HGLibA_06075 | 94 | -0.213204441 | C22orf39 | chromosome 22 open reading frame 39 [Source:HGNC Symbol;Acc:HGNC:27012] |
| ENSG00000242715 | HGLibA_07683 | 94 | -0.213204441 | CCDC169 | coiled-coil domain containing 169 [Source:HGNC Symbol;Acc:HGNC:34361] |
| ENSG00000255374 | HGLibA_48411 | 94 | -0.213204441 | TAS2R43 | taste 2 receptor member 43 [Source:HGNC Symbol;Acc:HGNC:18875] |
| ENSG00000257335 | HGLibA_29127 | 94 | -0.213204441 | MGAM | maltase-glucoamylase [Source:HGNC Symbol;Acc:HGNC:7043] |
| ENSG00000264364 | HGLibA_14255 | 94 | -0.213204441 | DYNLL2 | dynein light chain LC8-type 2 [Source:HGNC Symbol;Acc:HGNC:24596] |
| ENSG00000266964 | HGLibA_18225 | 94 | -0.213204441 | FXYD1 | FXYD domain containing ion transport regulator 1 [Source:HGNC Symbol;Acc:HGNC:4025] |
| ENSG00000271271 | HGLibA_52906 | 94 | -0.213204441 | UGT2A2 | UDP glucuronosyltransferase family 2 member A2 [Source:HGNC Symbol;Acc:HGNC:28183] |
| ENSG00000275700 | HGLibA_00087 | 94 | -0.213204441 | AATF | apoptosis antagonizing transcription factor [Source:HGNC Symbol;Acc:HGNC:19235] |
| ENSG00000278619 | HGLibA_29861 | 94 | -0.213204441 | MRM1 | mitochondrial rRNA methyltransferase 1 [Source:HGNC Symbol;Acc:HGNC:26202] |
| HIST1H3J | HGLibA_21483 | 94 | -0.213204441 | HIST1H3J | - |
| hsa-mir-1-1 | HGLibA_57143 | 94 | -0.213204441 | hsa-mir-1-1 | - |
| hsa-mir-1185-2 | HGLibA_57246 | 94 | -0.213204441 | hsa-mir-1185-2 | - |
| hsa-mir-1228 | HGLibA_57310 | 94 | -0.213204441 | hsa-mir-1228 | - |
| hsa-mir-1288 | HGLibA_57615 | 94 | -0.213204441 | hsa-mir-1288 | - |
| hsa-mir-1322 | HGLibA_57762 | 94 | -0.213204441 | hsa-mir-1322 | - |
| hsa-mir-1911 | HGLibA_58050 | 94 | -0.213204441 | hsa-mir-1911 | - |
| hsa-mir-21 | HGLibA_58208 | 94 | -0.213204441 | hsa-mir-21 | - |
| hsa-mir-4251 | HGLibA_59712 | 94 | -0.213204441 | hsa-mir-4251 | - |
| hsa-mir-4420 | HGLibA_60066 | 94 | -0.213204441 | hsa-mir-4420 | - |
| hsa-mir-4513 | HGLibA_60496 | 94 | -0.213204441 | hsa-mir-4513 | - |
| hsa-mir-4800 | HGLibA_61304 | 94 | -0.213204441 | hsa-mir-4800 | - |
| hsa-mir-511 | HGLibA_61555 | 94 | -0.213204441 | hsa-mir-511 | - |
| hsa-mir-548aa-1 | HGLibA_61847 | 94 | -0.213204441 | hsa-mir-548aa-1 | - |
| hsa-mir-548z | HGLibA_62089 | 94 | -0.213204441 | hsa-mir-548z | - |
| hsa-mir-5701-1 | HGLibA_62341 | 94 | -0.213204441 | hsa-mir-5701-1 | - |
| hsa-mir-5701-1 | HGLibA_62339 | 94 | -0.213204441 | hsa-mir-5701-1 | - |
| hsa-mir-629 | HGLibA_62755 | 94 | -0.213204441 | hsa-mir-629 | - |
| hsa-mir-6735 | HGLibA_63095 | 94 | -0.213204441 | hsa-mir-6735 | - |
| hsa-mir-6839 | HGLibA_63533 | 94 | -0.213204441 | hsa-mir-6839 | - |
| hsa-mir-769 | HGLibA_63934 | 94 | -0.213204441 | hsa-mir-769 | - |
| LOC100129361 | HGLibA_26760 | 94 | -0.213204441 | LOC100129361 | - |
| LOC100288255 | HGLibA_26828 | 94 | -0.213204441 | LOC100288255 | - |
| LOC100507003 | HGLibA_26868 | 94 | -0.213204441 | LOC100507003 | - |
| LOR | HGLibA_27030 | 94 | -0.213204441 | LOR | - |
| SUV420H1 | HGLibA_47866 | 94 | -0.213204441 | SUV420H1 | - |
| C11orf63 | HGLibA_05165 | 93 | -0.223155478 | C11orf63 | - |
| C12orf23 | HGLibA_05226 | 93 | -0.223155478 | C12orf23 | - |
| C14orf177 | HGLibA_05343 | 93 | -0.223155478 | C14orf177 | - |
| C7orf65 | HGLibA_06575 | 93 | -0.223155478 | C7orf65 | - |
| C8orf47 | HGLibA_06633 | 93 | -0.223155478 | C8orf47 | - |
| CASC4 | HGLibA_07278 | 93 | -0.223155478 | CASC4 | - |
| ENSG00000006788 | HGLibA_30712 | 93 | -0.223155478 | MYH13 | myosin heavy chain 13 [Source:HGNC Symbol;Acc:HGNC:7571] |
| ENSG00000007372 | HGLibA_35311 | 93 | -0.223155478 | PAX6 | paired box 6 [Source:HGNC Symbol;Acc:HGNC:8620] |
| ENSG00000010256 | HGLibA_53112 | 93 | -0.223155478 | UQCRC1 | ubiquinol-cytochrome c reductase core protein 1 [Source:HGNC Symbol;Acc:HGNC:12585] |
| ENSG00000017797 | HGLibA_40154 | 93 | -0.223155478 | RALBP1 | ralA binding protein 1 [Source:HGNC Symbol;Acc:HGNC:9841] |
| ENSG00000034152 | HGLibA_28145 | 93 | -0.223155478 | MAP2K3 | mitogen-activated protein kinase kinase 3 [Source:HGNC Symbol;Acc:HGNC:6843] |
| ENSG00000038945 | HGLibA_30234 | 93 | -0.223155478 | MSR1 | macrophage scavenger receptor 1 [Source:HGNC Symbol;Acc:HGNC:7376] |
| ENSG00000043591 | HGLibA_01055 | 93 | -0.223155478 | ADRB1 | adrenoceptor beta 1 [Source:HGNC Symbol;Acc:HGNC:285] |
| ENSG00000051523 | HGLibA_12015 | 93 | -0.223155478 | CYBA | cytochrome b-245 alpha chain [Source:HGNC Symbol;Acc:HGNC:2577] |
| ENSG00000067369 | HGLibA_51055 | 93 | -0.223155478 | TP53BP1 | tumor protein p53 binding protein 1 [Source:HGNC Symbol;Acc:HGNC:11999] |
| ENSG00000068971 | HGLibA_38172 | 93 | -0.223155478 | PPP2R5B | protein phosphatase 2 regulatory subunit B'beta [Source:HGNC Symbol;Acc:HGNC:9310] |
| ENSG00000073614 | HGLibA_24522 | 93 | -0.223155478 | KDM5A | lysine demethylase 5A [Source:HGNC Symbol;Acc:HGNC:9886] |
| ENSG00000077800 | HGLibA_17581 | 93 | -0.223155478 | FKBP6 | FK506 binding protein 6 [Source:HGNC Symbol;Acc:HGNC:3722] |
| ENSG00000079462 | HGLibA_34996 | 93 | -0.223155478 | PAFAH1B3 | platelet activating factor acetylhydrolase 1b catalytic subunit 3 [Source:HGNC Symbol;Acc:HGNC:8576] |
| ENSG00000080166 | HGLibA_12539 | 93 | -0.223155478 | DCT | dopachrome tautomerase [Source:HGNC Symbol;Acc:HGNC:2709] |
| ENSG00000080824 | HGLibA_22251 | 93 | -0.223155478 | HSP90AA1 | heat shock protein 90 alpha family class A member 1 [Source:HGNC Symbol;Acc:HGNC:5253] |
| ENSG00000081870 | HGLibA_22303 | 93 | -0.223155478 | HSPB11 | heat shock protein family B (small) member 11 [Source:HGNC Symbol;Acc:HGNC:25019] |
| ENSG00000087088 | HGLibA_04183 | 93 | -0.223155478 | BAX | apoptosis regulator [Source:HGNC Symbol;Acc:HGNC:959]" |
| ENSG00000088298 | HGLibA_14457 | 93 | -0.223155478 | EDEM2 | ER degradation enhancing alpha-mannosidase like protein 2 [Source:HGNC Symbol;Acc:HGNC:15877] |
| ENSG00000089116 | HGLibA_26460 | 93 | -0.223155478 | LHX5 | LIM homeobox 5 [Source:HGNC Symbol;Acc:HGNC:14216] |
| ENSG00000090924 | HGLibA_37105 | 93 | -0.223155478 | PLEKHG2 | pleckstrin homology and RhoGEF domain containing G2 [Source:HGNC Symbol;Acc:HGNC:29515] |
| ENSG00000092439 | HGLibA_51769 | 93 | -0.223155478 | TRPM7 | transient receptor potential cation channel subfamily M member 7 [Source:HGNC Symbol;Acc:HGNC:17994] |
| ENSG00000099721 | HGLibA_01770 | 93 | -0.223155478 | AMELY | amelogenin Y-linked [Source:HGNC Symbol;Acc:HGNC:462] |
| ENSG00000100031 | HGLibA_19011 | 93 | -0.223155478 | GGT1 | gamma-glutamyltransferase 1 [Source:HGNC Symbol;Acc:HGNC:4250] |
| ENSG00000101323 | HGLibA_20832 | 93 | -0.223155478 | HAO1 | hydroxyacid oxidase 1 [Source:HGNC Symbol;Acc:HGNC:4809] |
| ENSG00000101331 | HGLibA_08049 | 93 | -0.223155478 | CCM2L | CCM2 like scaffold protein [Source:HGNC Symbol;Acc:HGNC:16153] |
| ENSG00000102007 | HGLibA_37218 | 93 | -0.223155478 | PLP2 | proteolipid protein 2 [Source:HGNC Symbol;Acc:HGNC:9087] |
| ENSG00000102743 | HGLibA_44651 | 93 | -0.223155478 | SLC25A15 | solute carrier family 25 member 15 [Source:HGNC Symbol;Acc:HGNC:10985] |
| ENSG00000102870 | HGLibA_56431 | 93 | -0.223155478 | ZNF629 | zinc finger protein 629 [Source:HGNC Symbol;Acc:HGNC:29008] |
| ENSG00000102882 | HGLibA_28270 | 93 | -0.223155478 | MAPK3 | mitogen-activated protein kinase 3 [Source:HGNC Symbol;Acc:HGNC:6877] |
| ENSG00000102900 | HGLibA_33076 | 93 | -0.223155478 | NUP93 | nucleoporin 93 [Source:HGNC Symbol;Acc:HGNC:28958] |
| ENSG00000102984 | HGLibA_56818 | 93 | -0.223155478 | ZNF821 | zinc finger protein 821 [Source:HGNC Symbol;Acc:HGNC:28043] |
| ENSG00000104047 | HGLibA_14072 | 93 | -0.223155478 | DTWD1 | DTW domain containing 1 [Source:HGNC Symbol;Acc:HGNC:30926] |
| ENSG00000104321 | HGLibA_51724 | 93 | -0.223155478 | TRPA1 | transient receptor potential cation channel subfamily A member 1 [Source:HGNC Symbol;Acc:HGNC:497] |
| ENSG00000104343 | HGLibA_52671 | 93 | -0.223155478 | UBE2W | ubiquitin conjugating enzyme E2 W [Source:HGNC Symbol;Acc:HGNC:25616] |
| ENSG00000104432 | HGLibA_23190 | 93 | -0.223155478 | IL7 | interleukin 7 [Source:HGNC Symbol;Acc:HGNC:6023] |
| ENSG00000105127 | HGLibA_01444 | 93 | -0.223155478 | AKAP8 | A-kinase anchoring protein 8 [Source:HGNC Symbol;Acc:HGNC:378] |
| ENSG00000105402 | HGLibA_31127 | 93 | -0.223155478 | NAPA | NSF attachment protein alpha [Source:HGNC Symbol;Acc:HGNC:7641] |
| ENSG00000105997 | HGLibA_21907 | 93 | -0.223155478 | HOXA3 | homeobox A3 [Source:HGNC Symbol;Acc:HGNC:5104] |
| ENSG00000108379 | HGLibA_54365 | 93 | -0.223155478 | WNT3 | Wnt family member 3 [Source:HGNC Symbol;Acc:HGNC:12782] |
| ENSG00000108528 | HGLibA_44639 | 93 | -0.223155478 | SLC25A11 | solute carrier family 25 member 11 [Source:HGNC Symbol;Acc:HGNC:10981] |
| ENSG00000109181 | HGLibA_52911 | 93 | -0.223155478 | UGT2B10 | UDP glucuronosyltransferase family 2 member B10 [Source:HGNC Symbol;Acc:HGNC:12544] |
| ENSG00000109501 | HGLibA_54285 | 93 | -0.223155478 | WFS1 | wolframin ER transmembrane glycoprotein [Source:HGNC Symbol;Acc:HGNC:12762] |
| ENSG00000111537 | HGLibA_22707 | 93 | -0.223155478 | IFNG | interferon gamma [Source:HGNC Symbol;Acc:HGNC:5438] |
| ENSG00000112343 | HGLibA_51480 | 93 | -0.223155478 | TRIM38 | tripartite motif containing 38 [Source:HGNC Symbol;Acc:HGNC:10059] |
| ENSG00000113263 | HGLibA_23819 | 93 | -0.223155478 | ITK | IL2 inducible T cell kinase [Source:HGNC Symbol;Acc:HGNC:6171] |
| ENSG00000114115 | HGLibA_40630 | 93 | -0.223155478 | RBP1 | retinol binding protein 1 [Source:HGNC Symbol;Acc:HGNC:9919] |
| ENSG00000114742 | HGLibA_54109 | 93 | -0.223155478 | WDR48 | WD repeat domain 48 [Source:HGNC Symbol;Acc:HGNC:30914] |
| ENSG00000114790 | HGLibA_02839 | 93 | -0.223155478 | ARHGEF26 | Rho guanine nucleotide exchange factor 26 [Source:HGNC Symbol;Acc:HGNC:24490] |
| ENSG00000115091 | HGLibA_00658 | 93 | -0.223155478 | ACTR3 | ARP3 actin related protein 3 homolog [Source:HGNC Symbol;Acc:HGNC:170] |
| ENSG00000115425 | HGLibA_36021 | 93 | -0.223155478 | PECR | peroxisomal trans-2-enoyl-CoA reductase [Source:HGNC Symbol;Acc:HGNC:18281] |
| ENSG00000115839 | HGLibA_39919 | 93 | -0.223155478 | RAB3GAP1 | RAB3 GTPase activating protein catalytic subunit 1 [Source:HGNC Symbol;Acc:HGNC:17063] |
| ENSG00000116675 | HGLibA_13634 | 93 | -0.223155478 | DNAJC6 | DnaJ heat shock protein family (Hsp40) member C6 [Source:HGNC Symbol;Acc:HGNC:15469] |
| ENSG00000116729 | HGLibA_54331 | 93 | -0.223155478 | WLS | wntless Wnt ligand secretion mediator [Source:HGNC Symbol;Acc:HGNC:30238] |
| ENSG00000116830 | HGLibA_52170 | 93 | -0.223155478 | TTF2 | transcription termination factor 2 [Source:HGNC Symbol;Acc:HGNC:12398] |
| ENSG00000117419 | HGLibA_15484 | 93 | -0.223155478 | ERI3 | ERI1 exoribonuclease family member 3 [Source:HGNC Symbol;Acc:HGNC:17276] |
| ENSG00000119888 | HGLibA_15272 | 93 | -0.223155478 | EPCAM | epithelial cell adhesion molecule [Source:HGNC Symbol;Acc:HGNC:11529] |
| ENSG00000120328 | HGLibA_35478 | 93 | -0.223155478 | PCDHB12 | protocadherin beta 12 [Source:HGNC Symbol;Acc:HGNC:8683] |
| ENSG00000122224 | HGLibA_27689 | 93 | -0.223155478 | LY9 | lymphocyte antigen 9 [Source:HGNC Symbol;Acc:HGNC:6730] |
| ENSG00000124205 | HGLibA_14474 | 93 | -0.223155478 | EDN3 | endothelin 3 [Source:HGNC Symbol;Acc:HGNC:3178] |
| ENSG00000124237 | HGLibA_06029 | 93 | -0.223155478 | C20orf85 | chromosome 20 open reading frame 85 [Source:HGNC Symbol;Acc:HGNC:16216] |
| ENSG00000124562 | HGLibA_45973 | 93 | -0.223155478 | SNRPC | small nuclear ribonucleoprotein polypeptide C [Source:HGNC Symbol;Acc:HGNC:11157] |
| ENSG00000125247 | HGLibA_50680 | 93 | -0.223155478 | TMTC4 | transmembrane and tetratricopeptide repeat containing 4 [Source:HGNC Symbol;Acc:HGNC:25904] |
| ENSG00000125841 | HGLibA_32728 | 93 | -0.223155478 | NRSN2 | neurensin 2 [Source:HGNC Symbol;Acc:HGNC:16229] |
| ENSG00000125861 | HGLibA_18975 | 93 | -0.223155478 | GFRA4 | GDNF family receptor alpha 4 [Source:HGNC Symbol;Acc:HGNC:13821] |
| ENSG00000128276 | HGLibA_40935 | 93 | -0.223155478 | RFPL3 | ret finger protein like 3 [Source:HGNC Symbol;Acc:HGNC:9980] |
| ENSG00000128645 | HGLibA_21983 | 93 | -0.223155478 | HOXD1 | homeobox D1 [Source:HGNC Symbol;Acc:HGNC:5132] |
| ENSG00000129667 | HGLibA_41135 | 93 | -0.223155478 | RHBDF2 | rhomboid 5 homolog 2 [Source:HGNC Symbol;Acc:HGNC:20788] |
| ENSG00000130592 | HGLibA_27562 | 93 | -0.223155478 | LSP1 | lymphocyte specific protein 1 [Source:HGNC Symbol;Acc:HGNC:6707] |
| ENSG00000131473 | HGLibA_00453 | 93 | -0.223155478 | ACLY | ATP citrate lyase [Source:HGNC Symbol;Acc:HGNC:115] |
| ENSG00000134202 | HGLibA_20477 | 93 | -0.223155478 | GSTM3 | glutathione S-transferase mu 3 [Source:HGNC Symbol;Acc:HGNC:4635] |
| ENSG00000134330 | HGLibA_22467 | 93 | -0.223155478 | IAH1 | isoamyl acetate hydrolyzing esterase 1 (putative) [Source:HGNC Symbol;Acc:HGNC:27696] |

| | | | | | |
|---|---|---|---|---|---|
| ENSG00000134461 | HGLibA_02013 | 93 | -0.223155478 | ANKRD16 | ankyrin repeat domain 16 [Source:HGNC Symbol;Acc:HGNC:23471] |
| ENSG00000134882 | HGLibA_52537 | 93 | -0.223155478 | UBAC2 | UBA domain containing 2 [Source:HGNC Symbol;Acc:HGNC:20486] |
| ENSG00000134909 | HGLibA_02758 | 93 | -0.223155478 | ARHGAP32 | Rho GTPase activating protein 32 [Source:HGNC Symbol;Acc:HGNC:17399] |
| ENSG00000135773 | HGLibA_07180 | 93 | -0.223155478 | CAPN9 | calpain 9 [Source:HGNC Symbol;Acc:HGNC:1486] |
| ENSG00000135945 | HGLibA_40883 | 93 | -0.223155478 | REV1 | DNA directed polymerase [Source:HGNC Symbol;Acc:HGNC:14060]" |
| ENSG00000136490 | HGLibA_26533 | 93 | -0.223155478 | LIMD2 | LIM domain containing 2 [Source:HGNC Symbol;Acc:HGNC:28142] |
| ENSG00000137825 | HGLibA_23845 | 93 | -0.223155478 | ITPKA | inositol-trisphosphate 3-kinase A [Source:HGNC Symbol;Acc:HGNC:6178] |
| ENSG00000137936 | HGLibA_04247 | 93 | -0.223155478 | BCAR3 | NSP family adaptor protein [Source:HGNC Symbol;Acc:HGNC:973]" |
| ENSG00000140839 | HGLibA_09945 | 93 | -0.223155478 | CLEC18B | C-type lectin domain family 18 member B [Source:HGNC Symbol;Acc:HGNC:33849] |
| ENSG00000141034 | HGLibA_19056 | 93 | -0.223155478 | GID4 | GID complex subunit 4 homolog [Source:HGNC Symbol;Acc:HGNC:28453] |
| ENSG00000141741 | HGLibA_29249 | 93 | -0.223155478 | MIEN1 | migration and invasion enhancer 1 [Source:HGNC Symbol;Acc:HGNC:28230] |
| ENSG00000141933 | HGLibA_51122 | 93 | -0.223155478 | TPGS1 | tubulin polyglutamylase complex subunit 1 [Source:HGNC Symbol;Acc:HGNC:25058] |
| ENSG00000142330 | HGLibA_07146 | 93 | -0.223155478 | CAPN10 | calpain 10 [Source:HGNC Symbol;Acc:HGNC:1477] |
| ENSG00000143321 | HGLibA_21040 | 93 | -0.223155478 | HDGF | heparin binding growth factor [Source:HGNC Symbol;Acc:HGNC:4856] |
| ENSG00000143621 | HGLibA_23213 | 93 | -0.223155478 | ILF2 | interleukin enhancer binding factor 2 [Source:HGNC Symbol;Acc:HGNC:6037] |
| ENSG00000144218 | HGLibA_01111 | 93 | -0.223155478 | AFF3 | AF4/FMR2 family member 3 [Source:HGNC Symbol;Acc:HGNC:6473] |
| ENSG00000145826 | HGLibA_26269 | 93 | -0.223155478 | LECT2 | leukocyte cell derived chemotaxin 2 [Source:HGNC Symbol;Acc:HGNC:6550] |
| ENSG00000145833 | HGLibA_12720 | 93 | -0.223155478 | DDX46 | DEAD-box helicase 46 [Source:HGNC Symbol;Acc:HGNC:18681] |
| ENSG00000146039 | HGLibA_44467 | 93 | -0.223155478 | SLC17A4 | solute carrier family 17 member 4 [Source:HGNC Symbol;Acc:HGNC:10932] |
| ENSG00000146205 | HGLibA_02188 | 93 | -0.223155478 | ANO7 | anoctamin 7 [Source:HGNC Symbol;Acc:HGNC:31677] |
| ENSG00000146360 | HGLibA_20059 | 93 | -0.223155478 | GPR6 | G protein-coupled receptor 6 [Source:HGNC Symbol;Acc:HGNC:4515] |
| ENSG00000147650 | HGLibA_27206 | 93 | -0.223155478 | LRP12 | LDL receptor related protein 12 [Source:HGNC Symbol;Acc:HGNC:31708] |
| ENSG00000147872 | HGLibA_37173 | 93 | -0.223155478 | PLIN2 | perilipin 2 [Source:HGNC Symbol;Acc:HGNC:248] |
| ENSG00000149798 | HGLibA_08570 | 93 | -0.223155478 | CDC42EP2 | CDC42 effector protein 2 [Source:HGNC Symbol;Acc:HGNC:16263] |
| ENSG00000150347 | HGLibA_02908 | 93 | -0.223155478 | ARID5B | AT-rich interaction domain 5B [Source:HGNC Symbol;Acc:HGNC:17362] |
| ENSG00000151150 | HGLibA_01939 | 93 | -0.223155478 | ANK3 | ankyrin 3 [Source:HGNC Symbol;Acc:HGNC:494] |
| ENSG00000152642 | HGLibA_19785 | 93 | -0.223155478 | GPD1L | glycerol-3-phosphate dehydrogenase 1 like [Source:HGNC Symbol;Acc:HGNC:28956] |
| ENSG00000152700 | HGLibA_42740 | 93 | -0.223155478 | SAR1B | secretion associated Ras related GTPase 1B [Source:HGNC Symbol;Acc:HGNC:10535] |
| ENSG00000152785 | HGLibA_04604 | 93 | -0.223155478 | BMP3 | bone morphogenetic protein 3 [Source:HGNC Symbol;Acc:HGNC:1070] |
| ENSG00000152785 | HGLibA_04605 | 93 | -0.223155478 | BMP3 | bone morphogenetic protein 3 [Source:HGNC Symbol;Acc:HGNC:1070] |
| ENSG00000153208 | HGLibA_28907 | 93 | -0.223155478 | MERTK | tyrosine kinase [Source:HGNC Symbol;Acc:HGNC:7027]" |
| ENSG00000154309 | HGLibA_13281 | 93 | -0.223155478 | DISP1 | dispatched RND transporter family member 1 [Source:HGNC Symbol;Acc:HGNC:19711] |
| ENSG00000154328 | HGLibA_31632 | 93 | -0.223155478 | NEIL2 | nei like DNA glycosylase 2 [Source:HGNC Symbol;Acc:HGNC:18956] |
| ENSG00000155659 | HGLibA_53847 | 93 | -0.223155478 | VSIG4 | V-set and immunoglobulin domain containing 4 [Source:HGNC Symbol;Acc:HGNC:17032] |
| ENSG00000156232 | HGLibA_54289 | 93 | -0.223155478 | WHAMM | golgi membranes and microtubules [Source:HGNC Symbol;Acc:HGNC:30493]" |
| ENSG00000158186 | HGLibA_29813 | 93 | -0.223155478 | MRAS | muscle RAS oncogene homolog [Source:HGNC Symbol;Acc:HGNC:7227] |
| ENSG00000158402 | HGLibA_08533 | 93 | -0.223155478 | CDC25C | cell division cycle 25C [Source:HGNC Symbol;Acc:HGNC:1727] |
| ENSG00000161270 | HGLibA_32455 | 93 | -0.223155478 | NPHS1 | nephrin [Source:HGNC Symbol;Acc:HGNC:7908]" |
| ENSG00000161847 | HGLibA_40400 | 93 | -0.223155478 | RAVER1 | PTB binding 1 [Source:HGNC Symbol;Acc:HGNC:30296]" |
| ENSG00000162836 | HGLibA_00518 | 93 | -0.223155478 | ACP6 | lysophosphatidic [Source:HGNC Symbol;Acc:HGNC:29609]" |
| ENSG00000163932 | HGLibA_38540 | 93 | -0.223155478 | PRKCD | protein kinase C delta [Source:HGNC Symbol;Acc:HGNC:9399] |
| ENSG00000164031 | HGLibA_13535 | 93 | -0.223155478 | DNAJB14 | DnaJ heat shock protein family (Hsp40) member B14 [Source:HGNC Symbol;Acc:HGNC:25881] |
| ENSG00000164300 | HGLibA_43495 | 93 | -0.223155478 | SERINC5 | serine incorporator 5 [Source:HGNC Symbol;Acc:HGNC:18825] |
| ENSG00000165512 | HGLibA_55605 | 93 | -0.223155478 | ZNF22 | zinc finger protein 22 [Source:HGNC Symbol;Acc:HGNC:13012] |
| ENSG00000166111 | HGLibA_47892 | 93 | -0.223155478 | SVOP | SV2 related protein [Source:HGNC Symbol;Acc:HGNC:25417] |
| ENSG00000166510 | HGLibA_07842 | 93 | -0.223155478 | CCDC68 | coiled-coil domain containing 68 [Source:HGNC Symbol;Acc:HGNC:24350] |
| ENSG00000166685 | HGLibA_10470 | 93 | -0.223155478 | COG1 | component of oligomeric golgi complex 1 [Source:HGNC Symbol;Acc:HGNC:6545] |
| ENSG00000166704 | HGLibA_56367 | 93 | -0.223155478 | ZNF606 | zinc finger protein 606 [Source:HGNC Symbol;Acc:HGNC:25879] |
| ENSG00000166816 | HGLibA_26236 | 93 | -0.223155478 | LDHD | lactate dehydrogenase D [Source:HGNC Symbol;Acc:HGNC:19708] |
| ENSG00000166825 | HGLibA_02210 | 93 | -0.223155478 | ANPEP | membrane [Source:HGNC Symbol;Acc:HGNC:500]" |
| ENSG00000167642 | HGLibA_46716 | 93 | -0.223155478 | SPINT2 | Kunitz type 2 [Source:HGNC Symbol;Acc:HGNC:11247]" |
| ENSG00000167642 | HGLibA_46714 | 93 | -0.223155478 | SPINT2 | Kunitz type 2 [Source:HGNC Symbol;Acc:HGNC:11247]" |
| ENSG00000167702 | HGLibA_24974 | 93 | -0.223155478 | KIFC2 | kinesin family member C2 [Source:HGNC Symbol;Acc:HGNC:29530] |
| ENSG00000168612 | HGLibA_57037 | 93 | -0.223155478 | ZSWIM1 | zinc finger SWIM-type containing 1 [Source:HGNC Symbol;Acc:HGNC:16155] |
| ENSG00000170374 | HGLibA_46312 | 93 | -0.223155478 | SP7 | Sp7 transcription factor [Source:HGNC Symbol;Acc:HGNC:17321] |
| ENSG00000170948 | HGLibA_28472 | 93 | -0.223155478 | MBD3L1 | methyl-CpG binding domain protein 3 like 1 [Source:HGNC Symbol;Acc:HGNC:15774] |
| ENSG00000171124 | HGLibA_18191 | 93 | -0.223155478 | FUT3 | fucosyltransferase 3 (Lewis blood group) [Source:HGNC Symbol;Acc:HGNC:4014] |
| ENSG00000171320 | HGLibA_15560 | 93 | -0.223155478 | ESCO2 | establishment of sister chromatid cohesion N-acetyltransferase 2 [Source:HGNC Symbol;Acc:HGNC:27230] |
| ENSG00000171433 | HGLibA_19289 | 93 | -0.223155478 | GLOD5 | glyoxalase domain containing 5 [Source:HGNC Symbol;Acc:HGNC:33358] |
| ENSG00000171469 | HGLibA_56245 | 93 | -0.223155478 | ZNF561 | zinc finger protein 561 [Source:HGNC Symbol;Acc:HGNC:28684] |
| ENSG00000171793 | HGLibA_11667 | 93 | -0.223155478 | CTPS1 | CTP synthase 1 [Source:HGNC Symbol;Acc:HGNC:2519] |
| ENSG00000172156 | HGLibA_07963 | 93 | -0.223155478 | CCL11 | C-C motif chemokine ligand 11 [Source:HGNC Symbol;Acc:HGNC:10610] |
| ENSG00000172345 | HGLibA_47366 | 93 | -0.223155478 | STARD5 | StAR related lipid transfer domain containing 5 [Source:HGNC Symbol;Acc:HGNC:18065] |
| ENSG00000172543 | HGLibA_11735 | 93 | -0.223155478 | CTSW | cathepsin W [Source:HGNC Symbol;Acc:HGNC:2546] |
| ENSG00000172955 | HGLibA_00961 | 93 | -0.223155478 | ADH6 | alcohol dehydrogenase 6 (class V) [Source:HGNC Symbol;Acc:HGNC:255] |
| ENSG00000173473 | HGLibA_45622 | 93 | -0.223155478 | SMARCC1 | actin dependent regulator of chromatin subfamily c member 1 [Source:HGNC Symbol;Acc:HGNC:11104]" |
| ENSG00000174233 | HGLibA_00922 | 93 | -0.223155478 | ADCY6 | adenylate cyclase 6 [Source:HGNC Symbol;Acc:HGNC:237] |
| ENSG00000174483 | HGLibA_04205 | 93 | -0.223155478 | BBS1 | Bardet-Biedl syndrome 1 [Source:HGNC Symbol;Acc:HGNC:966] |
| ENSG00000175471 | HGLibA_28653 | 93 | -0.223155478 | MCTP1 | multiple C2 and transmembrane domain containing 1 [Source:HGNC Symbol;Acc:HGNC:26183] |
| ENSG00000175820 | HGLibA_07678 | 93 | -0.223155478 | CCDC168 | coiled-coil domain containing 168 [Source:HGNC Symbol;Acc:HGNC:26851] |
| ENSG00000175938 | HGLibA_34594 | 93 | -0.223155478 | ORAI3 | ORAI calcium release-activated calcium modulator 3 [Source:HGNC Symbol;Acc:HGNC:28185] |
| ENSG00000176058 | HGLibA_51190 | 93 | -0.223155478 | TPRN | taperin [Source:HGNC Symbol;Acc:HGNC:26894] |
| ENSG00000176438 | HGLibA_47982 | 93 | -0.223155478 | SYNE3 | spectrin repeat containing nuclear envelope family member 3 [Source:HGNC Symbol;Acc:HGNC:19861] |
| ENSG00000176927 | HGLibA_14547 | 93 | -0.223155478 | EFCAB5 | EF-hand calcium binding domain 5 [Source:HGNC Symbol;Acc:HGNC:24801] |
| ENSG00000178021 | HGLibA_51991 | 93 | -0.223155478 | TSPYL6 | TSPY like 6 [Source:HGNC Symbol;Acc:HGNC:14521] |
| ENSG00000178773 | HGLibA_10971 | 93 | -0.223155478 | CPNE7 | copine 7 [Source:HGNC Symbol;Acc:HGNC:2320] |
| ENSG00000178776 | HGLibA_06390 | 93 | -0.223155478 | C5orf46 | chromosome 5 open reading frame 46 [Source:HGNC Symbol;Acc:HGNC:33768] |
| ENSG00000179409 | HGLibA_18915 | 93 | -0.223155478 | GEMIN4 | gem nuclear organelle associated protein 4 [Source:HGNC Symbol;Acc:HGNC:15717] |
| ENSG00000180953 | HGLibA_47230 | 93 | -0.223155478 | ST20 | suppressor of tumorigenicity 20 [Source:HGNC Symbol;Acc:HGNC:33520] |
| ENSG00000182177 | HGLibA_03229 | 93 | -0.223155478 | ASB18 | ankyrin repeat and SOCS box containing 18 [Source:HGNC Symbol;Acc:HGNC:19770] |
| ENSG00000182318 | HGLibA_57004 | 93 | -0.223155478 | ZSCAN22 | zinc finger and SCAN domain containing 22 [Source:HGNC Symbol;Acc:HGNC:4929] |
| ENSG00000182747 | HGLibA_44982 | 93 | -0.223155478 | SLC35D3 | solute carrier family 35 member D3 [Source:HGNC Symbol;Acc:HGNC:15621] |
| ENSG00000182798 | HGLibA_27939 | 93 | -0.223155478 | MAGEB17 | MAGE family member B17 [Source:HGNC Symbol;Acc:HGNC:17418] |
| ENSG00000184047 | HGLibA_13213 | 93 | -0.223155478 | DIABLO | diablo IAP-binding mitochondrial protein [Source:HGNC Symbol;Acc:HGNC:21528] |
| ENSG00000184117 | HGLibA_31971 | 93 | -0.223155478 | NIPSNAP1 | nipsnap homolog 1 [Source:HGNC Symbol;Acc:HGNC:7827] |
| ENSG00000184500 | HGLibA_38690 | 93 | -0.223155478 | PROS1 | protein S [Source:HGNC Symbol;Acc:HGNC:9456] |
| ENSG00000184517 | HGLibA_55180 | 93 | -0.223155478 | ZFP1 | ZFP1 zinc finger protein [Source:HGNC Symbol;Acc:HGNC:23328] |
| ENSG00000185559 | HGLibA_13355 | 93 | -0.223155478 | DLK1 | delta like non-canonical Notch ligand 1 [Source:HGNC Symbol;Acc:HGNC:2907] |
| ENSG00000185946 | HGLibA_41723 | 93 | -0.223155478 | RNPC3 | RRM) containing 3 [Source:HGNC Symbol;Acc:HGNC:18666]" |
| ENSG00000186788 | HGLibA_46499 | 93 | -0.223155478 | SPATA31D3 | SPATA31 subfamily D member 3 [Source:HGNC Symbol;Acc:HGNC:38603] |
| ENSG00000187097 | HGLibA_15209 | 93 | -0.223155478 | ENTPD5 | ectonucleoside triphosphate diphosphohydrolase 5 [Source:HGNC Symbol;Acc:HGNC:3367] |
| ENSG00000187607 | HGLibA_55751 | 93 | -0.223155478 | ZNF286A | zinc finger protein 286A [Source:HGNC Symbol;Acc:HGNC:13501] |
| ENSG00000188981 | HGLibA_30170 | 93 | -0.223155478 | MSANTD1 | Myb/SANT DNA binding domain containing 1 [Source:HGNC Symbol;Acc:HGNC:33741] |
| ENSG00000196466 | HGLibA_56776 | 93 | -0.223155478 | ZNF799 | zinc finger protein 799 [Source:HGNC Symbol;Acc:HGNC:28071] |
| ENSG00000196704 | HGLibA_01858 | 93 | -0.223155478 | AMZ2 | archaelysin family metallopeptidase 2 [Source:HGNC Symbol;Acc:HGNC:28041] |
| ENSG00000196981 | HGLibA_54134 | 93 | -0.223155478 | WDR5B | WD repeat domain 5B [Source:HGNC Symbol;Acc:HGNC:17826] |
| ENSG00000197124 | HGLibA_56531 | 93 | -0.223155478 | ZNF682 | zinc finger protein 682 [Source:HGNC Symbol;Acc:HGNC:28857] |
| ENSG00000197324 | HGLibA_27201 | 93 | -0.223155478 | LRP10 | LDL receptor related protein 10 [Source:HGNC Symbol;Acc:HGNC:14553] |
| ENSG00000197448 | HGLibA_20470 | 93 | -0.223155478 | GSTK1 | glutathione S-transferase kappa 1 [Source:HGNC Symbol;Acc:HGNC:16906] |
| ENSG00000197506 | HGLibA_44837 | 93 | -0.223155478 | SLC28A3 | solute carrier family 28 member 3 [Source:HGNC Symbol;Acc:HGNC:16484] |
| ENSG00000197653 | HGLibA_13471 | 93 | -0.223155478 | DNAH10 | dynein axonemal heavy chain 10 [Source:HGNC Symbol;Acc:HGNC:2941] |
| ENSG00000197779 | HGLibA_56801 | 93 | -0.223155478 | ZNF81 | zinc finger protein 81 [Source:HGNC Symbol;Acc:HGNC:13156] |
| ENSG00000197943 | HGLibA_37006 | 93 | -0.223155478 | PLCG2 | phospholipase C gamma 2 [Source:HGNC Symbol;Acc:HGNC:9066] |
| ENSG00000198546 | HGLibA_56127 | 93 | -0.223155478 | ZNF511 | zinc finger protein 511 [Source:HGNC Symbol;Acc:HGNC:28445] |
| ENSG00000198765 | HGLibA_47927 | 93 | -0.223155478 | SYCP1 | synaptonemal complex protein 1 [Source:HGNC Symbol;Acc:HGNC:11487] |
| ENSG00000198821 | HGLibA_08310 | 93 | -0.223155478 | CD247 | CD247 molecule [Source:HGNC Symbol;Acc:HGNC:1677] |
| ENSG00000204385 | HGLibA_45165 | 93 | -0.223155478 | SLC44A4 | solute carrier family 44 member 4 [Source:HGNC Symbol;Acc:HGNC:13941] |
| ENSG00000204616 | HGLibA_51458 | 93 | -0.223155478 | TRIM31 | tripartite motif containing 31 [Source:HGNC Symbol;Acc:HGNC:16289] |
| ENSG00000205659 | HGLibA_26566 | 93 | -0.223155478 | LIN52 | lin-52 DREAM MuvB core complex component [Source:HGNC Symbol;Acc:HGNC:19856] |
| ENSG00000205858 | HGLibA_27421 | 93 | -0.223155478 | LRRC72 | leucine rich repeat containing 72 [Source:HGNC Symbol;Acc:HGNC:42972] |
| ENSG00000212657 | HGLibA_25657 | 93 | -0.223155478 | KRTAP16-1 | keratin associated protein 16-1 [Source:HGNC Symbol;Acc:HGNC:18916] |
| ENSG00000214944 | HGLibA_02843 | 93 | -0.223155478 | ARHGEF28 | Rho guanine nucleotide exchange factor 28 [Source:HGNC Symbol;Acc:HGNC:30322] |
| ENSG00000215018 | HGLibA_10558 | 93 | -0.223155478 | COL28A1 | collagen type XXVIII alpha 1 chain [Source:HGNC Symbol;Acc:HGNC:22442] |
| ENSG00000215301 | HGLibA_12702 | 93 | -0.223155478 | DDX3X | DEAD-box helicase 3 X-linked [Source:HGNC Symbol;Acc:HGNC:2745] |
| ENSG00000221933 | HGLibA_33732 | 93 | -0.223155478 | OR2A25 | olfactory receptor family 2 subfamily A member 25 [Source:HGNC Symbol;Acc:HGNC:19562] |
| ENSG00000225190 | HGLibA_37137 | 93 | -0.223155478 | PLEKHM1 | pleckstrin homology and RUN domain containing M1 [Source:HGNC Symbol;Acc:HGNC:29017] |
| ENSG00000237038 | HGLibA_53286 | 93 | -0.223155478 | USP17L8 | ubiquitin specific peptidase 17-like family member 8 [Source:HGNC Symbol;Acc:HGNC:37181] |
| ENSG00000237353 | HGLibA_35280 | 93 | -0.223155478 | PATE4 | prostate and testis expressed 4 [Source:HGNC Symbol;Acc:HGNC:35427] |
| ENSG00000240344 | HGLibA_37945 | 93 | -0.223155478 | PPIL3 | peptidylprolyl isomerase like 3 [Source:HGNC Symbol;Acc:HGNC:9262] |
| ENSG00000244395 | HGLibA_40618 | 93 | -0.223155478 | RBMY1D | member D [Source:HGNC Symbol;Acc:HGNC:23915]" |
| ENSG00000244734 | HGLibA_20914 | 93 | -0.223155478 | HBB | hemoglobin subunit beta [Source:HGNC Symbol;Acc:HGNC:4827] |
| ENSG00000255221 | HGLibA_07228 | 93 | -0.223155478 | CARD17 | caspase recruitment domain family member 17 [Source:HGNC Symbol;Acc:HGNC:33827] |
| ENSG00000258588 | HGLibA_51561 | 93 | -0.223155478 | TRIM6-TRIM34 | TRIM6-TRIM34 readthrough [Source:HGNC Symbol;Acc:HGNC:33440] |
| ENSG00000259571 | HGLibA_04543 | 93 | -0.223155478 | BLID | cell death inducer [Source:HGNC Symbol;Acc:HGNC:33495]" |
| ENSG00000260811 | HGLibA_33957 | 93 | -0.223155478 | OR4C45 | olfactory receptor family 4 subfamily C member 45 (gene/pseudogene) [Source:HGNC Symbol;Acc:HGNC:31270] |
| ENSG00000265190 | HGLibA_02252 | 93 | -0.223155478 | ANXA8 | annexin A8 [Source:HGNC Symbol;Acc:HGNC:546] |
| ENSG00000288722 | HGLibA_15886 | 93 | -0.223155478 | F8A1 | - |
| FTSJ2 | HGLibA_18137 | 93 | -0.223155478 | FTSJ2 | - |
| GPR125 | HGLibA_19880 | 93 | -0.223155478 | GPR125 | - |
| HIST1H4E | HGLibA_21498 | 93 | -0.223155478 | HIST1H4E | - |
| HIST4H4 | HGLibA_21563 | 93 | -0.223155478 | HIST4H4 | - |
| hsa-mir-103a-2 | HGLibA_57167 | 93 | -0.223155478 | hsa-mir-103a-2 | - |
| hsa-mir-135a-1 | HGLibA_57792 | 93 | -0.223155478 | hsa-mir-135a-1 | - |
| hsa-mir-147a | HGLibA_57871 | 93 | -0.223155478 | hsa-mir-147a | - |
| hsa-mir-181a-2 | HGLibA_57964 | 93 | -0.223155478 | hsa-mir-181a-2 | - |

| | | | | | |
|---|---|---|---|---|---|
| hsa-mir-300 | HGLibA_58427 | 93 | -0.223155478 | hsa-mir-300 | - |
| hsa-mir-3194 | HGLibA_58875 | 93 | -0.223155478 | hsa-mir-3194 | - |
| hsa-mir-378c | HGLibA_59432 | 93 | -0.223155478 | hsa-mir-378c | - |
| hsa-mir-3910-2 | HGLibA_59508 | 93 | -0.223155478 | hsa-mir-3910-2 | - |
| hsa-mir-3911 | HGLibA_59513 | 93 | -0.223155478 | hsa-mir-3911 | - |
| hsa-mir-3919 | HGLibA_59552 | 93 | -0.223155478 | hsa-mir-3919 | - |
| hsa-mir-4324 | HGLibA_60019 | 93 | -0.223155478 | hsa-mir-4324 | - |
| hsa-mir-4330 | HGLibA_60049 | 93 | -0.223155478 | hsa-mir-4330 | - |
| hsa-mir-4435-1 | HGLibA_60132 | 93 | -0.223155478 | hsa-mir-4435-1 | - |
| hsa-mir-4639 | HGLibA_60660 | 93 | -0.223155478 | hsa-mir-4639 | - |
| hsa-mir-4681 | HGLibA_60835 | 93 | -0.223155478 | hsa-mir-4681 | - |
| hsa-mir-4701 | HGLibA_60912 | 93 | -0.223155478 | hsa-mir-4701 | - |
| hsa-mir-4723 | HGLibA_60990 | 93 | -0.223155478 | hsa-mir-4723 | - |
| hsa-mir-4747 | HGLibA_61084 | 93 | -0.223155478 | hsa-mir-4747 | - |
| hsa-mir-4793 | HGLibA_61278 | 93 | -0.223155478 | hsa-mir-4793 | - |
| hsa-mir-5190 | HGLibA_61677 | 93 | -0.223155478 | hsa-mir-5190 | - |
| hsa-mir-548i-4 | HGLibA_62026 | 93 | -0.223155478 | hsa-mir-548i-4 | - |
| hsa-mir-5685 | HGLibA_62261 | 93 | -0.223155478 | hsa-mir-5685 | - |
| hsa-mir-5708 | HGLibA_62369 | 93 | -0.223155478 | hsa-mir-5708 | - |
| hsa-mir-6069 | HGLibA_62525 | 93 | -0.223155478 | hsa-mir-6069 | - |
| hsa-mir-6720 | HGLibA_63040 | 93 | -0.223155478 | hsa-mir-6720 | - |
| hsa-mir-6811 | HGLibA_63421 | 93 | -0.223155478 | hsa-mir-6811 | - |
| hsa-mir-6828 | HGLibA_63488 | 93 | -0.223155478 | hsa-mir-6828 | - |
| hsa-mir-6850 | HGLibA_63571 | 93 | -0.223155478 | hsa-mir-6850 | - |
| hsa-mir-7641-2 | HGLibA_63918 | 93 | -0.223155478 | hsa-mir-7641-2 | - |
| KDELC1 | HGLibA_24474 | 93 | -0.223155478 | KDELC1 | - |
| LEPREL1 | HGLibA_26317 | 93 | -0.223155478 | LEPREL1 | - |
| LOC100133267 | HGLibA_26809 | 93 | -0.223155478 | LOC100133267 | - |
| MLL | HGLibA_29394 | 93 | -0.223155478 | MLL | - |
| PRMT10 | HGLibA_38619 | 93 | -0.223155478 | PRMT10 | - |
| SOLH | HGLibA_46169 | 93 | -0.223155478 | SOLH | - |
| ZCCHC16 | HGLibA_55018 | 93 | -0.223155478 | ZCCHC16 | - |
| AGPAT6 | HGLibA_01233 | 92 | -0.233106516 | AGPAT6 | - |
| ANKRD20A4 | HGLibA_02037 | 92 | -0.233106516 | ANKRD20A4 | - |
| C17orf104 | HGLibA_05522 | 92 | -0.233106516 | C17orf104 | - |
| C21orf2 | HGLibA_06034 | 92 | -0.233106516 | C21orf2 | - |
| C7orf55 | HGLibA_06555 | 92 | -0.233106516 | C7orf55 | - |
| ENSG00000004799 | HGLibA_35903 | 92 | -0.233106516 | PDK4 | pyruvate dehydrogenase kinase 4 [Source:HGNC Symbol;Acc:HGNC:8812] |
| ENSG00000005175 | HGLibA_41821 | 92 | -0.233106516 | RPAP3 | RNA polymerase II associated protein 3 [Source:HGNC Symbol;Acc:HGNC:26151] |
| ENSG00000008438 | HGLibA_36248 | 92 | -0.233106516 | PGLYRP1 | peptidoglycan recognition protein 1 [Source:HGNC Symbol;Acc:HGNC:8904] |
| ENSG00000011566 | HGLibA_28217 | 92 | -0.233106516 | MAP4K3 | mitogen-activated protein kinase kinase kinase kinase 3 [Source:HGNC Symbol;Acc:HGNC:6865] |
| ENSG00000013441 | HGLibA_10055 | 92 | -0.233106516 | CLK1 | CDC like kinase 1 [Source:HGNC Symbol;Acc:HGNC:2068] |
| ENSG00000023318 | HGLibA_15529 | 92 | -0.233106516 | ERP44 | endoplasmic reticulum protein 44 [Source:HGNC Symbol;Acc:HGNC:18311] |
| ENSG00000033122 | HGLibA_27413 | 92 | -0.233106516 | LRRC7 | leucine rich repeat containing 7 [Source:HGNC Symbol;Acc:HGNC:18531] |
| ENSG00000034239 | HGLibA_14520 | 92 | -0.233106516 | EFCAB1 | EF-hand calcium binding domain 1 [Source:HGNC Symbol;Acc:HGNC:25678] |
| ENSG00000049130 | HGLibA_25018 | 92 | -0.233106516 | KITLG | KIT ligand [Source:HGNC Symbol;Acc:HGNC:6343] |
| ENSG00000060237 | HGLibA_54333 | 92 | -0.233106516 | WNK1 | WNK lysine deficient protein kinase 1 [Source:HGNC Symbol;Acc:HGNC:14540] |
| ENSG00000072310 | HGLibA_46948 | 92 | -0.233106516 | SREBF1 | sterol regulatory element binding transcription factor 1 [Source:HGNC Symbol;Acc:HGNC:11289] |
| ENSG00000072840 | HGLibA_15679 | 92 | -0.233106516 | EVC | EvC ciliary complex subunit 1 [Source:HGNC Symbol;Acc:HGNC:3497] |
| ENSG00000079313 | HGLibA_40891 | 92 | -0.233106516 | REXO1 | RNA exonuclease 1 homolog [Source:HGNC Symbol;Acc:HGNC:24616] |
| ENSG00000085644 | HGLibA_55590 | 92 | -0.233106516 | ZNF213 | zinc finger protein 213 [Source:HGNC Symbol;Acc:HGNC:13005] |
| ENSG00000090339 | HGLibA_22495 | 92 | -0.233106516 | ICAM1 | intercellular adhesion molecule 1 [Source:HGNC Symbol;Acc:HGNC:5344] |
| ENSG00000090621 | HGLibA_34935 | 92 | -0.233106516 | PABPC4 | poly(A) binding protein cytoplasmic 4 [Source:HGNC Symbol;Acc:HGNC:8557] |
| ENSG00000091428 | HGLibA_40254 | 92 | -0.233106516 | RAPGEF4 | Rap guanine nucleotide exchange factor 4 [Source:HGNC Symbol;Acc:HGNC:16626] |
| ENSG00000096264 | HGLibA_31366 | 92 | -0.233106516 | NCR2 | natural cytotoxicity triggering receptor 2 [Source:HGNC Symbol;Acc:HGNC:6732] |
| ENSG00000099219 | HGLibA_15508 | 92 | -0.233106516 | ERMP1 | endoplasmic reticulum metallopeptidase 1 [Source:HGNC Symbol;Acc:HGNC:23703] |
| ENSG00000100450 | HGLibA_20724 | 92 | -0.233106516 | GZMH | granzyme H [Source:HGNC Symbol;Acc:HGNC:4710] |
| ENSG00000101346 | HGLibA_37469 | 92 | -0.233106516 | POFUT1 | protein O-fucosyltransferase 1 [Source:HGNC Symbol;Acc:HGNC:14988] |
| ENSG00000104055 | HGLibA_49264 | 92 | -0.233106516 | TGM5 | transglutaminase 5 [Source:HGNC Symbol;Acc:HGNC:11781] |
| ENSG00000104885 | HGLibA_13805 | 92 | -0.233106516 | DOT1L | DOT1 like histone lysine methyltransferase [Source:HGNC Symbol;Acc:HGNC:24948] |
| ENSG00000105227 | HGLibA_38991 | 92 | -0.233106516 | PRX | periaxin [Source:HGNC Symbol;Acc:HGNC:13797] |
| ENSG00000106367 | HGLibA_02295 | 92 | -0.233106516 | AP1S1 | adaptor related protein complex 1 subunit sigma 1 [Source:HGNC Symbol;Acc:HGNC:559] |
| ENSG00000106628 | HGLibA_37496 | 92 | -0.233106516 | POLD2 | accessory subunit [Source:HGNC Symbol;Acc:HGNC:9176]" |
| ENSG00000106714 | HGLibA_10426 | 92 | -0.233106516 | CNTNAP3 | contactin associated protein like 3 [Source:HGNC Symbol;Acc:HGNC:13834] |
| ENSG00000107371 | HGLibA_15778 | 92 | -0.233106516 | EXOSC3 | exosome component 3 [Source:HGNC Symbol;Acc:HGNC:17944] |
| ENSG00000107771 | HGLibA_08192 | 92 | -0.233106516 | CCSER2 | coiled-coil serine rich protein 2 [Source:HGNC Symbol;Acc:HGNC:29197] |
| ENSG00000108474 | HGLibA_36563 | 92 | -0.233106516 | PIGL | phosphatidylinositol glycan anchor biosynthesis class L [Source:HGNC Symbol;Acc:HGNC:8966] |
| ENSG00000110002 | HGLibA_53907 | 92 | -0.233106516 | VWA5A | von Willebrand factor A domain containing 5A [Source:HGNC Symbol;Acc:HGNC:6658] |
| ENSG00000111087 | HGLibA_19246 | 92 | -0.233106516 | GLI1 | GLI family zinc finger 1 [Source:HGNC Symbol;Acc:HGNC:4317] |
| ENSG00000111245 | HGLibA_30756 | 92 | -0.233106516 | MYL2 | myosin light chain 2 [Source:HGNC Symbol;Acc:HGNC:7583] |
| ENSG00000111361 | HGLibA_14734 | 92 | -0.233106516 | EIF2B1 | eukaryotic translation initiation factor 2B subunit alpha [Source:HGNC Symbol;Acc:HGNC:3257] |
| ENSG00000113211 | HGLibA_35505 | 92 | -0.233106516 | PCDHB6 | protocadherin beta 6 [Source:HGNC Symbol;Acc:HGNC:8691] |
| ENSG00000113971 | HGLibA_32448 | 92 | -0.233106516 | NPHP3 | nephrocystin 3 [Source:HGNC Symbol;Acc:HGNC:7907] |
| ENSG00000115942 | HGLibA_34605 | 92 | -0.233106516 | ORC2 | origin recognition complex subunit 2 [Source:HGNC Symbol;Acc:HGNC:8488] |
| ENSG00000116183 | HGLibA_35147 | 92 | -0.233106516 | PAPPA2 | pappalysin 2 [Source:HGNC Symbol;Acc:HGNC:14615] |
| ENSG00000116353 | HGLibA_28729 | 92 | -0.233106516 | MECR | mitochondrial trans-2-enoyl-CoA reductase [Source:HGNC Symbol;Acc:HGNC:19691] |
| ENSG00000116785 | HGLibA_09270 | 92 | -0.233106516 | CFHR3 | complement factor H related 3 [Source:HGNC Symbol;Acc:HGNC:16980] |
| ENSG00000121005 | HGLibA_11175 | 92 | -0.233106516 | CRISPLD1 | cysteine rich secretory protein LCCL domain containing 1 [Source:HGNC Symbol;Acc:HGNC:18206] |
| ENSG00000121211 | HGLibA_29564 | 92 | -0.233106516 | MND1 | meiotic nuclear divisions 1 [Source:HGNC Symbol;Acc:HGNC:24839] |
| ENSG00000124107 | HGLibA_45545 | 92 | -0.233106516 | SLPI | secretory leukocyte peptidase inhibitor [Source:HGNC Symbol;Acc:HGNC:11092] |
| ENSG00000125657 | HGLibA_50830 | 92 | -0.233106516 | TNFSF9 | TNF superfamily member 9 [Source:HGNC Symbol;Acc:HGNC:11939] |
| ENSG00000125733 | HGLibA_51632 | 92 | -0.233106516 | TRIP10 | thyroid hormone receptor interactor 10 [Source:HGNC Symbol;Acc:HGNC:12304] |
| ENSG00000127954 | HGLibA_47432 | 92 | -0.233106516 | STEAP4 | STEAP4 metalloreductase [Source:HGNC Symbol;Acc:HGNC:21923] |
| ENSG00000129465 | HGLibA_41321 | 92 | -0.233106516 | RIPK3 | receptor interacting serine/threonine kinase 3 [Source:HGNC Symbol;Acc:HGNC:10021] |
| ENSG00000129636 | HGLibA_23707 | 92 | -0.233106516 | ITFG1 | integrin alpha FG-GAP repeat containing 1 [Source:HGNC Symbol;Acc:HGNC:30697] |
| ENSG00000130150 | HGLibA_29686 | 92 | -0.233106516 | MOSPD2 | motile sperm domain containing 2 [Source:HGNC Symbol;Acc:HGNC:28381] |
| ENSG00000130538 | HGLibA_33568 | 92 | -0.233106516 | OR11H1 | olfactory receptor family 11 subfamily H member 1 [Source:HGNC Symbol;Acc:HGNC:15404] |
| ENSG00000130558 | HGLibA_33345 | 92 | -0.233106516 | OLFM1 | olfactomedin 1 [Source:HGNC Symbol;Acc:HGNC:17187] |
| ENSG00000131097 | HGLibA_21310 | 92 | -0.233106516 | HIGD1B | HIG1 hypoxia inducible domain family member 1B [Source:HGNC Symbol;Acc:HGNC:24318] |
| ENSG00000132196 | HGLibA_22198 | 92 | -0.233106516 | HSD17B7 | hydroxysteroid 17-beta dehydrogenase 7 [Source:HGNC Symbol;Acc:HGNC:5215] |
| ENSG00000132305 | HGLibA_23235 | 92 | -0.233106516 | IMMT | inner membrane mitochondrial protein [Source:HGNC Symbol;Acc:HGNC:6047] |
| ENSG00000134198 | HGLibA_51921 | 92 | -0.233106516 | TSPAN2 | tetraspanin 2 [Source:HGNC Symbol;Acc:HGNC:20659] |
| ENSG00000134283 | HGLibA_37890 | 92 | -0.233106516 | PPHLN1 | periphilin 1 [Source:HGNC Symbol;Acc:HGNC:19369] |
| ENSG00000134321 | HGLibA_42300 | 92 | -0.233106516 | RSAD2 | radical S-adenosyl methionine domain containing 2 [Source:HGNC Symbol;Acc:HGNC:30908] |
| ENSG00000134330 | HGLibA_22466 | 92 | -0.233106516 | IAH1 | isoamyl acetate hydrolyzing esterase 1 (putative) [Source:HGNC Symbol;Acc:HGNC:27696] |
| ENSG00000135525 | HGLibA_28232 | 92 | -0.233106516 | MAP7 | microtubule associated protein 7 [Source:HGNC Symbol;Acc:HGNC:6869] |
| ENSG00000135679 | HGLibA_28698 | 92 | -0.233106516 | MDM2 | MDM2 proto-oncogene [Source:HGNC Symbol;Acc:HGNC:6973] |
| ENSG00000135801 | HGLibA_48244 | 92 | -0.233106516 | TAF5L | TATA-box binding protein associated factor 5 like [Source:HGNC Symbol;Acc:HGNC:17304] |
| ENSG00000136250 | HGLibA_02265 | 92 | -0.233106516 | AOAH | acyloxyacyl hydrolase [Source:HGNC Symbol;Acc:HGNC:548] |
| ENSG00000136573 | HGLibA_04546 | 92 | -0.233106516 | BLK | Src family tyrosine kinase [Source:HGNC Symbol;Acc:HGNC:1057]" |
| ENSG00000136631 | HGLibA_53794 | 92 | -0.233106516 | VPS45 | vacuolar protein sorting 45 homolog [Source:HGNC Symbol;Acc:HGNC:14579] |
| ENSG00000137098 | HGLibA_46370 | 92 | -0.233106516 | SPAG8 | sperm associated antigen 8 [Source:HGNC Symbol;Acc:HGNC:14105] |
| ENSG00000137522 | HGLibA_41484 | 92 | -0.233106516 | RNF121 | ring finger protein 121 [Source:HGNC Symbol;Acc:HGNC:21070] |
| ENSG00000139329 | HGLibA_27629 | 92 | -0.233106516 | LUM | lumican [Source:HGNC Symbol;Acc:HGNC:6724] |
| ENSG00000141744 | HGLibA_37389 | 92 | -0.233106516 | PNMT | phenylethanolamine N-methyltransferase [Source:HGNC Symbol;Acc:HGNC:9160] |
| ENSG00000143226 | HGLibA_17189 | 92 | -0.233106516 | FCGR2A | Fc fragment of IgG receptor IIa [Source:HGNC Symbol;Acc:HGNC:3616] |
| ENSG00000144021 | HGLibA_09677 | 92 | -0.233106516 | CIAO1 | cytosolic iron-sulfur assembly component 1 [Source:HGNC Symbol;Acc:HGNC:14280] |
| ENSG00000144566 | HGLibA_39961 | 92 | -0.233106516 | RAB5A | member RAS oncogene family [Source:HGNC Symbol;Acc:HGNC:9783]" |
| ENSG00000146094 | HGLibA_13774 | 92 | -0.233106516 | DOK3 | docking protein 3 [Source:HGNC Symbol;Acc:HGNC:24583] |
| ENSG00000146535 | HGLibA_19439 | 92 | -0.233106516 | GNA12 | G protein subunit alpha 12 [Source:HGNC Symbol;Acc:HGNC:4380] |
| ENSG00000148604 | HGLibA_41045 | 92 | -0.233106516 | RGR | retinal G protein coupled receptor [Source:HGNC Symbol;Acc:HGNC:9990] |
| ENSG00000148719 | HGLibA_13529 | 92 | -0.233106516 | DNAJB12 | DnaJ heat shock protein family (Hsp40) member B12 [Source:HGNC Symbol;Acc:HGNC:14891] |
| ENSG00000148834 | HGLibA_20486 | 92 | -0.233106516 | GSTO1 | glutathione S-transferase omega 1 [Source:HGNC Symbol;Acc:HGNC:13312] |
| ENSG00000151092 | HGLibA_31882 | 92 | -0.233106516 | NGLY1 | N-glycanase 1 [Source:HGNC Symbol;Acc:HGNC:17646] |
| ENSG00000151665 | HGLibA_36551 | 92 | -0.233106516 | PIGF | phosphatidylinositol glycan anchor biosynthesis class F [Source:HGNC Symbol;Acc:HGNC:8962] |
| ENSG00000152527 | HGLibA_37127 | 92 | -0.233106516 | PLEKHH2 | MyTH4 and FERM domain containing H2 [Source:HGNC Symbol;Acc:HGNC:30506]" |
| ENSG00000155111 | HGLibA_08751 | 92 | -0.233106516 | CDK19 | cyclin dependent kinase 19 [Source:HGNC Symbol;Acc:HGNC:19338] |
| ENSG00000155265 | HGLibA_19636 | 92 | -0.233106516 | GOLGA7B | golgin A7 family member B [Source:HGNC Symbol;Acc:HGNC:31668] |
| ENSG00000157111 | HGLibA_50061 | 92 | -0.233106516 | TMEM171 | transmembrane protein 171 [Source:HGNC Symbol;Acc:HGNC:27031] |
| ENSG00000158169 | HGLibA_16836 | 92 | -0.233106516 | FANCC | FA complementation group C [Source:HGNC Symbol;Acc:HGNC:3584] |
| ENSG00000159261 | HGLibA_09859 | 92 | -0.233106516 | CLDN14 | claudin 14 [Source:HGNC Symbol;Acc:HGNC:2035] |
| ENSG00000159346 | HGLibA_00979 | 92 | -0.233106516 | ADIPOR1 | adiponectin receptor 1 [Source:HGNC Symbol;Acc:HGNC:24040] |
| ENSG00000159763 | HGLibA_36702 | 92 | -0.233106516 | PIP | prolactin induced protein [Source:HGNC Symbol;Acc:HGNC:8993] |
| ENSG00000162426 | HGLibA_45171 | 92 | -0.233106516 | SLC45A1 | solute carrier family 45 member 1 [Source:HGNC Symbol;Acc:HGNC:17939] |
| ENSG00000162542 | HGLibA_49790 | 92 | -0.233106516 | TMCO4 | transmembrane and coiled-coil domains 4 [Source:HGNC Symbol;Acc:HGNC:27393] |
| ENSG00000162882 | HGLibA_20789 | 92 | -0.233106516 | HAAO | 4-dioxygenase [Source:HGNC Symbol;Acc:HGNC:4796]" |
| ENSG00000163217 | HGLibA_04593 | 92 | -0.233106516 | BMP10 | bone morphogenetic protein 10 [Source:HGNC Symbol;Acc:HGNC:20869] |
| ENSG00000163993 | HGLibA_42583 | 92 | -0.233106516 | S100P | S100 calcium binding protein P [Source:HGNC Symbol;Acc:HGNC:10504] |
| ENSG00000164062 | HGLibA_02405 | 92 | -0.233106516 | APEH | acylaminoacyl-peptide hydrolase [Source:HGNC Symbol;Acc:HGNC:586] |
| ENSG00000165806 | HGLibA_07322 | 92 | -0.233106516 | CASP7 | caspase 7 [Source:HGNC Symbol;Acc:HGNC:1508] |
| ENSG00000167332 | HGLibA_34099 | 92 | -0.233106516 | OR51E2 | olfactory receptor family 51 subfamily E member 2 [Source:HGNC Symbol;Acc:HGNC:15195] |
| ENSG00000168135 | HGLibA_24278 | 92 | -0.233106516 | KCNJ4 | potassium voltage-gated channel subfamily J member 4 [Source:HGNC Symbol;Acc:HGNC:6265] |
| ENSG00000169750 | HGLibA_40049 | 92 | -0.233106516 | RAC3 | Rac family small GTPase 3 [Source:HGNC Symbol;Acc:HGNC:9803] |
| ENSG00000169905 | HGLibA_51020 | 92 | -0.233106516 | TOR1AIP2 | torsin 1A interacting protein 2 [Source:HGNC Symbol;Acc:HGNC:24055] |
| ENSG00000169991 | HGLibA_22589 | 92 | -0.233106516 | IFFO2 | intermediate filament family orphan 2 [Source:HGNC Symbol;Acc:HGNC:27006] |

| | | | | | |
|---|---|---|---|---|---|
| ENSG00000170325 | HGLibA_38364 | 92 | -0.233106516 | PRDM10 | PR/SET domain 10 [Source:HGNC Symbol;Acc:HGNC:13995] |
| ENSG00000171451 | HGLibA_14020 | 92 | -0.233106516 | DSEL | dermatan sulfate epimerase like [Source:HGNC Symbol;Acc:HGNC:18144] |
| ENSG00000173597 | HGLibA_47742 | 92 | -0.233106516 | SULT1B1 | sulfotransferase family 1B member 1 [Source:HGNC Symbol;Acc:HGNC:17845] |
| ENSG00000173894 | HGLibA_07448 | 92 | -0.233106516 | CBX2 | chromobox 2 [Source:HGNC Symbol;Acc:HGNC:1552] |
| ENSG00000175065 | HGLibA_14032 | 92 | -0.233106516 | DSG4 | desmoglein 4 [Source:HGNC Symbol;Acc:HGNC:21307] |
| ENSG00000175985 | HGLibA_37093 | 92 | -0.233106516 | PLEKHD1 | pleckstrin homology and coiled-coil domain containing D1 [Source:HGNC Symbol;Acc:HGNC:20148] |
| ENSG00000176108 | HGLibA_09498 | 92 | -0.233106516 | CHMP6 | charged multivesicular body protein 6 [Source:HGNC Symbol;Acc:HGNC:25675] |
| ENSG00000176845 | HGLibA_28941 | 92 | -0.233106516 | METRNL | glial cell differentiation regulator [Source:HGNC Symbol;Acc:HGNC:27584]" |
| ENSG00000177427 | HGLibA_29245 | 92 | -0.233106516 | MIEF2 | mitochondrial elongation factor 2 [Source:HGNC Symbol;Acc:HGNC:17920] |
| ENSG00000178381 | HGLibA_55148 | 92 | -0.233106516 | ZFAND2A | zinc finger AN1-type containing 2A [Source:HGNC Symbol;Acc:HGNC:28073] |
| ENSG00000178386 | HGLibA_55614 | 92 | -0.233106516 | ZNF223 | zinc finger protein 223 [Source:HGNC Symbol;Acc:HGNC:13016] |
| ENSG00000179299 | HGLibA_32796 | 92 | -0.233106516 | NSUN7 | NOP2/Sun RNA methyltransferase family member 7 [Source:HGNC Symbol;Acc:HGNC:25857] |
| ENSG00000180198 | HGLibA_40684 | 92 | -0.233106516 | RCC1 | regulator of chromosome condensation 1 [Source:HGNC Symbol;Acc:HGNC:1913] |
| ENSG00000180370 | HGLibA_35042 | 92 | -0.233106516 | PAK2 | p21 (RAC1) activated kinase 2 [Source:HGNC Symbol;Acc:HGNC:8591] |
| ENSG00000180801 | HGLibA_03152 | 92 | -0.233106516 | ARSJ | arylsulfatase family member J [Source:HGNC Symbol;Acc:HGNC:26286] |
| ENSG00000181240 | HGLibA_44738 | 92 | -0.233106516 | SLC25A41 | solute carrier family 25 member 41 [Source:HGNC Symbol;Acc:HGNC:28533] |
| ENSG00000181817 | HGLibA_27520 | 92 | -0.233106516 | LSM10 | U7 small nuclear RNA associated [Source:HGNC Symbol;Acc:HGNC:17562]" |
| ENSG00000181826 | HGLibA_40819 | 92 | -0.233106516 | RELL1 | RELT like 1 [Source:HGNC Symbol;Acc:HGNC:27379] |
| ENSG00000182010 | HGLibA_42391 | 92 | -0.233106516 | RTKN2 | rhotekin 2 [Source:HGNC Symbol;Acc:HGNC:19364] |
| ENSG00000182450 | HGLibA_24323 | 92 | -0.233106516 | KCNK4 | potassium two pore domain channel subfamily K member 4 [Source:HGNC Symbol;Acc:HGNC:6279] |
| ENSG00000182511 | HGLibA_17303 | 92 | -0.233106516 | FES | tyrosine kinase [Source:HGNC Symbol;Acc:HGNC:3657]" |
| ENSG00000182634 | HGLibA_33497 | 92 | -0.233106516 | OR10G7 | olfactory receptor family 10 subfamily G member 7 [Source:HGNC Symbol;Acc:HGNC:14842] |
| ENSG00000183128 | HGLibA_07019 | 92 | -0.233106516 | CALHM3 | calcium homeostasis modulator 3 [Source:HGNC Symbol;Acc:HGNC:23458] |
| ENSG00000183287 | HGLibA_07493 | 92 | -0.233106516 | CCBE1 | collagen and calcium binding EGF domains 1 [Source:HGNC Symbol;Acc:HGNC:29426] |
| ENSG00000185963 | HGLibA_04497 | 92 | -0.233106516 | BICD2 | BICD cargo adaptor 2 [Source:HGNC Symbol;Acc:HGNC:17208] |
| ENSG00000185974 | HGLibA_20327 | 92 | -0.233106516 | GRK1 | G protein-coupled receptor kinase 1 [Source:HGNC Symbol;Acc:HGNC:10013] |
| ENSG00000186335 | HGLibA_45034 | 92 | -0.233106516 | SLC36A2 | solute carrier family 36 member 2 [Source:HGNC Symbol;Acc:HGNC:18762] |
| ENSG00000186350 | HGLibA_42505 | 92 | -0.233106516 | RXRA | retinoid X receptor alpha [Source:HGNC Symbol;Acc:HGNC:10477] |
| ENSG00000186628 | HGLibA_18083 | 92 | -0.233106516 | FSD2 | fibronectin type III and SPRY domain containing 2 [Source:HGNC Symbol;Acc:HGNC:18024] |
| ENSG00000188691 | HGLibA_34219 | 92 | -0.233106516 | OR56A5 | olfactory receptor family 56 subfamily A member 5 [Source:HGNC Symbol;Acc:HGNC:14792] |
| ENSG00000188783 | HGLibA_38434 | 92 | -0.233106516 | PRELP | proline and arginine rich end leucine rich repeat protein [Source:HGNC Symbol;Acc:HGNC:9357] |
| ENSG00000196350 | HGLibA_56642 | 92 | -0.233106516 | ZNF729 | zinc finger protein 729 [Source:HGNC Symbol;Acc:HGNC:32464] |
| ENSG00000196391 | HGLibA_56716 | 92 | -0.233106516 | ZNF774 | zinc finger protein 774 [Source:HGNC Symbol;Acc:HGNC:33108] |
| ENSG00000196476 | HGLibA_06031 | 92 | -0.233106516 | C20orf96 | chromosome 20 open reading frame 96 [Source:HGNC Symbol;Acc:HGNC:16227] |
| ENSG00000196814 | HGLibA_30592 | 92 | -0.233106516 | MVB12B | multivesicular body subunit 12B [Source:HGNC Symbol;Acc:HGNC:23368] |
| ENSG00000197479 | HGLibA_35474 | 92 | -0.233106516 | PCDHB11 | protocadherin beta 11 [Source:HGNC Symbol;Acc:HGNC:8682] |
| ENSG00000197586 | HGLibA_15213 | 92 | -0.233106516 | ENTPD6 | ectonucleoside triphosphate diphosphohydrolase 6 (putative) [Source:HGNC Symbol;Acc:HGNC:3368] |
| ENSG00000197841 | HGLibA_55541 | 92 | -0.233106516 | ZNF181 | zinc finger protein 181 [Source:HGNC Symbol;Acc:HGNC:12971] |
| ENSG00000198601 | HGLibA_33833 | 92 | -0.233106516 | OR2M2 | olfactory receptor family 2 subfamily M member 2 [Source:HGNC Symbol;Acc:HGNC:8268] |
| ENSG00000203995 | HGLibA_57082 | 92 | -0.233106516 | ZYG11A | cell cycle regulator [Source:HGNC Symbol;Acc:HGNC:32058]" |
| ENSG00000204291 | HGLibA_10514 | 92 | -0.233106516 | COL15A1 | collagen type XV alpha 1 chain [Source:HGNC Symbol;Acc:HGNC:2192] |
| ENSG00000204300 | HGLibA_50247 | 92 | -0.233106516 | TMEM225 | transmembrane protein 225 [Source:HGNC Symbol;Acc:HGNC:32390] |
| ENSG00000205111 | HGLibA_08815 | 92 | -0.233106516 | CDKL4 | cyclin dependent kinase like 4 [Source:HGNC Symbol;Acc:HGNC:19287] |
| ENSG00000212935 | HGLibA_25606 | 92 | -0.233106516 | KRTAP10-3 | keratin associated protein 10-3 [Source:HGNC Symbol;Acc:HGNC:22968] |
| ENSG00000214285 | HGLibA_32522 | 92 | -0.233106516 | NPS | neuropeptide S [Source:HGNC Symbol;Acc:HGNC:33940] |
| ENSG00000215218 | HGLibA_52651 | 92 | -0.233106516 | UBE2QL1 | ubiquitin conjugating enzyme E2 Q family like 1 [Source:HGNC Symbol;Acc:HGNC:37269] |
| ENSG00000221910 | HGLibA_33792 | 92 | -0.233106516 | OR2F2 | olfactory receptor family 2 subfamily F member 2 [Source:HGNC Symbol;Acc:HGNC:8247] |
| ENSG00000227059 | HGLibA_01930 | 92 | -0.233106516 | ANHX | anomalous homeobox [Source:HGNC Symbol;Acc:HGNC:40024] |
| ENSG00000228278 | HGLibA_34621 | 92 | -0.233106516 | ORM2 | orosomucoid 2 [Source:HGNC Symbol;Acc:HGNC:8499] |
| ENSG00000228927 | HGLibA_51969 | 92 | -0.233106516 | TSPY3 | testis specific protein Y-linked 3 [Source:HGNC Symbol;Acc:HGNC:33876] |
| ENSG00000232119 | HGLibA_28659 | 92 | -0.233106516 | MCTS1 | re-initiation and release factor [Source:HGNC Symbol;Acc:HGNC:23357]" |
| ENSG00000237190 | HGLibA_08835 | 92 | -0.233106516 | CDKN2AIPNL | CDKN2A interacting protein N-terminal like [Source:HGNC Symbol;Acc:HGNC:30545] |
| ENSG00000240720 | HGLibA_27447 | 92 | -0.233106516 | LRRD1 | leucine rich repeats and death domain containing 1 [Source:HGNC Symbol;Acc:HGNC:34300] |
| ENSG00000242220 | HGLibA_48836 | 92 | -0.233106516 | TCP10L | t-complex 10 like [Source:HGNC Symbol;Acc:HGNC:11657] |
| ENSG00000243989 | HGLibA_00705 | 92 | -0.233106516 | ACY1 | aminoacylase 1 [Source:HGNC Symbol;Acc:HGNC:177] |
| ENSG00000244122 | HGLibA_52895 | 92 | -0.233106516 | UGT1A7 | UDP glucuronosyltransferase family 1 member A7 [Source:HGNC Symbol;Acc:HGNC:12539] |
| ENSG00000253626 | HGLibA_14861 | 92 | -0.233106516 | EIF5AL1 | eukaryotic translation initiation factor 5A-like 1 [Source:HGNC Symbol;Acc:HGNC:17419] |
| ENSG00000254440 | HGLibA_35336 | 92 | -0.233106516 | PBOV1 | prostate and breast cancer overexpressed 1 [Source:HGNC Symbol;Acc:HGNC:21079] |
| ENSG00000256660 | HGLibA_09930 | 92 | -0.233106516 | CLEC12B | C-type lectin domain family 12 member B [Source:HGNC Symbol;Acc:HGNC:31966] |
| ENSG00000266094 | HGLibA_40385 | 92 | -0.233106516 | RASSF5 | Ras association domain family member 5 [Source:HGNC Symbol;Acc:HGNC:17609] |
| ENSG00000269404 | HGLibA_46655 | 92 | -0.233106516 | SPIB | Spi-B transcription factor [Source:HGNC Symbol;Acc:HGNC:11242] |
| ENSG00000271303 | HGLibA_47095 | 92 | -0.233106516 | SRXN1 | sulfiredoxin 1 [Source:HGNC Symbol;Acc:HGNC:16132] |
| ENSG00000273540 | HGLibA_01167 | 92 | -0.233106516 | AGBL1 | ATP/GTP binding protein like 1 [Source:HGNC Symbol;Acc:HGNC:26504] |
| ENSG00000276409 | HGLibA_07971 | 92 | -0.233106516 | CCL14 | C-C motif chemokine ligand 14 [Source:HGNC Symbol;Acc:HGNC:10612] |
| ENSG00000276747 | HGLibA_34980 | 92 | -0.233106516 | PADI6 | peptidyl arginine deiminase 6 [Source:HGNC Symbol;Acc:HGNC:20449] |
| GPR1 | HGLibA_19838 | 92 | -0.233106516 | GPR1 | - |
| GUCY1A3 | HGLibA_20655 | 92 | -0.233106516 | GUCY1A3 | - |
| H2AFB2 | HGLibA_20748 | 92 | -0.233106516 | H2AFB2 | - |
| HIST1H3A | HGLibA_21456 | 92 | -0.233106516 | HIST1H3A | - |
| hsa-mir-143 | HGLibA_57836 | 92 | -0.233106516 | hsa-mir-143 | - |
| hsa-mir-199b | HGLibA_58137 | 92 | -0.233106516 | hsa-mir-199b | - |
| hsa-mir-219b | HGLibA_58283 | 92 | -0.233106516 | hsa-mir-219b | - |
| hsa-mir-22 | HGLibA_58288 | 92 | -0.233106516 | hsa-mir-22 | - |
| hsa-mir-27b | HGLibA_58379 | 92 | -0.233106516 | hsa-mir-27b | - |
| hsa-mir-302a | HGLibA_58436 | 92 | -0.233106516 | hsa-mir-302a | - |
| hsa-mir-3158-2 | HGLibA_58708 | 92 | -0.233106516 | hsa-mir-3158-2 | - |
| hsa-mir-320c-2 | HGLibA_58938 | 92 | -0.233106516 | hsa-mir-320c-2 | - |
| hsa-mir-363 | HGLibA_59129 | 92 | -0.233106516 | hsa-mir-363 | - |
| hsa-mir-3664 | HGLibA_59209 | 92 | -0.233106516 | hsa-mir-3664 | - |
| hsa-mir-3680-1 | HGLibA_59271 | 92 | -0.233106516 | hsa-mir-3680-1 | - |
| hsa-mir-378a | HGLibA_59425 | 92 | -0.233106516 | hsa-mir-378a | - |
| hsa-mir-378i | HGLibA_59463 | 92 | -0.233106516 | hsa-mir-378i | - |
| hsa-mir-3925 | HGLibA_59571 | 92 | -0.233106516 | hsa-mir-3925 | - |
| hsa-mir-4315-2 | HGLibA_59979 | 92 | -0.233106516 | hsa-mir-4315-2 | - |
| hsa-mir-4420 | HGLibA_60069 | 92 | -0.233106516 | hsa-mir-4420 | - |
| hsa-mir-4478 | HGLibA_60327 | 92 | -0.233106516 | hsa-mir-4478 | - |
| hsa-mir-4735 | HGLibA_61036 | 92 | -0.233106516 | hsa-mir-4735 | - |
| hsa-mir-511 | HGLibA_61560 | 92 | -0.233106516 | hsa-mir-511 | - |
| hsa-mir-520g | HGLibA_61757 | 92 | -0.233106516 | hsa-mir-520g | - |
| hsa-mir-548o-2 | HGLibA_62051 | 92 | -0.233106516 | hsa-mir-548o-2 | - |
| hsa-mir-597 | HGLibA_62481 | 92 | -0.233106516 | hsa-mir-597 | - |
| hsa-mir-6514 | HGLibA_62930 | 92 | -0.233106516 | hsa-mir-6514 | - |
| hsa-mir-6780a | HGLibA_63292 | 92 | -0.233106516 | hsa-mir-6780a | - |
| hsa-mir-6782 | HGLibA_63304 | 92 | -0.233106516 | hsa-mir-6782 | - |
| hsa-mir-6802 | HGLibA_63384 | 92 | -0.233106516 | hsa-mir-6802 | - |
| hsa-mir-764 | HGLibA_63913 | 92 | -0.233106516 | hsa-mir-764 | - |
| LOC649330 | HGLibA_26982 | 92 | -0.233106516 | LOC649330 | - |
| LOC728819 | HGLibA_26993 | 92 | -0.233106516 | LOC728819 | - |
| PHF16 | HGLibA_36350 | 92 | -0.233106516 | PHF16 | - |
| PLCXD1_Y | HGLibA_37024 | 92 | -0.233106516 | PLCXD1_Y | - |
| SPRY3_X | HGLibA_46841 | 92 | -0.233106516 | SPRY3_X | - |
| ZCCHC5 | HGLibA_55038 | 92 | -0.233106516 | ZCCHC5 | - |
| C4orf29 | HGLibA_06315 | 91 | -0.243057553 | C4orf29 | - |
| ENSG00000008118 | HGLibA_07065 | 91 | -0.243057553 | CAMK1G | calcium/calmodulin dependent protein kinase IG [Source:HGNC Symbol;Acc:HGNC:14585] |
| ENSG00000009307 | HGLibA_11316 | 91 | -0.243057553 | CSDE1 | cold shock domain containing E1 [Source:HGNC Symbol;Acc:HGNC:29905] |
| ENSG00000009830 | HGLibA_37673 | 91 | -0.243057553 | POMT2 | protein O-mannosyltransferase 2 [Source:HGNC Symbol;Acc:HGNC:19743] |
| ENSG00000013583 | HGLibA_21083 | 91 | -0.243057553 | HEBP1 | heme binding protein 1 [Source:HGNC Symbol;Acc:HGNC:17176] |
| ENSG00000019144 | HGLibA_36427 | 91 | -0.243057553 | PHLDB1 | pleckstrin homology like domain family B member 1 [Source:HGNC Symbol;Acc:HGNC:23697] |
| ENSG00000050393 | HGLibA_28665 | 91 | -0.243057553 | MCUR1 | mitochondrial calcium uniporter regulator 1 [Source:HGNC Symbol;Acc:HGNC:21097] |
| ENSG00000064199 | HGLibA_46322 | 91 | -0.243057553 | SPA17 | sperm autoantigenic protein 17 [Source:HGNC Symbol;Acc:HGNC:11210] |
| ENSG00000064726 | HGLibA_04892 | 91 | -0.243057553 | BTBD1 | BTB domain containing 1 [Source:HGNC Symbol;Acc:HGNC:1120] |
| ENSG00000066468 | HGLibA_17445 | 91 | -0.243057553 | FGFR2 | fibroblast growth factor receptor 2 [Source:HGNC Symbol;Acc:HGNC:3689] |
| ENSG00000070087 | HGLibA_36177 | 91 | -0.243057553 | PFN2 | profilin 2 [Source:HGNC Symbol;Acc:HGNC:8882] |
| ENSG00000070748 | HGLibA_09356 | 91 | -0.243057553 | CHAT | choline O-acetyltransferase [Source:HGNC Symbol;Acc:HGNC:1912] |
| ENSG00000070886 | HGLibA_15308 | 91 | -0.243057553 | EPHA8 | EPH receptor A8 [Source:HGNC Symbol;Acc:HGNC:3391] |
| ENSG00000072864 | HGLibA_31386 | 91 | -0.243057553 | NDE1 | nudE neurodevelopment protein 1 [Source:HGNC Symbol;Acc:HGNC:17619] |
| ENSG00000074047 | HGLibA_19251 | 91 | -0.243057553 | GLI2 | GLI family zinc finger 2 [Source:HGNC Symbol;Acc:HGNC:4318] |
| ENSG00000075785 | HGLibA_39979 | 91 | -0.243057553 | RAB7A | member RAS oncogene family [Source:HGNC Symbol;Acc:HGNC:9788]" |
| ENSG00000076003 | HGLibA_28618 | 91 | -0.243057553 | MCM6 | minichromosome maintenance complex component 6 [Source:HGNC Symbol;Acc:HGNC:6949] |
| ENSG00000077984 | HGLibA_11465 | 91 | -0.243057553 | CST7 | cystatin F [Source:HGNC Symbol;Acc:HGNC:2479] |
| ENSG00000078081 | HGLibA_25983 | 91 | -0.243057553 | LAMP3 | lysosomal associated membrane protein 3 [Source:HGNC Symbol;Acc:HGNC:14582] |
| ENSG00000085265 | HGLibA_17217 | 91 | -0.243057553 | FCN1 | ficolin 1 [Source:HGNC Symbol;Acc:HGNC:3623] |
| ENSG00000085377 | HGLibA_38437 | 91 | -0.243057553 | PREP | prolyl endopeptidase [Source:HGNC Symbol;Acc:HGNC:9358] |
| ENSG00000088882 | HGLibA_11036 | 91 | -0.243057553 | CPXM1 | M14 family member 1 [Source:HGNC Symbol;Acc:HGNC:15771]" |
| ENSG00000094963 | HGLibA_17705 | 91 | -0.243057553 | FMO2 | flavin containing monooxygenase 2 [Source:HGNC Symbol;Acc:HGNC:3770] |
| ENSG00000099937 | HGLibA_43575 | 91 | -0.243057553 | SERPIND1 | serpin family D member 1 [Source:HGNC Symbol;Acc:HGNC:4838] |
| ENSG00000100461 | HGLibA_40511 | 91 | -0.243057553 | RBM23 | RNA binding motif protein 23 [Source:HGNC Symbol;Acc:HGNC:20155] |
| ENSG00000100934 | HGLibA_43221 | 91 | -0.243057553 | SEC23A | coat complex II component [Source:HGNC Symbol;Acc:HGNC:10701]" |
| ENSG00000101000 | HGLibA_38655 | 91 | -0.243057553 | PROCR | protein C receptor [Source:HGNC Symbol;Acc:HGNC:9452] |
| ENSG00000101079 | HGLibA_31418 | 91 | -0.243057553 | NDRG3 | NDRG family member 3 [Source:HGNC Symbol;Acc:HGNC:14462] |
| ENSG00000101197 | HGLibA_04528 | 91 | -0.243057553 | BIRC7 | baculoviral IAP repeat containing 7 [Source:HGNC Symbol;Acc:HGNC:13702] |
| ENSG00000102174 | HGLibA_36326 | 91 | -0.243057553 | PHEX | phosphate regulating endopeptidase homolog X-linked [Source:HGNC Symbol;Acc:HGNC:8918] |
| ENSG00000103021 | HGLibA_07547 | 91 | -0.243057553 | CCDC113 | coiled-coil domain containing 113 [Source:HGNC Symbol;Acc:HGNC:25002] |
| ENSG00000104177 | HGLibA_30687 | 91 | -0.243057553 | MYEF2 | myelin expression factor 2 [Source:HGNC Symbol;Acc:HGNC:17940] |
| ENSG00000105339 | HGLibA_12947 | 91 | -0.243057553 | DENND3 | DENN domain containing 3 [Source:HGNC Symbol;Acc:HGNC:29134] |

| | | | | | |
|---|---|---|---|---|---|
| ENSG00000107263 | HGLibA_40245 | 91 | -0.243057553 | RAPGEF1 | Rap guanine nucleotide exchange factor 1 [Source:HGNC Symbol;Acc:HGNC:4568] |
| ENSG00000107438 | HGLibA_35907 | 91 | -0.243057553 | PDLIM1 | PDZ and LIM domain 1 [Source:HGNC Symbol;Acc:HGNC:2067] |
| ENSG00000109466 | HGLibA_25148 | 91 | -0.243057553 | KLHL2 | kelch like family member 2 [Source:HGNC Symbol;Acc:HGNC:6353] |
| ENSG00000109787 | HGLibA_25070 | 91 | -0.243057553 | KLF3 | Kruppel like factor 3 [Source:HGNC Symbol;Acc:HGNC:16516] |
| ENSG00000111679 | HGLibA_39475 | 91 | -0.243057553 | PTPN6 | non-receptor type 6 [Source:HGNC Symbol;Acc:HGNC:9658]" |
| ENSG00000111834 | HGLibA_42334 | 91 | -0.243057553 | RSPH4A | radial spoke head component 4A [Source:HGNC Symbol;Acc:HGNC:21558] |
| ENSG00000112640 | HGLibA_38180 | 91 | -0.243057553 | PPP2R5D | protein phosphatase 2 regulatory subunit B'delta [Source:HGNC Symbol;Acc:HGNC:9312] |
| ENSG00000113249 | HGLibA_20899 | 91 | -0.243057553 | HAVCR1 | hepatitis A virus cellular receptor 1 [Source:HGNC Symbol;Acc:HGNC:17866] |
| ENSG00000113387 | HGLibA_47692 | 91 | -0.243057553 | SUB1 | transcriptional regulator [Source:HGNC Symbol;Acc:HGNC:19985]" |
| ENSG00000113578 | HGLibA_17360 | 91 | -0.243057553 | FGF1 | fibroblast growth factor 1 [Source:HGNC Symbol;Acc:HGNC:3665] |
| ENSG00000114859 | HGLibA_09822 | 91 | -0.243057553 | CLCN2 | chloride voltage-gated channel 2 [Source:HGNC Symbol;Acc:HGNC:2020] |
| ENSG00000114999 | HGLibA_52184 | 91 | -0.243057553 | TTL | tubulin tyrosine ligase [Source:HGNC Symbol;Acc:HGNC:21586] |
| ENSG00000115204 | HGLibA_29784 | 91 | -0.243057553 | MPV17 | mitochondrial inner membrane protein MPV17 [Source:HGNC Symbol;Acc:HGNC:7224] |
| ENSG00000116754 | HGLibA_47066 | 91 | -0.243057553 | SRSF11 | serine and arginine rich splicing factor 11 [Source:HGNC Symbol;Acc:HGNC:10782] |
| ENSG00000116981 | HGLibA_32803 | 91 | -0.243057553 | NT5C1A | cytosolic IA [Source:HGNC Symbol;Acc:HGNC:17819]" |
| ENSG00000117222 | HGLibA_40430 | 91 | -0.243057553 | RBBP5 | histone lysine methyltransferase complex subunit [Source:HGNC Symbol;Acc:HGNC:9888]" |
| ENSG00000117724 | HGLibA_09054 | 91 | -0.243057553 | CENPF | centromere protein F [Source:HGNC Symbol;Acc:HGNC:1857] |
| ENSG00000120742 | HGLibA_43498 | 91 | -0.243057553 | SERP1 | stress associated endoplasmic reticulum protein 1 [Source:HGNC Symbol;Acc:HGNC:10759] |
| ENSG00000121853 | HGLibA_19052 | 91 | -0.243057553 | GHSR | growth hormone secretagogue receptor [Source:HGNC Symbol;Acc:HGNC:4267] |
| ENSG00000124557 | HGLibA_04958 | 91 | -0.243057553 | BTN1A1 | butyrophilin subfamily 1 member A1 [Source:HGNC Symbol;Acc:HGNC:1135] |
| ENSG00000124767 | HGLibA_19283 | 91 | -0.243057553 | GLO1 | glyoxalase I [Source:HGNC Symbol;Acc:HGNC:4323] |
| ENSG00000125122 | HGLibA_27301 | 91 | -0.243057553 | LRRC29 | leucine rich repeat containing 29 [Source:HGNC Symbol;Acc:HGNC:13605] |
| ENSG00000129657 | HGLibA_43189 | 91 | -0.243057553 | SEC14L1 | SEC14 like lipid binding 1 [Source:HGNC Symbol;Acc:HGNC:10698] |
| ENSG00000130635 | HGLibA_10587 | 91 | -0.243057553 | COL5A1 | collagen type V alpha 1 chain [Source:HGNC Symbol;Acc:HGNC:2209] |
| ENSG00000130939 | HGLibA_52694 | 91 | -0.243057553 | UBE4B | ubiquitination factor E4B [Source:HGNC Symbol;Acc:HGNC:12500] |
| ENSG00000131477 | HGLibA_40184 | 91 | -0.243057553 | RAMP2 | receptor activity modifying protein 2 [Source:HGNC Symbol;Acc:HGNC:9844] |
| ENSG00000131849 | HGLibA_55462 | 91 | -0.243057553 | ZNF132 | zinc finger protein 132 [Source:HGNC Symbol;Acc:HGNC:12916] |
| ENSG00000132424 | HGLibA_37346 | 91 | -0.243057553 | PNISR | PNN interacting serine and arginine rich protein [Source:HGNC Symbol;Acc:HGNC:21222] |
| ENSG00000132970 | HGLibA_53959 | 91 | -0.243057553 | WASF3 | WAS protein family member 3 [Source:HGNC Symbol;Acc:HGNC:12734] |
| ENSG00000133142 | HGLibA_48714 | 91 | -0.243057553 | TCEAL4 | transcription elongation factor A like 4 [Source:HGNC Symbol;Acc:HGNC:26121] |
| ENSG00000133195 | HGLibA_45093 | 91 | -0.243057553 | SLC39A11 | solute carrier family 39 member 11 [Source:HGNC Symbol;Acc:HGNC:14463] |
| ENSG00000133398 | HGLibA_28735 | 91 | -0.243057553 | MED10 | mediator complex subunit 10 [Source:HGNC Symbol;Acc:HGNC:28760] |
| ENSG00000134030 | HGLibA_11632 | 91 | -0.243057553 | CTIF | cap binding complex dependent translation initiation factor [Source:HGNC Symbol;Acc:HGNC:23925] |
| ENSG00000135913 | HGLibA_53350 | 91 | -0.243057553 | USP37 | ubiquitin specific peptidase 37 [Source:HGNC Symbol;Acc:HGNC:20063] |
| ENSG00000136487 | HGLibA_19032 | 91 | -0.243057553 | GH2 | growth hormone 2 [Source:HGNC Symbol;Acc:HGNC:4262] |
| ENSG00000136868 | HGLibA_44927 | 91 | -0.243057553 | SLC31A1 | solute carrier family 31 member 1 [Source:HGNC Symbol;Acc:HGNC:11016] |
| ENSG00000137168 | HGLibA_37938 | 91 | -0.243057553 | PPIL1 | peptidylprolyl isomerase like 1 [Source:HGNC Symbol;Acc:HGNC:9260] |
| ENSG00000137504 | HGLibA_11117 | 91 | -0.243057553 | CREBZF | CREB/ATF bZIP transcription factor [Source:HGNC Symbol;Acc:HGNC:24905] |
| ENSG00000138030 | HGLibA_24576 | 91 | -0.243057553 | KHK | ketohexokinase [Source:HGNC Symbol;Acc:HGNC:6315] |
| ENSG00000139874 | HGLibA_47174 | 91 | -0.243057553 | SSTR1 | somatostatin receptor 1 [Source:HGNC Symbol;Acc:HGNC:11330] |
| ENSG00000143624 | HGLibA_23418 | 91 | -0.243057553 | INTS3 | integrator complex subunit 3 [Source:HGNC Symbol;Acc:HGNC:26153] |
| ENSG00000144031 | HGLibA_02120 | 91 | -0.243057553 | ANKRD53 | ankyrin repeat domain 53 [Source:HGNC Symbol;Acc:HGNC:25691] |
| ENSG00000146910 | HGLibA_10361 | 91 | -0.243057553 | CNPY1 | canopy FGF signaling regulator 1 [Source:HGNC Symbol;Acc:HGNC:27786] |
| ENSG00000148935 | HGLibA_18643 | 91 | -0.243057553 | GAS2 | growth arrest specific 2 [Source:HGNC Symbol;Acc:HGNC:4167] |
| ENSG00000149503 | HGLibA_23276 | 91 | -0.243057553 | INCENP | inner centromere protein [Source:HGNC Symbol;Acc:HGNC:6058] |
| ENSG00000153140 | HGLibA_09238 | 91 | -0.243057553 | CETN3 | centrin 3 [Source:HGNC Symbol;Acc:HGNC:1868] |
| ENSG00000155329 | HGLibA_55004 | 91 | -0.243057553 | ZCCHC10 | zinc finger CCHC-type containing 10 [Source:HGNC Symbol;Acc:HGNC:25954] |
| ENSG00000156463 | HGLibA_43974 | 91 | -0.243057553 | SH3RF2 | SH3 domain containing ring finger 2 [Source:HGNC Symbol;Acc:HGNC:26299] |
| ENSG00000158169 | HGLibA_16837 | 91 | -0.243057553 | FANCC | FA complementation group C [Source:HGNC Symbol;Acc:HGNC:3584] |
| ENSG00000158457 | HGLibA_51934 | 91 | -0.243057553 | TSPAN33 | tetraspanin 33 [Source:HGNC Symbol;Acc:HGNC:28743] |
| ENSG00000159259 | HGLibA_09350 | 91 | -0.243057553 | CHAF1B | chromatin assembly factor 1 subunit B [Source:HGNC Symbol;Acc:HGNC:1911] |
| ENSG00000160161 | HGLibA_09711 | 91 | -0.243057553 | CILP2 | cartilage intermediate layer protein 2 [Source:HGNC Symbol;Acc:HGNC:24213] |
| ENSG00000160716 | HGLibA_09592 | 91 | -0.243057553 | CHRNB2 | cholinergic receptor nicotinic beta 2 subunit [Source:HGNC Symbol;Acc:HGNC:1962] |
| ENSG00000162641 | HGLibA_01466 | 91 | -0.243057553 | AKNAD1 | AKNA domain containing 1 [Source:HGNC Symbol;Acc:HGNC:28398] |
| ENSG00000163082 | HGLibA_43854 | 91 | -0.243057553 | SGPP2 | sphingosine-1-phosphate phosphatase 2 [Source:HGNC Symbol;Acc:HGNC:19953] |
| ENSG00000163807 | HGLibA_24698 | 91 | -0.243057553 | KIAA1143 | KIAA1143 [Source:HGNC Symbol;Acc:HGNC:29198] |
| ENSG00000164078 | HGLibA_30260 | 91 | -0.243057553 | MST1R | macrophage stimulating 1 receptor [Source:HGNC Symbol;Acc:HGNC:7381] |
| ENSG00000164109 | HGLibA_27846 | 91 | -0.243057553 | MAD2L1 | mitotic arrest deficient 2 like 1 [Source:HGNC Symbol;Acc:HGNC:6763] |
| ENSG00000164181 | HGLibA_14974 | 91 | -0.243057553 | ELOVL7 | ELOVL fatty acid elongase 7 [Source:HGNC Symbol;Acc:HGNC:26292] |
| ENSG00000165553 | HGLibA_31863 | 91 | -0.243057553 | NGB | neuroglobin [Source:HGNC Symbol;Acc:HGNC:14077] |
| ENSG00000167080 | HGLibA_04014 | 91 | -0.243057553 | B4GALNT2 | 4-N-acetyl-galactosaminyltransferase 2 [Source:HGNC Symbol;Acc:HGNC:24136]" |
| ENSG00000167613 | HGLibA_25932 | 91 | -0.243057553 | LAIR1 | leukocyte associated immunoglobulin like receptor 1 [Source:HGNC Symbol;Acc:HGNC:6477] |
| ENSG00000168032 | HGLibA_15203 | 91 | -0.243057553 | ENTPD3 | ectonucleoside triphosphate diphosphohydrolase 3 [Source:HGNC Symbol;Acc:HGNC:3365] |
| ENSG00000168447 | HGLibA_43017 | 91 | -0.243057553 | SCNN1B | sodium channel epithelial 1 beta subunit [Source:HGNC Symbol;Acc:HGNC:10600] |
| ENSG00000168763 | HGLibA_10321 | 91 | -0.243057553 | CNNM3 | cyclin and CBS domain divalent metal cation transport mediator 3 [Source:HGNC Symbol;Acc:HGNC:104] |
| ENSG00000168843 | HGLibA_18109 | 91 | -0.243057553 | FSTL5 | follistatin like 5 [Source:HGNC Symbol;Acc:HGNC:21386] |
| ENSG00000169249 | HGLibA_56979 | 91 | -0.243057553 | ZRSR2 | RNA binding motif and serine/arginine rich 2 [Source:HGNC Symbol;Acc:HGNC:23019]" |
| ENSG00000170903 | HGLibA_30180 | 91 | -0.243057553 | MSANTD4 | Myb/SANT DNA binding domain containing 4 with coiled-coils [Source:HGNC Symbol;Acc:HGNC:29383] |
| ENSG00000171049 | HGLibA_17967 | 91 | -0.243057553 | FPR2 | formyl peptide receptor 2 [Source:HGNC Symbol;Acc:HGNC:3827] |
| ENSG00000171819 | HGLibA_01929 | 91 | -0.243057553 | ANGPTL7 | angiopoietin like 7 [Source:HGNC Symbol;Acc:HGNC:24078] |
| ENSG00000172037 | HGLibA_25960 | 91 | -0.243057553 | LAMB2 | laminin subunit beta 2 [Source:HGNC Symbol;Acc:HGNC:6487] |
| ENSG00000173157 | HGLibA_00824 | 91 | -0.243057553 | ADAMTS20 | ADAM metallopeptidase with thrombospondin type 1 motif 20 [Source:HGNC Symbol;Acc:HGNC:17178] |
| ENSG00000173578 | HGLibA_54498 | 91 | -0.243057553 | XCR1 | X-C motif chemokine receptor 1 [Source:HGNC Symbol;Acc:HGNC:1625] |
| ENSG00000173597 | HGLibA_47744 | 91 | -0.243057553 | SULT1B1 | sulfotransferase family 1B member 1 [Source:HGNC Symbol;Acc:HGNC:17845] |
| ENSG00000174173 | HGLibA_51665 | 91 | -0.243057553 | TRMT10C | mitochondrial RNase P subunit [Source:HGNC Symbol;Acc:HGNC:26022]" |
| ENSG00000174611 | HGLibA_25879 | 91 | -0.243057553 | KY | kyphoscoliosis peptidase [Source:HGNC Symbol;Acc:HGNC:26576] |
| ENSG00000175087 | HGLibA_35889 | 91 | -0.243057553 | PDIK1L | PDLIM1 interacting kinase 1 like [Source:HGNC Symbol;Acc:HGNC:18981] |
| ENSG00000175206 | HGLibA_32498 | 91 | -0.243057553 | NPPA | natriuretic peptide A [Source:HGNC Symbol;Acc:HGNC:7939] |
| ENSG00000175274 | HGLibA_51061 | 91 | -0.243057553 | TP53I11 | tumor protein p53 inducible protein 11 [Source:HGNC Symbol;Acc:HGNC:16842] |
| ENSG00000175354 | HGLibA_39447 | 91 | -0.243057553 | PTPN2 | non-receptor type 2 [Source:HGNC Symbol;Acc:HGNC:9650]" |
| ENSG00000177023 | HGLibA_12822 | 91 | -0.243057553 | DEFB104B | defensin beta 104B [Source:HGNC Symbol;Acc:HGNC:26165] |
| ENSG00000177076 | HGLibA_00430 | 91 | -0.243057553 | ACER2 | alkaline ceramidase 2 [Source:HGNC Symbol;Acc:HGNC:23675] |
| ENSG00000177106 | HGLibA_15379 | 91 | -0.243057553 | EPS8L2 | EPS8 like 2 [Source:HGNC Symbol;Acc:HGNC:21296] |
| ENSG00000177807 | HGLibA_24248 | 91 | -0.243057553 | KCNJ10 | potassium voltage-gated channel subfamily J member 10 [Source:HGNC Symbol;Acc:HGNC:6256] |
| ENSG00000179674 | HGLibA_02937 | 91 | -0.243057553 | ARL14 | ADP ribosylation factor like GTPase 14 [Source:HGNC Symbol;Acc:HGNC:22974] |
| ENSG00000179674 | HGLibA_02936 | 91 | -0.243057553 | ARL14 | ADP ribosylation factor like GTPase 14 [Source:HGNC Symbol;Acc:HGNC:22974] |
| ENSG00000181061 | HGLibA_21306 | 91 | -0.243057553 | HIGD1A | HIG1 hypoxia inducible domain family member 1A [Source:HGNC Symbol;Acc:HGNC:29527] |
| ENSG00000181767 | HGLibA_34530 | 91 | -0.243057553 | OR8H2 | olfactory receptor family 8 subfamily H member 2 [Source:HGNC Symbol;Acc:HGNC:15308] |
| ENSG00000183023 | HGLibA_45387 | 91 | -0.243057553 | SLC8A1 | solute carrier family 8 member A1 [Source:HGNC Symbol;Acc:HGNC:11068] |
| ENSG00000183114 | HGLibA_16590 | 91 | -0.243057553 | FAM43B | family with sequence similarity 43 member B [Source:HGNC Symbol;Acc:HGNC:31791] |
| ENSG00000183617 | HGLibA_30021 | 91 | -0.243057553 | MRPL54 | mitochondrial ribosomal protein L54 [Source:HGNC Symbol;Acc:HGNC:16685] |
| ENSG00000184507 | HGLibA_33103 | 91 | -0.243057553 | NUTM1 | NUT midline carcinoma family member 1 [Source:HGNC Symbol;Acc:HGNC:29919] |
| ENSG00000184575 | HGLibA_54576 | 91 | -0.243057553 | XPOT | exportin for tRNA [Source:HGNC Symbol;Acc:HGNC:12826] |
| ENSG00000184908 | HGLibA_09844 | 91 | -0.243057553 | CLCNKB | chloride voltage-gated channel Kb [Source:HGNC Symbol;Acc:HGNC:2027] |
| ENSG00000185873 | HGLibA_50615 | 91 | -0.243057553 | TMPRSS11B | transmembrane serine protease 11B [Source:HGNC Symbol;Acc:HGNC:25398] |
| ENSG00000185894 | HGLibA_04748 | 91 | -0.243057553 | BPY2C | basic charge Y-linked 2C [Source:HGNC Symbol;Acc:HGNC:18225] |
| ENSG00000186440 | HGLibA_34436 | 91 | -0.243057553 | OR6P1 | olfactory receptor family 6 subfamily P member 1 [Source:HGNC Symbol;Acc:HGNC:15036] |
| ENSG00000187105 | HGLibA_21074 | 91 | -0.243057553 | HEATR4 | HEAT repeat containing 4 [Source:HGNC Symbol;Acc:HGNC:16761] |
| ENSG00000188039 | HGLibA_33124 | 91 | -0.243057553 | NWD1 | NACHT and WD repeat domain containing 1 [Source:HGNC Symbol;Acc:HGNC:27619] |
| ENSG00000188782 | HGLibA_07363 | 91 | -0.243057553 | CATSPER4 | cation channel sperm associated 4 [Source:HGNC Symbol;Acc:HGNC:23220] |
| ENSG00000196123 | HGLibA_24662 | 91 | -0.243057553 | KIAA0895L | KIAA0895 like [Source:HGNC Symbol;Acc:HGNC:34408] |
| ENSG00000196944 | HGLibA_33884 | 91 | -0.243057553 | OR2T4 | olfactory receptor family 2 subfamily T member 4 [Source:HGNC Symbol;Acc:HGNC:15016] |
| ENSG00000196990 | HGLibA_16231 | 91 | -0.243057553 | FAM163B | family with sequence similarity 163 member B [Source:HGNC Symbol;Acc:HGNC:33277] |
| ENSG00000197016 | HGLibA_56052 | 91 | -0.243057553 | ZNF470 | zinc finger protein 470 [Source:HGNC Symbol;Acc:HGNC:22220] |
| ENSG00000197889 | HGLibA_28866 | 91 | -0.243057553 | MEIG1 | meiosis/spermiogenesis associated 1 [Source:HGNC Symbol;Acc:HGNC:23429] |
| ENSG00000197894 | HGLibA_00959 | 91 | -0.243057553 | ADH5 | chi polypeptide [Source:HGNC Symbol;Acc:HGNC:253]" |
| ENSG00000197933 | HGLibA_56820 | 91 | -0.243057553 | ZNF823 | zinc finger protein 823 [Source:HGNC Symbol;Acc:HGNC:30936] |
| ENSG00000198130 | HGLibA_21286 | 91 | -0.243057553 | HIBCH | 3-hydroxyisobutyryl-CoA hydrolase [Source:HGNC Symbol;Acc:HGNC:4908] |
| ENSG00000198205 | HGLibA_57074 | 91 | -0.243057553 | ZXDA | zinc finger X-linked duplicated A [Source:HGNC Symbol;Acc:HGNC:13198] |
| ENSG00000198440 | HGLibA_56313 | 91 | -0.243057553 | ZNF583 | zinc finger protein 583 [Source:HGNC Symbol;Acc:HGNC:26427] |
| ENSG00000198624 | HGLibA_07843 | 91 | -0.243057553 | CCDC69 | coiled-coil domain containing 69 [Source:HGNC Symbol;Acc:HGNC:24487] |
| ENSG00000198768 | HGLibA_02398 | 91 | -0.243057553 | APCDD1L | APC down-regulated 1 like [Source:HGNC Symbol;Acc:HGNC:26892] |
| ENSG00000198836 | HGLibA_33404 | 91 | -0.243057553 | OPA1 | mitochondrial dynamin like GTPase [Source:HGNC Symbol;Acc:HGNC:8140]" |
| ENSG00000198881 | HGLibA_03213 | 91 | -0.243057553 | ASB12 | ankyrin repeat and SOCS box containing 12 [Source:HGNC Symbol;Acc:HGNC:19763] |
| ENSG00000198967 | HGLibA_33561 | 91 | -0.243057553 | OR10Z1 | olfactory receptor family 10 subfamily Z member 1 [Source:HGNC Symbol;Acc:HGNC:14996] |
| ENSG00000204580 | HGLibA_12634 | 91 | -0.243057553 | DDR1 | discoidin domain receptor tyrosine kinase 1 [Source:HGNC Symbol;Acc:HGNC:2730] |
| ENSG00000205356 | HGLibA_48941 | 91 | -0.243057553 | TECPR1 | tectonin beta-propeller repeat containing 1 [Source:HGNC Symbol;Acc:HGNC:22214] |
| ENSG00000205809 | HGLibA_25287 | 91 | -0.243057553 | KLRC2 | killer cell lectin like receptor C2 [Source:HGNC Symbol;Acc:HGNC:6375] |
| ENSG00000206013 | HGLibA_22651 | 91 | -0.243057553 | IFITM5 | interferon induced transmembrane protein 5 [Source:HGNC Symbol;Acc:HGNC:16644] |
| ENSG00000213638 | HGLibA_00883 | 91 | -0.243057553 | ADAT3 | tRNA specific 3 [Source:HGNC Symbol;Acc:HGNC:25151]" |
| ENSG00000214941 | HGLibA_57054 | 91 | -0.243057553 | ZSWIM7 | zinc finger SWIM-type containing 7 [Source:HGNC Symbol;Acc:HGNC:26993] |
| ENSG00000221838 | HGLibA_02347 | 91 | -0.243057553 | AP4M1 | adaptor related protein complex 4 subunit mu 1 [Source:HGNC Symbol;Acc:HGNC:574] |
| ENSG00000221887 | HGLibA_21755 | 91 | -0.243057553 | HMSD | histocompatibility minor serpin domain containing [Source:HGNC Symbol;Acc:HGNC:23037] |
| ENSG00000221963 | HGLibA_02543 | 91 | -0.243057553 | APOL6 | apolipoprotein L6 [Source:HGNC Symbol;Acc:HGNC:14870] |
| ENSG00000233198 | HGLibA_41648 | 91 | -0.243057553 | RNF224 | ring finger protein 224 [Source:HGNC Symbol;Acc:HGNC:41912] |
| ENSG00000240563 | HGLibA_25888 | 91 | -0.243057553 | L1TD1 | LINE1 type transposase domain containing 1 [Source:HGNC Symbol;Acc:HGNC:25595] |
| ENSG00000241595 | HGLibA_25840 | 91 | -0.243057553 | KRTAP9-4 | keratin associated protein 9-4 [Source:HGNC Symbol;Acc:HGNC:18902] |
| ENSG00000244474 | HGLibA_52886 | 91 | -0.243057553 | UGT1A4 | UDP glucuronosyltransferase family 1 member A4 [Source:HGNC Symbol;Acc:HGNC:12536] |
| ENSG00000251369 | HGLibA_56212 | 91 | -0.243057553 | ZNF550 | zinc finger protein 550 [Source:HGNC Symbol;Acc:HGNC:28643] |
| ENSG00000253305 | HGLibA_35566 | 91 | -0.243057553 | PCDHGB6 | 6 [Source:HGNC Symbol;Acc:HGNC:8713]" |
| ENSG00000254505 | HGLibA_09487 | 91 | -0.243057553 | CHMP4A | charged multivesicular body protein 4A [Source:HGNC Symbol;Acc:HGNC:20274] |
| ENSG00000256394 | HGLibA_03310 | 91 | -0.243057553 | ASIC5 | acid sensing ion channel subunit family member 5 [Source:HGNC Symbol;Acc:HGNC:17537] |
| ENSG00000259120 | HGLibA_45763 | 91 | -0.243057553 | SMIM6 | small integral membrane protein 6 [Source:HGNC Symbol;Acc:HGNC:40032] |
| ENSG00000268447 | HGLibA_47202 | 91 | -0.243057553 | SSX2B | SSX family member 2B [Source:HGNC Symbol;Acc:HGNC:22263] |
| ENSG00000277443 | HGLibA_28356 | 91 | -0.243057553 | MARCKS | myristoylated alanine rich protein kinase C substrate [Source:HGNC Symbol;Acc:HGNC:6759] |

| | | | | | |
|---|---|---|---|---|---|
| ENSG00000278845 | HGLibA_29994 | 91 | -0.243057553 | MRPL45 | mitochondrial ribosomal protein L45 [Source:HGNC Symbol;Acc:HGNC:16651] |
| ENSG00000288709 | HGLibA_15889 | 91 | -0.243057553 | F8A2 | - |
| ENSG00000288867 | HGLibA_37903 | 91 | -0.243057553 | PPIAL4C | - |
| FAM19A1 | HGLibA_16396 | 91 | -0.243057553 | FAM19A1 | - |
| FAM19A4 | HGLibA_16406 | 91 | -0.243057553 | FAM19A4 | - |
| GPR75-ASB3 | HGLibA_20084 | 91 | -0.243057553 | GPR75-ASB3 | - |
| hsa-mir-107 | HGLibA_57195 | 91 | -0.243057553 | hsa-mir-107 | - |
| hsa-mir-10b | HGLibA_57203 | 91 | -0.243057553 | hsa-mir-10b | - |
| hsa-mir-1180 | HGLibA_57212 | 91 | -0.243057553 | hsa-mir-1180 | - |
| hsa-mir-1184-2 | HGLibA_57234 | 91 | -0.243057553 | hsa-mir-1184-2 | - |
| hsa-mir-1199 | HGLibA_57258 | 91 | -0.243057553 | hsa-mir-1199 | - |
| hsa-mir-193a | HGLibA_58072 | 91 | -0.243057553 | hsa-mir-193a | - |
| hsa-mir-2110 | HGLibA_58221 | 91 | -0.243057553 | hsa-mir-2110 | - |
| hsa-mir-3153 | HGLibA_58671 | 91 | -0.243057553 | hsa-mir-3153 | - |
| hsa-mir-3662 | HGLibA_59200 | 91 | -0.243057553 | hsa-mir-3662 | - |
| hsa-mir-449c | HGLibA_60425 | 91 | -0.243057553 | hsa-mir-449c | - |
| hsa-mir-4504 | HGLibA_60441 | 91 | -0.243057553 | hsa-mir-4504 | - |
| hsa-mir-4506 | HGLibA_60452 | 91 | -0.243057553 | hsa-mir-4506 | - |
| hsa-mir-4509-2 | HGLibA_60467 | 91 | -0.243057553 | hsa-mir-4509-2 | - |
| hsa-mir-4743 | HGLibA_61070 | 91 | -0.243057553 | hsa-mir-4743 | - |
| hsa-mir-509-2 | HGLibA_61510 | 91 | -0.243057553 | hsa-mir-509-2 | - |
| hsa-mir-591 | HGLibA_62463 | 91 | -0.243057553 | hsa-mir-591 | - |
| hsa-mir-609 | HGLibA_62625 | 91 | -0.243057553 | hsa-mir-609 | - |
| hsa-mir-614 | HGLibA_62692 | 91 | -0.243057553 | hsa-mir-614 | - |
| KIAA0195 | HGLibA_24600 | 91 | -0.243057553 | KIAA0195 | - |
| KIAA1456 | HGLibA_24761 | 91 | -0.243057553 | KIAA1456 | - |
| MTSS1L | HGLibA_30500 | 91 | -0.243057553 | MTSS1L | - |
| PNMAL1 | HGLibA_37383 | 91 | -0.243057553 | PNMAL1 | - |
| PTPLAD1 | HGLibA_39418 | 91 | -0.243057553 | PTPLAD1 | - |
| PVRL4 | HGLibA_39632 | 91 | -0.243057553 | PVRL4 | - |
| SMCR7 | HGLibA_45678 | 91 | -0.243057553 | SMCR7 | - |
| C1orf151-NBL1 | HGLibA_05777 | 90 | -0.253008591 | C1orf151-NBL1 | - |
| C20orf196 | HGLibA_06004 | 90 | -0.253008591 | C20orf196 | - |
| C5orf48 | HGLibA_06395 | 90 | -0.253008591 | C5orf48 | - |
| C6orf211 | HGLibA_06474 | 90 | -0.253008591 | C6orf211 | - |
| DIEXF | HGLibA_13232 | 90 | -0.253008591 | DIEXF | - |
| ENSG00000004961 | HGLibA_20956 | 90 | -0.253008591 | HCCS | holocytochrome c synthase [Source:HGNC Symbol;Acc:HGNC:4837] |
| ENSG00000006118 | HGLibA_49930 | 90 | -0.253008591 | TMEM132A | transmembrane protein 132A [Source:HGNC Symbol;Acc:HGNC:31092] |
| ENSG00000006282 | HGLibA_46446 | 90 | -0.253008591 | SPATA20 | spermatogenesis associated 20 [Source:HGNC Symbol;Acc:HGNC:26125] |
| ENSG00000006432 | HGLibA_28204 | 90 | -0.253008591 | MAP3K9 | mitogen-activated protein kinase kinase kinase 9 [Source:HGNC Symbol;Acc:HGNC:6861] |
| ENSG00000009780 | HGLibA_16723 | 90 | -0.253008591 | FAM76A | family with sequence similarity 76 member A [Source:HGNC Symbol;Acc:HGNC:28530] |
| ENSG00000013374 | HGLibA_32897 | 90 | -0.253008591 | NUB1 | negative regulator of ubiquitin like proteins 1 [Source:HGNC Symbol;Acc:HGNC:17623] |
| ENSG00000023516 | HGLibA_01408 | 90 | -0.253008591 | AKAP11 | A-kinase anchoring protein 11 [Source:HGNC Symbol;Acc:HGNC:369] |
| ENSG00000052126 | HGLibA_37075 | 90 | -0.253008591 | PLEKHA5 | pleckstrin homology domain containing A5 [Source:HGNC Symbol;Acc:HGNC:30036] |
| ENSG00000055211 | HGLibA_19101 | 90 | -0.253008591 | GINM1 | glycoprotein integral membrane 1 [Source:HGNC Symbol;Acc:HGNC:21074] |
| ENSG00000066230 | HGLibA_45406 | 90 | -0.253008591 | SLC9A3 | solute carrier family 9 member A3 [Source:HGNC Symbol;Acc:HGNC:11073] |
| ENSG00000073282 | HGLibA_51092 | 90 | -0.253008591 | TP63 | tumor protein p63 [Source:HGNC Symbol;Acc:HGNC:15979] |
| ENSG00000073969 | HGLibA_32752 | 90 | -0.253008591 | NSF | vesicle fusing ATPase [Source:HGNC Symbol;Acc:HGNC:8016]" |
| ENSG00000076924 | HGLibA_54460 | 90 | -0.253008591 | XAB2 | XPA binding protein 2 [Source:HGNC Symbol;Acc:HGNC:14089] |
| ENSG00000081842 | HGLibA_35451 | 90 | -0.253008591 | PCDHA6 | protocadherin alpha 6 [Source:HGNC Symbol;Acc:HGNC:8672] |
| ENSG00000082684 | HGLibA_43361 | 90 | -0.253008591 | SEMA5B | semaphorin 5B [Source:HGNC Symbol;Acc:HGNC:10737] |
| ENSG00000085721 | HGLibA_42260 | 90 | -0.253008591 | RRN3 | RNA polymerase I transcription factor [Source:HGNC Symbol;Acc:HGNC:30346]" |
| ENSG00000086102 | HGLibA_31847 | 90 | -0.253008591 | NFX1 | X-box binding 1 [Source:HGNC Symbol;Acc:HGNC:7803]" |
| ENSG00000088448 | HGLibA_01990 | 90 | -0.253008591 | ANKRD10 | ankyrin repeat domain 10 [Source:HGNC Symbol;Acc:HGNC:20265] |
| ENSG00000090534 | HGLibA_49384 | 90 | -0.253008591 | THPO | thrombopoietin [Source:HGNC Symbol;Acc:HGNC:11795] |
| ENSG00000092847 | HGLibA_01206 | 90 | -0.253008591 | AGO1 | RISC catalytic component [Source:HGNC Symbol;Acc:HGNC:3262]" |
| ENSG00000092978 | HGLibA_19738 | 90 | -0.253008591 | GPATCH2 | G-patch domain containing 2 [Source:HGNC Symbol;Acc:HGNC:25499] |
| ENSG00000095787 | HGLibA_53939 | 90 | -0.253008591 | WAC | WW domain containing adaptor with coiled-coil [Source:HGNC Symbol;Acc:HGNC:17327] |
| ENSG00000100084 | HGLibA_21351 | 90 | -0.253008591 | HIRA | histone cell cycle regulator [Source:HGNC Symbol;Acc:HGNC:4916] |
| ENSG00000101337 | HGLibA_49730 | 90 | -0.253008591 | TM9SF4 | transmembrane 9 superfamily member 4 [Source:HGNC Symbol;Acc:HGNC:30797] |
| ENSG00000101407 | HGLibA_52174 | 90 | -0.253008591 | TTI1 | TELO2 interacting protein 1 [Source:HGNC Symbol;Acc:HGNC:29029] |
| ENSG00000101457 | HGLibA_13720 | 90 | -0.253008591 | DNTTIP1 | deoxynucleotidyltransferase terminal interacting protein 1 [Source:HGNC Symbol;Acc:HGNC:16160] |
| ENSG00000102401 | HGLibA_03043 | 90 | -0.253008591 | ARMCX3 | armadillo repeat containing X-linked 3 [Source:HGNC Symbol;Acc:HGNC:24065] |
| ENSG00000103423 | HGLibA_13517 | 90 | -0.253008591 | DNAJA3 | DnaJ heat shock protein family (Hsp40) member A3 [Source:HGNC Symbol;Acc:HGNC:11808] |
| ENSG00000104918 | HGLibA_40875 | 90 | -0.253008591 | RETN | resistin [Source:HGNC Symbol;Acc:HGNC:20389] |
| ENSG00000105143 | HGLibA_44522 | 90 | -0.253008591 | SLC1A6 | solute carrier family 1 member 6 [Source:HGNC Symbol;Acc:HGNC:10944] |
| ENSG00000106144 | HGLibA_07306 | 90 | -0.253008591 | CASP2 | caspase 2 [Source:HGNC Symbol;Acc:HGNC:1503] |
| ENSG00000107731 | HGLibA_53016 | 90 | -0.253008591 | UNC5B | unc-5 netrin receptor B [Source:HGNC Symbol;Acc:HGNC:12568] |
| ENSG00000108001 | HGLibA_14354 | 90 | -0.253008591 | EBF3 | EBF transcription factor 3 [Source:HGNC Symbol;Acc:HGNC:19087] |
| ENSG00000108684 | HGLibA_03301 | 90 | -0.253008591 | ASIC2 | acid sensing ion channel subunit 2 [Source:HGNC Symbol;Acc:HGNC:99] |
| ENSG00000109991 | HGLibA_34853 | 90 | -0.253008591 | P2RX3 | purinergic receptor P2X 3 [Source:HGNC Symbol;Acc:HGNC:8534] |
| ENSG00000110931 | HGLibA_07090 | 90 | -0.253008591 | CAMKK2 | calcium/calmodulin dependent protein kinase kinase 2 [Source:HGNC Symbol;Acc:HGNC:1470] |
| ENSG00000111249 | HGLibA_11807 | 90 | -0.253008591 | CUX2 | cut like homeobox 2 [Source:HGNC Symbol;Acc:HGNC:19347] |
| ENSG00000111269 | HGLibA_11112 | 90 | -0.253008591 | CREBL2 | cAMP responsive element binding protein like 2 [Source:HGNC Symbol;Acc:HGNC:2350] |
| ENSG00000111832 | HGLibA_42480 | 90 | -0.253008591 | RWDD1 | RWD domain containing 1 [Source:HGNC Symbol;Acc:HGNC:20993] |
| ENSG00000111843 | HGLibA_49986 | 90 | -0.253008591 | TMEM14C | transmembrane protein 14C [Source:HGNC Symbol;Acc:HGNC:20952] |
| ENSG00000113073 | HGLibA_45232 | 90 | -0.253008591 | SLC4A9 | solute carrier family 4 member 9 [Source:HGNC Symbol;Acc:HGNC:11035] |
| ENSG00000114248 | HGLibA_27312 | 90 | -0.253008591 | LRRC31 | leucine rich repeat containing 31 [Source:HGNC Symbol;Acc:HGNC:26261] |
| ENSG00000114354 | HGLibA_49177 | 90 | -0.253008591 | TFG | TRK-fused gene [Source:HGNC Symbol;Acc:HGNC:11758] |
| ENSG00000115155 | HGLibA_34725 | 90 | -0.253008591 | OTOF | otoferlin [Source:HGNC Symbol;Acc:HGNC:8515] |
| ENSG00000115649 | HGLibA_10359 | 90 | -0.253008591 | CNPPD1 | cyclin Pas1/PHO80 domain containing 1 [Source:HGNC Symbol;Acc:HGNC:25220] |
| ENSG00000116260 | HGLibA_39754 | 90 | -0.253008591 | QSOX1 | quiescin sulfhydryl oxidase 1 [Source:HGNC Symbol;Acc:HGNC:9756] |
| ENSG00000116667 | HGLibA_05824 | 90 | -0.253008591 | C1orf21 | chromosome 1 open reading frame 21 [Source:HGNC Symbol;Acc:HGNC:15494] |
| ENSG00000118263 | HGLibA_25082 | 90 | -0.253008591 | KLF7 | Kruppel like factor 7 [Source:HGNC Symbol;Acc:HGNC:6350] |
| ENSG00000119242 | HGLibA_07927 | 90 | -0.253008591 | CCDC92 | coiled-coil domain containing 92 [Source:HGNC Symbol;Acc:HGNC:29563] |
| ENSG00000120215 | HGLibA_29364 | 90 | -0.253008591 | MLANA | melan-A [Source:HGNC Symbol;Acc:HGNC:7124] |
| ENSG00000120658 | HGLibA_15158 | 90 | -0.253008591 | ENOX1 | ecto-NOX disulfide-thiol exchanger 1 [Source:HGNC Symbol;Acc:HGNC:25474] |
| ENSG00000120913 | HGLibA_35910 | 90 | -0.253008591 | PDLIM2 | PDZ and LIM domain 2 [Source:HGNC Symbol;Acc:HGNC:13992] |
| ENSG00000120948 | HGLibA_48335 | 90 | -0.253008591 | TARDBP | TAR DNA binding protein [Source:HGNC Symbol;Acc:HGNC:11571] |
| ENSG00000121350 | HGLibA_39703 | 90 | -0.253008591 | PYROXD1 | pyridine nucleotide-disulphide oxidoreductase domain 1 [Source:HGNC Symbol;Acc:HGNC:26162] |
| ENSG00000121377 | HGLibA_48426 | 90 | -0.253008591 | TAS2R7 | taste 2 receptor member 7 [Source:HGNC Symbol;Acc:HGNC:14913] |
| ENSG00000122507 | HGLibA_04227 | 90 | -0.253008591 | BBS9 | Bardet-Biedl syndrome 9 [Source:HGNC Symbol;Acc:HGNC:30000] |
| ENSG00000123407 | HGLibA_21963 | 90 | -0.253008591 | HOXC12 | homeobox C12 [Source:HGNC Symbol;Acc:HGNC:5124] |
| ENSG00000124126 | HGLibA_38444 | 90 | -0.253008591 | PREX1 | 5-trisphosphate dependent Rac exchange factor 1 [Source:HGNC Symbol;Acc:HGNC:32594]" |
| ENSG00000124181 | HGLibA_37003 | 90 | -0.253008591 | PLCG1 | phospholipase C gamma 1 [Source:HGNC Symbol;Acc:HGNC:9065] |
| ENSG00000125285 | HGLibA_46255 | 90 | -0.253008591 | SOX21 | SRY-box 21 [Source:HGNC Symbol;Acc:HGNC:11197] |
| ENSG00000125903 | HGLibA_12892 | 90 | -0.253008591 | DEFB129 | defensin beta 129 [Source:HGNC Symbol;Acc:HGNC:16218] |
| ENSG00000126337 | HGLibA_25492 | 90 | -0.253008591 | KRT36 | keratin 36 [Source:HGNC Symbol;Acc:HGNC:6454] |
| ENSG00000127083 | HGLibA_33383 | 90 | -0.253008591 | OMD | osteomodulin [Source:HGNC Symbol;Acc:HGNC:8134] |
| ENSG00000127903 | HGLibA_56838 | 90 | -0.253008591 | ZNF835 | zinc finger protein 835 [Source:HGNC Symbol;Acc:HGNC:34332] |
| ENSG00000128709 | HGLibA_22007 | 90 | -0.253008591 | HOXD9 | homeobox D9 [Source:HGNC Symbol;Acc:HGNC:5140] |
| ENSG00000130427 | HGLibA_15349 | 90 | -0.253008591 | EPO | erythropoietin [Source:HGNC Symbol;Acc:HGNC:3415] |
| ENSG00000131067 | HGLibA_19019 | 90 | -0.253008591 | GGT7 | gamma-glutamyltransferase 7 [Source:HGNC Symbol;Acc:HGNC:4259] |
| ENSG00000133019 | HGLibA_09552 | 90 | -0.253008591 | CHRM3 | cholinergic receptor muscarinic 3 [Source:HGNC Symbol;Acc:HGNC:1952] |
| ENSG00000134323 | HGLibA_30679 | 90 | -0.253008591 | MYCN | bHLH transcription factor [Source:HGNC Symbol;Acc:HGNC:7559]" |
| ENSG00000134480 | HGLibA_08098 | 90 | -0.253008591 | CCNH | cyclin H [Source:HGNC Symbol;Acc:HGNC:1594] |
| ENSG00000135482 | HGLibA_54945 | 90 | -0.253008591 | ZC3H10 | zinc finger CCCH-type containing 10 [Source:HGNC Symbol;Acc:HGNC:25893] |
| ENSG00000137221 | HGLibA_49568 | 90 | -0.253008591 | TJAP1 | tight junction associated protein 1 [Source:HGNC Symbol;Acc:HGNC:17949] |
| ENSG00000137309 | HGLibA_21683 | 90 | -0.253008591 | HMGA1 | high mobility group AT-hook 1 [Source:HGNC Symbol;Acc:HGNC:5010] |
| ENSG00000137462 | HGLibA_49646 | 90 | -0.253008591 | TLR2 | toll like receptor 2 [Source:HGNC Symbol;Acc:HGNC:11848] |
| ENSG00000137491 | HGLibA_45462 | 90 | -0.253008591 | SLCO2B1 | solute carrier organic anion transporter family member 2B1 [Source:HGNC Symbol;Acc:HGNC:10962] |
| ENSG00000138459 | HGLibA_44960 | 90 | -0.253008591 | SLC35A5 | solute carrier family 35 member A5 [Source:HGNC Symbol;Acc:HGNC:20792] |
| ENSG00000140382 | HGLibA_21677 | 90 | -0.253008591 | HMG20A | high mobility group 20A [Source:HGNC Symbol;Acc:HGNC:5001] |
| ENSG00000140937 | HGLibA_08636 | 90 | -0.253008591 | CDH11 | cadherin 11 [Source:HGNC Symbol;Acc:HGNC:1750] |
| ENSG00000142684 | HGLibA_56341 | 90 | -0.253008591 | ZNF593 | zinc finger protein 593 [Source:HGNC Symbol;Acc:HGNC:30943] |
| ENSG00000142949 | HGLibA_39502 | 90 | -0.253008591 | PTPRF | receptor type F [Source:HGNC Symbol;Acc:HGNC:9670]" |
| ENSG00000143156 | HGLibA_32171 | 90 | -0.253008591 | NME7 | NME/NM23 family member 7 [Source:HGNC Symbol;Acc:HGNC:20461] |
| ENSG00000143437 | HGLibA_03061 | 90 | -0.253008591 | ARNT | aryl hydrocarbon receptor nuclear translocator [Source:HGNC Symbol;Acc:HGNC:700] |
| ENSG00000143450 | HGLibA_33220 | 90 | -0.253008591 | OAZ3 | ornithine decarboxylase antizyme 3 [Source:HGNC Symbol;Acc:HGNC:8097] |
| ENSG00000144749 | HGLibA_27176 | 90 | -0.253008591 | LRIG1 | leucine rich repeats and immunoglobulin like domains 1 [Source:HGNC Symbol;Acc:HGNC:17360] |
| ENSG00000148677 | HGLibA_01989 | 90 | -0.253008591 | ANKRD1 | ankyrin repeat domain 1 [Source:HGNC Symbol;Acc:HGNC:15819] |
| ENSG00000149636 | HGLibA_14033 | 90 | -0.253008591 | DSN1 | MIS12 kinetochore complex component [Source:HGNC Symbol;Acc:HGNC:16165]" |
| ENSG00000149970 | HGLibA_10298 | 90 | -0.253008591 | CNKSR2 | connector enhancer of kinase suppressor of Ras 2 [Source:HGNC Symbol;Acc:HGNC:19701] |
| ENSG00000150261 | HGLibA_34543 | 90 | -0.253008591 | OR8K1 | olfactory receptor family 8 subfamily K member 1 [Source:HGNC Symbol;Acc:HGNC:14831] |
| ENSG00000151229 | HGLibA_44865 | 90 | -0.253008591 | SLC2A13 | solute carrier family 2 member 13 [Source:HGNC Symbol;Acc:HGNC:15956] |
| ENSG00000151917 | HGLibA_04412 | 90 | -0.253008591 | BEND6 | BEN domain containing 6 [Source:HGNC Symbol;Acc:HGNC:20871] |
| ENSG00000152661 | HGLibA_19136 | 90 | -0.253008591 | GJA1 | gap junction protein alpha 1 [Source:HGNC Symbol;Acc:HGNC:4274] |
| ENSG00000152910 | HGLibA_10430 | 90 | -0.253008591 | CNTNAP4 | contactin associated protein like 4 [Source:HGNC Symbol;Acc:HGNC:18747] |
| ENSG00000155906 | HGLibA_41380 | 90 | -0.253008591 | RMND1 | required for meiotic nuclear division 1 homolog [Source:HGNC Symbol;Acc:HGNC:21176] |
| ENSG00000156599 | HGLibA_55119 | 90 | -0.253008591 | ZDHHC5 | zinc finger DHHC-type containing 5 [Source:HGNC Symbol;Acc:HGNC:18472] |
| ENSG00000157131 | HGLibA_06593 | 90 | -0.253008591 | C8A | complement C8 alpha chain [Source:HGNC Symbol;Acc:HGNC:1352] |
| ENSG00000157734 | HGLibA_46067 | 90 | -0.253008591 | SNX22 | sorting nexin 22 [Source:HGNC Symbol;Acc:HGNC:16315] |
| ENSG00000159166 | HGLibA_25923 | 90 | -0.253008591 | LAD1 | ladinin 1 [Source:HGNC Symbol;Acc:HGNC:6472] |
| ENSG00000159917 | HGLibA_55649 | 90 | -0.253008591 | ZNF235 | zinc finger protein 235 [Source:HGNC Symbol;Acc:HGNC:12866] |
| ENSG00000160194 | HGLibA_31576 | 90 | -0.253008591 | NDUFV3 | NADH:ubiquinone oxidoreductase subunit V3 [Source:HGNC Symbol;Acc:HGNC:7719] |

| | | | | | |
|---|---|---|---|---|---|
| ENSG00000160305 | HGLibA_13247 | 90 | -0.253008591 | DIP2A | disco interacting protein 2 homolog A [Source:HGNC Symbol;Acc:HGNC:17217] |
| ENSG00000160685 | HGLibA_54920 | 90 | -0.253008591 | ZBTB7B | zinc finger and BTB domain containing 7B [Source:HGNC Symbol;Acc:HGNC:18668] |
| ENSG00000161813 | HGLibA_26034 | 90 | -0.253008591 | LARP4 | La ribonucleoprotein domain family member 4 [Source:HGNC Symbol;Acc:HGNC:24320] |
| ENSG00000162804 | HGLibA_45923 | 90 | -0.253008591 | SNED1 | nidogen and EGF like domains 1 [Source:HGNC Symbol;Acc:HGNC:24696]" |
| ENSG00000163092 | HGLibA_54513 | 90 | -0.253008591 | XIRP2 | xin actin binding repeat containing 2 [Source:HGNC Symbol;Acc:HGNC:14303] |
| ENSG00000164023 | HGLibA_43839 | 90 | -0.253008591 | SGMS2 | sphingomyelin synthase 2 [Source:HGNC Symbol;Acc:HGNC:28395] |
| ENSG00000164035 | HGLibA_15030 | 90 | -0.253008591 | EMCN | endomucin [Source:HGNC Symbol;Acc:HGNC:16041] |
| ENSG00000164107 | HGLibA_20829 | 90 | -0.253008591 | HAND2 | heart and neural crest derivatives expressed 2 [Source:HGNC Symbol;Acc:HGNC:4808] |
| ENSG00000164123 | HGLibA_06332 | 90 | -0.253008591 | C4orf45 | chromosome 4 open reading frame 45 [Source:HGNC Symbol;Acc:HGNC:26342] |
| ENSG00000164318 | HGLibA_14634 | 90 | -0.253008591 | EGFLAM | fibronectin type III and laminin G domains [Source:HGNC Symbol;Acc:HGNC:26810]" |
| ENSG00000164465 | HGLibA_12468 | 90 | -0.253008591 | DCBLD1 | CUB and LCCL domain containing 1 [Source:HGNC Symbol;Acc:HGNC:21479]" |
| ENSG00000164683 | HGLibA_21217 | 90 | -0.253008591 | HEY1 | hes related family bHLH transcription factor with YRPW motif 1 [Source:HGNC Symbol;Acc:HGNC:4880] |
| ENSG00000165863 | HGLibA_05096 | 90 | -0.253008591 | C10orf82 | chromosome 10 open reading frame 82 [Source:HGNC Symbol;Acc:HGNC:28500] |
| ENSG00000166292 | HGLibA_49837 | 90 | -0.253008591 | TMEM100 | transmembrane protein 100 [Source:HGNC Symbol;Acc:HGNC:25607] |
| ENSG00000167315 | HGLibA_00343 | 90 | -0.253008591 | ACAA2 | acetyl-CoA acyltransferase 2 [Source:HGNC Symbol;Acc:HGNC:83] |
| ENSG00000167861 | HGLibA_21294 | 90 | -0.253008591 | HID1 | HID1 domain containing [Source:HGNC Symbol;Acc:HGNC:15736] |
| ENSG00000167967 | HGLibA_14330 | 90 | -0.253008591 | E4F1 | E4F transcription factor 1 [Source:HGNC Symbol;Acc:HGNC:3121] |
| ENSG00000167985 | HGLibA_43139 | 90 | -0.253008591 | SDHAF2 | succinate dehydrogenase complex assembly factor 2 [Source:HGNC Symbol;Acc:HGNC:26034] |
| ENSG00000168002 | HGLibA_37581 | 90 | -0.253008591 | POLR2G | RNA polymerase II subunit G [Source:HGNC Symbol;Acc:HGNC:9194] |
| ENSG00000168263 | HGLibA_24406 | 90 | -0.253008591 | KCNV2 | potassium voltage-gated channel modifier subfamily V member 2 [Source:HGNC Symbol;Acc:HGNC:19698] |
| ENSG00000168675 | HGLibA_26251 | 90 | -0.253008591 | LDLRAD4 | low density lipoprotein receptor class A domain containing 4 [Source:HGNC Symbol;Acc:HGNC:1224] |
| ENSG00000169019 | HGLibA_10677 | 90 | -0.253008591 | COMMD8 | COMM domain containing 8 [Source:HGNC Symbol;Acc:HGNC:26036] |
| ENSG00000169251 | HGLibA_32147 | 90 | -0.253008591 | NMD3 | NMD3 ribosome export adaptor [Source:HGNC Symbol;Acc:HGNC:24250] |
| ENSG00000169918 | HGLibA_34778 | 90 | -0.253008591 | OTUD7A | OTU deubiquitinase 7A [Source:HGNC Symbol;Acc:HGNC:20718] |
| ENSG00000171148 | HGLibA_48192 | 90 | -0.253008591 | TADA3 | transcriptional adaptor 3 [Source:HGNC Symbol;Acc:HGNC:19422] |
| ENSG00000171489 | HGLibA_46334 | 90 | -0.253008591 | SPACA5 | sperm acrosome associated 5 [Source:HGNC Symbol;Acc:HGNC:31353] |
| ENSG00000171763 | HGLibA_46520 | 90 | -0.253008591 | SPATA5L1 | spermatogenesis associated 5 like 1 [Source:HGNC Symbol;Acc:HGNC:28762] |
| ENSG00000172137 | HGLibA_06988 | 90 | -0.253008591 | CALB2 | calbindin 2 [Source:HGNC Symbol;Acc:HGNC:1435] |
| ENSG00000172150 | HGLibA_33643 | 90 | -0.253008591 | OR1A2 | olfactory receptor family 1 subfamily A member 2 [Source:HGNC Symbol;Acc:HGNC:8180] |
| ENSG00000172216 | HGLibA_08959 | 90 | -0.253008591 | CEBPB | CCAAT enhancer binding protein beta [Source:HGNC Symbol;Acc:HGNC:1834] |
| ENSG00000173253 | HGLibA_13420 | 90 | -0.253008591 | DMRT2 | doublesex and mab-3 related transcription factor 2 [Source:HGNC Symbol;Acc:HGNC:2935] |
| ENSG00000173846 | HGLibA_37195 | 90 | -0.253008591 | PLK3 | polo like kinase 3 [Source:HGNC Symbol;Acc:HGNC:2154] |
| ENSG00000174123 | HGLibA_49643 | 90 | -0.253008591 | TLR10 | toll like receptor 10 [Source:HGNC Symbol;Acc:HGNC:15634] |
| ENSG00000175106 | HGLibA_52363 | 90 | -0.253008591 | TVP23C | trans-golgi network vesicle protein 23 homolog C [Source:HGNC Symbol;Acc:HGNC:30453] |
| ENSG00000175697 | HGLibA_19944 | 90 | -0.253008591 | GPR156 | G protein-coupled receptor 156 [Source:HGNC Symbol;Acc:HGNC:20844] |
| ENSG00000176040 | HGLibA_50652 | 90 | -0.253008591 | TMPRSS7 | transmembrane serine protease 7 [Source:HGNC Symbol;Acc:HGNC:30846] |
| ENSG00000176177 | HGLibA_15190 | 90 | -0.253008591 | ENTHD1 | ENTH domain containing 1 [Source:HGNC Symbol;Acc:HGNC:26352] |
| ENSG00000176222 | HGLibA_55925 | 90 | -0.253008591 | ZNF404 | zinc finger protein 404 [Source:HGNC Symbol;Acc:HGNC:19417] |
| ENSG00000176269 | HGLibA_33999 | 90 | -0.253008591 | OR4F21 | olfactory receptor family 4 subfamily F member 21 [Source:HGNC Symbol;Acc:HGNC:19583] |
| ENSG00000176407 | HGLibA_24121 | 90 | -0.253008591 | KCMF1 | potassium channel modulatory factor 1 [Source:HGNC Symbol;Acc:HGNC:20589] |
| ENSG00000176771 | HGLibA_31317 | 90 | -0.253008591 | NCKAP5 | NCK associated protein 5 [Source:HGNC Symbol;Acc:HGNC:29847] |
| ENSG00000178796 | HGLibA_41246 | 90 | -0.253008591 | RIIAD1 | regulatory subunit of type II PKA R-subunit (RIIa) domain containing 1 [Source:HGNC Symbol;Acc:HGNC:26686] |
| ENSG00000179304 | HGLibA_16189 | 90 | -0.253008591 | FAM156B | family with sequence similarity 156 member B [Source:HGNC Symbol;Acc:HGNC:31962] |
| ENSG00000179476 | HGLibA_05360 | 90 | -0.253008591 | C14orf28 | chromosome 14 open reading frame 28 [Source:HGNC Symbol;Acc:HGNC:19834] |
| ENSG00000179914 | HGLibA_23822 | 90 | -0.253008591 | ITLN1 | intelectin 1 [Source:HGNC Symbol;Acc:HGNC:18259] |
| ENSG00000180083 | HGLibA_54252 | 90 | -0.253008591 | WFDC11 | WAP four-disulfide core domain 11 [Source:HGNC Symbol;Acc:HGNC:20478] |
| ENSG00000180509 | HGLibA_24182 | 90 | -0.253008591 | KCNE1 | potassium voltage-gated channel subfamily E regulatory subunit 1 [Source:HGNC Symbol;Acc:HGNC:6240] |
| ENSG00000181333 | HGLibA_21148 | 90 | -0.253008591 | HEPHL1 | hephaestin like 1 [Source:HGNC Symbol;Acc:HGNC:30477] |
| ENSG00000181458 | HGLibA_50407 | 90 | -0.253008591 | TMEM45A | transmembrane protein 45A [Source:HGNC Symbol;Acc:HGNC:25480] |
| ENSG00000184954 | HGLibA_34403 | 90 | -0.253008591 | OR6C70 | olfactory receptor family 6 subfamily C member 70 [Source:HGNC Symbol;Acc:HGNC:31299] |
| ENSG00000187833 | HGLibA_06184 | 90 | -0.253008591 | C2orf78 | chromosome 2 open reading frame 78 [Source:HGNC Symbol;Acc:HGNC:34349] |
| ENSG00000188243 | HGLibA_10672 | 90 | -0.253008591 | COMMD6 | COMM domain containing 6 [Source:HGNC Symbol;Acc:HGNC:24015] |
| ENSG00000188372 | HGLibA_56955 | 90 | -0.253008591 | ZP3 | zona pellucida glycoprotein 3 [Source:HGNC Symbol;Acc:HGNC:13189] |
| ENSG00000188483 | HGLibA_22584 | 90 | -0.253008591 | IER5L | immediate early response 5 like [Source:HGNC Symbol;Acc:HGNC:23679] |
| ENSG00000188730 | HGLibA_53923 | 90 | -0.253008591 | VWC2 | von Willebrand factor C domain containing 2 [Source:HGNC Symbol;Acc:HGNC:30200] |
| ENSG00000188938 | HGLibA_16057 | 90 | -0.253008591 | FAM120AOS | family with sequence similarity 120A opposite strand [Source:HGNC Symbol;Acc:HGNC:23389] |
| ENSG00000196171 | HGLibA_34417 | 90 | -0.253008591 | OR6K2 | olfactory receptor family 6 subfamily K member 2 [Source:HGNC Symbol;Acc:HGNC:15029] |
| ENSG00000196565 | HGLibA_20927 | 90 | -0.253008591 | HBG2 | hemoglobin subunit gamma 2 [Source:HGNC Symbol;Acc:HGNC:4832] |
| ENSG00000196600 | HGLibA_44578 | 90 | -0.253008591 | SLC22A25 | solute carrier family 22 member 25 [Source:HGNC Symbol;Acc:HGNC:32935] |
| ENSG00000196600 | HGLibA_44579 | 90 | -0.253008591 | SLC22A25 | solute carrier family 22 member 25 [Source:HGNC Symbol;Acc:HGNC:32935] |
| ENSG00000197785 | HGLibA_03410 | 90 | -0.253008591 | ATAD3A | AAA domain containing 3A [Source:HGNC Symbol;Acc:HGNC:25567]" |
| ENSG00000198231 | HGLibA_12714 | 90 | -0.253008591 | DDX42 | DEAD-box helicase 42 [Source:HGNC Symbol;Acc:HGNC:18676] |
| ENSG00000203734 | HGLibA_14428 | 90 | -0.253008591 | ECT2L | epithelial cell transforming 2 like [Source:HGNC Symbol;Acc:HGNC:21118] |
| ENSG00000204505 | HGLibA_38332 | 90 | -0.253008591 | PRAMEF9 | PRAME family member 9 [Source:HGNC Symbol;Acc:HGNC:27996] |
| ENSG00000204510 | HGLibA_38327 | 90 | -0.253008591 | PRAMEF7 | PRAME family member 7 [Source:HGNC Symbol;Acc:HGNC:28415] |
| ENSG00000204516 | HGLibA_29220 | 90 | -0.253008591 | MICB | MHC class I polypeptide-related sequence B [Source:HGNC Symbol;Acc:HGNC:7091] |
| ENSG00000211584 | HGLibA_45200 | 90 | -0.253008591 | SLC48A1 | solute carrier family 48 member 1 [Source:HGNC Symbol;Acc:HGNC:26035] |
| ENSG00000213614 | HGLibA_21202 | 90 | -0.253008591 | HEXA | hexosaminidase subunit alpha [Source:HGNC Symbol;Acc:HGNC:4878] |
| ENSG00000214128 | HGLibA_50212 | 90 | -0.253008591 | TMEM213 | transmembrane protein 213 [Source:HGNC Symbol;Acc:HGNC:27220] |
| ENSG00000214402 | HGLibA_26190 | 90 | -0.253008591 | LCNL1 | lipocalin like 1 [Source:HGNC Symbol;Acc:HGNC:34436] |
| ENSG00000221883 | HGLibA_02917 | 90 | -0.253008591 | ARIH2OS | ariadne RBR E3 ubiquitin protein ligase 2 opposite strand [Source:HGNC Symbol;Acc:HGNC:34425] |
| ENSG00000229544 | HGLibA_32028 | 90 | -0.253008591 | NKX1-2 | NK1 homeobox 2 [Source:HGNC Symbol;Acc:HGNC:31652] |
| ENSG00000237412 | HGLibA_38964 | 90 | -0.253008591 | PRSS56 | serine protease 56 [Source:HGNC Symbol;Acc:HGNC:39433] |
| ENSG00000254834 | HGLibA_34332 | 90 | -0.253008591 | OR5M10 | olfactory receptor family 5 subfamily M member 10 [Source:HGNC Symbol;Acc:HGNC:15290] |
| ENSG00000255181 | HGLibA_07674 | 90 | -0.253008591 | CCDC166 | coiled-coil domain containing 166 [Source:HGNC Symbol;Acc:HGNC:41910] |
| ENSG00000257520 | HGLibA_43762 | 90 | -0.253008591 | SFTA3 | surfactant associated 3 [Source:NCBI gene;Acc:253970] |
| ENSG00000259024 | HGLibA_52366 | 90 | -0.253008591 | TVP23C-CDRT4 | TVP23C-CDRT4 readthrough [Source:HGNC Symbol;Acc:HGNC:42961] |
| ENSG00000266964 | HGLibA_18224 | 90 | -0.253008591 | FXYD1 | FXYD domain containing ion transport regulator 1 [Source:HGNC Symbol;Acc:HGNC:4025] |
| ENSG00000269743 | HGLibA_44771 | 90 | -0.253008591 | SLC25A53 | solute carrier family 25 member 53 [Source:HGNC Symbol;Acc:HGNC:31894] |
| HIST1H2BC | HGLibA_21416 | 90 | -0.253008591 | HIST1H2BC | - |
| HIST4H4 | HGLibA_21564 | 90 | -0.253008591 | HIST4H4 | - |
| hsa-mir-1268a | HGLibA_57497 | 90 | -0.253008591 | hsa-mir-1268a | - |
| hsa-mir-1289-2 | HGLibA_57625 | 90 | -0.253008591 | hsa-mir-1289-2 | - |
| hsa-mir-1295b | HGLibA_57660 | 90 | -0.253008591 | hsa-mir-1295b | - |
| hsa-mir-184 | HGLibA_58000 | 90 | -0.253008591 | hsa-mir-184 | - |
| hsa-mir-199a-1 | HGLibA_58129 | 90 | -0.253008591 | hsa-mir-199a-1 | - |
| hsa-mir-3157 | HGLibA_58701 | 90 | -0.253008591 | hsa-mir-3157 | - |
| hsa-mir-3609 | HGLibA_59066 | 90 | -0.253008591 | hsa-mir-3609 | - |
| hsa-mir-3619 | HGLibA_59106 | 90 | -0.253008591 | hsa-mir-3619 | - |
| hsa-mir-4286 | HGLibA_59850 | 90 | -0.253008591 | hsa-mir-4286 | - |
| hsa-mir-4310 | HGLibA_59954 | 90 | -0.253008591 | hsa-mir-4310 | - |
| hsa-mir-4317 | HGLibA_59989 | 90 | -0.253008591 | hsa-mir-4317 | - |
| hsa-mir-4780 | HGLibA_61227 | 90 | -0.253008591 | hsa-mir-4780 | - |
| hsa-mir-500b | HGLibA_61448 | 90 | -0.253008591 | hsa-mir-500b | - |
| hsa-mir-593 | HGLibA_62467 | 90 | -0.253008591 | hsa-mir-593 | - |
| hsa-mir-595 | HGLibA_62474 | 90 | -0.253008591 | hsa-mir-595 | - |
| hsa-mir-6505 | HGLibA_62871 | 90 | -0.253008591 | hsa-mir-6505 | - |
| hsa-mir-6748 | HGLibA_63146 | 90 | -0.253008591 | hsa-mir-6748 | - |
| hsa-mir-6766 | HGLibA_63226 | 90 | -0.253008591 | hsa-mir-6766 | - |
| hsa-mir-6846 | HGLibA_63555 | 90 | -0.253008591 | hsa-mir-6846 | - |
| hsa-mir-7111 | HGLibA_63808 | 90 | -0.253008591 | hsa-mir-7111 | - |
| IL3RA_X | HGLibA_23161 | 90 | -0.253008591 | IL3RA_X | - |
| LOC389895 | HGLibA_26929 | 90 | -0.253008591 | LOC389895 | - |
| LPHN2 | HGLibA_27090 | 90 | -0.253008591 | LPHN2 | - |
| MFI2 | HGLibA_29065 | 90 | -0.253008591 | MFI2 | - |
| MURC | HGLibA_30571 | 90 | -0.253008591 | MURC | - |
| SGOL2 | HGLibA_43845 | 90 | -0.253008591 | SGOL2 | - |
| SMCR7 | HGLibA_45676 | 90 | -0.253008591 | SMCR7 | - |
| SPRY3_Y | HGLibA_46844 | 90 | -0.253008591 | SPRY3_Y | - |
| TMEM155 | HGLibA_50008 | 90 | -0.253008591 | TMEM155 | - |
| ZFP112 | HGLibA_55187 | 90 | -0.253008591 | ZFP112 | - |
| C10orf35 | HGLibA_05069 | 89 | -0.262959628 | C10orf35 | - |
| C3orf79 | HGLibA_06269 | 89 | -0.262959628 | C3orf79 | - |
| CIRH1A | HGLibA_09725 | 89 | -0.262959628 | CIRH1A | - |
| ENSG00000002834 | HGLibA_26054 | 89 | -0.262959628 | LASP1 | LIM and SH3 protein 1 [Source:HGNC Symbol;Acc:HGNC:6513] |
| ENSG00000004534 | HGLibA_40577 | 89 | -0.262959628 | RBM6 | RNA binding motif protein 6 [Source:HGNC Symbol;Acc:HGNC:9903] |
| ENSG00000005471 | HGLibA_00140 | 89 | -0.262959628 | ABCB4 | ATP binding cassette subfamily B member 4 [Source:HGNC Symbol;Acc:HGNC:45] |
| ENSG00000006638 | HGLibA_48679 | 89 | -0.262959628 | TBXA2R | thromboxane A2 receptor [Source:HGNC Symbol;Acc:HGNC:11608] |
| ENSG00000007174 | HGLibA_13500 | 89 | -0.262959628 | DNAH9 | dynein axonemal heavy chain 9 [Source:HGNC Symbol;Acc:HGNC:2953] |
| ENSG00000010295 | HGLibA_22586 | 89 | -0.262959628 | IFFO1 | intermediate filament family orphan 1 [Source:HGNC Symbol;Acc:HGNC:24970] |
| ENSG00000011258 | HGLibA_28531 | 89 | -0.262959628 | MBTD1 | mbt domain containing 1 [Source:HGNC Symbol;Acc:HGNC:19866] |
| ENSG00000013364 | HGLibA_30600 | 89 | -0.262959628 | MVP | major vault protein [Source:HGNC Symbol;Acc:HGNC:7531] |
| ENSG00000015153 | HGLibA_54627 | 89 | -0.262959628 | YAF2 | YY1 associated factor 2 [Source:HGNC Symbol;Acc:HGNC:17363] |
| ENSG00000055118 | HGLibA_24214 | 89 | -0.262959628 | KCNH2 | potassium voltage-gated channel subfamily H member 2 [Source:HGNC Symbol;Acc:HGNC:6251] |
| ENSG00000065491 | HGLibA_48507 | 89 | -0.262959628 | TBC1D22B | TBC1 domain family member 22B [Source:HGNC Symbol;Acc:HGNC:21602] |
| ENSG00000066382 | HGLibA_29773 | 89 | -0.262959628 | MPPED2 | metallophosphoesterase domain containing 2 [Source:HGNC Symbol;Acc:HGNC:1180] |
| ENSG00000069431 | HGLibA_00187 | 89 | -0.262959628 | ABCC9 | ATP binding cassette subfamily C member 9 [Source:HGNC Symbol;Acc:HGNC:60] |
| ENSG00000072609 | HGLibA_09427 | 89 | -0.262959628 | CHFR | checkpoint with forkhead and ring finger domains [Source:HGNC Symbol;Acc:HGNC:20455] |
| ENSG00000074590 | HGLibA_32892 | 89 | -0.262959628 | NUAK1 | NUAK family kinase 1 [Source:HGNC Symbol;Acc:HGNC:14311] |
| ENSG00000075618 | HGLibA_18067 | 89 | -0.262959628 | FSCN1 | fascin actin-bundling protein 1 [Source:HGNC Symbol;Acc:HGNC:11148] |
| ENSG00000077312 | HGLibA_45961 | 89 | -0.262959628 | SNRPA | small nuclear ribonucleoprotein polypeptide A [Source:HGNC Symbol;Acc:HGNC:11151] |
| ENSG00000082512 | HGLibA_51262 | 89 | -0.262959628 | TRAF5 | TNF receptor associated factor 5 [Source:HGNC Symbol;Acc:HGNC:12035] |
| ENSG00000083720 | HGLibA_34814 | 89 | -0.262959628 | OXCT1 | 3-oxoacid CoA-transferase 1 [Source:HGNC Symbol;Acc:HGNC:8527] |
| ENSG00000095015 | HGLibA_28161 | 89 | -0.262959628 | MAP3K1 | mitogen-activated protein kinase kinase kinase 1 [Source:HGNC Symbol;Acc:HGNC:6848] |
| ENSG00000095587 | HGLibA_49632 | 89 | -0.262959628 | TLL2 | tolloid like 2 [Source:HGNC Symbol;Acc:HGNC:11844] |

| | | | | | |
|---|---|---|---|---|---|
| ENSG00000100276 | HGLibA_40360 | 89 | -0.262959628 | RASL10A | RAS like family 10 member A [Source:HGNC Symbol;Acc:HGNC:16954] |
| ENSG00000100319 | HGLibA_55377 | 89 | -0.262959628 | ZMAT5 | zinc finger matrin-type 5 [Source:HGNC Symbol;Acc:HGNC:28046] |
| ENSG00000102401 | HGLibA_03045 | 89 | -0.262959628 | ARMCX3 | armadillo repeat containing X-linked 3 [Source:HGNC Symbol;Acc:HGNC:24065] |
| ENSG00000104805 | HGLibA_32909 | 89 | -0.262959628 | NUCB1 | nucleobindin 1 [Source:HGNC Symbol;Acc:HGNC:8043] |
| ENSG00000105202 | HGLibA_16936 | 89 | -0.262959628 | FBL | fibrillarin [Source:HGNC Symbol;Acc:HGNC:3599] |
| ENSG00000105374 | HGLibA_32012 | 89 | -0.262959628 | NKG7 | natural killer cell granule protein 7 [Source:HGNC Symbol;Acc:HGNC:7830] |
| ENSG00000105552 | HGLibA_04266 | 89 | -0.262959628 | BCAT2 | branched chain amino acid transaminase 2 [Source:HGNC Symbol;Acc:HGNC:977] |
| ENSG00000106178 | HGLibA_08002 | 89 | -0.262959628 | CCL24 | C-C motif chemokine ligand 24 [Source:HGNC Symbol;Acc:HGNC:10623] |
| ENSG00000106236 | HGLibA_32534 | 89 | -0.262959628 | NPTX2 | neuronal pentraxin 2 [Source:HGNC Symbol;Acc:HGNC:7953] |
| ENSG00000108176 | HGLibA_13569 | 89 | -0.262959628 | DNAJC12 | DnaJ heat shock protein family (Hsp40) member C12 [Source:HGNC Symbol;Acc:HGNC:28908] |
| ENSG00000108370 | HGLibA_41111 | 89 | -0.262959628 | RGS9 | regulator of G protein signaling 9 [Source:HGNC Symbol;Acc:HGNC:10004] |
| ENSG00000108825 | HGLibA_39337 | 89 | -0.262959628 | PTGES3L-AARSD1 | PTGES3L-AARSD1 readthrough [Source:HGNC Symbol;Acc:HGNC:43946] |
| ENSG00000109272 | HGLibA_36131 | 89 | -0.262959628 | PF4V1 | platelet factor 4 variant 1 [Source:HGNC Symbol;Acc:HGNC:8862] |
| ENSG00000109339 | HGLibA_28250 | 89 | -0.262959628 | MAPK10 | mitogen-activated protein kinase 10 [Source:HGNC Symbol;Acc:HGNC:6872] |
| ENSG00000111737 | HGLibA_39888 | 89 | -0.262959628 | RAB35 | member RAS oncogene family [Source:HGNC Symbol;Acc:HGNC:9774]" |
| ENSG00000112695 | HGLibA_10860 | 89 | -0.262959628 | COX7A2 | cytochrome c oxidase subunit 7A2 [Source:HGNC Symbol;Acc:HGNC:2288] |
| ENSG00000112837 | HGLibA_48649 | 89 | -0.262959628 | TBX18 | T-box 18 [Source:HGNC Symbol;Acc:HGNC:11595] |
| ENSG00000112983 | HGLibA_04785 | 89 | -0.262959628 | BRD8 | bromodomain containing 8 [Source:HGNC Symbol;Acc:HGNC:19874] |
| ENSG00000113916 | HGLibA_04332 | 89 | -0.262959628 | BCL6 | B cell CLL/lymphoma 6 [Source:HGNC Symbol;Acc:HGNC:1001] |
| ENSG00000114450 | HGLibA_19498 | 89 | -0.262959628 | GNB4 | G protein subunit beta 4 [Source:HGNC Symbol;Acc:HGNC:20731] |
| ENSG00000114491 | HGLibA_52984 | 89 | -0.262959628 | UMPS | uridine monophosphate synthetase [Source:HGNC Symbol;Acc:HGNC:12563] |
| ENSG00000115233 | HGLibA_39163 | 89 | -0.262959628 | PSMD14 | non-ATPase 14 [Source:HGNC Symbol;Acc:HGNC:16889]" |
| ENSG00000115616 | HGLibA_45403 | 89 | -0.262959628 | SLC9A2 | solute carrier family 9 member A2 [Source:HGNC Symbol;Acc:HGNC:11072] |
| ENSG00000116544 | HGLibA_13345 | 89 | -0.262959628 | DLGAP3 | DLG associated protein 3 [Source:HGNC Symbol;Acc:HGNC:30368] |
| ENSG00000116731 | HGLibA_38385 | 89 | -0.262959628 | PRDM2 | PR/SET domain 2 [Source:HGNC Symbol;Acc:HGNC:9347] |
| ENSG00000118094 | HGLibA_51352 | 89 | -0.262959628 | TREH | trehalase [Source:HGNC Symbol;Acc:HGNC:12266] |
| ENSG00000119688 | HGLibA_00201 | 89 | -0.262959628 | ABCD4 | ATP binding cassette subfamily D member 4 [Source:HGNC Symbol;Acc:HGNC:68] |
| ENSG00000121741 | HGLibA_55394 | 89 | -0.262959628 | ZMYM2 | zinc finger MYM-type containing 2 [Source:HGNC Symbol;Acc:HGNC:12989] |
| ENSG00000122873 | HGLibA_09728 | 89 | -0.262959628 | CISD1 | CDGSH iron sulfur domain 1 [Source:HGNC Symbol;Acc:HGNC:30880] |
| ENSG00000124818 | HGLibA_33441 | 89 | -0.262959628 | OPN5 | opsin 5 [Source:HGNC Symbol;Acc:HGNC:19992] |
| ENSG00000125735 | HGLibA_50814 | 89 | -0.262959628 | TNFSF14 | TNF superfamily member 14 [Source:HGNC Symbol;Acc:HGNC:11930] |
| ENSG00000125827 | HGLibA_50696 | 89 | -0.262959628 | TMX4 | thioredoxin related transmembrane protein 4 [Source:HGNC Symbol;Acc:HGNC:25237] |
| ENSG00000125850 | HGLibA_34804 | 89 | -0.262959628 | OVOL2 | ovo like zinc finger 2 [Source:HGNC Symbol;Acc:HGNC:15804] |
| ENSG00000126226 | HGLibA_35606 | 89 | -0.262959628 | PCID2 | PCI domain containing 2 [Source:HGNC Symbol;Acc:HGNC:25653] |
| ENSG00000126970 | HGLibA_54999 | 89 | -0.262959628 | ZC4H2 | zinc finger C4H2-type containing [Source:HGNC Symbol;Acc:HGNC:24931] |
| ENSG00000127415 | HGLibA_22569 | 89 | -0.262959628 | IDUA | alpha-L- [Source:HGNC Symbol;Acc:HGNC:5391]" |
| ENSG00000127533 | HGLibA_15871 | 89 | -0.262959628 | F2RL3 | F2R like thrombin or trypsin receptor 3 [Source:HGNC Symbol;Acc:HGNC:3540] |
| ENSG00000129473 | HGLibA_04323 | 89 | -0.262959628 | BCL2L2 | BCL2 like 2 [Source:HGNC Symbol;Acc:HGNC:995] |
| ENSG00000130528 | HGLibA_22085 | 89 | -0.262959628 | HRC | histidine rich calcium binding protein [Source:HGNC Symbol;Acc:HGNC:5178] |
| ENSG00000130700 | HGLibA_18676 | 89 | -0.262959628 | GATA5 | GATA binding protein 5 [Source:HGNC Symbol;Acc:HGNC:15802] |
| ENSG00000132406 | HGLibA_49918 | 89 | -0.262959628 | TMEM128 | transmembrane protein 128 [Source:HGNC Symbol;Acc:HGNC:28201] |
| ENSG00000134376 | HGLibA_11070 | 89 | -0.262959628 | CRB1 | cell polarity complex component [Source:HGNC Symbol;Acc:HGNC:2343]" |
| ENSG00000134461 | HGLibA_02012 | 89 | -0.262959628 | ANKRD16 | ankyrin repeat domain 16 [Source:HGNC Symbol;Acc:HGNC:23471] |
| ENSG00000134884 | HGLibA_02695 | 89 | -0.262959628 | ARGLU1 | arginine and glutamate rich 1 [Source:HGNC Symbol;Acc:HGNC:25482] |
| ENSG00000134917 | HGLibA_00843 | 89 | -0.262959628 | ADAMTS8 | ADAM metallopeptidase with thrombospondin type 1 motif 8 [Source:HGNC Symbol;Acc:HGNC:224] |
| ENSG00000134917 | HGLibA_00842 | 89 | -0.262959628 | ADAMTS8 | ADAM metallopeptidase with thrombospondin type 1 motif 8 [Source:HGNC Symbol;Acc:HGNC:224] |
| ENSG00000135355 | HGLibA_19138 | 89 | -0.262959628 | GJA10 | gap junction protein alpha 10 [Source:HGNC Symbol;Acc:HGNC:16995] |
| ENSG00000135365 | HGLibA_36370 | 89 | -0.262959628 | PHF21A | PHD finger protein 21A [Source:HGNC Symbol;Acc:HGNC:24156] |
| ENSG00000135624 | HGLibA_08213 | 89 | -0.262959628 | CCT7 | chaperonin containing TCP1 subunit 7 [Source:HGNC Symbol;Acc:HGNC:1622] |
| ENSG00000135636 | HGLibA_14286 | 89 | -0.262959628 | DYSF | dysferlin [Source:HGNC Symbol;Acc:HGNC:3097] |
| ENSG00000136630 | HGLibA_21660 | 89 | -0.262959628 | HLX | H2.0 like homeobox [Source:HGNC Symbol;Acc:HGNC:4978] |
| ENSG00000138435 | HGLibA_09561 | 89 | -0.262959628 | CHRNA1 | cholinergic receptor nicotinic alpha 1 subunit [Source:HGNC Symbol;Acc:HGNC:1955] |
| ENSG00000138757 | HGLibA_18303 | 89 | -0.262959628 | G3BP2 | G3BP stress granule assembly factor 2 [Source:HGNC Symbol;Acc:HGNC:30291] |
| ENSG00000139044 | HGLibA_04016 | 89 | -0.262959628 | B4GALNT3 | 4-N-acetyl-galactosaminyltransferase 3 [Source:HGNC Symbol;Acc:HGNC:24137]" |
| ENSG00000139193 | HGLibA_08314 | 89 | -0.262959628 | CD27 | CD27 molecule [Source:HGNC Symbol;Acc:HGNC:11922] |
| ENSG00000141098 | HGLibA_18957 | 89 | -0.262959628 | GFOD2 | glucose-fructose oxidoreductase domain containing 2 [Source:HGNC Symbol;Acc:HGNC:28159] |
| ENSG00000141127 | HGLibA_38767 | 89 | -0.262959628 | PRPSAP2 | phosphoribosyl pyrophosphate synthetase associated protein 2 [Source:HGNC Symbol;Acc:HGNC:9467] |
| ENSG00000141161 | HGLibA_53008 | 89 | -0.262959628 | UNC45B | unc-45 myosin chaperone B [Source:HGNC Symbol;Acc:HGNC:14304] |
| ENSG00000142619 | HGLibA_34976 | 89 | -0.262959628 | PADI3 | peptidyl arginine deiminase 3 [Source:HGNC Symbol;Acc:HGNC:18337] |
| ENSG00000143199 | HGLibA_00909 | 89 | -0.262959628 | ADCY10 | adenylate cyclase 10 [Source:HGNC Symbol;Acc:HGNC:21285] |
| ENSG00000143653 | HGLibA_42880 | 89 | -0.262959628 | SCCPDH | saccharopine dehydrogenase (putative) [Source:HGNC Symbol;Acc:HGNC:24275] |
| ENSG00000144015 | HGLibA_51502 | 89 | -0.262959628 | TRIM43 | tripartite motif containing 43 [Source:HGNC Symbol;Acc:HGNC:19015] |
| ENSG00000144229 | HGLibA_49409 | 89 | -0.262959628 | THSD7B | thrombospondin type 1 domain containing 7B [Source:HGNC Symbol;Acc:HGNC:29348] |
| ENSG00000144852 | HGLibA_32589 | 89 | -0.262959628 | NR1I2 | nuclear receptor subfamily 1 group I member 2 [Source:HGNC Symbol;Acc:HGNC:7968] |
| ENSG00000146530 | HGLibA_53931 | 89 | -0.262959628 | VWDE | von Willebrand factor D and EGF domains [Source:HGNC Symbol;Acc:HGNC:21897] |
| ENSG00000147206 | HGLibA_33135 | 89 | -0.262959628 | NXF3 | nuclear RNA export factor 3 [Source:HGNC Symbol;Acc:HGNC:8073] |
| ENSG00000147465 | HGLibA_47349 | 89 | -0.262959628 | STAR | steroidogenic acute regulatory protein [Source:HGNC Symbol;Acc:HGNC:11359] |
| ENSG00000148429 | HGLibA_53407 | 89 | -0.262959628 | USP6NL | USP6 N-terminal like [Source:HGNC Symbol;Acc:HGNC:16858] |
| ENSG00000149273 | HGLibA_42137 | 89 | -0.262959628 | RPS3 | ribosomal protein S3 [Source:HGNC Symbol;Acc:HGNC:10420] |
| ENSG00000150201 | HGLibA_18233 | 89 | -0.262959628 | FXYD4 | FXYD domain containing ion transport regulator 4 [Source:HGNC Symbol;Acc:HGNC:4028] |
| ENSG00000150977 | HGLibA_41256 | 89 | -0.262959628 | RILPL2 | Rab interacting lysosomal protein like 2 [Source:HGNC Symbol;Acc:HGNC:28787] |
| ENSG00000151023 | HGLibA_15138 | 89 | -0.262959628 | ENKUR | TRPC channel interacting protein [Source:HGNC Symbol;Acc:HGNC:28388]" |
| ENSG00000151474 | HGLibA_18013 | 89 | -0.262959628 | FRMD4A | FERM domain containing 4A [Source:HGNC Symbol;Acc:HGNC:25491] |
| ENSG00000152208 | HGLibA_20276 | 89 | -0.262959628 | GRID2 | glutamate ionotropic receptor delta type subunit 2 [Source:HGNC Symbol;Acc:HGNC:4576] |
| ENSG00000152348 | HGLibA_03461 | 89 | -0.262959628 | ATG10 | autophagy related 10 [Source:HGNC Symbol;Acc:HGNC:20315] |
| ENSG00000152413 | HGLibA_21863 | 89 | -0.262959628 | HOMER1 | homer scaffold protein 1 [Source:HGNC Symbol;Acc:HGNC:17512] |
| ENSG00000153029 | HGLibA_29803 | 89 | -0.262959628 | MR1 | class I-related [Source:HGNC Symbol;Acc:HGNC:4975]" |
| ENSG00000156113 | HGLibA_24339 | 89 | -0.262959628 | KCNMA1 | potassium calcium-activated channel subfamily M alpha 1 [Source:HGNC Symbol;Acc:HGNC:6284] |
| ENSG00000156170 | HGLibA_31496 | 89 | -0.262959628 | NDUFAF6 | NADH:ubiquinone oxidoreductase complex assembly factor 6 [Source:HGNC Symbol;Acc:HGNC:28625] |
| ENSG00000158828 | HGLibA_36690 | 89 | -0.262959628 | PINK1 | PTEN induced kinase 1 [Source:HGNC Symbol;Acc:HGNC:14581] |
| ENSG00000160233 | HGLibA_27305 | 89 | -0.262959628 | LRRC3 | leucine rich repeat containing 3 [Source:HGNC Symbol;Acc:HGNC:14965] |
| ENSG00000161204 | HGLibA_00212 | 89 | -0.262959628 | ABCF3 | ATP binding cassette subfamily F member 3 [Source:HGNC Symbol;Acc:HGNC:72] |
| ENSG00000162702 | HGLibA_55735 | 89 | -0.262959628 | ZNF281 | zinc finger protein 281 [Source:HGNC Symbol;Acc:HGNC:13075] |
| ENSG00000162999 | HGLibA_14153 | 89 | -0.262959628 | DUSP19 | dual specificity phosphatase 19 [Source:HGNC Symbol;Acc:HGNC:18894] |
| ENSG00000163110 | HGLibA_35920 | 89 | -0.262959628 | PDLIM5 | PDZ and LIM domain 5 [Source:HGNC Symbol;Acc:HGNC:17468] |
| ENSG00000163682 | HGLibA_42006 | 89 | -0.262959628 | RPL9 | ribosomal protein L9 [Source:HGNC Symbol;Acc:HGNC:10369] |
| ENSG00000163701 | HGLibA_23015 | 89 | -0.262959628 | IL17RE | interleukin 17 receptor E [Source:HGNC Symbol;Acc:HGNC:18439] |
| ENSG00000163762 | HGLibA_49693 | 89 | -0.262959628 | TM4SF18 | transmembrane 4 L six family member 18 [Source:HGNC Symbol;Acc:HGNC:25181] |
| ENSG00000163898 | HGLibA_26614 | 89 | -0.262959628 | LIPH | lipase H [Source:HGNC Symbol;Acc:HGNC:18483] |
| ENSG00000163918 | HGLibA_40911 | 89 | -0.262959628 | RFC4 | replication factor C subunit 4 [Source:HGNC Symbol;Acc:HGNC:9972] |
| ENSG00000165097 | HGLibA_24491 | 89 | -0.262959628 | KDM1B | lysine demethylase 1B [Source:HGNC Symbol;Acc:HGNC:21577] |
| ENSG00000165553 | HGLibA_31862 | 89 | -0.262959628 | NGB | neuroglobin [Source:HGNC Symbol;Acc:HGNC:14077] |
| ENSG00000166317 | HGLibA_48024 | 89 | -0.262959628 | SYNPO2L | synaptopodin 2 like [Source:HGNC Symbol;Acc:HGNC:23532] |
| ENSG00000166391 | HGLibA_29626 | 89 | -0.262959628 | MOGAT2 | monoacylglycerol O-acyltransferase 2 [Source:HGNC Symbol;Acc:HGNC:23248] |
| ENSG00000166455 | HGLibA_05449 | 89 | -0.262959628 | C16orf46 | chromosome 16 open reading frame 46 [Source:HGNC Symbol;Acc:HGNC:26525] |
| ENSG00000166471 | HGLibA_50395 | 89 | -0.262959628 | TMEM41B | transmembrane protein 41B [Source:HGNC Symbol;Acc:HGNC:28948] |
| ENSG00000167286 | HGLibA_08370 | 89 | -0.262959628 | CD3D | CD3d molecule [Source:HGNC Symbol;Acc:HGNC:1673] |
| ENSG00000169031 | HGLibA_10573 | 89 | -0.262959628 | COL4A3 | collagen type IV alpha 3 chain [Source:HGNC Symbol;Acc:HGNC:2204] |
| ENSG00000169946 | HGLibA_55255 | 89 | -0.262959628 | ZFPM2 | FOG family member 2 [Source:HGNC Symbol;Acc:HGNC:16700]" |
| ENSG00000170043 | HGLibA_51298 | 89 | -0.262959628 | TRAPPC1 | trafficking protein particle complex 1 [Source:HGNC Symbol;Acc:HGNC:19894] |
| ENSG00000170540 | HGLibA_02986 | 89 | -0.262959628 | ARL6IP1 | ADP ribosylation factor like GTPase 6 interacting protein 1 [Source:HGNC Symbol;Acc:HGNC:697] |
| ENSG00000170581 | HGLibA_47383 | 89 | -0.262959628 | STAT2 | signal transducer and activator of transcription 2 [Source:HGNC Symbol;Acc:HGNC:11363] |
| ENSG00000170802 | HGLibA_17911 | 89 | -0.262959628 | FOXN2 | forkhead box N2 [Source:HGNC Symbol;Acc:HGNC:5281] |
| ENSG00000171208 | HGLibA_31708 | 89 | -0.262959628 | NETO2 | neuropilin and tolloid like 2 [Source:HGNC Symbol;Acc:HGNC:14644] |
| ENSG00000171763 | HGLibA_46518 | 89 | -0.262959628 | SPATA5L1 | spermatogenesis associated 5 like 1 [Source:HGNC Symbol;Acc:HGNC:28762] |
| ENSG00000171806 | HGLibA_28969 | 89 | -0.262959628 | METTL18 | methyltransferase like 18 [Source:HGNC Symbol;Acc:HGNC:28793] |
| ENSG00000172340 | HGLibA_47703 | 89 | -0.262959628 | SUCLG2 | succinate-CoA ligase GDP-forming beta subunit [Source:HGNC Symbol;Acc:HGNC:11450] |
| ENSG00000173281 | HGLibA_38101 | 89 | -0.262959628 | PPP1R3B | protein phosphatase 1 regulatory subunit 3B [Source:HGNC Symbol;Acc:HGNC:14942] |
| ENSG00000173517 | HGLibA_36006 | 89 | -0.262959628 | PEAK1 | pseudopodium enriched atypical kinase 1 [Source:HGNC Symbol;Acc:HGNC:29431] |
| ENSG00000173714 | HGLibA_54282 | 89 | -0.262959628 | WFIKKN2 | kunitz and netrin domain containing 2 [Source:HGNC Symbol;Acc:HGNC:30916]" |
| ENSG00000174456 | HGLibA_05300 | 89 | -0.262959628 | C12orf76 | chromosome 12 open reading frame 76 [Source:HGNC Symbol;Acc:HGNC:33790] |
| ENSG00000175193 | HGLibA_35202 | 89 | -0.262959628 | PARL | presenilin associated rhomboid like [Source:HGNC Symbol;Acc:HGNC:18253] |
| ENSG00000175264 | HGLibA_09610 | 89 | -0.262959628 | CHST1 | carbohydrate sulfotransferase 1 [Source:HGNC Symbol;Acc:HGNC:1969] |
| ENSG00000175455 | HGLibA_07612 | 89 | -0.262959628 | CCDC14 | coiled-coil domain containing 14 [Source:HGNC Symbol;Acc:HGNC:25766] |
| ENSG00000176797 | HGLibA_12812 | 89 | -0.262959628 | DEFB103A | defensin beta 103A [Source:HGNC Symbol;Acc:HGNC:15967] |
| ENSG00000176834 | HGLibA_53840 | 89 | -0.262959628 | VSIG10 | V-set and immunoglobulin domain containing 10 [Source:HGNC Symbol;Acc:HGNC:26078] |
| ENSG00000178201 | HGLibA_53697 | 89 | -0.262959628 | VN1R1 | vomeronasal 1 receptor 1 [Source:HGNC Symbol;Acc:HGNC:13548] |
| ENSG00000178386 | HGLibA_55616 | 89 | -0.262959628 | ZNF223 | zinc finger protein 223 [Source:HGNC Symbol;Acc:HGNC:13016] |
| ENSG00000179241 | HGLibA_26246 | 89 | -0.262959628 | LDLRAD3 | low density lipoprotein receptor class A domain containing 3 [Source:HGNC Symbol;Acc:HGNC:27046] |
| ENSG00000179363 | HGLibA_50365 | 89 | -0.262959628 | TMEM31 | transmembrane protein 31 [Source:HGNC Symbol;Acc:HGNC:28601] |
| ENSG00000180884 | HGLibA_56770 | 89 | -0.262959628 | ZNF792 | zinc finger protein 792 [Source:HGNC Symbol;Acc:HGNC:24751] |
| ENSG00000181585 | HGLibA_50582 | 89 | -0.262959628 | TMIE | transmembrane inner ear [Source:HGNC Symbol;Acc:HGNC:30800] |
| ENSG00000182111 | HGLibA_56622 | 89 | -0.262959628 | ZNF716 | zinc finger protein 716 [Source:HGNC Symbol;Acc:HGNC:32458] |
| ENSG00000182645 | HGLibA_07698 | 89 | -0.262959628 | CCDC172 | coiled-coil domain containing 172 [Source:HGNC Symbol;Acc:HGNC:30524] |
| ENSG00000183246 | HGLibA_41268 | 89 | -0.262959628 | RIMBP3C | RIMS binding protein 3C [Source:HGNC Symbol;Acc:HGNC:33892] |
| ENSG00000183421 | HGLibA_41324 | 89 | -0.262959628 | RIPK4 | receptor interacting serine/threonine kinase 4 [Source:HGNC Symbol;Acc:HGNC:496] |
| ENSG00000184005 | HGLibA_47269 | 89 | -0.262959628 | ST6GALNAC3 | 6-sialyltransferase 3 [Source:HGNC Symbol;Acc:HGNC:19343]" |
| ENSG00000184368 | HGLibA_28239 | 89 | -0.262959628 | MAP7D2 | MAP7 domain containing 2 [Source:HGNC Symbol;Acc:HGNC:25899] |
| ENSG00000185231 | HGLibA_28544 | 89 | -0.262959628 | MC2R | melanocortin 2 receptor [Source:HGNC Symbol;Acc:HGNC:6930] |
| ENSG00000185482 | HGLibA_47318 | 89 | -0.262959628 | STAC3 | SH3 and cysteine rich domain 3 [Source:HGNC Symbol;Acc:HGNC:28423] |
| ENSG00000185585 | HGLibA_33359 | 89 | -0.262959628 | OLFML2A | olfactomedin like 2A [Source:HGNC Symbol;Acc:HGNC:27270] |
| ENSG00000186094 | HGLibA_01175 | 89 | -0.262959628 | AGBL4 | ATP/GTP binding protein like 4 [Source:HGNC Symbol;Acc:HGNC:25892] |
| ENSG00000186532 | HGLibA_45861 | 89 | -0.262959628 | SMYD4 | SET and MYND domain containing 4 [Source:HGNC Symbol;Acc:HGNC:21067] |
| ENSG00000186579 | HGLibA_12830 | 89 | -0.262959628 | DEFB106A | defensin beta 106A [Source:HGNC Symbol;Acc:HGNC:18088] |
| ENSG00000187003 | HGLibA_00626 | 89 | -0.262959628 | ACTL7A | actin like 7A [Source:HGNC Symbol;Acc:HGNC:161] |

| | | | | | |
|---|---|---|---|---|---|
| ENSG00000187223 | HGLibA_26127 | 89 | -0.262959628 | LCE2D | late cornified envelope 2D [Source:HGNC Symbol;Acc:HGNC:16518] |
| ENSG00000187569 | HGLibA_13890 | 89 | -0.262959628 | DPPA3 | developmental pluripotency associated 3 [Source:HGNC Symbol;Acc:HGNC:19199] |
| ENSG00000188175 | HGLibA_21141 | 89 | -0.262959628 | HEPACAM2 | HEPACAM family member 2 [Source:HGNC Symbol;Acc:HGNC:27364] |
| ENSG00000189367 | HGLibA_24636 | 89 | -0.262959628 | KIAA0408 | KIAA0408 [Source:HGNC Symbol;Acc:HGNC:21636] |
| ENSG00000196072 | HGLibA_04559 | 89 | -0.262959628 | BLOC1S2 | biogenesis of lysosomal organelles complex 1 subunit 2 [Source:HGNC Symbol;Acc:HGNC:20984] |
| ENSG00000196290 | HGLibA_31930 | 89 | -0.262959628 | NIF3L1 | NGG1 interacting factor 3 like 1 [Source:HGNC Symbol;Acc:HGNC:13390] |
| ENSG00000196296 | HGLibA_03605 | 89 | -0.262959628 | ATP2A1 | ATPase sarcoplasmic/endoplasmic reticulum Ca2+ transporting 1 [Source:HGNC Symbol;Acc:HGNC:811] |
| ENSG00000197019 | HGLibA_43605 | 89 | -0.262959628 | SERTAD1 | SERTA domain containing 1 [Source:HGNC Symbol;Acc:HGNC:17932] |
| ENSG00000197375 | HGLibA_44591 | 89 | -0.262959628 | SLC22A5 | solute carrier family 22 member 5 [Source:HGNC Symbol;Acc:HGNC:10969] |
| ENSG00000198728 | HGLibA_26211 | 89 | -0.262959628 | LDB1 | LIM domain binding 1 [Source:HGNC Symbol;Acc:HGNC:6532] |
| ENSG00000203727 | HGLibA_42690 | 89 | -0.262959628 | SAMD5 | sterile alpha motif domain containing 5 [Source:HGNC Symbol;Acc:HGNC:21180] |
| ENSG00000203877 | HGLibA_41331 | 89 | -0.262959628 | RIPPLY2 | ripply transcriptional repressor 2 [Source:HGNC Symbol;Acc:HGNC:21390] |
| ENSG00000204427 | HGLibA_00256 | 89 | -0.262959628 | ABHD16A | abhydrolase domain containing 16A [Source:HGNC Symbol;Acc:HGNC:13921] |
| ENSG00000204580 | HGLibA_12633 | 89 | -0.262959628 | DDR1 | discoidin domain receptor tyrosine kinase 1 [Source:HGNC Symbol;Acc:HGNC:2730] |
| ENSG00000204701 | HGLibA_33812 | 89 | -0.262959628 | OR2J3 | olfactory receptor family 2 subfamily J member 3 [Source:HGNC Symbol;Acc:HGNC:8261] |
| ENSG00000213022 | HGLibA_25272 | 89 | -0.262959628 | KLK9 | kallikrein related peptidase 9 [Source:HGNC Symbol;Acc:HGNC:6370] |
| ENSG00000213139 | HGLibA_11286 | 89 | -0.262959628 | CRYGS | crystallin gamma S [Source:HGNC Symbol;Acc:HGNC:2417] |
| ENSG00000213588 | HGLibA_54934 | 89 | -0.262959628 | ZBTB9 | zinc finger and BTB domain containing 9 [Source:HGNC Symbol;Acc:HGNC:28323] |
| ENSG00000221823 | HGLibA_38194 | 89 | -0.262959628 | PPP3R1 | alpha [Source:HGNC Symbol;Acc:HGNC:9317]" |
| ENSG00000221916 | HGLibA_05704 | 89 | -0.262959628 | C19orf73 | chromosome 19 open reading frame 73 [Source:HGNC Symbol;Acc:HGNC:25534] |
| ENSG00000226685 | HGLibA_11524 | 89 | -0.262959628 | CT47A12 | member A12 [Source:HGNC Symbol;Acc:HGNC:33292]" |
| ENSG00000234560 | HGLibA_33499 | 89 | -0.262959628 | OR10G8 | olfactory receptor family 10 subfamily G member 8 [Source:HGNC Symbol;Acc:HGNC:14845] |
| ENSG00000258484 | HGLibA_46628 | 89 | -0.262959628 | SPESP1 | sperm equatorial segment protein 1 [Source:HGNC Symbol;Acc:HGNC:15570] |
| ENSG00000271503 | HGLibA_08035 | 89 | -0.262959628 | CCL5 | C-C motif chemokine ligand 5 [Source:HGNC Symbol;Acc:HGNC:10632] |
| ENSG00000277481 | HGLibA_36795 | 89 | -0.262959628 | PKD1L3 | transient receptor potential channel interacting [Source:HGNC Symbol;Acc:HGNC:21716]" |
| ENSG00000286237 | HGLibA_03052 | 89 | -0.262959628 | ARMCX5-GPRASP2 | - |
| GATS | HGLibA_18697 | 89 | -0.262959628 | GATS | - |
| HNRPDL | HGLibA_21855 | 89 | -0.262959628 | HNRPDL | - |
| hsa-mir-136 | HGLibA_57806 | 89 | -0.262959628 | hsa-mir-136 | - |
| hsa-mir-1973 | HGLibA_58116 | 89 | -0.262959628 | hsa-mir-1973 | - |
| hsa-mir-2115 | HGLibA_58233 | 89 | -0.262959628 | hsa-mir-2115 | - |
| hsa-mir-216b | HGLibA_58259 | 89 | -0.262959628 | hsa-mir-216b | - |
| hsa-mir-3689d-1 | HGLibA_59327 | 89 | -0.262959628 | hsa-mir-3689d-1 | - |
| hsa-mir-3690-2 | HGLibA_59350 | 89 | -0.262959628 | hsa-mir-3690-2 | - |
| hsa-mir-3910-1 | HGLibA_59505 | 89 | -0.262959628 | hsa-mir-3910-1 | - |
| hsa-mir-4452 | HGLibA_60213 | 89 | -0.262959628 | hsa-mir-4452 | - |
| hsa-mir-4506 | HGLibA_60451 | 89 | -0.262959628 | hsa-mir-4506 | - |
| hsa-mir-4709 | HGLibA_60937 | 89 | -0.262959628 | hsa-mir-4709 | - |
| hsa-mir-4720 | HGLibA_60978 | 89 | -0.262959628 | hsa-mir-4720 | - |
| hsa-mir-524 | HGLibA_61782 | 89 | -0.262959628 | hsa-mir-524 | - |
| hsa-mir-6501 | HGLibA_62854 | 89 | -0.262959628 | hsa-mir-6501 | - |
| hsa-mir-6737 | HGLibA_63103 | 89 | -0.262959628 | hsa-mir-6737 | - |
| hsa-mir-6795 | HGLibA_63356 | 89 | -0.262959628 | hsa-mir-6795 | - |
| hsa-mir-6865 | HGLibA_63645 | 89 | -0.262959628 | hsa-mir-6865 | - |
| hsa-mir-942 | HGLibA_64355 | 89 | -0.262959628 | hsa-mir-942 | - |
| LOC81691 | HGLibA_27009 | 89 | -0.262959628 | LOC81691 | - |
| PPAPDC2 | HGLibA_37830 | 89 | -0.262959628 | PPAPDC2 | - |
| PTPLAD2 | HGLibA_39422 | 89 | -0.262959628 | PTPLAD2 | - |
| RGAG4 | HGLibA_40994 | 89 | -0.262959628 | RGAG4 | - |
| WDR60 | HGLibA_54138 | 89 | -0.262959628 | WDR60 | - |
| C1orf95 | HGLibA_05908 | 88 | -0.272910665 | C1orf95 | - |
| C20orf78 | HGLibA_06025 | 88 | -0.272910665 | C20orf78 | - |
| CT45A4 | HGLibA_11508 | 88 | -0.272910665 | CT45A4 | - |
| EFCAB4A | HGLibA_14540 | 88 | -0.272910665 | EFCAB4A | - |
| ENSG00000008516 | HGLibA_29521 | 88 | -0.272910665 | MMP25 | matrix metallopeptidase 25 [Source:HGNC Symbol;Acc:HGNC:14246] |
| ENSG00000017797 | HGLibA_40152 | 88 | -0.272910665 | RALBP1 | ralA binding protein 1 [Source:HGNC Symbol;Acc:HGNC:9841] |
| ENSG00000023318 | HGLibA_15531 | 88 | -0.272910665 | ERP44 | endoplasmic reticulum protein 44 [Source:HGNC Symbol;Acc:HGNC:18311] |
| ENSG00000034677 | HGLibA_41600 | 88 | -0.272910665 | RNF19A | RBR E3 ubiquitin protein ligase [Source:HGNC Symbol;Acc:HGNC:13432]" |
| ENSG00000048707 | HGLibA_53739 | 88 | -0.272910665 | VPS13D | vacuolar protein sorting 13 homolog D [Source:HGNC Symbol;Acc:HGNC:23595] |
| ENSG00000054277 | HGLibA_33435 | 88 | -0.272910665 | OPN3 | opsin 3 [Source:HGNC Symbol;Acc:HGNC:14007] |
| ENSG00000058404 | HGLibA_07069 | 88 | -0.272910665 | CAMK2B | calcium/calmodulin dependent protein kinase II beta [Source:HGNC Symbol;Acc:HGNC:1461] |
| ENSG00000065243 | HGLibA_36839 | 88 | -0.272910665 | PKN2 | protein kinase N2 [Source:HGNC Symbol;Acc:HGNC:9406] |
| ENSG00000066739 | HGLibA_03483 | 88 | -0.272910665 | ATG2B | autophagy related 2B [Source:HGNC Symbol;Acc:HGNC:20187] |
| ENSG00000068438 | HGLibA_18133 | 88 | -0.272910665 | FTSJ1 | FtsJ RNA methyltransferase homolog 1 [Source:HGNC Symbol;Acc:HGNC:13254] |
| ENSG00000069020 | HGLibA_28408 | 88 | -0.272910665 | MAST4 | microtubule associated serine/threonine kinase family member 4 [Source:HGNC Symbol;Acc:HGNC:19037] |
| ENSG00000069956 | HGLibA_28277 | 88 | -0.272910665 | MAPK6 | mitogen-activated protein kinase 6 [Source:HGNC Symbol;Acc:HGNC:6879] |
| ENSG00000073578 | HGLibA_43133 | 88 | -0.272910665 | SDHA | succinate dehydrogenase complex flavoprotein subunit A [Source:HGNC Symbol;Acc:HGNC:10680] |
| ENSG00000073712 | HGLibA_17296 | 88 | -0.272910665 | FERMT2 | fermitin family member 2 [Source:HGNC Symbol;Acc:HGNC:15767] |
| ENSG00000076248 | HGLibA_53044 | 88 | -0.272910665 | UNG | uracil DNA glycosylase [Source:HGNC Symbol;Acc:HGNC:12572] |
| ENSG00000078114 | HGLibA_31582 | 88 | -0.272910665 | NEBL | nebulette [Source:HGNC Symbol;Acc:HGNC:16932] |
| ENSG00000078124 | HGLibA_00433 | 88 | -0.272910665 | ACER3 | alkaline ceramidase 3 [Source:HGNC Symbol;Acc:HGNC:16066] |
| ENSG00000078674 | HGLibA_35623 | 88 | -0.272910665 | PCM1 | pericentriolar material 1 [Source:HGNC Symbol;Acc:HGNC:8727] |
| ENSG00000082805 | HGLibA_15426 | 88 | -0.272910665 | ERC1 | ELKS/RAB6-interacting/CAST family member 1 [Source:HGNC Symbol;Acc:HGNC:17072] |
| ENSG00000083750 | HGLibA_42227 | 88 | -0.272910665 | RRAGB | Ras related GTP binding B [Source:HGNC Symbol;Acc:HGNC:19901] |
| ENSG00000085514 | HGLibA_36668 | 88 | -0.272910665 | PILRA | paired immunoglobin like type 2 receptor alpha [Source:HGNC Symbol;Acc:HGNC:20396] |
| ENSG00000087263 | HGLibA_33307 | 88 | -0.272910665 | OGFOD1 | 2-oxoglutarate and iron dependent oxygenase domain containing 1 [Source:HGNC Symbol;Acc:HGNC:25585] |
| ENSG00000089159 | HGLibA_39665 | 88 | -0.272910665 | PXN | paxillin [Source:HGNC Symbol;Acc:HGNC:9718] |
| ENSG00000092096 | HGLibA_44558 | 88 | -0.272910665 | SLC22A17 | solute carrier family 22 member 17 [Source:HGNC Symbol;Acc:HGNC:23095] |
| ENSG00000095585 | HGLibA_04553 | 88 | -0.272910665 | BLNK | B cell linker [Source:HGNC Symbol;Acc:HGNC:14211] |
| ENSG00000100342 | HGLibA_02527 | 88 | -0.272910665 | APOL1 | apolipoprotein L1 [Source:HGNC Symbol;Acc:HGNC:618] |
| ENSG00000100362 | HGLibA_39612 | 88 | -0.272910665 | PVALB | parvalbumin [Source:HGNC Symbol;Acc:HGNC:9704] |
| ENSG00000100593 | HGLibA_23680 | 88 | -0.272910665 | ISM2 | isthmin 2 [Source:HGNC Symbol;Acc:HGNC:23176] |
| ENSG00000101448 | HGLibA_15355 | 88 | -0.272910665 | EPPIN | epididymal peptidase inhibitor [Source:HGNC Symbol;Acc:HGNC:15932] |
| ENSG00000101608 | HGLibA_30750 | 88 | -0.272910665 | MYL12A | myosin light chain 12A [Source:HGNC Symbol;Acc:HGNC:16701] |
| ENSG00000102312 | HGLibA_37707 | 88 | -0.272910665 | PORCN | porcupine O-acyltransferase [Source:HGNC Symbol;Acc:HGNC:17652] |
| ENSG00000102921 | HGLibA_30948 | 88 | -0.272910665 | N4BP1 | NEDD4 binding protein 1 [Source:HGNC Symbol;Acc:HGNC:29850] |
| ENSG00000102935 | HGLibA_55960 | 88 | -0.272910665 | ZNF423 | zinc finger protein 423 [Source:HGNC Symbol;Acc:HGNC:16762] |
| ENSG00000103248 | HGLibA_30395 | 88 | -0.272910665 | MTHFSD | methenyltetrahydrofolate synthetase domain containing [Source:HGNC Symbol;Acc:HGNC:25778] |
| ENSG00000104866 | HGLibA_38094 | 88 | -0.272910665 | PPP1R37 | protein phosphatase 1 regulatory subunit 37 [Source:HGNC Symbol;Acc:HGNC:27607] |
| ENSG00000107593 | HGLibA_36802 | 88 | -0.272910665 | PKD2L1 | transient receptor potential cation channel [Source:HGNC Symbol;Acc:HGNC:9011]" |
| ENSG00000108175 | HGLibA_55380 | 88 | -0.272910665 | ZMIZ1 | zinc finger MIZ-type containing 1 [Source:HGNC Symbol;Acc:HGNC:16493] |
| ENSG00000109171 | HGLibA_44310 | 88 | -0.272910665 | SLAIN2 | SLAIN motif family member 2 [Source:HGNC Symbol;Acc:HGNC:29282] |
| ENSG00000109272 | HGLibA_36132 | 88 | -0.272910665 | PF4V1 | platelet factor 4 variant 1 [Source:HGNC Symbol;Acc:HGNC:8862] |
| ENSG00000112062 | HGLibA_28263 | 88 | -0.272910665 | MAPK14 | mitogen-activated protein kinase 14 [Source:HGNC Symbol;Acc:HGNC:6876] |
| ENSG00000112367 | HGLibA_17511 | 88 | -0.272910665 | FIG4 | FIG4 phosphoinositide 5-phosphatase [Source:HGNC Symbol;Acc:HGNC:16873] |
| ENSG00000113282 | HGLibA_10038 | 88 | -0.272910665 | CLINT1 | clathrin interactor 1 [Source:HGNC Symbol;Acc:HGNC:23186] |
| ENSG00000113391 | HGLibA_16274 | 88 | -0.272910665 | FAM172A | family with sequence similarity 172 member A [Source:HGNC Symbol;Acc:HGNC:25365] |
| ENSG00000114956 | HGLibA_13087 | 88 | -0.272910665 | DGUOK | deoxyguanosine kinase [Source:HGNC Symbol;Acc:HGNC:2858] |
| ENSG00000115317 | HGLibA_22410 | 88 | -0.272910665 | HTRA2 | HtrA serine peptidase 2 [Source:HGNC Symbol;Acc:HGNC:14348] |
| ENSG00000115840 | HGLibA_44643 | 88 | -0.272910665 | SLC25A12 | solute carrier family 25 member 12 [Source:HGNC Symbol;Acc:HGNC:10982] |
| ENSG00000115944 | HGLibA_10864 | 88 | -0.272910665 | COX7A2L | cytochrome c oxidase subunit 7A2 like [Source:HGNC Symbol;Acc:HGNC:2289] |
| ENSG00000116117 | HGLibA_35181 | 88 | -0.272910665 | PARD3B | par-3 family cell polarity regulator beta [Source:HGNC Symbol;Acc:HGNC:14446] |
| ENSG00000116701 | HGLibA_31298 | 88 | -0.272910665 | NCF2 | neutrophil cytosolic factor 2 [Source:HGNC Symbol;Acc:HGNC:7661] |
| ENSG00000117411 | HGLibA_04025 | 88 | -0.272910665 | B4GALT2 | 4-galactosyltransferase 2 [Source:HGNC Symbol;Acc:HGNC:925]" |
| ENSG00000117834 | HGLibA_45286 | 88 | -0.272910665 | SLC5A9 | solute carrier family 5 member 9 [Source:HGNC Symbol;Acc:HGNC:22146] |
| ENSG00000119541 | HGLibA_53801 | 88 | -0.272910665 | VPS4B | vacuolar protein sorting 4 homolog B [Source:HGNC Symbol;Acc:HGNC:10895] |
| ENSG00000120329 | HGLibA_44666 | 88 | -0.272910665 | SLC25A2 | solute carrier family 25 member 2 [Source:HGNC Symbol;Acc:HGNC:22921] |
| ENSG00000120458 | HGLibA_30171 | 88 | -0.272910665 | MSANTD2 | Myb/SANT DNA binding domain containing 2 [Source:HGNC Symbol;Acc:HGNC:26266] |
| ENSG00000120498 | HGLibA_49053 | 88 | -0.272910665 | TEX11 | testis expressed 11 [Source:HGNC Symbol;Acc:HGNC:11733] |
| ENSG00000120662 | HGLibA_30467 | 88 | -0.272910665 | MTRF1 | mitochondrial translation release factor 1 [Source:HGNC Symbol;Acc:HGNC:7469] |
| ENSG00000120798 | HGLibA_32595 | 88 | -0.272910665 | NR2C1 | nuclear receptor subfamily 2 group C member 1 [Source:HGNC Symbol;Acc:HGNC:7971] |
| ENSG00000121068 | HGLibA_48657 | 88 | -0.272910665 | TBX2 | T-box 2 [Source:HGNC Symbol;Acc:HGNC:11597] |
| ENSG00000121289 | HGLibA_09175 | 88 | -0.272910665 | CEP89 | centrosomal protein 89 [Source:HGNC Symbol;Acc:HGNC:25907] |
| ENSG00000121900 | HGLibA_50440 | 88 | -0.272910665 | TMEM54 | transmembrane protein 54 [Source:HGNC Symbol;Acc:HGNC:24143] |
| ENSG00000122965 | HGLibA_40503 | 88 | -0.272910665 | RBM19 | RNA binding motif protein 19 [Source:HGNC Symbol;Acc:HGNC:29098] |
| ENSG00000124214 | HGLibA_47405 | 88 | -0.272910665 | STAU1 | staufen double-stranded RNA binding protein 1 [Source:HGNC Symbol;Acc:HGNC:11370] |
| ENSG00000124496 | HGLibA_51370 | 88 | -0.272910665 | TRERF1 | transcriptional regulating factor 1 [Source:HGNC Symbol;Acc:HGNC:18273] |
| ENSG00000124788 | HGLibA_03827 | 88 | -0.272910665 | ATXN1 | ataxin 1 [Source:HGNC Symbol;Acc:HGNC:10548] |
| ENSG00000125571 | HGLibA_23158 | 88 | -0.272910665 | IL37 | interleukin 37 [Source:HGNC Symbol;Acc:HGNC:15563] |
| ENSG00000126878 | HGLibA_01335 | 88 | -0.272910665 | AIF1L | allograft inflammatory factor 1 like [Source:HGNC Symbol;Acc:HGNC:28904] |
| ENSG00000128641 | HGLibA_30824 | 88 | -0.272910665 | MYO1B | myosin IB [Source:HGNC Symbol;Acc:HGNC:7596] |
| ENSG00000129048 | HGLibA_00450 | 88 | -0.272910665 | ACKR4 | atypical chemokine receptor 4 [Source:HGNC Symbol;Acc:HGNC:1611] |
| ENSG00000130300 | HGLibA_37250 | 88 | -0.272910665 | PLVAP | plasmalemma vesicle associated protein [Source:HGNC Symbol;Acc:HGNC:13635] |
| ENSG00000134365 | HGLibA_09272 | 88 | -0.272910665 | CFHR4 | complement factor H related 4 [Source:HGNC Symbol;Acc:HGNC:16979] |
| ENSG00000134668 | HGLibA_46745 | 88 | -0.272910665 | SPOCD1 | SPOC domain containing 1 [Source:HGNC Symbol;Acc:HGNC:26338] |
| ENSG00000135638 | HGLibA_15092 | 88 | -0.272910665 | EMX1 | empty spiracles homeobox 1 [Source:HGNC Symbol;Acc:HGNC:3340] |
| ENSG00000137770 | HGLibA_11618 | 88 | -0.272910665 | CTDSPL2 | CTD small phosphatase like 2 [Source:HGNC Symbol;Acc:HGNC:26936] |
| ENSG00000139133 | HGLibA_01603 | 88 | -0.272910665 | ALG10 | 2-glucosyltransferase [Source:HGNC Symbol;Acc:HGNC:23162]" |
| ENSG00000139223 | HGLibA_02205 | 88 | -0.272910665 | ANP32D | acidic nuclear phosphoprotein 32 family member D [Source:HGNC Symbol;Acc:HGNC:16676] |
| ENSG00000141371 | HGLibA_05559 | 88 | -0.272910665 | C17orf64 | chromosome 17 open reading frame 64 [Source:HGNC Symbol;Acc:HGNC:26990] |
| ENSG00000143756 | HGLibA_17061 | 88 | -0.272910665 | FBXO28 | F-box protein 28 [Source:HGNC Symbol;Acc:HGNC:29046] |
| ENSG00000143924 | HGLibA_15065 | 88 | -0.272910665 | EML4 | echinoderm microtubule associated protein like 4 [Source:HGNC Symbol;Acc:HGNC:1316] |
| ENSG00000143994 | HGLibA_00230 | 88 | -0.272910665 | ABHD1 | abhydrolase domain containing 1 [Source:HGNC Symbol;Acc:HGNC:17553] |
| ENSG00000144736 | HGLibA_44084 | 88 | -0.272910665 | SHQ1 | H/ACA ribonucleoprotein assembly factor [Source:HGNC Symbol;Acc:HGNC:25543]" |
| ENSG00000145476 | HGLibA_12198 | 88 | -0.272910665 | CYP4V2 | cytochrome P450 family 4 subfamily V member 2 [Source:HGNC Symbol;Acc:HGNC:23198] |

| | | | | | |
|---|---|---|---|---|---|
| ENSG00000145708 | HGLibA_11138 | 88 | -0.272910665 | CRHBP | corticotropin releasing hormone binding protein [Source:HGNC Symbol;Acc:HGNC:2356] |
| ENSG00000147400 | HGLibA_09236 | 88 | -0.272910665 | CETN2 | centrin 2 [Source:HGNC Symbol;Acc:HGNC:1867] |
| ENSG00000147789 | HGLibA_56570 | 88 | -0.272910665 | ZNF7 | zinc finger protein 7 [Source:HGNC Symbol;Acc:HGNC:13139] |
| ENSG00000152582 | HGLibA_46611 | 88 | -0.272910665 | SPEF2 | sperm flagellar 2 [Source:HGNC Symbol;Acc:HGNC:26293] |
| ENSG00000152583 | HGLibA_46421 | 88 | -0.272910665 | SPARCL1 | SPARC like 1 [Source:HGNC Symbol;Acc:HGNC:11220] |
| ENSG00000154479 | HGLibA_07700 | 88 | -0.272910665 | CCDC173 | coiled-coil domain containing 173 [Source:HGNC Symbol;Acc:HGNC:25064] |
| ENSG00000154654 | HGLibA_31256 | 88 | -0.272910665 | NCAM2 | neural cell adhesion molecule 2 [Source:HGNC Symbol;Acc:HGNC:7657] |
| ENSG00000156171 | HGLibA_13948 | 88 | -0.272910665 | DRAM2 | DNA damage regulated autophagy modulator 2 [Source:HGNC Symbol;Acc:HGNC:28769] |
| ENSG00000156958 | HGLibA_18517 | 88 | -0.272910665 | GALK2 | galactokinase 2 [Source:HGNC Symbol;Acc:HGNC:4119] |
| ENSG00000159217 | HGLibA_22799 | 88 | -0.272910665 | IGF2BP1 | insulin like growth factor 2 mRNA binding protein 1 [Source:HGNC Symbol;Acc:HGNC:28866] |
| ENSG00000159374 | HGLibA_27811 | 88 | -0.272910665 | M1AP | meiosis 1 associated protein [Source:HGNC Symbol;Acc:HGNC:25183] |
| ENSG00000159723 | HGLibA_01254 | 88 | -0.272910665 | AGRP | agouti related neuropeptide [Source:HGNC Symbol;Acc:HGNC:330] |
| ENSG00000160062 | HGLibA_54924 | 88 | -0.272910665 | ZBTB8A | zinc finger and BTB domain containing 8A [Source:HGNC Symbol;Acc:HGNC:24172] |
| ENSG00000160838 | HGLibA_27419 | 88 | -0.272910665 | LRRC71 | leucine rich repeat containing 71 [Source:HGNC Symbol;Acc:HGNC:26556] |
| ENSG00000160932 | HGLibA_27656 | 88 | -0.272910665 | LY6E | lymphocyte antigen 6 family member E [Source:HGNC Symbol;Acc:HGNC:6727] |
| ENSG00000161714 | HGLibA_36993 | 88 | -0.272910665 | PLCD3 | phospholipase C delta 3 [Source:HGNC Symbol;Acc:HGNC:9061] |
| ENSG00000162813 | HGLibA_04738 | 88 | -0.272910665 | BPNT1 | 5'-bisphosphate nucleotidase 1 [Source:HGNC Symbol;Acc:HGNC:1096]" |
| ENSG00000163624 | HGLibA_08880 | 88 | -0.272910665 | CDS1 | CDP-diacylglycerol synthase 1 [Source:HGNC Symbol;Acc:HGNC:1800] |
| ENSG00000163825 | HGLibA_42428 | 88 | -0.272910665 | RTP3 | receptor transporter protein 3 [Source:HGNC Symbol;Acc:HGNC:15572] |
| ENSG00000163956 | HGLibA_27237 | 88 | -0.272910665 | LRPAP1 | LDL receptor related protein associated protein 1 [Source:HGNC Symbol;Acc:HGNC:6701] |
| ENSG00000164256 | HGLibA_38405 | 88 | -0.272910665 | PRDM9 | PR/SET domain 9 [Source:HGNC Symbol;Acc:HGNC:13994] |
| ENSG00000164405 | HGLibA_53129 | 88 | -0.272910665 | UQCRQ | ubiquinol-cytochrome c reductase complex III subunit VII [Source:HGNC Symbol;Acc:HGNC:29594] |
| ENSG00000164687 | HGLibA_15923 | 88 | -0.272910665 | FABP5 | fatty acid binding protein 5 [Source:HGNC Symbol;Acc:HGNC:3560] |
| ENSG00000166266 | HGLibA_11787 | 88 | -0.272910665 | CUL5 | cullin 5 [Source:HGNC Symbol;Acc:HGNC:2556] |
| ENSG00000166710 | HGLibA_03946 | 88 | -0.272910665 | B2M | beta-2-microglobulin [Source:HGNC Symbol;Acc:HGNC:914] |
| ENSG00000166793 | HGLibA_54716 | 88 | -0.272910665 | YPEL4 | yippee like 4 [Source:HGNC Symbol;Acc:HGNC:18328] |
| ENSG00000166979 | HGLibA_15677 | 88 | -0.272910665 | EVA1C | eva-1 homolog C [Source:HGNC Symbol;Acc:HGNC:13239] |
| ENSG00000167384 | HGLibA_55536 | 88 | -0.272910665 | ZNF180 | zinc finger protein 180 [Source:HGNC Symbol;Acc:HGNC:12970] |
| ENSG00000167536 | HGLibA_13128 | 88 | -0.272910665 | DHRS13 | dehydrogenase/reductase 13 [Source:HGNC Symbol;Acc:HGNC:28326] |
| ENSG00000167733 | HGLibA_22162 | 88 | -0.272910665 | HSD11B1L | hydroxysteroid 11-beta dehydrogenase 1 like [Source:HGNC Symbol;Acc:HGNC:30419] |
| ENSG00000167754 | HGLibA_25262 | 88 | -0.272910665 | KLK5 | kallikrein related peptidase 5 [Source:HGNC Symbol;Acc:HGNC:6366] |
| ENSG00000168569 | HGLibA_50242 | 88 | -0.272910665 | TMEM223 | transmembrane protein 223 [Source:HGNC Symbol;Acc:HGNC:28464] |
| ENSG00000168610 | HGLibA_47386 | 88 | -0.272910665 | STAT3 | signal transducer and activator of transcription 3 [Source:HGNC Symbol;Acc:HGNC:11364] |
| ENSG00000169314 | HGLibA_06052 | 88 | -0.272910665 | C22orf15 | chromosome 22 open reading frame 15 [Source:HGNC Symbol;Acc:HGNC:15558] |
| ENSG00000169733 | HGLibA_40926 | 88 | -0.272910665 | RFNG | RFNG O-fucosylpeptide 3-beta-N-acetylglucosaminyltransferase [Source:HGNC Symbol;Acc:HGNC:9974] |
| ENSG00000171124 | HGLibA_18192 | 88 | -0.272910665 | FUT3 | fucosyltransferase 3 (Lewis blood group) [Source:HGNC Symbol;Acc:HGNC:4014] |
| ENSG00000171478 | HGLibA_46337 | 88 | -0.272910665 | SPACA5B | sperm acrosome associated 5B [Source:HGNC Symbol;Acc:HGNC:19142] |
| ENSG00000172073 | HGLibA_49105 | 88 | -0.272910665 | TEX37 | testis expressed 37 [Source:HGNC Symbol;Acc:HGNC:26341] |
| ENSG00000173040 | HGLibA_15684 | 88 | -0.272910665 | EVC2 | EvC ciliary complex subunit 2 [Source:HGNC Symbol;Acc:HGNC:19747] |
| ENSG00000173200 | HGLibA_35227 | 88 | -0.272910665 | PARP15 | poly(ADP-ribose) polymerase family member 15 [Source:HGNC Symbol;Acc:HGNC:26876] |
| ENSG00000173237 | HGLibA_05203 | 88 | -0.272910665 | C11orf86 | chromosome 11 open reading frame 86 [Source:HGNC Symbol;Acc:HGNC:34442] |
| ENSG00000173894 | HGLibA_07449 | 88 | -0.272910665 | CBX2 | chromobox 2 [Source:HGNC Symbol;Acc:HGNC:1552] |
| ENSG00000173918 | HGLibA_05935 | 88 | -0.272910665 | C1QTNF1 | C1q and TNF related 1 [Source:HGNC Symbol;Acc:HGNC:14324] |
| ENSG00000174238 | HGLibA_36740 | 88 | -0.272910665 | PITPNA | phosphatidylinositol transfer protein alpha [Source:HGNC Symbol;Acc:HGNC:9001] |
| ENSG00000174776 | HGLibA_54113 | 88 | -0.272910665 | WDR49 | WD repeat domain 49 [Source:HGNC Symbol;Acc:HGNC:26587] |
| ENSG00000174886 | HGLibA_31443 | 88 | -0.272910665 | NDUFA11 | NADH:ubiquinone oxidoreductase subunit A11 [Source:HGNC Symbol;Acc:HGNC:20371] |
| ENSG00000177932 | HGLibA_55868 | 88 | -0.272910665 | ZNF354C | zinc finger protein 354C [Source:HGNC Symbol;Acc:HGNC:16736] |
| ENSG00000178209 | HGLibA_37054 | 88 | -0.272910665 | PLEC | plectin [Source:HGNC Symbol;Acc:HGNC:9069] |
| ENSG00000180219 | HGLibA_16691 | 88 | -0.272910665 | FAM71C | family with sequence similarity 71 member C [Source:HGNC Symbol;Acc:HGNC:28594] |
| ENSG00000180475 | HGLibA_33539 | 88 | -0.272910665 | OR10Q1 | olfactory receptor family 10 subfamily Q member 1 [Source:HGNC Symbol;Acc:HGNC:15134] |
| ENSG00000180530 | HGLibA_32691 | 88 | -0.272910665 | NRIP1 | nuclear receptor interacting protein 1 [Source:HGNC Symbol;Acc:HGNC:8001] |
| ENSG00000180902 | HGLibA_12256 | 88 | -0.272910665 | D2HGDH | D-2-hydroxyglutarate dehydrogenase [Source:HGNC Symbol;Acc:HGNC:28358] |
| ENSG00000181027 | HGLibA_17596 | 88 | -0.272910665 | FKRP | fukutin related protein [Source:HGNC Symbol;Acc:HGNC:17997] |
| ENSG00000181061 | HGLibA_21307 | 88 | -0.272910665 | HIGD1A | HIG1 hypoxia inducible domain family member 1A [Source:HGNC Symbol;Acc:HGNC:29527] |
| ENSG00000182053 | HGLibA_51526 | 88 | -0.272910665 | TRIM49B | tripartite motif containing 49B [Source:HGNC Symbol;Acc:HGNC:42955] |
| ENSG00000182111 | HGLibA_56624 | 88 | -0.272910665 | ZNF716 | zinc finger protein 716 [Source:HGNC Symbol;Acc:HGNC:32458] |
| ENSG00000182170 | HGLibA_29842 | 88 | -0.272910665 | MRGPRG | MAS related GPR family member G [Source:HGNC Symbol;Acc:HGNC:24829] |
| ENSG00000182224 | HGLibA_11995 | 88 | -0.272910665 | CYB5D1 | cytochrome b5 domain containing 1 [Source:HGNC Symbol;Acc:HGNC:26516] |
| ENSG00000183186 | HGLibA_06101 | 88 | -0.272910665 | C2CD4C | C2 calcium dependent domain containing 4C [Source:HGNC Symbol;Acc:HGNC:29417] |
| ENSG00000183336 | HGLibA_04683 | 88 | -0.272910665 | BOLA2 | bolA family member 2 [Source:HGNC Symbol;Acc:HGNC:29488] |
| ENSG00000183647 | HGLibA_56176 | 88 | -0.272910665 | ZNF530 | zinc finger protein 530 [Source:HGNC Symbol;Acc:HGNC:29297] |
| ENSG00000185056 | HGLibA_06391 | 88 | -0.272910665 | C5orf47 | chromosome 5 open reading frame 47 [Source:HGNC Symbol;Acc:HGNC:27026] |
| ENSG00000185420 | HGLibA_45858 | 88 | -0.272910665 | SMYD3 | SET and MYND domain containing 3 [Source:HGNC Symbol;Acc:HGNC:15513] |
| ENSG00000186026 | HGLibA_55745 | 88 | -0.272910665 | ZNF284 | zinc finger protein 284 [Source:HGNC Symbol;Acc:HGNC:13078] |
| ENSG00000186732 | HGLibA_29770 | 88 | -0.272910665 | MPPED1 | metallophosphoesterase domain containing 1 [Source:HGNC Symbol;Acc:HGNC:1306] |
| ENSG00000187051 | HGLibA_42099 | 88 | -0.272910665 | RPS19BP1 | ribosomal protein S19 binding protein 1 [Source:HGNC Symbol;Acc:HGNC:28749] |
| ENSG00000187238 | HGLibA_26132 | 88 | -0.272910665 | LCE3B | late cornified envelope 3B [Source:HGNC Symbol;Acc:HGNC:29462] |
| ENSG00000188373 | HGLibA_05111 | 88 | -0.272910665 | C10orf99 | chromosome 10 open reading frame 99 [Source:HGNC Symbol;Acc:HGNC:31428] |
| ENSG00000188766 | HGLibA_46795 | 88 | -0.272910665 | SPRED3 | sprouty related EVH1 domain containing 3 [Source:HGNC Symbol;Acc:HGNC:31041] |
| ENSG00000196151 | HGLibA_54228 | 88 | -0.272910665 | WDSUB1 | sterile alpha motif and U-box domain containing 1 [Source:HGNC Symbol;Acc:HGNC:26697]" |
| ENSG00000196369 | HGLibA_46972 | 88 | -0.272910665 | SRGAP2B | SLIT-ROBO Rho GTPase activating protein 2B [Source:HGNC Symbol;Acc:HGNC:35237] |
| ENSG00000196376 | HGLibA_45001 | 88 | -0.272910665 | SLC35F1 | solute carrier family 35 member F1 [Source:HGNC Symbol;Acc:HGNC:21483] |
| ENSG00000196550 | HGLibA_16707 | 88 | -0.272910665 | FAM72A | family with sequence similarity 72 member A [Source:HGNC Symbol;Acc:HGNC:24044] |
| ENSG00000196660 | HGLibA_44901 | 88 | -0.272910665 | SLC30A10 | solute carrier family 30 member 10 [Source:HGNC Symbol;Acc:HGNC:25355] |
| ENSG00000197362 | HGLibA_56752 | 88 | -0.272910665 | ZNF786 | zinc finger protein 786 [Source:HGNC Symbol;Acc:HGNC:21806] |
| ENSG00000197385 | HGLibA_56879 | 88 | -0.272910665 | ZNF860 | zinc finger protein 860 [Source:HGNC Symbol;Acc:HGNC:34513] |
| ENSG00000197565 | HGLibA_10583 | 88 | -0.272910665 | COL4A6 | collagen type IV alpha 6 chain [Source:HGNC Symbol;Acc:HGNC:2208] |
| ENSG00000197956 | HGLibA_42564 | 88 | -0.272910665 | S100A6 | S100 calcium binding protein A6 [Source:HGNC Symbol;Acc:HGNC:10496] |
| ENSG00000198189 | HGLibA_22176 | 88 | -0.272910665 | HSD17B11 | hydroxysteroid 17-beta dehydrogenase 11 [Source:HGNC Symbol;Acc:HGNC:22960] |
| ENSG00000198246 | HGLibA_44847 | 88 | -0.272910665 | SLC29A3 | solute carrier family 29 member 3 [Source:HGNC Symbol;Acc:HGNC:23096] |
| ENSG00000198846 | HGLibA_51037 | 88 | -0.272910665 | TOX | thymocyte selection associated high mobility group box [Source:HGNC Symbol;Acc:HGNC:18988] |
| ENSG00000198908 | HGLibA_04473 | 88 | -0.272910665 | BHLHB9 | basic helix-loop-helix family member b9 [Source:HGNC Symbol;Acc:HGNC:29353] |
| ENSG00000204231 | HGLibA_42509 | 88 | -0.272910665 | RXRB | retinoid X receptor beta [Source:HGNC Symbol;Acc:HGNC:10478] |
| ENSG00000204449 | HGLibA_51529 | 88 | -0.272910665 | TRIM49C | tripartite motif containing 49C [Source:HGNC Symbol;Acc:HGNC:38877] |
| ENSG00000204450 | HGLibA_51576 | 88 | -0.272910665 | TRIM64 | tripartite motif containing 64 [Source:HGNC Symbol;Acc:HGNC:14663] |
| ENSG00000204571 | HGLibA_25787 | 88 | -0.272910665 | KRTAP5-11 | keratin associated protein 5-11 [Source:HGNC Symbol;Acc:HGNC:23606] |
| ENSG00000217236 | HGLibA_46318 | 88 | -0.272910665 | SP9 | Sp9 transcription factor [Source:HGNC Symbol;Acc:HGNC:30690] |
| ENSG00000220201 | HGLibA_55302 | 88 | -0.272910665 | ZGLP1 | GATA-like protein 1 [Source:HGNC Symbol;Acc:HGNC:37245]" |
| ENSG00000226929 | HGLibA_11521 | 88 | -0.272910665 | CT47A11 | member A11 [Source:HGNC Symbol;Acc:HGNC:27397]" |
| ENSG00000236032 | HGLibA_34293 | 88 | -0.272910665 | OR5H14 | olfactory receptor family 5 subfamily H member 14 [Source:HGNC Symbol;Acc:HGNC:31286] |
| ENSG00000242612 | HGLibA_12778 | 88 | -0.272910665 | DECR2 | 4-dienoyl-CoA reductase 2 [Source:HGNC Symbol;Acc:HGNC:2754]" |
| ENSG00000244474 | HGLibA_52885 | 88 | -0.272910665 | UGT1A4 | UDP glucuronosyltransferase family 1 member A4 [Source:HGNC Symbol;Acc:HGNC:12536] |
| ENSG00000249581 | HGLibA_10126 | 88 | -0.272910665 | CLRN2 | clarin 2 [Source:HGNC Symbol;Acc:HGNC:33939] |
| ENSG00000250510 | HGLibA_19961 | 88 | -0.272910665 | GPR162 | G protein-coupled receptor 162 [Source:HGNC Symbol;Acc:HGNC:16693] |
| ENSG00000251201 | HGLibA_49824 | 88 | -0.272910665 | TMED7-TICAM2 | TMED7-TICAM2 readthrough [Source:HGNC Symbol;Acc:HGNC:33945] |
| ENSG00000254636 | HGLibA_03060 | 88 | -0.272910665 | ARMS2 | age-related maculopathy susceptibility 2 [Source:HGNC Symbol;Acc:HGNC:32685] |
| ENSG00000255501 | HGLibA_07231 | 88 | -0.272910665 | CARD18 | caspase recruitment domain family member 18 [Source:HGNC Symbol;Acc:HGNC:28861] |
| ENSG00000256713 | HGLibA_36191 | 88 | -0.272910665 | PGA5 | group I (pepsinogen A) [Source:HGNC Symbol;Acc:HGNC:8887]" |
| ENSG00000256762 | HGLibA_47435 | 88 | -0.272910665 | STH | saitohin [Source:HGNC Symbol;Acc:HGNC:18839] |
| ENSG00000260027 | HGLibA_21947 | 88 | -0.272910665 | HOXB7 | homeobox B7 [Source:HGNC Symbol;Acc:HGNC:5118] |
| ENSG00000276085 | HGLibA_08023 | 88 | -0.272910665 | CCL3L3 | C-C motif chemokine ligand 3 like 1 [Source:HGNC Symbol;Acc:HGNC:10628] |
| FAM19A5 | HGLibA_16409 | 88 | -0.272910665 | FAM19A5 | - |
| FGFR1OP | HGLibA_17438 | 88 | -0.272910665 | FGFR1OP | - |
| HIST1H2BE | HGLibA_21423 | 88 | -0.272910665 | HIST1H2BE | - |
| hsa-mir-106b | HGLibA_57190 | 88 | -0.272910665 | hsa-mir-106b | - |
| hsa-mir-1238 | HGLibA_57344 | 88 | -0.272910665 | hsa-mir-1238 | - |
| hsa-mir-1243 | HGLibA_57357 | 88 | -0.272910665 | hsa-mir-1243 | - |
| hsa-mir-1273d | HGLibA_57539 | 88 | -0.272910665 | hsa-mir-1273d | - |
| hsa-mir-1278 | HGLibA_57567 | 88 | -0.272910665 | hsa-mir-1278 | - |
| hsa-mir-1285-2 | HGLibA_57603 | 88 | -0.272910665 | hsa-mir-1285-2 | - |
| hsa-mir-199a-1 | HGLibA_58127 | 88 | -0.272910665 | hsa-mir-199a-1 | - |
| hsa-mir-202 | HGLibA_58163 | 88 | -0.272910665 | hsa-mir-202 | - |
| hsa-mir-24-2 | HGLibA_58345 | 88 | -0.272910665 | hsa-mir-24-2 | - |
| hsa-mir-331 | HGLibA_58992 | 88 | -0.272910665 | hsa-mir-331 | - |
| hsa-mir-3659 | HGLibA_59180 | 88 | -0.272910665 | hsa-mir-3659 | - |
| hsa-mir-3689a | HGLibA_59314 | 88 | -0.272910665 | hsa-mir-3689a | - |
| hsa-mir-3690-2 | HGLibA_59349 | 88 | -0.272910665 | hsa-mir-3690-2 | - |
| hsa-mir-3934 | HGLibA_59597 | 88 | -0.272910665 | hsa-mir-3934 | - |
| hsa-mir-4268 | HGLibA_59778 | 88 | -0.272910665 | hsa-mir-4268 | - |
| hsa-mir-4299 | HGLibA_59909 | 88 | -0.272910665 | hsa-mir-4299 | - |
| hsa-mir-4475 | HGLibA_60311 | 88 | -0.272910665 | hsa-mir-4475 | - |
| hsa-mir-4521 | HGLibA_60542 | 88 | -0.272910665 | hsa-mir-4521 | - |
| hsa-mir-4661 | HGLibA_60755 | 88 | -0.272910665 | hsa-mir-4661 | - |
| hsa-mir-5009 | HGLibA_61438 | 88 | -0.272910665 | hsa-mir-5009 | - |
| hsa-mir-518d | HGLibA_61666 | 88 | -0.272910665 | hsa-mir-518d | - |
| hsa-mir-5191 | HGLibA_61682 | 88 | -0.272910665 | hsa-mir-5191 | - |
| hsa-mir-548x-2 | HGLibA_62084 | 88 | -0.272910665 | hsa-mir-548x-2 | - |
| hsa-mir-6772 | HGLibA_63263 | 88 | -0.272910665 | hsa-mir-6772 | - |
| hsa-mir-6891 | HGLibA_63748 | 88 | -0.272910665 | hsa-mir-6891 | - |
| hsa-mir-6893 | HGLibA_63755 | 88 | -0.272910665 | hsa-mir-6893 | - |
| hsa-mir-7845 | HGLibA_63969 | 88 | -0.272910665 | hsa-mir-7845 | - |
| hsa-mir-8057 | HGLibA_64068 | 88 | -0.272910665 | hsa-mir-8057 | - |
| hsa-mir-8067 | HGLibA_64106 | 88 | -0.272910665 | hsa-mir-8067 | - |
| MGEA5 | HGLibA_29157 | 88 | -0.272910665 | MGEA5 | - |
| RARS | HGLibA_40291 | 88 | -0.272910665 | RARS | - |

| | | | | | |
|---|---|---|---|---|---|
| C12orf66 | HGLibA_05283 | 87 | -0.282861703 | C12orf66 | - |
| C14orf79 | HGLibA_05370 | 87 | -0.282861703 | C14orf79 | - |
| C15orf27 | HGLibA_05380 | 87 | -0.282861703 | C15orf27 | - |
| C17orf70 | HGLibA_05565 | 87 | -0.282861703 | C17orf70 | - |
| C1orf64 | HGLibA_05884 | 87 | -0.282861703 | C1orf64 | - |
| C20orf195 | HGLibA_06003 | 87 | -0.282861703 | C20orf195 | - |
| C4orf40 | HGLibA_06329 | 87 | -0.282861703 | C4orf40 | - |
| CCDC109B | HGLibA_07532 | 87 | -0.282861703 | CCDC109B | - |
| CCL3L1 | HGLibA_08020 | 87 | -0.282861703 | CCL3L1 | - |
| DEFB130 | HGLibA_12893 | 87 | -0.282861703 | DEFB130 | - |
| DHFRL1 | HGLibA_13107 | 87 | -0.282861703 | DHFRL1 | - |
| ENSG00000004660 | HGLibA_07087 | 87 | -0.282861703 | CAMKK1 | calcium/calmodulin dependent protein kinase kinase 1 [Source:HGNC Symbol;Acc:HGNC:1469] |
| ENSG00000005238 | HGLibA_16480 | 87 | -0.282861703 | FAM214B | family with sequence similarity 214 member B [Source:HGNC Symbol;Acc:HGNC:25666] |
| ENSG00000010072 | HGLibA_46833 | 87 | -0.282861703 | SPRTN | SprT-like N-terminal domain [Source:HGNC Symbol;Acc:HGNC:25356] |
| ENSG00000011304 | HGLibA_39251 | 87 | -0.282861703 | PTBP1 | polypyrimidine tract binding protein 1 [Source:HGNC Symbol;Acc:HGNC:9583] |
| ENSG00000013016 | HGLibA_14673 | 87 | -0.282861703 | EHD3 | EH domain containing 3 [Source:HGNC Symbol;Acc:HGNC:3244] |
| ENSG00000017260 | HGLibA_03625 | 87 | -0.282861703 | ATP2C1 | ATPase secretory pathway Ca2+ transporting 1 [Source:HGNC Symbol;Acc:HGNC:13211] |
| ENSG00000053328 | HGLibA_28994 | 87 | -0.282861703 | METTL24 | methyltransferase like 24 [Source:HGNC Symbol;Acc:HGNC:21566] |
| ENSG00000058729 | HGLibA_41310 | 87 | -0.282861703 | RIOK2 | RIO kinase 2 [Source:HGNC Symbol;Acc:HGNC:18999] |
| ENSG00000064115 | HGLibA_49718 | 87 | -0.282861703 | TM7SF3 | transmembrane 7 superfamily member 3 [Source:HGNC Symbol;Acc:HGNC:23049] |
| ENSG00000064270 | HGLibA_03630 | 87 | -0.282861703 | ATP2C2 | ATPase secretory pathway Ca2+ transporting 2 [Source:HGNC Symbol;Acc:HGNC:29103] |
| ENSG00000066084 | HGLibA_13250 | 87 | -0.282861703 | DIP2B | disco interacting protein 2 homolog B [Source:HGNC Symbol;Acc:HGNC:29284] |
| ENSG00000068831 | HGLibA_40347 | 87 | -0.282861703 | RASGRP2 | RAS guanyl releasing protein 2 [Source:HGNC Symbol;Acc:HGNC:9879] |
| ENSG00000070190 | HGLibA_12334 | 87 | -0.282861703 | DAPP1 | dual adaptor of phosphotyrosine and 3-phosphoinositides 1 [Source:HGNC Symbol;Acc:HGNC:16500] |
| ENSG00000073598 | HGLibA_17760 | 87 | -0.282861703 | FNDC8 | fibronectin type III domain containing 8 [Source:HGNC Symbol;Acc:HGNC:25286] |
| ENSG00000077380 | HGLibA_14238 | 87 | -0.282861703 | DYNC1I2 | dynein cytoplasmic 1 intermediate chain 2 [Source:HGNC Symbol;Acc:HGNC:2964] |
| ENSG00000078699 | HGLibA_07384 | 87 | -0.282861703 | CBFA2T2 | CBFA2/RUNX1 translocation partner 2 [Source:HGNC Symbol;Acc:HGNC:1536] |
| ENSG00000089693 | HGLibA_29378 | 87 | -0.282861703 | MLF2 | myeloid leukemia factor 2 [Source:HGNC Symbol;Acc:HGNC:7126] |
| ENSG00000090470 | HGLibA_35745 | 87 | -0.282861703 | PDCD7 | programmed cell death 7 [Source:HGNC Symbol;Acc:HGNC:8767] |
| ENSG00000093183 | HGLibA_43218 | 87 | -0.282861703 | SEC22C | vesicle trafficking protein [Source:HGNC Symbol;Acc:HGNC:16828]" |
| ENSG00000100023 | HGLibA_37941 | 87 | -0.282861703 | PPIL2 | peptidylprolyl isomerase like 2 [Source:HGNC Symbol;Acc:HGNC:9261] |
| ENSG00000100300 | HGLibA_51957 | 87 | -0.282861703 | TSPO | translocator protein [Source:HGNC Symbol;Acc:HGNC:1158] |
| ENSG00000100335 | HGLibA_29243 | 87 | -0.282861703 | MIEF1 | mitochondrial elongation factor 1 [Source:HGNC Symbol;Acc:HGNC:25979] |
| ENSG00000100439 | HGLibA_00279 | 87 | -0.282861703 | ABHD4 | abhydrolase domain containing 4 [Source:HGNC Symbol;Acc:HGNC:20154] |
| ENSG00000103657 | HGLibA_21152 | 87 | -0.282861703 | HERC1 | HECT and RLD domain containing E3 ubiquitin protein ligase family member 1 [Source:HGNC Symbol;Acc:HGNC:4867] |
| ENSG00000104205 | HGLibA_43833 | 87 | -0.282861703 | SGK3 | serum/glucocorticoid regulated kinase family member 3 [Source:HGNC Symbol;Acc:HGNC:10812] |
| ENSG00000105135 | HGLibA_23225 | 87 | -0.282861703 | ILVBL | ilvB acetolactate synthase like [Source:HGNC Symbol;Acc:HGNC:6041] |
| ENSG00000105880 | HGLibA_13384 | 87 | -0.282861703 | DLX5 | distal-less homeobox 5 [Source:HGNC Symbol;Acc:HGNC:2918] |
| ENSG00000105963 | HGLibA_00864 | 87 | -0.282861703 | ADAP1 | ArfGAP with dual PH domains 1 [Source:HGNC Symbol;Acc:HGNC:16486] |
| ENSG00000106526 | HGLibA_00666 | 87 | -0.282861703 | ACTR3C | ARP3 actin related protein 3 homolog C [Source:HGNC Symbol;Acc:HGNC:37282] |
| ENSG00000106617 | HGLibA_38516 | 87 | -0.282861703 | PRKAG2 | protein kinase AMP-activated non-catalytic subunit gamma 2 [Source:HGNC Symbol;Acc:HGNC:9386] |
| ENSG00000106771 | HGLibA_50300 | 87 | -0.282861703 | TMEM245 | transmembrane protein 245 [Source:HGNC Symbol;Acc:HGNC:1363] |
| ENSG00000106799 | HGLibA_49222 | 87 | -0.282861703 | TGFBR1 | transforming growth factor beta receptor 1 [Source:HGNC Symbol;Acc:HGNC:11772] |
| ENSG00000110921 | HGLibA_30598 | 87 | -0.282861703 | MVK | mevalonate kinase [Source:HGNC Symbol;Acc:HGNC:7530] |
| ENSG00000111325 | HGLibA_33311 | 87 | -0.282861703 | OGFOD2 | 2-oxoglutarate and iron dependent oxygenase domain containing 2 [Source:HGNC Symbol;Acc:HGNC:25823] |
| ENSG00000111817 | HGLibA_14016 | 87 | -0.282861703 | DSE | dermatan sulfate epimerase [Source:HGNC Symbol;Acc:HGNC:21144] |
| ENSG00000112893 | HGLibA_28070 | 87 | -0.282861703 | MAN2A1 | mannosidase alpha class 2A member 1 [Source:HGNC Symbol;Acc:HGNC:6824] |
| ENSG00000113594 | HGLibA_26477 | 87 | -0.282861703 | LIFR | LIF receptor alpha [Source:HGNC Symbol;Acc:HGNC:6597] |
| ENSG00000115525 | HGLibA_47249 | 87 | -0.282861703 | ST3GAL5 | 3-sialyltransferase 5 [Source:HGNC Symbol;Acc:HGNC:10872]" |
| ENSG00000115694 | HGLibA_47474 | 87 | -0.282861703 | STK25 | serine/threonine kinase 25 [Source:HGNC Symbol;Acc:HGNC:11404] |
| ENSG00000117013 | HGLibA_24374 | 87 | -0.282861703 | KCNQ4 | potassium voltage-gated channel subfamily Q member 4 [Source:HGNC Symbol;Acc:HGNC:6298] |
| ENSG00000117298 | HGLibA_14382 | 87 | -0.282861703 | ECE1 | endothelin converting enzyme 1 [Source:HGNC Symbol;Acc:HGNC:3146] |
| ENSG00000117408 | HGLibA_23462 | 87 | -0.282861703 | IPO13 | importin 13 [Source:HGNC Symbol;Acc:HGNC:16853] |
| ENSG00000117640 | HGLibA_30365 | 87 | -0.282861703 | MTFR1L | mitochondrial fission regulator 1 like [Source:HGNC Symbol;Acc:HGNC:28836] |
| ENSG00000119614 | HGLibA_53878 | 87 | -0.282861703 | VSX2 | visual system homeobox 2 [Source:HGNC Symbol;Acc:HGNC:1975] |
| ENSG00000119684 | HGLibA_29385 | 87 | -0.282861703 | MLH3 | mutL homolog 3 [Source:HGNC Symbol;Acc:HGNC:7128] |
| ENSG00000119801 | HGLibA_54718 | 87 | -0.282861703 | YPEL5 | yippee like 5 [Source:HGNC Symbol;Acc:HGNC:18329] |
| ENSG00000119812 | HGLibA_16809 | 87 | -0.282861703 | FAM98A | family with sequence similarity 98 member A [Source:HGNC Symbol;Acc:HGNC:24520] |
| ENSG00000120265 | HGLibA_35625 | 87 | -0.282861703 | PCMT1 | protein-L-isoaspartate (D-aspartate) O-methyltransferase [Source:HGNC Symbol;Acc:HGNC:8728] |
| ENSG00000120329 | HGLibA_44665 | 87 | -0.282861703 | SLC25A2 | solute carrier family 25 member 2 [Source:HGNC Symbol;Acc:HGNC:22921] |
| ENSG00000121406 | HGLibA_56210 | 87 | -0.282861703 | ZNF549 | zinc finger protein 549 [Source:HGNC Symbol;Acc:HGNC:26632] |
| ENSG00000121440 | HGLibA_35997 | 87 | -0.282861703 | PDZRN3 | PDZ domain containing ring finger 3 [Source:HGNC Symbol;Acc:HGNC:17704] |
| ENSG00000121680 | HGLibA_36103 | 87 | -0.282861703 | PEX16 | peroxisomal biogenesis factor 16 [Source:HGNC Symbol;Acc:HGNC:8857] |
| ENSG00000122218 | HGLibA_10693 | 87 | -0.282861703 | COPA | coatomer protein complex subunit alpha [Source:HGNC Symbol;Acc:HGNC:2230] |
| ENSG00000123901 | HGLibA_20091 | 87 | -0.282861703 | GPR83 | G protein-coupled receptor 83 [Source:HGNC Symbol;Acc:HGNC:4523] |
| ENSG00000124678 | HGLibA_48841 | 87 | -0.282861703 | TCP11 | t-complex 11 [Source:HGNC Symbol;Acc:HGNC:11658] |
| ENSG00000125266 | HGLibA_14600 | 87 | -0.282861703 | EFNB2 | ephrin B2 [Source:HGNC Symbol;Acc:HGNC:3227] |
| ENSG00000126856 | HGLibA_38398 | 87 | -0.282861703 | PRDM7 | PR/SET domain 7 [Source:HGNC Symbol;Acc:HGNC:9351] |
| ENSG00000130158 | HGLibA_13753 | 87 | -0.282861703 | DOCK6 | dedicator of cytokinesis 6 [Source:HGNC Symbol;Acc:HGNC:19189] |
| ENSG00000130385 | HGLibA_04595 | 87 | -0.282861703 | BMP15 | bone morphogenetic protein 15 [Source:HGNC Symbol;Acc:HGNC:1068] |
| ENSG00000131737 | HGLibA_25487 | 87 | -0.282861703 | KRT34 | keratin 34 [Source:HGNC Symbol;Acc:HGNC:6452] |
| ENSG00000132196 | HGLibA_22200 | 87 | -0.282861703 | HSD17B7 | hydroxysteroid 17-beta dehydrogenase 7 [Source:HGNC Symbol;Acc:HGNC:5215] |
| ENSG00000135046 | HGLibA_02218 | 87 | -0.282861703 | ANXA1 | annexin A1 [Source:HGNC Symbol;Acc:HGNC:533] |
| ENSG00000135439 | HGLibA_01143 | 87 | -0.282861703 | AGAP2 | ankyrin repeat and PH domain 2 [Source:HGNC Symbol;Acc:HGNC:16921]" |
| ENSG00000135541 | HGLibA_01298 | 87 | -0.282861703 | AHI1 | Abelson helper integration site 1 [Source:HGNC Symbol;Acc:HGNC:21575] |
| ENSG00000137054 | HGLibA_37562 | 87 | -0.282861703 | POLR1E | RNA polymerase I subunit E [Source:HGNC Symbol;Acc:HGNC:17631] |
| ENSG00000137760 | HGLibA_01667 | 87 | -0.282861703 | ALKBH8 | tRNA methyltransferase [Source:HGNC Symbol;Acc:HGNC:25189]" |
| ENSG00000138136 | HGLibA_26084 | 87 | -0.282861703 | LBX1 | ladybird homeobox 1 [Source:HGNC Symbol;Acc:HGNC:16960] |
| ENSG00000138821 | HGLibA_45123 | 87 | -0.282861703 | SLC39A8 | solute carrier family 39 member 8 [Source:HGNC Symbol;Acc:HGNC:20862] |
| ENSG00000139044 | HGLibA_04018 | 87 | -0.282861703 | B4GALNT3 | 4-N-acetyl-galactosaminyltransferase 3 [Source:HGNC Symbol;Acc:HGNC:24137]" |
| ENSG00000139112 | HGLibA_18336 | 87 | -0.282861703 | GABARAPL1 | GABA type A receptor associated protein like 1 [Source:HGNC Symbol;Acc:HGNC:4068] |
| ENSG00000141867 | HGLibA_04780 | 87 | -0.282861703 | BRD4 | bromodomain containing 4 [Source:HGNC Symbol;Acc:HGNC:13575] |
| ENSG00000143126 | HGLibA_09024 | 87 | -0.282861703 | CELSR2 | cadherin EGF LAG seven-pass G-type receptor 2 [Source:HGNC Symbol;Acc:HGNC:3231] |
| ENSG00000143190 | HGLibA_37755 | 87 | -0.282861703 | POU2F1 | POU class 2 homeobox 1 [Source:HGNC Symbol;Acc:HGNC:9212] |
| ENSG00000143278 | HGLibA_15855 | 87 | -0.282861703 | F13B | coagulation factor XIII B chain [Source:HGNC Symbol;Acc:HGNC:3534] |
| ENSG00000143324 | HGLibA_54579 | 87 | -0.282861703 | XPR1 | xenotropic and polytropic retrovirus receptor 1 [Source:HGNC Symbol;Acc:HGNC:12827] |
| ENSG00000143543 | HGLibA_24001 | 87 | -0.282861703 | JTB | jumping translocation breakpoint [Source:HGNC Symbol;Acc:HGNC:6201] |
| ENSG00000144407 | HGLibA_39374 | 87 | -0.282861703 | PTH2R | parathyroid hormone 2 receptor [Source:HGNC Symbol;Acc:HGNC:9609] |
| ENSG00000144468 | HGLibA_41123 | 87 | -0.282861703 | RHBDD1 | rhomboid domain containing 1 [Source:HGNC Symbol;Acc:HGNC:23081] |
| ENSG00000145901 | HGLibA_50834 | 87 | -0.282861703 | TNIP1 | TNFAIP3 interacting protein 1 [Source:HGNC Symbol;Acc:HGNC:16903] |
| ENSG00000145912 | HGLibA_31909 | 87 | -0.282861703 | NHP2 | NHP2 ribonucleoprotein [Source:HGNC Symbol;Acc:HGNC:14377] |
| ENSG00000147883 | HGLibA_08838 | 87 | -0.282861703 | CDKN2B | cyclin dependent kinase inhibitor 2B [Source:HGNC Symbol;Acc:HGNC:1788] |
| ENSG00000148584 | HGLibA_00004 | 87 | -0.282861703 | A1CF | APOBEC1 complementation factor [Source:HGNC Symbol;Acc:HGNC:24086] |
| ENSG00000148735 | HGLibA_37155 | 87 | -0.282861703 | PLEKHS1 | pleckstrin homology domain containing S1 [Source:HGNC Symbol;Acc:HGNC:26285] |
| ENSG00000149136 | HGLibA_47166 | 87 | -0.282861703 | SSRP1 | structure specific recognition protein 1 [Source:HGNC Symbol;Acc:HGNC:11327] |
| ENSG00000151617 | HGLibA_14479 | 87 | -0.282861703 | EDNRA | endothelin receptor type A [Source:HGNC Symbol;Acc:HGNC:3179] |
| ENSG00000155629 | HGLibA_36618 | 87 | -0.282861703 | PIK3AP1 | phosphoinositide-3-kinase adaptor protein 1 [Source:HGNC Symbol;Acc:HGNC:30034] |
| ENSG00000155827 | HGLibA_41611 | 87 | -0.282861703 | RNF20 | ring finger protein 20 [Source:HGNC Symbol;Acc:HGNC:10062] |
| ENSG00000156265 | HGLibA_28200 | 87 | -0.282861703 | MAP3K7CL | MAP3K7 C-terminal like [Source:HGNC Symbol;Acc:HGNC:16457] |
| ENSG00000156804 | HGLibA_17071 | 87 | -0.282861703 | FBXO32 | F-box protein 32 [Source:HGNC Symbol;Acc:HGNC:16731] |
| ENSG00000158485 | HGLibA_08266 | 87 | -0.282861703 | CD1B | CD1b molecule [Source:HGNC Symbol;Acc:HGNC:1635] |
| ENSG00000160219 | HGLibA_18327 | 87 | -0.282861703 | GAB3 | GRB2 associated binding protein 3 [Source:HGNC Symbol;Acc:HGNC:17515] |
| ENSG00000160305 | HGLibA_13246 | 87 | -0.282861703 | DIP2A | disco interacting protein 2 homolog A [Source:HGNC Symbol;Acc:HGNC:17217] |
| ENSG00000160785 | HGLibA_44748 | 87 | -0.282861703 | SLC25A44 | solute carrier family 25 member 44 [Source:HGNC Symbol;Acc:HGNC:29036] |
| ENSG00000160993 | HGLibA_01655 | 87 | -0.282861703 | ALKBH4 | lysine demethylase [Source:HGNC Symbol;Acc:HGNC:21900]" |
| ENSG00000162975 | HGLibA_24196 | 87 | -0.282861703 | KCNF1 | potassium voltage-gated channel modifier subfamily F member 1 [Source:HGNC Symbol;Acc:HGNC:6246] |
| ENSG00000162975 | HGLibA_24195 | 87 | -0.282861703 | KCNF1 | potassium voltage-gated channel modifier subfamily F member 1 [Source:HGNC Symbol;Acc:HGNC:6246] |
| ENSG00000163132 | HGLibA_30272 | 87 | -0.282861703 | MSX1 | msh homeobox 1 [Source:HGNC Symbol;Acc:HGNC:7391] |
| ENSG00000163214 | HGLibA_13203 | 87 | -0.282861703 | DHX57 | DExH-box helicase 57 [Source:HGNC Symbol;Acc:HGNC:20086] |
| ENSG00000163221 | HGLibA_42538 | 87 | -0.282861703 | S100A12 | S100 calcium binding protein A12 [Source:HGNC Symbol;Acc:HGNC:10489] |
| ENSG00000163399 | HGLibA_03580 | 87 | -0.282861703 | ATP1A1 | ATPase Na+/K+ transporting subunit alpha 1 [Source:HGNC Symbol;Acc:HGNC:799] |
| ENSG00000164342 | HGLibA_49648 | 87 | -0.282861703 | TLR3 | toll like receptor 3 [Source:HGNC Symbol;Acc:HGNC:11849] |
| ENSG00000164414 | HGLibA_44946 | 87 | -0.282861703 | SLC35A1 | solute carrier family 35 member A1 [Source:HGNC Symbol;Acc:HGNC:11021] |
| ENSG00000165185 | HGLibA_24829 | 87 | -0.282861703 | KIAA1958 | KIAA1958 [Source:HGNC Symbol;Acc:HGNC:23427] |
| ENSG00000165516 | HGLibA_25095 | 87 | -0.282861703 | KLHDC2 | kelch domain containing 2 [Source:HGNC Symbol;Acc:HGNC:20231] |
| ENSG00000165688 | HGLibA_37326 | 87 | -0.282861703 | PMPCA | mitochondrial processing alpha subunit [Source:HGNC Symbol;Acc:HGNC:18667]" |
| ENSG00000166484 | HGLibA_28280 | 87 | -0.282861703 | MAPK7 | mitogen-activated protein kinase 7 [Source:HGNC Symbol;Acc:HGNC:6880] |
| ENSG00000166816 | HGLibA_26234 | 87 | -0.282861703 | LDHD | lactate dehydrogenase D [Source:HGNC Symbol;Acc:HGNC:19708] |
| ENSG00000166920 | HGLibA_05405 | 87 | -0.282861703 | C15orf48 | chromosome 15 open reading frame 48 [Source:HGNC Symbol;Acc:HGNC:29898] |
| ENSG00000167315 | HGLibA_00344 | 87 | -0.282861703 | ACAA2 | acetyl-CoA acyltransferase 2 [Source:HGNC Symbol;Acc:HGNC:83] |
| ENSG00000167601 | HGLibA_03927 | 87 | -0.282861703 | AXL | AXL receptor tyrosine kinase [Source:HGNC Symbol;Acc:HGNC:905] |
| ENSG00000167646 | HGLibA_13465 | 87 | -0.282861703 | DNAAF3 | dynein axonemal assembly factor 3 [Source:HGNC Symbol;Acc:HGNC:30492] |
| ENSG00000167772 | HGLibA_01920 | 87 | -0.282861703 | ANGPTL4 | angiopoietin like 4 [Source:HGNC Symbol;Acc:HGNC:16039] |
| ENSG00000168267 | HGLibA_39301 | 87 | -0.282861703 | PTF1A | pancreas associated transcription factor 1a [Source:HGNC Symbol;Acc:HGNC:23734] |
| ENSG00000169214 | HGLibA_34414 | 87 | -0.282861703 | OR6F1 | olfactory receptor family 6 subfamily F member 1 [Source:HGNC Symbol;Acc:HGNC:15027] |
| ENSG00000169302 | HGLibA_47484 | 87 | -0.282861703 | STK32A | serine/threonine kinase 32A [Source:HGNC Symbol;Acc:HGNC:28317] |
| ENSG00000169515 | HGLibA_07875 | 87 | -0.282861703 | CCDC8 | coiled-coil domain containing 8 [Source:HGNC Symbol;Acc:HGNC:25367] |
| ENSG00000169627 | HGLibA_04686 | 87 | -0.282861703 | BOLA2B | bolA family member 2B [Source:HGNC Symbol;Acc:HGNC:32479] |
| ENSG00000169871 | HGLibA_51549 | 87 | -0.282861703 | TRIM56 | tripartite motif containing 56 [Source:HGNC Symbol;Acc:HGNC:19028] |
| ENSG00000170037 | HGLibA_10441 | 87 | -0.282861703 | CNTROB | centriole duplication and spindle assembly protein [Source:HGNC Symbol;Acc:HGNC:29616]" |
| ENSG00000170498 | HGLibA_25010 | 87 | -0.282861703 | KISS1 | KiSS-1 metastasis suppressor [Source:HGNC Symbol;Acc:HGNC:6341] |
| ENSG00000170627 | HGLibA_20629 | 87 | -0.282861703 | GTSF1 | gametocyte specific factor 1 [Source:HGNC Symbol;Acc:HGNC:26565] |
| ENSG00000171102 | HGLibA_33228 | 87 | -0.282861703 | OBP2B | odorant binding protein 2B [Source:HGNC Symbol;Acc:HGNC:23381] |
| ENSG00000171217 | HGLibA_09881 | 87 | -0.282861703 | CLDN20 | claudin 20 [Source:HGNC Symbol;Acc:HGNC:2042] |
| ENSG00000171459 | HGLibA_33696 | 87 | -0.282861703 | OR1L6 | olfactory receptor family 1 subfamily L member 6 [Source:HGNC Symbol;Acc:HGNC:8218] |

| | | | | | |
|---|---|---|---|---|---|
| ENSG00000171492 | HGLibA_27438 | 87 | -0.282861703 | LRRC8D | leucine rich repeat containing 8 VRAC subunit D [Source:HGNC Symbol;Acc:HGNC:16992] |
| ENSG00000172673 | HGLibA_49349 | 87 | -0.282861703 | THEMIS | thymocyte selection associated [Source:HGNC Symbol;Acc:HGNC:21569] |
| ENSG00000173933 | HGLibA_40544 | 87 | -0.282861703 | RBM4 | RNA binding motif protein 4 [Source:HGNC Symbol;Acc:HGNC:9901] |
| ENSG00000173950 | HGLibA_54614 | 87 | -0.282861703 | XXYLT1 | xyloside xylosyltransferase 1 [Source:HGNC Symbol;Acc:HGNC:26639] |
| ENSG00000174373 | HGLibA_40156 | 87 | -0.282861703 | RALGAPA1 | Ral GTPase activating protein catalytic alpha subunit 1 [Source:HGNC Symbol;Acc:HGNC:17770] |
| ENSG00000174628 | HGLibA_23528 | 87 | -0.282861703 | IQCK | IQ motif containing K [Source:HGNC Symbol;Acc:HGNC:28556] |
| ENSG00000176946 | HGLibA_49302 | 87 | -0.282861703 | THAP4 | THAP domain containing 4 [Source:HGNC Symbol;Acc:HGNC:23187] |
| ENSG00000177732 | HGLibA_46234 | 87 | -0.282861703 | SOX12 | SRY-box 12 [Source:HGNC Symbol;Acc:HGNC:11198] |
| ENSG00000179085 | HGLibA_13864 | 87 | -0.282861703 | DPM3 | dolichyl-phosphate mannosyltransferase subunit 3 [Source:HGNC Symbol;Acc:HGNC:3007] |
| ENSG00000179363 | HGLibA_50366 | 87 | -0.282861703 | TMEM31 | transmembrane protein 31 [Source:HGNC Symbol;Acc:HGNC:28601] |
| ENSG00000180383 | HGLibA_12875 | 87 | -0.282861703 | DEFB124 | defensin beta 124 [Source:HGNC Symbol;Acc:HGNC:18104] |
| ENSG00000180919 | HGLibA_34225 | 87 | -0.282861703 | OR56B4 | olfactory receptor family 56 subfamily B member 4 [Source:HGNC Symbol;Acc:HGNC:15248] |
| ENSG00000181704 | HGLibA_54686 | 87 | -0.282861703 | YIPF6 | Yip1 domain family member 6 [Source:HGNC Symbol;Acc:HGNC:28304] |
| ENSG00000181789 | HGLibA_10705 | 87 | -0.282861703 | COPG1 | coatomer protein complex subunit gamma 1 [Source:HGNC Symbol;Acc:HGNC:2236] |
| ENSG00000181804 | HGLibA_45431 | 87 | -0.282861703 | SLC9A9 | solute carrier family 9 member A9 [Source:HGNC Symbol;Acc:HGNC:20653] |
| ENSG00000182393 | HGLibA_22718 | 87 | -0.282861703 | IFNL1 | interferon lambda 1 [Source:HGNC Symbol;Acc:HGNC:18363] |
| ENSG00000182783 | HGLibA_33868 | 87 | -0.282861703 | OR2T29 | olfactory receptor family 2 subfamily T member 29 [Source:HGNC Symbol;Acc:HGNC:31253] |
| ENSG00000183828 | HGLibA_32945 | 87 | -0.282861703 | NUDT14 | nudix hydrolase 14 [Source:HGNC Symbol;Acc:HGNC:20141] |
| ENSG00000184271 | HGLibA_37796 | 87 | -0.282861703 | POU6F1 | POU class 6 homeobox 1 [Source:HGNC Symbol;Acc:HGNC:9224] |
| ENSG00000184916 | HGLibA_23907 | 87 | -0.282861703 | JAG2 | jagged 2 [Source:HGNC Symbol;Acc:HGNC:6189] |
| ENSG00000184922 | HGLibA_17692 | 87 | -0.282861703 | FMNL1 | formin like 1 [Source:HGNC Symbol;Acc:HGNC:1212] |
| ENSG00000184999 | HGLibA_44538 | 87 | -0.282861703 | SLC22A10 | solute carrier family 22 member 10 [Source:HGNC Symbol;Acc:HGNC:18057] |
| ENSG00000185917 | HGLibA_43648 | 87 | -0.282861703 | SETD4 | SET domain containing 4 [Source:HGNC Symbol;Acc:HGNC:1258] |
| ENSG00000186448 | HGLibA_55562 | 87 | -0.282861703 | ZNF197 | zinc finger protein 197 [Source:HGNC Symbol;Acc:HGNC:12988] |
| ENSG00000186803 | HGLibA_22660 | 87 | -0.282861703 | IFNA10 | interferon alpha 10 [Source:HGNC Symbol;Acc:HGNC:5418] |
| ENSG00000186806 | HGLibA_53841 | 87 | -0.282861703 | VSIG10L | V-set and immunoglobulin domain containing 10 like [Source:HGNC Symbol;Acc:HGNC:27111] |
| ENSG00000186924 | HGLibA_25717 | 87 | -0.282861703 | KRTAP22-1 | keratin associated protein 22-1 [Source:HGNC Symbol;Acc:HGNC:18947] |
| ENSG00000187082 | HGLibA_12835 | 87 | -0.282861703 | DEFB106B | defensin beta 106B [Source:HGNC Symbol;Acc:HGNC:28879] |
| ENSG00000187546 | HGLibA_01203 | 87 | -0.282861703 | AGMO | alkylglycerol monooxygenase [Source:HGNC Symbol;Acc:HGNC:33784] |
| ENSG00000188566 | HGLibA_31408 | 87 | -0.282861703 | NDOR1 | NADPH dependent diflavin oxidoreductase 1 [Source:HGNC Symbol;Acc:HGNC:29838] |
| ENSG00000189139 | HGLibA_18065 | 87 | -0.282861703 | FSCB | fibrous sheath CABYR binding protein [Source:HGNC Symbol;Acc:HGNC:20494] |
| ENSG00000196110 | HGLibA_56567 | 87 | -0.282861703 | ZNF699 | zinc finger protein 699 [Source:HGNC Symbol;Acc:HGNC:24750] |
| ENSG00000196504 | HGLibA_38733 | 87 | -0.282861703 | PRPF40A | pre-mRNA processing factor 40 homolog A [Source:HGNC Symbol;Acc:HGNC:16463] |
| ENSG00000197302 | HGLibA_56627 | 87 | -0.282861703 | ZNF720 | zinc finger protein 720 [Source:HGNC Symbol;Acc:HGNC:26987] |
| ENSG00000198326 | HGLibA_50282 | 87 | -0.282861703 | TMEM239 | transmembrane protein 239 [Source:HGNC Symbol;Acc:HGNC:40044] |
| ENSG00000198513 | HGLibA_03514 | 87 | -0.282861703 | ATL1 | atlastin GTPase 1 [Source:HGNC Symbol;Acc:HGNC:11231] |
| ENSG00000198590 | HGLibA_06232 | 87 | -0.282861703 | C3orf35 | chromosome 3 open reading frame 35 [Source:HGNC Symbol;Acc:HGNC:24082] |
| ENSG00000198937 | HGLibA_07676 | 87 | -0.282861703 | CCDC167 | coiled-coil domain containing 167 [Source:HGNC Symbol;Acc:HGNC:21239] |
| ENSG00000204335 | HGLibA_46306 | 87 | -0.282861703 | SP5 | Sp5 transcription factor [Source:HGNC Symbol;Acc:HGNC:14529] |
| ENSG00000204345 | HGLibA_08339 | 87 | -0.282861703 | CD300LD | CD300 molecule like family member d [Source:HGNC Symbol;Acc:HGNC:16848] |
| ENSG00000204392 | HGLibA_27536 | 87 | -0.282861703 | LSM2 | U6 small nuclear RNA and mRNA degradation associated [Source:HGNC Symbol;Acc:HGNC:13940]" |
| ENSG00000204482 | HGLibA_27571 | 87 | -0.282861703 | LST1 | leukocyte specific transcript 1 [Source:HGNC Symbol;Acc:HGNC:14189] |
| ENSG00000204928 | HGLibA_20392 | 87 | -0.282861703 | GRXCR2 | glutaredoxin and cysteine rich domain containing 2 [Source:HGNC Symbol;Acc:HGNC:33862] |
| ENSG00000205710 | HGLibA_05529 | 87 | -0.282861703 | C17orf107 | chromosome 17 open reading frame 107 [Source:HGNC Symbol;Acc:HGNC:37238] |
| ENSG00000213023 | HGLibA_48074 | 87 | -0.282861703 | SYT3 | synaptotagmin 3 [Source:HGNC Symbol;Acc:HGNC:11511] |
| ENSG00000213030 | HGLibA_09315 | 87 | -0.282861703 | CGB8 | chorionic gonadotropin subunit beta 8 [Source:HGNC Symbol;Acc:HGNC:16453] |
| ENSG00000213928 | HGLibA_23603 | 87 | -0.282861703 | IRF9 | interferon regulatory factor 9 [Source:HGNC Symbol;Acc:HGNC:6131] |
| ENSG00000214819 | HGLibA_08878 | 87 | -0.282861703 | CDRT15L2 | CMT1A duplicated region transcript 15 like 2 [Source:HGNC Symbol;Acc:HGNC:34075] |
| ENSG00000219607 | HGLibA_38117 | 87 | -0.282861703 | PPP1R3G | protein phosphatase 1 regulatory subunit 3G [Source:HGNC Symbol;Acc:HGNC:14945] |
| ENSG00000221926 | HGLibA_51417 | 87 | -0.282861703 | TRIM16 | tripartite motif containing 16 [Source:HGNC Symbol;Acc:HGNC:17241] |
| ENSG00000226174 | HGLibA_49077 | 87 | -0.282861703 | TEX22 | testis expressed 22 [Source:HGNC Symbol;Acc:HGNC:40026] |
| ENSG00000228567 | HGLibA_53703 | 87 | -0.282861703 | VN1R4 | vomeronasal 1 receptor 4 [Source:HGNC Symbol;Acc:HGNC:19871] |
| ENSG00000229183 | HGLibA_36188 | 87 | -0.282861703 | PGA4 | group I (pepsinogen A) [Source:HGNC Symbol;Acc:HGNC:8886]" |
| ENSG00000236032 | HGLibA_34292 | 87 | -0.282861703 | OR5H14 | olfactory receptor family 5 subfamily H member 14 [Source:HGNC Symbol;Acc:HGNC:31286] |
| ENSG00000242366 | HGLibA_52897 | 87 | -0.282861703 | UGT1A8 | UDP glucuronosyltransferase family 1 member A8 [Source:HGNC Symbol;Acc:HGNC:12540] |
| ENSG00000255833 | HGLibA_49461 | 87 | -0.282861703 | TIFAB | TIFA inhibitor [Source:HGNC Symbol;Acc:HGNC:34024] |
| ENSG00000257365 | HGLibA_17774 | 87 | -0.282861703 | FNTB | beta [Source:HGNC Symbol;Acc:HGNC:3785]" |
| ENSG00000260811 | HGLibA_33956 | 87 | -0.282861703 | OR4C45 | olfactory receptor family 4 subfamily C member 45 (gene/pseudogene) [Source:HGNC Symbol;Acc:HGNC:31270] |
| ENSG00000274102 | HGLibA_34043 | 87 | -0.282861703 | OR4M2 | olfactory receptor family 4 subfamily M member 2 [Source:HGNC Symbol;Acc:HGNC:15373] |
| ENSG00000276045 | HGLibA_34590 | 87 | -0.282861703 | ORAI1 | ORAI calcium release-activated calcium modulator 1 [Source:HGNC Symbol;Acc:HGNC:25896] |
| hsa-mir-101-1 | HGLibA_57153 | 87 | -0.282861703 | hsa-mir-101-1 | - |
| hsa-mir-1234 | HGLibA_57331 | 87 | -0.282861703 | hsa-mir-1234 | - |
| hsa-mir-1587 | HGLibA_57936 | 87 | -0.282861703 | hsa-mir-1587 | - |
| hsa-mir-2053 | HGLibA_58186 | 87 | -0.282861703 | hsa-mir-2053 | - |
| hsa-mir-208b | HGLibA_58200 | 87 | -0.282861703 | hsa-mir-208b | - |
| hsa-mir-20b | HGLibA_58206 | 87 | -0.282861703 | hsa-mir-20b | - |
| hsa-mir-3180-3 | HGLibA_58809 | 87 | -0.282861703 | hsa-mir-3180-3 | - |
| hsa-mir-3674 | HGLibA_59251 | 87 | -0.282861703 | hsa-mir-3674 | - |
| hsa-mir-371a | HGLibA_59373 | 87 | -0.282861703 | hsa-mir-371a | - |
| hsa-mir-383 | HGLibA_59486 | 87 | -0.282861703 | hsa-mir-383 | - |
| hsa-mir-3910-2 | HGLibA_59509 | 87 | -0.282861703 | hsa-mir-3910-2 | - |
| hsa-mir-432 | HGLibA_59999 | 87 | -0.282861703 | hsa-mir-432 | - |
| hsa-mir-4327 | HGLibA_60032 | 87 | -0.282861703 | hsa-mir-4327 | - |
| hsa-mir-4453 | HGLibA_60217 | 87 | -0.282861703 | hsa-mir-4453 | - |
| hsa-mir-4463 | HGLibA_60260 | 87 | -0.282861703 | hsa-mir-4463 | - |
| hsa-mir-4538 | HGLibA_60612 | 87 | -0.282861703 | hsa-mir-4538 | - |
| hsa-mir-4711 | HGLibA_60944 | 87 | -0.282861703 | hsa-mir-4711 | - |
| hsa-mir-4753 | HGLibA_61111 | 87 | -0.282861703 | hsa-mir-4753 | - |
| hsa-mir-514b | HGLibA_61597 | 87 | -0.282861703 | hsa-mir-514b | - |
| hsa-mir-5588 | HGLibA_62193 | 87 | -0.282861703 | hsa-mir-5588 | - |
| hsa-mir-6134 | HGLibA_62687 | 87 | -0.282861703 | hsa-mir-6134 | - |
| hsa-mir-6726 | HGLibA_63056 | 87 | -0.282861703 | hsa-mir-6726 | - |
| hsa-mir-6750 | HGLibA_63158 | 87 | -0.282861703 | hsa-mir-6750 | - |
| hsa-mir-6795 | HGLibA_63358 | 87 | -0.282861703 | hsa-mir-6795 | - |
| hsa-mir-6823 | HGLibA_63468 | 87 | -0.282861703 | hsa-mir-6823 | - |
| hsa-mir-6827 | HGLibA_63487 | 87 | -0.282861703 | hsa-mir-6827 | - |
| hsa-mir-744 | HGLibA_63884 | 87 | -0.282861703 | hsa-mir-744 | - |
| hsa-mir-7975 | HGLibA_64031 | 87 | -0.282861703 | hsa-mir-7975 | - |
| hsa-mir-943 | HGLibA_64359 | 87 | -0.282861703 | hsa-mir-943 | - |
| KIAA1211 | HGLibA_24716 | 87 | -0.282861703 | KIAA1211 | - |
| KIAA1967 | HGLibA_24833 | 87 | -0.282861703 | KIAA1967 | - |
| KIRREL | HGLibA_24999 | 87 | -0.282861703 | KIRREL | - |
| MLL2 | HGLibA_29396 | 87 | -0.282861703 | MLL2 | - |
| PALM2 | HGLibA_35071 | 87 | -0.282861703 | PALM2 | - |
| SPATA31A4 | HGLibA_46477 | 87 | -0.282861703 | SPATA31A4 | - |
| WDR52 | HGLibA_54118 | 87 | -0.282861703 | WDR52 | - |
| C16orf52 | HGLibA_05452 | 86 | -0.29281274 | C16orf52 | - |
| C9orf170 | HGLibA_06706 | 86 | -0.29281274 | C9orf170 | - |
| ENSG00000004779 | HGLibA_31480 | 86 | -0.29281274 | NDUFAB1 | NADH:ubiquinone oxidoreductase subunit AB1 [Source:HGNC Symbol;Acc:HGNC:7694] |
| ENSG00000006740 | HGLibA_02781 | 86 | -0.29281274 | ARHGAP44 | Rho GTPase activating protein 44 [Source:HGNC Symbol;Acc:HGNC:29096] |
| ENSG00000008083 | HGLibA_23938 | 86 | -0.29281274 | JARID2 | jumonji and AT-rich interaction domain containing 2 [Source:HGNC Symbol;Acc:HGNC:6196] |
| ENSG00000009709 | HGLibA_35313 | 86 | -0.29281274 | PAX7 | paired box 7 [Source:HGNC Symbol;Acc:HGNC:8621] |
| ENSG00000010030 | HGLibA_15669 | 86 | -0.29281274 | ETV7 | ETS variant 7 [Source:HGNC Symbol;Acc:HGNC:18160] |
| ENSG00000023287 | HGLibA_40416 | 86 | -0.29281274 | RB1CC1 | RB1 inducible coiled-coil 1 [Source:HGNC Symbol;Acc:HGNC:15574] |
| ENSG00000029559 | HGLibA_22482 | 86 | -0.29281274 | IBSP | integrin binding sialoprotein [Source:HGNC Symbol;Acc:HGNC:5341] |
| ENSG00000051382 | HGLibA_36636 | 86 | -0.29281274 | PIK3CB | 5-bisphosphate 3-kinase catalytic subunit beta [Source:HGNC Symbol;Acc:HGNC:8976]" |
| ENSG00000056487 | HGLibA_36372 | 86 | -0.29281274 | PHF21B | PHD finger protein 21B [Source:HGNC Symbol;Acc:HGNC:25161] |
| ENSG00000058799 | HGLibA_54669 | 86 | -0.29281274 | YIPF1 | Yip1 domain family member 1 [Source:HGNC Symbol;Acc:HGNC:25231] |
| ENSG00000066044 | HGLibA_14878 | 86 | -0.29281274 | ELAVL1 | ELAV like RNA binding protein 1 [Source:HGNC Symbol;Acc:HGNC:3312] |
| ENSG00000066651 | HGLibA_51669 | 86 | -0.29281274 | TRMT11 | tRNA methyltransferase 11 homolog [Source:HGNC Symbol;Acc:HGNC:21080] |
| ENSG00000067840 | HGLibA_35979 | 86 | -0.29281274 | PDZD4 | PDZ domain containing 4 [Source:HGNC Symbol;Acc:HGNC:21167] |
| ENSG00000068394 | HGLibA_19814 | 86 | -0.29281274 | GPKOW | G-patch domain and KOW motifs [Source:HGNC Symbol;Acc:HGNC:30677] |
| ENSG00000072958 | HGLibA_02290 | 86 | -0.29281274 | AP1M1 | adaptor related protein complex 1 subunit mu 1 [Source:HGNC Symbol;Acc:HGNC:13667] |
| ENSG00000080824 | HGLibA_22250 | 86 | -0.29281274 | HSP90AA1 | heat shock protein 90 alpha family class A member 1 [Source:HGNC Symbol;Acc:HGNC:5253] |
| ENSG00000081087 | HGLibA_34713 | 86 | -0.29281274 | OSTM1 | osteoclastogenesis associated transmembrane protein 1 [Source:HGNC Symbol;Acc:HGNC:21652] |
| ENSG00000096717 | HGLibA_44215 | 86 | -0.29281274 | SIRT1 | sirtuin 1 [Source:HGNC Symbol;Acc:HGNC:14929] |
| ENSG00000099326 | HGLibA_30937 | 86 | -0.29281274 | MZF1 | myeloid zinc finger 1 [Source:HGNC Symbol;Acc:HGNC:13108] |
| ENSG00000100139 | HGLibA_29213 | 86 | -0.29281274 | MICALL1 | MICAL like 1 [Source:HGNC Symbol;Acc:HGNC:29804] |
| ENSG00000100227 | HGLibA_37508 | 86 | -0.29281274 | POLDIP3 | DNA polymerase delta interacting protein 3 [Source:HGNC Symbol;Acc:HGNC:23782] |
| ENSG00000100427 | HGLibA_29367 | 86 | -0.29281274 | MLC1 | megalencephalic leukoencephalopathy with subcortical cysts 1 [Source:HGNC Symbol;Acc:HGNC:17082] |
| ENSG00000100592 | HGLibA_12259 | 86 | -0.29281274 | DAAM1 | dishevelled associated activator of morphogenesis 1 [Source:HGNC Symbol;Acc:HGNC:18142] |
| ENSG00000101935 | HGLibA_01802 | 86 | -0.29281274 | AMMECR1 | midface hypoplasia and elliptocytosis chromosomal region gene 1 [Source:HGNC Symbol;Acc:HGNC:467]" |
| ENSG00000102010 | HGLibA_04640 | 86 | -0.29281274 | BMX | BMX non-receptor tyrosine kinase [Source:HGNC Symbol;Acc:HGNC:1079] |
| ENSG00000102239 | HGLibA_04845 | 86 | -0.29281274 | BRS3 | bombesin receptor subtype 3 [Source:HGNC Symbol;Acc:HGNC:1113] |
| ENSG00000102886 | HGLibA_18897 | 86 | -0.29281274 | GDPD3 | glycerophosphodiester phosphodiesterase domain containing 3 [Source:HGNC Symbol;Acc:HGNC:28638] |
| ENSG00000103194 | HGLibA_53193 | 86 | -0.29281274 | USP10 | ubiquitin specific peptidase 10 [Source:HGNC Symbol;Acc:HGNC:12608] |
| ENSG00000103313 | HGLibA_28843 | 86 | -0.29281274 | MEFV | pyrin innate immunity regulator [Source:HGNC Symbol;Acc:HGNC:6998]" |
| ENSG00000104267 | HGLibA_06804 | 86 | -0.29281274 | CA2 | carbonic anhydrase 2 [Source:HGNC Symbol;Acc:HGNC:1373] |
| ENSG00000104361 | HGLibA_31960 | 86 | -0.29281274 | NIPAL2 | NIPA like domain containing 2 [Source:HGNC Symbol;Acc:HGNC:25854] |
| ENSG00000104497 | HGLibA_46046 | 86 | -0.29281274 | SNX16 | sorting nexin 16 [Source:HGNC Symbol;Acc:HGNC:14980] |
| ENSG00000104938 | HGLibA_09997 | 86 | -0.29281274 | CLEC4M | C-type lectin domain family 4 member M [Source:HGNC Symbol;Acc:HGNC:13523] |
| ENSG00000105371 | HGLibA_22502 | 86 | -0.29281274 | ICAM4 | intercellular adhesion molecule 4 (Landsteiner-Wiener blood group) [Source:HGNC Symbol;Acc:HGNC:5347] |
| ENSG00000106443 | HGLibA_36344 | 86 | -0.29281274 | PHF14 | PHD finger protein 14 [Source:HGNC Symbol;Acc:HGNC:22203] |
| ENSG00000107105 | HGLibA_14881 | 86 | -0.29281274 | ELAVL2 | ELAV like RNA binding protein 2 [Source:HGNC Symbol;Acc:HGNC:3313] |
| ENSG00000107371 | HGLibA_15779 | 86 | -0.29281274 | EXOSC3 | exosome component 3 [Source:HGNC Symbol;Acc:HGNC:17944] |

| | | | | | |
|---|---|---|---|---|---|
| ENSG00000107796 | HGLibA_00600 | 86 | -0.29281274 | ACTA2 | aorta [Source:HGNC Symbol;Acc:HGNC:130]" |
| ENSG00000108557 | HGLibA_40137 | 86 | -0.29281274 | RAI1 | retinoic acid induced 1 [Source:HGNC Symbol;Acc:HGNC:9834] |
| ENSG00000111731 | HGLibA_06107 | 86 | -0.29281274 | C2CD5 | C2 calcium dependent domain containing 5 [Source:HGNC Symbol;Acc:HGNC:29062] |
| ENSG00000112238 | HGLibA_38375 | 86 | -0.29281274 | PRDM13 | PR/SET domain 13 [Source:HGNC Symbol;Acc:HGNC:13998] |
| ENSG00000112305 | HGLibA_45596 | 86 | -0.29281274 | SMAP1 | small ArfGAP 1 [Source:HGNC Symbol;Acc:HGNC:19651] |
| ENSG00000114473 | HGLibA_23518 | 86 | -0.29281274 | IQCG | IQ motif containing G [Source:HGNC Symbol;Acc:HGNC:25251] |
| ENSG00000114956 | HGLibA_13088 | 86 | -0.29281274 | DGUOK | deoxyguanosine kinase [Source:HGNC Symbol;Acc:HGNC:2858] |
| ENSG00000117533 | HGLibA_53497 | 86 | -0.29281274 | VAMP4 | vesicle associated membrane protein 4 [Source:HGNC Symbol;Acc:HGNC:12645] |
| ENSG00000120217 | HGLibA_08319 | 86 | -0.29281274 | CD274 | CD274 molecule [Source:HGNC Symbol;Acc:HGNC:17635] |
| ENSG00000121236 | HGLibA_51559 | 86 | -0.29281274 | TRIM6 | tripartite motif containing 6 [Source:HGNC Symbol;Acc:HGNC:16277] |
| ENSG00000121900 | HGLibA_50442 | 86 | -0.29281274 | TMEM54 | transmembrane protein 54 [Source:HGNC Symbol;Acc:HGNC:24143] |
| ENSG00000122034 | HGLibA_20578 | 86 | -0.29281274 | GTF3A | general transcription factor IIIA [Source:HGNC Symbol;Acc:HGNC:4662] |
| ENSG00000124701 | HGLibA_02469 | 86 | -0.29281274 | APOBEC2 | apolipoprotein B mRNA editing enzyme catalytic subunit 2 [Source:HGNC Symbol;Acc:HGNC:605] |
| ENSG00000125735 | HGLibA_50813 | 86 | -0.29281274 | TNFSF14 | TNF superfamily member 14 [Source:HGNC Symbol;Acc:HGNC:11930] |
| ENSG00000127948 | HGLibA_37705 | 86 | -0.29281274 | POR | cytochrome p450 oxidoreductase [Source:HGNC Symbol;Acc:HGNC:9208] |
| ENSG00000127990 | HGLibA_43805 | 86 | -0.29281274 | SGCE | sarcoglycan epsilon [Source:HGNC Symbol;Acc:HGNC:10808] |
| ENSG00000128973 | HGLibA_10082 | 86 | -0.29281274 | CLN6 | transmembrane ER protein [Source:HGNC Symbol;Acc:HGNC:2077]" |
| ENSG00000129422 | HGLibA_30505 | 86 | -0.29281274 | MTUS1 | microtubule associated scaffold protein 1 [Source:HGNC Symbol;Acc:HGNC:29789] |
| ENSG00000130164 | HGLibA_26237 | 86 | -0.29281274 | LDLR | low density lipoprotein receptor [Source:HGNC Symbol;Acc:HGNC:6547] |
| ENSG00000130477 | HGLibA_52992 | 86 | -0.29281274 | UNC13A | unc-13 homolog A [Source:HGNC Symbol;Acc:HGNC:23150] |
| ENSG00000130675 | HGLibA_29579 | 86 | -0.29281274 | MNX1 | motor neuron and pancreas homeobox 1 [Source:HGNC Symbol;Acc:HGNC:4979] |
| ENSG00000130762 | HGLibA_02822 | 86 | -0.29281274 | ARHGEF16 | Rho guanine nucleotide exchange factor 16 [Source:HGNC Symbol;Acc:HGNC:15515] |
| ENSG00000130821 | HGLibA_45340 | 86 | -0.29281274 | SLC6A8 | solute carrier family 6 member 8 [Source:HGNC Symbol;Acc:HGNC:11055] |
| ENSG00000132437 | HGLibA_12599 | 86 | -0.29281274 | DDC | dopa decarboxylase [Source:HGNC Symbol;Acc:HGNC:2719] |
| ENSG00000134058 | HGLibA_08794 | 86 | -0.29281274 | CDK7 | cyclin dependent kinase 7 [Source:HGNC Symbol;Acc:HGNC:1778] |
| ENSG00000134371 | HGLibA_08599 | 86 | -0.29281274 | CDC73 | cell division cycle 73 [Source:HGNC Symbol;Acc:HGNC:16783] |
| ENSG00000135372 | HGLibA_31168 | 86 | -0.29281274 | NAT10 | N-acetyltransferase 10 [Source:HGNC Symbol;Acc:HGNC:29830] |
| ENSG00000136237 | HGLibA_40257 | 86 | -0.29281274 | RAPGEF5 | Rap guanine nucleotide exchange factor 5 [Source:HGNC Symbol;Acc:HGNC:16862] |
| ENSG00000136688 | HGLibA_23152 | 86 | -0.29281274 | IL36G | interleukin 36 gamma [Source:HGNC Symbol;Acc:HGNC:15741] |
| ENSG00000137959 | HGLibA_22613 | 86 | -0.29281274 | IFI44L | interferon induced protein 44 like [Source:HGNC Symbol;Acc:HGNC:17817] |
| ENSG00000138796 | HGLibA_20805 | 86 | -0.29281274 | HADH | hydroxyacyl-CoA dehydrogenase [Source:HGNC Symbol;Acc:HGNC:4799] |
| ENSG00000139132 | HGLibA_17351 | 86 | -0.29281274 | FGD4 | RhoGEF and PH domain containing 4 [Source:HGNC Symbol;Acc:HGNC:19125]" |
| ENSG00000139200 | HGLibA_36505 | 86 | -0.29281274 | PIANP | PILR alpha associated neural protein [Source:HGNC Symbol;Acc:HGNC:25338] |
| ENSG00000139324 | HGLibA_50676 | 86 | -0.29281274 | TMTC3 | transmembrane and tetratricopeptide repeat containing 3 [Source:HGNC Symbol;Acc:HGNC:26899] |
| ENSG00000139988 | HGLibA_40738 | 86 | -0.29281274 | RDH12 | retinol dehydrogenase 12 [Source:HGNC Symbol;Acc:HGNC:19977] |
| ENSG00000140015 | HGLibA_24223 | 86 | -0.29281274 | KCNH5 | potassium voltage-gated channel subfamily H member 5 [Source:HGNC Symbol;Acc:HGNC:6254] |
| ENSG00000140451 | HGLibA_36536 | 86 | -0.29281274 | PIF1 | PIF1 5'-to-3' DNA helicase [Source:HGNC Symbol;Acc:HGNC:26220] |
| ENSG00000141295 | HGLibA_43052 | 86 | -0.29281274 | SCRN2 | secernin 2 [Source:HGNC Symbol;Acc:HGNC:30381] |
| ENSG00000141519 | HGLibA_07776 | 86 | -0.29281274 | CCDC40 | coiled-coil domain containing 40 [Source:HGNC Symbol;Acc:HGNC:26090] |
| ENSG00000142173 | HGLibA_10600 | 86 | -0.29281274 | COL6A2 | collagen type VI alpha 2 chain [Source:HGNC Symbol;Acc:HGNC:2212] |
| ENSG00000142185 | HGLibA_51755 | 86 | -0.29281274 | TRPM2 | transient receptor potential cation channel subfamily M member 2 [Source:HGNC Symbol;Acc:HGNC:12339] |
| ENSG00000142784 | HGLibA_54232 | 86 | -0.29281274 | WDTC1 | WD and tetratricopeptide repeats 1 [Source:HGNC Symbol;Acc:HGNC:29175] |
| ENSG00000142961 | HGLibA_29601 | 86 | -0.29281274 | MOB3C | MOB kinase activator 3C [Source:HGNC Symbol;Acc:HGNC:29800] |
| ENSG00000143318 | HGLibA_07333 | 86 | -0.29281274 | CASQ1 | calsequestrin 1 [Source:HGNC Symbol;Acc:HGNC:1512] |
| ENSG00000144868 | HGLibA_49864 | 86 | -0.29281274 | TMEM108 | transmembrane protein 108 [Source:HGNC Symbol;Acc:HGNC:28451] |
| ENSG00000145526 | HGLibA_08652 | 86 | -0.29281274 | CDH18 | cadherin 18 [Source:HGNC Symbol;Acc:HGNC:1757] |
| ENSG00000146038 | HGLibA_12483 | 86 | -0.29281274 | DCDC2 | doublecortin domain containing 2 [Source:HGNC Symbol;Acc:HGNC:18141] |
| ENSG00000146374 | HGLibA_42350 | 86 | -0.29281274 | RSPO3 | R-spondin 3 [Source:HGNC Symbol;Acc:HGNC:20866] |
| ENSG00000147614 | HGLibA_03722 | 86 | -0.29281274 | ATP6V0D2 | ATPase H+ transporting V0 subunit d2 [Source:HGNC Symbol;Acc:HGNC:18266] |
| ENSG00000147885 | HGLibA_22668 | 86 | -0.29281274 | IFNA16 | interferon alpha 16 [Source:HGNC Symbol;Acc:HGNC:5421] |
| ENSG00000148942 | HGLibA_45261 | 86 | -0.29281274 | SLC5A12 | solute carrier family 5 member 12 [Source:HGNC Symbol;Acc:HGNC:28750] |
| ENSG00000149742 | HGLibA_44603 | 86 | -0.29281274 | SLC22A9 | solute carrier family 22 member 9 [Source:HGNC Symbol;Acc:HGNC:16261] |
| ENSG00000150477 | HGLibA_24742 | 86 | -0.29281274 | KIAA1328 | KIAA1328 [Source:HGNC Symbol;Acc:HGNC:29248] |
| ENSG00000151914 | HGLibA_14042 | 86 | -0.29281274 | DST | dystonin [Source:HGNC Symbol;Acc:HGNC:1090] |
| ENSG00000152422 | HGLibA_54592 | 86 | -0.29281274 | XRCC4 | X-ray repair cross complementing 4 [Source:HGNC Symbol;Acc:HGNC:12831] |
| ENSG00000152518 | HGLibA_55211 | 86 | -0.29281274 | ZFP36L2 | ZFP36 ring finger protein like 2 [Source:HGNC Symbol;Acc:HGNC:1108] |
| ENSG00000153113 | HGLibA_07346 | 86 | -0.29281274 | CAST | calpastatin [Source:HGNC Symbol;Acc:HGNC:1515] |
| ENSG00000155506 | HGLibA_26027 | 86 | -0.29281274 | LARP1 | La ribonucleoprotein domain family member 1 [Source:HGNC Symbol;Acc:HGNC:29531] |
| ENSG00000155886 | HGLibA_44617 | 86 | -0.29281274 | SLC24A2 | solute carrier family 24 member 2 [Source:HGNC Symbol;Acc:HGNC:10976] |
| ENSG00000155903 | HGLibA_40300 | 86 | -0.29281274 | RASA2 | RAS p21 protein activator 2 [Source:HGNC Symbol;Acc:HGNC:9872] |
| ENSG00000156687 | HGLibA_53025 | 86 | -0.29281274 | UNC5D | unc-5 netrin receptor D [Source:HGNC Symbol;Acc:HGNC:18634] |
| ENSG00000156802 | HGLibA_03403 | 86 | -0.29281274 | ATAD2 | AAA domain containing 2 [Source:HGNC Symbol;Acc:HGNC:30123]" |
| ENSG00000159216 | HGLibA_42456 | 86 | -0.29281274 | RUNX1 | runt related transcription factor 1 [Source:HGNC Symbol;Acc:HGNC:10471] |
| ENSG00000160868 | HGLibA_12154 | 86 | -0.29281274 | CYP3A4 | cytochrome P450 family 3 subfamily A member 4 [Source:HGNC Symbol;Acc:HGNC:2637] |
| ENSG00000162641 | HGLibA_01467 | 86 | -0.29281274 | AKNAD1 | AKNA domain containing 1 [Source:HGNC Symbol;Acc:HGNC:28398] |
| ENSG00000163006 | HGLibA_07610 | 86 | -0.29281274 | CCDC138 | coiled-coil domain containing 138 [Source:HGNC Symbol;Acc:HGNC:26531] |
| ENSG00000163126 | HGLibA_02043 | 86 | -0.29281274 | ANKRD23 | ankyrin repeat domain 23 [Source:HGNC Symbol;Acc:HGNC:24470] |
| ENSG00000163157 | HGLibA_50603 | 86 | -0.29281274 | TMOD4 | tropomodulin 4 [Source:HGNC Symbol;Acc:HGNC:11874] |
| ENSG00000163501 | HGLibA_22907 | 86 | -0.29281274 | IHH | indian hedgehog [Source:HGNC Symbol;Acc:HGNC:5956] |
| ENSG00000163960 | HGLibA_52794 | 86 | -0.29281274 | UBXN7 | UBX domain protein 7 [Source:HGNC Symbol;Acc:HGNC:29119] |
| ENSG00000164120 | HGLibA_22031 | 86 | -0.29281274 | HPGD | 15-hydroxyprostaglandin dehydrogenase [Source:HGNC Symbol;Acc:HGNC:5154] |
| ENSG00000164308 | HGLibA_15407 | 86 | -0.29281274 | ERAP2 | endoplasmic reticulum aminopeptidase 2 [Source:HGNC Symbol;Acc:HGNC:29499] |
| ENSG00000164483 | HGLibA_42681 | 86 | -0.29281274 | SAMD3 | sterile alpha motif domain containing 3 [Source:HGNC Symbol;Acc:HGNC:21574] |
| ENSG00000164754 | HGLibA_40062 | 86 | -0.29281274 | RAD21 | RAD21 cohesin complex component [Source:HGNC Symbol;Acc:HGNC:9811] |
| ENSG00000164776 | HGLibA_36410 | 86 | -0.29281274 | PHKG1 | phosphorylase kinase catalytic subunit gamma 1 [Source:HGNC Symbol;Acc:HGNC:8930] |
| ENSG00000164920 | HGLibA_34703 | 86 | -0.29281274 | OSR2 | odd-skipped related transciption factor 2 [Source:HGNC Symbol;Acc:HGNC:15830] |
| ENSG00000165102 | HGLibA_21250 | 86 | -0.29281274 | HGSNAT | heparan-alpha-glucosaminide N-acetyltransferase [Source:HGNC Symbol;Acc:HGNC:26527] |
| ENSG00000165609 | HGLibA_32982 | 86 | -0.29281274 | NUDT5 | nudix hydrolase 5 [Source:HGNC Symbol;Acc:HGNC:8052] |
| ENSG00000166266 | HGLibA_11789 | 86 | -0.29281274 | CUL5 | cullin 5 [Source:HGNC Symbol;Acc:HGNC:2556] |
| ENSG00000166569 | HGLibA_10940 | 86 | -0.29281274 | CPLX4 | complexin 4 [Source:HGNC Symbol;Acc:HGNC:24330] |
| ENSG00000167107 | HGLibA_00548 | 86 | -0.29281274 | ACSF2 | acyl-CoA synthetase family member 2 [Source:HGNC Symbol;Acc:HGNC:26101] |
| ENSG00000167967 | HGLibA_14331 | 86 | -0.29281274 | E4F1 | E4F transcription factor 1 [Source:HGNC Symbol;Acc:HGNC:3121] |
| ENSG00000168016 | HGLibA_51290 | 86 | -0.29281274 | TRANK1 | tetratricopeptide repeat and ankyrin repeat containing 1 [Source:HGNC Symbol;Acc:HGNC:29011] |
| ENSG00000168795 | HGLibA_54911 | 86 | -0.29281274 | ZBTB5 | zinc finger and BTB domain containing 5 [Source:HGNC Symbol;Acc:HGNC:23836] |
| ENSG00000169507 | HGLibA_45061 | 86 | -0.29281274 | SLC38A11 | solute carrier family 38 member 11 [Source:HGNC Symbol;Acc:HGNC:26836] |
| ENSG00000169876 | HGLibA_30531 | 86 | -0.29281274 | MUC17 | cell surface associated [Source:HGNC Symbol;Acc:HGNC:16800]" |
| ENSG00000169906 | HGLibA_42581 | 86 | -0.29281274 | S100G | S100 calcium binding protein G [Source:HGNC Symbol;Acc:HGNC:1436] |
| ENSG00000170581 | HGLibA_47385 | 86 | -0.29281274 | STAT2 | signal transducer and activator of transcription 2 [Source:HGNC Symbol;Acc:HGNC:11363] |
| ENSG00000171033 | HGLibA_36818 | 86 | -0.29281274 | PKIA | cAMP-dependent protein kinase inhibitor alpha [Source:HGNC Symbol;Acc:HGNC:9017] |
| ENSG00000171777 | HGLibA_40352 | 86 | -0.29281274 | RASGRP4 | RAS guanyl releasing protein 4 [Source:HGNC Symbol;Acc:HGNC:18958] |
| ENSG00000171791 | HGLibA_04295 | 86 | -0.29281274 | BCL2 | apoptosis regulator [Source:HGNC Symbol;Acc:HGNC:990]" |
| ENSG00000171824 | HGLibA_15771 | 86 | -0.29281274 | EXOSC10 | exosome component 10 [Source:HGNC Symbol;Acc:HGNC:9138] |
| ENSG00000172289 | HGLibA_33552 | 86 | -0.29281274 | OR10V1 | olfactory receptor family 10 subfamily V member 1 [Source:HGNC Symbol;Acc:HGNC:15136] |
| ENSG00000172724 | HGLibA_07985 | 86 | -0.29281274 | CCL19 | C-C motif chemokine ligand 19 [Source:HGNC Symbol;Acc:HGNC:10617] |
| ENSG00000174948 | HGLibA_19924 | 86 | -0.29281274 | GPR149 | G protein-coupled receptor 149 [Source:HGNC Symbol;Acc:HGNC:23627] |
| ENSG00000174992 | HGLibA_55296 | 86 | -0.29281274 | ZG16 | zymogen granule protein 16 [Source:HGNC Symbol;Acc:HGNC:30961] |
| ENSG00000176087 | HGLibA_44956 | 86 | -0.29281274 | SLC35A4 | solute carrier family 35 member A4 [Source:HGNC Symbol;Acc:HGNC:20753] |
| ENSG00000176531 | HGLibA_36431 | 86 | -0.29281274 | PHLDB3 | pleckstrin homology like domain family B member 3 [Source:HGNC Symbol;Acc:HGNC:30499] |
| ENSG00000176658 | HGLibA_30830 | 86 | -0.29281274 | MYO1D | myosin ID [Source:HGNC Symbol;Acc:HGNC:7598] |
| ENSG00000177992 | HGLibA_46505 | 86 | -0.29281274 | SPATA31E1 | SPATA31 subfamily E member 1 [Source:HGNC Symbol;Acc:HGNC:26672] |
| ENSG00000178053 | HGLibA_29371 | 86 | -0.29281274 | MLF1 | myeloid leukemia factor 1 [Source:HGNC Symbol;Acc:HGNC:7125] |
| ENSG00000178127 | HGLibA_31571 | 86 | -0.29281274 | NDUFV2 | NADH:ubiquinone oxidoreductase core subunit V2 [Source:HGNC Symbol;Acc:HGNC:7717] |
| ENSG00000179083 | HGLibA_16117 | 86 | -0.29281274 | FAM133A | family with sequence similarity 133 member A [Source:HGNC Symbol;Acc:HGNC:26748] |
| ENSG00000179627 | HGLibA_54885 | 86 | -0.29281274 | ZBTB42 | zinc finger and BTB domain containing 42 [Source:HGNC Symbol;Acc:HGNC:32550] |
| ENSG00000180071 | HGLibA_02018 | 86 | -0.29281274 | ANKRD18A | ankyrin repeat domain 18A [Source:HGNC Symbol;Acc:HGNC:23643] |
| ENSG00000181007 | HGLibA_55238 | 86 | -0.29281274 | ZFP82 | ZFP82 zinc finger protein [Source:HGNC Symbol;Acc:HGNC:28682] |
| ENSG00000182866 | HGLibA_26153 | 86 | -0.29281274 | LCK | Src family tyrosine kinase [Source:HGNC Symbol;Acc:HGNC:6524]" |
| ENSG00000183313 | HGLibA_34188 | 86 | -0.29281274 | OR52L1 | olfactory receptor family 52 subfamily L member 1 [Source:HGNC Symbol;Acc:HGNC:14785] |
| ENSG00000183723 | HGLibA_10217 | 86 | -0.29281274 | CMTM4 | CKLF like MARVEL transmembrane domain containing 4 [Source:HGNC Symbol;Acc:HGNC:19175] |
| ENSG00000183753 | HGLibA_04743 | 86 | -0.29281274 | BPY2 | basic charge Y-linked 2 [Source:HGNC Symbol;Acc:HGNC:13508] |
| ENSG00000184210 | HGLibA_13040 | 86 | -0.29281274 | DGAT2L6 | diacylglycerol O-acyltransferase 2 like 6 [Source:HGNC Symbol;Acc:HGNC:23250] |
| ENSG00000184371 | HGLibA_11321 | 86 | -0.29281274 | CSF1 | colony stimulating factor 1 [Source:HGNC Symbol;Acc:HGNC:2432] |
| ENSG00000185149 | HGLibA_32554 | 86 | -0.29281274 | NPY2R | neuropeptide Y receptor Y2 [Source:HGNC Symbol;Acc:HGNC:7957] |
| ENSG00000186350 | HGLibA_42507 | 86 | -0.29281274 | RXRA | retinoid X receptor alpha [Source:HGNC Symbol;Acc:HGNC:10477] |
| ENSG00000186471 | HGLibA_01419 | 86 | -0.29281274 | AKAP14 | A-kinase anchoring protein 14 [Source:HGNC Symbol;Acc:HGNC:24061] |
| ENSG00000187372 | HGLibA_35481 | 86 | -0.29281274 | PCDHB13 | protocadherin beta 13 [Source:HGNC Symbol;Acc:HGNC:8684] |
| ENSG00000188107 | HGLibA_15830 | 86 | -0.29281274 | EYS | eyes shut homolog [Source:HGNC Symbol;Acc:HGNC:21555] |
| ENSG00000188175 | HGLibA_21142 | 86 | -0.29281274 | HEPACAM2 | HEPACAM family member 2 [Source:HGNC Symbol;Acc:HGNC:27364] |
| ENSG00000188306 | HGLibA_27460 | 86 | -0.29281274 | LRRIQ4 | leucine rich repeats and IQ motif containing 4 [Source:HGNC Symbol;Acc:HGNC:34298] |
| ENSG00000188508 | HGLibA_25860 | 86 | -0.29281274 | KRTDAP | keratinocyte differentiation associated protein [Source:HGNC Symbol;Acc:HGNC:16313] |
| ENSG00000188779 | HGLibA_44291 | 86 | -0.29281274 | SKOR1 | SKI family transcriptional corepressor 1 [Source:HGNC Symbol;Acc:HGNC:21326] |
| ENSG00000189120 | HGLibA_46308 | 86 | -0.29281274 | SP6 | Sp6 transcription factor [Source:HGNC Symbol;Acc:HGNC:14530] |
| ENSG00000197013 | HGLibA_55971 | 86 | -0.29281274 | ZNF429 | zinc finger protein 429 [Source:HGNC Symbol;Acc:HGNC:20817] |
| ENSG00000197079 | HGLibA_25489 | 86 | -0.29281274 | KRT35 | keratin 35 [Source:HGNC Symbol;Acc:HGNC:6453] |
| ENSG00000197363 | HGLibA_56147 | 86 | -0.29281274 | ZNF517 | zinc finger protein 517 [Source:HGNC Symbol;Acc:HGNC:27984] |
| ENSG00000204501 | HGLibA_38293 | 86 | -0.29281274 | PRAMEF15 | PRAME family member 15 [Source:HGNC Symbol;Acc:HGNC:26764] |
| ENSG00000204599 | HGLibA_51483 | 86 | -0.29281274 | TRIM39 | tripartite motif containing 39 [Source:HGNC Symbol;Acc:HGNC:10065] |
| ENSG00000204859 | HGLibA_54905 | 86 | -0.29281274 | ZBTB48 | zinc finger and BTB domain containing 48 [Source:HGNC Symbol;Acc:HGNC:4930] |
| ENSG00000204963 | HGLibA_35454 | 86 | -0.29281274 | PCDHA7 | protocadherin alpha 7 [Source:HGNC Symbol;Acc:HGNC:8673] |
| ENSG00000206075 | HGLibA_43557 | 86 | -0.29281274 | SERPINB5 | serpin family B member 5 [Source:HGNC Symbol;Acc:HGNC:8949] |
| ENSG00000213931 | HGLibA_20919 | 86 | -0.29281274 | HBE1 | hemoglobin subunit epsilon 1 [Source:HGNC Symbol;Acc:HGNC:4830] |
| ENSG00000214736 | HGLibA_50975 | 86 | -0.29281274 | TOMM6 | translocase of outer mitochondrial membrane 6 [Source:HGNC Symbol;Acc:HGNC:34528] |
| ENSG00000235109 | HGLibA_57020 | 86 | -0.29281274 | ZSCAN31 | zinc finger and SCAN domain containing 31 [Source:HGNC Symbol;Acc:HGNC:14097] |
| ENSG00000236311 | HGLibA_49673 | 86 | -0.29281274 | TLX1NB | TLX1 neighbor [Source:HGNC Symbol;Acc:HGNC:37183] |
| ENSG00000237541 | HGLibA_21624 | 86 | -0.29281274 | HLA-DQA2 | DQ alpha 2 [Source:HGNC Symbol;Acc:HGNC:4943]" |

| | | | | | |
|---|---|---|---|---|---|
| ENSG00000240871 | HGLibA_25772 | 86 | -0.29281274 | KRTAP4-7 | keratin associated protein 4-7 [Source:HGNC Symbol;Acc:HGNC:18898] |
| ENSG00000242362 | HGLibA_11527 | 86 | -0.29281274 | CT47A2 | member A2 [Source:HGNC Symbol;Acc:HGNC:33283]" |
| ENSG00000242875 | HGLibA_40615 | 86 | -0.29281274 | RBMY1B | member B [Source:HGNC Symbol;Acc:HGNC:23914]" |
| ENSG00000243279 | HGLibA_38270 | 86 | -0.29281274 | PRAF2 | PRA1 domain family member 2 [Source:HGNC Symbol;Acc:HGNC:28911] |
| ENSG00000249158 | HGLibA_35430 | 86 | -0.29281274 | PCDHA11 | protocadherin alpha 11 [Source:HGNC Symbol;Acc:HGNC:8665] |
| ENSG00000254726 | HGLibA_29026 | 86 | -0.29281274 | MEX3A | mex-3 RNA binding family member A [Source:HGNC Symbol;Acc:HGNC:33482] |
| ENSG00000255526 | HGLibA_31611 | 86 | -0.29281274 | NEDD8-MDP1 | NEDD8-MDP1 readthrough [Source:HGNC Symbol;Acc:HGNC:39551] |
| ENSG00000256453 | HGLibA_13671 | 86 | -0.29281274 | DND1 | DND microRNA-mediated repression inhibitor 1 [Source:HGNC Symbol;Acc:HGNC:23799] |
| ENSG00000260458 | HGLibA_24271 | 86 | -0.29281274 | KCNJ18 | potassium voltage-gated channel subfamily J member 18 [Source:HGNC Symbol;Acc:HGNC:39080] |
| ENSG00000276070 | HGLibA_08034 | 86 | -0.29281274 | CCL4L2 | C-C motif chemokine ligand 4 like 2 [Source:HGNC Symbol;Acc:HGNC:24066] |
| FAM84A | HGLibA_16764 | 86 | -0.29281274 | FAM84A | - |
| FLJ22184 | HGLibA_17620 | 86 | -0.29281274 | FLJ22184 | - |
| H2BFM | HGLibA_20771 | 86 | -0.29281274 | H2BFM | - |
| hsa-let-7i | HGLibA_57137 | 86 | -0.29281274 | hsa-let-7i | - |
| hsa-mir-1178 | HGLibA_57207 | 86 | -0.29281274 | hsa-mir-1178 | - |
| hsa-mir-126 | HGLibA_57455 | 86 | -0.29281274 | hsa-mir-126 | - |
| hsa-mir-1307 | HGLibA_57742 | 86 | -0.29281274 | hsa-mir-1307 | - |
| hsa-mir-141 | HGLibA_57829 | 86 | -0.29281274 | hsa-mir-141 | - |
| hsa-mir-153-2 | HGLibA_57913 | 86 | -0.29281274 | hsa-mir-153-2 | - |
| hsa-mir-1538 | HGLibA_57919 | 86 | -0.29281274 | hsa-mir-1538 | - |
| hsa-mir-190a | HGLibA_58035 | 86 | -0.29281274 | hsa-mir-190a | - |
| hsa-mir-222 | HGLibA_58297 | 86 | -0.29281274 | hsa-mir-222 | - |
| hsa-mir-33b | HGLibA_59014 | 86 | -0.29281274 | hsa-mir-33b | - |
| hsa-mir-362 | HGLibA_59111 | 86 | -0.29281274 | hsa-mir-362 | - |
| hsa-mir-3620 | HGLibA_59114 | 86 | -0.29281274 | hsa-mir-3620 | - |
| hsa-mir-3677 | HGLibA_59257 | 86 | -0.29281274 | hsa-mir-3677 | - |
| hsa-mir-3680-2 | HGLibA_59274 | 86 | -0.29281274 | hsa-mir-3680-2 | - |
| hsa-mir-374c | HGLibA_59397 | 86 | -0.29281274 | hsa-mir-374c | - |
| hsa-mir-3917 | HGLibA_59545 | 86 | -0.29281274 | hsa-mir-3917 | - |
| hsa-mir-3942 | HGLibA_59629 | 86 | -0.29281274 | hsa-mir-3942 | - |
| hsa-mir-4325 | HGLibA_60022 | 86 | -0.29281274 | hsa-mir-4325 | - |
| hsa-mir-4436b-2 | HGLibA_60145 | 86 | -0.29281274 | hsa-mir-4436b-2 | - |
| hsa-mir-4437 | HGLibA_60150 | 86 | -0.29281274 | hsa-mir-4437 | - |
| hsa-mir-4673 | HGLibA_60810 | 86 | -0.29281274 | hsa-mir-4673 | - |
| hsa-mir-4770 | HGLibA_61176 | 86 | -0.29281274 | hsa-mir-4770 | - |
| hsa-mir-4776-1 | HGLibA_61209 | 86 | -0.29281274 | hsa-mir-4776-1 | - |
| hsa-mir-4798 | HGLibA_61298 | 86 | -0.29281274 | hsa-mir-4798 | - |
| hsa-mir-548a-2 | HGLibA_61839 | 86 | -0.29281274 | hsa-mir-548a-2 | - |
| hsa-mir-548q | HGLibA_62060 | 86 | -0.29281274 | hsa-mir-548q | - |
| hsa-mir-555 | HGLibA_62135 | 86 | -0.29281274 | hsa-mir-555 | - |
| hsa-mir-562 | HGLibA_62211 | 86 | -0.29281274 | hsa-mir-562 | - |
| hsa-mir-641 | HGLibA_62805 | 86 | -0.29281274 | hsa-mir-641 | - |
| hsa-mir-6511b-2 | HGLibA_62919 | 86 | -0.29281274 | hsa-mir-6511b-2 | - |
| hsa-mir-6753 | HGLibA_63170 | 86 | -0.29281274 | hsa-mir-6753 | - |
| hsa-mir-6798 | HGLibA_63369 | 86 | -0.29281274 | hsa-mir-6798 | - |
| hsa-mir-7112-1 | HGLibA_63811 | 86 | -0.29281274 | hsa-mir-7112-1 | - |
| hsa-mir-766 | HGLibA_63926 | 86 | -0.29281274 | hsa-mir-766 | - |
| KCNE1L | HGLibA_24187 | 86 | -0.29281274 | KCNE1L | - |
| KIAA0317 | HGLibA_24617 | 86 | -0.29281274 | KIAA0317 | - |
| LOC388849 | HGLibA_26927 | 86 | -0.29281274 | LOC388849 | - |
| NARS | HGLibA_31155 | 86 | -0.29281274 | NARS | - |
| P2RY8_X | HGLibA_34894 | 86 | -0.29281274 | P2RY8_X | - |
| ADCK4 | HGLibA_00900 | 85 | -0.302763778 | ADCK4 | - |
| ATP5S | HGLibA_03689 | 85 | -0.302763778 | ATP5S | - |
| C1orf111 | HGLibA_05748 | 85 | -0.302763778 | C1orf111 | - |
| C1orf61 | HGLibA_05880 | 85 | -0.302763778 | C1orf61 | - |
| C20orf24 | HGLibA_06016 | 85 | -0.302763778 | C20orf24 | - |
| C9orf169 | HGLibA_06704 | 85 | -0.302763778 | C9orf169 | - |
| CCDC67 | HGLibA_07838 | 85 | -0.302763778 | CCDC67 | - |
| ENSG00000000938 | HGLibA_17467 | 85 | -0.302763778 | FGR | Src family tyrosine kinase [Source:HGNC Symbol;Acc:HGNC:3697]" |
| ENSG00000002549 | HGLibA_26013 | 85 | -0.302763778 | LAP3 | leucine aminopeptidase 3 [Source:HGNC Symbol;Acc:HGNC:18449] |
| ENSG00000005020 | HGLibA_44274 | 85 | -0.302763778 | SKAP2 | src kinase associated phosphoprotein 2 [Source:HGNC Symbol;Acc:HGNC:15687] |
| ENSG00000005801 | HGLibA_55558 | 85 | -0.302763778 | ZNF195 | zinc finger protein 195 [Source:HGNC Symbol;Acc:HGNC:12986] |
| ENSG00000006611 | HGLibA_53173 | 85 | -0.302763778 | USH1C | USH1 protein network component harmonin [Source:HGNC Symbol;Acc:HGNC:12597] |
| ENSG00000021488 | HGLibA_45386 | 85 | -0.302763778 | SLC7A9 | solute carrier family 7 member 9 [Source:HGNC Symbol;Acc:HGNC:11067] |
| ENSG00000025796 | HGLibA_43261 | 85 | -0.302763778 | SEC63 | protein translocation regulator [Source:HGNC Symbol;Acc:HGNC:21082]" |
| ENSG00000033170 | HGLibA_18206 | 85 | -0.302763778 | FUT8 | fucosyltransferase 8 [Source:HGNC Symbol;Acc:HGNC:4019] |
| ENSG00000049540 | HGLibA_14951 | 85 | -0.302763778 | ELN | elastin [Source:HGNC Symbol;Acc:HGNC:3327] |
| ENSG00000050393 | HGLibA_28664 | 85 | -0.302763778 | MCUR1 | mitochondrial calcium uniporter regulator 1 [Source:HGNC Symbol;Acc:HGNC:21097] |
| ENSG00000063244 | HGLibA_52498 | 85 | -0.302763778 | U2AF2 | U2 small nuclear RNA auxiliary factor 2 [Source:HGNC Symbol;Acc:HGNC:23156] |
| ENSG00000074370 | HGLibA_03612 | 85 | -0.302763778 | ATP2A3 | ATPase sarcoplasmic/endoplasmic reticulum Ca2+ transporting 3 [Source:HGNC Symbol;Acc:HGNC:813] |
| ENSG00000075651 | HGLibA_37036 | 85 | -0.302763778 | PLD1 | phospholipase D1 [Source:HGNC Symbol;Acc:HGNC:9067] |
| ENSG00000079150 | HGLibA_17585 | 85 | -0.302763778 | FKBP7 | FK506 binding protein 7 [Source:HGNC Symbol;Acc:HGNC:3723] |
| ENSG00000079385 | HGLibA_08923 | 85 | -0.302763778 | CEACAM1 | carcinoembryonic antigen related cell adhesion molecule 1 [Source:HGNC Symbol;Acc:HGNC:1814] |
| ENSG00000087085 | HGLibA_00436 | 85 | -0.302763778 | ACHE | acetylcholinesterase (Cartwright blood group) [Source:HGNC Symbol;Acc:HGNC:108] |
| ENSG00000087495 | HGLibA_36302 | 85 | -0.302763778 | PHACTR3 | phosphatase and actin regulator 3 [Source:HGNC Symbol;Acc:HGNC:15833] |
| ENSG00000088386 | HGLibA_44406 | 85 | -0.302763778 | SLC15A1 | solute carrier family 15 member 1 [Source:HGNC Symbol;Acc:HGNC:10920] |
| ENSG00000091073 | HGLibA_14081 | 85 | -0.302763778 | DTX2 | deltex E3 ubiquitin ligase 2 [Source:HGNC Symbol;Acc:HGNC:15973] |
| ENSG00000099617 | HGLibA_14586 | 85 | -0.302763778 | EFNA2 | ephrin A2 [Source:HGNC Symbol;Acc:HGNC:3222] |
| ENSG00000100106 | HGLibA_51631 | 85 | -0.302763778 | TRIOBP | TRIO and F-actin binding protein [Source:HGNC Symbol;Acc:HGNC:17009] |
| ENSG00000100412 | HGLibA_00464 | 85 | -0.302763778 | ACO2 | aconitase 2 [Source:HGNC Symbol;Acc:HGNC:118] |
| ENSG00000100558 | HGLibA_37060 | 85 | -0.302763778 | PLEK2 | pleckstrin 2 [Source:HGNC Symbol;Acc:HGNC:19238] |
| ENSG00000101280 | HGLibA_01908 | 85 | -0.302763778 | ANGPT4 | angiopoietin 4 [Source:HGNC Symbol;Acc:HGNC:487] |
| ENSG00000101292 | HGLibA_38673 | 85 | -0.302763778 | PROKR2 | prokineticin receptor 2 [Source:HGNC Symbol;Acc:HGNC:15836] |
| ENSG00000101310 | HGLibA_43222 | 85 | -0.302763778 | SEC23B | coat complex II component [Source:HGNC Symbol;Acc:HGNC:10702]" |
| ENSG00000101955 | HGLibA_47032 | 85 | -0.302763778 | SRPX | sushi repeat containing protein X-linked [Source:HGNC Symbol;Acc:HGNC:11309] |
| ENSG00000102908 | HGLibA_31770 | 85 | -0.302763778 | NFAT5 | nuclear factor of activated T cells 5 [Source:HGNC Symbol;Acc:HGNC:7774] |
| ENSG00000103550 | HGLibA_25345 | 85 | -0.302763778 | KNOP1 | lysine rich nucleolar protein 1 [Source:HGNC Symbol;Acc:HGNC:34404] |
| ENSG00000104164 | HGLibA_04572 | 85 | -0.302763778 | BLOC1S6 | biogenesis of lysosomal organelles complex 1 subunit 6 [Source:HGNC Symbol;Acc:HGNC:8549] |
| ENSG00000104863 | HGLibA_26577 | 85 | -0.302763778 | LIN7B | crumbs cell polarity complex component [Source:HGNC Symbol;Acc:HGNC:17788]" |
| ENSG00000105583 | HGLibA_54195 | 85 | -0.302763778 | WDR83OS | WD repeat domain 83 opposite strand [Source:HGNC Symbol;Acc:HGNC:30203] |
| ENSG00000106089 | HGLibA_47635 | 85 | -0.302763778 | STX1A | syntaxin 1A [Source:HGNC Symbol;Acc:HGNC:11433] |
| ENSG00000106302 | HGLibA_22446 | 85 | -0.302763778 | HYAL4 | hyaluronidase 4 [Source:HGNC Symbol;Acc:HGNC:5323] |
| ENSG00000106330 | HGLibA_29690 | 85 | -0.302763778 | MOSPD3 | motile sperm domain containing 3 [Source:HGNC Symbol;Acc:HGNC:25078] |
| ENSG00000106692 | HGLibA_17601 | 85 | -0.302763778 | FKTN | fukutin [Source:HGNC Symbol;Acc:HGNC:3622] |
| ENSG00000106733 | HGLibA_32195 | 85 | -0.302763778 | NMRK1 | nicotinamide riboside kinase 1 [Source:HGNC Symbol;Acc:HGNC:26057] |
| ENSG00000107104 | HGLibA_24024 | 85 | -0.302763778 | KANK1 | KN motif and ankyrin repeat domains 1 [Source:HGNC Symbol;Acc:HGNC:19309] |
| ENSG00000108479 | HGLibA_18515 | 85 | -0.302763778 | GALK1 | galactokinase 1 [Source:HGNC Symbol;Acc:HGNC:4118] |
| ENSG00000108819 | HGLibA_38132 | 85 | -0.302763778 | PPP1R9B | protein phosphatase 1 regulatory subunit 9B [Source:HGNC Symbol;Acc:HGNC:9298] |
| ENSG00000111605 | HGLibA_11012 | 85 | -0.302763778 | CPSF6 | cleavage and polyadenylation specific factor 6 [Source:HGNC Symbol;Acc:HGNC:13871] |
| ENSG00000112276 | HGLibA_05003 | 85 | -0.302763778 | BVES | blood vessel epicardial substance [Source:HGNC Symbol;Acc:HGNC:1152] |
| ENSG00000112308 | HGLibA_06503 | 85 | -0.302763778 | C6orf62 | chromosome 6 open reading frame 62 [Source:HGNC Symbol;Acc:HGNC:20998] |
| ENSG00000112699 | HGLibA_19393 | 85 | -0.302763778 | GMDS | 6-dehydratase [Source:HGNC Symbol;Acc:HGNC:4369]" |
| ENSG00000113916 | HGLibA_04333 | 85 | -0.302763778 | BCL6 | B cell CLL/lymphoma 6 [Source:HGNC Symbol;Acc:HGNC:1001] |
| ENSG00000115392 | HGLibA_16858 | 85 | -0.302763778 | FANCL | FA complementation group L [Source:HGNC Symbol;Acc:HGNC:20748] |
| ENSG00000115425 | HGLibA_36022 | 85 | -0.302763778 | PECR | peroxisomal trans-2-enoyl-CoA reductase [Source:HGNC Symbol;Acc:HGNC:18281] |
| ENSG00000117020 | HGLibA_01513 | 85 | -0.302763778 | AKT3 | AKT serine/threonine kinase 3 [Source:HGNC Symbol;Acc:HGNC:393] |
| ENSG00000117152 | HGLibA_41095 | 85 | -0.302763778 | RGS4 | regulator of G protein signaling 4 [Source:HGNC Symbol;Acc:HGNC:10000] |
| ENSG00000118292 | HGLibA_05873 | 85 | -0.302763778 | C1orf54 | chromosome 1 open reading frame 54 [Source:HGNC Symbol;Acc:HGNC:26258] |
| ENSG00000120251 | HGLibA_20263 | 85 | -0.302763778 | GRIA2 | glutamate ionotropic receptor AMPA type subunit 2 [Source:HGNC Symbol;Acc:HGNC:4572] |
| ENSG00000121207 | HGLibA_27132 | 85 | -0.302763778 | LRAT | lecithin retinol acyltransferase [Source:HGNC Symbol;Acc:HGNC:6685] |
| ENSG00000121774 | HGLibA_24567 | 85 | -0.302763778 | KHDRBS1 | signal transduction associated 1 [Source:HGNC Symbol;Acc:HGNC:18116]" |
| ENSG00000122696 | HGLibA_44765 | 85 | -0.302763778 | SLC25A51 | solute carrier family 25 member 51 [Source:HGNC Symbol;Acc:HGNC:23323] |
| ENSG00000122861 | HGLibA_36964 | 85 | -0.302763778 | PLAU | urokinase [Source:HGNC Symbol;Acc:HGNC:9052]" |
| ENSG00000123933 | HGLibA_30616 | 85 | -0.302763778 | MXD4 | MAX dimerization protein 4 [Source:HGNC Symbol;Acc:HGNC:13906] |
| ENSG00000124422 | HGLibA_53305 | 85 | -0.302763778 | USP22 | ubiquitin specific peptidase 22 [Source:HGNC Symbol;Acc:HGNC:12621] |
| ENSG00000124440 | HGLibA_21303 | 85 | -0.302763778 | HIF3A | hypoxia inducible factor 3 subunit alpha [Source:HGNC Symbol;Acc:HGNC:15825] |
| ENSG00000124678 | HGLibA_48842 | 85 | -0.302763778 | TCP11 | t-complex 11 [Source:HGNC Symbol;Acc:HGNC:11658] |
| ENSG00000125458 | HGLibA_32799 | 85 | -0.302763778 | NT5C | cytosolic [Source:HGNC Symbol;Acc:HGNC:17144]" |
| ENSG00000126838 | HGLibA_39714 | 85 | -0.302763778 | PZP | alpha-2-macroglobulin like [Source:HGNC Symbol;Acc:HGNC:9750]" |
| ENSG00000128284 | HGLibA_02533 | 85 | -0.302763778 | APOL3 | apolipoprotein L3 [Source:HGNC Symbol;Acc:HGNC:14868] |
| ENSG00000130227 | HGLibA_54574 | 85 | -0.302763778 | XPO7 | exportin 7 [Source:HGNC Symbol;Acc:HGNC:14108] |
| ENSG00000131126 | HGLibA_49048 | 85 | -0.302763778 | TEX101 | testis expressed 101 [Source:HGNC Symbol;Acc:HGNC:30722] |
| ENSG00000131584 | HGLibA_00388 | 85 | -0.302763778 | ACAP3 | ankyrin repeat and PH domains 3 [Source:HGNC Symbol;Acc:HGNC:16754]" |
| ENSG00000133103 | HGLibA_10484 | 85 | -0.302763778 | COG6 | component of oligomeric golgi complex 6 [Source:HGNC Symbol;Acc:HGNC:18621] |
| ENSG00000133313 | HGLibA_10253 | 85 | -0.302763778 | CNDP2 | carnosine dipeptidase 2 [Source:HGNC Symbol;Acc:HGNC:24437] |
| ENSG00000133641 | HGLibA_05230 | 85 | -0.302763778 | C12orf29 | chromosome 12 open reading frame 29 [Source:HGNC Symbol;Acc:HGNC:25322] |
| ENSG00000133739 | HGLibA_27442 | 85 | -0.302763778 | LRRCC1 | leucine rich repeat and coiled-coil centrosomal protein 1 [Source:HGNC Symbol;Acc:HGNC:29373] |
| ENSG00000135317 | HGLibA_46040 | 85 | -0.302763778 | SNX14 | sorting nexin 14 [Source:HGNC Symbol;Acc:HGNC:14977] |
| ENSG00000136205 | HGLibA_50926 | 85 | -0.302763778 | TNS3 | tensin 3 [Source:HGNC Symbol;Acc:HGNC:21616] |
| ENSG00000136213 | HGLibA_09618 | 85 | -0.302763778 | CHST12 | carbohydrate sulfotransferase 12 [Source:HGNC Symbol;Acc:HGNC:17423] |
| ENSG00000137076 | HGLibA_49635 | 85 | -0.302763778 | TLN1 | talin 1 [Source:HGNC Symbol;Acc:HGNC:11845] |
| ENSG00000137077 | HGLibA_07995 | 85 | -0.302763778 | CCL21 | C-C motif chemokine ligand 21 [Source:HGNC Symbol;Acc:HGNC:10620] |
| ENSG00000137275 | HGLibA_41316 | 85 | -0.302763778 | RIPK1 | receptor interacting serine/threonine kinase 1 [Source:HGNC Symbol;Acc:HGNC:10019] |
| ENSG00000137752 | HGLibA_07296 | 85 | -0.302763778 | CASP1 | caspase 1 [Source:HGNC Symbol;Acc:HGNC:1499] |

| | | | | | |
|---|---|---|---|---|---|
| ENSG00000141447 | HGLibA_34648 | 85 | -0.302763778 | OSBPL1A | oxysterol binding protein like 1A [Source:HGNC Symbol;Acc:HGNC:16398] |
| ENSG00000142910 | HGLibA_49550 | 85 | -0.302763778 | TINAGL1 | tubulointerstitial nephritis antigen like 1 [Source:HGNC Symbol;Acc:HGNC:19168] |
| ENSG00000143158 | HGLibA_29707 | 85 | -0.302763778 | MPC2 | mitochondrial pyruvate carrier 2 [Source:HGNC Symbol;Acc:HGNC:24515] |
| ENSG00000143365 | HGLibA_41781 | 85 | -0.302763778 | RORC | RAR related orphan receptor C [Source:HGNC Symbol;Acc:HGNC:10260] |
| ENSG00000145428 | HGLibA_41577 | 85 | -0.302763778 | RNF175 | ring finger protein 175 [Source:HGNC Symbol;Acc:HGNC:27735] |
| ENSG00000145536 | HGLibA_00808 | 85 | -0.302763778 | ADAMTS16 | ADAM metallopeptidase with thrombospondin type 1 motif 16 [Source:HGNC Symbol;Acc:HGNC:17108] |
| ENSG00000146066 | HGLibA_21316 | 85 | -0.302763778 | HIGD2A | HIG1 hypoxia inducible domain family member 2A [Source:HGNC Symbol;Acc:HGNC:28311] |
| ENSG00000149187 | HGLibA_09005 | 85 | -0.302763778 | CELF1 | CUGBP Elav-like family member 1 [Source:HGNC Symbol;Acc:HGNC:2549] |
| ENSG00000149547 | HGLibA_14690 | 85 | -0.302763778 | EI24 | autophagy associated transmembrane protein [Source:HGNC Symbol;Acc:HGNC:13276]" |
| ENSG00000150045 | HGLibA_25304 | 85 | -0.302763778 | KLRF1 | killer cell lectin like receptor F1 [Source:HGNC Symbol;Acc:HGNC:13342] |
| ENSG00000150628 | HGLibA_46513 | 85 | -0.302763778 | SPATA4 | spermatogenesis associated 4 [Source:HGNC Symbol;Acc:HGNC:17333] |
| ENSG00000150636 | HGLibA_07511 | 85 | -0.302763778 | CCDC102B | coiled-coil domain containing 102B [Source:HGNC Symbol;Acc:HGNC:26295] |
| ENSG00000152147 | HGLibA_18919 | 85 | -0.302763778 | GEMIN6 | gem nuclear organelle associated protein 6 [Source:HGNC Symbol;Acc:HGNC:20044] |
| ENSG00000152214 | HGLibA_41341 | 85 | -0.302763778 | RIT2 | Ras like without CAAX 2 [Source:HGNC Symbol;Acc:HGNC:10017] |
| ENSG00000152669 | HGLibA_08122 | 85 | -0.302763778 | CCNO | cyclin O [Source:HGNC Symbol;Acc:HGNC:18576] |
| ENSG00000152944 | HGLibA_28775 | 85 | -0.302763778 | MED21 | mediator complex subunit 21 [Source:HGNC Symbol;Acc:HGNC:11473] |
| ENSG00000153347 | HGLibA_16736 | 85 | -0.302763778 | FAM81B | family with sequence similarity 81 member B [Source:HGNC Symbol;Acc:HGNC:26335] |
| ENSG00000155368 | HGLibA_12386 | 85 | -0.302763778 | DBI | acyl-CoA binding protein [Source:HGNC Symbol;Acc:HGNC:2690]" |
| ENSG00000156096 | HGLibA_52928 | 85 | -0.302763778 | UGT2B4 | UDP glucuronosyltransferase family 2 member B4 [Source:HGNC Symbol;Acc:HGNC:12553] |
| ENSG00000156261 | HGLibA_08217 | 85 | -0.302763778 | CCT8 | chaperonin containing TCP1 subunit 8 [Source:HGNC Symbol;Acc:HGNC:1623] |
| ENSG00000158488 | HGLibA_08276 | 85 | -0.302763778 | CD1E | CD1e molecule [Source:HGNC Symbol;Acc:HGNC:1638] |
| ENSG00000158710 | HGLibA_48274 | 85 | -0.302763778 | TAGLN2 | transgelin 2 [Source:HGNC Symbol;Acc:HGNC:11554] |
| ENSG00000158985 | HGLibA_08584 | 85 | -0.302763778 | CDC42SE2 | CDC42 small effector 2 [Source:HGNC Symbol;Acc:HGNC:18547] |
| ENSG00000159200 | HGLibA_40668 | 85 | -0.302763778 | RCAN1 | regulator of calcineurin 1 [Source:HGNC Symbol;Acc:HGNC:3040] |
| ENSG00000161649 | HGLibA_08345 | 85 | -0.302763778 | CD300LG | CD300 molecule like family member g [Source:HGNC Symbol;Acc:HGNC:30455] |
| ENSG00000161914 | HGLibA_56463 | 85 | -0.302763778 | ZNF653 | zinc finger protein 653 [Source:HGNC Symbol;Acc:HGNC:25196] |
| ENSG00000161955 | HGLibA_50809 | 85 | -0.302763778 | TNFSF13 | TNF superfamily member 13 [Source:HGNC Symbol;Acc:HGNC:11928] |
| ENSG00000162630 | HGLibA_03957 | 85 | -0.302763778 | B3GALT2 | 3-galactosyltransferase 2 [Source:HGNC Symbol;Acc:HGNC:917]" |
| ENSG00000162746 | HGLibA_17245 | 85 | -0.302763778 | FCRLB | Fc receptor like B [Source:HGNC Symbol;Acc:HGNC:26431] |
| ENSG00000162980 | HGLibA_02974 | 85 | -0.302763778 | ARL5A | ADP ribosylation factor like GTPase 5A [Source:HGNC Symbol;Acc:HGNC:696] |
| ENSG00000163064 | HGLibA_15099 | 85 | -0.302763778 | EN1 | engrailed homeobox 1 [Source:HGNC Symbol;Acc:HGNC:3342] |
| ENSG00000163291 | HGLibA_35156 | 85 | -0.302763778 | PAQR3 | progestin and adipoQ receptor family member 3 [Source:HGNC Symbol;Acc:HGNC:30130] |
| ENSG00000163481 | HGLibA_41654 | 85 | -0.302763778 | RNF25 | ring finger protein 25 [Source:HGNC Symbol;Acc:HGNC:14662] |
| ENSG00000163683 | HGLibA_45732 | 85 | -0.302763778 | SMIM14 | small integral membrane protein 14 [Source:HGNC Symbol;Acc:HGNC:27321] |
| ENSG00000164087 | HGLibA_37442 | 85 | -0.302763778 | POC1A | POC1 centriolar protein A [Source:HGNC Symbol;Acc:HGNC:24488] |
| ENSG00000165092 | HGLibA_01541 | 85 | -0.302763778 | ALDH1A1 | aldehyde dehydrogenase 1 family member A1 [Source:HGNC Symbol;Acc:HGNC:402] |
| ENSG00000165752 | HGLibA_47489 | 85 | -0.302763778 | STK32C | serine/threonine kinase 32C [Source:HGNC Symbol;Acc:HGNC:21332] |
| ENSG00000165861 | HGLibA_55271 | 85 | -0.302763778 | ZFYVE1 | zinc finger FYVE-type containing 1 [Source:HGNC Symbol;Acc:HGNC:13180] |
| ENSG00000166922 | HGLibA_42904 | 85 | -0.302763778 | SCG5 | secretogranin V [Source:HGNC Symbol;Acc:HGNC:10816] |
| ENSG00000168291 | HGLibA_35867 | 85 | -0.302763778 | PDHB | pyruvate dehydrogenase E1 beta subunit [Source:HGNC Symbol;Acc:HGNC:8808] |
| ENSG00000168781 | HGLibA_37953 | 85 | -0.302763778 | PPIP5K1 | diphosphoinositol pentakisphosphate kinase 1 [Source:HGNC Symbol;Acc:HGNC:29023] |
| ENSG00000169605 | HGLibA_19209 | 85 | -0.302763778 | GKN1 | gastrokine 1 [Source:HGNC Symbol;Acc:HGNC:23217] |
| ENSG00000170242 | HGLibA_53379 | 85 | -0.302763778 | USP47 | ubiquitin specific peptidase 47 [Source:HGNC Symbol;Acc:HGNC:20076] |
| ENSG00000171234 | HGLibA_52929 | 85 | -0.302763778 | UGT2B7 | UDP glucuronosyltransferase family 2 member B7 [Source:HGNC Symbol;Acc:HGNC:12554] |
| ENSG00000171295 | HGLibA_56001 | 85 | -0.302763778 | ZNF440 | zinc finger protein 440 [Source:HGNC Symbol;Acc:HGNC:20874] |
| ENSG00000171385 | HGLibA_24179 | 85 | -0.302763778 | KCND3 | potassium voltage-gated channel subfamily D member 3 [Source:HGNC Symbol;Acc:HGNC:6239] |
| ENSG00000172465 | HGLibA_48705 | 85 | -0.302763778 | TCEAL1 | transcription elongation factor A like 1 [Source:HGNC Symbol;Acc:HGNC:11616] |
| ENSG00000172938 | HGLibA_29837 | 85 | -0.302763778 | MRGPRD | MAS related GPR family member D [Source:HGNC Symbol;Acc:HGNC:29626] |
| ENSG00000173473 | HGLibA_45624 | 85 | -0.302763778 | SMARCC1 | actin dependent regulator of chromatin subfamily c member 1 [Source:HGNC Symbol;Acc:HGNC:11104]" |
| ENSG00000173548 | HGLibA_46092 | 85 | -0.302763778 | SNX33 | sorting nexin 33 [Source:HGNC Symbol;Acc:HGNC:28468] |
| ENSG00000173933 | HGLibA_40545 | 85 | -0.302763778 | RBM4 | RNA binding motif protein 4 [Source:HGNC Symbol;Acc:HGNC:9901] |
| ENSG00000174669 | HGLibA_44844 | 85 | -0.302763778 | SLC29A2 | solute carrier family 29 member 2 [Source:HGNC Symbol;Acc:HGNC:11004] |
| ENSG00000175264 | HGLibA_09611 | 85 | -0.302763778 | CHST1 | carbohydrate sulfotransferase 1 [Source:HGNC Symbol;Acc:HGNC:1969] |
| ENSG00000176253 | HGLibA_34019 | 85 | -0.302763778 | OR4K13 | olfactory receptor family 4 subfamily K member 13 [Source:HGNC Symbol;Acc:HGNC:15351] |
| ENSG00000176393 | HGLibA_41727 | 85 | -0.302763778 | RNPEP | arginyl aminopeptidase [Source:HGNC Symbol;Acc:HGNC:10078] |
| ENSG00000178226 | HGLibA_38933 | 85 | -0.302763778 | PRSS36 | serine protease 36 [Source:HGNC Symbol;Acc:HGNC:26906] |
| ENSG00000179546 | HGLibA_22361 | 85 | -0.302763778 | HTR1D | 5-hydroxytryptamine receptor 1D [Source:HGNC Symbol;Acc:HGNC:5289] |
| ENSG00000180228 | HGLibA_38585 | 85 | -0.302763778 | PRKRA | protein activator of interferon induced protein kinase EIF2AK2 [Source:HGNC Symbol;Acc:HGNC:9438] |
| ENSG00000180549 | HGLibA_18204 | 85 | -0.302763778 | FUT7 | fucosyltransferase 7 [Source:HGNC Symbol;Acc:HGNC:4018] |
| ENSG00000180626 | HGLibA_56345 | 85 | -0.302763778 | ZNF594 | zinc finger protein 594 [Source:HGNC Symbol;Acc:HGNC:29392] |
| ENSG00000181847 | HGLibA_49483 | 85 | -0.302763778 | TIGIT | T cell immunoreceptor with Ig and ITIM domains [Source:HGNC Symbol;Acc:HGNC:26838] |
| ENSG00000182141 | HGLibA_56603 | 85 | -0.302763778 | ZNF708 | zinc finger protein 708 [Source:HGNC Symbol;Acc:HGNC:12945] |
| ENSG00000182150 | HGLibA_15453 | 85 | -0.302763778 | ERCC6L2 | ERCC excision repair 6 like 2 [Source:HGNC Symbol;Acc:HGNC:26922] |
| ENSG00000182415 | HGLibA_08911 | 85 | -0.302763778 | CDY2A | chromodomain Y-linked 2A [Source:HGNC Symbol;Acc:HGNC:1810] |
| ENSG00000182578 | HGLibA_11325 | 85 | -0.302763778 | CSF1R | colony stimulating factor 1 receptor [Source:HGNC Symbol;Acc:HGNC:2433] |
| ENSG00000182652 | HGLibA_34058 | 85 | -0.302763778 | OR4Q3 | olfactory receptor family 4 subfamily Q member 3 [Source:HGNC Symbol;Acc:HGNC:15426] |
| ENSG00000183018 | HGLibA_46737 | 85 | -0.302763778 | SPNS2 | sphingolipid transporter 2 [Source:HGNC Symbol;Acc:HGNC:26992] |
| ENSG00000183032 | HGLibA_44673 | 85 | -0.302763778 | SLC25A21 | solute carrier family 25 member 21 [Source:HGNC Symbol;Acc:HGNC:14411] |
| ENSG00000184083 | HGLibA_16063 | 85 | -0.302763778 | FAM120C | family with sequence similarity 120C [Source:HGNC Symbol;Acc:HGNC:16949] |
| ENSG00000184575 | HGLibA_54578 | 85 | -0.302763778 | XPOT | exportin for tRNA [Source:HGNC Symbol;Acc:HGNC:12826] |
| ENSG00000185896 | HGLibA_25976 | 85 | -0.302763778 | LAMP1 | lysosomal associated membrane protein 1 [Source:HGNC Symbol;Acc:HGNC:6499] |
| ENSG00000186766 | HGLibA_17878 | 85 | -0.302763778 | FOXI2 | forkhead box I2 [Source:HGNC Symbol;Acc:HGNC:32448] |
| ENSG00000187325 | HGLibA_48265 | 85 | -0.302763778 | TAF9B | TATA-box binding protein associated factor 9b [Source:HGNC Symbol;Acc:HGNC:17306] |
| ENSG00000187607 | HGLibA_55750 | 85 | -0.302763778 | ZNF286A | zinc finger protein 286A [Source:HGNC Symbol;Acc:HGNC:13501] |
| ENSG00000188674 | HGLibA_06188 | 85 | -0.302763778 | C2orf80 | chromosome 2 open reading frame 80 [Source:HGNC Symbol;Acc:HGNC:34352] |
| ENSG00000196189 | HGLibA_43341 | 85 | -0.302763778 | SEMA4A | semaphorin 4A [Source:HGNC Symbol;Acc:HGNC:10729] |
| ENSG00000196498 | HGLibA_31359 | 85 | -0.302763778 | NCOR2 | nuclear receptor corepressor 2 [Source:HGNC Symbol;Acc:HGNC:7673] |
| ENSG00000196542 | HGLibA_46903 | 85 | -0.302763778 | SPTSSB | serine palmitoyltransferase small subunit B [Source:HGNC Symbol;Acc:HGNC:24045] |
| ENSG00000196782 | HGLibA_28050 | 85 | -0.302763778 | MAML3 | mastermind like transcriptional coactivator 3 [Source:HGNC Symbol;Acc:HGNC:16272] |
| ENSG00000197147 | HGLibA_27432 | 85 | -0.302763778 | LRRC8B | leucine rich repeat containing 8 VRAC subunit B [Source:HGNC Symbol;Acc:HGNC:30692] |
| ENSG00000197299 | HGLibA_04547 | 85 | -0.302763778 | BLM | BLM RecQ like helicase [Source:HGNC Symbol;Acc:HGNC:1058] |
| ENSG00000197712 | HGLibA_16030 | 85 | -0.302763778 | FAM114A1 | family with sequence similarity 114 member A1 [Source:HGNC Symbol;Acc:HGNC:25087] |
| ENSG00000197714 | HGLibA_56036 | 85 | -0.302763778 | ZNF460 | zinc finger protein 460 [Source:HGNC Symbol;Acc:HGNC:21628] |
| ENSG00000197838 | HGLibA_12104 | 85 | -0.302763778 | CYP2A13 | cytochrome P450 family 2 subfamily A member 13 [Source:HGNC Symbol;Acc:HGNC:2608] |
| ENSG00000197980 | HGLibA_26281 | 85 | -0.302763778 | LEKR1 | glutamate and lysine rich 1 [Source:HGNC Symbol;Acc:HGNC:33765]" |
| ENSG00000198019 | HGLibA_17187 | 85 | -0.302763778 | FCGR1B | Fc fragment of IgG receptor Ib [Source:HGNC Symbol;Acc:HGNC:3614] |
| ENSG00000198271 | HGLibA_25766 | 85 | -0.302763778 | KRTAP4-5 | keratin associated protein 4-5 [Source:HGNC Symbol;Acc:HGNC:18899] |
| ENSG00000198286 | HGLibA_07219 | 85 | -0.302763778 | CARD11 | caspase recruitment domain family member 11 [Source:HGNC Symbol;Acc:HGNC:16393] |
| ENSG00000198798 | HGLibA_27948 | 85 | -0.302763778 | MAGEB3 | MAGE family member B3 [Source:HGNC Symbol;Acc:HGNC:6810] |
| ENSG00000198848 | HGLibA_09217 | 85 | -0.302763778 | CES1 | carboxylesterase 1 [Source:HGNC Symbol;Acc:HGNC:1863] |
| ENSG00000198964 | HGLibA_43834 | 85 | -0.302763778 | SGMS1 | sphingomyelin synthase 1 [Source:HGNC Symbol;Acc:HGNC:29799] |
| ENSG00000203908 | HGLibA_24563 | 85 | -0.302763778 | KHDC3L | subcortical maternal complex member [Source:HGNC Symbol;Acc:HGNC:33699]" |
| ENSG00000204381 | HGLibA_26074 | 85 | -0.302763778 | LAYN | layilin [Source:HGNC Symbol;Acc:HGNC:29471] |
| ENSG00000204463 | HGLibA_04088 | 85 | -0.302763778 | BAG6 | BCL2 associated athanogene 6 [Source:HGNC Symbol;Acc:HGNC:13919] |
| ENSG00000204612 | HGLibA_17819 | 85 | -0.302763778 | FOXB2 | forkhead box B2 [Source:HGNC Symbol;Acc:HGNC:23315] |
| ENSG00000204704 | HGLibA_33903 | 85 | -0.302763778 | OR2W1 | olfactory receptor family 2 subfamily W member 1 [Source:HGNC Symbol;Acc:HGNC:8281] |
| ENSG00000205089 | HGLibA_08104 | 85 | -0.302763778 | CCNI2 | cyclin I family member 2 [Source:HGNC Symbol;Acc:HGNC:33869] |
| ENSG00000213934 | HGLibA_20924 | 85 | -0.302763778 | HBG1 | hemoglobin subunit gamma 1 [Source:HGNC Symbol;Acc:HGNC:4831] |
| ENSG00000216588 | HGLibA_22889 | 85 | -0.302763778 | IGSF23 | immunoglobulin superfamily member 23 [Source:HGNC Symbol;Acc:HGNC:40040] |
| ENSG00000237330 | HGLibA_41645 | 85 | -0.302763778 | RNF223 | ring finger protein 223 [Source:HGNC Symbol;Acc:HGNC:40020] |
| ENSG00000241598 | HGLibA_25796 | 85 | -0.302763778 | KRTAP5-4 | keratin associated protein 5-4 [Source:HGNC Symbol;Acc:HGNC:23599] |
| ENSG00000248099 | HGLibA_23381 | 85 | -0.302763778 | INSL3 | insulin like 3 [Source:HGNC Symbol;Acc:HGNC:6086] |
| ENSG00000251655 | HGLibA_38339 | 85 | -0.302763778 | PRB1 | proline rich protein BstNI subfamily 1 [Source:HGNC Symbol;Acc:HGNC:9337] |
| ENSG00000253731 | HGLibA_35538 | 85 | -0.302763778 | PCDHGA6 | 6 [Source:HGNC Symbol;Acc:HGNC:8704]" |
| ENSG00000258453 | HGLibA_33576 | 85 | -0.302763778 | OR11H2 | olfactory receptor family 11 subfamily H member 2 [Source:HGNC Symbol;Acc:HGNC:14716] |
| ENSG00000267534 | HGLibA_42597 | 85 | -0.302763778 | S1PR2 | sphingosine-1-phosphate receptor 2 [Source:HGNC Symbol;Acc:HGNC:3169] |
| ENSG00000273899 | HGLibA_32262 | 85 | -0.302763778 | NOL12 | nucleolar protein 12 [Source:HGNC Symbol;Acc:HGNC:28585] |
| FAM195A | HGLibA_16375 | 85 | -0.302763778 | FAM195A | - |
| HIST1H2AC | HGLibA_21380 | 85 | -0.302763778 | HIST1H2AC | - |
| HN1 | HGLibA_21767 | 85 | -0.302763778 | HN1 | - |
| hsa-mir-1587 | HGLibA_57933 | 85 | -0.302763778 | hsa-mir-1587 | - |
| hsa-mir-3118-6 | HGLibA_58521 | 85 | -0.302763778 | hsa-mir-3118-6 | - |
| hsa-mir-3162 | HGLibA_58729 | 85 | -0.302763778 | hsa-mir-3162 | - |
| hsa-mir-324 | HGLibA_58965 | 85 | -0.302763778 | hsa-mir-324 | - |
| hsa-mir-3692 | HGLibA_59358 | 85 | -0.302763778 | hsa-mir-3692 | - |
| hsa-mir-384 | HGLibA_59489 | 85 | -0.302763778 | hsa-mir-384 | - |
| hsa-mir-3945 | HGLibA_59638 | 85 | -0.302763778 | hsa-mir-3945 | - |
| hsa-mir-421 | HGLibA_59690 | 85 | -0.302763778 | hsa-mir-421 | - |
| hsa-mir-4711 | HGLibA_60945 | 85 | -0.302763778 | hsa-mir-4711 | - |
| hsa-mir-491 | HGLibA_61363 | 85 | -0.302763778 | hsa-mir-491 | - |
| hsa-mir-511 | HGLibA_61556 | 85 | -0.302763778 | hsa-mir-511 | - |
| hsa-mir-518e | HGLibA_61670 | 85 | -0.302763778 | hsa-mir-518e | - |
| hsa-mir-572 | HGLibA_62380 | 85 | -0.302763778 | hsa-mir-572 | - |
| hsa-mir-6133 | HGLibA_62684 | 85 | -0.302763778 | hsa-mir-6133 | - |
| hsa-mir-6780b | HGLibA_63296 | 85 | -0.302763778 | hsa-mir-6780b | - |
| hsa-mir-8071-1 | HGLibA_64122 | 85 | -0.302763778 | hsa-mir-8071-1 | - |
| ICK | HGLibA_22508 | 85 | -0.302763778 | ICK | - |
| LOC283710 | HGLibA_26912 | 85 | -0.302763778 | LOC283710 | - |
| PALM2-AKAP2 | HGLibA_35074 | 85 | -0.302763778 | PALM2-AKAP2 | - |
| PPAPDC1B | HGLibA_37829 | 85 | -0.302763778 | PPAPDC1B | - |
| PRAMEF22 | HGLibA_38313 | 85 | -0.302763778 | PRAMEF22 | - |
| SLMO2 | HGLibA_45539 | 85 | -0.302763778 | SLMO2 | - |
| WBSCR28 | HGLibA_54002 | 85 | -0.302763778 | WBSCR28 | - |
| ADSSL1 | HGLibA_01080 | 84 | -0.312714815 | ADSSL1 | - |
| AKAP17A_X | HGLibA_01420 | 84 | -0.312714815 | AKAP17A_X | - |

| | | | | | |
|---|---|---|---|---|---|
| C16orf97 | HGLibA_05513 | 84 | -0.312714815 | C16orf97 | - |
| C19orf60 | HGLibA_05689 | 84 | -0.312714815 | C19orf60 | - |
| C19orf66 | HGLibA_05692 | 84 | -0.312714815 | C19orf66 | - |
| C6orf170 | HGLibA_06462 | 84 | -0.312714815 | C6orf170 | - |
| C7orf65 | HGLibA_06576 | 84 | -0.312714815 | C7orf65 | - |
| CCDC155 | HGLibA_07658 | 84 | -0.312714815 | CCDC155 | - |
| ENSG00000002016 | HGLibA_40096 | 84 | -0.312714815 | RAD52 | DNA repair protein [Source:HGNC Symbol;Acc:HGNC:9824]" |
| ENSG00000006283 | HGLibA_06893 | 84 | -0.312714815 | CACNA1G | calcium voltage-gated channel subunit alpha1 G [Source:HGNC Symbol;Acc:HGNC:1394] |
| ENSG00000023572 | HGLibA_19315 | 84 | -0.312714815 | GLRX2 | glutaredoxin 2 [Source:HGNC Symbol;Acc:HGNC:16065] |
| ENSG00000035862 | HGLibA_49537 | 84 | -0.312714815 | TIMP2 | TIMP metallopeptidase inhibitor 2 [Source:HGNC Symbol;Acc:HGNC:11821] |
| ENSG00000037965 | HGLibA_21978 | 84 | -0.312714815 | HOXC8 | homeobox C8 [Source:HGNC Symbol;Acc:HGNC:5129] |
| ENSG00000040731 | HGLibA_08633 | 84 | -0.312714815 | CDH10 | cadherin 10 [Source:HGNC Symbol;Acc:HGNC:1749] |
| ENSG00000065485 | HGLibA_35882 | 84 | -0.312714815 | PDIA5 | protein disulfide isomerase family A member 5 [Source:HGNC Symbol;Acc:HGNC:24811] |
| ENSG00000066583 | HGLibA_23681 | 84 | -0.312714815 | ISOC1 | isochorismatase domain containing 1 [Source:HGNC Symbol;Acc:HGNC:24254] |
| ENSG00000066697 | HGLibA_30175 | 84 | -0.312714815 | MSANTD3 | Myb/SANT DNA binding domain containing 3 [Source:HGNC Symbol;Acc:HGNC:23370] |
| ENSG00000070770 | HGLibA_11406 | 84 | -0.312714815 | CSNK2A2 | casein kinase 2 alpha 2 [Source:HGNC Symbol;Acc:HGNC:2459] |
| ENSG00000071564 | HGLibA_48786 | 84 | -0.312714815 | TCF3 | transcription factor 3 [Source:HGNC Symbol;Acc:HGNC:11633] |
| ENSG00000072274 | HGLibA_49197 | 84 | -0.312714815 | TFRC | transferrin receptor [Source:HGNC Symbol;Acc:HGNC:11763] |
| ENSG00000079462 | HGLibA_34994 | 84 | -0.312714815 | PAFAH1B3 | platelet activating factor acetylhydrolase 1b catalytic subunit 3 [Source:HGNC Symbol;Acc:HGNC:8576] |
| ENSG00000083799 | HGLibA_12049 | 84 | -0.312714815 | CYLD | CYLD lysine 63 deubiquitinase [Source:HGNC Symbol;Acc:HGNC:2584] |
| ENSG00000084453 | HGLibA_45446 | 84 | -0.312714815 | SLCO1A2 | solute carrier organic anion transporter family member 1A2 [Source:HGNC Symbol;Acc:HGNC:10956] |
| ENSG00000087077 | HGLibA_51647 | 84 | -0.312714815 | TRIP6 | thyroid hormone receptor interactor 6 [Source:HGNC Symbol;Acc:HGNC:12311] |
| ENSG00000090382 | HGLibA_27781 | 84 | -0.312714815 | LYZ | lysozyme [Source:HGNC Symbol;Acc:HGNC:6740] |
| ENSG00000095321 | HGLibA_11065 | 84 | -0.312714815 | CRAT | carnitine O-acetyltransferase [Source:HGNC Symbol;Acc:HGNC:2342] |
| ENSG00000099814 | HGLibA_09120 | 84 | -0.312714815 | CEP170B | centrosomal protein 170B [Source:HGNC Symbol;Acc:HGNC:20362] |
| ENSG00000100003 | HGLibA_43192 | 84 | -0.312714815 | SEC14L2 | SEC14 like lipid binding 2 [Source:HGNC Symbol;Acc:HGNC:10699] |
| ENSG00000100629 | HGLibA_09103 | 84 | -0.312714815 | CEP128 | centrosomal protein 128 [Source:HGNC Symbol;Acc:HGNC:20359] |
| ENSG00000101104 | HGLibA_34925 | 84 | -0.312714815 | PABPC1L | poly(A) binding protein cytoplasmic 1 like [Source:HGNC Symbol;Acc:HGNC:15797] |
| ENSG00000101230 | HGLibA_23676 | 84 | -0.312714815 | ISM1 | isthmin 1 [Source:HGNC Symbol;Acc:HGNC:16213] |
| ENSG00000101265 | HGLibA_40378 | 84 | -0.312714815 | RASSF2 | Ras association domain family member 2 [Source:HGNC Symbol;Acc:HGNC:9883] |
| ENSG00000102024 | HGLibA_37228 | 84 | -0.312714815 | PLS3 | plastin 3 [Source:HGNC Symbol;Acc:HGNC:9091] |
| ENSG00000102265 | HGLibA_49535 | 84 | -0.312714815 | TIMP1 | TIMP metallopeptidase inhibitor 1 [Source:HGNC Symbol;Acc:HGNC:11820] |
| ENSG00000102524 | HGLibA_50812 | 84 | -0.312714815 | TNFSF13B | TNF superfamily member 13b [Source:HGNC Symbol;Acc:HGNC:11929] |
| ENSG00000103111 | HGLibA_29641 | 84 | -0.312714815 | MON1B | secretory trafficking associated [Source:HGNC Symbol;Acc:HGNC:25020]" |
| ENSG00000103356 | HGLibA_14344 | 84 | -0.312714815 | EARS2 | mitochondrial [Source:HGNC Symbol;Acc:HGNC:29419]" |
| ENSG00000104290 | HGLibA_18268 | 84 | -0.312714815 | FZD3 | frizzled class receptor 3 [Source:HGNC Symbol;Acc:HGNC:4041] |
| ENSG00000104435 | HGLibA_47520 | 84 | -0.312714815 | STMN2 | stathmin 2 [Source:HGNC Symbol;Acc:HGNC:10577] |
| ENSG00000105011 | HGLibA_03285 | 84 | -0.312714815 | ASF1B | anti-silencing function 1B histone chaperone [Source:HGNC Symbol;Acc:HGNC:20996] |
| ENSG00000105428 | HGLibA_56946 | 84 | -0.312714815 | ZNRF4 | zinc and ring finger 4 [Source:HGNC Symbol;Acc:HGNC:17726] |
| ENSG00000106648 | HGLibA_18577 | 84 | -0.312714815 | GALNTL5 | polypeptide N-acetylgalactosaminyltransferase like 5 [Source:HGNC Symbol;Acc:HGNC:21725] |
| ENSG00000107099 | HGLibA_13759 | 84 | -0.312714815 | DOCK8 | dedicator of cytokinesis 8 [Source:HGNC Symbol;Acc:HGNC:19191] |
| ENSG00000107863 | HGLibA_02725 | 84 | -0.312714815 | ARHGAP21 | Rho GTPase activating protein 21 [Source:HGNC Symbol;Acc:HGNC:23725] |
| ENSG00000111335 | HGLibA_33201 | 84 | -0.312714815 | OAS2 | 2'-5'-oligoadenylate synthetase 2 [Source:HGNC Symbol;Acc:HGNC:8087] |
| ENSG00000111667 | HGLibA_53390 | 84 | -0.312714815 | USP5 | ubiquitin specific peptidase 5 [Source:HGNC Symbol;Acc:HGNC:12628] |
| ENSG00000111701 | HGLibA_02466 | 84 | -0.312714815 | APOBEC1 | apolipoprotein B mRNA editing enzyme catalytic subunit 1 [Source:HGNC Symbol;Acc:HGNC:604] |
| ENSG00000112159 | HGLibA_28703 | 84 | -0.312714815 | MDN1 | midasin AAA ATPase 1 [Source:HGNC Symbol;Acc:HGNC:18302] |
| ENSG00000112208 | HGLibA_04077 | 84 | -0.312714815 | BAG2 | BCL2 associated athanogene 2 [Source:HGNC Symbol;Acc:HGNC:938] |
| ENSG00000113810 | HGLibA_45653 | 84 | -0.312714815 | SMC4 | structural maintenance of chromosomes 4 [Source:HGNC Symbol;Acc:HGNC:14013] |
| ENSG00000114541 | HGLibA_18017 | 84 | -0.312714815 | FRMD4B | FERM domain containing 4B [Source:HGNC Symbol;Acc:HGNC:24886] |
| ENSG00000114853 | HGLibA_54901 | 84 | -0.312714815 | ZBTB47 | zinc finger and BTB domain containing 47 [Source:HGNC Symbol;Acc:HGNC:26955] |
| ENSG00000115594 | HGLibA_23047 | 84 | -0.312714815 | IL1R1 | interleukin 1 receptor type 1 [Source:HGNC Symbol;Acc:HGNC:5993] |
| ENSG00000115944 | HGLibA_10862 | 84 | -0.312714815 | COX7A2L | cytochrome c oxidase subunit 7A2 like [Source:HGNC Symbol;Acc:HGNC:2289] |
| ENSG00000118482 | HGLibA_36378 | 84 | -0.312714815 | PHF3 | PHD finger protein 3 [Source:HGNC Symbol;Acc:HGNC:8921] |
| ENSG00000118526 | HGLibA_48773 | 84 | -0.312714815 | TCF21 | transcription factor 21 [Source:HGNC Symbol;Acc:HGNC:11632] |
| ENSG00000119414 | HGLibA_38217 | 84 | -0.312714815 | PPP6C | protein phosphatase 6 catalytic subunit [Source:HGNC Symbol;Acc:HGNC:9323] |
| ENSG00000119718 | HGLibA_14737 | 84 | -0.312714815 | EIF2B2 | eukaryotic translation initiation factor 2B subunit beta [Source:HGNC Symbol;Acc:HGNC:3258] |
| ENSG00000119915 | HGLibA_14963 | 84 | -0.312714815 | ELOVL3 | ELOVL fatty acid elongase 3 [Source:HGNC Symbol;Acc:HGNC:18047] |
| ENSG00000120210 | HGLibA_23391 | 84 | -0.312714815 | INSL6 | insulin like 6 [Source:HGNC Symbol;Acc:HGNC:6089] |
| ENSG00000123159 | HGLibA_19118 | 84 | -0.312714815 | GIPC1 | GIPC PDZ domain containing family member 1 [Source:HGNC Symbol;Acc:HGNC:1226] |
| ENSG00000123505 | HGLibA_01758 | 84 | -0.312714815 | AMD1 | adenosylmethionine decarboxylase 1 [Source:HGNC Symbol;Acc:HGNC:457] |
| ENSG00000124209 | HGLibA_39840 | 84 | -0.312714815 | RAB22A | member RAS oncogene family [Source:HGNC Symbol;Acc:HGNC:9764]" |
| ENSG00000124212 | HGLibA_39349 | 84 | -0.312714815 | PTGIS | prostaglandin I2 synthase [Source:HGNC Symbol;Acc:HGNC:9603] |
| ENSG00000124243 | HGLibA_04260 | 84 | -0.312714815 | BCAS4 | breast carcinoma amplified sequence 4 [Source:HGNC Symbol;Acc:HGNC:14367] |
| ENSG00000126259 | HGLibA_25004 | 84 | -0.312714815 | KIRREL2 | kirre like nephrin family adhesion molecule 2 [Source:HGNC Symbol;Acc:HGNC:18816] |
| ENSG00000127955 | HGLibA_19451 | 84 | -0.312714815 | GNAI1 | G protein subunit alpha i1 [Source:HGNC Symbol;Acc:HGNC:4384] |
| ENSG00000128656 | HGLibA_09506 | 84 | -0.312714815 | CHN1 | chimerin 1 [Source:HGNC Symbol;Acc:HGNC:1943] |
| ENSG00000130224 | HGLibA_27139 | 84 | -0.312714815 | LRCH2 | leucine rich repeats and calponin homology domain containing 2 [Source:HGNC Symbol;Acc:HGNC:29292] |
| ENSG00000130775 | HGLibA_49351 | 84 | -0.312714815 | THEMIS2 | thymocyte selection associated family member 2 [Source:HGNC Symbol;Acc:HGNC:16839] |
| ENSG00000131459 | HGLibA_18962 | 84 | -0.312714815 | GFPT2 | glutamine-fructose-6-phosphate transaminase 2 [Source:HGNC Symbol;Acc:HGNC:4242] |
| ENSG00000132912 | HGLibA_12552 | 84 | -0.312714815 | DCTN4 | dynactin subunit 4 [Source:HGNC Symbol;Acc:HGNC:15518] |
| ENSG00000133835 | HGLibA_22192 | 84 | -0.312714815 | HSD17B4 | hydroxysteroid 17-beta dehydrogenase 4 [Source:HGNC Symbol;Acc:HGNC:5213] |
| ENSG00000134028 | HGLibA_00787 | 84 | -0.312714815 | ADAMDEC1 | ADAM like decysin 1 [Source:HGNC Symbol;Acc:HGNC:16299] |
| ENSG00000136205 | HGLibA_50924 | 84 | -0.312714815 | TNS3 | tensin 3 [Source:HGNC Symbol;Acc:HGNC:21616] |
| ENSG00000136371 | HGLibA_30390 | 84 | -0.312714815 | MTHFS | methenyltetrahydrofolate synthetase [Source:HGNC Symbol;Acc:HGNC:7437] |
| ENSG00000136827 | HGLibA_51014 | 84 | -0.312714815 | TOR1A | torsin family 1 member A [Source:HGNC Symbol;Acc:HGNC:3098] |
| ENSG00000137857 | HGLibA_14098 | 84 | -0.312714815 | DUOX1 | dual oxidase 1 [Source:HGNC Symbol;Acc:HGNC:3062] |
| ENSG00000138119 | HGLibA_30883 | 84 | -0.312714815 | MYOF | myoferlin [Source:HGNC Symbol;Acc:HGNC:3656] |
| ENSG00000138641 | HGLibA_21159 | 84 | -0.312714815 | HERC3 | HECT and RLD domain containing E3 ubiquitin protein ligase 3 [Source:HGNC Symbol;Acc:HGNC:4876] |
| ENSG00000139344 | HGLibA_01761 | 84 | -0.312714815 | AMDHD1 | amidohydrolase domain containing 1 [Source:HGNC Symbol;Acc:HGNC:28577] |
| ENSG00000142025 | HGLibA_13441 | 84 | -0.312714815 | DMRTC2 | DMRT like family C2 [Source:HGNC Symbol;Acc:HGNC:13911] |
| ENSG00000142173 | HGLibA_10599 | 84 | -0.312714815 | COL6A2 | collagen type VI alpha 2 chain [Source:HGNC Symbol;Acc:HGNC:2212] |
| ENSG00000143842 | HGLibA_46238 | 84 | -0.312714815 | SOX13 | SRY-box 13 [Source:HGNC Symbol;Acc:HGNC:11192] |
| ENSG00000146090 | HGLibA_40336 | 84 | -0.312714815 | RASGEF1C | RasGEF domain family member 1C [Source:HGNC Symbol;Acc:HGNC:27400] |
| ENSG00000146858 | HGLibA_54994 | 84 | -0.312714815 | ZC3HAV1L | antiviral 1 like [Source:HGNC Symbol;Acc:HGNC:22423]" |
| ENSG00000147119 | HGLibA_09647 | 84 | -0.312714815 | CHST7 | carbohydrate sulfotransferase 7 [Source:HGNC Symbol;Acc:HGNC:13817] |
| ENSG00000147854 | HGLibA_52954 | 84 | -0.312714815 | UHRF2 | ubiquitin like with PHD and ring finger domains 2 [Source:HGNC Symbol;Acc:HGNC:12557] |
| ENSG00000148735 | HGLibA_37156 | 84 | -0.312714815 | PLEKHS1 | pleckstrin homology domain containing S1 [Source:HGNC Symbol;Acc:HGNC:26285] |
| ENSG00000149100 | HGLibA_14800 | 84 | -0.312714815 | EIF3M | eukaryotic translation initiation factor 3 subunit M [Source:HGNC Symbol;Acc:HGNC:24460] |
| ENSG00000149499 | HGLibA_15061 | 84 | -0.312714815 | EML3 | echinoderm microtubule associated protein like 3 [Source:HGNC Symbol;Acc:HGNC:26666] |
| ENSG00000149527 | HGLibA_37012 | 84 | -0.312714815 | PLCH2 | phospholipase C eta 2 [Source:HGNC Symbol;Acc:HGNC:29037] |
| ENSG00000151150 | HGLibA_01941 | 84 | -0.312714815 | ANK3 | ankyrin 3 [Source:HGNC Symbol;Acc:HGNC:494] |
| ENSG00000151490 | HGLibA_39526 | 84 | -0.312714815 | PTPRO | receptor type O [Source:HGNC Symbol;Acc:HGNC:9678]" |
| ENSG00000151923 | HGLibA_49436 | 84 | -0.312714815 | TIAL1 | TIA1 cytotoxic granule associated RNA binding protein like 1 [Source:HGNC Symbol;Acc:HGNC:11804] |
| ENSG00000154146 | HGLibA_32688 | 84 | -0.312714815 | NRGN | neurogranin [Source:HGNC Symbol;Acc:HGNC:8000] |
| ENSG00000154380 | HGLibA_15104 | 84 | -0.312714815 | ENAH | actin regulator [Source:HGNC Symbol;Acc:HGNC:18271]" |
| ENSG00000155890 | HGLibA_51499 | 84 | -0.312714815 | TRIM42 | tripartite motif containing 42 [Source:HGNC Symbol;Acc:HGNC:19014] |
| ENSG00000157330 | HGLibA_05781 | 84 | -0.312714815 | C1orf158 | chromosome 1 open reading frame 158 [Source:HGNC Symbol;Acc:HGNC:28567] |
| ENSG00000157540 | HGLibA_14272 | 84 | -0.312714815 | DYRK1A | dual specificity tyrosine phosphorylation regulated kinase 1A [Source:HGNC Symbol;Acc:HGNC:3091] |
| ENSG00000157927 | HGLibA_40114 | 84 | -0.312714815 | RADIL | Rap associating with DIL domain [Source:HGNC Symbol;Acc:HGNC:22226] |
| ENSG00000158747 | HGLibA_31214 | 84 | -0.312714815 | NBL1 | DAN family BMP antagonist [Source:HGNC Symbol;Acc:HGNC:7650]" |
| ENSG00000159248 | HGLibA_19186 | 84 | -0.312714815 | GJD2 | gap junction protein delta 2 [Source:HGNC Symbol;Acc:HGNC:19154] |
| ENSG00000159459 | HGLibA_52746 | 84 | -0.312714815 | UBR1 | ubiquitin protein ligase E3 component n-recognin 1 [Source:HGNC Symbol;Acc:HGNC:16808] |
| ENSG00000160179 | HGLibA_00214 | 84 | -0.312714815 | ABCG1 | ATP binding cassette subfamily G member 1 [Source:HGNC Symbol;Acc:HGNC:73] |
| ENSG00000161179 | HGLibA_54652 | 84 | -0.312714815 | YDJC | YdjC chitooligosaccharide deacetylase homolog [Source:HGNC Symbol;Acc:HGNC:27158] |
| ENSG00000162032 | HGLibA_46866 | 84 | -0.312714815 | SPSB3 | splA/ryanodine receptor domain and SOCS box containing 3 [Source:HGNC Symbol;Acc:HGNC:30629] |
| ENSG00000162723 | HGLibA_44325 | 84 | -0.312714815 | SLAMF9 | SLAM family member 9 [Source:HGNC Symbol;Acc:HGNC:18430] |
| ENSG00000162931 | HGLibA_51423 | 84 | -0.312714815 | TRIM17 | tripartite motif containing 17 [Source:HGNC Symbol;Acc:HGNC:13430] |
| ENSG00000163132 | HGLibA_30271 | 84 | -0.312714815 | MSX1 | msh homeobox 1 [Source:HGNC Symbol;Acc:HGNC:7391] |
| ENSG00000163875 | HGLibA_28721 | 84 | -0.312714815 | MEAF6 | MYST/Esa1 associated factor 6 [Source:HGNC Symbol;Acc:HGNC:25674] |
| ENSG00000164008 | HGLibA_05860 | 84 | -0.312714815 | C1orf50 | chromosome 1 open reading frame 50 [Source:HGNC Symbol;Acc:HGNC:28795] |
| ENSG00000164068 | HGLibA_41489 | 84 | -0.312714815 | RNF123 | ring finger protein 123 [Source:HGNC Symbol;Acc:HGNC:21148] |
| ENSG00000165060 | HGLibA_18215 | 84 | -0.312714815 | FXN | frataxin [Source:HGNC Symbol;Acc:HGNC:3951] |
| ENSG00000165209 | HGLibA_47581 | 84 | -0.312714815 | STRBP | spermatid perinuclear RNA binding protein [Source:HGNC Symbol;Acc:HGNC:16462] |
| ENSG00000165443 | HGLibA_36476 | 84 | -0.312714815 | PHYHIPL | phytanoyl-CoA 2-hydroxylase interacting protein like [Source:HGNC Symbol;Acc:HGNC:29378] |
| ENSG00000166049 | HGLibA_35265 | 84 | -0.312714815 | PASD1 | PAS domain containing repressor 1 [Source:HGNC Symbol;Acc:HGNC:20686] |
| ENSG00000166323 | HGLibA_05168 | 84 | -0.312714815 | C11orf65 | chromosome 11 open reading frame 65 [Source:HGNC Symbol;Acc:HGNC:28519] |
| ENSG00000166402 | HGLibA_52240 | 84 | -0.312714815 | TUB | tubby bipartite transcription factor [Source:HGNC Symbol;Acc:HGNC:12406] |
| ENSG00000166478 | HGLibA_55492 | 84 | -0.312714815 | ZNF143 | zinc finger protein 143 [Source:HGNC Symbol;Acc:HGNC:12928] |
| ENSG00000166750 | HGLibA_45496 | 84 | -0.312714815 | SLFN5 | schlafen family member 5 [Source:HGNC Symbol;Acc:HGNC:28286] |
| ENSG00000166889 | HGLibA_35283 | 84 | -0.312714815 | PATL1 | processing body mRNA decay factor [Source:HGNC Symbol;Acc:HGNC:26721]" |
| ENSG00000166902 | HGLibA_29908 | 84 | -0.312714815 | MRPL16 | mitochondrial ribosomal protein L16 [Source:HGNC Symbol;Acc:HGNC:14476] |
| ENSG00000167165 | HGLibA_52891 | 84 | -0.312714815 | UGT1A6 | UDP glucuronosyltransferase family 1 member A6 [Source:HGNC Symbol;Acc:HGNC:12538] |
| ENSG00000167553 | HGLibA_52249 | 84 | -0.312714815 | TUBA1C | tubulin alpha 1c [Source:HGNC Symbol;Acc:HGNC:20768] |
| ENSG00000168263 | HGLibA_24405 | 84 | -0.312714815 | KCNV2 | potassium voltage-gated channel modifier subfamily V member 2 [Source:HGNC Symbol;Acc:HGNC:19698] |
| ENSG00000168517 | HGLibA_21213 | 84 | -0.312714815 | HEXIM2 | hexamethylene bisacetamide inducible 2 [Source:HGNC Symbol;Acc:HGNC:28591] |
| ENSG00000168658 | HGLibA_53905 | 84 | -0.312714815 | VWA3B | von Willebrand factor A domain containing 3B [Source:HGNC Symbol;Acc:HGNC:28385] |
| ENSG00000168884 | HGLibA_50837 | 84 | -0.312714815 | TNIP2 | TNFAIP3 interacting protein 2 [Source:HGNC Symbol;Acc:HGNC:19118] |
| ENSG00000169045 | HGLibA_21823 | 84 | -0.312714815 | HNRNPH1 | heterogeneous nuclear ribonucleoprotein H1 [Source:HGNC Symbol;Acc:HGNC:5041] |
| ENSG00000169900 | HGLibA_39683 | 84 | -0.312714815 | PYDC1 | pyrin domain containing 1 [Source:HGNC Symbol;Acc:HGNC:30261] |
| ENSG00000169989 | HGLibA_49472 | 84 | -0.312714815 | TIGD4 | tigger transposable element derived 4 [Source:HGNC Symbol;Acc:HGNC:18335] |
| ENSG00000170584 | HGLibA_32924 | 84 | -0.312714815 | NUDCD2 | NudC domain containing 2 [Source:HGNC Symbol;Acc:HGNC:30535] |
| ENSG00000170605 | HGLibA_34580 | 84 | -0.312714815 | OR9K2 | olfactory receptor family 9 subfamily K member 2 [Source:HGNC Symbol;Acc:HGNC:15339] |
| ENSG00000171403 | HGLibA_25577 | 84 | -0.312714815 | KRT9 | keratin 9 [Source:HGNC Symbol;Acc:HGNC:6447] |
| ENSG00000172794 | HGLibA_39896 | 84 | -0.312714815 | RAB37 | member RAS oncogene family [Source:HGNC Symbol;Acc:HGNC:30268]" |
| ENSG00000172943 | HGLibA_36390 | 84 | -0.312714815 | PHF8 | PHD finger protein 8 [Source:HGNC Symbol;Acc:HGNC:20672] |

| | | | | | |
|---|---|---|---|---|---|
| ENSG00000173157 | HGLibA_00825 | 84 | -0.312714815 | ADAMTS20 | ADAM metallopeptidase with thrombospondin type 1 motif 20 [Source:HGNC Symbol;Acc:HGNC:17178] |
| ENSG00000173456 | HGLibA_41658 | 84 | -0.312714815 | RNF26 | ring finger protein 26 [Source:HGNC Symbol;Acc:HGNC:14646] |
| ENSG00000174417 | HGLibA_51383 | 84 | -0.312714815 | TRHR | thyrotropin releasing hormone receptor [Source:HGNC Symbol;Acc:HGNC:12299] |
| ENSG00000175104 | HGLibA_51264 | 84 | -0.312714815 | TRAF6 | TNF receptor associated factor 6 [Source:HGNC Symbol;Acc:HGNC:12036] |
| ENSG00000175262 | HGLibA_05765 | 84 | -0.312714815 | C1orf127 | chromosome 1 open reading frame 127 [Source:HGNC Symbol;Acc:HGNC:26730] |
| ENSG00000175294 | HGLibA_07354 | 84 | -0.312714815 | CATSPER1 | cation channel sperm associated 1 [Source:HGNC Symbol;Acc:HGNC:17116] |
| ENSG00000175895 | HGLibA_37098 | 84 | -0.312714815 | PLEKHF2 | pleckstrin homology and FYVE domain containing 2 [Source:HGNC Symbol;Acc:HGNC:20757] |
| ENSG00000176595 | HGLibA_24092 | 84 | -0.312714815 | KBTBD11 | kelch repeat and BTB domain containing 11 [Source:HGNC Symbol;Acc:HGNC:29104] |
| ENSG00000176746 | HGLibA_27956 | 84 | -0.312714815 | MAGEB6 | MAGE family member B6 [Source:HGNC Symbol;Acc:HGNC:23796] |
| ENSG00000177324 | HGLibA_04400 | 84 | -0.312714815 | BEND2 | BEN domain containing 2 [Source:HGNC Symbol;Acc:HGNC:28509] |
| ENSG00000177504 | HGLibA_53583 | 84 | -0.312714815 | VCX2 | variable charge X-linked 2 [Source:HGNC Symbol;Acc:HGNC:18158] |
| ENSG00000178662 | HGLibA_11429 | 84 | -0.312714815 | CSRNP3 | cysteine and serine rich nuclear protein 3 [Source:HGNC Symbol;Acc:HGNC:30729] |
| ENSG00000179528 | HGLibA_26089 | 84 | -0.312714815 | LBX2 | ladybird homeobox 2 [Source:HGNC Symbol;Acc:HGNC:15525] |
| ENSG00000181817 | HGLibA_27521 | 84 | -0.312714815 | LSM10 | U7 small nuclear RNA associated [Source:HGNC Symbol;Acc:HGNC:17562]" |
| ENSG00000182083 | HGLibA_34375 | 84 | -0.312714815 | OR6B2 | olfactory receptor family 6 subfamily B member 2 [Source:HGNC Symbol;Acc:HGNC:15041] |
| ENSG00000182156 | HGLibA_15185 | 84 | -0.312714815 | ENPP7 | ectonucleotide pyrophosphatase/phosphodiesterase 7 [Source:HGNC Symbol;Acc:HGNC:23764] |
| ENSG00000182447 | HGLibA_34731 | 84 | -0.312714815 | OTOL1 | otolin 1 [Source:HGNC Symbol;Acc:HGNC:34071] |
| ENSG00000182521 | HGLibA_48628 | 84 | -0.312714815 | TBPL2 | TATA-box binding protein like 2 [Source:HGNC Symbol;Acc:HGNC:19841] |
| ENSG00000182541 | HGLibA_26542 | 84 | -0.312714815 | LIMK2 | LIM domain kinase 2 [Source:HGNC Symbol;Acc:HGNC:6614] |
| ENSG00000182950 | HGLibA_33291 | 84 | -0.312714815 | ODF3L1 | outer dense fiber of sperm tails 3 like 1 [Source:HGNC Symbol;Acc:HGNC:28735] |
| ENSG00000184471 | HGLibA_05958 | 84 | -0.312714815 | C1QTNF8 | C1q and TNF related 8 [Source:HGNC Symbol;Acc:HGNC:31374] |
| ENSG00000185527 | HGLibA_35818 | 84 | -0.312714815 | PDE6G | phosphodiesterase 6G [Source:HGNC Symbol;Acc:HGNC:8789] |
| ENSG00000185800 | HGLibA_13451 | 84 | -0.312714815 | DMWD | WD repeat containing [Source:HGNC Symbol;Acc:HGNC:2936]" |
| ENSG00000185973 | HGLibA_50591 | 84 | -0.312714815 | TMLHE | epsilon [Source:HGNC Symbol;Acc:HGNC:18308]" |
| ENSG00000186714 | HGLibA_07860 | 84 | -0.312714815 | CCDC73 | coiled-coil domain containing 73 [Source:HGNC Symbol;Acc:HGNC:23261] |
| ENSG00000188315 | HGLibA_06256 | 84 | -0.312714815 | C3orf62 | chromosome 3 open reading frame 62 [Source:HGNC Symbol;Acc:HGNC:24771] |
| ENSG00000188389 | HGLibA_35716 | 84 | -0.312714815 | PDCD1 | programmed cell death 1 [Source:HGNC Symbol;Acc:HGNC:8760] |
| ENSG00000189042 | HGLibA_56263 | 84 | -0.312714815 | ZNF567 | zinc finger protein 567 [Source:HGNC Symbol;Acc:HGNC:28696] |
| ENSG00000196417 | HGLibA_56692 | 84 | -0.312714815 | ZNF765 | zinc finger protein 765 [Source:HGNC Symbol;Acc:HGNC:25092] |
| ENSG00000196628 | HGLibA_48787 | 84 | -0.312714815 | TCF4 | transcription factor 4 [Source:HGNC Symbol;Acc:HGNC:11634] |
| ENSG00000197110 | HGLibA_22724 | 84 | -0.312714815 | IFNL3 | interferon lambda 3 [Source:HGNC Symbol;Acc:HGNC:18365] |
| ENSG00000197408 | HGLibA_12111 | 84 | -0.312714815 | CYP2B6 | cytochrome P450 family 2 subfamily B member 6 [Source:HGNC Symbol;Acc:HGNC:2615] |
| ENSG00000197591 | HGLibA_33583 | 84 | -0.312714815 | OR11L1 | olfactory receptor family 11 subfamily L member 1 [Source:HGNC Symbol;Acc:HGNC:14998] |
| ENSG00000197958 | HGLibA_41866 | 84 | -0.312714815 | RPL12 | ribosomal protein L12 [Source:HGNC Symbol;Acc:HGNC:10302] |
| ENSG00000198021 | HGLibA_46378 | 84 | -0.312714815 | SPANXA1 | family member A1 [Source:HGNC Symbol;Acc:HGNC:11218]" |
| ENSG00000198077 | HGLibA_12108 | 84 | -0.312714815 | CYP2A7 | cytochrome P450 family 2 subfamily A member 7 [Source:HGNC Symbol;Acc:HGNC:2611] |
| ENSG00000198573 | HGLibA_46389 | 84 | -0.312714815 | SPANXC | SPANX family member C [Source:HGNC Symbol;Acc:HGNC:14331] |
| ENSG00000198818 | HGLibA_43750 | 84 | -0.312714815 | SFT2D1 | SFT2 domain containing 1 [Source:HGNC Symbol;Acc:HGNC:21102] |
| ENSG00000198914 | HGLibA_37770 | 84 | -0.312714815 | POU3F3 | POU class 3 homeobox 3 [Source:HGNC Symbol;Acc:HGNC:9216] |
| ENSG00000198925 | HGLibA_03506 | 84 | -0.312714815 | ATG9A | autophagy related 9A [Source:HGNC Symbol;Acc:HGNC:22408] |
| ENSG00000205356 | HGLibA_48940 | 84 | -0.312714815 | TECPR1 | tectonin beta-propeller repeat containing 1 [Source:HGNC Symbol;Acc:HGNC:22214] |
| ENSG00000206104 | HGLibA_25705 | 84 | -0.312714815 | KRTAP20-3 | keratin associated protein 20-3 [Source:HGNC Symbol;Acc:HGNC:34001] |
| ENSG00000212123 | HGLibA_38817 | 84 | -0.312714815 | PRR22 | proline rich 22 [Source:HGNC Symbol;Acc:HGNC:28354] |
| ENSG00000213024 | HGLibA_33062 | 84 | -0.312714815 | NUP62 | nucleoporin 62 [Source:HGNC Symbol;Acc:HGNC:8066] |
| ENSG00000213809 | HGLibA_25316 | 84 | -0.312714815 | KLRK1 | killer cell lectin like receptor K1 [Source:HGNC Symbol;Acc:HGNC:18788] |
| ENSG00000214711 | HGLibA_07158 | 84 | -0.312714815 | CAPN14 | calpain 14 [Source:HGNC Symbol;Acc:HGNC:16664] |
| ENSG00000215397 | HGLibA_43062 | 84 | -0.312714815 | SCRT2 | scratch family transcriptional repressor 2 [Source:HGNC Symbol;Acc:HGNC:15952] |
| ENSG00000219626 | HGLibA_16536 | 84 | -0.312714815 | FAM228B | family with sequence similarity 228 member B [Source:HGNC Symbol;Acc:HGNC:24736] |
| ENSG00000221864 | HGLibA_25633 | 84 | -0.312714815 | KRTAP12-2 | keratin associated protein 12-2 [Source:HGNC Symbol;Acc:HGNC:20530] |
| ENSG00000221996 | HGLibA_34148 | 84 | -0.312714815 | OR52B4 | olfactory receptor family 52 subfamily B member 4 (gene/pseudogene) [Source:HGNC Symbol;Acc:HGNC:15209] |
| ENSG00000227057 | HGLibA_54102 | 84 | -0.312714815 | WDR46 | WD repeat domain 46 [Source:HGNC Symbol;Acc:HGNC:13923] |
| ENSG00000228198 | HGLibA_33835 | 84 | -0.312714815 | OR2M3 | olfactory receptor family 2 subfamily M member 3 [Source:HGNC Symbol;Acc:HGNC:8269] |
| ENSG00000228672 | HGLibA_38648 | 84 | -0.312714815 | PROB1 | proline rich basic protein 1 [Source:HGNC Symbol;Acc:HGNC:41906] |
| ENSG00000230453 | HGLibA_02021 | 84 | -0.312714815 | ANKRD18B | ankyrin repeat domain 18B [Source:HGNC Symbol;Acc:HGNC:23644] |
| ENSG00000239672 | HGLibA_32150 | 84 | -0.312714815 | NME1 | NME/NM23 nucleoside diphosphate kinase 1 [Source:HGNC Symbol;Acc:HGNC:7849] |
| ENSG00000239732 | HGLibA_49666 | 84 | -0.312714815 | TLR9 | toll like receptor 9 [Source:HGNC Symbol;Acc:HGNC:15633] |
| ENSG00000241553 | HGLibA_03086 | 84 | -0.312714815 | ARPC4 | actin related protein 2/3 complex subunit 4 [Source:HGNC Symbol;Acc:HGNC:707] |
| ENSG00000253313 | HGLibA_05827 | 84 | -0.312714815 | C1orf210 | chromosome 1 open reading frame 210 [Source:HGNC Symbol;Acc:HGNC:28755] |
| ENSG00000253485 | HGLibA_35536 | 84 | -0.312714815 | PCDHGA5 | 5 [Source:HGNC Symbol;Acc:HGNC:8703]" |
| ENSG00000266173 | HGLibA_47573 | 84 | -0.312714815 | STRADA | STE20-related kinase adaptor alpha [Source:HGNC Symbol;Acc:HGNC:30172] |
| ENSG00000266967 | HGLibA_00073 | 84 | -0.312714815 | AARSD1 | alanyl-tRNA synthetase domain containing 1 [Source:HGNC Symbol;Acc:HGNC:28417] |
| ENSG00000268964 | HGLibA_15549 | 84 | -0.312714815 | ERVV-2 | envelope [Source:HGNC Symbol;Acc:HGNC:39051]" |
| ENSG00000269226 | HGLibA_50662 | 84 | -0.312714815 | TMSB15B | thymosin beta 15B [Source:NCBI gene;Acc:286527] |
| ENSG00000272920 | HGLibA_57408 | 84 | -0.312714815 | hsa-mir-1253 | hsa-mir-1253 [Source:miRBase;Acc:MI0006387] |
| FAM21C | HGLibA_16511 | 84 | -0.312714815 | FAM21C | - |
| FAM65A | HGLibA_16667 | 84 | -0.312714815 | FAM65A | - |
| FAM69A | HGLibA_16676 | 84 | -0.312714815 | FAM69A | - |
| HIST1H2AE | HGLibA_21386 | 84 | -0.312714815 | HIST1H2AE | - |
| hsa-mir-1225 | HGLibA_57299 | 84 | -0.312714815 | hsa-mir-1225 | - |
| hsa-mir-1298 | HGLibA_57673 | 84 | -0.312714815 | hsa-mir-1298 | - |
| hsa-mir-187 | HGLibA_58012 | 84 | -0.312714815 | hsa-mir-187 | - |
| hsa-mir-1911 | HGLibA_58051 | 84 | -0.312714815 | hsa-mir-1911 | - |
| hsa-mir-3147 | HGLibA_58647 | 84 | -0.312714815 | hsa-mir-3147 | - |
| hsa-mir-320d-1 | HGLibA_58944 | 84 | -0.312714815 | hsa-mir-320d-1 | - |
| hsa-mir-378d-2 | HGLibA_59442 | 84 | -0.312714815 | hsa-mir-378d-2 | - |
| hsa-mir-4253 | HGLibA_59719 | 84 | -0.312714815 | hsa-mir-4253 | - |
| hsa-mir-4461 | HGLibA_60249 | 84 | -0.312714815 | hsa-mir-4461 | - |
| hsa-mir-4494 | HGLibA_60394 | 84 | -0.312714815 | hsa-mir-4494 | - |
| hsa-mir-4636 | HGLibA_60649 | 84 | -0.312714815 | hsa-mir-4636 | - |
| hsa-mir-4640 | HGLibA_60664 | 84 | -0.312714815 | hsa-mir-4640 | - |
| hsa-mir-4689 | HGLibA_60865 | 84 | -0.312714815 | hsa-mir-4689 | - |
| hsa-mir-4776-2 | HGLibA_61213 | 84 | -0.312714815 | hsa-mir-4776-2 | - |
| hsa-mir-606 | HGLibA_62518 | 84 | -0.312714815 | hsa-mir-606 | - |
| hsa-mir-648 | HGLibA_62836 | 84 | -0.312714815 | hsa-mir-648 | - |
| hsa-mir-6870 | HGLibA_63666 | 84 | -0.312714815 | hsa-mir-6870 | - |
| hsa-mir-7150 | HGLibA_63830 | 84 | -0.312714815 | hsa-mir-7150 | - |
| hsa-mir-8061 | HGLibA_64081 | 84 | -0.312714815 | hsa-mir-8061 | - |
| hsa-mir-8065 | HGLibA_64099 | 84 | -0.312714815 | hsa-mir-8065 | - |
| IL9R_Y | HGLibA_23206 | 84 | -0.312714815 | IL9R_Y | - |
| LOC100130705 | HGLibA_26795 | 84 | -0.312714815 | LOC100130705 | - |
| SMCR7L | HGLibA_45679 | 84 | -0.312714815 | SMCR7L | - |
| ASMT_Y | HGLibA_03322 | 83 | -0.322665852 | ASMT_Y | - |
| ATP5H | HGLibA_03664 | 83 | -0.322665852 | ATP5H | - |
| C10orf12 | HGLibA_05042 | 83 | -0.322665852 | C10orf12 | - |
| C10orf68 | HGLibA_05089 | 83 | -0.322665852 | C10orf68 | - |
| C1orf233 | HGLibA_05846 | 83 | -0.322665852 | C1orf233 | - |
| C7orf34 | HGLibA_06539 | 83 | -0.322665852 | C7orf34 | - |
| ENSG00000005243 | HGLibA_10741 | 83 | -0.322665852 | COPZ2 | coatomer protein complex subunit zeta 2 [Source:HGNC Symbol;Acc:HGNC:19356] |
| ENSG00000005339 | HGLibA_11108 | 83 | -0.322665852 | CREBBP | CREB binding protein [Source:HGNC Symbol;Acc:HGNC:2348] |
| ENSG00000011422 | HGLibA_36967 | 83 | -0.322665852 | PLAUR | urokinase receptor [Source:HGNC Symbol;Acc:HGNC:9053]" |
| ENSG00000012223 | HGLibA_27607 | 83 | -0.322665852 | LTF | lactotransferrin [Source:HGNC Symbol;Acc:HGNC:6720] |
| ENSG00000029364 | HGLibA_45127 | 83 | -0.322665852 | SLC39A9 | solute carrier family 39 member 9 [Source:HGNC Symbol;Acc:HGNC:20182] |
| ENSG00000038358 | HGLibA_14445 | 83 | -0.322665852 | EDC4 | enhancer of mRNA decapping 4 [Source:HGNC Symbol;Acc:HGNC:17157] |
| ENSG00000044115 | HGLibA_11638 | 83 | -0.322665852 | CTNNA1 | catenin alpha 1 [Source:HGNC Symbol;Acc:HGNC:2509] |
| ENSG00000054690 | HGLibA_37124 | 83 | -0.322665852 | PLEKHH1 | MyTH4 and FERM domain containing H1 [Source:HGNC Symbol;Acc:HGNC:17733]" |
| ENSG00000056558 | HGLibA_51241 | 83 | -0.322665852 | TRAF1 | TNF receptor associated factor 1 [Source:HGNC Symbol;Acc:HGNC:12031] |
| ENSG00000062038 | HGLibA_08678 | 83 | -0.322665852 | CDH3 | cadherin 3 [Source:HGNC Symbol;Acc:HGNC:1762] |
| ENSG00000065809 | HGLibA_16004 | 83 | -0.322665852 | FAM107B | family with sequence similarity 107 member B [Source:HGNC Symbol;Acc:HGNC:23726] |
| ENSG00000066855 | HGLibA_30361 | 83 | -0.322665852 | MTFR1 | mitochondrial fission regulator 1 [Source:HGNC Symbol;Acc:HGNC:29510] |
| ENSG00000070729 | HGLibA_10275 | 83 | -0.322665852 | CNGB1 | cyclic nucleotide gated channel beta 1 [Source:HGNC Symbol;Acc:HGNC:2151] |
| ENSG00000075340 | HGLibA_00944 | 83 | -0.322665852 | ADD2 | adducin 2 [Source:HGNC Symbol;Acc:HGNC:244] |
| ENSG00000076928 | HGLibA_02803 | 83 | -0.322665852 | ARHGEF1 | Rho guanine nucleotide exchange factor 1 [Source:HGNC Symbol;Acc:HGNC:681] |
| ENSG00000077152 | HGLibA_52660 | 83 | -0.322665852 | UBE2T | ubiquitin conjugating enzyme E2 T [Source:HGNC Symbol;Acc:HGNC:25009] |
| ENSG00000080618 | HGLibA_10907 | 83 | -0.322665852 | CPB2 | carboxypeptidase B2 [Source:HGNC Symbol;Acc:HGNC:2300] |
| ENSG00000090674 | HGLibA_28638 | 83 | -0.322665852 | MCOLN1 | mucolipin 1 [Source:HGNC Symbol;Acc:HGNC:13356] |
| ENSG00000093167 | HGLibA_27453 | 83 | -0.322665852 | LRRFIP2 | LRR binding FLII interacting protein 2 [Source:HGNC Symbol;Acc:HGNC:6703] |
| ENSG00000100201 | HGLibA_12658 | 83 | -0.322665852 | DDX17 | DEAD-box helicase 17 [Source:HGNC Symbol;Acc:HGNC:2740] |
| ENSG00000100372 | HGLibA_44656 | 83 | -0.322665852 | SLC25A17 | solute carrier family 25 member 17 [Source:HGNC Symbol;Acc:HGNC:10987] |
| ENSG00000101977 | HGLibA_28577 | 83 | -0.322665852 | MCF2 | MCF.2 cell line derived transforming sequence [Source:HGNC Symbol;Acc:HGNC:6940] |
| ENSG00000103005 | HGLibA_53162 | 83 | -0.322665852 | USB1 | U6 snRNA biogenesis phosphodiesterase 1 [Source:HGNC Symbol;Acc:HGNC:25792] |
| ENSG00000103489 | HGLibA_54619 | 83 | -0.322665852 | XYLT1 | xylosyltransferase 1 [Source:HGNC Symbol;Acc:HGNC:15516] |
| ENSG00000103591 | HGLibA_00046 | 83 | -0.322665852 | AAGAB | alpha and gamma adaptin binding protein [Source:HGNC Symbol;Acc:HGNC:25662] |
| ENSG00000104164 | HGLibA_04571 | 83 | -0.322665852 | BLOC1S6 | biogenesis of lysosomal organelles complex 1 subunit 6 [Source:HGNC Symbol;Acc:HGNC:8549] |
| ENSG00000106809 | HGLibA_33326 | 83 | -0.322665852 | OGN | osteoglycin [Source:HGNC Symbol;Acc:HGNC:8126] |
| ENSG00000108819 | HGLibA_38130 | 83 | -0.322665852 | PPP1R9B | protein phosphatase 1 regulatory subunit 9B [Source:HGNC Symbol;Acc:HGNC:9298] |
| ENSG00000109046 | HGLibA_54415 | 83 | -0.322665852 | WSB1 | WD repeat and SOCS box containing 1 [Source:HGNC Symbol;Acc:HGNC:19221] |
| ENSG00000109511 | HGLibA_02223 | 83 | -0.322665852 | ANXA10 | annexin A10 [Source:HGNC Symbol;Acc:HGNC:534] |
| ENSG00000111846 | HGLibA_18811 | 83 | -0.322665852 | GCNT2 | glucosaminyl (N-acetyl) transferase 2 (I blood group) [Source:HGNC Symbol;Acc:HGNC:4204] |
| ENSG00000111863 | HGLibA_01082 | 83 | -0.322665852 | ADTRP | androgen dependent TFPI regulating protein [Source:HGNC Symbol;Acc:HGNC:21214] |
| ENSG00000112273 | HGLibA_21041 | 83 | -0.322665852 | HDGFL1 | HDGF like 1 [Source:HGNC Symbol;Acc:HGNC:21095] |
| ENSG00000112419 | HGLibA_36299 | 83 | -0.322665852 | PHACTR2 | phosphatase and actin regulator 2 [Source:HGNC Symbol;Acc:HGNC:20956] |
| ENSG00000114742 | HGLibA_54108 | 83 | -0.322665852 | WDR48 | WD repeat domain 48 [Source:HGNC Symbol;Acc:HGNC:30914] |
| ENSG00000114850 | HGLibA_47158 | 83 | -0.322665852 | SSR3 | signal sequence receptor subunit 3 [Source:HGNC Symbol;Acc:HGNC:11325] |
| ENSG00000115009 | HGLibA_07991 | 83 | -0.322665852 | CCL20 | C-C motif chemokine ligand 20 [Source:HGNC Symbol;Acc:HGNC:10619] |
| ENSG00000115364 | HGLibA_29918 | 83 | -0.322665852 | MRPL19 | mitochondrial ribosomal protein L19 [Source:HGNC Symbol;Acc:HGNC:14052] |

| | | | | | |
|---|---|---|---|---|---|
| ENSG00000115657 | HGLibA_00147 | 83 | -0.322665852 | ABCB6 | ATP binding cassette subfamily B member 6 (Langereis blood group) [Source:HGNC Symbol;Acc:HGNC:47] |
| ENSG00000115850 | HGLibA_26204 | 83 | -0.322665852 | LCT | lactase [Source:HGNC Symbol;Acc:HGNC:6530] |
| ENSG00000116199 | HGLibA_16447 | 83 | -0.322665852 | FAM20B | glycosaminoglycan xylosylkinase [Source:HGNC Symbol;Acc:HGNC:23017]" |
| ENSG00000116678 | HGLibA_26312 | 83 | -0.322665852 | LEPR | leptin receptor [Source:HGNC Symbol;Acc:HGNC:6554] |
| ENSG00000117143 | HGLibA_52507 | 83 | -0.322665852 | UAP1 | UDP-N-acetylglucosamine pyrophosphorylase 1 [Source:HGNC Symbol;Acc:HGNC:12457] |
| ENSG00000118777 | HGLibA_00219 | 83 | -0.322665852 | ABCG2 | ATP binding cassette subfamily G member 2 (Junior blood group) [Source:HGNC Symbol;Acc:HGNC:74] |
| ENSG00000119737 | HGLibA_20079 | 83 | -0.322665852 | GPR75 | G protein-coupled receptor 75 [Source:HGNC Symbol;Acc:HGNC:4526] |
| ENSG00000119737 | HGLibA_20081 | 83 | -0.322665852 | GPR75 | G protein-coupled receptor 75 [Source:HGNC Symbol;Acc:HGNC:4526] |
| ENSG00000120451 | HGLibA_46055 | 83 | -0.322665852 | SNX19 | sorting nexin 19 [Source:HGNC Symbol;Acc:HGNC:21532] |
| ENSG00000120686 | HGLibA_52852 | 83 | -0.322665852 | UFM1 | ubiquitin fold modifier 1 [Source:HGNC Symbol;Acc:HGNC:20597] |
| ENSG00000120913 | HGLibA_35911 | 83 | -0.322665852 | PDLIM2 | PDZ and LIM domain 2 [Source:HGNC Symbol;Acc:HGNC:13992] |
| ENSG00000121577 | HGLibA_37699 | 83 | -0.322665852 | POPDC2 | popeye domain containing 2 [Source:HGNC Symbol;Acc:HGNC:17648] |
| ENSG00000125347 | HGLibA_23571 | 83 | -0.322665852 | IRF1 | interferon regulatory factor 1 [Source:HGNC Symbol;Acc:HGNC:6116] |
| ENSG00000125944 | HGLibA_21843 | 83 | -0.322665852 | HNRNPR | heterogeneous nuclear ribonucleoprotein R [Source:HGNC Symbol;Acc:HGNC:5047] |
| ENSG00000127528 | HGLibA_25066 | 83 | -0.322665852 | KLF2 | Kruppel like factor 2 [Source:HGNC Symbol;Acc:HGNC:6347] |
| ENSG00000127774 | HGLibA_15016 | 83 | -0.322665852 | EMC6 | ER membrane protein complex subunit 6 [Source:HGNC Symbol;Acc:HGNC:28430] |
| ENSG00000127952 | HGLibA_47689 | 83 | -0.322665852 | STYXL1 | serine/threonine/tyrosine interacting like 1 [Source:HGNC Symbol;Acc:HGNC:18165] |
| ENSG00000128039 | HGLibA_46945 | 83 | -0.322665852 | SRD5A3 | steroid 5 alpha-reductase 3 [Source:HGNC Symbol;Acc:HGNC:25812] |
| ENSG00000128165 | HGLibA_00996 | 83 | -0.322665852 | ADM2 | adrenomedullin 2 [Source:HGNC Symbol;Acc:HGNC:28898] |
| ENSG00000130522 | HGLibA_24010 | 83 | -0.322665852 | JUND | AP-1 transcription factor subunit [Source:HGNC Symbol;Acc:HGNC:6206]" |
| ENSG00000130726 | HGLibA_51451 | 83 | -0.322665852 | TRIM28 | tripartite motif containing 28 [Source:HGNC Symbol;Acc:HGNC:16384] |
| ENSG00000132005 | HGLibA_40963 | 83 | -0.322665852 | RFX1 | regulatory factor X1 [Source:HGNC Symbol;Acc:HGNC:9982] |
| ENSG00000132436 | HGLibA_17523 | 83 | -0.322665852 | FIGNL1 | fidgetin like 1 [Source:HGNC Symbol;Acc:HGNC:13286] |
| ENSG00000132681 | HGLibA_03589 | 83 | -0.322665852 | ATP1A4 | ATPase Na+/K+ transporting subunit alpha 4 [Source:HGNC Symbol;Acc:HGNC:14073] |
| ENSG00000134996 | HGLibA_34710 | 83 | -0.322665852 | OSTF1 | osteoclast stimulating factor 1 [Source:HGNC Symbol;Acc:HGNC:8510] |
| ENSG00000135549 | HGLibA_36822 | 83 | -0.322665852 | PKIB | cAMP-dependent protein kinase inhibitor beta [Source:HGNC Symbol;Acc:HGNC:9018] |
| ENSG00000136159 | HGLibA_32948 | 83 | -0.322665852 | NUDT15 | nudix hydrolase 15 [Source:HGNC Symbol;Acc:HGNC:23063] |
| ENSG00000136488 | HGLibA_11350 | 83 | -0.322665852 | CSH1 | chorionic somatomammotropin hormone 1 [Source:HGNC Symbol;Acc:HGNC:2440] |
| ENSG00000136824 | HGLibA_45648 | 83 | -0.322665852 | SMC2 | structural maintenance of chromosomes 2 [Source:HGNC Symbol;Acc:HGNC:14011] |
| ENSG00000137269 | HGLibA_27245 | 83 | -0.322665852 | LRRC1 | leucine rich repeat containing 1 [Source:HGNC Symbol;Acc:HGNC:14307] |
| ENSG00000137522 | HGLibA_41485 | 83 | -0.322665852 | RNF121 | ring finger protein 121 [Source:HGNC Symbol;Acc:HGNC:21070] |
| ENSG00000137709 | HGLibA_37761 | 83 | -0.322665852 | POU2F3 | POU class 2 homeobox 3 [Source:HGNC Symbol;Acc:HGNC:19864] |
| ENSG00000137877 | HGLibA_46889 | 83 | -0.322665852 | SPTBN5 | non-erythrocytic 5 [Source:HGNC Symbol;Acc:HGNC:15680]" |
| ENSG00000138660 | HGLibA_02279 | 83 | -0.322665852 | AP1AR | adaptor related protein complex 1 associated regulatory protein [Source:HGNC Symbol;Acc:HGNC:28808] |
| ENSG00000139174 | HGLibA_38470 | 83 | -0.322665852 | PRICKLE1 | prickle planar cell polarity protein 1 [Source:HGNC Symbol;Acc:HGNC:17019] |
| ENSG00000139364 | HGLibA_49935 | 83 | -0.322665852 | TMEM132B | transmembrane protein 132B [Source:HGNC Symbol;Acc:HGNC:29397] |
| ENSG00000139970 | HGLibA_42398 | 83 | -0.322665852 | RTN1 | reticulon 1 [Source:HGNC Symbol;Acc:HGNC:10467] |
| ENSG00000140382 | HGLibA_21678 | 83 | -0.322665852 | HMG20A | high mobility group 20A [Source:HGNC Symbol;Acc:HGNC:5001] |
| ENSG00000140563 | HGLibA_28655 | 83 | -0.322665852 | MCTP2 | multiple C2 and transmembrane domain containing 2 [Source:HGNC Symbol;Acc:HGNC:25636] |
| ENSG00000140931 | HGLibA_10215 | 83 | -0.322665852 | CMTM3 | CKLF like MARVEL transmembrane domain containing 3 [Source:HGNC Symbol;Acc:HGNC:19174] |
| ENSG00000142230 | HGLibA_42633 | 83 | -0.322665852 | SAE1 | SUMO1 activating enzyme subunit 1 [Source:HGNC Symbol;Acc:HGNC:30660] |
| ENSG00000143353 | HGLibA_27743 | 83 | -0.322665852 | LYPLAL1 | lysophospholipase like 1 [Source:HGNC Symbol;Acc:HGNC:20440] |
| ENSG00000143442 | HGLibA_37479 | 83 | -0.322665852 | POGZ | pogo transposable element derived with ZNF domain [Source:HGNC Symbol;Acc:HGNC:18801] |
| ENSG00000144026 | HGLibA_56141 | 83 | -0.322665852 | ZNF514 | zinc finger protein 514 [Source:HGNC Symbol;Acc:HGNC:25894] |
| ENSG00000144043 | HGLibA_49083 | 83 | -0.322665852 | TEX261 | testis expressed 261 [Source:HGNC Symbol;Acc:HGNC:30712] |
| ENSG00000145428 | HGLibA_41578 | 83 | -0.322665852 | RNF175 | ring finger protein 175 [Source:HGNC Symbol;Acc:HGNC:27735] |
| ENSG00000145592 | HGLibA_41965 | 83 | -0.322665852 | RPL37 | ribosomal protein L37 [Source:HGNC Symbol;Acc:HGNC:10347] |
| ENSG00000145723 | HGLibA_19098 | 83 | -0.322665852 | GIN1 | gypsy retrotransposon integrase 1 [Source:HGNC Symbol;Acc:HGNC:25959] |
| ENSG00000145725 | HGLibA_37956 | 83 | -0.322665852 | PPIP5K2 | diphosphoinositol pentakisphosphate kinase 2 [Source:HGNC Symbol;Acc:HGNC:29035] |
| ENSG00000145979 | HGLibA_48562 | 83 | -0.322665852 | TBC1D7 | TBC1 domain family member 7 [Source:HGNC Symbol;Acc:HGNC:21066] |
| ENSG00000147164 | HGLibA_46032 | 83 | -0.322665852 | SNX12 | sorting nexin 12 [Source:HGNC Symbol;Acc:HGNC:14976] |
| ENSG00000147256 | HGLibA_02768 | 83 | -0.322665852 | ARHGAP36 | Rho GTPase activating protein 36 [Source:HGNC Symbol;Acc:HGNC:26388] |
| ENSG00000147873 | HGLibA_22683 | 83 | -0.322665852 | IFNA5 | interferon alpha 5 [Source:HGNC Symbol;Acc:HGNC:5426] |
| ENSG00000148143 | HGLibA_56042 | 83 | -0.322665852 | ZNF462 | zinc finger protein 462 [Source:HGNC Symbol;Acc:HGNC:21684] |
| ENSG00000148180 | HGLibA_20437 | 83 | -0.322665852 | GSN | gelsolin [Source:HGNC Symbol;Acc:HGNC:4620] |
| ENSG00000148291 | HGLibA_47834 | 83 | -0.322665852 | SURF2 | surfeit 2 [Source:HGNC Symbol;Acc:HGNC:11475] |
| ENSG00000150556 | HGLibA_27732 | 83 | -0.322665852 | LYPD6B | LY6/PLAUR domain containing 6B [Source:HGNC Symbol;Acc:HGNC:27018] |
| ENSG00000150750 | HGLibA_05154 | 83 | -0.322665852 | C11orf53 | chromosome 11 open reading frame 53 [Source:HGNC Symbol;Acc:HGNC:30527] |
| ENSG00000151023 | HGLibA_15137 | 83 | -0.322665852 | ENKUR | TRPC channel interacting protein [Source:HGNC Symbol;Acc:HGNC:28388]" |
| ENSG00000151498 | HGLibA_00360 | 83 | -0.322665852 | ACAD8 | acyl-CoA dehydrogenase family member 8 [Source:HGNC Symbol;Acc:HGNC:87] |
| ENSG00000151704 | HGLibA_24247 | 83 | -0.322665852 | KCNJ1 | potassium voltage-gated channel subfamily J member 1 [Source:HGNC Symbol;Acc:HGNC:6255] |
| ENSG00000152284 | HGLibA_48793 | 83 | -0.322665852 | TCF7L1 | transcription factor 7 like 1 [Source:HGNC Symbol;Acc:HGNC:11640] |
| ENSG00000152465 | HGLibA_32208 | 83 | -0.322665852 | NMT2 | N-myristoyltransferase 2 [Source:HGNC Symbol;Acc:HGNC:7858] |
| ENSG00000153933 | HGLibA_13068 | 83 | -0.322665852 | DGKE | diacylglycerol kinase epsilon [Source:HGNC Symbol;Acc:HGNC:2852] |
| ENSG00000153993 | HGLibA_43327 | 83 | -0.322665852 | SEMA3D | semaphorin 3D [Source:HGNC Symbol;Acc:HGNC:10726] |
| ENSG00000156284 | HGLibA_09910 | 83 | -0.322665852 | CLDN8 | claudin 8 [Source:HGNC Symbol;Acc:HGNC:2050] |
| ENSG00000157303 | HGLibA_47849 | 83 | -0.322665852 | SUSD3 | sushi domain containing 3 [Source:HGNC Symbol;Acc:HGNC:28391] |
| ENSG00000157343 | HGLibA_03010 | 83 | -0.322665852 | ARMC12 | armadillo repeat containing 12 [Source:HGNC Symbol;Acc:HGNC:21099] |
| ENSG00000157379 | HGLibA_13119 | 83 | -0.322665852 | DHRS1 | dehydrogenase/reductase 1 [Source:HGNC Symbol;Acc:HGNC:16445] |
| ENSG00000157778 | HGLibA_39211 | 83 | -0.322665852 | PSMG3 | proteasome assembly chaperone 3 [Source:HGNC Symbol;Acc:HGNC:22420] |
| ENSG00000159184 | HGLibA_21930 | 83 | -0.322665852 | HOXB13 | homeobox B13 [Source:HGNC Symbol;Acc:HGNC:5112] |
| ENSG00000159374 | HGLibA_27813 | 83 | -0.322665852 | M1AP | meiosis 1 associated protein [Source:HGNC Symbol;Acc:HGNC:25183] |
| ENSG00000159388 | HGLibA_04945 | 83 | -0.322665852 | BTG2 | BTG anti-proliferation factor 2 [Source:HGNC Symbol;Acc:HGNC:1131] |
| ENSG00000160396 | HGLibA_21349 | 83 | -0.322665852 | HIPK4 | homeodomain interacting protein kinase 4 [Source:HGNC Symbol;Acc:HGNC:19007] |
| ENSG00000162104 | HGLibA_00933 | 83 | -0.322665852 | ADCY9 | adenylate cyclase 9 [Source:HGNC Symbol;Acc:HGNC:240] |
| ENSG00000162711 | HGLibA_32119 | 83 | -0.322665852 | NLRP3 | NLR family pyrin domain containing 3 [Source:HGNC Symbol;Acc:HGNC:16400] |
| ENSG00000163285 | HGLibA_18388 | 83 | -0.322665852 | GABRG1 | gamma-aminobutyric acid type A receptor gamma1 subunit [Source:HGNC Symbol;Acc:HGNC:4086] |
| ENSG00000163510 | HGLibA_11816 | 83 | -0.322665852 | CWC22 | CWC22 spliceosome associated protein homolog [Source:HGNC Symbol;Acc:HGNC:29322] |
| ENSG00000164007 | HGLibA_09874 | 83 | -0.322665852 | CLDN19 | claudin 19 [Source:HGNC Symbol;Acc:HGNC:2040] |
| ENSG00000164056 | HGLibA_46836 | 83 | -0.322665852 | SPRY1 | sprouty RTK signaling antagonist 1 [Source:HGNC Symbol;Acc:HGNC:11269] |
| ENSG00000164076 | HGLibA_07098 | 83 | -0.322665852 | CAMKV | CaM kinase like vesicle associated [Source:HGNC Symbol;Acc:HGNC:28788] |
| ENSG00000164270 | HGLibA_22396 | 83 | -0.322665852 | HTR4 | 5-hydroxytryptamine receptor 4 [Source:HGNC Symbol;Acc:HGNC:5299] |
| ENSG00000164400 | HGLibA_11327 | 83 | -0.322665852 | CSF2 | colony stimulating factor 2 [Source:HGNC Symbol;Acc:HGNC:2434] |
| ENSG00000164434 | HGLibA_15929 | 83 | -0.322665852 | FABP7 | fatty acid binding protein 7 [Source:HGNC Symbol;Acc:HGNC:3562] |
| ENSG00000164742 | HGLibA_00905 | 83 | -0.322665852 | ADCY1 | adenylate cyclase 1 [Source:HGNC Symbol;Acc:HGNC:232] |
| ENSG00000165275 | HGLibA_51662 | 83 | -0.322665852 | TRMT10B | tRNA methyltransferase 10B [Source:HGNC Symbol;Acc:HGNC:26454] |
| ENSG00000165879 | HGLibA_17978 | 83 | -0.322665852 | FRAT1 | WNT signaling pathway regulator [Source:HGNC Symbol;Acc:HGNC:3944]" |
| ENSG00000166401 | HGLibA_43564 | 83 | -0.322665852 | SERPINB8 | serpin family B member 8 [Source:HGNC Symbol;Acc:HGNC:8952] |
| ENSG00000166479 | HGLibA_50693 | 83 | -0.322665852 | TMX3 | thioredoxin related transmembrane protein 3 [Source:HGNC Symbol;Acc:HGNC:24718] |
| ENSG00000166822 | HGLibA_50053 | 83 | -0.322665852 | TMEM170A | transmembrane protein 170A [Source:HGNC Symbol;Acc:HGNC:29577] |
| ENSG00000167535 | HGLibA_06924 | 83 | -0.322665852 | CACNB3 | calcium voltage-gated channel auxiliary subunit beta 3 [Source:HGNC Symbol;Acc:HGNC:1403] |
| ENSG00000167566 | HGLibA_31320 | 83 | -0.322665852 | NCKAP5L | NCK associated protein 5 like [Source:HGNC Symbol;Acc:HGNC:29321] |
| ENSG00000167851 | HGLibA_08334 | 83 | -0.322665852 | CD300A | CD300a molecule [Source:HGNC Symbol;Acc:HGNC:19319] |
| ENSG00000167874 | HGLibA_50537 | 83 | -0.322665852 | TMEM88 | transmembrane protein 88 [Source:HGNC Symbol;Acc:HGNC:32371] |
| ENSG00000168702 | HGLibA_27208 | 83 | -0.322665852 | LRP1B | LDL receptor related protein 1B [Source:HGNC Symbol;Acc:HGNC:6693] |
| ENSG00000169087 | HGLibA_22324 | 83 | -0.322665852 | HSPBAP1 | HSPB1 associated protein 1 [Source:HGNC Symbol;Acc:HGNC:16389] |
| ENSG00000169105 | HGLibA_09624 | 83 | -0.322665852 | CHST14 | carbohydrate sulfotransferase 14 [Source:HGNC Symbol;Acc:HGNC:24464] |
| ENSG00000169410 | HGLibA_39480 | 83 | -0.322665852 | PTPN9 | non-receptor type 9 [Source:HGNC Symbol;Acc:HGNC:9661]" |
| ENSG00000169800 | HGLibA_40624 | 83 | -0.322665852 | RBMY1F | member F [Source:HGNC Symbol;Acc:HGNC:23974]" |
| ENSG00000169919 | HGLibA_20678 | 83 | -0.322665852 | GUSB | glucuronidase beta [Source:HGNC Symbol;Acc:HGNC:4696] |
| ENSG00000170242 | HGLibA_53381 | 83 | -0.322665852 | USP47 | ubiquitin specific peptidase 47 [Source:HGNC Symbol;Acc:HGNC:20076] |
| ENSG00000170325 | HGLibA_38366 | 83 | -0.322665852 | PRDM10 | PR/SET domain 10 [Source:HGNC Symbol;Acc:HGNC:13995] |
| ENSG00000170412 | HGLibA_20132 | 83 | -0.322665852 | GPRC5C | G protein-coupled receptor class C group 5 member C [Source:HGNC Symbol;Acc:HGNC:13309] |
| ENSG00000170482 | HGLibA_44607 | 83 | -0.322665852 | SLC23A1 | solute carrier family 23 member 1 [Source:HGNC Symbol;Acc:HGNC:10974] |
| ENSG00000171103 | HGLibA_51701 | 83 | -0.322665852 | TRMT61B | tRNA methyltransferase 61B [Source:HGNC Symbol;Acc:HGNC:26070] |
| ENSG00000171135 | HGLibA_23909 | 83 | -0.322665852 | JAGN1 | jagunal homolog 1 [Source:HGNC Symbol;Acc:HGNC:26926] |
| ENSG00000171163 | HGLibA_56555 | 83 | -0.322665852 | ZNF692 | zinc finger protein 692 [Source:HGNC Symbol;Acc:HGNC:26049] |
| ENSG00000171408 | HGLibA_35825 | 83 | -0.322665852 | PDE7B | phosphodiesterase 7B [Source:HGNC Symbol;Acc:HGNC:8792] |
| ENSG00000171806 | HGLibA_28970 | 83 | -0.322665852 | METTL18 | methyltransferase like 18 [Source:HGNC Symbol;Acc:HGNC:28793] |
| ENSG00000171864 | HGLibA_38641 | 83 | -0.322665852 | PRND | prion like protein doppel [Source:HGNC Symbol;Acc:HGNC:15748] |
| ENSG00000172915 | HGLibA_31207 | 83 | -0.322665852 | NBEA | neurobeachin [Source:HGNC Symbol;Acc:HGNC:7648] |
| ENSG00000173156 | HGLibA_41183 | 83 | -0.322665852 | RHOD | ras homolog family member D [Source:HGNC Symbol;Acc:HGNC:670] |
| ENSG00000173626 | HGLibA_51320 | 83 | -0.322665852 | TRAPPC3L | trafficking protein particle complex 3 like [Source:HGNC Symbol;Acc:HGNC:21090] |
| ENSG00000174292 | HGLibA_50843 | 83 | -0.322665852 | TNK1 | tyrosine kinase non receptor 1 [Source:HGNC Symbol;Acc:HGNC:11940] |
| ENSG00000174989 | HGLibA_17157 | 83 | -0.322665852 | FBXW8 | F-box and WD repeat domain containing 8 [Source:HGNC Symbol;Acc:HGNC:13597] |
| ENSG00000175164 | HGLibA_00317 | 83 | -0.322665852 | ABO | alpha 1-3-N-acetylgalactosaminyltransferase and alpha 1-3-galactosyltransferase [Source:HGNC Symbol;Acc:HGNC:79]" |
| ENSG00000175203 | HGLibA_12547 | 83 | -0.322665852 | DCTN2 | dynactin subunit 2 [Source:HGNC Symbol;Acc:HGNC:2712] |
| ENSG00000175826 | HGLibA_11603 | 83 | -0.322665852 | CTDNEP1 | CTD nuclear envelope phosphatase 1 [Source:HGNC Symbol;Acc:HGNC:19085] |
| ENSG00000177201 | HGLibA_33859 | 83 | -0.322665852 | OR2T12 | olfactory receptor family 2 subfamily T member 12 [Source:HGNC Symbol;Acc:HGNC:19592] |
| ENSG00000177426 | HGLibA_49238 | 83 | -0.322665852 | TGIF1 | TGFB induced factor homeobox 1 [Source:HGNC Symbol;Acc:HGNC:11776] |
| ENSG00000179817 | HGLibA_29856 | 83 | -0.322665852 | MRGPRX4 | MAS related GPR family member X4 [Source:HGNC Symbol;Acc:HGNC:17617] |
| ENSG00000180219 | HGLibA_16692 | 83 | -0.322665852 | FAM71C | family with sequence similarity 71 member C [Source:HGNC Symbol;Acc:HGNC:28594] |
| ENSG00000180329 | HGLibA_07788 | 83 | -0.322665852 | CCDC43 | coiled-coil domain containing 43 [Source:HGNC Symbol;Acc:HGNC:26472] |
| ENSG00000181609 | HGLibA_34154 | 83 | -0.322665852 | OR52D1 | olfactory receptor family 52 subfamily D member 1 [Source:HGNC Symbol;Acc:HGNC:15212] |
| ENSG00000182393 | HGLibA_22720 | 83 | -0.322665852 | IFNL1 | interferon lambda 1 [Source:HGNC Symbol;Acc:HGNC:18363] |
| ENSG00000182566 | HGLibA_09993 | 83 | -0.322665852 | CLEC4G | C-type lectin domain family 4 member G [Source:HGNC Symbol;Acc:HGNC:24591] |
| ENSG00000184434 | HGLibA_27274 | 83 | -0.322665852 | LRRC19 | leucine rich repeat containing 19 [Source:HGNC Symbol;Acc:HGNC:23379] |
| ENSG00000186113 | HGLibA_34276 | 83 | -0.322665852 | OR5D14 | olfactory receptor family 5 subfamily D member 14 [Source:HGNC Symbol;Acc:HGNC:15281] |
| ENSG00000186943 | HGLibA_33609 | 83 | -0.322665852 | OR13C8 | olfactory receptor family 13 subfamily C member 8 [Source:HGNC Symbol;Acc:HGNC:15103] |
| ENSG00000187650 | HGLibA_53688 | 83 | -0.322665852 | VMAC | vimentin type intermediate filament associated coiled-coil protein [Source:HGNC Symbol;Acc:HGNC:33803] |
| ENSG00000188315 | HGLibA_06258 | 83 | -0.322665852 | C3orf62 | chromosome 3 open reading frame 62 [Source:HGNC Symbol;Acc:HGNC:24771] |
| ENSG00000189157 | HGLibA_16616 | 83 | -0.322665852 | FAM47E | family with sequence similarity 47 member E [Source:HGNC Symbol;Acc:HGNC:34343] |
| ENSG00000196090 | HGLibA_39539 | 83 | -0.322665852 | PTPRT | receptor type T [Source:HGNC Symbol;Acc:HGNC:9682]" |
| ENSG00000196260 | HGLibA_43761 | 83 | -0.322665852 | SFTA2 | surfactant associated 2 [Source:HGNC Symbol;Acc:HGNC:18386] |
| ENSG00000196458 | HGLibA_56366 | 83 | -0.322665852 | ZNF605 | zinc finger protein 605 [Source:HGNC Symbol;Acc:HGNC:28068] |

| | | | | | |
|---|---|---|---|---|---|
| ENSG00000196482 | HGLibA_15600 | 83 | -0.322665852 | ESRRG | estrogen related receptor gamma [Source:HGNC Symbol;Acc:HGNC:3474] |
| ENSG00000197471 | HGLibA_46730 | 83 | -0.322665852 | SPN | sialophorin [Source:HGNC Symbol;Acc:HGNC:11249] |
| ENSG00000197568 | HGLibA_21274 | 83 | -0.322665852 | HHLA3 | HERV-H LTR-associating 3 [Source:HGNC Symbol;Acc:HGNC:4906] |
| ENSG00000197779 | HGLibA_56799 | 83 | -0.322665852 | ZNF81 | zinc finger protein 81 [Source:HGNC Symbol;Acc:HGNC:13156] |
| ENSG00000198028 | HGLibA_56241 | 83 | -0.322665852 | ZNF560 | zinc finger protein 560 [Source:HGNC Symbol;Acc:HGNC:26484] |
| ENSG00000198478 | HGLibA_43918 | 83 | -0.322665852 | SH3BGRL2 | SH3 domain binding glutamate rich protein like 2 [Source:HGNC Symbol;Acc:HGNC:15567] |
| ENSG00000198670 | HGLibA_27049 | 83 | -0.322665852 | LPA | lipoprotein(a) [Source:HGNC Symbol;Acc:HGNC:6667] |
| ENSG00000198914 | HGLibA_37772 | 83 | -0.322665852 | POU3F3 | POU class 3 homeobox 3 [Source:HGNC Symbol;Acc:HGNC:9216] |
| ENSG00000204577 | HGLibA_26514 | 83 | -0.322665852 | LILRB3 | leukocyte immunoglobulin like receptor B3 [Source:HGNC Symbol;Acc:HGNC:6607] |
| ENSG00000204640 | HGLibA_32202 | 83 | -0.322665852 | NMS | neuromedin S [Source:HGNC Symbol;Acc:HGNC:32203] |
| ENSG00000204659 | HGLibA_07471 | 83 | -0.322665852 | CBY3 | chibby family member 3 [Source:HGNC Symbol;Acc:HGNC:33278] |
| ENSG00000204661 | HGLibA_06417 | 83 | -0.322665852 | C5orf60 | chromosome 5 open reading frame 60 [Source:HGNC Symbol;Acc:HGNC:27753] |
| ENSG00000204913 | HGLibA_27348 | 83 | -0.322665852 | LRRC3C | leucine rich repeat containing 3C [Source:HGNC Symbol;Acc:HGNC:40034] |
| ENSG00000204967 | HGLibA_35445 | 83 | -0.322665852 | PCDHA4 | protocadherin alpha 4 [Source:HGNC Symbol;Acc:HGNC:8670] |
| ENSG00000205220 | HGLibA_39096 | 83 | -0.322665852 | PSMB10 | proteasome subunit beta 10 [Source:HGNC Symbol;Acc:HGNC:9538] |
| ENSG00000205560 | HGLibA_11020 | 83 | -0.322665852 | CPT1B | carnitine palmitoyltransferase 1B [Source:HGNC Symbol;Acc:HGNC:2329] |
| ENSG00000206102 | HGLibA_25683 | 83 | -0.322665852 | KRTAP19-8 | keratin associated protein 19-8 [Source:HGNC Symbol;Acc:HGNC:33898] |
| ENSG00000213918 | HGLibA_13652 | 83 | -0.322665852 | DNASE1 | deoxyribonuclease 1 [Source:HGNC Symbol;Acc:HGNC:2956] |
| ENSG00000213996 | HGLibA_49711 | 83 | -0.322665852 | TM6SF2 | transmembrane 6 superfamily member 2 [Source:HGNC Symbol;Acc:HGNC:11861] |
| ENSG00000214435 | HGLibA_03182 | 83 | -0.322665852 | AS3MT | arsenite methyltransferase [Source:HGNC Symbol;Acc:HGNC:17452] |
| ENSG00000214595 | HGLibA_15070 | 83 | -0.322665852 | EML6 | echinoderm microtubule associated protein like 6 [Source:HGNC Symbol;Acc:HGNC:35412] |
| ENSG00000221836 | HGLibA_33740 | 83 | -0.322665852 | OR2A5 | olfactory receptor family 2 subfamily A member 5 [Source:HGNC Symbol;Acc:HGNC:8232] |
| ENSG00000231738 | HGLibA_51918 | 83 | -0.322665852 | TSPAN19 | tetraspanin 19 [Source:HGNC Symbol;Acc:HGNC:31886] |
| ENSG00000232382 | HGLibA_34311 | 83 | -0.322665852 | OR5K1 | olfactory receptor family 5 subfamily K member 1 [Source:HGNC Symbol;Acc:HGNC:8349] |
| ENSG00000235699 | HGLibA_11940 | 83 | -0.322665852 | CXorf51B | chromosome X open reading frame 51B [Source:HGNC Symbol;Acc:HGNC:42787] |
| ENSG00000237289 | HGLibA_09782 | 83 | -0.322665852 | CKMT1B | mitochondrial 1B [Source:HGNC Symbol;Acc:HGNC:1995]" |
| ENSG00000237440 | HGLibA_56657 | 83 | -0.322665852 | ZNF737 | zinc finger protein 737 [Source:HGNC Symbol;Acc:HGNC:32468] |
| ENSG00000243279 | HGLibA_38268 | 83 | -0.322665852 | PRAF2 | PRA1 domain family member 2 [Source:HGNC Symbol;Acc:HGNC:28911] |
| ENSG00000243725 | HGLibA_52142 | 83 | -0.322665852 | TTC4 | tetratricopeptide repeat domain 4 [Source:HGNC Symbol;Acc:HGNC:12394] |
| ENSG00000243927 | HGLibA_30108 | 83 | -0.322665852 | MRPS6 | mitochondrial ribosomal protein S6 [Source:HGNC Symbol;Acc:HGNC:14051] |
| ENSG00000249471 | HGLibA_55804 | 83 | -0.322665852 | ZNF324B | zinc finger protein 324B [Source:HGNC Symbol;Acc:HGNC:33107] |
| ENSG00000256762 | HGLibA_47434 | 83 | -0.322665852 | STH | saitohin [Source:HGNC Symbol;Acc:HGNC:18839] |
| ENSG00000280236 | HGLibA_33587 | 83 | -0.322665852 | OR12D2 | olfactory receptor family 12 subfamily D member 2 (gene/pseudogene) [Source:HGNC Symbol;Acc:HGNC:8178] |
| FAM127A | HGLibA_16087 | 83 | -0.322665852 | FAM127A | - |
| FAM21B | HGLibA_16507 | 83 | -0.322665852 | FAM21B | - |
| FAM21B | HGLibA_16508 | 83 | -0.322665852 | FAM21B | - |
| GRAMD3 | HGLibA_20206 | 83 | -0.322665852 | GRAMD3 | - |
| HIST1H2BM | HGLibA_21448 | 83 | -0.322665852 | HIST1H2BM | - |
| HMP19 | HGLibA_21754 | 83 | -0.322665852 | HMP19 | - |
| hsa-mir-190a | HGLibA_58033 | 83 | -0.322665852 | hsa-mir-190a | - |
| hsa-mir-3064 | HGLibA_58460 | 83 | -0.322665852 | hsa-mir-3064 | - |
| hsa-mir-346 | HGLibA_59032 | 83 | -0.322665852 | hsa-mir-346 | - |
| hsa-mir-3615 | HGLibA_59091 | 83 | -0.322665852 | hsa-mir-3615 | - |
| hsa-mir-375 | HGLibA_59401 | 83 | -0.322665852 | hsa-mir-375 | - |
| hsa-mir-4321 | HGLibA_60008 | 83 | -0.322665852 | hsa-mir-4321 | - |
| hsa-mir-4490 | HGLibA_60377 | 83 | -0.322665852 | hsa-mir-4490 | - |
| hsa-mir-4500 | HGLibA_60427 | 83 | -0.322665852 | hsa-mir-4500 | - |
| hsa-mir-4513 | HGLibA_60497 | 83 | -0.322665852 | hsa-mir-4513 | - |
| hsa-mir-4516 | HGLibA_60509 | 83 | -0.322665852 | hsa-mir-4516 | - |
| hsa-mir-4662b | HGLibA_60762 | 83 | -0.322665852 | hsa-mir-4662b | - |
| hsa-mir-520a | HGLibA_61734 | 83 | -0.322665852 | hsa-mir-520a | - |
| hsa-mir-5695 | HGLibA_62315 | 83 | -0.322665852 | hsa-mir-5695 | - |
| hsa-mir-6811 | HGLibA_63420 | 83 | -0.322665852 | hsa-mir-6811 | - |
| hsa-mir-6888 | HGLibA_63735 | 83 | -0.322665852 | hsa-mir-6888 | - |
| KIAA1107 | HGLibA_24691 | 83 | -0.322665852 | KIAA1107 | - |
| KIAA2022 | HGLibA_24843 | 83 | -0.322665852 | KIAA2022 | - |
| LOC256021 | HGLibA_26906 | 83 | -0.322665852 | LOC256021 | - |
| ORAOV1 | HGLibA_34598 | 83 | -0.322665852 | ORAOV1 | - |
| RLTPR | HGLibA_41361 | 83 | -0.322665852 | RLTPR | - |
| RQCD1 | HGLibA_42217 | 83 | -0.322665852 | RQCD1 | - |
| SCAND3 | HGLibA_42854 | 83 | -0.322665852 | SCAND3 | - |
| SGOL1 | HGLibA_43842 | 83 | -0.322665852 | SGOL1 | - |
| TMEM261 | HGLibA_50353 | 83 | -0.322665852 | TMEM261 | - |
| TMEM35 | HGLibA_50371 | 83 | -0.322665852 | TMEM35 | - |
| TSSC1 | HGLibA_52003 | 83 | -0.322665852 | TSSC1 | - |
| WBP5 | HGLibA_53987 | 83 | -0.322665852 | WBP5 | - |
| C10orf91 | HGLibA_05107 | 82 | -0.33261689 | C10orf91 | - |
| C19orf52 | HGLibA_05675 | 82 | -0.33261689 | C19orf52 | - |
| CCDC135 | HGLibA_07600 | 82 | -0.33261689 | CCDC135 | - |
| DFNA5 | HGLibA_13025 | 82 | -0.33261689 | DFNA5 | - |
| ENSG00000007047 | HGLibA_28373 | 82 | -0.33261689 | MARK4 | microtubule affinity regulating kinase 4 [Source:HGNC Symbol;Acc:HGNC:13538] |
| ENSG00000012171 | HGLibA_43323 | 82 | -0.33261689 | SEMA3B | semaphorin 3B [Source:HGNC Symbol;Acc:HGNC:10724] |
| ENSG00000023171 | HGLibA_20198 | 82 | -0.33261689 | GRAMD1B | GRAM domain containing 1B [Source:HGNC Symbol;Acc:HGNC:29214] |
| ENSG00000061273 | HGLibA_21021 | 82 | -0.33261689 | HDAC7 | histone deacetylase 7 [Source:HGNC Symbol;Acc:HGNC:14067] |
| ENSG00000067842 | HGLibA_03619 | 82 | -0.33261689 | ATP2B3 | ATPase plasma membrane Ca2+ transporting 3 [Source:HGNC Symbol;Acc:HGNC:816] |
| ENSG00000067900 | HGLibA_41746 | 82 | -0.33261689 | ROCK1 | Rho associated coiled-coil containing protein kinase 1 [Source:HGNC Symbol;Acc:HGNC:10251] |
| ENSG00000068976 | HGLibA_39692 | 82 | -0.33261689 | PYGM | muscle associated [Source:HGNC Symbol;Acc:HGNC:9726]" |
| ENSG00000071073 | HGLibA_29141 | 82 | -0.33261689 | MGAT4A | 3-mannosyl-glycoprotein 4-beta-N-acetylglucosaminyltransferase A [Source:HGNC Symbol;Acc:HGNC:7047]" |
| ENSG00000075415 | HGLibA_44700 | 82 | -0.33261689 | SLC25A3 | solute carrier family 25 member 3 [Source:HGNC Symbol;Acc:HGNC:10989] |
| ENSG00000075914 | HGLibA_15790 | 82 | -0.33261689 | EXOSC7 | exosome component 7 [Source:HGNC Symbol;Acc:HGNC:28112] |
| ENSG00000079974 | HGLibA_40031 | 82 | -0.33261689 | RABL2B | member of RAS oncogene family like 2B [Source:HGNC Symbol;Acc:HGNC:9800]" |
| ENSG00000086827 | HGLibA_57065 | 82 | -0.33261689 | ZW10 | zw10 kinetochore protein [Source:HGNC Symbol;Acc:HGNC:13194] |
| ENSG00000088832 | HGLibA_17564 | 82 | -0.33261689 | FKBP1A | FK506 binding protein 1A [Source:HGNC Symbol;Acc:HGNC:3711] |
| ENSG00000088876 | HGLibA_55844 | 82 | -0.33261689 | ZNF343 | zinc finger protein 343 [Source:HGNC Symbol;Acc:HGNC:16017] |
| ENSG00000089060 | HGLibA_45398 | 82 | -0.33261689 | SLC8B1 | solute carrier family 8 member B1 [Source:HGNC Symbol;Acc:HGNC:26175] |
| ENSG00000095713 | HGLibA_11218 | 82 | -0.33261689 | CRTAC1 | cartilage acidic protein 1 [Source:HGNC Symbol;Acc:HGNC:14882] |
| ENSG00000096401 | HGLibA_08591 | 82 | -0.33261689 | CDC5L | cell division cycle 5 like [Source:HGNC Symbol;Acc:HGNC:1743] |
| ENSG00000099308 | HGLibA_28406 | 82 | -0.33261689 | MAST3 | microtubule associated serine/threonine kinase 3 [Source:HGNC Symbol;Acc:HGNC:19036] |
| ENSG00000099958 | HGLibA_13001 | 82 | -0.33261689 | DERL3 | derlin 3 [Source:HGNC Symbol;Acc:HGNC:14236] |
| ENSG00000100030 | HGLibA_28247 | 82 | -0.33261689 | MAPK1 | mitogen-activated protein kinase 1 [Source:HGNC Symbol;Acc:HGNC:6871] |
| ENSG00000100079 | HGLibA_26359 | 82 | -0.33261689 | LGALS2 | galectin 2 [Source:HGNC Symbol;Acc:HGNC:6562] |
| ENSG00000100324 | HGLibA_48140 | 82 | -0.33261689 | TAB1 | TGF-beta activated kinase 1 (MAP3K7) binding protein 1 [Source:HGNC Symbol;Acc:HGNC:18157] |
| ENSG00000100592 | HGLibA_12260 | 82 | -0.33261689 | DAAM1 | dishevelled associated activator of morphogenesis 1 [Source:HGNC Symbol;Acc:HGNC:18142] |
| ENSG00000101251 | HGLibA_43279 | 82 | -0.33261689 | SEL1L2 | ERAD E3 ligase adaptor subunit [Source:HGNC Symbol;Acc:HGNC:15897]" |
| ENSG00000101596 | HGLibA_45663 | 82 | -0.33261689 | SMCHD1 | structural maintenance of chromosomes flexible hinge domain containing 1 [Source:HGNC Symbol;Acc:HGNC:29090] |
| ENSG00000102781 | HGLibA_24074 | 82 | -0.33261689 | KATNAL1 | katanin catalytic subunit A1 like 1 [Source:HGNC Symbol;Acc:HGNC:28361] |
| ENSG00000103066 | HGLibA_36875 | 82 | -0.33261689 | PLA2G15 | phospholipase A2 group XV [Source:HGNC Symbol;Acc:HGNC:17163] |
| ENSG00000103723 | HGLibA_02324 | 82 | -0.33261689 | AP3B2 | adaptor related protein complex 3 subunit beta 2 [Source:HGNC Symbol;Acc:HGNC:567] |
| ENSG00000104381 | HGLibA_18840 | 82 | -0.33261689 | GDAP1 | ganglioside induced differentiation associated protein 1 [Source:HGNC Symbol;Acc:HGNC:15968] |
| ENSG00000104833 | HGLibA_52286 | 82 | -0.33261689 | TUBB4A | tubulin beta 4A class IVa [Source:HGNC Symbol;Acc:HGNC:20774] |
| ENSG00000105223 | HGLibA_37041 | 82 | -0.33261689 | PLD3 | phospholipase D family member 3 [Source:HGNC Symbol;Acc:HGNC:17158] |
| ENSG00000105647 | HGLibA_36650 | 82 | -0.33261689 | PIK3R2 | phosphoinositide-3-kinase regulatory subunit 2 [Source:HGNC Symbol;Acc:HGNC:8980] |
| ENSG00000105825 | HGLibA_49188 | 82 | -0.33261689 | TFPI2 | tissue factor pathway inhibitor 2 [Source:HGNC Symbol;Acc:HGNC:11761] |
| ENSG00000107521 | HGLibA_22045 | 82 | -0.33261689 | HPS1 | biogenesis of lysosomal organelles complex 3 subunit 1 [Source:HGNC Symbol;Acc:HGNC:5163]" |
| ENSG00000107957 | HGLibA_43964 | 82 | -0.33261689 | SH3PXD2A | SH3 and PX domains 2A [Source:HGNC Symbol;Acc:HGNC:23664] |
| ENSG00000108219 | HGLibA_51903 | 82 | -0.33261689 | TSPAN14 | tetraspanin 14 [Source:HGNC Symbol;Acc:HGNC:23303] |
| ENSG00000108381 | HGLibA_03341 | 82 | -0.33261689 | ASPA | aspartoacylase [Source:HGNC Symbol;Acc:HGNC:756] |
| ENSG00000108599 | HGLibA_01405 | 82 | -0.33261689 | AKAP10 | A-kinase anchoring protein 10 [Source:HGNC Symbol;Acc:HGNC:368] |
| ENSG00000109220 | HGLibA_09449 | 82 | -0.33261689 | CHIC2 | cysteine rich hydrophobic domain 2 [Source:HGNC Symbol;Acc:HGNC:1935] |
| ENSG00000109323 | HGLibA_28086 | 82 | -0.33261689 | MANBA | mannosidase beta [Source:HGNC Symbol;Acc:HGNC:6831] |
| ENSG00000109458 | HGLibA_18321 | 82 | -0.33261689 | GAB1 | GRB2 associated binding protein 1 [Source:HGNC Symbol;Acc:HGNC:4066] |
| ENSG00000112079 | HGLibA_47501 | 82 | -0.33261689 | STK38 | serine/threonine kinase 38 [Source:HGNC Symbol;Acc:HGNC:17847] |
| ENSG00000114626 | HGLibA_00334 | 82 | -0.33261689 | ABTB1 | ankyrin repeat and BTB domain containing 1 [Source:HGNC Symbol;Acc:HGNC:18275] |
| ENSG00000114779 | HGLibA_00251 | 82 | -0.33261689 | ABHD14B | abhydrolase domain containing 14B [Source:HGNC Symbol;Acc:HGNC:28235] |
| ENSG00000115286 | HGLibA_31562 | 82 | -0.33261689 | NDUFS7 | NADH:ubiquinone oxidoreductase core subunit S7 [Source:HGNC Symbol;Acc:HGNC:7714] |
| ENSG00000116205 | HGLibA_48731 | 82 | -0.33261689 | TCEANC2 | transcription elongation factor A N-terminal and central domain containing 2 [Source:HGNC Symbol;Acc:HGNC:26494] |
| ENSG00000117519 | HGLibA_10310 | 82 | -0.33261689 | CNN3 | calponin 3 [Source:HGNC Symbol;Acc:HGNC:2157] |
| ENSG00000119396 | HGLibA_39815 | 82 | -0.33261689 | RAB14 | member RAS oncogene family [Source:HGNC Symbol;Acc:HGNC:16524]" |
| ENSG00000119688 | HGLibA_00200 | 82 | -0.33261689 | ABCD4 | ATP binding cassette subfamily D member 4 [Source:HGNC Symbol;Acc:HGNC:68] |
| ENSG00000120253 | HGLibA_33054 | 82 | -0.33261689 | NUP43 | nucleoporin 43 [Source:HGNC Symbol;Acc:HGNC:21182] |
| ENSG00000120306 | HGLibA_12232 | 82 | -0.33261689 | CYSTM1 | cysteine rich transmembrane module containing 1 [Source:HGNC Symbol;Acc:HGNC:30239] |
| ENSG00000121775 | HGLibA_50388 | 82 | -0.33261689 | TMEM39B | transmembrane protein 39B [Source:HGNC Symbol;Acc:HGNC:25510] |
| ENSG00000122507 | HGLibA_04226 | 82 | -0.33261689 | BBS9 | Bardet-Biedl syndrome 9 [Source:HGNC Symbol;Acc:HGNC:30000] |
| ENSG00000123165 | HGLibA_00676 | 82 | -0.33261689 | ACTRT1 | actin related protein T1 [Source:HGNC Symbol;Acc:HGNC:24027] |
| ENSG00000123165 | HGLibA_00678 | 82 | -0.33261689 | ACTRT1 | actin related protein T1 [Source:HGNC Symbol;Acc:HGNC:24027] |
| ENSG00000124701 | HGLibA_02470 | 82 | -0.33261689 | APOBEC2 | apolipoprotein B mRNA editing enzyme catalytic subunit 2 [Source:HGNC Symbol;Acc:HGNC:605] |
| ENSG00000125355 | HGLibA_50330 | 82 | -0.33261689 | TMEM255A | transmembrane protein 255A [Source:HGNC Symbol;Acc:HGNC:26086] |
| ENSG00000125676 | HGLibA_49368 | 82 | -0.33261689 | THOC2 | THO complex 2 [Source:HGNC Symbol;Acc:HGNC:19073] |
| ENSG00000126583 | HGLibA_38549 | 82 | -0.33261689 | PRKCG | protein kinase C gamma [Source:HGNC Symbol;Acc:HGNC:9402] |
| ENSG00000129038 | HGLibA_27038 | 82 | -0.33261689 | LOXL1 | lysyl oxidase like 1 [Source:HGNC Symbol;Acc:HGNC:6665] |
| ENSG00000129187 | HGLibA_12542 | 82 | -0.33261689 | DCTD | dCMP deaminase [Source:HGNC Symbol;Acc:HGNC:2710] |
| ENSG00000130717 | HGLibA_52811 | 82 | -0.33261689 | UCK1 | uridine-cytidine kinase 1 [Source:HGNC Symbol;Acc:HGNC:14859] |
| ENSG00000130764 | HGLibA_27372 | 82 | -0.33261689 | LRRC47 | leucine rich repeat containing 47 [Source:HGNC Symbol;Acc:HGNC:29207] |
| ENSG00000131188 | HGLibA_38852 | 82 | -0.33261689 | PRR7 | synaptic [Source:HGNC Symbol;Acc:HGNC:28130]" |
| ENSG00000132275 | HGLibA_42283 | 82 | -0.33261689 | RRP8 | ribosomal RNA processing 8 [Source:HGNC Symbol;Acc:HGNC:29030] |

| | | | | | |
|---|---|---|---|---|---|
| ENSG00000132953 | HGLibA_54565 | 82 | -0.33261689 | XPO4 | exportin 4 [Source:HGNC Symbol;Acc:HGNC:17796] |
| ENSG00000134330 | HGLibA_22468 | 82 | -0.33261689 | IAH1 | isoamyl acetate hydrolyzing esterase 1 (putative) [Source:HGNC Symbol;Acc:HGNC:27696] |
| ENSG00000134551 | HGLibA_38464 | 82 | -0.33261689 | PRH2 | proline rich protein HaeIII subfamily 2 [Source:HGNC Symbol;Acc:HGNC:9367] |
| ENSG00000134970 | HGLibA_49820 | 82 | -0.33261689 | TMED7 | transmembrane p24 trafficking protein 7 [Source:HGNC Symbol;Acc:HGNC:24253] |
| ENSG00000135094 | HGLibA_43173 | 82 | -0.33261689 | SDS | serine dehydratase [Source:HGNC Symbol;Acc:HGNC:10691] |
| ENSG00000135824 | HGLibA_41108 | 82 | -0.33261689 | RGS8 | regulator of G protein signaling 8 [Source:HGNC Symbol;Acc:HGNC:16810] |
| ENSG00000137251 | HGLibA_49548 | 82 | -0.33261689 | TINAG | tubulointerstitial nephritis antigen [Source:HGNC Symbol;Acc:HGNC:14599] |
| ENSG00000137960 | HGLibA_19120 | 82 | -0.33261689 | GIPC2 | GIPC PDZ domain containing family member 2 [Source:HGNC Symbol;Acc:HGNC:18177] |
| ENSG00000137975 | HGLibA_09807 | 82 | -0.33261689 | CLCA2 | chloride channel accessory 2 [Source:HGNC Symbol;Acc:HGNC:2016] |
| ENSG00000138311 | HGLibA_55875 | 82 | -0.33261689 | ZNF365 | zinc finger protein 365 [Source:HGNC Symbol;Acc:HGNC:18194] |
| ENSG00000139370 | HGLibA_44415 | 82 | -0.33261689 | SLC15A4 | solute carrier family 15 member 4 [Source:HGNC Symbol;Acc:HGNC:23090] |
| ENSG00000141179 | HGLibA_35691 | 82 | -0.33261689 | PCTP | phosphatidylcholine transfer protein [Source:HGNC Symbol;Acc:HGNC:8752] |
| ENSG00000141314 | HGLibA_41143 | 82 | -0.33261689 | RHBDL3 | rhomboid like 3 [Source:HGNC Symbol;Acc:HGNC:16502] |
| ENSG00000141428 | HGLibA_05605 | 82 | -0.33261689 | C18orf21 | chromosome 18 open reading frame 21 [Source:HGNC Symbol;Acc:HGNC:28802] |
| ENSG00000142534 | HGLibA_42064 | 82 | -0.33261689 | RPS11 | ribosomal protein S11 [Source:HGNC Symbol;Acc:HGNC:10384] |
| ENSG00000142875 | HGLibA_38505 | 82 | -0.33261689 | PRKACB | protein kinase cAMP-activated catalytic subunit beta [Source:HGNC Symbol;Acc:HGNC:9381] |
| ENSG00000143278 | HGLibA_15857 | 82 | -0.33261689 | F13B | coagulation factor XIII B chain [Source:HGNC Symbol;Acc:HGNC:3534] |
| ENSG00000143319 | HGLibA_23661 | 82 | -0.33261689 | ISG20L2 | interferon stimulated exonuclease gene 20 like 2 [Source:HGNC Symbol;Acc:HGNC:25745] |
| ENSG00000143418 | HGLibA_09203 | 82 | -0.33261689 | CERS2 | ceramide synthase 2 [Source:HGNC Symbol;Acc:HGNC:14076] |
| ENSG00000143493 | HGLibA_23430 | 82 | -0.33261689 | INTS7 | integrator complex subunit 7 [Source:HGNC Symbol;Acc:HGNC:24484] |
| ENSG00000143498 | HGLibA_48212 | 82 | -0.33261689 | TAF1A | RNA polymerase I subunit A [Source:HGNC Symbol;Acc:HGNC:11532]" |
| ENSG00000144035 | HGLibA_31182 | 82 | -0.33261689 | NAT8 | N-acetyltransferase 8 (putative) [Source:HGNC Symbol;Acc:HGNC:18069] |
| ENSG00000145808 | HGLibA_00817 | 82 | -0.33261689 | ADAMTS19 | ADAM metallopeptidase with thrombospondin type 1 motif 19 [Source:HGNC Symbol;Acc:HGNC:17111] |
| ENSG00000146067 | HGLibA_16367 | 82 | -0.33261689 | FAM193B | family with sequence similarity 193 member B [Source:HGNC Symbol;Acc:HGNC:25524] |
| ENSG00000147119 | HGLibA_09646 | 82 | -0.33261689 | CHST7 | carbohydrate sulfotransferase 7 [Source:HGNC Symbol;Acc:HGNC:13817] |
| ENSG00000147570 | HGLibA_13628 | 82 | -0.33261689 | DNAJC5B | DnaJ heat shock protein family (Hsp40) member C5 beta [Source:HGNC Symbol;Acc:HGNC:24138] |
| ENSG00000148337 | HGLibA_09750 | 82 | -0.33261689 | CIZ1 | CDKN1A interacting zinc finger protein 1 [Source:HGNC Symbol;Acc:HGNC:16744] |
| ENSG00000149212 | HGLibA_43625 | 82 | -0.33261689 | SESN3 | sestrin 3 [Source:HGNC Symbol;Acc:HGNC:23060] |
| ENSG00000149654 | HGLibA_08666 | 82 | -0.33261689 | CDH22 | cadherin 22 [Source:HGNC Symbol;Acc:HGNC:13251] |
| ENSG00000154856 | HGLibA_02396 | 82 | -0.33261689 | APCDD1 | APC down-regulated 1 [Source:HGNC Symbol;Acc:HGNC:15718] |
| ENSG00000156127 | HGLibA_04174 | 82 | -0.33261689 | BATF | basic leucine zipper ATF-like transcription factor [Source:HGNC Symbol;Acc:HGNC:958] |
| ENSG00000156453 | HGLibA_35384 | 82 | -0.33261689 | PCDH1 | protocadherin 1 [Source:HGNC Symbol;Acc:HGNC:8655] |
| ENSG00000156689 | HGLibA_19376 | 82 | -0.33261689 | GLYATL2 | glycine-N-acyltransferase like 2 [Source:HGNC Symbol;Acc:HGNC:24178] |
| ENSG00000156735 | HGLibA_04082 | 82 | -0.33261689 | BAG4 | BCL2 associated athanogene 4 [Source:HGNC Symbol;Acc:HGNC:940] |
| ENSG00000159176 | HGLibA_11431 | 82 | -0.33261689 | CSRP1 | cysteine and glycine rich protein 1 [Source:HGNC Symbol;Acc:HGNC:2469] |
| ENSG00000160218 | HGLibA_51300 | 82 | -0.33261689 | TRAPPC10 | trafficking protein particle complex 10 [Source:HGNC Symbol;Acc:HGNC:11868] |
| ENSG00000160796 | HGLibA_31212 | 82 | -0.33261689 | NBEAL2 | neurobeachin like 2 [Source:HGNC Symbol;Acc:HGNC:31928] |
| ENSG00000160877 | HGLibA_31040 | 82 | -0.33261689 | NACC1 | nucleus accumbens associated 1 [Source:HGNC Symbol;Acc:HGNC:20967] |
| ENSG00000161381 | HGLibA_37251 | 82 | -0.33261689 | PLXDC1 | plexin domain containing 1 [Source:HGNC Symbol;Acc:HGNC:20945] |
| ENSG00000161929 | HGLibA_42944 | 82 | -0.33261689 | SCIMP | SLP adaptor and CSK interacting membrane protein [Source:HGNC Symbol;Acc:HGNC:33504] |
| ENSG00000162645 | HGLibA_18732 | 82 | -0.33261689 | GBP2 | guanylate binding protein 2 [Source:HGNC Symbol;Acc:HGNC:4183] |
| ENSG00000162885 | HGLibA_03951 | 82 | -0.33261689 | B3GALNT2 | 3-N-acetylgalactosaminyltransferase 2 [Source:HGNC Symbol;Acc:HGNC:28596]" |
| ENSG00000163239 | HGLibA_48898 | 82 | -0.33261689 | TDRD10 | tudor domain containing 10 [Source:HGNC Symbol;Acc:HGNC:25316] |
| ENSG00000165496 | HGLibA_41860 | 82 | -0.33261689 | RPL10L | ribosomal protein L10 like [Source:HGNC Symbol;Acc:HGNC:17976] |
| ENSG00000167377 | HGLibA_55632 | 82 | -0.33261689 | ZNF23 | zinc finger protein 23 [Source:HGNC Symbol;Acc:HGNC:13023] |
| ENSG00000167889 | HGLibA_29153 | 82 | -0.33261689 | MGAT5B | 6-mannosylglycoprotein 6-beta-N-acetylglucosaminyltransferase B [Source:HGNC Symbol;Acc:HGNC:24140]" |
| ENSG00000169306 | HGLibA_23056 | 82 | -0.33261689 | IL1RAPL1 | interleukin 1 receptor accessory protein like 1 [Source:HGNC Symbol;Acc:HGNC:5996] |
| ENSG00000169750 | HGLibA_40048 | 82 | -0.33261689 | RAC3 | Rac family small GTPase 3 [Source:HGNC Symbol;Acc:HGNC:9803] |
| ENSG00000170236 | HGLibA_53391 | 82 | -0.33261689 | USP50 | ubiquitin specific peptidase 50 [Source:HGNC Symbol;Acc:HGNC:20079] |
| ENSG00000170369 | HGLibA_11449 | 82 | -0.33261689 | CST2 | cystatin SA [Source:HGNC Symbol;Acc:HGNC:2474] |
| ENSG00000171403 | HGLibA_25576 | 82 | -0.33261689 | KRT9 | keratin 9 [Source:HGNC Symbol;Acc:HGNC:6447] |
| ENSG00000171503 | HGLibA_15627 | 82 | -0.33261689 | ETFDH | electron transfer flavoprotein dehydrogenase [Source:HGNC Symbol;Acc:HGNC:3483] |
| ENSG00000172000 | HGLibA_56228 | 82 | -0.33261689 | ZNF556 | zinc finger protein 556 [Source:HGNC Symbol;Acc:HGNC:25669] |
| ENSG00000172817 | HGLibA_12213 | 82 | -0.33261689 | CYP7B1 | cytochrome P450 family 7 subfamily B member 1 [Source:HGNC Symbol;Acc:HGNC:2652] |
| ENSG00000172867 | HGLibA_25445 | 82 | -0.33261689 | KRT2 | keratin 2 [Source:HGNC Symbol;Acc:HGNC:6439] |
| ENSG00000173480 | HGLibA_55947 | 82 | -0.33261689 | ZNF417 | zinc finger protein 417 [Source:HGNC Symbol;Acc:HGNC:20646] |
| ENSG00000173992 | HGLibA_08183 | 82 | -0.33261689 | CCS | copper chaperone for superoxide dismutase [Source:HGNC Symbol;Acc:HGNC:1613] |
| ENSG00000174099 | HGLibA_30248 | 82 | -0.33261689 | MSRB3 | methionine sulfoxide reductase B3 [Source:HGNC Symbol;Acc:HGNC:27375] |
| ENSG00000174348 | HGLibA_37453 | 82 | -0.33261689 | PODN | podocan [Source:HGNC Symbol;Acc:HGNC:23174] |
| ENSG00000174498 | HGLibA_22784 | 82 | -0.33261689 | IGDCC3 | immunoglobulin superfamily DCC subclass member 3 [Source:HGNC Symbol;Acc:HGNC:9700] |
| ENSG00000174839 | HGLibA_12965 | 82 | -0.33261689 | DENND6A | DENN domain containing 6A [Source:HGNC Symbol;Acc:HGNC:26635] |
| ENSG00000176723 | HGLibA_56858 | 82 | -0.33261689 | ZNF843 | zinc finger protein 843 [Source:HGNC Symbol;Acc:HGNC:28710] |
| ENSG00000177951 | HGLibA_04434 | 82 | -0.33261689 | BET1L | Bet1 golgi vesicular membrane trafficking protein like [Source:HGNC Symbol;Acc:HGNC:19348] |
| ENSG00000178343 | HGLibA_44037 | 82 | -0.33261689 | SHISA3 | shisa family member 3 [Source:HGNC Symbol;Acc:HGNC:25159] |
| ENSG00000180155 | HGLibA_27710 | 82 | -0.33261689 | LYNX1 | Ly6/neurotoxin 1 [Source:HGNC Symbol;Acc:HGNC:29604] |
| ENSG00000181562 | HGLibA_14447 | 82 | -0.33261689 | EDDM3A | epididymal protein 3A [Source:HGNC Symbol;Acc:HGNC:16978] |
| ENSG00000182287 | HGLibA_02298 | 82 | -0.33261689 | AP1S2 | adaptor related protein complex 1 subunit sigma 2 [Source:HGNC Symbol;Acc:HGNC:560] |
| ENSG00000183873 | HGLibA_42999 | 82 | -0.33261689 | SCN5A | sodium voltage-gated channel alpha subunit 5 [Source:HGNC Symbol;Acc:HGNC:10593] |
| ENSG00000184032 | HGLibA_25703 | 82 | -0.33261689 | KRTAP20-2 | keratin associated protein 20-2 [Source:HGNC Symbol;Acc:HGNC:18944] |
| ENSG00000184985 | HGLibA_46186 | 82 | -0.33261689 | SORCS2 | sortilin related VPS10 domain containing receptor 2 [Source:HGNC Symbol;Acc:HGNC:16698] |
| ENSG00000185379 | HGLibA_40094 | 82 | -0.33261689 | RAD51D | RAD51 paralog D [Source:HGNC Symbol;Acc:HGNC:9823] |
| ENSG00000185985 | HGLibA_45516 | 82 | -0.33261689 | SLITRK2 | SLIT and NTRK like family member 2 [Source:HGNC Symbol;Acc:HGNC:13449] |
| ENSG00000186198 | HGLibA_45242 | 82 | -0.33261689 | SLC51B | solute carrier family 51 beta subunit [Source:HGNC Symbol;Acc:HGNC:29956] |
| ENSG00000186907 | HGLibA_42419 | 82 | -0.33261689 | RTN4RL2 | reticulon 4 receptor like 2 [Source:HGNC Symbol;Acc:HGNC:23053] |
| ENSG00000186930 | HGLibA_25818 | 82 | -0.33261689 | KRTAP6-2 | keratin associated protein 6-2 [Source:HGNC Symbol;Acc:HGNC:18932] |
| ENSG00000187005 | HGLibA_25709 | 82 | -0.33261689 | KRTAP21-1 | keratin associated protein 21-1 [Source:HGNC Symbol;Acc:HGNC:18945] |
| ENSG00000188060 | HGLibA_39947 | 82 | -0.33261689 | RAB42 | member RAS oncogene family [Source:HGNC Symbol;Acc:HGNC:28702]" |
| ENSG00000189241 | HGLibA_51979 | 82 | -0.33261689 | TSPYL1 | TSPY like 1 [Source:HGNC Symbol;Acc:HGNC:12382] |
| ENSG00000196437 | HGLibA_56268 | 82 | -0.33261689 | ZNF569 | zinc finger protein 569 [Source:HGNC Symbol;Acc:HGNC:24737] |
| ENSG00000197172 | HGLibA_27917 | 82 | -0.33261689 | MAGEA6 | MAGE family member A6 [Source:HGNC Symbol;Acc:HGNC:6804] |
| ENSG00000197403 | HGLibA_34430 | 82 | -0.33261689 | OR6N1 | olfactory receptor family 6 subfamily N member 1 [Source:HGNC Symbol;Acc:HGNC:15034] |
| ENSG00000197408 | HGLibA_12113 | 82 | -0.33261689 | CYP2B6 | cytochrome P450 family 2 subfamily B member 6 [Source:HGNC Symbol;Acc:HGNC:2615] |
| ENSG00000197465 | HGLibA_20705 | 82 | -0.33261689 | GYPE | glycophorin E (MNS blood group) [Source:HGNC Symbol;Acc:HGNC:4705] |
| ENSG00000204323 | HGLibA_45761 | 82 | -0.33261689 | SMIM5 | small integral membrane protein 5 [Source:HGNC Symbol;Acc:HGNC:40030] |
| ENSG00000204595 | HGLibA_13899 | 82 | -0.33261689 | DPRX | divergent-paired related homeobox [Source:HGNC Symbol;Acc:HGNC:32166] |
| ENSG00000204866 | HGLibA_22842 | 82 | -0.33261689 | IGFL2 | IGF like family member 2 [Source:HGNC Symbol;Acc:HGNC:32929] |
| ENSG00000213967 | HGLibA_56631 | 82 | -0.33261689 | ZNF726 | zinc finger protein 726 [Source:HGNC Symbol;Acc:HGNC:32462] |
| ENSG00000222046 | HGLibA_12487 | 82 | -0.33261689 | DCDC2B | doublecortin domain containing 2B [Source:HGNC Symbol;Acc:HGNC:32576] |
| ENSG00000229474 | HGLibA_35285 | 82 | -0.33261689 | PATL2 | PAT1 homolog 2 [Source:HGNC Symbol;Acc:HGNC:33630] |
| ENSG00000237330 | HGLibA_41646 | 82 | -0.33261689 | RNF223 | ring finger protein 223 [Source:HGNC Symbol;Acc:HGNC:40020] |
| ENSG00000242950 | HGLibA_15550 | 82 | -0.33261689 | ERVW-1 | envelope [Source:HGNC Symbol;Acc:HGNC:13525]" |
| ENSG00000243156 | HGLibA_29206 | 82 | -0.33261689 | MICAL3 | calponin and LIM domain containing 3 [Source:HGNC Symbol;Acc:HGNC:24694]" |
| ENSG00000248487 | HGLibA_00248 | 82 | -0.33261689 | ABHD14A | abhydrolase domain containing 14A [Source:HGNC Symbol;Acc:HGNC:24538] |
| ENSG00000253485 | HGLibA_35535 | 82 | -0.33261689 | PCDHGA5 | 5 [Source:HGNC Symbol;Acc:HGNC:8703]" |
| ENSG00000254788 | HGLibA_09772 | 82 | -0.33261689 | CKLF-CMTM1 | CKLF-CMTM1 readthrough [Source:HGNC Symbol;Acc:HGNC:39977] |
| ENSG00000254827 | HGLibA_44563 | 82 | -0.33261689 | SLC22A18AS | solute carrier family 22 member 18 antisense [Source:HGNC Symbol;Acc:HGNC:10965] |
| ENSG00000256043 | HGLibA_11726 | 82 | -0.33261689 | CTSO | cathepsin O [Source:HGNC Symbol;Acc:HGNC:2542] |
| ENSG00000261609 | HGLibA_18603 | 82 | -0.33261689 | GAN | gigaxonin [Source:HGNC Symbol;Acc:HGNC:4137] |
| ENSG00000265972 | HGLibA_52431 | 82 | -0.33261689 | TXNIP | thioredoxin interacting protein [Source:HGNC Symbol;Acc:HGNC:16952] |
| ENSG00000272395 | HGLibA_22728 | 82 | -0.33261689 | IFNL4 | interferon lambda 4 (gene/pseudogene) [Source:HGNC Symbol;Acc:HGNC:44480] |
| ENSG00000276547 | HGLibA_35562 | 82 | -0.33261689 | PCDHGB5 | 5 [Source:HGNC Symbol;Acc:HGNC:8712]" |
| ENSG00000288705 | HGLibA_52889 | 82 | -0.33261689 | UGT1A5 | - |
| FAM46D | HGLibA_16604 | 82 | -0.33261689 | FAM46D | - |
| FAM63B | HGLibA_16661 | 82 | -0.33261689 | FAM63B | - |
| GPR116 | HGLibA_19864 | 82 | -0.33261689 | GPR116 | - |
| HIST1H2BO | HGLibA_21452 | 82 | -0.33261689 | HIST1H2BO | - |
| HIST2H3D | HGLibA_21546 | 82 | -0.33261689 | HIST2H3D | - |
| hsa-mir-126 | HGLibA_57457 | 82 | -0.33261689 | hsa-mir-126 | - |
| hsa-mir-1261 | HGLibA_57466 | 82 | -0.33261689 | hsa-mir-1261 | - |
| hsa-mir-147b | HGLibA_57878 | 82 | -0.33261689 | hsa-mir-147b | - |
| hsa-mir-195 | HGLibA_58087 | 82 | -0.33261689 | hsa-mir-195 | - |
| hsa-mir-200b | HGLibA_58156 | 82 | -0.33261689 | hsa-mir-200b | - |
| hsa-mir-3171 | HGLibA_58763 | 82 | -0.33261689 | hsa-mir-3171 | - |
| hsa-mir-3622a | HGLibA_59123 | 82 | -0.33261689 | hsa-mir-3622a | - |
| hsa-mir-3974 | HGLibA_59655 | 82 | -0.33261689 | hsa-mir-3974 | - |
| hsa-mir-425 | HGLibA_59709 | 82 | -0.33261689 | hsa-mir-425 | - |
| hsa-mir-4254 | HGLibA_59725 | 82 | -0.33261689 | hsa-mir-4254 | - |
| hsa-mir-4260 | HGLibA_59746 | 82 | -0.33261689 | hsa-mir-4260 | - |
| hsa-mir-4268 | HGLibA_59780 | 82 | -0.33261689 | hsa-mir-4268 | - |
| hsa-mir-4280 | HGLibA_59824 | 82 | -0.33261689 | hsa-mir-4280 | - |
| hsa-mir-4295 | HGLibA_59891 | 82 | -0.33261689 | hsa-mir-4295 | - |
| hsa-mir-4310 | HGLibA_59957 | 82 | -0.33261689 | hsa-mir-4310 | - |
| hsa-mir-4315-1 | HGLibA_59977 | 82 | -0.33261689 | hsa-mir-4315-1 | - |
| hsa-mir-4327 | HGLibA_60031 | 82 | -0.33261689 | hsa-mir-4327 | - |
| hsa-mir-4435-2 | HGLibA_60136 | 82 | -0.33261689 | hsa-mir-4435-2 | - |
| hsa-mir-4649 | HGLibA_60698 | 82 | -0.33261689 | hsa-mir-4649 | - |
| hsa-mir-4714 | HGLibA_60955 | 82 | -0.33261689 | hsa-mir-4714 | - |
| hsa-mir-4739 | HGLibA_61053 | 82 | -0.33261689 | hsa-mir-4739 | - |
| hsa-mir-4750 | HGLibA_61098 | 82 | -0.33261689 | hsa-mir-4750 | - |
| hsa-mir-4760 | HGLibA_61135 | 82 | -0.33261689 | hsa-mir-4760 | - |
| hsa-mir-4796 | HGLibA_61290 | 82 | -0.33261689 | hsa-mir-4796 | - |
| hsa-mir-4804 | HGLibA_61320 | 82 | -0.33261689 | hsa-mir-4804 | - |
| hsa-mir-525 | HGLibA_61785 | 82 | -0.33261689 | hsa-mir-525 | - |
| hsa-mir-526a-1 | HGLibA_61789 | 82 | -0.33261689 | hsa-mir-526a-1 | - |
| hsa-mir-548a-1 | HGLibA_61837 | 82 | -0.33261689 | hsa-mir-548a-1 | - |

| | | | | | |
|---|---|---|---|---|---|
| hsa-mir-548h-3 | HGLibA_62003 | 82 | -0.33261689 | hsa-mir-548h-3 | - |
| hsa-mir-548o | HGLibA_62048 | 82 | -0.33261689 | hsa-mir-548o | - |
| hsa-mir-582 | HGLibA_62425 | 82 | -0.33261689 | hsa-mir-582 | - |
| hsa-mir-6071 | HGLibA_62537 | 82 | -0.33261689 | hsa-mir-6071 | - |
| hsa-mir-6125 | HGLibA_62649 | 82 | -0.33261689 | hsa-mir-6125 | - |
| hsa-mir-664b | HGLibA_62996 | 82 | -0.33261689 | hsa-mir-664b | - |
| hsa-mir-6807 | HGLibA_63406 | 82 | -0.33261689 | hsa-mir-6807 | - |
| hsa-mir-6864 | HGLibA_63642 | 82 | -0.33261689 | hsa-mir-6864 | - |
| hsa-mir-8083 | HGLibA_64176 | 82 | -0.33261689 | hsa-mir-8083 | - |
| ICT1 | HGLibA_22522 | 82 | -0.33261689 | ICT1 | - |
| IL3RA_Y | HGLibA_23164 | 82 | -0.33261689 | IL3RA_Y | - |
| LOC100507003 | HGLibA_26866 | 82 | -0.33261689 | LOC100507003 | - |
| OR8G2 | HGLibA_34520 | 82 | -0.33261689 | OR8G2 | - |
| VIMP | HGLibA_53659 | 82 | -0.33261689 | VIMP | - |
| WDYHV1 | HGLibA_54236 | 82 | -0.33261689 | WDYHV1 | - |
| ZAK | HGLibA_54772 | 82 | -0.33261689 | ZAK | - |
| ASMTL_X | HGLibA_03327 | 81 | -0.342567927 | ASMTL_X | - |
| ASUN | HGLibA_03385 | 81 | -0.342567927 | ASUN | - |
| C10orf68 | HGLibA_05088 | 81 | -0.342567927 | C10orf68 | - |
| C19orf57 | HGLibA_05684 | 81 | -0.342567927 | C19orf57 | - |
| C1orf27 | HGLibA_05852 | 81 | -0.342567927 | C1orf27 | - |
| ENSG00000005194 | HGLibA_09679 | 81 | -0.342567927 | CIAPIN1 | cytokine induced apoptosis inhibitor 1 [Source:HGNC Symbol;Acc:HGNC:28050] |
| ENSG00000006606 | HGLibA_08009 | 81 | -0.342567927 | CCL26 | C-C motif chemokine ligand 26 [Source:HGNC Symbol;Acc:HGNC:10625] |
| ENSG00000009724 | HGLibA_28398 | 81 | -0.342567927 | MASP2 | mannan binding lectin serine peptidase 2 [Source:HGNC Symbol;Acc:HGNC:6902] |
| ENSG00000009954 | HGLibA_04189 | 81 | -0.342567927 | BAZ1B | bromodomain adjacent to zinc finger domain 1B [Source:HGNC Symbol;Acc:HGNC:961] |
| ENSG00000027001 | HGLibA_29294 | 81 | -0.342567927 | MIPEP | mitochondrial intermediate peptidase [Source:HGNC Symbol;Acc:HGNC:7104] |
| ENSG00000042429 | HGLibA_28764 | 81 | -0.342567927 | MED17 | mediator complex subunit 17 [Source:HGNC Symbol;Acc:HGNC:2375] |
| ENSG00000064012 | HGLibA_07325 | 81 | -0.342567927 | CASP8 | caspase 8 [Source:HGNC Symbol;Acc:HGNC:1509] |
| ENSG00000066557 | HGLibA_27352 | 81 | -0.342567927 | LRRC40 | leucine rich repeat containing 40 [Source:HGNC Symbol;Acc:HGNC:26004] |
| ENSG00000068028 | HGLibA_40374 | 81 | -0.342567927 | RASSF1 | Ras association domain family member 1 [Source:HGNC Symbol;Acc:HGNC:9882] |
| ENSG00000075213 | HGLibA_43319 | 81 | -0.342567927 | SEMA3A | semaphorin 3A [Source:HGNC Symbol;Acc:HGNC:10723] |
| ENSG00000076662 | HGLibA_22499 | 81 | -0.342567927 | ICAM3 | intercellular adhesion molecule 3 [Source:HGNC Symbol;Acc:HGNC:5346] |
| ENSG00000080815 | HGLibA_39025 | 81 | -0.342567927 | PSEN1 | presenilin 1 [Source:HGNC Symbol;Acc:HGNC:9508] |
| ENSG00000080823 | HGLibA_29636 | 81 | -0.342567927 | MOK | MOK protein kinase [Source:HGNC Symbol;Acc:HGNC:9833] |
| ENSG00000081923 | HGLibA_03780 | 81 | -0.342567927 | ATP8B1 | ATPase phospholipid transporting 8B1 [Source:HGNC Symbol;Acc:HGNC:3706] |
| ENSG00000083838 | HGLibA_56019 | 81 | -0.342567927 | ZNF446 | zinc finger protein 446 [Source:HGNC Symbol;Acc:HGNC:21036] |
| ENSG00000085276 | HGLibA_28724 | 81 | -0.342567927 | MECOM | MDS1 and EVI1 complex locus [Source:HGNC Symbol;Acc:HGNC:3498] |
| ENSG00000088035 | HGLibA_01634 | 81 | -0.342567927 | ALG6 | 3-glucosyltransferase [Source:HGNC Symbol;Acc:HGNC:23157]" |
| ENSG00000088930 | HGLibA_54608 | 81 | -0.342567927 | XRN2 | 5'-3' exoribonuclease 2 [Source:HGNC Symbol;Acc:HGNC:12836] |
| ENSG00000089169 | HGLibA_41843 | 81 | -0.342567927 | RPH3A | rabphilin 3A [Source:HGNC Symbol;Acc:HGNC:17056] |
| ENSG00000091039 | HGLibA_34667 | 81 | -0.342567927 | OSBPL8 | oxysterol binding protein like 8 [Source:HGNC Symbol;Acc:HGNC:16396] |
| ENSG00000095139 | HGLibA_02638 | 81 | -0.342567927 | ARCN1 | archain 1 [Source:HGNC Symbol;Acc:HGNC:649] |
| ENSG00000096092 | HGLibA_49978 | 81 | -0.342567927 | TMEM14A | transmembrane protein 14A [Source:HGNC Symbol;Acc:HGNC:21076] |
| ENSG00000096433 | HGLibA_23860 | 81 | -0.342567927 | ITPR3 | 5-trisphosphate receptor type 3 [Source:HGNC Symbol;Acc:HGNC:6182]" |
| ENSG00000099365 | HGLibA_47639 | 81 | -0.342567927 | STX1B | syntaxin 1B [Source:HGNC Symbol;Acc:HGNC:18539] |
| ENSG00000100060 | HGLibA_29075 | 81 | -0.342567927 | MFNG | MFNG O-fucosylpeptide 3-beta-N-acetylglucosaminyltransferase [Source:HGNC Symbol;Acc:HGNC:7038] |
| ENSG00000100239 | HGLibA_38223 | 81 | -0.342567927 | PPP6R2 | protein phosphatase 6 regulatory subunit 2 [Source:HGNC Symbol;Acc:HGNC:19253] |
| ENSG00000100243 | HGLibA_12007 | 81 | -0.342567927 | CYB5R3 | cytochrome b5 reductase 3 [Source:HGNC Symbol;Acc:HGNC:2873] |
| ENSG00000100568 | HGLibA_53891 | 81 | -0.342567927 | VTI1B | vesicle transport through interaction with t-SNAREs 1B [Source:HGNC Symbol;Acc:HGNC:17793] |
| ENSG00000100592 | HGLibA_12258 | 81 | -0.342567927 | DAAM1 | dishevelled associated activator of morphogenesis 1 [Source:HGNC Symbol;Acc:HGNC:18142] |
| ENSG00000100968 | HGLibA_31784 | 81 | -0.342567927 | NFATC4 | nuclear factor of activated T cells 4 [Source:HGNC Symbol;Acc:HGNC:7778] |
| ENSG00000103037 | HGLibA_43656 | 81 | -0.342567927 | SETD6 | SET domain containing 6 [Source:HGNC Symbol;Acc:HGNC:26116] |
| ENSG00000103043 | HGLibA_53484 | 81 | -0.342567927 | VAC14 | PIKFYVE complex component [Source:HGNC Symbol;Acc:HGNC:25507]" |
| ENSG00000103152 | HGLibA_29721 | 81 | -0.342567927 | MPG | N-methylpurine DNA glycosylase [Source:HGNC Symbol;Acc:HGNC:7211] |
| ENSG00000104856 | HGLibA_40814 | 81 | -0.342567927 | RELB | NF-kB subunit [Source:HGNC Symbol;Acc:HGNC:9956]" |
| ENSG00000105402 | HGLibA_31125 | 81 | -0.342567927 | NAPA | NSF attachment protein alpha [Source:HGNC Symbol;Acc:HGNC:7641] |
| ENSG00000107140 | HGLibA_49028 | 81 | -0.342567927 | TESK1 | testis associated actin remodelling kinase 1 [Source:HGNC Symbol;Acc:HGNC:11731] |
| ENSG00000107159 | HGLibA_06828 | 81 | -0.342567927 | CA9 | carbonic anhydrase 9 [Source:HGNC Symbol;Acc:HGNC:1383] |
| ENSG00000108465 | HGLibA_08789 | 81 | -0.342567927 | CDK5RAP3 | CDK5 regulatory subunit associated protein 3 [Source:HGNC Symbol;Acc:HGNC:18673] |
| ENSG00000110925 | HGLibA_11427 | 81 | -0.342567927 | CSRNP2 | cysteine and serine rich nuclear protein 2 [Source:HGNC Symbol;Acc:HGNC:16006] |
| ENSG00000111249 | HGLibA_11806 | 81 | -0.342567927 | CUX2 | cut like homeobox 2 [Source:HGNC Symbol;Acc:HGNC:19347] |
| ENSG00000112234 | HGLibA_17008 | 81 | -0.342567927 | FBXL4 | F-box and leucine rich repeat protein 4 [Source:HGNC Symbol;Acc:HGNC:13601] |
| ENSG00000112333 | HGLibA_32603 | 81 | -0.342567927 | NR2E1 | nuclear receptor subfamily 2 group E member 1 [Source:HGNC Symbol;Acc:HGNC:7973] |
| ENSG00000113504 | HGLibA_44376 | 81 | -0.342567927 | SLC12A7 | solute carrier family 12 member 7 [Source:HGNC Symbol;Acc:HGNC:10915] |
| ENSG00000114626 | HGLibA_00335 | 81 | -0.342567927 | ABTB1 | ankyrin repeat and BTB domain containing 1 [Source:HGNC Symbol;Acc:HGNC:18275] |
| ENSG00000114686 | HGLibA_29946 | 81 | -0.342567927 | MRPL3 | mitochondrial ribosomal protein L3 [Source:HGNC Symbol;Acc:HGNC:10379] |
| ENSG00000115271 | HGLibA_18758 | 81 | -0.342567927 | GCA | grancalcin [Source:HGNC Symbol;Acc:HGNC:15990] |
| ENSG00000115808 | HGLibA_47594 | 81 | -0.342567927 | STRN | striatin [Source:HGNC Symbol;Acc:HGNC:11424] |
| ENSG00000115844 | HGLibA_13376 | 81 | -0.342567927 | DLX2 | distal-less homeobox 2 [Source:HGNC Symbol;Acc:HGNC:2915] |
| ENSG00000119013 | HGLibA_31514 | 81 | -0.342567927 | NDUFB3 | NADH:ubiquinone oxidoreductase subunit B3 [Source:HGNC Symbol;Acc:HGNC:7698] |
| ENSG00000120337 | HGLibA_50821 | 81 | -0.342567927 | TNFSF18 | TNF superfamily member 18 [Source:HGNC Symbol;Acc:HGNC:11932] |
| ENSG00000123329 | HGLibA_02792 | 81 | -0.342567927 | ARHGAP9 | Rho GTPase activating protein 9 [Source:HGNC Symbol;Acc:HGNC:14130] |
| ENSG00000123374 | HGLibA_08754 | 81 | -0.342567927 | CDK2 | cyclin dependent kinase 2 [Source:HGNC Symbol;Acc:HGNC:1771] |
| ENSG00000124713 | HGLibA_19560 | 81 | -0.342567927 | GNMT | glycine N-methyltransferase [Source:HGNC Symbol;Acc:HGNC:4415] |
| ENSG00000128268 | HGLibA_29139 | 81 | -0.342567927 | MGAT3 | 4-N-acetylglucosaminyltransferase [Source:HGNC Symbol;Acc:HGNC:7046]" |
| ENSG00000128524 | HGLibA_03755 | 81 | -0.342567927 | ATP6V1F | ATPase H+ transporting V1 subunit F [Source:HGNC Symbol;Acc:HGNC:16832] |
| ENSG00000129083 | HGLibA_10694 | 81 | -0.342567927 | COPB1 | coatomer protein complex subunit beta 1 [Source:HGNC Symbol;Acc:HGNC:2231] |
| ENSG00000130714 | HGLibA_37668 | 81 | -0.342567927 | POMT1 | protein O-mannosyltransferase 1 [Source:HGNC Symbol;Acc:HGNC:9202] |
| ENSG00000131375 | HGLibA_07174 | 81 | -0.342567927 | CAPN7 | calpain 7 [Source:HGNC Symbol;Acc:HGNC:1484] |
| ENSG00000131620 | HGLibA_02172 | 81 | -0.342567927 | ANO1 | anoctamin 1 [Source:HGNC Symbol;Acc:HGNC:21625] |
| ENSG00000132286 | HGLibA_49497 | 81 | -0.342567927 | TIMM10B | translocase of inner mitochondrial membrane 10B [Source:HGNC Symbol;Acc:HGNC:4022] |
| ENSG00000132406 | HGLibA_49920 | 81 | -0.342567927 | TMEM128 | transmembrane protein 128 [Source:HGNC Symbol;Acc:HGNC:28201] |
| ENSG00000132518 | HGLibA_20663 | 81 | -0.342567927 | GUCY2D | retinal [Source:HGNC Symbol;Acc:HGNC:4689]" |
| ENSG00000132698 | HGLibA_39851 | 81 | -0.342567927 | RAB25 | member RAS oncogene family [Source:HGNC Symbol;Acc:HGNC:18238]" |
| ENSG00000133067 | HGLibA_26414 | 81 | -0.342567927 | LGR6 | leucine rich repeat containing G protein-coupled receptor 6 [Source:HGNC Symbol;Acc:HGNC:19719] |
| ENSG00000133114 | HGLibA_19724 | 81 | -0.342567927 | GPALPP1 | GPALPP motifs containing 1 [Source:HGNC Symbol;Acc:HGNC:20298] |
| ENSG00000133858 | HGLibA_55169 | 81 | -0.342567927 | ZFC3H1 | zinc finger C3H1-type containing [Source:HGNC Symbol;Acc:HGNC:28328] |
| ENSG00000134765 | HGLibA_13988 | 81 | -0.342567927 | DSC1 | desmocollin 1 [Source:HGNC Symbol;Acc:HGNC:3035] |
| ENSG00000135454 | HGLibA_04012 | 81 | -0.342567927 | B4GALNT1 | 4-N-acetyl-galactosaminyltransferase 1 [Source:HGNC Symbol;Acc:HGNC:4117]" |
| ENSG00000135778 | HGLibA_32871 | 81 | -0.342567927 | NTPCR | cancer-related [Source:HGNC Symbol;Acc:HGNC:28204]" |
| ENSG00000136811 | HGLibA_33278 | 81 | -0.342567927 | ODF2 | outer dense fiber of sperm tails 2 [Source:HGNC Symbol;Acc:HGNC:8114] |
| ENSG00000136834 | HGLibA_33674 | 81 | -0.342567927 | OR1J1 | olfactory receptor family 1 subfamily J member 1 [Source:HGNC Symbol;Acc:HGNC:8208] |
| ENSG00000136877 | HGLibA_17961 | 81 | -0.342567927 | FPGS | folylpolyglutamate synthase [Source:HGNC Symbol;Acc:HGNC:3824] |
| ENSG00000137310 | HGLibA_48766 | 81 | -0.342567927 | TCF19 | transcription factor 19 [Source:HGNC Symbol;Acc:HGNC:11629] |
| ENSG00000137478 | HGLibA_17212 | 81 | -0.342567927 | FCHSD2 | FCH and double SH3 domains 2 [Source:HGNC Symbol;Acc:HGNC:29114] |
| ENSG00000137817 | HGLibA_35241 | 81 | -0.342567927 | PARP6 | poly(ADP-ribose) polymerase family member 6 [Source:HGNC Symbol;Acc:HGNC:26921] |
| ENSG00000138286 | HGLibA_16153 | 81 | -0.342567927 | FAM149B1 | family with sequence similarity 149 member B1 [Source:HGNC Symbol;Acc:HGNC:29162] |
| ENSG00000139629 | HGLibA_18567 | 81 | -0.342567927 | GALNT6 | polypeptide N-acetylgalactosaminyltransferase 6 [Source:HGNC Symbol;Acc:HGNC:4128] |
| ENSG00000142082 | HGLibA_44223 | 81 | -0.342567927 | SIRT3 | sirtuin 3 [Source:HGNC Symbol;Acc:HGNC:14931] |
| ENSG00000143951 | HGLibA_54019 | 81 | -0.342567927 | WDPCP | WD repeat containing planar cell polarity effector [Source:HGNC Symbol;Acc:HGNC:28027] |
| ENSG00000144736 | HGLibA_44085 | 81 | -0.342567927 | SHQ1 | H/ACA ribonucleoprotein assembly factor [Source:HGNC Symbol;Acc:HGNC:25543]" |
| ENSG00000145113 | HGLibA_30546 | 81 | -0.342567927 | MUC4 | cell surface associated [Source:HGNC Symbol;Acc:HGNC:7514]" |
| ENSG00000145808 | HGLibA_00819 | 81 | -0.342567927 | ADAMTS19 | ADAM metallopeptidase with thrombospondin type 1 motif 19 [Source:HGNC Symbol;Acc:HGNC:17111] |
| ENSG00000146918 | HGLibA_31273 | 81 | -0.342567927 | NCAPG2 | non-SMC condensin II complex subunit G2 [Source:HGNC Symbol;Acc:HGNC:21904] |
| ENSG00000147481 | HGLibA_46007 | 81 | -0.342567927 | SNTG1 | syntrophin gamma 1 [Source:HGNC Symbol;Acc:HGNC:13740] |
| ENSG00000147588 | HGLibA_37322 | 81 | -0.342567927 | PMP2 | peripheral myelin protein 2 [Source:HGNC Symbol;Acc:HGNC:9117] |
| ENSG00000147654 | HGLibA_14346 | 81 | -0.342567927 | EBAG9 | 9 [Source:HGNC Symbol;Acc:HGNC:3123]" |
| ENSG00000149124 | HGLibA_19371 | 81 | -0.342567927 | GLYAT | glycine-N-acyltransferase [Source:HGNC Symbol;Acc:HGNC:13734] |
| ENSG00000149403 | HGLibA_20289 | 81 | -0.342567927 | GRIK4 | glutamate ionotropic receptor kainate type subunit 4 [Source:HGNC Symbol;Acc:HGNC:4582] |
| ENSG00000149506 | HGLibA_56951 | 81 | -0.342567927 | ZP1 | zona pellucida glycoprotein 1 [Source:HGNC Symbol;Acc:HGNC:13187] |
| ENSG00000151164 | HGLibA_40111 | 81 | -0.342567927 | RAD9B | RAD9 checkpoint clamp component B [Source:HGNC Symbol;Acc:HGNC:21700] |
| ENSG00000151778 | HGLibA_43502 | 81 | -0.342567927 | SERP2 | stress associated endoplasmic reticulum protein family member 2 [Source:HGNC Symbol;Acc:HGNC:20607] |
| ENSG00000151789 | HGLibA_55904 | 81 | -0.342567927 | ZNF385D | zinc finger protein 385D [Source:HGNC Symbol;Acc:HGNC:26191] |
| ENSG00000151967 | HGLibA_42942 | 81 | -0.342567927 | SCHIP1 | schwannomin interacting protein 1 [Source:HGNC Symbol;Acc:HGNC:15678] |
| ENSG00000154027 | HGLibA_01389 | 81 | -0.342567927 | AK5 | adenylate kinase 5 [Source:HGNC Symbol;Acc:HGNC:365] |
| ENSG00000154734 | HGLibA_00790 | 81 | -0.342567927 | ADAMTS1 | ADAM metallopeptidase with thrombospondin type 1 motif 1 [Source:HGNC Symbol;Acc:HGNC:217] |
| ENSG00000154783 | HGLibA_17354 | 81 | -0.342567927 | FGD5 | RhoGEF and PH domain containing 5 [Source:HGNC Symbol;Acc:HGNC:19117]" |
| ENSG00000154957 | HGLibA_55534 | 81 | -0.342567927 | ZNF18 | zinc finger protein 18 [Source:HGNC Symbol;Acc:HGNC:12969] |
| ENSG00000157445 | HGLibA_06912 | 81 | -0.342567927 | CACNA2D3 | calcium voltage-gated channel auxiliary subunit alpha2delta 3 [Source:HGNC Symbol;Acc:HGNC:15460] |
| ENSG00000158714 | HGLibA_44320 | 81 | -0.342567927 | SLAMF8 | SLAM family member 8 [Source:HGNC Symbol;Acc:HGNC:21391] |
| ENSG00000159176 | HGLibA_11433 | 81 | -0.342567927 | CSRP1 | cysteine and glycine rich protein 1 [Source:HGNC Symbol;Acc:HGNC:2469] |
| ENSG00000159189 | HGLibA_05921 | 81 | -0.342567927 | C1QC | complement C1q C chain [Source:HGNC Symbol;Acc:HGNC:1245] |
| ENSG00000161958 | HGLibA_17367 | 81 | -0.342567927 | FGF11 | fibroblast growth factor 11 [Source:HGNC Symbol;Acc:HGNC:3667] |
| ENSG00000163737 | HGLibA_36130 | 81 | -0.342567927 | PF4 | platelet factor 4 [Source:HGNC Symbol;Acc:HGNC:8861] |
| ENSG00000164236 | HGLibA_02071 | 81 | -0.342567927 | ANKRD33B | ankyrin repeat domain 33B [Source:HGNC Symbol;Acc:HGNC:35240] |
| ENSG00000164708 | HGLibA_36198 | 81 | -0.342567927 | PGAM2 | phosphoglycerate mutase 2 [Source:HGNC Symbol;Acc:HGNC:8889] |
| ENSG00000165370 | HGLibA_19839 | 81 | -0.342567927 | GPR101 | G protein-coupled receptor 101 [Source:HGNC Symbol;Acc:HGNC:14963] |
| ENSG00000165623 | HGLibA_52820 | 81 | -0.342567927 | UCMA | upper zone of growth plate and cartilage matrix associated [Source:HGNC Symbol;Acc:HGNC:25205] |
| ENSG00000165650 | HGLibA_35984 | 81 | -0.342567927 | PDZD8 | PDZ domain containing 8 [Source:HGNC Symbol;Acc:HGNC:26974] |
| ENSG00000166069 | HGLibA_49792 | 81 | -0.342567927 | TMCO5A | transmembrane and coiled-coil domains 5A [Source:HGNC Symbol;Acc:HGNC:28558] |
| ENSG00000166128 | HGLibA_39987 | 81 | -0.342567927 | RAB8B | member RAS oncogene family [Source:HGNC Symbol;Acc:HGNC:30273]" |
| ENSG00000166825 | HGLibA_02211 | 81 | -0.342567927 | ANPEP | membrane [Source:HGNC Symbol;Acc:HGNC:500]" |
| ENSG00000167077 | HGLibA_28861 | 81 | -0.342567927 | MEI1 | meiotic double-stranded break formation protein 1 [Source:HGNC Symbol;Acc:HGNC:28613] |
| ENSG00000167081 | HGLibA_35350 | 81 | -0.342567927 | PBX3 | PBX homeobox 3 [Source:HGNC Symbol;Acc:HGNC:8634] |
| ENSG00000167110 | HGLibA_19594 | 81 | -0.342567927 | GOLGA2 | golgin A2 [Source:HGNC Symbol;Acc:HGNC:4425] |

| | | | | | |
|---|---|---|---|---|---|
| ENSG00000168026 | HGLibA_52068 | 81 | -0.342567927 | TTC21A | tetratricopeptide repeat domain 21A [Source:HGNC Symbol;Acc:HGNC:30761] |
| ENSG00000168243 | HGLibA_19527 | 81 | -0.342567927 | GNG4 | G protein subunit gamma 4 [Source:HGNC Symbol;Acc:HGNC:4407] |
| ENSG00000168515 | HGLibA_42913 | 81 | -0.342567927 | SCGB1D1 | secretoglobin family 1D member 1 [Source:HGNC Symbol;Acc:HGNC:18395] |
| ENSG00000169594 | HGLibA_04643 | 81 | -0.342567927 | BNC1 | basonuclin 1 [Source:HGNC Symbol;Acc:HGNC:1081] |
| ENSG00000170180 | HGLibA_20698 | 81 | -0.342567927 | GYPA | glycophorin A (MNS blood group) [Source:HGNC Symbol;Acc:HGNC:4702] |
| ENSG00000172000 | HGLibA_56226 | 81 | -0.342567927 | ZNF556 | zinc finger protein 556 [Source:HGNC Symbol;Acc:HGNC:25669] |
| ENSG00000172500 | HGLibA_17505 | 81 | -0.342567927 | FIBP | FGF1 intracellular binding protein [Source:HGNC Symbol;Acc:HGNC:3705] |
| ENSG00000172757 | HGLibA_09281 | 81 | -0.342567927 | CFL1 | cofilin 1 [Source:HGNC Symbol;Acc:HGNC:1874] |
| ENSG00000172819 | HGLibA_40277 | 81 | -0.342567927 | RARG | retinoic acid receptor gamma [Source:HGNC Symbol;Acc:HGNC:9866] |
| ENSG00000173575 | HGLibA_09390 | 81 | -0.342567927 | CHD2 | chromodomain helicase DNA binding protein 2 [Source:HGNC Symbol;Acc:HGNC:1917] |
| ENSG00000173578 | HGLibA_54500 | 81 | -0.342567927 | XCR1 | X-C motif chemokine receptor 1 [Source:HGNC Symbol;Acc:HGNC:1625] |
| ENSG00000173818 | HGLibA_15122 | 81 | -0.342567927 | ENDOV | endonuclease V [Source:HGNC Symbol;Acc:HGNC:26640] |
| ENSG00000174339 | HGLibA_33908 | 81 | -0.342567927 | OR2Y1 | olfactory receptor family 2 subfamily Y member 1 [Source:HGNC Symbol;Acc:HGNC:14837] |
| ENSG00000175097 | HGLibA_40135 | 81 | -0.342567927 | RAG2 | recombination activating 2 [Source:HGNC Symbol;Acc:HGNC:9832] |
| ENSG00000176396 | HGLibA_14696 | 81 | -0.342567927 | EID2 | EP300 interacting inhibitor of differentiation 2 [Source:HGNC Symbol;Acc:HGNC:28292] |
| ENSG00000178449 | HGLibA_10808 | 81 | -0.342567927 | COX14 | cytochrome c oxidase assembly factor COX14 [Source:HGNC Symbol;Acc:HGNC:28216] |
| ENSG00000178934 | HGLibA_26376 | 81 | -0.342567927 | LGALS7B | galectin 7B [Source:HGNC Symbol;Acc:HGNC:34447] |
| ENSG00000179222 | HGLibA_27969 | 81 | -0.342567927 | MAGED1 | MAGE family member D1 [Source:HGNC Symbol;Acc:HGNC:6813] |
| ENSG00000179454 | HGLibA_25170 | 81 | -0.342567927 | KLHL28 | kelch like family member 28 [Source:HGNC Symbol;Acc:HGNC:19741] |
| ENSG00000179909 | HGLibA_55501 | 81 | -0.342567927 | ZNF154 | zinc finger protein 154 [Source:HGNC Symbol;Acc:HGNC:12939] |
| ENSG00000180210 | HGLibA_15859 | 81 | -0.342567927 | F2 | thrombin [Source:HGNC Symbol;Acc:HGNC:3535]" |
| ENSG00000181029 | HGLibA_51328 | 81 | -0.342567927 | TRAPPC5 | trafficking protein particle complex 5 [Source:HGNC Symbol;Acc:HGNC:23067] |
| ENSG00000181396 | HGLibA_33314 | 81 | -0.342567927 | OGFOD3 | 2-oxoglutarate and iron dependent oxygenase domain containing 3 [Source:HGNC Symbol;Acc:HGNC:26174] |
| ENSG00000181444 | HGLibA_56043 | 81 | -0.342567927 | ZNF467 | zinc finger protein 467 [Source:HGNC Symbol;Acc:HGNC:23154] |
| ENSG00000182348 | HGLibA_56790 | 81 | -0.342567927 | ZNF804B | zinc finger protein 804B [Source:HGNC Symbol;Acc:HGNC:21958] |
| ENSG00000182463 | HGLibA_51865 | 81 | -0.342567927 | TSHZ2 | teashirt zinc finger homeobox 2 [Source:HGNC Symbol;Acc:HGNC:13010] |
| ENSG00000182628 | HGLibA_44265 | 81 | -0.342567927 | SKA2 | spindle and kinetochore associated complex subunit 2 [Source:HGNC Symbol;Acc:HGNC:28006] |
| ENSG00000182798 | HGLibA_27938 | 81 | -0.342567927 | MAGEB17 | MAGE family member B17 [Source:HGNC Symbol;Acc:HGNC:17418] |
| ENSG00000183207 | HGLibA_42475 | 81 | -0.342567927 | RUVBL2 | RuvB like AAA ATPase 2 [Source:HGNC Symbol;Acc:HGNC:10475] |
| ENSG00000183337 | HGLibA_04362 | 81 | -0.342567927 | BCOR | BCL6 corepressor [Source:HGNC Symbol;Acc:HGNC:20893] |
| ENSG00000184675 | HGLibA_01773 | 81 | -0.342567927 | AMER1 | APC membrane recruitment protein 1 [Source:HGNC Symbol;Acc:HGNC:26837] |
| ENSG00000184905 | HGLibA_48707 | 81 | -0.342567927 | TCEAL2 | transcription elongation factor A like 2 [Source:HGNC Symbol;Acc:HGNC:29818] |
| ENSG00000185236 | HGLibA_39791 | 81 | -0.342567927 | RAB11B | member RAS oncogene family [Source:HGNC Symbol;Acc:HGNC:9761]" |
| ENSG00000185668 | HGLibA_37764 | 81 | -0.342567927 | POU3F1 | POU class 3 homeobox 1 [Source:HGNC Symbol;Acc:HGNC:9214] |
| ENSG00000185689 | HGLibA_06467 | 81 | -0.342567927 | C6orf201 | chromosome 6 open reading frame 201 [Source:HGNC Symbol;Acc:HGNC:21620] |
| ENSG00000185924 | HGLibA_42416 | 81 | -0.342567927 | RTN4RL1 | reticulon 4 receptor like 1 [Source:HGNC Symbol;Acc:HGNC:21329] |
| ENSG00000187005 | HGLibA_25708 | 81 | -0.342567927 | KRTAP21-1 | keratin associated protein 21-1 [Source:HGNC Symbol;Acc:HGNC:18945] |
| ENSG00000187492 | HGLibA_08706 | 81 | -0.342567927 | CDHR4 | cadherin related family member 4 [Source:HGNC Symbol;Acc:HGNC:34527] |
| ENSG00000188467 | HGLibA_44627 | 81 | -0.342567927 | SLC24A5 | solute carrier family 24 member 5 [Source:HGNC Symbol;Acc:HGNC:20611] |
| ENSG00000196497 | HGLibA_23466 | 81 | -0.342567927 | IPO4 | importin 4 [Source:HGNC Symbol;Acc:HGNC:19426] |
| ENSG00000196505 | HGLibA_18845 | 81 | -0.342567927 | GDAP2 | ganglioside induced differentiation associated protein 2 [Source:HGNC Symbol;Acc:HGNC:18010] |
| ENSG00000196632 | HGLibA_54337 | 81 | -0.342567927 | WNK3 | WNK lysine deficient protein kinase 3 [Source:HGNC Symbol;Acc:HGNC:14543] |
| ENSG00000196670 | HGLibA_55226 | 81 | -0.342567927 | ZFP62 | ZFP62 zinc finger protein [Source:HGNC Symbol;Acc:HGNC:23241] |
| ENSG00000197256 | HGLibA_24026 | 81 | -0.342567927 | KANK2 | KN motif and ankyrin repeat domains 2 [Source:HGNC Symbol;Acc:HGNC:29300] |
| ENSG00000197769 | HGLibA_28129 | 81 | -0.342567927 | MAP1LC3C | microtubule associated protein 1 light chain 3 gamma [Source:HGNC Symbol;Acc:HGNC:13353] |
| ENSG00000198088 | HGLibA_33065 | 81 | -0.342567927 | NUP62CL | nucleoporin 62 C-terminal like [Source:HGNC Symbol;Acc:HGNC:25960] |
| ENSG00000198128 | HGLibA_33825 | 81 | -0.342567927 | OR2L3 | olfactory receptor family 2 subfamily L member 3 [Source:HGNC Symbol;Acc:HGNC:15009] |
| ENSG00000198739 | HGLibA_27492 | 81 | -0.342567927 | LRRTM3 | leucine rich repeat transmembrane neuronal 3 [Source:HGNC Symbol;Acc:HGNC:19410] |
| ENSG00000198918 | HGLibA_41972 | 81 | -0.342567927 | RPL39 | ribosomal protein L39 [Source:HGNC Symbol;Acc:HGNC:10350] |
| ENSG00000203710 | HGLibA_11045 | 81 | -0.342567927 | CR1 | complement C3b/C4b receptor 1 (Knops blood group) [Source:HGNC Symbol;Acc:HGNC:2334] |
| ENSG00000204941 | HGLibA_39049 | 81 | -0.342567927 | PSG5 | pregnancy specific beta-1-glycoprotein 5 [Source:HGNC Symbol;Acc:HGNC:9522] |
| ENSG00000205078 | HGLibA_47916 | 81 | -0.342567927 | SYCE1L | synaptonemal complex central element protein 1 like [Source:HGNC Symbol;Acc:HGNC:37236] |
| ENSG00000205439 | HGLibA_25637 | 81 | -0.342567927 | KRTAP12-3 | keratin associated protein 12-3 [Source:HGNC Symbol;Acc:HGNC:20531] |
| ENSG00000205495 | HGLibA_34178 | 81 | -0.342567927 | OR52J3 | olfactory receptor family 52 subfamily J member 3 [Source:HGNC Symbol;Acc:HGNC:14799] |
| ENSG00000206560 | HGLibA_02055 | 81 | -0.342567927 | ANKRD28 | ankyrin repeat domain 28 [Source:HGNC Symbol;Acc:HGNC:29024] |
| ENSG00000213190 | HGLibA_29415 | 81 | -0.342567927 | MLLT11 | transcription factor 7 cofactor [Source:HGNC Symbol;Acc:HGNC:16997]" |
| ENSG00000214336 | HGLibA_17881 | 81 | -0.342567927 | FOXI3 | forkhead box I3 [Source:HGNC Symbol;Acc:HGNC:35123] |
| ENSG00000214414 | HGLibA_51612 | 81 | -0.342567927 | TRIM77 | tripartite motif containing 77 [Source:HGNC Symbol;Acc:HGNC:34228] |
| ENSG00000215029 | HGLibA_48851 | 81 | -0.342567927 | TCP11X2 | X-linked 2 [Source:HGNC Symbol;Acc:HGNC:48335]" |
| ENSG00000239389 | HGLibA_35437 | 81 | -0.342567927 | PCDHA13 | protocadherin alpha 13 [Source:HGNC Symbol;Acc:HGNC:8667] |
| ENSG00000254996 | HGLibA_01968 | 81 | -0.342567927 | ANKHD1-EIF4EBP3 | ANKHD1-EIF4EBP3 readthrough [Source:HGNC Symbol;Acc:HGNC:33530] |
| ENSG00000256087 | HGLibA_55984 | 81 | -0.342567927 | ZNF432 | zinc finger protein 432 [Source:HGNC Symbol;Acc:HGNC:20810] |
| ENSG00000256087 | HGLibA_55983 | 81 | -0.342567927 | ZNF432 | zinc finger protein 432 [Source:HGNC Symbol;Acc:HGNC:20810] |
| ENSG00000258083 | HGLibA_34566 | 81 | -0.342567927 | OR9A4 | olfactory receptor family 9 subfamily A member 4 [Source:HGNC Symbol;Acc:HGNC:15095] |
| ENSG00000278289 | HGLibA_11514 | 81 | -0.342567927 | CT45A6 | cancer/testis antigen family 45 member A6 [Source:HGNC Symbol;Acc:HGNC:33271] |
| EPB49 | HGLibA_15263 | 81 | -0.342567927 | EPB49 | - |
| GAREM | HGLibA_18627 | 81 | -0.342567927 | GAREM | - |
| hsa-let-7c | HGLibA_57114 | 81 | -0.342567927 | hsa-let-7c | - |
| hsa-let-7d | HGLibA_57117 | 81 | -0.342567927 | hsa-let-7d | - |
| hsa-mir-1184-1 | HGLibA_57230 | 81 | -0.342567927 | hsa-mir-1184-1 | - |
| hsa-mir-1289-1 | HGLibA_57619 | 81 | -0.342567927 | hsa-mir-1289-1 | - |
| hsa-mir-3119-2 | HGLibA_58529 | 81 | -0.342567927 | hsa-mir-3119-2 | - |
| hsa-mir-3139 | HGLibA_58615 | 81 | -0.342567927 | hsa-mir-3139 | - |
| hsa-mir-3159 | HGLibA_58710 | 81 | -0.342567927 | hsa-mir-3159 | - |
| hsa-mir-3663 | HGLibA_59203 | 81 | -0.342567927 | hsa-mir-3663 | - |
| hsa-mir-3689d-1 | HGLibA_59325 | 81 | -0.342567927 | hsa-mir-3689d-1 | - |
| hsa-mir-3689f | HGLibA_59336 | 81 | -0.342567927 | hsa-mir-3689f | - |
| hsa-mir-3922 | HGLibA_59566 | 81 | -0.342567927 | hsa-mir-3922 | - |
| hsa-mir-3976 | HGLibA_59662 | 81 | -0.342567927 | hsa-mir-3976 | - |
| hsa-mir-4294 | HGLibA_59888 | 81 | -0.342567927 | hsa-mir-4294 | - |
| hsa-mir-4301 | HGLibA_59914 | 81 | -0.342567927 | hsa-mir-4301 | - |
| hsa-mir-4435-1 | HGLibA_60129 | 81 | -0.342567927 | hsa-mir-4435-1 | - |
| hsa-mir-4467 | HGLibA_60275 | 81 | -0.342567927 | hsa-mir-4467 | - |
| hsa-mir-4698 | HGLibA_60901 | 81 | -0.342567927 | hsa-mir-4698 | - |
| hsa-mir-4785 | HGLibA_61249 | 81 | -0.342567927 | hsa-mir-4785 | - |
| hsa-mir-500a | HGLibA_61445 | 81 | -0.342567927 | hsa-mir-500a | - |
| hsa-mir-511 | HGLibA_61559 | 81 | -0.342567927 | hsa-mir-511 | - |
| hsa-mir-518a-1 | HGLibA_61650 | 81 | -0.342567927 | hsa-mir-518a-1 | - |
| hsa-mir-548c | HGLibA_61955 | 81 | -0.342567927 | hsa-mir-548c | - |
| hsa-mir-6130 | HGLibA_62671 | 81 | -0.342567927 | hsa-mir-6130 | - |
| hsa-mir-6503 | HGLibA_62861 | 81 | -0.342567927 | hsa-mir-6503 | - |
| hsa-mir-6506 | HGLibA_62873 | 81 | -0.342567927 | hsa-mir-6506 | - |
| hsa-mir-656 | HGLibA_62956 | 81 | -0.342567927 | hsa-mir-656 | - |
| hsa-mir-6739 | HGLibA_63111 | 81 | -0.342567927 | hsa-mir-6739 | - |
| hsa-mir-6770-3 | HGLibA_63255 | 81 | -0.342567927 | hsa-mir-6770-3 | - |
| hsa-mir-6790 | HGLibA_63339 | 81 | -0.342567927 | hsa-mir-6790 | - |
| hsa-mir-6861 | HGLibA_63623 | 81 | -0.342567927 | hsa-mir-6861 | - |
| hsa-mir-7976 | HGLibA_64033 | 81 | -0.342567927 | hsa-mir-7976 | - |
| IRG1 | HGLibA_23607 | 81 | -0.342567927 | IRG1 | - |
| PVRL4 | HGLibA_39631 | 81 | -0.342567927 | PVRL4 | - |
| SELT | HGLibA_43312 | 81 | -0.342567927 | SELT | - |
| TARSL2 | HGLibA_48352 | 81 | -0.342567927 | TARSL2 | - |
| ASMTL_Y | HGLibA_03329 | 80 | -0.352518965 | ASMTL_Y | - |
| ATP5C1 | HGLibA_03644 | 80 | -0.352518965 | ATP5C1 | - |
| C10orf76 | HGLibA_05095 | 80 | -0.352518965 | C10orf76 | - |
| C15orf26 | HGLibA_05378 | 80 | -0.352518965 | C15orf26 | - |
| C19orf26 | HGLibA_05646 | 80 | -0.352518965 | C19orf26 | - |
| C8orf22 | HGLibA_06602 | 80 | -0.352518965 | C8orf22 | - |
| CASC10 | HGLibA_07272 | 80 | -0.352518965 | CASC10 | - |
| CXorf23 | HGLibA_11909 | 80 | -0.352518965 | CXorf23 | - |
| DYX1C1 | HGLibA_14293 | 80 | -0.352518965 | DYX1C1 | - |
| ENSG00000007314 | HGLibA_42992 | 80 | -0.352518965 | SCN4A | sodium voltage-gated channel alpha subunit 4 [Source:HGNC Symbol;Acc:HGNC:10591] |
| ENSG00000008988 | HGLibA_42103 | 80 | -0.352518965 | RPS20 | ribosomal protein S20 [Source:HGNC Symbol;Acc:HGNC:10405] |
| ENSG00000013297 | HGLibA_09853 | 80 | -0.352518965 | CLDN11 | claudin 11 [Source:HGNC Symbol;Acc:HGNC:8514] |
| ENSG00000034053 | HGLibA_02371 | 80 | -0.352518965 | APBA2 | amyloid beta precursor protein binding family A member 2 [Source:HGNC Symbol;Acc:HGNC:579] |
| ENSG00000041353 | HGLibA_39858 | 80 | -0.352518965 | RAB27B | member RAS oncogene family [Source:HGNC Symbol;Acc:HGNC:9767]" |
| ENSG00000051620 | HGLibA_21087 | 80 | -0.352518965 | HEBP2 | heme binding protein 2 [Source:HGNC Symbol;Acc:HGNC:15716] |
| ENSG00000060688 | HGLibA_45954 | 80 | -0.352518965 | SNRNP40 | small nuclear ribonucleoprotein U5 subunit 40 [Source:HGNC Symbol;Acc:HGNC:30857] |
| ENSG00000064309 | HGLibA_08856 | 80 | -0.352518965 | CDON | oncogene regulated [Source:HGNC Symbol;Acc:HGNC:17104]" |
| ENSG00000065060 | HGLibA_52949 | 80 | -0.352518965 | UHRF1BP1 | UHRF1 binding protein 1 [Source:HGNC Symbol;Acc:HGNC:21216] |
| ENSG00000065457 | HGLibA_00879 | 80 | -0.352518965 | ADAT1 | tRNA specific 1 [Source:HGNC Symbol;Acc:HGNC:228]" |
| ENSG00000066405 | HGLibA_09871 | 80 | -0.352518965 | CLDN18 | claudin 18 [Source:HGNC Symbol;Acc:HGNC:2039] |
| ENSG00000075975 | HGLibA_29350 | 80 | -0.352518965 | MKRN2 | makorin ring finger protein 2 [Source:HGNC Symbol;Acc:HGNC:7113] |
| ENSG00000079435 | HGLibA_26606 | 80 | -0.352518965 | LIPE | hormone sensitive type [Source:HGNC Symbol;Acc:HGNC:6621]" |
| ENSG00000084754 | HGLibA_20807 | 80 | -0.352518965 | HADHA | hydroxyacyl-CoA dehydrogenase trifunctional multienzyme complex subunit alpha [Source:HGNC Symbol;Acc:HGNC:4801] |
| ENSG00000086712 | HGLibA_52397 | 80 | -0.352518965 | TXLNG | taxilin gamma [Source:HGNC Symbol;Acc:HGNC:18578] |
| ENSG00000087460 | HGLibA_19469 | 80 | -0.352518965 | GNAS | GNAS complex locus [Source:HGNC Symbol;Acc:HGNC:4392] |
| ENSG00000088756 | HGLibA_02746 | 80 | -0.352518965 | ARHGAP28 | Rho GTPase activating protein 28 [Source:HGNC Symbol;Acc:HGNC:25509] |
| ENSG00000088756 | HGLibA_02747 | 80 | -0.352518965 | ARHGAP28 | Rho GTPase activating protein 28 [Source:HGNC Symbol;Acc:HGNC:25509] |
| ENSG00000090372 | HGLibA_47601 | 80 | -0.352518965 | STRN4 | striatin 4 [Source:HGNC Symbol;Acc:HGNC:15721] |
| ENSG00000090554 | HGLibA_17670 | 80 | -0.352518965 | FLT3LG | fms related tyrosine kinase 3 ligand [Source:HGNC Symbol;Acc:HGNC:3766] |
| ENSG00000094755 | HGLibA_18399 | 80 | -0.352518965 | GABRP | gamma-aminobutyric acid type A receptor pi subunit [Source:HGNC Symbol;Acc:HGNC:4089] |
| ENSG00000099940 | HGLibA_45881 | 80 | -0.352518965 | SNAP29 | synaptosome associated protein 29 [Source:HGNC Symbol;Acc:HGNC:11133] |
| ENSG00000100399 | HGLibA_09344 | 80 | -0.352518965 | CHADL | chondroadherin like [Source:HGNC Symbol;Acc:HGNC:25165] |
| ENSG00000100583 | HGLibA_42678 | 80 | -0.352518965 | SAMD15 | sterile alpha motif domain containing 15 [Source:HGNC Symbol;Acc:HGNC:18631] |

| | | | | | |
|---|---|---|---|---|---|
| ENSG00000101977 | HGLibA_28578 | 80 | -0.352518965 | MCF2 | MCF.2 cell line derived transforming sequence [Source:HGNC Symbol;Acc:HGNC:6940] |
| ENSG00000102119 | HGLibA_15033 | 80 | -0.352518965 | EMD | emerin [Source:HGNC Symbol;Acc:HGNC:3331] |
| ENSG00000103534 | HGLibA_49760 | 80 | -0.352518965 | TMC5 | transmembrane channel like 5 [Source:HGNC Symbol;Acc:HGNC:22999] |
| ENSG00000103546 | HGLibA_45318 | 80 | -0.352518965 | SLC6A2 | solute carrier family 6 member 2 [Source:HGNC Symbol;Acc:HGNC:11048] |
| ENSG00000104731 | HGLibA_25103 | 80 | -0.352518965 | KLHDC4 | kelch domain containing 4 [Source:HGNC Symbol;Acc:HGNC:25272] |
| ENSG00000104859 | HGLibA_09800 | 80 | -0.352518965 | CLASRP | CLK4 associating serine/arginine rich protein [Source:HGNC Symbol;Acc:HGNC:17731] |
| ENSG00000105143 | HGLibA_44523 | 80 | -0.352518965 | SLC1A6 | solute carrier family 1 member 6 [Source:HGNC Symbol;Acc:HGNC:10944] |
| ENSG00000105325 | HGLibA_18289 | 80 | -0.352518965 | FZR1 | fizzy and cell division cycle 20 related 1 [Source:HGNC Symbol;Acc:HGNC:24824] |
| ENSG00000105726 | HGLibA_03565 | 80 | -0.352518965 | ATP13A1 | ATPase 13A1 [Source:HGNC Symbol;Acc:HGNC:24215] |
| ENSG00000106341 | HGLibA_38059 | 80 | -0.352518965 | PPP1R17 | protein phosphatase 1 regulatory subunit 17 [Source:HGNC Symbol;Acc:HGNC:16973] |
| ENSG00000106560 | HGLibA_19077 | 80 | -0.352518965 | GIMAP2 | IMAP family member 2 [Source:HGNC Symbol;Acc:HGNC:21789]" |
| ENSG00000109534 | HGLibA_18622 | 80 | -0.352518965 | GAR1 | GAR1 ribonucleoprotein [Source:HGNC Symbol;Acc:HGNC:14264] |
| ENSG00000110046 | HGLibA_03479 | 80 | -0.352518965 | ATG2A | autophagy related 2A [Source:HGNC Symbol;Acc:HGNC:29028] |
| ENSG00000111224 | HGLibA_35216 | 80 | -0.352518965 | PARP11 | poly(ADP-ribose) polymerase family member 11 [Source:HGNC Symbol;Acc:HGNC:1186] |
| ENSG00000111877 | HGLibA_28627 | 80 | -0.352518965 | MCM9 | minichromosome maintenance 9 homologous recombination repair factor [Source:HGNC Symbol;Acc:HGNC:21484] |
| ENSG00000112273 | HGLibA_21043 | 80 | -0.352518965 | HDGFL1 | HDGF like 1 [Source:HGNC Symbol;Acc:HGNC:21095] |
| ENSG00000112837 | HGLibA_48650 | 80 | -0.352518965 | TBX18 | T-box 18 [Source:HGNC Symbol;Acc:HGNC:11595] |
| ENSG00000113391 | HGLibA_16273 | 80 | -0.352518965 | FAM172A | family with sequence similarity 172 member A [Source:HGNC Symbol;Acc:HGNC:25365] |
| ENSG00000116176 | HGLibA_51201 | 80 | -0.352518965 | TPSG1 | tryptase gamma 1 [Source:HGNC Symbol;Acc:HGNC:14134] |
| ENSG00000116667 | HGLibA_05825 | 80 | -0.352518965 | C1orf21 | chromosome 1 open reading frame 21 [Source:HGNC Symbol;Acc:HGNC:15494] |
| ENSG00000116985 | HGLibA_04623 | 80 | -0.352518965 | BMP8B | bone morphogenetic protein 8b [Source:HGNC Symbol;Acc:HGNC:1075] |
| ENSG00000117090 | HGLibA_44311 | 80 | -0.352518965 | SLAMF1 | signaling lymphocytic activation molecule family member 1 [Source:HGNC Symbol;Acc:HGNC:10903] |
| ENSG00000117298 | HGLibA_14381 | 80 | -0.352518965 | ECE1 | endothelin converting enzyme 1 [Source:HGNC Symbol;Acc:HGNC:3146] |
| ENSG00000118260 | HGLibA_11088 | 80 | -0.352518965 | CREB1 | cAMP responsive element binding protein 1 [Source:HGNC Symbol;Acc:HGNC:2345] |
| ENSG00000118407 | HGLibA_17528 | 80 | -0.352518965 | FILIP1 | filamin A interacting protein 1 [Source:HGNC Symbol;Acc:HGNC:21015] |
| ENSG00000119929 | HGLibA_11800 | 80 | -0.352518965 | CUTC | cutC copper transporter [Source:HGNC Symbol;Acc:HGNC:24271] |
| ENSG00000120563 | HGLibA_27784 | 80 | -0.352518965 | LYZL1 | lysozyme like 1 [Source:HGNC Symbol;Acc:HGNC:30502] |
| ENSG00000120889 | HGLibA_50737 | 80 | -0.352518965 | TNFRSF10B | TNF receptor superfamily member 10b [Source:HGNC Symbol;Acc:HGNC:11905] |
| ENSG00000120910 | HGLibA_38190 | 80 | -0.352518965 | PPP3CC | protein phosphatase 3 catalytic subunit gamma [Source:HGNC Symbol;Acc:HGNC:9316] |
| ENSG00000122224 | HGLibA_27690 | 80 | -0.352518965 | LY9 | lymphocyte antigen 9 [Source:HGNC Symbol;Acc:HGNC:6730] |
| ENSG00000122641 | HGLibA_23300 | 80 | -0.352518965 | INHBA | inhibin subunit beta A [Source:HGNC Symbol;Acc:HGNC:6066] |
| ENSG00000123179 | HGLibA_14376 | 80 | -0.352518965 | EBPL | EBP like [Source:HGNC Symbol;Acc:HGNC:18061] |
| ENSG00000123268 | HGLibA_03432 | 80 | -0.352518965 | ATF1 | activating transcription factor 1 [Source:HGNC Symbol;Acc:HGNC:783] |
| ENSG00000124486 | HGLibA_53417 | 80 | -0.352518965 | USP9X | ubiquitin specific peptidase 9 X-linked [Source:HGNC Symbol;Acc:HGNC:12632] |
| ENSG00000126709 | HGLibA_22618 | 80 | -0.352518965 | IFI6 | interferon alpha inducible protein 6 [Source:HGNC Symbol;Acc:HGNC:4054] |
| ENSG00000127318 | HGLibA_23087 | 80 | -0.352518965 | IL22 | interleukin 22 [Source:HGNC Symbol;Acc:HGNC:14900] |
| ENSG00000127980 | HGLibA_36079 | 80 | -0.352518965 | PEX1 | peroxisomal biogenesis factor 1 [Source:HGNC Symbol;Acc:HGNC:8850] |
| ENSG00000128591 | HGLibA_17644 | 80 | -0.352518965 | FLNC | filamin C [Source:HGNC Symbol;Acc:HGNC:3756] |
| ENSG00000128731 | HGLibA_21157 | 80 | -0.352518965 | HERC2 | HECT and RLD domain containing E3 ubiquitin protein ligase 2 [Source:HGNC Symbol;Acc:HGNC:4868] |
| ENSG00000129116 | HGLibA_35065 | 80 | -0.352518965 | PALLD | cytoskeletal associated protein [Source:HGNC Symbol;Acc:HGNC:17068]" |
| ENSG00000133026 | HGLibA_30707 | 80 | -0.352518965 | MYH10 | myosin heavy chain 10 [Source:HGNC Symbol;Acc:HGNC:7568] |
| ENSG00000133056 | HGLibA_36624 | 80 | -0.352518965 | PIK3C2B | phosphatidylinositol-4-phosphate 3-kinase catalytic subunit type 2 beta [Source:HGNC Symbol;Acc:HGNC:8972] |
| ENSG00000133937 | HGLibA_20398 | 80 | -0.352518965 | GSC | goosecoid homeobox [Source:HGNC Symbol;Acc:HGNC:4612] |
| ENSG00000134539 | HGLibA_25299 | 80 | -0.352518965 | KLRD1 | killer cell lectin like receptor D1 [Source:HGNC Symbol;Acc:HGNC:6378] |
| ENSG00000135392 | HGLibA_13577 | 80 | -0.352518965 | DNAJC14 | DnaJ heat shock protein family (Hsp40) member C14 [Source:HGNC Symbol;Acc:HGNC:24581] |
| ENSG00000135679 | HGLibA_28696 | 80 | -0.352518965 | MDM2 | MDM2 proto-oncogene [Source:HGNC Symbol;Acc:HGNC:6973] |
| ENSG00000136048 | HGLibA_13945 | 80 | -0.352518965 | DRAM1 | DNA damage regulated autophagy modulator 1 [Source:HGNC Symbol;Acc:HGNC:25645] |
| ENSG00000136295 | HGLibA_52236 | 80 | -0.352518965 | TTYH3 | tweety family member 3 [Source:HGNC Symbol;Acc:HGNC:22222] |
| ENSG00000136908 | HGLibA_13859 | 80 | -0.352518965 | DPM2 | regulatory [Source:HGNC Symbol;Acc:HGNC:3006]" |
| ENSG00000137393 | HGLibA_41529 | 80 | -0.352518965 | RNF144B | ring finger protein 144B [Source:HGNC Symbol;Acc:HGNC:21578] |
| ENSG00000138166 | HGLibA_14183 | 80 | -0.352518965 | DUSP5 | dual specificity phosphatase 5 [Source:HGNC Symbol;Acc:HGNC:3071] |
| ENSG00000139626 | HGLibA_23795 | 80 | -0.352518965 | ITGB7 | integrin subunit beta 7 [Source:HGNC Symbol;Acc:HGNC:6162] |
| ENSG00000140092 | HGLibA_16949 | 80 | -0.352518965 | FBLN5 | fibulin 5 [Source:HGNC Symbol;Acc:HGNC:3602] |
| ENSG00000140832 | HGLibA_28384 | 80 | -0.352518965 | MARVELD3 | MARVEL domain containing 3 [Source:HGNC Symbol;Acc:HGNC:30525] |
| ENSG00000141198 | HGLibA_50948 | 80 | -0.352518965 | TOM1L1 | target of myb1 like 1 membrane trafficking protein [Source:HGNC Symbol;Acc:HGNC:11983] |
| ENSG00000141579 | HGLibA_56674 | 80 | -0.352518965 | ZNF750 | zinc finger protein 750 [Source:HGNC Symbol;Acc:HGNC:25843] |
| ENSG00000142686 | HGLibA_05830 | 80 | -0.352518965 | C1orf216 | chromosome 1 open reading frame 216 [Source:HGNC Symbol;Acc:HGNC:26800] |
| ENSG00000143149 | HGLibA_01589 | 80 | -0.352518965 | ALDH9A1 | aldehyde dehydrogenase 9 family member A1 [Source:HGNC Symbol;Acc:HGNC:412] |
| ENSG00000143314 | HGLibA_29936 | 80 | -0.352518965 | MRPL24 | mitochondrial ribosomal protein L24 [Source:HGNC Symbol;Acc:HGNC:14037] |
| ENSG00000143318 | HGLibA_07334 | 80 | -0.352518965 | CASQ1 | calsequestrin 1 [Source:HGNC Symbol;Acc:HGNC:1512] |
| ENSG00000143797 | HGLibA_28520 | 80 | -0.352518965 | MBOAT2 | membrane bound O-acyltransferase domain containing 2 [Source:HGNC Symbol;Acc:HGNC:25193] |
| ENSG00000145220 | HGLibA_27695 | 80 | -0.352518965 | LYAR | Ly1 antibody reactive [Source:HGNC Symbol;Acc:HGNC:26021] |
| ENSG00000145388 | HGLibA_28957 | 80 | -0.352518965 | METTL14 | methyltransferase like 14 [Source:HGNC Symbol;Acc:HGNC:29330] |
| ENSG00000147481 | HGLibA_46006 | 80 | -0.352518965 | SNTG1 | syntrophin gamma 1 [Source:HGNC Symbol;Acc:HGNC:13740] |
| ENSG00000147896 | HGLibA_22717 | 80 | -0.352518965 | IFNK | interferon kappa [Source:HGNC Symbol;Acc:HGNC:21714] |
| ENSG00000151320 | HGLibA_01440 | 80 | -0.352518965 | AKAP6 | A-kinase anchoring protein 6 [Source:HGNC Symbol;Acc:HGNC:376] |
| ENSG00000152954 | HGLibA_32725 | 80 | -0.352518965 | NRSN1 | neurensin 1 [Source:HGNC Symbol;Acc:HGNC:17881] |
| ENSG00000155011 | HGLibA_13297 | 80 | -0.352518965 | DKK2 | dickkopf WNT signaling pathway inhibitor 2 [Source:HGNC Symbol;Acc:HGNC:2892] |
| ENSG00000155252 | HGLibA_36493 | 80 | -0.352518965 | PI4K2A | phosphatidylinositol 4-kinase type 2 alpha [Source:HGNC Symbol;Acc:HGNC:30031] |
| ENSG00000155833 | HGLibA_12047 | 80 | -0.352518965 | CYLC2 | cylicin 2 [Source:HGNC Symbol;Acc:HGNC:2583] |
| ENSG00000155897 | HGLibA_00930 | 80 | -0.352518965 | ADCY8 | adenylate cyclase 8 [Source:HGNC Symbol;Acc:HGNC:239] |
| ENSG00000156253 | HGLibA_42486 | 80 | -0.352518965 | RWDD2B | RWD domain containing 2B [Source:HGNC Symbol;Acc:HGNC:1302] |
| ENSG00000157315 | HGLibA_49817 | 80 | -0.352518965 | TMED6 | transmembrane p24 trafficking protein 6 [Source:HGNC Symbol;Acc:HGNC:28331] |
| ENSG00000158473 | HGLibA_08272 | 80 | -0.352518965 | CD1D | CD1d molecule [Source:HGNC Symbol;Acc:HGNC:1637] |
| ENSG00000158717 | HGLibA_41558 | 80 | -0.352518965 | RNF166 | ring finger protein 166 [Source:HGNC Symbol;Acc:HGNC:28856] |
| ENSG00000161544 | HGLibA_12037 | 80 | -0.352518965 | CYGB | cytoglobin [Source:HGNC Symbol;Acc:HGNC:16505] |
| ENSG00000162236 | HGLibA_47652 | 80 | -0.352518965 | STX5 | syntaxin 5 [Source:HGNC Symbol;Acc:HGNC:11440] |
| ENSG00000162413 | HGLibA_25153 | 80 | -0.352518965 | KLHL21 | kelch like family member 21 [Source:HGNC Symbol;Acc:HGNC:29041] |
| ENSG00000162552 | HGLibA_54371 | 80 | -0.352518965 | WNT4 | Wnt family member 4 [Source:HGNC Symbol;Acc:HGNC:12783] |
| ENSG00000163285 | HGLibA_18389 | 80 | -0.352518965 | GABRG1 | gamma-aminobutyric acid type A receptor gamma1 subunit [Source:HGNC Symbol;Acc:HGNC:4086] |
| ENSG00000163297 | HGLibA_02216 | 80 | -0.352518965 | ANTXR2 | ANTXR cell adhesion molecule 2 [Source:HGNC Symbol;Acc:HGNC:21732] |
| ENSG00000163947 | HGLibA_02846 | 80 | -0.352518965 | ARHGEF3 | Rho guanine nucleotide exchange factor 3 [Source:HGNC Symbol;Acc:HGNC:683] |
| ENSG00000163995 | HGLibA_00310 | 80 | -0.352518965 | ABLIM2 | actin binding LIM protein family member 2 [Source:HGNC Symbol;Acc:HGNC:19195] |
| ENSG00000165066 | HGLibA_32060 | 80 | -0.352518965 | NKX6-3 | NK6 homeobox 3 [Source:HGNC Symbol;Acc:HGNC:26328] |
| ENSG00000165630 | HGLibA_38711 | 80 | -0.352518965 | PRPF18 | pre-mRNA processing factor 18 [Source:HGNC Symbol;Acc:HGNC:17351] |
| ENSG00000166750 | HGLibA_45497 | 80 | -0.352518965 | SLFN5 | schlafen family member 5 [Source:HGNC Symbol;Acc:HGNC:28286] |
| ENSG00000166984 | HGLibA_48838 | 80 | -0.352518965 | TCP10L2 | t-complex 10 like 2 [Source:HGNC Symbol;Acc:HGNC:21254] |
| ENSG00000167434 | HGLibA_06810 | 80 | -0.352518965 | CA4 | carbonic anhydrase 4 [Source:HGNC Symbol;Acc:HGNC:1375] |
| ENSG00000168061 | HGLibA_42623 | 80 | -0.352518965 | SAC3D1 | SAC3 domain containing 1 [Source:HGNC Symbol;Acc:HGNC:30179] |
| ENSG00000168653 | HGLibA_31556 | 80 | -0.352518965 | NDUFS5 | NADH:ubiquinone oxidoreductase subunit S5 [Source:HGNC Symbol;Acc:HGNC:7712] |
| ENSG00000168675 | HGLibA_26249 | 80 | -0.352518965 | LDLRAD4 | low density lipoprotein receptor class A domain containing 4 [Source:HGNC Symbol;Acc:HGNC:1224] |
| ENSG00000169618 | HGLibA_38671 | 80 | -0.352518965 | PROKR1 | prokineticin receptor 1 [Source:HGNC Symbol;Acc:HGNC:4524] |
| ENSG00000169862 | HGLibA_11661 | 80 | -0.352518965 | CTNND2 | catenin delta 2 [Source:HGNC Symbol;Acc:HGNC:2516] |
| ENSG00000170890 | HGLibA_36882 | 80 | -0.352518965 | PLA2G1B | phospholipase A2 group IB [Source:HGNC Symbol;Acc:HGNC:9030] |
| ENSG00000171772 | HGLibA_47914 | 80 | -0.352518965 | SYCE1 | synaptonemal complex central element protein 1 [Source:HGNC Symbol;Acc:HGNC:28852] |
| ENSG00000172590 | HGLibA_30014 | 80 | -0.352518965 | MRPL52 | mitochondrial ribosomal protein L52 [Source:HGNC Symbol;Acc:HGNC:16655] |
| ENSG00000173011 | HGLibA_48188 | 80 | -0.352518965 | TADA2B | transcriptional adaptor 2B [Source:HGNC Symbol;Acc:HGNC:30781] |
| ENSG00000173013 | HGLibA_07938 | 80 | -0.352518965 | CCDC96 | coiled-coil domain containing 96 [Source:HGNC Symbol;Acc:HGNC:26900] |
| ENSG00000174292 | HGLibA_50844 | 80 | -0.352518965 | TNK1 | tyrosine kinase non receptor 1 [Source:HGNC Symbol;Acc:HGNC:11940] |
| ENSG00000174460 | HGLibA_55008 | 80 | -0.352518965 | ZCCHC12 | zinc finger CCHC-type containing 12 [Source:HGNC Symbol;Acc:HGNC:27273] |
| ENSG00000174938 | HGLibA_43684 | 80 | -0.352518965 | SEZ6L2 | seizure related 6 homolog like 2 [Source:HGNC Symbol;Acc:HGNC:30844] |
| ENSG00000176900 | HGLibA_34134 | 80 | -0.352518965 | OR51T1 | olfactory receptor family 51 subfamily T member 1 [Source:HGNC Symbol;Acc:HGNC:15205] |
| ENSG00000177483 | HGLibA_40556 | 80 | -0.352518965 | RBM44 | RNA binding motif protein 44 [Source:HGNC Symbol;Acc:HGNC:24756] |
| ENSG00000178031 | HGLibA_00847 | 80 | -0.352518965 | ADAMTSL1 | ADAMTS like 1 [Source:HGNC Symbol;Acc:HGNC:14632] |
| ENSG00000178607 | HGLibA_15513 | 80 | -0.352518965 | ERN1 | endoplasmic reticulum to nucleus signaling 1 [Source:HGNC Symbol;Acc:HGNC:3449] |
| ENSG00000179055 | HGLibA_33615 | 80 | -0.352518965 | OR13D1 | olfactory receptor family 13 subfamily D member 1 [Source:HGNC Symbol;Acc:HGNC:14695] |
| ENSG00000180353 | HGLibA_20970 | 80 | -0.352518965 | HCLS1 | hematopoietic cell-specific Lyn substrate 1 [Source:HGNC Symbol;Acc:HGNC:4844] |
| ENSG00000181896 | HGLibA_55434 | 80 | -0.352518965 | ZNF101 | zinc finger protein 101 [Source:HGNC Symbol;Acc:HGNC:12881] |
| ENSG00000182333 | HGLibA_26608 | 80 | -0.352518965 | LIPF | gastric type [Source:HGNC Symbol;Acc:HGNC:6622]" |
| ENSG00000183313 | HGLibA_34187 | 80 | -0.352518965 | OR52L1 | olfactory receptor family 52 subfamily L member 1 [Source:HGNC Symbol;Acc:HGNC:14785] |
| ENSG00000183513 | HGLibA_10452 | 80 | -0.352518965 | COA5 | cytochrome c oxidase assembly factor 5 [Source:HGNC Symbol;Acc:HGNC:33848] |
| ENSG00000184845 | HGLibA_13960 | 80 | -0.352518965 | DRD1 | dopamine receptor D1 [Source:HGNC Symbol;Acc:HGNC:3020] |
| ENSG00000184867 | HGLibA_03041 | 80 | -0.352518965 | ARMCX2 | armadillo repeat containing X-linked 2 [Source:HGNC Symbol;Acc:HGNC:16869] |
| ENSG00000184905 | HGLibA_48706 | 80 | -0.352518965 | TCEAL2 | transcription elongation factor A like 2 [Source:HGNC Symbol;Acc:HGNC:29818] |
| ENSG00000184954 | HGLibA_34404 | 80 | -0.352518965 | OR6C70 | olfactory receptor family 6 subfamily C member 70 [Source:HGNC Symbol;Acc:HGNC:31299] |
| ENSG00000185008 | HGLibA_41736 | 80 | -0.352518965 | ROBO2 | roundabout guidance receptor 2 [Source:HGNC Symbol;Acc:HGNC:10250] |
| ENSG00000185262 | HGLibA_52542 | 80 | -0.352518965 | UBALD2 | UBA like domain containing 2 [Source:HGNC Symbol;Acc:HGNC:28438] |
| ENSG00000185482 | HGLibA_47319 | 80 | -0.352518965 | STAC3 | SH3 and cysteine rich domain 3 [Source:HGNC Symbol;Acc:HGNC:28423] |
| ENSG00000185697 | HGLibA_30644 | 80 | -0.352518965 | MYBL1 | MYB proto-oncogene like 1 [Source:HGNC Symbol;Acc:HGNC:7547] |
| ENSG00000186088 | HGLibA_20396 | 80 | -0.352518965 | GSAP | gamma-secretase activating protein [Source:HGNC Symbol;Acc:HGNC:28042] |
| ENSG00000187513 | HGLibA_19146 | 80 | -0.352518965 | GJA4 | gap junction protein alpha 4 [Source:HGNC Symbol;Acc:HGNC:4278] |
| ENSG00000187601 | HGLibA_27988 | 80 | -0.352518965 | MAGEH1 | MAGE family member H1 [Source:HGNC Symbol;Acc:HGNC:24092] |
| ENSG00000187833 | HGLibA_06185 | 80 | -0.352518965 | C2orf78 | chromosome 2 open reading frame 78 [Source:HGNC Symbol;Acc:HGNC:34349] |
| ENSG00000187997 | HGLibA_05602 | 80 | -0.352518965 | C17orf99 | chromosome 17 open reading frame 99 [Source:HGNC Symbol;Acc:HGNC:34490] |
| ENSG00000188811 | HGLibA_31903 | 80 | -0.352518965 | NHLRC3 | NHL repeat containing 3 [Source:HGNC Symbol;Acc:HGNC:33751] |
| ENSG00000188827 | HGLibA_45562 | 80 | -0.352518965 | SLX4 | SLX4 structure-specific endonuclease subunit [Source:HGNC Symbol;Acc:HGNC:23845] |
| ENSG00000188917 | HGLibA_51685 | 80 | -0.352518965 | TRMT2B | tRNA methyltransferase 2 homolog B [Source:HGNC Symbol;Acc:HGNC:25748] |
| ENSG00000196132 | HGLibA_30924 | 80 | -0.352518965 | MYT1 | myelin transcription factor 1 [Source:HGNC Symbol;Acc:HGNC:7622] |
| ENSG00000196876 | HGLibA_43005 | 80 | -0.352518965 | SCN8A | sodium voltage-gated channel alpha subunit 8 [Source:HGNC Symbol;Acc:HGNC:10596] |
| ENSG00000198492 | HGLibA_54734 | 80 | -0.352518965 | YTHDF2 | YTH N6-methyladenosine RNA binding protein 2 [Source:HGNC Symbol;Acc:HGNC:31675] |
| ENSG00000198746 | HGLibA_19745 | 80 | -0.352518965 | GPATCH3 | G-patch domain containing 3 [Source:HGNC Symbol;Acc:HGNC:25720] |
| ENSG00000198892 | HGLibA_44039 | 80 | -0.352518965 | SHISA4 | shisa family member 4 [Source:HGNC Symbol;Acc:HGNC:27139] |
| ENSG00000198919 | HGLibA_14303 | 80 | -0.352518965 | DZIP3 | DAZ interacting zinc finger protein 3 [Source:HGNC Symbol;Acc:HGNC:30938] |
| ENSG00000198932 | HGLibA_20119 | 80 | -0.352518965 | GPRASP1 | G protein-coupled receptor associated sorting protein 1 [Source:HGNC Symbol;Acc:HGNC:24834] |
| ENSG00000204138 | HGLibA_36305 | 80 | -0.352518965 | PHACTR4 | phosphatase and actin regulator 4 [Source:HGNC Symbol;Acc:HGNC:25793] |

| | | | | | |
|---|---|---|---|---|---|
| ENSG00000204165 | HGLibA_11960 | 80 | -0.352518965 | CXorf65 | chromosome X open reading frame 65 [Source:HGNC Symbol;Acc:HGNC:33713] |
| ENSG00000206102 | HGLibA_25685 | 80 | -0.352518965 | KRTAP19-8 | keratin associated protein 19-8 [Source:HGNC Symbol;Acc:HGNC:33898] |
| ENSG00000213988 | HGLibA_56901 | 80 | -0.352518965 | ZNF90 | zinc finger protein 90 [Source:HGNC Symbol;Acc:HGNC:13165] |
| ENSG00000215475 | HGLibA_44110 | 80 | -0.352518965 | SIAH3 | siah E3 ubiquitin protein ligase family member 3 [Source:HGNC Symbol;Acc:HGNC:30553] |
| ENSG00000215545 | HGLibA_12860 | 80 | -0.352518965 | DEFB116 | defensin beta 116 [Source:HGNC Symbol;Acc:HGNC:18097] |
| ENSG00000219481 | HGLibA_31220 | 80 | -0.352518965 | NBPF1 | NBPF member 1 [Source:HGNC Symbol;Acc:HGNC:26088] |
| ENSG00000220201 | HGLibA_55303 | 80 | -0.352518965 | ZGLP1 | GATA-like protein 1 [Source:HGNC Symbol;Acc:HGNC:37245]" |
| ENSG00000221994 | HGLibA_56435 | 80 | -0.352518965 | ZNF630 | zinc finger protein 630 [Source:HGNC Symbol;Acc:HGNC:28855] |
| ENSG00000227345 | HGLibA_35192 | 80 | -0.352518965 | PARG | poly(ADP-ribose) glycohydrolase [Source:HGNC Symbol;Acc:HGNC:8605] |
| ENSG00000228517 | HGLibA_11542 | 80 | -0.352518965 | CT47A7 | member A7 [Source:HGNC Symbol;Acc:HGNC:33288]" |
| ENSG00000230873 | HGLibA_47527 | 80 | -0.352518965 | STMND1 | stathmin domain containing 1 [Source:HGNC Symbol;Acc:HGNC:44668] |
| ENSG00000234965 | HGLibA_44051 | 80 | -0.352518965 | SHISA8 | shisa family member 8 [Source:HGNC Symbol;Acc:HGNC:18351] |
| ENSG00000236279 | HGLibA_09969 | 80 | -0.352518965 | CLEC2L | C-type lectin domain family 2 member L [Source:HGNC Symbol;Acc:HGNC:21969] |
| ENSG00000236761 | HGLibA_11582 | 80 | -0.352518965 | CTAGE9 | CTAGE family member 9 [Source:HGNC Symbol;Acc:HGNC:37275] |
| ENSG00000237441 | HGLibA_41004 | 80 | -0.352518965 | RGL2 | ral guanine nucleotide dissociation stimulator like 2 [Source:HGNC Symbol;Acc:HGNC:9769] |
| ENSG00000239389 | HGLibA_35436 | 80 | -0.352518965 | PCDHA13 | protocadherin alpha 13 [Source:HGNC Symbol;Acc:HGNC:8667] |
| ENSG00000241119 | HGLibA_52900 | 80 | -0.352518965 | UGT1A9 | UDP glucuronosyltransferase family 1 member A9 [Source:HGNC Symbol;Acc:HGNC:12541] |
| ENSG00000241635 | HGLibA_52876 | 80 | -0.352518965 | UGT1A1 | UDP glucuronosyltransferase family 1 member A1 [Source:HGNC Symbol;Acc:HGNC:12530] |
| ENSG00000243232 | HGLibA_35467 | 80 | -0.352518965 | PCDHAC2 | 2 [Source:HGNC Symbol;Acc:HGNC:8677]" |
| ENSG00000250361 | HGLibA_20701 | 80 | -0.352518965 | GYPB | glycophorin B (MNS blood group) [Source:HGNC Symbol;Acc:HGNC:4703] |
| ENSG00000253846 | HGLibA_35519 | 80 | -0.352518965 | PCDHGA10 | 10 [Source:HGNC Symbol;Acc:HGNC:8697]" |
| ENSG00000253910 | HGLibA_35553 | 80 | -0.352518965 | PCDHGB2 | 2 [Source:HGNC Symbol;Acc:HGNC:8709]" |
| ENSG00000253953 | HGLibA_35558 | 80 | -0.352518965 | PCDHGB4 | 4 [Source:HGNC Symbol;Acc:HGNC:8711]" |
| ENSG00000254087 | HGLibA_27708 | 80 | -0.352518965 | LYN | Src family tyrosine kinase [Source:HGNC Symbol;Acc:HGNC:6735]" |
| ENSG00000256045 | HGLibA_30474 | 80 | -0.352518965 | MTRNR2L10 | MT-RNR2 like 10 [Source:HGNC Symbol;Acc:HGNC:37167] |
| ENSG00000257008 | HGLibA_19912 | 80 | -0.352518965 | GPR142 | G protein-coupled receptor 142 [Source:HGNC Symbol;Acc:HGNC:20088] |
| ENSG00000258890 | HGLibA_09178 | 80 | -0.352518965 | CEP95 | centrosomal protein 95 [Source:HGNC Symbol;Acc:HGNC:25141] |
| ENSG00000261652 | HGLibA_05434 | 80 | -0.352518965 | C15orf65 | chromosome 15 open reading frame 65 [Source:HGNC Symbol;Acc:HGNC:44654] |
| ENSG00000267795 | HGLibA_45753 | 80 | -0.352518965 | SMIM22 | small integral membrane protein 22 [Source:HGNC Symbol;Acc:HGNC:48329] |
| ENSG00000272398 | HGLibA_08304 | 80 | -0.352518965 | CD24 | CD24 molecule [Source:HGNC Symbol;Acc:HGNC:1645] |
| ENSG00000276023 | HGLibA_14141 | 80 | -0.352518965 | DUSP14 | dual specificity phosphatase 14 [Source:HGNC Symbol;Acc:HGNC:17007] |
| ENSG00000279051 | HGLibA_34439 | 80 | -0.352518965 | OR6Q1 | olfactory receptor family 6 subfamily Q member 1 (gene/pseudogene) [Source:HGNC Symbol;Acc:HGNC:15302] |
| ENSG00000279270 | HGLibA_34205 | 80 | -0.352518965 | OR52R1 | olfactory receptor family 52 subfamily R member 1 (gene/pseudogene) [Source:HGNC Symbol;Acc:HGNC:15235] |
| FAM19A5 | HGLibA_16407 | 80 | -0.352518965 | FAM19A5 | - |
| FAM213B | HGLibA_16474 | 80 | -0.352518965 | FAM213B | - |
| GLTSCR1L | HGLibA_19356 | 80 | -0.352518965 | GLTSCR1L | - |
| GPR1 | HGLibA_19836 | 80 | -0.352518965 | GPR1 | - |
| H1FOO | HGLibA_20739 | 80 | -0.352518965 | H1FOO | - |
| H2AFX | HGLibA_20760 | 80 | -0.352518965 | H2AFX | - |
| hsa-let-7c | HGLibA_57113 | 80 | -0.352518965 | hsa-let-7c | - |
| hsa-mir-25 | HGLibA_58353 | 80 | -0.352518965 | hsa-mir-25 | - |
| hsa-mir-3118-4 | HGLibA_58517 | 80 | -0.352518965 | hsa-mir-3118-4 | - |
| hsa-mir-3160-2 | HGLibA_58720 | 80 | -0.352518965 | hsa-mir-3160-2 | - |
| hsa-mir-3186 | HGLibA_58841 | 80 | -0.352518965 | hsa-mir-3186 | - |
| hsa-mir-3529 | HGLibA_59048 | 80 | -0.352518965 | hsa-mir-3529 | - |
| hsa-mir-363 | HGLibA_59130 | 80 | -0.352518965 | hsa-mir-363 | - |
| hsa-mir-3679 | HGLibA_59267 | 80 | -0.352518965 | hsa-mir-3679 | - |
| hsa-mir-4309 | HGLibA_59946 | 80 | -0.352518965 | hsa-mir-4309 | - |
| hsa-mir-4427 | HGLibA_60093 | 80 | -0.352518965 | hsa-mir-4427 | - |
| hsa-mir-4442 | HGLibA_60171 | 80 | -0.352518965 | hsa-mir-4442 | - |
| hsa-mir-4463 | HGLibA_60259 | 80 | -0.352518965 | hsa-mir-4463 | - |
| hsa-mir-4481 | HGLibA_60344 | 80 | -0.352518965 | hsa-mir-4481 | - |
| hsa-mir-4535 | HGLibA_60601 | 80 | -0.352518965 | hsa-mir-4535 | - |
| hsa-mir-455 | HGLibA_60630 | 80 | -0.352518965 | hsa-mir-455 | - |
| hsa-mir-4654 | HGLibA_60722 | 80 | -0.352518965 | hsa-mir-4654 | - |
| hsa-mir-4696 | HGLibA_60891 | 80 | -0.352518965 | hsa-mir-4696 | - |
| hsa-mir-4796 | HGLibA_61291 | 80 | -0.352518965 | hsa-mir-4796 | - |
| hsa-mir-4803 | HGLibA_61318 | 80 | -0.352518965 | hsa-mir-4803 | - |
| hsa-mir-5006 | HGLibA_61426 | 80 | -0.352518965 | hsa-mir-5006 | - |
| hsa-mir-520h | HGLibA_61762 | 80 | -0.352518965 | hsa-mir-520h | - |
| hsa-mir-600 | HGLibA_62491 | 80 | -0.352518965 | hsa-mir-600 | - |
| hsa-mir-6503 | HGLibA_62864 | 80 | -0.352518965 | hsa-mir-6503 | - |
| hsa-mir-6511a-1 | HGLibA_62896 | 80 | -0.352518965 | hsa-mir-6511a-1 | - |
| hsa-mir-660 | HGLibA_62972 | 80 | -0.352518965 | hsa-mir-660 | - |
| hsa-mir-7153 | HGLibA_63841 | 80 | -0.352518965 | hsa-mir-7153 | - |
| hsa-mir-873 | HGLibA_64203 | 80 | -0.352518965 | hsa-mir-873 | - |
| IL3RA_X | HGLibA_23160 | 80 | -0.352518965 | IL3RA_X | - |
| NUTMG | HGLibA_33118 | 80 | -0.352518965 | NUTMG | - |
| SCXB | HGLibA_43083 | 80 | -0.352518965 | SCXB | - |
| AQPEP | HGLibA_02614 | 79 | -0.362470002 | AQPEP | - |
| ASMTL_Y | HGLibA_03330 | 79 | -0.362470002 | ASMTL_Y | - |
| C14orf169 | HGLibA_05340 | 79 | -0.362470002 | C14orf169 | - |
| C1orf110 | HGLibA_05745 | 79 | -0.362470002 | C1orf110 | - |
| CXorf21 | HGLibA_11901 | 79 | -0.362470002 | CXorf21 | - |
| ENSG00000013563 | HGLibA_13657 | 79 | -0.362470002 | DNASE1L1 | deoxyribonuclease 1 like 1 [Source:HGNC Symbol;Acc:HGNC:2957] |
| ENSG00000037241 | HGLibA_41917 | 79 | -0.362470002 | RPL26L1 | ribosomal protein L26 like 1 [Source:HGNC Symbol;Acc:HGNC:17050] |
| ENSG00000046889 | HGLibA_38446 | 79 | -0.362470002 | PREX2 | 5-trisphosphate dependent Rac exchange factor 2 [Source:HGNC Symbol;Acc:HGNC:22950]" |
| ENSG00000047644 | HGLibA_54445 | 79 | -0.362470002 | WWC3 | WWC family member 3 [Source:HGNC Symbol;Acc:HGNC:29237] |
| ENSG00000053108 | HGLibA_18108 | 79 | -0.362470002 | FSTL4 | follistatin like 4 [Source:HGNC Symbol;Acc:HGNC:21389] |
| ENSG00000054356 | HGLibA_39520 | 79 | -0.362470002 | PTPRN | receptor type N [Source:HGNC Symbol;Acc:HGNC:9676]" |
| ENSG00000063438 | HGLibA_01309 | 79 | -0.362470002 | AHRR | aryl-hydrocarbon receptor repressor [Source:HGNC Symbol;Acc:HGNC:346] |
| ENSG00000064999 | HGLibA_02150 | 79 | -0.362470002 | ANKS1A | ankyrin repeat and sterile alpha motif domain containing 1A [Source:HGNC Symbol;Acc:HGNC:20961] |
| ENSG00000065970 | HGLibA_17889 | 79 | -0.362470002 | FOXJ2 | forkhead box J2 [Source:HGNC Symbol;Acc:HGNC:24818] |
| ENSG00000070882 | HGLibA_34655 | 79 | -0.362470002 | OSBPL3 | oxysterol binding protein like 3 [Source:HGNC Symbol;Acc:HGNC:16370] |
| ENSG00000076706 | HGLibA_28557 | 79 | -0.362470002 | MCAM | melanoma cell adhesion molecule [Source:HGNC Symbol;Acc:HGNC:6934] |
| ENSG00000080819 | HGLibA_10981 | 79 | -0.362470002 | CPOX | coproporphyrinogen oxidase [Source:HGNC Symbol;Acc:HGNC:2321] |
| ENSG00000087088 | HGLibA_04181 | 79 | -0.362470002 | BAX | apoptosis regulator [Source:HGNC Symbol;Acc:HGNC:959]" |
| ENSG00000089472 | HGLibA_21144 | 79 | -0.362470002 | HEPH | hephaestin [Source:HGNC Symbol;Acc:HGNC:4866] |
| ENSG00000089820 | HGLibA_02774 | 79 | -0.362470002 | ARHGAP4 | Rho GTPase activating protein 4 [Source:HGNC Symbol;Acc:HGNC:674] |
| ENSG00000095066 | HGLibA_21879 | 79 | -0.362470002 | HOOK2 | hook microtubule tethering protein 2 [Source:HGNC Symbol;Acc:HGNC:19885] |
| ENSG00000095637 | HGLibA_46175 | 79 | -0.362470002 | SORBS1 | sorbin and SH3 domain containing 1 [Source:HGNC Symbol;Acc:HGNC:14565] |
| ENSG00000100362 | HGLibA_39613 | 79 | -0.362470002 | PVALB | parvalbumin [Source:HGNC Symbol;Acc:HGNC:9704] |
| ENSG00000100368 | HGLibA_11335 | 79 | -0.362470002 | CSF2RB | colony stimulating factor 2 receptor beta common subunit [Source:HGNC Symbol;Acc:HGNC:2436] |
| ENSG00000100523 | HGLibA_12604 | 79 | -0.362470002 | DDHD1 | DDHD domain containing 1 [Source:HGNC Symbol;Acc:HGNC:19714] |
| ENSG00000101187 | HGLibA_45469 | 79 | -0.362470002 | SLCO4A1 | solute carrier organic anion transporter family member 4A1 [Source:HGNC Symbol;Acc:HGNC:10953] |
| ENSG00000101557 | HGLibA_53207 | 79 | -0.362470002 | USP14 | ubiquitin specific peptidase 14 [Source:HGNC Symbol;Acc:HGNC:12612] |
| ENSG00000103326 | HGLibA_07160 | 79 | -0.362470002 | CAPN15 | calpain 15 [Source:HGNC Symbol;Acc:HGNC:11182] |
| ENSG00000105258 | HGLibA_37587 | 79 | -0.362470002 | POLR2I | RNA polymerase II subunit I [Source:HGNC Symbol;Acc:HGNC:9196] |
| ENSG00000105376 | HGLibA_22506 | 79 | -0.362470002 | ICAM5 | intercellular adhesion molecule 5 [Source:HGNC Symbol;Acc:HGNC:5348] |
| ENSG00000105655 | HGLibA_23701 | 79 | -0.362470002 | ISYNA1 | inositol-3-phosphate synthase 1 [Source:HGNC Symbol;Acc:HGNC:29821] |
| ENSG00000106366 | HGLibA_43578 | 79 | -0.362470002 | SERPINE1 | serpin family E member 1 [Source:HGNC Symbol;Acc:HGNC:8583] |
| ENSG00000107020 | HGLibA_37168 | 79 | -0.362470002 | PLGRKT | plasminogen receptor with a C-terminal lysine [Source:HGNC Symbol;Acc:HGNC:23633] |
| ENSG00000107186 | HGLibA_29714 | 79 | -0.362470002 | MPDZ | multiple PDZ domain crumbs cell polarity complex component [Source:HGNC Symbol;Acc:HGNC:7208] |
| ENSG00000108702 | HGLibA_07960 | 79 | -0.362470002 | CCL1 | C-C motif chemokine ligand 1 [Source:HGNC Symbol;Acc:HGNC:10609] |
| ENSG00000108799 | HGLibA_15831 | 79 | -0.362470002 | EZH1 | enhancer of zeste 1 polycomb repressive complex 2 subunit [Source:HGNC Symbol;Acc:HGNC:3526] |
| ENSG00000109943 | HGLibA_11222 | 79 | -0.362470002 | CRTAM | cytotoxic and regulatory T cell molecule [Source:HGNC Symbol;Acc:HGNC:24313] |
| ENSG00000111716 | HGLibA_26229 | 79 | -0.362470002 | LDHB | lactate dehydrogenase B [Source:HGNC Symbol;Acc:HGNC:6541] |
| ENSG00000112186 | HGLibA_07136 | 79 | -0.362470002 | CAP2 | cyclase associated actin cytoskeleton regulatory protein 2 [Source:HGNC Symbol;Acc:HGNC:20039] |
| ENSG00000113302 | HGLibA_22963 | 79 | -0.362470002 | IL12B | interleukin 12B [Source:HGNC Symbol;Acc:HGNC:5970] |
| ENSG00000114854 | HGLibA_50865 | 79 | -0.362470002 | TNNC1 | slow skeletal and cardiac type [Source:HGNC Symbol;Acc:HGNC:11943]" |
| ENSG00000115758 | HGLibA_33271 | 79 | -0.362470002 | ODC1 | ornithine decarboxylase 1 [Source:HGNC Symbol;Acc:HGNC:8109] |
| ENSG00000116521 | HGLibA_42842 | 79 | -0.362470002 | SCAMP3 | secretory carrier membrane protein 3 [Source:HGNC Symbol;Acc:HGNC:10565] |
| ENSG00000116580 | HGLibA_19668 | 79 | -0.362470002 | GON4L | gon-4 like [Source:HGNC Symbol;Acc:HGNC:25973] |
| ENSG00000117595 | HGLibA_23595 | 79 | -0.362470002 | IRF6 | interferon regulatory factor 6 [Source:HGNC Symbol;Acc:HGNC:6121] |
| ENSG00000118276 | HGLibA_04037 | 79 | -0.362470002 | B4GALT6 | 4-galactosyltransferase 6 [Source:HGNC Symbol;Acc:HGNC:929]" |
| ENSG00000118369 | HGLibA_53345 | 79 | -0.362470002 | USP35 | ubiquitin specific peptidase 35 [Source:HGNC Symbol;Acc:HGNC:20061] |
| ENSG00000118520 | HGLibA_02686 | 79 | -0.362470002 | ARG1 | arginase 1 [Source:HGNC Symbol;Acc:HGNC:663] |
| ENSG00000119725 | HGLibA_55937 | 79 | -0.362470002 | ZNF410 | zinc finger protein 410 [Source:HGNC Symbol;Acc:HGNC:20144] |
| ENSG00000121377 | HGLibA_48425 | 79 | -0.362470002 | TAS2R7 | taste 2 receptor member 7 [Source:HGNC Symbol;Acc:HGNC:14913] |
| ENSG00000121417 | HGLibA_55586 | 79 | -0.362470002 | ZNF211 | zinc finger protein 211 [Source:HGNC Symbol;Acc:HGNC:13003] |
| ENSG00000123843 | HGLibA_06292 | 79 | -0.362470002 | C4BPB | complement component 4 binding protein beta [Source:HGNC Symbol;Acc:HGNC:1328] |
| ENSG00000124827 | HGLibA_18802 | 79 | -0.362470002 | GCM2 | glial cells missing homolog 2 [Source:HGNC Symbol;Acc:HGNC:4198] |
| ENSG00000125337 | HGLibA_24912 | 79 | -0.362470002 | KIF25 | kinesin family member 25 [Source:HGNC Symbol;Acc:HGNC:6390] |
| ENSG00000125885 | HGLibA_28624 | 79 | -0.362470002 | MCM8 | minichromosome maintenance 8 homologous recombination repair factor [Source:HGNC Symbol;Acc:HGNC:16147] |
| ENSG00000125945 | HGLibA_55991 | 79 | -0.362470002 | ZNF436 | zinc finger protein 436 [Source:HGNC Symbol;Acc:HGNC:20814] |
| ENSG00000126107 | HGLibA_21100 | 79 | -0.362470002 | HECTD3 | HECT domain E3 ubiquitin protein ligase 3 [Source:HGNC Symbol;Acc:HGNC:26117] |
| ENSG00000126368 | HGLibA_32573 | 79 | -0.362470002 | NR1D1 | nuclear receptor subfamily 1 group D member 1 [Source:HGNC Symbol;Acc:HGNC:7962] |
| ENSG00000126890 | HGLibA_11564 | 79 | -0.362470002 | CTAG2 | cancer/testis antigen 2 [Source:HGNC Symbol;Acc:HGNC:2492] |
| ENSG00000127311 | HGLibA_21111 | 79 | -0.362470002 | HELB | DNA helicase B [Source:HGNC Symbol;Acc:HGNC:17196] |
| ENSG00000127947 | HGLibA_39436 | 79 | -0.362470002 | PTPN12 | non-receptor type 12 [Source:HGNC Symbol;Acc:HGNC:9645]" |
| ENSG00000128040 | HGLibA_46691 | 79 | -0.362470002 | SPINK2 | Kazal type 2 [Source:HGNC Symbol;Acc:HGNC:11245]" |
| ENSG00000129292 | HGLibA_36366 | 79 | -0.362470002 | PHF20L1 | PHD finger protein 20 like 1 [Source:HGNC Symbol;Acc:HGNC:24280] |
| ENSG00000130024 | HGLibA_36333 | 79 | -0.362470002 | PHF10 | PHD finger protein 10 [Source:HGNC Symbol;Acc:HGNC:18250] |
| ENSG00000130158 | HGLibA_13751 | 79 | -0.362470002 | DOCK6 | dedicator of cytokinesis 6 [Source:HGNC Symbol;Acc:HGNC:19189] |
| ENSG00000130176 | HGLibA_10306 | 79 | -0.362470002 | CNN1 | calponin 1 [Source:HGNC Symbol;Acc:HGNC:2155] |

| | | | | | |
|---|---|---|---|---|---|
| ENSG00000130584 | HGLibA_54899 | 79 | -0.362470002 | ZBTB46 | zinc finger and BTB domain containing 46 [Source:HGNC Symbol;Acc:HGNC:16094] |
| ENSG00000131791 | HGLibA_38500 | 79 | -0.362470002 | PRKAB2 | protein kinase AMP-activated non-catalytic subunit beta 2 [Source:HGNC Symbol;Acc:HGNC:9379] |
| ENSG00000132530 | HGLibA_54463 | 79 | -0.362470002 | XAF1 | XIAP associated factor 1 [Source:HGNC Symbol;Acc:HGNC:30932] |
| ENSG00000134686 | HGLibA_36320 | 79 | -0.362470002 | PHC2 | polyhomeotic homolog 2 [Source:HGNC Symbol;Acc:HGNC:3183] |
| ENSG00000135338 | HGLibA_26091 | 79 | -0.362470002 | LCA5 | lebercilin [Source:HGNC Symbol;Acc:HGNC:31923]" |
| ENSG00000135346 | HGLibA_09296 | 79 | -0.362470002 | CGA | alpha polypeptide [Source:HGNC Symbol;Acc:HGNC:1885]" |
| ENSG00000135365 | HGLibA_36368 | 79 | -0.362470002 | PHF21A | PHD finger protein 21A [Source:HGNC Symbol;Acc:HGNC:24156] |
| ENSG00000135424 | HGLibA_23743 | 79 | -0.362470002 | ITGA7 | integrin subunit alpha 7 [Source:HGNC Symbol;Acc:HGNC:6143] |
| ENSG00000135698 | HGLibA_29726 | 79 | -0.362470002 | MPHOSPH6 | M-phase phosphoprotein 6 [Source:HGNC Symbol;Acc:HGNC:7214] |
| ENSG00000137338 | HGLibA_36215 | 79 | -0.362470002 | PGBD1 | piggyBac transposable element derived 1 [Source:HGNC Symbol;Acc:HGNC:19398] |
| ENSG00000137474 | HGLibA_30863 | 79 | -0.362470002 | MYO7A | myosin VIIA [Source:HGNC Symbol;Acc:HGNC:7606] |
| ENSG00000137727 | HGLibA_02722 | 79 | -0.362470002 | ARHGAP20 | Rho GTPase activating protein 20 [Source:HGNC Symbol;Acc:HGNC:18357] |
| ENSG00000137880 | HGLibA_18786 | 79 | -0.362470002 | GCHFR | GTP cyclohydrolase I feedback regulator [Source:HGNC Symbol;Acc:HGNC:4194] |
| ENSG00000138101 | HGLibA_14067 | 79 | -0.362470002 | DTNB | dystrobrevin beta [Source:HGNC Symbol;Acc:HGNC:3058] |
| ENSG00000138685 | HGLibA_17390 | 79 | -0.362470002 | FGF2 | fibroblast growth factor 2 [Source:HGNC Symbol;Acc:HGNC:3676] |
| ENSG00000140368 | HGLibA_39237 | 79 | -0.362470002 | PSTPIP1 | proline-serine-threonine phosphatase interacting protein 1 [Source:HGNC Symbol;Acc:HGNC:9580] |
| ENSG00000140749 | HGLibA_22900 | 79 | -0.362470002 | IGSF6 | immunoglobulin superfamily member 6 [Source:HGNC Symbol;Acc:HGNC:5953] |
| ENSG00000140839 | HGLibA_09947 | 79 | -0.362470002 | CLEC18B | C-type lectin domain family 18 member B [Source:HGNC Symbol;Acc:HGNC:33849] |
| ENSG00000141026 | HGLibA_28819 | 79 | -0.362470002 | MED9 | mediator complex subunit 9 [Source:HGNC Symbol;Acc:HGNC:25487] |
| ENSG00000141084 | HGLibA_40194 | 79 | -0.362470002 | RANBP10 | RAN binding protein 10 [Source:HGNC Symbol;Acc:HGNC:29285] |
| ENSG00000141429 | HGLibA_18527 | 79 | -0.362470002 | GALNT1 | polypeptide N-acetylgalactosaminyltransferase 1 [Source:HGNC Symbol;Acc:HGNC:4123] |
| ENSG00000142484 | HGLibA_49707 | 79 | -0.362470002 | TM4SF5 | transmembrane 4 L six family member 5 [Source:HGNC Symbol;Acc:HGNC:11857] |
| ENSG00000142856 | HGLibA_23784 | 79 | -0.362470002 | ITGB3BP | integrin subunit beta 3 binding protein [Source:HGNC Symbol;Acc:HGNC:6157] |
| ENSG00000144306 | HGLibA_43055 | 79 | -0.362470002 | SCRN3 | secernin 3 [Source:HGNC Symbol;Acc:HGNC:30382] |
| ENSG00000144559 | HGLibA_48288 | 79 | -0.362470002 | TAMM41 | TAM41 mitochondrial translocator assembly and maintenance homolog [Source:HGNC Symbol;Acc:HGNC:25187] |
| ENSG00000144867 | HGLibA_47031 | 79 | -0.362470002 | SRPRB | SRP receptor subunit beta [Source:HGNC Symbol;Acc:HGNC:24085] |
| ENSG00000146386 | HGLibA_00328 | 79 | -0.362470002 | ABRACL | ABRA C-terminal like [Source:HGNC Symbol;Acc:HGNC:21230] |
| ENSG00000146477 | HGLibA_44581 | 79 | -0.362470002 | SLC22A3 | solute carrier family 22 member 3 [Source:HGNC Symbol;Acc:HGNC:10967] |
| ENSG00000148303 | HGLibA_41996 | 79 | -0.362470002 | RPL7A | ribosomal protein L7a [Source:HGNC Symbol;Acc:HGNC:10364] |
| ENSG00000149548 | HGLibA_07640 | 79 | -0.362470002 | CCDC15 | coiled-coil domain containing 15 [Source:HGNC Symbol;Acc:HGNC:25798] |
| ENSG00000151640 | HGLibA_13931 | 79 | -0.362470002 | DPYSL4 | dihydropyrimidinase like 4 [Source:HGNC Symbol;Acc:HGNC:3016] |
| ENSG00000151929 | HGLibA_04080 | 79 | -0.362470002 | BAG3 | BCL2 associated athanogene 3 [Source:HGNC Symbol;Acc:HGNC:939] |
| ENSG00000152822 | HGLibA_20341 | 79 | -0.362470002 | GRM1 | glutamate metabotropic receptor 1 [Source:HGNC Symbol;Acc:HGNC:4593] |
| ENSG00000153201 | HGLibA_40202 | 79 | -0.362470002 | RANBP2 | RAN binding protein 2 [Source:HGNC Symbol;Acc:HGNC:9848] |
| ENSG00000153814 | HGLibA_23939 | 79 | -0.362470002 | JAZF1 | JAZF zinc finger 1 [Source:HGNC Symbol;Acc:HGNC:28917] |
| ENSG00000154240 | HGLibA_09098 | 79 | -0.362470002 | CEP112 | centrosomal protein 112 [Source:HGNC Symbol;Acc:HGNC:28514] |
| ENSG00000154814 | HGLibA_34827 | 79 | -0.362470002 | OXNAD1 | oxidoreductase NAD binding domain containing 1 [Source:HGNC Symbol;Acc:HGNC:25128] |
| ENSG00000155744 | HGLibA_16084 | 79 | -0.362470002 | FAM126B | family with sequence similarity 126 member B [Source:HGNC Symbol;Acc:HGNC:28593] |
| ENSG00000155886 | HGLibA_44619 | 79 | -0.362470002 | SLC24A2 | solute carrier family 24 member 2 [Source:HGNC Symbol;Acc:HGNC:10976] |
| ENSG00000157637 | HGLibA_45059 | 79 | -0.362470002 | SLC38A10 | solute carrier family 38 member 10 [Source:HGNC Symbol;Acc:HGNC:28237] |
| ENSG00000159363 | HGLibA_03568 | 79 | -0.362470002 | ATP13A2 | ATPase cation transporting 13A2 [Source:HGNC Symbol;Acc:HGNC:30213] |
| ENSG00000159388 | HGLibA_04943 | 79 | -0.362470002 | BTG2 | BTG anti-proliferation factor 2 [Source:HGNC Symbol;Acc:HGNC:1131] |
| ENSG00000159714 | HGLibA_55067 | 79 | -0.362470002 | ZDHHC1 | zinc finger DHHC-type containing 1 [Source:HGNC Symbol;Acc:HGNC:17916] |
| ENSG00000160305 | HGLibA_13248 | 79 | -0.362470002 | DIP2A | disco interacting protein 2 homolog A [Source:HGNC Symbol;Acc:HGNC:17217] |
| ENSG00000162490 | HGLibA_13954 | 79 | -0.362470002 | DRAXIN | dorsal inhibitory axon guidance protein [Source:HGNC Symbol;Acc:HGNC:25054] |
| ENSG00000162654 | HGLibA_18736 | 79 | -0.362470002 | GBP4 | guanylate binding protein 4 [Source:HGNC Symbol;Acc:HGNC:20480] |
| ENSG00000163254 | HGLibA_11277 | 79 | -0.362470002 | CRYGC | crystallin gamma C [Source:HGNC Symbol;Acc:HGNC:2410] |
| ENSG00000163739 | HGLibA_11841 | 79 | -0.362470002 | CXCL1 | C-X-C motif chemokine ligand 1 [Source:HGNC Symbol;Acc:HGNC:4602] |
| ENSG00000164627 | HGLibA_24957 | 79 | -0.362470002 | KIF6 | kinesin family member 6 [Source:HGNC Symbol;Acc:HGNC:21202] |
| ENSG00000165060 | HGLibA_18216 | 79 | -0.362470002 | FXN | frataxin [Source:HGNC Symbol;Acc:HGNC:3951] |
| ENSG00000165092 | HGLibA_01542 | 79 | -0.362470002 | ALDH1A1 | aldehyde dehydrogenase 1 family member A1 [Source:HGNC Symbol;Acc:HGNC:402] |
| ENSG00000166579 | HGLibA_31388 | 79 | -0.362470002 | NDEL1 | nudE neurodevelopment protein 1 like 1 [Source:HGNC Symbol;Acc:HGNC:17620] |
| ENSG00000166908 | HGLibA_36710 | 79 | -0.362470002 | PIP4K2C | phosphatidylinositol-5-phosphate 4-kinase type 2 gamma [Source:HGNC Symbol;Acc:HGNC:23786] |
| ENSG00000167077 | HGLibA_28863 | 79 | -0.362470002 | MEI1 | meiotic double-stranded break formation protein 1 [Source:HGNC Symbol;Acc:HGNC:28613] |
| ENSG00000167523 | HGLibA_46511 | 79 | -0.362470002 | SPATA33 | spermatogenesis associated 33 [Source:HGNC Symbol;Acc:HGNC:26463] |
| ENSG00000169245 | HGLibA_11846 | 79 | -0.362470002 | CXCL10 | C-X-C motif chemokine ligand 10 [Source:HGNC Symbol;Acc:HGNC:10637] |
| ENSG00000169733 | HGLibA_40927 | 79 | -0.362470002 | RFNG | RFNG O-fucosylpeptide 3-beta-N-acetylglucosaminyltransferase [Source:HGNC Symbol;Acc:HGNC:9974] |
| ENSG00000169862 | HGLibA_11663 | 79 | -0.362470002 | CTNND2 | catenin delta 2 [Source:HGNC Symbol;Acc:HGNC:2516] |
| ENSG00000170777 | HGLibA_51120 | 79 | -0.362470002 | TPD52L3 | TPD52 like 3 [Source:HGNC Symbol;Acc:HGNC:23382] |
| ENSG00000170852 | HGLibA_24101 | 79 | -0.362470002 | KBTBD2 | kelch repeat and BTB domain containing 2 [Source:HGNC Symbol;Acc:HGNC:21751] |
| ENSG00000171053 | HGLibA_35271 | 79 | -0.362470002 | PATE1 | prostate and testis expressed 1 [Source:HGNC Symbol;Acc:HGNC:24664] |
| ENSG00000171054 | HGLibA_33622 | 79 | -0.362470002 | OR13H1 | olfactory receptor family 13 subfamily H member 1 [Source:HGNC Symbol;Acc:HGNC:14755] |
| ENSG00000171453 | HGLibA_37554 | 79 | -0.362470002 | POLR1C | RNA polymerase I and III subunit C [Source:HGNC Symbol;Acc:HGNC:20194] |
| ENSG00000171467 | HGLibA_55786 | 79 | -0.362470002 | ZNF318 | zinc finger protein 318 [Source:HGNC Symbol;Acc:HGNC:13578] |
| ENSG00000171566 | HGLibA_37221 | 79 | -0.362470002 | PLRG1 | pleiotropic regulator 1 [Source:HGNC Symbol;Acc:HGNC:9089] |
| ENSG00000173258 | HGLibA_56074 | 79 | -0.362470002 | ZNF483 | zinc finger protein 483 [Source:HGNC Symbol;Acc:HGNC:23384] |
| ENSG00000174876 | HGLibA_01843 | 79 | -0.362470002 | AMY1B | alpha 1B (salivary) [Source:HGNC Symbol;Acc:HGNC:475]" |
| ENSG00000175197 | HGLibA_12615 | 79 | -0.362470002 | DDIT3 | DNA damage inducible transcript 3 [Source:HGNC Symbol;Acc:HGNC:2726] |
| ENSG00000175514 | HGLibA_19936 | 79 | -0.362470002 | GPR152 | G protein-coupled receptor 152 [Source:HGNC Symbol;Acc:HGNC:23622] |
| ENSG00000175727 | HGLibA_29440 | 79 | -0.362470002 | MLXIP | MLX interacting protein [Source:HGNC Symbol;Acc:HGNC:17055] |
| ENSG00000176148 | HGLibA_48845 | 79 | -0.362470002 | TCP11L1 | t-complex 11 like 1 [Source:HGNC Symbol;Acc:HGNC:25655] |
| ENSG00000176463 | HGLibA_45467 | 79 | -0.362470002 | SLCO3A1 | solute carrier organic anion transporter family member 3A1 [Source:HGNC Symbol;Acc:HGNC:10952] |
| ENSG00000177511 | HGLibA_47294 | 79 | -0.362470002 | ST8SIA3 | 8-sialyltransferase 3 [Source:HGNC Symbol;Acc:HGNC:14269]" |
| ENSG00000178074 | HGLibA_06168 | 79 | -0.362470002 | C2orf69 | chromosome 2 open reading frame 69 [Source:HGNC Symbol;Acc:HGNC:26799] |
| ENSG00000179364 | HGLibA_34956 | 79 | -0.362470002 | PACS2 | phosphofurin acidic cluster sorting protein 2 [Source:HGNC Symbol;Acc:HGNC:23794] |
| ENSG00000179526 | HGLibA_43992 | 79 | -0.362470002 | SHARPIN | SHANK associated RH domain interactor [Source:HGNC Symbol;Acc:HGNC:25321] |
| ENSG00000179921 | HGLibA_19752 | 79 | -0.362470002 | GPBAR1 | G protein-coupled bile acid receptor 1 [Source:HGNC Symbol;Acc:HGNC:19680] |
| ENSG00000180083 | HGLibA_54254 | 79 | -0.362470002 | WFDC11 | WAP four-disulfide core domain 11 [Source:HGNC Symbol;Acc:HGNC:20478] |
| ENSG00000180818 | HGLibA_21956 | 79 | -0.362470002 | HOXC10 | homeobox C10 [Source:HGNC Symbol;Acc:HGNC:5122] |
| ENSG00000180828 | HGLibA_04477 | 79 | -0.362470002 | BHLHE22 | basic helix-loop-helix family member e22 [Source:HGNC Symbol;Acc:HGNC:11963] |
| ENSG00000181778 | HGLibA_50320 | 79 | -0.362470002 | TMEM252 | transmembrane protein 252 [Source:HGNC Symbol;Acc:HGNC:28537] |
| ENSG00000183762 | HGLibA_25403 | 79 | -0.362470002 | KREMEN1 | kringle containing transmembrane protein 1 [Source:HGNC Symbol;Acc:HGNC:17550] |
| ENSG00000183779 | HGLibA_56580 | 79 | -0.362470002 | ZNF703 | zinc finger protein 703 [Source:HGNC Symbol;Acc:HGNC:25883] |
| ENSG00000184305 | HGLibA_08189 | 79 | -0.362470002 | CCSER1 | coiled-coil serine rich protein 1 [Source:HGNC Symbol;Acc:HGNC:29349] |
| ENSG00000184371 | HGLibA_11320 | 79 | -0.362470002 | CSF1 | colony stimulating factor 1 [Source:HGNC Symbol;Acc:HGNC:2432] |
| ENSG00000184635 | HGLibA_56912 | 79 | -0.362470002 | ZNF93 | zinc finger protein 93 [Source:HGNC Symbol;Acc:HGNC:13169] |
| ENSG00000184949 | HGLibA_16528 | 79 | -0.362470002 | FAM227A | family with sequence similarity 227 member A [Source:HGNC Symbol;Acc:HGNC:44197] |
| ENSG00000185105 | HGLibA_30635 | 79 | -0.362470002 | MYADML2 | myeloid associated differentiation marker like 2 [Source:HGNC Symbol;Acc:HGNC:34548] |
| ENSG00000185640 | HGLibA_25550 | 79 | -0.362470002 | KRT79 | keratin 79 [Source:HGNC Symbol;Acc:HGNC:28930] |
| ENSG00000187144 | HGLibA_46451 | 79 | -0.362470002 | SPATA21 | spermatogenesis associated 21 [Source:HGNC Symbol;Acc:HGNC:28026] |
| ENSG00000187258 | HGLibA_32526 | 79 | -0.362470002 | NPSR1 | neuropeptide S receptor 1 [Source:HGNC Symbol;Acc:HGNC:23631] |
| ENSG00000187605 | HGLibA_49042 | 79 | -0.362470002 | TET3 | tet methylcytosine dioxygenase 3 [Source:HGNC Symbol;Acc:HGNC:28313] |
| ENSG00000187754 | HGLibA_47220 | 79 | -0.362470002 | SSX7 | SSX family member 7 [Source:HGNC Symbol;Acc:HGNC:19653] |
| ENSG00000188859 | HGLibA_16730 | 79 | -0.362470002 | FAM78B | family with sequence similarity 78 member B [Source:HGNC Symbol;Acc:HGNC:13495] |
| ENSG00000196371 | HGLibA_18194 | 79 | -0.362470002 | FUT4 | fucosyltransferase 4 [Source:HGNC Symbol;Acc:HGNC:4015] |
| ENSG00000197165 | HGLibA_47734 | 79 | -0.362470002 | SULT1A2 | sulfotransferase family 1A member 2 [Source:HGNC Symbol;Acc:HGNC:11454] |
| ENSG00000197702 | HGLibA_35256 | 79 | -0.362470002 | PARVA | parvin alpha [Source:HGNC Symbol;Acc:HGNC:14652] |
| ENSG00000197888 | HGLibA_52920 | 79 | -0.362470002 | UGT2B17 | UDP glucuronosyltransferase family 2 member B17 [Source:HGNC Symbol;Acc:HGNC:12547] |
| ENSG00000197888 | HGLibA_52922 | 79 | -0.362470002 | UGT2B17 | UDP glucuronosyltransferase family 2 member B17 [Source:HGNC Symbol;Acc:HGNC:12547] |
| ENSG00000198088 | HGLibA_33066 | 79 | -0.362470002 | NUP62CL | nucleoporin 62 C-terminal like [Source:HGNC Symbol;Acc:HGNC:25960] |
| ENSG00000198826 | HGLibA_02701 | 79 | -0.362470002 | ARHGAP11A | Rho GTPase activating protein 11A [Source:HGNC Symbol;Acc:HGNC:15783] |
| ENSG00000198951 | HGLibA_31064 | 79 | -0.362470002 | NAGA | alpha-N-acetylgalactosaminidase [Source:HGNC Symbol;Acc:HGNC:7631] |
| ENSG00000198964 | HGLibA_43835 | 79 | -0.362470002 | SGMS1 | sphingomyelin synthase 1 [Source:HGNC Symbol;Acc:HGNC:29799] |
| ENSG00000204138 | HGLibA_36307 | 79 | -0.362470002 | PHACTR4 | phosphatase and actin regulator 4 [Source:HGNC Symbol;Acc:HGNC:25793] |
| ENSG00000204604 | HGLibA_56048 | 79 | -0.362470002 | ZNF468 | zinc finger protein 468 [Source:HGNC Symbol;Acc:HGNC:33105] |
| ENSG00000204872 | HGLibA_31185 | 79 | -0.362470002 | NAT8B | gene/pseudogene) [Source:HGNC Symbol;Acc:HGNC:30235]" |
| ENSG00000204965 | HGLibA_35449 | 79 | -0.362470002 | PCDHA5 | protocadherin alpha 5 [Source:HGNC Symbol;Acc:HGNC:8671] |
| ENSG00000204970 | HGLibA_35424 | 79 | -0.362470002 | PCDHA1 | protocadherin alpha 1 [Source:HGNC Symbol;Acc:HGNC:8663] |
| ENSG00000205002 | HGLibA_00066 | 79 | -0.362470002 | AARD | alanine and arginine rich domain containing protein [Source:HGNC Symbol;Acc:HGNC:33842] |
| ENSG00000205277 | HGLibA_30523 | 79 | -0.362470002 | MUC12 | cell surface associated [Source:HGNC Symbol;Acc:HGNC:7510]" |
| ENSG00000205307 | HGLibA_42719 | 79 | -0.362470002 | SAP25 | Sin3A associated protein 25 [Source:HGNC Symbol;Acc:HGNC:41908] |
| ENSG00000205899 | HGLibA_04470 | 79 | -0.362470002 | BHLHA9 | basic helix-loop-helix family member a9 [Source:HGNC Symbol;Acc:HGNC:35126] |
| ENSG00000221867 | HGLibA_27911 | 79 | -0.362470002 | MAGEA3 | MAGE family member A3 [Source:HGNC Symbol;Acc:HGNC:6801] |
| ENSG00000226941 | HGLibA_40627 | 79 | -0.362470002 | RBMY1J | member J [Source:HGNC Symbol;Acc:HGNC:23917]" |
| ENSG00000232196 | HGLibA_30481 | 79 | -0.362470002 | MTRNR2L4 | MT-RNR2 like 4 [Source:HGNC Symbol;Acc:HGNC:37161] |
| ENSG00000244623 | HGLibA_33745 | 79 | -0.362470002 | OR2AE1 | olfactory receptor family 2 subfamily AE member 1 [Source:HGNC Symbol;Acc:HGNC:15087] |
| ENSG00000255223 | HGLibA_34335 | 79 | -0.362470002 | OR5M11 | olfactory receptor family 5 subfamily M member 11 [Source:HGNC Symbol;Acc:HGNC:15291] |
| ENSG00000261236 | HGLibA_04695 | 79 | -0.362470002 | BOP1 | block of proliferation 1 [Source:HGNC Symbol;Acc:HGNC:15519] |
| ENSG00000266967 | HGLibA_00075 | 79 | -0.362470002 | AARSD1 | alanyl-tRNA synthetase domain containing 1 [Source:HGNC Symbol;Acc:HGNC:28417] |
| GPR98 | HGLibA_20117 | 79 | -0.362470002 | GPR98 | - |
| HIST1H3E | HGLibA_21468 | 79 | -0.362470002 | HIST1H3E | - |
| hsa-mir-1288 | HGLibA_57618 | 79 | -0.362470002 | hsa-mir-1288 | - |
| hsa-mir-1909 | HGLibA_58032 | 79 | -0.362470002 | hsa-mir-1909 | - |
| hsa-mir-196a-1 | HGLibA_58091 | 79 | -0.362470002 | hsa-mir-196a-1 | - |
| hsa-mir-208a | HGLibA_58194 | 79 | -0.362470002 | hsa-mir-208a | - |
| hsa-mir-3117 | HGLibA_58511 | 79 | -0.362470002 | hsa-mir-3117 | - |
| hsa-mir-3686 | HGLibA_59298 | 79 | -0.362470002 | hsa-mir-3686 | - |
| hsa-mir-4251 | HGLibA_59713 | 79 | -0.362470002 | hsa-mir-4251 | - |
| hsa-mir-4276 | HGLibA_59809 | 79 | -0.362470002 | hsa-mir-4276 | - |
| hsa-mir-4302 | HGLibA_59919 | 79 | -0.362470002 | hsa-mir-4302 | - |
| hsa-mir-4468 | HGLibA_60279 | 79 | -0.362470002 | hsa-mir-4468 | - |
| hsa-mir-4520b | HGLibA_60539 | 79 | -0.362470002 | hsa-mir-4520b | - |
| hsa-mir-4747 | HGLibA_61087 | 79 | -0.362470002 | hsa-mir-4747 | - |
| hsa-mir-501 | HGLibA_61452 | 79 | -0.362470002 | hsa-mir-501 | - |
| hsa-mir-6511b-1 | HGLibA_62915 | 79 | -0.362470002 | hsa-mir-6511b-1 | - |

| | | | | | |
|---|---|---|---|---|---|
| hsa-mir-6511b-2 | HGLibA_62918 | 79 | -0.362470002 | hsa-mir-6511b-2 | - |
| hsa-mir-6512 | HGLibA_62920 | 79 | -0.362470002 | hsa-mir-6512 | - |
| hsa-mir-6729 | HGLibA_63068 | 79 | -0.362470002 | hsa-mir-6729 | - |
| hsa-mir-6842 | HGLibA_63545 | 79 | -0.362470002 | hsa-mir-6842 | - |
| hsa-mir-769 | HGLibA_63935 | 79 | -0.362470002 | hsa-mir-769 | - |
| KIAA1211L | HGLibA_24717 | 79 | -0.362470002 | KIAA1211L | - |
| LOC730183 | HGLibA_27006 | 79 | -0.362470002 | LOC730183 | - |
| RNMTL1 | HGLibA_41722 | 79 | -0.362470002 | RNMTL1 | - |
| T | HGLibA_48121 | 79 | -0.362470002 | T | - |
| WBSCR17 | HGLibA_53992 | 79 | -0.362470002 | WBSCR17 | - |
| WTH3DI | HGLibA_54432 | 79 | -0.362470002 | WTH3DI | - |
| ASNA1 | HGLibA_03331 | 78 | -0.37242104 | ASNA1 | - |
| ATP5E | HGLibA_03650 | 78 | -0.37242104 | ATP5E | - |
| C10orf35 | HGLibA_05071 | 78 | -0.37242104 | C10orf35 | - |
| C11orf88 | HGLibA_05209 | 78 | -0.37242104 | C11orf88 | - |
| C15orf27 | HGLibA_05382 | 78 | -0.37242104 | C15orf27 | - |
| C19orf59 | HGLibA_05688 | 78 | -0.37242104 | C19orf59 | - |
| C20orf26 | HGLibA_06021 | 78 | -0.37242104 | C20orf26 | - |
| C6orf70 | HGLibA_06509 | 78 | -0.37242104 | C6orf70 | - |
| C9orf156 | HGLibA_06694 | 78 | -0.37242104 | C9orf156 | - |
| ENSG00000005022 | HGLibA_44763 | 78 | -0.37242104 | SLC25A5 | solute carrier family 25 member 5 [Source:HGNC Symbol;Acc:HGNC:10991] |
| ENSG00000026950 | HGLibA_04969 | 78 | -0.37242104 | BTN3A1 | butyrophilin subfamily 3 member A1 [Source:HGNC Symbol;Acc:HGNC:1138] |
| ENSG00000048991 | HGLibA_39771 | 78 | -0.37242104 | R3HDM1 | R3H domain containing 1 [Source:HGNC Symbol;Acc:HGNC:9757] |
| ENSG00000055070 | HGLibA_48114 | 78 | -0.37242104 | SZRD1 | SUZ RNA binding domain containing 1 [Source:HGNC Symbol;Acc:HGNC:30232] |
| ENSG00000067066 | HGLibA_46284 | 78 | -0.37242104 | SP100 | SP100 nuclear antigen [Source:HGNC Symbol;Acc:HGNC:11206] |
| ENSG00000068383 | HGLibA_23344 | 78 | -0.37242104 | INPP5A | inositol polyphosphate-5-phosphatase A [Source:HGNC Symbol;Acc:HGNC:6076] |
| ENSG00000068724 | HGLibA_52149 | 78 | -0.37242104 | TTC7A | tetratricopeptide repeat domain 7A [Source:HGNC Symbol;Acc:HGNC:19750] |
| ENSG00000070814 | HGLibA_48826 | 78 | -0.37242104 | TCOF1 | treacle ribosome biogenesis factor 1 [Source:HGNC Symbol;Acc:HGNC:11654] |
| ENSG00000073111 | HGLibA_28604 | 78 | -0.37242104 | MCM2 | minichromosome maintenance complex component 2 [Source:HGNC Symbol;Acc:HGNC:6944] |
| ENSG00000075073 | HGLibA_48175 | 78 | -0.37242104 | TACR2 | tachykinin receptor 2 [Source:HGNC Symbol;Acc:HGNC:11527] |
| ENSG00000075914 | HGLibA_15791 | 78 | -0.37242104 | EXOSC7 | exosome component 7 [Source:HGNC Symbol;Acc:HGNC:28112] |
| ENSG00000089012 | HGLibA_44214 | 78 | -0.37242104 | SIRPG | signal regulatory protein gamma [Source:HGNC Symbol;Acc:HGNC:15757] |
| ENSG00000089041 | HGLibA_34864 | 78 | -0.37242104 | P2RX7 | purinergic receptor P2X 7 [Source:HGNC Symbol;Acc:HGNC:8537] |
| ENSG00000095564 | HGLibA_04890 | 78 | -0.37242104 | BTAF1 | B-TFIID TATA-box binding protein associated factor 1 [Source:HGNC Symbol;Acc:HGNC:17307] |
| ENSG00000095574 | HGLibA_22940 | 78 | -0.37242104 | IKZF5 | IKAROS family zinc finger 5 [Source:HGNC Symbol;Acc:HGNC:14283] |
| ENSG00000095794 | HGLibA_11132 | 78 | -0.37242104 | CREM | cAMP responsive element modulator [Source:HGNC Symbol;Acc:HGNC:2352] |
| ENSG00000099998 | HGLibA_19013 | 78 | -0.37242104 | GGT5 | gamma-glutamyltransferase 5 [Source:HGNC Symbol;Acc:HGNC:4260] |
| ENSG00000100078 | HGLibA_36897 | 78 | -0.37242104 | PLA2G3 | phospholipase A2 group III [Source:HGNC Symbol;Acc:HGNC:17934] |
| ENSG00000100253 | HGLibA_29288 | 78 | -0.37242104 | MIOX | myo-inositol oxygenase [Source:HGNC Symbol;Acc:HGNC:14522] |
| ENSG00000100665 | HGLibA_43520 | 78 | -0.37242104 | SERPINA4 | serpin family A member 4 [Source:HGNC Symbol;Acc:HGNC:8948] |
| ENSG00000101216 | HGLibA_19401 | 78 | -0.37242104 | GMEB2 | glucocorticoid modulatory element binding protein 2 [Source:HGNC Symbol;Acc:HGNC:4371] |
| ENSG00000102595 | HGLibA_52869 | 78 | -0.37242104 | UGGT2 | UDP-glucose glycoprotein glucosyltransferase 2 [Source:HGNC Symbol;Acc:HGNC:15664] |
| ENSG00000102606 | HGLibA_02877 | 78 | -0.37242104 | ARHGEF7 | Rho guanine nucleotide exchange factor 7 [Source:HGNC Symbol;Acc:HGNC:15607] |
| ENSG00000102699 | HGLibA_35237 | 78 | -0.37242104 | PARP4 | poly(ADP-ribose) polymerase family member 4 [Source:HGNC Symbol;Acc:HGNC:271] |
| ENSG00000102924 | HGLibA_07405 | 78 | -0.37242104 | CBLN1 | cerebellin 1 precursor [Source:HGNC Symbol;Acc:HGNC:1543] |
| ENSG00000103490 | HGLibA_39670 | 78 | -0.37242104 | PYCARD | PYD and CARD domain containing [Source:HGNC Symbol;Acc:HGNC:16608] |
| ENSG00000103653 | HGLibA_11359 | 78 | -0.37242104 | CSK | C-terminal Src kinase [Source:HGNC Symbol;Acc:HGNC:2444] |
| ENSG00000104356 | HGLibA_37688 | 78 | -0.37242104 | POP1 | ribonuclease P/MRP subunit [Source:HGNC Symbol;Acc:HGNC:30129]" |
| ENSG00000106571 | HGLibA_19254 | 78 | -0.37242104 | GLI3 | GLI family zinc finger 3 [Source:HGNC Symbol;Acc:HGNC:4319] |
| ENSG00000106588 | HGLibA_39072 | 78 | -0.37242104 | PSMA2 | proteasome subunit alpha 2 [Source:HGNC Symbol;Acc:HGNC:9531] |
| ENSG00000107262 | HGLibA_04075 | 78 | -0.37242104 | BAG1 | BCL2 associated athanogene 1 [Source:HGNC Symbol;Acc:HGNC:937] |
| ENSG00000107554 | HGLibA_13695 | 78 | -0.37242104 | DNMBP | dynamin binding protein [Source:HGNC Symbol;Acc:HGNC:30373] |
| ENSG00000107742 | HGLibA_46752 | 78 | -0.37242104 | SPOCK2 | cwcv and kazal like domains proteoglycan 2 [Source:HGNC Symbol;Acc:HGNC:13564]" |
| ENSG00000108094 | HGLibA_11777 | 78 | -0.37242104 | CUL2 | cullin 2 [Source:HGNC Symbol;Acc:HGNC:2552] |
| ENSG00000111203 | HGLibA_23709 | 78 | -0.37242104 | ITFG2 | integrin alpha FG-GAP repeat containing 2 [Source:HGNC Symbol;Acc:HGNC:30879] |
| ENSG00000111224 | HGLibA_35218 | 78 | -0.37242104 | PARP11 | poly(ADP-ribose) polymerase family member 11 [Source:HGNC Symbol;Acc:HGNC:1186] |
| ENSG00000111912 | HGLibA_31354 | 78 | -0.37242104 | NCOA7 | nuclear receptor coactivator 7 [Source:HGNC Symbol;Acc:HGNC:21081] |
| ENSG00000112992 | HGLibA_32225 | 78 | -0.37242104 | NNT | nicotinamide nucleotide transhydrogenase [Source:HGNC Symbol;Acc:HGNC:7863] |
| ENSG00000113048 | HGLibA_30081 | 78 | -0.37242104 | MRPS27 | mitochondrial ribosomal protein S27 [Source:HGNC Symbol;Acc:HGNC:14512] |
| ENSG00000113303 | HGLibA_04981 | 78 | -0.37242104 | BTNL8 | butyrophilin like 8 [Source:HGNC Symbol;Acc:HGNC:26131] |
| ENSG00000114948 | HGLibA_00762 | 78 | -0.37242104 | ADAM23 | ADAM metallopeptidase domain 23 [Source:HGNC Symbol;Acc:HGNC:202] |
| ENSG00000116815 | HGLibA_08414 | 78 | -0.37242104 | CD58 | CD58 molecule [Source:HGNC Symbol;Acc:HGNC:1688] |
| ENSG00000116903 | HGLibA_15764 | 78 | -0.37242104 | EXOC8 | exocyst complex component 8 [Source:HGNC Symbol;Acc:HGNC:24659] |
| ENSG00000118197 | HGLibA_12756 | 78 | -0.37242104 | DDX59 | DEAD-box helicase 59 [Source:HGNC Symbol;Acc:HGNC:25360] |
| ENSG00000118655 | HGLibA_12510 | 78 | -0.37242104 | DCLRE1B | DNA cross-link repair 1B [Source:HGNC Symbol;Acc:HGNC:17641] |
| ENSG00000119314 | HGLibA_39255 | 78 | -0.37242104 | PTBP3 | polypyrimidine tract binding protein 3 [Source:HGNC Symbol;Acc:HGNC:10253] |
| ENSG00000119487 | HGLibA_28297 | 78 | -0.37242104 | MAPKAP1 | mitogen-activated protein kinase associated protein 1 [Source:HGNC Symbol;Acc:HGNC:18752] |
| ENSG00000119725 | HGLibA_55936 | 78 | -0.37242104 | ZNF410 | zinc finger protein 410 [Source:HGNC Symbol;Acc:HGNC:20144] |
| ENSG00000120008 | HGLibA_54026 | 78 | -0.37242104 | WDR11 | WD repeat domain 11 [Source:HGNC Symbol;Acc:HGNC:13831] |
| ENSG00000120328 | HGLibA_35479 | 78 | -0.37242104 | PCDHB12 | protocadherin beta 12 [Source:HGNC Symbol;Acc:HGNC:8683] |
| ENSG00000121067 | HGLibA_46763 | 78 | -0.37242104 | SPOP | speckle type BTB/POZ protein [Source:HGNC Symbol;Acc:HGNC:11254] |
| ENSG00000121898 | HGLibA_11040 | 78 | -0.37242104 | CPXM2 | M14 family member 2 [Source:HGNC Symbol;Acc:HGNC:26977]" |
| ENSG00000122557 | HGLibA_21175 | 78 | -0.37242104 | HERPUD2 | HERPUD family member 2 [Source:HGNC Symbol;Acc:HGNC:21915] |
| ENSG00000123685 | HGLibA_04178 | 78 | -0.37242104 | BATF3 | basic leucine zipper ATF-like transcription factor 3 [Source:HGNC Symbol;Acc:HGNC:28915] |
| ENSG00000124181 | HGLibA_37001 | 78 | -0.37242104 | PLCG1 | phospholipase C gamma 1 [Source:HGNC Symbol;Acc:HGNC:9065] |
| ENSG00000124256 | HGLibA_54809 | 78 | -0.37242104 | ZBP1 | Z-DNA binding protein 1 [Source:HGNC Symbol;Acc:HGNC:16176] |
| ENSG00000125454 | HGLibA_44663 | 78 | -0.37242104 | SLC25A19 | solute carrier family 25 member 19 [Source:HGNC Symbol;Acc:HGNC:14409] |
| ENSG00000126012 | HGLibA_24526 | 78 | -0.37242104 | KDM5C | lysine demethylase 5C [Source:HGNC Symbol;Acc:HGNC:11114] |
| ENSG00000126602 | HGLibA_51295 | 78 | -0.37242104 | TRAP1 | TNF receptor associated protein 1 [Source:HGNC Symbol;Acc:HGNC:16264] |
| ENSG00000127325 | HGLibA_04425 | 78 | -0.37242104 | BEST3 | bestrophin 3 [Source:HGNC Symbol;Acc:HGNC:17105] |
| ENSG00000128563 | HGLibA_38587 | 78 | -0.37242104 | PRKRIP1 | PRKR interacting protein 1 [Source:HGNC Symbol;Acc:HGNC:21894] |
| ENSG00000129675 | HGLibA_02873 | 78 | -0.37242104 | ARHGEF6 | Rac/Cdc42 guanine nucleotide exchange factor 6 [Source:HGNC Symbol;Acc:HGNC:685] |
| ENSG00000131653 | HGLibA_51266 | 78 | -0.37242104 | TRAF7 | TNF receptor associated factor 7 [Source:HGNC Symbol;Acc:HGNC:20456] |
| ENSG00000132718 | HGLibA_48051 | 78 | -0.37242104 | SYT11 | synaptotagmin 11 [Source:HGNC Symbol;Acc:HGNC:19239] |
| ENSG00000133138 | HGLibA_48569 | 78 | -0.37242104 | TBC1D8B | TBC1 domain family member 8B [Source:HGNC Symbol;Acc:HGNC:24715] |
| ENSG00000134294 | HGLibA_45064 | 78 | -0.37242104 | SLC38A2 | solute carrier family 38 member 2 [Source:HGNC Symbol;Acc:HGNC:13448] |
| ENSG00000134824 | HGLibA_15937 | 78 | -0.37242104 | FADS2 | fatty acid desaturase 2 [Source:HGNC Symbol;Acc:HGNC:3575] |
| ENSG00000134954 | HGLibA_15641 | 78 | -0.37242104 | ETS1 | transcription factor [Source:HGNC Symbol;Acc:HGNC:3488]" |
| ENSG00000135299 | HGLibA_02129 | 78 | -0.37242104 | ANKRD6 | ankyrin repeat domain 6 [Source:HGNC Symbol;Acc:HGNC:17280] |
| ENSG00000138316 | HGLibA_00803 | 78 | -0.37242104 | ADAMTS14 | ADAM metallopeptidase with thrombospondin type 1 motif 14 [Source:HGNC Symbol;Acc:HGNC:14899] |
| ENSG00000138750 | HGLibA_33061 | 78 | -0.37242104 | NUP54 | nucleoporin 54 [Source:HGNC Symbol;Acc:HGNC:17359] |
| ENSG00000138814 | HGLibA_38186 | 78 | -0.37242104 | PPP3CA | protein phosphatase 3 catalytic subunit alpha [Source:HGNC Symbol;Acc:HGNC:9314] |
| ENSG00000139793 | HGLibA_28512 | 78 | -0.37242104 | MBNL2 | muscleblind like splicing regulator 2 [Source:HGNC Symbol;Acc:HGNC:16746] |
| ENSG00000140153 | HGLibA_54047 | 78 | -0.37242104 | WDR20 | WD repeat domain 20 [Source:HGNC Symbol;Acc:HGNC:19667] |
| ENSG00000141380 | HGLibA_47103 | 78 | -0.37242104 | SS18 | nBAF chromatin remodeling complex subunit [Source:HGNC Symbol;Acc:HGNC:11340]" |
| ENSG00000142700 | HGLibA_13428 | 78 | -0.37242104 | DMRTA2 | DMRT like family A2 [Source:HGNC Symbol;Acc:HGNC:13908] |
| ENSG00000143320 | HGLibA_11057 | 78 | -0.37242104 | CRABP2 | cellular retinoic acid binding protein 2 [Source:HGNC Symbol;Acc:HGNC:2339] |
| ENSG00000143507 | HGLibA_14131 | 78 | -0.37242104 | DUSP10 | dual specificity phosphatase 10 [Source:HGNC Symbol;Acc:HGNC:3065] |
| ENSG00000143947 | HGLibA_42124 | 78 | -0.37242104 | RPS27A | ribosomal protein S27a [Source:HGNC Symbol;Acc:HGNC:10417] |
| ENSG00000144285 | HGLibA_42974 | 78 | -0.37242104 | SCN1A | sodium voltage-gated channel alpha subunit 1 [Source:HGNC Symbol;Acc:HGNC:10585] |
| ENSG00000147224 | HGLibA_38755 | 78 | -0.37242104 | PRPS1 | phosphoribosyl pyrophosphate synthetase 1 [Source:HGNC Symbol;Acc:HGNC:9462] |
| ENSG00000147364 | HGLibA_17053 | 78 | -0.37242104 | FBXO25 | F-box protein 25 [Source:HGNC Symbol;Acc:HGNC:13596] |
| ENSG00000148411 | HGLibA_31043 | 78 | -0.37242104 | NACC2 | NACC family member 2 [Source:HGNC Symbol;Acc:HGNC:23846] |
| ENSG00000148843 | HGLibA_35720 | 78 | -0.37242104 | PDCD11 | programmed cell death 11 [Source:HGNC Symbol;Acc:HGNC:13408] |
| ENSG00000149599 | HGLibA_14144 | 78 | -0.37242104 | DUSP15 | dual specificity phosphatase 15 [Source:HGNC Symbol;Acc:HGNC:16236] |
| ENSG00000149929 | HGLibA_21353 | 78 | -0.37242104 | HIRIP3 | HIRA interacting protein 3 [Source:HGNC Symbol;Acc:HGNC:4917] |
| ENSG00000152093 | HGLibA_09251 | 78 | -0.37242104 | CFC1B | cryptic family 1B [Source:HGNC Symbol;Acc:HGNC:33983]" |
| ENSG00000152207 | HGLibA_12230 | 78 | -0.37242104 | CYSLTR2 | cysteinyl leukotriene receptor 2 [Source:HGNC Symbol;Acc:HGNC:18274] |
| ENSG00000154263 | HGLibA_00097 | 78 | -0.37242104 | ABCA10 | ATP binding cassette subfamily A member 10 [Source:HGNC Symbol;Acc:HGNC:30] |
| ENSG00000155980 | HGLibA_24949 | 78 | -0.37242104 | KIF5A | kinesin family member 5A [Source:HGNC Symbol;Acc:HGNC:6323] |
| ENSG00000156009 | HGLibA_27919 | 78 | -0.37242104 | MAGEA8 | MAGE family member A8 [Source:HGNC Symbol;Acc:HGNC:6806] |
| ENSG00000156170 | HGLibA_31497 | 78 | -0.37242104 | NDUFAF6 | NADH:ubiquinone oxidoreductase complex assembly factor 6 [Source:HGNC Symbol;Acc:HGNC:28625] |
| ENSG00000156831 | HGLibA_32770 | 78 | -0.37242104 | NSMCE2 | SMC5-SMC6 complex SUMO ligase [Source:HGNC Symbol;Acc:HGNC:26513]" |
| ENSG00000157106 | HGLibA_45697 | 78 | -0.37242104 | SMG1 | nonsense mediated mRNA decay associated PI3K related kinase [Source:HGNC Symbol;Acc:HGNC:30045]" |
| ENSG00000157992 | HGLibA_25857 | 78 | -0.37242104 | KRTCAP3 | keratinocyte associated protein 3 [Source:HGNC Symbol;Acc:HGNC:28943] |
| ENSG00000158062 | HGLibA_52781 | 78 | -0.37242104 | UBXN11 | UBX domain protein 11 [Source:HGNC Symbol;Acc:HGNC:30600] |
| ENSG00000158578 | HGLibA_01527 | 78 | -0.37242104 | ALAS2 | 5'-aminolevulinate synthase 2 [Source:HGNC Symbol;Acc:HGNC:397] |
| ENSG00000159873 | HGLibA_07558 | 78 | -0.37242104 | CCDC117 | coiled-coil domain containing 117 [Source:HGNC Symbol;Acc:HGNC:26599] |
| ENSG00000160410 | HGLibA_44057 | 78 | -0.37242104 | SHKBP1 | SH3KBP1 binding protein 1 [Source:HGNC Symbol;Acc:HGNC:19214] |
| ENSG00000160973 | HGLibA_17872 | 78 | -0.37242104 | FOXH1 | forkhead box H1 [Source:HGNC Symbol;Acc:HGNC:3814] |
| ENSG00000161921 | HGLibA_11861 | 78 | -0.37242104 | CXCL16 | C-X-C motif chemokine ligand 16 [Source:HGNC Symbol;Acc:HGNC:16642] |
| ENSG00000162174 | HGLibA_03369 | 78 | -0.37242104 | ASRGL1 | asparaginase like 1 [Source:HGNC Symbol;Acc:HGNC:16448] |
| ENSG00000162598 | HGLibA_05902 | 78 | -0.37242104 | C1orf87 | chromosome 1 open reading frame 87 [Source:HGNC Symbol;Acc:HGNC:28547] |
| ENSG00000162601 | HGLibA_30922 | 78 | -0.37242104 | MYSM1 | SWIRM and MPN domains 1 [Source:HGNC Symbol;Acc:HGNC:29401]" |
| ENSG00000162722 | HGLibA_51553 | 78 | -0.37242104 | TRIM58 | tripartite motif containing 58 [Source:HGNC Symbol;Acc:HGNC:24150] |
| ENSG00000162729 | HGLibA_22901 | 78 | -0.37242104 | IGSF8 | immunoglobulin superfamily member 8 [Source:HGNC Symbol;Acc:HGNC:17813] |
| ENSG00000162892 | HGLibA_23103 | 78 | -0.37242104 | IL24 | interleukin 24 [Source:HGNC Symbol;Acc:HGNC:11346] |
| ENSG00000163435 | HGLibA_14896 | 78 | -0.37242104 | ELF3 | E74 like ETS transcription factor 3 [Source:HGNC Symbol;Acc:HGNC:3318] |
| ENSG00000163472 | HGLibA_50515 | 78 | -0.37242104 | TMEM79 | transmembrane protein 79 [Source:HGNC Symbol;Acc:HGNC:28196] |
| ENSG00000163554 | HGLibA_46870 | 78 | -0.37242104 | SPTA1 | erythrocytic 1 [Source:HGNC Symbol;Acc:HGNC:11272]" |
| ENSG00000164346 | HGLibA_32742 | 78 | -0.37242104 | NSA2 | ribosome biogenesis homolog [Source:HGNC Symbol;Acc:HGNC:30728]" |
| ENSG00000164398 | HGLibA_00565 | 78 | -0.37242104 | ACSL6 | acyl-CoA synthetase long chain family member 6 [Source:HGNC Symbol;Acc:HGNC:16496] |
| ENSG00000164442 | HGLibA_09746 | 78 | -0.37242104 | CITED2 | Cbp/p300 interacting transactivator with Glu/Asp rich carboxy-terminal domain 2 [Source:HGNC Symbol;Acc:HGNC:1987] |
| ENSG00000164548 | HGLibA_51223 | 78 | -0.37242104 | TRA2A | transformer 2 alpha homolog [Source:HGNC Symbol;Acc:HGNC:16645] |
| ENSG00000164920 | HGLibA_34702 | 78 | -0.37242104 | OSR2 | odd-skipped related transciption factor 2 [Source:HGNC Symbol;Acc:HGNC:15830] |

| | | | | | |
|---|---|---|---|---|---|
| ENSG00000164946 | HGLibA_17985 | 78 | -0.37242104 | FREM1 | FRAS1 related extracellular matrix 1 [Source:HGNC Symbol;Acc:HGNC:23399] |
| ENSG00000165105 | HGLibA_40327 | 78 | -0.37242104 | RASEF | RAS and EF-hand domain containing [Source:HGNC Symbol;Acc:HGNC:26464] |
| ENSG00000165914 | HGLibA_52152 | 78 | -0.37242104 | TTC7B | tetratricopeptide repeat domain 7B [Source:HGNC Symbol;Acc:HGNC:19858] |
| ENSG00000166869 | HGLibA_09520 | 78 | -0.37242104 | CHP2 | calcineurin like EF-hand protein 2 [Source:HGNC Symbol;Acc:HGNC:24927] |
| ENSG00000167280 | HGLibA_15129 | 78 | -0.37242104 | ENGASE | endo-beta-N-acetylglucosaminidase [Source:HGNC Symbol;Acc:HGNC:24622] |
| ENSG00000168003 | HGLibA_45133 | 78 | -0.37242104 | SLC3A2 | solute carrier family 3 member 2 [Source:HGNC Symbol;Acc:HGNC:11026] |
| ENSG00000168484 | HGLibA_43776 | 78 | -0.37242104 | SFTPC | surfactant protein C [Source:HGNC Symbol;Acc:HGNC:10802] |
| ENSG00000168779 | HGLibA_44074 | 78 | -0.37242104 | SHOX2 | short stature homeobox 2 [Source:HGNC Symbol;Acc:HGNC:10854] |
| ENSG00000169031 | HGLibA_10571 | 78 | -0.37242104 | COL4A3 | collagen type IV alpha 3 chain [Source:HGNC Symbol;Acc:HGNC:2204] |
| ENSG00000169442 | HGLibA_08405 | 78 | -0.37242104 | CD52 | CD52 molecule [Source:HGNC Symbol;Acc:HGNC:1804] |
| ENSG00000169629 | HGLibA_41044 | 78 | -0.37242104 | RGPD8 | RANBP2-like and GRIP domain containing 8 [Source:HGNC Symbol;Acc:HGNC:9849] |
| ENSG00000170085 | HGLibA_44179 | 78 | -0.37242104 | SIMC1 | SUMO interacting motifs containing 1 [Source:HGNC Symbol;Acc:HGNC:24779] |
| ENSG00000170791 | HGLibA_09381 | 78 | -0.37242104 | CHCHD7 | coiled-coil-helix-coiled-coil-helix domain containing 7 [Source:HGNC Symbol;Acc:HGNC:28314] |
| ENSG00000170819 | HGLibA_04457 | 78 | -0.37242104 | BFSP2 | beaded filament structural protein 2 [Source:HGNC Symbol;Acc:HGNC:1041] |
| ENSG00000172172 | HGLibA_29901 | 78 | -0.37242104 | MRPL13 | mitochondrial ribosomal protein L13 [Source:HGNC Symbol;Acc:HGNC:14278] |
| ENSG00000173137 | HGLibA_00903 | 78 | -0.37242104 | ADCK5 | aarF domain containing kinase 5 [Source:HGNC Symbol;Acc:HGNC:21738] |
| ENSG00000173137 | HGLibA_00902 | 78 | -0.37242104 | ADCK5 | aarF domain containing kinase 5 [Source:HGNC Symbol;Acc:HGNC:21738] |
| ENSG00000173464 | HGLibA_41394 | 78 | -0.37242104 | RNASE11 | ribonuclease A family member 11 (inactive) [Source:HGNC Symbol;Acc:HGNC:19269] |
| ENSG00000174502 | HGLibA_44811 | 78 | -0.37242104 | SLC26A9 | solute carrier family 26 member 9 [Source:HGNC Symbol;Acc:HGNC:14469] |
| ENSG00000174529 | HGLibA_50518 | 78 | -0.37242104 | TMEM81 | transmembrane protein 81 [Source:HGNC Symbol;Acc:HGNC:32349] |
| ENSG00000176018 | HGLibA_27771 | 78 | -0.37242104 | LYSMD3 | LysM domain containing 3 [Source:HGNC Symbol;Acc:HGNC:26969] |
| ENSG00000177556 | HGLibA_03542 | 78 | -0.37242104 | ATOX1 | antioxidant 1 copper chaperone [Source:HGNC Symbol;Acc:HGNC:798] |
| ENSG00000177752 | HGLibA_54689 | 78 | -0.37242104 | YIPF7 | Yip1 domain family member 7 [Source:HGNC Symbol;Acc:HGNC:26825] |
| ENSG00000177885 | HGLibA_20231 | 78 | -0.37242104 | GRB2 | growth factor receptor bound protein 2 [Source:HGNC Symbol;Acc:HGNC:4566] |
| ENSG00000178105 | HGLibA_12650 | 78 | -0.37242104 | DDX10 | DEAD-box helicase 10 [Source:HGNC Symbol;Acc:HGNC:2735] |
| ENSG00000178814 | HGLibA_33418 | 78 | -0.37242104 | OPLAH | ATP-hydrolysing [Source:HGNC Symbol;Acc:HGNC:8149]" |
| ENSG00000180549 | HGLibA_18202 | 78 | -0.37242104 | FUT7 | fucosyltransferase 7 [Source:HGNC Symbol;Acc:HGNC:4018] |
| ENSG00000181035 | HGLibA_44740 | 78 | -0.37242104 | SLC25A42 | solute carrier family 25 member 42 [Source:HGNC Symbol;Acc:HGNC:28380] |
| ENSG00000181626 | HGLibA_02134 | 78 | -0.37242104 | ANKRD62 | ankyrin repeat domain 62 [Source:HGNC Symbol;Acc:HGNC:35241] |
| ENSG00000182379 | HGLibA_33171 | 78 | -0.37242104 | NXPH4 | neurexophilin 4 [Source:HGNC Symbol;Acc:HGNC:8078] |
| ENSG00000183439 | HGLibA_51568 | 78 | -0.37242104 | TRIM61 | tripartite motif containing 61 [Source:HGNC Symbol;Acc:HGNC:24339] |
| ENSG00000184661 | HGLibA_08602 | 78 | -0.37242104 | CDCA2 | cell division cycle associated 2 [Source:HGNC Symbol;Acc:HGNC:14623] |
| ENSG00000185033 | HGLibA_43342 | 78 | -0.37242104 | SEMA4B | semaphorin 4B [Source:HGNC Symbol;Acc:HGNC:10730] |
| ENSG00000185043 | HGLibA_09683 | 78 | -0.37242104 | CIB1 | calcium and integrin binding 1 [Source:HGNC Symbol;Acc:HGNC:16920] |
| ENSG00000185069 | HGLibA_25540 | 78 | -0.37242104 | KRT76 | keratin 76 [Source:HGNC Symbol;Acc:HGNC:24430] |
| ENSG00000185385 | HGLibA_34460 | 78 | -0.37242104 | OR7A17 | olfactory receptor family 7 subfamily A member 17 [Source:HGNC Symbol;Acc:HGNC:8363] |
| ENSG00000185551 | HGLibA_32612 | 78 | -0.37242104 | NR2F2 | nuclear receptor subfamily 2 group F member 2 [Source:HGNC Symbol;Acc:HGNC:7976] |
| ENSG00000185787 | HGLibA_29660 | 78 | -0.37242104 | MORF4L1 | mortality factor 4 like 1 [Source:HGNC Symbol;Acc:HGNC:16989] |
| ENSG00000185894 | HGLibA_04749 | 78 | -0.37242104 | BPY2C | basic charge Y-linked 2C [Source:HGNC Symbol;Acc:HGNC:18225] |
| ENSG00000186049 | HGLibA_25531 | 78 | -0.37242104 | KRT73 | keratin 73 [Source:HGNC Symbol;Acc:HGNC:28928] |
| ENSG00000186395 | HGLibA_25418 | 78 | -0.37242104 | KRT10 | keratin 10 [Source:HGNC Symbol;Acc:HGNC:6413] |
| ENSG00000186767 | HGLibA_46680 | 78 | -0.37242104 | SPIN4 | spindlin family member 4 [Source:HGNC Symbol;Acc:HGNC:27040] |
| ENSG00000187079 | HGLibA_48925 | 78 | -0.37242104 | TEAD1 | TEA domain transcription factor 1 [Source:HGNC Symbol;Acc:HGNC:11714] |
| ENSG00000187268 | HGLibA_16825 | 78 | -0.37242104 | FAM9C | family with sequence similarity 9 member C [Source:HGNC Symbol;Acc:HGNC:18405] |
| ENSG00000188428 | HGLibA_04570 | 78 | -0.37242104 | BLOC1S5 | biogenesis of lysosomal organelles complex 1 subunit 5 [Source:HGNC Symbol;Acc:HGNC:18561] |
| ENSG00000188641 | HGLibA_13919 | 78 | -0.37242104 | DPYD | dihydropyrimidine dehydrogenase [Source:HGNC Symbol;Acc:HGNC:3012] |
| ENSG00000188738 | HGLibA_18096 | 78 | -0.37242104 | FSIP2 | fibrous sheath interacting protein 2 [Source:HGNC Symbol;Acc:HGNC:21675] |
| ENSG00000188817 | HGLibA_46011 | 78 | -0.37242104 | SNTN | cilia apical structure protein [Source:HGNC Symbol;Acc:HGNC:33706]" |
| ENSG00000189052 | HGLibA_09308 | 78 | -0.37242104 | CGB5 | chorionic gonadotropin subunit beta 5 [Source:HGNC Symbol;Acc:HGNC:16452] |
| ENSG00000189266 | HGLibA_37437 | 78 | -0.37242104 | PNRC2 | proline rich nuclear receptor coactivator 2 [Source:HGNC Symbol;Acc:HGNC:23158] |
| ENSG00000189376 | HGLibA_06648 | 78 | -0.37242104 | C8orf76 | chromosome 8 open reading frame 76 [Source:HGNC Symbol;Acc:HGNC:25924] |
| ENSG00000196227 | HGLibA_16493 | 78 | -0.37242104 | FAM217B | family with sequence similarity 217 member B [Source:HGNC Symbol;Acc:HGNC:16170] |
| ENSG00000196437 | HGLibA_56269 | 78 | -0.37242104 | ZNF569 | zinc finger protein 569 [Source:HGNC Symbol;Acc:HGNC:24737] |
| ENSG00000196975 | HGLibA_02240 | 78 | -0.37242104 | ANXA4 | annexin A4 [Source:HGNC Symbol;Acc:HGNC:542] |
| ENSG00000197241 | HGLibA_44888 | 78 | -0.37242104 | SLC2A7 | solute carrier family 2 member 7 [Source:HGNC Symbol;Acc:HGNC:13445] |
| ENSG00000197437 | HGLibA_33620 | 78 | -0.37242104 | OR13G1 | olfactory receptor family 13 subfamily G member 1 [Source:HGNC Symbol;Acc:HGNC:14999] |
| ENSG00000197763 | HGLibA_52450 | 78 | -0.37242104 | TXNRD3 | thioredoxin reductase 3 [Source:HGNC Symbol;Acc:HGNC:20667] |
| ENSG00000197951 | HGLibA_56609 | 78 | -0.37242104 | ZNF71 | zinc finger protein 71 [Source:HGNC Symbol;Acc:HGNC:13141] |
| ENSG00000197965 | HGLibA_29796 | 78 | -0.37242104 | MPZL1 | myelin protein zero like 1 [Source:HGNC Symbol;Acc:HGNC:7226] |
| ENSG00000204634 | HGLibA_48565 | 78 | -0.37242104 | TBC1D8 | TBC1 domain family member 8 [Source:HGNC Symbol;Acc:HGNC:17791] |
| ENSG00000204764 | HGLibA_40198 | 78 | -0.37242104 | RANBP17 | RAN binding protein 17 [Source:HGNC Symbol;Acc:HGNC:14428] |
| ENSG00000205856 | HGLibA_06078 | 78 | -0.37242104 | C22orf42 | chromosome 22 open reading frame 42 [Source:HGNC Symbol;Acc:HGNC:27160] |
| ENSG00000206474 | HGLibA_33486 | 78 | -0.37242104 | OR10C1 | olfactory receptor family 10 subfamily C member 1 (gene/pseudogene) [Source:HGNC Symbol;Acc:HGNC:8165] |
| ENSG00000213759 | HGLibA_52914 | 78 | -0.37242104 | UGT2B11 | UDP glucuronosyltransferase family 2 member B11 [Source:HGNC Symbol;Acc:HGNC:12545] |
| ENSG00000214063 | HGLibA_51936 | 78 | -0.37242104 | TSPAN4 | tetraspanin 4 [Source:HGNC Symbol;Acc:HGNC:11859] |
| ENSG00000233932 | HGLibA_11758 | 78 | -0.37242104 | CTXN2 | cortexin 2 [Source:HGNC Symbol;Acc:HGNC:31109] |
| ENSG00000236126 | HGLibA_11530 | 78 | -0.37242104 | CT47A3 | member A3 [Source:HGNC Symbol;Acc:HGNC:33284]" |
| ENSG00000239590 | HGLibA_33680 | 78 | -0.37242104 | OR1J4 | olfactory receptor family 1 subfamily J member 4 [Source:HGNC Symbol;Acc:HGNC:8211] |
| ENSG00000240432 | HGLibA_25648 | 78 | -0.37242104 | KRTAP13-3 | keratin associated protein 13-3 [Source:HGNC Symbol;Acc:HGNC:18925] |
| ENSG00000241852 | HGLibA_06639 | 78 | -0.37242104 | C8orf58 | chromosome 8 open reading frame 58 [Source:HGNC Symbol;Acc:HGNC:32233] |
| ENSG00000243137 | HGLibA_39045 | 78 | -0.37242104 | PSG4 | pregnancy specific beta-1-glycoprotein 4 [Source:HGNC Symbol;Acc:HGNC:9521] |
| ENSG00000250298 | HGLibA_19094 | 78 | -0.37242104 | GIMD1 | GIMAP family P-loop NTPase domain containing 1 [Source:HGNC Symbol;Acc:HGNC:44141] |
| ENSG00000254521 | HGLibA_44131 | 78 | -0.37242104 | SIGLEC12 | sialic acid binding Ig like lectin 12 (gene/pseudogene) [Source:HGNC Symbol;Acc:HGNC:15482] |
| ENSG00000261796 | HGLibA_23697 | 78 | -0.37242104 | ISY1-RAB43 | ISY1-RAB43 readthrough [Source:HGNC Symbol;Acc:HGNC:42969] |
| ENSG00000267060 | HGLibA_39333 | 78 | -0.37242104 | PTGES3L | prostaglandin E synthase 3 like [Source:HGNC Symbol;Acc:HGNC:43943] |
| ENSG00000269713 | HGLibA_31244 | 78 | -0.37242104 | NBPF9 | NBPF member 9 [Source:HGNC Symbol;Acc:HGNC:31991] |
| ENSG00000278318 | HGLibA_55629 | 78 | -0.37242104 | ZNF229 | zinc finger protein 229 [Source:HGNC Symbol;Acc:HGNC:13022] |
| FOPNL | HGLibA_17793 | 78 | -0.37242104 | FOPNL | - |
| G6PC | HGLibA_18306 | 78 | -0.37242104 | G6PC | - |
| HIST3H2BB | HGLibA_21558 | 78 | -0.37242104 | HIST3H2BB | - |
| hsa-mir-1255a | HGLibA_57421 | 78 | -0.37242104 | hsa-mir-1255a | - |
| hsa-mir-205 | HGLibA_58177 | 78 | -0.37242104 | hsa-mir-205 | - |
| hsa-mir-320c-1 | HGLibA_58934 | 78 | -0.37242104 | hsa-mir-320c-1 | - |
| hsa-mir-367 | HGLibA_59229 | 78 | -0.37242104 | hsa-mir-367 | - |
| hsa-mir-376b | HGLibA_59414 | 78 | -0.37242104 | hsa-mir-376b | - |
| hsa-mir-410 | HGLibA_59678 | 78 | -0.37242104 | hsa-mir-410 | - |
| hsa-mir-4687 | HGLibA_60859 | 78 | -0.37242104 | hsa-mir-4687 | - |
| hsa-mir-4774 | HGLibA_61202 | 78 | -0.37242104 | hsa-mir-4774 | - |
| hsa-mir-505 | HGLibA_61480 | 78 | -0.37242104 | hsa-mir-505 | - |
| hsa-mir-5192 | HGLibA_61687 | 78 | -0.37242104 | hsa-mir-5192 | - |
| hsa-mir-520d | HGLibA_61745 | 78 | -0.37242104 | hsa-mir-520d | - |
| hsa-mir-527 | HGLibA_61803 | 78 | -0.37242104 | hsa-mir-527 | - |
| hsa-mir-527 | HGLibA_61801 | 78 | -0.37242104 | hsa-mir-527 | - |
| hsa-mir-542 | HGLibA_61819 | 78 | -0.37242104 | hsa-mir-542 | - |
| hsa-mir-548h-1 | HGLibA_61993 | 78 | -0.37242104 | hsa-mir-548h-1 | - |
| hsa-mir-548x-2 | HGLibA_62083 | 78 | -0.37242104 | hsa-mir-548x-2 | - |
| hsa-mir-567 | HGLibA_62226 | 78 | -0.37242104 | hsa-mir-567 | - |
| hsa-mir-630 | HGLibA_62761 | 78 | -0.37242104 | hsa-mir-630 | - |
| hsa-mir-6722 | HGLibA_63048 | 78 | -0.37242104 | hsa-mir-6722 | - |
| hsa-mir-6753 | HGLibA_63171 | 78 | -0.37242104 | hsa-mir-6753 | - |
| hsa-mir-6762 | HGLibA_63208 | 78 | -0.37242104 | hsa-mir-6762 | - |
| hsa-mir-6770-2 | HGLibA_63250 | 78 | -0.37242104 | hsa-mir-6770-2 | - |
| hsa-mir-7977 | HGLibA_64038 | 78 | -0.37242104 | hsa-mir-7977 | - |
| hsa-mir-8062 | HGLibA_64086 | 78 | -0.37242104 | hsa-mir-8062 | - |
| hsa-mir-8076 | HGLibA_64145 | 78 | -0.37242104 | hsa-mir-8076 | - |
| hsa-mir-8079 | HGLibA_64159 | 78 | -0.37242104 | hsa-mir-8079 | - |
| hsa-mir-888 | HGLibA_64232 | 78 | -0.37242104 | hsa-mir-888 | - |
| hsa-mir-891b | HGLibA_64245 | 78 | -0.37242104 | hsa-mir-891b | - |
| LOC100288814 | HGLibA_26836 | 78 | -0.37242104 | LOC100288814 | - |
| OCLM | HGLibA_33251 | 78 | -0.37242104 | OCLM | - |
| PHF15 | HGLibA_36348 | 78 | -0.37242104 | PHF15 | - |
| PVRL1 | HGLibA_39622 | 78 | -0.37242104 | PVRL1 | - |
| RARRES3 | HGLibA_40287 | 78 | -0.37242104 | RARRES3 | - |
| TXNRD3NB | HGLibA_52453 | 78 | -0.37242104 | TXNRD3NB | - |
| ACPT | HGLibA_00528 | 77 | -0.382372077 | ACPT | - |
| ACPT | HGLibA_00526 | 77 | -0.382372077 | ACPT | - |
| AGXT2L1 | HGLibA_01278 | 77 | -0.382372077 | AGXT2L1 | - |
| ANKRD32 | HGLibA_02066 | 77 | -0.382372077 | ANKRD32 | - |
| ASMTL_X | HGLibA_03326 | 77 | -0.382372077 | ASMTL_X | - |
| ATP5D | HGLibA_03648 | 77 | -0.382372077 | ATP5D | - |
| BET3L | HGLibA_04436 | 77 | -0.382372077 | BET3L | - |
| BRE | HGLibA_04795 | 77 | -0.382372077 | BRE | - |
| C6orf203 | HGLibA_06470 | 77 | -0.382372077 | C6orf203 | - |
| C9orf66 | HGLibA_06751 | 77 | -0.382372077 | C9orf66 | - |
| CCDC108 | HGLibA_07530 | 77 | -0.382372077 | CCDC108 | - |
| CCDC37 | HGLibA_07767 | 77 | -0.382372077 | CCDC37 | - |
| CSB-PGBD3 | HGLibA_11310 | 77 | -0.382372077 | CSB-PGBD3 | - |
| ENSG00000004455 | HGLibA_01378 | 77 | -0.382372077 | AK2 | adenylate kinase 2 [Source:HGNC Symbol;Acc:HGNC:362] |
| ENSG00000004866 | HGLibA_47282 | 77 | -0.382372077 | ST7 | suppression of tumorigenicity 7 [Source:HGNC Symbol;Acc:HGNC:11351] |
| ENSG00000005238 | HGLibA_16481 | 77 | -0.382372077 | FAM214B | family with sequence similarity 214 member B [Source:HGNC Symbol;Acc:HGNC:25666] |
| ENSG00000007933 | HGLibA_17709 | 77 | -0.382372077 | FMO3 | flavin containing monooxygenase 3 [Source:HGNC Symbol;Acc:HGNC:3771] |
| ENSG00000010282 | HGLibA_21254 | 77 | -0.382372077 | HHATL | hedgehog acyltransferase like [Source:HGNC Symbol;Acc:HGNC:13242] |
| ENSG00000010310 | HGLibA_19127 | 77 | -0.382372077 | GIPR | gastric inhibitory polypeptide receptor [Source:HGNC Symbol;Acc:HGNC:4271] |
| ENSG00000026508 | HGLibA_08389 | 77 | -0.382372077 | CD44 | CD44 molecule (Indian blood group) [Source:HGNC Symbol;Acc:HGNC:1681] |
| ENSG00000057149 | HGLibA_43549 | 77 | -0.382372077 | SERPINB3 | serpin family B member 3 [Source:HGNC Symbol;Acc:HGNC:10569] |

| | | | | | |
|---|---|---|---|---|---|
| ENSG00000060709 | HGLibA_41259 | 77 | -0.382372077 | RIMBP2 | RIMS binding protein 2 [Source:HGNC Symbol;Acc:HGNC:30339] |
| ENSG00000072506 | HGLibA_22173 | 77 | -0.382372077 | HSD17B10 | hydroxysteroid 17-beta dehydrogenase 10 [Source:HGNC Symbol;Acc:HGNC:4800] |
| ENSG00000072657 | HGLibA_51382 | 77 | -0.382372077 | TRHDE | thyrotropin releasing hormone degrading enzyme [Source:HGNC Symbol;Acc:HGNC:30748] |
| ENSG00000081803 | HGLibA_06979 | 77 | -0.382372077 | CADPS2 | calcium dependent secretion activator 2 [Source:HGNC Symbol;Acc:HGNC:16018] |
| ENSG00000083290 | HGLibA_52971 | 77 | -0.382372077 | ULK2 | unc-51 like autophagy activating kinase 2 [Source:HGNC Symbol;Acc:HGNC:13480] |
| ENSG00000084636 | HGLibA_10517 | 77 | -0.382372077 | COL16A1 | collagen type XVI alpha 1 chain [Source:HGNC Symbol;Acc:HGNC:2193] |
| ENSG00000092094 | HGLibA_34682 | 77 | -0.382372077 | OSGEP | O-sialoglycoprotein endopeptidase [Source:HGNC Symbol;Acc:HGNC:18028] |
| ENSG00000092847 | HGLibA_01204 | 77 | -0.382372077 | AGO1 | RISC catalytic component [Source:HGNC Symbol;Acc:HGNC:3262]" |
| ENSG00000099860 | HGLibA_18418 | 77 | -0.382372077 | GADD45B | growth arrest and DNA damage inducible beta [Source:HGNC Symbol;Acc:HGNC:4096] |
| ENSG00000099974 | HGLibA_12645 | 77 | -0.382372077 | DDTL | D-dopachrome tautomerase like [Source:HGNC Symbol;Acc:HGNC:33446] |
| ENSG00000100109 | HGLibA_49182 | 77 | -0.382372077 | TFIP11 | tuftelin interacting protein 11 [Source:HGNC Symbol;Acc:HGNC:17165] |
| ENSG00000100241 | HGLibA_42793 | 77 | -0.382372077 | SBF1 | SET binding factor 1 [Source:HGNC Symbol;Acc:HGNC:10542] |
| ENSG00000100242 | HGLibA_47792 | 77 | -0.382372077 | SUN2 | Sad1 and UNC84 domain containing 2 [Source:HGNC Symbol;Acc:HGNC:14210] |
| ENSG00000100445 | HGLibA_43162 | 77 | -0.382372077 | SDR39U1 | short chain dehydrogenase/reductase family 39U member 1 [Source:HGNC Symbol;Acc:HGNC:20275] |
| ENSG00000100601 | HGLibA_01646 | 77 | -0.382372077 | ALKBH1 | histone H2A dioxygenase [Source:HGNC Symbol;Acc:HGNC:17911]" |
| ENSG00000100614 | HGLibA_37963 | 77 | -0.382372077 | PPM1A | Mg2+/Mn2+ dependent 1A [Source:HGNC Symbol;Acc:HGNC:9275]" |
| ENSG00000100811 | HGLibA_54756 | 77 | -0.382372077 | YY1 | YY1 transcription factor [Source:HGNC Symbol;Acc:HGNC:12856] |
| ENSG00000100949 | HGLibA_40019 | 77 | -0.382372077 | RABGGTA | Rab geranylgeranyltransferase subunit alpha [Source:HGNC Symbol;Acc:HGNC:9795] |
| ENSG00000101236 | HGLibA_41652 | 77 | -0.382372077 | RNF24 | ring finger protein 24 [Source:HGNC Symbol;Acc:HGNC:13779] |
| ENSG00000101417 | HGLibA_39660 | 77 | -0.382372077 | PXMP4 | peroxisomal membrane protein 4 [Source:HGNC Symbol;Acc:HGNC:15920] |
| ENSG00000102241 | HGLibA_22350 | 77 | -0.382372077 | HTATSF1 | HIV-1 Tat specific factor 1 [Source:HGNC Symbol;Acc:HGNC:5276] |
| ENSG00000105261 | HGLibA_34807 | 77 | -0.382372077 | OVOL3 | ovo like zinc finger 3 [Source:HGNC Symbol;Acc:HGNC:14186] |
| ENSG00000105509 | HGLibA_20862 | 77 | -0.382372077 | HAS1 | hyaluronan synthase 1 [Source:HGNC Symbol;Acc:HGNC:4818] |
| ENSG00000105699 | HGLibA_27565 | 77 | -0.382372077 | LSR | lipolysis stimulated lipoprotein receptor [Source:HGNC Symbol;Acc:HGNC:29572] |
| ENSG00000105929 | HGLibA_03708 | 77 | -0.382372077 | ATP6V0A4 | ATPase H+ transporting V0 subunit a4 [Source:HGNC Symbol;Acc:HGNC:866] |
| ENSG00000106331 | HGLibA_35305 | 77 | -0.382372077 | PAX4 | paired box 4 [Source:HGNC Symbol;Acc:HGNC:8618] |
| ENSG00000106484 | HGLibA_28921 | 77 | -0.382372077 | MEST | mesoderm specific transcript [Source:HGNC Symbol;Acc:HGNC:7028] |
| ENSG00000111445 | HGLibA_40914 | 77 | -0.382372077 | RFC5 | replication factor C subunit 5 [Source:HGNC Symbol;Acc:HGNC:9973] |
| ENSG00000111554 | HGLibA_28695 | 77 | -0.382372077 | MDM1 | Mdm1 nuclear protein [Source:HGNC Symbol;Acc:HGNC:29917] |
| ENSG00000112062 | HGLibA_28262 | 77 | -0.382372077 | MAPK14 | mitogen-activated protein kinase 14 [Source:HGNC Symbol;Acc:HGNC:6876] |
| ENSG00000112299 | HGLibA_53710 | 77 | -0.382372077 | VNN1 | vanin 1 [Source:HGNC Symbol;Acc:HGNC:12705] |
| ENSG00000113196 | HGLibA_20827 | 77 | -0.382372077 | HAND1 | heart and neural crest derivatives expressed 1 [Source:HGNC Symbol;Acc:HGNC:4807] |
| ENSG00000113492 | HGLibA_01274 | 77 | -0.382372077 | AGXT2 | alanine--glyoxylate aminotransferase 2 [Source:HGNC Symbol;Acc:HGNC:14412] |
| ENSG00000113569 | HGLibA_33028 | 77 | -0.382372077 | NUP155 | nucleoporin 155 [Source:HGNC Symbol;Acc:HGNC:8063] |
| ENSG00000114354 | HGLibA_49179 | 77 | -0.382372077 | TFG | TRK-fused gene [Source:HGNC Symbol;Acc:HGNC:11758] |
| ENSG00000114739 | HGLibA_00698 | 77 | -0.382372077 | ACVR2B | activin A receptor type 2B [Source:HGNC Symbol;Acc:HGNC:174] |
| ENSG00000115085 | HGLibA_54775 | 77 | -0.382372077 | ZAP70 | zeta chain of T cell receptor associated protein kinase 70 [Source:HGNC Symbol;Acc:HGNC:12858] |
| ENSG00000115677 | HGLibA_21061 | 77 | -0.382372077 | HDLBP | high density lipoprotein binding protein [Source:HGNC Symbol;Acc:HGNC:4857] |
| ENSG00000117601 | HGLibA_43570 | 77 | -0.382372077 | SERPINC1 | serpin family C member 1 [Source:HGNC Symbol;Acc:HGNC:775] |
| ENSG00000117650 | HGLibA_31646 | 77 | -0.382372077 | NEK2 | NIMA related kinase 2 [Source:HGNC Symbol;Acc:HGNC:7745] |
| ENSG00000119682 | HGLibA_02644 | 77 | -0.382372077 | AREL1 | apoptosis resistant E3 ubiquitin protein ligase 1 [Source:HGNC Symbol;Acc:HGNC:20363] |
| ENSG00000121578 | HGLibA_04031 | 77 | -0.382372077 | B4GALT4 | 4-galactosyltransferase 4 [Source:HGNC Symbol;Acc:HGNC:927]" |
| ENSG00000121594 | HGLibA_08452 | 77 | -0.382372077 | CD80 | CD80 molecule [Source:HGNC Symbol;Acc:HGNC:1700] |
| ENSG00000123119 | HGLibA_31585 | 77 | -0.382372077 | NECAB1 | N-terminal EF-hand calcium binding protein 1 [Source:HGNC Symbol;Acc:HGNC:20983] |
| ENSG00000123360 | HGLibA_35773 | 77 | -0.382372077 | PDE1B | phosphodiesterase 1B [Source:HGNC Symbol;Acc:HGNC:8775] |
| ENSG00000123576 | HGLibA_15602 | 77 | -0.382372077 | ESX1 | ESX homeobox 1 [Source:HGNC Symbol;Acc:HGNC:14865] |
| ENSG00000124157 | HGLibA_43382 | 77 | -0.382372077 | SEMG2 | semenogelin 2 [Source:HGNC Symbol;Acc:HGNC:10743] |
| ENSG00000124216 | HGLibA_45865 | 77 | -0.382372077 | SNAI1 | snail family transcriptional repressor 1 [Source:HGNC Symbol;Acc:HGNC:11128] |
| ENSG00000124570 | HGLibA_43558 | 77 | -0.382372077 | SERPINB6 | serpin family B member 6 [Source:HGNC Symbol;Acc:HGNC:8950] |
| ENSG00000125798 | HGLibA_17811 | 77 | -0.382372077 | FOXA2 | forkhead box A2 [Source:HGNC Symbol;Acc:HGNC:5022] |
| ENSG00000128218 | HGLibA_53724 | 77 | -0.382372077 | VPREB3 | V-set pre-B cell surrogate light chain 3 [Source:HGNC Symbol;Acc:HGNC:12710] |
| ENSG00000130713 | HGLibA_15776 | 77 | -0.382372077 | EXOSC2 | exosome component 2 [Source:HGNC Symbol;Acc:HGNC:17097] |
| ENSG00000132323 | HGLibA_23222 | 77 | -0.382372077 | ILKAP | ILK associated serine/threonine phosphatase [Source:HGNC Symbol;Acc:HGNC:15566] |
| ENSG00000132975 | HGLibA_19869 | 77 | -0.382372077 | GPR12 | G protein-coupled receptor 12 [Source:HGNC Symbol;Acc:HGNC:4466] |
| ENSG00000133401 | HGLibA_35972 | 77 | -0.382372077 | PDZD2 | PDZ domain containing 2 [Source:HGNC Symbol;Acc:HGNC:18486] |
| ENSG00000133636 | HGLibA_32882 | 77 | -0.382372077 | NTS | neurotensin [Source:HGNC Symbol;Acc:HGNC:8038] |
| ENSG00000135441 | HGLibA_04556 | 77 | -0.382372077 | BLOC1S1 | biogenesis of lysosomal organelles complex 1 subunit 1 [Source:HGNC Symbol;Acc:HGNC:4200] |
| ENSG00000135919 | HGLibA_43580 | 77 | -0.382372077 | SERPINE2 | serpin family E member 2 [Source:HGNC Symbol;Acc:HGNC:8951] |
| ENSG00000136280 | HGLibA_08045 | 77 | -0.382372077 | CCM2 | CCM2 scaffold protein [Source:HGNC Symbol;Acc:HGNC:21708] |
| ENSG00000137080 | HGLibA_22678 | 77 | -0.382372077 | IFNA21 | interferon alpha 21 [Source:HGNC Symbol;Acc:HGNC:5424] |
| ENSG00000137707 | HGLibA_04951 | 77 | -0.382372077 | BTG4 | BTG anti-proliferation factor 4 [Source:HGNC Symbol;Acc:HGNC:13862] |
| ENSG00000137713 | HGLibA_38143 | 77 | -0.382372077 | PPP2R1B | protein phosphatase 2 scaffold subunit Abeta [Source:HGNC Symbol;Acc:HGNC:9303] |
| ENSG00000138381 | HGLibA_03338 | 77 | -0.382372077 | ASNSD1 | asparagine synthetase domain containing 1 [Source:HGNC Symbol;Acc:HGNC:24910] |
| ENSG00000138688 | HGLibA_24693 | 77 | -0.382372077 | KIAA1109 | KIAA1109 [Source:HGNC Symbol;Acc:HGNC:26953] |
| ENSG00000138813 | HGLibA_06296 | 77 | -0.382372077 | C4orf17 | chromosome 4 open reading frame 17 [Source:HGNC Symbol;Acc:HGNC:25274] |
| ENSG00000139330 | HGLibA_24555 | 77 | -0.382372077 | KERA | keratocan [Source:HGNC Symbol;Acc:HGNC:6309] |
| ENSG00000140259 | HGLibA_29039 | 77 | -0.382372077 | MFAP1 | microfibril associated protein 1 [Source:HGNC Symbol;Acc:HGNC:7032] |
| ENSG00000140396 | HGLibA_31337 | 77 | -0.382372077 | NCOA2 | nuclear receptor coactivator 2 [Source:HGNC Symbol;Acc:HGNC:7669] |
| ENSG00000140987 | HGLibA_57022 | 77 | -0.382372077 | ZSCAN32 | zinc finger and SCAN domain containing 32 [Source:HGNC Symbol;Acc:HGNC:20812] |
| ENSG00000142405 | HGLibA_32106 | 77 | -0.382372077 | NLRP12 | NLR family pyrin domain containing 12 [Source:HGNC Symbol;Acc:HGNC:22938] |
| ENSG00000143195 | HGLibA_23212 | 77 | -0.382372077 | ILDR2 | immunoglobulin like domain containing receptor 2 [Source:HGNC Symbol;Acc:HGNC:18131] |
| ENSG00000143373 | HGLibA_56540 | 77 | -0.382372077 | ZNF687 | zinc finger protein 687 [Source:HGNC Symbol;Acc:HGNC:29277] |
| ENSG00000144214 | HGLibA_27698 | 77 | -0.382372077 | LYG1 | lysozyme g1 [Source:HGNC Symbol;Acc:HGNC:27014] |
| ENSG00000144476 | HGLibA_00447 | 77 | -0.382372077 | ACKR3 | atypical chemokine receptor 3 [Source:HGNC Symbol;Acc:HGNC:23692] |
| ENSG00000144645 | HGLibA_34643 | 77 | -0.382372077 | OSBPL10 | oxysterol binding protein like 10 [Source:HGNC Symbol;Acc:HGNC:16395] |
| ENSG00000144962 | HGLibA_46431 | 77 | -0.382372077 | SPATA16 | spermatogenesis associated 16 [Source:HGNC Symbol;Acc:HGNC:29935] |
| ENSG00000146021 | HGLibA_25178 | 77 | -0.382372077 | KLHL3 | kelch like family member 3 [Source:HGNC Symbol;Acc:HGNC:6354] |
| ENSG00000146648 | HGLibA_14636 | 77 | -0.382372077 | EGFR | epidermal growth factor receptor [Source:HGNC Symbol;Acc:HGNC:3236] |
| ENSG00000147036 | HGLibA_26011 | 77 | -0.382372077 | LANCL3 | LanC like 3 [Source:HGNC Symbol;Acc:HGNC:24767] |
| ENSG00000147041 | HGLibA_48109 | 77 | -0.382372077 | SYTL5 | synaptotagmin like 5 [Source:HGNC Symbol;Acc:HGNC:15589] |
| ENSG00000147592 | HGLibA_25919 | 77 | -0.382372077 | LACTB2 | lactamase beta 2 [Source:HGNC Symbol;Acc:HGNC:18512] |
| ENSG00000148498 | HGLibA_35177 | 77 | -0.382372077 | PARD3 | par-3 family cell polarity regulator [Source:HGNC Symbol;Acc:HGNC:16051] |
| ENSG00000149292 | HGLibA_52048 | 77 | -0.382372077 | TTC12 | tetratricopeptide repeat domain 12 [Source:HGNC Symbol;Acc:HGNC:23700] |
| ENSG00000150551 | HGLibA_27712 | 77 | -0.382372077 | LYPD1 | LY6/PLAUR domain containing 1 [Source:HGNC Symbol;Acc:HGNC:28431] |
| ENSG00000152127 | HGLibA_29151 | 77 | -0.382372077 | MGAT5 | 6-mannosylglycoprotein 6-beta-N-acetylglucosaminyltransferase [Source:HGNC Symbol;Acc:HGNC:7049]" |
| ENSG00000152527 | HGLibA_37129 | 77 | -0.382372077 | PLEKHH2 | MyTH4 and FERM domain containing H2 [Source:HGNC Symbol;Acc:HGNC:30506]" |
| ENSG00000154642 | HGLibA_06051 | 77 | -0.382372077 | C21orf91 | chromosome 21 open reading frame 91 [Source:HGNC Symbol;Acc:HGNC:16459] |
| ENSG00000155016 | HGLibA_12146 | 77 | -0.382372077 | CYP2U1 | cytochrome P450 family 2 subfamily U member 1 [Source:HGNC Symbol;Acc:HGNC:20582] |
| ENSG00000159128 | HGLibA_22713 | 77 | -0.382372077 | IFNGR2 | interferon gamma receptor 2 [Source:HGNC Symbol;Acc:HGNC:5440] |
| ENSG00000160633 | HGLibA_42636 | 77 | -0.382372077 | SAFB | scaffold attachment factor B [Source:HGNC Symbol;Acc:HGNC:10520] |
| ENSG00000161405 | HGLibA_22936 | 77 | -0.382372077 | IKZF3 | IKAROS family zinc finger 3 [Source:HGNC Symbol;Acc:HGNC:13178] |
| ENSG00000161642 | HGLibA_55897 | 77 | -0.382372077 | ZNF385A | zinc finger protein 385A [Source:HGNC Symbol;Acc:HGNC:17521] |
| ENSG00000162009 | HGLibA_47185 | 77 | -0.382372077 | SSTR5 | somatostatin receptor 5 [Source:HGNC Symbol;Acc:HGNC:11334] |
| ENSG00000162692 | HGLibA_53566 | 77 | -0.382372077 | VCAM1 | vascular cell adhesion molecule 1 [Source:HGNC Symbol;Acc:HGNC:12663] |
| ENSG00000162739 | HGLibA_44314 | 77 | -0.382372077 | SLAMF6 | SLAM family member 6 [Source:HGNC Symbol;Acc:HGNC:21392] |
| ENSG00000162745 | HGLibA_33362 | 77 | -0.382372077 | OLFML2B | olfactomedin like 2B [Source:HGNC Symbol;Acc:HGNC:24558] |
| ENSG00000162975 | HGLibA_24194 | 77 | -0.382372077 | KCNF1 | potassium voltage-gated channel modifier subfamily F member 1 [Source:HGNC Symbol;Acc:HGNC:6246] |
| ENSG00000163069 | HGLibA_43800 | 77 | -0.382372077 | SGCB | sarcoglycan beta [Source:HGNC Symbol;Acc:HGNC:10806] |
| ENSG00000163072 | HGLibA_32337 | 77 | -0.382372077 | NOSTRIN | nitric oxide synthase trafficking [Source:HGNC Symbol;Acc:HGNC:20203] |
| ENSG00000163884 | HGLibA_25058 | 77 | -0.382372077 | KLF15 | Kruppel like factor 15 [Source:HGNC Symbol;Acc:HGNC:14536] |
| ENSG00000163958 | HGLibA_55093 | 77 | -0.382372077 | ZDHHC19 | zinc finger DHHC-type containing 19 [Source:HGNC Symbol;Acc:HGNC:20713] |
| ENSG00000164039 | HGLibA_04378 | 77 | -0.382372077 | BDH2 | 3-hydroxybutyrate dehydrogenase 2 [Source:HGNC Symbol;Acc:HGNC:32389] |
| ENSG00000165474 | HGLibA_19160 | 77 | -0.382372077 | GJB2 | gap junction protein beta 2 [Source:HGNC Symbol;Acc:HGNC:4284] |
| ENSG00000165646 | HGLibA_44489 | 77 | -0.382372077 | SLC18A2 | solute carrier family 18 member A2 [Source:HGNC Symbol;Acc:HGNC:10935] |
| ENSG00000166351 | HGLibA_37726 | 77 | -0.382372077 | POTED | POTE ankyrin domain family member D [Source:HGNC Symbol;Acc:HGNC:23822] |
| ENSG00000167536 | HGLibA_13126 | 77 | -0.382372077 | DHRS13 | dehydrogenase/reductase 13 [Source:HGNC Symbol;Acc:HGNC:28326] |
| ENSG00000167562 | HGLibA_56578 | 77 | -0.382372077 | ZNF701 | zinc finger protein 701 [Source:HGNC Symbol;Acc:HGNC:25597] |
| ENSG00000167740 | HGLibA_11999 | 77 | -0.382372077 | CYB5D2 | cytochrome b5 domain containing 2 [Source:HGNC Symbol;Acc:HGNC:28471] |
| ENSG00000167755 | HGLibA_25263 | 77 | -0.382372077 | KLK6 | kallikrein related peptidase 6 [Source:HGNC Symbol;Acc:HGNC:6367] |
| ENSG00000168481 | HGLibA_26399 | 77 | -0.382372077 | LGI3 | leucine rich repeat LGI family member 3 [Source:HGNC Symbol;Acc:HGNC:18711] |
| ENSG00000168575 | HGLibA_44531 | 77 | -0.382372077 | SLC20A2 | solute carrier family 20 member 2 [Source:HGNC Symbol;Acc:HGNC:10947] |
| ENSG00000168826 | HGLibA_54906 | 77 | -0.382372077 | ZBTB49 | zinc finger and BTB domain containing 49 [Source:HGNC Symbol;Acc:HGNC:19883] |
| ENSG00000169299 | HGLibA_36263 | 77 | -0.382372077 | PGM2 | phosphoglucomutase 2 [Source:HGNC Symbol;Acc:HGNC:8906] |
| ENSG00000169548 | HGLibA_55722 | 77 | -0.382372077 | ZNF280A | zinc finger protein 280A [Source:HGNC Symbol;Acc:HGNC:18597] |
| ENSG00000169906 | HGLibA_42582 | 77 | -0.382372077 | S100G | S100 calcium binding protein G [Source:HGNC Symbol;Acc:HGNC:1436] |
| ENSG00000170044 | HGLibA_56969 | 77 | -0.382372077 | ZPLD1 | zona pellucida like domain containing 1 [Source:HGNC Symbol;Acc:HGNC:27022] |
| ENSG00000170049 | HGLibA_24153 | 77 | -0.382372077 | KCNAB3 | potassium voltage-gated channel subfamily A regulatory beta subunit 3 [Source:HGNC Symbol;Acc:HGNC:6230] |
| ENSG00000170092 | HGLibA_46598 | 77 | -0.382372077 | SPDYE5 | speedy/RINGO cell cycle regulator family member E5 [Source:HGNC Symbol;Acc:HGNC:35464] |
| ENSG00000170234 | HGLibA_39639 | 77 | -0.382372077 | PWWP2A | PWWP domain containing 2A [Source:HGNC Symbol;Acc:HGNC:29406] |
| ENSG00000170465 | HGLibA_25520 | 77 | -0.382372077 | KRT6C | keratin 6C [Source:HGNC Symbol;Acc:HGNC:20406] |
| ENSG00000170791 | HGLibA_09380 | 77 | -0.382372077 | CHCHD7 | coiled-coil-helix-coiled-coil-helix domain containing 7 [Source:HGNC Symbol;Acc:HGNC:28314] |
| ENSG00000170835 | HGLibA_08986 | 77 | -0.382372077 | CEL | carboxyl ester lipase [Source:HGNC Symbol;Acc:HGNC:1848] |
| ENSG00000171475 | HGLibA_54307 | 77 | -0.382372077 | WIPF2 | WAS/WASL interacting protein family member 2 [Source:HGNC Symbol;Acc:HGNC:30923] |
| ENSG00000173226 | HGLibA_23491 | 77 | -0.382372077 | IQCB1 | IQ motif containing B1 [Source:HGNC Symbol;Acc:HGNC:28949] |
| ENSG00000173908 | HGLibA_25468 | 77 | -0.382372077 | KRT28 | keratin 28 [Source:HGNC Symbol;Acc:HGNC:30842] |
| ENSG00000174586 | HGLibA_56103 | 77 | -0.382372077 | ZNF497 | zinc finger protein 497 [Source:HGNC Symbol;Acc:HGNC:23714] |
| ENSG00000174842 | HGLibA_19280 | 77 | -0.382372077 | GLMN | FKBP associated protein [Source:HGNC Symbol;Acc:HGNC:14373]" |
| ENSG00000174951 | HGLibA_18178 | 77 | -0.382372077 | FUT1 | fucosyltransferase 1 (H blood group) [Source:HGNC Symbol;Acc:HGNC:4012] |
| ENSG00000174992 | HGLibA_55297 | 77 | -0.382372077 | ZG16 | zymogen granule protein 16 [Source:HGNC Symbol;Acc:HGNC:30961] |
| ENSG00000175536 | HGLibA_26635 | 77 | -0.382372077 | LIPT2 | lipoyl(octanoyl) transferase 2 [Source:HGNC Symbol;Acc:HGNC:37216] |
| ENSG00000176101 | HGLibA_47145 | 77 | -0.382372077 | SSNA1 | SS nuclear autoantigen 1 [Source:HGNC Symbol;Acc:HGNC:11321] |
| ENSG00000177106 | HGLibA_15381 | 77 | -0.382372077 | EPS8L2 | EPS8 like 2 [Source:HGNC Symbol;Acc:HGNC:21296] |
| ENSG00000177303 | HGLibA_07292 | 77 | -0.382372077 | CASKIN2 | CASK interacting protein 2 [Source:HGNC Symbol;Acc:HGNC:18200] |
| ENSG00000177409 | HGLibA_42701 | 77 | -0.382372077 | SAMD9L | sterile alpha motif domain containing 9 like [Source:HGNC Symbol;Acc:HGNC:1349] |
| ENSG00000178372 | HGLibA_07038 | 77 | -0.382372077 | CALML5 | calmodulin like 5 [Source:HGNC Symbol;Acc:HGNC:18180] |

| | | | | | |
|---|---|---|---|---|---|
| ENSG00000178586 | HGLibA_34379 | 77 | -0.382372077 | OR6B3 | olfactory receptor family 6 subfamily B member 3 [Source:HGNC Symbol;Acc:HGNC:15042] |
| ENSG00000179934 | HGLibA_08169 | 77 | -0.382372077 | CCR8 | C-C motif chemokine receptor 8 [Source:HGNC Symbol;Acc:HGNC:1609] |
| ENSG00000183775 | HGLibA_24433 | 77 | -0.382372077 | KCTD16 | potassium channel tetramerization domain containing 16 [Source:HGNC Symbol;Acc:HGNC:29244] |
| ENSG00000184785 | HGLibA_45720 | 77 | -0.382372077 | SMIM10 | small integral membrane protein 10 [Source:HGNC Symbol;Acc:HGNC:41913] |
| ENSG00000185133 | HGLibA_23357 | 77 | -0.382372077 | INPP5J | inositol polyphosphate-5-phosphatase J [Source:HGNC Symbol;Acc:HGNC:8956] |
| ENSG00000186047 | HGLibA_13322 | 77 | -0.382372077 | DLEU7 | deleted in lymphocytic leukemia 7 [Source:HGNC Symbol;Acc:HGNC:17567] |
| ENSG00000187753 | HGLibA_06693 | 77 | -0.382372077 | C9orf153 | chromosome 9 open reading frame 153 [Source:HGNC Symbol;Acc:HGNC:31456] |
| ENSG00000188277 | HGLibA_05431 | 77 | -0.382372077 | C15orf62 | chromosome 15 open reading frame 62 [Source:HGNC Symbol;Acc:HGNC:34489] |
| ENSG00000188859 | HGLibA_16731 | 77 | -0.382372077 | FAM78B | family with sequence similarity 78 member B [Source:HGNC Symbol;Acc:HGNC:13495] |
| ENSG00000188992 | HGLibA_26617 | 77 | -0.382372077 | LIPI | lipase I [Source:HGNC Symbol;Acc:HGNC:18821] |
| ENSG00000189357 | HGLibA_46501 | 77 | -0.382372077 | SPATA31D4 | SPATA31 subfamily D member 4 [Source:HGNC Symbol;Acc:HGNC:38601] |
| ENSG00000196119 | HGLibA_34489 | 77 | -0.382372077 | OR8A1 | olfactory receptor family 8 subfamily A member 1 [Source:HGNC Symbol;Acc:HGNC:8469] |
| ENSG00000196154 | HGLibA_42557 | 77 | -0.382372077 | S100A4 | S100 calcium binding protein A4 [Source:HGNC Symbol;Acc:HGNC:10494] |
| ENSG00000196431 | HGLibA_11257 | 77 | -0.382372077 | CRYBA4 | crystallin beta A4 [Source:HGNC Symbol;Acc:HGNC:2396] |
| ENSG00000196565 | HGLibA_20929 | 77 | -0.382372077 | HBG2 | hemoglobin subunit gamma 2 [Source:HGNC Symbol;Acc:HGNC:4832] |
| ENSG00000196642 | HGLibA_40038 | 77 | -0.382372077 | RABL6 | member RAS oncogene family like 6 [Source:HGNC Symbol;Acc:HGNC:24703]" |
| ENSG00000197261 | HGLibA_06450 | 77 | -0.382372077 | C6orf141 | chromosome 6 open reading frame 141 [Source:HGNC Symbol;Acc:HGNC:21351] |
| ENSG00000197568 | HGLibA_21273 | 77 | -0.382372077 | HHLA3 | HERV-H LTR-associating 3 [Source:HGNC Symbol;Acc:HGNC:4906] |
| ENSG00000197599 | HGLibA_07654 | 77 | -0.382372077 | CCDC154 | coiled-coil domain containing 154 [Source:HGNC Symbol;Acc:HGNC:34454] |
| ENSG00000197724 | HGLibA_36361 | 77 | -0.382372077 | PHF2 | PHD finger protein 2 [Source:HGNC Symbol;Acc:HGNC:8920] |
| ENSG00000198756 | HGLibA_10644 | 77 | -0.382372077 | COLGALT2 | collagen beta(1-O)galactosyltransferase 2 [Source:HGNC Symbol;Acc:HGNC:16790] |
| ENSG00000204176 | HGLibA_48064 | 77 | -0.382372077 | SYT15 | synaptotagmin 15 [Source:HGNC Symbol;Acc:HGNC:17167] |
| ENSG00000213022 | HGLibA_25274 | 77 | -0.382372077 | KLK9 | kallikrein related peptidase 9 [Source:HGNC Symbol;Acc:HGNC:6370] |
| ENSG00000226023 | HGLibA_11539 | 77 | -0.382372077 | CT47A6 | member A6 [Source:HGNC Symbol;Acc:HGNC:33287]" |
| ENSG00000228836 | HGLibA_11510 | 77 | -0.382372077 | CT45A5 | cancer/testis antigen family 45 member A5 [Source:HGNC Symbol;Acc:HGNC:33270] |
| ENSG00000239590 | HGLibA_33681 | 77 | -0.382372077 | OR1J4 | olfactory receptor family 1 subfamily J member 4 [Source:HGNC Symbol;Acc:HGNC:8211] |
| ENSG00000244405 | HGLibA_15663 | 77 | -0.382372077 | ETV5 | ETS variant 5 [Source:HGNC Symbol;Acc:HGNC:3494] |
| ENSG00000249437 | HGLibA_31082 | 77 | -0.382372077 | NAIP | NLR family apoptosis inhibitory protein [Source:HGNC Symbol;Acc:HGNC:7634] |
| ENSG00000250120 | HGLibA_35426 | 77 | -0.382372077 | PCDHA10 | protocadherin alpha 10 [Source:HGNC Symbol;Acc:HGNC:8664] |
| ENSG00000261272 | HGLibA_30544 | 77 | -0.382372077 | MUC22 | mucin 22 [Source:HGNC Symbol;Acc:HGNC:39755] |
| ENSG00000268964 | HGLibA_15548 | 77 | -0.382372077 | ERVV-2 | envelope [Source:HGNC Symbol;Acc:HGNC:39051]" |
| ENSG00000284680 | HGLibA_34497 | 77 | -0.382372077 | OR8B2 | olfactory receptor family 8 subfamily B member 2 [Source:HGNC Symbol;Acc:HGNC:8471] |
| ENSG00000284701 | HGLibA_50306 | 77 | -0.382372077 | TMEM247 | transmembrane protein 247 [Source:HGNC Symbol;Acc:HGNC:42967] |
| FAM175A | HGLibA_16288 | 77 | -0.382372077 | FAM175A | - |
| FAM26D | HGLibA_16562 | 77 | -0.382372077 | FAM26D | - |
| H2BFWT | HGLibA_20776 | 77 | -0.382372077 | H2BFWT | - |
| HIST1H2AL | HGLibA_21406 | 77 | -0.382372077 | HIST1H2AL | - |
| HIST1H3H | HGLibA_21477 | 77 | -0.382372077 | HIST1H3H | - |
| hsa-mir-1290 | HGLibA_57638 | 77 | -0.382372077 | hsa-mir-1290 | - |
| hsa-mir-1296 | HGLibA_57665 | 77 | -0.382372077 | hsa-mir-1296 | - |
| hsa-mir-1302-6 | HGLibA_57713 | 77 | -0.382372077 | hsa-mir-1302-6 | - |
| hsa-mir-216a | HGLibA_58258 | 77 | -0.382372077 | hsa-mir-216a | - |
| hsa-mir-3135a | HGLibA_58595 | 77 | -0.382372077 | hsa-mir-3135a | - |
| hsa-mir-3141 | HGLibA_58623 | 77 | -0.382372077 | hsa-mir-3141 | - |
| hsa-mir-33b | HGLibA_59017 | 77 | -0.382372077 | hsa-mir-33b | - |
| hsa-mir-3612 | HGLibA_59078 | 77 | -0.382372077 | hsa-mir-3612 | - |
| hsa-mir-4264 | HGLibA_59764 | 77 | -0.382372077 | hsa-mir-4264 | - |
| hsa-mir-4278 | HGLibA_59817 | 77 | -0.382372077 | hsa-mir-4278 | - |
| hsa-mir-4298 | HGLibA_59904 | 77 | -0.382372077 | hsa-mir-4298 | - |
| hsa-mir-4690 | HGLibA_60871 | 77 | -0.382372077 | hsa-mir-4690 | - |
| hsa-mir-6088 | HGLibA_62612 | 77 | -0.382372077 | hsa-mir-6088 | - |
| hsa-mir-6089-1 | HGLibA_62617 | 77 | -0.382372077 | hsa-mir-6089-1 | - |
| hsa-mir-6089-2 | HGLibA_62621 | 77 | -0.382372077 | hsa-mir-6089-2 | - |
| hsa-mir-6516 | HGLibA_62938 | 77 | -0.382372077 | hsa-mir-6516 | - |
| hsa-mir-6831 | HGLibA_63500 | 77 | -0.382372077 | hsa-mir-6831 | - |
| hsa-mir-7162 | HGLibA_63875 | 77 | -0.382372077 | hsa-mir-7162 | - |
| hsa-mir-7973-1 | HGLibA_64018 | 77 | -0.382372077 | hsa-mir-7973-1 | - |
| hsa-mir-8067 | HGLibA_64105 | 77 | -0.382372077 | hsa-mir-8067 | - |
| hsa-mir-8080 | HGLibA_64163 | 77 | -0.382372077 | hsa-mir-8080 | - |
| hsa-mir-8081 | HGLibA_64168 | 77 | -0.382372077 | hsa-mir-8081 | - |
| LOC100129636 | HGLibA_26767 | 77 | -0.382372077 | LOC100129636 | - |
| PPP2R3B_Y | HGLibA_38162 | 77 | -0.382372077 | PPP2R3B_Y | - |
| SDPR | HGLibA_43156 | 77 | -0.382372077 | SDPR | - |
| SELK | HGLibA_43292 | 77 | -0.382372077 | SELK | - |
| SELM | HGLibA_43297 | 77 | -0.382372077 | SELM | - |
| TMEM194B | HGLibA_50157 | 77 | -0.382372077 | TMEM194B | - |
| APOA1BP | HGLibA_02451 | 76 | -0.392323114 | APOA1BP | - |
| C11orf83 | HGLibA_05192 | 76 | -0.392323114 | C11orf83 | - |
| C17orf112 | HGLibA_05530 | 76 | -0.392323114 | C17orf112 | - |
| C19orf35 | HGLibA_05652 | 76 | -0.392323114 | C19orf35 | - |
| C5orf48 | HGLibA_06396 | 76 | -0.392323114 | C5orf48 | - |
| C9orf139 | HGLibA_06682 | 76 | -0.392323114 | C9orf139 | - |
| ENSG00000005844 | HGLibA_23758 | 76 | -0.392323114 | ITGAL | integrin subunit alpha L [Source:HGNC Symbol;Acc:HGNC:6148] |
| ENSG00000008517 | HGLibA_23135 | 76 | -0.392323114 | IL32 | interleukin 32 [Source:HGNC Symbol;Acc:HGNC:16830] |
| ENSG00000013275 | HGLibA_39138 | 76 | -0.392323114 | PSMC4 | ATPase 4 [Source:HGNC Symbol;Acc:HGNC:9551]" |
| ENSG00000015676 | HGLibA_32929 | 76 | -0.392323114 | NUDCD3 | NudC domain containing 3 [Source:HGNC Symbol;Acc:HGNC:22208] |
| ENSG00000016864 | HGLibA_19337 | 76 | -0.392323114 | GLT8D1 | glycosyltransferase 8 domain containing 1 [Source:HGNC Symbol;Acc:HGNC:24870] |
| ENSG00000040608 | HGLibA_42414 | 76 | -0.392323114 | RTN4R | reticulon 4 receptor [Source:HGNC Symbol;Acc:HGNC:18601] |
| ENSG00000044090 | HGLibA_11791 | 76 | -0.392323114 | CUL7 | cullin 7 [Source:HGNC Symbol;Acc:HGNC:21024] |
| ENSG00000048028 | HGLibA_53320 | 76 | -0.392323114 | USP28 | ubiquitin specific peptidase 28 [Source:HGNC Symbol;Acc:HGNC:12625] |
| ENSG00000049283 | HGLibA_15347 | 76 | -0.392323114 | EPN3 | epsin 3 [Source:HGNC Symbol;Acc:HGNC:18235] |
| ENSG00000054611 | HGLibA_48502 | 76 | -0.392323114 | TBC1D22A | TBC1 domain family member 22A [Source:HGNC Symbol;Acc:HGNC:1309] |
| ENSG00000056277 | HGLibA_55730 | 76 | -0.392323114 | ZNF280C | zinc finger protein 280C [Source:HGNC Symbol;Acc:HGNC:25955] |
| ENSG00000064886 | HGLibA_09439 | 76 | -0.392323114 | CHI3L2 | chitinase 3 like 2 [Source:HGNC Symbol;Acc:HGNC:1933] |
| ENSG00000069424 | HGLibA_24150 | 76 | -0.392323114 | KCNAB2 | potassium voltage-gated channel subfamily A regulatory beta subunit 2 [Source:HGNC Symbol;Acc:HGNC:6229] |
| ENSG00000070759 | HGLibA_49031 | 76 | -0.392323114 | TESK2 | testis associated actin remodelling kinase 2 [Source:HGNC Symbol;Acc:HGNC:11732] |
| ENSG00000071282 | HGLibA_26681 | 76 | -0.392323114 | LMCD1 | LIM and cysteine rich domains 1 [Source:HGNC Symbol;Acc:HGNC:6633] |
| ENSG00000074211 | HGLibA_38152 | 76 | -0.392323114 | PPP2R2C | protein phosphatase 2 regulatory subunit Bgamma [Source:HGNC Symbol;Acc:HGNC:9306] |
| ENSG00000078579 | HGLibA_17394 | 76 | -0.392323114 | FGF20 | fibroblast growth factor 20 [Source:HGNC Symbol;Acc:HGNC:3677] |
| ENSG00000079482 | HGLibA_33417 | 76 | -0.392323114 | OPHN1 | oligophrenin 1 [Source:HGNC Symbol;Acc:HGNC:8148] |
| ENSG00000080293 | HGLibA_43067 | 76 | -0.392323114 | SCTR | secretin receptor [Source:HGNC Symbol;Acc:HGNC:10608] |
| ENSG00000084733 | HGLibA_39785 | 76 | -0.392323114 | RAB10 | member RAS oncogene family [Source:HGNC Symbol;Acc:HGNC:9759]" |
| ENSG00000100147 | HGLibA_07599 | 76 | -0.392323114 | CCDC134 | coiled-coil domain containing 134 [Source:HGNC Symbol;Acc:HGNC:26185] |
| ENSG00000100722 | HGLibA_54965 | 76 | -0.392323114 | ZC3H14 | zinc finger CCCH-type containing 14 [Source:HGNC Symbol;Acc:HGNC:20509] |
| ENSG00000101017 | HGLibA_08385 | 76 | -0.392323114 | CD40 | CD40 molecule [Source:HGNC Symbol;Acc:HGNC:11919] |
| ENSG00000101187 | HGLibA_45468 | 76 | -0.392323114 | SLCO4A1 | solute carrier organic anion transporter family member 4A1 [Source:HGNC Symbol;Acc:HGNC:10953] |
| ENSG00000101251 | HGLibA_43281 | 76 | -0.392323114 | SEL1L2 | ERAD E3 ligase adaptor subunit [Source:HGNC Symbol;Acc:HGNC:15897]" |
| ENSG00000102034 | HGLibA_14900 | 76 | -0.392323114 | ELF4 | E74 like ETS transcription factor 4 [Source:HGNC Symbol;Acc:HGNC:3319] |
| ENSG00000104044 | HGLibA_33240 | 76 | -0.392323114 | OCA2 | OCA2 melanosomal transmembrane protein [Source:HGNC Symbol;Acc:HGNC:8101] |
| ENSG00000104435 | HGLibA_47518 | 76 | -0.392323114 | STMN2 | stathmin 2 [Source:HGNC Symbol;Acc:HGNC:10577] |
| ENSG00000105879 | HGLibA_07402 | 76 | -0.392323114 | CBLL1 | Cbl proto-oncogene like 1 [Source:HGNC Symbol;Acc:HGNC:21225] |
| ENSG00000106113 | HGLibA_11143 | 76 | -0.392323114 | CRHR2 | corticotropin releasing hormone receptor 2 [Source:HGNC Symbol;Acc:HGNC:2358] |
| ENSG00000106648 | HGLibA_18579 | 76 | -0.392323114 | GALNTL5 | polypeptide N-acetylgalactosaminyltransferase like 5 [Source:HGNC Symbol;Acc:HGNC:21725] |
| ENSG00000107447 | HGLibA_13715 | 76 | -0.392323114 | DNTT | DNA nucleotidylexotransferase [Source:HGNC Symbol;Acc:HGNC:2983] |
| ENSG00000108375 | HGLibA_41684 | 76 | -0.392323114 | RNF43 | ring finger protein 43 [Source:HGNC Symbol;Acc:HGNC:18505] |
| ENSG00000108561 | HGLibA_05919 | 76 | -0.392323114 | C1QBP | complement C1q binding protein [Source:HGNC Symbol;Acc:HGNC:1243] |
| ENSG00000109771 | HGLibA_27216 | 76 | -0.392323114 | LRP2BP | LRP2 binding protein [Source:HGNC Symbol;Acc:HGNC:25434] |
| ENSG00000110076 | HGLibA_32735 | 76 | -0.392323114 | NRXN2 | neurexin 2 [Source:HGNC Symbol;Acc:HGNC:8009] |
| ENSG00000110274 | HGLibA_09113 | 76 | -0.392323114 | CEP164 | centrosomal protein 164 [Source:HGNC Symbol;Acc:HGNC:29182] |
| ENSG00000112337 | HGLibA_44462 | 76 | -0.392323114 | SLC17A2 | solute carrier family 17 member 2 [Source:HGNC Symbol;Acc:HGNC:10930] |
| ENSG00000113013 | HGLibA_22299 | 76 | -0.392323114 | HSPA9 | heat shock protein family A (Hsp70) member 9 [Source:HGNC Symbol;Acc:HGNC:5244] |
| ENSG00000113327 | HGLibA_18393 | 76 | -0.392323114 | GABRG2 | gamma-aminobutyric acid type A receptor gamma2 subunit [Source:HGNC Symbol;Acc:HGNC:4087] |
| ENSG00000113722 | HGLibA_08895 | 76 | -0.392323114 | CDX1 | caudal type homeobox 1 [Source:HGNC Symbol;Acc:HGNC:1805] |
| ENSG00000114767 | HGLibA_42288 | 76 | -0.392323114 | RRP9 | U3 small nucleolar RNA binding protein [Source:HGNC Symbol;Acc:HGNC:16829]" |
| ENSG00000115523 | HGLibA_19556 | 76 | -0.392323114 | GNLY | granulysin [Source:HGNC Symbol;Acc:HGNC:4414] |
| ENSG00000115590 | HGLibA_23048 | 76 | -0.392323114 | IL1R2 | interleukin 1 receptor type 2 [Source:HGNC Symbol;Acc:HGNC:5994] |
| ENSG00000115677 | HGLibA_21059 | 76 | -0.392323114 | HDLBP | high density lipoprotein binding protein [Source:HGNC Symbol;Acc:HGNC:4857] |
| ENSG00000116095 | HGLibA_37068 | 76 | -0.392323114 | PLEKHA3 | pleckstrin homology domain containing A3 [Source:HGNC Symbol;Acc:HGNC:14338] |
| ENSG00000118689 | HGLibA_17924 | 76 | -0.392323114 | FOXO3 | forkhead box O3 [Source:HGNC Symbol;Acc:HGNC:3821] |
| ENSG00000119720 | HGLibA_32668 | 76 | -0.392323114 | NRDE2 | domain containing [Source:HGNC Symbol;Acc:HGNC:20186]" |
| ENSG00000122711 | HGLibA_46694 | 76 | -0.392323114 | SPINK4 | Kazal type 4 [Source:HGNC Symbol;Acc:HGNC:16646]" |
| ENSG00000122779 | HGLibA_51437 | 76 | -0.392323114 | TRIM24 | tripartite motif containing 24 [Source:HGNC Symbol;Acc:HGNC:11812] |
| ENSG00000122873 | HGLibA_09727 | 76 | -0.392323114 | CISD1 | CDGSH iron sulfur domain 1 [Source:HGNC Symbol;Acc:HGNC:30880] |
| ENSG00000123143 | HGLibA_36837 | 76 | -0.392323114 | PKN1 | protein kinase N1 [Source:HGNC Symbol;Acc:HGNC:9405] |
| ENSG00000123453 | HGLibA_42744 | 76 | -0.392323114 | SARDH | sarcosine dehydrogenase [Source:HGNC Symbol;Acc:HGNC:10536] |
| ENSG00000124126 | HGLibA_38443 | 76 | -0.392323114 | PREX1 | 5-trisphosphate dependent Rac exchange factor 1 [Source:HGNC Symbol;Acc:HGNC:32594]" |
| ENSG00000124523 | HGLibA_44228 | 76 | -0.392323114 | SIRT5 | sirtuin 5 [Source:HGNC Symbol;Acc:HGNC:14933] |
| ENSG00000125772 | HGLibA_19779 | 76 | -0.392323114 | GPCPD1 | glycerophosphocholine phosphodiesterase 1 [Source:HGNC Symbol;Acc:HGNC:26957] |
| ENSG00000125895 | HGLibA_50513 | 76 | -0.392323114 | TMEM74B | transmembrane protein 74B [Source:HGNC Symbol;Acc:HGNC:15893] |
| ENSG00000126561 | HGLibA_47394 | 76 | -0.392323114 | STAT5A | signal transducer and activator of transcription 5A [Source:HGNC Symbol;Acc:HGNC:11366] |
| ENSG00000128272 | HGLibA_03439 | 76 | -0.392323114 | ATF4 | activating transcription factor 4 [Source:HGNC Symbol;Acc:HGNC:786] |
| ENSG00000128313 | HGLibA_02538 | 76 | -0.392323114 | APOL5 | apolipoprotein L5 [Source:HGNC Symbol;Acc:HGNC:14869] |
| ENSG00000130414 | HGLibA_31441 | 76 | -0.392323114 | NDUFA10 | NADH:ubiquinone oxidoreductase subunit A10 [Source:HGNC Symbol;Acc:HGNC:7684] |
| ENSG00000130638 | HGLibA_03831 | 76 | -0.392323114 | ATXN10 | ataxin 10 [Source:HGNC Symbol;Acc:HGNC:10549] |
| ENSG00000130748 | HGLibA_50020 | 76 | -0.392323114 | TMEM160 | transmembrane protein 160 [Source:HGNC Symbol;Acc:HGNC:26042] |

| | | | | | |
|---|---|---|---|---|---|
| ENSG00000130816 | HGLibA_13698 | 76 | -0.392323114 | DNMT1 | DNA methyltransferase 1 [Source:HGNC Symbol;Acc:HGNC:2976] |
| ENSG00000130939 | HGLibA_52693 | 76 | -0.392323114 | UBE4B | ubiquitination factor E4B [Source:HGNC Symbol;Acc:HGNC:12500] |
| ENSG00000131019 | HGLibA_52966 | 76 | -0.392323114 | ULBP3 | UL16 binding protein 3 [Source:HGNC Symbol;Acc:HGNC:14895] |
| ENSG00000131370 | HGLibA_43935 | 76 | -0.392323114 | SH3BP5 | SH3 domain binding protein 5 [Source:HGNC Symbol;Acc:HGNC:10827] |
| ENSG00000131771 | HGLibA_38069 | 76 | -0.392323114 | PPP1R1B | protein phosphatase 1 regulatory inhibitor subunit 1B [Source:HGNC Symbol;Acc:HGNC:9287] |
| ENSG00000133027 | HGLibA_36045 | 76 | -0.392323114 | PEMT | phosphatidylethanolamine N-methyltransferase [Source:HGNC Symbol;Acc:HGNC:8830] |
| ENSG00000134028 | HGLibA_00788 | 76 | -0.392323114 | ADAMDEC1 | ADAM like decysin 1 [Source:HGNC Symbol;Acc:HGNC:16299] |
| ENSG00000134200 | HGLibA_51855 | 76 | -0.392323114 | TSHB | thyroid stimulating hormone beta [Source:HGNC Symbol;Acc:HGNC:12372] |
| ENSG00000134202 | HGLibA_20478 | 76 | -0.392323114 | GSTM3 | glutathione S-transferase mu 3 [Source:HGNC Symbol;Acc:HGNC:4635] |
| ENSG00000134873 | HGLibA_09850 | 76 | -0.392323114 | CLDN10 | claudin 10 [Source:HGNC Symbol;Acc:HGNC:2033] |
| ENSG00000135441 | HGLibA_04558 | 76 | -0.392323114 | BLOC1S1 | biogenesis of lysosomal organelles complex 1 subunit 1 [Source:HGNC Symbol;Acc:HGNC:4200] |
| ENSG00000135482 | HGLibA_54946 | 76 | -0.392323114 | ZC3H10 | zinc finger CCCH-type containing 10 [Source:HGNC Symbol;Acc:HGNC:25893] |
| ENSG00000136044 | HGLibA_02569 | 76 | -0.392323114 | APPL2 | phosphotyrosine interacting with PH domain and leucine zipper 2 [Source:HGNC Symbol;Acc:HGNC:18242]" |
| ENSG00000137073 | HGLibA_52551 | 76 | -0.392323114 | UBAP2 | ubiquitin associated protein 2 [Source:HGNC Symbol;Acc:HGNC:14185] |
| ENSG00000138622 | HGLibA_20983 | 76 | -0.392323114 | HCN4 | hyperpolarization activated cyclic nucleotide gated potassium channel 4 [Source:HGNC Symbol;Acc:HGNC:16882] |
| ENSG00000139445 | HGLibA_17917 | 76 | -0.392323114 | FOXN4 | forkhead box N4 [Source:HGNC Symbol;Acc:HGNC:21399] |
| ENSG00000139618 | HGLibA_04764 | 76 | -0.392323114 | BRCA2 | DNA repair associated [Source:HGNC Symbol;Acc:HGNC:1101]" |
| ENSG00000141540 | HGLibA_52234 | 76 | -0.392323114 | TTYH2 | tweety family member 2 [Source:HGNC Symbol;Acc:HGNC:13877] |
| ENSG00000141646 | HGLibA_45578 | 76 | -0.392323114 | SMAD4 | SMAD family member 4 [Source:HGNC Symbol;Acc:HGNC:6770] |
| ENSG00000141959 | HGLibA_36165 | 76 | -0.392323114 | PFKL | liver type [Source:HGNC Symbol;Acc:HGNC:8876]" |
| ENSG00000142002 | HGLibA_13885 | 76 | -0.392323114 | DPP9 | dipeptidyl peptidase 9 [Source:HGNC Symbol;Acc:HGNC:18648] |
| ENSG00000143318 | HGLibA_07335 | 76 | -0.392323114 | CASQ1 | calsequestrin 1 [Source:HGNC Symbol;Acc:HGNC:1512] |
| ENSG00000143340 | HGLibA_16229 | 76 | -0.392323114 | FAM163A | family with sequence similarity 163 member A [Source:HGNC Symbol;Acc:HGNC:28274] |
| ENSG00000143977 | HGLibA_45992 | 76 | -0.392323114 | SNRPG | small nuclear ribonucleoprotein polypeptide G [Source:HGNC Symbol;Acc:HGNC:11163] |
| ENSG00000144644 | HGLibA_18427 | 76 | -0.392323114 | GADL1 | glutamate decarboxylase like 1 [Source:HGNC Symbol;Acc:HGNC:27949] |
| ENSG00000144736 | HGLibA_44083 | 76 | -0.392323114 | SHQ1 | H/ACA ribonucleoprotein assembly factor [Source:HGNC Symbol;Acc:HGNC:25543]" |
| ENSG00000144895 | HGLibA_14720 | 76 | -0.392323114 | EIF2A | eukaryotic translation initiation factor 2A [Source:HGNC Symbol;Acc:HGNC:3254] |
| ENSG00000145246 | HGLibA_03550 | 76 | -0.392323114 | ATP10D | ATPase phospholipid transporting 10D (putative) [Source:HGNC Symbol;Acc:HGNC:13549] |
| ENSG00000145879 | HGLibA_46704 | 76 | -0.392323114 | SPINK7 | Kazal type 7 (putative) [Source:HGNC Symbol;Acc:HGNC:24643]" |
| ENSG00000146143 | HGLibA_38486 | 76 | -0.392323114 | PRIM2 | DNA primase subunit 2 [Source:HGNC Symbol;Acc:HGNC:9370] |
| ENSG00000146197 | HGLibA_43077 | 76 | -0.392323114 | SCUBE3 | CUB domain and EGF like domain containing 3 [Source:HGNC Symbol;Acc:HGNC:13655]" |
| ENSG00000146425 | HGLibA_14266 | 76 | -0.392323114 | DYNLT1 | dynein light chain Tctex-type 1 [Source:HGNC Symbol;Acc:HGNC:11697] |
| ENSG00000147459 | HGLibA_13749 | 76 | -0.392323114 | DOCK5 | dedicator of cytokinesis 5 [Source:HGNC Symbol;Acc:HGNC:23476] |
| ENSG00000148488 | HGLibA_47302 | 76 | -0.392323114 | ST8SIA6 | 8-sialyltransferase 6 [Source:HGNC Symbol;Acc:HGNC:23317]" |
| ENSG00000148848 | HGLibA_00734 | 76 | -0.392323114 | ADAM12 | ADAM metallopeptidase domain 12 [Source:HGNC Symbol;Acc:HGNC:190] |
| ENSG00000149150 | HGLibA_45148 | 76 | -0.392323114 | SLC43A1 | solute carrier family 43 member 1 [Source:HGNC Symbol;Acc:HGNC:9225] |
| ENSG00000149506 | HGLibA_56950 | 76 | -0.392323114 | ZP1 | zona pellucida glycoprotein 1 [Source:HGNC Symbol;Acc:HGNC:13187] |
| ENSG00000149591 | HGLibA_48271 | 76 | -0.392323114 | TAGLN | transgelin [Source:HGNC Symbol;Acc:HGNC:11553] |
| ENSG00000151503 | HGLibA_31267 | 76 | -0.392323114 | NCAPD3 | non-SMC condensin II complex subunit D3 [Source:HGNC Symbol;Acc:HGNC:28952] |
| ENSG00000151665 | HGLibA_36553 | 76 | -0.392323114 | PIGF | phosphatidylinositol glycan anchor biosynthesis class F [Source:HGNC Symbol;Acc:HGNC:8962] |
| ENSG00000151693 | HGLibA_03197 | 76 | -0.392323114 | ASAP2 | ankyrin repeat and PH domain 2 [Source:HGNC Symbol;Acc:HGNC:2721]" |
| ENSG00000151882 | HGLibA_08014 | 76 | -0.392323114 | CCL28 | C-C motif chemokine ligand 28 [Source:HGNC Symbol;Acc:HGNC:17700] |
| ENSG00000155659 | HGLibA_53848 | 76 | -0.392323114 | VSIG4 | V-set and immunoglobulin domain containing 4 [Source:HGNC Symbol;Acc:HGNC:17032] |
| ENSG00000158869 | HGLibA_17171 | 76 | -0.392323114 | FCER1G | Fc fragment of IgE receptor Ig [Source:HGNC Symbol;Acc:HGNC:3611] |
| ENSG00000159189 | HGLibA_05920 | 76 | -0.392323114 | C1QC | complement C1q C chain [Source:HGNC Symbol;Acc:HGNC:1245] |
| ENSG00000160339 | HGLibA_17218 | 76 | -0.392323114 | FCN2 | ficolin 2 [Source:HGNC Symbol;Acc:HGNC:3624] |
| ENSG00000160753 | HGLibA_42468 | 76 | -0.392323114 | RUSC1 | RUN and SH3 domain containing 1 [Source:HGNC Symbol;Acc:HGNC:17153] |
| ENSG00000162494 | HGLibA_27338 | 76 | -0.392323114 | LRRC38 | leucine rich repeat containing 38 [Source:HGNC Symbol;Acc:HGNC:27005] |
| ENSG00000162782 | HGLibA_48909 | 76 | -0.392323114 | TDRD5 | tudor domain containing 5 [Source:HGNC Symbol;Acc:HGNC:20614] |
| ENSG00000163012 | HGLibA_57039 | 76 | -0.392323114 | ZSWIM2 | zinc finger SWIM-type containing 2 [Source:HGNC Symbol;Acc:HGNC:30990] |
| ENSG00000163518 | HGLibA_17233 | 76 | -0.392323114 | FCRL4 | Fc receptor like 4 [Source:HGNC Symbol;Acc:HGNC:18507] |
| ENSG00000164010 | HGLibA_15500 | 76 | -0.392323114 | ERMAP | erythroblast membrane associated protein (Scianna blood group) [Source:HGNC Symbol;Acc:HGNC:15743] |
| ENSG00000164185 | HGLibA_56062 | 76 | -0.392323114 | ZNF474 | zinc finger protein 474 [Source:HGNC Symbol;Acc:HGNC:23245] |
| ENSG00000164509 | HGLibA_23132 | 76 | -0.392323114 | IL31RA | interleukin 31 receptor A [Source:HGNC Symbol;Acc:HGNC:18969] |
| ENSG00000164879 | HGLibA_06806 | 76 | -0.392323114 | CA3 | carbonic anhydrase 3 [Source:HGNC Symbol;Acc:HGNC:1374] |
| ENSG00000165669 | HGLibA_16419 | 76 | -0.392323114 | FAM204A | family with sequence similarity 204 member A [Source:HGNC Symbol;Acc:HGNC:25794] |
| ENSG00000166407 | HGLibA_26702 | 76 | -0.392323114 | LMO1 | LIM domain only 1 [Source:HGNC Symbol;Acc:HGNC:6641] |
| ENSG00000166477 | HGLibA_26306 | 76 | -0.392323114 | LEO1 | Paf1/RNA polymerase II complex component [Source:HGNC Symbol;Acc:HGNC:30401]" |
| ENSG00000167216 | HGLibA_24078 | 76 | -0.392323114 | KATNAL2 | katanin catalytic subunit A1 like 2 [Source:HGNC Symbol;Acc:HGNC:25387] |
| ENSG00000167733 | HGLibA_22163 | 76 | -0.392323114 | HSD11B1L | hydroxysteroid 11-beta dehydrogenase 1 like [Source:HGNC Symbol;Acc:HGNC:30419] |
| ENSG00000168930 | HGLibA_51523 | 76 | -0.392323114 | TRIM49 | tripartite motif containing 49 [Source:HGNC Symbol;Acc:HGNC:13431] |
| ENSG00000169258 | HGLibA_20140 | 76 | -0.392323114 | GPRIN1 | G protein regulated inducer of neurite outgrowth 1 [Source:HGNC Symbol;Acc:HGNC:24835] |
| ENSG00000169599 | HGLibA_31844 | 76 | -0.392323114 | NFU1 | NFU1 iron-sulfur cluster scaffold [Source:HGNC Symbol;Acc:HGNC:16287] |
| ENSG00000170310 | HGLibA_47661 | 76 | -0.392323114 | STX8 | syntaxin 8 [Source:HGNC Symbol;Acc:HGNC:11443] |
| ENSG00000171302 | HGLibA_07128 | 76 | -0.392323114 | CANT1 | calcium activated nucleotidase 1 [Source:HGNC Symbol;Acc:HGNC:19721] |
| ENSG00000171659 | HGLibA_20025 | 76 | -0.392323114 | GPR34 | G protein-coupled receptor 34 [Source:HGNC Symbol;Acc:HGNC:4490] |
| ENSG00000172154 | HGLibA_34535 | 76 | -0.392323114 | OR8I2 | olfactory receptor family 8 subfamily I member 2 [Source:HGNC Symbol;Acc:HGNC:15310] |
| ENSG00000173237 | HGLibA_05202 | 76 | -0.392323114 | C11orf86 | chromosome 11 open reading frame 86 [Source:HGNC Symbol;Acc:HGNC:34442] |
| ENSG00000173930 | HGLibA_45471 | 76 | -0.392323114 | SLCO4C1 | solute carrier organic anion transporter family member 4C1 [Source:HGNC Symbol;Acc:HGNC:23612] |
| ENSG00000174004 | HGLibA_32721 | 76 | -0.392323114 | NRROS | negative regulator of reactive oxygen species [Source:HGNC Symbol;Acc:HGNC:24613] |
| ENSG00000175213 | HGLibA_55929 | 76 | -0.392323114 | ZNF408 | zinc finger protein 408 [Source:HGNC Symbol;Acc:HGNC:20041] |
| ENSG00000175455 | HGLibA_07614 | 76 | -0.392323114 | CCDC14 | coiled-coil domain containing 14 [Source:HGNC Symbol;Acc:HGNC:25766] |
| ENSG00000175536 | HGLibA_26637 | 76 | -0.392323114 | LIPT2 | lipoyl(octanoyl) transferase 2 [Source:HGNC Symbol;Acc:HGNC:37216] |
| ENSG00000176177 | HGLibA_15192 | 76 | -0.392323114 | ENTHD1 | ENTH domain containing 1 [Source:HGNC Symbol;Acc:HGNC:26352] |
| ENSG00000176732 | HGLibA_36183 | 76 | -0.392323114 | PFN4 | profilin family member 4 [Source:HGNC Symbol;Acc:HGNC:31103] |
| ENSG00000176749 | HGLibA_08775 | 76 | -0.392323114 | CDK5R1 | cyclin dependent kinase 5 regulatory subunit 1 [Source:HGNC Symbol;Acc:HGNC:1775] |
| ENSG00000176994 | HGLibA_45684 | 76 | -0.392323114 | SMCR8 | candidate 8 [Source:HGNC Symbol;Acc:HGNC:17921]" |
| ENSG00000177556 | HGLibA_03543 | 76 | -0.392323114 | ATOX1 | antioxidant 1 copper chaperone [Source:HGNC Symbol;Acc:HGNC:798] |
| ENSG00000179088 | HGLibA_05242 | 76 | -0.392323114 | C12orf42 | chromosome 12 open reading frame 42 [Source:HGNC Symbol;Acc:HGNC:24729] |
| ENSG00000179407 | HGLibA_13554 | 76 | -0.392323114 | DNAJB8 | DnaJ heat shock protein family (Hsp40) member B8 [Source:HGNC Symbol;Acc:HGNC:23699] |
| ENSG00000179855 | HGLibA_19123 | 76 | -0.392323114 | GIPC3 | GIPC PDZ domain containing family member 3 [Source:HGNC Symbol;Acc:HGNC:18183] |
| ENSG00000179869 | HGLibA_00104 | 76 | -0.392323114 | ABCA13 | ATP binding cassette subfamily A member 13 [Source:HGNC Symbol;Acc:HGNC:14638] |
| ENSG00000179909 | HGLibA_55500 | 76 | -0.392323114 | ZNF154 | zinc finger protein 154 [Source:HGNC Symbol;Acc:HGNC:12939] |
| ENSG00000180318 | HGLibA_01733 | 76 | -0.392323114 | ALX1 | ALX homeobox 1 [Source:HGNC Symbol;Acc:HGNC:1494] |
| ENSG00000180340 | HGLibA_18267 | 76 | -0.392323114 | FZD2 | frizzled class receptor 2 [Source:HGNC Symbol;Acc:HGNC:4040] |
| ENSG00000180745 | HGLibA_10127 | 76 | -0.392323114 | CLRN3 | clarin 3 [Source:HGNC Symbol;Acc:HGNC:20795] |
| ENSG00000180938 | HGLibA_56282 | 76 | -0.392323114 | ZNF572 | zinc finger protein 572 [Source:HGNC Symbol;Acc:HGNC:26758] |
| ENSG00000181903 | HGLibA_33961 | 76 | -0.392323114 | OR4C6 | olfactory receptor family 4 subfamily C member 6 [Source:HGNC Symbol;Acc:HGNC:14743] |
| ENSG00000182534 | HGLibA_30625 | 76 | -0.392323114 | MXRA7 | matrix remodeling associated 7 [Source:HGNC Symbol;Acc:HGNC:7541] |
| ENSG00000182898 | HGLibA_48805 | 76 | -0.392323114 | TCHHL1 | trichohyalin like 1 [Source:HGNC Symbol;Acc:HGNC:31796] |
| ENSG00000183513 | HGLibA_10453 | 76 | -0.392323114 | COA5 | cytochrome c oxidase assembly factor 5 [Source:HGNC Symbol;Acc:HGNC:33848] |
| ENSG00000183597 | HGLibA_48296 | 76 | -0.392323114 | TANGO2 | transport and golgi organization 2 homolog [Source:HGNC Symbol;Acc:HGNC:25439] |
| ENSG00000183715 | HGLibA_33414 | 76 | -0.392323114 | OPCML | opioid binding protein/cell adhesion molecule like [Source:HGNC Symbol;Acc:HGNC:8143] |
| ENSG00000184209 | HGLibA_45949 | 76 | -0.392323114 | SNRNP35 | small nuclear ribonucleoprotein U11/U12 subunit 35 [Source:HGNC Symbol;Acc:HGNC:30852] |
| ENSG00000184650 | HGLibA_33295 | 76 | -0.392323114 | ODF4 | outer dense fiber of sperm tails 4 [Source:HGNC Symbol;Acc:HGNC:19056] |
| ENSG00000184672 | HGLibA_40178 | 76 | -0.392323114 | RALYL | RALY RNA binding protein like [Source:HGNC Symbol;Acc:HGNC:27036] |
| ENSG00000185187 | HGLibA_44120 | 76 | -0.392323114 | SIGIRR | single Ig and TIR domain containing [Source:HGNC Symbol;Acc:HGNC:30575] |
| ENSG00000185737 | HGLibA_32682 | 76 | -0.392323114 | NRG3 | neuregulin 3 [Source:HGNC Symbol;Acc:HGNC:7999] |
| ENSG00000186010 | HGLibA_31448 | 76 | -0.392323114 | NDUFA13 | NADH:ubiquinone oxidoreductase subunit A13 [Source:HGNC Symbol;Acc:HGNC:17194] |
| ENSG00000186197 | HGLibA_14439 | 76 | -0.392323114 | EDARADD | EDAR associated death domain [Source:HGNC Symbol;Acc:HGNC:14341] |
| ENSG00000187821 | HGLibA_21120 | 76 | -0.392323114 | HELT | helt bHLH transcription factor [Source:HGNC Symbol;Acc:HGNC:33783] |
| ENSG00000188015 | HGLibA_42555 | 76 | -0.392323114 | S100A3 | S100 calcium binding protein A3 [Source:HGNC Symbol;Acc:HGNC:10493] |
| ENSG00000196090 | HGLibA_39537 | 76 | -0.392323114 | PTPRT | receptor type T [Source:HGNC Symbol;Acc:HGNC:9682]" |
| ENSG00000196616 | HGLibA_00952 | 76 | -0.392323114 | ADH1B | beta polypeptide [Source:HGNC Symbol;Acc:HGNC:250]" |
| ENSG00000196678 | HGLibA_15483 | 76 | -0.392323114 | ERI2 | ERI1 exoribonuclease family member 2 [Source:HGNC Symbol;Acc:HGNC:30541] |
| ENSG00000197140 | HGLibA_00772 | 76 | -0.392323114 | ADAM32 | ADAM metallopeptidase domain 32 [Source:HGNC Symbol;Acc:HGNC:15479] |
| ENSG00000197776 | HGLibA_25091 | 76 | -0.392323114 | KLHDC1 | kelch domain containing 1 [Source:HGNC Symbol;Acc:HGNC:19836] |
| ENSG00000198133 | HGLibA_50251 | 76 | -0.392323114 | TMEM229B | transmembrane protein 229B [Source:HGNC Symbol;Acc:HGNC:20130] |
| ENSG00000198182 | HGLibA_56371 | 76 | -0.392323114 | ZNF607 | zinc finger protein 607 [Source:HGNC Symbol;Acc:HGNC:28192] |
| ENSG00000198355 | HGLibA_36680 | 76 | -0.392323114 | PIM3 | serine/threonine kinase [Source:HGNC Symbol;Acc:HGNC:19310]" |
| ENSG00000198380 | HGLibA_18959 | 76 | -0.392323114 | GFPT1 | glutamine--fructose-6-phosphate transaminase 1 [Source:HGNC Symbol;Acc:HGNC:4241] |
| ENSG00000198626 | HGLibA_42525 | 76 | -0.392323114 | RYR2 | ryanodine receptor 2 [Source:HGNC Symbol;Acc:HGNC:10484] |
| ENSG00000204116 | HGLibA_09445 | 76 | -0.392323114 | CHIC1 | cysteine rich hydrophobic domain 1 [Source:HGNC Symbol;Acc:HGNC:1934] |
| ENSG00000204396 | HGLibA_53914 | 76 | -0.392323114 | VWA7 | von Willebrand factor A domain containing 7 [Source:HGNC Symbol;Acc:HGNC:13939] |
| ENSG00000204538 | HGLibA_39220 | 76 | -0.392323114 | PSORS1C2 | psoriasis susceptibility 1 candidate 2 [Source:HGNC Symbol;Acc:HGNC:17199] |
| ENSG00000204599 | HGLibA_51484 | 76 | -0.392323114 | TRIM39 | tripartite motif containing 39 [Source:HGNC Symbol;Acc:HGNC:10065] |
| ENSG00000205250 | HGLibA_14315 | 76 | -0.392323114 | E2F4 | E2F transcription factor 4 [Source:HGNC Symbol;Acc:HGNC:3118] |
| ENSG00000213714 | HGLibA_16442 | 76 | -0.392323114 | FAM209B | family with sequence similarity 209 member B [Source:HGNC Symbol;Acc:HGNC:16101] |
| ENSG00000221855 | HGLibA_48406 | 76 | -0.392323114 | TAS2R41 | taste 2 receptor member 41 [Source:HGNC Symbol;Acc:HGNC:18883] |
| ENSG00000234829 | HGLibA_22670 | 76 | -0.392323114 | IFNA17 | interferon alpha 17 [Source:HGNC Symbol;Acc:HGNC:5422] |
| ENSG00000237957 | HGLibA_11536 | 76 | -0.392323114 | CT47A5 | member A5 [Source:HGNC Symbol;Acc:HGNC:33286]" |
| ENSG00000239264 | HGLibA_52423 | 76 | -0.392323114 | TXNDC5 | thioredoxin domain containing 5 [Source:HGNC Symbol;Acc:HGNC:21073] |
| ENSG00000239474 | HGLibA_25210 | 76 | -0.392323114 | KLHL41 | kelch like family member 41 [Source:HGNC Symbol;Acc:HGNC:16905] |
| ENSG00000242252 | HGLibA_04460 | 76 | -0.392323114 | BGLAP | bone gamma-carboxyglutamate protein [Source:HGNC Symbol;Acc:HGNC:1043] |
| ENSG00000244687 | HGLibA_52667 | 76 | -0.392323114 | UBE2V1 | ubiquitin conjugating enzyme E2 V1 [Source:HGNC Symbol;Acc:HGNC:12494] |
| ENSG00000253293 | HGLibA_21896 | 76 | -0.392323114 | HOXA10 | homeobox A10 [Source:HGNC Symbol;Acc:HGNC:5100] |
| ENSG00000261934 | HGLibA_35546 | 76 | -0.392323114 | PCDHGA9 | 9 [Source:HGNC Symbol;Acc:HGNC:8707]" |
| ENSG00000271271 | HGLibA_52905 | 76 | -0.392323114 | UGT2A2 | UDP glucuronosyltransferase family 2 member A2 [Source:HGNC Symbol;Acc:HGNC:28183] |
| ENSG00000276430 | HGLibA_16555 | 76 | -0.392323114 | FAM25C | family with sequence similarity 25 member C [Source:HGNC Symbol;Acc:HGNC:23586] |
| ENSG00000278535 | HGLibA_13120 | 76 | -0.392323114 | DHRS11 | dehydrogenase/reductase 11 [Source:HGNC Symbol;Acc:HGNC:28639] |
| FAM129B | HGLibA_16100 | 76 | -0.392323114 | FAM129B | - |
| FAM19A3 | HGLibA_16402 | 76 | -0.392323114 | FAM19A3 | - |
| FAM206A | HGLibA_16425 | 76 | -0.392323114 | FAM206A | - |
| hsa-let-7e | HGLibA_57121 | 76 | -0.392323114 | hsa-let-7e | - |
| hsa-mir-105-2 | HGLibA_57181 | 76 | -0.392323114 | hsa-mir-105-2 | - |

| | | | | | |
|---|---|---|---|---|---|
| hsa-mir-1182 | HGLibA_57223 | 76 | -0.392323114 | hsa-mir-1182 | - |
| hsa-mir-133a-2 | HGLibA_57775 | 76 | -0.392323114 | hsa-mir-133a-2 | - |
| hsa-mir-1908 | HGLibA_58026 | 76 | -0.392323114 | hsa-mir-1908 | - |
| hsa-mir-210 | HGLibA_58212 | 76 | -0.392323114 | hsa-mir-210 | - |
| hsa-mir-2113 | HGLibA_58223 | 76 | -0.392323114 | hsa-mir-2113 | - |
| hsa-mir-3160-1 | HGLibA_58716 | 76 | -0.392323114 | hsa-mir-3160-1 | - |
| hsa-mir-320d-2 | HGLibA_58949 | 76 | -0.392323114 | hsa-mir-320d-2 | - |
| hsa-mir-365a | HGLibA_59184 | 76 | -0.392323114 | hsa-mir-365a | - |
| hsa-mir-4252 | HGLibA_59714 | 76 | -0.392323114 | hsa-mir-4252 | - |
| hsa-mir-4641 | HGLibA_60667 | 76 | -0.392323114 | hsa-mir-4641 | - |
| hsa-mir-4753 | HGLibA_61109 | 76 | -0.392323114 | hsa-mir-4753 | - |
| hsa-mir-4799 | HGLibA_61303 | 76 | -0.392323114 | hsa-mir-4799 | - |
| hsa-mir-5089 | HGLibA_61504 | 76 | -0.392323114 | hsa-mir-5089 | - |
| hsa-mir-513b | HGLibA_61578 | 76 | -0.392323114 | hsa-mir-513b | - |
| hsa-mir-516a-1 | HGLibA_61607 | 76 | -0.392323114 | hsa-mir-516a-1 | - |
| hsa-mir-548h-1 | HGLibA_61992 | 76 | -0.392323114 | hsa-mir-548h-1 | - |
| hsa-mir-5579 | HGLibA_62154 | 76 | -0.392323114 | hsa-mir-5579 | - |
| hsa-mir-577 | HGLibA_62404 | 76 | -0.392323114 | hsa-mir-577 | - |
| hsa-mir-587 | HGLibA_62446 | 76 | -0.392323114 | hsa-mir-587 | - |
| hsa-mir-6070 | HGLibA_62531 | 76 | -0.392323114 | hsa-mir-6070 | - |
| hsa-mir-6071 | HGLibA_62535 | 76 | -0.392323114 | hsa-mir-6071 | - |
| hsa-mir-6077-1 | HGLibA_62560 | 76 | -0.392323114 | hsa-mir-6077-1 | - |
| hsa-mir-6499 | HGLibA_62845 | 76 | -0.392323114 | hsa-mir-6499 | - |
| hsa-mir-650 | HGLibA_62849 | 76 | -0.392323114 | hsa-mir-650 | - |
| hsa-mir-6716 | HGLibA_63026 | 76 | -0.392323114 | hsa-mir-6716 | - |
| hsa-mir-6770-1 | HGLibA_63246 | 76 | -0.392323114 | hsa-mir-6770-1 | - |
| hsa-mir-889 | HGLibA_64236 | 76 | -0.392323114 | hsa-mir-889 | - |
| hsa-mir-892b | HGLibA_64256 | 76 | -0.392323114 | hsa-mir-892b | - |
| KIAA1324L | HGLibA_24739 | 76 | -0.392323114 | KIAA1324L | - |
| ZCCHC11 | HGLibA_55007 | 76 | -0.392323114 | ZCCHC11 | - |
| APITD1-CORT | HGLibA_02428 | 75 | -0.402274152 | APITD1-CORT | - |
| C13orf45 | HGLibA_05312 | 75 | -0.402274152 | C13orf45 | - |
| C14orf37 | HGLibA_05364 | 75 | -0.402274152 | C14orf37 | - |
| C6orf1 | HGLibA_06428 | 75 | -0.402274152 | C6orf1 | - |
| CCDC176 | HGLibA_07710 | 75 | -0.402274152 | CCDC176 | - |
| ENSG00000006576 | HGLibA_36464 | 75 | -0.402274152 | PHTF2 | putative homeodomain transcription factor 2 [Source:HGNC Symbol;Acc:HGNC:13411] |
| ENSG00000010539 | HGLibA_55569 | 75 | -0.402274152 | ZNF200 | zinc finger protein 200 [Source:HGNC Symbol;Acc:HGNC:12993] |
| ENSG00000023171 | HGLibA_20197 | 75 | -0.402274152 | GRAMD1B | GRAM domain containing 1B [Source:HGNC Symbol;Acc:HGNC:29214] |
| ENSG00000031691 | HGLibA_09083 | 75 | -0.402274152 | CENPQ | centromere protein Q [Source:HGNC Symbol;Acc:HGNC:21347] |
| ENSG00000042317 | HGLibA_46527 | 75 | -0.402274152 | SPATA7 | spermatogenesis associated 7 [Source:HGNC Symbol;Acc:HGNC:20423] |
| ENSG00000050555 | HGLibA_25975 | 75 | -0.402274152 | LAMC3 | laminin subunit gamma 3 [Source:HGNC Symbol;Acc:HGNC:6494] |
| ENSG00000054267 | HGLibA_02902 | 75 | -0.402274152 | ARID4B | AT-rich interaction domain 4B [Source:HGNC Symbol;Acc:HGNC:15550] |
| ENSG00000054690 | HGLibA_37125 | 75 | -0.402274152 | PLEKHH1 | MyTH4 and FERM domain containing H1 [Source:HGNC Symbol;Acc:HGNC:17733]" |
| ENSG00000061794 | HGLibA_30098 | 75 | -0.402274152 | MRPS35 | mitochondrial ribosomal protein S35 [Source:HGNC Symbol;Acc:HGNC:16635] |
| ENSG00000069011 | HGLibA_36763 | 75 | -0.402274152 | PITX1 | paired like homeodomain 1 [Source:HGNC Symbol;Acc:HGNC:9004] |
| ENSG00000071994 | HGLibA_35727 | 75 | -0.402274152 | PDCD2 | programmed cell death 2 [Source:HGNC Symbol;Acc:HGNC:8762] |
| ENSG00000075131 | HGLibA_49559 | 75 | -0.402274152 | TIPIN | TIMELESS interacting protein [Source:HGNC Symbol;Acc:HGNC:30750] |
| ENSG00000077721 | HGLibA_52572 | 75 | -0.402274152 | UBE2A | ubiquitin conjugating enzyme E2 A [Source:HGNC Symbol;Acc:HGNC:12472] |
| ENSG00000081320 | HGLibA_47466 | 75 | -0.402274152 | STK17B | serine/threonine kinase 17b [Source:HGNC Symbol;Acc:HGNC:11396] |
| ENSG00000081320 | HGLibA_47465 | 75 | -0.402274152 | STK17B | serine/threonine kinase 17b [Source:HGNC Symbol;Acc:HGNC:11396] |
| ENSG00000081913 | HGLibA_36436 | 75 | -0.402274152 | PHLPP1 | PH domain and leucine rich repeat protein phosphatase 1 [Source:HGNC Symbol;Acc:HGNC:20610] |
| ENSG00000087502 | HGLibA_15470 | 75 | -0.402274152 | ERGIC2 | ERGIC and golgi 2 [Source:HGNC Symbol;Acc:HGNC:30208] |
| ENSG00000091536 | HGLibA_30805 | 75 | -0.402274152 | MYO15A | myosin XVA [Source:HGNC Symbol;Acc:HGNC:7594] |
| ENSG00000102893 | HGLibA_36409 | 75 | -0.402274152 | PHKB | phosphorylase kinase regulatory subunit beta [Source:HGNC Symbol;Acc:HGNC:8927] |
| ENSG00000102962 | HGLibA_07997 | 75 | -0.402274152 | CCL22 | C-C motif chemokine ligand 22 [Source:HGNC Symbol;Acc:HGNC:10621] |
| ENSG00000103248 | HGLibA_30394 | 75 | -0.402274152 | MTHFSD | methenyltetrahydrofolate synthetase domain containing [Source:HGNC Symbol;Acc:HGNC:25778] |
| ENSG00000105976 | HGLibA_28925 | 75 | -0.402274152 | MET | receptor tyrosine kinase [Source:HGNC Symbol;Acc:HGNC:7029]" |
| ENSG00000106006 | HGLibA_21918 | 75 | -0.402274152 | HOXA6 | homeobox A6 [Source:HGNC Symbol;Acc:HGNC:5107] |
| ENSG00000107281 | HGLibA_32428 | 75 | -0.402274152 | NPDC1 | differentiation and control 1 [Source:HGNC Symbol;Acc:HGNC:7899]" |
| ENSG00000107731 | HGLibA_53018 | 75 | -0.402274152 | UNC5B | unc-5 netrin receptor B [Source:HGNC Symbol;Acc:HGNC:12568] |
| ENSG00000108953 | HGLibA_54741 | 75 | -0.402274152 | YWHAE | tyrosine 3-monooxygenase/tryptophan 5-monooxygenase activation protein epsilon [Source:HGNC Symbol;Acc:HGNC:12851] |
| ENSG00000109133 | HGLibA_50370 | 75 | -0.402274152 | TMEM33 | transmembrane protein 33 [Source:HGNC Symbol;Acc:HGNC:25541] |
| ENSG00000109832 | HGLibA_12680 | 75 | -0.402274152 | DDX25 | DEAD-box helicase 25 [Source:HGNC Symbol;Acc:HGNC:18698] |
| ENSG00000110497 | HGLibA_01753 | 75 | -0.402274152 | AMBRA1 | autophagy and beclin 1 regulator 1 [Source:HGNC Symbol;Acc:HGNC:25990] |
| ENSG00000111344 | HGLibA_40311 | 75 | -0.402274152 | RASAL1 | RAS protein activator like 1 [Source:HGNC Symbol;Acc:HGNC:9873] |
| ENSG00000111490 | HGLibA_48534 | 75 | -0.402274152 | TBC1D30 | TBC1 domain family member 30 [Source:HGNC Symbol;Acc:HGNC:29164] |
| ENSG00000111490 | HGLibA_48532 | 75 | -0.402274152 | TBC1D30 | TBC1 domain family member 30 [Source:HGNC Symbol;Acc:HGNC:29164] |
| ENSG00000112149 | HGLibA_08462 | 75 | -0.402274152 | CD83 | CD83 molecule [Source:HGNC Symbol;Acc:HGNC:1703] |
| ENSG00000112200 | HGLibA_56029 | 75 | -0.402274152 | ZNF451 | zinc finger protein 451 [Source:HGNC Symbol;Acc:HGNC:21091] |
| ENSG00000113595 | HGLibA_51435 | 75 | -0.402274152 | TRIM23 | tripartite motif containing 23 [Source:HGNC Symbol;Acc:HGNC:660] |
| ENSG00000114904 | HGLibA_31654 | 75 | -0.402274152 | NEK4 | NIMA related kinase 4 [Source:HGNC Symbol;Acc:HGNC:11399] |
| ENSG00000115239 | HGLibA_03236 | 75 | -0.402274152 | ASB3 | ankyrin repeat and SOCS box containing 3 [Source:HGNC Symbol;Acc:HGNC:16013] |
| ENSG00000115364 | HGLibA_29919 | 75 | -0.402274152 | MRPL19 | mitochondrial ribosomal protein L19 [Source:HGNC Symbol;Acc:HGNC:14052] |
| ENSG00000116663 | HGLibA_17122 | 75 | -0.402274152 | FBXO6 | F-box protein 6 [Source:HGNC Symbol;Acc:HGNC:13585] |
| ENSG00000116871 | HGLibA_28236 | 75 | -0.402274152 | MAP7D1 | MAP7 domain containing 1 [Source:HGNC Symbol;Acc:HGNC:25514] |
| ENSG00000118898 | HGLibA_37961 | 75 | -0.402274152 | PPL | periplakin [Source:HGNC Symbol;Acc:HGNC:9273] |
| ENSG00000119782 | HGLibA_17567 | 75 | -0.402274152 | FKBP1B | FK506 binding protein 1B [Source:HGNC Symbol;Acc:HGNC:3712] |
| ENSG00000121552 | HGLibA_11476 | 75 | -0.402274152 | CSTA | cystatin A [Source:HGNC Symbol;Acc:HGNC:2481] |
| ENSG00000121644 | HGLibA_13009 | 75 | -0.402274152 | DESI2 | desumoylating isopeptidase 2 [Source:HGNC Symbol;Acc:HGNC:24264] |
| ENSG00000122696 | HGLibA_44764 | 75 | -0.402274152 | SLC25A51 | solute carrier family 25 member 51 [Source:HGNC Symbol;Acc:HGNC:23323] |
| ENSG00000123594 | HGLibA_03846 | 75 | -0.402274152 | ATXN3L | ataxin 3 like [Source:HGNC Symbol;Acc:HGNC:24173] |
| ENSG00000124003 | HGLibA_29623 | 75 | -0.402274152 | MOGAT1 | monoacylglycerol O-acyltransferase 1 [Source:HGNC Symbol;Acc:HGNC:18210] |
| ENSG00000124102 | HGLibA_36489 | 75 | -0.402274152 | PI3 | peptidase inhibitor 3 [Source:HGNC Symbol;Acc:HGNC:8947] |
| ENSG00000124177 | HGLibA_09402 | 75 | -0.402274152 | CHD6 | chromodomain helicase DNA binding protein 6 [Source:HGNC Symbol;Acc:HGNC:19057] |
| ENSG00000125676 | HGLibA_49366 | 75 | -0.402274152 | THOC2 | THO complex 2 [Source:HGNC Symbol;Acc:HGNC:19073] |
| ENSG00000126010 | HGLibA_20377 | 75 | -0.402274152 | GRPR | gastrin releasing peptide receptor [Source:HGNC Symbol;Acc:HGNC:4609] |
| ENSG00000126464 | HGLibA_38772 | 75 | -0.402274152 | PRR12 | proline rich 12 [Source:HGNC Symbol;Acc:HGNC:29217] |
| ENSG00000128694 | HGLibA_34683 | 75 | -0.402274152 | OSGEPL1 | O-sialoglycoprotein endopeptidase like 1 [Source:HGNC Symbol;Acc:HGNC:23075] |
| ENSG00000129654 | HGLibA_17886 | 75 | -0.402274152 | FOXJ1 | forkhead box J1 [Source:HGNC Symbol;Acc:HGNC:3816] |
| ENSG00000132004 | HGLibA_17159 | 75 | -0.402274152 | FBXW9 | F-box and WD repeat domain containing 9 [Source:HGNC Symbol;Acc:HGNC:28136] |
| ENSG00000132274 | HGLibA_51432 | 75 | -0.402274152 | TRIM22 | tripartite motif containing 22 [Source:HGNC Symbol;Acc:HGNC:16379] |
| ENSG00000132911 | HGLibA_32217 | 75 | -0.402274152 | NMUR2 | neuromedin U receptor 2 [Source:HGNC Symbol;Acc:HGNC:16454] |
| ENSG00000134909 | HGLibA_02760 | 75 | -0.402274152 | ARHGAP32 | Rho GTPase activating protein 32 [Source:HGNC Symbol;Acc:HGNC:17399] |
| ENSG00000135047 | HGLibA_11715 | 75 | -0.402274152 | CTSL | cathepsin L [Source:HGNC Symbol;Acc:HGNC:2537] |
| ENSG00000135535 | HGLibA_08249 | 75 | -0.402274152 | CD164 | CD164 molecule [Source:HGNC Symbol;Acc:HGNC:1632] |
| ENSG00000136383 | HGLibA_01705 | 75 | -0.402274152 | ALPK3 | alpha kinase 3 [Source:HGNC Symbol;Acc:HGNC:17574] |
| ENSG00000137776 | HGLibA_45548 | 75 | -0.402274152 | SLTM | SAFB like transcription modulator [Source:HGNC Symbol;Acc:HGNC:20709] |
| ENSG00000138083 | HGLibA_44248 | 75 | -0.402274152 | SIX3 | SIX homeobox 3 [Source:HGNC Symbol;Acc:HGNC:10889] |
| ENSG00000138136 | HGLibA_26085 | 75 | -0.402274152 | LBX1 | ladybird homeobox 1 [Source:HGNC Symbol;Acc:HGNC:16960] |
| ENSG00000138688 | HGLibA_24694 | 75 | -0.402274152 | KIAA1109 | KIAA1109 [Source:HGNC Symbol;Acc:HGNC:26953] |
| ENSG00000139323 | HGLibA_37443 | 75 | -0.402274152 | POC1B | POC1 centriolar protein B [Source:HGNC Symbol;Acc:HGNC:30836] |
| ENSG00000139946 | HGLibA_36036 | 75 | -0.402274152 | PELI2 | pellino E3 ubiquitin protein ligase family member 2 [Source:HGNC Symbol;Acc:HGNC:8828] |
| ENSG00000140521 | HGLibA_37522 | 75 | -0.402274152 | POLG | catalytic subunit [Source:HGNC Symbol;Acc:HGNC:9179]" |
| ENSG00000141965 | HGLibA_17273 | 75 | -0.402274152 | FEM1A | fem-1 homolog A [Source:HGNC Symbol;Acc:HGNC:16934] |
| ENSG00000142538 | HGLibA_39371 | 75 | -0.402274152 | PTH2 | parathyroid hormone 2 [Source:HGNC Symbol;Acc:HGNC:30828] |
| ENSG00000143224 | HGLibA_38002 | 75 | -0.402274152 | PPOX | protoporphyrinogen oxidase [Source:HGNC Symbol;Acc:HGNC:9280] |
| ENSG00000143553 | HGLibA_45904 | 75 | -0.402274152 | SNAPIN | SNAP associated protein [Source:HGNC Symbol;Acc:HGNC:17145] |
| ENSG00000143793 | HGLibA_05855 | 75 | -0.402274152 | C1orf35 | chromosome 1 open reading frame 35 [Source:HGNC Symbol;Acc:HGNC:19032] |
| ENSG00000145780 | HGLibA_17278 | 75 | -0.402274152 | FEM1C | fem-1 homolog C [Source:HGNC Symbol;Acc:HGNC:16933] |
| ENSG00000146013 | HGLibA_18970 | 75 | -0.402274152 | GFRA3 | GDNF family receptor alpha 3 [Source:HGNC Symbol;Acc:HGNC:4245] |
| ENSG00000146350 | HGLibA_48540 | 75 | -0.402274152 | TBC1D32 | TBC1 domain family member 32 [Source:HGNC Symbol;Acc:HGNC:21485] |
| ENSG00000146433 | HGLibA_50100 | 75 | -0.402274152 | TMEM181 | transmembrane protein 181 [Source:HGNC Symbol;Acc:HGNC:20958] |
| ENSG00000146918 | HGLibA_31272 | 75 | -0.402274152 | NCAPG2 | non-SMC condensin II complex subunit G2 [Source:HGNC Symbol;Acc:HGNC:21904] |
| ENSG00000149488 | HGLibA_49750 | 75 | -0.402274152 | TMC2 | transmembrane channel like 2 [Source:HGNC Symbol;Acc:HGNC:16527] |
| ENSG00000151491 | HGLibA_15374 | 75 | -0.402274152 | EPS8 | epidermal growth factor receptor pathway substrate 8 [Source:HGNC Symbol;Acc:HGNC:3420] |
| ENSG00000151615 | HGLibA_37781 | 75 | -0.402274152 | POU4F2 | POU class 4 homeobox 2 [Source:HGNC Symbol;Acc:HGNC:9219] |
| ENSG00000152254 | HGLibA_18307 | 75 | -0.402274152 | G6PC2 | glucose-6-phosphatase catalytic subunit 2 [Source:HGNC Symbol;Acc:HGNC:28906] |
| ENSG00000153823 | HGLibA_36527 | 75 | -0.402274152 | PID1 | phosphotyrosine interaction domain containing 1 [Source:HGNC Symbol;Acc:HGNC:26084] |
| ENSG00000153879 | HGLibA_08967 | 75 | -0.402274152 | CEBPG | CCAAT enhancer binding protein gamma [Source:HGNC Symbol;Acc:HGNC:1837] |
| ENSG00000154059 | HGLibA_23249 | 75 | -0.402274152 | IMPACT | impact RWD domain protein [Source:HGNC Symbol;Acc:HGNC:20387] |
| ENSG00000155034 | HGLibA_16991 | 75 | -0.402274152 | FBXL18 | F-box and leucine rich repeat protein 18 [Source:HGNC Symbol;Acc:HGNC:21874] |
| ENSG00000155111 | HGLibA_08752 | 75 | -0.402274152 | CDK19 | cyclin dependent kinase 19 [Source:HGNC Symbol;Acc:HGNC:19338] |
| ENSG00000155755 | HGLibA_50276 | 75 | -0.402274152 | TMEM237 | transmembrane protein 237 [Source:HGNC Symbol;Acc:HGNC:14432] |
| ENSG00000156968 | HGLibA_29787 | 75 | -0.402274152 | MPV17L | MPV17 mitochondrial inner membrane protein like [Source:HGNC Symbol;Acc:HGNC:26827] |
| ENSG00000157349 | HGLibA_12667 | 75 | -0.402274152 | DDX19B | DEAD-box helicase 19B [Source:HGNC Symbol;Acc:HGNC:2742] |
| ENSG00000157554 | HGLibA_15464 | 75 | -0.402274152 | ERG | ETS transcription factor ERG [Source:HGNC Symbol;Acc:HGNC:3446] |
| ENSG00000157637 | HGLibA_45057 | 75 | -0.402274152 | SLC38A10 | solute carrier family 38 member 10 [Source:HGNC Symbol;Acc:HGNC:28237] |
| ENSG00000158113 | HGLibA_27361 | 75 | -0.402274152 | LRRC43 | leucine rich repeat containing 43 [Source:HGNC Symbol;Acc:HGNC:28562] |
| ENSG00000158552 | HGLibA_55149 | 75 | -0.402274152 | ZFAND2B | zinc finger AN1-type containing 2B [Source:HGNC Symbol;Acc:HGNC:25206] |
| ENSG00000158552 | HGLibA_55151 | 75 | -0.402274152 | ZFAND2B | zinc finger AN1-type containing 2B [Source:HGNC Symbol;Acc:HGNC:25206] |
| ENSG00000158560 | HGLibA_14234 | 75 | -0.402274152 | DYNC1I1 | dynein cytoplasmic 1 intermediate chain 1 [Source:HGNC Symbol;Acc:HGNC:2963] |
| ENSG00000158711 | HGLibA_14918 | 75 | -0.402274152 | ELK4 | ETS transcription factor [Source:HGNC Symbol;Acc:HGNC:3326]" |
| ENSG00000158714 | HGLibA_44322 | 75 | -0.402274152 | SLAMF8 | SLAM family member 8 [Source:HGNC Symbol;Acc:HGNC:21391] |
| ENSG00000159495 | HGLibA_49271 | 75 | -0.402274152 | TGM7 | transglutaminase 7 [Source:HGNC Symbol;Acc:HGNC:30790] |
| ENSG00000160201 | HGLibA_52492 | 75 | -0.402274152 | U2AF1 | U2 small nuclear RNA auxiliary factor 1 [Source:HGNC Symbol;Acc:HGNC:12453] |

| | | | | | |
|---|---|---|---|---|---|
| ENSG00000160321 | HGLibA_55582 | 75 | -0.402274152 | ZNF208 | zinc finger protein 208 [Source:HGNC Symbol;Acc:HGNC:12999] |
| ENSG00000162399 | HGLibA_04871 | 75 | -0.402274152 | BSND | barttin CLCNK type accessory beta subunit [Source:HGNC Symbol;Acc:HGNC:16512] |
| ENSG00000162493 | HGLibA_35933 | 75 | -0.402274152 | PDPN | podoplanin [Source:HGNC Symbol;Acc:HGNC:29602] |
| ENSG00000162688 | HGLibA_01196 | 75 | -0.402274152 | AGL | 4-alpha-glucanotransferase [Source:HGNC Symbol;Acc:HGNC:321]" |
| ENSG00000162851 | HGLibA_49143 | 75 | -0.402274152 | TFB2M | mitochondrial [Source:HGNC Symbol;Acc:HGNC:18559]" |
| ENSG00000162927 | HGLibA_39597 | 75 | -0.402274152 | PUS10 | pseudouridine synthase 10 [Source:HGNC Symbol;Acc:HGNC:26505] |
| ENSG00000163155 | HGLibA_27764 | 75 | -0.402274152 | LYSMD1 | LysM domain containing 1 [Source:HGNC Symbol;Acc:HGNC:32070] |
| ENSG00000163249 | HGLibA_08134 | 75 | -0.402274152 | CCNYL1 | cyclin Y like 1 [Source:HGNC Symbol;Acc:HGNC:26868] |
| ENSG00000163491 | HGLibA_31641 | 75 | -0.402274152 | NEK10 | NIMA related kinase 10 [Source:HGNC Symbol;Acc:HGNC:18592] |
| ENSG00000164197 | HGLibA_41579 | 75 | -0.402274152 | RNF180 | ring finger protein 180 [Source:HGNC Symbol;Acc:HGNC:27752] |
| ENSG00000164542 | HGLibA_24657 | 75 | -0.402274152 | KIAA0895 | KIAA0895 [Source:HGNC Symbol;Acc:HGNC:22206] |
| ENSG00000164949 | HGLibA_18907 | 75 | -0.402274152 | GEM | GTP binding protein overexpressed in skeletal muscle [Source:HGNC Symbol;Acc:HGNC:4234] |
| ENSG00000165028 | HGLibA_31976 | 75 | -0.402274152 | NIPSNAP3B | nipsnap homolog 3B [Source:HGNC Symbol;Acc:HGNC:23641] |
| ENSG00000165283 | HGLibA_47537 | 75 | -0.402274152 | STOML2 | stomatin like 2 [Source:HGNC Symbol;Acc:HGNC:14559] |
| ENSG00000165309 | HGLibA_03017 | 75 | -0.402274152 | ARMC3 | armadillo repeat containing 3 [Source:HGNC Symbol;Acc:HGNC:30964] |
| ENSG00000166321 | HGLibA_32943 | 75 | -0.402274152 | NUDT13 | nudix hydrolase 13 [Source:HGNC Symbol;Acc:HGNC:18827] |
| ENSG00000166558 | HGLibA_45083 | 75 | -0.402274152 | SLC38A8 | solute carrier family 38 member 8 [Source:HGNC Symbol;Acc:HGNC:32434] |
| ENSG00000166670 | HGLibA_29481 | 75 | -0.402274152 | MMP10 | matrix metallopeptidase 10 [Source:HGNC Symbol;Acc:HGNC:7156] |
| ENSG00000167632 | HGLibA_51339 | 75 | -0.402274152 | TRAPPC9 | trafficking protein particle complex 9 [Source:HGNC Symbol;Acc:HGNC:30832] |
| ENSG00000167757 | HGLibA_25238 | 75 | -0.402274152 | KLK11 | kallikrein related peptidase 11 [Source:HGNC Symbol;Acc:HGNC:6359] |
| ENSG00000167916 | HGLibA_25455 | 75 | -0.402274152 | KRT24 | keratin 24 [Source:HGNC Symbol;Acc:HGNC:18527] |
| ENSG00000168259 | HGLibA_13635 | 75 | -0.402274152 | DNAJC7 | DnaJ heat shock protein family (Hsp40) member C7 [Source:HGNC Symbol;Acc:HGNC:12392] |
| ENSG00000168528 | HGLibA_43487 | 75 | -0.402274152 | SERINC2 | serine incorporator 2 [Source:HGNC Symbol;Acc:HGNC:23231] |
| ENSG00000168530 | HGLibA_30744 | 75 | -0.402274152 | MYL1 | myosin light chain 1 [Source:HGNC Symbol;Acc:HGNC:7582] |
| ENSG00000169105 | HGLibA_09625 | 75 | -0.402274152 | CHST14 | carbohydrate sulfotransferase 14 [Source:HGNC Symbol;Acc:HGNC:24464] |
| ENSG00000169857 | HGLibA_03887 | 75 | -0.402274152 | AVEN | apoptosis and caspase activation inhibitor [Source:HGNC Symbol;Acc:HGNC:13509] |
| ENSG00000170374 | HGLibA_46313 | 75 | -0.402274152 | SP7 | Sp7 transcription factor [Source:HGNC Symbol;Acc:HGNC:17321] |
| ENSG00000170454 | HGLibA_25537 | 75 | -0.402274152 | KRT75 | keratin 75 [Source:HGNC Symbol;Acc:HGNC:24431] |
| ENSG00000170627 | HGLibA_20628 | 75 | -0.402274152 | GTSF1 | gametocyte specific factor 1 [Source:HGNC Symbol;Acc:HGNC:26565] |
| ENSG00000171345 | HGLibA_25441 | 75 | -0.402274152 | KRT19 | keratin 19 [Source:HGNC Symbol;Acc:HGNC:6436] |
| ENSG00000171481 | HGLibA_33690 | 75 | -0.402274152 | OR1L3 | olfactory receptor family 1 subfamily L member 3 [Source:HGNC Symbol;Acc:HGNC:8215] |
| ENSG00000171551 | HGLibA_14388 | 75 | -0.402274152 | ECEL1 | endothelin converting enzyme like 1 [Source:HGNC Symbol;Acc:HGNC:3147] |
| ENSG00000171595 | HGLibA_13506 | 75 | -0.402274152 | DNAI2 | dynein axonemal intermediate chain 2 [Source:HGNC Symbol;Acc:HGNC:18744] |
| ENSG00000171916 | HGLibA_26388 | 75 | -0.402274152 | LGALS9C | galectin 9C [Source:HGNC Symbol;Acc:HGNC:33874] |
| ENSG00000171916 | HGLibA_26387 | 75 | -0.402274152 | LGALS9C | galectin 9C [Source:HGNC Symbol;Acc:HGNC:33874] |
| ENSG00000172156 | HGLibA_07964 | 75 | -0.402274152 | CCL11 | C-C motif chemokine ligand 11 [Source:HGNC Symbol;Acc:HGNC:10610] |
| ENSG00000172346 | HGLibA_11311 | 75 | -0.402274152 | CSDC2 | cold shock domain containing C2 [Source:HGNC Symbol;Acc:HGNC:30359] |
| ENSG00000172590 | HGLibA_30015 | 75 | -0.402274152 | MRPL52 | mitochondrial ribosomal protein L52 [Source:HGNC Symbol;Acc:HGNC:16655] |
| ENSG00000172840 | HGLibA_35928 | 75 | -0.402274152 | PDP2 | pyruvate dehyrogenase phosphatase catalytic subunit 2 [Source:HGNC Symbol;Acc:HGNC:30263] |
| ENSG00000173065 | HGLibA_16526 | 75 | -0.402274152 | FAM222B | family with sequence similarity 222 member B [Source:HGNC Symbol;Acc:HGNC:25563] |
| ENSG00000173406 | HGLibA_12266 | 75 | -0.402274152 | DAB1 | reelin adaptor protein [Source:HGNC Symbol;Acc:HGNC:2661]" |
| ENSG00000174428 | HGLibA_20575 | 75 | -0.402274152 | GTF2IRD2B | GTF2I repeat domain containing 2B [Source:HGNC Symbol;Acc:HGNC:33125] |
| ENSG00000174953 | HGLibA_13191 | 75 | -0.402274152 | DHX36 | DEAH-box helicase 36 [Source:HGNC Symbol;Acc:HGNC:14410] |
| ENSG00000175322 | HGLibA_56154 | 75 | -0.402274152 | ZNF519 | zinc finger protein 519 [Source:HGNC Symbol;Acc:HGNC:30574] |
| ENSG00000175344 | HGLibA_09583 | 75 | -0.402274152 | CHRNA7 | cholinergic receptor nicotinic alpha 7 subunit [Source:HGNC Symbol;Acc:HGNC:1960] |
| ENSG00000176787 | HGLibA_34158 | 75 | -0.402274152 | OR52E2 | olfactory receptor family 52 subfamily E member 2 [Source:HGNC Symbol;Acc:HGNC:14769] |
| ENSG00000177084 | HGLibA_37511 | 75 | -0.402274152 | POLE | catalytic subunit [Source:HGNC Symbol;Acc:HGNC:9177]" |
| ENSG00000178235 | HGLibA_45512 | 75 | -0.402274152 | SLITRK1 | SLIT and NTRK like family member 1 [Source:HGNC Symbol;Acc:HGNC:20297] |
| ENSG00000178295 | HGLibA_18929 | 75 | -0.402274152 | GEN1 | Holliday junction 5' flap endonuclease [Source:HGNC Symbol;Acc:HGNC:26881]" |
| ENSG00000179051 | HGLibA_40686 | 75 | -0.402274152 | RCC2 | regulator of chromosome condensation 2 [Source:HGNC Symbol;Acc:HGNC:30297] |
| ENSG00000181323 | HGLibA_46617 | 75 | -0.402274152 | SPEM1 | spermatid maturation 1 [Source:HGNC Symbol;Acc:HGNC:32429] |
| ENSG00000181552 | HGLibA_14451 | 75 | -0.402274152 | EDDM3B | epididymal protein 3B [Source:HGNC Symbol;Acc:HGNC:19223] |
| ENSG00000182400 | HGLibA_51332 | 75 | -0.402274152 | TRAPPC6B | trafficking protein particle complex 6B [Source:HGNC Symbol;Acc:HGNC:23066] |
| ENSG00000183317 | HGLibA_15287 | 75 | -0.402274152 | EPHA10 | EPH receptor A10 [Source:HGNC Symbol;Acc:HGNC:19987] |
| ENSG00000183628 | HGLibA_13048 | 75 | -0.402274152 | DGCR6 | DiGeorge syndrome critical region gene 6 [Source:HGNC Symbol;Acc:HGNC:2846] |
| ENSG00000184389 | HGLibA_00014 | 75 | -0.402274152 | A3GALT2 | 3-galactosyltransferase 2 [Source:HGNC Symbol;Acc:HGNC:30005]" |
| ENSG00000184613 | HGLibA_31687 | 75 | -0.402274152 | NELL2 | neural EGFL like 2 [Source:HGNC Symbol;Acc:HGNC:7751] |
| ENSG00000184716 | HGLibA_43493 | 75 | -0.402274152 | SERINC4 | serine incorporator 4 [Source:HGNC Symbol;Acc:HGNC:32237] |
| ENSG00000184730 | HGLibA_02501 | 75 | -0.402274152 | APOBR | apolipoprotein B receptor [Source:HGNC Symbol;Acc:HGNC:24087] |
| ENSG00000185189 | HGLibA_32659 | 75 | -0.402274152 | NRBP2 | nuclear receptor binding protein 2 [Source:HGNC Symbol;Acc:HGNC:19339] |
| ENSG00000185278 | HGLibA_54869 | 75 | -0.402274152 | ZBTB37 | zinc finger and BTB domain containing 37 [Source:HGNC Symbol;Acc:HGNC:28365] |
| ENSG00000185483 | HGLibA_41770 | 75 | -0.402274152 | ROR1 | receptor tyrosine kinase like orphan receptor 1 [Source:HGNC Symbol;Acc:HGNC:10256] |
| ENSG00000185499 | HGLibA_30520 | 75 | -0.402274152 | MUC1 | cell surface associated [Source:HGNC Symbol;Acc:HGNC:7508]" |
| ENSG00000187764 | HGLibA_43348 | 75 | -0.402274152 | SEMA4D | semaphorin 4D [Source:HGNC Symbol;Acc:HGNC:10732] |
| ENSG00000187954 | HGLibA_12039 | 75 | -0.402274152 | CYHR1 | cysteine and histidine rich 1 [Source:HGNC Symbol;Acc:HGNC:17806] |
| ENSG00000188095 | HGLibA_28920 | 75 | -0.402274152 | MESP2 | mesoderm posterior bHLH transcription factor 2 [Source:HGNC Symbol;Acc:HGNC:29659] |
| ENSG00000188157 | HGLibA_01249 | 75 | -0.402274152 | AGRN | agrin [Source:HGNC Symbol;Acc:HGNC:329] |
| ENSG00000188389 | HGLibA_35715 | 75 | -0.402274152 | PDCD1 | programmed cell death 1 [Source:HGNC Symbol;Acc:HGNC:8760] |
| ENSG00000189164 | HGLibA_56167 | 75 | -0.402274152 | ZNF527 | zinc finger protein 527 [Source:HGNC Symbol;Acc:HGNC:29385] |
| ENSG00000189369 | HGLibA_20443 | 75 | -0.402274152 | GSPT2 | G1 to S phase transition 2 [Source:HGNC Symbol;Acc:HGNC:4622] |
| ENSG00000196155 | HGLibA_37109 | 75 | -0.402274152 | PLEKHG4 | pleckstrin homology and RhoGEF domain containing G4 [Source:HGNC Symbol;Acc:HGNC:24501] |
| ENSG00000196199 | HGLibA_29728 | 75 | -0.402274152 | MPHOSPH8 | M-phase phosphoprotein 8 [Source:HGNC Symbol;Acc:HGNC:29810] |
| ENSG00000197006 | HGLibA_29025 | 75 | -0.402274152 | METTL9 | methyltransferase like 9 [Source:HGNC Symbol;Acc:HGNC:24586] |
| ENSG00000197070 | HGLibA_03114 | 75 | -0.402274152 | ARRDC1 | arrestin domain containing 1 [Source:HGNC Symbol;Acc:HGNC:28633] |
| ENSG00000197629 | HGLibA_29718 | 75 | -0.402274152 | MPEG1 | macrophage expressed 1 [Source:HGNC Symbol;Acc:HGNC:29619] |
| ENSG00000198390 | HGLibA_25642 | 75 | -0.402274152 | KRTAP13-1 | keratin associated protein 13-1 [Source:HGNC Symbol;Acc:HGNC:18924] |
| ENSG00000198440 | HGLibA_56315 | 75 | -0.402274152 | ZNF583 | zinc finger protein 583 [Source:HGNC Symbol;Acc:HGNC:26427] |
| ENSG00000204478 | HGLibA_38307 | 75 | -0.402274152 | PRAMEF20 | PRAME family member 20 [Source:HGNC Symbol;Acc:HGNC:25224] |
| ENSG00000204869 | HGLibA_22849 | 75 | -0.402274152 | IGFL4 | IGF like family member 4 [Source:HGNC Symbol;Acc:HGNC:32931] |
| ENSG00000213160 | HGLibA_25159 | 75 | -0.402274152 | KLHL23 | kelch like family member 23 [Source:HGNC Symbol;Acc:HGNC:27506] |
| ENSG00000213398 | HGLibA_26097 | 75 | -0.402274152 | LCAT | lecithin-cholesterol acyltransferase [Source:HGNC Symbol;Acc:HGNC:6522] |
| ENSG00000213799 | HGLibA_56862 | 75 | -0.402274152 | ZNF845 | zinc finger protein 845 [Source:HGNC Symbol;Acc:HGNC:25112] |
| ENSG00000221866 | HGLibA_37266 | 75 | -0.402274152 | PLXNA4 | plexin A4 [Source:HGNC Symbol;Acc:HGNC:9102] |
| ENSG00000221923 | HGLibA_56894 | 75 | -0.402274152 | ZNF880 | zinc finger protein 880 [Source:HGNC Symbol;Acc:HGNC:37249] |
| ENSG00000221955 | HGLibA_44377 | 75 | -0.402274152 | SLC12A8 | solute carrier family 12 member 8 [Source:HGNC Symbol;Acc:HGNC:15595] |
| ENSG00000221977 | HGLibA_33987 | 75 | -0.402274152 | OR4E2 | olfactory receptor family 4 subfamily E member 2 [Source:HGNC Symbol;Acc:HGNC:8297] |
| ENSG00000223572 | HGLibA_09779 | 75 | -0.402274152 | CKMT1A | mitochondrial 1A [Source:HGNC Symbol;Acc:HGNC:31736]" |
| ENSG00000225830 | HGLibA_15446 | 75 | -0.402274152 | ERCC6 | chromatin remodeling factor [Source:HGNC Symbol;Acc:HGNC:3438]" |
| ENSG00000229859 | HGLibA_36185 | 75 | -0.402274152 | PGA3 | group I (pepsinogen A) [Source:HGNC Symbol;Acc:HGNC:8885]" |
| ENSG00000230594 | HGLibA_11533 | 75 | -0.402274152 | CT47A4 | member A4 [Source:HGNC Symbol;Acc:HGNC:33285]" |
| ENSG00000237763 | HGLibA_01841 | 75 | -0.402274152 | AMY1A | alpha 1A (salivary) [Source:HGNC Symbol;Acc:HGNC:474]" |
| ENSG00000242019 | HGLibA_24998 | 75 | -0.402274152 | KIR3DL3 | three Ig domains and long cytoplasmic tail 3 [Source:HGNC Symbol;Acc:HGNC:16312]" |
| ENSG00000243708 | HGLibA_36903 | 75 | -0.402274152 | PLA2G4B | phospholipase A2 group IVB [Source:HGNC Symbol;Acc:HGNC:9036] |
| ENSG00000244122 | HGLibA_52894 | 75 | -0.402274152 | UGT1A7 | UDP glucuronosyltransferase family 1 member A7 [Source:HGNC Symbol;Acc:HGNC:12539] |
| ENSG00000253537 | HGLibA_35540 | 75 | -0.402274152 | PCDHGA7 | 7 [Source:HGNC Symbol;Acc:HGNC:8705]" |
| ENSG00000257218 | HGLibA_18691 | 75 | -0.402274152 | GATC | glutamyl-tRNA amidotransferase subunit C [Source:HGNC Symbol;Acc:HGNC:25068] |
| FAM179B | HGLibA_16309 | 75 | -0.402274152 | FAM179B | - |
| FGFR1OP | HGLibA_17437 | 75 | -0.402274152 | FGFR1OP | - |
| GPR113 | HGLibA_19854 | 75 | -0.402274152 | GPR113 | - |
| GUCY1B3 | HGLibA_20659 | 75 | -0.402274152 | GUCY1B3 | - |
| HN1L | HGLibA_21771 | 75 | -0.402274152 | HN1L | - |
| hsa-mir-122 | HGLibA_57292 | 75 | -0.402274152 | hsa-mir-122 | - |
| hsa-mir-1287 | HGLibA_57613 | 75 | -0.402274152 | hsa-mir-1287 | - |
| hsa-mir-1301 | HGLibA_57678 | 75 | -0.402274152 | hsa-mir-1301 | - |
| hsa-mir-219a-2 | HGLibA_58280 | 75 | -0.402274152 | hsa-mir-219a-2 | - |
| hsa-mir-3158-1 | HGLibA_58702 | 75 | -0.402274152 | hsa-mir-3158-1 | - |
| hsa-mir-4484 | HGLibA_60355 | 75 | -0.402274152 | hsa-mir-4484 | - |
| hsa-mir-4520b | HGLibA_60537 | 75 | -0.402274152 | hsa-mir-4520b | - |
| hsa-mir-4741 | HGLibA_61060 | 75 | -0.402274152 | hsa-mir-4741 | - |
| hsa-mir-4743 | HGLibA_61071 | 75 | -0.402274152 | hsa-mir-4743 | - |
| hsa-mir-4776-1 | HGLibA_61207 | 75 | -0.402274152 | hsa-mir-4776-1 | - |
| hsa-mir-5093 | HGLibA_61532 | 75 | -0.402274152 | hsa-mir-5093 | - |
| hsa-mir-548ae-1 | HGLibA_61867 | 75 | -0.402274152 | hsa-mir-548ae-1 | - |
| hsa-mir-548h-5 | HGLibA_62009 | 75 | -0.402274152 | hsa-mir-548h-5 | - |
| hsa-mir-561 | HGLibA_62206 | 75 | -0.402274152 | hsa-mir-561 | - |
| hsa-mir-583 | HGLibA_62430 | 75 | -0.402274152 | hsa-mir-583 | - |
| hsa-mir-631 | HGLibA_62764 | 75 | -0.402274152 | hsa-mir-631 | - |
| hsa-mir-6514 | HGLibA_62929 | 75 | -0.402274152 | hsa-mir-6514 | - |
| hsa-mir-6770-3 | HGLibA_63254 | 75 | -0.402274152 | hsa-mir-6770-3 | - |
| hsa-mir-6817 | HGLibA_63446 | 75 | -0.402274152 | hsa-mir-6817 | - |
| hsa-mir-6878 | HGLibA_63698 | 75 | -0.402274152 | hsa-mir-6878 | - |
| hsa-mir-8073 | HGLibA_64133 | 75 | -0.402274152 | hsa-mir-8073 | - |
| hsa-mir-888 | HGLibA_64231 | 75 | -0.402274152 | hsa-mir-888 | - |
| LOC100130301 | HGLibA_26775 | 75 | -0.402274152 | LOC100130301 | - |
| LOC100505549 | HGLibA_26849 | 75 | -0.402274152 | LOC100505549 | - |
| LOC100506688 | HGLibA_26864 | 75 | -0.402274152 | LOC100506688 | - |
| LOC100996485 | HGLibA_26886 | 75 | -0.402274152 | LOC100996485 | - |
| NHP2L1 | HGLibA_31911 | 75 | -0.402274152 | NHP2L1 | - |
| PPAPDC2 | HGLibA_37831 | 75 | -0.402274152 | PPAPDC2 | - |
| PTPLB | HGLibA_39424 | 75 | -0.402274152 | PTPLB | - |
| TBC1D29 | HGLibA_48525 | 75 | -0.402274152 | TBC1D29 | - |
| WBSCR27 | HGLibA_53999 | 75 | -0.402274152 | WBSCR27 | - |
| WDR34 | HGLibA_54069 | 75 | -0.402274152 | WDR34 | - |
| WDR85 | HGLibA_54199 | 75 | -0.402274152 | WDR85 | - |
| C10orf137 | HGLibA_05057 | 74 | -0.412225189 | C10orf137 | - |

| | | | | | |
|---|---|---|---|---|---|
| C10orf35 | HGLibA_05070 | 74 | -0.412225189 | C10orf35 | - |
| C12orf10 | HGLibA_05224 | 74 | -0.412225189 | C12orf10 | - |
| C1orf86 | HGLibA_05900 | 74 | -0.412225189 | C1orf86 | - |
| C3orf72 | HGLibA_06266 | 74 | -0.412225189 | C3orf72 | - |
| C7orf29 | HGLibA_06531 | 74 | -0.412225189 | C7orf29 | - |
| C7orf62 | HGLibA_06568 | 74 | -0.412225189 | C7orf62 | - |
| CGB | HGLibA_09300 | 74 | -0.412225189 | CGB | - |
| ENSG00000006282 | HGLibA_46448 | 74 | -0.412225189 | SPATA20 | spermatogenesis associated 20 [Source:HGNC Symbol;Acc:HGNC:26125] |
| ENSG00000006625 | HGLibA_18993 | 74 | -0.412225189 | GGCT | gamma-glutamylcyclotransferase [Source:HGNC Symbol;Acc:HGNC:21705] |
| ENSG00000007314 | HGLibA_42991 | 74 | -0.412225189 | SCN4A | sodium voltage-gated channel alpha subunit 4 [Source:HGNC Symbol;Acc:HGNC:10591] |
| ENSG00000008710 | HGLibA_36789 | 74 | -0.412225189 | PKD1 | transient receptor potential channel interacting [Source:HGNC Symbol;Acc:HGNC:9008]" |
| ENSG00000009765 | HGLibA_23889 | 74 | -0.412225189 | IYD | iodotyrosine deiodinase [Source:HGNC Symbol;Acc:HGNC:21071] |
| ENSG00000010404 | HGLibA_22567 | 74 | -0.412225189 | IDS | iduronate 2-sulfatase [Source:HGNC Symbol;Acc:HGNC:5389] |
| ENSG00000011295 | HGLibA_52066 | 74 | -0.412225189 | TTC19 | tetratricopeptide repeat domain 19 [Source:HGNC Symbol;Acc:HGNC:26006] |
| ENSG00000013288 | HGLibA_28078 | 74 | -0.412225189 | MAN2B2 | mannosidase alpha class 2B member 2 [Source:HGNC Symbol;Acc:HGNC:29623] |
| ENSG00000052723 | HGLibA_44168 | 74 | -0.412225189 | SIKE1 | suppressor of IKBKE 1 [Source:HGNC Symbol;Acc:HGNC:26119] |
| ENSG00000053108 | HGLibA_18106 | 74 | -0.412225189 | FSTL4 | follistatin like 4 [Source:HGNC Symbol;Acc:HGNC:21389] |
| ENSG00000053702 | HGLibA_32693 | 74 | -0.412225189 | NRIP2 | nuclear receptor interacting protein 2 [Source:HGNC Symbol;Acc:HGNC:23078] |
| ENSG00000055483 | HGLibA_53347 | 74 | -0.412225189 | USP36 | ubiquitin specific peptidase 36 [Source:HGNC Symbol;Acc:HGNC:20062] |
| ENSG00000060982 | HGLibA_04262 | 74 | -0.412225189 | BCAT1 | branched chain amino acid transaminase 1 [Source:HGNC Symbol;Acc:HGNC:976] |
| ENSG00000064989 | HGLibA_07007 | 74 | -0.412225189 | CALCRL | calcitonin receptor like receptor [Source:HGNC Symbol;Acc:HGNC:16709] |
| ENSG00000066405 | HGLibA_09872 | 74 | -0.412225189 | CLDN18 | claudin 18 [Source:HGNC Symbol;Acc:HGNC:2039] |
| ENSG00000066735 | HGLibA_24915 | 74 | -0.412225189 | KIF26A | kinesin family member 26A [Source:HGNC Symbol;Acc:HGNC:20226] |
| ENSG00000067048 | HGLibA_12706 | 74 | -0.412225189 | DDX3Y | DEAD-box helicase 3 Y-linked [Source:HGNC Symbol;Acc:HGNC:2699] |
| ENSG00000070501 | HGLibA_37488 | 74 | -0.412225189 | POLB | DNA polymerase beta [Source:HGNC Symbol;Acc:HGNC:9174] |
| ENSG00000071553 | HGLibA_03694 | 74 | -0.412225189 | ATP6AP1 | ATPase H+ transporting accessory protein 1 [Source:HGNC Symbol;Acc:HGNC:868] |
| ENSG00000073861 | HGLibA_48663 | 74 | -0.412225189 | TBX21 | T-box 21 [Source:HGNC Symbol;Acc:HGNC:11599] |
| ENSG00000076513 | HGLibA_02000 | 74 | -0.412225189 | ANKRD13A | ankyrin repeat domain 13A [Source:HGNC Symbol;Acc:HGNC:21268] |
| ENSG00000078898 | HGLibA_04722 | 74 | -0.412225189 | BPIFB2 | BPI fold containing family B member 2 [Source:HGNC Symbol;Acc:HGNC:16177] |
| ENSG00000081479 | HGLibA_27211 | 74 | -0.412225189 | LRP2 | LDL receptor related protein 2 [Source:HGNC Symbol;Acc:HGNC:6694] |
| ENSG00000081721 | HGLibA_14137 | 74 | -0.412225189 | DUSP12 | dual specificity phosphatase 12 [Source:HGNC Symbol;Acc:HGNC:3067] |
| ENSG00000087470 | HGLibA_13686 | 74 | -0.412225189 | DNM1L | dynamin 1 like [Source:HGNC Symbol;Acc:HGNC:2973] |
| ENSG00000089558 | HGLibA_24218 | 74 | -0.412225189 | KCNH4 | potassium voltage-gated channel subfamily H member 4 [Source:HGNC Symbol;Acc:HGNC:6253] |
| ENSG00000089692 | HGLibA_25926 | 74 | -0.412225189 | LAG3 | lymphocyte activating 3 [Source:HGNC Symbol;Acc:HGNC:6476] |
| ENSG00000091428 | HGLibA_40255 | 74 | -0.412225189 | RAPGEF4 | Rap guanine nucleotide exchange factor 4 [Source:HGNC Symbol;Acc:HGNC:16626] |
| ENSG00000092529 | HGLibA_07167 | 74 | -0.412225189 | CAPN3 | calpain 3 [Source:HGNC Symbol;Acc:HGNC:1480] |
| ENSG00000092841 | HGLibA_30770 | 74 | -0.412225189 | MYL6 | myosin light chain 6 [Source:HGNC Symbol;Acc:HGNC:7587] |
| ENSG00000095464 | HGLibA_35811 | 74 | -0.412225189 | PDE6C | phosphodiesterase 6C [Source:HGNC Symbol;Acc:HGNC:8787] |
| ENSG00000095906 | HGLibA_32903 | 74 | -0.412225189 | NUBP2 | nucleotide binding protein 2 [Source:HGNC Symbol;Acc:HGNC:8042] |
| ENSG00000099817 | HGLibA_37576 | 74 | -0.412225189 | POLR2E | RNA polymerase II subunit E [Source:HGNC Symbol;Acc:HGNC:9192] |
| ENSG00000100417 | HGLibA_37315 | 74 | -0.412225189 | PMM1 | phosphomannomutase 1 [Source:HGNC Symbol;Acc:HGNC:9114] |
| ENSG00000101076 | HGLibA_21779 | 74 | -0.412225189 | HNF4A | hepatocyte nuclear factor 4 alpha [Source:HGNC Symbol;Acc:HGNC:5024] |
| ENSG00000103449 | HGLibA_42648 | 74 | -0.412225189 | SALL1 | spalt like transcription factor 1 [Source:HGNC Symbol;Acc:HGNC:10524] |
| ENSG00000104450 | HGLibA_46341 | 74 | -0.412225189 | SPAG1 | sperm associated antigen 1 [Source:HGNC Symbol;Acc:HGNC:11212] |
| ENSG00000104679 | HGLibA_39765 | 74 | -0.412225189 | R3HCC1 | R3H domain and coiled-coil containing 1 [Source:HGNC Symbol;Acc:HGNC:27329] |
| ENSG00000104723 | HGLibA_52349 | 74 | -0.412225189 | TUSC3 | tumor suppressor candidate 3 [Source:HGNC Symbol;Acc:HGNC:30242] |
| ENSG00000104907 | HGLibA_51658 | 74 | -0.412225189 | TRMT1 | tRNA methyltransferase 1 [Source:HGNC Symbol;Acc:HGNC:25980] |
| ENSG00000105852 | HGLibA_37684 | 74 | -0.412225189 | PON3 | paraoxonase 3 [Source:HGNC Symbol;Acc:HGNC:9206] |
| ENSG00000105856 | HGLibA_20934 | 74 | -0.412225189 | HBP1 | HMG-box transcription factor 1 [Source:HGNC Symbol;Acc:HGNC:23200] |
| ENSG00000106436 | HGLibA_30749 | 74 | -0.412225189 | MYL10 | myosin light chain 10 [Source:HGNC Symbol;Acc:HGNC:29825] |
| ENSG00000106511 | HGLibA_28893 | 74 | -0.412225189 | MEOX2 | mesenchyme homeobox 2 [Source:HGNC Symbol;Acc:HGNC:7014] |
| ENSG00000107625 | HGLibA_12733 | 74 | -0.412225189 | DDX50 | DExD-box helicase 50 [Source:HGNC Symbol;Acc:HGNC:17906] |
| ENSG00000107771 | HGLibA_08193 | 74 | -0.412225189 | CCSER2 | coiled-coil serine rich protein 2 [Source:HGNC Symbol;Acc:HGNC:29197] |
| ENSG00000108344 | HGLibA_39169 | 74 | -0.412225189 | PSMD3 | non-ATPase 3 [Source:HGNC Symbol;Acc:HGNC:9560]" |
| ENSG00000108465 | HGLibA_08788 | 74 | -0.412225189 | CDK5RAP3 | CDK5 regulatory subunit associated protein 3 [Source:HGNC Symbol;Acc:HGNC:18673] |
| ENSG00000109381 | HGLibA_14895 | 74 | -0.412225189 | ELF2 | E74 like ETS transcription factor 2 [Source:HGNC Symbol;Acc:HGNC:3317] |
| ENSG00000110013 | HGLibA_44101 | 74 | -0.412225189 | SIAE | sialic acid acetylesterase [Source:HGNC Symbol;Acc:HGNC:18187] |
| ENSG00000110079 | HGLibA_30152 | 74 | -0.412225189 | MS4A4A | membrane spanning 4-domains A4A [Source:HGNC Symbol;Acc:HGNC:13371] |
| ENSG00000111058 | HGLibA_00593 | 74 | -0.412225189 | ACSS3 | acyl-CoA synthetase short chain family member 3 [Source:HGNC Symbol;Acc:HGNC:24723] |
| ENSG00000112782 | HGLibA_10034 | 74 | -0.412225189 | CLIC5 | chloride intracellular channel 5 [Source:HGNC Symbol;Acc:HGNC:13517] |
| ENSG00000113905 | HGLibA_22092 | 74 | -0.412225189 | HRG | histidine rich glycoprotein [Source:HGNC Symbol;Acc:HGNC:5181] |
| ENSG00000118162 | HGLibA_25381 | 74 | -0.412225189 | KPTN | actin binding protein [Source:HGNC Symbol;Acc:HGNC:6404]" |
| ENSG00000119446 | HGLibA_40499 | 74 | -0.412225189 | RBM18 | RNA binding motif protein 18 [Source:HGNC Symbol;Acc:HGNC:28413] |
| ENSG00000119844 | HGLibA_01129 | 74 | -0.412225189 | AFTPH | aftiphilin [Source:HGNC Symbol;Acc:HGNC:25951] |
| ENSG00000121634 | HGLibA_19151 | 74 | -0.412225189 | GJA8 | gap junction protein alpha 8 [Source:HGNC Symbol;Acc:HGNC:4281] |
| ENSG00000121749 | HGLibA_48482 | 74 | -0.412225189 | TBC1D15 | TBC1 domain family member 15 [Source:HGNC Symbol;Acc:HGNC:25694] |
| ENSG00000122194 | HGLibA_37157 | 74 | -0.412225189 | PLG | plasminogen [Source:HGNC Symbol;Acc:HGNC:9071] |
| ENSG00000122863 | HGLibA_09635 | 74 | -0.412225189 | CHST3 | carbohydrate sulfotransferase 3 [Source:HGNC Symbol;Acc:HGNC:1971] |
| ENSG00000123739 | HGLibA_36871 | 74 | -0.412225189 | PLA2G12A | phospholipase A2 group XIIA [Source:HGNC Symbol;Acc:HGNC:18554] |
| ENSG00000123977 | HGLibA_12346 | 74 | -0.412225189 | DAW1 | dynein assembly factor with WD repeats 1 [Source:HGNC Symbol;Acc:HGNC:26383] |
| ENSG00000124374 | HGLibA_35035 | 74 | -0.412225189 | PAIP2B | poly(A) binding protein interacting protein 2B [Source:HGNC Symbol;Acc:HGNC:29200] |
| ENSG00000124882 | HGLibA_15458 | 74 | -0.412225189 | EREG | epiregulin [Source:HGNC Symbol;Acc:HGNC:3443] |
| ENSG00000124935 | HGLibA_42918 | 74 | -0.412225189 | SCGB1D2 | secretoglobin family 1D member 2 [Source:HGNC Symbol;Acc:HGNC:18396] |
| ENSG00000126432 | HGLibA_38418 | 74 | -0.412225189 | PRDX5 | peroxiredoxin 5 [Source:HGNC Symbol;Acc:HGNC:9355] |
| ENSG00000126522 | HGLibA_03316 | 74 | -0.412225189 | ASL | argininosuccinate lyase [Source:HGNC Symbol;Acc:HGNC:746] |
| ENSG00000129317 | HGLibA_39606 | 74 | -0.412225189 | PUS7L | pseudouridine synthase 7 like [Source:HGNC Symbol;Acc:HGNC:25276] |
| ENSG00000131233 | HGLibA_19154 | 74 | -0.412225189 | GJA9 | gap junction protein alpha 9 [Source:HGNC Symbol;Acc:HGNC:19155] |
| ENSG00000131242 | HGLibA_39802 | 74 | -0.412225189 | RAB11FIP4 | RAB11 family interacting protein 4 [Source:HGNC Symbol;Acc:HGNC:30267] |
| ENSG00000131697 | HGLibA_32450 | 74 | -0.412225189 | NPHP4 | nephrocystin 4 [Source:HGNC Symbol;Acc:HGNC:19104] |
| ENSG00000131732 | HGLibA_55050 | 74 | -0.412225189 | ZCCHC9 | zinc finger CCHC-type containing 9 [Source:HGNC Symbol;Acc:HGNC:25424] |
| ENSG00000132010 | HGLibA_55566 | 74 | -0.412225189 | ZNF20 | zinc finger protein 20 [Source:HGNC Symbol;Acc:HGNC:12992] |
| ENSG00000132017 | HGLibA_12429 | 74 | -0.412225189 | DCAF15 | DDB1 and CUL4 associated factor 15 [Source:HGNC Symbol;Acc:HGNC:25095] |
| ENSG00000134042 | HGLibA_29864 | 74 | -0.412225189 | MRO | maestro [Source:HGNC Symbol;Acc:HGNC:24121] |
| ENSG00000134748 | HGLibA_38721 | 74 | -0.412225189 | PRPF38A | pre-mRNA processing factor 38A [Source:HGNC Symbol;Acc:HGNC:25930] |
| ENSG00000135334 | HGLibA_01459 | 74 | -0.412225189 | AKIRIN2 | akirin 2 [Source:HGNC Symbol;Acc:HGNC:21407] |
| ENSG00000135521 | HGLibA_27618 | 74 | -0.412225189 | LTV1 | LTV1 ribosome biogenesis factor [Source:HGNC Symbol;Acc:HGNC:21173] |
| ENSG00000136697 | HGLibA_23042 | 74 | -0.412225189 | IL1F10 | interleukin 1 family member 10 [Source:HGNC Symbol;Acc:HGNC:15552] |
| ENSG00000136854 | HGLibA_47664 | 74 | -0.412225189 | STXBP1 | syntaxin binding protein 1 [Source:HGNC Symbol;Acc:HGNC:11444] |
| ENSG00000136986 | HGLibA_12995 | 74 | -0.412225189 | DERL1 | derlin 1 [Source:HGNC Symbol;Acc:HGNC:28454] |
| ENSG00000137494 | HGLibA_02100 | 74 | -0.412225189 | ANKRD42 | ankyrin repeat domain 42 [Source:HGNC Symbol;Acc:HGNC:26752] |
| ENSG00000137942 | HGLibA_17739 | 74 | -0.412225189 | FNBP1L | formin binding protein 1 like [Source:HGNC Symbol;Acc:HGNC:20851] |
| ENSG00000138600 | HGLibA_46776 | 74 | -0.412225189 | SPPL2A | signal peptide peptidase like 2A [Source:HGNC Symbol;Acc:HGNC:30227] |
| ENSG00000138741 | HGLibA_51730 | 74 | -0.412225189 | TRPC3 | transient receptor potential cation channel subfamily C member 3 [Source:HGNC Symbol;Acc:HGNC:12335] |
| ENSG00000139648 | HGLibA_25526 | 74 | -0.412225189 | KRT71 | keratin 71 [Source:HGNC Symbol;Acc:HGNC:28927] |
| ENSG00000139874 | HGLibA_47173 | 74 | -0.412225189 | SSTR1 | somatostatin receptor 1 [Source:HGNC Symbol;Acc:HGNC:11330] |
| ENSG00000140025 | HGLibA_14522 | 74 | -0.412225189 | EFCAB11 | EF-hand calcium binding domain 11 [Source:HGNC Symbol;Acc:HGNC:20357] |
| ENSG00000140519 | HGLibA_41147 | 74 | -0.412225189 | RHCG | Rh family C glycoprotein [Source:HGNC Symbol;Acc:HGNC:18140] |
| ENSG00000140835 | HGLibA_09637 | 74 | -0.412225189 | CHST4 | carbohydrate sulfotransferase 4 [Source:HGNC Symbol;Acc:HGNC:1972] |
| ENSG00000141376 | HGLibA_04256 | 74 | -0.412225189 | BCAS3 | microtubule associated cell migration factor [Source:HGNC Symbol;Acc:HGNC:14347]" |
| ENSG00000141510 | HGLibA_51047 | 74 | -0.412225189 | TP53 | tumor protein p53 [Source:HGNC Symbol;Acc:HGNC:11998] |
| ENSG00000141569 | HGLibA_51584 | 74 | -0.412225189 | TRIM65 | tripartite motif containing 65 [Source:HGNC Symbol;Acc:HGNC:27316] |
| ENSG00000143315 | HGLibA_36567 | 74 | -0.412225189 | PIGM | phosphatidylinositol glycan anchor biosynthesis class M [Source:HGNC Symbol;Acc:HGNC:18858] |
| ENSG00000143570 | HGLibA_45089 | 74 | -0.412225189 | SLC39A1 | solute carrier family 39 member 1 [Source:HGNC Symbol;Acc:HGNC:12876] |
| ENSG00000143862 | HGLibA_02995 | 74 | -0.412225189 | ARL8A | ADP ribosylation factor like GTPase 8A [Source:HGNC Symbol;Acc:HGNC:25192] |
| ENSG00000144214 | HGLibA_27697 | 74 | -0.412225189 | LYG1 | lysozyme g1 [Source:HGNC Symbol;Acc:HGNC:27014] |
| ENSG00000145780 | HGLibA_17279 | 74 | -0.412225189 | FEM1C | fem-1 homolog C [Source:HGNC Symbol;Acc:HGNC:16933] |
| ENSG00000146426 | HGLibA_49443 | 74 | -0.412225189 | TIAM2 | T cell lymphoma invasion and metastasis 2 [Source:HGNC Symbol;Acc:HGNC:11806] |
| ENSG00000147862 | HGLibA_31804 | 74 | -0.412225189 | NFIB | nuclear factor I B [Source:HGNC Symbol;Acc:HGNC:7785] |
| ENSG00000148248 | HGLibA_47838 | 74 | -0.412225189 | SURF4 | surfeit 4 [Source:HGNC Symbol;Acc:HGNC:11476] |
| ENSG00000148516 | HGLibA_55134 | 74 | -0.412225189 | ZEB1 | zinc finger E-box binding homeobox 1 [Source:HGNC Symbol;Acc:HGNC:11642] |
| ENSG00000148942 | HGLibA_45262 | 74 | -0.412225189 | SLC5A12 | solute carrier family 5 member 12 [Source:HGNC Symbol;Acc:HGNC:28750] |
| ENSG00000149357 | HGLibA_25988 | 74 | -0.412225189 | LAMTOR1 | MAPK and MTOR activator 1 [Source:HGNC Symbol;Acc:HGNC:26068]" |
| ENSG00000149806 | HGLibA_16924 | 74 | -0.412225189 | FAU | ubiquitin like and ribosomal protein S30 fusion [Source:HGNC Symbol;Acc:HGNC:3597]" |
| ENSG00000149927 | HGLibA_13725 | 74 | -0.412225189 | DOC2A | double C2 domain alpha [Source:HGNC Symbol;Acc:HGNC:2985] |
| ENSG00000150760 | HGLibA_13730 | 74 | -0.412225189 | DOCK1 | dedicator of cytokinesis 1 [Source:HGNC Symbol;Acc:HGNC:2987] |
| ENSG00000150783 | HGLibA_49056 | 74 | -0.412225189 | TEX12 | testis expressed 12 [Source:HGNC Symbol;Acc:HGNC:11734] |
| ENSG00000151623 | HGLibA_32622 | 74 | -0.412225189 | NR3C2 | nuclear receptor subfamily 3 group C member 2 [Source:HGNC Symbol;Acc:HGNC:7979] |
| ENSG00000152580 | HGLibA_22879 | 74 | -0.412225189 | IGSF10 | immunoglobulin superfamily member 10 [Source:HGNC Symbol;Acc:HGNC:26384] |
| ENSG00000154642 | HGLibA_06049 | 74 | -0.412225189 | C21orf91 | chromosome 21 open reading frame 91 [Source:HGNC Symbol;Acc:HGNC:16459] |
| ENSG00000155657 | HGLibA_52219 | 74 | -0.412225189 | TTN | titin [Source:HGNC Symbol;Acc:HGNC:12403] |
| ENSG00000156983 | HGLibA_04838 | 74 | -0.412225189 | BRPF1 | bromodomain and PHD finger containing 1 [Source:HGNC Symbol;Acc:HGNC:14255] |
| ENSG00000157240 | HGLibA_18260 | 74 | -0.412225189 | FZD1 | frizzled class receptor 1 [Source:HGNC Symbol;Acc:HGNC:4038] |
| ENSG00000158604 | HGLibA_49812 | 74 | -0.412225189 | TMED4 | transmembrane p24 trafficking protein 4 [Source:HGNC Symbol;Acc:HGNC:22301] |
| ENSG00000158966 | HGLibA_06873 | 74 | -0.412225189 | CACHD1 | cache domain containing 1 [Source:HGNC Symbol;Acc:HGNC:29314] |
| ENSG00000159231 | HGLibA_07422 | 74 | -0.412225189 | CBR3 | carbonyl reductase 3 [Source:HGNC Symbol;Acc:HGNC:1549] |
| ENSG00000160633 | HGLibA_42634 | 74 | -0.412225189 | SAFB | scaffold attachment factor B [Source:HGNC Symbol;Acc:HGNC:10520] |
| ENSG00000160688 | HGLibA_17603 | 74 | -0.412225189 | FLAD1 | flavin adenine dinucleotide synthetase 1 [Source:HGNC Symbol;Acc:HGNC:24671] |
| ENSG00000161180 | HGLibA_07556 | 74 | -0.412225189 | CCDC116 | coiled-coil domain containing 116 [Source:HGNC Symbol;Acc:HGNC:26688] |
| ENSG00000161652 | HGLibA_23896 | 74 | -0.412225189 | IZUMO2 | IZUMO family member 2 [Source:HGNC Symbol;Acc:HGNC:28518] |
| ENSG00000161671 | HGLibA_15006 | 74 | -0.412225189 | EMC10 | ER membrane protein complex subunit 10 [Source:HGNC Symbol;Acc:HGNC:27609] |
| ENSG00000161791 | HGLibA_17698 | 74 | -0.412225189 | FMNL3 | formin like 3 [Source:HGNC Symbol;Acc:HGNC:23698] |
| ENSG00000161807 | HGLibA_34482 | 74 | -0.412225189 | OR7G1 | olfactory receptor family 7 subfamily G member 1 [Source:HGNC Symbol;Acc:HGNC:8465] |
| ENSG00000162522 | HGLibA_24773 | 74 | -0.412225189 | KIAA1522 | KIAA1522 [Source:HGNC Symbol;Acc:HGNC:29301] |
| ENSG00000162623 | HGLibA_52486 | 74 | -0.412225189 | TYW3 | tRNA-yW synthesizing protein 3 homolog [Source:HGNC Symbol;Acc:HGNC:24757] |
| ENSG00000163389 | HGLibA_37478 | 74 | -0.412225189 | POGLUT1 | protein O-glucosyltransferase 1 [Source:HGNC Symbol;Acc:HGNC:22954] |

| | | | | | |
|---|---|---|---|---|---|
| ENSG00000163703 | HGLibA_11127 | 74 | -0.412225189 | CRELD1 | cysteine rich with EGF like domains 1 [Source:HGNC Symbol;Acc:HGNC:14630] |
| ENSG00000163743 | HGLibA_40694 | 74 | -0.412225189 | RCHY1 | ring finger and CHY zinc finger domain containing 1 [Source:HGNC Symbol;Acc:HGNC:17479] |
| ENSG00000164221 | HGLibA_07544 | 74 | -0.412225189 | CCDC112 | coiled-coil domain containing 112 [Source:HGNC Symbol;Acc:HGNC:28599] |
| ENSG00000164604 | HGLibA_20099 | 74 | -0.412225189 | GPR85 | G protein-coupled receptor 85 [Source:HGNC Symbol;Acc:HGNC:4536] |
| ENSG00000164889 | HGLibA_45214 | 74 | -0.412225189 | SLC4A2 | solute carrier family 4 member 2 [Source:HGNC Symbol;Acc:HGNC:11028] |
| ENSG00000165023 | HGLibA_13259 | 74 | -0.412225189 | DIRAS2 | DIRAS family GTPase 2 [Source:HGNC Symbol;Acc:HGNC:19323] |
| ENSG00000166348 | HGLibA_53402 | 74 | -0.412225189 | USP54 | ubiquitin specific peptidase 54 [Source:HGNC Symbol;Acc:HGNC:23513] |
| ENSG00000166507 | HGLibA_31429 | 74 | -0.412225189 | NDST2 | N-deacetylase and N-sulfotransferase 2 [Source:HGNC Symbol;Acc:HGNC:7681] |
| ENSG00000166664 | HGLibA_09544 | 74 | -0.412225189 | CHRFAM7A | CHRNA7 (exons 5-10) and FAM7A (exons A-E) fusion [Source:HGNC Symbol;Acc:HGNC:15781] |
| ENSG00000166669 | HGLibA_03458 | 74 | -0.412225189 | ATF7IP2 | activating transcription factor 7 interacting protein 2 [Source:HGNC Symbol;Acc:HGNC:20397] |
| ENSG00000166676 | HGLibA_52357 | 74 | -0.412225189 | TVP23A | trans-golgi network vesicle protein 23 homolog A [Source:HGNC Symbol;Acc:HGNC:20398] |
| ENSG00000167664 | HGLibA_50589 | 74 | -0.412225189 | TMIGD2 | transmembrane and immunoglobulin domain containing 2 [Source:HGNC Symbol;Acc:HGNC:28324] |
| ENSG00000168158 | HGLibA_33775 | 74 | -0.412225189 | OR2C1 | olfactory receptor family 2 subfamily C member 1 [Source:HGNC Symbol;Acc:HGNC:8242] |
| ENSG00000168228 | HGLibA_55035 | 74 | -0.412225189 | ZCCHC4 | zinc finger CCHC-type containing 4 [Source:HGNC Symbol;Acc:HGNC:22917] |
| ENSG00000168309 | HGLibA_16001 | 74 | -0.412225189 | FAM107A | family with sequence similarity 107 member A [Source:HGNC Symbol;Acc:HGNC:30827] |
| ENSG00000168522 | HGLibA_17772 | 74 | -0.412225189 | FNTA | alpha [Source:HGNC Symbol;Acc:HGNC:3782]" |
| ENSG00000168556 | HGLibA_23287 | 74 | -0.412225189 | ING2 | inhibitor of growth family member 2 [Source:HGNC Symbol;Acc:HGNC:6063] |
| ENSG00000169242 | HGLibA_14581 | 74 | -0.412225189 | EFNA1 | ephrin A1 [Source:HGNC Symbol;Acc:HGNC:3221] |
| ENSG00000169789 | HGLibA_38995 | 74 | -0.412225189 | PRY | PTPN13 like Y-linked [Source:HGNC Symbol;Acc:HGNC:14024] |
| ENSG00000169877 | HGLibA_01322 | 74 | -0.412225189 | AHSP | alpha hemoglobin stabilizing protein [Source:HGNC Symbol;Acc:HGNC:18075] |
| ENSG00000170421 | HGLibA_25553 | 74 | -0.412225189 | KRT8 | keratin 8 [Source:HGNC Symbol;Acc:HGNC:6446] |
| ENSG00000170484 | HGLibA_25534 | 74 | -0.412225189 | KRT74 | keratin 74 [Source:HGNC Symbol;Acc:HGNC:28929] |
| ENSG00000170677 | HGLibA_46139 | 74 | -0.412225189 | SOCS6 | suppressor of cytokine signaling 6 [Source:HGNC Symbol;Acc:HGNC:16833] |
| ENSG00000171055 | HGLibA_17314 | 74 | -0.412225189 | FEZ2 | fasciculation and elongation protein zeta 2 [Source:HGNC Symbol;Acc:HGNC:3660] |
| ENSG00000171133 | HGLibA_33816 | 74 | -0.412225189 | OR2K2 | olfactory receptor family 2 subfamily K member 2 [Source:HGNC Symbol;Acc:HGNC:8264] |
| ENSG00000171368 | HGLibA_51166 | 74 | -0.412225189 | TPPP | tubulin polymerization promoting protein [Source:HGNC Symbol;Acc:HGNC:24164] |
| ENSG00000171914 | HGLibA_49636 | 74 | -0.412225189 | TLN2 | talin 2 [Source:HGNC Symbol;Acc:HGNC:15447] |
| ENSG00000171960 | HGLibA_37936 | 74 | -0.412225189 | PPIH | peptidylprolyl isomerase H [Source:HGNC Symbol;Acc:HGNC:14651] |
| ENSG00000172717 | HGLibA_16696 | 74 | -0.412225189 | FAM71D | family with sequence similarity 71 member D [Source:HGNC Symbol;Acc:HGNC:20101] |
| ENSG00000173535 | HGLibA_50738 | 74 | -0.412225189 | TNFRSF10C | TNF receptor superfamily member 10c [Source:HGNC Symbol;Acc:HGNC:11906] |
| ENSG00000173706 | HGLibA_21109 | 74 | -0.412225189 | HEG1 | heart development protein with EGF like domains 1 [Source:HGNC Symbol;Acc:HGNC:29227] |
| ENSG00000173905 | HGLibA_19651 | 74 | -0.412225189 | GOLIM4 | golgi integral membrane protein 4 [Source:HGNC Symbol;Acc:HGNC:15448] |
| ENSG00000173908 | HGLibA_25467 | 74 | -0.412225189 | KRT28 | keratin 28 [Source:HGNC Symbol;Acc:HGNC:30842] |
| ENSG00000174579 | HGLibA_30215 | 74 | -0.412225189 | MSL2 | MSL complex subunit 2 [Source:HGNC Symbol;Acc:HGNC:25544] |
| ENSG00000175398 | HGLibA_33537 | 74 | -0.412225189 | OR10P1 | olfactory receptor family 10 subfamily P member 1 [Source:HGNC Symbol;Acc:HGNC:15378] |
| ENSG00000175398 | HGLibA_33536 | 74 | -0.412225189 | OR10P1 | olfactory receptor family 10 subfamily P member 1 [Source:HGNC Symbol;Acc:HGNC:15378] |
| ENSG00000175806 | HGLibA_30237 | 74 | -0.412225189 | MSRA | methionine sulfoxide reductase A [Source:HGNC Symbol;Acc:HGNC:7377] |
| ENSG00000177689 | HGLibA_27933 | 74 | -0.412225189 | MAGEB10 | MAGE family member B10 [Source:HGNC Symbol;Acc:HGNC:25377] |
| ENSG00000178860 | HGLibA_30185 | 74 | -0.412225189 | MSC | musculin [Source:HGNC Symbol;Acc:HGNC:7321] |
| ENSG00000179603 | HGLibA_20363 | 74 | -0.412225189 | GRM8 | glutamate metabotropic receptor 8 [Source:HGNC Symbol;Acc:HGNC:4600] |
| ENSG00000179695 | HGLibA_34386 | 74 | -0.412225189 | OR6C2 | olfactory receptor family 6 subfamily C member 2 [Source:HGNC Symbol;Acc:HGNC:15436] |
| ENSG00000181408 | HGLibA_53467 | 74 | -0.412225189 | UTS2R | urotensin 2 receptor [Source:HGNC Symbol;Acc:HGNC:4468] |
| ENSG00000181718 | HGLibA_34359 | 74 | -0.412225189 | OR5T2 | olfactory receptor family 5 subfamily T member 2 [Source:HGNC Symbol;Acc:HGNC:15296] |
| ENSG00000181781 | HGLibA_33294 | 74 | -0.412225189 | ODF3L2 | outer dense fiber of sperm tails 3 like 2 [Source:HGNC Symbol;Acc:HGNC:26841] |
| ENSG00000182698 | HGLibA_40867 | 74 | -0.412225189 | RESP18 | regulated endocrine specific protein 18 [Source:HGNC Symbol;Acc:HGNC:33762] |
| ENSG00000183559 | HGLibA_05045 | 74 | -0.412225189 | C10orf120 | chromosome 10 open reading frame 120 [Source:HGNC Symbol;Acc:HGNC:25707] |
| ENSG00000183795 | HGLibA_04745 | 74 | -0.412225189 | BPY2B | basic charge Y-linked 2B [Source:HGNC Symbol;Acc:HGNC:25449] |
| ENSG00000186026 | HGLibA_55743 | 74 | -0.412225189 | ZNF284 | zinc finger protein 284 [Source:HGNC Symbol;Acc:HGNC:13078] |
| ENSG00000186038 | HGLibA_22391 | 74 | -0.412225189 | HTR3E | 5-hydroxytryptamine receptor 3E [Source:HGNC Symbol;Acc:HGNC:24005] |
| ENSG00000186431 | HGLibA_17164 | 74 | -0.412225189 | FCAR | Fc fragment of IgA receptor [Source:HGNC Symbol;Acc:HGNC:3608] |
| ENSG00000187242 | HGLibA_25420 | 74 | -0.412225189 | KRT12 | keratin 12 [Source:HGNC Symbol;Acc:HGNC:6414] |
| ENSG00000187801 | HGLibA_55233 | 74 | -0.412225189 | ZFP69B | ZFP69 zinc finger protein B [Source:HGNC Symbol;Acc:HGNC:28053] |
| ENSG00000187806 | HGLibA_50182 | 74 | -0.412225189 | TMEM202 | transmembrane protein 202 [Source:HGNC Symbol;Acc:HGNC:33733] |
| ENSG00000188186 | HGLibA_25998 | 74 | -0.412225189 | LAMTOR4 | MAPK and MTOR activator 4 [Source:HGNC Symbol;Acc:HGNC:33772]" |
| ENSG00000188611 | HGLibA_03189 | 74 | -0.412225189 | ASAH2 | N-acylsphingosine amidohydrolase 2 [Source:HGNC Symbol;Acc:HGNC:18860] |
| ENSG00000196417 | HGLibA_56691 | 74 | -0.412225189 | ZNF765 | zinc finger protein 765 [Source:HGNC Symbol;Acc:HGNC:25092] |
| ENSG00000196632 | HGLibA_54336 | 74 | -0.412225189 | WNK3 | WNK lysine deficient protein kinase 3 [Source:HGNC Symbol;Acc:HGNC:14543] |
| ENSG00000197050 | HGLibA_55957 | 74 | -0.412225189 | ZNF420 | zinc finger protein 420 [Source:HGNC Symbol;Acc:HGNC:20649] |
| ENSG00000197472 | HGLibA_56556 | 74 | -0.412225189 | ZNF695 | zinc finger protein 695 [Source:HGNC Symbol;Acc:HGNC:30954] |
| ENSG00000198271 | HGLibA_25767 | 74 | -0.412225189 | KRTAP4-5 | keratin associated protein 4-5 [Source:HGNC Symbol;Acc:HGNC:18899] |
| ENSG00000198498 | HGLibA_49732 | 74 | -0.412225189 | TMA16 | translation machinery associated 16 homolog [Source:HGNC Symbol;Acc:HGNC:25638] |
| ENSG00000198854 | HGLibA_05891 | 74 | -0.412225189 | C1orf68 | chromosome 1 open reading frame 68 [Source:HGNC Symbol;Acc:HGNC:29468] |
| ENSG00000204469 | HGLibA_38859 | 74 | -0.412225189 | PRRC2A | proline rich coiled-coil 2A [Source:HGNC Symbol;Acc:HGNC:13918] |
| ENSG00000204711 | HGLibA_06680 | 74 | -0.412225189 | C9orf135 | chromosome 9 open reading frame 135 [Source:HGNC Symbol;Acc:HGNC:31422] |
| ENSG00000204961 | HGLibA_35460 | 74 | -0.412225189 | PCDHA9 | protocadherin alpha 9 [Source:HGNC Symbol;Acc:HGNC:8675] |
| ENSG00000213689 | HGLibA_51373 | 74 | -0.412225189 | TREX1 | three prime repair exonuclease 1 [Source:HGNC Symbol;Acc:HGNC:12269] |
| ENSG00000213782 | HGLibA_12724 | 74 | -0.412225189 | DDX47 | DEAD-box helicase 47 [Source:HGNC Symbol;Acc:HGNC:18682] |
| ENSG00000213999 | HGLibA_28830 | 74 | -0.412225189 | MEF2B | myocyte enhancer factor 2B [Source:HGNC Symbol;Acc:HGNC:6995] |
| ENSG00000214360 | HGLibA_14556 | 74 | -0.412225189 | EFCAB9 | EF-hand calcium binding domain 9 [Source:HGNC Symbol;Acc:HGNC:34530] |
| ENSG00000221932 | HGLibA_21150 | 74 | -0.412225189 | HEPN1 | down-regulated 1 [Source:HGNC Symbol;Acc:HGNC:34400]" |
| ENSG00000221986 | HGLibA_30662 | 74 | -0.412225189 | MYBPHL | myosin binding protein H like [Source:HGNC Symbol;Acc:HGNC:30434] |
| ENSG00000224440 | HGLibA_11938 | 74 | -0.412225189 | CXorf51A | chromosome X open reading frame 51A [Source:HGNC Symbol;Acc:HGNC:30533] |
| ENSG00000227551 | HGLibA_53222 | 74 | -0.412225189 | USP17L12 | ubiquitin specific peptidase 17-like family member 12 [Source:HGNC Symbol;Acc:HGNC:44440] |
| ENSG00000228075 | HGLibA_04675 | 74 | -0.412225189 | BOD1L2 | biorientation of chromosomes in cell division 1 like 2 [Source:HGNC Symbol;Acc:HGNC:28505] |
| ENSG00000243943 | HGLibA_56130 | 74 | -0.412225189 | ZNF512 | zinc finger protein 512 [Source:HGNC Symbol;Acc:HGNC:29380] |
| ENSG00000244165 | HGLibA_34874 | 74 | -0.412225189 | P2RY11 | purinergic receptor P2Y11 [Source:HGNC Symbol;Acc:HGNC:8540] |
| ENSG00000266472 | HGLibA_30061 | 74 | -0.412225189 | MRPS21 | mitochondrial ribosomal protein S21 [Source:HGNC Symbol;Acc:HGNC:14046] |
| ENSG00000269526 | HGLibA_15544 | 74 | -0.412225189 | ERVV-1 | envelope [Source:HGNC Symbol;Acc:HGNC:26501]" |
| ENSG00000276119 | HGLibA_33595 | 74 | -0.412225189 | OR13C2 | olfactory receptor family 13 subfamily C member 2 [Source:HGNC Symbol;Acc:HGNC:14701] |
| ENSG00000276234 | HGLibA_48186 | 74 | -0.412225189 | TADA2A | transcriptional adaptor 2A [Source:HGNC Symbol;Acc:HGNC:11531] |
| ENSG00000288702 | HGLibA_52883 | 74 | -0.412225189 | UGT1A3 | - |
| FAM60A | HGLibA_16656 | 74 | -0.412225189 | FAM60A | - |
| FAM69B | HGLibA_16680 | 74 | -0.412225189 | FAM69B | - |
| GATSL2 | HGLibA_18703 | 74 | -0.412225189 | GATSL2 | - |
| H1FX | HGLibA_20741 | 74 | -0.412225189 | H1FX | - |
| H2AFB1 | HGLibA_20746 | 74 | -0.412225189 | H2AFB1 | - |
| HIST1H2AB | HGLibA_21379 | 74 | -0.412225189 | HIST1H2AB | - |
| hsa-mir-1-1 | HGLibA_57140 | 74 | -0.412225189 | hsa-mir-1-1 | - |
| hsa-mir-1238 | HGLibA_57342 | 74 | -0.412225189 | hsa-mir-1238 | - |
| hsa-mir-1250 | HGLibA_57400 | 74 | -0.412225189 | hsa-mir-1250 | - |
| hsa-mir-133b | HGLibA_57781 | 74 | -0.412225189 | hsa-mir-133b | - |
| hsa-mir-150 | HGLibA_57890 | 74 | -0.412225189 | hsa-mir-150 | - |
| hsa-mir-1914 | HGLibA_58061 | 74 | -0.412225189 | hsa-mir-1914 | - |
| hsa-mir-194-2 | HGLibA_58086 | 74 | -0.412225189 | hsa-mir-194-2 | - |
| hsa-mir-3188 | HGLibA_58848 | 74 | -0.412225189 | hsa-mir-3188 | - |
| hsa-mir-325 | HGLibA_58967 | 74 | -0.412225189 | hsa-mir-325 | - |
| hsa-mir-3675 | HGLibA_59253 | 74 | -0.412225189 | hsa-mir-3675 | - |
| hsa-mir-3713 | HGLibA_59367 | 74 | -0.412225189 | hsa-mir-3713 | - |
| hsa-mir-374b | HGLibA_59393 | 74 | -0.412225189 | hsa-mir-374b | - |
| hsa-mir-4298 | HGLibA_59903 | 74 | -0.412225189 | hsa-mir-4298 | - |
| hsa-mir-4425 | HGLibA_60088 | 74 | -0.412225189 | hsa-mir-4425 | - |
| hsa-mir-4491 | HGLibA_60383 | 74 | -0.412225189 | hsa-mir-4491 | - |
| hsa-mir-449a | HGLibA_60417 | 74 | -0.412225189 | hsa-mir-449a | - |
| hsa-mir-500b | HGLibA_61449 | 74 | -0.412225189 | hsa-mir-500b | - |
| hsa-mir-509-1 | HGLibA_61506 | 74 | -0.412225189 | hsa-mir-509-1 | - |
| hsa-mir-514a-3 | HGLibA_61594 | 74 | -0.412225189 | hsa-mir-514a-3 | - |
| hsa-mir-5579 | HGLibA_62157 | 74 | -0.412225189 | hsa-mir-5579 | - |
| hsa-mir-579 | HGLibA_62413 | 74 | -0.412225189 | hsa-mir-579 | - |
| hsa-mir-6077-2 | HGLibA_62564 | 74 | -0.412225189 | hsa-mir-6077-2 | - |
| hsa-mir-6127 | HGLibA_62655 | 74 | -0.412225189 | hsa-mir-6127 | - |
| hsa-mir-6506 | HGLibA_62874 | 74 | -0.412225189 | hsa-mir-6506 | - |
| hsa-mir-6511a-4 | HGLibA_62910 | 74 | -0.412225189 | hsa-mir-6511a-4 | - |
| hsa-mir-6738 | HGLibA_63104 | 74 | -0.412225189 | hsa-mir-6738 | - |
| hsa-mir-6777 | HGLibA_63282 | 74 | -0.412225189 | hsa-mir-6777 | - |
| hsa-mir-6855 | HGLibA_63593 | 74 | -0.412225189 | hsa-mir-6855 | - |
| hsa-mir-6873 | HGLibA_63675 | 74 | -0.412225189 | hsa-mir-6873 | - |
| hsa-mir-7107 | HGLibA_63789 | 74 | -0.412225189 | hsa-mir-7107 | - |
| hsa-mir-8068 | HGLibA_64109 | 74 | -0.412225189 | hsa-mir-8068 | - |
| KIAA0430 | HGLibA_24639 | 74 | -0.412225189 | KIAA0430 | - |
| LOC100130480 | HGLibA_26790 | 74 | -0.412225189 | LOC100130480 | - |
| LOC100131094 | HGLibA_26802 | 74 | -0.412225189 | LOC100131094 | - |
| PLK1S1 | HGLibA_37187 | 74 | -0.412225189 | PLK1S1 | - |
| PQLC3 | HGLibA_38257 | 74 | -0.412225189 | PQLC3 | - |
| C9orf123 | HGLibA_06672 | 73 | -0.422176227 | C9orf123 | - |
| CASC4 | HGLibA_07277 | 73 | -0.422176227 | CASC4 | - |
| CCDC109B | HGLibA_07533 | 73 | -0.422176227 | CCDC109B | - |
| CCDC114 | HGLibA_07549 | 73 | -0.422176227 | CCDC114 | - |
| CSB-PGBD3 | HGLibA_11309 | 73 | -0.422176227 | CSB-PGBD3 | - |
| ENSG00000001561 | HGLibA_15176 | 73 | -0.422176227 | ENPP4 | ectonucleotide pyrophosphatase/phosphodiesterase 4 [Source:HGNC Symbol;Acc:HGNC:3359] |
| ENSG00000003393 | HGLibA_01717 | 73 | -0.422176227 | ALS2 | alsin Rho guanine nucleotide exchange factor [Source:HGNC Symbol;Acc:HGNC:443]" |
| ENSG00000008196 | HGLibA_49123 | 73 | -0.422176227 | TFAP2B | transcription factor AP-2 beta [Source:HGNC Symbol;Acc:HGNC:11743] |
| ENSG00000010165 | HGLibA_28956 | 73 | -0.422176227 | METTL13 | eEF1A lysine and N-terminal methyltransferase [Source:HGNC Symbol;Acc:HGNC:24248] |
| ENSG00000014123 | HGLibA_52850 | 73 | -0.422176227 | UFL1 | UFM1 specific ligase 1 [Source:HGNC Symbol;Acc:HGNC:23039] |
| ENSG00000028277 | HGLibA_37760 | 73 | -0.422176227 | POU2F2 | POU class 2 homeobox 2 [Source:HGNC Symbol;Acc:HGNC:9213] |
| ENSG00000047457 | HGLibA_10882 | 73 | -0.422176227 | CP | ceruloplasmin [Source:HGNC Symbol;Acc:HGNC:2295] |

| | | | | | |
|---|---|---|---|---|---|
| ENSG00000053747 | HGLibA_25947 | 73 | -0.422176227 | LAMA3 | laminin subunit alpha 3 [Source:HGNC Symbol;Acc:HGNC:6483] |
| ENSG00000066654 | HGLibA_49415 | 73 | -0.422176227 | THUMPD1 | THUMP domain containing 1 [Source:HGNC Symbol;Acc:HGNC:23807] |
| ENSG00000075142 | HGLibA_46988 | 73 | -0.422176227 | SRI | sorcin [Source:HGNC Symbol;Acc:HGNC:11292] |
| ENSG00000075539 | HGLibA_18055 | 73 | -0.422176227 | FRYL | FRY like transcription coactivator [Source:HGNC Symbol;Acc:HGNC:29127] |
| ENSG00000078487 | HGLibA_55056 | 73 | -0.422176227 | ZCWPW1 | zinc finger CW-type and PWWP domain containing 1 [Source:HGNC Symbol;Acc:HGNC:23486] |
| ENSG00000078808 | HGLibA_43130 | 73 | -0.422176227 | SDF4 | stromal cell derived factor 4 [Source:HGNC Symbol;Acc:HGNC:24188] |
| ENSG00000086712 | HGLibA_52395 | 73 | -0.422176227 | TXLNG | taxilin gamma [Source:HGNC Symbol;Acc:HGNC:18578] |
| ENSG00000087495 | HGLibA_36303 | 73 | -0.422176227 | PHACTR3 | phosphatase and actin regulator 3 [Source:HGNC Symbol;Acc:HGNC:15833] |
| ENSG00000089225 | HGLibA_48675 | 73 | -0.422176227 | TBX5 | T-box 5 [Source:HGNC Symbol;Acc:HGNC:11604] |
| ENSG00000092148 | HGLibA_21094 | 73 | -0.422176227 | HECTD1 | HECT domain E3 ubiquitin protein ligase 1 [Source:HGNC Symbol;Acc:HGNC:20157] |
| ENSG00000092929 | HGLibA_53001 | 73 | -0.422176227 | UNC13D | unc-13 homolog D [Source:HGNC Symbol;Acc:HGNC:23147] |
| ENSG00000095585 | HGLibA_04555 | 73 | -0.422176227 | BLNK | B cell linker [Source:HGNC Symbol;Acc:HGNC:14211] |
| ENSG00000096433 | HGLibA_23859 | 73 | -0.422176227 | ITPR3 | 5-trisphosphate receptor type 3 [Source:HGNC Symbol;Acc:HGNC:6182]" |
| ENSG00000100029 | HGLibA_36067 | 73 | -0.422176227 | PES1 | pescadillo ribosomal biogenesis factor 1 [Source:HGNC Symbol;Acc:HGNC:8848] |
| ENSG00000100227 | HGLibA_37507 | 73 | -0.422176227 | POLDIP3 | DNA polymerase delta interacting protein 3 [Source:HGNC Symbol;Acc:HGNC:23782] |
| ENSG00000100678 | HGLibA_45393 | 73 | -0.422176227 | SLC8A3 | solute carrier family 8 member A3 [Source:HGNC Symbol;Acc:HGNC:11070] |
| ENSG00000101003 | HGLibA_19102 | 73 | -0.422176227 | GINS1 | GINS complex subunit 1 [Source:HGNC Symbol;Acc:HGNC:28980] |
| ENSG00000101391 | HGLibA_08782 | 73 | -0.422176227 | CDK5RAP1 | CDK5 regulatory subunit associated protein 1 [Source:HGNC Symbol;Acc:HGNC:15880] |
| ENSG00000103043 | HGLibA_53486 | 73 | -0.422176227 | VAC14 | PIKFYVE complex component [Source:HGNC Symbol;Acc:HGNC:25507]" |
| ENSG00000103343 | HGLibA_55525 | 73 | -0.422176227 | ZNF174 | zinc finger protein 174 [Source:HGNC Symbol;Acc:HGNC:12963] |
| ENSG00000103494 | HGLibA_41841 | 73 | -0.422176227 | RPGRIP1L | RPGRIP1 like [Source:HGNC Symbol;Acc:HGNC:29168] |
| ENSG00000104140 | HGLibA_41211 | 73 | -0.422176227 | RHOV | ras homolog family member V [Source:HGNC Symbol;Acc:HGNC:18313] |
| ENSG00000104765 | HGLibA_04658 | 73 | -0.422176227 | BNIP3L | BCL2 interacting protein 3 like [Source:HGNC Symbol;Acc:HGNC:1085] |
| ENSG00000104805 | HGLibA_32911 | 73 | -0.422176227 | NUCB1 | nucleobindin 1 [Source:HGNC Symbol;Acc:HGNC:8043] |
| ENSG00000105193 | HGLibA_42083 | 73 | -0.422176227 | RPS16 | ribosomal protein S16 [Source:HGNC Symbol;Acc:HGNC:10396] |
| ENSG00000105397 | HGLibA_52455 | 73 | -0.422176227 | TYK2 | tyrosine kinase 2 [Source:HGNC Symbol;Acc:HGNC:12440] |
| ENSG00000105672 | HGLibA_15651 | 73 | -0.422176227 | ETV2 | ETS variant 2 [Source:HGNC Symbol;Acc:HGNC:3491] |
| ENSG00000106355 | HGLibA_27544 | 73 | -0.422176227 | LSM5 | U6 small nuclear RNA and mRNA degradation associated [Source:HGNC Symbol;Acc:HGNC:17162]" |
| ENSG00000106537 | HGLibA_51902 | 73 | -0.422176227 | TSPAN13 | tetraspanin 13 [Source:HGNC Symbol;Acc:HGNC:21643] |
| ENSG00000106785 | HGLibA_51410 | 73 | -0.422176227 | TRIM14 | tripartite motif containing 14 [Source:HGNC Symbol;Acc:HGNC:16283] |
| ENSG00000107242 | HGLibA_36718 | 73 | -0.422176227 | PIP5K1B | phosphatidylinositol-4-phosphate 5-kinase type 1 beta [Source:HGNC Symbol;Acc:HGNC:8995] |
| ENSG00000107317 | HGLibA_39311 | 73 | -0.422176227 | PTGDS | prostaglandin D2 synthase [Source:HGNC Symbol;Acc:HGNC:9592] |
| ENSG00000108556 | HGLibA_09603 | 73 | -0.422176227 | CHRNE | cholinergic receptor nicotinic epsilon subunit [Source:HGNC Symbol;Acc:HGNC:1966] |
| ENSG00000110080 | HGLibA_47245 | 73 | -0.422176227 | ST3GAL4 | 3-sialyltransferase 4 [Source:HGNC Symbol;Acc:HGNC:10864]" |
| ENSG00000111199 | HGLibA_51790 | 73 | -0.422176227 | TRPV4 | transient receptor potential cation channel subfamily V member 4 [Source:HGNC Symbol;Acc:HGNC:18083] |
| ENSG00000113575 | HGLibA_38133 | 73 | -0.422176227 | PPP2CA | protein phosphatase 2 catalytic subunit alpha [Source:HGNC Symbol;Acc:HGNC:9299] |
| ENSG00000114026 | HGLibA_33322 | 73 | -0.422176227 | OGG1 | 8-oxoguanine DNA glycosylase [Source:HGNC Symbol;Acc:HGNC:8125] |
| ENSG00000114745 | HGLibA_19679 | 73 | -0.422176227 | GORASP1 | golgi reassembly stacking protein 1 [Source:HGNC Symbol;Acc:HGNC:16769] |
| ENSG00000116675 | HGLibA_13632 | 73 | -0.422176227 | DNAJC6 | DnaJ heat shock protein family (Hsp40) member C6 [Source:HGNC Symbol;Acc:HGNC:15469] |
| ENSG00000117215 | HGLibA_36887 | 73 | -0.422176227 | PLA2G2D | phospholipase A2 group IID [Source:HGNC Symbol;Acc:HGNC:9033] |
| ENSG00000117906 | HGLibA_40705 | 73 | -0.422176227 | RCN2 | reticulocalbin 2 [Source:HGNC Symbol;Acc:HGNC:9935] |
| ENSG00000119537 | HGLibA_24545 | 73 | -0.422176227 | KDSR | 3-ketodihydrosphingosine reductase [Source:HGNC Symbol;Acc:HGNC:4021] |
| ENSG00000124383 | HGLibA_29722 | 73 | -0.422176227 | MPHOSPH10 | M-phase phosphoprotein 10 [Source:HGNC Symbol;Acc:HGNC:7213] |
| ENSG00000124486 | HGLibA_53416 | 73 | -0.422176227 | USP9X | ubiquitin specific peptidase 9 X-linked [Source:HGNC Symbol;Acc:HGNC:12632] |
| ENSG00000125257 | HGLibA_00175 | 73 | -0.422176227 | ABCC4 | ATP binding cassette subfamily C member 4 [Source:HGNC Symbol;Acc:HGNC:55] |
| ENSG00000125851 | HGLibA_35673 | 73 | -0.422176227 | PCSK2 | proprotein convertase subtilisin/kexin type 2 [Source:HGNC Symbol;Acc:HGNC:8744] |
| ENSG00000127564 | HGLibA_36835 | 73 | -0.422176227 | PKMYT1 | membrane associated tyrosine/threonine 1 [Source:HGNC Symbol;Acc:HGNC:29650]" |
| ENSG00000128394 | HGLibA_02488 | 73 | -0.422176227 | APOBEC3F | apolipoprotein B mRNA editing enzyme catalytic subunit 3F [Source:HGNC Symbol;Acc:HGNC:17356] |
| ENSG00000128617 | HGLibA_33432 | 73 | -0.422176227 | OPN1SW | short wave sensitive [Source:HGNC Symbol;Acc:HGNC:1012]" |
| ENSG00000128708 | HGLibA_20871 | 73 | -0.422176227 | HAT1 | histone acetyltransferase 1 [Source:HGNC Symbol;Acc:HGNC:4821] |
| ENSG00000129214 | HGLibA_43998 | 73 | -0.422176227 | SHBG | sex hormone binding globulin [Source:HGNC Symbol;Acc:HGNC:10839] |
| ENSG00000130164 | HGLibA_26238 | 73 | -0.422176227 | LDLR | low density lipoprotein receptor [Source:HGNC Symbol;Acc:HGNC:6547] |
| ENSG00000130208 | HGLibA_02502 | 73 | -0.422176227 | APOC1 | apolipoprotein C1 [Source:HGNC Symbol;Acc:HGNC:607] |
| ENSG00000130748 | HGLibA_50021 | 73 | -0.422176227 | TMEM160 | transmembrane protein 160 [Source:HGNC Symbol;Acc:HGNC:26042] |
| ENSG00000131373 | HGLibA_20801 | 73 | -0.422176227 | HACL1 | 2-hydroxyacyl-CoA lyase 1 [Source:HGNC Symbol;Acc:HGNC:17856] |
| ENSG00000131446 | HGLibA_29132 | 73 | -0.422176227 | MGAT1 | 2-N-acetylglucosaminyltransferase [Source:HGNC Symbol;Acc:HGNC:7044]" |
| ENSG00000131844 | HGLibA_28567 | 73 | -0.422176227 | MCCC2 | methylcrotonoyl-CoA carboxylase 2 [Source:HGNC Symbol;Acc:HGNC:6937] |
| ENSG00000132357 | HGLibA_07236 | 73 | -0.422176227 | CARD6 | caspase recruitment domain family member 6 [Source:HGNC Symbol;Acc:HGNC:16394] |
| ENSG00000132698 | HGLibA_39850 | 73 | -0.422176227 | RAB25 | member RAS oncogene family [Source:HGNC Symbol;Acc:HGNC:18238]" |
| ENSG00000133111 | HGLibA_40989 | 73 | -0.422176227 | RFXAP | regulatory factor X associated protein [Source:HGNC Symbol;Acc:HGNC:9988] |
| ENSG00000134538 | HGLibA_45448 | 73 | -0.422176227 | SLCO1B1 | solute carrier organic anion transporter family member 1B1 [Source:HGNC Symbol;Acc:HGNC:10959] |
| ENSG00000135045 | HGLibA_06729 | 73 | -0.422176227 | C9orf40 | chromosome 9 open reading frame 40 [Source:HGNC Symbol;Acc:HGNC:23433] |
| ENSG00000135090 | HGLibA_48312 | 73 | -0.422176227 | TAOK3 | TAO kinase 3 [Source:HGNC Symbol;Acc:HGNC:18133] |
| ENSG00000135540 | HGLibA_31918 | 73 | -0.422176227 | NHSL1 | NHS like 1 [Source:HGNC Symbol;Acc:HGNC:21021] |
| ENSG00000135622 | HGLibA_43351 | 73 | -0.422176227 | SEMA4F | ssemaphorin 4F [Source:HGNC Symbol;Acc:HGNC:10734] |
| ENSG00000135625 | HGLibA_14657 | 73 | -0.422176227 | EGR4 | early growth response 4 [Source:HGNC Symbol;Acc:HGNC:3241] |
| ENSG00000136231 | HGLibA_22805 | 73 | -0.422176227 | IGF2BP3 | insulin like growth factor 2 mRNA binding protein 3 [Source:HGNC Symbol;Acc:HGNC:28868] |
| ENSG00000136560 | HGLibA_48303 | 73 | -0.422176227 | TANK | TRAF family member associated NFKB activator [Source:HGNC Symbol;Acc:HGNC:11562] |
| ENSG00000136698 | HGLibA_09249 | 73 | -0.422176227 | CFC1 | cryptic family 1 [Source:HGNC Symbol;Acc:HGNC:18292]" |
| ENSG00000136738 | HGLibA_47329 | 73 | -0.422176227 | STAM | signal transducing adaptor molecule [Source:HGNC Symbol;Acc:HGNC:11357] |
| ENSG00000137090 | HGLibA_13418 | 73 | -0.422176227 | DMRT1 | doublesex and mab-3 related transcription factor 1 [Source:HGNC Symbol;Acc:HGNC:2934] |
| ENSG00000137185 | HGLibA_57033 | 73 | -0.422176227 | ZSCAN9 | zinc finger and SCAN domain containing 9 [Source:HGNC Symbol;Acc:HGNC:12984] |
| ENSG00000137200 | HGLibA_10232 | 73 | -0.422176227 | CMTR1 | cap methyltransferase 1 [Source:HGNC Symbol;Acc:HGNC:21077] |
| ENSG00000137200 | HGLibA_10233 | 73 | -0.422176227 | CMTR1 | cap methyltransferase 1 [Source:HGNC Symbol;Acc:HGNC:21077] |
| ENSG00000137573 | HGLibA_47724 | 73 | -0.422176227 | SULF1 | sulfatase 1 [Source:HGNC Symbol;Acc:HGNC:20391] |
| ENSG00000137720 | HGLibA_05115 | 73 | -0.422176227 | C11orf1 | chromosome 11 open reading frame 1 [Source:HGNC Symbol;Acc:HGNC:1163] |
| ENSG00000137834 | HGLibA_45583 | 73 | -0.422176227 | SMAD6 | SMAD family member 6 [Source:HGNC Symbol;Acc:HGNC:6772] |
| ENSG00000138092 | HGLibA_09076 | 73 | -0.422176227 | CENPO | centromere protein O [Source:HGNC Symbol;Acc:HGNC:28152] |
| ENSG00000138778 | HGLibA_09052 | 73 | -0.422176227 | CENPE | centromere protein E [Source:HGNC Symbol;Acc:HGNC:1856] |
| ENSG00000138834 | HGLibA_28292 | 73 | -0.422176227 | MAPK8IP3 | mitogen-activated protein kinase 8 interacting protein 3 [Source:HGNC Symbol;Acc:HGNC:6884] |
| ENSG00000139726 | HGLibA_12971 | 73 | -0.422176227 | DENR | density regulated re-initiation and release factor [Source:HGNC Symbol;Acc:HGNC:2769] |
| ENSG00000139890 | HGLibA_40833 | 73 | -0.422176227 | REM2 | RRAD and GEM like GTPase 2 [Source:HGNC Symbol;Acc:HGNC:20248] |
| ENSG00000140416 | HGLibA_51140 | 73 | -0.422176227 | TPM1 | tropomyosin 1 [Source:HGNC Symbol;Acc:HGNC:12010] |
| ENSG00000140939 | HGLibA_32266 | 73 | -0.422176227 | NOL3 | nucleolar protein 3 [Source:HGNC Symbol;Acc:HGNC:7869] |
| ENSG00000141956 | HGLibA_38380 | 73 | -0.422176227 | PRDM15 | PR/SET domain 15 [Source:HGNC Symbol;Acc:HGNC:13999] |
| ENSG00000142694 | HGLibA_15673 | 73 | -0.422176227 | EVA1B | eva-1 homolog B [Source:HGNC Symbol;Acc:HGNC:25558] |
| ENSG00000143199 | HGLibA_00908 | 73 | -0.422176227 | ADCY10 | adenylate cyclase 10 [Source:HGNC Symbol;Acc:HGNC:21285] |
| ENSG00000143374 | HGLibA_48349 | 73 | -0.422176227 | TARS2 | mitochondrial [Source:HGNC Symbol;Acc:HGNC:30740]" |
| ENSG00000143633 | HGLibA_05768 | 73 | -0.422176227 | C1orf131 | chromosome 1 open reading frame 131 [Source:HGNC Symbol;Acc:HGNC:25332] |
| ENSG00000143815 | HGLibA_26082 | 73 | -0.422176227 | LBR | lamin B receptor [Source:HGNC Symbol;Acc:HGNC:6518] |
| ENSG00000144045 | HGLibA_13939 | 73 | -0.422176227 | DQX1 | DEAQ-box RNA dependent ATPase 1 [Source:HGNC Symbol;Acc:HGNC:20410] |
| ENSG00000144579 | HGLibA_11608 | 73 | -0.422176227 | CTDSP1 | CTD small phosphatase 1 [Source:HGNC Symbol;Acc:HGNC:21614] |
| ENSG00000144834 | HGLibA_48275 | 73 | -0.422176227 | TAGLN3 | transgelin 3 [Source:HGNC Symbol;Acc:HGNC:29868] |
| ENSG00000145332 | HGLibA_25224 | 73 | -0.422176227 | KLHL8 | kelch like family member 8 [Source:HGNC Symbol;Acc:HGNC:18644] |
| ENSG00000145868 | HGLibA_17083 | 73 | -0.422176227 | FBXO38 | F-box protein 38 [Source:HGNC Symbol;Acc:HGNC:28844] |
| ENSG00000147443 | HGLibA_13771 | 73 | -0.422176227 | DOK2 | docking protein 2 [Source:HGNC Symbol;Acc:HGNC:2991] |
| ENSG00000148225 | HGLibA_54063 | 73 | -0.422176227 | WDR31 | WD repeat domain 31 [Source:HGNC Symbol;Acc:HGNC:21421] |
| ENSG00000148334 | HGLibA_39327 | 73 | -0.422176227 | PTGES2 | prostaglandin E synthase 2 [Source:HGNC Symbol;Acc:HGNC:17822] |
| ENSG00000149932 | HGLibA_50232 | 73 | -0.422176227 | TMEM219 | transmembrane protein 219 [Source:HGNC Symbol;Acc:HGNC:25201] |
| ENSG00000150773 | HGLibA_36613 | 73 | -0.422176227 | PIH1D2 | PIH1 domain containing 2 [Source:HGNC Symbol;Acc:HGNC:25210] |
| ENSG00000151151 | HGLibA_23456 | 73 | -0.422176227 | IPMK | inositol polyphosphate multikinase [Source:HGNC Symbol;Acc:HGNC:20739] |
| ENSG00000151208 | HGLibA_13335 | 73 | -0.422176227 | DLG5 | discs large MAGUK scaffold protein 5 [Source:HGNC Symbol;Acc:HGNC:2904] |
| ENSG00000151292 | HGLibA_11398 | 73 | -0.422176227 | CSNK1G3 | casein kinase 1 gamma 3 [Source:HGNC Symbol;Acc:HGNC:2456] |
| ENSG00000151552 | HGLibA_39722 | 73 | -0.422176227 | QDPR | quinoid dihydropteridine reductase [Source:HGNC Symbol;Acc:HGNC:9752] |
| ENSG00000151790 | HGLibA_48888 | 73 | -0.422176227 | TDO2 | 3-dioxygenase [Source:HGNC Symbol;Acc:HGNC:11708]" |
| ENSG00000151923 | HGLibA_49437 | 73 | -0.422176227 | TIAL1 | TIA1 cytotoxic granule associated RNA binding protein like 1 [Source:HGNC Symbol;Acc:HGNC:11804] |
| ENSG00000153237 | HGLibA_07634 | 73 | -0.422176227 | CCDC148 | coiled-coil domain containing 148 [Source:HGNC Symbol;Acc:HGNC:25191] |
| ENSG00000155666 | HGLibA_24538 | 73 | -0.422176227 | KDM8 | lysine demethylase 8 [Source:HGNC Symbol;Acc:HGNC:25840] |
| ENSG00000156256 | HGLibA_53212 | 73 | -0.422176227 | USP16 | ubiquitin specific peptidase 16 [Source:HGNC Symbol;Acc:HGNC:12614] |
| ENSG00000156510 | HGLibA_21589 | 73 | -0.422176227 | HKDC1 | hexokinase domain containing 1 [Source:HGNC Symbol;Acc:HGNC:23302] |
| ENSG00000156968 | HGLibA_29785 | 73 | -0.422176227 | MPV17L | MPV17 mitochondrial inner membrane protein like [Source:HGNC Symbol;Acc:HGNC:26827] |
| ENSG00000157045 | HGLibA_32834 | 73 | -0.422176227 | NTAN1 | N-terminal asparagine amidase [Source:HGNC Symbol;Acc:HGNC:29909] |
| ENSG00000157778 | HGLibA_39212 | 73 | -0.422176227 | PSMG3 | proteasome assembly chaperone 3 [Source:HGNC Symbol;Acc:HGNC:22420] |
| ENSG00000157954 | HGLibA_54316 | 73 | -0.422176227 | WIPI2 | phosphoinositide interacting 2 [Source:HGNC Symbol;Acc:HGNC:32225]" |
| ENSG00000158079 | HGLibA_39412 | 73 | -0.422176227 | PTPDC1 | protein tyrosine phosphatase domain containing 1 [Source:HGNC Symbol;Acc:HGNC:30184] |
| ENSG00000158555 | HGLibA_18903 | 73 | -0.422176227 | GDPD5 | glycerophosphodiester phosphodiesterase domain containing 5 [Source:HGNC Symbol;Acc:HGNC:28804] |
| ENSG00000158825 | HGLibA_08499 | 73 | -0.422176227 | CDA | cytidine deaminase [Source:HGNC Symbol;Acc:HGNC:1712] |
| ENSG00000160870 | HGLibA_12164 | 73 | -0.422176227 | CYP3A7 | cytochrome P450 family 3 subfamily A member 7 [Source:HGNC Symbol;Acc:HGNC:2640] |
| ENSG00000162517 | HGLibA_36024 | 73 | -0.422176227 | PEF1 | penta-EF-hand domain containing 1 [Source:HGNC Symbol;Acc:HGNC:30009] |
| ENSG00000162576 | HGLibA_30627 | 73 | -0.422176227 | MXRA8 | matrix remodeling associated 8 [Source:HGNC Symbol;Acc:HGNC:7542] |
| ENSG00000162959 | HGLibA_28883 | 73 | -0.422176227 | MEMO1 | mediator of cell motility 1 [Source:HGNC Symbol;Acc:HGNC:14014] |
| ENSG00000163347 | HGLibA_09846 | 73 | -0.422176227 | CLDN1 | claudin 1 [Source:HGNC Symbol;Acc:HGNC:2032] |
| ENSG00000163792 | HGLibA_48775 | 73 | -0.422176227 | TCF23 | transcription factor 23 [Source:HGNC Symbol;Acc:HGNC:18602] |
| ENSG00000164051 | HGLibA_07797 | 73 | -0.422176227 | CCDC51 | coiled-coil domain containing 51 [Source:HGNC Symbol;Acc:HGNC:25714] |
| ENSG00000164168 | HGLibA_50118 | 73 | -0.422176227 | TMEM184C | transmembrane protein 184C [Source:HGNC Symbol;Acc:HGNC:25587] |
| ENSG00000164299 | HGLibA_46909 | 73 | -0.422176227 | SPZ1 | spermatogenic leucine zipper 1 [Source:HGNC Symbol;Acc:HGNC:30721] |
| ENSG00000164713 | HGLibA_04803 | 73 | -0.422176227 | BRI3 | brain protein I3 [Source:HGNC Symbol;Acc:HGNC:1109] |
| ENSG00000164855 | HGLibA_50111 | 73 | -0.422176227 | TMEM184A | transmembrane protein 184A [Source:HGNC Symbol;Acc:HGNC:28797] |
| ENSG00000165799 | HGLibA_41415 | 73 | -0.422176227 | RNASE7 | ribonuclease A family member 7 [Source:HGNC Symbol;Acc:HGNC:19278] |
| ENSG00000165949 | HGLibA_22597 | 73 | -0.422176227 | IFI27 | interferon alpha inducible protein 27 [Source:HGNC Symbol;Acc:HGNC:5397] |
| ENSG00000166025 | HGLibA_01818 | 73 | -0.422176227 | AMOTL1 | angiomotin like 1 [Source:HGNC Symbol;Acc:HGNC:17811] |
| ENSG00000166197 | HGLibA_32284 | 73 | -0.422176227 | NOLC1 | nucleolar and coiled-body phosphoprotein 1 [Source:HGNC Symbol;Acc:HGNC:15608] |
| ENSG00000166341 | HGLibA_12491 | 73 | -0.422176227 | DCHS1 | dachsous cadherin-related 1 [Source:HGNC Symbol;Acc:HGNC:13681] |
| ENSG00000166501 | HGLibA_38535 | 73 | -0.422176227 | PRKCB | protein kinase C beta [Source:HGNC Symbol;Acc:HGNC:9395] |
| ENSG00000166558 | HGLibA_45082 | 73 | -0.422176227 | SLC38A8 | solute carrier family 38 member 8 [Source:HGNC Symbol;Acc:HGNC:32434] |

| | | | | | |
|---|---|---|---|---|---|
| ENSG00000166927 | HGLibA_30163 | 73 | -0.422176227 | MS4A7 | membrane spanning 4-domains A7 [Source:HGNC Symbol;Acc:HGNC:13378] |
| ENSG00000166979 | HGLibA_15676 | 73 | -0.422176227 | EVA1C | eva-1 homolog C [Source:HGNC Symbol;Acc:HGNC:13239] |
| ENSG00000167700 | HGLibA_29098 | 73 | -0.422176227 | MFSD3 | major facilitator superfamily domain containing 3 [Source:HGNC Symbol;Acc:HGNC:25157] |
| ENSG00000167767 | HGLibA_25556 | 73 | -0.422176227 | KRT80 | keratin 80 [Source:HGNC Symbol;Acc:HGNC:27056] |
| ENSG00000167914 | HGLibA_20405 | 73 | -0.422176227 | GSDMA | gasdermin A [Source:HGNC Symbol;Acc:HGNC:13311] |
| ENSG00000168216 | HGLibA_26674 | 73 | -0.422176227 | LMBRD1 | LMBR1 domain containing 1 [Source:HGNC Symbol;Acc:HGNC:23038] |
| ENSG00000168356 | HGLibA_42970 | 73 | -0.422176227 | SCN11A | sodium voltage-gated channel alpha subunit 11 [Source:HGNC Symbol;Acc:HGNC:10583] |
| ENSG00000168671 | HGLibA_52936 | 73 | -0.422176227 | UGT3A2 | UDP glycosyltransferase family 3 member A2 [Source:HGNC Symbol;Acc:HGNC:27266] |
| ENSG00000169957 | HGLibA_56699 | 73 | -0.422176227 | ZNF768 | zinc finger protein 768 [Source:HGNC Symbol;Acc:HGNC:26273] |
| ENSG00000171201 | HGLibA_45827 | 73 | -0.422176227 | SMR3B | submaxillary gland androgen regulated protein 3B [Source:HGNC Symbol;Acc:HGNC:17326] |
| ENSG00000172188 | HGLibA_33939 | 73 | -0.422176227 | OR4C11 | olfactory receptor family 4 subfamily C member 11 [Source:HGNC Symbol;Acc:HGNC:15167] |
| ENSG00000172322 | HGLibA_09927 | 73 | -0.422176227 | CLEC12A | C-type lectin domain family 12 member A [Source:HGNC Symbol;Acc:HGNC:31713] |
| ENSG00000172680 | HGLibA_29681 | 73 | -0.422176227 | MOS | serine/threonine kinase [Source:HGNC Symbol;Acc:HGNC:7199]" |
| ENSG00000173335 | HGLibA_11471 | 73 | -0.422176227 | CST9 | cystatin 9 [Source:HGNC Symbol;Acc:HGNC:13261] |
| ENSG00000173699 | HGLibA_46466 | 73 | -0.422176227 | SPATA3 | spermatogenesis associated 3 [Source:HGNC Symbol;Acc:HGNC:17884] |
| ENSG00000174799 | HGLibA_09108 | 73 | -0.422176227 | CEP135 | centrosomal protein 135 [Source:HGNC Symbol;Acc:HGNC:29086] |
| ENSG00000174957 | HGLibA_34308 | 73 | -0.422176227 | OR5J2 | olfactory receptor family 5 subfamily J member 2 [Source:HGNC Symbol;Acc:HGNC:19612] |
| ENSG00000175315 | HGLibA_11462 | 73 | -0.422176227 | CST6 | cystatin E/M [Source:HGNC Symbol;Acc:HGNC:2478] |
| ENSG00000175352 | HGLibA_32696 | 73 | -0.422176227 | NRIP3 | nuclear receptor interacting protein 3 [Source:HGNC Symbol;Acc:HGNC:1167] |
| ENSG00000176371 | HGLibA_56994 | 73 | -0.422176227 | ZSCAN2 | zinc finger and SCAN domain containing 2 [Source:HGNC Symbol;Acc:HGNC:20994] |
| ENSG00000176595 | HGLibA_24093 | 73 | -0.422176227 | KBTBD11 | kelch repeat and BTB domain containing 11 [Source:HGNC Symbol;Acc:HGNC:29104] |
| ENSG00000176749 | HGLibA_08777 | 73 | -0.422176227 | CDK5R1 | cyclin dependent kinase 5 regulatory subunit 1 [Source:HGNC Symbol;Acc:HGNC:1775] |
| ENSG00000176978 | HGLibA_13878 | 73 | -0.422176227 | DPP7 | dipeptidyl peptidase 7 [Source:HGNC Symbol;Acc:HGNC:14892] |
| ENSG00000177125 | HGLibA_54866 | 73 | -0.422176227 | ZBTB34 | zinc finger and BTB domain containing 34 [Source:HGNC Symbol;Acc:HGNC:31446] |
| ENSG00000179403 | HGLibA_53897 | 73 | -0.422176227 | VWA1 | von Willebrand factor A domain containing 1 [Source:HGNC Symbol;Acc:HGNC:30910] |
| ENSG00000179630 | HGLibA_25908 | 73 | -0.422176227 | LACC1 | laccase domain containing 1 [Source:HGNC Symbol;Acc:HGNC:26789] |
| ENSG00000179833 | HGLibA_43606 | 73 | -0.422176227 | SERTAD2 | SERTA domain containing 2 [Source:HGNC Symbol;Acc:HGNC:30784] |
| ENSG00000181752 | HGLibA_34550 | 73 | -0.422176227 | OR8K5 | olfactory receptor family 8 subfamily K member 5 [Source:HGNC Symbol;Acc:HGNC:15315] |
| ENSG00000181852 | HGLibA_41683 | 73 | -0.422176227 | RNF41 | ring finger protein 41 [Source:HGNC Symbol;Acc:HGNC:18401] |
| ENSG00000183077 | HGLibA_01125 | 73 | -0.422176227 | AFMID | arylformamidase [Source:HGNC Symbol;Acc:HGNC:20910] |
| ENSG00000183735 | HGLibA_48601 | 73 | -0.422176227 | TBK1 | TANK binding kinase 1 [Source:HGNC Symbol;Acc:HGNC:11584] |
| ENSG00000184226 | HGLibA_35422 | 73 | -0.422176227 | PCDH9 | protocadherin 9 [Source:HGNC Symbol;Acc:HGNC:8661] |
| ENSG00000185305 | HGLibA_02944 | 73 | -0.422176227 | ARL15 | ADP ribosylation factor like GTPase 15 [Source:HGNC Symbol;Acc:HGNC:25945] |
| ENSG00000185527 | HGLibA_35817 | 73 | -0.422176227 | PDE6G | phosphodiesterase 6G [Source:HGNC Symbol;Acc:HGNC:8789] |
| ENSG00000186174 | HGLibA_04349 | 73 | -0.422176227 | BCL9L | B cell CLL/lymphoma 9 like [Source:HGNC Symbol;Acc:HGNC:23688] |
| ENSG00000186297 | HGLibA_18367 | 73 | -0.422176227 | GABRA5 | gamma-aminobutyric acid type A receptor alpha5 subunit [Source:HGNC Symbol;Acc:HGNC:4079] |
| ENSG00000186628 | HGLibA_18082 | 73 | -0.422176227 | FSD2 | fibronectin type III and SPRY domain containing 2 [Source:HGNC Symbol;Acc:HGNC:18024] |
| ENSG00000186652 | HGLibA_38452 | 73 | -0.422176227 | PRG2 | pro eosinophil major basic protein [Source:HGNC Symbol;Acc:HGNC:9362]" |
| ENSG00000186918 | HGLibA_55911 | 73 | -0.422176227 | ZNF395 | zinc finger protein 395 [Source:HGNC Symbol;Acc:HGNC:18737] |
| ENSG00000187556 | HGLibA_31101 | 73 | -0.422176227 | NANOS3 | nanos C2HC-type zinc finger 3 [Source:HGNC Symbol;Acc:HGNC:22048] |
| ENSG00000188089 | HGLibA_36913 | 73 | -0.422176227 | PLA2G4E | phospholipase A2 group IVE [Source:HGNC Symbol;Acc:HGNC:24791] |
| ENSG00000188313 | HGLibA_37230 | 73 | -0.422176227 | PLSCR1 | phospholipid scramblase 1 [Source:HGNC Symbol;Acc:HGNC:9092] |
| ENSG00000189337 | HGLibA_24090 | 73 | -0.422176227 | KAZN | periplakin interacting protein [Source:HGNC Symbol;Acc:HGNC:29173]" |
| ENSG00000196072 | HGLibA_04560 | 73 | -0.422176227 | BLOC1S2 | biogenesis of lysosomal organelles complex 1 subunit 2 [Source:HGNC Symbol;Acc:HGNC:20984] |
| ENSG00000196159 | HGLibA_16919 | 73 | -0.422176227 | FAT4 | FAT atypical cadherin 4 [Source:HGNC Symbol;Acc:HGNC:23109] |
| ENSG00000196388 | HGLibA_23274 | 73 | -0.422176227 | INCA1 | cyclin A1 interacting protein 1 [Source:HGNC Symbol;Acc:HGNC:32224]" |
| ENSG00000196693 | HGLibA_55833 | 73 | -0.422176227 | ZNF33B | zinc finger protein 33B [Source:HGNC Symbol;Acc:HGNC:13097] |
| ENSG00000196735 | HGLibA_21620 | 73 | -0.422176227 | HLA-DQA1 | DQ alpha 1 [Source:HGNC Symbol;Acc:HGNC:4942]" |
| ENSG00000197410 | HGLibA_12492 | 73 | -0.422176227 | DCHS2 | dachsous cadherin-related 2 [Source:HGNC Symbol;Acc:HGNC:23111] |
| ENSG00000197658 | HGLibA_44577 | 73 | -0.422176227 | SLC22A24 | solute carrier family 22 member 24 [Source:HGNC Symbol;Acc:HGNC:28542] |
| ENSG00000197893 | HGLibA_32643 | 73 | -0.422176227 | NRAP | nebulin related anchoring protein [Source:HGNC Symbol;Acc:HGNC:7988] |
| ENSG00000197933 | HGLibA_56821 | 73 | -0.422176227 | ZNF823 | zinc finger protein 823 [Source:HGNC Symbol;Acc:HGNC:30936] |
| ENSG00000198075 | HGLibA_47753 | 73 | -0.422176227 | SULT1C4 | sulfotransferase family 1C member 4 [Source:HGNC Symbol;Acc:HGNC:11457] |
| ENSG00000198612 | HGLibA_10734 | 73 | -0.422176227 | COPS8 | COP9 signalosome subunit 8 [Source:HGNC Symbol;Acc:HGNC:24335] |
| ENSG00000204450 | HGLibA_51575 | 73 | -0.422176227 | TRIM64 | tripartite motif containing 64 [Source:HGNC Symbol;Acc:HGNC:14663] |
| ENSG00000204681 | HGLibA_18341 | 73 | -0.422176227 | GABBR1 | gamma-aminobutyric acid type B receptor subunit 1 [Source:HGNC Symbol;Acc:HGNC:4070] |
| ENSG00000204695 | HGLibA_33638 | 73 | -0.422176227 | OR14J1 | olfactory receptor family 14 subfamily J member 1 [Source:HGNC Symbol;Acc:HGNC:13971] |
| ENSG00000204918 | HGLibA_38803 | 73 | -0.422176227 | PRR20B | proline rich 20B [Source:HGNC Symbol;Acc:HGNC:37220] |
| ENSG00000205108 | HGLibA_16423 | 73 | -0.422176227 | FAM205A | family with sequence similarity 205 member A [Source:HGNC Symbol;Acc:HGNC:41911] |
| ENSG00000213265 | HGLibA_51854 | 73 | -0.422176227 | TSGA13 | testis specific 13 [Source:HGNC Symbol;Acc:HGNC:12369] |
| ENSG00000214753 | HGLibA_21853 | 73 | -0.422176227 | HNRNPUL2 | heterogeneous nuclear ribonucleoprotein U like 2 [Source:HGNC Symbol;Acc:HGNC:25451] |
| ENSG00000226650 | HGLibA_24947 | 73 | -0.422176227 | KIF4B | kinesin family member 4B [Source:HGNC Symbol;Acc:HGNC:6322] |
| ENSG00000234560 | HGLibA_33501 | 73 | -0.422176227 | OR10G8 | olfactory receptor family 10 subfamily G member 8 [Source:HGNC Symbol;Acc:HGNC:14845] |
| ENSG00000240682 | HGLibA_23694 | 73 | -0.422176227 | ISY1 | ISY1 splicing factor homolog [Source:HGNC Symbol;Acc:HGNC:29201] |
| ENSG00000241106 | HGLibA_21613 | 73 | -0.422176227 | HLA-DOB | DO beta [Source:HGNC Symbol;Acc:HGNC:4937]" |
| ENSG00000242110 | HGLibA_01744 | 73 | -0.422176227 | AMACR | alpha-methylacyl-CoA racemase [Source:HGNC Symbol;Acc:HGNC:451] |
| ENSG00000253304 | HGLibA_50175 | 73 | -0.422176227 | TMEM200B | transmembrane protein 200B [Source:HGNC Symbol;Acc:HGNC:33785] |
| ENSG00000258644 | HGLibA_48012 | 73 | -0.422176227 | SYNJ2BP-COX16 | SYNJ2BP-COX16 readthrough [Source:HGNC Symbol;Acc:HGNC:48350] |
| ENSG00000269113 | HGLibA_51233 | 73 | -0.422176227 | TRABD2B | TraB domain containing 2B [Source:HGNC Symbol;Acc:HGNC:44200] |
| ENSG00000271447 | HGLibA_29532 | 73 | -0.422176227 | MMP28 | matrix metallopeptidase 28 [Source:HGNC Symbol;Acc:HGNC:14366] |
| FAM159A | HGLibA_16198 | 73 | -0.422176227 | FAM159A | - |
| hsa-mir-10a | HGLibA_57196 | 73 | -0.422176227 | hsa-mir-10a | - |
| hsa-mir-1204 | HGLibA_57273 | 73 | -0.422176227 | hsa-mir-1204 | - |
| hsa-mir-1251 | HGLibA_57404 | 73 | -0.422176227 | hsa-mir-1251 | - |
| hsa-mir-1289-1 | HGLibA_57622 | 73 | -0.422176227 | hsa-mir-1289-1 | - |
| hsa-mir-3144 | HGLibA_58633 | 73 | -0.422176227 | hsa-mir-3144 | - |
| hsa-mir-3683 | HGLibA_59286 | 73 | -0.422176227 | hsa-mir-3683 | - |
| hsa-mir-379 | HGLibA_59469 | 73 | -0.422176227 | hsa-mir-379 | - |
| hsa-mir-3917 | HGLibA_59543 | 73 | -0.422176227 | hsa-mir-3917 | - |
| hsa-mir-3918 | HGLibA_59547 | 73 | -0.422176227 | hsa-mir-3918 | - |
| hsa-mir-3936 | HGLibA_59603 | 73 | -0.422176227 | hsa-mir-3936 | - |
| hsa-mir-3943 | HGLibA_59631 | 73 | -0.422176227 | hsa-mir-3943 | - |
| hsa-mir-3977 | HGLibA_59669 | 73 | -0.422176227 | hsa-mir-3977 | - |
| hsa-mir-3978 | HGLibA_59671 | 73 | -0.422176227 | hsa-mir-3978 | - |
| hsa-mir-4254 | HGLibA_59723 | 73 | -0.422176227 | hsa-mir-4254 | - |
| hsa-mir-4524a | HGLibA_60552 | 73 | -0.422176227 | hsa-mir-4524a | - |
| hsa-mir-4534 | HGLibA_60597 | 73 | -0.422176227 | hsa-mir-4534 | - |
| hsa-mir-4648 | HGLibA_60697 | 73 | -0.422176227 | hsa-mir-4648 | - |
| hsa-mir-4652 | HGLibA_60717 | 73 | -0.422176227 | hsa-mir-4652 | - |
| hsa-mir-4794 | HGLibA_61281 | 73 | -0.422176227 | hsa-mir-4794 | - |
| hsa-mir-499b | HGLibA_61403 | 73 | -0.422176227 | hsa-mir-499b | - |
| hsa-mir-526a-2 | HGLibA_61795 | 73 | -0.422176227 | hsa-mir-526a-2 | - |
| hsa-mir-5701-2 | HGLibA_62343 | 73 | -0.422176227 | hsa-mir-5701-2 | - |
| hsa-mir-6089-1 | HGLibA_62615 | 73 | -0.422176227 | hsa-mir-6089-1 | - |
| hsa-mir-610 | HGLibA_62630 | 73 | -0.422176227 | hsa-mir-610 | - |
| hsa-mir-623 | HGLibA_62730 | 73 | -0.422176227 | hsa-mir-623 | - |
| hsa-mir-641 | HGLibA_62802 | 73 | -0.422176227 | hsa-mir-641 | - |
| hsa-mir-6727 | HGLibA_63063 | 73 | -0.422176227 | hsa-mir-6727 | - |
| hsa-mir-6824 | HGLibA_63472 | 73 | -0.422176227 | hsa-mir-6824 | - |
| hsa-mir-6863 | HGLibA_63636 | 73 | -0.422176227 | hsa-mir-6863 | - |
| hsa-mir-8071-1 | HGLibA_64124 | 73 | -0.422176227 | hsa-mir-8071-1 | - |
| hsa-mir-8071-2 | HGLibA_64128 | 73 | -0.422176227 | hsa-mir-8071-2 | - |
| hsa-mir-922 | HGLibA_64283 | 73 | -0.422176227 | hsa-mir-922 | - |
| hsa-mir-934 | HGLibA_64309 | 73 | -0.422176227 | hsa-mir-934 | - |
| hsa-mir-95 | HGLibA_64366 | 73 | -0.422176227 | hsa-mir-95 | - |
| MARS | HGLibA_28377 | 73 | -0.422176227 | MARS | - |
| PIH1D3 | HGLibA_36614 | 73 | -0.422176227 | PIH1D3 | - |
| PYCRL | HGLibA_39680 | 73 | -0.422176227 | PYCRL | - |
| TUSC5 | HGLibA_52351 | 73 | -0.422176227 | TUSC5 | - |
| ZMYM6NB | HGLibA_55409 | 73 | -0.422176227 | ZMYM6NB | - |
| C10orf76 | HGLibA_05094 | 72 | -0.432127264 | C10orf76 | - |
| C14orf159 | HGLibA_05330 | 72 | -0.432127264 | C14orf159 | - |
| C15orf38 | HGLibA_05386 | 72 | -0.432127264 | C15orf38 | - |
| C15orf53 | HGLibA_05410 | 72 | -0.432127264 | C15orf53 | - |
| C21orf2 | HGLibA_06035 | 72 | -0.432127264 | C21orf2 | - |
| C4orf27 | HGLibA_06310 | 72 | -0.432127264 | C4orf27 | - |
| COL4A3BP | HGLibA_10576 | 72 | -0.432127264 | COL4A3BP | - |
| CXorf40A | HGLibA_11923 | 72 | -0.432127264 | CXorf40A | - |
| ENSG00000001497 | HGLibA_26053 | 72 | -0.432127264 | LAS1L | ribosome biogenesis factor [Source:HGNC Symbol;Acc:HGNC:25726]" |
| ENSG00000001629 | HGLibA_01970 | 72 | -0.432127264 | ANKIB1 | ankyrin repeat and IBR domain containing 1 [Source:HGNC Symbol;Acc:HGNC:22215] |
| ENSG00000002726 | HGLibA_02266 | 72 | -0.432127264 | AOC1 | copper containing 1 [Source:HGNC Symbol;Acc:HGNC:80]" |
| ENSG00000008086 | HGLibA_08819 | 72 | -0.432127264 | CDKL5 | cyclin dependent kinase like 5 [Source:HGNC Symbol;Acc:HGNC:11411] |
| ENSG00000008282 | HGLibA_48035 | 72 | -0.432127264 | SYPL1 | synaptophysin like 1 [Source:HGNC Symbol;Acc:HGNC:11507] |
| ENSG00000010704 | HGLibA_21226 | 72 | -0.432127264 | HFE | homeostatic iron regulator [Source:HGNC Symbol;Acc:HGNC:4886] |
| ENSG00000013573 | HGLibA_12655 | 72 | -0.432127264 | DDX11 | DEAD/H-box helicase 11 [Source:HGNC Symbol;Acc:HGNC:2736] |
| ENSG00000014641 | HGLibA_28681 | 72 | -0.432127264 | MDH1 | malate dehydrogenase 1 [Source:HGNC Symbol;Acc:HGNC:6970] |
| ENSG00000017483 | HGLibA_45074 | 72 | -0.432127264 | SLC38A5 | solute carrier family 38 member 5 [Source:HGNC Symbol;Acc:HGNC:18070] |
| ENSG00000018510 | HGLibA_01242 | 72 | -0.432127264 | AGPS | alkylglycerone phosphate synthase [Source:HGNC Symbol;Acc:HGNC:327] |
| ENSG00000025772 | HGLibA_50963 | 72 | -0.432127264 | TOMM34 | translocase of outer mitochondrial membrane 34 [Source:HGNC Symbol;Acc:HGNC:15746] |
| ENSG00000033170 | HGLibA_18205 | 72 | -0.432127264 | FUT8 | fucosyltransferase 8 [Source:HGNC Symbol;Acc:HGNC:4019] |
| ENSG00000034239 | HGLibA_14519 | 72 | -0.432127264 | EFCAB1 | EF-hand calcium binding domain 1 [Source:HGNC Symbol;Acc:HGNC:25678] |
| ENSG00000039600 | HGLibA_46261 | 72 | -0.432127264 | SOX30 | SRY-box 30 [Source:HGNC Symbol;Acc:HGNC:30635] |
| ENSG00000049449 | HGLibA_40701 | 72 | -0.432127264 | RCN1 | reticulocalbin 1 [Source:HGNC Symbol;Acc:HGNC:9934] |
| ENSG00000049656 | HGLibA_10115 | 72 | -0.432127264 | CLPTM1L | CLPTM1 like [Source:HGNC Symbol;Acc:HGNC:24308] |
| ENSG00000055483 | HGLibA_53348 | 72 | -0.432127264 | USP36 | ubiquitin specific peptidase 36 [Source:HGNC Symbol;Acc:HGNC:20062] |

| | | | | | |
|---|---|---|---|---|---|
| ENSG00000061656 | HGLibA_46357 | 72 | -0.432127264 | SPAG4 | sperm associated antigen 4 [Source:HGNC Symbol;Acc:HGNC:11214] |
| ENSG00000062725 | HGLibA_02563 | 72 | -0.432127264 | APPBP2 | amyloid beta precursor protein binding protein 2 [Source:HGNC Symbol;Acc:HGNC:622] |
| ENSG00000063601 | HGLibA_30408 | 72 | -0.432127264 | MTMR1 | myotubularin related protein 1 [Source:HGNC Symbol;Acc:HGNC:7449] |
| ENSG00000064989 | HGLibA_07008 | 72 | -0.432127264 | CALCRL | calcitonin receptor like receptor [Source:HGNC Symbol;Acc:HGNC:16709] |
| ENSG00000065559 | HGLibA_28149 | 72 | -0.432127264 | MAP2K4 | mitogen-activated protein kinase kinase 4 [Source:HGNC Symbol;Acc:HGNC:6844] |
| ENSG00000065833 | HGLibA_28708 | 72 | -0.432127264 | ME1 | malic enzyme 1 [Source:HGNC Symbol;Acc:HGNC:6983] |
| ENSG00000065970 | HGLibA_17887 | 72 | -0.432127264 | FOXJ2 | forkhead box J2 [Source:HGNC Symbol;Acc:HGNC:24818] |
| ENSG00000069399 | HGLibA_04328 | 72 | -0.432127264 | BCL3 | B cell CLL/lymphoma 3 [Source:HGNC Symbol;Acc:HGNC:998] |
| ENSG00000071242 | HGLibA_42161 | 72 | -0.432127264 | RPS6KA2 | ribosomal protein S6 kinase A2 [Source:HGNC Symbol;Acc:HGNC:10431] |
| ENSG00000071539 | HGLibA_51643 | 72 | -0.432127264 | TRIP13 | thyroid hormone receptor interactor 13 [Source:HGNC Symbol;Acc:HGNC:12307] |
| ENSG00000072110 | HGLibA_00639 | 72 | -0.432127264 | ACTN1 | actinin alpha 1 [Source:HGNC Symbol;Acc:HGNC:163] |
| ENSG00000072195 | HGLibA_46615 | 72 | -0.432127264 | SPEG | SPEG complex locus [Source:HGNC Symbol;Acc:HGNC:16901] |
| ENSG00000074317 | HGLibA_45915 | 72 | -0.432127264 | SNCB | synuclein beta [Source:HGNC Symbol;Acc:HGNC:11140] |
| ENSG00000076053 | HGLibA_40581 | 72 | -0.432127264 | RBM7 | RNA binding motif protein 7 [Source:HGNC Symbol;Acc:HGNC:9904] |
| ENSG00000077063 | HGLibA_11744 | 72 | -0.432127264 | CTTNBP2 | cortactin binding protein 2 [Source:HGNC Symbol;Acc:HGNC:15679] |
| ENSG00000079459 | HGLibA_17253 | 72 | -0.432127264 | FDFT1 | farnesyl-diphosphate farnesyltransferase 1 [Source:HGNC Symbol;Acc:HGNC:3629] |
| ENSG00000080224 | HGLibA_15302 | 72 | -0.432127264 | EPHA6 | EPH receptor A6 [Source:HGNC Symbol;Acc:HGNC:19296] |
| ENSG00000080644 | HGLibA_09571 | 72 | -0.432127264 | CHRNA3 | cholinergic receptor nicotinic alpha 3 subunit [Source:HGNC Symbol;Acc:HGNC:1957] |
| ENSG00000081237 | HGLibA_39489 | 72 | -0.432127264 | PTPRC | receptor type C [Source:HGNC Symbol;Acc:HGNC:9666]" |
| ENSG00000083814 | HGLibA_56504 | 72 | -0.432127264 | ZNF671 | zinc finger protein 671 [Source:HGNC Symbol;Acc:HGNC:26279] |
| ENSG00000085449 | HGLibA_54004 | 72 | -0.432127264 | WDFY1 | WD repeat and FYVE domain containing 1 [Source:HGNC Symbol;Acc:HGNC:20451] |
| ENSG00000087510 | HGLibA_49128 | 72 | -0.432127264 | TFAP2C | transcription factor AP-2 gamma [Source:HGNC Symbol;Acc:HGNC:11744] |
| ENSG00000088538 | HGLibA_13744 | 72 | -0.432127264 | DOCK3 | dedicator of cytokinesis 3 [Source:HGNC Symbol;Acc:HGNC:2989] |
| ENSG00000090470 | HGLibA_35746 | 72 | -0.432127264 | PDCD7 | programmed cell death 7 [Source:HGNC Symbol;Acc:HGNC:8767] |
| ENSG00000091986 | HGLibA_07876 | 72 | -0.432127264 | CCDC80 | coiled-coil domain containing 80 [Source:HGNC Symbol;Acc:HGNC:30649] |
| ENSG00000100211 | HGLibA_07469 | 72 | -0.432127264 | CBY1 | beta catenin antagonist [Source:HGNC Symbol;Acc:HGNC:1307]" |
| ENSG00000100298 | HGLibA_02494 | 72 | -0.432127264 | APOBEC3H | apolipoprotein B mRNA editing enzyme catalytic subunit 3H [Source:HGNC Symbol;Acc:HGNC:24100] |
| ENSG00000100612 | HGLibA_13145 | 72 | -0.432127264 | DHRS7 | dehydrogenase/reductase 7 [Source:HGNC Symbol;Acc:HGNC:21524] |
| ENSG00000101193 | HGLibA_19057 | 72 | -0.432127264 | GID8 | GID complex subunit 8 homolog [Source:HGNC Symbol;Acc:HGNC:15857] |
| ENSG00000101752 | HGLibA_29193 | 72 | -0.432127264 | MIB1 | mindbomb E3 ubiquitin protein ligase 1 [Source:HGNC Symbol;Acc:HGNC:21086] |
| ENSG00000102003 | HGLibA_48033 | 72 | -0.432127264 | SYP | synaptophysin [Source:HGNC Symbol;Acc:HGNC:11506] |
| ENSG00000102241 | HGLibA_22348 | 72 | -0.432127264 | HTATSF1 | HIV-1 Tat specific factor 1 [Source:HGNC Symbol;Acc:HGNC:5276] |
| ENSG00000103876 | HGLibA_15953 | 72 | -0.432127264 | FAH | fumarylacetoacetate hydrolase [Source:HGNC Symbol;Acc:HGNC:3579] |
| ENSG00000105088 | HGLibA_33348 | 72 | -0.432127264 | OLFM2 | olfactomedin 2 [Source:HGNC Symbol;Acc:HGNC:17189] |
| ENSG00000105227 | HGLibA_38993 | 72 | -0.432127264 | PRX | periaxin [Source:HGNC Symbol;Acc:HGNC:13797] |
| ENSG00000105852 | HGLibA_37685 | 72 | -0.432127264 | PON3 | paraoxonase 3 [Source:HGNC Symbol;Acc:HGNC:9206] |
| ENSG00000108509 | HGLibA_07118 | 72 | -0.432127264 | CAMTA2 | calmodulin binding transcription activator 2 [Source:HGNC Symbol;Acc:HGNC:18807] |
| ENSG00000108798 | HGLibA_00296 | 72 | -0.432127264 | ABI3 | ABI family member 3 [Source:HGNC Symbol;Acc:HGNC:29859] |
| ENSG00000108839 | HGLibA_01676 | 72 | -0.432127264 | ALOX12 | 12S type [Source:HGNC Symbol;Acc:HGNC:429]" |
| ENSG00000109046 | HGLibA_54416 | 72 | -0.432127264 | WSB1 | WD repeat and SOCS box containing 1 [Source:HGNC Symbol;Acc:HGNC:19221] |
| ENSG00000109062 | HGLibA_45408 | 72 | -0.432127264 | SLC9A3R1 | SLC9A3 regulator 1 [Source:HGNC Symbol;Acc:HGNC:11075] |
| ENSG00000109670 | HGLibA_17153 | 72 | -0.432127264 | FBXW7 | F-box and WD repeat domain containing 7 [Source:HGNC Symbol;Acc:HGNC:16712] |
| ENSG00000110723 | HGLibA_15800 | 72 | -0.432127264 | EXPH5 | exophilin 5 [Source:HGNC Symbol;Acc:HGNC:30578] |
| ENSG00000111245 | HGLibA_30757 | 72 | -0.432127264 | MYL2 | myosin light chain 2 [Source:HGNC Symbol;Acc:HGNC:7583] |
| ENSG00000111364 | HGLibA_12746 | 72 | -0.432127264 | DDX55 | DEAD-box helicase 55 [Source:HGNC Symbol;Acc:HGNC:20085] |
| ENSG00000112309 | HGLibA_03974 | 72 | -0.432127264 | B3GAT2 | 3-glucuronyltransferase 2 [Source:HGNC Symbol;Acc:HGNC:922]" |
| ENSG00000113108 | HGLibA_02385 | 72 | -0.432127264 | APBB3 | amyloid beta precursor protein binding family B member 3 [Source:HGNC Symbol;Acc:HGNC:20708] |
| ENSG00000115084 | HGLibA_45009 | 72 | -0.432127264 | SLC35F5 | solute carrier family 35 member F5 [Source:HGNC Symbol;Acc:HGNC:23617] |
| ENSG00000115207 | HGLibA_20583 | 72 | -0.432127264 | GTF3C2 | general transcription factor IIIC subunit 2 [Source:HGNC Symbol;Acc:HGNC:4665] |
| ENSG00000115339 | HGLibA_18557 | 72 | -0.432127264 | GALNT3 | polypeptide N-acetylgalactosaminyltransferase 3 [Source:HGNC Symbol;Acc:HGNC:4125] |
| ENSG00000115461 | HGLibA_22826 | 72 | -0.432127264 | IGFBP5 | insulin like growth factor binding protein 5 [Source:HGNC Symbol;Acc:HGNC:5474] |
| ENSG00000115884 | HGLibA_43098 | 72 | -0.432127264 | SDC1 | syndecan 1 [Source:HGNC Symbol;Acc:HGNC:10658] |
| ENSG00000116035 | HGLibA_53561 | 72 | -0.432127264 | VAX2 | ventral anterior homeobox 2 [Source:HGNC Symbol;Acc:HGNC:12661] |
| ENSG00000116691 | HGLibA_29268 | 72 | -0.432127264 | MIIP | migration and invasion inhibitory protein [Source:HGNC Symbol;Acc:HGNC:25715] |
| ENSG00000116774 | HGLibA_33366 | 72 | -0.432127264 | OLFML3 | olfactomedin like 3 [Source:HGNC Symbol;Acc:HGNC:24956] |
| ENSG00000118160 | HGLibA_45390 | 72 | -0.432127264 | SLC8A2 | solute carrier family 8 member A2 [Source:HGNC Symbol;Acc:HGNC:11069] |
| ENSG00000119004 | HGLibA_12076 | 72 | -0.432127264 | CYP20A1 | cytochrome P450 family 20 subfamily A member 1 [Source:HGNC Symbol;Acc:HGNC:20576] |
| ENSG00000120333 | HGLibA_30038 | 72 | -0.432127264 | MRPS14 | mitochondrial ribosomal protein S14 [Source:HGNC Symbol;Acc:HGNC:14049] |
| ENSG00000120440 | HGLibA_52199 | 72 | -0.432127264 | TTLL2 | tubulin tyrosine ligase like 2 [Source:HGNC Symbol;Acc:HGNC:21211] |
| ENSG00000120645 | HGLibA_23547 | 72 | -0.432127264 | IQSEC3 | IQ motif and Sec7 domain 3 [Source:HGNC Symbol;Acc:HGNC:29193] |
| ENSG00000123388 | HGLibA_21958 | 72 | -0.432127264 | HOXC11 | homeobox C11 [Source:HGNC Symbol;Acc:HGNC:5123] |
| ENSG00000124818 | HGLibA_33440 | 72 | -0.432127264 | OPN5 | opsin 5 [Source:HGNC Symbol;Acc:HGNC:19992] |
| ENSG00000126391 | HGLibA_18029 | 72 | -0.432127264 | FRMD8 | FERM domain containing 8 [Source:HGNC Symbol;Acc:HGNC:25462] |
| ENSG00000127588 | HGLibA_19516 | 72 | -0.432127264 | GNG13 | G protein subunit gamma 13 [Source:HGNC Symbol;Acc:HGNC:14131] |
| ENSG00000127616 | HGLibA_45609 | 72 | -0.432127264 | SMARCA4 | member 4 [Source:HGNC Symbol;Acc:HGNC:11100]" |
| ENSG00000128185 | HGLibA_13053 | 72 | -0.432127264 | DGCR6L | DiGeorge syndrome critical region gene 6 like [Source:HGNC Symbol;Acc:HGNC:18551] |
| ENSG00000128274 | HGLibA_00018 | 72 | -0.432127264 | A4GALT | 4-galactosyltransferase (P blood group) [Source:HGNC Symbol;Acc:HGNC:18149]" |
| ENSG00000129244 | HGLibA_03597 | 72 | -0.432127264 | ATP1B2 | ATPase Na+/K+ transporting subunit beta 2 [Source:HGNC Symbol;Acc:HGNC:805] |
| ENSG00000130560 | HGLibA_52535 | 72 | -0.432127264 | UBAC1 | UBA domain containing 1 [Source:HGNC Symbol;Acc:HGNC:30221] |
| ENSG00000130707 | HGLibA_03371 | 72 | -0.432127264 | ASS1 | argininosuccinate synthase 1 [Source:HGNC Symbol;Acc:HGNC:758] |
| ENSG00000131668 | HGLibA_04163 | 72 | -0.432127264 | BARX1 | BARX homeobox 1 [Source:HGNC Symbol;Acc:HGNC:955] |
| ENSG00000132005 | HGLibA_40962 | 72 | -0.432127264 | RFX1 | regulatory factor X1 [Source:HGNC Symbol;Acc:HGNC:9982] |
| ENSG00000134077 | HGLibA_49421 | 72 | -0.432127264 | THUMPD3 | THUMP domain containing 3 [Source:HGNC Symbol;Acc:HGNC:24493] |
| ENSG00000134318 | HGLibA_41748 | 72 | -0.432127264 | ROCK2 | Rho associated coiled-coil containing protein kinase 2 [Source:HGNC Symbol;Acc:HGNC:10252] |
| ENSG00000134438 | HGLibA_40407 | 72 | -0.432127264 | RAX | retina and anterior neural fold homeobox [Source:HGNC Symbol;Acc:HGNC:18662] |
| ENSG00000134716 | HGLibA_12136 | 72 | -0.432127264 | CYP2J2 | cytochrome P450 family 2 subfamily J member 2 [Source:HGNC Symbol;Acc:HGNC:2634] |
| ENSG00000135148 | HGLibA_51271 | 72 | -0.432127264 | TRAFD1 | TRAF-type zinc finger domain containing 1 [Source:HGNC Symbol;Acc:HGNC:24808] |
| ENSG00000135404 | HGLibA_08425 | 72 | -0.432127264 | CD63 | CD63 molecule [Source:HGNC Symbol;Acc:HGNC:1692] |
| ENSG00000135974 | HGLibA_06139 | 72 | -0.432127264 | C2orf49 | chromosome 2 open reading frame 49 [Source:HGNC Symbol;Acc:HGNC:28772] |
| ENSG00000136457 | HGLibA_09340 | 72 | -0.432127264 | CHAD | chondroadherin [Source:HGNC Symbol;Acc:HGNC:1909] |
| ENSG00000137764 | HGLibA_28152 | 72 | -0.432127264 | MAP2K5 | mitogen-activated protein kinase kinase 5 [Source:HGNC Symbol;Acc:HGNC:6845] |
| ENSG00000137871 | HGLibA_55731 | 72 | -0.432127264 | ZNF280D | zinc finger protein 280D [Source:HGNC Symbol;Acc:HGNC:25953] |
| ENSG00000137936 | HGLibA_04248 | 72 | -0.432127264 | BCAR3 | NSP family adaptor protein [Source:HGNC Symbol;Acc:HGNC:973]" |
| ENSG00000138134 | HGLibA_47340 | 72 | -0.432127264 | STAMBPL1 | STAM binding protein like 1 [Source:HGNC Symbol;Acc:HGNC:24105] |
| ENSG00000138593 | HGLibA_43269 | 72 | -0.432127264 | SECISBP2L | SECIS binding protein 2 like [Source:HGNC Symbol;Acc:HGNC:28997] |
| ENSG00000139154 | HGLibA_01089 | 72 | -0.432127264 | AEBP2 | AE binding protein 2 [Source:HGNC Symbol;Acc:HGNC:24051] |
| ENSG00000139323 | HGLibA_37444 | 72 | -0.432127264 | POC1B | POC1 centriolar protein B [Source:HGNC Symbol;Acc:HGNC:30836] |
| ENSG00000140694 | HGLibA_35208 | 72 | -0.432127264 | PARN | poly(A)-specific ribonuclease [Source:HGNC Symbol;Acc:HGNC:8609] |
| ENSG00000140955 | HGLibA_00721 | 72 | -0.432127264 | ADAD2 | adenosine deaminase domain containing 2 [Source:HGNC Symbol;Acc:HGNC:30714] |
| ENSG00000141510 | HGLibA_51049 | 72 | -0.432127264 | TP53 | tumor protein p53 [Source:HGNC Symbol;Acc:HGNC:11998] |
| ENSG00000143340 | HGLibA_16227 | 72 | -0.432127264 | FAM163A | family with sequence similarity 163 member A [Source:HGNC Symbol;Acc:HGNC:28274] |
| ENSG00000143493 | HGLibA_23431 | 72 | -0.432127264 | INTS7 | integrator complex subunit 7 [Source:HGNC Symbol;Acc:HGNC:24484] |
| ENSG00000143554 | HGLibA_44820 | 72 | -0.432127264 | SLC27A3 | solute carrier family 27 member 3 [Source:HGNC Symbol;Acc:HGNC:10997] |
| ENSG00000144283 | HGLibA_36860 | 72 | -0.432127264 | PKP4 | plakophilin 4 [Source:HGNC Symbol;Acc:HGNC:9026] |
| ENSG00000144857 | HGLibA_04664 | 72 | -0.432127264 | BOC | oncogene regulated [Source:HGNC Symbol;Acc:HGNC:17173]" |
| ENSG00000152684 | HGLibA_36043 | 72 | -0.432127264 | PELO | pelota mRNA surveillance and ribosome rescue factor [Source:HGNC Symbol;Acc:HGNC:8829] |
| ENSG00000153094 | HGLibA_04309 | 72 | -0.432127264 | BCL2L11 | BCL2 like 11 [Source:HGNC Symbol;Acc:HGNC:994] |
| ENSG00000153558 | HGLibA_16998 | 72 | -0.432127264 | FBXL2 | F-box and leucine rich repeat protein 2 [Source:HGNC Symbol;Acc:HGNC:13598] |
| ENSG00000153814 | HGLibA_23941 | 72 | -0.432127264 | JAZF1 | JAZF zinc finger 1 [Source:HGNC Symbol;Acc:HGNC:28917] |
| ENSG00000155256 | HGLibA_55287 | 72 | -0.432127264 | ZFYVE27 | zinc finger FYVE-type containing 27 [Source:HGNC Symbol;Acc:HGNC:26559] |
| ENSG00000155495 | HGLibA_27958 | 72 | -0.432127264 | MAGEC1 | MAGE family member C1 [Source:HGNC Symbol;Acc:HGNC:6812] |
| ENSG00000156515 | HGLibA_21580 | 72 | -0.432127264 | HK1 | hexokinase 1 [Source:HGNC Symbol;Acc:HGNC:4922] |
| ENSG00000157379 | HGLibA_13117 | 72 | -0.432127264 | DHRS1 | dehydrogenase/reductase 1 [Source:HGNC Symbol;Acc:HGNC:16445] |
| ENSG00000157600 | HGLibA_50034 | 72 | -0.432127264 | TMEM164 | transmembrane protein 164 [Source:HGNC Symbol;Acc:HGNC:26217] |
| ENSG00000157680 | HGLibA_13077 | 72 | -0.432127264 | DGKI | diacylglycerol kinase iota [Source:HGNC Symbol;Acc:HGNC:2855] |
| ENSG00000158423 | HGLibA_41228 | 72 | -0.432127264 | RIBC1 | RIB43A domain with coiled-coils 1 [Source:HGNC Symbol;Acc:HGNC:26537] |
| ENSG00000158864 | HGLibA_31547 | 72 | -0.432127264 | NDUFS2 | NADH:ubiquinone oxidoreductase core subunit S2 [Source:HGNC Symbol;Acc:HGNC:7708] |
| ENSG00000159592 | HGLibA_19760 | 72 | -0.432127264 | GPBP1L1 | GC-rich promoter binding protein 1 like 1 [Source:HGNC Symbol;Acc:HGNC:28843] |
| ENSG00000159842 | HGLibA_00324 | 72 | -0.432127264 | ABR | RhoGEF and GTPase activating protein [Source:HGNC Symbol;Acc:HGNC:81]" |
| ENSG00000161849 | HGLibA_25567 | 72 | -0.432127264 | KRT84 | keratin 84 [Source:HGNC Symbol;Acc:HGNC:6461] |
| ENSG00000161958 | HGLibA_17365 | 72 | -0.432127264 | FGF11 | fibroblast growth factor 11 [Source:HGNC Symbol;Acc:HGNC:3667] |
| ENSG00000162063 | HGLibA_08089 | 72 | -0.432127264 | CCNF | cyclin F [Source:HGNC Symbol;Acc:HGNC:1591] |
| ENSG00000162300 | HGLibA_55248 | 72 | -0.432127264 | ZFPL1 | zinc finger protein like 1 [Source:HGNC Symbol;Acc:HGNC:12868] |
| ENSG00000162383 | HGLibA_44524 | 72 | -0.432127264 | SLC1A7 | solute carrier family 1 member 7 [Source:HGNC Symbol;Acc:HGNC:10945] |
| ENSG00000162490 | HGLibA_13952 | 72 | -0.432127264 | DRAXIN | dorsal inhibitory axon guidance protein [Source:HGNC Symbol;Acc:HGNC:25054] |
| ENSG00000162783 | HGLibA_22582 | 72 | -0.432127264 | IER5 | immediate early response 5 [Source:HGNC Symbol;Acc:HGNC:5393] |
| ENSG00000162931 | HGLibA_51422 | 72 | -0.432127264 | TRIM17 | tripartite motif containing 17 [Source:HGNC Symbol;Acc:HGNC:13430] |
| ENSG00000163867 | HGLibA_55406 | 72 | -0.432127264 | ZMYM6 | zinc finger MYM-type containing 6 [Source:HGNC Symbol;Acc:HGNC:13050] |
| ENSG00000164674 | HGLibA_48101 | 72 | -0.432127264 | SYTL3 | synaptotagmin like 3 [Source:HGNC Symbol;Acc:HGNC:15587] |
| ENSG00000164896 | HGLibA_16898 | 72 | -0.432127264 | FASTK | Fas activated serine/threonine kinase [Source:HGNC Symbol;Acc:HGNC:24676] |
| ENSG00000165182 | HGLibA_11951 | 72 | -0.432127264 | CXorf58 | chromosome X open reading frame 58 [Source:HGNC Symbol;Acc:HGNC:26356] |
| ENSG00000165271 | HGLibA_32270 | 72 | -0.432127264 | NOL6 | nucleolar protein 6 [Source:HGNC Symbol;Acc:HGNC:19910] |
| ENSG00000165409 | HGLibA_51860 | 72 | -0.432127264 | TSHR | thyroid stimulating hormone receptor [Source:HGNC Symbol;Acc:HGNC:12373] |
| ENSG00000165475 | HGLibA_11288 | 72 | -0.432127264 | CRYL1 | crystallin lambda 1 [Source:HGNC Symbol;Acc:HGNC:18246] |
| ENSG00000165970 | HGLibA_45332 | 72 | -0.432127264 | SLC6A5 | solute carrier family 6 member 5 [Source:HGNC Symbol;Acc:HGNC:11051] |
| ENSG00000166311 | HGLibA_45803 | 72 | -0.432127264 | SMPD1 | sphingomyelin phosphodiesterase 1 [Source:HGNC Symbol;Acc:HGNC:11120] |
| ENSG00000166359 | HGLibA_54208 | 72 | -0.432127264 | WDR88 | WD repeat domain 88 [Source:HGNC Symbol;Acc:HGNC:26999] |
| ENSG00000166569 | HGLibA_10942 | 72 | -0.432127264 | CPLX4 | complexin 4 [Source:HGNC Symbol;Acc:HGNC:24330] |
| ENSG00000166965 | HGLibA_40690 | 72 | -0.432127264 | RCCD1 | RCC1 domain containing 1 [Source:HGNC Symbol;Acc:HGNC:30457] |
| ENSG00000167207 | HGLibA_32248 | 72 | -0.432127264 | NOD2 | nucleotide binding oligomerization domain containing 2 [Source:HGNC Symbol;Acc:HGNC:5331] |
| ENSG00000167257 | HGLibA_41626 | 72 | -0.432127264 | RNF214 | ring finger protein 214 [Source:HGNC Symbol;Acc:HGNC:25335] |
| ENSG00000167851 | HGLibA_08332 | 72 | -0.432127264 | CD300A | CD300a molecule [Source:HGNC Symbol;Acc:HGNC:19319] |
| ENSG00000168060 | HGLibA_31012 | 72 | -0.432127264 | NAALADL1 | N-acetylated alpha-linked acidic dipeptidase like 1 [Source:HGNC Symbol;Acc:HGNC:23536] |
| ENSG00000168067 | HGLibA_28213 | 72 | -0.432127264 | MAP4K2 | mitogen-activated protein kinase kinase kinase kinase 2 [Source:HGNC Symbol;Acc:HGNC:6864] |
| ENSG00000168229 | HGLibA_39305 | 72 | -0.432127264 | PTGDR | prostaglandin D2 receptor [Source:HGNC Symbol;Acc:HGNC:9591] |

| | | | | | |
|---|---|---|---|---|---|
| ENSG00000168421 | HGLibA_41192 | 72 | -0.432127264 | RHOH | ras homolog family member H [Source:HGNC Symbol;Acc:HGNC:686] |
| ENSG00000168427 | HGLibA_25179 | 72 | -0.432127264 | KLHL30 | kelch like family member 30 [Source:HGNC Symbol;Acc:HGNC:24770] |
| ENSG00000168575 | HGLibA_44532 | 72 | -0.432127264 | SLC20A2 | solute carrier family 20 member 2 [Source:HGNC Symbol;Acc:HGNC:10947] |
| ENSG00000168994 | HGLibA_39646 | 72 | -0.432127264 | PXDC1 | PX domain containing 1 [Source:HGNC Symbol;Acc:HGNC:21361] |
| ENSG00000169180 | HGLibA_54570 | 72 | -0.432127264 | XPO6 | exportin 6 [Source:HGNC Symbol;Acc:HGNC:19733] |
| ENSG00000169953 | HGLibA_22243 | 72 | -0.432127264 | HSFY2 | heat shock transcription factor Y-linked 2 [Source:HGNC Symbol;Acc:HGNC:23950] |
| ENSG00000170442 | HGLibA_25572 | 72 | -0.432127264 | KRT86 | keratin 86 [Source:HGNC Symbol;Acc:HGNC:6463] |
| ENSG00000171428 | HGLibA_31164 | 72 | -0.432127264 | NAT1 | N-acetyltransferase 1 [Source:HGNC Symbol;Acc:HGNC:7645] |
| ENSG00000171448 | HGLibA_54853 | 72 | -0.432127264 | ZBTB26 | zinc finger and BTB domain containing 26 [Source:HGNC Symbol;Acc:HGNC:23383] |
| ENSG00000171772 | HGLibA_47913 | 72 | -0.432127264 | SYCE1 | synaptonemal complex central element protein 1 [Source:HGNC Symbol;Acc:HGNC:28852] |
| ENSG00000172113 | HGLibA_32169 | 72 | -0.432127264 | NME6 | NME/NM23 nucleoside diphosphate kinase 6 [Source:HGNC Symbol;Acc:HGNC:20567] |
| ENSG00000172409 | HGLibA_10096 | 72 | -0.432127264 | CLP1 | cleavage and polyadenylation factor I subunit 1 [Source:HGNC Symbol;Acc:HGNC:16999] |
| ENSG00000172508 | HGLibA_07256 | 72 | -0.432127264 | CARNS1 | carnosine synthase 1 [Source:HGNC Symbol;Acc:HGNC:29268] |
| ENSG00000175334 | HGLibA_04139 | 72 | -0.432127264 | BANF1 | barrier to autointegration factor 1 [Source:HGNC Symbol;Acc:HGNC:17397] |
| ENSG00000175387 | HGLibA_45572 | 72 | -0.432127264 | SMAD2 | SMAD family member 2 [Source:HGNC Symbol;Acc:HGNC:6768] |
| ENSG00000175395 | HGLibA_55662 | 72 | -0.432127264 | ZNF25 | zinc finger protein 25 [Source:HGNC Symbol;Acc:HGNC:13043] |
| ENSG00000176563 | HGLibA_10387 | 72 | -0.432127264 | CNTD1 | cyclin N-terminal domain containing 1 [Source:HGNC Symbol;Acc:HGNC:26847] |
| ENSG00000177300 | HGLibA_09882 | 72 | -0.432127264 | CLDN22 | claudin 22 [Source:HGNC Symbol;Acc:HGNC:2044] |
| ENSG00000178462 | HGLibA_52268 | 72 | -0.432127264 | TUBAL3 | tubulin alpha like 3 [Source:HGNC Symbol;Acc:HGNC:23534] |
| ENSG00000179615 | HGLibA_33758 | 72 | -0.432127264 | OR2AP1 | olfactory receptor family 2 subfamily AP member 1 [Source:HGNC Symbol;Acc:HGNC:15335] |
| ENSG00000179796 | HGLibA_27345 | 72 | -0.432127264 | LRRC3B | leucine rich repeat containing 3B [Source:HGNC Symbol;Acc:HGNC:28105] |
| ENSG00000180613 | HGLibA_20510 | 72 | -0.432127264 | GSX2 | GS homeobox 2 [Source:HGNC Symbol;Acc:HGNC:24959] |
| ENSG00000180785 | HGLibA_34096 | 72 | -0.432127264 | OR51E1 | olfactory receptor family 51 subfamily E member 1 [Source:HGNC Symbol;Acc:HGNC:15194] |
| ENSG00000180974 | HGLibA_34159 | 72 | -0.432127264 | OR52E4 | olfactory receptor family 52 subfamily E member 4 [Source:HGNC Symbol;Acc:HGNC:15213] |
| ENSG00000181333 | HGLibA_21147 | 72 | -0.432127264 | HEPHL1 | hephaestin like 1 [Source:HGNC Symbol;Acc:HGNC:30477] |
| ENSG00000182287 | HGLibA_02299 | 72 | -0.432127264 | AP1S2 | adaptor related protein complex 1 subunit sigma 2 [Source:HGNC Symbol;Acc:HGNC:560] |
| ENSG00000182551 | HGLibA_00972 | 72 | -0.432127264 | ADI1 | acireductone dioxygenase 1 [Source:HGNC Symbol;Acc:HGNC:30576] |
| ENSG00000183208 | HGLibA_18906 | 72 | -0.432127264 | GDPGP1 | GDP-D-glucose phosphorylase 1 [Source:HGNC Symbol;Acc:HGNC:34360] |
| ENSG00000183255 | HGLibA_39565 | 72 | -0.432127264 | PTTG1IP | PTTG1 interacting protein [Source:HGNC Symbol;Acc:HGNC:13524] |
| ENSG00000184271 | HGLibA_37794 | 72 | -0.432127264 | POU6F1 | POU class 6 homeobox 1 [Source:HGNC Symbol;Acc:HGNC:9224] |
| ENSG00000184436 | HGLibA_49310 | 72 | -0.432127264 | THAP7 | THAP domain containing 7 [Source:HGNC Symbol;Acc:HGNC:23190] |
| ENSG00000184515 | HGLibA_04449 | 72 | -0.432127264 | BEX5 | brain expressed X-linked 5 [Source:HGNC Symbol;Acc:HGNC:27990] |
| ENSG00000184530 | HGLibA_06499 | 72 | -0.432127264 | C6orf58 | chromosome 6 open reading frame 58 [Source:HGNC Symbol;Acc:HGNC:20960] |
| ENSG00000184564 | HGLibA_45528 | 72 | -0.432127264 | SLITRK6 | SLIT and NTRK like family member 6 [Source:HGNC Symbol;Acc:HGNC:23503] |
| ENSG00000184613 | HGLibA_31686 | 72 | -0.432127264 | NELL2 | neural EGFL like 2 [Source:HGNC Symbol;Acc:HGNC:7751] |
| ENSG00000184675 | HGLibA_01771 | 72 | -0.432127264 | AMER1 | APC membrane recruitment protein 1 [Source:HGNC Symbol;Acc:HGNC:26837] |
| ENSG00000185532 | HGLibA_38578 | 72 | -0.432127264 | PRKG1 | protein kinase cGMP-dependent 1 [Source:HGNC Symbol;Acc:HGNC:9414] |
| ENSG00000185823 | HGLibA_32392 | 72 | -0.432127264 | NPAP1 | nuclear pore associated protein 1 [Source:HGNC Symbol;Acc:HGNC:1190] |
| ENSG00000188316 | HGLibA_15149 | 72 | -0.432127264 | ENO4 | enolase 4 [Source:HGNC Symbol;Acc:HGNC:31670] |
| ENSG00000188610 | HGLibA_16710 | 72 | -0.432127264 | FAM72B | family with sequence similarity 72 member B [Source:HGNC Symbol;Acc:HGNC:24805] |
| ENSG00000188655 | HGLibA_41421 | 72 | -0.432127264 | RNASE9 | ribonuclease A family member 9 (inactive) [Source:HGNC Symbol;Acc:HGNC:20673] |
| ENSG00000196230 | HGLibA_52271 | 72 | -0.432127264 | TUBB | tubulin beta class I [Source:HGNC Symbol;Acc:HGNC:20778] |
| ENSG00000198498 | HGLibA_49733 | 72 | -0.432127264 | TMA16 | translation machinery associated 16 homolog [Source:HGNC Symbol;Acc:HGNC:25638] |
| ENSG00000198909 | HGLibA_28184 | 72 | -0.432127264 | MAP3K3 | mitogen-activated protein kinase kinase kinase 3 [Source:HGNC Symbol;Acc:HGNC:6855] |
| ENSG00000204130 | HGLibA_42441 | 72 | -0.432127264 | RUFY2 | RUN and FYVE domain containing 2 [Source:HGNC Symbol;Acc:HGNC:19761] |
| ENSG00000204217 | HGLibA_04635 | 72 | -0.432127264 | BMPR2 | bone morphogenetic protein receptor type 2 [Source:HGNC Symbol;Acc:HGNC:1078] |
| ENSG00000204611 | HGLibA_56395 | 72 | -0.432127264 | ZNF616 | zinc finger protein 616 [Source:HGNC Symbol;Acc:HGNC:28062] |
| ENSG00000204849 | HGLibA_46468 | 72 | -0.432127264 | SPATA31A1 | SPATA31 subfamily A member 1 [Source:HGNC Symbol;Acc:HGNC:23394] |
| ENSG00000204983 | HGLibA_38899 | 72 | -0.432127264 | PRSS1 | serine protease 1 [Source:HGNC Symbol;Acc:HGNC:9475] |
| ENSG00000211460 | HGLibA_51879 | 72 | -0.432127264 | TSN | translin [Source:HGNC Symbol;Acc:HGNC:12379] |
| ENSG00000212658 | HGLibA_25736 | 72 | -0.432127264 | KRTAP29-1 | keratin associated protein 29-1 [Source:HGNC Symbol;Acc:HGNC:34211] |
| ENSG00000213760 | HGLibA_03761 | 72 | -0.432127264 | ATP6V1G2 | ATPase H+ transporting V1 subunit G2 [Source:HGNC Symbol;Acc:HGNC:862] |
| ENSG00000213780 | HGLibA_20558 | 72 | -0.432127264 | GTF2H4 | general transcription factor IIH subunit 4 [Source:HGNC Symbol;Acc:HGNC:4658] |
| ENSG00000213927 | HGLibA_08012 | 72 | -0.432127264 | CCL27 | C-C motif chemokine ligand 27 [Source:HGNC Symbol;Acc:HGNC:10626] |
| ENSG00000214029 | HGLibA_56899 | 72 | -0.432127264 | ZNF891 | zinc finger protein 891 [Source:HGNC Symbol;Acc:HGNC:38709] |
| ENSG00000225697 | HGLibA_44802 | 72 | -0.432127264 | SLC26A6 | solute carrier family 26 member 6 [Source:HGNC Symbol;Acc:HGNC:14472] |
| ENSG00000236027 | HGLibA_35277 | 72 | -0.432127264 | PATE3 | prostate and testis expressed 3 [Source:HGNC Symbol;Acc:HGNC:35426] |
| ENSG00000236334 | HGLibA_37914 | 72 | -0.432127264 | PPIAL4G | peptidylprolyl isomerase A like 4G [Source:HGNC Symbol;Acc:HGNC:33996] |
| ENSG00000244067 | HGLibA_20453 | 72 | -0.432127264 | GSTA2 | glutathione S-transferase alpha 2 [Source:HGNC Symbol;Acc:HGNC:4627] |
| ENSG00000247596 | HGLibA_52372 | 72 | -0.432127264 | TWF2 | twinfilin actin binding protein 2 [Source:HGNC Symbol;Acc:HGNC:9621] |
| ENSG00000253159 | HGLibA_35526 | 72 | -0.432127264 | PCDHGA12 | 12 [Source:HGNC Symbol;Acc:HGNC:8699]" |
| ENSG00000254999 | HGLibA_04827 | 72 | -0.432127264 | BRK1 | SCAR/WAVE actin nucleating complex subunit [Source:HGNC Symbol;Acc:HGNC:23057]" |
| ENSG00000258839 | HGLibA_28541 | 72 | -0.432127264 | MC1R | melanocortin 1 receptor [Source:HGNC Symbol;Acc:HGNC:6929] |
| ENSG00000261652 | HGLibA_05436 | 72 | -0.432127264 | C15orf65 | chromosome 15 open reading frame 65 [Source:HGNC Symbol;Acc:HGNC:44654] |
| ENSG00000265107 | HGLibA_19147 | 72 | -0.432127264 | GJA5 | gap junction protein alpha 5 [Source:HGNC Symbol;Acc:HGNC:4279] |
| ENSG00000266524 | HGLibA_18853 | 72 | -0.432127264 | GDF10 | growth differentiation factor 10 [Source:HGNC Symbol;Acc:HGNC:4215] |
| ENSG00000274600 | HGLibA_41266 | 72 | -0.432127264 | RIMBP3B | RIMS binding protein 3B [Source:HGNC Symbol;Acc:HGNC:33891] |
| ENSG00000276085 | HGLibA_08024 | 72 | -0.432127264 | CCL3L3 | C-C motif chemokine ligand 3 like 1 [Source:HGNC Symbol;Acc:HGNC:10628] |
| ENSG00000278311 | HGLibA_19003 | 72 | -0.432127264 | GGNBP2 | gametogenetin binding protein 2 [Source:HGNC Symbol;Acc:HGNC:19357] |
| FAM134A | HGLibA_16123 | 72 | -0.432127264 | FAM134A | - |
| FAM96B | HGLibA_16806 | 72 | -0.432127264 | FAM96B | - |
| GATSL1 | HGLibA_18701 | 72 | -0.432127264 | GATSL1 | - |
| GRAMD2 | HGLibA_20202 | 72 | -0.432127264 | GRAMD2 | - |
| H2BFWT | HGLibA_20774 | 72 | -0.432127264 | H2BFWT | - |
| HIST1H3B | HGLibA_21460 | 72 | -0.432127264 | HIST1H3B | - |
| HRASLS | HGLibA_22076 | 72 | -0.432127264 | HRASLS | - |
| hsa-mir-103a-2 | HGLibA_57166 | 72 | -0.432127264 | hsa-mir-103a-2 | - |
| hsa-mir-1184-3 | HGLibA_57238 | 72 | -0.432127264 | hsa-mir-1184-3 | - |
| hsa-mir-1207 | HGLibA_57284 | 72 | -0.432127264 | hsa-mir-1207 | - |
| hsa-mir-1277 | HGLibA_57563 | 72 | -0.432127264 | hsa-mir-1277 | - |
| hsa-mir-153-1 | HGLibA_57908 | 72 | -0.432127264 | hsa-mir-153-1 | - |
| hsa-mir-1911 | HGLibA_58052 | 72 | -0.432127264 | hsa-mir-1911 | - |
| hsa-mir-215 | HGLibA_58253 | 72 | -0.432127264 | hsa-mir-215 | - |
| hsa-mir-302a | HGLibA_58435 | 72 | -0.432127264 | hsa-mir-302a | - |
| hsa-mir-3064 | HGLibA_58461 | 72 | -0.432127264 | hsa-mir-3064 | - |
| hsa-mir-3158-1 | HGLibA_58704 | 72 | -0.432127264 | hsa-mir-3158-1 | - |
| hsa-mir-3173 | HGLibA_58765 | 72 | -0.432127264 | hsa-mir-3173 | - |
| hsa-mir-34b | HGLibA_59040 | 72 | -0.432127264 | hsa-mir-34b | - |
| hsa-mir-3609 | HGLibA_59065 | 72 | -0.432127264 | hsa-mir-3609 | - |
| hsa-mir-3678 | HGLibA_59261 | 72 | -0.432127264 | hsa-mir-3678 | - |
| hsa-mir-4315-1 | HGLibA_59975 | 72 | -0.432127264 | hsa-mir-4315-1 | - |
| hsa-mir-4494 | HGLibA_60396 | 72 | -0.432127264 | hsa-mir-4494 | - |
| hsa-mir-4521 | HGLibA_60543 | 72 | -0.432127264 | hsa-mir-4521 | - |
| hsa-mir-4663 | HGLibA_60765 | 72 | -0.432127264 | hsa-mir-4663 | - |
| hsa-mir-4692 | HGLibA_60880 | 72 | -0.432127264 | hsa-mir-4692 | - |
| hsa-mir-4735 | HGLibA_61039 | 72 | -0.432127264 | hsa-mir-4735 | - |
| hsa-mir-4761 | HGLibA_61139 | 72 | -0.432127264 | hsa-mir-4761 | - |
| hsa-mir-4999 | HGLibA_61397 | 72 | -0.432127264 | hsa-mir-4999 | - |
| hsa-mir-509-3 | HGLibA_61514 | 72 | -0.432127264 | hsa-mir-509-3 | - |
| hsa-mir-510 | HGLibA_61545 | 72 | -0.432127264 | hsa-mir-510 | - |
| hsa-mir-514a-1 | HGLibA_61587 | 72 | -0.432127264 | hsa-mir-514a-1 | - |
| hsa-mir-514a-2 | HGLibA_61591 | 72 | -0.432127264 | hsa-mir-514a-2 | - |
| hsa-mir-514a-2 | HGLibA_61590 | 72 | -0.432127264 | hsa-mir-514a-2 | - |
| hsa-mir-664a | HGLibA_62995 | 72 | -0.432127264 | hsa-mir-664a | - |
| hsa-mir-6715a | HGLibA_63019 | 72 | -0.432127264 | hsa-mir-6715a | - |
| hsa-mir-6723 | HGLibA_63054 | 72 | -0.432127264 | hsa-mir-6723 | - |
| hsa-mir-6863 | HGLibA_63637 | 72 | -0.432127264 | hsa-mir-6863 | - |
| hsa-mir-6873 | HGLibA_63676 | 72 | -0.432127264 | hsa-mir-6873 | - |
| hsa-mir-6886 | HGLibA_63730 | 72 | -0.432127264 | hsa-mir-6886 | - |
| hsa-mir-7702 | HGLibA_63944 | 72 | -0.432127264 | hsa-mir-7702 | - |
| hsa-mir-892a | HGLibA_64251 | 72 | -0.432127264 | hsa-mir-892a | - |
| hsa-mir-92a-2 | HGLibA_64296 | 72 | -0.432127264 | hsa-mir-92a-2 | - |
| JHDM1D | HGLibA_23945 | 72 | -0.432127264 | JHDM1D | - |
| KIAA1429 | HGLibA_24750 | 72 | -0.432127264 | KIAA1429 | - |
| MLF1IP | HGLibA_29375 | 72 | -0.432127264 | MLF1IP | - |
| MUM1L1 | HGLibA_30567 | 72 | -0.432127264 | MUM1L1 | - |
| NIM1 | HGLibA_31933 | 72 | -0.432127264 | NIM1 | - |
| PIDD | HGLibA_36532 | 72 | -0.432127264 | PIDD | - |
| SELO | HGLibA_43302 | 72 | -0.432127264 | SELO | - |
| SPANXF1 | HGLibA_46398 | 72 | -0.432127264 | SPANXF1 | - |
| TCEB3CL | HGLibA_48749 | 72 | -0.432127264 | TCEB3CL | - |
| TMEM14E | HGLibA_49988 | 72 | -0.432127264 | TMEM14E | - |
| ABP1 | HGLibA_00321 | 71 | -0.442078302 | ABP1 | - |
| C15orf57 | HGLibA_05421 | 71 | -0.442078302 | C15orf57 | - |
| C3orf43 | HGLibA_06246 | 71 | -0.442078302 | C3orf43 | - |
| CRAMP1L | HGLibA_11062 | 71 | -0.442078302 | CRAMP1L | - |
| CXorf30 | HGLibA_11915 | 71 | -0.442078302 | CXorf30 | - |
| ENSG00000004059 | HGLibA_02654 | 71 | -0.442078302 | ARF5 | ADP ribosylation factor 5 [Source:HGNC Symbol;Acc:HGNC:658] |
| ENSG00000006210 | HGLibA_11833 | 71 | -0.442078302 | CX3CL1 | C-X3-C motif chemokine ligand 1 [Source:HGNC Symbol;Acc:HGNC:10647] |
| ENSG00000007372 | HGLibA_35310 | 71 | -0.442078302 | PAX6 | paired box 6 [Source:HGNC Symbol;Acc:HGNC:8620] |
| ENSG00000008394 | HGLibA_29175 | 71 | -0.442078302 | MGST1 | microsomal glutathione S-transferase 1 [Source:HGNC Symbol;Acc:HGNC:7061] |
| ENSG00000009765 | HGLibA_23890 | 71 | -0.442078302 | IYD | iodotyrosine deiodinase [Source:HGNC Symbol;Acc:HGNC:21071] |
| ENSG00000011028 | HGLibA_29819 | 71 | -0.442078302 | MRC2 | mannose receptor C type 2 [Source:HGNC Symbol;Acc:HGNC:16875] |
| ENSG00000028277 | HGLibA_37759 | 71 | -0.442078302 | POU2F2 | POU class 2 homeobox 2 [Source:HGNC Symbol;Acc:HGNC:9213] |
| ENSG00000048462 | HGLibA_50763 | 71 | -0.442078302 | TNFRSF17 | TNF receptor superfamily member 17 [Source:HGNC Symbol;Acc:HGNC:11913] |

| | | | | | |
|---|---|---|---|---|---|
| ENSG00000049768 | HGLibA_17936 | 71 | -0.442078302 | FOXP3 | forkhead box P3 [Source:HGNC Symbol;Acc:HGNC:6106] |
| ENSG00000055208 | HGLibA_48144 | 71 | -0.442078302 | TAB2 | TGF-beta activated kinase 1 (MAP3K7) binding protein 2 [Source:HGNC Symbol;Acc:HGNC:17075] |
| ENSG00000064313 | HGLibA_48229 | 71 | -0.442078302 | TAF2 | TATA-box binding protein associated factor 2 [Source:HGNC Symbol;Acc:HGNC:11536] |
| ENSG00000064835 | HGLibA_37751 | 71 | -0.442078302 | POU1F1 | POU class 1 homeobox 1 [Source:HGNC Symbol;Acc:HGNC:9210] |
| ENSG00000067048 | HGLibA_12705 | 71 | -0.442078302 | DDX3Y | DEAD-box helicase 3 Y-linked [Source:HGNC Symbol;Acc:HGNC:2699] |
| ENSG00000069812 | HGLibA_21181 | 71 | -0.442078302 | HES2 | hes family bHLH transcription factor 2 [Source:HGNC Symbol;Acc:HGNC:16005] |
| ENSG00000070718 | HGLibA_02332 | 71 | -0.442078302 | AP3M2 | adaptor related protein complex 3 subunit mu 2 [Source:HGNC Symbol;Acc:HGNC:570] |
| ENSG00000072954 | HGLibA_50379 | 71 | -0.442078302 | TMEM38A | transmembrane protein 38A [Source:HGNC Symbol;Acc:HGNC:28462] |
| ENSG00000073008 | HGLibA_39617 | 71 | -0.442078302 | PVR | poliovirus receptor [Source:HGNC Symbol;Acc:HGNC:9705] |
| ENSG00000084731 | HGLibA_24939 | 71 | -0.442078302 | KIF3C | kinesin family member 3C [Source:HGNC Symbol;Acc:HGNC:6321] |
| ENSG00000086065 | HGLibA_09496 | 71 | -0.442078302 | CHMP5 | charged multivesicular body protein 5 [Source:HGNC Symbol;Acc:HGNC:26942] |
| ENSG00000087494 | HGLibA_39375 | 71 | -0.442078302 | PTHLH | parathyroid hormone like hormone [Source:HGNC Symbol;Acc:HGNC:9607] |
| ENSG00000087884 | HGLibA_00053 | 71 | -0.442078302 | AAMDC | adipogenesis associated Mth938 domain containing [Source:HGNC Symbol;Acc:HGNC:30205] |
| ENSG00000088543 | HGLibA_06216 | 71 | -0.442078302 | C3orf18 | chromosome 3 open reading frame 18 [Source:HGNC Symbol;Acc:HGNC:24837] |
| ENSG00000089682 | HGLibA_40547 | 71 | -0.442078302 | RBM41 | RNA binding motif protein 41 [Source:HGNC Symbol;Acc:HGNC:25617] |
| ENSG00000091262 | HGLibA_00182 | 71 | -0.442078302 | ABCC6 | ATP binding cassette subfamily C member 6 [Source:HGNC Symbol;Acc:HGNC:57] |
| ENSG00000091704 | HGLibA_10883 | 71 | -0.442078302 | CPA1 | carboxypeptidase A1 [Source:HGNC Symbol;Acc:HGNC:2296] |
| ENSG00000100024 | HGLibA_53052 | 71 | -0.442078302 | UPB1 | beta-ureidopropionase 1 [Source:HGNC Symbol;Acc:HGNC:16297] |
| ENSG00000100028 | HGLibA_45983 | 71 | -0.442078302 | SNRPD3 | small nuclear ribonucleoprotein D3 polypeptide [Source:HGNC Symbol;Acc:HGNC:11160] |
| ENSG00000100297 | HGLibA_28615 | 71 | -0.442078302 | MCM5 | minichromosome maintenance complex component 5 [Source:HGNC Symbol;Acc:HGNC:6948] |
| ENSG00000100324 | HGLibA_48142 | 71 | -0.442078302 | TAB1 | TGF-beta activated kinase 1 (MAP3K7) binding protein 1 [Source:HGNC Symbol;Acc:HGNC:18157] |
| ENSG00000100453 | HGLibA_20720 | 71 | -0.442078302 | GZMB | granzyme B [Source:HGNC Symbol;Acc:HGNC:4709] |
| ENSG00000101017 | HGLibA_08384 | 71 | -0.442078302 | CD40 | CD40 molecule [Source:HGNC Symbol;Acc:HGNC:11919] |
| ENSG00000101333 | HGLibA_36988 | 71 | -0.442078302 | PLCB4 | phospholipase C beta 4 [Source:HGNC Symbol;Acc:HGNC:9059] |
| ENSG00000101361 | HGLibA_32313 | 71 | -0.442078302 | NOP56 | NOP56 ribonucleoprotein [Source:HGNC Symbol;Acc:HGNC:15911] |
| ENSG00000101439 | HGLibA_11454 | 71 | -0.442078302 | CST3 | cystatin C [Source:HGNC Symbol;Acc:HGNC:2475] |
| ENSG00000101843 | HGLibA_39150 | 71 | -0.442078302 | PSMD10 | non-ATPase 10 [Source:HGNC Symbol;Acc:HGNC:9555]" |
| ENSG00000101974 | HGLibA_03559 | 71 | -0.442078302 | ATP11C | ATPase phospholipid transporting 11C [Source:HGNC Symbol;Acc:HGNC:13554] |
| ENSG00000102760 | HGLibA_40999 | 71 | -0.442078302 | RGCC | regulator of cell cycle [Source:HGNC Symbol;Acc:HGNC:20369] |
| ENSG00000102805 | HGLibA_10079 | 71 | -0.442078302 | CLN5 | intracellular trafficking protein [Source:HGNC Symbol;Acc:HGNC:2076]" |
| ENSG00000102901 | HGLibA_09087 | 71 | -0.442078302 | CENPT | centromere protein T [Source:HGNC Symbol;Acc:HGNC:25787] |
| ENSG00000103175 | HGLibA_54244 | 71 | -0.442078302 | WFDC1 | WAP four-disulfide core domain 1 [Source:HGNC Symbol;Acc:HGNC:15466] |
| ENSG00000103257 | HGLibA_45370 | 71 | -0.442078302 | SLC7A5 | solute carrier family 7 member 5 [Source:HGNC Symbol;Acc:HGNC:11063] |
| ENSG00000104218 | HGLibA_11421 | 71 | -0.442078302 | CSPP1 | centrosome and spindle pole associated protein 1 [Source:HGNC Symbol;Acc:HGNC:26193] |
| ENSG00000104388 | HGLibA_39865 | 71 | -0.442078302 | RAB2A | member RAS oncogene family [Source:HGNC Symbol;Acc:HGNC:9763]" |
| ENSG00000104818 | HGLibA_09304 | 71 | -0.442078302 | CGB2 | chorionic gonadotropin subunit beta 2 [Source:HGNC Symbol;Acc:HGNC:16722] |
| ENSG00000105398 | HGLibA_47757 | 71 | -0.442078302 | SULT2A1 | sulfotransferase family 2A member 1 [Source:HGNC Symbol;Acc:HGNC:11458] |
| ENSG00000105697 | HGLibA_20824 | 71 | -0.442078302 | HAMP | hepcidin antimicrobial peptide [Source:HGNC Symbol;Acc:HGNC:15598] |
| ENSG00000106070 | HGLibA_20223 | 71 | -0.442078302 | GRB10 | growth factor receptor bound protein 10 [Source:HGNC Symbol;Acc:HGNC:4564] |
| ENSG00000106333 | HGLibA_35651 | 71 | -0.442078302 | PCOLCE | procollagen C-endopeptidase enhancer [Source:HGNC Symbol;Acc:HGNC:8738] |
| ENSG00000106948 | HGLibA_01463 | 71 | -0.442078302 | AKNA | AT-hook transcription factor [Source:HGNC Symbol;Acc:HGNC:24108] |
| ENSG00000107223 | HGLibA_14463 | 71 | -0.442078302 | EDF1 | endothelial differentiation related factor 1 [Source:HGNC Symbol;Acc:HGNC:3164] |
| ENSG00000109101 | HGLibA_17909 | 71 | -0.442078302 | FOXN1 | forkhead box N1 [Source:HGNC Symbol;Acc:HGNC:12765] |
| ENSG00000110042 | HGLibA_14090 | 71 | -0.442078302 | DTX4 | deltex E3 ubiquitin ligase 4 [Source:HGNC Symbol;Acc:HGNC:29151] |
| ENSG00000110047 | HGLibA_14666 | 71 | -0.442078302 | EHD1 | EH domain containing 1 [Source:HGNC Symbol;Acc:HGNC:3242] |
| ENSG00000110536 | HGLibA_39427 | 71 | -0.442078302 | PTPMT1 | mitochondrial 1 [Source:HGNC Symbol;Acc:HGNC:26965]" |
| ENSG00000111049 | HGLibA_30696 | 71 | -0.442078302 | MYF5 | myogenic factor 5 [Source:HGNC Symbol;Acc:HGNC:7565] |
| ENSG00000111266 | HGLibA_14147 | 71 | -0.442078302 | DUSP16 | dual specificity phosphatase 16 [Source:HGNC Symbol;Acc:HGNC:17909] |
| ENSG00000112246 | HGLibA_44174 | 71 | -0.442078302 | SIM1 | SIM bHLH transcription factor 1 [Source:HGNC Symbol;Acc:HGNC:10882] |
| ENSG00000112667 | HGLibA_13712 | 71 | -0.442078302 | DNPH1 | 2'-deoxynucleoside 5'-phosphate N-hydrolase 1 [Source:HGNC Symbol;Acc:HGNC:21218] |
| ENSG00000114120 | HGLibA_44720 | 71 | -0.442078302 | SLC25A36 | solute carrier family 25 member 36 [Source:HGNC Symbol;Acc:HGNC:25554] |
| ENSG00000114383 | HGLibA_52345 | 71 | -0.442078302 | TUSC2 | mitochondrial calcium regulator [Source:HGNC Symbol;Acc:HGNC:17034]" |
| ENSG00000114638 | HGLibA_53072 | 71 | -0.442078302 | UPK1B | uroplakin 1B [Source:HGNC Symbol;Acc:HGNC:12578] |
| ENSG00000115526 | HGLibA_09612 | 71 | -0.442078302 | CHST10 | carbohydrate sulfotransferase 10 [Source:HGNC Symbol;Acc:HGNC:19650] |
| ENSG00000115935 | HGLibA_54304 | 71 | -0.442078302 | WIPF1 | WAS/WASL interacting protein family member 1 [Source:HGNC Symbol;Acc:HGNC:12736] |
| ENSG00000115998 | HGLibA_06125 | 71 | -0.442078302 | C2orf42 | chromosome 2 open reading frame 42 [Source:HGNC Symbol;Acc:HGNC:26056] |
| ENSG00000116273 | HGLibA_36341 | 71 | -0.442078302 | PHF13 | PHD finger protein 13 [Source:HGNC Symbol;Acc:HGNC:22983] |
| ENSG00000116748 | HGLibA_01824 | 71 | -0.442078302 | AMPD1 | adenosine monophosphate deaminase 1 [Source:HGNC Symbol;Acc:HGNC:468] |
| ENSG00000117751 | HGLibA_38124 | 71 | -0.442078302 | PPP1R8 | protein phosphatase 1 regulatory subunit 8 [Source:HGNC Symbol;Acc:HGNC:9296] |
| ENSG00000118972 | HGLibA_17403 | 71 | -0.442078302 | FGF23 | fibroblast growth factor 23 [Source:HGNC Symbol;Acc:HGNC:3680] |
| ENSG00000119682 | HGLibA_02642 | 71 | -0.442078302 | AREL1 | apoptosis resistant E3 ubiquitin protein ligase 1 [Source:HGNC Symbol;Acc:HGNC:20363] |
| ENSG00000119812 | HGLibA_16808 | 71 | -0.442078302 | FAM98A | family with sequence similarity 98 member A [Source:HGNC Symbol;Acc:HGNC:24520] |
| ENSG00000120471 | HGLibA_51050 | 71 | -0.442078302 | TP53AIP1 | tumor protein p53 regulated apoptosis inducing protein 1 [Source:HGNC Symbol;Acc:HGNC:29984] |
| ENSG00000120925 | HGLibA_41575 | 71 | -0.442078302 | RNF170 | ring finger protein 170 [Source:HGNC Symbol;Acc:HGNC:25358] |
| ENSG00000122694 | HGLibA_19267 | 71 | -0.442078302 | GLIPR2 | GLI pathogenesis related 2 [Source:HGNC Symbol;Acc:HGNC:18007] |
| ENSG00000123342 | HGLibA_29503 | 71 | -0.442078302 | MMP19 | matrix metallopeptidase 19 [Source:HGNC Symbol;Acc:HGNC:7165] |
| ENSG00000124831 | HGLibA_27450 | 71 | -0.442078302 | LRRFIP1 | LRR binding FLII interacting protein 1 [Source:HGNC Symbol;Acc:HGNC:6702] |
| ENSG00000125753 | HGLibA_53539 | 71 | -0.442078302 | VASP | vasodilator stimulated phosphoprotein [Source:HGNC Symbol;Acc:HGNC:12652] |
| ENSG00000129214 | HGLibA_43996 | 71 | -0.442078302 | SHBG | sex hormone binding globulin [Source:HGNC Symbol;Acc:HGNC:10839] |
| ENSG00000129465 | HGLibA_41322 | 71 | -0.442078302 | RIPK3 | receptor interacting serine/threonine kinase 3 [Source:HGNC Symbol;Acc:HGNC:10021] |
| ENSG00000131386 | HGLibA_18544 | 71 | -0.442078302 | GALNT15 | polypeptide N-acetylgalactosaminyltransferase 15 [Source:HGNC Symbol;Acc:HGNC:21531] |
| ENSG00000131914 | HGLibA_26559 | 71 | -0.442078302 | LIN28A | lin-28 homolog A [Source:HGNC Symbol;Acc:HGNC:15986] |
| ENSG00000132405 | HGLibA_48478 | 71 | -0.442078302 | TBC1D14 | TBC1 domain family member 14 [Source:HGNC Symbol;Acc:HGNC:29246] |
| ENSG00000133895 | HGLibA_28886 | 71 | -0.442078302 | MEN1 | menin 1 [Source:HGNC Symbol;Acc:HGNC:7010] |
| ENSG00000134253 | HGLibA_51509 | 71 | -0.442078302 | TRIM45 | tripartite motif containing 45 [Source:HGNC Symbol;Acc:HGNC:19018] |
| ENSG00000135503 | HGLibA_00690 | 71 | -0.442078302 | ACVR1B | activin A receptor type 1B [Source:HGNC Symbol;Acc:HGNC:172] |
| ENSG00000135930 | HGLibA_14822 | 71 | -0.442078302 | EIF4E2 | eukaryotic translation initiation factor 4E family member 2 [Source:HGNC Symbol;Acc:HGNC:3293] |
| ENSG00000135953 | HGLibA_29120 | 71 | -0.442078302 | MFSD9 | major facilitator superfamily domain containing 9 [Source:HGNC Symbol;Acc:HGNC:28158] |
| ENSG00000136003 | HGLibA_23653 | 71 | -0.442078302 | ISCU | iron-sulfur cluster assembly enzyme [Source:HGNC Symbol;Acc:HGNC:29882] |
| ENSG00000136059 | HGLibA_53654 | 71 | -0.442078302 | VILL | villin like [Source:HGNC Symbol;Acc:HGNC:30906] |
| ENSG00000136156 | HGLibA_23833 | 71 | -0.442078302 | ITM2B | integral membrane protein 2B [Source:HGNC Symbol;Acc:HGNC:6174] |
| ENSG00000136327 | HGLibA_32047 | 71 | -0.442078302 | NKX2-8 | NK2 homeobox 8 [Source:HGNC Symbol;Acc:HGNC:16364] |
| ENSG00000137491 | HGLibA_45464 | 71 | -0.442078302 | SLCO2B1 | solute carrier organic anion transporter family member 2B1 [Source:HGNC Symbol;Acc:HGNC:10962] |
| ENSG00000137494 | HGLibA_02099 | 71 | -0.442078302 | ANKRD42 | ankyrin repeat domain 42 [Source:HGNC Symbol;Acc:HGNC:26752] |
| ENSG00000137815 | HGLibA_42382 | 71 | -0.442078302 | RTF1 | Paf1/RNA polymerase II complex component [Source:HGNC Symbol;Acc:HGNC:28996]" |
| ENSG00000139537 | HGLibA_07831 | 71 | -0.442078302 | CCDC65 | coiled-coil domain containing 65 [Source:HGNC Symbol;Acc:HGNC:29937] |
| ENSG00000139618 | HGLibA_04765 | 71 | -0.442078302 | BRCA2 | DNA repair associated [Source:HGNC Symbol;Acc:HGNC:1101]" |
| ENSG00000140262 | HGLibA_48760 | 71 | -0.442078302 | TCF12 | transcription factor 12 [Source:HGNC Symbol;Acc:HGNC:11623] |
| ENSG00000140481 | HGLibA_07757 | 71 | -0.442078302 | CCDC33 | coiled-coil domain containing 33 [Source:HGNC Symbol;Acc:HGNC:26552] |
| ENSG00000140506 | HGLibA_26661 | 71 | -0.442078302 | LMAN1L | mannose binding 1 like [Source:HGNC Symbol;Acc:HGNC:6632]" |
| ENSG00000142789 | HGLibA_08999 | 71 | -0.442078302 | CELA3A | chymotrypsin like elastase family member 3A [Source:HGNC Symbol;Acc:HGNC:15944] |
| ENSG00000143006 | HGLibA_13433 | 71 | -0.442078302 | DMRTB1 | DMRT like family B with proline rich C-terminal 1 [Source:HGNC Symbol;Acc:HGNC:13913] |
| ENSG00000143552 | HGLibA_33043 | 71 | -0.442078302 | NUP210L | nucleoporin 210 like [Source:HGNC Symbol;Acc:HGNC:29915] |
| ENSG00000144063 | HGLibA_28027 | 71 | -0.442078302 | MALL | T cell differentiation protein like [Source:HGNC Symbol;Acc:HGNC:6818]" |
| ENSG00000145287 | HGLibA_36941 | 71 | -0.442078302 | PLAC8 | placenta specific 8 [Source:HGNC Symbol;Acc:HGNC:19254] |
| ENSG00000145864 | HGLibA_18376 | 71 | -0.442078302 | GABRB2 | gamma-aminobutyric acid type A receptor beta2 subunit [Source:HGNC Symbol;Acc:HGNC:4082] |
| ENSG00000145916 | HGLibA_41384 | 71 | -0.442078302 | RMND5B | required for meiotic nuclear division 5 homolog B [Source:HGNC Symbol;Acc:HGNC:26181] |
| ENSG00000147649 | HGLibA_30334 | 71 | -0.442078302 | MTDH | metadherin [Source:HGNC Symbol;Acc:HGNC:29608] |
| ENSG00000147804 | HGLibA_45113 | 71 | -0.442078302 | SLC39A4 | solute carrier family 39 member 4 [Source:HGNC Symbol;Acc:HGNC:17129] |
| ENSG00000148215 | HGLibA_34270 | 71 | -0.442078302 | OR5C1 | olfactory receptor family 5 subfamily C member 1 [Source:HGNC Symbol;Acc:HGNC:8331] |
| ENSG00000148229 | HGLibA_37516 | 71 | -0.442078302 | POLE3 | accessory subunit [Source:HGNC Symbol;Acc:HGNC:13546]" |
| ENSG00000148985 | HGLibA_36211 | 71 | -0.442078302 | PGAP2 | post-GPI attachment to proteins 2 [Source:HGNC Symbol;Acc:HGNC:17893] |
| ENSG00000149609 | HGLibA_05990 | 71 | -0.442078302 | C20orf144 | chromosome 20 open reading frame 144 [Source:HGNC Symbol;Acc:HGNC:16137] |
| ENSG00000150201 | HGLibA_18234 | 71 | -0.442078302 | FXYD4 | FXYD domain containing ion transport regulator 4 [Source:HGNC Symbol;Acc:HGNC:4028] |
| ENSG00000151322 | HGLibA_32401 | 71 | -0.442078302 | NPAS3 | neuronal PAS domain protein 3 [Source:HGNC Symbol;Acc:HGNC:19311] |
| ENSG00000154134 | HGLibA_41740 | 71 | -0.442078302 | ROBO3 | roundabout guidance receptor 3 [Source:HGNC Symbol;Acc:HGNC:13433] |
| ENSG00000154229 | HGLibA_38532 | 71 | -0.442078302 | PRKCA | protein kinase C alpha [Source:HGNC Symbol;Acc:HGNC:9393] |
| ENSG00000156171 | HGLibA_13946 | 71 | -0.442078302 | DRAM2 | DNA damage regulated autophagy modulator 2 [Source:HGNC Symbol;Acc:HGNC:28769] |
| ENSG00000156414 | HGLibA_48918 | 71 | -0.442078302 | TDRD9 | tudor domain containing 9 [Source:HGNC Symbol;Acc:HGNC:20122] |
| ENSG00000156575 | HGLibA_38455 | 71 | -0.442078302 | PRG3 | pro eosinophil major basic protein 2 [Source:HGNC Symbol;Acc:HGNC:9363]" |
| ENSG00000156575 | HGLibA_38456 | 71 | -0.442078302 | PRG3 | pro eosinophil major basic protein 2 [Source:HGNC Symbol;Acc:HGNC:9363]" |
| ENSG00000157510 | HGLibA_01100 | 71 | -0.442078302 | AFAP1L1 | actin filament associated protein 1 like 1 [Source:HGNC Symbol;Acc:HGNC:26714] |
| ENSG00000157823 | HGLibA_02337 | 71 | -0.442078302 | AP3S2 | adaptor related protein complex 3 subunit sigma 2 [Source:HGNC Symbol;Acc:HGNC:571] |
| ENSG00000159224 | HGLibA_19114 | 71 | -0.442078302 | GIP | gastric inhibitory polypeptide [Source:HGNC Symbol;Acc:HGNC:4270] |
| ENSG00000159640 | HGLibA_00421 | 71 | -0.442078302 | ACE | angiotensin I converting enzyme [Source:HGNC Symbol;Acc:HGNC:2707] |
| ENSG00000160113 | HGLibA_32616 | 71 | -0.442078302 | NR2F6 | nuclear receptor subfamily 2 group F member 6 [Source:HGNC Symbol;Acc:HGNC:7977] |
| ENSG00000160469 | HGLibA_04847 | 71 | -0.442078302 | BRSK1 | BR serine/threonine kinase 1 [Source:HGNC Symbol;Acc:HGNC:18994] |
| ENSG00000160505 | HGLibA_32120 | 71 | -0.442078302 | NLRP4 | NLR family pyrin domain containing 4 [Source:HGNC Symbol;Acc:HGNC:22943] |
| ENSG00000161179 | HGLibA_54651 | 71 | -0.442078302 | YDJC | YdjC chitooligosaccharide deacetylase homolog [Source:HGNC Symbol;Acc:HGNC:27158] |
| ENSG00000161653 | HGLibA_31074 | 71 | -0.442078302 | NAGS | N-acetylglutamate synthase [Source:HGNC Symbol;Acc:HGNC:17996] |
| ENSG00000162458 | HGLibA_16941 | 71 | -0.442078302 | FBLIM1 | filamin binding LIM protein 1 [Source:HGNC Symbol;Acc:HGNC:24686] |
| ENSG00000162761 | HGLibA_26733 | 71 | -0.442078302 | LMX1A | LIM homeobox transcription factor 1 alpha [Source:HGNC Symbol;Acc:HGNC:6653] |
| ENSG00000163235 | HGLibA_49206 | 71 | -0.442078302 | TGFA | transforming growth factor alpha [Source:HGNC Symbol;Acc:HGNC:11765] |
| ENSG00000163273 | HGLibA_32504 | 71 | -0.442078302 | NPPC | natriuretic peptide C [Source:HGNC Symbol;Acc:HGNC:7941] |
| ENSG00000163430 | HGLibA_18102 | 71 | -0.442078302 | FSTL1 | follistatin like 1 [Source:HGNC Symbol;Acc:HGNC:3972] |
| ENSG00000163430 | HGLibA_18101 | 71 | -0.442078302 | FSTL1 | follistatin like 1 [Source:HGNC Symbol;Acc:HGNC:3972] |
| ENSG00000163467 | HGLibA_51807 | 71 | -0.442078302 | TSACC | TSSK6 activating cochaperone [Source:HGNC Symbol;Acc:HGNC:30636] |
| ENSG00000163636 | HGLibA_39179 | 71 | -0.442078302 | PSMD6 | non-ATPase 6 [Source:HGNC Symbol;Acc:HGNC:9564]" |
| ENSG00000163646 | HGLibA_10122 | 71 | -0.442078302 | CLRN1 | clarin 1 [Source:HGNC Symbol;Acc:HGNC:12605] |
| ENSG00000163900 | HGLibA_50392 | 71 | -0.442078302 | TMEM41A | transmembrane protein 41A [Source:HGNC Symbol;Acc:HGNC:30544] |
| ENSG00000164031 | HGLibA_13534 | 71 | -0.442078302 | DNAJB14 | DnaJ heat shock protein family (Hsp40) member B14 [Source:HGNC Symbol;Acc:HGNC:25881] |
| ENSG00000164209 | HGLibA_44754 | 71 | -0.442078302 | SLC25A46 | solute carrier family 25 member 46 [Source:HGNC Symbol;Acc:HGNC:25198] |
| ENSG00000164877 | HGLibA_29216 | 71 | -0.442078302 | MICALL2 | MICAL like 2 [Source:HGNC Symbol;Acc:HGNC:29672] |
| ENSG00000164879 | HGLibA_06807 | 71 | -0.442078302 | CA3 | carbonic anhydrase 3 [Source:HGNC Symbol;Acc:HGNC:1374] |
| ENSG00000165023 | HGLibA_13260 | 71 | -0.442078302 | DIRAS2 | DIRAS family GTPase 2 [Source:HGNC Symbol;Acc:HGNC:19323] |

| | | | | | |
|---|---|---|---|---|---|
| ENSG00000165271 | HGLibA_32272 | 71 | -0.442078302 | NOL6 | nucleolar protein 6 [Source:HGNC Symbol;Acc:HGNC:19910] |
| ENSG00000165410 | HGLibA_09285 | 71 | -0.442078302 | CFL2 | cofilin 2 [Source:HGNC Symbol;Acc:HGNC:1875] |
| ENSG00000165669 | HGLibA_16420 | 71 | -0.442078302 | FAM204A | family with sequence similarity 204 member A [Source:HGNC Symbol;Acc:HGNC:25794] |
| ENSG00000165775 | HGLibA_18168 | 71 | -0.442078302 | FUNDC2 | FUN14 domain containing 2 [Source:HGNC Symbol;Acc:HGNC:24925] |
| ENSG00000166160 | HGLibA_33427 | 71 | -0.442078302 | OPN1MW2 | medium wave sensitive 2 [Source:HGNC Symbol;Acc:HGNC:26952]" |
| ENSG00000166348 | HGLibA_53401 | 71 | -0.442078302 | USP54 | ubiquitin specific peptidase 54 [Source:HGNC Symbol;Acc:HGNC:23513] |
| ENSG00000167414 | HGLibA_19534 | 71 | -0.442078302 | GNG8 | G protein subunit gamma 8 [Source:HGNC Symbol;Acc:HGNC:19664] |
| ENSG00000168259 | HGLibA_13636 | 71 | -0.442078302 | DNAJC7 | DnaJ heat shock protein family (Hsp40) member C7 [Source:HGNC Symbol;Acc:HGNC:12392] |
| ENSG00000168329 | HGLibA_11837 | 71 | -0.442078302 | CX3CR1 | C-X3-C motif chemokine receptor 1 [Source:HGNC Symbol;Acc:HGNC:2558] |
| ENSG00000169193 | HGLibA_07580 | 71 | -0.442078302 | CCDC126 | coiled-coil domain containing 126 [Source:HGNC Symbol;Acc:HGNC:22398] |
| ENSG00000169715 | HGLibA_30284 | 71 | -0.442078302 | MT1E | metallothionein 1E [Source:HGNC Symbol;Acc:HGNC:7397] |
| ENSG00000169914 | HGLibA_34761 | 71 | -0.442078302 | OTUD3 | OTU deubiquitinase 3 [Source:HGNC Symbol;Acc:HGNC:29038] |
| ENSG00000170011 | HGLibA_30920 | 71 | -0.442078302 | MYRIP | myosin VIIA and Rab interacting protein [Source:HGNC Symbol;Acc:HGNC:19156] |
| ENSG00000170365 | HGLibA_45570 | 71 | -0.442078302 | SMAD1 | SMAD family member 1 [Source:HGNC Symbol;Acc:HGNC:6767] |
| ENSG00000170471 | HGLibA_40162 | 71 | -0.442078302 | RALGAPB | Ral GTPase activating protein non-catalytic beta subunit [Source:HGNC Symbol;Acc:HGNC:29221] |
| ENSG00000170915 | HGLibA_35173 | 71 | -0.442078302 | PAQR8 | progestin and adipoQ receptor family member 8 [Source:HGNC Symbol;Acc:HGNC:15708] |
| ENSG00000170953 | HGLibA_34492 | 71 | -0.442078302 | OR8B12 | olfactory receptor family 8 subfamily B member 12 [Source:HGNC Symbol;Acc:HGNC:15307] |
| ENSG00000171509 | HGLibA_42493 | 71 | -0.442078302 | RXFP1 | relaxin family peptide receptor 1 [Source:HGNC Symbol;Acc:HGNC:19718] |
| ENSG00000172468 | HGLibA_22241 | 71 | -0.442078302 | HSFY1 | heat shock transcription factor Y-linked 1 [Source:HGNC Symbol;Acc:HGNC:18568] |
| ENSG00000172519 | HGLibA_33518 | 71 | -0.442078302 | OR10H5 | olfactory receptor family 10 subfamily H member 5 [Source:HGNC Symbol;Acc:HGNC:15389] |
| ENSG00000173207 | HGLibA_09786 | 71 | -0.442078302 | CKS1B | CDC28 protein kinase regulatory subunit 1B [Source:HGNC Symbol;Acc:HGNC:19083] |
| ENSG00000174886 | HGLibA_31444 | 71 | -0.442078302 | NDUFA11 | NADH:ubiquinone oxidoreductase subunit A11 [Source:HGNC Symbol;Acc:HGNC:20371] |
| ENSG00000175471 | HGLibA_28652 | 71 | -0.442078302 | MCTP1 | multiple C2 and transmembrane domain containing 1 [Source:HGNC Symbol;Acc:HGNC:26183] |
| ENSG00000176945 | HGLibA_30537 | 71 | -0.442078302 | MUC20 | cell surface associated [Source:HGNC Symbol;Acc:HGNC:23282]" |
| ENSG00000177303 | HGLibA_07293 | 71 | -0.442078302 | CASKIN2 | CASK interacting protein 2 [Source:HGNC Symbol;Acc:HGNC:18200] |
| ENSG00000177917 | HGLibA_02992 | 71 | -0.442078302 | ARL6IP6 | ADP ribosylation factor like GTPase 6 interacting protein 6 [Source:HGNC Symbol;Acc:HGNC:24048] |
| ENSG00000179520 | HGLibA_44481 | 71 | -0.442078302 | SLC17A8 | solute carrier family 17 member 8 [Source:HGNC Symbol;Acc:HGNC:20151] |
| ENSG00000179918 | HGLibA_43414 | 71 | -0.442078302 | SEPHS2 | selenophosphate synthetase 2 [Source:HGNC Symbol;Acc:HGNC:19686] |
| ENSG00000181374 | HGLibA_07967 | 71 | -0.442078302 | CCL13 | C-C motif chemokine ligand 13 [Source:HGNC Symbol;Acc:HGNC:10611] |
| ENSG00000181991 | HGLibA_30031 | 71 | -0.442078302 | MRPS11 | mitochondrial ribosomal protein S11 [Source:HGNC Symbol;Acc:HGNC:14050] |
| ENSG00000182636 | HGLibA_31398 | 71 | -0.442078302 | NDN | MAGE family member [Source:HGNC Symbol;Acc:HGNC:7675]" |
| ENSG00000183569 | HGLibA_43481 | 71 | -0.442078302 | SERHL2 | serine hydrolase like 2 [Source:HGNC Symbol;Acc:HGNC:29446] |
| ENSG00000183770 | HGLibA_17902 | 71 | -0.442078302 | FOXL2 | forkhead box L2 [Source:HGNC Symbol;Acc:HGNC:1092] |
| ENSG00000183828 | HGLibA_32947 | 71 | -0.442078302 | NUDT14 | nudix hydrolase 14 [Source:HGNC Symbol;Acc:HGNC:20141] |
| ENSG00000184378 | HGLibA_00683 | 71 | -0.442078302 | ACTRT3 | actin related protein T3 [Source:HGNC Symbol;Acc:HGNC:24022] |
| ENSG00000185894 | HGLibA_04750 | 71 | -0.442078302 | BPY2C | basic charge Y-linked 2C [Source:HGNC Symbol;Acc:HGNC:18225] |
| ENSG00000185989 | HGLibA_40301 | 71 | -0.442078302 | RASA3 | RAS p21 protein activator 3 [Source:HGNC Symbol;Acc:HGNC:20331] |
| ENSG00000186517 | HGLibA_02754 | 71 | -0.442078302 | ARHGAP30 | Rho GTPase activating protein 30 [Source:HGNC Symbol;Acc:HGNC:27414] |
| ENSG00000187713 | HGLibA_50187 | 71 | -0.442078302 | TMEM203 | transmembrane protein 203 [Source:HGNC Symbol;Acc:HGNC:28217] |
| ENSG00000188100 | HGLibA_16551 | 71 | -0.442078302 | FAM25A | family with sequence similarity 25 member A [Source:HGNC Symbol;Acc:HGNC:23436] |
| ENSG00000188293 | HGLibA_22839 | 71 | -0.442078302 | IGFL1 | IGF like family member 1 [Source:HGNC Symbol;Acc:HGNC:24093] |
| ENSG00000188655 | HGLibA_41420 | 71 | -0.442078302 | RNASE9 | ribonuclease A family member 9 (inactive) [Source:HGNC Symbol;Acc:HGNC:20673] |
| ENSG00000188878 | HGLibA_16934 | 71 | -0.442078302 | FBF1 | Fas binding factor 1 [Source:HGNC Symbol;Acc:HGNC:24674] |
| ENSG00000189134 | HGLibA_32003 | 71 | -0.442078302 | NKAPL | NFKB activating protein like [Source:HGNC Symbol;Acc:HGNC:21584] |
| ENSG00000189299 | HGLibA_17949 | 71 | -0.442078302 | FOXR2 | forkhead box R2 [Source:HGNC Symbol;Acc:HGNC:30469] |
| ENSG00000197063 | HGLibA_27882 | 71 | -0.442078302 | MAFG | MAF bZIP transcription factor G [Source:HGNC Symbol;Acc:HGNC:6781] |
| ENSG00000197915 | HGLibA_22110 | 71 | -0.442078302 | HRNR | hornerin [Source:HGNC Symbol;Acc:HGNC:20846] |
| ENSG00000198034 | HGLibA_42143 | 71 | -0.442078302 | RPS4X | ribosomal protein S4 X-linked [Source:HGNC Symbol;Acc:HGNC:10424] |
| ENSG00000198853 | HGLibA_42471 | 71 | -0.442078302 | RUSC2 | RUN and SH3 domain containing 2 [Source:HGNC Symbol;Acc:HGNC:23625] |
| ENSG00000204130 | HGLibA_42440 | 71 | -0.442078302 | RUFY2 | RUN and FYVE domain containing 2 [Source:HGNC Symbol;Acc:HGNC:19761] |
| ENSG00000204248 | HGLibA_10503 | 71 | -0.442078302 | COL11A2 | collagen type XI alpha 2 chain [Source:HGNC Symbol;Acc:HGNC:2187] |
| ENSG00000204308 | HGLibA_41692 | 71 | -0.442078302 | RNF5 | ring finger protein 5 [Source:HGNC Symbol;Acc:HGNC:10068] |
| ENSG00000204390 | HGLibA_22278 | 71 | -0.442078302 | HSPA1L | heat shock protein family A (Hsp70) member 1 like [Source:HGNC Symbol;Acc:HGNC:5234] |
| ENSG00000204510 | HGLibA_38326 | 71 | -0.442078302 | PRAMEF7 | PRAME family member 7 [Source:HGNC Symbol;Acc:HGNC:28415] |
| ENSG00000204969 | HGLibA_35439 | 71 | -0.442078302 | PCDHA2 | protocadherin alpha 2 [Source:HGNC Symbol;Acc:HGNC:8668] |
| ENSG00000205327 | HGLibA_34401 | 71 | -0.442078302 | OR6C68 | olfactory receptor family 6 subfamily C member 68 [Source:HGNC Symbol;Acc:HGNC:31297] |
| ENSG00000206075 | HGLibA_43555 | 71 | -0.442078302 | SERPINB5 | serpin family B member 5 [Source:HGNC Symbol;Acc:HGNC:8949] |
| ENSG00000213809 | HGLibA_25315 | 71 | -0.442078302 | KLRK1 | killer cell lectin like receptor K1 [Source:HGNC Symbol;Acc:HGNC:18788] |
| ENSG00000232112 | HGLibA_49735 | 71 | -0.442078302 | TMA7 | translation machinery associated 7 homolog [Source:HGNC Symbol;Acc:HGNC:26932] |
| ENSG00000234414 | HGLibA_40612 | 71 | -0.442078302 | RBMY1A1 | member A1 [Source:HGNC Symbol;Acc:HGNC:9912]" |
| ENSG00000236637 | HGLibA_22679 | 71 | -0.442078302 | IFNA4 | interferon alpha 4 [Source:HGNC Symbol;Acc:HGNC:5425] |
| ENSG00000240654 | HGLibA_05960 | 71 | -0.442078302 | C1QTNF9 | C1q and TNF related 9 [Source:HGNC Symbol;Acc:HGNC:28732] |
| ENSG00000244588 | HGLibA_40066 | 71 | -0.442078302 | RAD21L1 | RAD21 cohesin complex component like 1 [Source:HGNC Symbol;Acc:HGNC:16271] |
| ENSG00000250298 | HGLibA_19095 | 71 | -0.442078302 | GIMD1 | GIMAP family P-loop NTPase domain containing 1 [Source:HGNC Symbol;Acc:HGNC:44141] |
| ENSG00000253767 | HGLibA_35543 | 71 | -0.442078302 | PCDHGA8 | 8 [Source:HGNC Symbol;Acc:HGNC:8706]" |
| ENSG00000253831 | HGLibA_15655 | 71 | -0.442078302 | ETV3L | ETS variant 3 like [Source:HGNC Symbol;Acc:HGNC:33834] |
| ENSG00000254245 | HGLibA_35532 | 71 | -0.442078302 | PCDHGA3 | 3 [Source:HGNC Symbol;Acc:HGNC:8701]" |
| ENSG00000254598 | HGLibA_11409 | 71 | -0.442078302 | CSNK2A3 | casein kinase 2 alpha 3 [Source:HGNC Symbol;Acc:HGNC:2458] |
| ENSG00000261678 | HGLibA_43058 | 71 | -0.442078302 | SCRT1 | scratch family transcriptional repressor 1 [Source:HGNC Symbol;Acc:HGNC:15950] |
| ENSG00000268964 | HGLibA_15547 | 71 | -0.442078302 | ERVV-2 | envelope [Source:HGNC Symbol;Acc:HGNC:39051]" |
| ENSG00000274611 | HGLibA_48530 | 71 | -0.442078302 | TBC1D3 | TBC1 domain family member 3 [Source:HGNC Symbol;Acc:HGNC:19031] |
| ENSG00000276547 | HGLibA_35563 | 71 | -0.442078302 | PCDHGB5 | 5 [Source:HGNC Symbol;Acc:HGNC:8712]" |
| ENSG00000284609 | HGLibA_34499 | 71 | -0.442078302 | OR8B3 | olfactory receptor family 8 subfamily B member 3 [Source:HGNC Symbol;Acc:HGNC:8472] |
| ENSG00000288702 | HGLibA_52882 | 71 | -0.442078302 | UGT1A3 | - |
| FAM129C | HGLibA_16102 | 71 | -0.442078302 | FAM129C | - |
| FAM160B1 | HGLibA_16210 | 71 | -0.442078302 | FAM160B1 | - |
| H2AFB3 | HGLibA_20750 | 71 | -0.442078302 | H2AFB3 | - |
| H2BFM | HGLibA_20772 | 71 | -0.442078302 | H2BFM | - |
| HIST1H2AL | HGLibA_21404 | 71 | -0.442078302 | HIST1H2AL | - |
| HIST1H2BN | HGLibA_21449 | 71 | -0.442078302 | HIST1H2BN | - |
| hsa-mir-1264 | HGLibA_57478 | 71 | -0.442078302 | hsa-mir-1264 | - |
| hsa-mir-1271 | HGLibA_57523 | 71 | -0.442078302 | hsa-mir-1271 | - |
| hsa-mir-1273f | HGLibA_57543 | 71 | -0.442078302 | hsa-mir-1273f | - |
| hsa-mir-1275 | HGLibA_57554 | 71 | -0.442078302 | hsa-mir-1275 | - |
| hsa-mir-1295a | HGLibA_57658 | 71 | -0.442078302 | hsa-mir-1295a | - |
| hsa-mir-138-1 | HGLibA_57814 | 71 | -0.442078302 | hsa-mir-138-1 | - |
| hsa-mir-187 | HGLibA_58010 | 71 | -0.442078302 | hsa-mir-187 | - |
| hsa-mir-190a | HGLibA_58034 | 71 | -0.442078302 | hsa-mir-190a | - |
| hsa-mir-2052 | HGLibA_58181 | 71 | -0.442078302 | hsa-mir-2052 | - |
| hsa-mir-27a | HGLibA_58375 | 71 | -0.442078302 | hsa-mir-27a | - |
| hsa-mir-3118-5 | HGLibA_58519 | 71 | -0.442078302 | hsa-mir-3118-5 | - |
| hsa-mir-3126 | HGLibA_58557 | 71 | -0.442078302 | hsa-mir-3126 | - |
| hsa-mir-3177 | HGLibA_58782 | 71 | -0.442078302 | hsa-mir-3177 | - |
| hsa-mir-330 | HGLibA_58986 | 71 | -0.442078302 | hsa-mir-330 | - |
| hsa-mir-335 | HGLibA_58996 | 71 | -0.442078302 | hsa-mir-335 | - |
| hsa-mir-379 | HGLibA_59468 | 71 | -0.442078302 | hsa-mir-379 | - |
| hsa-mir-3939 | HGLibA_59617 | 71 | -0.442078302 | hsa-mir-3939 | - |
| hsa-mir-4300 | HGLibA_59913 | 71 | -0.442078302 | hsa-mir-4300 | - |
| hsa-mir-4301 | HGLibA_59917 | 71 | -0.442078302 | hsa-mir-4301 | - |
| hsa-mir-4310 | HGLibA_59956 | 71 | -0.442078302 | hsa-mir-4310 | - |
| hsa-mir-4443 | HGLibA_60175 | 71 | -0.442078302 | hsa-mir-4443 | - |
| hsa-mir-451b | HGLibA_60529 | 71 | -0.442078302 | hsa-mir-451b | - |
| hsa-mir-4526 | HGLibA_60565 | 71 | -0.442078302 | hsa-mir-4526 | - |
| hsa-mir-4659b | HGLibA_60745 | 71 | -0.442078302 | hsa-mir-4659b | - |
| hsa-mir-4745 | HGLibA_61078 | 71 | -0.442078302 | hsa-mir-4745 | - |
| hsa-mir-4802 | HGLibA_61313 | 71 | -0.442078302 | hsa-mir-4802 | - |
| hsa-mir-519a-2 | HGLibA_61713 | 71 | -0.442078302 | hsa-mir-519a-2 | - |
| hsa-mir-548a-3 | HGLibA_61843 | 71 | -0.442078302 | hsa-mir-548a-3 | - |
| hsa-mir-548u | HGLibA_62072 | 71 | -0.442078302 | hsa-mir-548u | - |
| hsa-mir-644a | HGLibA_62820 | 71 | -0.442078302 | hsa-mir-644a | - |
| hsa-mir-6500 | HGLibA_62853 | 71 | -0.442078302 | hsa-mir-6500 | - |
| hsa-mir-6792 | HGLibA_63347 | 71 | -0.442078302 | hsa-mir-6792 | - |
| hsa-mir-6820 | HGLibA_63459 | 71 | -0.442078302 | hsa-mir-6820 | - |
| hsa-mir-6861 | HGLibA_63625 | 71 | -0.442078302 | hsa-mir-6861 | - |
| hsa-mir-891a | HGLibA_64242 | 71 | -0.442078302 | hsa-mir-891a | - |
| KIAA1009 | HGLibA_24676 | 71 | -0.442078302 | KIAA1009 | - |
| PROL1 | HGLibA_38676 | 71 | -0.442078302 | PROL1 | - |
| PTPN20B | HGLibA_39454 | 71 | -0.442078302 | PTPN20B | - |
| ZFYVE20 | HGLibA_55278 | 71 | -0.442078302 | ZFYVE20 | - |
| ASMT_X | HGLibA_03321 | 70 | -0.452029339 | ASMT_X | - |
| C11orf73 | HGLibA_05180 | 70 | -0.452029339 | C11orf73 | - |
| C1orf86 | HGLibA_05899 | 70 | -0.452029339 | C1orf86 | - |
| C5orf55 | HGLibA_06409 | 70 | -0.452029339 | C5orf55 | - |
| CXorf61 | HGLibA_11952 | 70 | -0.452029339 | CXorf61 | - |
| ENSG00000003056 | HGLibA_27814 | 70 | -0.452029339 | M6PR | cation dependent [Source:HGNC Symbol;Acc:HGNC:6752]" |
| ENSG00000007306 | HGLibA_08951 | 70 | -0.452029339 | CEACAM7 | carcinoembryonic antigen related cell adhesion molecule 7 [Source:HGNC Symbol;Acc:HGNC:1819] |
| ENSG00000007341 | HGLibA_47286 | 70 | -0.452029339 | ST7L | suppression of tumorigenicity 7 like [Source:HGNC Symbol;Acc:HGNC:18441] |
| ENSG00000019995 | HGLibA_56972 | 70 | -0.452029339 | ZRANB1 | zinc finger RANBP2-type containing 1 [Source:HGNC Symbol;Acc:HGNC:18224] |
| ENSG00000065135 | HGLibA_19457 | 70 | -0.452029339 | GNAI3 | G protein subunit alpha i3 [Source:HGNC Symbol;Acc:HGNC:4387] |
| ENSG00000065268 | HGLibA_54041 | 70 | -0.452029339 | WDR18 | WD repeat domain 18 [Source:HGNC Symbol;Acc:HGNC:17956] |
| ENSG00000067715 | HGLibA_48044 | 70 | -0.452029339 | SYT1 | synaptotagmin 1 [Source:HGNC Symbol;Acc:HGNC:11509] |
| ENSG00000070669 | HGLibA_03336 | 70 | -0.452029339 | ASNS | asparagine synthetase (glutamine-hydrolyzing) [Source:HGNC Symbol;Acc:HGNC:753] |
| ENSG00000071677 | HGLibA_38598 | 70 | -0.452029339 | PRLH | prolactin releasing hormone [Source:HGNC Symbol;Acc:HGNC:17945] |
| ENSG00000078140 | HGLibA_52623 | 70 | -0.452029339 | UBE2K | ubiquitin conjugating enzyme E2 K [Source:HGNC Symbol;Acc:HGNC:4914] |
| ENSG00000078401 | HGLibA_14469 | 70 | -0.452029339 | EDN1 | endothelin 1 [Source:HGNC Symbol;Acc:HGNC:3176] |

| | | | | | |
|---|---|---|---|---|---|
| ENSG00000078814 | HGLibA_30737 | 70 | -0.452029339 | MYH7B | myosin heavy chain 7B [Source:HGNC Symbol;Acc:HGNC:15906] |
| ENSG00000078902 | HGLibA_50944 | 70 | -0.452029339 | TOLLIP | toll interacting protein [Source:HGNC Symbol;Acc:HGNC:16476] |
| ENSG00000084092 | HGLibA_32230 | 70 | -0.452029339 | NOA1 | nitric oxide associated 1 [Source:HGNC Symbol;Acc:HGNC:28473] |
| ENSG00000086570 | HGLibA_16917 | 70 | -0.452029339 | FAT2 | FAT atypical cadherin 2 [Source:HGNC Symbol;Acc:HGNC:3596] |
| ENSG00000087263 | HGLibA_33308 | 70 | -0.452029339 | OGFOD1 | 2-oxoglutarate and iron dependent oxygenase domain containing 1 [Source:HGNC Symbol;Acc:HGNC:25585] |
| ENSG00000091138 | HGLibA_44792 | 70 | -0.452029339 | SLC26A3 | solute carrier family 26 member 3 [Source:HGNC Symbol;Acc:HGNC:3018] |
| ENSG00000093009 | HGLibA_08587 | 70 | -0.452029339 | CDC45 | cell division cycle 45 [Source:HGNC Symbol;Acc:HGNC:1739] |
| ENSG00000099769 | HGLibA_22812 | 70 | -0.452029339 | IGFALS | insulin like growth factor binding protein acid labile subunit [Source:HGNC Symbol;Acc:HGNC:5468] |
| ENSG00000100578 | HGLibA_24647 | 70 | -0.452029339 | KIAA0586 | KIAA0586 [Source:HGNC Symbol;Acc:HGNC:19960] |
| ENSG00000100644 | HGLibA_21296 | 70 | -0.452029339 | HIF1A | hypoxia inducible factor 1 subunit alpha [Source:HGNC Symbol;Acc:HGNC:4910] |
| ENSG00000100906 | HGLibA_31820 | 70 | -0.452029339 | NFKBIA | NFKB inhibitor alpha [Source:HGNC Symbol;Acc:HGNC:7797] |
| ENSG00000101342 | HGLibA_49604 | 70 | -0.452029339 | TLDC2 | TBC/LysM-associated domain containing 2 [Source:HGNC Symbol;Acc:HGNC:16112] |
| ENSG00000101846 | HGLibA_47602 | 70 | -0.452029339 | STS | steroid sulfatase [Source:HGNC Symbol;Acc:HGNC:11425] |
| ENSG00000101849 | HGLibA_48609 | 70 | -0.452029339 | TBL1X | transducin beta like 1 X-linked [Source:HGNC Symbol;Acc:HGNC:11585] |
| ENSG00000102524 | HGLibA_50810 | 70 | -0.452029339 | TNFSF13B | TNF superfamily member 13b [Source:HGNC Symbol;Acc:HGNC:11929] |
| ENSG00000102796 | HGLibA_13123 | 70 | -0.452029339 | DHRS12 | dehydrogenase/reductase 12 [Source:HGNC Symbol;Acc:HGNC:25832] |
| ENSG00000103126 | HGLibA_03920 | 70 | -0.452029339 | AXIN1 | axin 1 [Source:HGNC Symbol;Acc:HGNC:903] |
| ENSG00000103495 | HGLibA_28451 | 70 | -0.452029339 | MAZ | MYC associated zinc finger protein [Source:HGNC Symbol;Acc:HGNC:6914] |
| ENSG00000103769 | HGLibA_39788 | 70 | -0.452029339 | RAB11A | member RAS oncogene family [Source:HGNC Symbol;Acc:HGNC:9760]" |
| ENSG00000104760 | HGLibA_17463 | 70 | -0.452029339 | FGL1 | fibrinogen like 1 [Source:HGNC Symbol;Acc:HGNC:3695] |
| ENSG00000104998 | HGLibA_23114 | 70 | -0.452029339 | IL27RA | interleukin 27 receptor subunit alpha [Source:HGNC Symbol;Acc:HGNC:17290] |
| ENSG00000105398 | HGLibA_47758 | 70 | -0.452029339 | SULT2A1 | sulfotransferase family 2A member 1 [Source:HGNC Symbol;Acc:HGNC:11458] |
| ENSG00000105726 | HGLibA_03566 | 70 | -0.452029339 | ATP13A1 | ATPase 13A1 [Source:HGNC Symbol;Acc:HGNC:24215] |
| ENSG00000105866 | HGLibA_46302 | 70 | -0.452029339 | SP4 | Sp4 transcription factor [Source:HGNC Symbol;Acc:HGNC:11209] |
| ENSG00000106211 | HGLibA_22302 | 70 | -0.452029339 | HSPB1 | heat shock protein family B (small) member 1 [Source:HGNC Symbol;Acc:HGNC:5246] |
| ENSG00000107816 | HGLibA_27810 | 70 | -0.452029339 | LZTS2 | leucine zipper tumor suppressor 2 [Source:HGNC Symbol;Acc:HGNC:29381] |
| ENSG00000107929 | HGLibA_26037 | 70 | -0.452029339 | LARP4B | La ribonucleoprotein domain family member 4B [Source:HGNC Symbol;Acc:HGNC:28987] |
| ENSG00000108262 | HGLibA_19129 | 70 | -0.452029339 | GIT1 | GIT ArfGAP 1 [Source:HGNC Symbol;Acc:HGNC:4272] |
| ENSG00000109062 | HGLibA_45410 | 70 | -0.452029339 | SLC9A3R1 | SLC9A3 regulator 1 [Source:HGNC Symbol;Acc:HGNC:11075] |
| ENSG00000109118 | HGLibA_36340 | 70 | -0.452029339 | PHF12 | PHD finger protein 12 [Source:HGNC Symbol;Acc:HGNC:20816] |
| ENSG00000109255 | HGLibA_32210 | 70 | -0.452029339 | NMU | neuromedin U [Source:HGNC Symbol;Acc:HGNC:7859] |
| ENSG00000109320 | HGLibA_31814 | 70 | -0.452029339 | NFKB1 | nuclear factor kappa B subunit 1 [Source:HGNC Symbol;Acc:HGNC:7794] |
| ENSG00000109323 | HGLibA_28084 | 70 | -0.452029339 | MANBA | mannosidase beta [Source:HGNC Symbol;Acc:HGNC:6831] |
| ENSG00000109424 | HGLibA_52831 | 70 | -0.452029339 | UCP1 | uncoupling protein 1 [Source:HGNC Symbol;Acc:HGNC:12517] |
| ENSG00000111647 | HGLibA_52951 | 70 | -0.452029339 | UHRF1BP1L | UHRF1 binding protein 1 like [Source:HGNC Symbol;Acc:HGNC:29102] |
| ENSG00000111674 | HGLibA_15142 | 70 | -0.452029339 | ENO2 | enolase 2 [Source:HGNC Symbol;Acc:HGNC:3353] |
| ENSG00000111790 | HGLibA_17442 | 70 | -0.452029339 | FGFR1OP2 | FGFR1 oncogene partner 2 [Source:HGNC Symbol;Acc:HGNC:23098] |
| ENSG00000111834 | HGLibA_42335 | 70 | -0.452029339 | RSPH4A | radial spoke head component 4A [Source:HGNC Symbol;Acc:HGNC:21558] |
| ENSG00000112984 | HGLibA_24893 | 70 | -0.452029339 | KIF20A | kinesin family member 20A [Source:HGNC Symbol;Acc:HGNC:9787] |
| ENSG00000113597 | HGLibA_51308 | 70 | -0.452029339 | TRAPPC13 | trafficking protein particle complex 13 [Source:HGNC Symbol;Acc:HGNC:25828] |
| ENSG00000114541 | HGLibA_18016 | 70 | -0.452029339 | FRMD4B | FERM domain containing 4B [Source:HGNC Symbol;Acc:HGNC:24886] |
| ENSG00000114956 | HGLibA_13089 | 70 | -0.452029339 | DGUOK | deoxyguanosine kinase [Source:HGNC Symbol;Acc:HGNC:2858] |
| ENSG00000117724 | HGLibA_09053 | 70 | -0.452029339 | CENPF | centromere protein F [Source:HGNC Symbol;Acc:HGNC:1857] |
| ENSG00000119865 | HGLibA_10381 | 70 | -0.452029339 | CNRIP1 | cannabinoid receptor interacting protein 1 [Source:HGNC Symbol;Acc:HGNC:24546] |
| ENSG00000122861 | HGLibA_36962 | 70 | -0.452029339 | PLAU | urokinase [Source:HGNC Symbol;Acc:HGNC:9052]" |
| ENSG00000123575 | HGLibA_16394 | 70 | -0.452029339 | FAM199X | X-linked [Source:HGNC Symbol;Acc:HGNC:25195]" |
| ENSG00000123595 | HGLibA_39992 | 70 | -0.452029339 | RAB9A | member RAS oncogene family [Source:HGNC Symbol;Acc:HGNC:9792]" |
| ENSG00000125434 | HGLibA_44716 | 70 | -0.452029339 | SLC25A35 | solute carrier family 25 member 35 [Source:HGNC Symbol;Acc:HGNC:31921] |
| ENSG00000125520 | HGLibA_44880 | 70 | -0.452029339 | SLC2A4RG | SLC2A4 regulator [Source:HGNC Symbol;Acc:HGNC:15930] |
| ENSG00000126218 | HGLibA_15841 | 70 | -0.452029339 | F10 | coagulation factor X [Source:HGNC Symbol;Acc:HGNC:3528] |
| ENSG00000126953 | HGLibA_49522 | 70 | -0.452029339 | TIMM8A | translocase of inner mitochondrial membrane 8A [Source:HGNC Symbol;Acc:HGNC:11817] |
| ENSG00000128050 | HGLibA_35026 | 70 | -0.452029339 | PAICS | phosphoribosylaminoimidazole carboxylase and phosphoribosylaminoimidazolesuccinocarboxamide synthase [Source:HGNC Symbol;Acc:HGNC:8587] |
| ENSG00000130762 | HGLibA_02823 | 70 | -0.452029339 | ARHGEF16 | Rho guanine nucleotide exchange factor 16 [Source:HGNC Symbol;Acc:HGNC:15515] |
| ENSG00000132554 | HGLibA_41087 | 70 | -0.452029339 | RGS22 | regulator of G protein signaling 22 [Source:HGNC Symbol;Acc:HGNC:24499] |
| ENSG00000133460 | HGLibA_44859 | 70 | -0.452029339 | SLC2A11 | solute carrier family 2 member 11 [Source:HGNC Symbol;Acc:HGNC:14239] |
| ENSG00000133878 | HGLibA_14170 | 70 | -0.452029339 | DUSP26 | dual specificity phosphatase 26 [Source:HGNC Symbol;Acc:HGNC:28161] |
| ENSG00000133895 | HGLibA_28885 | 70 | -0.452029339 | MEN1 | menin 1 [Source:HGNC Symbol;Acc:HGNC:7010] |
| ENSG00000134595 | HGLibA_46259 | 70 | -0.452029339 | SOX3 | SRY-box 3 [Source:HGNC Symbol;Acc:HGNC:11199] |
| ENSG00000134987 | HGLibA_54077 | 70 | -0.452029339 | WDR36 | WD repeat domain 36 [Source:HGNC Symbol;Acc:HGNC:30696] |
| ENSG00000135097 | HGLibA_30205 | 70 | -0.452029339 | MSI1 | musashi RNA binding protein 1 [Source:HGNC Symbol;Acc:HGNC:7330] |
| ENSG00000135596 | HGLibA_29200 | 70 | -0.452029339 | MICAL1 | calponin and LIM domain containing 1 [Source:HGNC Symbol;Acc:HGNC:20619]" |
| ENSG00000135953 | HGLibA_29119 | 70 | -0.452029339 | MFSD9 | major facilitator superfamily domain containing 9 [Source:HGNC Symbol;Acc:HGNC:28158] |
| ENSG00000136531 | HGLibA_42981 | 70 | -0.452029339 | SCN2A | sodium voltage-gated channel alpha subunit 2 [Source:HGNC Symbol;Acc:HGNC:10588] |
| ENSG00000138036 | HGLibA_14251 | 70 | -0.452029339 | DYNC2LI1 | dynein cytoplasmic 2 light intermediate chain 1 [Source:HGNC Symbol;Acc:HGNC:24595] |
| ENSG00000138050 | HGLibA_49417 | 70 | -0.452029339 | THUMPD2 | THUMP domain containing 2 [Source:HGNC Symbol;Acc:HGNC:14890] |
| ENSG00000138138 | HGLibA_03401 | 70 | -0.452029339 | ATAD1 | AAA domain containing 1 [Source:HGNC Symbol;Acc:HGNC:25903]" |
| ENSG00000138433 | HGLibA_09717 | 70 | -0.452029339 | CIR1 | 1 [Source:HGNC Symbol;Acc:HGNC:24217]" |
| ENSG00000138650 | HGLibA_35387 | 70 | -0.452029339 | PCDH10 | protocadherin 10 [Source:HGNC Symbol;Acc:HGNC:13404] |
| ENSG00000138755 | HGLibA_11878 | 70 | -0.452029339 | CXCL9 | C-X-C motif chemokine ligand 9 [Source:HGNC Symbol;Acc:HGNC:7098] |
| ENSG00000139044 | HGLibA_04017 | 70 | -0.452029339 | B4GALNT3 | 4-N-acetyl-galactosaminyltransferase 3 [Source:HGNC Symbol;Acc:HGNC:24137]" |
| ENSG00000139289 | HGLibA_36416 | 70 | -0.452029339 | PHLDA1 | pleckstrin homology like domain family A member 1 [Source:HGNC Symbol;Acc:HGNC:8933] |
| ENSG00000139505 | HGLibA_30433 | 70 | -0.452029339 | MTMR6 | myotubularin related protein 6 [Source:HGNC Symbol;Acc:HGNC:7453] |
| ENSG00000139926 | HGLibA_18024 | 70 | -0.452029339 | FRMD6 | FERM domain containing 6 [Source:HGNC Symbol;Acc:HGNC:19839] |
| ENSG00000140022 | HGLibA_47548 | 70 | -0.452029339 | STON2 | stonin 2 [Source:HGNC Symbol;Acc:HGNC:30652] |
| ENSG00000140374 | HGLibA_15619 | 70 | -0.452029339 | ETFA | electron transfer flavoprotein subunit alpha [Source:HGNC Symbol;Acc:HGNC:3481] |
| ENSG00000140545 | HGLibA_29060 | 70 | -0.452029339 | MFGE8 | milk fat globule-EGF factor 8 protein [Source:HGNC Symbol;Acc:HGNC:7036] |
| ENSG00000140553 | HGLibA_53004 | 70 | -0.452029339 | UNC45A | unc-45 myosin chaperone A [Source:HGNC Symbol;Acc:HGNC:30594] |
| ENSG00000140961 | HGLibA_34687 | 70 | -0.452029339 | OSGIN1 | oxidative stress induced growth inhibitor 1 [Source:HGNC Symbol;Acc:HGNC:30093] |
| ENSG00000141194 | HGLibA_33965 | 70 | -0.452029339 | OR4D1 | olfactory receptor family 4 subfamily D member 1 [Source:HGNC Symbol;Acc:HGNC:8293] |
| ENSG00000141503 | HGLibA_29277 | 70 | -0.452029339 | MINK1 | misshapen like kinase 1 [Source:HGNC Symbol;Acc:HGNC:17565] |
| ENSG00000144152 | HGLibA_16952 | 70 | -0.452029339 | FBLN7 | fibulin 7 [Source:HGNC Symbol;Acc:HGNC:26740] |
| ENSG00000145087 | HGLibA_47679 | 70 | -0.452029339 | STXBP5L | syntaxin binding protein 5 like [Source:HGNC Symbol;Acc:HGNC:30757] |
| ENSG00000145734 | HGLibA_04390 | 70 | -0.452029339 | BDP1 | subunit of RNA polymerase III transcription initiation factor IIIB [Source:HGNC Symbol;Acc:HGNC:13652]" |
| ENSG00000146192 | HGLibA_17346 | 70 | -0.452029339 | FGD2 | RhoGEF and PH domain containing 2 [Source:HGNC Symbol;Acc:HGNC:3664]" |
| ENSG00000146574 | HGLibA_08226 | 70 | -0.452029339 | CCZ1B | vacuolar protein trafficking and biogenesis associated [Source:HGNC Symbol;Acc:HGNC:21717]" |
| ENSG00000147676 | HGLibA_28025 | 70 | -0.452029339 | MAL2 | T cell differentiation protein 2 (gene/pseudogene) [Source:HGNC Symbol;Acc:HGNC:13634]" |
| ENSG00000148291 | HGLibA_47835 | 70 | -0.452029339 | SURF2 | surfeit 2 [Source:HGNC Symbol;Acc:HGNC:11475] |
| ENSG00000148346 | HGLibA_26179 | 70 | -0.452029339 | LCN2 | lipocalin 2 [Source:HGNC Symbol;Acc:HGNC:6526] |
| ENSG00000148704 | HGLibA_53556 | 70 | -0.452029339 | VAX1 | ventral anterior homeobox 1 [Source:HGNC Symbol;Acc:HGNC:12660] |
| ENSG00000148842 | HGLibA_10318 | 70 | -0.452029339 | CNNM2 | cyclin and CBS domain divalent metal cation transport mediator 2 [Source:HGNC Symbol;Acc:HGNC:103] |
| ENSG00000148925 | HGLibA_04895 | 70 | -0.452029339 | BTBD10 | BTB domain containing 10 [Source:HGNC Symbol;Acc:HGNC:21445] |
| ENSG00000148950 | HGLibA_23229 | 70 | -0.452029339 | IMMP1L | inner mitochondrial membrane peptidase subunit 1 [Source:HGNC Symbol;Acc:HGNC:26317] |
| ENSG00000149972 | HGLibA_10412 | 70 | -0.452029339 | CNTN5 | contactin 5 [Source:HGNC Symbol;Acc:HGNC:2175] |
| ENSG00000151364 | HGLibA_24426 | 70 | -0.452029339 | KCTD14 | potassium channel tetramerization domain containing 14 [Source:HGNC Symbol;Acc:HGNC:23295] |
| ENSG00000151475 | HGLibA_44705 | 70 | -0.452029339 | SLC25A31 | solute carrier family 25 member 31 [Source:HGNC Symbol;Acc:HGNC:25319] |
| ENSG00000152104 | HGLibA_39443 | 70 | -0.452029339 | PTPN14 | non-receptor type 14 [Source:HGNC Symbol;Acc:HGNC:9647]" |
| ENSG00000153721 | HGLibA_10302 | 70 | -0.452029339 | CNKSR3 | CNKSR family member 3 [Source:HGNC Symbol;Acc:HGNC:23034] |
| ENSG00000153902 | HGLibA_26403 | 70 | -0.452029339 | LGI4 | leucine rich repeat LGI family member 4 [Source:HGNC Symbol;Acc:HGNC:18712] |
| ENSG00000154478 | HGLibA_20008 | 70 | -0.452029339 | GPR26 | G protein-coupled receptor 26 [Source:HGNC Symbol;Acc:HGNC:4481] |
| ENSG00000156222 | HGLibA_44830 | 70 | -0.452029339 | SLC28A1 | solute carrier family 28 member 1 [Source:HGNC Symbol;Acc:HGNC:11001] |
| ENSG00000156475 | HGLibA_38150 | 70 | -0.452029339 | PPP2R2B | protein phosphatase 2 regulatory subunit Bbeta [Source:HGNC Symbol;Acc:HGNC:9305] |
| ENSG00000156482 | HGLibA_41934 | 70 | -0.452029339 | RPL30 | ribosomal protein L30 [Source:HGNC Symbol;Acc:HGNC:10333] |
| ENSG00000157322 | HGLibA_09943 | 70 | -0.452029339 | CLEC18A | C-type lectin domain family 18 member A [Source:HGNC Symbol;Acc:HGNC:30388] |
| ENSG00000157540 | HGLibA_14270 | 70 | -0.452029339 | DYRK1A | dual specificity tyrosine phosphorylation regulated kinase 1A [Source:HGNC Symbol;Acc:HGNC:3091] |
| ENSG00000158050 | HGLibA_14157 | 70 | -0.452029339 | DUSP2 | dual specificity phosphatase 2 [Source:HGNC Symbol;Acc:HGNC:3068] |
| ENSG00000161692 | HGLibA_12380 | 70 | -0.452029339 | DBF4B | DBF4 zinc finger B [Source:HGNC Symbol;Acc:HGNC:17883] |
| ENSG00000161896 | HGLibA_23450 | 70 | -0.452029339 | IP6K3 | inositol hexakisphosphate kinase 3 [Source:HGNC Symbol;Acc:HGNC:17269] |
| ENSG00000161960 | HGLibA_14805 | 70 | -0.452029339 | EIF4A1 | eukaryotic translation initiation factor 4A1 [Source:HGNC Symbol;Acc:HGNC:3282] |
| ENSG00000162104 | HGLibA_00932 | 70 | -0.452029339 | ADCY9 | adenylate cyclase 9 [Source:HGNC Symbol;Acc:HGNC:240] |
| ENSG00000163288 | HGLibA_18373 | 70 | -0.452029339 | GABRB1 | gamma-aminobutyric acid type A receptor beta1 subunit [Source:HGNC Symbol;Acc:HGNC:4081] |
| ENSG00000163736 | HGLibA_37855 | 70 | -0.452029339 | PPBP | pro-platelet basic protein [Source:HGNC Symbol;Acc:HGNC:9240] |
| ENSG00000164161 | HGLibA_21260 | 70 | -0.452029339 | HHIP | hedgehog interacting protein [Source:HGNC Symbol;Acc:HGNC:14866] |
| ENSG00000164284 | HGLibA_20375 | 70 | -0.452029339 | GRPEL2 | mitochondrial [Source:HGNC Symbol;Acc:HGNC:21060]" |
| ENSG00000164484 | HGLibA_50171 | 70 | -0.452029339 | TMEM200A | transmembrane protein 200A [Source:HGNC Symbol;Acc:HGNC:21075] |
| ENSG00000164741 | HGLibA_13313 | 70 | -0.452029339 | DLC1 | DLC1 Rho GTPase activating protein [Source:HGNC Symbol;Acc:HGNC:2897] |
| ENSG00000165215 | HGLibA_09896 | 70 | -0.452029339 | CLDN3 | claudin 3 [Source:HGNC Symbol;Acc:HGNC:2045] |
| ENSG00000167769 | HGLibA_00429 | 70 | -0.452029339 | ACER1 | alkaline ceramidase 1 [Source:HGNC Symbol;Acc:HGNC:18356] |
| ENSG00000168143 | HGLibA_16744 | 70 | -0.452029339 | FAM83B | family with sequence similarity 83 member B [Source:HGNC Symbol;Acc:HGNC:21357] |
| ENSG00000168301 | HGLibA_24462 | 70 | -0.452029339 | KCTD6 | potassium channel tetramerization domain containing 6 [Source:HGNC Symbol;Acc:HGNC:22235] |
| ENSG00000168487 | HGLibA_04590 | 70 | -0.452029339 | BMP1 | bone morphogenetic protein 1 [Source:HGNC Symbol;Acc:HGNC:1067] |
| ENSG00000169255 | HGLibA_03948 | 70 | -0.452029339 | B3GALNT1 | 3-N-acetylgalactosaminyltransferase 1 (globoside blood group) [Source:HGNC Symbol;Acc:HGNC:918]" |
| ENSG00000170439 | HGLibA_29021 | 70 | -0.452029339 | METTL7B | methyltransferase like 7B [Source:HGNC Symbol;Acc:HGNC:28276] |
| ENSG00000170989 | HGLibA_42593 | 70 | -0.452029339 | S1PR1 | sphingosine-1-phosphate receptor 1 [Source:HGNC Symbol;Acc:HGNC:3165] |
| ENSG00000171219 | HGLibA_08563 | 70 | -0.452029339 | CDC42BPG | CDC42 binding protein kinase gamma [Source:HGNC Symbol;Acc:HGNC:29829] |
| ENSG00000171476 | HGLibA_21884 | 70 | -0.452029339 | HOPX | HOP homeobox [Source:HGNC Symbol;Acc:HGNC:24961] |
| ENSG00000171587 | HGLibA_13999 | 70 | -0.452029339 | DSCAM | DS cell adhesion molecule [Source:HGNC Symbol;Acc:HGNC:3039] |
| ENSG00000171793 | HGLibA_11668 | 70 | -0.452029339 | CTPS1 | CTP synthase 1 [Source:HGNC Symbol;Acc:HGNC:2519] |
| ENSG00000171928 | HGLibA_52359 | 70 | -0.452029339 | TVP23B | trans-golgi network vesicle protein 23 homolog B [Source:HGNC Symbol;Acc:HGNC:20399] |
| ENSG00000172172 | HGLibA_29899 | 70 | -0.452029339 | MRPL13 | mitochondrial ribosomal protein L13 [Source:HGNC Symbol;Acc:HGNC:14278] |
| ENSG00000172489 | HGLibA_34362 | 70 | -0.452029339 | OR5T3 | olfactory receptor family 5 subfamily T member 3 [Source:HGNC Symbol;Acc:HGNC:15297] |
| ENSG00000173679 | HGLibA_33687 | 70 | -0.452029339 | OR1L1 | olfactory receptor family 1 subfamily L member 1 [Source:HGNC Symbol;Acc:HGNC:8213] |
| ENSG00000173805 | HGLibA_20839 | 70 | -0.452029339 | HAP1 | huntingtin associated protein 1 [Source:HGNC Symbol;Acc:HGNC:4812] |
| ENSG00000174130 | HGLibA_49659 | 70 | -0.452029339 | TLR6 | toll like receptor 6 [Source:HGNC Symbol;Acc:HGNC:16711] |
| ENSG00000174132 | HGLibA_16283 | 70 | -0.452029339 | FAM174A | family with sequence similarity 174 member A [Source:HGNC Symbol;Acc:HGNC:24943] |
| ENSG00000175518 | HGLibA_52744 | 70 | -0.452029339 | UBQLNL | ubiquilin like [Source:HGNC Symbol;Acc:HGNC:28294] |

| | | | | | |
|---|---|---|---|---|---|
| ENSG00000176845 | HGLibA_28940 | 70 | -0.452029339 | METRNL | glial cell differentiation regulator [Source:HGNC Symbol;Acc:HGNC:27584]" |
| ENSG00000176922 | HGLibA_34129 | 70 | -0.452029339 | OR51S1 | olfactory receptor family 51 subfamily S member 1 [Source:HGNC Symbol;Acc:HGNC:15204] |
| ENSG00000177054 | HGLibA_55076 | 70 | -0.452029339 | ZDHHC13 | zinc finger DHHC-type containing 13 [Source:HGNC Symbol;Acc:HGNC:18413] |
| ENSG00000177508 | HGLibA_23634 | 70 | -0.452029339 | IRX3 | iroquois homeobox 3 [Source:HGNC Symbol;Acc:HGNC:14360] |
| ENSG00000177839 | HGLibA_35515 | 70 | -0.452029339 | PCDHB9 | protocadherin beta 9 [Source:HGNC Symbol;Acc:HGNC:8694] |
| ENSG00000178403 | HGLibA_31749 | 70 | -0.452029339 | NEUROG2 | neurogenin 2 [Source:HGNC Symbol;Acc:HGNC:13805] |
| ENSG00000180447 | HGLibA_18642 | 70 | -0.452029339 | GAS1 | growth arrest specific 1 [Source:HGNC Symbol;Acc:HGNC:4165] |
| ENSG00000182179 | HGLibA_52531 | 70 | -0.452029339 | UBA7 | ubiquitin like modifier activating enzyme 7 [Source:HGNC Symbol;Acc:HGNC:12471] |
| ENSG00000182518 | HGLibA_15992 | 70 | -0.452029339 | FAM104B | family with sequence similarity 104 member B [Source:HGNC Symbol;Acc:HGNC:25085] |
| ENSG00000182742 | HGLibA_21937 | 70 | -0.452029339 | HOXB4 | homeobox B4 [Source:HGNC Symbol;Acc:HGNC:5115] |
| ENSG00000183914 | HGLibA_13482 | 70 | -0.452029339 | DNAH2 | dynein axonemal heavy chain 2 [Source:HGNC Symbol;Acc:HGNC:2948] |
| ENSG00000184345 | HGLibA_23504 | 70 | -0.452029339 | IQCF2 | IQ motif containing F2 [Source:HGNC Symbol;Acc:HGNC:31815] |
| ENSG00000185681 | HGLibA_29679 | 70 | -0.452029339 | MORN5 | MORN repeat containing 5 [Source:HGNC Symbol;Acc:HGNC:17841] |
| ENSG00000185842 | HGLibA_13479 | 70 | -0.452029339 | DNAH14 | dynein axonemal heavy chain 14 [Source:HGNC Symbol;Acc:HGNC:2945] |
| ENSG00000186452 | HGLibA_50627 | 70 | -0.452029339 | TMPRSS12 | transmembrane serine protease 12 [Source:HGNC Symbol;Acc:HGNC:28779] |
| ENSG00000186471 | HGLibA_01418 | 70 | -0.452029339 | AKAP14 | A-kinase anchoring protein 14 [Source:HGNC Symbol;Acc:HGNC:24061] |
| ENSG00000187151 | HGLibA_01923 | 70 | -0.452029339 | ANGPTL5 | angiopoietin like 5 [Source:HGNC Symbol;Acc:HGNC:19705] |
| ENSG00000187726 | HGLibA_13532 | 70 | -0.452029339 | DNAJB13 | DnaJ heat shock protein family (Hsp40) member B13 [Source:HGNC Symbol;Acc:HGNC:30718] |
| ENSG00000196177 | HGLibA_00375 | 70 | -0.452029339 | ACADSB | acyl-CoA dehydrogenase short/branched chain [Source:HGNC Symbol;Acc:HGNC:91] |
| ENSG00000196547 | HGLibA_28074 | 70 | -0.452029339 | MAN2A2 | mannosidase alpha class 2A member 2 [Source:HGNC Symbol;Acc:HGNC:6825] |
| ENSG00000196859 | HGLibA_25500 | 70 | -0.452029339 | KRT39 | keratin 39 [Source:HGNC Symbol;Acc:HGNC:32971] |
| ENSG00000197380 | HGLibA_12286 | 70 | -0.452029339 | DACT3 | dishevelled binding antagonist of beta catenin 3 [Source:HGNC Symbol;Acc:HGNC:30745] |
| ENSG00000197386 | HGLibA_22420 | 70 | -0.452029339 | HTT | huntingtin [Source:HGNC Symbol;Acc:HGNC:4851] |
| ENSG00000197415 | HGLibA_53622 | 70 | -0.452029339 | VEPH1 | ventricular zone expressed PH domain containing 1 [Source:HGNC Symbol;Acc:HGNC:25735] |
| ENSG00000197937 | HGLibA_55852 | 70 | -0.452029339 | ZNF347 | zinc finger protein 347 [Source:HGNC Symbol;Acc:HGNC:16447] |
| ENSG00000198171 | HGLibA_12640 | 70 | -0.452029339 | DDRGK1 | DDRGK domain containing 1 [Source:HGNC Symbol;Acc:HGNC:16110] |
| ENSG00000198848 | HGLibA_09218 | 70 | -0.452029339 | CES1 | carboxylesterase 1 [Source:HGNC Symbol;Acc:HGNC:1863] |
| ENSG00000204482 | HGLibA_27572 | 70 | -0.452029339 | LST1 | leukocyte specific transcript 1 [Source:HGNC Symbol;Acc:HGNC:14189] |
| ENSG00000204544 | HGLibA_30541 | 70 | -0.452029339 | MUC21 | cell surface associated [Source:HGNC Symbol;Acc:HGNC:21661]" |
| ENSG00000204887 | HGLibA_25586 | 70 | -0.452029339 | KRTAP1-4 | keratin associated protein 1-4 [Source:HGNC Symbol;Acc:HGNC:18904] |
| ENSG00000206384 | HGLibA_10607 | 70 | -0.452029339 | COL6A6 | collagen type VI alpha 6 chain [Source:HGNC Symbol;Acc:HGNC:27023] |
| ENSG00000214140 | HGLibA_38357 | 70 | -0.452029339 | PRCD | photoreceptor disc component [Source:HGNC Symbol;Acc:HGNC:32528] |
| ENSG00000214376 | HGLibA_53873 | 70 | -0.452029339 | VSTM5 | V-set and transmembrane domain containing 5 [Source:HGNC Symbol;Acc:HGNC:34443] |
| ENSG00000215041 | HGLibA_31730 | 70 | -0.452029339 | NEURL4 | neuralized E3 ubiquitin protein ligase 4 [Source:HGNC Symbol;Acc:HGNC:34410] |
| ENSG00000215274 | HGLibA_18433 | 70 | -0.452029339 | GAGE10 | G antigen 10 [Source:HGNC Symbol;Acc:HGNC:30968] |
| ENSG00000215644 | HGLibA_18780 | 70 | -0.452029339 | GCGR | glucagon receptor [Source:HGNC Symbol;Acc:HGNC:4192] |
| ENSG00000221914 | HGLibA_38145 | 70 | -0.452029339 | PPP2R2A | protein phosphatase 2 regulatory subunit Balpha [Source:HGNC Symbol;Acc:HGNC:9304] |
| ENSG00000221978 | HGLibA_08121 | 70 | -0.452029339 | CCNL2 | cyclin L2 [Source:HGNC Symbol;Acc:HGNC:20570] |
| ENSG00000226887 | HGLibA_15541 | 70 | -0.452029339 | ERVMER34-1 | envelope [Source:HGNC Symbol;Acc:HGNC:42970]" |
| ENSG00000230347 | HGLibA_11545 | 70 | -0.452029339 | CT47A8 | member A8 [Source:HGNC Symbol;Acc:HGNC:33289]" |
| ENSG00000231637 | HGLibA_53270 | 70 | -0.452029339 | USP17L29 | ubiquitin specific peptidase 17-like family member 29 [Source:HGNC Symbol;Acc:HGNC:44457] |
| ENSG00000232196 | HGLibA_30480 | 70 | -0.452029339 | MTRNR2L4 | MT-RNR2 like 4 [Source:HGNC Symbol;Acc:HGNC:37161] |
| ENSG00000247315 | HGLibA_55032 | 70 | -0.452029339 | ZCCHC3 | zinc finger CCHC-type containing 3 [Source:HGNC Symbol;Acc:HGNC:16230] |
| ENSG00000249861 | HGLibA_26357 | 70 | -0.452029339 | LGALS16 | galectin 16 [Source:HGNC Symbol;Acc:HGNC:40039] |
| ENSG00000249915 | HGLibA_35740 | 70 | -0.452029339 | PDCD6 | programmed cell death 6 [Source:HGNC Symbol;Acc:HGNC:8765] |
| ENSG00000250844 | HGLibA_53234 | 70 | -0.452029339 | USP17L18 | ubiquitin specific peptidase 17-like family member 18 [Source:HGNC Symbol;Acc:HGNC:44446] |
| ENSG00000253719 | HGLibA_03861 | 70 | -0.452029339 | ATXN7L3B | ataxin 7 like 3B [Source:HGNC Symbol;Acc:HGNC:37931] |
| ENSG00000255833 | HGLibA_49460 | 70 | -0.452029339 | TIFAB | TIFA inhibitor [Source:HGNC Symbol;Acc:HGNC:34024] |
| ENSG00000256235 | HGLibA_45755 | 70 | -0.452029339 | SMIM3 | small integral membrane protein 3 [Source:HGNC Symbol;Acc:HGNC:30248] |
| ENSG00000257108 | HGLibA_31906 | 70 | -0.452029339 | NHLRC4 | NHL repeat containing 4 [Source:HGNC Symbol;Acc:HGNC:26700] |
| ENSG00000265491 | HGLibA_41480 | 70 | -0.452029339 | RNF115 | ring finger protein 115 [Source:HGNC Symbol;Acc:HGNC:18154] |
| ENSG00000268221 | HGLibA_33424 | 70 | -0.452029339 | OPN1MW | medium wave sensitive [Source:HGNC Symbol;Acc:HGNC:4206]" |
| ENSG00000276119 | HGLibA_33596 | 70 | -0.452029339 | OR13C2 | olfactory receptor family 13 subfamily C member 2 [Source:HGNC Symbol;Acc:HGNC:14701] |
| ENSG00000278299 | HGLibA_48545 | 70 | -0.452029339 | TBC1D3C | TBC1 domain family member 3C [Source:HGNC Symbol;Acc:HGNC:24889] |
| GLTSCR1L | HGLibA_19354 | 70 | -0.452029339 | GLTSCR1L | - |
| HIST1H2AJ | HGLibA_21398 | 70 | -0.452029339 | HIST1H2AJ | - |
| hsa-mir-1229 | HGLibA_57313 | 70 | -0.452029339 | hsa-mir-1229 | - |
| hsa-mir-26a-1 | HGLibA_58365 | 70 | -0.452029339 | hsa-mir-26a-1 | - |
| hsa-mir-3116-1 | HGLibA_58504 | 70 | -0.452029339 | hsa-mir-3116-1 | - |
| hsa-mir-3151 | HGLibA_58662 | 70 | -0.452029339 | hsa-mir-3151 | - |
| hsa-mir-3159 | HGLibA_58712 | 70 | -0.452029339 | hsa-mir-3159 | - |
| hsa-mir-361 | HGLibA_59069 | 70 | -0.452029339 | hsa-mir-361 | - |
| hsa-mir-3680-1 | HGLibA_59270 | 70 | -0.452029339 | hsa-mir-3680-1 | - |
| hsa-mir-3928 | HGLibA_59588 | 70 | -0.452029339 | hsa-mir-3928 | - |
| hsa-mir-424 | HGLibA_59702 | 70 | -0.452029339 | hsa-mir-424 | - |
| hsa-mir-4274 | HGLibA_59800 | 70 | -0.452029339 | hsa-mir-4274 | - |
| hsa-mir-4294 | HGLibA_59889 | 70 | -0.452029339 | hsa-mir-4294 | - |
| hsa-mir-4436a | HGLibA_60140 | 70 | -0.452029339 | hsa-mir-4436a | - |
| hsa-mir-4647 | HGLibA_60691 | 70 | -0.452029339 | hsa-mir-4647 | - |
| hsa-mir-4728 | HGLibA_61010 | 70 | -0.452029339 | hsa-mir-4728 | - |
| hsa-mir-4778 | HGLibA_61220 | 70 | -0.452029339 | hsa-mir-4778 | - |
| hsa-mir-4795 | HGLibA_61285 | 70 | -0.452029339 | hsa-mir-4795 | - |
| hsa-mir-4800 | HGLibA_61307 | 70 | -0.452029339 | hsa-mir-4800 | - |
| hsa-mir-513c | HGLibA_61583 | 70 | -0.452029339 | hsa-mir-513c | - |
| hsa-mir-5189 | HGLibA_61646 | 70 | -0.452029339 | hsa-mir-5189 | - |
| hsa-mir-548ap | HGLibA_61912 | 70 | -0.452029339 | hsa-mir-548ap | - |
| hsa-mir-548q | HGLibA_62057 | 70 | -0.452029339 | hsa-mir-548q | - |
| hsa-mir-5591 | HGLibA_62204 | 70 | -0.452029339 | hsa-mir-5591 | - |
| hsa-mir-581 | HGLibA_62422 | 70 | -0.452029339 | hsa-mir-581 | - |
| hsa-mir-6089-2 | HGLibA_62619 | 70 | -0.452029339 | hsa-mir-6089-2 | - |
| hsa-mir-6511a-2 | HGLibA_62900 | 70 | -0.452029339 | hsa-mir-6511a-2 | - |
| hsa-mir-6771 | HGLibA_63257 | 70 | -0.452029339 | hsa-mir-6771 | - |
| hsa-mir-6803 | HGLibA_63391 | 70 | -0.452029339 | hsa-mir-6803 | - |
| hsa-mir-6819 | HGLibA_63453 | 70 | -0.452029339 | hsa-mir-6819 | - |
| hsa-mir-6851 | HGLibA_63577 | 70 | -0.452029339 | hsa-mir-6851 | - |
| LOC100288814 | HGLibA_26837 | 70 | -0.452029339 | LOC100288814 | - |
| LOC643355 | HGLibA_26966 | 70 | -0.452029339 | LOC643355 | - |
| MEF2BNB | HGLibA_28832 | 70 | -0.452029339 | MEF2BNB | - |
| MTERFD3 | HGLibA_30347 | 70 | -0.452029339 | MTERFD3 | - |
| MUM1 | HGLibA_30565 | 70 | -0.452029339 | MUM1 | - |
| PION | HGLibA_36699 | 70 | -0.452029339 | PION | - |
| PPP2R3B_X | HGLibA_38159 | 70 | -0.452029339 | PPP2R3B_X | - |
| SEPW1 | HGLibA_43460 | 70 | -0.452029339 | SEPW1 | - |
| USP17L1P | HGLibA_53240 | 70 | -0.452029339 | USP17L1P | - |
| WBP5 | HGLibA_53986 | 70 | -0.452029339 | WBP5 | - |
| ZCCHC11 | HGLibA_55005 | 70 | -0.452029339 | ZCCHC11 | - |
| ZFYVE20 | HGLibA_55280 | 70 | -0.452029339 | ZFYVE20 | - |
| APOPT1 | HGLibA_02557 | 69 | -0.461980376 | APOPT1 | - |
| ARMC4 | HGLibA_03021 | 69 | -0.461980376 | ARMC4 | - |
| ATP5I | HGLibA_03666 | 69 | -0.461980376 | ATP5I | - |
| C2orf61 | HGLibA_06154 | 69 | -0.461980376 | C2orf61 | - |
| CT45A4 | HGLibA_11507 | 69 | -0.461980376 | CT45A4 | - |
| ENSG00000005175 | HGLibA_41820 | 69 | -0.461980376 | RPAP3 | RNA polymerase II associated protein 3 [Source:HGNC Symbol;Acc:HGNC:26151] |
| ENSG00000005436 | HGLibA_18774 | 69 | -0.461980376 | GCFC2 | GC-rich sequence DNA-binding factor 2 [Source:HGNC Symbol;Acc:HGNC:1317] |
| ENSG00000005882 | HGLibA_35899 | 69 | -0.461980376 | PDK2 | pyruvate dehydrogenase kinase 2 [Source:HGNC Symbol;Acc:HGNC:8810] |
| ENSG00000007237 | HGLibA_18657 | 69 | -0.461980376 | GAS7 | growth arrest specific 7 [Source:HGNC Symbol;Acc:HGNC:4169] |
| ENSG00000010671 | HGLibA_04954 | 69 | -0.461980376 | BTK | Bruton tyrosine kinase [Source:HGNC Symbol;Acc:HGNC:1133] |
| ENSG00000011243 | HGLibA_01449 | 69 | -0.461980376 | AKAP8L | A-kinase anchoring protein 8 like [Source:HGNC Symbol;Acc:HGNC:29857] |
| ENSG00000015520 | HGLibA_32421 | 69 | -0.461980376 | NPC1L1 | NPC1 like intracellular cholesterol transporter 1 [Source:HGNC Symbol;Acc:HGNC:7898] |
| ENSG00000018869 | HGLibA_56311 | 69 | -0.461980376 | ZNF582 | zinc finger protein 582 [Source:HGNC Symbol;Acc:HGNC:26421] |
| ENSG00000025293 | HGLibA_36364 | 69 | -0.461980376 | PHF20 | PHD finger protein 20 [Source:HGNC Symbol;Acc:HGNC:16098] |
| ENSG00000047936 | HGLibA_41785 | 69 | -0.461980376 | ROS1 | receptor tyrosine kinase [Source:HGNC Symbol;Acc:HGNC:10261]" |
| ENSG00000048740 | HGLibA_09007 | 69 | -0.461980376 | CELF2 | CUGBP Elav-like family member 2 [Source:HGNC Symbol;Acc:HGNC:2550] |
| ENSG00000050130 | HGLibA_23949 | 69 | -0.461980376 | JKAMP | JNK1/MAPK8 associated membrane protein [Source:HGNC Symbol;Acc:HGNC:20184] |
| ENSG00000050748 | HGLibA_28294 | 69 | -0.461980376 | MAPK9 | mitogen-activated protein kinase 9 [Source:HGNC Symbol;Acc:HGNC:6886] |
| ENSG00000057294 | HGLibA_36854 | 69 | -0.461980376 | PKP2 | plakophilin 2 [Source:HGNC Symbol;Acc:HGNC:9024] |
| ENSG00000075131 | HGLibA_49560 | 69 | -0.461980376 | TIPIN | TIMELESS interacting protein [Source:HGNC Symbol;Acc:HGNC:30750] |
| ENSG00000082497 | HGLibA_43612 | 69 | -0.461980376 | SERTAD4 | SERTA domain containing 4 [Source:HGNC Symbol;Acc:HGNC:25236] |
| ENSG00000087269 | HGLibA_32303 | 69 | -0.461980376 | NOP14 | NOP14 nucleolar protein [Source:HGNC Symbol;Acc:HGNC:16821] |
| ENSG00000089775 | HGLibA_54849 | 69 | -0.461980376 | ZBTB25 | zinc finger and BTB domain containing 25 [Source:HGNC Symbol;Acc:HGNC:13112] |
| ENSG00000100249 | HGLibA_06071 | 69 | -0.461980376 | C22orf31 | chromosome 22 open reading frame 31 [Source:HGNC Symbol;Acc:HGNC:26931] |
| ENSG00000101150 | HGLibA_51116 | 69 | -0.461980376 | TPD52L2 | TPD52 like 2 [Source:HGNC Symbol;Acc:HGNC:12007] |
| ENSG00000101182 | HGLibA_39088 | 69 | -0.461980376 | PSMA7 | proteasome subunit alpha 7 [Source:HGNC Symbol;Acc:HGNC:9536] |
| ENSG00000101363 | HGLibA_28087 | 69 | -0.461980376 | MANBAL | mannosidase beta like [Source:HGNC Symbol;Acc:HGNC:15799] |
| ENSG00000101986 | HGLibA_00192 | 69 | -0.461980376 | ABCD1 | ATP binding cassette subfamily D member 1 [Source:HGNC Symbol;Acc:HGNC:61] |
| ENSG00000102100 | HGLibA_44949 | 69 | -0.461980376 | SLC35A2 | solute carrier family 35 member A2 [Source:HGNC Symbol;Acc:HGNC:11022] |
| ENSG00000103342 | HGLibA_20440 | 69 | -0.461980376 | GSPT1 | G1 to S phase transition 1 [Source:HGNC Symbol;Acc:HGNC:4621] |
| ENSG00000104313 | HGLibA_15816 | 69 | -0.461980376 | EYA1 | EYA transcriptional coactivator and phosphatase 1 [Source:HGNC Symbol;Acc:HGNC:3519] |
| ENSG00000104635 | HGLibA_45103 | 69 | -0.461980376 | SLC39A14 | solute carrier family 39 member 14 [Source:HGNC Symbol;Acc:HGNC:20858] |
| ENSG00000104979 | HGLibA_05678 | 69 | -0.461980376 | C19orf53 | chromosome 19 open reading frame 53 [Source:HGNC Symbol;Acc:HGNC:24991] |
| ENSG00000105507 | HGLibA_06857 | 69 | -0.461980376 | CABP5 | calcium binding protein 5 [Source:HGNC Symbol;Acc:HGNC:13714] |
| ENSG00000105723 | HGLibA_20428 | 69 | -0.461980376 | GSK3A | glycogen synthase kinase 3 alpha [Source:HGNC Symbol;Acc:HGNC:4616] |
| ENSG00000105738 | HGLibA_44199 | 69 | -0.461980376 | SIPA1L3 | signal induced proliferation associated 1 like 3 [Source:HGNC Symbol;Acc:HGNC:23801] |
| ENSG00000105877 | HGLibA_13474 | 69 | -0.461980376 | DNAH11 | dynein axonemal heavy chain 11 [Source:HGNC Symbol;Acc:HGNC:2942] |
| ENSG00000106615 | HGLibA_41154 | 69 | -0.461980376 | RHEB | mTORC1 binding [Source:HGNC Symbol;Acc:HGNC:10011]" |

| | | | | | |
|---|---|---|---|---|---|
| ENSG00000106633 | HGLibA_18787 | 69 | -0.461980376 | GCK | glucokinase [Source:HGNC Symbol;Acc:HGNC:4195] |
| ENSG00000107758 | HGLibA_38187 | 69 | -0.461980376 | PPP3CB | protein phosphatase 3 catalytic subunit beta [Source:HGNC Symbol;Acc:HGNC:9315] |
| ENSG00000108523 | HGLibA_41562 | 69 | -0.461980376 | RNF167 | ring finger protein 167 [Source:HGNC Symbol;Acc:HGNC:24544] |
| ENSG00000108788 | HGLibA_29439 | 69 | -0.461980376 | MLX | MAX dimerization protein [Source:HGNC Symbol;Acc:HGNC:11645]" |
| ENSG00000108852 | HGLibA_29750 | 69 | -0.461980376 | MPP2 | membrane palmitoylated protein 2 [Source:HGNC Symbol;Acc:HGNC:7220] |
| ENSG00000108963 | HGLibA_13836 | 69 | -0.461980376 | DPH1 | diphthamide biosynthesis 1 [Source:HGNC Symbol;Acc:HGNC:3003] |
| ENSG00000109758 | HGLibA_21243 | 69 | -0.461980376 | HGFAC | HGF activator [Source:HGNC Symbol;Acc:HGNC:4894] |
| ENSG00000110492 | HGLibA_28691 | 69 | -0.461980376 | MDK | midkine [Source:HGNC Symbol;Acc:HGNC:6972] |
| ENSG00000111262 | HGLibA_24124 | 69 | -0.461980376 | KCNA1 | potassium voltage-gated channel subfamily A member 1 [Source:HGNC Symbol;Acc:HGNC:6218] |
| ENSG00000111328 | HGLibA_08760 | 69 | -0.461980376 | CDK2AP1 | cyclin dependent kinase 2 associated protein 1 [Source:HGNC Symbol;Acc:HGNC:14002] |
| ENSG00000111602 | HGLibA_49491 | 69 | -0.461980376 | TIMELESS | timeless circadian regulator [Source:HGNC Symbol;Acc:HGNC:11813] |
| ENSG00000111775 | HGLibA_10843 | 69 | -0.461980376 | COX6A1 | cytochrome c oxidase subunit 6A1 [Source:HGNC Symbol;Acc:HGNC:2277] |
| ENSG00000112195 | HGLibA_51364 | 69 | -0.461980376 | TREML2 | triggering receptor expressed on myeloid cells like 2 [Source:HGNC Symbol;Acc:HGNC:21092] |
| ENSG00000112578 | HGLibA_05008 | 69 | -0.461980376 | BYSL | bystin like [Source:HGNC Symbol;Acc:HGNC:1157] |
| ENSG00000112659 | HGLibA_11795 | 69 | -0.461980376 | CUL9 | cullin 9 [Source:HGNC Symbol;Acc:HGNC:15982] |
| ENSG00000114812 | HGLibA_53667 | 69 | -0.461980376 | VIPR1 | vasoactive intestinal peptide receptor 1 [Source:HGNC Symbol;Acc:HGNC:12694] |
| ENSG00000115234 | HGLibA_46049 | 69 | -0.461980376 | SNX17 | sorting nexin 17 [Source:HGNC Symbol;Acc:HGNC:14979] |
| ENSG00000115593 | HGLibA_45850 | 69 | -0.461980376 | SMYD1 | SET and MYND domain containing 1 [Source:HGNC Symbol;Acc:HGNC:20986] |
| ENSG00000115808 | HGLibA_47595 | 69 | -0.461980376 | STRN | striatin [Source:HGNC Symbol;Acc:HGNC:11424] |
| ENSG00000116035 | HGLibA_53559 | 69 | -0.461980376 | VAX2 | ventral anterior homeobox 2 [Source:HGNC Symbol;Acc:HGNC:12661] |
| ENSG00000116254 | HGLibA_09397 | 69 | -0.461980376 | CHD5 | chromodomain helicase DNA binding protein 5 [Source:HGNC Symbol;Acc:HGNC:16816] |
| ENSG00000116698 | HGLibA_45707 | 69 | -0.461980376 | SMG7 | nonsense mediated mRNA decay factor [Source:HGNC Symbol;Acc:HGNC:16792]" |
| ENSG00000116852 | HGLibA_24902 | 69 | -0.461980376 | KIF21B | kinesin family member 21B [Source:HGNC Symbol;Acc:HGNC:29442] |
| ENSG00000116871 | HGLibA_28235 | 69 | -0.461980376 | MAP7D1 | MAP7 domain containing 1 [Source:HGNC Symbol;Acc:HGNC:25514] |
| ENSG00000118271 | HGLibA_52229 | 69 | -0.461980376 | TTR | transthyretin [Source:HGNC Symbol;Acc:HGNC:12405] |
| ENSG00000119392 | HGLibA_19240 | 69 | -0.461980376 | GLE1 | RNA export mediator [Source:HGNC Symbol;Acc:HGNC:4315]" |
| ENSG00000119408 | HGLibA_31658 | 69 | -0.461980376 | NEK6 | NIMA related kinase 6 [Source:HGNC Symbol;Acc:HGNC:7749] |
| ENSG00000120262 | HGLibA_07690 | 69 | -0.461980376 | CCDC170 | coiled-coil domain containing 170 [Source:HGNC Symbol;Acc:HGNC:21177] |
| ENSG00000120440 | HGLibA_52198 | 69 | -0.461980376 | TTLL2 | tubulin tyrosine ligase like 2 [Source:HGNC Symbol;Acc:HGNC:21211] |
| ENSG00000120798 | HGLibA_32594 | 69 | -0.461980376 | NR2C1 | nuclear receptor subfamily 2 group C member 1 [Source:HGNC Symbol;Acc:HGNC:7971] |
| ENSG00000123064 | HGLibA_12743 | 69 | -0.461980376 | DDX54 | DEAD-box helicase 54 [Source:HGNC Symbol;Acc:HGNC:20084] |
| ENSG00000124302 | HGLibA_09648 | 69 | -0.461980376 | CHST8 | carbohydrate sulfotransferase 8 [Source:HGNC Symbol;Acc:HGNC:15993] |
| ENSG00000124766 | HGLibA_46265 | 69 | -0.461980376 | SOX4 | SRY-box 4 [Source:HGNC Symbol;Acc:HGNC:11200] |
| ENSG00000124780 | HGLibA_24311 | 69 | -0.461980376 | KCNK17 | potassium two pore domain channel subfamily K member 17 [Source:HGNC Symbol;Acc:HGNC:14465] |
| ENSG00000125257 | HGLibA_00176 | 69 | -0.461980376 | ABCC4 | ATP binding cassette subfamily C member 4 [Source:HGNC Symbol;Acc:HGNC:55] |
| ENSG00000127824 | HGLibA_52260 | 69 | -0.461980376 | TUBA4A | tubulin alpha 4a [Source:HGNC Symbol;Acc:HGNC:12407] |
| ENSG00000128789 | HGLibA_39209 | 69 | -0.461980376 | PSMG2 | proteasome assembly chaperone 2 [Source:HGNC Symbol;Acc:HGNC:24929] |
| ENSG00000128789 | HGLibA_39207 | 69 | -0.461980376 | PSMG2 | proteasome assembly chaperone 2 [Source:HGNC Symbol;Acc:HGNC:24929] |
| ENSG00000130175 | HGLibA_38561 | 69 | -0.461980376 | PRKCSH | protein kinase C substrate 80K-H [Source:HGNC Symbol;Acc:HGNC:9411] |
| ENSG00000130707 | HGLibA_03372 | 69 | -0.461980376 | ASS1 | argininosuccinate synthase 1 [Source:HGNC Symbol;Acc:HGNC:758] |
| ENSG00000130948 | HGLibA_22189 | 69 | -0.461980376 | HSD17B3 | hydroxysteroid 17-beta dehydrogenase 3 [Source:HGNC Symbol;Acc:HGNC:5212] |
| ENSG00000131374 | HGLibA_48559 | 69 | -0.461980376 | TBC1D5 | TBC1 domain family member 5 [Source:HGNC Symbol;Acc:HGNC:19166] |
| ENSG00000131379 | HGLibA_06218 | 69 | -0.461980376 | C3orf20 | chromosome 3 open reading frame 20 [Source:HGNC Symbol;Acc:HGNC:25320] |
| ENSG00000131408 | HGLibA_32579 | 69 | -0.461980376 | NR1H2 | nuclear receptor subfamily 1 group H member 2 [Source:HGNC Symbol;Acc:HGNC:7965] |
| ENSG00000132205 | HGLibA_15050 | 69 | -0.461980376 | EMILIN2 | elastin microfibril interfacer 2 [Source:HGNC Symbol;Acc:HGNC:19881] |
| ENSG00000133116 | HGLibA_25020 | 69 | -0.461980376 | KL | klotho [Source:HGNC Symbol;Acc:HGNC:6344] |
| ENSG00000133433 | HGLibA_20502 | 69 | -0.461980376 | GSTT2B | glutathione S-transferase theta 2B (gene/pseudogene) [Source:HGNC Symbol;Acc:HGNC:33437] |
| ENSG00000134352 | HGLibA_23186 | 69 | -0.461980376 | IL6ST | interleukin 6 signal transducer [Source:HGNC Symbol;Acc:HGNC:6021] |
| ENSG00000134758 | HGLibA_41515 | 69 | -0.461980376 | RNF138 | ring finger protein 138 [Source:HGNC Symbol;Acc:HGNC:17765] |
| ENSG00000135299 | HGLibA_02130 | 69 | -0.461980376 | ANKRD6 | ankyrin repeat domain 6 [Source:HGNC Symbol;Acc:HGNC:17280] |
| ENSG00000135476 | HGLibA_15573 | 69 | -0.461980376 | ESPL1 | separase [Source:HGNC Symbol;Acc:HGNC:16856]" |
| ENSG00000138193 | HGLibA_36998 | 69 | -0.461980376 | PLCE1 | phospholipase C epsilon 1 [Source:HGNC Symbol;Acc:HGNC:17175] |
| ENSG00000138207 | HGLibA_40638 | 69 | -0.461980376 | RBP4 | retinol binding protein 4 [Source:HGNC Symbol;Acc:HGNC:9922] |
| ENSG00000138606 | HGLibA_44025 | 69 | -0.461980376 | SHF | Src homology 2 domain containing F [Source:HGNC Symbol;Acc:HGNC:25116] |
| ENSG00000138622 | HGLibA_20981 | 69 | -0.461980376 | HCN4 | hyperpolarization activated cyclic nucleotide gated potassium channel 4 [Source:HGNC Symbol;Acc:HGNC:16882] |
| ENSG00000139151 | HGLibA_37031 | 69 | -0.461980376 | PLCZ1 | phospholipase C zeta 1 [Source:HGNC Symbol;Acc:HGNC:19218] |
| ENSG00000140465 | HGLibA_12066 | 69 | -0.461980376 | CYP1A1 | cytochrome P450 family 1 subfamily A member 1 [Source:HGNC Symbol;Acc:HGNC:2595] |
| ENSG00000142528 | HGLibA_56060 | 69 | -0.461980376 | ZNF473 | zinc finger protein 473 [Source:HGNC Symbol;Acc:HGNC:23239] |
| ENSG00000142661 | HGLibA_30896 | 69 | -0.461980376 | MYOM3 | myomesin 3 [Source:HGNC Symbol;Acc:HGNC:26679] |
| ENSG00000143751 | HGLibA_43121 | 69 | -0.461980376 | SDE2 | SDE2 telomere maintenance homolog [Source:HGNC Symbol;Acc:HGNC:26643] |
| ENSG00000144401 | HGLibA_28975 | 69 | -0.461980376 | METTL21A | methyltransferase like 21A [Source:HGNC Symbol;Acc:HGNC:30476] |
| ENSG00000145217 | HGLibA_44780 | 69 | -0.461980376 | SLC26A1 | solute carrier family 26 member 1 [Source:HGNC Symbol;Acc:HGNC:10993] |
| ENSG00000145332 | HGLibA_25226 | 69 | -0.461980376 | KLHL8 | kelch like family member 8 [Source:HGNC Symbol;Acc:HGNC:18644] |
| ENSG00000145741 | HGLibA_04936 | 69 | -0.461980376 | BTF3 | basic transcription factor 3 [Source:HGNC Symbol;Acc:HGNC:1125] |
| ENSG00000145936 | HGLibA_24341 | 69 | -0.461980376 | KCNMB1 | potassium calcium-activated channel subfamily M regulatory beta subunit 1 [Source:HGNC Symbol;Acc:HGNC:6285] |
| ENSG00000148335 | HGLibA_32851 | 69 | -0.461980376 | NTMT1 | N-terminal Xaa-Pro-Lys N-methyltransferase 1 [Source:HGNC Symbol;Acc:HGNC:23373] |
| ENSG00000148584 | HGLibA_00006 | 69 | -0.461980376 | A1CF | APOBEC1 complementation factor [Source:HGNC Symbol;Acc:HGNC:24086] |
| ENSG00000148950 | HGLibA_23228 | 69 | -0.461980376 | IMMP1L | inner mitochondrial membrane peptidase subunit 1 [Source:HGNC Symbol;Acc:HGNC:26317] |
| ENSG00000154016 | HGLibA_20213 | 69 | -0.461980376 | GRAP | GRB2 related adaptor protein [Source:HGNC Symbol;Acc:HGNC:4562] |
| ENSG00000154217 | HGLibA_36747 | 69 | -0.461980376 | PITPNC1 | phosphatidylinositol transfer protein cytoplasmic 1 [Source:HGNC Symbol;Acc:HGNC:21045] |
| ENSG00000154548 | HGLibA_47069 | 69 | -0.461980376 | SRSF12 | serine and arginine rich splicing factor 12 [Source:HGNC Symbol;Acc:HGNC:21220] |
| ENSG00000154639 | HGLibA_11839 | 69 | -0.461980376 | CXADR | Ig-like cell adhesion molecule [Source:HGNC Symbol;Acc:HGNC:2559]" |
| ENSG00000156097 | HGLibA_20063 | 69 | -0.461980376 | GPR61 | G protein-coupled receptor 61 [Source:HGNC Symbol;Acc:HGNC:13300] |
| ENSG00000157404 | HGLibA_25015 | 69 | -0.461980376 | KIT | KIT proto-oncogene receptor tyrosine kinase [Source:HGNC Symbol;Acc:HGNC:6342] |
| ENSG00000157593 | HGLibA_44963 | 69 | -0.461980376 | SLC35B2 | solute carrier family 35 member B2 [Source:HGNC Symbol;Acc:HGNC:16872] |
| ENSG00000158417 | HGLibA_14863 | 69 | -0.461980376 | EIF5B | eukaryotic translation initiation factor 5B [Source:HGNC Symbol;Acc:HGNC:30793] |
| ENSG00000158435 | HGLibA_10331 | 69 | -0.461980376 | CNOT11 | CCR4-NOT transcription complex subunit 11 [Source:HGNC Symbol;Acc:HGNC:25217] |
| ENSG00000158773 | HGLibA_53168 | 69 | -0.461980376 | USF1 | upstream transcription factor 1 [Source:HGNC Symbol;Acc:HGNC:12593] |
| ENSG00000159445 | HGLibA_49340 | 69 | -0.461980376 | THEM4 | thioesterase superfamily member 4 [Source:HGNC Symbol;Acc:HGNC:17947] |
| ENSG00000159527 | HGLibA_36254 | 69 | -0.461980376 | PGLYRP3 | peptidoglycan recognition protein 3 [Source:HGNC Symbol;Acc:HGNC:30014] |
| ENSG00000160439 | HGLibA_40740 | 69 | -0.461980376 | RDH13 | retinol dehydrogenase 13 [Source:HGNC Symbol;Acc:HGNC:19978] |
| ENSG00000162595 | HGLibA_13261 | 69 | -0.461980376 | DIRAS3 | DIRAS family GTPase 3 [Source:HGNC Symbol;Acc:HGNC:687] |
| ENSG00000162604 | HGLibA_49683 | 69 | -0.461980376 | TM2D1 | TM2 domain containing 1 [Source:HGNC Symbol;Acc:HGNC:24142] |
| ENSG00000162885 | HGLibA_03950 | 69 | -0.461980376 | B3GALNT2 | 3-N-acetylgalactosaminyltransferase 2 [Source:HGNC Symbol;Acc:HGNC:28596]" |
| ENSG00000162946 | HGLibA_13279 | 69 | -0.461980376 | DISC1 | DISC1 scaffold protein [Source:HGNC Symbol;Acc:HGNC:2888] |
| ENSG00000163539 | HGLibA_09795 | 69 | -0.461980376 | CLASP2 | cytoplasmic linker associated protein 2 [Source:HGNC Symbol;Acc:HGNC:17078] |
| ENSG00000163702 | HGLibA_23009 | 69 | -0.461980376 | IL17RC | interleukin 17 receptor C [Source:HGNC Symbol;Acc:HGNC:18358] |
| ENSG00000163864 | HGLibA_32191 | 69 | -0.461980376 | NMNAT3 | nicotinamide nucleotide adenylyltransferase 3 [Source:HGNC Symbol;Acc:HGNC:20989] |
| ENSG00000164048 | HGLibA_56336 | 69 | -0.461980376 | ZNF589 | zinc finger protein 589 [Source:HGNC Symbol;Acc:HGNC:16747] |
| ENSG00000164588 | HGLibA_20972 | 69 | -0.461980376 | HCN1 | hyperpolarization activated cyclic nucleotide gated potassium channel 1 [Source:HGNC Symbol;Acc:HGNC:4845] |
| ENSG00000164626 | HGLibA_24326 | 69 | -0.461980376 | KCNK5 | potassium two pore domain channel subfamily K member 5 [Source:HGNC Symbol;Acc:HGNC:6280] |
| ENSG00000164975 | HGLibA_45896 | 69 | -0.461980376 | SNAPC3 | small nuclear RNA activating complex polypeptide 3 [Source:HGNC Symbol;Acc:HGNC:11136] |
| ENSG00000165240 | HGLibA_03770 | 69 | -0.461980376 | ATP7A | ATPase copper transporting alpha [Source:HGNC Symbol;Acc:HGNC:869] |
| ENSG00000166441 | HGLibA_41922 | 69 | -0.461980376 | RPL27A | ribosomal protein L27a [Source:HGNC Symbol;Acc:HGNC:10329] |
| ENSG00000167522 | HGLibA_01994 | 69 | -0.461980376 | ANKRD11 | ankyrin repeat domain 11 [Source:HGNC Symbol;Acc:HGNC:21316] |
| ENSG00000167685 | HGLibA_56015 | 69 | -0.461980376 | ZNF444 | zinc finger protein 444 [Source:HGNC Symbol;Acc:HGNC:16052] |
| ENSG00000168679 | HGLibA_44441 | 69 | -0.461980376 | SLC16A4 | solute carrier family 16 member 4 [Source:HGNC Symbol;Acc:HGNC:10925] |
| ENSG00000169016 | HGLibA_14321 | 69 | -0.461980376 | E2F6 | E2F transcription factor 6 [Source:HGNC Symbol;Acc:HGNC:3120] |
| ENSG00000169499 | HGLibA_37065 | 69 | -0.461980376 | PLEKHA2 | pleckstrin homology domain containing A2 [Source:HGNC Symbol;Acc:HGNC:14336] |
| ENSG00000170485 | HGLibA_32398 | 69 | -0.461980376 | NPAS2 | neuronal PAS domain protein 2 [Source:HGNC Symbol;Acc:HGNC:7895] |
| ENSG00000170558 | HGLibA_08659 | 69 | -0.461980376 | CDH2 | cadherin 2 [Source:HGNC Symbol;Acc:HGNC:1759] |
| ENSG00000171450 | HGLibA_08778 | 69 | -0.461980376 | CDK5R2 | cyclin dependent kinase 5 regulatory subunit 2 [Source:HGNC Symbol;Acc:HGNC:1776] |
| ENSG00000171469 | HGLibA_56246 | 69 | -0.461980376 | ZNF561 | zinc finger protein 561 [Source:HGNC Symbol;Acc:HGNC:28684] |
| ENSG00000171490 | HGLibA_42318 | 69 | -0.461980376 | RSL1D1 | ribosomal L1 domain containing 1 [Source:HGNC Symbol;Acc:HGNC:24534] |
| ENSG00000172046 | HGLibA_53294 | 69 | -0.461980376 | USP19 | ubiquitin specific peptidase 19 [Source:HGNC Symbol;Acc:HGNC:12617] |
| ENSG00000172081 | HGLibA_29595 | 69 | -0.461980376 | MOB3A | MOB kinase activator 3A [Source:HGNC Symbol;Acc:HGNC:29802] |
| ENSG00000172955 | HGLibA_00962 | 69 | -0.461980376 | ADH6 | alcohol dehydrogenase 6 (class V) [Source:HGNC Symbol;Acc:HGNC:255] |
| ENSG00000173273 | HGLibA_50851 | 69 | -0.461980376 | TNKS | tankyrase [Source:HGNC Symbol;Acc:HGNC:11941] |
| ENSG00000173627 | HGLibA_02497 | 69 | -0.461980376 | APOBEC4 | apolipoprotein B mRNA editing enzyme catalytic polypeptide like 4 [Source:HGNC Symbol;Acc:HGNC:32152] |
| ENSG00000176083 | HGLibA_56532 | 69 | -0.461980376 | ZNF683 | zinc finger protein 683 [Source:HGNC Symbol;Acc:HGNC:28495] |
| ENSG00000177200 | HGLibA_09411 | 69 | -0.461980376 | CHD9 | chromodomain helicase DNA binding protein 9 [Source:HGNC Symbol;Acc:HGNC:25701] |
| ENSG00000178188 | HGLibA_43875 | 69 | -0.461980376 | SH2B1 | SH2B adaptor protein 1 [Source:HGNC Symbol;Acc:HGNC:30417] |
| ENSG00000179168 | HGLibA_19001 | 69 | -0.461980376 | GGN | gametogenetin [Source:HGNC Symbol;Acc:HGNC:18869] |
| ENSG00000179532 | HGLibA_13676 | 69 | -0.461980376 | DNHD1 | dynein heavy chain domain 1 [Source:HGNC Symbol;Acc:HGNC:26532] |
| ENSG00000179709 | HGLibA_32132 | 69 | -0.461980376 | NLRP8 | NLR family pyrin domain containing 8 [Source:HGNC Symbol;Acc:HGNC:22940] |
| ENSG00000181092 | HGLibA_00977 | 69 | -0.461980376 | ADIPOQ | C1Q and collagen domain containing [Source:HGNC Symbol;Acc:HGNC:13633]" |
| ENSG00000182224 | HGLibA_11996 | 69 | -0.461980376 | CYB5D1 | cytochrome b5 domain containing 1 [Source:HGNC Symbol;Acc:HGNC:26516] |
| ENSG00000182983 | HGLibA_56478 | 69 | -0.461980376 | ZNF662 | zinc finger protein 662 [Source:HGNC Symbol;Acc:HGNC:31930] |
| ENSG00000183066 | HGLibA_53980 | 69 | -0.461980376 | WBP2NL | WBP2 N-terminal like [Source:HGNC Symbol;Acc:HGNC:28389] |
| ENSG00000183072 | HGLibA_32044 | 69 | -0.461980376 | NKX2-5 | NK2 homeobox 5 [Source:HGNC Symbol;Acc:HGNC:2488] |
| ENSG00000183454 | HGLibA_20298 | 69 | -0.461980376 | GRIN2A | glutamate ionotropic receptor NMDA type subunit 2A [Source:HGNC Symbol;Acc:HGNC:4585] |
| ENSG00000184557 | HGLibA_46129 | 69 | -0.461980376 | SOCS3 | suppressor of cytokine signaling 3 [Source:HGNC Symbol;Acc:HGNC:19391] |
| ENSG00000184933 | HGLibA_34369 | 69 | -0.461980376 | OR6A2 | olfactory receptor family 6 subfamily A member 2 [Source:HGNC Symbol;Acc:HGNC:15301] |
| ENSG00000184949 | HGLibA_16529 | 69 | -0.461980376 | FAM227A | family with sequence similarity 227 member A [Source:HGNC Symbol;Acc:HGNC:44197] |
| ENSG00000185219 | HGLibA_56017 | 69 | -0.461980376 | ZNF445 | zinc finger protein 445 [Source:HGNC Symbol;Acc:HGNC:21018] |
| ENSG00000185303 | HGLibA_43769 | 69 | -0.461980376 | SFTPA2 | surfactant protein A2 [Source:HGNC Symbol;Acc:HGNC:10799] |
| ENSG00000185803 | HGLibA_45247 | 69 | -0.461980376 | SLC52A2 | solute carrier family 52 member 2 [Source:HGNC Symbol;Acc:HGNC:30224] |
| ENSG00000185958 | HGLibA_16336 | 69 | -0.461980376 | FAM186A | family with sequence similarity 186 member A [Source:HGNC Symbol;Acc:HGNC:26980] |
| ENSG00000187581 | HGLibA_10877 | 69 | -0.461980376 | COX8C | cytochrome c oxidase subunit 8C [Source:HGNC Symbol;Acc:HGNC:24382] |
| ENSG00000187735 | HGLibA_48696 | 69 | -0.461980376 | TCEA1 | transcription elongation factor A1 [Source:HGNC Symbol;Acc:HGNC:11612] |
| ENSG00000187742 | HGLibA_43265 | 69 | -0.461980376 | SECISBP2 | SECIS binding protein 2 [Source:HGNC Symbol;Acc:HGNC:30972] |
| ENSG00000188368 | HGLibA_38796 | 69 | -0.461980376 | PRR19 | proline rich 19 [Source:HGNC Symbol;Acc:HGNC:33728] |
| ENSG00000188487 | HGLibA_23374 | 69 | -0.461980376 | INSC | spindle orientation adaptor protein [Source:HGNC Symbol;Acc:HGNC:33116]" |
| ENSG00000188542 | HGLibA_14174 | 69 | -0.461980376 | DUSP28 | dual specificity phosphatase 28 [Source:HGNC Symbol;Acc:HGNC:33237] |
| ENSG00000188603 | HGLibA_10075 | 69 | -0.461980376 | CLN3 | battenin [Source:HGNC Symbol;Acc:HGNC:2074]" |

| | | | | | |
|---|---|---|---|---|---|
| ENSG00000188706 | HGLibA_55131 | 69 | -0.461980376 | ZDHHC9 | zinc finger DHHC-type containing 9 [Source:HGNC Symbol;Acc:HGNC:18475] |
| ENSG00000188770 | HGLibA_33456 | 69 | -0.461980376 | OPTC | opticin [Source:HGNC Symbol;Acc:HGNC:8158] |
| ENSG00000188869 | HGLibA_49755 | 69 | -0.461980376 | TMC3 | transmembrane channel like 3 [Source:HGNC Symbol;Acc:HGNC:22995] |
| ENSG00000197296 | HGLibA_17542 | 69 | -0.461980376 | FITM2 | fat storage inducing transmembrane protein 2 [Source:HGNC Symbol;Acc:HGNC:16135] |
| ENSG00000197706 | HGLibA_34407 | 69 | -0.461980376 | OR6C74 | olfactory receptor family 6 subfamily C member 74 [Source:HGNC Symbol;Acc:HGNC:31303] |
| ENSG00000197892 | HGLibA_24858 | 69 | -0.461980376 | KIF13B | kinesin family member 13B [Source:HGNC Symbol;Acc:HGNC:14405] |
| ENSG00000197937 | HGLibA_55853 | 69 | -0.461980376 | ZNF347 | zinc finger protein 347 [Source:HGNC Symbol;Acc:HGNC:16447] |
| ENSG00000198053 | HGLibA_44200 | 69 | -0.461980376 | SIRPA | signal regulatory protein alpha [Source:HGNC Symbol;Acc:HGNC:9662] |
| ENSG00000198208 | HGLibA_42185 | 69 | -0.461980376 | RPS6KL1 | ribosomal protein S6 kinase like 1 [Source:HGNC Symbol;Acc:HGNC:20222] |
| ENSG00000198563 | HGLibA_12700 | 69 | -0.461980376 | DDX39B | DExD-box helicase 39B [Source:HGNC Symbol;Acc:HGNC:13917] |
| ENSG00000203485 | HGLibA_23281 | 69 | -0.461980376 | INF2 | FH2 and WH2 domain containing [Source:HGNC Symbol;Acc:HGNC:23791]" |
| ENSG00000203943 | HGLibA_42671 | 69 | -0.461980376 | SAMD13 | sterile alpha motif domain containing 13 [Source:HGNC Symbol;Acc:HGNC:24582] |
| ENSG00000204022 | HGLibA_26622 | 69 | -0.461980376 | LIPJ | lipase family member J [Source:HGNC Symbol;Acc:HGNC:21773] |
| ENSG00000204421 | HGLibA_27665 | 69 | -0.461980376 | LY6G6C | lymphocyte antigen 6 family member G6C [Source:HGNC Symbol;Acc:HGNC:13936] |
| ENSG00000204956 | HGLibA_35516 | 69 | -0.461980376 | PCDHGA1 | 1 [Source:HGNC Symbol;Acc:HGNC:8696]" |
| ENSG00000213625 | HGLibA_26328 | 69 | -0.461980376 | LEPROT | leptin receptor overlapping transcript [Source:HGNC Symbol;Acc:HGNC:29477] |
| ENSG00000223865 | HGLibA_21618 | 69 | -0.461980376 | HLA-DPB1 | DP beta 1 [Source:HGNC Symbol;Acc:HGNC:4940]" |
| ENSG00000225828 | HGLibA_16539 | 69 | -0.461980376 | FAM229A | family with sequence similarity 229 member A [Source:HGNC Symbol;Acc:HGNC:44652] |
| ENSG00000227345 | HGLibA_35194 | 69 | -0.461980376 | PARG | poly(ADP-ribose) glycohydrolase [Source:HGNC Symbol;Acc:HGNC:8605] |
| ENSG00000231852 | HGLibA_12080 | 69 | -0.461980376 | CYP21A2 | cytochrome P450 family 21 subfamily A member 2 [Source:HGNC Symbol;Acc:HGNC:2600] |
| ENSG00000232119 | HGLibA_28657 | 69 | -0.461980376 | MCTS1 | re-initiation and release factor [Source:HGNC Symbol;Acc:HGNC:23357]" |
| ENSG00000244588 | HGLibA_40067 | 69 | -0.461980376 | RAD21L1 | RAD21 cohesin complex component like 1 [Source:HGNC Symbol;Acc:HGNC:16271] |
| ENSG00000249115 | HGLibA_20885 | 69 | -0.461980376 | HAUS5 | HAUS augmin like complex subunit 5 [Source:HGNC Symbol;Acc:HGNC:29130] |
| ENSG00000250120 | HGLibA_35428 | 69 | -0.461980376 | PCDHA10 | protocadherin alpha 10 [Source:HGNC Symbol;Acc:HGNC:8664] |
| ENSG00000251664 | HGLibA_35433 | 69 | -0.461980376 | PCDHA12 | protocadherin alpha 12 [Source:HGNC Symbol;Acc:HGNC:8666] |
| ENSG00000253767 | HGLibA_35544 | 69 | -0.461980376 | PCDHGA8 | 8 [Source:HGNC Symbol;Acc:HGNC:8706]" |
| ENSG00000270765 | HGLibA_18648 | 69 | -0.461980376 | GAS2L2 | growth arrest specific 2 like 2 [Source:HGNC Symbol;Acc:HGNC:24846] |
| ENSG00000277893 | HGLibA_46942 | 69 | -0.461980376 | SRD5A2 | steroid 5 alpha-reductase 2 [Source:HGNC Symbol;Acc:HGNC:11285] |
| ENSG00000278129 | HGLibA_56780 | 69 | -0.461980376 | ZNF8 | zinc finger protein 8 [Source:HGNC Symbol;Acc:HGNC:13154] |
| ENSG00000278311 | HGLibA_19004 | 69 | -0.461980376 | GGNBP2 | gametogenetin binding protein 2 [Source:HGNC Symbol;Acc:HGNC:19357] |
| ENSG00000278505 | HGLibA_05578 | 69 | -0.461980376 | C17orf78 | chromosome 17 open reading frame 78 [Source:HGNC Symbol;Acc:HGNC:26831] |
| hsa-mir-138-2 | HGLibA_57815 | 69 | -0.461980376 | hsa-mir-138-2 | - |
| hsa-mir-2467 | HGLibA_58347 | 69 | -0.461980376 | hsa-mir-2467 | - |
| hsa-mir-3124 | HGLibA_58549 | 69 | -0.461980376 | hsa-mir-3124 | - |
| hsa-mir-3913-1 | HGLibA_59521 | 69 | -0.461980376 | hsa-mir-3913-1 | - |
| hsa-mir-3920 | HGLibA_59557 | 69 | -0.461980376 | hsa-mir-3920 | - |
| hsa-mir-3937 | HGLibA_59609 | 69 | -0.461980376 | hsa-mir-3937 | - |
| hsa-mir-411 | HGLibA_59685 | 69 | -0.461980376 | hsa-mir-411 | - |
| hsa-mir-4293 | HGLibA_59885 | 69 | -0.461980376 | hsa-mir-4293 | - |
| hsa-mir-4447 | HGLibA_60195 | 69 | -0.461980376 | hsa-mir-4447 | - |
| hsa-mir-4495 | HGLibA_60398 | 69 | -0.461980376 | hsa-mir-4495 | - |
| hsa-mir-4669 | HGLibA_60795 | 69 | -0.461980376 | hsa-mir-4669 | - |
| hsa-mir-504 | HGLibA_61472 | 69 | -0.461980376 | hsa-mir-504 | - |
| hsa-mir-5087 | HGLibA_61495 | 69 | -0.461980376 | hsa-mir-5087 | - |
| hsa-mir-5196 | HGLibA_61701 | 69 | -0.461980376 | hsa-mir-5196 | - |
| hsa-mir-5689 | HGLibA_62270 | 69 | -0.461980376 | hsa-mir-5689 | - |
| hsa-mir-5787 | HGLibA_62411 | 69 | -0.461980376 | hsa-mir-5787 | - |
| hsa-mir-587 | HGLibA_62447 | 69 | -0.461980376 | hsa-mir-587 | - |
| hsa-mir-613 | HGLibA_62669 | 69 | -0.461980376 | hsa-mir-613 | - |
| hsa-mir-760 | HGLibA_63901 | 69 | -0.461980376 | hsa-mir-760 | - |
| LOC391322 | HGLibA_26932 | 69 | -0.461980376 | LOC391322 | - |
| NPIPL3 | HGLibA_32477 | 69 | -0.461980376 | NPIPL3 | - |
| NUPR1L | HGLibA_33089 | 69 | -0.461980376 | NUPR1L | - |
| ACPP | HGLibA_00525 | 68 | -0.471931414 | ACPP | - |
| ATP5O | HGLibA_03684 | 68 | -0.471931414 | ATP5O | - |
| C14orf159 | HGLibA_05329 | 68 | -0.471931414 | C14orf159 | - |
| C1orf173 | HGLibA_05795 | 68 | -0.471931414 | C1orf173 | - |
| ENSG00000004897 | HGLibA_08540 | 68 | -0.471931414 | CDC27 | cell division cycle 27 [Source:HGNC Symbol;Acc:HGNC:1728] |
| ENSG00000004975 | HGLibA_14211 | 68 | -0.471931414 | DVL2 | dishevelled segment polarity protein 2 [Source:HGNC Symbol;Acc:HGNC:3086] |
| ENSG00000005421 | HGLibA_37678 | 68 | -0.471931414 | PON1 | paraoxonase 1 [Source:HGNC Symbol;Acc:HGNC:9204] |
| ENSG00000006025 | HGLibA_34665 | 68 | -0.471931414 | OSBPL7 | oxysterol binding protein like 7 [Source:HGNC Symbol;Acc:HGNC:16387] |
| ENSG00000006128 | HGLibA_48151 | 68 | -0.471931414 | TAC1 | tachykinin precursor 1 [Source:HGNC Symbol;Acc:HGNC:11517] |
| ENSG00000009724 | HGLibA_28397 | 68 | -0.471931414 | MASP2 | mannan binding lectin serine peptidase 2 [Source:HGNC Symbol;Acc:HGNC:6902] |
| ENSG00000012171 | HGLibA_43321 | 68 | -0.471931414 | SEMA3B | semaphorin 3B [Source:HGNC Symbol;Acc:HGNC:10724] |
| ENSG00000018189 | HGLibA_42442 | 68 | -0.471931414 | RUFY3 | RUN and FYVE domain containing 3 [Source:HGNC Symbol;Acc:HGNC:30285] |
| ENSG00000018510 | HGLibA_01241 | 68 | -0.471931414 | AGPS | alkylglycerone phosphate synthase [Source:HGNC Symbol;Acc:HGNC:327] |
| ENSG00000054118 | HGLibA_49391 | 68 | -0.471931414 | THRAP3 | thyroid hormone receptor associated protein 3 [Source:HGNC Symbol;Acc:HGNC:22964] |
| ENSG00000054967 | HGLibA_40826 | 68 | -0.471931414 | RELT | TNF receptor [Source:HGNC Symbol;Acc:HGNC:13764]" |
| ENSG00000070950 | HGLibA_40060 | 68 | -0.471931414 | RAD18 | E3 ubiquitin protein ligase [Source:HGNC Symbol;Acc:HGNC:18278]" |
| ENSG00000072694 | HGLibA_17192 | 68 | -0.471931414 | FCGR2B | Fc fragment of IgG receptor IIb [Source:HGNC Symbol;Acc:HGNC:3618] |
| ENSG00000075391 | HGLibA_40314 | 68 | -0.471931414 | RASAL2 | RAS protein activator like 2 [Source:HGNC Symbol;Acc:HGNC:9874] |
| ENSG00000077232 | HGLibA_13565 | 68 | -0.471931414 | DNAJC10 | DnaJ heat shock protein family (Hsp40) member C10 [Source:HGNC Symbol;Acc:HGNC:24637] |
| ENSG00000077238 | HGLibA_23171 | 68 | -0.471931414 | IL4R | interleukin 4 receptor [Source:HGNC Symbol;Acc:HGNC:6015] |
| ENSG00000078725 | HGLibA_04813 | 68 | -0.471931414 | BRINP1 | BMP/retinoic acid inducible neural specific 1 [Source:HGNC Symbol;Acc:HGNC:2687] |
| ENSG00000078808 | HGLibA_43129 | 68 | -0.471931414 | SDF4 | stromal cell derived factor 4 [Source:HGNC Symbol;Acc:HGNC:24188] |
| ENSG00000079150 | HGLibA_17586 | 68 | -0.471931414 | FKBP7 | FK506 binding protein 7 [Source:HGNC Symbol;Acc:HGNC:3723] |
| ENSG00000084693 | HGLibA_01179 | 68 | -0.471931414 | AGBL5 | ATP/GTP binding protein like 5 [Source:HGNC Symbol;Acc:HGNC:26147] |
| ENSG00000085433 | HGLibA_54107 | 68 | -0.471931414 | WDR47 | WD repeat domain 47 [Source:HGNC Symbol;Acc:HGNC:29141] |
| ENSG00000087077 | HGLibA_51648 | 68 | -0.471931414 | TRIP6 | thyroid hormone receptor interactor 6 [Source:HGNC Symbol;Acc:HGNC:12311] |
| ENSG00000092871 | HGLibA_40920 | 68 | -0.471931414 | RFFL | ring finger and FYVE like domain containing E3 ubiquitin protein ligase [Source:HGNC Symbol;Acc:HGNC:24821] |
| ENSG00000093144 | HGLibA_14394 | 68 | -0.471931414 | ECHDC1 | ethylmalonyl-CoA decarboxylase 1 [Source:HGNC Symbol;Acc:HGNC:21489] |
| ENSG00000095209 | HGLibA_50381 | 68 | -0.471931414 | TMEM38B | transmembrane protein 38B [Source:HGNC Symbol;Acc:HGNC:25535] |
| ENSG00000096070 | HGLibA_04843 | 68 | -0.471931414 | BRPF3 | bromodomain and PHD finger containing 3 [Source:HGNC Symbol;Acc:HGNC:14256] |
| ENSG00000099999 | HGLibA_41627 | 68 | -0.471931414 | RNF215 | ring finger protein 215 [Source:HGNC Symbol;Acc:HGNC:33434] |
| ENSG00000100053 | HGLibA_11267 | 68 | -0.471931414 | CRYBB3 | crystallin beta B3 [Source:HGNC Symbol;Acc:HGNC:2400] |
| ENSG00000100219 | HGLibA_54490 | 68 | -0.471931414 | XBP1 | X-box binding protein 1 [Source:HGNC Symbol;Acc:HGNC:12801] |
| ENSG00000100380 | HGLibA_47223 | 68 | -0.471931414 | ST13 | Hsp70 interacting protein [Source:HGNC Symbol;Acc:HGNC:11343]" |
| ENSG00000100583 | HGLibA_42676 | 68 | -0.471931414 | SAMD15 | sterile alpha motif domain containing 15 [Source:HGNC Symbol;Acc:HGNC:18631] |
| ENSG00000101213 | HGLibA_39386 | 68 | -0.471931414 | PTK6 | protein tyrosine kinase 6 [Source:HGNC Symbol;Acc:HGNC:9617] |
| ENSG00000101460 | HGLibA_28120 | 68 | -0.471931414 | MAP1LC3A | microtubule associated protein 1 light chain 3 alpha [Source:HGNC Symbol;Acc:HGNC:6838] |
| ENSG00000101846 | HGLibA_47603 | 68 | -0.471931414 | STS | steroid sulfatase [Source:HGNC Symbol;Acc:HGNC:11425] |
| ENSG00000102362 | HGLibA_48106 | 68 | -0.471931414 | SYTL4 | synaptotagmin like 4 [Source:HGNC Symbol;Acc:HGNC:15588] |
| ENSG00000102871 | HGLibA_51236 | 68 | -0.471931414 | TRADD | TNFRSF1A associated via death domain [Source:HGNC Symbol;Acc:HGNC:12030] |
| ENSG00000103051 | HGLibA_10479 | 68 | -0.471931414 | COG4 | component of oligomeric golgi complex 4 [Source:HGNC Symbol;Acc:HGNC:18620] |
| ENSG00000103647 | HGLibA_10786 | 68 | -0.471931414 | CORO2B | coronin 2B [Source:HGNC Symbol;Acc:HGNC:2256] |
| ENSG00000104213 | HGLibA_35860 | 68 | -0.471931414 | PDGFRL | platelet derived growth factor receptor like [Source:HGNC Symbol;Acc:HGNC:8805] |
| ENSG00000104549 | HGLibA_46912 | 68 | -0.471931414 | SQLE | squalene epoxidase [Source:HGNC Symbol;Acc:HGNC:11279] |
| ENSG00000104825 | HGLibA_31825 | 68 | -0.471931414 | NFKBIB | NFKB inhibitor beta [Source:HGNC Symbol;Acc:HGNC:7798] |
| ENSG00000104853 | HGLibA_10112 | 68 | -0.471931414 | CLPTM1 | transmembrane protein [Source:HGNC Symbol;Acc:HGNC:2087]" |
| ENSG00000104960 | HGLibA_39400 | 68 | -0.471931414 | PTOV1 | prostate tumor overexpressed 1 [Source:HGNC Symbol;Acc:HGNC:9632] |
| ENSG00000105609 | HGLibA_26520 | 68 | -0.471931414 | LILRB5 | leukocyte immunoglobulin like receptor B5 [Source:HGNC Symbol;Acc:HGNC:6609] |
| ENSG00000105676 | HGLibA_03026 | 68 | -0.471931414 | ARMC6 | armadillo repeat containing 6 [Source:HGNC Symbol;Acc:HGNC:25049] |
| ENSG00000105708 | HGLibA_55481 | 68 | -0.471931414 | ZNF14 | zinc finger protein 14 [Source:HGNC Symbol;Acc:HGNC:12924] |
| ENSG00000105971 | HGLibA_07379 | 68 | -0.471931414 | CAV2 | caveolin 2 [Source:HGNC Symbol;Acc:HGNC:1528] |
| ENSG00000106484 | HGLibA_28922 | 68 | -0.471931414 | MEST | mesoderm specific transcript [Source:HGNC Symbol;Acc:HGNC:7028] |
| ENSG00000107447 | HGLibA_13716 | 68 | -0.471931414 | DNTT | DNA nucleotidylexotransferase [Source:HGNC Symbol;Acc:HGNC:2983] |
| ENSG00000107537 | HGLibA_36467 | 68 | -0.471931414 | PHYH | phytanoyl-CoA 2-hydroxylase [Source:HGNC Symbol;Acc:HGNC:8940] |
| ENSG00000108001 | HGLibA_14355 | 68 | -0.471931414 | EBF3 | EBF transcription factor 3 [Source:HGNC Symbol;Acc:HGNC:19087] |
| ENSG00000108551 | HGLibA_40319 | 68 | -0.471931414 | RASD1 | ras related dexamethasone induced 1 [Source:HGNC Symbol;Acc:HGNC:15828] |
| ENSG00000108641 | HGLibA_04044 | 68 | -0.471931414 | B9D1 | B9 domain containing 1 [Source:HGNC Symbol;Acc:HGNC:24123] |
| ENSG00000109099 | HGLibA_37325 | 68 | -0.471931414 | PMP22 | peripheral myelin protein 22 [Source:HGNC Symbol;Acc:HGNC:9118] |
| ENSG00000110090 | HGLibA_11017 | 68 | -0.471931414 | CPT1A | carnitine palmitoyltransferase 1A [Source:HGNC Symbol;Acc:HGNC:2328] |
| ENSG00000110172 | HGLibA_09515 | 68 | -0.471931414 | CHORDC1 | cysteine and histidine rich domain containing 1 [Source:HGNC Symbol;Acc:HGNC:14525] |
| ENSG00000112294 | HGLibA_01576 | 68 | -0.471931414 | ALDH5A1 | aldehyde dehydrogenase 5 family member A1 [Source:HGNC Symbol;Acc:HGNC:408] |
| ENSG00000112305 | HGLibA_45597 | 68 | -0.471931414 | SMAP1 | small ArfGAP 1 [Source:HGNC Symbol;Acc:HGNC:19651] |
| ENSG00000112739 | HGLibA_38740 | 68 | -0.471931414 | PRPF4B | pre-mRNA processing factor 4B [Source:HGNC Symbol;Acc:HGNC:17346] |
| ENSG00000113460 | HGLibA_04824 | 68 | -0.471931414 | BRIX1 | biogenesis of ribosomes [Source:HGNC Symbol;Acc:HGNC:24170]" |
| ENSG00000113522 | HGLibA_40074 | 68 | -0.471931414 | RAD50 | RAD50 double strand break repair protein [Source:HGNC Symbol;Acc:HGNC:9816] |
| ENSG00000113552 | HGLibA_19566 | 68 | -0.471931414 | GNPDA1 | glucosamine-6-phosphate deaminase 1 [Source:HGNC Symbol;Acc:HGNC:4417] |
| ENSG00000114648 | HGLibA_25144 | 68 | -0.471931414 | KLHL18 | kelch like family member 18 [Source:HGNC Symbol;Acc:HGNC:29120] |
| ENSG00000115221 | HGLibA_23793 | 68 | -0.471931414 | ITGB6 | integrin subunit beta 6 [Source:HGNC Symbol;Acc:HGNC:6161] |
| ENSG00000115592 | HGLibA_38517 | 68 | -0.471931414 | PRKAG3 | protein kinase AMP-activated non-catalytic subunit gamma 3 [Source:HGNC Symbol;Acc:HGNC:9387] |
| ENSG00000115652 | HGLibA_53478 | 68 | -0.471931414 | UXS1 | UDP-glucuronate decarboxylase 1 [Source:HGNC Symbol;Acc:HGNC:17729] |
| ENSG00000116649 | HGLibA_46995 | 68 | -0.471931414 | SRM | spermidine synthase [Source:HGNC Symbol;Acc:HGNC:11296] |
| ENSG00000117139 | HGLibA_24525 | 68 | -0.471931414 | KDM5B | lysine demethylase 5B [Source:HGNC Symbol;Acc:HGNC:18039] |
| ENSG00000117862 | HGLibA_52408 | 68 | -0.471931414 | TXNDC12 | thioredoxin domain containing 12 [Source:HGNC Symbol;Acc:HGNC:24626] |
| ENSG00000118689 | HGLibA_17923 | 68 | -0.471931414 | FOXO3 | forkhead box O3 [Source:HGNC Symbol;Acc:HGNC:3821] |
| ENSG00000120594 | HGLibA_37254 | 68 | -0.471931414 | PLXDC2 | plexin domain containing 2 [Source:HGNC Symbol;Acc:HGNC:21013] |
| ENSG00000122741 | HGLibA_12413 | 68 | -0.471931414 | DCAF10 | DDB1 and CUL4 associated factor 10 [Source:HGNC Symbol;Acc:HGNC:23686] |
| ENSG00000124006 | HGLibA_33233 | 68 | -0.471931414 | OBSL1 | obscurin like 1 [Source:HGNC Symbol;Acc:HGNC:29092] |
| ENSG00000124507 | HGLibA_34959 | 68 | -0.471931414 | PACSIN1 | protein kinase C and casein kinase substrate in neurons 1 [Source:HGNC Symbol;Acc:HGNC:8570] |
| ENSG00000124602 | HGLibA_53024 | 68 | -0.471931414 | UNC5CL | unc-5 family C-terminal like [Source:HGNC Symbol;Acc:HGNC:21203] |
| ENSG00000125510 | HGLibA_33448 | 68 | -0.471931414 | OPRL1 | opioid related nociceptin receptor 1 [Source:HGNC Symbol;Acc:HGNC:8155] |
| ENSG00000125869 | HGLibA_25987 | 68 | -0.471931414 | LAMP5 | lysosomal associated membrane protein family member 5 [Source:HGNC Symbol;Acc:HGNC:16097] |
| ENSG00000125962 | HGLibA_03050 | 68 | -0.471931414 | ARMCX5 | armadillo repeat containing X-linked 5 [Source:HGNC Symbol;Acc:HGNC:25772] |

| | | | | | |
|---|---|---|---|---|---|
| ENSG00000127561 | HGLibA_47998 | 68 | -0.471931414 | SYNGR3 | synaptogyrin 3 [Source:HGNC Symbol;Acc:HGNC:11501] |
| ENSG00000127720 | HGLibA_28998 | 68 | -0.471931414 | METTL25 | methyltransferase like 25 [Source:HGNC Symbol;Acc:HGNC:26228] |
| ENSG00000128242 | HGLibA_18498 | 68 | -0.471931414 | GAL3ST1 | galactose-3-O-sulfotransferase 1 [Source:HGNC Symbol;Acc:HGNC:24240] |
| ENSG00000128250 | HGLibA_40928 | 68 | -0.471931414 | RFPL1 | ret finger protein like 1 [Source:HGNC Symbol;Acc:HGNC:9977] |
| ENSG00000128266 | HGLibA_19481 | 68 | -0.471931414 | GNAZ | G protein subunit alpha z [Source:HGNC Symbol;Acc:HGNC:4395] |
| ENSG00000128833 | HGLibA_30855 | 68 | -0.471931414 | MYO5C | myosin VC [Source:HGNC Symbol;Acc:HGNC:7604] |
| ENSG00000129474 | HGLibA_01373 | 68 | -0.471931414 | AJUBA | ajuba LIM protein [Source:HGNC Symbol;Acc:HGNC:20250] |
| ENSG00000130816 | HGLibA_13699 | 68 | -0.471931414 | DNMT1 | DNA methyltransferase 1 [Source:HGNC Symbol;Acc:HGNC:2976] |
| ENSG00000132153 | HGLibA_13174 | 68 | -0.471931414 | DHX30 | DExH-box helicase 30 [Source:HGNC Symbol;Acc:HGNC:16716] |
| ENSG00000132821 | HGLibA_53865 | 68 | -0.471931414 | VSTM2L | V-set and transmembrane domain containing 2 like [Source:HGNC Symbol;Acc:HGNC:16096] |
| ENSG00000132872 | HGLibA_48077 | 68 | -0.471931414 | SYT4 | synaptotagmin 4 [Source:HGNC Symbol;Acc:HGNC:11512] |
| ENSG00000133627 | HGLibA_00662 | 68 | -0.471931414 | ACTR3B | ARP3 actin related protein 3 homolog B [Source:HGNC Symbol;Acc:HGNC:17256] |
| ENSG00000133710 | HGLibA_46696 | 68 | -0.471931414 | SPINK5 | Kazal type 5 [Source:HGNC Symbol;Acc:HGNC:15464]" |
| ENSG00000134363 | HGLibA_18099 | 68 | -0.471931414 | FST | follistatin [Source:HGNC Symbol;Acc:HGNC:3971] |
| ENSG00000136206 | HGLibA_46580 | 68 | -0.471931414 | SPDYE1 | speedy/RINGO cell cycle regulator family member E1 [Source:HGNC Symbol;Acc:HGNC:16408] |
| ENSG00000136274 | HGLibA_31037 | 68 | -0.471931414 | NACAD | NAC alpha domain containing [Source:HGNC Symbol;Acc:HGNC:22196] |
| ENSG00000136297 | HGLibA_29464 | 68 | -0.471931414 | MMD2 | monocyte to macrophage differentiation associated 2 [Source:HGNC Symbol;Acc:HGNC:30133] |
| ENSG00000136935 | HGLibA_19591 | 68 | -0.471931414 | GOLGA1 | golgin A1 [Source:HGNC Symbol;Acc:HGNC:4424] |
| ENSG00000138594 | HGLibA_50599 | 68 | -0.471931414 | TMOD3 | tropomodulin 3 [Source:HGNC Symbol;Acc:HGNC:11873] |
| ENSG00000138769 | HGLibA_08809 | 68 | -0.471931414 | CDKL2 | cyclin dependent kinase like 2 [Source:HGNC Symbol;Acc:HGNC:1782] |
| ENSG00000138834 | HGLibA_28291 | 68 | -0.471931414 | MAPK8IP3 | mitogen-activated protein kinase 8 interacting protein 3 [Source:HGNC Symbol;Acc:HGNC:6884] |
| ENSG00000139144 | HGLibA_36626 | 68 | -0.471931414 | PIK3C2G | phosphatidylinositol-4-phosphate 3-kinase catalytic subunit type 2 gamma [Source:HGNC Symbol;Acc:HGNC:8973] |
| ENSG00000139438 | HGLibA_16523 | 68 | -0.471931414 | FAM222A | family with sequence similarity 222 member A [Source:HGNC Symbol;Acc:HGNC:25915] |
| ENSG00000139767 | HGLibA_47051 | 68 | -0.471931414 | SRRM4 | serine/arginine repetitive matrix 4 [Source:HGNC Symbol;Acc:HGNC:29389] |
| ENSG00000140326 | HGLibA_08504 | 68 | -0.471931414 | CDAN1 | codanin 1 [Source:HGNC Symbol;Acc:HGNC:1713] |
| ENSG00000141200 | HGLibA_24927 | 68 | -0.471931414 | KIF2B | kinesin family member 2B [Source:HGNC Symbol;Acc:HGNC:29443] |
| ENSG00000142544 | HGLibA_11748 | 68 | -0.471931414 | CTU1 | cytosolic thiouridylase subunit 1 [Source:HGNC Symbol;Acc:HGNC:29590] |
| ENSG00000142748 | HGLibA_17221 | 68 | -0.471931414 | FCN3 | ficolin 3 [Source:HGNC Symbol;Acc:HGNC:3625] |
| ENSG00000143499 | HGLibA_45853 | 68 | -0.471931414 | SMYD2 | SET and MYND domain containing 2 [Source:HGNC Symbol;Acc:HGNC:20982] |
| ENSG00000143753 | HGLibA_12920 | 68 | -0.471931414 | DEGS1 | sphingolipid 1 [Source:HGNC Symbol;Acc:HGNC:13709]" |
| ENSG00000144136 | HGLibA_44528 | 68 | -0.471931414 | SLC20A1 | solute carrier family 20 member 1 [Source:HGNC Symbol;Acc:HGNC:10946] |
| ENSG00000144199 | HGLibA_15962 | 68 | -0.471931414 | FAHD2B | fumarylacetoacetate hydrolase domain containing 2B [Source:HGNC Symbol;Acc:HGNC:25318] |
| ENSG00000144566 | HGLibA_39960 | 68 | -0.471931414 | RAB5A | member RAS oncogene family [Source:HGNC Symbol;Acc:HGNC:9783]" |
| ENSG00000145626 | HGLibA_52933 | 68 | -0.471931414 | UGT3A1 | UDP glycosyltransferase family 3 member A1 [Source:HGNC Symbol;Acc:HGNC:26625] |
| ENSG00000147144 | HGLibA_07566 | 68 | -0.471931414 | CCDC120 | coiled-coil domain containing 120 [Source:HGNC Symbol;Acc:HGNC:28910] |
| ENSG00000147416 | HGLibA_03736 | 68 | -0.471931414 | ATP6V1B2 | ATPase H+ transporting V1 subunit B2 [Source:HGNC Symbol;Acc:HGNC:854] |
| ENSG00000147592 | HGLibA_25921 | 68 | -0.471931414 | LACTB2 | lactamase beta 2 [Source:HGNC Symbol;Acc:HGNC:18512] |
| ENSG00000148468 | HGLibA_16264 | 68 | -0.471931414 | FAM171A1 | family with sequence similarity 171 member A1 [Source:HGNC Symbol;Acc:HGNC:23522] |
| ENSG00000150459 | HGLibA_42715 | 68 | -0.471931414 | SAP18 | Sin3A associated protein 18 [Source:HGNC Symbol;Acc:HGNC:10530] |
| ENSG00000150540 | HGLibA_21785 | 68 | -0.471931414 | HNMT | histamine N-methyltransferase [Source:HGNC Symbol;Acc:HGNC:5028] |
| ENSG00000150593 | HGLibA_35734 | 68 | -0.471931414 | PDCD4 | programmed cell death 4 [Source:HGNC Symbol;Acc:HGNC:8763] |
| ENSG00000151929 | HGLibA_04079 | 68 | -0.471931414 | BAG3 | BCL2 associated athanogene 3 [Source:HGNC Symbol;Acc:HGNC:939] |
| ENSG00000152439 | HGLibA_56714 | 68 | -0.471931414 | ZNF773 | zinc finger protein 773 [Source:HGNC Symbol;Acc:HGNC:30487] |
| ENSG00000152684 | HGLibA_36042 | 68 | -0.471931414 | PELO | pelota mRNA surveillance and ribosome rescue factor [Source:HGNC Symbol;Acc:HGNC:8829] |
| ENSG00000153404 | HGLibA_37112 | 68 | -0.471931414 | PLEKHG4B | pleckstrin homology and RhoGEF domain containing G4B [Source:HGNC Symbol;Acc:HGNC:29399] |
| ENSG00000156384 | HGLibA_43732 | 68 | -0.471931414 | SFR1 | SWI5 dependent homologous recombination repair protein 1 [Source:HGNC Symbol;Acc:HGNC:29574] |
| ENSG00000156502 | HGLibA_47830 | 68 | -0.471931414 | SUPV3L1 | Suv3 like RNA helicase [Source:HGNC Symbol;Acc:HGNC:11471] |
| ENSG00000156853 | HGLibA_56546 | 68 | -0.471931414 | ZNF689 | zinc finger protein 689 [Source:HGNC Symbol;Acc:HGNC:25173] |
| ENSG00000157326 | HGLibA_13136 | 68 | -0.471931414 | DHRS4 | dehydrogenase/reductase 4 [Source:HGNC Symbol;Acc:HGNC:16985] |
| ENSG00000160111 | HGLibA_10903 | 68 | -0.471931414 | CPAMD8 | alpha-2-macroglobulin domain containing 8 [Source:HGNC Symbol;Acc:HGNC:23228]" |
| ENSG00000160972 | HGLibA_38054 | 68 | -0.471931414 | PPP1R16A | protein phosphatase 1 regulatory subunit 16A [Source:HGNC Symbol;Acc:HGNC:14941] |
| ENSG00000161888 | HGLibA_46552 | 68 | -0.471931414 | SPC24 | NDC80 kinetochore complex component [Source:HGNC Symbol;Acc:HGNC:26913]" |
| ENSG00000162086 | HGLibA_56678 | 68 | -0.471931414 | ZNF75A | zinc finger protein 75a [Source:HGNC Symbol;Acc:HGNC:13146] |
| ENSG00000162341 | HGLibA_51107 | 68 | -0.471931414 | TPCN2 | two pore segment channel 2 [Source:HGNC Symbol;Acc:HGNC:20820] |
| ENSG00000162650 | HGLibA_03854 | 68 | -0.471931414 | ATXN7L2 | ataxin 7 like 2 [Source:HGNC Symbol;Acc:HGNC:28713] |
| ENSG00000163468 | HGLibA_08197 | 68 | -0.471931414 | CCT3 | chaperonin containing TCP1 subunit 3 [Source:HGNC Symbol;Acc:HGNC:1616] |
| ENSG00000163705 | HGLibA_16841 | 68 | -0.471931414 | FANCD2OS | FANCD2 opposite strand [Source:HGNC Symbol;Acc:HGNC:28623] |
| ENSG00000163918 | HGLibA_40912 | 68 | -0.471931414 | RFC4 | replication factor C subunit 4 [Source:HGNC Symbol;Acc:HGNC:9972] |
| ENSG00000164078 | HGLibA_30259 | 68 | -0.471931414 | MST1R | macrophage stimulating 1 receptor [Source:HGNC Symbol;Acc:HGNC:7381] |
| ENSG00000164082 | HGLibA_20345 | 68 | -0.471931414 | GRM2 | glutamate metabotropic receptor 2 [Source:HGNC Symbol;Acc:HGNC:4594] |
| ENSG00000164211 | HGLibA_47362 | 68 | -0.471931414 | STARD4 | StAR related lipid transfer domain containing 4 [Source:HGNC Symbol;Acc:HGNC:18058] |
| ENSG00000165389 | HGLibA_46902 | 68 | -0.471931414 | SPTSSA | serine palmitoyltransferase small subunit A [Source:HGNC Symbol;Acc:HGNC:20361] |
| ENSG00000166676 | HGLibA_52356 | 68 | -0.471931414 | TVP23A | trans-golgi network vesicle protein 23 homolog A [Source:HGNC Symbol;Acc:HGNC:20398] |
| ENSG00000166762 | HGLibA_07359 | 68 | -0.471931414 | CATSPER2 | cation channel sperm associated 2 [Source:HGNC Symbol;Acc:HGNC:18810] |
| ENSG00000166794 | HGLibA_37919 | 68 | -0.471931414 | PPIB | peptidylprolyl isomerase B [Source:HGNC Symbol;Acc:HGNC:9255] |
| ENSG00000166839 | HGLibA_01945 | 68 | -0.471931414 | ANKDD1A | ankyrin repeat and death domain containing 1A [Source:HGNC Symbol;Acc:HGNC:28002] |
| ENSG00000168280 | HGLibA_24955 | 68 | -0.471931414 | KIF5C | kinesin family member 5C [Source:HGNC Symbol;Acc:HGNC:6325] |
| ENSG00000168374 | HGLibA_02653 | 68 | -0.471931414 | ARF4 | ADP ribosylation factor 4 [Source:HGNC Symbol;Acc:HGNC:655] |
| ENSG00000168883 | HGLibA_53357 | 68 | -0.471931414 | USP39 | ubiquitin specific peptidase 39 [Source:HGNC Symbol;Acc:HGNC:20071] |
| ENSG00000169302 | HGLibA_47482 | 68 | -0.471931414 | STK32A | serine/threonine kinase 32A [Source:HGNC Symbol;Acc:HGNC:28317] |
| ENSG00000169550 | HGLibA_30527 | 68 | -0.471931414 | MUC15 | cell surface associated [Source:HGNC Symbol;Acc:HGNC:14956]" |
| ENSG00000169891 | HGLibA_40850 | 68 | -0.471931414 | REPS2 | RALBP1 associated Eps domain containing 2 [Source:HGNC Symbol;Acc:HGNC:9963] |
| ENSG00000169955 | HGLibA_56669 | 68 | -0.471931414 | ZNF747 | zinc finger protein 747 [Source:HGNC Symbol;Acc:HGNC:28350] |
| ENSG00000170293 | HGLibA_10229 | 68 | -0.471931414 | CMTM8 | CKLF like MARVEL transmembrane domain containing 8 [Source:HGNC Symbol;Acc:HGNC:19179] |
| ENSG00000170677 | HGLibA_46137 | 68 | -0.471931414 | SOCS6 | suppressor of cytokine signaling 6 [Source:HGNC Symbol;Acc:HGNC:16833] |
| ENSG00000170782 | HGLibA_33466 | 68 | -0.471931414 | OR10A4 | olfactory receptor family 10 subfamily A member 4 [Source:HGNC Symbol;Acc:HGNC:15130] |
| ENSG00000171488 | HGLibA_27434 | 68 | -0.471931414 | LRRC8C | leucine rich repeat containing 8 VRAC subunit C [Source:HGNC Symbol;Acc:HGNC:25075] |
| ENSG00000171552 | HGLibA_04303 | 68 | -0.471931414 | BCL2L1 | BCL2 like 1 [Source:HGNC Symbol;Acc:HGNC:992] |
| ENSG00000171793 | HGLibA_11669 | 68 | -0.471931414 | CTPS1 | CTP synthase 1 [Source:HGNC Symbol;Acc:HGNC:2519] |
| ENSG00000172061 | HGLibA_27259 | 68 | -0.471931414 | LRRC15 | leucine rich repeat containing 15 [Source:HGNC Symbol;Acc:HGNC:20818] |
| ENSG00000172139 | HGLibA_45440 | 68 | -0.471931414 | SLC9C1 | solute carrier family 9 member C1 [Source:HGNC Symbol;Acc:HGNC:31401] |
| ENSG00000172238 | HGLibA_03534 | 68 | -0.471931414 | ATOH1 | atonal bHLH transcription factor 1 [Source:HGNC Symbol;Acc:HGNC:797] |
| ENSG00000172476 | HGLibA_39931 | 68 | -0.471931414 | RAB40A | member RAS oncogene family [Source:HGNC Symbol;Acc:HGNC:18283]" |
| ENSG00000172748 | HGLibA_56351 | 68 | -0.471931414 | ZNF596 | zinc finger protein 596 [Source:HGNC Symbol;Acc:HGNC:27268] |
| ENSG00000172795 | HGLibA_12523 | 68 | -0.471931414 | DCP2 | decapping mRNA 2 [Source:HGNC Symbol;Acc:HGNC:24452] |
| ENSG00000174370 | HGLibA_05143 | 68 | -0.471931414 | C11orf45 | chromosome 11 open reading frame 45 [Source:HGNC Symbol;Acc:HGNC:28584] |
| ENSG00000176555 | HGLibA_34060 | 68 | -0.471931414 | OR4S1 | olfactory receptor family 4 subfamily S member 1 [Source:HGNC Symbol;Acc:HGNC:14705] |
| ENSG00000178741 | HGLibA_10835 | 68 | -0.471931414 | COX5A | cytochrome c oxidase subunit 5A [Source:HGNC Symbol;Acc:HGNC:2267] |
| ENSG00000179178 | HGLibA_49907 | 68 | -0.471931414 | TMEM125 | transmembrane protein 125 [Source:HGNC Symbol;Acc:HGNC:28275] |
| ENSG00000179295 | HGLibA_39434 | 68 | -0.471931414 | PTPN11 | non-receptor type 11 [Source:HGNC Symbol;Acc:HGNC:9644]" |
| ENSG00000179314 | HGLibA_54421 | 68 | -0.471931414 | WSCD1 | WSC domain containing 1 [Source:HGNC Symbol;Acc:HGNC:29060] |
| ENSG00000179627 | HGLibA_54887 | 68 | -0.471931414 | ZBTB42 | zinc finger and BTB domain containing 42 [Source:HGNC Symbol;Acc:HGNC:32550] |
| ENSG00000180316 | HGLibA_37406 | 68 | -0.471931414 | PNPLA1 | patatin like phospholipase domain containing 1 [Source:HGNC Symbol;Acc:HGNC:21246] |
| ENSG00000180433 | HGLibA_34423 | 68 | -0.471931414 | OR6K6 | olfactory receptor family 6 subfamily K member 6 [Source:HGNC Symbol;Acc:HGNC:15033] |
| ENSG00000180475 | HGLibA_33538 | 68 | -0.471931414 | OR10Q1 | olfactory receptor family 10 subfamily Q member 1 [Source:HGNC Symbol;Acc:HGNC:15134] |
| ENSG00000180919 | HGLibA_34227 | 68 | -0.471931414 | OR56B4 | olfactory receptor family 56 subfamily B member 4 [Source:HGNC Symbol;Acc:HGNC:15248] |
| ENSG00000181009 | HGLibA_34202 | 68 | -0.471931414 | OR52N5 | olfactory receptor family 52 subfamily N member 5 [Source:HGNC Symbol;Acc:HGNC:15231] |
| ENSG00000181610 | HGLibA_30069 | 68 | -0.471931414 | MRPS23 | mitochondrial ribosomal protein S23 [Source:HGNC Symbol;Acc:HGNC:14509] |
| ENSG00000181693 | HGLibA_34525 | 68 | -0.471931414 | OR8H1 | olfactory receptor family 8 subfamily H member 1 [Source:HGNC Symbol;Acc:HGNC:14824] |
| ENSG00000182585 | HGLibA_15280 | 68 | -0.471931414 | EPGN | epithelial mitogen [Source:HGNC Symbol;Acc:HGNC:17470] |
| ENSG00000182899 | HGLibA_41949 | 68 | -0.471931414 | RPL35A | ribosomal protein L35a [Source:HGNC Symbol;Acc:HGNC:10345] |
| ENSG00000183463 | HGLibA_53132 | 68 | -0.471931414 | URAD | ureidoimidazoline (2-oxo-4-hydroxy-4-carboxy-5-) decarboxylase [Source:HGNC Symbol;Acc:HGNC:17785] |
| ENSG00000183495 | HGLibA_15234 | 68 | -0.471931414 | EP400 | E1A binding protein p400 [Source:HGNC Symbol;Acc:HGNC:11958] |
| ENSG00000183706 | HGLibA_34050 | 68 | -0.471931414 | OR4N4 | olfactory receptor family 4 subfamily N member 4 [Source:HGNC Symbol;Acc:HGNC:15375] |
| ENSG00000184110 | HGLibA_14768 | 68 | -0.471931414 | EIF3C | eukaryotic translation initiation factor 3 subunit C [Source:HGNC Symbol;Acc:HGNC:3279] |
| ENSG00000184619 | HGLibA_25390 | 68 | -0.471931414 | KRBA2 | KRAB-A domain containing 2 [Source:HGNC Symbol;Acc:HGNC:26989] |
| ENSG00000184956 | HGLibA_30553 | 68 | -0.471931414 | MUC6 | oligomeric mucus/gel-forming [Source:HGNC Symbol;Acc:HGNC:7517]" |
| ENSG00000184979 | HGLibA_53289 | 68 | -0.471931414 | USP18 | ubiquitin specific peptidase 18 [Source:HGNC Symbol;Acc:HGNC:12616] |
| ENSG00000185442 | HGLibA_16286 | 68 | -0.471931414 | FAM174B | family with sequence similarity 174 member B [Source:HGNC Symbol;Acc:HGNC:34339] |
| ENSG00000185818 | HGLibA_31188 | 68 | -0.471931414 | NAT8L | N-acetyltransferase 8 like [Source:HGNC Symbol;Acc:HGNC:26742] |
| ENSG00000186136 | HGLibA_48409 | 68 | -0.471931414 | TAS2R42 | taste 2 receptor member 42 [Source:HGNC Symbol;Acc:HGNC:18888] |
| ENSG00000186654 | HGLibA_38841 | 68 | -0.471931414 | PRR5 | proline rich 5 [Source:HGNC Symbol;Acc:HGNC:31682] |
| ENSG00000187098 | HGLibA_29319 | 68 | -0.471931414 | MITF | melanocyte inducing transcription factor [Source:HGNC Symbol;Acc:HGNC:7105] |
| ENSG00000187479 | HGLibA_05219 | 68 | -0.471931414 | C11orf96 | chromosome 11 open reading frame 96 [Source:HGNC Symbol;Acc:HGNC:38675] |
| ENSG00000187550 | HGLibA_42803 | 68 | -0.471931414 | SBK2 | SH3 domain binding kinase family member 2 [Source:HGNC Symbol;Acc:HGNC:34416] |
| ENSG00000189042 | HGLibA_56264 | 68 | -0.471931414 | ZNF567 | zinc finger protein 567 [Source:HGNC Symbol;Acc:HGNC:28696] |
| ENSG00000196150 | HGLibA_55666 | 68 | -0.471931414 | ZNF250 | zinc finger protein 250 [Source:HGNC Symbol;Acc:HGNC:13044] |
| ENSG00000196396 | HGLibA_39429 | 68 | -0.471931414 | PTPN1 | non-receptor type 1 [Source:HGNC Symbol;Acc:HGNC:9642]" |
| ENSG00000196511 | HGLibA_51139 | 68 | -0.471931414 | TPK1 | thiamin pyrophosphokinase 1 [Source:HGNC Symbol;Acc:HGNC:17358] |
| ENSG00000196570 | HGLibA_36181 | 68 | -0.471931414 | PFN3 | profilin 3 [Source:HGNC Symbol;Acc:HGNC:18627] |
| ENSG00000196739 | HGLibA_10555 | 68 | -0.471931414 | COL27A1 | collagen type XXVII alpha 1 chain [Source:HGNC Symbol;Acc:HGNC:22986] |
| ENSG00000196754 | HGLibA_42550 | 68 | -0.471931414 | S100A2 | S100 calcium binding protein A2 [Source:HGNC Symbol;Acc:HGNC:10492] |
| ENSG00000196878 | HGLibA_25963 | 68 | -0.471931414 | LAMB3 | laminin subunit beta 3 [Source:HGNC Symbol;Acc:HGNC:6490] |
| ENSG00000197056 | HGLibA_55389 | 68 | -0.471931414 | ZMYM1 | zinc finger MYM-type containing 1 [Source:HGNC Symbol;Acc:HGNC:26253] |
| ENSG00000198445 | HGLibA_08218 | 68 | -0.471931414 | CCT8L2 | chaperonin containing TCP1 subunit 8 like 2 [Source:HGNC Symbol;Acc:HGNC:15553] |
| ENSG00000198818 | HGLibA_43752 | 68 | -0.471931414 | SFT2D1 | SFT2 domain containing 1 [Source:HGNC Symbol;Acc:HGNC:21102] |
| ENSG00000203870 | HGLibA_45774 | 68 | -0.471931414 | SMIM9 | small integral membrane protein 9 [Source:HGNC Symbol;Acc:HGNC:41915] |
| ENSG00000203942 | HGLibA_05083 | 68 | -0.471931414 | C10orf62 | chromosome 10 open reading frame 62 [Source:HGNC Symbol;Acc:HGNC:23294] |
| ENSG00000204104 | HGLibA_51249 | 68 | -0.471931414 | TRAF3IP1 | TRAF3 interacting protein 1 [Source:HGNC Symbol;Acc:HGNC:17861] |
| ENSG00000204356 | HGLibA_31681 | 68 | -0.471931414 | NELFE | negative elongation factor complex member E [Source:HGNC Symbol;Acc:HGNC:13974] |
| ENSG00000205045 | HGLibA_45488 | 68 | -0.471931414 | SLFN12L | schlafen family member 12 like [Source:HGNC Symbol;Acc:HGNC:33920] |
| ENSG00000205560 | HGLibA_11021 | 68 | -0.471931414 | CPT1B | carnitine palmitoyltransferase 1B [Source:HGNC Symbol;Acc:HGNC:2329] |
| ENSG00000206579 | HGLibA_54524 | 68 | -0.471931414 | XKR4 | XK related 4 [Source:HGNC Symbol;Acc:HGNC:29394] |
| ENSG00000213022 | HGLibA_25273 | 68 | -0.471931414 | KLK9 | kallikrein related peptidase 9 [Source:HGNC Symbol;Acc:HGNC:6370] |
| ENSG00000215883 | HGLibA_12013 | 68 | -0.471931414 | CYB5RL | cytochrome b5 reductase like [Source:HGNC Symbol;Acc:HGNC:32220] |

| | | | | | |
|---|---|---|---|---|---|
| ENSG00000221968 | HGLibA_15940 | 68 | -0.471931414 | FADS3 | fatty acid desaturase 3 [Source:HGNC Symbol;Acc:HGNC:3576] |
| ENSG00000227802 | HGLibA_13541 | 68 | -0.471931414 | DNAJB3 | DnaJ heat shock protein family (Hsp40) member B3 [Source:HGNC Symbol;Acc:HGNC:32397] |
| ENSG00000228741 | HGLibA_46429 | 68 | -0.471931414 | SPATA13 | spermatogenesis associated 13 [Source:NCBI gene;Acc:221178] |
| ENSG00000230062 | HGLibA_02141 | 68 | -0.471931414 | ANKRD66 | ankyrin repeat domain 66 [Source:HGNC Symbol;Acc:HGNC:44669] |
| ENSG00000230453 | HGLibA_02022 | 68 | -0.471931414 | ANKRD18B | ankyrin repeat domain 18B [Source:HGNC Symbol;Acc:HGNC:23644] |
| ENSG00000241119 | HGLibA_52901 | 68 | -0.471931414 | UGT1A9 | UDP glucuronosyltransferase family 1 member A9 [Source:HGNC Symbol;Acc:HGNC:12541] |
| ENSG00000248920 | HGLibA_53237 | 68 | -0.471931414 | USP17L19 | ubiquitin specific peptidase 17-like family member 19 [Source:HGNC Symbol;Acc:HGNC:44447] |
| ENSG00000255072 | HGLibA_36602 | 68 | -0.471931414 | PIGY | phosphatidylinositol glycan anchor biosynthesis class Y [Source:HGNC Symbol;Acc:HGNC:28213] |
| ENSG00000255374 | HGLibA_48410 | 68 | -0.471931414 | TAS2R43 | taste 2 receptor member 43 [Source:HGNC Symbol;Acc:HGNC:18875] |
| ENSG00000261371 | HGLibA_36018 | 68 | -0.471931414 | PECAM1 | platelet and endothelial cell adhesion molecule 1 [Source:HGNC Symbol;Acc:HGNC:8823] |
| ENSG00000274349 | HGLibA_56472 | 68 | -0.471931414 | ZNF658 | zinc finger protein 658 [Source:HGNC Symbol;Acc:HGNC:25226] |
| ENSG00000278289 | HGLibA_11513 | 68 | -0.471931414 | CT45A6 | cancer/testis antigen family 45 member A6 [Source:HGNC Symbol;Acc:HGNC:33271] |
| FAM122A | HGLibA_16066 | 68 | -0.471931414 | FAM122A | - |
| FAM65C | HGLibA_16674 | 68 | -0.471931414 | FAM65C | - |
| GTPBP6_Y | HGLibA_20620 | 68 | -0.471931414 | GTPBP6_Y | - |
| hsa-mir-105-2 | HGLibA_57182 | 68 | -0.471931414 | hsa-mir-105-2 | - |
| hsa-mir-132 | HGLibA_57757 | 68 | -0.471931414 | hsa-mir-132 | - |
| hsa-mir-154 | HGLibA_57927 | 68 | -0.471931414 | hsa-mir-154 | - |
| hsa-mir-196b | HGLibA_58101 | 68 | -0.471931414 | hsa-mir-196b | - |
| hsa-mir-22 | HGLibA_58290 | 68 | -0.471931414 | hsa-mir-22 | - |
| hsa-mir-2681 | HGLibA_58356 | 68 | -0.471931414 | hsa-mir-2681 | - |
| hsa-mir-3199-1 | HGLibA_58899 | 68 | -0.471931414 | hsa-mir-3199-1 | - |
| hsa-mir-370 | HGLibA_59362 | 68 | -0.471931414 | hsa-mir-370 | - |
| hsa-mir-377 | HGLibA_59420 | 68 | -0.471931414 | hsa-mir-377 | - |
| hsa-mir-4292 | HGLibA_59881 | 68 | -0.471931414 | hsa-mir-4292 | - |
| hsa-mir-4435-2 | HGLibA_60134 | 68 | -0.471931414 | hsa-mir-4435-2 | - |
| hsa-mir-4443 | HGLibA_60173 | 68 | -0.471931414 | hsa-mir-4443 | - |
| hsa-mir-4525 | HGLibA_60561 | 68 | -0.471931414 | hsa-mir-4525 | - |
| hsa-mir-4670 | HGLibA_60798 | 68 | -0.471931414 | hsa-mir-4670 | - |
| hsa-mir-4675 | HGLibA_60817 | 68 | -0.471931414 | hsa-mir-4675 | - |
| hsa-mir-4758 | HGLibA_61131 | 68 | -0.471931414 | hsa-mir-4758 | - |
| hsa-mir-5003 | HGLibA_61420 | 68 | -0.471931414 | hsa-mir-5003 | - |
| hsa-mir-5089 | HGLibA_61502 | 68 | -0.471931414 | hsa-mir-5089 | - |
| hsa-mir-517a | HGLibA_61623 | 68 | -0.471931414 | hsa-mir-517a | - |
| hsa-mir-548aw | HGLibA_61935 | 68 | -0.471931414 | hsa-mir-548aw | - |
| hsa-mir-548h-1 | HGLibA_61995 | 68 | -0.471931414 | hsa-mir-548h-1 | - |
| hsa-mir-639 | HGLibA_62794 | 68 | -0.471931414 | hsa-mir-639 | - |
| hsa-mir-652 | HGLibA_62941 | 68 | -0.471931414 | hsa-mir-652 | - |
| hsa-mir-6736 | HGLibA_63098 | 68 | -0.471931414 | hsa-mir-6736 | - |
| hsa-mir-6822 | HGLibA_63464 | 68 | -0.471931414 | hsa-mir-6822 | - |
| hsa-mir-6876 | HGLibA_63690 | 68 | -0.471931414 | hsa-mir-6876 | - |
| hsa-mir-6883 | HGLibA_63715 | 68 | -0.471931414 | hsa-mir-6883 | - |
| IKBKAP | HGLibA_22916 | 68 | -0.471931414 | IKBKAP | - |
| KDELC2 | HGLibA_24476 | 68 | -0.471931414 | KDELC2 | - |
| LOC389895 | HGLibA_26930 | 68 | -0.471931414 | LOC389895 | - |
| 2-Mar | HGLibA_28324 | 68 | -0.471931414 | 2-Mar | - |
| WDR60 | HGLibA_54140 | 68 | -0.471931414 | WDR60 | - |
| ATP5S | HGLibA_03687 | 67 | -0.481882451 | ATP5S | - |
| C19orf40 | HGLibA_05658 | 67 | -0.481882451 | C19orf40 | - |
| DFNB59 | HGLibA_13032 | 67 | -0.481882451 | DFNB59 | - |
| DPCR1 | HGLibA_13815 | 67 | -0.481882451 | DPCR1 | - |
| ENSG00000001630 | HGLibA_12207 | 67 | -0.481882451 | CYP51A1 | cytochrome P450 family 51 subfamily A member 1 [Source:HGNC Symbol;Acc:HGNC:2649] |
| ENSG00000006062 | HGLibA_28175 | 67 | -0.481882451 | MAP3K14 | mitogen-activated protein kinase kinase kinase 14 [Source:HGNC Symbol;Acc:HGNC:6853] |
| ENSG00000008018 | HGLibA_39093 | 67 | -0.481882451 | PSMB1 | proteasome subunit beta 1 [Source:HGNC Symbol;Acc:HGNC:9537] |
| ENSG00000010072 | HGLibA_46831 | 67 | -0.481882451 | SPRTN | SprT-like N-terminal domain [Source:HGNC Symbol;Acc:HGNC:25356] |
| ENSG00000010539 | HGLibA_55571 | 67 | -0.481882451 | ZNF200 | zinc finger protein 200 [Source:HGNC Symbol;Acc:HGNC:12993] |
| ENSG00000011426 | HGLibA_02167 | 67 | -0.481882451 | ANLN | anillin actin binding protein [Source:HGNC Symbol;Acc:HGNC:14082] |
| ENSG00000013016 | HGLibA_14671 | 67 | -0.481882451 | EHD3 | EH domain containing 3 [Source:HGNC Symbol;Acc:HGNC:3244] |
| ENSG00000037749 | HGLibA_29044 | 67 | -0.481882451 | MFAP3 | microfibril associated protein 3 [Source:HGNC Symbol;Acc:HGNC:7034] |
| ENSG00000049769 | HGLibA_38114 | 67 | -0.481882451 | PPP1R3F | protein phosphatase 1 regulatory subunit 3F [Source:HGNC Symbol;Acc:HGNC:14944] |
| ENSG00000060656 | HGLibA_39540 | 67 | -0.481882451 | PTPRU | receptor type U [Source:HGNC Symbol;Acc:HGNC:9683]" |
| ENSG00000061273 | HGLibA_21022 | 67 | -0.481882451 | HDAC7 | histone deacetylase 7 [Source:HGNC Symbol;Acc:HGNC:14067] |
| ENSG00000063978 | HGLibA_41676 | 67 | -0.481882451 | RNF4 | ring finger protein 4 [Source:HGNC Symbol;Acc:HGNC:10067] |
| ENSG00000066827 | HGLibA_55164 | 67 | -0.481882451 | ZFAT | zinc finger and AT-hook domain containing [Source:HGNC Symbol;Acc:HGNC:19899] |
| ENSG00000070269 | HGLibA_50350 | 67 | -0.481882451 | TMEM260 | transmembrane protein 260 [Source:HGNC Symbol;Acc:HGNC:20185] |
| ENSG00000070476 | HGLibA_57080 | 67 | -0.481882451 | ZXDC | ZXD family zinc finger C [Source:HGNC Symbol;Acc:HGNC:28160] |
| ENSG00000074047 | HGLibA_19250 | 67 | -0.481882451 | GLI2 | GLI family zinc finger 2 [Source:HGNC Symbol;Acc:HGNC:4318] |
| ENSG00000075292 | HGLibA_56438 | 67 | -0.481882451 | ZNF638 | zinc finger protein 638 [Source:HGNC Symbol;Acc:HGNC:17894] |
| ENSG00000076662 | HGLibA_22501 | 67 | -0.481882451 | ICAM3 | intercellular adhesion molecule 3 [Source:HGNC Symbol;Acc:HGNC:5346] |
| ENSG00000076770 | HGLibA_28515 | 67 | -0.481882451 | MBNL3 | muscleblind like splicing regulator 3 [Source:HGNC Symbol;Acc:HGNC:20564] |
| ENSG00000077147 | HGLibA_49727 | 67 | -0.481882451 | TM9SF3 | transmembrane 9 superfamily member 3 [Source:HGNC Symbol;Acc:HGNC:21529] |
| ENSG00000077616 | HGLibA_31009 | 67 | -0.481882451 | NAALAD2 | N-acetylated alpha-linked acidic dipeptidase 2 [Source:HGNC Symbol;Acc:HGNC:14526] |
| ENSG00000078061 | HGLibA_02623 | 67 | -0.481882451 | ARAF | serine/threonine kinase [Source:HGNC Symbol;Acc:HGNC:646]" |
| ENSG00000080561 | HGLibA_29238 | 67 | -0.481882451 | MID2 | midline 2 [Source:HGNC Symbol;Acc:HGNC:7096] |
| ENSG00000084628 | HGLibA_31989 | 67 | -0.481882451 | NKAIN1 | sodium/potassium transporting ATPase interacting 1 [Source:HGNC Symbol;Acc:HGNC:25743] |
| ENSG00000087494 | HGLibA_39377 | 67 | -0.481882451 | PTHLH | parathyroid hormone like hormone [Source:HGNC Symbol;Acc:HGNC:9607] |
| ENSG00000092094 | HGLibA_34680 | 67 | -0.481882451 | OSGEP | O-sialoglycoprotein endopeptidase [Source:HGNC Symbol;Acc:HGNC:18028] |
| ENSG00000092820 | HGLibA_15839 | 67 | -0.481882451 | EZR | ezrin [Source:HGNC Symbol;Acc:HGNC:12691] |
| ENSG00000100097 | HGLibA_26345 | 67 | -0.481882451 | LGALS1 | galectin 1 [Source:HGNC Symbol;Acc:HGNC:6561] |
| ENSG00000100191 | HGLibA_45270 | 67 | -0.481882451 | SLC5A4 | solute carrier family 5 member 4 [Source:HGNC Symbol;Acc:HGNC:11039] |
| ENSG00000100889 | HGLibA_35616 | 67 | -0.481882451 | PCK2 | mitochondrial [Source:HGNC Symbol;Acc:HGNC:8725]" |
| ENSG00000101109 | HGLibA_47511 | 67 | -0.481882451 | STK4 | serine/threonine kinase 4 [Source:HGNC Symbol;Acc:HGNC:11408] |
| ENSG00000101343 | HGLibA_11204 | 67 | -0.481882451 | CRNKL1 | crooked neck pre-mRNA splicing factor 1 [Source:HGNC Symbol;Acc:HGNC:15762] |
| ENSG00000101811 | HGLibA_11486 | 67 | -0.481882451 | CSTF2 | cleavage stimulation factor subunit 2 [Source:HGNC Symbol;Acc:HGNC:2484] |
| ENSG00000102053 | HGLibA_54954 | 67 | -0.481882451 | ZC3H12B | zinc finger CCCH-type containing 12B [Source:HGNC Symbol;Acc:HGNC:17407] |
| ENSG00000102390 | HGLibA_35328 | 67 | -0.481882451 | PBDC1 | polysaccharide biosynthesis domain containing 1 [Source:HGNC Symbol;Acc:HGNC:28790] |
| ENSG00000102393 | HGLibA_19213 | 67 | -0.481882451 | GLA | galactosidase alpha [Source:HGNC Symbol;Acc:HGNC:4296] |
| ENSG00000102871 | HGLibA_51237 | 67 | -0.481882451 | TRADD | TNFRSF1A associated via death domain [Source:HGNC Symbol;Acc:HGNC:12030] |
| ENSG00000103044 | HGLibA_20868 | 67 | -0.481882451 | HAS3 | hyaluronan synthase 3 [Source:HGNC Symbol;Acc:HGNC:4820] |
| ENSG00000103241 | HGLibA_17865 | 67 | -0.481882451 | FOXF1 | forkhead box F1 [Source:HGNC Symbol;Acc:HGNC:3809] |
| ENSG00000104517 | HGLibA_52758 | 67 | -0.481882451 | UBR5 | ubiquitin protein ligase E3 component n-recognin 5 [Source:HGNC Symbol;Acc:HGNC:16806] |
| ENSG00000104853 | HGLibA_10114 | 67 | -0.481882451 | CLPTM1 | transmembrane protein [Source:HGNC Symbol;Acc:HGNC:2087]" |
| ENSG00000105708 | HGLibA_55479 | 67 | -0.481882451 | ZNF14 | zinc finger protein 14 [Source:HGNC Symbol;Acc:HGNC:12924] |
| ENSG00000105717 | HGLibA_35352 | 67 | -0.481882451 | PBX4 | PBX homeobox 4 [Source:HGNC Symbol;Acc:HGNC:13403] |
| ENSG00000105737 | HGLibA_20293 | 67 | -0.481882451 | GRIK5 | glutamate ionotropic receptor kainate type subunit 5 [Source:HGNC Symbol;Acc:HGNC:4583] |
| ENSG00000106034 | HGLibA_10930 | 67 | -0.481882451 | CPED1 | cadherin like and PC-esterase domain containing 1 [Source:HGNC Symbol;Acc:HGNC:26159] |
| ENSG00000107281 | HGLibA_32426 | 67 | -0.481882451 | NPDC1 | differentiation and control 1 [Source:HGNC Symbol;Acc:HGNC:7899]" |
| ENSG00000109205 | HGLibA_33270 | 67 | -0.481882451 | ODAM | ameloblast associated [Source:HGNC Symbol;Acc:HGNC:26043]" |
| ENSG00000110876 | HGLibA_43308 | 67 | -0.481882451 | SELPLG | selectin P ligand [Source:HGNC Symbol;Acc:HGNC:10722] |
| ENSG00000112208 | HGLibA_04078 | 67 | -0.481882451 | BAG2 | BCL2 associated athanogene 2 [Source:HGNC Symbol;Acc:HGNC:938] |
| ENSG00000112306 | HGLibA_42069 | 67 | -0.481882451 | RPS12 | ribosomal protein S12 [Source:HGNC Symbol;Acc:HGNC:10385] |
| ENSG00000112701 | HGLibA_43396 | 67 | -0.481882451 | SENP6 | SUMO specific peptidase 6 [Source:HGNC Symbol;Acc:HGNC:20944] |
| ENSG00000113600 | HGLibA_06660 | 67 | -0.481882451 | C9 | complement C9 [Source:HGNC Symbol;Acc:HGNC:1358] |
| ENSG00000114423 | HGLibA_07396 | 67 | -0.481882451 | CBLB | Cbl proto-oncogene B [Source:HGNC Symbol;Acc:HGNC:1542] |
| ENSG00000115138 | HGLibA_37653 | 67 | -0.481882451 | POMC | proopiomelanocortin [Source:HGNC Symbol;Acc:HGNC:9201] |
| ENSG00000115525 | HGLibA_47248 | 67 | -0.481882451 | ST3GAL5 | 3-sialyltransferase 5 [Source:HGNC Symbol;Acc:HGNC:10872]" |
| ENSG00000116254 | HGLibA_09398 | 67 | -0.481882451 | CHD5 | chromodomain helicase DNA binding protein 5 [Source:HGNC Symbol;Acc:HGNC:16816] |
| ENSG00000117115 | HGLibA_34971 | 67 | -0.481882451 | PADI2 | peptidyl arginine deiminase 2 [Source:HGNC Symbol;Acc:HGNC:18341] |
| ENSG00000117139 | HGLibA_24523 | 67 | -0.481882451 | KDM5B | lysine demethylase 5B [Source:HGNC Symbol;Acc:HGNC:18039] |
| ENSG00000117676 | HGLibA_42157 | 67 | -0.481882451 | RPS6KA1 | ribosomal protein S6 kinase A1 [Source:HGNC Symbol;Acc:HGNC:10430] |
| ENSG00000117682 | HGLibA_13098 | 67 | -0.481882451 | DHDDS | dehydrodolichyl diphosphate synthase subunit [Source:HGNC Symbol;Acc:HGNC:20603] |
| ENSG00000119414 | HGLibA_38218 | 67 | -0.481882451 | PPP6C | protein phosphatase 6 catalytic subunit [Source:HGNC Symbol;Acc:HGNC:9323] |
| ENSG00000119596 | HGLibA_54697 | 67 | -0.481882451 | YLPM1 | YLP motif containing 1 [Source:HGNC Symbol;Acc:HGNC:17798] |
| ENSG00000119725 | HGLibA_55935 | 67 | -0.481882451 | ZNF410 | zinc finger protein 410 [Source:HGNC Symbol;Acc:HGNC:20144] |
| ENSG00000120708 | HGLibA_49219 | 67 | -0.481882451 | TGFBI | transforming growth factor beta induced [Source:HGNC Symbol;Acc:HGNC:11771] |
| ENSG00000120899 | HGLibA_39382 | 67 | -0.481882451 | PTK2B | protein tyrosine kinase 2 beta [Source:HGNC Symbol;Acc:HGNC:9612] |
| ENSG00000120952 | HGLibA_38306 | 67 | -0.481882451 | PRAMEF2 | PRAME family member 2 [Source:HGNC Symbol;Acc:HGNC:28841] |
| ENSG00000120963 | HGLibA_56595 | 67 | -0.481882451 | ZNF706 | zinc finger protein 706 [Source:HGNC Symbol;Acc:HGNC:24992] |
| ENSG00000121350 | HGLibA_39702 | 67 | -0.481882451 | PYROXD1 | pyridine nucleotide-disulphide oxidoreductase domain 1 [Source:HGNC Symbol;Acc:HGNC:26162] |
| ENSG00000121895 | HGLibA_50011 | 67 | -0.481882451 | TMEM156 | transmembrane protein 156 [Source:HGNC Symbol;Acc:HGNC:26260] |
| ENSG00000123552 | HGLibA_53374 | 67 | -0.481882451 | USP45 | ubiquitin specific peptidase 45 [Source:HGNC Symbol;Acc:HGNC:20080] |
| ENSG00000124299 | HGLibA_36050 | 67 | -0.481882451 | PEPD | peptidase D [Source:HGNC Symbol;Acc:HGNC:8840] |
| ENSG00000127445 | HGLibA_36683 | 67 | -0.481882451 | PIN1 | NIMA-interacting 1 [Source:HGNC Symbol;Acc:HGNC:8988]" |
| ENSG00000129518 | HGLibA_14339 | 67 | -0.481882451 | EAPP | E2F associated phosphoprotein [Source:HGNC Symbol;Acc:HGNC:19312] |
| ENSG00000129596 | HGLibA_08855 | 67 | -0.481882451 | CDO1 | cysteine dioxygenase type 1 [Source:HGNC Symbol;Acc:HGNC:1795] |
| ENSG00000129864 | HGLibA_53593 | 67 | -0.481882451 | VCY | variable charge Y-linked [Source:HGNC Symbol;Acc:HGNC:12668] |
| ENSG00000132507 | HGLibA_14856 | 67 | -0.481882451 | EIF5A | eukaryotic translation initiation factor 5A [Source:HGNC Symbol;Acc:HGNC:3300] |
| ENSG00000132514 | HGLibA_09921 | 67 | -0.481882451 | CLEC10A | C-type lectin domain containing 10A [Source:HGNC Symbol;Acc:HGNC:16916] |
| ENSG00000133101 | HGLibA_08051 | 67 | -0.481882451 | CCNA1 | cyclin A1 [Source:HGNC Symbol;Acc:HGNC:1577] |
| ENSG00000133574 | HGLibA_19079 | 67 | -0.481882451 | GIMAP4 | IMAP family member 4 [Source:HGNC Symbol;Acc:HGNC:21872]" |
| ENSG00000134070 | HGLibA_23559 | 67 | -0.481882451 | IRAK2 | interleukin 1 receptor associated kinase 2 [Source:HGNC Symbol;Acc:HGNC:6113] |
| ENSG00000134121 | HGLibA_09464 | 67 | -0.481882451 | CHL1 | cell adhesion molecule L1 like [Source:HGNC Symbol;Acc:HGNC:1939] |
| ENSG00000134910 | HGLibA_47606 | 67 | -0.481882451 | STT3A | catalytic subunit of the oligosaccharyltransferase complex [Source:HGNC Symbol;Acc:HGNC:6172]" |
| ENSG00000135069 | HGLibA_39008 | 67 | -0.481882451 | PSAT1 | phosphoserine aminotransferase 1 [Source:HGNC Symbol;Acc:HGNC:19129] |
| ENSG00000135248 | HGLibA_16700 | 67 | -0.481882451 | FAM71F1 | family with sequence similarity 71 member F1 [Source:HGNC Symbol;Acc:HGNC:30704] |

| | | | | | |
|---|---|---|---|---|---|
| ENSG00000135903 | HGLibA_35301 | 67 | -0.481882451 | PAX3 | paired box 3 [Source:HGNC Symbol;Acc:HGNC:8617] |
| ENSG00000136144 | HGLibA_40677 | 67 | -0.481882451 | RCBTB1 | RCC1 and BTB domain containing protein 1 [Source:HGNC Symbol;Acc:HGNC:18243] |
| ENSG00000136636 | HGLibA_24453 | 67 | -0.481882451 | KCTD3 | potassium channel tetramerization domain containing 3 [Source:HGNC Symbol;Acc:HGNC:21305] |
| ENSG00000136925 | HGLibA_52032 | 67 | -0.481882451 | TSTD2 | thiosulfate sulfurtransferase like domain containing 2 [Source:HGNC Symbol;Acc:HGNC:30087] |
| ENSG00000137491 | HGLibA_45463 | 67 | -0.481882451 | SLCO2B1 | solute carrier organic anion transporter family member 2B1 [Source:HGNC Symbol;Acc:HGNC:10962] |
| ENSG00000138071 | HGLibA_00656 | 67 | -0.481882451 | ACTR2 | ARP2 actin related protein 2 homolog [Source:HGNC Symbol;Acc:HGNC:169] |
| ENSG00000138079 | HGLibA_45130 | 67 | -0.481882451 | SLC3A1 | solute carrier family 3 member 1 [Source:HGNC Symbol;Acc:HGNC:11025] |
| ENSG00000138180 | HGLibA_09143 | 67 | -0.481882451 | CEP55 | centrosomal protein 55 [Source:HGNC Symbol;Acc:HGNC:1161] |
| ENSG00000138483 | HGLibA_07802 | 67 | -0.481882451 | CCDC54 | coiled-coil domain containing 54 [Source:HGNC Symbol;Acc:HGNC:30703] |
| ENSG00000138684 | HGLibA_23083 | 67 | -0.481882451 | IL21 | interleukin 21 [Source:HGNC Symbol;Acc:HGNC:6005] |
| ENSG00000138722 | HGLibA_29545 | 67 | -0.481882451 | MMRN1 | multimerin 1 [Source:HGNC Symbol;Acc:HGNC:7178] |
| ENSG00000139508 | HGLibA_45191 | 67 | -0.481882451 | SLC46A3 | solute carrier family 46 member 3 [Source:HGNC Symbol;Acc:HGNC:27501] |
| ENSG00000143537 | HGLibA_00736 | 67 | -0.481882451 | ADAM15 | ADAM metallopeptidase domain 15 [Source:HGNC Symbol;Acc:HGNC:193] |
| ENSG00000143933 | HGLibA_07025 | 67 | -0.481882451 | CALM2 | calmodulin 2 [Source:HGNC Symbol;Acc:HGNC:1445] |
| ENSG00000143952 | HGLibA_53811 | 67 | -0.481882451 | VPS54 | GARP complex subunit [Source:HGNC Symbol;Acc:HGNC:18652]" |
| ENSG00000144504 | HGLibA_01980 | 67 | -0.481882451 | ANKMY1 | ankyrin repeat and MYND domain containing 1 [Source:HGNC Symbol;Acc:HGNC:20987] |
| ENSG00000145192 | HGLibA_01320 | 67 | -0.481882451 | AHSG | alpha 2-HS glycoprotein [Source:HGNC Symbol;Acc:HGNC:349] |
| ENSG00000145604 | HGLibA_44298 | 67 | -0.481882451 | SKP2 | S-phase kinase associated protein 2 [Source:HGNC Symbol;Acc:HGNC:10901] |
| ENSG00000145692 | HGLibA_04484 | 67 | -0.481882451 | BHMT | betaine--homocysteine S-methyltransferase [Source:HGNC Symbol;Acc:HGNC:1047] |
| ENSG00000145734 | HGLibA_04389 | 67 | -0.481882451 | BDP1 | subunit of RNA polymerase III transcription initiation factor IIIB [Source:HGNC Symbol;Acc:HGNC:13652]" |
| ENSG00000145920 | HGLibA_10935 | 67 | -0.481882451 | CPLX2 | complexin 2 [Source:HGNC Symbol;Acc:HGNC:2310] |
| ENSG00000146425 | HGLibA_14265 | 67 | -0.481882451 | DYNLT1 | dynein light chain Tctex-type 1 [Source:HGNC Symbol;Acc:HGNC:11697] |
| ENSG00000146733 | HGLibA_39227 | 67 | -0.481882451 | PSPH | phosphoserine phosphatase [Source:HGNC Symbol;Acc:HGNC:9577] |
| ENSG00000146834 | HGLibA_28901 | 67 | -0.481882451 | MEPCE | methylphosphate capping enzyme [Source:HGNC Symbol;Acc:HGNC:20247] |
| ENSG00000147251 | HGLibA_13737 | 67 | -0.481882451 | DOCK11 | dedicator of cytokinesis 11 [Source:HGNC Symbol;Acc:HGNC:23483] |
| ENSG00000148737 | HGLibA_48796 | 67 | -0.481882451 | TCF7L2 | transcription factor 7 like 2 [Source:HGNC Symbol;Acc:HGNC:11641] |
| ENSG00000149634 | HGLibA_46460 | 67 | -0.481882451 | SPATA25 | spermatogenesis associated 25 [Source:HGNC Symbol;Acc:HGNC:16158] |
| ENSG00000149634 | HGLibA_46459 | 67 | -0.481882451 | SPATA25 | spermatogenesis associated 25 [Source:HGNC Symbol;Acc:HGNC:16158] |
| ENSG00000149679 | HGLibA_06844 | 67 | -0.481882451 | CABLES2 | Cdk5 and Abl enzyme substrate 2 [Source:HGNC Symbol;Acc:HGNC:16143] |
| ENSG00000150401 | HGLibA_12568 | 67 | -0.481882451 | DCUN1D2 | defective in cullin neddylation 1 domain containing 2 [Source:HGNC Symbol;Acc:HGNC:20328] |
| ENSG00000151079 | HGLibA_24142 | 67 | -0.481882451 | KCNA6 | potassium voltage-gated channel subfamily A member 6 [Source:HGNC Symbol;Acc:HGNC:6225] |
| ENSG00000152578 | HGLibA_20268 | 67 | -0.481882451 | GRIA4 | glutamate ionotropic receptor AMPA type subunit 4 [Source:HGNC Symbol;Acc:HGNC:4574] |
| ENSG00000152932 | HGLibA_39913 | 67 | -0.481882451 | RAB3C | member RAS oncogene family [Source:HGNC Symbol;Acc:HGNC:30269]" |
| ENSG00000153832 | HGLibA_17080 | 67 | -0.481882451 | FBXO36 | F-box protein 36 [Source:HGNC Symbol;Acc:HGNC:27020] |
| ENSG00000154473 | HGLibA_04996 | 67 | -0.481882451 | BUB3 | mitotic checkpoint protein [Source:HGNC Symbol;Acc:HGNC:1151]" |
| ENSG00000154839 | HGLibA_44261 | 67 | -0.481882451 | SKA1 | spindle and kinetochore associated complex subunit 1 [Source:HGNC Symbol;Acc:HGNC:28109] |
| ENSG00000155542 | HGLibA_43663 | 67 | -0.481882451 | SETD9 | SET domain containing 9 [Source:HGNC Symbol;Acc:HGNC:28508] |
| ENSG00000156298 | HGLibA_51947 | 67 | -0.481882451 | TSPAN7 | tetraspanin 7 [Source:HGNC Symbol;Acc:HGNC:11854] |
| ENSG00000156463 | HGLibA_43973 | 67 | -0.481882451 | SH3RF2 | SH3 domain containing ring finger 2 [Source:HGNC Symbol;Acc:HGNC:26299] |
| ENSG00000157259 | HGLibA_18682 | 67 | -0.481882451 | GATAD1 | GATA zinc finger domain containing 1 [Source:HGNC Symbol;Acc:HGNC:29941] |
| ENSG00000157554 | HGLibA_15463 | 67 | -0.481882451 | ERG | ETS transcription factor ERG [Source:HGNC Symbol;Acc:HGNC:3446] |
| ENSG00000159921 | HGLibA_19505 | 67 | -0.481882451 | GNE | glucosamine (UDP-N-acetyl)-2-epimerase/N-acetylmannosamine kinase [Source:HGNC Symbol;Acc:HGNC:23657] |
| ENSG00000161594 | HGLibA_25123 | 67 | -0.481882451 | KLHL10 | kelch like family member 10 [Source:HGNC Symbol;Acc:HGNC:18829] |
| ENSG00000162571 | HGLibA_52190 | 67 | -0.481882451 | TTLL10 | tubulin tyrosine ligase like 10 [Source:HGNC Symbol;Acc:HGNC:26693] |
| ENSG00000163206 | HGLibA_45674 | 67 | -0.481882451 | SMCP | sperm mitochondria associated cysteine rich protein [Source:HGNC Symbol;Acc:HGNC:6962] |
| ENSG00000163249 | HGLibA_08136 | 67 | -0.481882451 | CCNYL1 | cyclin Y like 1 [Source:HGNC Symbol;Acc:HGNC:26868] |
| ENSG00000163346 | HGLibA_35354 | 67 | -0.481882451 | PBXIP1 | PBX homeobox interacting protein 1 [Source:HGNC Symbol;Acc:HGNC:21199] |
| ENSG00000164068 | HGLibA_41490 | 67 | -0.481882451 | RNF123 | ring finger protein 123 [Source:HGNC Symbol;Acc:HGNC:21148] |
| ENSG00000164256 | HGLibA_38404 | 67 | -0.481882451 | PRDM9 | PR/SET domain 9 [Source:HGNC Symbol;Acc:HGNC:13994] |
| ENSG00000164794 | HGLibA_24402 | 67 | -0.481882451 | KCNV1 | potassium voltage-gated channel modifier subfamily V member 1 [Source:HGNC Symbol;Acc:HGNC:18861] |
| ENSG00000164853 | HGLibA_53042 | 67 | -0.481882451 | UNCX | UNC homeobox [Source:HGNC Symbol;Acc:HGNC:33194] |
| ENSG00000165409 | HGLibA_51858 | 67 | -0.481882451 | TSHR | thyroid stimulating hormone receptor [Source:HGNC Symbol;Acc:HGNC:12373] |
| ENSG00000165644 | HGLibA_10690 | 67 | -0.481882451 | COMTD1 | catechol-O-methyltransferase domain containing 1 [Source:HGNC Symbol;Acc:HGNC:26309] |
| ENSG00000166265 | HGLibA_12253 | 67 | -0.481882451 | CYYR1 | cysteine and tyrosine rich 1 [Source:HGNC Symbol;Acc:HGNC:16274] |
| ENSG00000166333 | HGLibA_23220 | 67 | -0.481882451 | ILK | integrin linked kinase [Source:HGNC Symbol;Acc:HGNC:6040] |
| ENSG00000166455 | HGLibA_05451 | 67 | -0.481882451 | C16orf46 | chromosome 16 open reading frame 46 [Source:HGNC Symbol;Acc:HGNC:26525] |
| ENSG00000166507 | HGLibA_31428 | 67 | -0.481882451 | NDST2 | N-deacetylase and N-sulfotransferase 2 [Source:HGNC Symbol;Acc:HGNC:7681] |
| ENSG00000166558 | HGLibA_45081 | 67 | -0.481882451 | SLC38A8 | solute carrier family 38 member 8 [Source:HGNC Symbol;Acc:HGNC:32434] |
| ENSG00000167281 | HGLibA_40461 | 67 | -0.481882451 | RBFOX3 | RNA binding fox-1 homolog 3 [Source:HGNC Symbol;Acc:HGNC:27097] |
| ENSG00000167721 | HGLibA_51995 | 67 | -0.481882451 | TSR1 | ribosome maturation factor [Source:HGNC Symbol;Acc:HGNC:25542]" |
| ENSG00000167825 | HGLibA_34305 | 67 | -0.481882451 | OR5I1 | olfactory receptor family 5 subfamily I member 1 [Source:HGNC Symbol;Acc:HGNC:8347] |
| ENSG00000168077 | HGLibA_42864 | 67 | -0.481882451 | SCARA3 | scavenger receptor class A member 3 [Source:HGNC Symbol;Acc:HGNC:19000] |
| ENSG00000168090 | HGLibA_10725 | 67 | -0.481882451 | COPS6 | COP9 signalosome subunit 6 [Source:HGNC Symbol;Acc:HGNC:21749] |
| ENSG00000168246 | HGLibA_52767 | 67 | -0.481882451 | UBTD2 | ubiquitin domain containing 2 [Source:HGNC Symbol;Acc:HGNC:24463] |
| ENSG00000168589 | HGLibA_14263 | 67 | -0.481882451 | DYNLRB2 | dynein light chain roadblock-type 2 [Source:HGNC Symbol;Acc:HGNC:15467] |
| ENSG00000168612 | HGLibA_57036 | 67 | -0.481882451 | ZSWIM1 | zinc finger SWIM-type containing 1 [Source:HGNC Symbol;Acc:HGNC:16155] |
| ENSG00000168658 | HGLibA_53904 | 67 | -0.481882451 | VWA3B | von Willebrand factor A domain containing 3B [Source:HGNC Symbol;Acc:HGNC:28385] |
| ENSG00000168701 | HGLibA_50202 | 67 | -0.481882451 | TMEM208 | transmembrane protein 208 [Source:HGNC Symbol;Acc:HGNC:25015] |
| ENSG00000169059 | HGLibA_53588 | 67 | -0.481882451 | VCX3A | variable charge X-linked 3A [Source:HGNC Symbol;Acc:HGNC:18159] |
| ENSG00000170260 | HGLibA_55587 | 67 | -0.481882451 | ZNF212 | zinc finger protein 212 [Source:HGNC Symbol;Acc:HGNC:13004] |
| ENSG00000170439 | HGLibA_29020 | 67 | -0.481882451 | METTL7B | methyltransferase like 7B [Source:HGNC Symbol;Acc:HGNC:28276] |
| ENSG00000171150 | HGLibA_46135 | 67 | -0.481882451 | SOCS5 | suppressor of cytokine signaling 5 [Source:HGNC Symbol;Acc:HGNC:16852] |
| ENSG00000171611 | HGLibA_39285 | 67 | -0.481882451 | PTCRA | pre T cell antigen receptor alpha [Source:HGNC Symbol;Acc:HGNC:21290] |
| ENSG00000171847 | HGLibA_16791 | 67 | -0.481882451 | FAM90A1 | family with sequence similarity 90 member A1 [Source:HGNC Symbol;Acc:HGNC:25526] |
| ENSG00000171944 | HGLibA_34142 | 67 | -0.481882451 | OR52A5 | olfactory receptor family 52 subfamily A member 5 [Source:HGNC Symbol;Acc:HGNC:19580] |
| ENSG00000172016 | HGLibA_40799 | 67 | -0.481882451 | REG3A | regenerating family member 3 alpha [Source:HGNC Symbol;Acc:HGNC:8601] |
| ENSG00000172062 | HGLibA_45781 | 67 | -0.481882451 | SMN1 | telomeric [Source:HGNC Symbol;Acc:HGNC:11117]" |
| ENSG00000172345 | HGLibA_47367 | 67 | -0.481882451 | STARD5 | StAR related lipid transfer domain containing 5 [Source:HGNC Symbol;Acc:HGNC:18065] |
| ENSG00000172602 | HGLibA_41446 | 67 | -0.481882451 | RND1 | Rho family GTPase 1 [Source:HGNC Symbol;Acc:HGNC:18314] |
| ENSG00000173597 | HGLibA_47743 | 67 | -0.481882451 | SULT1B1 | sulfotransferase family 1B member 1 [Source:HGNC Symbol;Acc:HGNC:17845] |
| ENSG00000173673 | HGLibA_21183 | 67 | -0.481882451 | HES3 | hes family bHLH transcription factor 3 [Source:HGNC Symbol;Acc:HGNC:26226] |
| ENSG00000173960 | HGLibA_52784 | 67 | -0.481882451 | UBXN2A | UBX domain protein 2A [Source:HGNC Symbol;Acc:HGNC:27265] |
| ENSG00000174007 | HGLibA_09121 | 67 | -0.481882451 | CEP19 | centrosomal protein 19 [Source:HGNC Symbol;Acc:HGNC:28209] |
| ENSG00000174667 | HGLibA_34476 | 67 | -0.481882451 | OR7D4 | olfactory receptor family 7 subfamily D member 4 [Source:HGNC Symbol;Acc:HGNC:8380] |
| ENSG00000176893 | HGLibA_34113 | 67 | -0.481882451 | OR51G2 | olfactory receptor family 51 subfamily G member 2 [Source:HGNC Symbol;Acc:HGNC:15198] |
| ENSG00000179709 | HGLibA_32134 | 67 | -0.481882451 | NLRP8 | NLR family pyrin domain containing 8 [Source:HGNC Symbol;Acc:HGNC:22940] |
| ENSG00000180440 | HGLibA_43617 | 67 | -0.481882451 | SERTM1 | serine rich and transmembrane domain containing 1 [Source:HGNC Symbol;Acc:HGNC:33792] |
| ENSG00000180999 | HGLibA_05735 | 67 | -0.481882451 | C1orf105 | chromosome 1 open reading frame 105 [Source:HGNC Symbol;Acc:HGNC:29591] |
| ENSG00000181915 | HGLibA_01008 | 67 | -0.481882451 | ADO | 2-aminoethanethiol dioxygenase [Source:HGNC Symbol;Acc:HGNC:23506] |
| ENSG00000181938 | HGLibA_19110 | 67 | -0.481882451 | GINS3 | GINS complex subunit 3 [Source:HGNC Symbol;Acc:HGNC:25851] |
| ENSG00000182545 | HGLibA_41392 | 67 | -0.481882451 | RNASE10 | ribonuclease A family member 10 (inactive) [Source:HGNC Symbol;Acc:HGNC:19275] |
| ENSG00000183155 | HGLibA_40024 | 67 | -0.481882451 | RABIF | RAB interacting factor [Source:HGNC Symbol;Acc:HGNC:9797] |
| ENSG00000183747 | HGLibA_00572 | 67 | -0.481882451 | ACSM2A | acyl-CoA synthetase medium chain family member 2A [Source:HGNC Symbol;Acc:HGNC:32017] |
| ENSG00000184661 | HGLibA_08601 | 67 | -0.481882451 | CDCA2 | cell division cycle associated 2 [Source:HGNC Symbol;Acc:HGNC:14623] |
| ENSG00000185436 | HGLibA_22731 | 67 | -0.481882451 | IFNLR1 | interferon lambda receptor 1 [Source:HGNC Symbol;Acc:HGNC:18584] |
| ENSG00000185594 | HGLibA_46532 | 67 | -0.481882451 | SPATA8 | spermatogenesis associated 8 [Source:HGNC Symbol;Acc:HGNC:28676] |
| ENSG00000186193 | HGLibA_42734 | 67 | -0.481882451 | SAPCD2 | suppressor APC domain containing 2 [Source:HGNC Symbol;Acc:HGNC:28055] |
| ENSG00000186777 | HGLibA_56646 | 67 | -0.481882451 | ZNF732 | zinc finger protein 732 [Source:HGNC Symbol;Acc:HGNC:37138] |
| ENSG00000187118 | HGLibA_10186 | 67 | -0.481882451 | CMC1 | C-X9-C motif containing 1 [Source:HGNC Symbol;Acc:HGNC:28783] |
| ENSG00000187187 | HGLibA_56200 | 67 | -0.481882451 | ZNF546 | zinc finger protein 546 [Source:HGNC Symbol;Acc:HGNC:28671] |
| ENSG00000187486 | HGLibA_24253 | 67 | -0.481882451 | KCNJ11 | potassium voltage-gated channel subfamily J member 11 [Source:HGNC Symbol;Acc:HGNC:6257] |
| ENSG00000187627 | HGLibA_41025 | 67 | -0.481882451 | RGPD1 | RANBP2-like and GRIP domain containing 1 [Source:HGNC Symbol;Acc:HGNC:32414] |
| ENSG00000187855 | HGLibA_03276 | 67 | -0.481882451 | ASCL4 | achaete-scute family bHLH transcription factor 4 [Source:HGNC Symbol;Acc:HGNC:24311] |
| ENSG00000187997 | HGLibA_05603 | 67 | -0.481882451 | C17orf99 | chromosome 17 open reading frame 99 [Source:HGNC Symbol;Acc:HGNC:34490] |
| ENSG00000188000 | HGLibA_34472 | 67 | -0.481882451 | OR7D2 | olfactory receptor family 7 subfamily D member 2 [Source:HGNC Symbol;Acc:HGNC:8378] |
| ENSG00000188691 | HGLibA_34220 | 67 | -0.481882451 | OR56A5 | olfactory receptor family 56 subfamily A member 5 [Source:HGNC Symbol;Acc:HGNC:14792] |
| ENSG00000188725 | HGLibA_45733 | 67 | -0.481882451 | SMIM15 | small integral membrane protein 15 [Source:HGNC Symbol;Acc:HGNC:33861] |
| ENSG00000189043 | HGLibA_31459 | 67 | -0.481882451 | NDUFA4 | mitochondrial complex associated [Source:HGNC Symbol;Acc:HGNC:7687]" |
| ENSG00000189108 | HGLibA_23058 | 67 | -0.481882451 | IL1RAPL2 | interleukin 1 receptor accessory protein like 2 [Source:HGNC Symbol;Acc:HGNC:5997] |
| ENSG00000196337 | HGLibA_09310 | 67 | -0.481882451 | CGB7 | chorionic gonadotropin subunit beta 7 [Source:HGNC Symbol;Acc:HGNC:16451] |
| ENSG00000196476 | HGLibA_06032 | 67 | -0.481882451 | C20orf96 | chromosome 20 open reading frame 96 [Source:HGNC Symbol;Acc:HGNC:16227] |
| ENSG00000196839 | HGLibA_00716 | 67 | -0.481882451 | ADA | adenosine deaminase [Source:HGNC Symbol;Acc:HGNC:186] |
| ENSG00000196998 | HGLibA_54098 | 67 | -0.481882451 | WDR45 | WD repeat domain 45 [Source:HGNC Symbol;Acc:HGNC:28912] |
| ENSG00000197415 | HGLibA_53623 | 67 | -0.481882451 | VEPH1 | ventricular zone expressed PH domain containing 1 [Source:HGNC Symbol;Acc:HGNC:25735] |
| ENSG00000197586 | HGLibA_15212 | 67 | -0.481882451 | ENTPD6 | ectonucleoside triphosphate diphosphohydrolase 6 (putative) [Source:HGNC Symbol;Acc:HGNC:3368] |
| ENSG00000197891 | HGLibA_44544 | 67 | -0.481882451 | SLC22A12 | solute carrier family 22 member 12 [Source:HGNC Symbol;Acc:HGNC:17989] |
| ENSG00000198081 | HGLibA_54824 | 67 | -0.481882451 | ZBTB14 | zinc finger and BTB domain containing 14 [Source:HGNC Symbol;Acc:HGNC:12860] |
| ENSG00000198642 | HGLibA_25228 | 67 | -0.481882451 | KLHL9 | kelch like family member 9 [Source:HGNC Symbol;Acc:HGNC:18732] |
| ENSG00000198690 | HGLibA_16827 | 67 | -0.481882451 | FAN1 | FANCD2 and FANCI associated nuclease 1 [Source:HGNC Symbol;Acc:HGNC:29170] |
| ENSG00000198862 | HGLibA_27614 | 67 | -0.481882451 | LTN1 | listerin E3 ubiquitin protein ligase 1 [Source:HGNC Symbol;Acc:HGNC:13082] |
| ENSG00000198863 | HGLibA_42446 | 67 | -0.481882451 | RUNDC1 | RUN domain containing 1 [Source:HGNC Symbol;Acc:HGNC:25418] |
| ENSG00000204220 | HGLibA_36149 | 67 | -0.481882451 | PFDN6 | prefoldin subunit 6 [Source:HGNC Symbol;Acc:HGNC:4926] |
| ENSG00000205442 | HGLibA_23899 | 67 | -0.481882451 | IZUMO3 | IZUMO family member 3 [Source:HGNC Symbol;Acc:HGNC:31421] |
| ENSG00000205442 | HGLibA_23898 | 67 | -0.481882451 | IZUMO3 | IZUMO family member 3 [Source:HGNC Symbol;Acc:HGNC:31421] |
| ENSG00000205710 | HGLibA_05527 | 67 | -0.481882451 | C17orf107 | chromosome 17 open reading frame 107 [Source:HGNC Symbol;Acc:HGNC:37238] |
| ENSG00000206052 | HGLibA_13781 | 67 | -0.481882451 | DOK6 | docking protein 6 [Source:HGNC Symbol;Acc:HGNC:28301] |
| ENSG00000206531 | HGLibA_08289 | 67 | -0.481882451 | CD200R1L | CD200 receptor 1 like [Source:HGNC Symbol;Acc:HGNC:24665] |
| ENSG00000206538 | HGLibA_53642 | 67 | -0.481882451 | VGLL3 | vestigial like family member 3 [Source:HGNC Symbol;Acc:HGNC:24327] |
| ENSG00000213901 | HGLibA_44613 | 67 | -0.481882451 | SLC23A3 | solute carrier family 23 member 3 [Source:HGNC Symbol;Acc:HGNC:20601] |
| ENSG00000214013 | HGLibA_18608 | 67 | -0.481882451 | GANC | neutral C [Source:HGNC Symbol;Acc:HGNC:4139]" |
| ENSG00000214042 | HGLibA_22688 | 67 | -0.481882451 | IFNA7 | interferon alpha 7 [Source:HGNC Symbol;Acc:HGNC:5428] |
| ENSG00000214290 | HGLibA_10630 | 67 | -0.481882451 | COLCA2 | colorectal cancer associated 2 [Source:HGNC Symbol;Acc:HGNC:26978] |
| ENSG00000221829 | HGLibA_16851 | 67 | -0.481882451 | FANCG | FA complementation group G [Source:HGNC Symbol;Acc:HGNC:3588] |
| ENSG00000221900 | HGLibA_37648 | 67 | -0.481882451 | POM121L12 | POM121 transmembrane nucleoporin like 12 [Source:HGNC Symbol;Acc:HGNC:25369] |
| ENSG00000224712 | HGLibA_32468 | 67 | -0.481882451 | NPIPA3 | nuclear pore complex interacting protein family member A3 [Source:HGNC Symbol;Acc:HGNC:41978] |

| | | | | | |
|---|---|---|---|---|---|
| ENSG00000226479 | HGLibA_50123 | 67 | -0.481882451 | TMEM185B | transmembrane protein 185B [Source:HGNC Symbol;Acc:HGNC:18896] |
| ENSG00000229579 | HGLibA_53261 | 67 | -0.481882451 | USP17L26 | ubiquitin specific peptidase 17-like family member 26 [Source:HGNC Symbol;Acc:HGNC:44454] |
| ENSG00000229665 | HGLibA_38806 | 67 | -0.481882451 | PRR20C | proline rich 20C [Source:HGNC Symbol;Acc:HGNC:37221] |
| ENSG00000233816 | HGLibA_22661 | 67 | -0.481882451 | IFNA13 | interferon alpha 13 [Source:HGNC Symbol;Acc:HGNC:5419] |
| ENSG00000242419 | HGLibA_35573 | 67 | -0.481882451 | PCDHGC4 | 4 [Source:HGNC Symbol;Acc:HGNC:8717]" |
| ENSG00000244607 | HGLibA_07589 | 67 | -0.481882451 | CCDC13 | coiled-coil domain containing 13 [Source:HGNC Symbol;Acc:HGNC:26358] |
| ENSG00000255298 | HGLibA_34522 | 67 | -0.481882451 | OR8G5 | olfactory receptor family 8 subfamily G member 5 [Source:HGNC Symbol;Acc:HGNC:19622] |
| ENSG00000275410 | HGLibA_21776 | 67 | -0.481882451 | HNF1B | HNF1 homeobox B [Source:HGNC Symbol;Acc:HGNC:11630] |
| ENSG00000278259 | HGLibA_30816 | 67 | -0.481882451 | MYO19 | myosin XIX [Source:HGNC Symbol;Acc:HGNC:26234] |
| ENSG00000279169 | HGLibA_38290 | 67 | -0.481882451 | PRAMEF13 | PRAME family member 13 [Source:HGNC Symbol;Acc:HGNC:13262] |
| FAM109B | HGLibA_16010 | 67 | -0.481882451 | FAM109B | - |
| FAM175B | HGLibA_16290 | 67 | -0.481882451 | FAM175B | - |
| H2AFX | HGLibA_20759 | 67 | -0.481882451 | H2AFX | - |
| hsa-mir-1269b | HGLibA_57509 | 67 | -0.481882451 | hsa-mir-1269b | - |
| hsa-mir-181a-2 | HGLibA_57963 | 67 | -0.481882451 | hsa-mir-181a-2 | - |
| hsa-mir-217 | HGLibA_58263 | 67 | -0.481882451 | hsa-mir-217 | - |
| hsa-mir-219a-2 | HGLibA_58282 | 67 | -0.481882451 | hsa-mir-219a-2 | - |
| hsa-mir-3122 | HGLibA_58541 | 67 | -0.481882451 | hsa-mir-3122 | - |
| hsa-mir-3199-2 | HGLibA_58903 | 67 | -0.481882451 | hsa-mir-3199-2 | - |
| hsa-mir-3620 | HGLibA_59113 | 67 | -0.481882451 | hsa-mir-3620 | - |
| hsa-mir-3689e | HGLibA_59335 | 67 | -0.481882451 | hsa-mir-3689e | - |
| hsa-mir-3907 | HGLibA_59491 | 67 | -0.481882451 | hsa-mir-3907 | - |
| hsa-mir-3913-2 | HGLibA_59525 | 67 | -0.481882451 | hsa-mir-3913-2 | - |
| hsa-mir-4269 | HGLibA_59783 | 67 | -0.481882451 | hsa-mir-4269 | - |
| hsa-mir-4322 | HGLibA_60011 | 67 | -0.481882451 | hsa-mir-4322 | - |
| hsa-mir-4447 | HGLibA_60194 | 67 | -0.481882451 | hsa-mir-4447 | - |
| hsa-mir-4498 | HGLibA_60407 | 67 | -0.481882451 | hsa-mir-4498 | - |
| hsa-mir-4536-2 | HGLibA_60607 | 67 | -0.481882451 | hsa-mir-4536-2 | - |
| hsa-mir-4635 | HGLibA_60646 | 67 | -0.481882451 | hsa-mir-4635 | - |
| hsa-mir-4672 | HGLibA_60805 | 67 | -0.481882451 | hsa-mir-4672 | - |
| hsa-mir-4803 | HGLibA_61315 | 67 | -0.481882451 | hsa-mir-4803 | - |
| hsa-mir-514a-3 | HGLibA_61595 | 67 | -0.481882451 | hsa-mir-514a-3 | - |
| hsa-mir-521-1 | HGLibA_61765 | 67 | -0.481882451 | hsa-mir-521-1 | - |
| hsa-mir-548ad | HGLibA_61866 | 67 | -0.481882451 | hsa-mir-548ad | - |
| hsa-mir-559 | HGLibA_62198 | 67 | -0.481882451 | hsa-mir-559 | - |
| hsa-mir-6088 | HGLibA_62613 | 67 | -0.481882451 | hsa-mir-6088 | - |
| hsa-mir-6735 | HGLibA_63093 | 67 | -0.481882451 | hsa-mir-6735 | - |
| hsa-mir-6766 | HGLibA_63225 | 67 | -0.481882451 | hsa-mir-6766 | - |
| hsa-mir-6832 | HGLibA_63506 | 67 | -0.481882451 | hsa-mir-6832 | - |
| hsa-mir-6839 | HGLibA_63530 | 67 | -0.481882451 | hsa-mir-6839 | - |
| hsa-mir-6858 | HGLibA_63605 | 67 | -0.481882451 | hsa-mir-6858 | - |
| hsa-mir-6875 | HGLibA_63685 | 67 | -0.481882451 | hsa-mir-6875 | - |
| hsa-mir-7-1 | HGLibA_63770 | 67 | -0.481882451 | hsa-mir-7-1 | - |
| hsa-mir-8052 | HGLibA_64048 | 67 | -0.481882451 | hsa-mir-8052 | - |
| hsa-mir-8054 | HGLibA_64055 | 67 | -0.481882451 | hsa-mir-8054 | - |
| hsa-mir-8088 | HGLibA_64194 | 67 | -0.481882451 | hsa-mir-8088 | - |
| hsa-mir-874 | HGLibA_64205 | 67 | -0.481882451 | hsa-mir-874 | - |
| KIAA0226 | HGLibA_24605 | 67 | -0.481882451 | KIAA0226 | - |
| KIAA1468 | HGLibA_24769 | 67 | -0.481882451 | KIAA1468 | - |
| LPHN2 | HGLibA_27088 | 67 | -0.481882451 | LPHN2 | - |
| MEF2BNB-MEF2B | HGLibA_28835 | 67 | -0.481882451 | MEF2BNB-MEF2B | - |
| MICALCL | HGLibA_29211 | 67 | -0.481882451 | MICALCL | - |
| RRNAD1 | HGLibA_42262 | 67 | -0.481882451 | RRNAD1 | - |
| SLC25A6_X | HGLibA_44773 | 67 | -0.481882451 | SLC25A6_X | - |
| SPANXE | HGLibA_46396 | 67 | -0.481882451 | SPANXE | - |
| TMEM2 | HGLibA_50167 | 67 | -0.481882451 | TMEM2 | - |
| 6-Mar | HGLibA_28342 | 66 | -0.491833489 | 6-Mar | - |
| BAI2 | HGLibA_04115 | 66 | -0.491833489 | BAI2 | - |
| C10orf107 | HGLibA_05026 | 66 | -0.491833489 | C10orf107 | - |
| C10orf118 | HGLibA_05041 | 66 | -0.491833489 | C10orf118 | - |
| C11orf85 | HGLibA_05200 | 66 | -0.491833489 | C11orf85 | - |
| C14orf169 | HGLibA_05338 | 66 | -0.491833489 | C14orf169 | - |
| C19orf57 | HGLibA_05685 | 66 | -0.491833489 | C19orf57 | - |
| C4orf26 | HGLibA_06307 | 66 | -0.491833489 | C4orf26 | - |
| C7orf71 | HGLibA_06585 | 66 | -0.491833489 | C7orf71 | - |
| DAK | HGLibA_12300 | 66 | -0.491833489 | DAK | - |
| DEFB130 | HGLibA_12894 | 66 | -0.491833489 | DEFB130 | - |
| ENSG00000004975 | HGLibA_14212 | 66 | -0.491833489 | DVL2 | dishevelled segment polarity protein 2 [Source:HGNC Symbol;Acc:HGNC:3086] |
| ENSG00000010818 | HGLibA_21571 | 66 | -0.491833489 | HIVEP2 | human immunodeficiency virus type I enhancer binding protein 2 [Source:HGNC Symbol;Acc:HGNC:4921] |
| ENSG00000012211 | HGLibA_38477 | 66 | -0.491833489 | PRICKLE3 | prickle planar cell polarity protein 3 [Source:HGNC Symbol;Acc:HGNC:6645] |
| ENSG00000014824 | HGLibA_44923 | 66 | -0.491833489 | SLC30A9 | solute carrier family 30 member 9 [Source:HGNC Symbol;Acc:HGNC:1329] |
| ENSG00000021645 | HGLibA_32740 | 66 | -0.491833489 | NRXN3 | neurexin 3 [Source:HGNC Symbol;Acc:HGNC:8010] |
| ENSG00000024862 | HGLibA_07745 | 66 | -0.491833489 | CCDC28A | coiled-coil domain containing 28A [Source:HGNC Symbol;Acc:HGNC:21098] |
| ENSG00000043093 | HGLibA_12565 | 66 | -0.491833489 | DCUN1D1 | defective in cullin neddylation 1 domain containing 1 [Source:HGNC Symbol;Acc:HGNC:18184] |
| ENSG00000064652 | HGLibA_46069 | 66 | -0.491833489 | SNX24 | sorting nexin 24 [Source:HGNC Symbol;Acc:HGNC:21533] |
| ENSG00000065615 | HGLibA_12009 | 66 | -0.491833489 | CYB5R4 | cytochrome b5 reductase 4 [Source:HGNC Symbol;Acc:HGNC:20147] |
| ENSG00000070808 | HGLibA_07066 | 66 | -0.491833489 | CAMK2A | calcium/calmodulin dependent protein kinase II alpha [Source:HGNC Symbol;Acc:HGNC:1460] |
| ENSG00000070985 | HGLibA_51764 | 66 | -0.491833489 | TRPM5 | transient receptor potential cation channel subfamily M member 5 [Source:HGNC Symbol;Acc:HGNC:14323] |
| ENSG00000075539 | HGLibA_18056 | 66 | -0.491833489 | FRYL | FRY like transcription coactivator [Source:HGNC Symbol;Acc:HGNC:29127] |
| ENSG00000075856 | HGLibA_42757 | 66 | -0.491833489 | SART3 | U4/U6 recycling protein [Source:HGNC Symbol;Acc:HGNC:16860]" |
| ENSG00000076706 | HGLibA_28556 | 66 | -0.491833489 | MCAM | melanoma cell adhesion molecule [Source:HGNC Symbol;Acc:HGNC:6934] |
| ENSG00000077420 | HGLibA_02381 | 66 | -0.491833489 | APBB1IP | amyloid beta precursor protein binding family B member 1 interacting protein [Source:HGNC Symbol;Acc:HGNC:17379] |
| ENSG00000077713 | HGLibA_44744 | 66 | -0.491833489 | SLC25A43 | solute carrier family 25 member 43 [Source:HGNC Symbol;Acc:HGNC:30557] |
| ENSG00000079785 | HGLibA_12649 | 66 | -0.491833489 | DDX1 | DEAD-box helicase 1 [Source:HGNC Symbol;Acc:HGNC:2734] |
| ENSG00000083845 | HGLibA_42151 | 66 | -0.491833489 | RPS5 | ribosomal protein S5 [Source:HGNC Symbol;Acc:HGNC:10426] |
| ENSG00000097046 | HGLibA_08597 | 66 | -0.491833489 | CDC7 | cell division cycle 7 [Source:HGNC Symbol;Acc:HGNC:1745] |
| ENSG00000100429 | HGLibA_21000 | 66 | -0.491833489 | HDAC10 | histone deacetylase 10 [Source:HGNC Symbol;Acc:HGNC:18128] |
| ENSG00000100433 | HGLibA_24298 | 66 | -0.491833489 | KCNK10 | potassium two pore domain channel subfamily K member 10 [Source:HGNC Symbol;Acc:HGNC:6273] |
| ENSG00000100526 | HGLibA_08849 | 66 | -0.491833489 | CDKN3 | cyclin dependent kinase inhibitor 3 [Source:HGNC Symbol;Acc:HGNC:1791] |
| ENSG00000100934 | HGLibA_43220 | 66 | -0.491833489 | SEC23A | coat complex II component [Source:HGNC Symbol;Acc:HGNC:10701]" |
| ENSG00000100979 | HGLibA_37247 | 66 | -0.491833489 | PLTP | phospholipid transfer protein [Source:HGNC Symbol;Acc:HGNC:9093] |
| ENSG00000101222 | HGLibA_46610 | 66 | -0.491833489 | SPEF1 | sperm flagellar 1 [Source:HGNC Symbol;Acc:HGNC:15874] |
| ENSG00000102524 | HGLibA_50811 | 66 | -0.491833489 | TNFSF13B | TNF superfamily member 13b [Source:HGNC Symbol;Acc:HGNC:11929] |
| ENSG00000102981 | HGLibA_35183 | 66 | -0.491833489 | PARD6A | par-6 family cell polarity regulator alpha [Source:HGNC Symbol;Acc:HGNC:15943] |
| ENSG00000103319 | HGLibA_14510 | 66 | -0.491833489 | EEF2K | eukaryotic elongation factor 2 kinase [Source:HGNC Symbol;Acc:HGNC:24615] |
| ENSG00000104112 | HGLibA_42902 | 66 | -0.491833489 | SCG3 | secretogranin III [Source:HGNC Symbol;Acc:HGNC:13707] |
| ENSG00000104221 | HGLibA_04801 | 66 | -0.491833489 | BRF2 | RNA polymerase III transcription initiation factor subunit [Source:HGNC Symbol;Acc:HGNC:17298]" |
| ENSG00000105146 | HGLibA_03883 | 66 | -0.491833489 | AURKC | aurora kinase C [Source:HGNC Symbol;Acc:HGNC:11391] |
| ENSG00000105246 | HGLibA_14361 | 66 | -0.491833489 | EBI3 | Epstein-Barr virus induced 3 [Source:HGNC Symbol;Acc:HGNC:3129] |
| ENSG00000106392 | HGLibA_05724 | 66 | -0.491833489 | C1GALT1 | glycoprotein-N-acetylgalactosamine 3-beta-galactosyltransferase 1 [Source:HGNC Symbol;Acc:HGNC:24337]" |
| ENSG00000107104 | HGLibA_24025 | 66 | -0.491833489 | KANK1 | KN motif and ankyrin repeat domains 1 [Source:HGNC Symbol;Acc:HGNC:19309] |
| ENSG00000108468 | HGLibA_07446 | 66 | -0.491833489 | CBX1 | chromobox 1 [Source:HGNC Symbol;Acc:HGNC:1551] |
| ENSG00000110203 | HGLibA_17789 | 66 | -0.491833489 | FOLR3 | folate receptor 3 [Source:HGNC Symbol;Acc:HGNC:3795] |
| ENSG00000110315 | HGLibA_41522 | 66 | -0.491833489 | RNF141 | ring finger protein 141 [Source:HGNC Symbol;Acc:HGNC:21159] |
| ENSG00000113209 | HGLibA_35502 | 66 | -0.491833489 | PCDHB5 | protocadherin beta 5 [Source:HGNC Symbol;Acc:HGNC:8690] |
| ENSG00000114439 | HGLibA_04231 | 66 | -0.491833489 | BBX | HMG-box containing [Source:HGNC Symbol;Acc:HGNC:14422]" |
| ENSG00000115137 | HGLibA_13609 | 66 | -0.491833489 | DNAJC27 | DnaJ heat shock protein family (Hsp40) member C27 [Source:HGNC Symbol;Acc:HGNC:30290] |
| ENSG00000115596 | HGLibA_54378 | 66 | -0.491833489 | WNT6 | Wnt family member 6 [Source:HGNC Symbol;Acc:HGNC:12785] |
| ENSG00000116641 | HGLibA_13756 | 66 | -0.491833489 | DOCK7 | dedicator of cytokinesis 7 [Source:HGNC Symbol;Acc:HGNC:19190] |
| ENSG00000116690 | HGLibA_38459 | 66 | -0.491833489 | PRG4 | proteoglycan 4 [Source:HGNC Symbol;Acc:HGNC:9364] |
| ENSG00000116984 | HGLibA_30462 | 66 | -0.491833489 | MTR | 5-methyltetrahydrofolate-homocysteine methyltransferase [Source:HGNC Symbol;Acc:HGNC:7468] |
| ENSG00000117016 | HGLibA_41282 | 66 | -0.491833489 | RIMS3 | regulating synaptic membrane exocytosis 3 [Source:HGNC Symbol;Acc:HGNC:21292] |
| ENSG00000117713 | HGLibA_02883 | 66 | -0.491833489 | ARID1A | AT-rich interaction domain 1A [Source:HGNC Symbol;Acc:HGNC:11110] |
| ENSG00000119669 | HGLibA_23584 | 66 | -0.491833489 | IRF2BPL | interferon regulatory factor 2 binding protein like [Source:HGNC Symbol;Acc:HGNC:14282] |
| ENSG00000120645 | HGLibA_23546 | 66 | -0.491833489 | IQSEC3 | IQ motif and Sec7 domain 3 [Source:HGNC Symbol;Acc:HGNC:29193] |
| ENSG00000121542 | HGLibA_43214 | 66 | -0.491833489 | SEC22A | vesicle trafficking protein [Source:HGNC Symbol;Acc:HGNC:20260]" |
| ENSG00000122299 | HGLibA_54981 | 66 | -0.491833489 | ZC3H7A | zinc finger CCCH-type containing 7A [Source:HGNC Symbol;Acc:HGNC:30959] |
| ENSG00000122591 | HGLibA_16081 | 66 | -0.491833489 | FAM126A | family with sequence similarity 126 member A [Source:HGNC Symbol;Acc:HGNC:24587] |
| ENSG00000123091 | HGLibA_41463 | 66 | -0.491833489 | RNF11 | ring finger protein 11 [Source:HGNC Symbol;Acc:HGNC:10056] |
| ENSG00000123171 | HGLibA_07851 | 66 | -0.491833489 | CCDC70 | coiled-coil domain containing 70 [Source:HGNC Symbol;Acc:HGNC:25303] |
| ENSG00000124104 | HGLibA_46062 | 66 | -0.491833489 | SNX21 | sorting nexin family member 21 [Source:HGNC Symbol;Acc:HGNC:16154] |
| ENSG00000124134 | HGLibA_24383 | 66 | -0.491833489 | KCNS1 | potassium voltage-gated channel modifier subfamily S member 1 [Source:HGNC Symbol;Acc:HGNC:6300] |
| ENSG00000124134 | HGLibA_24385 | 66 | -0.491833489 | KCNS1 | potassium voltage-gated channel modifier subfamily S member 1 [Source:HGNC Symbol;Acc:HGNC:6300] |
| ENSG00000125814 | HGLibA_31128 | 66 | -0.491833489 | NAPB | NSF attachment protein beta [Source:HGNC Symbol;Acc:HGNC:15751] |
| ENSG00000125823 | HGLibA_11494 | 66 | -0.491833489 | CSTL1 | cystatin like 1 [Source:HGNC Symbol;Acc:HGNC:15958] |
| ENSG00000125879 | HGLibA_34744 | 66 | -0.491833489 | OTOR | otoraplin [Source:HGNC Symbol;Acc:HGNC:8517] |
| ENSG00000125900 | HGLibA_44211 | 66 | -0.491833489 | SIRPD | signal regulatory protein delta [Source:HGNC Symbol;Acc:HGNC:16248] |
| ENSG00000126226 | HGLibA_35607 | 66 | -0.491833489 | PCID2 | PCI domain containing 2 [Source:HGNC Symbol;Acc:HGNC:25653] |
| ENSG00000126945 | HGLibA_21826 | 66 | -0.491833489 | HNRNPH2 | heterogeneous nuclear ribonucleoprotein H2 [Source:HGNC Symbol;Acc:HGNC:5042] |
| ENSG00000127527 | HGLibA_15370 | 66 | -0.491833489 | EPS15L1 | epidermal growth factor receptor pathway substrate 15 like 1 [Source:HGNC Symbol;Acc:HGNC:24634] |
| ENSG00000128739 | HGLibA_45993 | 66 | -0.491833489 | SNRPN | small nuclear ribonucleoprotein polypeptide N [Source:HGNC Symbol;Acc:HGNC:11164] |
| ENSG00000128815 | HGLibA_54014 | 66 | -0.491833489 | WDFY4 | WDFY family member 4 [Source:HGNC Symbol;Acc:HGNC:29323] |
| ENSG00000129862 | HGLibA_53595 | 66 | -0.491833489 | VCY1B | variable charge Y-linked 1B [Source:HGNC Symbol;Acc:HGNC:31751] |
| ENSG00000129873 | HGLibA_08915 | 66 | -0.491833489 | CDY2B | chromodomain Y-linked 2B [Source:HGNC Symbol;Acc:HGNC:23921] |
| ENSG00000130255 | HGLibA_41952 | 66 | -0.491833489 | RPL36 | ribosomal protein L36 [Source:HGNC Symbol;Acc:HGNC:13631] |

| | | | | | |
|---|---|---|---|---|---|
| ENSG00000130311 | HGLibA_12585 | 66 | -0.491833489 | DDA1 | DET1 and DDB1 associated 1 [Source:HGNC Symbol;Acc:HGNC:28360] |
| ENSG00000130649 | HGLibA_12131 | 66 | -0.491833489 | CYP2E1 | cytochrome P450 family 2 subfamily E member 1 [Source:HGNC Symbol;Acc:HGNC:2631] |
| ENSG00000130956 | HGLibA_20797 | 66 | -0.491833489 | HABP4 | hyaluronan binding protein 4 [Source:HGNC Symbol;Acc:HGNC:17062] |
| ENSG00000131373 | HGLibA_20802 | 66 | -0.491833489 | HACL1 | 2-hydroxyacyl-CoA lyase 1 [Source:HGNC Symbol;Acc:HGNC:17856] |
| ENSG00000131931 | HGLibA_49287 | 66 | -0.491833489 | THAP1 | THAP domain containing 1 [Source:HGNC Symbol;Acc:HGNC:20856] |
| ENSG00000132801 | HGLibA_57044 | 66 | -0.491833489 | ZSWIM3 | zinc finger SWIM-type containing 3 [Source:HGNC Symbol;Acc:HGNC:16157] |
| ENSG00000133997 | HGLibA_28810 | 66 | -0.491833489 | MED6 | mediator complex subunit 6 [Source:HGNC Symbol;Acc:HGNC:19970] |
| ENSG00000134532 | HGLibA_46268 | 66 | -0.491833489 | SOX5 | SRY-box 5 [Source:HGNC Symbol;Acc:HGNC:11201] |
| ENSG00000135218 | HGLibA_08361 | 66 | -0.491833489 | CD36 | CD36 molecule [Source:HGNC Symbol;Acc:HGNC:1663] |
| ENSG00000135222 | HGLibA_11374 | 66 | -0.491833489 | CSN2 | casein beta [Source:HGNC Symbol;Acc:HGNC:2447] |
| ENSG00000135387 | HGLibA_07189 | 66 | -0.491833489 | CAPRIN1 | cell cycle associated protein 1 [Source:HGNC Symbol;Acc:HGNC:6743] |
| ENSG00000135469 | HGLibA_10743 | 66 | -0.491833489 | COQ10A | coenzyme Q10A [Source:HGNC Symbol;Acc:HGNC:26515] |
| ENSG00000135643 | HGLibA_24351 | 66 | -0.491833489 | KCNMB4 | potassium calcium-activated channel subfamily M regulatory beta subunit 4 [Source:HGNC Symbol;Acc:HGNC:6289] |
| ENSG00000135966 | HGLibA_49235 | 66 | -0.491833489 | TGFBRAP1 | transforming growth factor beta receptor associated protein 1 [Source:HGNC Symbol;Acc:HGNC:16836] |
| ENSG00000135973 | HGLibA_20043 | 66 | -0.491833489 | GPR45 | G protein-coupled receptor 45 [Source:HGNC Symbol;Acc:HGNC:4503] |
| ENSG00000136305 | HGLibA_09701 | 66 | -0.491833489 | CIDEB | cell death-inducing DFFA-like effector b [Source:HGNC Symbol;Acc:HGNC:1977] |
| ENSG00000137135 | HGLibA_02861 | 66 | -0.491833489 | ARHGEF39 | Rho guanine nucleotide exchange factor 39 [Source:HGNC Symbol;Acc:HGNC:25909] |
| ENSG00000138722 | HGLibA_29546 | 66 | -0.491833489 | MMRN1 | multimerin 1 [Source:HGNC Symbol;Acc:HGNC:7178] |
| ENSG00000139263 | HGLibA_27183 | 66 | -0.491833489 | LRIG3 | leucine rich repeats and immunoglobulin like domains 3 [Source:HGNC Symbol;Acc:HGNC:30991] |
| ENSG00000139537 | HGLibA_07832 | 66 | -0.491833489 | CCDC65 | coiled-coil domain containing 65 [Source:HGNC Symbol;Acc:HGNC:29937] |
| ENSG00000141013 | HGLibA_18660 | 66 | -0.491833489 | GAS8 | growth arrest specific 8 [Source:HGNC Symbol;Acc:HGNC:4166] |
| ENSG00000141378 | HGLibA_39552 | 66 | -0.491833489 | PTRH2 | peptidyl-tRNA hydrolase 2 [Source:HGNC Symbol;Acc:HGNC:24265] |
| ENSG00000141437 | HGLibA_44768 | 66 | -0.491833489 | SLC25A52 | solute carrier family 25 member 52 [Source:HGNC Symbol;Acc:HGNC:23324] |
| ENSG00000142002 | HGLibA_13884 | 66 | -0.491833489 | DPP9 | dipeptidyl peptidase 9 [Source:HGNC Symbol;Acc:HGNC:18648] |
| ENSG00000143970 | HGLibA_03391 | 66 | -0.491833489 | ASXL2 | ASXL transcriptional regulator 2 [Source:HGNC Symbol;Acc:HGNC:23805] |
| ENSG00000144659 | HGLibA_44727 | 66 | -0.491833489 | SLC25A38 | solute carrier family 25 member 38 [Source:HGNC Symbol;Acc:HGNC:26054] |
| ENSG00000145685 | HGLibA_26432 | 66 | -0.491833489 | LHFPL2 | LHFPL tetraspan subfamily member 2 [Source:HGNC Symbol;Acc:HGNC:6588] |
| ENSG00000146678 | HGLibA_22816 | 66 | -0.491833489 | IGFBP1 | insulin like growth factor binding protein 1 [Source:HGNC Symbol;Acc:HGNC:5469] |
| ENSG00000146833 | HGLibA_51490 | 66 | -0.491833489 | TRIM4 | tripartite motif containing 4 [Source:HGNC Symbol;Acc:HGNC:16275] |
| ENSG00000146963 | HGLibA_27624 | 66 | -0.491833489 | LUC7L2 | pre-mRNA splicing factor [Source:HGNC Symbol;Acc:HGNC:21608]" |
| ENSG00000147164 | HGLibA_46034 | 66 | -0.491833489 | SNX12 | sorting nexin 12 [Source:HGNC Symbol;Acc:HGNC:14976] |
| ENSG00000149743 | HGLibA_51778 | 66 | -0.491833489 | TRPT1 | tRNA phosphotransferase 1 [Source:HGNC Symbol;Acc:HGNC:20316] |
| ENSG00000154133 | HGLibA_41741 | 66 | -0.491833489 | ROBO4 | roundabout guidance receptor 4 [Source:HGNC Symbol;Acc:HGNC:17985] |
| ENSG00000156006 | HGLibA_31175 | 66 | -0.491833489 | NAT2 | N-acetyltransferase 2 [Source:HGNC Symbol;Acc:HGNC:7646] |
| ENSG00000157911 | HGLibA_36081 | 66 | -0.491833489 | PEX10 | peroxisomal biogenesis factor 10 [Source:HGNC Symbol;Acc:HGNC:8851] |
| ENSG00000158195 | HGLibA_53957 | 66 | -0.491833489 | WASF2 | WAS protein family member 2 [Source:HGNC Symbol;Acc:HGNC:12733] |
| ENSG00000158717 | HGLibA_41559 | 66 | -0.491833489 | RNF166 | ring finger protein 166 [Source:HGNC Symbol;Acc:HGNC:28856] |
| ENSG00000159692 | HGLibA_11585 | 66 | -0.491833489 | CTBP1 | C-terminal binding protein 1 [Source:HGNC Symbol;Acc:HGNC:2494] |
| ENSG00000160781 | HGLibA_35167 | 66 | -0.491833489 | PAQR6 | progestin and adipoQ receptor family member 6 [Source:HGNC Symbol;Acc:HGNC:30132] |
| ENSG00000162408 | HGLibA_32281 | 66 | -0.491833489 | NOL9 | nucleolar protein 9 [Source:HGNC Symbol;Acc:HGNC:26265] |
| ENSG00000162836 | HGLibA_00519 | 66 | -0.491833489 | ACP6 | lysophosphatidic [Source:HGNC Symbol;Acc:HGNC:29609]" |
| ENSG00000163376 | HGLibA_24117 | 66 | -0.491833489 | KBTBD8 | kelch repeat and BTB domain containing 8 [Source:HGNC Symbol;Acc:HGNC:30691] |
| ENSG00000163528 | HGLibA_09370 | 66 | -0.491833489 | CHCHD4 | coiled-coil-helix-coiled-coil-helix domain containing 4 [Source:HGNC Symbol;Acc:HGNC:26467] |
| ENSG00000163870 | HGLibA_51177 | 66 | -0.491833489 | TPRA1 | transmembrane protein adipocyte associated 1 [Source:HGNC Symbol;Acc:HGNC:30413] |
| ENSG00000163913 | HGLibA_22744 | 66 | -0.491833489 | IFT122 | intraflagellar transport 122 [Source:HGNC Symbol;Acc:HGNC:13556] |
| ENSG00000164171 | HGLibA_23724 | 66 | -0.491833489 | ITGA2 | integrin subunit alpha 2 [Source:HGNC Symbol;Acc:HGNC:6137] |
| ENSG00000164414 | HGLibA_44948 | 66 | -0.491833489 | SLC35A1 | solute carrier family 35 member A1 [Source:HGNC Symbol;Acc:HGNC:11021] |
| ENSG00000164535 | HGLibA_12299 | 66 | -0.491833489 | DAGLB | diacylglycerol lipase beta [Source:HGNC Symbol;Acc:HGNC:28923] |
| ENSG00000164744 | HGLibA_47795 | 66 | -0.491833489 | SUN3 | Sad1 and UNC84 domain containing 3 [Source:HGNC Symbol;Acc:HGNC:22429] |
| ENSG00000165071 | HGLibA_50503 | 66 | -0.491833489 | TMEM71 | transmembrane protein 71 [Source:HGNC Symbol;Acc:HGNC:26572] |
| ENSG00000165138 | HGLibA_02162 | 66 | -0.491833489 | ANKS6 | ankyrin repeat and sterile alpha motif domain containing 6 [Source:HGNC Symbol;Acc:HGNC:26724] |
| ENSG00000165502 | HGLibA_41961 | 66 | -0.491833489 | RPL36AL | ribosomal protein L36a like [Source:HGNC Symbol;Acc:HGNC:10346] |
| ENSG00000165506 | HGLibA_13461 | 66 | -0.491833489 | DNAAF2 | dynein axonemal assembly factor 2 [Source:HGNC Symbol;Acc:HGNC:20188] |
| ENSG00000165688 | HGLibA_37327 | 66 | -0.491833489 | PMPCA | mitochondrial processing alpha subunit [Source:HGNC Symbol;Acc:HGNC:18667]" |
| ENSG00000165912 | HGLibA_34967 | 66 | -0.491833489 | PACSIN3 | protein kinase C and casein kinase substrate in neurons 3 [Source:HGNC Symbol;Acc:HGNC:8572] |
| ENSG00000166035 | HGLibA_26604 | 66 | -0.491833489 | LIPC | hepatic type [Source:HGNC Symbol;Acc:HGNC:6619]" |
| ENSG00000166170 | HGLibA_04086 | 66 | -0.491833489 | BAG5 | BCL2 associated athanogene 5 [Source:HGNC Symbol;Acc:HGNC:941] |
| ENSG00000166736 | HGLibA_22379 | 66 | -0.491833489 | HTR3A | 5-hydroxytryptamine receptor 3A [Source:HGNC Symbol;Acc:HGNC:5297] |
| ENSG00000166987 | HGLibA_28493 | 66 | -0.491833489 | MBD6 | methyl-CpG binding domain protein 6 [Source:HGNC Symbol;Acc:HGNC:20445] |
| ENSG00000167196 | HGLibA_17047 | 66 | -0.491833489 | FBXO22 | F-box protein 22 [Source:HGNC Symbol;Acc:HGNC:13593] |
| ENSG00000167658 | HGLibA_14509 | 66 | -0.491833489 | EEF2 | eukaryotic translation elongation factor 2 [Source:HGNC Symbol;Acc:HGNC:3214] |
| ENSG00000167780 | HGLibA_46118 | 66 | -0.491833489 | SOAT2 | sterol O-acyltransferase 2 [Source:HGNC Symbol;Acc:HGNC:11178] |
| ENSG00000167981 | HGLibA_56354 | 66 | -0.491833489 | ZNF597 | zinc finger protein 597 [Source:HGNC Symbol;Acc:HGNC:26573] |
| ENSG00000168461 | HGLibA_39873 | 66 | -0.491833489 | RAB31 | member RAS oncogene family [Source:HGNC Symbol;Acc:HGNC:9771]" |
| ENSG00000169139 | HGLibA_52669 | 66 | -0.491833489 | UBE2V2 | ubiquitin conjugating enzyme E2 V2 [Source:HGNC Symbol;Acc:HGNC:12495] |
| ENSG00000169224 | HGLibA_18830 | 66 | -0.491833489 | GCSAML | germinal center associated signaling and motility like [Source:HGNC Symbol;Acc:HGNC:29583] |
| ENSG00000170290 | HGLibA_45543 | 66 | -0.491833489 | SLN | sarcolipin [Source:HGNC Symbol;Acc:HGNC:11089] |
| ENSG00000170852 | HGLibA_24102 | 66 | -0.491833489 | KBTBD2 | kelch repeat and BTB domain containing 2 [Source:HGNC Symbol;Acc:HGNC:21751] |
| ENSG00000171219 | HGLibA_08562 | 66 | -0.491833489 | CDC42BPG | CDC42 binding protein kinase gamma [Source:HGNC Symbol;Acc:HGNC:29829] |
| ENSG00000171346 | HGLibA_25430 | 66 | -0.491833489 | KRT15 | keratin 15 [Source:HGNC Symbol;Acc:HGNC:6421] |
| ENSG00000171487 | HGLibA_32125 | 66 | -0.491833489 | NLRP5 | NLR family pyrin domain containing 5 [Source:HGNC Symbol;Acc:HGNC:21269] |
| ENSG00000171759 | HGLibA_35023 | 66 | -0.491833489 | PAH | phenylalanine hydroxylase [Source:HGNC Symbol;Acc:HGNC:8582] |
| ENSG00000172367 | HGLibA_35976 | 66 | -0.491833489 | PDZD3 | PDZ domain containing 3 [Source:HGNC Symbol;Acc:HGNC:19891] |
| ENSG00000172554 | HGLibA_46008 | 66 | -0.491833489 | SNTG2 | syntrophin gamma 2 [Source:HGNC Symbol;Acc:HGNC:13741] |
| ENSG00000172594 | HGLibA_45814 | 66 | -0.491833489 | SMPDL3A | sphingomyelin phosphodiesterase acid like 3A [Source:HGNC Symbol;Acc:HGNC:17389] |
| ENSG00000173660 | HGLibA_53121 | 66 | -0.491833489 | UQCRH | ubiquinol-cytochrome c reductase hinge protein [Source:HGNC Symbol;Acc:HGNC:12590] |
| ENSG00000173757 | HGLibA_47395 | 66 | -0.491833489 | STAT5B | signal transducer and activator of transcription 5B [Source:HGNC Symbol;Acc:HGNC:11367] |
| ENSG00000174080 | HGLibA_11703 | 66 | -0.491833489 | CTSF | cathepsin F [Source:HGNC Symbol;Acc:HGNC:2531] |
| ENSG00000174132 | HGLibA_16282 | 66 | -0.491833489 | FAM174A | family with sequence similarity 174 member A [Source:HGNC Symbol;Acc:HGNC:24943] |
| ENSG00000175514 | HGLibA_19937 | 66 | -0.491833489 | GPR152 | G protein-coupled receptor 152 [Source:HGNC Symbol;Acc:HGNC:23622] |
| ENSG00000175662 | HGLibA_50952 | 66 | -0.491833489 | TOM1L2 | target of myb1 like 2 membrane trafficking protein [Source:HGNC Symbol;Acc:HGNC:11984] |
| ENSG00000177479 | HGLibA_02915 | 66 | -0.491833489 | ARIH2 | ariadne RBR E3 ubiquitin protein ligase 2 [Source:HGNC Symbol;Acc:HGNC:690] |
| ENSG00000177984 | HGLibA_26174 | 66 | -0.491833489 | LCN15 | lipocalin 15 [Source:HGNC Symbol;Acc:HGNC:33777] |
| ENSG00000179817 | HGLibA_29855 | 66 | -0.491833489 | MRGPRX4 | MAS related GPR family member X4 [Source:HGNC Symbol;Acc:HGNC:17617] |
| ENSG00000180329 | HGLibA_07787 | 66 | -0.491833489 | CCDC43 | coiled-coil domain containing 43 [Source:HGNC Symbol;Acc:HGNC:26472] |
| ENSG00000180773 | HGLibA_45039 | 66 | -0.491833489 | SLC36A4 | solute carrier family 36 member 4 [Source:HGNC Symbol;Acc:HGNC:19660] |
| ENSG00000181827 | HGLibA_40981 | 66 | -0.491833489 | RFX7 | regulatory factor X7 [Source:HGNC Symbol;Acc:HGNC:25777] |
| ENSG00000182836 | HGLibA_37030 | 66 | -0.491833489 | PLCXD3 | phosphatidylinositol specific phospholipase C X domain containing 3 [Source:HGNC Symbol;Acc:HGNC:31822] |
| ENSG00000183196 | HGLibA_09643 | 66 | -0.491833489 | CHST6 | carbohydrate sulfotransferase 6 [Source:HGNC Symbol;Acc:HGNC:6938] |
| ENSG00000183206 | HGLibA_37724 | 66 | -0.491833489 | POTEC | POTE ankyrin domain family member C [Source:HGNC Symbol;Acc:HGNC:33894] |
| ENSG00000183753 | HGLibA_04742 | 66 | -0.491833489 | BPY2 | basic charge Y-linked 2 [Source:HGNC Symbol;Acc:HGNC:13508] |
| ENSG00000185163 | HGLibA_12736 | 66 | -0.491833489 | DDX51 | DEAD-box helicase 51 [Source:HGNC Symbol;Acc:HGNC:20082] |
| ENSG00000185189 | HGLibA_32657 | 66 | -0.491833489 | NRBP2 | nuclear receptor binding protein 2 [Source:HGNC Symbol;Acc:HGNC:19339] |
| ENSG00000186104 | HGLibA_12140 | 66 | -0.491833489 | CYP2R1 | cytochrome P450 family 2 subfamily R member 1 [Source:HGNC Symbol;Acc:HGNC:20580] |
| ENSG00000186583 | HGLibA_46536 | 66 | -0.491833489 | SPATC1 | spermatogenesis and centriole associated 1 [Source:HGNC Symbol;Acc:HGNC:30510] |
| ENSG00000186599 | HGLibA_12827 | 66 | -0.491833489 | DEFB105B | defensin beta 105B [Source:HGNC Symbol;Acc:HGNC:29930] |
| ENSG00000186803 | HGLibA_22658 | 66 | -0.491833489 | IFNA10 | interferon alpha 10 [Source:HGNC Symbol;Acc:HGNC:5418] |
| ENSG00000188732 | HGLibA_16515 | 66 | -0.491833489 | FAM221A | family with sequence similarity 221 member A [Source:HGNC Symbol;Acc:HGNC:27977] |
| ENSG00000189099 | HGLibA_38950 | 66 | -0.491833489 | PRSS48 | serine protease 48 [Source:HGNC Symbol;Acc:HGNC:24635] |
| ENSG00000196188 | HGLibA_11702 | 66 | -0.491833489 | CTSE | cathepsin E [Source:HGNC Symbol;Acc:HGNC:2530] |
| ENSG00000196266 | HGLibA_33523 | 66 | -0.491833489 | OR10J3 | olfactory receptor family 10 subfamily J member 3 [Source:HGNC Symbol;Acc:HGNC:14992] |
| ENSG00000196358 | HGLibA_32868 | 66 | -0.491833489 | NTNG2 | netrin G2 [Source:HGNC Symbol;Acc:HGNC:14288] |
| ENSG00000196427 | HGLibA_31237 | 66 | -0.491833489 | NBPF4 | NBPF member 4 [Source:HGNC Symbol;Acc:HGNC:26550] |
| ENSG00000196678 | HGLibA_15482 | 66 | -0.491833489 | ERI2 | ERI1 exoribonuclease family member 2 [Source:HGNC Symbol;Acc:HGNC:30541] |
| ENSG00000197312 | HGLibA_12611 | 66 | -0.491833489 | DDI2 | DNA damage inducible 1 homolog 2 [Source:HGNC Symbol;Acc:HGNC:24578] |
| ENSG00000197323 | HGLibA_51466 | 66 | -0.491833489 | TRIM33 | tripartite motif containing 33 [Source:HGNC Symbol;Acc:HGNC:16290] |
| ENSG00000197561 | HGLibA_14875 | 66 | -0.491833489 | ELANE | neutrophil expressed [Source:HGNC Symbol;Acc:HGNC:3309]" |
| ENSG00000197584 | HGLibA_24346 | 66 | -0.491833489 | KCNMB2 | potassium calcium-activated channel subfamily M regulatory beta subunit 2 [Source:HGNC Symbol;Acc:HGNC:6286] |
| ENSG00000197587 | HGLibA_13395 | 66 | -0.491833489 | DMBX1 | diencephalon/mesencephalon homeobox 1 [Source:HGNC Symbol;Acc:HGNC:19026] |
| ENSG00000197921 | HGLibA_21188 | 66 | -0.491833489 | HES5 | hes family bHLH transcription factor 5 [Source:HGNC Symbol;Acc:HGNC:19764] |
| ENSG00000197935 | HGLibA_55781 | 66 | -0.491833489 | ZNF311 | zinc finger protein 311 [Source:HGNC Symbol;Acc:HGNC:13847] |
| ENSG00000197937 | HGLibA_55851 | 66 | -0.491833489 | ZNF347 | zinc finger protein 347 [Source:HGNC Symbol;Acc:HGNC:16447] |
| ENSG00000198074 | HGLibA_01475 | 66 | -0.491833489 | AKR1B10 | aldo-keto reductase family 1 member B10 [Source:HGNC Symbol;Acc:HGNC:382] |
| ENSG00000198793 | HGLibA_30453 | 66 | -0.491833489 | MTOR | mechanistic target of rapamycin kinase [Source:HGNC Symbol;Acc:HGNC:3942] |
| ENSG00000198885 | HGLibA_23866 | 66 | -0.491833489 | ITPRIPL1 | ITPRIP like 1 [Source:HGNC Symbol;Acc:HGNC:29371] |
| ENSG00000198939 | HGLibA_55193 | 66 | -0.491833489 | ZFP2 | ZFP2 zinc finger protein [Source:HGNC Symbol;Acc:HGNC:26138] |
| ENSG00000198945 | HGLibA_25902 | 66 | -0.491833489 | L3MBTL3 | histone methyl-lysine binding protein [Source:HGNC Symbol;Acc:HGNC:23035]" |
| ENSG00000198960 | HGLibA_03056 | 66 | -0.491833489 | ARMCX6 | armadillo repeat containing X-linked 6 [Source:HGNC Symbol;Acc:HGNC:26094] |
| ENSG00000203747 | HGLibA_17194 | 66 | -0.491833489 | FCGR3A | Fc fragment of IgG receptor IIIa [Source:HGNC Symbol;Acc:HGNC:3619] |
| ENSG00000204065 | HGLibA_48716 | 66 | -0.491833489 | TCEAL5 | transcription elongation factor A like 5 [Source:HGNC Symbol;Acc:HGNC:22282] |
| ENSG00000204128 | HGLibA_06176 | 66 | -0.491833489 | C2orf72 | chromosome 2 open reading frame 72 [Source:HGNC Symbol;Acc:HGNC:27418] |
| ENSG00000205143 | HGLibA_02896 | 66 | -0.491833489 | ARID3C | AT-rich interaction domain 3C [Source:HGNC Symbol;Acc:HGNC:21209] |
| ENSG00000205364 | HGLibA_30294 | 66 | -0.491833489 | MT1M | metallothionein 1M [Source:HGNC Symbol;Acc:HGNC:14296] |
| ENSG00000205929 | HGLibA_06046 | 66 | -0.491833489 | C21orf62 | chromosome 21 open reading frame 62 [Source:HGNC Symbol;Acc:HGNC:1305] |
| ENSG00000212901 | HGLibA_25739 | 66 | -0.491833489 | KRTAP3-1 | keratin associated protein 3-1 [Source:HGNC Symbol;Acc:HGNC:16778] |
| ENSG00000213203 | HGLibA_19070 | 66 | -0.491833489 | GIMAP1 | IMAP family member 1 [Source:HGNC Symbol;Acc:HGNC:23237]" |
| ENSG00000213626 | HGLibA_26077 | 66 | -0.491833489 | LBH | limb bud and heart development [Source:HGNC Symbol;Acc:HGNC:29532] |
| ENSG00000214891 | HGLibA_51583 | 66 | -0.491833489 | TRIM64C | tripartite motif containing 64C [Source:HGNC Symbol;Acc:HGNC:37148] |
| ENSG00000221869 | HGLibA_08963 | 66 | -0.491833489 | CEBPD | CCAAT enhancer binding protein delta [Source:HGNC Symbol;Acc:HGNC:1835] |
| ENSG00000225830 | HGLibA_15447 | 66 | -0.491833489 | ERCC6 | chromatin remodeling factor [Source:HGNC Symbol;Acc:HGNC:3438]" |
| ENSG00000229937 | HGLibA_38758 | 66 | -0.491833489 | PRPS1L1 | phosphoribosyl pyrophosphate synthetase 1-like 1 [Source:HGNC Symbol;Acc:HGNC:9463] |
| ENSG00000236311 | HGLibA_49672 | 66 | -0.491833489 | TLX1NB | TLX1 neighbor [Source:HGNC Symbol;Acc:HGNC:37183] |
| ENSG00000239961 | HGLibA_26499 | 66 | -0.491833489 | LILRA4 | leukocyte immunoglobulin like receptor A4 [Source:HGNC Symbol;Acc:HGNC:15503] |
| ENSG00000242173 | HGLibA_02802 | 66 | -0.491833489 | ARHGDIG | Rho GDP dissociation inhibitor gamma [Source:HGNC Symbol;Acc:HGNC:680] |
| ENSG00000243709 | HGLibA_26274 | 66 | -0.491833489 | LEFTY1 | left-right determination factor 1 [Source:HGNC Symbol;Acc:HGNC:6552] |

| | | | | | |
|---|---|---|---|---|---|
| ENSG00000243902 | HGLibA_14909 | 66 | -0.491833489 | ELFN2 | extracellular leucine rich repeat and fibronectin type III domain containing 2 [Source:NCBI gene;Acc:114794] |
| ENSG00000247077 | HGLibA_36204 | 66 | -0.491833489 | PGAM5 | mitochondrial serine/threonine protein phosphatase [Source:HGNC Symbol;Acc:HGNC:28763]" |
| ENSG00000249709 | HGLibA_56254 | 66 | -0.491833489 | ZNF564 | zinc finger protein 564 [Source:HGNC Symbol;Acc:HGNC:31106] |
| ENSG00000254122 | HGLibA_35567 | 66 | -0.491833489 | PCDHGB7 | 7 [Source:HGNC Symbol;Acc:HGNC:8714]" |
| ENSG00000254772 | HGLibA_14506 | 66 | -0.491833489 | EEF1G | eukaryotic translation elongation factor 1 gamma [Source:HGNC Symbol;Acc:HGNC:3213] |
| ENSG00000256797 | HGLibA_25305 | 66 | -0.491833489 | KLRF2 | killer cell lectin like receptor F2 [Source:HGNC Symbol;Acc:HGNC:37646] |
| ENSG00000258947 | HGLibA_52283 | 66 | -0.491833489 | TUBB3 | tubulin beta 3 class III [Source:HGNC Symbol;Acc:HGNC:20772] |
| ENSG00000274588 | HGLibA_13080 | 66 | -0.491833489 | DGKK | diacylglycerol kinase kappa [Source:HGNC Symbol;Acc:HGNC:32395] |
| ENSG00000275954 | HGLibA_48549 | 66 | -0.491833489 | TBC1D3F | TBC1 domain family member 3F [Source:HGNC Symbol;Acc:HGNC:18257] |
| ENSG00000280021 | HGLibA_34104 | 66 | -0.491833489 | OR51F1 | olfactory receptor family 51 subfamily F member 1 (gene/pseudogene) [Source:HGNC Symbol;Acc:HGNC:15196] |
| FAM173A | HGLibA_16276 | 66 | -0.491833489 | FAM173A | - |
| FAM69B | HGLibA_16681 | 66 | -0.491833489 | FAM69B | - |
| FLJ27352 | HGLibA_17627 | 66 | -0.491833489 | FLJ27352 | - |
| GPR125 | HGLibA_19878 | 66 | -0.491833489 | GPR125 | - |
| hsa-mir-1255a | HGLibA_57422 | 66 | -0.491833489 | hsa-mir-1255a | - |
| hsa-mir-137 | HGLibA_57810 | 66 | -0.491833489 | hsa-mir-137 | - |
| hsa-mir-200c | HGLibA_58158 | 66 | -0.491833489 | hsa-mir-200c | - |
| hsa-mir-27b | HGLibA_58382 | 66 | -0.491833489 | hsa-mir-27b | - |
| hsa-mir-3168 | HGLibA_58753 | 66 | -0.491833489 | hsa-mir-3168 | - |
| hsa-mir-3910-2 | HGLibA_59510 | 66 | -0.491833489 | hsa-mir-3910-2 | - |
| hsa-mir-421 | HGLibA_59692 | 66 | -0.491833489 | hsa-mir-421 | - |
| hsa-mir-4288 | HGLibA_59859 | 66 | -0.491833489 | hsa-mir-4288 | - |
| hsa-mir-4536-1 | HGLibA_60602 | 66 | -0.491833489 | hsa-mir-4536-1 | - |
| hsa-mir-4633 | HGLibA_60637 | 66 | -0.491833489 | hsa-mir-4633 | - |
| hsa-mir-4657 | HGLibA_60734 | 66 | -0.491833489 | hsa-mir-4657 | - |
| hsa-mir-4733 | HGLibA_61029 | 66 | -0.491833489 | hsa-mir-4733 | - |
| hsa-mir-4763 | HGLibA_61150 | 66 | -0.491833489 | hsa-mir-4763 | - |
| hsa-mir-4789 | HGLibA_61265 | 66 | -0.491833489 | hsa-mir-4789 | - |
| hsa-mir-487a | HGLibA_61346 | 66 | -0.491833489 | hsa-mir-487a | - |
| hsa-mir-548an | HGLibA_61905 | 66 | -0.491833489 | hsa-mir-548an | - |
| hsa-mir-596 | HGLibA_62478 | 66 | -0.491833489 | hsa-mir-596 | - |
| hsa-mir-626 | HGLibA_62743 | 66 | -0.491833489 | hsa-mir-626 | - |
| hsa-mir-6508 | HGLibA_62882 | 66 | -0.491833489 | hsa-mir-6508 | - |
| hsa-mir-6511a-3 | HGLibA_62904 | 66 | -0.491833489 | hsa-mir-6511a-3 | - |
| hsa-mir-6774 | HGLibA_63269 | 66 | -0.491833489 | hsa-mir-6774 | - |
| hsa-mir-6801 | HGLibA_63382 | 66 | -0.491833489 | hsa-mir-6801 | - |
| hsa-mir-6826 | HGLibA_63482 | 66 | -0.491833489 | hsa-mir-6826 | - |
| hsa-mir-6831 | HGLibA_63503 | 66 | -0.491833489 | hsa-mir-6831 | - |
| hsa-mir-6882 | HGLibA_63712 | 66 | -0.491833489 | hsa-mir-6882 | - |
| hsa-mir-6893 | HGLibA_63756 | 66 | -0.491833489 | hsa-mir-6893 | - |
| hsa-mir-7114 | HGLibA_63824 | 66 | -0.491833489 | hsa-mir-7114 | - |
| hsa-mir-770 | HGLibA_63938 | 66 | -0.491833489 | hsa-mir-770 | - |
| LOC100862671 | HGLibA_26878 | 66 | -0.491833489 | LOC100862671 | - |
| PRHOXNB | HGLibA_38468 | 66 | -0.491833489 | PRHOXNB | - |
| RBAK-LOC389458 | HGLibA_40421 | 66 | -0.491833489 | RBAK-LOC389458 | - |
| SSFA2 | HGLibA_47128 | 66 | -0.491833489 | SSFA2 | - |
| C11orf93 | HGLibA_05215 | 65 | -0.501784526 | C11orf93 | - |
| C1orf192 | HGLibA_05814 | 65 | -0.501784526 | C1orf192 | - |
| C1orf86 | HGLibA_05901 | 65 | -0.501784526 | C1orf86 | - |
| C7orf66 | HGLibA_06578 | 65 | -0.501784526 | C7orf66 | - |
| DARS | HGLibA_12341 | 65 | -0.501784526 | DARS | - |
| ENSG00000001561 | HGLibA_15175 | 65 | -0.501784526 | ENPP4 | ectonucleotide pyrophosphatase/phosphodiesterase 4 [Source:HGNC Symbol;Acc:HGNC:3359] |
| ENSG00000004777 | HGLibA_02763 | 65 | -0.501784526 | ARHGAP33 | Rho GTPase activating protein 33 [Source:HGNC Symbol;Acc:HGNC:23085] |
| ENSG00000006128 | HGLibA_48150 | 65 | -0.501784526 | TAC1 | tachykinin precursor 1 [Source:HGNC Symbol;Acc:HGNC:11517] |
| ENSG00000008226 | HGLibA_13317 | 65 | -0.501784526 | DLEC1 | cilia and flagella associated protein [Source:HGNC Symbol;Acc:HGNC:2899]" |
| ENSG00000013810 | HGLibA_48165 | 65 | -0.501784526 | TACC3 | transforming acidic coiled-coil containing protein 3 [Source:HGNC Symbol;Acc:HGNC:11524] |
| ENSG00000016490 | HGLibA_09804 | 65 | -0.501784526 | CLCA1 | chloride channel accessory 1 [Source:HGNC Symbol;Acc:HGNC:2015] |
| ENSG00000033011 | HGLibA_01600 | 65 | -0.501784526 | ALG1 | chitobiosyldiphosphodolichol beta-mannosyltransferase [Source:HGNC Symbol;Acc:HGNC:18294]" |
| ENSG00000050327 | HGLibA_02870 | 65 | -0.501784526 | ARHGEF5 | Rho guanine nucleotide exchange factor 5 [Source:HGNC Symbol;Acc:HGNC:13209] |
| ENSG00000058056 | HGLibA_53204 | 65 | -0.501784526 | USP13 | ubiquitin specific peptidase 13 [Source:HGNC Symbol;Acc:HGNC:12611] |
| ENSG00000060749 | HGLibA_39751 | 65 | -0.501784526 | QSER1 | glutamine and serine rich 1 [Source:HGNC Symbol;Acc:HGNC:26154] |
| ENSG00000065357 | HGLibA_13058 | 65 | -0.501784526 | DGKA | diacylglycerol kinase alpha [Source:HGNC Symbol;Acc:HGNC:2849] |
| ENSG00000067445 | HGLibA_51715 | 65 | -0.501784526 | TRO | trophinin [Source:HGNC Symbol;Acc:HGNC:12326] |
| ENSG00000068001 | HGLibA_22439 | 65 | -0.501784526 | HYAL2 | hyaluronidase 2 [Source:HGNC Symbol;Acc:HGNC:5321] |
| ENSG00000072210 | HGLibA_01564 | 65 | -0.501784526 | ALDH3A2 | aldehyde dehydrogenase 3 family member A2 [Source:HGNC Symbol;Acc:HGNC:403] |
| ENSG00000077254 | HGLibA_53339 | 65 | -0.501784526 | USP33 | ubiquitin specific peptidase 33 [Source:HGNC Symbol;Acc:HGNC:20059] |
| ENSG00000084628 | HGLibA_31990 | 65 | -0.501784526 | NKAIN1 | sodium/potassium transporting ATPase interacting 1 [Source:HGNC Symbol;Acc:HGNC:25743] |
| ENSG00000086289 | HGLibA_15275 | 65 | -0.501784526 | EPDR1 | ependymin related 1 [Source:HGNC Symbol;Acc:HGNC:17572] |
| ENSG00000088002 | HGLibA_47761 | 65 | -0.501784526 | SULT2B1 | sulfotransferase family 2B member 1 [Source:HGNC Symbol;Acc:HGNC:11459] |
| ENSG00000088930 | HGLibA_54606 | 65 | -0.501784526 | XRN2 | 5'-3' exoribonuclease 2 [Source:HGNC Symbol;Acc:HGNC:12836] |
| ENSG00000090060 | HGLibA_35137 | 65 | -0.501784526 | PAPOLA | poly(A) polymerase alpha [Source:HGNC Symbol;Acc:HGNC:14981] |
| ENSG00000090520 | HGLibA_13524 | 65 | -0.501784526 | DNAJB11 | DnaJ heat shock protein family (Hsp40) member B11 [Source:HGNC Symbol;Acc:HGNC:14889] |
| ENSG00000092108 | HGLibA_42893 | 65 | -0.501784526 | SCFD1 | sec1 family domain containing 1 [Source:HGNC Symbol;Acc:HGNC:20726] |
| ENSG00000092529 | HGLibA_07166 | 65 | -0.501784526 | CAPN3 | calpain 3 [Source:HGNC Symbol;Acc:HGNC:1480] |
| ENSG00000096060 | HGLibA_17578 | 65 | -0.501784526 | FKBP5 | FK506 binding protein 5 [Source:HGNC Symbol;Acc:HGNC:3721] |
| ENSG00000096070 | HGLibA_04842 | 65 | -0.501784526 | BRPF3 | bromodomain and PHD finger containing 3 [Source:HGNC Symbol;Acc:HGNC:14256] |
| ENSG00000100299 | HGLibA_03127 | 65 | -0.501784526 | ARSA | arylsulfatase A [Source:HGNC Symbol;Acc:HGNC:713] |
| ENSG00000100523 | HGLibA_12603 | 65 | -0.501784526 | DDHD1 | DDHD domain containing 1 [Source:HGNC Symbol;Acc:HGNC:19714] |
| ENSG00000104221 | HGLibA_04799 | 65 | -0.501784526 | BRF2 | RNA polymerase III transcription initiation factor subunit [Source:HGNC Symbol;Acc:HGNC:17298]" |
| ENSG00000104312 | HGLibA_41319 | 65 | -0.501784526 | RIPK2 | receptor interacting serine/threonine kinase 2 [Source:HGNC Symbol;Acc:HGNC:10020] |
| ENSG00000104313 | HGLibA_15818 | 65 | -0.501784526 | EYA1 | EYA transcriptional coactivator and phosphatase 1 [Source:HGNC Symbol;Acc:HGNC:3519] |
| ENSG00000104549 | HGLibA_46913 | 65 | -0.501784526 | SQLE | squalene epoxidase [Source:HGNC Symbol;Acc:HGNC:11279] |
| ENSG00000105352 | HGLibA_08942 | 65 | -0.501784526 | CEACAM4 | carcinoembryonic antigen related cell adhesion molecule 4 [Source:HGNC Symbol;Acc:HGNC:1816] |
| ENSG00000105371 | HGLibA_22504 | 65 | -0.501784526 | ICAM4 | intercellular adhesion molecule 4 (Landsteiner-Wiener blood group) [Source:HGNC Symbol;Acc:HGNC:5347] |
| ENSG00000105472 | HGLibA_09925 | 65 | -0.501784526 | CLEC11A | C-type lectin domain containing 11A [Source:HGNC Symbol;Acc:HGNC:10576] |
| ENSG00000105732 | HGLibA_56288 | 65 | -0.501784526 | ZNF574 | zinc finger protein 574 [Source:HGNC Symbol;Acc:HGNC:26166] |
| ENSG00000105880 | HGLibA_13383 | 65 | -0.501784526 | DLX5 | distal-less homeobox 5 [Source:HGNC Symbol;Acc:HGNC:2918] |
| ENSG00000106992 | HGLibA_01377 | 65 | -0.501784526 | AK1 | adenylate kinase 1 [Source:HGNC Symbol;Acc:HGNC:361] |
| ENSG00000107201 | HGLibA_12752 | 65 | -0.501784526 | DDX58 | DExD/H-box helicase 58 [Source:HGNC Symbol;Acc:HGNC:19102] |
| ENSG00000108375 | HGLibA_41685 | 65 | -0.501784526 | RNF43 | ring finger protein 43 [Source:HGNC Symbol;Acc:HGNC:18505] |
| ENSG00000108587 | HGLibA_19683 | 65 | -0.501784526 | GOSR1 | golgi SNAP receptor complex member 1 [Source:HGNC Symbol;Acc:HGNC:4430] |
| ENSG00000108821 | HGLibA_10529 | 65 | -0.501784526 | COL1A1 | collagen type I alpha 1 chain [Source:HGNC Symbol;Acc:HGNC:2197] |
| ENSG00000110237 | HGLibA_02824 | 65 | -0.501784526 | ARHGEF17 | Rho guanine nucleotide exchange factor 17 [Source:HGNC Symbol;Acc:HGNC:21726] |
| ENSG00000110330 | HGLibA_04516 | 65 | -0.501784526 | BIRC2 | baculoviral IAP repeat containing 2 [Source:HGNC Symbol;Acc:HGNC:590] |
| ENSG00000111596 | HGLibA_10336 | 65 | -0.501784526 | CNOT2 | CCR4-NOT transcription complex subunit 2 [Source:HGNC Symbol;Acc:HGNC:7878] |
| ENSG00000111879 | HGLibA_16327 | 65 | -0.501784526 | FAM184A | family with sequence similarity 184 member A [Source:HGNC Symbol;Acc:HGNC:20991] |
| ENSG00000112306 | HGLibA_42068 | 65 | -0.501784526 | RPS12 | ribosomal protein S12 [Source:HGNC Symbol;Acc:HGNC:10385] |
| ENSG00000114796 | HGLibA_25163 | 65 | -0.501784526 | KLHL24 | kelch like family member 24 [Source:HGNC Symbol;Acc:HGNC:25947] |
| ENSG00000115607 | HGLibA_23031 | 65 | -0.501784526 | IL18RAP | interleukin 18 receptor accessory protein [Source:HGNC Symbol;Acc:HGNC:5989] |
| ENSG00000115694 | HGLibA_47475 | 65 | -0.501784526 | STK25 | serine/threonine kinase 25 [Source:HGNC Symbol;Acc:HGNC:11404] |
| ENSG00000115942 | HGLibA_34604 | 65 | -0.501784526 | ORC2 | origin recognition complex subunit 2 [Source:HGNC Symbol;Acc:HGNC:8488] |
| ENSG00000115956 | HGLibA_37056 | 65 | -0.501784526 | PLEK | pleckstrin [Source:HGNC Symbol;Acc:HGNC:9070] |
| ENSG00000119777 | HGLibA_50217 | 65 | -0.501784526 | TMEM214 | transmembrane protein 214 [Source:HGNC Symbol;Acc:HGNC:25983] |
| ENSG00000119977 | HGLibA_48877 | 65 | -0.501784526 | TCTN3 | tectonic family member 3 [Source:HGNC Symbol;Acc:HGNC:24519] |
| ENSG00000120337 | HGLibA_50820 | 65 | -0.501784526 | TNFSF18 | TNF superfamily member 18 [Source:HGNC Symbol;Acc:HGNC:11932] |
| ENSG00000121879 | HGLibA_36633 | 65 | -0.501784526 | PIK3CA | 5-bisphosphate 3-kinase catalytic subunit alpha [Source:HGNC Symbol;Acc:HGNC:8975]" |
| ENSG00000122512 | HGLibA_37337 | 65 | -0.501784526 | PMS2 | mismatch repair system component [Source:HGNC Symbol;Acc:HGNC:9122]" |
| ENSG00000122574 | HGLibA_54311 | 65 | -0.501784526 | WIPF3 | WAS/WASL interacting protein family member 3 [Source:HGNC Symbol;Acc:HGNC:22004] |
| ENSG00000122585 | HGLibA_32548 | 65 | -0.501784526 | NPY | neuropeptide Y [Source:HGNC Symbol;Acc:HGNC:7955] |
| ENSG00000122787 | HGLibA_01493 | 65 | -0.501784526 | AKR1D1 | aldo-keto reductase family 1 member D1 [Source:HGNC Symbol;Acc:HGNC:388] |
| ENSG00000123416 | HGLibA_52247 | 65 | -0.501784526 | TUBA1B | tubulin alpha 1b [Source:HGNC Symbol;Acc:HGNC:18809] |
| ENSG00000124103 | HGLibA_16438 | 65 | -0.501784526 | FAM209A | family with sequence similarity 209 member A [Source:HGNC Symbol;Acc:HGNC:16100] |
| ENSG00000124535 | HGLibA_54411 | 65 | -0.501784526 | WRNIP1 | Werner helicase interacting protein 1 [Source:HGNC Symbol;Acc:HGNC:20876] |
| ENSG00000125818 | HGLibA_39202 | 65 | -0.501784526 | PSMF1 | proteasome inhibitor subunit 1 [Source:HGNC Symbol;Acc:HGNC:9571] |
| ENSG00000126733 | HGLibA_12278 | 65 | -0.501784526 | DACH2 | dachshund family transcription factor 2 [Source:HGNC Symbol;Acc:HGNC:16814] |
| ENSG00000126785 | HGLibA_41197 | 65 | -0.501784526 | RHOJ | ras homolog family member J [Source:HGNC Symbol;Acc:HGNC:688] |
| ENSG00000127870 | HGLibA_41695 | 65 | -0.501784526 | RNF6 | ring finger protein 6 [Source:HGNC Symbol;Acc:HGNC:10069] |
| ENSG00000127951 | HGLibA_17465 | 65 | -0.501784526 | FGL2 | fibrinogen like 2 [Source:HGNC Symbol;Acc:HGNC:3696] |
| ENSG00000130005 | HGLibA_18598 | 65 | -0.501784526 | GAMT | guanidinoacetate N-methyltransferase [Source:HGNC Symbol;Acc:HGNC:4136] |
| ENSG00000131848 | HGLibA_57028 | 65 | -0.501784526 | ZSCAN5A | zinc finger and SCAN domain containing 5A [Source:HGNC Symbol;Acc:HGNC:23710] |
| ENSG00000132031 | HGLibA_28432 | 65 | -0.501784526 | MATN3 | matrilin 3 [Source:HGNC Symbol;Acc:HGNC:6909] |
| ENSG00000132965 | HGLibA_01691 | 65 | -0.501784526 | ALOX5AP | arachidonate 5-lipoxygenase activating protein [Source:HGNC Symbol;Acc:HGNC:436] |
| ENSG00000133275 | HGLibA_11397 | 65 | -0.501784526 | CSNK1G2 | casein kinase 1 gamma 2 [Source:HGNC Symbol;Acc:HGNC:2455] |
| ENSG00000134490 | HGLibA_50289 | 65 | -0.501784526 | TMEM241 | transmembrane protein 241 [Source:HGNC Symbol;Acc:HGNC:31723] |
| ENSG00000134815 | HGLibA_13183 | 65 | -0.501784526 | DHX34 | DExH-box helicase 34 [Source:HGNC Symbol;Acc:HGNC:16719] |
| ENSG00000135205 | HGLibA_07629 | 65 | -0.501784526 | CCDC146 | coiled-coil domain containing 146 [Source:HGNC Symbol;Acc:HGNC:29296] |
| ENSG00000136828 | HGLibA_40168 | 65 | -0.501784526 | RALGPS1 | Ral GEF with PH domain and SH3 binding motif 1 [Source:HGNC Symbol;Acc:HGNC:16851] |
| ENSG00000136929 | HGLibA_21129 | 65 | -0.501784526 | HEMGN | hemogen [Source:HGNC Symbol;Acc:HGNC:17509] |
| ENSG00000137210 | HGLibA_49982 | 65 | -0.501784526 | TMEM14B | transmembrane protein 14B [Source:HGNC Symbol;Acc:HGNC:21384] |
| ENSG00000137766 | HGLibA_52998 | 65 | -0.501784526 | UNC13C | unc-13 homolog C [Source:HGNC Symbol;Acc:HGNC:23149] |
| ENSG00000137806 | HGLibA_31483 | 65 | -0.501784526 | NDUFAF1 | NADH:ubiquinone oxidoreductase complex assembly factor 1 [Source:HGNC Symbol;Acc:HGNC:18828] |
| ENSG00000138035 | HGLibA_37432 | 65 | -0.501784526 | PNPT1 | polyribonucleotide nucleotidyltransferase 1 [Source:HGNC Symbol;Acc:HGNC:23166] |
| ENSG00000138172 | HGLibA_07015 | 65 | -0.501784526 | CALHM2 | calcium homeostasis modulator family member 2 [Source:HGNC Symbol;Acc:HGNC:23493] |
| ENSG00000139180 | HGLibA_31475 | 65 | -0.501784526 | NDUFA9 | NADH:ubiquinone oxidoreductase subunit A9 [Source:HGNC Symbol;Acc:HGNC:7693] |
| ENSG00000139323 | HGLibA_37445 | 65 | -0.501784526 | POC1B | POC1 centriolar protein B [Source:HGNC Symbol;Acc:HGNC:30836] |

| | | | | | |
|---|---|---|---|---|---|
| ENSG00000140465 | HGLibA_12067 | 65 | -0.501784526 | CYP1A1 | cytochrome P450 family 1 subfamily A member 1 [Source:HGNC Symbol;Acc:HGNC:2595] |
| ENSG00000140740 | HGLibA_53117 | 65 | -0.501784526 | UQCRC2 | ubiquinol-cytochrome c reductase core protein 2 [Source:HGNC Symbol;Acc:HGNC:12586] |
| ENSG00000141002 | HGLibA_48782 | 65 | -0.501784526 | TCF25 | transcription factor 25 [Source:HGNC Symbol;Acc:HGNC:29181] |
| ENSG00000142530 | HGLibA_16699 | 65 | -0.501784526 | FAM71E1 | family with sequence similarity 71 member E1 [Source:HGNC Symbol;Acc:HGNC:25107] |
| ENSG00000143107 | HGLibA_17755 | 65 | -0.501784526 | FNDC7 | fibronectin type III domain containing 7 [Source:HGNC Symbol;Acc:HGNC:26668] |
| ENSG00000143184 | HGLibA_54493 | 65 | -0.501784526 | XCL1 | X-C motif chemokine ligand 1 [Source:HGNC Symbol;Acc:HGNC:10645] |
| ENSG00000143416 | HGLibA_43290 | 65 | -0.501784526 | SELENBP1 | selenium binding protein 1 [Source:HGNC Symbol;Acc:HGNC:10719] |
| ENSG00000144406 | HGLibA_53033 | 65 | -0.501784526 | UNC80 | NALCN channel complex subunit [Source:HGNC Symbol;Acc:HGNC:26582]" |
| ENSG00000144560 | HGLibA_53644 | 65 | -0.501784526 | VGLL4 | vestigial like family member 4 [Source:HGNC Symbol;Acc:HGNC:28966] |
| ENSG00000144791 | HGLibA_26532 | 65 | -0.501784526 | LIMD1 | LIM domains containing 1 [Source:HGNC Symbol;Acc:HGNC:6612] |
| ENSG00000144891 | HGLibA_01263 | 65 | -0.501784526 | AGTR1 | angiotensin II receptor type 1 [Source:HGNC Symbol;Acc:HGNC:336] |
| ENSG00000145214 | HGLibA_13081 | 65 | -0.501784526 | DGKQ | diacylglycerol kinase theta [Source:HGNC Symbol;Acc:HGNC:2856] |
| ENSG00000145451 | HGLibA_19303 | 65 | -0.501784526 | GLRA3 | glycine receptor alpha 3 [Source:HGNC Symbol;Acc:HGNC:4328] |
| ENSG00000145491 | HGLibA_41765 | 65 | -0.501784526 | ROPN1L | rhophilin associated tail protein 1 like [Source:HGNC Symbol;Acc:HGNC:24060] |
| ENSG00000145781 | HGLibA_10652 | 65 | -0.501784526 | COMMD10 | COMM domain containing 10 [Source:HGNC Symbol;Acc:HGNC:30201] |
| ENSG00000146955 | HGLibA_39825 | 65 | -0.501784526 | RAB19 | member RAS oncogene family [Source:HGNC Symbol;Acc:HGNC:19982]" |
| ENSG00000147799 | HGLibA_02771 | 65 | -0.501784526 | ARHGAP39 | Rho GTPase activating protein 39 [Source:HGNC Symbol;Acc:HGNC:29351] |
| ENSG00000147889 | HGLibA_08830 | 65 | -0.501784526 | CDKN2A | cyclin dependent kinase inhibitor 2A [Source:HGNC Symbol;Acc:HGNC:1787] |
| ENSG00000149021 | HGLibA_42908 | 65 | -0.501784526 | SCGB1A1 | secretoglobin family 1A member 1 [Source:HGNC Symbol;Acc:HGNC:12523] |
| ENSG00000152270 | HGLibA_35783 | 65 | -0.501784526 | PDE3B | phosphodiesterase 3B [Source:HGNC Symbol;Acc:HGNC:8779] |
| ENSG00000153094 | HGLibA_04308 | 65 | -0.501784526 | BCL2L11 | BCL2 like 11 [Source:HGNC Symbol;Acc:HGNC:994] |
| ENSG00000153113 | HGLibA_07345 | 65 | -0.501784526 | CAST | calpastatin [Source:HGNC Symbol;Acc:HGNC:1515] |
| ENSG00000153234 | HGLibA_32628 | 65 | -0.501784526 | NR4A2 | nuclear receptor subfamily 4 group A member 2 [Source:HGNC Symbol;Acc:HGNC:7981] |
| ENSG00000153786 | HGLibA_55125 | 65 | -0.501784526 | ZDHHC7 | zinc finger DHHC-type containing 7 [Source:HGNC Symbol;Acc:HGNC:18459] |
| ENSG00000156787 | HGLibA_48537 | 65 | -0.501784526 | TBC1D31 | TBC1 domain family member 31 [Source:HGNC Symbol;Acc:HGNC:30888] |
| ENSG00000158715 | HGLibA_45179 | 65 | -0.501784526 | SLC45A3 | solute carrier family 45 member 3 [Source:HGNC Symbol;Acc:HGNC:8642] |
| ENSG00000159131 | HGLibA_18638 | 65 | -0.501784526 | GART | phosphoribosylaminoimidazole synthetase [Source:HGNC Symbol;Acc:HGNC:4163]" |
| ENSG00000160183 | HGLibA_50639 | 65 | -0.501784526 | TMPRSS3 | transmembrane serine protease 3 [Source:HGNC Symbol;Acc:HGNC:11877] |
| ENSG00000160710 | HGLibA_00868 | 65 | -0.501784526 | ADAR | RNA specific [Source:HGNC Symbol;Acc:HGNC:225]" |
| ENSG00000160994 | HGLibA_07519 | 65 | -0.501784526 | CCDC105 | coiled-coil domain containing 105 [Source:HGNC Symbol;Acc:HGNC:26866] |
| ENSG00000162817 | HGLibA_05754 | 65 | -0.501784526 | C1orf115 | chromosome 1 open reading frame 115 [Source:HGNC Symbol;Acc:HGNC:25873] |
| ENSG00000163125 | HGLibA_42050 | 65 | -0.501784526 | RPRD2 | regulation of nuclear pre-mRNA domain containing 2 [Source:HGNC Symbol;Acc:HGNC:29039] |
| ENSG00000163399 | HGLibA_03581 | 65 | -0.501784526 | ATP1A1 | ATPase Na+/K+ transporting subunit alpha 1 [Source:HGNC Symbol;Acc:HGNC:799] |
| ENSG00000163534 | HGLibA_17224 | 65 | -0.501784526 | FCRL1 | Fc receptor like 1 [Source:HGNC Symbol;Acc:HGNC:18509] |
| ENSG00000165192 | HGLibA_03210 | 65 | -0.501784526 | ASB11 | ankyrin repeat and SOCS box containing 11 [Source:HGNC Symbol;Acc:HGNC:17186] |
| ENSG00000166012 | HGLibA_48222 | 65 | -0.501784526 | TAF1D | RNA polymerase I subunit D [Source:HGNC Symbol;Acc:HGNC:28759]" |
| ENSG00000166377 | HGLibA_03795 | 65 | -0.501784526 | ATP9B | ATPase phospholipid transporting 9B (putative) [Source:HGNC Symbol;Acc:HGNC:13541] |
| ENSG00000166913 | HGLibA_54738 | 65 | -0.501784526 | YWHAB | tyrosine 3-monooxygenase/tryptophan 5-monooxygenase activation protein beta [Source:HGNC Symbol;Acc:HGNC:12849] |
| ENSG00000167098 | HGLibA_47797 | 65 | -0.501784526 | SUN5 | Sad1 and UNC84 domain containing 5 [Source:HGNC Symbol;Acc:HGNC:16252] |
| ENSG00000167397 | HGLibA_53676 | 65 | -0.501784526 | VKORC1 | vitamin K epoxide reductase complex subunit 1 [Source:HGNC Symbol;Acc:HGNC:23663] |
| ENSG00000167601 | HGLibA_03928 | 65 | -0.501784526 | AXL | AXL receptor tyrosine kinase [Source:HGNC Symbol;Acc:HGNC:905] |
| ENSG00000167608 | HGLibA_49757 | 65 | -0.501784526 | TMC4 | transmembrane channel like 4 [Source:HGNC Symbol;Acc:HGNC:22998] |
| ENSG00000167716 | HGLibA_54188 | 65 | -0.501784526 | WDR81 | WD repeat domain 81 [Source:HGNC Symbol;Acc:HGNC:26600] |
| ENSG00000168291 | HGLibA_35869 | 65 | -0.501784526 | PDHB | pyruvate dehydrogenase E1 beta subunit [Source:HGNC Symbol;Acc:HGNC:8808] |
| ENSG00000169951 | HGLibA_56690 | 65 | -0.501784526 | ZNF764 | zinc finger protein 764 [Source:HGNC Symbol;Acc:HGNC:28200] |
| ENSG00000170516 | HGLibA_10869 | 65 | -0.501784526 | COX7B2 | cytochrome c oxidase subunit 7B2 [Source:HGNC Symbol;Acc:HGNC:24381] |
| ENSG00000170638 | HGLibA_51227 | 65 | -0.501784526 | TRABD | TraB domain containing [Source:HGNC Symbol;Acc:HGNC:28805] |
| ENSG00000171105 | HGLibA_23401 | 65 | -0.501784526 | INSR | insulin receptor [Source:HGNC Symbol;Acc:HGNC:6091] |
| ENSG00000171126 | HGLibA_24205 | 65 | -0.501784526 | KCNG3 | potassium voltage-gated channel modifier subfamily G member 3 [Source:HGNC Symbol;Acc:HGNC:18306] |
| ENSG00000171208 | HGLibA_31706 | 65 | -0.501784526 | NETO2 | neuropilin and tolloid like 2 [Source:HGNC Symbol;Acc:HGNC:14644] |
| ENSG00000171208 | HGLibA_31707 | 65 | -0.501784526 | NETO2 | neuropilin and tolloid like 2 [Source:HGNC Symbol;Acc:HGNC:14644] |
| ENSG00000172377 | HGLibA_34577 | 65 | -0.501784526 | OR9I1 | olfactory receptor family 9 subfamily I member 1 [Source:HGNC Symbol;Acc:HGNC:14718] |
| ENSG00000172717 | HGLibA_16694 | 65 | -0.501784526 | FAM71D | family with sequence similarity 71 member D [Source:HGNC Symbol;Acc:HGNC:20101] |
| ENSG00000172954 | HGLibA_26158 | 65 | -0.501784526 | LCLAT1 | lysocardiolipin acyltransferase 1 [Source:HGNC Symbol;Acc:HGNC:26756] |
| ENSG00000173120 | HGLibA_24494 | 65 | -0.501784526 | KDM2A | lysine demethylase 2A [Source:HGNC Symbol;Acc:HGNC:13606] |
| ENSG00000173200 | HGLibA_35225 | 65 | -0.501784526 | PARP15 | poly(ADP-ribose) polymerase family member 15 [Source:HGNC Symbol;Acc:HGNC:26876] |
| ENSG00000173914 | HGLibA_40572 | 65 | -0.501784526 | RBM4B | RNA binding motif protein 4B [Source:HGNC Symbol;Acc:HGNC:28842] |
| ENSG00000175920 | HGLibA_13784 | 65 | -0.501784526 | DOK7 | docking protein 7 [Source:HGNC Symbol;Acc:HGNC:26594] |
| ENSG00000178217 | HGLibA_43901 | 65 | -0.501784526 | SH2D4B | SH2 domain containing 4B [Source:HGNC Symbol;Acc:HGNC:31440] |
| ENSG00000179604 | HGLibA_08576 | 65 | -0.501784526 | CDC42EP4 | CDC42 effector protein 4 [Source:HGNC Symbol;Acc:HGNC:17147] |
| ENSG00000180263 | HGLibA_17356 | 65 | -0.501784526 | FGD6 | RhoGEF and PH domain containing 6 [Source:HGNC Symbol;Acc:HGNC:21740]" |
| ENSG00000180318 | HGLibA_01734 | 65 | -0.501784526 | ALX1 | ALX homeobox 1 [Source:HGNC Symbol;Acc:HGNC:1494] |
| ENSG00000180433 | HGLibA_34425 | 65 | -0.501784526 | OR6K6 | olfactory receptor family 6 subfamily K member 6 [Source:HGNC Symbol;Acc:HGNC:15033] |
| ENSG00000180483 | HGLibA_12866 | 65 | -0.501784526 | DEFB119 | defensin beta 119 [Source:HGNC Symbol;Acc:HGNC:18099] |
| ENSG00000181023 | HGLibA_34223 | 65 | -0.501784526 | OR56B1 | olfactory receptor family 56 subfamily B member 1 [Source:HGNC Symbol;Acc:HGNC:15245] |
| ENSG00000181481 | HGLibA_41511 | 65 | -0.501784526 | RNF135 | ring finger protein 135 [Source:HGNC Symbol;Acc:HGNC:21158] |
| ENSG00000181939 | HGLibA_33946 | 65 | -0.501784526 | OR4C15 | olfactory receptor family 4 subfamily C member 15 [Source:HGNC Symbol;Acc:HGNC:15171] |
| ENSG00000182583 | HGLibA_53582 | 65 | -0.501784526 | VCX | variable charge X-linked [Source:HGNC Symbol;Acc:HGNC:12667] |
| ENSG00000182985 | HGLibA_06965 | 65 | -0.501784526 | CADM1 | cell adhesion molecule 1 [Source:HGNC Symbol;Acc:HGNC:5951] |
| ENSG00000183785 | HGLibA_52264 | 65 | -0.501784526 | TUBA8 | tubulin alpha 8 [Source:HGNC Symbol;Acc:HGNC:12410] |
| ENSG00000184677 | HGLibA_54881 | 65 | -0.501784526 | ZBTB40 | zinc finger and BTB domain containing 40 [Source:HGNC Symbol;Acc:HGNC:29045] |
| ENSG00000184863 | HGLibA_40532 | 65 | -0.501784526 | RBM33 | RNA binding motif protein 33 [Source:HGNC Symbol;Acc:HGNC:27223] |
| ENSG00000185085 | HGLibA_23425 | 65 | -0.501784526 | INTS5 | integrator complex subunit 5 [Source:HGNC Symbol;Acc:HGNC:29352] |
| ENSG00000185264 | HGLibA_49096 | 65 | -0.501784526 | TEX33 | testis expressed 33 [Source:HGNC Symbol;Acc:HGNC:28568] |
| ENSG00000185652 | HGLibA_32839 | 65 | -0.501784526 | NTF3 | neurotrophin 3 [Source:HGNC Symbol;Acc:HGNC:8023] |
| ENSG00000186638 | HGLibA_24909 | 65 | -0.501784526 | KIF24 | kinesin family member 24 [Source:HGNC Symbol;Acc:HGNC:19916] |
| ENSG00000189233 | HGLibA_33006 | 65 | -0.501784526 | NUGGC | germinal center associated [Source:HGNC Symbol;Acc:HGNC:33550]" |
| ENSG00000189298 | HGLibA_55352 | 65 | -0.501784526 | ZKSCAN3 | zinc finger with KRAB and SCAN domains 3 [Source:HGNC Symbol;Acc:HGNC:13853] |
| ENSG00000196169 | HGLibA_24880 | 65 | -0.501784526 | KIF19 | kinesin family member 19 [Source:HGNC Symbol;Acc:HGNC:26735] |
| ENSG00000197555 | HGLibA_44192 | 65 | -0.501784526 | SIPA1L1 | signal induced proliferation associated 1 like 1 [Source:HGNC Symbol;Acc:HGNC:20284] |
| ENSG00000197780 | HGLibA_48207 | 65 | -0.501784526 | TAF13 | TATA-box binding protein associated factor 13 [Source:HGNC Symbol;Acc:HGNC:11546] |
| ENSG00000197786 | HGLibA_34258 | 65 | -0.501784526 | OR5B17 | olfactory receptor family 5 subfamily B member 17 [Source:HGNC Symbol;Acc:HGNC:15267] |
| ENSG00000198934 | HGLibA_27979 | 65 | -0.501784526 | MAGEE1 | MAGE family member E1 [Source:HGNC Symbol;Acc:HGNC:24934] |
| ENSG00000203943 | HGLibA_42670 | 65 | -0.501784526 | SAMD13 | sterile alpha motif domain containing 13 [Source:HGNC Symbol;Acc:HGNC:24582] |
| ENSG00000204138 | HGLibA_36306 | 65 | -0.501784526 | PHACTR4 | phosphatase and actin regulator 4 [Source:HGNC Symbol;Acc:HGNC:25793] |
| ENSG00000204424 | HGLibA_27672 | 65 | -0.501784526 | LY6G6F | lymphocyte antigen 6 family member G6F [Source:HGNC Symbol;Acc:HGNC:13933] |
| ENSG00000205364 | HGLibA_30295 | 65 | -0.501784526 | MT1M | metallothionein 1M [Source:HGNC Symbol;Acc:HGNC:14296] |
| ENSG00000205496 | HGLibA_34074 | 65 | -0.501784526 | OR51A2 | olfactory receptor family 51 subfamily A member 2 [Source:HGNC Symbol;Acc:HGNC:14764] |
| ENSG00000206073 | HGLibA_43553 | 65 | -0.501784526 | SERPINB4 | serpin family B member 4 [Source:HGNC Symbol;Acc:HGNC:10570] |
| ENSG00000213015 | HGLibA_56306 | 65 | -0.501784526 | ZNF580 | zinc finger protein 580 [Source:HGNC Symbol;Acc:HGNC:29473] |
| ENSG00000235194 | HGLibA_38109 | 65 | -0.501784526 | PPP1R3E | protein phosphatase 1 regulatory subunit 3E [Source:HGNC Symbol;Acc:HGNC:14943] |
| ENSG00000239810 | HGLibA_38285 | 65 | -0.501784526 | PRAMEF11 | PRAME family member 11 [Source:HGNC Symbol;Acc:HGNC:14086] |
| ENSG00000240184 | HGLibA_35572 | 65 | -0.501784526 | PCDHGC3 | 3 [Source:HGNC Symbol;Acc:HGNC:8716]" |
| ENSG00000248871 | HGLibA_50804 | 65 | -0.501784526 | TNFSF12-TNFSF13 | TNFSF12-TNFSF13 readthrough [Source:HGNC Symbol;Acc:HGNC:33537] |
| ENSG00000250745 | HGLibA_53246 | 65 | -0.501784526 | USP17L20 | ubiquitin specific peptidase 17-like family member 20 [Source:HGNC Symbol;Acc:HGNC:44448] |
| ENSG00000251655 | HGLibA_38337 | 65 | -0.501784526 | PRB1 | proline rich protein BstNI subfamily 1 [Source:HGNC Symbol;Acc:HGNC:9337] |
| ENSG00000254440 | HGLibA_35337 | 65 | -0.501784526 | PBOV1 | prostate and breast cancer overexpressed 1 [Source:HGNC Symbol;Acc:HGNC:21079] |
| ENSG00000254585 | HGLibA_27993 | 65 | -0.501784526 | MAGEL2 | MAGE family member L2 [Source:HGNC Symbol;Acc:HGNC:6814] |
| ENSG00000265763 | HGLibA_56085 | 65 | -0.501784526 | ZNF488 | zinc finger protein 488 [Source:HGNC Symbol;Acc:HGNC:23535] |
| ENSG00000276430 | HGLibA_16556 | 65 | -0.501784526 | FAM25C | family with sequence similarity 25 member C [Source:HGNC Symbol;Acc:HGNC:23586] |
| ENSG00000279111 | HGLibA_33557 | 65 | -0.501784526 | OR10X1 | olfactory receptor family 10 subfamily X member 1 (gene/pseudogene) [Source:HGNC Symbol;Acc:HGNC:14995] |
| ENSG00000281887 | HGLibA_19072 | 65 | -0.501784526 | GIMAP1-GIMAP5 | GIMAP1-GIMAP5 readthrough [Source:HGNC Symbol;Acc:HGNC:51257] |
| GPR113 | HGLibA_19856 | 65 | -0.501784526 | GPR113 | - |
| HDGFRP2 | HGLibA_21045 | 65 | -0.501784526 | HDGFRP2 | - |
| HIST1H2BO | HGLibA_21453 | 65 | -0.501784526 | HIST1H2BO | - |
| hsa-mir-126 | HGLibA_57454 | 65 | -0.501784526 | hsa-mir-126 | - |
| hsa-mir-135a-2 | HGLibA_57797 | 65 | -0.501784526 | hsa-mir-135a-2 | - |
| hsa-mir-3125 | HGLibA_58554 | 65 | -0.501784526 | hsa-mir-3125 | - |
| hsa-mir-3152 | HGLibA_58666 | 65 | -0.501784526 | hsa-mir-3152 | - |
| hsa-mir-3179-1 | HGLibA_58789 | 65 | -0.501784526 | hsa-mir-3179-1 | - |
| hsa-mir-3187 | HGLibA_58846 | 65 | -0.501784526 | hsa-mir-3187 | - |
| hsa-mir-3200 | HGLibA_58906 | 65 | -0.501784526 | hsa-mir-3200 | - |
| hsa-mir-340 | HGLibA_59018 | 65 | -0.501784526 | hsa-mir-340 | - |
| hsa-mir-3621 | HGLibA_59118 | 65 | -0.501784526 | hsa-mir-3621 | - |
| hsa-mir-375 | HGLibA_59402 | 65 | -0.501784526 | hsa-mir-375 | - |
| hsa-mir-3908 | HGLibA_59498 | 65 | -0.501784526 | hsa-mir-3908 | - |
| hsa-mir-4266 | HGLibA_59771 | 65 | -0.501784526 | hsa-mir-4266 | - |
| hsa-mir-4315-1 | HGLibA_59974 | 65 | -0.501784526 | hsa-mir-4315-1 | - |
| hsa-mir-4459 | HGLibA_60242 | 65 | -0.501784526 | hsa-mir-4459 | - |
| hsa-mir-4654 | HGLibA_60725 | 65 | -0.501784526 | hsa-mir-4654 | - |
| hsa-mir-4697 | HGLibA_60898 | 65 | -0.501784526 | hsa-mir-4697 | - |
| hsa-mir-518a-2 | HGLibA_61657 | 65 | -0.501784526 | hsa-mir-518a-2 | - |
| hsa-mir-548f-3 | HGLibA_61980 | 65 | -0.501784526 | hsa-mir-548f-3 | - |
| hsa-mir-548i-2 | HGLibA_62017 | 65 | -0.501784526 | hsa-mir-548i-2 | - |
| hsa-mir-6715b | HGLibA_63023 | 65 | -0.501784526 | hsa-mir-6715b | - |
| hsa-mir-6770-2 | HGLibA_63251 | 65 | -0.501784526 | hsa-mir-6770-2 | - |
| hsa-mir-6774 | HGLibA_63271 | 65 | -0.501784526 | hsa-mir-6774 | - |
| hsa-mir-6833 | HGLibA_63508 | 65 | -0.501784526 | hsa-mir-6833 | - |
| hsa-mir-6836 | HGLibA_63518 | 65 | -0.501784526 | hsa-mir-6836 | - |
| hsa-mir-8065 | HGLibA_64097 | 65 | -0.501784526 | hsa-mir-8065 | - |
| KIAA1984 | HGLibA_24834 | 65 | -0.501784526 | KIAA1984 | - |
| PTPN20A | HGLibA_39451 | 65 | -0.501784526 | PTPN20A | - |
| TAZ | HGLibA_48455 | 65 | -0.501784526 | TAZ | - |
| TMEM110 | HGLibA_49874 | 65 | -0.501784526 | TMEM110 | - |
| TMEM110 | HGLibA_49875 | 65 | -0.501784526 | TMEM110 | - |

| | | | | | |
|---|---|---|---|---|---|
| 15-Sep | HGLibA_43408 | 64 | -0.511735564 | 15-Sep | - |
| ATP5L2 | HGLibA_03683 | 64 | -0.511735564 | ATP5L2 | - |
| C2orf53 | HGLibA_06145 | 64 | -0.511735564 | C2orf53 | - |
| C3orf43 | HGLibA_06245 | 64 | -0.511735564 | C3orf43 | - |
| C4orf21 | HGLibA_06302 | 64 | -0.511735564 | C4orf21 | - |
| C9orf41 | HGLibA_06731 | 64 | -0.511735564 | C9orf41 | - |
| CRAMP1L | HGLibA_11063 | 64 | -0.511735564 | CRAMP1L | - |
| ENSG00000032444 | HGLibA_37421 | 64 | -0.511735564 | PNPLA6 | patatin like phospholipase domain containing 6 [Source:HGNC Symbol;Acc:HGNC:16268] |
| ENSG00000039319 | HGLibA_55274 | 64 | -0.511735564 | ZFYVE16 | zinc finger FYVE-type containing 16 [Source:HGNC Symbol;Acc:HGNC:20756] |
| ENSG00000048828 | HGLibA_16053 | 64 | -0.511735564 | FAM120A | family with sequence similarity 120A [Source:HGNC Symbol;Acc:HGNC:13247] |
| ENSG00000051341 | HGLibA_37546 | 64 | -0.511735564 | POLQ | DNA polymerase theta [Source:HGNC Symbol;Acc:HGNC:9186] |
| ENSG00000053900 | HGLibA_01884 | 64 | -0.511735564 | ANAPC4 | anaphase promoting complex subunit 4 [Source:HGNC Symbol;Acc:HGNC:19990] |
| ENSG00000054965 | HGLibA_16246 | 64 | -0.511735564 | FAM168A | family with sequence similarity 168 member A [Source:HGNC Symbol;Acc:HGNC:28999] |
| ENSG00000055917 | HGLibA_39582 | 64 | -0.511735564 | PUM2 | pumilio RNA binding family member 2 [Source:HGNC Symbol;Acc:HGNC:14958] |
| ENSG00000065057 | HGLibA_32843 | 64 | -0.511735564 | NTHL1 | nth like DNA glycosylase 1 [Source:HGNC Symbol;Acc:HGNC:8028] |
| ENSG00000068796 | HGLibA_24924 | 64 | -0.511735564 | KIF2A | kinesin family member 2A [Source:HGNC Symbol;Acc:HGNC:6318] |
| ENSG00000075043 | HGLibA_24369 | 64 | -0.511735564 | KCNQ2 | potassium voltage-gated channel subfamily Q member 2 [Source:HGNC Symbol;Acc:HGNC:6296] |
| ENSG00000075388 | HGLibA_17409 | 64 | -0.511735564 | FGF4 | fibroblast growth factor 4 [Source:HGNC Symbol;Acc:HGNC:3682] |
| ENSG00000078053 | HGLibA_01831 | 64 | -0.511735564 | AMPH | amphiphysin [Source:HGNC Symbol;Acc:HGNC:471] |
| ENSG00000081842 | HGLibA_35452 | 64 | -0.511735564 | PCDHA6 | protocadherin alpha 6 [Source:HGNC Symbol;Acc:HGNC:8672] |
| ENSG00000086544 | HGLibA_23849 | 64 | -0.511735564 | ITPKC | inositol-trisphosphate 3-kinase C [Source:HGNC Symbol;Acc:HGNC:14897] |
| ENSG00000087111 | HGLibA_36584 | 64 | -0.511735564 | PIGS | phosphatidylinositol glycan anchor biosynthesis class S [Source:HGNC Symbol;Acc:HGNC:14937] |
| ENSG00000088926 | HGLibA_15845 | 64 | -0.511735564 | F11 | coagulation factor XI [Source:HGNC Symbol;Acc:HGNC:3529] |
| ENSG00000094796 | HGLibA_25475 | 64 | -0.511735564 | KRT31 | keratin 31 [Source:HGNC Symbol;Acc:HGNC:6448] |
| ENSG00000099810 | HGLibA_30320 | 64 | -0.511735564 | MTAP | methylthioadenosine phosphorylase [Source:HGNC Symbol;Acc:HGNC:7413] |
| ENSG00000100490 | HGLibA_08807 | 64 | -0.511735564 | CDKL1 | cyclin dependent kinase like 1 [Source:HGNC Symbol;Acc:HGNC:1781] |
| ENSG00000101203 | HGLibA_10536 | 64 | -0.511735564 | COL20A1 | collagen type XX alpha 1 chain [Source:HGNC Symbol;Acc:HGNC:14670] |
| ENSG00000101246 | HGLibA_02681 | 64 | -0.511735564 | ARFRP1 | ADP ribosylation factor related protein 1 [Source:HGNC Symbol;Acc:HGNC:662] |
| ENSG00000101282 | HGLibA_42354 | 64 | -0.511735564 | RSPO4 | R-spondin 4 [Source:HGNC Symbol;Acc:HGNC:16175] |
| ENSG00000101695 | HGLibA_41493 | 64 | -0.511735564 | RNF125 | ring finger protein 125 [Source:HGNC Symbol;Acc:HGNC:21150] |
| ENSG00000102001 | HGLibA_06889 | 64 | -0.511735564 | CACNA1F | calcium voltage-gated channel subunit alpha1 F [Source:HGNC Symbol;Acc:HGNC:1393] |
| ENSG00000102057 | HGLibA_24175 | 64 | -0.511735564 | KCND1 | potassium voltage-gated channel subfamily D member 1 [Source:HGNC Symbol;Acc:HGNC:6237] |
| ENSG00000102145 | HGLibA_18664 | 64 | -0.511735564 | GATA1 | GATA binding protein 1 [Source:HGNC Symbol;Acc:HGNC:4170] |
| ENSG00000102890 | HGLibA_14936 | 64 | -0.511735564 | ELMO3 | engulfment and cell motility 3 [Source:HGNC Symbol;Acc:HGNC:17289] |
| ENSG00000102898 | HGLibA_33100 | 64 | -0.511735564 | NUTF2 | nuclear transport factor 2 [Source:HGNC Symbol;Acc:HGNC:13722] |
| ENSG00000103064 | HGLibA_45372 | 64 | -0.511735564 | SLC7A6 | solute carrier family 7 member 6 [Source:HGNC Symbol;Acc:HGNC:11064] |
| ENSG00000103351 | HGLibA_10159 | 64 | -0.511735564 | CLUAP1 | clusterin associated protein 1 [Source:HGNC Symbol;Acc:HGNC:19009] |
| ENSG00000104131 | HGLibA_14792 | 64 | -0.511735564 | EIF3J | eukaryotic translation initiation factor 3 subunit J [Source:HGNC Symbol;Acc:HGNC:3270] |
| ENSG00000104814 | HGLibA_28211 | 64 | -0.511735564 | MAP4K1 | mitogen-activated protein kinase kinase kinase kinase 1 [Source:HGNC Symbol;Acc:HGNC:6863] |
| ENSG00000104941 | HGLibA_42337 | 64 | -0.511735564 | RSPH6A | radial spoke head 6 homolog A [Source:HGNC Symbol;Acc:HGNC:14241] |
| ENSG00000104953 | HGLibA_49620 | 64 | -0.511735564 | TLE6 | transducin like enhancer of split 6 [Source:HGNC Symbol;Acc:HGNC:30788] |
| ENSG00000105447 | HGLibA_20387 | 64 | -0.511735564 | GRWD1 | glutamate rich WD repeat containing 1 [Source:HGNC Symbol;Acc:HGNC:21270] |
| ENSG00000105696 | HGLibA_50463 | 64 | -0.511735564 | TMEM59L | transmembrane protein 59 like [Source:HGNC Symbol;Acc:HGNC:13237] |
| ENSG00000105821 | HGLibA_13593 | 64 | -0.511735564 | DNAJC2 | DnaJ heat shock protein family (Hsp40) member C2 [Source:HGNC Symbol;Acc:HGNC:13192] |
| ENSG00000106686 | HGLibA_46526 | 64 | -0.511735564 | SPATA6L | spermatogenesis associated 6 like [Source:HGNC Symbol;Acc:HGNC:25472] |
| ENSG00000108559 | HGLibA_33071 | 64 | -0.511735564 | NUP88 | nucleoporin 88 [Source:HGNC Symbol;Acc:HGNC:8067] |
| ENSG00000108691 | HGLibA_07988 | 64 | -0.511735564 | CCL2 | C-C motif chemokine ligand 2 [Source:HGNC Symbol;Acc:HGNC:10618] |
| ENSG00000108825 | HGLibA_39338 | 64 | -0.511735564 | PTGES3L-AARSD1 | PTGES3L-AARSD1 readthrough [Source:HGNC Symbol;Acc:HGNC:43946] |
| ENSG00000108878 | HGLibA_06929 | 64 | -0.511735564 | CACNG1 | calcium voltage-gated channel auxiliary subunit gamma 1 [Source:HGNC Symbol;Acc:HGNC:1405] |
| ENSG00000109738 | HGLibA_19310 | 64 | -0.511735564 | GLRB | glycine receptor beta [Source:HGNC Symbol;Acc:HGNC:4329] |
| ENSG00000110435 | HGLibA_35872 | 64 | -0.511735564 | PDHX | pyruvate dehydrogenase complex component X [Source:HGNC Symbol;Acc:HGNC:21350] |
| ENSG00000112799 | HGLibA_27685 | 64 | -0.511735564 | LY86 | lymphocyte antigen 86 [Source:HGNC Symbol;Acc:HGNC:16837] |
| ENSG00000112964 | HGLibA_19039 | 64 | -0.511735564 | GHR | growth hormone receptor [Source:HGNC Symbol;Acc:HGNC:4263] |
| ENSG00000114279 | HGLibA_17369 | 64 | -0.511735564 | FGF12 | fibroblast growth factor 12 [Source:HGNC Symbol;Acc:HGNC:3668] |
| ENSG00000115155 | HGLibA_34726 | 64 | -0.511735564 | OTOF | otoferlin [Source:HGNC Symbol;Acc:HGNC:8515] |
| ENSG00000115649 | HGLibA_10358 | 64 | -0.511735564 | CNPPD1 | cyclin Pas1/PHO80 domain containing 1 [Source:HGNC Symbol;Acc:HGNC:25220] |
| ENSG00000116031 | HGLibA_08290 | 64 | -0.511735564 | CD207 | CD207 molecule [Source:HGNC Symbol;Acc:HGNC:17935] |
| ENSG00000116127 | HGLibA_01673 | 64 | -0.511735564 | ALMS1 | centrosome and basal body associated protein [Source:HGNC Symbol;Acc:HGNC:428]" |
| ENSG00000116183 | HGLibA_35148 | 64 | -0.511735564 | PAPPA2 | pappalysin 2 [Source:HGNC Symbol;Acc:HGNC:14615] |
| ENSG00000116996 | HGLibA_56959 | 64 | -0.511735564 | ZP4 | zona pellucida glycoprotein 4 [Source:HGNC Symbol;Acc:HGNC:15770] |
| ENSG00000117480 | HGLibA_15901 | 64 | -0.511735564 | FAAH | fatty acid amide hydrolase [Source:HGNC Symbol;Acc:HGNC:3553] |
| ENSG00000118369 | HGLibA_53343 | 64 | -0.511735564 | USP35 | ubiquitin specific peptidase 35 [Source:HGNC Symbol;Acc:HGNC:20061] |
| ENSG00000119632 | HGLibA_22601 | 64 | -0.511735564 | IFI27L2 | interferon alpha inducible protein 27 like 2 [Source:HGNC Symbol;Acc:HGNC:19753] |
| ENSG00000120798 | HGLibA_32596 | 64 | -0.511735564 | NR2C1 | nuclear receptor subfamily 2 group C member 1 [Source:HGNC Symbol;Acc:HGNC:7971] |
| ENSG00000121871 | HGLibA_45518 | 64 | -0.511735564 | SLITRK3 | SLIT and NTRK like family member 3 [Source:HGNC Symbol;Acc:HGNC:23501] |
| ENSG00000121895 | HGLibA_50012 | 64 | -0.511735564 | TMEM156 | transmembrane protein 156 [Source:HGNC Symbol;Acc:HGNC:26260] |
| ENSG00000124006 | HGLibA_33234 | 64 | -0.511735564 | OBSL1 | obscurin like 1 [Source:HGNC Symbol;Acc:HGNC:29092] |
| ENSG00000126878 | HGLibA_01334 | 64 | -0.511735564 | AIF1L | allograft inflammatory factor 1 like [Source:HGNC Symbol;Acc:HGNC:28904] |
| ENSG00000127530 | HGLibA_34467 | 64 | -0.511735564 | OR7C1 | olfactory receptor family 7 subfamily C member 1 [Source:HGNC Symbol;Acc:HGNC:8373] |
| ENSG00000128487 | HGLibA_46604 | 64 | -0.511735564 | SPECC1 | sperm antigen with calponin homology and coiled-coil domains 1 [Source:HGNC Symbol;Acc:HGNC:30615] |
| ENSG00000129083 | HGLibA_10695 | 64 | -0.511735564 | COPB1 | coatomer protein complex subunit beta 1 [Source:HGNC Symbol;Acc:HGNC:2231] |
| ENSG00000129467 | HGLibA_00918 | 64 | -0.511735564 | ADCY4 | adenylate cyclase 4 [Source:HGNC Symbol;Acc:HGNC:235] |
| ENSG00000129744 | HGLibA_03157 | 64 | -0.511735564 | ART1 | ADP-ribosyltransferase 1 [Source:HGNC Symbol;Acc:HGNC:723] |
| ENSG00000129749 | HGLibA_09566 | 64 | -0.511735564 | CHRNA10 | cholinergic receptor nicotinic alpha 10 subunit [Source:HGNC Symbol;Acc:HGNC:13800] |
| ENSG00000130052 | HGLibA_47374 | 64 | -0.511735564 | STARD8 | StAR related lipid transfer domain containing 8 [Source:HGNC Symbol;Acc:HGNC:19161] |
| ENSG00000130733 | HGLibA_54674 | 64 | -0.511735564 | YIPF2 | Yip1 domain family member 2 [Source:HGNC Symbol;Acc:HGNC:28476] |
| ENSG00000131115 | HGLibA_55628 | 64 | -0.511735564 | ZNF227 | zinc finger protein 227 [Source:HGNC Symbol;Acc:HGNC:13020] |
| ENSG00000131686 | HGLibA_06818 | 64 | -0.511735564 | CA6 | carbonic anhydrase 6 [Source:HGNC Symbol;Acc:HGNC:1380] |
| ENSG00000135002 | HGLibA_40922 | 64 | -0.511735564 | RFK | riboflavin kinase [Source:HGNC Symbol;Acc:HGNC:30324] |
| ENSG00000135316 | HGLibA_47967 | 64 | -0.511735564 | SYNCRIP | synaptotagmin binding cytoplasmic RNA interacting protein [Source:HGNC Symbol;Acc:HGNC:16918] |
| ENSG00000135447 | HGLibA_38064 | 64 | -0.511735564 | PPP1R1A | protein phosphatase 1 regulatory inhibitor subunit 1A [Source:HGNC Symbol;Acc:HGNC:9286] |
| ENSG00000135597 | HGLibA_40848 | 64 | -0.511735564 | REPS1 | RALBP1 associated Eps domain containing 1 [Source:HGNC Symbol;Acc:HGNC:15578] |
| ENSG00000136634 | HGLibA_22943 | 64 | -0.511735564 | IL10 | interleukin 10 [Source:HGNC Symbol;Acc:HGNC:5962] |
| ENSG00000136819 | HGLibA_06761 | 64 | -0.511735564 | C9orf78 | chromosome 9 open reading frame 78 [Source:HGNC Symbol;Acc:HGNC:24932] |
| ENSG00000137941 | HGLibA_52212 | 64 | -0.511735564 | TTLL7 | tubulin tyrosine ligase like 7 [Source:HGNC Symbol;Acc:HGNC:26242] |
| ENSG00000137962 | HGLibA_02751 | 64 | -0.511735564 | ARHGAP29 | Rho GTPase activating protein 29 [Source:HGNC Symbol;Acc:HGNC:30207] |
| ENSG00000138069 | HGLibA_39829 | 64 | -0.511735564 | RAB1A | member RAS oncogene family [Source:HGNC Symbol;Acc:HGNC:9758]" |
| ENSG00000139154 | HGLibA_01087 | 64 | -0.511735564 | AEBP2 | AE binding protein 2 [Source:HGNC Symbol;Acc:HGNC:24051] |
| ENSG00000139182 | HGLibA_10141 | 64 | -0.511735564 | CLSTN3 | calsyntenin 3 [Source:HGNC Symbol;Acc:HGNC:18371] |
| ENSG00000139899 | HGLibA_07412 | 64 | -0.511735564 | CBLN3 | cerebellin 3 precursor [Source:HGNC Symbol;Acc:HGNC:20146] |
| ENSG00000141519 | HGLibA_07774 | 64 | -0.511735564 | CCDC40 | coiled-coil domain containing 40 [Source:HGNC Symbol;Acc:HGNC:26090] |
| ENSG00000142530 | HGLibA_16698 | 64 | -0.511735564 | FAM71E1 | family with sequence similarity 71 member E1 [Source:HGNC Symbol;Acc:HGNC:25107] |
| ENSG00000143167 | HGLibA_19716 | 64 | -0.511735564 | GPA33 | glycoprotein A33 [Source:HGNC Symbol;Acc:HGNC:4445] |
| ENSG00000145335 | HGLibA_45908 | 64 | -0.511735564 | SNCA | synuclein alpha [Source:HGNC Symbol;Acc:HGNC:11138] |
| ENSG00000145743 | HGLibA_16989 | 64 | -0.511735564 | FBXL17 | F-box and leucine rich repeat protein 17 [Source:HGNC Symbol;Acc:HGNC:13615] |
| ENSG00000145832 | HGLibA_44758 | 64 | -0.511735564 | SLC25A48 | solute carrier family 25 member 48 [Source:HGNC Symbol;Acc:HGNC:30451] |
| ENSG00000146910 | HGLibA_10362 | 64 | -0.511735564 | CNPY1 | canopy FGF signaling regulator 1 [Source:HGNC Symbol;Acc:HGNC:27786] |
| ENSG00000148331 | HGLibA_03246 | 64 | -0.511735564 | ASB6 | ankyrin repeat and SOCS box containing 6 [Source:HGNC Symbol;Acc:HGNC:17181] |
| ENSG00000149761 | HGLibA_32972 | 64 | -0.511735564 | NUDT22 | nudix hydrolase 22 [Source:HGNC Symbol;Acc:HGNC:28189] |
| ENSG00000151033 | HGLibA_27789 | 64 | -0.511735564 | LYZL2 | lysozyme like 2 [Source:HGNC Symbol;Acc:HGNC:29613] |
| ENSG00000151079 | HGLibA_24140 | 64 | -0.511735564 | KCNA6 | potassium voltage-gated channel subfamily A member 6 [Source:HGNC Symbol;Acc:HGNC:6225] |
| ENSG00000151490 | HGLibA_39525 | 64 | -0.511735564 | PTPRO | receptor type O [Source:HGNC Symbol;Acc:HGNC:9678]" |
| ENSG00000151689 | HGLibA_23333 | 64 | -0.511735564 | INPP1 | inositol polyphosphate-1-phosphatase [Source:HGNC Symbol;Acc:HGNC:6071] |
| ENSG00000152591 | HGLibA_14040 | 64 | -0.511735564 | DSPP | dentin sialophosphoprotein [Source:HGNC Symbol;Acc:HGNC:3054] |
| ENSG00000153234 | HGLibA_32629 | 64 | -0.511735564 | NR4A2 | nuclear receptor subfamily 4 group A member 2 [Source:HGNC Symbol;Acc:HGNC:7981] |
| ENSG00000153406 | HGLibA_32192 | 64 | -0.511735564 | NMRAL1 | NmrA like redox sensor 1 [Source:HGNC Symbol;Acc:HGNC:24987] |
| ENSG00000154429 | HGLibA_08187 | 64 | -0.511735564 | CCSAP | cilia and spindle associated protein [Source:HGNC Symbol;Acc:HGNC:29578]" |
| ENSG00000154845 | HGLibA_38203 | 64 | -0.511735564 | PPP4R1 | protein phosphatase 4 regulatory subunit 1 [Source:HGNC Symbol;Acc:HGNC:9320] |
| ENSG00000155906 | HGLibA_41378 | 64 | -0.511735564 | RMND1 | required for meiotic nuclear division 1 homolog [Source:HGNC Symbol;Acc:HGNC:21176] |
| ENSG00000157335 | HGLibA_09949 | 64 | -0.511735564 | CLEC18C | C-type lectin domain family 18 member C [Source:HGNC Symbol;Acc:HGNC:28538] |
| ENSG00000157954 | HGLibA_54317 | 64 | -0.511735564 | WIPI2 | phosphoinositide interacting 2 [Source:HGNC Symbol;Acc:HGNC:32225]" |
| ENSG00000158234 | HGLibA_15963 | 64 | -0.511735564 | FAIM | Fas apoptotic inhibitory molecule [Source:HGNC Symbol;Acc:HGNC:18703] |
| ENSG00000158865 | HGLibA_45259 | 64 | -0.511735564 | SLC5A11 | solute carrier family 5 member 11 [Source:HGNC Symbol;Acc:HGNC:23091] |
| ENSG00000159197 | HGLibA_24189 | 64 | -0.511735564 | KCNE2 | potassium voltage-gated channel subfamily E regulatory subunit 2 [Source:HGNC Symbol;Acc:HGNC:6242] |
| ENSG00000160185 | HGLibA_52557 | 64 | -0.511735564 | UBASH3A | ubiquitin associated and SH3 domain containing A [Source:HGNC Symbol;Acc:HGNC:12462] |
| ENSG00000160948 | HGLibA_53758 | 64 | -0.511735564 | VPS28 | ESCRT-I subunit [Source:HGNC Symbol;Acc:HGNC:18178]" |
| ENSG00000163092 | HGLibA_54515 | 64 | -0.511735564 | XIRP2 | xin actin binding repeat containing 2 [Source:HGNC Symbol;Acc:HGNC:14303] |
| ENSG00000163380 | HGLibA_26724 | 64 | -0.511735564 | LMOD3 | leiomodin 3 [Source:HGNC Symbol;Acc:HGNC:6649] |
| ENSG00000163810 | HGLibA_49263 | 64 | -0.511735564 | TGM4 | transglutaminase 4 [Source:HGNC Symbol;Acc:HGNC:11780] |
| ENSG00000164093 | HGLibA_36764 | 64 | -0.511735564 | PITX2 | paired like homeodomain 2 [Source:HGNC Symbol;Acc:HGNC:9005] |
| ENSG00000164162 | HGLibA_01866 | 64 | -0.511735564 | ANAPC10 | anaphase promoting complex subunit 10 [Source:HGNC Symbol;Acc:HGNC:24077] |
| ENSG00000164334 | HGLibA_16258 | 64 | -0.511735564 | FAM170A | family with sequence similarity 170 member A [Source:HGNC Symbol;Acc:HGNC:27963] |
| ENSG00000164695 | HGLibA_09494 | 64 | -0.511735564 | CHMP4C | charged multivesicular body protein 4C [Source:HGNC Symbol;Acc:HGNC:30599] |
| ENSG00000165304 | HGLibA_28881 | 64 | -0.511735564 | MELK | maternal embryonic leucine zipper kinase [Source:HGNC Symbol;Acc:HGNC:16870] |
| ENSG00000166049 | HGLibA_35266 | 64 | -0.511735564 | PASD1 | PAS domain containing repressor 1 [Source:HGNC Symbol;Acc:HGNC:20686] |
| ENSG00000166323 | HGLibA_05169 | 64 | -0.511735564 | C11orf65 | chromosome 11 open reading frame 65 [Source:HGNC Symbol;Acc:HGNC:28519] |
| ENSG00000166368 | HGLibA_33781 | 64 | -0.511735564 | OR2D2 | olfactory receptor family 2 subfamily D member 2 [Source:HGNC Symbol;Acc:HGNC:8244] |
| ENSG00000166578 | HGLibA_23495 | 64 | -0.511735564 | IQCD | IQ motif containing D [Source:HGNC Symbol;Acc:HGNC:25168] |
| ENSG00000166924 | HGLibA_33181 | 64 | -0.511735564 | NYAP1 | neuronal tyrosine phosphorylated phosphoinositide-3-kinase adaptor 1 [Source:HGNC Symbol;Acc:HGNC:22009] |
| ENSG00000166987 | HGLibA_28494 | 64 | -0.511735564 | MBD6 | methyl-CpG binding domain protein 6 [Source:HGNC Symbol;Acc:HGNC:20445] |
| ENSG00000167333 | HGLibA_51590 | 64 | -0.511735564 | TRIM68 | tripartite motif containing 68 [Source:HGNC Symbol;Acc:HGNC:21161] |
| ENSG00000167633 | HGLibA_24990 | 64 | -0.511735564 | KIR3DL1 | three Ig domains and long cytoplasmic tail 1 [Source:HGNC Symbol;Acc:HGNC:6338]" |
| ENSG00000168066 | HGLibA_43688 | 64 | -0.511735564 | SF1 | splicing factor 1 [Source:HGNC Symbol;Acc:HGNC:12950] |
| ENSG00000168434 | HGLibA_10489 | 64 | -0.511735564 | COG7 | component of oligomeric golgi complex 7 [Source:HGNC Symbol;Acc:HGNC:18622] |
| ENSG00000168959 | HGLibA_20353 | 64 | -0.511735564 | GRM5 | glutamate metabotropic receptor 5 [Source:HGNC Symbol;Acc:HGNC:4597] |

| | | | | | |
|---|---|---|---|---|---|
| ENSG00000169062 | HGLibA_53063 | 64 | -0.511735564 | UPF3A | regulator of nonsense mediated mRNA decay [Source:HGNC Symbol;Acc:HGNC:20332]" |
| ENSG00000169217 | HGLibA_08329 | 64 | -0.511735564 | CD2BP2 | CD2 cytoplasmic tail binding protein 2 [Source:HGNC Symbol;Acc:HGNC:1656] |
| ENSG00000169992 | HGLibA_32069 | 64 | -0.511735564 | NLGN2 | neuroligin 2 [Source:HGNC Symbol;Acc:HGNC:14290] |
| ENSG00000169994 | HGLibA_30866 | 64 | -0.511735564 | MYO7B | myosin VIIB [Source:HGNC Symbol;Acc:HGNC:7607] |
| ENSG00000170276 | HGLibA_22308 | 64 | -0.511735564 | HSPB2 | heat shock protein family B (small) member 2 [Source:HGNC Symbol;Acc:HGNC:5247] |
| ENSG00000170417 | HGLibA_50101 | 64 | -0.511735564 | TMEM182 | transmembrane protein 182 [Source:HGNC Symbol;Acc:HGNC:26391] |
| ENSG00000170604 | HGLibA_23576 | 64 | -0.511735564 | IRF2BP1 | interferon regulatory factor 2 binding protein 1 [Source:HGNC Symbol;Acc:HGNC:21728] |
| ENSG00000170634 | HGLibA_00712 | 64 | -0.511735564 | ACYP2 | acylphosphatase 2 [Source:HGNC Symbol;Acc:HGNC:180] |
| ENSG00000170745 | HGLibA_24390 | 64 | -0.511735564 | KCNS3 | potassium voltage-gated channel modifier subfamily S member 3 [Source:HGNC Symbol;Acc:HGNC:6302] |
| ENSG00000170891 | HGLibA_12251 | 64 | -0.511735564 | CYTL1 | cytokine like 1 [Source:HGNC Symbol;Acc:HGNC:24435] |
| ENSG00000172209 | HGLibA_20002 | 64 | -0.511735564 | GPR22 | G protein-coupled receptor 22 [Source:HGNC Symbol;Acc:HGNC:4477] |
| ENSG00000172889 | HGLibA_14626 | 64 | -0.511735564 | EGFL7 | EGF like domain multiple 7 [Source:HGNC Symbol;Acc:HGNC:20594] |
| ENSG00000172922 | HGLibA_41433 | 64 | -0.511735564 | RNASEH2C | ribonuclease H2 subunit C [Source:HGNC Symbol;Acc:HGNC:24116] |
| ENSG00000173511 | HGLibA_53615 | 64 | -0.511735564 | VEGFB | vascular endothelial growth factor B [Source:HGNC Symbol;Acc:HGNC:12681] |
| ENSG00000174748 | HGLibA_41878 | 64 | -0.511735564 | RPL15 | ribosomal protein L15 [Source:HGNC Symbol;Acc:HGNC:10306] |
| ENSG00000174953 | HGLibA_13189 | 64 | -0.511735564 | DHX36 | DEAH-box helicase 36 [Source:HGNC Symbol;Acc:HGNC:14410] |
| ENSG00000175564 | HGLibA_52836 | 64 | -0.511735564 | UCP3 | uncoupling protein 3 [Source:HGNC Symbol;Acc:HGNC:12519] |
| ENSG00000176623 | HGLibA_41363 | 64 | -0.511735564 | RMDN1 | regulator of microtubule dynamics 1 [Source:HGNC Symbol;Acc:HGNC:24285] |
| ENSG00000176678 | HGLibA_17901 | 64 | -0.511735564 | FOXL1 | forkhead box L1 [Source:HGNC Symbol;Acc:HGNC:3817] |
| ENSG00000177476 | HGLibA_33796 | 64 | -0.511735564 | OR2G3 | olfactory receptor family 2 subfamily G member 3 [Source:HGNC Symbol;Acc:HGNC:15008] |
| ENSG00000178935 | HGLibA_56218 | 64 | -0.511735564 | ZNF552 | zinc finger protein 552 [Source:HGNC Symbol;Acc:HGNC:26135] |
| ENSG00000181396 | HGLibA_33313 | 64 | -0.511735564 | OGFOD3 | 2-oxoglutarate and iron dependent oxygenase domain containing 3 [Source:HGNC Symbol;Acc:HGNC:26174] |
| ENSG00000181785 | HGLibA_34250 | 64 | -0.511735564 | OR5AS1 | olfactory receptor family 5 subfamily AS member 1 [Source:HGNC Symbol;Acc:HGNC:15261] |
| ENSG00000182518 | HGLibA_15991 | 64 | -0.511735564 | FAM104B | family with sequence similarity 104 member B [Source:HGNC Symbol;Acc:HGNC:25085] |
| ENSG00000184208 | HGLibA_06083 | 64 | -0.511735564 | C22orf46 | chromosome 22 open reading frame 46 [Source:HGNC Symbol;Acc:HGNC:26294] |
| ENSG00000184208 | HGLibA_06084 | 64 | -0.511735564 | C22orf46 | chromosome 22 open reading frame 46 [Source:HGNC Symbol;Acc:HGNC:26294] |
| ENSG00000184305 | HGLibA_08188 | 64 | -0.511735564 | CCSER1 | coiled-coil serine rich protein 1 [Source:HGNC Symbol;Acc:HGNC:29349] |
| ENSG00000184659 | HGLibA_17849 | 64 | -0.511735564 | FOXD4L4 | forkhead box D4 like 4 [Source:HGNC Symbol;Acc:HGNC:23762] |
| ENSG00000185739 | HGLibA_46991 | 64 | -0.511735564 | SRL | sarcalumenin [Source:HGNC Symbol;Acc:HGNC:11295] |
| ENSG00000185798 | HGLibA_54122 | 64 | -0.511735564 | WDR53 | WD repeat domain 53 [Source:HGNC Symbol;Acc:HGNC:28786] |
| ENSG00000185873 | HGLibA_50616 | 64 | -0.511735564 | TMPRSS11B | transmembrane serine protease 11B [Source:HGNC Symbol;Acc:HGNC:25398] |
| ENSG00000185942 | HGLibA_31995 | 64 | -0.511735564 | NKAIN3 | sodium/potassium transporting ATPase interacting 3 [Source:HGNC Symbol;Acc:HGNC:26829] |
| ENSG00000187180 | HGLibA_26123 | 64 | -0.511735564 | LCE2C | late cornified envelope 2C [Source:HGNC Symbol;Acc:HGNC:29460] |
| ENSG00000187908 | HGLibA_13392 | 64 | -0.511735564 | DMBT1 | deleted in malignant brain tumors 1 [Source:HGNC Symbol;Acc:HGNC:2926] |
| ENSG00000187959 | HGLibA_11009 | 64 | -0.511735564 | CPSF4L | cleavage and polyadenylation specific factor 4 like [Source:HGNC Symbol;Acc:HGNC:33632] |
| ENSG00000189377 | HGLibA_11863 | 64 | -0.511735564 | CXCL17 | C-X-C motif chemokine ligand 17 [Source:HGNC Symbol;Acc:HGNC:19232] |
| ENSG00000196188 | HGLibA_11701 | 64 | -0.511735564 | CTSE | cathepsin E [Source:HGNC Symbol;Acc:HGNC:2530] |
| ENSG00000196586 | HGLibA_30858 | 64 | -0.511735564 | MYO6 | myosin VI [Source:HGNC Symbol;Acc:HGNC:7605] |
| ENSG00000196748 | HGLibA_10110 | 64 | -0.511735564 | CLPSL2 | colipase like 2 [Source:HGNC Symbol;Acc:HGNC:21250] |
| ENSG00000196961 | HGLibA_02306 | 64 | -0.511735564 | AP2A1 | adaptor related protein complex 2 subunit alpha 1 [Source:HGNC Symbol;Acc:HGNC:561] |
| ENSG00000197079 | HGLibA_25488 | 64 | -0.511735564 | KRT35 | keratin 35 [Source:HGNC Symbol;Acc:HGNC:6453] |
| ENSG00000197125 | HGLibA_34506 | 64 | -0.511735564 | OR8B8 | olfactory receptor family 8 subfamily B member 8 [Source:HGNC Symbol;Acc:HGNC:8477] |
| ENSG00000197948 | HGLibA_17210 | 64 | -0.511735564 | FCHSD1 | FCH and double SH3 domains 1 [Source:HGNC Symbol;Acc:HGNC:25463] |
| ENSG00000198000 | HGLibA_32278 | 64 | -0.511735564 | NOL8 | nucleolar protein 8 [Source:HGNC Symbol;Acc:HGNC:23387] |
| ENSG00000198471 | HGLibA_42424 | 64 | -0.511735564 | RTP2 | receptor transporter protein 2 [Source:HGNC Symbol;Acc:HGNC:32486] |
| ENSG00000198517 | HGLibA_27885 | 64 | -0.511735564 | MAFK | MAF bZIP transcription factor K [Source:HGNC Symbol;Acc:HGNC:6782] |
| ENSG00000198874 | HGLibA_52481 | 64 | -0.511735564 | TYW1 | tRNA-yW synthesizing protein 1 homolog [Source:HGNC Symbol;Acc:HGNC:25598] |
| ENSG00000198901 | HGLibA_38349 | 64 | -0.511735564 | PRC1 | protein regulator of cytokinesis 1 [Source:HGNC Symbol;Acc:HGNC:9341] |
| ENSG00000204071 | HGLibA_48719 | 64 | -0.511735564 | TCEAL6 | transcription elongation factor A like 6 [Source:HGNC Symbol;Acc:HGNC:24553] |
| ENSG00000204351 | HGLibA_44284 | 64 | -0.511735564 | SKIV2L | Ski2 like RNA helicase [Source:HGNC Symbol;Acc:HGNC:10898] |
| ENSG00000205177 | HGLibA_05212 | 64 | -0.511735564 | C11orf91 | chromosome 11 open reading frame 91 [Source:HGNC Symbol;Acc:HGNC:34444] |
| ENSG00000213512 | HGLibA_18746 | 64 | -0.511735564 | GBP7 | guanylate binding protein 7 [Source:HGNC Symbol;Acc:HGNC:29606] |
| ENSG00000215193 | HGLibA_36111 | 64 | -0.511735564 | PEX26 | peroxisomal biogenesis factor 26 [Source:HGNC Symbol;Acc:HGNC:22965] |
| ENSG00000215912 | HGLibA_52121 | 64 | -0.511735564 | TTC34 | tetratricopeptide repeat domain 34 [Source:HGNC Symbol;Acc:HGNC:34297] |
| ENSG00000218891 | HGLibA_56301 | 64 | -0.511735564 | ZNF579 | zinc finger protein 579 [Source:HGNC Symbol;Acc:HGNC:26646] |
| ENSG00000221900 | HGLibA_37647 | 64 | -0.511735564 | POM121L12 | POM121 transmembrane nucleoporin like 12 [Source:HGNC Symbol;Acc:HGNC:25369] |
| ENSG00000221909 | HGLibA_16411 | 64 | -0.511735564 | FAM200A | family with sequence similarity 200 member A [Source:HGNC Symbol;Acc:HGNC:25401] |
| ENSG00000228623 | HGLibA_56897 | 64 | -0.511735564 | ZNF883 | zinc finger protein 883 [Source:HGNC Symbol;Acc:HGNC:27271] |
| ENSG00000229474 | HGLibA_35286 | 64 | -0.511735564 | PATL2 | PAT1 homolog 2 [Source:HGNC Symbol;Acc:HGNC:33630] |
| ENSG00000230031 | HGLibA_37719 | 64 | -0.511735564 | POTEB2 | POTE ankyrin domain family member B2 [Source:HGNC Symbol;Acc:HGNC:48327] |
| ENSG00000232264 | HGLibA_53255 | 64 | -0.511735564 | USP17L24 | ubiquitin specific peptidase 17-like family member 24 [Source:HGNC Symbol;Acc:HGNC:44453] |
| ENSG00000234438 | HGLibA_24098 | 64 | -0.511735564 | KBTBD13 | kelch repeat and BTB domain containing 13 [Source:HGNC Symbol;Acc:HGNC:37227] |
| ENSG00000249811 | HGLibA_53249 | 64 | -0.511735564 | USP17L21 | ubiquitin specific peptidase 17-like family member 21 [Source:HGNC Symbol;Acc:HGNC:44449] |
| ENSG00000253305 | HGLibA_35564 | 64 | -0.511735564 | PCDHGB6 | 6 [Source:HGNC Symbol;Acc:HGNC:8713]" |
| ENSG00000253873 | HGLibA_35522 | 64 | -0.511735564 | PCDHGA11 | 11 [Source:HGNC Symbol;Acc:HGNC:8698]" |
| ENSG00000253958 | HGLibA_09886 | 64 | -0.511735564 | CLDN23 | claudin 23 [Source:HGNC Symbol;Acc:HGNC:17591] |
| ENSG00000255346 | HGLibA_32378 | 64 | -0.511735564 | NOX5 | NADPH oxidase 5 [Source:HGNC Symbol;Acc:HGNC:14874] |
| ENSG00000256525 | HGLibA_37526 | 64 | -0.511735564 | POLG2 | accessory subunit [Source:HGNC Symbol;Acc:HGNC:9180]" |
| ENSG00000257446 | HGLibA_56888 | 64 | -0.511735564 | ZNF878 | zinc finger protein 878 [Source:HGNC Symbol;Acc:HGNC:37246] |
| ENSG00000257594 | HGLibA_18559 | 64 | -0.511735564 | GALNT4 | polypeptide N-acetylgalactosaminyltransferase 4 [Source:HGNC Symbol;Acc:HGNC:4126] |
| ENSG00000258429 | HGLibA_35837 | 64 | -0.511735564 | PDF | mitochondrial [Source:HGNC Symbol;Acc:HGNC:30012]" |
| ENSG00000266265 | HGLibA_25053 | 64 | -0.511735564 | KLF14 | Kruppel like factor 14 [Source:HGNC Symbol;Acc:HGNC:23025] |
| ENSG00000270757 | HGLibA_22337 | 64 | -0.511735564 | HSPE1-MOB4 | HSPE1-MOB4 readthrough [Source:HGNC Symbol;Acc:HGNC:49184] |
| ENSG00000279486 | HGLibA_33750 | 64 | -0.511735564 | OR2AG1 | olfactory receptor family 2 subfamily AG member 1 (gene/pseudogene) [Source:HGNC Symbol;Acc:HGNC:15142] |
| ENSG00000280165 | HGLibA_35413 | 64 | -0.511735564 | PCDH20 | protocadherin 20 [Source:HGNC Symbol;Acc:HGNC:14257] |
| ENSG00000288867 | HGLibA_37902 | 64 | -0.511735564 | PPIAL4C | - |
| FAM5C | HGLibA_16652 | 64 | -0.511735564 | FAM5C | - |
| HIST1H4B | HGLibA_21488 | 64 | -0.511735564 | HIST1H4B | - |
| hsa-mir-1182 | HGLibA_57221 | 64 | -0.511735564 | hsa-mir-1182 | - |
| hsa-mir-1184-1 | HGLibA_57231 | 64 | -0.511735564 | hsa-mir-1184-1 | - |
| hsa-mir-1206 | HGLibA_57280 | 64 | -0.511735564 | hsa-mir-1206 | - |
| hsa-mir-1251 | HGLibA_57401 | 64 | -0.511735564 | hsa-mir-1251 | - |
| hsa-mir-1275 | HGLibA_57557 | 64 | -0.511735564 | hsa-mir-1275 | - |
| hsa-mir-1302-1 | HGLibA_57684 | 64 | -0.511735564 | hsa-mir-1302-1 | - |
| hsa-mir-154 | HGLibA_57928 | 64 | -0.511735564 | hsa-mir-154 | - |
| hsa-mir-19a | HGLibA_58140 | 64 | -0.511735564 | hsa-mir-19a | - |
| hsa-mir-2052 | HGLibA_58182 | 64 | -0.511735564 | hsa-mir-2052 | - |
| hsa-mir-218-2 | HGLibA_58272 | 64 | -0.511735564 | hsa-mir-218-2 | - |
| hsa-mir-30d | HGLibA_58488 | 64 | -0.511735564 | hsa-mir-30d | - |
| hsa-mir-3162 | HGLibA_58727 | 64 | -0.511735564 | hsa-mir-3162 | - |
| hsa-mir-326 | HGLibA_58973 | 64 | -0.511735564 | hsa-mir-326 | - |
| hsa-mir-3591 | HGLibA_59053 | 64 | -0.511735564 | hsa-mir-3591 | - |
| hsa-mir-3689a | HGLibA_59315 | 64 | -0.511735564 | hsa-mir-3689a | - |
| hsa-mir-3921 | HGLibA_59561 | 64 | -0.511735564 | hsa-mir-3921 | - |
| hsa-mir-4436b-2 | HGLibA_60147 | 64 | -0.511735564 | hsa-mir-4436b-2 | - |
| hsa-mir-4443 | HGLibA_60174 | 64 | -0.511735564 | hsa-mir-4443 | - |
| hsa-mir-4464 | HGLibA_60261 | 64 | -0.511735564 | hsa-mir-4464 | - |
| hsa-mir-4657 | HGLibA_60737 | 64 | -0.511735564 | hsa-mir-4657 | - |
| hsa-mir-515-2 | HGLibA_61604 | 64 | -0.511735564 | hsa-mir-515-2 | - |
| hsa-mir-5192 | HGLibA_61685 | 64 | -0.511735564 | hsa-mir-5192 | - |
| hsa-mir-520a | HGLibA_61736 | 64 | -0.511735564 | hsa-mir-520a | - |
| hsa-mir-527 | HGLibA_61804 | 64 | -0.511735564 | hsa-mir-527 | - |
| hsa-mir-548aq | HGLibA_61915 | 64 | -0.511735564 | hsa-mir-548aq | - |
| hsa-mir-5708 | HGLibA_62371 | 64 | -0.511735564 | hsa-mir-5708 | - |
| hsa-mir-6090 | HGLibA_62628 | 64 | -0.511735564 | hsa-mir-6090 | - |
| hsa-mir-6729 | HGLibA_63070 | 64 | -0.511735564 | hsa-mir-6729 | - |
| hsa-mir-6843 | HGLibA_63549 | 64 | -0.511735564 | hsa-mir-6843 | - |
| hsa-mir-6886 | HGLibA_63728 | 64 | -0.511735564 | hsa-mir-6886 | - |
| hsa-mir-7160 | HGLibA_63869 | 64 | -0.511735564 | hsa-mir-7160 | - |
| LOC100130539 | HGLibA_26793 | 64 | -0.511735564 | LOC100130539 | - |
| LOC100505549 | HGLibA_26850 | 64 | -0.511735564 | LOC100505549 | - |
| LOC650293 | HGLibA_26983 | 64 | -0.511735564 | LOC650293 | - |
| MST4 | HGLibA_30263 | 64 | -0.511735564 | MST4 | - |
| PNMAL1 | HGLibA_37384 | 64 | -0.511735564 | PNMAL1 | - |
| PRKCDBP | HGLibA_38543 | 64 | -0.511735564 | PRKCDBP | - |
| PRR25 | HGLibA_38834 | 64 | -0.511735564 | PRR25 | - |
| RNF219 | HGLibA_41636 | 64 | -0.511735564 | RNF219 | - |
| SELV | HGLibA_43317 | 64 | -0.511735564 | SELV | - |
| SEPP1 | HGLibA_43420 | 64 | -0.511735564 | SEPP1 | - |
| 9-Sep | HGLibA_43458 | 64 | -0.511735564 | 9-Sep | - |
| TOMM70A | HGLibA_50981 | 64 | -0.511735564 | TOMM70A | - |
| AGPAT9 | HGLibA_01234 | 63 | -0.521686601 | AGPAT9 | - |
| AGXT2L1 | HGLibA_01277 | 63 | -0.521686601 | AGXT2L1 | - |
| C11orf82 | HGLibA_05190 | 63 | -0.521686601 | C11orf82 | - |
| C17orf102 | HGLibA_05519 | 63 | -0.521686601 | C17orf102 | - |
| C7orf29 | HGLibA_06530 | 63 | -0.521686601 | C7orf29 | - |
| CCDC64B | HGLibA_07830 | 63 | -0.521686601 | CCDC64B | - |
| CCRL1 | HGLibA_08173 | 63 | -0.521686601 | CCRL1 | - |
| ENSG00000004455 | HGLibA_01379 | 63 | -0.521686601 | AK2 | adenylate kinase 2 [Source:HGNC Symbol;Acc:HGNC:362] |
| ENSG00000005007 | HGLibA_53055 | 63 | -0.521686601 | UPF1 | RNA helicase and ATPase [Source:HGNC Symbol;Acc:HGNC:9962]" |
| ENSG00000005700 | HGLibA_22486 | 63 | -0.521686601 | IBTK | inhibitor of Bruton tyrosine kinase [Source:HGNC Symbol;Acc:HGNC:17853] |
| ENSG00000006125 | HGLibA_02310 | 63 | -0.521686601 | AP2B1 | adaptor related protein complex 2 subunit beta 1 [Source:HGNC Symbol;Acc:HGNC:563] |
| ENSG00000008256 | HGLibA_12240 | 63 | -0.521686601 | CYTH3 | cytohesin 3 [Source:HGNC Symbol;Acc:HGNC:9504] |
| ENSG00000027644 | HGLibA_23402 | 63 | -0.521686601 | INSRR | insulin receptor related receptor [Source:HGNC Symbol;Acc:HGNC:6093] |
| ENSG00000049167 | HGLibA_15456 | 63 | -0.521686601 | ERCC8 | CSA ubiquitin ligase complex subunit [Source:HGNC Symbol;Acc:HGNC:3439]" |

| | | | | | |
|---|---|---|---|---|---|
| ENSG00000053254 | HGLibA_17914 | 63 | -0.521686601 | FOXN3 | forkhead box N3 [Source:HGNC Symbol;Acc:HGNC:1928] |
| ENSG00000063660 | HGLibA_19762 | 63 | -0.521686601 | GPC1 | glypican 1 [Source:HGNC Symbol;Acc:HGNC:4449] |
| ENSG00000069535 | HGLibA_28110 | 63 | -0.521686601 | MAOB | monoamine oxidase B [Source:HGNC Symbol;Acc:HGNC:6834] |
| ENSG00000072041 | HGLibA_45303 | 63 | -0.521686601 | SLC6A15 | solute carrier family 6 member 15 [Source:HGNC Symbol;Acc:HGNC:13621] |
| ENSG00000074416 | HGLibA_29159 | 63 | -0.521686601 | MGLL | monoglyceride lipase [Source:HGNC Symbol;Acc:HGNC:17038] |
| ENSG00000075399 | HGLibA_53822 | 63 | -0.521686601 | VPS9D1 | VPS9 domain containing 1 [Source:HGNC Symbol;Acc:HGNC:13526] |
| ENSG00000077498 | HGLibA_52465 | 63 | -0.521686601 | TYR | tyrosinase [Source:HGNC Symbol;Acc:HGNC:12442] |
| ENSG00000078098 | HGLibA_16865 | 63 | -0.521686601 | FAP | fibroblast activation protein alpha [Source:HGNC Symbol;Acc:HGNC:3590] |
| ENSG00000082014 | HGLibA_45635 | 63 | -0.521686601 | SMARCD3 | member 3 [Source:HGNC Symbol;Acc:HGNC:11108]" |
| ENSG00000087191 | HGLibA_39142 | 63 | -0.521686601 | PSMC5 | ATPase 5 [Source:HGNC Symbol;Acc:HGNC:9552]" |
| ENSG00000089916 | HGLibA_19740 | 63 | -0.521686601 | GPATCH2L | G-patch domain containing 2 like [Source:HGNC Symbol;Acc:HGNC:20210] |
| ENSG00000091513 | HGLibA_49114 | 63 | -0.521686601 | TF | transferrin [Source:HGNC Symbol;Acc:HGNC:11740] |
| ENSG00000092098 | HGLibA_41661 | 63 | -0.521686601 | RNF31 | ring finger protein 31 [Source:HGNC Symbol;Acc:HGNC:16031] |
| ENSG00000095587 | HGLibA_49630 | 63 | -0.521686601 | TLL2 | tolloid like 2 [Source:HGNC Symbol;Acc:HGNC:11844] |
| ENSG00000096093 | HGLibA_14569 | 63 | -0.521686601 | EFHC1 | EF-hand domain containing 1 [Source:HGNC Symbol;Acc:HGNC:16406] |
| ENSG00000099331 | HGLibA_30870 | 63 | -0.521686601 | MYO9B | myosin IXB [Source:HGNC Symbol;Acc:HGNC:7609] |
| ENSG00000100083 | HGLibA_18979 | 63 | -0.521686601 | GGA1 | ARF binding protein 1 [Source:HGNC Symbol;Acc:HGNC:17842]" |
| ENSG00000100201 | HGLibA_12657 | 63 | -0.521686601 | DDX17 | DEAD-box helicase 17 [Source:HGNC Symbol;Acc:HGNC:2740] |
| ENSG00000100867 | HGLibA_13130 | 63 | -0.521686601 | DHRS2 | dehydrogenase/reductase 2 [Source:HGNC Symbol;Acc:HGNC:18349] |
| ENSG00000101955 | HGLibA_47034 | 63 | -0.521686601 | SRPX | sushi repeat containing protein X-linked [Source:HGNC Symbol;Acc:HGNC:11309] |
| ENSG00000103152 | HGLibA_29720 | 63 | -0.521686601 | MPG | N-methylpurine DNA glycosylase [Source:HGNC Symbol;Acc:HGNC:7211] |
| ENSG00000104413 | HGLibA_15587 | 63 | -0.521686601 | ESRP1 | epithelial splicing regulatory protein 1 [Source:HGNC Symbol;Acc:HGNC:25966] |
| ENSG00000104938 | HGLibA_09998 | 63 | -0.521686601 | CLEC4M | C-type lectin domain family 4 member M [Source:HGNC Symbol;Acc:HGNC:13523] |
| ENSG00000105583 | HGLibA_54196 | 63 | -0.521686601 | WDR83OS | WD repeat domain 83 opposite strand [Source:HGNC Symbol;Acc:HGNC:30203] |
| ENSG00000106025 | HGLibA_51897 | 63 | -0.521686601 | TSPAN12 | tetraspanin 12 [Source:HGNC Symbol;Acc:HGNC:21641] |
| ENSG00000107968 | HGLibA_28202 | 63 | -0.521686601 | MAP3K8 | mitogen-activated protein kinase kinase kinase 8 [Source:HGNC Symbol;Acc:HGNC:6860] |
| ENSG00000108349 | HGLibA_07273 | 63 | -0.521686601 | CASC3 | exon junction complex subunit [Source:HGNC Symbol;Acc:HGNC:17040]" |
| ENSG00000109775 | HGLibA_52859 | 63 | -0.521686601 | UFSP2 | UFM1 specific peptidase 2 [Source:HGNC Symbol;Acc:HGNC:25640] |
| ENSG00000109832 | HGLibA_12682 | 63 | -0.521686601 | DDX25 | DEAD-box helicase 25 [Source:HGNC Symbol;Acc:HGNC:18698] |
| ENSG00000109846 | HGLibA_11250 | 63 | -0.521686601 | CRYAB | crystallin alpha B [Source:HGNC Symbol;Acc:HGNC:2389] |
| ENSG00000109881 | HGLibA_07759 | 63 | -0.521686601 | CCDC34 | coiled-coil domain containing 34 [Source:HGNC Symbol;Acc:HGNC:25079] |
| ENSG00000110107 | HGLibA_38713 | 63 | -0.521686601 | PRPF19 | pre-mRNA processing factor 19 [Source:HGNC Symbol;Acc:HGNC:17896] |
| ENSG00000112304 | HGLibA_00477 | 63 | -0.521686601 | ACOT13 | acyl-CoA thioesterase 13 [Source:HGNC Symbol;Acc:HGNC:20999] |
| ENSG00000112335 | HGLibA_46080 | 63 | -0.521686601 | SNX3 | sorting nexin 3 [Source:HGNC Symbol;Acc:HGNC:11174] |
| ENSG00000113638 | HGLibA_52118 | 63 | -0.521686601 | TTC33 | tetratricopeptide repeat domain 33 [Source:HGNC Symbol;Acc:HGNC:29959] |
| ENSG00000113658 | HGLibA_45580 | 63 | -0.521686601 | SMAD5 | SMAD family member 5 [Source:HGNC Symbol;Acc:HGNC:6771] |
| ENSG00000115520 | HGLibA_10745 | 63 | -0.521686601 | COQ10B | coenzyme Q10B [Source:HGNC Symbol;Acc:HGNC:25819] |
| ENSG00000115548 | HGLibA_24500 | 63 | -0.521686601 | KDM3A | lysine demethylase 3A [Source:HGNC Symbol;Acc:HGNC:20815] |
| ENSG00000115738 | HGLibA_22526 | 63 | -0.521686601 | ID2 | inhibitor of DNA binding 2 [Source:HGNC Symbol;Acc:HGNC:5361] |
| ENSG00000115875 | HGLibA_47087 | 63 | -0.521686601 | SRSF7 | serine and arginine rich splicing factor 7 [Source:HGNC Symbol;Acc:HGNC:10789] |
| ENSG00000116726 | HGLibA_38286 | 63 | -0.521686601 | PRAMEF12 | PRAME family member 12 [Source:HGNC Symbol;Acc:HGNC:22125] |
| ENSG00000117151 | HGLibA_11591 | 63 | -0.521686601 | CTBS | chitobiase [Source:HGNC Symbol;Acc:HGNC:2496] |
| ENSG00000117400 | HGLibA_29735 | 63 | -0.521686601 | MPL | thrombopoietin receptor [Source:HGNC Symbol;Acc:HGNC:7217]" |
| ENSG00000118096 | HGLibA_22760 | 63 | -0.521686601 | IFT46 | intraflagellar transport 46 [Source:HGNC Symbol;Acc:HGNC:26146] |
| ENSG00000122512 | HGLibA_37335 | 63 | -0.521686601 | PMS2 | mismatch repair system component [Source:HGNC Symbol;Acc:HGNC:9122]" |
| ENSG00000122543 | HGLibA_33258 | 63 | -0.521686601 | OCM | oncomodulin [Source:HGNC Symbol;Acc:HGNC:8105] |
| ENSG00000122644 | HGLibA_02967 | 63 | -0.521686601 | ARL4A | ADP ribosylation factor like GTPase 4A [Source:HGNC Symbol;Acc:HGNC:695] |
| ENSG00000123983 | HGLibA_00557 | 63 | -0.521686601 | ACSL3 | acyl-CoA synthetase long chain family member 3 [Source:HGNC Symbol;Acc:HGNC:3570] |
| ENSG00000124225 | HGLibA_37300 | 63 | -0.521686601 | PMEPA1 | androgen induced 1 [Source:HGNC Symbol;Acc:HGNC:14107]" |
| ENSG00000124466 | HGLibA_27719 | 63 | -0.521686601 | LYPD3 | LY6/PLAUR domain containing 3 [Source:HGNC Symbol;Acc:HGNC:24880] |
| ENSG00000125820 | HGLibA_32033 | 63 | -0.521686601 | NKX2-2 | NK2 homeobox 2 [Source:HGNC Symbol;Acc:HGNC:7835] |
| ENSG00000127241 | HGLibA_28395 | 63 | -0.521686601 | MASP1 | mannan binding lectin serine peptidase 1 [Source:HGNC Symbol;Acc:HGNC:6901] |
| ENSG00000127884 | HGLibA_14404 | 63 | -0.521686601 | ECHS1 | short chain 1 [Source:HGNC Symbol;Acc:HGNC:3151]" |
| ENSG00000127884 | HGLibA_14402 | 63 | -0.521686601 | ECHS1 | short chain 1 [Source:HGNC Symbol;Acc:HGNC:3151]" |
| ENSG00000127928 | HGLibA_19539 | 63 | -0.521686601 | GNGT1 | G protein subunit gamma transducin 1 [Source:HGNC Symbol;Acc:HGNC:4411] |
| ENSG00000128463 | HGLibA_15013 | 63 | -0.521686601 | EMC4 | ER membrane protein complex subunit 4 [Source:HGNC Symbol;Acc:HGNC:28032] |
| ENSG00000128791 | HGLibA_52385 | 63 | -0.521686601 | TWSG1 | twisted gastrulation BMP signaling modulator 1 [Source:HGNC Symbol;Acc:HGNC:12429] |
| ENSG00000131018 | HGLibA_47976 | 63 | -0.521686601 | SYNE1 | spectrin repeat containing nuclear envelope protein 1 [Source:HGNC Symbol;Acc:HGNC:17089] |
| ENSG00000132702 | HGLibA_20843 | 63 | -0.521686601 | HAPLN2 | hyaluronan and proteoglycan link protein 2 [Source:HGNC Symbol;Acc:HGNC:17410] |
| ENSG00000132825 | HGLibA_38107 | 63 | -0.521686601 | PPP1R3D | protein phosphatase 1 regulatory subunit 3D [Source:HGNC Symbol;Acc:HGNC:9294] |
| ENSG00000134070 | HGLibA_23558 | 63 | -0.521686601 | IRAK2 | interleukin 1 receptor associated kinase 2 [Source:HGNC Symbol;Acc:HGNC:6113] |
| ENSG00000134917 | HGLibA_00841 | 63 | -0.521686601 | ADAMTS8 | ADAM metallopeptidase with thrombospondin type 1 motif 8 [Source:HGNC Symbol;Acc:HGNC:224] |
| ENSG00000135245 | HGLibA_21318 | 63 | -0.521686601 | HILPDA | hypoxia inducible lipid droplet associated [Source:HGNC Symbol;Acc:HGNC:28859] |
| ENSG00000135451 | HGLibA_51720 | 63 | -0.521686601 | TROAP | trophinin associated protein [Source:HGNC Symbol;Acc:HGNC:12327] |
| ENSG00000135486 | HGLibA_21792 | 63 | -0.521686601 | HNRNPA1 | heterogeneous nuclear ribonucleoprotein A1 [Source:HGNC Symbol;Acc:HGNC:5031] |
| ENSG00000136100 | HGLibA_53774 | 63 | -0.521686601 | VPS36 | vacuolar protein sorting 36 homolog [Source:HGNC Symbol;Acc:HGNC:20312] |
| ENSG00000136238 | HGLibA_40041 | 63 | -0.521686601 | RAC1 | Rac family small GTPase 1 [Source:HGNC Symbol;Acc:HGNC:9801] |
| ENSG00000136267 | HGLibA_13060 | 63 | -0.521686601 | DGKB | diacylglycerol kinase beta [Source:HGNC Symbol;Acc:HGNC:2850] |
| ENSG00000136367 | HGLibA_55171 | 63 | -0.521686601 | ZFHX2 | zinc finger homeobox 2 [Source:HGNC Symbol;Acc:HGNC:20152] |
| ENSG00000137210 | HGLibA_49981 | 63 | -0.521686601 | TMEM14B | transmembrane protein 14B [Source:HGNC Symbol;Acc:HGNC:21384] |
| ENSG00000137265 | HGLibA_23589 | 63 | -0.521686601 | IRF4 | interferon regulatory factor 4 [Source:HGNC Symbol;Acc:HGNC:6119] |
| ENSG00000138032 | HGLibA_37966 | 63 | -0.521686601 | PPM1B | Mg2+/Mn2+ dependent 1B [Source:HGNC Symbol;Acc:HGNC:9276]" |
| ENSG00000138756 | HGLibA_04601 | 63 | -0.521686601 | BMP2K | BMP2 inducible kinase [Source:HGNC Symbol;Acc:HGNC:18041] |
| ENSG00000138785 | HGLibA_23413 | 63 | -0.521686601 | INTS12 | integrator complex subunit 12 [Source:HGNC Symbol;Acc:HGNC:25067] |
| ENSG00000138942 | HGLibA_41593 | 63 | -0.521686601 | RNF185 | ring finger protein 185 [Source:HGNC Symbol;Acc:HGNC:26783] |
| ENSG00000141506 | HGLibA_36659 | 63 | -0.521686601 | PIK3R5 | phosphoinositide-3-kinase regulatory subunit 5 [Source:HGNC Symbol;Acc:HGNC:30035] |
| ENSG00000141985 | HGLibA_43947 | 63 | -0.521686601 | SH3GL1 | endophilin A2 [Source:HGNC Symbol;Acc:HGNC:10830]" |
| ENSG00000143315 | HGLibA_36568 | 63 | -0.521686601 | PIGM | phosphatidylinositol glycan anchor biosynthesis class M [Source:HGNC Symbol;Acc:HGNC:18858] |
| ENSG00000144182 | HGLibA_26632 | 63 | -0.521686601 | LIPT1 | lipoyltransferase 1 [Source:HGNC Symbol;Acc:HGNC:29569] |
| ENSG00000144642 | HGLibA_40593 | 63 | -0.521686601 | RBMS3 | RNA binding motif single stranded interacting protein 3 [Source:HGNC Symbol;Acc:HGNC:13427] |
| ENSG00000144712 | HGLibA_07123 | 63 | -0.521686601 | CAND2 | cullin associated and neddylation dissociated 2 (putative) [Source:HGNC Symbol;Acc:HGNC:30689] |
| ENSG00000145194 | HGLibA_14386 | 63 | -0.521686601 | ECE2 | endothelin converting enzyme 2 [Source:HGNC Symbol;Acc:HGNC:13275] |
| ENSG00000145692 | HGLibA_04486 | 63 | -0.521686601 | BHMT | betaine--homocysteine S-methyltransferase [Source:HGNC Symbol;Acc:HGNC:1047] |
| ENSG00000146242 | HGLibA_51098 | 63 | -0.521686601 | TPBG | trophoblast glycoprotein [Source:HGNC Symbol;Acc:HGNC:12004] |
| ENSG00000147383 | HGLibA_32749 | 63 | -0.521686601 | NSDHL | NAD(P) dependent steroid dehydrogenase-like [Source:HGNC Symbol;Acc:HGNC:13398] |
| ENSG00000148634 | HGLibA_21161 | 63 | -0.521686601 | HERC4 | HECT and RLD domain containing E3 ubiquitin protein ligase 4 [Source:HGNC Symbol;Acc:HGNC:24521] |
| ENSG00000150275 | HGLibA_35401 | 63 | -0.521686601 | PCDH15 | protocadherin related 15 [Source:HGNC Symbol;Acc:HGNC:14674] |
| ENSG00000152620 | HGLibA_31048 | 63 | -0.521686601 | NADK2 | mitochondrial [Source:HGNC Symbol;Acc:HGNC:26404]" |
| ENSG00000152749 | HGLibA_19985 | 63 | -0.521686601 | GPR180 | G protein-coupled receptor 180 [Source:HGNC Symbol;Acc:HGNC:28899] |
| ENSG00000153187 | HGLibA_21845 | 63 | -0.521686601 | HNRNPU | heterogeneous nuclear ribonucleoprotein U [Source:HGNC Symbol;Acc:HGNC:5048] |
| ENSG00000153233 | HGLibA_39531 | 63 | -0.521686601 | PTPRR | receptor type R [Source:HGNC Symbol;Acc:HGNC:9680]" |
| ENSG00000153820 | HGLibA_46649 | 63 | -0.521686601 | SPHKAP | AKAP domain containing [Source:HGNC Symbol;Acc:HGNC:30619]" |
| ENSG00000153904 | HGLibA_12587 | 63 | -0.521686601 | DDAH1 | dimethylarginine dimethylaminohydrolase 1 [Source:HGNC Symbol;Acc:HGNC:2715] |
| ENSG00000155265 | HGLibA_19637 | 63 | -0.521686601 | GOLGA7B | golgin A7 family member B [Source:HGNC Symbol;Acc:HGNC:31668] |
| ENSG00000157429 | HGLibA_55555 | 63 | -0.521686601 | ZNF19 | zinc finger protein 19 [Source:HGNC Symbol;Acc:HGNC:12981] |
| ENSG00000159128 | HGLibA_22714 | 63 | -0.521686601 | IFNGR2 | interferon gamma receptor 2 [Source:HGNC Symbol;Acc:HGNC:5440] |
| ENSG00000159164 | HGLibA_47874 | 63 | -0.521686601 | SV2A | synaptic vesicle glycoprotein 2A [Source:HGNC Symbol;Acc:HGNC:20566] |
| ENSG00000160014 | HGLibA_07028 | 63 | -0.521686601 | CALM3 | calmodulin 3 [Source:HGNC Symbol;Acc:HGNC:1449] |
| ENSG00000160075 | HGLibA_47189 | 63 | -0.521686601 | SSU72 | RNA polymerase II CTD phosphatase [Source:HGNC Symbol;Acc:HGNC:25016]" |
| ENSG00000160570 | HGLibA_12784 | 63 | -0.521686601 | DEDD2 | death effector domain containing 2 [Source:HGNC Symbol;Acc:HGNC:24450] |
| ENSG00000160791 | HGLibA_08160 | 63 | -0.521686601 | CCR5 | C-C motif chemokine receptor 5 (gene/pseudogene) [Source:HGNC Symbol;Acc:HGNC:1606] |
| ENSG00000160796 | HGLibA_31211 | 63 | -0.521686601 | NBEAL2 | neurobeachin like 2 [Source:HGNC Symbol;Acc:HGNC:31928] |
| ENSG00000162576 | HGLibA_30629 | 63 | -0.521686601 | MXRA8 | matrix remodeling associated 8 [Source:HGNC Symbol;Acc:HGNC:7542] |
| ENSG00000162676 | HGLibA_18940 | 63 | -0.521686601 | GFI1 | growth factor independent 1 transcriptional repressor [Source:HGNC Symbol;Acc:HGNC:4237] |
| ENSG00000162851 | HGLibA_49141 | 63 | -0.521686601 | TFB2M | mitochondrial [Source:HGNC Symbol;Acc:HGNC:18559]" |
| ENSG00000163348 | HGLibA_39698 | 63 | -0.521686601 | PYGO2 | pygopus family PHD finger 2 [Source:HGNC Symbol;Acc:HGNC:30257] |
| ENSG00000163466 | HGLibA_03079 | 63 | -0.521686601 | ARPC2 | actin related protein 2/3 complex subunit 2 [Source:HGNC Symbol;Acc:HGNC:705] |
| ENSG00000164187 | HGLibA_26677 | 63 | -0.521686601 | LMBRD2 | LMBR1 domain containing 2 [Source:HGNC Symbol;Acc:HGNC:25287] |
| ENSG00000164695 | HGLibA_09492 | 63 | -0.521686601 | CHMP4C | charged multivesicular body protein 4C [Source:HGNC Symbol;Acc:HGNC:30599] |
| ENSG00000164929 | HGLibA_04049 | 63 | -0.521686601 | BAALC | MAP3K1 and KLF4 binding [Source:HGNC Symbol;Acc:HGNC:14333]" |
| ENSG00000165076 | HGLibA_38934 | 63 | -0.521686601 | PRSS37 | serine protease 37 [Source:HGNC Symbol;Acc:HGNC:29211] |
| ENSG00000165182 | HGLibA_11949 | 63 | -0.521686601 | CXorf58 | chromosome X open reading frame 58 [Source:HGNC Symbol;Acc:HGNC:26356] |
| ENSG00000165632 | HGLibA_48231 | 63 | -0.521686601 | TAF3 | TATA-box binding protein associated factor 3 [Source:HGNC Symbol;Acc:HGNC:17303] |
| ENSG00000165879 | HGLibA_17979 | 63 | -0.521686601 | FRAT1 | WNT signaling pathway regulator [Source:HGNC Symbol;Acc:HGNC:3944]" |
| ENSG00000166589 | HGLibA_08648 | 63 | -0.521686601 | CDH16 | cadherin 16 [Source:HGNC Symbol;Acc:HGNC:1755] |
| ENSG00000166959 | HGLibA_30166 | 63 | -0.521686601 | MS4A8 | membrane spanning 4-domains A8 [Source:HGNC Symbol;Acc:HGNC:13380] |
| ENSG00000167244 | HGLibA_22798 | 63 | -0.521686601 | IGF2 | insulin like growth factor 2 [Source:HGNC Symbol;Acc:HGNC:5466] |
| ENSG00000168397 | HGLibA_03490 | 63 | -0.521686601 | ATG4B | autophagy related 4B cysteine peptidase [Source:HGNC Symbol;Acc:HGNC:20790] |
| ENSG00000168878 | HGLibA_43772 | 63 | -0.521686601 | SFTPB | surfactant protein B [Source:HGNC Symbol;Acc:HGNC:10801] |
| ENSG00000169208 | HGLibA_33492 | 63 | -0.521686601 | OR10G3 | olfactory receptor family 10 subfamily G member 3 [Source:HGNC Symbol;Acc:HGNC:8171] |
| ENSG00000170502 | HGLibA_32995 | 63 | -0.521686601 | NUDT9 | nudix hydrolase 9 [Source:HGNC Symbol;Acc:HGNC:8056] |
| ENSG00000171016 | HGLibA_39695 | 63 | -0.521686601 | PYGO1 | pygopus family PHD finger 1 [Source:HGNC Symbol;Acc:HGNC:30256] |
| ENSG00000171408 | HGLibA_35826 | 63 | -0.521686601 | PDE7B | phosphodiesterase 7B [Source:HGNC Symbol;Acc:HGNC:8792] |
| ENSG00000171862 | HGLibA_39296 | 63 | -0.521686601 | PTEN | phosphatase and tensin homolog [Source:HGNC Symbol;Acc:HGNC:9588] |
| ENSG00000172349 | HGLibA_22986 | 63 | -0.521686601 | IL16 | interleukin 16 [Source:HGNC Symbol;Acc:HGNC:5980] |
| ENSG00000172845 | HGLibA_46299 | 63 | -0.521686601 | SP3 | Sp3 transcription factor [Source:HGNC Symbol;Acc:HGNC:11208] |
| ENSG00000172954 | HGLibA_26156 | 63 | -0.521686601 | LCLAT1 | lysocardiolipin acyltransferase 1 [Source:HGNC Symbol;Acc:HGNC:26756] |
| ENSG00000173769 | HGLibA_51005 | 63 | -0.521686601 | TOPAZ1 | testis and ovary specific PAZ domain containing 1 [Source:HGNC Symbol;Acc:HGNC:24746] |
| ENSG00000174740 | HGLibA_34938 | 63 | -0.521686601 | PABPC5 | poly(A) binding protein cytoplasmic 5 [Source:HGNC Symbol;Acc:HGNC:13629] |
| ENSG00000174796 | HGLibA_49306 | 63 | -0.521686601 | THAP6 | THAP domain containing 6 [Source:HGNC Symbol;Acc:HGNC:23189] |
| ENSG00000174885 | HGLibA_32128 | 63 | -0.521686601 | NLRP6 | NLR family pyrin domain containing 6 [Source:HGNC Symbol;Acc:HGNC:22944] |
| ENSG00000174938 | HGLibA_43685 | 63 | -0.521686601 | SEZ6L2 | seizure related 6 homolog like 2 [Source:HGNC Symbol;Acc:HGNC:30844] |
| ENSG00000175063 | HGLibA_52580 | 63 | -0.521686601 | UBE2C | ubiquitin conjugating enzyme E2 C [Source:HGNC Symbol;Acc:HGNC:15937] |
| ENSG00000175309 | HGLibA_36481 | 63 | -0.521686601 | PHYKPL | 5-phosphohydroxy-L-lysine phospho-lyase [Source:HGNC Symbol;Acc:HGNC:28249] |
| ENSG00000175826 | HGLibA_11601 | 63 | -0.521686601 | CTDNEP1 | CTD nuclear envelope phosphatase 1 [Source:HGNC Symbol;Acc:HGNC:19085] |

| | | | | | |
|---|---|---|---|---|---|
| ENSG00000175874 | HGLibA_11123 | 63 | -0.521686601 | CREG2 | cellular repressor of E1A stimulated genes 2 [Source:HGNC Symbol;Acc:HGNC:14272] |
| ENSG00000175985 | HGLibA_37092 | 63 | -0.521686601 | PLEKHD1 | pleckstrin homology and coiled-coil domain containing D1 [Source:HGNC Symbol;Acc:HGNC:20148] |
| ENSG00000176230 | HGLibA_34027 | 63 | -0.521686601 | OR4K17 | olfactory receptor family 4 subfamily K member 17 [Source:HGNC Symbol;Acc:HGNC:15355] |
| ENSG00000176239 | HGLibA_34091 | 63 | -0.521686601 | OR51B6 | olfactory receptor family 51 subfamily B member 6 [Source:HGNC Symbol;Acc:HGNC:19600] |
| ENSG00000176635 | HGLibA_21889 | 63 | -0.521686601 | HORMAD2 | HORMA domain containing 2 [Source:HGNC Symbol;Acc:HGNC:28383] |
| ENSG00000176697 | HGLibA_04386 | 63 | -0.521686601 | BDNF | brain derived neurotrophic factor [Source:HGNC Symbol;Acc:HGNC:1033] |
| ENSG00000176714 | HGLibA_07568 | 63 | -0.521686601 | CCDC121 | coiled-coil domain containing 121 [Source:HGNC Symbol;Acc:HGNC:25833] |
| ENSG00000176922 | HGLibA_34131 | 63 | -0.521686601 | OR51S1 | olfactory receptor family 51 subfamily S member 1 [Source:HGNC Symbol;Acc:HGNC:15204] |
| ENSG00000178498 | HGLibA_14085 | 63 | -0.521686601 | DTX3 | deltex E3 ubiquitin ligase 3 [Source:HGNC Symbol;Acc:HGNC:24457] |
| ENSG00000182054 | HGLibA_22543 | 63 | -0.521686601 | IDH2 | mitochondrial [Source:HGNC Symbol;Acc:HGNC:5383]" |
| ENSG00000182108 | HGLibA_13017 | 63 | -0.521686601 | DEXI | Dexi homolog [Source:HGNC Symbol;Acc:HGNC:13267] |
| ENSG00000182170 | HGLibA_29843 | 63 | -0.521686601 | MRGPRG | MAS related GPR family member G [Source:HGNC Symbol;Acc:HGNC:24829] |
| ENSG00000182199 | HGLibA_44062 | 63 | -0.521686601 | SHMT2 | serine hydroxymethyltransferase 2 [Source:HGNC Symbol;Acc:HGNC:10852] |
| ENSG00000182389 | HGLibA_06927 | 63 | -0.521686601 | CACNB4 | calcium voltage-gated channel auxiliary subunit beta 4 [Source:HGNC Symbol;Acc:HGNC:1404] |
| ENSG00000182446 | HGLibA_32485 | 63 | -0.521686601 | NPLOC4 | ubiquitin recognition factor [Source:HGNC Symbol;Acc:HGNC:18261]" |
| ENSG00000182631 | HGLibA_42500 | 63 | -0.521686601 | RXFP3 | relaxin family peptide receptor 3 [Source:HGNC Symbol;Acc:HGNC:24883] |
| ENSG00000183196 | HGLibA_09642 | 63 | -0.521686601 | CHST6 | carbohydrate sulfotransferase 6 [Source:HGNC Symbol;Acc:HGNC:6938] |
| ENSG00000183340 | HGLibA_23994 | 63 | -0.521686601 | JRKL | JRK like [Source:HGNC Symbol;Acc:HGNC:6200] |
| ENSG00000184471 | HGLibA_05957 | 63 | -0.521686601 | C1QTNF8 | C1q and TNF related 8 [Source:HGNC Symbol;Acc:HGNC:31374] |
| ENSG00000185070 | HGLibA_17657 | 63 | -0.521686601 | FLRT2 | fibronectin leucine rich transmembrane protein 2 [Source:HGNC Symbol;Acc:HGNC:3761] |
| ENSG00000186020 | HGLibA_56172 | 63 | -0.521686601 | ZNF529 | zinc finger protein 529 [Source:HGNC Symbol;Acc:HGNC:29328] |
| ENSG00000186088 | HGLibA_20394 | 63 | -0.521686601 | GSAP | gamma-secretase activating protein [Source:HGNC Symbol;Acc:HGNC:28042] |
| ENSG00000186562 | HGLibA_12825 | 63 | -0.521686601 | DEFB105A | defensin beta 105A [Source:HGNC Symbol;Acc:HGNC:18087] |
| ENSG00000186591 | HGLibA_52612 | 63 | -0.521686601 | UBE2H | ubiquitin conjugating enzyme E2 H [Source:HGNC Symbol;Acc:HGNC:12484] |
| ENSG00000187416 | HGLibA_26436 | 63 | -0.521686601 | LHFPL3 | LHFPL tetraspan subfamily member 3 [Source:HGNC Symbol;Acc:HGNC:6589] |
| ENSG00000187682 | HGLibA_15411 | 63 | -0.521686601 | ERAS | ES cell expressed Ras [Source:HGNC Symbol;Acc:HGNC:5174] |
| ENSG00000188322 | HGLibA_42799 | 63 | -0.521686601 | SBK1 | SH3 domain binding kinase 1 [Source:HGNC Symbol;Acc:HGNC:17699] |
| ENSG00000196187 | HGLibA_50475 | 63 | -0.521686601 | TMEM63A | transmembrane protein 63A [Source:HGNC Symbol;Acc:HGNC:29118] |
| ENSG00000196517 | HGLibA_45342 | 63 | -0.521686601 | SLC6A9 | solute carrier family 6 member 9 [Source:HGNC Symbol;Acc:HGNC:11056] |
| ENSG00000196539 | HGLibA_33873 | 63 | -0.521686601 | OR2T3 | olfactory receptor family 2 subfamily T member 3 [Source:HGNC Symbol;Acc:HGNC:14727] |
| ENSG00000197111 | HGLibA_35371 | 63 | -0.521686601 | PCBP2 | poly(rC) binding protein 2 [Source:HGNC Symbol;Acc:HGNC:8648] |
| ENSG00000197816 | HGLibA_07722 | 63 | -0.521686601 | CCDC180 | coiled-coil domain containing 180 [Source:HGNC Symbol;Acc:HGNC:29303] |
| ENSG00000198160 | HGLibA_29253 | 63 | -0.521686601 | MIER1 | MIER1 transcriptional regulator [Source:HGNC Symbol;Acc:HGNC:29657] |
| ENSG00000198205 | HGLibA_57073 | 63 | -0.521686601 | ZXDA | zinc finger X-linked duplicated A [Source:HGNC Symbol;Acc:HGNC:13198] |
| ENSG00000198363 | HGLibA_03350 | 63 | -0.521686601 | ASPH | aspartate beta-hydroxylase [Source:HGNC Symbol;Acc:HGNC:757] |
| ENSG00000198399 | HGLibA_23874 | 63 | -0.521686601 | ITSN2 | intersectin 2 [Source:HGNC Symbol;Acc:HGNC:6184] |
| ENSG00000198443 | HGLibA_25750 | 63 | -0.521686601 | KRTAP4-1 | keratin associated protein 4-1 [Source:HGNC Symbol;Acc:HGNC:18907] |
| ENSG00000198488 | HGLibA_03995 | 63 | -0.521686601 | B3GNT6 | 3-N-acetylglucosaminyltransferase 6 [Source:HGNC Symbol;Acc:HGNC:24141]" |
| ENSG00000203772 | HGLibA_46800 | 63 | -0.521686601 | SPRN | shadow of prion protein [Source:HGNC Symbol;Acc:HGNC:16871] |
| ENSG00000203989 | HGLibA_41220 | 63 | -0.521686601 | RHOXF2B | Rhox homeobox family member 2B [Source:HGNC Symbol;Acc:HGNC:33519] |
| ENSG00000204084 | HGLibA_23347 | 63 | -0.521686601 | INPP5B | inositol polyphosphate-5-phosphatase B [Source:HGNC Symbol;Acc:HGNC:6077] |
| ENSG00000204403 | HGLibA_07300 | 63 | -0.521686601 | CASP12 | caspase 12 (gene/pseudogene) [Source:HGNC Symbol;Acc:HGNC:19004] |
| ENSG00000204524 | HGLibA_56793 | 63 | -0.521686601 | ZNF805 | zinc finger protein 805 [Source:HGNC Symbol;Acc:HGNC:23272] |
| ENSG00000204583 | HGLibA_27149 | 63 | -0.521686601 | LRCOL1 | leucine rich colipase like 1 [Source:HGNC Symbol;Acc:HGNC:44160] |
| ENSG00000204897 | HGLibA_25460 | 63 | -0.521686601 | KRT25 | keratin 25 [Source:HGNC Symbol;Acc:HGNC:30839] |
| ENSG00000204936 | HGLibA_08254 | 63 | -0.521686601 | CD177 | CD177 molecule [Source:HGNC Symbol;Acc:HGNC:30072] |
| ENSG00000212123 | HGLibA_38819 | 63 | -0.521686601 | PRR22 | proline rich 22 [Source:HGNC Symbol;Acc:HGNC:28354] |
| ENSG00000213463 | HGLibA_48009 | 63 | -0.521686601 | SYNJ2BP | synaptojanin 2 binding protein [Source:HGNC Symbol;Acc:HGNC:18955] |
| ENSG00000214360 | HGLibA_14555 | 63 | -0.521686601 | EFCAB9 | EF-hand calcium binding domain 9 [Source:HGNC Symbol;Acc:HGNC:34530] |
| ENSG00000215454 | HGLibA_25610 | 63 | -0.521686601 | KRTAP10-4 | keratin associated protein 10-4 [Source:HGNC Symbol;Acc:HGNC:20521] |
| ENSG00000220008 | HGLibA_26592 | 63 | -0.521686601 | LINGO3 | leucine rich repeat and Ig domain containing 3 [Source:HGNC Symbol;Acc:HGNC:21206] |
| ENSG00000221955 | HGLibA_44378 | 63 | -0.521686601 | SLC12A8 | solute carrier family 12 member 8 [Source:HGNC Symbol;Acc:HGNC:15595] |
| ENSG00000221978 | HGLibA_08119 | 63 | -0.521686601 | CCNL2 | cyclin L2 [Source:HGNC Symbol;Acc:HGNC:20570] |
| ENSG00000223443 | HGLibA_53241 | 63 | -0.521686601 | USP17L2 | ubiquitin specific peptidase 17-like family member 2 [Source:HGNC Symbol;Acc:HGNC:34434] |
| ENSG00000234438 | HGLibA_24099 | 63 | -0.521686601 | KBTBD13 | kelch repeat and BTB domain containing 13 [Source:HGNC Symbol;Acc:HGNC:37227] |
| ENSG00000241878 | HGLibA_36734 | 63 | -0.521686601 | PISD | phosphatidylserine decarboxylase [Source:HGNC Symbol;Acc:HGNC:8999] |
| ENSG00000255359 | HGLibA_07717 | 63 | -0.521686601 | CCDC179 | coiled-coil domain containing 179 [Source:HGNC Symbol;Acc:HGNC:44653] |
| ENSG00000260458 | HGLibA_24270 | 63 | -0.521686601 | KCNJ18 | potassium voltage-gated channel subfamily J member 18 [Source:HGNC Symbol;Acc:HGNC:39080] |
| ENSG00000267508 | HGLibA_55747 | 63 | -0.521686601 | ZNF285 | zinc finger protein 285 [Source:HGNC Symbol;Acc:HGNC:13079] |
| ENSG00000277150 | HGLibA_15893 | 63 | -0.521686601 | F8A3 | coagulation factor VIII associated 3 [Source:HGNC Symbol;Acc:HGNC:31850] |
| ENSG00000277494 | HGLibA_19811 | 63 | -0.521686601 | GPIHBP1 | glycosylphosphatidylinositol anchored high density lipoprotein binding protein 1 [Source:HGNC Symbol;Acc:HGNC:24945] |
| ENSG00000280109 | HGLibA_36940 | 63 | -0.521686601 | PLAC4 | placenta specific 4 [Source:HGNC Symbol;Acc:HGNC:14616] |
| ENSG00000288602 | HGLibA_06626 | 63 | -0.521686601 | C8orf44-SGK3 | - |
| ENSG00000288705 | HGLibA_52888 | 63 | -0.521686601 | UGT1A5 | - |
| FAM122B | HGLibA_16070 | 63 | -0.521686601 | FAM122B | - |
| H1FNT | HGLibA_20735 | 63 | -0.521686601 | H1FNT | - |
| HGC6.3 | HGLibA_21233 | 63 | -0.521686601 | HGC6.3 | - |
| hsa-mir-1283-2 | HGLibA_57592 | 63 | -0.521686601 | hsa-mir-1283-2 | - |
| hsa-mir-1289-2 | HGLibA_57624 | 63 | -0.521686601 | hsa-mir-1289-2 | - |
| hsa-mir-1468 | HGLibA_57848 | 63 | -0.521686601 | hsa-mir-1468 | - |
| hsa-mir-1471 | HGLibA_57867 | 63 | -0.521686601 | hsa-mir-1471 | - |
| hsa-mir-1537 | HGLibA_57916 | 63 | -0.521686601 | hsa-mir-1537 | - |
| hsa-mir-203b | HGLibA_58171 | 63 | -0.521686601 | hsa-mir-203b | - |
| hsa-mir-2355 | HGLibA_58322 | 63 | -0.521686601 | hsa-mir-2355 | - |
| hsa-mir-3179-2 | HGLibA_58793 | 63 | -0.521686601 | hsa-mir-3179-2 | - |
| hsa-mir-3915 | HGLibA_59536 | 63 | -0.521686601 | hsa-mir-3915 | - |
| hsa-mir-4259 | HGLibA_59745 | 63 | -0.521686601 | hsa-mir-4259 | - |
| hsa-mir-4444-1 | HGLibA_60180 | 63 | -0.521686601 | hsa-mir-4444-1 | - |
| hsa-mir-4470 | HGLibA_60286 | 63 | -0.521686601 | hsa-mir-4470 | - |
| hsa-mir-4520a | HGLibA_60535 | 63 | -0.521686601 | hsa-mir-4520a | - |
| hsa-mir-4534 | HGLibA_60595 | 63 | -0.521686601 | hsa-mir-4534 | - |
| hsa-mir-4741 | HGLibA_61062 | 63 | -0.521686601 | hsa-mir-4741 | - |
| hsa-mir-4804 | HGLibA_61319 | 63 | -0.521686601 | hsa-mir-4804 | - |
| hsa-mir-520e | HGLibA_61749 | 63 | -0.521686601 | hsa-mir-520e | - |
| hsa-mir-5698 | HGLibA_62323 | 63 | -0.521686601 | hsa-mir-5698 | - |
| hsa-mir-670 | HGLibA_63009 | 63 | -0.521686601 | hsa-mir-670 | - |
| hsa-mir-6741 | HGLibA_63116 | 63 | -0.521686601 | hsa-mir-6741 | - |
| hsa-mir-6827 | HGLibA_63484 | 63 | -0.521686601 | hsa-mir-6827 | - |
| hsa-mir-6843 | HGLibA_63546 | 63 | -0.521686601 | hsa-mir-6843 | - |
| hsa-mir-6873 | HGLibA_63677 | 63 | -0.521686601 | hsa-mir-6873 | - |
| hsa-mir-6886 | HGLibA_63729 | 63 | -0.521686601 | hsa-mir-6886 | - |
| hsa-mir-765 | HGLibA_63925 | 63 | -0.521686601 | hsa-mir-765 | - |
| hsa-mir-7702 | HGLibA_63945 | 63 | -0.521686601 | hsa-mir-7702 | - |
| hsa-mir-892c | HGLibA_64259 | 63 | -0.521686601 | hsa-mir-892c | - |
| KIAA1737 | HGLibA_24813 | 63 | -0.521686601 | KIAA1737 | - |
| MUM1L1 | HGLibA_30569 | 63 | -0.521686601 | MUM1L1 | - |
| PHOSPHO2-KLHL23 | HGLibA_36447 | 63 | -0.521686601 | PHOSPHO2-KLHL23 | - |
| TARP | HGLibA_48341 | 63 | -0.521686601 | TARP | - |
| TMEM206 | HGLibA_50196 | 63 | -0.521686601 | TMEM206 | - |
| WBP5 | HGLibA_53985 | 63 | -0.521686601 | WBP5 | - |
| WBSCR27 | HGLibA_53998 | 63 | -0.521686601 | WBSCR27 | - |
| WDR67 | HGLibA_54156 | 63 | -0.521686601 | WDR67 | - |
| ZHX1-C8ORF76 | HGLibA_55313 | 63 | -0.521686601 | ZHX1-C8ORF76 | - |
| AGPHD1 | HGLibA_01239 | 62 | -0.531637638 | AGPHD1 | - |
| ANXA8L2 | HGLibA_02258 | 62 | -0.531637638 | ANXA8L2 | - |
| ASMT_X | HGLibA_03319 | 62 | -0.531637638 | ASMT_X | - |
| C10orf25 | HGLibA_05065 | 62 | -0.531637638 | C10orf25 | - |
| C16orf93 | HGLibA_05504 | 62 | -0.531637638 | C16orf93 | - |
| CRLF2_X | HGLibA_11188 | 62 | -0.531637638 | CRLF2_X | - |
| CSRP2BP | HGLibA_11439 | 62 | -0.531637638 | CSRP2BP | - |
| DUX2 | HGLibA_14202 | 62 | -0.531637638 | DUX2 | - |
| EFCAB4A | HGLibA_14541 | 62 | -0.531637638 | EFCAB4A | - |
| ENSG00000002919 | HGLibA_46029 | 62 | -0.531637638 | SNX11 | sorting nexin 11 [Source:HGNC Symbol;Acc:HGNC:14975] |
| ENSG00000005243 | HGLibA_10740 | 62 | -0.531637638 | COPZ2 | coatomer protein complex subunit zeta 2 [Source:HGNC Symbol;Acc:HGNC:19356] |
| ENSG00000006831 | HGLibA_00984 | 62 | -0.531637638 | ADIPOR2 | adiponectin receptor 2 [Source:HGNC Symbol;Acc:HGNC:24041] |
| ENSG00000007944 | HGLibA_30780 | 62 | -0.531637638 | MYLIP | myosin regulatory light chain interacting protein [Source:HGNC Symbol;Acc:HGNC:21155] |
| ENSG00000008952 | HGLibA_43259 | 62 | -0.531637638 | SEC62 | preprotein translocation factor [Source:HGNC Symbol;Acc:HGNC:11846]" |
| ENSG00000021574 | HGLibA_46424 | 62 | -0.531637638 | SPAST | spastin [Source:HGNC Symbol;Acc:HGNC:11233] |
| ENSG00000031003 | HGLibA_16145 | 62 | -0.531637638 | FAM13B | family with sequence similarity 13 member B [Source:HGNC Symbol;Acc:HGNC:1335] |
| ENSG00000040341 | HGLibA_47407 | 62 | -0.531637638 | STAU2 | staufen double-stranded RNA binding protein 2 [Source:HGNC Symbol;Acc:HGNC:11371] |
| ENSG00000043591 | HGLibA_01056 | 62 | -0.531637638 | ADRB1 | adrenoceptor beta 1 [Source:HGNC Symbol;Acc:HGNC:285] |
| ENSG00000059804 | HGLibA_44873 | 62 | -0.531637638 | SLC2A3 | solute carrier family 2 member 3 [Source:HGNC Symbol;Acc:HGNC:11007] |
| ENSG00000061337 | HGLibA_27807 | 62 | -0.531637638 | LZTS1 | leucine zipper tumor suppressor 1 [Source:HGNC Symbol;Acc:HGNC:13861] |
| ENSG00000062716 | HGLibA_53696 | 62 | -0.531637638 | VMP1 | vacuole membrane protein 1 [Source:HGNC Symbol;Acc:HGNC:29559] |
| ENSG00000063176 | HGLibA_46647 | 62 | -0.531637638 | SPHK2 | sphingosine kinase 2 [Source:HGNC Symbol;Acc:HGNC:18859] |
| ENSG00000065357 | HGLibA_13059 | 62 | -0.531637638 | DGKA | diacylglycerol kinase alpha [Source:HGNC Symbol;Acc:HGNC:2849] |
| ENSG00000068323 | HGLibA_49160 | 62 | -0.531637638 | TFE3 | transcription factor binding to IGHM enhancer 3 [Source:HGNC Symbol;Acc:HGNC:11752] |
| ENSG00000070031 | HGLibA_43063 | 62 | -0.531637638 | SCT | secretin [Source:HGNC Symbol;Acc:HGNC:10607] |
| ENSG00000072062 | HGLibA_38503 | 62 | -0.531637638 | PRKACA | protein kinase cAMP-activated catalytic subunit alpha [Source:HGNC Symbol;Acc:HGNC:9380] |
| ENSG00000075073 | HGLibA_48174 | 62 | -0.531637638 | TACR2 | tachykinin receptor 2 [Source:HGNC Symbol;Acc:HGNC:11527] |
| ENSG00000075223 | HGLibA_43326 | 62 | -0.531637638 | SEMA3C | semaphorin 3C [Source:HGNC Symbol;Acc:HGNC:10725] |
| ENSG00000075303 | HGLibA_44735 | 62 | -0.531637638 | SLC25A40 | solute carrier family 25 member 40 [Source:HGNC Symbol;Acc:HGNC:29680] |
| ENSG00000081138 | HGLibA_08689 | 62 | -0.531637638 | CDH7 | cadherin 7 [Source:HGNC Symbol;Acc:HGNC:1766] |
| ENSG00000083457 | HGLibA_23755 | 62 | -0.531637638 | ITGAE | integrin subunit alpha E [Source:HGNC Symbol;Acc:HGNC:6147] |
| ENSG00000083544 | HGLibA_48906 | 62 | -0.531637638 | TDRD3 | tudor domain containing 3 [Source:HGNC Symbol;Acc:HGNC:20612] |

| | | | | | |
|---|---|---|---|---|---|
| ENSG00000085231 | HGLibA_01391 | 62 | -0.531637638 | AK6 | adenylate kinase 6 [Source:HGNC Symbol;Acc:HGNC:49151] |
| ENSG00000087589 | HGLibA_07342 | 62 | -0.531637638 | CASS4 | Cas scaffold protein family member 4 [Source:HGNC Symbol;Acc:HGNC:15878] |
| ENSG00000091137 | HGLibA_44795 | 62 | -0.531637638 | SLC26A4 | solute carrier family 26 member 4 [Source:HGNC Symbol;Acc:HGNC:8818] |
| ENSG00000092010 | HGLibA_39191 | 62 | -0.531637638 | PSME1 | proteasome activator subunit 1 [Source:HGNC Symbol;Acc:HGNC:9568] |
| ENSG00000092208 | HGLibA_18912 | 62 | -0.531637638 | GEMIN2 | gem nuclear organelle associated protein 2 [Source:HGNC Symbol;Acc:HGNC:10884] |
| ENSG00000092345 | HGLibA_12369 | 62 | -0.531637638 | DAZL | deleted in azoospermia like [Source:HGNC Symbol;Acc:HGNC:2685] |
| ENSG00000096070 | HGLibA_04841 | 62 | -0.531637638 | BRPF3 | bromodomain and PHD finger containing 3 [Source:HGNC Symbol;Acc:HGNC:14256] |
| ENSG00000100341 | HGLibA_37417 | 62 | -0.531637638 | PNPLA5 | patatin like phospholipase domain containing 5 [Source:HGNC Symbol;Acc:HGNC:24888] |
| ENSG00000100346 | HGLibA_06900 | 62 | -0.531637638 | CACNA1I | calcium voltage-gated channel subunit alpha1 I [Source:HGNC Symbol;Acc:HGNC:1396] |
| ENSG00000100380 | HGLibA_47221 | 62 | -0.531637638 | ST13 | Hsp70 interacting protein [Source:HGNC Symbol;Acc:HGNC:11343]" |
| ENSG00000101224 | HGLibA_08529 | 62 | -0.531637638 | CDC25B | cell division cycle 25B [Source:HGNC Symbol;Acc:HGNC:1726] |
| ENSG00000101474 | HGLibA_02446 | 62 | -0.531637638 | APMAP | adipocyte plasma membrane associated protein [Source:HGNC Symbol;Acc:HGNC:13238] |
| ENSG00000102910 | HGLibA_27016 | 62 | -0.531637638 | LONP2 | peroxisomal [Source:HGNC Symbol;Acc:HGNC:20598]" |
| ENSG00000104946 | HGLibA_48488 | 62 | -0.531637638 | TBC1D17 | TBC1 domain family member 17 [Source:HGNC Symbol;Acc:HGNC:25699] |
| ENSG00000104946 | HGLibA_48489 | 62 | -0.531637638 | TBC1D17 | TBC1 domain family member 17 [Source:HGNC Symbol;Acc:HGNC:25699] |
| ENSG00000105085 | HGLibA_28790 | 62 | -0.531637638 | MED26 | mediator complex subunit 26 [Source:HGNC Symbol;Acc:HGNC:2376] |
| ENSG00000105141 | HGLibA_07303 | 62 | -0.531637638 | CASP14 | caspase 14 [Source:HGNC Symbol;Acc:HGNC:1502] |
| ENSG00000105641 | HGLibA_45273 | 62 | -0.531637638 | SLC5A5 | solute carrier family 5 member 5 [Source:HGNC Symbol;Acc:HGNC:11040] |
| ENSG00000105982 | HGLibA_41663 | 62 | -0.531637638 | RNF32 | ring finger protein 32 [Source:HGNC Symbol;Acc:HGNC:17118] |
| ENSG00000106367 | HGLibA_02297 | 62 | -0.531637638 | AP1S1 | adaptor related protein complex 1 subunit sigma 1 [Source:HGNC Symbol;Acc:HGNC:559] |
| ENSG00000106733 | HGLibA_32197 | 62 | -0.531637638 | NMRK1 | nicotinamide riboside kinase 1 [Source:HGNC Symbol;Acc:HGNC:26057] |
| ENSG00000107625 | HGLibA_12731 | 62 | -0.531637638 | DDX50 | DExD-box helicase 50 [Source:HGNC Symbol;Acc:HGNC:17906] |
| ENSG00000108256 | HGLibA_33002 | 62 | -0.531637638 | NUFIP2 | nuclear FMR1 interacting protein 2 [Source:HGNC Symbol;Acc:HGNC:17634] |
| ENSG00000108342 | HGLibA_11340 | 62 | -0.531637638 | CSF3 | colony stimulating factor 3 [Source:HGNC Symbol;Acc:HGNC:2438] |
| ENSG00000108433 | HGLibA_19686 | 62 | -0.531637638 | GOSR2 | golgi SNAP receptor complex member 2 [Source:HGNC Symbol;Acc:HGNC:4431] |
| ENSG00000108622 | HGLibA_22498 | 62 | -0.531637638 | ICAM2 | intercellular adhesion molecule 2 [Source:HGNC Symbol;Acc:HGNC:5345] |
| ENSG00000108821 | HGLibA_10530 | 62 | -0.531637638 | COL1A1 | collagen type I alpha 1 chain [Source:HGNC Symbol;Acc:HGNC:2197] |
| ENSG00000109255 | HGLibA_32212 | 62 | -0.531637638 | NMU | neuromedin U [Source:HGNC Symbol;Acc:HGNC:7859] |
| ENSG00000109339 | HGLibA_28251 | 62 | -0.531637638 | MAPK10 | mitogen-activated protein kinase 10 [Source:HGNC Symbol;Acc:HGNC:6872] |
| ENSG00000109390 | HGLibA_31537 | 62 | -0.531637638 | NDUFC1 | NADH:ubiquinone oxidoreductase subunit C1 [Source:HGNC Symbol;Acc:HGNC:7705] |
| ENSG00000111726 | HGLibA_10178 | 62 | -0.531637638 | CMAS | cytidine monophosphate N-acetylneuraminic acid synthetase [Source:HGNC Symbol;Acc:HGNC:18290] |
| ENSG00000112282 | HGLibA_28782 | 62 | -0.531637638 | MED23 | mediator complex subunit 23 [Source:HGNC Symbol;Acc:HGNC:2372] |
| ENSG00000114166 | HGLibA_24053 | 62 | -0.531637638 | KAT2B | lysine acetyltransferase 2B [Source:HGNC Symbol;Acc:HGNC:8638] |
| ENSG00000114999 | HGLibA_52182 | 62 | -0.531637638 | TTL | tubulin tyrosine ligase [Source:HGNC Symbol;Acc:HGNC:21586] |
| ENSG00000115257 | HGLibA_35675 | 62 | -0.531637638 | PCSK4 | proprotein convertase subtilisin/kexin type 4 [Source:HGNC Symbol;Acc:HGNC:8746] |
| ENSG00000115592 | HGLibA_38519 | 62 | -0.531637638 | PRKAG3 | protein kinase AMP-activated non-catalytic subunit gamma 3 [Source:HGNC Symbol;Acc:HGNC:9387] |
| ENSG00000115919 | HGLibA_25881 | 62 | -0.531637638 | KYNU | kynureninase [Source:HGNC Symbol;Acc:HGNC:6469] |
| ENSG00000116396 | HGLibA_24171 | 62 | -0.531637638 | KCNC4 | potassium voltage-gated channel subfamily C member 4 [Source:HGNC Symbol;Acc:HGNC:6236] |
| ENSG00000116793 | HGLibA_36462 | 62 | -0.531637638 | PHTF1 | putative homeodomain transcription factor 1 [Source:HGNC Symbol;Acc:HGNC:8939] |
| ENSG00000116981 | HGLibA_32801 | 62 | -0.531637638 | NT5C1A | cytosolic IA [Source:HGNC Symbol;Acc:HGNC:17819]" |
| ENSG00000117602 | HGLibA_40675 | 62 | -0.531637638 | RCAN3 | RCAN family member 3 [Source:HGNC Symbol;Acc:HGNC:3042] |
| ENSG00000117862 | HGLibA_52407 | 62 | -0.531637638 | TXNDC12 | thioredoxin domain containing 12 [Source:HGNC Symbol;Acc:HGNC:24626] |
| ENSG00000118520 | HGLibA_02684 | 62 | -0.531637638 | ARG1 | arginase 1 [Source:HGNC Symbol;Acc:HGNC:663] |
| ENSG00000119403 | HGLibA_36356 | 62 | -0.531637638 | PHF19 | PHD finger protein 19 [Source:HGNC Symbol;Acc:HGNC:24566] |
| ENSG00000119541 | HGLibA_53799 | 62 | -0.531637638 | VPS4B | vacuolar protein sorting 4 homolog B [Source:HGNC Symbol;Acc:HGNC:10895] |
| ENSG00000119865 | HGLibA_10380 | 62 | -0.531637638 | CNRIP1 | cannabinoid receptor interacting protein 1 [Source:HGNC Symbol;Acc:HGNC:24546] |
| ENSG00000119899 | HGLibA_44472 | 62 | -0.531637638 | SLC17A5 | solute carrier family 17 member 5 [Source:HGNC Symbol;Acc:HGNC:10933] |
| ENSG00000120279 | HGLibA_30682 | 62 | -0.531637638 | MYCT1 | MYC target 1 [Source:HGNC Symbol;Acc:HGNC:23172] |
| ENSG00000122359 | HGLibA_02225 | 62 | -0.531637638 | ANXA11 | annexin A11 [Source:HGNC Symbol;Acc:HGNC:535] |
| ENSG00000123191 | HGLibA_03773 | 62 | -0.531637638 | ATP7B | ATPase copper transporting beta [Source:HGNC Symbol;Acc:HGNC:870] |
| ENSG00000123352 | HGLibA_46547 | 62 | -0.531637638 | SPATS2 | spermatogenesis associated serine rich 2 [Source:HGNC Symbol;Acc:HGNC:18650] |
| ENSG00000125347 | HGLibA_23572 | 62 | -0.531637638 | IRF1 | interferon regulatory factor 1 [Source:HGNC Symbol;Acc:HGNC:6116] |
| ENSG00000125944 | HGLibA_21844 | 62 | -0.531637638 | HNRNPR | heterogeneous nuclear ribonucleoprotein R [Source:HGNC Symbol;Acc:HGNC:5047] |
| ENSG00000126457 | HGLibA_38618 | 62 | -0.531637638 | PRMT1 | protein arginine methyltransferase 1 [Source:HGNC Symbol;Acc:HGNC:5187] |
| ENSG00000126561 | HGLibA_47393 | 62 | -0.531637638 | STAT5A | signal transducer and activator of transcription 5A [Source:HGNC Symbol;Acc:HGNC:11366] |
| ENSG00000128610 | HGLibA_17319 | 62 | -0.531637638 | FEZF1 | FEZ family zinc finger 1 [Source:HGNC Symbol;Acc:HGNC:22788] |
| ENSG00000128881 | HGLibA_52041 | 62 | -0.531637638 | TTBK2 | tau tubulin kinase 2 [Source:HGNC Symbol;Acc:HGNC:19141] |
| ENSG00000129009 | HGLibA_23670 | 62 | -0.531637638 | ISLR | immunoglobulin superfamily containing leucine rich repeat [Source:HGNC Symbol;Acc:HGNC:6133] |
| ENSG00000129028 | HGLibA_49289 | 62 | -0.531637638 | THAP10 | THAP domain containing 10 [Source:HGNC Symbol;Acc:HGNC:23193] |
| ENSG00000129128 | HGLibA_46565 | 62 | -0.531637638 | SPCS3 | signal peptidase complex subunit 3 [Source:HGNC Symbol;Acc:HGNC:26212] |
| ENSG00000129667 | HGLibA_41137 | 62 | -0.531637638 | RHBDF2 | rhomboid 5 homolog 2 [Source:HGNC Symbol;Acc:HGNC:20788] |
| ENSG00000130227 | HGLibA_54575 | 62 | -0.531637638 | XPO7 | exportin 7 [Source:HGNC Symbol;Acc:HGNC:14108] |
| ENSG00000131437 | HGLibA_24935 | 62 | -0.531637638 | KIF3A | kinesin family member 3A [Source:HGNC Symbol;Acc:HGNC:6319] |
| ENSG00000132773 | HGLibA_50939 | 62 | -0.531637638 | TOE1 | exonuclease [Source:HGNC Symbol;Acc:HGNC:15954]" |
| ENSG00000133317 | HGLibA_26347 | 62 | -0.531637638 | LGALS12 | galectin 12 [Source:HGNC Symbol;Acc:HGNC:15788] |
| ENSG00000134183 | HGLibA_19476 | 62 | -0.531637638 | GNAT2 | G protein subunit alpha transducin 2 [Source:HGNC Symbol;Acc:HGNC:4394] |
| ENSG00000134644 | HGLibA_39579 | 62 | -0.531637638 | PUM1 | pumilio RNA binding family member 1 [Source:HGNC Symbol;Acc:HGNC:14957] |
| ENSG00000135164 | HGLibA_13444 | 62 | -0.531637638 | DMTF1 | cyclin D binding myb like transcription factor 1 [Source:HGNC Symbol;Acc:HGNC:14603] |
| ENSG00000135912 | HGLibA_52204 | 62 | -0.531637638 | TTLL4 | tubulin tyrosine ligase like 4 [Source:HGNC Symbol;Acc:HGNC:28976] |
| ENSG00000136478 | HGLibA_49073 | 62 | -0.531637638 | TEX2 | testis expressed 2 [Source:HGNC Symbol;Acc:HGNC:30884] |
| ENSG00000136504 | HGLibA_24065 | 62 | -0.531637638 | KAT7 | lysine acetyltransferase 7 [Source:HGNC Symbol;Acc:HGNC:17016] |
| ENSG00000136891 | HGLibA_49045 | 62 | -0.531637638 | TEX10 | testis expressed 10 [Source:HGNC Symbol;Acc:HGNC:25988] |
| ENSG00000136895 | HGLibA_18632 | 62 | -0.531637638 | GARNL3 | GTPase activating Rap/RanGAP domain like 3 [Source:HGNC Symbol;Acc:HGNC:25425] |
| ENSG00000137601 | HGLibA_31638 | 62 | -0.531637638 | NEK1 | NIMA related kinase 1 [Source:HGNC Symbol;Acc:HGNC:7744] |
| ENSG00000137648 | HGLibA_50642 | 62 | -0.531637638 | TMPRSS4 | transmembrane serine protease 4 [Source:HGNC Symbol;Acc:HGNC:11878] |
| ENSG00000138448 | HGLibA_23763 | 62 | -0.531637638 | ITGAV | integrin subunit alpha V [Source:HGNC Symbol;Acc:HGNC:6150] |
| ENSG00000139163 | HGLibA_15631 | 62 | -0.531637638 | ETNK1 | ethanolamine kinase 1 [Source:HGNC Symbol;Acc:HGNC:24649] |
| ENSG00000139163 | HGLibA_15632 | 62 | -0.531637638 | ETNK1 | ethanolamine kinase 1 [Source:HGNC Symbol;Acc:HGNC:24649] |
| ENSG00000140988 | HGLibA_42102 | 62 | -0.531637638 | RPS2 | ribosomal protein S2 [Source:HGNC Symbol;Acc:HGNC:10404] |
| ENSG00000142002 | HGLibA_13883 | 62 | -0.531637638 | DPP9 | dipeptidyl peptidase 9 [Source:HGNC Symbol;Acc:HGNC:18648] |
| ENSG00000142025 | HGLibA_13442 | 62 | -0.531637638 | DMRTC2 | DMRT like family C2 [Source:HGNC Symbol;Acc:HGNC:13911] |
| ENSG00000142408 | HGLibA_06949 | 62 | -0.531637638 | CACNG8 | calcium voltage-gated channel auxiliary subunit gamma 8 [Source:HGNC Symbol;Acc:HGNC:13628] |
| ENSG00000143155 | HGLibA_49562 | 62 | -0.531637638 | TIPRL | TOR signaling pathway regulator [Source:HGNC Symbol;Acc:HGNC:30231] |
| ENSG00000143198 | HGLibA_29179 | 62 | -0.531637638 | MGST3 | microsomal glutathione S-transferase 3 [Source:HGNC Symbol;Acc:HGNC:7064] |
| ENSG00000143437 | HGLibA_03062 | 62 | -0.531637638 | ARNT | aryl hydrocarbon receptor nuclear translocator [Source:HGNC Symbol;Acc:HGNC:700] |
| ENSG00000144827 | HGLibA_00234 | 62 | -0.531637638 | ABHD10 | abhydrolase domain containing 10 [Source:HGNC Symbol;Acc:HGNC:25656] |
| ENSG00000145242 | HGLibA_15298 | 62 | -0.531637638 | EPHA5 | EPH receptor A5 [Source:HGNC Symbol;Acc:HGNC:3389] |
| ENSG00000145721 | HGLibA_26642 | 62 | -0.531637638 | LIX1 | limb and CNS expressed 1 [Source:HGNC Symbol;Acc:HGNC:18581] |
| ENSG00000148826 | HGLibA_32057 | 62 | -0.531637638 | NKX6-2 | NK6 homeobox 2 [Source:HGNC Symbol;Acc:HGNC:19321] |
| ENSG00000148925 | HGLibA_04897 | 62 | -0.531637638 | BTBD10 | BTB domain containing 10 [Source:HGNC Symbol;Acc:HGNC:21445] |
| ENSG00000149516 | HGLibA_30147 | 62 | -0.531637638 | MS4A3 | membrane spanning 4-domains A3 [Source:HGNC Symbol;Acc:HGNC:7317] |
| ENSG00000149541 | HGLibA_03979 | 62 | -0.531637638 | B3GAT3 | 3-glucuronyltransferase 3 [Source:HGNC Symbol;Acc:HGNC:923]" |
| ENSG00000149582 | HGLibA_50315 | 62 | -0.531637638 | TMEM25 | transmembrane protein 25 [Source:HGNC Symbol;Acc:HGNC:25890] |
| ENSG00000150779 | HGLibA_49527 | 62 | -0.531637638 | TIMM8B | translocase of inner mitochondrial membrane 8 homolog B [Source:HGNC Symbol;Acc:HGNC:11818] |
| ENSG00000150967 | HGLibA_00154 | 62 | -0.531637638 | ABCB9 | ATP binding cassette subfamily B member 9 [Source:HGNC Symbol;Acc:HGNC:50] |
| ENSG00000152518 | HGLibA_55210 | 62 | -0.531637638 | ZFP36L2 | ZFP36 ring finger protein like 2 [Source:HGNC Symbol;Acc:HGNC:1108] |
| ENSG00000152942 | HGLibA_40056 | 62 | -0.531637638 | RAD17 | RAD17 checkpoint clamp loader component [Source:HGNC Symbol;Acc:HGNC:9807] |
| ENSG00000152953 | HGLibA_47485 | 62 | -0.531637638 | STK32B | serine/threonine kinase 32B [Source:HGNC Symbol;Acc:HGNC:14217] |
| ENSG00000152977 | HGLibA_55322 | 62 | -0.531637638 | ZIC1 | Zic family member 1 [Source:HGNC Symbol;Acc:HGNC:12872] |
| ENSG00000153802 | HGLibA_50618 | 62 | -0.531637638 | TMPRSS11D | transmembrane serine protease 11D [Source:HGNC Symbol;Acc:HGNC:24059] |
| ENSG00000154889 | HGLibA_29767 | 62 | -0.531637638 | MPPE1 | metallophosphoesterase 1 [Source:HGNC Symbol;Acc:HGNC:15988] |
| ENSG00000154975 | HGLibA_06788 | 62 | -0.531637638 | CA10 | carbonic anhydrase 10 [Source:HGNC Symbol;Acc:HGNC:1369] |
| ENSG00000157542 | HGLibA_24284 | 62 | -0.531637638 | KCNJ6 | potassium voltage-gated channel subfamily J member 6 [Source:HGNC Symbol;Acc:HGNC:6267] |
| ENSG00000157978 | HGLibA_26252 | 62 | -0.531637638 | LDLRAP1 | low density lipoprotein receptor adaptor protein 1 [Source:HGNC Symbol;Acc:HGNC:18640] |
| ENSG00000158528 | HGLibA_38129 | 62 | -0.531637638 | PPP1R9A | protein phosphatase 1 regulatory subunit 9A [Source:HGNC Symbol;Acc:HGNC:14946] |
| ENSG00000160049 | HGLibA_13020 | 62 | -0.531637638 | DFFA | DNA fragmentation factor subunit alpha [Source:HGNC Symbol;Acc:HGNC:2772] |
| ENSG00000161638 | HGLibA_23737 | 62 | -0.531637638 | ITGA5 | integrin subunit alpha 5 [Source:HGNC Symbol;Acc:HGNC:6141] |
| ENSG00000162396 | HGLibA_35254 | 62 | -0.531637638 | PARS2 | mitochondrial [Source:HGNC Symbol;Acc:HGNC:30563]" |
| ENSG00000162482 | HGLibA_01503 | 62 | -0.531637638 | AKR7A3 | aldo-keto reductase family 7 member A3 [Source:HGNC Symbol;Acc:HGNC:390] |
| ENSG00000162604 | HGLibA_49681 | 62 | -0.531637638 | TM2D1 | TM2 domain containing 1 [Source:HGNC Symbol;Acc:HGNC:24142] |
| ENSG00000162664 | HGLibA_55808 | 62 | -0.531637638 | ZNF326 | zinc finger protein 326 [Source:HGNC Symbol;Acc:HGNC:14104] |
| ENSG00000162769 | HGLibA_17675 | 62 | -0.531637638 | FLVCR1 | feline leukemia virus subgroup C cellular receptor 1 [Source:HGNC Symbol;Acc:HGNC:24682] |
| ENSG00000162814 | HGLibA_46436 | 62 | -0.531637638 | SPATA17 | spermatogenesis associated 17 [Source:HGNC Symbol;Acc:HGNC:25184] |
| ENSG00000163032 | HGLibA_53854 | 62 | -0.531637638 | VSNL1 | visinin like 1 [Source:HGNC Symbol;Acc:HGNC:12722] |
| ENSG00000163220 | HGLibA_42576 | 62 | -0.531637638 | S100A9 | S100 calcium binding protein A9 [Source:HGNC Symbol;Acc:HGNC:10499] |
| ENSG00000163565 | HGLibA_22593 | 62 | -0.531637638 | IFI16 | interferon gamma inducible protein 16 [Source:HGNC Symbol;Acc:HGNC:5395] |
| ENSG00000163636 | HGLibA_39178 | 62 | -0.531637638 | PSMD6 | non-ATPase 6 [Source:HGNC Symbol;Acc:HGNC:9564]" |
| ENSG00000163655 | HGLibA_19432 | 62 | -0.531637638 | GMPS | guanine monophosphate synthase [Source:HGNC Symbol;Acc:HGNC:4378] |
| ENSG00000164088 | HGLibA_37992 | 62 | -0.531637638 | PPM1M | Mg2+/Mn2+ dependent 1M [Source:HGNC Symbol;Acc:HGNC:26506]" |
| ENSG00000164967 | HGLibA_42031 | 62 | -0.531637638 | RPP25L | ribonuclease P/MRP subunit p25 like [Source:HGNC Symbol;Acc:HGNC:19909] |
| ENSG00000165929 | HGLibA_48687 | 62 | -0.531637638 | TC2N | nuclear [Source:HGNC Symbol;Acc:HGNC:19859]" |
| ENSG00000165970 | HGLibA_45331 | 62 | -0.531637638 | SLC6A5 | solute carrier family 6 member 5 [Source:HGNC Symbol;Acc:HGNC:11051] |
| ENSG00000166508 | HGLibA_28622 | 62 | -0.531637638 | MCM7 | minichromosome maintenance complex component 7 [Source:HGNC Symbol;Acc:HGNC:6950] |
| ENSG00000166741 | HGLibA_32222 | 62 | -0.531637638 | NNMT | nicotinamide N-methyltransferase [Source:HGNC Symbol;Acc:HGNC:7861] |
| ENSG00000166848 | HGLibA_49015 | 62 | -0.531637638 | TERF2IP | TERF2 interacting protein [Source:HGNC Symbol;Acc:HGNC:19246] |
| ENSG00000166869 | HGLibA_09521 | 62 | -0.531637638 | CHP2 | calcineurin like EF-hand protein 2 [Source:HGNC Symbol;Acc:HGNC:24927] |
| ENSG00000166923 | HGLibA_20243 | 62 | -0.531637638 | GREM1 | DAN family BMP antagonist [Source:HGNC Symbol;Acc:HGNC:2001]" |
| ENSG00000166928 | HGLibA_30140 | 62 | -0.531637638 | MS4A14 | membrane spanning 4-domains A14 [Source:HGNC Symbol;Acc:HGNC:30706] |
| ENSG00000167196 | HGLibA_17049 | 62 | -0.531637638 | FBXO22 | F-box protein 22 [Source:HGNC Symbol;Acc:HGNC:13593] |
| ENSG00000167653 | HGLibA_39011 | 62 | -0.531637638 | PSCA | prostate stem cell antigen [Source:HGNC Symbol;Acc:HGNC:9500] |
| ENSG00000167699 | HGLibA_19286 | 62 | -0.531637638 | GLOD4 | glyoxalase domain containing 4 [Source:HGNC Symbol;Acc:HGNC:14111] |
| ENSG00000167780 | HGLibA_46117 | 62 | -0.531637638 | SOAT2 | sterol O-acyltransferase 2 [Source:HGNC Symbol;Acc:HGNC:11178] |
| ENSG00000170262 | HGLibA_29807 | 62 | -0.531637638 | MRAP | melanocortin 2 receptor accessory protein [Source:HGNC Symbol;Acc:HGNC:1304] |
| ENSG00000170390 | HGLibA_12501 | 62 | -0.531637638 | DCLK2 | doublecortin like kinase 2 [Source:HGNC Symbol;Acc:HGNC:19002] |
| ENSG00000171121 | HGLibA_24348 | 62 | -0.531637638 | KCNMB3 | potassium calcium-activated channel subfamily M regulatory beta subunit 3 [Source:HGNC Symbol;Acc:HGNC:6287] |

| | | | | | |
|---|---|---|---|---|---|
| ENSG00000171532 | HGLibA_31736 | 62 | -0.531637638 | NEUROD2 | neuronal differentiation 2 [Source:HGNC Symbol;Acc:HGNC:7763] |
| ENSG00000171903 | HGLibA_12180 | 62 | -0.531637638 | CYP4F11 | cytochrome P450 family 4 subfamily F member 11 [Source:HGNC Symbol;Acc:HGNC:13265] |
| ENSG00000172244 | HGLibA_06378 | 62 | -0.531637638 | C5orf34 | chromosome 5 open reading frame 34 [Source:HGNC Symbol;Acc:HGNC:24738] |
| ENSG00000172262 | HGLibA_55459 | 62 | -0.531637638 | ZNF131 | zinc finger protein 131 [Source:HGNC Symbol;Acc:HGNC:12915] |
| ENSG00000172769 | HGLibA_34267 | 62 | -0.531637638 | OR5B3 | olfactory receptor family 5 subfamily B member 3 [Source:HGNC Symbol;Acc:HGNC:8324] |
| ENSG00000172969 | HGLibA_18001 | 62 | -0.531637638 | FRG2C | FSHD region gene 2 family member C [Source:HGNC Symbol;Acc:HGNC:33626] |
| ENSG00000173221 | HGLibA_19312 | 62 | -0.531637638 | GLRX | glutaredoxin [Source:HGNC Symbol;Acc:HGNC:4330] |
| ENSG00000173275 | HGLibA_56023 | 62 | -0.531637638 | ZNF449 | zinc finger protein 449 [Source:HGNC Symbol;Acc:HGNC:21039] |
| ENSG00000174137 | HGLibA_16634 | 62 | -0.531637638 | FAM53A | family with sequence similarity 53 member A [Source:HGNC Symbol;Acc:HGNC:31860] |
| ENSG00000174576 | HGLibA_32404 | 62 | -0.531637638 | NPAS4 | neuronal PAS domain protein 4 [Source:HGNC Symbol;Acc:HGNC:18983] |
| ENSG00000174788 | HGLibA_35657 | 62 | -0.531637638 | PCP2 | Purkinje cell protein 2 [Source:HGNC Symbol;Acc:HGNC:30209] |
| ENSG00000175315 | HGLibA_11461 | 62 | -0.531637638 | CST6 | cystatin E/M [Source:HGNC Symbol;Acc:HGNC:2478] |
| ENSG00000175467 | HGLibA_42754 | 62 | -0.531637638 | SART1 | recruiter of U4/U6.U5 tri-snRNP [Source:HGNC Symbol;Acc:HGNC:10538]" |
| ENSG00000175664 | HGLibA_49078 | 62 | -0.531637638 | TEX26 | testis expressed 26 [Source:HGNC Symbol;Acc:HGNC:28622] |
| ENSG00000176058 | HGLibA_51188 | 62 | -0.531637638 | TPRN | taperin [Source:HGNC Symbol;Acc:HGNC:26894] |
| ENSG00000176246 | HGLibA_34036 | 62 | -0.531637638 | OR4L1 | olfactory receptor family 4 subfamily L member 1 [Source:HGNC Symbol;Acc:HGNC:15356] |
| ENSG00000176945 | HGLibA_30538 | 62 | -0.531637638 | MUC20 | cell surface associated [Source:HGNC Symbol;Acc:HGNC:23282]" |
| ENSG00000176994 | HGLibA_45682 | 62 | -0.531637638 | SMCR8 | candidate 8 [Source:HGNC Symbol;Acc:HGNC:17921]" |
| ENSG00000177238 | HGLibA_51602 | 62 | -0.531637638 | TRIM72 | tripartite motif containing 72 [Source:HGNC Symbol;Acc:HGNC:32671] |
| ENSG00000177646 | HGLibA_00362 | 62 | -0.531637638 | ACAD9 | acyl-CoA dehydrogenase family member 9 [Source:HGNC Symbol;Acc:HGNC:21497] |
| ENSG00000177947 | HGLibA_33283 | 62 | -0.531637638 | ODF3 | outer dense fiber of sperm tails 3 [Source:HGNC Symbol;Acc:HGNC:19905] |
| ENSG00000178928 | HGLibA_51191 | 62 | -0.531637638 | TPRX1 | tetrapeptide repeat homeobox 1 [Source:HGNC Symbol;Acc:HGNC:32174] |
| ENSG00000179104 | HGLibA_50673 | 62 | -0.531637638 | TMTC2 | transmembrane and tetratricopeptide repeat containing 2 [Source:HGNC Symbol;Acc:HGNC:25440] |
| ENSG00000179639 | HGLibA_17168 | 62 | -0.531637638 | FCER1A | Fc fragment of IgE receptor Ia [Source:HGNC Symbol;Acc:HGNC:3609] |
| ENSG00000179988 | HGLibA_39236 | 62 | -0.531637638 | PSTK | phosphoseryl-tRNA kinase [Source:HGNC Symbol;Acc:HGNC:28578] |
| ENSG00000180957 | HGLibA_36745 | 62 | -0.531637638 | PITPNB | phosphatidylinositol transfer protein beta [Source:HGNC Symbol;Acc:HGNC:9002] |
| ENSG00000181609 | HGLibA_34153 | 62 | -0.531637638 | OR52D1 | olfactory receptor family 52 subfamily D member 1 [Source:HGNC Symbol;Acc:HGNC:15212] |
| ENSG00000181965 | HGLibA_31746 | 62 | -0.531637638 | NEUROG1 | neurogenin 1 [Source:HGNC Symbol;Acc:HGNC:7764] |
| ENSG00000181991 | HGLibA_30033 | 62 | -0.531637638 | MRPS11 | mitochondrial ribosomal protein S11 [Source:HGNC Symbol;Acc:HGNC:14050] |
| ENSG00000182054 | HGLibA_22542 | 62 | -0.531637638 | IDH2 | mitochondrial [Source:HGNC Symbol;Acc:HGNC:5383]" |
| ENSG00000182613 | HGLibA_33900 | 62 | -0.531637638 | OR2V2 | olfactory receptor family 2 subfamily V member 2 [Source:HGNC Symbol;Acc:HGNC:15341] |
| ENSG00000182732 | HGLibA_41101 | 62 | -0.531637638 | RGS6 | regulator of G protein signaling 6 [Source:HGNC Symbol;Acc:HGNC:10002] |
| ENSG00000183153 | HGLibA_19189 | 62 | -0.531637638 | GJD3 | gap junction protein delta 3 [Source:HGNC Symbol;Acc:HGNC:19147] |
| ENSG00000183807 | HGLibA_16225 | 62 | -0.531637638 | FAM162B | family with sequence similarity 162 member B [Source:HGNC Symbol;Acc:HGNC:21549] |
| ENSG00000184182 | HGLibA_52602 | 62 | -0.531637638 | UBE2F | ubiquitin conjugating enzyme E2 F (putative) [Source:HGNC Symbol;Acc:HGNC:12480] |
| ENSG00000184601 | HGLibA_05347 | 62 | -0.531637638 | C14orf180 | chromosome 14 open reading frame 180 [Source:HGNC Symbol;Acc:HGNC:33795] |
| ENSG00000184792 | HGLibA_34640 | 62 | -0.531637638 | OSBP2 | oxysterol binding protein 2 [Source:HGNC Symbol;Acc:HGNC:8504] |
| ENSG00000184992 | HGLibA_04807 | 62 | -0.531637638 | BRI3BP | BRI3 binding protein [Source:HGNC Symbol;Acc:HGNC:14251] |
| ENSG00000185013 | HGLibA_32804 | 62 | -0.531637638 | NT5C1B | cytosolic IB [Source:HGNC Symbol;Acc:HGNC:17818]" |
| ENSG00000185745 | HGLibA_22624 | 62 | -0.531637638 | IFIT1 | interferon induced protein with tetratricopeptide repeats 1 [Source:HGNC Symbol;Acc:HGNC:5407] |
| ENSG00000186001 | HGLibA_27142 | 62 | -0.531637638 | LRCH3 | leucine rich repeats and calponin homology domain containing 3 [Source:HGNC Symbol;Acc:HGNC:28637] |
| ENSG00000186184 | HGLibA_37559 | 62 | -0.531637638 | POLR1D | RNA polymerase I and III subunit D [Source:HGNC Symbol;Acc:HGNC:20422] |
| ENSG00000186329 | HGLibA_50211 | 62 | -0.531637638 | TMEM212 | transmembrane protein 212 [Source:HGNC Symbol;Acc:HGNC:34295] |
| ENSG00000186501 | HGLibA_50240 | 62 | -0.531637638 | TMEM222 | transmembrane protein 222 [Source:HGNC Symbol;Acc:HGNC:25363] |
| ENSG00000186943 | HGLibA_33607 | 62 | -0.531637638 | OR13C8 | olfactory receptor family 13 subfamily C member 8 [Source:HGNC Symbol;Acc:HGNC:15103] |
| ENSG00000187713 | HGLibA_50185 | 62 | -0.531637638 | TMEM203 | transmembrane protein 203 [Source:HGNC Symbol;Acc:HGNC:28217] |
| ENSG00000188493 | HGLibA_05680 | 62 | -0.531637638 | C19orf54 | chromosome 19 open reading frame 54 [Source:HGNC Symbol;Acc:HGNC:24758] |
| ENSG00000188641 | HGLibA_13920 | 62 | -0.531637638 | DPYD | dihydropyrimidine dehydrogenase [Source:HGNC Symbol;Acc:HGNC:3012] |
| ENSG00000188895 | HGLibA_30212 | 62 | -0.531637638 | MSL1 | male specific lethal 1 homolog [Source:HGNC Symbol;Acc:HGNC:27905] |
| ENSG00000188986 | HGLibA_31674 | 62 | -0.531637638 | NELFB | negative elongation factor complex member B [Source:HGNC Symbol;Acc:HGNC:24324] |
| ENSG00000189252 | HGLibA_46408 | 62 | -0.531637638 | SPANXN3 | SPANX family member N3 [Source:HGNC Symbol;Acc:HGNC:33176] |
| ENSG00000196132 | HGLibA_30926 | 62 | -0.531637638 | MYT1 | myelin transcription factor 1 [Source:HGNC Symbol;Acc:HGNC:7622] |
| ENSG00000196172 | HGLibA_56528 | 62 | -0.531637638 | ZNF681 | zinc finger protein 681 [Source:HGNC Symbol;Acc:HGNC:26457] |
| ENSG00000196405 | HGLibA_15698 | 62 | -0.531637638 | EVL | Enah/Vasp-like [Source:HGNC Symbol;Acc:HGNC:20234] |
| ENSG00000196420 | HGLibA_42559 | 62 | -0.531637638 | S100A5 | S100 calcium binding protein A5 [Source:HGNC Symbol;Acc:HGNC:10495] |
| ENSG00000196778 | HGLibA_34180 | 62 | -0.531637638 | OR52K1 | olfactory receptor family 52 subfamily K member 1 [Source:HGNC Symbol;Acc:HGNC:15222] |
| ENSG00000196843 | HGLibA_02906 | 62 | -0.531637638 | ARID5A | AT-rich interaction domain 5A [Source:HGNC Symbol;Acc:HGNC:17361] |
| ENSG00000197045 | HGLibA_19403 | 62 | -0.531637638 | GMFB | glia maturation factor beta [Source:HGNC Symbol;Acc:HGNC:4373] |
| ENSG00000197355 | HGLibA_52509 | 62 | -0.531637638 | UAP1L1 | UDP-N-acetylglucosamine pyrophosphorylase 1 like 1 [Source:HGNC Symbol;Acc:HGNC:28082] |
| ENSG00000198189 | HGLibA_22175 | 62 | -0.531637638 | HSD17B11 | hydroxysteroid 17-beta dehydrogenase 11 [Source:HGNC Symbol;Acc:HGNC:22960] |
| ENSG00000198205 | HGLibA_57072 | 62 | -0.531637638 | ZXDA | zinc finger X-linked duplicated A [Source:HGNC Symbol;Acc:HGNC:13198] |
| ENSG00000198690 | HGLibA_16828 | 62 | -0.531637638 | FAN1 | FANCD2 and FANCI associated nuclease 1 [Source:HGNC Symbol;Acc:HGNC:29170] |
| ENSG00000198729 | HGLibA_38040 | 62 | -0.531637638 | PPP1R14C | protein phosphatase 1 regulatory inhibitor subunit 14C [Source:HGNC Symbol;Acc:HGNC:14952] |
| ENSG00000198856 | HGLibA_34707 | 62 | -0.531637638 | OSTC | oligosaccharyltransferase complex non-catalytic subunit [Source:HGNC Symbol;Acc:HGNC:24448] |
| ENSG00000198873 | HGLibA_20333 | 62 | -0.531637638 | GRK5 | G protein-coupled receptor kinase 5 [Source:HGNC Symbol;Acc:HGNC:4544] |
| ENSG00000203837 | HGLibA_37363 | 62 | -0.531637638 | PNLIPRP3 | pancreatic lipase related protein 3 [Source:HGNC Symbol;Acc:HGNC:23492] |
| ENSG00000204195 | HGLibA_03913 | 62 | -0.531637638 | AWAT1 | acyl-CoA wax alcohol acyltransferase 1 [Source:HGNC Symbol;Acc:HGNC:23252] |
| ENSG00000204869 | HGLibA_22848 | 62 | -0.531637638 | IGFL4 | IGF like family member 4 [Source:HGNC Symbol;Acc:HGNC:32931] |
| ENSG00000205133 | HGLibA_51651 | 62 | -0.531637638 | TRIQK | triple QxxK/R motif containing [Source:HGNC Symbol;Acc:HGNC:27828] |
| ENSG00000205571 | HGLibA_45786 | 62 | -0.531637638 | SMN2 | centromeric [Source:HGNC Symbol;Acc:HGNC:11118]" |
| ENSG00000212127 | HGLibA_48372 | 62 | -0.531637638 | TAS2R14 | taste 2 receptor member 14 [Source:HGNC Symbol;Acc:HGNC:14920] |
| ENSG00000213390 | HGLibA_02720 | 62 | -0.531637638 | ARHGAP19 | Rho GTPase activating protein 19 [Source:HGNC Symbol;Acc:HGNC:23724] |
| ENSG00000215218 | HGLibA_52652 | 62 | -0.531637638 | UBE2QL1 | ubiquitin conjugating enzyme E2 Q family like 1 [Source:HGNC Symbol;Acc:HGNC:37269] |
| ENSG00000220205 | HGLibA_53492 | 62 | -0.531637638 | VAMP2 | vesicle associated membrane protein 2 [Source:HGNC Symbol;Acc:HGNC:12643] |
| ENSG00000221995 | HGLibA_49433 | 62 | -0.531637638 | TIAF1 | TGFB1-induced anti-apoptotic factor 1 [Source:HGNC Symbol;Acc:HGNC:11803] |
| ENSG00000228727 | HGLibA_42731 | 62 | -0.531637638 | SAPCD1 | suppressor APC domain containing 1 [Source:HGNC Symbol;Acc:HGNC:13938] |
| ENSG00000233136 | HGLibA_53219 | 62 | -0.531637638 | USP17L11 | ubiquitin specific peptidase 17-like family member 11 [Source:HGNC Symbol;Acc:HGNC:44439] |
| ENSG00000233493 | HGLibA_50280 | 62 | -0.531637638 | TMEM238 | transmembrane protein 238 [Source:HGNC Symbol;Acc:HGNC:40042] |
| ENSG00000240583 | HGLibA_02579 | 62 | -0.531637638 | AQP1 | aquaporin 1 (Colton blood group) [Source:HGNC Symbol;Acc:HGNC:633] |
| ENSG00000258429 | HGLibA_35839 | 62 | -0.531637638 | PDF | mitochondrial [Source:HGNC Symbol;Acc:HGNC:30012]" |
| ENSG00000258713 | HGLibA_05987 | 62 | -0.531637638 | C20orf141 | chromosome 20 open reading frame 141 [Source:HGNC Symbol;Acc:HGNC:16134] |
| ENSG00000261609 | HGLibA_18601 | 62 | -0.531637638 | GAN | gigaxonin [Source:HGNC Symbol;Acc:HGNC:4137] |
| ENSG00000262209 | HGLibA_35556 | 62 | -0.531637638 | PCDHGB3 | 3 [Source:HGNC Symbol;Acc:HGNC:8710]" |
| ENSG00000267060 | HGLibA_39334 | 62 | -0.531637638 | PTGES3L | prostaglandin E synthase 3 like [Source:HGNC Symbol;Acc:HGNC:43943] |
| ENSG00000271043 | HGLibA_30476 | 62 | -0.531637638 | MTRNR2L2 | MT-RNR2 like 2 [Source:HGNC Symbol;Acc:HGNC:37156] |
| ENSG00000273173 | HGLibA_46018 | 62 | -0.531637638 | SNURF | SNRPN upstream reading frame [Source:HGNC Symbol;Acc:HGNC:11171] |
| FAM105A | HGLibA_15995 | 62 | -0.531637638 | FAM105A | - |
| GPR123 | HGLibA_19873 | 62 | -0.531637638 | GPR123 | - |
| HIST1H2BK | HGLibA_21440 | 62 | -0.531637638 | HIST1H2BK | - |
| HIST4H4 | HGLibA_21565 | 62 | -0.531637638 | HIST4H4 | - |
| hsa-mir-1233-2 | HGLibA_57328 | 62 | -0.531637638 | hsa-mir-1233-2 | - |
| hsa-mir-1273a | HGLibA_57532 | 62 | -0.531637638 | hsa-mir-1273a | - |
| hsa-mir-1283-1 | HGLibA_57590 | 62 | -0.531637638 | hsa-mir-1283-1 | - |
| hsa-mir-1302-7 | HGLibA_57714 | 62 | -0.531637638 | hsa-mir-1302-7 | - |
| hsa-mir-137 | HGLibA_57808 | 62 | -0.531637638 | hsa-mir-137 | - |
| hsa-mir-150 | HGLibA_57892 | 62 | -0.531637638 | hsa-mir-150 | - |
| hsa-mir-16-1 | HGLibA_57948 | 62 | -0.531637638 | hsa-mir-16-1 | - |
| hsa-mir-3156-3 | HGLibA_58694 | 62 | -0.531637638 | hsa-mir-3156-3 | - |
| hsa-mir-3160-2 | HGLibA_58718 | 62 | -0.531637638 | hsa-mir-3160-2 | - |
| hsa-mir-3175 | HGLibA_58773 | 62 | -0.531637638 | hsa-mir-3175 | - |
| hsa-mir-33b | HGLibA_59015 | 62 | -0.531637638 | hsa-mir-33b | - |
| hsa-mir-3614 | HGLibA_59087 | 62 | -0.531637638 | hsa-mir-3614 | - |
| hsa-mir-3658 | HGLibA_59177 | 62 | -0.531637638 | hsa-mir-3658 | - |
| hsa-mir-3912 | HGLibA_59518 | 62 | -0.531637638 | hsa-mir-3912 | - |
| hsa-mir-3915 | HGLibA_59537 | 62 | -0.531637638 | hsa-mir-3915 | - |
| hsa-mir-4296 | HGLibA_59894 | 62 | -0.531637638 | hsa-mir-4296 | - |
| hsa-mir-4315-2 | HGLibA_59981 | 62 | -0.531637638 | hsa-mir-4315-2 | - |
| hsa-mir-4322 | HGLibA_60012 | 62 | -0.531637638 | hsa-mir-4322 | - |
| hsa-mir-4659a | HGLibA_60742 | 62 | -0.531637638 | hsa-mir-4659a | - |
| hsa-mir-4668 | HGLibA_60791 | 62 | -0.531637638 | hsa-mir-4668 | - |
| hsa-mir-4778 | HGLibA_61219 | 62 | -0.531637638 | hsa-mir-4778 | - |
| hsa-mir-4791 | HGLibA_61269 | 62 | -0.531637638 | hsa-mir-4791 | - |
| hsa-mir-503 | HGLibA_61468 | 62 | -0.531637638 | hsa-mir-503 | - |
| hsa-mir-509-3 | HGLibA_61515 | 62 | -0.531637638 | hsa-mir-509-3 | - |
| hsa-mir-5692b | HGLibA_62297 | 62 | -0.531637638 | hsa-mir-5692b | - |
| hsa-mir-580 | HGLibA_62418 | 62 | -0.531637638 | hsa-mir-580 | - |
| hsa-mir-6071 | HGLibA_62538 | 62 | -0.531637638 | hsa-mir-6071 | - |
| hsa-mir-6740 | HGLibA_63112 | 62 | -0.531637638 | hsa-mir-6740 | - |
| hsa-mir-6756 | HGLibA_63182 | 62 | -0.531637638 | hsa-mir-6756 | - |
| hsa-mir-6760 | HGLibA_63202 | 62 | -0.531637638 | hsa-mir-6760 | - |
| hsa-mir-6780b | HGLibA_63299 | 62 | -0.531637638 | hsa-mir-6780b | - |
| hsa-mir-6793 | HGLibA_63351 | 62 | -0.531637638 | hsa-mir-6793 | - |
| hsa-mir-6829 | HGLibA_63494 | 62 | -0.531637638 | hsa-mir-6829 | - |
| hsa-mir-6856 | HGLibA_63598 | 62 | -0.531637638 | hsa-mir-6856 | - |
| hsa-mir-7112-2 | HGLibA_63815 | 62 | -0.531637638 | hsa-mir-7112-2 | - |
| hsa-mir-7159 | HGLibA_63864 | 62 | -0.531637638 | hsa-mir-7159 | - |
| hsa-mir-760 | HGLibA_63900 | 62 | -0.531637638 | hsa-mir-760 | - |
| hsa-mir-7975 | HGLibA_64030 | 62 | -0.531637638 | hsa-mir-7975 | - |
| hsa-mir-96 | HGLibA_64370 | 62 | -0.531637638 | hsa-mir-96 | - |
| LOC152586 | HGLibA_26895 | 62 | -0.531637638 | LOC152586 | - |
| LOC650293 | HGLibA_26985 | 62 | -0.531637638 | LOC650293 | - |
| MLF1IP | HGLibA_29374 | 62 | -0.531637638 | MLF1IP | - |
| ORAOV1 | HGLibA_34599 | 62 | -0.531637638 | ORAOV1 | - |
| PAPD7 | HGLibA_35127 | 62 | -0.531637638 | PAPD7 | - |
| PLK1S1 | HGLibA_37189 | 62 | -0.531637638 | PLK1S1 | - |

| | | | | | |
|---|---|---|---|---|---|
| RABL5 | HGLibA_40036 | 62 | -0.531637638 | RABL5 | - |
| SRPR | HGLibA_47026 | 62 | -0.531637638 | SRPR | - |
| TCEB1 | HGLibA_48734 | 62 | -0.531637638 | TCEB1 | - |
| TMEM261 | HGLibA_50355 | 62 | -0.531637638 | TMEM261 | - |
| TTC18 | HGLibA_52063 | 62 | -0.531637638 | TTC18 | - |
| 4-Mar | HGLibA_28336 | 61 | -0.541588676 | 4-Mar | - |
| BCMO1 | HGLibA_04357 | 61 | -0.541588676 | BCMO1 | - |
| C15orf38 | HGLibA_05388 | 61 | -0.541588676 | C15orf38 | - |
| CCDC111 | HGLibA_07540 | 61 | -0.541588676 | CCDC111 | - |
| CCL3L1 | HGLibA_08021 | 61 | -0.541588676 | CCL3L1 | - |
| CXorf22 | HGLibA_11906 | 61 | -0.541588676 | CXorf22 | - |
| CXorf56 | HGLibA_11943 | 61 | -0.541588676 | CXorf56 | - |
| DBC1 | HGLibA_12372 | 61 | -0.541588676 | DBC1 | - |
| ENSG00000006128 | HGLibA_48149 | 61 | -0.541588676 | TAC1 | tachykinin precursor 1 [Source:HGNC Symbol;Acc:HGNC:11517] |
| ENSG00000008300 | HGLibA_09027 | 61 | -0.541588676 | CELSR3 | cadherin EGF LAG seven-pass G-type receptor 3 [Source:HGNC Symbol;Acc:HGNC:3230] |
| ENSG00000010017 | HGLibA_40211 | 61 | -0.541588676 | RANBP9 | RAN binding protein 9 [Source:HGNC Symbol;Acc:HGNC:13727] |
| ENSG00000014164 | HGLibA_54974 | 61 | -0.541588676 | ZC3H3 | zinc finger CCCH-type containing 3 [Source:HGNC Symbol;Acc:HGNC:28972] |
| ENSG00000039139 | HGLibA_13490 | 61 | -0.541588676 | DNAH5 | dynein axonemal heavy chain 5 [Source:HGNC Symbol;Acc:HGNC:2950] |
| ENSG00000064012 | HGLibA_07326 | 61 | -0.541588676 | CASP8 | caspase 8 [Source:HGNC Symbol;Acc:HGNC:1509] |
| ENSG00000067606 | HGLibA_38563 | 61 | -0.541588676 | PRKCZ | protein kinase C zeta [Source:HGNC Symbol;Acc:HGNC:9412] |
| ENSG00000069206 | HGLibA_00778 | 61 | -0.541588676 | ADAM7 | ADAM metallopeptidase domain 7 [Source:HGNC Symbol;Acc:HGNC:214] |
| ENSG00000069206 | HGLibA_00780 | 61 | -0.541588676 | ADAM7 | ADAM metallopeptidase domain 7 [Source:HGNC Symbol;Acc:HGNC:214] |
| ENSG00000080618 | HGLibA_10909 | 61 | -0.541588676 | CPB2 | carboxypeptidase B2 [Source:HGNC Symbol;Acc:HGNC:2300] |
| ENSG00000086696 | HGLibA_22187 | 61 | -0.541588676 | HSD17B2 | hydroxysteroid 17-beta dehydrogenase 2 [Source:HGNC Symbol;Acc:HGNC:5211] |
| ENSG00000087303 | HGLibA_31927 | 61 | -0.541588676 | NID2 | nidogen 2 [Source:HGNC Symbol;Acc:HGNC:13389] |
| ENSG00000088179 | HGLibA_39470 | 61 | -0.541588676 | PTPN4 | non-receptor type 4 [Source:HGNC Symbol;Acc:HGNC:9656]" |
| ENSG00000088538 | HGLibA_13743 | 61 | -0.541588676 | DOCK3 | dedicator of cytokinesis 3 [Source:HGNC Symbol;Acc:HGNC:2989] |
| ENSG00000088826 | HGLibA_45799 | 61 | -0.541588676 | SMOX | spermine oxidase [Source:HGNC Symbol;Acc:HGNC:15862] |
| ENSG00000089472 | HGLibA_21143 | 61 | -0.541588676 | HEPH | hephaestin [Source:HGNC Symbol;Acc:HGNC:4866] |
| ENSG00000091622 | HGLibA_36757 | 61 | -0.541588676 | PITPNM3 | PITPNM family member 3 [Source:HGNC Symbol;Acc:HGNC:21043] |
| ENSG00000095739 | HGLibA_04138 | 61 | -0.541588676 | BAMBI | BMP and activin membrane bound inhibitor [Source:HGNC Symbol;Acc:HGNC:30251] |
| ENSG00000099849 | HGLibA_40393 | 61 | -0.541588676 | RASSF7 | Ras association domain family member 7 [Source:HGNC Symbol;Acc:HGNC:1166] |
| ENSG00000099999 | HGLibA_41629 | 61 | -0.541588676 | RNF215 | ring finger protein 215 [Source:HGNC Symbol;Acc:HGNC:33434] |
| ENSG00000100519 | HGLibA_39146 | 61 | -0.541588676 | PSMC6 | ATPase 6 [Source:HGNC Symbol;Acc:HGNC:9553]" |
| ENSG00000101200 | HGLibA_03896 | 61 | -0.541588676 | AVP | arginine vasopressin [Source:HGNC Symbol;Acc:HGNC:894] |
| ENSG00000101246 | HGLibA_02682 | 61 | -0.541588676 | ARFRP1 | ADP ribosylation factor related protein 1 [Source:HGNC Symbol;Acc:HGNC:662] |
| ENSG00000101292 | HGLibA_38674 | 61 | -0.541588676 | PROKR2 | prokineticin receptor 2 [Source:HGNC Symbol;Acc:HGNC:15836] |
| ENSG00000102119 | HGLibA_15032 | 61 | -0.541588676 | EMD | emerin [Source:HGNC Symbol;Acc:HGNC:3331] |
| ENSG00000102218 | HGLibA_41793 | 61 | -0.541588676 | RP2 | ARL3 GTPase activating protein [Source:HGNC Symbol;Acc:HGNC:10274]" |
| ENSG00000102974 | HGLibA_11595 | 61 | -0.541588676 | CTCF | CCCTC-binding factor [Source:HGNC Symbol;Acc:HGNC:13723] |
| ENSG00000103091 | HGLibA_54130 | 61 | -0.541588676 | WDR59 | WD repeat domain 59 [Source:HGNC Symbol;Acc:HGNC:25706] |
| ENSG00000103145 | HGLibA_20961 | 61 | -0.541588676 | HCFC1R1 | host cell factor C1 regulator 1 [Source:HGNC Symbol;Acc:HGNC:21198] |
| ENSG00000105894 | HGLibA_39396 | 61 | -0.541588676 | PTN | pleiotrophin [Source:HGNC Symbol;Acc:HGNC:9630] |
| ENSG00000106018 | HGLibA_53670 | 61 | -0.541588676 | VIPR2 | vasoactive intestinal peptide receptor 2 [Source:HGNC Symbol;Acc:HGNC:12695] |
| ENSG00000106304 | HGLibA_46375 | 61 | -0.541588676 | SPAM1 | sperm adhesion molecule 1 [Source:HGNC Symbol;Acc:HGNC:11217] |
| ENSG00000106538 | HGLibA_40283 | 61 | -0.541588676 | RARRES2 | retinoic acid receptor responder 2 [Source:HGNC Symbol;Acc:HGNC:9868] |
| ENSG00000107077 | HGLibA_24513 | 61 | -0.541588676 | KDM4C | lysine demethylase 4C [Source:HGNC Symbol;Acc:HGNC:17071] |
| ENSG00000107872 | HGLibA_16982 | 61 | -0.541588676 | FBXL15 | F-box and leucine rich repeat protein 15 [Source:HGNC Symbol;Acc:HGNC:28155] |
| ENSG00000107951 | HGLibA_30458 | 61 | -0.541588676 | MTPAP | mitochondrial poly(A) polymerase [Source:HGNC Symbol;Acc:HGNC:25532] |
| ENSG00000108604 | HGLibA_45631 | 61 | -0.541588676 | SMARCD2 | member 2 [Source:HGNC Symbol;Acc:HGNC:11107]" |
| ENSG00000109321 | HGLibA_02640 | 61 | -0.541588676 | AREG | amphiregulin [Source:HGNC Symbol;Acc:HGNC:651] |
| ENSG00000111271 | HGLibA_00354 | 61 | -0.541588676 | ACAD10 | acyl-CoA dehydrogenase family member 10 [Source:HGNC Symbol;Acc:HGNC:21597] |
| ENSG00000111596 | HGLibA_10334 | 61 | -0.541588676 | CNOT2 | CCR4-NOT transcription complex subunit 2 [Source:HGNC Symbol;Acc:HGNC:7878] |
| ENSG00000111875 | HGLibA_03282 | 61 | -0.541588676 | ASF1A | anti-silencing function 1A histone chaperone [Source:HGNC Symbol;Acc:HGNC:20995] |
| ENSG00000112294 | HGLibA_01577 | 61 | -0.541588676 | ALDH5A1 | aldehyde dehydrogenase 5 family member A1 [Source:HGNC Symbol;Acc:HGNC:408] |
| ENSG00000112706 | HGLibA_23261 | 61 | -0.541588676 | IMPG1 | interphotoreceptor matrix proteoglycan 1 [Source:HGNC Symbol;Acc:HGNC:6055] |
| ENSG00000113396 | HGLibA_44827 | 61 | -0.541588676 | SLC27A6 | solute carrier family 27 member 6 [Source:HGNC Symbol;Acc:HGNC:11000] |
| ENSG00000113722 | HGLibA_08896 | 61 | -0.541588676 | CDX1 | caudal type homeobox 1 [Source:HGNC Symbol;Acc:HGNC:1805] |
| ENSG00000113889 | HGLibA_25343 | 61 | -0.541588676 | KNG1 | kininogen 1 [Source:HGNC Symbol;Acc:HGNC:6383] |
| ENSG00000114439 | HGLibA_04230 | 61 | -0.541588676 | BBX | HMG-box containing [Source:HGNC Symbol;Acc:HGNC:14422]" |
| ENSG00000114573 | HGLibA_03729 | 61 | -0.541588676 | ATP6V1A | ATPase H+ transporting V1 subunit A [Source:HGNC Symbol;Acc:HGNC:851] |
| ENSG00000116035 | HGLibA_53560 | 61 | -0.541588676 | VAX2 | ventral anterior homeobox 2 [Source:HGNC Symbol;Acc:HGNC:12661] |
| ENSG00000118855 | HGLibA_29080 | 61 | -0.541588676 | MFSD1 | major facilitator superfamily domain containing 1 [Source:HGNC Symbol;Acc:HGNC:25874] |
| ENSG00000119285 | HGLibA_21066 | 61 | -0.541588676 | HEATR1 | HEAT repeat containing 1 [Source:HGNC Symbol;Acc:HGNC:25517] |
| ENSG00000119630 | HGLibA_36233 | 61 | -0.541588676 | PGF | placental growth factor [Source:HGNC Symbol;Acc:HGNC:8893] |
| ENSG00000119888 | HGLibA_15273 | 61 | -0.541588676 | EPCAM | epithelial cell adhesion molecule [Source:HGNC Symbol;Acc:HGNC:11529] |
| ENSG00000121417 | HGLibA_55584 | 61 | -0.541588676 | ZNF211 | zinc finger protein 211 [Source:HGNC Symbol;Acc:HGNC:13003] |
| ENSG00000122481 | HGLibA_42488 | 61 | -0.541588676 | RWDD3 | RWD domain containing 3 [Source:HGNC Symbol;Acc:HGNC:21393] |
| ENSG00000122884 | HGLibA_34900 | 61 | -0.541588676 | P4HA1 | prolyl 4-hydroxylase subunit alpha 1 [Source:HGNC Symbol;Acc:HGNC:8546] |
| ENSG00000123064 | HGLibA_12745 | 61 | -0.541588676 | DDX54 | DEAD-box helicase 54 [Source:HGNC Symbol;Acc:HGNC:20084] |
| ENSG00000123066 | HGLibA_28751 | 61 | -0.541588676 | MED13L | mediator complex subunit 13 like [Source:HGNC Symbol;Acc:HGNC:22962] |
| ENSG00000123374 | HGLibA_08755 | 61 | -0.541588676 | CDK2 | cyclin dependent kinase 2 [Source:HGNC Symbol;Acc:HGNC:1771] |
| ENSG00000123843 | HGLibA_06293 | 61 | -0.541588676 | C4BPB | complement component 4 binding protein beta [Source:HGNC Symbol;Acc:HGNC:1328] |
| ENSG00000125388 | HGLibA_20329 | 61 | -0.541588676 | GRK4 | G protein-coupled receptor kinase 4 [Source:HGNC Symbol;Acc:HGNC:4543] |
| ENSG00000126262 | HGLibA_17327 | 61 | -0.541588676 | FFAR2 | free fatty acid receptor 2 [Source:HGNC Symbol;Acc:HGNC:4501] |
| ENSG00000128052 | HGLibA_24541 | 61 | -0.541588676 | KDR | kinase insert domain receptor [Source:HGNC Symbol;Acc:HGNC:6307] |
| ENSG00000129048 | HGLibA_00449 | 61 | -0.541588676 | ACKR4 | atypical chemokine receptor 4 [Source:HGNC Symbol;Acc:HGNC:1611] |
| ENSG00000130024 | HGLibA_36332 | 61 | -0.541588676 | PHF10 | PHD finger protein 10 [Source:HGNC Symbol;Acc:HGNC:18250] |
| ENSG00000130475 | HGLibA_17205 | 61 | -0.541588676 | FCHO1 | FCH domain only 1 [Source:HGNC Symbol;Acc:HGNC:29002] |
| ENSG00000131969 | HGLibA_00241 | 61 | -0.541588676 | ABHD12B | abhydrolase domain containing 12B [Source:HGNC Symbol;Acc:HGNC:19837] |
| ENSG00000132478 | HGLibA_53048 | 61 | -0.541588676 | UNK | unkempt family zinc finger [Source:HGNC Symbol;Acc:HGNC:29369] |
| ENSG00000132570 | HGLibA_35363 | 61 | -0.541588676 | PCBD2 | pterin-4 alpha-carbinolamine dehydratase 2 [Source:HGNC Symbol;Acc:HGNC:24474] |
| ENSG00000132692 | HGLibA_04237 | 61 | -0.541588676 | BCAN | brevican [Source:HGNC Symbol;Acc:HGNC:23059] |
| ENSG00000133962 | HGLibA_07367 | 61 | -0.541588676 | CATSPERB | cation channel sperm associated auxiliary subunit beta [Source:HGNC Symbol;Acc:HGNC:20500] |
| ENSG00000136114 | HGLibA_49399 | 61 | -0.541588676 | THSD1 | thrombospondin type 1 domain containing 1 [Source:HGNC Symbol;Acc:HGNC:17754] |
| ENSG00000136839 | HGLibA_33611 | 61 | -0.541588676 | OR13C9 | olfactory receptor family 13 subfamily C member 9 [Source:HGNC Symbol;Acc:HGNC:15104] |
| ENSG00000136960 | HGLibA_15171 | 61 | -0.541588676 | ENPP2 | ectonucleotide pyrophosphatase/phosphodiesterase 2 [Source:HGNC Symbol;Acc:HGNC:3357] |
| ENSG00000137265 | HGLibA_23590 | 61 | -0.541588676 | IRF4 | interferon regulatory factor 4 [Source:HGNC Symbol;Acc:HGNC:6119] |
| ENSG00000137266 | HGLibA_44574 | 61 | -0.541588676 | SLC22A23 | solute carrier family 22 member 23 [Source:HGNC Symbol;Acc:HGNC:21106] |
| ENSG00000137561 | HGLibA_52223 | 61 | -0.541588676 | TTPA | alpha tocopherol transfer protein [Source:HGNC Symbol;Acc:HGNC:12404] |
| ENSG00000138385 | HGLibA_47110 | 61 | -0.541588676 | SSB | Sjogren syndrome antigen B [Source:HGNC Symbol;Acc:HGNC:11316] |
| ENSG00000138744 | HGLibA_31006 | 61 | -0.541588676 | NAAA | N-acylethanolamine acid amidase [Source:HGNC Symbol;Acc:HGNC:736] |
| ENSG00000139880 | HGLibA_08672 | 61 | -0.541588676 | CDH24 | cadherin 24 [Source:HGNC Symbol;Acc:HGNC:14265] |
| ENSG00000140522 | HGLibA_41343 | 61 | -0.541588676 | RLBP1 | retinaldehyde binding protein 1 [Source:HGNC Symbol;Acc:HGNC:10024] |
| ENSG00000140553 | HGLibA_53006 | 61 | -0.541588676 | UNC45A | unc-45 myosin chaperone A [Source:HGNC Symbol;Acc:HGNC:30594] |
| ENSG00000140678 | HGLibA_23766 | 61 | -0.541588676 | ITGAX | integrin subunit alpha X [Source:HGNC Symbol;Acc:HGNC:6152] |
| ENSG00000140682 | HGLibA_49210 | 61 | -0.541588676 | TGFB1I1 | transforming growth factor beta 1 induced transcript 1 [Source:HGNC Symbol;Acc:HGNC:11767] |
| ENSG00000141161 | HGLibA_53009 | 61 | -0.541588676 | UNC45B | unc-45 myosin chaperone B [Source:HGNC Symbol;Acc:HGNC:14304] |
| ENSG00000142208 | HGLibA_01505 | 61 | -0.541588676 | AKT1 | AKT serine/threonine kinase 1 [Source:HGNC Symbol;Acc:HGNC:391] |
| ENSG00000144407 | HGLibA_39372 | 61 | -0.541588676 | PTH2R | parathyroid hormone 2 receptor [Source:HGNC Symbol;Acc:HGNC:9609] |
| ENSG00000144468 | HGLibA_41124 | 61 | -0.541588676 | RHBDD1 | rhomboid domain containing 1 [Source:HGNC Symbol;Acc:HGNC:23081] |
| ENSG00000145911 | HGLibA_30961 | 61 | -0.541588676 | N4BP3 | NEDD4 binding protein 3 [Source:HGNC Symbol;Acc:HGNC:29852] |
| ENSG00000147099 | HGLibA_21023 | 61 | -0.541588676 | HDAC8 | histone deacetylase 8 [Source:HGNC Symbol;Acc:HGNC:13315] |
| ENSG00000147127 | HGLibA_39944 | 61 | -0.541588676 | RAB41 | member RAS oncogene family [Source:HGNC Symbol;Acc:HGNC:18293]" |
| ENSG00000150054 | HGLibA_29764 | 61 | -0.541588676 | MPP7 | membrane palmitoylated protein 7 [Source:HGNC Symbol;Acc:HGNC:26542] |
| ENSG00000151445 | HGLibA_53666 | 61 | -0.541588676 | VIPAS39 | spe-39 homolog [Source:HGNC Symbol;Acc:HGNC:20347]" |
| ENSG00000151461 | HGLibA_53060 | 61 | -0.541588676 | UPF2 | regulator of nonsense mediated mRNA decay [Source:HGNC Symbol;Acc:HGNC:17854]" |
| ENSG00000151893 | HGLibA_06955 | 61 | -0.541588676 | CACUL1 | CDK2 associated cullin domain 1 [Source:HGNC Symbol;Acc:HGNC:23727] |
| ENSG00000152102 | HGLibA_16248 | 61 | -0.541588676 | FAM168B | family with sequence similarity 168 member B [Source:HGNC Symbol;Acc:HGNC:27016] |
| ENSG00000154438 | HGLibA_03397 | 61 | -0.541588676 | ASZ1 | SAM and basic leucine zipper domain containing 1 [Source:HGNC Symbol;Acc:HGNC:1350]" |
| ENSG00000154493 | HGLibA_05103 | 61 | -0.541588676 | C10orf90 | chromosome 10 open reading frame 90 [Source:HGNC Symbol;Acc:HGNC:26563] |
| ENSG00000154957 | HGLibA_55535 | 61 | -0.541588676 | ZNF18 | zinc finger protein 18 [Source:HGNC Symbol;Acc:HGNC:12969] |
| ENSG00000155660 | HGLibA_35879 | 61 | -0.541588676 | PDIA4 | protein disulfide isomerase family A member 4 [Source:HGNC Symbol;Acc:HGNC:30167] |
| ENSG00000155959 | HGLibA_53564 | 61 | -0.541588676 | VBP1 | VHL binding protein 1 [Source:HGNC Symbol;Acc:HGNC:12662] |
| ENSG00000156162 | HGLibA_13914 | 61 | -0.541588676 | DPY19L4 | dpy-19 like 4 [Source:HGNC Symbol;Acc:HGNC:27829] |
| ENSG00000156886 | HGLibA_23750 | 61 | -0.541588676 | ITGAD | integrin subunit alpha D [Source:HGNC Symbol;Acc:HGNC:6146] |
| ENSG00000158042 | HGLibA_29912 | 61 | -0.541588676 | MRPL17 | mitochondrial ribosomal protein L17 [Source:HGNC Symbol;Acc:HGNC:14053] |
| ENSG00000158077 | HGLibA_32112 | 61 | -0.541588676 | NLRP14 | NLR family pyrin domain containing 14 [Source:HGNC Symbol;Acc:HGNC:22939] |
| ENSG00000158089 | HGLibA_18541 | 61 | -0.541588676 | GALNT14 | polypeptide N-acetylgalactosaminyltransferase 14 [Source:HGNC Symbol;Acc:HGNC:22946] |
| ENSG00000158859 | HGLibA_00830 | 61 | -0.541588676 | ADAMTS4 | ADAM metallopeptidase with thrombospondin type 1 motif 4 [Source:HGNC Symbol;Acc:HGNC:220] |
| ENSG00000159399 | HGLibA_21582 | 61 | -0.541588676 | HK2 | hexokinase 2 [Source:HGNC Symbol;Acc:HGNC:4923] |
| ENSG00000159556 | HGLibA_23667 | 61 | -0.541588676 | ISL2 | ISL LIM homeobox 2 [Source:HGNC Symbol;Acc:HGNC:18524] |
| ENSG00000160049 | HGLibA_13019 | 61 | -0.541588676 | DFFA | DNA fragmentation factor subunit alpha [Source:HGNC Symbol;Acc:HGNC:2772] |
| ENSG00000160202 | HGLibA_11245 | 61 | -0.541588676 | CRYAA | crystallin alpha A [Source:HGNC Symbol;Acc:HGNC:2388] |
| ENSG00000160408 | HGLibA_47278 | 61 | -0.541588676 | ST6GALNAC6 | 6-sialyltransferase 6 [Source:HGNC Symbol;Acc:HGNC:23364]" |
| ENSG00000160602 | HGLibA_31666 | 61 | -0.541588676 | NEK8 | NIMA related kinase 8 [Source:HGNC Symbol;Acc:HGNC:13387] |
| ENSG00000160741 | HGLibA_11232 | 61 | -0.541588676 | CRTC2 | CREB regulated transcription coactivator 2 [Source:HGNC Symbol;Acc:HGNC:27301] |
| ENSG00000162148 | HGLibA_38087 | 61 | -0.541588676 | PPP1R32 | protein phosphatase 1 regulatory subunit 32 [Source:HGNC Symbol;Acc:HGNC:28869] |
| ENSG00000162227 | HGLibA_48250 | 61 | -0.541588676 | TAF6L | TATA-box binding protein associated factor 6 like [Source:HGNC Symbol;Acc:HGNC:17305] |
| ENSG00000162413 | HGLibA_25154 | 61 | -0.541588676 | KLHL21 | kelch like family member 21 [Source:HGNC Symbol;Acc:HGNC:29041] |
| ENSG00000162543 | HGLibA_52777 | 61 | -0.541588676 | UBXN10 | UBX domain protein 10 [Source:HGNC Symbol;Acc:HGNC:26354] |
| ENSG00000163291 | HGLibA_35158 | 61 | -0.541588676 | PAQR3 | progestin and adipoQ receptor family member 3 [Source:HGNC Symbol;Acc:HGNC:30130] |
| ENSG00000163694 | HGLibA_40565 | 61 | -0.541588676 | RBM47 | RNA binding motif protein 47 [Source:HGNC Symbol;Acc:HGNC:30358] |
| ENSG00000164134 | HGLibA_30975 | 61 | -0.541588676 | NAA15 | NatA auxiliary subunit [Source:HGNC Symbol;Acc:HGNC:30782]" |
| ENSG00000164258 | HGLibA_31555 | 61 | -0.541588676 | NDUFS4 | NADH:ubiquinone oxidoreductase subunit S4 [Source:HGNC Symbol;Acc:HGNC:7711] |

| | | | | | |
|---|---|---|---|---|---|
| ENSG00000165474 | HGLibA_19161 | 61 | -0.541588676 | GJB2 | gap junction protein beta 2 [Source:HGNC Symbol;Acc:HGNC:4284] |
| ENSG00000166828 | HGLibA_43023 | 61 | -0.541588676 | SCNN1G | sodium channel epithelial 1 gamma subunit [Source:HGNC Symbol;Acc:HGNC:10602] |
| ENSG00000167363 | HGLibA_17730 | 61 | -0.541588676 | FN3K | fructosamine 3 kinase [Source:HGNC Symbol;Acc:HGNC:24822] |
| ENSG00000167377 | HGLibA_55633 | 61 | -0.541588676 | ZNF23 | zinc finger protein 23 [Source:HGNC Symbol;Acc:HGNC:13023] |
| ENSG00000167693 | HGLibA_33140 | 61 | -0.541588676 | NXN | nucleoredoxin [Source:HGNC Symbol;Acc:HGNC:18008] |
| ENSG00000167741 | HGLibA_19017 | 61 | -0.541588676 | GGT6 | gamma-glutamyltransferase 6 [Source:HGNC Symbol;Acc:HGNC:26891] |
| ENSG00000169344 | HGLibA_52977 | 61 | -0.541588676 | UMOD | uromodulin [Source:HGNC Symbol;Acc:HGNC:12559] |
| ENSG00000169375 | HGLibA_44182 | 61 | -0.541588676 | SIN3A | SIN3 transcription regulator family member A [Source:HGNC Symbol;Acc:HGNC:19353] |
| ENSG00000169435 | HGLibA_40390 | 61 | -0.541588676 | RASSF6 | Ras association domain family member 6 [Source:HGNC Symbol;Acc:HGNC:20796] |
| ENSG00000169604 | HGLibA_02213 | 61 | -0.541588676 | ANTXR1 | ANTXR cell adhesion molecule 1 [Source:HGNC Symbol;Acc:HGNC:21014] |
| ENSG00000169717 | HGLibA_00680 | 61 | -0.541588676 | ACTRT2 | actin related protein T2 [Source:HGNC Symbol;Acc:HGNC:24026] |
| ENSG00000169902 | HGLibA_51205 | 61 | -0.541588676 | TPST1 | tyrosylprotein sulfotransferase 1 [Source:HGNC Symbol;Acc:HGNC:12020] |
| ENSG00000169957 | HGLibA_56698 | 61 | -0.541588676 | ZNF768 | zinc finger protein 768 [Source:HGNC Symbol;Acc:HGNC:26273] |
| ENSG00000170801 | HGLibA_22412 | 61 | -0.541588676 | HTRA3 | HtrA serine peptidase 3 [Source:HGNC Symbol;Acc:HGNC:30406] |
| ENSG00000171360 | HGLibA_25499 | 61 | -0.541588676 | KRT38 | keratin 38 [Source:HGNC Symbol;Acc:HGNC:6456] |
| ENSG00000171564 | HGLibA_17340 | 61 | -0.541588676 | FGB | fibrinogen beta chain [Source:HGNC Symbol;Acc:HGNC:3662] |
| ENSG00000173041 | HGLibA_56524 | 61 | -0.541588676 | ZNF680 | zinc finger protein 680 [Source:HGNC Symbol;Acc:HGNC:26897] |
| ENSG00000173261 | HGLibA_36946 | 61 | -0.541588676 | PLAC8L1 | PLAC8 like 1 [Source:HGNC Symbol;Acc:HGNC:31746] |
| ENSG00000173262 | HGLibA_44868 | 61 | -0.541588676 | SLC2A14 | solute carrier family 2 member 14 [Source:HGNC Symbol;Acc:HGNC:18301] |
| ENSG00000176009 | HGLibA_03273 | 61 | -0.541588676 | ASCL3 | achaete-scute family bHLH transcription factor 3 [Source:HGNC Symbol;Acc:HGNC:740] |
| ENSG00000176402 | HGLibA_19183 | 61 | -0.541588676 | GJC3 | gap junction protein gamma 3 [Source:HGNC Symbol;Acc:HGNC:17495] |
| ENSG00000177243 | HGLibA_12815 | 61 | -0.541588676 | DEFB103B | defensin beta 103B [Source:HGNC Symbol;Acc:HGNC:31702] |
| ENSG00000178694 | HGLibA_32783 | 61 | -0.541588676 | NSUN3 | NOP2/Sun RNA methyltransferase family member 3 [Source:HGNC Symbol;Acc:HGNC:26208] |
| ENSG00000178732 | HGLibA_19709 | 61 | -0.541588676 | GP5 | glycoprotein V platelet [Source:HGNC Symbol;Acc:HGNC:4443] |
| ENSG00000179010 | HGLibA_29827 | 61 | -0.541588676 | MRFAP1 | Morf4 family associated protein 1 [Source:HGNC Symbol;Acc:HGNC:24549] |
| ENSG00000179023 | HGLibA_25106 | 61 | -0.541588676 | KLHDC7A | kelch domain containing 7A [Source:HGNC Symbol;Acc:HGNC:26791] |
| ENSG00000179222 | HGLibA_27967 | 61 | -0.541588676 | MAGED1 | MAGE family member D1 [Source:HGNC Symbol;Acc:HGNC:6813] |
| ENSG00000179304 | HGLibA_16190 | 61 | -0.541588676 | FAM156B | family with sequence similarity 156 member B [Source:HGNC Symbol;Acc:HGNC:31962] |
| ENSG00000179546 | HGLibA_22363 | 61 | -0.541588676 | HTR1D | 5-hydroxytryptamine receptor 1D [Source:HGNC Symbol;Acc:HGNC:5289] |
| ENSG00000180098 | HGLibA_51707 | 61 | -0.541588676 | TRNAU1AP | tRNA selenocysteine 1 associated protein 1 [Source:HGNC Symbol;Acc:HGNC:30813] |
| ENSG00000180817 | HGLibA_37803 | 61 | -0.541588676 | PPA1 | pyrophosphatase (inorganic) 1 [Source:HGNC Symbol;Acc:HGNC:9226] |
| ENSG00000181773 | HGLibA_20013 | 61 | -0.541588676 | GPR3 | G protein-coupled receptor 3 [Source:HGNC Symbol;Acc:HGNC:4484] |
| ENSG00000181788 | HGLibA_44109 | 61 | -0.541588676 | SIAH2 | siah E3 ubiquitin protein ligase 2 [Source:HGNC Symbol;Acc:HGNC:10858] |
| ENSG00000181856 | HGLibA_44875 | 61 | -0.541588676 | SLC2A4 | solute carrier family 2 member 4 [Source:HGNC Symbol;Acc:HGNC:11009] |
| ENSG00000182580 | HGLibA_15318 | 61 | -0.541588676 | EPHB3 | EPH receptor B3 [Source:HGNC Symbol;Acc:HGNC:3394] |
| ENSG00000183054 | HGLibA_41041 | 61 | -0.541588676 | RGPD6 | RANBP2-like and GRIP domain containing 6 [Source:HGNC Symbol;Acc:HGNC:32419] |
| ENSG00000183751 | HGLibA_48620 | 61 | -0.541588676 | TBL3 | transducin beta like 3 [Source:HGNC Symbol;Acc:HGNC:11587] |
| ENSG00000183977 | HGLibA_37800 | 61 | -0.541588676 | PP2D1 | protein phosphatase 2C like domain containing 1 [Source:HGNC Symbol;Acc:HGNC:28406] |
| ENSG00000184113 | HGLibA_09900 | 61 | -0.541588676 | CLDN5 | claudin 5 [Source:HGNC Symbol;Acc:HGNC:2047] |
| ENSG00000184194 | HGLibA_19968 | 61 | -0.541588676 | GPR173 | G protein-coupled receptor 173 [Source:HGNC Symbol;Acc:HGNC:18186] |
| ENSG00000184292 | HGLibA_48181 | 61 | -0.541588676 | TACSTD2 | tumor associated calcium signal transducer 2 [Source:HGNC Symbol;Acc:HGNC:11530] |
| ENSG00000184313 | HGLibA_29877 | 61 | -0.541588676 | MROH7 | maestro heat like repeat family member 7 [Source:HGNC Symbol;Acc:HGNC:24802] |
| ENSG00000184828 | HGLibA_54923 | 61 | -0.541588676 | ZBTB7C | zinc finger and BTB domain containing 7C [Source:HGNC Symbol;Acc:HGNC:31700] |
| ENSG00000185304 | HGLibA_41029 | 61 | -0.541588676 | RGPD2 | RANBP2-like and GRIP domain containing 2 [Source:HGNC Symbol;Acc:HGNC:32415] |
| ENSG00000185372 | HGLibA_33897 | 61 | -0.541588676 | OR2V1 | olfactory receptor family 2 subfamily V member 1 [Source:HGNC Symbol;Acc:HGNC:8280] |
| ENSG00000185436 | HGLibA_22730 | 61 | -0.541588676 | IFNLR1 | interferon lambda receptor 1 [Source:HGNC Symbol;Acc:HGNC:18584] |
| ENSG00000185813 | HGLibA_35707 | 61 | -0.541588676 | PCYT2 | ethanolamine [Source:HGNC Symbol;Acc:HGNC:8756]" |
| ENSG00000186185 | HGLibA_24876 | 61 | -0.541588676 | KIF18B | kinesin family member 18B [Source:HGNC Symbol;Acc:HGNC:27102] |
| ENSG00000187871 | HGLibA_18978 | 61 | -0.541588676 | GFRAL | GDNF family receptor alpha like [Source:HGNC Symbol;Acc:HGNC:32789] |
| ENSG00000188266 | HGLibA_22455 | 61 | -0.541588676 | HYKK | hydroxylysine kinase [Source:HGNC Symbol;Acc:HGNC:34403] |
| ENSG00000188505 | HGLibA_31286 | 61 | -0.541588676 | NCCRP1 | non-specific cytotoxic cell receptor protein 1 homolog (zebrafish) [Source:HGNC Symbol;Acc:HGNC:33739] |
| ENSG00000188770 | HGLibA_33455 | 61 | -0.541588676 | OPTC | opticin [Source:HGNC Symbol;Acc:HGNC:8158] |
| ENSG00000189195 | HGLibA_04924 | 61 | -0.541588676 | BTBD8 | BTB domain containing 8 [Source:HGNC Symbol;Acc:HGNC:21019] |
| ENSG00000196449 | HGLibA_54720 | 61 | -0.541588676 | YRDC | yrdC N6-threonylcarbamoyltransferase domain containing [Source:HGNC Symbol;Acc:HGNC:28905] |
| ENSG00000196458 | HGLibA_56365 | 61 | -0.541588676 | ZNF605 | zinc finger protein 605 [Source:HGNC Symbol;Acc:HGNC:28068] |
| ENSG00000196646 | HGLibA_55474 | 61 | -0.541588676 | ZNF136 | zinc finger protein 136 [Source:HGNC Symbol;Acc:HGNC:12920] |
| ENSG00000196859 | HGLibA_25502 | 61 | -0.541588676 | KRT39 | keratin 39 [Source:HGNC Symbol;Acc:HGNC:32971] |
| ENSG00000197162 | HGLibA_56749 | 61 | -0.541588676 | ZNF785 | zinc finger protein 785 [Source:HGNC Symbol;Acc:HGNC:26496] |
| ENSG00000197457 | HGLibA_47522 | 61 | -0.541588676 | STMN3 | stathmin 3 [Source:HGNC Symbol;Acc:HGNC:15926] |
| ENSG00000198482 | HGLibA_56797 | 61 | -0.541588676 | ZNF808 | zinc finger protein 808 [Source:HGNC Symbol;Acc:HGNC:33230] |
| ENSG00000198793 | HGLibA_30455 | 61 | -0.541588676 | MTOR | mechanistic target of rapamycin kinase [Source:HGNC Symbol;Acc:HGNC:3942] |
| ENSG00000198900 | HGLibA_50987 | 61 | -0.541588676 | TOP1 | DNA topoisomerase I [Source:HGNC Symbol;Acc:HGNC:11986] |
| ENSG00000198915 | HGLibA_40328 | 61 | -0.541588676 | RASGEF1A | RasGEF domain family member 1A [Source:HGNC Symbol;Acc:HGNC:24246] |
| ENSG00000204237 | HGLibA_34825 | 61 | -0.541588676 | OXLD1 | oxidoreductase like domain containing 1 [Source:HGNC Symbol;Acc:HGNC:27901] |
| ENSG00000204962 | HGLibA_35458 | 61 | -0.541588676 | PCDHA8 | protocadherin alpha 8 [Source:HGNC Symbol;Acc:HGNC:8674] |
| ENSG00000205531 | HGLibA_31120 | 61 | -0.541588676 | NAP1L4 | nucleosome assembly protein 1 like 4 [Source:HGNC Symbol;Acc:HGNC:7640] |
| ENSG00000206073 | HGLibA_43554 | 61 | -0.541588676 | SERPINB4 | serpin family B member 4 [Source:HGNC Symbol;Acc:HGNC:10570] |
| ENSG00000213185 | HGLibA_16548 | 61 | -0.541588676 | FAM24B | family with sequence similarity 24 member B [Source:HGNC Symbol;Acc:HGNC:23475] |
| ENSG00000213889 | HGLibA_37995 | 61 | -0.541588676 | PPM1N | Mg2+/Mn2+ dependent 1N (putative) [Source:HGNC Symbol;Acc:HGNC:26845]" |
| ENSG00000215305 | HGLibA_53742 | 61 | -0.541588676 | VPS16 | CORVET/HOPS core subunit [Source:HGNC Symbol;Acc:HGNC:14584]" |
| ENSG00000223614 | HGLibA_56650 | 61 | -0.541588676 | ZNF735 | zinc finger protein 735 [Source:HGNC Symbol;Acc:HGNC:32466] |
| ENSG00000227151 | HGLibA_38809 | 61 | -0.541588676 | PRR20D | proline rich 20D [Source:HGNC Symbol;Acc:HGNC:37222] |
| ENSG00000227507 | HGLibA_27580 | 61 | -0.541588676 | LTB | lymphotoxin beta [Source:HGNC Symbol;Acc:HGNC:6711] |
| ENSG00000229117 | HGLibA_41985 | 61 | -0.541588676 | RPL41 | ribosomal protein L41 [Source:HGNC Symbol;Acc:HGNC:10354] |
| ENSG00000233932 | HGLibA_11757 | 61 | -0.541588676 | CTXN2 | cortexin 2 [Source:HGNC Symbol;Acc:HGNC:31109] |
| ENSG00000234511 | HGLibA_06412 | 61 | -0.541588676 | C5orf58 | chromosome 5 open reading frame 58 [Source:HGNC Symbol;Acc:HGNC:37272] |
| ENSG00000234602 | HGLibA_28595 | 61 | -0.541588676 | MCIDAS | multiciliate differentiation and DNA synthesis associated cell cycle protein [Source:HGNC Symbol;Acc:HGNC:40050] |
| ENSG00000242515 | HGLibA_52879 | 61 | -0.541588676 | UGT1A10 | UDP glucuronosyltransferase family 1 member A10 [Source:HGNC Symbol;Acc:HGNC:12531] |
| ENSG00000243364 | HGLibA_14592 | 61 | -0.541588676 | EFNA4 | ephrin A4 [Source:HGNC Symbol;Acc:HGNC:3224] |
| ENSG00000248933 | HGLibA_53252 | 61 | -0.541588676 | USP17L22 | ubiquitin specific peptidase 17-like family member 22 [Source:HGNC Symbol;Acc:HGNC:44450] |
| ENSG00000250486 | HGLibA_16495 | 61 | -0.541588676 | FAM218A | family with sequence similarity 218 member A [Source:HGNC Symbol;Acc:HGNC:26466] |
| ENSG00000265763 | HGLibA_56087 | 61 | -0.541588676 | ZNF488 | zinc finger protein 488 [Source:HGNC Symbol;Acc:HGNC:23535] |
| ENSG00000270800 | HGLibA_42063 | 61 | -0.541588676 | RPS10-NUDT3 | RPS10-NUDT3 readthrough [Source:HGNC Symbol;Acc:HGNC:49181] |
| ENSG00000271449 | HGLibA_11502 | 61 | -0.541588676 | CT45A2 | cancer/testis antigen family 45 member A2 [Source:HGNC Symbol;Acc:HGNC:28400] |
| ENSG00000274226 | HGLibA_48554 | 61 | -0.541588676 | TBC1D3H | TBC1 domain family member 3H [Source:HGNC Symbol;Acc:HGNC:30708] |
| ENSG00000275111 | HGLibA_55564 | 61 | -0.541588676 | ZNF2 | zinc finger protein 2 [Source:HGNC Symbol;Acc:HGNC:12991] |
| ENSG00000276119 | HGLibA_33597 | 61 | -0.541588676 | OR13C2 | olfactory receptor family 13 subfamily C member 2 [Source:HGNC Symbol;Acc:HGNC:14701] |
| ENSG00000278889 | HGLibA_33847 | 61 | -0.541588676 | OR2S2 | olfactory receptor family 2 subfamily S member 2 (gene/pseudogene) [Source:HGNC Symbol;Acc:HGNC:8276] |
| ENTHD2 | HGLibA_15194 | 61 | -0.541588676 | ENTHD2 | - |
| FAM129B | HGLibA_16099 | 61 | -0.541588676 | FAM129B | - |
| FAM160A1 | HGLibA_16204 | 61 | -0.541588676 | FAM160A1 | - |
| FAM179A | HGLibA_16305 | 61 | -0.541588676 | FAM179A | - |
| GLTSCR2 | HGLibA_19357 | 61 | -0.541588676 | GLTSCR2 | - |
| GPR64 | HGLibA_20070 | 61 | -0.541588676 | GPR64 | - |
| hsa-let-7f-1 | HGLibA_57124 | 61 | -0.541588676 | hsa-let-7f-1 | - |
| hsa-mir-103b-2 | HGLibA_57174 | 61 | -0.541588676 | hsa-mir-103b-2 | - |
| hsa-mir-10a | HGLibA_57198 | 61 | -0.541588676 | hsa-mir-10a | - |
| hsa-mir-128-1 | HGLibA_57572 | 61 | -0.541588676 | hsa-mir-128-1 | - |
| hsa-mir-1282 | HGLibA_57586 | 61 | -0.541588676 | hsa-mir-1282 | - |
| hsa-mir-1295b | HGLibA_57662 | 61 | -0.541588676 | hsa-mir-1295b | - |
| hsa-mir-145 | HGLibA_57844 | 61 | -0.541588676 | hsa-mir-145 | - |
| hsa-mir-218-1 | HGLibA_58270 | 61 | -0.541588676 | hsa-mir-218-1 | - |
| hsa-mir-28 | HGLibA_58385 | 61 | -0.541588676 | hsa-mir-28 | - |
| hsa-mir-3130-1 | HGLibA_58570 | 61 | -0.541588676 | hsa-mir-3130-1 | - |
| hsa-mir-3156-1 | HGLibA_58687 | 61 | -0.541588676 | hsa-mir-3156-1 | - |
| hsa-mir-3160-1 | HGLibA_58714 | 61 | -0.541588676 | hsa-mir-3160-1 | - |
| hsa-mir-3183 | HGLibA_58830 | 61 | -0.541588676 | hsa-mir-3183 | - |
| hsa-mir-3186 | HGLibA_58843 | 61 | -0.541588676 | hsa-mir-3186 | - |
| hsa-mir-3192 | HGLibA_58865 | 61 | -0.541588676 | hsa-mir-3192 | - |
| hsa-mir-320e | HGLibA_58950 | 61 | -0.541588676 | hsa-mir-320e | - |
| hsa-mir-3912 | HGLibA_59516 | 61 | -0.541588676 | hsa-mir-3912 | - |
| hsa-mir-4282 | HGLibA_59833 | 61 | -0.541588676 | hsa-mir-4282 | - |
| hsa-mir-4466 | HGLibA_60269 | 61 | -0.541588676 | hsa-mir-4466 | - |
| hsa-mir-4520a | HGLibA_60532 | 61 | -0.541588676 | hsa-mir-4520a | - |
| hsa-mir-4646 | HGLibA_60687 | 61 | -0.541588676 | hsa-mir-4646 | - |
| hsa-mir-4705 | HGLibA_60921 | 61 | -0.541588676 | hsa-mir-4705 | - |
| hsa-mir-4707 | HGLibA_60929 | 61 | -0.541588676 | hsa-mir-4707 | - |
| hsa-mir-4762 | HGLibA_61144 | 61 | -0.541588676 | hsa-mir-4762 | - |
| hsa-mir-4768 | HGLibA_61169 | 61 | -0.541588676 | hsa-mir-4768 | - |
| hsa-mir-5000 | HGLibA_61409 | 61 | -0.541588676 | hsa-mir-5000 | - |
| hsa-mir-510 | HGLibA_61548 | 61 | -0.541588676 | hsa-mir-510 | - |
| hsa-mir-525 | HGLibA_61788 | 61 | -0.541588676 | hsa-mir-525 | - |
| hsa-mir-548k | HGLibA_62033 | 61 | -0.541588676 | hsa-mir-548k | - |
| hsa-mir-5588 | HGLibA_62192 | 61 | -0.541588676 | hsa-mir-5588 | - |
| hsa-mir-602 | HGLibA_62501 | 61 | -0.541588676 | hsa-mir-602 | - |
| hsa-mir-6089-1 | HGLibA_62614 | 61 | -0.541588676 | hsa-mir-6089-1 | - |
| hsa-mir-640 | HGLibA_62798 | 61 | -0.541588676 | hsa-mir-640 | - |
| hsa-mir-6504 | HGLibA_62865 | 61 | -0.541588676 | hsa-mir-6504 | - |
| hsa-mir-6770-2 | HGLibA_63249 | 61 | -0.541588676 | hsa-mir-6770-2 | - |
| hsa-mir-6866 | HGLibA_63647 | 61 | -0.541588676 | hsa-mir-6866 | - |
| hsa-mir-7151 | HGLibA_63832 | 61 | -0.541588676 | hsa-mir-7151 | - |
| hsa-mir-7973-2 | HGLibA_64022 | 61 | -0.541588676 | hsa-mir-7973-2 | - |
| hsa-mir-8081 | HGLibA_64167 | 61 | -0.541588676 | hsa-mir-8081 | - |
| hsa-mir-924 | HGLibA_64287 | 61 | -0.541588676 | hsa-mir-924 | - |
| LINS | HGLibA_26598 | 61 | -0.541588676 | LINS | - |

| | | | | | |
|---|---|---|---|---|---|
| P2RY8_X | HGLibA_34895 | 61 | -0.541588676 | P2RY8_X | - |
| PCNXL3 | HGLibA_35649 | 61 | -0.541588676 | PCNXL3 | - |
| SLC25A6_Y | HGLibA_44776 | 61 | -0.541588676 | SLC25A6_Y | - |
| WBSCR17 | HGLibA_53991 | 61 | -0.541588676 | WBSCR17 | - |
| ACPP | HGLibA_00524 | 60 | -0.551539713 | ACPP | - |
| C10orf118 | HGLibA_05040 | 60 | -0.551539713 | C10orf118 | - |
| C17orf85 | HGLibA_05587 | 60 | -0.551539713 | C17orf85 | - |
| C1orf227 | HGLibA_05838 | 60 | -0.551539713 | C1orf227 | - |
| DEFB131 | HGLibA_12896 | 60 | -0.551539713 | DEFB131 | - |
| ENSG00000004478 | HGLibA_17576 | 60 | -0.551539713 | FKBP4 | FK506 binding protein 4 [Source:HGNC Symbol;Acc:HGNC:3720] |
| ENSG00000006756 | HGLibA_03134 | 60 | -0.551539713 | ARSD | arylsulfatase D [Source:HGNC Symbol;Acc:HGNC:717] |
| ENSG00000010219 | HGLibA_14284 | 60 | -0.551539713 | DYRK4 | dual specificity tyrosine phosphorylation regulated kinase 4 [Source:HGNC Symbol;Acc:HGNC:3095] |
| ENSG00000011198 | HGLibA_00281 | 60 | -0.551539713 | ABHD5 | abhydrolase domain containing 5 [Source:HGNC Symbol;Acc:HGNC:21396] |
| ENSG00000012963 | HGLibA_52762 | 60 | -0.551539713 | UBR7 | ubiquitin protein ligase E3 component n-recognin 7 (putative) [Source:HGNC Symbol;Acc:HGNC:20344] |
| ENSG00000013275 | HGLibA_39139 | 60 | -0.551539713 | PSMC4 | ATPase 4 [Source:HGNC Symbol;Acc:HGNC:9551]" |
| ENSG00000021355 | HGLibA_43536 | 60 | -0.551539713 | SERPINB1 | serpin family B member 1 [Source:HGNC Symbol;Acc:HGNC:3311] |
| ENSG00000026950 | HGLibA_04968 | 60 | -0.551539713 | BTN3A1 | butyrophilin subfamily 3 member A1 [Source:HGNC Symbol;Acc:HGNC:1138] |
| ENSG00000028203 | HGLibA_53630 | 60 | -0.551539713 | VEZT | adherens junctions transmembrane protein [Source:HGNC Symbol;Acc:HGNC:18258]" |
| ENSG00000037757 | HGLibA_29858 | 60 | -0.551539713 | MRI1 | methylthioribose-1-phosphate isomerase 1 [Source:HGNC Symbol;Acc:HGNC:28469] |
| ENSG00000050130 | HGLibA_23948 | 60 | -0.551539713 | JKAMP | JNK1/MAPK8 associated membrane protein [Source:HGNC Symbol;Acc:HGNC:20184] |
| ENSG00000054803 | HGLibA_07416 | 60 | -0.551539713 | CBLN4 | cerebellin 4 precursor [Source:HGNC Symbol;Acc:HGNC:16231] |
| ENSG00000059378 | HGLibA_35219 | 60 | -0.551539713 | PARP12 | poly(ADP-ribose) polymerase family member 12 [Source:HGNC Symbol;Acc:HGNC:21919] |
| ENSG00000067141 | HGLibA_31694 | 60 | -0.551539713 | NEO1 | neogenin 1 [Source:HGNC Symbol;Acc:HGNC:7754] |
| ENSG00000072080 | HGLibA_46773 | 60 | -0.551539713 | SPP2 | secreted phosphoprotein 2 [Source:HGNC Symbol;Acc:HGNC:11256] |
| ENSG00000072310 | HGLibA_46950 | 60 | -0.551539713 | SREBF1 | sterol regulatory element binding transcription factor 1 [Source:HGNC Symbol;Acc:HGNC:11289] |
| ENSG00000072849 | HGLibA_12997 | 60 | -0.551539713 | DERL2 | derlin 2 [Source:HGNC Symbol;Acc:HGNC:17943] |
| ENSG00000073536 | HGLibA_32064 | 60 | -0.551539713 | NLE1 | notchless homolog 1 [Source:HGNC Symbol;Acc:HGNC:19889] |
| ENSG00000079689 | HGLibA_42939 | 60 | -0.551539713 | SCGN | EF-hand calcium binding protein [Source:HGNC Symbol;Acc:HGNC:16941]" |
| ENSG00000081853 | HGLibA_35529 | 60 | -0.551539713 | PCDHGA2 | 2 [Source:HGNC Symbol;Acc:HGNC:8700]" |
| ENSG00000088038 | HGLibA_10339 | 60 | -0.551539713 | CNOT3 | CCR4-NOT transcription complex subunit 3 [Source:HGNC Symbol;Acc:HGNC:7879] |
| ENSG00000089012 | HGLibA_44213 | 60 | -0.551539713 | SIRPG | signal regulatory protein gamma [Source:HGNC Symbol;Acc:HGNC:15757] |
| ENSG00000095627 | HGLibA_48896 | 60 | -0.551539713 | TDRD1 | tudor domain containing 1 [Source:HGNC Symbol;Acc:HGNC:11712] |
| ENSG00000099834 | HGLibA_08711 | 60 | -0.551539713 | CDHR5 | cadherin related family member 5 [Source:HGNC Symbol;Acc:HGNC:7521] |
| ENSG00000099860 | HGLibA_18420 | 60 | -0.551539713 | GADD45B | growth arrest and DNA damage inducible beta [Source:HGNC Symbol;Acc:HGNC:4096] |
| ENSG00000099899 | HGLibA_51682 | 60 | -0.551539713 | TRMT2A | tRNA methyltransferase 2 homolog A [Source:HGNC Symbol;Acc:HGNC:24974] |
| ENSG00000100625 | HGLibA_44252 | 60 | -0.551539713 | SIX4 | SIX homeobox 4 [Source:HGNC Symbol;Acc:HGNC:10890] |
| ENSG00000100804 | HGLibA_39113 | 60 | -0.551539713 | PSMB5 | proteasome subunit beta 5 [Source:HGNC Symbol;Acc:HGNC:9542] |
| ENSG00000100983 | HGLibA_20449 | 60 | -0.551539713 | GSS | glutathione synthetase [Source:HGNC Symbol;Acc:HGNC:4624] |
| ENSG00000101384 | HGLibA_23905 | 60 | -0.551539713 | JAG1 | jagged 1 [Source:HGNC Symbol;Acc:HGNC:6188] |
| ENSG00000101745 | HGLibA_01996 | 60 | -0.551539713 | ANKRD12 | ankyrin repeat domain 12 [Source:HGNC Symbol;Acc:HGNC:29135] |
| ENSG00000102021 | HGLibA_27644 | 60 | -0.551539713 | LUZP4 | leucine zipper protein 4 [Source:HGNC Symbol;Acc:HGNC:24971] |
| ENSG00000103184 | HGLibA_43201 | 60 | -0.551539713 | SEC14L5 | SEC14 like lipid binding 5 [Source:HGNC Symbol;Acc:HGNC:29032] |
| ENSG00000103257 | HGLibA_45371 | 60 | -0.551539713 | SLC7A5 | solute carrier family 7 member 5 [Source:HGNC Symbol;Acc:HGNC:11063] |
| ENSG00000103599 | HGLibA_23520 | 60 | -0.551539713 | IQCH | IQ motif containing H [Source:HGNC Symbol;Acc:HGNC:25721] |
| ENSG00000104321 | HGLibA_51726 | 60 | -0.551539713 | TRPA1 | transient receptor potential cation channel subfamily A member 1 [Source:HGNC Symbol;Acc:HGNC:497] |
| ENSG00000105053 | HGLibA_53831 | 60 | -0.551539713 | VRK3 | VRK serine/threonine kinase 3 [Source:HGNC Symbol;Acc:HGNC:18996] |
| ENSG00000106263 | HGLibA_14766 | 60 | -0.551539713 | EIF3B | eukaryotic translation initiation factor 3 subunit B [Source:HGNC Symbol;Acc:HGNC:3280] |
| ENSG00000106462 | HGLibA_15834 | 60 | -0.551539713 | EZH2 | enhancer of zeste 2 polycomb repressive complex 2 subunit [Source:HGNC Symbol;Acc:HGNC:3527] |
| ENSG00000107175 | HGLibA_11091 | 60 | -0.551539713 | CREB3 | cAMP responsive element binding protein 3 [Source:HGNC Symbol;Acc:HGNC:2347] |
| ENSG00000107175 | HGLibA_11089 | 60 | -0.551539713 | CREB3 | cAMP responsive element binding protein 3 [Source:HGNC Symbol;Acc:HGNC:2347] |
| ENSG00000107290 | HGLibA_43676 | 60 | -0.551539713 | SETX | senataxin [Source:HGNC Symbol;Acc:HGNC:445] |
| ENSG00000107371 | HGLibA_15777 | 60 | -0.551539713 | EXOSC3 | exosome component 3 [Source:HGNC Symbol;Acc:HGNC:17944] |
| ENSG00000107719 | HGLibA_35060 | 60 | -0.551539713 | PALD1 | phosphatase domain containing paladin 1 [Source:HGNC Symbol;Acc:HGNC:23530] |
| ENSG00000107890 | HGLibA_02049 | 60 | -0.551539713 | ANKRD26 | ankyrin repeat domain 26 [Source:HGNC Symbol;Acc:HGNC:29186] |
| ENSG00000108622 | HGLibA_22496 | 60 | -0.551539713 | ICAM2 | intercellular adhesion molecule 2 [Source:HGNC Symbol;Acc:HGNC:5345] |
| ENSG00000110047 | HGLibA_14665 | 60 | -0.551539713 | EHD1 | EH domain containing 1 [Source:HGNC Symbol;Acc:HGNC:3242] |
| ENSG00000110245 | HGLibA_02508 | 60 | -0.551539713 | APOC3 | apolipoprotein C3 [Source:HGNC Symbol;Acc:HGNC:610] |
| ENSG00000111319 | HGLibA_43013 | 60 | -0.551539713 | SCNN1A | sodium channel epithelial 1 alpha subunit [Source:HGNC Symbol;Acc:HGNC:10599] |
| ENSG00000111581 | HGLibA_33018 | 60 | -0.551539713 | NUP107 | nucleoporin 107 [Source:HGNC Symbol;Acc:HGNC:29914] |
| ENSG00000111674 | HGLibA_15144 | 60 | -0.551539713 | ENO2 | enolase 2 [Source:HGNC Symbol;Acc:HGNC:3353] |
| ENSG00000111731 | HGLibA_06106 | 60 | -0.551539713 | C2CD5 | C2 calcium dependent domain containing 5 [Source:HGNC Symbol;Acc:HGNC:29062] |
| ENSG00000111850 | HGLibA_45771 | 60 | -0.551539713 | SMIM8 | small integral membrane protein 8 [Source:HGNC Symbol;Acc:HGNC:21401] |
| ENSG00000112159 | HGLibA_28704 | 60 | -0.551539713 | MDN1 | midasin AAA ATPase 1 [Source:HGNC Symbol;Acc:HGNC:18302] |
| ENSG00000112936 | HGLibA_06519 | 60 | -0.551539713 | C7 | complement C7 [Source:HGNC Symbol;Acc:HGNC:1346] |
| ENSG00000113749 | HGLibA_22097 | 60 | -0.551539713 | HRH2 | histamine receptor H2 [Source:HGNC Symbol;Acc:HGNC:5183] |
| ENSG00000115665 | HGLibA_45280 | 60 | -0.551539713 | SLC5A7 | solute carrier family 5 member 7 [Source:HGNC Symbol;Acc:HGNC:14025] |
| ENSG00000115850 | HGLibA_26206 | 60 | -0.551539713 | LCT | lactase [Source:HGNC Symbol;Acc:HGNC:6530] |
| ENSG00000119185 | HGLibA_23771 | 60 | -0.551539713 | ITGB1BP1 | integrin subunit beta 1 binding protein 1 [Source:HGNC Symbol;Acc:HGNC:23927] |
| ENSG00000119616 | HGLibA_17177 | 60 | -0.551539713 | FCF1 | rRNA-processing protein [Source:HGNC Symbol;Acc:HGNC:20220]" |
| ENSG00000120159 | HGLibA_06831 | 60 | -0.551539713 | CAAP1 | caspase activity and apoptosis inhibitor 1 [Source:HGNC Symbol;Acc:HGNC:25834] |
| ENSG00000120458 | HGLibA_30172 | 60 | -0.551539713 | MSANTD2 | Myb/SANT DNA binding domain containing 2 [Source:HGNC Symbol;Acc:HGNC:26266] |
| ENSG00000120526 | HGLibA_32921 | 60 | -0.551539713 | NUDCD1 | NudC domain containing 1 [Source:HGNC Symbol;Acc:HGNC:24306] |
| ENSG00000120942 | HGLibA_52699 | 60 | -0.551539713 | UBIAD1 | UbiA prenyltransferase domain containing 1 [Source:HGNC Symbol;Acc:HGNC:30791] |
| ENSG00000121905 | HGLibA_22016 | 60 | -0.551539713 | HPCA | hippocalcin [Source:HGNC Symbol;Acc:HGNC:5144] |
| ENSG00000122390 | HGLibA_31002 | 60 | -0.551539713 | NAA60 | NatF catalytic subunit [Source:HGNC Symbol;Acc:HGNC:25875]" |
| ENSG00000123485 | HGLibA_21577 | 60 | -0.551539713 | HJURP | Holliday junction recognition protein [Source:HGNC Symbol;Acc:HGNC:25444] |
| ENSG00000124098 | HGLibA_16455 | 60 | -0.551539713 | FAM210B | family with sequence similarity 210 member B [Source:HGNC Symbol;Acc:HGNC:16102] |
| ENSG00000124493 | HGLibA_20350 | 60 | -0.551539713 | GRM4 | glutamate metabotropic receptor 4 [Source:HGNC Symbol;Acc:HGNC:4596] |
| ENSG00000124568 | HGLibA_44458 | 60 | -0.551539713 | SLC17A1 | solute carrier family 17 member 1 [Source:HGNC Symbol;Acc:HGNC:10929] |
| ENSG00000126456 | HGLibA_23586 | 60 | -0.551539713 | IRF3 | interferon regulatory factor 3 [Source:HGNC Symbol;Acc:HGNC:6118] |
| ENSG00000126500 | HGLibA_17654 | 60 | -0.551539713 | FLRT1 | fibronectin leucine rich transmembrane protein 1 [Source:HGNC Symbol;Acc:HGNC:3760] |
| ENSG00000129562 | HGLibA_12290 | 60 | -0.551539713 | DAD1 | defender against cell death 1 [Source:HGNC Symbol;Acc:HGNC:2664] |
| ENSG00000129673 | HGLibA_00058 | 60 | -0.551539713 | AANAT | aralkylamine N-acetyltransferase [Source:HGNC Symbol;Acc:HGNC:19] |
| ENSG00000131845 | HGLibA_55777 | 60 | -0.551539713 | ZNF304 | zinc finger protein 304 [Source:HGNC Symbol;Acc:HGNC:13505] |
| ENSG00000132155 | HGLibA_40128 | 60 | -0.551539713 | RAF1 | serine/threonine kinase [Source:HGNC Symbol;Acc:HGNC:9829]" |
| ENSG00000132563 | HGLibA_40780 | 60 | -0.551539713 | REEP2 | receptor accessory protein 2 [Source:HGNC Symbol;Acc:HGNC:17975] |
| ENSG00000133316 | HGLibA_54172 | 60 | -0.551539713 | WDR74 | WD repeat domain 74 [Source:HGNC Symbol;Acc:HGNC:25529] |
| ENSG00000133958 | HGLibA_53030 | 60 | -0.551539713 | UNC79 | NALCN channel complex subunit [Source:HGNC Symbol;Acc:HGNC:19966]" |
| ENSG00000134014 | HGLibA_14981 | 60 | -0.551539713 | ELP3 | elongator acetyltransferase complex subunit 3 [Source:HGNC Symbol;Acc:HGNC:20696] |
| ENSG00000134046 | HGLibA_28466 | 60 | -0.551539713 | MBD2 | methyl-CpG binding domain protein 2 [Source:HGNC Symbol;Acc:HGNC:6917] |
| ENSG00000134160 | HGLibA_51753 | 60 | -0.551539713 | TRPM1 | transient receptor potential cation channel subfamily M member 1 [Source:HGNC Symbol;Acc:HGNC:7146] |
| ENSG00000134291 | HGLibA_49859 | 60 | -0.551539713 | TMEM106C | transmembrane protein 106C [Source:HGNC Symbol;Acc:HGNC:28775] |
| ENSG00000134755 | HGLibA_13992 | 60 | -0.551539713 | DSC2 | desmocollin 2 [Source:HGNC Symbol;Acc:HGNC:3036] |
| ENSG00000134910 | HGLibA_47607 | 60 | -0.551539713 | STT3A | catalytic subunit of the oligosaccharyltransferase complex [Source:HGNC Symbol;Acc:HGNC:6172]" |
| ENSG00000135124 | HGLibA_34855 | 60 | -0.551539713 | P2RX4 | purinergic receptor P2X 4 [Source:HGNC Symbol;Acc:HGNC:8535] |
| ENSG00000135931 | HGLibA_03035 | 60 | -0.551539713 | ARMC9 | armadillo repeat containing 9 [Source:HGNC Symbol;Acc:HGNC:20730] |
| ENSG00000136521 | HGLibA_31520 | 60 | -0.551539713 | NDUFB5 | NADH:ubiquinone oxidoreductase subunit B5 [Source:HGNC Symbol;Acc:HGNC:7700] |
| ENSG00000137200 | HGLibA_10234 | 60 | -0.551539713 | CMTR1 | cap methyltransferase 1 [Source:HGNC Symbol;Acc:HGNC:21077] |
| ENSG00000137331 | HGLibA_22574 | 60 | -0.551539713 | IER3 | immediate early response 3 [Source:HGNC Symbol;Acc:HGNC:5392] |
| ENSG00000137674 | HGLibA_29509 | 60 | -0.551539713 | MMP20 | matrix metallopeptidase 20 [Source:HGNC Symbol;Acc:HGNC:7167] |
| ENSG00000138134 | HGLibA_47339 | 60 | -0.551539713 | STAMBPL1 | STAM binding protein like 1 [Source:HGNC Symbol;Acc:HGNC:24105] |
| ENSG00000138279 | HGLibA_02250 | 60 | -0.551539713 | ANXA7 | annexin A7 [Source:HGNC Symbol;Acc:HGNC:545] |
| ENSG00000139517 | HGLibA_26748 | 60 | -0.551539713 | LNX2 | ligand of numb-protein X 2 [Source:HGNC Symbol;Acc:HGNC:20421] |
| ENSG00000139714 | HGLibA_29671 | 60 | -0.551539713 | MORN3 | MORN repeat containing 3 [Source:HGNC Symbol;Acc:HGNC:29807] |
| ENSG00000140398 | HGLibA_31629 | 60 | -0.551539713 | NEIL1 | nei like DNA glycosylase 1 [Source:HGNC Symbol;Acc:HGNC:18448] |
| ENSG00000141582 | HGLibA_07453 | 60 | -0.551539713 | CBX4 | chromobox 4 [Source:HGNC Symbol;Acc:HGNC:1554] |
| ENSG00000142864 | HGLibA_43466 | 60 | -0.551539713 | SERBP1 | SERPINE1 mRNA binding protein 1 [Source:HGNC Symbol;Acc:HGNC:17860] |
| ENSG00000143013 | HGLibA_26710 | 60 | -0.551539713 | LMO4 | LIM domain only 4 [Source:HGNC Symbol;Acc:HGNC:6644] |
| ENSG00000143105 | HGLibA_24126 | 60 | -0.551539713 | KCNA10 | potassium voltage-gated channel subfamily A member 10 [Source:HGNC Symbol;Acc:HGNC:6219] |
| ENSG00000143164 | HGLibA_12451 | 60 | -0.551539713 | DCAF6 | DDB1 and CUL4 associated factor 6 [Source:HGNC Symbol;Acc:HGNC:30002] |
| ENSG00000143502 | HGLibA_47854 | 60 | -0.551539713 | SUSD4 | sushi domain containing 4 [Source:HGNC Symbol;Acc:HGNC:25470] |
| ENSG00000143512 | HGLibA_21267 | 60 | -0.551539713 | HHIPL2 | HHIP like 2 [Source:HGNC Symbol;Acc:HGNC:25842] |
| ENSG00000143515 | HGLibA_03782 | 60 | -0.551539713 | ATP8B2 | ATPase phospholipid transporting 8B2 [Source:HGNC Symbol;Acc:HGNC:13534] |
| ENSG00000143889 | HGLibA_21837 | 60 | -0.551539713 | HNRNPLL | heterogeneous nuclear ribonucleoprotein L like [Source:HGNC Symbol;Acc:HGNC:25127] |
| ENSG00000144040 | HGLibA_43793 | 60 | -0.551539713 | SFXN5 | sideroflexin 5 [Source:HGNC Symbol;Acc:HGNC:16073] |
| ENSG00000145020 | HGLibA_01834 | 60 | -0.551539713 | AMT | aminomethyltransferase [Source:HGNC Symbol;Acc:HGNC:473] |
| ENSG00000145050 | HGLibA_28097 | 60 | -0.551539713 | MANF | mesencephalic astrocyte derived neurotrophic factor [Source:HGNC Symbol;Acc:HGNC:15461] |
| ENSG00000145147 | HGLibA_45508 | 60 | -0.551539713 | SLIT2 | slit guidance ligand 2 [Source:HGNC Symbol;Acc:HGNC:11086] |
| ENSG00000145626 | HGLibA_52934 | 60 | -0.551539713 | UGT3A1 | UDP glycosyltransferase family 3 member A1 [Source:HGNC Symbol;Acc:HGNC:26625] |
| ENSG00000147381 | HGLibA_27913 | 60 | -0.551539713 | MAGEA4 | MAGE family member A4 [Source:HGNC Symbol;Acc:HGNC:6802] |
| ENSG00000148386 | HGLibA_26187 | 60 | -0.551539713 | LCN9 | lipocalin 9 [Source:HGNC Symbol;Acc:HGNC:17442] |
| ENSG00000149257 | HGLibA_43594 | 60 | -0.551539713 | SERPINH1 | serpin family H member 1 [Source:HGNC Symbol;Acc:HGNC:1546] |
| ENSG00000151532 | HGLibA_53888 | 60 | -0.551539713 | VTI1A | vesicle transport through interaction with t-SNAREs 1A [Source:HGNC Symbol;Acc:HGNC:17792] |
| ENSG00000152749 | HGLibA_19983 | 60 | -0.551539713 | GPR180 | G protein-coupled receptor 180 [Source:HGNC Symbol;Acc:HGNC:28899] |
| ENSG00000154059 | HGLibA_23251 | 60 | -0.551539713 | IMPACT | impact RWD domain protein [Source:HGNC Symbol;Acc:HGNC:20387] |
| ENSG00000154258 | HGLibA_00127 | 60 | -0.551539713 | ABCA9 | ATP binding cassette subfamily A member 9 [Source:HGNC Symbol;Acc:HGNC:39] |
| ENSG00000156374 | HGLibA_35603 | 60 | -0.551539713 | PCGF6 | polycomb group ring finger 6 [Source:HGNC Symbol;Acc:HGNC:21156] |
| ENSG00000157483 | HGLibA_30832 | 60 | -0.551539713 | MYO1E | myosin IE [Source:HGNC Symbol;Acc:HGNC:7599] |
| ENSG00000158748 | HGLibA_22402 | 60 | -0.551539713 | HTR6 | 5-hydroxytryptamine receptor 6 [Source:HGNC Symbol;Acc:HGNC:5301] |
| ENSG00000158966 | HGLibA_06872 | 60 | -0.551539713 | CACHD1 | cache domain containing 1 [Source:HGNC Symbol;Acc:HGNC:29314] |
| ENSG00000159023 | HGLibA_15240 | 60 | -0.551539713 | EPB41 | erythrocyte membrane protein band 4.1 [Source:HGNC Symbol;Acc:HGNC:3377] |
| ENSG00000160396 | HGLibA_21347 | 60 | -0.551539713 | HIPK4 | homeodomain interacting protein kinase 4 [Source:HGNC Symbol;Acc:HGNC:19007] |
| ENSG00000160446 | HGLibA_55073 | 60 | -0.551539713 | ZDHHC12 | zinc finger DHHC-type containing 12 [Source:HGNC Symbol;Acc:HGNC:19159] |
| ENSG00000163283 | HGLibA_01713 | 60 | -0.551539713 | ALPP | placental [Source:HGNC Symbol;Acc:HGNC:439]" |
| ENSG00000163468 | HGLibA_08199 | 60 | -0.551539713 | CCT3 | chaperonin containing TCP1 subunit 3 [Source:HGNC Symbol;Acc:HGNC:1616] |

| | | | | | |
|---|---|---|---|---|---|
| ENSG00000163482 | HGLibA_47497 | 60 | -0.551539713 | STK36 | serine/threonine kinase 36 [Source:HGNC Symbol;Acc:HGNC:17209] |
| ENSG00000163515 | HGLibA_40877 | 60 | -0.551539713 | RETNLB | resistin like beta [Source:HGNC Symbol;Acc:HGNC:20388] |
| ENSG00000163581 | HGLibA_44869 | 60 | -0.551539713 | SLC2A2 | solute carrier family 2 member 2 [Source:HGNC Symbol;Acc:HGNC:11006] |
| ENSG00000163705 | HGLibA_16843 | 60 | -0.551539713 | FANCD2OS | FANCD2 opposite strand [Source:HGNC Symbol;Acc:HGNC:28623] |
| ENSG00000163904 | HGLibA_43389 | 60 | -0.551539713 | SENP2 | SUMO specific peptidase 2 [Source:HGNC Symbol;Acc:HGNC:23116] |
| ENSG00000164080 | HGLibA_40104 | 60 | -0.551539713 | RAD54L2 | RAD54 like 2 [Source:HGNC Symbol;Acc:HGNC:29123] |
| ENSG00000164082 | HGLibA_20344 | 60 | -0.551539713 | GRM2 | glutamate metabotropic receptor 2 [Source:HGNC Symbol;Acc:HGNC:4594] |
| ENSG00000164237 | HGLibA_10182 | 60 | -0.551539713 | CMBL | carboxymethylenebutenolidase homolog [Source:HGNC Symbol;Acc:HGNC:25090] |
| ENSG00000164609 | HGLibA_45552 | 60 | -0.551539713 | SLU7 | splicing factor [Source:HGNC Symbol;Acc:HGNC:16939]" |
| ENSG00000164904 | HGLibA_01582 | 60 | -0.551539713 | ALDH7A1 | aldehyde dehydrogenase 7 family member A1 [Source:HGNC Symbol;Acc:HGNC:877] |
| ENSG00000165476 | HGLibA_40782 | 60 | -0.551539713 | REEP3 | receptor accessory protein 3 [Source:HGNC Symbol;Acc:HGNC:23711] |
| ENSG00000165804 | HGLibA_55602 | 60 | -0.551539713 | ZNF219 | zinc finger protein 219 [Source:HGNC Symbol;Acc:HGNC:13011] |
| ENSG00000165995 | HGLibA_06919 | 60 | -0.551539713 | CACNB2 | calcium voltage-gated channel auxiliary subunit beta 2 [Source:HGNC Symbol;Acc:HGNC:1402] |
| ENSG00000166006 | HGLibA_24164 | 60 | -0.551539713 | KCNC2 | potassium voltage-gated channel subfamily C member 2 [Source:HGNC Symbol;Acc:HGNC:6234] |
| ENSG00000166411 | HGLibA_22545 | 60 | -0.551539713 | IDH3A | isocitrate dehydrogenase 3 (NAD(+)) alpha [Source:HGNC Symbol;Acc:HGNC:5384] |
| ENSG00000166441 | HGLibA_41923 | 60 | -0.551539713 | RPL27A | ribosomal protein L27a [Source:HGNC Symbol;Acc:HGNC:10329] |
| ENSG00000166912 | HGLibA_30411 | 60 | -0.551539713 | MTMR10 | myotubularin related protein 10 [Source:HGNC Symbol;Acc:HGNC:25999] |
| ENSG00000167565 | HGLibA_43611 | 60 | -0.551539713 | SERTAD3 | SERTA domain containing 3 [Source:HGNC Symbol;Acc:HGNC:17931] |
| ENSG00000168874 | HGLibA_03538 | 60 | -0.551539713 | ATOH8 | atonal bHLH transcription factor 8 [Source:HGNC Symbol;Acc:HGNC:24126] |
| ENSG00000169045 | HGLibA_21821 | 60 | -0.551539713 | HNRNPH1 | heterogeneous nuclear ribonucleoprotein H1 [Source:HGNC Symbol;Acc:HGNC:5041] |
| ENSG00000169359 | HGLibA_44936 | 60 | -0.551539713 | SLC33A1 | solute carrier family 33 member 1 [Source:HGNC Symbol;Acc:HGNC:95] |
| ENSG00000170262 | HGLibA_29806 | 60 | -0.551539713 | MRAP | melanocortin 2 receptor accessory protein [Source:HGNC Symbol;Acc:HGNC:1304] |
| ENSG00000170558 | HGLibA_08658 | 60 | -0.551539713 | CDH2 | cadherin 2 [Source:HGNC Symbol;Acc:HGNC:1759] |
| ENSG00000170677 | HGLibA_46138 | 60 | -0.551539713 | SOCS6 | suppressor of cytokine signaling 6 [Source:HGNC Symbol;Acc:HGNC:16833] |
| ENSG00000170748 | HGLibA_40606 | 60 | -0.551539713 | RBMXL2 | RBMX like 2 [Source:HGNC Symbol;Acc:HGNC:17886] |
| ENSG00000171060 | HGLibA_06644 | 60 | -0.551539713 | C8orf74 | chromosome 8 open reading frame 74 [Source:HGNC Symbol;Acc:HGNC:32296] |
| ENSG00000171189 | HGLibA_20280 | 60 | -0.551539713 | GRIK1 | glutamate ionotropic receptor kainate type subunit 1 [Source:HGNC Symbol;Acc:HGNC:4579] |
| ENSG00000172301 | HGLibA_10710 | 60 | -0.551539713 | COPRS | coordinator of PRMT5 and differentiation stimulator [Source:HGNC Symbol;Acc:HGNC:28848] |
| ENSG00000172578 | HGLibA_25219 | 60 | -0.551539713 | KLHL6 | kelch like family member 6 [Source:HGNC Symbol;Acc:HGNC:18653] |
| ENSG00000172667 | HGLibA_55372 | 60 | -0.551539713 | ZMAT3 | zinc finger matrin-type 3 [Source:HGNC Symbol;Acc:HGNC:29983] |
| ENSG00000172987 | HGLibA_22063 | 60 | -0.551539713 | HPSE2 | heparanase 2 (inactive) [Source:HGNC Symbol;Acc:HGNC:18374] |
| ENSG00000173611 | HGLibA_42834 | 60 | -0.551539713 | SCAI | suppressor of cancer cell invasion [Source:HGNC Symbol;Acc:HGNC:26709] |
| ENSG00000173626 | HGLibA_51322 | 60 | -0.551539713 | TRAPPC3L | trafficking protein particle complex 3 like [Source:HGNC Symbol;Acc:HGNC:21090] |
| ENSG00000173769 | HGLibA_51006 | 60 | -0.551539713 | TOPAZ1 | testis and ovary specific PAZ domain containing 1 [Source:HGNC Symbol;Acc:HGNC:24746] |
| ENSG00000176454 | HGLibA_27079 | 60 | -0.551539713 | LPCAT4 | lysophosphatidylcholine acyltransferase 4 [Source:HGNC Symbol;Acc:HGNC:30059] |
| ENSG00000177455 | HGLibA_08261 | 60 | -0.551539713 | CD19 | CD19 molecule [Source:HGNC Symbol;Acc:HGNC:1633] |
| ENSG00000177599 | HGLibA_56091 | 60 | -0.551539713 | ZNF491 | zinc finger protein 491 [Source:HGNC Symbol;Acc:HGNC:23706] |
| ENSG00000180011 | HGLibA_54768 | 60 | -0.551539713 | ZADH2 | zinc binding alcohol dehydrogenase domain containing 2 [Source:HGNC Symbol;Acc:HGNC:28697] |
| ENSG00000180098 | HGLibA_51708 | 60 | -0.551539713 | TRNAU1AP | tRNA selenocysteine 1 associated protein 1 [Source:HGNC Symbol;Acc:HGNC:30813] |
| ENSG00000180398 | HGLibA_28587 | 60 | -0.551539713 | MCFD2 | multiple coagulation factor deficiency 2 [Source:HGNC Symbol;Acc:HGNC:18451] |
| ENSG00000180626 | HGLibA_56343 | 60 | -0.551539713 | ZNF594 | zinc finger protein 594 [Source:HGNC Symbol;Acc:HGNC:29392] |
| ENSG00000180745 | HGLibA_10129 | 60 | -0.551539713 | CLRN3 | clarin 3 [Source:HGNC Symbol;Acc:HGNC:20795] |
| ENSG00000180817 | HGLibA_37804 | 60 | -0.551539713 | PPA1 | pyrophosphatase (inorganic) 1 [Source:HGNC Symbol;Acc:HGNC:9226] |
| ENSG00000181016 | HGLibA_27556 | 60 | -0.551539713 | LSMEM1 | leucine rich single-pass membrane protein 1 [Source:HGNC Symbol;Acc:HGNC:22036] |
| ENSG00000181418 | HGLibA_12624 | 60 | -0.551539713 | DDN | dendrin [Source:HGNC Symbol;Acc:HGNC:24458] |
| ENSG00000181786 | HGLibA_00636 | 60 | -0.551539713 | ACTL9 | actin like 9 [Source:HGNC Symbol;Acc:HGNC:28494] |
| ENSG00000182264 | HGLibA_23891 | 60 | -0.551539713 | IZUMO1 | izumo sperm-egg fusion 1 [Source:HGNC Symbol;Acc:HGNC:28539] |
| ENSG00000182993 | HGLibA_05273 | 60 | -0.551539713 | C12orf60 | chromosome 12 open reading frame 60 [Source:HGNC Symbol;Acc:HGNC:28726] |
| ENSG00000183166 | HGLibA_07043 | 60 | -0.551539713 | CALN1 | calneuron 1 [Source:HGNC Symbol;Acc:HGNC:13248] |
| ENSG00000183580 | HGLibA_17019 | 60 | -0.551539713 | FBXL7 | F-box and leucine rich repeat protein 7 [Source:HGNC Symbol;Acc:HGNC:13604] |
| ENSG00000183682 | HGLibA_04619 | 60 | -0.551539713 | BMP8A | bone morphogenetic protein 8a [Source:HGNC Symbol;Acc:HGNC:21650] |
| ENSG00000183741 | HGLibA_07459 | 60 | -0.551539713 | CBX6 | chromobox 6 [Source:HGNC Symbol;Acc:HGNC:1556] |
| ENSG00000184571 | HGLibA_36778 | 60 | -0.551539713 | PIWIL3 | piwi like RNA-mediated gene silencing 3 [Source:HGNC Symbol;Acc:HGNC:18443] |
| ENSG00000186197 | HGLibA_14440 | 60 | -0.551539713 | EDARADD | EDAR associated death domain [Source:HGNC Symbol;Acc:HGNC:14341] |
| ENSG00000187024 | HGLibA_39549 | 60 | -0.551539713 | PTRH1 | peptidyl-tRNA hydrolase 1 homolog [Source:HGNC Symbol;Acc:HGNC:27039] |
| ENSG00000187546 | HGLibA_01201 | 60 | -0.551539713 | AGMO | alkylglycerol monooxygenase [Source:HGNC Symbol;Acc:HGNC:33784] |
| ENSG00000187855 | HGLibA_03275 | 60 | -0.551539713 | ASCL4 | achaete-scute family bHLH transcription factor 4 [Source:HGNC Symbol;Acc:HGNC:24311] |
| ENSG00000187969 | HGLibA_55011 | 60 | -0.551539713 | ZCCHC13 | zinc finger CCHC-type containing 13 [Source:HGNC Symbol;Acc:HGNC:31749] |
| ENSG00000188064 | HGLibA_54385 | 60 | -0.551539713 | WNT7B | Wnt family member 7B [Source:HGNC Symbol;Acc:HGNC:12787] |
| ENSG00000188177 | HGLibA_54979 | 60 | -0.551539713 | ZC3H6 | zinc finger CCCH-type containing 6 [Source:HGNC Symbol;Acc:HGNC:24762] |
| ENSG00000188694 | HGLibA_25726 | 60 | -0.551539713 | KRTAP24-1 | keratin associated protein 24-1 [Source:HGNC Symbol;Acc:HGNC:33902] |
| ENSG00000188707 | HGLibA_54805 | 60 | -0.551539713 | ZBED6CL | ZBED6 C-terminal like [Source:HGNC Symbol;Acc:HGNC:21720] |
| ENSG00000188770 | HGLibA_33454 | 60 | -0.551539713 | OPTC | opticin [Source:HGNC Symbol;Acc:HGNC:8158] |
| ENSG00000188846 | HGLibA_41874 | 60 | -0.551539713 | RPL14 | ribosomal protein L14 [Source:HGNC Symbol;Acc:HGNC:10305] |
| ENSG00000189152 | HGLibA_20218 | 60 | -0.551539713 | GRAPL | GRB2 related adaptor protein like [Source:HGNC Symbol;Acc:HGNC:37240] |
| ENSG00000189283 | HGLibA_17476 | 60 | -0.551539713 | FHIT | fragile histidine triad [Source:HGNC Symbol;Acc:HGNC:3701] |
| ENSG00000189326 | HGLibA_46412 | 60 | -0.551539713 | SPANXN4 | SPANX family member N4 [Source:HGNC Symbol;Acc:HGNC:33177] |
| ENSG00000196267 | HGLibA_56841 | 60 | -0.551539713 | ZNF836 | zinc finger protein 836 [Source:HGNC Symbol;Acc:HGNC:34333] |
| ENSG00000196923 | HGLibA_35921 | 60 | -0.551539713 | PDLIM7 | PDZ and LIM domain 7 [Source:HGNC Symbol;Acc:HGNC:22958] |
| ENSG00000197147 | HGLibA_27431 | 60 | -0.551539713 | LRRC8B | leucine rich repeat containing 8 VRAC subunit B [Source:HGNC Symbol;Acc:HGNC:30692] |
| ENSG00000197386 | HGLibA_22418 | 60 | -0.551539713 | HTT | huntingtin [Source:HGNC Symbol;Acc:HGNC:4851] |
| ENSG00000197483 | HGLibA_56429 | 60 | -0.551539713 | ZNF628 | zinc finger protein 628 [Source:HGNC Symbol;Acc:HGNC:28054] |
| ENSG00000198794 | HGLibA_42847 | 60 | -0.551539713 | SCAMP5 | secretory carrier membrane protein 5 [Source:HGNC Symbol;Acc:HGNC:30386] |
| ENSG00000203926 | HGLibA_46380 | 60 | -0.551539713 | SPANXA2 | SPANX family member A2 [Source:HGNC Symbol;Acc:HGNC:14328] |
| ENSG00000205129 | HGLibA_06337 | 60 | -0.551539713 | C4orf47 | chromosome 4 open reading frame 47 [Source:HGNC Symbol;Acc:HGNC:34346] |
| ENSG00000205413 | HGLibA_42697 | 60 | -0.551539713 | SAMD9 | sterile alpha motif domain containing 9 [Source:HGNC Symbol;Acc:HGNC:1348] |
| ENSG00000205869 | HGLibA_25782 | 60 | -0.551539713 | KRTAP5-1 | keratin associated protein 5-1 [Source:HGNC Symbol;Acc:HGNC:23596] |
| ENSG00000206140 | HGLibA_50147 | 60 | -0.551539713 | TMEM191C | transmembrane protein 191C [Source:HGNC Symbol;Acc:HGNC:33601] |
| ENSG00000212721 | HGLibA_25751 | 60 | -0.551539713 | KRTAP4-11 | keratin associated protein 4-11 [Source:HGNC Symbol;Acc:HGNC:18911] |
| ENSG00000212993 | HGLibA_37788 | 60 | -0.551539713 | POU5F1B | POU class 5 homeobox 1B [Source:HGNC Symbol;Acc:HGNC:9223] |
| ENSG00000213676 | HGLibA_03448 | 60 | -0.551539713 | ATF6B | activating transcription factor 6 beta [Source:HGNC Symbol;Acc:HGNC:2349] |
| ENSG00000213934 | HGLibA_20926 | 60 | -0.551539713 | HBG1 | hemoglobin subunit gamma 1 [Source:HGNC Symbol;Acc:HGNC:4831] |
| ENSG00000214357 | HGLibA_31726 | 60 | -0.551539713 | NEURL1B | neuralized E3 ubiquitin protein ligase 1B [Source:HGNC Symbol;Acc:HGNC:35422] |
| ENSG00000219607 | HGLibA_38116 | 60 | -0.551539713 | PPP1R3G | protein phosphatase 1 regulatory subunit 3G [Source:HGNC Symbol;Acc:HGNC:14945] |
| ENSG00000221888 | HGLibA_33649 | 60 | -0.551539713 | OR1C1 | olfactory receptor family 1 subfamily C member 1 [Source:HGNC Symbol;Acc:HGNC:8182] |
| ENSG00000223443 | HGLibA_53243 | 60 | -0.551539713 | USP17L2 | ubiquitin specific peptidase 17-like family member 2 [Source:HGNC Symbol;Acc:HGNC:34434] |
| ENSG00000224531 | HGLibA_45728 | 60 | -0.551539713 | SMIM13 | small integral membrane protein 13 [Source:HGNC Symbol;Acc:HGNC:27356] |
| ENSG00000227151 | HGLibA_38808 | 60 | -0.551539713 | PRR20D | proline rich 20D [Source:HGNC Symbol;Acc:HGNC:37222] |
| ENSG00000231925 | HGLibA_48322 | 60 | -0.551539713 | TAPBP | TAP binding protein [Source:HGNC Symbol;Acc:HGNC:11566] |
| ENSG00000234278 | HGLibA_38811 | 60 | -0.551539713 | PRR20E | proline rich 20E [Source:HGNC Symbol;Acc:HGNC:37223] |
| ENSG00000235169 | HGLibA_45715 | 60 | -0.551539713 | SMIM1 | small integral membrane protein 1 (Vel blood group) [Source:HGNC Symbol;Acc:HGNC:44204] |
| ENSG00000240247 | HGLibA_12795 | 60 | -0.551539713 | DEFA1B | defensin alpha 1B [Source:HGNC Symbol;Acc:HGNC:33596] |
| ENSG00000240432 | HGLibA_25649 | 60 | -0.551539713 | KRTAP13-3 | keratin associated protein 13-3 [Source:HGNC Symbol;Acc:HGNC:18925] |
| ENSG00000242485 | HGLibA_29925 | 60 | -0.551539713 | MRPL20 | mitochondrial ribosomal protein L20 [Source:HGNC Symbol;Acc:HGNC:14478] |
| ENSG00000244025 | HGLibA_25668 | 60 | -0.551539713 | KRTAP19-3 | keratin associated protein 19-3 [Source:HGNC Symbol;Acc:HGNC:18938] |
| ENSG00000259431 | HGLibA_49412 | 60 | -0.551539713 | THTPA | thiamine triphosphatase [Source:HGNC Symbol;Acc:HGNC:18987] |
| ENSG00000260230 | HGLibA_18044 | 60 | -0.551539713 | FRRS1L | ferric chelate reductase 1 like [Source:HGNC Symbol;Acc:HGNC:1362] |
| ENSG00000272325 | HGLibA_32977 | 60 | -0.551539713 | NUDT3 | nudix hydrolase 3 [Source:HGNC Symbol;Acc:HGNC:8050] |
| ENSG00000275722 | HGLibA_27793 | 60 | -0.551539713 | LYZL6 | lysozyme like 6 [Source:HGNC Symbol;Acc:HGNC:29614] |
| ENSG00000281887 | HGLibA_19073 | 60 | -0.551539713 | GIMAP1-GIMAP5 | GIMAP1-GIMAP5 readthrough [Source:HGNC Symbol;Acc:HGNC:51257] |
| ENSG00000288596 | HGLibA_06622 | 60 | -0.551539713 | C8orf44 | - |
| ENSG00000289549 | HGLibA_37905 | 60 | -0.551539713 | PPIAL4D | - |
| FOXD4L2 | HGLibA_17842 | 60 | -0.551539713 | FOXD4L2 | - |
| H3F3C | HGLibA_20785 | 60 | -0.551539713 | H3F3C | - |
| HIST1H2AM | HGLibA_21407 | 60 | -0.551539713 | HIST1H2AM | - |
| HIST1H2BA | HGLibA_21411 | 60 | -0.551539713 | HIST1H2BA | - |
| hsa-mir-1268b | HGLibA_57498 | 60 | -0.551539713 | hsa-mir-1268b | - |
| hsa-mir-133a-1 | HGLibA_57773 | 60 | -0.551539713 | hsa-mir-133a-1 | - |
| hsa-mir-135a-2 | HGLibA_57795 | 60 | -0.551539713 | hsa-mir-135a-2 | - |
| hsa-mir-152 | HGLibA_57902 | 60 | -0.551539713 | hsa-mir-152 | - |
| hsa-mir-15b | HGLibA_57941 | 60 | -0.551539713 | hsa-mir-15b | - |
| hsa-mir-21 | HGLibA_58210 | 60 | -0.551539713 | hsa-mir-21 | - |
| hsa-mir-3137 | HGLibA_58607 | 60 | -0.551539713 | hsa-mir-3137 | - |
| hsa-mir-3156-2 | HGLibA_58691 | 60 | -0.551539713 | hsa-mir-3156-2 | - |
| hsa-mir-3529 | HGLibA_59046 | 60 | -0.551539713 | hsa-mir-3529 | - |
| hsa-mir-3660 | HGLibA_59194 | 60 | -0.551539713 | hsa-mir-3660 | - |
| hsa-mir-3940 | HGLibA_59619 | 60 | -0.551539713 | hsa-mir-3940 | - |
| hsa-mir-411 | HGLibA_59684 | 60 | -0.551539713 | hsa-mir-411 | - |
| hsa-mir-4329 | HGLibA_60039 | 60 | -0.551539713 | hsa-mir-4329 | - |
| hsa-mir-450b | HGLibA_60481 | 60 | -0.551539713 | hsa-mir-450b | - |
| hsa-mir-450b | HGLibA_60480 | 60 | -0.551539713 | hsa-mir-450b | - |
| hsa-mir-4675 | HGLibA_60816 | 60 | -0.551539713 | hsa-mir-4675 | - |
| hsa-mir-4696 | HGLibA_60894 | 60 | -0.551539713 | hsa-mir-4696 | - |
| hsa-mir-4701 | HGLibA_60914 | 60 | -0.551539713 | hsa-mir-4701 | - |
| hsa-mir-4732 | HGLibA_61026 | 60 | -0.551539713 | hsa-mir-4732 | - |
| hsa-mir-5186 | HGLibA_61637 | 60 | -0.551539713 | hsa-mir-5186 | - |
| hsa-mir-5195 | HGLibA_61698 | 60 | -0.551539713 | hsa-mir-5195 | - |
| hsa-mir-548i-4 | HGLibA_62023 | 60 | -0.551539713 | hsa-mir-548i-4 | - |
| hsa-mir-548o-2 | HGLibA_62054 | 60 | -0.551539713 | hsa-mir-548o-2 | - |
| hsa-mir-5585 | HGLibA_62181 | 60 | -0.551539713 | hsa-mir-5585 | - |
| hsa-mir-588 | HGLibA_62452 | 60 | -0.551539713 | hsa-mir-588 | - |
| hsa-mir-612 | HGLibA_62641 | 60 | -0.551539713 | hsa-mir-612 | - |
| hsa-mir-6769a | HGLibA_63237 | 60 | -0.551539713 | hsa-mir-6769a | - |
| hsa-mir-6770-1 | HGLibA_63245 | 60 | -0.551539713 | hsa-mir-6770-1 | - |
| hsa-mir-6851 | HGLibA_63578 | 60 | -0.551539713 | hsa-mir-6851 | - |

| | | | | | |
|---|---|---|---|---|---|
| hsa-mir-711 | HGLibA_63800 | 60 | -0.551539713 | hsa-mir-711 | - |
| hsa-mir-7152 | HGLibA_63837 | 60 | -0.551539713 | hsa-mir-7152 | - |
| KDELC1 | HGLibA_24473 | 60 | -0.551539713 | KDELC1 | - |
| LOC100505679 | HGLibA_26853 | 60 | -0.551539713 | LOC100505679 | - |
| P2RY8_Y | HGLibA_34898 | 60 | -0.551539713 | P2RY8_Y | - |
| PRAMEF22 | HGLibA_38315 | 60 | -0.551539713 | PRAMEF22 | - |
| PTRF | HGLibA_39548 | 60 | -0.551539713 | PTRF | - |
| RBAK-LOC389458 | HGLibA_40422 | 60 | -0.551539713 | RBAK-LOC389458 | - |
| SRPR | HGLibA_47027 | 60 | -0.551539713 | SRPR | - |
| C3orf37 | HGLibA_06240 | 59 | -0.561490751 | C3orf37 | - |
| CXorf30 | HGLibA_11914 | 59 | -0.561490751 | CXorf30 | - |
| ENSG00000002745 | HGLibA_54356 | 59 | -0.561490751 | WNT16 | Wnt family member 16 [Source:HGNC Symbol;Acc:HGNC:16267] |
| ENSG00000004777 | HGLibA_02762 | 59 | -0.561490751 | ARHGAP33 | Rho GTPase activating protein 33 [Source:HGNC Symbol;Acc:HGNC:23085] |
| ENSG00000008083 | HGLibA_23937 | 59 | -0.561490751 | JARID2 | jumonji and AT-rich interaction domain containing 2 [Source:HGNC Symbol;Acc:HGNC:6196] |
| ENSG00000008128 | HGLibA_08725 | 59 | -0.561490751 | CDK11A | cyclin dependent kinase 11A [Source:HGNC Symbol;Acc:HGNC:1730] |
| ENSG00000008838 | HGLibA_28783 | 59 | -0.561490751 | MED24 | mediator complex subunit 24 [Source:HGNC Symbol;Acc:HGNC:22963] |
| ENSG00000009709 | HGLibA_35314 | 59 | -0.561490751 | PAX7 | paired box 7 [Source:HGNC Symbol;Acc:HGNC:8621] |
| ENSG00000022567 | HGLibA_45182 | 59 | -0.561490751 | SLC45A4 | solute carrier family 45 member 4 [Source:HGNC Symbol;Acc:HGNC:29196] |
| ENSG00000036672 | HGLibA_53297 | 59 | -0.561490751 | USP2 | ubiquitin specific peptidase 2 [Source:HGNC Symbol;Acc:HGNC:12618] |
| ENSG00000044012 | HGLibA_20645 | 59 | -0.561490751 | GUCA2B | guanylate cyclase activator 2B [Source:HGNC Symbol;Acc:HGNC:4683] |
| ENSG00000044574 | HGLibA_22290 | 59 | -0.561490751 | HSPA5 | heat shock protein family A (Hsp70) member 5 [Source:HGNC Symbol;Acc:HGNC:5238] |
| ENSG00000048162 | HGLibA_32308 | 59 | -0.561490751 | NOP16 | NOP16 nucleolar protein [Source:HGNC Symbol;Acc:HGNC:26934] |
| ENSG00000054654 | HGLibA_47980 | 59 | -0.561490751 | SYNE2 | spectrin repeat containing nuclear envelope protein 2 [Source:HGNC Symbol;Acc:HGNC:17084] |
| ENSG00000063322 | HGLibA_28798 | 59 | -0.561490751 | MED29 | mediator complex subunit 29 [Source:HGNC Symbol;Acc:HGNC:23074] |
| ENSG00000068024 | HGLibA_21012 | 59 | -0.561490751 | HDAC4 | histone deacetylase 4 [Source:HGNC Symbol;Acc:HGNC:14063] |
| ENSG00000073754 | HGLibA_08420 | 59 | -0.561490751 | CD5L | CD5 molecule like [Source:HGNC Symbol;Acc:HGNC:1690] |
| ENSG00000074621 | HGLibA_44616 | 59 | -0.561490751 | SLC24A1 | solute carrier family 24 member 1 [Source:HGNC Symbol;Acc:HGNC:10975] |
| ENSG00000075785 | HGLibA_39980 | 59 | -0.561490751 | RAB7A | member RAS oncogene family [Source:HGNC Symbol;Acc:HGNC:9788]" |
| ENSG00000076641 | HGLibA_35000 | 59 | -0.561490751 | PAG1 | phosphoprotein membrane anchor with glycosphingolipid microdomains 1 [Source:HGNC Symbol;Acc:HGNC:30043] |
| ENSG00000077463 | HGLibA_44232 | 59 | -0.561490751 | SIRT6 | sirtuin 6 [Source:HGNC Symbol;Acc:HGNC:14934] |
| ENSG00000079459 | HGLibA_17252 | 59 | -0.561490751 | FDFT1 | farnesyl-diphosphate farnesyltransferase 1 [Source:HGNC Symbol;Acc:HGNC:3629] |
| ENSG00000079739 | HGLibA_36261 | 59 | -0.561490751 | PGM1 | phosphoglucomutase 1 [Source:HGNC Symbol;Acc:HGNC:8905] |
| ENSG00000090447 | HGLibA_49135 | 59 | -0.561490751 | TFAP4 | transcription factor AP-4 [Source:HGNC Symbol;Acc:HGNC:11745] |
| ENSG00000090554 | HGLibA_17668 | 59 | -0.561490751 | FLT3LG | fms related tyrosine kinase 3 ligand [Source:HGNC Symbol;Acc:HGNC:3766] |
| ENSG00000092607 | HGLibA_48646 | 59 | -0.561490751 | TBX15 | T-box 15 [Source:HGNC Symbol;Acc:HGNC:11594] |
| ENSG00000094880 | HGLibA_08523 | 59 | -0.561490751 | CDC23 | cell division cycle 23 [Source:HGNC Symbol;Acc:HGNC:1724] |
| ENSG00000096654 | HGLibA_55546 | 59 | -0.561490751 | ZNF184 | zinc finger protein 184 [Source:HGNC Symbol;Acc:HGNC:12975] |
| ENSG00000099721 | HGLibA_01769 | 59 | -0.561490751 | AMELY | amelogenin Y-linked [Source:HGNC Symbol;Acc:HGNC:462] |
| ENSG00000099968 | HGLibA_04315 | 59 | -0.561490751 | BCL2L13 | BCL2 like 13 [Source:HGNC Symbol;Acc:HGNC:17164] |
| ENSG00000100075 | HGLibA_44634 | 59 | -0.561490751 | SLC25A1 | solute carrier family 25 member 1 [Source:HGNC Symbol;Acc:HGNC:10979] |
| ENSG00000100129 | HGLibA_14798 | 59 | -0.561490751 | EIF3L | eukaryotic translation initiation factor 3 subunit L [Source:HGNC Symbol;Acc:HGNC:18138] |
| ENSG00000100207 | HGLibA_48770 | 59 | -0.561490751 | TCF20 | transcription factor 20 [Source:HGNC Symbol;Acc:HGNC:11631] |
| ENSG00000100433 | HGLibA_24297 | 59 | -0.561490751 | KCNK10 | potassium two pore domain channel subfamily K member 10 [Source:HGNC Symbol;Acc:HGNC:6273] |
| ENSG00000100503 | HGLibA_31936 | 59 | -0.561490751 | NIN | ninein [Source:HGNC Symbol;Acc:HGNC:14906] |
| ENSG00000100811 | HGLibA_54757 | 59 | -0.561490751 | YY1 | YY1 transcription factor [Source:HGNC Symbol;Acc:HGNC:12856] |
| ENSG00000101665 | HGLibA_45588 | 59 | -0.561490751 | SMAD7 | SMAD family member 7 [Source:HGNC Symbol;Acc:HGNC:6773] |
| ENSG00000101842 | HGLibA_53836 | 59 | -0.561490751 | VSIG1 | V-set and immunoglobulin domain containing 1 [Source:HGNC Symbol;Acc:HGNC:28675] |
| ENSG00000103326 | HGLibA_07161 | 59 | -0.561490751 | CAPN15 | calpain 15 [Source:HGNC Symbol;Acc:HGNC:11182] |
| ENSG00000103426 | HGLibA_10795 | 59 | -0.561490751 | CORO7-PAM16 | CORO7-PAM16 readthrough [Source:HGNC Symbol;Acc:HGNC:44424] |
| ENSG00000105641 | HGLibA_45275 | 59 | -0.561490751 | SLC5A5 | solute carrier family 5 member 5 [Source:HGNC Symbol;Acc:HGNC:11040] |
| ENSG00000106258 | HGLibA_12160 | 59 | -0.561490751 | CYP3A5 | cytochrome P450 family 3 subfamily A member 5 [Source:HGNC Symbol;Acc:HGNC:2638] |
| ENSG00000106278 | HGLibA_39543 | 59 | -0.561490751 | PTPRZ1 | receptor type Z1 [Source:HGNC Symbol;Acc:HGNC:9685]" |
| ENSG00000106341 | HGLibA_38058 | 59 | -0.561490751 | PPP1R17 | protein phosphatase 1 regulatory subunit 17 [Source:HGNC Symbol;Acc:HGNC:16973] |
| ENSG00000106355 | HGLibA_27545 | 59 | -0.561490751 | LSM5 | U6 small nuclear RNA and mRNA degradation associated [Source:HGNC Symbol;Acc:HGNC:17162]" |
| ENSG00000106415 | HGLibA_19228 | 59 | -0.561490751 | GLCCI1 | glucocorticoid induced 1 [Source:HGNC Symbol;Acc:HGNC:18713] |
| ENSG00000106628 | HGLibA_37494 | 59 | -0.561490751 | POLD2 | accessory subunit [Source:HGNC Symbol;Acc:HGNC:9176]" |
| ENSG00000107281 | HGLibA_32427 | 59 | -0.561490751 | NPDC1 | differentiation and control 1 [Source:HGNC Symbol;Acc:HGNC:7899]" |
| ENSG00000108590 | HGLibA_28805 | 59 | -0.561490751 | MED31 | mediator complex subunit 31 [Source:HGNC Symbol;Acc:HGNC:24260] |
| ENSG00000109814 | HGLibA_52863 | 59 | -0.561490751 | UGDH | UDP-glucose 6-dehydrogenase [Source:HGNC Symbol;Acc:HGNC:12525] |
| ENSG00000109819 | HGLibA_37847 | 59 | -0.561490751 | PPARGC1A | PPARG coactivator 1 alpha [Source:HGNC Symbol;Acc:HGNC:9237] |
| ENSG00000111145 | HGLibA_14916 | 59 | -0.561490751 | ELK3 | ETS transcription factor [Source:HGNC Symbol;Acc:HGNC:3325]" |
| ENSG00000111196 | HGLibA_28011 | 59 | -0.561490751 | MAGOHB | exon junction complex subunit [Source:HGNC Symbol;Acc:HGNC:25504]" |
| ENSG00000112081 | HGLibA_47076 | 59 | -0.561490751 | SRSF3 | serine and arginine rich splicing factor 3 [Source:HGNC Symbol;Acc:HGNC:10785] |
| ENSG00000112167 | HGLibA_42787 | 59 | -0.561490751 | SAYSD1 | SAYSVFN motif domain containing 1 [Source:HGNC Symbol;Acc:HGNC:21025] |
| ENSG00000112902 | HGLibA_43358 | 59 | -0.561490751 | SEMA5A | semaphorin 5A [Source:HGNC Symbol;Acc:HGNC:10736] |
| ENSG00000113273 | HGLibA_03130 | 59 | -0.561490751 | ARSB | arylsulfatase B [Source:HGNC Symbol;Acc:HGNC:714] |
| ENSG00000114113 | HGLibA_40631 | 59 | -0.561490751 | RBP2 | retinol binding protein 2 [Source:HGNC Symbol;Acc:HGNC:9920] |
| ENSG00000116771 | HGLibA_01200 | 59 | -0.561490751 | AGMAT | agmatinase [Source:HGNC Symbol;Acc:HGNC:18407] |
| ENSG00000117475 | HGLibA_04580 | 59 | -0.561490751 | BLZF1 | basic leucine zipper nuclear factor 1 [Source:HGNC Symbol;Acc:HGNC:1065] |
| ENSG00000118689 | HGLibA_17925 | 59 | -0.561490751 | FOXO3 | forkhead box O3 [Source:HGNC Symbol;Acc:HGNC:3821] |
| ENSG00000119777 | HGLibA_50215 | 59 | -0.561490751 | TMEM214 | transmembrane protein 214 [Source:HGNC Symbol;Acc:HGNC:25983] |
| ENSG00000124103 | HGLibA_16439 | 59 | -0.561490751 | FAM209A | family with sequence similarity 209 member A [Source:HGNC Symbol;Acc:HGNC:16100] |
| ENSG00000124356 | HGLibA_47335 | 59 | -0.561490751 | STAMBP | STAM binding protein [Source:HGNC Symbol;Acc:HGNC:16950] |
| ENSG00000124762 | HGLibA_08822 | 59 | -0.561490751 | CDKN1A | cyclin dependent kinase inhibitor 1A [Source:HGNC Symbol;Acc:HGNC:1784] |
| ENSG00000124827 | HGLibA_18803 | 59 | -0.561490751 | GCM2 | glial cells missing homolog 2 [Source:HGNC Symbol;Acc:HGNC:4198] |
| ENSG00000125037 | HGLibA_15010 | 59 | -0.561490751 | EMC3 | ER membrane protein complex subunit 3 [Source:HGNC Symbol;Acc:HGNC:23999] |
| ENSG00000125046 | HGLibA_47193 | 59 | -0.561490751 | SSUH2 | ssu-2 homolog (C. elegans) [Source:HGNC Symbol;Acc:HGNC:24809] |
| ENSG00000125618 | HGLibA_35315 | 59 | -0.561490751 | PAX8 | paired box 8 [Source:HGNC Symbol;Acc:HGNC:8622] |
| ENSG00000126456 | HGLibA_23585 | 59 | -0.561490751 | IRF3 | interferon regulatory factor 3 [Source:HGNC Symbol;Acc:HGNC:6118] |
| ENSG00000126777 | HGLibA_25872 | 59 | -0.561490751 | KTN1 | kinectin 1 [Source:HGNC Symbol;Acc:HGNC:6467] |
| ENSG00000128833 | HGLibA_30856 | 59 | -0.561490751 | MYO5C | myosin VC [Source:HGNC Symbol;Acc:HGNC:7604] |
| ENSG00000130037 | HGLibA_24138 | 59 | -0.561490751 | KCNA5 | potassium voltage-gated channel subfamily A member 5 [Source:HGNC Symbol;Acc:HGNC:6224] |
| ENSG00000130254 | HGLibA_42637 | 59 | -0.561490751 | SAFB2 | scaffold attachment factor B2 [Source:HGNC Symbol;Acc:HGNC:21605] |
| ENSG00000130377 | HGLibA_00545 | 59 | -0.561490751 | ACSBG2 | acyl-CoA synthetase bubblegum family member 2 [Source:HGNC Symbol;Acc:HGNC:24174] |
| ENSG00000131480 | HGLibA_02270 | 59 | -0.561490751 | AOC2 | copper containing 2 [Source:HGNC Symbol;Acc:HGNC:549]" |
| ENSG00000132388 | HGLibA_52605 | 59 | -0.561490751 | UBE2G1 | ubiquitin conjugating enzyme E2 G1 [Source:HGNC Symbol;Acc:HGNC:12482] |
| ENSG00000135632 | HGLibA_45863 | 59 | -0.561490751 | SMYD5 | SMYD family member 5 [Source:HGNC Symbol;Acc:HGNC:16258] |
| ENSG00000135775 | HGLibA_10472 | 59 | -0.561490751 | COG2 | component of oligomeric golgi complex 2 [Source:HGNC Symbol;Acc:HGNC:6546] |
| ENSG00000135972 | HGLibA_30112 | 59 | -0.561490751 | MRPS9 | mitochondrial ribosomal protein S9 [Source:HGNC Symbol;Acc:HGNC:14501] |
| ENSG00000136327 | HGLibA_32045 | 59 | -0.561490751 | NKX2-8 | NK2 homeobox 8 [Source:HGNC Symbol;Acc:HGNC:16364] |
| ENSG00000137261 | HGLibA_24619 | 59 | -0.561490751 | KIAA0319 | KIAA0319 [Source:HGNC Symbol;Acc:HGNC:21580] |
| ENSG00000137274 | HGLibA_04704 | 59 | -0.561490751 | BPHL | biphenyl hydrolase like [Source:HGNC Symbol;Acc:HGNC:1094] |
| ENSG00000137731 | HGLibA_18226 | 59 | -0.561490751 | FXYD2 | FXYD domain containing ion transport regulator 2 [Source:HGNC Symbol;Acc:HGNC:4026] |
| ENSG00000138028 | HGLibA_09327 | 59 | -0.561490751 | CGREF1 | cell growth regulator with EF-hand domain 1 [Source:HGNC Symbol;Acc:HGNC:16962] |
| ENSG00000138083 | HGLibA_44249 | 59 | -0.561490751 | SIX3 | SIX homeobox 3 [Source:HGNC Symbol;Acc:HGNC:10889] |
| ENSG00000138162 | HGLibA_48161 | 59 | -0.561490751 | TACC2 | transforming acidic coiled-coil containing protein 2 [Source:HGNC Symbol;Acc:HGNC:11523] |
| ENSG00000138613 | HGLibA_02416 | 59 | -0.561490751 | APH1B | gamma-secretase subunit [Source:HGNC Symbol;Acc:HGNC:24080]" |
| ENSG00000138663 | HGLibA_10720 | 59 | -0.561490751 | COPS4 | COP9 signalosome subunit 4 [Source:HGNC Symbol;Acc:HGNC:16702] |
| ENSG00000138722 | HGLibA_29547 | 59 | -0.561490751 | MMRN1 | multimerin 1 [Source:HGNC Symbol;Acc:HGNC:7178] |
| ENSG00000140600 | HGLibA_43953 | 59 | -0.561490751 | SH3GL3 | endophilin A3 [Source:HGNC Symbol;Acc:HGNC:10832]" |
| ENSG00000142065 | HGLibA_55189 | 59 | -0.561490751 | ZFP14 | ZFP14 zinc finger protein [Source:HGNC Symbol;Acc:HGNC:29312] |
| ENSG00000143127 | HGLibA_23719 | 59 | -0.561490751 | ITGA10 | integrin subunit alpha 10 [Source:HGNC Symbol;Acc:HGNC:6135] |
| ENSG00000143621 | HGLibA_23214 | 59 | -0.561490751 | ILF2 | interleukin enhancer binding factor 2 [Source:HGNC Symbol;Acc:HGNC:6037] |
| ENSG00000147027 | HGLibA_50415 | 59 | -0.561490751 | TMEM47 | transmembrane protein 47 [Source:HGNC Symbol;Acc:HGNC:18515] |
| ENSG00000147869 | HGLibA_09189 | 59 | -0.561490751 | CER1 | DAN family BMP antagonist [Source:HGNC Symbol;Acc:HGNC:1862]" |
| ENSG00000149300 | HGLibA_05152 | 59 | -0.561490751 | C11orf52 | chromosome 11 open reading frame 52 [Source:HGNC Symbol;Acc:HGNC:30531] |
| ENSG00000149596 | HGLibA_23981 | 59 | -0.561490751 | JPH2 | junctophilin 2 [Source:HGNC Symbol;Acc:HGNC:14202] |
| ENSG00000150628 | HGLibA_46514 | 59 | -0.561490751 | SPATA4 | spermatogenesis associated 4 [Source:HGNC Symbol;Acc:HGNC:17333] |
| ENSG00000151702 | HGLibA_17614 | 59 | -0.561490751 | FLI1 | ETS transcription factor [Source:HGNC Symbol;Acc:HGNC:3749]" |
| ENSG00000153214 | HGLibA_50534 | 59 | -0.561490751 | TMEM87B | transmembrane protein 87B [Source:HGNC Symbol;Acc:HGNC:25913] |
| ENSG00000153291 | HGLibA_44691 | 59 | -0.561490751 | SLC25A27 | solute carrier family 25 member 27 [Source:HGNC Symbol;Acc:HGNC:21065] |
| ENSG00000154001 | HGLibA_38182 | 59 | -0.561490751 | PPP2R5E | protein phosphatase 2 regulatory subunit B'epsilon [Source:HGNC Symbol;Acc:HGNC:9313] |
| ENSG00000154305 | HGLibA_29189 | 59 | -0.561490751 | MIA3 | MIA SH3 domain ER export factor 3 [Source:HGNC Symbol;Acc:HGNC:24008] |
| ENSG00000154611 | HGLibA_39092 | 59 | -0.561490751 | PSMA8 | proteasome subunit alpha 8 [Source:HGNC Symbol;Acc:HGNC:22985] |
| ENSG00000157343 | HGLibA_03011 | 59 | -0.561490751 | ARMC12 | armadillo repeat containing 12 [Source:HGNC Symbol;Acc:HGNC:21099] |
| ENSG00000157851 | HGLibA_13935 | 59 | -0.561490751 | DPYSL5 | dihydropyrimidinase like 5 [Source:HGNC Symbol;Acc:HGNC:20637] |
| ENSG00000158793 | HGLibA_31984 | 59 | -0.561490751 | NIT1 | nitrilase 1 [Source:HGNC Symbol;Acc:HGNC:7828] |
| ENSG00000160200 | HGLibA_07428 | 59 | -0.561490751 | CBS | cystathionine-beta-synthase [Source:HGNC Symbol;Acc:HGNC:1550] |
| ENSG00000160606 | HGLibA_49594 | 59 | -0.561490751 | TLCD1 | TLC domain containing 1 [Source:HGNC Symbol;Acc:HGNC:25177] |
| ENSG00000162571 | HGLibA_52188 | 59 | -0.561490751 | TTLL10 | tubulin tyrosine ligase like 10 [Source:HGNC Symbol;Acc:HGNC:26693] |
| ENSG00000162650 | HGLibA_03856 | 59 | -0.561490751 | ATXN7L2 | ataxin 7 like 2 [Source:HGNC Symbol;Acc:HGNC:28713] |
| ENSG00000163093 | HGLibA_04220 | 59 | -0.561490751 | BBS5 | Bardet-Biedl syndrome 5 [Source:HGNC Symbol;Acc:HGNC:970] |
| ENSG00000163216 | HGLibA_46813 | 59 | -0.561490751 | SPRR2D | small proline rich protein 2D [Source:HGNC Symbol;Acc:HGNC:11264] |
| ENSG00000163297 | HGLibA_02215 | 59 | -0.561490751 | ANTXR2 | ANTXR cell adhesion molecule 2 [Source:HGNC Symbol;Acc:HGNC:21732] |
| ENSG00000163499 | HGLibA_11255 | 59 | -0.561490751 | CRYBA2 | crystallin beta A2 [Source:HGNC Symbol;Acc:HGNC:2395] |
| ENSG00000164128 | HGLibA_32551 | 59 | -0.561490751 | NPY1R | neuropeptide Y receptor Y1 [Source:HGNC Symbol;Acc:HGNC:7956] |
| ENSG00000164483 | HGLibA_42679 | 59 | -0.561490751 | SAMD3 | sterile alpha motif domain containing 3 [Source:HGNC Symbol;Acc:HGNC:21574] |
| ENSG00000164542 | HGLibA_24658 | 59 | -0.561490751 | KIAA0895 | KIAA0895 [Source:HGNC Symbol;Acc:HGNC:22206] |
| ENSG00000164751 | HGLibA_36107 | 59 | -0.561490751 | PEX2 | peroxisomal biogenesis factor 2 [Source:HGNC Symbol;Acc:HGNC:9717] |
| ENSG00000164821 | HGLibA_12800 | 59 | -0.561490751 | DEFA4 | defensin alpha 4 [Source:HGNC Symbol;Acc:HGNC:2763] |
| ENSG00000166016 | HGLibA_00339 | 59 | -0.561490751 | ABTB2 | ankyrin repeat and BTB domain containing 2 [Source:HGNC Symbol;Acc:HGNC:23842] |
| ENSG00000166261 | HGLibA_55574 | 59 | -0.561490751 | ZNF202 | zinc finger protein 202 [Source:HGNC Symbol;Acc:HGNC:12994] |
| ENSG00000166509 | HGLibA_09974 | 59 | -0.561490751 | CLEC3A | C-type lectin domain family 3 member A [Source:HGNC Symbol;Acc:HGNC:2052] |
| ENSG00000167881 | HGLibA_47008 | 59 | -0.561490751 | SRP68 | signal recognition particle 68 [Source:HGNC Symbol;Acc:HGNC:11302] |
| ENSG00000168014 | HGLibA_06092 | 59 | -0.561490751 | C2CD3 | C2 calcium dependent domain containing 3 [Source:HGNC Symbol;Acc:HGNC:24564] |

| | | | | | |
|---|---|---|---|---|---|
| ENSG00000168079 | HGLibA_42867 | 59 | -0.561490751 | SCARA5 | scavenger receptor class A member 5 [Source:HGNC Symbol;Acc:HGNC:28701] |
| ENSG00000168438 | HGLibA_08551 | 59 | -0.561490751 | CDC40 | cell division cycle 40 [Source:HGNC Symbol;Acc:HGNC:17350] |
| ENSG00000170166 | HGLibA_22000 | 59 | -0.561490751 | HOXD4 | homeobox D4 [Source:HGNC Symbol;Acc:HGNC:5138] |
| ENSG00000170209 | HGLibA_01972 | 59 | -0.561490751 | ANKK1 | ankyrin repeat and kinase domain containing 1 [Source:HGNC Symbol;Acc:HGNC:21027] |
| ENSG00000170465 | HGLibA_25518 | 59 | -0.561490751 | KRT6C | keratin 6C [Source:HGNC Symbol;Acc:HGNC:20406] |
| ENSG00000170540 | HGLibA_02987 | 59 | -0.561490751 | ARL6IP1 | ADP ribosylation factor like GTPase 6 interacting protein 1 [Source:HGNC Symbol;Acc:HGNC:697] |
| ENSG00000171903 | HGLibA_12182 | 59 | -0.561490751 | CYP4F11 | cytochrome P450 family 4 subfamily F member 11 [Source:HGNC Symbol;Acc:HGNC:13265] |
| ENSG00000172016 | HGLibA_40800 | 59 | -0.561490751 | REG3A | regenerating family member 3 alpha [Source:HGNC Symbol;Acc:HGNC:8601] |
| ENSG00000172023 | HGLibA_40796 | 59 | -0.561490751 | REG1B | regenerating family member 1 beta [Source:HGNC Symbol;Acc:HGNC:9952] |
| ENSG00000172115 | HGLibA_12028 | 59 | -0.561490751 | CYCS | somatic [Source:HGNC Symbol;Acc:HGNC:19986]" |
| ENSG00000173376 | HGLibA_31402 | 59 | -0.561490751 | NDNF | neuron derived neurotrophic factor [Source:HGNC Symbol;Acc:HGNC:26256] |
| ENSG00000173545 | HGLibA_56410 | 59 | -0.561490751 | ZNF622 | zinc finger protein 622 [Source:HGNC Symbol;Acc:HGNC:30958] |
| ENSG00000174799 | HGLibA_09106 | 59 | -0.561490751 | CEP135 | centrosomal protein 135 [Source:HGNC Symbol;Acc:HGNC:29086] |
| ENSG00000175054 | HGLibA_03809 | 59 | -0.561490751 | ATR | ATR serine/threonine kinase [Source:HGNC Symbol;Acc:HGNC:882] |
| ENSG00000175229 | HGLibA_18503 | 59 | -0.561490751 | GAL3ST3 | galactose-3-O-sulfotransferase 3 [Source:HGNC Symbol;Acc:HGNC:24144] |
| ENSG00000175582 | HGLibA_39969 | 59 | -0.561490751 | RAB6A | member RAS oncogene family [Source:HGNC Symbol;Acc:HGNC:9786]" |
| ENSG00000175691 | HGLibA_56702 | 59 | -0.561490751 | ZNF77 | zinc finger protein 77 [Source:HGNC Symbol;Acc:HGNC:13150] |
| ENSG00000175792 | HGLibA_42472 | 59 | -0.561490751 | RUVBL1 | RuvB like AAA ATPase 1 [Source:HGNC Symbol;Acc:HGNC:10474] |
| ENSG00000177291 | HGLibA_19193 | 59 | -0.561490751 | GJD4 | gap junction protein delta 4 [Source:HGNC Symbol;Acc:HGNC:23296] |
| ENSG00000178951 | HGLibA_54917 | 59 | -0.561490751 | ZBTB7A | zinc finger and BTB domain containing 7A [Source:HGNC Symbol;Acc:HGNC:18078] |
| ENSG00000179388 | HGLibA_14654 | 59 | -0.561490751 | EGR3 | early growth response 3 [Source:HGNC Symbol;Acc:HGNC:3240] |
| ENSG00000181004 | HGLibA_04211 | 59 | -0.561490751 | BBS12 | Bardet-Biedl syndrome 12 [Source:HGNC Symbol;Acc:HGNC:26648] |
| ENSG00000181074 | HGLibA_34198 | 59 | -0.561490751 | OR52N4 | olfactory receptor family 52 subfamily N member 4 (gene/pseudogene) [Source:HGNC Symbol;Acc:HGNC:15230] |
| ENSG00000181104 | HGLibA_15861 | 59 | -0.561490751 | F2R | coagulation factor II thrombin receptor [Source:HGNC Symbol;Acc:HGNC:3537] |
| ENSG00000182628 | HGLibA_44264 | 59 | -0.561490751 | SKA2 | spindle and kinetochore associated complex subunit 2 [Source:HGNC Symbol;Acc:HGNC:28006] |
| ENSG00000182831 | HGLibA_05476 | 59 | -0.561490751 | C16orf72 | chromosome 16 open reading frame 72 [Source:HGNC Symbol;Acc:HGNC:30103] |
| ENSG00000183269 | HGLibA_34166 | 59 | -0.561490751 | OR52E8 | olfactory receptor family 52 subfamily E member 8 [Source:HGNC Symbol;Acc:HGNC:15217] |
| ENSG00000185324 | HGLibA_08721 | 59 | -0.561490751 | CDK10 | cyclin dependent kinase 10 [Source:HGNC Symbol;Acc:HGNC:1770] |
| ENSG00000185480 | HGLibA_35250 | 59 | -0.561490751 | PARPBP | PARP1 binding protein [Source:HGNC Symbol;Acc:HGNC:26074] |
| ENSG00000185966 | HGLibA_26142 | 59 | -0.561490751 | LCE3E | late cornified envelope 3E [Source:HGNC Symbol;Acc:HGNC:29463] |
| ENSG00000186469 | HGLibA_19519 | 59 | -0.561490751 | GNG2 | G protein subunit gamma 2 [Source:HGNC Symbol;Acc:HGNC:4404] |
| ENSG00000187010 | HGLibA_41152 | 59 | -0.561490751 | RHD | Rh blood group D antigen [Source:HGNC Symbol;Acc:HGNC:10009] |
| ENSG00000187109 | HGLibA_31111 | 59 | -0.561490751 | NAP1L1 | nucleosome assembly protein 1 like 1 [Source:HGNC Symbol;Acc:HGNC:7637] |
| ENSG00000187955 | HGLibA_10513 | 59 | -0.561490751 | COL14A1 | collagen type XIV alpha 1 chain [Source:HGNC Symbol;Acc:HGNC:2191] |
| ENSG00000188152 | HGLibA_33112 | 59 | -0.561490751 | NUTM2G | NUT family member 2G [Source:HGNC Symbol;Acc:HGNC:23449] |
| ENSG00000188425 | HGLibA_31098 | 59 | -0.561490751 | NANOS2 | nanos C2HC-type zinc finger 2 [Source:HGNC Symbol;Acc:HGNC:23292] |
| ENSG00000188786 | HGLibA_30350 | 59 | -0.561490751 | MTF1 | metal regulatory transcription factor 1 [Source:HGNC Symbol;Acc:HGNC:7428] |
| ENSG00000189181 | HGLibA_33635 | 59 | -0.561490751 | OR14I1 | olfactory receptor family 14 subfamily I member 1 [Source:HGNC Symbol;Acc:HGNC:19575] |
| ENSG00000196166 | HGLibA_06652 | 59 | -0.561490751 | C8orf86 | chromosome 8 open reading frame 86 [Source:HGNC Symbol;Acc:HGNC:33774] |
| ENSG00000196396 | HGLibA_39430 | 59 | -0.561490751 | PTPN1 | non-receptor type 1 [Source:HGNC Symbol;Acc:HGNC:9642]" |
| ENSG00000197557 | HGLibA_52105 | 59 | -0.561490751 | TTC30A | tetratricopeptide repeat domain 30A [Source:HGNC Symbol;Acc:HGNC:25853] |
| ENSG00000198455 | HGLibA_57076 | 59 | -0.561490751 | ZXDB | zinc finger X-linked duplicated B [Source:HGNC Symbol;Acc:HGNC:13199] |
| ENSG00000198648 | HGLibA_47506 | 59 | -0.561490751 | STK39 | serine/threonine kinase 39 [Source:HGNC Symbol;Acc:HGNC:17717] |
| ENSG00000198740 | HGLibA_56461 | 59 | -0.561490751 | ZNF652 | zinc finger protein 652 [Source:HGNC Symbol;Acc:HGNC:29147] |
| ENSG00000198931 | HGLibA_02571 | 59 | -0.561490751 | APRT | adenine phosphoribosyltransferase [Source:HGNC Symbol;Acc:HGNC:626] |
| ENSG00000203837 | HGLibA_37362 | 59 | -0.561490751 | PNLIPRP3 | pancreatic lipase related protein 3 [Source:HGNC Symbol;Acc:HGNC:23492] |
| ENSG00000204403 | HGLibA_07302 | 59 | -0.561490751 | CASP12 | caspase 12 (gene/pseudogene) [Source:HGNC Symbol;Acc:HGNC:19004] |
| ENSG00000204542 | HGLibA_06452 | 59 | -0.561490751 | C6orf15 | chromosome 6 open reading frame 15 [Source:HGNC Symbol;Acc:HGNC:13927] |
| ENSG00000204640 | HGLibA_32201 | 59 | -0.561490751 | NMS | neuromedin S [Source:HGNC Symbol;Acc:HGNC:32203] |
| ENSG00000204872 | HGLibA_31184 | 59 | -0.561490751 | NAT8B | gene/pseudogene) [Source:HGNC Symbol;Acc:HGNC:30235]" |
| ENSG00000205403 | HGLibA_09279 | 59 | -0.561490751 | CFI | complement factor I [Source:HGNC Symbol;Acc:HGNC:5394] |
| ENSG00000205409 | HGLibA_34164 | 59 | -0.561490751 | OR52E6 | olfactory receptor family 52 subfamily E member 6 [Source:HGNC Symbol;Acc:HGNC:15215] |
| ENSG00000205669 | HGLibA_00485 | 59 | -0.561490751 | ACOT6 | acyl-CoA thioesterase 6 [Source:HGNC Symbol;Acc:HGNC:33159] |
| ENSG00000205678 | HGLibA_48951 | 59 | -0.561490751 | TECRL | 3-enoyl-CoA reductase like [Source:HGNC Symbol;Acc:HGNC:27365]" |
| ENSG00000213639 | HGLibA_38008 | 59 | -0.561490751 | PPP1CB | protein phosphatase 1 catalytic subunit beta [Source:HGNC Symbol;Acc:HGNC:9282] |
| ENSG00000214274 | HGLibA_01893 | 59 | -0.561490751 | ANG | angiogenin [Source:HGNC Symbol;Acc:HGNC:483] |
| ENSG00000223569 | HGLibA_53228 | 59 | -0.561490751 | USP17L15 | ubiquitin specific peptidase 17-like family member 15 [Source:HGNC Symbol;Acc:HGNC:44443] |
| ENSG00000230453 | HGLibA_02020 | 59 | -0.561490751 | ANKRD18B | ankyrin repeat domain 18B [Source:HGNC Symbol;Acc:HGNC:23644] |
| ENSG00000235780 | HGLibA_53264 | 59 | -0.561490751 | USP17L27 | ubiquitin specific peptidase 17-like family member 27 [Source:HGNC Symbol;Acc:HGNC:44455] |
| ENSG00000240764 | HGLibA_35576 | 59 | -0.561490751 | PCDHGC5 | 5 [Source:HGNC Symbol;Acc:HGNC:8718]" |
| ENSG00000241685 | HGLibA_03073 | 59 | -0.561490751 | ARPC1A | actin related protein 2/3 complex subunit 1A [Source:HGNC Symbol;Acc:HGNC:703] |
| ENSG00000244482 | HGLibA_26505 | 59 | -0.561490751 | LILRA6 | leukocyte immunoglobulin like receptor A6 [Source:HGNC Symbol;Acc:HGNC:15495] |
| ENSG00000250151 | HGLibA_03089 | 59 | -0.561490751 | ARPC4-TTLL3 | ARPC4-TTLL3 readthrough [Source:HGNC Symbol;Acc:HGNC:38830] |
| ENSG00000253117 | HGLibA_33238 | 59 | -0.561490751 | OC90 | otoconin 90 [Source:HGNC Symbol;Acc:HGNC:8100] |
| ENSG00000254466 | HGLibA_33969 | 59 | -0.561490751 | OR4D10 | olfactory receptor family 4 subfamily D member 10 [Source:HGNC Symbol;Acc:HGNC:15173] |
| ENSG00000258817 | HGLibA_33943 | 59 | -0.561490751 | OR4C13 | olfactory receptor family 4 subfamily C member 13 [Source:HGNC Symbol;Acc:HGNC:15169] |
| ENSG00000261150 | HGLibA_15363 | 59 | -0.561490751 | EPPK1 | epiplakin 1 [Source:HGNC Symbol;Acc:HGNC:15577] |
| ENSG00000273841 | HGLibA_48260 | 59 | -0.561490751 | TAF9 | TATA-box binding protein associated factor 9 [Source:HGNC Symbol;Acc:HGNC:11542] |
| ENSG00000275385 | HGLibA_07982 | 59 | -0.561490751 | CCL18 | C-C motif chemokine ligand 18 [Source:HGNC Symbol;Acc:HGNC:10616] |
| ENSG00000276430 | HGLibA_16554 | 59 | -0.561490751 | FAM25C | family with sequence similarity 25 member C [Source:HGNC Symbol;Acc:HGNC:23586] |
| ENSG00000284701 | HGLibA_50307 | 59 | -0.561490751 | TMEM247 | transmembrane protein 247 [Source:HGNC Symbol;Acc:HGNC:42967] |
| ERO1L | HGLibA_15517 | 59 | -0.561490751 | ERO1L | - |
| FLJ45513 | HGLibA_17636 | 59 | -0.561490751 | FLJ45513 | - |
| FTSJD2 | HGLibA_18146 | 59 | -0.561490751 | FTSJD2 | - |
| H2AFB2 | HGLibA_20749 | 59 | -0.561490751 | H2AFB2 | - |
| hsa-mir-105-1 | HGLibA_57178 | 59 | -0.561490751 | hsa-mir-105-1 | - |
| hsa-mir-1233-1 | HGLibA_57324 | 59 | -0.561490751 | hsa-mir-1233-1 | - |
| hsa-mir-1293 | HGLibA_57650 | 59 | -0.561490751 | hsa-mir-1293 | - |
| hsa-mir-1305 | HGLibA_57737 | 59 | -0.561490751 | hsa-mir-1305 | - |
| hsa-mir-1321 | HGLibA_57761 | 59 | -0.561490751 | hsa-mir-1321 | - |
| hsa-mir-143 | HGLibA_57837 | 59 | -0.561490751 | hsa-mir-143 | - |
| hsa-mir-145 | HGLibA_57846 | 59 | -0.561490751 | hsa-mir-145 | - |
| hsa-mir-1539 | HGLibA_57923 | 59 | -0.561490751 | hsa-mir-1539 | - |
| hsa-mir-2681 | HGLibA_58355 | 59 | -0.561490751 | hsa-mir-2681 | - |
| hsa-mir-28 | HGLibA_58384 | 59 | -0.561490751 | hsa-mir-28 | - |
| hsa-mir-3179-1 | HGLibA_58790 | 59 | -0.561490751 | hsa-mir-3179-1 | - |
| hsa-mir-3185 | HGLibA_58837 | 59 | -0.561490751 | hsa-mir-3185 | - |
| hsa-mir-3613 | HGLibA_59083 | 59 | -0.561490751 | hsa-mir-3613 | - |
| hsa-mir-381 | HGLibA_59478 | 59 | -0.561490751 | hsa-mir-381 | - |
| hsa-mir-382 | HGLibA_59480 | 59 | -0.561490751 | hsa-mir-382 | - |
| hsa-mir-4329 | HGLibA_60041 | 59 | -0.561490751 | hsa-mir-4329 | - |
| hsa-mir-4478 | HGLibA_60326 | 59 | -0.561490751 | hsa-mir-4478 | - |
| hsa-mir-4491 | HGLibA_60382 | 59 | -0.561490751 | hsa-mir-4491 | - |
| hsa-mir-4496 | HGLibA_60399 | 59 | -0.561490751 | hsa-mir-4496 | - |
| hsa-mir-4536-1 | HGLibA_60603 | 59 | -0.561490751 | hsa-mir-4536-1 | - |
| hsa-mir-4745 | HGLibA_61076 | 59 | -0.561490751 | hsa-mir-4745 | - |
| hsa-mir-4749 | HGLibA_61094 | 59 | -0.561490751 | hsa-mir-4749 | - |
| hsa-mir-4758 | HGLibA_61130 | 59 | -0.561490751 | hsa-mir-4758 | - |
| hsa-mir-4763 | HGLibA_61148 | 59 | -0.561490751 | hsa-mir-4763 | - |
| hsa-mir-515-1 | HGLibA_61601 | 59 | -0.561490751 | hsa-mir-515-1 | - |
| hsa-mir-5191 | HGLibA_61681 | 59 | -0.561490751 | hsa-mir-5191 | - |
| hsa-mir-548g | HGLibA_61990 | 59 | -0.561490751 | hsa-mir-548g | - |
| hsa-mir-5579 | HGLibA_62155 | 59 | -0.561490751 | hsa-mir-5579 | - |
| hsa-mir-653 | HGLibA_62944 | 59 | -0.561490751 | hsa-mir-653 | - |
| hsa-mir-6800 | HGLibA_63379 | 59 | -0.561490751 | hsa-mir-6800 | - |
| hsa-mir-6831 | HGLibA_63501 | 59 | -0.561490751 | hsa-mir-6831 | - |
| hsa-mir-8072 | HGLibA_64129 | 59 | -0.561490751 | hsa-mir-8072 | - |
| hsa-mir-875 | HGLibA_64212 | 59 | -0.561490751 | hsa-mir-875 | - |
| LOC100130539 | HGLibA_26791 | 59 | -0.561490751 | LOC100130539 | - |
| LOC100862671 | HGLibA_26880 | 59 | -0.561490751 | LOC100862671 | - |
| MEF2BNB-MEF2B | HGLibA_28836 | 59 | -0.561490751 | MEF2BNB-MEF2B | - |
| P2RY8_X | HGLibA_34893 | 59 | -0.561490751 | P2RY8_X | - |
| PCNX | HGLibA_35643 | 59 | -0.561490751 | PCNX | - |
| SELM | HGLibA_43298 | 59 | -0.561490751 | SELM | - |
| TARP | HGLibA_48343 | 59 | -0.561490751 | TARP | - |
| WRB | HGLibA_54405 | 59 | -0.561490751 | WRB | - |
| AAED1 | HGLibA_00043 | 58 | -0.571441788 | AAED1 | - |
| AGXT2L2 | HGLibA_01281 | 58 | -0.571441788 | AGXT2L2 | - |
| APITD1 | HGLibA_02424 | 58 | -0.571441788 | APITD1 | - |
| C19orf10 | HGLibA_05627 | 58 | -0.571441788 | C19orf10 | - |
| C19orf82 | HGLibA_05717 | 58 | -0.571441788 | C19orf82 | - |
| C5orf28 | HGLibA_06370 | 58 | -0.571441788 | C5orf28 | - |
| C9orf37 | HGLibA_06724 | 58 | -0.571441788 | C9orf37 | - |
| CCDC108 | HGLibA_07528 | 58 | -0.571441788 | CCDC108 | - |
| CNTD2 | HGLibA_10388 | 58 | -0.571441788 | CNTD2 | - |
| CRLF2_Y | HGLibA_11193 | 58 | -0.571441788 | CRLF2_Y | - |
| CRLF2_Y | HGLibA_11191 | 58 | -0.571441788 | CRLF2_Y | - |
| ENSG00000004468 | HGLibA_08367 | 58 | -0.571441788 | CD38 | CD38 molecule [Source:HGNC Symbol;Acc:HGNC:1667] |
| ENSG00000008277 | HGLibA_00757 | 58 | -0.571441788 | ADAM22 | ADAM metallopeptidase domain 22 [Source:HGNC Symbol;Acc:HGNC:201] |
| ENSG00000010610 | HGLibA_08381 | 58 | -0.571441788 | CD4 | CD4 molecule [Source:HGNC Symbol;Acc:HGNC:1678] |
| ENSG00000019991 | HGLibA_21240 | 58 | -0.571441788 | HGF | hepatocyte growth factor [Source:HGNC Symbol;Acc:HGNC:4893] |
| ENSG00000023445 | HGLibA_04517 | 58 | -0.571441788 | BIRC3 | baculoviral IAP repeat containing 3 [Source:HGNC Symbol;Acc:HGNC:591] |
| ENSG00000025423 | HGLibA_22196 | 58 | -0.571441788 | HSD17B6 | hydroxysteroid 17-beta dehydrogenase 6 [Source:HGNC Symbol;Acc:HGNC:23316] |
| ENSG00000026751 | HGLibA_44317 | 58 | -0.571441788 | SLAMF7 | SLAM family member 7 [Source:HGNC Symbol;Acc:HGNC:21394] |
| ENSG00000036565 | HGLibA_44486 | 58 | -0.571441788 | SLC18A1 | solute carrier family 18 member A1 [Source:HGNC Symbol;Acc:HGNC:10934] |

| | | | | | |
|---|---|---|---|---|---|
| ENSG00000047188 | HGLibA_54727 | 58 | -0.571441788 | YTHDC2 | YTH domain containing 2 [Source:HGNC Symbol;Acc:HGNC:24721] |
| ENSG00000055732 | HGLibA_28643 | 58 | -0.571441788 | MCOLN3 | mucolipin 3 [Source:HGNC Symbol;Acc:HGNC:13358] |
| ENSG00000056586 | HGLibA_40665 | 58 | -0.571441788 | RC3H2 | ring finger and CCCH-type domains 2 [Source:HGNC Symbol;Acc:HGNC:21461] |
| ENSG00000057935 | HGLibA_30315 | 58 | -0.571441788 | MTA3 | metastasis associated 1 family member 3 [Source:HGNC Symbol;Acc:HGNC:23784] |
| ENSG00000067248 | HGLibA_13173 | 58 | -0.571441788 | DHX29 | DExH-box helicase 29 [Source:HGNC Symbol;Acc:HGNC:15815] |
| ENSG00000067369 | HGLibA_51053 | 58 | -0.571441788 | TP53BP1 | tumor protein p53 binding protein 1 [Source:HGNC Symbol;Acc:HGNC:11999] |
| ENSG00000074855 | HGLibA_02193 | 58 | -0.571441788 | ANO8 | anoctamin 8 [Source:HGNC Symbol;Acc:HGNC:29329] |
| ENSG00000074935 | HGLibA_52301 | 58 | -0.571441788 | TUBE1 | tubulin epsilon 1 [Source:HGNC Symbol;Acc:HGNC:20775] |
| ENSG00000076924 | HGLibA_54459 | 58 | -0.571441788 | XAB2 | XPA binding protein 2 [Source:HGNC Symbol;Acc:HGNC:14089] |
| ENSG00000077348 | HGLibA_15784 | 58 | -0.571441788 | EXOSC5 | exosome component 5 [Source:HGNC Symbol;Acc:HGNC:24662] |
| ENSG00000078596 | HGLibA_23828 | 58 | -0.571441788 | ITM2A | integral membrane protein 2A [Source:HGNC Symbol;Acc:HGNC:6173] |
| ENSG00000080007 | HGLibA_12716 | 58 | -0.571441788 | DDX43 | DEAD-box helicase 43 [Source:HGNC Symbol;Acc:HGNC:18677] |
| ENSG00000080493 | HGLibA_45220 | 58 | -0.571441788 | SLC4A4 | solute carrier family 4 member 4 [Source:HGNC Symbol;Acc:HGNC:11030] |
| ENSG00000085788 | HGLibA_12605 | 58 | -0.571441788 | DDHD2 | DDHD domain containing 2 [Source:HGNC Symbol;Acc:HGNC:29106] |
| ENSG00000087301 | HGLibA_52413 | 58 | -0.571441788 | TXNDC16 | thioredoxin domain containing 16 [Source:HGNC Symbol;Acc:HGNC:19965] |
| ENSG00000091039 | HGLibA_34668 | 58 | -0.571441788 | OSBPL8 | oxysterol binding protein like 8 [Source:HGNC Symbol;Acc:HGNC:16396] |
| ENSG00000091138 | HGLibA_44791 | 58 | -0.571441788 | SLC26A3 | solute carrier family 26 member 3 [Source:HGNC Symbol;Acc:HGNC:3018] |
| ENSG00000094796 | HGLibA_25474 | 58 | -0.571441788 | KRT31 | keratin 31 [Source:HGNC Symbol;Acc:HGNC:6448] |
| ENSG00000100095 | HGLibA_43682 | 58 | -0.571441788 | SEZ6L | seizure related 6 homolog like [Source:HGNC Symbol;Acc:HGNC:10763] |
| ENSG00000100207 | HGLibA_48771 | 58 | -0.571441788 | TCF20 | transcription factor 20 [Source:HGNC Symbol;Acc:HGNC:11631] |
| ENSG00000100442 | HGLibA_17574 | 58 | -0.571441788 | FKBP3 | FK506 binding protein 3 [Source:HGNC Symbol;Acc:HGNC:3719] |
| ENSG00000100614 | HGLibA_37964 | 58 | -0.571441788 | PPM1A | Mg2+/Mn2+ dependent 1A [Source:HGNC Symbol;Acc:HGNC:9275]" |
| ENSG00000100711 | HGLibA_55281 | 58 | -0.571441788 | ZFYVE21 | zinc finger FYVE-type containing 21 [Source:HGNC Symbol;Acc:HGNC:20760] |
| ENSG00000101574 | HGLibA_29008 | 58 | -0.571441788 | METTL4 | methyltransferase like 4 [Source:HGNC Symbol;Acc:HGNC:24726] |
| ENSG00000102967 | HGLibA_13112 | 58 | -0.571441788 | DHODH | dihydroorotate dehydrogenase (quinone) [Source:HGNC Symbol;Acc:HGNC:2867] |
| ENSG00000103111 | HGLibA_29643 | 58 | -0.571441788 | MON1B | secretory trafficking associated [Source:HGNC Symbol;Acc:HGNC:25020]" |
| ENSG00000104442 | HGLibA_03004 | 58 | -0.571441788 | ARMC1 | armadillo repeat containing 1 [Source:HGNC Symbol;Acc:HGNC:17684] |
| ENSG00000104490 | HGLibA_31251 | 58 | -0.571441788 | NCALD | neurocalcin delta [Source:HGNC Symbol;Acc:HGNC:7655] |
| ENSG00000105355 | HGLibA_37176 | 58 | -0.571441788 | PLIN3 | perilipin 3 [Source:HGNC Symbol;Acc:HGNC:16893] |
| ENSG00000105464 | HGLibA_20308 | 58 | -0.571441788 | GRIN2D | glutamate ionotropic receptor NMDA type subunit 2D [Source:HGNC Symbol;Acc:HGNC:4588] |
| ENSG00000105671 | HGLibA_12727 | 58 | -0.571441788 | DDX49 | DEAD-box helicase 49 [Source:HGNC Symbol;Acc:HGNC:18684] |
| ENSG00000106571 | HGLibA_19252 | 58 | -0.571441788 | GLI3 | GLI family zinc finger 3 [Source:HGNC Symbol;Acc:HGNC:4319] |
| ENSG00000107882 | HGLibA_47713 | 58 | -0.571441788 | SUFU | SUFU negative regulator of hedgehog signaling [Source:HGNC Symbol;Acc:HGNC:16466] |
| ENSG00000108255 | HGLibA_11253 | 58 | -0.571441788 | CRYBA1 | crystallin beta A1 [Source:HGNC Symbol;Acc:HGNC:2394] |
| ENSG00000109066 | HGLibA_49847 | 58 | -0.571441788 | TMEM104 | transmembrane protein 104 [Source:HGNC Symbol;Acc:HGNC:25984] |
| ENSG00000109819 | HGLibA_37846 | 58 | -0.571441788 | PPARGC1A | PPARG coactivator 1 alpha [Source:HGNC Symbol;Acc:HGNC:9237] |
| ENSG00000110171 | HGLibA_51455 | 58 | -0.571441788 | TRIM3 | tripartite motif containing 3 [Source:HGNC Symbol;Acc:HGNC:10064] |
| ENSG00000110723 | HGLibA_15798 | 58 | -0.571441788 | EXPH5 | exophilin 5 [Source:HGNC Symbol;Acc:HGNC:30578] |
| ENSG00000111728 | HGLibA_47287 | 58 | -0.571441788 | ST8SIA1 | 8-sialyltransferase 1 [Source:HGNC Symbol;Acc:HGNC:10869]" |
| ENSG00000111786 | HGLibA_47094 | 58 | -0.571441788 | SRSF9 | serine and arginine rich splicing factor 9 [Source:HGNC Symbol;Acc:HGNC:10791] |
| ENSG00000111846 | HGLibA_18813 | 58 | -0.571441788 | GCNT2 | glucosaminyl (N-acetyl) transferase 2 (I blood group) [Source:HGNC Symbol;Acc:HGNC:4204] |
| ENSG00000112473 | HGLibA_45121 | 58 | -0.571441788 | SLC39A7 | solute carrier family 39 member 7 [Source:HGNC Symbol;Acc:HGNC:4927] |
| ENSG00000113318 | HGLibA_30192 | 58 | -0.571441788 | MSH3 | mutS homolog 3 [Source:HGNC Symbol;Acc:HGNC:7326] |
| ENSG00000113742 | HGLibA_10925 | 58 | -0.571441788 | CPEB4 | cytoplasmic polyadenylation element binding protein 4 [Source:HGNC Symbol;Acc:HGNC:21747] |
| ENSG00000113924 | HGLibA_21236 | 58 | -0.571441788 | HGD | 2-dioxygenase [Source:HGNC Symbol;Acc:HGNC:4892]" |
| ENSG00000115183 | HGLibA_48292 | 58 | -0.571441788 | TANC1 | ankyrin repeat and coiled-coil containing 1 [Source:HGNC Symbol;Acc:HGNC:29364]" |
| ENSG00000115252 | HGLibA_35770 | 58 | -0.571441788 | PDE1A | phosphodiesterase 1A [Source:HGNC Symbol;Acc:HGNC:8774] |
| ENSG00000115762 | HGLibA_37089 | 58 | -0.571441788 | PLEKHB2 | pleckstrin homology domain containing B2 [Source:HGNC Symbol;Acc:HGNC:19236] |
| ENSG00000116001 | HGLibA_49429 | 58 | -0.571441788 | TIA1 | TIA1 cytotoxic granule associated RNA binding protein [Source:HGNC Symbol;Acc:HGNC:11802] |
| ENSG00000116096 | HGLibA_46786 | 58 | -0.571441788 | SPR | sepiapterin reductase [Source:HGNC Symbol;Acc:HGNC:11257] |
| ENSG00000116729 | HGLibA_54332 | 58 | -0.571441788 | WLS | wntless Wnt ligand secretion mediator [Source:HGNC Symbol;Acc:HGNC:30238] |
| ENSG00000117298 | HGLibA_14383 | 58 | -0.571441788 | ECE1 | endothelin converting enzyme 1 [Source:HGNC Symbol;Acc:HGNC:3146] |
| ENSG00000119559 | HGLibA_05643 | 58 | -0.571441788 | C19orf25 | chromosome 19 open reading frame 25 [Source:HGNC Symbol;Acc:HGNC:26711] |
| ENSG00000121716 | HGLibA_36673 | 58 | -0.571441788 | PILRB | paired immunoglobin-like type 2 receptor beta [Source:HGNC Symbol;Acc:HGNC:18297] |
| ENSG00000122375 | HGLibA_33437 | 58 | -0.571441788 | OPN4 | opsin 4 [Source:HGNC Symbol;Acc:HGNC:14449] |
| ENSG00000122691 | HGLibA_52375 | 58 | -0.571441788 | TWIST1 | twist family bHLH transcription factor 1 [Source:HGNC Symbol;Acc:HGNC:12428] |
| ENSG00000123297 | HGLibA_51841 | 58 | -0.571441788 | TSFM | mitochondrial [Source:HGNC Symbol;Acc:HGNC:12367]" |
| ENSG00000123838 | HGLibA_06291 | 58 | -0.571441788 | C4BPA | complement component 4 binding protein alpha [Source:HGNC Symbol;Acc:HGNC:1325] |
| ENSG00000124508 | HGLibA_04966 | 58 | -0.571441788 | BTN2A2 | butyrophilin subfamily 2 member A2 [Source:HGNC Symbol;Acc:HGNC:1137] |
| ENSG00000126756 | HGLibA_53483 | 58 | -0.571441788 | UXT | ubiquitously expressed prefoldin like chaperone [Source:HGNC Symbol;Acc:HGNC:12641] |
| ENSG00000129566 | HGLibA_49006 | 58 | -0.571441788 | TEP1 | telomerase associated protein 1 [Source:HGNC Symbol;Acc:HGNC:11726] |
| ENSG00000130203 | HGLibA_02519 | 58 | -0.571441788 | APOE | apolipoprotein E [Source:HGNC Symbol;Acc:HGNC:613] |
| ENSG00000130649 | HGLibA_12130 | 58 | -0.571441788 | CYP2E1 | cytochrome P450 family 2 subfamily E member 1 [Source:HGNC Symbol;Acc:HGNC:2631] |
| ENSG00000131264 | HGLibA_08901 | 58 | -0.571441788 | CDX4 | caudal type homeobox 4 [Source:HGNC Symbol;Acc:HGNC:1808] |
| ENSG00000131470 | HGLibA_39136 | 58 | -0.571441788 | PSMC3IP | PSMC3 interacting protein [Source:HGNC Symbol;Acc:HGNC:17928] |
| ENSG00000132005 | HGLibA_40961 | 58 | -0.571441788 | RFX1 | regulatory factor X1 [Source:HGNC Symbol;Acc:HGNC:9982] |
| ENSG00000132164 | HGLibA_45293 | 58 | -0.571441788 | SLC6A11 | solute carrier family 6 member 11 [Source:HGNC Symbol;Acc:HGNC:11044] |
| ENSG00000132275 | HGLibA_42284 | 58 | -0.571441788 | RRP8 | ribosomal RNA processing 8 [Source:HGNC Symbol;Acc:HGNC:29030] |
| ENSG00000132386 | HGLibA_43585 | 58 | -0.571441788 | SERPINF1 | serpin family F member 1 [Source:HGNC Symbol;Acc:HGNC:8824] |
| ENSG00000132470 | HGLibA_23788 | 58 | -0.571441788 | ITGB4 | integrin subunit beta 4 [Source:HGNC Symbol;Acc:HGNC:6158] |
| ENSG00000132563 | HGLibA_40779 | 58 | -0.571441788 | REEP2 | receptor accessory protein 2 [Source:HGNC Symbol;Acc:HGNC:17975] |
| ENSG00000134240 | HGLibA_21715 | 58 | -0.571441788 | HMGCS2 | 3-hydroxy-3-methylglutaryl-CoA synthase 2 [Source:HGNC Symbol;Acc:HGNC:5008] |
| ENSG00000134333 | HGLibA_26221 | 58 | -0.571441788 | LDHA | lactate dehydrogenase A [Source:HGNC Symbol;Acc:HGNC:6535] |
| ENSG00000134508 | HGLibA_06842 | 58 | -0.571441788 | CABLES1 | Cdk5 and Abl enzyme substrate 1 [Source:HGNC Symbol;Acc:HGNC:25097] |
| ENSG00000134987 | HGLibA_54075 | 58 | -0.571441788 | WDR36 | WD repeat domain 36 [Source:HGNC Symbol;Acc:HGNC:30696] |
| ENSG00000136451 | HGLibA_53625 | 58 | -0.571441788 | VEZF1 | vascular endothelial zinc finger 1 [Source:HGNC Symbol;Acc:HGNC:12949] |
| ENSG00000136982 | HGLibA_14005 | 58 | -0.571441788 | DSCC1 | DNA replication and sister chromatid cohesion 1 [Source:HGNC Symbol;Acc:HGNC:24453] |
| ENSG00000137261 | HGLibA_24621 | 58 | -0.571441788 | KIAA0319 | KIAA0319 [Source:HGNC Symbol;Acc:HGNC:21580] |
| ENSG00000138078 | HGLibA_38441 | 58 | -0.571441788 | PREPL | prolyl endopeptidase like [Source:HGNC Symbol;Acc:HGNC:30228] |
| ENSG00000138600 | HGLibA_46774 | 58 | -0.571441788 | SPPL2A | signal peptide peptidase like 2A [Source:HGNC Symbol;Acc:HGNC:30227] |
| ENSG00000138606 | HGLibA_44024 | 58 | -0.571441788 | SHF | Src homology 2 domain containing F [Source:HGNC Symbol;Acc:HGNC:25116] |
| ENSG00000138759 | HGLibA_17974 | 58 | -0.571441788 | FRAS1 | Fraser extracellular matrix complex subunit 1 [Source:HGNC Symbol;Acc:HGNC:19185] |
| ENSG00000139579 | HGLibA_31028 | 58 | -0.571441788 | NABP2 | nucleic acid binding protein 2 [Source:HGNC Symbol;Acc:HGNC:28412] |
| ENSG00000140057 | HGLibA_01394 | 58 | -0.571441788 | AK7 | adenylate kinase 7 [Source:HGNC Symbol;Acc:HGNC:20091] |
| ENSG00000140937 | HGLibA_08634 | 58 | -0.571441788 | CDH11 | cadherin 11 [Source:HGNC Symbol;Acc:HGNC:1750] |
| ENSG00000141012 | HGLibA_18524 | 58 | -0.571441788 | GALNS | galactosamine (N-acetyl)-6-sulfatase [Source:HGNC Symbol;Acc:HGNC:4122] |
| ENSG00000141026 | HGLibA_28821 | 58 | -0.571441788 | MED9 | mediator complex subunit 9 [Source:HGNC Symbol;Acc:HGNC:25487] |
| ENSG00000141034 | HGLibA_19055 | 58 | -0.571441788 | GID4 | GID complex subunit 4 homolog [Source:HGNC Symbol;Acc:HGNC:28453] |
| ENSG00000141198 | HGLibA_50950 | 58 | -0.571441788 | TOM1L1 | target of myb1 like 1 membrane trafficking protein [Source:HGNC Symbol;Acc:HGNC:11983] |
| ENSG00000141639 | HGLibA_28274 | 58 | -0.571441788 | MAPK4 | mitogen-activated protein kinase 4 [Source:HGNC Symbol;Acc:HGNC:6878] |
| ENSG00000142623 | HGLibA_34970 | 58 | -0.571441788 | PADI1 | peptidyl arginine deiminase 1 [Source:HGNC Symbol;Acc:HGNC:18367] |
| ENSG00000142731 | HGLibA_37198 | 58 | -0.571441788 | PLK4 | polo like kinase 4 [Source:HGNC Symbol;Acc:HGNC:11397] |
| ENSG00000143156 | HGLibA_32173 | 58 | -0.571441788 | NME7 | NME/NM23 family member 7 [Source:HGNC Symbol;Acc:HGNC:20461] |
| ENSG00000143256 | HGLibA_36142 | 58 | -0.571441788 | PFDN2 | prefoldin subunit 2 [Source:HGNC Symbol;Acc:HGNC:8867] |
| ENSG00000143748 | HGLibA_33121 | 58 | -0.571441788 | NVL | nuclear VCP-like [Source:HGNC Symbol;Acc:HGNC:8070] |
| ENSG00000143924 | HGLibA_15066 | 58 | -0.571441788 | EML4 | echinoderm microtubule associated protein like 4 [Source:HGNC Symbol;Acc:HGNC:1316] |
| ENSG00000144357 | HGLibA_52752 | 58 | -0.571441788 | UBR3 | ubiquitin protein ligase E3 component n-recognin 3 [Source:HGNC Symbol;Acc:HGNC:30467] |
| ENSG00000145414 | HGLibA_31061 | 58 | -0.571441788 | NAF1 | nuclear assembly factor 1 ribonucleoprotein [Source:HGNC Symbol;Acc:HGNC:25126] |
| ENSG00000146938 | HGLibA_32076 | 58 | -0.571441788 | NLGN4X | neuroligin 4 X-linked [Source:HGNC Symbol;Acc:HGNC:14287] |
| ENSG00000147955 | HGLibA_44160 | 58 | -0.571441788 | SIGMAR1 | sigma non-opioid intracellular receptor 1 [Source:HGNC Symbol;Acc:HGNC:8157] |
| ENSG00000148488 | HGLibA_47303 | 58 | -0.571441788 | ST8SIA6 | 8-sialyltransferase 6 [Source:HGNC Symbol;Acc:HGNC:23317]" |
| ENSG00000148803 | HGLibA_18169 | 58 | -0.571441788 | FUOM | fucose mutarotase [Source:HGNC Symbol;Acc:HGNC:24733] |
| ENSG00000148835 | HGLibA_48240 | 58 | -0.571441788 | TAF5 | TATA-box binding protein associated factor 5 [Source:HGNC Symbol;Acc:HGNC:11539] |
| ENSG00000149131 | HGLibA_43591 | 58 | -0.571441788 | SERPING1 | serpin family G member 1 [Source:HGNC Symbol;Acc:HGNC:1228] |
| ENSG00000149930 | HGLibA_48308 | 58 | -0.571441788 | TAOK2 | TAO kinase 2 [Source:HGNC Symbol;Acc:HGNC:16835] |
| ENSG00000150051 | HGLibA_29360 | 58 | -0.571441788 | MKX | mohawk homeobox [Source:HGNC Symbol;Acc:HGNC:23729] |
| ENSG00000150773 | HGLibA_36612 | 58 | -0.571441788 | PIH1D2 | PIH1 domain containing 2 [Source:HGNC Symbol;Acc:HGNC:25210] |
| ENSG00000151224 | HGLibA_28416 | 58 | -0.571441788 | MAT1A | methionine adenosyltransferase 1A [Source:HGNC Symbol;Acc:HGNC:6903] |
| ENSG00000151338 | HGLibA_29297 | 58 | -0.571441788 | MIPOL1 | mirror-image polydactyly 1 [Source:HGNC Symbol;Acc:HGNC:21460] |
| ENSG00000151690 | HGLibA_29109 | 58 | -0.571441788 | MFSD6 | major facilitator superfamily domain containing 6 [Source:HGNC Symbol;Acc:HGNC:24711] |
| ENSG00000153246 | HGLibA_36927 | 58 | -0.571441788 | PLA2R1 | phospholipase A2 receptor 1 [Source:HGNC Symbol;Acc:HGNC:9042] |
| ENSG00000153790 | HGLibA_06534 | 58 | -0.571441788 | C7orf31 | chromosome 7 open reading frame 31 [Source:HGNC Symbol;Acc:HGNC:21722] |
| ENSG00000153904 | HGLibA_12589 | 58 | -0.571441788 | DDAH1 | dimethylarginine dimethylaminohydrolase 1 [Source:HGNC Symbol;Acc:HGNC:2715] |
| ENSG00000154978 | HGLibA_53715 | 58 | -0.571441788 | VOPP1 | WBP1/VOPP1 family member [Source:HGNC Symbol;Acc:HGNC:34518]" |
| ENSG00000155269 | HGLibA_20086 | 58 | -0.571441788 | GPR78 | G protein-coupled receptor 78 [Source:HGNC Symbol;Acc:HGNC:4528] |
| ENSG00000155508 | HGLibA_10354 | 58 | -0.571441788 | CNOT8 | CCR4-NOT transcription complex subunit 8 [Source:HGNC Symbol;Acc:HGNC:9207] |
| ENSG00000157330 | HGLibA_05780 | 58 | -0.571441788 | C1orf158 | chromosome 1 open reading frame 158 [Source:HGNC Symbol;Acc:HGNC:28567] |
| ENSG00000158050 | HGLibA_14158 | 58 | -0.571441788 | DUSP2 | dual specificity phosphatase 2 [Source:HGNC Symbol;Acc:HGNC:3068] |
| ENSG00000159588 | HGLibA_07687 | 58 | -0.571441788 | CCDC17 | coiled-coil domain containing 17 [Source:HGNC Symbol;Acc:HGNC:26574] |
| ENSG00000159871 | HGLibA_27725 | 58 | -0.571441788 | LYPD5 | LY6/PLAUR domain containing 5 [Source:HGNC Symbol;Acc:HGNC:26397] |
| ENSG00000162298 | HGLibA_48110 | 58 | -0.571441788 | SYVN1 | synoviolin 1 [Source:HGNC Symbol;Acc:HGNC:20738] |
| ENSG00000162869 | HGLibA_38076 | 58 | -0.571441788 | PPP1R21 | protein phosphatase 1 regulatory subunit 21 [Source:HGNC Symbol;Acc:HGNC:30595] |
| ENSG00000162989 | HGLibA_24276 | 58 | -0.571441788 | KCNJ3 | potassium voltage-gated channel subfamily J member 3 [Source:HGNC Symbol;Acc:HGNC:6264] |
| ENSG00000163166 | HGLibA_23885 | 58 | -0.571441788 | IWS1 | SUPT6H interacting protein [Source:HGNC Symbol;Acc:HGNC:25467]" |
| ENSG00000163501 | HGLibA_22909 | 58 | -0.571441788 | IHH | indian hedgehog [Source:HGNC Symbol;Acc:HGNC:5956] |
| ENSG00000164610 | HGLibA_41797 | 58 | -0.571441788 | RP9 | pre-mRNA splicing factor [Source:HGNC Symbol;Acc:HGNC:10288]" |
| ENSG00000165029 | HGLibA_00096 | 58 | -0.571441788 | ABCA1 | ATP binding cassette subfamily A member 1 [Source:HGNC Symbol;Acc:HGNC:29] |
| ENSG00000165682 | HGLibA_09957 | 58 | -0.571441788 | CLEC1B | C-type lectin domain family 1 member B [Source:HGNC Symbol;Acc:HGNC:24356] |
| ENSG00000166181 | HGLibA_02420 | 58 | -0.571441788 | API5 | apoptosis inhibitor 5 [Source:HGNC Symbol;Acc:HGNC:594] |
| ENSG00000166261 | HGLibA_55572 | 58 | -0.571441788 | ZNF202 | zinc finger protein 202 [Source:HGNC Symbol;Acc:HGNC:12994] |
| ENSG00000166263 | HGLibA_47673 | 58 | -0.571441788 | STXBP4 | syntaxin binding protein 4 [Source:HGNC Symbol;Acc:HGNC:19694] |
| ENSG00000166704 | HGLibA_56368 | 58 | -0.571441788 | ZNF606 | zinc finger protein 606 [Source:HGNC Symbol;Acc:HGNC:25879] |
| ENSG00000166959 | HGLibA_30167 | 58 | -0.571441788 | MS4A8 | membrane spanning 4-domains A8 [Source:HGNC Symbol;Acc:HGNC:13380] |
| ENSG00000167508 | HGLibA_30596 | 58 | -0.571441788 | MVD | mevalonate diphosphate decarboxylase [Source:HGNC Symbol;Acc:HGNC:7529] |
| ENSG00000167548 | HGLibA_25330 | 58 | -0.571441788 | KMT2D | lysine methyltransferase 2D [Source:HGNC Symbol;Acc:HGNC:7133] |

| | | | | | |
|---|---|---|---|---|---|
| ENSG00000167653 | HGLibA_39010 | 58 | -0.571441788 | PSCA | prostate stem cell antigen [Source:HGNC Symbol;Acc:HGNC:9500] |
| ENSG00000167705 | HGLibA_41251 | 58 | -0.571441788 | RILP | Rab interacting lysosomal protein [Source:HGNC Symbol;Acc:HGNC:30266] |
| ENSG00000167770 | HGLibA_34752 | 58 | -0.571441788 | OTUB1 | ubiquitin aldehyde binding 1 [Source:HGNC Symbol;Acc:HGNC:23077]" |
| ENSG00000169435 | HGLibA_40389 | 58 | -0.571441788 | RASSF6 | Ras association domain family member 6 [Source:HGNC Symbol;Acc:HGNC:20796] |
| ENSG00000170035 | HGLibA_52599 | 58 | -0.571441788 | UBE2E3 | ubiquitin conjugating enzyme E2 E3 [Source:HGNC Symbol;Acc:HGNC:12479] |
| ENSG00000170929 | HGLibA_33700 | 58 | -0.571441788 | OR1M1 | olfactory receptor family 1 subfamily M member 1 [Source:HGNC Symbol;Acc:HGNC:8220] |
| ENSG00000171944 | HGLibA_34141 | 58 | -0.571441788 | OR52A5 | olfactory receptor family 52 subfamily A member 5 [Source:HGNC Symbol;Acc:HGNC:19580] |
| ENSG00000172530 | HGLibA_04148 | 58 | -0.571441788 | BANP | BTG3 associated nuclear protein [Source:HGNC Symbol;Acc:HGNC:13450] |
| ENSG00000173442 | HGLibA_14662 | 58 | -0.571441788 | EHBP1L1 | EH domain binding protein 1 like 1 [Source:HGNC Symbol;Acc:HGNC:30682] |
| ENSG00000173486 | HGLibA_17570 | 58 | -0.571441788 | FKBP2 | FK506 binding protein 2 [Source:HGNC Symbol;Acc:HGNC:3718] |
| ENSG00000173762 | HGLibA_08436 | 58 | -0.571441788 | CD7 | CD7 molecule [Source:HGNC Symbol;Acc:HGNC:1695] |
| ENSG00000174943 | HGLibA_24422 | 58 | -0.571441788 | KCTD13 | potassium channel tetramerization domain containing 13 [Source:HGNC Symbol;Acc:HGNC:22234] |
| ENSG00000176294 | HGLibA_34047 | 58 | -0.571441788 | OR4N2 | olfactory receptor family 4 subfamily N member 2 [Source:HGNC Symbol;Acc:HGNC:14742] |
| ENSG00000177370 | HGLibA_49511 | 58 | -0.571441788 | TIMM22 | translocase of inner mitochondrial membrane 22 [Source:HGNC Symbol;Acc:HGNC:17317] |
| ENSG00000177830 | HGLibA_09451 | 58 | -0.571441788 | CHID1 | chitinase domain containing 1 [Source:HGNC Symbol;Acc:HGNC:28474] |
| ENSG00000178726 | HGLibA_49318 | 58 | -0.571441788 | THBD | thrombomodulin [Source:HGNC Symbol;Acc:HGNC:11784] |
| ENSG00000179772 | HGLibA_17956 | 58 | -0.571441788 | FOXS1 | forkhead box S1 [Source:HGNC Symbol;Acc:HGNC:3735] |
| ENSG00000180061 | HGLibA_49993 | 58 | -0.571441788 | TMEM150B | transmembrane protein 150B [Source:HGNC Symbol;Acc:HGNC:34415] |
| ENSG00000180398 | HGLibA_28586 | 58 | -0.571441788 | MCFD2 | multiple coagulation factor deficiency 2 [Source:HGNC Symbol;Acc:HGNC:18451] |
| ENSG00000180611 | HGLibA_28461 | 58 | -0.571441788 | MB21D2 | Mab-21 domain containing 2 [Source:HGNC Symbol;Acc:HGNC:30438] |
| ENSG00000180644 | HGLibA_38450 | 58 | -0.571441788 | PRF1 | perforin 1 [Source:HGNC Symbol;Acc:HGNC:9360] |
| ENSG00000180891 | HGLibA_11767 | 58 | -0.571441788 | CUEDC1 | CUE domain containing 1 [Source:HGNC Symbol;Acc:HGNC:31350] |
| ENSG00000181552 | HGLibA_14450 | 58 | -0.571441788 | EDDM3B | epididymal protein 3B [Source:HGNC Symbol;Acc:HGNC:19223] |
| ENSG00000182010 | HGLibA_42392 | 58 | -0.571441788 | RTKN2 | rhotekin 2 [Source:HGNC Symbol;Acc:HGNC:19364] |
| ENSG00000182208 | HGLibA_29590 | 58 | -0.571441788 | MOB2 | MOB kinase activator 2 [Source:HGNC Symbol;Acc:HGNC:24904] |
| ENSG00000182330 | HGLibA_38328 | 58 | -0.571441788 | PRAMEF8 | PRAME family member 8 [Source:HGNC Symbol;Acc:HGNC:24074] |
| ENSG00000183023 | HGLibA_45389 | 58 | -0.571441788 | SLC8A1 | solute carrier family 8 member A1 [Source:HGNC Symbol;Acc:HGNC:11068] |
| ENSG00000183032 | HGLibA_44672 | 58 | -0.571441788 | SLC25A21 | solute carrier family 25 member 21 [Source:HGNC Symbol;Acc:HGNC:14411] |
| ENSG00000184307 | HGLibA_55108 | 58 | -0.571441788 | ZDHHC23 | zinc finger DHHC-type containing 23 [Source:HGNC Symbol;Acc:HGNC:28654] |
| ENSG00000184381 | HGLibA_36920 | 58 | -0.571441788 | PLA2G6 | phospholipase A2 group VI [Source:HGNC Symbol;Acc:HGNC:9039] |
| ENSG00000184517 | HGLibA_55179 | 58 | -0.571441788 | ZFP1 | ZFP1 zinc finger protein [Source:HGNC Symbol;Acc:HGNC:23328] |
| ENSG00000184661 | HGLibA_08603 | 58 | -0.571441788 | CDCA2 | cell division cycle associated 2 [Source:HGNC Symbol;Acc:HGNC:14623] |
| ENSG00000184719 | HGLibA_41714 | 58 | -0.571441788 | RNLS | FAD dependent amine oxidase [Source:HGNC Symbol;Acc:HGNC:25641]" |
| ENSG00000185053 | HGLibA_43810 | 58 | -0.571441788 | SGCZ | sarcoglycan zeta [Source:HGNC Symbol;Acc:HGNC:14075] |
| ENSG00000185420 | HGLibA_45856 | 58 | -0.571441788 | SMYD3 | SET and MYND domain containing 3 [Source:HGNC Symbol;Acc:HGNC:15513] |
| ENSG00000185507 | HGLibA_23597 | 58 | -0.571441788 | IRF7 | interferon regulatory factor 7 [Source:HGNC Symbol;Acc:HGNC:6122] |
| ENSG00000185567 | HGLibA_01303 | 58 | -0.571441788 | AHNAK2 | AHNAK nucleoprotein 2 [Source:HGNC Symbol;Acc:HGNC:20125] |
| ENSG00000186197 | HGLibA_14438 | 58 | -0.571441788 | EDARADD | EDAR associated death domain [Source:HGNC Symbol;Acc:HGNC:14341] |
| ENSG00000186329 | HGLibA_50209 | 58 | -0.571441788 | TMEM212 | transmembrane protein 212 [Source:HGNC Symbol;Acc:HGNC:34295] |
| ENSG00000186795 | HGLibA_24315 | 58 | -0.571441788 | KCNK18 | potassium two pore domain channel subfamily K member 18 [Source:HGNC Symbol;Acc:HGNC:19439] |
| ENSG00000186806 | HGLibA_53842 | 58 | -0.571441788 | VSIG10L | V-set and immunoglobulin domain containing 10 like [Source:HGNC Symbol;Acc:HGNC:27111] |
| ENSG00000187257 | HGLibA_42305 | 58 | -0.571441788 | RSBN1L | round spermatid basic protein 1 like [Source:HGNC Symbol;Acc:HGNC:24765] |
| ENSG00000187682 | HGLibA_15410 | 58 | -0.571441788 | ERAS | ES cell expressed Ras [Source:HGNC Symbol;Acc:HGNC:5174] |
| ENSG00000188133 | HGLibA_50220 | 58 | -0.571441788 | TMEM215 | transmembrane protein 215 [Source:HGNC Symbol;Acc:HGNC:33816] |
| ENSG00000196341 | HGLibA_34507 | 58 | -0.571441788 | OR8D1 | olfactory receptor family 8 subfamily D member 1 [Source:HGNC Symbol;Acc:HGNC:8481] |
| ENSG00000196475 | HGLibA_19200 | 58 | -0.571441788 | GK2 | glycerol kinase 2 [Source:HGNC Symbol;Acc:HGNC:4291] |
| ENSG00000196510 | HGLibA_01890 | 58 | -0.571441788 | ANAPC7 | anaphase promoting complex subunit 7 [Source:HGNC Symbol;Acc:HGNC:17380] |
| ENSG00000196923 | HGLibA_35922 | 58 | -0.571441788 | PDLIM7 | PDZ and LIM domain 7 [Source:HGNC Symbol;Acc:HGNC:22958] |
| ENSG00000197728 | HGLibA_42118 | 58 | -0.571441788 | RPS26 | ribosomal protein S26 [Source:HGNC Symbol;Acc:HGNC:10414] |
| ENSG00000197863 | HGLibA_56764 | 58 | -0.571441788 | ZNF790 | zinc finger protein 790 [Source:HGNC Symbol;Acc:HGNC:33114] |
| ENSG00000204849 | HGLibA_46469 | 58 | -0.571441788 | SPATA31A1 | SPATA31 subfamily A member 1 [Source:HGNC Symbol;Acc:HGNC:23394] |
| ENSG00000204869 | HGLibA_22847 | 58 | -0.571441788 | IGFL4 | IGF like family member 4 [Source:HGNC Symbol;Acc:HGNC:32931] |
| ENSG00000213689 | HGLibA_51372 | 58 | -0.571441788 | TREX1 | three prime repair exonuclease 1 [Source:HGNC Symbol;Acc:HGNC:12269] |
| ENSG00000214946 | HGLibA_48517 | 58 | -0.571441788 | TBC1D26 | TBC1 domain family member 26 [Source:HGNC Symbol;Acc:HGNC:28745] |
| ENSG00000215193 | HGLibA_36112 | 58 | -0.571441788 | PEX26 | peroxisomal biogenesis factor 26 [Source:HGNC Symbol;Acc:HGNC:22965] |
| ENSG00000223417 | HGLibA_51532 | 58 | -0.571441788 | TRIM49D1 | tripartite motif containing 49D1 [Source:HGNC Symbol;Acc:HGNC:43973] |
| ENSG00000231051 | HGLibA_53267 | 58 | -0.571441788 | USP17L28 | ubiquitin specific peptidase 17-like family member 28 [Source:HGNC Symbol;Acc:HGNC:44456] |
| ENSG00000233412 | HGLibA_34295 | 58 | -0.571441788 | OR5H15 | olfactory receptor family 5 subfamily H member 15 [Source:HGNC Symbol;Acc:HGNC:31287] |
| ENSG00000234602 | HGLibA_28596 | 58 | -0.571441788 | MCIDAS | multiciliate differentiation and DNA synthesis associated cell cycle protein [Source:HGNC Symbol;Acc:HGNC:40050] |
| ENSG00000235631 | HGLibA_41538 | 58 | -0.571441788 | RNF148 | ring finger protein 148 [Source:HGNC Symbol;Acc:HGNC:22411] |
| ENSG00000239839 | HGLibA_12798 | 58 | -0.571441788 | DEFA3 | defensin alpha 3 [Source:HGNC Symbol;Acc:HGNC:2762] |
| ENSG00000249811 | HGLibA_53248 | 58 | -0.571441788 | USP17L21 | ubiquitin specific peptidase 17-like family member 21 [Source:HGNC Symbol;Acc:HGNC:44449] |
| ENSG00000261221 | HGLibA_56884 | 58 | -0.571441788 | ZNF865 | zinc finger protein 865 [Source:HGNC Symbol;Acc:HGNC:38705] |
| ENSG00000266074 | HGLibA_04106 | 58 | -0.571441788 | BAHCC1 | BAH domain and coiled-coil containing 1 [Source:HGNC Symbol;Acc:HGNC:29279] |
| ENSG00000280204 | HGLibA_33713 | 58 | -0.571441788 | OR1S1 | olfactory receptor family 1 subfamily S member 1 (gene/pseudogene) [Source:HGNC Symbol;Acc:HGNC:8227] |
| GPR75-ASB3 | HGLibA_20082 | 58 | -0.571441788 | GPR75-ASB3 | - |
| HIST1H3I | HGLibA_21481 | 58 | -0.571441788 | HIST1H3I | - |
| hsa-mir-1252 | HGLibA_57405 | 58 | -0.571441788 | hsa-mir-1252 | - |
| hsa-mir-1283-2 | HGLibA_57593 | 58 | -0.571441788 | hsa-mir-1283-2 | - |
| hsa-mir-1286 | HGLibA_57608 | 58 | -0.571441788 | hsa-mir-1286 | - |
| hsa-mir-130b | HGLibA_57751 | 58 | -0.571441788 | hsa-mir-130b | - |
| hsa-mir-3116-2 | HGLibA_58508 | 58 | -0.571441788 | hsa-mir-3116-2 | - |
| hsa-mir-320b-2 | HGLibA_58931 | 58 | -0.571441788 | hsa-mir-320b-2 | - |
| hsa-mir-330 | HGLibA_58989 | 58 | -0.571441788 | hsa-mir-330 | - |
| hsa-mir-338 | HGLibA_59003 | 58 | -0.571441788 | hsa-mir-338 | - |
| hsa-mir-3689d-2 | HGLibA_59330 | 58 | -0.571441788 | hsa-mir-3689d-2 | - |
| hsa-mir-378d-1 | HGLibA_59437 | 58 | -0.571441788 | hsa-mir-378d-1 | - |
| hsa-mir-3960 | HGLibA_59642 | 58 | -0.571441788 | hsa-mir-3960 | - |
| hsa-mir-4305 | HGLibA_59933 | 58 | -0.571441788 | hsa-mir-4305 | - |
| hsa-mir-4453 | HGLibA_60220 | 58 | -0.571441788 | hsa-mir-4453 | - |
| hsa-mir-4491 | HGLibA_60384 | 58 | -0.571441788 | hsa-mir-4491 | - |
| hsa-mir-4536-2 | HGLibA_60605 | 58 | -0.571441788 | hsa-mir-4536-2 | - |
| hsa-mir-4727 | HGLibA_61004 | 58 | -0.571441788 | hsa-mir-4727 | - |
| hsa-mir-4744 | HGLibA_61075 | 58 | -0.571441788 | hsa-mir-4744 | - |
| hsa-mir-4762 | HGLibA_61145 | 58 | -0.571441788 | hsa-mir-4762 | - |
| hsa-mir-488 | HGLibA_61353 | 58 | -0.571441788 | hsa-mir-488 | - |
| hsa-mir-489 | HGLibA_61356 | 58 | -0.571441788 | hsa-mir-489 | - |
| hsa-mir-5089 | HGLibA_61503 | 58 | -0.571441788 | hsa-mir-5089 | - |
| hsa-mir-517c | HGLibA_61631 | 58 | -0.571441788 | hsa-mir-517c | - |
| hsa-mir-518c | HGLibA_61664 | 58 | -0.571441788 | hsa-mir-518c | - |
| hsa-mir-548an | HGLibA_61903 | 58 | -0.571441788 | hsa-mir-548an | - |
| hsa-mir-5692a-1 | HGLibA_62286 | 58 | -0.571441788 | hsa-mir-5692a-1 | - |
| hsa-mir-593 | HGLibA_62470 | 58 | -0.571441788 | hsa-mir-593 | - |
| hsa-mir-6078 | HGLibA_62568 | 58 | -0.571441788 | hsa-mir-6078 | - |
| hsa-mir-6124 | HGLibA_62645 | 58 | -0.571441788 | hsa-mir-6124 | - |
| hsa-mir-638 | HGLibA_62793 | 58 | -0.571441788 | hsa-mir-638 | - |
| hsa-mir-6511a-3 | HGLibA_62906 | 58 | -0.571441788 | hsa-mir-6511a-3 | - |
| hsa-mir-655 | HGLibA_62952 | 58 | -0.571441788 | hsa-mir-655 | - |
| hsa-mir-6716 | HGLibA_63025 | 58 | -0.571441788 | hsa-mir-6716 | - |
| hsa-mir-6787 | HGLibA_63326 | 58 | -0.571441788 | hsa-mir-6787 | - |
| hsa-mir-6885 | HGLibA_63726 | 58 | -0.571441788 | hsa-mir-6885 | - |
| hsa-mir-7109 | HGLibA_63796 | 58 | -0.571441788 | hsa-mir-7109 | - |
| LEPRE1 | HGLibA_26316 | 58 | -0.571441788 | LEPRE1 | - |
| LOC100131094 | HGLibA_26801 | 58 | -0.571441788 | LOC100131094 | - |
| PAK7 | HGLibA_35055 | 58 | -0.571441788 | PAK7 | - |
| PNMAL2 | HGLibA_37388 | 58 | -0.571441788 | PNMAL2 | - |
| PVRL3 | HGLibA_39629 | 58 | -0.571441788 | PVRL3 | - |
| TCP10 | HGLibA_48834 | 58 | -0.571441788 | TCP10 | - |
| WHSC1L1 | HGLibA_54295 | 58 | -0.571441788 | WHSC1L1 | - |
| 15-Sep | HGLibA_43409 | 57 | -0.581392826 | 15-Sep | - |
| C12orf77 | HGLibA_05302 | 57 | -0.581392826 | C12orf77 | - |
| C17orf51 | HGLibA_05542 | 57 | -0.581392826 | C17orf51 | - |
| C17orf74 | HGLibA_05569 | 57 | -0.581392826 | C17orf74 | - |
| C19orf40 | HGLibA_05656 | 57 | -0.581392826 | C19orf40 | - |
| CCBP2 | HGLibA_07503 | 57 | -0.581392826 | CCBP2 | - |
| ENSG00000004864 | HGLibA_44644 | 57 | -0.581392826 | SLC25A13 | solute carrier family 25 member 13 [Source:HGNC Symbol;Acc:HGNC:10983] |
| ENSG00000005513 | HGLibA_46277 | 57 | -0.581392826 | SOX8 | SRY-box 8 [Source:HGNC Symbol;Acc:HGNC:11203] |
| ENSG00000006016 | HGLibA_11186 | 57 | -0.581392826 | CRLF1 | cytokine receptor like factor 1 [Source:HGNC Symbol;Acc:HGNC:2364] |
| ENSG00000007216 | HGLibA_44386 | 57 | -0.581392826 | SLC13A2 | solute carrier family 13 member 2 [Source:HGNC Symbol;Acc:HGNC:10917] |
| ENSG00000008130 | HGLibA_31045 | 57 | -0.581392826 | NADK | NAD kinase [Source:HGNC Symbol;Acc:HGNC:29831] |
| ENSG00000008300 | HGLibA_09029 | 57 | -0.581392826 | CELSR3 | cadherin EGF LAG seven-pass G-type receptor 3 [Source:HGNC Symbol;Acc:HGNC:3230] |
| ENSG00000013293 | HGLibA_45357 | 57 | -0.581392826 | SLC7A14 | solute carrier family 7 member 14 [Source:HGNC Symbol;Acc:HGNC:29326] |
| ENSG00000023445 | HGLibA_04518 | 57 | -0.581392826 | BIRC3 | baculoviral IAP repeat containing 3 [Source:HGNC Symbol;Acc:HGNC:591] |
| ENSG00000028839 | HGLibA_48626 | 57 | -0.581392826 | TBPL1 | TATA-box binding protein like 1 [Source:HGNC Symbol;Acc:HGNC:11589] |
| ENSG00000030582 | HGLibA_20365 | 57 | -0.581392826 | GRN | granulin precursor [Source:HGNC Symbol;Acc:HGNC:4601] |
| ENSG00000037897 | HGLibA_28944 | 57 | -0.581392826 | METTL1 | methyltransferase like 1 [Source:HGNC Symbol;Acc:HGNC:7030] |
| ENSG00000056998 | HGLibA_20692 | 57 | -0.581392826 | GYG2 | glycogenin 2 [Source:HGNC Symbol;Acc:HGNC:4700] |
| ENSG00000058404 | HGLibA_07070 | 57 | -0.581392826 | CAMK2B | calcium/calmodulin dependent protein kinase II beta [Source:HGNC Symbol;Acc:HGNC:1461] |
| ENSG00000058404 | HGLibA_07071 | 57 | -0.581392826 | CAMK2B | calcium/calmodulin dependent protein kinase II beta [Source:HGNC Symbol;Acc:HGNC:1461] |
| ENSG00000060491 | HGLibA_33318 | 57 | -0.581392826 | OGFR | opioid growth factor receptor [Source:HGNC Symbol;Acc:HGNC:15768] |
| ENSG00000067842 | HGLibA_03621 | 57 | -0.581392826 | ATP2B3 | ATPase plasma membrane Ca2+ transporting 3 [Source:HGNC Symbol;Acc:HGNC:816] |
| ENSG00000068615 | HGLibA_40776 | 57 | -0.581392826 | REEP1 | receptor accessory protein 1 [Source:HGNC Symbol;Acc:HGNC:25786] |
| ENSG00000075618 | HGLibA_18069 | 57 | -0.581392826 | FSCN1 | fascin actin-bundling protein 1 [Source:HGNC Symbol;Acc:HGNC:11148] |
| ENSG00000079931 | HGLibA_29700 | 57 | -0.581392826 | MOXD1 | monooxygenase DBH like 1 [Source:HGNC Symbol;Acc:HGNC:21063] |
| ENSG00000081052 | HGLibA_10579 | 57 | -0.581392826 | COL4A4 | collagen type IV alpha 4 chain [Source:HGNC Symbol;Acc:HGNC:2206] |

| | | | | | |
|---|---|---|---|---|---|
| ENSG00000081059 | HGLibA_48791 | 57 | -0.581392826 | TCF7 | transcription factor 7 [Source:HGNC Symbol;Acc:HGNC:11639] |
| ENSG00000081377 | HGLibA_08511 | 57 | -0.581392826 | CDC14B | cell division cycle 14B [Source:HGNC Symbol;Acc:HGNC:1719] |
| ENSG00000083838 | HGLibA_56020 | 57 | -0.581392826 | ZNF446 | zinc finger protein 446 [Source:HGNC Symbol;Acc:HGNC:21036] |
| ENSG00000085831 | HGLibA_52132 | 57 | -0.581392826 | TTC39A | tetratricopeptide repeat domain 39A [Source:HGNC Symbol;Acc:HGNC:18657] |
| ENSG00000086717 | HGLibA_37866 | 57 | -0.581392826 | PPEF1 | protein phosphatase with EF-hand domain 1 [Source:HGNC Symbol;Acc:HGNC:9243] |
| ENSG00000087206 | HGLibA_52958 | 57 | -0.581392826 | UIMC1 | ubiquitin interaction motif containing 1 [Source:HGNC Symbol;Acc:HGNC:30298] |
| ENSG00000088305 | HGLibA_13703 | 57 | -0.581392826 | DNMT3B | DNA methyltransferase 3 beta [Source:HGNC Symbol;Acc:HGNC:2979] |
| ENSG00000089250 | HGLibA_32323 | 57 | -0.581392826 | NOS1 | nitric oxide synthase 1 [Source:HGNC Symbol;Acc:HGNC:7872] |
| ENSG00000090487 | HGLibA_46635 | 57 | -0.581392826 | SPG21 | maspardin [Source:HGNC Symbol;Acc:HGNC:20373]" |
| ENSG00000092201 | HGLibA_47803 | 57 | -0.581392826 | SUPT16H | facilitates chromatin remodeling subunit [Source:HGNC Symbol;Acc:HGNC:11465]" |
| ENSG00000092208 | HGLibA_18910 | 57 | -0.581392826 | GEMIN2 | gem nuclear organelle associated protein 2 [Source:HGNC Symbol;Acc:HGNC:10884] |
| ENSG00000097021 | HGLibA_00489 | 57 | -0.581392826 | ACOT7 | acyl-CoA thioesterase 7 [Source:HGNC Symbol;Acc:HGNC:24157] |
| ENSG00000100341 | HGLibA_37416 | 57 | -0.581392826 | PNPLA5 | patatin like phospholipase domain containing 5 [Source:HGNC Symbol;Acc:HGNC:24888] |
| ENSG00000100532 | HGLibA_09330 | 57 | -0.581392826 | CGRRF1 | cell growth regulator with ring finger domain 1 [Source:HGNC Symbol;Acc:HGNC:15528] |
| ENSG00000101327 | HGLibA_35966 | 57 | -0.581392826 | PDYN | prodynorphin [Source:HGNC Symbol;Acc:HGNC:8820] |
| ENSG00000102243 | HGLibA_53635 | 57 | -0.581392826 | VGLL1 | vestigial like family member 1 [Source:HGNC Symbol;Acc:HGNC:20985] |
| ENSG00000102471 | HGLibA_31395 | 57 | -0.581392826 | NDFIP2 | Nedd4 family interacting protein 2 [Source:HGNC Symbol;Acc:HGNC:18537] |
| ENSG00000103018 | HGLibA_11993 | 57 | -0.581392826 | CYB5B | cytochrome b5 type B [Source:HGNC Symbol;Acc:HGNC:24374] |
| ENSG00000103042 | HGLibA_45079 | 57 | -0.581392826 | SLC38A7 | solute carrier family 38 member 7 [Source:HGNC Symbol;Acc:HGNC:25582] |
| ENSG00000103496 | HGLibA_47649 | 57 | -0.581392826 | STX4 | syntaxin 4 [Source:HGNC Symbol;Acc:HGNC:11439] |
| ENSG00000104154 | HGLibA_44910 | 57 | -0.581392826 | SLC30A4 | solute carrier family 30 member 4 [Source:HGNC Symbol;Acc:HGNC:11015] |
| ENSG00000104879 | HGLibA_09776 | 57 | -0.581392826 | CKM | M-type [Source:HGNC Symbol;Acc:HGNC:1994]" |
| ENSG00000104972 | HGLibA_26506 | 57 | -0.581392826 | LILRB1 | leukocyte immunoglobulin like receptor B1 [Source:HGNC Symbol;Acc:HGNC:6605] |
| ENSG00000105516 | HGLibA_12401 | 57 | -0.581392826 | DBP | D-box binding PAR bZIP transcription factor [Source:HGNC Symbol;Acc:HGNC:2697] |
| ENSG00000105723 | HGLibA_20429 | 57 | -0.581392826 | GSK3A | glycogen synthase kinase 3 alpha [Source:HGNC Symbol;Acc:HGNC:4616] |
| ENSG00000105877 | HGLibA_13475 | 57 | -0.581392826 | DNAH11 | dynein axonemal heavy chain 11 [Source:HGNC Symbol;Acc:HGNC:2942] |
| ENSG00000105879 | HGLibA_07404 | 57 | -0.581392826 | CBLL1 | Cbl proto-oncogene like 1 [Source:HGNC Symbol;Acc:HGNC:21225] |
| ENSG00000106086 | HGLibA_37083 | 57 | -0.581392826 | PLEKHA8 | pleckstrin homology domain containing A8 [Source:HGNC Symbol;Acc:HGNC:30037] |
| ENSG00000106346 | HGLibA_53366 | 57 | -0.581392826 | USP42 | ubiquitin specific peptidase 42 [Source:HGNC Symbol;Acc:HGNC:20068] |
| ENSG00000106554 | HGLibA_09368 | 57 | -0.581392826 | CHCHD3 | coiled-coil-helix-coiled-coil-helix domain containing 3 [Source:HGNC Symbol;Acc:HGNC:21906] |
| ENSG00000107902 | HGLibA_26443 | 57 | -0.581392826 | LHPP | phospholysine phosphohistidine inorganic pyrophosphate phosphatase [Source:HGNC Symbol;Acc:HGNC:30042] |
| ENSG00000108342 | HGLibA_11339 | 57 | -0.581392826 | CSF3 | colony stimulating factor 3 [Source:HGNC Symbol;Acc:HGNC:2438] |
| ENSG00000108684 | HGLibA_03303 | 57 | -0.581392826 | ASIC2 | acid sensing ion channel subunit 2 [Source:HGNC Symbol;Acc:HGNC:99] |
| ENSG00000108733 | HGLibA_36093 | 57 | -0.581392826 | PEX12 | peroxisomal biogenesis factor 12 [Source:HGNC Symbol;Acc:HGNC:8854] |
| ENSG00000109472 | HGLibA_10913 | 57 | -0.581392826 | CPE | carboxypeptidase E [Source:HGNC Symbol;Acc:HGNC:2303] |
| ENSG00000109686 | HGLibA_43941 | 57 | -0.581392826 | SH3D19 | SH3 domain containing 19 [Source:HGNC Symbol;Acc:HGNC:30418] |
| ENSG00000109756 | HGLibA_40248 | 57 | -0.581392826 | RAPGEF2 | Rap guanine nucleotide exchange factor 2 [Source:HGNC Symbol;Acc:HGNC:16854] |
| ENSG00000109771 | HGLibA_27215 | 57 | -0.581392826 | LRP2BP | LRP2 binding protein [Source:HGNC Symbol;Acc:HGNC:25434] |
| ENSG00000111052 | HGLibA_26572 | 57 | -0.581392826 | LIN7A | crumbs cell polarity complex component [Source:HGNC Symbol;Acc:HGNC:17787]" |
| ENSG00000112218 | HGLibA_20067 | 57 | -0.581392826 | GPR63 | G protein-coupled receptor 63 [Source:HGNC Symbol;Acc:HGNC:13302] |
| ENSG00000112249 | HGLibA_03263 | 57 | -0.581392826 | ASCC3 | activating signal cointegrator 1 complex subunit 3 [Source:HGNC Symbol;Acc:HGNC:18697] |
| ENSG00000113441 | HGLibA_26742 | 57 | -0.581392826 | LNPEP | leucyl and cystinyl aminopeptidase [Source:HGNC Symbol;Acc:HGNC:6656] |
| ENSG00000113522 | HGLibA_40076 | 57 | -0.581392826 | RAD50 | RAD50 double strand break repair protein [Source:HGNC Symbol;Acc:HGNC:9816] |
| ENSG00000114491 | HGLibA_52983 | 57 | -0.581392826 | UMPS | uridine monophosphate synthetase [Source:HGNC Symbol;Acc:HGNC:12563] |
| ENSG00000115263 | HGLibA_18775 | 57 | -0.581392826 | GCG | glucagon [Source:HGNC Symbol;Acc:HGNC:4191] |
| ENSG00000115839 | HGLibA_39920 | 57 | -0.581392826 | RAB3GAP1 | RAB3 GTPase activating protein catalytic subunit 1 [Source:HGNC Symbol;Acc:HGNC:17063] |
| ENSG00000115935 | HGLibA_54305 | 57 | -0.581392826 | WIPF1 | WAS/WASL interacting protein family member 1 [Source:HGNC Symbol;Acc:HGNC:12736] |
| ENSG00000115946 | HGLibA_37396 | 57 | -0.581392826 | PNO1 | partner of NOB1 homolog [Source:HGNC Symbol;Acc:HGNC:32790] |
| ENSG00000116663 | HGLibA_17123 | 57 | -0.581392826 | FBXO6 | F-box protein 6 [Source:HGNC Symbol;Acc:HGNC:13585] |
| ENSG00000117335 | HGLibA_08393 | 57 | -0.581392826 | CD46 | CD46 molecule [Source:HGNC Symbol;Acc:HGNC:6953] |
| ENSG00000117360 | HGLibA_38716 | 57 | -0.581392826 | PRPF3 | pre-mRNA processing factor 3 [Source:HGNC Symbol;Acc:HGNC:17348] |
| ENSG00000119396 | HGLibA_39813 | 57 | -0.581392826 | RAB14 | member RAS oncogene family [Source:HGNC Symbol;Acc:HGNC:16524]" |
| ENSG00000120332 | HGLibA_50861 | 57 | -0.581392826 | TNN | tenascin N [Source:HGNC Symbol;Acc:HGNC:22942] |
| ENSG00000120805 | HGLibA_02922 | 57 | -0.581392826 | ARL1 | ADP ribosylation factor like GTPase 1 [Source:HGNC Symbol;Acc:HGNC:692] |
| ENSG00000120949 | HGLibA_50791 | 57 | -0.581392826 | TNFRSF8 | TNF receptor superfamily member 8 [Source:HGNC Symbol;Acc:HGNC:11923] |
| ENSG00000122861 | HGLibA_36963 | 57 | -0.581392826 | PLAU | urokinase [Source:HGNC Symbol;Acc:HGNC:9052]" |
| ENSG00000123473 | HGLibA_47439 | 57 | -0.581392826 | STIL | centriolar assembly protein [Source:HGNC Symbol;Acc:HGNC:10879]" |
| ENSG00000123843 | HGLibA_06294 | 57 | -0.581392826 | C4BPB | complement component 4 binding protein beta [Source:HGNC Symbol;Acc:HGNC:1328] |
| ENSG00000124157 | HGLibA_43381 | 57 | -0.581392826 | SEMG2 | semenogelin 2 [Source:HGNC Symbol;Acc:HGNC:10743] |
| ENSG00000124212 | HGLibA_39348 | 57 | -0.581392826 | PTGIS | prostaglandin I2 synthase [Source:HGNC Symbol;Acc:HGNC:9603] |
| ENSG00000124257 | HGLibA_31729 | 57 | -0.581392826 | NEURL2 | neuralized E3 ubiquitin protein ligase 2 [Source:HGNC Symbol;Acc:HGNC:16156] |
| ENSG00000125089 | HGLibA_43978 | 57 | -0.581392826 | SH3TC1 | SH3 domain and tetratricopeptide repeats 1 [Source:HGNC Symbol;Acc:HGNC:26009] |
| ENSG00000125864 | HGLibA_04455 | 57 | -0.581392826 | BFSP1 | beaded filament structural protein 1 [Source:HGNC Symbol;Acc:HGNC:1040] |
| ENSG00000126698 | HGLibA_13638 | 57 | -0.581392826 | DNAJC8 | DnaJ heat shock protein family (Hsp40) member C8 [Source:HGNC Symbol;Acc:HGNC:15470] |
| ENSG00000126804 | HGLibA_54811 | 57 | -0.581392826 | ZBTB1 | zinc finger and BTB domain containing 1 [Source:HGNC Symbol;Acc:HGNC:20259] |
| ENSG00000127946 | HGLibA_21334 | 57 | -0.581392826 | HIP1 | huntingtin interacting protein 1 [Source:HGNC Symbol;Acc:HGNC:4913] |
| ENSG00000128699 | HGLibA_34625 | 57 | -0.581392826 | ORMDL1 | ORMDL sphingolipid biosynthesis regulator 1 [Source:HGNC Symbol;Acc:HGNC:16036] |
| ENSG00000128886 | HGLibA_14927 | 57 | -0.581392826 | ELL3 | elongation factor for RNA polymerase II 3 [Source:HGNC Symbol;Acc:HGNC:23113] |
| ENSG00000129460 | HGLibA_31867 | 57 | -0.581392826 | NGDN | neuroguidin [Source:HGNC Symbol;Acc:HGNC:20271] |
| ENSG00000129965 | HGLibA_23370 | 57 | -0.581392826 | INS-IGF2 | INS-IGF2 readthrough [Source:HGNC Symbol;Acc:HGNC:33527] |
| ENSG00000130222 | HGLibA_18423 | 57 | -0.581392826 | GADD45G | growth arrest and DNA damage inducible gamma [Source:HGNC Symbol;Acc:HGNC:4097] |
| ENSG00000130772 | HGLibA_28767 | 57 | -0.581392826 | MED18 | mediator complex subunit 18 [Source:HGNC Symbol;Acc:HGNC:25944] |
| ENSG00000132746 | HGLibA_01571 | 57 | -0.581392826 | ALDH3B2 | aldehyde dehydrogenase 3 family member B2 [Source:HGNC Symbol;Acc:HGNC:411] |
| ENSG00000133019 | HGLibA_09554 | 57 | -0.581392826 | CHRM3 | cholinergic receptor muscarinic 3 [Source:HGNC Symbol;Acc:HGNC:1952] |
| ENSG00000133315 | HGLibA_27835 | 57 | -0.581392826 | MACROD1 | MACRO domain containing 1 [Source:HGNC Symbol;Acc:HGNC:29598] |
| ENSG00000133858 | HGLibA_55168 | 57 | -0.581392826 | ZFC3H1 | zinc finger C3H1-type containing [Source:HGNC Symbol;Acc:HGNC:28328] |
| ENSG00000134717 | HGLibA_04939 | 57 | -0.581392826 | BTF3L4 | basic transcription factor 3 like 4 [Source:HGNC Symbol;Acc:HGNC:30547] |
| ENSG00000135083 | HGLibA_08111 | 57 | -0.581392826 | CCNJL | cyclin J like [Source:HGNC Symbol;Acc:HGNC:25876] |
| ENSG00000135387 | HGLibA_07191 | 57 | -0.581392826 | CAPRIN1 | cell cycle associated protein 1 [Source:HGNC Symbol;Acc:HGNC:6743] |
| ENSG00000135457 | HGLibA_49146 | 57 | -0.581392826 | TFCP2 | transcription factor CP2 [Source:HGNC Symbol;Acc:HGNC:11748] |
| ENSG00000135541 | HGLibA_01297 | 57 | -0.581392826 | AHI1 | Abelson helper integration site 1 [Source:HGNC Symbol;Acc:HGNC:21575] |
| ENSG00000135951 | HGLibA_51848 | 57 | -0.581392826 | TSGA10 | testis specific 10 [Source:HGNC Symbol;Acc:HGNC:14927] |
| ENSG00000136874 | HGLibA_47627 | 57 | -0.581392826 | STX17 | syntaxin 17 [Source:HGNC Symbol;Acc:HGNC:11432] |
| ENSG00000137070 | HGLibA_22956 | 57 | -0.581392826 | IL11RA | interleukin 11 receptor subunit alpha [Source:HGNC Symbol;Acc:HGNC:5967] |
| ENSG00000137080 | HGLibA_22677 | 57 | -0.581392826 | IFNA21 | interferon alpha 21 [Source:HGNC Symbol;Acc:HGNC:5424] |
| ENSG00000137252 | HGLibA_20992 | 57 | -0.581392826 | HCRTR2 | hypocretin receptor 2 [Source:HGNC Symbol;Acc:HGNC:4849] |
| ENSG00000137710 | HGLibA_40757 | 57 | -0.581392826 | RDX | radixin [Source:HGNC Symbol;Acc:HGNC:9944] |
| ENSG00000138246 | HGLibA_13574 | 57 | -0.581392826 | DNAJC13 | DnaJ heat shock protein family (Hsp40) member C13 [Source:HGNC Symbol;Acc:HGNC:30343] |
| ENSG00000138376 | HGLibA_04154 | 57 | -0.581392826 | BARD1 | BRCA1 associated RING domain 1 [Source:HGNC Symbol;Acc:HGNC:952] |
| ENSG00000138443 | HGLibA_00294 | 57 | -0.581392826 | ABI2 | abl interactor 2 [Source:HGNC Symbol;Acc:HGNC:24011] |
| ENSG00000140350 | HGLibA_02197 | 57 | -0.581392826 | ANP32A | acidic nuclear phosphoprotein 32 family member A [Source:HGNC Symbol;Acc:HGNC:13233] |
| ENSG00000140400 | HGLibA_28081 | 57 | -0.581392826 | MAN2C1 | mannosidase alpha class 2C member 1 [Source:HGNC Symbol;Acc:HGNC:6827] |
| ENSG00000141449 | HGLibA_20239 | 57 | -0.581392826 | GREB1L | GREB1 like retinoic acid receptor coactivator [Source:HGNC Symbol;Acc:HGNC:31042] |
| ENSG00000141524 | HGLibA_49762 | 57 | -0.581392826 | TMC6 | transmembrane channel like 6 [Source:HGNC Symbol;Acc:HGNC:18021] |
| ENSG00000142606 | HGLibA_29472 | 57 | -0.581392826 | MMEL1 | membrane metalloendopeptidase like 1 [Source:HGNC Symbol;Acc:HGNC:14668] |
| ENSG00000143258 | HGLibA_53303 | 57 | -0.581392826 | USP21 | ubiquitin specific peptidase 21 [Source:HGNC Symbol;Acc:HGNC:12620] |
| ENSG00000143355 | HGLibA_26469 | 57 | -0.581392826 | LHX9 | LIM homeobox 9 [Source:HGNC Symbol;Acc:HGNC:14222] |
| ENSG00000143641 | HGLibA_18554 | 57 | -0.581392826 | GALNT2 | polypeptide N-acetylgalactosaminyltransferase 2 [Source:HGNC Symbol;Acc:HGNC:4124] |
| ENSG00000145794 | HGLibA_28846 | 57 | -0.581392826 | MEGF10 | multiple EGF like domains 10 [Source:HGNC Symbol;Acc:HGNC:29634] |
| ENSG00000145934 | HGLibA_48999 | 57 | -0.581392826 | TENM2 | teneurin transmembrane protein 2 [Source:HGNC Symbol;Acc:HGNC:29943] |
| ENSG00000146197 | HGLibA_43075 | 57 | -0.581392826 | SCUBE3 | CUB domain and EGF like domain containing 3 [Source:HGNC Symbol;Acc:HGNC:13655]" |
| ENSG00000146278 | HGLibA_37434 | 57 | -0.581392826 | PNRC1 | proline rich nuclear receptor coactivator 1 [Source:HGNC Symbol;Acc:HGNC:17278] |
| ENSG00000149735 | HGLibA_19797 | 57 | -0.581392826 | GPHA2 | glycoprotein hormone alpha 2 [Source:HGNC Symbol;Acc:HGNC:18054] |
| ENSG00000151148 | HGLibA_52682 | 57 | -0.581392826 | UBE3B | ubiquitin protein ligase E3B [Source:HGNC Symbol;Acc:HGNC:13478] |
| ENSG00000151693 | HGLibA_03198 | 57 | -0.581392826 | ASAP2 | ankyrin repeat and PH domain 2 [Source:HGNC Symbol;Acc:HGNC:2721]" |
| ENSG00000152592 | HGLibA_13410 | 57 | -0.581392826 | DMP1 | dentin matrix acidic phosphoprotein 1 [Source:HGNC Symbol;Acc:HGNC:2932] |
| ENSG00000154917 | HGLibA_39972 | 57 | -0.581392826 | RAB6B | member RAS oncogene family [Source:HGNC Symbol;Acc:HGNC:14902]" |
| ENSG00000156299 | HGLibA_49438 | 57 | -0.581392826 | TIAM1 | T cell lymphoma invasion and metastasis 1 [Source:HGNC Symbol;Acc:HGNC:11805] |
| ENSG00000156738 | HGLibA_30127 | 57 | -0.581392826 | MS4A1 | membrane spanning 4-domains A1 [Source:HGNC Symbol;Acc:HGNC:7315] |
| ENSG00000157045 | HGLibA_32835 | 57 | -0.581392826 | NTAN1 | N-terminal asparagine amidase [Source:HGNC Symbol;Acc:HGNC:29909] |
| ENSG00000159346 | HGLibA_00981 | 57 | -0.581392826 | ADIPOR1 | adiponectin receptor 1 [Source:HGNC Symbol;Acc:HGNC:24040] |
| ENSG00000160570 | HGLibA_12782 | 57 | -0.581392826 | DEDD2 | death effector domain containing 2 [Source:HGNC Symbol;Acc:HGNC:24450] |
| ENSG00000163093 | HGLibA_04222 | 57 | -0.581392826 | BBS5 | Bardet-Biedl syndrome 5 [Source:HGNC Symbol;Acc:HGNC:970] |
| ENSG00000163273 | HGLibA_32505 | 57 | -0.581392826 | NPPC | natriuretic peptide C [Source:HGNC Symbol;Acc:HGNC:7941] |
| ENSG00000163349 | HGLibA_21338 | 57 | -0.581392826 | HIPK1 | homeodomain interacting protein kinase 1 [Source:HGNC Symbol;Acc:HGNC:19006] |
| ENSG00000163577 | HGLibA_14859 | 57 | -0.581392826 | EIF5A2 | eukaryotic translation initiation factor 5A2 [Source:HGNC Symbol;Acc:HGNC:3301] |
| ENSG00000163683 | HGLibA_45731 | 57 | -0.581392826 | SMIM14 | small integral membrane protein 14 [Source:HGNC Symbol;Acc:HGNC:27321] |
| ENSG00000163812 | HGLibA_55115 | 57 | -0.581392826 | ZDHHC3 | zinc finger DHHC-type containing 3 [Source:HGNC Symbol;Acc:HGNC:18470] |
| ENSG00000163959 | HGLibA_45239 | 57 | -0.581392826 | SLC51A | solute carrier family 51 alpha subunit [Source:HGNC Symbol;Acc:HGNC:29955] |
| ENSG00000163961 | HGLibA_41564 | 57 | -0.581392826 | RNF168 | ring finger protein 168 [Source:HGNC Symbol;Acc:HGNC:26661] |
| ENSG00000164299 | HGLibA_46911 | 57 | -0.581392826 | SPZ1 | spermatogenic leucine zipper 1 [Source:HGNC Symbol;Acc:HGNC:30721] |
| ENSG00000164855 | HGLibA_50110 | 57 | -0.581392826 | TMEM184A | transmembrane protein 184A [Source:HGNC Symbol;Acc:HGNC:28797] |
| ENSG00000165204 | HGLibA_33684 | 57 | -0.581392826 | OR1K1 | olfactory receptor family 1 subfamily K member 1 [Source:HGNC Symbol;Acc:HGNC:8212] |
| ENSG00000165376 | HGLibA_09876 | 57 | -0.581392826 | CLDN2 | claudin 2 [Source:HGNC Symbol;Acc:HGNC:2041] |
| ENSG00000165494 | HGLibA_35590 | 57 | -0.581392826 | PCF11 | cleavage and polyadenylation factor subunit [Source:HGNC Symbol;Acc:HGNC:30097]" |
| ENSG00000165792 | HGLibA_28967 | 57 | -0.581392826 | METTL17 | methyltransferase like 17 [Source:HGNC Symbol;Acc:HGNC:19280] |
| ENSG00000166452 | HGLibA_01453 | 57 | -0.581392826 | AKIP1 | A-kinase interacting protein 1 [Source:HGNC Symbol;Acc:HGNC:1170] |
| ENSG00000166535 | HGLibA_00011 | 57 | -0.581392826 | A2ML1 | alpha-2-macroglobulin like 1 [Source:HGNC Symbol;Acc:HGNC:23336] |
| ENSG00000166821 | HGLibA_36084 | 57 | -0.581392826 | PEX11A | peroxisomal biogenesis factor 11 alpha [Source:HGNC Symbol;Acc:HGNC:8852] |
| ENSG00000167065 | HGLibA_14150 | 57 | -0.581392826 | DUSP18 | dual specificity phosphatase 18 [Source:HGNC Symbol;Acc:HGNC:18484] |
| ENSG00000167103 | HGLibA_36724 | 57 | -0.581392826 | PIP5KL1 | phosphatidylinositol-4-phosphate 5-kinase like 1 [Source:HGNC Symbol;Acc:HGNC:28711] |
| ENSG00000167332 | HGLibA_34100 | 57 | -0.581392826 | OR51E2 | olfactory receptor family 51 subfamily E member 2 [Source:HGNC Symbol;Acc:HGNC:15195] |
| ENSG00000167612 | HGLibA_02070 | 57 | -0.581392826 | ANKRD33 | ankyrin repeat domain 33 [Source:HGNC Symbol;Acc:HGNC:13788] |
| ENSG00000167740 | HGLibA_11997 | 57 | -0.581392826 | CYB5D2 | cytochrome b5 domain containing 2 [Source:HGNC Symbol;Acc:HGNC:28471] |
| ENSG00000167779 | HGLibA_22829 | 57 | -0.581392826 | IGFBP6 | insulin like growth factor binding protein 6 [Source:HGNC Symbol;Acc:HGNC:5475] |

| | | | | | |
|---|---|---|---|---|---|
| ENSG00000168306 | HGLibA_00500 | 57 | -0.581392826 | ACOX2 | acyl-CoA oxidase 2 [Source:HGNC Symbol;Acc:HGNC:120] |
| ENSG00000168961 | HGLibA_26380 | 57 | -0.581392826 | LGALS9 | galectin 9 [Source:HGNC Symbol;Acc:HGNC:6570] |
| ENSG00000169247 | HGLibA_43981 | 57 | -0.581392826 | SH3TC2 | SH3 domain and tetratricopeptide repeats 2 [Source:HGNC Symbol;Acc:HGNC:29427] |
| ENSG00000169683 | HGLibA_27365 | 57 | -0.581392826 | LRRC45 | leucine rich repeat containing 45 [Source:HGNC Symbol;Acc:HGNC:28302] |
| ENSG00000170037 | HGLibA_10440 | 57 | -0.581392826 | CNTROB | centriole duplication and spindle assembly protein [Source:HGNC Symbol;Acc:HGNC:29616]" |
| ENSG00000170382 | HGLibA_27472 | 57 | -0.581392826 | LRRN2 | leucine rich repeat neuronal 2 [Source:HGNC Symbol;Acc:HGNC:16914] |
| ENSG00000170396 | HGLibA_56788 | 57 | -0.581392826 | ZNF804A | zinc finger protein 804A [Source:HGNC Symbol;Acc:HGNC:21711] |
| ENSG00000170486 | HGLibA_25528 | 57 | -0.581392826 | KRT72 | keratin 72 [Source:HGNC Symbol;Acc:HGNC:28932] |
| ENSG00000170917 | HGLibA_32984 | 57 | -0.581392826 | NUDT6 | nudix hydrolase 6 [Source:HGNC Symbol;Acc:HGNC:8053] |
| ENSG00000171365 | HGLibA_09833 | 57 | -0.581392826 | CLCN5 | chloride voltage-gated channel 5 [Source:HGNC Symbol;Acc:HGNC:2023] |
| ENSG00000172037 | HGLibA_25958 | 57 | -0.581392826 | LAMB2 | laminin subunit beta 2 [Source:HGNC Symbol;Acc:HGNC:6487] |
| ENSG00000172939 | HGLibA_34837 | 57 | -0.581392826 | OXSR1 | oxidative stress responsive 1 [Source:HGNC Symbol;Acc:HGNC:8508] |
| ENSG00000173302 | HGLibA_19920 | 57 | -0.581392826 | GPR148 | G protein-coupled receptor 148 [Source:HGNC Symbol;Acc:HGNC:23623] |
| ENSG00000173825 | HGLibA_49468 | 57 | -0.581392826 | TIGD3 | tigger transposable element derived 3 [Source:HGNC Symbol;Acc:HGNC:18334] |
| ENSG00000176083 | HGLibA_56534 | 57 | -0.581392826 | ZNF683 | zinc finger protein 683 [Source:HGNC Symbol;Acc:HGNC:28495] |
| ENSG00000176956 | HGLibA_27675 | 57 | -0.581392826 | LY6H | lymphocyte antigen 6 family member H [Source:HGNC Symbol;Acc:HGNC:6728] |
| ENSG00000177181 | HGLibA_41270 | 57 | -0.581392826 | RIMKLA | ribosomal modification protein rimK like family member A [Source:HGNC Symbol;Acc:HGNC:28725] |
| ENSG00000178172 | HGLibA_46701 | 57 | -0.581392826 | SPINK6 | Kazal type 6 [Source:HGNC Symbol;Acc:HGNC:29486]" |
| ENSG00000178188 | HGLibA_43874 | 57 | -0.581392826 | SH2B1 | SH2B adaptor protein 1 [Source:HGNC Symbol;Acc:HGNC:30417] |
| ENSG00000178719 | HGLibA_20317 | 57 | -0.581392826 | GRINA | glutamate ionotropic receptor NMDA type subunit associated protein 1 [Source:HGNC Symbol;Acc:HGNC:4589] |
| ENSG00000179002 | HGLibA_48356 | 57 | -0.581392826 | TAS1R2 | taste 1 receptor member 2 [Source:HGNC Symbol;Acc:HGNC:14905] |
| ENSG00000179195 | HGLibA_56483 | 57 | -0.581392826 | ZNF664 | zinc finger protein 664 [Source:HGNC Symbol;Acc:HGNC:25406] |
| ENSG00000179256 | HGLibA_45667 | 57 | -0.581392826 | SMCO3 | single-pass membrane protein with coiled-coil domains 3 [Source:HGNC Symbol;Acc:HGNC:34401] |
| ENSG00000181656 | HGLibA_20105 | 57 | -0.581392826 | GPR88 | G protein-coupled receptor 88 [Source:HGNC Symbol;Acc:HGNC:4539] |
| ENSG00000182040 | HGLibA_53177 | 57 | -0.581392826 | USH1G | USH1 protein network component sans [Source:HGNC Symbol;Acc:HGNC:16356] |
| ENSG00000182405 | HGLibA_36224 | 57 | -0.581392826 | PGBD4 | piggyBac transposable element derived 4 [Source:HGNC Symbol;Acc:HGNC:19401] |
| ENSG00000182871 | HGLibA_10524 | 57 | -0.581392826 | COL18A1 | collagen type XVIII alpha 1 chain [Source:HGNC Symbol;Acc:HGNC:2195] |
| ENSG00000183549 | HGLibA_00584 | 57 | -0.581392826 | ACSM5 | acyl-CoA synthetase medium chain family member 5 [Source:HGNC Symbol;Acc:HGNC:26060] |
| ENSG00000183696 | HGLibA_53087 | 57 | -0.581392826 | UPP1 | uridine phosphorylase 1 [Source:HGNC Symbol;Acc:HGNC:12576] |
| ENSG00000184221 | HGLibA_33367 | 57 | -0.581392826 | OLIG1 | oligodendrocyte transcription factor 1 [Source:HGNC Symbol;Acc:HGNC:16983] |
| ENSG00000184330 | HGLibA_42570 | 57 | -0.581392826 | S100A7A | S100 calcium binding protein A7A [Source:HGNC Symbol;Acc:HGNC:21657] |
| ENSG00000184502 | HGLibA_18662 | 57 | -0.581392826 | GAST | gastrin [Source:HGNC Symbol;Acc:HGNC:4164] |
| ENSG00000184867 | HGLibA_03042 | 57 | -0.581392826 | ARMCX2 | armadillo repeat containing X-linked 2 [Source:HGNC Symbol;Acc:HGNC:16869] |
| ENSG00000185897 | HGLibA_17329 | 57 | -0.581392826 | FFAR3 | free fatty acid receptor 3 [Source:HGNC Symbol;Acc:HGNC:4499] |
| ENSG00000186222 | HGLibA_04566 | 57 | -0.581392826 | BLOC1S4 | biogenesis of lysosomal organelles complex 1 subunit 4 [Source:HGNC Symbol;Acc:HGNC:24206] |
| ENSG00000186566 | HGLibA_19750 | 57 | -0.581392826 | GPATCH8 | G-patch domain containing 8 [Source:HGNC Symbol;Acc:HGNC:29066] |
| ENSG00000186907 | HGLibA_42418 | 57 | -0.581392826 | RTN4RL2 | reticulon 4 receptor like 2 [Source:HGNC Symbol;Acc:HGNC:23053] |
| ENSG00000186912 | HGLibA_34889 | 57 | -0.581392826 | P2RY4 | pyrimidinergic receptor P2Y4 [Source:HGNC Symbol;Acc:HGNC:8542] |
| ENSG00000186973 | HGLibA_16324 | 57 | -0.581392826 | FAM183A | family with sequence similarity 183 member A [Source:HGNC Symbol;Acc:HGNC:34347] |
| ENSG00000187531 | HGLibA_44234 | 57 | -0.581392826 | SIRT7 | sirtuin 7 [Source:HGNC Symbol;Acc:HGNC:14935] |
| ENSG00000187658 | HGLibA_06403 | 57 | -0.581392826 | C5orf52 | chromosome 5 open reading frame 52 [Source:HGNC Symbol;Acc:HGNC:35121] |
| ENSG00000187961 | HGLibA_25140 | 57 | -0.581392826 | KLHL17 | kelch like family member 17 [Source:HGNC Symbol;Acc:HGNC:24023] |
| ENSG00000196659 | HGLibA_52109 | 57 | -0.581392826 | TTC30B | tetratricopeptide repeat domain 30B [Source:HGNC Symbol;Acc:HGNC:26425] |
| ENSG00000196712 | HGLibA_31759 | 57 | -0.581392826 | NF1 | neurofibromin 1 [Source:HGNC Symbol;Acc:HGNC:7765] |
| ENSG00000196730 | HGLibA_12322 | 57 | -0.581392826 | DAPK1 | death associated protein kinase 1 [Source:HGNC Symbol;Acc:HGNC:2674] |
| ENSG00000197381 | HGLibA_00872 | 57 | -0.581392826 | ADARB1 | RNA specific B1 [Source:HGNC Symbol;Acc:HGNC:226]" |
| ENSG00000198129 | HGLibA_12841 | 57 | -0.581392826 | DEFB107B | defensin beta 107B [Source:HGNC Symbol;Acc:HGNC:31918] |
| ENSG00000204174 | HGLibA_32557 | 57 | -0.581392826 | NPY4R | neuropeptide Y receptor Y4 [Source:HGNC Symbol;Acc:HGNC:9329] |
| ENSG00000204361 | HGLibA_33152 | 57 | -0.581392826 | NXPE2 | neurexophilin and PC-esterase domain family member 2 [Source:HGNC Symbol;Acc:HGNC:26331] |
| ENSG00000204852 | HGLibA_48871 | 57 | -0.581392826 | TCTN1 | tectonic family member 1 [Source:HGNC Symbol;Acc:HGNC:26113] |
| ENSG00000205250 | HGLibA_14316 | 57 | -0.581392826 | E2F4 | E2F transcription factor 4 [Source:HGNC Symbol;Acc:HGNC:3118] |
| ENSG00000205339 | HGLibA_23473 | 57 | -0.581392826 | IPO7 | importin 7 [Source:HGNC Symbol;Acc:HGNC:9852] |
| ENSG00000205364 | HGLibA_30296 | 57 | -0.581392826 | MT1M | metallothionein 1M [Source:HGNC Symbol;Acc:HGNC:14296] |
| ENSG00000205718 | HGLibA_28480 | 57 | -0.581392826 | MBD3L4 | methyl-CpG binding domain protein 3 like 4 [Source:HGNC Symbol;Acc:HGNC:37206] |
| ENSG00000205726 | HGLibA_23870 | 57 | -0.581392826 | ITSN1 | intersectin 1 [Source:HGNC Symbol;Acc:HGNC:6183] |
| ENSG00000206474 | HGLibA_33485 | 57 | -0.581392826 | OR10C1 | olfactory receptor family 10 subfamily C member 1 (gene/pseudogene) [Source:HGNC Symbol;Acc:HGNC:8165] |
| ENSG00000213983 | HGLibA_02286 | 57 | -0.581392826 | AP1G2 | adaptor related protein complex 1 subunit gamma 2 [Source:HGNC Symbol;Acc:HGNC:556] |
| ENSG00000224982 | HGLibA_50264 | 57 | -0.581392826 | TMEM233 | transmembrane protein 233 [Source:HGNC Symbol;Acc:HGNC:37219] |
| ENSG00000226761 | HGLibA_48413 | 57 | -0.581392826 | TAS2R46 | taste 2 receptor member 46 [Source:HGNC Symbol;Acc:HGNC:18877] |
| ENSG00000228517 | HGLibA_11543 | 57 | -0.581392826 | CT47A7 | member A7 [Source:HGNC Symbol;Acc:HGNC:33288]" |
| ENSG00000234278 | HGLibA_38812 | 57 | -0.581392826 | PRR20E | proline rich 20E [Source:HGNC Symbol;Acc:HGNC:37223] |
| ENSG00000239264 | HGLibA_52422 | 57 | -0.581392826 | TXNDC5 | thioredoxin domain containing 5 [Source:HGNC Symbol;Acc:HGNC:21073] |
| ENSG00000241878 | HGLibA_36736 | 57 | -0.581392826 | PISD | phosphatidylserine decarboxylase [Source:HGNC Symbol;Acc:HGNC:8999] |
| ENSG00000242362 | HGLibA_11528 | 57 | -0.581392826 | CT47A2 | member A2 [Source:HGNC Symbol;Acc:HGNC:33283]" |
| ENSG00000244646 | HGLibA_54544 | 57 | -0.581392826 | XKRY2 | Y-linked 2 [Source:HGNC Symbol;Acc:HGNC:23922]" |
| ENSG00000247077 | HGLibA_36203 | 57 | -0.581392826 | PGAM5 | mitochondrial serine/threonine protein phosphatase [Source:HGNC Symbol;Acc:HGNC:28763]" |
| ENSG00000249139 | HGLibA_15360 | 57 | -0.581392826 | EPPIN-WFDC6 | EPPIN-WFDC6 readthrough [Source:HGNC Symbol;Acc:HGNC:38825] |
| ENSG00000251201 | HGLibA_49822 | 57 | -0.581392826 | TMED7-TICAM2 | TMED7-TICAM2 readthrough [Source:HGNC Symbol;Acc:HGNC:33945] |
| ENSG00000251493 | HGLibA_17829 | 57 | -0.581392826 | FOXD1 | forkhead box D1 [Source:HGNC Symbol;Acc:HGNC:3802] |
| ENSG00000254221 | HGLibA_35549 | 57 | -0.581392826 | PCDHGB1 | 1 [Source:HGNC Symbol;Acc:HGNC:8708]" |
| ENSG00000255823 | HGLibA_30491 | 57 | -0.581392826 | MTRNR2L8 | MT-RNR2 like 8 [Source:HGNC Symbol;Acc:HGNC:37165] |
| ENSG00000260287 | HGLibA_48551 | 57 | -0.581392826 | TBC1D3G | TBC1 domain family member 3G [Source:HGNC Symbol;Acc:HGNC:29860] |
| ENSG00000261857 | HGLibA_29182 | 57 | -0.581392826 | MIA | MIA SH3 domain containing [Source:HGNC Symbol;Acc:HGNC:7076] |
| ENSG00000269502 | HGLibA_13435 | 57 | -0.581392826 | DMRTC1 | DMRT like family C1 [Source:HGNC Symbol;Acc:HGNC:13910] |
| ENSG00000273274 | HGLibA_54929 | 57 | -0.581392826 | ZBTB8B | zinc finger and BTB domain containing 8B [Source:HGNC Symbol;Acc:HGNC:37057] |
| ENSG00000279170 | HGLibA_52036 | 57 | -0.581392826 | TSTD3 | TEC |
| ENSG00000284723 | HGLibA_34553 | 57 | -0.581392826 | OR8S1 | olfactory receptor family 8 subfamily S member 1 [Source:HGNC Symbol;Acc:HGNC:19628] |
| FAM178A | HGLibA_16299 | 57 | -0.581392826 | FAM178A | - |
| HGC6.3 | HGLibA_21234 | 57 | -0.581392826 | HGC6.3 | - |
| HIST3H2BB | HGLibA_21559 | 57 | -0.581392826 | HIST3H2BB | - |
| hsa-mir-181a-1 | HGLibA_57957 | 57 | -0.581392826 | hsa-mir-181a-1 | - |
| hsa-mir-224 | HGLibA_58306 | 57 | -0.581392826 | hsa-mir-224 | - |
| hsa-mir-3130-2 | HGLibA_58574 | 57 | -0.581392826 | hsa-mir-3130-2 | - |
| hsa-mir-3136 | HGLibA_58602 | 57 | -0.581392826 | hsa-mir-3136 | - |
| hsa-mir-3139 | HGLibA_58616 | 57 | -0.581392826 | hsa-mir-3139 | - |
| hsa-mir-3146 | HGLibA_58641 | 57 | -0.581392826 | hsa-mir-3146 | - |
| hsa-mir-320a | HGLibA_58922 | 57 | -0.581392826 | hsa-mir-320a | - |
| hsa-mir-367 | HGLibA_59228 | 57 | -0.581392826 | hsa-mir-367 | - |
| hsa-mir-4280 | HGLibA_59823 | 57 | -0.581392826 | hsa-mir-4280 | - |
| hsa-mir-4425 | HGLibA_60087 | 57 | -0.581392826 | hsa-mir-4425 | - |
| hsa-mir-4518 | HGLibA_60517 | 57 | -0.581392826 | hsa-mir-4518 | - |
| hsa-mir-4672 | HGLibA_60806 | 57 | -0.581392826 | hsa-mir-4672 | - |
| hsa-mir-4752 | HGLibA_61107 | 57 | -0.581392826 | hsa-mir-4752 | - |
| hsa-mir-4796 | HGLibA_61288 | 57 | -0.581392826 | hsa-mir-4796 | - |
| hsa-mir-493 | HGLibA_61374 | 57 | -0.581392826 | hsa-mir-493 | - |
| hsa-mir-563 | HGLibA_62216 | 57 | -0.581392826 | hsa-mir-563 | - |
| hsa-mir-563 | HGLibA_62214 | 57 | -0.581392826 | hsa-mir-563 | - |
| hsa-mir-5706 | HGLibA_62364 | 57 | -0.581392826 | hsa-mir-5706 | - |
| hsa-mir-583 | HGLibA_62431 | 57 | -0.581392826 | hsa-mir-583 | - |
| hsa-mir-592 | HGLibA_62465 | 57 | -0.581392826 | hsa-mir-592 | - |
| hsa-mir-6089-2 | HGLibA_62618 | 57 | -0.581392826 | hsa-mir-6089-2 | - |
| hsa-mir-647 | HGLibA_62833 | 57 | -0.581392826 | hsa-mir-647 | - |
| hsa-mir-6511a-1 | HGLibA_62898 | 57 | -0.581392826 | hsa-mir-6511a-1 | - |
| hsa-mir-6746 | HGLibA_63136 | 57 | -0.581392826 | hsa-mir-6746 | - |
| hsa-mir-6844 | HGLibA_63550 | 57 | -0.581392826 | hsa-mir-6844 | - |
| hsa-mir-7515 | HGLibA_63890 | 57 | -0.581392826 | hsa-mir-7515 | - |
| hsa-mir-7976 | HGLibA_64032 | 57 | -0.581392826 | hsa-mir-7976 | - |
| hsa-mir-8087 | HGLibA_64192 | 57 | -0.581392826 | hsa-mir-8087 | - |
| KIAA1430 | HGLibA_24753 | 57 | -0.581392826 | KIAA1430 | - |
| LOC100287399 | HGLibA_26821 | 57 | -0.581392826 | LOC100287399 | - |
| PLK1S1 | HGLibA_37188 | 57 | -0.581392826 | PLK1S1 | - |
| PPAP2A | HGLibA_37816 | 57 | -0.581392826 | PPAP2A | - |
| PPAP2B | HGLibA_37819 | 57 | -0.581392826 | PPAP2B | - |
| RTDR1 | HGLibA_42376 | 57 | -0.581392826 | RTDR1 | - |
| B3GNT1 | HGLibA_03981 | 56 | -0.591343863 | B3GNT1 | - |
| C1orf101 | HGLibA_05732 | 56 | -0.591343863 | C1orf101 | - |
| C1orf27 | HGLibA_05851 | 56 | -0.591343863 | C1orf27 | - |
| CD99_Y | HGLibA_08492 | 56 | -0.591343863 | CD99_Y | - |
| CXorf40B | HGLibA_11927 | 56 | -0.591343863 | CXorf40B | - |
| ENSG00000015475 | HGLibA_04501 | 56 | -0.591343863 | BID | BH3 interacting domain death agonist [Source:HGNC Symbol;Acc:HGNC:1050] |
| ENSG00000023839 | HGLibA_00169 | 56 | -0.591343863 | ABCC2 | ATP binding cassette subfamily C member 2 [Source:HGNC Symbol;Acc:HGNC:53] |
| ENSG00000033800 | HGLibA_36507 | 56 | -0.591343863 | PIAS1 | protein inhibitor of activated STAT 1 [Source:HGNC Symbol;Acc:HGNC:2752] |
| ENSG00000038002 | HGLibA_01134 | 56 | -0.591343863 | AGA | aspartylglucosaminidase [Source:HGNC Symbol;Acc:HGNC:318] |
| ENSG00000064933 | HGLibA_37333 | 56 | -0.591343863 | PMS1 | mismatch repair system component [Source:HGNC Symbol;Acc:HGNC:9121]" |
| ENSG00000066027 | HGLibA_38169 | 56 | -0.591343863 | PPP2R5A | protein phosphatase 2 regulatory subunit B'alpha [Source:HGNC Symbol;Acc:HGNC:9309] |
| ENSG00000066855 | HGLibA_30360 | 56 | -0.591343863 | MTFR1 | mitochondrial fission regulator 1 [Source:HGNC Symbol;Acc:HGNC:29510] |
| ENSG00000069956 | HGLibA_28276 | 56 | -0.591343863 | MAPK6 | mitogen-activated protein kinase 6 [Source:HGNC Symbol;Acc:HGNC:6879] |
| ENSG00000072133 | HGLibA_42172 | 56 | -0.591343863 | RPS6KA6 | ribosomal protein S6 kinase A6 [Source:HGNC Symbol;Acc:HGNC:10435] |
| ENSG00000075420 | HGLibA_17749 | 56 | -0.591343863 | FNDC3B | fibronectin type III domain containing 3B [Source:HGNC Symbol;Acc:HGNC:24670] |
| ENSG00000082996 | HGLibA_41503 | 56 | -0.591343863 | RNF13 | ring finger protein 13 [Source:HGNC Symbol;Acc:HGNC:10057] |
| ENSG00000084652 | HGLibA_52389 | 56 | -0.591343863 | TXLNA | taxilin alpha [Source:HGNC Symbol;Acc:HGNC:30685] |
| ENSG00000087245 | HGLibA_29507 | 56 | -0.591343863 | MMP2 | matrix metallopeptidase 2 [Source:HGNC Symbol;Acc:HGNC:7166] |
| ENSG00000089916 | HGLibA_19742 | 56 | -0.591343863 | GPATCH2L | G-patch domain containing 2 like [Source:HGNC Symbol;Acc:HGNC:20210] |
| ENSG00000092051 | HGLibA_23989 | 56 | -0.591343863 | JPH4 | junctophilin 4 [Source:HGNC Symbol;Acc:HGNC:20156] |
| ENSG00000099385 | HGLibA_04344 | 56 | -0.591343863 | BCL7C | BCL tumor suppressor 7C [Source:HGNC Symbol;Acc:HGNC:1006] |

| | | | | | |
|---|---|---|---|---|---|
| ENSG00000099901 | HGLibA_40192 | 56 | -0.591343863 | RANBP1 | RAN binding protein 1 [Source:HGNC Symbol;Acc:HGNC:9847] |
| ENSG00000100124 | HGLibA_02123 | 56 | -0.591343863 | ANKRD54 | ankyrin repeat domain 54 [Source:HGNC Symbol;Acc:HGNC:25185] |
| ENSG00000100150 | HGLibA_12983 | 56 | -0.591343863 | DEPDC5 | DEP domain containing 5 [Source:HGNC Symbol;Acc:HGNC:18423] |
| ENSG00000100216 | HGLibA_50960 | 56 | -0.591343863 | TOMM22 | translocase of outer mitochondrial membrane 22 [Source:HGNC Symbol;Acc:HGNC:18002] |
| ENSG00000100417 | HGLibA_37316 | 56 | -0.591343863 | PMM1 | phosphomannomutase 1 [Source:HGNC Symbol;Acc:HGNC:9114] |
| ENSG00000100485 | HGLibA_46203 | 56 | -0.591343863 | SOS2 | SOS Ras/Rho guanine nucleotide exchange factor 2 [Source:HGNC Symbol;Acc:HGNC:11188] |
| ENSG00000100926 | HGLibA_49720 | 56 | -0.591343863 | TM9SF1 | transmembrane 9 superfamily member 1 [Source:HGNC Symbol;Acc:HGNC:11864] |
| ENSG00000101132 | HGLibA_36145 | 56 | -0.591343863 | PFDN4 | prefoldin subunit 4 [Source:HGNC Symbol;Acc:HGNC:8868] |
| ENSG00000101306 | HGLibA_30786 | 56 | -0.591343863 | MYLK2 | myosin light chain kinase 2 [Source:HGNC Symbol;Acc:HGNC:16243] |
| ENSG00000101695 | HGLibA_41494 | 56 | -0.591343863 | RNF125 | ring finger protein 125 [Source:HGNC Symbol;Acc:HGNC:21150] |
| ENSG00000102290 | HGLibA_35390 | 56 | -0.591343863 | PCDH11X | protocadherin 11 X-linked [Source:HGNC Symbol;Acc:HGNC:8656] |
| ENSG00000103241 | HGLibA_17864 | 56 | -0.591343863 | FOXF1 | forkhead box F1 [Source:HGNC Symbol;Acc:HGNC:3809] |
| ENSG00000103769 | HGLibA_39787 | 56 | -0.591343863 | RAB11A | member RAS oncogene family [Source:HGNC Symbol;Acc:HGNC:9760]" |
| ENSG00000104131 | HGLibA_14793 | 56 | -0.591343863 | EIF3J | eukaryotic translation initiation factor 3 subunit J [Source:HGNC Symbol;Acc:HGNC:3270] |
| ENSG00000104529 | HGLibA_14498 | 56 | -0.591343863 | EEF1D | eukaryotic translation elongation factor 1 delta [Source:HGNC Symbol;Acc:HGNC:3211] |
| ENSG00000104812 | HGLibA_20709 | 56 | -0.591343863 | GYS1 | glycogen synthase 1 [Source:HGNC Symbol;Acc:HGNC:4706] |
| ENSG00000105058 | HGLibA_16570 | 56 | -0.591343863 | FAM32A | family with sequence similarity 32 member A [Source:HGNC Symbol;Acc:HGNC:24563] |
| ENSG00000105281 | HGLibA_44520 | 56 | -0.591343863 | SLC1A5 | solute carrier family 1 member 5 [Source:HGNC Symbol;Acc:HGNC:10943] |
| ENSG00000105605 | HGLibA_06946 | 56 | -0.591343863 | CACNG7 | calcium voltage-gated channel auxiliary subunit gamma 7 [Source:HGNC Symbol;Acc:HGNC:13626] |
| ENSG00000105617 | HGLibA_26298 | 56 | -0.591343863 | LENG1 | leukocyte receptor cluster member 1 [Source:HGNC Symbol;Acc:HGNC:15502] |
| ENSG00000105732 | HGLibA_56287 | 56 | -0.591343863 | ZNF574 | zinc finger protein 574 [Source:HGNC Symbol;Acc:HGNC:26166] |
| ENSG00000105929 | HGLibA_03710 | 56 | -0.591343863 | ATP6V0A4 | ATPase H+ transporting V0 subunit a4 [Source:HGNC Symbol;Acc:HGNC:866] |
| ENSG00000105971 | HGLibA_07380 | 56 | -0.591343863 | CAV2 | caveolin 2 [Source:HGNC Symbol;Acc:HGNC:1528] |
| ENSG00000106100 | HGLibA_32243 | 56 | -0.591343863 | NOD1 | nucleotide binding oligomerization domain containing 1 [Source:HGNC Symbol;Acc:HGNC:16390] |
| ENSG00000106384 | HGLibA_29629 | 56 | -0.591343863 | MOGAT3 | monoacylglycerol O-acyltransferase 3 [Source:HGNC Symbol;Acc:HGNC:23249] |
| ENSG00000107742 | HGLibA_46750 | 56 | -0.591343863 | SPOCK2 | cwcv and kazal like domains proteoglycan 2 [Source:HGNC Symbol;Acc:HGNC:13564]" |
| ENSG00000107874 | HGLibA_11769 | 56 | -0.591343863 | CUEDC2 | CUE domain containing 2 [Source:HGNC Symbol;Acc:HGNC:28352] |
| ENSG00000108219 | HGLibA_51904 | 56 | -0.591343863 | TSPAN14 | tetraspanin 14 [Source:HGNC Symbol;Acc:HGNC:23303] |
| ENSG00000108797 | HGLibA_10420 | 56 | -0.591343863 | CNTNAP1 | contactin associated protein 1 [Source:HGNC Symbol;Acc:HGNC:8011] |
| ENSG00000108924 | HGLibA_21654 | 56 | -0.591343863 | HLF | PAR bZIP transcription factor [Source:HGNC Symbol;Acc:HGNC:4977]" |
| ENSG00000110492 | HGLibA_28692 | 56 | -0.591343863 | MDK | midkine [Source:HGNC Symbol;Acc:HGNC:6972] |
| ENSG00000112242 | HGLibA_14313 | 56 | -0.591343863 | E2F3 | E2F transcription factor 3 [Source:HGNC Symbol;Acc:HGNC:3115] |
| ENSG00000113209 | HGLibA_35501 | 56 | -0.591343863 | PCDHB5 | protocadherin beta 5 [Source:HGNC Symbol;Acc:HGNC:8690] |
| ENSG00000113361 | HGLibA_08685 | 56 | -0.591343863 | CDH6 | cadherin 6 [Source:HGNC Symbol;Acc:HGNC:1765] |
| ENSG00000115073 | HGLibA_00653 | 56 | -0.591343863 | ACTR1B | ARP1 actin related protein 1 homolog B [Source:HGNC Symbol;Acc:HGNC:168] |
| ENSG00000115211 | HGLibA_14745 | 56 | -0.591343863 | EIF2B4 | eukaryotic translation initiation factor 2B subunit delta [Source:HGNC Symbol;Acc:HGNC:3260] |
| ENSG00000115268 | HGLibA_42077 | 56 | -0.591343863 | RPS15 | ribosomal protein S15 [Source:HGNC Symbol;Acc:HGNC:10388] |
| ENSG00000116260 | HGLibA_39753 | 56 | -0.591343863 | QSOX1 | quiescin sulfhydryl oxidase 1 [Source:HGNC Symbol;Acc:HGNC:9756] |
| ENSG00000116288 | HGLibA_35198 | 56 | -0.591343863 | PARK7 | Parkinsonism associated deglycase [Source:HGNC Symbol;Acc:HGNC:16369] |
| ENSG00000116898 | HGLibA_30042 | 56 | -0.591343863 | MRPS15 | mitochondrial ribosomal protein S15 [Source:HGNC Symbol;Acc:HGNC:14504] |
| ENSG00000117533 | HGLibA_53496 | 56 | -0.591343863 | VAMP4 | vesicle associated membrane protein 4 [Source:HGNC Symbol;Acc:HGNC:12645] |
| ENSG00000117586 | HGLibA_50824 | 56 | -0.591343863 | TNFSF4 | TNF superfamily member 4 [Source:HGNC Symbol;Acc:HGNC:11934] |
| ENSG00000118197 | HGLibA_12757 | 56 | -0.591343863 | DDX59 | DEAD-box helicase 59 [Source:HGNC Symbol;Acc:HGNC:25360] |
| ENSG00000118971 | HGLibA_08076 | 56 | -0.591343863 | CCND2 | cyclin D2 [Source:HGNC Symbol;Acc:HGNC:1583] |
| ENSG00000119760 | HGLibA_47826 | 56 | -0.591343863 | SUPT7L | STAGA complex gamma subunit [Source:HGNC Symbol;Acc:HGNC:30632]" |
| ENSG00000120699 | HGLibA_15793 | 56 | -0.591343863 | EXOSC8 | exosome component 8 [Source:HGNC Symbol;Acc:HGNC:17035] |
| ENSG00000120705 | HGLibA_15617 | 56 | -0.591343863 | ETF1 | eukaryotic translation termination factor 1 [Source:HGNC Symbol;Acc:HGNC:3477] |
| ENSG00000120875 | HGLibA_14181 | 56 | -0.591343863 | DUSP4 | dual specificity phosphatase 4 [Source:HGNC Symbol;Acc:HGNC:3070] |
| ENSG00000121897 | HGLibA_26470 | 56 | -0.591343863 | LIAS | lipoic acid synthetase [Source:HGNC Symbol;Acc:HGNC:16429] |
| ENSG00000123416 | HGLibA_52245 | 56 | -0.591343863 | TUBA1B | tubulin alpha 1b [Source:HGNC Symbol;Acc:HGNC:18809] |
| ENSG00000123989 | HGLibA_09524 | 56 | -0.591343863 | CHPF | chondroitin polymerizing factor [Source:HGNC Symbol;Acc:HGNC:24291] |
| ENSG00000124557 | HGLibA_04960 | 56 | -0.591343863 | BTN1A1 | butyrophilin subfamily 1 member A1 [Source:HGNC Symbol;Acc:HGNC:1135] |
| ENSG00000125458 | HGLibA_32800 | 56 | -0.591343863 | NT5C | cytosolic [Source:HGNC Symbol;Acc:HGNC:17144]" |
| ENSG00000126062 | HGLibA_49882 | 56 | -0.591343863 | TMEM115 | transmembrane protein 115 [Source:HGNC Symbol;Acc:HGNC:30055] |
| ENSG00000126217 | HGLibA_28580 | 56 | -0.591343863 | MCF2L | MCF.2 cell line derived transforming sequence like [Source:HGNC Symbol;Acc:HGNC:14576] |
| ENSG00000126746 | HGLibA_55894 | 56 | -0.591343863 | ZNF384 | zinc finger protein 384 [Source:HGNC Symbol;Acc:HGNC:11955] |
| ENSG00000127074 | HGLibA_41060 | 56 | -0.591343863 | RGS13 | regulator of G protein signaling 13 [Source:HGNC Symbol;Acc:HGNC:9995] |
| ENSG00000127191 | HGLibA_51244 | 56 | -0.591343863 | TRAF2 | TNF receptor associated factor 2 [Source:HGNC Symbol;Acc:HGNC:12032] |
| ENSG00000128165 | HGLibA_00994 | 56 | -0.591343863 | ADM2 | adrenomedullin 2 [Source:HGNC Symbol;Acc:HGNC:28898] |
| ENSG00000128573 | HGLibA_17934 | 56 | -0.591343863 | FOXP2 | forkhead box P2 [Source:HGNC Symbol;Acc:HGNC:13875] |
| ENSG00000131242 | HGLibA_39801 | 56 | -0.591343863 | RAB11FIP4 | RAB11 family interacting protein 4 [Source:HGNC Symbol;Acc:HGNC:30267] |
| ENSG00000131737 | HGLibA_25486 | 56 | -0.591343863 | KRT34 | keratin 34 [Source:HGNC Symbol;Acc:HGNC:6452] |
| ENSG00000132000 | HGLibA_37457 | 56 | -0.591343863 | PODNL1 | podocan like 1 [Source:HGNC Symbol;Acc:HGNC:26275] |
| ENSG00000134107 | HGLibA_04480 | 56 | -0.591343863 | BHLHE40 | basic helix-loop-helix family member e40 [Source:HGNC Symbol;Acc:HGNC:1046] |
| ENSG00000135541 | HGLibA_01299 | 56 | -0.591343863 | AHI1 | Abelson helper integration site 1 [Source:HGNC Symbol;Acc:HGNC:21575] |
| ENSG00000136237 | HGLibA_40258 | 56 | -0.591343863 | RAPGEF5 | Rap guanine nucleotide exchange factor 5 [Source:HGNC Symbol;Acc:HGNC:16862] |
| ENSG00000136636 | HGLibA_24452 | 56 | -0.591343863 | KCTD3 | potassium channel tetramerization domain containing 3 [Source:HGNC Symbol;Acc:HGNC:21305] |
| ENSG00000137133 | HGLibA_21328 | 56 | -0.591343863 | HINT2 | histidine triad nucleotide binding protein 2 [Source:HGNC Symbol;Acc:HGNC:18344] |
| ENSG00000138709 | HGLibA_26030 | 56 | -0.591343863 | LARP1B | La ribonucleoprotein domain family member 1B [Source:HGNC Symbol;Acc:HGNC:24704] |
| ENSG00000139278 | HGLibA_19260 | 56 | -0.591343863 | GLIPR1 | GLI pathogenesis related 1 [Source:HGNC Symbol;Acc:HGNC:17001] |
| ENSG00000140093 | HGLibA_43508 | 56 | -0.591343863 | SERPINA10 | serpin family A member 10 [Source:HGNC Symbol;Acc:HGNC:15996] |
| ENSG00000140743 | HGLibA_08867 | 56 | -0.591343863 | CDR2 | cerebellar degeneration related protein 2 [Source:HGNC Symbol;Acc:HGNC:1799] |
| ENSG00000140941 | HGLibA_28123 | 56 | -0.591343863 | MAP1LC3B | microtubule associated protein 1 light chain 3 beta [Source:HGNC Symbol;Acc:HGNC:13352] |
| ENSG00000141434 | HGLibA_28899 | 56 | -0.591343863 | MEP1B | meprin A subunit beta [Source:HGNC Symbol;Acc:HGNC:7020] |
| ENSG00000144228 | HGLibA_46765 | 56 | -0.591343863 | SPOPL | speckle type BTB/POZ protein like [Source:HGNC Symbol;Acc:HGNC:27934] |
| ENSG00000144451 | HGLibA_46352 | 56 | -0.591343863 | SPAG16 | sperm associated antigen 16 [Source:HGNC Symbol;Acc:HGNC:23225] |
| ENSG00000144746 | HGLibA_02991 | 56 | -0.591343863 | ARL6IP5 | ADP ribosylation factor like GTPase 6 interacting protein 5 [Source:HGNC Symbol;Acc:HGNC:16937] |
| ENSG00000145982 | HGLibA_16879 | 56 | -0.591343863 | FARS2 | mitochondrial [Source:HGNC Symbol;Acc:HGNC:21062]" |
| ENSG00000146243 | HGLibA_23556 | 56 | -0.591343863 | IRAK1BP1 | interleukin 1 receptor associated kinase 1 binding protein 1 [Source:HGNC Symbol;Acc:HGNC:17368] |
| ENSG00000146859 | HGLibA_49961 | 56 | -0.591343863 | TMEM140 | transmembrane protein 140 [Source:HGNC Symbol;Acc:HGNC:21870] |
| ENSG00000147164 | HGLibA_46033 | 56 | -0.591343863 | SNX12 | sorting nexin 12 [Source:HGNC Symbol;Acc:HGNC:14976] |
| ENSG00000147324 | HGLibA_29063 | 56 | -0.591343863 | MFHAS1 | malignant fibrous histiocytoma amplified sequence 1 [Source:HGNC Symbol;Acc:HGNC:16982] |
| ENSG00000149428 | HGLibA_22461 | 56 | -0.591343863 | HYOU1 | hypoxia up-regulated 1 [Source:HGNC Symbol;Acc:HGNC:16931] |
| ENSG00000150594 | HGLibA_01045 | 56 | -0.591343863 | ADRA2A | adrenoceptor alpha 2A [Source:HGNC Symbol;Acc:HGNC:281] |
| ENSG00000150779 | HGLibA_49526 | 56 | -0.591343863 | TIMM8B | translocase of inner mitochondrial membrane 8 homolog B [Source:HGNC Symbol;Acc:HGNC:11818] |
| ENSG00000150787 | HGLibA_39559 | 56 | -0.591343863 | PTS | 6-pyruvoyltetrahydropterin synthase [Source:HGNC Symbol;Acc:HGNC:9689] |
| ENSG00000150893 | HGLibA_17988 | 56 | -0.591343863 | FREM2 | FRAS1 related extracellular matrix protein 2 [Source:HGNC Symbol;Acc:HGNC:25396] |
| ENSG00000151065 | HGLibA_12521 | 56 | -0.591343863 | DCP1B | decapping mRNA 1B [Source:HGNC Symbol;Acc:HGNC:24451] |
| ENSG00000151611 | HGLibA_29451 | 56 | -0.591343863 | MMAA | metabolism of cobalamin associated A [Source:HGNC Symbol;Acc:HGNC:18871] |
| ENSG00000151846 | HGLibA_34932 | 56 | -0.591343863 | PABPC3 | poly(A) binding protein cytoplasmic 3 [Source:HGNC Symbol;Acc:HGNC:8556] |
| ENSG00000153006 | HGLibA_46958 | 56 | -0.591343863 | SREK1IP1 | SREK1 interacting protein 1 [Source:HGNC Symbol;Acc:HGNC:26716] |
| ENSG00000154277 | HGLibA_52800 | 56 | -0.591343863 | UCHL1 | ubiquitin C-terminal hydrolase L1 [Source:HGNC Symbol;Acc:HGNC:12513] |
| ENSG00000154767 | HGLibA_54549 | 56 | -0.591343863 | XPC | DNA damage recognition and repair factor [Source:HGNC Symbol;Acc:HGNC:12816]" |
| ENSG00000155886 | HGLibA_44618 | 56 | -0.591343863 | SLC24A2 | solute carrier family 24 member 2 [Source:HGNC Symbol;Acc:HGNC:10976] |
| ENSG00000155957 | HGLibA_49743 | 56 | -0.591343863 | TMBIM4 | transmembrane BAX inhibitor motif containing 4 [Source:HGNC Symbol;Acc:HGNC:24257] |
| ENSG00000156150 | HGLibA_01735 | 56 | -0.591343863 | ALX3 | ALX homeobox 3 [Source:HGNC Symbol;Acc:HGNC:449] |
| ENSG00000156587 | HGLibA_52630 | 56 | -0.591343863 | UBE2L6 | ubiquitin conjugating enzyme E2 L6 [Source:HGNC Symbol;Acc:HGNC:12490] |
| ENSG00000157036 | HGLibA_15765 | 56 | -0.591343863 | EXOG | exo/endonuclease G [Source:HGNC Symbol;Acc:HGNC:3347] |
| ENSG00000158901 | HGLibA_54273 | 56 | -0.591343863 | WFDC8 | WAP four-disulfide core domain 8 [Source:HGNC Symbol;Acc:HGNC:16163] |
| ENSG00000160207 | HGLibA_22227 | 56 | -0.591343863 | HSF2BP | heat shock transcription factor 2 binding protein [Source:HGNC Symbol;Acc:HGNC:5226] |
| ENSG00000160917 | HGLibA_11006 | 56 | -0.591343863 | CPSF4 | cleavage and polyadenylation specific factor 4 [Source:HGNC Symbol;Acc:HGNC:2327] |
| ENSG00000160949 | HGLibA_50985 | 56 | -0.591343863 | TONSL | DNA repair protein [Source:HGNC Symbol;Acc:HGNC:7801]" |
| ENSG00000161243 | HGLibA_17058 | 56 | -0.591343863 | FBXO27 | F-box protein 27 [Source:HGNC Symbol;Acc:HGNC:18753] |
| ENSG00000162521 | HGLibA_40427 | 56 | -0.591343863 | RBBP4 | chromatin remodeling factor [Source:HGNC Symbol;Acc:HGNC:9887]" |
| ENSG00000162627 | HGLibA_46105 | 56 | -0.591343863 | SNX7 | sorting nexin 7 [Source:HGNC Symbol;Acc:HGNC:14971] |
| ENSG00000162746 | HGLibA_17247 | 56 | -0.591343863 | FCRLB | Fc receptor like B [Source:HGNC Symbol;Acc:HGNC:26431] |
| ENSG00000162755 | HGLibA_25118 | 56 | -0.591343863 | KLHDC9 | kelch domain containing 9 [Source:HGNC Symbol;Acc:HGNC:28489] |
| ENSG00000163281 | HGLibA_19569 | 56 | -0.591343863 | GNPDA2 | glucosamine-6-phosphate deaminase 2 [Source:HGNC Symbol;Acc:HGNC:21526] |
| ENSG00000163898 | HGLibA_26615 | 56 | -0.591343863 | LIPH | lipase H [Source:HGNC Symbol;Acc:HGNC:18483] |
| ENSG00000164916 | HGLibA_17894 | 56 | -0.591343863 | FOXK1 | forkhead box K1 [Source:HGNC Symbol;Acc:HGNC:23480] |
| ENSG00000165661 | HGLibA_39757 | 56 | -0.591343863 | QSOX2 | quiescin sulfhydryl oxidase 2 [Source:HGNC Symbol;Acc:HGNC:30249] |
| ENSG00000165806 | HGLibA_07323 | 56 | -0.591343863 | CASP7 | caspase 7 [Source:HGNC Symbol;Acc:HGNC:1508] |
| ENSG00000166592 | HGLibA_42221 | 56 | -0.591343863 | RRAD | Ras related glycolysis inhibitor and calcium channel regulator [Source:HGNC Symbol;Acc:HGNC:10446]" |
| ENSG00000167210 | HGLibA_27034 | 56 | -0.591343863 | LOXHD1 | lipoxygenase homology domains 1 [Source:HGNC Symbol;Acc:HGNC:26521] |
| ENSG00000167657 | HGLibA_12329 | 56 | -0.591343863 | DAPK3 | death associated protein kinase 3 [Source:HGNC Symbol;Acc:HGNC:2676] |
| ENSG00000167657 | HGLibA_12327 | 56 | -0.591343863 | DAPK3 | death associated protein kinase 3 [Source:HGNC Symbol;Acc:HGNC:2676] |
| ENSG00000167815 | HGLibA_38410 | 56 | -0.591343863 | PRDX2 | peroxiredoxin 2 [Source:HGNC Symbol;Acc:HGNC:9353] |
| ENSG00000167996 | HGLibA_18116 | 56 | -0.591343863 | FTH1 | ferritin heavy chain 1 [Source:HGNC Symbol;Acc:HGNC:3976] |
| ENSG00000168273 | HGLibA_45757 | 56 | -0.591343863 | SMIM4 | small integral membrane protein 4 [Source:HGNC Symbol;Acc:HGNC:37257] |
| ENSG00000168575 | HGLibA_44530 | 56 | -0.591343863 | SLC20A2 | solute carrier family 20 member 2 [Source:HGNC Symbol;Acc:HGNC:10947] |
| ENSG00000168743 | HGLibA_32496 | 56 | -0.591343863 | NPNT | nephronectin [Source:HGNC Symbol;Acc:HGNC:27405] |
| ENSG00000168874 | HGLibA_03540 | 56 | -0.591343863 | ATOH8 | atonal bHLH transcription factor 8 [Source:HGNC Symbol;Acc:HGNC:24126] |
| ENSG00000169188 | HGLibA_02409 | 56 | -0.591343863 | APEX2 | apurinic/apyrimidinic endodeoxyribonuclease 2 [Source:HGNC Symbol;Acc:HGNC:17889] |
| ENSG00000169575 | HGLibA_53723 | 56 | -0.591343863 | VPREB1 | V-set pre-B cell surrogate light chain 1 [Source:HGNC Symbol;Acc:HGNC:12709] |
| ENSG00000170100 | HGLibA_56729 | 56 | -0.591343863 | ZNF778 | zinc finger protein 778 [Source:HGNC Symbol;Acc:HGNC:26479] |
| ENSG00000170385 | HGLibA_44896 | 56 | -0.591343863 | SLC30A1 | solute carrier family 30 member 1 [Source:HGNC Symbol;Acc:HGNC:11012] |
| ENSG00000170581 | HGLibA_47384 | 56 | -0.591343863 | STAT2 | signal transducer and activator of transcription 2 [Source:HGNC Symbol;Acc:HGNC:11363] |
| ENSG00000170633 | HGLibA_41666 | 56 | -0.591343863 | RNF34 | ring finger protein 34 [Source:HGNC Symbol;Acc:HGNC:17297] |
| ENSG00000170734 | HGLibA_37528 | 56 | -0.591343863 | POLH | DNA polymerase eta [Source:HGNC Symbol;Acc:HGNC:9181] |
| ENSG00000170786 | HGLibA_43159 | 56 | -0.591343863 | SDR16C5 | short chain dehydrogenase/reductase family 16C member 5 [Source:HGNC Symbol;Acc:HGNC:30311] |
| ENSG00000170890 | HGLibA_36883 | 56 | -0.591343863 | PLA2G1B | phospholipase A2 group IB [Source:HGNC Symbol;Acc:HGNC:9030] |
| ENSG00000170899 | HGLibA_20461 | 56 | -0.591343863 | GSTA4 | glutathione S-transferase alpha 4 [Source:HGNC Symbol;Acc:HGNC:4629] |
| ENSG00000171262 | HGLibA_16812 | 56 | -0.591343863 | FAM98B | family with sequence similarity 98 member B [Source:HGNC Symbol;Acc:HGNC:26773] |
| ENSG00000172380 | HGLibA_19515 | 56 | -0.591343863 | GNG12 | G protein subunit gamma 12 [Source:HGNC Symbol;Acc:HGNC:19663] |
| ENSG00000173456 | HGLibA_41659 | 56 | -0.591343863 | RNF26 | ring finger protein 26 [Source:HGNC Symbol;Acc:HGNC:14646] |

| | | | | | |
|---|---|---|---|---|---|
| ENSG00000174238 | HGLibA_36742 | 56 | -0.591343863 | PITPNA | phosphatidylinositol transfer protein alpha [Source:HGNC Symbol;Acc:HGNC:9001] |
| ENSG00000174327 | HGLibA_44428 | 56 | -0.591343863 | SLC16A13 | solute carrier family 16 member 13 [Source:HGNC Symbol;Acc:HGNC:31037] |
| ENSG00000175322 | HGLibA_56156 | 56 | -0.591343863 | ZNF519 | zinc finger protein 519 [Source:HGNC Symbol;Acc:HGNC:30574] |
| ENSG00000175785 | HGLibA_38489 | 56 | -0.591343863 | PRIMA1 | proline rich membrane anchor 1 [Source:HGNC Symbol;Acc:HGNC:18319] |
| ENSG00000175874 | HGLibA_11122 | 56 | -0.591343863 | CREG2 | cellular repressor of E1A stimulated genes 2 [Source:HGNC Symbol;Acc:HGNC:14272] |
| ENSG00000175946 | HGLibA_25200 | 56 | -0.591343863 | KLHL38 | kelch like family member 38 [Source:HGNC Symbol;Acc:HGNC:34435] |
| ENSG00000175984 | HGLibA_12940 | 56 | -0.591343863 | DENND2C | DENN domain containing 2C [Source:HGNC Symbol;Acc:HGNC:24748] |
| ENSG00000176927 | HGLibA_14546 | 56 | -0.591343863 | EFCAB5 | EF-hand calcium binding domain 5 [Source:HGNC Symbol;Acc:HGNC:24801] |
| ENSG00000177463 | HGLibA_32597 | 56 | -0.591343863 | NR2C2 | nuclear receptor subfamily 2 group C member 2 [Source:HGNC Symbol;Acc:HGNC:7972] |
| ENSG00000177565 | HGLibA_48611 | 56 | -0.591343863 | TBL1XR1 | transducin beta like 1 X-linked receptor 1 [Source:HGNC Symbol;Acc:HGNC:29529] |
| ENSG00000178096 | HGLibA_04679 | 56 | -0.591343863 | BOLA1 | bolA family member 1 [Source:HGNC Symbol;Acc:HGNC:24263] |
| ENSG00000178586 | HGLibA_34378 | 56 | -0.591343863 | OR6B3 | olfactory receptor family 6 subfamily B member 3 [Source:HGNC Symbol;Acc:HGNC:15042] |
| ENSG00000178773 | HGLibA_10970 | 56 | -0.591343863 | CPNE7 | copine 7 [Source:HGNC Symbol;Acc:HGNC:2320] |
| ENSG00000179431 | HGLibA_17548 | 56 | -0.591343863 | FJX1 | four-jointed box kinase 1 [Source:HGNC Symbol;Acc:HGNC:17166] |
| ENSG00000179933 | HGLibA_05321 | 56 | -0.591343863 | C14orf119 | chromosome 14 open reading frame 119 [Source:HGNC Symbol;Acc:HGNC:20270] |
| ENSG00000179941 | HGLibA_04210 | 56 | -0.591343863 | BBS10 | Bardet-Biedl syndrome 10 [Source:HGNC Symbol;Acc:HGNC:26291] |
| ENSG00000180535 | HGLibA_04466 | 56 | -0.591343863 | BHLHA15 | basic helix-loop-helix family member a15 [Source:HGNC Symbol;Acc:HGNC:22265] |
| ENSG00000180806 | HGLibA_21981 | 56 | -0.591343863 | HOXC9 | homeobox C9 [Source:HGNC Symbol;Acc:HGNC:5130] |
| ENSG00000180828 | HGLibA_04475 | 56 | -0.591343863 | BHLHE22 | basic helix-loop-helix family member e22 [Source:HGNC Symbol;Acc:HGNC:11963] |
| ENSG00000181588 | HGLibA_29036 | 56 | -0.591343863 | MEX3D | mex-3 RNA binding family member D [Source:HGNC Symbol;Acc:HGNC:16734] |
| ENSG00000182400 | HGLibA_51333 | 56 | -0.591343863 | TRAPPC6B | trafficking protein particle complex 6B [Source:HGNC Symbol;Acc:HGNC:23066] |
| ENSG00000183963 | HGLibA_45832 | 56 | -0.591343863 | SMTN | smoothelin [Source:HGNC Symbol;Acc:HGNC:11126] |
| ENSG00000184508 | HGLibA_21036 | 56 | -0.591343863 | HDDC3 | HD domain containing 3 [Source:HGNC Symbol;Acc:HGNC:30522] |
| ENSG00000185437 | HGLibA_43913 | 56 | -0.591343863 | SH3BGR | SH3 domain binding glutamate rich protein [Source:HGNC Symbol;Acc:HGNC:10822] |
| ENSG00000185630 | HGLibA_35344 | 56 | -0.591343863 | PBX1 | PBX homeobox 1 [Source:HGNC Symbol;Acc:HGNC:8632] |
| ENSG00000185924 | HGLibA_42417 | 56 | -0.591343863 | RTN4RL1 | reticulon 4 receptor like 1 [Source:HGNC Symbol;Acc:HGNC:21329] |
| ENSG00000186334 | HGLibA_45037 | 56 | -0.591343863 | SLC36A3 | solute carrier family 36 member 3 [Source:HGNC Symbol;Acc:HGNC:19659] |
| ENSG00000186564 | HGLibA_17831 | 56 | -0.591343863 | FOXD2 | forkhead box D2 [Source:HGNC Symbol;Acc:HGNC:3803] |
| ENSG00000186579 | HGLibA_12832 | 56 | -0.591343863 | DEFB106A | defensin beta 106A [Source:HGNC Symbol;Acc:HGNC:18088] |
| ENSG00000186654 | HGLibA_38842 | 56 | -0.591343863 | PRR5 | proline rich 5 [Source:HGNC Symbol;Acc:HGNC:31682] |
| ENSG00000186965 | HGLibA_25666 | 56 | -0.591343863 | KRTAP19-2 | keratin associated protein 19-2 [Source:HGNC Symbol;Acc:HGNC:18937] |
| ENSG00000186994 | HGLibA_24029 | 56 | -0.591343863 | KANK3 | KN motif and ankyrin repeat domains 3 [Source:HGNC Symbol;Acc:HGNC:24796] |
| ENSG00000187147 | HGLibA_41641 | 56 | -0.591343863 | RNF220 | ring finger protein 220 [Source:HGNC Symbol;Acc:HGNC:25552] |
| ENSG00000187151 | HGLibA_01922 | 56 | -0.591343863 | ANGPTL5 | angiopoietin like 5 [Source:HGNC Symbol;Acc:HGNC:19705] |
| ENSG00000187474 | HGLibA_17968 | 56 | -0.591343863 | FPR3 | formyl peptide receptor 3 [Source:HGNC Symbol;Acc:HGNC:3828] |
| ENSG00000187790 | HGLibA_16860 | 56 | -0.591343863 | FANCM | FA complementation group M [Source:HGNC Symbol;Acc:HGNC:23168] |
| ENSG00000188171 | HGLibA_56422 | 56 | -0.591343863 | ZNF626 | zinc finger protein 626 [Source:HGNC Symbol;Acc:HGNC:30461] |
| ENSG00000188312 | HGLibA_09081 | 56 | -0.591343863 | CENPP | centromere protein P [Source:HGNC Symbol;Acc:HGNC:32933] |
| ENSG00000188580 | HGLibA_31991 | 56 | -0.591343863 | NKAIN2 | sodium/potassium transporting ATPase interacting 2 [Source:HGNC Symbol;Acc:HGNC:16443] |
| ENSG00000188710 | HGLibA_39737 | 56 | -0.591343863 | QRFP | pyroglutamylated RFamide peptide [Source:HGNC Symbol;Acc:HGNC:29982] |
| ENSG00000189339 | HGLibA_44991 | 56 | -0.591343863 | SLC35E2B | solute carrier family 35 member E2B [Source:HGNC Symbol;Acc:HGNC:33941] |
| ENSG00000189420 | HGLibA_55245 | 56 | -0.591343863 | ZFP92 | ZFP92 zinc finger protein [Source:HGNC Symbol;Acc:HGNC:12865] |
| ENSG00000196497 | HGLibA_23467 | 56 | -0.591343863 | IPO4 | importin 4 [Source:HGNC Symbol;Acc:HGNC:19426] |
| ENSG00000196693 | HGLibA_55835 | 56 | -0.591343863 | ZNF33B | zinc finger protein 33B [Source:HGNC Symbol;Acc:HGNC:13097] |
| ENSG00000196734 | HGLibA_26102 | 56 | -0.591343863 | LCE1B | late cornified envelope 1B [Source:HGNC Symbol;Acc:HGNC:16611] |
| ENSG00000196937 | HGLibA_16581 | 56 | -0.591343863 | FAM3C | family with sequence similarity 3 member C [Source:HGNC Symbol;Acc:HGNC:18664] |
| ENSG00000197324 | HGLibA_27199 | 56 | -0.591343863 | LRP10 | LDL receptor related protein 10 [Source:HGNC Symbol;Acc:HGNC:14553] |
| ENSG00000198130 | HGLibA_21285 | 56 | -0.591343863 | HIBCH | 3-hydroxyisobutyryl-CoA hydrolase [Source:HGNC Symbol;Acc:HGNC:4908] |
| ENSG00000198967 | HGLibA_33560 | 56 | -0.591343863 | OR10Z1 | olfactory receptor family 10 subfamily Z member 1 [Source:HGNC Symbol;Acc:HGNC:14996] |
| ENSG00000204006 | HGLibA_05804 | 56 | -0.591343863 | C1orf185 | chromosome 1 open reading frame 185 [Source:HGNC Symbol;Acc:HGNC:28096] |
| ENSG00000204519 | HGLibA_56216 | 56 | -0.591343863 | ZNF551 | zinc finger protein 551 [Source:HGNC Symbol;Acc:HGNC:25108] |
| ENSG00000204919 | HGLibA_38800 | 56 | -0.591343863 | PRR20A | proline rich 20A [Source:HGNC Symbol;Acc:HGNC:24754] |
| ENSG00000205138 | HGLibA_43136 | 56 | -0.591343863 | SDHAF1 | succinate dehydrogenase complex assembly factor 1 [Source:HGNC Symbol;Acc:HGNC:33867] |
| ENSG00000212721 | HGLibA_25753 | 56 | -0.591343863 | KRTAP4-11 | keratin associated protein 4-11 [Source:HGNC Symbol;Acc:HGNC:18911] |
| ENSG00000213203 | HGLibA_19069 | 56 | -0.591343863 | GIMAP1 | IMAP family member 1 [Source:HGNC Symbol;Acc:HGNC:23237]" |
| ENSG00000213999 | HGLibA_28828 | 56 | -0.591343863 | MEF2B | myocyte enhancer factor 2B [Source:HGNC Symbol;Acc:HGNC:6995] |
| ENSG00000221874 | HGLibA_56816 | 56 | -0.591343863 | ZNF816-ZNF321P | ZNF816-ZNF321P readthrough [Source:HGNC Symbol;Acc:HGNC:38879] |
| ENSG00000227551 | HGLibA_53221 | 56 | -0.591343863 | USP17L12 | ubiquitin specific peptidase 17-like family member 12 [Source:HGNC Symbol;Acc:HGNC:44440] |
| ENSG00000230989 | HGLibA_22150 | 56 | -0.591343863 | HSBP1 | heat shock factor binding protein 1 [Source:HGNC Symbol;Acc:HGNC:5203] |
| ENSG00000232859 | HGLibA_27762 | 56 | -0.591343863 | LYRM9 | LYR motif containing 9 [Source:HGNC Symbol;Acc:HGNC:27314] |
| ENSG00000233701 | HGLibA_38828 | 56 | -0.591343863 | PRR23C | proline rich 23C [Source:HGNC Symbol;Acc:HGNC:37173] |
| ENSG00000234278 | HGLibA_38813 | 56 | -0.591343863 | PRR20E | proline rich 20E [Source:HGNC Symbol;Acc:HGNC:37223] |
| ENSG00000234465 | HGLibA_36692 | 56 | -0.591343863 | PINLYP | phospholipase A2 inhibitor and LY6/PLAUR domain containing [Source:HGNC Symbol;Acc:HGNC:44206] |
| ENSG00000255245 | HGLibA_18243 | 56 | -0.591343863 | FXYD6-FXYD2 | FXYD6-FXYD2 readthrough [Source:HGNC Symbol;Acc:HGNC:39978] |
| ENSG00000258873 | HGLibA_14204 | 56 | -0.591343863 | DUXA | double homeobox A [Source:HGNC Symbol;Acc:HGNC:32179] |
| ENSG00000259956 | HGLibA_40495 | 56 | -0.591343863 | RBM15B | RNA binding motif protein 15B [Source:HGNC Symbol;Acc:HGNC:24303] |
| ENSG00000263353 | HGLibA_37896 | 56 | -0.591343863 | PPIAL4A | peptidylprolyl isomerase A like 4A [Source:HGNC Symbol;Acc:HGNC:24369] |
| ENSG00000269343 | HGLibA_56333 | 56 | -0.591343863 | ZNF587B | zinc finger protein 587B [Source:HGNC Symbol;Acc:HGNC:37142] |
| FAM179B | HGLibA_16310 | 56 | -0.591343863 | FAM179B | - |
| HNRPLL | HGLibA_21859 | 56 | -0.591343863 | HNRPLL | - |
| hsa-mir-1200 | HGLibA_57263 | 56 | -0.591343863 | hsa-mir-1200 | - |
| hsa-mir-1471 | HGLibA_57870 | 56 | -0.591343863 | hsa-mir-1471 | - |
| hsa-mir-182 | HGLibA_57983 | 56 | -0.591343863 | hsa-mir-182 | - |
| hsa-mir-2117 | HGLibA_58241 | 56 | -0.591343863 | hsa-mir-2117 | - |
| hsa-mir-218-1 | HGLibA_58267 | 56 | -0.591343863 | hsa-mir-218-1 | - |
| hsa-mir-3120 | HGLibA_58533 | 56 | -0.591343863 | hsa-mir-3120 | - |
| hsa-mir-3135a | HGLibA_58594 | 56 | -0.591343863 | hsa-mir-3135a | - |
| hsa-mir-3618 | HGLibA_59100 | 56 | -0.591343863 | hsa-mir-3618 | - |
| hsa-mir-3622b | HGLibA_59127 | 56 | -0.591343863 | hsa-mir-3622b | - |
| hsa-mir-3686 | HGLibA_59299 | 56 | -0.591343863 | hsa-mir-3686 | - |
| hsa-mir-381 | HGLibA_59479 | 56 | -0.591343863 | hsa-mir-381 | - |
| hsa-mir-3910-1 | HGLibA_59506 | 56 | -0.591343863 | hsa-mir-3910-1 | - |
| hsa-mir-4315-2 | HGLibA_59978 | 56 | -0.591343863 | hsa-mir-4315-2 | - |
| hsa-mir-4424 | HGLibA_60085 | 56 | -0.591343863 | hsa-mir-4424 | - |
| hsa-mir-4761 | HGLibA_61140 | 56 | -0.591343863 | hsa-mir-4761 | - |
| hsa-mir-4762 | HGLibA_61146 | 56 | -0.591343863 | hsa-mir-4762 | - |
| hsa-mir-516a-1 | HGLibA_61609 | 56 | -0.591343863 | hsa-mir-516a-1 | - |
| hsa-mir-554 | HGLibA_62132 | 56 | -0.591343863 | hsa-mir-554 | - |
| hsa-mir-600 | HGLibA_62493 | 56 | -0.591343863 | hsa-mir-600 | - |
| hsa-mir-6082 | HGLibA_62589 | 56 | -0.591343863 | hsa-mir-6082 | - |
| hsa-mir-6499 | HGLibA_62844 | 56 | -0.591343863 | hsa-mir-6499 | - |
| hsa-mir-6515 | HGLibA_62933 | 56 | -0.591343863 | hsa-mir-6515 | - |
| hsa-mir-6734 | HGLibA_63088 | 56 | -0.591343863 | hsa-mir-6734 | - |
| hsa-mir-6737 | HGLibA_63102 | 56 | -0.591343863 | hsa-mir-6737 | - |
| hsa-mir-6766 | HGLibA_63224 | 56 | -0.591343863 | hsa-mir-6766 | - |
| hsa-mir-6770-1 | HGLibA_63247 | 56 | -0.591343863 | hsa-mir-6770-1 | - |
| hsa-mir-6771 | HGLibA_63256 | 56 | -0.591343863 | hsa-mir-6771 | - |
| hsa-mir-7107 | HGLibA_63790 | 56 | -0.591343863 | hsa-mir-7107 | - |
| hsa-mir-7515 | HGLibA_63887 | 56 | -0.591343863 | hsa-mir-7515 | - |
| hsa-mir-762 | HGLibA_63908 | 56 | -0.591343863 | hsa-mir-762 | - |
| hsa-mir-7853 | HGLibA_64003 | 56 | -0.591343863 | hsa-mir-7853 | - |
| hsa-mir-93 | HGLibA_64303 | 56 | -0.591343863 | hsa-mir-93 | - |
| KAL1 | HGLibA_24017 | 56 | -0.591343863 | KAL1 | - |
| KIAA0196 | HGLibA_24602 | 56 | -0.591343863 | KIAA0196 | - |
| LOC100129083 | HGLibA_26753 | 56 | -0.591343863 | LOC100129083 | - |
| LOC100996331 | HGLibA_26881 | 56 | -0.591343863 | LOC100996331 | - |
| LOC642778 | HGLibA_26956 | 56 | -0.591343863 | LOC642778 | - |
| PHF15 | HGLibA_36347 | 56 | -0.591343863 | PHF15 | - |
| PPIAL4B | HGLibA_37899 | 56 | -0.591343863 | PPIAL4B | - |
| TOMM70A | HGLibA_50982 | 56 | -0.591343863 | TOMM70A | - |
| ASMT_Y | HGLibA_03324 | 55 | -0.6012949 | ASMT_Y | - |
| C11orf63 | HGLibA_05167 | 55 | -0.6012949 | C11orf63 | - |
| C2orf43 | HGLibA_06127 | 55 | -0.6012949 | C2orf43 | - |
| C6orf7 | HGLibA_06506 | 55 | -0.6012949 | C6orf7 | - |
| CRLF2_X | HGLibA_11190 | 55 | -0.6012949 | CRLF2_X | - |
| CYP3A7-CYP3AP1 | HGLibA_12167 | 55 | -0.6012949 | CYP3A7-CYP3AP1 | - |
| ENSG00000003987 | HGLibA_30437 | 55 | -0.6012949 | MTMR7 | myotubularin related protein 7 [Source:HGNC Symbol;Acc:HGNC:7454] |
| ENSG00000006071 | HGLibA_00184 | 55 | -0.6012949 | ABCC8 | ATP binding cassette subfamily C member 8 [Source:HGNC Symbol;Acc:HGNC:59] |
| ENSG00000006283 | HGLibA_06894 | 55 | -0.6012949 | CACNA1G | calcium voltage-gated channel subunit alpha1 G [Source:HGNC Symbol;Acc:HGNC:1394] |
| ENSG00000008226 | HGLibA_13318 | 55 | -0.6012949 | DLEC1 | cilia and flagella associated protein [Source:HGNC Symbol;Acc:HGNC:2899]" |
| ENSG00000008300 | HGLibA_09028 | 55 | -0.6012949 | CELSR3 | cadherin EGF LAG seven-pass G-type receptor 3 [Source:HGNC Symbol;Acc:HGNC:3230] |
| ENSG00000012232 | HGLibA_15813 | 55 | -0.6012949 | EXTL3 | exostosin like glycosyltransferase 3 [Source:HGNC Symbol;Acc:HGNC:3518] |
| ENSG00000016402 | HGLibA_23077 | 55 | -0.6012949 | IL20RA | interleukin 20 receptor subunit alpha [Source:HGNC Symbol;Acc:HGNC:6003] |
| ENSG00000025770 | HGLibA_31279 | 55 | -0.6012949 | NCAPH2 | non-SMC condensin II complex subunit H2 [Source:HGNC Symbol;Acc:HGNC:25071] |
| ENSG00000037474 | HGLibA_32781 | 55 | -0.6012949 | NSUN2 | NOP2/Sun RNA methyltransferase family member 2 [Source:HGNC Symbol;Acc:HGNC:25994] |
| ENSG00000041982 | HGLibA_50699 | 55 | -0.6012949 | TNC | tenascin C [Source:HGNC Symbol;Acc:HGNC:5318] |
| ENSG00000049540 | HGLibA_14952 | 55 | -0.6012949 | ELN | elastin [Source:HGNC Symbol;Acc:HGNC:3327] |
| ENSG00000054219 | HGLibA_27679 | 55 | -0.6012949 | LY75 | lymphocyte antigen 75 [Source:HGNC Symbol;Acc:HGNC:6729] |
| ENSG00000062282 | HGLibA_13038 | 55 | -0.6012949 | DGAT2 | diacylglycerol O-acyltransferase 2 [Source:HGNC Symbol;Acc:HGNC:16940] |
| ENSG00000064655 | HGLibA_15819 | 55 | -0.6012949 | EYA2 | EYA transcriptional coactivator and phosphatase 2 [Source:HGNC Symbol;Acc:HGNC:3520] |
| ENSG00000065000 | HGLibA_02327 | 55 | -0.6012949 | AP3D1 | adaptor related protein complex 3 subunit delta 1 [Source:HGNC Symbol;Acc:HGNC:568] |
| ENSG00000065989 | HGLibA_35788 | 55 | -0.6012949 | PDE4A | phosphodiesterase 4A [Source:HGNC Symbol;Acc:HGNC:8780] |
| ENSG00000066322 | HGLibA_14957 | 55 | -0.6012949 | ELOVL1 | ELOVL fatty acid elongase 1 [Source:HGNC Symbol;Acc:HGNC:14418] |
| ENSG00000068305 | HGLibA_28827 | 55 | -0.6012949 | MEF2A | myocyte enhancer factor 2A [Source:HGNC Symbol;Acc:HGNC:6993] |
| ENSG00000069943 | HGLibA_36545 | 55 | -0.6012949 | PIGB | phosphatidylinositol glycan anchor biosynthesis class B [Source:HGNC Symbol;Acc:HGNC:8959] |

| | | | | | |
|---|---|---|---|---|---|
| ENSG00000070831 | HGLibA_08555 | 55 | -0.6012949 | CDC42 | cell division cycle 42 [Source:HGNC Symbol;Acc:HGNC:1736] |
| ENSG00000071889 | HGLibA_16577 | 55 | -0.6012949 | FAM3A | family with sequence similarity 3 member A [Source:HGNC Symbol;Acc:HGNC:13749] |
| ENSG00000073417 | HGLibA_35829 | 55 | -0.6012949 | PDE8A | phosphodiesterase 8A [Source:HGNC Symbol;Acc:HGNC:8793] |
| ENSG00000074590 | HGLibA_32893 | 55 | -0.6012949 | NUAK1 | NUAK family kinase 1 [Source:HGNC Symbol;Acc:HGNC:14311] |
| ENSG00000074803 | HGLibA_44357 | 55 | -0.6012949 | SLC12A1 | solute carrier family 12 member 1 [Source:HGNC Symbol;Acc:HGNC:10910] |
| ENSG00000076344 | HGLibA_41054 | 55 | -0.6012949 | RGS11 | regulator of G protein signaling 11 [Source:HGNC Symbol;Acc:HGNC:9993] |
| ENSG00000080031 | HGLibA_39509 | 55 | -0.6012949 | PTPRH | receptor type H [Source:HGNC Symbol;Acc:HGNC:9672]" |
| ENSG00000080822 | HGLibA_09915 | 55 | -0.6012949 | CLDND1 | claudin domain containing 1 [Source:HGNC Symbol;Acc:HGNC:1322] |
| ENSG00000083642 | HGLibA_35947 | 55 | -0.6012949 | PDS5B | PDS5 cohesin associated factor B [Source:HGNC Symbol;Acc:HGNC:20418] |
| ENSG00000085063 | HGLibA_08417 | 55 | -0.6012949 | CD59 | CD59 molecule (CD59 blood group) [Source:HGNC Symbol;Acc:HGNC:1689] |
| ENSG00000085382 | HGLibA_20798 | 55 | -0.6012949 | HACE1 | HECT domain and ankyrin repeat containing E3 ubiquitin protein ligase 1 [Source:HGNC Symbol;Acc:HGNC:21033] |
| ENSG00000088876 | HGLibA_55843 | 55 | -0.6012949 | ZNF343 | zinc finger protein 343 [Source:HGNC Symbol;Acc:HGNC:16017] |
| ENSG00000090905 | HGLibA_50912 | 55 | -0.6012949 | TNRC6A | trinucleotide repeat containing 6A [Source:HGNC Symbol;Acc:HGNC:11969] |
| ENSG00000095752 | HGLibA_22952 | 55 | -0.6012949 | IL11 | interleukin 11 [Source:HGNC Symbol;Acc:HGNC:5966] |
| ENSG00000100079 | HGLibA_26360 | 55 | -0.6012949 | LGALS2 | galectin 2 [Source:HGNC Symbol;Acc:HGNC:6562] |
| ENSG00000100249 | HGLibA_06072 | 55 | -0.6012949 | C22orf31 | chromosome 22 open reading frame 31 [Source:HGNC Symbol;Acc:HGNC:26931] |
| ENSG00000100462 | HGLibA_38628 | 55 | -0.6012949 | PRMT5 | protein arginine methyltransferase 5 [Source:HGNC Symbol;Acc:HGNC:10894] |
| ENSG00000100884 | HGLibA_10968 | 55 | -0.6012949 | CPNE6 | copine 6 [Source:HGNC Symbol;Acc:HGNC:2319] |
| ENSG00000102580 | HGLibA_13615 | 55 | -0.6012949 | DNAJC3 | DnaJ heat shock protein family (Hsp40) member C3 [Source:HGNC Symbol;Acc:HGNC:9439] |
| ENSG00000103061 | HGLibA_45375 | 55 | -0.6012949 | SLC7A6OS | solute carrier family 7 member 6 opposite strand [Source:HGNC Symbol;Acc:HGNC:25807] |
| ENSG00000103485 | HGLibA_39734 | 55 | -0.6012949 | QPRT | quinolinate phosphoribosyltransferase [Source:HGNC Symbol;Acc:HGNC:9755] |
| ENSG00000104343 | HGLibA_52673 | 55 | -0.6012949 | UBE2W | ubiquitin conjugating enzyme E2 W [Source:HGNC Symbol;Acc:HGNC:25616] |
| ENSG00000105429 | HGLibA_28856 | 55 | -0.6012949 | MEGF8 | multiple EGF like domains 8 [Source:HGNC Symbol;Acc:HGNC:3233] |
| ENSG00000105974 | HGLibA_07375 | 55 | -0.6012949 | CAV1 | caveolin 1 [Source:HGNC Symbol;Acc:HGNC:1527] |
| ENSG00000106789 | HGLibA_10781 | 55 | -0.6012949 | CORO2A | coronin 2A [Source:HGNC Symbol;Acc:HGNC:2255] |
| ENSG00000107021 | HGLibA_48476 | 55 | -0.6012949 | TBC1D13 | TBC1 domain family member 13 [Source:HGNC Symbol;Acc:HGNC:25571] |
| ENSG00000108829 | HGLibA_27400 | 55 | -0.6012949 | LRRC59 | leucine rich repeat containing 59 [Source:HGNC Symbol;Acc:HGNC:28817] |
| ENSG00000109270 | HGLibA_25995 | 55 | -0.6012949 | LAMTOR3 | MAPK and MTOR activator 3 [Source:HGNC Symbol;Acc:HGNC:15606]" |
| ENSG00000109927 | HGLibA_48952 | 55 | -0.6012949 | TECTA | tectorin alpha [Source:HGNC Symbol;Acc:HGNC:11720] |
| ENSG00000111237 | HGLibA_53760 | 55 | -0.6012949 | VPS29 | retromer complex component [Source:HGNC Symbol;Acc:HGNC:14340]" |
| ENSG00000111361 | HGLibA_14735 | 55 | -0.6012949 | EIF2B1 | eukaryotic translation initiation factor 2B subunit alpha [Source:HGNC Symbol;Acc:HGNC:3257] |
| ENSG00000111670 | HGLibA_19573 | 55 | -0.6012949 | GNPTAB | N-acetylglucosamine-1-phosphate transferase subunits alpha and beta [Source:HGNC Symbol;Acc:HGNC:29670] |
| ENSG00000112214 | HGLibA_17488 | 55 | -0.6012949 | FHL5 | four and a half LIM domains 5 [Source:HGNC Symbol;Acc:HGNC:17371] |
| ENSG00000112561 | HGLibA_49164 | 55 | -0.6012949 | TFEB | transcription factor EB [Source:HGNC Symbol;Acc:HGNC:11753] |
| ENSG00000114054 | HGLibA_35381 | 55 | -0.6012949 | PCCB | propionyl-CoA carboxylase subunit beta [Source:HGNC Symbol;Acc:HGNC:8654] |
| ENSG00000115368 | HGLibA_54175 | 55 | -0.6012949 | WDR75 | WD repeat domain 75 [Source:HGNC Symbol;Acc:HGNC:25725] |
| ENSG00000115421 | HGLibA_35143 | 55 | -0.6012949 | PAPOLG | poly(A) polymerase gamma [Source:HGNC Symbol;Acc:HGNC:14982] |
| ENSG00000115998 | HGLibA_06124 | 55 | -0.6012949 | C2orf42 | chromosome 2 open reading frame 42 [Source:HGNC Symbol;Acc:HGNC:26056] |
| ENSG00000116525 | HGLibA_51570 | 55 | -0.6012949 | TRIM62 | tripartite motif containing 62 [Source:HGNC Symbol;Acc:HGNC:25574] |
| ENSG00000117691 | HGLibA_31691 | 55 | -0.6012949 | NENF | neudesin neurotrophic factor [Source:HGNC Symbol;Acc:HGNC:30384] |
| ENSG00000118160 | HGLibA_45391 | 55 | -0.6012949 | SLC8A2 | solute carrier family 8 member A2 [Source:HGNC Symbol;Acc:HGNC:11069] |
| ENSG00000118407 | HGLibA_17529 | 55 | -0.6012949 | FILIP1 | filamin A interacting protein 1 [Source:HGNC Symbol;Acc:HGNC:21015] |
| ENSG00000119698 | HGLibA_38210 | 55 | -0.6012949 | PPP4R4 | protein phosphatase 4 regulatory subunit 4 [Source:HGNC Symbol;Acc:HGNC:23788] |
| ENSG00000119801 | HGLibA_54717 | 55 | -0.6012949 | YPEL5 | yippee like 5 [Source:HGNC Symbol;Acc:HGNC:18329] |
| ENSG00000119844 | HGLibA_01130 | 55 | -0.6012949 | AFTPH | aftiphilin [Source:HGNC Symbol;Acc:HGNC:25951] |
| ENSG00000120729 | HGLibA_30897 | 55 | -0.6012949 | MYOT | myotilin [Source:HGNC Symbol;Acc:HGNC:12399] |
| ENSG00000121022 | HGLibA_10722 | 55 | -0.6012949 | COPS5 | COP9 signalosome subunit 5 [Source:HGNC Symbol;Acc:HGNC:2240] |
| ENSG00000122033 | HGLibA_30401 | 55 | -0.6012949 | MTIF3 | mitochondrial translational initiation factor 3 [Source:HGNC Symbol;Acc:HGNC:29788] |
| ENSG00000124193 | HGLibA_47083 | 55 | -0.6012949 | SRSF6 | serine and arginine rich splicing factor 6 [Source:HGNC Symbol;Acc:HGNC:10788] |
| ENSG00000125037 | HGLibA_15012 | 55 | -0.6012949 | EMC3 | ER membrane protein complex subunit 3 [Source:HGNC Symbol;Acc:HGNC:23999] |
| ENSG00000125740 | HGLibA_17799 | 55 | -0.6012949 | FOSB | AP-1 transcription factor subunit [Source:HGNC Symbol;Acc:HGNC:3797]" |
| ENSG00000127528 | HGLibA_25065 | 55 | -0.6012949 | KLF2 | Kruppel like factor 2 [Source:HGNC Symbol;Acc:HGNC:6347] |
| ENSG00000127990 | HGLibA_43806 | 55 | -0.6012949 | SGCE | sarcoglycan epsilon [Source:HGNC Symbol;Acc:HGNC:10808] |
| ENSG00000129636 | HGLibA_23705 | 55 | -0.6012949 | ITFG1 | integrin alpha FG-GAP repeat containing 1 [Source:HGNC Symbol;Acc:HGNC:30697] |
| ENSG00000130382 | HGLibA_29407 | 55 | -0.6012949 | MLLT1 | super elongation complex subunit [Source:HGNC Symbol;Acc:HGNC:7134]" |
| ENSG00000130829 | HGLibA_14195 | 55 | -0.6012949 | DUSP9 | dual specificity phosphatase 9 [Source:HGNC Symbol;Acc:HGNC:3076] |
| ENSG00000130988 | HGLibA_41019 | 55 | -0.6012949 | RGN | regucalcin [Source:HGNC Symbol;Acc:HGNC:9989] |
| ENSG00000131165 | HGLibA_09471 | 55 | -0.6012949 | CHMP1A | charged multivesicular body protein 1A [Source:HGNC Symbol;Acc:HGNC:8740] |
| ENSG00000131183 | HGLibA_44939 | 55 | -0.6012949 | SLC34A1 | solute carrier family 34 member 1 [Source:HGNC Symbol;Acc:HGNC:11019] |
| ENSG00000131435 | HGLibA_35915 | 55 | -0.6012949 | PDLIM4 | PDZ and LIM domain 4 [Source:HGNC Symbol;Acc:HGNC:16501] |
| ENSG00000131477 | HGLibA_40182 | 55 | -0.6012949 | RAMP2 | receptor activity modifying protein 2 [Source:HGNC Symbol;Acc:HGNC:9844] |
| ENSG00000131910 | HGLibA_32570 | 55 | -0.6012949 | NR0B2 | nuclear receptor subfamily 0 group B member 2 [Source:HGNC Symbol;Acc:HGNC:7961] |
| ENSG00000132274 | HGLibA_51433 | 55 | -0.6012949 | TRIM22 | tripartite motif containing 22 [Source:HGNC Symbol;Acc:HGNC:16379] |
| ENSG00000132963 | HGLibA_37665 | 55 | -0.6012949 | POMP | proteasome maturation protein [Source:HGNC Symbol;Acc:HGNC:20330] |
| ENSG00000133083 | HGLibA_12499 | 55 | -0.6012949 | DCLK1 | doublecortin like kinase 1 [Source:HGNC Symbol;Acc:HGNC:2700] |
| ENSG00000133773 | HGLibA_07808 | 55 | -0.6012949 | CCDC59 | coiled-coil domain containing 59 [Source:HGNC Symbol;Acc:HGNC:25005] |
| ENSG00000133997 | HGLibA_28812 | 55 | -0.6012949 | MED6 | mediator complex subunit 6 [Source:HGNC Symbol;Acc:HGNC:19970] |
| ENSG00000134775 | HGLibA_17494 | 55 | -0.6012949 | FHOD3 | formin homology 2 domain containing 3 [Source:HGNC Symbol;Acc:HGNC:26178] |
| ENSG00000134825 | HGLibA_50343 | 55 | -0.6012949 | TMEM258 | transmembrane protein 258 [Source:HGNC Symbol;Acc:HGNC:1164] |
| ENSG00000135318 | HGLibA_32828 | 55 | -0.6012949 | NT5E | 5'-nucleotidase ecto [Source:HGNC Symbol;Acc:HGNC:8021] |
| ENSG00000135677 | HGLibA_19589 | 55 | -0.6012949 | GNS | glucosamine (N-acetyl)-6-sulfatase [Source:HGNC Symbol;Acc:HGNC:4422] |
| ENSG00000135914 | HGLibA_22373 | 55 | -0.6012949 | HTR2B | 5-hydroxytryptamine receptor 2B [Source:HGNC Symbol;Acc:HGNC:5294] |
| ENSG00000137098 | HGLibA_46368 | 55 | -0.6012949 | SPAG8 | sperm associated antigen 8 [Source:HGNC Symbol;Acc:HGNC:14105] |
| ENSG00000137478 | HGLibA_17213 | 55 | -0.6012949 | FCHSD2 | FCH and double SH3 domains 2 [Source:HGNC Symbol;Acc:HGNC:29114] |
| ENSG00000137814 | HGLibA_20877 | 55 | -0.6012949 | HAUS2 | HAUS augmin like complex subunit 2 [Source:HGNC Symbol;Acc:HGNC:25530] |
| ENSG00000137948 | HGLibA_04791 | 55 | -0.6012949 | BRDT | bromodomain testis associated [Source:HGNC Symbol;Acc:HGNC:1105] |
| ENSG00000138435 | HGLibA_09562 | 55 | -0.6012949 | CHRNA1 | cholinergic receptor nicotinic alpha 1 subunit [Source:HGNC Symbol;Acc:HGNC:1955] |
| ENSG00000138767 | HGLibA_10346 | 55 | -0.6012949 | CNOT6L | CCR4-NOT transcription complex subunit 6 like [Source:HGNC Symbol;Acc:HGNC:18042] |
| ENSG00000140025 | HGLibA_14524 | 55 | -0.6012949 | EFCAB11 | EF-hand calcium binding domain 11 [Source:HGNC Symbol;Acc:HGNC:20357] |
| ENSG00000140527 | HGLibA_54224 | 55 | -0.6012949 | WDR93 | WD repeat domain 93 [Source:HGNC Symbol;Acc:HGNC:26924] |
| ENSG00000140830 | HGLibA_52442 | 55 | -0.6012949 | TXNL4B | thioredoxin like 4B [Source:HGNC Symbol;Acc:HGNC:26041] |
| ENSG00000143196 | HGLibA_13901 | 55 | -0.6012949 | DPT | dermatopontin [Source:HGNC Symbol;Acc:HGNC:3011] |
| ENSG00000143341 | HGLibA_21674 | 55 | -0.6012949 | HMCN1 | hemicentin 1 [Source:HGNC Symbol;Acc:HGNC:19194] |
| ENSG00000143889 | HGLibA_21838 | 55 | -0.6012949 | HNRNPLL | heterogeneous nuclear ribonucleoprotein L like [Source:HGNC Symbol;Acc:HGNC:25127] |
| ENSG00000143971 | HGLibA_15614 | 55 | -0.6012949 | ETAA1 | ATR kinase activator [Source:HGNC Symbol;Acc:HGNC:24648]" |
| ENSG00000144395 | HGLibA_07644 | 55 | -0.6012949 | CCDC150 | coiled-coil domain containing 150 [Source:HGNC Symbol;Acc:HGNC:26834] |
| ENSG00000144713 | HGLibA_41940 | 55 | -0.6012949 | RPL32 | ribosomal protein L32 [Source:HGNC Symbol;Acc:HGNC:10336] |
| ENSG00000145423 | HGLibA_43740 | 55 | -0.6012949 | SFRP2 | secreted frizzled related protein 2 [Source:HGNC Symbol;Acc:HGNC:10777] |
| ENSG00000145681 | HGLibA_20840 | 55 | -0.6012949 | HAPLN1 | hyaluronan and proteoglycan link protein 1 [Source:HGNC Symbol;Acc:HGNC:2380] |
| ENSG00000146285 | HGLibA_42964 | 55 | -0.6012949 | SCML4 | Scm polycomb group protein like 4 [Source:HGNC Symbol;Acc:HGNC:21397] |
| ENSG00000146463 | HGLibA_55400 | 55 | -0.6012949 | ZMYM4 | zinc finger MYM-type containing 4 [Source:HGNC Symbol;Acc:HGNC:13055] |
| ENSG00000146731 | HGLibA_08207 | 55 | -0.6012949 | CCT6A | chaperonin containing TCP1 subunit 6A [Source:HGNC Symbol;Acc:HGNC:1620] |
| ENSG00000147117 | HGLibA_55508 | 55 | -0.6012949 | ZNF157 | zinc finger protein 157 [Source:HGNC Symbol;Acc:HGNC:12942] |
| ENSG00000147324 | HGLibA_29062 | 55 | -0.6012949 | MFHAS1 | malignant fibrous histiocytoma amplified sequence 1 [Source:HGNC Symbol;Acc:HGNC:16982] |
| ENSG00000147955 | HGLibA_44159 | 55 | -0.6012949 | SIGMAR1 | sigma non-opioid intracellular receptor 1 [Source:HGNC Symbol;Acc:HGNC:8157] |
| ENSG00000148948 | HGLibA_27382 | 55 | -0.6012949 | LRRC4C | leucine rich repeat containing 4C [Source:HGNC Symbol;Acc:HGNC:29317] |
| ENSG00000149380 | HGLibA_34906 | 55 | -0.6012949 | P4HA3 | prolyl 4-hydroxylase subunit alpha 3 [Source:HGNC Symbol;Acc:HGNC:30135] |
| ENSG00000149571 | HGLibA_25006 | 55 | -0.6012949 | KIRREL3 | kirre like nephrin family adhesion molecule 3 [Source:HGNC Symbol;Acc:HGNC:23204] |
| ENSG00000149646 | HGLibA_10245 | 55 | -0.6012949 | CNBD2 | cyclic nucleotide binding domain containing 2 [Source:HGNC Symbol;Acc:HGNC:16145] |
| ENSG00000150045 | HGLibA_25303 | 55 | -0.6012949 | KLRF1 | killer cell lectin like receptor F1 [Source:HGNC Symbol;Acc:HGNC:13342] |
| ENSG00000151846 | HGLibA_34934 | 55 | -0.6012949 | PABPC3 | poly(A) binding protein cytoplasmic 3 [Source:HGNC Symbol;Acc:HGNC:8556] |
| ENSG00000152409 | HGLibA_23971 | 55 | -0.6012949 | JMY | p53 cofactor [Source:HGNC Symbol;Acc:HGNC:28916]" |
| ENSG00000152661 | HGLibA_19137 | 55 | -0.6012949 | GJA1 | gap junction protein alpha 1 [Source:HGNC Symbol;Acc:HGNC:4274] |
| ENSG00000153395 | HGLibA_27072 | 55 | -0.6012949 | LPCAT1 | lysophosphatidylcholine acyltransferase 1 [Source:HGNC Symbol;Acc:HGNC:25718] |
| ENSG00000154175 | HGLibA_00300 | 55 | -0.6012949 | ABI3BP | ABI family member 3 binding protein [Source:HGNC Symbol;Acc:HGNC:17265] |
| ENSG00000154473 | HGLibA_04995 | 55 | -0.6012949 | BUB3 | mitotic checkpoint protein [Source:HGNC Symbol;Acc:HGNC:1151]" |
| ENSG00000155097 | HGLibA_03738 | 55 | -0.6012949 | ATP6V1C1 | ATPase H+ transporting V1 subunit C1 [Source:HGNC Symbol;Acc:HGNC:856] |
| ENSG00000156466 | HGLibA_18872 | 55 | -0.6012949 | GDF6 | growth differentiation factor 6 [Source:HGNC Symbol;Acc:HGNC:4221] |
| ENSG00000157869 | HGLibA_39863 | 55 | -0.6012949 | RAB28 | member RAS oncogene family [Source:HGNC Symbol;Acc:HGNC:9768]" |
| ENSG00000159792 | HGLibA_39065 | 55 | -0.6012949 | PSKH1 | protein serine kinase H1 [Source:HGNC Symbol;Acc:HGNC:9529] |
| ENSG00000160161 | HGLibA_09712 | 55 | -0.6012949 | CILP2 | cartilage intermediate layer protein 2 [Source:HGNC Symbol;Acc:HGNC:24213] |
| ENSG00000160218 | HGLibA_51299 | 55 | -0.6012949 | TRAPPC10 | trafficking protein particle complex 10 [Source:HGNC Symbol;Acc:HGNC:11868] |
| ENSG00000160551 | HGLibA_48306 | 55 | -0.6012949 | TAOK1 | TAO kinase 1 [Source:HGNC Symbol;Acc:HGNC:29259] |
| ENSG00000160752 | HGLibA_17254 | 55 | -0.6012949 | FDPS | farnesyl diphosphate synthase [Source:HGNC Symbol;Acc:HGNC:3631] |
| ENSG00000162244 | HGLibA_41929 | 55 | -0.6012949 | RPL29 | ribosomal protein L29 [Source:HGNC Symbol;Acc:HGNC:10331] |
| ENSG00000162641 | HGLibA_01465 | 55 | -0.6012949 | AKNAD1 | AKNA domain containing 1 [Source:HGNC Symbol;Acc:HGNC:28398] |
| ENSG00000162994 | HGLibA_10017 | 55 | -0.6012949 | CLHC1 | clathrin heavy chain linker domain containing 1 [Source:HGNC Symbol;Acc:HGNC:26453] |
| ENSG00000163166 | HGLibA_23886 | 55 | -0.6012949 | IWS1 | SUPT6H interacting protein [Source:HGNC Symbol;Acc:HGNC:25467]" |
| ENSG00000163273 | HGLibA_32506 | 55 | -0.6012949 | NPPC | natriuretic peptide C [Source:HGNC Symbol;Acc:HGNC:7941] |
| ENSG00000163499 | HGLibA_11254 | 55 | -0.6012949 | CRYBA2 | crystallin beta A2 [Source:HGNC Symbol;Acc:HGNC:2395] |
| ENSG00000164051 | HGLibA_07795 | 55 | -0.6012949 | CCDC51 | coiled-coil domain containing 51 [Source:HGNC Symbol;Acc:HGNC:25714] |
| ENSG00000164466 | HGLibA_43780 | 55 | -0.6012949 | SFXN1 | sideroflexin 1 [Source:HGNC Symbol;Acc:HGNC:16085] |
| ENSG00000164506 | HGLibA_47676 | 55 | -0.6012949 | STXBP5 | syntaxin binding protein 5 [Source:HGNC Symbol;Acc:HGNC:19665] |
| ENSG00000164808 | HGLibA_46664 | 55 | -0.6012949 | SPIDR | scaffold protein involved in DNA repair [Source:HGNC Symbol;Acc:HGNC:28971] |
| ENSG00000164849 | HGLibA_19917 | 55 | -0.6012949 | GPR146 | G protein-coupled receptor 146 [Source:HGNC Symbol;Acc:HGNC:21718] |
| ENSG00000164920 | HGLibA_34701 | 55 | -0.6012949 | OSR2 | odd-skipped related transciption factor 2 [Source:HGNC Symbol;Acc:HGNC:15830] |
| ENSG00000165494 | HGLibA_35589 | 55 | -0.6012949 | PCF11 | cleavage and polyadenylation factor subunit [Source:HGNC Symbol;Acc:HGNC:30097]" |
| ENSG00000166573 | HGLibA_18588 | 55 | -0.6012949 | GALR1 | galanin receptor 1 [Source:HGNC Symbol;Acc:HGNC:4132] |
| ENSG00000168395 | HGLibA_23295 | 55 | -0.6012949 | ING5 | inhibitor of growth family member 5 [Source:HGNC Symbol;Acc:HGNC:19421] |
| ENSG00000170113 | HGLibA_31950 | 55 | -0.6012949 | NIPA1 | NIPA magnesium transporter 1 [Source:HGNC Symbol;Acc:HGNC:17043] |
| ENSG00000170191 | HGLibA_31104 | 55 | -0.6012949 | NANP | N-acetylneuraminic acid phosphatase [Source:HGNC Symbol;Acc:HGNC:16140] |
| ENSG00000170248 | HGLibA_35741 | 55 | -0.6012949 | PDCD6IP | programmed cell death 6 interacting protein [Source:HGNC Symbol;Acc:HGNC:8766] |
| ENSG00000170385 | HGLibA_44898 | 55 | -0.6012949 | SLC30A1 | solute carrier family 30 member 1 [Source:HGNC Symbol;Acc:HGNC:11012] |
| ENSG00000171450 | HGLibA_08779 | 55 | -0.6012949 | CDK5R2 | cyclin dependent kinase 5 regulatory subunit 2 [Source:HGNC Symbol;Acc:HGNC:1776] |
| ENSG00000171794 | HGLibA_53429 | 55 | -0.6012949 | UTF1 | undifferentiated embryonic cell transcription factor 1 [Source:HGNC Symbol;Acc:HGNC:12634] |

| | | | | | |
|---|---|---|---|---|---|
| ENSG00000171804 | HGLibA_54204 | 55 | -0.6012949 | WDR87 | WD repeat domain 87 [Source:HGNC Symbol;Acc:HGNC:29934] |
| ENSG00000172057 | HGLibA_34632 | 55 | -0.6012949 | ORMDL3 | ORMDL sphingolipid biosynthesis regulator 3 [Source:HGNC Symbol;Acc:HGNC:16038] |
| ENSG00000172733 | HGLibA_39591 | 55 | -0.6012949 | PURG | purine rich element binding protein G [Source:HGNC Symbol;Acc:HGNC:17930] |
| ENSG00000173083 | HGLibA_22061 | 55 | -0.6012949 | HPSE | heparanase [Source:HGNC Symbol;Acc:HGNC:5164] |
| ENSG00000173110 | HGLibA_22291 | 55 | -0.6012949 | HSPA6 | heat shock protein family A (Hsp70) member 6 [Source:HGNC Symbol;Acc:HGNC:5239] |
| ENSG00000173269 | HGLibA_29548 | 55 | -0.6012949 | MMRN2 | multimerin 2 [Source:HGNC Symbol;Acc:HGNC:19888] |
| ENSG00000173559 | HGLibA_31024 | 55 | -0.6012949 | NABP1 | nucleic acid binding protein 1 [Source:HGNC Symbol;Acc:HGNC:26232] |
| ENSG00000173674 | HGLibA_14710 | 55 | -0.6012949 | EIF1AX | eukaryotic translation initiation factor 1A X-linked [Source:HGNC Symbol;Acc:HGNC:3250] |
| ENSG00000173744 | HGLibA_01183 | 55 | -0.6012949 | AGFG1 | ArfGAP with FG repeats 1 [Source:HGNC Symbol;Acc:HGNC:5175] |
| ENSG00000174502 | HGLibA_44809 | 55 | -0.6012949 | SLC26A9 | solute carrier family 26 member 9 [Source:HGNC Symbol;Acc:HGNC:14469] |
| ENSG00000175189 | HGLibA_23306 | 55 | -0.6012949 | INHBC | inhibin subunit beta C [Source:HGNC Symbol;Acc:HGNC:6068] |
| ENSG00000175602 | HGLibA_07896 | 55 | -0.6012949 | CCDC85B | coiled-coil domain containing 85B [Source:HGNC Symbol;Acc:HGNC:24926] |
| ENSG00000175820 | HGLibA_07680 | 55 | -0.6012949 | CCDC168 | coiled-coil domain containing 168 [Source:HGNC Symbol;Acc:HGNC:26851] |
| ENSG00000176239 | HGLibA_34092 | 55 | -0.6012949 | OR51B6 | olfactory receptor family 51 subfamily B member 6 [Source:HGNC Symbol;Acc:HGNC:19600] |
| ENSG00000176399 | HGLibA_13426 | 55 | -0.6012949 | DMRTA1 | DMRT like family A1 [Source:HGNC Symbol;Acc:HGNC:13826] |
| ENSG00000177082 | HGLibA_54170 | 55 | -0.6012949 | WDR73 | WD repeat domain 73 [Source:HGNC Symbol;Acc:HGNC:25928] |
| ENSG00000177151 | HGLibA_33882 | 55 | -0.6012949 | OR2T35 | olfactory receptor family 2 subfamily T member 35 [Source:HGNC Symbol;Acc:HGNC:31257] |
| ENSG00000177192 | HGLibA_39595 | 55 | -0.6012949 | PUS1 | pseudouridine synthase 1 [Source:HGNC Symbol;Acc:HGNC:15508] |
| ENSG00000177551 | HGLibA_31892 | 55 | -0.6012949 | NHLH2 | nescient helix-loop-helix 2 [Source:HGNC Symbol;Acc:HGNC:7818] |
| ENSG00000178462 | HGLibA_52267 | 55 | -0.6012949 | TUBAL3 | tubulin alpha like 3 [Source:HGNC Symbol;Acc:HGNC:23534] |
| ENSG00000178531 | HGLibA_11754 | 55 | -0.6012949 | CTXN1 | cortexin 1 [Source:HGNC Symbol;Acc:HGNC:31108] |
| ENSG00000178665 | HGLibA_56616 | 55 | -0.6012949 | ZNF713 | zinc finger protein 713 [Source:HGNC Symbol;Acc:HGNC:22043] |
| ENSG00000178809 | HGLibA_51605 | 55 | -0.6012949 | TRIM73 | tripartite motif containing 73 [Source:HGNC Symbol;Acc:HGNC:18162] |
| ENSG00000178934 | HGLibA_26374 | 55 | -0.6012949 | LGALS7B | galectin 7B [Source:HGNC Symbol;Acc:HGNC:34447] |
| ENSG00000179055 | HGLibA_33614 | 55 | -0.6012949 | OR13D1 | olfactory receptor family 13 subfamily D member 1 [Source:HGNC Symbol;Acc:HGNC:14695] |
| ENSG00000179331 | HGLibA_39900 | 55 | -0.6012949 | RAB39A | member RAS oncogene family [Source:HGNC Symbol;Acc:HGNC:16521]" |
| ENSG00000179950 | HGLibA_39576 | 55 | -0.6012949 | PUF60 | poly(U) binding splicing factor 60 [Source:HGNC Symbol;Acc:HGNC:17042] |
| ENSG00000179950 | HGLibA_39578 | 55 | -0.6012949 | PUF60 | poly(U) binding splicing factor 60 [Source:HGNC Symbol;Acc:HGNC:17042] |
| ENSG00000180720 | HGLibA_09556 | 55 | -0.6012949 | CHRM4 | cholinergic receptor muscarinic 4 [Source:HGNC Symbol;Acc:HGNC:1953] |
| ENSG00000182896 | HGLibA_50564 | 55 | -0.6012949 | TMEM95 | transmembrane protein 95 [Source:HGNC Symbol;Acc:HGNC:27898] |
| ENSG00000183010 | HGLibA_39673 | 55 | -0.6012949 | PYCR1 | pyrroline-5-carboxylate reductase 1 [Source:HGNC Symbol;Acc:HGNC:9721] |
| ENSG00000183318 | HGLibA_46595 | 55 | -0.6012949 | SPDYE4 | speedy/RINGO cell cycle regulator family member E4 [Source:HGNC Symbol;Acc:HGNC:35463] |
| ENSG00000183559 | HGLibA_05047 | 55 | -0.6012949 | C10orf120 | chromosome 10 open reading frame 120 [Source:HGNC Symbol;Acc:HGNC:25707] |
| ENSG00000183963 | HGLibA_45834 | 55 | -0.6012949 | SMTN | smoothelin [Source:HGNC Symbol;Acc:HGNC:11126] |
| ENSG00000184185 | HGLibA_24255 | 55 | -0.6012949 | KCNJ12 | potassium voltage-gated channel subfamily J member 12 [Source:HGNC Symbol;Acc:HGNC:6258] |
| ENSG00000184313 | HGLibA_29875 | 55 | -0.6012949 | MROH7 | maestro heat like repeat family member 7 [Source:HGNC Symbol;Acc:HGNC:24802] |
| ENSG00000184371 | HGLibA_11322 | 55 | -0.6012949 | CSF1 | colony stimulating factor 1 [Source:HGNC Symbol;Acc:HGNC:2432] |
| ENSG00000184735 | HGLibA_12740 | 55 | -0.6012949 | DDX53 | DEAD-box helicase 53 [Source:HGNC Symbol;Acc:HGNC:20083] |
| ENSG00000184911 | HGLibA_13439 | 55 | -0.6012949 | DMRTC1B | DMRT like family C1B [Source:HGNC Symbol;Acc:HGNC:31686] |
| ENSG00000185730 | HGLibA_56561 | 55 | -0.6012949 | ZNF696 | zinc finger protein 696 [Source:HGNC Symbol;Acc:HGNC:25872] |
| ENSG00000186017 | HGLibA_56260 | 55 | -0.6012949 | ZNF566 | zinc finger protein 566 [Source:HGNC Symbol;Acc:HGNC:25919] |
| ENSG00000186815 | HGLibA_51104 | 55 | -0.6012949 | TPCN1 | two pore segment channel 1 [Source:HGNC Symbol;Acc:HGNC:18182] |
| ENSG00000186952 | HGLibA_50261 | 55 | -0.6012949 | TMEM232 | transmembrane protein 232 [Source:HGNC Symbol;Acc:HGNC:37270] |
| ENSG00000187626 | HGLibA_55354 | 55 | -0.6012949 | ZKSCAN4 | zinc finger with KRAB and SCAN domains 4 [Source:HGNC Symbol;Acc:HGNC:13854] |
| ENSG00000187766 | HGLibA_25621 | 55 | -0.6012949 | KRTAP10-8 | keratin associated protein 10-8 [Source:HGNC Symbol;Acc:HGNC:20525] |
| ENSG00000187833 | HGLibA_06186 | 55 | -0.6012949 | C2orf78 | chromosome 2 open reading frame 78 [Source:HGNC Symbol;Acc:HGNC:34349] |
| ENSG00000189045 | HGLibA_01949 | 55 | -0.6012949 | ANKDD1B | ankyrin repeat and death domain containing 1B [Source:HGNC Symbol;Acc:HGNC:32525] |
| ENSG00000189090 | HGLibA_16557 | 55 | -0.6012949 | FAM25G | family with sequence similarity 25 member G [Source:HGNC Symbol;Acc:HGNC:23590] |
| ENSG00000196139 | HGLibA_01486 | 55 | -0.6012949 | AKR1C3 | aldo-keto reductase family 1 member C3 [Source:HGNC Symbol;Acc:HGNC:386] |
| ENSG00000196267 | HGLibA_56842 | 55 | -0.6012949 | ZNF836 | zinc finger protein 836 [Source:HGNC Symbol;Acc:HGNC:34333] |
| ENSG00000196406 | HGLibA_46393 | 55 | -0.6012949 | SPANXD | SPANX family member D [Source:HGNC Symbol;Acc:HGNC:14332] |
| ENSG00000196597 | HGLibA_56740 | 55 | -0.6012949 | ZNF782 | zinc finger protein 782 [Source:HGNC Symbol;Acc:HGNC:33110] |
| ENSG00000196683 | HGLibA_50978 | 55 | -0.6012949 | TOMM7 | translocase of outer mitochondrial membrane 7 [Source:HGNC Symbol;Acc:HGNC:21648] |
| ENSG00000196739 | HGLibA_10553 | 55 | -0.6012949 | COL27A1 | collagen type XXVII alpha 1 chain [Source:HGNC Symbol;Acc:HGNC:22986] |
| ENSG00000197747 | HGLibA_42532 | 55 | -0.6012949 | S100A10 | S100 calcium binding protein A10 [Source:HGNC Symbol;Acc:HGNC:10487] |
| ENSG00000197782 | HGLibA_56732 | 55 | -0.6012949 | ZNF780A | zinc finger protein 780A [Source:HGNC Symbol;Acc:HGNC:27603] |
| ENSG00000197919 | HGLibA_22656 | 55 | -0.6012949 | IFNA1 | interferon alpha 1 [Source:HGNC Symbol;Acc:HGNC:5417] |
| ENSG00000198466 | HGLibA_56330 | 55 | -0.6012949 | ZNF587 | zinc finger protein 587 [Source:HGNC Symbol;Acc:HGNC:30955] |
| ENSG00000198774 | HGLibA_40397 | 55 | -0.6012949 | RASSF9 | Ras association domain family member 9 [Source:HGNC Symbol;Acc:HGNC:15739] |
| ENSG00000198792 | HGLibA_50115 | 55 | -0.6012949 | TMEM184B | transmembrane protein 184B [Source:HGNC Symbol;Acc:HGNC:1310] |
| ENSG00000198887 | HGLibA_45657 | 55 | -0.6012949 | SMC5 | structural maintenance of chromosomes 5 [Source:HGNC Symbol;Acc:HGNC:20465] |
| ENSG00000204539 | HGLibA_08888 | 55 | -0.6012949 | CDSN | corneodesmosin [Source:HGNC Symbol;Acc:HGNC:1802] |
| ENSG00000204962 | HGLibA_35456 | 55 | -0.6012949 | PCDHA8 | protocadherin alpha 8 [Source:HGNC Symbol;Acc:HGNC:8674] |
| ENSG00000205439 | HGLibA_25636 | 55 | -0.6012949 | KRTAP12-3 | keratin associated protein 12-3 [Source:HGNC Symbol;Acc:HGNC:20531] |
| ENSG00000205718 | HGLibA_28482 | 55 | -0.6012949 | MBD3L4 | methyl-CpG binding domain protein 3 like 4 [Source:HGNC Symbol;Acc:HGNC:37206] |
| ENSG00000206559 | HGLibA_55059 | 55 | -0.6012949 | ZCWPW2 | zinc finger CW-type and PWWP domain containing 2 [Source:HGNC Symbol;Acc:HGNC:23574] |
| ENSG00000212933 | HGLibA_25638 | 55 | -0.6012949 | KRTAP12-4 | keratin associated protein 12-4 [Source:HGNC Symbol;Acc:HGNC:20532] |
| ENSG00000213625 | HGLibA_26326 | 55 | -0.6012949 | LEPROT | leptin receptor overlapping transcript [Source:HGNC Symbol;Acc:HGNC:29477] |
| ENSG00000213626 | HGLibA_26076 | 55 | -0.6012949 | LBH | limb bud and heart development [Source:HGNC Symbol;Acc:HGNC:29532] |
| ENSG00000213672 | HGLibA_31322 | 55 | -0.6012949 | NCKIPSD | NCK interacting protein with SH3 domain [Source:HGNC Symbol;Acc:HGNC:15486] |
| ENSG00000214415 | HGLibA_19477 | 55 | -0.6012949 | GNAT3 | G protein subunit alpha transducin 3 [Source:HGNC Symbol;Acc:HGNC:22800] |
| ENSG00000214530 | HGLibA_47350 | 55 | -0.6012949 | STARD10 | StAR related lipid transfer domain containing 10 [Source:HGNC Symbol;Acc:HGNC:10666] |
| ENSG00000215126 | HGLibA_07442 | 55 | -0.6012949 | CBWD6 | COBW domain containing 6 [Source:HGNC Symbol;Acc:HGNC:31978] |
| ENSG00000215595 | HGLibA_06013 | 55 | -0.6012949 | C20orf202 | chromosome 20 open reading frame 202 [Source:HGNC Symbol;Acc:HGNC:37254] |
| ENSG00000221880 | HGLibA_25583 | 55 | -0.6012949 | KRTAP1-3 | keratin associated protein 1-3 [Source:HGNC Symbol;Acc:HGNC:16771] |
| ENSG00000230594 | HGLibA_11534 | 55 | -0.6012949 | CT47A4 | member A4 [Source:HGNC Symbol;Acc:HGNC:33285]" |
| ENSG00000248405 | HGLibA_38844 | 55 | -0.6012949 | PRR5-ARHGAP8 | PRR5-ARHGAP8 readthrough [Source:HGNC Symbol;Acc:HGNC:34512] |
| ENSG00000248483 | HGLibA_37791 | 55 | -0.6012949 | POU5F2 | transcription factor 2 [Source:HGNC Symbol;Acc:HGNC:26367]" |
| ENSG00000248920 | HGLibA_53236 | 55 | -0.6012949 | USP17L19 | ubiquitin specific peptidase 17-like family member 19 [Source:HGNC Symbol;Acc:HGNC:44447] |
| ENSG00000250868 | HGLibA_54541 | 55 | -0.6012949 | XKRY | Y-linked [Source:HGNC Symbol;Acc:HGNC:18571]" |
| ENSG00000253293 | HGLibA_21895 | 55 | -0.6012949 | HOXA10 | homeobox A10 [Source:HGNC Symbol;Acc:HGNC:5100] |
| ENSG00000253537 | HGLibA_35542 | 55 | -0.6012949 | PCDHGA7 | 7 [Source:HGNC Symbol;Acc:HGNC:8705]" |
| ENSG00000254834 | HGLibA_34331 | 55 | -0.6012949 | OR5M10 | olfactory receptor family 5 subfamily M member 10 [Source:HGNC Symbol;Acc:HGNC:15290] |
| ENSG00000258588 | HGLibA_51562 | 55 | -0.6012949 | TRIM6-TRIM34 | TRIM6-TRIM34 readthrough [Source:HGNC Symbol;Acc:HGNC:33440] |
| ENSG00000259075 | HGLibA_37447 | 55 | -0.6012949 | POC1B-GALNT4 | POC1B-GALNT4 readthrough [Source:HGNC Symbol;Acc:HGNC:42957] |
| ENSG00000262664 | HGLibA_34790 | 55 | -0.6012949 | OVCA2 | serine hydrolase domain containing [Source:HGNC Symbol;Acc:HGNC:24203]" |
| ENSG00000264230 | HGLibA_02254 | 55 | -0.6012949 | ANXA8L1 | annexin A8 like 1 [Source:HGNC Symbol;Acc:HGNC:23334] |
| ENSG00000270885 | HGLibA_40361 | 55 | -0.6012949 | RASL10B | RAS like family 10 member B [Source:HGNC Symbol;Acc:HGNC:30295] |
| ENSG00000272636 | HGLibA_13727 | 55 | -0.6012949 | DOC2B | double C2 domain beta [Source:HGNC Symbol;Acc:HGNC:2986] |
| ENSG00000277556 | HGLibA_33604 | 55 | -0.6012949 | OR13C5 | olfactory receptor family 13 subfamily C member 5 [Source:HGNC Symbol;Acc:HGNC:15100] |
| ENSG00000284723 | HGLibA_34554 | 55 | -0.6012949 | OR8S1 | olfactory receptor family 8 subfamily S member 1 [Source:HGNC Symbol;Acc:HGNC:19628] |
| GLTSCR1 | HGLibA_19353 | 55 | -0.6012949 | GLTSCR1 | - |
| H1FNT | HGLibA_20736 | 55 | -0.6012949 | H1FNT | - |
| HIST1H2AA | HGLibA_21376 | 55 | -0.6012949 | HIST1H2AA | - |
| hsa-mir-103b-1 | HGLibA_57169 | 55 | -0.6012949 | hsa-mir-103b-1 | - |
| hsa-mir-107 | HGLibA_57193 | 55 | -0.6012949 | hsa-mir-107 | - |
| hsa-mir-1179 | HGLibA_57210 | 55 | -0.6012949 | hsa-mir-1179 | - |
| hsa-mir-1229 | HGLibA_57314 | 55 | -0.6012949 | hsa-mir-1229 | - |
| hsa-mir-1231 | HGLibA_57317 | 55 | -0.6012949 | hsa-mir-1231 | - |
| hsa-mir-1236 | HGLibA_57333 | 55 | -0.6012949 | hsa-mir-1236 | - |
| hsa-mir-1244-2 | HGLibA_57367 | 55 | -0.6012949 | hsa-mir-1244-2 | - |
| hsa-mir-1275 | HGLibA_57555 | 55 | -0.6012949 | hsa-mir-1275 | - |
| hsa-mir-1289-2 | HGLibA_57623 | 55 | -0.6012949 | hsa-mir-1289-2 | - |
| hsa-mir-1321 | HGLibA_57758 | 55 | -0.6012949 | hsa-mir-1321 | - |
| hsa-mir-204 | HGLibA_58174 | 55 | -0.6012949 | hsa-mir-204 | - |
| hsa-mir-212 | HGLibA_58246 | 55 | -0.6012949 | hsa-mir-212 | - |
| hsa-mir-3117 | HGLibA_58512 | 55 | -0.6012949 | hsa-mir-3117 | - |
| hsa-mir-4271 | HGLibA_59793 | 55 | -0.6012949 | hsa-mir-4271 | - |
| hsa-mir-4287 | HGLibA_59857 | 55 | -0.6012949 | hsa-mir-4287 | - |
| hsa-mir-4509-2 | HGLibA_60466 | 55 | -0.6012949 | hsa-mir-4509-2 | - |
| hsa-mir-4757 | HGLibA_61124 | 55 | -0.6012949 | hsa-mir-4757 | - |
| hsa-mir-489 | HGLibA_61355 | 55 | -0.6012949 | hsa-mir-489 | - |
| hsa-mir-513a-1 | HGLibA_61569 | 55 | -0.6012949 | hsa-mir-513a-1 | - |
| hsa-mir-600 | HGLibA_62492 | 55 | -0.6012949 | hsa-mir-600 | - |
| hsa-mir-6132 | HGLibA_62681 | 55 | -0.6012949 | hsa-mir-6132 | - |
| hsa-mir-6511b-1 | HGLibA_62914 | 55 | -0.6012949 | hsa-mir-6511b-1 | - |
| hsa-mir-654 | HGLibA_62950 | 55 | -0.6012949 | hsa-mir-654 | - |
| hsa-mir-6736 | HGLibA_63099 | 55 | -0.6012949 | hsa-mir-6736 | - |
| hsa-mir-675 | HGLibA_63154 | 55 | -0.6012949 | hsa-mir-675 | - |
| hsa-mir-6779 | HGLibA_63290 | 55 | -0.6012949 | hsa-mir-6779 | - |
| hsa-mir-6834 | HGLibA_63512 | 55 | -0.6012949 | hsa-mir-6834 | - |
| hsa-mir-8068 | HGLibA_64110 | 55 | -0.6012949 | hsa-mir-8068 | - |
| LOC284385 | HGLibA_26916 | 55 | -0.6012949 | LOC284385 | - |
| MAATS1 | HGLibA_27818 | 55 | -0.6012949 | MAATS1 | - |
| MEF2BNB-MEF2B | HGLibA_28834 | 55 | -0.6012949 | MEF2BNB-MEF2B | - |
| MFSD7 | HGLibA_29113 | 55 | -0.6012949 | MFSD7 | - |
| STRA13 | HGLibA_47563 | 55 | -0.6012949 | STRA13 | - |
| UTP11L | HGLibA_53432 | 55 | -0.6012949 | UTP11L | - |
| WDR96 | HGLibA_54226 | 55 | -0.6012949 | WDR96 | - |
| C2orf47 | HGLibA_06134 | 54 | -0.611245938 | C2orf47 | - |
| EMR1 | HGLibA_15082 | 54 | -0.611245938 | EMR1 | - |
| ENSG00000002587 | HGLibA_22121 | 54 | -0.611245938 | HS3ST1 | heparan sulfate-glucosamine 3-sulfotransferase 1 [Source:HGNC Symbol;Acc:HGNC:5194] |
| ENSG00000004487 | HGLibA_24487 | 54 | -0.611245938 | KDM1A | lysine demethylase 1A [Source:HGNC Symbol;Acc:HGNC:29079] |
| ENSG00000010256 | HGLibA_53114 | 54 | -0.611245938 | UQCRC1 | ubiquinol-cytochrome c reductase core protein 1 [Source:HGNC Symbol;Acc:HGNC:12585] |
| ENSG00000011478 | HGLibA_39729 | 54 | -0.611245938 | QPCTL | glutaminyl-peptide cyclotransferase like [Source:HGNC Symbol;Acc:HGNC:25952] |

| | | | | | |
|---|---|---|---|---|---|
| ENSG00000012171 | HGLibA_43322 | 54 | -0.611245938 | SEMA3B | semaphorin 3B [Source:HGNC Symbol;Acc:HGNC:10724] |
| ENSG00000013392 | HGLibA_42481 | 54 | -0.611245938 | RWDD2A | RWD domain containing 2A [Source:HGNC Symbol;Acc:HGNC:21385] |
| ENSG00000038295 | HGLibA_49628 | 54 | -0.611245938 | TLL1 | tolloid like 1 [Source:HGNC Symbol;Acc:HGNC:11843] |
| ENSG00000044090 | HGLibA_11792 | 54 | -0.611245938 | CUL7 | cullin 7 [Source:HGNC Symbol;Acc:HGNC:21024] |
| ENSG00000065183 | HGLibA_54062 | 54 | -0.611245938 | WDR3 | WD repeat domain 3 [Source:HGNC Symbol;Acc:HGNC:12755] |
| ENSG00000065883 | HGLibA_08735 | 54 | -0.611245938 | CDK13 | cyclin dependent kinase 13 [Source:HGNC Symbol;Acc:HGNC:1733] |
| ENSG00000066248 | HGLibA_31869 | 54 | -0.611245938 | NGEF | neuronal guanine nucleotide exchange factor [Source:HGNC Symbol;Acc:HGNC:7807] |
| ENSG00000069956 | HGLibA_28278 | 54 | -0.611245938 | MAPK6 | mitogen-activated protein kinase 6 [Source:HGNC Symbol;Acc:HGNC:6879] |
| ENSG00000070423 | HGLibA_41495 | 54 | -0.611245938 | RNF126 | ring finger protein 126 [Source:HGNC Symbol;Acc:HGNC:21151] |
| ENSG00000070731 | HGLibA_47268 | 54 | -0.611245938 | ST6GALNAC2 | 6-sialyltransferase 2 [Source:HGNC Symbol;Acc:HGNC:10867]" |
| ENSG00000070961 | HGLibA_03615 | 54 | -0.611245938 | ATP2B1 | ATPase plasma membrane Ca2+ transporting 1 [Source:HGNC Symbol;Acc:HGNC:814] |
| ENSG00000072310 | HGLibA_46949 | 54 | -0.611245938 | SREBF1 | sterol regulatory element binding transcription factor 1 [Source:HGNC Symbol;Acc:HGNC:11289] |
| ENSG00000072803 | HGLibA_17137 | 54 | -0.611245938 | FBXW11 | F-box and WD repeat domain containing 11 [Source:HGNC Symbol;Acc:HGNC:13607] |
| ENSG00000072840 | HGLibA_15681 | 54 | -0.611245938 | EVC | EvC ciliary complex subunit 1 [Source:HGNC Symbol;Acc:HGNC:3497] |
| ENSG00000075643 | HGLibA_29610 | 54 | -0.611245938 | MOCOS | molybdenum cofactor sulfurase [Source:HGNC Symbol;Acc:HGNC:18234] |
| ENSG00000078725 | HGLibA_04812 | 54 | -0.611245938 | BRINP1 | BMP/retinoic acid inducible neural specific 1 [Source:HGNC Symbol;Acc:HGNC:2687] |
| ENSG00000079387 | HGLibA_43386 | 54 | -0.611245938 | SENP1 | SUMO specific peptidase 1 [Source:HGNC Symbol;Acc:HGNC:17927] |
| ENSG00000079462 | HGLibA_34995 | 54 | -0.611245938 | PAFAH1B3 | platelet activating factor acetylhydrolase 1b catalytic subunit 3 [Source:HGNC Symbol;Acc:HGNC:8576] |
| ENSG00000080644 | HGLibA_09572 | 54 | -0.611245938 | CHRNA3 | cholinergic receptor nicotinic alpha 3 subunit [Source:HGNC Symbol;Acc:HGNC:1957] |
| ENSG00000081386 | HGLibA_56124 | 54 | -0.611245938 | ZNF510 | zinc finger protein 510 [Source:HGNC Symbol;Acc:HGNC:29161] |
| ENSG00000081665 | HGLibA_56119 | 54 | -0.611245938 | ZNF506 | zinc finger protein 506 [Source:HGNC Symbol;Acc:HGNC:23780] |
| ENSG00000086717 | HGLibA_37867 | 54 | -0.611245938 | PPEF1 | protein phosphatase with EF-hand domain 1 [Source:HGNC Symbol;Acc:HGNC:9243] |
| ENSG00000087842 | HGLibA_36728 | 54 | -0.611245938 | PIR | pirin [Source:HGNC Symbol;Acc:HGNC:30048] |
| ENSG00000092203 | HGLibA_51045 | 54 | -0.611245938 | TOX4 | TOX high mobility group box family member 4 [Source:HGNC Symbol;Acc:HGNC:20161] |
| ENSG00000100243 | HGLibA_12006 | 54 | -0.611245938 | CYB5R3 | cytochrome b5 reductase 3 [Source:HGNC Symbol;Acc:HGNC:2873] |
| ENSG00000101842 | HGLibA_53835 | 54 | -0.611245938 | VSIG1 | V-set and immunoglobulin domain containing 1 [Source:HGNC Symbol;Acc:HGNC:28675] |
| ENSG00000101850 | HGLibA_19916 | 54 | -0.611245938 | GPR143 | G protein-coupled receptor 143 [Source:HGNC Symbol;Acc:HGNC:20145] |
| ENSG00000103351 | HGLibA_10157 | 54 | -0.611245938 | CLUAP1 | clusterin associated protein 1 [Source:HGNC Symbol;Acc:HGNC:19009] |
| ENSG00000103381 | HGLibA_10986 | 54 | -0.611245938 | CPPED1 | calcineurin like phosphoesterase domain containing 1 [Source:HGNC Symbol;Acc:HGNC:25632] |
| ENSG00000104499 | HGLibA_19411 | 54 | -0.611245938 | GML | glycosylphosphatidylinositol anchored molecule like [Source:HGNC Symbol;Acc:HGNC:4375] |
| ENSG00000105193 | HGLibA_42084 | 54 | -0.611245938 | RPS16 | ribosomal protein S16 [Source:HGNC Symbol;Acc:HGNC:10396] |
| ENSG00000105245 | HGLibA_33016 | 54 | -0.611245938 | NUMBL | endocytic adaptor protein [Source:HGNC Symbol;Acc:HGNC:8061]" |
| ENSG00000105617 | HGLibA_26296 | 54 | -0.611245938 | LENG1 | leukocyte receptor cluster member 1 [Source:HGNC Symbol;Acc:HGNC:15502] |
| ENSG00000105755 | HGLibA_15629 | 54 | -0.611245938 | ETHE1 | persulfide dioxygenase [Source:HGNC Symbol;Acc:HGNC:23287]" |
| ENSG00000105887 | HGLibA_30460 | 54 | -0.611245938 | MTPN | myotrophin [Source:HGNC Symbol;Acc:HGNC:15667] |
| ENSG00000107338 | HGLibA_43993 | 54 | -0.611245938 | SHB | SH2 domain containing adaptor protein B [Source:HGNC Symbol;Acc:HGNC:10838] |
| ENSG00000108773 | HGLibA_24052 | 54 | -0.611245938 | KAT2A | lysine acetyltransferase 2A [Source:HGNC Symbol;Acc:HGNC:4201] |
| ENSG00000108821 | HGLibA_10531 | 54 | -0.611245938 | COL1A1 | collagen type I alpha 1 chain [Source:HGNC Symbol;Acc:HGNC:2197] |
| ENSG00000109111 | HGLibA_47824 | 54 | -0.611245938 | SUPT6H | histone chaperone [Source:HGNC Symbol;Acc:HGNC:11470]" |
| ENSG00000109606 | HGLibA_13166 | 54 | -0.611245938 | DHX15 | DEAH-box helicase 15 [Source:HGNC Symbol;Acc:HGNC:2738] |
| ENSG00000109756 | HGLibA_40249 | 54 | -0.611245938 | RAPGEF2 | Rap guanine nucleotide exchange factor 2 [Source:HGNC Symbol;Acc:HGNC:16854] |
| ENSG00000109794 | HGLibA_16150 | 54 | -0.611245938 | FAM149A | family with sequence similarity 149 member A [Source:HGNC Symbol;Acc:HGNC:24527] |
| ENSG00000110321 | HGLibA_14843 | 54 | -0.611245938 | EIF4G2 | eukaryotic translation initiation factor 4 gamma 2 [Source:HGNC Symbol;Acc:HGNC:3297] |
| ENSG00000111181 | HGLibA_45294 | 54 | -0.611245938 | SLC6A12 | solute carrier family 6 member 12 [Source:HGNC Symbol;Acc:HGNC:11045] |
| ENSG00000111666 | HGLibA_09528 | 54 | -0.611245938 | CHPT1 | choline phosphotransferase 1 [Source:HGNC Symbol;Acc:HGNC:17852] |
| ENSG00000111845 | HGLibA_35041 | 54 | -0.611245938 | PAK1IP1 | PAK1 interacting protein 1 [Source:HGNC Symbol;Acc:HGNC:20882] |
| ENSG00000111907 | HGLibA_51115 | 54 | -0.611245938 | TPD52L1 | TPD52 like 1 [Source:HGNC Symbol;Acc:HGNC:12006] |
| ENSG00000114353 | HGLibA_19454 | 54 | -0.611245938 | GNAI2 | G protein subunit alpha i2 [Source:HGNC Symbol;Acc:HGNC:4385] |
| ENSG00000115282 | HGLibA_52110 | 54 | -0.611245938 | TTC31 | tetratricopeptide repeat domain 31 [Source:HGNC Symbol;Acc:HGNC:25759] |
| ENSG00000115295 | HGLibA_10051 | 54 | -0.611245938 | CLIP4 | CAP-Gly domain containing linker protein family member 4 [Source:HGNC Symbol;Acc:HGNC:26108] |
| ENSG00000116584 | HGLibA_02833 | 54 | -0.611245938 | ARHGEF2 | Rho/Rac guanine nucleotide exchange factor 2 [Source:HGNC Symbol;Acc:HGNC:682] |
| ENSG00000117245 | HGLibA_24871 | 54 | -0.611245938 | KIF17 | kinesin family member 17 [Source:HGNC Symbol;Acc:HGNC:19167] |
| ENSG00000117983 | HGLibA_30550 | 54 | -0.611245938 | MUC5B | oligomeric mucus/gel-forming [Source:HGNC Symbol;Acc:HGNC:7516]" |
| ENSG00000118322 | HGLibA_03548 | 54 | -0.611245938 | ATP10B | ATPase phospholipid transporting 10B (putative) [Source:HGNC Symbol;Acc:HGNC:13543] |
| ENSG00000119638 | HGLibA_31667 | 54 | -0.611245938 | NEK9 | NIMA related kinase 9 [Source:HGNC Symbol;Acc:HGNC:18591] |
| ENSG00000119900 | HGLibA_33320 | 54 | -0.611245938 | OGFRL1 | opioid growth factor receptor like 1 [Source:HGNC Symbol;Acc:HGNC:21378] |
| ENSG00000121775 | HGLibA_50387 | 54 | -0.611245938 | TMEM39B | transmembrane protein 39B [Source:HGNC Symbol;Acc:HGNC:25510] |
| ENSG00000121904 | HGLibA_11366 | 54 | -0.611245938 | CSMD2 | CUB and Sushi multiple domains 2 [Source:HGNC Symbol;Acc:HGNC:19290] |
| ENSG00000122952 | HGLibA_57070 | 54 | -0.611245938 | ZWINT | ZW10 interacting kinetochore protein [Source:HGNC Symbol;Acc:HGNC:13195] |
| ENSG00000123171 | HGLibA_07849 | 54 | -0.611245938 | CCDC70 | coiled-coil domain containing 70 [Source:HGNC Symbol;Acc:HGNC:25303] |
| ENSG00000123200 | HGLibA_54961 | 54 | -0.611245938 | ZC3H13 | zinc finger CCCH-type containing 13 [Source:HGNC Symbol;Acc:HGNC:20368] |
| ENSG00000124226 | HGLibA_41477 | 54 | -0.611245938 | RNF114 | ring finger protein 114 [Source:HGNC Symbol;Acc:HGNC:13094] |
| ENSG00000125246 | HGLibA_10173 | 54 | -0.611245938 | CLYBL | citrate lyase beta like [Source:HGNC Symbol;Acc:HGNC:18355] |
| ENSG00000125459 | HGLibA_30269 | 54 | -0.611245938 | MSTO1 | mitochondrial distribution and morphology regulator [Source:HGNC Symbol;Acc:HGNC:29678]" |
| ENSG00000125503 | HGLibA_38026 | 54 | -0.611245938 | PPP1R12C | protein phosphatase 1 regulatory subunit 12C [Source:HGNC Symbol;Acc:HGNC:14947] |
| ENSG00000125845 | HGLibA_04599 | 54 | -0.611245938 | BMP2 | bone morphogenetic protein 2 [Source:HGNC Symbol;Acc:HGNC:1069] |
| ENSG00000126062 | HGLibA_49884 | 54 | -0.611245938 | TMEM115 | transmembrane protein 115 [Source:HGNC Symbol;Acc:HGNC:30055] |
| ENSG00000127124 | HGLibA_21573 | 54 | -0.611245938 | HIVEP3 | human immunodeficiency virus type I enhancer binding protein 3 [Source:HGNC Symbol;Acc:HGNC:13561] |
| ENSG00000127377 | HGLibA_11282 | 54 | -0.611245938 | CRYGN | crystallin gamma N [Source:HGNC Symbol;Acc:HGNC:20458] |
| ENSG00000128641 | HGLibA_30822 | 54 | -0.611245938 | MYO1B | myosin IB [Source:HGNC Symbol;Acc:HGNC:7596] |
| ENSG00000129245 | HGLibA_18222 | 54 | -0.611245938 | FXR2 | FMR1 autosomal homolog 2 [Source:HGNC Symbol;Acc:HGNC:4024] |
| ENSG00000130226 | HGLibA_13875 | 54 | -0.611245938 | DPP6 | dipeptidyl peptidase like 6 [Source:HGNC Symbol;Acc:HGNC:3010] |
| ENSG00000131848 | HGLibA_57029 | 54 | -0.611245938 | ZSCAN5A | zinc finger and SCAN domain containing 5A [Source:HGNC Symbol;Acc:HGNC:23710] |
| ENSG00000132321 | HGLibA_23488 | 54 | -0.611245938 | IQCA1 | IQ motif containing with AAA domain 1 [Source:HGNC Symbol;Acc:HGNC:26195] |
| ENSG00000133250 | HGLibA_55940 | 54 | -0.611245938 | ZNF414 | zinc finger protein 414 [Source:HGNC Symbol;Acc:HGNC:20630] |
| ENSG00000135409 | HGLibA_01787 | 54 | -0.611245938 | AMHR2 | anti-Mullerian hormone receptor type 2 [Source:HGNC Symbol;Acc:HGNC:465] |
| ENSG00000135587 | HGLibA_45806 | 54 | -0.611245938 | SMPD2 | sphingomyelin phosphodiesterase 2 [Source:HGNC Symbol;Acc:HGNC:11121] |
| ENSG00000135837 | HGLibA_09133 | 54 | -0.611245938 | CEP350 | centrosomal protein 350 [Source:HGNC Symbol;Acc:HGNC:24238] |
| ENSG00000136052 | HGLibA_45143 | 54 | -0.611245938 | SLC41A2 | solute carrier family 41 member 2 [Source:HGNC Symbol;Acc:HGNC:31045] |
| ENSG00000136731 | HGLibA_52867 | 54 | -0.611245938 | UGGT1 | UDP-glucose glycoprotein glucosyltransferase 1 [Source:HGNC Symbol;Acc:HGNC:15663] |
| ENSG00000137124 | HGLibA_01549 | 54 | -0.611245938 | ALDH1B1 | aldehyde dehydrogenase 1 family member B1 [Source:HGNC Symbol;Acc:HGNC:407] |
| ENSG00000137198 | HGLibA_19426 | 54 | -0.611245938 | GMPR | guanosine monophosphate reductase [Source:HGNC Symbol;Acc:HGNC:4376] |
| ENSG00000137221 | HGLibA_49567 | 54 | -0.611245938 | TJAP1 | tight junction associated protein 1 [Source:HGNC Symbol;Acc:HGNC:17949] |
| ENSG00000138757 | HGLibA_18301 | 54 | -0.611245938 | G3BP2 | G3BP stress granule assembly factor 2 [Source:HGNC Symbol;Acc:HGNC:30291] |
| ENSG00000140459 | HGLibA_12052 | 54 | -0.611245938 | CYP11A1 | cytochrome P450 family 11 subfamily A member 1 [Source:HGNC Symbol;Acc:HGNC:2590] |
| ENSG00000142182 | HGLibA_13706 | 54 | -0.611245938 | DNMT3L | DNA methyltransferase 3 like [Source:HGNC Symbol;Acc:HGNC:2980] |
| ENSG00000143469 | HGLibA_48059 | 54 | -0.611245938 | SYT14 | synaptotagmin 14 [Source:HGNC Symbol;Acc:HGNC:23143] |
| ENSG00000144230 | HGLibA_19962 | 54 | -0.611245938 | GPR17 | G protein-coupled receptor 17 [Source:HGNC Symbol;Acc:HGNC:4471] |
| ENSG00000144357 | HGLibA_52753 | 54 | -0.611245938 | UBR3 | ubiquitin protein ligase E3 component n-recognin 3 [Source:HGNC Symbol;Acc:HGNC:30467] |
| ENSG00000145198 | HGLibA_53912 | 54 | -0.611245938 | VWA5B2 | von Willebrand factor A domain containing 5B2 [Source:HGNC Symbol;Acc:HGNC:25144] |
| ENSG00000145248 | HGLibA_44339 | 54 | -0.611245938 | SLC10A4 | solute carrier family 10 member 4 [Source:HGNC Symbol;Acc:HGNC:22980] |
| ENSG00000145545 | HGLibA_46940 | 54 | -0.611245938 | SRD5A1 | steroid 5 alpha-reductase 1 [Source:HGNC Symbol;Acc:HGNC:11284] |
| ENSG00000146063 | HGLibA_51496 | 54 | -0.611245938 | TRIM41 | tripartite motif containing 41 [Source:HGNC Symbol;Acc:HGNC:19013] |
| ENSG00000146856 | HGLibA_01171 | 54 | -0.611245938 | AGBL3 | ATP/GTP binding protein like 3 [Source:HGNC Symbol;Acc:HGNC:27981] |
| ENSG00000147647 | HGLibA_13924 | 54 | -0.611245938 | DPYS | dihydropyrimidinase [Source:HGNC Symbol;Acc:HGNC:3013] |
| ENSG00000148377 | HGLibA_22558 | 54 | -0.611245938 | IDI2 | isopentenyl-diphosphate delta isomerase 2 [Source:HGNC Symbol;Acc:HGNC:23487] |
| ENSG00000150433 | HGLibA_50228 | 54 | -0.611245938 | TMEM218 | transmembrane protein 218 [Source:HGNC Symbol;Acc:HGNC:27344] |
| ENSG00000150433 | HGLibA_50229 | 54 | -0.611245938 | TMEM218 | transmembrane protein 218 [Source:HGNC Symbol;Acc:HGNC:27344] |
| ENSG00000150787 | HGLibA_39558 | 54 | -0.611245938 | PTS | 6-pyruvoyltetrahydropterin synthase [Source:HGNC Symbol;Acc:HGNC:9689] |
| ENSG00000150873 | HGLibA_06143 | 54 | -0.611245938 | C2orf50 | chromosome 2 open reading frame 50 [Source:HGNC Symbol;Acc:HGNC:26324] |
| ENSG00000151834 | HGLibA_18359 | 54 | -0.611245938 | GABRA2 | gamma-aminobutyric acid type A receptor alpha2 subunit [Source:HGNC Symbol;Acc:HGNC:4076] |
| ENSG00000152455 | HGLibA_47863 | 54 | -0.611245938 | SUV39H2 | suppressor of variegation 3-9 homolog 2 [Source:HGNC Symbol;Acc:HGNC:17287] |
| ENSG00000153132 | HGLibA_10016 | 54 | -0.611245938 | CLGN | calmegin [Source:HGNC Symbol;Acc:HGNC:2060] |
| ENSG00000154553 | HGLibA_35914 | 54 | -0.611245938 | PDLIM3 | PDZ and LIM domain 3 [Source:HGNC Symbol;Acc:HGNC:20767] |
| ENSG00000155428 | HGLibA_51609 | 54 | -0.611245938 | TRIM74 | tripartite motif containing 74 [Source:HGNC Symbol;Acc:HGNC:17453] |
| ENSG00000155545 | HGLibA_29257 | 54 | -0.611245938 | MIER3 | MIER family member 3 [Source:HGNC Symbol;Acc:HGNC:26678] |
| ENSG00000156642 | HGLibA_32530 | 54 | -0.611245938 | NPTN | neuroplastin [Source:HGNC Symbol;Acc:HGNC:17867] |
| ENSG00000156735 | HGLibA_04083 | 54 | -0.611245938 | BAG4 | BCL2 associated athanogene 4 [Source:HGNC Symbol;Acc:HGNC:940] |
| ENSG00000157216 | HGLibA_47120 | 54 | -0.611245938 | SSBP3 | single stranded DNA binding protein 3 [Source:HGNC Symbol;Acc:HGNC:15674] |
| ENSG00000157335 | HGLibA_09950 | 54 | -0.611245938 | CLEC18C | C-type lectin domain family 18 member C [Source:HGNC Symbol;Acc:HGNC:28538] |
| ENSG00000158008 | HGLibA_15809 | 54 | -0.611245938 | EXTL1 | exostosin like glycosyltransferase 1 [Source:HGNC Symbol;Acc:HGNC:3515] |
| ENSG00000158158 | HGLibA_10322 | 54 | -0.611245938 | CNNM4 | cyclin and CBS domain divalent metal cation transport mediator 4 [Source:HGNC Symbol;Acc:HGNC:105] |
| ENSG00000160199 | HGLibA_36847 | 54 | -0.611245938 | PKNOX1 | PBX/knotted 1 homeobox 1 [Source:HGNC Symbol;Acc:HGNC:9022] |
| ENSG00000160211 | HGLibA_18313 | 54 | -0.611245938 | G6PD | glucose-6-phosphate dehydrogenase [Source:HGNC Symbol;Acc:HGNC:4057] |
| ENSG00000160803 | HGLibA_52741 | 54 | -0.611245938 | UBQLN4 | ubiquilin 4 [Source:HGNC Symbol;Acc:HGNC:1237] |
| ENSG00000160870 | HGLibA_12163 | 54 | -0.611245938 | CYP3A7 | cytochrome P450 family 3 subfamily A member 7 [Source:HGNC Symbol;Acc:HGNC:2640] |
| ENSG00000160957 | HGLibA_40769 | 54 | -0.611245938 | RECQL4 | RecQ like helicase 4 [Source:HGNC Symbol;Acc:HGNC:9949] |
| ENSG00000162490 | HGLibA_13953 | 54 | -0.611245938 | DRAXIN | dorsal inhibitory axon guidance protein [Source:HGNC Symbol;Acc:HGNC:25054] |
| ENSG00000162757 | HGLibA_05894 | 54 | -0.611245938 | C1orf74 | chromosome 1 open reading frame 74 [Source:HGNC Symbol;Acc:HGNC:26319] |
| ENSG00000163155 | HGLibA_27765 | 54 | -0.611245938 | LYSMD1 | LysM domain containing 1 [Source:HGNC Symbol;Acc:HGNC:32070] |
| ENSG00000163754 | HGLibA_20689 | 54 | -0.611245938 | GYG1 | glycogenin 1 [Source:HGNC Symbol;Acc:HGNC:4699] |
| ENSG00000163874 | HGLibA_54952 | 54 | -0.611245938 | ZC3H12A | zinc finger CCCH-type containing 12A [Source:HGNC Symbol;Acc:HGNC:26259] |
| ENSG00000163930 | HGLibA_04152 | 54 | -0.611245938 | BAP1 | BRCA1 associated protein 1 [Source:HGNC Symbol;Acc:HGNC:950] |
| ENSG00000163933 | HGLibA_40948 | 54 | -0.611245938 | RFT1 | RFT1 homolog [Source:HGNC Symbol;Acc:HGNC:30220] |
| ENSG00000164219 | HGLibA_36238 | 54 | -0.611245938 | PGGT1B | protein geranylgeranyltransferase type I subunit beta [Source:HGNC Symbol;Acc:HGNC:8895] |
| ENSG00000164270 | HGLibA_22395 | 54 | -0.611245938 | HTR4 | 5-hydroxytryptamine receptor 4 [Source:HGNC Symbol;Acc:HGNC:5299] |
| ENSG00000164535 | HGLibA_12298 | 54 | -0.611245938 | DAGLB | diacylglycerol lipase beta [Source:HGNC Symbol;Acc:HGNC:28923] |
| ENSG00000164626 | HGLibA_24327 | 54 | -0.611245938 | KCNK5 | potassium two pore domain channel subfamily K member 5 [Source:HGNC Symbol;Acc:HGNC:6280] |
| ENSG00000165102 | HGLibA_21248 | 54 | -0.611245938 | HGSNAT | heparan-alpha-glucosaminide N-acetyltransferase [Source:HGNC Symbol;Acc:HGNC:26527] |
| ENSG00000166741 | HGLibA_32223 | 54 | -0.611245938 | NNMT | nicotinamide N-methyltransferase [Source:HGNC Symbol;Acc:HGNC:7861] |
| ENSG00000167642 | HGLibA_46715 | 54 | -0.611245938 | SPINT2 | Kunitz type 2 [Source:HGNC Symbol;Acc:HGNC:11247]" |
| ENSG00000167766 | HGLibA_56831 | 54 | -0.611245938 | ZNF83 | zinc finger protein 83 [Source:HGNC Symbol;Acc:HGNC:13158] |
| ENSG00000167910 | HGLibA_12211 | 54 | -0.611245938 | CYP7A1 | cytochrome P450 family 7 subfamily A member 1 [Source:HGNC Symbol;Acc:HGNC:2651] |
| ENSG00000168028 | HGLibA_42196 | 54 | -0.611245938 | RPSA | ribosomal protein SA [Source:HGNC Symbol;Acc:HGNC:6502] |
| ENSG00000169432 | HGLibA_43006 | 54 | -0.611245938 | SCN9A | sodium voltage-gated channel alpha subunit 9 [Source:HGNC Symbol;Acc:HGNC:10597] |

| | | | | | |
|---|---|---|---|---|---|
| ENSG00000169504 | HGLibA_10030 | 54 | -0.611245938 | CLIC4 | chloride intracellular channel 4 [Source:HGNC Symbol;Acc:HGNC:13518] |
| ENSG00000169683 | HGLibA_27366 | 54 | -0.611245938 | LRRC45 | leucine rich repeat containing 45 [Source:HGNC Symbol;Acc:HGNC:28302] |
| ENSG00000169919 | HGLibA_20680 | 54 | -0.611245938 | GUSB | glucuronidase beta [Source:HGNC Symbol;Acc:HGNC:4696] |
| ENSG00000169953 | HGLibA_22244 | 54 | -0.611245938 | HSFY2 | heat shock transcription factor Y-linked 2 [Source:HGNC Symbol;Acc:HGNC:23950] |
| ENSG00000170293 | HGLibA_10230 | 54 | -0.611245938 | CMTM8 | CKLF like MARVEL transmembrane domain containing 8 [Source:HGNC Symbol;Acc:HGNC:19179] |
| ENSG00000171346 | HGLibA_25428 | 54 | -0.611245938 | KRT15 | keratin 15 [Source:HGNC Symbol;Acc:HGNC:6421] |
| ENSG00000171540 | HGLibA_34750 | 54 | -0.611245938 | OTP | orthopedia homeobox [Source:HGNC Symbol;Acc:HGNC:8518] |
| ENSG00000171786 | HGLibA_31889 | 54 | -0.611245938 | NHLH1 | nescient helix-loop-helix 1 [Source:HGNC Symbol;Acc:HGNC:7817] |
| ENSG00000173039 | HGLibA_40813 | 54 | -0.611245938 | RELA | NF-kB subunit [Source:HGNC Symbol;Acc:HGNC:9955]" |
| ENSG00000173110 | HGLibA_22293 | 54 | -0.611245938 | HSPA6 | heat shock protein family A (Hsp70) member 6 [Source:HGNC Symbol;Acc:HGNC:5239] |
| ENSG00000173120 | HGLibA_24493 | 54 | -0.611245938 | KDM2A | lysine demethylase 2A [Source:HGNC Symbol;Acc:HGNC:13606] |
| ENSG00000173166 | HGLibA_40265 | 54 | -0.611245938 | RAPH1 | Ras association (RalGDS/AF-6) and pleckstrin homology domains 1 [Source:HGNC Symbol;Acc:HGNC:14436] |
| ENSG00000173599 | HGLibA_35357 | 54 | -0.611245938 | PC | pyruvate carboxylase [Source:HGNC Symbol;Acc:HGNC:8636] |
| ENSG00000174446 | HGLibA_45903 | 54 | -0.611245938 | SNAPC5 | small nuclear RNA activating complex polypeptide 5 [Source:HGNC Symbol;Acc:HGNC:15484] |
| ENSG00000174607 | HGLibA_52939 | 54 | -0.611245938 | UGT8 | UDP glycosyltransferase 8 [Source:HGNC Symbol;Acc:HGNC:12555] |
| ENSG00000174705 | HGLibA_43967 | 54 | -0.611245938 | SH3PXD2B | SH3 and PX domains 2B [Source:HGNC Symbol;Acc:HGNC:29242] |
| ENSG00000174748 | HGLibA_41876 | 54 | -0.611245938 | RPL15 | ribosomal protein L15 [Source:HGNC Symbol;Acc:HGNC:10306] |
| ENSG00000174946 | HGLibA_19966 | 54 | -0.611245938 | GPR171 | G protein-coupled receptor 171 [Source:HGNC Symbol;Acc:HGNC:30057] |
| ENSG00000175155 | HGLibA_54708 | 54 | -0.611245938 | YPEL2 | yippee like 2 [Source:HGNC Symbol;Acc:HGNC:18326] |
| ENSG00000175505 | HGLibA_09817 | 54 | -0.611245938 | CLCF1 | cardiotrophin like cytokine factor 1 [Source:HGNC Symbol;Acc:HGNC:17412] |
| ENSG00000176253 | HGLibA_34020 | 54 | -0.611245938 | OR4K13 | olfactory receptor family 4 subfamily K member 13 [Source:HGNC Symbol;Acc:HGNC:15351] |
| ENSG00000176281 | HGLibA_34033 | 54 | -0.611245938 | OR4K5 | olfactory receptor family 4 subfamily K member 5 [Source:HGNC Symbol;Acc:HGNC:14745] |
| ENSG00000176358 | HGLibA_48155 | 54 | -0.611245938 | TAC4 | tachykinin 4 [Source:HGNC Symbol;Acc:HGNC:16641] |
| ENSG00000176435 | HGLibA_09935 | 54 | -0.611245938 | CLEC14A | C-type lectin domain containing 14A [Source:HGNC Symbol;Acc:HGNC:19832] |
| ENSG00000176641 | HGLibA_41549 | 54 | -0.611245938 | RNF152 | ring finger protein 152 [Source:HGNC Symbol;Acc:HGNC:26811] |
| ENSG00000177300 | HGLibA_09883 | 54 | -0.611245938 | CLDN22 | claudin 22 [Source:HGNC Symbol;Acc:HGNC:2044] |
| ENSG00000178597 | HGLibA_39005 | 54 | -0.611245938 | PSAPL1 | prosaposin like 1 (gene/pseudogene) [Source:HGNC Symbol;Acc:HGNC:33131] |
| ENSG00000178852 | HGLibA_14530 | 54 | -0.611245938 | EFCAB13 | EF-hand calcium binding domain 13 [Source:HGNC Symbol;Acc:HGNC:26864] |
| ENSG00000178896 | HGLibA_15781 | 54 | -0.611245938 | EXOSC4 | exosome component 4 [Source:HGNC Symbol;Acc:HGNC:18189] |
| ENSG00000179058 | HGLibA_06741 | 54 | -0.611245938 | C9orf50 | chromosome 9 open reading frame 50 [Source:HGNC Symbol;Acc:HGNC:23677] |
| ENSG00000179144 | HGLibA_19089 | 54 | -0.611245938 | GIMAP7 | IMAP family member 7 [Source:HGNC Symbol;Acc:HGNC:22404]" |
| ENSG00000179583 | HGLibA_09706 | 54 | -0.611245938 | CIITA | class II major histocompatibility complex transactivator [Source:HGNC Symbol;Acc:HGNC:7067] |
| ENSG00000179600 | HGLibA_19800 | 54 | -0.611245938 | GPHB5 | glycoprotein hormone beta 5 [Source:HGNC Symbol;Acc:HGNC:18055] |
| ENSG00000179889 | HGLibA_35957 | 54 | -0.611245938 | PDXDC1 | pyridoxal dependent decarboxylase domain containing 1 [Source:HGNC Symbol;Acc:HGNC:28995] |
| ENSG00000180900 | HGLibA_43047 | 54 | -0.611245938 | SCRIB | scribbled planar cell polarity protein [Source:HGNC Symbol;Acc:HGNC:30377] |
| ENSG00000181690 | HGLibA_36951 | 54 | -0.611245938 | PLAG1 | PLAG1 zinc finger [Source:HGNC Symbol;Acc:HGNC:9045] |
| ENSG00000182533 | HGLibA_07383 | 54 | -0.611245938 | CAV3 | caveolin 3 [Source:HGNC Symbol;Acc:HGNC:1529] |
| ENSG00000182557 | HGLibA_46740 | 54 | -0.611245938 | SPNS3 | sphingolipid transporter 3 (putative) [Source:HGNC Symbol;Acc:HGNC:28433] |
| ENSG00000182985 | HGLibA_06966 | 54 | -0.611245938 | CADM1 | cell adhesion molecule 1 [Source:HGNC Symbol;Acc:HGNC:5951] |
| ENSG00000183206 | HGLibA_37722 | 54 | -0.611245938 | POTEC | POTE ankyrin domain family member C [Source:HGNC Symbol;Acc:HGNC:33894] |
| ENSG00000184349 | HGLibA_14594 | 54 | -0.611245938 | EFNA5 | ephrin A5 [Source:HGNC Symbol;Acc:HGNC:3225] |
| ENSG00000184432 | HGLibA_10697 | 54 | -0.611245938 | COPB2 | coatomer protein complex subunit beta 2 [Source:HGNC Symbol;Acc:HGNC:2232] |
| ENSG00000184470 | HGLibA_52446 | 54 | -0.611245938 | TXNRD2 | thioredoxin reductase 2 [Source:HGNC Symbol;Acc:HGNC:18155] |
| ENSG00000184481 | HGLibA_17928 | 54 | -0.611245938 | FOXO4 | forkhead box O4 [Source:HGNC Symbol;Acc:HGNC:7139] |
| ENSG00000184489 | HGLibA_39409 | 54 | -0.611245938 | PTP4A3 | member 3 [Source:HGNC Symbol;Acc:HGNC:9636]" |
| ENSG00000185187 | HGLibA_44121 | 54 | -0.611245938 | SIGIRR | single Ig and TIR domain containing [Source:HGNC Symbol;Acc:HGNC:30575] |
| ENSG00000186868 | HGLibA_28321 | 54 | -0.611245938 | MAPT | microtubule associated protein tau [Source:HGNC Symbol;Acc:HGNC:6893] |
| ENSG00000187550 | HGLibA_42804 | 54 | -0.611245938 | SBK2 | SH3 domain binding kinase family member 2 [Source:HGNC Symbol;Acc:HGNC:34416] |
| ENSG00000187790 | HGLibA_16861 | 54 | -0.611245938 | FANCM | FA complementation group M [Source:HGNC Symbol;Acc:HGNC:23168] |
| ENSG00000187800 | HGLibA_36008 | 54 | -0.611245938 | PEAR1 | platelet endothelial aggregation receptor 1 [Source:HGNC Symbol;Acc:HGNC:33631] |
| ENSG00000189090 | HGLibA_16558 | 54 | -0.611245938 | FAM25G | family with sequence similarity 25 member G [Source:HGNC Symbol;Acc:HGNC:23590] |
| ENSG00000189114 | HGLibA_04563 | 54 | -0.611245938 | BLOC1S3 | biogenesis of lysosomal organelles complex 1 subunit 3 [Source:HGNC Symbol;Acc:HGNC:20914] |
| ENSG00000189182 | HGLibA_25543 | 54 | -0.611245938 | KRT77 | keratin 77 [Source:HGNC Symbol;Acc:HGNC:20411] |
| ENSG00000189366 | HGLibA_01622 | 54 | -0.611245938 | ALG1L | chitobiosyldiphosphodolichol beta-mannosyltransferase like [Source:HGNC Symbol;Acc:HGNC:33721]" |
| ENSG00000196235 | HGLibA_47819 | 54 | -0.611245938 | SUPT5H | DSIF elongation factor subunit [Source:HGNC Symbol;Acc:HGNC:11469]" |
| ENSG00000196323 | HGLibA_54891 | 54 | -0.611245938 | ZBTB44 | zinc finger and BTB domain containing 44 [Source:HGNC Symbol;Acc:HGNC:25001] |
| ENSG00000196542 | HGLibA_46905 | 54 | -0.611245938 | SPTSSB | serine palmitoyltransferase small subunit B [Source:HGNC Symbol;Acc:HGNC:24045] |
| ENSG00000196562 | HGLibA_47729 | 54 | -0.611245938 | SULF2 | sulfatase 2 [Source:HGNC Symbol;Acc:HGNC:20392] |
| ENSG00000196569 | HGLibA_25943 | 54 | -0.611245938 | LAMA2 | laminin subunit alpha 2 [Source:HGNC Symbol;Acc:HGNC:6482] |
| ENSG00000196811 | HGLibA_09607 | 54 | -0.611245938 | CHRNG | cholinergic receptor nicotinic gamma subunit [Source:HGNC Symbol;Acc:HGNC:1967] |
| ENSG00000196924 | HGLibA_17638 | 54 | -0.611245938 | FLNA | filamin A [Source:HGNC Symbol;Acc:HGNC:3754] |
| ENSG00000197594 | HGLibA_15168 | 54 | -0.611245938 | ENPP1 | ectonucleotide pyrophosphatase/phosphodiesterase 1 [Source:HGNC Symbol;Acc:HGNC:3356] |
| ENSG00000197608 | HGLibA_56855 | 54 | -0.611245938 | ZNF841 | zinc finger protein 841 [Source:HGNC Symbol;Acc:HGNC:27611] |
| ENSG00000198400 | HGLibA_32875 | 54 | -0.611245938 | NTRK1 | neurotrophic receptor tyrosine kinase 1 [Source:HGNC Symbol;Acc:HGNC:8031] |
| ENSG00000198838 | HGLibA_42526 | 54 | -0.611245938 | RYR3 | ryanodine receptor 3 [Source:HGNC Symbol;Acc:HGNC:10485] |
| ENSG00000204052 | HGLibA_27425 | 54 | -0.611245938 | LRRC73 | leucine rich repeat containing 73 [Source:HGNC Symbol;Acc:HGNC:21375] |
| ENSG00000204681 | HGLibA_18342 | 54 | -0.611245938 | GABBR1 | gamma-aminobutyric acid type B receptor subunit 1 [Source:HGNC Symbol;Acc:HGNC:4070] |
| ENSG00000213339 | HGLibA_39761 | 54 | -0.611245938 | QTRT1 | queuine tRNA-ribosyltransferase catalytic subunit 1 [Source:HGNC Symbol;Acc:HGNC:23797] |
| ENSG00000213780 | HGLibA_20560 | 54 | -0.611245938 | GTF2H4 | general transcription factor IIH subunit 4 [Source:HGNC Symbol;Acc:HGNC:4658] |
| ENSG00000214046 | HGLibA_45766 | 54 | -0.611245938 | SMIM7 | small integral membrane protein 7 [Source:HGNC Symbol;Acc:HGNC:28419] |
| ENSG00000215343 | HGLibA_56594 | 54 | -0.611245938 | ZNF705D | zinc finger protein 705D [Source:HGNC Symbol;Acc:HGNC:33202] |
| ENSG00000226685 | HGLibA_11525 | 54 | -0.611245938 | CT47A12 | member A12 [Source:HGNC Symbol;Acc:HGNC:33292]" |
| ENSG00000232399 | HGLibA_53225 | 54 | -0.611245938 | USP17L13 | ubiquitin specific peptidase 17-like family member 13 [Source:HGNC Symbol;Acc:HGNC:44441] |
| ENSG00000233917 | HGLibA_37716 | 54 | -0.611245938 | POTEB | POTE ankyrin domain family member B [Source:HGNC Symbol;Acc:HGNC:33734] |
| ENSG00000244242 | HGLibA_22641 | 54 | -0.611245938 | IFITM10 | interferon induced transmembrane protein 10 [Source:HGNC Symbol;Acc:HGNC:40022] |
| ENSG00000250067 | HGLibA_54691 | 54 | -0.611245938 | YJEFN3 | YjeF N-terminal domain containing 3 [Source:HGNC Symbol;Acc:HGNC:24785] |
| ENSG00000251247 | HGLibA_55845 | 54 | -0.611245938 | ZNF345 | zinc finger protein 345 [Source:HGNC Symbol;Acc:HGNC:16367] |
| ENSG00000254221 | HGLibA_35550 | 54 | -0.611245938 | PCDHGB1 | 1 [Source:HGNC Symbol;Acc:HGNC:8708]" |
| ENSG00000261150 | HGLibA_15362 | 54 | -0.611245938 | EPPK1 | epiplakin 1 [Source:HGNC Symbol;Acc:HGNC:15577] |
| ENSG00000262628 | HGLibA_33655 | 54 | -0.611245938 | OR1D5 | olfactory receptor family 1 subfamily D member 5 [Source:HGNC Symbol;Acc:HGNC:8186] |
| ENSG00000267978 | HGLibA_27927 | 54 | -0.611245938 | MAGEA9B | MAGE family member A9B [Source:HGNC Symbol;Acc:HGNC:31909] |
| ENSG00000268940 | HGLibA_11497 | 54 | -0.611245938 | CT45A1 | cancer/testis antigen family 45 member A1 [Source:HGNC Symbol;Acc:HGNC:33267] |
| ENSG00000274736 | HGLibA_08000 | 54 | -0.611245938 | CCL23 | C-C motif chemokine ligand 23 [Source:HGNC Symbol;Acc:HGNC:10622] |
| ENSG00000278023 | HGLibA_40754 | 54 | -0.611245938 | RDM1 | RAD52 motif containing 1 [Source:HGNC Symbol;Acc:HGNC:19950] |
| FAM194B | HGLibA_16373 | 54 | -0.611245938 | FAM194B | - |
| HIST1H1D | HGLibA_21367 | 54 | -0.611245938 | HIST1H1D | - |
| HN1L | HGLibA_21772 | 54 | -0.611245938 | HN1L | - |
| hsa-mir-1182 | HGLibA_57222 | 54 | -0.611245938 | hsa-mir-1182 | - |
| hsa-mir-1184-2 | HGLibA_57235 | 54 | -0.611245938 | hsa-mir-1184-2 | - |
| hsa-mir-1227 | HGLibA_57306 | 54 | -0.611245938 | hsa-mir-1227 | - |
| hsa-mir-1244-1 | HGLibA_57362 | 54 | -0.611245938 | hsa-mir-1244-1 | - |
| hsa-mir-130b | HGLibA_57750 | 54 | -0.611245938 | hsa-mir-130b | - |
| hsa-mir-1537 | HGLibA_57915 | 54 | -0.611245938 | hsa-mir-1537 | - |
| hsa-mir-203a | HGLibA_58168 | 54 | -0.611245938 | hsa-mir-203a | - |
| hsa-mir-25 | HGLibA_58352 | 54 | -0.611245938 | hsa-mir-25 | - |
| hsa-mir-2861 | HGLibA_58389 | 54 | -0.611245938 | hsa-mir-2861 | - |
| hsa-mir-320d-1 | HGLibA_58943 | 54 | -0.611245938 | hsa-mir-320d-1 | - |
| hsa-mir-34b | HGLibA_59041 | 54 | -0.611245938 | hsa-mir-34b | - |
| hsa-mir-378f | HGLibA_59448 | 54 | -0.611245938 | hsa-mir-378f | - |
| hsa-mir-3973 | HGLibA_59653 | 54 | -0.611245938 | hsa-mir-3973 | - |
| hsa-mir-4465 | HGLibA_60265 | 54 | -0.611245938 | hsa-mir-4465 | - |
| hsa-mir-454 | HGLibA_60620 | 54 | -0.611245938 | hsa-mir-454 | - |
| hsa-mir-4664 | HGLibA_60770 | 54 | -0.611245938 | hsa-mir-4664 | - |
| hsa-mir-4706 | HGLibA_60923 | 54 | -0.611245938 | hsa-mir-4706 | - |
| hsa-mir-4737 | HGLibA_61045 | 54 | -0.611245938 | hsa-mir-4737 | - |
| hsa-mir-511 | HGLibA_61553 | 54 | -0.611245938 | hsa-mir-511 | - |
| hsa-mir-521-1 | HGLibA_61767 | 54 | -0.611245938 | hsa-mir-521-1 | - |
| hsa-mir-548av | HGLibA_61933 | 54 | -0.611245938 | hsa-mir-548av | - |
| hsa-mir-5683 | HGLibA_62253 | 54 | -0.611245938 | hsa-mir-5683 | - |
| hsa-mir-5688 | HGLibA_62266 | 54 | -0.611245938 | hsa-mir-5688 | - |
| hsa-mir-5702 | HGLibA_62348 | 54 | -0.611245938 | hsa-mir-5702 | - |
| hsa-mir-6740 | HGLibA_63113 | 54 | -0.611245938 | hsa-mir-6740 | - |
| hsa-mir-6762 | HGLibA_63211 | 54 | -0.611245938 | hsa-mir-6762 | - |
| hsa-mir-6807 | HGLibA_63404 | 54 | -0.611245938 | hsa-mir-6807 | - |
| hsa-mir-6821 | HGLibA_63463 | 54 | -0.611245938 | hsa-mir-6821 | - |
| hsa-mir-6874 | HGLibA_63679 | 54 | -0.611245938 | hsa-mir-6874 | - |
| hsa-mir-7109 | HGLibA_63797 | 54 | -0.611245938 | hsa-mir-7109 | - |
| hsa-mir-7114 | HGLibA_63826 | 54 | -0.611245938 | hsa-mir-7114 | - |
| hsa-mir-770 | HGLibA_63941 | 54 | -0.611245938 | hsa-mir-770 | - |
| hsa-mir-8073 | HGLibA_64135 | 54 | -0.611245938 | hsa-mir-8073 | - |
| hsa-mir-892b | HGLibA_64254 | 54 | -0.611245938 | hsa-mir-892b | - |
| hsa-mir-892c | HGLibA_64257 | 54 | -0.611245938 | hsa-mir-892c | - |
| hsa-mir-922 | HGLibA_64284 | 54 | -0.611245938 | hsa-mir-922 | - |
| KIAA0020 | HGLibA_24583 | 54 | -0.611245938 | KIAA0020 | - |
| PVRL1 | HGLibA_39621 | 54 | -0.611245938 | PVRL1 | - |
| SKIV2L2 | HGLibA_44287 | 54 | -0.611245938 | SKIV2L2 | - |
| C15orf56 | HGLibA_05417 | 53 | -0.621196975 | C15orf56 | - |
| C19orf21 | HGLibA_05637 | 53 | -0.621196975 | C19orf21 | - |
| C1orf151-NBL1 | HGLibA_05776 | 53 | -0.621196975 | C1orf151-NBL1 | - |
| C20orf197 | HGLibA_06008 | 53 | -0.621196975 | C20orf197 | - |
| C2orf62 | HGLibA_06157 | 53 | -0.621196975 | C2orf62 | - |
| C7orf60 | HGLibA_06563 | 53 | -0.621196975 | C7orf60 | - |
| C8orf47 | HGLibA_06632 | 53 | -0.621196975 | C8orf47 | - |
| CECR1 | HGLibA_08973 | 53 | -0.621196975 | CECR1 | - |
| CNIH | HGLibA_10281 | 53 | -0.621196975 | CNIH | - |

| | | | | | |
|---|---|---|---|---|---|
| ENSG00000003249 | HGLibA_12392 | 53 | -0.621196975 | DBNDD1 | dysbindin domain containing 1 [Source:HGNC Symbol;Acc:HGNC:28455] |
| ENSG00000006712 | HGLibA_34987 | 53 | -0.621196975 | PAF1 | Paf1/RNA polymerase II complex component [Source:HGNC Symbol;Acc:HGNC:25459]" |
| ENSG00000006788 | HGLibA_30711 | 53 | -0.621196975 | MYH13 | myosin heavy chain 13 [Source:HGNC Symbol;Acc:HGNC:7571] |
| ENSG00000007520 | HGLibA_52000 | 53 | -0.621196975 | TSR3 | acp transferase ribosome maturation factor [Source:HGNC Symbol;Acc:HGNC:14175]" |
| ENSG00000042088 | HGLibA_48891 | 53 | -0.621196975 | TDP1 | tyrosyl-DNA phosphodiesterase 1 [Source:HGNC Symbol;Acc:HGNC:18884] |
| ENSG00000048540 | HGLibA_26707 | 53 | -0.621196975 | LMO3 | LIM domain only 3 [Source:HGNC Symbol;Acc:HGNC:6643] |
| ENSG00000054179 | HGLibA_15200 | 53 | -0.621196975 | ENTPD2 | ectonucleoside triphosphate diphosphohydrolase 2 [Source:HGNC Symbol;Acc:HGNC:3364] |
| ENSG00000055917 | HGLibA_39584 | 53 | -0.621196975 | PUM2 | pumilio RNA binding family member 2 [Source:HGNC Symbol;Acc:HGNC:14958] |
| ENSG00000059728 | HGLibA_30609 | 53 | -0.621196975 | MXD1 | MAX dimerization protein 1 [Source:HGNC Symbol;Acc:HGNC:6761] |
| ENSG00000065357 | HGLibA_13057 | 53 | -0.621196975 | DGKA | diacylglycerol kinase alpha [Source:HGNC Symbol;Acc:HGNC:2849] |
| ENSG00000067182 | HGLibA_50773 | 53 | -0.621196975 | TNFRSF1A | TNF receptor superfamily member 1A [Source:HGNC Symbol;Acc:HGNC:11916] |
| ENSG00000070495 | HGLibA_23958 | 53 | -0.621196975 | JMJD6 | arginine demethylase and lysine hydroxylase [Source:HGNC Symbol;Acc:HGNC:19355]" |
| ENSG00000075089 | HGLibA_00670 | 53 | -0.621196975 | ACTR6 | ARP6 actin related protein 6 homolog [Source:HGNC Symbol;Acc:HGNC:24025] |
| ENSG00000077063 | HGLibA_11743 | 53 | -0.621196975 | CTTNBP2 | cortactin binding protein 2 [Source:HGNC Symbol;Acc:HGNC:15679] |
| ENSG00000083857 | HGLibA_16912 | 53 | -0.621196975 | FAT1 | FAT atypical cadherin 1 [Source:HGNC Symbol;Acc:HGNC:3595] |
| ENSG00000085433 | HGLibA_54105 | 53 | -0.621196975 | WDR47 | WD repeat domain 47 [Source:HGNC Symbol;Acc:HGNC:29141] |
| ENSG00000085563 | HGLibA_00131 | 53 | -0.621196975 | ABCB1 | ATP binding cassette subfamily B member 1 [Source:HGNC Symbol;Acc:HGNC:40] |
| ENSG00000087903 | HGLibA_40965 | 53 | -0.621196975 | RFX2 | regulatory factor X2 [Source:HGNC Symbol;Acc:HGNC:9983] |
| ENSG00000088053 | HGLibA_19712 | 53 | -0.621196975 | GP6 | glycoprotein VI platelet [Source:HGNC Symbol;Acc:HGNC:14388] |
| ENSG00000091490 | HGLibA_43283 | 53 | -0.621196975 | SEL1L3 | SEL1L family member 3 [Source:HGNC Symbol;Acc:HGNC:29108] |
| ENSG00000099399 | HGLibA_27944 | 53 | -0.621196975 | MAGEB2 | MAGE family member B2 [Source:HGNC Symbol;Acc:HGNC:6809] |
| ENSG00000099954 | HGLibA_08978 | 53 | -0.621196975 | CECR2 | histone acetyl-lysine reader [Source:HGNC Symbol;Acc:HGNC:1840]" |
| ENSG00000099992 | HGLibA_48465 | 53 | -0.621196975 | TBC1D10A | TBC1 domain family member 10A [Source:HGNC Symbol;Acc:HGNC:23609] |
| ENSG00000101266 | HGLibA_11403 | 53 | -0.621196975 | CSNK2A1 | casein kinase 2 alpha 1 [Source:HGNC Symbol;Acc:HGNC:2457] |
| ENSG00000102580 | HGLibA_13616 | 53 | -0.621196975 | DNAJC3 | DnaJ heat shock protein family (Hsp40) member C3 [Source:HGNC Symbol;Acc:HGNC:9439] |
| ENSG00000104763 | HGLibA_03185 | 53 | -0.621196975 | ASAH1 | N-acylsphingosine amidohydrolase 1 [Source:HGNC Symbol;Acc:HGNC:735] |
| ENSG00000105402 | HGLibA_31126 | 53 | -0.621196975 | NAPA | NSF attachment protein alpha [Source:HGNC Symbol;Acc:HGNC:7641] |
| ENSG00000106331 | HGLibA_35304 | 53 | -0.621196975 | PAX4 | paired box 4 [Source:HGNC Symbol;Acc:HGNC:8618] |
| ENSG00000106991 | HGLibA_15126 | 53 | -0.621196975 | ENG | endoglin [Source:HGNC Symbol;Acc:HGNC:3349] |
| ENSG00000107443 | HGLibA_08108 | 53 | -0.621196975 | CCNJ | cyclin J [Source:HGNC Symbol;Acc:HGNC:23434] |
| ENSG00000110665 | HGLibA_05120 | 53 | -0.621196975 | C11orf21 | chromosome 11 open reading frame 21 [Source:HGNC Symbol;Acc:HGNC:13231] |
| ENSG00000111145 | HGLibA_14914 | 53 | -0.621196975 | ELK3 | ETS transcription factor [Source:HGNC Symbol;Acc:HGNC:3325]" |
| ENSG00000111639 | HGLibA_30010 | 53 | -0.621196975 | MRPL51 | mitochondrial ribosomal protein L51 [Source:HGNC Symbol;Acc:HGNC:14044] |
| ENSG00000111732 | HGLibA_01325 | 53 | -0.621196975 | AICDA | activation induced cytidine deaminase [Source:HGNC Symbol;Acc:HGNC:13203] |
| ENSG00000111879 | HGLibA_16328 | 53 | -0.621196975 | FAM184A | family with sequence similarity 184 member A [Source:HGNC Symbol;Acc:HGNC:20991] |
| ENSG00000112115 | HGLibA_22988 | 53 | -0.621196975 | IL17A | interleukin 17A [Source:HGNC Symbol;Acc:HGNC:5981] |
| ENSG00000112130 | HGLibA_41701 | 53 | -0.621196975 | RNF8 | ring finger protein 8 [Source:HGNC Symbol;Acc:HGNC:10071] |
| ENSG00000112339 | HGLibA_20941 | 53 | -0.621196975 | HBS1L | HBS1 like translational GTPase [Source:HGNC Symbol;Acc:HGNC:4834] |
| ENSG00000112984 | HGLibA_24891 | 53 | -0.621196975 | KIF20A | kinesin family member 20A [Source:HGNC Symbol;Acc:HGNC:9787] |
| ENSG00000114374 | HGLibA_53420 | 53 | -0.621196975 | USP9Y | ubiquitin specific peptidase 9 Y-linked [Source:HGNC Symbol;Acc:HGNC:12633] |
| ENSG00000115556 | HGLibA_36997 | 53 | -0.621196975 | PLCD4 | phospholipase C delta 4 [Source:HGNC Symbol;Acc:HGNC:9062] |
| ENSG00000115649 | HGLibA_10360 | 53 | -0.621196975 | CNPPD1 | cyclin Pas1/PHO80 domain containing 1 [Source:HGNC Symbol;Acc:HGNC:25220] |
| ENSG00000116032 | HGLibA_20315 | 53 | -0.621196975 | GRIN3B | glutamate ionotropic receptor NMDA type subunit 3B [Source:HGNC Symbol;Acc:HGNC:16768] |
| ENSG00000116703 | HGLibA_35712 | 53 | -0.621196975 | PDC | phosducin [Source:HGNC Symbol;Acc:HGNC:8759] |
| ENSG00000118242 | HGLibA_29826 | 53 | -0.621196975 | MREG | melanoregulin [Source:HGNC Symbol;Acc:HGNC:25478] |
| ENSG00000118473 | HGLibA_43814 | 53 | -0.621196975 | SGIP1 | SH3 domain GRB2 like endophilin interacting protein 1 [Source:HGNC Symbol;Acc:HGNC:25412] |
| ENSG00000119537 | HGLibA_24546 | 53 | -0.621196975 | KDSR | 3-ketodihydrosphingosine reductase [Source:HGNC Symbol;Acc:HGNC:4021] |
| ENSG00000119596 | HGLibA_54698 | 53 | -0.621196975 | YLPM1 | YLP motif containing 1 [Source:HGNC Symbol;Acc:HGNC:17798] |
| ENSG00000119685 | HGLibA_52208 | 53 | -0.621196975 | TTLL5 | tubulin tyrosine ligase like 5 [Source:HGNC Symbol;Acc:HGNC:19963] |
| ENSG00000120471 | HGLibA_51051 | 53 | -0.621196975 | TP53AIP1 | tumor protein p53 regulated apoptosis inducing protein 1 [Source:HGNC Symbol;Acc:HGNC:29984] |
| ENSG00000120658 | HGLibA_15157 | 53 | -0.621196975 | ENOX1 | ecto-NOX disulfide-thiol exchanger 1 [Source:HGNC Symbol;Acc:HGNC:25474] |
| ENSG00000120693 | HGLibA_45590 | 53 | -0.621196975 | SMAD9 | SMAD family member 9 [Source:HGNC Symbol;Acc:HGNC:6774] |
| ENSG00000121797 | HGLibA_08176 | 53 | -0.621196975 | CCRL2 | C-C motif chemokine receptor like 2 [Source:HGNC Symbol;Acc:HGNC:1612] |
| ENSG00000123561 | HGLibA_43529 | 53 | -0.621196975 | SERPINA7 | serpin family A member 7 [Source:HGNC Symbol;Acc:HGNC:11583] |
| ENSG00000123901 | HGLibA_20092 | 53 | -0.621196975 | GPR83 | G protein-coupled receptor 83 [Source:HGNC Symbol;Acc:HGNC:4523] |
| ENSG00000124422 | HGLibA_53304 | 53 | -0.621196975 | USP22 | ubiquitin specific peptidase 22 [Source:HGNC Symbol;Acc:HGNC:12621] |
| ENSG00000124882 | HGLibA_15457 | 53 | -0.621196975 | EREG | epiregulin [Source:HGNC Symbol;Acc:HGNC:3443] |
| ENSG00000125388 | HGLibA_20328 | 53 | -0.621196975 | GRK4 | G protein-coupled receptor kinase 4 [Source:HGNC Symbol;Acc:HGNC:4543] |
| ENSG00000125966 | HGLibA_29518 | 53 | -0.621196975 | MMP24 | matrix metallopeptidase 24 [Source:HGNC Symbol;Acc:HGNC:7172] |
| ENSG00000125967 | HGLibA_31589 | 53 | -0.621196975 | NECAB3 | N-terminal EF-hand calcium binding protein 3 [Source:HGNC Symbol;Acc:HGNC:15851] |
| ENSG00000127084 | HGLibA_17347 | 53 | -0.621196975 | FGD3 | RhoGEF and PH domain containing 3 [Source:HGNC Symbol;Acc:HGNC:16027]" |
| ENSG00000127124 | HGLibA_21572 | 53 | -0.621196975 | HIVEP3 | human immunodeficiency virus type I enhancer binding protein 3 [Source:HGNC Symbol;Acc:HGNC:13561] |
| ENSG00000127311 | HGLibA_21112 | 53 | -0.621196975 | HELB | DNA helicase B [Source:HGNC Symbol;Acc:HGNC:17196] |
| ENSG00000129173 | HGLibA_14327 | 53 | -0.621196975 | E2F8 | E2F transcription factor 8 [Source:HGNC Symbol;Acc:HGNC:24727] |
| ENSG00000129862 | HGLibA_53596 | 53 | -0.621196975 | VCY1B | variable charge Y-linked 1B [Source:HGNC Symbol;Acc:HGNC:31751] |
| ENSG00000129946 | HGLibA_44002 | 53 | -0.621196975 | SHC2 | SHC adaptor protein 2 [Source:HGNC Symbol;Acc:HGNC:29869] |
| ENSG00000130635 | HGLibA_10586 | 53 | -0.621196975 | COL5A1 | collagen type V alpha 1 chain [Source:HGNC Symbol;Acc:HGNC:2209] |
| ENSG00000130826 | HGLibA_13292 | 53 | -0.621196975 | DKC1 | dyskerin pseudouridine synthase 1 [Source:HGNC Symbol;Acc:HGNC:2890] |
| ENSG00000131238 | HGLibA_38233 | 53 | -0.621196975 | PPT1 | palmitoyl-protein thioesterase 1 [Source:HGNC Symbol;Acc:HGNC:9325] |
| ENSG00000131941 | HGLibA_41227 | 53 | -0.621196975 | RHPN2 | rhophilin Rho GTPase binding protein 2 [Source:HGNC Symbol;Acc:HGNC:19974] |
| ENSG00000132466 | HGLibA_02016 | 53 | -0.621196975 | ANKRD17 | ankyrin repeat domain 17 [Source:HGNC Symbol;Acc:HGNC:23575] |
| ENSG00000132664 | HGLibA_37623 | 53 | -0.621196975 | POLR3F | RNA polymerase III subunit F [Source:HGNC Symbol;Acc:HGNC:15763] |
| ENSG00000132740 | HGLibA_22857 | 53 | -0.621196975 | IGHMBP2 | immunoglobulin mu DNA binding protein 2 [Source:HGNC Symbol;Acc:HGNC:5542] |
| ENSG00000133937 | HGLibA_20397 | 53 | -0.621196975 | GSC | goosecoid homeobox [Source:HGNC Symbol;Acc:HGNC:4612] |
| ENSG00000134827 | HGLibA_48822 | 53 | -0.621196975 | TCN1 | transcobalamin 1 [Source:HGNC Symbol;Acc:HGNC:11652] |
| ENSG00000135063 | HGLibA_16354 | 53 | -0.621196975 | FAM189A2 | family with sequence similarity 189 member A2 [Source:HGNC Symbol;Acc:HGNC:24820] |
| ENSG00000135720 | HGLibA_14243 | 53 | -0.621196975 | DYNC1LI2 | dynein cytoplasmic 1 light intermediate chain 2 [Source:HGNC Symbol;Acc:HGNC:2966] |
| ENSG00000135776 | HGLibA_00134 | 53 | -0.621196975 | ABCB10 | ATP binding cassette subfamily B member 10 [Source:HGNC Symbol;Acc:HGNC:41] |
| ENSG00000136371 | HGLibA_30391 | 53 | -0.621196975 | MTHFS | methenyltetrahydrofolate synthetase [Source:HGNC Symbol;Acc:HGNC:7437] |
| ENSG00000136872 | HGLibA_01595 | 53 | -0.621196975 | ALDOB | fructose-bisphosphate B [Source:HGNC Symbol;Acc:HGNC:417]" |
| ENSG00000137054 | HGLibA_37561 | 53 | -0.621196975 | POLR1E | RNA polymerase I subunit E [Source:HGNC Symbol;Acc:HGNC:17631] |
| ENSG00000138449 | HGLibA_45136 | 53 | -0.621196975 | SLC40A1 | solute carrier family 40 member 1 [Source:HGNC Symbol;Acc:HGNC:10909] |
| ENSG00000138802 | HGLibA_43233 | 53 | -0.621196975 | SEC24B | COPII coat complex component [Source:HGNC Symbol;Acc:HGNC:10704]" |
| ENSG00000139508 | HGLibA_45189 | 53 | -0.621196975 | SLC46A3 | solute carrier family 46 member 3 [Source:HGNC Symbol;Acc:HGNC:27501] |
| ENSG00000139549 | HGLibA_13109 | 53 | -0.621196975 | DHH | desert hedgehog [Source:HGNC Symbol;Acc:HGNC:2865] |
| ENSG00000139636 | HGLibA_26673 | 53 | -0.621196975 | LMBR1L | limb development membrane protein 1 like [Source:HGNC Symbol;Acc:HGNC:18268] |
| ENSG00000140153 | HGLibA_54046 | 53 | -0.621196975 | WDR20 | WD repeat domain 20 [Source:HGNC Symbol;Acc:HGNC:19667] |
| ENSG00000140478 | HGLibA_19617 | 53 | -0.621196975 | GOLGA6D | golgin A6 family member D [Source:HGNC Symbol;Acc:HGNC:32204] |
| ENSG00000140675 | HGLibA_45264 | 53 | -0.621196975 | SLC5A2 | solute carrier family 5 member 2 [Source:HGNC Symbol;Acc:HGNC:11037] |
| ENSG00000141098 | HGLibA_18955 | 53 | -0.621196975 | GFOD2 | glucose-fructose oxidoreductase domain containing 2 [Source:HGNC Symbol;Acc:HGNC:28159] |
| ENSG00000143106 | HGLibA_39082 | 53 | -0.621196975 | PSMA5 | proteasome subunit alpha 5 [Source:HGNC Symbol;Acc:HGNC:9534] |
| ENSG00000143126 | HGLibA_09025 | 53 | -0.621196975 | CELSR2 | cadherin EGF LAG seven-pass G-type receptor 2 [Source:HGNC Symbol;Acc:HGNC:3231] |
| ENSG00000143543 | HGLibA_23999 | 53 | -0.621196975 | JTB | jumping translocation breakpoint [Source:HGNC Symbol;Acc:HGNC:6201] |
| ENSG00000144445 | HGLibA_24038 | 53 | -0.621196975 | KANSL1L | KAT8 regulatory NSL complex subunit 1 like [Source:HGNC Symbol;Acc:HGNC:26310] |
| ENSG00000144724 | HGLibA_39505 | 53 | -0.621196975 | PTPRG | receptor type G [Source:HGNC Symbol;Acc:HGNC:9671]" |
| ENSG00000145247 | HGLibA_33250 | 53 | -0.621196975 | OCIAD2 | OCIA domain containing 2 [Source:HGNC Symbol;Acc:HGNC:28685] |
| ENSG00000145908 | HGLibA_55772 | 53 | -0.621196975 | ZNF300 | zinc finger protein 300 [Source:HGNC Symbol;Acc:HGNC:13091] |
| ENSG00000146247 | HGLibA_36398 | 53 | -0.621196975 | PHIP | pleckstrin homology domain interacting protein [Source:HGNC Symbol;Acc:HGNC:15673] |
| ENSG00000147596 | HGLibA_38377 | 53 | -0.621196975 | PRDM14 | PR/SET domain 14 [Source:HGNC Symbol;Acc:HGNC:14001] |
| ENSG00000148308 | HGLibA_20591 | 53 | -0.621196975 | GTF3C5 | general transcription factor IIIC subunit 5 [Source:HGNC Symbol;Acc:HGNC:4668] |
| ENSG00000149182 | HGLibA_02663 | 53 | -0.621196975 | ARFGAP2 | ADP ribosylation factor GTPase activating protein 2 [Source:HGNC Symbol;Acc:HGNC:13504] |
| ENSG00000149532 | HGLibA_11014 | 53 | -0.621196975 | CPSF7 | cleavage and polyadenylation specific factor 7 [Source:HGNC Symbol;Acc:HGNC:30098] |
| ENSG00000151655 | HGLibA_23808 | 53 | -0.621196975 | ITIH2 | inter-alpha-trypsin inhibitor heavy chain 2 [Source:HGNC Symbol;Acc:HGNC:6167] |
| ENSG00000152578 | HGLibA_20269 | 53 | -0.621196975 | GRIA4 | glutamate ionotropic receptor AMPA type subunit 4 [Source:HGNC Symbol;Acc:HGNC:4574] |
| ENSG00000152822 | HGLibA_20340 | 53 | -0.621196975 | GRM1 | glutamate metabotropic receptor 1 [Source:HGNC Symbol;Acc:HGNC:4593] |
| ENSG00000153002 | HGLibA_10904 | 53 | -0.621196975 | CPB1 | carboxypeptidase B1 [Source:HGNC Symbol;Acc:HGNC:2299] |
| ENSG00000154134 | HGLibA_41739 | 53 | -0.621196975 | ROBO3 | roundabout guidance receptor 3 [Source:HGNC Symbol;Acc:HGNC:13433] |
| ENSG00000154165 | HGLibA_19926 | 53 | -0.621196975 | GPR15 | G protein-coupled receptor 15 [Source:HGNC Symbol;Acc:HGNC:4469] |
| ENSG00000156509 | HGLibA_17102 | 53 | -0.621196975 | FBXO43 | F-box protein 43 [Source:HGNC Symbol;Acc:HGNC:28521] |
| ENSG00000156804 | HGLibA_17073 | 53 | -0.621196975 | FBXO32 | F-box protein 32 [Source:HGNC Symbol;Acc:HGNC:16731] |
| ENSG00000157764 | HGLibA_04753 | 53 | -0.621196975 | BRAF | serine/threonine kinase [Source:HGNC Symbol;Acc:HGNC:1097]" |
| ENSG00000158022 | HGLibA_51572 | 53 | -0.621196975 | TRIM63 | tripartite motif containing 63 [Source:HGNC Symbol;Acc:HGNC:16007] |
| ENSG00000158604 | HGLibA_49811 | 53 | -0.621196975 | TMED4 | transmembrane p24 trafficking protein 4 [Source:HGNC Symbol;Acc:HGNC:22301] |
| ENSG00000158786 | HGLibA_36895 | 53 | -0.621196975 | PLA2G2F | phospholipase A2 group IIF [Source:HGNC Symbol;Acc:HGNC:30040] |
| ENSG00000159140 | HGLibA_46172 | 53 | -0.621196975 | SON | SON DNA binding protein [Source:HGNC Symbol;Acc:HGNC:11183] |
| ENSG00000159596 | HGLibA_50497 | 53 | -0.621196975 | TMEM69 | transmembrane protein 69 [Source:HGNC Symbol;Acc:HGNC:28035] |
| ENSG00000160917 | HGLibA_11007 | 53 | -0.621196975 | CPSF4 | cleavage and polyadenylation specific factor 4 [Source:HGNC Symbol;Acc:HGNC:2327] |
| ENSG00000161996 | HGLibA_54213 | 53 | -0.621196975 | WDR90 | WD repeat domain 90 [Source:HGNC Symbol;Acc:HGNC:26960] |
| ENSG00000162873 | HGLibA_25112 | 53 | -0.621196975 | KLHDC8A | kelch domain containing 8A [Source:HGNC Symbol;Acc:HGNC:25573] |
| ENSG00000163462 | HGLibA_51512 | 53 | -0.621196975 | TRIM46 | tripartite motif containing 46 [Source:HGNC Symbol;Acc:HGNC:19019] |
| ENSG00000164190 | HGLibA_31969 | 53 | -0.621196975 | NIPBL | cohesin loading factor [Source:HGNC Symbol;Acc:HGNC:28862]" |
| ENSG00000164619 | HGLibA_04625 | 53 | -0.621196975 | BMPER | BMP binding endothelial regulator [Source:HGNC Symbol;Acc:HGNC:24154] |
| ENSG00000164823 | HGLibA_34689 | 53 | -0.621196975 | OSGIN2 | oxidative stress induced growth inhibitor family member 2 [Source:HGNC Symbol;Acc:HGNC:1355] |
| ENSG00000165072 | HGLibA_28036 | 53 | -0.621196975 | MAMDC2 | MAM domain containing 2 [Source:HGNC Symbol;Acc:HGNC:23673] |
| ENSG00000165555 | HGLibA_32388 | 53 | -0.621196975 | NOXRED1 | NADP dependent oxidoreductase domain containing 1 [Source:HGNC Symbol;Acc:HGNC:20487] |
| ENSG00000166342 | HGLibA_31703 | 53 | -0.621196975 | NETO1 | neuropilin and tolloid like 1 [Source:HGNC Symbol;Acc:HGNC:13823] |
| ENSG00000167491 | HGLibA_18686 | 53 | -0.621196975 | GATAD2A | GATA zinc finger domain containing 2A [Source:HGNC Symbol;Acc:HGNC:29989] |
| ENSG00000167825 | HGLibA_34303 | 53 | -0.621196975 | OR5I1 | olfactory receptor family 5 subfamily I member 1 [Source:HGNC Symbol;Acc:HGNC:8347] |
| ENSG00000168490 | HGLibA_36474 | 53 | -0.621196975 | PHYHIP | phytanoyl-CoA 2-hydroxylase interacting protein [Source:HGNC Symbol;Acc:HGNC:16865] |
| ENSG00000168566 | HGLibA_45955 | 53 | -0.621196975 | SNRNP48 | small nuclear ribonucleoprotein U11/U12 subunit 48 [Source:HGNC Symbol;Acc:HGNC:21368] |
| ENSG00000169057 | HGLibA_28727 | 53 | -0.621196975 | MECP2 | methyl-CpG binding protein 2 [Source:HGNC Symbol;Acc:HGNC:6990] |
| ENSG00000169223 | HGLibA_26664 | 53 | -0.621196975 | LMAN2 | mannose binding 2 [Source:HGNC Symbol;Acc:HGNC:16986]" |
| ENSG00000169247 | HGLibA_43982 | 53 | -0.621196975 | SH3TC2 | SH3 domain and tetratricopeptide repeats 2 [Source:HGNC Symbol;Acc:HGNC:29427] |
| ENSG00000169484 | HGLibA_34023 | 53 | -0.621196975 | OR4K14 | olfactory receptor family 4 subfamily K member 14 [Source:HGNC Symbol;Acc:HGNC:15352] |
| ENSG00000169570 | HGLibA_14076 | 53 | -0.621196975 | DTWD2 | DTW domain containing 2 [Source:HGNC Symbol;Acc:HGNC:19334] |

| | | | | | |
|---|---|---|---|---|---|
| ENSG00000169752 | HGLibA_32686 | 53 | -0.621196975 | NRG4 | neuregulin 4 [Source:HGNC Symbol;Acc:HGNC:29862] |
| ENSG00000170577 | HGLibA_44245 | 53 | -0.621196975 | SIX2 | SIX homeobox 2 [Source:HGNC Symbol;Acc:HGNC:10888] |
| ENSG00000171402 | HGLibA_54484 | 53 | -0.621196975 | XAGE3 | X antigen family member 3 [Source:HGNC Symbol;Acc:HGNC:14618] |
| ENSG00000171496 | HGLibA_33697 | 53 | -0.621196975 | OR1L8 | olfactory receptor family 1 subfamily L member 8 [Source:HGNC Symbol;Acc:HGNC:15110] |
| ENSG00000171942 | HGLibA_33509 | 53 | -0.621196975 | OR10H2 | olfactory receptor family 10 subfamily H member 2 [Source:HGNC Symbol;Acc:HGNC:8173] |
| ENSG00000172538 | HGLibA_16262 | 53 | -0.621196975 | FAM170B | family with sequence similarity 170 member B [Source:HGNC Symbol;Acc:HGNC:19736] |
| ENSG00000173431 | HGLibA_41419 | 53 | -0.621196975 | RNASE8 | ribonuclease A family member 8 [Source:HGNC Symbol;Acc:HGNC:19277] |
| ENSG00000174255 | HGLibA_56782 | 53 | -0.621196975 | ZNF80 | zinc finger protein 80 [Source:HGNC Symbol;Acc:HGNC:13155] |
| ENSG00000174937 | HGLibA_34336 | 53 | -0.621196975 | OR5M3 | olfactory receptor family 5 subfamily M member 3 [Source:HGNC Symbol;Acc:HGNC:14806] |
| ENSG00000175215 | HGLibA_11610 | 53 | -0.621196975 | CTDSP2 | CTD small phosphatase 2 [Source:HGNC Symbol;Acc:HGNC:17077] |
| ENSG00000175352 | HGLibA_32698 | 53 | -0.621196975 | NRIP3 | nuclear receptor interacting protein 3 [Source:HGNC Symbol;Acc:HGNC:1167] |
| ENSG00000177476 | HGLibA_33797 | 53 | -0.621196975 | OR2G3 | olfactory receptor family 2 subfamily G member 3 [Source:HGNC Symbol;Acc:HGNC:15008] |
| ENSG00000177752 | HGLibA_54688 | 53 | -0.621196975 | YIPF7 | Yip1 domain family member 7 [Source:HGNC Symbol;Acc:HGNC:26825] |
| ENSG00000178035 | HGLibA_23260 | 53 | -0.621196975 | IMPDH2 | inosine monophosphate dehydrogenase 2 [Source:HGNC Symbol;Acc:HGNC:6053] |
| ENSG00000178084 | HGLibA_22385 | 53 | -0.621196975 | HTR3C | 5-hydroxytryptamine receptor 3C [Source:HGNC Symbol;Acc:HGNC:24003] |
| ENSG00000178397 | HGLibA_16513 | 53 | -0.621196975 | FAM220A | family with sequence similarity 220 member A [Source:HGNC Symbol;Acc:HGNC:22422] |
| ENSG00000179344 | HGLibA_21627 | 53 | -0.621196975 | HLA-DQB1 | DQ beta 1 [Source:HGNC Symbol;Acc:HGNC:4944]" |
| ENSG00000179455 | HGLibA_29353 | 53 | -0.621196975 | MKRN3 | makorin ring finger protein 3 [Source:HGNC Symbol;Acc:HGNC:7114] |
| ENSG00000180777 | HGLibA_02064 | 53 | -0.621196975 | ANKRD30B | ankyrin repeat domain 30B [Source:HGNC Symbol;Acc:HGNC:24165] |
| ENSG00000181523 | HGLibA_43855 | 53 | -0.621196975 | SGSH | N-sulfoglucosamine sulfohydrolase [Source:HGNC Symbol;Acc:HGNC:10818] |
| ENSG00000181544 | HGLibA_16834 | 53 | -0.621196975 | FANCB | FA complementation group B [Source:HGNC Symbol;Acc:HGNC:3583] |
| ENSG00000181656 | HGLibA_20103 | 53 | -0.621196975 | GPR88 | G protein-coupled receptor 88 [Source:HGNC Symbol;Acc:HGNC:4539] |
| ENSG00000182362 | HGLibA_54639 | 53 | -0.621196975 | YBEY | ybeY metalloendoribonuclease [Source:HGNC Symbol;Acc:HGNC:1299] |
| ENSG00000182472 | HGLibA_07150 | 53 | -0.621196975 | CAPN12 | calpain 12 [Source:HGNC Symbol;Acc:HGNC:13249] |
| ENSG00000183150 | HGLibA_19994 | 53 | -0.621196975 | GPR19 | G protein-coupled receptor 19 [Source:HGNC Symbol;Acc:HGNC:4473] |
| ENSG00000183395 | HGLibA_37293 | 53 | -0.621196975 | PMCH | pro-melanin concentrating hormone [Source:HGNC Symbol;Acc:HGNC:9109] |
| ENSG00000183621 | HGLibA_55992 | 53 | -0.621196975 | ZNF438 | zinc finger protein 438 [Source:HGNC Symbol;Acc:HGNC:21029] |
| ENSG00000183668 | HGLibA_39059 | 53 | -0.621196975 | PSG9 | pregnancy specific beta-1-glycoprotein 9 [Source:HGNC Symbol;Acc:HGNC:9526] |
| ENSG00000183706 | HGLibA_34049 | 53 | -0.621196975 | OR4N4 | olfactory receptor family 4 subfamily N member 4 [Source:HGNC Symbol;Acc:HGNC:15375] |
| ENSG00000184032 | HGLibA_25702 | 53 | -0.621196975 | KRTAP20-2 | keratin associated protein 20-2 [Source:HGNC Symbol;Acc:HGNC:18944] |
| ENSG00000184058 | HGLibA_48641 | 53 | -0.621196975 | TBX1 | T-box 1 [Source:HGNC Symbol;Acc:HGNC:11592] |
| ENSG00000184207 | HGLibA_36275 | 53 | -0.621196975 | PGP | phosphoglycolate phosphatase [Source:HGNC Symbol;Acc:HGNC:8909] |
| ENSG00000184330 | HGLibA_42569 | 53 | -0.621196975 | S100A7A | S100 calcium binding protein A7A [Source:HGNC Symbol;Acc:HGNC:21657] |
| ENSG00000184492 | HGLibA_17839 | 53 | -0.621196975 | FOXD4L1 | forkhead box D4 like 1 [Source:HGNC Symbol;Acc:HGNC:18521] |
| ENSG00000184508 | HGLibA_21037 | 53 | -0.621196975 | HDDC3 | HD domain containing 3 [Source:HGNC Symbol;Acc:HGNC:30522] |
| ENSG00000184708 | HGLibA_14837 | 53 | -0.621196975 | EIF4ENIF1 | eukaryotic translation initiation factor 4E nuclear import factor 1 [Source:HGNC Symbol;Acc:HGNC:16687] |
| ENSG00000185028 | HGLibA_27256 | 53 | -0.621196975 | LRRC14B | leucine rich repeat containing 14B [Source:HGNC Symbol;Acc:HGNC:37268] |
| ENSG00000185404 | HGLibA_46295 | 53 | -0.621196975 | SP140L | SP140 nuclear body protein like [Source:HGNC Symbol;Acc:HGNC:25105] |
| ENSG00000185669 | HGLibA_45873 | 53 | -0.621196975 | SNAI3 | snail family transcriptional repressor 3 [Source:HGNC Symbol;Acc:HGNC:18411] |
| ENSG00000185896 | HGLibA_25978 | 53 | -0.621196975 | LAMP1 | lysosomal associated membrane protein 1 [Source:HGNC Symbol;Acc:HGNC:6499] |
| ENSG00000186603 | HGLibA_22027 | 53 | -0.621196975 | HPDL | 4-hydroxyphenylpyruvate dioxygenase like [Source:HGNC Symbol;Acc:HGNC:28242] |
| ENSG00000187730 | HGLibA_18384 | 53 | -0.621196975 | GABRD | gamma-aminobutyric acid type A receptor delta subunit [Source:HGNC Symbol;Acc:HGNC:4084] |
| ENSG00000188100 | HGLibA_16552 | 53 | -0.621196975 | FAM25A | family with sequence similarity 25 member A [Source:HGNC Symbol;Acc:HGNC:23436] |
| ENSG00000188321 | HGLibA_56237 | 53 | -0.621196975 | ZNF559 | zinc finger protein 559 [Source:HGNC Symbol;Acc:HGNC:28197] |
| ENSG00000188493 | HGLibA_05682 | 53 | -0.621196975 | C19orf54 | chromosome 19 open reading frame 54 [Source:HGNC Symbol;Acc:HGNC:24758] |
| ENSG00000188566 | HGLibA_31406 | 53 | -0.621196975 | NDOR1 | NADPH dependent diflavin oxidoreductase 1 [Source:HGNC Symbol;Acc:HGNC:29838] |
| ENSG00000188611 | HGLibA_03187 | 53 | -0.621196975 | ASAH2 | N-acylsphingosine amidohydrolase 2 [Source:HGNC Symbol;Acc:HGNC:18860] |
| ENSG00000189152 | HGLibA_20219 | 53 | -0.621196975 | GRAPL | GRB2 related adaptor protein like [Source:HGNC Symbol;Acc:HGNC:37240] |
| ENSG00000196228 | HGLibA_47748 | 53 | -0.621196975 | SULT1C3 | sulfotransferase family 1C member 3 [Source:HGNC Symbol;Acc:HGNC:33543] |
| ENSG00000196378 | HGLibA_55836 | 53 | -0.621196975 | ZNF34 | zinc finger protein 34 [Source:HGNC Symbol;Acc:HGNC:13098] |
| ENSG00000196453 | HGLibA_56725 | 53 | -0.621196975 | ZNF777 | zinc finger protein 777 [Source:HGNC Symbol;Acc:HGNC:22213] |
| ENSG00000196549 | HGLibA_29467 | 53 | -0.621196975 | MME | membrane metalloendopeptidase [Source:HGNC Symbol;Acc:HGNC:7154] |
| ENSG00000196976 | HGLibA_25929 | 53 | -0.621196975 | LAGE3 | L antigen family member 3 [Source:HGNC Symbol;Acc:HGNC:26058] |
| ENSG00000197249 | HGLibA_43506 | 53 | -0.621196975 | SERPINA1 | serpin family A member 1 [Source:HGNC Symbol;Acc:HGNC:8941] |
| ENSG00000197444 | HGLibA_33304 | 53 | -0.621196975 | OGDHL | oxoglutarate dehydrogenase like [Source:HGNC Symbol;Acc:HGNC:25590] |
| ENSG00000197520 | HGLibA_16298 | 53 | -0.621196975 | FAM177B | family with sequence similarity 177 member B [Source:HGNC Symbol;Acc:HGNC:34395] |
| ENSG00000197928 | HGLibA_56519 | 53 | -0.621196975 | ZNF677 | zinc finger protein 677 [Source:HGNC Symbol;Acc:HGNC:28730] |
| ENSG00000198901 | HGLibA_38350 | 53 | -0.621196975 | PRC1 | protein regulator of cytokinesis 1 [Source:HGNC Symbol;Acc:HGNC:9341] |
| ENSG00000203797 | HGLibA_12627 | 53 | -0.621196975 | DDO | D-aspartate oxidase [Source:HGNC Symbol;Acc:HGNC:2727] |
| ENSG00000203942 | HGLibA_05081 | 53 | -0.621196975 | C10orf62 | chromosome 10 open reading frame 62 [Source:HGNC Symbol;Acc:HGNC:23294] |
| ENSG00000204001 | HGLibA_26185 | 53 | -0.621196975 | LCN8 | lipocalin 8 [Source:HGNC Symbol;Acc:HGNC:27038] |
| ENSG00000204688 | HGLibA_33803 | 53 | -0.621196975 | OR2H1 | olfactory receptor family 2 subfamily H member 1 [Source:HGNC Symbol;Acc:HGNC:8252] |
| ENSG00000205038 | HGLibA_36815 | 53 | -0.621196975 | PKHD1L1 | PKHD1 like 1 [Source:HGNC Symbol;Acc:HGNC:20313] |
| ENSG00000205108 | HGLibA_16422 | 53 | -0.621196975 | FAM205A | family with sequence similarity 205 member A [Source:HGNC Symbol;Acc:HGNC:41911] |
| ENSG00000205882 | HGLibA_12905 | 53 | -0.621196975 | DEFB134 | defensin beta 134 [Source:HGNC Symbol;Acc:HGNC:32399] |
| ENSG00000205929 | HGLibA_06048 | 53 | -0.621196975 | C21orf62 | chromosome 21 open reading frame 62 [Source:HGNC Symbol;Acc:HGNC:1305] |
| ENSG00000213171 | HGLibA_26594 | 53 | -0.621196975 | LINGO4 | leucine rich repeat and Ig domain containing 4 [Source:HGNC Symbol;Acc:HGNC:31814] |
| ENSG00000213920 | HGLibA_28707 | 53 | -0.621196975 | MDP1 | magnesium dependent phosphatase 1 [Source:HGNC Symbol;Acc:HGNC:28781] |
| ENSG00000214022 | HGLibA_40845 | 53 | -0.621196975 | REPIN1 | replication initiator 1 [Source:HGNC Symbol;Acc:HGNC:17922] |
| ENSG00000221869 | HGLibA_08961 | 53 | -0.621196975 | CEBPD | CCAAT enhancer binding protein delta [Source:HGNC Symbol;Acc:HGNC:1835] |
| ENSG00000229809 | HGLibA_56542 | 53 | -0.621196975 | ZNF688 | zinc finger protein 688 [Source:HGNC Symbol;Acc:HGNC:30489] |
| ENSG00000233041 | HGLibA_36397 | 53 | -0.621196975 | PHGR1 | histidine and glycine rich 1 [Source:HGNC Symbol;Acc:HGNC:37226]" |
| ENSG00000233436 | HGLibA_04908 | 53 | -0.621196975 | BTBD18 | BTB domain containing 18 [Source:HGNC Symbol;Acc:HGNC:37214] |
| ENSG00000236609 | HGLibA_56874 | 53 | -0.621196975 | ZNF853 | zinc finger protein 853 [Source:HGNC Symbol;Acc:HGNC:21767] |
| ENSG00000248643 | HGLibA_40488 | 53 | -0.621196975 | RBM14-RBM4 | RBM14-RBM4 readthrough [Source:HGNC Symbol;Acc:HGNC:38840] |
| ENSG00000249751 | HGLibA_14417 | 53 | -0.621196975 | ECSCR | endothelial cell surface expressed chemotaxis and apoptosis regulator [Source:HGNC Symbol;Acc:HGNC:35454] |
| ENSG00000253148 | HGLibA_41084 | 53 | -0.621196975 | RGS21 | regulator of G protein signaling 21 [Source:HGNC Symbol;Acc:HGNC:26839] |
| ENSG00000253958 | HGLibA_09885 | 53 | -0.621196975 | CLDN23 | claudin 23 [Source:HGNC Symbol;Acc:HGNC:17591] |
| ENSG00000254521 | HGLibA_44133 | 53 | -0.621196975 | SIGLEC12 | sialic acid binding Ig like lectin 12 (gene/pseudogene) [Source:HGNC Symbol;Acc:HGNC:15482] |
| ENSG00000257949 | HGLibA_48990 | 53 | -0.621196975 | TEN1 | CST complex subunit [Source:HGNC Symbol;Acc:HGNC:37242]" |
| ENSG00000258986 | HGLibA_50086 | 53 | -0.621196975 | TMEM179 | transmembrane protein 179 [Source:HGNC Symbol;Acc:HGNC:20137] |
| ENSG00000268738 | HGLibA_22239 | 53 | -0.621196975 | HSFX2 | X-linked 2 [Source:HGNC Symbol;Acc:HGNC:32701]" |
| ENSG00000271321 | HGLibA_11579 | 53 | -0.621196975 | CTAGE6 | CTAGE family member 6 [Source:HGNC Symbol;Acc:HGNC:28644] |
| ENSG00000277462 | HGLibA_56501 | 53 | -0.621196975 | ZNF670 | zinc finger protein 670 [Source:HGNC Symbol;Acc:HGNC:28167] |
| ENSG00000280987 | HGLibA_28438 | 53 | -0.621196975 | MATR3 | matrin 3 [Source:NCBI gene;Acc:9782] |
| FAM69B | HGLibA_16679 | 53 | -0.621196975 | FAM69B | - |
| GAGE7 | HGLibA_18488 | 53 | -0.621196975 | GAGE7 | - |
| GPR112 | HGLibA_19853 | 53 | -0.621196975 | GPR112 | - |
| HIST1H4J | HGLibA_21512 | 53 | -0.621196975 | HIST1H4J | - |
| hsa-mir-1204 | HGLibA_57275 | 53 | -0.621196975 | hsa-mir-1204 | - |
| hsa-mir-1538 | HGLibA_57920 | 53 | -0.621196975 | hsa-mir-1538 | - |
| hsa-mir-15b | HGLibA_57944 | 53 | -0.621196975 | hsa-mir-15b | - |
| hsa-mir-300 | HGLibA_58426 | 53 | -0.621196975 | hsa-mir-300 | - |
| hsa-mir-3175 | HGLibA_58772 | 53 | -0.621196975 | hsa-mir-3175 | - |
| hsa-mir-3610 | HGLibA_59072 | 53 | -0.621196975 | hsa-mir-3610 | - |
| hsa-mir-3670-2 | HGLibA_59236 | 53 | -0.621196975 | hsa-mir-3670-2 | - |
| hsa-mir-376b | HGLibA_59412 | 53 | -0.621196975 | hsa-mir-376b | - |
| hsa-mir-3975 | HGLibA_59659 | 53 | -0.621196975 | hsa-mir-3975 | - |
| hsa-mir-4422 | HGLibA_60075 | 53 | -0.621196975 | hsa-mir-4422 | - |
| hsa-mir-4428 | HGLibA_60097 | 53 | -0.621196975 | hsa-mir-4428 | - |
| hsa-mir-4465 | HGLibA_60267 | 53 | -0.621196975 | hsa-mir-4465 | - |
| hsa-mir-4472-1 | HGLibA_60293 | 53 | -0.621196975 | hsa-mir-4472-1 | - |
| hsa-mir-4538 | HGLibA_60613 | 53 | -0.621196975 | hsa-mir-4538 | - |
| hsa-mir-4798 | HGLibA_61297 | 53 | -0.621196975 | hsa-mir-4798 | - |
| hsa-mir-503 | HGLibA_61466 | 53 | -0.621196975 | hsa-mir-503 | - |
| hsa-mir-548d-1 | HGLibA_61960 | 53 | -0.621196975 | hsa-mir-548d-1 | - |
| hsa-mir-548f-1 | HGLibA_61971 | 53 | -0.621196975 | hsa-mir-548f-1 | - |
| hsa-mir-548j | HGLibA_62030 | 53 | -0.621196975 | hsa-mir-548j | - |
| hsa-mir-550a-3 | HGLibA_62108 | 53 | -0.621196975 | hsa-mir-550a-3 | - |
| hsa-mir-5587 | HGLibA_62188 | 53 | -0.621196975 | hsa-mir-5587 | - |
| hsa-mir-5689 | HGLibA_62273 | 53 | -0.621196975 | hsa-mir-5689 | - |
| hsa-mir-6134 | HGLibA_62688 | 53 | -0.621196975 | hsa-mir-6134 | - |
| hsa-mir-6765 | HGLibA_63221 | 53 | -0.621196975 | hsa-mir-6765 | - |
| hsa-mir-6781 | HGLibA_63301 | 53 | -0.621196975 | hsa-mir-6781 | - |
| hsa-mir-6808 | HGLibA_63411 | 53 | -0.621196975 | hsa-mir-6808 | - |
| hsa-mir-6810 | HGLibA_63419 | 53 | -0.621196975 | hsa-mir-6810 | - |
| hsa-mir-6857 | HGLibA_63601 | 53 | -0.621196975 | hsa-mir-6857 | - |
| hsa-mir-6870 | HGLibA_63665 | 53 | -0.621196975 | hsa-mir-6870 | - |
| hsa-mir-887 | HGLibA_64226 | 53 | -0.621196975 | hsa-mir-887 | - |
| KAL1 | HGLibA_24018 | 53 | -0.621196975 | KAL1 | - |
| KIAA1456 | HGLibA_24759 | 53 | -0.621196975 | KIAA1456 | - |
| LEPREL4 | HGLibA_26324 | 53 | -0.621196975 | LEPREL4 | - |
| LOC100133267 | HGLibA_26810 | 53 | -0.621196975 | LOC100133267 | - |
| LOC158434 | HGLibA_26900 | 53 | -0.621196975 | LOC158434 | - |
| LOC730159 | HGLibA_27001 | 53 | -0.621196975 | LOC730159 | - |
| NIM1 | HGLibA_31931 | 53 | -0.621196975 | NIM1 | - |
| SDCCAG3 | HGLibA_43115 | 53 | -0.621196975 | SDCCAG3 | - |
| SLC22A20 | HGLibA_44571 | 53 | -0.621196975 | SLC22A20 | - |
| WDR85 | HGLibA_54198 | 53 | -0.621196975 | WDR85 | - |
| ZFP106 | HGLibA_55182 | 53 | -0.621196975 | ZFP106 | - |
| ATP5B | HGLibA_03641 | 52 | -0.631148013 | ATP5B | - |
| C4B_2 | HGLibA_06286 | 52 | -0.631148013 | C4B_2 | - |
| C8orf46 | HGLibA_06629 | 52 | -0.631148013 | C8orf46 | - |
| C9orf172 | HGLibA_06712 | 52 | -0.631148013 | C9orf172 | - |
| CD3EAP | HGLibA_08375 | 52 | -0.631148013 | CD3EAP | - |

| | | | | | |
|---|---|---|---|---|---|
| CXorf64 | HGLibA_11955 | 52 | -0.631148013 | CXorf64 | - |
| ENSG00000001461 | HGLibA_31961 | 52 | -0.631148013 | NIPAL3 | NIPA like domain containing 3 [Source:HGNC Symbol;Acc:HGNC:25233] |
| ENSG00000005513 | HGLibA_46275 | 52 | -0.631148013 | SOX8 | SRY-box 8 [Source:HGNC Symbol;Acc:HGNC:11203] |
| ENSG00000006744 | HGLibA_14874 | 52 | -0.631148013 | ELAC2 | elaC ribonuclease Z 2 [Source:HGNC Symbol;Acc:HGNC:14198] |
| ENSG00000008311 | HGLibA_00082 | 52 | -0.631148013 | AASS | aminoadipate-semialdehyde synthase [Source:HGNC Symbol;Acc:HGNC:17366] |
| ENSG00000008516 | HGLibA_29523 | 52 | -0.631148013 | MMP25 | matrix metallopeptidase 25 [Source:HGNC Symbol;Acc:HGNC:14246] |
| ENSG00000010322 | HGLibA_31981 | 52 | -0.631148013 | NISCH | nischarin [Source:HGNC Symbol;Acc:HGNC:18006] |
| ENSG00000011275 | HGLibA_41632 | 52 | -0.631148013 | RNF216 | ring finger protein 216 [Source:HGNC Symbol;Acc:HGNC:21698] |
| ENSG00000023445 | HGLibA_04519 | 52 | -0.631148013 | BIRC3 | baculoviral IAP repeat containing 3 [Source:HGNC Symbol;Acc:HGNC:591] |
| ENSG00000049247 | HGLibA_53461 | 52 | -0.631148013 | UTS2 | urotensin 2 [Source:HGNC Symbol;Acc:HGNC:12636] |
| ENSG00000050730 | HGLibA_50842 | 52 | -0.631148013 | TNIP3 | TNFAIP3 interacting protein 3 [Source:HGNC Symbol;Acc:HGNC:19315] |
| ENSG00000054267 | HGLibA_02903 | 52 | -0.631148013 | ARID4B | AT-rich interaction domain 4B [Source:HGNC Symbol;Acc:HGNC:15550] |
| ENSG00000054392 | HGLibA_21251 | 52 | -0.631148013 | HHAT | hedgehog acyltransferase [Source:HGNC Symbol;Acc:HGNC:18270] |
| ENSG00000054793 | HGLibA_03793 | 52 | -0.631148013 | ATP9A | ATPase phospholipid transporting 9A (putative) [Source:HGNC Symbol;Acc:HGNC:13540] |
| ENSG00000054965 | HGLibA_16247 | 52 | -0.631148013 | FAM168A | family with sequence similarity 168 member A [Source:HGNC Symbol;Acc:HGNC:28999] |
| ENSG00000066027 | HGLibA_38170 | 52 | -0.631148013 | PPP2R5A | protein phosphatase 2 regulatory subunit B'alpha [Source:HGNC Symbol;Acc:HGNC:9309] |
| ENSG00000066382 | HGLibA_29775 | 52 | -0.631148013 | MPPED2 | metallophosphoesterase domain containing 2 [Source:HGNC Symbol;Acc:HGNC:1180] |
| ENSG00000068079 | HGLibA_22608 | 52 | -0.631148013 | IFI35 | interferon induced protein 35 [Source:HGNC Symbol;Acc:HGNC:5399] |
| ENSG00000069206 | HGLibA_00779 | 52 | -0.631148013 | ADAM7 | ADAM metallopeptidase domain 7 [Source:HGNC Symbol;Acc:HGNC:214] |
| ENSG00000069493 | HGLibA_09967 | 52 | -0.631148013 | CLEC2D | C-type lectin domain family 2 member D [Source:HGNC Symbol;Acc:HGNC:14351] |
| ENSG00000070961 | HGLibA_03614 | 52 | -0.631148013 | ATP2B1 | ATPase plasma membrane Ca2+ transporting 1 [Source:HGNC Symbol;Acc:HGNC:814] |
| ENSG00000080166 | HGLibA_12538 | 52 | -0.631148013 | DCT | dopachrome tautomerase [Source:HGNC Symbol;Acc:HGNC:2709] |
| ENSG00000084093 | HGLibA_40870 | 52 | -0.631148013 | REST | RE1 silencing transcription factor [Source:HGNC Symbol;Acc:HGNC:9966] |
| ENSG00000086570 | HGLibA_16916 | 52 | -0.631148013 | FAT2 | FAT atypical cadherin 2 [Source:HGNC Symbol;Acc:HGNC:3596] |
| ENSG00000092054 | HGLibA_30732 | 52 | -0.631148013 | MYH7 | myosin heavy chain 7 [Source:HGNC Symbol;Acc:HGNC:7577] |
| ENSG00000092621 | HGLibA_36394 | 52 | -0.631148013 | PHGDH | phosphoglycerate dehydrogenase [Source:HGNC Symbol;Acc:HGNC:8923] |
| ENSG00000092853 | HGLibA_10132 | 52 | -0.631148013 | CLSPN | claspin [Source:HGNC Symbol;Acc:HGNC:19715] |
| ENSG00000099338 | HGLibA_07373 | 52 | -0.631148013 | CATSPERG | cation channel sperm associated auxiliary subunit gamma [Source:HGNC Symbol;Acc:HGNC:25243] |
| ENSG00000099365 | HGLibA_47640 | 52 | -0.631148013 | STX1B | syntaxin 1B [Source:HGNC Symbol;Acc:HGNC:18539] |
| ENSG00000099399 | HGLibA_27943 | 52 | -0.631148013 | MAGEB2 | MAGE family member B2 [Source:HGNC Symbol;Acc:HGNC:6809] |
| ENSG00000099860 | HGLibA_18419 | 52 | -0.631148013 | GADD45B | growth arrest and DNA damage inducible beta [Source:HGNC Symbol;Acc:HGNC:4096] |
| ENSG00000100099 | HGLibA_22053 | 52 | -0.631148013 | HPS4 | biogenesis of lysosomal organelles complex 3 subunit 2 [Source:HGNC Symbol;Acc:HGNC:15844]" |
| ENSG00000101040 | HGLibA_55426 | 52 | -0.631148013 | ZMYND8 | zinc finger MYND-type containing 8 [Source:HGNC Symbol;Acc:HGNC:9397] |
| ENSG00000101236 | HGLibA_41651 | 52 | -0.631148013 | RNF24 | ring finger protein 24 [Source:HGNC Symbol;Acc:HGNC:13779] |
| ENSG00000101473 | HGLibA_00492 | 52 | -0.631148013 | ACOT8 | acyl-CoA thioesterase 8 [Source:HGNC Symbol;Acc:HGNC:15919] |
| ENSG00000101751 | HGLibA_37530 | 52 | -0.631148013 | POLI | DNA polymerase iota [Source:HGNC Symbol;Acc:HGNC:9182] |
| ENSG00000102048 | HGLibA_03253 | 52 | -0.631148013 | ASB9 | ankyrin repeat and SOCS box containing 9 [Source:HGNC Symbol;Acc:HGNC:17184] |
| ENSG00000103507 | HGLibA_04284 | 52 | -0.631148013 | BCKDK | branched chain ketoacid dehydrogenase kinase [Source:HGNC Symbol;Acc:HGNC:16902] |
| ENSG00000105404 | HGLibA_39997 | 52 | -0.631148013 | RABAC1 | Rab acceptor 1 [Source:HGNC Symbol;Acc:HGNC:9794] |
| ENSG00000105612 | HGLibA_13664 | 52 | -0.631148013 | DNASE2 | lysosomal [Source:HGNC Symbol;Acc:HGNC:2960]" |
| ENSG00000105617 | HGLibA_26297 | 52 | -0.631148013 | LENG1 | leukocyte receptor cluster member 1 [Source:HGNC Symbol;Acc:HGNC:15502] |
| ENSG00000105676 | HGLibA_03027 | 52 | -0.631148013 | ARMC6 | armadillo repeat containing 6 [Source:HGNC Symbol;Acc:HGNC:25049] |
| ENSG00000105993 | HGLibA_13550 | 52 | -0.631148013 | DNAJB6 | DnaJ heat shock protein family (Hsp40) member B6 [Source:HGNC Symbol;Acc:HGNC:14888] |
| ENSG00000106004 | HGLibA_21915 | 52 | -0.631148013 | HOXA5 | homeobox A5 [Source:HGNC Symbol;Acc:HGNC:5106] |
| ENSG00000108255 | HGLibA_11251 | 52 | -0.631148013 | CRYBA1 | crystallin beta A1 [Source:HGNC Symbol;Acc:HGNC:2394] |
| ENSG00000108528 | HGLibA_44640 | 52 | -0.631148013 | SLC25A11 | solute carrier family 25 member 11 [Source:HGNC Symbol;Acc:HGNC:10981] |
| ENSG00000109132 | HGLibA_36453 | 52 | -0.631148013 | PHOX2B | paired like homeobox 2B [Source:HGNC Symbol;Acc:HGNC:9143] |
| ENSG00000109762 | HGLibA_46072 | 52 | -0.631148013 | SNX25 | sorting nexin 25 [Source:HGNC Symbol;Acc:HGNC:21883] |
| ENSG00000110330 | HGLibA_04515 | 52 | -0.631148013 | BIRC2 | baculoviral IAP repeat containing 2 [Source:HGNC Symbol;Acc:HGNC:590] |
| ENSG00000112539 | HGLibA_06436 | 52 | -0.631148013 | C6orf118 | chromosome 6 open reading frame 118 [Source:HGNC Symbol;Acc:HGNC:21233] |
| ENSG00000113212 | HGLibA_35509 | 52 | -0.631148013 | PCDHB7 | protocadherin beta 7 [Source:HGNC Symbol;Acc:HGNC:8692] |
| ENSG00000114019 | HGLibA_01820 | 52 | -0.631148013 | AMOTL2 | angiomotin like 2 [Source:HGNC Symbol;Acc:HGNC:17812] |
| ENSG00000114302 | HGLibA_38526 | 52 | -0.631148013 | PRKAR2A | protein kinase cAMP-dependent type II regulatory subunit alpha [Source:HGNC Symbol;Acc:HGNC:9391] |
| ENSG00000116704 | HGLibA_44978 | 52 | -0.631148013 | SLC35D1 | solute carrier family 35 member D1 [Source:HGNC Symbol;Acc:HGNC:20800] |
| ENSG00000116824 | HGLibA_08278 | 52 | -0.631148013 | CD2 | CD2 molecule [Source:HGNC Symbol;Acc:HGNC:1639] |
| ENSG00000116996 | HGLibA_56960 | 52 | -0.631148013 | ZP4 | zona pellucida glycoprotein 4 [Source:HGNC Symbol;Acc:HGNC:15770] |
| ENSG00000120211 | HGLibA_23384 | 52 | -0.631148013 | INSL4 | insulin like 4 [Source:HGNC Symbol;Acc:HGNC:6087] |
| ENSG00000120322 | HGLibA_35512 | 52 | -0.631148013 | PCDHB8 | protocadherin beta 8 [Source:HGNC Symbol;Acc:HGNC:8693] |
| ENSG00000121851 | HGLibA_37631 | 52 | -0.631148013 | POLR3GL | RNA polymerase III subunit G like [Source:HGNC Symbol;Acc:HGNC:28466] |
| ENSG00000122140 | HGLibA_30060 | 52 | -0.631148013 | MRPS2 | mitochondrial ribosomal protein S2 [Source:HGNC Symbol;Acc:HGNC:14495] |
| ENSG00000122507 | HGLibA_04228 | 52 | -0.631148013 | BBS9 | Bardet-Biedl syndrome 9 [Source:HGNC Symbol;Acc:HGNC:30000] |
| ENSG00000122779 | HGLibA_51439 | 52 | -0.631148013 | TRIM24 | tripartite motif containing 24 [Source:HGNC Symbol;Acc:HGNC:11812] |
| ENSG00000123124 | HGLibA_54450 | 52 | -0.631148013 | WWP1 | WW domain containing E3 ubiquitin protein ligase 1 [Source:HGNC Symbol;Acc:HGNC:17004] |
| ENSG00000124391 | HGLibA_22996 | 52 | -0.631148013 | IL17C | interleukin 17C [Source:HGNC Symbol;Acc:HGNC:5983] |
| ENSG00000125304 | HGLibA_49725 | 52 | -0.631148013 | TM9SF2 | transmembrane 9 superfamily member 2 [Source:HGNC Symbol;Acc:HGNC:11865] |
| ENSG00000125743 | HGLibA_45979 | 52 | -0.631148013 | SNRPD2 | small nuclear ribonucleoprotein D2 polypeptide [Source:HGNC Symbol;Acc:HGNC:11159] |
| ENSG00000125744 | HGLibA_42402 | 52 | -0.631148013 | RTN2 | reticulon 2 [Source:HGNC Symbol;Acc:HGNC:10468] |
| ENSG00000125816 | HGLibA_32041 | 52 | -0.631148013 | NKX2-4 | NK2 homeobox 4 [Source:HGNC Symbol;Acc:HGNC:7837] |
| ENSG00000126749 | HGLibA_15042 | 52 | -0.631148013 | EMG1 | N1-specific pseudouridine methyltransferase [Source:HGNC Symbol;Acc:HGNC:16912]" |
| ENSG00000127526 | HGLibA_44986 | 52 | -0.631148013 | SLC35E1 | solute carrier family 35 member E1 [Source:HGNC Symbol;Acc:HGNC:20803] |
| ENSG00000128342 | HGLibA_26473 | 52 | -0.631148013 | LIF | interleukin 6 family cytokine [Source:HGNC Symbol;Acc:HGNC:6596]" |
| ENSG00000129864 | HGLibA_53592 | 52 | -0.631148013 | VCY | variable charge Y-linked [Source:HGNC Symbol;Acc:HGNC:12668] |
| ENSG00000132950 | HGLibA_55403 | 52 | -0.631148013 | ZMYM5 | zinc finger MYM-type containing 5 [Source:HGNC Symbol;Acc:HGNC:13029] |
| ENSG00000133884 | HGLibA_13829 | 52 | -0.631148013 | DPF2 | double PHD fingers 2 [Source:HGNC Symbol;Acc:HGNC:9964] |
| ENSG00000134014 | HGLibA_14980 | 52 | -0.631148013 | ELP3 | elongator acetyltransferase complex subunit 3 [Source:HGNC Symbol;Acc:HGNC:20696] |
| ENSG00000134864 | HGLibA_18989 | 52 | -0.631148013 | GGACT | gamma-glutamylamine cyclotransferase [Source:HGNC Symbol;Acc:HGNC:25100] |
| ENSG00000135148 | HGLibA_51269 | 52 | -0.631148013 | TRAFD1 | TRAF-type zinc finger domain containing 1 [Source:HGNC Symbol;Acc:HGNC:24808] |
| ENSG00000136697 | HGLibA_23043 | 52 | -0.631148013 | IL1F10 | interleukin 1 family member 10 [Source:HGNC Symbol;Acc:HGNC:15552] |
| ENSG00000137145 | HGLibA_12955 | 52 | -0.631148013 | DENND4C | DENN domain containing 4C [Source:HGNC Symbol;Acc:HGNC:26079] |
| ENSG00000138080 | HGLibA_15048 | 52 | -0.631148013 | EMILIN1 | elastin microfibril interfacer 1 [Source:HGNC Symbol;Acc:HGNC:19880] |
| ENSG00000138315 | HGLibA_33334 | 52 | -0.631148013 | OIT3 | oncoprotein induced transcript 3 [Source:HGNC Symbol;Acc:HGNC:29953] |
| ENSG00000138386 | HGLibA_31018 | 52 | -0.631148013 | NAB1 | NGFI-A binding protein 1 [Source:HGNC Symbol;Acc:HGNC:7626] |
| ENSG00000138386 | HGLibA_31019 | 52 | -0.631148013 | NAB1 | NGFI-A binding protein 1 [Source:HGNC Symbol;Acc:HGNC:7626] |
| ENSG00000138496 | HGLibA_35248 | 52 | -0.631148013 | PARP9 | poly(ADP-ribose) polymerase family member 9 [Source:HGNC Symbol;Acc:HGNC:24118] |
| ENSG00000138622 | HGLibA_20982 | 52 | -0.631148013 | HCN4 | hyperpolarization activated cyclic nucleotide gated potassium channel 4 [Source:HGNC Symbol;Acc:HGNC:16882] |
| ENSG00000138684 | HGLibA_23082 | 52 | -0.631148013 | IL21 | interleukin 21 [Source:HGNC Symbol;Acc:HGNC:6005] |
| ENSG00000139687 | HGLibA_40414 | 52 | -0.631148013 | RB1 | RB transcriptional corepressor 1 [Source:HGNC Symbol;Acc:HGNC:9884] |
| ENSG00000140876 | HGLibA_32989 | 52 | -0.631148013 | NUDT7 | nudix hydrolase 7 [Source:HGNC Symbol;Acc:HGNC:8054] |
| ENSG00000141448 | HGLibA_18681 | 52 | -0.631148013 | GATA6 | GATA binding protein 6 [Source:HGNC Symbol;Acc:HGNC:4174] |
| ENSG00000141480 | HGLibA_03111 | 52 | -0.631148013 | ARRB2 | arrestin beta 2 [Source:HGNC Symbol;Acc:HGNC:712] |
| ENSG00000141522 | HGLibA_02794 | 52 | -0.631148013 | ARHGDIA | Rho GDP dissociation inhibitor alpha [Source:HGNC Symbol;Acc:HGNC:678] |
| ENSG00000141564 | HGLibA_42203 | 52 | -0.631148013 | RPTOR | regulatory associated protein of MTOR complex 1 [Source:HGNC Symbol;Acc:HGNC:30287] |
| ENSG00000143156 | HGLibA_32172 | 52 | -0.631148013 | NME7 | NME/NM23 family member 7 [Source:HGNC Symbol;Acc:HGNC:20461] |
| ENSG00000143178 | HGLibA_48652 | 52 | -0.631148013 | TBX19 | T-box 19 [Source:HGNC Symbol;Acc:HGNC:11596] |
| ENSG00000143546 | HGLibA_42571 | 52 | -0.631148013 | S100A8 | S100 calcium binding protein A8 [Source:HGNC Symbol;Acc:HGNC:10498] |
| ENSG00000143631 | HGLibA_17609 | 52 | -0.631148013 | FLG | filaggrin [Source:HGNC Symbol;Acc:HGNC:3748] |
| ENSG00000143882 | HGLibA_03743 | 52 | -0.631148013 | ATP6V1C2 | ATPase H+ transporting V1 subunit C2 [Source:HGNC Symbol;Acc:HGNC:18264] |
| ENSG00000143921 | HGLibA_00228 | 52 | -0.631148013 | ABCG8 | ATP binding cassette subfamily G member 8 [Source:HGNC Symbol;Acc:HGNC:13887] |
| ENSG00000144824 | HGLibA_36430 | 52 | -0.631148013 | PHLDB2 | pleckstrin homology like domain family B member 2 [Source:HGNC Symbol;Acc:HGNC:29573] |
| ENSG00000144827 | HGLibA_00232 | 52 | -0.631148013 | ABHD10 | abhydrolase domain containing 10 [Source:HGNC Symbol;Acc:HGNC:25656] |
| ENSG00000145506 | HGLibA_32011 | 52 | -0.631148013 | NKD2 | naked cuticle homolog 2 [Source:HGNC Symbol;Acc:HGNC:17046] |
| ENSG00000145824 | HGLibA_11857 | 52 | -0.631148013 | CXCL14 | C-X-C motif chemokine ligand 14 [Source:HGNC Symbol;Acc:HGNC:10640] |
| ENSG00000151572 | HGLibA_02180 | 52 | -0.631148013 | ANO4 | anoctamin 4 [Source:HGNC Symbol;Acc:HGNC:23837] |
| ENSG00000151640 | HGLibA_13932 | 52 | -0.631148013 | DPYSL4 | dihydropyrimidinase like 4 [Source:HGNC Symbol;Acc:HGNC:3016] |
| ENSG00000151876 | HGLibA_17091 | 52 | -0.631148013 | FBXO4 | F-box protein 4 [Source:HGNC Symbol;Acc:HGNC:13583] |
| ENSG00000151962 | HGLibA_40563 | 52 | -0.631148013 | RBM46 | RNA binding motif protein 46 [Source:HGNC Symbol;Acc:HGNC:28401] |
| ENSG00000153944 | HGLibA_30208 | 52 | -0.631148013 | MSI2 | musashi RNA binding protein 2 [Source:HGNC Symbol;Acc:HGNC:18585] |
| ENSG00000153982 | HGLibA_18890 | 52 | -0.631148013 | GDPD1 | glycerophosphodiester phosphodiesterase domain containing 1 [Source:HGNC Symbol;Acc:HGNC:20883] |
| ENSG00000155366 | HGLibA_41180 | 52 | -0.631148013 | RHOC | ras homolog family member C [Source:HGNC Symbol;Acc:HGNC:669] |
| ENSG00000155367 | HGLibA_37984 | 52 | -0.631148013 | PPM1J | Mg2+/Mn2+ dependent 1J [Source:HGNC Symbol;Acc:HGNC:20785]" |
| ENSG00000156162 | HGLibA_13915 | 52 | -0.631148013 | DPY19L4 | dpy-19 like 4 [Source:HGNC Symbol;Acc:HGNC:27829] |
| ENSG00000157106 | HGLibA_45699 | 52 | -0.631148013 | SMG1 | nonsense mediated mRNA decay associated PI3K related kinase [Source:HGNC Symbol;Acc:HGNC:30045]" |
| ENSG00000157456 | HGLibA_08064 | 52 | -0.631148013 | CCNB2 | cyclin B2 [Source:HGNC Symbol;Acc:HGNC:1580] |
| ENSG00000157933 | HGLibA_44275 | 52 | -0.631148013 | SKI | SKI proto-oncogene [Source:HGNC Symbol;Acc:HGNC:10896] |
| ENSG00000158022 | HGLibA_51573 | 52 | -0.631148013 | TRIM63 | tripartite motif containing 63 [Source:HGNC Symbol;Acc:HGNC:16007] |
| ENSG00000158473 | HGLibA_08274 | 52 | -0.631148013 | CD1D | CD1d molecule [Source:HGNC Symbol;Acc:HGNC:1637] |
| ENSG00000159588 | HGLibA_07688 | 52 | -0.631148013 | CCDC17 | coiled-coil domain containing 17 [Source:HGNC Symbol;Acc:HGNC:26574] |
| ENSG00000159882 | HGLibA_55635 | 52 | -0.631148013 | ZNF230 | zinc finger protein 230 [Source:HGNC Symbol;Acc:HGNC:13024] |
| ENSG00000159905 | HGLibA_55610 | 52 | -0.631148013 | ZNF221 | zinc finger protein 221 [Source:HGNC Symbol;Acc:HGNC:13014] |
| ENSG00000162688 | HGLibA_01197 | 52 | -0.631148013 | AGL | 4-alpha-glucanotransferase [Source:HGNC Symbol;Acc:HGNC:321]" |
| ENSG00000162695 | HGLibA_44918 | 52 | -0.631148013 | SLC30A7 | solute carrier family 30 member 7 [Source:HGNC Symbol;Acc:HGNC:19306] |
| ENSG00000163624 | HGLibA_08882 | 52 | -0.631148013 | CDS1 | CDP-diacylglycerol synthase 1 [Source:HGNC Symbol;Acc:HGNC:1800] |
| ENSG00000163736 | HGLibA_37856 | 52 | -0.631148013 | PPBP | pro-platelet basic protein [Source:HGNC Symbol;Acc:HGNC:9240] |
| ENSG00000164117 | HGLibA_17129 | 52 | -0.631148013 | FBXO8 | F-box protein 8 [Source:HGNC Symbol;Acc:HGNC:13587] |
| ENSG00000164318 | HGLibA_14633 | 52 | -0.631148013 | EGFLAM | fibronectin type III and laminin G domains [Source:HGNC Symbol;Acc:HGNC:26810]" |
| ENSG00000164949 | HGLibA_18909 | 52 | -0.631148013 | GEM | GTP binding protein overexpressed in skeletal muscle [Source:HGNC Symbol;Acc:HGNC:4234] |
| ENSG00000165379 | HGLibA_27163 | 52 | -0.631148013 | LRFN5 | leucine rich repeat and fibronectin type III domain containing 5 [Source:HGNC Symbol;Acc:HGNC:20360] |
| ENSG00000165671 | HGLibA_32745 | 52 | -0.631148013 | NSD1 | nuclear receptor binding SET domain protein 1 [Source:HGNC Symbol;Acc:HGNC:14234] |
| ENSG00000165704 | HGLibA_22043 | 52 | -0.631148013 | HPRT1 | hypoxanthine phosphoribosyltransferase 1 [Source:HGNC Symbol;Acc:HGNC:5157] |
| ENSG00000165810 | HGLibA_04983 | 52 | -0.631148013 | BTNL9 | butyrophilin like 9 [Source:HGNC Symbol;Acc:HGNC:24176] |
| ENSG00000165861 | HGLibA_55270 | 52 | -0.631148013 | ZFYVE1 | zinc finger FYVE-type containing 1 [Source:HGNC Symbol;Acc:HGNC:13180] |
| ENSG00000166197 | HGLibA_32282 | 52 | -0.631148013 | NOLC1 | nucleolar and coiled-body phosphoprotein 1 [Source:HGNC Symbol;Acc:HGNC:15608] |
| ENSG00000166582 | HGLibA_09090 | 52 | -0.631148013 | CENPV | centromere protein V [Source:HGNC Symbol;Acc:HGNC:29920] |
| ENSG00000166619 | HGLibA_04539 | 52 | -0.631148013 | BLCAP | apoptosis inducing factor [Source:HGNC Symbol;Acc:HGNC:1055]" |
| ENSG00000166800 | HGLibA_26223 | 52 | -0.631148013 | LDHAL6A | lactate dehydrogenase A like 6A [Source:HGNC Symbol;Acc:HGNC:28335] |

| | | | | | |
|---|---|---|---|---|---|
| ENSG00000166887 | HGLibA_53788 | 52 | -0.631148013 | VPS39 | HOPS complex subunit [Source:HGNC Symbol;Acc:HGNC:20593]" |
| ENSG00000167768 | HGLibA_25415 | 52 | -0.631148013 | KRT1 | keratin 1 [Source:HGNC Symbol;Acc:HGNC:6412] |
| ENSG00000168958 | HGLibA_29058 | 52 | -0.631148013 | MFF | mitochondrial fission factor [Source:HGNC Symbol;Acc:HGNC:24858] |
| ENSG00000168959 | HGLibA_20354 | 52 | -0.631148013 | GRM5 | glutamate metabotropic receptor 5 [Source:HGNC Symbol;Acc:HGNC:4597] |
| ENSG00000169554 | HGLibA_55137 | 52 | -0.631148013 | ZEB2 | zinc finger E-box binding homeobox 2 [Source:HGNC Symbol;Acc:HGNC:14881] |
| ENSG00000169862 | HGLibA_11662 | 52 | -0.631148013 | CTNND2 | catenin delta 2 [Source:HGNC Symbol;Acc:HGNC:2516] |
| ENSG00000169896 | HGLibA_23759 | 52 | -0.631148013 | ITGAM | integrin subunit alpha M [Source:HGNC Symbol;Acc:HGNC:6149] |
| ENSG00000170037 | HGLibA_10439 | 52 | -0.631148013 | CNTROB | centriole duplication and spindle assembly protein [Source:HGNC Symbol;Acc:HGNC:29616]" |
| ENSG00000170522 | HGLibA_14971 | 52 | -0.631148013 | ELOVL6 | ELOVL fatty acid elongase 6 [Source:HGNC Symbol;Acc:HGNC:15829] |
| ENSG00000170743 | HGLibA_48093 | 52 | -0.631148013 | SYT9 | synaptotagmin 9 [Source:HGNC Symbol;Acc:HGNC:19265] |
| ENSG00000171206 | HGLibA_51614 | 52 | -0.631148013 | TRIM8 | tripartite motif containing 8 [Source:HGNC Symbol;Acc:HGNC:15579] |
| ENSG00000171757 | HGLibA_27321 | 52 | -0.631148013 | LRRC34 | leucine rich repeat containing 34 [Source:HGNC Symbol;Acc:HGNC:28408] |
| ENSG00000172208 | HGLibA_34070 | 52 | -0.631148013 | OR4X2 | olfactory receptor family 4 subfamily X member 2 (gene/pseudogene) [Source:HGNC Symbol;Acc:HGNC:15184] |
| ENSG00000172403 | HGLibA_48020 | 52 | -0.631148013 | SYNPO2 | synaptopodin 2 [Source:HGNC Symbol;Acc:HGNC:17732] |
| ENSG00000172840 | HGLibA_35929 | 52 | -0.631148013 | PDP2 | pyruvate dehyrogenase phosphatase catalytic subunit 2 [Source:HGNC Symbol;Acc:HGNC:30263] |
| ENSG00000172890 | HGLibA_31055 | 52 | -0.631148013 | NADSYN1 | NAD synthetase 1 [Source:HGNC Symbol;Acc:HGNC:29832] |
| ENSG00000172893 | HGLibA_13095 | 52 | -0.631148013 | DHCR7 | 7-dehydrocholesterol reductase [Source:HGNC Symbol;Acc:HGNC:2860] |
| ENSG00000173285 | HGLibA_33529 | 52 | -0.631148013 | OR10K1 | olfactory receptor family 10 subfamily K member 1 [Source:HGNC Symbol;Acc:HGNC:14693] |
| ENSG00000173540 | HGLibA_19425 | 52 | -0.631148013 | GMPPB | GDP-mannose pyrophosphorylase B [Source:HGNC Symbol;Acc:HGNC:22932] |
| ENSG00000173699 | HGLibA_46465 | 52 | -0.631148013 | SPATA3 | spermatogenesis associated 3 [Source:HGNC Symbol;Acc:HGNC:17884] |
| ENSG00000174844 | HGLibA_13477 | 52 | -0.631148013 | DNAH12 | dynein axonemal heavy chain 12 [Source:HGNC Symbol;Acc:HGNC:2943] |
| ENSG00000174915 | HGLibA_39293 | 52 | -0.631148013 | PTDSS2 | phosphatidylserine synthase 2 [Source:HGNC Symbol;Acc:HGNC:15463] |
| ENSG00000174950 | HGLibA_08251 | 52 | -0.631148013 | CD164L2 | CD164 molecule like 2 [Source:HGNC Symbol;Acc:HGNC:32043] |
| ENSG00000175161 | HGLibA_06967 | 52 | -0.631148013 | CADM2 | cell adhesion molecule 2 [Source:HGNC Symbol;Acc:HGNC:29849] |
| ENSG00000176746 | HGLibA_27957 | 52 | -0.631148013 | MAGEB6 | MAGE family member B6 [Source:HGNC Symbol;Acc:HGNC:23796] |
| ENSG00000177352 | HGLibA_07853 | 52 | -0.631148013 | CCDC71 | coiled-coil domain containing 71 [Source:HGNC Symbol;Acc:HGNC:25760] |
| ENSG00000177938 | HGLibA_07212 | 52 | -0.631148013 | CAPZA3 | capping actin protein of muscle Z-line subunit alpha 3 [Source:HGNC Symbol;Acc:HGNC:24205] |
| ENSG00000177946 | HGLibA_09043 | 52 | -0.631148013 | CENPBD1 | CENPB DNA-binding domain containing 1 [Source:HGNC Symbol;Acc:HGNC:28272] |
| ENSG00000181104 | HGLibA_15862 | 52 | -0.631148013 | F2R | coagulation factor II thrombin receptor [Source:HGNC Symbol;Acc:HGNC:3537] |
| ENSG00000182315 | HGLibA_28479 | 52 | -0.631148013 | MBD3L3 | methyl-CpG binding domain protein 3 like 3 [Source:HGNC Symbol;Acc:HGNC:37205] |
| ENSG00000182712 | HGLibA_10190 | 52 | -0.631148013 | CMC4 | C-X9-C motif containing 4 [Source:HGNC Symbol;Acc:HGNC:35428] |
| ENSG00000183098 | HGLibA_19776 | 52 | -0.631148013 | GPC6 | glypican 6 [Source:HGNC Symbol;Acc:HGNC:4454] |
| ENSG00000183378 | HGLibA_34794 | 52 | -0.631148013 | OVCH2 | ovochymase 2 (gene/pseudogene) [Source:HGNC Symbol;Acc:HGNC:29970] |
| ENSG00000183378 | HGLibA_34796 | 52 | -0.631148013 | OVCH2 | ovochymase 2 (gene/pseudogene) [Source:HGNC Symbol;Acc:HGNC:29970] |
| ENSG00000183475 | HGLibA_03248 | 52 | -0.631148013 | ASB7 | ankyrin repeat and SOCS box containing 7 [Source:HGNC Symbol;Acc:HGNC:17182] |
| ENSG00000183624 | HGLibA_21672 | 52 | -0.631148013 | HMCES | ES cell specific [Source:HGNC Symbol;Acc:HGNC:24446]" |
| ENSG00000183773 | HGLibA_01344 | 52 | -0.631148013 | AIFM3 | mitochondria associated 3 [Source:HGNC Symbol;Acc:HGNC:26398]" |
| ENSG00000184384 | HGLibA_28045 | 52 | -0.631148013 | MAML2 | mastermind like transcriptional coactivator 2 [Source:HGNC Symbol;Acc:HGNC:16259] |
| ENSG00000185043 | HGLibA_09682 | 52 | -0.631148013 | CIB1 | calcium and integrin binding 1 [Source:HGNC Symbol;Acc:HGNC:16920] |
| ENSG00000186187 | HGLibA_56939 | 52 | -0.631148013 | ZNRF1 | zinc and ring finger 1 [Source:HGNC Symbol;Acc:HGNC:18452] |
| ENSG00000186635 | HGLibA_02625 | 52 | -0.631148013 | ARAP1 | ankyrin repeat and PH domain 1 [Source:HGNC Symbol;Acc:HGNC:16925]" |
| ENSG00000188050 | HGLibA_41509 | 52 | -0.631148013 | RNF133 | ring finger protein 133 [Source:HGNC Symbol;Acc:HGNC:21154] |
| ENSG00000188385 | HGLibA_23927 | 52 | -0.631148013 | JAKMIP3 | Janus kinase and microtubule interacting protein 3 [Source:HGNC Symbol;Acc:HGNC:23523] |
| ENSG00000196136 | HGLibA_43518 | 52 | -0.631148013 | SERPINA3 | serpin family A member 3 [Source:HGNC Symbol;Acc:HGNC:16] |
| ENSG00000196664 | HGLibA_49660 | 52 | -0.631148013 | TLR7 | toll like receptor 7 [Source:HGNC Symbol;Acc:HGNC:15631] |
| ENSG00000196873 | HGLibA_07436 | 52 | -0.631148013 | CBWD3 | COBW domain containing 3 [Source:HGNC Symbol;Acc:HGNC:18519] |
| ENSG00000197020 | HGLibA_55432 | 52 | -0.631148013 | ZNF100 | zinc finger protein 100 [Source:HGNC Symbol;Acc:HGNC:12880] |
| ENSG00000197479 | HGLibA_35475 | 52 | -0.631148013 | PCDHB11 | protocadherin beta 11 [Source:HGNC Symbol;Acc:HGNC:8682] |
| ENSG00000197905 | HGLibA_48934 | 52 | -0.631148013 | TEAD4 | TEA domain transcription factor 4 [Source:HGNC Symbol;Acc:HGNC:11717] |
| ENSG00000197956 | HGLibA_42563 | 52 | -0.631148013 | S100A6 | S100 calcium binding protein A6 [Source:HGNC Symbol;Acc:HGNC:10496] |
| ENSG00000198034 | HGLibA_42144 | 52 | -0.631148013 | RPS4X | ribosomal protein S4 X-linked [Source:HGNC Symbol;Acc:HGNC:10424] |
| ENSG00000198173 | HGLibA_16613 | 52 | -0.631148013 | FAM47C | family with sequence similarity 47 member C [Source:HGNC Symbol;Acc:HGNC:25301] |
| ENSG00000198203 | HGLibA_47747 | 52 | -0.631148013 | SULT1C2 | sulfotransferase family 1C member 2 [Source:HGNC Symbol;Acc:HGNC:11456] |
| ENSG00000198301 | HGLibA_43093 | 52 | -0.631148013 | SDAD1 | SDA1 domain containing 1 [Source:HGNC Symbol;Acc:HGNC:25537] |
| ENSG00000198399 | HGLibA_23875 | 52 | -0.631148013 | ITSN2 | intersectin 2 [Source:HGNC Symbol;Acc:HGNC:6184] |
| ENSG00000198785 | HGLibA_20310 | 52 | -0.631148013 | GRIN3A | glutamate ionotropic receptor NMDA type subunit 3A [Source:HGNC Symbol;Acc:HGNC:16767] |
| ENSG00000198925 | HGLibA_03507 | 52 | -0.631148013 | ATG9A | autophagy related 9A [Source:HGNC Symbol;Acc:HGNC:22408] |
| ENSG00000198952 | HGLibA_45700 | 52 | -0.631148013 | SMG5 | nonsense mediated mRNA decay factor [Source:HGNC Symbol;Acc:HGNC:24644]" |
| ENSG00000204065 | HGLibA_48717 | 52 | -0.631148013 | TCEAL5 | transcription elongation factor A like 5 [Source:HGNC Symbol;Acc:HGNC:22282] |
| ENSG00000204086 | HGLibA_41807 | 52 | -0.631148013 | RPA4 | replication protein A4 [Source:HGNC Symbol;Acc:HGNC:30305] |
| ENSG00000204140 | HGLibA_10108 | 52 | -0.631148013 | CLPSL1 | colipase like 1 [Source:HGNC Symbol;Acc:HGNC:21251] |
| ENSG00000204919 | HGLibA_38801 | 52 | -0.631148013 | PRR20A | proline rich 20A [Source:HGNC Symbol;Acc:HGNC:24754] |
| ENSG00000204946 | HGLibA_56742 | 52 | -0.631148013 | ZNF783 | zinc finger family member 783 [Source:HGNC Symbol;Acc:HGNC:27222] |
| ENSG00000205138 | HGLibA_43137 | 52 | -0.631148013 | SDHAF1 | succinate dehydrogenase complex assembly factor 1 [Source:HGNC Symbol;Acc:HGNC:33867] |
| ENSG00000205883 | HGLibA_12907 | 52 | -0.631148013 | DEFB135 | defensin beta 135 [Source:HGNC Symbol;Acc:HGNC:32400] |
| ENSG00000205883 | HGLibA_12909 | 52 | -0.631148013 | DEFB135 | defensin beta 135 [Source:HGNC Symbol;Acc:HGNC:32400] |
| ENSG00000206047 | HGLibA_12792 | 52 | -0.631148013 | DEFA1 | defensin alpha 1 [Source:HGNC Symbol;Acc:HGNC:2761] |
| ENSG00000213889 | HGLibA_37997 | 52 | -0.631148013 | PPM1N | Mg2+/Mn2+ dependent 1N (putative) [Source:HGNC Symbol;Acc:HGNC:26845]" |
| ENSG00000214102 | HGLibA_54242 | 52 | -0.631148013 | WEE2 | WEE1 homolog 2 [Source:HGNC Symbol;Acc:HGNC:19684] |
| ENSG00000215217 | HGLibA_06397 | 52 | -0.631148013 | C5orf49 | chromosome 5 open reading frame 49 [Source:HGNC Symbol;Acc:HGNC:27028] |
| ENSG00000216937 | HGLibA_07848 | 52 | -0.631148013 | CCDC7 | coiled-coil domain containing 7 [Source:HGNC Symbol;Acc:HGNC:26533] |
| ENSG00000220205 | HGLibA_53491 | 52 | -0.631148013 | VAMP2 | vesicle associated membrane protein 2 [Source:HGNC Symbol;Acc:HGNC:12643] |
| ENSG00000229665 | HGLibA_38805 | 52 | -0.631148013 | PRR20C | proline rich 20C [Source:HGNC Symbol;Acc:HGNC:37221] |
| ENSG00000239887 | HGLibA_05835 | 52 | -0.631148013 | C1orf226 | chromosome 1 open reading frame 226 [Source:HGNC Symbol;Acc:HGNC:34351] |
| ENSG00000240386 | HGLibA_26114 | 52 | -0.631148013 | LCE1F | late cornified envelope 1F [Source:HGNC Symbol;Acc:HGNC:29467] |
| ENSG00000242550 | HGLibA_43537 | 52 | -0.631148013 | SERPINB10 | serpin family B member 10 [Source:HGNC Symbol;Acc:HGNC:8942] |
| ENSG00000248383 | HGLibA_35462 | 52 | -0.631148013 | PCDHAC1 | 1 [Source:HGNC Symbol;Acc:HGNC:8676]" |
| ENSG00000250741 | HGLibA_32807 | 52 | -0.631148013 | NT5C1B-RDH14 | NT5C1B-RDH14 readthrough [Source:HGNC Symbol;Acc:HGNC:38831] |
| ENSG00000261011 | HGLibA_01138 | 52 | -0.631148013 | AGAP11 | ankyrin repeat and PH domain family (AGAP) pseudogene" |
| ENSG00000264364 | HGLibA_14257 | 52 | -0.631148013 | DYNLL2 | dynein light chain LC8-type 2 [Source:HGNC Symbol;Acc:HGNC:24596] |
| ENSG00000265681 | HGLibA_41879 | 52 | -0.631148013 | RPL17 | ribosomal protein L17 [Source:HGNC Symbol;Acc:HGNC:10307] |
| ENSG00000267060 | HGLibA_39335 | 52 | -0.631148013 | PTGES3L | prostaglandin E synthase 3 like [Source:HGNC Symbol;Acc:HGNC:43943] |
| ENSG00000276045 | HGLibA_34589 | 52 | -0.631148013 | ORAI1 | ORAI calcium release-activated calcium modulator 1 [Source:HGNC Symbol;Acc:HGNC:25896] |
| ENSG00000280165 | HGLibA_35411 | 52 | -0.631148013 | PCDH20 | protocadherin 20 [Source:HGNC Symbol;Acc:HGNC:14257] |
| ENSG00000288642 | HGLibA_08863 | 52 | -0.631148013 | CDR1 | - |
| FAM115C | HGLibA_16038 | 52 | -0.631148013 | FAM115C | - |
| FAM179A | HGLibA_16306 | 52 | -0.631148013 | FAM179A | - |
| FAM63A | HGLibA_16659 | 52 | -0.631148013 | FAM63A | - |
| HIST1H1B | HGLibA_21361 | 52 | -0.631148013 | HIST1H1B | - |
| HIST1H2AC | HGLibA_21382 | 52 | -0.631148013 | HIST1H2AC | - |
| HIST1H4B | HGLibA_21490 | 52 | -0.631148013 | HIST1H4B | - |
| hsa-mir-148a | HGLibA_57879 | 52 | -0.631148013 | hsa-mir-148a | - |
| hsa-mir-1973 | HGLibA_58115 | 52 | -0.631148013 | hsa-mir-1973 | - |
| hsa-mir-2861 | HGLibA_58387 | 52 | -0.631148013 | hsa-mir-2861 | - |
| hsa-mir-296 | HGLibA_58397 | 52 | -0.631148013 | hsa-mir-296 | - |
| hsa-mir-30b | HGLibA_58474 | 52 | -0.631148013 | hsa-mir-30b | - |
| hsa-mir-3142 | HGLibA_58627 | 52 | -0.631148013 | hsa-mir-3142 | - |
| hsa-mir-34c | HGLibA_59042 | 52 | -0.631148013 | hsa-mir-34c | - |
| hsa-mir-3689b | HGLibA_59317 | 52 | -0.631148013 | hsa-mir-3689b | - |
| hsa-mir-3934 | HGLibA_59596 | 52 | -0.631148013 | hsa-mir-3934 | - |
| hsa-mir-4440 | HGLibA_60164 | 52 | -0.631148013 | hsa-mir-4440 | - |
| hsa-mir-4476 | HGLibA_60314 | 52 | -0.631148013 | hsa-mir-4476 | - |
| hsa-mir-4523 | HGLibA_60549 | 52 | -0.631148013 | hsa-mir-4523 | - |
| hsa-mir-4524b | HGLibA_60556 | 52 | -0.631148013 | hsa-mir-4524b | - |
| hsa-mir-4647 | HGLibA_60690 | 52 | -0.631148013 | hsa-mir-4647 | - |
| hsa-mir-4736 | HGLibA_61042 | 52 | -0.631148013 | hsa-mir-4736 | - |
| hsa-mir-484 | HGLibA_61330 | 52 | -0.631148013 | hsa-mir-484 | - |
| hsa-mir-516a-2 | HGLibA_61613 | 52 | -0.631148013 | hsa-mir-516a-2 | - |
| hsa-mir-5703 | HGLibA_62352 | 52 | -0.631148013 | hsa-mir-5703 | - |
| hsa-mir-580 | HGLibA_62417 | 52 | -0.631148013 | hsa-mir-580 | - |
| hsa-mir-6732 | HGLibA_63080 | 52 | -0.631148013 | hsa-mir-6732 | - |
| hsa-mir-6883 | HGLibA_63717 | 52 | -0.631148013 | hsa-mir-6883 | - |
| hsa-mir-7703 | HGLibA_63946 | 52 | -0.631148013 | hsa-mir-7703 | - |
| KIAA0020 | HGLibA_24584 | 52 | -0.631148013 | KIAA0020 | - |
| LOC388849 | HGLibA_26926 | 52 | -0.631148013 | LOC388849 | - |
| NARR | HGLibA_31153 | 52 | -0.631148013 | NARR | - |
| 11-Sep | HGLibA_43429 | 52 | -0.631148013 | 11-Sep | - |
| SOLH | HGLibA_46167 | 52 | -0.631148013 | SOLH | - |
| ABP1 | HGLibA_00319 | 51 | -0.64109905 | ABP1 | - |
| AHSA2 | HGLibA_01315 | 51 | -0.64109905 | AHSA2 | - |
| C12orf77 | HGLibA_05304 | 51 | -0.64109905 | C12orf77 | - |
| C14orf142 | HGLibA_05328 | 51 | -0.64109905 | C14orf142 | - |
| C17orf77 | HGLibA_05576 | 51 | -0.64109905 | C17orf77 | - |
| C20orf194 | HGLibA_05999 | 51 | -0.64109905 | C20orf194 | - |
| C5orf30 | HGLibA_06375 | 51 | -0.64109905 | C5orf30 | - |
| C6orf1 | HGLibA_06427 | 51 | -0.64109905 | C6orf1 | - |
| C6orf106 | HGLibA_06434 | 51 | -0.64109905 | C6orf106 | - |
| C7orf10 | HGLibA_06522 | 51 | -0.64109905 | C7orf10 | - |
| CRIPAK | HGLibA_11158 | 51 | -0.64109905 | CRIPAK | - |
| ENSG00000005381 | HGLibA_29744 | 51 | -0.64109905 | MPO | myeloperoxidase [Source:HGNC Symbol;Acc:HGNC:7218] |
| ENSG00000030419 | HGLibA_22931 | 51 | -0.64109905 | IKZF2 | IKAROS family zinc finger 2 [Source:HGNC Symbol;Acc:HGNC:13177] |
| ENSG00000040199 | HGLibA_36439 | 51 | -0.64109905 | PHLPP2 | PH domain and leucine rich repeat protein phosphatase 2 [Source:HGNC Symbol;Acc:HGNC:29149] |
| ENSG00000050820 | HGLibA_04245 | 51 | -0.64109905 | BCAR1 | Cas family scaffold protein [Source:HGNC Symbol;Acc:HGNC:971]" |
| ENSG00000058729 | HGLibA_41309 | 51 | -0.64109905 | RIOK2 | RIO kinase 2 [Source:HGNC Symbol;Acc:HGNC:18999] |
| ENSG00000066654 | HGLibA_49416 | 51 | -0.64109905 | THUMPD1 | THUMP domain containing 1 [Source:HGNC Symbol;Acc:HGNC:23807] |

| | | | | | |
|---|---|---|---|---|---|
| ENSG00000076685 | HGLibA_32810 | 51 | -0.64109905 | NT5C2 | cytosolic II [Source:HGNC Symbol;Acc:HGNC:8022]" |
| ENSG00000077152 | HGLibA_52661 | 51 | -0.64109905 | UBE2T | ubiquitin conjugating enzyme E2 T [Source:HGNC Symbol;Acc:HGNC:25009] |
| ENSG00000080031 | HGLibA_39508 | 51 | -0.64109905 | PTPRH | receptor type H [Source:HGNC Symbol;Acc:HGNC:9672]" |
| ENSG00000081386 | HGLibA_56126 | 51 | -0.64109905 | ZNF510 | zinc finger protein 510 [Source:HGNC Symbol;Acc:HGNC:29161] |
| ENSG00000083290 | HGLibA_52973 | 51 | -0.64109905 | ULK2 | unc-51 like autophagy activating kinase 2 [Source:HGNC Symbol;Acc:HGNC:13480] |
| ENSG00000090339 | HGLibA_22494 | 51 | -0.64109905 | ICAM1 | intercellular adhesion molecule 1 [Source:HGNC Symbol;Acc:HGNC:5344] |
| ENSG00000099998 | HGLibA_19014 | 51 | -0.64109905 | GGT5 | gamma-glutamyltransferase 5 [Source:HGNC Symbol;Acc:HGNC:4260] |
| ENSG00000100068 | HGLibA_27228 | 51 | -0.64109905 | LRP5L | LDL receptor related protein 5 like [Source:HGNC Symbol;Acc:HGNC:25323] |
| ENSG00000100883 | HGLibA_47007 | 51 | -0.64109905 | SRP54 | signal recognition particle 54 [Source:HGNC Symbol;Acc:HGNC:11301] |
| ENSG00000101335 | HGLibA_30779 | 51 | -0.64109905 | MYL9 | myosin light chain 9 [Source:HGNC Symbol;Acc:HGNC:15754] |
| ENSG00000101342 | HGLibA_49603 | 51 | -0.64109905 | TLDC2 | TBC/LysM-associated domain containing 2 [Source:HGNC Symbol;Acc:HGNC:16112] |
| ENSG00000101457 | HGLibA_13719 | 51 | -0.64109905 | DNTTIP1 | deoxynucleotidyltransferase terminal interacting protein 1 [Source:HGNC Symbol;Acc:HGNC:16160] |
| ENSG00000101577 | HGLibA_27097 | 51 | -0.64109905 | LPIN2 | lipin 2 [Source:HGNC Symbol;Acc:HGNC:14450] |
| ENSG00000102743 | HGLibA_44650 | 51 | -0.64109905 | SLC25A15 | solute carrier family 25 member 15 [Source:HGNC Symbol;Acc:HGNC:10985] |
| ENSG00000103056 | HGLibA_45810 | 51 | -0.64109905 | SMPD3 | sphingomyelin phosphodiesterase 3 [Source:HGNC Symbol;Acc:HGNC:14240] |
| ENSG00000103742 | HGLibA_22787 | 51 | -0.64109905 | IGDCC4 | immunoglobulin superfamily DCC subclass member 4 [Source:HGNC Symbol;Acc:HGNC:13770] |
| ENSG00000104231 | HGLibA_55145 | 51 | -0.64109905 | ZFAND1 | zinc finger AN1-type containing 1 [Source:HGNC Symbol;Acc:HGNC:25858] |
| ENSG00000104442 | HGLibA_03005 | 51 | -0.64109905 | ARMC1 | armadillo repeat containing 1 [Source:HGNC Symbol;Acc:HGNC:17684] |
| ENSG00000104953 | HGLibA_49619 | 51 | -0.64109905 | TLE6 | transducin like enhancer of split 6 [Source:HGNC Symbol;Acc:HGNC:30788] |
| ENSG00000105974 | HGLibA_07377 | 51 | -0.64109905 | CAV1 | caveolin 1 [Source:HGNC Symbol;Acc:HGNC:1527] |
| ENSG00000105983 | HGLibA_26670 | 51 | -0.64109905 | LMBR1 | limb development membrane protein 1 [Source:HGNC Symbol;Acc:HGNC:13243] |
| ENSG00000107338 | HGLibA_43994 | 51 | -0.64109905 | SHB | SH2 domain containing adaptor protein B [Source:HGNC Symbol;Acc:HGNC:10838] |
| ENSG00000107485 | HGLibA_18671 | 51 | -0.64109905 | GATA3 | GATA binding protein 3 [Source:HGNC Symbol;Acc:HGNC:4172] |
| ENSG00000108039 | HGLibA_54552 | 51 | -0.64109905 | XPNPEP1 | X-prolyl aminopeptidase 1 [Source:HGNC Symbol;Acc:HGNC:12822] |
| ENSG00000108239 | HGLibA_48473 | 51 | -0.64109905 | TBC1D12 | TBC1 domain family member 12 [Source:HGNC Symbol;Acc:HGNC:29082] |
| ENSG00000108306 | HGLibA_17001 | 51 | -0.64109905 | FBXL20 | F-box and leucine rich repeat protein 20 [Source:HGNC Symbol;Acc:HGNC:24679] |
| ENSG00000109084 | HGLibA_50568 | 51 | -0.64109905 | TMEM97 | transmembrane protein 97 [Source:HGNC Symbol;Acc:HGNC:28106] |
| ENSG00000109736 | HGLibA_29085 | 51 | -0.64109905 | MFSD10 | major facilitator superfamily domain containing 10 [Source:HGNC Symbol;Acc:HGNC:16894] |
| ENSG00000110881 | HGLibA_03299 | 51 | -0.64109905 | ASIC1 | acid sensing ion channel subunit 1 [Source:HGNC Symbol;Acc:HGNC:100] |
| ENSG00000110906 | HGLibA_24414 | 51 | -0.64109905 | KCTD10 | potassium channel tetramerization domain containing 10 [Source:HGNC Symbol;Acc:HGNC:23236] |
| ENSG00000111321 | HGLibA_27602 | 51 | -0.64109905 | LTBR | lymphotoxin beta receptor [Source:HGNC Symbol;Acc:HGNC:6718] |
| ENSG00000111339 | HGLibA_03165 | 51 | -0.64109905 | ART4 | ADP-ribosyltransferase 4 (Dombrock blood group) [Source:HGNC Symbol;Acc:HGNC:726] |
| ENSG00000111732 | HGLibA_01324 | 51 | -0.64109905 | AICDA | activation induced cytidine deaminase [Source:HGNC Symbol;Acc:HGNC:13203] |
| ENSG00000112796 | HGLibA_15180 | 51 | -0.64109905 | ENPP5 | ectonucleotide pyrophosphatase/phosphodiesterase 5 (putative) [Source:HGNC Symbol;Acc:HGNC:13717] |
| ENSG00000113739 | HGLibA_47417 | 51 | -0.64109905 | STC2 | stanniocalcin 2 [Source:HGNC Symbol;Acc:HGNC:11374] |
| ENSG00000114648 | HGLibA_25143 | 51 | -0.64109905 | KLHL18 | kelch like family member 18 [Source:HGNC Symbol;Acc:HGNC:29120] |
| ENSG00000116521 | HGLibA_42843 | 51 | -0.64109905 | SCAMP3 | secretory carrier membrane protein 3 [Source:HGNC Symbol;Acc:HGNC:10565] |
| ENSG00000116996 | HGLibA_56958 | 51 | -0.64109905 | ZP4 | zona pellucida glycoprotein 4 [Source:HGNC Symbol;Acc:HGNC:15770] |
| ENSG00000117697 | HGLibA_32759 | 51 | -0.64109905 | NSL1 | MIS12 kinetochore complex component [Source:HGNC Symbol;Acc:HGNC:24548]" |
| ENSG00000118596 | HGLibA_44449 | 51 | -0.64109905 | SLC16A7 | solute carrier family 16 member 7 [Source:HGNC Symbol;Acc:HGNC:10928] |
| ENSG00000119125 | HGLibA_18837 | 51 | -0.64109905 | GDA | guanine deaminase [Source:HGNC Symbol;Acc:HGNC:4212] |
| ENSG00000119973 | HGLibA_38603 | 51 | -0.64109905 | PRLHR | prolactin releasing hormone receptor [Source:HGNC Symbol;Acc:HGNC:4464] |
| ENSG00000121236 | HGLibA_51558 | 51 | -0.64109905 | TRIM6 | tripartite motif containing 6 [Source:HGNC Symbol;Acc:HGNC:16277] |
| ENSG00000121594 | HGLibA_08453 | 51 | -0.64109905 | CD80 | CD80 molecule [Source:HGNC Symbol;Acc:HGNC:1700] |
| ENSG00000121966 | HGLibA_11891 | 51 | -0.64109905 | CXCR4 | C-X-C motif chemokine receptor 4 [Source:HGNC Symbol;Acc:HGNC:2561] |
| ENSG00000122824 | HGLibA_32935 | 51 | -0.64109905 | NUDT10 | nudix hydrolase 10 [Source:HGNC Symbol;Acc:HGNC:17621] |
| ENSG00000124233 | HGLibA_43379 | 51 | -0.64109905 | SEMG1 | semenogelin 1 [Source:HGNC Symbol;Acc:HGNC:10742] |
| ENSG00000124406 | HGLibA_03776 | 51 | -0.64109905 | ATP8A1 | ATPase phospholipid transporting 8A1 [Source:HGNC Symbol;Acc:HGNC:13531] |
| ENSG00000126233 | HGLibA_45555 | 51 | -0.64109905 | SLURP1 | secreted LY6/PLAUR domain containing 1 [Source:HGNC Symbol;Acc:HGNC:18746] |
| ENSG00000126460 | HGLibA_38873 | 51 | -0.64109905 | PRRG2 | proline rich and Gla domain 2 [Source:HGNC Symbol;Acc:HGNC:9470] |
| ENSG00000129028 | HGLibA_49290 | 51 | -0.64109905 | THAP10 | THAP domain containing 10 [Source:HGNC Symbol;Acc:HGNC:23193] |
| ENSG00000129657 | HGLibA_43191 | 51 | -0.64109905 | SEC14L1 | SEC14 like lipid binding 1 [Source:HGNC Symbol;Acc:HGNC:10698] |
| ENSG00000130311 | HGLibA_12586 | 51 | -0.64109905 | DDA1 | DET1 and DDB1 associated 1 [Source:HGNC Symbol;Acc:HGNC:28360] |
| ENSG00000130695 | HGLibA_09169 | 51 | -0.64109905 | CEP85 | centrosomal protein 85 [Source:HGNC Symbol;Acc:HGNC:25309] |
| ENSG00000131781 | HGLibA_17715 | 51 | -0.64109905 | FMO5 | flavin containing monooxygenase 5 [Source:HGNC Symbol;Acc:HGNC:3773] |
| ENSG00000132434 | HGLibA_26006 | 51 | -0.64109905 | LANCL2 | LanC like 2 [Source:HGNC Symbol;Acc:HGNC:6509] |
| ENSG00000132950 | HGLibA_55401 | 51 | -0.64109905 | ZMYM5 | zinc finger MYM-type containing 5 [Source:HGNC Symbol;Acc:HGNC:13029] |
| ENSG00000133739 | HGLibA_27443 | 51 | -0.64109905 | LRRCC1 | leucine rich repeat and coiled-coil centrosomal protein 1 [Source:HGNC Symbol;Acc:HGNC:29373] |
| ENSG00000134326 | HGLibA_10204 | 51 | -0.64109905 | CMPK2 | cytidine/uridine monophosphate kinase 2 [Source:HGNC Symbol;Acc:HGNC:27015] |
| ENSG00000134389 | HGLibA_09274 | 51 | -0.64109905 | CFHR5 | complement factor H related 5 [Source:HGNC Symbol;Acc:HGNC:24668] |
| ENSG00000134508 | HGLibA_06841 | 51 | -0.64109905 | CABLES1 | Cdk5 and Abl enzyme substrate 1 [Source:HGNC Symbol;Acc:HGNC:25097] |
| ENSG00000135108 | HGLibA_17046 | 51 | -0.64109905 | FBXO21 | F-box protein 21 [Source:HGNC Symbol;Acc:HGNC:13592] |
| ENSG00000135363 | HGLibA_26705 | 51 | -0.64109905 | LMO2 | LIM domain only 2 [Source:HGNC Symbol;Acc:HGNC:6642] |
| ENSG00000135736 | HGLibA_07507 | 51 | -0.64109905 | CCDC102A | coiled-coil domain containing 102A [Source:HGNC Symbol;Acc:HGNC:28097] |
| ENSG00000136244 | HGLibA_23182 | 51 | -0.64109905 | IL6 | interleukin 6 [Source:HGNC Symbol;Acc:HGNC:6018] |
| ENSG00000136518 | HGLibA_00621 | 51 | -0.64109905 | ACTL6A | actin like 6A [Source:HGNC Symbol;Acc:HGNC:24124] |
| ENSG00000136574 | HGLibA_18674 | 51 | -0.64109905 | GATA4 | GATA binding protein 4 [Source:HGNC Symbol;Acc:HGNC:4173] |
| ENSG00000136783 | HGLibA_31975 | 51 | -0.64109905 | NIPSNAP3A | nipsnap homolog 3A [Source:HGNC Symbol;Acc:HGNC:23619] |
| ENSG00000136868 | HGLibA_44926 | 51 | -0.64109905 | SLC31A1 | solute carrier family 31 member 1 [Source:HGNC Symbol;Acc:HGNC:11016] |
| ENSG00000137274 | HGLibA_04705 | 51 | -0.64109905 | BPHL | biphenyl hydrolase like [Source:HGNC Symbol;Acc:HGNC:1094] |
| ENSG00000137497 | HGLibA_33009 | 51 | -0.64109905 | NUMA1 | nuclear mitotic apparatus protein 1 [Source:HGNC Symbol;Acc:HGNC:8059] |
| ENSG00000137692 | HGLibA_12577 | 51 | -0.64109905 | DCUN1D5 | defective in cullin neddylation 1 domain containing 5 [Source:HGNC Symbol;Acc:HGNC:28409] |
| ENSG00000137817 | HGLibA_35240 | 51 | -0.64109905 | PARP6 | poly(ADP-ribose) polymerase family member 6 [Source:HGNC Symbol;Acc:HGNC:26921] |
| ENSG00000137959 | HGLibA_22614 | 51 | -0.64109905 | IFI44L | interferon induced protein 44 like [Source:HGNC Symbol;Acc:HGNC:17817] |
| ENSG00000138443 | HGLibA_00292 | 51 | -0.64109905 | ABI2 | abl interactor 2 [Source:HGNC Symbol;Acc:HGNC:24011] |
| ENSG00000139055 | HGLibA_15524 | 51 | -0.64109905 | ERP27 | endoplasmic reticulum protein 27 [Source:HGNC Symbol;Acc:HGNC:26495] |
| ENSG00000139620 | HGLibA_24043 | 51 | -0.64109905 | KANSL2 | KAT8 regulatory NSL complex subunit 2 [Source:HGNC Symbol;Acc:HGNC:26024] |
| ENSG00000139625 | HGLibA_28169 | 51 | -0.64109905 | MAP3K12 | mitogen-activated protein kinase kinase kinase 12 [Source:HGNC Symbol;Acc:HGNC:6851] |
| ENSG00000139910 | HGLibA_32365 | 51 | -0.64109905 | NOVA1 | NOVA alternative splicing regulator 1 [Source:HGNC Symbol;Acc:HGNC:7886] |
| ENSG00000140365 | HGLibA_10665 | 51 | -0.64109905 | COMMD4 | COMM domain containing 4 [Source:HGNC Symbol;Acc:HGNC:26027] |
| ENSG00000140470 | HGLibA_00812 | 51 | -0.64109905 | ADAMTS17 | ADAM metallopeptidase with thrombospondin type 1 motif 17 [Source:HGNC Symbol;Acc:HGNC:17109] |
| ENSG00000141030 | HGLibA_10715 | 51 | -0.64109905 | COPS3 | COP9 signalosome subunit 3 [Source:HGNC Symbol;Acc:HGNC:2239] |
| ENSG00000141294 | HGLibA_27369 | 51 | -0.64109905 | LRRC46 | leucine rich repeat containing 46 [Source:HGNC Symbol;Acc:HGNC:25047] |
| ENSG00000141506 | HGLibA_36660 | 51 | -0.64109905 | PIK3R5 | phosphoinositide-3-kinase regulatory subunit 5 [Source:HGNC Symbol;Acc:HGNC:30035] |
| ENSG00000141582 | HGLibA_07455 | 51 | -0.64109905 | CBX4 | chromobox 4 [Source:HGNC Symbol;Acc:HGNC:1554] |
| ENSG00000141736 | HGLibA_15412 | 51 | -0.64109905 | ERBB2 | erb-b2 receptor tyrosine kinase 2 [Source:HGNC Symbol;Acc:HGNC:3430] |
| ENSG00000142449 | HGLibA_16962 | 51 | -0.64109905 | FBN3 | fibrillin 3 [Source:HGNC Symbol;Acc:HGNC:18794] |
| ENSG00000143157 | HGLibA_37475 | 51 | -0.64109905 | POGK | pogo transposable element derived with KRAB domain [Source:HGNC Symbol;Acc:HGNC:18800] |
| ENSG00000144048 | HGLibA_14133 | 51 | -0.64109905 | DUSP11 | dual specificity phosphatase 11 [Source:HGNC Symbol;Acc:HGNC:3066] |
| ENSG00000146143 | HGLibA_38485 | 51 | -0.64109905 | PRIM2 | DNA primase subunit 2 [Source:HGNC Symbol;Acc:HGNC:9370] |
| ENSG00000147099 | HGLibA_21025 | 51 | -0.64109905 | HDAC8 | histone deacetylase 8 [Source:HGNC Symbol;Acc:HGNC:13315] |
| ENSG00000147274 | HGLibA_40597 | 51 | -0.64109905 | RBMX | RNA binding motif protein X-linked [Source:HGNC Symbol;Acc:HGNC:9910] |
| ENSG00000147439 | HGLibA_04512 | 51 | -0.64109905 | BIN3 | bridging integrator 3 [Source:HGNC Symbol;Acc:HGNC:1054] |
| ENSG00000148090 | HGLibA_03864 | 51 | -0.64109905 | AUH | AU RNA binding methylglutaconyl-CoA hydratase [Source:HGNC Symbol;Acc:HGNC:890] |
| ENSG00000148841 | HGLibA_23863 | 51 | -0.64109905 | ITPRIP | 5-trisphosphate receptor interacting protein [Source:HGNC Symbol;Acc:HGNC:29370]" |
| ENSG00000149212 | HGLibA_43624 | 51 | -0.64109905 | SESN3 | sestrin 3 [Source:HGNC Symbol;Acc:HGNC:23060] |
| ENSG00000150051 | HGLibA_29359 | 51 | -0.64109905 | MKX | mohawk homeobox [Source:HGNC Symbol;Acc:HGNC:23729] |
| ENSG00000150316 | HGLibA_11813 | 51 | -0.64109905 | CWC15 | CWC15 spliceosome associated protein homolog [Source:HGNC Symbol;Acc:HGNC:26939] |
| ENSG00000150722 | HGLibA_38070 | 51 | -0.64109905 | PPP1R1C | protein phosphatase 1 regulatory inhibitor subunit 1C [Source:HGNC Symbol;Acc:HGNC:14940] |
| ENSG00000152952 | HGLibA_37211 | 51 | -0.64109905 | PLOD2 | 2-oxoglutarate 5-dioxygenase 2 [Source:HGNC Symbol;Acc:HGNC:9082]" |
| ENSG00000153046 | HGLibA_08918 | 51 | -0.64109905 | CDYL | chromodomain Y like [Source:HGNC Symbol;Acc:HGNC:1811] |
| ENSG00000153317 | HGLibA_03194 | 51 | -0.64109905 | ASAP1 | ankyrin repeat and PH domain 1 [Source:HGNC Symbol;Acc:HGNC:2720]" |
| ENSG00000154222 | HGLibA_07479 | 51 | -0.64109905 | CC2D1B | coiled-coil and C2 domain containing 1B [Source:HGNC Symbol;Acc:HGNC:29386] |
| ENSG00000154529 | HGLibA_10429 | 51 | -0.64109905 | CNTNAP3B | contactin associated protein like 3B [Source:HGNC Symbol;Acc:HGNC:32035] |
| ENSG00000154928 | HGLibA_15312 | 51 | -0.64109905 | EPHB1 | EPH receptor B1 [Source:HGNC Symbol;Acc:HGNC:3392] |
| ENSG00000155158 | HGLibA_52135 | 51 | -0.64109905 | TTC39B | tetratricopeptide repeat domain 39B [Source:HGNC Symbol;Acc:HGNC:23704] |
| ENSG00000156232 | HGLibA_54288 | 51 | -0.64109905 | WHAMM | golgi membranes and microtubules [Source:HGNC Symbol;Acc:HGNC:30493]" |
| ENSG00000156711 | HGLibA_28260 | 51 | -0.64109905 | MAPK13 | mitogen-activated protein kinase 13 [Source:HGNC Symbol;Acc:HGNC:6875] |
| ENSG00000157600 | HGLibA_50033 | 51 | -0.64109905 | TMEM164 | transmembrane protein 164 [Source:HGNC Symbol;Acc:HGNC:26217] |
| ENSG00000158055 | HGLibA_20255 | 51 | -0.64109905 | GRHL3 | grainyhead like transcription factor 3 [Source:HGNC Symbol;Acc:HGNC:25839] |
| ENSG00000160325 | HGLibA_06868 | 51 | -0.64109905 | CACFD1 | calcium channel flower domain containing 1 [Source:HGNC Symbol;Acc:HGNC:1365] |
| ENSG00000161642 | HGLibA_55898 | 51 | -0.64109905 | ZNF385A | zinc finger protein 385A [Source:HGNC Symbol;Acc:HGNC:17521] |
| ENSG00000162073 | HGLibA_35160 | 51 | -0.64109905 | PAQR4 | progestin and adipoQ receptor family member 4 [Source:HGNC Symbol;Acc:HGNC:26386] |
| ENSG00000162437 | HGLibA_40405 | 51 | -0.64109905 | RAVER2 | PTB binding 2 [Source:HGNC Symbol;Acc:HGNC:25577]" |
| ENSG00000162779 | HGLibA_03917 | 51 | -0.64109905 | AXDND1 | axonemal dynein light chain domain containing 1 [Source:HGNC Symbol;Acc:HGNC:26564] |
| ENSG00000163631 | HGLibA_01529 | 51 | -0.64109905 | ALB | albumin [Source:HGNC Symbol;Acc:HGNC:399] |
| ENSG00000163913 | HGLibA_22742 | 51 | -0.64109905 | IFT122 | intraflagellar transport 122 [Source:HGNC Symbol;Acc:HGNC:13556] |
| ENSG00000164010 | HGLibA_15499 | 51 | -0.64109905 | ERMAP | erythroblast membrane associated protein (Scianna blood group) [Source:HGNC Symbol;Acc:HGNC:15743] |
| ENSG00000164035 | HGLibA_15028 | 51 | -0.64109905 | EMCN | endomucin [Source:HGNC Symbol;Acc:HGNC:16041] |
| ENSG00000164054 | HGLibA_44043 | 51 | -0.64109905 | SHISA5 | shisa family member 5 [Source:HGNC Symbol;Acc:HGNC:30376] |
| ENSG00000164106 | HGLibA_43043 | 51 | -0.64109905 | SCRG1 | stimulator of chondrogenesis 1 [Source:HGNC Symbol;Acc:HGNC:17036] |
| ENSG00000164751 | HGLibA_36109 | 51 | -0.64109905 | PEX2 | peroxisomal biogenesis factor 2 [Source:HGNC Symbol;Acc:HGNC:9717] |
| ENSG00000165588 | HGLibA_34785 | 51 | -0.64109905 | OTX2 | orthodenticle homeobox 2 [Source:HGNC Symbol;Acc:HGNC:8522] |
| ENSG00000165997 | HGLibA_02978 | 51 | -0.64109905 | ARL5B | ADP ribosylation factor like GTPase 5B [Source:HGNC Symbol;Acc:HGNC:23052] |
| ENSG00000166166 | HGLibA_51697 | 51 | -0.64109905 | TRMT61A | tRNA methyltransferase 61A [Source:HGNC Symbol;Acc:HGNC:23790] |
| ENSG00000166796 | HGLibA_26231 | 51 | -0.64109905 | LDHC | lactate dehydrogenase C [Source:HGNC Symbol;Acc:HGNC:6544] |
| ENSG00000167291 | HGLibA_48486 | 51 | -0.64109905 | TBC1D16 | TBC1 domain family member 16 [Source:HGNC Symbol;Acc:HGNC:28356] |
| ENSG00000167720 | HGLibA_47038 | 51 | -0.64109905 | SRR | serine racemase [Source:HGNC Symbol;Acc:HGNC:14398] |
| ENSG00000167840 | HGLibA_55640 | 51 | -0.64109905 | ZNF232 | zinc finger protein 232 [Source:HGNC Symbol;Acc:HGNC:13026] |
| ENSG00000167910 | HGLibA_12212 | 51 | -0.64109905 | CYP7A1 | cytochrome P450 family 7 subfamily A member 1 [Source:HGNC Symbol;Acc:HGNC:2651] |
| ENSG00000168003 | HGLibA_45134 | 51 | -0.64109905 | SLC3A2 | solute carrier family 3 member 2 [Source:HGNC Symbol;Acc:HGNC:11026] |
| ENSG00000168610 | HGLibA_47387 | 51 | -0.64109905 | STAT3 | signal transducer and activator of transcription 3 [Source:HGNC Symbol;Acc:HGNC:11364] |
| ENSG00000169059 | HGLibA_53587 | 51 | -0.64109905 | VCX3A | variable charge X-linked 3A [Source:HGNC Symbol;Acc:HGNC:18159] |
| ENSG00000169507 | HGLibA_45060 | 51 | -0.64109905 | SLC38A11 | solute carrier family 38 member 11 [Source:HGNC Symbol;Acc:HGNC:26836] |
| ENSG00000169575 | HGLibA_53722 | 51 | -0.64109905 | VPREB1 | V-set pre-B cell surrogate light chain 1 [Source:HGNC Symbol;Acc:HGNC:12709] |

| | | | | | |
|---|---|---|---|---|---|
| ENSG00000170209 | HGLibA_01974 | 51 | -0.64109905 | ANKK1 | ankyrin repeat and kinase domain containing 1 [Source:HGNC Symbol;Acc:HGNC:21027] |
| ENSG00000170458 | HGLibA_08234 | 51 | -0.64109905 | CD14 | CD14 molecule [Source:HGNC Symbol;Acc:HGNC:1628] |
| ENSG00000170579 | HGLibA_13338 | 51 | -0.64109905 | DLGAP1 | DLG associated protein 1 [Source:HGNC Symbol;Acc:HGNC:2905] |
| ENSG00000170606 | HGLibA_22284 | 51 | -0.64109905 | HSPA4 | heat shock protein family A (Hsp70) member 4 [Source:HGNC Symbol;Acc:HGNC:5237] |
| ENSG00000170667 | HGLibA_40309 | 51 | -0.64109905 | RASA4B | RAS p21 protein activator 4B [Source:HGNC Symbol;Acc:HGNC:35202] |
| ENSG00000170819 | HGLibA_04459 | 51 | -0.64109905 | BFSP2 | beaded filament structural protein 2 [Source:HGNC Symbol;Acc:HGNC:1041] |
| ENSG00000171495 | HGLibA_29870 | 51 | -0.64109905 | MROH2B | maestro heat like repeat family member 2B [Source:HGNC Symbol;Acc:HGNC:26857] |
| ENSG00000171503 | HGLibA_15625 | 51 | -0.64109905 | ETFDH | electron transfer flavoprotein dehydrogenase [Source:HGNC Symbol;Acc:HGNC:3483] |
| ENSG00000171720 | HGLibA_21009 | 51 | -0.64109905 | HDAC3 | histone deacetylase 3 [Source:HGNC Symbol;Acc:HGNC:4854] |
| ENSG00000172059 | HGLibA_25046 | 51 | -0.64109905 | KLF11 | Kruppel like factor 11 [Source:HGNC Symbol;Acc:HGNC:11811] |
| ENSG00000172379 | HGLibA_03065 | 51 | -0.64109905 | ARNT2 | aryl hydrocarbon receptor nuclear translocator 2 [Source:HGNC Symbol;Acc:HGNC:16876] |
| ENSG00000172409 | HGLibA_10094 | 51 | -0.64109905 | CLP1 | cleavage and polyadenylation factor I subunit 1 [Source:HGNC Symbol;Acc:HGNC:16999] |
| ENSG00000172466 | HGLibA_55658 | 51 | -0.64109905 | ZNF24 | zinc finger protein 24 [Source:HGNC Symbol;Acc:HGNC:13032] |
| ENSG00000172938 | HGLibA_29836 | 51 | -0.64109905 | MRGPRD | MAS related GPR family member D [Source:HGNC Symbol;Acc:HGNC:29626] |
| ENSG00000173389 | HGLibA_23503 | 51 | -0.64109905 | IQCF1 | IQ motif containing F1 [Source:HGNC Symbol;Acc:HGNC:28607] |
| ENSG00000174032 | HGLibA_44702 | 51 | -0.64109905 | SLC25A30 | solute carrier family 25 member 30 [Source:HGNC Symbol;Acc:HGNC:27371] |
| ENSG00000175143 | HGLibA_33851 | 51 | -0.64109905 | OR2T1 | olfactory receptor family 2 subfamily T member 1 [Source:HGNC Symbol;Acc:HGNC:8277] |
| ENSG00000175793 | HGLibA_43727 | 51 | -0.64109905 | SFN | stratifin [Source:HGNC Symbol;Acc:HGNC:10773] |
| ENSG00000176171 | HGLibA_04655 | 51 | -0.64109905 | BNIP3 | BCL2 interacting protein 3 [Source:HGNC Symbol;Acc:HGNC:1084] |
| ENSG00000176383 | HGLibA_03990 | 51 | -0.64109905 | B3GNT4 | 3-N-acetylglucosaminyltransferase 4 [Source:HGNC Symbol;Acc:HGNC:15683]" |
| ENSG00000176386 | HGLibA_08537 | 51 | -0.64109905 | CDC26 | cell division cycle 26 [Source:HGNC Symbol;Acc:HGNC:17839] |
| ENSG00000176567 | HGLibA_34066 | 51 | -0.64109905 | OR4X1 | olfactory receptor family 4 subfamily X member 1 (gene/pseudogene) [Source:HGNC Symbol;Acc:HGNC:14854] |
| ENSG00000177675 | HGLibA_08246 | 51 | -0.64109905 | CD163L1 | CD163 molecule like 1 [Source:HGNC Symbol;Acc:HGNC:30375] |
| ENSG00000178235 | HGLibA_45513 | 51 | -0.64109905 | SLITRK1 | SLIT and NTRK like family member 1 [Source:HGNC Symbol;Acc:HGNC:20297] |
| ENSG00000178522 | HGLibA_01749 | 51 | -0.64109905 | AMBN | ameloblastin [Source:HGNC Symbol;Acc:HGNC:452] |
| ENSG00000179532 | HGLibA_13677 | 51 | -0.64109905 | DNHD1 | dynein heavy chain domain 1 [Source:HGNC Symbol;Acc:HGNC:26532] |
| ENSG00000181273 | HGLibA_34238 | 51 | -0.64109905 | OR5AK2 | olfactory receptor family 5 subfamily AK member 2 [Source:HGNC Symbol;Acc:HGNC:15251] |
| ENSG00000181904 | HGLibA_06368 | 51 | -0.64109905 | C5orf24 | chromosome 5 open reading frame 24 [Source:HGNC Symbol;Acc:HGNC:26746] |
| ENSG00000182481 | HGLibA_25358 | 51 | -0.64109905 | KPNA2 | karyopherin subunit alpha 2 [Source:HGNC Symbol;Acc:HGNC:6395] |
| ENSG00000183397 | HGLibA_05701 | 51 | -0.64109905 | C19orf71 | chromosome 19 open reading frame 71 [Source:HGNC Symbol;Acc:HGNC:34496] |
| ENSG00000183426 | HGLibA_32462 | 51 | -0.64109905 | NPIPA1 | nuclear pore complex interacting protein family member A1 [Source:HGNC Symbol;Acc:HGNC:7909] |
| ENSG00000183542 | HGLibA_25294 | 51 | -0.64109905 | KLRC4 | killer cell lectin like receptor C4 [Source:HGNC Symbol;Acc:HGNC:6377] |
| ENSG00000184032 | HGLibA_25701 | 51 | -0.64109905 | KRTAP20-2 | keratin associated protein 20-2 [Source:HGNC Symbol;Acc:HGNC:18944] |
| ENSG00000184155 | HGLibA_33526 | 51 | -0.64109905 | OR10J5 | olfactory receptor family 10 subfamily J member 5 [Source:HGNC Symbol;Acc:HGNC:14993] |
| ENSG00000184160 | HGLibA_01053 | 51 | -0.64109905 | ADRA2C | adrenoceptor alpha 2C [Source:HGNC Symbol;Acc:HGNC:283] |
| ENSG00000184933 | HGLibA_34371 | 51 | -0.64109905 | OR6A2 | olfactory receptor family 6 subfamily A member 2 [Source:HGNC Symbol;Acc:HGNC:15301] |
| ENSG00000185567 | HGLibA_01305 | 51 | -0.64109905 | AHNAK2 | AHNAK nucleoprotein 2 [Source:HGNC Symbol;Acc:HGNC:20125] |
| ENSG00000185973 | HGLibA_50590 | 51 | -0.64109905 | TMLHE | epsilon [Source:HGNC Symbol;Acc:HGNC:18308]" |
| ENSG00000186230 | HGLibA_56670 | 51 | -0.64109905 | ZNF749 | zinc finger protein 749 [Source:HGNC Symbol;Acc:HGNC:32783] |
| ENSG00000186306 | HGLibA_33548 | 51 | -0.64109905 | OR10T2 | olfactory receptor family 10 subfamily T member 2 [Source:HGNC Symbol;Acc:HGNC:14816] |
| ENSG00000186318 | HGLibA_04059 | 51 | -0.64109905 | BACE1 | beta-secretase 1 [Source:HGNC Symbol;Acc:HGNC:933] |
| ENSG00000186501 | HGLibA_50239 | 51 | -0.64109905 | TMEM222 | transmembrane protein 222 [Source:HGNC Symbol;Acc:HGNC:25363] |
| ENSG00000186908 | HGLibA_55087 | 51 | -0.64109905 | ZDHHC17 | zinc finger DHHC-type containing 17 [Source:HGNC Symbol;Acc:HGNC:18412] |
| ENSG00000187486 | HGLibA_24251 | 51 | -0.64109905 | KCNJ11 | potassium voltage-gated channel subfamily J member 11 [Source:HGNC Symbol;Acc:HGNC:6257] |
| ENSG00000189057 | HGLibA_16026 | 51 | -0.64109905 | FAM111B | family with sequence similarity 111 member B [Source:HGNC Symbol;Acc:HGNC:24200] |
| ENSG00000189120 | HGLibA_46309 | 51 | -0.64109905 | SP6 | Sp6 transcription factor [Source:HGNC Symbol;Acc:HGNC:14530] |
| ENSG00000196171 | HGLibA_34419 | 51 | -0.64109905 | OR6K2 | olfactory receptor family 6 subfamily K member 2 [Source:HGNC Symbol;Acc:HGNC:15029] |
| ENSG00000196705 | HGLibA_55980 | 51 | -0.64109905 | ZNF431 | zinc finger protein 431 [Source:HGNC Symbol;Acc:HGNC:20809] |
| ENSG00000196776 | HGLibA_08397 | 51 | -0.64109905 | CD47 | CD47 molecule [Source:HGNC Symbol;Acc:HGNC:1682] |
| ENSG00000197446 | HGLibA_12134 | 51 | -0.64109905 | CYP2F1 | cytochrome P450 family 2 subfamily F member 1 [Source:HGNC Symbol;Acc:HGNC:2632] |
| ENSG00000197548 | HGLibA_03504 | 51 | -0.64109905 | ATG7 | autophagy related 7 [Source:HGNC Symbol;Acc:HGNC:16935] |
| ENSG00000197798 | HGLibA_16051 | 51 | -0.64109905 | FAM118B | family with sequence similarity 118 member B [Source:HGNC Symbol;Acc:HGNC:26110] |
| ENSG00000198183 | HGLibA_04709 | 51 | -0.64109905 | BPIFA1 | BPI fold containing family A member 1 [Source:HGNC Symbol;Acc:HGNC:15749] |
| ENSG00000198198 | HGLibA_48116 | 51 | -0.64109905 | SZT2 | KICSTOR complex subunit [Source:HGNC Symbol;Acc:HGNC:29040]" |
| ENSG00000198252 | HGLibA_47686 | 51 | -0.64109905 | STYX | serine/threonine/tyrosine interacting protein [Source:HGNC Symbol;Acc:HGNC:11447] |
| ENSG00000198498 | HGLibA_49734 | 51 | -0.64109905 | TMA16 | translation machinery associated 16 homolog [Source:HGNC Symbol;Acc:HGNC:25638] |
| ENSG00000198663 | HGLibA_06513 | 51 | -0.64109905 | C6orf89 | chromosome 6 open reading frame 89 [Source:HGNC Symbol;Acc:HGNC:21114] |
| ENSG00000198844 | HGLibA_02819 | 51 | -0.64109905 | ARHGEF15 | Rho guanine nucleotide exchange factor 15 [Source:HGNC Symbol;Acc:HGNC:15590] |
| ENSG00000198860 | HGLibA_51828 | 51 | -0.64109905 | TSEN15 | tRNA splicing endonuclease subunit 15 [Source:HGNC Symbol;Acc:HGNC:16791] |
| ENSG00000198915 | HGLibA_40330 | 51 | -0.64109905 | RASGEF1A | RasGEF domain family member 1A [Source:HGNC Symbol;Acc:HGNC:24246] |
| ENSG00000205029 | HGLibA_34279 | 51 | -0.64109905 | OR5D16 | olfactory receptor family 5 subfamily D member 16 [Source:HGNC Symbol;Acc:HGNC:15283] |
| ENSG00000205045 | HGLibA_45487 | 51 | -0.64109905 | SLFN12L | schlafen family member 12 like [Source:HGNC Symbol;Acc:HGNC:33920] |
| ENSG00000205213 | HGLibA_26408 | 51 | -0.64109905 | LGR4 | leucine rich repeat containing G protein-coupled receptor 4 [Source:HGNC Symbol;Acc:HGNC:13299] |
| ENSG00000205899 | HGLibA_04469 | 51 | -0.64109905 | BHLHA9 | basic helix-loop-helix family member a9 [Source:HGNC Symbol;Acc:HGNC:35126] |
| ENSG00000213215 | HGLibA_33787 | 51 | -0.64109905 | OR2F1 | olfactory receptor family 2 subfamily F member 1 (gene/pseudogene) [Source:HGNC Symbol;Acc:HGNC:8246] |
| ENSG00000213402 | HGLibA_39493 | 51 | -0.64109905 | PTPRCAP | receptor type C associated protein [Source:HGNC Symbol;Acc:HGNC:9667]" |
| ENSG00000215115 | HGLibA_11932 | 51 | -0.64109905 | CXorf49 | chromosome X open reading frame 49 [Source:HGNC Symbol;Acc:HGNC:30891] |
| ENSG00000227151 | HGLibA_38810 | 51 | -0.64109905 | PRR20D | proline rich 20D [Source:HGNC Symbol;Acc:HGNC:37222] |
| ENSG00000227471 | HGLibA_01479 | 51 | -0.64109905 | AKR1B15 | aldo-keto reductase family 1 member B15 [Source:HGNC Symbol;Acc:HGNC:37281] |
| ENSG00000228856 | HGLibA_53276 | 51 | -0.64109905 | USP17L30 | ubiquitin specific peptidase 17-like family member 30 [Source:HGNC Symbol;Acc:HGNC:44458] |
| ENSG00000229676 | HGLibA_56094 | 51 | -0.64109905 | ZNF492 | zinc finger protein 492 [Source:HGNC Symbol;Acc:HGNC:23707] |
| ENSG00000230657 | HGLibA_38346 | 51 | -0.64109905 | PRB4 | proline rich protein BstNI subfamily 4 [Source:HGNC Symbol;Acc:HGNC:9340] |
| ENSG00000239857 | HGLibA_18932 | 51 | -0.64109905 | GET4 | golgi to ER traffic protein 4 [Source:HGNC Symbol;Acc:HGNC:21690] |
| ENSG00000240344 | HGLibA_37944 | 51 | -0.64109905 | PPIL3 | peptidylprolyl isomerase like 3 [Source:HGNC Symbol;Acc:HGNC:9262] |
| ENSG00000241973 | HGLibA_36497 | 51 | -0.64109905 | PI4KA | phosphatidylinositol 4-kinase alpha [Source:HGNC Symbol;Acc:HGNC:8983] |
| ENSG00000250506 | HGLibA_08767 | 51 | -0.64109905 | CDK3 | cyclin dependent kinase 3 [Source:HGNC Symbol;Acc:HGNC:1772] |
| ENSG00000254087 | HGLibA_27706 | 51 | -0.64109905 | LYN | Src family tyrosine kinase [Source:HGNC Symbol;Acc:HGNC:6735]" |
| ENSG00000255150 | HGLibA_14702 | 51 | -0.64109905 | EID3 | EP300 interacting inhibitor of differentiation 3 [Source:HGNC Symbol;Acc:HGNC:32961] |
| ENSG00000260456 | HGLibA_05507 | 51 | -0.64109905 | C16orf95 | chromosome 16 open reading frame 95 [Source:HGNC Symbol;Acc:HGNC:40033] |
| ENSG00000261857 | HGLibA_29184 | 51 | -0.64109905 | MIA | MIA SH3 domain containing [Source:HGNC Symbol;Acc:HGNC:7076] |
| ENSG00000267508 | HGLibA_55746 | 51 | -0.64109905 | ZNF285 | zinc finger protein 285 [Source:HGNC Symbol;Acc:HGNC:13079] |
| ENSG00000268350 | HGLibA_16187 | 51 | -0.64109905 | FAM156A | family with sequence similarity 156 member A [Source:HGNC Symbol;Acc:HGNC:30114] |
| ENSG00000269405 | HGLibA_33129 | 51 | -0.64109905 | NXF2 | nuclear RNA export factor 2 [Source:HGNC Symbol;Acc:HGNC:8072] |
| ENSG00000269556 | HGLibA_50119 | 51 | -0.64109905 | TMEM185A | transmembrane protein 185A [Source:HGNC Symbol;Acc:HGNC:17125] |
| ENSG00000275969 | HGLibA_46474 | 51 | -0.64109905 | SPATA31A3 | SPATA31 subfamily A member 3 [Source:HGNC Symbol;Acc:HGNC:32003] |
| ENSG00000284862 | HGLibA_07773 | 51 | -0.64109905 | CCDC39 | coiled-coil domain containing 39 [Source:NCBI gene;Acc:339829] |
| FAM132A | HGLibA_16113 | 51 | -0.64109905 | FAM132A | - |
| GLTPD1 | HGLibA_19346 | 51 | -0.64109905 | GLTPD1 | - |
| HIST1H3J | HGLibA_21482 | 51 | -0.64109905 | HIST1H3J | - |
| hsa-mir-1251 | HGLibA_57402 | 51 | -0.64109905 | hsa-mir-1251 | - |
| hsa-mir-1270-2 | HGLibA_57521 | 51 | -0.64109905 | hsa-mir-1270-2 | - |
| hsa-mir-1281 | HGLibA_57582 | 51 | -0.64109905 | hsa-mir-1281 | - |
| hsa-mir-181d | HGLibA_57977 | 51 | -0.64109905 | hsa-mir-181d | - |
| hsa-mir-1908 | HGLibA_58027 | 51 | -0.64109905 | hsa-mir-1908 | - |
| hsa-mir-193b | HGLibA_58075 | 51 | -0.64109905 | hsa-mir-193b | - |
| hsa-mir-199a-2 | HGLibA_58133 | 51 | -0.64109905 | hsa-mir-199a-2 | - |
| hsa-mir-19a | HGLibA_58139 | 51 | -0.64109905 | hsa-mir-19a | - |
| hsa-mir-324 | HGLibA_58963 | 51 | -0.64109905 | hsa-mir-324 | - |
| hsa-mir-3616 | HGLibA_59093 | 51 | -0.64109905 | hsa-mir-3616 | - |
| hsa-mir-3663 | HGLibA_59204 | 51 | -0.64109905 | hsa-mir-3663 | - |
| hsa-mir-374b | HGLibA_59395 | 51 | -0.64109905 | hsa-mir-374b | - |
| hsa-mir-3907 | HGLibA_59494 | 51 | -0.64109905 | hsa-mir-3907 | - |
| hsa-mir-3974 | HGLibA_59654 | 51 | -0.64109905 | hsa-mir-3974 | - |
| hsa-mir-4480 | HGLibA_60339 | 51 | -0.64109905 | hsa-mir-4480 | - |
| hsa-mir-4527 | HGLibA_60568 | 51 | -0.64109905 | hsa-mir-4527 | - |
| hsa-mir-4536-1 | HGLibA_60604 | 51 | -0.64109905 | hsa-mir-4536-1 | - |
| hsa-mir-4657 | HGLibA_60735 | 51 | -0.64109905 | hsa-mir-4657 | - |
| hsa-mir-4712 | HGLibA_60948 | 51 | -0.64109905 | hsa-mir-4712 | - |
| hsa-mir-4754 | HGLibA_61114 | 51 | -0.64109905 | hsa-mir-4754 | - |
| hsa-mir-516a-2 | HGLibA_61611 | 51 | -0.64109905 | hsa-mir-516a-2 | - |
| hsa-mir-5194 | HGLibA_61694 | 51 | -0.64109905 | hsa-mir-5194 | - |
| hsa-mir-5684 | HGLibA_62257 | 51 | -0.64109905 | hsa-mir-5684 | - |
| hsa-mir-5692b | HGLibA_62294 | 51 | -0.64109905 | hsa-mir-5692b | - |
| hsa-mir-5699 | HGLibA_62330 | 51 | -0.64109905 | hsa-mir-5699 | - |
| hsa-mir-5699 | HGLibA_62328 | 51 | -0.64109905 | hsa-mir-5699 | - |
| hsa-mir-602 | HGLibA_62502 | 51 | -0.64109905 | hsa-mir-602 | - |
| hsa-mir-615 | HGLibA_62694 | 51 | -0.64109905 | hsa-mir-615 | - |
| hsa-mir-620 | HGLibA_62720 | 51 | -0.64109905 | hsa-mir-620 | - |
| hsa-mir-648 | HGLibA_62837 | 51 | -0.64109905 | hsa-mir-648 | - |
| hsa-mir-6758 | HGLibA_63188 | 51 | -0.64109905 | hsa-mir-6758 | - |
| hsa-mir-6778 | HGLibA_63285 | 51 | -0.64109905 | hsa-mir-6778 | - |
| hsa-mir-6794 | HGLibA_63355 | 51 | -0.64109905 | hsa-mir-6794 | - |
| hsa-mir-7155 | HGLibA_63847 | 51 | -0.64109905 | hsa-mir-7155 | - |
| hsa-mir-7850 | HGLibA_63989 | 51 | -0.64109905 | hsa-mir-7850 | - |
| hsa-mir-7975 | HGLibA_64028 | 51 | -0.64109905 | hsa-mir-7975 | - |
| hsa-mir-8077 | HGLibA_64151 | 51 | -0.64109905 | hsa-mir-8077 | - |
| hsa-mir-889 | HGLibA_64234 | 51 | -0.64109905 | hsa-mir-889 | - |
| LOC100130357 | HGLibA_26781 | 51 | -0.64109905 | LOC100130357 | - |
| MRE11A | HGLibA_29823 | 51 | -0.64109905 | MRE11A | - |
| NUPL2 | HGLibA_33085 | 51 | -0.64109905 | NUPL2 | - |
| PRAMEF16 | HGLibA_38297 | 51 | -0.64109905 | PRAMEF16 | - |
| PRAMEF22 | HGLibA_38314 | 51 | -0.64109905 | PRAMEF22 | - |
| PRSS46 | HGLibA_38946 | 51 | -0.64109905 | PRSS46 | - |
| 4-Sep | HGLibA_43441 | 51 | -0.64109905 | 4-Sep | - |
| TMEM2 | HGLibA_50169 | 51 | -0.64109905 | TMEM2 | - |

| | | | | | |
|---|---|---|---|---|---|
| APITD1 | HGLibA_02425 | 50 | -0.651050088 | APITD1 | - |
| ATP5J2 | HGLibA_03673 | 50 | -0.651050088 | ATP5J2 | - |
| AZI1 | HGLibA_03934 | 50 | -0.651050088 | AZI1 | - |
| C12orf49 | HGLibA_05252 | 50 | -0.651050088 | C12orf49 | - |
| C16orf13 | HGLibA_05441 | 50 | -0.651050088 | C16orf13 | - |
| C21orf33 | HGLibA_06038 | 50 | -0.651050088 | C21orf33 | - |
| C4orf21 | HGLibA_06303 | 50 | -0.651050088 | C4orf21 | - |
| CD99_X | HGLibA_08489 | 50 | -0.651050088 | CD99_X | - |
| CXorf36 | HGLibA_11917 | 50 | -0.651050088 | CXorf36 | - |
| ENSG00000004866 | HGLibA_47283 | 50 | -0.651050088 | ST7 | suppression of tumorigenicity 7 [Source:HGNC Symbol;Acc:HGNC:11351] |
| ENSG00000006534 | HGLibA_01568 | 50 | -0.651050088 | ALDH3B1 | aldehyde dehydrogenase 3 family member B1 [Source:HGNC Symbol;Acc:HGNC:410] |
| ENSG00000007001 | HGLibA_53088 | 50 | -0.651050088 | UPP2 | uridine phosphorylase 2 [Source:HGNC Symbol;Acc:HGNC:23061] |
| ENSG00000008118 | HGLibA_07063 | 50 | -0.651050088 | CAMK1G | calcium/calmodulin dependent protein kinase IG [Source:HGNC Symbol;Acc:HGNC:14585] |
| ENSG00000008405 | HGLibA_11241 | 50 | -0.651050088 | CRY1 | cryptochrome circadian regulator 1 [Source:HGNC Symbol;Acc:HGNC:2384] |
| ENSG00000016082 | HGLibA_23665 | 50 | -0.651050088 | ISL1 | ISL LIM homeobox 1 [Source:HGNC Symbol;Acc:HGNC:6132] |
| ENSG00000035720 | HGLibA_47342 | 50 | -0.651050088 | STAP1 | signal transducing adaptor family member 1 [Source:HGNC Symbol;Acc:HGNC:24133] |
| ENSG00000047346 | HGLibA_16477 | 50 | -0.651050088 | FAM214A | family with sequence similarity 214 member A [Source:HGNC Symbol;Acc:HGNC:25609] |
| ENSG00000047932 | HGLibA_19671 | 50 | -0.651050088 | GOPC | golgi associated PDZ and coiled-coil motif containing [Source:HGNC Symbol;Acc:HGNC:17643] |
| ENSG00000053770 | HGLibA_02356 | 50 | -0.651050088 | AP5M1 | adaptor related protein complex 5 subunit mu 1 [Source:HGNC Symbol;Acc:HGNC:20192] |
| ENSG00000058335 | HGLibA_40337 | 50 | -0.651050088 | RASGRF1 | Ras protein specific guanine nucleotide releasing factor 1 [Source:HGNC Symbol;Acc:HGNC:9875] |
| ENSG00000058673 | HGLibA_54949 | 50 | -0.651050088 | ZC3H11A | zinc finger CCCH-type containing 11A [Source:HGNC Symbol;Acc:HGNC:29093] |
| ENSG00000061676 | HGLibA_31311 | 50 | -0.651050088 | NCKAP1 | NCK associated protein 1 [Source:HGNC Symbol;Acc:HGNC:7666] |
| ENSG00000063978 | HGLibA_41677 | 50 | -0.651050088 | RNF4 | ring finger protein 4 [Source:HGNC Symbol;Acc:HGNC:10067] |
| ENSG00000064490 | HGLibA_40985 | 50 | -0.651050088 | RFXANK | regulatory factor X associated ankyrin containing protein [Source:HGNC Symbol;Acc:HGNC:9987] |
| ENSG00000070915 | HGLibA_44362 | 50 | -0.651050088 | SLC12A3 | solute carrier family 12 member 3 [Source:HGNC Symbol;Acc:HGNC:10912] |
| ENSG00000071991 | HGLibA_08655 | 50 | -0.651050088 | CDH19 | cadherin 19 [Source:HGNC Symbol;Acc:HGNC:1758] |
| ENSG00000072364 | HGLibA_01115 | 50 | -0.651050088 | AFF4 | AF4/FMR2 family member 4 [Source:HGNC Symbol;Acc:HGNC:17869] |
| ENSG00000073060 | HGLibA_42868 | 50 | -0.651050088 | SCARB1 | scavenger receptor class B member 1 [Source:HGNC Symbol;Acc:HGNC:1664] |
| ENSG00000073060 | HGLibA_42869 | 50 | -0.651050088 | SCARB1 | scavenger receptor class B member 1 [Source:HGNC Symbol;Acc:HGNC:1664] |
| ENSG00000077157 | HGLibA_38024 | 50 | -0.651050088 | PPP1R12B | protein phosphatase 1 regulatory subunit 12B [Source:HGNC Symbol;Acc:HGNC:7619] |
| ENSG00000077274 | HGLibA_07172 | 50 | -0.651050088 | CAPN6 | calpain 6 [Source:HGNC Symbol;Acc:HGNC:1483] |
| ENSG00000084733 | HGLibA_39783 | 50 | -0.651050088 | RAB10 | member RAS oncogene family [Source:HGNC Symbol;Acc:HGNC:9759]" |
| ENSG00000087884 | HGLibA_00054 | 50 | -0.651050088 | AAMDC | adipogenesis associated Mth938 domain containing [Source:HGNC Symbol;Acc:HGNC:30205] |
| ENSG00000088726 | HGLibA_50390 | 50 | -0.651050088 | TMEM40 | transmembrane protein 40 [Source:HGNC Symbol;Acc:HGNC:25620] |
| ENSG00000090857 | HGLibA_35937 | 50 | -0.651050088 | PDPR | pyruvate dehydrogenase phosphatase regulatory subunit [Source:HGNC Symbol;Acc:HGNC:30264] |
| ENSG00000092421 | HGLibA_43363 | 50 | -0.651050088 | SEMA6A | semaphorin 6A [Source:HGNC Symbol;Acc:HGNC:10738] |
| ENSG00000095380 | HGLibA_31109 | 50 | -0.651050088 | NANS | N-acetylneuraminate synthase [Source:HGNC Symbol;Acc:HGNC:19237] |
| ENSG00000099875 | HGLibA_29345 | 50 | -0.651050088 | MKNK2 | MAP kinase interacting serine/threonine kinase 2 [Source:HGNC Symbol;Acc:HGNC:7111] |
| ENSG00000099889 | HGLibA_03176 | 50 | -0.651050088 | ARVCF | delta catenin family member [Source:HGNC Symbol;Acc:HGNC:728]" |
| ENSG00000100362 | HGLibA_39614 | 50 | -0.651050088 | PVALB | parvalbumin [Source:HGNC Symbol;Acc:HGNC:9704] |
| ENSG00000100802 | HGLibA_05375 | 50 | -0.651050088 | C14orf93 | chromosome 14 open reading frame 93 [Source:HGNC Symbol;Acc:HGNC:20162] |
| ENSG00000100865 | HGLibA_09715 | 50 | -0.651050088 | CINP | cyclin dependent kinase 2 interacting protein [Source:HGNC Symbol;Acc:HGNC:23789] |
| ENSG00000101670 | HGLibA_26611 | 50 | -0.651050088 | LIPG | endothelial type [Source:HGNC Symbol;Acc:HGNC:6623]" |
| ENSG00000101746 | HGLibA_32268 | 50 | -0.651050088 | NOL4 | nucleolar protein 4 [Source:HGNC Symbol;Acc:HGNC:7870] |
| ENSG00000102038 | HGLibA_45603 | 50 | -0.651050088 | SMARCA1 | member 1 [Source:HGNC Symbol;Acc:HGNC:11097]" |
| ENSG00000102048 | HGLibA_03255 | 50 | -0.651050088 | ASB9 | ankyrin repeat and SOCS box containing 9 [Source:HGNC Symbol;Acc:HGNC:17184] |
| ENSG00000103978 | HGLibA_50532 | 50 | -0.651050088 | TMEM87A | transmembrane protein 87A [Source:HGNC Symbol;Acc:HGNC:24522] |
| ENSG00000104888 | HGLibA_44478 | 50 | -0.651050088 | SLC17A7 | solute carrier family 17 member 7 [Source:HGNC Symbol;Acc:HGNC:16704] |
| ENSG00000105516 | HGLibA_12400 | 50 | -0.651050088 | DBP | D-box binding PAR bZIP transcription factor [Source:HGNC Symbol;Acc:HGNC:2697] |
| ENSG00000105655 | HGLibA_23699 | 50 | -0.651050088 | ISYNA1 | inositol-3-phosphate synthase 1 [Source:HGNC Symbol;Acc:HGNC:29821] |
| ENSG00000105875 | HGLibA_54216 | 50 | -0.651050088 | WDR91 | WD repeat domain 91 [Source:HGNC Symbol;Acc:HGNC:24997] |
| ENSG00000109991 | HGLibA_34852 | 50 | -0.651050088 | P2RX3 | purinergic receptor P2X 3 [Source:HGNC Symbol;Acc:HGNC:8534] |
| ENSG00000110852 | HGLibA_09965 | 50 | -0.651050088 | CLEC2B | C-type lectin domain family 2 member B [Source:HGNC Symbol;Acc:HGNC:2053] |
| ENSG00000111711 | HGLibA_19665 | 50 | -0.651050088 | GOLT1B | golgi transport 1B [Source:HGNC Symbol;Acc:HGNC:20175] |
| ENSG00000113140 | HGLibA_46416 | 50 | -0.651050088 | SPARC | secreted protein acidic and cysteine rich [Source:HGNC Symbol;Acc:HGNC:11219] |
| ENSG00000114021 | HGLibA_31986 | 50 | -0.651050088 | NIT2 | nitrilase family member 2 [Source:HGNC Symbol;Acc:HGNC:29878] |
| ENSG00000115145 | HGLibA_47334 | 50 | -0.651050088 | STAM2 | signal transducing adaptor molecule 2 [Source:HGNC Symbol;Acc:HGNC:11358] |
| ENSG00000116117 | HGLibA_35182 | 50 | -0.651050088 | PARD3B | par-3 family cell polarity regulator beta [Source:HGNC Symbol;Acc:HGNC:14446] |
| ENSG00000116251 | HGLibA_41899 | 50 | -0.651050088 | RPL22 | ribosomal protein L22 [Source:HGNC Symbol;Acc:HGNC:10315] |
| ENSG00000116977 | HGLibA_26377 | 50 | -0.651050088 | LGALS8 | galectin 8 [Source:HGNC Symbol;Acc:HGNC:6569] |
| ENSG00000117408 | HGLibA_23464 | 50 | -0.651050088 | IPO13 | importin 13 [Source:HGNC Symbol;Acc:HGNC:16853] |
| ENSG00000118194 | HGLibA_50886 | 50 | -0.651050088 | TNNT2 | cardiac type [Source:HGNC Symbol;Acc:HGNC:11949]" |
| ENSG00000119673 | HGLibA_00478 | 50 | -0.651050088 | ACOT2 | acyl-CoA thioesterase 2 [Source:HGNC Symbol;Acc:HGNC:18431] |
| ENSG00000119681 | HGLibA_27593 | 50 | -0.651050088 | LTBP2 | latent transforming growth factor beta binding protein 2 [Source:HGNC Symbol;Acc:HGNC:6715] |
| ENSG00000120675 | HGLibA_13579 | 50 | -0.651050088 | DNAJC15 | DnaJ heat shock protein family (Hsp40) member C15 [Source:HGNC Symbol;Acc:HGNC:20325] |
| ENSG00000120756 | HGLibA_37225 | 50 | -0.651050088 | PLS1 | plastin 1 [Source:HGNC Symbol;Acc:HGNC:9090] |
| ENSG00000120784 | HGLibA_55201 | 50 | -0.651050088 | ZFP30 | ZFP30 zinc finger protein [Source:HGNC Symbol;Acc:HGNC:29555] |
| ENSG00000120885 | HGLibA_10154 | 50 | -0.651050088 | CLU | clusterin [Source:HGNC Symbol;Acc:HGNC:2095] |
| ENSG00000121207 | HGLibA_27131 | 50 | -0.651050088 | LRAT | lecithin retinol acyltransferase [Source:HGNC Symbol;Acc:HGNC:6685] |
| ENSG00000122741 | HGLibA_12412 | 50 | -0.651050088 | DCAF10 | DDB1 and CUL4 associated factor 10 [Source:HGNC Symbol;Acc:HGNC:23686] |
| ENSG00000123338 | HGLibA_31314 | 50 | -0.651050088 | NCKAP1L | NCK associated protein 1 like [Source:HGNC Symbol;Acc:HGNC:4862] |
| ENSG00000123737 | HGLibA_15797 | 50 | -0.651050088 | EXOSC9 | exosome component 9 [Source:HGNC Symbol;Acc:HGNC:9137] |
| ENSG00000125755 | HGLibA_47951 | 50 | -0.651050088 | SYMPK | symplekin [Source:HGNC Symbol;Acc:HGNC:22935] |
| ENSG00000125975 | HGLibA_05996 | 50 | -0.651050088 | C20orf173 | chromosome 20 open reading frame 173 [Source:HGNC Symbol;Acc:HGNC:16166] |
| ENSG00000126215 | HGLibA_54590 | 50 | -0.651050088 | XRCC3 | X-ray repair cross complementing 3 [Source:HGNC Symbol;Acc:HGNC:12830] |
| ENSG00000127184 | HGLibA_10872 | 50 | -0.651050088 | COX7C | cytochrome c oxidase subunit 7C [Source:HGNC Symbol;Acc:HGNC:2292] |
| ENSG00000127870 | HGLibA_41693 | 50 | -0.651050088 | RNF6 | ring finger protein 6 [Source:HGNC Symbol;Acc:HGNC:10069] |
| ENSG00000127920 | HGLibA_19512 | 50 | -0.651050088 | GNG11 | G protein subunit gamma 11 [Source:HGNC Symbol;Acc:HGNC:4403] |
| ENSG00000128285 | HGLibA_28589 | 50 | -0.651050088 | MCHR1 | melanin concentrating hormone receptor 1 [Source:HGNC Symbol;Acc:HGNC:4479] |
| ENSG00000129354 | HGLibA_02293 | 50 | -0.651050088 | AP1M2 | adaptor related protein complex 1 subunit mu 2 [Source:HGNC Symbol;Acc:HGNC:558] |
| ENSG00000132141 | HGLibA_08211 | 50 | -0.651050088 | CCT6B | chaperonin containing TCP1 subunit 6B [Source:HGNC Symbol;Acc:HGNC:1621] |
| ENSG00000132846 | HGLibA_54795 | 50 | -0.651050088 | ZBED3 | zinc finger BED-type containing 3 [Source:HGNC Symbol;Acc:HGNC:20711] |
| ENSG00000134371 | HGLibA_08598 | 50 | -0.651050088 | CDC73 | cell division cycle 73 [Source:HGNC Symbol;Acc:HGNC:16783] |
| ENSG00000134748 | HGLibA_38722 | 50 | -0.651050088 | PRPF38A | pre-mRNA processing factor 38A [Source:HGNC Symbol;Acc:HGNC:25930] |
| ENSG00000134900 | HGLibA_51163 | 50 | -0.651050088 | TPP2 | tripeptidyl peptidase 2 [Source:HGNC Symbol;Acc:HGNC:12016] |
| ENSG00000136274 | HGLibA_31035 | 50 | -0.651050088 | NACAD | NAC alpha domain containing [Source:HGNC Symbol;Acc:HGNC:22196] |
| ENSG00000136897 | HGLibA_30007 | 50 | -0.651050088 | MRPL50 | mitochondrial ribosomal protein L50 [Source:HGNC Symbol;Acc:HGNC:16654] |
| ENSG00000136933 | HGLibA_40007 | 50 | -0.651050088 | RABEPK | Rab9 effector protein with kelch motifs [Source:HGNC Symbol;Acc:HGNC:16896] |
| ENSG00000137463 | HGLibA_29128 | 50 | -0.651050088 | MGARP | mitochondria localized glutamic acid rich protein [Source:HGNC Symbol;Acc:HGNC:29969] |
| ENSG00000137494 | HGLibA_02098 | 50 | -0.651050088 | ANKRD42 | ankyrin repeat domain 42 [Source:HGNC Symbol;Acc:HGNC:26752] |
| ENSG00000138161 | HGLibA_11809 | 50 | -0.651050088 | CUZD1 | CUB and zona pellucida like domains 1 [Source:HGNC Symbol;Acc:HGNC:17937] |
| ENSG00000139985 | HGLibA_00756 | 50 | -0.651050088 | ADAM21 | ADAM metallopeptidase domain 21 [Source:HGNC Symbol;Acc:HGNC:200] |
| ENSG00000140479 | HGLibA_35682 | 50 | -0.651050088 | PCSK6 | proprotein convertase subtilisin/kexin type 6 [Source:HGNC Symbol;Acc:HGNC:8569] |
| ENSG00000140968 | HGLibA_23600 | 50 | -0.651050088 | IRF8 | interferon regulatory factor 8 [Source:HGNC Symbol;Acc:HGNC:5358] |
| ENSG00000141431 | HGLibA_03396 | 50 | -0.651050088 | ASXL3 | ASXL transcriptional regulator 3 [Source:HGNC Symbol;Acc:HGNC:29357] |
| ENSG00000141551 | HGLibA_11387 | 50 | -0.651050088 | CSNK1D | casein kinase 1 delta [Source:HGNC Symbol;Acc:HGNC:2452] |
| ENSG00000142207 | HGLibA_53135 | 50 | -0.651050088 | URB1 | URB1 ribosome biogenesis homolog [Source:HGNC Symbol;Acc:HGNC:17344] |
| ENSG00000142684 | HGLibA_56342 | 50 | -0.651050088 | ZNF593 | zinc finger protein 593 [Source:HGNC Symbol;Acc:HGNC:30943] |
| ENSG00000143390 | HGLibA_40973 | 50 | -0.651050088 | RFX5 | regulatory factor X5 [Source:HGNC Symbol;Acc:HGNC:9986] |
| ENSG00000143434 | HGLibA_43371 | 50 | -0.651050088 | SEMA6C | semaphorin 6C [Source:HGNC Symbol;Acc:HGNC:10740] |
| ENSG00000143643 | HGLibA_52051 | 50 | -0.651050088 | TTC13 | tetratricopeptide repeat domain 13 [Source:HGNC Symbol;Acc:HGNC:26204] |
| ENSG00000143768 | HGLibA_26278 | 50 | -0.651050088 | LEFTY2 | left-right determination factor 2 [Source:HGNC Symbol;Acc:HGNC:3122] |
| ENSG00000143921 | HGLibA_00226 | 50 | -0.651050088 | ABCG8 | ATP binding cassette subfamily G member 8 [Source:HGNC Symbol;Acc:HGNC:13887] |
| ENSG00000144120 | HGLibA_50079 | 50 | -0.651050088 | TMEM177 | transmembrane protein 177 [Source:HGNC Symbol;Acc:HGNC:28143] |
| ENSG00000145439 | HGLibA_07423 | 50 | -0.651050088 | CBR4 | carbonyl reductase 4 [Source:HGNC Symbol;Acc:HGNC:25891] |
| ENSG00000146399 | HGLibA_48123 | 50 | -0.651050088 | TAAR1 | trace amine associated receptor 1 [Source:HGNC Symbol;Acc:HGNC:17734] |
| ENSG00000147144 | HGLibA_07565 | 50 | -0.651050088 | CCDC120 | coiled-coil domain containing 120 [Source:HGNC Symbol;Acc:HGNC:28910] |
| ENSG00000147650 | HGLibA_27205 | 50 | -0.651050088 | LRP12 | LDL receptor related protein 12 [Source:HGNC Symbol;Acc:HGNC:31708] |
| ENSG00000148660 | HGLibA_07075 | 50 | -0.651050088 | CAMK2G | calcium/calmodulin dependent protein kinase II gamma [Source:HGNC Symbol;Acc:HGNC:1463] |
| ENSG00000149256 | HGLibA_49005 | 50 | -0.651050088 | TENM4 | teneurin transmembrane protein 4 [Source:HGNC Symbol;Acc:HGNC:29945] |
| ENSG00000149328 | HGLibA_19223 | 50 | -0.651050088 | GLB1L2 | galactosidase beta 1 like 2 [Source:HGNC Symbol;Acc:HGNC:25129] |
| ENSG00000150712 | HGLibA_30418 | 50 | -0.651050088 | MTMR12 | myotubularin related protein 12 [Source:HGNC Symbol;Acc:HGNC:18191] |
| ENSG00000152404 | HGLibA_11828 | 50 | -0.651050088 | CWF19L2 | CWF19 like cell cycle control factor 2 [Source:HGNC Symbol;Acc:HGNC:26508] |
| ENSG00000153086 | HGLibA_00454 | 50 | -0.651050088 | ACMSD | aminocarboxymuconate semialdehyde decarboxylase [Source:HGNC Symbol;Acc:HGNC:19288] |
| ENSG00000154237 | HGLibA_27465 | 50 | -0.651050088 | LRRK1 | leucine rich repeat kinase 1 [Source:HGNC Symbol;Acc:HGNC:18608] |
| ENSG00000156427 | HGLibA_17385 | 50 | -0.651050088 | FGF18 | fibroblast growth factor 18 [Source:HGNC Symbol;Acc:HGNC:3674] |
| ENSG00000159784 | HGLibA_16108 | 50 | -0.651050088 | FAM131B | family with sequence similarity 131 member B [Source:HGNC Symbol;Acc:HGNC:22202] |
| ENSG00000160284 | HGLibA_46539 | 50 | -0.651050088 | SPATC1L | spermatogenesis and centriole associated 1 like [Source:HGNC Symbol;Acc:HGNC:1298] |
| ENSG00000162461 | HGLibA_44715 | 50 | -0.651050088 | SLC25A34 | solute carrier family 25 member 34 [Source:HGNC Symbol;Acc:HGNC:27653] |
| ENSG00000162482 | HGLibA_01501 | 50 | -0.651050088 | AKR7A3 | aldo-keto reductase family 7 member A3 [Source:HGNC Symbol;Acc:HGNC:390] |
| ENSG00000163501 | HGLibA_22908 | 50 | -0.651050088 | IHH | indian hedgehog [Source:HGNC Symbol;Acc:HGNC:5956] |
| ENSG00000163697 | HGLibA_02382 | 50 | -0.651050088 | APBB2 | amyloid beta precursor protein binding family B member 2 [Source:HGNC Symbol;Acc:HGNC:582] |
| ENSG00000164185 | HGLibA_56063 | 50 | -0.651050088 | ZNF474 | zinc finger protein 474 [Source:HGNC Symbol;Acc:HGNC:23245] |
| ENSG00000164619 | HGLibA_04626 | 50 | -0.651050088 | BMPER | BMP binding endothelial regulator [Source:HGNC Symbol;Acc:HGNC:24154] |
| ENSG00000164695 | HGLibA_09493 | 50 | -0.651050088 | CHMP4C | charged multivesicular body protein 4C [Source:HGNC Symbol;Acc:HGNC:30599] |
| ENSG00000165059 | HGLibA_38510 | 50 | -0.651050088 | PRKACG | protein kinase cAMP-activated catalytic subunit gamma [Source:HGNC Symbol;Acc:HGNC:9382] |
| ENSG00000165092 | HGLibA_01540 | 50 | -0.651050088 | ALDH1A1 | aldehyde dehydrogenase 1 family member A1 [Source:HGNC Symbol;Acc:HGNC:402] |
| ENSG00000165457 | HGLibA_17786 | 50 | -0.651050088 | FOLR2 | folate receptor beta [Source:HGNC Symbol;Acc:HGNC:3793] |
| ENSG00000165568 | HGLibA_01495 | 50 | -0.651050088 | AKR1E2 | aldo-keto reductase family 1 member E2 [Source:HGNC Symbol;Acc:HGNC:23437] |
| ENSG00000165644 | HGLibA_10689 | 50 | -0.651050088 | COMTD1 | catechol-O-methyltransferase domain containing 1 [Source:HGNC Symbol;Acc:HGNC:26309] |
| ENSG00000166704 | HGLibA_56369 | 50 | -0.651050088 | ZNF606 | zinc finger protein 606 [Source:HGNC Symbol;Acc:HGNC:25879] |
| ENSG00000166845 | HGLibA_05618 | 50 | -0.651050088 | C18orf54 | chromosome 18 open reading frame 54 [Source:HGNC Symbol;Acc:HGNC:13796] |
| ENSG00000167711 | HGLibA_43589 | 50 | -0.651050088 | SERPINF2 | serpin family F member 2 [Source:HGNC Symbol;Acc:HGNC:9075] |
| ENSG00000167757 | HGLibA_25237 | 50 | -0.651050088 | KLK11 | kallikrein related peptidase 11 [Source:HGNC Symbol;Acc:HGNC:6359] |
| ENSG00000167984 | HGLibA_32088 | 50 | -0.651050088 | NLRC3 | NLR family CARD domain containing 3 [Source:HGNC Symbol;Acc:HGNC:29889] |

| | | | | | |
|---|---|---|---|---|---|
| ENSG00000168243 | HGLibA_19525 | 50 | -0.651050088 | GNG4 | G protein subunit gamma 4 [Source:HGNC Symbol;Acc:HGNC:4407] |
| ENSG00000168267 | HGLibA_39302 | 50 | -0.651050088 | PTF1A | pancreas associated transcription factor 1a [Source:HGNC Symbol;Acc:HGNC:23734] |
| ENSG00000168938 | HGLibA_37920 | 50 | -0.651050088 | PPIC | peptidylprolyl isomerase C [Source:HGNC Symbol;Acc:HGNC:9256] |
| ENSG00000169682 | HGLibA_46733 | 50 | -0.651050088 | SPNS1 | sphingolipid transporter 1 (putative) [Source:HGNC Symbol;Acc:HGNC:30621] |
| ENSG00000170075 | HGLibA_20036 | 50 | -0.651050088 | GPR37L1 | G protein-coupled receptor 37 like 1 [Source:HGNC Symbol;Acc:HGNC:14923] |
| ENSG00000170296 | HGLibA_18333 | 50 | -0.651050088 | GABARAP | GABA type A receptor-associated protein [Source:HGNC Symbol;Acc:HGNC:4067] |
| ENSG00000170454 | HGLibA_25536 | 50 | -0.651050088 | KRT75 | keratin 75 [Source:HGNC Symbol;Acc:HGNC:24431] |
| ENSG00000170748 | HGLibA_40605 | 50 | -0.651050088 | RBMXL2 | RBMX like 2 [Source:HGNC Symbol;Acc:HGNC:17886] |
| ENSG00000170836 | HGLibA_37969 | 50 | -0.651050088 | PPM1D | Mg2+/Mn2+ dependent 1D [Source:HGNC Symbol;Acc:HGNC:9277]" |
| ENSG00000170860 | HGLibA_27539 | 50 | -0.651050088 | LSM3 | U6 small nuclear RNA and mRNA degradation associated [Source:HGNC Symbol;Acc:HGNC:17874]" |
| ENSG00000170899 | HGLibA_20459 | 50 | -0.651050088 | GSTA4 | glutathione S-transferase alpha 4 [Source:HGNC Symbol;Acc:HGNC:4629] |
| ENSG00000171014 | HGLibA_33976 | 50 | -0.651050088 | OR4D5 | olfactory receptor family 4 subfamily D member 5 [Source:HGNC Symbol;Acc:HGNC:14852] |
| ENSG00000171219 | HGLibA_08564 | 50 | -0.651050088 | CDC42BPG | CDC42 binding protein kinase gamma [Source:HGNC Symbol;Acc:HGNC:29829] |
| ENSG00000171307 | HGLibA_55085 | 50 | -0.651050088 | ZDHHC16 | zinc finger DHHC-type containing 16 [Source:HGNC Symbol;Acc:HGNC:20714] |
| ENSG00000171357 | HGLibA_27633 | 50 | -0.651050088 | LURAP1 | leucine rich adaptor protein 1 [Source:HGNC Symbol;Acc:HGNC:32327] |
| ENSG00000171873 | HGLibA_01043 | 50 | -0.651050088 | ADRA1D | adrenoceptor alpha 1D [Source:HGNC Symbol;Acc:HGNC:280] |
| ENSG00000172382 | HGLibA_38920 | 50 | -0.651050088 | PRSS27 | serine protease 27 [Source:HGNC Symbol;Acc:HGNC:15475] |
| ENSG00000172468 | HGLibA_22240 | 50 | -0.651050088 | HSFY1 | heat shock transcription factor Y-linked 1 [Source:HGNC Symbol;Acc:HGNC:18568] |
| ENSG00000173598 | HGLibA_32980 | 50 | -0.651050088 | NUDT4 | nudix hydrolase 4 [Source:HGNC Symbol;Acc:HGNC:8051] |
| ENSG00000174669 | HGLibA_44843 | 50 | -0.651050088 | SLC29A2 | solute carrier family 29 member 2 [Source:HGNC Symbol;Acc:HGNC:11004] |
| ENSG00000176390 | HGLibA_11195 | 50 | -0.651050088 | CRLF3 | cytokine receptor like factor 3 [Source:HGNC Symbol;Acc:HGNC:17177] |
| ENSG00000177324 | HGLibA_04401 | 50 | -0.651050088 | BEND2 | BEN domain containing 2 [Source:HGNC Symbol;Acc:HGNC:28509] |
| ENSG00000177462 | HGLibA_33893 | 50 | -0.651050088 | OR2T8 | olfactory receptor family 2 subfamily T member 8 [Source:HGNC Symbol;Acc:HGNC:15020] |
| ENSG00000177504 | HGLibA_53585 | 50 | -0.651050088 | VCX2 | variable charge X-linked 2 [Source:HGNC Symbol;Acc:HGNC:18158] |
| ENSG00000177910 | HGLibA_46491 | 50 | -0.651050088 | SPATA31C2 | SPATA31 subfamily C member 2 [Source:HGNC Symbol;Acc:HGNC:24508] |
| ENSG00000178257 | HGLibA_38613 | 50 | -0.651050088 | PRM3 | protamine 3 [Source:HGNC Symbol;Acc:HGNC:13732] |
| ENSG00000178522 | HGLibA_01747 | 50 | -0.651050088 | AMBN | ameloblastin [Source:HGNC Symbol;Acc:HGNC:452] |
| ENSG00000178913 | HGLibA_48253 | 50 | -0.651050088 | TAF7 | TATA-box binding protein associated factor 7 [Source:HGNC Symbol;Acc:HGNC:11541] |
| ENSG00000178997 | HGLibA_15717 | 50 | -0.651050088 | EXD1 | exonuclease 3'-5' domain containing 1 [Source:HGNC Symbol;Acc:HGNC:28507] |
| ENSG00000179528 | HGLibA_26087 | 50 | -0.651050088 | LBX2 | ladybird homeobox 2 [Source:HGNC Symbol;Acc:HGNC:15525] |
| ENSG00000181009 | HGLibA_34203 | 50 | -0.651050088 | OR52N5 | olfactory receptor family 52 subfamily N member 5 [Source:HGNC Symbol;Acc:HGNC:15231] |
| ENSG00000181778 | HGLibA_50322 | 50 | -0.651050088 | TMEM252 | transmembrane protein 252 [Source:HGNC Symbol;Acc:HGNC:28537] |
| ENSG00000182177 | HGLibA_03230 | 50 | -0.651050088 | ASB18 | ankyrin repeat and SOCS box containing 18 [Source:HGNC Symbol;Acc:HGNC:19770] |
| ENSG00000182223 | HGLibA_54779 | 50 | -0.651050088 | ZAR1 | zygote arrest 1 [Source:HGNC Symbol;Acc:HGNC:20436] |
| ENSG00000182687 | HGLibA_18591 | 50 | -0.651050088 | GALR2 | galanin receptor 2 [Source:HGNC Symbol;Acc:HGNC:4133] |
| ENSG00000183035 | HGLibA_12044 | 50 | -0.651050088 | CYLC1 | cylicin 1 [Source:HGNC Symbol;Acc:HGNC:2582] |
| ENSG00000183260 | HGLibA_00259 | 50 | -0.651050088 | ABHD16B | abhydrolase domain containing 16B [Source:HGNC Symbol;Acc:HGNC:16128] |
| ENSG00000183709 | HGLibA_22723 | 50 | -0.651050088 | IFNL2 | interferon lambda 2 [Source:HGNC Symbol;Acc:HGNC:18364] |
| ENSG00000184007 | HGLibA_39406 | 50 | -0.651050088 | PTP4A2 | member 2 [Source:HGNC Symbol;Acc:HGNC:9635]" |
| ENSG00000184148 | HGLibA_46828 | 50 | -0.651050088 | SPRR4 | small proline rich protein 4 [Source:HGNC Symbol;Acc:HGNC:23173] |
| ENSG00000184178 | HGLibA_42895 | 50 | -0.651050088 | SCFD2 | sec1 family domain containing 2 [Source:HGNC Symbol;Acc:HGNC:30676] |
| ENSG00000185189 | HGLibA_32658 | 50 | -0.651050088 | NRBP2 | nuclear receptor binding protein 2 [Source:HGNC Symbol;Acc:HGNC:19339] |
| ENSG00000185818 | HGLibA_31189 | 50 | -0.651050088 | NAT8L | N-acetyltransferase 8 like [Source:HGNC Symbol;Acc:HGNC:26742] |
| ENSG00000186009 | HGLibA_03636 | 50 | -0.651050088 | ATP4B | ATPase H+/K+ transporting subunit beta [Source:HGNC Symbol;Acc:HGNC:820] |
| ENSG00000186432 | HGLibA_25363 | 50 | -0.651050088 | KPNA4 | karyopherin subunit alpha 4 [Source:HGNC Symbol;Acc:HGNC:6397] |
| ENSG00000186567 | HGLibA_08930 | 50 | -0.651050088 | CEACAM19 | carcinoembryonic antigen related cell adhesion molecule 19 [Source:HGNC Symbol;Acc:HGNC:31951] |
| ENSG00000186810 | HGLibA_11887 | 50 | -0.651050088 | CXCR3 | C-X-C motif chemokine receptor 3 [Source:HGNC Symbol;Acc:HGNC:4540] |
| ENSG00000187257 | HGLibA_42306 | 50 | -0.651050088 | RSBN1L | round spermatid basic protein 1 like [Source:HGNC Symbol;Acc:HGNC:24765] |
| ENSG00000188558 | HGLibA_33800 | 50 | -0.651050088 | OR2G6 | olfactory receptor family 2 subfamily G member 6 [Source:HGNC Symbol;Acc:HGNC:27019] |
| ENSG00000197632 | HGLibA_43547 | 50 | -0.651050088 | SERPINB2 | serpin family B member 2 [Source:HGNC Symbol;Acc:HGNC:8584] |
| ENSG00000198162 | HGLibA_28062 | 50 | -0.651050088 | MAN1A2 | mannosidase alpha class 1A member 2 [Source:HGNC Symbol;Acc:HGNC:6822] |
| ENSG00000198183 | HGLibA_04710 | 50 | -0.651050088 | BPIFA1 | BPI fold containing family A member 1 [Source:HGNC Symbol;Acc:HGNC:15749] |
| ENSG00000198874 | HGLibA_52480 | 50 | -0.651050088 | TYW1 | tRNA-yW synthesizing protein 1 homolog [Source:HGNC Symbol;Acc:HGNC:25598] |
| ENSG00000204428 | HGLibA_27662 | 50 | -0.651050088 | LY6G5C | lymphocyte antigen 6 family member G5C [Source:HGNC Symbol;Acc:HGNC:13932] |
| ENSG00000204560 | HGLibA_13168 | 50 | -0.651050088 | DHX16 | DEAH-box helicase 16 [Source:HGNC Symbol;Acc:HGNC:2739] |
| ENSG00000204843 | HGLibA_12545 | 50 | -0.651050088 | DCTN1 | dynactin subunit 1 [Source:HGNC Symbol;Acc:HGNC:2711] |
| ENSG00000212725 | HGLibA_25688 | 50 | -0.651050088 | KRTAP2-1 | keratin associated protein 2-1 [Source:HGNC Symbol;Acc:HGNC:16775] |
| ENSG00000213246 | HGLibA_47818 | 50 | -0.651050088 | SUPT4H1 | DSIF elongation factor subunit [Source:HGNC Symbol;Acc:HGNC:11467]" |
| ENSG00000217930 | HGLibA_35084 | 50 | -0.651050088 | PAM16 | presequence translocase associated motor 16 [Source:HGNC Symbol;Acc:HGNC:29679] |
| ENSG00000223865 | HGLibA_21619 | 50 | -0.651050088 | HLA-DPB1 | DP beta 1 [Source:HGNC Symbol;Acc:HGNC:4940]" |
| ENSG00000231738 | HGLibA_51920 | 50 | -0.651050088 | TSPAN19 | tetraspanin 19 [Source:HGNC Symbol;Acc:HGNC:31886] |
| ENSG00000233608 | HGLibA_52378 | 50 | -0.651050088 | TWIST2 | twist family bHLH transcription factor 2 [Source:HGNC Symbol;Acc:HGNC:20670] |
| ENSG00000239900 | HGLibA_01072 | 50 | -0.651050088 | ADSL | adenylosuccinate lyase [Source:HGNC Symbol;Acc:HGNC:291] |
| ENSG00000241839 | HGLibA_37152 | 50 | -0.651050088 | PLEKHO2 | pleckstrin homology domain containing O2 [Source:HGNC Symbol;Acc:HGNC:30026] |
| ENSG00000243414 | HGLibA_49447 | 50 | -0.651050088 | TICAM2 | toll like receptor adaptor molecule 2 [Source:HGNC Symbol;Acc:HGNC:21354] |
| ENSG00000249104 | HGLibA_53231 | 50 | -0.651050088 | USP17L17 | ubiquitin specific peptidase 17-like family member 17 [Source:HGNC Symbol;Acc:HGNC:44445] |
| ENSG00000250799 | HGLibA_38663 | 50 | -0.651050088 | PRODH2 | proline dehydrogenase 2 [Source:HGNC Symbol;Acc:HGNC:17325] |
| ENSG00000255298 | HGLibA_34523 | 50 | -0.651050088 | OR8G5 | olfactory receptor family 8 subfamily G member 5 [Source:HGNC Symbol;Acc:HGNC:19622] |
| ENSG00000259332 | HGLibA_47234 | 50 | -0.651050088 | ST20-MTHFS | ST20-MTHFS readthrough [Source:HGNC Symbol;Acc:HGNC:44655] |
| ENSG00000273173 | HGLibA_46019 | 50 | -0.651050088 | SNURF | SNRPN upstream reading frame [Source:HGNC Symbol;Acc:HGNC:11171] |
| ENSG00000273749 | HGLibA_12032 | 50 | -0.651050088 | CYFIP1 | cytoplasmic FMR1 interacting protein 1 [Source:HGNC Symbol;Acc:HGNC:13759] |
| ENSG00000273899 | HGLibA_32263 | 50 | -0.651050088 | NOL12 | nucleolar protein 12 [Source:HGNC Symbol;Acc:HGNC:28585] |
| ENSG00000278259 | HGLibA_30818 | 50 | -0.651050088 | MYO19 | myosin XIX [Source:HGNC Symbol;Acc:HGNC:26234] |
| ENSG00000280204 | HGLibA_33712 | 50 | -0.651050088 | OR1S1 | olfactory receptor family 1 subfamily S member 1 (gene/pseudogene) [Source:HGNC Symbol;Acc:HGNC:8227] |
| FAM194B | HGLibA_16371 | 50 | -0.651050088 | FAM194B | - |
| FAM207A | HGLibA_16428 | 50 | -0.651050088 | FAM207A | - |
| HIST2H2AA4 | HGLibA_21524 | 50 | -0.651050088 | HIST2H2AA4 | - |
| HIST3H2A | HGLibA_21554 | 50 | -0.651050088 | HIST3H2A | - |
| hsa-mir-1224 | HGLibA_57296 | 50 | -0.651050088 | hsa-mir-1224 | - |
| hsa-mir-1226 | HGLibA_57304 | 50 | -0.651050088 | hsa-mir-1226 | - |
| hsa-mir-1244-3 | HGLibA_57372 | 50 | -0.651050088 | hsa-mir-1244-3 | - |
| hsa-mir-1302-2 | HGLibA_57694 | 50 | -0.651050088 | hsa-mir-1302-2 | - |
| hsa-mir-153-1 | HGLibA_57909 | 50 | -0.651050088 | hsa-mir-153-1 | - |
| hsa-mir-217 | HGLibA_58264 | 50 | -0.651050088 | hsa-mir-217 | - |
| hsa-mir-29b-1 | HGLibA_58412 | 50 | -0.651050088 | hsa-mir-29b-1 | - |
| hsa-mir-3142 | HGLibA_58625 | 50 | -0.651050088 | hsa-mir-3142 | - |
| hsa-mir-3174 | HGLibA_58771 | 50 | -0.651050088 | hsa-mir-3174 | - |
| hsa-mir-3668 | HGLibA_59222 | 50 | -0.651050088 | hsa-mir-3668 | - |
| hsa-mir-3670-2 | HGLibA_59238 | 50 | -0.651050088 | hsa-mir-3670-2 | - |
| hsa-mir-3677 | HGLibA_59259 | 50 | -0.651050088 | hsa-mir-3677 | - |
| hsa-mir-4444-2 | HGLibA_60184 | 50 | -0.651050088 | hsa-mir-4444-2 | - |
| hsa-mir-4518 | HGLibA_60515 | 50 | -0.651050088 | hsa-mir-4518 | - |
| hsa-mir-4673 | HGLibA_60808 | 50 | -0.651050088 | hsa-mir-4673 | - |
| hsa-mir-4676 | HGLibA_60822 | 50 | -0.651050088 | hsa-mir-4676 | - |
| hsa-mir-4686 | HGLibA_60853 | 50 | -0.651050088 | hsa-mir-4686 | - |
| hsa-mir-4726 | HGLibA_61001 | 50 | -0.651050088 | hsa-mir-4726 | - |
| hsa-mir-4756 | HGLibA_61123 | 50 | -0.651050088 | hsa-mir-4756 | - |
| hsa-mir-5010 | HGLibA_61456 | 50 | -0.651050088 | hsa-mir-5010 | - |
| hsa-mir-517b | HGLibA_61626 | 50 | -0.651050088 | hsa-mir-517b | - |
| hsa-mir-520d | HGLibA_61748 | 50 | -0.651050088 | hsa-mir-520d | - |
| hsa-mir-539 | HGLibA_61809 | 50 | -0.651050088 | hsa-mir-539 | - |
| hsa-mir-539 | HGLibA_61811 | 50 | -0.651050088 | hsa-mir-539 | - |
| hsa-mir-548ag-1 | HGLibA_61875 | 50 | -0.651050088 | hsa-mir-548ag-1 | - |
| hsa-mir-558 | HGLibA_62159 | 50 | -0.651050088 | hsa-mir-558 | - |
| hsa-mir-5680 | HGLibA_62235 | 50 | -0.651050088 | hsa-mir-5680 | - |
| hsa-mir-649 | HGLibA_62838 | 50 | -0.651050088 | hsa-mir-649 | - |
| hsa-mir-654 | HGLibA_62951 | 50 | -0.651050088 | hsa-mir-654 | - |
| hsa-mir-661 | HGLibA_62977 | 50 | -0.651050088 | hsa-mir-661 | - |
| hsa-mir-6803 | HGLibA_63389 | 50 | -0.651050088 | hsa-mir-6803 | - |
| hsa-mir-6849 | HGLibA_63569 | 50 | -0.651050088 | hsa-mir-6849 | - |
| hsa-mir-6892 | HGLibA_63754 | 50 | -0.651050088 | hsa-mir-6892 | - |
| hsa-mir-8057 | HGLibA_64065 | 50 | -0.651050088 | hsa-mir-8057 | - |
| hsa-mir-9-3 | HGLibA_64269 | 50 | -0.651050088 | hsa-mir-9-3 | - |
| LOC642799 | HGLibA_26959 | 50 | -0.651050088 | LOC642799 | - |
| LOH12CR1 | HGLibA_27011 | 50 | -0.651050088 | LOH12CR1 | - |
| MESDC2 | HGLibA_28914 | 50 | -0.651050088 | MESDC2 | - |
| SETD8 | HGLibA_43662 | 50 | -0.651050088 | SETD8 | - |
| SGOL2 | HGLibA_43843 | 50 | -0.651050088 | SGOL2 | - |
| TMEM136 | HGLibA_49951 | 50 | -0.651050088 | TMEM136 | - |
| WDR52 | HGLibA_54117 | 50 | -0.651050088 | WDR52 | - |
| ZUFSP | HGLibA_57061 | 50 | -0.651050088 | ZUFSP | - |
| APOA1BP | HGLibA_02452 | 49 | -0.661001125 | APOA1BP | - |
| C11orf85 | HGLibA_05198 | 49 | -0.661001125 | C11orf85 | - |
| C6orf106 | HGLibA_06435 | 49 | -0.661001125 | C6orf106 | - |
| CECR6 | HGLibA_08984 | 49 | -0.661001125 | CECR6 | - |
| CYP3A7-CYP3AP1 | HGLibA_12165 | 49 | -0.661001125 | CYP3A7-CYP3AP1 | - |
| ENSG00000005075 | HGLibA_37591 | 49 | -0.661001125 | POLR2J | RNA polymerase II subunit J [Source:HGNC Symbol;Acc:HGNC:9197] |
| ENSG00000005889 | HGLibA_55264 | 49 | -0.661001125 | ZFX | zinc finger protein X-linked [Source:HGNC Symbol;Acc:HGNC:12869] |
| ENSG00000007062 | HGLibA_38679 | 49 | -0.661001125 | PROM1 | prominin 1 [Source:HGNC Symbol;Acc:HGNC:9454] |
| ENSG00000010270 | HGLibA_47359 | 49 | -0.661001125 | STARD3NL | STARD3 N-terminal like [Source:HGNC Symbol;Acc:HGNC:19169] |
| ENSG00000010318 | HGLibA_36387 | 49 | -0.661001125 | PHF7 | PHD finger protein 7 [Source:HGNC Symbol;Acc:HGNC:18458] |
| ENSG00000015532 | HGLibA_54621 | 49 | -0.661001125 | XYLT2 | xylosyltransferase 2 [Source:HGNC Symbol;Acc:HGNC:15517] |
| ENSG00000021826 | HGLibA_10992 | 49 | -0.661001125 | CPS1 | carbamoyl-phosphate synthase 1 [Source:HGNC Symbol;Acc:HGNC:2323] |
| ENSG00000023041 | HGLibA_55123 | 49 | -0.661001125 | ZDHHC6 | zinc finger DHHC-type containing 6 [Source:HGNC Symbol;Acc:HGNC:19160] |
| ENSG00000024422 | HGLibA_14668 | 49 | -0.661001125 | EHD2 | EH domain containing 2 [Source:HGNC Symbol;Acc:HGNC:3243] |

| | | | | | |
|---|---|---|---|---|---|
| ENSG00000030066 | HGLibA_33029 | 49 | -0.661001125 | NUP160 | nucleoporin 160 [Source:HGNC Symbol;Acc:HGNC:18017] |
| ENSG00000047410 | HGLibA_51173 | 49 | -0.661001125 | TPR | nuclear basket protein [Source:HGNC Symbol;Acc:HGNC:12017]" |
| ENSG00000048405 | HGLibA_56785 | 49 | -0.661001125 | ZNF800 | zinc finger protein 800 [Source:HGNC Symbol;Acc:HGNC:27267] |
| ENSG00000062485 | HGLibA_11300 | 49 | -0.661001125 | CS | citrate synthase [Source:HGNC Symbol;Acc:HGNC:2422] |
| ENSG00000064547 | HGLibA_27056 | 49 | -0.661001125 | LPAR2 | lysophosphatidic acid receptor 2 [Source:HGNC Symbol;Acc:HGNC:3168] |
| ENSG00000064666 | HGLibA_10308 | 49 | -0.661001125 | CNN2 | calponin 2 [Source:HGNC Symbol;Acc:HGNC:2156] |
| ENSG00000072571 | HGLibA_21744 | 49 | -0.661001125 | HMMR | hyaluronan mediated motility receptor [Source:HGNC Symbol;Acc:HGNC:5012] |
| ENSG00000072657 | HGLibA_51381 | 49 | -0.661001125 | TRHDE | thyrotropin releasing hormone degrading enzyme [Source:HGNC Symbol;Acc:HGNC:30748] |
| ENSG00000075856 | HGLibA_42758 | 49 | -0.661001125 | SART3 | U4/U6 recycling protein [Source:HGNC Symbol;Acc:HGNC:16860]" |
| ENSG00000075945 | HGLibA_24966 | 49 | -0.661001125 | KIFAP3 | kinesin associated protein 3 [Source:HGNC Symbol;Acc:HGNC:17060] |
| ENSG00000077800 | HGLibA_17583 | 49 | -0.661001125 | FKBP6 | FK506 binding protein 6 [Source:HGNC Symbol;Acc:HGNC:3722] |
| ENSG00000078804 | HGLibA_51072 | 49 | -0.661001125 | TP53INP2 | tumor protein p53 inducible nuclear protein 2 [Source:HGNC Symbol;Acc:HGNC:16104] |
| ENSG00000080345 | HGLibA_41245 | 49 | -0.661001125 | RIF1 | replication timing regulatory factor 1 [Source:HGNC Symbol;Acc:HGNC:23207] |
| ENSG00000081923 | HGLibA_03781 | 49 | -0.661001125 | ATP8B1 | ATPase phospholipid transporting 8B1 [Source:HGNC Symbol;Acc:HGNC:3706] |
| ENSG00000083817 | HGLibA_55944 | 49 | -0.661001125 | ZNF416 | zinc finger protein 416 [Source:HGNC Symbol;Acc:HGNC:20645] |
| ENSG00000084112 | HGLibA_47132 | 49 | -0.661001125 | SSH1 | slingshot protein phosphatase 1 [Source:HGNC Symbol;Acc:HGNC:30579] |
| ENSG00000085377 | HGLibA_38438 | 49 | -0.661001125 | PREP | prolyl endopeptidase [Source:HGNC Symbol;Acc:HGNC:9358] |
| ENSG00000085662 | HGLibA_01472 | 49 | -0.661001125 | AKR1B1 | aldo-keto reductase family 1 member B [Source:HGNC Symbol;Acc:HGNC:381] |
| ENSG00000086205 | HGLibA_17779 | 49 | -0.661001125 | FOLH1 | folate hydrolase 1 [Source:HGNC Symbol;Acc:HGNC:3788] |
| ENSG00000086666 | HGLibA_55162 | 49 | -0.661001125 | ZFAND6 | zinc finger AN1-type containing 6 [Source:HGNC Symbol;Acc:HGNC:30164] |
| ENSG00000088836 | HGLibA_45209 | 49 | -0.661001125 | SLC4A11 | solute carrier family 4 member 11 [Source:HGNC Symbol;Acc:HGNC:16438] |
| ENSG00000091009 | HGLibA_40523 | 49 | -0.661001125 | RBM27 | RNA binding motif protein 27 [Source:HGNC Symbol;Acc:HGNC:29243] |
| ENSG00000091656 | HGLibA_55177 | 49 | -0.661001125 | ZFHX4 | zinc finger homeobox 4 [Source:HGNC Symbol;Acc:HGNC:30939] |
| ENSG00000094914 | HGLibA_00024 | 49 | -0.661001125 | AAAS | aladin WD repeat nucleoporin [Source:HGNC Symbol;Acc:HGNC:13666] |
| ENSG00000095981 | HGLibA_24310 | 49 | -0.661001125 | KCNK16 | potassium two pore domain channel subfamily K member 16 [Source:HGNC Symbol;Acc:HGNC:14464] |
| ENSG00000099377 | HGLibA_22212 | 49 | -0.661001125 | HSD3B7 | 3 beta- and steroid delta-isomerase 7 [Source:HGNC Symbol;Acc:HGNC:18324]" |
| ENSG00000100023 | HGLibA_37943 | 49 | -0.661001125 | PPIL2 | peptidylprolyl isomerase like 2 [Source:HGNC Symbol;Acc:HGNC:9261] |
| ENSG00000100030 | HGLibA_28246 | 49 | -0.661001125 | MAPK1 | mitogen-activated protein kinase 1 [Source:HGNC Symbol;Acc:HGNC:6871] |
| ENSG00000100678 | HGLibA_45394 | 49 | -0.661001125 | SLC8A3 | solute carrier family 8 member A3 [Source:HGNC Symbol;Acc:HGNC:11070] |
| ENSG00000100842 | HGLibA_14612 | 49 | -0.661001125 | EFS | embryonal Fyn-associated substrate [Source:HGNC Symbol;Acc:HGNC:16898] |
| ENSG00000101004 | HGLibA_31944 | 49 | -0.661001125 | NINL | ninein like [Source:HGNC Symbol;Acc:HGNC:29163] |
| ENSG00000101052 | HGLibA_22764 | 49 | -0.661001125 | IFT52 | intraflagellar transport 52 [Source:HGNC Symbol;Acc:HGNC:15901] |
| ENSG00000101210 | HGLibA_14494 | 49 | -0.661001125 | EEF1A2 | eukaryotic translation elongation factor 1 alpha 2 [Source:HGNC Symbol;Acc:HGNC:3192] |
| ENSG00000101868 | HGLibA_37483 | 49 | -0.661001125 | POLA1 | catalytic subunit [Source:HGNC Symbol;Acc:HGNC:9173]" |
| ENSG00000102595 | HGLibA_52870 | 49 | -0.661001125 | UGGT2 | UDP-glucose glycoprotein glucosyltransferase 2 [Source:HGNC Symbol;Acc:HGNC:15664] |
| ENSG00000103089 | HGLibA_15899 | 49 | -0.661001125 | FA2H | fatty acid 2-hydroxylase [Source:HGNC Symbol;Acc:HGNC:21197] |
| ENSG00000104738 | HGLibA_28614 | 49 | -0.661001125 | MCM4 | minichromosome maintenance complex component 4 [Source:HGNC Symbol;Acc:HGNC:6947] |
| ENSG00000104885 | HGLibA_13806 | 49 | -0.661001125 | DOT1L | DOT1 like histone lysine methyltransferase [Source:HGNC Symbol;Acc:HGNC:24948] |
| ENSG00000105668 | HGLibA_53068 | 49 | -0.661001125 | UPK1A | uroplakin 1A [Source:HGNC Symbol;Acc:HGNC:12577] |
| ENSG00000105854 | HGLibA_37682 | 49 | -0.661001125 | PON2 | paraoxonase 2 [Source:HGNC Symbol;Acc:HGNC:9205] |
| ENSG00000107077 | HGLibA_24512 | 49 | -0.661001125 | KDM4C | lysine demethylase 4C [Source:HGNC Symbol;Acc:HGNC:17071] |
| ENSG00000107099 | HGLibA_13757 | 49 | -0.661001125 | DOCK8 | dedicator of cytokinesis 8 [Source:HGNC Symbol;Acc:HGNC:19191] |
| ENSG00000107164 | HGLibA_18153 | 49 | -0.661001125 | FUBP3 | far upstream element binding protein 3 [Source:HGNC Symbol;Acc:HGNC:4005] |
| ENSG00000107485 | HGLibA_18670 | 49 | -0.661001125 | GATA3 | GATA binding protein 3 [Source:HGNC Symbol;Acc:HGNC:4172] |
| ENSG00000108774 | HGLibA_39968 | 49 | -0.661001125 | RAB5C | member RAS oncogene family [Source:HGNC Symbol;Acc:HGNC:9785]" |
| ENSG00000109132 | HGLibA_36452 | 49 | -0.661001125 | PHOX2B | paired like homeobox 2B [Source:HGNC Symbol;Acc:HGNC:9143] |
| ENSG00000110074 | HGLibA_17950 | 49 | -0.661001125 | FOXRED1 | FAD dependent oxidoreductase domain containing 1 [Source:HGNC Symbol;Acc:HGNC:26927] |
| ENSG00000110881 | HGLibA_03298 | 49 | -0.661001125 | ASIC1 | acid sensing ion channel subunit 1 [Source:HGNC Symbol;Acc:HGNC:100] |
| ENSG00000111110 | HGLibA_37982 | 49 | -0.661001125 | PPM1H | Mg2+/Mn2+ dependent 1H [Source:HGNC Symbol;Acc:HGNC:18583]" |
| ENSG00000111540 | HGLibA_39964 | 49 | -0.661001125 | RAB5B | member RAS oncogene family [Source:HGNC Symbol;Acc:HGNC:9784]" |
| ENSG00000111642 | HGLibA_09394 | 49 | -0.661001125 | CHD4 | chromodomain helicase DNA binding protein 4 [Source:HGNC Symbol;Acc:HGNC:1919] |
| ENSG00000111678 | HGLibA_05270 | 49 | -0.661001125 | C12orf57 | chromosome 12 open reading frame 57 [Source:HGNC Symbol;Acc:HGNC:29521] |
| ENSG00000111727 | HGLibA_20965 | 49 | -0.661001125 | HCFC2 | host cell factor C2 [Source:HGNC Symbol;Acc:HGNC:24972] |
| ENSG00000112116 | HGLibA_23000 | 49 | -0.661001125 | IL17F | interleukin 17F [Source:HGNC Symbol;Acc:HGNC:16404] |
| ENSG00000113387 | HGLibA_47694 | 49 | -0.661001125 | SUB1 | transcriptional regulator [Source:HGNC Symbol;Acc:HGNC:19985]" |
| ENSG00000114547 | HGLibA_41762 | 49 | -0.661001125 | ROPN1B | rhophilin associated tail protein 1B [Source:HGNC Symbol;Acc:HGNC:31927] |
| ENSG00000115289 | HGLibA_35593 | 49 | -0.661001125 | PCGF1 | polycomb group ring finger 1 [Source:HGNC Symbol;Acc:HGNC:17615] |
| ENSG00000115738 | HGLibA_22527 | 49 | -0.661001125 | ID2 | inhibitor of DNA binding 2 [Source:HGNC Symbol;Acc:HGNC:5361] |
| ENSG00000116147 | HGLibA_50906 | 49 | -0.661001125 | TNR | tenascin R [Source:HGNC Symbol;Acc:HGNC:11953] |
| ENSG00000116580 | HGLibA_19670 | 49 | -0.661001125 | GON4L | gon-4 like [Source:HGNC Symbol;Acc:HGNC:25973] |
| ENSG00000116584 | HGLibA_02835 | 49 | -0.661001125 | ARHGEF2 | Rho/Rac guanine nucleotide exchange factor 2 [Source:HGNC Symbol;Acc:HGNC:682] |
| ENSG00000116678 | HGLibA_26311 | 49 | -0.661001125 | LEPR | leptin receptor [Source:HGNC Symbol;Acc:HGNC:6554] |
| ENSG00000116748 | HGLibA_01822 | 49 | -0.661001125 | AMPD1 | adenosine monophosphate deaminase 1 [Source:HGNC Symbol;Acc:HGNC:468] |
| ENSG00000117262 | HGLibA_20107 | 49 | -0.661001125 | GPR89A | G protein-coupled receptor 89A [Source:HGNC Symbol;Acc:HGNC:31984] |
| ENSG00000118245 | HGLibA_50891 | 49 | -0.661001125 | TNP1 | transition protein 1 [Source:HGNC Symbol;Acc:HGNC:11951] |
| ENSG00000118507 | HGLibA_01441 | 49 | -0.661001125 | AKAP7 | A-kinase anchoring protein 7 [Source:HGNC Symbol;Acc:HGNC:377] |
| ENSG00000119616 | HGLibA_17178 | 49 | -0.661001125 | FCF1 | rRNA-processing protein [Source:HGNC Symbol;Acc:HGNC:20220]" |
| ENSG00000119699 | HGLibA_49217 | 49 | -0.661001125 | TGFB3 | transforming growth factor beta 3 [Source:HGNC Symbol;Acc:HGNC:11769] |
| ENSG00000120149 | HGLibA_30274 | 49 | -0.661001125 | MSX2 | msh homeobox 2 [Source:HGNC Symbol;Acc:HGNC:7392] |
| ENSG00000121289 | HGLibA_09177 | 49 | -0.661001125 | CEP89 | centrosomal protein 89 [Source:HGNC Symbol;Acc:HGNC:25907] |
| ENSG00000123576 | HGLibA_15603 | 49 | -0.661001125 | ESX1 | ESX homeobox 1 [Source:HGNC Symbol;Acc:HGNC:14865] |
| ENSG00000124203 | HGLibA_56837 | 49 | -0.661001125 | ZNF831 | zinc finger protein 831 [Source:HGNC Symbol;Acc:HGNC:16167] |
| ENSG00000124459 | HGLibA_56026 | 49 | -0.661001125 | ZNF45 | zinc finger protein 45 [Source:HGNC Symbol;Acc:HGNC:13111] |
| ENSG00000125505 | HGLibA_28525 | 49 | -0.661001125 | MBOAT7 | membrane bound O-acyltransferase domain containing 7 [Source:HGNC Symbol;Acc:HGNC:15505] |
| ENSG00000125820 | HGLibA_32035 | 49 | -0.661001125 | NKX2-2 | NK2 homeobox 2 [Source:HGNC Symbol;Acc:HGNC:7835] |
| ENSG00000125952 | HGLibA_28449 | 49 | -0.661001125 | MAX | MYC associated factor X [Source:HGNC Symbol;Acc:HGNC:6913] |
| ENSG00000126246 | HGLibA_22852 | 49 | -0.661001125 | IGFLR1 | IGF like family receptor 1 [Source:HGNC Symbol;Acc:HGNC:23620] |
| ENSG00000126583 | HGLibA_38548 | 49 | -0.661001125 | PRKCG | protein kinase C gamma [Source:HGNC Symbol;Acc:HGNC:9402] |
| ENSG00000126787 | HGLibA_13352 | 49 | -0.661001125 | DLGAP5 | DLG associated protein 5 [Source:HGNC Symbol;Acc:HGNC:16864] |
| ENSG00000127152 | HGLibA_04293 | 49 | -0.661001125 | BCL11B | B cell CLL/lymphoma 11B [Source:HGNC Symbol;Acc:HGNC:13222] |
| ENSG00000127530 | HGLibA_34466 | 49 | -0.661001125 | OR7C1 | olfactory receptor family 7 subfamily C member 1 [Source:HGNC Symbol;Acc:HGNC:8373] |
| ENSG00000129521 | HGLibA_14644 | 49 | -0.661001125 | EGLN3 | egl-9 family hypoxia inducible factor 3 [Source:HGNC Symbol;Acc:HGNC:14661] |
| ENSG00000130307 | HGLibA_53183 | 49 | -0.661001125 | USHBP1 | USH1 protein network component harmonin binding protein 1 [Source:HGNC Symbol;Acc:HGNC:24058] |
| ENSG00000130513 | HGLibA_18860 | 49 | -0.661001125 | GDF15 | growth differentiation factor 15 [Source:HGNC Symbol;Acc:HGNC:30142] |
| ENSG00000131711 | HGLibA_28118 | 49 | -0.661001125 | MAP1B | microtubule associated protein 1B [Source:HGNC Symbol;Acc:HGNC:6836] |
| ENSG00000131914 | HGLibA_26558 | 49 | -0.661001125 | LIN28A | lin-28 homolog A [Source:HGNC Symbol;Acc:HGNC:15986] |
| ENSG00000132361 | HGLibA_10161 | 49 | -0.661001125 | CLUH | clustered mitochondria homolog [Source:HGNC Symbol;Acc:HGNC:29094] |
| ENSG00000132463 | HGLibA_20380 | 49 | -0.661001125 | GRSF1 | G-rich RNA sequence binding factor 1 [Source:HGNC Symbol;Acc:HGNC:4610] |
| ENSG00000133466 | HGLibA_05951 | 49 | -0.661001125 | C1QTNF6 | C1q and TNF related 6 [Source:HGNC Symbol;Acc:HGNC:14343] |
| ENSG00000133816 | HGLibA_29205 | 49 | -0.661001125 | MICAL2 | calponin and LIM domain containing 2 [Source:HGNC Symbol;Acc:HGNC:24693]" |
| ENSG00000135473 | HGLibA_35091 | 49 | -0.661001125 | PAN2 | poly(A) specific ribonuclease subunit PAN2 [Source:HGNC Symbol;Acc:HGNC:20074] |
| ENSG00000135638 | HGLibA_15093 | 49 | -0.661001125 | EMX1 | empty spiracles homeobox 1 [Source:HGNC Symbol;Acc:HGNC:3340] |
| ENSG00000135999 | HGLibA_15268 | 49 | -0.661001125 | EPC2 | enhancer of polycomb homolog 2 [Source:HGNC Symbol;Acc:HGNC:24543] |
| ENSG00000136770 | HGLibA_13560 | 49 | -0.661001125 | DNAJC1 | DnaJ heat shock protein family (Hsp40) member C1 [Source:HGNC Symbol;Acc:HGNC:20090] |
| ENSG00000137314 | HGLibA_27314 | 49 | -0.661001125 | LRRC32 | leucine rich repeat containing 32 [Source:HGNC Symbol;Acc:HGNC:4161] |
| ENSG00000137558 | HGLibA_36483 | 49 | -0.661001125 | PI15 | peptidase inhibitor 15 [Source:HGNC Symbol;Acc:HGNC:8946] |
| ENSG00000137628 | HGLibA_12761 | 49 | -0.661001125 | DDX60 | DExD/H-box helicase 60 [Source:HGNC Symbol;Acc:HGNC:25942] |
| ENSG00000138759 | HGLibA_17975 | 49 | -0.661001125 | FRAS1 | Fraser extracellular matrix complex subunit 1 [Source:HGNC Symbol;Acc:HGNC:19185] |
| ENSG00000139219 | HGLibA_10560 | 49 | -0.661001125 | COL2A1 | collagen type II alpha 1 chain [Source:HGNC Symbol;Acc:HGNC:2200] |
| ENSG00000139514 | HGLibA_45345 | 49 | -0.661001125 | SLC7A1 | solute carrier family 7 member 1 [Source:HGNC Symbol;Acc:HGNC:11057] |
| ENSG00000139985 | HGLibA_00754 | 49 | -0.661001125 | ADAM21 | ADAM metallopeptidase domain 21 [Source:HGNC Symbol;Acc:HGNC:200] |
| ENSG00000140600 | HGLibA_43951 | 49 | -0.661001125 | SH3GL3 | endophilin A3 [Source:HGNC Symbol;Acc:HGNC:10832]" |
| ENSG00000141232 | HGLibA_50934 | 49 | -0.661001125 | TOB1 | 1 [Source:HGNC Symbol;Acc:HGNC:11979]" |
| ENSG00000141433 | HGLibA_00934 | 49 | -0.661001125 | ADCYAP1 | adenylate cyclase activating polypeptide 1 [Source:HGNC Symbol;Acc:HGNC:241] |
| ENSG00000141905 | HGLibA_31805 | 49 | -0.661001125 | NFIC | nuclear factor I C [Source:HGNC Symbol;Acc:HGNC:7786] |
| ENSG00000142065 | HGLibA_55188 | 49 | -0.661001125 | ZFP14 | ZFP14 zinc finger protein [Source:HGNC Symbol;Acc:HGNC:29312] |
| ENSG00000142089 | HGLibA_22646 | 49 | -0.661001125 | IFITM3 | interferon induced transmembrane protein 3 [Source:HGNC Symbol;Acc:HGNC:5414] |
| ENSG00000142611 | HGLibA_38382 | 49 | -0.661001125 | PRDM16 | PR/SET domain 16 [Source:HGNC Symbol;Acc:HGNC:14000] |
| ENSG00000142875 | HGLibA_38506 | 49 | -0.661001125 | PRKACB | protein kinase cAMP-activated catalytic subunit beta [Source:HGNC Symbol;Acc:HGNC:9381] |
| ENSG00000143106 | HGLibA_39083 | 49 | -0.661001125 | PSMA5 | proteasome subunit alpha 5 [Source:HGNC Symbol;Acc:HGNC:9534] |
| ENSG00000145244 | HGLibA_10769 | 49 | -0.661001125 | CORIN | serine peptidase [Source:HGNC Symbol;Acc:HGNC:19012]" |
| ENSG00000145337 | HGLibA_39708 | 49 | -0.661001125 | PYURF | PIGY upstream reading frame [Source:HGNC Symbol;Acc:HGNC:44317] |
| ENSG00000145428 | HGLibA_41576 | 49 | -0.661001125 | RNF175 | ring finger protein 175 [Source:HGNC Symbol;Acc:HGNC:27735] |
| ENSG00000145912 | HGLibA_31908 | 49 | -0.661001125 | NHP2 | NHP2 ribonucleoprotein [Source:HGNC Symbol;Acc:HGNC:14377] |
| ENSG00000146021 | HGLibA_25176 | 49 | -0.661001125 | KLHL3 | kelch like family member 3 [Source:HGNC Symbol;Acc:HGNC:6354] |
| ENSG00000149100 | HGLibA_14801 | 49 | -0.661001125 | EIF3M | eukaryotic translation initiation factor 3 subunit M [Source:HGNC Symbol;Acc:HGNC:24460] |
| ENSG00000149311 | HGLibA_03523 | 49 | -0.661001125 | ATM | ATM serine/threonine kinase [Source:HGNC Symbol;Acc:HGNC:795] |
| ENSG00000149346 | HGLibA_45565 | 49 | -0.661001125 | SLX4IP | SLX4 interacting protein [Source:HGNC Symbol;Acc:HGNC:16225] |
| ENSG00000152433 | HGLibA_56203 | 49 | -0.661001125 | ZNF547 | zinc finger protein 547 [Source:HGNC Symbol;Acc:HGNC:26432] |
| ENSG00000152556 | HGLibA_36169 | 49 | -0.661001125 | PFKM | muscle [Source:HGNC Symbol;Acc:HGNC:8877]" |
| ENSG00000152778 | HGLibA_22636 | 49 | -0.661001125 | IFIT5 | interferon induced protein with tetratricopeptide repeats 5 [Source:HGNC Symbol;Acc:HGNC:13328] |
| ENSG00000153446 | HGLibA_05494 | 49 | -0.661001125 | C16orf89 | chromosome 16 open reading frame 89 [Source:HGNC Symbol;Acc:HGNC:28687] |
| ENSG00000153982 | HGLibA_18891 | 49 | -0.661001125 | GDPD1 | glycerophosphodiester phosphodiesterase domain containing 1 [Source:HGNC Symbol;Acc:HGNC:20883] |
| ENSG00000154222 | HGLibA_07478 | 49 | -0.661001125 | CC2D1B | coiled-coil and C2 domain containing 1B [Source:HGNC Symbol;Acc:HGNC:29386] |
| ENSG00000155760 | HGLibA_18280 | 49 | -0.661001125 | FZD7 | frizzled class receptor 7 [Source:HGNC Symbol;Acc:HGNC:4045] |
| ENSG00000155849 | HGLibA_14930 | 49 | -0.661001125 | ELMO1 | engulfment and cell motility 1 [Source:HGNC Symbol;Acc:HGNC:16286] |
| ENSG00000157093 | HGLibA_27790 | 49 | -0.661001125 | LYZL4 | lysozyme like 4 [Source:HGNC Symbol;Acc:HGNC:28387] |
| ENSG00000157106 | HGLibA_45698 | 49 | -0.661001125 | SMG1 | nonsense mediated mRNA decay associated PI3K related kinase [Source:HGNC Symbol;Acc:HGNC:30045]" |
| ENSG00000157470 | HGLibA_16734 | 49 | -0.661001125 | FAM81A | family with sequence similarity 81 member A [Source:HGNC Symbol;Acc:HGNC:28379] |
| ENSG00000158488 | HGLibA_08275 | 49 | -0.661001125 | CD1E | CD1e molecule [Source:HGNC Symbol;Acc:HGNC:1638] |
| ENSG00000158716 | HGLibA_14167 | 49 | -0.661001125 | DUSP23 | dual specificity phosphatase 23 [Source:HGNC Symbol;Acc:HGNC:21480] |
| ENSG00000159455 | HGLibA_26120 | 49 | -0.661001125 | LCE2B | late cornified envelope 2B [Source:HGNC Symbol;Acc:HGNC:16610] |
| ENSG00000160058 | HGLibA_04864 | 49 | -0.661001125 | BSDC1 | BSD domain containing 1 [Source:HGNC Symbol;Acc:HGNC:25501] |
| ENSG00000160445 | HGLibA_55141 | 49 | -0.661001125 | ZER1 | zyg-11 related cell cycle regulator [Source:HGNC Symbol;Acc:HGNC:30960] |
| ENSG00000160991 | HGLibA_34591 | 49 | -0.661001125 | ORAI2 | ORAI calcium release-activated calcium modulator 2 [Source:HGNC Symbol;Acc:HGNC:21667] |

| | | | | | |
|---|---|---|---|---|---|
| ENSG00000161082 | HGLibA_09015 | 49 | -0.661001125 | CELF5 | CUGBP Elav-like family member 5 [Source:HGNC Symbol;Acc:HGNC:14058] |
| ENSG00000162493 | HGLibA_35935 | 49 | -0.661001125 | PDPN | podoplanin [Source:HGNC Symbol;Acc:HGNC:29602] |
| ENSG00000164118 | HGLibA_09140 | 49 | -0.661001125 | CEP44 | centrosomal protein 44 [Source:HGNC Symbol;Acc:HGNC:29356] |
| ENSG00000164244 | HGLibA_38856 | 49 | -0.661001125 | PRRC1 | proline rich coiled-coil 1 [Source:HGNC Symbol;Acc:HGNC:28164] |
| ENSG00000164896 | HGLibA_16899 | 49 | -0.661001125 | FASTK | Fas activated serine/threonine kinase [Source:HGNC Symbol;Acc:HGNC:24676] |
| ENSG00000164930 | HGLibA_18278 | 49 | -0.661001125 | FZD6 | frizzled class receptor 6 [Source:HGNC Symbol;Acc:HGNC:4044] |
| ENSG00000165478 | HGLibA_21137 | 49 | -0.661001125 | HEPACAM | hepatic and glial cell adhesion molecule [Source:HGNC Symbol;Acc:HGNC:26361] |
| ENSG00000165626 | HGLibA_04417 | 49 | -0.661001125 | BEND7 | BEN domain containing 7 [Source:HGNC Symbol;Acc:HGNC:23514] |
| ENSG00000165702 | HGLibA_18944 | 49 | -0.661001125 | GFI1B | growth factor independent 1B transcriptional repressor [Source:HGNC Symbol;Acc:HGNC:4238] |
| ENSG00000165775 | HGLibA_18166 | 49 | -0.661001125 | FUNDC2 | FUN14 domain containing 2 [Source:HGNC Symbol;Acc:HGNC:24925] |
| ENSG00000165828 | HGLibA_38336 | 49 | -0.661001125 | PRAP1 | proline rich acidic protein 1 [Source:HGNC Symbol;Acc:HGNC:23304] |
| ENSG00000166262 | HGLibA_16532 | 49 | -0.661001125 | FAM227B | family with sequence similarity 227 member B [Source:HGNC Symbol;Acc:HGNC:26543] |
| ENSG00000166377 | HGLibA_03796 | 49 | -0.661001125 | ATP9B | ATPase phospholipid transporting 9B (putative) [Source:HGNC Symbol;Acc:HGNC:13541] |
| ENSG00000167094 | HGLibA_52058 | 49 | -0.661001125 | TTC16 | tetratricopeptide repeat domain 16 [Source:HGNC Symbol;Acc:HGNC:26536] |
| ENSG00000167460 | HGLibA_51150 | 49 | -0.661001125 | TPM4 | tropomyosin 4 [Source:HGNC Symbol;Acc:HGNC:12013] |
| ENSG00000167613 | HGLibA_25931 | 49 | -0.661001125 | LAIR1 | leukocyte associated immunoglobulin like receptor 1 [Source:HGNC Symbol;Acc:HGNC:6477] |
| ENSG00000167671 | HGLibA_52793 | 49 | -0.661001125 | UBXN6 | UBX domain protein 6 [Source:HGNC Symbol;Acc:HGNC:14928] |
| ENSG00000168676 | HGLibA_24440 | 49 | -0.661001125 | KCTD19 | potassium channel tetramerization domain containing 19 [Source:HGNC Symbol;Acc:HGNC:24753] |
| ENSG00000168917 | HGLibA_45020 | 49 | -0.661001125 | SLC35G2 | solute carrier family 35 member G2 [Source:HGNC Symbol;Acc:HGNC:28480] |
| ENSG00000168995 | HGLibA_44146 | 49 | -0.661001125 | SIGLEC7 | sialic acid binding Ig like lectin 7 [Source:HGNC Symbol;Acc:HGNC:10876] |
| ENSG00000169129 | HGLibA_01102 | 49 | -0.661001125 | AFAP1L2 | actin filament associated protein 1 like 2 [Source:HGNC Symbol;Acc:HGNC:25901] |
| ENSG00000169251 | HGLibA_32148 | 49 | -0.661001125 | NMD3 | NMD3 ribosome export adaptor [Source:HGNC Symbol;Acc:HGNC:24250] |
| ENSG00000169764 | HGLibA_52874 | 49 | -0.661001125 | UGP2 | UDP-glucose pyrophosphorylase 2 [Source:HGNC Symbol;Acc:HGNC:12527] |
| ENSG00000169800 | HGLibA_40623 | 49 | -0.661001125 | RBMY1F | member F [Source:HGNC Symbol;Acc:HGNC:23974]" |
| ENSG00000170191 | HGLibA_31106 | 49 | -0.661001125 | NANP | N-acetylneuraminic acid phosphatase [Source:HGNC Symbol;Acc:HGNC:16140] |
| ENSG00000170236 | HGLibA_53393 | 49 | -0.661001125 | USP50 | ubiquitin specific peptidase 50 [Source:HGNC Symbol;Acc:HGNC:20079] |
| ENSG00000170364 | HGLibA_43672 | 49 | -0.661001125 | SETMAR | SET domain and mariner transposase fusion gene [Source:HGNC Symbol;Acc:HGNC:10762] |
| ENSG00000171033 | HGLibA_36820 | 49 | -0.661001125 | PKIA | cAMP-dependent protein kinase inhibitor alpha [Source:HGNC Symbol;Acc:HGNC:9017] |
| ENSG00000171262 | HGLibA_16811 | 49 | -0.661001125 | FAM98B | family with sequence similarity 98 member B [Source:HGNC Symbol;Acc:HGNC:26773] |
| ENSG00000171428 | HGLibA_31165 | 49 | -0.661001125 | NAT1 | N-acetyltransferase 1 [Source:HGNC Symbol;Acc:HGNC:7645] |
| ENSG00000171848 | HGLibA_42253 | 49 | -0.661001125 | RRM2 | ribonucleotide reductase regulatory subunit M2 [Source:HGNC Symbol;Acc:HGNC:10452] |
| ENSG00000172236 | HGLibA_51195 | 49 | -0.661001125 | TPSAB1 | tryptase alpha/beta 1 [Source:HGNC Symbol;Acc:HGNC:12019] |
| ENSG00000172469 | HGLibA_28092 | 49 | -0.661001125 | MANEA | mannosidase endo-alpha [Source:HGNC Symbol;Acc:HGNC:21072] |
| ENSG00000173041 | HGLibA_56523 | 49 | -0.661001125 | ZNF680 | zinc finger protein 680 [Source:HGNC Symbol;Acc:HGNC:26897] |
| ENSG00000173452 | HGLibA_50160 | 49 | -0.661001125 | TMEM196 | transmembrane protein 196 [Source:HGNC Symbol;Acc:HGNC:22431] |
| ENSG00000173786 | HGLibA_10356 | 49 | -0.661001125 | CNP | 3'-cyclic nucleotide 3' phosphodiesterase [Source:HGNC Symbol;Acc:HGNC:2158]" |
| ENSG00000175161 | HGLibA_06968 | 49 | -0.661001125 | CADM2 | cell adhesion molecule 2 [Source:HGNC Symbol;Acc:HGNC:29849] |
| ENSG00000175220 | HGLibA_02698 | 49 | -0.661001125 | ARHGAP1 | Rho GTPase activating protein 1 [Source:HGNC Symbol;Acc:HGNC:673] |
| ENSG00000175467 | HGLibA_42756 | 49 | -0.661001125 | SART1 | recruiter of U4/U6.U5 tri-snRNP [Source:HGNC Symbol;Acc:HGNC:10538]" |
| ENSG00000175793 | HGLibA_43728 | 49 | -0.661001125 | SFN | stratifin [Source:HGNC Symbol;Acc:HGNC:10773] |
| ENSG00000176055 | HGLibA_28505 | 49 | -0.661001125 | MBLAC2 | metallo-beta-lactamase domain containing 2 [Source:HGNC Symbol;Acc:HGNC:33711] |
| ENSG00000176393 | HGLibA_41726 | 49 | -0.661001125 | RNPEP | arginyl aminopeptidase [Source:HGNC Symbol;Acc:HGNC:10078] |
| ENSG00000176809 | HGLibA_27333 | 49 | -0.661001125 | LRRC37A3 | leucine rich repeat containing 37 member A3 [Source:HGNC Symbol;Acc:HGNC:32427] |
| ENSG00000177082 | HGLibA_54169 | 49 | -0.661001125 | WDR73 | WD repeat domain 73 [Source:HGNC Symbol;Acc:HGNC:25928] |
| ENSG00000178473 | HGLibA_52829 | 49 | -0.661001125 | UCN3 | urocortin 3 [Source:HGNC Symbol;Acc:HGNC:17781] |
| ENSG00000178602 | HGLibA_34746 | 49 | -0.661001125 | OTOS | otospiralin [Source:HGNC Symbol;Acc:HGNC:22644] |
| ENSG00000178996 | HGLibA_46051 | 49 | -0.661001125 | SNX18 | sorting nexin 18 [Source:HGNC Symbol;Acc:HGNC:19245] |
| ENSG00000179314 | HGLibA_54422 | 49 | -0.661001125 | WSCD1 | WSC domain containing 1 [Source:HGNC Symbol;Acc:HGNC:29060] |
| ENSG00000179526 | HGLibA_43990 | 49 | -0.661001125 | SHARPIN | SHANK associated RH domain interactor [Source:HGNC Symbol;Acc:HGNC:25321] |
| ENSG00000180287 | HGLibA_37047 | 49 | -0.661001125 | PLD5 | phospholipase D family member 5 [Source:HGNC Symbol;Acc:HGNC:26879] |
| ENSG00000180730 | HGLibA_44033 | 49 | -0.661001125 | SHISA2 | shisa family member 2 [Source:HGNC Symbol;Acc:HGNC:20366] |
| ENSG00000180777 | HGLibA_02063 | 49 | -0.661001125 | ANKRD30B | ankyrin repeat domain 30B [Source:HGNC Symbol;Acc:HGNC:24165] |
| ENSG00000180806 | HGLibA_21979 | 49 | -0.661001125 | HOXC9 | homeobox C9 [Source:HGNC Symbol;Acc:HGNC:5130] |
| ENSG00000181588 | HGLibA_29037 | 49 | -0.661001125 | MEX3D | mex-3 RNA binding family member D [Source:HGNC Symbol;Acc:HGNC:16734] |
| ENSG00000181873 | HGLibA_22479 | 49 | -0.661001125 | IBA57 | iron-sulfur cluster assembly [Source:HGNC Symbol;Acc:HGNC:27302]" |
| ENSG00000182040 | HGLibA_53176 | 49 | -0.661001125 | USH1G | USH1 protein network component sans [Source:HGNC Symbol;Acc:HGNC:16356] |
| ENSG00000182103 | HGLibA_16320 | 49 | -0.661001125 | FAM181B | family with sequence similarity 181 member B [Source:HGNC Symbol;Acc:HGNC:28512] |
| ENSG00000183087 | HGLibA_18654 | 49 | -0.661001125 | GAS6 | growth arrest specific 6 [Source:HGNC Symbol;Acc:HGNC:4168] |
| ENSG00000183283 | HGLibA_12368 | 49 | -0.661001125 | DAZAP2 | DAZ associated protein 2 [Source:HGNC Symbol;Acc:HGNC:2684] |
| ENSG00000183943 | HGLibA_38593 | 49 | -0.661001125 | PRKX | protein kinase X-linked [Source:HGNC Symbol;Acc:HGNC:9441] |
| ENSG00000184304 | HGLibA_38566 | 49 | -0.661001125 | PRKD1 | protein kinase D1 [Source:HGNC Symbol;Acc:HGNC:9407] |
| ENSG00000184304 | HGLibA_38565 | 49 | -0.661001125 | PRKD1 | protein kinase D1 [Source:HGNC Symbol;Acc:HGNC:9407] |
| ENSG00000184465 | HGLibA_54058 | 49 | -0.661001125 | WDR27 | WD repeat domain 27 [Source:HGNC Symbol;Acc:HGNC:21248] |
| ENSG00000184478 | HGLibA_34213 | 49 | -0.661001125 | OR56A3 | olfactory receptor family 56 subfamily A member 3 [Source:HGNC Symbol;Acc:HGNC:14786] |
| ENSG00000184601 | HGLibA_05348 | 49 | -0.661001125 | C14orf180 | chromosome 14 open reading frame 180 [Source:HGNC Symbol;Acc:HGNC:33795] |
| ENSG00000184984 | HGLibA_09559 | 49 | -0.661001125 | CHRM5 | cholinergic receptor muscarinic 5 [Source:HGNC Symbol;Acc:HGNC:1954] |
| ENSG00000185028 | HGLibA_27257 | 49 | -0.661001125 | LRRC14B | leucine rich repeat containing 14B [Source:HGNC Symbol;Acc:HGNC:37268] |
| ENSG00000185306 | HGLibA_05268 | 49 | -0.661001125 | C12orf56 | chromosome 12 open reading frame 56 [Source:HGNC Symbol;Acc:HGNC:26967] |
| ENSG00000185379 | HGLibA_40092 | 49 | -0.661001125 | RAD51D | RAD51 paralog D [Source:HGNC Symbol;Acc:HGNC:9823] |
| ENSG00000185862 | HGLibA_15690 | 49 | -0.661001125 | EVI2B | ecotropic viral integration site 2B [Source:HGNC Symbol;Acc:HGNC:3500] |
| ENSG00000185974 | HGLibA_20325 | 49 | -0.661001125 | GRK1 | G protein-coupled receptor kinase 1 [Source:HGNC Symbol;Acc:HGNC:10013] |
| ENSG00000187122 | HGLibA_45504 | 49 | -0.661001125 | SLIT1 | slit guidance ligand 1 [Source:HGNC Symbol;Acc:HGNC:11085] |
| ENSG00000188076 | HGLibA_42911 | 49 | -0.661001125 | SCGB1C1 | secretoglobin family 1C member 1 [Source:HGNC Symbol;Acc:HGNC:18394] |
| ENSG00000188655 | HGLibA_41422 | 49 | -0.661001125 | RNASE9 | ribonuclease A family member 9 (inactive) [Source:HGNC Symbol;Acc:HGNC:20673] |
| ENSG00000196437 | HGLibA_56270 | 49 | -0.661001125 | ZNF569 | zinc finger protein 569 [Source:HGNC Symbol;Acc:HGNC:24737] |
| ENSG00000196734 | HGLibA_26104 | 49 | -0.661001125 | LCE1B | late cornified envelope 1B [Source:HGNC Symbol;Acc:HGNC:16611] |
| ENSG00000196735 | HGLibA_21622 | 49 | -0.661001125 | HLA-DQA1 | DQ alpha 1 [Source:HGNC Symbol;Acc:HGNC:4942]" |
| ENSG00000197683 | HGLibA_25731 | 49 | -0.661001125 | KRTAP26-1 | keratin associated protein 26-1 [Source:HGNC Symbol;Acc:HGNC:33760] |
| ENSG00000198099 | HGLibA_00957 | 49 | -0.661001125 | ADH4 | pi polypeptide [Source:HGNC Symbol;Acc:HGNC:252]" |
| ENSG00000198483 | HGLibA_02083 | 49 | -0.661001125 | ANKRD35 | ankyrin repeat domain 35 [Source:HGNC Symbol;Acc:HGNC:26323] |
| ENSG00000198646 | HGLibA_31350 | 49 | -0.661001125 | NCOA6 | nuclear receptor coactivator 6 [Source:HGNC Symbol;Acc:HGNC:15936] |
| ENSG00000198829 | HGLibA_47704 | 49 | -0.661001125 | SUCNR1 | succinate receptor 1 [Source:HGNC Symbol;Acc:HGNC:4542] |
| ENSG00000203666 | HGLibA_14534 | 49 | -0.661001125 | EFCAB2 | EF-hand calcium binding domain 2 [Source:HGNC Symbol;Acc:HGNC:28166] |
| ENSG00000203933 | HGLibA_11961 | 49 | -0.661001125 | CXorf66 | chromosome X open reading frame 66 [Source:HGNC Symbol;Acc:HGNC:33743] |
| ENSG00000204070 | HGLibA_48043 | 49 | -0.661001125 | SYS1 | golgi trafficking protein [Source:HGNC Symbol;Acc:HGNC:16162]" |
| ENSG00000204228 | HGLibA_22201 | 49 | -0.661001125 | HSD17B8 | hydroxysteroid 17-beta dehydrogenase 8 [Source:HGNC Symbol;Acc:HGNC:3554] |
| ENSG00000204882 | HGLibA_19995 | 49 | -0.661001125 | GPR20 | G protein-coupled receptor 20 [Source:HGNC Symbol;Acc:HGNC:4475] |
| ENSG00000204889 | HGLibA_25506 | 49 | -0.661001125 | KRT40 | keratin 40 [Source:HGNC Symbol;Acc:HGNC:26707] |
| ENSG00000204919 | HGLibA_38799 | 49 | -0.661001125 | PRR20A | proline rich 20A [Source:HGNC Symbol;Acc:HGNC:24754] |
| ENSG00000205531 | HGLibA_31119 | 49 | -0.661001125 | NAP1L4 | nucleosome assembly protein 1 like 4 [Source:HGNC Symbol;Acc:HGNC:7640] |
| ENSG00000205835 | HGLibA_19416 | 49 | -0.661001125 | GMNC | geminin coiled-coil domain containing [Source:HGNC Symbol;Acc:HGNC:40049] |
| ENSG00000206106 | HGLibA_25719 | 49 | -0.661001125 | KRTAP22-2 | keratin associated protein 22-2 [Source:HGNC Symbol;Acc:HGNC:37091] |
| ENSG00000214336 | HGLibA_17883 | 49 | -0.661001125 | FOXI3 | forkhead box I3 [Source:HGNC Symbol;Acc:HGNC:35123] |
| ENSG00000214491 | HGLibA_43205 | 49 | -0.661001125 | SEC14L6 | SEC14 like lipid binding 6 [Source:HGNC Symbol;Acc:HGNC:40047] |
| ENSG00000215788 | HGLibA_50781 | 49 | -0.661001125 | TNFRSF25 | TNF receptor superfamily member 25 [Source:HGNC Symbol;Acc:HGNC:11910] |
| ENSG00000228567 | HGLibA_53705 | 49 | -0.661001125 | VN1R4 | vomeronasal 1 receptor 4 [Source:HGNC Symbol;Acc:HGNC:19871] |
| ENSG00000230430 | HGLibA_53258 | 49 | -0.661001125 | USP17L25 | ubiquitin specific peptidase 17-like family member 25 [Source:HGNC Symbol;Acc:HGNC:44452] |
| ENSG00000233816 | HGLibA_22663 | 49 | -0.661001125 | IFNA13 | interferon alpha 13 [Source:HGNC Symbol;Acc:HGNC:5419] |
| ENSG00000235268 | HGLibA_24519 | 49 | -0.661001125 | KDM4E | lysine demethylase 4E [Source:HGNC Symbol;Acc:HGNC:37098] |
| ENSG00000236737 | HGLibA_18437 | 49 | -0.661001125 | GAGE12B | G antigen 12B [Source:HGNC Symbol;Acc:HGNC:26779] |
| ENSG00000243927 | HGLibA_30107 | 49 | -0.661001125 | MRPS6 | mitochondrial ribosomal protein S6 [Source:HGNC Symbol;Acc:HGNC:14051] |
| ENSG00000248487 | HGLibA_00249 | 49 | -0.661001125 | ABHD14A | abhydrolase domain containing 14A [Source:HGNC Symbol;Acc:HGNC:24538] |
| ENSG00000251258 | HGLibA_40943 | 49 | -0.661001125 | RFPL4B | ret finger protein like 4B [Source:HGNC Symbol;Acc:HGNC:33264] |
| ENSG00000253506 | HGLibA_31032 | 49 | -0.661001125 | NACA2 | nascent polypeptide associated complex subunit alpha 2 [Source:HGNC Symbol;Acc:HGNC:23290] |
| ENSG00000256771 | HGLibA_55672 | 49 | -0.661001125 | ZNF253 | zinc finger protein 253 [Source:HGNC Symbol;Acc:HGNC:13497] |
| ENSG00000271447 | HGLibA_29531 | 49 | -0.661001125 | MMP28 | matrix metallopeptidase 28 [Source:HGNC Symbol;Acc:HGNC:14366] |
| ENSG00000273777 | HGLibA_08931 | 49 | -0.661001125 | CEACAM20 | carcinoembryonic antigen related cell adhesion molecule 20 [Source:HGNC Symbol;Acc:HGNC:24879] |
| FAM115C | HGLibA_16039 | 49 | -0.661001125 | FAM115C | - |
| FAM160A2 | HGLibA_16207 | 49 | -0.661001125 | FAM160A2 | - |
| FAM46C | HGLibA_16603 | 49 | -0.661001125 | FAM46C | - |
| GPER | HGLibA_19791 | 49 | -0.661001125 | GPER | - |
| H1F0 | HGLibA_20733 | 49 | -0.661001125 | H1F0 | - |
| HIST1H3A | HGLibA_21455 | 49 | -0.661001125 | HIST1H3A | - |
| HIST1H4K | HGLibA_21516 | 49 | -0.661001125 | HIST1H4K | - |
| hsa-mir-1256 | HGLibA_57430 | 49 | -0.661001125 | hsa-mir-1256 | - |
| hsa-mir-1298 | HGLibA_57670 | 49 | -0.661001125 | hsa-mir-1298 | - |
| hsa-mir-182 | HGLibA_57981 | 49 | -0.661001125 | hsa-mir-182 | - |
| hsa-mir-22 | HGLibA_58289 | 49 | -0.661001125 | hsa-mir-22 | - |
| hsa-mir-301b | HGLibA_58432 | 49 | -0.661001125 | hsa-mir-301b | - |
| hsa-mir-30c-2 | HGLibA_58482 | 49 | -0.661001125 | hsa-mir-30c-2 | - |
| hsa-mir-3181 | HGLibA_58823 | 49 | -0.661001125 | hsa-mir-3181 | - |
| hsa-mir-3651 | HGLibA_59148 | 49 | -0.661001125 | hsa-mir-3651 | - |
| hsa-mir-3670-1 | HGLibA_59234 | 49 | -0.661001125 | hsa-mir-3670-1 | - |
| hsa-mir-3681 | HGLibA_59276 | 49 | -0.661001125 | hsa-mir-3681 | - |
| hsa-mir-3685 | HGLibA_59292 | 49 | -0.661001125 | hsa-mir-3685 | - |
| hsa-mir-3688-1 | HGLibA_59305 | 49 | -0.661001125 | hsa-mir-3688-1 | - |
| hsa-mir-369 | HGLibA_59342 | 49 | -0.661001125 | hsa-mir-369 | - |
| hsa-mir-371a | HGLibA_59375 | 49 | -0.661001125 | hsa-mir-371a | - |
| hsa-mir-3919 | HGLibA_59551 | 49 | -0.661001125 | hsa-mir-3919 | - |
| hsa-mir-433 | HGLibA_60042 | 49 | -0.661001125 | hsa-mir-433 | - |
| hsa-mir-4427 | HGLibA_60096 | 49 | -0.661001125 | hsa-mir-4427 | - |
| hsa-mir-4458 | HGLibA_60237 | 49 | -0.661001125 | hsa-mir-4458 | - |
| hsa-mir-4524a | HGLibA_60554 | 49 | -0.661001125 | hsa-mir-4524a | - |
| hsa-mir-4641 | HGLibA_60669 | 49 | -0.661001125 | hsa-mir-4641 | - |
| hsa-mir-4684 | HGLibA_60847 | 49 | -0.661001125 | hsa-mir-4684 | - |

| | | | | | |
|---|---|---|---|---|---|
| hsa-mir-4695 | HGLibA_60888 | 49 | -0.661001125 | hsa-mir-4695 | - |
| hsa-mir-4720 | HGLibA_60976 | 49 | -0.661001125 | hsa-mir-4720 | - |
| hsa-mir-4758 | HGLibA_61129 | 49 | -0.661001125 | hsa-mir-4758 | - |
| hsa-mir-4787 | HGLibA_61257 | 49 | -0.661001125 | hsa-mir-4787 | - |
| hsa-mir-5196 | HGLibA_61703 | 49 | -0.661001125 | hsa-mir-5196 | - |
| hsa-mir-520c | HGLibA_61742 | 49 | -0.661001125 | hsa-mir-520c | - |
| hsa-mir-524 | HGLibA_61783 | 49 | -0.661001125 | hsa-mir-524 | - |
| hsa-mir-548ap | HGLibA_61914 | 49 | -0.661001125 | hsa-mir-548ap | - |
| hsa-mir-548av | HGLibA_61932 | 49 | -0.661001125 | hsa-mir-548av | - |
| hsa-mir-548av | HGLibA_61931 | 49 | -0.661001125 | hsa-mir-548av | - |
| hsa-mir-548f-2 | HGLibA_61978 | 49 | -0.661001125 | hsa-mir-548f-2 | - |
| hsa-mir-5682 | HGLibA_62248 | 49 | -0.661001125 | hsa-mir-5682 | - |
| hsa-mir-601 | HGLibA_62497 | 49 | -0.661001125 | hsa-mir-601 | - |
| hsa-mir-6125 | HGLibA_62648 | 49 | -0.661001125 | hsa-mir-6125 | - |
| hsa-mir-654 | HGLibA_62949 | 49 | -0.661001125 | hsa-mir-654 | - |
| hsa-mir-6754 | HGLibA_63173 | 49 | -0.661001125 | hsa-mir-6754 | - |
| hsa-mir-6804 | HGLibA_63394 | 49 | -0.661001125 | hsa-mir-6804 | - |
| LOC283710 | HGLibA_26911 | 49 | -0.661001125 | LOC283710 | - |
| MST4 | HGLibA_30262 | 49 | -0.661001125 | MST4 | - |
| NUPL2 | HGLibA_33084 | 49 | -0.661001125 | NUPL2 | - |
| P2RY8_Y | HGLibA_34896 | 49 | -0.661001125 | P2RY8_Y | - |
| RFWD2 | HGLibA_40957 | 49 | -0.661001125 | RFWD2 | - |
| 2-Sep | HGLibA_43437 | 49 | -0.661001125 | 2-Sep | - |
| WRB | HGLibA_54407 | 49 | -0.661001125 | WRB | - |
| ZFYVE20 | HGLibA_55279 | 49 | -0.661001125 | ZFYVE20 | - |
| AMICA1 | HGLibA_01790 | 48 | -0.670952162 | AMICA1 | - |
| ANKRD32 | HGLibA_02065 | 48 | -0.670952162 | ANKRD32 | - |
| APITD1-CORT | HGLibA_02429 | 48 | -0.670952162 | APITD1-CORT | - |
| C19orf40 | HGLibA_05657 | 48 | -0.670952162 | C19orf40 | - |
| CCDC94 | HGLibA_07935 | 48 | -0.670952162 | CCDC94 | - |
| ENSG00000013503 | HGLibA_37612 | 48 | -0.670952162 | POLR3B | RNA polymerase III subunit B [Source:HGNC Symbol;Acc:HGNC:30348] |
| ENSG00000016391 | HGLibA_09416 | 48 | -0.670952162 | CHDH | choline dehydrogenase [Source:HGNC Symbol;Acc:HGNC:24288] |
| ENSG00000023839 | HGLibA_00171 | 48 | -0.670952162 | ABCC2 | ATP binding cassette subfamily C member 2 [Source:HGNC Symbol;Acc:HGNC:53] |
| ENSG00000030110 | HGLibA_04134 | 48 | -0.670952162 | BAK1 | BCL2 antagonist/killer 1 [Source:HGNC Symbol;Acc:HGNC:949] |
| ENSG00000054611 | HGLibA_48504 | 48 | -0.670952162 | TBC1D22A | TBC1 domain family member 22A [Source:HGNC Symbol;Acc:HGNC:1309] |
| ENSG00000054938 | HGLibA_09540 | 48 | -0.670952162 | CHRDL2 | chordin like 2 [Source:HGNC Symbol;Acc:HGNC:24168] |
| ENSG00000061455 | HGLibA_38395 | 48 | -0.670952162 | PRDM6 | PR/SET domain 6 [Source:HGNC Symbol;Acc:HGNC:9350] |
| ENSG00000068097 | HGLibA_21081 | 48 | -0.670952162 | HEATR6 | HEAT repeat containing 6 [Source:HGNC Symbol;Acc:HGNC:24076] |
| ENSG00000071082 | HGLibA_41937 | 48 | -0.670952162 | RPL31 | ribosomal protein L31 [Source:HGNC Symbol;Acc:HGNC:10334] |
| ENSG00000072518 | HGLibA_28368 | 48 | -0.670952162 | MARK2 | microtubule affinity regulating kinase 2 [Source:HGNC Symbol;Acc:HGNC:3332] |
| ENSG00000075461 | HGLibA_06939 | 48 | -0.670952162 | CACNG4 | calcium voltage-gated channel auxiliary subunit gamma 4 [Source:HGNC Symbol;Acc:HGNC:1408] |
| ENSG00000080819 | HGLibA_10982 | 48 | -0.670952162 | CPOX | coproporphyrinogen oxidase [Source:HGNC Symbol;Acc:HGNC:2321] |
| ENSG00000081148 | HGLibA_23264 | 48 | -0.670952162 | IMPG2 | interphotoreceptor matrix proteoglycan 2 [Source:HGNC Symbol;Acc:HGNC:18362] |
| ENSG00000082515 | HGLibA_29929 | 48 | -0.670952162 | MRPL22 | mitochondrial ribosomal protein L22 [Source:HGNC Symbol;Acc:HGNC:14480] |
| ENSG00000087074 | HGLibA_38047 | 48 | -0.670952162 | PPP1R15A | protein phosphatase 1 regulatory subunit 15A [Source:HGNC Symbol;Acc:HGNC:14375] |
| ENSG00000088766 | HGLibA_11198 | 48 | -0.670952162 | CRLS1 | cardiolipin synthase 1 [Source:HGNC Symbol;Acc:HGNC:16148] |
| ENSG00000092439 | HGLibA_51770 | 48 | -0.670952162 | TRPM7 | transient receptor potential cation channel subfamily M member 7 [Source:HGNC Symbol;Acc:HGNC:17994] |
| ENSG00000092978 | HGLibA_19737 | 48 | -0.670952162 | GPATCH2 | G-patch domain containing 2 [Source:HGNC Symbol;Acc:HGNC:25499] |
| ENSG00000100578 | HGLibA_24646 | 48 | -0.670952162 | KIAA0586 | KIAA0586 [Source:HGNC Symbol;Acc:HGNC:19960] |
| ENSG00000101079 | HGLibA_31420 | 48 | -0.670952162 | NDRG3 | NDRG family member 3 [Source:HGNC Symbol;Acc:HGNC:14462] |
| ENSG00000101082 | HGLibA_44304 | 48 | -0.670952162 | SLA2 | Src like adaptor 2 [Source:HGNC Symbol;Acc:HGNC:17329] |
| ENSG00000101266 | HGLibA_11401 | 48 | -0.670952162 | CSNK2A1 | casein kinase 2 alpha 1 [Source:HGNC Symbol;Acc:HGNC:2457] |
| ENSG00000101363 | HGLibA_28089 | 48 | -0.670952162 | MANBAL | mannosidase beta like [Source:HGNC Symbol;Acc:HGNC:15799] |
| ENSG00000102595 | HGLibA_52871 | 48 | -0.670952162 | UGGT2 | UDP-glucose glycoprotein glucosyltransferase 2 [Source:HGNC Symbol;Acc:HGNC:15664] |
| ENSG00000103199 | HGLibA_56108 | 48 | -0.670952162 | ZNF500 | zinc finger protein 500 [Source:HGNC Symbol;Acc:HGNC:23716] |
| ENSG00000104731 | HGLibA_25101 | 48 | -0.670952162 | KLHDC4 | kelch domain containing 4 [Source:HGNC Symbol;Acc:HGNC:25272] |
| ENSG00000104941 | HGLibA_42338 | 48 | -0.670952162 | RSPH6A | radial spoke head 6 homolog A [Source:HGNC Symbol;Acc:HGNC:14241] |
| ENSG00000105143 | HGLibA_44521 | 48 | -0.670952162 | SLC1A6 | solute carrier family 1 member 6 [Source:HGNC Symbol;Acc:HGNC:10944] |
| ENSG00000105221 | HGLibA_01511 | 48 | -0.670952162 | AKT2 | AKT serine/threonine kinase 2 [Source:HGNC Symbol;Acc:HGNC:392] |
| ENSG00000105255 | HGLibA_18078 | 48 | -0.670952162 | FSD1 | fibronectin type III and SPRY domain containing 1 [Source:HGNC Symbol;Acc:HGNC:13745] |
| ENSG00000105393 | HGLibA_04055 | 48 | -0.670952162 | BABAM1 | BRISC and BRCA1 A complex member 1 [Source:HGNC Symbol;Acc:HGNC:25008] |
| ENSG00000105675 | HGLibA_03632 | 48 | -0.670952162 | ATP4A | ATPase H+/K+ transporting subunit alpha [Source:HGNC Symbol;Acc:HGNC:819] |
| ENSG00000105963 | HGLibA_00863 | 48 | -0.670952162 | ADAP1 | ArfGAP with dual PH domains 1 [Source:HGNC Symbol;Acc:HGNC:16486] |
| ENSG00000107679 | HGLibA_37061 | 48 | -0.670952162 | PLEKHA1 | pleckstrin homology domain containing A1 [Source:HGNC Symbol;Acc:HGNC:14335] |
| ENSG00000107779 | HGLibA_04629 | 48 | -0.670952162 | BMPR1A | bone morphogenetic protein receptor type 1A [Source:HGNC Symbol;Acc:HGNC:1076] |
| ENSG00000108242 | HGLibA_12115 | 48 | -0.670952162 | CYP2C18 | cytochrome P450 family 2 subfamily C member 18 [Source:HGNC Symbol;Acc:HGNC:2620] |
| ENSG00000109471 | HGLibA_23070 | 48 | -0.670952162 | IL2 | interleukin 2 [Source:HGNC Symbol;Acc:HGNC:6001] |
| ENSG00000110042 | HGLibA_14092 | 48 | -0.670952162 | DTX4 | deltex E3 ubiquitin ligase 4 [Source:HGNC Symbol;Acc:HGNC:29151] |
| ENSG00000112078 | HGLibA_24448 | 48 | -0.670952162 | KCTD20 | potassium channel tetramerization domain containing 20 [Source:HGNC Symbol;Acc:HGNC:21052] |
| ENSG00000112304 | HGLibA_00476 | 48 | -0.670952162 | ACOT13 | acyl-CoA thioesterase 13 [Source:HGNC Symbol;Acc:HGNC:20999] |
| ENSG00000112365 | HGLibA_54846 | 48 | -0.670952162 | ZBTB24 | zinc finger and BTB domain containing 24 [Source:HGNC Symbol;Acc:HGNC:21143] |
| ENSG00000112514 | HGLibA_11797 | 48 | -0.670952162 | CUTA | cutA divalent cation tolerance homolog [Source:HGNC Symbol;Acc:HGNC:21101] |
| ENSG00000112874 | HGLibA_32939 | 48 | -0.670952162 | NUDT12 | nudix hydrolase 12 [Source:HGNC Symbol;Acc:HGNC:18826] |
| ENSG00000112996 | HGLibA_30085 | 48 | -0.670952162 | MRPS30 | mitochondrial ribosomal protein S30 [Source:HGNC Symbol;Acc:HGNC:8769] |
| ENSG00000113583 | HGLibA_06359 | 48 | -0.670952162 | C5orf15 | chromosome 5 open reading frame 15 [Source:HGNC Symbol;Acc:HGNC:20656] |
| ENSG00000114739 | HGLibA_00699 | 48 | -0.670952162 | ACVR2B | activin A receptor type 2B [Source:HGNC Symbol;Acc:HGNC:174] |
| ENSG00000115041 | HGLibA_24239 | 48 | -0.670952162 | KCNIP3 | potassium voltage-gated channel interacting protein 3 [Source:HGNC Symbol;Acc:HGNC:15523] |
| ENSG00000116133 | HGLibA_13091 | 48 | -0.670952162 | DHCR24 | 24-dehydrocholesterol reductase [Source:HGNC Symbol;Acc:HGNC:2859] |
| ENSG00000117122 | HGLibA_29043 | 48 | -0.670952162 | MFAP2 | microfibril associated protein 2 [Source:HGNC Symbol;Acc:HGNC:7033] |
| ENSG00000117983 | HGLibA_30549 | 48 | -0.670952162 | MUC5B | oligomeric mucus/gel-forming [Source:HGNC Symbol;Acc:HGNC:7516]" |
| ENSG00000118292 | HGLibA_05874 | 48 | -0.670952162 | C1orf54 | chromosome 1 open reading frame 54 [Source:HGNC Symbol;Acc:HGNC:26258] |
| ENSG00000118729 | HGLibA_07338 | 48 | -0.670952162 | CASQ2 | calsequestrin 2 [Source:HGNC Symbol;Acc:HGNC:1513] |
| ENSG00000119737 | HGLibA_20080 | 48 | -0.670952162 | GPR75 | G protein-coupled receptor 75 [Source:HGNC Symbol;Acc:HGNC:4526] |
| ENSG00000119760 | HGLibA_47825 | 48 | -0.670952162 | SUPT7L | STAGA complex gamma subunit [Source:HGNC Symbol;Acc:HGNC:30632]" |
| ENSG00000119938 | HGLibA_38104 | 48 | -0.670952162 | PPP1R3C | protein phosphatase 1 regulatory subunit 3C [Source:HGNC Symbol;Acc:HGNC:9293] |
| ENSG00000120549 | HGLibA_24720 | 48 | -0.670952162 | KIAA1217 | KIAA1217 [Source:HGNC Symbol;Acc:HGNC:25428] |
| ENSG00000120563 | HGLibA_27786 | 48 | -0.670952162 | LYZL1 | lysozyme like 1 [Source:HGNC Symbol;Acc:HGNC:30502] |
| ENSG00000120925 | HGLibA_41573 | 48 | -0.670952162 | RNF170 | ring finger protein 170 [Source:HGNC Symbol;Acc:HGNC:25358] |
| ENSG00000122140 | HGLibA_30058 | 48 | -0.670952162 | MRPS2 | mitochondrial ribosomal protein S2 [Source:HGNC Symbol;Acc:HGNC:14495] |
| ENSG00000123500 | HGLibA_10498 | 48 | -0.670952162 | COL10A1 | collagen type X alpha 1 chain [Source:HGNC Symbol;Acc:HGNC:2185] |
| ENSG00000124207 | HGLibA_11318 | 48 | -0.670952162 | CSE1L | chromosome segregation 1 like [Source:HGNC Symbol;Acc:HGNC:2431] |
| ENSG00000124657 | HGLibA_33773 | 48 | -0.670952162 | OR2B6 | olfactory receptor family 2 subfamily B member 6 [Source:HGNC Symbol;Acc:HGNC:8241] |
| ENSG00000124939 | HGLibA_42922 | 48 | -0.670952162 | SCGB2A1 | secretoglobin family 2A member 1 [Source:HGNC Symbol;Acc:HGNC:7051] |
| ENSG00000125485 | HGLibA_12692 | 48 | -0.670952162 | DDX31 | DEAD-box helicase 31 [Source:HGNC Symbol;Acc:HGNC:16715] |
| ENSG00000125703 | HGLibA_03493 | 48 | -0.670952162 | ATG4C | autophagy related 4C cysteine peptidase [Source:HGNC Symbol;Acc:HGNC:16040] |
| ENSG00000125821 | HGLibA_14053 | 48 | -0.670952162 | DTD1 | D-tyrosyl-tRNA deacylase 1 [Source:HGNC Symbol;Acc:HGNC:16219] |
| ENSG00000128309 | HGLibA_29781 | 48 | -0.670952162 | MPST | mercaptopyruvate sulfurtransferase [Source:HGNC Symbol;Acc:HGNC:7223] |
| ENSG00000128383 | HGLibA_02473 | 48 | -0.670952162 | APOBEC3A | apolipoprotein B mRNA editing enzyme catalytic subunit 3A [Source:HGNC Symbol;Acc:HGNC:17343] |
| ENSG00000129235 | HGLibA_52418 | 48 | -0.670952162 | TXNDC17 | thioredoxin domain containing 17 [Source:HGNC Symbol;Acc:HGNC:28218] |
| ENSG00000129480 | HGLibA_14055 | 48 | -0.670952162 | DTD2 | D-tyrosyl-tRNA deacylase 2 (putative) [Source:HGNC Symbol;Acc:HGNC:20277] |
| ENSG00000130348 | HGLibA_39747 | 48 | -0.670952162 | QRSL1 | glutaminyl-tRNA amidotransferase subunit A [Source:HGNC Symbol;Acc:HGNC:21020]" |
| ENSG00000130711 | HGLibA_38370 | 48 | -0.670952162 | PRDM12 | PR/SET domain 12 [Source:HGNC Symbol;Acc:HGNC:13997] |
| ENSG00000131378 | HGLibA_40950 | 48 | -0.670952162 | RFTN1 | lipid raft linker 1 [Source:HGNC Symbol;Acc:HGNC:30278]" |
| ENSG00000131711 | HGLibA_28119 | 48 | -0.670952162 | MAP1B | microtubule associated protein 1B [Source:HGNC Symbol;Acc:HGNC:6836] |
| ENSG00000132932 | HGLibA_03777 | 48 | -0.670952162 | ATP8A2 | ATPase phospholipid transporting 8A2 [Source:HGNC Symbol;Acc:HGNC:13533] |
| ENSG00000134242 | HGLibA_39459 | 48 | -0.670952162 | PTPN22 | non-receptor type 22 [Source:HGNC Symbol;Acc:HGNC:9652]" |
| ENSG00000134285 | HGLibA_17556 | 48 | -0.670952162 | FKBP11 | FK506 binding protein 11 [Source:HGNC Symbol;Acc:HGNC:18624] |
| ENSG00000135094 | HGLibA_43172 | 48 | -0.670952162 | SDS | serine dehydratase [Source:HGNC Symbol;Acc:HGNC:10691] |
| ENSG00000135404 | HGLibA_08427 | 48 | -0.670952162 | CD63 | CD63 molecule [Source:HGNC Symbol;Acc:HGNC:1692] |
| ENSG00000136754 | HGLibA_00289 | 48 | -0.670952162 | ABI1 | abl interactor 1 [Source:HGNC Symbol;Acc:HGNC:11320] |
| ENSG00000136840 | HGLibA_47272 | 48 | -0.670952162 | ST6GALNAC4 | 6-sialyltransferase 4 [Source:HGNC Symbol;Acc:HGNC:17846]" |
| ENSG00000136883 | HGLibA_24852 | 48 | -0.670952162 | KIF12 | kinesin family member 12 [Source:HGNC Symbol;Acc:HGNC:21495] |
| ENSG00000137124 | HGLibA_01550 | 48 | -0.670952162 | ALDH1B1 | aldehyde dehydrogenase 1 family member B1 [Source:HGNC Symbol;Acc:HGNC:407] |
| ENSG00000137843 | HGLibA_35053 | 48 | -0.670952162 | PAK6 | p21 (RAC1) activated kinase 6 [Source:HGNC Symbol;Acc:HGNC:16061] |
| ENSG00000138031 | HGLibA_00914 | 48 | -0.670952162 | ADCY3 | adenylate cyclase 3 [Source:HGNC Symbol;Acc:HGNC:234] |
| ENSG00000138231 | HGLibA_12404 | 48 | -0.670952162 | DBR1 | debranching RNA lariats 1 [Source:HGNC Symbol;Acc:HGNC:15594] |
| ENSG00000138686 | HGLibA_04223 | 48 | -0.670952162 | BBS7 | Bardet-Biedl syndrome 7 [Source:HGNC Symbol;Acc:HGNC:18758] |
| ENSG00000138709 | HGLibA_26031 | 48 | -0.670952162 | LARP1B | La ribonucleoprotein domain family member 1B [Source:HGNC Symbol;Acc:HGNC:24704] |
| ENSG00000138764 | HGLibA_08096 | 48 | -0.670952162 | CCNG2 | cyclin G2 [Source:HGNC Symbol;Acc:HGNC:1593] |
| ENSG00000139645 | HGLibA_02116 | 48 | -0.670952162 | ANKRD52 | ankyrin repeat domain 52 [Source:HGNC Symbol;Acc:HGNC:26614] |
| ENSG00000139767 | HGLibA_47052 | 48 | -0.670952162 | SRRM4 | serine/arginine repetitive matrix 4 [Source:HGNC Symbol;Acc:HGNC:29389] |
| ENSG00000140067 | HGLibA_16318 | 48 | -0.670952162 | FAM181A | family with sequence similarity 181 member A [Source:HGNC Symbol;Acc:HGNC:20491] |
| ENSG00000140157 | HGLibA_31953 | 48 | -0.670952162 | NIPA2 | NIPA magnesium transporter 2 [Source:HGNC Symbol;Acc:HGNC:17044] |
| ENSG00000140332 | HGLibA_49614 | 48 | -0.670952162 | TLE3 | transducin like enhancer of split 3 [Source:HGNC Symbol;Acc:HGNC:11839] |
| ENSG00000140395 | HGLibA_54141 | 48 | -0.670952162 | WDR61 | WD repeat domain 61 [Source:HGNC Symbol;Acc:HGNC:30300] |
| ENSG00000142511 | HGLibA_20019 | 48 | -0.670952162 | GPR32 | G protein-coupled receptor 32 [Source:HGNC Symbol;Acc:HGNC:4487] |
| ENSG00000142655 | HGLibA_36098 | 48 | -0.670952162 | PEX14 | peroxisomal biogenesis factor 14 [Source:HGNC Symbol;Acc:HGNC:8856] |
| ENSG00000142892 | HGLibA_36562 | 48 | -0.670952162 | PIGK | phosphatidylinositol glycan anchor biosynthesis class K [Source:HGNC Symbol;Acc:HGNC:8965] |
| ENSG00000143578 | HGLibA_11101 | 48 | -0.670952162 | CREB3L4 | cAMP responsive element binding protein 3 like 4 [Source:HGNC Symbol;Acc:HGNC:18854] |
| ENSG00000143614 | HGLibA_18690 | 48 | -0.670952162 | GATAD2B | GATA zinc finger domain containing 2B [Source:HGNC Symbol;Acc:HGNC:30778] |
| ENSG00000143751 | HGLibA_43120 | 48 | -0.670952162 | SDE2 | SDE2 telomere maintenance homolog [Source:HGNC Symbol;Acc:HGNC:26643] |
| ENSG00000143952 | HGLibA_53812 | 48 | -0.670952162 | VPS54 | GARP complex subunit [Source:HGNC Symbol;Acc:HGNC:18652]" |
| ENSG00000144837 | HGLibA_36864 | 48 | -0.670952162 | PLA1A | phospholipase A1 member A [Source:HGNC Symbol;Acc:HGNC:17661] |
| ENSG00000145632 | HGLibA_37192 | 48 | -0.670952162 | PLK2 | polo like kinase 2 [Source:HGNC Symbol;Acc:HGNC:19699] |
| ENSG00000146469 | HGLibA_53663 | 48 | -0.670952162 | VIP | vasoactive intestinal peptide [Source:HGNC Symbol;Acc:HGNC:12693] |

| | | | | | |
|---|---|---|---|---|---|
| ENSG00000146707 | HGLibA_37675 | 48 | -0.670952162 | POMZP3 | POM121 and ZP3 fusion [Source:HGNC Symbol;Acc:HGNC:9203] |
| ENSG00000147408 | HGLibA_11345 | 48 | -0.670952162 | CSGALNACT1 | chondroitin sulfate N-acetylgalactosaminyltransferase 1 [Source:HGNC Symbol;Acc:HGNC:24290] |
| ENSG00000148204 | HGLibA_11072 | 48 | -0.670952162 | CRB2 | cell polarity complex component [Source:HGNC Symbol;Acc:HGNC:18688]" |
| ENSG00000148450 | HGLibA_30245 | 48 | -0.670952162 | MSRB2 | methionine sulfoxide reductase B2 [Source:HGNC Symbol;Acc:HGNC:17061] |
| ENSG00000149289 | HGLibA_54959 | 48 | -0.670952162 | ZC3H12C | zinc finger CCCH-type containing 12C [Source:HGNC Symbol;Acc:HGNC:29362] |
| ENSG00000150593 | HGLibA_35732 | 48 | -0.670952162 | PDCD4 | programmed cell death 4 [Source:HGNC Symbol;Acc:HGNC:8763] |
| ENSG00000150630 | HGLibA_53616 | 48 | -0.670952162 | VEGFC | vascular endothelial growth factor C [Source:HGNC Symbol;Acc:HGNC:12682] |
| ENSG00000151690 | HGLibA_29107 | 48 | -0.670952162 | MFSD6 | major facilitator superfamily domain containing 6 [Source:HGNC Symbol;Acc:HGNC:24711] |
| ENSG00000151773 | HGLibA_07571 | 48 | -0.670952162 | CCDC122 | coiled-coil domain containing 122 [Source:HGNC Symbol;Acc:HGNC:26478] |
| ENSG00000152253 | HGLibA_46554 | 48 | -0.670952162 | SPC25 | NDC80 kinetochore complex component [Source:HGNC Symbol;Acc:HGNC:24031]" |
| ENSG00000154016 | HGLibA_20211 | 48 | -0.670952162 | GRAP | GRB2 related adaptor protein [Source:HGNC Symbol;Acc:HGNC:4562] |
| ENSG00000154370 | HGLibA_51406 | 48 | -0.670952162 | TRIM11 | tripartite motif containing 11 [Source:HGNC Symbol;Acc:HGNC:16281] |
| ENSG00000155097 | HGLibA_03739 | 48 | -0.670952162 | ATP6V1C1 | ATPase H+ transporting V1 subunit C1 [Source:HGNC Symbol;Acc:HGNC:856] |
| ENSG00000155363 | HGLibA_29693 | 48 | -0.670952162 | MOV10 | Mov10 RISC complex RNA helicase [Source:HGNC Symbol;Acc:HGNC:7200] |
| ENSG00000155380 | HGLibA_44418 | 48 | -0.670952162 | SLC16A1 | solute carrier family 16 member 1 [Source:HGNC Symbol;Acc:HGNC:10922] |
| ENSG00000156395 | HGLibA_46189 | 48 | -0.670952162 | SORCS3 | sortilin related VPS10 domain containing receptor 3 [Source:HGNC Symbol;Acc:HGNC:16699] |
| ENSG00000156931 | HGLibA_53819 | 48 | -0.670952162 | VPS8 | CORVET complex subunit [Source:HGNC Symbol;Acc:HGNC:29122]" |
| ENSG00000158352 | HGLibA_44096 | 48 | -0.670952162 | SHROOM4 | shroom family member 4 [Source:HGNC Symbol;Acc:HGNC:29215] |
| ENSG00000159387 | HGLibA_23642 | 48 | -0.670952162 | IRX6 | iroquois homeobox 6 [Source:HGNC Symbol;Acc:HGNC:14675] |
| ENSG00000159496 | HGLibA_41011 | 48 | -0.670952162 | RGL4 | ral guanine nucleotide dissociation stimulator like 4 [Source:HGNC Symbol;Acc:HGNC:31911] |
| ENSG00000159915 | HGLibA_55642 | 48 | -0.670952162 | ZNF233 | zinc finger protein 233 [Source:HGNC Symbol;Acc:HGNC:30946] |
| ENSG00000160113 | HGLibA_32615 | 48 | -0.670952162 | NR2F6 | nuclear receptor subfamily 2 group F member 6 [Source:HGNC Symbol;Acc:HGNC:7977] |
| ENSG00000161036 | HGLibA_27509 | 48 | -0.670952162 | LRWD1 | leucine rich repeats and WD repeat domain containing 1 [Source:HGNC Symbol;Acc:HGNC:21769] |
| ENSG00000162365 | HGLibA_12175 | 48 | -0.670952162 | CYP4A22 | cytochrome P450 family 4 subfamily A member 22 [Source:HGNC Symbol;Acc:HGNC:20575] |
| ENSG00000162909 | HGLibA_07163 | 48 | -0.670952162 | CAPN2 | calpain 2 [Source:HGNC Symbol;Acc:HGNC:1479] |
| ENSG00000163518 | HGLibA_17234 | 48 | -0.670952162 | FCRL4 | Fc receptor like 4 [Source:HGNC Symbol;Acc:HGNC:18507] |
| ENSG00000163599 | HGLibA_11634 | 48 | -0.670952162 | CTLA4 | cytotoxic T-lymphocyte associated protein 4 [Source:HGNC Symbol;Acc:HGNC:2505] |
| ENSG00000163807 | HGLibA_24696 | 48 | -0.670952162 | KIAA1143 | KIAA1143 [Source:HGNC Symbol;Acc:HGNC:29198] |
| ENSG00000163848 | HGLibA_55497 | 48 | -0.670952162 | ZNF148 | zinc finger protein 148 [Source:HGNC Symbol;Acc:HGNC:12933] |
| ENSG00000163931 | HGLibA_49586 | 48 | -0.670952162 | TKT | transketolase [Source:HGNC Symbol;Acc:HGNC:11834] |
| ENSG00000164123 | HGLibA_06331 | 48 | -0.670952162 | C4orf45 | chromosome 4 open reading frame 45 [Source:HGNC Symbol;Acc:HGNC:26342] |
| ENSG00000164326 | HGLibA_07265 | 48 | -0.670952162 | CARTPT | CART prepropeptide [Source:HGNC Symbol;Acc:HGNC:24323] |
| ENSG00000164924 | HGLibA_54755 | 48 | -0.670952162 | YWHAZ | tyrosine 3-monooxygenase/tryptophan 5-monooxygenase activation protein zeta [Source:HGNC Symbol;Acc:HGNC:12855] |
| ENSG00000165046 | HGLibA_26335 | 48 | -0.670952162 | LETM2 | leucine zipper and EF-hand containing transmembrane protein 2 [Source:HGNC Symbol;Acc:HGNC:14648] |
| ENSG00000165584 | HGLibA_47206 | 48 | -0.670952162 | SSX3 | SSX family member 3 [Source:HGNC Symbol;Acc:HGNC:11337] |
| ENSG00000165644 | HGLibA_10688 | 48 | -0.670952162 | COMTD1 | catechol-O-methyltransferase domain containing 1 [Source:HGNC Symbol;Acc:HGNC:26309] |
| ENSG00000165695 | HGLibA_01397 | 48 | -0.670952162 | AK8 | adenylate kinase 8 [Source:HGNC Symbol;Acc:HGNC:26526] |
| ENSG00000166135 | HGLibA_21300 | 48 | -0.670952162 | HIF1AN | hypoxia inducible factor 1 subunit alpha inhibitor [Source:HGNC Symbol;Acc:HGNC:17113] |
| ENSG00000167195 | HGLibA_19614 | 48 | -0.670952162 | GOLGA6C | golgin A6 family member C [Source:HGNC Symbol;Acc:HGNC:32206] |
| ENSG00000167261 | HGLibA_13820 | 48 | -0.670952162 | DPEP2 | dipeptidase 2 [Source:HGNC Symbol;Acc:HGNC:23028] |
| ENSG00000167535 | HGLibA_06922 | 48 | -0.670952162 | CACNB3 | calcium voltage-gated channel auxiliary subunit beta 3 [Source:HGNC Symbol;Acc:HGNC:1403] |
| ENSG00000167562 | HGLibA_56577 | 48 | -0.670952162 | ZNF701 | zinc finger protein 701 [Source:HGNC Symbol;Acc:HGNC:25597] |
| ENSG00000167618 | HGLibA_25936 | 48 | -0.670952162 | LAIR2 | leukocyte associated immunoglobulin like receptor 2 [Source:HGNC Symbol;Acc:HGNC:6478] |
| ENSG00000167778 | HGLibA_46849 | 48 | -0.670952162 | SPRYD3 | SPRY domain containing 3 [Source:HGNC Symbol;Acc:HGNC:25920] |
| ENSG00000168010 | HGLibA_03475 | 48 | -0.670952162 | ATG16L2 | autophagy related 16 like 2 [Source:HGNC Symbol;Acc:HGNC:25464] |
| ENSG00000168434 | HGLibA_10487 | 48 | -0.670952162 | COG7 | component of oligomeric golgi complex 7 [Source:HGNC Symbol;Acc:HGNC:18622] |
| ENSG00000168724 | HGLibA_13597 | 48 | -0.670952162 | DNAJC21 | DnaJ heat shock protein family (Hsp40) member C21 [Source:HGNC Symbol;Acc:HGNC:27030] |
| ENSG00000169255 | HGLibA_03947 | 48 | -0.670952162 | B3GALNT1 | 3-N-acetylgalactosaminyltransferase 1 (globoside blood group) [Source:HGNC Symbol;Acc:HGNC:918]" |
| ENSG00000169621 | HGLibA_02431 | 48 | -0.670952162 | APLF | aprataxin and PNKP like factor [Source:HGNC Symbol;Acc:HGNC:28724] |
| ENSG00000169783 | HGLibA_26584 | 48 | -0.670952162 | LINGO1 | leucine rich repeat and Ig domain containing 1 [Source:HGNC Symbol;Acc:HGNC:21205] |
| ENSG00000170315 | HGLibA_52563 | 48 | -0.670952162 | UBB | ubiquitin B [Source:HGNC Symbol;Acc:HGNC:12463] |
| ENSG00000170412 | HGLibA_20130 | 48 | -0.670952162 | GPRC5C | G protein-coupled receptor class C group 5 member C [Source:HGNC Symbol;Acc:HGNC:13309] |
| ENSG00000170542 | HGLibA_43568 | 48 | -0.670952162 | SERPINB9 | serpin family B member 9 [Source:HGNC Symbol;Acc:HGNC:8955] |
| ENSG00000170608 | HGLibA_17812 | 48 | -0.670952162 | FOXA3 | forkhead box A3 [Source:HGNC Symbol;Acc:HGNC:5023] |
| ENSG00000170734 | HGLibA_37529 | 48 | -0.670952162 | POLH | DNA polymerase eta [Source:HGNC Symbol;Acc:HGNC:9181] |
| ENSG00000170920 | HGLibA_34488 | 48 | -0.670952162 | OR7G3 | olfactory receptor family 7 subfamily G member 3 [Source:HGNC Symbol;Acc:HGNC:8467] |
| ENSG00000171100 | HGLibA_30405 | 48 | -0.670952162 | MTM1 | myotubularin 1 [Source:HGNC Symbol;Acc:HGNC:7448] |
| ENSG00000171126 | HGLibA_24204 | 48 | -0.670952162 | KCNG3 | potassium voltage-gated channel modifier subfamily G member 3 [Source:HGNC Symbol;Acc:HGNC:18306] |
| ENSG00000171150 | HGLibA_46136 | 48 | -0.670952162 | SOCS5 | suppressor of cytokine signaling 5 [Source:HGNC Symbol;Acc:HGNC:16852] |
| ENSG00000171209 | HGLibA_11378 | 48 | -0.670952162 | CSN3 | casein kappa [Source:HGNC Symbol;Acc:HGNC:2446] |
| ENSG00000171804 | HGLibA_54206 | 48 | -0.670952162 | WDR87 | WD repeat domain 87 [Source:HGNC Symbol;Acc:HGNC:29934] |
| ENSG00000172568 | HGLibA_17761 | 48 | -0.670952162 | FNDC9 | fibronectin type III domain containing 9 [Source:HGNC Symbol;Acc:HGNC:33547] |
| ENSG00000172716 | HGLibA_45482 | 48 | -0.670952162 | SLFN11 | schlafen family member 11 [Source:HGNC Symbol;Acc:HGNC:26633] |
| ENSG00000174444 | HGLibA_41981 | 48 | -0.670952162 | RPL4 | ribosomal protein L4 [Source:HGNC Symbol;Acc:HGNC:10353] |
| ENSG00000175764 | HGLibA_52191 | 48 | -0.670952162 | TTLL11 | tubulin tyrosine ligase like 11 [Source:HGNC Symbol;Acc:HGNC:18113] |
| ENSG00000177575 | HGLibA_08242 | 48 | -0.670952162 | CD163 | CD163 molecule [Source:HGNC Symbol;Acc:HGNC:1631] |
| ENSG00000178127 | HGLibA_31572 | 48 | -0.670952162 | NDUFV2 | NADH:ubiquinone oxidoreductase core subunit V2 [Source:HGNC Symbol;Acc:HGNC:7717] |
| ENSG00000178279 | HGLibA_50894 | 48 | -0.670952162 | TNP2 | transition protein 2 [Source:HGNC Symbol;Acc:HGNC:11952] |
| ENSG00000178685 | HGLibA_35215 | 48 | -0.670952162 | PARP10 | poly(ADP-ribose) polymerase family member 10 [Source:HGNC Symbol;Acc:HGNC:25895] |
| ENSG00000178690 | HGLibA_14230 | 48 | -0.670952162 | DYNAP | dynactin associated protein [Source:HGNC Symbol;Acc:HGNC:26808] |
| ENSG00000178795 | HGLibA_18899 | 48 | -0.670952162 | GDPD4 | glycerophosphodiester phosphodiesterase domain containing 4 [Source:HGNC Symbol;Acc:HGNC:24849] |
| ENSG00000179284 | HGLibA_12308 | 48 | -0.670952162 | DAND5 | DAN domain BMP antagonist family member 5 [Source:HGNC Symbol;Acc:HGNC:26780] |
| ENSG00000180090 | HGLibA_33913 | 48 | -0.670952162 | OR3A1 | olfactory receptor family 3 subfamily A member 1 [Source:HGNC Symbol;Acc:HGNC:8282] |
| ENSG00000180263 | HGLibA_17358 | 48 | -0.670952162 | FGD6 | RhoGEF and PH domain containing 6 [Source:HGNC Symbol;Acc:HGNC:21740]" |
| ENSG00000180785 | HGLibA_34098 | 48 | -0.670952162 | OR51E1 | olfactory receptor family 51 subfamily E member 1 [Source:HGNC Symbol;Acc:HGNC:15194] |
| ENSG00000180999 | HGLibA_05734 | 48 | -0.670952162 | C1orf105 | chromosome 1 open reading frame 105 [Source:HGNC Symbol;Acc:HGNC:29591] |
| ENSG00000181392 | HGLibA_47986 | 48 | -0.670952162 | SYNE4 | spectrin repeat containing nuclear envelope family member 4 [Source:HGNC Symbol;Acc:HGNC:26703] |
| ENSG00000181958 | HGLibA_33924 | 48 | -0.670952162 | OR4A15 | olfactory receptor family 4 subfamily A member 15 [Source:HGNC Symbol;Acc:HGNC:15152] |
| ENSG00000182050 | HGLibA_29147 | 48 | -0.670952162 | MGAT4C | MGAT4 family member C [Source:HGNC Symbol;Acc:HGNC:30871] |
| ENSG00000182362 | HGLibA_54640 | 48 | -0.670952162 | YBEY | ybeY metalloendoribonuclease [Source:HGNC Symbol;Acc:HGNC:1299] |
| ENSG00000182793 | HGLibA_20464 | 48 | -0.670952162 | GSTA5 | glutathione S-transferase alpha 5 [Source:HGNC Symbol;Acc:HGNC:19662] |
| ENSG00000183569 | HGLibA_43480 | 48 | -0.670952162 | SERHL2 | serine hydrolase like 2 [Source:HGNC Symbol;Acc:HGNC:29446] |
| ENSG00000183578 | HGLibA_50730 | 48 | -0.670952162 | TNFAIP8L3 | TNF alpha induced protein 8 like 3 [Source:HGNC Symbol;Acc:HGNC:20620] |
| ENSG00000183856 | HGLibA_23537 | 48 | -0.670952162 | IQGAP3 | IQ motif containing GTPase activating protein 3 [Source:HGNC Symbol;Acc:HGNC:20669] |
| ENSG00000184831 | HGLibA_02550 | 48 | -0.670952162 | APOO | apolipoprotein O [Source:HGNC Symbol;Acc:HGNC:28727] |
| ENSG00000185507 | HGLibA_23599 | 48 | -0.670952162 | IRF7 | interferon regulatory factor 7 [Source:HGNC Symbol;Acc:HGNC:6122] |
| ENSG00000185621 | HGLibA_26690 | 48 | -0.670952162 | LMLN | leishmanolysin like peptidase [Source:HGNC Symbol;Acc:HGNC:15991] |
| ENSG00000185670 | HGLibA_54855 | 48 | -0.670952162 | ZBTB3 | zinc finger and BTB domain containing 3 [Source:HGNC Symbol;Acc:HGNC:22918] |
| ENSG00000185721 | HGLibA_13974 | 48 | -0.670952162 | DRG1 | developmentally regulated GTP binding protein 1 [Source:HGNC Symbol;Acc:HGNC:3029] |
| ENSG00000185721 | HGLibA_13973 | 48 | -0.670952162 | DRG1 | developmentally regulated GTP binding protein 1 [Source:HGNC Symbol;Acc:HGNC:3029] |
| ENSG00000186103 | HGLibA_02690 | 48 | -0.670952162 | ARGFX | arginine-fifty homeobox [Source:HGNC Symbol;Acc:HGNC:30146] |
| ENSG00000186479 | HGLibA_41107 | 48 | -0.670952162 | RGS7BP | regulator of G protein signaling 7 binding protein [Source:HGNC Symbol;Acc:HGNC:23271] |
| ENSG00000186564 | HGLibA_17832 | 48 | -0.670952162 | FOXD2 | forkhead box D2 [Source:HGNC Symbol;Acc:HGNC:3803] |
| ENSG00000186687 | HGLibA_27758 | 48 | -0.670952162 | LYRM7 | LYR motif containing 7 [Source:HGNC Symbol;Acc:HGNC:28072] |
| ENSG00000186895 | HGLibA_17406 | 48 | -0.670952162 | FGF3 | fibroblast growth factor 3 [Source:HGNC Symbol;Acc:HGNC:3681] |
| ENSG00000187238 | HGLibA_26133 | 48 | -0.670952162 | LCE3B | late cornified envelope 3B [Source:HGNC Symbol;Acc:HGNC:29462] |
| ENSG00000187944 | HGLibA_06160 | 48 | -0.670952162 | C2orf66 | chromosome 2 open reading frame 66 [Source:HGNC Symbol;Acc:HGNC:33809] |
| ENSG00000188324 | HGLibA_34395 | 48 | -0.670952162 | OR6C6 | olfactory receptor family 6 subfamily C member 6 [Source:HGNC Symbol;Acc:HGNC:31293] |
| ENSG00000188710 | HGLibA_39736 | 48 | -0.670952162 | QRFP | pyroglutamylated RFamide peptide [Source:HGNC Symbol;Acc:HGNC:29982] |
| ENSG00000189430 | HGLibA_31362 | 48 | -0.670952162 | NCR1 | natural cytotoxicity triggering receptor 1 [Source:HGNC Symbol;Acc:HGNC:6731] |
| ENSG00000196576 | HGLibA_37273 | 48 | -0.670952162 | PLXNB2 | plexin B2 [Source:HGNC Symbol;Acc:HGNC:9104] |
| ENSG00000197043 | HGLibA_02245 | 48 | -0.670952162 | ANXA6 | annexin A6 [Source:HGNC Symbol;Acc:HGNC:544] |
| ENSG00000197451 | HGLibA_21803 | 48 | -0.670952162 | HNRNPAB | heterogeneous nuclear ribonucleoprotein A/B [Source:HGNC Symbol;Acc:HGNC:5034] |
| ENSG00000197769 | HGLibA_28130 | 48 | -0.670952162 | MAP1LC3C | microtubule associated protein 1 light chain 3 gamma [Source:HGNC Symbol;Acc:HGNC:13353] |
| ENSG00000198046 | HGLibA_56490 | 48 | -0.670952162 | ZNF667 | zinc finger protein 667 [Source:HGNC Symbol;Acc:HGNC:28854] |
| ENSG00000204252 | HGLibA_21608 | 48 | -0.670952162 | HLA-DOA | DO alpha [Source:HGNC Symbol;Acc:HGNC:4936]" |
| ENSG00000204518 | HGLibA_00037 | 48 | -0.670952162 | AADACL4 | arylacetamide deacetylase like 4 [Source:HGNC Symbol;Acc:HGNC:32038] |
| ENSG00000204520 | HGLibA_29198 | 48 | -0.670952162 | MICA | MHC class I polypeptide-related sequence A [Source:HGNC Symbol;Acc:HGNC:7090] |
| ENSG00000204613 | HGLibA_51402 | 48 | -0.670952162 | TRIM10 | tripartite motif containing 10 [Source:HGNC Symbol;Acc:HGNC:10072] |
| ENSG00000205329 | HGLibA_34388 | 48 | -0.670952162 | OR6C3 | olfactory receptor family 6 subfamily C member 3 [Source:HGNC Symbol;Acc:HGNC:15437] |
| ENSG00000213930 | HGLibA_18595 | 48 | -0.670952162 | GALT | galactose-1-phosphate uridylyltransferase [Source:HGNC Symbol;Acc:HGNC:4135] |
| ENSG00000221882 | HGLibA_33918 | 48 | -0.670952162 | OR3A2 | olfactory receptor family 3 subfamily A member 2 [Source:HGNC Symbol;Acc:HGNC:8283] |
| ENSG00000228607 | HGLibA_09891 | 48 | -0.670952162 | CLDN25 | claudin 25 [Source:HGNC Symbol;Acc:HGNC:37218] |
| ENSG00000228672 | HGLibA_38646 | 48 | -0.670952162 | PROB1 | proline rich basic protein 1 [Source:HGNC Symbol;Acc:HGNC:41906] |
| ENSG00000229183 | HGLibA_36190 | 48 | -0.670952162 | PGA4 | group I (pepsinogen A) [Source:HGNC Symbol;Acc:HGNC:8886]" |
| ENSG00000230347 | HGLibA_11546 | 48 | -0.670952162 | CT47A8 | member A8 [Source:HGNC Symbol;Acc:HGNC:33289]" |
| ENSG00000230430 | HGLibA_53257 | 48 | -0.670952162 | USP17L25 | ubiquitin specific peptidase 17-like family member 25 [Source:HGNC Symbol;Acc:HGNC:44452] |
| ENSG00000234616 | HGLibA_23991 | 48 | -0.670952162 | JRK | Jrk helix-turn-helix protein [Source:HGNC Symbol;Acc:HGNC:6199] |
| ENSG00000236371 | HGLibA_11516 | 48 | -0.670952162 | CT47A1 | member A1 [Source:HGNC Symbol;Acc:HGNC:33282]" |
| ENSG00000240505 | HGLibA_50754 | 48 | -0.670952162 | TNFRSF13B | TNF receptor superfamily member 13B [Source:HGNC Symbol;Acc:HGNC:18153] |
| ENSG00000242221 | HGLibA_39041 | 48 | -0.670952162 | PSG2 | pregnancy specific beta-1-glycoprotein 2 [Source:HGNC Symbol;Acc:HGNC:9519] |
| ENSG00000244482 | HGLibA_26503 | 48 | -0.670952162 | LILRA6 | leukocyte immunoglobulin like receptor A6 [Source:HGNC Symbol;Acc:HGNC:15495] |
| ENSG00000244588 | HGLibA_40065 | 48 | -0.670952162 | RAD21L1 | RAD21 cohesin complex component like 1 [Source:HGNC Symbol;Acc:HGNC:16271] |
| ENSG00000248405 | HGLibA_38846 | 48 | -0.670952162 | PRR5-ARHGAP8 | PRR5-ARHGAP8 readthrough [Source:HGNC Symbol;Acc:HGNC:34512] |
| ENSG00000248905 | HGLibA_17687 | 48 | -0.670952162 | FMN1 | formin 1 [Source:HGNC Symbol;Acc:HGNC:3768] |
| ENSG00000250510 | HGLibA_19960 | 48 | -0.670952162 | GPR162 | G protein-coupled receptor 162 [Source:HGNC Symbol;Acc:HGNC:16693] |
| ENSG00000254834 | HGLibA_34330 | 48 | -0.670952162 | OR5M10 | olfactory receptor family 5 subfamily M member 10 [Source:HGNC Symbol;Acc:HGNC:15290] |
| ENSG00000255974 | HGLibA_12105 | 48 | -0.670952162 | CYP2A6 | cytochrome P450 family 2 subfamily A member 6 [Source:HGNC Symbol;Acc:HGNC:2610] |
| ENSG00000258839 | HGLibA_28542 | 48 | -0.670952162 | MC1R | melanocortin 1 receptor [Source:HGNC Symbol;Acc:HGNC:6929] |
| ENSG00000273045 | HGLibA_06110 | 48 | -0.670952162 | C2orf15 | chromosome 2 open reading frame 15 [Source:HGNC Symbol;Acc:HGNC:28436] |
| ENSG00000273173 | HGLibA_46017 | 48 | -0.670952162 | SNURF | SNRPN upstream reading frame [Source:HGNC Symbol;Acc:HGNC:11171] |
| FAM109B | HGLibA_16008 | 48 | -0.670952162 | FAM109B | - |
| GNB2L1 | HGLibA_19493 | 48 | -0.670952162 | GNB2L1 | - |
| GTPBP6_X | HGLibA_20617 | 48 | -0.670952162 | GTPBP6_X | - |
| HIST1H2AD | HGLibA_21385 | 48 | -0.670952162 | HIST1H2AD | - |

| | | | | | |
|---|---|---|---|---|---|
| HIST1H2BG | HGLibA_21428 | 48 | -0.670952162 | HIST1H2BG | - |
| HIST1H4D | HGLibA_21495 | 48 | -0.670952162 | HIST1H4D | - |
| hsa-mir-1233-1 | HGLibA_57321 | 48 | -0.670952162 | hsa-mir-1233-1 | - |
| hsa-mir-1270-1 | HGLibA_57515 | 48 | -0.670952162 | hsa-mir-1270-1 | - |
| hsa-mir-1270-2 | HGLibA_57519 | 48 | -0.670952162 | hsa-mir-1270-2 | - |
| hsa-mir-1282 | HGLibA_57583 | 48 | -0.670952162 | hsa-mir-1282 | - |
| hsa-mir-1294 | HGLibA_57654 | 48 | -0.670952162 | hsa-mir-1294 | - |
| hsa-mir-133a-1 | HGLibA_57772 | 48 | -0.670952162 | hsa-mir-133a-1 | - |
| hsa-mir-1470 | HGLibA_57865 | 48 | -0.670952162 | hsa-mir-1470 | - |
| hsa-mir-17 | HGLibA_57953 | 48 | -0.670952162 | hsa-mir-17 | - |
| hsa-mir-192 | HGLibA_58067 | 48 | -0.670952162 | hsa-mir-192 | - |
| hsa-mir-203b | HGLibA_58169 | 48 | -0.670952162 | hsa-mir-203b | - |
| hsa-mir-210 | HGLibA_58213 | 48 | -0.670952162 | hsa-mir-210 | - |
| hsa-mir-30b | HGLibA_58477 | 48 | -0.670952162 | hsa-mir-30b | - |
| hsa-mir-30c-1 | HGLibA_58479 | 48 | -0.670952162 | hsa-mir-30c-1 | - |
| hsa-mir-3168 | HGLibA_58751 | 48 | -0.670952162 | hsa-mir-3168 | - |
| hsa-mir-3606 | HGLibA_59061 | 48 | -0.670952162 | hsa-mir-3606 | - |
| hsa-mir-3670-1 | HGLibA_59232 | 48 | -0.670952162 | hsa-mir-3670-1 | - |
| hsa-mir-3683 | HGLibA_59287 | 48 | -0.670952162 | hsa-mir-3683 | - |
| hsa-mir-3688-1 | HGLibA_59304 | 48 | -0.670952162 | hsa-mir-3688-1 | - |
| hsa-mir-373 | HGLibA_59384 | 48 | -0.670952162 | hsa-mir-373 | - |
| hsa-mir-3927 | HGLibA_59585 | 48 | -0.670952162 | hsa-mir-3927 | - |
| hsa-mir-4466 | HGLibA_60271 | 48 | -0.670952162 | hsa-mir-4466 | - |
| hsa-mir-4476 | HGLibA_60315 | 48 | -0.670952162 | hsa-mir-4476 | - |
| hsa-mir-4482 | HGLibA_60345 | 48 | -0.670952162 | hsa-mir-4482 | - |
| hsa-mir-4657 | HGLibA_60736 | 48 | -0.670952162 | hsa-mir-4657 | - |
| hsa-mir-4795 | HGLibA_61286 | 48 | -0.670952162 | hsa-mir-4795 | - |
| hsa-mir-4797 | HGLibA_61295 | 48 | -0.670952162 | hsa-mir-4797 | - |
| hsa-mir-548aj-2 | HGLibA_61892 | 48 | -0.670952162 | hsa-mir-548aj-2 | - |
| hsa-mir-556 | HGLibA_62139 | 48 | -0.670952162 | hsa-mir-556 | - |
| hsa-mir-6511a-2 | HGLibA_62902 | 48 | -0.670952162 | hsa-mir-6511a-2 | - |
| hsa-mir-6511a-3 | HGLibA_62907 | 48 | -0.670952162 | hsa-mir-6511a-3 | - |
| hsa-mir-6715b | HGLibA_63022 | 48 | -0.670952162 | hsa-mir-6715b | - |
| hsa-mir-6784 | HGLibA_63315 | 48 | -0.670952162 | hsa-mir-6784 | - |
| hsa-mir-6854 | HGLibA_63588 | 48 | -0.670952162 | hsa-mir-6854 | - |
| hsa-mir-7111 | HGLibA_63807 | 48 | -0.670952162 | hsa-mir-7111 | - |
| hsa-mir-7158 | HGLibA_63861 | 48 | -0.670952162 | hsa-mir-7158 | - |
| hsa-mir-888 | HGLibA_64229 | 48 | -0.670952162 | hsa-mir-888 | - |
| KIAA0317 | HGLibA_24616 | 48 | -0.670952162 | KIAA0317 | - |
| PRHOXNB | HGLibA_38467 | 48 | -0.670952162 | PRHOXNB | - |
| 11-Sep | HGLibA_43430 | 48 | -0.670952162 | 11-Sep | - |
| SKIV2L2 | HGLibA_44289 | 48 | -0.670952162 | SKIV2L2 | - |
| TMEM173 | HGLibA_50064 | 48 | -0.670952162 | TMEM173 | - |
| WASH1 | HGLibA_53961 | 48 | -0.670952162 | WASH1 | - |
| WRB | HGLibA_54406 | 48 | -0.670952162 | WRB | - |
| ACPL2 | HGLibA_00521 | 47 | -0.6809032 | ACPL2 | - |
| ATP5A1 | HGLibA_03637 | 47 | -0.6809032 | ATP5A1 | - |
| C15orf38-AP3S2 | HGLibA_05389 | 47 | -0.6809032 | C15orf38-AP3S2 | - |
| C20orf78 | HGLibA_06026 | 47 | -0.6809032 | C20orf78 | - |
| C2orf44 | HGLibA_06130 | 47 | -0.6809032 | C2orf44 | - |
| C3orf55 | HGLibA_06252 | 47 | -0.6809032 | C3orf55 | - |
| C7orf63 | HGLibA_06573 | 47 | -0.6809032 | C7orf63 | - |
| CD99_Y | HGLibA_08493 | 47 | -0.6809032 | CD99_Y | - |
| CSF2RA_X | HGLibA_11330 | 47 | -0.6809032 | CSF2RA_X | - |
| ENSG00000003147 | HGLibA_22488 | 47 | -0.6809032 | ICA1 | islet cell autoantigen 1 [Source:HGNC Symbol;Acc:HGNC:5343] |
| ENSG00000003509 | HGLibA_31500 | 47 | -0.6809032 | NDUFAF7 | NADH:ubiquinone oxidoreductase complex assembly factor 7 [Source:HGNC Symbol;Acc:HGNC:28816] |
| ENSG00000005156 | HGLibA_26482 | 47 | -0.6809032 | LIG3 | DNA ligase 3 [Source:HGNC Symbol;Acc:HGNC:6600] |
| ENSG00000006747 | HGLibA_42948 | 47 | -0.6809032 | SCIN | scinderin [Source:HGNC Symbol;Acc:HGNC:21695] |
| ENSG00000006747 | HGLibA_42946 | 47 | -0.6809032 | SCIN | scinderin [Source:HGNC Symbol;Acc:HGNC:21695] |
| ENSG00000007866 | HGLibA_48933 | 47 | -0.6809032 | TEAD3 | TEA domain transcription factor 3 [Source:HGNC Symbol;Acc:HGNC:11716] |
| ENSG00000011426 | HGLibA_02169 | 47 | -0.6809032 | ANLN | anillin actin binding protein [Source:HGNC Symbol;Acc:HGNC:14082] |
| ENSG00000011451 | HGLibA_54327 | 47 | -0.6809032 | WIZ | widely interspaced zinc finger motifs [Source:HGNC Symbol;Acc:HGNC:30917] |
| ENSG00000012124 | HGLibA_08298 | 47 | -0.6809032 | CD22 | CD22 molecule [Source:HGNC Symbol;Acc:HGNC:1643] |
| ENSG00000015532 | HGLibA_54623 | 47 | -0.6809032 | XYLT2 | xylosyltransferase 2 [Source:HGNC Symbol;Acc:HGNC:15517] |
| ENSG00000034677 | HGLibA_41601 | 47 | -0.6809032 | RNF19A | RBR E3 ubiquitin protein ligase [Source:HGNC Symbol;Acc:HGNC:13432]" |
| ENSG00000036828 | HGLibA_07340 | 47 | -0.6809032 | CASR | calcium sensing receptor [Source:HGNC Symbol;Acc:HGNC:1514] |
| ENSG00000043514 | HGLibA_51654 | 47 | -0.6809032 | TRIT1 | tRNA isopentenyltransferase 1 [Source:HGNC Symbol;Acc:HGNC:20286] |
| ENSG00000049239 | HGLibA_20786 | 47 | -0.6809032 | H6PD | hexose-6-phosphate dehydrogenase/glucose 1-dehydrogenase [Source:HGNC Symbol;Acc:HGNC:4795] |
| ENSG00000050438 | HGLibA_45230 | 47 | -0.6809032 | SLC4A8 | solute carrier family 4 member 8 [Source:HGNC Symbol;Acc:HGNC:11034] |
| ENSG00000050748 | HGLibA_28296 | 47 | -0.6809032 | MAPK9 | mitogen-activated protein kinase 9 [Source:HGNC Symbol;Acc:HGNC:6886] |
| ENSG00000054523 | HGLibA_24887 | 47 | -0.6809032 | KIF1B | kinesin family member 1B [Source:HGNC Symbol;Acc:HGNC:16636] |
| ENSG00000064195 | HGLibA_13379 | 47 | -0.6809032 | DLX3 | distal-less homeobox 3 [Source:HGNC Symbol;Acc:HGNC:2916] |
| ENSG00000069018 | HGLibA_51749 | 47 | -0.6809032 | TRPC7 | transient receptor potential cation channel subfamily C member 7 [Source:HGNC Symbol;Acc:HGNC:20754] |
| ENSG00000069869 | HGLibA_31601 | 47 | -0.6809032 | NEDD4 | E3 ubiquitin protein ligase [Source:HGNC Symbol;Acc:HGNC:7727]" |
| ENSG00000070444 | HGLibA_29577 | 47 | -0.6809032 | MNT | MAX network transcriptional repressor [Source:HGNC Symbol;Acc:HGNC:7188] |
| ENSG00000072315 | HGLibA_51741 | 47 | -0.6809032 | TRPC5 | transient receptor potential cation channel subfamily C member 5 [Source:HGNC Symbol;Acc:HGNC:12337] |
| ENSG00000074181 | HGLibA_32348 | 47 | -0.6809032 | NOTCH3 | notch 3 [Source:HGNC Symbol;Acc:HGNC:7883] |
| ENSG00000076067 | HGLibA_40590 | 47 | -0.6809032 | RBMS2 | RNA binding motif single stranded interacting protein 2 [Source:HGNC Symbol;Acc:HGNC:9909] |
| ENSG00000080345 | HGLibA_41244 | 47 | -0.6809032 | RIF1 | replication timing regulatory factor 1 [Source:HGNC Symbol;Acc:HGNC:23207] |
| ENSG00000080822 | HGLibA_09916 | 47 | -0.6809032 | CLDND1 | claudin domain containing 1 [Source:HGNC Symbol;Acc:HGNC:1322] |
| ENSG00000081019 | HGLibA_42301 | 47 | -0.6809032 | RSBN1 | round spermatid basic protein 1 [Source:HGNC Symbol;Acc:HGNC:25642] |
| ENSG00000082153 | HGLibA_05013 | 47 | -0.6809032 | BZW1 | basic leucine zipper and W2 domains 1 [Source:HGNC Symbol;Acc:HGNC:18380] |
| ENSG00000085491 | HGLibA_44682 | 47 | -0.6809032 | SLC25A24 | solute carrier family 25 member 24 [Source:HGNC Symbol;Acc:HGNC:20662] |
| ENSG00000086200 | HGLibA_23461 | 47 | -0.6809032 | IPO11 | importin 11 [Source:HGNC Symbol;Acc:HGNC:20628] |
| ENSG00000089234 | HGLibA_04755 | 47 | -0.6809032 | BRAP | BRCA1 associated protein [Source:HGNC Symbol;Acc:HGNC:1099] |
| ENSG00000089818 | HGLibA_31594 | 47 | -0.6809032 | NECAP1 | NECAP endocytosis associated 1 [Source:HGNC Symbol;Acc:HGNC:24539] |
| ENSG00000091513 | HGLibA_49115 | 47 | -0.6809032 | TF | transferrin [Source:HGNC Symbol;Acc:HGNC:11740] |
| ENSG00000095970 | HGLibA_51357 | 47 | -0.6809032 | TREM2 | triggering receptor expressed on myeloid cells 2 [Source:HGNC Symbol;Acc:HGNC:17761] |
| ENSG00000100288 | HGLibA_09460 | 47 | -0.6809032 | CHKB | choline kinase beta [Source:HGNC Symbol;Acc:HGNC:1938] |
| ENSG00000100629 | HGLibA_09104 | 47 | -0.6809032 | CEP128 | centrosomal protein 128 [Source:HGNC Symbol;Acc:HGNC:20359] |
| ENSG00000101337 | HGLibA_49731 | 47 | -0.6809032 | TM9SF4 | transmembrane 9 superfamily member 4 [Source:HGNC Symbol;Acc:HGNC:30797] |
| ENSG00000101901 | HGLibA_01617 | 47 | -0.6809032 | ALG13 | UDP-N-acetylglucosaminyltransferase subunit [Source:HGNC Symbol;Acc:HGNC:30881]" |
| ENSG00000102034 | HGLibA_14899 | 47 | -0.6809032 | ELF4 | E74 like ETS transcription factor 4 [Source:HGNC Symbol;Acc:HGNC:3319] |
| ENSG00000102743 | HGLibA_44652 | 47 | -0.6809032 | SLC25A15 | solute carrier family 25 member 15 [Source:HGNC Symbol;Acc:HGNC:10985] |
| ENSG00000103264 | HGLibA_17068 | 47 | -0.6809032 | FBXO31 | F-box protein 31 [Source:HGNC Symbol;Acc:HGNC:16510] |
| ENSG00000103266 | HGLibA_47613 | 47 | -0.6809032 | STUB1 | STIP1 homology and U-box containing protein 1 [Source:HGNC Symbol;Acc:HGNC:11427] |
| ENSG00000104299 | HGLibA_23435 | 47 | -0.6809032 | INTS9 | integrator complex subunit 9 [Source:HGNC Symbol;Acc:HGNC:25592] |
| ENSG00000104714 | HGLibA_15488 | 47 | -0.6809032 | ERICH1 | glutamate rich 1 [Source:HGNC Symbol;Acc:HGNC:27234] |
| ENSG00000104774 | HGLibA_28076 | 47 | -0.6809032 | MAN2B1 | mannosidase alpha class 2B member 1 [Source:HGNC Symbol;Acc:HGNC:6826] |
| ENSG00000104884 | HGLibA_15435 | 47 | -0.6809032 | ERCC2 | TFIIH core complex helicase subunit [Source:HGNC Symbol;Acc:HGNC:3434]" |
| ENSG00000104886 | HGLibA_37135 | 47 | -0.6809032 | PLEKHJ1 | pleckstrin homology domain containing J1 [Source:HGNC Symbol;Acc:HGNC:18211] |
| ENSG00000105141 | HGLibA_07304 | 47 | -0.6809032 | CASP14 | caspase 14 [Source:HGNC Symbol;Acc:HGNC:1502] |
| ENSG00000105176 | HGLibA_53146 | 47 | -0.6809032 | URI1 | prefoldin like chaperone [Source:HGNC Symbol;Acc:HGNC:13236]" |
| ENSG00000105516 | HGLibA_12399 | 47 | -0.6809032 | DBP | D-box binding PAR bZIP transcription factor [Source:HGNC Symbol;Acc:HGNC:2697] |
| ENSG00000105993 | HGLibA_13549 | 47 | -0.6809032 | DNAJB6 | DnaJ heat shock protein family (Hsp40) member B6 [Source:HGNC Symbol;Acc:HGNC:14888] |
| ENSG00000106066 | HGLibA_11031 | 47 | -0.6809032 | CPVL | vitellogenic like [Source:HGNC Symbol;Acc:HGNC:14399]" |
| ENSG00000106615 | HGLibA_41155 | 47 | -0.6809032 | RHEB | mTORC1 binding [Source:HGNC Symbol;Acc:HGNC:10011]" |
| ENSG00000106665 | HGLibA_10046 | 47 | -0.6809032 | CLIP2 | CAP-Gly domain containing linker protein 2 [Source:HGNC Symbol;Acc:HGNC:2586] |
| ENSG00000106803 | HGLibA_43253 | 47 | -0.6809032 | SEC61B | Sec61 translocon beta subunit [Source:HGNC Symbol;Acc:HGNC:16993] |
| ENSG00000108100 | HGLibA_08131 | 47 | -0.6809032 | CCNY | cyclin Y [Source:HGNC Symbol;Acc:HGNC:23354] |
| ENSG00000109072 | HGLibA_53893 | 47 | -0.6809032 | VTN | vitronectin [Source:HGNC Symbol;Acc:HGNC:12724] |
| ENSG00000109180 | HGLibA_33245 | 47 | -0.6809032 | OCIAD1 | OCIA domain containing 1 [Source:HGNC Symbol;Acc:HGNC:16074] |
| ENSG00000110172 | HGLibA_09514 | 47 | -0.6809032 | CHORDC1 | cysteine and histidine rich domain containing 1 [Source:HGNC Symbol;Acc:HGNC:14525] |
| ENSG00000110455 | HGLibA_00412 | 47 | -0.6809032 | ACCS | 1-aminocyclopropane-1-carboxylate synthase homolog (inactive) [Source:HGNC Symbol;Acc:HGNC:23989] |
| ENSG00000110851 | HGLibA_38389 | 47 | -0.6809032 | PRDM4 | PR/SET domain 4 [Source:HGNC Symbol;Acc:HGNC:9348] |
| ENSG00000111644 | HGLibA_00532 | 47 | -0.6809032 | ACRBP | acrosin binding protein [Source:HGNC Symbol;Acc:HGNC:17195] |
| ENSG00000112562 | HGLibA_45798 | 47 | -0.6809032 | SMOC2 | SPARC related modular calcium binding 2 [Source:HGNC Symbol;Acc:HGNC:20323] |
| ENSG00000114346 | HGLibA_14424 | 47 | -0.6809032 | ECT2 | epithelial cell transforming 2 [Source:HGNC Symbol;Acc:HGNC:3155] |
| ENSG00000115204 | HGLibA_29782 | 47 | -0.6809032 | MPV17 | mitochondrial inner membrane protein MPV17 [Source:HGNC Symbol;Acc:HGNC:7224] |
| ENSG00000115233 | HGLibA_39162 | 47 | -0.6809032 | PSMD14 | non-ATPase 14 [Source:HGNC Symbol;Acc:HGNC:16889]" |
| ENSG00000115307 | HGLibA_03870 | 47 | -0.6809032 | AUP1 | lipid droplet regulating VLDL assembly factor [Source:HGNC Symbol;Acc:HGNC:891]" |
| ENSG00000116106 | HGLibA_15297 | 47 | -0.6809032 | EPHA4 | EPH receptor A4 [Source:HGNC Symbol;Acc:HGNC:3388] |
| ENSG00000118420 | HGLibA_52686 | 47 | -0.6809032 | UBE3D | ubiquitin protein ligase E3D [Source:HGNC Symbol;Acc:HGNC:21381] |
| ENSG00000119523 | HGLibA_01626 | 47 | -0.6809032 | ALG2 | 6-mannosyltransferase [Source:HGNC Symbol;Acc:HGNC:23159]" |
| ENSG00000119946 | HGLibA_10313 | 47 | -0.6809032 | CNNM1 | cyclin and CBS domain divalent metal cation transport mediator 1 [Source:HGNC Symbol;Acc:HGNC:102] |
| ENSG00000120063 | HGLibA_19443 | 47 | -0.6809032 | GNA13 | G protein subunit alpha 13 [Source:HGNC Symbol;Acc:HGNC:4381] |
| ENSG00000120696 | HGLibA_24115 | 47 | -0.6809032 | KBTBD7 | kelch repeat and BTB domain containing 7 [Source:HGNC Symbol;Acc:HGNC:25266] |
| ENSG00000121897 | HGLibA_26471 | 47 | -0.6809032 | LIAS | lipoic acid synthetase [Source:HGNC Symbol;Acc:HGNC:16429] |
| ENSG00000122121 | HGLibA_54557 | 47 | -0.6809032 | XPNPEP2 | X-prolyl aminopeptidase 2 [Source:HGNC Symbol;Acc:HGNC:12823] |
| ENSG00000122188 | HGLibA_26071 | 47 | -0.6809032 | LAX1 | lymphocyte transmembrane adaptor 1 [Source:HGNC Symbol;Acc:HGNC:26005] |
| ENSG00000124251 | HGLibA_51090 | 47 | -0.6809032 | TP53TG5 | TP53 target 5 [Source:HGNC Symbol;Acc:HGNC:15856] |
| ENSG00000124479 | HGLibA_31411 | 47 | -0.6809032 | NDP | norrin cystine knot growth factor [Source:HGNC Symbol;Acc:HGNC:7678]" |
| ENSG00000124733 | HGLibA_28719 | 47 | -0.6809032 | MEA1 | male-enhanced antigen 1 [Source:HGNC Symbol;Acc:HGNC:6986] |
| ENSG00000125355 | HGLibA_50329 | 47 | -0.6809032 | TMEM255A | transmembrane protein 255A [Source:HGNC Symbol;Acc:HGNC:26086] |
| ENSG00000125741 | HGLibA_33407 | 47 | -0.6809032 | OPA3 | outer mitochondrial membrane lipid metabolism regulator [Source:HGNC Symbol;Acc:HGNC:8142]" |

| | | | | | |
|---|---|---|---|---|---|
| ENSG00000126500 | HGLibA_17653 | 47 | -0.6809032 | FLRT1 | fibronectin leucine rich transmembrane protein 1 [Source:HGNC Symbol;Acc:HGNC:3760] |
| ENSG00000126861 | HGLibA_33386 | 47 | -0.6809032 | OMG | oligodendrocyte myelin glycoprotein [Source:HGNC Symbol;Acc:HGNC:8135] |
| ENSG00000127884 | HGLibA_14403 | 47 | -0.6809032 | ECHS1 | short chain 1 [Source:HGNC Symbol;Acc:HGNC:3151]" |
| ENSG00000127993 | HGLibA_40568 | 47 | -0.6809032 | RBM48 | RNA binding motif protein 48 [Source:HGNC Symbol;Acc:HGNC:21785] |
| ENSG00000130066 | HGLibA_42770 | 47 | -0.6809032 | SAT1 | spermidine/spermine N1-acetyltransferase 1 [Source:HGNC Symbol;Acc:HGNC:10540] |
| ENSG00000130294 | HGLibA_24883 | 47 | -0.6809032 | KIF1A | kinesin family member 1A [Source:HGNC Symbol;Acc:HGNC:888] |
| ENSG00000131375 | HGLibA_07175 | 47 | -0.6809032 | CAPN7 | calpain 7 [Source:HGNC Symbol;Acc:HGNC:1484] |
| ENSG00000131721 | HGLibA_41216 | 47 | -0.6809032 | RHOXF2 | Rhox homeobox family member 2 [Source:HGNC Symbol;Acc:HGNC:30011] |
| ENSG00000133433 | HGLibA_20501 | 47 | -0.6809032 | GSTT2B | glutathione S-transferase theta 2B (gene/pseudogene) [Source:HGNC Symbol;Acc:HGNC:33437] |
| ENSG00000133983 | HGLibA_10815 | 47 | -0.6809032 | COX16 | cytochrome c oxidase assembly factor COX16 [Source:HGNC Symbol;Acc:HGNC:20213] |
| ENSG00000134250 | HGLibA_32343 | 47 | -0.6809032 | NOTCH2 | notch 2 [Source:HGNC Symbol;Acc:HGNC:7882] |
| ENSG00000134830 | HGLibA_06356 | 47 | -0.6809032 | C5AR2 | complement component 5a receptor 2 [Source:HGNC Symbol;Acc:HGNC:4527] |
| ENSG00000135378 | HGLibA_38878 | 47 | -0.6809032 | PRRG4 | proline rich and Gla domain 4 [Source:HGNC Symbol;Acc:HGNC:30799] |
| ENSG00000135605 | HGLibA_48937 | 47 | -0.6809032 | TEC | tec protein tyrosine kinase [Source:HGNC Symbol;Acc:HGNC:11719] |
| ENSG00000136696 | HGLibA_23149 | 47 | -0.6809032 | IL36B | interleukin 36 beta [Source:HGNC Symbol;Acc:HGNC:15564] |
| ENSG00000136872 | HGLibA_01596 | 47 | -0.6809032 | ALDOB | fructose-bisphosphate B [Source:HGNC Symbol;Acc:HGNC:417]" |
| ENSG00000137962 | HGLibA_02750 | 47 | -0.6809032 | ARHGAP29 | Rho GTPase activating protein 29 [Source:HGNC Symbol;Acc:HGNC:30207] |
| ENSG00000137996 | HGLibA_42370 | 47 | -0.6809032 | RTCA | RNA 3'-terminal phosphate cyclase [Source:HGNC Symbol;Acc:HGNC:17981] |
| ENSG00000138378 | HGLibA_47389 | 47 | -0.6809032 | STAT4 | signal transducer and activator of transcription 4 [Source:HGNC Symbol;Acc:HGNC:11365] |
| ENSG00000139155 | HGLibA_45458 | 47 | -0.6809032 | SLCO1C1 | solute carrier organic anion transporter family member 1C1 [Source:HGNC Symbol;Acc:HGNC:13819] |
| ENSG00000139352 | HGLibA_03266 | 47 | -0.6809032 | ASCL1 | achaete-scute family bHLH transcription factor 1 [Source:HGNC Symbol;Acc:HGNC:738] |
| ENSG00000139973 | HGLibA_48065 | 47 | -0.6809032 | SYT16 | synaptotagmin 16 [Source:HGNC Symbol;Acc:HGNC:23142] |
| ENSG00000140463 | HGLibA_04218 | 47 | -0.6809032 | BBS4 | Bardet-Biedl syndrome 4 [Source:HGNC Symbol;Acc:HGNC:969] |
| ENSG00000141027 | HGLibA_31356 | 47 | -0.6809032 | NCOR1 | nuclear receptor corepressor 1 [Source:HGNC Symbol;Acc:HGNC:7672] |
| ENSG00000143079 | HGLibA_11747 | 47 | -0.6809032 | CTTNBP2NL | CTTNBP2 N-terminal like [Source:HGNC Symbol;Acc:HGNC:25330] |
| ENSG00000143105 | HGLibA_24125 | 47 | -0.6809032 | KCNA10 | potassium voltage-gated channel subfamily A member 10 [Source:HGNC Symbol;Acc:HGNC:6219] |
| ENSG00000143183 | HGLibA_49780 | 47 | -0.6809032 | TMCO1 | transmembrane and coiled-coil domains 1 [Source:HGNC Symbol;Acc:HGNC:18188] |
| ENSG00000143556 | HGLibA_42565 | 47 | -0.6809032 | S100A7 | S100 calcium binding protein A7 [Source:HGNC Symbol;Acc:HGNC:10497] |
| ENSG00000145743 | HGLibA_16987 | 47 | -0.6809032 | FBXL17 | F-box and leucine rich repeat protein 17 [Source:HGNC Symbol;Acc:HGNC:13615] |
| ENSG00000146383 | HGLibA_48133 | 47 | -0.6809032 | TAAR6 | trace amine associated receptor 6 [Source:HGNC Symbol;Acc:HGNC:20978] |
| ENSG00000147041 | HGLibA_48108 | 47 | -0.6809032 | SYTL5 | synaptotagmin like 5 [Source:HGNC Symbol;Acc:HGNC:15589] |
| ENSG00000147604 | HGLibA_41993 | 47 | -0.6809032 | RPL7 | ribosomal protein L7 [Source:HGNC Symbol;Acc:HGNC:10363] |
| ENSG00000147669 | HGLibA_37600 | 47 | -0.6809032 | POLR2K | RNA polymerase II subunit K [Source:HGNC Symbol;Acc:HGNC:9198] |
| ENSG00000149483 | HGLibA_49956 | 47 | -0.6809032 | TMEM138 | transmembrane protein 138 [Source:HGNC Symbol;Acc:HGNC:26944] |
| ENSG00000149582 | HGLibA_50314 | 47 | -0.6809032 | TMEM25 | transmembrane protein 25 [Source:HGNC Symbol;Acc:HGNC:25890] |
| ENSG00000150556 | HGLibA_27730 | 47 | -0.6809032 | LYPD6B | LY6/PLAUR domain containing 6B [Source:HGNC Symbol;Acc:HGNC:27018] |
| ENSG00000151376 | HGLibA_28714 | 47 | -0.6809032 | ME3 | malic enzyme 3 [Source:HGNC Symbol;Acc:HGNC:6985] |
| ENSG00000151445 | HGLibA_53664 | 47 | -0.6809032 | VIPAS39 | spe-39 homolog [Source:HGNC Symbol;Acc:HGNC:20347]" |
| ENSG00000154134 | HGLibA_41738 | 47 | -0.6809032 | ROBO3 | roundabout guidance receptor 3 [Source:HGNC Symbol;Acc:HGNC:13433] |
| ENSG00000154781 | HGLibA_07703 | 47 | -0.6809032 | CCDC174 | coiled-coil domain containing 174 [Source:HGNC Symbol;Acc:HGNC:28033] |
| ENSG00000154889 | HGLibA_29768 | 47 | -0.6809032 | MPPE1 | metallophosphoesterase 1 [Source:HGNC Symbol;Acc:HGNC:15988] |
| ENSG00000155229 | HGLibA_29552 | 47 | -0.6809032 | MMS19 | cytosolic iron-sulfur assembly component [Source:HGNC Symbol;Acc:HGNC:13824]" |
| ENSG00000155380 | HGLibA_44417 | 47 | -0.6809032 | SLC16A1 | solute carrier family 16 member 1 [Source:HGNC Symbol;Acc:HGNC:10922] |
| ENSG00000156509 | HGLibA_17103 | 47 | -0.6809032 | FBXO43 | F-box protein 43 [Source:HGNC Symbol;Acc:HGNC:28521] |
| ENSG00000156510 | HGLibA_21588 | 47 | -0.6809032 | HKDC1 | hexokinase domain containing 1 [Source:HGNC Symbol;Acc:HGNC:23302] |
| ENSG00000157680 | HGLibA_13075 | 47 | -0.6809032 | DGKI | diacylglycerol kinase iota [Source:HGNC Symbol;Acc:HGNC:2855] |
| ENSG00000157999 | HGLibA_02132 | 47 | -0.6809032 | ANKRD61 | ankyrin repeat domain 61 [Source:HGNC Symbol;Acc:HGNC:22467] |
| ENSG00000158683 | HGLibA_36792 | 47 | -0.6809032 | PKD1L1 | transient receptor potential channel interacting [Source:HGNC Symbol;Acc:HGNC:18053]" |
| ENSG00000158955 | HGLibA_54396 | 47 | -0.6809032 | WNT9B | Wnt family member 9B [Source:HGNC Symbol;Acc:HGNC:12779] |
| ENSG00000160062 | HGLibA_54925 | 47 | -0.6809032 | ZBTB8A | zinc finger and BTB domain containing 8A [Source:HGNC Symbol;Acc:HGNC:24172] |
| ENSG00000160131 | HGLibA_53685 | 47 | -0.6809032 | VMA21 | vacuolar ATPase assembly factor [Source:HGNC Symbol;Acc:HGNC:22082]" |
| ENSG00000160194 | HGLibA_31574 | 47 | -0.6809032 | NDUFV3 | NADH:ubiquinone oxidoreductase subunit V3 [Source:HGNC Symbol;Acc:HGNC:7719] |
| ENSG00000160326 | HGLibA_44885 | 47 | -0.6809032 | SLC2A6 | solute carrier family 2 member 6 [Source:HGNC Symbol;Acc:HGNC:11011] |
| ENSG00000160326 | HGLibA_44884 | 47 | -0.6809032 | SLC2A6 | solute carrier family 2 member 6 [Source:HGNC Symbol;Acc:HGNC:11011] |
| ENSG00000161270 | HGLibA_32454 | 47 | -0.6809032 | NPHS1 | nephrin [Source:HGNC Symbol;Acc:HGNC:7908]" |
| ENSG00000161692 | HGLibA_12378 | 47 | -0.6809032 | DBF4B | DBF4 zinc finger B [Source:HGNC Symbol;Acc:HGNC:17883] |
| ENSG00000162066 | HGLibA_01763 | 47 | -0.6809032 | AMDHD2 | amidohydrolase domain containing 2 [Source:HGNC Symbol;Acc:HGNC:24262] |
| ENSG00000162385 | HGLibA_28008 | 47 | -0.6809032 | MAGOH | exon junction complex subunit [Source:HGNC Symbol;Acc:HGNC:6815]" |
| ENSG00000162639 | HGLibA_21134 | 47 | -0.6809032 | HENMT1 | HEN methyltransferase 1 [Source:HGNC Symbol;Acc:HGNC:26400] |
| ENSG00000162654 | HGLibA_18737 | 47 | -0.6809032 | GBP4 | guanylate binding protein 4 [Source:HGNC Symbol;Acc:HGNC:20480] |
| ENSG00000162664 | HGLibA_55807 | 47 | -0.6809032 | ZNF326 | zinc finger protein 326 [Source:HGNC Symbol;Acc:HGNC:14104] |
| ENSG00000163563 | HGLibA_29566 | 47 | -0.6809032 | MNDA | myeloid cell nuclear differentiation antigen [Source:HGNC Symbol;Acc:HGNC:7183] |
| ENSG00000163630 | HGLibA_48026 | 47 | -0.6809032 | SYNPR | synaptoporin [Source:HGNC Symbol;Acc:HGNC:16507] |
| ENSG00000163781 | HGLibA_51008 | 47 | -0.6809032 | TOPBP1 | DNA topoisomerase II binding protein 1 [Source:HGNC Symbol;Acc:HGNC:17008] |
| ENSG00000163864 | HGLibA_32190 | 47 | -0.6809032 | NMNAT3 | nicotinamide nucleotide adenylyltransferase 3 [Source:HGNC Symbol;Acc:HGNC:20989] |
| ENSG00000164068 | HGLibA_41491 | 47 | -0.6809032 | RNF123 | ring finger protein 123 [Source:HGNC Symbol;Acc:HGNC:21148] |
| ENSG00000164849 | HGLibA_19919 | 47 | -0.6809032 | GPR146 | G protein-coupled receptor 146 [Source:HGNC Symbol;Acc:HGNC:21718] |
| ENSG00000164904 | HGLibA_01583 | 47 | -0.6809032 | ALDH7A1 | aldehyde dehydrogenase 7 family member A1 [Source:HGNC Symbol;Acc:HGNC:877] |
| ENSG00000165194 | HGLibA_35409 | 47 | -0.6809032 | PCDH19 | protocadherin 19 [Source:HGNC Symbol;Acc:HGNC:14270] |
| ENSG00000165526 | HGLibA_42215 | 47 | -0.6809032 | RPUSD4 | RNA pseudouridine synthase D4 [Source:HGNC Symbol;Acc:HGNC:25898] |
| ENSG00000165953 | HGLibA_43514 | 47 | -0.6809032 | SERPINA12 | serpin family A member 12 [Source:HGNC Symbol;Acc:HGNC:18359] |
| ENSG00000167195 | HGLibA_19612 | 47 | -0.6809032 | GOLGA6C | golgin A6 family member C [Source:HGNC Symbol;Acc:HGNC:32206] |
| ENSG00000167202 | HGLibA_48527 | 47 | -0.6809032 | TBC1D2B | TBC1 domain family member 2B [Source:HGNC Symbol;Acc:HGNC:29183] |
| ENSG00000167641 | HGLibA_38034 | 47 | -0.6809032 | PPP1R14A | protein phosphatase 1 regulatory inhibitor subunit 14A [Source:HGNC Symbol;Acc:HGNC:14871] |
| ENSG00000167685 | HGLibA_56014 | 47 | -0.6809032 | ZNF444 | zinc finger protein 444 [Source:HGNC Symbol;Acc:HGNC:16052] |
| ENSG00000167771 | HGLibA_40713 | 47 | -0.6809032 | RCOR2 | REST corepressor 2 [Source:HGNC Symbol;Acc:HGNC:27455] |
| ENSG00000167962 | HGLibA_56357 | 47 | -0.6809032 | ZNF598 | zinc finger protein 598 [Source:HGNC Symbol;Acc:HGNC:28079] |
| ENSG00000168288 | HGLibA_29459 | 47 | -0.6809032 | MMADHC | metabolism of cobalamin associated D [Source:HGNC Symbol;Acc:HGNC:25221] |
| ENSG00000169189 | HGLibA_32765 | 47 | -0.6809032 | NSMCE1 | SMC5-SMC6 complex component [Source:HGNC Symbol;Acc:HGNC:29897]" |
| ENSG00000169217 | HGLibA_08331 | 47 | -0.6809032 | CD2BP2 | CD2 cytoplasmic tail binding protein 2 [Source:HGNC Symbol;Acc:HGNC:1656] |
| ENSG00000169777 | HGLibA_48362 | 47 | -0.6809032 | TAS2R1 | taste 2 receptor member 1 [Source:HGNC Symbol;Acc:HGNC:14909] |
| ENSG00000170549 | HGLibA_23627 | 47 | -0.6809032 | IRX1 | iroquois homeobox 1 [Source:HGNC Symbol;Acc:HGNC:14358] |
| ENSG00000170890 | HGLibA_36881 | 47 | -0.6809032 | PLA2G1B | phospholipase A2 group IB [Source:HGNC Symbol;Acc:HGNC:9030] |
| ENSG00000170959 | HGLibA_12482 | 47 | -0.6809032 | DCDC1 | doublecortin domain containing 1 [Source:HGNC Symbol;Acc:HGNC:20625] |
| ENSG00000171094 | HGLibA_01643 | 47 | -0.6809032 | ALK | ALK receptor tyrosine kinase [Source:HGNC Symbol;Acc:HGNC:427] |
| ENSG00000171608 | HGLibA_36640 | 47 | -0.6809032 | PIK3CD | 5-bisphosphate 3-kinase catalytic subunit delta [Source:HGNC Symbol;Acc:HGNC:8977]" |
| ENSG00000172352 | HGLibA_08909 | 47 | -0.6809032 | CDY1B | chromodomain Y-linked 1B [Source:HGNC Symbol;Acc:HGNC:23920] |
| ENSG00000173011 | HGLibA_48190 | 47 | -0.6809032 | TADA2B | transcriptional adaptor 2B [Source:HGNC Symbol;Acc:HGNC:30781] |
| ENSG00000173442 | HGLibA_14664 | 47 | -0.6809032 | EHBP1L1 | EH domain binding protein 1 like 1 [Source:HGNC Symbol;Acc:HGNC:30682] |
| ENSG00000173559 | HGLibA_31025 | 47 | -0.6809032 | NABP1 | nucleic acid binding protein 1 [Source:HGNC Symbol;Acc:HGNC:26232] |
| ENSG00000173610 | HGLibA_52902 | 47 | -0.6809032 | UGT2A1 | UDP glucuronosyltransferase family 2 member A1 complex locus [Source:HGNC Symbol;Acc:HGNC:12542] |
| ENSG00000174442 | HGLibA_57066 | 47 | -0.6809032 | ZWILCH | zwilch kinetochore protein [Source:HGNC Symbol;Acc:HGNC:25468] |
| ENSG00000174903 | HGLibA_39832 | 47 | -0.6809032 | RAB1B | member RAS oncogene family [Source:HGNC Symbol;Acc:HGNC:18370]" |
| ENSG00000175097 | HGLibA_40134 | 47 | -0.6809032 | RAG2 | recombination activating 2 [Source:HGNC Symbol;Acc:HGNC:9832] |
| ENSG00000175155 | HGLibA_54710 | 47 | -0.6809032 | YPEL2 | yippee like 2 [Source:HGNC Symbol;Acc:HGNC:18326] |
| ENSG00000175538 | HGLibA_24191 | 47 | -0.6809032 | KCNE3 | potassium voltage-gated channel subfamily E regulatory subunit 3 [Source:HGNC Symbol;Acc:HGNC:6243] |
| ENSG00000175544 | HGLibA_06855 | 47 | -0.6809032 | CABP4 | calcium binding protein 4 [Source:HGNC Symbol;Acc:HGNC:1386] |
| ENSG00000176406 | HGLibA_41281 | 47 | -0.6809032 | RIMS2 | regulating synaptic membrane exocytosis 2 [Source:HGNC Symbol;Acc:HGNC:17283] |
| ENSG00000177202 | HGLibA_46331 | 47 | -0.6809032 | SPACA4 | sperm acrosome associated 4 [Source:HGNC Symbol;Acc:HGNC:16441] |
| ENSG00000177212 | HGLibA_33875 | 47 | -0.6809032 | OR2T33 | olfactory receptor family 2 subfamily T member 33 [Source:HGNC Symbol;Acc:HGNC:31255] |
| ENSG00000177494 | HGLibA_54793 | 47 | -0.6809032 | ZBED2 | zinc finger BED-type containing 2 [Source:HGNC Symbol;Acc:HGNC:20710] |
| ENSG00000177599 | HGLibA_56092 | 47 | -0.6809032 | ZNF491 | zinc finger protein 491 [Source:HGNC Symbol;Acc:HGNC:23706] |
| ENSG00000178445 | HGLibA_19236 | 47 | -0.6809032 | GLDC | glycine decarboxylase [Source:HGNC Symbol;Acc:HGNC:4313] |
| ENSG00000179085 | HGLibA_13862 | 47 | -0.6809032 | DPM3 | dolichyl-phosphate mannosyltransferase subunit 3 [Source:HGNC Symbol;Acc:HGNC:3007] |
| ENSG00000179593 | HGLibA_01684 | 47 | -0.6809032 | ALOX15B | type B [Source:HGNC Symbol;Acc:HGNC:434]" |
| ENSG00000180543 | HGLibA_51988 | 47 | -0.6809032 | TSPYL5 | TSPY like 5 [Source:HGNC Symbol;Acc:HGNC:29367] |
| ENSG00000181029 | HGLibA_51327 | 47 | -0.6809032 | TRAPPC5 | trafficking protein particle complex 5 [Source:HGNC Symbol;Acc:HGNC:23067] |
| ENSG00000181499 | HGLibA_34446 | 47 | -0.6809032 | OR6T1 | olfactory receptor family 6 subfamily T member 1 [Source:HGNC Symbol;Acc:HGNC:14848] |
| ENSG00000182095 | HGLibA_50909 | 47 | -0.6809032 | TNRC18 | trinucleotide repeat containing 18 [Source:HGNC Symbol;Acc:HGNC:11962] |
| ENSG00000182359 | HGLibA_24106 | 47 | -0.6809032 | KBTBD3 | kelch repeat and BTB domain containing 3 [Source:HGNC Symbol;Acc:HGNC:22934] |
| ENSG00000182534 | HGLibA_30624 | 47 | -0.6809032 | MXRA7 | matrix remodeling associated 7 [Source:HGNC Symbol;Acc:HGNC:7541] |
| ENSG00000183011 | HGLibA_30993 | 47 | -0.6809032 | NAA38 | NatC auxiliary subunit [Source:HGNC Symbol;Acc:HGNC:28212]" |
| ENSG00000183160 | HGLibA_49891 | 47 | -0.6809032 | TMEM119 | transmembrane protein 119 [Source:HGNC Symbol;Acc:HGNC:27884] |
| ENSG00000183691 | HGLibA_32253 | 47 | -0.6809032 | NOG | noggin [Source:HGNC Symbol;Acc:HGNC:7866] |
| ENSG00000183709 | HGLibA_22721 | 47 | -0.6809032 | IFNL2 | interferon lambda 2 [Source:HGNC Symbol;Acc:HGNC:18364] |
| ENSG00000183734 | HGLibA_03269 | 47 | -0.6809032 | ASCL2 | achaete-scute family bHLH transcription factor 2 [Source:HGNC Symbol;Acc:HGNC:739] |
| ENSG00000183780 | HGLibA_45006 | 47 | -0.6809032 | SLC35F3 | solute carrier family 35 member F3 [Source:HGNC Symbol;Acc:HGNC:23616] |
| ENSG00000183826 | HGLibA_04925 | 47 | -0.6809032 | BTBD9 | BTB domain containing 9 [Source:HGNC Symbol;Acc:HGNC:21228] |
| ENSG00000184863 | HGLibA_40534 | 47 | -0.6809032 | RBM33 | RNA binding motif protein 33 [Source:HGNC Symbol;Acc:HGNC:27223] |
| ENSG00000185792 | HGLibA_32135 | 47 | -0.6809032 | NLRP9 | NLR family pyrin domain containing 9 [Source:HGNC Symbol;Acc:HGNC:22941] |
| ENSG00000187242 | HGLibA_25421 | 47 | -0.6809032 | KRT12 | keratin 12 [Source:HGNC Symbol;Acc:HGNC:6414] |
| ENSG00000188092 | HGLibA_20109 | 47 | -0.6809032 | GPR89B | G protein-coupled receptor 89B [Source:HGNC Symbol;Acc:HGNC:13840] |
| ENSG00000196151 | HGLibA_54229 | 47 | -0.6809032 | WDSUB1 | sterile alpha motif and U-box domain containing 1 [Source:HGNC Symbol;Acc:HGNC:26697]" |
| ENSG00000196844 | HGLibA_35273 | 47 | -0.6809032 | PATE2 | prostate and testis expressed 2 [Source:HGNC Symbol;Acc:HGNC:32249] |
| ENSG00000197272 | HGLibA_23112 | 47 | -0.6809032 | IL27 | interleukin 27 [Source:HGNC Symbol;Acc:HGNC:19157] |
| ENSG00000197619 | HGLibA_56393 | 47 | -0.6809032 | ZNF615 | zinc finger protein 615 [Source:HGNC Symbol;Acc:HGNC:24740] |
| ENSG00000198286 | HGLibA_07221 | 47 | -0.6809032 | CARD11 | caspase recruitment domain family member 11 [Source:HGNC Symbol;Acc:HGNC:16393] |
| ENSG00000198400 | HGLibA_32874 | 47 | -0.6809032 | NTRK1 | neurotrophic receptor tyrosine kinase 1 [Source:HGNC Symbol;Acc:HGNC:8031] |
| ENSG00000198740 | HGLibA_56462 | 47 | -0.6809032 | ZNF652 | zinc finger protein 652 [Source:HGNC Symbol;Acc:HGNC:29147] |
| ENSG00000198799 | HGLibA_27179 | 47 | -0.6809032 | LRIG2 | leucine rich repeats and immunoglobulin like domains 2 [Source:HGNC Symbol;Acc:HGNC:20889] |
| ENSG00000198825 | HGLibA_23354 | 47 | -0.6809032 | INPP5F | inositol polyphosphate-5-phosphatase F [Source:HGNC Symbol;Acc:HGNC:17054] |
| ENSG00000198944 | HGLibA_46214 | 47 | -0.6809032 | SOWAHA | sosondowah ankyrin repeat domain family member A [Source:HGNC Symbol;Acc:HGNC:27033] |
| ENSG00000203697 | HGLibA_07179 | 47 | -0.6809032 | CAPN8 | calpain 8 [Source:HGNC Symbol;Acc:HGNC:1485] |
| ENSG00000204531 | HGLibA_37786 | 47 | -0.6809032 | POU5F1 | POU class 5 homeobox 1 [Source:HGNC Symbol;Acc:HGNC:9221] |
| ENSG00000205279 | HGLibA_11760 | 47 | -0.6809032 | CTXN3 | cortexin 3 [Source:HGNC Symbol;Acc:HGNC:31110] |

| | | | | | |
|---|---|---|---|---|---|
| ENSG00000205439 | HGLibA_25635 | 47 | -0.6809032 | KRTAP12-3 | keratin associated protein 12-3 [Source:HGNC Symbol;Acc:HGNC:20531] |
| ENSG00000206073 | HGLibA_43552 | 47 | -0.6809032 | SERPINB4 | serpin family B member 4 [Source:HGNC Symbol;Acc:HGNC:10570] |
| ENSG00000206432 | HGLibA_50176 | 47 | -0.6809032 | TMEM200C | transmembrane protein 200C [Source:HGNC Symbol;Acc:HGNC:37208] |
| ENSG00000213214 | HGLibA_02852 | 47 | -0.6809032 | ARHGEF35 | Rho guanine nucleotide exchange factor 35 [Source:HGNC Symbol;Acc:HGNC:33846] |
| ENSG00000215114 | HGLibA_52785 | 47 | -0.6809032 | UBXN2B | UBX domain protein 2B [Source:HGNC Symbol;Acc:HGNC:27035] |
| ENSG00000215114 | HGLibA_52787 | 47 | -0.6809032 | UBXN2B | UBX domain protein 2B [Source:HGNC Symbol;Acc:HGNC:27035] |
| ENSG00000215788 | HGLibA_50780 | 47 | -0.6809032 | TNFRSF25 | TNF receptor superfamily member 25 [Source:HGNC Symbol;Acc:HGNC:11910] |
| ENSG00000217442 | HGLibA_47922 | 47 | -0.6809032 | SYCE3 | synaptonemal complex central element protein 3 [Source:HGNC Symbol;Acc:HGNC:35245] |
| ENSG00000221866 | HGLibA_37267 | 47 | -0.6809032 | PLXNA4 | plexin A4 [Source:HGNC Symbol;Acc:HGNC:9102] |
| ENSG00000229665 | HGLibA_38807 | 47 | -0.6809032 | PRR20C | proline rich 20C [Source:HGNC Symbol;Acc:HGNC:37221] |
| ENSG00000233927 | HGLibA_42130 | 47 | -0.6809032 | RPS28 | ribosomal protein S28 [Source:HGNC Symbol;Acc:HGNC:10418] |
| ENSG00000240432 | HGLibA_25647 | 47 | -0.6809032 | KRTAP13-3 | keratin associated protein 13-3 [Source:HGNC Symbol;Acc:HGNC:18925] |
| ENSG00000248712 | HGLibA_07652 | 47 | -0.6809032 | CCDC153 | coiled-coil domain containing 153 [Source:HGNC Symbol;Acc:HGNC:27446] |
| ENSG00000253953 | HGLibA_35560 | 47 | -0.6809032 | PCDHGB4 | 4 [Source:HGNC Symbol;Acc:HGNC:8711]" |
| ENSG00000254004 | HGLibA_55691 | 47 | -0.6809032 | ZNF260 | zinc finger protein 260 [Source:HGNC Symbol;Acc:HGNC:13499] |
| ENSG00000255713 | HGLibA_33975 | 47 | -0.6809032 | OR4D2 | olfactory receptor family 4 subfamily D member 2 [Source:HGNC Symbol;Acc:HGNC:8294] |
| ENSG00000255713 | HGLibA_33974 | 47 | -0.6809032 | OR4D2 | olfactory receptor family 4 subfamily D member 2 [Source:HGNC Symbol;Acc:HGNC:8294] |
| ENSG00000256162 | HGLibA_45779 | 47 | -0.6809032 | SMLR1 | small leucine rich protein 1 [Source:HGNC Symbol;Acc:HGNC:44670] |
| ENSG00000257017 | HGLibA_22010 | 47 | -0.6809032 | HP | haptoglobin [Source:HGNC Symbol;Acc:HGNC:5141] |
| ENSG00000258223 | HGLibA_38970 | 47 | -0.6809032 | PRSS58 | serine protease 58 [Source:HGNC Symbol;Acc:HGNC:39125] |
| ENSG00000259075 | HGLibA_37446 | 47 | -0.6809032 | POC1B-GALNT4 | POC1B-GALNT4 readthrough [Source:HGNC Symbol;Acc:HGNC:42957] |
| ENSG00000267680 | HGLibA_55617 | 47 | -0.6809032 | ZNF224 | zinc finger protein 224 [Source:HGNC Symbol;Acc:HGNC:13017] |
| ENSG00000270181 | HGLibA_04537 | 47 | -0.6809032 | BIVM-ERCC5 | BIVM-ERCC5 readthrough [Source:HGNC Symbol;Acc:HGNC:43690] |
| GAGE5 | HGLibA_18482 | 47 | -0.6809032 | GAGE5 | - |
| H3F3B | HGLibA_20782 | 47 | -0.6809032 | H3F3B | - |
| HIST1H2BF | HGLibA_21425 | 47 | -0.6809032 | HIST1H2BF | - |
| HIST2H2BE | HGLibA_21533 | 47 | -0.6809032 | HIST2H2BE | - |
| HIST2H3C | HGLibA_21544 | 47 | -0.6809032 | HIST2H3C | - |
| hsa-let-7d | HGLibA_57119 | 47 | -0.6809032 | hsa-let-7d | - |
| hsa-mir-1225 | HGLibA_57297 | 47 | -0.6809032 | hsa-mir-1225 | - |
| hsa-mir-1269a | HGLibA_57503 | 47 | -0.6809032 | hsa-mir-1269a | - |
| hsa-mir-1302-11 | HGLibA_57690 | 47 | -0.6809032 | hsa-mir-1302-11 | - |
| hsa-mir-29a | HGLibA_58408 | 47 | -0.6809032 | hsa-mir-29a | - |
| hsa-mir-3179-3 | HGLibA_58798 | 47 | -0.6809032 | hsa-mir-3179-3 | - |
| hsa-mir-3198-1 | HGLibA_58891 | 47 | -0.6809032 | hsa-mir-3198-1 | - |
| hsa-mir-338 | HGLibA_59002 | 47 | -0.6809032 | hsa-mir-338 | - |
| hsa-mir-3907 | HGLibA_59492 | 47 | -0.6809032 | hsa-mir-3907 | - |
| hsa-mir-3920 | HGLibA_59558 | 47 | -0.6809032 | hsa-mir-3920 | - |
| hsa-mir-4289 | HGLibA_59864 | 47 | -0.6809032 | hsa-mir-4289 | - |
| hsa-mir-4314 | HGLibA_59971 | 47 | -0.6809032 | hsa-mir-4314 | - |
| hsa-mir-4457 | HGLibA_60235 | 47 | -0.6809032 | hsa-mir-4457 | - |
| hsa-mir-450a-2 | HGLibA_60476 | 47 | -0.6809032 | hsa-mir-450a-2 | - |
| hsa-mir-4536-2 | HGLibA_60606 | 47 | -0.6809032 | hsa-mir-4536-2 | - |
| hsa-mir-455 | HGLibA_60629 | 47 | -0.6809032 | hsa-mir-455 | - |
| hsa-mir-4638 | HGLibA_60656 | 47 | -0.6809032 | hsa-mir-4638 | - |
| hsa-mir-4648 | HGLibA_60696 | 47 | -0.6809032 | hsa-mir-4648 | - |
| hsa-mir-4742 | HGLibA_61064 | 47 | -0.6809032 | hsa-mir-4742 | - |
| hsa-mir-4772 | HGLibA_61190 | 47 | -0.6809032 | hsa-mir-4772 | - |
| hsa-mir-4999 | HGLibA_61396 | 47 | -0.6809032 | hsa-mir-4999 | - |
| hsa-mir-509-3 | HGLibA_61516 | 47 | -0.6809032 | hsa-mir-509-3 | - |
| hsa-mir-518a-2 | HGLibA_61656 | 47 | -0.6809032 | hsa-mir-518a-2 | - |
| hsa-mir-548m | HGLibA_62039 | 47 | -0.6809032 | hsa-mir-548m | - |
| hsa-mir-570 | HGLibA_62332 | 47 | -0.6809032 | hsa-mir-570 | - |
| hsa-mir-6743 | HGLibA_63124 | 47 | -0.6809032 | hsa-mir-6743 | - |
| hsa-mir-6758 | HGLibA_63191 | 47 | -0.6809032 | hsa-mir-6758 | - |
| hsa-mir-6830 | HGLibA_63499 | 47 | -0.6809032 | hsa-mir-6830 | - |
| hsa-mir-6882 | HGLibA_63711 | 47 | -0.6809032 | hsa-mir-6882 | - |
| hsa-mir-6890 | HGLibA_63745 | 47 | -0.6809032 | hsa-mir-6890 | - |
| hsa-mir-7706 | HGLibA_63959 | 47 | -0.6809032 | hsa-mir-7706 | - |
| hsa-mir-7843 | HGLibA_63963 | 47 | -0.6809032 | hsa-mir-7843 | - |
| KIAA1984 | HGLibA_24836 | 47 | -0.6809032 | KIAA1984 | - |
| LOC100288332 | HGLibA_26831 | 47 | -0.6809032 | LOC100288332 | - |
| LPPR1 | HGLibA_27114 | 47 | -0.6809032 | LPPR1 | - |
| MLL2 | HGLibA_29397 | 47 | -0.6809032 | MLL2 | - |
| PQLC2 | HGLibA_38255 | 47 | -0.6809032 | PQLC2 | - |
| SSSCA1 | HGLibA_47169 | 47 | -0.6809032 | SSSCA1 | - |
| TWISTNB | HGLibA_52380 | 47 | -0.6809032 | TWISTNB | - |
| ATP5L2 | HGLibA_03681 | 46 | -0.690854237 | ATP5L2 | - |
| C11orf84 | HGLibA_05197 | 46 | -0.690854237 | C11orf84 | - |
| C13orf35 | HGLibA_05308 | 46 | -0.690854237 | C13orf35 | - |
| CASC1 | HGLibA_07267 | 46 | -0.690854237 | CASC1 | - |
| DUPD1 | HGLibA_14109 | 46 | -0.690854237 | DUPD1 | - |
| ENSG00000005700 | HGLibA_22485 | 46 | -0.690854237 | IBTK | inhibitor of Bruton tyrosine kinase [Source:HGNC Symbol;Acc:HGNC:17853] |
| ENSG00000008838 | HGLibA_28784 | 46 | -0.690854237 | MED24 | mediator complex subunit 24 [Source:HGNC Symbol;Acc:HGNC:22963] |
| ENSG00000011275 | HGLibA_41631 | 46 | -0.690854237 | RNF216 | ring finger protein 216 [Source:HGNC Symbol;Acc:HGNC:21698] |
| ENSG00000023608 | HGLibA_45891 | 46 | -0.690854237 | SNAPC1 | small nuclear RNA activating complex polypeptide 1 [Source:HGNC Symbol;Acc:HGNC:11134] |
| ENSG00000042445 | HGLibA_40881 | 46 | -0.690854237 | RETSAT | retinol saturase [Source:HGNC Symbol;Acc:HGNC:25991] |
| ENSG00000047188 | HGLibA_54726 | 46 | -0.690854237 | YTHDC2 | YTH domain containing 2 [Source:HGNC Symbol;Acc:HGNC:24721] |
| ENSG00000049283 | HGLibA_15348 | 46 | -0.690854237 | EPN3 | epsin 3 [Source:HGNC Symbol;Acc:HGNC:18235] |
| ENSG00000054118 | HGLibA_49390 | 46 | -0.690854237 | THRAP3 | thyroid hormone receptor associated protein 3 [Source:HGNC Symbol;Acc:HGNC:22964] |
| ENSG00000055955 | HGLibA_23814 | 46 | -0.690854237 | ITIH4 | inter-alpha-trypsin inhibitor heavy chain family member 4 [Source:HGNC Symbol;Acc:HGNC:6169] |
| ENSG00000057252 | HGLibA_46114 | 46 | -0.690854237 | SOAT1 | sterol O-acyltransferase 1 [Source:HGNC Symbol;Acc:HGNC:11177] |
| ENSG00000059573 | HGLibA_01538 | 46 | -0.690854237 | ALDH18A1 | aldehyde dehydrogenase 18 family member A1 [Source:HGNC Symbol;Acc:HGNC:9722] |
| ENSG00000062194 | HGLibA_19756 | 46 | -0.690854237 | GPBP1 | GC-rich promoter binding protein 1 [Source:HGNC Symbol;Acc:HGNC:29520] |
| ENSG00000062598 | HGLibA_14934 | 46 | -0.690854237 | ELMO2 | engulfment and cell motility 2 [Source:HGNC Symbol;Acc:HGNC:17233] |
| ENSG00000065150 | HGLibA_23468 | 46 | -0.690854237 | IPO5 | importin 5 [Source:HGNC Symbol;Acc:HGNC:6402] |
| ENSG00000072163 | HGLibA_26548 | 46 | -0.690854237 | LIMS2 | LIM zinc finger domain containing 2 [Source:HGNC Symbol;Acc:HGNC:16084] |
| ENSG00000072682 | HGLibA_34903 | 46 | -0.690854237 | P4HA2 | prolyl 4-hydroxylase subunit alpha 2 [Source:HGNC Symbol;Acc:HGNC:8547] |
| ENSG00000075891 | HGLibA_35298 | 46 | -0.690854237 | PAX2 | paired box 2 [Source:HGNC Symbol;Acc:HGNC:8616] |
| ENSG00000077713 | HGLibA_44745 | 46 | -0.690854237 | SLC25A43 | solute carrier family 25 member 43 [Source:HGNC Symbol;Acc:HGNC:30557] |
| ENSG00000079150 | HGLibA_17584 | 46 | -0.690854237 | FKBP7 | FK506 binding protein 7 [Source:HGNC Symbol;Acc:HGNC:3723] |
| ENSG00000083520 | HGLibA_13271 | 46 | -0.690854237 | DIS3 | exosome endoribonuclease and 3'-5' exoribonuclease [Source:HGNC Symbol;Acc:HGNC:20604]" |
| ENSG00000084110 | HGLibA_20819 | 46 | -0.690854237 | HAL | histidine ammonia-lyase [Source:HGNC Symbol;Acc:HGNC:4806] |
| ENSG00000092140 | HGLibA_18296 | 46 | -0.690854237 | G2E3 | G2/M-phase specific E3 ubiquitin protein ligase [Source:HGNC Symbol;Acc:HGNC:20338] |
| ENSG00000093010 | HGLibA_10685 | 46 | -0.690854237 | COMT | catechol-O-methyltransferase [Source:HGNC Symbol;Acc:HGNC:2228] |
| ENSG00000095981 | HGLibA_24309 | 46 | -0.690854237 | KCNK16 | potassium two pore domain channel subfamily K member 16 [Source:HGNC Symbol;Acc:HGNC:14464] |
| ENSG00000099953 | HGLibA_29483 | 46 | -0.690854237 | MMP11 | matrix metallopeptidase 11 [Source:HGNC Symbol;Acc:HGNC:7157] |
| ENSG00000100142 | HGLibA_37579 | 46 | -0.690854237 | POLR2F | RNA polymerase II subunit F [Source:HGNC Symbol;Acc:HGNC:9193] |
| ENSG00000100325 | HGLibA_03261 | 46 | -0.690854237 | ASCC2 | activating signal cointegrator 1 complex subunit 2 [Source:HGNC Symbol;Acc:HGNC:24103] |
| ENSG00000100504 | HGLibA_39689 | 46 | -0.690854237 | PYGL | glycogen phosphorylase L [Source:HGNC Symbol;Acc:HGNC:9725] |
| ENSG00000100889 | HGLibA_35617 | 46 | -0.690854237 | PCK2 | mitochondrial [Source:HGNC Symbol;Acc:HGNC:8725]" |
| ENSG00000100902 | HGLibA_39085 | 46 | -0.690854237 | PSMA6 | proteasome subunit alpha 6 [Source:HGNC Symbol;Acc:HGNC:9535] |
| ENSG00000101074 | HGLibA_39782 | 46 | -0.690854237 | R3HDML | R3H domain containing like [Source:HGNC Symbol;Acc:HGNC:16249] |
| ENSG00000101220 | HGLibA_06024 | 46 | -0.690854237 | C20orf27 | chromosome 20 open reading frame 27 [Source:HGNC Symbol;Acc:HGNC:15873] |
| ENSG00000101421 | HGLibA_09491 | 46 | -0.690854237 | CHMP4B | charged multivesicular body protein 4B [Source:HGNC Symbol;Acc:HGNC:16171] |
| ENSG00000101425 | HGLibA_04706 | 46 | -0.690854237 | BPI | bactericidal permeability increasing protein [Source:HGNC Symbol;Acc:HGNC:1095] |
| ENSG00000102098 | HGLibA_42963 | 46 | -0.690854237 | SCML2 | Scm polycomb group protein like 2 [Source:HGNC Symbol;Acc:HGNC:10581] |
| ENSG00000103356 | HGLibA_14343 | 46 | -0.690854237 | EARS2 | mitochondrial [Source:HGNC Symbol;Acc:HGNC:29419]" |
| ENSG00000103599 | HGLibA_23521 | 46 | -0.690854237 | IQCH | IQ motif containing H [Source:HGNC Symbol;Acc:HGNC:25721] |
| ENSG00000104129 | HGLibA_13585 | 46 | -0.690854237 | DNAJC17 | DnaJ heat shock protein family (Hsp40) member C17 [Source:HGNC Symbol;Acc:HGNC:25556] |
| ENSG00000104783 | HGLibA_24362 | 46 | -0.690854237 | KCNN4 | potassium calcium-activated channel subfamily N member 4 [Source:HGNC Symbol;Acc:HGNC:6293] |
| ENSG00000105376 | HGLibA_22507 | 46 | -0.690854237 | ICAM5 | intercellular adhesion molecule 5 [Source:HGNC Symbol;Acc:HGNC:5348] |
| ENSG00000105518 | HGLibA_50193 | 46 | -0.690854237 | TMEM205 | transmembrane protein 205 [Source:HGNC Symbol;Acc:HGNC:29631] |
| ENSG00000105568 | HGLibA_38139 | 46 | -0.690854237 | PPP2R1A | protein phosphatase 2 scaffold subunit Aalpha [Source:HGNC Symbol;Acc:HGNC:9302] |
| ENSG00000105642 | HGLibA_24355 | 46 | -0.690854237 | KCNN1 | potassium calcium-activated channel subfamily N member 1 [Source:HGNC Symbol;Acc:HGNC:6290] |
| ENSG00000105851 | HGLibA_36642 | 46 | -0.690854237 | PIK3CG | 5-bisphosphate 3-kinase catalytic subunit gamma [Source:HGNC Symbol;Acc:HGNC:8978]" |
| ENSG00000106336 | HGLibA_17050 | 46 | -0.690854237 | FBXO24 | F-box protein 24 [Source:HGNC Symbol;Acc:HGNC:13595] |
| ENSG00000108255 | HGLibA_11252 | 46 | -0.690854237 | CRYBA1 | crystallin beta A1 [Source:HGNC Symbol;Acc:HGNC:2394] |
| ENSG00000108384 | HGLibA_40089 | 46 | -0.690854237 | RAD51C | RAD51 paralog C [Source:HGNC Symbol;Acc:HGNC:9820] |
| ENSG00000109272 | HGLibA_36133 | 46 | -0.690854237 | PF4V1 | platelet factor 4 variant 1 [Source:HGNC Symbol;Acc:HGNC:8862] |
| ENSG00000109667 | HGLibA_44893 | 46 | -0.690854237 | SLC2A9 | solute carrier family 2 member 9 [Source:HGNC Symbol;Acc:HGNC:13446] |
| ENSG00000109680 | HGLibA_48491 | 46 | -0.690854237 | TBC1D19 | TBC1 domain family member 19 [Source:HGNC Symbol;Acc:HGNC:25624] |
| ENSG00000110786 | HGLibA_39473 | 46 | -0.690854237 | PTPN5 | non-receptor type 5 [Source:HGNC Symbol;Acc:HGNC:9657]" |
| ENSG00000111262 | HGLibA_24122 | 46 | -0.690854237 | KCNA1 | potassium voltage-gated channel subfamily A member 1 [Source:HGNC Symbol;Acc:HGNC:6218] |
| ENSG00000111300 | HGLibA_30984 | 46 | -0.690854237 | NAA25 | NatB auxiliary subunit [Source:HGNC Symbol;Acc:HGNC:25783]" |
| ENSG00000111615 | HGLibA_25410 | 46 | -0.690854237 | KRR1 | small subunit processome component homolog [Source:HGNC Symbol;Acc:HGNC:5176]" |
| ENSG00000111850 | HGLibA_45770 | 46 | -0.690854237 | SMIM8 | small integral membrane protein 8 [Source:HGNC Symbol;Acc:HGNC:21401] |
| ENSG00000111961 | HGLibA_42760 | 46 | -0.690854237 | SASH1 | SAM and SH3 domain containing 1 [Source:HGNC Symbol;Acc:HGNC:19182] |
| ENSG00000111961 | HGLibA_42761 | 46 | -0.690854237 | SASH1 | SAM and SH3 domain containing 1 [Source:HGNC Symbol;Acc:HGNC:19182] |
| ENSG00000112293 | HGLibA_19815 | 46 | -0.690854237 | GPLD1 | glycosylphosphatidylinositol specific phospholipase D1 [Source:HGNC Symbol;Acc:HGNC:4459] |
| ENSG00000113889 | HGLibA_25341 | 46 | -0.690854237 | KNG1 | kininogen 1 [Source:HGNC Symbol;Acc:HGNC:6383] |
| ENSG00000114978 | HGLibA_29585 | 46 | -0.690854237 | MOB1A | MOB kinase activator 1A [Source:HGNC Symbol;Acc:HGNC:16015] |
| ENSG00000115073 | HGLibA_00652 | 46 | -0.690854237 | ACTR1B | ARP1 actin related protein 1 homolog B [Source:HGNC Symbol;Acc:HGNC:168] |
| ENSG00000115275 | HGLibA_29633 | 46 | -0.690854237 | MOGS | mannosyl-oligosaccharide glucosidase [Source:HGNC Symbol;Acc:HGNC:24862] |
| ENSG00000115392 | HGLibA_16856 | 46 | -0.690854237 | FANCL | FA complementation group L [Source:HGNC Symbol;Acc:HGNC:20748] |

| | | | | | |
|---|---|---|---|---|---|
| ENSG00000115806 | HGLibA_19680 | 46 | -0.690854237 | GORASP2 | golgi reassembly stacking protein 2 [Source:HGNC Symbol;Acc:HGNC:17500] |
| ENSG00000115828 | HGLibA_39727 | 46 | -0.690854237 | QPCT | glutaminyl-peptide cyclotransferase [Source:HGNC Symbol;Acc:HGNC:9753] |
| ENSG00000116127 | HGLibA_01674 | 46 | -0.690854237 | ALMS1 | centrosome and basal body associated protein [Source:HGNC Symbol;Acc:HGNC:428]" |
| ENSG00000116731 | HGLibA_38387 | 46 | -0.690854237 | PRDM2 | PR/SET domain 2 [Source:HGNC Symbol;Acc:HGNC:9347] |
| ENSG00000116741 | HGLibA_41080 | 46 | -0.690854237 | RGS2 | regulator of G protein signaling 2 [Source:HGNC Symbol;Acc:HGNC:9998] |
| ENSG00000116857 | HGLibA_50556 | 46 | -0.690854237 | TMEM9 | transmembrane protein 9 [Source:HGNC Symbol;Acc:HGNC:18823] |
| ENSG00000119778 | HGLibA_03408 | 46 | -0.690854237 | ATAD2B | AAA domain containing 2B [Source:HGNC Symbol;Acc:HGNC:29230]" |
| ENSG00000120162 | HGLibA_29598 | 46 | -0.690854237 | MOB3B | MOB kinase activator 3B [Source:HGNC Symbol;Acc:HGNC:23825] |
| ENSG00000120594 | HGLibA_37256 | 46 | -0.690854237 | PLXDC2 | plexin domain containing 2 [Source:HGNC Symbol;Acc:HGNC:21013] |
| ENSG00000121634 | HGLibA_19152 | 46 | -0.690854237 | GJA8 | gap junction protein alpha 8 [Source:HGNC Symbol;Acc:HGNC:4281] |
| ENSG00000122223 | HGLibA_08306 | 46 | -0.690854237 | CD244 | CD244 molecule [Source:HGNC Symbol;Acc:HGNC:18171] |
| ENSG00000124659 | HGLibA_48585 | 46 | -0.690854237 | TBCC | tubulin folding cofactor C [Source:HGNC Symbol;Acc:HGNC:11580] |
| ENSG00000124749 | HGLibA_10539 | 46 | -0.690854237 | COL21A1 | collagen type XXI alpha 1 chain [Source:HGNC Symbol;Acc:HGNC:17025] |
| ENSG00000124787 | HGLibA_42040 | 46 | -0.690854237 | RPP40 | ribonuclease P/MRP subunit p40 [Source:HGNC Symbol;Acc:HGNC:20992] |
| ENSG00000125107 | HGLibA_10325 | 46 | -0.690854237 | CNOT1 | CCR4-NOT transcription complex subunit 1 [Source:HGNC Symbol;Acc:HGNC:7877] |
| ENSG00000125571 | HGLibA_23156 | 46 | -0.690854237 | IL37 | interleukin 37 [Source:HGNC Symbol;Acc:HGNC:15563] |
| ENSG00000125952 | HGLibA_28447 | 46 | -0.690854237 | MAX | MYC associated factor X [Source:HGNC Symbol;Acc:HGNC:6913] |
| ENSG00000126467 | HGLibA_51872 | 46 | -0.690854237 | TSKS | testis specific serine kinase substrate [Source:HGNC Symbol;Acc:HGNC:30719] |
| ENSG00000126882 | HGLibA_16727 | 46 | -0.690854237 | FAM78A | family with sequence similarity 78 member A [Source:HGNC Symbol;Acc:HGNC:25465] |
| ENSG00000128040 | HGLibA_46690 | 46 | -0.690854237 | SPINK2 | Kazal type 2 [Source:HGNC Symbol;Acc:HGNC:11245]" |
| ENSG00000128283 | HGLibA_08567 | 46 | -0.690854237 | CDC42EP1 | CDC42 effector protein 1 [Source:HGNC Symbol;Acc:HGNC:17014] |
| ENSG00000131096 | HGLibA_39712 | 46 | -0.690854237 | PYY | peptide YY [Source:HGNC Symbol;Acc:HGNC:9748] |
| ENSG00000131153 | HGLibA_19106 | 46 | -0.690854237 | GINS2 | GINS complex subunit 2 [Source:HGNC Symbol;Acc:HGNC:24575] |
| ENSG00000131386 | HGLibA_18546 | 46 | -0.690854237 | GALNT15 | polypeptide N-acetylgalactosaminyltransferase 15 [Source:HGNC Symbol;Acc:HGNC:21531] |
| ENSG00000133678 | HGLibA_50327 | 46 | -0.690854237 | TMEM254 | transmembrane protein 254 [Source:HGNC Symbol;Acc:HGNC:25804] |
| ENSG00000134160 | HGLibA_51752 | 46 | -0.690854237 | TRPM1 | transient receptor potential cation channel subfamily M member 1 [Source:HGNC Symbol;Acc:HGNC:7146] |
| ENSG00000134201 | HGLibA_20484 | 46 | -0.690854237 | GSTM5 | glutathione S-transferase mu 5 [Source:HGNC Symbol;Acc:HGNC:4637] |
| ENSG00000134243 | HGLibA_46199 | 46 | -0.690854237 | SORT1 | sortilin 1 [Source:HGNC Symbol;Acc:HGNC:11186] |
| ENSG00000134376 | HGLibA_11068 | 46 | -0.690854237 | CRB1 | cell polarity complex component [Source:HGNC Symbol;Acc:HGNC:2343]" |
| ENSG00000134760 | HGLibA_14023 | 46 | -0.690854237 | DSG1 | desmoglein 1 [Source:HGNC Symbol;Acc:HGNC:3048] |
| ENSG00000135314 | HGLibA_24556 | 46 | -0.690854237 | KHDC1 | KH domain containing 1 [Source:HGNC Symbol;Acc:HGNC:21366] |
| ENSG00000135637 | HGLibA_07619 | 46 | -0.690854237 | CCDC142 | coiled-coil domain containing 142 [Source:HGNC Symbol;Acc:HGNC:25889] |
| ENSG00000135905 | HGLibA_13735 | 46 | -0.690854237 | DOCK10 | dedicator of cytokinesis 10 [Source:HGNC Symbol;Acc:HGNC:23479] |
| ENSG00000136156 | HGLibA_23832 | 46 | -0.690854237 | ITM2B | integral membrane protein 2B [Source:HGNC Symbol;Acc:HGNC:6174] |
| ENSG00000136371 | HGLibA_30392 | 46 | -0.690854237 | MTHFS | methenyltetrahydrofolate synthetase [Source:HGNC Symbol;Acc:HGNC:7437] |
| ENSG00000137672 | HGLibA_51745 | 46 | -0.690854237 | TRPC6 | transient receptor potential cation channel subfamily C member 6 [Source:HGNC Symbol;Acc:HGNC:12338] |
| ENSG00000139675 | HGLibA_21794 | 46 | -0.690854237 | HNRNPA1L2 | heterogeneous nuclear ribonucleoprotein A1-like 2 [Source:HGNC Symbol;Acc:HGNC:27067] |
| ENSG00000140199 | HGLibA_44371 | 46 | -0.690854237 | SLC12A6 | solute carrier family 12 member 6 [Source:HGNC Symbol;Acc:HGNC:10914] |
| ENSG00000141338 | HGLibA_00125 | 46 | -0.690854237 | ABCA8 | ATP binding cassette subfamily A member 8 [Source:HGNC Symbol;Acc:HGNC:38] |
| ENSG00000141568 | HGLibA_17897 | 46 | -0.690854237 | FOXK2 | forkhead box K2 [Source:HGNC Symbol;Acc:HGNC:6036] |
| ENSG00000142789 | HGLibA_08997 | 46 | -0.690854237 | CELA3A | chymotrypsin like elastase family member 3A [Source:HGNC Symbol;Acc:HGNC:15944] |
| ENSG00000143110 | HGLibA_05786 | 46 | -0.690854237 | C1orf162 | chromosome 1 open reading frame 162 [Source:HGNC Symbol;Acc:HGNC:28344] |
| ENSG00000143536 | HGLibA_11207 | 46 | -0.690854237 | CRNN | cornulin [Source:HGNC Symbol;Acc:HGNC:1230] |
| ENSG00000143774 | HGLibA_20672 | 46 | -0.690854237 | GUK1 | guanylate kinase 1 [Source:HGNC Symbol;Acc:HGNC:4693] |
| ENSG00000145242 | HGLibA_15299 | 46 | -0.690854237 | EPHA5 | EPH receptor A5 [Source:HGNC Symbol;Acc:HGNC:3389] |
| ENSG00000146278 | HGLibA_37436 | 46 | -0.690854237 | PNRC1 | proline rich nuclear receptor coactivator 1 [Source:HGNC Symbol;Acc:HGNC:17278] |
| ENSG00000147127 | HGLibA_39943 | 46 | -0.690854237 | RAB41 | member RAS oncogene family [Source:HGNC Symbol;Acc:HGNC:18293]" |
| ENSG00000147316 | HGLibA_28645 | 46 | -0.690854237 | MCPH1 | microcephalin 1 [Source:HGNC Symbol;Acc:HGNC:6954] |
| ENSG00000147536 | HGLibA_19111 | 46 | -0.690854237 | GINS4 | GINS complex subunit 4 [Source:HGNC Symbol;Acc:HGNC:28226] |
| ENSG00000147647 | HGLibA_13922 | 46 | -0.690854237 | DPYS | dihydropyrimidinase [Source:HGNC Symbol;Acc:HGNC:3013] |
| ENSG00000150433 | HGLibA_50227 | 46 | -0.690854237 | TMEM218 | transmembrane protein 218 [Source:HGNC Symbol;Acc:HGNC:27344] |
| ENSG00000150510 | HGLibA_16076 | 46 | -0.690854237 | FAM124A | family with sequence similarity 124 member A [Source:HGNC Symbol;Acc:HGNC:26413] |
| ENSG00000151846 | HGLibA_34933 | 46 | -0.690854237 | PABPC3 | poly(A) binding protein cytoplasmic 3 [Source:HGNC Symbol;Acc:HGNC:8556] |
| ENSG00000151952 | HGLibA_49937 | 46 | -0.690854237 | TMEM132D | transmembrane protein 132D [Source:HGNC Symbol;Acc:HGNC:29411] |
| ENSG00000152705 | HGLibA_07360 | 46 | -0.690854237 | CATSPER3 | cation channel sperm associated 3 [Source:HGNC Symbol;Acc:HGNC:20819] |
| ENSG00000154721 | HGLibA_23931 | 46 | -0.690854237 | JAM2 | junctional adhesion molecule 2 [Source:HGNC Symbol;Acc:HGNC:14686] |
| ENSG00000155959 | HGLibA_53563 | 46 | -0.690854237 | VBP1 | VHL binding protein 1 [Source:HGNC Symbol;Acc:HGNC:12662] |
| ENSG00000157933 | HGLibA_44276 | 46 | -0.690854237 | SKI | SKI proto-oncogene [Source:HGNC Symbol;Acc:HGNC:10896] |
| ENSG00000158477 | HGLibA_08264 | 46 | -0.690854237 | CD1A | CD1a molecule [Source:HGNC Symbol;Acc:HGNC:1634] |
| ENSG00000160062 | HGLibA_54926 | 46 | -0.690854237 | ZBTB8A | zinc finger and BTB domain containing 8A [Source:HGNC Symbol;Acc:HGNC:24172] |
| ENSG00000160255 | HGLibA_23778 | 46 | -0.690854237 | ITGB2 | integrin subunit beta 2 [Source:HGNC Symbol;Acc:HGNC:6155] |
| ENSG00000160310 | HGLibA_38623 | 46 | -0.690854237 | PRMT2 | protein arginine methyltransferase 2 [Source:HGNC Symbol;Acc:HGNC:5186] |
| ENSG00000160352 | HGLibA_56619 | 46 | -0.690854237 | ZNF714 | zinc finger protein 714 [Source:HGNC Symbol;Acc:HGNC:27124] |
| ENSG00000160472 | HGLibA_50140 | 46 | -0.690854237 | TMEM190 | transmembrane protein 190 [Source:HGNC Symbol;Acc:HGNC:29632] |
| ENSG00000160785 | HGLibA_44747 | 46 | -0.690854237 | SLC25A44 | solute carrier family 25 member 44 [Source:HGNC Symbol;Acc:HGNC:29036] |
| ENSG00000161914 | HGLibA_56464 | 46 | -0.690854237 | ZNF653 | zinc finger protein 653 [Source:HGNC Symbol;Acc:HGNC:25196] |
| ENSG00000162598 | HGLibA_05903 | 46 | -0.690854237 | C1orf87 | chromosome 1 open reading frame 87 [Source:HGNC Symbol;Acc:HGNC:28547] |
| ENSG00000162733 | HGLibA_12636 | 46 | -0.690854237 | DDR2 | discoidin domain receptor tyrosine kinase 2 [Source:HGNC Symbol;Acc:HGNC:2731] |
| ENSG00000162896 | HGLibA_36583 | 46 | -0.690854237 | PIGR | polymeric immunoglobulin receptor [Source:HGNC Symbol;Acc:HGNC:8968] |
| ENSG00000162944 | HGLibA_40953 | 46 | -0.690854237 | RFTN2 | raftlin family member 2 [Source:HGNC Symbol;Acc:HGNC:26402] |
| ENSG00000163209 | HGLibA_46825 | 46 | -0.690854237 | SPRR3 | small proline rich protein 3 [Source:HGNC Symbol;Acc:HGNC:11268] |
| ENSG00000163394 | HGLibA_07956 | 46 | -0.690854237 | CCKAR | cholecystokinin A receptor [Source:HGNC Symbol;Acc:HGNC:1570] |
| ENSG00000163527 | HGLibA_47610 | 46 | -0.690854237 | STT3B | catalytic subunit of the oligosaccharyltransferase complex [Source:HGNC Symbol;Acc:HGNC:30611]" |
| ENSG00000164022 | HGLibA_01356 | 46 | -0.690854237 | AIMP1 | aminoacyl tRNA synthetase complex interacting multifunctional protein 1 [Source:HGNC Symbol;Acc:HGNC:10648] |
| ENSG00000164076 | HGLibA_07097 | 46 | -0.690854237 | CAMKV | CaM kinase like vesicle associated [Source:HGNC Symbol;Acc:HGNC:28788] |
| ENSG00000164325 | HGLibA_50065 | 46 | -0.690854237 | TMEM174 | transmembrane protein 174 [Source:HGNC Symbol;Acc:HGNC:28187] |
| ENSG00000164822 | HGLibA_12807 | 46 | -0.690854237 | DEFA6 | defensin alpha 6 [Source:HGNC Symbol;Acc:HGNC:2765] |
| ENSG00000164830 | HGLibA_34830 | 46 | -0.690854237 | OXR1 | oxidation resistance 1 [Source:HGNC Symbol;Acc:HGNC:15822] |
| ENSG00000164953 | HGLibA_50493 | 46 | -0.690854237 | TMEM67 | transmembrane protein 67 [Source:HGNC Symbol;Acc:HGNC:28396] |
| ENSG00000165526 | HGLibA_42214 | 46 | -0.690854237 | RPUSD4 | RNA pseudouridine synthase D4 [Source:HGNC Symbol;Acc:HGNC:25898] |
| ENSG00000165915 | HGLibA_45101 | 46 | -0.690854237 | SLC39A13 | solute carrier family 39 member 13 [Source:HGNC Symbol;Acc:HGNC:20859] |
| ENSG00000166069 | HGLibA_49794 | 46 | -0.690854237 | TMCO5A | transmembrane and coiled-coil domains 5A [Source:HGNC Symbol;Acc:HGNC:28558] |
| ENSG00000166160 | HGLibA_33428 | 46 | -0.690854237 | OPN1MW2 | medium wave sensitive 2 [Source:HGNC Symbol;Acc:HGNC:26952]" |
| ENSG00000166446 | HGLibA_08919 | 46 | -0.690854237 | CDYL2 | chromodomain Y like 2 [Source:HGNC Symbol;Acc:HGNC:23030] |
| ENSG00000166840 | HGLibA_19373 | 46 | -0.690854237 | GLYATL1 | glycine-N-acyltransferase like 1 [Source:HGNC Symbol;Acc:HGNC:30519] |
| ENSG00000167414 | HGLibA_19535 | 46 | -0.690854237 | GNG8 | G protein subunit gamma 8 [Source:HGNC Symbol;Acc:HGNC:19664] |
| ENSG00000168710 | HGLibA_01288 | 46 | -0.690854237 | AHCYL1 | adenosylhomocysteinase like 1 [Source:HGNC Symbol;Acc:HGNC:344] |
| ENSG00000168769 | HGLibA_49041 | 46 | -0.690854237 | TET2 | tet methylcytosine dioxygenase 2 [Source:HGNC Symbol;Acc:HGNC:25941] |
| ENSG00000169241 | HGLibA_45236 | 46 | -0.690854237 | SLC50A1 | solute carrier family 50 member 1 [Source:HGNC Symbol;Acc:HGNC:30657] |
| ENSG00000169288 | HGLibA_29888 | 46 | -0.690854237 | MRPL1 | mitochondrial ribosomal protein L1 [Source:HGNC Symbol;Acc:HGNC:14275] |
| ENSG00000169635 | HGLibA_21292 | 46 | -0.690854237 | HIC2 | HIC ZBTB transcriptional repressor 2 [Source:HGNC Symbol;Acc:HGNC:18595] |
| ENSG00000170017 | HGLibA_01533 | 46 | -0.690854237 | ALCAM | activated leukocyte cell adhesion molecule [Source:HGNC Symbol;Acc:HGNC:400] |
| ENSG00000170522 | HGLibA_14973 | 46 | -0.690854237 | ELOVL6 | ELOVL fatty acid elongase 6 [Source:HGNC Symbol;Acc:HGNC:15829] |
| ENSG00000170549 | HGLibA_23629 | 46 | -0.690854237 | IRX1 | iroquois homeobox 1 [Source:HGNC Symbol;Acc:HGNC:14358] |
| ENSG00000170561 | HGLibA_23631 | 46 | -0.690854237 | IRX2 | iroquois homeobox 2 [Source:HGNC Symbol;Acc:HGNC:14359] |
| ENSG00000170891 | HGLibA_12250 | 46 | -0.690854237 | CYTL1 | cytokine like 1 [Source:HGNC Symbol;Acc:HGNC:24435] |
| ENSG00000171606 | HGLibA_55710 | 46 | -0.690854237 | ZNF274 | zinc finger protein 274 [Source:HGNC Symbol;Acc:HGNC:13068] |
| ENSG00000171729 | HGLibA_50428 | 46 | -0.690854237 | TMEM51 | transmembrane protein 51 [Source:HGNC Symbol;Acc:HGNC:25488] |
| ENSG00000172215 | HGLibA_11897 | 46 | -0.690854237 | CXCR6 | C-X-C motif chemokine receptor 6 [Source:HGNC Symbol;Acc:HGNC:16647] |
| ENSG00000172365 | HGLibA_34263 | 46 | -0.690854237 | OR5B2 | olfactory receptor family 5 subfamily B member 2 [Source:HGNC Symbol;Acc:HGNC:8323] |
| ENSG00000173039 | HGLibA_40812 | 46 | -0.690854237 | RELA | NF-kB subunit [Source:HGNC Symbol;Acc:HGNC:9955]" |
| ENSG00000173821 | HGLibA_41621 | 46 | -0.690854237 | RNF213 | ring finger protein 213 [Source:HGNC Symbol;Acc:HGNC:14539] |
| ENSG00000175315 | HGLibA_11463 | 46 | -0.690854237 | CST6 | cystatin E/M [Source:HGNC Symbol;Acc:HGNC:2478] |
| ENSG00000175928 | HGLibA_27471 | 46 | -0.690854237 | LRRN1 | leucine rich repeat neuronal 1 [Source:HGNC Symbol;Acc:HGNC:20980] |
| ENSG00000176438 | HGLibA_47981 | 46 | -0.690854237 | SYNE3 | spectrin repeat containing nuclear envelope family member 3 [Source:HGNC Symbol;Acc:HGNC:19861] |
| ENSG00000176641 | HGLibA_41551 | 46 | -0.690854237 | RNF152 | ring finger protein 152 [Source:HGNC Symbol;Acc:HGNC:26811] |
| ENSG00000177047 | HGLibA_22734 | 46 | -0.690854237 | IFNW1 | interferon omega 1 [Source:HGNC Symbol;Acc:HGNC:5448] |
| ENSG00000177098 | HGLibA_42995 | 46 | -0.690854237 | SCN4B | sodium voltage-gated channel beta subunit 4 [Source:HGNC Symbol;Acc:HGNC:10592] |
| ENSG00000177688 | HGLibA_47785 | 46 | -0.690854237 | SUMO4 | small ubiquitin-like modifier 4 [Source:HGNC Symbol;Acc:HGNC:21181] |
| ENSG00000177721 | HGLibA_02233 | 46 | -0.690854237 | ANXA2R | annexin A2 receptor [Source:HGNC Symbol;Acc:HGNC:33463] |
| ENSG00000177888 | HGLibA_54883 | 46 | -0.690854237 | ZBTB41 | zinc finger and BTB domain containing 41 [Source:HGNC Symbol;Acc:HGNC:24819] |
| ENSG00000178287 | HGLibA_46345 | 46 | -0.690854237 | SPAG11A | sperm associated antigen 11A [Source:HGNC Symbol;Acc:HGNC:33342] |
| ENSG00000178401 | HGLibA_13600 | 46 | -0.690854237 | DNAJC22 | DnaJ heat shock protein family (Hsp40) member C22 [Source:HGNC Symbol;Acc:HGNC:25802] |
| ENSG00000179059 | HGLibA_55220 | 46 | -0.690854237 | ZFP42 | ZFP42 zinc finger protein [Source:HGNC Symbol;Acc:HGNC:30949] |
| ENSG00000181915 | HGLibA_01007 | 46 | -0.690854237 | ADO | 2-aminoethanethiol dioxygenase [Source:HGNC Symbol;Acc:HGNC:23506] |
| ENSG00000182551 | HGLibA_00971 | 46 | -0.690854237 | ADI1 | acireductone dioxygenase 1 [Source:HGNC Symbol;Acc:HGNC:30576] |
| ENSG00000183186 | HGLibA_06102 | 46 | -0.690854237 | C2CD4C | C2 calcium dependent domain containing 4C [Source:HGNC Symbol;Acc:HGNC:29417] |
| ENSG00000183463 | HGLibA_53131 | 46 | -0.690854237 | URAD | ureidoimidazoline (2-oxo-4-hydroxy-4-carboxy-5-) decarboxylase [Source:HGNC Symbol;Acc:HGNC:17785] |
| ENSG00000183476 | HGLibA_43911 | 46 | -0.690854237 | SH2D7 | SH2 domain containing 7 [Source:HGNC Symbol;Acc:HGNC:34549] |
| ENSG00000183625 | HGLibA_08153 | 46 | -0.690854237 | CCR3 | C-C motif chemokine receptor 3 [Source:HGNC Symbol;Acc:HGNC:1604] |
| ENSG00000183625 | HGLibA_08154 | 46 | -0.690854237 | CCR3 | C-C motif chemokine receptor 3 [Source:HGNC Symbol;Acc:HGNC:1604] |
| ENSG00000184076 | HGLibA_53104 | 46 | -0.690854237 | UQCR10 | complex III subunit X [Source:HGNC Symbol;Acc:HGNC:30863]" |
| ENSG00000184293 | HGLibA_10013 | 46 | -0.690854237 | CLECL1 | C-type lectin like 1 [Source:HGNC Symbol;Acc:HGNC:24462] |
| ENSG00000185298 | HGLibA_07607 | 46 | -0.690854237 | CCDC137 | coiled-coil domain containing 137 [Source:HGNC Symbol;Acc:HGNC:33451] |
| ENSG00000185404 | HGLibA_46293 | 46 | -0.690854237 | SP140L | SP140 nuclear body protein like [Source:HGNC Symbol;Acc:HGNC:25105] |
| ENSG00000185591 | HGLibA_46282 | 46 | -0.690854237 | SP1 | Sp1 transcription factor [Source:HGNC Symbol;Acc:HGNC:11205] |
| ENSG00000185681 | HGLibA_29677 | 46 | -0.690854237 | MORN5 | MORN repeat containing 5 [Source:HGNC Symbol;Acc:HGNC:17841] |
| ENSG00000185758 | HGLibA_09889 | 46 | -0.690854237 | CLDN24 | claudin 24 [Source:HGNC Symbol;Acc:HGNC:37200] |
| ENSG00000185888 | HGLibA_38939 | 46 | -0.690854237 | PRSS38 | serine protease 38 [Source:HGNC Symbol;Acc:HGNC:29625] |
| ENSG00000185946 | HGLibA_41725 | 46 | -0.690854237 | RNPC3 | RRM) containing 3 [Source:HGNC Symbol;Acc:HGNC:18666]" |
| ENSG00000186453 | HGLibA_16535 | 46 | -0.690854237 | FAM228A | family with sequence similarity 228 member A [Source:HGNC Symbol;Acc:HGNC:34418] |
| ENSG00000186566 | HGLibA_19751 | 46 | -0.690854237 | GPATCH8 | G-patch domain containing 8 [Source:HGNC Symbol;Acc:HGNC:29066] |
| ENSG00000186818 | HGLibA_26517 | 46 | -0.690854237 | LILRB4 | leukocyte immunoglobulin like receptor B4 [Source:HGNC Symbol;Acc:HGNC:6608] |
| ENSG00000186889 | HGLibA_50050 | 46 | -0.690854237 | TMEM17 | transmembrane protein 17 [Source:HGNC Symbol;Acc:HGNC:26623] |
| ENSG00000187535 | HGLibA_22746 | 46 | -0.690854237 | IFT140 | intraflagellar transport 140 [Source:HGNC Symbol;Acc:HGNC:29077] |

| | | | | | |
|---|---|---|---|---|---|
| ENSG00000187555 | HGLibA_53411 | 46 | -0.690854237 | USP7 | ubiquitin specific peptidase 7 [Source:HGNC Symbol;Acc:HGNC:12630] |
| ENSG00000187715 | HGLibA_24095 | 46 | -0.690854237 | KBTBD12 | kelch repeat and BTB domain containing 12 [Source:HGNC Symbol;Acc:HGNC:25731] |
| ENSG00000187821 | HGLibA_21119 | 46 | -0.690854237 | HELT | helt bHLH transcription factor [Source:HGNC Symbol;Acc:HGNC:33783] |
| ENSG00000188026 | HGLibA_41252 | 46 | -0.690854237 | RILPL1 | Rab interacting lysosomal protein like 1 [Source:HGNC Symbol;Acc:HGNC:26814] |
| ENSG00000188227 | HGLibA_56772 | 46 | -0.690854237 | ZNF793 | zinc finger protein 793 [Source:HGNC Symbol;Acc:HGNC:33115] |
| ENSG00000189099 | HGLibA_38949 | 46 | -0.690854237 | PRSS48 | serine protease 48 [Source:HGNC Symbol;Acc:HGNC:24635] |
| ENSG00000189134 | HGLibA_32004 | 46 | -0.690854237 | NKAPL | NFKB activating protein like [Source:HGNC Symbol;Acc:HGNC:21584] |
| ENSG00000196150 | HGLibA_55667 | 46 | -0.690854237 | ZNF250 | zinc finger protein 250 [Source:HGNC Symbol;Acc:HGNC:13044] |
| ENSG00000196418 | HGLibA_55457 | 46 | -0.690854237 | ZNF124 | zinc finger protein 124 [Source:HGNC Symbol;Acc:HGNC:12907] |
| ENSG00000196704 | HGLibA_01860 | 46 | -0.690854237 | AMZ2 | archaelysin family metallopeptidase 2 [Source:HGNC Symbol;Acc:HGNC:28041] |
| ENSG00000196967 | HGLibA_56320 | 46 | -0.690854237 | ZNF585A | zinc finger protein 585A [Source:HGNC Symbol;Acc:HGNC:26305] |
| ENSG00000197245 | HGLibA_16020 | 46 | -0.690854237 | FAM110D | family with sequence similarity 110 member D [Source:HGNC Symbol;Acc:HGNC:25860] |
| ENSG00000197965 | HGLibA_29794 | 46 | -0.690854237 | MPZL1 | myelin protein zero like 1 [Source:HGNC Symbol;Acc:HGNC:7226] |
| ENSG00000198018 | HGLibA_15216 | 46 | -0.690854237 | ENTPD7 | ectonucleoside triphosphate diphosphohydrolase 7 [Source:HGNC Symbol;Acc:HGNC:19745] |
| ENSG00000198521 | HGLibA_55975 | 46 | -0.690854237 | ZNF43 | zinc finger protein 43 [Source:HGNC Symbol;Acc:HGNC:13109] |
| ENSG00000203797 | HGLibA_12626 | 46 | -0.690854237 | DDO | D-aspartate oxidase [Source:HGNC Symbol;Acc:HGNC:2727] |
| ENSG00000204052 | HGLibA_27424 | 46 | -0.690854237 | LRRC73 | leucine rich repeat containing 73 [Source:HGNC Symbol;Acc:HGNC:21375] |
| ENSG00000204392 | HGLibA_27537 | 46 | -0.690854237 | LSM2 | U6 small nuclear RNA and mRNA degradation associated [Source:HGNC Symbol;Acc:HGNC:13940]" |
| ENSG00000204619 | HGLibA_38017 | 46 | -0.690854237 | PPP1R11 | protein phosphatase 1 regulatory inhibitor subunit 11 [Source:HGNC Symbol;Acc:HGNC:9285] |
| ENSG00000204880 | HGLibA_25776 | 46 | -0.690854237 | KRTAP4-8 | keratin associated protein 4-8 [Source:HGNC Symbol;Acc:HGNC:17230] |
| ENSG00000205683 | HGLibA_13832 | 46 | -0.690854237 | DPF3 | double PHD fingers 3 [Source:HGNC Symbol;Acc:HGNC:17427] |
| ENSG00000205884 | HGLibA_12912 | 46 | -0.690854237 | DEFB136 | defensin beta 136 [Source:HGNC Symbol;Acc:HGNC:34433] |
| ENSG00000213079 | HGLibA_42830 | 46 | -0.690854237 | SCAF8 | SR-related CTD associated factor 8 [Source:HGNC Symbol;Acc:HGNC:20959] |
| ENSG00000213619 | HGLibA_31552 | 46 | -0.690854237 | NDUFS3 | NADH:ubiquinone oxidoreductase core subunit S3 [Source:HGNC Symbol;Acc:HGNC:7710] |
| ENSG00000214511 | HGLibA_21312 | 46 | -0.690854237 | HIGD1C | HIG1 hypoxia inducible domain family member 1C [Source:HGNC Symbol;Acc:HGNC:28044] |
| ENSG00000214819 | HGLibA_08877 | 46 | -0.690854237 | CDRT15L2 | CMT1A duplicated region transcript 15 like 2 [Source:HGNC Symbol;Acc:HGNC:34075] |
| ENSG00000215375 | HGLibA_30766 | 46 | -0.690854237 | MYL5 | myosin light chain 5 [Source:HGNC Symbol;Acc:HGNC:7586] |
| ENSG00000226600 | HGLibA_11549 | 46 | -0.690854237 | CT47A9 | member A9 [Source:HGNC Symbol;Acc:HGNC:33290]" |
| ENSG00000236334 | HGLibA_37916 | 46 | -0.690854237 | PPIAL4G | peptidylprolyl isomerase A like 4G [Source:HGNC Symbol;Acc:HGNC:33996] |
| ENSG00000237957 | HGLibA_11537 | 46 | -0.690854237 | CT47A5 | member A5 [Source:HGNC Symbol;Acc:HGNC:33286]" |
| ENSG00000239886 | HGLibA_25834 | 46 | -0.690854237 | KRTAP9-2 | keratin associated protein 9-2 [Source:HGNC Symbol;Acc:HGNC:16926] |
| ENSG00000240344 | HGLibA_37946 | 46 | -0.690854237 | PPIL3 | peptidylprolyl isomerase like 3 [Source:HGNC Symbol;Acc:HGNC:9262] |
| ENSG00000242612 | HGLibA_12777 | 46 | -0.690854237 | DECR2 | 4-dienoyl-CoA reductase 2 [Source:HGNC Symbol;Acc:HGNC:2754]" |
| ENSG00000249915 | HGLibA_35739 | 46 | -0.690854237 | PDCD6 | programmed cell death 6 [Source:HGNC Symbol;Acc:HGNC:8765] |
| ENSG00000253873 | HGLibA_35523 | 46 | -0.690854237 | PCDHGA11 | 11 [Source:HGNC Symbol;Acc:HGNC:8698]" |
| ENSG00000273559 | HGLibA_11818 | 46 | -0.690854237 | CWC25 | CWC25 spliceosome associated protein homolog [Source:HGNC Symbol;Acc:HGNC:25989] |
| ENSG00000274529 | HGLibA_43177 | 46 | -0.690854237 | SEBOX | SEBOX homeobox [Source:HGNC Symbol;Acc:HGNC:32942] |
| ENSG00000278311 | HGLibA_19005 | 46 | -0.690854237 | GGNBP2 | gametogenetin binding protein 2 [Source:HGNC Symbol;Acc:HGNC:19357] |
| ENSG00000278662 | HGLibA_19621 | 46 | -0.690854237 | GOLGA6L10 | golgin A6 family-like 10 [Source:HGNC Symbol;Acc:HGNC:37228] |
| FAM122A | HGLibA_16067 | 46 | -0.690854237 | FAM122A | - |
| FAM194A | HGLibA_16368 | 46 | -0.690854237 | FAM194A | - |
| FAM19A5 | HGLibA_16408 | 46 | -0.690854237 | FAM19A5 | - |
| HRASLS5 | HGLibA_22083 | 46 | -0.690854237 | HRASLS5 | - |
| hsa-mir-101-1 | HGLibA_57154 | 46 | -0.690854237 | hsa-mir-101-1 | - |
| hsa-mir-1243 | HGLibA_57358 | 46 | -0.690854237 | hsa-mir-1243 | - |
| hsa-mir-1276 | HGLibA_57559 | 46 | -0.690854237 | hsa-mir-1276 | - |
| hsa-mir-1291 | HGLibA_57642 | 46 | -0.690854237 | hsa-mir-1291 | - |
| hsa-mir-147a | HGLibA_57873 | 46 | -0.690854237 | hsa-mir-147a | - |
| hsa-mir-1910 | HGLibA_58045 | 46 | -0.690854237 | hsa-mir-1910 | - |
| hsa-mir-2115 | HGLibA_58232 | 46 | -0.690854237 | hsa-mir-2115 | - |
| hsa-mir-216a | HGLibA_58256 | 46 | -0.690854237 | hsa-mir-216a | - |
| hsa-mir-3065 | HGLibA_58463 | 46 | -0.690854237 | hsa-mir-3065 | - |
| hsa-mir-3116-1 | HGLibA_58502 | 46 | -0.690854237 | hsa-mir-3116-1 | - |
| hsa-mir-3156-2 | HGLibA_58693 | 46 | -0.690854237 | hsa-mir-3156-2 | - |
| hsa-mir-3185 | HGLibA_58836 | 46 | -0.690854237 | hsa-mir-3185 | - |
| hsa-mir-378d-2 | HGLibA_59443 | 46 | -0.690854237 | hsa-mir-378d-2 | - |
| hsa-mir-4296 | HGLibA_59895 | 46 | -0.690854237 | hsa-mir-4296 | - |
| hsa-mir-450a-1 | HGLibA_60474 | 46 | -0.690854237 | hsa-mir-450a-1 | - |
| hsa-mir-4635 | HGLibA_60644 | 46 | -0.690854237 | hsa-mir-4635 | - |
| hsa-mir-4719 | HGLibA_60975 | 46 | -0.690854237 | hsa-mir-4719 | - |
| hsa-mir-4774 | HGLibA_61199 | 46 | -0.690854237 | hsa-mir-4774 | - |
| hsa-mir-5187 | HGLibA_61641 | 46 | -0.690854237 | hsa-mir-5187 | - |
| hsa-mir-5194 | HGLibA_61695 | 46 | -0.690854237 | hsa-mir-5194 | - |
| hsa-mir-543 | HGLibA_61821 | 46 | -0.690854237 | hsa-mir-543 | - |
| hsa-mir-548i-2 | HGLibA_62018 | 46 | -0.690854237 | hsa-mir-548i-2 | - |
| hsa-mir-548y | HGLibA_62085 | 46 | -0.690854237 | hsa-mir-548y | - |
| hsa-mir-5572 | HGLibA_62152 | 46 | -0.690854237 | hsa-mir-5572 | - |
| hsa-mir-5684 | HGLibA_62254 | 46 | -0.690854237 | hsa-mir-5684 | - |
| hsa-mir-5701-2 | HGLibA_62344 | 46 | -0.690854237 | hsa-mir-5701-2 | - |
| hsa-mir-6070 | HGLibA_62532 | 46 | -0.690854237 | hsa-mir-6070 | - |
| hsa-mir-643 | HGLibA_62814 | 46 | -0.690854237 | hsa-mir-643 | - |
| hsa-mir-6513 | HGLibA_62927 | 46 | -0.690854237 | hsa-mir-6513 | - |
| hsa-mir-6721 | HGLibA_63045 | 46 | -0.690854237 | hsa-mir-6721 | - |
| hsa-mir-6745 | HGLibA_63132 | 46 | -0.690854237 | hsa-mir-6745 | - |
| hsa-mir-6819 | HGLibA_63452 | 46 | -0.690854237 | hsa-mir-6819 | - |
| hsa-mir-6840 | HGLibA_63536 | 46 | -0.690854237 | hsa-mir-6840 | - |
| hsa-mir-7843 | HGLibA_63962 | 46 | -0.690854237 | hsa-mir-7843 | - |
| hsa-mir-7974 | HGLibA_64024 | 46 | -0.690854237 | hsa-mir-7974 | - |
| hsa-mir-8062 | HGLibA_64085 | 46 | -0.690854237 | hsa-mir-8062 | - |
| hsa-mir-938 | HGLibA_64325 | 46 | -0.690854237 | hsa-mir-938 | - |
| KIAA1737 | HGLibA_24814 | 46 | -0.690854237 | KIAA1737 | - |
| LEPREL1 | HGLibA_26318 | 46 | -0.690854237 | LEPREL1 | - |
| METTL20 | HGLibA_28972 | 46 | -0.690854237 | METTL20 | - |
| VAMP7_Y | HGLibA_53507 | 46 | -0.690854237 | VAMP7_Y | - |
| VAMP7_Y | HGLibA_53505 | 46 | -0.690854237 | VAMP7_Y | - |
| ACPT | HGLibA_00527 | 45 | -0.700805275 | ACPT | - |
| APOBEC3A_B | HGLibA_02476 | 45 | -0.700805275 | APOBEC3A_B | - |
| BAI1 | HGLibA_04113 | 45 | -0.700805275 | BAI1 | - |
| C12orf68 | HGLibA_05284 | 45 | -0.700805275 | C12orf68 | - |
| C19orf80 | HGLibA_05712 | 45 | -0.700805275 | C19orf80 | - |
| C7orf69 | HGLibA_06581 | 45 | -0.700805275 | C7orf69 | - |
| EFCAB4B | HGLibA_14543 | 45 | -0.700805275 | EFCAB4B | - |
| ENSG00000010327 | HGLibA_47305 | 45 | -0.700805275 | STAB1 | stabilin 1 [Source:HGNC Symbol;Acc:HGNC:18628] |
| ENSG00000013583 | HGLibA_21084 | 45 | -0.700805275 | HEBP1 | heme binding protein 1 [Source:HGNC Symbol;Acc:HGNC:17176] |
| ENSG00000015153 | HGLibA_54629 | 45 | -0.700805275 | YAF2 | YY1 associated factor 2 [Source:HGNC Symbol;Acc:HGNC:17363] |
| ENSG00000021488 | HGLibA_45385 | 45 | -0.700805275 | SLC7A9 | solute carrier family 7 member 9 [Source:HGNC Symbol;Acc:HGNC:11067] |
| ENSG00000032219 | HGLibA_02899 | 45 | -0.700805275 | ARID4A | AT-rich interaction domain 4A [Source:HGNC Symbol;Acc:HGNC:9885] |
| ENSG00000034971 | HGLibA_30873 | 45 | -0.700805275 | MYOC | myocilin [Source:HGNC Symbol;Acc:HGNC:7610] |
| ENSG00000038274 | HGLibA_28421 | 45 | -0.700805275 | MAT2B | methionine adenosyltransferase 2B [Source:HGNC Symbol;Acc:HGNC:6905] |
| ENSG00000040341 | HGLibA_47408 | 45 | -0.700805275 | STAU2 | staufen double-stranded RNA binding protein 2 [Source:HGNC Symbol;Acc:HGNC:11371] |
| ENSG00000048392 | HGLibA_42257 | 45 | -0.700805275 | RRM2B | ribonucleotide reductase regulatory TP53 inducible subunit M2B [Source:HGNC Symbol;Acc:HGNC:17296] |
| ENSG00000049247 | HGLibA_53460 | 45 | -0.700805275 | UTS2 | urotensin 2 [Source:HGNC Symbol;Acc:HGNC:12636] |
| ENSG00000055147 | HGLibA_16033 | 45 | -0.700805275 | FAM114A2 | family with sequence similarity 114 member A2 [Source:HGNC Symbol;Acc:HGNC:1333] |
| ENSG00000059122 | HGLibA_17682 | 45 | -0.700805275 | FLYWCH1 | FLYWCH-type zinc finger 1 [Source:HGNC Symbol;Acc:HGNC:25404] |
| ENSG00000062598 | HGLibA_14933 | 45 | -0.700805275 | ELMO2 | engulfment and cell motility 2 [Source:HGNC Symbol;Acc:HGNC:17233] |
| ENSG00000076826 | HGLibA_07113 | 45 | -0.700805275 | CAMSAP3 | calmodulin regulated spectrin associated protein family member 3 [Source:HGNC Symbol;Acc:HGNC:29307] |
| ENSG00000080910 | HGLibA_09267 | 45 | -0.700805275 | CFHR2 | complement factor H related 2 [Source:HGNC Symbol;Acc:HGNC:4890] |
| ENSG00000082497 | HGLibA_43613 | 45 | -0.700805275 | SERTAD4 | SERTA domain containing 4 [Source:HGNC Symbol;Acc:HGNC:25236] |
| ENSG00000085063 | HGLibA_08418 | 45 | -0.700805275 | CD59 | CD59 molecule (CD59 blood group) [Source:HGNC Symbol;Acc:HGNC:1689] |
| ENSG00000086200 | HGLibA_23460 | 45 | -0.700805275 | IPO11 | importin 11 [Source:HGNC Symbol;Acc:HGNC:20628] |
| ENSG00000087995 | HGLibA_29000 | 45 | -0.700805275 | METTL2A | methyltransferase like 2A [Source:HGNC Symbol;Acc:HGNC:25755] |
| ENSG00000088992 | HGLibA_49024 | 45 | -0.700805275 | TESC | tescalcin [Source:HGNC Symbol;Acc:HGNC:26065] |
| ENSG00000091164 | HGLibA_52436 | 45 | -0.700805275 | TXNL1 | thioredoxin like 1 [Source:HGNC Symbol;Acc:HGNC:12436] |
| ENSG00000092345 | HGLibA_12371 | 45 | -0.700805275 | DAZL | deleted in azoospermia like [Source:HGNC Symbol;Acc:HGNC:2685] |
| ENSG00000096060 | HGLibA_17580 | 45 | -0.700805275 | FKBP5 | FK506 binding protein 5 [Source:HGNC Symbol;Acc:HGNC:3721] |
| ENSG00000100099 | HGLibA_22052 | 45 | -0.700805275 | HPS4 | biogenesis of lysosomal organelles complex 3 subunit 2 [Source:HGNC Symbol;Acc:HGNC:15844]" |
| ENSG00000100109 | HGLibA_49180 | 45 | -0.700805275 | TFIP11 | tuftelin interacting protein 11 [Source:HGNC Symbol;Acc:HGNC:17165] |
| ENSG00000100263 | HGLibA_41131 | 45 | -0.700805275 | RHBDD3 | rhomboid domain containing 3 [Source:HGNC Symbol;Acc:HGNC:1308] |
| ENSG00000100320 | HGLibA_40458 | 45 | -0.700805275 | RBFOX2 | RNA binding fox-1 homolog 2 [Source:HGNC Symbol;Acc:HGNC:9906] |
| ENSG00000100580 | HGLibA_49827 | 45 | -0.700805275 | TMED8 | transmembrane p24 trafficking protein family member 8 [Source:HGNC Symbol;Acc:HGNC:18633] |
| ENSG00000100601 | HGLibA_01647 | 45 | -0.700805275 | ALKBH1 | histone H2A dioxygenase [Source:HGNC Symbol;Acc:HGNC:17911]" |
| ENSG00000101057 | HGLibA_30646 | 45 | -0.700805275 | MYBL2 | MYB proto-oncogene like 2 [Source:HGNC Symbol;Acc:HGNC:7548] |
| ENSG00000101442 | HGLibA_00669 | 45 | -0.700805275 | ACTR5 | ARP5 actin related protein 5 homolog [Source:HGNC Symbol;Acc:HGNC:14671] |
| ENSG00000101844 | HGLibA_03489 | 45 | -0.700805275 | ATG4A | autophagy related 4A cysteine peptidase [Source:HGNC Symbol;Acc:HGNC:16489] |
| ENSG00000101890 | HGLibA_20666 | 45 | -0.700805275 | GUCY2F | retinal [Source:HGNC Symbol;Acc:HGNC:4691]" |
| ENSG00000102858 | HGLibA_29171 | 45 | -0.700805275 | MGRN1 | mahogunin ring finger 1 [Source:HGNC Symbol;Acc:HGNC:20254] |
| ENSG00000102858 | HGLibA_29170 | 45 | -0.700805275 | MGRN1 | mahogunin ring finger 1 [Source:HGNC Symbol;Acc:HGNC:20254] |
| ENSG00000103528 | HGLibA_48070 | 45 | -0.700805275 | SYT17 | synaptotagmin 17 [Source:HGNC Symbol;Acc:HGNC:24119] |
| ENSG00000104093 | HGLibA_13456 | 45 | -0.700805275 | DMXL2 | Dmx like 2 [Source:HGNC Symbol;Acc:HGNC:2938] |
| ENSG00000104129 | HGLibA_13584 | 45 | -0.700805275 | DNAJC17 | DnaJ heat shock protein family (Hsp40) member C17 [Source:HGNC Symbol;Acc:HGNC:25556] |
| ENSG00000104870 | HGLibA_17202 | 45 | -0.700805275 | FCGRT | Fc fragment of IgG receptor and transporter [Source:HGNC Symbol;Acc:HGNC:3621] |
| ENSG00000104974 | HGLibA_26488 | 45 | -0.700805275 | LILRA1 | leukocyte immunoglobulin like receptor A1 [Source:HGNC Symbol;Acc:HGNC:6602] |
| ENSG00000105137 | HGLibA_47940 | 45 | -0.700805275 | SYDE1 | synapse defective Rho GTPase homolog 1 [Source:HGNC Symbol;Acc:HGNC:25824] |
| ENSG00000105889 | HGLibA_47422 | 45 | -0.700805275 | STEAP1B | STEAP family member 1B [Source:HGNC Symbol;Acc:HGNC:41907] |
| ENSG00000106336 | HGLibA_17052 | 45 | -0.700805275 | FBXO24 | F-box protein 24 [Source:HGNC Symbol;Acc:HGNC:13595] |

| | | | | | |
|---|---|---|---|---|---|
| ENSG00000107185 | HGLibA_41023 | 45 | -0.700805275 | RGP1 | RAB6A GEF complex partner 1 [Source:HGNC Symbol;Acc:HGNC:21965]" |
| ENSG00000109189 | HGLibA_53376 | 45 | -0.700805275 | USP46 | ubiquitin specific peptidase 46 [Source:HGNC Symbol;Acc:HGNC:20075] |
| ENSG00000111653 | HGLibA_23293 | 45 | -0.700805275 | ING4 | inhibitor of growth family member 4 [Source:HGNC Symbol;Acc:HGNC:19423] |
| ENSG00000111665 | HGLibA_08606 | 45 | -0.700805275 | CDCA3 | cell division cycle associated 3 [Source:HGNC Symbol;Acc:HGNC:14624] |
| ENSG00000111906 | HGLibA_21032 | 45 | -0.700805275 | HDDC2 | HD domain containing 2 [Source:HGNC Symbol;Acc:HGNC:21078] |
| ENSG00000112183 | HGLibA_40514 | 45 | -0.700805275 | RBM24 | RNA binding motif protein 24 [Source:HGNC Symbol;Acc:HGNC:21539] |
| ENSG00000112852 | HGLibA_35493 | 45 | -0.700805275 | PCDHB2 | protocadherin beta 2 [Source:HGNC Symbol;Acc:HGNC:8687] |
| ENSG00000113161 | HGLibA_21707 | 45 | -0.700805275 | HMGCR | 3-hydroxy-3-methylglutaryl-CoA reductase [Source:HGNC Symbol;Acc:HGNC:5006] |
| ENSG00000113971 | HGLibA_32447 | 45 | -0.700805275 | NPHP3 | nephrocystin 3 [Source:HGNC Symbol;Acc:HGNC:7907] |
| ENSG00000114209 | HGLibA_35719 | 45 | -0.700805275 | PDCD10 | programmed cell death 10 [Source:HGNC Symbol;Acc:HGNC:8761] |
| ENSG00000115648 | HGLibA_29431 | 45 | -0.700805275 | MLPH | melanophilin [Source:HGNC Symbol;Acc:HGNC:29643] |
| ENSG00000115963 | HGLibA_41450 | 45 | -0.700805275 | RND3 | Rho family GTPase 3 [Source:HGNC Symbol;Acc:HGNC:671] |
| ENSG00000116199 | HGLibA_16446 | 45 | -0.700805275 | FAM20B | glycosaminoglycan xylosylkinase [Source:HGNC Symbol;Acc:HGNC:23017]" |
| ENSG00000116745 | HGLibA_41825 | 45 | -0.700805275 | RPE65 | retinoid isomerohydrolase [Source:HGNC Symbol;Acc:HGNC:10294]" |
| ENSG00000116906 | HGLibA_19563 | 45 | -0.700805275 | GNPAT | glyceronephosphate O-acyltransferase [Source:HGNC Symbol;Acc:HGNC:4416] |
| ENSG00000117758 | HGLibA_47620 | 45 | -0.700805275 | STX12 | syntaxin 12 [Source:HGNC Symbol;Acc:HGNC:11430] |
| ENSG00000117984 | HGLibA_11699 | 45 | -0.700805275 | CTSD | cathepsin D [Source:HGNC Symbol;Acc:HGNC:2529] |
| ENSG00000119771 | HGLibA_25174 | 45 | -0.700805275 | KLHL29 | kelch like family member 29 [Source:HGNC Symbol;Acc:HGNC:29404] |
| ENSG00000121335 | HGLibA_38340 | 45 | -0.700805275 | PRB2 | proline rich protein BstNI subfamily 2 [Source:HGNC Symbol;Acc:HGNC:9338] |
| ENSG00000121410 | HGLibA_00002 | 45 | -0.700805275 | A1BG | alpha-1-B glycoprotein [Source:HGNC Symbol;Acc:HGNC:5] |
| ENSG00000121653 | HGLibA_28285 | 45 | -0.700805275 | MAPK8IP1 | mitogen-activated protein kinase 8 interacting protein 1 [Source:HGNC Symbol;Acc:HGNC:6882] |
| ENSG00000123143 | HGLibA_36838 | 45 | -0.700805275 | PKN1 | protein kinase N1 [Source:HGNC Symbol;Acc:HGNC:9405] |
| ENSG00000124217 | HGLibA_29618 | 45 | -0.700805275 | MOCS3 | molybdenum cofactor synthesis 3 [Source:HGNC Symbol;Acc:HGNC:15765] |
| ENSG00000124233 | HGLibA_43378 | 45 | -0.700805275 | SEMG1 | semenogelin 1 [Source:HGNC Symbol;Acc:HGNC:10742] |
| ENSG00000124343 | HGLibA_54506 | 45 | -0.700805275 | XG | Xg glycoprotein (Xg blood group) [Source:HGNC Symbol;Acc:HGNC:12806] |
| ENSG00000124570 | HGLibA_43559 | 45 | -0.700805275 | SERPINB6 | serpin family B member 6 [Source:HGNC Symbol;Acc:HGNC:8950] |
| ENSG00000125815 | HGLibA_11469 | 45 | -0.700805275 | CST8 | cystatin 8 [Source:HGNC Symbol;Acc:HGNC:2480] |
| ENSG00000130283 | HGLibA_18851 | 45 | -0.700805275 | GDF1 | growth differentiation factor 1 [Source:HGNC Symbol;Acc:HGNC:4214] |
| ENSG00000130309 | HGLibA_10641 | 45 | -0.700805275 | COLGALT1 | collagen beta(1-O)galactosyltransferase 1 [Source:HGNC Symbol;Acc:HGNC:26182] |
| ENSG00000130427 | HGLibA_15350 | 45 | -0.700805275 | EPO | erythropoietin [Source:HGNC Symbol;Acc:HGNC:3415] |
| ENSG00000130726 | HGLibA_51449 | 45 | -0.700805275 | TRIM28 | tripartite motif containing 28 [Source:HGNC Symbol;Acc:HGNC:16384] |
| ENSG00000131023 | HGLibA_26063 | 45 | -0.700805275 | LATS1 | large tumor suppressor kinase 1 [Source:HGNC Symbol;Acc:HGNC:6514] |
| ENSG00000131778 | HGLibA_09386 | 45 | -0.700805275 | CHD1L | chromodomain helicase DNA binding protein 1 like [Source:HGNC Symbol;Acc:HGNC:1916] |
| ENSG00000131808 | HGLibA_18087 | 45 | -0.700805275 | FSHB | follicle stimulating hormone subunit beta [Source:HGNC Symbol;Acc:HGNC:3964] |
| ENSG00000132386 | HGLibA_43586 | 45 | -0.700805275 | SERPINF1 | serpin family F member 1 [Source:HGNC Symbol;Acc:HGNC:8824] |
| ENSG00000134007 | HGLibA_00752 | 45 | -0.700805275 | ADAM20 | ADAM metallopeptidase domain 20 [Source:HGNC Symbol;Acc:HGNC:199] |
| ENSG00000134242 | HGLibA_39460 | 45 | -0.700805275 | PTPN22 | non-receptor type 22 [Source:HGNC Symbol;Acc:HGNC:9652]" |
| ENSG00000135164 | HGLibA_13443 | 45 | -0.700805275 | DMTF1 | cyclin D binding myb like transcription factor 1 [Source:HGNC Symbol;Acc:HGNC:14603] |
| ENSG00000135164 | HGLibA_13445 | 45 | -0.700805275 | DMTF1 | cyclin D binding myb like transcription factor 1 [Source:HGNC Symbol;Acc:HGNC:14603] |
| ENSG00000135346 | HGLibA_09297 | 45 | -0.700805275 | CGA | alpha polypeptide [Source:HGNC Symbol;Acc:HGNC:1885]" |
| ENSG00000136807 | HGLibA_08801 | 45 | -0.700805275 | CDK9 | cyclin dependent kinase 9 [Source:HGNC Symbol;Acc:HGNC:1780] |
| ENSG00000137413 | HGLibA_48257 | 45 | -0.700805275 | TAF8 | TATA-box binding protein associated factor 8 [Source:HGNC Symbol;Acc:HGNC:17300] |
| ENSG00000138039 | HGLibA_26422 | 45 | -0.700805275 | LHCGR | luteinizing hormone/choriogonadotropin receptor [Source:HGNC Symbol;Acc:HGNC:6585] |
| ENSG00000138172 | HGLibA_07016 | 45 | -0.700805275 | CALHM2 | calcium homeostasis modulator family member 2 [Source:HGNC Symbol;Acc:HGNC:23493] |
| ENSG00000138379 | HGLibA_30265 | 45 | -0.700805275 | MSTN | myostatin [Source:HGNC Symbol;Acc:HGNC:4223] |
| ENSG00000138468 | HGLibA_43400 | 45 | -0.700805275 | SENP7 | SUMO specific peptidase 7 [Source:HGNC Symbol;Acc:HGNC:30402] |
| ENSG00000138675 | HGLibA_17411 | 45 | -0.700805275 | FGF5 | fibroblast growth factor 5 [Source:HGNC Symbol;Acc:HGNC:3683] |
| ENSG00000139352 | HGLibA_03267 | 45 | -0.700805275 | ASCL1 | achaete-scute family bHLH transcription factor 1 [Source:HGNC Symbol;Acc:HGNC:738] |
| ENSG00000139697 | HGLibA_42807 | 45 | -0.700805275 | SBNO1 | strawberry notch homolog 1 [Source:HGNC Symbol;Acc:HGNC:22973] |
| ENSG00000140280 | HGLibA_27768 | 45 | -0.700805275 | LYSMD2 | LysM domain containing 2 [Source:HGNC Symbol;Acc:HGNC:28571] |
| ENSG00000140986 | HGLibA_41979 | 45 | -0.700805275 | RPL3L | ribosomal protein L3 like [Source:HGNC Symbol;Acc:HGNC:10351] |
| ENSG00000141316 | HGLibA_46326 | 45 | -0.700805275 | SPACA3 | sperm acrosome associated 3 [Source:HGNC Symbol;Acc:HGNC:16260] |
| ENSG00000141371 | HGLibA_05557 | 45 | -0.700805275 | C17orf64 | chromosome 17 open reading frame 64 [Source:HGNC Symbol;Acc:HGNC:26990] |
| ENSG00000141741 | HGLibA_29248 | 45 | -0.700805275 | MIEN1 | migration and invasion enhancer 1 [Source:HGNC Symbol;Acc:HGNC:28230] |
| ENSG00000142025 | HGLibA_13440 | 45 | -0.700805275 | DMRTC2 | DMRT like family C2 [Source:HGNC Symbol;Acc:HGNC:13911] |
| ENSG00000142188 | HGLibA_50427 | 45 | -0.700805275 | TMEM50B | transmembrane protein 50B [Source:HGNC Symbol;Acc:HGNC:1280] |
| ENSG00000143614 | HGLibA_18689 | 45 | -0.700805275 | GATAD2B | GATA zinc finger domain containing 2B [Source:HGNC Symbol;Acc:HGNC:30778] |
| ENSG00000143643 | HGLibA_52050 | 45 | -0.700805275 | TTC13 | tetratricopeptide repeat domain 13 [Source:HGNC Symbol;Acc:HGNC:26204] |
| ENSG00000144852 | HGLibA_32590 | 45 | -0.700805275 | NR1I2 | nuclear receptor subfamily 1 group I member 2 [Source:HGNC Symbol;Acc:HGNC:7968] |
| ENSG00000145703 | HGLibA_23534 | 45 | -0.700805275 | IQGAP2 | IQ motif containing GTPase activating protein 2 [Source:HGNC Symbol;Acc:HGNC:6111] |
| ENSG00000146216 | HGLibA_52039 | 45 | -0.700805275 | TTBK1 | tau tubulin kinase 1 [Source:HGNC Symbol;Acc:HGNC:19140] |
| ENSG00000146409 | HGLibA_44495 | 45 | -0.700805275 | SLC18B1 | solute carrier family 18 member B1 [Source:HGNC Symbol;Acc:HGNC:21573] |
| ENSG00000146909 | HGLibA_32287 | 45 | -0.700805275 | NOM1 | nucleolar protein with MIF4G domain 1 [Source:HGNC Symbol;Acc:HGNC:13244] |
| ENSG00000147874 | HGLibA_20888 | 45 | -0.700805275 | HAUS6 | HAUS augmin like complex subunit 6 [Source:HGNC Symbol;Acc:HGNC:25948] |
| ENSG00000148484 | HGLibA_42365 | 45 | -0.700805275 | RSU1 | Ras suppressor protein 1 [Source:HGNC Symbol;Acc:HGNC:10464] |
| ENSG00000148842 | HGLibA_10316 | 45 | -0.700805275 | CNNM2 | cyclin and CBS domain divalent metal cation transport mediator 2 [Source:HGNC Symbol;Acc:HGNC:103] |
| ENSG00000151005 | HGLibA_49593 | 45 | -0.700805275 | TKTL2 | transketolase like 2 [Source:HGNC Symbol;Acc:HGNC:25313] |
| ENSG00000151491 | HGLibA_15375 | 45 | -0.700805275 | EPS8 | epidermal growth factor receptor pathway substrate 8 [Source:HGNC Symbol;Acc:HGNC:3420] |
| ENSG00000151502 | HGLibA_53754 | 45 | -0.700805275 | VPS26B | retromer complex component B [Source:HGNC Symbol;Acc:HGNC:28119]" |
| ENSG00000152092 | HGLibA_03381 | 45 | -0.700805275 | ASTN1 | astrotactin 1 [Source:HGNC Symbol;Acc:HGNC:773] |
| ENSG00000152430 | HGLibA_04692 | 45 | -0.700805275 | BOLL | RNA binding protein [Source:HGNC Symbol;Acc:HGNC:14273]" |
| ENSG00000152932 | HGLibA_39912 | 45 | -0.700805275 | RAB3C | member RAS oncogene family [Source:HGNC Symbol;Acc:HGNC:30269]" |
| ENSG00000153786 | HGLibA_55126 | 45 | -0.700805275 | ZDHHC7 | zinc finger DHHC-type containing 7 [Source:HGNC Symbol;Acc:HGNC:18459] |
| ENSG00000154118 | HGLibA_23986 | 45 | -0.700805275 | JPH3 | junctophilin 3 [Source:HGNC Symbol;Acc:HGNC:14203] |
| ENSG00000154640 | HGLibA_04948 | 45 | -0.700805275 | BTG3 | BTG anti-proliferation factor 3 [Source:HGNC Symbol;Acc:HGNC:1132] |
| ENSG00000157184 | HGLibA_11026 | 45 | -0.700805275 | CPT2 | carnitine palmitoyltransferase 2 [Source:HGNC Symbol;Acc:HGNC:2330] |
| ENSG00000157343 | HGLibA_03012 | 45 | -0.700805275 | ARMC12 | armadillo repeat containing 12 [Source:HGNC Symbol;Acc:HGNC:21099] |
| ENSG00000157368 | HGLibA_23143 | 45 | -0.700805275 | IL34 | interleukin 34 [Source:HGNC Symbol;Acc:HGNC:28529] |
| ENSG00000158290 | HGLibA_11786 | 45 | -0.700805275 | CUL4B | cullin 4B [Source:HGNC Symbol;Acc:HGNC:2555] |
| ENSG00000158805 | HGLibA_55713 | 45 | -0.700805275 | ZNF276 | zinc finger protein 276 [Source:HGNC Symbol;Acc:HGNC:23330] |
| ENSG00000159337 | HGLibA_36910 | 45 | -0.700805275 | PLA2G4D | phospholipase A2 group IVD [Source:HGNC Symbol;Acc:HGNC:30038] |
| ENSG00000159648 | HGLibA_49011 | 45 | -0.700805275 | TEPP | prostate and placenta expressed [Source:HGNC Symbol;Acc:HGNC:33745]" |
| ENSG00000160214 | HGLibA_42267 | 45 | -0.700805275 | RRP1 | ribosomal RNA processing 1 [Source:HGNC Symbol;Acc:HGNC:18785] |
| ENSG00000160746 | HGLibA_02174 | 45 | -0.700805275 | ANO10 | anoctamin 10 [Source:HGNC Symbol;Acc:HGNC:25519] |
| ENSG00000160867 | HGLibA_17451 | 45 | -0.700805275 | FGFR4 | fibroblast growth factor receptor 4 [Source:HGNC Symbol;Acc:HGNC:3691] |
| ENSG00000160882 | HGLibA_12056 | 45 | -0.700805275 | CYP11B1 | cytochrome P450 family 11 subfamily B member 1 [Source:HGNC Symbol;Acc:HGNC:2591] |
| ENSG00000161944 | HGLibA_03291 | 45 | -0.700805275 | ASGR2 | asialoglycoprotein receptor 2 [Source:HGNC Symbol;Acc:HGNC:743] |
| ENSG00000162073 | HGLibA_35161 | 45 | -0.700805275 | PAQR4 | progestin and adipoQ receptor family member 4 [Source:HGNC Symbol;Acc:HGNC:26386] |
| ENSG00000163029 | HGLibA_45660 | 45 | -0.700805275 | SMC6 | structural maintenance of chromosomes 6 [Source:HGNC Symbol;Acc:HGNC:20466] |
| ENSG00000163145 | HGLibA_05955 | 45 | -0.700805275 | C1QTNF7 | C1q and TNF related 7 [Source:HGNC Symbol;Acc:HGNC:14342] |
| ENSG00000163159 | HGLibA_53816 | 45 | -0.700805275 | VPS72 | vacuolar protein sorting 72 homolog [Source:HGNC Symbol;Acc:HGNC:11644] |
| ENSG00000163516 | HGLibA_02165 | 45 | -0.700805275 | ANKZF1 | ankyrin repeat and zinc finger domain containing 1 [Source:HGNC Symbol;Acc:HGNC:25527] |
| ENSG00000163568 | HGLibA_01352 | 45 | -0.700805275 | AIM2 | absent in melanoma 2 [Source:HGNC Symbol;Acc:HGNC:357] |
| ENSG00000163798 | HGLibA_45212 | 45 | -0.700805275 | SLC4A1AP | solute carrier family 4 member 1 adaptor protein [Source:HGNC Symbol;Acc:HGNC:13813] |
| ENSG00000163939 | HGLibA_35339 | 45 | -0.700805275 | PBRM1 | polybromo 1 [Source:HGNC Symbol;Acc:HGNC:30064] |
| ENSG00000164187 | HGLibA_26678 | 45 | -0.700805275 | LMBRD2 | LMBR1 domain containing 2 [Source:HGNC Symbol;Acc:HGNC:25287] |
| ENSG00000164219 | HGLibA_36236 | 45 | -0.700805275 | PGGT1B | protein geranylgeranyltransferase type I subunit beta [Source:HGNC Symbol;Acc:HGNC:8895] |
| ENSG00000164707 | HGLibA_44393 | 45 | -0.700805275 | SLC13A4 | solute carrier family 13 member 4 [Source:HGNC Symbol;Acc:HGNC:15827] |
| ENSG00000164885 | HGLibA_08772 | 45 | -0.700805275 | CDK5 | cyclin dependent kinase 5 [Source:HGNC Symbol;Acc:HGNC:1774] |
| ENSG00000165046 | HGLibA_26337 | 45 | -0.700805275 | LETM2 | leucine zipper and EF-hand containing transmembrane protein 2 [Source:HGNC Symbol;Acc:HGNC:14648] |
| ENSG00000165349 | HGLibA_45364 | 45 | -0.700805275 | SLC7A3 | solute carrier family 7 member 3 [Source:HGNC Symbol;Acc:HGNC:11061] |
| ENSG00000165487 | HGLibA_29226 | 45 | -0.700805275 | MICU2 | mitochondrial calcium uptake 2 [Source:HGNC Symbol;Acc:HGNC:31830] |
| ENSG00000165733 | HGLibA_04637 | 45 | -0.700805275 | BMS1 | ribosome biogenesis factor [Source:HGNC Symbol;Acc:HGNC:23505]" |
| ENSG00000165868 | HGLibA_22259 | 45 | -0.700805275 | HSPA12A | heat shock protein family A (Hsp70) member 12A [Source:HGNC Symbol;Acc:HGNC:19022] |
| ENSG00000166432 | HGLibA_55366 | 45 | -0.700805275 | ZMAT1 | zinc finger matrin-type 1 [Source:HGNC Symbol;Acc:HGNC:29377] |
| ENSG00000167194 | HGLibA_05500 | 45 | -0.700805275 | C16orf92 | chromosome 16 open reading frame 92 [Source:HGNC Symbol;Acc:HGNC:26346] |
| ENSG00000167208 | HGLibA_46061 | 45 | -0.700805275 | SNX20 | sorting nexin 20 [Source:HGNC Symbol;Acc:HGNC:30390] |
| ENSG00000167261 | HGLibA_13822 | 45 | -0.700805275 | DPEP2 | dipeptidase 2 [Source:HGNC Symbol;Acc:HGNC:23028] |
| ENSG00000167523 | HGLibA_46510 | 45 | -0.700805275 | SPATA33 | spermatogenesis associated 33 [Source:HGNC Symbol;Acc:HGNC:26463] |
| ENSG00000167772 | HGLibA_01918 | 45 | -0.700805275 | ANGPTL4 | angiopoietin like 4 [Source:HGNC Symbol;Acc:HGNC:16039] |
| ENSG00000168876 | HGLibA_02111 | 45 | -0.700805275 | ANKRD49 | ankyrin repeat domain 49 [Source:HGNC Symbol;Acc:HGNC:25970] |
| ENSG00000169509 | HGLibA_11084 | 45 | -0.700805275 | CRCT1 | cysteine rich C-terminal 1 [Source:HGNC Symbol;Acc:HGNC:29875] |
| ENSG00000169621 | HGLibA_02430 | 45 | -0.700805275 | APLF | aprataxin and PNKP like factor [Source:HGNC Symbol;Acc:HGNC:28724] |
| ENSG00000169627 | HGLibA_04687 | 45 | -0.700805275 | BOLA2B | bolA family member 2B [Source:HGNC Symbol;Acc:HGNC:32479] |
| ENSG00000169851 | HGLibA_35415 | 45 | -0.700805275 | PCDH7 | protocadherin 7 [Source:HGNC Symbol;Acc:HGNC:8659] |
| ENSG00000170006 | HGLibA_50007 | 45 | -0.700805275 | TMEM154 | transmembrane protein 154 [Source:HGNC Symbol;Acc:HGNC:26489] |
| ENSG00000170049 | HGLibA_24152 | 45 | -0.700805275 | KCNAB3 | potassium voltage-gated channel subfamily A regulatory beta subunit 3 [Source:HGNC Symbol;Acc:HGNC:6230] |
| ENSG00000170989 | HGLibA_42594 | 45 | -0.700805275 | S1PR1 | sphingosine-1-phosphate receptor 1 [Source:HGNC Symbol;Acc:HGNC:3165] |
| ENSG00000171481 | HGLibA_33688 | 45 | -0.700805275 | OR1L3 | olfactory receptor family 1 subfamily L member 3 [Source:HGNC Symbol;Acc:HGNC:8215] |
| ENSG00000171759 | HGLibA_35021 | 45 | -0.700805275 | PAH | phenylalanine hydroxylase [Source:HGNC Symbol;Acc:HGNC:8582] |
| ENSG00000171931 | HGLibA_17135 | 45 | -0.700805275 | FBXW10 | F-box and WD repeat domain containing 10 [Source:HGNC Symbol;Acc:HGNC:1211] |
| ENSG00000172260 | HGLibA_31626 | 45 | -0.700805275 | NEGR1 | neuronal growth regulator 1 [Source:HGNC Symbol;Acc:HGNC:17302] |
| ENSG00000172940 | HGLibA_44546 | 45 | -0.700805275 | SLC22A13 | solute carrier family 22 member 13 [Source:HGNC Symbol;Acc:HGNC:8494] |
| ENSG00000173193 | HGLibA_35223 | 45 | -0.700805275 | PARP14 | poly(ADP-ribose) polymerase family member 14 [Source:HGNC Symbol;Acc:HGNC:29232] |
| ENSG00000174946 | HGLibA_19965 | 45 | -0.700805275 | GPR171 | G protein-coupled receptor 171 [Source:HGNC Symbol;Acc:HGNC:30057] |
| ENSG00000176624 | HGLibA_29034 | 45 | -0.700805275 | MEX3C | mex-3 RNA binding family member C [Source:HGNC Symbol;Acc:HGNC:28040] |
| ENSG00000177051 | HGLibA_17112 | 45 | -0.700805275 | FBXO46 | F-box protein 46 [Source:HGNC Symbol;Acc:HGNC:25069] |
| ENSG00000178999 | HGLibA_03878 | 45 | -0.700805275 | AURKB | aurora kinase B [Source:HGNC Symbol;Acc:HGNC:11390] |
| ENSG00000179833 | HGLibA_43608 | 45 | -0.700805275 | SERTAD2 | SERTA domain containing 2 [Source:HGNC Symbol;Acc:HGNC:30784] |
| ENSG00000180098 | HGLibA_51706 | 45 | -0.700805275 | TRNAU1AP | tRNA selenocysteine 1 associated protein 1 [Source:HGNC Symbol;Acc:HGNC:30813] |
| ENSG00000180251 | HGLibA_45415 | 45 | -0.700805275 | SLC9A4 | solute carrier family 9 member A4 [Source:HGNC Symbol;Acc:HGNC:11077] |
| ENSG00000180667 | HGLibA_54702 | 45 | -0.700805275 | YOD1 | YOD1 deubiquitinase [Source:HGNC Symbol;Acc:HGNC:25035] |
| ENSG00000181001 | HGLibA_34194 | 45 | -0.700805275 | OR52N1 | olfactory receptor family 52 subfamily N member 1 [Source:HGNC Symbol;Acc:HGNC:14853] |
| ENSG00000181135 | HGLibA_56600 | 45 | -0.700805275 | ZNF707 | zinc finger protein 707 [Source:HGNC Symbol;Acc:HGNC:27815] |
| ENSG00000182551 | HGLibA_00970 | 45 | -0.700805275 | ADI1 | acireductone dioxygenase 1 [Source:HGNC Symbol;Acc:HGNC:30576] |

| | | | | | |
|---|---|---|---|---|---|
| ENSG00000182896 | HGLibA_50563 | 45 | -0.700805275 | TMEM95 | transmembrane protein 95 [Source:HGNC Symbol;Acc:HGNC:27898] |
| ENSG00000183336 | HGLibA_04682 | 45 | -0.700805275 | BOLA2 | bolA family member 2 [Source:HGNC Symbol;Acc:HGNC:29488] |
| ENSG00000183527 | HGLibA_39205 | 45 | -0.700805275 | PSMG1 | proteasome assembly chaperone 1 [Source:HGNC Symbol;Acc:HGNC:3043] |
| ENSG00000183837 | HGLibA_37371 | 45 | -0.700805275 | PNMA3 | PNMA family member 3 [Source:HGNC Symbol;Acc:HGNC:18742] |
| ENSG00000183918 | HGLibA_43882 | 45 | -0.700805275 | SH2D1A | SH2 domain containing 1A [Source:HGNC Symbol;Acc:HGNC:10820] |
| ENSG00000184033 | HGLibA_11561 | 45 | -0.700805275 | CTAG1B | cancer/testis antigen 1B [Source:HGNC Symbol;Acc:HGNC:2491] |
| ENSG00000184428 | HGLibA_50990 | 45 | -0.700805275 | TOP1MT | DNA topoisomerase I mitochondrial [Source:HGNC Symbol;Acc:HGNC:29787] |
| ENSG00000184564 | HGLibA_45529 | 45 | -0.700805275 | SLITRK6 | SLIT and NTRK like family member 6 [Source:HGNC Symbol;Acc:HGNC:23503] |
| ENSG00000184916 | HGLibA_23906 | 45 | -0.700805275 | JAG2 | jagged 2 [Source:HGNC Symbol;Acc:HGNC:6189] |
| ENSG00000185019 | HGLibA_52725 | 45 | -0.700805275 | UBOX5 | U-box domain containing 5 [Source:HGNC Symbol;Acc:HGNC:17777] |
| ENSG00000185133 | HGLibA_23358 | 45 | -0.700805275 | INPP5J | inositol polyphosphate-5-phosphatase J [Source:HGNC Symbol;Acc:HGNC:8956] |
| ENSG00000185519 | HGLibA_16111 | 45 | -0.700805275 | FAM131C | family with sequence similarity 131 member C [Source:HGNC Symbol;Acc:HGNC:26717] |
| ENSG00000186185 | HGLibA_24878 | 45 | -0.700805275 | KIF18B | kinesin family member 18B [Source:HGNC Symbol;Acc:HGNC:27102] |
| ENSG00000186190 | HGLibA_04726 | 45 | -0.700805275 | BPIFB3 | BPI fold containing family B member 3 [Source:HGNC Symbol;Acc:HGNC:16178] |
| ENSG00000186440 | HGLibA_34435 | 45 | -0.700805275 | OR6P1 | olfactory receptor family 6 subfamily P member 1 [Source:HGNC Symbol;Acc:HGNC:15036] |
| ENSG00000187922 | HGLibA_26169 | 45 | -0.700805275 | LCN10 | lipocalin 10 [Source:HGNC Symbol;Acc:HGNC:20892] |
| ENSG00000188032 | HGLibA_05697 | 45 | -0.700805275 | C19orf67 | chromosome 19 open reading frame 67 [Source:HGNC Symbol;Acc:HGNC:34354] |
| ENSG00000188191 | HGLibA_38525 | 45 | -0.700805275 | PRKAR1B | protein kinase cAMP-dependent type I regulatory subunit beta [Source:HGNC Symbol;Acc:HGNC:9390] |
| ENSG00000188649 | HGLibA_07485 | 45 | -0.700805275 | CC2D2B | coiled-coil and C2 domain containing 2B [Source:HGNC Symbol;Acc:HGNC:31666] |
| ENSG00000188766 | HGLibA_46797 | 45 | -0.700805275 | SPRED3 | sprouty related EVH1 domain containing 3 [Source:HGNC Symbol;Acc:HGNC:31041] |
| ENSG00000189433 | HGLibA_19166 | 45 | -0.700805275 | GJB4 | gap junction protein beta 4 [Source:HGNC Symbol;Acc:HGNC:4286] |
| ENSG00000196098 | HGLibA_34318 | 45 | -0.700805275 | OR5K4 | olfactory receptor family 5 subfamily K member 4 [Source:HGNC Symbol;Acc:HGNC:31291] |
| ENSG00000196104 | HGLibA_46754 | 45 | -0.700805275 | SPOCK3 | cwcv and kazal like domains proteoglycan 3 [Source:HGNC Symbol;Acc:HGNC:13565]" |
| ENSG00000196776 | HGLibA_08395 | 45 | -0.700805275 | CD47 | CD47 molecule [Source:HGNC Symbol;Acc:HGNC:1682] |
| ENSG00000196950 | HGLibA_45092 | 45 | -0.700805275 | SLC39A10 | solute carrier family 39 member 10 [Source:HGNC Symbol;Acc:HGNC:20861] |
| ENSG00000197084 | HGLibA_26107 | 45 | -0.700805275 | LCE1C | late cornified envelope 1C [Source:HGNC Symbol;Acc:HGNC:29464] |
| ENSG00000197181 | HGLibA_36774 | 45 | -0.700805275 | PIWIL2 | piwi like RNA-mediated gene silencing 2 [Source:HGNC Symbol;Acc:HGNC:17644] |
| ENSG00000197444 | HGLibA_33305 | 45 | -0.700805275 | OGDHL | oxoglutarate dehydrogenase like [Source:HGNC Symbol;Acc:HGNC:25590] |
| ENSG00000197859 | HGLibA_00852 | 45 | -0.700805275 | ADAMTSL2 | ADAMTS like 2 [Source:HGNC Symbol;Acc:HGNC:14631] |
| ENSG00000198576 | HGLibA_02633 | 45 | -0.700805275 | ARC | activity regulated cytoskeleton associated protein [Source:HGNC Symbol;Acc:HGNC:648] |
| ENSG00000198625 | HGLibA_28700 | 45 | -0.700805275 | MDM4 | p53 regulator [Source:HGNC Symbol;Acc:HGNC:6974]" |
| ENSG00000198890 | HGLibA_38632 | 45 | -0.700805275 | PRMT6 | protein arginine methyltransferase 6 [Source:HGNC Symbol;Acc:HGNC:18241] |
| ENSG00000203667 | HGLibA_10827 | 45 | -0.700805275 | COX20 | cytochrome c oxidase assembly factor COX20 [Source:HGNC Symbol;Acc:HGNC:26970] |
| ENSG00000204248 | HGLibA_10504 | 45 | -0.700805275 | COL11A2 | collagen type XI alpha 2 chain [Source:HGNC Symbol;Acc:HGNC:2187] |
| ENSG00000204655 | HGLibA_29622 | 45 | -0.700805275 | MOG | myelin oligodendrocyte glycoprotein [Source:HGNC Symbol;Acc:HGNC:7197] |
| ENSG00000204852 | HGLibA_48873 | 45 | -0.700805275 | TCTN1 | tectonic family member 1 [Source:HGNC Symbol;Acc:HGNC:26113] |
| ENSG00000204918 | HGLibA_38804 | 45 | -0.700805275 | PRR20B | proline rich 20B [Source:HGNC Symbol;Acc:HGNC:37220] |
| ENSG00000205730 | HGLibA_23868 | 45 | -0.700805275 | ITPRIPL2 | ITPRIP like 2 [Source:HGNC Symbol;Acc:HGNC:27257] |
| ENSG00000206043 | HGLibA_05621 | 45 | -0.700805275 | C18orf63 | chromosome 18 open reading frame 63 [Source:HGNC Symbol;Acc:HGNC:40037] |
| ENSG00000206150 | HGLibA_41401 | 45 | -0.700805275 | RNASE13 | ribonuclease A family member 13 (inactive) [Source:HGNC Symbol;Acc:HGNC:25285] |
| ENSG00000206177 | HGLibA_20931 | 45 | -0.700805275 | HBM | hemoglobin subunit mu [Source:HGNC Symbol;Acc:HGNC:4826] |
| ENSG00000212901 | HGLibA_25741 | 45 | -0.700805275 | KRTAP3-1 | keratin associated protein 3-1 [Source:HGNC Symbol;Acc:HGNC:16778] |
| ENSG00000215269 | HGLibA_18452 | 45 | -0.700805275 | GAGE12G | G antigen 12G [Source:HGNC Symbol;Acc:HGNC:31907] |
| ENSG00000221931 | HGLibA_34452 | 45 | -0.700805275 | OR6X1 | olfactory receptor family 6 subfamily X member 1 [Source:HGNC Symbol;Acc:HGNC:14737] |
| ENSG00000221988 | HGLibA_38237 | 45 | -0.700805275 | PPT2 | palmitoyl-protein thioesterase 2 [Source:HGNC Symbol;Acc:HGNC:9326] |
| ENSG00000225766 | HGLibA_13139 | 45 | -0.700805275 | DHRS4L1 | dehydrogenase/reductase 4 like 1 [Source:HGNC Symbol;Acc:HGNC:19732] |
| ENSG00000230124 | HGLibA_00408 | 45 | -0.700805275 | ACBD6 | acyl-CoA binding domain containing 6 [Source:HGNC Symbol;Acc:HGNC:23339] |
| ENSG00000231051 | HGLibA_53266 | 45 | -0.700805275 | USP17L28 | ubiquitin specific peptidase 17-like family member 28 [Source:HGNC Symbol;Acc:HGNC:44456] |
| ENSG00000235780 | HGLibA_53263 | 45 | -0.700805275 | USP17L27 | ubiquitin specific peptidase 17-like family member 27 [Source:HGNC Symbol;Acc:HGNC:44455] |
| ENSG00000237649 | HGLibA_24970 | 45 | -0.700805275 | KIFC1 | kinesin family member C1 [Source:HGNC Symbol;Acc:HGNC:6389] |
| ENSG00000240654 | HGLibA_05961 | 45 | -0.700805275 | C1QTNF9 | C1q and TNF related 9 [Source:HGNC Symbol;Acc:HGNC:28732] |
| ENSG00000240857 | HGLibA_40742 | 45 | -0.700805275 | RDH14 | retinol dehydrogenase 14 [Source:HGNC Symbol;Acc:HGNC:19979] |
| ENSG00000242019 | HGLibA_24996 | 45 | -0.700805275 | KIR3DL3 | three Ig domains and long cytoplasmic tail 3 [Source:HGNC Symbol;Acc:HGNC:16312]" |
| ENSG00000244025 | HGLibA_25670 | 45 | -0.700805275 | KRTAP19-3 | keratin associated protein 19-3 [Source:HGNC Symbol;Acc:HGNC:18938] |
| ENSG00000244476 | HGLibA_15538 | 45 | -0.700805275 | ERVFRD-1 | envelope [Source:HGNC Symbol;Acc:HGNC:33823]" |
| ENSG00000248871 | HGLibA_50805 | 45 | -0.700805275 | TNFSF12-TNFSF13 | TNFSF12-TNFSF13 readthrough [Source:HGNC Symbol;Acc:HGNC:33537] |
| ENSG00000255819 | HGLibA_25298 | 45 | -0.700805275 | KLRC4-KLRK1 | KLRC4-KLRK1 readthrough [Source:HGNC Symbol;Acc:HGNC:48357] |
| ENSG00000267206 | HGLibA_26181 | 45 | -0.700805275 | LCN6 | lipocalin 6 [Source:HGNC Symbol;Acc:HGNC:17337] |
| ENSG00000268221 | HGLibA_33425 | 45 | -0.700805275 | OPN1MW | medium wave sensitive [Source:HGNC Symbol;Acc:HGNC:4206]" |
| ENSG00000274808 | HGLibA_48542 | 45 | -0.700805275 | TBC1D3B | TBC1 domain family member 3B [Source:HGNC Symbol;Acc:HGNC:27011] |
| ENSG00000288649 | HGLibA_00616 | 45 | -0.700805275 | ACTL10 | - |
| ENTHD2 | HGLibA_15195 | 45 | -0.700805275 | ENTHD2 | - |
| GARS | HGLibA_18636 | 45 | -0.700805275 | GARS | - |
| GRASP | HGLibA_20222 | 45 | -0.700805275 | GRASP | - |
| HIST2H3C | HGLibA_21542 | 45 | -0.700805275 | HIST2H3C | - |
| hsa-let-7b | HGLibA_57108 | 45 | -0.700805275 | hsa-let-7b | - |
| hsa-mir-1244-1 | HGLibA_57364 | 45 | -0.700805275 | hsa-mir-1244-1 | - |
| hsa-mir-198 | HGLibA_58125 | 45 | -0.700805275 | hsa-mir-198 | - |
| hsa-mir-2909 | HGLibA_58392 | 45 | -0.700805275 | hsa-mir-2909 | - |
| hsa-mir-302f | HGLibA_58457 | 45 | -0.700805275 | hsa-mir-302f | - |
| hsa-mir-3131 | HGLibA_58581 | 45 | -0.700805275 | hsa-mir-3131 | - |
| hsa-mir-3135a | HGLibA_58593 | 45 | -0.700805275 | hsa-mir-3135a | - |
| hsa-mir-3158-2 | HGLibA_58706 | 45 | -0.700805275 | hsa-mir-3158-2 | - |
| hsa-mir-3161 | HGLibA_58722 | 45 | -0.700805275 | hsa-mir-3161 | - |
| hsa-mir-3162 | HGLibA_58728 | 45 | -0.700805275 | hsa-mir-3162 | - |
| hsa-mir-3612 | HGLibA_59076 | 45 | -0.700805275 | hsa-mir-3612 | - |
| hsa-mir-365a | HGLibA_59187 | 45 | -0.700805275 | hsa-mir-365a | - |
| hsa-mir-377 | HGLibA_59423 | 45 | -0.700805275 | hsa-mir-377 | - |
| hsa-mir-4282 | HGLibA_59832 | 45 | -0.700805275 | hsa-mir-4282 | - |
| hsa-mir-4460 | HGLibA_60245 | 45 | -0.700805275 | hsa-mir-4460 | - |
| hsa-mir-4492 | HGLibA_60387 | 45 | -0.700805275 | hsa-mir-4492 | - |
| hsa-mir-4651 | HGLibA_60711 | 45 | -0.700805275 | hsa-mir-4651 | - |
| hsa-mir-4698 | HGLibA_60899 | 45 | -0.700805275 | hsa-mir-4698 | - |
| hsa-mir-4715 | HGLibA_60959 | 45 | -0.700805275 | hsa-mir-4715 | - |
| hsa-mir-4999 | HGLibA_61395 | 45 | -0.700805275 | hsa-mir-4999 | - |
| hsa-mir-5193 | HGLibA_61690 | 45 | -0.700805275 | hsa-mir-5193 | - |
| hsa-mir-521-2 | HGLibA_61771 | 45 | -0.700805275 | hsa-mir-521-2 | - |
| hsa-mir-548ao | HGLibA_61910 | 45 | -0.700805275 | hsa-mir-548ao | - |
| hsa-mir-5701-1 | HGLibA_62340 | 45 | -0.700805275 | hsa-mir-5701-1 | - |
| hsa-mir-6165 | HGLibA_62705 | 45 | -0.700805275 | hsa-mir-6165 | - |
| hsa-mir-640 | HGLibA_62800 | 45 | -0.700805275 | hsa-mir-640 | - |
| hsa-mir-652 | HGLibA_62942 | 45 | -0.700805275 | hsa-mir-652 | - |
| hsa-mir-6757 | HGLibA_63186 | 45 | -0.700805275 | hsa-mir-6757 | - |
| hsa-mir-6850 | HGLibA_63574 | 45 | -0.700805275 | hsa-mir-6850 | - |
| hsa-mir-6880 | HGLibA_63703 | 45 | -0.700805275 | hsa-mir-6880 | - |
| hsa-mir-7-3 | HGLibA_63777 | 45 | -0.700805275 | hsa-mir-7-3 | - |
| hsa-mir-943 | HGLibA_64358 | 45 | -0.700805275 | hsa-mir-943 | - |
| METTL10 | HGLibA_28947 | 45 | -0.700805275 | METTL10 | - |
| PRUNE | HGLibA_38987 | 45 | -0.700805275 | PRUNE | - |
| SPG20 | HGLibA_46634 | 45 | -0.700805275 | SPG20 | - |
| SSFA2 | HGLibA_47130 | 45 | -0.700805275 | SSFA2 | - |
| ATP5D | HGLibA_03646 | 44 | -0.710756312 | ATP5D | - |
| C10orf111 | HGLibA_05032 | 44 | -0.710756312 | C10orf111 | - |
| C19orf43 | HGLibA_05661 | 44 | -0.710756312 | C19orf43 | - |
| CTAGE5 | HGLibA_11575 | 44 | -0.710756312 | CTAGE5 | - |
| DHRSX_Y | HGLibA_13160 | 44 | -0.710756312 | DHRSX_Y | - |
| DYX1C1 | HGLibA_14292 | 44 | -0.710756312 | DYX1C1 | - |
| ENSG00000010278 | HGLibA_08477 | 44 | -0.710756312 | CD9 | CD9 molecule [Source:HGNC Symbol;Acc:HGNC:1709] |
| ENSG00000010292 | HGLibA_31262 | 44 | -0.710756312 | NCAPD2 | non-SMC condensin I complex subunit D2 [Source:HGNC Symbol;Acc:HGNC:24305] |
| ENSG00000014123 | HGLibA_52849 | 44 | -0.710756312 | UFL1 | UFM1 specific ligase 1 [Source:HGNC Symbol;Acc:HGNC:23039] |
| ENSG00000015285 | HGLibA_53950 | 44 | -0.710756312 | WAS | Wiskott-Aldrich syndrome [Source:HGNC Symbol;Acc:HGNC:12731] |
| ENSG00000021762 | HGLibA_34658 | 44 | -0.710756312 | OSBPL5 | oxysterol binding protein like 5 [Source:HGNC Symbol;Acc:HGNC:16392] |
| ENSG00000024422 | HGLibA_14670 | 44 | -0.710756312 | EHD2 | EH domain containing 2 [Source:HGNC Symbol;Acc:HGNC:3243] |
| ENSG00000028137 | HGLibA_50774 | 44 | -0.710756312 | TNFRSF1B | TNF receptor superfamily member 1B [Source:HGNC Symbol;Acc:HGNC:11917] |
| ENSG00000050628 | HGLibA_39320 | 44 | -0.710756312 | PTGER3 | prostaglandin E receptor 3 [Source:HGNC Symbol;Acc:HGNC:9595] |
| ENSG00000053372 | HGLibA_30122 | 44 | -0.710756312 | MRTO4 | ribosome maturation factor [Source:HGNC Symbol;Acc:HGNC:18477]" |
| ENSG00000057468 | HGLibA_30197 | 44 | -0.710756312 | MSH4 | mutS homolog 4 [Source:HGNC Symbol;Acc:HGNC:7327] |
| ENSG00000058091 | HGLibA_08736 | 44 | -0.710756312 | CDK14 | cyclin dependent kinase 14 [Source:HGNC Symbol;Acc:HGNC:8883] |
| ENSG00000074755 | HGLibA_57090 | 44 | -0.710756312 | ZZEF1 | zinc finger ZZ-type and EF-hand domain containing 1 [Source:HGNC Symbol;Acc:HGNC:29027] |
| ENSG00000077348 | HGLibA_15785 | 44 | -0.710756312 | EXOSC5 | exosome component 5 [Source:HGNC Symbol;Acc:HGNC:24662] |
| ENSG00000080986 | HGLibA_31382 | 44 | -0.710756312 | NDC80 | kinetochore complex component [Source:HGNC Symbol;Acc:HGNC:16909]" |
| ENSG00000083845 | HGLibA_42153 | 44 | -0.710756312 | RPS5 | ribosomal protein S5 [Source:HGNC Symbol;Acc:HGNC:10426] |
| ENSG00000087269 | HGLibA_32304 | 44 | -0.710756312 | NOP14 | NOP14 nucleolar protein [Source:HGNC Symbol;Acc:HGNC:16821] |
| ENSG00000090432 | HGLibA_30561 | 44 | -0.710756312 | MUL1 | mitochondrial E3 ubiquitin protein ligase 1 [Source:HGNC Symbol;Acc:HGNC:25762] |
| ENSG00000091136 | HGLibA_25957 | 44 | -0.710756312 | LAMB1 | laminin subunit beta 1 [Source:HGNC Symbol;Acc:HGNC:6486] |
| ENSG00000091947 | HGLibA_49840 | 44 | -0.710756312 | TMEM101 | transmembrane protein 101 [Source:HGNC Symbol;Acc:HGNC:28653] |
| ENSG00000095319 | HGLibA_33032 | 44 | -0.710756312 | NUP188 | nucleoporin 188 [Source:HGNC Symbol;Acc:HGNC:17859] |
| ENSG00000100055 | HGLibA_12243 | 44 | -0.710756312 | CYTH4 | cytohesin 4 [Source:HGNC Symbol;Acc:HGNC:9505] |
| ENSG00000100055 | HGLibA_12244 | 44 | -0.710756312 | CYTH4 | cytohesin 4 [Source:HGNC Symbol;Acc:HGNC:9505] |
| ENSG00000100316 | HGLibA_41931 | 44 | -0.710756312 | RPL3 | ribosomal protein L3 [Source:HGNC Symbol;Acc:HGNC:10332] |
| ENSG00000101138 | HGLibA_11483 | 44 | -0.710756312 | CSTF1 | cleavage stimulation factor subunit 1 [Source:HGNC Symbol;Acc:HGNC:2483] |
| ENSG00000101204 | HGLibA_09574 | 44 | -0.710756312 | CHRNA4 | cholinergic receptor nicotinic alpha 4 subunit [Source:HGNC Symbol;Acc:HGNC:1958] |
| ENSG00000101367 | HGLibA_28313 | 44 | -0.710756312 | MAPRE1 | microtubule associated protein RP/EB family member 1 [Source:HGNC Symbol;Acc:HGNC:6890] |
| ENSG00000101843 | HGLibA_39152 | 44 | -0.710756312 | PSMD10 | non-ATPase 10 [Source:HGNC Symbol;Acc:HGNC:9555]" |
| ENSG00000101972 | HGLibA_47323 | 44 | -0.710756312 | STAG2 | stromal antigen 2 [Source:HGNC Symbol;Acc:HGNC:11355] |

| | | | | | |
|---|---|---|---|---|---|
| ENSG00000102471 | HGLibA_31394 | 44 | -0.710756312 | NDFIP2 | Nedd4 family interacting protein 2 [Source:HGNC Symbol;Acc:HGNC:18537] |
| ENSG00000103187 | HGLibA_10799 | 44 | -0.710756312 | COTL1 | coactosin like F-actin binding protein 1 [Source:HGNC Symbol;Acc:HGNC:18304] |
| ENSG00000103479 | HGLibA_40469 | 44 | -0.710756312 | RBL2 | RB transcriptional corepressor like 2 [Source:HGNC Symbol;Acc:HGNC:9894] |
| ENSG00000103490 | HGLibA_39671 | 44 | -0.710756312 | PYCARD | PYD and CARD domain containing [Source:HGNC Symbol;Acc:HGNC:16608] |
| ENSG00000104142 | HGLibA_53745 | 44 | -0.710756312 | VPS18 | CORVET/HOPS core subunit [Source:HGNC Symbol;Acc:HGNC:15972]" |
| ENSG00000104783 | HGLibA_24364 | 44 | -0.710756312 | KCNN4 | potassium calcium-activated channel subfamily N member 4 [Source:HGNC Symbol;Acc:HGNC:6293] |
| ENSG00000104814 | HGLibA_28212 | 44 | -0.710756312 | MAP4K1 | mitogen-activated protein kinase kinase kinase kinase 1 [Source:HGNC Symbol;Acc:HGNC:6863] |
| ENSG00000104852 | HGLibA_45958 | 44 | -0.710756312 | SNRNP70 | small nuclear ribonucleoprotein U1 subunit 70 [Source:HGNC Symbol;Acc:HGNC:11150] |
| ENSG00000104859 | HGLibA_09798 | 44 | -0.710756312 | CLASRP | CLK4 associating serine/arginine rich protein [Source:HGNC Symbol;Acc:HGNC:17731] |
| ENSG00000104936 | HGLibA_13413 | 44 | -0.710756312 | DMPK | DM1 protein kinase [Source:HGNC Symbol;Acc:HGNC:2933] |
| ENSG00000105647 | HGLibA_36651 | 44 | -0.710756312 | PIK3R2 | phosphoinositide-3-kinase regulatory subunit 2 [Source:HGNC Symbol;Acc:HGNC:8980] |
| ENSG00000105778 | HGLibA_03895 | 44 | -0.710756312 | AVL9 | AVL9 cell migration associated [Source:HGNC Symbol;Acc:HGNC:28994] |
| ENSG00000105829 | HGLibA_04430 | 44 | -0.710756312 | BET1 | Bet1 golgi vesicular membrane trafficking protein [Source:HGNC Symbol;Acc:HGNC:14562] |
| ENSG00000106009 | HGLibA_04759 | 44 | -0.710756312 | BRAT1 | BRCA1 associated ATM activator 1 [Source:HGNC Symbol;Acc:HGNC:21701] |
| ENSG00000106266 | HGLibA_46108 | 44 | -0.710756312 | SNX8 | sorting nexin 8 [Source:HGNC Symbol;Acc:HGNC:14972] |
| ENSG00000106351 | HGLibA_01188 | 44 | -0.710756312 | AGFG2 | ArfGAP with FG repeats 2 [Source:HGNC Symbol;Acc:HGNC:5177] |
| ENSG00000107020 | HGLibA_37167 | 44 | -0.710756312 | PLGRKT | plasminogen receptor with a C-terminal lysine [Source:HGNC Symbol;Acc:HGNC:23633] |
| ENSG00000107719 | HGLibA_35062 | 44 | -0.710756312 | PALD1 | phosphatase domain containing paladin 1 [Source:HGNC Symbol;Acc:HGNC:23530] |
| ENSG00000108511 | HGLibA_21943 | 44 | -0.710756312 | HOXB6 | homeobox B6 [Source:HGNC Symbol;Acc:HGNC:5117] |
| ENSG00000109099 | HGLibA_37323 | 44 | -0.710756312 | PMP22 | peripheral myelin protein 22 [Source:HGNC Symbol;Acc:HGNC:9118] |
| ENSG00000109163 | HGLibA_19587 | 44 | -0.710756312 | GNRHR | gonadotropin releasing hormone receptor [Source:HGNC Symbol;Acc:HGNC:4421] |
| ENSG00000110171 | HGLibA_51456 | 44 | -0.710756312 | TRIM3 | tripartite motif containing 3 [Source:HGNC Symbol;Acc:HGNC:10064] |
| ENSG00000110427 | HGLibA_24781 | 44 | -0.710756312 | KIAA1549L | KIAA1549 like [Source:HGNC Symbol;Acc:HGNC:24836] |
| ENSG00000110696 | HGLibA_05162 | 44 | -0.710756312 | C11orf58 | chromosome 11 open reading frame 58 [Source:HGNC Symbol;Acc:HGNC:16990] |
| ENSG00000111261 | HGLibA_28101 | 44 | -0.710756312 | MANSC1 | MANSC domain containing 1 [Source:HGNC Symbol;Acc:HGNC:25505] |
| ENSG00000112110 | HGLibA_29916 | 44 | -0.710756312 | MRPL18 | mitochondrial ribosomal protein L18 [Source:HGNC Symbol;Acc:HGNC:14477] |
| ENSG00000112146 | HGLibA_17131 | 44 | -0.710756312 | FBXO9 | F-box protein 9 [Source:HGNC Symbol;Acc:HGNC:13588] |
| ENSG00000113296 | HGLibA_49330 | 44 | -0.710756312 | THBS4 | thrombospondin 4 [Source:HGNC Symbol;Acc:HGNC:11788] |
| ENSG00000113368 | HGLibA_26697 | 44 | -0.710756312 | LMNB1 | lamin B1 [Source:HGNC Symbol;Acc:HGNC:6637] |
| ENSG00000113594 | HGLibA_26476 | 44 | -0.710756312 | LIFR | LIF receptor alpha [Source:HGNC Symbol;Acc:HGNC:6597] |
| ENSG00000114923 | HGLibA_45217 | 44 | -0.710756312 | SLC4A3 | solute carrier family 4 member 3 [Source:HGNC Symbol;Acc:HGNC:11029] |
| ENSG00000115085 | HGLibA_54776 | 44 | -0.710756312 | ZAP70 | zeta chain of T cell receptor associated protein kinase 70 [Source:HGNC Symbol;Acc:HGNC:12858] |
| ENSG00000115286 | HGLibA_31564 | 44 | -0.710756312 | NDUFS7 | NADH:ubiquinone oxidoreductase core subunit S7 [Source:HGNC Symbol;Acc:HGNC:7714] |
| ENSG00000118276 | HGLibA_04039 | 44 | -0.710756312 | B4GALT6 | 4-galactosyltransferase 6 [Source:HGNC Symbol;Acc:HGNC:929]" |
| ENSG00000118420 | HGLibA_52687 | 44 | -0.710756312 | UBE3D | ubiquitin protein ligase E3D [Source:HGNC Symbol;Acc:HGNC:21381] |
| ENSG00000120053 | HGLibA_19689 | 44 | -0.710756312 | GOT1 | glutamic-oxaloacetic transaminase 1 [Source:HGNC Symbol;Acc:HGNC:4432] |
| ENSG00000125611 | HGLibA_09374 | 44 | -0.710756312 | CHCHD5 | coiled-coil-helix-coiled-coil-helix domain containing 5 [Source:HGNC Symbol;Acc:HGNC:17840] |
| ENSG00000125755 | HGLibA_47952 | 44 | -0.710756312 | SYMPK | symplekin [Source:HGNC Symbol;Acc:HGNC:22935] |
| ENSG00000125965 | HGLibA_18870 | 44 | -0.710756312 | GDF5 | growth differentiation factor 5 [Source:HGNC Symbol;Acc:HGNC:4220] |
| ENSG00000126091 | HGLibA_47244 | 44 | -0.710756312 | ST3GAL3 | 3-sialyltransferase 3 [Source:HGNC Symbol;Acc:HGNC:10866]" |
| ENSG00000126231 | HGLibA_38708 | 44 | -0.710756312 | PROZ | vitamin K dependent plasma glycoprotein [Source:HGNC Symbol;Acc:HGNC:9460]" |
| ENSG00000126861 | HGLibA_33385 | 44 | -0.710756312 | OMG | oligodendrocyte myelin glycoprotein [Source:HGNC Symbol;Acc:HGNC:8135] |
| ENSG00000128918 | HGLibA_01545 | 44 | -0.710756312 | ALDH1A2 | aldehyde dehydrogenase 1 family member A2 [Source:HGNC Symbol;Acc:HGNC:15472] |
| ENSG00000129009 | HGLibA_23669 | 44 | -0.710756312 | ISLR | immunoglobulin superfamily containing leucine rich repeat [Source:HGNC Symbol;Acc:HGNC:6133] |
| ENSG00000129691 | HGLibA_03295 | 44 | -0.710756312 | ASH2L | histone lysine methyltransferase complex subunit [Source:HGNC Symbol;Acc:HGNC:744]" |
| ENSG00000130165 | HGLibA_14953 | 44 | -0.710756312 | ELOF1 | elongation factor 1 homolog [Source:HGNC Symbol;Acc:HGNC:28691] |
| ENSG00000130300 | HGLibA_37248 | 44 | -0.710756312 | PLVAP | plasmalemma vesicle associated protein [Source:HGNC Symbol;Acc:HGNC:13635] |
| ENSG00000132286 | HGLibA_49496 | 44 | -0.710756312 | TIMM10B | translocase of inner mitochondrial membrane 10B [Source:HGNC Symbol;Acc:HGNC:4022] |
| ENSG00000135473 | HGLibA_35090 | 44 | -0.710756312 | PAN2 | poly(A) specific ribonuclease subunit PAN2 [Source:HGNC Symbol;Acc:HGNC:20074] |
| ENSG00000135945 | HGLibA_40884 | 44 | -0.710756312 | REV1 | DNA directed polymerase [Source:HGNC Symbol;Acc:HGNC:14060]" |
| ENSG00000136573 | HGLibA_04545 | 44 | -0.710756312 | BLK | Src family tyrosine kinase [Source:HGNC Symbol;Acc:HGNC:1057]" |
| ENSG00000136603 | HGLibA_44283 | 44 | -0.710756312 | SKIL | SKI like proto-oncogene [Source:HGNC Symbol;Acc:HGNC:10897] |
| ENSG00000136950 | HGLibA_03094 | 44 | -0.710756312 | ARPC5L | actin related protein 2/3 complex subunit 5 like [Source:HGNC Symbol;Acc:HGNC:23366] |
| ENSG00000137710 | HGLibA_40759 | 44 | -0.710756312 | RDX | radixin [Source:HGNC Symbol;Acc:HGNC:9944] |
| ENSG00000137960 | HGLibA_19121 | 44 | -0.710756312 | GIPC2 | GIPC PDZ domain containing family member 2 [Source:HGNC Symbol;Acc:HGNC:18177] |
| ENSG00000138207 | HGLibA_40639 | 44 | -0.710756312 | RBP4 | retinol binding protein 4 [Source:HGNC Symbol;Acc:HGNC:9922] |
| ENSG00000138698 | HGLibA_40232 | 44 | -0.710756312 | RAP1GDS1 | Rap1 GTPase-GDP dissociation stimulator 1 [Source:HGNC Symbol;Acc:HGNC:9859] |
| ENSG00000138942 | HGLibA_41592 | 44 | -0.710756312 | RNF185 | ring finger protein 185 [Source:HGNC Symbol;Acc:HGNC:26783] |
| ENSG00000139220 | HGLibA_37875 | 44 | -0.710756312 | PPFIA2 | PTPRF interacting protein alpha 2 [Source:HGNC Symbol;Acc:HGNC:9246] |
| ENSG00000140548 | HGLibA_56610 | 44 | -0.710756312 | ZNF710 | zinc finger protein 710 [Source:HGNC Symbol;Acc:HGNC:25352] |
| ENSG00000140743 | HGLibA_08865 | 44 | -0.710756312 | CDR2 | cerebellar degeneration related protein 2 [Source:HGNC Symbol;Acc:HGNC:1799] |
| ENSG00000140749 | HGLibA_22899 | 44 | -0.710756312 | IGSF6 | immunoglobulin superfamily member 6 [Source:HGNC Symbol;Acc:HGNC:5953] |
| ENSG00000141378 | HGLibA_39553 | 44 | -0.710756312 | PTRH2 | peptidyl-tRNA hydrolase 2 [Source:HGNC Symbol;Acc:HGNC:24265] |
| ENSG00000141497 | HGLibA_55421 | 44 | -0.710756312 | ZMYND15 | zinc finger MYND-type containing 15 [Source:HGNC Symbol;Acc:HGNC:20997] |
| ENSG00000141639 | HGLibA_28275 | 44 | -0.710756312 | MAPK4 | mitogen-activated protein kinase 4 [Source:HGNC Symbol;Acc:HGNC:6878] |
| ENSG00000142615 | HGLibA_08992 | 44 | -0.710756312 | CELA2A | chymotrypsin like elastase family member 2A [Source:HGNC Symbol;Acc:HGNC:24609] |
| ENSG00000142959 | HGLibA_04428 | 44 | -0.710756312 | BEST4 | bestrophin 4 [Source:HGNC Symbol;Acc:HGNC:17106] |
| ENSG00000143727 | HGLibA_00509 | 44 | -0.710756312 | ACP1 | acid phosphatase 1 [Source:HGNC Symbol;Acc:HGNC:122] |
| ENSG00000146006 | HGLibA_27489 | 44 | -0.710756312 | LRRTM2 | leucine rich repeat transmembrane neuronal 2 [Source:HGNC Symbol;Acc:HGNC:19409] |
| ENSG00000147124 | HGLibA_55932 | 44 | -0.710756312 | ZNF41 | zinc finger protein 41 [Source:HGNC Symbol;Acc:HGNC:13107] |
| ENSG00000147432 | HGLibA_09596 | 44 | -0.710756312 | CHRNB3 | cholinergic receptor nicotinic beta 3 subunit [Source:HGNC Symbol;Acc:HGNC:1963] |
| ENSG00000148702 | HGLibA_20794 | 44 | -0.710756312 | HABP2 | hyaluronan binding protein 2 [Source:HGNC Symbol;Acc:HGNC:4798] |
| ENSG00000149050 | HGLibA_55594 | 44 | -0.710756312 | ZNF214 | zinc finger protein 214 [Source:HGNC Symbol;Acc:HGNC:13006] |
| ENSG00000149923 | HGLibA_38200 | 44 | -0.710756312 | PPP4C | protein phosphatase 4 catalytic subunit [Source:HGNC Symbol;Acc:HGNC:9319] |
| ENSG00000150477 | HGLibA_24741 | 44 | -0.710756312 | KIAA1328 | KIAA1328 [Source:HGNC Symbol;Acc:HGNC:29248] |
| ENSG00000151116 | HGLibA_52841 | 44 | -0.710756312 | UEVLD | UEV and lactate/malate dehyrogenase domains [Source:HGNC Symbol;Acc:HGNC:30866] |
| ENSG00000151117 | HGLibA_50526 | 44 | -0.710756312 | TMEM86A | transmembrane protein 86A [Source:HGNC Symbol;Acc:HGNC:26890] |
| ENSG00000152056 | HGLibA_02303 | 44 | -0.710756312 | AP1S3 | adaptor related protein complex 1 subunit sigma 3 [Source:HGNC Symbol;Acc:HGNC:18971] |
| ENSG00000152154 | HGLibA_50080 | 44 | -0.710756312 | TMEM178A | transmembrane protein 178A [Source:HGNC Symbol;Acc:HGNC:28517] |
| ENSG00000152766 | HGLibA_02040 | 44 | -0.710756312 | ANKRD22 | ankyrin repeat domain 22 [Source:HGNC Symbol;Acc:HGNC:28321] |
| ENSG00000153443 | HGLibA_52539 | 44 | -0.710756312 | UBALD1 | UBA like domain containing 1 [Source:HGNC Symbol;Acc:HGNC:29576] |
| ENSG00000154143 | HGLibA_35114 | 44 | -0.710756312 | PANX3 | pannexin 3 [Source:HGNC Symbol;Acc:HGNC:20573] |
| ENSG00000154330 | HGLibA_36274 | 44 | -0.710756312 | PGM5 | phosphoglucomutase 5 [Source:HGNC Symbol;Acc:HGNC:8908] |
| ENSG00000154767 | HGLibA_54550 | 44 | -0.710756312 | XPC | DNA damage recognition and repair factor [Source:HGNC Symbol;Acc:HGNC:12816]" |
| ENSG00000155115 | HGLibA_20594 | 44 | -0.710756312 | GTF3C6 | general transcription factor IIIC subunit 6 [Source:HGNC Symbol;Acc:HGNC:20872] |
| ENSG00000155890 | HGLibA_51497 | 44 | -0.710756312 | TRIM42 | tripartite motif containing 42 [Source:HGNC Symbol;Acc:HGNC:19014] |
| ENSG00000156687 | HGLibA_53026 | 44 | -0.710756312 | UNC5D | unc-5 netrin receptor D [Source:HGNC Symbol;Acc:HGNC:18634] |
| ENSG00000156958 | HGLibA_18518 | 44 | -0.710756312 | GALK2 | galactokinase 2 [Source:HGNC Symbol;Acc:HGNC:4119] |
| ENSG00000157796 | HGLibA_54043 | 44 | -0.710756312 | WDR19 | WD repeat domain 19 [Source:HGNC Symbol;Acc:HGNC:18340] |
| ENSG00000158077 | HGLibA_32113 | 44 | -0.710756312 | NLRP14 | NLR family pyrin domain containing 14 [Source:HGNC Symbol;Acc:HGNC:22939] |
| ENSG00000158552 | HGLibA_55150 | 44 | -0.710756312 | ZFAND2B | zinc finger AN1-type containing 2B [Source:HGNC Symbol;Acc:HGNC:25206] |
| ENSG00000162139 | HGLibA_31717 | 44 | -0.710756312 | NEU3 | neuraminidase 3 [Source:HGNC Symbol;Acc:HGNC:7760] |
| ENSG00000163017 | HGLibA_00615 | 44 | -0.710756312 | ACTG2 | enteric [Source:HGNC Symbol;Acc:HGNC:145]" |
| ENSG00000164597 | HGLibA_10481 | 44 | -0.710756312 | COG5 | component of oligomeric golgi complex 5 [Source:HGNC Symbol;Acc:HGNC:14857] |
| ENSG00000164764 | HGLibA_42816 | 44 | -0.710756312 | SBSPON | somatomedin B and thrombospondin type 1 domain containing [Source:HGNC Symbol;Acc:HGNC:30362] |
| ENSG00000165272 | HGLibA_02592 | 44 | -0.710756312 | AQP3 | aquaporin 3 (Gill blood group) [Source:HGNC Symbol;Acc:HGNC:636] |
| ENSG00000165762 | HGLibA_34032 | 44 | -0.710756312 | OR4K2 | olfactory receptor family 4 subfamily K member 2 [Source:HGNC Symbol;Acc:HGNC:14728] |
| ENSG00000166165 | HGLibA_09765 | 44 | -0.710756312 | CKB | creatine kinase B [Source:HGNC Symbol;Acc:HGNC:1991] |
| ENSG00000166171 | HGLibA_13811 | 44 | -0.710756312 | DPCD | deleted in primary ciliary dyskinesia homolog (mouse) [Source:HGNC Symbol;Acc:HGNC:24542] |
| ENSG00000167113 | HGLibA_10755 | 44 | -0.710756312 | COQ4 | coenzyme Q4 [Source:HGNC Symbol;Acc:HGNC:19693] |
| ENSG00000167996 | HGLibA_18117 | 44 | -0.710756312 | FTH1 | ferritin heavy chain 1 [Source:HGNC Symbol;Acc:HGNC:3976] |
| ENSG00000168427 | HGLibA_25181 | 44 | -0.710756312 | KLHL30 | kelch like family member 30 [Source:HGNC Symbol;Acc:HGNC:24770] |
| ENSG00000168802 | HGLibA_09663 | 44 | -0.710756312 | CHTF8 | chromosome transmission fidelity factor 8 [Source:HGNC Symbol;Acc:HGNC:24353] |
| ENSG00000169826 | HGLibA_11347 | 44 | -0.710756312 | CSGALNACT2 | chondroitin sulfate N-acetylgalactosaminyltransferase 2 [Source:HGNC Symbol;Acc:HGNC:24292] |
| ENSG00000169926 | HGLibA_25052 | 44 | -0.710756312 | KLF13 | Kruppel like factor 13 [Source:HGNC Symbol;Acc:HGNC:13672] |
| ENSG00000170917 | HGLibA_32986 | 44 | -0.710756312 | NUDT6 | nudix hydrolase 6 [Source:HGNC Symbol;Acc:HGNC:8053] |
| ENSG00000171132 | HGLibA_38544 | 44 | -0.710756312 | PRKCE | protein kinase C epsilon [Source:HGNC Symbol;Acc:HGNC:9401] |
| ENSG00000171180 | HGLibA_33840 | 44 | -0.710756312 | OR2M4 | olfactory receptor family 2 subfamily M member 4 [Source:HGNC Symbol;Acc:HGNC:8270] |
| ENSG00000171517 | HGLibA_27059 | 44 | -0.710756312 | LPAR3 | lysophosphatidic acid receptor 3 [Source:HGNC Symbol;Acc:HGNC:14298] |
| ENSG00000171522 | HGLibA_39323 | 44 | -0.710756312 | PTGER4 | prostaglandin E receptor 4 [Source:HGNC Symbol;Acc:HGNC:9596] |
| ENSG00000171681 | HGLibA_03455 | 44 | -0.710756312 | ATF7IP | activating transcription factor 7 interacting protein [Source:HGNC Symbol;Acc:HGNC:20092] |
| ENSG00000171791 | HGLibA_04297 | 44 | -0.710756312 | BCL2 | apoptosis regulator [Source:HGNC Symbol;Acc:HGNC:990]" |
| ENSG00000171867 | HGLibA_38644 | 44 | -0.710756312 | PRNP | prion protein [Source:HGNC Symbol;Acc:HGNC:9449] |
| ENSG00000171956 | HGLibA_17815 | 44 | -0.710756312 | FOXB1 | forkhead box B1 [Source:HGNC Symbol;Acc:HGNC:3799] |
| ENSG00000172667 | HGLibA_55371 | 44 | -0.710756312 | ZMAT3 | zinc finger matrin-type 3 [Source:HGNC Symbol;Acc:HGNC:29983] |
| ENSG00000173163 | HGLibA_10651 | 44 | -0.710756312 | COMMD1 | copper metabolism domain containing 1 [Source:HGNC Symbol;Acc:HGNC:23024] |
| ENSG00000174672 | HGLibA_04852 | 44 | -0.710756312 | BRSK2 | BR serine/threonine kinase 2 [Source:HGNC Symbol;Acc:HGNC:11405] |
| ENSG00000174721 | HGLibA_17433 | 44 | -0.710756312 | FGFBP3 | fibroblast growth factor binding protein 3 [Source:HGNC Symbol;Acc:HGNC:23428] |
| ENSG00000177854 | HGLibA_50129 | 44 | -0.710756312 | TMEM187 | transmembrane protein 187 [Source:HGNC Symbol;Acc:HGNC:13705] |
| ENSG00000178150 | HGLibA_55443 | 44 | -0.710756312 | ZNF114 | zinc finger protein 114 [Source:HGNC Symbol;Acc:HGNC:12894] |
| ENSG00000178394 | HGLibA_22356 | 44 | -0.710756312 | HTR1A | 5-hydroxytryptamine receptor 1A [Source:HGNC Symbol;Acc:HGNC:5286] |
| ENSG00000179213 | HGLibA_44156 | 44 | -0.710756312 | SIGLECL1 | SIGLEC family like 1 [Source:HGNC Symbol;Acc:HGNC:26856] |
| ENSG00000179832 | HGLibA_29867 | 44 | -0.710756312 | MROH1 | maestro heat like repeat family member 1 [Source:HGNC Symbol;Acc:HGNC:26958] |
| ENSG00000179941 | HGLibA_04209 | 44 | -0.710756312 | BBS10 | Bardet-Biedl syndrome 10 [Source:HGNC Symbol;Acc:HGNC:26291] |
| ENSG00000180190 | HGLibA_48923 | 44 | -0.710756312 | TDRP | testis development related protein [Source:HGNC Symbol;Acc:HGNC:26951] |
| ENSG00000180872 | HGLibA_12849 | 44 | -0.710756312 | DEFB112 | defensin beta 112 [Source:HGNC Symbol;Acc:HGNC:18093] |
| ENSG00000180974 | HGLibA_34160 | 44 | -0.710756312 | OR52E4 | olfactory receptor family 52 subfamily E member 4 [Source:HGNC Symbol;Acc:HGNC:15213] |
| ENSG00000181315 | HGLibA_55797 | 44 | -0.710756312 | ZNF322 | zinc finger protein 322 [Source:HGNC Symbol;Acc:HGNC:23640] |
| ENSG00000182095 | HGLibA_50911 | 44 | -0.710756312 | TNRC18 | trinucleotide repeat containing 18 [Source:HGNC Symbol;Acc:HGNC:11962] |
| ENSG00000182333 | HGLibA_26610 | 44 | -0.710756312 | LIPF | gastric type [Source:HGNC Symbol;Acc:HGNC:6622]" |
| ENSG00000182552 | HGLibA_42491 | 44 | -0.710756312 | RWDD4 | RWD domain containing 4 [Source:HGNC Symbol;Acc:HGNC:23750] |
| ENSG00000182583 | HGLibA_53580 | 44 | -0.710756312 | VCX | variable charge X-linked [Source:HGNC Symbol;Acc:HGNC:12667] |
| ENSG00000182809 | HGLibA_11152 | 44 | -0.710756312 | CRIP2 | cysteine rich protein 2 [Source:HGNC Symbol;Acc:HGNC:2361] |
| ENSG00000183035 | HGLibA_12043 | 44 | -0.710756312 | CYLC1 | cylicin 1 [Source:HGNC Symbol;Acc:HGNC:2582] |
| ENSG00000183044 | HGLibA_00091 | 44 | -0.710756312 | ABAT | 4-aminobutyrate aminotransferase [Source:HGNC Symbol;Acc:HGNC:23] |

| | | | | | |
|---|---|---|---|---|---|
| ENSG00000184515 | HGLibA_04450 | 44 | -0.710756312 | BEX5 | brain expressed X-linked 5 [Source:HGNC Symbol;Acc:HGNC:27990] |
| ENSG00000184524 | HGLibA_09034 | 44 | -0.710756312 | CEND1 | cell cycle exit and neuronal differentiation 1 [Source:HGNC Symbol;Acc:HGNC:24153] |
| ENSG00000185198 | HGLibA_38968 | 44 | -0.710756312 | PRSS57 | serine protease 57 [Source:HGNC Symbol;Acc:HGNC:31397] |
| ENSG00000186132 | HGLibA_06181 | 44 | -0.710756312 | C2orf76 | chromosome 2 open reading frame 76 [Source:HGNC Symbol;Acc:HGNC:27017] |
| ENSG00000186451 | HGLibA_46427 | 44 | -0.710756312 | SPATA12 | spermatogenesis associated 12 [Source:HGNC Symbol;Acc:HGNC:23221] |
| ENSG00000186509 | HGLibA_34582 | 44 | -0.710756312 | OR9Q1 | olfactory receptor family 9 subfamily Q member 1 [Source:HGNC Symbol;Acc:HGNC:14724] |
| ENSG00000186628 | HGLibA_18084 | 44 | -0.710756312 | FSD2 | fibronectin type III and SPRY domain containing 2 [Source:HGNC Symbol;Acc:HGNC:18024] |
| ENSG00000186897 | HGLibA_05932 | 44 | -0.710756312 | C1QL4 | complement C1q like 4 [Source:HGNC Symbol;Acc:HGNC:31416] |
| ENSG00000187080 | HGLibA_33756 | 44 | -0.710756312 | OR2AK2 | olfactory receptor family 2 subfamily AK member 2 [Source:HGNC Symbol;Acc:HGNC:19569] |
| ENSG00000187922 | HGLibA_26170 | 44 | -0.710756312 | LCN10 | lipocalin 10 [Source:HGNC Symbol;Acc:HGNC:20892] |
| ENSG00000188761 | HGLibA_04319 | 44 | -0.710756312 | BCL2L15 | BCL2 like 15 [Source:HGNC Symbol;Acc:HGNC:33624] |
| ENSG00000188883 | HGLibA_25313 | 44 | -0.710756312 | KLRG2 | killer cell lectin like receptor G2 [Source:HGNC Symbol;Acc:HGNC:24778] |
| ENSG00000188981 | HGLibA_30169 | 44 | -0.710756312 | MSANTD1 | Myb/SANT DNA binding domain containing 1 [Source:HGNC Symbol;Acc:HGNC:33741] |
| ENSG00000189144 | HGLibA_56284 | 44 | -0.710756312 | ZNF573 | zinc finger protein 573 [Source:HGNC Symbol;Acc:HGNC:26420] |
| ENSG00000189241 | HGLibA_51980 | 44 | -0.710756312 | TSPYL1 | TSPY like 1 [Source:HGNC Symbol;Acc:HGNC:12382] |
| ENSG00000189308 | HGLibA_26569 | 44 | -0.710756312 | LIN54 | lin-54 DREAM MuvB core complex component [Source:HGNC Symbol;Acc:HGNC:25397] |
| ENSG00000196268 | HGLibA_56098 | 44 | -0.710756312 | ZNF493 | zinc finger protein 493 [Source:HGNC Symbol;Acc:HGNC:23708] |
| ENSG00000196586 | HGLibA_30860 | 44 | -0.710756312 | MYO6 | myosin VI [Source:HGNC Symbol;Acc:HGNC:7605] |
| ENSG00000196781 | HGLibA_49607 | 44 | -0.710756312 | TLE1 | transducin like enhancer of split 1 [Source:HGNC Symbol;Acc:HGNC:11837] |
| ENSG00000196917 | HGLibA_20946 | 44 | -0.710756312 | HCAR1 | hydroxycarboxylic acid receptor 1 [Source:HGNC Symbol;Acc:HGNC:4532] |
| ENSG00000197428 | HGLibA_34094 | 44 | -0.710756312 | OR51D1 | olfactory receptor family 51 subfamily D member 1 [Source:HGNC Symbol;Acc:HGNC:15193] |
| ENSG00000197619 | HGLibA_56391 | 44 | -0.710756312 | ZNF615 | zinc finger protein 615 [Source:HGNC Symbol;Acc:HGNC:24740] |
| ENSG00000197921 | HGLibA_21189 | 44 | -0.710756312 | HES5 | hes family bHLH transcription factor 5 [Source:HGNC Symbol;Acc:HGNC:19764] |
| ENSG00000197978 | HGLibA_19630 | 44 | -0.710756312 | GOLGA6L9 | golgin A6 family-like 9 [Source:HGNC Symbol;Acc:HGNC:37229] |
| ENSG00000198668 | HGLibA_07022 | 44 | -0.710756312 | CALM1 | calmodulin 1 [Source:HGNC Symbol;Acc:HGNC:1442] |
| ENSG00000203730 | HGLibA_48959 | 44 | -0.710756312 | TEDDM1 | transmembrane epididymal protein 1 [Source:HGNC Symbol;Acc:HGNC:30233] |
| ENSG00000204231 | HGLibA_42510 | 44 | -0.710756312 | RXRB | retinoid X receptor beta [Source:HGNC Symbol;Acc:HGNC:10478] |
| ENSG00000204291 | HGLibA_10516 | 44 | -0.710756312 | COL15A1 | collagen type XV alpha 1 chain [Source:HGNC Symbol;Acc:HGNC:2192] |
| ENSG00000204439 | HGLibA_06487 | 44 | -0.710756312 | C6orf47 | chromosome 6 open reading frame 47 [Source:HGNC Symbol;Acc:HGNC:19076] |
| ENSG00000204856 | HGLibA_16482 | 44 | -0.710756312 | FAM216A | family with sequence similarity 216 member A [Source:HGNC Symbol;Acc:HGNC:30180] |
| ENSG00000205030 | HGLibA_34325 | 44 | -0.710756312 | OR5L2 | olfactory receptor family 5 subfamily L member 2 [Source:HGNC Symbol;Acc:HGNC:8351] |
| ENSG00000205502 | HGLibA_06097 | 44 | -0.710756312 | C2CD4B | C2 calcium dependent domain containing 4B [Source:HGNC Symbol;Acc:HGNC:33628] |
| ENSG00000213551 | HGLibA_13643 | 44 | -0.710756312 | DNAJC9 | DnaJ heat shock protein family (Hsp40) member C9 [Source:HGNC Symbol;Acc:HGNC:19123] |
| ENSG00000216490 | HGLibA_22604 | 44 | -0.710756312 | IFI30 | lysosomal thiol reductase [Source:HGNC Symbol;Acc:HGNC:5398]" |
| ENSG00000221995 | HGLibA_49432 | 44 | -0.710756312 | TIAF1 | TGFB1-induced anti-apoptotic factor 1 [Source:HGNC Symbol;Acc:HGNC:11803] |
| ENSG00000232112 | HGLibA_49737 | 44 | -0.710756312 | TMA7 | translation machinery associated 7 homolog [Source:HGNC Symbol;Acc:HGNC:26932] |
| ENSG00000236126 | HGLibA_11531 | 44 | -0.710756312 | CT47A3 | member A3 [Source:HGNC Symbol;Acc:HGNC:33284]" |
| ENSG00000238205 | HGLibA_29705 | 44 | -0.710756312 | MPC1L | mitochondrial pyruvate carrier 1 like [Source:HGNC Symbol;Acc:HGNC:44205] |
| ENSG00000242114 | HGLibA_30358 | 44 | -0.710756312 | MTFP1 | mitochondrial fission process 1 [Source:HGNC Symbol;Acc:HGNC:26945] |
| ENSG00000243056 | HGLibA_14835 | 44 | -0.710756312 | EIF4EBP3 | eukaryotic translation initiation factor 4E binding protein 3 [Source:HGNC Symbol;Acc:HGNC:3290] |
| ENSG00000243943 | HGLibA_56132 | 44 | -0.710756312 | ZNF512 | zinc finger protein 512 [Source:HGNC Symbol;Acc:HGNC:29380] |
| ENSG00000251493 | HGLibA_17827 | 44 | -0.710756312 | FOXD1 | forkhead box D1 [Source:HGNC Symbol;Acc:HGNC:3802] |
| ENSG00000254852 | HGLibA_32465 | 44 | -0.710756312 | NPIPA2 | nuclear pore complex interacting protein family member A2 [Source:HGNC Symbol;Acc:HGNC:41979] |
| ENSG00000255072 | HGLibA_36603 | 44 | -0.710756312 | PIGY | phosphatidylinositol glycan anchor biosynthesis class Y [Source:HGNC Symbol;Acc:HGNC:28213] |
| ENSG00000256812 | HGLibA_07186 | 44 | -0.710756312 | CAPNS2 | calpain small subunit 2 [Source:HGNC Symbol;Acc:HGNC:16371] |
| ENSG00000258102 | HGLibA_28126 | 44 | -0.710756312 | MAP1LC3B2 | microtubule associated protein 1 light chain 3 beta 2 [Source:HGNC Symbol;Acc:HGNC:34390] |
| ENSG00000261150 | HGLibA_15361 | 44 | -0.710756312 | EPPK1 | epiplakin 1 [Source:HGNC Symbol;Acc:HGNC:15577] |
| ENSG00000265107 | HGLibA_19148 | 44 | -0.710756312 | GJA5 | gap junction protein alpha 5 [Source:HGNC Symbol;Acc:HGNC:4279] |
| ENSG00000265241 | HGLibA_40584 | 44 | -0.710756312 | RBM8A | RNA binding motif protein 8A [Source:HGNC Symbol;Acc:HGNC:9905] |
| ENSG00000265972 | HGLibA_52432 | 44 | -0.710756312 | TXNIP | thioredoxin interacting protein [Source:HGNC Symbol;Acc:HGNC:16952] |
| ENSG00000269067 | HGLibA_56637 | 44 | -0.710756312 | ZNF728 | zinc finger protein 728 [Source:HGNC Symbol;Acc:HGNC:32463] |
| ENSG00000274736 | HGLibA_07999 | 44 | -0.710756312 | CCL23 | C-C motif chemokine ligand 23 [Source:HGNC Symbol;Acc:HGNC:10622] |
| ENSG00000274749 | HGLibA_25824 | 44 | -0.710756312 | KRTAP7-1 | keratin associated protein 7-1 (gene/pseudogene) [Source:HGNC Symbol;Acc:HGNC:18934] |
| FAM86C1 | HGLibA_16780 | 44 | -0.710756312 | FAM86C1 | - |
| HIST1H3E | HGLibA_21469 | 44 | -0.710756312 | HIST1H3E | - |
| HIST2H3A | HGLibA_21540 | 44 | -0.710756312 | HIST2H3A | - |
| hsa-mir-1260a | HGLibA_57460 | 44 | -0.710756312 | hsa-mir-1260a | - |
| hsa-mir-1276 | HGLibA_57561 | 44 | -0.710756312 | hsa-mir-1276 | - |
| hsa-mir-1292 | HGLibA_57646 | 44 | -0.710756312 | hsa-mir-1292 | - |
| hsa-mir-3140 | HGLibA_58619 | 44 | -0.710756312 | hsa-mir-3140 | - |
| hsa-mir-3622a | HGLibA_59122 | 44 | -0.710756312 | hsa-mir-3622a | - |
| hsa-mir-4297 | HGLibA_59901 | 44 | -0.710756312 | hsa-mir-4297 | - |
| hsa-mir-4433b | HGLibA_60122 | 44 | -0.710756312 | hsa-mir-4433b | - |
| hsa-mir-4436b-1 | HGLibA_60144 | 44 | -0.710756312 | hsa-mir-4436b-1 | - |
| hsa-mir-4520a | HGLibA_60534 | 44 | -0.710756312 | hsa-mir-4520a | - |
| hsa-mir-454 | HGLibA_60623 | 44 | -0.710756312 | hsa-mir-454 | - |
| hsa-mir-4663 | HGLibA_60766 | 44 | -0.710756312 | hsa-mir-4663 | - |
| hsa-mir-4691 | HGLibA_60874 | 44 | -0.710756312 | hsa-mir-4691 | - |
| hsa-mir-4711 | HGLibA_60946 | 44 | -0.710756312 | hsa-mir-4711 | - |
| hsa-mir-4715 | HGLibA_60961 | 44 | -0.710756312 | hsa-mir-4715 | - |
| hsa-mir-4729 | HGLibA_61015 | 44 | -0.710756312 | hsa-mir-4729 | - |
| hsa-mir-4799 | HGLibA_61300 | 44 | -0.710756312 | hsa-mir-4799 | - |
| hsa-mir-489 | HGLibA_61358 | 44 | -0.710756312 | hsa-mir-489 | - |
| hsa-mir-493 | HGLibA_61373 | 44 | -0.710756312 | hsa-mir-493 | - |
| hsa-mir-5002 | HGLibA_61414 | 44 | -0.710756312 | hsa-mir-5002 | - |
| hsa-mir-5186 | HGLibA_61636 | 44 | -0.710756312 | hsa-mir-5186 | - |
| hsa-mir-548e | HGLibA_61968 | 44 | -0.710756312 | hsa-mir-548e | - |
| hsa-mir-550a-2 | HGLibA_62103 | 44 | -0.710756312 | hsa-mir-550a-2 | - |
| hsa-mir-5692a-2 | HGLibA_62290 | 44 | -0.710756312 | hsa-mir-5692a-2 | - |
| hsa-mir-584 | HGLibA_62435 | 44 | -0.710756312 | hsa-mir-584 | - |
| hsa-mir-661 | HGLibA_62978 | 44 | -0.710756312 | hsa-mir-661 | - |
| hsa-mir-6715a | HGLibA_63018 | 44 | -0.710756312 | hsa-mir-6715a | - |
| hsa-mir-6762 | HGLibA_63209 | 44 | -0.710756312 | hsa-mir-6762 | - |
| hsa-mir-6783 | HGLibA_63308 | 44 | -0.710756312 | hsa-mir-6783 | - |
| hsa-mir-6818 | HGLibA_63450 | 44 | -0.710756312 | hsa-mir-6818 | - |
| hsa-mir-6820 | HGLibA_63456 | 44 | -0.710756312 | hsa-mir-6820 | - |
| hsa-mir-6836 | HGLibA_63520 | 44 | -0.710756312 | hsa-mir-6836 | - |
| hsa-mir-6892 | HGLibA_63751 | 44 | -0.710756312 | hsa-mir-6892 | - |
| hsa-mir-765 | HGLibA_63923 | 44 | -0.710756312 | hsa-mir-765 | - |
| hsa-mir-8067 | HGLibA_64107 | 44 | -0.710756312 | hsa-mir-8067 | - |
| hsa-mir-8073 | HGLibA_64134 | 44 | -0.710756312 | hsa-mir-8073 | - |
| hsa-mir-889 | HGLibA_64235 | 44 | -0.710756312 | hsa-mir-889 | - |
| hsa-mir-920 | HGLibA_64274 | 44 | -0.710756312 | hsa-mir-920 | - |
| IL9R_X | HGLibA_23201 | 44 | -0.710756312 | IL9R_X | - |
| KIAA1244 | HGLibA_24726 | 44 | -0.710756312 | KIAA1244 | - |
| LEPREL1 | HGLibA_26319 | 44 | -0.710756312 | LEPREL1 | - |
| LOC100130301 | HGLibA_26774 | 44 | -0.710756312 | LOC100130301 | - |
| LOC154872 | HGLibA_26898 | 44 | -0.710756312 | LOC154872 | - |
| LOC653486 | HGLibA_26988 | 44 | -0.710756312 | LOC653486 | - |
| NBPF16 | HGLibA_31227 | 44 | -0.710756312 | NBPF16 | - |
| PLCXD1_X | HGLibA_37019 | 44 | -0.710756312 | PLCXD1_X | - |
| PTPLA | HGLibA_39416 | 44 | -0.710756312 | PTPLA | - |
| SPG20 | HGLibA_46633 | 44 | -0.710756312 | SPG20 | - |
| 1-Dec | HGLibA_12772 | 43 | -0.720707349 | 1-Dec | - |
| ALS2CR8 | HGLibA_01730 | 43 | -0.720707349 | ALS2CR8 | - |
| C11orf48 | HGLibA_05145 | 43 | -0.720707349 | C11orf48 | - |
| C14orf105 | HGLibA_05318 | 43 | -0.720707349 | C14orf105 | - |
| C16orf71 | HGLibA_05475 | 43 | -0.720707349 | C16orf71 | - |
| C1orf172 | HGLibA_05793 | 43 | -0.720707349 | C1orf172 | - |
| C9orf139 | HGLibA_06684 | 43 | -0.720707349 | C9orf139 | - |
| CCDC108 | HGLibA_07529 | 43 | -0.720707349 | CCDC108 | - |
| ENSG00000005882 | HGLibA_35898 | 43 | -0.720707349 | PDK2 | pyruvate dehydrogenase kinase 2 [Source:HGNC Symbol;Acc:HGNC:8810] |
| ENSG00000006283 | HGLibA_06892 | 43 | -0.720707349 | CACNA1G | calcium voltage-gated channel subunit alpha1 G [Source:HGNC Symbol;Acc:HGNC:1394] |
| ENSG00000007312 | HGLibA_08449 | 43 | -0.720707349 | CD79B | CD79b molecule [Source:HGNC Symbol;Acc:HGNC:1699] |
| ENSG00000007944 | HGLibA_30781 | 43 | -0.720707349 | MYLIP | myosin regulatory light chain interacting protein [Source:HGNC Symbol;Acc:HGNC:21155] |
| ENSG00000012504 | HGLibA_32586 | 43 | -0.720707349 | NR1H4 | nuclear receptor subfamily 1 group H member 4 [Source:HGNC Symbol;Acc:HGNC:7967] |
| ENSG00000036672 | HGLibA_53296 | 43 | -0.720707349 | USP2 | ubiquitin specific peptidase 2 [Source:HGNC Symbol;Acc:HGNC:12618] |
| ENSG00000041353 | HGLibA_39859 | 43 | -0.720707349 | RAB27B | member RAS oncogene family [Source:HGNC Symbol;Acc:HGNC:9767]" |
| ENSG00000048342 | HGLibA_07480 | 43 | -0.720707349 | CC2D2A | coiled-coil and C2 domain containing 2A [Source:HGNC Symbol;Acc:HGNC:29253] |
| ENSG00000058729 | HGLibA_41311 | 43 | -0.720707349 | RIOK2 | RIO kinase 2 [Source:HGNC Symbol;Acc:HGNC:18999] |
| ENSG00000065618 | HGLibA_10521 | 43 | -0.720707349 | COL17A1 | collagen type XVII alpha 1 chain [Source:HGNC Symbol;Acc:HGNC:2194] |
| ENSG00000066279 | HGLibA_03358 | 43 | -0.720707349 | ASPM | abnormal spindle microtubule assembly [Source:HGNC Symbol;Acc:HGNC:19048] |
| ENSG00000069509 | HGLibA_18164 | 43 | -0.720707349 | FUNDC1 | FUN14 domain containing 1 [Source:HGNC Symbol;Acc:HGNC:28746] |
| ENSG00000073734 | HGLibA_00136 | 43 | -0.720707349 | ABCB11 | ATP binding cassette subfamily B member 11 [Source:HGNC Symbol;Acc:HGNC:42] |
| ENSG00000075891 | HGLibA_35297 | 43 | -0.720707349 | PAX2 | paired box 2 [Source:HGNC Symbol;Acc:HGNC:8616] |
| ENSG00000079257 | HGLibA_27650 | 43 | -0.720707349 | LXN | latexin [Source:HGNC Symbol;Acc:HGNC:13347] |
| ENSG00000086475 | HGLibA_43412 | 43 | -0.720707349 | SEPHS1 | selenophosphate synthetase 1 [Source:HGNC Symbol;Acc:HGNC:19685] |
| ENSG00000087152 | HGLibA_03857 | 43 | -0.720707349 | ATXN7L3 | ataxin 7 like 3 [Source:HGNC Symbol;Acc:HGNC:25416] |
| ENSG00000087250 | HGLibA_30303 | 43 | -0.720707349 | MT3 | metallothionein 3 [Source:HGNC Symbol;Acc:HGNC:7408] |
| ENSG00000088682 | HGLibA_10766 | 43 | -0.720707349 | COQ9 | coenzyme Q9 [Source:HGNC Symbol;Acc:HGNC:25302] |
| ENSG00000089916 | HGLibA_19741 | 43 | -0.720707349 | GPATCH2L | G-patch domain containing 2 like [Source:HGNC Symbol;Acc:HGNC:20210] |
| ENSG00000090382 | HGLibA_27783 | 43 | -0.720707349 | LYZ | lysozyme [Source:HGNC Symbol;Acc:HGNC:6740] |
| ENSG00000090674 | HGLibA_28637 | 43 | -0.720707349 | MCOLN1 | mucolipin 1 [Source:HGNC Symbol;Acc:HGNC:13356] |
| ENSG00000091073 | HGLibA_14082 | 43 | -0.720707349 | DTX2 | deltex E3 ubiquitin ligase 2 [Source:HGNC Symbol;Acc:HGNC:15973] |
| ENSG00000092531 | HGLibA_45875 | 43 | -0.720707349 | SNAP23 | synaptosome associated protein 23 [Source:HGNC Symbol;Acc:HGNC:11131] |

| | | | | | |
|---|---|---|---|---|---|
| ENSG00000092758 | HGLibA_10627 | 43 | -0.720707349 | COL9A3 | collagen type IX alpha 3 chain [Source:HGNC Symbol;Acc:HGNC:2219] |
| ENSG00000094804 | HGLibA_08593 | 43 | -0.720707349 | CDC6 | cell division cycle 6 [Source:HGNC Symbol;Acc:HGNC:1744] |
| ENSG00000099282 | HGLibA_51907 | 43 | -0.720707349 | TSPAN15 | tetraspanin 15 [Source:HGNC Symbol;Acc:HGNC:23298] |
| ENSG00000099822 | HGLibA_20977 | 43 | -0.720707349 | HCN2 | hyperpolarization activated cyclic nucleotide gated potassium and sodium channel 2 [Source:HGNC Symbol;Acc:HGNC:4846] |
| ENSG00000100065 | HGLibA_07216 | 43 | -0.720707349 | CARD10 | caspase recruitment domain family member 10 [Source:HGNC Symbol;Acc:HGNC:16422] |
| ENSG00000100206 | HGLibA_13399 | 43 | -0.720707349 | DMC1 | DNA meiotic recombinase 1 [Source:HGNC Symbol;Acc:HGNC:2927] |
| ENSG00000100564 | HGLibA_36557 | 43 | -0.720707349 | PIGH | phosphatidylinositol glycan anchor biosynthesis class H [Source:HGNC Symbol;Acc:HGNC:8964] |
| ENSG00000100575 | HGLibA_49528 | 43 | -0.720707349 | TIMM9 | translocase of inner mitochondrial membrane 9 [Source:HGNC Symbol;Acc:HGNC:11819] |
| ENSG00000100897 | HGLibA_12414 | 43 | -0.720707349 | DCAF11 | DDB1 and CUL4 associated factor 11 [Source:HGNC Symbol;Acc:HGNC:20258] |
| ENSG00000101638 | HGLibA_47299 | 43 | -0.720707349 | ST8SIA5 | 8-sialyltransferase 5 [Source:HGNC Symbol;Acc:HGNC:17827]" |
| ENSG00000102144 | HGLibA_36241 | 43 | -0.720707349 | PGK1 | phosphoglycerate kinase 1 [Source:HGNC Symbol;Acc:HGNC:8896] |
| ENSG00000103353 | HGLibA_52696 | 43 | -0.720707349 | UBFD1 | ubiquitin family domain containing 1 [Source:HGNC Symbol;Acc:HGNC:30565] |
| ENSG00000104643 | HGLibA_30443 | 43 | -0.720707349 | MTMR9 | myotubularin related protein 9 [Source:HGNC Symbol;Acc:HGNC:14596] |
| ENSG00000104897 | HGLibA_43695 | 43 | -0.720707349 | SF3A2 | splicing factor 3a subunit 2 [Source:HGNC Symbol;Acc:HGNC:10766] |
| ENSG00000105808 | HGLibA_40305 | 43 | -0.720707349 | RASA4 | RAS p21 protein activator 4 [Source:HGNC Symbol;Acc:HGNC:23181] |
| ENSG00000106038 | HGLibA_15706 | 43 | -0.720707349 | EVX1 | even-skipped homeobox 1 [Source:HGNC Symbol;Acc:HGNC:3506] |
| ENSG00000107317 | HGLibA_39310 | 43 | -0.720707349 | PTGDS | prostaglandin D2 synthase [Source:HGNC Symbol;Acc:HGNC:9592] |
| ENSG00000108018 | HGLibA_46183 | 43 | -0.720707349 | SORCS1 | sortilin related VPS10 domain containing receptor 1 [Source:HGNC Symbol;Acc:HGNC:16697] |
| ENSG00000108576 | HGLibA_45327 | 43 | -0.720707349 | SLC6A4 | solute carrier family 6 member 4 [Source:HGNC Symbol;Acc:HGNC:11050] |
| ENSG00000108578 | HGLibA_04551 | 43 | -0.720707349 | BLMH | bleomycin hydrolase [Source:HGNC Symbol;Acc:HGNC:1059] |
| ENSG00000108639 | HGLibA_47995 | 43 | -0.720707349 | SYNGR2 | synaptogyrin 2 [Source:HGNC Symbol;Acc:HGNC:11499] |
| ENSG00000108829 | HGLibA_27401 | 43 | -0.720707349 | LRRC59 | leucine rich repeat containing 59 [Source:HGNC Symbol;Acc:HGNC:28817] |
| ENSG00000109061 | HGLibA_30702 | 43 | -0.720707349 | MYH1 | myosin heavy chain 1 [Source:HGNC Symbol;Acc:HGNC:7567] |
| ENSG00000109079 | HGLibA_50707 | 43 | -0.720707349 | TNFAIP1 | TNF alpha induced protein 1 [Source:HGNC Symbol;Acc:HGNC:11894] |
| ENSG00000109775 | HGLibA_52858 | 43 | -0.720707349 | UFSP2 | UFM1 specific peptidase 2 [Source:HGNC Symbol;Acc:HGNC:25640] |
| ENSG00000110880 | HGLibA_10780 | 43 | -0.720707349 | CORO1C | coronin 1C [Source:HGNC Symbol;Acc:HGNC:2254] |
| ENSG00000111276 | HGLibA_08824 | 43 | -0.720707349 | CDKN1B | cyclin dependent kinase inhibitor 1B [Source:HGNC Symbol;Acc:HGNC:1785] |
| ENSG00000112130 | HGLibA_41699 | 43 | -0.720707349 | RNF8 | ring finger protein 8 [Source:HGNC Symbol;Acc:HGNC:10071] |
| ENSG00000112309 | HGLibA_03976 | 43 | -0.720707349 | B3GAT2 | 3-glucuronyltransferase 2 [Source:HGNC Symbol;Acc:HGNC:922]" |
| ENSG00000112739 | HGLibA_38739 | 43 | -0.720707349 | PRPF4B | pre-mRNA processing factor 4B [Source:HGNC Symbol;Acc:HGNC:17346] |
| ENSG00000113088 | HGLibA_20727 | 43 | -0.720707349 | GZMK | granzyme K [Source:HGNC Symbol;Acc:HGNC:4711] |
| ENSG00000113522 | HGLibA_40075 | 43 | -0.720707349 | RAD50 | RAD50 double strand break repair protein [Source:HGNC Symbol;Acc:HGNC:9816] |
| ENSG00000114346 | HGLibA_14425 | 43 | -0.720707349 | ECT2 | epithelial cell transforming 2 [Source:HGNC Symbol;Acc:HGNC:3155] |
| ENSG00000114503 | HGLibA_31284 | 43 | -0.720707349 | NCBP2 | nuclear cap binding protein subunit 2 [Source:HGNC Symbol;Acc:HGNC:7659] |
| ENSG00000115145 | HGLibA_47333 | 43 | -0.720707349 | STAM2 | signal transducing adaptor molecule 2 [Source:HGNC Symbol;Acc:HGNC:11358] |
| ENSG00000116584 | HGLibA_02834 | 43 | -0.720707349 | ARHGEF2 | Rho/Rac guanine nucleotide exchange factor 2 [Source:HGNC Symbol;Acc:HGNC:682] |
| ENSG00000117152 | HGLibA_41094 | 43 | -0.720707349 | RGS4 | regulator of G protein signaling 4 [Source:HGNC Symbol;Acc:HGNC:10000] |
| ENSG00000117707 | HGLibA_38700 | 43 | -0.720707349 | PROX1 | prospero homeobox 1 [Source:HGNC Symbol;Acc:HGNC:9459] |
| ENSG00000118363 | HGLibA_46561 | 43 | -0.720707349 | SPCS2 | signal peptidase complex subunit 2 [Source:HGNC Symbol;Acc:HGNC:28962] |
| ENSG00000118785 | HGLibA_46769 | 43 | -0.720707349 | SPP1 | secreted phosphoprotein 1 [Source:HGNC Symbol;Acc:HGNC:11255] |
| ENSG00000118816 | HGLibA_08102 | 43 | -0.720707349 | CCNI | cyclin I [Source:HGNC Symbol;Acc:HGNC:1595] |
| ENSG00000119929 | HGLibA_11799 | 43 | -0.720707349 | CUTC | cutC copper transporter [Source:HGNC Symbol;Acc:HGNC:24271] |
| ENSG00000120235 | HGLibA_22686 | 43 | -0.720707349 | IFNA6 | interferon alpha 6 [Source:HGNC Symbol;Acc:HGNC:5427] |
| ENSG00000121570 | HGLibA_13892 | 43 | -0.720707349 | DPPA4 | developmental pluripotency associated 4 [Source:HGNC Symbol;Acc:HGNC:19200] |
| ENSG00000121903 | HGLibA_56999 | 43 | -0.720707349 | ZSCAN20 | zinc finger and SCAN domain containing 20 [Source:HGNC Symbol;Acc:HGNC:13093] |
| ENSG00000123219 | HGLibA_09066 | 43 | -0.720707349 | CENPK | centromere protein K [Source:HGNC Symbol;Acc:HGNC:29479] |
| ENSG00000123485 | HGLibA_21576 | 43 | -0.720707349 | HJURP | Holliday junction recognition protein [Source:HGNC Symbol;Acc:HGNC:25444] |
| ENSG00000123562 | HGLibA_29662 | 43 | -0.720707349 | MORF4L2 | mortality factor 4 like 2 [Source:HGNC Symbol;Acc:HGNC:16849] |
| ENSG00000124074 | HGLibA_15135 | 43 | -0.720707349 | ENKD1 | enkurin domain containing 1 [Source:HGNC Symbol;Acc:HGNC:25246] |
| ENSG00000124203 | HGLibA_56835 | 43 | -0.720707349 | ZNF831 | zinc finger protein 831 [Source:HGNC Symbol;Acc:HGNC:16167] |
| ENSG00000124228 | HGLibA_12686 | 43 | -0.720707349 | DDX27 | DEAD-box helicase 27 [Source:HGNC Symbol;Acc:HGNC:15837] |
| ENSG00000124493 | HGLibA_20351 | 43 | -0.720707349 | GRM4 | glutamate metabotropic receptor 4 [Source:HGNC Symbol;Acc:HGNC:4596] |
| ENSG00000126016 | HGLibA_01815 | 43 | -0.720707349 | AMOT | angiomotin [Source:HGNC Symbol;Acc:HGNC:17810] |
| ENSG00000126016 | HGLibA_01814 | 43 | -0.720707349 | AMOT | angiomotin [Source:HGNC Symbol;Acc:HGNC:17810] |
| ENSG00000126266 | HGLibA_17325 | 43 | -0.720707349 | FFAR1 | free fatty acid receptor 1 [Source:HGNC Symbol;Acc:HGNC:4498] |
| ENSG00000126524 | HGLibA_42790 | 43 | -0.720707349 | SBDS | ribosome maturation factor [Source:HGNC Symbol;Acc:HGNC:19440]" |
| ENSG00000129116 | HGLibA_35063 | 43 | -0.720707349 | PALLD | cytoskeletal associated protein [Source:HGNC Symbol;Acc:HGNC:17068]" |
| ENSG00000130724 | HGLibA_09479 | 43 | -0.720707349 | CHMP2A | charged multivesicular body protein 2A [Source:HGNC Symbol;Acc:HGNC:30216] |
| ENSG00000130755 | HGLibA_19406 | 43 | -0.720707349 | GMFG | glia maturation factor gamma [Source:HGNC Symbol;Acc:HGNC:4374] |
| ENSG00000131503 | HGLibA_01965 | 43 | -0.720707349 | ANKHD1 | ankyrin repeat and KH domain containing 1 [Source:HGNC Symbol;Acc:HGNC:24714] |
| ENSG00000131969 | HGLibA_00242 | 43 | -0.720707349 | ABHD12B | abhydrolase domain containing 12B [Source:HGNC Symbol;Acc:HGNC:19837] |
| ENSG00000132718 | HGLibA_48050 | 43 | -0.720707349 | SYT11 | synaptotagmin 11 [Source:HGNC Symbol;Acc:HGNC:19239] |
| ENSG00000132972 | HGLibA_41572 | 43 | -0.720707349 | RNF17 | ring finger protein 17 [Source:HGNC Symbol;Acc:HGNC:10060] |
| ENSG00000133794 | HGLibA_03067 | 43 | -0.720707349 | ARNTL | aryl hydrocarbon receptor nuclear translocator like [Source:HGNC Symbol;Acc:HGNC:701] |
| ENSG00000134762 | HGLibA_13996 | 43 | -0.720707349 | DSC3 | desmocollin 3 [Source:HGNC Symbol;Acc:HGNC:3037] |
| ENSG00000134815 | HGLibA_13184 | 43 | -0.720707349 | DHX34 | DExH-box helicase 34 [Source:HGNC Symbol;Acc:HGNC:16719] |
| ENSG00000135956 | HGLibA_49917 | 43 | -0.720707349 | TMEM127 | transmembrane protein 127 [Source:HGNC Symbol;Acc:HGNC:26038] |
| ENSG00000136244 | HGLibA_23181 | 43 | -0.720707349 | IL6 | interleukin 6 [Source:HGNC Symbol;Acc:HGNC:6018] |
| ENSG00000136720 | HGLibA_22143 | 43 | -0.720707349 | HS6ST1 | heparan sulfate 6-O-sulfotransferase 1 [Source:HGNC Symbol;Acc:HGNC:5201] |
| ENSG00000136758 | HGLibA_54700 | 43 | -0.720707349 | YME1L1 | YME1 like 1 ATPase [Source:HGNC Symbol;Acc:HGNC:12843] |
| ENSG00000137404 | HGLibA_32705 | 43 | -0.720707349 | NRM | nurim [Source:HGNC Symbol;Acc:HGNC:8003] |
| ENSG00000137700 | HGLibA_45053 | 43 | -0.720707349 | SLC37A4 | solute carrier family 37 member 4 [Source:HGNC Symbol;Acc:HGNC:4061] |
| ENSG00000137809 | HGLibA_23720 | 43 | -0.720707349 | ITGA11 | integrin subunit alpha 11 [Source:HGNC Symbol;Acc:HGNC:6136] |
| ENSG00000138615 | HGLibA_09710 | 43 | -0.720707349 | CILP | cartilage intermediate layer protein [Source:HGNC Symbol;Acc:HGNC:1980] |
| ENSG00000139540 | HGLibA_45116 | 43 | -0.720707349 | SLC39A5 | solute carrier family 39 member 5 [Source:HGNC Symbol;Acc:HGNC:20502] |
| ENSG00000140262 | HGLibA_48762 | 43 | -0.720707349 | TCF12 | transcription factor 12 [Source:HGNC Symbol;Acc:HGNC:11623] |
| ENSG00000140675 | HGLibA_45266 | 43 | -0.720707349 | SLC5A2 | solute carrier family 5 member 2 [Source:HGNC Symbol;Acc:HGNC:11037] |
| ENSG00000141627 | HGLibA_14225 | 43 | -0.720707349 | DYM | dymeclin [Source:HGNC Symbol;Acc:HGNC:21317] |
| ENSG00000142252 | HGLibA_18923 | 43 | -0.720707349 | GEMIN7 | gem nuclear organelle associated protein 7 [Source:HGNC Symbol;Acc:HGNC:20045] |
| ENSG00000143036 | HGLibA_45162 | 43 | -0.720707349 | SLC44A3 | solute carrier family 44 member 3 [Source:HGNC Symbol;Acc:HGNC:28689] |
| ENSG00000143157 | HGLibA_37474 | 43 | -0.720707349 | POGK | pogo transposable element derived with KRAB domain [Source:HGNC Symbol;Acc:HGNC:18800] |
| ENSG00000143552 | HGLibA_33041 | 43 | -0.720707349 | NUP210L | nucleoporin 210 like [Source:HGNC Symbol;Acc:HGNC:29915] |
| ENSG00000144115 | HGLibA_49362 | 43 | -0.720707349 | THNSL2 | threonine synthase like 2 [Source:HGNC Symbol;Acc:HGNC:25602] |
| ENSG00000145107 | HGLibA_49697 | 43 | -0.720707349 | TM4SF19 | transmembrane 4 L six family member 19 [Source:HGNC Symbol;Acc:HGNC:25167] |
| ENSG00000145191 | HGLibA_14748 | 43 | -0.720707349 | EIF2B5 | eukaryotic translation initiation factor 2B subunit epsilon [Source:HGNC Symbol;Acc:HGNC:3261] |
| ENSG00000145194 | HGLibA_14384 | 43 | -0.720707349 | ECE2 | endothelin converting enzyme 2 [Source:HGNC Symbol;Acc:HGNC:13275] |
| ENSG00000145860 | HGLibA_41531 | 43 | -0.720707349 | RNF145 | ring finger protein 145 [Source:HGNC Symbol;Acc:HGNC:20853] |
| ENSG00000146904 | HGLibA_15284 | 43 | -0.720707349 | EPHA1 | EPH receptor A1 [Source:HGNC Symbol;Acc:HGNC:3385] |
| ENSG00000147684 | HGLibA_31534 | 43 | -0.720707349 | NDUFB9 | NADH:ubiquinone oxidoreductase subunit B9 [Source:HGNC Symbol;Acc:HGNC:7704] |
| ENSG00000151458 | HGLibA_02113 | 43 | -0.720707349 | ANKRD50 | ankyrin repeat domain 50 [Source:HGNC Symbol;Acc:HGNC:29223] |
| ENSG00000151702 | HGLibA_17615 | 43 | -0.720707349 | FLI1 | ETS transcription factor [Source:HGNC Symbol;Acc:HGNC:3749]" |
| ENSG00000151849 | HGLibA_09062 | 43 | -0.720707349 | CENPJ | centromere protein J [Source:HGNC Symbol;Acc:HGNC:17272] |
| ENSG00000152583 | HGLibA_46420 | 43 | -0.720707349 | SPARCL1 | SPARC like 1 [Source:HGNC Symbol;Acc:HGNC:11220] |
| ENSG00000153820 | HGLibA_46650 | 43 | -0.720707349 | SPHKAP | AKAP domain containing [Source:HGNC Symbol;Acc:HGNC:30619]" |
| ENSG00000154143 | HGLibA_35116 | 43 | -0.720707349 | PANX3 | pannexin 3 [Source:HGNC Symbol;Acc:HGNC:20573] |
| ENSG00000154265 | HGLibA_00116 | 43 | -0.720707349 | ABCA5 | ATP binding cassette subfamily A member 5 [Source:HGNC Symbol;Acc:HGNC:35] |
| ENSG00000154309 | HGLibA_13283 | 43 | -0.720707349 | DISP1 | dispatched RND transporter family member 1 [Source:HGNC Symbol;Acc:HGNC:19711] |
| ENSG00000154485 | HGLibA_29513 | 43 | -0.720707349 | MMP21 | matrix metallopeptidase 21 [Source:HGNC Symbol;Acc:HGNC:14357] |
| ENSG00000154545 | HGLibA_27973 | 43 | -0.720707349 | MAGED4 | MAGE family member D4 [Source:HGNC Symbol;Acc:HGNC:23793] |
| ENSG00000155189 | HGLibA_01230 | 43 | -0.720707349 | AGPAT5 | 1-acylglycerol-3-phosphate O-acyltransferase 5 [Source:HGNC Symbol;Acc:HGNC:20886] |
| ENSG00000155962 | HGLibA_10025 | 43 | -0.720707349 | CLIC2 | chloride intracellular channel 2 [Source:HGNC Symbol;Acc:HGNC:2063] |
| ENSG00000157224 | HGLibA_09855 | 43 | -0.720707349 | CLDN12 | claudin 12 [Source:HGNC Symbol;Acc:HGNC:2034] |
| ENSG00000158292 | HGLibA_19938 | 43 | -0.720707349 | GPR153 | G protein-coupled receptor 153 [Source:HGNC Symbol;Acc:HGNC:23618] |
| ENSG00000159259 | HGLibA_09351 | 43 | -0.720707349 | CHAF1B | chromatin assembly factor 1 subunit B [Source:HGNC Symbol;Acc:HGNC:1911] |
| ENSG00000160193 | HGLibA_54086 | 43 | -0.720707349 | WDR4 | WD repeat domain 4 [Source:HGNC Symbol;Acc:HGNC:12756] |
| ENSG00000161036 | HGLibA_27510 | 43 | -0.720707349 | LRWD1 | leucine rich repeats and WD repeat domain containing 1 [Source:HGNC Symbol;Acc:HGNC:21769] |
| ENSG00000161091 | HGLibA_29091 | 43 | -0.720707349 | MFSD12 | major facilitator superfamily domain containing 12 [Source:HGNC Symbol;Acc:HGNC:28299] |
| ENSG00000161298 | HGLibA_55888 | 43 | -0.720707349 | ZNF382 | zinc finger protein 382 [Source:HGNC Symbol;Acc:HGNC:17409] |
| ENSG00000161654 | HGLibA_27528 | 43 | -0.720707349 | LSM12 | LSM12 homolog [Source:HGNC Symbol;Acc:HGNC:26407] |
| ENSG00000163081 | HGLibA_07616 | 43 | -0.720707349 | CCDC140 | coiled-coil domain containing 140 [Source:HGNC Symbol;Acc:HGNC:26514] |
| ENSG00000163584 | HGLibA_41902 | 43 | -0.720707349 | RPL22L1 | ribosomal protein L22 like 1 [Source:HGNC Symbol;Acc:HGNC:27610] |
| ENSG00000163606 | HGLibA_08284 | 43 | -0.720707349 | CD200R1 | CD200 receptor 1 [Source:HGNC Symbol;Acc:HGNC:24235] |
| ENSG00000163635 | HGLibA_03850 | 43 | -0.720707349 | ATXN7 | ataxin 7 [Source:HGNC Symbol;Acc:HGNC:10560] |
| ENSG00000163661 | HGLibA_39572 | 43 | -0.720707349 | PTX3 | pentraxin 3 [Source:HGNC Symbol;Acc:HGNC:9692] |
| ENSG00000163739 | HGLibA_11843 | 43 | -0.720707349 | CXCL1 | C-X-C motif chemokine ligand 1 [Source:HGNC Symbol;Acc:HGNC:4602] |
| ENSG00000163930 | HGLibA_04151 | 43 | -0.720707349 | BAP1 | BRCA1 associated protein 1 [Source:HGNC Symbol;Acc:HGNC:950] |
| ENSG00000163938 | HGLibA_19550 | 43 | -0.720707349 | GNL3 | G protein nucleolar 3 [Source:HGNC Symbol;Acc:HGNC:29931] |
| ENSG00000164047 | HGLibA_07103 | 43 | -0.720707349 | CAMP | cathelicidin antimicrobial peptide [Source:HGNC Symbol;Acc:HGNC:1472] |
| ENSG00000164485 | HGLibA_23094 | 43 | -0.720707349 | IL22RA2 | interleukin 22 receptor subunit alpha 2 [Source:HGNC Symbol;Acc:HGNC:14901] |
| ENSG00000164743 | HGLibA_06635 | 43 | -0.720707349 | C8orf48 | chromosome 8 open reading frame 48 [Source:HGNC Symbol;Acc:HGNC:26345] |
| ENSG00000165804 | HGLibA_55603 | 43 | -0.720707349 | ZNF219 | zinc finger protein 219 [Source:HGNC Symbol;Acc:HGNC:13011] |
| ENSG00000165810 | HGLibA_04982 | 43 | -0.720707349 | BTNL9 | butyrophilin like 9 [Source:HGNC Symbol;Acc:HGNC:24176] |
| ENSG00000166136 | HGLibA_31530 | 43 | -0.720707349 | NDUFB8 | NADH:ubiquinone oxidoreductase subunit B8 [Source:HGNC Symbol;Acc:HGNC:7703] |
| ENSG00000166886 | HGLibA_31021 | 43 | -0.720707349 | NAB2 | NGFI-A binding protein 2 [Source:HGNC Symbol;Acc:HGNC:7627] |
| ENSG00000167123 | HGLibA_09191 | 43 | -0.720707349 | CERCAM | cerebral endothelial cell adhesion molecule [Source:HGNC Symbol;Acc:HGNC:23723] |
| ENSG00000167549 | HGLibA_10787 | 43 | -0.720707349 | CORO6 | coronin 6 [Source:HGNC Symbol;Acc:HGNC:21356] |
| ENSG00000167754 | HGLibA_25261 | 43 | -0.720707349 | KLK5 | kallikrein related peptidase 5 [Source:HGNC Symbol;Acc:HGNC:6366] |
| ENSG00000168010 | HGLibA_03476 | 43 | -0.720707349 | ATG16L2 | autophagy related 16 like 2 [Source:HGNC Symbol;Acc:HGNC:25464] |
| ENSG00000168140 | HGLibA_53536 | 43 | -0.720707349 | VASN | vasorin [Source:HGNC Symbol;Acc:HGNC:18517] |
| ENSG00000168454 | HGLibA_52421 | 43 | -0.720707349 | TXNDC2 | thioredoxin domain containing 2 [Source:HGNC Symbol;Acc:HGNC:16470] |
| ENSG00000168542 | HGLibA_10564 | 43 | -0.720707349 | COL3A1 | collagen type III alpha 1 chain [Source:HGNC Symbol;Acc:HGNC:2201] |
| ENSG00000168621 | HGLibA_18886 | 43 | -0.720707349 | GDNF | glial cell derived neurotrophic factor [Source:HGNC Symbol;Acc:HGNC:4232] |
| ENSG00000169282 | HGLibA_24147 | 43 | -0.720707349 | KCNAB1 | potassium voltage-gated channel subfamily A member regulatory beta subunit 1 [Source:HGNC Symbol;Acc:HGNC:6228] |
| ENSG00000169397 | HGLibA_41406 | 43 | -0.720707349 | RNASE3 | ribonuclease A family member 3 [Source:HGNC Symbol;Acc:HGNC:10046] |
| ENSG00000169607 | HGLibA_09757 | 43 | -0.720707349 | CKAP2L | cytoskeleton associated protein 2 like [Source:HGNC Symbol;Acc:HGNC:26877] |

| | | | | | |
|---|---|---|---|---|---|
| ENSG00000169857 | HGLibA_03888 | 43 | -0.720707349 | AVEN | apoptosis and caspase activation inhibitor [Source:HGNC Symbol;Acc:HGNC:13509] |
| ENSG00000170745 | HGLibA_24391 | 43 | -0.720707349 | KCNS3 | potassium voltage-gated channel modifier subfamily S member 3 [Source:HGNC Symbol;Acc:HGNC:6302] |
| ENSG00000170836 | HGLibA_37968 | 43 | -0.720707349 | PPM1D | Mg2+/Mn2+ dependent 1D [Source:HGNC Symbol;Acc:HGNC:9277]" |
| ENSG00000170923 | HGLibA_34485 | 43 | -0.720707349 | OR7G2 | olfactory receptor family 7 subfamily G member 2 [Source:HGNC Symbol;Acc:HGNC:8466] |
| ENSG00000171320 | HGLibA_15561 | 43 | -0.720707349 | ESCO2 | establishment of sister chromatid cohesion N-acetyltransferase 2 [Source:HGNC Symbol;Acc:HGNC:27230] |
| ENSG00000171425 | HGLibA_56307 | 43 | -0.720707349 | ZNF581 | zinc finger protein 581 [Source:HGNC Symbol;Acc:HGNC:25017] |
| ENSG00000171495 | HGLibA_29871 | 43 | -0.720707349 | MROH2B | maestro heat like repeat family member 2B [Source:HGNC Symbol;Acc:HGNC:26857] |
| ENSG00000171517 | HGLibA_27058 | 43 | -0.720707349 | LPAR3 | lysophosphatidic acid receptor 3 [Source:HGNC Symbol;Acc:HGNC:14298] |
| ENSG00000172239 | HGLibA_35028 | 43 | -0.720707349 | PAIP1 | poly(A) binding protein interacting protein 1 [Source:HGNC Symbol;Acc:HGNC:16945] |
| ENSG00000172380 | HGLibA_19513 | 43 | -0.720707349 | GNG12 | G protein subunit gamma 12 [Source:HGNC Symbol;Acc:HGNC:19663] |
| ENSG00000172425 | HGLibA_52122 | 43 | -0.720707349 | TTC36 | tetratricopeptide repeat domain 36 [Source:HGNC Symbol;Acc:HGNC:33708] |
| ENSG00000173221 | HGLibA_19314 | 43 | -0.720707349 | GLRX | glutaredoxin [Source:HGNC Symbol;Acc:HGNC:4330] |
| ENSG00000173812 | HGLibA_14705 | 43 | -0.720707349 | EIF1 | eukaryotic translation initiation factor 1 [Source:HGNC Symbol;Acc:HGNC:3249] |
| ENSG00000173826 | HGLibA_24225 | 43 | -0.720707349 | KCNH6 | potassium voltage-gated channel subfamily H member 6 [Source:HGNC Symbol;Acc:HGNC:18862] |
| ENSG00000174151 | HGLibA_11983 | 43 | -0.720707349 | CYB561D1 | cytochrome b561 family member D1 [Source:HGNC Symbol;Acc:HGNC:26804] |
| ENSG00000174429 | HGLibA_00325 | 43 | -0.720707349 | ABRA | actin binding Rho activating protein [Source:HGNC Symbol;Acc:HGNC:30655] |
| ENSG00000174611 | HGLibA_25877 | 43 | -0.720707349 | KY | kyphoscoliosis peptidase [Source:HGNC Symbol;Acc:HGNC:26576] |
| ENSG00000174740 | HGLibA_34939 | 43 | -0.720707349 | PABPC5 | poly(A) binding protein cytoplasmic 5 [Source:HGNC Symbol;Acc:HGNC:13629] |
| ENSG00000174871 | HGLibA_10287 | 43 | -0.720707349 | CNIH2 | cornichon family AMPA receptor auxiliary protein 2 [Source:HGNC Symbol;Acc:HGNC:28744] |
| ENSG00000175265 | HGLibA_19641 | 43 | -0.720707349 | GOLGA8A | golgin A8 family member A [Source:HGNC Symbol;Acc:HGNC:31972] |
| ENSG00000175768 | HGLibA_50973 | 43 | -0.720707349 | TOMM5 | translocase of outer mitochondrial membrane 5 [Source:HGNC Symbol;Acc:HGNC:31369] |
| ENSG00000176692 | HGLibA_17824 | 43 | -0.720707349 | FOXC2 | forkhead box C2 [Source:HGNC Symbol;Acc:HGNC:3801] |
| ENSG00000177294 | HGLibA_17086 | 43 | -0.720707349 | FBXO39 | F-box protein 39 [Source:HGNC Symbol;Acc:HGNC:28565] |
| ENSG00000177558 | HGLibA_16343 | 43 | -0.720707349 | FAM187B | family with sequence similarity 187 member B [Source:HGNC Symbol;Acc:HGNC:26366] |
| ENSG00000177853 | HGLibA_56149 | 43 | -0.720707349 | ZNF518A | zinc finger protein 518A [Source:HGNC Symbol;Acc:HGNC:29009] |
| ENSG00000178473 | HGLibA_52827 | 43 | -0.720707349 | UCN3 | urocortin 3 [Source:HGNC Symbol;Acc:HGNC:17781] |
| ENSG00000178952 | HGLibA_52324 | 43 | -0.720707349 | TUFM | mitochondrial [Source:HGNC Symbol;Acc:HGNC:12420]" |
| ENSG00000180644 | HGLibA_38448 | 43 | -0.720707349 | PRF1 | perforin 1 [Source:HGNC Symbol;Acc:HGNC:9360] |
| ENSG00000181381 | HGLibA_12766 | 43 | -0.720707349 | DDX60L | DExD/H-box 60 like [Source:HGNC Symbol;Acc:HGNC:26429] |
| ENSG00000183283 | HGLibA_12366 | 43 | -0.720707349 | DAZAP2 | DAZ associated protein 2 [Source:HGNC Symbol;Acc:HGNC:2684] |
| ENSG00000183640 | HGLibA_25828 | 43 | -0.720707349 | KRTAP8-1 | keratin associated protein 8-1 [Source:HGNC Symbol;Acc:HGNC:18935] |
| ENSG00000183747 | HGLibA_00571 | 43 | -0.720707349 | ACSM2A | acyl-CoA synthetase medium chain family member 2A [Source:HGNC Symbol;Acc:HGNC:32017] |
| ENSG00000183908 | HGLibA_27389 | 43 | -0.720707349 | LRRC55 | leucine rich repeat containing 55 [Source:HGNC Symbol;Acc:HGNC:32324] |
| ENSG00000184588 | HGLibA_35789 | 43 | -0.720707349 | PDE4B | phosphodiesterase 4B [Source:HGNC Symbol;Acc:HGNC:8781] |
| ENSG00000185252 | HGLibA_56659 | 43 | -0.720707349 | ZNF74 | zinc finger protein 74 [Source:HGNC Symbol;Acc:HGNC:13144] |
| ENSG00000185988 | HGLibA_37199 | 43 | -0.720707349 | PLK5 | polo like kinase 5 [Source:HGNC Symbol;Acc:HGNC:27001] |
| ENSG00000186306 | HGLibA_33547 | 43 | -0.720707349 | OR10T2 | olfactory receptor family 10 subfamily T member 2 [Source:HGNC Symbol;Acc:HGNC:14816] |
| ENSG00000186625 | HGLibA_24071 | 43 | -0.720707349 | KATNA1 | katanin catalytic subunit A1 [Source:HGNC Symbol;Acc:HGNC:6216] |
| ENSG00000186827 | HGLibA_50785 | 43 | -0.720707349 | TNFRSF4 | TNF receptor superfamily member 4 [Source:HGNC Symbol;Acc:HGNC:11918] |
| ENSG00000186834 | HGLibA_21210 | 43 | -0.720707349 | HEXIM1 | hexamethylene bisacetamide inducible 1 [Source:HGNC Symbol;Acc:HGNC:24953] |
| ENSG00000186912 | HGLibA_34887 | 43 | -0.720707349 | P2RY4 | pyrimidinergic receptor P2Y4 [Source:HGNC Symbol;Acc:HGNC:8542] |
| ENSG00000186970 | HGLibA_25653 | 43 | -0.720707349 | KRTAP15-1 | keratin associated protein 15-1 [Source:HGNC Symbol;Acc:HGNC:18927] |
| ENSG00000187630 | HGLibA_13142 | 43 | -0.720707349 | DHRS4L2 | dehydrogenase/reductase 4 like 2 [Source:HGNC Symbol;Acc:HGNC:19731] |
| ENSG00000187957 | HGLibA_13673 | 43 | -0.720707349 | DNER | delta/notch like EGF repeat containing [Source:HGNC Symbol;Acc:HGNC:24456] |
| ENSG00000188488 | HGLibA_43523 | 43 | -0.720707349 | SERPINA5 | serpin family A member 5 [Source:HGNC Symbol;Acc:HGNC:8723] |
| ENSG00000188827 | HGLibA_45564 | 43 | -0.720707349 | SLX4 | SLX4 structure-specific endonuclease subunit [Source:HGNC Symbol;Acc:HGNC:23845] |
| ENSG00000189339 | HGLibA_44993 | 43 | -0.720707349 | SLC35E2B | solute carrier family 35 member E2B [Source:HGNC Symbol;Acc:HGNC:33941] |
| ENSG00000197312 | HGLibA_12612 | 43 | -0.720707349 | DDI2 | DNA damage inducible 1 homolog 2 [Source:HGNC Symbol;Acc:HGNC:24578] |
| ENSG00000197894 | HGLibA_00958 | 43 | -0.720707349 | ADH5 | chi polypeptide [Source:HGNC Symbol;Acc:HGNC:253]" |
| ENSG00000197961 | HGLibA_55452 | 43 | -0.720707349 | ZNF121 | zinc finger protein 121 [Source:HGNC Symbol;Acc:HGNC:12904] |
| ENSG00000198815 | HGLibA_17891 | 43 | -0.720707349 | FOXJ3 | forkhead box J3 [Source:HGNC Symbol;Acc:HGNC:29178] |
| ENSG00000203747 | HGLibA_17195 | 43 | -0.720707349 | FCGR3A | Fc fragment of IgG receptor IIIa [Source:HGNC Symbol;Acc:HGNC:3619] |
| ENSG00000203952 | HGLibA_07669 | 43 | -0.720707349 | CCDC160 | coiled-coil domain containing 160 [Source:HGNC Symbol;Acc:HGNC:37286] |
| ENSG00000204033 | HGLibA_27189 | 43 | -0.720707349 | LRIT2 | Ig-like and transmembrane domains 2 [Source:HGNC Symbol;Acc:HGNC:23443]" |
| ENSG00000204267 | HGLibA_48318 | 43 | -0.720707349 | TAP2 | ATP binding cassette subfamily B member [Source:HGNC Symbol;Acc:HGNC:44]" |
| ENSG00000206561 | HGLibA_10648 | 43 | -0.720707349 | COLQ | collagen like tail subunit of asymmetric acetylcholinesterase [Source:HGNC Symbol;Acc:HGNC:2226] |
| ENSG00000213281 | HGLibA_32649 | 43 | -0.720707349 | NRAS | GTPase [Source:HGNC Symbol;Acc:HGNC:7989]" |
| ENSG00000213417 | HGLibA_25695 | 43 | -0.720707349 | KRTAP2-4 | keratin associated protein 2-4 [Source:HGNC Symbol;Acc:HGNC:18891] |
| ENSG00000213420 | HGLibA_19766 | 43 | -0.720707349 | GPC2 | glypican 2 [Source:HGNC Symbol;Acc:HGNC:4450] |
| ENSG00000214309 | HGLibA_28503 | 43 | -0.720707349 | MBLAC1 | metallo-beta-lactamase domain containing 1 [Source:HGNC Symbol;Acc:HGNC:22180] |
| ENSG00000215271 | HGLibA_21872 | 43 | -0.720707349 | HOMEZ | homeobox and leucine zipper encoding [Source:HGNC Symbol;Acc:HGNC:20164] |
| ENSG00000231637 | HGLibA_53269 | 43 | -0.720707349 | USP17L29 | ubiquitin specific peptidase 17-like family member 29 [Source:HGNC Symbol;Acc:HGNC:44457] |
| ENSG00000235631 | HGLibA_41539 | 43 | -0.720707349 | RNF148 | ring finger protein 148 [Source:HGNC Symbol;Acc:HGNC:22411] |
| ENSG00000236362 | HGLibA_18449 | 43 | -0.720707349 | GAGE12F | G antigen 12F [Source:HGNC Symbol;Acc:HGNC:31906] |
| ENSG00000237247 | HGLibA_28484 | 43 | -0.720707349 | MBD3L5 | methyl-CpG binding domain protein 3 like 5 [Source:HGNC Symbol;Acc:HGNC:37204] |
| ENSG00000240972 | HGLibA_29262 | 43 | -0.720707349 | MIF | macrophage migration inhibitory factor [Source:HGNC Symbol;Acc:HGNC:7097] |
| ENSG00000244038 | HGLibA_12630 | 43 | -0.720707349 | DDOST | dolichyl-diphosphooligosaccharide--protein glycosyltransferase non-catalytic subunit [Source:HGNC Symbol;Acc:HGNC:2728] |
| ENSG00000250361 | HGLibA_20700 | 43 | -0.720707349 | GYPB | glycophorin B (MNS blood group) [Source:HGNC Symbol;Acc:HGNC:4703] |
| ENSG00000254996 | HGLibA_01966 | 43 | -0.720707349 | ANKHD1-EIF4EBP3 | ANKHD1-EIF4EBP3 readthrough [Source:HGNC Symbol;Acc:HGNC:33530] |
| ENSG00000255582 | HGLibA_33488 | 43 | -0.720707349 | OR10G2 | olfactory receptor family 10 subfamily G member 2 [Source:HGNC Symbol;Acc:HGNC:8170] |
| ENSG00000261587 | HGLibA_50311 | 43 | -0.720707349 | TMEM249 | transmembrane protein 249 [Source:HGNC Symbol;Acc:HGNC:44155] |
| ENSG00000263001 | HGLibA_20565 | 43 | -0.720707349 | GTF2I | general transcription factor IIi [Source:HGNC Symbol;Acc:HGNC:4659] |
| ENSG00000267909 | HGLibA_07712 | 43 | -0.720707349 | CCDC177 | coiled-coil domain containing 177 [Source:HGNC Symbol;Acc:HGNC:23243] |
| ENSG00000268651 | HGLibA_11557 | 43 | -0.720707349 | CTAG1A | cancer/testis antigen 1A [Source:HGNC Symbol;Acc:HGNC:24198] |
| ENSG00000277399 | HGLibA_19977 | 43 | -0.720707349 | GPR179 | G protein-coupled receptor 179 [Source:HGNC Symbol;Acc:HGNC:31371] |
| ENSG00000279263 | HGLibA_33829 | 43 | -0.720707349 | OR2L8 | olfactory receptor family 2 subfamily L member 8 (gene/pseudogene) [Source:HGNC Symbol;Acc:HGNC:15014] |
| ENSG00000288611 | HGLibA_32412 | 43 | -0.720707349 | NPBWR1 | - |
| FAM115A | HGLibA_16035 | 43 | -0.720707349 | FAM115A | - |
| FAM175A | HGLibA_16287 | 43 | -0.720707349 | FAM175A | - |
| HFE2 | HGLibA_21228 | 43 | -0.720707349 | HFE2 | - |
| hsa-mir-124-1 | HGLibA_57347 | 43 | -0.720707349 | hsa-mir-124-1 | - |
| hsa-mir-1302-9 | HGLibA_57722 | 43 | -0.720707349 | hsa-mir-1302-9 | - |
| hsa-mir-130a | HGLibA_57749 | 43 | -0.720707349 | hsa-mir-130a | - |
| hsa-mir-141 | HGLibA_57830 | 43 | -0.720707349 | hsa-mir-141 | - |
| hsa-mir-181b-2 | HGLibA_57970 | 43 | -0.720707349 | hsa-mir-181b-2 | - |
| hsa-mir-3122 | HGLibA_58542 | 43 | -0.720707349 | hsa-mir-3122 | - |
| hsa-mir-3156-3 | HGLibA_58697 | 43 | -0.720707349 | hsa-mir-3156-3 | - |
| hsa-mir-3620 | HGLibA_59115 | 43 | -0.720707349 | hsa-mir-3620 | - |
| hsa-mir-3689c | HGLibA_59323 | 43 | -0.720707349 | hsa-mir-3689c | - |
| hsa-mir-3926-1 | HGLibA_59577 | 43 | -0.720707349 | hsa-mir-3926-1 | - |
| hsa-mir-4439 | HGLibA_60157 | 43 | -0.720707349 | hsa-mir-4439 | - |
| hsa-mir-454 | HGLibA_60622 | 43 | -0.720707349 | hsa-mir-454 | - |
| hsa-mir-4686 | HGLibA_60854 | 43 | -0.720707349 | hsa-mir-4686 | - |
| hsa-mir-4779 | HGLibA_61224 | 43 | -0.720707349 | hsa-mir-4779 | - |
| hsa-mir-493 | HGLibA_61371 | 43 | -0.720707349 | hsa-mir-493 | - |
| hsa-mir-526b | HGLibA_61798 | 43 | -0.720707349 | hsa-mir-526b | - |
| hsa-mir-548f-5 | HGLibA_61988 | 43 | -0.720707349 | hsa-mir-548f-5 | - |
| hsa-mir-548i-1 | HGLibA_62013 | 43 | -0.720707349 | hsa-mir-548i-1 | - |
| hsa-mir-5685 | HGLibA_62259 | 43 | -0.720707349 | hsa-mir-5685 | - |
| hsa-mir-5701-1 | HGLibA_62342 | 43 | -0.720707349 | hsa-mir-5701-1 | - |
| hsa-mir-598 | HGLibA_62483 | 43 | -0.720707349 | hsa-mir-598 | - |
| hsa-mir-6090 | HGLibA_62627 | 43 | -0.720707349 | hsa-mir-6090 | - |
| hsa-mir-616 | HGLibA_62701 | 43 | -0.720707349 | hsa-mir-616 | - |
| hsa-mir-6782 | HGLibA_63305 | 43 | -0.720707349 | hsa-mir-6782 | - |
| hsa-mir-6812 | HGLibA_63427 | 43 | -0.720707349 | hsa-mir-6812 | - |
| hsa-mir-6843 | HGLibA_63547 | 43 | -0.720707349 | hsa-mir-6843 | - |
| hsa-mir-6854 | HGLibA_63589 | 43 | -0.720707349 | hsa-mir-6854 | - |
| hsa-mir-6889 | HGLibA_63742 | 43 | -0.720707349 | hsa-mir-6889 | - |
| hsa-mir-7-1 | HGLibA_63768 | 43 | -0.720707349 | hsa-mir-7-1 | - |
| hsa-mir-7852 | HGLibA_63997 | 43 | -0.720707349 | hsa-mir-7852 | - |
| hsa-mir-8053 | HGLibA_64050 | 43 | -0.720707349 | hsa-mir-8053 | - |
| hsa-mir-8055 | HGLibA_64059 | 43 | -0.720707349 | hsa-mir-8055 | - |
| hsa-mir-8063 | HGLibA_64089 | 43 | -0.720707349 | hsa-mir-8063 | - |
| IL3RA_X | HGLibA_23159 | 43 | -0.720707349 | IL3RA_X | - |
| KIAA1407 | HGLibA_24747 | 43 | -0.720707349 | KIAA1407 | - |
| LOC100287036 | HGLibA_26815 | 43 | -0.720707349 | LOC100287036 | - |
| PCDP1 | HGLibA_35580 | 43 | -0.720707349 | PCDP1 | - |
| PCNXL2 | HGLibA_35647 | 43 | -0.720707349 | PCNXL2 | - |
| PPAP2A | HGLibA_37815 | 43 | -0.720707349 | PPAP2A | - |
| TSTA3 | HGLibA_52027 | 43 | -0.720707349 | TSTA3 | - |
| VPRBP | HGLibA_53718 | 43 | -0.720707349 | VPRBP | - |
| ZNF664-FAM101A | HGLibA_56486 | 43 | -0.720707349 | ZNF664-FAM101A | - |
| 3-Mar | HGLibA_28333 | 42 | -0.730658387 | 3-Mar | - |
| C11orf88 | HGLibA_05208 | 42 | -0.730658387 | C11orf88 | - |
| C17orf51 | HGLibA_05543 | 42 | -0.730658387 | C17orf51 | - |
| C1orf65 | HGLibA_05888 | 42 | -0.730658387 | C1orf65 | - |
| C5orf55 | HGLibA_06410 | 42 | -0.730658387 | C5orf55 | - |
| ENSG00000004660 | HGLibA_07089 | 42 | -0.730658387 | CAMKK1 | calcium/calmodulin dependent protein kinase kinase 1 [Source:HGNC Symbol;Acc:HGNC:1469] |
| ENSG00000007516 | HGLibA_04132 | 42 | -0.730658387 | BAIAP3 | BAI1 associated protein 3 [Source:HGNC Symbol;Acc:HGNC:948] |
| ENSG00000009413 | HGLibA_40886 | 42 | -0.730658387 | REV3L | DNA directed polymerase zeta catalytic subunit [Source:HGNC Symbol;Acc:HGNC:9968]" |
| ENSG00000013619 | HGLibA_28051 | 42 | -0.730658387 | MAMLD1 | mastermind like domain containing 1 [Source:HGNC Symbol;Acc:HGNC:2568] |
| ENSG00000025293 | HGLibA_36362 | 42 | -0.730658387 | PHF20 | PHD finger protein 20 [Source:HGNC Symbol;Acc:HGNC:16098] |
| ENSG00000028839 | HGLibA_48625 | 42 | -0.730658387 | TBPL1 | TATA-box binding protein like 1 [Source:HGNC Symbol;Acc:HGNC:11589] |
| ENSG00000031691 | HGLibA_09082 | 42 | -0.730658387 | CENPQ | centromere protein Q [Source:HGNC Symbol;Acc:HGNC:21347] |
| ENSG00000047365 | HGLibA_02627 | 42 | -0.730658387 | ARAP2 | ankyrin repeat and PH domain 2 [Source:HGNC Symbol;Acc:HGNC:16924]" |

| | | | | | |
|---|---|---|---|---|---|
| ENSG00000051108 | HGLibA_21172 | 42 | -0.730658387 | HERPUD1 | homocysteine inducible ER protein with ubiquitin like domain 1 [Source:HGNC Symbol;Acc:HGNC:13744] |
| ENSG00000051180 | HGLibA_40078 | 42 | -0.730658387 | RAD51 | RAD51 recombinase [Source:HGNC Symbol;Acc:HGNC:9817] |
| ENSG00000051523 | HGLibA_12017 | 42 | -0.730658387 | CYBA | cytochrome b-245 alpha chain [Source:HGNC Symbol;Acc:HGNC:2577] |
| ENSG00000060718 | HGLibA_10500 | 42 | -0.730658387 | COL11A1 | collagen type XI alpha 1 chain [Source:HGNC Symbol;Acc:HGNC:2186] |
| ENSG00000068366 | HGLibA_00561 | 42 | -0.730658387 | ACSL4 | acyl-CoA synthetase long chain family member 4 [Source:HGNC Symbol;Acc:HGNC:3571] |
| ENSG00000069431 | HGLibA_00188 | 42 | -0.730658387 | ABCC9 | ATP binding cassette subfamily C member 9 [Source:HGNC Symbol;Acc:HGNC:60] |
| ENSG00000073350 | HGLibA_26651 | 42 | -0.730658387 | LLGL2 | scribble cell polarity complex component [Source:HGNC Symbol;Acc:HGNC:6629]" |
| ENSG00000075420 | HGLibA_17751 | 42 | -0.730658387 | FNDC3B | fibronectin type III domain containing 3B [Source:HGNC Symbol;Acc:HGNC:24670] |
| ENSG00000077522 | HGLibA_00640 | 42 | -0.730658387 | ACTN2 | actinin alpha 2 [Source:HGNC Symbol;Acc:HGNC:164] |
| ENSG00000079246 | HGLibA_54594 | 42 | -0.730658387 | XRCC5 | X-ray repair cross complementing 5 [Source:HGNC Symbol;Acc:HGNC:12833] |
| ENSG00000080802 | HGLibA_10340 | 42 | -0.730658387 | CNOT4 | CCR4-NOT transcription complex subunit 4 [Source:HGNC Symbol;Acc:HGNC:7880] |
| ENSG00000083123 | HGLibA_04280 | 42 | -0.730658387 | BCKDHB | branched chain keto acid dehydrogenase E1 subunit beta [Source:HGNC Symbol;Acc:HGNC:987] |
| ENSG00000088451 | HGLibA_49201 | 42 | -0.730658387 | TGDS | 6-dehydratase [Source:HGNC Symbol;Acc:HGNC:20324]" |
| ENSG00000089169 | HGLibA_41844 | 42 | -0.730658387 | RPH3A | rabphilin 3A [Source:HGNC Symbol;Acc:HGNC:17056] |
| ENSG00000089289 | HGLibA_22783 | 42 | -0.730658387 | IGBP1 | immunoglobulin binding protein 1 [Source:HGNC Symbol;Acc:HGNC:5461] |
| ENSG00000090061 | HGLibA_08113 | 42 | -0.730658387 | CCNK | cyclin K [Source:HGNC Symbol;Acc:HGNC:1596] |
| ENSG00000091527 | HGLibA_08893 | 42 | -0.730658387 | CDV3 | CDV3 homolog [Source:HGNC Symbol;Acc:HGNC:26928] |
| ENSG00000099917 | HGLibA_28758 | 42 | -0.730658387 | MED15 | mediator complex subunit 15 [Source:HGNC Symbol;Acc:HGNC:14248] |
| ENSG00000101282 | HGLibA_42353 | 42 | -0.730658387 | RSPO4 | R-spondin 4 [Source:HGNC Symbol;Acc:HGNC:16175] |
| ENSG00000101310 | HGLibA_43224 | 42 | -0.730658387 | SEC23B | coat complex II component [Source:HGNC Symbol;Acc:HGNC:10702]" |
| ENSG00000103121 | HGLibA_10189 | 42 | -0.730658387 | CMC2 | C-X9-C motif containing 2 [Source:HGNC Symbol;Acc:HGNC:24447] |
| ENSG00000104369 | HGLibA_23980 | 42 | -0.730658387 | JPH1 | junctophilin 1 [Source:HGNC Symbol;Acc:HGNC:14201] |
| ENSG00000106991 | HGLibA_15125 | 42 | -0.730658387 | ENG | endoglin [Source:HGNC Symbol;Acc:HGNC:3349] |
| ENSG00000107223 | HGLibA_14462 | 42 | -0.730658387 | EDF1 | endothelial differentiation related factor 1 [Source:HGNC Symbol;Acc:HGNC:3164] |
| ENSG00000107984 | HGLibA_13295 | 42 | -0.730658387 | DKK1 | dickkopf WNT signaling pathway inhibitor 1 [Source:HGNC Symbol;Acc:HGNC:2891] |
| ENSG00000108010 | HGLibA_19319 | 42 | -0.730658387 | GLRX3 | glutaredoxin 3 [Source:HGNC Symbol;Acc:HGNC:15987] |
| ENSG00000108433 | HGLibA_19687 | 42 | -0.730658387 | GOSR2 | golgi SNAP receptor complex member 2 [Source:HGNC Symbol;Acc:HGNC:4431] |
| ENSG00000108932 | HGLibA_44448 | 42 | -0.730658387 | SLC16A6 | solute carrier family 16 member 6 [Source:HGNC Symbol;Acc:HGNC:10927] |
| ENSG00000110060 | HGLibA_39602 | 42 | -0.730658387 | PUS3 | pseudouridine synthase 3 [Source:HGNC Symbol;Acc:HGNC:25461] |
| ENSG00000110395 | HGLibA_07395 | 42 | -0.730658387 | CBL | Cbl proto-oncogene [Source:HGNC Symbol;Acc:HGNC:1541] |
| ENSG00000112237 | HGLibA_08070 | 42 | -0.730658387 | CCNC | cyclin C [Source:HGNC Symbol;Acc:HGNC:1581] |
| ENSG00000114503 | HGLibA_31285 | 42 | -0.730658387 | NCBP2 | nuclear cap binding protein subunit 2 [Source:HGNC Symbol;Acc:HGNC:7659] |
| ENSG00000114735 | HGLibA_21132 | 42 | -0.730658387 | HEMK1 | HemK methyltransferase family member 1 [Source:HGNC Symbol;Acc:HGNC:24923] |
| ENSG00000115414 | HGLibA_17725 | 42 | -0.730658387 | FN1 | fibronectin 1 [Source:HGNC Symbol;Acc:HGNC:3778] |
| ENSG00000115468 | HGLibA_14577 | 42 | -0.730658387 | EFHD1 | EF-hand domain family member D1 [Source:HGNC Symbol;Acc:HGNC:29556] |
| ENSG00000115514 | HGLibA_52428 | 42 | -0.730658387 | TXNDC9 | thioredoxin domain containing 9 [Source:HGNC Symbol;Acc:HGNC:24110] |
| ENSG00000117118 | HGLibA_43142 | 42 | -0.730658387 | SDHB | succinate dehydrogenase complex iron sulfur subunit B [Source:HGNC Symbol;Acc:HGNC:10681] |
| ENSG00000117155 | HGLibA_47204 | 42 | -0.730658387 | SSX2IP | SSX family member 2 interacting protein [Source:HGNC Symbol;Acc:HGNC:16509] |
| ENSG00000118197 | HGLibA_12755 | 42 | -0.730658387 | DDX59 | DEAD-box helicase 59 [Source:HGNC Symbol;Acc:HGNC:25360] |
| ENSG00000118777 | HGLibA_00217 | 42 | -0.730658387 | ABCG2 | ATP binding cassette subfamily G member 2 (Junior blood group) [Source:HGNC Symbol;Acc:HGNC:74] |
| ENSG00000119723 | HGLibA_10760 | 42 | -0.730658387 | COQ6 | monooxygenase [Source:HGNC Symbol;Acc:HGNC:20233]" |
| ENSG00000120910 | HGLibA_38192 | 42 | -0.730658387 | PPP3CC | protein phosphatase 3 catalytic subunit gamma [Source:HGNC Symbol;Acc:HGNC:9316] |
| ENSG00000123388 | HGLibA_21960 | 42 | -0.730658387 | HOXC11 | homeobox C11 [Source:HGNC Symbol;Acc:HGNC:5123] |
| ENSG00000123689 | HGLibA_18292 | 42 | -0.730658387 | G0S2 | G0/G1 switch 2 [Source:HGNC Symbol;Acc:HGNC:30229] |
| ENSG00000124151 | HGLibA_31342 | 42 | -0.730658387 | NCOA3 | nuclear receptor coactivator 3 [Source:HGNC Symbol;Acc:HGNC:7670] |
| ENSG00000124615 | HGLibA_29613 | 42 | -0.730658387 | MOCS1 | molybdenum cofactor synthesis 1 [Source:HGNC Symbol;Acc:HGNC:7190] |
| ENSG00000126733 | HGLibA_12276 | 42 | -0.730658387 | DACH2 | dachshund family transcription factor 2 [Source:HGNC Symbol;Acc:HGNC:16814] |
| ENSG00000126952 | HGLibA_33139 | 42 | -0.730658387 | NXF5 | nuclear RNA export factor 5 [Source:HGNC Symbol;Acc:HGNC:8075] |
| ENSG00000128585 | HGLibA_29339 | 42 | -0.730658387 | MKLN1 | muskelin 1 [Source:HGNC Symbol;Acc:HGNC:7109] |
| ENSG00000128918 | HGLibA_01544 | 42 | -0.730658387 | ALDH1A2 | aldehyde dehydrogenase 1 family member A2 [Source:HGNC Symbol;Acc:HGNC:15472] |
| ENSG00000129167 | HGLibA_51130 | 42 | -0.730658387 | TPH1 | tryptophan hydroxylase 1 [Source:HGNC Symbol;Acc:HGNC:12008] |
| ENSG00000129187 | HGLibA_12541 | 42 | -0.730658387 | DCTD | dCMP deaminase [Source:HGNC Symbol;Acc:HGNC:2710] |
| ENSG00000131389 | HGLibA_45333 | 42 | -0.730658387 | SLC6A6 | solute carrier family 6 member 6 [Source:HGNC Symbol;Acc:HGNC:11052] |
| ENSG00000131435 | HGLibA_35916 | 42 | -0.730658387 | PDLIM4 | PDZ and LIM domain 4 [Source:HGNC Symbol;Acc:HGNC:16501] |
| ENSG00000131747 | HGLibA_50994 | 42 | -0.730658387 | TOP2A | DNA topoisomerase II alpha [Source:HGNC Symbol;Acc:HGNC:11989] |
| ENSG00000132321 | HGLibA_23486 | 42 | -0.730658387 | IQCA1 | IQ motif containing with AAA domain 1 [Source:HGNC Symbol;Acc:HGNC:26195] |
| ENSG00000132581 | HGLibA_43124 | 42 | -0.730658387 | SDF2 | stromal cell derived factor 2 [Source:HGNC Symbol;Acc:HGNC:10675] |
| ENSG00000133627 | HGLibA_00661 | 42 | -0.730658387 | ACTR3B | ARP3 actin related protein 3 homolog B [Source:HGNC Symbol;Acc:HGNC:17256] |
| ENSG00000133641 | HGLibA_05229 | 42 | -0.730658387 | C12orf29 | chromosome 12 open reading frame 29 [Source:HGNC Symbol;Acc:HGNC:25322] |
| ENSG00000133687 | HGLibA_50669 | 42 | -0.730658387 | TMTC1 | transmembrane and tetratricopeptide repeat containing 1 [Source:HGNC Symbol;Acc:HGNC:24099] |
| ENSG00000134247 | HGLibA_39343 | 42 | -0.730658387 | PTGFRN | prostaglandin F2 receptor inhibitor [Source:HGNC Symbol;Acc:HGNC:9601] |
| ENSG00000134755 | HGLibA_13991 | 42 | -0.730658387 | DSC2 | desmocollin 2 [Source:HGNC Symbol;Acc:HGNC:3036] |
| ENSG00000134802 | HGLibA_45153 | 42 | -0.730658387 | SLC43A3 | solute carrier family 43 member 3 [Source:HGNC Symbol;Acc:HGNC:17466] |
| ENSG00000135116 | HGLibA_22105 | 42 | -0.730658387 | HRK | BCL2 interacting protein [Source:HGNC Symbol;Acc:HGNC:5185]" |
| ENSG00000135829 | HGLibA_13210 | 42 | -0.730658387 | DHX9 | DExH-box helicase 9 [Source:HGNC Symbol;Acc:HGNC:2750] |
| ENSG00000135898 | HGLibA_20052 | 42 | -0.730658387 | GPR55 | G protein-coupled receptor 55 [Source:HGNC Symbol;Acc:HGNC:4511] |
| ENSG00000136111 | HGLibA_48557 | 42 | -0.730658387 | TBC1D4 | TBC1 domain family member 4 [Source:HGNC Symbol;Acc:HGNC:19165] |
| ENSG00000136514 | HGLibA_42432 | 42 | -0.730658387 | RTP4 | receptor transporter protein 4 [Source:HGNC Symbol;Acc:HGNC:23992] |
| ENSG00000137312 | HGLibA_17648 | 42 | -0.730658387 | FLOT1 | flotillin 1 [Source:HGNC Symbol;Acc:HGNC:3757] |
| ENSG00000137414 | HGLibA_16788 | 42 | -0.730658387 | FAM8A1 | family with sequence similarity 8 member A1 [Source:HGNC Symbol;Acc:HGNC:16372] |
| ENSG00000138079 | HGLibA_45129 | 42 | -0.730658387 | SLC3A1 | solute carrier family 3 member 1 [Source:HGNC Symbol;Acc:HGNC:11025] |
| ENSG00000138398 | HGLibA_37933 | 42 | -0.730658387 | PPIG | peptidylprolyl isomerase G [Source:HGNC Symbol;Acc:HGNC:14650] |
| ENSG00000138617 | HGLibA_35230 | 42 | -0.730658387 | PARP16 | poly(ADP-ribose) polymerase family member 16 [Source:HGNC Symbol;Acc:HGNC:26040] |
| ENSG00000139921 | HGLibA_50687 | 42 | -0.730658387 | TMX1 | thioredoxin related transmembrane protein 1 [Source:HGNC Symbol;Acc:HGNC:15487] |
| ENSG00000140564 | HGLibA_18174 | 42 | -0.730658387 | FURIN | paired basic amino acid cleaving enzyme [Source:HGNC Symbol;Acc:HGNC:8568]" |
| ENSG00000140682 | HGLibA_49212 | 42 | -0.730658387 | TGFB1I1 | transforming growth factor beta 1 induced transcript 1 [Source:HGNC Symbol;Acc:HGNC:11767] |
| ENSG00000140795 | HGLibA_30791 | 42 | -0.730658387 | MYLK3 | myosin light chain kinase 3 [Source:HGNC Symbol;Acc:HGNC:29826] |
| ENSG00000141404 | HGLibA_19459 | 42 | -0.730658387 | GNAL | G protein subunit alpha L [Source:HGNC Symbol;Acc:HGNC:4388] |
| ENSG00000141542 | HGLibA_39936 | 42 | -0.730658387 | RAB40B | member RAS oncogene family [Source:HGNC Symbol;Acc:HGNC:18284]" |
| ENSG00000141655 | HGLibA_50745 | 42 | -0.730658387 | TNFRSF11A | TNF receptor superfamily member 11a [Source:HGNC Symbol;Acc:HGNC:11908] |
| ENSG00000142192 | HGLibA_02561 | 42 | -0.730658387 | APP | amyloid beta precursor protein [Source:HGNC Symbol;Acc:HGNC:620] |
| ENSG00000142224 | HGLibA_23034 | 42 | -0.730658387 | IL19 | interleukin 19 [Source:HGNC Symbol;Acc:HGNC:5990] |
| ENSG00000143036 | HGLibA_45163 | 42 | -0.730658387 | SLC44A3 | solute carrier family 44 member 3 [Source:HGNC Symbol;Acc:HGNC:28689] |
| ENSG00000143061 | HGLibA_22894 | 42 | -0.730658387 | IGSF3 | immunoglobulin superfamily member 3 [Source:HGNC Symbol;Acc:HGNC:5950] |
| ENSG00000143324 | HGLibA_54580 | 42 | -0.730658387 | XPR1 | xenotropic and polytropic retrovirus receptor 1 [Source:HGNC Symbol;Acc:HGNC:12827] |
| ENSG00000143333 | HGLibA_41068 | 42 | -0.730658387 | RGS16 | regulator of G protein signaling 16 [Source:HGNC Symbol;Acc:HGNC:9997] |
| ENSG00000143839 | HGLibA_40835 | 42 | -0.730658387 | REN | renin [Source:HGNC Symbol;Acc:HGNC:9958] |
| ENSG00000144455 | HGLibA_47771 | 42 | -0.730658387 | SUMF1 | sulfatase modifying factor 1 [Source:HGNC Symbol;Acc:HGNC:20376] |
| ENSG00000145002 | HGLibA_16775 | 42 | -0.730658387 | FAM86B2 | family with sequence similarity 86 member B2 [Source:HGNC Symbol;Acc:HGNC:32222] |
| ENSG00000145439 | HGLibA_07424 | 42 | -0.730658387 | CBR4 | carbonyl reductase 4 [Source:HGNC Symbol;Acc:HGNC:25891] |
| ENSG00000149090 | HGLibA_35087 | 42 | -0.730658387 | PAMR1 | peptidase domain containing associated with muscle regeneration 1 [Source:HGNC Symbol;Acc:HGNC:24554] |
| ENSG00000149124 | HGLibA_19369 | 42 | -0.730658387 | GLYAT | glycine-N-acyltransferase [Source:HGNC Symbol;Acc:HGNC:13734] |
| ENSG00000149133 | HGLibA_34285 | 42 | -0.730658387 | OR5F1 | olfactory receptor family 5 subfamily F member 1 [Source:HGNC Symbol;Acc:HGNC:8343] |
| ENSG00000151176 | HGLibA_36975 | 42 | -0.730658387 | PLBD2 | phospholipase B domain containing 2 [Source:HGNC Symbol;Acc:HGNC:27283] |
| ENSG00000152229 | HGLibA_39241 | 42 | -0.730658387 | PSTPIP2 | proline-serine-threonine phosphatase interacting protein 2 [Source:HGNC Symbol;Acc:HGNC:9581] |
| ENSG00000153046 | HGLibA_08917 | 42 | -0.730658387 | CDYL | chromodomain Y like [Source:HGNC Symbol;Acc:HGNC:1811] |
| ENSG00000153339 | HGLibA_51337 | 42 | -0.730658387 | TRAPPC8 | trafficking protein particle complex 8 [Source:HGNC Symbol;Acc:HGNC:29169] |
| ENSG00000153485 | HGLibA_50317 | 42 | -0.730658387 | TMEM251 | transmembrane protein 251 [Source:HGNC Symbol;Acc:HGNC:20218] |
| ENSG00000153574 | HGLibA_41850 | 42 | -0.730658387 | RPIA | ribose 5-phosphate isomerase A [Source:HGNC Symbol;Acc:HGNC:10297] |
| ENSG00000154277 | HGLibA_52801 | 42 | -0.730658387 | UCHL1 | ubiquitin C-terminal hydrolase L1 [Source:HGNC Symbol;Acc:HGNC:12513] |
| ENSG00000154736 | HGLibA_00834 | 42 | -0.730658387 | ADAMTS5 | ADAM metallopeptidase with thrombospondin type 1 motif 5 [Source:HGNC Symbol;Acc:HGNC:221] |
| ENSG00000154914 | HGLibA_53369 | 42 | -0.730658387 | USP43 | ubiquitin specific peptidase 43 [Source:HGNC Symbol;Acc:HGNC:20072] |
| ENSG00000154914 | HGLibA_53368 | 42 | -0.730658387 | USP43 | ubiquitin specific peptidase 43 [Source:HGNC Symbol;Acc:HGNC:20072] |
| ENSG00000155465 | HGLibA_45380 | 42 | -0.730658387 | SLC7A7 | solute carrier family 7 member 7 [Source:HGNC Symbol;Acc:HGNC:11065] |
| ENSG00000155975 | HGLibA_53775 | 42 | -0.730658387 | VPS37A | ESCRT-I subunit [Source:HGNC Symbol;Acc:HGNC:24928]" |
| ENSG00000156650 | HGLibA_24062 | 42 | -0.730658387 | KAT6B | lysine acetyltransferase 6B [Source:HGNC Symbol;Acc:HGNC:17582] |
| ENSG00000157911 | HGLibA_36082 | 42 | -0.730658387 | PEX10 | peroxisomal biogenesis factor 10 [Source:HGNC Symbol;Acc:HGNC:8851] |
| ENSG00000157999 | HGLibA_02133 | 42 | -0.730658387 | ANKRD61 | ankyrin repeat domain 61 [Source:HGNC Symbol;Acc:HGNC:22467] |
| ENSG00000158201 | HGLibA_00276 | 42 | -0.730658387 | ABHD3 | abhydrolase domain containing 3 [Source:HGNC Symbol;Acc:HGNC:18718] |
| ENSG00000159248 | HGLibA_19187 | 42 | -0.730658387 | GJD2 | gap junction protein delta 2 [Source:HGNC Symbol;Acc:HGNC:19154] |
| ENSG00000159496 | HGLibA_41009 | 42 | -0.730658387 | RGL4 | ral guanine nucleotide dissociation stimulator like 4 [Source:HGNC Symbol;Acc:HGNC:31911] |
| ENSG00000160813 | HGLibA_38089 | 42 | -0.730658387 | PPP1R35 | protein phosphatase 1 regulatory subunit 35 [Source:HGNC Symbol;Acc:HGNC:28320] |
| ENSG00000161860 | HGLibA_47918 | 42 | -0.730658387 | SYCE2 | synaptonemal complex central element protein 2 [Source:HGNC Symbol;Acc:HGNC:27411] |
| ENSG00000162231 | HGLibA_33125 | 42 | -0.730658387 | NXF1 | nuclear RNA export factor 1 [Source:HGNC Symbol;Acc:HGNC:8071] |
| ENSG00000163125 | HGLibA_42051 | 42 | -0.730658387 | RPRD2 | regulation of nuclear pre-mRNA domain containing 2 [Source:HGNC Symbol;Acc:HGNC:29039] |
| ENSG00000163141 | HGLibA_04662 | 42 | -0.730658387 | BNIPL | BCL2 interacting protein like [Source:HGNC Symbol;Acc:HGNC:16976] |
| ENSG00000163202 | HGLibA_26138 | 42 | -0.730658387 | LCE3D | late cornified envelope 3D [Source:HGNC Symbol;Acc:HGNC:16615] |
| ENSG00000163531 | HGLibA_31766 | 42 | -0.730658387 | NFASC | neurofascin [Source:HGNC Symbol;Acc:HGNC:29866] |
| ENSG00000163755 | HGLibA_22049 | 42 | -0.730658387 | HPS3 | biogenesis of lysosomal organelles complex 2 subunit 1 [Source:HGNC Symbol;Acc:HGNC:15597]" |
| ENSG00000163947 | HGLibA_02847 | 42 | -0.730658387 | ARHGEF3 | Rho guanine nucleotide exchange factor 3 [Source:HGNC Symbol;Acc:HGNC:683] |
| ENSG00000164038 | HGLibA_45437 | 42 | -0.730658387 | SLC9B2 | solute carrier family 9 member B2 [Source:HGNC Symbol;Acc:HGNC:25143] |
| ENSG00000164265 | HGLibA_42936 | 42 | -0.730658387 | SCGB3A2 | secretoglobin family 3A member 2 [Source:HGNC Symbol;Acc:HGNC:18391] |
| ENSG00000164332 | HGLibA_52718 | 42 | -0.730658387 | UBLCP1 | ubiquitin like domain containing CTD phosphatase 1 [Source:HGNC Symbol;Acc:HGNC:28110] |
| ENSG00000164867 | HGLibA_32332 | 42 | -0.730658387 | NOS3 | nitric oxide synthase 3 [Source:HGNC Symbol;Acc:HGNC:7876] |
| ENSG00000164877 | HGLibA_29215 | 42 | -0.730658387 | MICALL2 | MICAL like 2 [Source:HGNC Symbol;Acc:HGNC:29672] |
| ENSG00000164941 | HGLibA_23432 | 42 | -0.730658387 | INTS8 | integrator complex subunit 8 [Source:HGNC Symbol;Acc:HGNC:26048] |
| ENSG00000165028 | HGLibA_31978 | 42 | -0.730658387 | NIPSNAP3B | nipsnap homolog 3B [Source:HGNC Symbol;Acc:HGNC:23641] |
| ENSG00000165169 | HGLibA_14269 | 42 | -0.730658387 | DYNLT3 | dynein light chain Tctex-type 3 [Source:HGNC Symbol;Acc:HGNC:11694] |
| ENSG00000165209 | HGLibA_47582 | 42 | -0.730658387 | STRBP | spermatid perinuclear RNA binding protein [Source:HGNC Symbol;Acc:HGNC:16462] |
| ENSG00000166143 | HGLibA_38044 | 42 | -0.730658387 | PPP1R14D | protein phosphatase 1 regulatory inhibitor subunit 14D [Source:HGNC Symbol;Acc:HGNC:14953] |
| ENSG00000166603 | HGLibA_28549 | 42 | -0.730658387 | MC4R | melanocortin 4 receptor [Source:HGNC Symbol;Acc:HGNC:6932] |
| ENSG00000166788 | HGLibA_42621 | 42 | -0.730658387 | SAAL1 | serum amyloid A like 1 [Source:HGNC Symbol;Acc:HGNC:25158] |
| ENSG00000166884 | HGLibA_33981 | 42 | -0.730658387 | OR4D6 | olfactory receptor family 4 subfamily D member 6 [Source:HGNC Symbol;Acc:HGNC:15175] |
| ENSG00000167378 | HGLibA_23616 | 42 | -0.730658387 | IRGQ | immunity related GTPase Q [Source:HGNC Symbol;Acc:HGNC:24868] |
| ENSG00000167723 | HGLibA_51787 | 42 | -0.730658387 | TRPV3 | transient receptor potential cation channel subfamily V member 3 [Source:HGNC Symbol;Acc:HGNC:18084] |

| | | | | | |
|---|---|---|---|---|---|
| ENSG00000167748 | HGLibA_25230 | 42 | -0.730658387 | KLK1 | kallikrein 1 [Source:HGNC Symbol;Acc:HGNC:6357] |
| ENSG00000167769 | HGLibA_00428 | 42 | -0.730658387 | ACER1 | alkaline ceramidase 1 [Source:HGNC Symbol;Acc:HGNC:18356] |
| ENSG00000167772 | HGLibA_01919 | 42 | -0.730658387 | ANGPTL4 | angiopoietin like 4 [Source:HGNC Symbol;Acc:HGNC:16039] |
| ENSG00000168530 | HGLibA_30745 | 42 | -0.730658387 | MYL1 | myosin light chain 1 [Source:HGNC Symbol;Acc:HGNC:7582] |
| ENSG00000168872 | HGLibA_12663 | 42 | -0.730658387 | DDX19A | DEAD-box helicase 19A [Source:HGNC Symbol;Acc:HGNC:25628] |
| ENSG00000169327 | HGLibA_34254 | 42 | -0.730658387 | OR5AU1 | olfactory receptor family 5 subfamily AU member 1 [Source:HGNC Symbol;Acc:HGNC:15362] |
| ENSG00000169439 | HGLibA_43100 | 42 | -0.730658387 | SDC2 | syndecan 2 [Source:HGNC Symbol;Acc:HGNC:10659] |
| ENSG00000169583 | HGLibA_10027 | 42 | -0.730658387 | CLIC3 | chloride intracellular channel 3 [Source:HGNC Symbol;Acc:HGNC:2064] |
| ENSG00000169826 | HGLibA_11348 | 42 | -0.730658387 | CSGALNACT2 | chondroitin sulfate N-acetylgalactosaminyltransferase 2 [Source:HGNC Symbol;Acc:HGNC:24292] |
| ENSG00000169962 | HGLibA_48359 | 42 | -0.730658387 | TAS1R3 | taste 1 receptor member 3 [Source:HGNC Symbol;Acc:HGNC:15661] |
| ENSG00000169976 | HGLibA_43716 | 42 | -0.730658387 | SF3B5 | splicing factor 3b subunit 5 [Source:HGNC Symbol;Acc:HGNC:21083] |
| ENSG00000170477 | HGLibA_25504 | 42 | -0.730658387 | KRT4 | keratin 4 [Source:HGNC Symbol;Acc:HGNC:6441] |
| ENSG00000170860 | HGLibA_27540 | 42 | -0.730658387 | LSM3 | U6 small nuclear RNA and mRNA degradation associated [Source:HGNC Symbol;Acc:HGNC:17874]" |
| ENSG00000170962 | HGLibA_35850 | 42 | -0.730658387 | PDGFD | platelet derived growth factor D [Source:HGNC Symbol;Acc:HGNC:30620] |
| ENSG00000171478 | HGLibA_46335 | 42 | -0.730658387 | SPACA5B | sperm acrosome associated 5B [Source:HGNC Symbol;Acc:HGNC:19142] |
| ENSG00000172362 | HGLibA_34257 | 42 | -0.730658387 | OR5B12 | olfactory receptor family 5 subfamily B member 12 [Source:HGNC Symbol;Acc:HGNC:15432] |
| ENSG00000172757 | HGLibA_09282 | 42 | -0.730658387 | CFL1 | cofilin 1 [Source:HGNC Symbol;Acc:HGNC:1874] |
| ENSG00000172765 | HGLibA_49772 | 42 | -0.730658387 | TMCC1 | transmembrane and coiled-coil domain family 1 [Source:HGNC Symbol;Acc:HGNC:29116] |
| ENSG00000172771 | HGLibA_14526 | 42 | -0.730658387 | EFCAB12 | EF-hand calcium binding domain 12 [Source:HGNC Symbol;Acc:HGNC:28061] |
| ENSG00000172878 | HGLibA_28932 | 42 | -0.730658387 | METAP1D | mitochondrial [Source:HGNC Symbol;Acc:HGNC:32583]" |
| ENSG00000173715 | HGLibA_05187 | 42 | -0.730658387 | C11orf80 | chromosome 11 open reading frame 80 [Source:HGNC Symbol;Acc:HGNC:26197] |
| ENSG00000173908 | HGLibA_25469 | 42 | -0.730658387 | KRT28 | keratin 28 [Source:HGNC Symbol;Acc:HGNC:30842] |
| ENSG00000174238 | HGLibA_36741 | 42 | -0.730658387 | PITPNA | phosphatidylinositol transfer protein alpha [Source:HGNC Symbol;Acc:HGNC:9001] |
| ENSG00000174243 | HGLibA_12675 | 42 | -0.730658387 | DDX23 | DEAD-box helicase 23 [Source:HGNC Symbol;Acc:HGNC:17347] |
| ENSG00000174339 | HGLibA_33907 | 42 | -0.730658387 | OR2Y1 | olfactory receptor family 2 subfamily Y member 1 [Source:HGNC Symbol;Acc:HGNC:14837] |
| ENSG00000174928 | HGLibA_06231 | 42 | -0.730658387 | C3orf33 | chromosome 3 open reading frame 33 [Source:HGNC Symbol;Acc:HGNC:26434] |
| ENSG00000175294 | HGLibA_07356 | 42 | -0.730658387 | CATSPER1 | cation channel sperm associated 1 [Source:HGNC Symbol;Acc:HGNC:17116] |
| ENSG00000175711 | HGLibA_04008 | 42 | -0.730658387 | B3GNTL1 | 3-N-acetylglucosaminyltransferase like 1 [Source:HGNC Symbol;Acc:HGNC:21727]" |
| ENSG00000176208 | HGLibA_03418 | 42 | -0.730658387 | ATAD5 | AAA domain containing 5 [Source:HGNC Symbol;Acc:HGNC:25752]" |
| ENSG00000176797 | HGLibA_12813 | 42 | -0.730658387 | DEFB103A | defensin beta 103A [Source:HGNC Symbol;Acc:HGNC:15967] |
| ENSG00000176809 | HGLibA_27331 | 42 | -0.730658387 | LRRC37A3 | leucine rich repeat containing 37 member A3 [Source:HGNC Symbol;Acc:HGNC:32427] |
| ENSG00000177138 | HGLibA_16820 | 42 | -0.730658387 | FAM9B | family with sequence similarity 9 member B [Source:HGNC Symbol;Acc:HGNC:18404] |
| ENSG00000177425 | HGLibA_35291 | 42 | -0.730658387 | PAWR | pro-apoptotic WT1 regulator [Source:HGNC Symbol;Acc:HGNC:8614] |
| ENSG00000178078 | HGLibA_47346 | 42 | -0.730658387 | STAP2 | signal transducing adaptor family member 2 [Source:HGNC Symbol;Acc:HGNC:30430] |
| ENSG00000178573 | HGLibA_27865 | 42 | -0.730658387 | MAF | MAF bZIP transcription factor [Source:HGNC Symbol;Acc:HGNC:6776] |
| ENSG00000179348 | HGLibA_18667 | 42 | -0.730658387 | GATA2 | GATA binding protein 2 [Source:HGNC Symbol;Acc:HGNC:4171] |
| ENSG00000179546 | HGLibA_22362 | 42 | -0.730658387 | HTR1D | 5-hydroxytryptamine receptor 1D [Source:HGNC Symbol;Acc:HGNC:5289] |
| ENSG00000179626 | HGLibA_34391 | 42 | -0.730658387 | OR6C4 | olfactory receptor family 6 subfamily C member 4 [Source:HGNC Symbol;Acc:HGNC:19632] |
| ENSG00000179832 | HGLibA_29868 | 42 | -0.730658387 | MROH1 | maestro heat like repeat family member 1 [Source:HGNC Symbol;Acc:HGNC:26958] |
| ENSG00000180209 | HGLibA_30797 | 42 | -0.730658387 | MYLPF | fast skeletal muscle [Source:HGNC Symbol;Acc:HGNC:29824]" |
| ENSG00000180767 | HGLibA_09622 | 42 | -0.730658387 | CHST13 | carbohydrate sulfotransferase 13 [Source:HGNC Symbol;Acc:HGNC:21755] |
| ENSG00000181409 | HGLibA_00088 | 42 | -0.730658387 | AATK | apoptosis associated tyrosine kinase [Source:HGNC Symbol;Acc:HGNC:21] |
| ENSG00000181804 | HGLibA_45429 | 42 | -0.730658387 | SLC9A9 | solute carrier family 9 member A9 [Source:HGNC Symbol;Acc:HGNC:20653] |
| ENSG00000181963 | HGLibA_34183 | 42 | -0.730658387 | OR52K2 | olfactory receptor family 52 subfamily K member 2 [Source:HGNC Symbol;Acc:HGNC:15223] |
| ENSG00000182179 | HGLibA_52530 | 42 | -0.730658387 | UBA7 | ubiquitin like modifier activating enzyme 7 [Source:HGNC Symbol;Acc:HGNC:12471] |
| ENSG00000182973 | HGLibA_10328 | 42 | -0.730658387 | CNOT10 | CCR4-NOT transcription complex subunit 10 [Source:HGNC Symbol;Acc:HGNC:23817] |
| ENSG00000183765 | HGLibA_09421 | 42 | -0.730658387 | CHEK2 | checkpoint kinase 2 [Source:HGNC Symbol;Acc:HGNC:16627] |
| ENSG00000185267 | HGLibA_08852 | 42 | -0.730658387 | CDNF | cerebral dopamine neurotrophic factor [Source:HGNC Symbol;Acc:HGNC:24913] |
| ENSG00000185519 | HGLibA_16110 | 42 | -0.730658387 | FAM131C | family with sequence similarity 131 member C [Source:HGNC Symbol;Acc:HGNC:26717] |
| ENSG00000185739 | HGLibA_46992 | 42 | -0.730658387 | SRL | sarcalumenin [Source:HGNC Symbol;Acc:HGNC:11295] |
| ENSG00000185838 | HGLibA_19488 | 42 | -0.730658387 | GNB1L | G protein subunit beta 1 like [Source:HGNC Symbol;Acc:HGNC:4397] |
| ENSG00000186952 | HGLibA_50262 | 42 | -0.730658387 | TMEM232 | transmembrane protein 232 [Source:HGNC Symbol;Acc:HGNC:37270] |
| ENSG00000186971 | HGLibA_25651 | 42 | -0.730658387 | KRTAP13-4 | keratin associated protein 13-4 [Source:HGNC Symbol;Acc:HGNC:18926] |
| ENSG00000188807 | HGLibA_50181 | 42 | -0.730658387 | TMEM201 | transmembrane protein 201 [Source:HGNC Symbol;Acc:HGNC:33719] |
| ENSG00000188992 | HGLibA_26618 | 42 | -0.730658387 | LIPI | lipase I [Source:HGNC Symbol;Acc:HGNC:18821] |
| ENSG00000189334 | HGLibA_42545 | 42 | -0.730658387 | S100A14 | S100 calcium binding protein A14 [Source:HGNC Symbol;Acc:HGNC:18901] |
| ENSG00000189410 | HGLibA_43904 | 42 | -0.730658387 | SH2D5 | SH2 domain containing 5 [Source:HGNC Symbol;Acc:HGNC:28819] |
| ENSG00000196275 | HGLibA_20571 | 42 | -0.730658387 | GTF2IRD2 | GTF2I repeat domain containing 2 [Source:HGNC Symbol;Acc:HGNC:30775] |
| ENSG00000196352 | HGLibA_08410 | 42 | -0.730658387 | CD55 | CD55 molecule (Cromer blood group) [Source:HGNC Symbol;Acc:HGNC:2665] |
| ENSG00000196455 | HGLibA_36656 | 42 | -0.730658387 | PIK3R4 | phosphoinositide-3-kinase regulatory subunit 4 [Source:HGNC Symbol;Acc:HGNC:8982] |
| ENSG00000196664 | HGLibA_49662 | 42 | -0.730658387 | TLR7 | toll like receptor 7 [Source:HGNC Symbol;Acc:HGNC:15631] |
| ENSG00000197208 | HGLibA_44587 | 42 | -0.730658387 | SLC22A4 | solute carrier family 22 member 4 [Source:HGNC Symbol;Acc:HGNC:10968] |
| ENSG00000197416 | HGLibA_15910 | 42 | -0.730658387 | FABP12 | fatty acid binding protein 12 [Source:HGNC Symbol;Acc:HGNC:34524] |
| ENSG00000197728 | HGLibA_42119 | 42 | -0.730658387 | RPS26 | ribosomal protein S26 [Source:HGNC Symbol;Acc:HGNC:10414] |
| ENSG00000198231 | HGLibA_12713 | 42 | -0.730658387 | DDX42 | DEAD-box helicase 42 [Source:HGNC Symbol;Acc:HGNC:18676] |
| ENSG00000198690 | HGLibA_16826 | 42 | -0.730658387 | FAN1 | FANCD2 and FANCI associated nuclease 1 [Source:HGNC Symbol;Acc:HGNC:29170] |
| ENSG00000198739 | HGLibA_27490 | 42 | -0.730658387 | LRRTM3 | leucine rich repeat transmembrane neuronal 3 [Source:HGNC Symbol;Acc:HGNC:19410] |
| ENSG00000198788 | HGLibA_30536 | 42 | -0.730658387 | MUC2 | oligomeric mucus/gel-forming [Source:HGNC Symbol;Acc:HGNC:7512]" |
| ENSG00000198788 | HGLibA_30534 | 42 | -0.730658387 | MUC2 | oligomeric mucus/gel-forming [Source:HGNC Symbol;Acc:HGNC:7512]" |
| ENSG00000198825 | HGLibA_23355 | 42 | -0.730658387 | INPP5F | inositol polyphosphate-5-phosphatase F [Source:HGNC Symbol;Acc:HGNC:17054] |
| ENSG00000198839 | HGLibA_55716 | 42 | -0.730658387 | ZNF277 | zinc finger protein 277 [Source:HGNC Symbol;Acc:HGNC:13070] |
| ENSG00000198892 | HGLibA_44038 | 42 | -0.730658387 | SHISA4 | shisa family member 4 [Source:HGNC Symbol;Acc:HGNC:27139] |
| ENSG00000198929 | HGLibA_32325 | 42 | -0.730658387 | NOS1AP | nitric oxide synthase 1 adaptor protein [Source:HGNC Symbol;Acc:HGNC:16859] |
| ENSG00000203972 | HGLibA_19378 | 42 | -0.730658387 | GLYATL3 | glycine-N-acyltransferase like 3 [Source:HGNC Symbol;Acc:HGNC:21349] |
| ENSG00000203972 | HGLibA_19380 | 42 | -0.730658387 | GLYATL3 | glycine-N-acyltransferase like 3 [Source:HGNC Symbol;Acc:HGNC:21349] |
| ENSG00000204544 | HGLibA_30540 | 42 | -0.730658387 | MUC21 | cell surface associated [Source:HGNC Symbol;Acc:HGNC:21661]" |
| ENSG00000205129 | HGLibA_06338 | 42 | -0.730658387 | C4orf47 | chromosome 4 open reading frame 47 [Source:HGNC Symbol;Acc:HGNC:34346] |
| ENSG00000205777 | HGLibA_18431 | 42 | -0.730658387 | GAGE1 | G antigen 1 [Source:HGNC Symbol;Acc:HGNC:4098] |
| ENSG00000211452 | HGLibA_13237 | 42 | -0.730658387 | DIO1 | iodothyronine deiodinase 1 [Source:HGNC Symbol;Acc:HGNC:2883] |
| ENSG00000212124 | HGLibA_48378 | 42 | -0.730658387 | TAS2R19 | taste 2 receptor member 19 [Source:HGNC Symbol;Acc:HGNC:19108] |
| ENSG00000213401 | HGLibA_27902 | 42 | -0.730658387 | MAGEA12 | MAGE family member A12 [Source:HGNC Symbol;Acc:HGNC:6799] |
| ENSG00000213417 | HGLibA_25697 | 42 | -0.730658387 | KRTAP2-4 | keratin associated protein 2-4 [Source:HGNC Symbol;Acc:HGNC:18891] |
| ENSG00000213923 | HGLibA_11389 | 42 | -0.730658387 | CSNK1E | casein kinase 1 epsilon [Source:HGNC Symbol;Acc:HGNC:2453] |
| ENSG00000215186 | HGLibA_19611 | 42 | -0.730658387 | GOLGA6B | golgin A6 family member B [Source:HGNC Symbol;Acc:HGNC:32205] |
| ENSG00000227488 | HGLibA_18443 | 42 | -0.730658387 | GAGE12D | G antigen 12D [Source:HGNC Symbol;Acc:HGNC:31904] |
| ENSG00000232264 | HGLibA_53254 | 42 | -0.730658387 | USP17L24 | ubiquitin specific peptidase 17-like family member 24 [Source:HGNC Symbol;Acc:HGNC:44453] |
| ENSG00000253719 | HGLibA_03860 | 42 | -0.730658387 | ATXN7L3B | ataxin 7 like 3B [Source:HGNC Symbol;Acc:HGNC:37931] |
| ENSG00000254550 | HGLibA_33389 | 42 | -0.730658387 | OMP | olfactory marker protein [Source:HGNC Symbol;Acc:HGNC:8136] |
| ENSG00000254585 | HGLibA_27991 | 42 | -0.730658387 | MAGEL2 | MAGE family member L2 [Source:HGNC Symbol;Acc:HGNC:6814] |
| ENSG00000255150 | HGLibA_14703 | 42 | -0.730658387 | EID3 | EP300 interacting inhibitor of differentiation 3 [Source:HGNC Symbol;Acc:HGNC:32961] |
| ENSG00000256269 | HGLibA_21669 | 42 | -0.730658387 | HMBS | hydroxymethylbilane synthase [Source:HGNC Symbol;Acc:HGNC:4982] |
| ENSG00000257218 | HGLibA_18693 | 42 | -0.730658387 | GATC | glutamyl-tRNA amidotransferase subunit C [Source:HGNC Symbol;Acc:HGNC:25068] |
| ENSG00000259112 | HGLibA_31542 | 42 | -0.730658387 | NDUFC2-KCTD14 | NDUFC2-KCTD14 readthrough [Source:HGNC Symbol;Acc:HGNC:42956] |
| ENSG00000269096 | HGLibA_11505 | 42 | -0.730658387 | CT45A3 | cancer/testis antigen family 45 member A3 [Source:HGNC Symbol;Acc:HGNC:33268] |
| ENSG00000269437 | HGLibA_33133 | 42 | -0.730658387 | NXF2B | nuclear RNA export factor 2B [Source:HGNC Symbol;Acc:HGNC:23984] |
| ENSG00000272391 | HGLibA_37644 | 42 | -0.730658387 | POM121C | POM121 transmembrane nucleoporin C [Source:HGNC Symbol;Acc:HGNC:34005] |
| ENSG00000273611 | HGLibA_56930 | 42 | -0.730658387 | ZNHIT3 | zinc finger HIT-type containing 3 [Source:HGNC Symbol;Acc:HGNC:12309] |
| ENSG00000282608 | HGLibA_01019 | 42 | -0.730658387 | ADORA3 | adenosine A3 receptor [Source:HGNC Symbol;Acc:HGNC:268] |
| FLJ22184 | HGLibA_17622 | 42 | -0.730658387 | FLJ22184 | - |
| GUCY1B3 | HGLibA_20657 | 42 | -0.730658387 | GUCY1B3 | - |
| hsa-mir-103a-1 | HGLibA_57162 | 42 | -0.730658387 | hsa-mir-103a-1 | - |
| hsa-mir-1202 | HGLibA_57264 | 42 | -0.730658387 | hsa-mir-1202 | - |
| hsa-mir-1236 | HGLibA_57334 | 42 | -0.730658387 | hsa-mir-1236 | - |
| hsa-mir-1244-3 | HGLibA_57370 | 42 | -0.730658387 | hsa-mir-1244-3 | - |
| hsa-mir-1298 | HGLibA_57671 | 42 | -0.730658387 | hsa-mir-1298 | - |
| hsa-mir-1302-10 | HGLibA_57686 | 42 | -0.730658387 | hsa-mir-1302-10 | - |
| hsa-mir-146a | HGLibA_57855 | 42 | -0.730658387 | hsa-mir-146a | - |
| hsa-mir-188 | HGLibA_58013 | 42 | -0.730658387 | hsa-mir-188 | - |
| hsa-mir-224 | HGLibA_58304 | 42 | -0.730658387 | hsa-mir-224 | - |
| hsa-mir-3116-2 | HGLibA_58506 | 42 | -0.730658387 | hsa-mir-3116-2 | - |
| hsa-mir-3119-1 | HGLibA_58526 | 42 | -0.730658387 | hsa-mir-3119-1 | - |
| hsa-mir-3193 | HGLibA_58868 | 42 | -0.730658387 | hsa-mir-3193 | - |
| hsa-mir-320c-1 | HGLibA_58936 | 42 | -0.730658387 | hsa-mir-320c-1 | - |
| hsa-mir-3692 | HGLibA_59357 | 42 | -0.730658387 | hsa-mir-3692 | - |
| hsa-mir-371b | HGLibA_59378 | 42 | -0.730658387 | hsa-mir-371b | - |
| hsa-mir-3928 | HGLibA_59589 | 42 | -0.730658387 | hsa-mir-3928 | - |
| hsa-mir-4267 | HGLibA_59776 | 42 | -0.730658387 | hsa-mir-4267 | - |
| hsa-mir-4423 | HGLibA_60079 | 42 | -0.730658387 | hsa-mir-4423 | - |
| hsa-mir-4430 | HGLibA_60105 | 42 | -0.730658387 | hsa-mir-4430 | - |
| hsa-mir-4433 | HGLibA_60118 | 42 | -0.730658387 | hsa-mir-4433 | - |
| hsa-mir-4520b | HGLibA_60538 | 42 | -0.730658387 | hsa-mir-4520b | - |
| hsa-mir-4520b | HGLibA_60536 | 42 | -0.730658387 | hsa-mir-4520b | - |
| hsa-mir-4706 | HGLibA_60925 | 42 | -0.730658387 | hsa-mir-4706 | - |
| hsa-mir-5006 | HGLibA_61428 | 42 | -0.730658387 | hsa-mir-5006 | - |
| hsa-mir-5088 | HGLibA_61498 | 42 | -0.730658387 | hsa-mir-5088 | - |
| hsa-mir-514a-1 | HGLibA_61588 | 42 | -0.730658387 | hsa-mir-514a-1 | - |
| hsa-mir-5586 | HGLibA_62186 | 42 | -0.730658387 | hsa-mir-5586 | - |
| hsa-mir-6165 | HGLibA_62702 | 42 | -0.730658387 | hsa-mir-6165 | - |
| hsa-mir-629 | HGLibA_62756 | 42 | -0.730658387 | hsa-mir-629 | - |
| hsa-mir-629 | HGLibA_62757 | 42 | -0.730658387 | hsa-mir-629 | - |
| hsa-mir-6511a-2 | HGLibA_62903 | 42 | -0.730658387 | hsa-mir-6511a-2 | - |
| hsa-mir-6511b-2 | HGLibA_62917 | 42 | -0.730658387 | hsa-mir-6511b-2 | - |
| hsa-mir-6515 | HGLibA_62934 | 42 | -0.730658387 | hsa-mir-6515 | - |
| hsa-mir-6719 | HGLibA_63037 | 42 | -0.730658387 | hsa-mir-6719 | - |
| hsa-mir-6769b | HGLibA_63243 | 42 | -0.730658387 | hsa-mir-6769b | - |

| | | | | | |
|---|---|---|---|---|---|
| hsa-mir-6830 | HGLibA_63497 | 42 | -0.730658387 | hsa-mir-6830 | - |
| hsa-mir-941-1 | HGLibA_64337 | 42 | -0.730658387 | hsa-mir-941-1 | - |
| SUV420H2 | HGLibA_47867 | 42 | -0.730658387 | SUV420H2 | - |
| TMEM257 | HGLibA_50340 | 42 | -0.730658387 | TMEM257 | - |
| WDR78 | HGLibA_54184 | 42 | -0.730658387 | WDR78 | - |
| BZRAP1 | HGLibA_05009 | 41 | -0.740609424 | BZRAP1 | - |
| C10orf118 | HGLibA_05039 | 41 | -0.740609424 | C10orf118 | - |
| C11orf70 | HGLibA_05175 | 41 | -0.740609424 | C11orf70 | - |
| C14orf105 | HGLibA_05319 | 41 | -0.740609424 | C14orf105 | - |
| C15orf57 | HGLibA_05419 | 41 | -0.740609424 | C15orf57 | - |
| C16orf45 | HGLibA_05448 | 41 | -0.740609424 | C16orf45 | - |
| C19orf59 | HGLibA_05686 | 41 | -0.740609424 | C19orf59 | - |
| C1orf110 | HGLibA_05743 | 41 | -0.740609424 | C1orf110 | - |
| CCDC41 | HGLibA_07778 | 41 | -0.740609424 | CCDC41 | - |
| CPSF3L | HGLibA_11002 | 41 | -0.740609424 | CPSF3L | - |
| DHRSX_X | HGLibA_13157 | 41 | -0.740609424 | DHRSX_X | - |
| ENSG00000012817 | HGLibA_24530 | 41 | -0.740609424 | KDM5D | lysine demethylase 5D [Source:HGNC Symbol;Acc:HGNC:11115] |
| ENSG00000032444 | HGLibA_37419 | 41 | -0.740609424 | PNPLA6 | patatin like phospholipase domain containing 6 [Source:HGNC Symbol;Acc:HGNC:16268] |
| ENSG00000037749 | HGLibA_29045 | 41 | -0.740609424 | MFAP3 | microfibril associated protein 3 [Source:HGNC Symbol;Acc:HGNC:7034] |
| ENSG00000048828 | HGLibA_16054 | 41 | -0.740609424 | FAM120A | family with sequence similarity 120A [Source:HGNC Symbol;Acc:HGNC:13247] |
| ENSG00000053438 | HGLibA_32219 | 41 | -0.740609424 | NNAT | neuronatin [Source:HGNC Symbol;Acc:HGNC:7860] |
| ENSG00000057468 | HGLibA_30195 | 41 | -0.740609424 | MSH4 | mutS homolog 4 [Source:HGNC Symbol;Acc:HGNC:7327] |
| ENSG00000060339 | HGLibA_07488 | 41 | -0.740609424 | CCAR1 | cell division cycle and apoptosis regulator 1 [Source:HGNC Symbol;Acc:HGNC:24236] |
| ENSG00000061656 | HGLibA_46358 | 41 | -0.740609424 | SPAG4 | sperm associated antigen 4 [Source:HGNC Symbol;Acc:HGNC:11214] |
| ENSG00000063180 | HGLibA_06790 | 41 | -0.740609424 | CA11 | carbonic anhydrase 11 [Source:HGNC Symbol;Acc:HGNC:1370] |
| ENSG00000065183 | HGLibA_54060 | 41 | -0.740609424 | WDR3 | WD repeat domain 3 [Source:HGNC Symbol;Acc:HGNC:12755] |
| ENSG00000074706 | HGLibA_23454 | 41 | -0.740609424 | IPCEF1 | interaction protein for cytohesin exchange factors 1 [Source:HGNC Symbol;Acc:HGNC:21204] |
| ENSG00000075568 | HGLibA_49928 | 41 | -0.740609424 | TMEM131 | transmembrane protein 131 [Source:HGNC Symbol;Acc:HGNC:30366] |
| ENSG00000075886 | HGLibA_52254 | 41 | -0.740609424 | TUBA3D | tubulin alpha 3d [Source:HGNC Symbol;Acc:HGNC:24071] |
| ENSG00000075975 | HGLibA_29351 | 41 | -0.740609424 | MKRN2 | makorin ring finger protein 2 [Source:HGNC Symbol;Acc:HGNC:7113] |
| ENSG00000080603 | HGLibA_46930 | 41 | -0.740609424 | SRCAP | Snf2 related CREBBP activator protein [Source:HGNC Symbol;Acc:HGNC:16974] |
| ENSG00000080845 | HGLibA_13347 | 41 | -0.740609424 | DLGAP4 | DLG associated protein 4 [Source:HGNC Symbol;Acc:HGNC:24476] |
| ENSG00000082014 | HGLibA_45634 | 41 | -0.740609424 | SMARCD3 | member 3 [Source:HGNC Symbol;Acc:HGNC:11108]" |
| ENSG00000088205 | HGLibA_12659 | 41 | -0.740609424 | DDX18 | DEAD-box helicase 18 [Source:HGNC Symbol;Acc:HGNC:2741] |
| ENSG00000095539 | HGLibA_43354 | 41 | -0.740609424 | SEMA4G | semaphorin 4G [Source:HGNC Symbol;Acc:HGNC:10735] |
| ENSG00000099834 | HGLibA_08709 | 41 | -0.740609424 | CDHR5 | cadherin related family member 5 [Source:HGNC Symbol;Acc:HGNC:7521] |
| ENSG00000100225 | HGLibA_17127 | 41 | -0.740609424 | FBXO7 | F-box protein 7 [Source:HGNC Symbol;Acc:HGNC:13586] |
| ENSG00000100346 | HGLibA_06899 | 41 | -0.740609424 | CACNA1I | calcium voltage-gated channel subunit alpha1 I [Source:HGNC Symbol;Acc:HGNC:1396] |
| ENSG00000101160 | HGLibA_11736 | 41 | -0.740609424 | CTSZ | cathepsin Z [Source:HGNC Symbol;Acc:HGNC:2547] |
| ENSG00000101463 | HGLibA_47969 | 41 | -0.740609424 | SYNDIG1 | synapse differentiation inducing 1 [Source:HGNC Symbol;Acc:HGNC:15885] |
| ENSG00000101542 | HGLibA_08661 | 41 | -0.740609424 | CDH20 | cadherin 20 [Source:HGNC Symbol;Acc:HGNC:1760] |
| ENSG00000101745 | HGLibA_01997 | 41 | -0.740609424 | ANKRD12 | ankyrin repeat domain 12 [Source:HGNC Symbol;Acc:HGNC:29135] |
| ENSG00000102796 | HGLibA_13124 | 41 | -0.740609424 | DHRS12 | dehydrogenase/reductase 12 [Source:HGNC Symbol;Acc:HGNC:25832] |
| ENSG00000103202 | HGLibA_32163 | 41 | -0.740609424 | NME4 | NME/NM23 nucleoside diphosphate kinase 4 [Source:HGNC Symbol;Acc:HGNC:7852] |
| ENSG00000103507 | HGLibA_04283 | 41 | -0.740609424 | BCKDK | branched chain ketoacid dehydrogenase kinase [Source:HGNC Symbol;Acc:HGNC:16902] |
| ENSG00000103642 | HGLibA_25916 | 41 | -0.740609424 | LACTB | lactamase beta [Source:HGNC Symbol;Acc:HGNC:16468] |
| ENSG00000105321 | HGLibA_07919 | 41 | -0.740609424 | CCDC9 | coiled-coil domain containing 9 [Source:HGNC Symbol;Acc:HGNC:24560] |
| ENSG00000106290 | HGLibA_48246 | 41 | -0.740609424 | TAF6 | TATA-box binding protein associated factor 6 [Source:HGNC Symbol;Acc:HGNC:11540] |
| ENSG00000110200 | HGLibA_01873 | 41 | -0.740609424 | ANAPC15 | anaphase promoting complex subunit 15 [Source:HGNC Symbol;Acc:HGNC:24531] |
| ENSG00000110848 | HGLibA_08432 | 41 | -0.740609424 | CD69 | CD69 molecule [Source:HGNC Symbol;Acc:HGNC:1694] |
| ENSG00000112118 | HGLibA_28606 | 41 | -0.740609424 | MCM3 | minichromosome maintenance complex component 3 [Source:HGNC Symbol;Acc:HGNC:6945] |
| ENSG00000112146 | HGLibA_17132 | 41 | -0.740609424 | FBXO9 | F-box protein 9 [Source:HGNC Symbol;Acc:HGNC:13588] |
| ENSG00000112237 | HGLibA_08069 | 41 | -0.740609424 | CCNC | cyclin C [Source:HGNC Symbol;Acc:HGNC:1581] |
| ENSG00000112578 | HGLibA_05006 | 41 | -0.740609424 | BYSL | bystin like [Source:HGNC Symbol;Acc:HGNC:1157] |
| ENSG00000112619 | HGLibA_38752 | 41 | -0.740609424 | PRPH2 | peripherin 2 [Source:HGNC Symbol;Acc:HGNC:9942] |
| ENSG00000112655 | HGLibA_39388 | 41 | -0.740609424 | PTK7 | protein tyrosine kinase 7 (inactive) [Source:HGNC Symbol;Acc:HGNC:9618] |
| ENSG00000113212 | HGLibA_35507 | 41 | -0.740609424 | PCDHB7 | protocadherin beta 7 [Source:HGNC Symbol;Acc:HGNC:8692] |
| ENSG00000115325 | HGLibA_13768 | 41 | -0.740609424 | DOK1 | docking protein 1 [Source:HGNC Symbol;Acc:HGNC:2990] |
| ENSG00000115661 | HGLibA_47460 | 41 | -0.740609424 | STK16 | serine/threonine kinase 16 [Source:HGNC Symbol;Acc:HGNC:11394] |
| ENSG00000116679 | HGLibA_23882 | 41 | -0.740609424 | IVNS1ABP | influenza virus NS1A binding protein [Source:HGNC Symbol;Acc:HGNC:16951] |
| ENSG00000116691 | HGLibA_29267 | 41 | -0.740609424 | MIIP | migration and invasion inhibitory protein [Source:HGNC Symbol;Acc:HGNC:25715] |
| ENSG00000117020 | HGLibA_01515 | 41 | -0.740609424 | AKT3 | AKT serine/threonine kinase 3 [Source:HGNC Symbol;Acc:HGNC:393] |
| ENSG00000117020 | HGLibA_01514 | 41 | -0.740609424 | AKT3 | AKT serine/threonine kinase 3 [Source:HGNC Symbol;Acc:HGNC:393] |
| ENSG00000117139 | HGLibA_24524 | 41 | -0.740609424 | KDM5B | lysine demethylase 5B [Source:HGNC Symbol;Acc:HGNC:18039] |
| ENSG00000117523 | HGLibA_38867 | 41 | -0.740609424 | PRRC2C | proline rich coiled-coil 2C [Source:HGNC Symbol;Acc:HGNC:24903] |
| ENSG00000117697 | HGLibA_32761 | 41 | -0.740609424 | NSL1 | MIS12 kinetochore complex component [Source:HGNC Symbol;Acc:HGNC:24548]" |
| ENSG00000117906 | HGLibA_40704 | 41 | -0.740609424 | RCN2 | reticulocalbin 2 [Source:HGNC Symbol;Acc:HGNC:9935] |
| ENSG00000118137 | HGLibA_02448 | 41 | -0.740609424 | APOA1 | apolipoprotein A1 [Source:HGNC Symbol;Acc:HGNC:600] |
| ENSG00000119042 | HGLibA_42779 | 41 | -0.740609424 | SATB2 | SATB homeobox 2 [Source:HGNC Symbol;Acc:HGNC:21637] |
| ENSG00000119048 | HGLibA_52575 | 41 | -0.740609424 | UBE2B | ubiquitin conjugating enzyme E2 B [Source:HGNC Symbol;Acc:HGNC:12473] |
| ENSG00000122966 | HGLibA_09740 | 41 | -0.740609424 | CIT | citron rho-interacting serine/threonine kinase [Source:HGNC Symbol;Acc:HGNC:1985] |
| ENSG00000123992 | HGLibA_13711 | 41 | -0.740609424 | DNPEP | aspartyl aminopeptidase [Source:HGNC Symbol;Acc:HGNC:2981] |
| ENSG00000125122 | HGLibA_27302 | 41 | -0.740609424 | LRRC29 | leucine rich repeat containing 29 [Source:HGNC Symbol;Acc:HGNC:13605] |
| ENSG00000125266 | HGLibA_14599 | 41 | -0.740609424 | EFNB2 | ephrin B2 [Source:HGNC Symbol;Acc:HGNC:3227] |
| ENSG00000125787 | HGLibA_19583 | 41 | -0.740609424 | GNRH2 | gonadotropin releasing hormone 2 [Source:HGNC Symbol;Acc:HGNC:4420] |
| ENSG00000126010 | HGLibA_20378 | 41 | -0.740609424 | GRPR | gastrin releasing peptide receptor [Source:HGNC Symbol;Acc:HGNC:4609] |
| ENSG00000126775 | HGLibA_03470 | 41 | -0.740609424 | ATG14 | autophagy related 14 [Source:HGNC Symbol;Acc:HGNC:19962] |
| ENSG00000126803 | HGLibA_22279 | 41 | -0.740609424 | HSPA2 | heat shock protein family A (Hsp70) member 2 [Source:HGNC Symbol;Acc:HGNC:5235] |
| ENSG00000127586 | HGLibA_09662 | 41 | -0.740609424 | CHTF18 | chromosome transmission fidelity factor 18 [Source:HGNC Symbol;Acc:HGNC:18435] |
| ENSG00000128487 | HGLibA_46602 | 41 | -0.740609424 | SPECC1 | sperm antigen with calponin homology and coiled-coil domains 1 [Source:HGNC Symbol;Acc:HGNC:30615] |
| ENSG00000128709 | HGLibA_22006 | 41 | -0.740609424 | HOXD9 | homeobox D9 [Source:HGNC Symbol;Acc:HGNC:5140] |
| ENSG00000129055 | HGLibA_01870 | 41 | -0.740609424 | ANAPC13 | anaphase promoting complex subunit 13 [Source:HGNC Symbol;Acc:HGNC:24540] |
| ENSG00000129245 | HGLibA_18221 | 41 | -0.740609424 | FXR2 | FMR1 autosomal homolog 2 [Source:HGNC Symbol;Acc:HGNC:4024] |
| ENSG00000129968 | HGLibA_00263 | 41 | -0.740609424 | ABHD17A | abhydrolase domain containing 17A [Source:HGNC Symbol;Acc:HGNC:28756] |
| ENSG00000130032 | HGLibA_38875 | 41 | -0.740609424 | PRRG3 | proline rich and Gla domain 3 [Source:HGNC Symbol;Acc:HGNC:30798] |
| ENSG00000130167 | HGLibA_51909 | 41 | -0.740609424 | TSPAN16 | tetraspanin 16 [Source:HGNC Symbol;Acc:HGNC:30725] |
| ENSG00000130540 | HGLibA_47765 | 41 | -0.740609424 | SULT4A1 | sulfotransferase family 4A member 1 [Source:HGNC Symbol;Acc:HGNC:14903] |
| ENSG00000131016 | HGLibA_01412 | 41 | -0.740609424 | AKAP12 | A-kinase anchoring protein 12 [Source:HGNC Symbol;Acc:HGNC:370] |
| ENSG00000131043 | HGLibA_00062 | 41 | -0.740609424 | AAR2 | AAR2 splicing factor homolog [Source:HGNC Symbol;Acc:HGNC:15886] |
| ENSG00000131446 | HGLibA_29133 | 41 | -0.740609424 | MGAT1 | 2-N-acetylglucosaminyltransferase [Source:HGNC Symbol;Acc:HGNC:7044]" |
| ENSG00000131779 | HGLibA_36088 | 41 | -0.740609424 | PEX11B | peroxisomal biogenesis factor 11 beta [Source:HGNC Symbol;Acc:HGNC:8853] |
| ENSG00000132405 | HGLibA_48480 | 41 | -0.740609424 | TBC1D14 | TBC1 domain family member 14 [Source:HGNC Symbol;Acc:HGNC:29246] |
| ENSG00000133105 | HGLibA_42496 | 41 | -0.740609424 | RXFP2 | relaxin family peptide receptor 2 [Source:HGNC Symbol;Acc:HGNC:17318] |
| ENSG00000133639 | HGLibA_04940 | 41 | -0.740609424 | BTG1 | BTG anti-proliferation factor 1 [Source:HGNC Symbol;Acc:HGNC:1130] |
| ENSG00000134245 | HGLibA_54362 | 41 | -0.740609424 | WNT2B | Wnt family member 2B [Source:HGNC Symbol;Acc:HGNC:12781] |
| ENSG00000134245 | HGLibA_54360 | 41 | -0.740609424 | WNT2B | Wnt family member 2B [Source:HGNC Symbol;Acc:HGNC:12781] |
| ENSG00000134489 | HGLibA_22104 | 41 | -0.740609424 | HRH4 | histamine receptor H4 [Source:HGNC Symbol;Acc:HGNC:17383] |
| ENSG00000134824 | HGLibA_15936 | 41 | -0.740609424 | FADS2 | fatty acid desaturase 2 [Source:HGNC Symbol;Acc:HGNC:3575] |
| ENSG00000134882 | HGLibA_52536 | 41 | -0.740609424 | UBAC2 | UBA domain containing 2 [Source:HGNC Symbol;Acc:HGNC:20486] |
| ENSG00000135677 | HGLibA_19588 | 41 | -0.740609424 | GNS | glucosamine (N-acetyl)-6-sulfatase [Source:HGNC Symbol;Acc:HGNC:4422] |
| ENSG00000135828 | HGLibA_41438 | 41 | -0.740609424 | RNASEL | ribonuclease L [Source:HGNC Symbol;Acc:HGNC:10050] |
| ENSG00000135931 | HGLibA_03034 | 41 | -0.740609424 | ARMC9 | armadillo repeat containing 9 [Source:HGNC Symbol;Acc:HGNC:20730] |
| ENSG00000136206 | HGLibA_46578 | 41 | -0.740609424 | SPDYE1 | speedy/RINGO cell cycle regulator family member E1 [Source:HGNC Symbol;Acc:HGNC:16408] |
| ENSG00000137103 | HGLibA_50548 | 41 | -0.740609424 | TMEM8B | transmembrane protein 8B [Source:HGNC Symbol;Acc:HGNC:21427] |
| ENSG00000138653 | HGLibA_31435 | 41 | -0.740609424 | NDST4 | N-deacetylase and N-sulfotransferase 4 [Source:HGNC Symbol;Acc:HGNC:20779] |
| ENSG00000139168 | HGLibA_55054 | 41 | -0.740609424 | ZCRB1 | zinc finger CCHC-type and RNA binding motif containing 1 [Source:HGNC Symbol;Acc:HGNC:29620] |
| ENSG00000140367 | HGLibA_52649 | 41 | -0.740609424 | UBE2Q2 | ubiquitin conjugating enzyme E2 Q2 [Source:HGNC Symbol;Acc:HGNC:19248] |
| ENSG00000140526 | HGLibA_00272 | 41 | -0.740609424 | ABHD2 | abhydrolase domain containing 2 [Source:HGNC Symbol;Acc:HGNC:18717] |
| ENSG00000140854 | HGLibA_24082 | 41 | -0.740609424 | KATNB1 | katanin regulatory subunit B1 [Source:HGNC Symbol;Acc:HGNC:6217] |
| ENSG00000141933 | HGLibA_51124 | 41 | -0.740609424 | TPGS1 | tubulin polyglutamylase complex subunit 1 [Source:HGNC Symbol;Acc:HGNC:25058] |
| ENSG00000141977 | HGLibA_09688 | 41 | -0.740609424 | CIB3 | calcium and integrin binding family member 3 [Source:HGNC Symbol;Acc:HGNC:24580] |
| ENSG00000143190 | HGLibA_37756 | 41 | -0.740609424 | POU2F1 | POU class 2 homeobox 1 [Source:HGNC Symbol;Acc:HGNC:9212] |
| ENSG00000143546 | HGLibA_42573 | 41 | -0.740609424 | S100A8 | S100 calcium binding protein A8 [Source:HGNC Symbol;Acc:HGNC:10498] |
| ENSG00000144445 | HGLibA_24039 | 41 | -0.740609424 | KANSL1L | KAT8 regulatory NSL complex subunit 1 like [Source:HGNC Symbol;Acc:HGNC:26310] |
| ENSG00000144468 | HGLibA_41125 | 41 | -0.740609424 | RHBDD1 | rhomboid domain containing 1 [Source:HGNC Symbol;Acc:HGNC:23081] |
| ENSG00000145349 | HGLibA_07074 | 41 | -0.740609424 | CAMK2D | calcium/calmodulin dependent protein kinase II delta [Source:HGNC Symbol;Acc:HGNC:1462] |
| ENSG00000145777 | HGLibA_51876 | 41 | -0.740609424 | TSLP | thymic stromal lymphopoietin [Source:HGNC Symbol;Acc:HGNC:30743] |
| ENSG00000146166 | HGLibA_26418 | 41 | -0.740609424 | LGSN | lens protein with glutamine synthetase domain [Source:HGNC Symbol;Acc:HGNC:21016]" |
| ENSG00000147118 | HGLibA_55542 | 41 | -0.740609424 | ZNF182 | zinc finger protein 182 [Source:HGNC Symbol;Acc:HGNC:13001] |
| ENSG00000147274 | HGLibA_40595 | 41 | -0.740609424 | RBMX | RNA binding motif protein X-linked [Source:HGNC Symbol;Acc:HGNC:9910] |
| ENSG00000147885 | HGLibA_22667 | 41 | -0.740609424 | IFNA16 | interferon alpha 16 [Source:HGNC Symbol;Acc:HGNC:5421] |
| ENSG00000147894 | HGLibA_06759 | 41 | -0.740609424 | C9orf72 | chromosome 9 open reading frame 72 [Source:HGNC Symbol;Acc:HGNC:28337] |
| ENSG00000148400 | HGLibA_32341 | 41 | -0.740609424 | NOTCH1 | notch 1 [Source:HGNC Symbol;Acc:HGNC:7881] |
| ENSG00000149054 | HGLibA_55596 | 41 | -0.740609424 | ZNF215 | zinc finger protein 215 [Source:HGNC Symbol;Acc:HGNC:13007] |
| ENSG00000149177 | HGLibA_39510 | 41 | -0.740609424 | PTPRJ | receptor type J [Source:HGNC Symbol;Acc:HGNC:9673]" |
| ENSG00000149782 | HGLibA_36983 | 41 | -0.740609424 | PLCB3 | phospholipase C beta 3 [Source:HGNC Symbol;Acc:HGNC:9056] |
| ENSG00000151413 | HGLibA_32906 | 41 | -0.740609424 | NUBPL | nucleotide binding protein like [Source:HGNC Symbol;Acc:HGNC:20278] |
| ENSG00000151849 | HGLibA_09063 | 41 | -0.740609424 | CENPJ | centromere protein J [Source:HGNC Symbol;Acc:HGNC:17272] |
| ENSG00000152315 | HGLibA_24302 | 41 | -0.740609424 | KCNK13 | potassium two pore domain channel subfamily K member 13 [Source:HGNC Symbol;Acc:HGNC:6275] |
| ENSG00000152672 | HGLibA_09990 | 41 | -0.740609424 | CLEC4F | C-type lectin domain family 4 member F [Source:HGNC Symbol;Acc:HGNC:25357] |
| ENSG00000152784 | HGLibA_38402 | 41 | -0.740609424 | PRDM8 | PR/SET domain 8 [Source:HGNC Symbol;Acc:HGNC:13993] |
| ENSG00000153015 | HGLibA_11821 | 41 | -0.740609424 | CWC27 | CWC27 spliceosome associated protein homolog [Source:HGNC Symbol;Acc:HGNC:10664] |
| ENSG00000155158 | HGLibA_52134 | 41 | -0.740609424 | TTC39B | tetratricopeptide repeat domain 39B [Source:HGNC Symbol;Acc:HGNC:23704] |
| ENSG00000156931 | HGLibA_53818 | 41 | -0.740609424 | VPS8 | CORVET complex subunit [Source:HGNC Symbol;Acc:HGNC:29122]" |
| ENSG00000156959 | HGLibA_26438 | 41 | -0.740609424 | LHFPL4 | LHFPL tetraspan subfamily member 4 [Source:HGNC Symbol;Acc:HGNC:29568] |

| | | | | | |
|---|---|---|---|---|---|
| ENSG00000157837 | HGLibA_46785 | 41 | -0.740609424 | SPPL3 | signal peptide peptidase like 3 [Source:HGNC Symbol;Acc:HGNC:30424] |
| ENSG00000158828 | HGLibA_36689 | 41 | -0.740609424 | PINK1 | PTEN induced kinase 1 [Source:HGNC Symbol;Acc:HGNC:14581] |
| ENSG00000159055 | HGLibA_29307 | 41 | -0.740609424 | MIS18A | MIS18 kinetochore protein A [Source:HGNC Symbol;Acc:HGNC:1286] |
| ENSG00000159166 | HGLibA_25924 | 41 | -0.740609424 | LAD1 | ladinin 1 [Source:HGNC Symbol;Acc:HGNC:6472] |
| ENSG00000159202 | HGLibA_52674 | 41 | -0.740609424 | UBE2Z | ubiquitin conjugating enzyme E2 Z [Source:HGNC Symbol;Acc:HGNC:25847] |
| ENSG00000159579 | HGLibA_42355 | 41 | -0.740609424 | RSPRY1 | ring finger and SPRY domain containing 1 [Source:HGNC Symbol;Acc:HGNC:29420] |
| ENSG00000160188 | HGLibA_42324 | 41 | -0.740609424 | RSPH1 | radial spoke head component 1 [Source:HGNC Symbol;Acc:HGNC:12371] |
| ENSG00000160460 | HGLibA_46886 | 41 | -0.740609424 | SPTBN4 | non-erythrocytic 4 [Source:HGNC Symbol;Acc:HGNC:14896]" |
| ENSG00000160460 | HGLibA_46885 | 41 | -0.740609424 | SPTBN4 | non-erythrocytic 4 [Source:HGNC Symbol;Acc:HGNC:14896]" |
| ENSG00000161533 | HGLibA_00498 | 41 | -0.740609424 | ACOX1 | acyl-CoA oxidase 1 [Source:HGNC Symbol;Acc:HGNC:119] |
| ENSG00000162066 | HGLibA_01764 | 41 | -0.740609424 | AMDHD2 | amidohydrolase domain containing 2 [Source:HGNC Symbol;Acc:HGNC:24262] |
| ENSG00000162068 | HGLibA_32856 | 41 | -0.740609424 | NTN3 | netrin 3 [Source:HGNC Symbol;Acc:HGNC:8030] |
| ENSG00000162341 | HGLibA_51109 | 41 | -0.740609424 | TPCN2 | two pore segment channel 2 [Source:HGNC Symbol;Acc:HGNC:20820] |
| ENSG00000162511 | HGLibA_26021 | 41 | -0.740609424 | LAPTM5 | lysosomal protein transmembrane 5 [Source:HGNC Symbol;Acc:HGNC:29612] |
| ENSG00000162542 | HGLibA_49791 | 41 | -0.740609424 | TMCO4 | transmembrane and coiled-coil domains 4 [Source:HGNC Symbol;Acc:HGNC:27393] |
| ENSG00000163251 | HGLibA_18276 | 41 | -0.740609424 | FZD5 | frizzled class receptor 5 [Source:HGNC Symbol;Acc:HGNC:4043] |
| ENSG00000164266 | HGLibA_46683 | 41 | -0.740609424 | SPINK1 | Kazal type 1 [Source:HGNC Symbol;Acc:HGNC:11244]" |
| ENSG00000164609 | HGLibA_45550 | 41 | -0.740609424 | SLU7 | splicing factor [Source:HGNC Symbol;Acc:HGNC:16939]" |
| ENSG00000164649 | HGLibA_08617 | 41 | -0.740609424 | CDCA7L | cell division cycle associated 7 like [Source:HGNC Symbol;Acc:HGNC:30777] |
| ENSG00000165271 | HGLibA_32271 | 41 | -0.740609424 | NOL6 | nucleolar protein 6 [Source:HGNC Symbol;Acc:HGNC:19910] |
| ENSG00000165487 | HGLibA_29225 | 41 | -0.740609424 | MICU2 | mitochondrial calcium uptake 2 [Source:HGNC Symbol;Acc:HGNC:31830] |
| ENSG00000165828 | HGLibA_38335 | 41 | -0.740609424 | PRAP1 | proline rich acidic protein 1 [Source:HGNC Symbol;Acc:HGNC:23304] |
| ENSG00000165916 | HGLibA_39134 | 41 | -0.740609424 | PSMC3 | ATPase 3 [Source:HGNC Symbol;Acc:HGNC:9549]" |
| ENSG00000166148 | HGLibA_03904 | 41 | -0.740609424 | AVPR1A | arginine vasopressin receptor 1A [Source:HGNC Symbol;Acc:HGNC:895] |
| ENSG00000166920 | HGLibA_05406 | 41 | -0.740609424 | C15orf48 | chromosome 15 open reading frame 48 [Source:HGNC Symbol;Acc:HGNC:29898] |
| ENSG00000167858 | HGLibA_48971 | 41 | -0.740609424 | TEKT1 | tektin 1 [Source:HGNC Symbol;Acc:HGNC:15534] |
| ENSG00000168118 | HGLibA_39956 | 41 | -0.740609424 | RAB4A | member RAS oncogene family [Source:HGNC Symbol;Acc:HGNC:9781]" |
| ENSG00000168118 | HGLibA_39955 | 41 | -0.740609424 | RAB4A | member RAS oncogene family [Source:HGNC Symbol;Acc:HGNC:9781]" |
| ENSG00000168461 | HGLibA_39874 | 41 | -0.740609424 | RAB31 | member RAS oncogene family [Source:HGNC Symbol;Acc:HGNC:9771]" |
| ENSG00000171060 | HGLibA_06643 | 41 | -0.740609424 | C8orf74 | chromosome 8 open reading frame 74 [Source:HGNC Symbol;Acc:HGNC:32296] |
| ENSG00000171116 | HGLibA_22234 | 41 | -0.740609424 | HSFX1 | X-linked 1 [Source:HGNC Symbol;Acc:HGNC:29603]" |
| ENSG00000171246 | HGLibA_32531 | 41 | -0.740609424 | NPTX1 | neuronal pentraxin 1 [Source:HGNC Symbol;Acc:HGNC:7952] |
| ENSG00000171408 | HGLibA_35827 | 41 | -0.740609424 | PDE7B | phosphodiesterase 7B [Source:HGNC Symbol;Acc:HGNC:8792] |
| ENSG00000172399 | HGLibA_30903 | 41 | -0.740609424 | MYOZ2 | myozenin 2 [Source:HGNC Symbol;Acc:HGNC:1330] |
| ENSG00000172943 | HGLibA_36391 | 41 | -0.740609424 | PHF8 | PHD finger protein 8 [Source:HGNC Symbol;Acc:HGNC:20672] |
| ENSG00000173065 | HGLibA_16524 | 41 | -0.740609424 | FAM222B | family with sequence similarity 222 member B [Source:HGNC Symbol;Acc:HGNC:25563] |
| ENSG00000173080 | HGLibA_42503 | 41 | -0.740609424 | RXFP4 | relaxin family peptide/INSL5 receptor 4 [Source:HGNC Symbol;Acc:HGNC:14666] |
| ENSG00000173272 | HGLibA_30943 | 41 | -0.740609424 | MZT2A | mitotic spindle organizing protein 2A [Source:HGNC Symbol;Acc:HGNC:33187] |
| ENSG00000173406 | HGLibA_12265 | 41 | -0.740609424 | DAB1 | reelin adaptor protein [Source:HGNC Symbol;Acc:HGNC:2661]" |
| ENSG00000173545 | HGLibA_56409 | 41 | -0.740609424 | ZNF622 | zinc finger protein 622 [Source:HGNC Symbol;Acc:HGNC:30958] |
| ENSG00000173626 | HGLibA_51321 | 41 | -0.740609424 | TRAPPC3L | trafficking protein particle complex 3 like [Source:HGNC Symbol;Acc:HGNC:21090] |
| ENSG00000174564 | HGLibA_23079 | 41 | -0.740609424 | IL20RB | interleukin 20 receptor subunit beta [Source:HGNC Symbol;Acc:HGNC:6004] |
| ENSG00000174600 | HGLibA_10197 | 41 | -0.740609424 | CMKLR1 | chemerin chemokine-like receptor 1 [Source:HGNC Symbol;Acc:HGNC:2121] |
| ENSG00000176200 | HGLibA_33971 | 41 | -0.740609424 | OR4D11 | olfactory receptor family 4 subfamily D member 11 [Source:HGNC Symbol;Acc:HGNC:15174] |
| ENSG00000176783 | HGLibA_42438 | 41 | -0.740609424 | RUFY1 | RUN and FYVE domain containing 1 [Source:HGNC Symbol;Acc:HGNC:19760] |
| ENSG00000179826 | HGLibA_29851 | 41 | -0.740609424 | MRGPRX3 | MAS related GPR family member X3 [Source:HGNC Symbol;Acc:HGNC:17980] |
| ENSG00000180116 | HGLibA_05239 | 41 | -0.740609424 | C12orf40 | chromosome 12 open reading frame 40 [Source:HGNC Symbol;Acc:HGNC:26846] |
| ENSG00000180616 | HGLibA_47178 | 41 | -0.740609424 | SSTR2 | somatostatin receptor 2 [Source:HGNC Symbol;Acc:HGNC:11331] |
| ENSG00000180694 | HGLibA_50482 | 41 | -0.740609424 | TMEM64 | transmembrane protein 64 [Source:HGNC Symbol;Acc:HGNC:25441] |
| ENSG00000180998 | HGLibA_19904 | 41 | -0.740609424 | GPR137C | G protein-coupled receptor 137C [Source:HGNC Symbol;Acc:HGNC:25445] |
| ENSG00000181733 | HGLibA_33911 | 41 | -0.740609424 | OR2Z1 | olfactory receptor family 2 subfamily Z member 1 [Source:HGNC Symbol;Acc:HGNC:15391] |
| ENSG00000182185 | HGLibA_40088 | 41 | -0.740609424 | RAD51B | RAD51 paralog B [Source:HGNC Symbol;Acc:HGNC:9822] |
| ENSG00000182521 | HGLibA_48630 | 41 | -0.740609424 | TBPL2 | TATA-box binding protein like 2 [Source:HGNC Symbol;Acc:HGNC:19841] |
| ENSG00000183067 | HGLibA_22896 | 41 | -0.740609424 | IGSF5 | immunoglobulin superfamily member 5 [Source:HGNC Symbol;Acc:HGNC:5952] |
| ENSG00000183258 | HGLibA_12710 | 41 | -0.740609424 | DDX41 | DEAD-box helicase 41 [Source:HGNC Symbol;Acc:HGNC:18674] |
| ENSG00000183733 | HGLibA_17515 | 41 | -0.740609424 | FIGLA | folliculogenesis specific bHLH transcription factor [Source:HGNC Symbol;Acc:HGNC:24669] |
| ENSG00000184445 | HGLibA_25352 | 41 | -0.740609424 | KNTC1 | kinetochore associated 1 [Source:HGNC Symbol;Acc:HGNC:17255] |
| ENSG00000184787 | HGLibA_52609 | 41 | -0.740609424 | UBE2G2 | ubiquitin conjugating enzyme E2 G2 [Source:HGNC Symbol;Acc:HGNC:12483] |
| ENSG00000185215 | HGLibA_50708 | 41 | -0.740609424 | TNFAIP2 | TNF alpha induced protein 2 [Source:HGNC Symbol;Acc:HGNC:11895] |
| ENSG00000185619 | HGLibA_35598 | 41 | -0.740609424 | PCGF3 | polycomb group ring finger 3 [Source:HGNC Symbol;Acc:HGNC:10066] |
| ENSG00000186204 | HGLibA_12185 | 41 | -0.740609424 | CYP4F12 | cytochrome P450 family 4 subfamily F member 12 [Source:HGNC Symbol;Acc:HGNC:18857] |
| ENSG00000186792 | HGLibA_22444 | 41 | -0.740609424 | HYAL3 | hyaluronidase 3 [Source:HGNC Symbol;Acc:HGNC:5322] |
| ENSG00000187243 | HGLibA_27976 | 41 | -0.740609424 | MAGED4B | MAGE family member D4B [Source:HGNC Symbol;Acc:HGNC:22880] |
| ENSG00000187498 | HGLibA_10566 | 41 | -0.740609424 | COL4A1 | collagen type IV alpha 1 chain [Source:HGNC Symbol;Acc:HGNC:2202] |
| ENSG00000187730 | HGLibA_18383 | 41 | -0.740609424 | GABRD | gamma-aminobutyric acid type A receptor delta subunit [Source:HGNC Symbol;Acc:HGNC:4084] |
| ENSG00000188010 | HGLibA_29670 | 41 | -0.740609424 | MORN2 | MORN repeat containing 2 [Source:HGNC Symbol;Acc:HGNC:30166] |
| ENSG00000188408 | HGLibA_27953 | 41 | -0.740609424 | MAGEB5 | MAGE family member B5 [Source:HGNC Symbol;Acc:HGNC:23795] |
| ENSG00000188828 | HGLibA_19307 | 41 | -0.740609424 | GLRA4 | glycine receptor alpha 4 [Source:HGNC Symbol;Acc:HGNC:31715] |
| ENSG00000188909 | HGLibA_04886 | 41 | -0.740609424 | BSX | brain specific homeobox [Source:HGNC Symbol;Acc:HGNC:20450] |
| ENSG00000189023 | HGLibA_27935 | 41 | -0.740609424 | MAGEB16 | MAGE family member B16 [Source:HGNC Symbol;Acc:HGNC:21188] |
| ENSG00000189108 | HGLibA_23059 | 41 | -0.740609424 | IL1RAPL2 | interleukin 1 receptor accessory protein like 2 [Source:HGNC Symbol;Acc:HGNC:5997] |
| ENSG00000197020 | HGLibA_55431 | 41 | -0.740609424 | ZNF100 | zinc finger protein 100 [Source:HGNC Symbol;Acc:HGNC:12880] |
| ENSG00000197121 | HGLibA_36206 | 41 | -0.740609424 | PGAP1 | post-GPI attachment to proteins 1 [Source:HGNC Symbol;Acc:HGNC:25712] |
| ENSG00000197591 | HGLibA_33584 | 41 | -0.740609424 | OR11L1 | olfactory receptor family 11 subfamily L member 1 [Source:HGNC Symbol;Acc:HGNC:14998] |
| ENSG00000197601 | HGLibA_16868 | 41 | -0.740609424 | FAR1 | fatty acyl-CoA reductase 1 [Source:HGNC Symbol;Acc:HGNC:26222] |
| ENSG00000198276 | HGLibA_52815 | 41 | -0.740609424 | UCKL1 | uridine-cytidine kinase 1 like 1 [Source:HGNC Symbol;Acc:HGNC:15938] |
| ENSG00000198326 | HGLibA_50283 | 41 | -0.740609424 | TMEM239 | transmembrane protein 239 [Source:HGNC Symbol;Acc:HGNC:40044] |
| ENSG00000198373 | HGLibA_54454 | 41 | -0.740609424 | WWP2 | WW domain containing E3 ubiquitin protein ligase 2 [Source:HGNC Symbol;Acc:HGNC:16804] |
| ENSG00000198574 | HGLibA_43887 | 41 | -0.740609424 | SH2D1B | SH2 domain containing 1B [Source:HGNC Symbol;Acc:HGNC:30416] |
| ENSG00000198721 | HGLibA_14409 | 41 | -0.740609424 | ECI2 | enoyl-CoA delta isomerase 2 [Source:HGNC Symbol;Acc:HGNC:14601] |
| ENSG00000198833 | HGLibA_52618 | 41 | -0.740609424 | UBE2J1 | ubiquitin conjugating enzyme E2 J1 [Source:HGNC Symbol;Acc:HGNC:17598] |
| ENSG00000198932 | HGLibA_20118 | 41 | -0.740609424 | GPRASP1 | G protein-coupled receptor associated sorting protein 1 [Source:HGNC Symbol;Acc:HGNC:24834] |
| ENSG00000198961 | HGLibA_36785 | 41 | -0.740609424 | PJA2 | praja ring finger ubiquitin ligase 2 [Source:HGNC Symbol;Acc:HGNC:17481] |
| ENSG00000204568 | HGLibA_30052 | 41 | -0.740609424 | MRPS18B | mitochondrial ribosomal protein S18B [Source:HGNC Symbol;Acc:HGNC:14516] |
| ENSG00000204918 | HGLibA_38802 | 41 | -0.740609424 | PRR20B | proline rich 20B [Source:HGNC Symbol;Acc:HGNC:37220] |
| ENSG00000205029 | HGLibA_34280 | 41 | -0.740609424 | OR5D16 | olfactory receptor family 5 subfamily D member 16 [Source:HGNC Symbol;Acc:HGNC:15283] |
| ENSG00000205549 | HGLibA_06780 | 41 | -0.740609424 | C9orf92 | chromosome 9 open reading frame 92 [Source:HGNC Symbol;Acc:HGNC:19054] |
| ENSG00000205858 | HGLibA_27423 | 41 | -0.740609424 | LRRC72 | leucine rich repeat containing 72 [Source:HGNC Symbol;Acc:HGNC:42972] |
| ENSG00000212724 | HGLibA_25693 | 41 | -0.740609424 | KRTAP2-3 | keratin associated protein 2-3 [Source:HGNC Symbol;Acc:HGNC:18906] |
| ENSG00000213588 | HGLibA_54933 | 41 | -0.740609424 | ZBTB9 | zinc finger and BTB domain containing 9 [Source:HGNC Symbol;Acc:HGNC:28323] |
| ENSG00000213593 | HGLibA_50692 | 41 | -0.740609424 | TMX2 | thioredoxin related transmembrane protein 2 [Source:HGNC Symbol;Acc:HGNC:30739] |
| ENSG00000213799 | HGLibA_56863 | 41 | -0.740609424 | ZNF845 | zinc finger protein 845 [Source:HGNC Symbol;Acc:HGNC:25112] |
| ENSG00000225327 | HGLibA_53272 | 41 | -0.740609424 | USP17L3 | ubiquitin specific peptidase 17-like family member 3 [Source:HGNC Symbol;Acc:HGNC:37175] |
| ENSG00000230937 | HGLibA_29300 | 41 | -0.740609424 | MIR205HG | MIR205 host gene [Source:HGNC Symbol;Acc:HGNC:43562] |
| ENSG00000231925 | HGLibA_48321 | 41 | -0.740609424 | TAPBP | TAP binding protein [Source:HGNC Symbol;Acc:HGNC:11566] |
| ENSG00000234444 | HGLibA_56653 | 41 | -0.740609424 | ZNF736 | zinc finger protein 736 [Source:HGNC Symbol;Acc:HGNC:32467] |
| ENSG00000234745 | HGLibA_21598 | 41 | -0.740609424 | HLA-B | B [Source:HGNC Symbol;Acc:HGNC:4932]" |
| ENSG00000238083 | HGLibA_27330 | 41 | -0.740609424 | LRRC37A2 | leucine rich repeat containing 37 member A2 [Source:HGNC Symbol;Acc:HGNC:32404] |
| ENSG00000240053 | HGLibA_27659 | 41 | -0.740609424 | LY6G5B | lymphocyte antigen 6 family member G5B [Source:HGNC Symbol;Acc:HGNC:13931] |
| ENSG00000240771 | HGLibA_02836 | 41 | -0.740609424 | ARHGEF25 | Rho guanine nucleotide exchange factor 25 [Source:HGNC Symbol;Acc:HGNC:30275] |
| ENSG00000241322 | HGLibA_08873 | 41 | -0.740609424 | CDRT1 | CMT1A duplicated region transcript 1 [Source:HGNC Symbol;Acc:HGNC:14379] |
| ENSG00000244057 | HGLibA_26137 | 41 | -0.740609424 | LCE3C | late cornified envelope 3C [Source:HGNC Symbol;Acc:HGNC:16612] |
| ENSG00000248485 | HGLibA_35663 | 41 | -0.740609424 | PCP4L1 | Purkinje cell protein 4 like 1 [Source:HGNC Symbol;Acc:HGNC:20448] |
| ENSG00000256294 | HGLibA_55621 | 41 | -0.740609424 | ZNF225 | zinc finger protein 225 [Source:HGNC Symbol;Acc:HGNC:13018] |
| ENSG00000260903 | HGLibA_54528 | 41 | -0.740609424 | XKR7 | XK related 7 [Source:HGNC Symbol;Acc:HGNC:23062] |
| ENSG00000274349 | HGLibA_56473 | 41 | -0.740609424 | ZNF658 | zinc finger protein 658 [Source:HGNC Symbol;Acc:HGNC:25226] |
| ENSG00000279395 | HGLibA_34321 | 41 | -0.740609424 | OR5L1 | olfactory receptor family 5 subfamily L member 1 (gene/pseudogene) [Source:HGNC Symbol;Acc:HGNC:8350] |
| ENSG00000288867 | HGLibA_37904 | 41 | -0.740609424 | PPIAL4C | - |
| FLJ27352 | HGLibA_17628 | 41 | -0.740609424 | FLJ27352 | - |
| GAGE6 | HGLibA_18485 | 41 | -0.740609424 | GAGE6 | - |
| GPR64 | HGLibA_20072 | 41 | -0.740609424 | GPR64 | - |
| hsa-mir-1203 | HGLibA_57270 | 41 | -0.740609424 | hsa-mir-1203 | - |
| hsa-mir-1233-2 | HGLibA_57325 | 41 | -0.740609424 | hsa-mir-1233-2 | - |
| hsa-mir-1285-2 | HGLibA_57606 | 41 | -0.740609424 | hsa-mir-1285-2 | - |
| hsa-mir-1287 | HGLibA_57611 | 41 | -0.740609424 | hsa-mir-1287 | - |
| hsa-mir-1295a | HGLibA_57655 | 41 | -0.740609424 | hsa-mir-1295a | - |
| hsa-mir-1976 | HGLibA_58120 | 41 | -0.740609424 | hsa-mir-1976 | - |
| hsa-mir-208a | HGLibA_58196 | 41 | -0.740609424 | hsa-mir-208a | - |
| hsa-mir-23b | HGLibA_58331 | 41 | -0.740609424 | hsa-mir-23b | - |
| hsa-mir-299 | HGLibA_58404 | 41 | -0.740609424 | hsa-mir-299 | - |
| hsa-mir-3155b | HGLibA_58684 | 41 | -0.740609424 | hsa-mir-3155b | - |
| hsa-mir-3156-3 | HGLibA_58696 | 41 | -0.740609424 | hsa-mir-3156-3 | - |
| hsa-mir-3614 | HGLibA_59084 | 41 | -0.740609424 | hsa-mir-3614 | - |
| hsa-mir-4423 | HGLibA_60078 | 41 | -0.740609424 | hsa-mir-4423 | - |
| hsa-mir-4436a | HGLibA_60137 | 41 | -0.740609424 | hsa-mir-4436a | - |
| hsa-mir-4500 | HGLibA_60429 | 41 | -0.740609424 | hsa-mir-4500 | - |
| hsa-mir-4638 | HGLibA_60658 | 41 | -0.740609424 | hsa-mir-4638 | - |
| hsa-mir-4733 | HGLibA_61028 | 41 | -0.740609424 | hsa-mir-4733 | - |
| hsa-mir-4785 | HGLibA_61246 | 41 | -0.740609424 | hsa-mir-4785 | - |
| hsa-mir-548aw | HGLibA_61936 | 41 | -0.740609424 | hsa-mir-548aw | - |
| hsa-mir-548az | HGLibA_61948 | 41 | -0.740609424 | hsa-mir-548az | - |
| hsa-mir-550b-2 | HGLibA_62115 | 41 | -0.740609424 | hsa-mir-550b-2 | - |
| hsa-mir-5586 | HGLibA_62185 | 41 | -0.740609424 | hsa-mir-5586 | - |
| hsa-mir-5589 | HGLibA_62196 | 41 | -0.740609424 | hsa-mir-5589 | - |
| hsa-mir-578 | HGLibA_62406 | 41 | -0.740609424 | hsa-mir-578 | - |

| | | | | | |
|---|---|---|---|---|---|
| hsa-mir-588 | HGLibA_62449 | 41 | -0.740609424 | hsa-mir-588 | - |
| hsa-mir-6125 | HGLibA_62646 | 41 | -0.740609424 | hsa-mir-6125 | - |
| hsa-mir-651 | HGLibA_62890 | 41 | -0.740609424 | hsa-mir-651 | - |
| hsa-mir-6743 | HGLibA_63125 | 41 | -0.740609424 | hsa-mir-6743 | - |
| hsa-mir-6768 | HGLibA_63235 | 41 | -0.740609424 | hsa-mir-6768 | - |
| hsa-mir-6776 | HGLibA_63278 | 41 | -0.740609424 | hsa-mir-6776 | - |
| hsa-mir-6796 | HGLibA_63362 | 41 | -0.740609424 | hsa-mir-6796 | - |
| hsa-mir-6828 | HGLibA_63489 | 41 | -0.740609424 | hsa-mir-6828 | - |
| hsa-mir-6871 | HGLibA_63668 | 41 | -0.740609424 | hsa-mir-6871 | - |
| KIAA1024 | HGLibA_24678 | 41 | -0.740609424 | KIAA1024 | - |
| LOC147646 | HGLibA_26888 | 41 | -0.740609424 | LOC147646 | - |
| METTL12 | HGLibA_28953 | 41 | -0.740609424 | METTL12 | - |
| NAPRT1 | HGLibA_31136 | 41 | -0.740609424 | NAPRT1 | - |
| PRSS42 | HGLibA_38942 | 41 | -0.740609424 | PRSS42 | - |
| PVRL2 | HGLibA_39625 | 41 | -0.740609424 | PVRL2 | - |
| RNMTL1 | HGLibA_41720 | 41 | -0.740609424 | RNMTL1 | - |
| BAI2 | HGLibA_04116 | 40 | -0.750560462 | BAI2 | - |
| C16orf11 | HGLibA_05439 | 40 | -0.750560462 | C16orf11 | - |
| C16orf93 | HGLibA_05505 | 40 | -0.750560462 | C16orf93 | - |
| C8orf37 | HGLibA_06614 | 40 | -0.750560462 | C8orf37 | - |
| CSF2RA_Y | HGLibA_11333 | 40 | -0.750560462 | CSF2RA_Y | - |
| CXXC11 | HGLibA_11968 | 40 | -0.750560462 | CXXC11 | - |
| ENSG00000005469 | HGLibA_11214 | 40 | -0.750560462 | CROT | carnitine O-octanoyltransferase [Source:HGNC Symbol;Acc:HGNC:2366] |
| ENSG00000006712 | HGLibA_34988 | 40 | -0.750560462 | PAF1 | Paf1/RNA polymerase II complex component [Source:HGNC Symbol;Acc:HGNC:25459]" |
| ENSG00000012822 | HGLibA_06999 | 40 | -0.750560462 | CALCOCO1 | calcium binding and coiled-coil domain 1 [Source:HGNC Symbol;Acc:HGNC:29306] |
| ENSG00000014824 | HGLibA_44924 | 40 | -0.750560462 | SLC30A9 | solute carrier family 30 member 9 [Source:HGNC Symbol;Acc:HGNC:1329] |
| ENSG00000023516 | HGLibA_01410 | 40 | -0.750560462 | AKAP11 | A-kinase anchoring protein 11 [Source:HGNC Symbol;Acc:HGNC:369] |
| ENSG00000023734 | HGLibA_47578 | 40 | -0.750560462 | STRAP | serine/threonine kinase receptor associated protein [Source:HGNC Symbol;Acc:HGNC:30796] |
| ENSG00000037042 | HGLibA_52305 | 40 | -0.750560462 | TUBG2 | tubulin gamma 2 [Source:HGNC Symbol;Acc:HGNC:12419] |
| ENSG00000038219 | HGLibA_04670 | 40 | -0.750560462 | BOD1L1 | biorientation of chromosomes in cell division 1 like 1 [Source:HGNC Symbol;Acc:HGNC:31792] |
| ENSG00000048392 | HGLibA_42258 | 40 | -0.750560462 | RRM2B | ribonucleotide reductase regulatory TP53 inducible subunit M2B [Source:HGNC Symbol;Acc:HGNC:17296] |
| ENSG00000048540 | HGLibA_26709 | 40 | -0.750560462 | LMO3 | LIM domain only 3 [Source:HGNC Symbol;Acc:HGNC:6643] |
| ENSG00000056736 | HGLibA_23008 | 40 | -0.750560462 | IL17RB | interleukin 17 receptor B [Source:HGNC Symbol;Acc:HGNC:18015] |
| ENSG00000064999 | HGLibA_02151 | 40 | -0.750560462 | ANKS1A | ankyrin repeat and sterile alpha motif domain containing 1A [Source:HGNC Symbol;Acc:HGNC:20961] |
| ENSG00000068323 | HGLibA_49159 | 40 | -0.750560462 | TFE3 | transcription factor binding to IGHM enhancer 3 [Source:HGNC Symbol;Acc:HGNC:11752] |
| ENSG00000068615 | HGLibA_40775 | 40 | -0.750560462 | REEP1 | receptor accessory protein 1 [Source:HGNC Symbol;Acc:HGNC:25786] |
| ENSG00000070366 | HGLibA_45703 | 40 | -0.750560462 | SMG6 | nonsense mediated mRNA decay factor [Source:HGNC Symbol;Acc:HGNC:17809]" |
| ENSG00000070614 | HGLibA_31426 | 40 | -0.750560462 | NDST1 | N-deacetylase and N-sulfotransferase 1 [Source:HGNC Symbol;Acc:HGNC:7680] |
| ENSG00000072121 | HGLibA_55284 | 40 | -0.750560462 | ZFYVE26 | zinc finger FYVE-type containing 26 [Source:HGNC Symbol;Acc:HGNC:20761] |
| ENSG00000072818 | HGLibA_00384 | 40 | -0.750560462 | ACAP1 | ankyrin repeat and PH domains 1 [Source:HGNC Symbol;Acc:HGNC:16467]" |
| ENSG00000077264 | HGLibA_35047 | 40 | -0.750560462 | PAK3 | p21 (RAC1) activated kinase 3 [Source:HGNC Symbol;Acc:HGNC:8592] |
| ENSG00000078177 | HGLibA_30953 | 40 | -0.750560462 | N4BP2 | NEDD4 binding protein 2 [Source:HGNC Symbol;Acc:HGNC:29851] |
| ENSG00000081692 | HGLibA_23954 | 40 | -0.750560462 | JMJD4 | jumonji domain containing 4 [Source:HGNC Symbol;Acc:HGNC:25724] |
| ENSG00000082516 | HGLibA_18916 | 40 | -0.750560462 | GEMIN5 | gem nuclear organelle associated protein 5 [Source:HGNC Symbol;Acc:HGNC:20043] |
| ENSG00000089053 | HGLibA_01886 | 40 | -0.750560462 | ANAPC5 | anaphase promoting complex subunit 5 [Source:HGNC Symbol;Acc:HGNC:15713] |
| ENSG00000090376 | HGLibA_23562 | 40 | -0.750560462 | IRAK3 | interleukin 1 receptor associated kinase 3 [Source:HGNC Symbol;Acc:HGNC:17020] |
| ENSG00000095485 | HGLibA_11823 | 40 | -0.750560462 | CWF19L1 | CWF19 like cell cycle control factor 1 [Source:HGNC Symbol;Acc:HGNC:25613] |
| ENSG00000095596 | HGLibA_12086 | 40 | -0.750560462 | CYP26A1 | cytochrome P450 family 26 subfamily A member 1 [Source:HGNC Symbol;Acc:HGNC:2603] |
| ENSG00000099204 | HGLibA_00307 | 40 | -0.750560462 | ABLIM1 | actin binding LIM protein 1 [Source:HGNC Symbol;Acc:HGNC:78] |
| ENSG00000100106 | HGLibA_51629 | 40 | -0.750560462 | TRIOBP | TRIO and F-actin binding protein [Source:HGNC Symbol;Acc:HGNC:17009] |
| ENSG00000100298 | HGLibA_02493 | 40 | -0.750560462 | APOBEC3H | apolipoprotein B mRNA editing enzyme catalytic subunit 3H [Source:HGNC Symbol;Acc:HGNC:24100] |
| ENSG00000100564 | HGLibA_36559 | 40 | -0.750560462 | PIGH | phosphatidylinositol glycan anchor biosynthesis class H [Source:HGNC Symbol;Acc:HGNC:8964] |
| ENSG00000101323 | HGLibA_20831 | 40 | -0.750560462 | HAO1 | hydroxyacid oxidase 1 [Source:HGNC Symbol;Acc:HGNC:4809] |
| ENSG00000102021 | HGLibA_27645 | 40 | -0.750560462 | LUZP4 | leucine zipper protein 4 [Source:HGNC Symbol;Acc:HGNC:24971] |
| ENSG00000102753 | HGLibA_25360 | 40 | -0.750560462 | KPNA3 | karyopherin subunit alpha 3 [Source:HGNC Symbol;Acc:HGNC:6396] |
| ENSG00000102802 | HGLibA_28823 | 40 | -0.750560462 | MEDAG | mesenteric estrogen dependent adipogenesis [Source:HGNC Symbol;Acc:HGNC:25926] |
| ENSG00000102805 | HGLibA_10078 | 40 | -0.750560462 | CLN5 | intracellular trafficking protein [Source:HGNC Symbol;Acc:HGNC:2076]" |
| ENSG00000103710 | HGLibA_40372 | 40 | -0.750560462 | RASL12 | RAS like family 12 [Source:HGNC Symbol;Acc:HGNC:30289] |
| ENSG00000104879 | HGLibA_09774 | 40 | -0.750560462 | CKM | M-type [Source:HGNC Symbol;Acc:HGNC:1994]" |
| ENSG00000105176 | HGLibA_53145 | 40 | -0.750560462 | URI1 | prefoldin like chaperone [Source:HGNC Symbol;Acc:HGNC:13236]" |
| ENSG00000105221 | HGLibA_01512 | 40 | -0.750560462 | AKT2 | AKT serine/threonine kinase 2 [Source:HGNC Symbol;Acc:HGNC:392] |
| ENSG00000105281 | HGLibA_44519 | 40 | -0.750560462 | SLC1A5 | solute carrier family 1 member 5 [Source:HGNC Symbol;Acc:HGNC:10943] |
| ENSG00000105618 | HGLibA_38718 | 40 | -0.750560462 | PRPF31 | pre-mRNA processing factor 31 [Source:HGNC Symbol;Acc:HGNC:15446] |
| ENSG00000105618 | HGLibA_38720 | 40 | -0.750560462 | PRPF31 | pre-mRNA processing factor 31 [Source:HGNC Symbol;Acc:HGNC:15446] |
| ENSG00000105722 | HGLibA_15462 | 40 | -0.750560462 | ERF | ETS2 repressor factor [Source:HGNC Symbol;Acc:HGNC:3444] |
| ENSG00000106144 | HGLibA_07307 | 40 | -0.750560462 | CASP2 | caspase 2 [Source:HGNC Symbol;Acc:HGNC:1503] |
| ENSG00000109158 | HGLibA_18366 | 40 | -0.750560462 | GABRA4 | gamma-aminobutyric acid type A receptor alpha4 subunit [Source:HGNC Symbol;Acc:HGNC:4078] |
| ENSG00000109424 | HGLibA_52830 | 40 | -0.750560462 | UCP1 | uncoupling protein 1 [Source:HGNC Symbol;Acc:HGNC:12517] |
| ENSG00000110717 | HGLibA_31565 | 40 | -0.750560462 | NDUFS8 | NADH:ubiquinone oxidoreductase core subunit S8 [Source:HGNC Symbol;Acc:HGNC:7715] |
| ENSG00000111644 | HGLibA_00533 | 40 | -0.750560462 | ACRBP | acrosin binding protein [Source:HGNC Symbol;Acc:HGNC:17195] |
| ENSG00000112290 | HGLibA_53954 | 40 | -0.750560462 | WASF1 | WAS protein family member 1 [Source:HGNC Symbol;Acc:HGNC:12732] |
| ENSG00000112695 | HGLibA_10859 | 40 | -0.750560462 | COX7A2 | cytochrome c oxidase subunit 7A2 [Source:HGNC Symbol;Acc:HGNC:2288] |
| ENSG00000112981 | HGLibA_32166 | 40 | -0.750560462 | NME5 | NME/NM23 family member 5 [Source:HGNC Symbol;Acc:HGNC:7853] |
| ENSG00000114744 | HGLibA_10655 | 40 | -0.750560462 | COMMD2 | COMM domain containing 2 [Source:HGNC Symbol;Acc:HGNC:24993] |
| ENSG00000115138 | HGLibA_37654 | 40 | -0.750560462 | POMC | proopiomelanocortin [Source:HGNC Symbol;Acc:HGNC:9201] |
| ENSG00000115353 | HGLibA_48171 | 40 | -0.750560462 | TACR1 | tachykinin receptor 1 [Source:HGNC Symbol;Acc:HGNC:11526] |
| ENSG00000115423 | HGLibA_13493 | 40 | -0.750560462 | DNAH6 | dynein axonemal heavy chain 6 [Source:HGNC Symbol;Acc:HGNC:2951] |
| ENSG00000115464 | HGLibA_53341 | 40 | -0.750560462 | USP34 | ubiquitin specific peptidase 34 [Source:HGNC Symbol;Acc:HGNC:20066] |
| ENSG00000116157 | HGLibA_20188 | 40 | -0.750560462 | GPX7 | glutathione peroxidase 7 [Source:HGNC Symbol;Acc:HGNC:4559] |
| ENSG00000116353 | HGLibA_28730 | 40 | -0.750560462 | MECR | mitochondrial trans-2-enoyl-CoA reductase [Source:HGNC Symbol;Acc:HGNC:19691] |
| ENSG00000116704 | HGLibA_44977 | 40 | -0.750560462 | SLC35D1 | solute carrier family 35 member D1 [Source:HGNC Symbol;Acc:HGNC:20800] |
| ENSG00000119865 | HGLibA_10379 | 40 | -0.750560462 | CNRIP1 | cannabinoid receptor interacting protein 1 [Source:HGNC Symbol;Acc:HGNC:24546] |
| ENSG00000120709 | HGLibA_16642 | 40 | -0.750560462 | FAM53C | family with sequence similarity 53 member C [Source:HGNC Symbol;Acc:HGNC:1336] |
| ENSG00000120907 | HGLibA_01037 | 40 | -0.750560462 | ADRA1A | adrenoceptor alpha 1A [Source:HGNC Symbol;Acc:HGNC:277] |
| ENSG00000122786 | HGLibA_07011 | 40 | -0.750560462 | CALD1 | caldesmon 1 [Source:HGNC Symbol;Acc:HGNC:1441] |
| ENSG00000123131 | HGLibA_38417 | 40 | -0.750560462 | PRDX4 | peroxiredoxin 4 [Source:HGNC Symbol;Acc:HGNC:17169] |
| ENSG00000123154 | HGLibA_54194 | 40 | -0.750560462 | WDR83 | WD repeat domain 83 [Source:HGNC Symbol;Acc:HGNC:32672] |
| ENSG00000123179 | HGLibA_14377 | 40 | -0.750560462 | EBPL | EBP like [Source:HGNC Symbol;Acc:HGNC:18061] |
| ENSG00000123364 | HGLibA_21965 | 40 | -0.750560462 | HOXC13 | homeobox C13 [Source:HGNC Symbol;Acc:HGNC:5125] |
| ENSG00000124251 | HGLibA_51091 | 40 | -0.750560462 | TP53TG5 | TP53 target 5 [Source:HGNC Symbol;Acc:HGNC:15856] |
| ENSG00000124275 | HGLibA_30494 | 40 | -0.750560462 | MTRR | 5-methyltetrahydrofolate-homocysteine methyltransferase reductase [Source:HGNC Symbol;Acc:HGNC:7473] |
| ENSG00000124490 | HGLibA_11168 | 40 | -0.750560462 | CRISP2 | cysteine rich secretory protein 2 [Source:HGNC Symbol;Acc:HGNC:12024] |
| ENSG00000124615 | HGLibA_29612 | 40 | -0.750560462 | MOCS1 | molybdenum cofactor synthesis 1 [Source:HGNC Symbol;Acc:HGNC:7190] |
| ENSG00000125900 | HGLibA_44210 | 40 | -0.750560462 | SIRPD | signal regulatory protein delta [Source:HGNC Symbol;Acc:HGNC:16248] |
| ENSG00000125910 | HGLibA_42601 | 40 | -0.750560462 | S1PR4 | sphingosine-1-phosphate receptor 4 [Source:HGNC Symbol;Acc:HGNC:3170] |
| ENSG00000127554 | HGLibA_18938 | 40 | -0.750560462 | GFER | augmenter of liver regeneration [Source:HGNC Symbol;Acc:HGNC:4236]" |
| ENSG00000129167 | HGLibA_51128 | 40 | -0.750560462 | TPH1 | tryptophan hydroxylase 1 [Source:HGNC Symbol;Acc:HGNC:12008] |
| ENSG00000129654 | HGLibA_17885 | 40 | -0.750560462 | FOXJ1 | forkhead box J1 [Source:HGNC Symbol;Acc:HGNC:3816] |
| ENSG00000130150 | HGLibA_29687 | 40 | -0.750560462 | MOSPD2 | motile sperm domain containing 2 [Source:HGNC Symbol;Acc:HGNC:28381] |
| ENSG00000130204 | HGLibA_50966 | 40 | -0.750560462 | TOMM40 | translocase of outer mitochondrial membrane 40 [Source:HGNC Symbol;Acc:HGNC:18001] |
| ENSG00000130287 | HGLibA_31259 | 40 | -0.750560462 | NCAN | neurocan [Source:HGNC Symbol;Acc:HGNC:2465] |
| ENSG00000130363 | HGLibA_42332 | 40 | -0.750560462 | RSPH3 | radial spoke head 3 homolog [Source:HGNC Symbol;Acc:HGNC:21054] |
| ENSG00000132664 | HGLibA_37625 | 40 | -0.750560462 | POLR3F | RNA polymerase III subunit F [Source:HGNC Symbol;Acc:HGNC:15763] |
| ENSG00000133059 | HGLibA_14048 | 40 | -0.750560462 | DSTYK | dual serine/threonine and tyrosine protein kinase [Source:HGNC Symbol;Acc:HGNC:29043] |
| ENSG00000134028 | HGLibA_00789 | 40 | -0.750560462 | ADAMDEC1 | ADAM like decysin 1 [Source:HGNC Symbol;Acc:HGNC:16299] |
| ENSG00000134802 | HGLibA_45154 | 40 | -0.750560462 | SLC43A3 | solute carrier family 43 member 3 [Source:HGNC Symbol;Acc:HGNC:17466] |
| ENSG00000135093 | HGLibA_53329 | 40 | -0.750560462 | USP30 | ubiquitin specific peptidase 30 [Source:HGNC Symbol;Acc:HGNC:20065] |
| ENSG00000135925 | HGLibA_54345 | 40 | -0.750560462 | WNT10A | Wnt family member 10A [Source:HGNC Symbol;Acc:HGNC:13829] |
| ENSG00000136100 | HGLibA_53772 | 40 | -0.750560462 | VPS36 | vacuolar protein sorting 36 homolog [Source:HGNC Symbol;Acc:HGNC:20312] |
| ENSG00000136270 | HGLibA_48638 | 40 | -0.750560462 | TBRG4 | transforming growth factor beta regulator 4 [Source:HGNC Symbol;Acc:HGNC:17443] |
| ENSG00000137875 | HGLibA_04305 | 40 | -0.750560462 | BCL2L10 | BCL2 like 10 [Source:HGNC Symbol;Acc:HGNC:993] |
| ENSG00000137942 | HGLibA_17738 | 40 | -0.750560462 | FNBP1L | formin binding protein 1 like [Source:HGNC Symbol;Acc:HGNC:20851] |
| ENSG00000138175 | HGLibA_02963 | 40 | -0.750560462 | ARL3 | ADP ribosylation factor like GTPase 3 [Source:HGNC Symbol;Acc:HGNC:694] |
| ENSG00000138347 | HGLibA_30909 | 40 | -0.750560462 | MYPN | myopalladin [Source:HGNC Symbol;Acc:HGNC:23246] |
| ENSG00000138356 | HGLibA_02277 | 40 | -0.750560462 | AOX1 | aldehyde oxidase 1 [Source:HGNC Symbol;Acc:HGNC:553] |
| ENSG00000138660 | HGLibA_02278 | 40 | -0.750560462 | AP1AR | adaptor related protein complex 1 associated regulatory protein [Source:HGNC Symbol;Acc:HGNC:28808] |
| ENSG00000138757 | HGLibA_18302 | 40 | -0.750560462 | G3BP2 | G3BP stress granule assembly factor 2 [Source:HGNC Symbol;Acc:HGNC:30291] |
| ENSG00000140274 | HGLibA_14106 | 40 | -0.750560462 | DUOXA2 | dual oxidase maturation factor 2 [Source:HGNC Symbol;Acc:HGNC:32698] |
| ENSG00000141279 | HGLibA_32432 | 40 | -0.750560462 | NPEPPS | aminopeptidase puromycin sensitive [Source:HGNC Symbol;Acc:HGNC:7900] |
| ENSG00000141458 | HGLibA_32418 | 40 | -0.750560462 | NPC1 | NPC intracellular cholesterol transporter 1 [Source:HGNC Symbol;Acc:HGNC:7897] |
| ENSG00000142661 | HGLibA_30895 | 40 | -0.750560462 | MYOM3 | myomesin 3 [Source:HGNC Symbol;Acc:HGNC:26679] |
| ENSG00000143061 | HGLibA_22893 | 40 | -0.750560462 | IGSF3 | immunoglobulin superfamily member 3 [Source:HGNC Symbol;Acc:HGNC:5950] |
| ENSG00000143321 | HGLibA_21039 | 40 | -0.750560462 | HDGF | heparin binding growth factor [Source:HGNC Symbol;Acc:HGNC:4856] |
| ENSG00000144061 | HGLibA_32444 | 40 | -0.750560462 | NPHP1 | nephrocystin 1 [Source:HGNC Symbol;Acc:HGNC:7905] |
| ENSG00000144535 | HGLibA_13277 | 40 | -0.750560462 | DIS3L2 | DIS3 like 3'-5' exoribonuclease 2 [Source:HGNC Symbol;Acc:HGNC:28648] |
| ENSG00000145604 | HGLibA_44296 | 40 | -0.750560462 | SKP2 | S-phase kinase associated protein 2 [Source:HGNC Symbol;Acc:HGNC:10901] |
| ENSG00000145908 | HGLibA_55770 | 40 | -0.750560462 | ZNF300 | zinc finger protein 300 [Source:HGNC Symbol;Acc:HGNC:13091] |
| ENSG00000146221 | HGLibA_48858 | 40 | -0.750560462 | TCTE1 | t-complex-associated-testis-expressed 1 [Source:HGNC Symbol;Acc:HGNC:11693] |
| ENSG00000147100 | HGLibA_44434 | 40 | -0.750560462 | SLC16A2 | solute carrier family 16 member 2 [Source:HGNC Symbol;Acc:HGNC:10923] |
| ENSG00000147872 | HGLibA_37174 | 40 | -0.750560462 | PLIN2 | perilipin 2 [Source:HGNC Symbol;Acc:HGNC:248] |
| ENSG00000148248 | HGLibA_47839 | 40 | -0.750560462 | SURF4 | surfeit 4 [Source:HGNC Symbol;Acc:HGNC:11476] |
| ENSG00000148248 | HGLibA_47837 | 40 | -0.750560462 | SURF4 | surfeit 4 [Source:HGNC Symbol;Acc:HGNC:11476] |
| ENSG00000148541 | HGLibA_16146 | 40 | -0.750560462 | FAM13C | family with sequence similarity 13 member C [Source:HGNC Symbol;Acc:HGNC:19371] |
| ENSG00000148600 | HGLibA_08697 | 40 | -0.750560462 | CDHR1 | cadherin related family member 1 [Source:HGNC Symbol;Acc:HGNC:14550] |
| ENSG00000149131 | HGLibA_43592 | 40 | -0.750560462 | SERPING1 | serpin family G member 1 [Source:HGNC Symbol;Acc:HGNC:1228] |

| | | | | | |
|---|---|---|---|---|---|
| ENSG00000150051 | HGLibA_29361 | 40 | -0.750560462 | MKX | mohawk homeobox [Source:HGNC Symbol;Acc:HGNC:23729] |
| ENSG00000151006 | HGLibA_38957 | 40 | -0.750560462 | PRSS53 | serine protease 53 [Source:HGNC Symbol;Acc:HGNC:34407] |
| ENSG00000151062 | HGLibA_06914 | 40 | -0.750560462 | CACNA2D4 | calcium voltage-gated channel auxiliary subunit alpha2delta 4 [Source:HGNC Symbol;Acc:HGNC:20202] |
| ENSG00000151687 | HGLibA_01942 | 40 | -0.750560462 | ANKAR | ankyrin and armadillo repeat containing [Source:HGNC Symbol;Acc:HGNC:26350] |
| ENSG00000152348 | HGLibA_03460 | 40 | -0.750560462 | ATG10 | autophagy related 10 [Source:HGNC Symbol;Acc:HGNC:20315] |
| ENSG00000152977 | HGLibA_55320 | 40 | -0.750560462 | ZIC1 | Zic family member 1 [Source:HGNC Symbol;Acc:HGNC:12872] |
| ENSG00000154007 | HGLibA_03226 | 40 | -0.750560462 | ASB17 | ankyrin repeat and SOCS box containing 17 [Source:HGNC Symbol;Acc:HGNC:19769] |
| ENSG00000154269 | HGLibA_15172 | 40 | -0.750560462 | ENPP3 | ectonucleotide pyrophosphatase/phosphodiesterase 3 [Source:HGNC Symbol;Acc:HGNC:3358] |
| ENSG00000155008 | HGLibA_02555 | 40 | -0.750560462 | APOOL | apolipoprotein O like [Source:HGNC Symbol;Acc:HGNC:24009] |
| ENSG00000155287 | HGLibA_44693 | 40 | -0.750560462 | SLC25A28 | solute carrier family 25 member 28 [Source:HGNC Symbol;Acc:HGNC:23472] |
| ENSG00000155876 | HGLibA_42223 | 40 | -0.750560462 | RRAGA | Ras related GTP binding A [Source:HGNC Symbol;Acc:HGNC:16963] |
| ENSG00000156304 | HGLibA_42827 | 40 | -0.750560462 | SCAF4 | SR-related CTD associated factor 4 [Source:HGNC Symbol;Acc:HGNC:19304] |
| ENSG00000157119 | HGLibA_25208 | 40 | -0.750560462 | KLHL40 | kelch like family member 40 [Source:HGNC Symbol;Acc:HGNC:30372] |
| ENSG00000158571 | HGLibA_36154 | 40 | -0.750560462 | PFKFB1 | 6-biphosphatase 1 [Source:HGNC Symbol;Acc:HGNC:8872]" |
| ENSG00000159720 | HGLibA_03717 | 40 | -0.750560462 | ATP6V0D1 | ATPase H+ transporting V0 subunit d1 [Source:HGNC Symbol;Acc:HGNC:13724] |
| ENSG00000159840 | HGLibA_57089 | 40 | -0.750560462 | ZYX | zyxin [Source:HGNC Symbol;Acc:HGNC:13200] |
| ENSG00000160683 | HGLibA_11893 | 40 | -0.750560462 | CXCR5 | C-X-C motif chemokine receptor 5 [Source:HGNC Symbol;Acc:HGNC:1060] |
| ENSG00000161664 | HGLibA_03223 | 40 | -0.750560462 | ASB16 | ankyrin repeat and SOCS box containing 16 [Source:HGNC Symbol;Acc:HGNC:19768] |
| ENSG00000162592 | HGLibA_07743 | 40 | -0.750560462 | CCDC27 | coiled-coil domain containing 27 [Source:HGNC Symbol;Acc:HGNC:26546] |
| ENSG00000162836 | HGLibA_00517 | 40 | -0.750560462 | ACP6 | lysophosphatidic [Source:HGNC Symbol;Acc:HGNC:29609]" |
| ENSG00000163673 | HGLibA_12505 | 40 | -0.750560462 | DCLK3 | doublecortin like kinase 3 [Source:HGNC Symbol;Acc:HGNC:19005] |
| ENSG00000163719 | HGLibA_30421 | 40 | -0.750560462 | MTMR14 | myotubularin related protein 14 [Source:HGNC Symbol;Acc:HGNC:26190] |
| ENSG00000163832 | HGLibA_14989 | 40 | -0.750560462 | ELP6 | elongator acetyltransferase complex subunit 6 [Source:HGNC Symbol;Acc:HGNC:25976] |
| ENSG00000164463 | HGLibA_11114 | 40 | -0.750560462 | CREBRF | CREB3 regulatory factor [Source:HGNC Symbol;Acc:HGNC:24050] |
| ENSG00000164736 | HGLibA_46247 | 40 | -0.750560462 | SOX17 | SRY-box 17 [Source:HGNC Symbol;Acc:HGNC:18122] |
| ENSG00000164983 | HGLibA_50486 | 40 | -0.750560462 | TMEM65 | transmembrane protein 65 [Source:HGNC Symbol;Acc:HGNC:25203] |
| ENSG00000165792 | HGLibA_28968 | 40 | -0.750560462 | METTL17 | methyltransferase like 17 [Source:HGNC Symbol;Acc:HGNC:19280] |
| ENSG00000166145 | HGLibA_46713 | 40 | -0.750560462 | SPINT1 | Kunitz type 1 [Source:HGNC Symbol;Acc:HGNC:11246]" |
| ENSG00000166523 | HGLibA_09987 | 40 | -0.750560462 | CLEC4E | C-type lectin domain family 4 member E [Source:HGNC Symbol;Acc:HGNC:14555] |
| ENSG00000166716 | HGLibA_56339 | 40 | -0.750560462 | ZNF592 | zinc finger protein 592 [Source:HGNC Symbol;Acc:HGNC:28986] |
| ENSG00000166961 | HGLibA_30141 | 40 | -0.750560462 | MS4A15 | membrane spanning 4-domains A15 [Source:HGNC Symbol;Acc:HGNC:28573] |
| ENSG00000167658 | HGLibA_14507 | 40 | -0.750560462 | EEF2 | eukaryotic translation elongation factor 2 [Source:HGNC Symbol;Acc:HGNC:3214] |
| ENSG00000167685 | HGLibA_56013 | 40 | -0.750560462 | ZNF444 | zinc finger protein 444 [Source:HGNC Symbol;Acc:HGNC:16052] |
| ENSG00000167992 | HGLibA_53928 | 40 | -0.750560462 | VWCE | von Willebrand factor C and EGF domains [Source:HGNC Symbol;Acc:HGNC:26487] |
| ENSG00000168806 | HGLibA_26163 | 40 | -0.750560462 | LCMT2 | leucine carboxyl methyltransferase 2 [Source:HGNC Symbol;Acc:HGNC:17558] |
| ENSG00000169548 | HGLibA_55724 | 40 | -0.750560462 | ZNF280A | zinc finger protein 280A [Source:HGNC Symbol;Acc:HGNC:18597] |
| ENSG00000169567 | HGLibA_21324 | 40 | -0.750560462 | HINT1 | histidine triad nucleotide binding protein 1 [Source:HGNC Symbol;Acc:HGNC:4912] |
| ENSG00000169704 | HGLibA_19715 | 40 | -0.750560462 | GP9 | glycoprotein IX platelet [Source:HGNC Symbol;Acc:HGNC:4444] |
| ENSG00000169877 | HGLibA_01321 | 40 | -0.750560462 | AHSP | alpha hemoglobin stabilizing protein [Source:HGNC Symbol;Acc:HGNC:18075] |
| ENSG00000170255 | HGLibA_29846 | 40 | -0.750560462 | MRGPRX1 | MAS related GPR family member X1 [Source:HGNC Symbol;Acc:HGNC:17962] |
| ENSG00000171320 | HGLibA_15559 | 40 | -0.750560462 | ESCO2 | establishment of sister chromatid cohesion N-acetyltransferase 2 [Source:HGNC Symbol;Acc:HGNC:27230] |
| ENSG00000171466 | HGLibA_56248 | 40 | -0.750560462 | ZNF562 | zinc finger protein 562 [Source:HGNC Symbol;Acc:HGNC:25950] |
| ENSG00000171847 | HGLibA_16792 | 40 | -0.750560462 | FAM90A1 | family with sequence similarity 90 member A1 [Source:HGNC Symbol;Acc:HGNC:25526] |
| ENSG00000172301 | HGLibA_10711 | 40 | -0.750560462 | COPRS | coordinator of PRMT5 and differentiation stimulator [Source:HGNC Symbol;Acc:HGNC:28848] |
| ENSG00000172348 | HGLibA_40672 | 40 | -0.750560462 | RCAN2 | regulator of calcineurin 2 [Source:HGNC Symbol;Acc:HGNC:3041] |
| ENSG00000172519 | HGLibA_33517 | 40 | -0.750560462 | OR10H5 | olfactory receptor family 10 subfamily H member 5 [Source:HGNC Symbol;Acc:HGNC:15389] |
| ENSG00000172992 | HGLibA_12465 | 40 | -0.750560462 | DCAKD | dephospho-CoA kinase domain containing [Source:HGNC Symbol;Acc:HGNC:26238] |
| ENSG00000173281 | HGLibA_38100 | 40 | -0.750560462 | PPP1R3B | protein phosphatase 1 regulatory subunit 3B [Source:HGNC Symbol;Acc:HGNC:14942] |
| ENSG00000174405 | HGLibA_26486 | 40 | -0.750560462 | LIG4 | DNA ligase 4 [Source:HGNC Symbol;Acc:HGNC:6601] |
| ENSG00000175213 | HGLibA_55930 | 40 | -0.750560462 | ZNF408 | zinc finger protein 408 [Source:HGNC Symbol;Acc:HGNC:20041] |
| ENSG00000176890 | HGLibA_52462 | 40 | -0.750560462 | TYMS | thymidylate synthetase [Source:HGNC Symbol;Acc:HGNC:12441] |
| ENSG00000177468 | HGLibA_33374 | 40 | -0.750560462 | OLIG3 | oligodendrocyte transcription factor 3 [Source:HGNC Symbol;Acc:HGNC:18003] |
| ENSG00000178149 | HGLibA_12305 | 40 | -0.750560462 | DALRD3 | DALR anticodon binding domain containing 3 [Source:HGNC Symbol;Acc:HGNC:25536] |
| ENSG00000178222 | HGLibA_41619 | 40 | -0.750560462 | RNF212 | ring finger protein 212 [Source:HGNC Symbol;Acc:HGNC:27729] |
| ENSG00000178662 | HGLibA_11428 | 40 | -0.750560462 | CSRNP3 | cysteine and serine rich nuclear protein 3 [Source:HGNC Symbol;Acc:HGNC:30729] |
| ENSG00000179477 | HGLibA_01679 | 40 | -0.750560462 | ALOX12B | 12R type [Source:HGNC Symbol;Acc:HGNC:430]" |
| ENSG00000180875 | HGLibA_20244 | 40 | -0.750560462 | GREM2 | DAN family BMP antagonist [Source:HGNC Symbol;Acc:HGNC:17655]" |
| ENSG00000181371 | HGLibA_34340 | 40 | -0.750560462 | OR5M8 | olfactory receptor family 5 subfamily M member 8 [Source:HGNC Symbol;Acc:HGNC:14846] |
| ENSG00000181698 | HGLibA_34354 | 40 | -0.750560462 | OR5T1 | olfactory receptor family 5 subfamily T member 1 [Source:HGNC Symbol;Acc:HGNC:14821] |
| ENSG00000182327 | HGLibA_19350 | 40 | -0.750560462 | GLTPD2 | glycolipid transfer protein domain containing 2 [Source:HGNC Symbol;Acc:HGNC:33756] |
| ENSG00000183024 | HGLibA_33668 | 40 | -0.750560462 | OR1G1 | olfactory receptor family 1 subfamily G member 1 [Source:HGNC Symbol;Acc:HGNC:8204] |
| ENSG00000185033 | HGLibA_43343 | 40 | -0.750560462 | SEMA4B | semaphorin 4B [Source:HGNC Symbol;Acc:HGNC:10730] |
| ENSG00000185669 | HGLibA_45871 | 40 | -0.750560462 | SNAI3 | snail family transcriptional repressor 3 [Source:HGNC Symbol;Acc:HGNC:18411] |
| ENSG00000185792 | HGLibA_32136 | 40 | -0.750560462 | NLRP9 | NLR family pyrin domain containing 9 [Source:HGNC Symbol;Acc:HGNC:22941] |
| ENSG00000186103 | HGLibA_02692 | 40 | -0.750560462 | ARGFX | arginine-fifty homeobox [Source:HGNC Symbol;Acc:HGNC:30146] |
| ENSG00000187151 | HGLibA_01921 | 40 | -0.750560462 | ANGPTL5 | angiopoietin like 5 [Source:HGNC Symbol;Acc:HGNC:19705] |
| ENSG00000187242 | HGLibA_25419 | 40 | -0.750560462 | KRT12 | keratin 12 [Source:HGNC Symbol;Acc:HGNC:6414] |
| ENSG00000187605 | HGLibA_49043 | 40 | -0.750560462 | TET3 | tet methylcytosine dioxygenase 3 [Source:HGNC Symbol;Acc:HGNC:28313] |
| ENSG00000187778 | HGLibA_28649 | 40 | -0.750560462 | MCRS1 | microspherule protein 1 [Source:HGNC Symbol;Acc:HGNC:6960] |
| ENSG00000188229 | HGLibA_52288 | 40 | -0.750560462 | TUBB4B | tubulin beta 4B class IVb [Source:HGNC Symbol;Acc:HGNC:20771] |
| ENSG00000188408 | HGLibA_27952 | 40 | -0.750560462 | MAGEB5 | MAGE family member B5 [Source:HGNC Symbol;Acc:HGNC:23795] |
| ENSG00000189430 | HGLibA_31363 | 40 | -0.750560462 | NCR1 | natural cytotoxicity triggering receptor 1 [Source:HGNC Symbol;Acc:HGNC:6731] |
| ENSG00000197223 | HGLibA_05720 | 40 | -0.750560462 | C1D | C1D nuclear receptor corepressor [Source:HGNC Symbol;Acc:HGNC:29911] |
| ENSG00000197734 | HGLibA_05346 | 40 | -0.750560462 | C14orf178 | chromosome 14 open reading frame 178 [Source:HGNC Symbol;Acc:HGNC:26385] |
| ENSG00000197978 | HGLibA_19631 | 40 | -0.750560462 | GOLGA6L9 | golgin A6 family-like 9 [Source:HGNC Symbol;Acc:HGNC:37229] |
| ENSG00000198056 | HGLibA_38482 | 40 | -0.750560462 | PRIM1 | DNA primase subunit 1 [Source:HGNC Symbol;Acc:HGNC:9369] |
| ENSG00000198198 | HGLibA_48118 | 40 | -0.750560462 | SZT2 | KICSTOR complex subunit [Source:HGNC Symbol;Acc:HGNC:29040]" |
| ENSG00000198203 | HGLibA_47746 | 40 | -0.750560462 | SULT1C2 | sulfotransferase family 1C member 2 [Source:HGNC Symbol;Acc:HGNC:11456] |
| ENSG00000198765 | HGLibA_47929 | 40 | -0.750560462 | SYCP1 | synaptonemal complex protein 1 [Source:HGNC Symbol;Acc:HGNC:11487] |
| ENSG00000198780 | HGLibA_16253 | 40 | -0.750560462 | FAM169A | family with sequence similarity 169 member A [Source:HGNC Symbol;Acc:HGNC:29138] |
| ENSG00000205302 | HGLibA_46057 | 40 | -0.750560462 | SNX2 | sorting nexin 2 [Source:HGNC Symbol;Acc:HGNC:11173] |
| ENSG00000206069 | HGLibA_50208 | 40 | -0.750560462 | TMEM211 | transmembrane protein 211 [Source:HGNC Symbol;Acc:HGNC:33725] |
| ENSG00000206203 | HGLibA_52012 | 40 | -0.750560462 | TSSK2 | testis specific serine kinase 2 [Source:HGNC Symbol;Acc:HGNC:11401] |
| ENSG00000214336 | HGLibA_17882 | 40 | -0.750560462 | FOXI3 | forkhead box I3 [Source:HGNC Symbol;Acc:HGNC:35123] |
| ENSG00000215018 | HGLibA_10557 | 40 | -0.750560462 | COL28A1 | collagen type XXVIII alpha 1 chain [Source:HGNC Symbol;Acc:HGNC:22442] |
| ENSG00000215029 | HGLibA_48852 | 40 | -0.750560462 | TCP11X2 | X-linked 2 [Source:HGNC Symbol;Acc:HGNC:48335]" |
| ENSG00000215113 | HGLibA_11935 | 40 | -0.750560462 | CXorf49B | chromosome X open reading frame 49B [Source:HGNC Symbol;Acc:HGNC:34229] |
| ENSG00000219626 | HGLibA_16538 | 40 | -0.750560462 | FAM228B | family with sequence similarity 228 member B [Source:HGNC Symbol;Acc:HGNC:24736] |
| ENSG00000228856 | HGLibA_53275 | 40 | -0.750560462 | USP17L30 | ubiquitin specific peptidase 17-like family member 30 [Source:HGNC Symbol;Acc:HGNC:44458] |
| ENSG00000232258 | HGLibA_49881 | 40 | -0.750560462 | TMEM114 | transmembrane protein 114 [Source:HGNC Symbol;Acc:HGNC:33227] |
| ENSG00000233954 | HGLibA_53126 | 40 | -0.750560462 | UQCRHL | ubiquinol-cytochrome c reductase hinge protein like [Source:HGNC Symbol;Acc:HGNC:51714] |
| ENSG00000240184 | HGLibA_35570 | 40 | -0.750560462 | PCDHGC3 | 3 [Source:HGNC Symbol;Acc:HGNC:8716]" |
| ENSG00000242485 | HGLibA_29924 | 40 | -0.750560462 | MRPL20 | mitochondrial ribosomal protein L20 [Source:HGNC Symbol;Acc:HGNC:14478] |
| ENSG00000259571 | HGLibA_04542 | 40 | -0.750560462 | BLID | cell death inducer [Source:HGNC Symbol;Acc:HGNC:33495]" |
| EPT1 | HGLibA_15389 | 40 | -0.750560462 | EPT1 | - |
| FLJ25363 | HGLibA_17624 | 40 | -0.750560462 | FLJ25363 | - |
| FLJ44635 | HGLibA_17632 | 40 | -0.750560462 | FLJ44635 | - |
| FTSJD1 | HGLibA_18143 | 40 | -0.750560462 | FTSJD1 | - |
| GAGE2B | HGLibA_18470 | 40 | -0.750560462 | GAGE2B | - |
| HIST1H1D | HGLibA_21366 | 40 | -0.750560462 | HIST1H1D | - |
| HIST1H2BA | HGLibA_21412 | 40 | -0.750560462 | HIST1H2BA | - |
| HIST1H3C | HGLibA_21461 | 40 | -0.750560462 | HIST1H3C | - |
| hsa-mir-1200 | HGLibA_57260 | 40 | -0.750560462 | hsa-mir-1200 | - |
| hsa-mir-1255a | HGLibA_57420 | 40 | -0.750560462 | hsa-mir-1255a | - |
| hsa-mir-125b-2 | HGLibA_57450 | 40 | -0.750560462 | hsa-mir-125b-2 | - |
| hsa-mir-1268b | HGLibA_57501 | 40 | -0.750560462 | hsa-mir-1268b | - |
| hsa-mir-1295b | HGLibA_57659 | 40 | -0.750560462 | hsa-mir-1295b | - |
| hsa-mir-135b | HGLibA_57801 | 40 | -0.750560462 | hsa-mir-135b | - |
| hsa-mir-181a-2 | HGLibA_57962 | 40 | -0.750560462 | hsa-mir-181a-2 | - |
| hsa-mir-1914 | HGLibA_58060 | 40 | -0.750560462 | hsa-mir-1914 | - |
| hsa-mir-223 | HGLibA_58301 | 40 | -0.750560462 | hsa-mir-223 | - |
| hsa-mir-300 | HGLibA_58425 | 40 | -0.750560462 | hsa-mir-300 | - |
| hsa-mir-3153 | HGLibA_58672 | 40 | -0.750560462 | hsa-mir-3153 | - |
| hsa-mir-3684 | HGLibA_59290 | 40 | -0.750560462 | hsa-mir-3684 | - |
| hsa-mir-378i | HGLibA_59461 | 40 | -0.750560462 | hsa-mir-378i | - |
| hsa-mir-4281 | HGLibA_59827 | 40 | -0.750560462 | hsa-mir-4281 | - |
| hsa-mir-4437 | HGLibA_60152 | 40 | -0.750560462 | hsa-mir-4437 | - |
| hsa-mir-4476 | HGLibA_60313 | 40 | -0.750560462 | hsa-mir-4476 | - |
| hsa-mir-4490 | HGLibA_60378 | 40 | -0.750560462 | hsa-mir-4490 | - |
| hsa-mir-4697 | HGLibA_60897 | 40 | -0.750560462 | hsa-mir-4697 | - |
| hsa-mir-4735 | HGLibA_61038 | 40 | -0.750560462 | hsa-mir-4735 | - |
| hsa-mir-4765 | HGLibA_61158 | 40 | -0.750560462 | hsa-mir-4765 | - |
| hsa-mir-4776-1 | HGLibA_61206 | 40 | -0.750560462 | hsa-mir-4776-1 | - |
| hsa-mir-484 | HGLibA_61329 | 40 | -0.750560462 | hsa-mir-484 | - |
| hsa-mir-511 | HGLibA_61557 | 40 | -0.750560462 | hsa-mir-511 | - |
| hsa-mir-5190 | HGLibA_61679 | 40 | -0.750560462 | hsa-mir-5190 | - |
| hsa-mir-544b | HGLibA_61829 | 40 | -0.750560462 | hsa-mir-544b | - |
| hsa-mir-548i-4 | HGLibA_62025 | 40 | -0.750560462 | hsa-mir-548i-4 | - |
| hsa-mir-5701-2 | HGLibA_62346 | 40 | -0.750560462 | hsa-mir-5701-2 | - |
| hsa-mir-576 | HGLibA_62399 | 40 | -0.750560462 | hsa-mir-576 | - |
| hsa-mir-603 | HGLibA_62505 | 40 | -0.750560462 | hsa-mir-603 | - |
| hsa-mir-606 | HGLibA_62517 | 40 | -0.750560462 | hsa-mir-606 | - |
| hsa-mir-607 | HGLibA_62529 | 40 | -0.750560462 | hsa-mir-607 | - |
| hsa-mir-6770-3 | HGLibA_63252 | 40 | -0.750560462 | hsa-mir-6770-3 | - |
| hsa-mir-6867 | HGLibA_63653 | 40 | -0.750560462 | hsa-mir-6867 | - |

| | | | | | |
|---|---|---|---|---|---|
| hsa-mir-6883 | HGLibA_63718 | 40 | -0.750560462 | hsa-mir-6883 | - |
| hsa-mir-7847 | HGLibA_63977 | 40 | -0.750560462 | hsa-mir-7847 | - |
| hsa-mir-8056 | HGLibA_64062 | 40 | -0.750560462 | hsa-mir-8056 | - |
| hsa-mir-924 | HGLibA_64285 | 40 | -0.750560462 | hsa-mir-924 | - |
| hsa-mir-944 | HGLibA_64363 | 40 | -0.750560462 | hsa-mir-944 | - |
| IL9R_Y | HGLibA_23204 | 40 | -0.750560462 | IL9R_Y | - |
| LOC653486 | HGLibA_26987 | 40 | -0.750560462 | LOC653486 | - |
| LOH12CR1 | HGLibA_27012 | 40 | -0.750560462 | LOH12CR1 | - |
| LYRM5 | HGLibA_27756 | 40 | -0.750560462 | LYRM5 | - |
| NPIP | HGLibA_32459 | 40 | -0.750560462 | NPIP | - |
| PCNXL3 | HGLibA_35648 | 40 | -0.750560462 | PCNXL3 | - |
| PTPLAD1 | HGLibA_39417 | 40 | -0.750560462 | PTPLAD1 | - |
| TCEB3CL | HGLibA_48748 | 40 | -0.750560462 | TCEB3CL | - |
| VAMP7_X | HGLibA_53504 | 40 | -0.750560462 | VAMP7_X | - |
| C10orf113 | HGLibA_05034 | 39 | -0.760511499 | C10orf113 | - |
| C12orf52 | HGLibA_05262 | 39 | -0.760511499 | C12orf52 | - |
| C16orf13 | HGLibA_05442 | 39 | -0.760511499 | C16orf13 | - |
| C17orf66 | HGLibA_05561 | 39 | -0.760511499 | C17orf66 | - |
| C20orf26 | HGLibA_06019 | 39 | -0.760511499 | C20orf26 | - |
| CD99_X | HGLibA_08490 | 39 | -0.760511499 | CD99_X | - |
| CYP3A7-CYP3AP1 | HGLibA_12166 | 39 | -0.760511499 | CYP3A7-CYP3AP1 | - |
| ENSG00000001626 | HGLibA_09292 | 39 | -0.760511499 | CFTR | cystic fibrosis transmembrane conductance regulator [Source:HGNC Symbol;Acc:HGNC:1884] |
| ENSG00000004961 | HGLibA_20955 | 39 | -0.760511499 | HCCS | holocytochrome c synthase [Source:HGNC Symbol;Acc:HGNC:4837] |
| ENSG00000005884 | HGLibA_23729 | 39 | -0.760511499 | ITGA3 | integrin subunit alpha 3 [Source:HGNC Symbol;Acc:HGNC:6139] |
| ENSG00000006715 | HGLibA_53790 | 39 | -0.760511499 | VPS41 | HOPS complex subunit [Source:HGNC Symbol;Acc:HGNC:12713]" |
| ENSG00000008056 | HGLibA_47954 | 39 | -0.760511499 | SYN1 | synapsin I [Source:HGNC Symbol;Acc:HGNC:11494] |
| ENSG00000010322 | HGLibA_31980 | 39 | -0.760511499 | NISCH | nischarin [Source:HGNC Symbol;Acc:HGNC:18006] |
| ENSG00000012963 | HGLibA_52763 | 39 | -0.760511499 | UBR7 | ubiquitin protein ligase E3 component n-recognin 7 (putative) [Source:HGNC Symbol;Acc:HGNC:20344] |
| ENSG00000013523 | HGLibA_01896 | 39 | -0.760511499 | ANGEL1 | angel homolog 1 [Source:HGNC Symbol;Acc:HGNC:19961] |
| ENSG00000019505 | HGLibA_48058 | 39 | -0.760511499 | SYT13 | synaptotagmin 13 [Source:HGNC Symbol;Acc:HGNC:14962] |
| ENSG00000023892 | HGLibA_12787 | 39 | -0.760511499 | DEF6 | guanine nucleotide exchange factor [Source:HGNC Symbol;Acc:HGNC:2760]" |
| ENSG00000025772 | HGLibA_50965 | 39 | -0.760511499 | TOMM34 | translocase of outer mitochondrial membrane 34 [Source:HGNC Symbol;Acc:HGNC:15746] |
| ENSG00000034693 | HGLibA_36115 | 39 | -0.760511499 | PEX3 | peroxisomal biogenesis factor 3 [Source:HGNC Symbol;Acc:HGNC:8858] |
| ENSG00000038274 | HGLibA_28422 | 39 | -0.760511499 | MAT2B | methionine adenosyltransferase 2B [Source:HGNC Symbol;Acc:HGNC:6905] |
| ENSG00000039987 | HGLibA_04423 | 39 | -0.760511499 | BEST2 | bestrophin 2 [Source:HGNC Symbol;Acc:HGNC:17107] |
| ENSG00000051108 | HGLibA_21170 | 39 | -0.760511499 | HERPUD1 | homocysteine inducible ER protein with ubiquitin like domain 1 [Source:HGNC Symbol;Acc:HGNC:13744] |
| ENSG00000073417 | HGLibA_35828 | 39 | -0.760511499 | PDE8A | phosphodiesterase 8A [Source:HGNC Symbol;Acc:HGNC:8793] |
| ENSG00000074771 | HGLibA_32372 | 39 | -0.760511499 | NOX3 | NADPH oxidase 3 [Source:HGNC Symbol;Acc:HGNC:7890] |
| ENSG00000076826 | HGLibA_07111 | 39 | -0.760511499 | CAMSAP3 | calmodulin regulated spectrin associated protein family member 3 [Source:HGNC Symbol;Acc:HGNC:29307] |
| ENSG00000079313 | HGLibA_40890 | 39 | -0.760511499 | REXO1 | RNA exonuclease 1 homolog [Source:HGNC Symbol;Acc:HGNC:24616] |
| ENSG00000079785 | HGLibA_12647 | 39 | -0.760511499 | DDX1 | DEAD-box helicase 1 [Source:HGNC Symbol;Acc:HGNC:2734] |
| ENSG00000090565 | HGLibA_39800 | 39 | -0.760511499 | RAB11FIP3 | RAB11 family interacting protein 3 [Source:HGNC Symbol;Acc:HGNC:17224] |
| ENSG00000095002 | HGLibA_30190 | 39 | -0.760511499 | MSH2 | mutS homolog 2 [Source:HGNC Symbol;Acc:HGNC:7325] |
| ENSG00000097007 | HGLibA_00301 | 39 | -0.760511499 | ABL1 | non-receptor tyrosine kinase [Source:HGNC Symbol;Acc:HGNC:76]" |
| ENSG00000099326 | HGLibA_30938 | 39 | -0.760511499 | MZF1 | myeloid zinc finger 1 [Source:HGNC Symbol;Acc:HGNC:13108] |
| ENSG00000099341 | HGLibA_39185 | 39 | -0.760511499 | PSMD8 | non-ATPase 8 [Source:HGNC Symbol;Acc:HGNC:9566]" |
| ENSG00000100652 | HGLibA_44329 | 39 | -0.760511499 | SLC10A1 | solute carrier family 10 member 1 [Source:HGNC Symbol;Acc:HGNC:10905] |
| ENSG00000100987 | HGLibA_53876 | 39 | -0.760511499 | VSX1 | visual system homeobox 1 [Source:HGNC Symbol;Acc:HGNC:12723] |
| ENSG00000101052 | HGLibA_22765 | 39 | -0.760511499 | IFT52 | intraflagellar transport 52 [Source:HGNC Symbol;Acc:HGNC:15901] |
| ENSG00000101210 | HGLibA_14493 | 39 | -0.760511499 | EEF1A2 | eukaryotic translation elongation factor 1 alpha 2 [Source:HGNC Symbol;Acc:HGNC:3192] |
| ENSG00000103423 | HGLibA_13516 | 39 | -0.760511499 | DNAJA3 | DnaJ heat shock protein family (Hsp40) member A3 [Source:HGNC Symbol;Acc:HGNC:11808] |
| ENSG00000103966 | HGLibA_14675 | 39 | -0.760511499 | EHD4 | EH domain containing 4 [Source:HGNC Symbol;Acc:HGNC:3245] |
| ENSG00000104427 | HGLibA_54938 | 39 | -0.760511499 | ZC2HC1A | zinc finger C2HC-type containing 1A [Source:HGNC Symbol;Acc:HGNC:24277] |
| ENSG00000104976 | HGLibA_45893 | 39 | -0.760511499 | SNAPC2 | small nuclear RNA activating complex polypeptide 2 [Source:HGNC Symbol;Acc:HGNC:11135] |
| ENSG00000105287 | HGLibA_38568 | 39 | -0.760511499 | PRKD2 | protein kinase D2 [Source:HGNC Symbol;Acc:HGNC:17293] |
| ENSG00000105409 | HGLibA_03586 | 39 | -0.760511499 | ATP1A3 | ATPase Na+/K+ transporting subunit alpha 3 [Source:HGNC Symbol;Acc:HGNC:801] |
| ENSG00000107021 | HGLibA_48475 | 39 | -0.760511499 | TBC1D13 | TBC1 domain family member 13 [Source:HGNC Symbol;Acc:HGNC:25571] |
| ENSG00000107331 | HGLibA_00108 | 39 | -0.760511499 | ABCA2 | ATP binding cassette subfamily A member 2 [Source:HGNC Symbol;Acc:HGNC:32] |
| ENSG00000107562 | HGLibA_11850 | 39 | -0.760511499 | CXCL12 | C-X-C motif chemokine ligand 12 [Source:HGNC Symbol;Acc:HGNC:10672] |
| ENSG00000107672 | HGLibA_32771 | 39 | -0.760511499 | NSMCE4A | SMC5-SMC6 complex component [Source:HGNC Symbol;Acc:HGNC:25935]" |
| ENSG00000107863 | HGLibA_02726 | 39 | -0.760511499 | ARHGAP21 | Rho GTPase activating protein 21 [Source:HGNC Symbol;Acc:HGNC:23725] |
| ENSG00000107874 | HGLibA_11771 | 39 | -0.760511499 | CUEDC2 | CUE domain containing 2 [Source:HGNC Symbol;Acc:HGNC:28352] |
| ENSG00000108344 | HGLibA_39168 | 39 | -0.760511499 | PSMD3 | non-ATPase 3 [Source:HGNC Symbol;Acc:HGNC:9560]" |
| ENSG00000109911 | HGLibA_14985 | 39 | -0.760511499 | ELP4 | elongator acetyltransferase complex subunit 4 [Source:HGNC Symbol;Acc:HGNC:1171] |
| ENSG00000110723 | HGLibA_15799 | 39 | -0.760511499 | EXPH5 | exophilin 5 [Source:HGNC Symbol;Acc:HGNC:30578] |
| ENSG00000110887 | HGLibA_12311 | 39 | -0.760511499 | DAO | D-amino acid oxidase [Source:HGNC Symbol;Acc:HGNC:2671] |
| ENSG00000111186 | HGLibA_54377 | 39 | -0.760511499 | WNT5B | Wnt family member 5B [Source:HGNC Symbol;Acc:HGNC:16265] |
| ENSG00000111229 | HGLibA_03084 | 39 | -0.760511499 | ARPC3 | actin related protein 2/3 complex subunit 3 [Source:HGNC Symbol;Acc:HGNC:706] |
| ENSG00000111530 | HGLibA_07122 | 39 | -0.760511499 | CAND1 | cullin associated and neddylation dissociated 1 [Source:HGNC Symbol;Acc:HGNC:30688] |
| ENSG00000112592 | HGLibA_48623 | 39 | -0.760511499 | TBP | TATA-box binding protein [Source:HGNC Symbol;Acc:HGNC:11588] |
| ENSG00000112977 | HGLibA_12315 | 39 | -0.760511499 | DAP | death associated protein [Source:HGNC Symbol;Acc:HGNC:2672] |
| ENSG00000113552 | HGLibA_19564 | 39 | -0.760511499 | GNPDA1 | glucosamine-6-phosphate deaminase 1 [Source:HGNC Symbol;Acc:HGNC:4417] |
| ENSG00000113658 | HGLibA_45582 | 39 | -0.760511499 | SMAD5 | SMAD family member 5 [Source:HGNC Symbol;Acc:HGNC:6771] |
| ENSG00000113851 | HGLibA_11077 | 39 | -0.760511499 | CRBN | cereblon [Source:HGNC Symbol;Acc:HGNC:30185] |
| ENSG00000114646 | HGLibA_11416 | 39 | -0.760511499 | CSPG5 | chondroitin sulfate proteoglycan 5 [Source:HGNC Symbol;Acc:HGNC:2467] |
| ENSG00000114861 | HGLibA_17930 | 39 | -0.760511499 | FOXP1 | forkhead box P1 [Source:HGNC Symbol;Acc:HGNC:3823] |
| ENSG00000115275 | HGLibA_29632 | 39 | -0.760511499 | MOGS | mannosyl-oligosaccharide glucosidase [Source:HGNC Symbol;Acc:HGNC:24862] |
| ENSG00000116954 | HGLibA_42230 | 39 | -0.760511499 | RRAGC | Ras related GTP binding C [Source:HGNC Symbol;Acc:HGNC:19902] |
| ENSG00000116991 | HGLibA_44194 | 39 | -0.760511499 | SIPA1L2 | signal induced proliferation associated 1 like 2 [Source:HGNC Symbol;Acc:HGNC:23800] |
| ENSG00000117569 | HGLibA_39254 | 39 | -0.760511499 | PTBP2 | polypyrimidine tract binding protein 2 [Source:HGNC Symbol;Acc:HGNC:17662] |
| ENSG00000117632 | HGLibA_47517 | 39 | -0.760511499 | STMN1 | stathmin 1 [Source:HGNC Symbol;Acc:HGNC:6510] |
| ENSG00000118058 | HGLibA_25321 | 39 | -0.760511499 | KMT2A | lysine methyltransferase 2A [Source:HGNC Symbol;Acc:HGNC:7132] |
| ENSG00000118181 | HGLibA_42117 | 39 | -0.760511499 | RPS25 | ribosomal protein S25 [Source:HGNC Symbol;Acc:HGNC:10413] |
| ENSG00000119041 | HGLibA_20585 | 39 | -0.760511499 | GTF3C3 | general transcription factor IIIC subunit 3 [Source:HGNC Symbol;Acc:HGNC:4666] |
| ENSG00000119608 | HGLibA_38705 | 39 | -0.760511499 | PROX2 | prospero homeobox 2 [Source:HGNC Symbol;Acc:HGNC:26715] |
| ENSG00000119614 | HGLibA_53877 | 39 | -0.760511499 | VSX2 | visual system homeobox 2 [Source:HGNC Symbol;Acc:HGNC:1975] |
| ENSG00000119723 | HGLibA_10762 | 39 | -0.760511499 | COQ6 | monooxygenase [Source:HGNC Symbol;Acc:HGNC:20233]" |
| ENSG00000120963 | HGLibA_56596 | 39 | -0.760511499 | ZNF706 | zinc finger protein 706 [Source:HGNC Symbol;Acc:HGNC:24992] |
| ENSG00000123612 | HGLibA_00691 | 39 | -0.760511499 | ACVR1C | activin A receptor type 1C [Source:HGNC Symbol;Acc:HGNC:18123] |
| ENSG00000123728 | HGLibA_40243 | 39 | -0.760511499 | RAP2C | member of RAS oncogene family [Source:HGNC Symbol;Acc:HGNC:21165]" |
| ENSG00000124102 | HGLibA_36488 | 39 | -0.760511499 | PI3 | peptidase inhibitor 3 [Source:HGNC Symbol;Acc:HGNC:8947] |
| ENSG00000124702 | HGLibA_25098 | 39 | -0.760511499 | KLHDC3 | kelch domain containing 3 [Source:HGNC Symbol;Acc:HGNC:20704] |
| ENSG00000124713 | HGLibA_19559 | 39 | -0.760511499 | GNMT | glycine N-methyltransferase [Source:HGNC Symbol;Acc:HGNC:4415] |
| ENSG00000125492 | HGLibA_04159 | 39 | -0.760511499 | BARHL1 | BarH like homeobox 1 [Source:HGNC Symbol;Acc:HGNC:953] |
| ENSG00000125730 | HGLibA_06204 | 39 | -0.760511499 | C3 | complement C3 [Source:HGNC Symbol;Acc:HGNC:1318] |
| ENSG00000125741 | HGLibA_33406 | 39 | -0.760511499 | OPA3 | outer mitochondrial membrane lipid metabolism regulator [Source:HGNC Symbol;Acc:HGNC:8142]" |
| ENSG00000126860 | HGLibA_15686 | 39 | -0.760511499 | EVI2A | ecotropic viral integration site 2A [Source:HGNC Symbol;Acc:HGNC:3499] |
| ENSG00000128594 | HGLibA_27349 | 39 | -0.760511499 | LRRC4 | leucine rich repeat containing 4 [Source:HGNC Symbol;Acc:HGNC:15586] |
| ENSG00000130055 | HGLibA_18894 | 39 | -0.760511499 | GDPD2 | glycerophosphodiester phosphodiesterase domain containing 2 [Source:HGNC Symbol;Acc:HGNC:25974] |
| ENSG00000130167 | HGLibA_51911 | 39 | -0.760511499 | TSPAN16 | tetraspanin 16 [Source:HGNC Symbol;Acc:HGNC:30725] |
| ENSG00000130347 | HGLibA_42410 | 39 | -0.760511499 | RTN4IP1 | reticulon 4 interacting protein 1 [Source:HGNC Symbol;Acc:HGNC:18647] |
| ENSG00000130413 | HGLibA_47493 | 39 | -0.760511499 | STK33 | serine/threonine kinase 33 [Source:HGNC Symbol;Acc:HGNC:14568] |
| ENSG00000131171 | HGLibA_43916 | 39 | -0.760511499 | SH3BGRL | SH3 domain binding glutamate rich protein like [Source:HGNC Symbol;Acc:HGNC:10823] |
| ENSG00000131409 | HGLibA_27379 | 39 | -0.760511499 | LRRC4B | leucine rich repeat containing 4B [Source:HGNC Symbol;Acc:HGNC:25042] |
| ENSG00000133863 | HGLibA_49068 | 39 | -0.760511499 | TEX15 | meiosis and synapsis associated [Source:HGNC Symbol;Acc:HGNC:11738]" |
| ENSG00000134697 | HGLibA_19547 | 39 | -0.760511499 | GNL2 | G protein nucleolar 2 [Source:HGNC Symbol;Acc:HGNC:29925] |
| ENSG00000134757 | HGLibA_14028 | 39 | -0.760511499 | DSG3 | desmoglein 3 [Source:HGNC Symbol;Acc:HGNC:3050] |
| ENSG00000136052 | HGLibA_45141 | 39 | -0.760511499 | SLC41A2 | solute carrier family 41 member 2 [Source:HGNC Symbol;Acc:HGNC:31045] |
| ENSG00000136696 | HGLibA_23147 | 39 | -0.760511499 | IL36B | interleukin 36 beta [Source:HGNC Symbol;Acc:HGNC:15564] |
| ENSG00000136810 | HGLibA_52399 | 39 | -0.760511499 | TXN | thioredoxin [Source:HGNC Symbol;Acc:HGNC:12435] |
| ENSG00000136811 | HGLibA_33279 | 39 | -0.760511499 | ODF2 | outer dense fiber of sperm tails 2 [Source:HGNC Symbol;Acc:HGNC:8114] |
| ENSG00000137819 | HGLibA_35162 | 39 | -0.760511499 | PAQR5 | progestin and adipoQ receptor family member 5 [Source:HGNC Symbol;Acc:HGNC:29645] |
| ENSG00000137992 | HGLibA_12407 | 39 | -0.760511499 | DBT | dihydrolipoamide branched chain transacylase E2 [Source:HGNC Symbol;Acc:HGNC:2698] |
| ENSG00000138430 | HGLibA_33337 | 39 | -0.760511499 | OLA1 | Obg like ATPase 1 [Source:HGNC Symbol;Acc:HGNC:28833] |
| ENSG00000138670 | HGLibA_40331 | 39 | -0.760511499 | RASGEF1B | RasGEF domain family member 1B [Source:HGNC Symbol;Acc:HGNC:24881] |
| ENSG00000138771 | HGLibA_44092 | 39 | -0.760511499 | SHROOM3 | shroom family member 3 [Source:HGNC Symbol;Acc:HGNC:30422] |
| ENSG00000139144 | HGLibA_36627 | 39 | -0.760511499 | PIK3C2G | phosphatidylinositol-4-phosphate 3-kinase catalytic subunit type 2 gamma [Source:HGNC Symbol;Acc:HGNC:8973] |
| ENSG00000139173 | HGLibA_49890 | 39 | -0.760511499 | TMEM117 | transmembrane protein 117 [Source:HGNC Symbol;Acc:HGNC:25308] |
| ENSG00000139318 | HGLibA_14187 | 39 | -0.760511499 | DUSP6 | dual specificity phosphatase 6 [Source:HGNC Symbol;Acc:HGNC:3072] |
| ENSG00000139998 | HGLibA_39816 | 39 | -0.760511499 | RAB15 | member RAS oncogene family [Source:HGNC Symbol;Acc:HGNC:20150]" |
| ENSG00000140470 | HGLibA_00813 | 39 | -0.760511499 | ADAMTS17 | ADAM metallopeptidase with thrombospondin type 1 motif 17 [Source:HGNC Symbol;Acc:HGNC:17109] |
| ENSG00000140955 | HGLibA_00722 | 39 | -0.760511499 | ADAD2 | adenosine deaminase domain containing 2 [Source:HGNC Symbol;Acc:HGNC:30714] |
| ENSG00000140995 | HGLibA_12789 | 39 | -0.760511499 | DEF8 | differentially expressed in FDCP 8 homolog [Source:HGNC Symbol;Acc:HGNC:25969] |
| ENSG00000142330 | HGLibA_07144 | 39 | -0.760511499 | CAPN10 | calpain 10 [Source:HGNC Symbol;Acc:HGNC:1477] |
| ENSG00000143171 | HGLibA_42513 | 39 | -0.760511499 | RXRG | retinoid X receptor gamma [Source:HGNC Symbol;Acc:HGNC:10479] |
| ENSG00000143384 | HGLibA_28599 | 39 | -0.760511499 | MCL1 | BCL2 family apoptosis regulator [Source:HGNC Symbol;Acc:HGNC:6943]" |
| ENSG00000143553 | HGLibA_45905 | 39 | -0.760511499 | SNAPIN | SNAP associated protein [Source:HGNC Symbol;Acc:HGNC:17145] |
| ENSG00000143995 | HGLibA_28870 | 39 | -0.760511499 | MEIS1 | Meis homeobox 1 [Source:HGNC Symbol;Acc:HGNC:7000] |
| ENSG00000145022 | HGLibA_48855 | 39 | -0.760511499 | TCTA | T cell leukemia translocation altered [Source:HGNC Symbol;Acc:HGNC:11692] |
| ENSG00000146122 | HGLibA_12262 | 39 | -0.760511499 | DAAM2 | dishevelled associated activator of morphogenesis 2 [Source:HGNC Symbol;Acc:HGNC:18143] |
| ENSG00000147124 | HGLibA_55933 | 39 | -0.760511499 | ZNF41 | zinc finger protein 41 [Source:HGNC Symbol;Acc:HGNC:13107] |
| ENSG00000148290 | HGLibA_47832 | 39 | -0.760511499 | SURF1 | cytochrome c oxidase assembly factor [Source:HGNC Symbol;Acc:HGNC:11474]" |
| ENSG00000148339 | HGLibA_44683 | 39 | -0.760511499 | SLC25A25 | solute carrier family 25 member 25 [Source:HGNC Symbol;Acc:HGNC:20663] |
| ENSG00000148429 | HGLibA_53408 | 39 | -0.760511499 | USP6NL | USP6 N-terminal like [Source:HGNC Symbol;Acc:HGNC:16858] |
| ENSG00000150656 | HGLibA_10252 | 39 | -0.760511499 | CNDP1 | carnosine dipeptidase 1 [Source:HGNC Symbol;Acc:HGNC:20675] |

| | | | | | |
|---|---|---|---|---|---|
| ENSG00000150867 | HGLibA_36706 | 39 | -0.760511499 | PIP4K2A | phosphatidylinositol-5-phosphate 4-kinase type 2 alpha [Source:HGNC Symbol;Acc:HGNC:8997] |
| ENSG00000151322 | HGLibA_32399 | 39 | -0.760511499 | NPAS3 | neuronal PAS domain protein 3 [Source:HGNC Symbol;Acc:HGNC:19311] |
| ENSG00000151491 | HGLibA_15373 | 39 | -0.760511499 | EPS8 | epidermal growth factor receptor pathway substrate 8 [Source:HGNC Symbol;Acc:HGNC:3420] |
| ENSG00000151615 | HGLibA_37779 | 39 | -0.760511499 | POU4F2 | POU class 4 homeobox 2 [Source:HGNC Symbol;Acc:HGNC:9219] |
| ENSG00000153551 | HGLibA_10226 | 39 | -0.760511499 | CMTM7 | CKLF like MARVEL transmembrane domain containing 7 [Source:HGNC Symbol;Acc:HGNC:19178] |
| ENSG00000155966 | HGLibA_01109 | 39 | -0.760511499 | AFF2 | AF4/FMR2 family member 2 [Source:HGNC Symbol;Acc:HGNC:3776] |
| ENSG00000156885 | HGLibA_10845 | 39 | -0.760511499 | COX6A2 | cytochrome c oxidase subunit 6A2 [Source:HGNC Symbol;Acc:HGNC:2279] |
| ENSG00000157214 | HGLibA_47427 | 39 | -0.760511499 | STEAP2 | STEAP2 metalloreductase [Source:HGNC Symbol;Acc:HGNC:17885] |
| ENSG00000157227 | HGLibA_29491 | 39 | -0.760511499 | MMP14 | matrix metallopeptidase 14 [Source:HGNC Symbol;Acc:HGNC:7160] |
| ENSG00000157554 | HGLibA_15465 | 39 | -0.760511499 | ERG | ETS transcription factor ERG [Source:HGNC Symbol;Acc:HGNC:3446] |
| ENSG00000159086 | HGLibA_35322 | 39 | -0.760511499 | PAXBP1 | PAX3 and PAX7 binding protein 1 [Source:HGNC Symbol;Acc:HGNC:13579] |
| ENSG00000159182 | HGLibA_38263 | 39 | -0.760511499 | PRAC1 | PRAC1 small nuclear protein [Source:HGNC Symbol;Acc:HGNC:30591] |
| ENSG00000159212 | HGLibA_10036 | 39 | -0.760511499 | CLIC6 | chloride intracellular channel 6 [Source:HGNC Symbol;Acc:HGNC:2065] |
| ENSG00000159388 | HGLibA_04944 | 39 | -0.760511499 | BTG2 | BTG anti-proliferation factor 2 [Source:HGNC Symbol;Acc:HGNC:1131] |
| ENSG00000160294 | HGLibA_28610 | 39 | -0.760511499 | MCM3AP | minichromosome maintenance complex component 3 associated protein [Source:HGNC Symbol;Acc:HGNC:6946] |
| ENSG00000160703 | HGLibA_32139 | 39 | -0.760511499 | NLRX1 | NLR family member X1 [Source:HGNC Symbol;Acc:HGNC:29890] |
| ENSG00000160856 | HGLibA_17230 | 39 | -0.760511499 | FCRL3 | Fc receptor like 3 [Source:HGNC Symbol;Acc:HGNC:18506] |
| ENSG00000163214 | HGLibA_13202 | 39 | -0.760511499 | DHX57 | DExH-box helicase 57 [Source:HGNC Symbol;Acc:HGNC:20086] |
| ENSG00000163328 | HGLibA_19941 | 39 | -0.760511499 | GPR155 | G protein-coupled receptor 155 [Source:HGNC Symbol;Acc:HGNC:22951] |
| ENSG00000163349 | HGLibA_21340 | 39 | -0.760511499 | HIPK1 | homeodomain interacting protein kinase 1 [Source:HGNC Symbol;Acc:HGNC:19006] |
| ENSG00000163564 | HGLibA_39699 | 39 | -0.760511499 | PYHIN1 | pyrin and HIN domain family member 1 [Source:HGNC Symbol;Acc:HGNC:28894] |
| ENSG00000163798 | HGLibA_45210 | 39 | -0.760511499 | SLC4A1AP | solute carrier family 4 member 1 adaptor protein [Source:HGNC Symbol;Acc:HGNC:13813] |
| ENSG00000164346 | HGLibA_32741 | 39 | -0.760511499 | NSA2 | ribosome biogenesis homolog [Source:HGNC Symbol;Acc:HGNC:30728]" |
| ENSG00000164440 | HGLibA_52392 | 39 | -0.760511499 | TXLNB | taxilin beta [Source:HGNC Symbol;Acc:HGNC:21617] |
| ENSG00000164494 | HGLibA_35952 | 39 | -0.760511499 | PDSS2 | decaprenyl diphosphate synthase subunit 2 [Source:HGNC Symbol;Acc:HGNC:23041] |
| ENSG00000164764 | HGLibA_42814 | 39 | -0.760511499 | SBSPON | somatomedin B and thrombospondin type 1 domain containing [Source:HGNC Symbol;Acc:HGNC:30362] |
| ENSG00000165006 | HGLibA_52546 | 39 | -0.760511499 | UBAP1 | ubiquitin associated protein 1 [Source:HGNC Symbol;Acc:HGNC:12461] |
| ENSG00000165091 | HGLibA_49749 | 39 | -0.760511499 | TMC1 | transmembrane channel like 1 [Source:HGNC Symbol;Acc:HGNC:16513] |
| ENSG00000165458 | HGLibA_23363 | 39 | -0.760511499 | INPPL1 | inositol polyphosphate phosphatase like 1 [Source:HGNC Symbol;Acc:HGNC:6080] |
| ENSG00000165983 | HGLibA_39299 | 39 | -0.760511499 | PTER | phosphotriesterase related [Source:HGNC Symbol;Acc:HGNC:9590] |
| ENSG00000166226 | HGLibA_08196 | 39 | -0.760511499 | CCT2 | chaperonin containing TCP1 subunit 2 [Source:HGNC Symbol;Acc:HGNC:1615] |
| ENSG00000166432 | HGLibA_55365 | 39 | -0.760511499 | ZMAT1 | zinc finger matrin-type 1 [Source:HGNC Symbol;Acc:HGNC:29377] |
| ENSG00000166548 | HGLibA_49584 | 39 | -0.760511499 | TK2 | mitochondrial [Source:HGNC Symbol;Acc:HGNC:11831]" |
| ENSG00000166796 | HGLibA_26232 | 39 | -0.760511499 | LDHC | lactate dehydrogenase C [Source:HGNC Symbol;Acc:HGNC:6544] |
| ENSG00000167325 | HGLibA_42250 | 39 | -0.760511499 | RRM1 | ribonucleotide reductase catalytic subunit M1 [Source:HGNC Symbol;Acc:HGNC:10451] |
| ENSG00000167346 | HGLibA_29524 | 39 | -0.760511499 | MMP26 | matrix metallopeptidase 26 [Source:HGNC Symbol;Acc:HGNC:14249] |
| ENSG00000167355 | HGLibA_34089 | 39 | -0.760511499 | OR51B5 | olfactory receptor family 51 subfamily B member 5 [Source:HGNC Symbol;Acc:HGNC:19599] |
| ENSG00000167359 | HGLibA_34116 | 39 | -0.760511499 | OR51I1 | olfactory receptor family 51 subfamily I member 1 [Source:HGNC Symbol;Acc:HGNC:15200] |
| ENSG00000167747 | HGLibA_05673 | 39 | -0.760511499 | C19orf48 | chromosome 19 open reading frame 48 [Source:HGNC Symbol;Acc:HGNC:29667] |
| ENSG00000167840 | HGLibA_55638 | 39 | -0.760511499 | ZNF232 | zinc finger protein 232 [Source:HGNC Symbol;Acc:HGNC:13026] |
| ENSG00000168135 | HGLibA_24280 | 39 | -0.760511499 | KCNJ4 | potassium voltage-gated channel subfamily J member 4 [Source:HGNC Symbol;Acc:HGNC:6265] |
| ENSG00000168264 | HGLibA_23580 | 39 | -0.760511499 | IRF2BP2 | interferon regulatory factor 2 binding protein 2 [Source:HGNC Symbol;Acc:HGNC:21729] |
| ENSG00000168275 | HGLibA_10454 | 39 | -0.760511499 | COA6 | cytochrome c oxidase assembly factor 6 [Source:HGNC Symbol;Acc:HGNC:18025] |
| ENSG00000169241 | HGLibA_45234 | 39 | -0.760511499 | SLC50A1 | solute carrier family 50 member 1 [Source:HGNC Symbol;Acc:HGNC:30657] |
| ENSG00000169855 | HGLibA_41734 | 39 | -0.760511499 | ROBO1 | roundabout guidance receptor 1 [Source:HGNC Symbol;Acc:HGNC:10249] |
| ENSG00000170160 | HGLibA_07621 | 39 | -0.760511499 | CCDC144A | coiled-coil domain containing 144A [Source:HGNC Symbol;Acc:HGNC:29072] |
| ENSG00000170469 | HGLibA_46457 | 39 | -0.760511499 | SPATA24 | spermatogenesis associated 24 [Source:HGNC Symbol;Acc:HGNC:27322] |
| ENSG00000170950 | HGLibA_36243 | 39 | -0.760511499 | PGK2 | phosphoglycerate kinase 2 [Source:HGNC Symbol;Acc:HGNC:8898] |
| ENSG00000171105 | HGLibA_23399 | 39 | -0.760511499 | INSR | insulin receptor [Source:HGNC Symbol;Acc:HGNC:6091] |
| ENSG00000172243 | HGLibA_10006 | 39 | -0.760511499 | CLEC7A | C-type lectin domain containing 7A [Source:HGNC Symbol;Acc:HGNC:14558] |
| ENSG00000172663 | HGLibA_49947 | 39 | -0.760511499 | TMEM134 | transmembrane protein 134 [Source:HGNC Symbol;Acc:HGNC:26142] |
| ENSG00000172731 | HGLibA_27281 | 39 | -0.760511499 | LRRC20 | leucine rich repeat containing 20 [Source:HGNC Symbol;Acc:HGNC:23421] |
| ENSG00000172819 | HGLibA_40279 | 39 | -0.760511499 | RARG | retinoic acid receptor gamma [Source:HGNC Symbol;Acc:HGNC:9866] |
| ENSG00000173848 | HGLibA_31700 | 39 | -0.760511499 | NET1 | neuroepithelial cell transforming 1 [Source:HGNC Symbol;Acc:HGNC:14592] |
| ENSG00000174243 | HGLibA_12676 | 39 | -0.760511499 | DDX23 | DEAD-box helicase 23 [Source:HGNC Symbol;Acc:HGNC:17347] |
| ENSG00000174799 | HGLibA_09107 | 39 | -0.760511499 | CEP135 | centrosomal protein 135 [Source:HGNC Symbol;Acc:HGNC:29086] |
| ENSG00000174945 | HGLibA_01857 | 39 | -0.760511499 | AMZ1 | archaelysin family metallopeptidase 1 [Source:HGNC Symbol;Acc:HGNC:22231] |
| ENSG00000175309 | HGLibA_36479 | 39 | -0.760511499 | PHYKPL | 5-phosphohydroxy-L-lysine phospho-lyase [Source:HGNC Symbol;Acc:HGNC:28249] |
| ENSG00000175606 | HGLibA_50501 | 39 | -0.760511499 | TMEM70 | transmembrane protein 70 [Source:HGNC Symbol;Acc:HGNC:26050] |
| ENSG00000175766 | HGLibA_14818 | 39 | -0.760511499 | EIF4E1B | eukaryotic translation initiation factor 4E family member 1B [Source:HGNC Symbol;Acc:HGNC:33179] |
| ENSG00000175868 | HGLibA_06994 | 39 | -0.760511499 | CALCB | calcitonin related polypeptide beta [Source:HGNC Symbol;Acc:HGNC:1438] |
| ENSG00000177243 | HGLibA_12816 | 39 | -0.760511499 | DEFB103B | defensin beta 103B [Source:HGNC Symbol;Acc:HGNC:31702] |
| ENSG00000177853 | HGLibA_56148 | 39 | -0.760511499 | ZNF518A | zinc finger protein 518A [Source:HGNC Symbol;Acc:HGNC:29009] |
| ENSG00000177994 | HGLibA_06179 | 39 | -0.760511499 | C2orf73 | chromosome 2 open reading frame 73 [Source:HGNC Symbol;Acc:HGNC:26861] |
| ENSG00000178201 | HGLibA_53699 | 39 | -0.760511499 | VN1R1 | vomeronasal 1 receptor 1 [Source:HGNC Symbol;Acc:HGNC:13548] |
| ENSG00000178338 | HGLibA_55865 | 39 | -0.760511499 | ZNF354B | zinc finger protein 354B [Source:HGNC Symbol;Acc:HGNC:17197] |
| ENSG00000178860 | HGLibA_30184 | 39 | -0.760511499 | MSC | musculin [Source:HGNC Symbol;Acc:HGNC:7321] |
| ENSG00000179796 | HGLibA_27344 | 39 | -0.760511499 | LRRC3B | leucine rich repeat containing 3B [Source:HGNC Symbol;Acc:HGNC:28105] |
| ENSG00000179954 | HGLibA_47127 | 39 | -0.760511499 | SSC5D | scavenger receptor cysteine rich family member with 5 domains [Source:HGNC Symbol;Acc:HGNC:26641] |
| ENSG00000179958 | HGLibA_12561 | 39 | -0.760511499 | DCTPP1 | dCTP pyrophosphatase 1 [Source:HGNC Symbol;Acc:HGNC:28777] |
| ENSG00000180872 | HGLibA_12850 | 39 | -0.760511499 | DEFB112 | defensin beta 112 [Source:HGNC Symbol;Acc:HGNC:18093] |
| ENSG00000180917 | HGLibA_10235 | 39 | -0.760511499 | CMTR2 | cap methyltransferase 2 [Source:HGNC Symbol;Acc:HGNC:25635] |
| ENSG00000182134 | HGLibA_48920 | 39 | -0.760511499 | TDRKH | tudor and KH domain containing [Source:HGNC Symbol;Acc:HGNC:11713] |
| ENSG00000182346 | HGLibA_12313 | 39 | -0.760511499 | DAOA | D-amino acid oxidase activator [Source:HGNC Symbol;Acc:HGNC:21191] |
| ENSG00000183336 | HGLibA_04684 | 39 | -0.760511499 | BOLA2 | bolA family member 2 [Source:HGNC Symbol;Acc:HGNC:29488] |
| ENSG00000183878 | HGLibA_53471 | 39 | -0.760511499 | UTY | Y-linked [Source:HGNC Symbol;Acc:HGNC:12638]" |
| ENSG00000183943 | HGLibA_38594 | 39 | -0.760511499 | PRKX | protein kinase X-linked [Source:HGNC Symbol;Acc:HGNC:9441] |
| ENSG00000183979 | HGLibA_32409 | 39 | -0.760511499 | NPB | neuropeptide B [Source:HGNC Symbol;Acc:HGNC:30099] |
| ENSG00000184014 | HGLibA_12958 | 39 | -0.760511499 | DENND5A | DENN domain containing 5A [Source:HGNC Symbol;Acc:HGNC:19344] |
| ENSG00000184887 | HGLibA_04917 | 39 | -0.760511499 | BTBD6 | BTB domain containing 6 [Source:HGNC Symbol;Acc:HGNC:19897] |
| ENSG00000185338 | HGLibA_46123 | 39 | -0.760511499 | SOCS1 | suppressor of cytokine signaling 1 [Source:HGNC Symbol;Acc:HGNC:19383] |
| ENSG00000185372 | HGLibA_33895 | 39 | -0.760511499 | OR2V1 | olfactory receptor family 2 subfamily V member 1 [Source:HGNC Symbol;Acc:HGNC:8280] |
| ENSG00000185736 | HGLibA_00876 | 39 | -0.760511499 | ADARB2 | RNA specific B2 (inactive) [Source:HGNC Symbol;Acc:HGNC:227]" |
| ENSG00000186191 | HGLibA_04727 | 39 | -0.760511499 | BPIFB4 | BPI fold containing family B member 4 [Source:HGNC Symbol;Acc:HGNC:16179] |
| ENSG00000186377 | HGLibA_12203 | 39 | -0.760511499 | CYP4X1 | cytochrome P450 family 4 subfamily X member 1 [Source:HGNC Symbol;Acc:HGNC:20244] |
| ENSG00000186417 | HGLibA_19237 | 39 | -0.760511499 | GLDN | gliomedin [Source:HGNC Symbol;Acc:HGNC:29514] |
| ENSG00000186470 | HGLibA_04972 | 39 | -0.760511499 | BTN3A2 | butyrophilin subfamily 3 member A2 [Source:HGNC Symbol;Acc:HGNC:1139] |
| ENSG00000186675 | HGLibA_27983 | 39 | -0.760511499 | MAGEE2 | MAGE family member E2 [Source:HGNC Symbol;Acc:HGNC:24935] |
| ENSG00000186910 | HGLibA_43512 | 39 | -0.760511499 | SERPINA11 | serpin family A member 11 [Source:HGNC Symbol;Acc:HGNC:19193] |
| ENSG00000186952 | HGLibA_50260 | 39 | -0.760511499 | TMEM232 | transmembrane protein 232 [Source:HGNC Symbol;Acc:HGNC:37270] |
| ENSG00000187024 | HGLibA_39551 | 39 | -0.760511499 | PTRH1 | peptidyl-tRNA hydrolase 1 homolog [Source:HGNC Symbol;Acc:HGNC:27039] |
| ENSG00000188015 | HGLibA_42554 | 39 | -0.760511499 | S100A3 | S100 calcium binding protein A3 [Source:HGNC Symbol;Acc:HGNC:10493] |
| ENSG00000188038 | HGLibA_32712 | 39 | -0.760511499 | NRN1L | neuritin 1 like [Source:HGNC Symbol;Acc:HGNC:29811] |
| ENSG00000188191 | HGLibA_38523 | 39 | -0.760511499 | PRKAR1B | protein kinase cAMP-dependent type I regulatory subunit beta [Source:HGNC Symbol;Acc:HGNC:9390] |
| ENSG00000196139 | HGLibA_01487 | 39 | -0.760511499 | AKR1C3 | aldo-keto reductase family 1 member C3 [Source:HGNC Symbol;Acc:HGNC:386] |
| ENSG00000196466 | HGLibA_56775 | 39 | -0.760511499 | ZNF799 | zinc finger protein 799 [Source:HGNC Symbol;Acc:HGNC:28071] |
| ENSG00000196620 | HGLibA_52918 | 39 | -0.760511499 | UGT2B15 | UDP glucuronosyltransferase family 2 member B15 [Source:HGNC Symbol;Acc:HGNC:12546] |
| ENSG00000196712 | HGLibA_31757 | 39 | -0.760511499 | NF1 | neurofibromin 1 [Source:HGNC Symbol;Acc:HGNC:7765] |
| ENSG00000197077 | HGLibA_24800 | 39 | -0.760511499 | KIAA1671 | KIAA1671 [Source:HGNC Symbol;Acc:HGNC:29345] |
| ENSG00000197562 | HGLibA_39940 | 39 | -0.760511499 | RAB40C | member RAS oncogene family [Source:HGNC Symbol;Acc:HGNC:18285]" |
| ENSG00000197658 | HGLibA_44576 | 39 | -0.760511499 | SLC22A24 | solute carrier family 22 member 24 [Source:HGNC Symbol;Acc:HGNC:28542] |
| ENSG00000198113 | HGLibA_51034 | 39 | -0.760511499 | TOR4A | torsin family 4 member A [Source:HGNC Symbol;Acc:HGNC:25981] |
| ENSG00000198216 | HGLibA_06888 | 39 | -0.760511499 | CACNA1E | calcium voltage-gated channel subunit alpha1 E [Source:HGNC Symbol;Acc:HGNC:1392] |
| ENSG00000198218 | HGLibA_39742 | 39 | -0.760511499 | QRICH1 | glutamine rich 1 [Source:HGNC Symbol;Acc:HGNC:24713] |
| ENSG00000198301 | HGLibA_43094 | 39 | -0.760511499 | SDAD1 | SDA1 domain containing 1 [Source:HGNC Symbol;Acc:HGNC:25537] |
| ENSG00000198354 | HGLibA_12423 | 39 | -0.760511499 | DCAF12L2 | DDB1 and CUL4 associated factor 12 like 2 [Source:HGNC Symbol;Acc:HGNC:32950] |
| ENSG00000198417 | HGLibA_30286 | 39 | -0.760511499 | MT1F | metallothionein 1F [Source:HGNC Symbol;Acc:HGNC:7398] |
| ENSG00000198440 | HGLibA_56314 | 39 | -0.760511499 | ZNF583 | zinc finger protein 583 [Source:HGNC Symbol;Acc:HGNC:26427] |
| ENSG00000198604 | HGLibA_04184 | 39 | -0.760511499 | BAZ1A | bromodomain adjacent to zinc finger domain 1A [Source:HGNC Symbol;Acc:HGNC:960] |
| ENSG00000198642 | HGLibA_25229 | 39 | -0.760511499 | KLHL9 | kelch like family member 9 [Source:HGNC Symbol;Acc:HGNC:18732] |
| ENSG00000204310 | HGLibA_01217 | 39 | -0.760511499 | AGPAT1 | 1-acylglycerol-3-phosphate O-acyltransferase 1 [Source:HGNC Symbol;Acc:HGNC:324] |
| ENSG00000204363 | HGLibA_46415 | 39 | -0.760511499 | SPANXN5 | SPANX family member N5 [Source:HGNC Symbol;Acc:HGNC:33178] |
| ENSG00000204516 | HGLibA_29219 | 39 | -0.760511499 | MICB | MHC class I polypeptide-related sequence B [Source:HGNC Symbol;Acc:HGNC:7091] |
| ENSG00000204540 | HGLibA_39218 | 39 | -0.760511499 | PSORS1C1 | psoriasis susceptibility 1 candidate 1 [Source:HGNC Symbol;Acc:HGNC:17202] |
| ENSG00000204604 | HGLibA_56046 | 39 | -0.760511499 | ZNF468 | zinc finger protein 468 [Source:HGNC Symbol;Acc:HGNC:33105] |
| ENSG00000205060 | HGLibA_44969 | 39 | -0.760511499 | SLC35B4 | solute carrier family 35 member B4 [Source:HGNC Symbol;Acc:HGNC:20584] |
| ENSG00000205542 | HGLibA_50666 | 39 | -0.760511499 | TMSB4X | thymosin beta 4 X-linked [Source:HGNC Symbol;Acc:HGNC:11881] |
| ENSG00000205643 | HGLibA_08859 | 39 | -0.760511499 | CDPF1 | cysteine rich DPF motif domain containing 1 [Source:HGNC Symbol;Acc:HGNC:33710] |
| ENSG00000213020 | HGLibA_56384 | 39 | -0.760511499 | ZNF611 | zinc finger protein 611 [Source:HGNC Symbol;Acc:HGNC:28766] |
| ENSG00000213398 | HGLibA_26096 | 39 | -0.760511499 | LCAT | lecithin-cholesterol acyltransferase [Source:HGNC Symbol;Acc:HGNC:6522] |
| ENSG00000214642 | HGLibA_12852 | 39 | -0.760511499 | DEFB113 | defensin beta 113 [Source:HGNC Symbol;Acc:HGNC:18094] |
| ENSG00000215421 | HGLibA_55926 | 39 | -0.760511499 | ZNF407 | zinc finger protein 407 [Source:HGNC Symbol;Acc:HGNC:19904] |
| ENSG00000215712 | HGLibA_50290 | 39 | -0.760511499 | TMEM242 | transmembrane protein 242 [Source:HGNC Symbol;Acc:HGNC:17206] |
| ENSG00000221858 | HGLibA_33722 | 39 | -0.760511499 | OR2A12 | olfactory receptor family 2 subfamily A member 12 [Source:HGNC Symbol;Acc:HGNC:15082] |
| ENSG00000224470 | HGLibA_03834 | 39 | -0.760511499 | ATXN1L | ataxin 1 like [Source:HGNC Symbol;Acc:HGNC:33279] |
| ENSG00000229579 | HGLibA_53260 | 39 | -0.760511499 | USP17L26 | ubiquitin specific peptidase 17-like family member 26 [Source:HGNC Symbol;Acc:HGNC:44454] |
| ENSG00000237671 | HGLibA_18440 | 39 | -0.760511499 | GAGE12C | G antigen 12C [Source:HGNC Symbol;Acc:HGNC:28402] |
| ENSG00000240184 | HGLibA_35571 | 39 | -0.760511499 | PCDHGC3 | 3 [Source:HGNC Symbol;Acc:HGNC:8716]" |
| ENSG00000244462 | HGLibA_40480 | 39 | -0.760511499 | RBM12 | RNA binding motif protein 12 [Source:HGNC Symbol;Acc:HGNC:9898] |
| ENSG00000244476 | HGLibA_15539 | 39 | -0.760511499 | ERVFRD-1 | envelope [Source:HGNC Symbol;Acc:HGNC:33823]" |
| ENSG00000244754 | HGLibA_30958 | 39 | -0.760511499 | N4BP2L2 | NEDD4 binding protein 2 like 2 [Source:HGNC Symbol;Acc:HGNC:26916] |
| ENSG00000248333 | HGLibA_08729 | 39 | -0.760511499 | CDK11B | cyclin dependent kinase 11B [Source:HGNC Symbol;Acc:HGNC:1729] |
| ENSG00000248385 | HGLibA_48340 | 39 | -0.760511499 | TARM1 | activating receptor on myeloid cells 1 [Source:HGNC Symbol;Acc:HGNC:37250]" |
| ENSG00000250709 | HGLibA_07685 | 39 | -0.760511499 | CCDC169-SOHLH2 | CCDC169-SOHLH2 readthrough [Source:HGNC Symbol;Acc:HGNC:38866] |
| ENSG00000257103 | HGLibA_27529 | 39 | -0.760511499 | LSM14A | mRNA processing body assembly factor [Source:HGNC Symbol;Acc:HGNC:24489]" |

| | | | | | |
|---|---|---|---|---|---|
| ENSG00000270765 | HGLibA_18647 | 39 | -0.760511499 | GAS2L2 | growth arrest specific 2 like 2 [Source:HGNC Symbol;Acc:HGNC:24846] |
| ENSG00000271503 | HGLibA_08037 | 39 | -0.760511499 | CCL5 | C-C motif chemokine ligand 5 [Source:HGNC Symbol;Acc:HGNC:10632] |
| ENSG00000272602 | HGLibA_56346 | 39 | -0.760511499 | ZNF595 | zinc finger protein 595 [Source:HGNC Symbol;Acc:HGNC:27196] |
| HIST1H2BJ | HGLibA_21437 | 39 | -0.760511499 | HIST1H2BJ | - |
| HIST1H4D | HGLibA_21494 | 39 | -0.760511499 | HIST1H4D | - |
| hsa-mir-101-2 | HGLibA_57159 | 39 | -0.760511499 | hsa-mir-101-2 | - |
| hsa-mir-1184-3 | HGLibA_57236 | 39 | -0.760511499 | hsa-mir-1184-3 | - |
| hsa-mir-1197 | HGLibA_57253 | 39 | -0.760511499 | hsa-mir-1197 | - |
| hsa-mir-1243 | HGLibA_57359 | 39 | -0.760511499 | hsa-mir-1243 | - |
| hsa-mir-134 | HGLibA_57785 | 39 | -0.760511499 | hsa-mir-134 | - |
| hsa-mir-3064 | HGLibA_58458 | 39 | -0.760511499 | hsa-mir-3064 | - |
| hsa-mir-3127 | HGLibA_58561 | 39 | -0.760511499 | hsa-mir-3127 | - |
| hsa-mir-3149 | HGLibA_58653 | 39 | -0.760511499 | hsa-mir-3149 | - |
| hsa-mir-374c | HGLibA_59399 | 39 | -0.760511499 | hsa-mir-374c | - |
| hsa-mir-3922 | HGLibA_59565 | 39 | -0.760511499 | hsa-mir-3922 | - |
| hsa-mir-4283-1 | HGLibA_59835 | 39 | -0.760511499 | hsa-mir-4283-1 | - |
| hsa-mir-4420 | HGLibA_60067 | 39 | -0.760511499 | hsa-mir-4420 | - |
| hsa-mir-4529 | HGLibA_60574 | 39 | -0.760511499 | hsa-mir-4529 | - |
| hsa-mir-4673 | HGLibA_60807 | 39 | -0.760511499 | hsa-mir-4673 | - |
| hsa-mir-4707 | HGLibA_60927 | 39 | -0.760511499 | hsa-mir-4707 | - |
| hsa-mir-4709 | HGLibA_60935 | 39 | -0.760511499 | hsa-mir-4709 | - |
| hsa-mir-4788 | HGLibA_61258 | 39 | -0.760511499 | hsa-mir-4788 | - |
| hsa-mir-520b | HGLibA_61739 | 39 | -0.760511499 | hsa-mir-520b | - |
| hsa-mir-522 | HGLibA_61776 | 39 | -0.760511499 | hsa-mir-522 | - |
| hsa-mir-548aa-1 | HGLibA_61848 | 39 | -0.760511499 | hsa-mir-548aa-1 | - |
| hsa-mir-548f-4 | HGLibA_61982 | 39 | -0.760511499 | hsa-mir-548f-4 | - |
| hsa-mir-550a-3 | HGLibA_62106 | 39 | -0.760511499 | hsa-mir-550a-3 | - |
| hsa-mir-5681a | HGLibA_62239 | 39 | -0.760511499 | hsa-mir-5681a | - |
| hsa-mir-5692a-2 | HGLibA_62292 | 39 | -0.760511499 | hsa-mir-5692a-2 | - |
| hsa-mir-585 | HGLibA_62439 | 39 | -0.760511499 | hsa-mir-585 | - |
| hsa-mir-6076 | HGLibA_62556 | 39 | -0.760511499 | hsa-mir-6076 | - |
| hsa-mir-615 | HGLibA_62697 | 39 | -0.760511499 | hsa-mir-615 | - |
| hsa-mir-625 | HGLibA_62740 | 39 | -0.760511499 | hsa-mir-625 | - |
| hsa-mir-638 | HGLibA_62792 | 39 | -0.760511499 | hsa-mir-638 | - |
| hsa-mir-6809 | HGLibA_63413 | 39 | -0.760511499 | hsa-mir-6809 | - |
| hsa-mir-7150 | HGLibA_63829 | 39 | -0.760511499 | hsa-mir-7150 | - |
| hsa-mir-7705 | HGLibA_63954 | 39 | -0.760511499 | hsa-mir-7705 | - |
| hsa-mir-7974 | HGLibA_64026 | 39 | -0.760511499 | hsa-mir-7974 | - |
| KIAA1430 | HGLibA_24754 | 39 | -0.760511499 | KIAA1430 | - |
| LECT1 | HGLibA_26264 | 39 | -0.760511499 | LECT1 | - |
| LOC440563 | HGLibA_26947 | 39 | -0.760511499 | LOC440563 | - |
| LRRC33 | HGLibA_27317 | 39 | -0.760511499 | LRRC33 | - |
| N6AMT2 | HGLibA_30967 | 39 | -0.760511499 | N6AMT2 | - |
| SMEK2 | HGLibA_45695 | 39 | -0.760511499 | SMEK2 | - |
| TBC1D29 | HGLibA_48524 | 39 | -0.760511499 | TBC1D29 | - |
| C10orf111 | HGLibA_05030 | 38 | -0.770462537 | C10orf111 | - |
| C16orf58 | HGLibA_05461 | 38 | -0.770462537 | C16orf58 | - |
| C19orf70 | HGLibA_05700 | 38 | -0.770462537 | C19orf70 | - |
| C3orf58 | HGLibA_06255 | 38 | -0.770462537 | C3orf58 | - |
| C8orf37 | HGLibA_06613 | 38 | -0.770462537 | C8orf37 | - |
| CXorf36 | HGLibA_11918 | 38 | -0.770462537 | CXorf36 | - |
| CXorf40B | HGLibA_11925 | 38 | -0.770462537 | CXorf40B | - |
| ENSG00000004809 | HGLibA_44554 | 38 | -0.770462537 | SLC22A16 | solute carrier family 22 member 16 [Source:HGNC Symbol;Acc:HGNC:20302] |
| ENSG00000029153 | HGLibA_03070 | 38 | -0.770462537 | ARNTL2 | aryl hydrocarbon receptor nuclear translocator like 2 [Source:HGNC Symbol;Acc:HGNC:18984] |
| ENSG00000029993 | HGLibA_21695 | 38 | -0.770462537 | HMGB3 | high mobility group box 3 [Source:HGNC Symbol;Acc:HGNC:5004] |
| ENSG00000047634 | HGLibA_42960 | 38 | -0.770462537 | SCML1 | Scm polycomb group protein like 1 [Source:HGNC Symbol;Acc:HGNC:10580] |
| ENSG00000049089 | HGLibA_10623 | 38 | -0.770462537 | COL9A2 | collagen type IX alpha 2 chain [Source:HGNC Symbol;Acc:HGNC:2218] |
| ENSG00000058056 | HGLibA_53203 | 38 | -0.770462537 | USP13 | ubiquitin specific peptidase 13 [Source:HGNC Symbol;Acc:HGNC:12611] |
| ENSG00000058272 | HGLibA_38019 | 38 | -0.770462537 | PPP1R12A | protein phosphatase 1 regulatory subunit 12A [Source:HGNC Symbol;Acc:HGNC:7618] |
| ENSG00000060642 | HGLibA_36595 | 38 | -0.770462537 | PIGV | phosphatidylinositol glycan anchor biosynthesis class V [Source:HGNC Symbol;Acc:HGNC:26031] |
| ENSG00000060718 | HGLibA_10501 | 38 | -0.770462537 | COL11A1 | collagen type XI alpha 1 chain [Source:HGNC Symbol;Acc:HGNC:2186] |
| ENSG00000061492 | HGLibA_54387 | 38 | -0.770462537 | WNT8A | Wnt family member 8A [Source:HGNC Symbol;Acc:HGNC:12788] |
| ENSG00000064225 | HGLibA_47253 | 38 | -0.770462537 | ST3GAL6 | 3-sialyltransferase 6 [Source:HGNC Symbol;Acc:HGNC:18080]" |
| ENSG00000068305 | HGLibA_28826 | 38 | -0.770462537 | MEF2A | myocyte enhancer factor 2A [Source:HGNC Symbol;Acc:HGNC:6993] |
| ENSG00000073711 | HGLibA_38155 | 38 | -0.770462537 | PPP2R3A | protein phosphatase 2 regulatory subunit B''alpha [Source:HGNC Symbol;Acc:HGNC:9307] |
| ENSG00000075651 | HGLibA_37035 | 38 | -0.770462537 | PLD1 | phospholipase D1 [Source:HGNC Symbol;Acc:HGNC:9067] |
| ENSG00000076258 | HGLibA_17710 | 38 | -0.770462537 | FMO4 | flavin containing monooxygenase 4 [Source:HGNC Symbol;Acc:HGNC:3772] |
| ENSG00000077063 | HGLibA_11742 | 38 | -0.770462537 | CTTNBP2 | cortactin binding protein 2 [Source:HGNC Symbol;Acc:HGNC:15679] |
| ENSG00000081307 | HGLibA_52522 | 38 | -0.770462537 | UBA5 | ubiquitin like modifier activating enzyme 5 [Source:HGNC Symbol;Acc:HGNC:23230] |
| ENSG00000085872 | HGLibA_09424 | 38 | -0.770462537 | CHERP | calcium homeostasis endoplasmic reticulum protein [Source:HGNC Symbol;Acc:HGNC:16930] |
| ENSG00000085999 | HGLibA_40103 | 38 | -0.770462537 | RAD54L | RAD54 like [Source:HGNC Symbol;Acc:HGNC:9826] |
| ENSG00000086506 | HGLibA_20936 | 38 | -0.770462537 | HBQ1 | hemoglobin subunit theta 1 [Source:HGNC Symbol;Acc:HGNC:4833] |
| ENSG00000087470 | HGLibA_13685 | 38 | -0.770462537 | DNM1L | dynamin 1 like [Source:HGNC Symbol;Acc:HGNC:2973] |
| ENSG00000090097 | HGLibA_35375 | 38 | -0.770462537 | PCBP4 | poly(rC) binding protein 4 [Source:HGNC Symbol;Acc:HGNC:8652] |
| ENSG00000099864 | HGLibA_35068 | 38 | -0.770462537 | PALM | paralemmin [Source:HGNC Symbol;Acc:HGNC:8594] |
| ENSG00000100030 | HGLibA_28248 | 38 | -0.770462537 | MAPK1 | mitogen-activated protein kinase 1 [Source:HGNC Symbol;Acc:HGNC:6871] |
| ENSG00000100239 | HGLibA_38225 | 38 | -0.770462537 | PPP6R2 | protein phosphatase 6 regulatory subunit 2 [Source:HGNC Symbol;Acc:HGNC:19253] |
| ENSG00000100296 | HGLibA_49373 | 38 | -0.770462537 | THOC5 | THO complex 5 [Source:HGNC Symbol;Acc:HGNC:19074] |
| ENSG00000102271 | HGLibA_25205 | 38 | -0.770462537 | KLHL4 | kelch like family member 4 [Source:HGNC Symbol;Acc:HGNC:6355] |
| ENSG00000102755 | HGLibA_17664 | 38 | -0.770462537 | FLT1 | fms related tyrosine kinase 1 [Source:HGNC Symbol;Acc:HGNC:3763] |
| ENSG00000102931 | HGLibA_02961 | 38 | -0.770462537 | ARL2BP | ADP ribosylation factor like GTPase 2 binding protein [Source:HGNC Symbol;Acc:HGNC:17146] |
| ENSG00000104529 | HGLibA_14500 | 38 | -0.770462537 | EEF1D | eukaryotic translation elongation factor 1 delta [Source:HGNC Symbol;Acc:HGNC:3211] |
| ENSG00000104818 | HGLibA_09305 | 38 | -0.770462537 | CGB2 | chorionic gonadotropin subunit beta 2 [Source:HGNC Symbol;Acc:HGNC:16722] |
| ENSG00000104859 | HGLibA_09799 | 38 | -0.770462537 | CLASRP | CLK4 associating serine/arginine rich protein [Source:HGNC Symbol;Acc:HGNC:17731] |
| ENSG00000104979 | HGLibA_05679 | 38 | -0.770462537 | C19orf53 | chromosome 19 open reading frame 53 [Source:HGNC Symbol;Acc:HGNC:24991] |
| ENSG00000105205 | HGLibA_09803 | 38 | -0.770462537 | CLC | Charcot-Leyden crystal galectin [Source:HGNC Symbol;Acc:HGNC:2014] |
| ENSG00000105419 | HGLibA_28877 | 38 | -0.770462537 | MEIS3 | Meis homeobox 3 [Source:HGNC Symbol;Acc:HGNC:29537] |
| ENSG00000106636 | HGLibA_54694 | 38 | -0.770462537 | YKT6 | YKT6 v-SNARE homolog [Source:HGNC Symbol;Acc:HGNC:16959] |
| ENSG00000106853 | HGLibA_39352 | 38 | -0.770462537 | PTGR1 | prostaglandin reductase 1 [Source:HGNC Symbol;Acc:HGNC:18429] |
| ENSG00000107643 | HGLibA_28284 | 38 | -0.770462537 | MAPK8 | mitogen-activated protein kinase 8 [Source:HGNC Symbol;Acc:HGNC:6881] |
| ENSG00000108018 | HGLibA_46182 | 38 | -0.770462537 | SORCS1 | sortilin related VPS10 domain containing receptor 1 [Source:HGNC Symbol;Acc:HGNC:16697] |
| ENSG00000108395 | HGLibA_51478 | 38 | -0.770462537 | TRIM37 | tripartite motif containing 37 [Source:HGNC Symbol;Acc:HGNC:7523] |
| ENSG00000108423 | HGLibA_52298 | 38 | -0.770462537 | TUBD1 | tubulin delta 1 [Source:HGNC Symbol;Acc:HGNC:16811] |
| ENSG00000108786 | HGLibA_22168 | 38 | -0.770462537 | HSD17B1 | hydroxysteroid 17-beta dehydrogenase 1 [Source:HGNC Symbol;Acc:HGNC:5210] |
| ENSG00000109061 | HGLibA_30704 | 38 | -0.770462537 | MYH1 | myosin heavy chain 1 [Source:HGNC Symbol;Acc:HGNC:7567] |
| ENSG00000109689 | HGLibA_47445 | 38 | -0.770462537 | STIM2 | stromal interaction molecule 2 [Source:HGNC Symbol;Acc:HGNC:19205] |
| ENSG00000109775 | HGLibA_52857 | 38 | -0.770462537 | UFSP2 | UFM1 specific peptidase 2 [Source:HGNC Symbol;Acc:HGNC:25640] |
| ENSG00000110514 | HGLibA_27858 | 38 | -0.770462537 | MADD | MAP kinase activating death domain [Source:HGNC Symbol;Acc:HGNC:6766] |
| ENSG00000111215 | HGLibA_38839 | 38 | -0.770462537 | PRR4 | proline rich 4 [Source:HGNC Symbol;Acc:HGNC:18020] |
| ENSG00000111716 | HGLibA_26230 | 38 | -0.770462537 | LDHB | lactate dehydrogenase B [Source:HGNC Symbol;Acc:HGNC:6541] |
| ENSG00000112041 | HGLibA_52329 | 38 | -0.770462537 | TULP1 | tubby like protein 1 [Source:HGNC Symbol;Acc:HGNC:12423] |
| ENSG00000112640 | HGLibA_38178 | 38 | -0.770462537 | PPP2R5D | protein phosphatase 2 regulatory subunit B'delta [Source:HGNC Symbol;Acc:HGNC:9312] |
| ENSG00000112742 | HGLibA_52181 | 38 | -0.770462537 | TTK | TTK protein kinase [Source:HGNC Symbol;Acc:HGNC:12401] |
| ENSG00000112964 | HGLibA_19040 | 38 | -0.770462537 | GHR | growth hormone receptor [Source:HGNC Symbol;Acc:HGNC:4263] |
| ENSG00000113621 | HGLibA_52410 | 38 | -0.770462537 | TXNDC15 | thioredoxin domain containing 15 [Source:HGNC Symbol;Acc:HGNC:20652] |
| ENSG00000113645 | HGLibA_54440 | 38 | -0.770462537 | WWC1 | WW and C2 domain containing 1 [Source:HGNC Symbol;Acc:HGNC:29435] |
| ENSG00000114405 | HGLibA_06209 | 38 | -0.770462537 | C3orf14 | chromosome 3 open reading frame 14 [Source:HGNC Symbol;Acc:HGNC:25024] |
| ENSG00000114473 | HGLibA_23516 | 38 | -0.770462537 | IQCG | IQ motif containing G [Source:HGNC Symbol;Acc:HGNC:25251] |
| ENSG00000114698 | HGLibA_37240 | 38 | -0.770462537 | PLSCR4 | phospholipid scramblase 4 [Source:HGNC Symbol;Acc:HGNC:16497] |
| ENSG00000116580 | HGLibA_19669 | 38 | -0.770462537 | GON4L | gon-4 like [Source:HGNC Symbol;Acc:HGNC:25973] |
| ENSG00000118402 | HGLibA_14966 | 38 | -0.770462537 | ELOVL4 | ELOVL fatty acid elongase 4 [Source:HGNC Symbol;Acc:HGNC:14415] |
| ENSG00000118473 | HGLibA_43815 | 38 | -0.770462537 | SGIP1 | SH3 domain GRB2 like endophilin interacting protein 1 [Source:HGNC Symbol;Acc:HGNC:25412] |
| ENSG00000118579 | HGLibA_28797 | 38 | -0.770462537 | MED28 | mediator complex subunit 28 [Source:HGNC Symbol;Acc:HGNC:24628] |
| ENSG00000118690 | HGLibA_03013 | 38 | -0.770462537 | ARMC2 | armadillo repeat containing 2 [Source:HGNC Symbol;Acc:HGNC:23045] |
| ENSG00000119711 | HGLibA_01580 | 38 | -0.770462537 | ALDH6A1 | aldehyde dehydrogenase 6 family member A1 [Source:HGNC Symbol;Acc:HGNC:7179] |
| ENSG00000120156 | HGLibA_48967 | 38 | -0.770462537 | TEK | TEK receptor tyrosine kinase [Source:HGNC Symbol;Acc:HGNC:11724] |
| ENSG00000121486 | HGLibA_51681 | 38 | -0.770462537 | TRMT1L | tRNA methyltransferase 1 like [Source:HGNC Symbol;Acc:HGNC:16782] |
| ENSG00000122012 | HGLibA_47881 | 38 | -0.770462537 | SV2C | synaptic vesicle glycoprotein 2C [Source:HGNC Symbol;Acc:HGNC:30670] |
| ENSG00000123240 | HGLibA_33459 | 38 | -0.770462537 | OPTN | optineurin [Source:HGNC Symbol;Acc:HGNC:17142] |
| ENSG00000123892 | HGLibA_39899 | 38 | -0.770462537 | RAB38 | member RAS oncogene family [Source:HGNC Symbol;Acc:HGNC:9776]" |
| ENSG00000124237 | HGLibA_06028 | 38 | -0.770462537 | C20orf85 | chromosome 20 open reading frame 85 [Source:HGNC Symbol;Acc:HGNC:16216] |
| ENSG00000124789 | HGLibA_33025 | 38 | -0.770462537 | NUP153 | nucleoporin 153 [Source:HGNC Symbol;Acc:HGNC:8062] |
| ENSG00000125409 | HGLibA_48977 | 38 | -0.770462537 | TEKT3 | tektin 3 [Source:HGNC Symbol;Acc:HGNC:14293] |
| ENSG00000128694 | HGLibA_34684 | 38 | -0.770462537 | OSGEPL1 | O-sialoglycoprotein endopeptidase like 1 [Source:HGNC Symbol;Acc:HGNC:23075] |
| ENSG00000129214 | HGLibA_43997 | 38 | -0.770462537 | SHBG | sex hormone binding globulin [Source:HGNC Symbol;Acc:HGNC:10839] |
| ENSG00000130119 | HGLibA_19552 | 38 | -0.770462537 | GNL3L | G protein nucleolar 3 like [Source:HGNC Symbol;Acc:HGNC:25553] |
| ENSG00000130159 | HGLibA_14420 | 38 | -0.770462537 | ECSIT | ECSIT signalling integrator [Source:HGNC Symbol;Acc:HGNC:29548] |
| ENSG00000130299 | HGLibA_20606 | 38 | -0.770462537 | GTPBP3 | mitochondrial [Source:HGNC Symbol;Acc:HGNC:14880]" |
| ENSG00000130520 | HGLibA_27542 | 38 | -0.770462537 | LSM4 | U6 small nuclear RNA and mRNA degradation associated [Source:HGNC Symbol;Acc:HGNC:17259]" |
| ENSG00000130985 | HGLibA_52513 | 38 | -0.770462537 | UBA1 | ubiquitin like modifier activating enzyme 1 [Source:HGNC Symbol;Acc:HGNC:12469] |
| ENSG00000133019 | HGLibA_09553 | 38 | -0.770462537 | CHRM3 | cholinergic receptor muscarinic 3 [Source:HGNC Symbol;Acc:HGNC:1952] |
| ENSG00000133962 | HGLibA_07368 | 38 | -0.770462537 | CATSPERB | cation channel sperm associated auxiliary subunit beta [Source:HGNC Symbol;Acc:HGNC:20500] |
| ENSG00000134258 | HGLibA_53883 | 38 | -0.770462537 | VTCN1 | V-set domain containing T cell activation inhibitor 1 [Source:HGNC Symbol;Acc:HGNC:28873] |
| ENSG00000134575 | HGLibA_00512 | 38 | -0.770462537 | ACP2 | lysosomal [Source:HGNC Symbol;Acc:HGNC:123]" |
| ENSG00000136286 | HGLibA_30839 | 38 | -0.770462537 | MYO1G | myosin IG [Source:HGNC Symbol;Acc:HGNC:13880] |

| | | | | | |
|---|---|---|---|---|---|
| ENSG00000136709 | HGLibA_54067 | 38 | -0.770462537 | WDR33 | WD repeat domain 33 [Source:HGNC Symbol;Acc:HGNC:25651] |
| ENSG00000136816 | HGLibA_51024 | 38 | -0.770462537 | TOR1B | torsin family 1 member B [Source:HGNC Symbol;Acc:HGNC:11995] |
| ENSG00000137273 | HGLibA_17867 | 38 | -0.770462537 | FOXF2 | forkhead box F2 [Source:HGNC Symbol;Acc:HGNC:3810] |
| ENSG00000137571 | HGLibA_45474 | 38 | -0.770462537 | SLCO5A1 | solute carrier organic anion transporter family member 5A1 [Source:HGNC Symbol;Acc:HGNC:19046] |
| ENSG00000138646 | HGLibA_21164 | 38 | -0.770462537 | HERC5 | HECT and RLD domain containing E3 ubiquitin protein ligase 5 [Source:HGNC Symbol;Acc:HGNC:24368] |
| ENSG00000138709 | HGLibA_26032 | 38 | -0.770462537 | LARP1B | La ribonucleoprotein domain family member 1B [Source:HGNC Symbol;Acc:HGNC:24704] |
| ENSG00000140287 | HGLibA_21030 | 38 | -0.770462537 | HDC | histidine decarboxylase [Source:HGNC Symbol;Acc:HGNC:4855] |
| ENSG00000140525 | HGLibA_16855 | 38 | -0.770462537 | FANCI | FA complementation group I [Source:HGNC Symbol;Acc:HGNC:25568] |
| ENSG00000140853 | HGLibA_32094 | 38 | -0.770462537 | NLRC5 | NLR family CARD domain containing 5 [Source:HGNC Symbol;Acc:HGNC:29933] |
| ENSG00000141698 | HGLibA_32818 | 38 | -0.770462537 | NT5C3B | cytosolic IIIB [Source:HGNC Symbol;Acc:HGNC:28300]" |
| ENSG00000142599 | HGLibA_40858 | 38 | -0.770462537 | RERE | arginine-glutamic acid dipeptide repeats [Source:HGNC Symbol;Acc:HGNC:9965] |
| ENSG00000142892 | HGLibA_36561 | 38 | -0.770462537 | PIGK | phosphatidylinositol glycan anchor biosynthesis class K [Source:HGNC Symbol;Acc:HGNC:8965] |
| ENSG00000142961 | HGLibA_29599 | 38 | -0.770462537 | MOB3C | MOB kinase activator 3C [Source:HGNC Symbol;Acc:HGNC:29800] |
| ENSG00000143514 | HGLibA_51057 | 38 | -0.770462537 | TP53BP2 | tumor protein p53 binding protein 2 [Source:HGNC Symbol;Acc:HGNC:12000] |
| ENSG00000145715 | HGLibA_40295 | 38 | -0.770462537 | RASA1 | RAS p21 protein activator 1 [Source:HGNC Symbol;Acc:HGNC:9871] |
| ENSG00000145725 | HGLibA_37957 | 38 | -0.770462537 | PPIP5K2 | diphosphoinositol pentakisphosphate kinase 2 [Source:HGNC Symbol;Acc:HGNC:29035] |
| ENSG00000147081 | HGLibA_01432 | 38 | -0.770462537 | AKAP4 | A-kinase anchoring protein 4 [Source:HGNC Symbol;Acc:HGNC:374] |
| ENSG00000147082 | HGLibA_08066 | 38 | -0.770462537 | CCNB3 | cyclin B3 [Source:HGNC Symbol;Acc:HGNC:18709] |
| ENSG00000147155 | HGLibA_14372 | 38 | -0.770462537 | EBP | cholestenol delta-isomerase [Source:HGNC Symbol;Acc:HGNC:3133]" |
| ENSG00000148090 | HGLibA_03863 | 38 | -0.770462537 | AUH | AU RNA binding methylglutaconyl-CoA hydratase [Source:HGNC Symbol;Acc:HGNC:890] |
| ENSG00000148229 | HGLibA_37515 | 38 | -0.770462537 | POLE3 | accessory subunit [Source:HGNC Symbol;Acc:HGNC:13546]" |
| ENSG00000148672 | HGLibA_19362 | 38 | -0.770462537 | GLUD1 | glutamate dehydrogenase 1 [Source:HGNC Symbol;Acc:HGNC:4335] |
| ENSG00000151093 | HGLibA_34834 | 38 | -0.770462537 | OXSM | mitochondrial [Source:HGNC Symbol;Acc:HGNC:26063]" |
| ENSG00000151746 | HGLibA_04493 | 38 | -0.770462537 | BICD1 | BICD cargo adaptor 1 [Source:HGNC Symbol;Acc:HGNC:1049] |
| ENSG00000152455 | HGLibA_47862 | 38 | -0.770462537 | SUV39H2 | suppressor of variegation 3-9 homolog 2 [Source:HGNC Symbol;Acc:HGNC:17287] |
| ENSG00000152954 | HGLibA_32724 | 38 | -0.770462537 | NRSN1 | neurensin 1 [Source:HGNC Symbol;Acc:HGNC:17881] |
| ENSG00000153006 | HGLibA_46959 | 38 | -0.770462537 | SREK1IP1 | SREK1 interacting protein 1 [Source:HGNC Symbol;Acc:HGNC:26716] |
| ENSG00000158477 | HGLibA_08263 | 38 | -0.770462537 | CD1A | CD1a molecule [Source:HGNC Symbol;Acc:HGNC:1634] |
| ENSG00000160349 | HGLibA_26166 | 38 | -0.770462537 | LCN1 | lipocalin 1 [Source:HGNC Symbol;Acc:HGNC:6525] |
| ENSG00000161551 | HGLibA_56296 | 38 | -0.770462537 | ZNF577 | zinc finger protein 577 [Source:HGNC Symbol;Acc:HGNC:28673] |
| ENSG00000161905 | HGLibA_01681 | 38 | -0.770462537 | ALOX15 | arachidonate 15-lipoxygenase [Source:HGNC Symbol;Acc:HGNC:433] |
| ENSG00000162374 | HGLibA_14889 | 38 | -0.770462537 | ELAVL4 | ELAV like RNA binding protein 4 [Source:HGNC Symbol;Acc:HGNC:3315] |
| ENSG00000162959 | HGLibA_28882 | 38 | -0.770462537 | MEMO1 | mediator of cell motility 1 [Source:HGNC Symbol;Acc:HGNC:14014] |
| ENSG00000163002 | HGLibA_33048 | 38 | -0.770462537 | NUP35 | nucleoporin 35 [Source:HGNC Symbol;Acc:HGNC:29797] |
| ENSG00000163161 | HGLibA_15437 | 38 | -0.770462537 | ERCC3 | TFIIH core complex helicase subunit [Source:HGNC Symbol;Acc:HGNC:3435]" |
| ENSG00000163320 | HGLibA_09316 | 38 | -0.770462537 | CGGBP1 | CGG triplet repeat binding protein 1 [Source:HGNC Symbol;Acc:HGNC:1888] |
| ENSG00000163331 | HGLibA_12331 | 38 | -0.770462537 | DAPL1 | death associated protein like 1 [Source:HGNC Symbol;Acc:HGNC:21490] |
| ENSG00000163605 | HGLibA_38205 | 38 | -0.770462537 | PPP4R2 | protein phosphatase 4 regulatory subunit 2 [Source:HGNC Symbol;Acc:HGNC:18296] |
| ENSG00000163661 | HGLibA_39571 | 38 | -0.770462537 | PTX3 | pentraxin 3 [Source:HGNC Symbol;Acc:HGNC:9692] |
| ENSG00000163705 | HGLibA_16842 | 38 | -0.770462537 | FANCD2OS | FANCD2 opposite strand [Source:HGNC Symbol;Acc:HGNC:28623] |
| ENSG00000164168 | HGLibA_50117 | 38 | -0.770462537 | TMEM184C | transmembrane protein 184C [Source:HGNC Symbol;Acc:HGNC:25587] |
| ENSG00000164176 | HGLibA_14466 | 38 | -0.770462537 | EDIL3 | EGF like repeats and discoidin domains 3 [Source:HGNC Symbol;Acc:HGNC:3173] |
| ENSG00000164729 | HGLibA_45022 | 38 | -0.770462537 | SLC35G3 | solute carrier family 35 member G3 [Source:HGNC Symbol;Acc:HGNC:26848] |
| ENSG00000165055 | HGLibA_29003 | 38 | -0.770462537 | METTL2B | methyltransferase like 2B [Source:HGNC Symbol;Acc:HGNC:18272] |
| ENSG00000165060 | HGLibA_18214 | 38 | -0.770462537 | FXN | frataxin [Source:HGNC Symbol;Acc:HGNC:3951] |
| ENSG00000165462 | HGLibA_36451 | 38 | -0.770462537 | PHOX2A | paired like homeobox 2A [Source:HGNC Symbol;Acc:HGNC:691] |
| ENSG00000165526 | HGLibA_42216 | 38 | -0.770462537 | RPUSD4 | RNA pseudouridine synthase D4 [Source:HGNC Symbol;Acc:HGNC:25898] |
| ENSG00000166250 | HGLibA_10073 | 38 | -0.770462537 | CLMP | CXADR like membrane protein [Source:HGNC Symbol;Acc:HGNC:24039] |
| ENSG00000166965 | HGLibA_40688 | 38 | -0.770462537 | RCCD1 | RCC1 domain containing 1 [Source:HGNC Symbol;Acc:HGNC:30457] |
| ENSG00000167332 | HGLibA_34101 | 38 | -0.770462537 | OR51E2 | olfactory receptor family 51 subfamily E member 2 [Source:HGNC Symbol;Acc:HGNC:15195] |
| ENSG00000167555 | HGLibA_56170 | 38 | -0.770462537 | ZNF528 | zinc finger protein 528 [Source:HGNC Symbol;Acc:HGNC:29384] |
| ENSG00000167969 | HGLibA_14406 | 38 | -0.770462537 | ECI1 | enoyl-CoA delta isomerase 1 [Source:HGNC Symbol;Acc:HGNC:2703] |
| ENSG00000168591 | HGLibA_50685 | 38 | -0.770462537 | TMUB2 | transmembrane and ubiquitin like domain containing 2 [Source:HGNC Symbol;Acc:HGNC:28459] |
| ENSG00000168619 | HGLibA_00744 | 38 | -0.770462537 | ADAM18 | ADAM metallopeptidase domain 18 [Source:HGNC Symbol;Acc:HGNC:196] |
| ENSG00000168785 | HGLibA_51941 | 38 | -0.770462537 | TSPAN5 | tetraspanin 5 [Source:HGNC Symbol;Acc:HGNC:17753] |
| ENSG00000168936 | HGLibA_49922 | 38 | -0.770462537 | TMEM129 | transmembrane protein 129 [Source:HGNC Symbol;Acc:HGNC:25137] |
| ENSG00000169057 | HGLibA_28726 | 38 | -0.770462537 | MECP2 | methyl-CpG binding protein 2 [Source:HGNC Symbol;Acc:HGNC:6990] |
| ENSG00000169976 | HGLibA_43714 | 38 | -0.770462537 | SF3B5 | splicing factor 3b subunit 5 [Source:HGNC Symbol;Acc:HGNC:21083] |
| ENSG00000170085 | HGLibA_44180 | 38 | -0.770462537 | SIMC1 | SUMO interacting motifs containing 1 [Source:HGNC Symbol;Acc:HGNC:24779] |
| ENSG00000170802 | HGLibA_17912 | 38 | -0.770462537 | FOXN2 | forkhead box N2 [Source:HGNC Symbol;Acc:HGNC:5281] |
| ENSG00000171016 | HGLibA_39693 | 38 | -0.770462537 | PYGO1 | pygopus family PHD finger 1 [Source:HGNC Symbol;Acc:HGNC:30256] |
| ENSG00000171051 | HGLibA_17963 | 38 | -0.770462537 | FPR1 | formyl peptide receptor 1 [Source:HGNC Symbol;Acc:HGNC:3826] |
| ENSG00000171657 | HGLibA_20088 | 38 | -0.770462537 | GPR82 | G protein-coupled receptor 82 [Source:HGNC Symbol;Acc:HGNC:4533] |
| ENSG00000172020 | HGLibA_18612 | 38 | -0.770462537 | GAP43 | growth associated protein 43 [Source:HGNC Symbol;Acc:HGNC:4140] |
| ENSG00000172061 | HGLibA_27261 | 38 | -0.770462537 | LRRC15 | leucine rich repeat containing 15 [Source:HGNC Symbol;Acc:HGNC:20818] |
| ENSG00000172346 | HGLibA_11312 | 38 | -0.770462537 | CSDC2 | cold shock domain containing C2 [Source:HGNC Symbol;Acc:HGNC:30359] |
| ENSG00000173171 | HGLibA_30511 | 38 | -0.770462537 | MTX1 | metaxin 1 [Source:HGNC Symbol;Acc:HGNC:7504] |
| ENSG00000173302 | HGLibA_19921 | 38 | -0.770462537 | GPR148 | G protein-coupled receptor 148 [Source:HGNC Symbol;Acc:HGNC:23623] |
| ENSG00000173991 | HGLibA_48692 | 38 | -0.770462537 | TCAP | titin-cap [Source:HGNC Symbol;Acc:HGNC:11610] |
| ENSG00000174567 | HGLibA_19663 | 38 | -0.770462537 | GOLT1A | golgi transport 1A [Source:HGNC Symbol;Acc:HGNC:24766] |
| ENSG00000174937 | HGLibA_34338 | 38 | -0.770462537 | OR5M3 | olfactory receptor family 5 subfamily M member 3 [Source:HGNC Symbol;Acc:HGNC:14806] |
| ENSG00000175121 | HGLibA_54269 | 38 | -0.770462537 | WFDC5 | WAP four-disulfide core domain 5 [Source:HGNC Symbol;Acc:HGNC:20477] |
| ENSG00000176386 | HGLibA_08536 | 38 | -0.770462537 | CDC26 | cell division cycle 26 [Source:HGNC Symbol;Acc:HGNC:17839] |
| ENSG00000176533 | HGLibA_19532 | 38 | -0.770462537 | GNG7 | G protein subunit gamma 7 [Source:HGNC Symbol;Acc:HGNC:4410] |
| ENSG00000176635 | HGLibA_21891 | 38 | -0.770462537 | HORMAD2 | HORMA domain containing 2 [Source:HGNC Symbol;Acc:HGNC:28383] |
| ENSG00000176920 | HGLibA_18187 | 38 | -0.770462537 | FUT2 | fucosyltransferase 2 [Source:HGNC Symbol;Acc:HGNC:4013] |
| ENSG00000177174 | HGLibA_33632 | 38 | -0.770462537 | OR14C36 | olfactory receptor family 14 subfamily C member 36 [Source:HGNC Symbol;Acc:HGNC:15026] |
| ENSG00000177721 | HGLibA_02235 | 38 | -0.770462537 | ANXA2R | annexin A2 receptor [Source:HGNC Symbol;Acc:HGNC:33463] |
| ENSG00000178084 | HGLibA_22387 | 38 | -0.770462537 | HTR3C | 5-hydroxytryptamine receptor 3C [Source:HGNC Symbol;Acc:HGNC:24003] |
| ENSG00000178403 | HGLibA_31750 | 38 | -0.770462537 | NEUROG2 | neurogenin 2 [Source:HGNC Symbol;Acc:HGNC:13805] |
| ENSG00000179930 | HGLibA_56454 | 38 | -0.770462537 | ZNF648 | zinc finger protein 648 [Source:HGNC Symbol;Acc:HGNC:18190] |
| ENSG00000180182 | HGLibA_28755 | 38 | -0.770462537 | MED14 | mediator complex subunit 14 [Source:HGNC Symbol;Acc:HGNC:2370] |
| ENSG00000180205 | HGLibA_54278 | 38 | -0.770462537 | WFDC9 | WAP four-disulfide core domain 9 [Source:HGNC Symbol;Acc:HGNC:20380] |
| ENSG00000180822 | HGLibA_39214 | 38 | -0.770462537 | PSMG4 | proteasome assembly chaperone 4 [Source:HGNC Symbol;Acc:HGNC:21108] |
| ENSG00000181481 | HGLibA_41512 | 38 | -0.770462537 | RNF135 | ring finger protein 135 [Source:HGNC Symbol;Acc:HGNC:21158] |
| ENSG00000182512 | HGLibA_19323 | 38 | -0.770462537 | GLRX5 | glutaredoxin 5 [Source:HGNC Symbol;Acc:HGNC:20134] |
| ENSG00000183036 | HGLibA_35662 | 38 | -0.770462537 | PCP4 | Purkinje cell protein 4 [Source:HGNC Symbol;Acc:HGNC:8742] |
| ENSG00000183161 | HGLibA_16848 | 38 | -0.770462537 | FANCF | FA complementation group F [Source:HGNC Symbol;Acc:HGNC:3587] |
| ENSG00000184752 | HGLibA_31445 | 38 | -0.770462537 | NDUFA12 | NADH:ubiquinone oxidoreductase subunit A12 [Source:HGNC Symbol;Acc:HGNC:23987] |
| ENSG00000185163 | HGLibA_12734 | 38 | -0.770462537 | DDX51 | DEAD-box helicase 51 [Source:HGNC Symbol;Acc:HGNC:20082] |
| ENSG00000185610 | HGLibA_12409 | 38 | -0.770462537 | DBX2 | developing brain homeobox 2 [Source:HGNC Symbol;Acc:HGNC:33186] |
| ENSG00000186007 | HGLibA_26287 | 38 | -0.770462537 | LEMD1 | LEM domain containing 1 [Source:HGNC Symbol;Acc:HGNC:18725] |
| ENSG00000186230 | HGLibA_56671 | 38 | -0.770462537 | ZNF749 | zinc finger protein 749 [Source:HGNC Symbol;Acc:HGNC:32783] |
| ENSG00000187094 | HGLibA_07953 | 38 | -0.770462537 | CCK | cholecystokinin [Source:HGNC Symbol;Acc:HGNC:1569] |
| ENSG00000187172 | HGLibA_04095 | 38 | -0.770462537 | BAGE2 | BAGE family member 2 [Source:HGNC Symbol;Acc:HGNC:15723] |
| ENSG00000187231 | HGLibA_43627 | 38 | -0.770462537 | SESTD1 | SEC14 and spectrin domain containing 1 [Source:HGNC Symbol;Acc:HGNC:18379] |
| ENSG00000187736 | HGLibA_31887 | 38 | -0.770462537 | NHEJ1 | non-homologous end joining factor 1 [Source:HGNC Symbol;Acc:HGNC:25737] |
| ENSG00000188352 | HGLibA_17778 | 38 | -0.770462537 | FOCAD | focadhesin [Source:HGNC Symbol;Acc:HGNC:23377] |
| ENSG00000188408 | HGLibA_27954 | 38 | -0.770462537 | MAGEB5 | MAGE family member B5 [Source:HGNC Symbol;Acc:HGNC:23795] |
| ENSG00000188629 | HGLibA_55530 | 38 | -0.770462537 | ZNF177 | zinc finger protein 177 [Source:HGNC Symbol;Acc:HGNC:12966] |
| ENSG00000189001 | HGLibA_42813 | 38 | -0.770462537 | SBSN | suprabasin [Source:HGNC Symbol;Acc:HGNC:24950] |
| ENSG00000189308 | HGLibA_26571 | 38 | -0.770462537 | LIN54 | lin-54 DREAM MuvB core complex component [Source:HGNC Symbol;Acc:HGNC:25397] |
| ENSG00000196083 | HGLibA_23051 | 38 | -0.770462537 | IL1RAP | interleukin 1 receptor accessory protein [Source:HGNC Symbol;Acc:HGNC:5995] |
| ENSG00000196136 | HGLibA_43516 | 38 | -0.770462537 | SERPINA3 | serpin family A member 3 [Source:HGNC Symbol;Acc:HGNC:16] |
| ENSG00000196263 | HGLibA_56057 | 38 | -0.770462537 | ZNF471 | zinc finger protein 471 [Source:HGNC Symbol;Acc:HGNC:23226] |
| ENSG00000196531 | HGLibA_31029 | 38 | -0.770462537 | NACA | nascent polypeptide associated complex subunit alpha [Source:HGNC Symbol;Acc:HGNC:7629] |
| ENSG00000196632 | HGLibA_54338 | 38 | -0.770462537 | WNK3 | WNK lysine deficient protein kinase 3 [Source:HGNC Symbol;Acc:HGNC:14543] |
| ENSG00000196705 | HGLibA_55981 | 38 | -0.770462537 | ZNF431 | zinc finger protein 431 [Source:HGNC Symbol;Acc:HGNC:20809] |
| ENSG00000196832 | HGLibA_33565 | 38 | -0.770462537 | OR11G2 | olfactory receptor family 11 subfamily G member 2 [Source:HGNC Symbol;Acc:HGNC:15346] |
| ENSG00000197054 | HGLibA_56686 | 38 | -0.770462537 | ZNF763 | zinc finger protein 763 [Source:HGNC Symbol;Acc:HGNC:27614] |
| ENSG00000197150 | HGLibA_00151 | 38 | -0.770462537 | ABCB8 | ATP binding cassette subfamily B member 8 [Source:HGNC Symbol;Acc:HGNC:49] |
| ENSG00000197461 | HGLibA_35842 | 38 | -0.770462537 | PDGFA | platelet derived growth factor subunit A [Source:HGNC Symbol;Acc:HGNC:8799] |
| ENSG00000197576 | HGLibA_21910 | 38 | -0.770462537 | HOXA4 | homeobox A4 [Source:HGNC Symbol;Acc:HGNC:5105] |
| ENSG00000198353 | HGLibA_21967 | 38 | -0.770462537 | HOXC4 | homeobox C4 [Source:HGNC Symbol;Acc:HGNC:5126] |
| ENSG00000198692 | HGLibA_14713 | 38 | -0.770462537 | EIF1AY | eukaryotic translation initiation factor 1A Y-linked [Source:HGNC Symbol;Acc:HGNC:3252] |
| ENSG00000198839 | HGLibA_55717 | 38 | -0.770462537 | ZNF277 | zinc finger protein 277 [Source:HGNC Symbol;Acc:HGNC:13070] |
| ENSG00000198915 | HGLibA_40329 | 38 | -0.770462537 | RASGEF1A | RasGEF domain family member 1A [Source:HGNC Symbol;Acc:HGNC:24246] |
| ENSG00000198933 | HGLibA_48606 | 38 | -0.770462537 | TBKBP1 | TBK1 binding protein 1 [Source:HGNC Symbol;Acc:HGNC:30140] |
| ENSG00000198963 | HGLibA_41777 | 38 | -0.770462537 | RORB | RAR related orphan receptor B [Source:HGNC Symbol;Acc:HGNC:10259] |
| ENSG00000203663 | HGLibA_33820 | 38 | -0.770462537 | OR2L2 | olfactory receptor family 2 subfamily L member 2 [Source:HGNC Symbol;Acc:HGNC:8266] |
| ENSG00000203785 | HGLibA_46817 | 38 | -0.770462537 | SPRR2E | small proline rich protein 2E [Source:HGNC Symbol;Acc:HGNC:11265] |
| ENSG00000204956 | HGLibA_35518 | 38 | -0.770462537 | PCDHGA1 | 1 [Source:HGNC Symbol;Acc:HGNC:8696]" |
| ENSG00000206106 | HGLibA_25720 | 38 | -0.770462537 | KRTAP22-2 | keratin associated protein 22-2 [Source:HGNC Symbol;Acc:HGNC:37091] |
| ENSG00000206190 | HGLibA_03544 | 38 | -0.770462537 | ATP10A | ATPase phospholipid transporting 10A (putative) [Source:HGNC Symbol;Acc:HGNC:13542] |
| ENSG00000212938 | HGLibA_25821 | 38 | -0.770462537 | KRTAP6-3 | keratin associated protein 6-3 [Source:HGNC Symbol;Acc:HGNC:18933] |
| ENSG00000214160 | HGLibA_01628 | 38 | -0.770462537 | ALG3 | 3- mannosyltransferase [Source:HGNC Symbol;Acc:HGNC:23056]" |
| ENSG00000214511 | HGLibA_21311 | 38 | -0.770462537 | HIGD1C | HIG1 hypoxia inducible domain family member 1C [Source:HGNC Symbol;Acc:HGNC:28044] |
| ENSG00000214655 | HGLibA_57057 | 38 | -0.770462537 | ZSWIM8 | zinc finger SWIM-type containing 8 [Source:HGNC Symbol;Acc:HGNC:23528] |
| ENSG00000214706 | HGLibA_22739 | 38 | -0.770462537 | IFRD2 | interferon related developmental regulator 2 [Source:HGNC Symbol;Acc:HGNC:5457] |
| ENSG00000215045 | HGLibA_20277 | 38 | -0.770462537 | GRID2IP | Grid2 interacting protein [Source:HGNC Symbol;Acc:HGNC:18464] |
| ENSG00000215545 | HGLibA_12861 | 38 | -0.770462537 | DEFB116 | defensin beta 116 [Source:HGNC Symbol;Acc:HGNC:18097] |
| ENSG00000226174 | HGLibA_49076 | 38 | -0.770462537 | TEX22 | testis expressed 22 [Source:HGNC Symbol;Acc:HGNC:40026] |
| ENSG00000226929 | HGLibA_11522 | 38 | -0.770462537 | CT47A11 | member A11 [Source:HGNC Symbol;Acc:HGNC:27397]" |
| ENSG00000227140 | HGLibA_53282 | 38 | -0.770462537 | USP17L5 | ubiquitin specific peptidase 17-like family member 5 [Source:HGNC Symbol;Acc:HGNC:37177] |
| ENSG00000227500 | HGLibA_42845 | 38 | -0.770462537 | SCAMP4 | secretory carrier membrane protein 4 [Source:HGNC Symbol;Acc:HGNC:30385] |

| | | | | | |
|---|---|---|---|---|---|
| ENSG00000232423 | HGLibA_38322 | 38 | -0.770462537 | PRAMEF6 | PRAME family member 6 [Source:HGNC Symbol;Acc:HGNC:30583] |
| ENSG00000240038 | HGLibA_01852 | 38 | -0.770462537 | AMY2B | alpha 2B (pancreatic) [Source:HGNC Symbol;Acc:HGNC:478]" |
| ENSG00000240386 | HGLibA_26116 | 38 | -0.770462537 | LCE1F | late cornified envelope 1F [Source:HGNC Symbol;Acc:HGNC:29467] |
| ENSG00000244094 | HGLibA_46821 | 38 | -0.770462537 | SPRR2F | small proline rich protein 2F [Source:HGNC Symbol;Acc:HGNC:11266] |
| ENSG00000244274 | HGLibA_12395 | 38 | -0.770462537 | DBNDD2 | dysbindin domain containing 2 [Source:HGNC Symbol;Acc:HGNC:15881] |
| ENSG00000251192 | HGLibA_56509 | 38 | -0.770462537 | ZNF674 | zinc finger protein 674 [Source:HGNC Symbol;Acc:HGNC:17625] |
| ENSG00000253457 | HGLibA_45739 | 38 | -0.770462537 | SMIM18 | small integral membrane protein 18 [Source:HGNC Symbol;Acc:HGNC:42973] |
| ENSG00000254415 | HGLibA_44135 | 38 | -0.770462537 | SIGLEC14 | sialic acid binding Ig like lectin 14 [Source:HGNC Symbol;Acc:HGNC:32926] |
| ENSG00000256229 | HGLibA_56083 | 38 | -0.770462537 | ZNF486 | zinc finger protein 486 [Source:HGNC Symbol;Acc:HGNC:20807] |
| ENSG00000256229 | HGLibA_56082 | 38 | -0.770462537 | ZNF486 | zinc finger protein 486 [Source:HGNC Symbol;Acc:HGNC:20807] |
| ENSG00000258643 | HGLibA_04326 | 38 | -0.770462537 | BCL2L2-PABPN1 | BCL2L2-PABPN1 readthrough [Source:HGNC Symbol;Acc:HGNC:42959] |
| ENSG00000263528 | HGLibA_22923 | 38 | -0.770462537 | IKBKE | inhibitor of nuclear factor kappa B kinase subunit epsilon [Source:HGNC Symbol;Acc:HGNC:14552] |
| ENSG00000266173 | HGLibA_47574 | 38 | -0.770462537 | STRADA | STE20-related kinase adaptor alpha [Source:HGNC Symbol;Acc:HGNC:30172] |
| ENSG00000275113 | HGLibA_18479 | 38 | -0.770462537 | GAGE2E | G antigen 2E [Source:HGNC Symbol;Acc:HGNC:31960] |
| ENSG00000284922 | HGLibA_27507 | 38 | -0.770462537 | LRTOMT | novel protein |
| FAM65B | HGLibA_16672 | 38 | -0.770462537 | FAM65B | - |
| FAM96A | HGLibA_16802 | 38 | -0.770462537 | FAM96A | - |
| H1FOO | HGLibA_20740 | 38 | -0.770462537 | H1FOO | - |
| HIST1H2AM | HGLibA_21409 | 38 | -0.770462537 | HIST1H2AM | - |
| HIST1H4J | HGLibA_21513 | 38 | -0.770462537 | HIST1H4J | - |
| hsa-let-7e | HGLibA_57123 | 38 | -0.770462537 | hsa-let-7e | - |
| hsa-mir-1261 | HGLibA_57467 | 38 | -0.770462537 | hsa-mir-1261 | - |
| hsa-mir-1302-3 | HGLibA_57700 | 38 | -0.770462537 | hsa-mir-1302-3 | - |
| hsa-mir-17 | HGLibA_57954 | 38 | -0.770462537 | hsa-mir-17 | - |
| hsa-mir-3065 | HGLibA_58462 | 38 | -0.770462537 | hsa-mir-3065 | - |
| hsa-mir-3119-2 | HGLibA_58530 | 38 | -0.770462537 | hsa-mir-3119-2 | - |
| hsa-mir-3138 | HGLibA_58609 | 38 | -0.770462537 | hsa-mir-3138 | - |
| hsa-mir-3143 | HGLibA_58630 | 38 | -0.770462537 | hsa-mir-3143 | - |
| hsa-mir-3146 | HGLibA_58640 | 38 | -0.770462537 | hsa-mir-3146 | - |
| hsa-mir-3156-2 | HGLibA_58692 | 38 | -0.770462537 | hsa-mir-3156-2 | - |
| hsa-mir-3160-1 | HGLibA_58717 | 38 | -0.770462537 | hsa-mir-3160-1 | - |
| hsa-mir-3179-2 | HGLibA_58794 | 38 | -0.770462537 | hsa-mir-3179-2 | - |
| hsa-mir-34a | HGLibA_59037 | 38 | -0.770462537 | hsa-mir-34a | - |
| hsa-mir-3605 | HGLibA_59057 | 38 | -0.770462537 | hsa-mir-3605 | - |
| hsa-mir-3610 | HGLibA_59073 | 38 | -0.770462537 | hsa-mir-3610 | - |
| hsa-mir-3654 | HGLibA_59160 | 38 | -0.770462537 | hsa-mir-3654 | - |
| hsa-mir-3688-2 | HGLibA_59308 | 38 | -0.770462537 | hsa-mir-3688-2 | - |
| hsa-mir-3688-2 | HGLibA_59311 | 38 | -0.770462537 | hsa-mir-3688-2 | - |
| hsa-mir-4265 | HGLibA_59768 | 38 | -0.770462537 | hsa-mir-4265 | - |
| hsa-mir-4279 | HGLibA_59819 | 38 | -0.770462537 | hsa-mir-4279 | - |
| hsa-mir-4284 | HGLibA_59845 | 38 | -0.770462537 | hsa-mir-4284 | - |
| hsa-mir-4326 | HGLibA_60028 | 38 | -0.770462537 | hsa-mir-4326 | - |
| hsa-mir-4422 | HGLibA_60074 | 38 | -0.770462537 | hsa-mir-4422 | - |
| hsa-mir-4452 | HGLibA_60215 | 38 | -0.770462537 | hsa-mir-4452 | - |
| hsa-mir-4512 | HGLibA_60492 | 38 | -0.770462537 | hsa-mir-4512 | - |
| hsa-mir-4632 | HGLibA_60632 | 38 | -0.770462537 | hsa-mir-4632 | - |
| hsa-mir-4634 | HGLibA_60643 | 38 | -0.770462537 | hsa-mir-4634 | - |
| hsa-mir-4732 | HGLibA_61024 | 38 | -0.770462537 | hsa-mir-4732 | - |
| hsa-mir-518e | HGLibA_61671 | 38 | -0.770462537 | hsa-mir-518e | - |
| hsa-mir-550a-1 | HGLibA_62099 | 38 | -0.770462537 | hsa-mir-550a-1 | - |
| hsa-mir-566 | HGLibA_62223 | 38 | -0.770462537 | hsa-mir-566 | - |
| hsa-mir-5694 | HGLibA_62309 | 38 | -0.770462537 | hsa-mir-5694 | - |
| hsa-mir-663a | HGLibA_62985 | 38 | -0.770462537 | hsa-mir-663a | - |
| hsa-mir-6729 | HGLibA_63069 | 38 | -0.770462537 | hsa-mir-6729 | - |
| hsa-mir-6730 | HGLibA_63074 | 38 | -0.770462537 | hsa-mir-6730 | - |
| hsa-mir-6773 | HGLibA_63264 | 38 | -0.770462537 | hsa-mir-6773 | - |
| hsa-mir-6799 | HGLibA_63373 | 38 | -0.770462537 | hsa-mir-6799 | - |
| hsa-mir-6853 | HGLibA_63584 | 38 | -0.770462537 | hsa-mir-6853 | - |
| hsa-mir-873 | HGLibA_64201 | 38 | -0.770462537 | hsa-mir-873 | - |
| KIAA0020 | HGLibA_24585 | 38 | -0.770462537 | KIAA0020 | - |
| KIAA1967 | HGLibA_24831 | 38 | -0.770462537 | KIAA1967 | - |
| LPPR4 | HGLibA_27122 | 38 | -0.770462537 | LPPR4 | - |
| NOV | HGLibA_32360 | 38 | -0.770462537 | NOV | - |
| PALM2-AKAP2 | HGLibA_35073 | 38 | -0.770462537 | PALM2-AKAP2 | - |
| PAPD5 | HGLibA_35124 | 38 | -0.770462537 | PAPD5 | - |
| SF3B14 | HGLibA_43703 | 38 | -0.770462537 | SF3B14 | - |
| TCEB3CL2 | HGLibA_48753 | 38 | -0.770462537 | TCEB3CL2 | - |
| XRCC6BP1 | HGLibA_54602 | 38 | -0.770462537 | XRCC6BP1 | - |
| XRCC6BP1 | HGLibA_54601 | 38 | -0.770462537 | XRCC6BP1 | - |
| 1-Dec | HGLibA_12771 | 37 | -0.780413574 | 1-Dec | - |
| ATP5D | HGLibA_03647 | 37 | -0.780413574 | ATP5D | - |
| C16orf52 | HGLibA_05454 | 37 | -0.780413574 | C16orf52 | - |
| C18orf8 | HGLibA_05625 | 37 | -0.780413574 | C18orf8 | - |
| C3orf55 | HGLibA_06251 | 37 | -0.780413574 | C3orf55 | - |
| DOPEY1 | HGLibA_13799 | 37 | -0.780413574 | DOPEY1 | - |
| ENSG00000000457 | HGLibA_43090 | 37 | -0.780413574 | SCYL3 | SCY1 like pseudokinase 3 [Source:HGNC Symbol;Acc:HGNC:19285] |
| ENSG00000005844 | HGLibA_23757 | 37 | -0.780413574 | ITGAL | integrin subunit alpha L [Source:HGNC Symbol;Acc:HGNC:6148] |
| ENSG00000006837 | HGLibA_08812 | 37 | -0.780413574 | CDKL3 | cyclin dependent kinase like 3 [Source:HGNC Symbol;Acc:HGNC:15483] |
| ENSG00000009844 | HGLibA_53882 | 37 | -0.780413574 | VTA1 | vesicle trafficking 1 [Source:HGNC Symbol;Acc:HGNC:20954] |
| ENSG00000010282 | HGLibA_21255 | 37 | -0.780413574 | HHATL | hedgehog acyltransferase like [Source:HGNC Symbol;Acc:HGNC:13242] |
| ENSG00000011566 | HGLibA_28216 | 37 | -0.780413574 | MAP4K3 | mitogen-activated protein kinase kinase kinase kinase 3 [Source:HGNC Symbol;Acc:HGNC:6865] |
| ENSG00000025434 | HGLibA_32584 | 37 | -0.780413574 | NR1H3 | nuclear receptor subfamily 1 group H member 3 [Source:HGNC Symbol;Acc:HGNC:7966] |
| ENSG00000031823 | HGLibA_40205 | 37 | -0.780413574 | RANBP3 | RAN binding protein 3 [Source:HGNC Symbol;Acc:HGNC:9850] |
| ENSG00000036565 | HGLibA_44487 | 37 | -0.780413574 | SLC18A1 | solute carrier family 18 member A1 [Source:HGNC Symbol;Acc:HGNC:10934] |
| ENSG00000055211 | HGLibA_19100 | 37 | -0.780413574 | GINM1 | glycoprotein integral membrane 1 [Source:HGNC Symbol;Acc:HGNC:21074] |
| ENSG00000061656 | HGLibA_46356 | 37 | -0.780413574 | SPAG4 | sperm associated antigen 4 [Source:HGNC Symbol;Acc:HGNC:11214] |
| ENSG00000069974 | HGLibA_39855 | 37 | -0.780413574 | RAB27A | member RAS oncogene family [Source:HGNC Symbol;Acc:HGNC:9766]" |
| ENSG00000070159 | HGLibA_39467 | 37 | -0.780413574 | PTPN3 | non-receptor type 3 [Source:HGNC Symbol;Acc:HGNC:9655]" |
| ENSG00000074181 | HGLibA_32350 | 37 | -0.780413574 | NOTCH3 | notch 3 [Source:HGNC Symbol;Acc:HGNC:7883] |
| ENSG00000075218 | HGLibA_20626 | 37 | -0.780413574 | GTSE1 | G2 and S-phase expressed 1 [Source:HGNC Symbol;Acc:HGNC:13698] |
| ENSG00000076924 | HGLibA_54461 | 37 | -0.780413574 | XAB2 | XPA binding protein 2 [Source:HGNC Symbol;Acc:HGNC:14089] |
| ENSG00000077279 | HGLibA_12579 | 37 | -0.780413574 | DCX | doublecortin [Source:HGNC Symbol;Acc:HGNC:2714] |
| ENSG00000080371 | HGLibA_39839 | 37 | -0.780413574 | RAB21 | member RAS oncogene family [Source:HGNC Symbol;Acc:HGNC:18263]" |
| ENSG00000086300 | HGLibA_46028 | 37 | -0.780413574 | SNX10 | sorting nexin 10 [Source:HGNC Symbol;Acc:HGNC:14974] |
| ENSG00000087250 | HGLibA_30305 | 37 | -0.780413574 | MT3 | metallothionein 3 [Source:HGNC Symbol;Acc:HGNC:7408] |
| ENSG00000088367 | HGLibA_15243 | 37 | -0.780413574 | EPB41L1 | erythrocyte membrane protein band 4.1 like 1 [Source:HGNC Symbol;Acc:HGNC:3378] |
| ENSG00000088387 | HGLibA_13761 | 37 | -0.780413574 | DOCK9 | dedicator of cytokinesis 9 [Source:HGNC Symbol;Acc:HGNC:14132] |
| ENSG00000088782 | HGLibA_12886 | 37 | -0.780413574 | DEFB127 | defensin beta 127 [Source:HGNC Symbol;Acc:HGNC:16206] |
| ENSG00000089041 | HGLibA_34863 | 37 | -0.780413574 | P2RX7 | purinergic receptor P2X 7 [Source:HGNC Symbol;Acc:HGNC:8537] |
| ENSG00000089351 | HGLibA_20194 | 37 | -0.780413574 | GRAMD1A | GRAM domain containing 1A [Source:HGNC Symbol;Acc:HGNC:29305] |
| ENSG00000090889 | HGLibA_24944 | 37 | -0.780413574 | KIF4A | kinesin family member 4A [Source:HGNC Symbol;Acc:HGNC:13339] |
| ENSG00000092969 | HGLibA_49215 | 37 | -0.780413574 | TGFB2 | transforming growth factor beta 2 [Source:HGNC Symbol;Acc:HGNC:11768] |
| ENSG00000095380 | HGLibA_31107 | 37 | -0.780413574 | NANS | N-acetylneuraminate synthase [Source:HGNC Symbol;Acc:HGNC:19237] |
| ENSG00000099940 | HGLibA_45882 | 37 | -0.780413574 | SNAP29 | synaptosome associated protein 29 [Source:HGNC Symbol;Acc:HGNC:11133] |
| ENSG00000100031 | HGLibA_19009 | 37 | -0.780413574 | GGT1 | gamma-glutamyltransferase 1 [Source:HGNC Symbol;Acc:HGNC:4250] |
| ENSG00000100084 | HGLibA_21350 | 37 | -0.780413574 | HIRA | histone cell cycle regulator [Source:HGNC Symbol;Acc:HGNC:4916] |
| ENSG00000100146 | HGLibA_46229 | 37 | -0.780413574 | SOX10 | SRY-box 10 [Source:HGNC Symbol;Acc:HGNC:11190] |
| ENSG00000100285 | HGLibA_31617 | 37 | -0.780413574 | NEFH | neurofilament heavy [Source:HGNC Symbol;Acc:HGNC:7737] |
| ENSG00000100376 | HGLibA_16047 | 37 | -0.780413574 | FAM118A | family with sequence similarity 118 member A [Source:HGNC Symbol;Acc:HGNC:1313] |
| ENSG00000100429 | HGLibA_20999 | 37 | -0.780413574 | HDAC10 | histone deacetylase 10 [Source:HGNC Symbol;Acc:HGNC:18128] |
| ENSG00000100918 | HGLibA_40761 | 37 | -0.780413574 | REC8 | REC8 meiotic recombination protein [Source:HGNC Symbol;Acc:HGNC:16879] |
| ENSG00000100949 | HGLibA_40018 | 37 | -0.780413574 | RABGGTA | Rab geranylgeranyltransferase subunit alpha [Source:HGNC Symbol;Acc:HGNC:9795] |
| ENSG00000101413 | HGLibA_42046 | 37 | -0.780413574 | RPRD1B | regulation of nuclear pre-mRNA domain containing 1B [Source:HGNC Symbol;Acc:HGNC:16209] |
| ENSG00000101624 | HGLibA_09163 | 37 | -0.780413574 | CEP76 | centrosomal protein 76 [Source:HGNC Symbol;Acc:HGNC:25727] |
| ENSG00000102897 | HGLibA_27745 | 37 | -0.780413574 | LYRM1 | LYR motif containing 1 [Source:HGNC Symbol;Acc:HGNC:25074] |
| ENSG00000103174 | HGLibA_31073 | 37 | -0.780413574 | NAGPA | N-acetylglucosamine-1-phosphodiester alpha-N-acetylglucosaminidase [Source:HGNC Symbol;Acc:HGNC:17378] |
| ENSG00000105392 | HGLibA_11237 | 37 | -0.780413574 | CRX | cone-rod homeobox [Source:HGNC Symbol;Acc:HGNC:2383] |
| ENSG00000105722 | HGLibA_15460 | 37 | -0.780413574 | ERF | ETS2 repressor factor [Source:HGNC Symbol;Acc:HGNC:3444] |
| ENSG00000105983 | HGLibA_26668 | 37 | -0.780413574 | LMBR1 | limb development membrane protein 1 [Source:HGNC Symbol;Acc:HGNC:13243] |
| ENSG00000107882 | HGLibA_47715 | 37 | -0.780413574 | SUFU | SUFU negative regulator of hedgehog signaling [Source:HGNC Symbol;Acc:HGNC:16466] |
| ENSG00000108666 | HGLibA_05573 | 37 | -0.780413574 | C17orf75 | chromosome 17 open reading frame 75 [Source:HGNC Symbol;Acc:HGNC:30173] |
| ENSG00000108953 | HGLibA_54742 | 37 | -0.780413574 | YWHAE | tyrosine 3-monooxygenase/tryptophan 5-monooxygenase activation protein epsilon [Source:HGNC Symbol;Acc:HGNC:12851] |
| ENSG00000110025 | HGLibA_46042 | 37 | -0.780413574 | SNX15 | sorting nexin 15 [Source:HGNC Symbol;Acc:HGNC:14978] |
| ENSG00000110048 | HGLibA_34636 | 37 | -0.780413574 | OSBP | oxysterol binding protein [Source:HGNC Symbol;Acc:HGNC:8503] |
| ENSG00000110768 | HGLibA_20545 | 37 | -0.780413574 | GTF2H1 | general transcription factor IIH subunit 1 [Source:HGNC Symbol;Acc:HGNC:4655] |
| ENSG00000111639 | HGLibA_30011 | 37 | -0.780413574 | MRPL51 | mitochondrial ribosomal protein L51 [Source:HGNC Symbol;Acc:HGNC:14044] |
| ENSG00000112357 | HGLibA_36125 | 37 | -0.780413574 | PEX7 | peroxisomal biogenesis factor 7 [Source:HGNC Symbol;Acc:HGNC:8860] |
| ENSG00000113205 | HGLibA_35497 | 37 | -0.780413574 | PCDHB3 | protocadherin beta 3 [Source:HGNC Symbol;Acc:HGNC:8688] |
| ENSG00000113209 | HGLibA_35503 | 37 | -0.780413574 | PCDHB5 | protocadherin beta 5 [Source:HGNC Symbol;Acc:HGNC:8690] |
| ENSG00000113269 | HGLibA_41506 | 37 | -0.780413574 | RNF130 | ring finger protein 130 [Source:HGNC Symbol;Acc:HGNC:18280] |
| ENSG00000114573 | HGLibA_03731 | 37 | -0.780413574 | ATP6V1A | ATPase H+ transporting V1 subunit A [Source:HGNC Symbol;Acc:HGNC:851] |
| ENSG00000115271 | HGLibA_18757 | 37 | -0.780413574 | GCA | grancalcin [Source:HGNC Symbol;Acc:HGNC:15990] |
| ENSG00000115290 | HGLibA_20227 | 37 | -0.780413574 | GRB14 | growth factor receptor bound protein 14 [Source:HGNC Symbol;Acc:HGNC:4565] |
| ENSG00000115665 | HGLibA_45279 | 37 | -0.780413574 | SLC5A7 | solute carrier family 5 member 7 [Source:HGNC Symbol;Acc:HGNC:14025] |
| ENSG00000116752 | HGLibA_04253 | 37 | -0.780413574 | BCAS2 | pre-mRNA processing factor [Source:HGNC Symbol;Acc:HGNC:975]" |

| | | | | | |
|---|---|---|---|---|---|
| ENSG00000116874 | HGLibA_53948 | 37 | -0.780413574 | WARS2 | mitochondrial [Source:HGNC Symbol;Acc:HGNC:12730]" |
| ENSG00000117009 | HGLibA_25319 | 37 | -0.780413574 | KMO | kynurenine 3-monooxygenase [Source:HGNC Symbol;Acc:HGNC:6381] |
| ENSG00000118491 | HGLibA_54940 | 37 | -0.780413574 | ZC2HC1B | zinc finger C2HC-type containing 1B [Source:HGNC Symbol;Acc:HGNC:21174] |
| ENSG00000118729 | HGLibA_07337 | 37 | -0.780413574 | CASQ2 | calsequestrin 2 [Source:HGNC Symbol;Acc:HGNC:1513] |
| ENSG00000119431 | HGLibA_21058 | 37 | -0.780413574 | HDHD3 | haloacid dehalogenase like hydrolase domain containing 3 [Source:HGNC Symbol;Acc:HGNC:28171] |
| ENSG00000119929 | HGLibA_11801 | 37 | -0.780413574 | CUTC | cutC copper transporter [Source:HGNC Symbol;Acc:HGNC:24271] |
| ENSG00000120156 | HGLibA_48968 | 37 | -0.780413574 | TEK | TEK receptor tyrosine kinase [Source:HGNC Symbol;Acc:HGNC:11724] |
| ENSG00000120949 | HGLibA_50790 | 37 | -0.780413574 | TNFRSF8 | TNF receptor superfamily member 8 [Source:HGNC Symbol;Acc:HGNC:11923] |
| ENSG00000121653 | HGLibA_28287 | 37 | -0.780413574 | MAPK8IP1 | mitogen-activated protein kinase 8 interacting protein 1 [Source:HGNC Symbol;Acc:HGNC:6882] |
| ENSG00000121741 | HGLibA_55392 | 37 | -0.780413574 | ZMYM2 | zinc finger MYM-type containing 2 [Source:HGNC Symbol;Acc:HGNC:12989] |
| ENSG00000123572 | HGLibA_32699 | 37 | -0.780413574 | NRK | Nik related kinase [Source:HGNC Symbol;Acc:HGNC:25391] |
| ENSG00000124243 | HGLibA_04259 | 37 | -0.780413574 | BCAS4 | breast carcinoma amplified sequence 4 [Source:HGNC Symbol;Acc:HGNC:14367] |
| ENSG00000125122 | HGLibA_27303 | 37 | -0.780413574 | LRRC29 | leucine rich repeat containing 29 [Source:HGNC Symbol;Acc:HGNC:13605] |
| ENSG00000125871 | HGLibA_29162 | 37 | -0.780413574 | MGME1 | mitochondrial genome maintenance exonuclease 1 [Source:HGNC Symbol;Acc:HGNC:16205] |
| ENSG00000125878 | HGLibA_48765 | 37 | -0.780413574 | TCF15 | transcription factor 15 [Source:HGNC Symbol;Acc:HGNC:11627] |
| ENSG00000126856 | HGLibA_38399 | 37 | -0.780413574 | PRDM7 | PR/SET domain 7 [Source:HGNC Symbol;Acc:HGNC:9351] |
| ENSG00000127399 | HGLibA_27407 | 37 | -0.780413574 | LRRC61 | leucine rich repeat containing 61 [Source:HGNC Symbol;Acc:HGNC:21704] |
| ENSG00000128191 | HGLibA_13056 | 37 | -0.780413574 | DGCR8 | microprocessor complex subunit [Source:HGNC Symbol;Acc:HGNC:2847]" |
| ENSG00000128710 | HGLibA_21986 | 37 | -0.780413574 | HOXD10 | homeobox D10 [Source:HGNC Symbol;Acc:HGNC:5133] |
| ENSG00000129451 | HGLibA_25234 | 37 | -0.780413574 | KLK10 | kallikrein related peptidase 10 [Source:HGNC Symbol;Acc:HGNC:6358] |
| ENSG00000129474 | HGLibA_01374 | 37 | -0.780413574 | AJUBA | ajuba LIM protein [Source:HGNC Symbol;Acc:HGNC:20250] |
| ENSG00000130158 | HGLibA_13752 | 37 | -0.780413574 | DOCK6 | dedicator of cytokinesis 6 [Source:HGNC Symbol;Acc:HGNC:19189] |
| ENSG00000130830 | HGLibA_29748 | 37 | -0.780413574 | MPP1 | membrane palmitoylated protein 1 [Source:HGNC Symbol;Acc:HGNC:7219] |
| ENSG00000131386 | HGLibA_18545 | 37 | -0.780413574 | GALNT15 | polypeptide N-acetylgalactosaminyltransferase 15 [Source:HGNC Symbol;Acc:HGNC:21531] |
| ENSG00000131475 | HGLibA_53750 | 37 | -0.780413574 | VPS25 | vacuolar protein sorting 25 homolog [Source:HGNC Symbol;Acc:HGNC:28122] |
| ENSG00000132952 | HGLibA_53423 | 37 | -0.780413574 | USPL1 | ubiquitin specific peptidase like 1 [Source:HGNC Symbol;Acc:HGNC:20294] |
| ENSG00000133110 | HGLibA_37712 | 37 | -0.780413574 | POSTN | periostin [Source:HGNC Symbol;Acc:HGNC:16953] |
| ENSG00000133256 | HGLibA_35807 | 37 | -0.780413574 | PDE6B | phosphodiesterase 6B [Source:HGNC Symbol;Acc:HGNC:8786] |
| ENSG00000134575 | HGLibA_00511 | 37 | -0.780413574 | ACP2 | lysosomal [Source:HGNC Symbol;Acc:HGNC:123]" |
| ENSG00000135999 | HGLibA_15269 | 37 | -0.780413574 | EPC2 | enhancer of polycomb homolog 2 [Source:HGNC Symbol;Acc:HGNC:24543] |
| ENSG00000136933 | HGLibA_40006 | 37 | -0.780413574 | RABEPK | Rab9 effector protein with kelch motifs [Source:HGNC Symbol;Acc:HGNC:16896] |
| ENSG00000137656 | HGLibA_04999 | 37 | -0.780413574 | BUD13 | BUD13 homolog [Source:HGNC Symbol;Acc:HGNC:28199] |
| ENSG00000137965 | HGLibA_22611 | 37 | -0.780413574 | IFI44 | interferon induced protein 44 [Source:HGNC Symbol;Acc:HGNC:16938] |
| ENSG00000138768 | HGLibA_53189 | 37 | -0.780413574 | USO1 | USO1 vesicle transport factor [Source:HGNC Symbol;Acc:HGNC:30904] |
| ENSG00000138823 | HGLibA_30503 | 37 | -0.780413574 | MTTP | microsomal triglyceride transfer protein [Source:HGNC Symbol;Acc:HGNC:7467] |
| ENSG00000139220 | HGLibA_37877 | 37 | -0.780413574 | PPFIA2 | PTPRF interacting protein alpha 2 [Source:HGNC Symbol;Acc:HGNC:9246] |
| ENSG00000139797 | HGLibA_41476 | 37 | -0.780413574 | RNF113B | ring finger protein 113B [Source:HGNC Symbol;Acc:HGNC:17267] |
| ENSG00000140650 | HGLibA_37318 | 37 | -0.780413574 | PMM2 | phosphomannomutase 2 [Source:HGNC Symbol;Acc:HGNC:9115] |
| ENSG00000141098 | HGLibA_18956 | 37 | -0.780413574 | GFOD2 | glucose-fructose oxidoreductase domain containing 2 [Source:HGNC Symbol;Acc:HGNC:28159] |
| ENSG00000142156 | HGLibA_10595 | 37 | -0.780413574 | COL6A1 | collagen type VI alpha 1 chain [Source:HGNC Symbol;Acc:HGNC:2211] |
| ENSG00000143368 | HGLibA_43713 | 37 | -0.780413574 | SF3B4 | splicing factor 3b subunit 4 [Source:HGNC Symbol;Acc:HGNC:10771] |
| ENSG00000143502 | HGLibA_47853 | 37 | -0.780413574 | SUSD4 | sushi domain containing 4 [Source:HGNC Symbol;Acc:HGNC:25470] |
| ENSG00000143669 | HGLibA_27775 | 37 | -0.780413574 | LYST | lysosomal trafficking regulator [Source:HGNC Symbol;Acc:HGNC:1968] |
| ENSG00000143870 | HGLibA_35886 | 37 | -0.780413574 | PDIA6 | protein disulfide isomerase family A member 6 [Source:HGNC Symbol;Acc:HGNC:30168] |
| ENSG00000147251 | HGLibA_13738 | 37 | -0.780413574 | DOCK11 | dedicator of cytokinesis 11 [Source:HGNC Symbol;Acc:HGNC:23483] |
| ENSG00000148399 | HGLibA_13855 | 37 | -0.780413574 | DPH7 | diphthamide biosynthesis 7 [Source:HGNC Symbol;Acc:HGNC:25199] |
| ENSG00000148483 | HGLibA_50273 | 37 | -0.780413574 | TMEM236 | transmembrane protein 236 [Source:HGNC Symbol;Acc:HGNC:23473] |
| ENSG00000149489 | HGLibA_41755 | 37 | -0.780413574 | ROM1 | retinal outer segment membrane protein 1 [Source:HGNC Symbol;Acc:HGNC:10254] |
| ENSG00000149541 | HGLibA_03977 | 37 | -0.780413574 | B3GAT3 | 3-glucuronyltransferase 3 [Source:HGNC Symbol;Acc:HGNC:923]" |
| ENSG00000151079 | HGLibA_24141 | 37 | -0.780413574 | KCNA6 | potassium voltage-gated channel subfamily A member 6 [Source:HGNC Symbol;Acc:HGNC:6225] |
| ENSG00000152192 | HGLibA_37777 | 37 | -0.780413574 | POU4F1 | POU class 4 homeobox 1 [Source:HGNC Symbol;Acc:HGNC:9218] |
| ENSG00000152778 | HGLibA_22635 | 37 | -0.780413574 | IFIT5 | interferon induced protein with tetratricopeptide repeats 5 [Source:HGNC Symbol;Acc:HGNC:13328] |
| ENSG00000153165 | HGLibA_41031 | 37 | -0.780413574 | RGPD3 | RANBP2-like and GRIP domain containing 3 [Source:HGNC Symbol;Acc:HGNC:32416] |
| ENSG00000153347 | HGLibA_16737 | 37 | -0.780413574 | FAM81B | family with sequence similarity 81 member B [Source:HGNC Symbol;Acc:HGNC:26335] |
| ENSG00000154645 | HGLibA_09512 | 37 | -0.780413574 | CHODL | chondrolectin [Source:HGNC Symbol;Acc:HGNC:17807] |
| ENSG00000154734 | HGLibA_00792 | 37 | -0.780413574 | ADAMTS1 | ADAM metallopeptidase with thrombospondin type 1 motif 1 [Source:HGNC Symbol;Acc:HGNC:217] |
| ENSG00000156639 | HGLibA_55153 | 37 | -0.780413574 | ZFAND3 | zinc finger AN1-type containing 3 [Source:HGNC Symbol;Acc:HGNC:18019] |
| ENSG00000158315 | HGLibA_41139 | 37 | -0.780413574 | RHBDL2 | rhomboid like 2 [Source:HGNC Symbol;Acc:HGNC:16083] |
| ENSG00000159713 | HGLibA_51172 | 37 | -0.780413574 | TPPP3 | tubulin polymerization promoting protein family member 3 [Source:HGNC Symbol;Acc:HGNC:24162] |
| ENSG00000160973 | HGLibA_17873 | 37 | -0.780413574 | FOXH1 | forkhead box H1 [Source:HGNC Symbol;Acc:HGNC:3814] |
| ENSG00000161714 | HGLibA_36994 | 37 | -0.780413574 | PLCD3 | phospholipase C delta 3 [Source:HGNC Symbol;Acc:HGNC:9061] |
| ENSG00000162009 | HGLibA_47186 | 37 | -0.780413574 | SSTR5 | somatostatin receptor 5 [Source:HGNC Symbol;Acc:HGNC:11334] |
| ENSG00000162727 | HGLibA_33843 | 37 | -0.780413574 | OR2M5 | olfactory receptor family 2 subfamily M member 5 [Source:HGNC Symbol;Acc:HGNC:19576] |
| ENSG00000163154 | HGLibA_50725 | 37 | -0.780413574 | TNFAIP8L2 | TNF alpha induced protein 8 like 2 [Source:HGNC Symbol;Acc:HGNC:26277] |
| ENSG00000163600 | HGLibA_22516 | 37 | -0.780413574 | ICOS | inducible T cell costimulator [Source:HGNC Symbol;Acc:HGNC:5351] |
| ENSG00000163935 | HGLibA_43720 | 37 | -0.780413574 | SFMBT1 | Scm like with four mbt domains 1 [Source:HGNC Symbol;Acc:HGNC:20255] |
| ENSG00000163993 | HGLibA_42584 | 37 | -0.780413574 | S100P | S100 calcium binding protein P [Source:HGNC Symbol;Acc:HGNC:10504] |
| ENSG00000164077 | HGLibA_29639 | 37 | -0.780413574 | MON1A | secretory trafficking associated [Source:HGNC Symbol;Acc:HGNC:28207]" |
| ENSG00000164253 | HGLibA_54087 | 37 | -0.780413574 | WDR41 | WD repeat domain 41 [Source:HGNC Symbol;Acc:HGNC:25601] |
| ENSG00000164287 | HGLibA_08520 | 37 | -0.780413574 | CDC20B | cell division cycle 20B [Source:HGNC Symbol;Acc:HGNC:24222] |
| ENSG00000165591 | HGLibA_15904 | 37 | -0.780413574 | FAAH2 | fatty acid amide hydrolase 2 [Source:HGNC Symbol;Acc:HGNC:26440] |
| ENSG00000166073 | HGLibA_19975 | 37 | -0.780413574 | GPR176 | G protein-coupled receptor 176 [Source:HGNC Symbol;Acc:HGNC:32370] |
| ENSG00000166090 | HGLibA_23105 | 37 | -0.780413574 | IL25 | interleukin 25 [Source:HGNC Symbol;Acc:HGNC:13765] |
| ENSG00000166793 | HGLibA_54715 | 37 | -0.780413574 | YPEL4 | yippee like 4 [Source:HGNC Symbol;Acc:HGNC:18328] |
| ENSG00000166947 | HGLibA_15261 | 37 | -0.780413574 | EPB42 | erythrocyte membrane protein band 4.2 [Source:HGNC Symbol;Acc:HGNC:3381] |
| ENSG00000167767 | HGLibA_25555 | 37 | -0.780413574 | KRT80 | keratin 80 [Source:HGNC Symbol;Acc:HGNC:27056] |
| ENSG00000167850 | HGLibA_08335 | 37 | -0.780413574 | CD300C | CD300c molecule [Source:HGNC Symbol;Acc:HGNC:19320] |
| ENSG00000167900 | HGLibA_49581 | 37 | -0.780413574 | TK1 | thymidine kinase 1 [Source:HGNC Symbol;Acc:HGNC:11830] |
| ENSG00000169213 | HGLibA_39911 | 37 | -0.780413574 | RAB3B | member RAS oncogene family [Source:HGNC Symbol;Acc:HGNC:9778]" |
| ENSG00000169271 | HGLibA_22309 | 37 | -0.780413574 | HSPB3 | heat shock protein family B (small) member 3 [Source:HGNC Symbol;Acc:HGNC:5248] |
| ENSG00000169403 | HGLibA_39245 | 37 | -0.780413574 | PTAFR | platelet activating factor receptor [Source:HGNC Symbol;Acc:HGNC:9582] |
| ENSG00000169442 | HGLibA_08404 | 37 | -0.780413574 | CD52 | CD52 molecule [Source:HGNC Symbol;Acc:HGNC:1804] |
| ENSG00000170396 | HGLibA_56787 | 37 | -0.780413574 | ZNF804A | zinc finger protein 804A [Source:HGNC Symbol;Acc:HGNC:21711] |
| ENSG00000170703 | HGLibA_52210 | 37 | -0.780413574 | TTLL6 | tubulin tyrosine ligase like 6 [Source:HGNC Symbol;Acc:HGNC:26664] |
| ENSG00000171103 | HGLibA_51700 | 37 | -0.780413574 | TRMT61B | tRNA methyltransferase 61B [Source:HGNC Symbol;Acc:HGNC:26070] |
| ENSG00000171368 | HGLibA_51165 | 37 | -0.780413574 | TPPP | tubulin polymerization promoting protein [Source:HGNC Symbol;Acc:HGNC:24164] |
| ENSG00000171476 | HGLibA_21885 | 37 | -0.780413574 | HOPX | HOP homeobox [Source:HGNC Symbol;Acc:HGNC:24961] |
| ENSG00000171940 | HGLibA_55599 | 37 | -0.780413574 | ZNF217 | zinc finger protein 217 [Source:HGNC Symbol;Acc:HGNC:13009] |
| ENSG00000172354 | HGLibA_19490 | 37 | -0.780413574 | GNB2 | G protein subunit beta 2 [Source:HGNC Symbol;Acc:HGNC:4398] |
| ENSG00000172845 | HGLibA_46301 | 37 | -0.780413574 | SP3 | Sp3 transcription factor [Source:HGNC Symbol;Acc:HGNC:11208] |
| ENSG00000173093 | HGLibA_07824 | 37 | -0.780413574 | CCDC63 | coiled-coil domain containing 63 [Source:HGNC Symbol;Acc:HGNC:26669] |
| ENSG00000174705 | HGLibA_43968 | 37 | -0.780413574 | SH3PXD2B | SH3 and PX domains 2B [Source:HGNC Symbol;Acc:HGNC:29242] |
| ENSG00000174945 | HGLibA_01856 | 37 | -0.780413574 | AMZ1 | archaelysin family metallopeptidase 1 [Source:HGNC Symbol;Acc:HGNC:22231] |
| ENSG00000175018 | HGLibA_49102 | 37 | -0.780413574 | TEX36 | testis expressed 36 [Source:HGNC Symbol;Acc:HGNC:31653] |
| ENSG00000176125 | HGLibA_52854 | 37 | -0.780413574 | UFSP1 | UFM1 specific peptidase 1 (inactive) [Source:HGNC Symbol;Acc:HGNC:33821] |
| ENSG00000176182 | HGLibA_30912 | 37 | -0.780413574 | MYPOP | partner of profilin [Source:HGNC Symbol;Acc:HGNC:20178]" |
| ENSG00000176406 | HGLibA_41280 | 37 | -0.780413574 | RIMS2 | regulating synaptic membrane exocytosis 2 [Source:HGNC Symbol;Acc:HGNC:17283] |
| ENSG00000176444 | HGLibA_10056 | 37 | -0.780413574 | CLK2 | CDC like kinase 2 [Source:HGNC Symbol;Acc:HGNC:2069] |
| ENSG00000177485 | HGLibA_54862 | 37 | -0.780413574 | ZBTB33 | zinc finger and BTB domain containing 33 [Source:HGNC Symbol;Acc:HGNC:16682] |
| ENSG00000177600 | HGLibA_42015 | 37 | -0.780413574 | RPLP2 | ribosomal protein lateral stalk subunit P2 [Source:HGNC Symbol;Acc:HGNC:10377] |
| ENSG00000178172 | HGLibA_46700 | 37 | -0.780413574 | SPINK6 | Kazal type 6 [Source:HGNC Symbol;Acc:HGNC:29486]" |
| ENSG00000178425 | HGLibA_32821 | 37 | -0.780413574 | NT5DC1 | 5'-nucleotidase domain containing 1 [Source:HGNC Symbol;Acc:HGNC:21556] |
| ENSG00000179010 | HGLibA_29829 | 37 | -0.780413574 | MRFAP1 | Morf4 family associated protein 1 [Source:HGNC Symbol;Acc:HGNC:24549] |
| ENSG00000179195 | HGLibA_56482 | 37 | -0.780413574 | ZNF664 | zinc finger protein 664 [Source:HGNC Symbol;Acc:HGNC:25406] |
| ENSG00000179988 | HGLibA_39235 | 37 | -0.780413574 | PSTK | phosphoseryl-tRNA kinase [Source:HGNC Symbol;Acc:HGNC:28578] |
| ENSG00000180257 | HGLibA_56813 | 37 | -0.780413574 | ZNF816 | zinc finger protein 816 [Source:HGNC Symbol;Acc:HGNC:26995] |
| ENSG00000181092 | HGLibA_00978 | 37 | -0.780413574 | ADIPOQ | C1Q and collagen domain containing [Source:HGNC Symbol;Acc:HGNC:13633]" |
| ENSG00000181873 | HGLibA_22478 | 37 | -0.780413574 | IBA57 | iron-sulfur cluster assembly [Source:HGNC Symbol;Acc:HGNC:27302]" |
| ENSG00000182117 | HGLibA_32301 | 37 | -0.780413574 | NOP10 | NOP10 ribonucleoprotein [Source:HGNC Symbol;Acc:HGNC:14378] |
| ENSG00000182950 | HGLibA_33289 | 37 | -0.780413574 | ODF3L1 | outer dense fiber of sperm tails 3 like 1 [Source:HGNC Symbol;Acc:HGNC:28735] |
| ENSG00000183340 | HGLibA_23995 | 37 | -0.780413574 | JRKL | JRK like [Source:HGNC Symbol;Acc:HGNC:6200] |
| ENSG00000184898 | HGLibA_40554 | 37 | -0.780413574 | RBM43 | RNA binding motif protein 43 [Source:HGNC Symbol;Acc:HGNC:24790] |
| ENSG00000185015 | HGLibA_06797 | 37 | -0.780413574 | CA13 | carbonic anhydrase 13 [Source:HGNC Symbol;Acc:HGNC:14914] |
| ENSG00000185689 | HGLibA_06468 | 37 | -0.780413574 | C6orf201 | chromosome 6 open reading frame 201 [Source:HGNC Symbol;Acc:HGNC:21620] |
| ENSG00000186222 | HGLibA_04565 | 37 | -0.780413574 | BLOC1S4 | biogenesis of lysosomal organelles complex 1 subunit 4 [Source:HGNC Symbol;Acc:HGNC:24206] |
| ENSG00000186432 | HGLibA_25364 | 37 | -0.780413574 | KPNA4 | karyopherin subunit alpha 4 [Source:HGNC Symbol;Acc:HGNC:6397] |
| ENSG00000186814 | HGLibA_57015 | 37 | -0.780413574 | ZSCAN30 | zinc finger and SCAN domain containing 30 [Source:HGNC Symbol;Acc:HGNC:33517] |
| ENSG00000187105 | HGLibA_21076 | 37 | -0.780413574 | HEATR4 | HEAT repeat containing 4 [Source:HGNC Symbol;Acc:HGNC:16761] |
| ENSG00000188021 | HGLibA_52736 | 37 | -0.780413574 | UBQLN2 | ubiquilin 2 [Source:HGNC Symbol;Acc:HGNC:12509] |
| ENSG00000188060 | HGLibA_39945 | 37 | -0.780413574 | RAB42 | member RAS oncogene family [Source:HGNC Symbol;Acc:HGNC:28702]" |
| ENSG00000188342 | HGLibA_20540 | 37 | -0.780413574 | GTF2F2 | general transcription factor IIF subunit 2 [Source:HGNC Symbol;Acc:HGNC:4653] |
| ENSG00000188404 | HGLibA_43296 | 37 | -0.780413574 | SELL | selectin L [Source:HGNC Symbol;Acc:HGNC:10720] |
| ENSG00000188803 | HGLibA_44045 | 37 | -0.780413574 | SHISA6 | shisa family member 6 [Source:HGNC Symbol;Acc:HGNC:34491] |
| ENSG00000189077 | HGLibA_49894 | 37 | -0.780413574 | TMEM120A | transmembrane protein 120A [Source:HGNC Symbol;Acc:HGNC:21697] |
| ENSG00000189369 | HGLibA_20441 | 37 | -0.780413574 | GSPT2 | G1 to S phase transition 2 [Source:HGNC Symbol;Acc:HGNC:4622] |
| ENSG00000196456 | HGLibA_56720 | 37 | -0.780413574 | ZNF775 | zinc finger protein 775 [Source:HGNC Symbol;Acc:HGNC:28501] |
| ENSG00000196772 | HGLibA_33628 | 37 | -0.780413574 | OR14A16 | olfactory receptor family 14 subfamily A member 16 [Source:HGNC Symbol;Acc:HGNC:15022] |
| ENSG00000196781 | HGLibA_49608 | 37 | -0.780413574 | TLE1 | transducin like enhancer of split 1 [Source:HGNC Symbol;Acc:HGNC:11837] |
| ENSG00000196793 | HGLibA_55654 | 37 | -0.780413574 | ZNF239 | zinc finger protein 239 [Source:HGNC Symbol;Acc:HGNC:13031] |
| ENSG00000197093 | HGLibA_18507 | 37 | -0.780413574 | GAL3ST4 | galactose-3-O-sulfotransferase 4 [Source:HGNC Symbol;Acc:HGNC:24145] |
| ENSG00000197617 | HGLibA_53707 | 37 | -0.780413574 | VN1R5 | vomeronasal 1 receptor 5 (gene/pseudogene) [Source:HGNC Symbol;Acc:HGNC:19870] |
| ENSG00000197863 | HGLibA_56763 | 37 | -0.780413574 | ZNF790 | zinc finger protein 790 [Source:HGNC Symbol;Acc:HGNC:33114] |
| ENSG00000198242 | HGLibA_41908 | 37 | -0.780413574 | RPL23A | ribosomal protein L23a [Source:HGNC Symbol;Acc:HGNC:10317] |
| ENSG00000198807 | HGLibA_35320 | 37 | -0.780413574 | PAX9 | paired box 9 [Source:HGNC Symbol;Acc:HGNC:8623] |
| ENSG00000198909 | HGLibA_28183 | 37 | -0.780413574 | MAP3K3 | mitogen-activated protein kinase kinase kinase 3 [Source:HGNC Symbol;Acc:HGNC:6855] |

| | | | | | |
|---|---|---|---|---|---|
| ENSG00000203926 | HGLibA_46382 | 37 | -0.780413574 | SPANXA2 | SPANX family member A2 [Source:HGNC Symbol;Acc:HGNC:14328] |
| ENSG00000204909 | HGLibA_46708 | 37 | -0.780413574 | SPINK9 | Kazal type 9 [Source:HGNC Symbol;Acc:HGNC:32951]" |
| ENSG00000206562 | HGLibA_29015 | 37 | -0.780413574 | METTL6 | methyltransferase like 6 [Source:HGNC Symbol;Acc:HGNC:28343] |
| ENSG00000213064 | HGLibA_43754 | 37 | -0.780413574 | SFT2D2 | SFT2 domain containing 2 [Source:HGNC Symbol;Acc:HGNC:25140] |
| ENSG00000235942 | HGLibA_26152 | 37 | -0.780413574 | LCE6A | late cornified envelope 6A [Source:HGNC Symbol;Acc:HGNC:31824] |
| ENSG00000237693 | HGLibA_23612 | 37 | -0.780413574 | IRGM | immunity related GTPase M [Source:HGNC Symbol;Acc:HGNC:29597] |
| ENSG00000240972 | HGLibA_29260 | 37 | -0.780413574 | MIF | macrophage migration inhibitory factor [Source:HGNC Symbol;Acc:HGNC:7097] |
| ENSG00000241399 | HGLibA_08349 | 37 | -0.780413574 | CD302 | CD302 molecule [Source:HGNC Symbol;Acc:HGNC:30843] |
| ENSG00000242372 | HGLibA_14867 | 37 | -0.780413574 | EIF6 | eukaryotic translation initiation factor 6 [Source:HGNC Symbol;Acc:HGNC:6159] |
| ENSG00000248643 | HGLibA_40487 | 37 | -0.780413574 | RBM14-RBM4 | RBM14-RBM4 readthrough [Source:HGNC Symbol;Acc:HGNC:38840] |
| ENSG00000251349 | HGLibA_30179 | 37 | -0.780413574 | MSANTD3-TMEFF1 | MSANTD3-TMEFF1 readthrough [Source:HGNC Symbol;Acc:HGNC:38838] |
| ENSG00000266338 | HGLibA_31224 | 37 | -0.780413574 | NBPF15 | NBPF member 15 [Source:HGNC Symbol;Acc:HGNC:28791] |
| ENSG00000270601 | HGLibA_38321 | 37 | -0.780413574 | PRAMEF5 | PRAME family member 5 [Source:HGNC Symbol;Acc:HGNC:27995] |
| FAM65C | HGLibA_16673 | 37 | -0.780413574 | FAM65C | - |
| HIST2H3D | HGLibA_21545 | 37 | -0.780413574 | HIST2H3D | - |
| HRASLS2 | HGLibA_22080 | 37 | -0.780413574 | HRASLS2 | - |
| hsa-mir-103b-2 | HGLibA_57173 | 37 | -0.780413574 | hsa-mir-103b-2 | - |
| hsa-mir-1234 | HGLibA_57330 | 37 | -0.780413574 | hsa-mir-1234 | - |
| hsa-mir-1262 | HGLibA_57472 | 37 | -0.780413574 | hsa-mir-1262 | - |
| hsa-mir-129-2 | HGLibA_57633 | 37 | -0.780413574 | hsa-mir-129-2 | - |
| hsa-mir-1295b | HGLibA_57661 | 37 | -0.780413574 | hsa-mir-1295b | - |
| hsa-mir-1302-9 | HGLibA_57724 | 37 | -0.780413574 | hsa-mir-1302-9 | - |
| hsa-mir-192 | HGLibA_58068 | 37 | -0.780413574 | hsa-mir-192 | - |
| hsa-mir-26a-1 | HGLibA_58363 | 37 | -0.780413574 | hsa-mir-26a-1 | - |
| hsa-mir-29b-1 | HGLibA_58413 | 37 | -0.780413574 | hsa-mir-29b-1 | - |
| hsa-mir-3152 | HGLibA_58668 | 37 | -0.780413574 | hsa-mir-3152 | - |
| hsa-mir-3202-2 | HGLibA_58918 | 37 | -0.780413574 | hsa-mir-3202-2 | - |
| hsa-mir-34a | HGLibA_59035 | 37 | -0.780413574 | hsa-mir-34a | - |
| hsa-mir-3591 | HGLibA_59051 | 37 | -0.780413574 | hsa-mir-3591 | - |
| hsa-mir-3689d-1 | HGLibA_59324 | 37 | -0.780413574 | hsa-mir-3689d-1 | - |
| hsa-mir-376a-1 | HGLibA_59404 | 37 | -0.780413574 | hsa-mir-376a-1 | - |
| hsa-mir-4251 | HGLibA_59711 | 37 | -0.780413574 | hsa-mir-4251 | - |
| hsa-mir-4263 | HGLibA_59759 | 37 | -0.780413574 | hsa-mir-4263 | - |
| hsa-mir-4438 | HGLibA_60154 | 37 | -0.780413574 | hsa-mir-4438 | - |
| hsa-mir-4445 | HGLibA_60188 | 37 | -0.780413574 | hsa-mir-4445 | - |
| hsa-mir-4741 | HGLibA_61063 | 37 | -0.780413574 | hsa-mir-4741 | - |
| hsa-mir-4773-2 | HGLibA_61197 | 37 | -0.780413574 | hsa-mir-4773-2 | - |
| hsa-mir-4793 | HGLibA_61279 | 37 | -0.780413574 | hsa-mir-4793 | - |
| hsa-mir-518c | HGLibA_61665 | 37 | -0.780413574 | hsa-mir-518c | - |
| hsa-mir-5194 | HGLibA_61696 | 37 | -0.780413574 | hsa-mir-5194 | - |
| hsa-mir-548aj-1 | HGLibA_61887 | 37 | -0.780413574 | hsa-mir-548aj-1 | - |
| hsa-mir-5689 | HGLibA_62271 | 37 | -0.780413574 | hsa-mir-5689 | - |
| hsa-mir-5692c-1 | HGLibA_62299 | 37 | -0.780413574 | hsa-mir-5692c-1 | - |
| hsa-mir-579 | HGLibA_62415 | 37 | -0.780413574 | hsa-mir-579 | - |
| hsa-mir-6075 | HGLibA_62553 | 37 | -0.780413574 | hsa-mir-6075 | - |
| hsa-mir-656 | HGLibA_62958 | 37 | -0.780413574 | hsa-mir-656 | - |
| hsa-mir-664b | HGLibA_62999 | 37 | -0.780413574 | hsa-mir-664b | - |
| hsa-mir-6742 | HGLibA_63123 | 37 | -0.780413574 | hsa-mir-6742 | - |
| hsa-mir-6833 | HGLibA_63509 | 37 | -0.780413574 | hsa-mir-6833 | - |
| hsa-mir-6879 | HGLibA_63702 | 37 | -0.780413574 | hsa-mir-6879 | - |
| hsa-mir-765 | HGLibA_63922 | 37 | -0.780413574 | hsa-mir-765 | - |
| IGJ | HGLibA_22863 | 37 | -0.780413574 | IGJ | - |
| LOC200726 | HGLibA_26902 | 37 | -0.780413574 | LOC200726 | - |
| LOC283403 | HGLibA_26908 | 37 | -0.780413574 | LOC283403 | - |
| LOC391322 | HGLibA_26933 | 37 | -0.780413574 | LOC391322 | - |
| NUTMG | HGLibA_33117 | 37 | -0.780413574 | NUTMG | - |
| SGK196 | HGLibA_43823 | 37 | -0.780413574 | SGK196 | - |
| SQRDL | HGLibA_46915 | 37 | -0.780413574 | SQRDL | - |
| TENC1 | HGLibA_48993 | 37 | -0.780413574 | TENC1 | - |
| TMEM110 | HGLibA_49873 | 37 | -0.780413574 | TMEM110 | - |
| TMEM257 | HGLibA_50338 | 37 | -0.780413574 | TMEM257 | - |
| AGPHD1 | HGLibA_01237 | 36 | -0.790364611 | AGPHD1 | - |
| C10orf25 | HGLibA_05064 | 36 | -0.790364611 | C10orf25 | - |
| C2orf57 | HGLibA_06151 | 36 | -0.790364611 | C2orf57 | - |
| C9orf84 | HGLibA_06764 | 36 | -0.790364611 | C9orf84 | - |
| CCDC111 | HGLibA_07541 | 36 | -0.790364611 | CCDC111 | - |
| CD99_Y | HGLibA_08491 | 36 | -0.790364611 | CD99_Y | - |
| ENSG00000008710 | HGLibA_36788 | 36 | -0.790364611 | PKD1 | transient receptor potential channel interacting [Source:HGNC Symbol;Acc:HGNC:9008]" |
| ENSG00000009335 | HGLibA_52683 | 36 | -0.790364611 | UBE3C | ubiquitin protein ligase E3C [Source:HGNC Symbol;Acc:HGNC:16803] |
| ENSG00000009335 | HGLibA_52685 | 36 | -0.790364611 | UBE3C | ubiquitin protein ligase E3C [Source:HGNC Symbol;Acc:HGNC:16803] |
| ENSG00000011405 | HGLibA_36621 | 36 | -0.790364611 | PIK3C2A | phosphatidylinositol-4-phosphate 3-kinase catalytic subunit type 2 alpha [Source:HGNC Symbol;Acc:HGNC:8971] |
| ENSG00000011523 | HGLibA_09154 | 36 | -0.790364611 | CEP68 | centrosomal protein 68 [Source:HGNC Symbol;Acc:HGNC:29076] |
| ENSG00000013375 | HGLibA_36270 | 36 | -0.790364611 | PGM3 | phosphoglucomutase 3 [Source:HGNC Symbol;Acc:HGNC:8907] |
| ENSG00000018189 | HGLibA_42443 | 36 | -0.790364611 | RUFY3 | RUN and FYVE domain containing 3 [Source:HGNC Symbol;Acc:HGNC:30285] |
| ENSG00000018280 | HGLibA_44352 | 36 | -0.790364611 | SLC11A1 | solute carrier family 11 member 1 [Source:HGNC Symbol;Acc:HGNC:10907] |
| ENSG00000019991 | HGLibA_21239 | 36 | -0.790364611 | HGF | hepatocyte growth factor [Source:HGNC Symbol;Acc:HGNC:4893] |
| ENSG00000021300 | HGLibA_37085 | 36 | -0.790364611 | PLEKHB1 | pleckstrin homology domain containing B1 [Source:HGNC Symbol;Acc:HGNC:19079] |
| ENSG00000024526 | HGLibA_12974 | 36 | -0.790364611 | DEPDC1 | DEP domain containing 1 [Source:HGNC Symbol;Acc:HGNC:22949] |
| ENSG00000057663 | HGLibA_03500 | 36 | -0.790364611 | ATG5 | autophagy related 5 [Source:HGNC Symbol;Acc:HGNC:589] |
| ENSG00000061936 | HGLibA_43748 | 36 | -0.790364611 | SFSWAP | splicing factor SWAP [Source:HGNC Symbol;Acc:HGNC:10790] |
| ENSG00000063854 | HGLibA_20813 | 36 | -0.790364611 | HAGH | hydroxyacylglutathione hydrolase [Source:HGNC Symbol;Acc:HGNC:4805] |
| ENSG00000065621 | HGLibA_20491 | 36 | -0.790364611 | GSTO2 | glutathione S-transferase omega 2 [Source:HGNC Symbol;Acc:HGNC:23064] |
| ENSG00000067606 | HGLibA_38564 | 36 | -0.790364611 | PRKCZ | protein kinase C zeta [Source:HGNC Symbol;Acc:HGNC:9412] |
| ENSG00000070476 | HGLibA_57079 | 36 | -0.790364611 | ZXDC | ZXD family zinc finger C [Source:HGNC Symbol;Acc:HGNC:28160] |
| ENSG00000071127 | HGLibA_54022 | 36 | -0.790364611 | WDR1 | WD repeat domain 1 [Source:HGNC Symbol;Acc:HGNC:12754] |
| ENSG00000075240 | HGLibA_20208 | 36 | -0.790364611 | GRAMD4 | GRAM domain containing 4 [Source:HGNC Symbol;Acc:HGNC:29113] |
| ENSG00000077097 | HGLibA_50996 | 36 | -0.790364611 | TOP2B | DNA topoisomerase II beta [Source:HGNC Symbol;Acc:HGNC:11990] |
| ENSG00000077147 | HGLibA_49726 | 36 | -0.790364611 | TM9SF3 | transmembrane 9 superfamily member 3 [Source:HGNC Symbol;Acc:HGNC:21529] |
| ENSG00000079277 | HGLibA_29343 | 36 | -0.790364611 | MKNK1 | MAP kinase interacting serine/threonine kinase 1 [Source:HGNC Symbol;Acc:HGNC:7110] |
| ENSG00000079557 | HGLibA_01120 | 36 | -0.790364611 | AFM | afamin [Source:HGNC Symbol;Acc:HGNC:316] |
| ENSG00000079689 | HGLibA_42937 | 36 | -0.790364611 | SCGN | EF-hand calcium binding protein [Source:HGNC Symbol;Acc:HGNC:16941]" |
| ENSG00000082213 | HGLibA_06366 | 36 | -0.790364611 | C5orf22 | chromosome 5 open reading frame 22 [Source:HGNC Symbol;Acc:HGNC:25639] |
| ENSG00000083520 | HGLibA_13270 | 36 | -0.790364611 | DIS3 | exosome endoribonuclease and 3'-5' exoribonuclease [Source:HGNC Symbol;Acc:HGNC:20604]" |
| ENSG00000086062 | HGLibA_04022 | 36 | -0.790364611 | B4GALT1 | 4-galactosyltransferase 1 [Source:HGNC Symbol;Acc:HGNC:924]" |
| ENSG00000087206 | HGLibA_52957 | 36 | -0.790364611 | UIMC1 | ubiquitin interaction motif containing 1 [Source:HGNC Symbol;Acc:HGNC:30298] |
| ENSG00000087303 | HGLibA_31926 | 36 | -0.790364611 | NID2 | nidogen 2 [Source:HGNC Symbol;Acc:HGNC:13389] |
| ENSG00000089356 | HGLibA_18230 | 36 | -0.790364611 | FXYD3 | FXYD domain containing ion transport regulator 3 [Source:HGNC Symbol;Acc:HGNC:4027] |
| ENSG00000091164 | HGLibA_52435 | 36 | -0.790364611 | TXNL1 | thioredoxin like 1 [Source:HGNC Symbol;Acc:HGNC:12436] |
| ENSG00000095574 | HGLibA_22942 | 36 | -0.790364611 | IKZF5 | IKAROS family zinc finger 5 [Source:HGNC Symbol;Acc:HGNC:14283] |
| ENSG00000099330 | HGLibA_33242 | 36 | -0.790364611 | OCEL1 | occludin/ELL domain containing 1 [Source:HGNC Symbol;Acc:HGNC:26221] |
| ENSG00000100395 | HGLibA_25898 | 36 | -0.790364611 | L3MBTL2 | polycomb repressive complex 1 subunit [Source:HGNC Symbol;Acc:HGNC:18594]" |
| ENSG00000100985 | HGLibA_29542 | 36 | -0.790364611 | MMP9 | matrix metallopeptidase 9 [Source:HGNC Symbol;Acc:HGNC:7176] |
| ENSG00000101213 | HGLibA_39384 | 36 | -0.790364611 | PTK6 | protein tyrosine kinase 6 [Source:HGNC Symbol;Acc:HGNC:9617] |
| ENSG00000101446 | HGLibA_46718 | 36 | -0.790364611 | SPINT3 | Kunitz type 3 [Source:HGNC Symbol;Acc:HGNC:11248]" |
| ENSG00000102245 | HGLibA_08388 | 36 | -0.790364611 | CD40LG | CD40 ligand [Source:HGNC Symbol;Acc:HGNC:11935] |
| ENSG00000102309 | HGLibA_36687 | 36 | -0.790364611 | PIN4 | NIMA-interacting 4 [Source:HGNC Symbol;Acc:HGNC:8992]" |
| ENSG00000102890 | HGLibA_14935 | 36 | -0.790364611 | ELMO3 | engulfment and cell motility 3 [Source:HGNC Symbol;Acc:HGNC:17289] |
| ENSG00000103550 | HGLibA_25344 | 36 | -0.790364611 | KNOP1 | lysine rich nucleolar protein 1 [Source:HGNC Symbol;Acc:HGNC:34404] |
| ENSG00000104365 | HGLibA_22921 | 36 | -0.790364611 | IKBKB | inhibitor of nuclear factor kappa B kinase subunit beta [Source:HGNC Symbol;Acc:HGNC:5960] |
| ENSG00000104863 | HGLibA_26576 | 36 | -0.790364611 | LIN7B | crumbs cell polarity complex component [Source:HGNC Symbol;Acc:HGNC:17788]" |
| ENSG00000105127 | HGLibA_01446 | 36 | -0.790364611 | AKAP8 | A-kinase anchoring protein 8 [Source:HGNC Symbol;Acc:HGNC:378] |
| ENSG00000105290 | HGLibA_02441 | 36 | -0.790364611 | APLP1 | amyloid beta precursor like protein 1 [Source:HGNC Symbol;Acc:HGNC:597] |
| ENSG00000106246 | HGLibA_39258 | 36 | -0.790364611 | PTCD1 | pentatricopeptide repeat domain 1 [Source:HGNC Symbol;Acc:HGNC:22198] |
| ENSG00000106686 | HGLibA_46524 | 36 | -0.790364611 | SPATA6L | spermatogenesis associated 6 like [Source:HGNC Symbol;Acc:HGNC:25472] |
| ENSG00000106789 | HGLibA_10783 | 36 | -0.790364611 | CORO2A | coronin 2A [Source:HGNC Symbol;Acc:HGNC:2255] |
| ENSG00000107187 | HGLibA_26454 | 36 | -0.790364611 | LHX3 | LIM homeobox 3 [Source:HGNC Symbol;Acc:HGNC:6595] |
| ENSG00000108559 | HGLibA_33073 | 36 | -0.790364611 | NUP88 | nucleoporin 88 [Source:HGNC Symbol;Acc:HGNC:8067] |
| ENSG00000108654 | HGLibA_12728 | 36 | -0.790364611 | DDX5 | DEAD-box helicase 5 [Source:HGNC Symbol;Acc:HGNC:2746] |
| ENSG00000108840 | HGLibA_21014 | 36 | -0.790364611 | HDAC5 | histone deacetylase 5 [Source:HGNC Symbol;Acc:HGNC:14068] |
| ENSG00000109576 | HGLibA_00041 | 36 | -0.790364611 | AADAT | aminoadipate aminotransferase [Source:HGNC Symbol;Acc:HGNC:17929] |
| ENSG00000111666 | HGLibA_09530 | 36 | -0.790364611 | CHPT1 | choline phosphotransferase 1 [Source:HGNC Symbol;Acc:HGNC:17852] |
| ENSG00000111837 | HGLibA_28015 | 36 | -0.790364611 | MAK | male germ cell associated kinase [Source:HGNC Symbol;Acc:HGNC:6816] |
| ENSG00000112964 | HGLibA_19041 | 36 | -0.790364611 | GHR | growth hormone receptor [Source:HGNC Symbol;Acc:HGNC:4263] |
| ENSG00000113318 | HGLibA_30193 | 36 | -0.790364611 | MSH3 | mutS homolog 3 [Source:HGNC Symbol;Acc:HGNC:7326] |
| ENSG00000114491 | HGLibA_52985 | 36 | -0.790364611 | UMPS | uridine monophosphate synthetase [Source:HGNC Symbol;Acc:HGNC:12563] |
| ENSG00000115194 | HGLibA_44906 | 36 | -0.790364611 | SLC30A3 | solute carrier family 30 member 3 [Source:HGNC Symbol;Acc:HGNC:11014] |
| ENSG00000115274 | HGLibA_23322 | 36 | -0.790364611 | INO80B | INO80 complex subunit B [Source:HGNC Symbol;Acc:HGNC:13324] |
| ENSG00000117154 | HGLibA_22884 | 36 | -0.790364611 | IGSF21 | immunoglobin superfamily member 21 [Source:HGNC Symbol;Acc:HGNC:28246] |
| ENSG00000117425 | HGLibA_39270 | 36 | -0.790364611 | PTCH2 | patched 2 [Source:HGNC Symbol;Acc:HGNC:9586] |
| ENSG00000117682 | HGLibA_13097 | 36 | -0.790364611 | DHDDS | dehydrodolichyl diphosphate synthase subunit [Source:HGNC Symbol;Acc:HGNC:20603] |
| ENSG00000118705 | HGLibA_42021 | 36 | -0.790364611 | RPN2 | ribophorin II [Source:HGNC Symbol;Acc:HGNC:10382] |
| ENSG00000119596 | HGLibA_54696 | 36 | -0.790364611 | YLPM1 | YLP motif containing 1 [Source:HGNC Symbol;Acc:HGNC:17798] |
| ENSG00000120049 | HGLibA_24237 | 36 | -0.790364611 | KCNIP2 | potassium voltage-gated channel interacting protein 2 [Source:HGNC Symbol;Acc:HGNC:15522] |
| ENSG00000120451 | HGLibA_46054 | 36 | -0.790364611 | SNX19 | sorting nexin 19 [Source:HGNC Symbol;Acc:HGNC:21532] |
| ENSG00000120509 | HGLibA_35971 | 36 | -0.790364611 | PDZD11 | PDZ domain containing 11 [Source:HGNC Symbol;Acc:HGNC:28034] |

| | | | | | |
|---|---|---|---|---|---|
| ENSG00000121807 | HGLibA_08150 | 36 | -0.790364611 | CCR2 | C-C motif chemokine receptor 2 [Source:HGNC Symbol;Acc:HGNC:1603] |
| ENSG00000122952 | HGLibA_57071 | 36 | -0.790364611 | ZWINT | ZW10 interacting kinetochore protein [Source:HGNC Symbol;Acc:HGNC:13195] |
| ENSG00000123095 | HGLibA_04481 | 36 | -0.790364611 | BHLHE41 | basic helix-loop-helix family member e41 [Source:HGNC Symbol;Acc:HGNC:16617] |
| ENSG00000123562 | HGLibA_29663 | 36 | -0.790364611 | MORF4L2 | mortality factor 4 like 2 [Source:HGNC Symbol;Acc:HGNC:16849] |
| ENSG00000123594 | HGLibA_03847 | 36 | -0.790364611 | ATXN3L | ataxin 3 like [Source:HGNC Symbol;Acc:HGNC:24173] |
| ENSG00000124788 | HGLibA_03828 | 36 | -0.790364611 | ATXN1 | ataxin 1 [Source:HGNC Symbol;Acc:HGNC:10548] |
| ENSG00000124935 | HGLibA_42917 | 36 | -0.790364611 | SCGB1D2 | secretoglobin family 1D member 2 [Source:HGNC Symbol;Acc:HGNC:18396] |
| ENSG00000125285 | HGLibA_46254 | 36 | -0.790364611 | SOX21 | SRY-box 21 [Source:HGNC Symbol;Acc:HGNC:11197] |
| ENSG00000125850 | HGLibA_34803 | 36 | -0.790364611 | OVOL2 | ovo like zinc finger 2 [Source:HGNC Symbol;Acc:HGNC:15804] |
| ENSG00000126215 | HGLibA_54589 | 36 | -0.790364611 | XRCC3 | X-ray repair cross complementing 3 [Source:HGNC Symbol;Acc:HGNC:12830] |
| ENSG00000126231 | HGLibA_38706 | 36 | -0.790364611 | PROZ | vitamin K dependent plasma glycoprotein [Source:HGNC Symbol;Acc:HGNC:9460]" |
| ENSG00000126337 | HGLibA_25491 | 36 | -0.790364611 | KRT36 | keratin 36 [Source:HGNC Symbol;Acc:HGNC:6454] |
| ENSG00000127377 | HGLibA_11283 | 36 | -0.790364611 | CRYGN | crystallin gamma N [Source:HGNC Symbol;Acc:HGNC:20458] |
| ENSG00000128573 | HGLibA_17932 | 36 | -0.790364611 | FOXP2 | forkhead box P2 [Source:HGNC Symbol;Acc:HGNC:13875] |
| ENSG00000128602 | HGLibA_45791 | 36 | -0.790364611 | SMO | frizzled class receptor [Source:HGNC Symbol;Acc:HGNC:11119]" |
| ENSG00000130725 | HGLibA_52634 | 36 | -0.790364611 | UBE2M | ubiquitin conjugating enzyme E2 M [Source:HGNC Symbol;Acc:HGNC:12491] |
| ENSG00000131165 | HGLibA_09472 | 36 | -0.790364611 | CHMP1A | charged multivesicular body protein 1A [Source:HGNC Symbol;Acc:HGNC:8740] |
| ENSG00000131495 | HGLibA_31453 | 36 | -0.790364611 | NDUFA2 | NADH:ubiquinone oxidoreductase subunit A2 [Source:HGNC Symbol;Acc:HGNC:7685] |
| ENSG00000132429 | HGLibA_37703 | 36 | -0.790364611 | POPDC3 | popeye domain containing 3 [Source:HGNC Symbol;Acc:HGNC:17649] |
| ENSG00000132481 | HGLibA_51516 | 36 | -0.790364611 | TRIM47 | tripartite motif containing 47 [Source:HGNC Symbol;Acc:HGNC:19020] |
| ENSG00000132622 | HGLibA_22262 | 36 | -0.790364611 | HSPA12B | heat shock protein family A (Hsp70) member 12B [Source:HGNC Symbol;Acc:HGNC:16193] |
| ENSG00000132698 | HGLibA_39849 | 36 | -0.790364611 | RAB25 | member RAS oncogene family [Source:HGNC Symbol;Acc:HGNC:18238]" |
| ENSG00000132825 | HGLibA_38106 | 36 | -0.790364611 | PPP1R3D | protein phosphatase 1 regulatory subunit 3D [Source:HGNC Symbol;Acc:HGNC:9294] |
| ENSG00000133110 | HGLibA_37711 | 36 | -0.790364611 | POSTN | periostin [Source:HGNC Symbol;Acc:HGNC:16953] |
| ENSG00000133121 | HGLibA_47353 | 36 | -0.790364611 | STARD13 | StAR related lipid transfer domain containing 13 [Source:HGNC Symbol;Acc:HGNC:19164] |
| ENSG00000134007 | HGLibA_00751 | 36 | -0.790364611 | ADAM20 | ADAM metallopeptidase domain 20 [Source:HGNC Symbol;Acc:HGNC:199] |
| ENSG00000134259 | HGLibA_31872 | 36 | -0.790364611 | NGF | nerve growth factor [Source:HGNC Symbol;Acc:HGNC:7808] |
| ENSG00000134278 | HGLibA_46725 | 36 | -0.790364611 | SPIRE1 | spire type actin nucleation factor 1 [Source:HGNC Symbol;Acc:HGNC:30622] |
| ENSG00000134758 | HGLibA_41514 | 36 | -0.790364611 | RNF138 | ring finger protein 138 [Source:HGNC Symbol;Acc:HGNC:17765] |
| ENSG00000135018 | HGLibA_52732 | 36 | -0.790364611 | UBQLN1 | ubiquilin 1 [Source:HGNC Symbol;Acc:HGNC:12508] |
| ENSG00000135766 | HGLibA_14638 | 36 | -0.790364611 | EGLN1 | egl-9 family hypoxia inducible factor 1 [Source:HGNC Symbol;Acc:HGNC:1232] |
| ENSG00000135900 | HGLibA_29989 | 36 | -0.790364611 | MRPL44 | mitochondrial ribosomal protein L44 [Source:HGNC Symbol;Acc:HGNC:16650] |
| ENSG00000136108 | HGLibA_09755 | 36 | -0.790364611 | CKAP2 | cytoskeleton associated protein 2 [Source:HGNC Symbol;Acc:HGNC:1990] |
| ENSG00000136155 | HGLibA_42890 | 36 | -0.790364611 | SCEL | sciellin [Source:HGNC Symbol;Acc:HGNC:10573] |
| ENSG00000136367 | HGLibA_55170 | 36 | -0.790364611 | ZFHX2 | zinc finger homeobox 2 [Source:HGNC Symbol;Acc:HGNC:20152] |
| ENSG00000137815 | HGLibA_42383 | 36 | -0.790364611 | RTF1 | Paf1/RNA polymerase II complex component [Source:HGNC Symbol;Acc:HGNC:28996]" |
| ENSG00000137860 | HGLibA_44835 | 36 | -0.790364611 | SLC28A2 | solute carrier family 28 member 2 [Source:HGNC Symbol;Acc:HGNC:11002] |
| ENSG00000138772 | HGLibA_02238 | 36 | -0.790364611 | ANXA3 | annexin A3 [Source:HGNC Symbol;Acc:HGNC:541] |
| ENSG00000139292 | HGLibA_26410 | 36 | -0.790364611 | LGR5 | leucine rich repeat containing G protein-coupled receptor 5 [Source:HGNC Symbol;Acc:HGNC:4504] |
| ENSG00000139437 | HGLibA_48810 | 36 | -0.790364611 | TCHP | trichoplein keratin filament binding [Source:HGNC Symbol;Acc:HGNC:28135] |
| ENSG00000139574 | HGLibA_32437 | 36 | -0.790364611 | NPFF | neuropeptide FF-amide peptide precursor [Source:HGNC Symbol;Acc:HGNC:7901] |
| ENSG00000140285 | HGLibA_17417 | 36 | -0.790364611 | FGF7 | fibroblast growth factor 7 [Source:HGNC Symbol;Acc:HGNC:3685] |
| ENSG00000140873 | HGLibA_00816 | 36 | -0.790364611 | ADAMTS18 | ADAM metallopeptidase with thrombospondin type 1 motif 18 [Source:HGNC Symbol;Acc:HGNC:17110] |
| ENSG00000141200 | HGLibA_24929 | 36 | -0.790364611 | KIF2B | kinesin family member 2B [Source:HGNC Symbol;Acc:HGNC:29443] |
| ENSG00000142082 | HGLibA_44221 | 36 | -0.790364611 | SIRT3 | sirtuin 3 [Source:HGNC Symbol;Acc:HGNC:14931] |
| ENSG00000143006 | HGLibA_13431 | 36 | -0.790364611 | DMRTB1 | DMRT like family B with proline rich C-terminal 1 [Source:HGNC Symbol;Acc:HGNC:13913] |
| ENSG00000143194 | HGLibA_27864 | 36 | -0.790364611 | MAEL | maelstrom spermatogenic transposon silencer [Source:HGNC Symbol;Acc:HGNC:25929] |
| ENSG00000143479 | HGLibA_14281 | 36 | -0.790364611 | DYRK3 | dual specificity tyrosine phosphorylation regulated kinase 3 [Source:HGNC Symbol;Acc:HGNC:3094] |
| ENSG00000143545 | HGLibA_39812 | 36 | -0.790364611 | RAB13 | member RAS oncogene family [Source:HGNC Symbol;Acc:HGNC:9762]" |
| ENSG00000143845 | HGLibA_15634 | 36 | -0.790364611 | ETNK2 | ethanolamine kinase 2 [Source:HGNC Symbol;Acc:HGNC:25575] |
| ENSG00000143995 | HGLibA_28871 | 36 | -0.790364611 | MEIS1 | Meis homeobox 1 [Source:HGNC Symbol;Acc:HGNC:7000] |
| ENSG00000144455 | HGLibA_47769 | 36 | -0.790364611 | SUMF1 | sulfatase modifying factor 1 [Source:HGNC Symbol;Acc:HGNC:20376] |
| ENSG00000144668 | HGLibA_23747 | 36 | -0.790364611 | ITGA9 | integrin subunit alpha 9 [Source:HGNC Symbol;Acc:HGNC:6145] |
| ENSG00000145721 | HGLibA_26641 | 36 | -0.790364611 | LIX1 | limb and CNS expressed 1 [Source:HGNC Symbol;Acc:HGNC:18581] |
| ENSG00000146066 | HGLibA_21315 | 36 | -0.790364611 | HIGD2A | HIG1 hypoxia inducible domain family member 2A [Source:HGNC Symbol;Acc:HGNC:28311] |
| ENSG00000147432 | HGLibA_09594 | 36 | -0.790364611 | CHRNB3 | cholinergic receptor nicotinic beta 3 subunit [Source:HGNC Symbol;Acc:HGNC:1963] |
| ENSG00000147488 | HGLibA_47229 | 36 | -0.790364611 | ST18 | C2H2C-type zinc finger [Source:HGNC Symbol;Acc:HGNC:18695]" |
| ENSG00000148700 | HGLibA_00946 | 36 | -0.790364611 | ADD3 | adducin 3 [Source:HGNC Symbol;Acc:HGNC:245] |
| ENSG00000149742 | HGLibA_44604 | 36 | -0.790364611 | SLC22A9 | solute carrier family 22 member 9 [Source:HGNC Symbol;Acc:HGNC:16261] |
| ENSG00000151376 | HGLibA_28716 | 36 | -0.790364611 | ME3 | malic enzyme 3 [Source:HGNC Symbol;Acc:HGNC:6985] |
| ENSG00000153237 | HGLibA_07635 | 36 | -0.790364611 | CCDC148 | coiled-coil domain containing 148 [Source:HGNC Symbol;Acc:HGNC:25191] |
| ENSG00000153956 | HGLibA_06905 | 36 | -0.790364611 | CACNA2D1 | calcium voltage-gated channel auxiliary subunit alpha2delta 1 [Source:HGNC Symbol;Acc:HGNC:1399] |
| ENSG00000154485 | HGLibA_29512 | 36 | -0.790364611 | MMP21 | matrix metallopeptidase 21 [Source:HGNC Symbol;Acc:HGNC:14357] |
| ENSG00000155465 | HGLibA_45378 | 36 | -0.790364611 | SLC7A7 | solute carrier family 7 member 7 [Source:HGNC Symbol;Acc:HGNC:11065] |
| ENSG00000155495 | HGLibA_27960 | 36 | -0.790364611 | MAGEC1 | MAGE family member C1 [Source:HGNC Symbol;Acc:HGNC:6812] |
| ENSG00000156113 | HGLibA_24338 | 36 | -0.790364611 | KCNMA1 | potassium calcium-activated channel subfamily M alpha 1 [Source:HGNC Symbol;Acc:HGNC:6284] |
| ENSG00000156475 | HGLibA_38149 | 36 | -0.790364611 | PPP2R2B | protein phosphatase 2 regulatory subunit Bbeta [Source:HGNC Symbol;Acc:HGNC:9305] |
| ENSG00000156983 | HGLibA_04840 | 36 | -0.790364611 | BRPF1 | bromodomain and PHD finger containing 1 [Source:HGNC Symbol;Acc:HGNC:14255] |
| ENSG00000157423 | HGLibA_22448 | 36 | -0.790364611 | HYDIN | axonemal central pair apparatus protein [Source:HGNC Symbol;Acc:HGNC:19368]" |
| ENSG00000158089 | HGLibA_18543 | 36 | -0.790364611 | GALNT14 | polypeptide N-acetylgalactosaminyltransferase 14 [Source:HGNC Symbol;Acc:HGNC:22946] |
| ENSG00000160183 | HGLibA_50640 | 36 | -0.790364611 | TMPRSS3 | transmembrane serine protease 3 [Source:HGNC Symbol;Acc:HGNC:11877] |
| ENSG00000160404 | HGLibA_51027 | 36 | -0.790364611 | TOR2A | torsin family 2 member A [Source:HGNC Symbol;Acc:HGNC:11996] |
| ENSG00000160472 | HGLibA_50141 | 36 | -0.790364611 | TMEM190 | transmembrane protein 190 [Source:HGNC Symbol;Acc:HGNC:29632] |
| ENSG00000160703 | HGLibA_32140 | 36 | -0.790364611 | NLRX1 | NLR family member X1 [Source:HGNC Symbol;Acc:HGNC:29890] |
| ENSG00000160856 | HGLibA_17232 | 36 | -0.790364611 | FCRL3 | Fc receptor like 3 [Source:HGNC Symbol;Acc:HGNC:18506] |
| ENSG00000160951 | HGLibA_39312 | 36 | -0.790364611 | PTGER1 | prostaglandin E receptor 1 [Source:HGNC Symbol;Acc:HGNC:9593] |
| ENSG00000162460 | HGLibA_50521 | 36 | -0.790364611 | TMEM82 | transmembrane protein 82 [Source:HGNC Symbol;Acc:HGNC:32350] |
| ENSG00000162687 | HGLibA_24396 | 36 | -0.790364611 | KCNT2 | potassium sodium-activated channel subfamily T member 2 [Source:HGNC Symbol;Acc:HGNC:18866] |
| ENSG00000163694 | HGLibA_40566 | 36 | -0.790364611 | RBM47 | RNA binding motif protein 47 [Source:HGNC Symbol;Acc:HGNC:30358] |
| ENSG00000164039 | HGLibA_04377 | 36 | -0.790364611 | BDH2 | 3-hydroxybutyrate dehydrogenase 2 [Source:HGNC Symbol;Acc:HGNC:32389] |
| ENSG00000164124 | HGLibA_49971 | 36 | -0.790364611 | TMEM144 | transmembrane protein 144 [Source:HGNC Symbol;Acc:HGNC:25633] |
| ENSG00000164181 | HGLibA_14976 | 36 | -0.790364611 | ELOVL7 | ELOVL fatty acid elongase 7 [Source:HGNC Symbol;Acc:HGNC:26292] |
| ENSG00000164363 | HGLibA_45312 | 36 | -0.790364611 | SLC6A18 | solute carrier family 6 member 18 [Source:HGNC Symbol;Acc:HGNC:26441] |
| ENSG00000165175 | HGLibA_29233 | 36 | -0.790364611 | MID1IP1 | MID1 interacting protein 1 [Source:HGNC Symbol;Acc:HGNC:20715] |
| ENSG00000165915 | HGLibA_45100 | 36 | -0.790364611 | SLC39A13 | solute carrier family 39 member 13 [Source:HGNC Symbol;Acc:HGNC:20859] |
| ENSG00000166295 | HGLibA_01876 | 36 | -0.790364611 | ANAPC16 | anaphase promoting complex subunit 16 [Source:HGNC Symbol;Acc:HGNC:26976] |
| ENSG00000166862 | HGLibA_06933 | 36 | -0.790364611 | CACNG2 | calcium voltage-gated channel auxiliary subunit gamma 2 [Source:HGNC Symbol;Acc:HGNC:1406] |
| ENSG00000167191 | HGLibA_20127 | 36 | -0.790364611 | GPRC5B | G protein-coupled receptor class C group 5 member B [Source:HGNC Symbol;Acc:HGNC:13308] |
| ENSG00000167280 | HGLibA_15128 | 36 | -0.790364611 | ENGASE | endo-beta-N-acetylglucosaminidase [Source:HGNC Symbol;Acc:HGNC:24622] |
| ENSG00000167633 | HGLibA_24992 | 36 | -0.790364611 | KIR3DL1 | three Ig domains and long cytoplasmic tail 1 [Source:HGNC Symbol;Acc:HGNC:6338]" |
| ENSG00000168214 | HGLibA_40648 | 36 | -0.790364611 | RBPJ | recombination signal binding protein for immunoglobulin kappa J region [Source:HGNC Symbol;Acc:HGNC:5724] |
| ENSG00000168484 | HGLibA_43775 | 36 | -0.790364611 | SFTPC | surfactant protein C [Source:HGNC Symbol;Acc:HGNC:10802] |
| ENSG00000168899 | HGLibA_53500 | 36 | -0.790364611 | VAMP5 | vesicle associated membrane protein 5 [Source:HGNC Symbol;Acc:HGNC:12646] |
| ENSG00000168907 | HGLibA_36914 | 36 | -0.790364611 | PLA2G4F | phospholipase A2 group IVF [Source:HGNC Symbol;Acc:HGNC:27396] |
| ENSG00000169439 | HGLibA_43101 | 36 | -0.790364611 | SDC2 | syndecan 2 [Source:HGNC Symbol;Acc:HGNC:10659] |
| ENSG00000169592 | HGLibA_23331 | 36 | -0.790364611 | INO80E | INO80 complex subunit E [Source:HGNC Symbol;Acc:HGNC:26905] |
| ENSG00000169679 | HGLibA_04988 | 36 | -0.790364611 | BUB1 | BUB1 mitotic checkpoint serine/threonine kinase [Source:HGNC Symbol;Acc:HGNC:1148] |
| ENSG00000169715 | HGLibA_30283 | 36 | -0.790364611 | MT1E | metallothionein 1E [Source:HGNC Symbol;Acc:HGNC:7397] |
| ENSG00000170430 | HGLibA_29166 | 36 | -0.790364611 | MGMT | O-6-methylguanine-DNA methyltransferase [Source:HGNC Symbol;Acc:HGNC:7059] |
| ENSG00000170542 | HGLibA_43569 | 36 | -0.790364611 | SERPINB9 | serpin family B member 9 [Source:HGNC Symbol;Acc:HGNC:8955] |
| ENSG00000170775 | HGLibA_20031 | 36 | -0.790364611 | GPR37 | G protein-coupled receptor 37 [Source:HGNC Symbol;Acc:HGNC:4494] |
| ENSG00000170777 | HGLibA_51119 | 36 | -0.790364611 | TPD52L3 | TPD52 like 3 [Source:HGNC Symbol;Acc:HGNC:23382] |
| ENSG00000171016 | HGLibA_39694 | 36 | -0.790364611 | PYGO1 | pygopus family PHD finger 1 [Source:HGNC Symbol;Acc:HGNC:30256] |
| ENSG00000171133 | HGLibA_33815 | 36 | -0.790364611 | OR2K2 | olfactory receptor family 2 subfamily K member 2 [Source:HGNC Symbol;Acc:HGNC:8264] |
| ENSG00000171310 | HGLibA_09617 | 36 | -0.790364611 | CHST11 | carbohydrate sulfotransferase 11 [Source:HGNC Symbol;Acc:HGNC:17422] |
| ENSG00000171703 | HGLibA_48698 | 36 | -0.790364611 | TCEA2 | transcription elongation factor A2 [Source:HGNC Symbol;Acc:HGNC:11614] |
| ENSG00000171988 | HGLibA_23951 | 36 | -0.790364611 | JMJD1C | jumonji domain containing 1C [Source:HGNC Symbol;Acc:HGNC:12313] |
| ENSG00000172216 | HGLibA_08960 | 36 | -0.790364611 | CEBPB | CCAAT enhancer binding protein beta [Source:HGNC Symbol;Acc:HGNC:1834] |
| ENSG00000172992 | HGLibA_12466 | 36 | -0.790364611 | DCAKD | dephospho-CoA kinase domain containing [Source:HGNC Symbol;Acc:HGNC:26238] |
| ENSG00000173193 | HGLibA_35224 | 36 | -0.790364611 | PARP14 | poly(ADP-ribose) polymerase family member 14 [Source:HGNC Symbol;Acc:HGNC:29232] |
| ENSG00000173442 | HGLibA_14663 | 36 | -0.790364611 | EHBP1L1 | EH domain binding protein 1 like 1 [Source:HGNC Symbol;Acc:HGNC:30682] |
| ENSG00000173728 | HGLibA_05729 | 36 | -0.790364611 | C1orf100 | chromosome 1 open reading frame 100 [Source:HGNC Symbol;Acc:HGNC:30435] |
| ENSG00000173825 | HGLibA_49470 | 36 | -0.790364611 | TIGD3 | tigger transposable element derived 3 [Source:HGNC Symbol;Acc:HGNC:18334] |
| ENSG00000174839 | HGLibA_12966 | 36 | -0.790364611 | DENND6A | DENN domain containing 6A [Source:HGNC Symbol;Acc:HGNC:26635] |
| ENSG00000174876 | HGLibA_01844 | 36 | -0.790364611 | AMY1B | alpha 1B (salivary) [Source:HGNC Symbol;Acc:HGNC:475]" |
| ENSG00000175344 | HGLibA_09584 | 36 | -0.790364611 | CHRNA7 | cholinergic receptor nicotinic alpha 7 subunit [Source:HGNC Symbol;Acc:HGNC:1960] |
| ENSG00000175518 | HGLibA_52745 | 36 | -0.790364611 | UBQLNL | ubiquilin like [Source:HGNC Symbol;Acc:HGNC:28294] |
| ENSG00000175606 | HGLibA_50500 | 36 | -0.790364611 | TMEM70 | transmembrane protein 70 [Source:HGNC Symbol;Acc:HGNC:26050] |
| ENSG00000175782 | HGLibA_44995 | 36 | -0.790364611 | SLC35E3 | solute carrier family 35 member E3 [Source:HGNC Symbol;Acc:HGNC:20864] |
| ENSG00000176383 | HGLibA_03989 | 36 | -0.790364611 | B3GNT4 | 3-N-acetylglucosaminyltransferase 4 [Source:HGNC Symbol;Acc:HGNC:15683]" |
| ENSG00000176407 | HGLibA_24119 | 36 | -0.790364611 | KCMF1 | potassium channel modulatory factor 1 [Source:HGNC Symbol;Acc:HGNC:20589] |
| ENSG00000176884 | HGLibA_20296 | 36 | -0.790364611 | GRIN1 | glutamate ionotropic receptor NMDA type subunit 1 [Source:HGNC Symbol;Acc:HGNC:4584] |
| ENSG00000176928 | HGLibA_18818 | 36 | -0.790364611 | GCNT4 | core 2 [Source:HGNC Symbol;Acc:HGNC:17973]" |
| ENSG00000177212 | HGLibA_33874 | 36 | -0.790364611 | OR2T33 | olfactory receptor family 2 subfamily T member 33 [Source:HGNC Symbol;Acc:HGNC:31255] |
| ENSG00000177463 | HGLibA_32599 | 36 | -0.790364611 | NR2C2 | nuclear receptor subfamily 2 group C member 2 [Source:HGNC Symbol;Acc:HGNC:7972] |
| ENSG00000177946 | HGLibA_09042 | 36 | -0.790364611 | CENPBD1 | CENPB DNA-binding domain containing 1 [Source:HGNC Symbol;Acc:HGNC:28272] |
| ENSG00000177990 | HGLibA_13909 | 36 | -0.790364611 | DPY19L2 | dpy-19 like 2 [Source:HGNC Symbol;Acc:HGNC:19414] |
| ENSG00000178562 | HGLibA_08324 | 36 | -0.790364611 | CD28 | CD28 molecule [Source:HGNC Symbol;Acc:HGNC:1653] |
| ENSG00000179041 | HGLibA_42290 | 36 | -0.790364611 | RRS1 | ribosome biogenesis regulator homolog [Source:HGNC Symbol;Acc:HGNC:17083] |
| ENSG00000179111 | HGLibA_21195 | 36 | -0.790364611 | HES7 | hes family bHLH transcription factor 7 [Source:HGNC Symbol;Acc:HGNC:15977] |
| ENSG00000181135 | HGLibA_56598 | 36 | -0.790364611 | ZNF707 | zinc finger protein 707 [Source:HGNC Symbol;Acc:HGNC:27815] |
| ENSG00000181284 | HGLibA_49843 | 36 | -0.790364611 | TMEM102 | transmembrane protein 102 [Source:HGNC Symbol;Acc:HGNC:26722] |
| ENSG00000182415 | HGLibA_08912 | 36 | -0.790364611 | CDY2A | chromodomain Y-linked 2A [Source:HGNC Symbol;Acc:HGNC:1810] |
| ENSG00000182621 | HGLibA_36977 | 36 | -0.790364611 | PLCB1 | phospholipase C beta 1 [Source:HGNC Symbol;Acc:HGNC:15917] |
| ENSG00000183397 | HGLibA_05703 | 36 | -0.790364611 | C19orf71 | chromosome 19 open reading frame 71 [Source:HGNC Symbol;Acc:HGNC:34496] |

| | | | | | |
|---|---|---|---|---|---|
| ENSG00000183527 | HGLibA_39204 | 36 | -0.790364611 | PSMG1 | proteasome assembly chaperone 1 [Source:HGNC Symbol;Acc:HGNC:3043] |
| ENSG00000183668 | HGLibA_39057 | 36 | -0.790364611 | PSG9 | pregnancy specific beta-1-glycoprotein 9 [Source:HGNC Symbol;Acc:HGNC:9526] |
| ENSG00000184307 | HGLibA_55109 | 36 | -0.790364611 | ZDHHC23 | zinc finger DHHC-type containing 23 [Source:HGNC Symbol;Acc:HGNC:28654] |
| ENSG00000185008 | HGLibA_41735 | 36 | -0.790364611 | ROBO2 | roundabout guidance receptor 2 [Source:HGNC Symbol;Acc:HGNC:10250] |
| ENSG00000185933 | HGLibA_07013 | 36 | -0.790364611 | CALHM1 | calcium homeostasis modulator 1 [Source:HGNC Symbol;Acc:HGNC:23494] |
| ENSG00000185989 | HGLibA_40302 | 36 | -0.790364611 | RASA3 | RAS p21 protein activator 3 [Source:HGNC Symbol;Acc:HGNC:20331] |
| ENSG00000186230 | HGLibA_56672 | 36 | -0.790364611 | ZNF749 | zinc finger protein 749 [Source:HGNC Symbol;Acc:HGNC:32783] |
| ENSG00000186765 | HGLibA_18071 | 36 | -0.790364611 | FSCN2 | retinal [Source:HGNC Symbol;Acc:HGNC:3960]" |
| ENSG00000186871 | HGLibA_15450 | 36 | -0.790364611 | ERCC6L | spindle assembly checkpoint helicase [Source:HGNC Symbol;Acc:HGNC:20794]" |
| ENSG00000186918 | HGLibA_55913 | 36 | -0.790364611 | ZNF395 | zinc finger protein 395 [Source:HGNC Symbol;Acc:HGNC:18737] |
| ENSG00000188033 | HGLibA_56090 | 36 | -0.790364611 | ZNF490 | zinc finger protein 490 [Source:HGNC Symbol;Acc:HGNC:23705] |
| ENSG00000188076 | HGLibA_42910 | 36 | -0.790364611 | SCGB1C1 | secretoglobin family 1C member 1 [Source:HGNC Symbol;Acc:HGNC:18394] |
| ENSG00000188404 | HGLibA_43294 | 36 | -0.790364611 | SELL | selectin L [Source:HGNC Symbol;Acc:HGNC:10720] |
| ENSG00000188580 | HGLibA_31993 | 36 | -0.790364611 | NKAIN2 | sodium/potassium transporting ATPase interacting 2 [Source:HGNC Symbol;Acc:HGNC:16443] |
| ENSG00000196344 | HGLibA_00964 | 36 | -0.790364611 | ADH7 | mu or sigma polypeptide [Source:HGNC Symbol;Acc:HGNC:256]" |
| ENSG00000196387 | HGLibA_55483 | 36 | -0.790364611 | ZNF140 | zinc finger protein 140 [Source:HGNC Symbol;Acc:HGNC:12925] |
| ENSG00000196468 | HGLibA_17378 | 36 | -0.790364611 | FGF16 | fibroblast growth factor 16 [Source:HGNC Symbol;Acc:HGNC:3672] |
| ENSG00000196862 | HGLibA_41034 | 36 | -0.790364611 | RGPD4 | RANBP2-like and GRIP domain containing 4 [Source:HGNC Symbol;Acc:HGNC:32417] |
| ENSG00000196932 | HGLibA_50349 | 36 | -0.790364611 | TMEM26 | transmembrane protein 26 [Source:HGNC Symbol;Acc:HGNC:28550] |
| ENSG00000197114 | HGLibA_55306 | 36 | -0.790364611 | ZGPAT | zinc finger CCCH-type and G-patch domain containing [Source:HGNC Symbol;Acc:HGNC:15948] |
| ENSG00000197712 | HGLibA_16029 | 36 | -0.790364611 | FAM114A1 | family with sequence similarity 114 member A1 [Source:HGNC Symbol;Acc:HGNC:25087] |
| ENSG00000197776 | HGLibA_25089 | 36 | -0.790364611 | KLHDC1 | kelch domain containing 1 [Source:HGNC Symbol;Acc:HGNC:19836] |
| ENSG00000197808 | HGLibA_56037 | 36 | -0.790364611 | ZNF461 | zinc finger protein 461 [Source:HGNC Symbol;Acc:HGNC:21629] |
| ENSG00000197860 | HGLibA_43870 | 36 | -0.790364611 | SGTB | small glutamine rich tetratricopeptide repeat containing beta [Source:HGNC Symbol;Acc:HGNC:23567] |
| ENSG00000198768 | HGLibA_02399 | 36 | -0.790364611 | APCDD1L | APC down-regulated 1 like [Source:HGNC Symbol;Acc:HGNC:26892] |
| ENSG00000204389 | HGLibA_22271 | 36 | -0.790364611 | HSPA1A | heat shock protein family A (Hsp70) member 1A [Source:HGNC Symbol;Acc:HGNC:5232] |
| ENSG00000204576 | HGLibA_38837 | 36 | -0.790364611 | PRR3 | proline rich 3 [Source:HGNC Symbol;Acc:HGNC:21149] |
| ENSG00000205089 | HGLibA_08106 | 36 | -0.790364611 | CCNI2 | cyclin I family member 2 [Source:HGNC Symbol;Acc:HGNC:33869] |
| ENSG00000214087 | HGLibA_02949 | 36 | -0.790364611 | ARL16 | ADP ribosylation factor like GTPase 16 [Source:HGNC Symbol;Acc:HGNC:27902] |
| ENSG00000214711 | HGLibA_07156 | 36 | -0.790364611 | CAPN14 | calpain 14 [Source:HGNC Symbol;Acc:HGNC:16664] |
| ENSG00000215472 | HGLibA_41882 | 36 | -0.790364611 | RPL17-C18orf32 | RPL17-C18orf32 readthrough [Source:HGNC Symbol;Acc:HGNC:44661] |
| ENSG00000226023 | HGLibA_11540 | 36 | -0.790364611 | CT47A6 | member A6 [Source:HGNC Symbol;Acc:HGNC:33287]" |
| ENSG00000234224 | HGLibA_50248 | 36 | -0.790364611 | TMEM229A | transmembrane protein 229A [Source:HGNC Symbol;Acc:HGNC:37279] |
| ENSG00000238243 | HGLibA_33906 | 36 | -0.790364611 | OR2W3 | olfactory receptor family 2 subfamily W member 3 [Source:HGNC Symbol;Acc:HGNC:15021] |
| ENSG00000241343 | HGLibA_41955 | 36 | -0.790364611 | RPL36A | ribosomal protein L36a [Source:HGNC Symbol;Acc:HGNC:10359] |
| ENSG00000241476 | HGLibA_47198 | 36 | -0.790364611 | SSX2 | SSX family member 2 [Source:HGNC Symbol;Acc:HGNC:11336] |
| ENSG00000242802 | HGLibA_02362 | 36 | -0.790364611 | AP5Z1 | adaptor related protein complex 5 subunit zeta 1 [Source:HGNC Symbol;Acc:HGNC:22197] |
| ENSG00000243156 | HGLibA_29208 | 36 | -0.790364611 | MICAL3 | calponin and LIM domain containing 3 [Source:HGNC Symbol;Acc:HGNC:24694]" |
| ENSG00000244537 | HGLibA_25757 | 36 | -0.790364611 | KRTAP4-2 | keratin associated protein 4-2 [Source:HGNC Symbol;Acc:HGNC:18900] |
| ENSG00000249437 | HGLibA_31081 | 36 | -0.790364611 | NAIP | NLR family apoptosis inhibitory protein [Source:HGNC Symbol;Acc:HGNC:7634] |
| ENSG00000249709 | HGLibA_56255 | 36 | -0.790364611 | ZNF564 | zinc finger protein 564 [Source:HGNC Symbol;Acc:HGNC:31106] |
| ENSG00000250571 | HGLibA_19255 | 36 | -0.790364611 | GLI4 | GLI family zinc finger 4 [Source:HGNC Symbol;Acc:HGNC:4320] |
| ENSG00000260916 | HGLibA_08140 | 36 | -0.790364611 | CCPG1 | cell cycle progression 1 [Source:HGNC Symbol;Acc:HGNC:24227] |
| ENSG00000268940 | HGLibA_11499 | 36 | -0.790364611 | CT45A1 | cancer/testis antigen family 45 member A1 [Source:HGNC Symbol;Acc:HGNC:33267] |
| ENSG00000269226 | HGLibA_50664 | 36 | -0.790364611 | TMSB15B | thymosin beta 15B [Source:NCBI gene;Acc:286527] |
| ENSG00000272333 | HGLibA_25325 | 36 | -0.790364611 | KMT2B | lysine methyltransferase 2B [Source:HGNC Symbol;Acc:HGNC:15840] |
| ENSG00000275183 | HGLibA_26302 | 36 | -0.790364611 | LENG9 | leukocyte receptor cluster member 9 [Source:HGNC Symbol;Acc:HGNC:16306] |
| ENSG00000277586 | HGLibA_31620 | 36 | -0.790364611 | NEFL | neurofilament light [Source:HGNC Symbol;Acc:HGNC:7739] |
| ENSG00000278662 | HGLibA_19622 | 36 | -0.790364611 | GOLGA6L10 | golgin A6 family-like 10 [Source:HGNC Symbol;Acc:HGNC:37228] |
| ENSG00000279301 | HGLibA_33858 | 36 | -0.790364611 | OR2T11 | olfactory receptor family 2 subfamily T member 11 (gene/pseudogene) [Source:HGNC Symbol;Acc:HGNC:19574] |
| ENSG00000288709 | HGLibA_15890 | 36 | -0.790364611 | F8A2 | - |
| HIST1H2AM | HGLibA_21408 | 36 | -0.790364611 | HIST1H2AM | - |
| HIST1H2BK | HGLibA_21442 | 36 | -0.790364611 | HIST1H2BK | - |
| hsa-mir-1185-1 | HGLibA_57242 | 36 | -0.790364611 | hsa-mir-1185-1 | - |
| hsa-mir-1249 | HGLibA_57393 | 36 | -0.790364611 | hsa-mir-1249 | - |
| hsa-mir-1270-1 | HGLibA_57517 | 36 | -0.790364611 | hsa-mir-1270-1 | - |
| hsa-mir-129-2 | HGLibA_57631 | 36 | -0.790364611 | hsa-mir-129-2 | - |
| hsa-mir-1302-3 | HGLibA_57698 | 36 | -0.790364611 | hsa-mir-1302-3 | - |
| hsa-mir-136 | HGLibA_57803 | 36 | -0.790364611 | hsa-mir-136 | - |
| hsa-mir-151a | HGLibA_57895 | 36 | -0.790364611 | hsa-mir-151a | - |
| hsa-mir-219a-1 | HGLibA_58278 | 36 | -0.790364611 | hsa-mir-219a-1 | - |
| hsa-mir-3133 | HGLibA_58588 | 36 | -0.790364611 | hsa-mir-3133 | - |
| hsa-mir-3166 | HGLibA_58742 | 36 | -0.790364611 | hsa-mir-3166 | - |
| hsa-mir-3187 | HGLibA_58845 | 36 | -0.790364611 | hsa-mir-3187 | - |
| hsa-mir-325 | HGLibA_58966 | 36 | -0.790364611 | hsa-mir-325 | - |
| hsa-mir-3664 | HGLibA_59206 | 36 | -0.790364611 | hsa-mir-3664 | - |
| hsa-mir-3686 | HGLibA_59296 | 36 | -0.790364611 | hsa-mir-3686 | - |
| hsa-mir-383 | HGLibA_59485 | 36 | -0.790364611 | hsa-mir-383 | - |
| hsa-mir-3974 | HGLibA_59657 | 36 | -0.790364611 | hsa-mir-3974 | - |
| hsa-mir-4270 | HGLibA_59786 | 36 | -0.790364611 | hsa-mir-4270 | - |
| hsa-mir-4521 | HGLibA_60540 | 36 | -0.790364611 | hsa-mir-4521 | - |
| hsa-mir-4705 | HGLibA_60920 | 36 | -0.790364611 | hsa-mir-4705 | - |
| hsa-mir-4746 | HGLibA_61080 | 36 | -0.790364611 | hsa-mir-4746 | - |
| hsa-mir-4758 | HGLibA_61128 | 36 | -0.790364611 | hsa-mir-4758 | - |
| hsa-mir-4772 | HGLibA_61189 | 36 | -0.790364611 | hsa-mir-4772 | - |
| hsa-mir-5092 | HGLibA_61525 | 36 | -0.790364611 | hsa-mir-5092 | - |
| hsa-mir-5684 | HGLibA_62255 | 36 | -0.790364611 | hsa-mir-5684 | - |
| hsa-mir-575 | HGLibA_62396 | 36 | -0.790364611 | hsa-mir-575 | - |
| hsa-mir-605 | HGLibA_62513 | 36 | -0.790364611 | hsa-mir-605 | - |
| hsa-mir-641 | HGLibA_62803 | 36 | -0.790364611 | hsa-mir-641 | - |
| hsa-mir-6718 | HGLibA_63032 | 36 | -0.790364611 | hsa-mir-6718 | - |
| hsa-mir-6816 | HGLibA_63440 | 36 | -0.790364611 | hsa-mir-6816 | - |
| hsa-mir-6854 | HGLibA_63590 | 36 | -0.790364611 | hsa-mir-6854 | - |
| hsa-mir-7-2 | HGLibA_63772 | 36 | -0.790364611 | hsa-mir-7-2 | - |
| hsa-mir-7855 | HGLibA_64008 | 36 | -0.790364611 | hsa-mir-7855 | - |
| hsa-mir-92a-2 | HGLibA_64294 | 36 | -0.790364611 | hsa-mir-92a-2 | - |
| KIAA0430 | HGLibA_24638 | 36 | -0.790364611 | KIAA0430 | - |
| METTL10 | HGLibA_28946 | 36 | -0.790364611 | METTL10 | - |
| PNMAL2 | HGLibA_37387 | 36 | -0.790364611 | PNMAL2 | - |
| PRR24 | HGLibA_38829 | 36 | -0.790364611 | PRR24 | - |
| SPANXE | HGLibA_46397 | 36 | -0.790364611 | SPANXE | - |
| C12orf23 | HGLibA_05225 | 35 | -0.800315649 | C12orf23 | - |
| C19orf57 | HGLibA_05683 | 35 | -0.800315649 | C19orf57 | - |
| ENSG00000006652 | HGLibA_22736 | 35 | -0.800315649 | IFRD1 | interferon related developmental regulator 1 [Source:HGNC Symbol;Acc:HGNC:5456] |
| ENSG00000006704 | HGLibA_20567 | 35 | -0.800315649 | GTF2IRD1 | GTF2I repeat domain containing 1 [Source:HGNC Symbol;Acc:HGNC:4661] |
| ENSG00000008056 | HGLibA_47956 | 35 | -0.800315649 | SYN1 | synapsin I [Source:HGNC Symbol;Acc:HGNC:11494] |
| ENSG00000013293 | HGLibA_45358 | 35 | -0.800315649 | SLC7A14 | solute carrier family 7 member 14 [Source:HGNC Symbol;Acc:HGNC:29326] |
| ENSG00000014824 | HGLibA_44922 | 35 | -0.800315649 | SLC30A9 | solute carrier family 30 member 9 [Source:HGNC Symbol;Acc:HGNC:1329] |
| ENSG00000015171 | HGLibA_55413 | 35 | -0.800315649 | ZMYND11 | zinc finger MYND-type containing 11 [Source:HGNC Symbol;Acc:HGNC:16966] |
| ENSG00000025434 | HGLibA_32582 | 35 | -0.800315649 | NR1H3 | nuclear receptor subfamily 1 group H member 3 [Source:HGNC Symbol;Acc:HGNC:7966] |
| ENSG00000037897 | HGLibA_28942 | 35 | -0.800315649 | METTL1 | methyltransferase like 1 [Source:HGNC Symbol;Acc:HGNC:7030] |
| ENSG00000038210 | HGLibA_36496 | 35 | -0.800315649 | PI4K2B | phosphatidylinositol 4-kinase type 2 beta [Source:HGNC Symbol;Acc:HGNC:18215] |
| ENSG00000043514 | HGLibA_51655 | 35 | -0.800315649 | TRIT1 | tRNA isopentenyltransferase 1 [Source:HGNC Symbol;Acc:HGNC:20286] |
| ENSG00000064545 | HGLibA_50023 | 35 | -0.800315649 | TMEM161A | transmembrane protein 161A [Source:HGNC Symbol;Acc:HGNC:26020] |
| ENSG00000065615 | HGLibA_12010 | 35 | -0.800315649 | CYB5R4 | cytochrome b5 reductase 4 [Source:HGNC Symbol;Acc:HGNC:20147] |
| ENSG00000069696 | HGLibA_13968 | 35 | -0.800315649 | DRD4 | dopamine receptor D4 [Source:HGNC Symbol;Acc:HGNC:3025] |
| ENSG00000070019 | HGLibA_20661 | 35 | -0.800315649 | GUCY2C | guanylate cyclase 2C [Source:HGNC Symbol;Acc:HGNC:4688] |
| ENSG00000070718 | HGLibA_02333 | 35 | -0.800315649 | AP3M2 | adaptor related protein complex 3 subunit mu 2 [Source:HGNC Symbol;Acc:HGNC:570] |
| ENSG00000071051 | HGLibA_31309 | 35 | -0.800315649 | NCK2 | NCK adaptor protein 2 [Source:HGNC Symbol;Acc:HGNC:7665] |
| ENSG00000075426 | HGLibA_17803 | 35 | -0.800315649 | FOSL2 | AP-1 transcription factor subunit [Source:HGNC Symbol;Acc:HGNC:3798]" |
| ENSG00000077157 | HGLibA_38022 | 35 | -0.800315649 | PPP1R12B | protein phosphatase 1 regulatory subunit 12B [Source:HGNC Symbol;Acc:HGNC:7619] |
| ENSG00000077454 | HGLibA_27146 | 35 | -0.800315649 | LRCH4 | leucine rich repeats and calponin homology domain containing 4 [Source:HGNC Symbol;Acc:HGNC:6691] |
| ENSG00000078403 | HGLibA_29412 | 35 | -0.800315649 | MLLT10 | histone lysine methyltransferase DOT1L cofactor [Source:HGNC Symbol;Acc:HGNC:16063]" |
| ENSG00000079335 | HGLibA_08510 | 35 | -0.800315649 | CDC14A | cell division cycle 14A [Source:HGNC Symbol;Acc:HGNC:1718] |
| ENSG00000083307 | HGLibA_20251 | 35 | -0.800315649 | GRHL2 | grainyhead like transcription factor 2 [Source:HGNC Symbol;Acc:HGNC:2799] |
| ENSG00000085465 | HGLibA_34798 | 35 | -0.800315649 | OVGP1 | oviductal glycoprotein 1 [Source:HGNC Symbol;Acc:HGNC:8524] |
| ENSG00000090054 | HGLibA_46893 | 35 | -0.800315649 | SPTLC1 | serine palmitoyltransferase long chain base subunit 1 [Source:HGNC Symbol;Acc:HGNC:11277] |
| ENSG00000092096 | HGLibA_44559 | 35 | -0.800315649 | SLC22A17 | solute carrier family 22 member 17 [Source:HGNC Symbol;Acc:HGNC:23095] |
| ENSG00000092200 | HGLibA_41838 | 35 | -0.800315649 | RPGRIP1 | RPGR interacting protein 1 [Source:HGNC Symbol;Acc:HGNC:13436] |
| ENSG00000092445 | HGLibA_52467 | 35 | -0.800315649 | TYRO3 | TYRO3 protein tyrosine kinase [Source:HGNC Symbol;Acc:HGNC:12446] |
| ENSG00000100219 | HGLibA_54491 | 35 | -0.800315649 | XBP1 | X-box binding protein 1 [Source:HGNC Symbol;Acc:HGNC:12801] |
| ENSG00000100258 | HGLibA_26686 | 35 | -0.800315649 | LMF2 | lipase maturation factor 2 [Source:HGNC Symbol;Acc:HGNC:25096] |
| ENSG00000100319 | HGLibA_55379 | 35 | -0.800315649 | ZMAT5 | zinc finger matrin-type 5 [Source:HGNC Symbol;Acc:HGNC:28046] |
| ENSG00000100632 | HGLibA_15476 | 35 | -0.800315649 | ERH | mRNA splicing and mitosis factor [Source:HGNC Symbol;Acc:HGNC:3447]" |
| ENSG00000100749 | HGLibA_53825 | 35 | -0.800315649 | VRK1 | VRK serine/threonine kinase 1 [Source:HGNC Symbol;Acc:HGNC:12718] |
| ENSG00000101353 | HGLibA_29879 | 35 | -0.800315649 | MROH8 | maestro heat like repeat family member 8 [Source:HGNC Symbol;Acc:HGNC:16125] |
| ENSG00000101473 | HGLibA_00491 | 35 | -0.800315649 | ACOT8 | acyl-CoA thioesterase 8 [Source:HGNC Symbol;Acc:HGNC:15919] |
| ENSG00000102755 | HGLibA_17663 | 35 | -0.800315649 | FLT1 | fms related tyrosine kinase 1 [Source:HGNC Symbol;Acc:HGNC:3763] |
| ENSG00000102879 | HGLibA_10772 | 35 | -0.800315649 | CORO1A | coronin 1A [Source:HGNC Symbol;Acc:HGNC:2252] |
| ENSG00000103264 | HGLibA_17070 | 35 | -0.800315649 | FBXO31 | F-box protein 31 [Source:HGNC Symbol;Acc:HGNC:16510] |
| ENSG00000104112 | HGLibA_42901 | 35 | -0.800315649 | SCG3 | secretogranin III [Source:HGNC Symbol;Acc:HGNC:13707] |
| ENSG00000104413 | HGLibA_15588 | 35 | -0.800315649 | ESRP1 | epithelial splicing regulatory protein 1 [Source:HGNC Symbol;Acc:HGNC:25966] |
| ENSG00000104824 | HGLibA_21833 | 35 | -0.800315649 | HNRNPL | heterogeneous nuclear ribonucleoprotein L [Source:HGNC Symbol;Acc:HGNC:5045] |
| ENSG00000105771 | HGLibA_45713 | 35 | -0.800315649 | SMG9 | nonsense mediated mRNA decay factor [Source:HGNC Symbol;Acc:HGNC:25763]" |
| ENSG00000105856 | HGLibA_20935 | 35 | -0.800315649 | HBP1 | HMG-box transcription factor 1 [Source:HGNC Symbol;Acc:HGNC:23200] |

| | | | | | |
|---|---|---|---|---|---|
| ENSG00000106013 | HGLibA_02143 | 35 | -0.800315649 | ANKRD7 | ankyrin repeat domain 7 [Source:HGNC Symbol;Acc:HGNC:18588] |
| ENSG00000107105 | HGLibA_14883 | 35 | -0.800315649 | ELAVL2 | ELAV like RNA binding protein 2 [Source:HGNC Symbol;Acc:HGNC:3313] |
| ENSG00000107625 | HGLibA_12732 | 35 | -0.800315649 | DDX50 | DExD-box helicase 50 [Source:HGNC Symbol;Acc:HGNC:17906] |
| ENSG00000107968 | HGLibA_28203 | 35 | -0.800315649 | MAP3K8 | mitogen-activated protein kinase kinase kinase 8 [Source:HGNC Symbol;Acc:HGNC:6860] |
| ENSG00000109118 | HGLibA_36339 | 35 | -0.800315649 | PHF12 | PHD finger protein 12 [Source:HGNC Symbol;Acc:HGNC:20816] |
| ENSG00000110367 | HGLibA_12760 | 35 | -0.800315649 | DDX6 | DEAD-box helicase 6 [Source:HGNC Symbol;Acc:HGNC:2747] |
| ENSG00000111087 | HGLibA_19248 | 35 | -0.800315649 | GLI1 | GLI family zinc finger 1 [Source:HGNC Symbol;Acc:HGNC:4317] |
| ENSG00000112195 | HGLibA_51363 | 35 | -0.800315649 | TREML2 | triggering receptor expressed on myeloid cells like 2 [Source:HGNC Symbol;Acc:HGNC:21092] |
| ENSG00000112212 | HGLibA_51961 | 35 | -0.800315649 | TSPO2 | translocator protein 2 [Source:HGNC Symbol;Acc:HGNC:21256] |
| ENSG00000112599 | HGLibA_20638 | 35 | -0.800315649 | GUCA1B | guanylate cyclase activator 1B [Source:HGNC Symbol;Acc:HGNC:4679] |
| ENSG00000113269 | HGLibA_41504 | 35 | -0.800315649 | RNF130 | ring finger protein 130 [Source:HGNC Symbol;Acc:HGNC:18280] |
| ENSG00000113758 | HGLibA_12387 | 35 | -0.800315649 | DBN1 | drebrin 1 [Source:HGNC Symbol;Acc:HGNC:2695] |
| ENSG00000114902 | HGLibA_46558 | 35 | -0.800315649 | SPCS1 | signal peptidase complex subunit 1 [Source:HGNC Symbol;Acc:HGNC:23401] |
| ENSG00000115008 | HGLibA_23038 | 35 | -0.800315649 | IL1A | interleukin 1 alpha [Source:HGNC Symbol;Acc:HGNC:5991] |
| ENSG00000115762 | HGLibA_37088 | 35 | -0.800315649 | PLEKHB2 | pleckstrin homology domain containing B2 [Source:HGNC Symbol;Acc:HGNC:19236] |
| ENSG00000116497 | HGLibA_42588 | 35 | -0.800315649 | S100PBP | S100P binding protein [Source:HGNC Symbol;Acc:HGNC:25768] |
| ENSG00000116809 | HGLibA_54829 | 35 | -0.800315649 | ZBTB17 | zinc finger and BTB domain containing 17 [Source:HGNC Symbol;Acc:HGNC:12936] |
| ENSG00000117122 | HGLibA_29041 | 35 | -0.800315649 | MFAP2 | microfibril associated protein 2 [Source:HGNC Symbol;Acc:HGNC:7033] |
| ENSG00000117592 | HGLibA_38423 | 35 | -0.800315649 | PRDX6 | peroxiredoxin 6 [Source:HGNC Symbol;Acc:HGNC:16753] |
| ENSG00000117868 | HGLibA_15608 | 35 | -0.800315649 | ESYT2 | extended synaptotagmin 2 [Source:HGNC Symbol;Acc:HGNC:22211] |
| ENSG00000120437 | HGLibA_00396 | 35 | -0.800315649 | ACAT2 | acetyl-CoA acetyltransferase 2 [Source:HGNC Symbol;Acc:HGNC:94] |
| ENSG00000120616 | HGLibA_15265 | 35 | -0.800315649 | EPC1 | enhancer of polycomb homolog 1 [Source:HGNC Symbol;Acc:HGNC:19876] |
| ENSG00000120733 | HGLibA_24504 | 35 | -0.800315649 | KDM3B | lysine demethylase 3B [Source:HGNC Symbol;Acc:HGNC:1337] |
| ENSG00000121075 | HGLibA_48671 | 35 | -0.800315649 | TBX4 | T-box 4 [Source:HGNC Symbol;Acc:HGNC:11603] |
| ENSG00000122679 | HGLibA_40186 | 35 | -0.800315649 | RAMP3 | receptor activity modifying protein 3 [Source:HGNC Symbol;Acc:HGNC:9845] |
| ENSG00000122728 | HGLibA_48224 | 35 | -0.800315649 | TAF1L | TATA-box binding protein associated factor 1 like [Source:HGNC Symbol;Acc:HGNC:18056] |
| ENSG00000123342 | HGLibA_29505 | 35 | -0.800315649 | MMP19 | matrix metallopeptidase 19 [Source:HGNC Symbol;Acc:HGNC:7165] |
| ENSG00000124140 | HGLibA_44369 | 35 | -0.800315649 | SLC12A5 | solute carrier family 12 member 5 [Source:HGNC Symbol;Acc:HGNC:13818] |
| ENSG00000125492 | HGLibA_04157 | 35 | -0.800315649 | BARHL1 | BarH like homeobox 1 [Source:HGNC Symbol;Acc:HGNC:953] |
| ENSG00000127184 | HGLibA_10871 | 35 | -0.800315649 | COX7C | cytochrome c oxidase subunit 7C [Source:HGNC Symbol;Acc:HGNC:2292] |
| ENSG00000128011 | HGLibA_27153 | 35 | -0.800315649 | LRFN1 | leucine rich repeat and fibronectin type III domain containing 1 [Source:HGNC Symbol;Acc:HGNC:29290] |
| ENSG00000128310 | HGLibA_18593 | 35 | -0.800315649 | GALR3 | galanin receptor 3 [Source:HGNC Symbol;Acc:HGNC:4134] |
| ENSG00000128965 | HGLibA_09335 | 35 | -0.800315649 | CHAC1 | ChaC glutathione specific gamma-glutamylcyclotransferase 1 [Source:HGNC Symbol;Acc:HGNC:28680] |
| ENSG00000130758 | HGLibA_28164 | 35 | -0.800315649 | MAP3K10 | mitogen-activated protein kinase kinase kinase 10 [Source:HGNC Symbol;Acc:HGNC:6849] |
| ENSG00000130997 | HGLibA_37544 | 35 | -0.800315649 | POLN | DNA polymerase nu [Source:HGNC Symbol;Acc:HGNC:18870] |
| ENSG00000131096 | HGLibA_39711 | 35 | -0.800315649 | PYY | peptide YY [Source:HGNC Symbol;Acc:HGNC:9748] |
| ENSG00000132294 | HGLibA_14605 | 35 | -0.800315649 | EFR3A | EFR3 homolog A [Source:HGNC Symbol;Acc:HGNC:28970] |
| ENSG00000132305 | HGLibA_23234 | 35 | -0.800315649 | IMMT | inner membrane mitochondrial protein [Source:HGNC Symbol;Acc:HGNC:6047] |
| ENSG00000132879 | HGLibA_17106 | 35 | -0.800315649 | FBXO44 | F-box protein 44 [Source:HGNC Symbol;Acc:HGNC:24847] |
| ENSG00000133731 | HGLibA_23245 | 35 | -0.800315649 | IMPA1 | inositol monophosphatase 1 [Source:HGNC Symbol;Acc:HGNC:6050] |
| ENSG00000133742 | HGLibA_06785 | 35 | -0.800315649 | CA1 | carbonic anhydrase 1 [Source:HGNC Symbol;Acc:HGNC:1368] |
| ENSG00000134115 | HGLibA_10416 | 35 | -0.800315649 | CNTN6 | contactin 6 [Source:HGNC Symbol;Acc:HGNC:2176] |
| ENSG00000135828 | HGLibA_41439 | 35 | -0.800315649 | RNASEL | ribonuclease L [Source:HGNC Symbol;Acc:HGNC:10050] |
| ENSG00000135976 | HGLibA_02086 | 35 | -0.800315649 | ANKRD36 | ankyrin repeat domain 36 [Source:HGNC Symbol;Acc:HGNC:24079] |
| ENSG00000137103 | HGLibA_50550 | 35 | -0.800315649 | TMEM8B | transmembrane protein 8B [Source:HGNC Symbol;Acc:HGNC:21427] |
| ENSG00000138379 | HGLibA_30266 | 35 | -0.800315649 | MSTN | myostatin [Source:HGNC Symbol;Acc:HGNC:4223] |
| ENSG00000138587 | HGLibA_29574 | 35 | -0.800315649 | MNS1 | meiosis specific nuclear structural 1 [Source:HGNC Symbol;Acc:HGNC:29636] |
| ENSG00000138668 | HGLibA_21814 | 35 | -0.800315649 | HNRNPD | heterogeneous nuclear ribonucleoprotein D [Source:HGNC Symbol;Acc:HGNC:5036] |
| ENSG00000138777 | HGLibA_37807 | 35 | -0.800315649 | PPA2 | pyrophosphatase (inorganic) 2 [Source:HGNC Symbol;Acc:HGNC:28883] |
| ENSG00000138835 | HGLibA_41092 | 35 | -0.800315649 | RGS3 | regulator of G protein signaling 3 [Source:HGNC Symbol;Acc:HGNC:9999] |
| ENSG00000139116 | HGLibA_24897 | 35 | -0.800315649 | KIF21A | kinesin family member 21A [Source:HGNC Symbol;Acc:HGNC:19349] |
| ENSG00000139194 | HGLibA_40641 | 35 | -0.800315649 | RBP5 | retinol binding protein 5 [Source:HGNC Symbol;Acc:HGNC:15847] |
| ENSG00000139626 | HGLibA_23796 | 35 | -0.800315649 | ITGB7 | integrin subunit beta 7 [Source:HGNC Symbol;Acc:HGNC:6162] |
| ENSG00000140299 | HGLibA_04654 | 35 | -0.800315649 | BNIP2 | BCL2 interacting protein 2 [Source:HGNC Symbol;Acc:HGNC:1083] |
| ENSG00000140307 | HGLibA_20527 | 35 | -0.800315649 | GTF2A2 | general transcription factor IIA subunit 2 [Source:HGNC Symbol;Acc:HGNC:4647] |
| ENSG00000140525 | HGLibA_16853 | 35 | -0.800315649 | FANCI | FA complementation group I [Source:HGNC Symbol;Acc:HGNC:25568] |
| ENSG00000140988 | HGLibA_42100 | 35 | -0.800315649 | RPS2 | ribosomal protein S2 [Source:HGNC Symbol;Acc:HGNC:10404] |
| ENSG00000141562 | HGLibA_31142 | 35 | -0.800315649 | NARF | nuclear prelamin A recognition factor [Source:HGNC Symbol;Acc:HGNC:29916] |
| ENSG00000143458 | HGLibA_18354 | 35 | -0.800315649 | GABPB2 | GA binding protein transcription factor subunit beta 2 [Source:HGNC Symbol;Acc:HGNC:28441] |
| ENSG00000143878 | HGLibA_41169 | 35 | -0.800315649 | RHOB | ras homolog family member B [Source:HGNC Symbol;Acc:HGNC:668] |
| ENSG00000144744 | HGLibA_52520 | 35 | -0.800315649 | UBA3 | ubiquitin like modifier activating enzyme 3 [Source:HGNC Symbol;Acc:HGNC:12470] |
| ENSG00000144895 | HGLibA_14721 | 35 | -0.800315649 | EIF2A | eukaryotic translation initiation factor 2A [Source:HGNC Symbol;Acc:HGNC:3254] |
| ENSG00000145014 | HGLibA_50406 | 35 | -0.800315649 | TMEM44 | transmembrane protein 44 [Source:HGNC Symbol;Acc:HGNC:25120] |
| ENSG00000146469 | HGLibA_53661 | 35 | -0.800315649 | VIP | vasoactive intestinal peptide [Source:HGNC Symbol;Acc:HGNC:12693] |
| ENSG00000146676 | HGLibA_39589 | 35 | -0.800315649 | PURB | purine rich element binding protein B [Source:HGNC Symbol;Acc:HGNC:9702] |
| ENSG00000147183 | HGLibA_11034 | 35 | -0.800315649 | CPXCR1 | candidate 1 [Source:HGNC Symbol;Acc:HGNC:2332]" |
| ENSG00000147224 | HGLibA_38754 | 35 | -0.800315649 | PRPS1 | phosphoribosyl pyrophosphate synthetase 1 [Source:HGNC Symbol;Acc:HGNC:9462] |
| ENSG00000148408 | HGLibA_06879 | 35 | -0.800315649 | CACNA1B | calcium voltage-gated channel subunit alpha1 B [Source:HGNC Symbol;Acc:HGNC:1389] |
| ENSG00000148814 | HGLibA_27297 | 35 | -0.800315649 | LRRC27 | leucine rich repeat containing 27 [Source:HGNC Symbol;Acc:HGNC:29346] |
| ENSG00000149179 | HGLibA_05147 | 35 | -0.800315649 | C11orf49 | chromosome 11 open reading frame 49 [Source:HGNC Symbol;Acc:HGNC:28720] |
| ENSG00000149380 | HGLibA_34905 | 35 | -0.800315649 | P4HA3 | prolyl 4-hydroxylase subunit alpha 3 [Source:HGNC Symbol;Acc:HGNC:30135] |
| ENSG00000149925 | HGLibA_01592 | 35 | -0.800315649 | ALDOA | fructose-bisphosphate A [Source:HGNC Symbol;Acc:HGNC:414]" |
| ENSG00000150593 | HGLibA_35733 | 35 | -0.800315649 | PDCD4 | programmed cell death 4 [Source:HGNC Symbol;Acc:HGNC:8763] |
| ENSG00000151475 | HGLibA_44706 | 35 | -0.800315649 | SLC25A31 | solute carrier family 25 member 31 [Source:HGNC Symbol;Acc:HGNC:25319] |
| ENSG00000151617 | HGLibA_14477 | 35 | -0.800315649 | EDNRA | endothelin receptor type A [Source:HGNC Symbol;Acc:HGNC:3179] |
| ENSG00000152464 | HGLibA_42038 | 35 | -0.800315649 | RPP38 | ribonuclease P/MRP subunit p38 [Source:HGNC Symbol;Acc:HGNC:30329] |
| ENSG00000152952 | HGLibA_37210 | 35 | -0.800315649 | PLOD2 | 2-oxoglutarate 5-dioxygenase 2 [Source:HGNC Symbol;Acc:HGNC:9082]" |
| ENSG00000153779 | HGLibA_49247 | 35 | -0.800315649 | TGIF2LX | TGFB induced factor homeobox 2 like X-linked [Source:HGNC Symbol;Acc:HGNC:18570] |
| ENSG00000155660 | HGLibA_35880 | 35 | -0.800315649 | PDIA4 | protein disulfide isomerase family A member 4 [Source:HGNC Symbol;Acc:HGNC:30167] |
| ENSG00000155980 | HGLibA_24950 | 35 | -0.800315649 | KIF5A | kinesin family member 5A [Source:HGNC Symbol;Acc:HGNC:6323] |
| ENSG00000158161 | HGLibA_15822 | 35 | -0.800315649 | EYA3 | EYA transcriptional coactivator and phosphatase 3 [Source:HGNC Symbol;Acc:HGNC:3521] |
| ENSG00000159409 | HGLibA_09010 | 35 | -0.800315649 | CELF3 | CUGBP Elav-like family member 3 [Source:HGNC Symbol;Acc:HGNC:11967] |
| ENSG00000159579 | HGLibA_42357 | 35 | -0.800315649 | RSPRY1 | ring finger and SPRY domain containing 1 [Source:HGNC Symbol;Acc:HGNC:29420] |
| ENSG00000160188 | HGLibA_42323 | 35 | -0.800315649 | RSPH1 | radial spoke head component 1 [Source:HGNC Symbol;Acc:HGNC:12371] |
| ENSG00000160282 | HGLibA_18113 | 35 | -0.800315649 | FTCD | formimidoyltransferase cyclodeaminase [Source:HGNC Symbol;Acc:HGNC:3974] |
| ENSG00000162434 | HGLibA_23913 | 35 | -0.800315649 | JAK1 | Janus kinase 1 [Source:HGNC Symbol;Acc:HGNC:6190] |
| ENSG00000162594 | HGLibA_23100 | 35 | -0.800315649 | IL23R | interleukin 23 receptor [Source:HGNC Symbol;Acc:HGNC:19100] |
| ENSG00000163719 | HGLibA_30420 | 35 | -0.800315649 | MTMR14 | myotubularin related protein 14 [Source:HGNC Symbol;Acc:HGNC:26190] |
| ENSG00000164163 | HGLibA_00202 | 35 | -0.800315649 | ABCE1 | ATP binding cassette subfamily E member 1 [Source:HGNC Symbol;Acc:HGNC:69] |
| ENSG00000164325 | HGLibA_50067 | 35 | -0.800315649 | TMEM174 | transmembrane protein 174 [Source:HGNC Symbol;Acc:HGNC:28187] |
| ENSG00000164684 | HGLibA_56584 | 35 | -0.800315649 | ZNF704 | zinc finger protein 704 [Source:HGNC Symbol;Acc:HGNC:32291] |
| ENSG00000165119 | HGLibA_21830 | 35 | -0.800315649 | HNRNPK | heterogeneous nuclear ribonucleoprotein K [Source:HGNC Symbol;Acc:HGNC:5044] |
| ENSG00000165810 | HGLibA_04984 | 35 | -0.800315649 | BTNL9 | butyrophilin like 9 [Source:HGNC Symbol;Acc:HGNC:24176] |
| ENSG00000166133 | HGLibA_42210 | 35 | -0.800315649 | RPUSD2 | RNA pseudouridine synthase domain containing 2 [Source:HGNC Symbol;Acc:HGNC:24180] |
| ENSG00000166188 | HGLibA_55790 | 35 | -0.800315649 | ZNF319 | zinc finger protein 319 [Source:HGNC Symbol;Acc:HGNC:13644] |
| ENSG00000166292 | HGLibA_49839 | 35 | -0.800315649 | TMEM100 | transmembrane protein 100 [Source:HGNC Symbol;Acc:HGNC:25607] |
| ENSG00000166435 | HGLibA_54609 | 35 | -0.800315649 | XRRA1 | X-ray radiation resistance associated 1 [Source:HGNC Symbol;Acc:HGNC:18868] |
| ENSG00000166471 | HGLibA_50397 | 35 | -0.800315649 | TMEM41B | transmembrane protein 41B [Source:HGNC Symbol;Acc:HGNC:28948] |
| ENSG00000166573 | HGLibA_18587 | 35 | -0.800315649 | GALR1 | galanin receptor 1 [Source:HGNC Symbol;Acc:HGNC:4132] |
| ENSG00000166578 | HGLibA_23497 | 35 | -0.800315649 | IQCD | IQ motif containing D [Source:HGNC Symbol;Acc:HGNC:25168] |
| ENSG00000167565 | HGLibA_43609 | 35 | -0.800315649 | SERTAD3 | SERTA domain containing 3 [Source:HGNC Symbol;Acc:HGNC:17931] |
| ENSG00000167619 | HGLibA_49973 | 35 | -0.800315649 | TMEM145 | transmembrane protein 145 [Source:HGNC Symbol;Acc:HGNC:26912] |
| ENSG00000168386 | HGLibA_17532 | 35 | -0.800315649 | FILIP1L | filamin A interacting protein 1 like [Source:HGNC Symbol;Acc:HGNC:24589] |
| ENSG00000168438 | HGLibA_08550 | 35 | -0.800315649 | CDC40 | cell division cycle 40 [Source:HGNC Symbol;Acc:HGNC:17350] |
| ENSG00000169032 | HGLibA_28139 | 35 | -0.800315649 | MAP2K1 | mitogen-activated protein kinase kinase 1 [Source:HGNC Symbol;Acc:HGNC:6840] |
| ENSG00000169214 | HGLibA_34416 | 35 | -0.800315649 | OR6F1 | olfactory receptor family 6 subfamily F member 1 [Source:HGNC Symbol;Acc:HGNC:15027] |
| ENSG00000169641 | HGLibA_27639 | 35 | -0.800315649 | LUZP1 | leucine zipper protein 1 [Source:HGNC Symbol;Acc:HGNC:14985] |
| ENSG00000169752 | HGLibA_32685 | 35 | -0.800315649 | NRG4 | neuregulin 4 [Source:HGNC Symbol;Acc:HGNC:29862] |
| ENSG00000169860 | HGLibA_34868 | 35 | -0.800315649 | P2RY1 | purinergic receptor P2Y1 [Source:HGNC Symbol;Acc:HGNC:8539] |
| ENSG00000169903 | HGLibA_49703 | 35 | -0.800315649 | TM4SF4 | transmembrane 4 L six family member 4 [Source:HGNC Symbol;Acc:HGNC:11856] |
| ENSG00000170255 | HGLibA_29847 | 35 | -0.800315649 | MRGPRX1 | MAS related GPR family member X1 [Source:HGNC Symbol;Acc:HGNC:17962] |
| ENSG00000170485 | HGLibA_32396 | 35 | -0.800315649 | NPAS2 | neuronal PAS domain protein 2 [Source:HGNC Symbol;Acc:HGNC:7895] |
| ENSG00000170515 | HGLibA_34916 | 35 | -0.800315649 | PA2G4 | proliferation-associated 2G4 [Source:HGNC Symbol;Acc:HGNC:8550] |
| ENSG00000170523 | HGLibA_25563 | 35 | -0.800315649 | KRT83 | keratin 83 [Source:HGNC Symbol;Acc:HGNC:6460] |
| ENSG00000171303 | HGLibA_24321 | 35 | -0.800315649 | KCNK3 | potassium two pore domain channel subfamily K member 3 [Source:HGNC Symbol;Acc:HGNC:6278] |
| ENSG00000171425 | HGLibA_56309 | 35 | -0.800315649 | ZNF581 | zinc finger protein 581 [Source:HGNC Symbol;Acc:HGNC:25017] |
| ENSG00000171532 | HGLibA_31737 | 35 | -0.800315649 | NEUROD2 | neuronal differentiation 2 [Source:HGNC Symbol;Acc:HGNC:7763] |
| ENSG00000171551 | HGLibA_14389 | 35 | -0.800315649 | ECEL1 | endothelin converting enzyme like 1 [Source:HGNC Symbol;Acc:HGNC:3147] |
| ENSG00000171777 | HGLibA_40354 | 35 | -0.800315649 | RASGRP4 | RAS guanyl releasing protein 4 [Source:HGNC Symbol;Acc:HGNC:18958] |
| ENSG00000171865 | HGLibA_41423 | 35 | -0.800315649 | RNASEH1 | ribonuclease H1 [Source:HGNC Symbol;Acc:HGNC:18466] |
| ENSG00000172031 | HGLibA_15332 | 35 | -0.800315649 | EPHX4 | epoxide hydrolase 4 [Source:HGNC Symbol;Acc:HGNC:23758] |
| ENSG00000172262 | HGLibA_55458 | 35 | -0.800315649 | ZNF131 | zinc finger protein 131 [Source:HGNC Symbol;Acc:HGNC:12915] |
| ENSG00000172728 | HGLibA_18182 | 35 | -0.800315649 | FUT10 | fucosyltransferase 10 [Source:HGNC Symbol;Acc:HGNC:19234] |
| ENSG00000172935 | HGLibA_29841 | 35 | -0.800315649 | MRGPRF | MAS related GPR family member F [Source:HGNC Symbol;Acc:HGNC:24828] |
| ENSG00000172943 | HGLibA_36389 | 35 | -0.800315649 | PHF8 | PHD finger protein 8 [Source:HGNC Symbol;Acc:HGNC:20672] |
| ENSG00000173269 | HGLibA_29550 | 35 | -0.800315649 | MMRN2 | multimerin 2 [Source:HGNC Symbol;Acc:HGNC:19888] |
| ENSG00000173402 | HGLibA_12291 | 35 | -0.800315649 | DAG1 | dystroglycan 1 [Source:HGNC Symbol;Acc:HGNC:2666] |
| ENSG00000174370 | HGLibA_05141 | 35 | -0.800315649 | C11orf45 | chromosome 11 open reading frame 45 [Source:HGNC Symbol;Acc:HGNC:28584] |
| ENSG00000175376 | HGLibA_14707 | 35 | -0.800315649 | EIF1AD | eukaryotic translation initiation factor 1A domain containing [Source:HGNC Symbol;Acc:HGNC:28147] |
| ENSG00000176597 | HGLibA_03993 | 35 | -0.800315649 | B3GNT5 | 3-N-acetylglucosaminyltransferase 5 [Source:HGNC Symbol;Acc:HGNC:15684]" |
| ENSG00000176624 | HGLibA_29033 | 35 | -0.800315649 | MEX3C | mex-3 RNA binding family member C [Source:HGNC Symbol;Acc:HGNC:28040] |
| ENSG00000176925 | HGLibA_34106 | 35 | -0.800315649 | OR51F2 | olfactory receptor family 51 subfamily F member 2 [Source:HGNC Symbol;Acc:HGNC:15197] |
| ENSG00000176979 | HGLibA_51565 | 35 | -0.800315649 | TRIM60 | tripartite motif containing 60 [Source:HGNC Symbol;Acc:HGNC:21162] |
| ENSG00000177463 | HGLibA_32598 | 35 | -0.800315649 | NR2C2 | nuclear receptor subfamily 2 group C member 2 [Source:HGNC Symbol;Acc:HGNC:7972] |
| ENSG00000177464 | HGLibA_20041 | 35 | -0.800315649 | GPR4 | G protein-coupled receptor 4 [Source:HGNC Symbol;Acc:HGNC:4497] |

| | | | | | |
|---|---|---|---|---|---|
| ENSG00000178761 | HGLibA_16501 | 35 | -0.800315649 | FAM219B | family with sequence similarity 219 member B [Source:HGNC Symbol;Acc:HGNC:24695] |
| ENSG00000179023 | HGLibA_25104 | 35 | -0.800315649 | KLHDC7A | kelch domain containing 7A [Source:HGNC Symbol;Acc:HGNC:26791] |
| ENSG00000180008 | HGLibA_46133 | 35 | -0.800315649 | SOCS4 | suppressor of cytokine signaling 4 [Source:HGNC Symbol;Acc:HGNC:19392] |
| ENSG00000180626 | HGLibA_56344 | 35 | -0.800315649 | ZNF594 | zinc finger protein 594 [Source:HGNC Symbol;Acc:HGNC:29392] |
| ENSG00000180787 | HGLibA_55199 | 35 | -0.800315649 | ZFP3 | ZFP3 zinc finger protein [Source:HGNC Symbol;Acc:HGNC:12861] |
| ENSG00000181371 | HGLibA_34339 | 35 | -0.800315649 | OR5M8 | olfactory receptor family 5 subfamily M member 8 [Source:HGNC Symbol;Acc:HGNC:14846] |
| ENSG00000181894 | HGLibA_55811 | 35 | -0.800315649 | ZNF329 | zinc finger protein 329 [Source:HGNC Symbol;Acc:HGNC:14209] |
| ENSG00000182324 | HGLibA_24260 | 35 | -0.800315649 | KCNJ14 | potassium voltage-gated channel subfamily J member 14 [Source:HGNC Symbol;Acc:HGNC:6260] |
| ENSG00000182901 | HGLibA_41104 | 35 | -0.800315649 | RGS7 | regulator of G protein signaling 7 [Source:HGNC Symbol;Acc:HGNC:10003] |
| ENSG00000183434 | HGLibA_49158 | 35 | -0.800315649 | TFDP3 | transcription factor Dp family member 3 [Source:HGNC Symbol;Acc:HGNC:24603] |
| ENSG00000183734 | HGLibA_03268 | 35 | -0.800315649 | ASCL2 | achaete-scute family bHLH transcription factor 2 [Source:HGNC Symbol;Acc:HGNC:739] |
| ENSG00000183918 | HGLibA_43883 | 35 | -0.800315649 | SH2D1A | SH2 domain containing 1A [Source:HGNC Symbol;Acc:HGNC:10820] |
| ENSG00000184389 | HGLibA_00013 | 35 | -0.800315649 | A3GALT2 | 3-galactosyltransferase 2 [Source:HGNC Symbol;Acc:HGNC:30005]" |
| ENSG00000184524 | HGLibA_09033 | 35 | -0.800315649 | CEND1 | cell cycle exit and neuronal differentiation 1 [Source:HGNC Symbol;Acc:HGNC:24153] |
| ENSG00000184787 | HGLibA_52608 | 35 | -0.800315649 | UBE2G2 | ubiquitin conjugating enzyme E2 G2 [Source:HGNC Symbol;Acc:HGNC:12483] |
| ENSG00000185475 | HGLibA_50090 | 35 | -0.800315649 | TMEM179B | transmembrane protein 179B [Source:HGNC Symbol;Acc:HGNC:33744] |
| ENSG00000185664 | HGLibA_37296 | 35 | -0.800315649 | PMEL | premelanosome protein [Source:HGNC Symbol;Acc:HGNC:10880] |
| ENSG00000185774 | HGLibA_24244 | 35 | -0.800315649 | KCNIP4 | potassium voltage-gated channel interacting protein 4 [Source:HGNC Symbol;Acc:HGNC:30083] |
| ENSG00000185813 | HGLibA_35705 | 35 | -0.800315649 | PCYT2 | ethanolamine [Source:HGNC Symbol;Acc:HGNC:8756]" |
| ENSG00000185905 | HGLibA_05455 | 35 | -0.800315649 | C16orf54 | chromosome 16 open reading frame 54 [Source:HGNC Symbol;Acc:HGNC:26649] |
| ENSG00000185909 | HGLibA_25114 | 35 | -0.800315649 | KLHDC8B | kelch domain containing 8B [Source:HGNC Symbol;Acc:HGNC:28557] |
| ENSG00000186130 | HGLibA_54913 | 35 | -0.800315649 | ZBTB6 | zinc finger and BTB domain containing 6 [Source:HGNC Symbol;Acc:HGNC:16764] |
| ENSG00000186288 | HGLibA_34928 | 35 | -0.800315649 | PABPC1L2A | poly(A) binding protein cytoplasmic 1 like 2A [Source:HGNC Symbol;Acc:HGNC:27989] |
| ENSG00000186480 | HGLibA_23375 | 35 | -0.800315649 | INSIG1 | insulin induced gene 1 [Source:HGNC Symbol;Acc:HGNC:6083] |
| ENSG00000186795 | HGLibA_24316 | 35 | -0.800315649 | KCNK18 | potassium two pore domain channel subfamily K member 18 [Source:HGNC Symbol;Acc:HGNC:19439] |
| ENSG00000188042 | HGLibA_02970 | 35 | -0.800315649 | ARL4C | ADP ribosylation factor like GTPase 4C [Source:HGNC Symbol;Acc:HGNC:698] |
| ENSG00000188313 | HGLibA_37231 | 35 | -0.800315649 | PLSCR1 | phospholipid scramblase 1 [Source:HGNC Symbol;Acc:HGNC:9092] |
| ENSG00000188425 | HGLibA_31100 | 35 | -0.800315649 | NANOS2 | nanos C2HC-type zinc finger 2 [Source:HGNC Symbol;Acc:HGNC:23292] |
| ENSG00000188690 | HGLibA_53157 | 35 | -0.800315649 | UROS | uroporphyrinogen III synthase [Source:HGNC Symbol;Acc:HGNC:12592] |
| ENSG00000188735 | HGLibA_49898 | 35 | -0.800315649 | TMEM120B | transmembrane protein 120B [Source:HGNC Symbol;Acc:HGNC:32008] |
| ENSG00000188869 | HGLibA_49754 | 35 | -0.800315649 | TMC3 | transmembrane channel like 3 [Source:HGNC Symbol;Acc:HGNC:22995] |
| ENSG00000188937 | HGLibA_33190 | 35 | -0.800315649 | NYX | nyctalopin [Source:HGNC Symbol;Acc:HGNC:8082] |
| ENSG00000189143 | HGLibA_09897 | 35 | -0.800315649 | CLDN4 | claudin 4 [Source:HGNC Symbol;Acc:HGNC:2046] |
| ENSG00000189164 | HGLibA_56168 | 35 | -0.800315649 | ZNF527 | zinc finger protein 527 [Source:HGNC Symbol;Acc:HGNC:29385] |
| ENSG00000196182 | HGLibA_47513 | 35 | -0.800315649 | STK40 | serine/threonine kinase 40 [Source:HGNC Symbol;Acc:HGNC:21373] |
| ENSG00000196313 | HGLibA_37642 | 35 | -0.800315649 | POM121 | POM121 transmembrane nucleoporin [Source:HGNC Symbol;Acc:HGNC:19702] |
| ENSG00000196678 | HGLibA_15481 | 35 | -0.800315649 | ERI2 | ERI1 exoribonuclease family member 2 [Source:HGNC Symbol;Acc:HGNC:30541] |
| ENSG00000196781 | HGLibA_49606 | 35 | -0.800315649 | TLE1 | transducin like enhancer of split 1 [Source:HGNC Symbol;Acc:HGNC:11837] |
| ENSG00000198315 | HGLibA_55364 | 35 | -0.800315649 | ZKSCAN8 | zinc finger with KRAB and SCAN domains 8 [Source:HGNC Symbol;Acc:HGNC:12983] |
| ENSG00000198390 | HGLibA_25643 | 35 | -0.800315649 | KRTAP13-1 | keratin associated protein 13-1 [Source:HGNC Symbol;Acc:HGNC:18924] |
| ENSG00000198515 | HGLibA_10263 | 35 | -0.800315649 | CNGA1 | cyclic nucleotide gated channel alpha 1 [Source:HGNC Symbol;Acc:HGNC:2148] |
| ENSG00000198722 | HGLibA_52997 | 35 | -0.800315649 | UNC13B | unc-13 homolog B [Source:HGNC Symbol;Acc:HGNC:12566] |
| ENSG00000204352 | HGLibA_06674 | 35 | -0.800315649 | C9orf129 | chromosome 9 open reading frame 129 [Source:HGNC Symbol;Acc:HGNC:31116] |
| ENSG00000204616 | HGLibA_51460 | 35 | -0.800315649 | TRIM31 | tripartite motif containing 31 [Source:HGNC Symbol;Acc:HGNC:16289] |
| ENSG00000204947 | HGLibA_55963 | 35 | -0.800315649 | ZNF425 | zinc finger protein 425 [Source:HGNC Symbol;Acc:HGNC:20690] |
| ENSG00000213185 | HGLibA_16550 | 35 | -0.800315649 | FAM24B | family with sequence similarity 24 member B [Source:HGNC Symbol;Acc:HGNC:23475] |
| ENSG00000213762 | HGLibA_55468 | 35 | -0.800315649 | ZNF134 | zinc finger protein 134 [Source:HGNC Symbol;Acc:HGNC:12918] |
| ENSG00000213923 | HGLibA_11390 | 35 | -0.800315649 | CSNK1E | casein kinase 1 epsilon [Source:HGNC Symbol;Acc:HGNC:2453] |
| ENSG00000216649 | HGLibA_18446 | 35 | -0.800315649 | GAGE12E | G antigen 12E [Source:HGNC Symbol;Acc:HGNC:31905] |
| ENSG00000225781 | HGLibA_34447 | 35 | -0.800315649 | OR6V1 | olfactory receptor family 6 subfamily V member 1 [Source:HGNC Symbol;Acc:HGNC:15090] |
| ENSG00000239474 | HGLibA_25209 | 35 | -0.800315649 | KLHL41 | kelch like family member 41 [Source:HGNC Symbol;Acc:HGNC:16905] |
| ENSG00000241058 | HGLibA_32792 | 35 | -0.800315649 | NSUN6 | NOP2/Sun RNA methyltransferase family member 6 [Source:HGNC Symbol;Acc:HGNC:23529] |
| ENSG00000242689 | HGLibA_10392 | 35 | -0.800315649 | CNTF | ciliary neurotrophic factor [Source:HGNC Symbol;Acc:HGNC:2169] |
| ENSG00000243646 | HGLibA_22951 | 35 | -0.800315649 | IL10RB | interleukin 10 receptor subunit beta [Source:HGNC Symbol;Acc:HGNC:5965] |
| ENSG00000244094 | HGLibA_46819 | 35 | -0.800315649 | SPRR2F | small proline rich protein 2F [Source:HGNC Symbol;Acc:HGNC:11266] |
| ENSG00000244405 | HGLibA_15662 | 35 | -0.800315649 | ETV5 | ETS variant 5 [Source:HGNC Symbol;Acc:HGNC:3494] |
| ENSG00000248098 | HGLibA_04279 | 35 | -0.800315649 | BCKDHA | alpha polypeptide [Source:HGNC Symbol;Acc:HGNC:986]" |
| ENSG00000253506 | HGLibA_31033 | 35 | -0.800315649 | NACA2 | nascent polypeptide associated complex subunit alpha 2 [Source:HGNC Symbol;Acc:HGNC:23290] |
| ENSG00000253767 | HGLibA_35545 | 35 | -0.800315649 | PCDHGA8 | 8 [Source:HGNC Symbol;Acc:HGNC:8706]" |
| ENSG00000255112 | HGLibA_09476 | 35 | -0.800315649 | CHMP1B | charged multivesicular body protein 1B [Source:HGNC Symbol;Acc:HGNC:24287] |
| ENSG00000258818 | HGLibA_41410 | 35 | -0.800315649 | RNASE4 | ribonuclease A family member 4 [Source:HGNC Symbol;Acc:HGNC:10047] |
| ENSG00000263465 | HGLibA_47091 | 35 | -0.800315649 | SRSF8 | serine and arginine rich splicing factor 8 [Source:HGNC Symbol;Acc:HGNC:16988] |
| ENSG00000273899 | HGLibA_32261 | 35 | -0.800315649 | NOL12 | nucleolar protein 12 [Source:HGNC Symbol;Acc:HGNC:28585] |
| ENSG00000275395 | HGLibA_17181 | 35 | -0.800315649 | FCGBP | Fc fragment of IgG binding protein [Source:HGNC Symbol;Acc:HGNC:13572] |
| ENSG00000277632 | HGLibA_08018 | 35 | -0.800315649 | CCL3 | C-C motif chemokine ligand 3 [Source:HGNC Symbol;Acc:HGNC:10627] |
| ENSG00000279486 | HGLibA_33749 | 35 | -0.800315649 | OR2AG1 | olfactory receptor family 2 subfamily AG member 1 (gene/pseudogene) [Source:HGNC Symbol;Acc:HGNC:15142] |
| FAM26E | HGLibA_16565 | 35 | -0.800315649 | FAM26E | - |
| GARS | HGLibA_18635 | 35 | -0.800315649 | GARS | - |
| HIST1H4C | HGLibA_21491 | 35 | -0.800315649 | HIST1H4C | - |
| HIST2H4A | HGLibA_21548 | 35 | -0.800315649 | HIST2H4A | - |
| hsa-let-7a-2 | HGLibA_57103 | 35 | -0.800315649 | hsa-let-7a-2 | - |
| hsa-let-7i | HGLibA_57138 | 35 | -0.800315649 | hsa-let-7i | - |
| hsa-mir-1185-2 | HGLibA_57247 | 35 | -0.800315649 | hsa-mir-1185-2 | - |
| hsa-mir-1283-2 | HGLibA_57594 | 35 | -0.800315649 | hsa-mir-1283-2 | - |
| hsa-mir-1295a | HGLibA_57657 | 35 | -0.800315649 | hsa-mir-1295a | - |
| hsa-mir-1469 | HGLibA_57853 | 35 | -0.800315649 | hsa-mir-1469 | - |
| hsa-mir-146b | HGLibA_57859 | 35 | -0.800315649 | hsa-mir-146b | - |
| hsa-mir-204 | HGLibA_58173 | 35 | -0.800315649 | hsa-mir-204 | - |
| hsa-mir-206 | HGLibA_58191 | 35 | -0.800315649 | hsa-mir-206 | - |
| hsa-mir-3174 | HGLibA_58770 | 35 | -0.800315649 | hsa-mir-3174 | - |
| hsa-mir-3179-2 | HGLibA_58795 | 35 | -0.800315649 | hsa-mir-3179-2 | - |
| hsa-mir-3195 | HGLibA_58879 | 35 | -0.800315649 | hsa-mir-3195 | - |
| hsa-mir-320d-2 | HGLibA_58947 | 35 | -0.800315649 | hsa-mir-320d-2 | - |
| hsa-mir-3653 | HGLibA_59157 | 35 | -0.800315649 | hsa-mir-3653 | - |
| hsa-mir-378c | HGLibA_59434 | 35 | -0.800315649 | hsa-mir-378c | - |
| hsa-mir-384 | HGLibA_59487 | 35 | -0.800315649 | hsa-mir-384 | - |
| hsa-mir-3926-2 | HGLibA_59580 | 35 | -0.800315649 | hsa-mir-3926-2 | - |
| hsa-mir-4434 | HGLibA_60126 | 35 | -0.800315649 | hsa-mir-4434 | - |
| hsa-mir-4436b-2 | HGLibA_60146 | 35 | -0.800315649 | hsa-mir-4436b-2 | - |
| hsa-mir-4677 | HGLibA_60823 | 35 | -0.800315649 | hsa-mir-4677 | - |
| hsa-mir-4688 | HGLibA_60864 | 35 | -0.800315649 | hsa-mir-4688 | - |
| hsa-mir-4739 | HGLibA_61055 | 35 | -0.800315649 | hsa-mir-4739 | - |
| hsa-mir-4764 | HGLibA_61152 | 35 | -0.800315649 | hsa-mir-4764 | - |
| hsa-mir-490 | HGLibA_61361 | 35 | -0.800315649 | hsa-mir-490 | - |
| hsa-mir-5095 | HGLibA_61537 | 35 | -0.800315649 | hsa-mir-5095 | - |
| hsa-mir-513a-2 | HGLibA_61573 | 35 | -0.800315649 | hsa-mir-513a-2 | - |
| hsa-mir-519b | HGLibA_61719 | 35 | -0.800315649 | hsa-mir-519b | - |
| hsa-mir-548aa-2 | HGLibA_61853 | 35 | -0.800315649 | hsa-mir-548aa-2 | - |
| hsa-mir-548am | HGLibA_61901 | 35 | -0.800315649 | hsa-mir-548am | - |
| hsa-mir-548d-2 | HGLibA_61965 | 35 | -0.800315649 | hsa-mir-548d-2 | - |
| hsa-mir-5571 | HGLibA_62146 | 35 | -0.800315649 | hsa-mir-5571 | - |
| hsa-mir-568 | HGLibA_62233 | 35 | -0.800315649 | hsa-mir-568 | - |
| hsa-mir-5687 | HGLibA_62262 | 35 | -0.800315649 | hsa-mir-5687 | - |
| hsa-mir-580 | HGLibA_62419 | 35 | -0.800315649 | hsa-mir-580 | - |
| hsa-mir-598 | HGLibA_62484 | 35 | -0.800315649 | hsa-mir-598 | - |
| hsa-mir-601 | HGLibA_62498 | 35 | -0.800315649 | hsa-mir-601 | - |
| hsa-mir-6082 | HGLibA_62587 | 35 | -0.800315649 | hsa-mir-6082 | - |
| hsa-mir-6723 | HGLibA_63055 | 35 | -0.800315649 | hsa-mir-6723 | - |
| hsa-mir-6732 | HGLibA_63082 | 35 | -0.800315649 | hsa-mir-6732 | - |
| hsa-mir-6756 | HGLibA_63180 | 35 | -0.800315649 | hsa-mir-6756 | - |
| hsa-mir-6776 | HGLibA_63279 | 35 | -0.800315649 | hsa-mir-6776 | - |
| hsa-mir-7111 | HGLibA_63809 | 35 | -0.800315649 | hsa-mir-7111 | - |
| hsa-mir-887 | HGLibA_64228 | 35 | -0.800315649 | hsa-mir-887 | - |
| hsa-mir-922 | HGLibA_64282 | 35 | -0.800315649 | hsa-mir-922 | - |
| LOC100505679 | HGLibA_26852 | 35 | -0.800315649 | LOC100505679 | - |
| LOC388813 | HGLibA_26924 | 35 | -0.800315649 | LOC388813 | - |
| LOC646862 | HGLibA_26979 | 35 | -0.800315649 | LOC646862 | - |
| MTERFD1 | HGLibA_30340 | 35 | -0.800315649 | MTERFD1 | - |
| PROSC | HGLibA_38693 | 35 | -0.800315649 | PROSC | - |
| PTPLAD2 | HGLibA_39420 | 35 | -0.800315649 | PTPLAD2 | - |
| SLC35E2 | HGLibA_44989 | 35 | -0.800315649 | SLC35E2 | - |
| C2orf91 | HGLibA_06199 | 34 | -0.810266686 | C2orf91 | - |
| EMR3 | HGLibA_15088 | 34 | -0.810266686 | EMR3 | - |
| ENSG00000002549 | HGLibA_26012 | 34 | -0.810266686 | LAP3 | leucine aminopeptidase 3 [Source:HGNC Symbol;Acc:HGNC:18449] |
| ENSG00000003989 | HGLibA_45360 | 34 | -0.810266686 | SLC7A2 | solute carrier family 7 member 2 [Source:HGNC Symbol;Acc:HGNC:11060] |
| ENSG00000013561 | HGLibA_41520 | 34 | -0.810266686 | RNF14 | ring finger protein 14 [Source:HGNC Symbol;Acc:HGNC:10058] |
| ENSG00000017483 | HGLibA_45072 | 34 | -0.810266686 | SLC38A5 | solute carrier family 38 member 5 [Source:HGNC Symbol;Acc:HGNC:18070] |
| ENSG00000018189 | HGLibA_42444 | 34 | -0.810266686 | RUFY3 | RUN and FYVE domain containing 3 [Source:HGNC Symbol;Acc:HGNC:30285] |
| ENSG00000019186 | HGLibA_12081 | 34 | -0.810266686 | CYP24A1 | cytochrome P450 family 24 subfamily A member 1 [Source:HGNC Symbol;Acc:HGNC:2602] |
| ENSG00000027847 | HGLibA_04040 | 34 | -0.810266686 | B4GALT7 | 4-galactosyltransferase 7 [Source:HGNC Symbol;Acc:HGNC:930]" |
| ENSG00000030304 | HGLibA_30577 | 34 | -0.810266686 | MUSK | muscle associated receptor tyrosine kinase [Source:HGNC Symbol;Acc:HGNC:7525] |
| ENSG00000047410 | HGLibA_51174 | 34 | -0.810266686 | TPR | nuclear basket protein [Source:HGNC Symbol;Acc:HGNC:12017]" |
| ENSG00000056558 | HGLibA_51240 | 34 | -0.810266686 | TRAF1 | TNF receptor associated factor 1 [Source:HGNC Symbol;Acc:HGNC:12031] |
| ENSG00000062716 | HGLibA_53694 | 34 | -0.810266686 | VMP1 | vacuole membrane protein 1 [Source:HGNC Symbol;Acc:HGNC:29559] |
| ENSG00000064201 | HGLibA_51932 | 34 | -0.810266686 | TSPAN32 | tetraspanin 32 [Source:HGNC Symbol;Acc:HGNC:13410] |

| | | | | | |
|---|---|---|---|---|---|
| ENSG00000067066 | HGLibA_46285 | 34 | -0.810266686 | SP100 | SP100 nuclear antigen [Source:HGNC Symbol;Acc:HGNC:11206] |
| ENSG00000067221 | HGLibA_47534 | 34 | -0.810266686 | STOML1 | stomatin like 1 [Source:HGNC Symbol;Acc:HGNC:14560] |
| ENSG00000068078 | HGLibA_17447 | 34 | -0.810266686 | FGFR3 | fibroblast growth factor receptor 3 [Source:HGNC Symbol;Acc:HGNC:3690] |
| ENSG00000070785 | HGLibA_14742 | 34 | -0.810266686 | EIF2B3 | eukaryotic translation initiation factor 2B subunit gamma [Source:HGNC Symbol;Acc:HGNC:3259] |
| ENSG00000071246 | HGLibA_53531 | 34 | -0.810266686 | VASH1 | vasohibin 1 [Source:HGNC Symbol;Acc:HGNC:19964] |
| ENSG00000071794 | HGLibA_21656 | 34 | -0.810266686 | HLTF | helicase like transcription factor [Source:HGNC Symbol;Acc:HGNC:11099] |
| ENSG00000077238 | HGLibA_23173 | 34 | -0.810266686 | IL4R | interleukin 4 receptor [Source:HGNC Symbol;Acc:HGNC:6015] |
| ENSG00000082068 | HGLibA_54163 | 34 | -0.810266686 | WDR70 | WD repeat domain 70 [Source:HGNC Symbol;Acc:HGNC:25495] |
| ENSG00000082175 | HGLibA_36285 | 34 | -0.810266686 | PGR | progesterone receptor [Source:HGNC Symbol;Acc:HGNC:8910] |
| ENSG00000082212 | HGLibA_28712 | 34 | -0.810266686 | ME2 | malic enzyme 2 [Source:HGNC Symbol;Acc:HGNC:6984] |
| ENSG00000083635 | HGLibA_33001 | 34 | -0.810266686 | NUFIP1 | nuclear FMR1 interacting protein 1 [Source:HGNC Symbol;Acc:HGNC:8057] |
| ENSG00000084628 | HGLibA_31988 | 34 | -0.810266686 | NKAIN1 | sodium/potassium transporting ATPase interacting 1 [Source:HGNC Symbol;Acc:HGNC:25743] |
| ENSG00000085978 | HGLibA_03472 | 34 | -0.810266686 | ATG16L1 | autophagy related 16 like 1 [Source:HGNC Symbol;Acc:HGNC:21498] |
| ENSG00000086065 | HGLibA_09495 | 34 | -0.810266686 | CHMP5 | charged multivesicular body protein 5 [Source:HGNC Symbol;Acc:HGNC:26942] |
| ENSG00000086589 | HGLibA_40509 | 34 | -0.810266686 | RBM22 | RNA binding motif protein 22 [Source:HGNC Symbol;Acc:HGNC:25503] |
| ENSG00000090581 | HGLibA_19578 | 34 | -0.810266686 | GNPTG | N-acetylglucosamine-1-phosphate transferase subunit gamma [Source:HGNC Symbol;Acc:HGNC:23026] |
| ENSG00000092010 | HGLibA_39190 | 34 | -0.810266686 | PSME1 | proteasome activator subunit 1 [Source:HGNC Symbol;Acc:HGNC:9568] |
| ENSG00000092094 | HGLibA_34681 | 34 | -0.810266686 | OSGEP | O-sialoglycoprotein endopeptidase [Source:HGNC Symbol;Acc:HGNC:18028] |
| ENSG00000095380 | HGLibA_31108 | 34 | -0.810266686 | NANS | N-acetylneuraminate synthase [Source:HGNC Symbol;Acc:HGNC:19237] |
| ENSG00000097096 | HGLibA_47943 | 34 | -0.810266686 | SYDE2 | synapse defective Rho GTPase homolog 2 [Source:HGNC Symbol;Acc:HGNC:25841] |
| ENSG00000099956 | HGLibA_45620 | 34 | -0.810266686 | SMARCB1 | member 1 [Source:HGNC Symbol;Acc:HGNC:11103]" |
| ENSG00000100078 | HGLibA_36898 | 34 | -0.810266686 | PLA2G3 | phospholipase A2 group III [Source:HGNC Symbol;Acc:HGNC:17934] |
| ENSG00000100220 | HGLibA_42374 | 34 | -0.810266686 | RTCB | 3'-cyclic phosphate and 5'-OH ligase [Source:HGNC Symbol;Acc:HGNC:26935]" |
| ENSG00000100836 | HGLibA_34942 | 34 | -0.810266686 | PABPN1 | poly(A) binding protein nuclear 1 [Source:HGNC Symbol;Acc:HGNC:8565] |
| ENSG00000101098 | HGLibA_41286 | 34 | -0.810266686 | RIMS4 | regulating synaptic membrane exocytosis 4 [Source:HGNC Symbol;Acc:HGNC:16183] |
| ENSG00000101391 | HGLibA_08781 | 34 | -0.810266686 | CDK5RAP1 | CDK5 regulatory subunit associated protein 1 [Source:HGNC Symbol;Acc:HGNC:15880] |
| ENSG00000101574 | HGLibA_29009 | 34 | -0.810266686 | METTL4 | methyltransferase like 4 [Source:HGNC Symbol;Acc:HGNC:24726] |
| ENSG00000101958 | HGLibA_19301 | 34 | -0.810266686 | GLRA2 | glycine receptor alpha 2 [Source:HGNC Symbol;Acc:HGNC:4327] |
| ENSG00000102043 | HGLibA_30440 | 34 | -0.810266686 | MTMR8 | myotubularin related protein 8 [Source:HGNC Symbol;Acc:HGNC:16825] |
| ENSG00000102554 | HGLibA_25076 | 34 | -0.810266686 | KLF5 | Kruppel like factor 5 [Source:HGNC Symbol;Acc:HGNC:6349] |
| ENSG00000103047 | HGLibA_48300 | 34 | -0.810266686 | TANGO6 | transport and golgi organization 6 homolog [Source:HGNC Symbol;Acc:HGNC:25749] |
| ENSG00000103485 | HGLibA_39733 | 34 | -0.810266686 | QPRT | quinolinate phosphoribosyltransferase [Source:HGNC Symbol;Acc:HGNC:9755] |
| ENSG00000104695 | HGLibA_38137 | 34 | -0.810266686 | PPP2CB | protein phosphatase 2 catalytic subunit beta [Source:HGNC Symbol;Acc:HGNC:9300] |
| ENSG00000104760 | HGLibA_17462 | 34 | -0.810266686 | FGL1 | fibrinogen like 1 [Source:HGNC Symbol;Acc:HGNC:3695] |
| ENSG00000104880 | HGLibA_02829 | 34 | -0.810266686 | ARHGEF18 | Rho/Rac guanine nucleotide exchange factor 18 [Source:HGNC Symbol;Acc:HGNC:17090] |
| ENSG00000104980 | HGLibA_49517 | 34 | -0.810266686 | TIMM44 | translocase of inner mitochondrial membrane 44 [Source:HGNC Symbol;Acc:HGNC:17316] |
| ENSG00000105220 | HGLibA_19808 | 34 | -0.810266686 | GPI | glucose-6-phosphate isomerase [Source:HGNC Symbol;Acc:HGNC:4458] |
| ENSG00000105298 | HGLibA_06953 | 34 | -0.810266686 | CACTIN | spliceosome C complex subunit [Source:HGNC Symbol;Acc:HGNC:29938]" |
| ENSG00000105374 | HGLibA_32014 | 34 | -0.810266686 | NKG7 | natural killer cell granule protein 7 [Source:HGNC Symbol;Acc:HGNC:7830] |
| ENSG00000105559 | HGLibA_37071 | 34 | -0.810266686 | PLEKHA4 | pleckstrin homology domain containing A4 [Source:HGNC Symbol;Acc:HGNC:14339] |
| ENSG00000105662 | HGLibA_11229 | 34 | -0.810266686 | CRTC1 | CREB regulated transcription coactivator 1 [Source:HGNC Symbol;Acc:HGNC:16062] |
| ENSG00000105971 | HGLibA_07378 | 34 | -0.810266686 | CAV2 | caveolin 2 [Source:HGNC Symbol;Acc:HGNC:1528] |
| ENSG00000105991 | HGLibA_21893 | 34 | -0.810266686 | HOXA1 | homeobox A1 [Source:HGNC Symbol;Acc:HGNC:5099] |
| ENSG00000106153 | HGLibA_09364 | 34 | -0.810266686 | CHCHD2 | coiled-coil-helix-coiled-coil-helix domain containing 2 [Source:HGNC Symbol;Acc:HGNC:21645] |
| ENSG00000106261 | HGLibA_55344 | 34 | -0.810266686 | ZKSCAN1 | zinc finger with KRAB and SCAN domains 1 [Source:HGNC Symbol;Acc:HGNC:13101] |
| ENSG00000108309 | HGLibA_42450 | 34 | -0.810266686 | RUNDC3A | RUN domain containing 3A [Source:HGNC Symbol;Acc:HGNC:16984] |
| ENSG00000108654 | HGLibA_12729 | 34 | -0.810266686 | DDX5 | DEAD-box helicase 5 [Source:HGNC Symbol;Acc:HGNC:2746] |
| ENSG00000109047 | HGLibA_40722 | 34 | -0.810266686 | RCVRN | recoverin [Source:HGNC Symbol;Acc:HGNC:9937] |
| ENSG00000110717 | HGLibA_31566 | 34 | -0.810266686 | NDUFS8 | NADH:ubiquinone oxidoreductase core subunit S8 [Source:HGNC Symbol;Acc:HGNC:7715] |
| ENSG00000111639 | HGLibA_30012 | 34 | -0.810266686 | MRPL51 | mitochondrial ribosomal protein L51 [Source:HGNC Symbol;Acc:HGNC:14044] |
| ENSG00000111704 | HGLibA_31090 | 34 | -0.810266686 | NANOG | Nanog homeobox [Source:HGNC Symbol;Acc:HGNC:20857] |
| ENSG00000111816 | HGLibA_18006 | 34 | -0.810266686 | FRK | fyn related Src family tyrosine kinase [Source:HGNC Symbol;Acc:HGNC:3955] |
| ENSG00000111816 | HGLibA_18005 | 34 | -0.810266686 | FRK | fyn related Src family tyrosine kinase [Source:HGNC Symbol;Acc:HGNC:3955] |
| ENSG00000112079 | HGLibA_47500 | 34 | -0.810266686 | STK38 | serine/threonine kinase 38 [Source:HGNC Symbol;Acc:HGNC:17847] |
| ENSG00000112146 | HGLibA_17133 | 34 | -0.810266686 | FBXO9 | F-box protein 9 [Source:HGNC Symbol;Acc:HGNC:13588] |
| ENSG00000112208 | HGLibA_04076 | 34 | -0.810266686 | BAG2 | BCL2 associated athanogene 2 [Source:HGNC Symbol;Acc:HGNC:938] |
| ENSG00000112992 | HGLibA_32226 | 34 | -0.810266686 | NNT | nicotinamide nucleotide transhydrogenase [Source:HGNC Symbol;Acc:HGNC:7863] |
| ENSG00000113013 | HGLibA_22297 | 34 | -0.810266686 | HSPA9 | heat shock protein family A (Hsp70) member 9 [Source:HGNC Symbol;Acc:HGNC:5244] |
| ENSG00000114771 | HGLibA_00030 | 34 | -0.810266686 | AADAC | arylacetamide deacetylase [Source:HGNC Symbol;Acc:HGNC:17] |
| ENSG00000115020 | HGLibA_36667 | 34 | -0.810266686 | PIKFYVE | FYVE-type zinc finger containing [Source:HGNC Symbol;Acc:HGNC:23785]" |
| ENSG00000115718 | HGLibA_38651 | 34 | -0.810266686 | PROC | inactivator of coagulation factors Va and VIIIa [Source:HGNC Symbol;Acc:HGNC:9451]" |
| ENSG00000115946 | HGLibA_37397 | 34 | -0.810266686 | PNO1 | partner of NOB1 homolog [Source:HGNC Symbol;Acc:HGNC:32790] |
| ENSG00000116001 | HGLibA_49430 | 34 | -0.810266686 | TIA1 | TIA1 cytotoxic granule associated RNA binding protein [Source:HGNC Symbol;Acc:HGNC:11802] |
| ENSG00000116171 | HGLibA_43034 | 34 | -0.810266686 | SCP2 | sterol carrier protein 2 [Source:HGNC Symbol;Acc:HGNC:10606] |
| ENSG00000116350 | HGLibA_47077 | 34 | -0.810266686 | SRSF4 | serine and arginine rich splicing factor 4 [Source:HGNC Symbol;Acc:HGNC:10786] |
| ENSG00000117000 | HGLibA_41346 | 34 | -0.810266686 | RLF | rearranged L-myc fusion [Source:HGNC Symbol;Acc:HGNC:10025] |
| ENSG00000118420 | HGLibA_52688 | 34 | -0.810266686 | UBE3D | ubiquitin protein ligase E3D [Source:HGNC Symbol;Acc:HGNC:21381] |
| ENSG00000118482 | HGLibA_36377 | 34 | -0.810266686 | PHF3 | PHD finger protein 3 [Source:HGNC Symbol;Acc:HGNC:8921] |
| ENSG00000119203 | HGLibA_11000 | 34 | -0.810266686 | CPSF3 | cleavage and polyadenylation specific factor 3 [Source:HGNC Symbol;Acc:HGNC:2326] |
| ENSG00000119599 | HGLibA_12439 | 34 | -0.810266686 | DCAF4 | DDB1 and CUL4 associated factor 4 [Source:HGNC Symbol;Acc:HGNC:20229] |
| ENSG00000120868 | HGLibA_02366 | 34 | -0.810266686 | APAF1 | apoptotic peptidase activating factor 1 [Source:HGNC Symbol;Acc:HGNC:576] |
| ENSG00000121310 | HGLibA_14396 | 34 | -0.810266686 | ECHDC2 | enoyl-CoA hydratase domain containing 2 [Source:HGNC Symbol;Acc:HGNC:23408] |
| ENSG00000122121 | HGLibA_54556 | 34 | -0.810266686 | XPNPEP2 | X-prolyl aminopeptidase 2 [Source:HGNC Symbol;Acc:HGNC:12823] |
| ENSG00000122566 | HGLibA_21799 | 34 | -0.810266686 | HNRNPA2B1 | heterogeneous nuclear ribonucleoprotein A2/B1 [Source:HGNC Symbol;Acc:HGNC:5033] |
| ENSG00000123728 | HGLibA_40241 | 34 | -0.810266686 | RAP2C | member of RAS oncogene family [Source:HGNC Symbol;Acc:HGNC:21165]" |
| ENSG00000124171 | HGLibA_35187 | 34 | -0.810266686 | PARD6B | par-6 family cell polarity regulator beta [Source:HGNC Symbol;Acc:HGNC:16245] |
| ENSG00000125084 | HGLibA_54343 | 34 | -0.810266686 | WNT1 | Wnt family member 1 [Source:HGNC Symbol;Acc:HGNC:12774] |
| ENSG00000125726 | HGLibA_08437 | 34 | -0.810266686 | CD70 | CD70 molecule [Source:HGNC Symbol;Acc:HGNC:11937] |
| ENSG00000128710 | HGLibA_21985 | 34 | -0.810266686 | HOXD10 | homeobox D10 [Source:HGNC Symbol;Acc:HGNC:5133] |
| ENSG00000128713 | HGLibA_21988 | 34 | -0.810266686 | HOXD11 | homeobox D11 [Source:HGNC Symbol;Acc:HGNC:5134] |
| ENSG00000128789 | HGLibA_39208 | 34 | -0.810266686 | PSMG2 | proteasome assembly chaperone 2 [Source:HGNC Symbol;Acc:HGNC:24929] |
| ENSG00000129682 | HGLibA_17372 | 34 | -0.810266686 | FGF13 | fibroblast growth factor 13 [Source:HGNC Symbol;Acc:HGNC:3670] |
| ENSG00000129744 | HGLibA_03158 | 34 | -0.810266686 | ART1 | ADP-ribosyltransferase 1 [Source:HGNC Symbol;Acc:HGNC:723] |
| ENSG00000130005 | HGLibA_18600 | 34 | -0.810266686 | GAMT | guanidinoacetate N-methyltransferase [Source:HGNC Symbol;Acc:HGNC:4136] |
| ENSG00000130304 | HGLibA_44812 | 34 | -0.810266686 | SLC27A1 | solute carrier family 27 member 1 [Source:HGNC Symbol;Acc:HGNC:10995] |
| ENSG00000130669 | HGLibA_35048 | 34 | -0.810266686 | PAK4 | p21 (RAC1) activated kinase 4 [Source:HGNC Symbol;Acc:HGNC:16059] |
| ENSG00000131042 | HGLibA_26509 | 34 | -0.810266686 | LILRB2 | leukocyte immunoglobulin like receptor B2 [Source:HGNC Symbol;Acc:HGNC:6606] |
| ENSG00000131459 | HGLibA_18963 | 34 | -0.810266686 | GFPT2 | glutamine-fructose-6-phosphate transaminase 2 [Source:HGNC Symbol;Acc:HGNC:4242] |
| ENSG00000133317 | HGLibA_26348 | 34 | -0.810266686 | LGALS12 | galectin 12 [Source:HGNC Symbol;Acc:HGNC:15788] |
| ENSG00000133561 | HGLibA_19085 | 34 | -0.810266686 | GIMAP6 | IMAP family member 6 [Source:HGNC Symbol;Acc:HGNC:21918]" |
| ENSG00000134864 | HGLibA_18990 | 34 | -0.810266686 | GGACT | gamma-glutamylamine cyclotransferase [Source:HGNC Symbol;Acc:HGNC:25100] |
| ENSG00000135821 | HGLibA_19366 | 34 | -0.810266686 | GLUL | glutamate-ammonia ligase [Source:HGNC Symbol;Acc:HGNC:4341] |
| ENSG00000135951 | HGLibA_51846 | 34 | -0.810266686 | TSGA10 | testis specific 10 [Source:HGNC Symbol;Acc:HGNC:14927] |
| ENSG00000136169 | HGLibA_43669 | 34 | -0.810266686 | SETDB2 | SET domain bifurcated 2 [Source:HGNC Symbol;Acc:HGNC:20263] |
| ENSG00000136247 | HGLibA_55118 | 34 | -0.810266686 | ZDHHC4 | zinc finger DHHC-type containing 4 [Source:HGNC Symbol;Acc:HGNC:18471] |
| ENSG00000136267 | HGLibA_13062 | 34 | -0.810266686 | DGKB | diacylglycerol kinase beta [Source:HGNC Symbol;Acc:HGNC:2850] |
| ENSG00000136527 | HGLibA_51225 | 34 | -0.810266686 | TRA2B | transformer 2 beta homolog [Source:HGNC Symbol;Acc:HGNC:10781] |
| ENSG00000136531 | HGLibA_42979 | 34 | -0.810266686 | SCN2A | sodium voltage-gated channel alpha subunit 2 [Source:HGNC Symbol;Acc:HGNC:10588] |
| ENSG00000137496 | HGLibA_23024 | 34 | -0.810266686 | IL18BP | interleukin 18 binding protein [Source:HGNC Symbol;Acc:HGNC:5987] |
| ENSG00000137674 | HGLibA_29510 | 34 | -0.810266686 | MMP20 | matrix metallopeptidase 20 [Source:HGNC Symbol;Acc:HGNC:7167] |
| ENSG00000138036 | HGLibA_14249 | 34 | -0.810266686 | DYNC2LI1 | dynein cytoplasmic 2 light intermediate chain 1 [Source:HGNC Symbol;Acc:HGNC:24595] |
| ENSG00000138336 | HGLibA_49037 | 34 | -0.810266686 | TET1 | tet methylcytosine dioxygenase 1 [Source:HGNC Symbol;Acc:HGNC:29484] |
| ENSG00000138356 | HGLibA_02276 | 34 | -0.810266686 | AOX1 | aldehyde oxidase 1 [Source:HGNC Symbol;Acc:HGNC:553] |
| ENSG00000138613 | HGLibA_02415 | 34 | -0.810266686 | APH1B | gamma-secretase subunit [Source:HGNC Symbol;Acc:HGNC:24080]" |
| ENSG00000139618 | HGLibA_04763 | 34 | -0.810266686 | BRCA2 | DNA repair associated [Source:HGNC Symbol;Acc:HGNC:1101]" |
| ENSG00000139645 | HGLibA_02117 | 34 | -0.810266686 | ANKRD52 | ankyrin repeat domain 52 [Source:HGNC Symbol;Acc:HGNC:26614] |
| ENSG00000139926 | HGLibA_18022 | 34 | -0.810266686 | FRMD6 | FERM domain containing 6 [Source:HGNC Symbol;Acc:HGNC:19839] |
| ENSG00000140043 | HGLibA_39354 | 34 | -0.810266686 | PTGR2 | prostaglandin reductase 2 [Source:HGNC Symbol;Acc:HGNC:20149] |
| ENSG00000140043 | HGLibA_39356 | 34 | -0.810266686 | PTGR2 | prostaglandin reductase 2 [Source:HGNC Symbol;Acc:HGNC:20149] |
| ENSG00000140798 | HGLibA_00167 | 34 | -0.810266686 | ABCC12 | ATP binding cassette subfamily C member 12 [Source:HGNC Symbol;Acc:HGNC:14640] |
| ENSG00000141499 | HGLibA_54401 | 34 | -0.810266686 | WRAP53 | WD repeat containing antisense to TP53 [Source:HGNC Symbol;Acc:HGNC:25522] |
| ENSG00000141971 | HGLibA_30590 | 34 | -0.810266686 | MVB12A | multivesicular body subunit 12A [Source:HGNC Symbol;Acc:HGNC:25153] |
| ENSG00000143119 | HGLibA_08407 | 34 | -0.810266686 | CD53 | CD53 molecule [Source:HGNC Symbol;Acc:HGNC:1686] |
| ENSG00000143520 | HGLibA_17612 | 34 | -0.810266686 | FLG2 | filaggrin family member 2 [Source:HGNC Symbol;Acc:HGNC:33276] |
| ENSG00000144589 | HGLibA_47455 | 34 | -0.810266686 | STK11IP | serine/threonine kinase 11 interacting protein [Source:HGNC Symbol;Acc:HGNC:19184] |
| ENSG00000144741 | HGLibA_44686 | 34 | -0.810266686 | SLC25A26 | solute carrier family 25 member 26 [Source:HGNC Symbol;Acc:HGNC:20661] |
| ENSG00000147044 | HGLibA_07287 | 34 | -0.810266686 | CASK | calcium/calmodulin dependent serine protein kinase [Source:HGNC Symbol;Acc:HGNC:1497] |
| ENSG00000147180 | HGLibA_56614 | 34 | -0.810266686 | ZNF711 | zinc finger protein 711 [Source:HGNC Symbol;Acc:HGNC:13128] |
| ENSG00000148290 | HGLibA_47831 | 34 | -0.810266686 | SURF1 | cytochrome c oxidase assembly factor [Source:HGNC Symbol;Acc:HGNC:11474]" |
| ENSG00000148344 | HGLibA_39325 | 34 | -0.810266686 | PTGES | prostaglandin E synthase [Source:HGNC Symbol;Acc:HGNC:9599] |
| ENSG00000148950 | HGLibA_23230 | 34 | -0.810266686 | IMMP1L | inner mitochondrial membrane peptidase subunit 1 [Source:HGNC Symbol;Acc:HGNC:26317] |
| ENSG00000149091 | HGLibA_13084 | 34 | -0.810266686 | DGKZ | diacylglycerol kinase zeta [Source:HGNC Symbol;Acc:HGNC:2857] |
| ENSG00000151379 | HGLibA_30187 | 34 | -0.810266686 | MSGN1 | mesogenin 1 [Source:HGNC Symbol;Acc:HGNC:14907] |
| ENSG00000152382 | HGLibA_48183 | 34 | -0.810266686 | TADA1 | transcriptional adaptor 1 [Source:HGNC Symbol;Acc:HGNC:30631] |
| ENSG00000152457 | HGLibA_12513 | 34 | -0.810266686 | DCLRE1C | DNA cross-link repair 1C [Source:HGNC Symbol;Acc:HGNC:17642] |
| ENSG00000152784 | HGLibA_38400 | 34 | -0.810266686 | PRDM8 | PR/SET domain 8 [Source:HGNC Symbol;Acc:HGNC:13993] |
| ENSG00000153707 | HGLibA_39496 | 34 | -0.810266686 | PTPRD | receptor type D [Source:HGNC Symbol;Acc:HGNC:9668]" |
| ENSG00000153714 | HGLibA_27635 | 34 | -0.810266686 | LURAP1L | leucine rich adaptor protein 1 like [Source:HGNC Symbol;Acc:HGNC:31452] |
| ENSG00000153898 | HGLibA_28640 | 34 | -0.810266686 | MCOLN2 | mucolipin 2 [Source:HGNC Symbol;Acc:HGNC:13357] |
| ENSG00000154001 | HGLibA_38183 | 34 | -0.810266686 | PPP2R5E | protein phosphatase 2 regulatory subunit B'epsilon [Source:HGNC Symbol;Acc:HGNC:9313] |
| ENSG00000154040 | HGLibA_06867 | 34 | -0.810266686 | CABYR | calcium binding tyrosine phosphorylation regulated [Source:HGNC Symbol;Acc:HGNC:15569] |
| ENSG00000154114 | HGLibA_48595 | 34 | -0.810266686 | TBCEL | tubulin folding cofactor E like [Source:HGNC Symbol;Acc:HGNC:28115] |
| ENSG00000155085 | HGLibA_01399 | 34 | -0.810266686 | AK9 | adenylate kinase 9 [Source:HGNC Symbol;Acc:HGNC:33814] |
| ENSG00000155906 | HGLibA_41379 | 34 | -0.810266686 | RMND1 | required for meiotic nuclear division 1 homolog [Source:HGNC Symbol;Acc:HGNC:21176] |
| ENSG00000158467 | HGLibA_01293 | 34 | -0.810266686 | AHCYL2 | adenosylhomocysteinase like 2 [Source:HGNC Symbol;Acc:HGNC:22204] |

| | | | | | |
|---|---|---|---|---|---|
| ENSG00000159346 | HGLibA_00980 | 34 | -0.810266686 | ADIPOR1 | adiponectin receptor 1 [Source:HGNC Symbol;Acc:HGNC:24040] |
| ENSG00000160282 | HGLibA_18114 | 34 | -0.810266686 | FTCD | formimidoyltransferase cyclodeaminase [Source:HGNC Symbol;Acc:HGNC:3974] |
| ENSG00000160870 | HGLibA_12162 | 34 | -0.810266686 | CYP3A7 | cytochrome P450 family 3 subfamily A member 7 [Source:HGNC Symbol;Acc:HGNC:2640] |
| ENSG00000161904 | HGLibA_26288 | 34 | -0.810266686 | LEMD2 | LEM domain containing 2 [Source:HGNC Symbol;Acc:HGNC:21244] |
| ENSG00000162426 | HGLibA_45172 | 34 | -0.810266686 | SLC45A1 | solute carrier family 45 member 1 [Source:HGNC Symbol;Acc:HGNC:17939] |
| ENSG00000162998 | HGLibA_18060 | 34 | -0.810266686 | FRZB | frizzled related protein [Source:HGNC Symbol;Acc:HGNC:3959] |
| ENSG00000163344 | HGLibA_37338 | 34 | -0.810266686 | PMVK | phosphomevalonate kinase [Source:HGNC Symbol;Acc:HGNC:9141] |
| ENSG00000163389 | HGLibA_37476 | 34 | -0.810266686 | POGLUT1 | protein O-glucosyltransferase 1 [Source:HGNC Symbol;Acc:HGNC:22954] |
| ENSG00000164587 | HGLibA_42073 | 34 | -0.810266686 | RPS14 | ribosomal protein S14 [Source:HGNC Symbol;Acc:HGNC:10387] |
| ENSG00000164687 | HGLibA_15921 | 34 | -0.810266686 | FABP5 | fatty acid binding protein 5 [Source:HGNC Symbol;Acc:HGNC:3560] |
| ENSG00000165006 | HGLibA_52547 | 34 | -0.810266686 | UBAP1 | ubiquitin associated protein 1 [Source:HGNC Symbol;Acc:HGNC:12461] |
| ENSG00000165168 | HGLibA_12018 | 34 | -0.810266686 | CYBB | cytochrome b-245 beta chain [Source:HGNC Symbol;Acc:HGNC:2578] |
| ENSG00000165434 | HGLibA_36266 | 34 | -0.810266686 | PGM2L1 | phosphoglucomutase 2 like 1 [Source:HGNC Symbol;Acc:HGNC:20898] |
| ENSG00000166167 | HGLibA_04986 | 34 | -0.810266686 | BTRC | beta-transducin repeat containing E3 ubiquitin protein ligase [Source:HGNC Symbol;Acc:HGNC:1144] |
| ENSG00000166220 | HGLibA_48459 | 34 | -0.810266686 | TBATA | brain and testes associated [Source:HGNC Symbol;Acc:HGNC:23511]" |
| ENSG00000166483 | HGLibA_54237 | 34 | -0.810266686 | WEE1 | WEE1 G2 checkpoint kinase [Source:HGNC Symbol;Acc:HGNC:12761] |
| ENSG00000166664 | HGLibA_09543 | 34 | -0.810266686 | CHRFAM7A | CHRNA7 (exons 5-10) and FAM7A (exons A-E) fusion [Source:HGNC Symbol;Acc:HGNC:15781] |
| ENSG00000166924 | HGLibA_33180 | 34 | -0.810266686 | NYAP1 | neuronal tyrosine phosphorylated phosphoinositide-3-kinase adaptor 1 [Source:HGNC Symbol;Acc:HGNC:22009] |
| ENSG00000166947 | HGLibA_15259 | 34 | -0.810266686 | EPB42 | erythrocyte membrane protein band 4.2 [Source:HGNC Symbol;Acc:HGNC:3381] |
| ENSG00000167460 | HGLibA_51151 | 34 | -0.810266686 | TPM4 | tropomyosin 4 [Source:HGNC Symbol;Acc:HGNC:12013] |
| ENSG00000167617 | HGLibA_08577 | 34 | -0.810266686 | CDC42EP5 | CDC42 effector protein 5 [Source:HGNC Symbol;Acc:HGNC:17408] |
| ENSG00000168491 | HGLibA_07537 | 34 | -0.810266686 | CCDC110 | coiled-coil domain containing 110 [Source:HGNC Symbol;Acc:HGNC:28504] |
| ENSG00000168495 | HGLibA_37618 | 34 | -0.810266686 | POLR3D | RNA polymerase III subunit D [Source:HGNC Symbol;Acc:HGNC:1080] |
| ENSG00000169252 | HGLibA_01059 | 34 | -0.810266686 | ADRB2 | adrenoceptor beta 2 [Source:HGNC Symbol;Acc:HGNC:286] |
| ENSG00000169393 | HGLibA_14993 | 34 | -0.810266686 | ELSPBP1 | epididymal sperm binding protein 1 [Source:HGNC Symbol;Acc:HGNC:14417] |
| ENSG00000169627 | HGLibA_04685 | 34 | -0.810266686 | BOLA2B | bolA family member 2B [Source:HGNC Symbol;Acc:HGNC:32479] |
| ENSG00000169860 | HGLibA_34867 | 34 | -0.810266686 | P2RY1 | purinergic receptor P2Y1 [Source:HGNC Symbol;Acc:HGNC:8539] |
| ENSG00000169902 | HGLibA_51204 | 34 | -0.810266686 | TPST1 | tyrosylprotein sulfotransferase 1 [Source:HGNC Symbol;Acc:HGNC:12020] |
| ENSG00000170043 | HGLibA_51297 | 34 | -0.810266686 | TRAPPC1 | trafficking protein particle complex 1 [Source:HGNC Symbol;Acc:HGNC:19894] |
| ENSG00000170113 | HGLibA_31949 | 34 | -0.810266686 | NIPA1 | NIPA magnesium transporter 1 [Source:HGNC Symbol;Acc:HGNC:17043] |
| ENSG00000170323 | HGLibA_15919 | 34 | -0.810266686 | FABP4 | fatty acid binding protein 4 [Source:HGNC Symbol;Acc:HGNC:3559] |
| ENSG00000170579 | HGLibA_13339 | 34 | -0.810266686 | DLGAP1 | DLG associated protein 1 [Source:HGNC Symbol;Acc:HGNC:2905] |
| ENSG00000170632 | HGLibA_03008 | 34 | -0.810266686 | ARMC10 | armadillo repeat containing 10 [Source:HGNC Symbol;Acc:HGNC:21706] |
| ENSG00000171345 | HGLibA_25440 | 34 | -0.810266686 | KRT19 | keratin 19 [Source:HGNC Symbol;Acc:HGNC:6436] |
| ENSG00000171724 | HGLibA_53546 | 34 | -0.810266686 | VAT1L | vesicle amine transport 1 like [Source:HGNC Symbol;Acc:HGNC:29315] |
| ENSG00000171872 | HGLibA_25062 | 34 | -0.810266686 | KLF17 | Kruppel like factor 17 [Source:HGNC Symbol;Acc:HGNC:18830] |
| ENSG00000171987 | HGLibA_05137 | 34 | -0.810266686 | C11orf40 | chromosome 11 open reading frame 40 [Source:HGNC Symbol;Acc:HGNC:23986] |
| ENSG00000172071 | HGLibA_14728 | 34 | -0.810266686 | EIF2AK3 | eukaryotic translation initiation factor 2 alpha kinase 3 [Source:HGNC Symbol;Acc:HGNC:3255] |
| ENSG00000172215 | HGLibA_11896 | 34 | -0.810266686 | CXCR6 | C-X-C motif chemokine receptor 6 [Source:HGNC Symbol;Acc:HGNC:16647] |
| ENSG00000172367 | HGLibA_35975 | 34 | -0.810266686 | PDZD3 | PDZ domain containing 3 [Source:HGNC Symbol;Acc:HGNC:19891] |
| ENSG00000172432 | HGLibA_20603 | 34 | -0.810266686 | GTPBP2 | GTP binding protein 2 [Source:HGNC Symbol;Acc:HGNC:4670] |
| ENSG00000172748 | HGLibA_56349 | 34 | -0.810266686 | ZNF596 | zinc finger protein 596 [Source:HGNC Symbol;Acc:HGNC:27268] |
| ENSG00000172803 | HGLibA_46090 | 34 | -0.810266686 | SNX32 | sorting nexin 32 [Source:HGNC Symbol;Acc:HGNC:26423] |
| ENSG00000172824 | HGLibA_09227 | 34 | -0.810266686 | CES4A | carboxylesterase 4A [Source:HGNC Symbol;Acc:HGNC:26741] |
| ENSG00000173391 | HGLibA_33376 | 34 | -0.810266686 | OLR1 | oxidized low density lipoprotein receptor 1 [Source:HGNC Symbol;Acc:HGNC:8133] |
| ENSG00000173947 | HGLibA_36540 | 34 | -0.810266686 | PIFO | primary cilia formation [Source:HGNC Symbol;Acc:HGNC:27009] |
| ENSG00000174236 | HGLibA_40841 | 34 | -0.810266686 | REP15 | RAB15 effector protein [Source:HGNC Symbol;Acc:HGNC:33748] |
| ENSG00000175164 | HGLibA_00316 | 34 | -0.810266686 | ABO | alpha 1-3-N-acetylgalactosaminyltransferase and alpha 1-3-galactosyltransferase [Source:HGNC Symbol;Acc:HGNC:79]" |
| ENSG00000175899 | HGLibA_00009 | 34 | -0.810266686 | A2M | alpha-2-macroglobulin [Source:HGNC Symbol;Acc:HGNC:7] |
| ENSG00000176695 | HGLibA_33994 | 34 | -0.810266686 | OR4F17 | olfactory receptor family 4 subfamily F member 17 [Source:HGNC Symbol;Acc:HGNC:15381] |
| ENSG00000176894 | HGLibA_39659 | 34 | -0.810266686 | PXMP2 | peroxisomal membrane protein 2 [Source:HGNC Symbol;Acc:HGNC:9716] |
| ENSG00000178913 | HGLibA_48251 | 34 | -0.810266686 | TAF7 | TATA-box binding protein associated factor 7 [Source:HGNC Symbol;Acc:HGNC:11541] |
| ENSG00000178922 | HGLibA_22452 | 34 | -0.810266686 | HYI | hydroxypyruvate isomerase (putative) [Source:HGNC Symbol;Acc:HGNC:26948] |
| ENSG00000178971 | HGLibA_11592 | 34 | -0.810266686 | CTC1 | CST telomere replication complex component 1 [Source:HGNC Symbol;Acc:HGNC:26169] |
| ENSG00000178999 | HGLibA_03879 | 34 | -0.810266686 | AURKB | aurora kinase B [Source:HGNC Symbol;Acc:HGNC:11390] |
| ENSG00000179921 | HGLibA_19754 | 34 | -0.810266686 | GPBAR1 | G protein-coupled bile acid receptor 1 [Source:HGNC Symbol;Acc:HGNC:19680] |
| ENSG00000180071 | HGLibA_02017 | 34 | -0.810266686 | ANKRD18A | ankyrin repeat domain 18A [Source:HGNC Symbol;Acc:HGNC:23643] |
| ENSG00000180185 | HGLibA_15955 | 34 | -0.810266686 | FAHD1 | fumarylacetoacetate hydrolase domain containing 1 [Source:HGNC Symbol;Acc:HGNC:14169] |
| ENSG00000180919 | HGLibA_34226 | 34 | -0.810266686 | OR56B4 | olfactory receptor family 56 subfamily B member 4 [Source:HGNC Symbol;Acc:HGNC:15248] |
| ENSG00000181634 | HGLibA_50817 | 34 | -0.810266686 | TNFSF15 | TNF superfamily member 15 [Source:HGNC Symbol;Acc:HGNC:11931] |
| ENSG00000181963 | HGLibA_34185 | 34 | -0.810266686 | OR52K2 | olfactory receptor family 52 subfamily K member 2 [Source:HGNC Symbol;Acc:HGNC:15223] |
| ENSG00000183066 | HGLibA_53979 | 34 | -0.810266686 | WBP2NL | WBP2 N-terminal like [Source:HGNC Symbol;Acc:HGNC:28389] |
| ENSG00000183770 | HGLibA_17903 | 34 | -0.810266686 | FOXL2 | forkhead box L2 [Source:HGNC Symbol;Acc:HGNC:1092] |
| ENSG00000183813 | HGLibA_08155 | 34 | -0.810266686 | CCR4 | C-C motif chemokine receptor 4 [Source:HGNC Symbol;Acc:HGNC:1605] |
| ENSG00000183814 | HGLibA_26581 | 34 | -0.810266686 | LIN9 | lin-9 DREAM MuvB core complex component [Source:HGNC Symbol;Acc:HGNC:30830] |
| ENSG00000184014 | HGLibA_12959 | 34 | -0.810266686 | DENND5A | DENN domain containing 5A [Source:HGNC Symbol;Acc:HGNC:19344] |
| ENSG00000184144 | HGLibA_10405 | 34 | -0.810266686 | CNTN2 | contactin 2 [Source:HGNC Symbol;Acc:HGNC:2172] |
| ENSG00000184302 | HGLibA_44258 | 34 | -0.810266686 | SIX6 | SIX homeobox 6 [Source:HGNC Symbol;Acc:HGNC:10892] |
| ENSG00000185043 | HGLibA_09681 | 34 | -0.810266686 | CIB1 | calcium and integrin binding 1 [Source:HGNC Symbol;Acc:HGNC:16920] |
| ENSG00000185313 | HGLibA_42968 | 34 | -0.810266686 | SCN10A | sodium voltage-gated channel alpha subunit 10 [Source:HGNC Symbol;Acc:HGNC:10582] |
| ENSG00000185532 | HGLibA_38577 | 34 | -0.810266686 | PRKG1 | protein kinase cGMP-dependent 1 [Source:HGNC Symbol;Acc:HGNC:9414] |
| ENSG00000185619 | HGLibA_35597 | 34 | -0.810266686 | PCGF3 | polycomb group ring finger 3 [Source:HGNC Symbol;Acc:HGNC:10066] |
| ENSG00000185880 | HGLibA_51595 | 34 | -0.810266686 | TRIM69 | tripartite motif containing 69 [Source:HGNC Symbol;Acc:HGNC:17857] |
| ENSG00000186452 | HGLibA_50628 | 34 | -0.810266686 | TMPRSS12 | transmembrane serine protease 12 [Source:HGNC Symbol;Acc:HGNC:28779] |
| ENSG00000186470 | HGLibA_04970 | 34 | -0.810266686 | BTN3A2 | butyrophilin subfamily 3 member A2 [Source:HGNC Symbol;Acc:HGNC:1139] |
| ENSG00000188868 | HGLibA_56252 | 34 | -0.810266686 | ZNF563 | zinc finger protein 563 [Source:HGNC Symbol;Acc:HGNC:30498] |
| ENSG00000196419 | HGLibA_54599 | 34 | -0.810266686 | XRCC6 | X-ray repair cross complementing 6 [Source:HGNC Symbol;Acc:HGNC:4055] |
| ENSG00000196526 | HGLibA_01098 | 34 | -0.810266686 | AFAP1 | actin filament associated protein 1 [Source:HGNC Symbol;Acc:HGNC:24017] |
| ENSG00000197563 | HGLibA_36569 | 34 | -0.810266686 | PIGN | phosphatidylinositol glycan anchor biosynthesis class N [Source:HGNC Symbol;Acc:HGNC:8967] |
| ENSG00000198879 | HGLibA_43723 | 34 | -0.810266686 | SFMBT2 | Scm like with four mbt domains 2 [Source:HGNC Symbol;Acc:HGNC:20256] |
| ENSG00000203880 | HGLibA_35632 | 34 | -0.810266686 | PCMTD2 | protein-L-isoaspartate (D-aspartate) O-methyltransferase domain containing 2 [Source:HGNC Symbol;Acc:HGNC:15882] |
| ENSG00000204033 | HGLibA_27187 | 34 | -0.810266686 | LRIT2 | Ig-like and transmembrane domains 2 [Source:HGNC Symbol;Acc:HGNC:23443]" |
| ENSG00000204217 | HGLibA_04634 | 34 | -0.810266686 | BMPR2 | bone morphogenetic protein receptor type 2 [Source:HGNC Symbol;Acc:HGNC:1078] |
| ENSG00000204315 | HGLibA_17594 | 34 | -0.810266686 | FKBPL | FK506 binding protein like [Source:HGNC Symbol;Acc:HGNC:13949] |
| ENSG00000204348 | HGLibA_14216 | 34 | -0.810266686 | DXO | decapping exoribonuclease [Source:HGNC Symbol;Acc:HGNC:2992] |
| ENSG00000204382 | HGLibA_54469 | 34 | -0.810266686 | XAGE1B | X antigen family member 1B [Source:HGNC Symbol;Acc:HGNC:25400] |
| ENSG00000204479 | HGLibA_38300 | 34 | -0.810266686 | PRAMEF17 | PRAME family member 17 [Source:HGNC Symbol;Acc:HGNC:29485] |
| ENSG00000204843 | HGLibA_12543 | 34 | -0.810266686 | DCTN1 | dynactin subunit 1 [Source:HGNC Symbol;Acc:HGNC:2711] |
| ENSG00000205030 | HGLibA_34324 | 34 | -0.810266686 | OR5L2 | olfactory receptor family 5 subfamily L member 2 [Source:HGNC Symbol;Acc:HGNC:8351] |
| ENSG00000212659 | HGLibA_25842 | 34 | -0.810266686 | KRTAP9-6 | keratin associated protein 9-6 [Source:HGNC Symbol;Acc:HGNC:18914] |
| ENSG00000213337 | HGLibA_02094 | 34 | -0.810266686 | ANKRD39 | ankyrin repeat domain 39 [Source:HGNC Symbol;Acc:HGNC:28640] |
| ENSG00000213390 | HGLibA_02721 | 34 | -0.810266686 | ARHGAP19 | Rho GTPase activating protein 19 [Source:HGNC Symbol;Acc:HGNC:23724] |
| ENSG00000213719 | HGLibA_10020 | 34 | -0.810266686 | CLIC1 | chloride intracellular channel 1 [Source:HGNC Symbol;Acc:HGNC:2062] |
| ENSG00000213920 | HGLibA_28706 | 34 | -0.810266686 | MDP1 | magnesium dependent phosphatase 1 [Source:HGNC Symbol;Acc:HGNC:28781] |
| ENSG00000214513 | HGLibA_32355 | 34 | -0.810266686 | NOTO | notochord homeobox [Source:HGNC Symbol;Acc:HGNC:31839] |
| ENSG00000214941 | HGLibA_57056 | 34 | -0.810266686 | ZSWIM7 | zinc finger SWIM-type containing 7 [Source:HGNC Symbol;Acc:HGNC:26993] |
| ENSG00000215472 | HGLibA_41884 | 34 | -0.810266686 | RPL17-C18orf32 | RPL17-C18orf32 readthrough [Source:HGNC Symbol;Acc:HGNC:44661] |
| ENSG00000215475 | HGLibA_44112 | 34 | -0.810266686 | SIAH3 | siah E3 ubiquitin protein ligase family member 3 [Source:HGNC Symbol;Acc:HGNC:30553] |
| ENSG00000221840 | HGLibA_33932 | 34 | -0.810266686 | OR4A5 | olfactory receptor family 4 subfamily A member 5 [Source:HGNC Symbol;Acc:HGNC:15162] |
| ENSG00000221983 | HGLibA_52526 | 34 | -0.810266686 | UBA52 | ubiquitin A-52 residue ribosomal protein fusion product 1 [Source:HGNC Symbol;Acc:HGNC:12458] |
| ENSG00000230657 | HGLibA_38348 | 34 | -0.810266686 | PRB4 | proline rich protein BstNI subfamily 4 [Source:HGNC Symbol;Acc:HGNC:9340] |
| ENSG00000243649 | HGLibA_09246 | 34 | -0.810266686 | CFB | complement factor B [Source:HGNC Symbol;Acc:HGNC:1037] |
| ENSG00000264522 | HGLibA_34779 | 34 | -0.810266686 | OTUD7B | OTU deubiquitinase 7B [Source:HGNC Symbol;Acc:HGNC:16683] |
| ENSG00000267796 | HGLibA_26565 | 34 | -0.810266686 | LIN37 | lin-37 DREAM MuvB core complex component [Source:HGNC Symbol;Acc:HGNC:33234] |
| ENSG00000268629 | HGLibA_49057 | 34 | -0.810266686 | TEX13A | testis expressed 13A [Source:HGNC Symbol;Acc:HGNC:11735] |
| ENSG00000279804 | HGLibA_38302 | 34 | -0.810266686 | PRAMEF18 | PRAME family member 18 [Source:HGNC Symbol;Acc:HGNC:30693] |
| ENSG00000280090 | HGLibA_34503 | 34 | -0.810266686 | OR8B4 | olfactory receptor family 8 subfamily B member 4 (gene/pseudogene) [Source:HGNC Symbol;Acc:HGNC:8473] |
| ENSG00000288722 | HGLibA_15887 | 34 | -0.810266686 | F8A1 | - |
| ENSG00000288784 | HGLibA_11574 | 34 | -0.810266686 | CTAGE4 | - |
| FAM60A | HGLibA_16657 | 34 | -0.810266686 | FAM60A | - |
| FAM65C | HGLibA_16675 | 34 | -0.810266686 | FAM65C | - |
| GAGE2C | HGLibA_18473 | 34 | -0.810266686 | GAGE2C | - |
| GTPBP6_Y | HGLibA_20619 | 34 | -0.810266686 | GTPBP6_Y | - |
| GYLTL1B | HGLibA_20695 | 34 | -0.810266686 | GYLTL1B | - |
| HIST2H2AA3 | HGLibA_21522 | 34 | -0.810266686 | HIST2H2AA3 | - |
| HIST2H4B | HGLibA_21552 | 34 | -0.810266686 | HIST2H4B | - |
| hsa-let-7f-2 | HGLibA_57131 | 34 | -0.810266686 | hsa-let-7f-2 | - |
| hsa-mir-122 | HGLibA_57290 | 34 | -0.810266686 | hsa-mir-122 | - |
| hsa-mir-2392 | HGLibA_58326 | 34 | -0.810266686 | hsa-mir-2392 | - |
| hsa-mir-26a-2 | HGLibA_58368 | 34 | -0.810266686 | hsa-mir-26a-2 | - |
| hsa-mir-3155a | HGLibA_58680 | 34 | -0.810266686 | hsa-mir-3155a | - |
| hsa-mir-3157 | HGLibA_58699 | 34 | -0.810266686 | hsa-mir-3157 | - |
| hsa-mir-3179-1 | HGLibA_58791 | 34 | -0.810266686 | hsa-mir-3179-1 | - |
| hsa-mir-3179-2 | HGLibA_58792 | 34 | -0.810266686 | hsa-mir-3179-2 | - |
| hsa-mir-33b | HGLibA_59016 | 34 | -0.810266686 | hsa-mir-33b | - |
| hsa-mir-3646 | HGLibA_59134 | 34 | -0.810266686 | hsa-mir-3646 | - |
| hsa-mir-374a | HGLibA_59390 | 34 | -0.810266686 | hsa-mir-374a | - |
| hsa-mir-3918 | HGLibA_59550 | 34 | -0.810266686 | hsa-mir-3918 | - |
| hsa-mir-3940 | HGLibA_59621 | 34 | -0.810266686 | hsa-mir-3940 | - |
| hsa-mir-4465 | HGLibA_60266 | 34 | -0.810266686 | hsa-mir-4465 | - |
| hsa-mir-4526 | HGLibA_60566 | 34 | -0.810266686 | hsa-mir-4526 | - |
| hsa-mir-455 | HGLibA_60631 | 34 | -0.810266686 | hsa-mir-455 | - |
| hsa-mir-4651 | HGLibA_60713 | 34 | -0.810266686 | hsa-mir-4651 | - |
| hsa-mir-4761 | HGLibA_61142 | 34 | -0.810266686 | hsa-mir-4761 | - |
| hsa-mir-5010 | HGLibA_61454 | 34 | -0.810266686 | hsa-mir-5010 | - |

| | | | | | |
|---|---|---|---|---|---|
| hsa-mir-5188 | HGLibA_61643 | 34 | -0.810266686 | hsa-mir-5188 | - |
| hsa-mir-5589 | HGLibA_62194 | 34 | -0.810266686 | hsa-mir-5589 | - |
| hsa-mir-5739 | HGLibA_62388 | 34 | -0.810266686 | hsa-mir-5739 | - |
| hsa-mir-637 | HGLibA_62788 | 34 | -0.810266686 | hsa-mir-637 | - |
| hsa-mir-6504 | HGLibA_62866 | 34 | -0.810266686 | hsa-mir-6504 | - |
| hsa-mir-7156 | HGLibA_63854 | 34 | -0.810266686 | hsa-mir-7156 | - |
| hsa-mir-874 | HGLibA_64208 | 34 | -0.810266686 | hsa-mir-874 | - |
| MESDC1 | HGLibA_28909 | 34 | -0.810266686 | MESDC1 | - |
| NARR | HGLibA_31151 | 34 | -0.810266686 | NARR | - |
| NRD1 | HGLibA_32665 | 34 | -0.810266686 | NRD1 | - |
| PALM2 | HGLibA_35070 | 34 | -0.810266686 | PALM2 | - |
| SPG20 | HGLibA_46632 | 34 | -0.810266686 | SPG20 | - |
| VAMP7_X | HGLibA_53502 | 34 | -0.810266686 | VAMP7_X | - |
| 5-Mar | HGLibA_28341 | 33 | -0.820217724 | 5-Mar | - |
| C11orf35 | HGLibA_05134 | 33 | -0.820217724 | C11orf35 | - |
| C16orf59 | HGLibA_05465 | 33 | -0.820217724 | C16orf59 | - |
| C16orf62 | HGLibA_05467 | 33 | -0.820217724 | C16orf62 | - |
| C22orf29 | HGLibA_06068 | 33 | -0.820217724 | C22orf29 | - |
| C6orf25 | HGLibA_06485 | 33 | -0.820217724 | C6orf25 | - |
| C8orf22 | HGLibA_06601 | 33 | -0.820217724 | C8orf22 | - |
| ENSG00000007933 | HGLibA_17707 | 33 | -0.820217724 | FMO3 | flavin containing monooxygenase 3 [Source:HGNC Symbol;Acc:HGNC:3771] |
| ENSG00000013561 | HGLibA_41521 | 33 | -0.820217724 | RNF14 | ring finger protein 14 [Source:HGNC Symbol;Acc:HGNC:10058] |
| ENSG00000013619 | HGLibA_28053 | 33 | -0.820217724 | MAMLD1 | mastermind like domain containing 1 [Source:HGNC Symbol;Acc:HGNC:2568] |
| ENSG00000023909 | HGLibA_18797 | 33 | -0.820217724 | GCLM | glutamate-cysteine ligase modifier subunit [Source:HGNC Symbol;Acc:HGNC:4312] |
| ENSG00000036549 | HGLibA_57095 | 33 | -0.820217724 | ZZZ3 | zinc finger ZZ-type containing 3 [Source:NCBI gene;Acc:26009] |
| ENSG00000037757 | HGLibA_29859 | 33 | -0.820217724 | MRI1 | methylthioribose-1-phosphate isomerase 1 [Source:HGNC Symbol;Acc:HGNC:28469] |
| ENSG00000042304 | HGLibA_06195 | 33 | -0.820217724 | C2orf83 | chromosome 2 open reading frame 83 [Source:HGNC Symbol;Acc:HGNC:25344] |
| ENSG00000042493 | HGLibA_07139 | 33 | -0.820217724 | CAPG | gelsolin like [Source:HGNC Symbol;Acc:HGNC:1474]" |
| ENSG00000050405 | HGLibA_26524 | 33 | -0.820217724 | LIMA1 | LIM domain and actin binding 1 [Source:HGNC Symbol;Acc:HGNC:24636] |
| ENSG00000064300 | HGLibA_31875 | 33 | -0.820217724 | NGFR | nerve growth factor receptor [Source:HGNC Symbol;Acc:HGNC:7809] |
| ENSG00000064313 | HGLibA_48227 | 33 | -0.820217724 | TAF2 | TATA-box binding protein associated factor 2 [Source:HGNC Symbol;Acc:HGNC:11536] |
| ENSG00000065911 | HGLibA_30382 | 33 | -0.820217724 | MTHFD2 | methenyltetrahydrofolate cyclohydrolase [Source:HGNC Symbol;Acc:HGNC:7434]" |
| ENSG00000068650 | HGLibA_03554 | 33 | -0.820217724 | ATP11A | ATPase phospholipid transporting 11A [Source:HGNC Symbol;Acc:HGNC:13552] |
| ENSG00000069764 | HGLibA_36866 | 33 | -0.820217724 | PLA2G10 | phospholipase A2 group X [Source:HGNC Symbol;Acc:HGNC:9029] |
| ENSG00000072134 | HGLibA_15344 | 33 | -0.820217724 | EPN2 | epsin 2 [Source:HGNC Symbol;Acc:HGNC:18639] |
| ENSG00000073605 | HGLibA_20408 | 33 | -0.820217724 | GSDMB | gasdermin B [Source:HGNC Symbol;Acc:HGNC:23690] |
| ENSG00000074054 | HGLibA_09793 | 33 | -0.820217724 | CLASP1 | cytoplasmic linker associated protein 1 [Source:HGNC Symbol;Acc:HGNC:17088] |
| ENSG00000075213 | HGLibA_43320 | 33 | -0.820217724 | SEMA3A | semaphorin 3A [Source:HGNC Symbol;Acc:HGNC:10723] |
| ENSG00000075702 | HGLibA_54146 | 33 | -0.820217724 | WDR62 | WD repeat domain 62 [Source:HGNC Symbol;Acc:HGNC:24502] |
| ENSG00000083099 | HGLibA_27750 | 33 | -0.820217724 | LYRM2 | LYR motif containing 2 [Source:HGNC Symbol;Acc:HGNC:25229] |
| ENSG00000083123 | HGLibA_04282 | 33 | -0.820217724 | BCKDHB | branched chain keto acid dehydrogenase E1 subunit beta [Source:HGNC Symbol;Acc:HGNC:987] |
| ENSG00000083312 | HGLibA_50898 | 33 | -0.820217724 | TNPO1 | transportin 1 [Source:HGNC Symbol;Acc:HGNC:6401] |
| ENSG00000085449 | HGLibA_54003 | 33 | -0.820217724 | WDFY1 | WD repeat and FYVE domain containing 1 [Source:HGNC Symbol;Acc:HGNC:20451] |
| ENSG00000086189 | HGLibA_13236 | 33 | -0.820217724 | DIMT1 | DIM1 dimethyladenosine transferase 1 homolog [Source:HGNC Symbol;Acc:HGNC:30217] |
| ENSG00000086200 | HGLibA_23459 | 33 | -0.820217724 | IPO11 | importin 11 [Source:HGNC Symbol;Acc:HGNC:20628] |
| ENSG00000095139 | HGLibA_02637 | 33 | -0.820217724 | ARCN1 | archain 1 [Source:HGNC Symbol;Acc:HGNC:649] |
| ENSG00000096063 | HGLibA_47017 | 33 | -0.820217724 | SRPK1 | SRSF protein kinase 1 [Source:HGNC Symbol;Acc:HGNC:11305] |
| ENSG00000096872 | HGLibA_22770 | 33 | -0.820217724 | IFT74 | intraflagellar transport 74 [Source:HGNC Symbol;Acc:HGNC:21424] |
| ENSG00000100385 | HGLibA_23121 | 33 | -0.820217724 | IL2RB | interleukin 2 receptor subunit beta [Source:HGNC Symbol;Acc:HGNC:6009] |
| ENSG00000101003 | HGLibA_19104 | 33 | -0.820217724 | GINS1 | GINS complex subunit 1 [Source:HGNC Symbol;Acc:HGNC:28980] |
| ENSG00000101198 | HGLibA_31999 | 33 | -0.820217724 | NKAIN4 | sodium/potassium transporting ATPase interacting 4 [Source:HGNC Symbol;Acc:HGNC:16191] |
| ENSG00000101247 | HGLibA_31494 | 33 | -0.820217724 | NDUFAF5 | NADH:ubiquinone oxidoreductase complex assembly factor 5 [Source:HGNC Symbol;Acc:HGNC:15899] |
| ENSG00000101883 | HGLibA_41215 | 33 | -0.820217724 | RHOXF1 | Rhox homeobox family member 1 [Source:HGNC Symbol;Acc:HGNC:29993] |
| ENSG00000102172 | HGLibA_45831 | 33 | -0.820217724 | SMS | spermine synthase [Source:HGNC Symbol;Acc:HGNC:11123] |
| ENSG00000103423 | HGLibA_13515 | 33 | -0.820217724 | DNAJA3 | DnaJ heat shock protein family (Hsp40) member A3 [Source:HGNC Symbol;Acc:HGNC:11808] |
| ENSG00000104472 | HGLibA_09533 | 33 | -0.820217724 | CHRAC1 | chromatin accessibility complex subunit 1 [Source:HGNC Symbol;Acc:HGNC:13544] |
| ENSG00000105426 | HGLibA_39534 | 33 | -0.820217724 | PTPRS | receptor type S [Source:HGNC Symbol;Acc:HGNC:9681]" |
| ENSG00000105639 | HGLibA_23919 | 33 | -0.820217724 | JAK3 | Janus kinase 3 [Source:HGNC Symbol;Acc:HGNC:6193] |
| ENSG00000105696 | HGLibA_50462 | 33 | -0.820217724 | TMEM59L | transmembrane protein 59 like [Source:HGNC Symbol;Acc:HGNC:13237] |
| ENSG00000106511 | HGLibA_28892 | 33 | -0.820217724 | MEOX2 | mesenchyme homeobox 2 [Source:HGNC Symbol;Acc:HGNC:7014] |
| ENSG00000106733 | HGLibA_32196 | 33 | -0.820217724 | NMRK1 | nicotinamide riboside kinase 1 [Source:HGNC Symbol;Acc:HGNC:26057] |
| ENSG00000107331 | HGLibA_00106 | 33 | -0.820217724 | ABCA2 | ATP binding cassette subfamily A member 2 [Source:HGNC Symbol;Acc:HGNC:32] |
| ENSG00000107731 | HGLibA_53017 | 33 | -0.820217724 | UNC5B | unc-5 netrin receptor B [Source:HGNC Symbol;Acc:HGNC:12568] |
| ENSG00000107864 | HGLibA_10923 | 33 | -0.820217724 | CPEB3 | cytoplasmic polyadenylation element binding protein 3 [Source:HGNC Symbol;Acc:HGNC:21746] |
| ENSG00000107984 | HGLibA_13293 | 33 | -0.820217724 | DKK1 | dickkopf WNT signaling pathway inhibitor 1 [Source:HGNC Symbol;Acc:HGNC:2891] |
| ENSG00000108231 | HGLibA_26394 | 33 | -0.820217724 | LGI1 | leucine rich glioma inactivated 1 [Source:HGNC Symbol;Acc:HGNC:6572] |
| ENSG00000108389 | HGLibA_30430 | 33 | -0.820217724 | MTMR4 | myotubularin related protein 4 [Source:HGNC Symbol;Acc:HGNC:7452] |
| ENSG00000108511 | HGLibA_21944 | 33 | -0.820217724 | HOXB6 | homeobox B6 [Source:HGNC Symbol;Acc:HGNC:5117] |
| ENSG00000108669 | HGLibA_12235 | 33 | -0.820217724 | CYTH1 | cytohesin 1 [Source:HGNC Symbol;Acc:HGNC:9501] |
| ENSG00000109113 | HGLibA_39886 | 33 | -0.820217724 | RAB34 | member RAS oncogene family [Source:HGNC Symbol;Acc:HGNC:16519]" |
| ENSG00000109205 | HGLibA_33269 | 33 | -0.820217724 | ODAM | ameloblast associated [Source:HGNC Symbol;Acc:HGNC:26043]" |
| ENSG00000109511 | HGLibA_02221 | 33 | -0.820217724 | ANXA10 | annexin A10 [Source:HGNC Symbol;Acc:HGNC:534] |
| ENSG00000110427 | HGLibA_24780 | 33 | -0.820217724 | KIAA1549L | KIAA1549 like [Source:HGNC Symbol;Acc:HGNC:24836] |
| ENSG00000110665 | HGLibA_05122 | 33 | -0.820217724 | C11orf21 | chromosome 11 open reading frame 21 [Source:HGNC Symbol;Acc:HGNC:13231] |
| ENSG00000111348 | HGLibA_02797 | 33 | -0.820217724 | ARHGDIB | Rho GDP dissociation inhibitor beta [Source:HGNC Symbol;Acc:HGNC:679] |
| ENSG00000111885 | HGLibA_28059 | 33 | -0.820217724 | MAN1A1 | mannosidase alpha class 1A member 1 [Source:HGNC Symbol;Acc:HGNC:6821] |
| ENSG00000112486 | HGLibA_08161 | 33 | -0.820217724 | CCR6 | C-C motif chemokine receptor 6 [Source:HGNC Symbol;Acc:HGNC:1607] |
| ENSG00000113119 | HGLibA_49796 | 33 | -0.820217724 | TMCO6 | transmembrane and coiled-coil domains 6 [Source:HGNC Symbol;Acc:HGNC:28814] |
| ENSG00000113525 | HGLibA_23175 | 33 | -0.820217724 | IL5 | interleukin 5 [Source:HGNC Symbol;Acc:HGNC:6016] |
| ENSG00000115297 | HGLibA_49676 | 33 | -0.820217724 | TLX2 | T cell leukemia homeobox 2 [Source:HGNC Symbol;Acc:HGNC:5057] |
| ENSG00000115970 | HGLibA_49283 | 33 | -0.820217724 | THADA | armadillo repeat containing [Source:HGNC Symbol;Acc:HGNC:19217]" |
| ENSG00000116209 | HGLibA_50459 | 33 | -0.820217724 | TMEM59 | transmembrane protein 59 [Source:HGNC Symbol;Acc:HGNC:1239] |
| ENSG00000117228 | HGLibA_18728 | 33 | -0.820217724 | GBP1 | guanylate binding protein 1 [Source:HGNC Symbol;Acc:HGNC:4182] |
| ENSG00000117479 | HGLibA_44500 | 33 | -0.820217724 | SLC19A2 | solute carrier family 19 member 2 [Source:HGNC Symbol;Acc:HGNC:10938] |
| ENSG00000117748 | HGLibA_41801 | 33 | -0.820217724 | RPA2 | replication protein A2 [Source:HGNC Symbol;Acc:HGNC:10290] |
| ENSG00000119185 | HGLibA_23773 | 33 | -0.820217724 | ITGB1BP1 | integrin subunit beta 1 binding protein 1 [Source:HGNC Symbol;Acc:HGNC:23927] |
| ENSG00000119707 | HGLibA_40519 | 33 | -0.820217724 | RBM25 | RNA binding motif protein 25 [Source:HGNC Symbol;Acc:HGNC:23244] |
| ENSG00000119919 | HGLibA_32037 | 33 | -0.820217724 | NKX2-3 | NK2 homeobox 3 [Source:HGNC Symbol;Acc:HGNC:7836] |
| ENSG00000119977 | HGLibA_48879 | 33 | -0.820217724 | TCTN3 | tectonic family member 3 [Source:HGNC Symbol;Acc:HGNC:24519] |
| ENSG00000120088 | HGLibA_11141 | 33 | -0.820217724 | CRHR1 | corticotropin releasing hormone receptor 1 [Source:HGNC Symbol;Acc:HGNC:2357] |
| ENSG00000120784 | HGLibA_55200 | 33 | -0.820217724 | ZFP30 | ZFP30 zinc finger protein [Source:HGNC Symbol;Acc:HGNC:29555] |
| ENSG00000121481 | HGLibA_41606 | 33 | -0.820217724 | RNF2 | ring finger protein 2 [Source:HGNC Symbol;Acc:HGNC:10061] |
| ENSG00000122877 | HGLibA_14651 | 33 | -0.820217724 | EGR2 | early growth response 2 [Source:HGNC Symbol;Acc:HGNC:3239] |
| ENSG00000124198 | HGLibA_02672 | 33 | -0.820217724 | ARFGEF2 | ADP ribosylation factor guanine nucleotide exchange factor 2 [Source:HGNC Symbol;Acc:HGNC:15853] |
| ENSG00000125384 | HGLibA_39315 | 33 | -0.820217724 | PTGER2 | prostaglandin E receptor 2 [Source:HGNC Symbol;Acc:HGNC:9594] |
| ENSG00000126895 | HGLibA_03909 | 33 | -0.820217724 | AVPR2 | arginine vasopressin receptor 2 [Source:HGNC Symbol;Acc:HGNC:897] |
| ENSG00000127616 | HGLibA_45608 | 33 | -0.820217724 | SMARCA4 | member 4 [Source:HGNC Symbol;Acc:HGNC:11100]" |
| ENSG00000127903 | HGLibA_56840 | 33 | -0.820217724 | ZNF835 | zinc finger protein 835 [Source:HGNC Symbol;Acc:HGNC:34332] |
| ENSG00000128203 | HGLibA_03357 | 33 | -0.820217724 | ASPHD2 | aspartate beta-hydroxylase domain containing 2 [Source:HGNC Symbol;Acc:HGNC:30437] |
| ENSG00000128253 | HGLibA_40932 | 33 | -0.820217724 | RFPL2 | ret finger protein like 2 [Source:HGNC Symbol;Acc:HGNC:9979] |
| ENSG00000129355 | HGLibA_08844 | 33 | -0.820217724 | CDKN2D | cyclin dependent kinase inhibitor 2D [Source:HGNC Symbol;Acc:HGNC:1790] |
| ENSG00000129864 | HGLibA_53594 | 33 | -0.820217724 | VCY | variable charge Y-linked [Source:HGNC Symbol;Acc:HGNC:12668] |
| ENSG00000130528 | HGLibA_22086 | 33 | -0.820217724 | HRC | histidine rich calcium binding protein [Source:HGNC Symbol;Acc:HGNC:5178] |
| ENSG00000130675 | HGLibA_29580 | 33 | -0.820217724 | MNX1 | motor neuron and pancreas homeobox 1 [Source:HGNC Symbol;Acc:HGNC:4979] |
| ENSG00000130950 | HGLibA_33109 | 33 | -0.820217724 | NUTM2F | NUT family member 2F [Source:HGNC Symbol;Acc:HGNC:23450] |
| ENSG00000131368 | HGLibA_30075 | 33 | -0.820217724 | MRPS25 | mitochondrial ribosomal protein S25 [Source:HGNC Symbol;Acc:HGNC:14511] |
| ENSG00000131653 | HGLibA_51268 | 33 | -0.820217724 | TRAF7 | TNF receptor associated factor 7 [Source:HGNC Symbol;Acc:HGNC:20456] |
| ENSG00000132341 | HGLibA_40190 | 33 | -0.820217724 | RAN | member RAS oncogene family [Source:HGNC Symbol;Acc:HGNC:9846]" |
| ENSG00000132510 | HGLibA_24536 | 33 | -0.820217724 | KDM6B | lysine demethylase 6B [Source:HGNC Symbol;Acc:HGNC:29012] |
| ENSG00000132855 | HGLibA_01916 | 33 | -0.820217724 | ANGPTL3 | angiopoietin like 3 [Source:HGNC Symbol;Acc:HGNC:491] |
| ENSG00000133703 | HGLibA_25385 | 33 | -0.820217724 | KRAS | GTPase [Source:HGNC Symbol;Acc:HGNC:6407]" |
| ENSG00000134825 | HGLibA_50342 | 33 | -0.820217724 | TMEM258 | transmembrane protein 258 [Source:HGNC Symbol;Acc:HGNC:1164] |
| ENSG00000135912 | HGLibA_52205 | 33 | -0.820217724 | TTLL4 | tubulin tyrosine ligase like 4 [Source:HGNC Symbol;Acc:HGNC:28976] |
| ENSG00000136100 | HGLibA_53773 | 33 | -0.820217724 | VPS36 | vacuolar protein sorting 36 homolog [Source:HGNC Symbol;Acc:HGNC:20312] |
| ENSG00000136153 | HGLibA_26715 | 33 | -0.820217724 | LMO7 | LIM domain 7 [Source:HGNC Symbol;Acc:HGNC:6646] |
| ENSG00000138068 | HGLibA_47767 | 33 | -0.820217724 | SULT6B1 | sulfotransferase family 6B member 1 [Source:HGNC Symbol;Acc:HGNC:33433] |
| ENSG00000138161 | HGLibA_11810 | 33 | -0.820217724 | CUZD1 | CUB and zona pellucida like domains 1 [Source:HGNC Symbol;Acc:HGNC:17937] |
| ENSG00000140254 | HGLibA_14104 | 33 | -0.820217724 | DUOXA1 | dual oxidase maturation factor 1 [Source:HGNC Symbol;Acc:HGNC:26507] |
| ENSG00000140284 | HGLibA_44816 | 33 | -0.820217724 | SLC27A2 | solute carrier family 27 member 2 [Source:HGNC Symbol;Acc:HGNC:10996] |
| ENSG00000142168 | HGLibA_46143 | 33 | -0.820217724 | SOD1 | superoxide dismutase 1 [Source:HGNC Symbol;Acc:HGNC:11179] |
| ENSG00000143199 | HGLibA_00907 | 33 | -0.820217724 | ADCY10 | adenylate cyclase 10 [Source:HGNC Symbol;Acc:HGNC:21285] |
| ENSG00000143942 | HGLibA_09339 | 33 | -0.820217724 | CHAC2 | ChaC cation transport regulator homolog 2 [Source:HGNC Symbol;Acc:HGNC:32363] |
| ENSG00000144554 | HGLibA_16840 | 33 | -0.820217724 | FANCD2 | FA complementation group D2 [Source:HGNC Symbol;Acc:HGNC:3585] |
| ENSG00000145730 | HGLibA_35081 | 33 | -0.820217724 | PAM | peptidylglycine alpha-amidating monooxygenase [Source:HGNC Symbol;Acc:HGNC:8596] |
| ENSG00000146066 | HGLibA_21314 | 33 | -0.820217724 | HIGD2A | HIG1 hypoxia inducible domain family member 2A [Source:HGNC Symbol;Acc:HGNC:28311] |
| ENSG00000146463 | HGLibA_55398 | 33 | -0.820217724 | ZMYM4 | zinc finger MYM-type containing 4 [Source:HGNC Symbol;Acc:HGNC:13055] |
| ENSG00000147202 | HGLibA_13221 | 33 | -0.820217724 | DIAPH2 | diaphanous related formin 2 [Source:HGNC Symbol;Acc:HGNC:2877] |
| ENSG00000147419 | HGLibA_07740 | 33 | -0.820217724 | CCDC25 | coiled-coil domain containing 25 [Source:HGNC Symbol;Acc:HGNC:25591] |
| ENSG00000147434 | HGLibA_09580 | 33 | -0.820217724 | CHRNA6 | cholinergic receptor nicotinic alpha 6 subunit [Source:HGNC Symbol;Acc:HGNC:15963] |
| ENSG00000147874 | HGLibA_20890 | 33 | -0.820217724 | HAUS6 | HAUS augmin like complex subunit 6 [Source:HGNC Symbol;Acc:HGNC:25948] |
| ENSG00000149806 | HGLibA_16925 | 33 | -0.820217724 | FAU | ubiquitin like and ribosomal protein S30 fusion [Source:HGNC Symbol;Acc:HGNC:3597]" |
| ENSG00000151338 | HGLibA_29298 | 33 | -0.820217724 | MIPOL1 | mirror-image polydactyly 1 [Source:HGNC Symbol;Acc:HGNC:21460] |
| ENSG00000151778 | HGLibA_43501 | 33 | -0.820217724 | SERP2 | stress associated endoplasmic reticulum protein family member 2 [Source:HGNC Symbol;Acc:HGNC:20607] |
| ENSG00000152242 | HGLibA_05608 | 33 | -0.820217724 | C18orf25 | chromosome 18 open reading frame 25 [Source:HGNC Symbol;Acc:HGNC:28172] |
| ENSG00000154040 | HGLibA_06865 | 33 | -0.820217724 | CABYR | calcium binding tyrosine phosphorylation regulated [Source:HGNC Symbol;Acc:HGNC:15569] |

| | | | | | |
|---|---|---|---|---|---|
| ENSG00000154059 | HGLibA_23250 | 33 | -0.820217724 | IMPACT | impact RWD domain protein [Source:HGNC Symbol;Acc:HGNC:20387] |
| ENSG00000154813 | HGLibA_13843 | 33 | -0.820217724 | DPH3 | diphthamide biosynthesis 3 [Source:HGNC Symbol;Acc:HGNC:27717] |
| ENSG00000155016 | HGLibA_12144 | 33 | -0.820217724 | CYP2U1 | cytochrome P450 family 2 subfamily U member 1 [Source:HGNC Symbol;Acc:HGNC:20582] |
| ENSG00000155868 | HGLibA_28815 | 33 | -0.820217724 | MED7 | mediator complex subunit 7 [Source:HGNC Symbol;Acc:HGNC:2378] |
| ENSG00000155918 | HGLibA_40126 | 33 | -0.820217724 | RAET1L | retinoic acid early transcript 1L [Source:HGNC Symbol;Acc:HGNC:16798] |
| ENSG00000156345 | HGLibA_08759 | 33 | -0.820217724 | CDK20 | cyclin dependent kinase 20 [Source:HGNC Symbol;Acc:HGNC:21420] |
| ENSG00000158445 | HGLibA_24156 | 33 | -0.820217724 | KCNB1 | potassium voltage-gated channel subfamily B member 1 [Source:HGNC Symbol;Acc:HGNC:6231] |
| ENSG00000159842 | HGLibA_00322 | 33 | -0.820217724 | ABR | RhoGEF and GTPase activating protein [Source:HGNC Symbol;Acc:HGNC:81]" |
| ENSG00000160218 | HGLibA_51301 | 33 | -0.820217724 | TRAPPC10 | trafficking protein particle complex 10 [Source:HGNC Symbol;Acc:HGNC:11868] |
| ENSG00000160801 | HGLibA_39366 | 33 | -0.820217724 | PTH1R | parathyroid hormone 1 receptor [Source:HGNC Symbol;Acc:HGNC:9608] |
| ENSG00000161860 | HGLibA_47920 | 33 | -0.820217724 | SYCE2 | synaptonemal complex central element protein 2 [Source:HGNC Symbol;Acc:HGNC:27411] |
| ENSG00000162004 | HGLibA_07870 | 33 | -0.820217724 | CCDC78 | coiled-coil domain containing 78 [Source:HGNC Symbol;Acc:HGNC:14153] |
| ENSG00000162522 | HGLibA_24772 | 33 | -0.820217724 | KIAA1522 | KIAA1522 [Source:HGNC Symbol;Acc:HGNC:29301] |
| ENSG00000162747 | HGLibA_17199 | 33 | -0.820217724 | FCGR3B | Fc fragment of IgG receptor IIIb [Source:HGNC Symbol;Acc:HGNC:3620] |
| ENSG00000162763 | HGLibA_27385 | 33 | -0.820217724 | LRRC52 | leucine rich repeat containing 52 [Source:HGNC Symbol;Acc:HGNC:32156] |
| ENSG00000163110 | HGLibA_35918 | 33 | -0.820217724 | PDLIM5 | PDZ and LIM domain 5 [Source:HGNC Symbol;Acc:HGNC:17468] |
| ENSG00000163145 | HGLibA_05953 | 33 | -0.820217724 | C1QTNF7 | C1q and TNF related 7 [Source:HGNC Symbol;Acc:HGNC:14342] |
| ENSG00000163466 | HGLibA_03080 | 33 | -0.820217724 | ARPC2 | actin related protein 2/3 complex subunit 2 [Source:HGNC Symbol;Acc:HGNC:705] |
| ENSG00000163517 | HGLibA_21002 | 33 | -0.820217724 | HDAC11 | histone deacetylase 11 [Source:HGNC Symbol;Acc:HGNC:19086] |
| ENSG00000163590 | HGLibA_37991 | 33 | -0.820217724 | PPM1L | Mg2+/Mn2+ dependent 1L [Source:HGNC Symbol;Acc:HGNC:16381]" |
| ENSG00000163605 | HGLibA_38206 | 33 | -0.820217724 | PPP4R2 | protein phosphatase 4 regulatory subunit 2 [Source:HGNC Symbol;Acc:HGNC:18296] |
| ENSG00000163681 | HGLibA_45534 | 33 | -0.820217724 | SLMAP | sarcolemma associated protein [Source:HGNC Symbol;Acc:HGNC:16643] |
| ENSG00000164105 | HGLibA_42721 | 33 | -0.820217724 | SAP30 | Sin3A associated protein 30 [Source:HGNC Symbol;Acc:HGNC:10532] |
| ENSG00000164816 | HGLibA_12803 | 33 | -0.820217724 | DEFA5 | defensin alpha 5 [Source:HGNC Symbol;Acc:HGNC:2764] |
| ENSG00000165283 | HGLibA_47538 | 33 | -0.820217724 | STOML2 | stomatin like 2 [Source:HGNC Symbol;Acc:HGNC:14559] |
| ENSG00000165819 | HGLibA_29007 | 33 | -0.820217724 | METTL3 | methyltransferase like 3 [Source:HGNC Symbol;Acc:HGNC:17563] |
| ENSG00000165916 | HGLibA_39133 | 33 | -0.820217724 | PSMC3 | ATPase 3 [Source:HGNC Symbol;Acc:HGNC:9549]" |
| ENSG00000166145 | HGLibA_46711 | 33 | -0.820217724 | SPINT1 | Kunitz type 1 [Source:HGNC Symbol;Acc:HGNC:11246]" |
| ENSG00000166192 | HGLibA_43404 | 33 | -0.820217724 | SENP8 | NEDD8 specific [Source:HGNC Symbol;Acc:HGNC:22992]" |
| ENSG00000166348 | HGLibA_53400 | 33 | -0.820217724 | USP54 | ubiquitin specific peptidase 54 [Source:HGNC Symbol;Acc:HGNC:23513] |
| ENSG00000167106 | HGLibA_15979 | 33 | -0.820217724 | FAM102A | family with sequence similarity 102 member A [Source:HGNC Symbol;Acc:HGNC:31419] |
| ENSG00000169239 | HGLibA_06814 | 33 | -0.820217724 | CA5B | carbonic anhydrase 5B [Source:HGNC Symbol;Acc:HGNC:1378] |
| ENSG00000169398 | HGLibA_39380 | 33 | -0.820217724 | PTK2 | protein tyrosine kinase 2 [Source:HGNC Symbol;Acc:HGNC:9611] |
| ENSG00000169469 | HGLibA_46804 | 33 | -0.820217724 | SPRR1B | small proline rich protein 1B [Source:HGNC Symbol;Acc:HGNC:11260] |
| ENSG00000169840 | HGLibA_20509 | 33 | -0.820217724 | GSX1 | GS homeobox 1 [Source:HGNC Symbol;Acc:HGNC:20374] |
| ENSG00000170604 | HGLibA_23577 | 33 | -0.820217724 | IRF2BP1 | interferon regulatory factor 2 binding protein 1 [Source:HGNC Symbol;Acc:HGNC:21728] |
| ENSG00000171916 | HGLibA_26386 | 33 | -0.820217724 | LGALS9C | galectin 9C [Source:HGNC Symbol;Acc:HGNC:33874] |
| ENSG00000172006 | HGLibA_56220 | 33 | -0.820217724 | ZNF554 | zinc finger protein 554 [Source:HGNC Symbol;Acc:HGNC:26629] |
| ENSG00000172175 | HGLibA_28033 | 33 | -0.820217724 | MALT1 | MALT1 paracaspase [Source:HGNC Symbol;Acc:HGNC:6819] |
| ENSG00000172288 | HGLibA_08904 | 33 | -0.820217724 | CDY1 | chromodomain Y-linked 1 [Source:HGNC Symbol;Acc:HGNC:1809] |
| ENSG00000173281 | HGLibA_38102 | 33 | -0.820217724 | PPP1R3B | protein phosphatase 1 regulatory subunit 3B [Source:HGNC Symbol;Acc:HGNC:14942] |
| ENSG00000174442 | HGLibA_57067 | 33 | -0.820217724 | ZWILCH | zwilch kinetochore protein [Source:HGNC Symbol;Acc:HGNC:25468] |
| ENSG00000174840 | HGLibA_35766 | 33 | -0.820217724 | PDE12 | phosphodiesterase 12 [Source:HGNC Symbol;Acc:HGNC:25386] |
| ENSG00000175643 | HGLibA_41377 | 33 | -0.820217724 | RMI2 | RecQ mediated genome instability 2 [Source:HGNC Symbol;Acc:HGNC:28349] |
| ENSG00000175764 | HGLibA_52193 | 33 | -0.820217724 | TTLL11 | tubulin tyrosine ligase like 11 [Source:HGNC Symbol;Acc:HGNC:18113] |
| ENSG00000175766 | HGLibA_14819 | 33 | -0.820217724 | EIF4E1B | eukaryotic translation initiation factor 4E family member 1B [Source:HGNC Symbol;Acc:HGNC:33179] |
| ENSG00000176020 | HGLibA_01799 | 33 | -0.820217724 | AMIGO3 | adhesion molecule with Ig like domain 3 [Source:HGNC Symbol;Acc:HGNC:24075] |
| ENSG00000178460 | HGLibA_28633 | 33 | -0.820217724 | MCMDC2 | minichromosome maintenance domain containing 2 [Source:HGNC Symbol;Acc:HGNC:26368] |
| ENSG00000178567 | HGLibA_15339 | 33 | -0.820217724 | EPM2AIP1 | EPM2A interacting protein 1 [Source:HGNC Symbol;Acc:HGNC:19735] |
| ENSG00000178761 | HGLibA_16500 | 33 | -0.820217724 | FAM219B | family with sequence similarity 219 member B [Source:HGNC Symbol;Acc:HGNC:24695] |
| ENSG00000178982 | HGLibA_14796 | 33 | -0.820217724 | EIF3K | eukaryotic translation initiation factor 3 subunit K [Source:HGNC Symbol;Acc:HGNC:24656] |
| ENSG00000180376 | HGLibA_07834 | 33 | -0.820217724 | CCDC66 | coiled-coil domain containing 66 [Source:HGNC Symbol;Acc:HGNC:27709] |
| ENSG00000181778 | HGLibA_50321 | 33 | -0.820217724 | TMEM252 | transmembrane protein 252 [Source:HGNC Symbol;Acc:HGNC:28537] |
| ENSG00000181867 | HGLibA_18128 | 33 | -0.820217724 | FTMT | ferritin mitochondrial [Source:HGNC Symbol;Acc:HGNC:17345] |
| ENSG00000182326 | HGLibA_05976 | 33 | -0.820217724 | C1S | complement C1s [Source:HGNC Symbol;Acc:HGNC:1247] |
| ENSG00000182771 | HGLibA_20272 | 33 | -0.820217724 | GRID1 | glutamate ionotropic receptor delta type subunit 1 [Source:HGNC Symbol;Acc:HGNC:4575] |
| ENSG00000183718 | HGLibA_51541 | 33 | -0.820217724 | TRIM52 | tripartite motif containing 52 [Source:HGNC Symbol;Acc:HGNC:19024] |
| ENSG00000183785 | HGLibA_52265 | 33 | -0.820217724 | TUBA8 | tubulin alpha 8 [Source:HGNC Symbol;Acc:HGNC:12410] |
| ENSG00000184708 | HGLibA_14838 | 33 | -0.820217724 | EIF4ENIF1 | eukaryotic translation initiation factor 4E nuclear import factor 1 [Source:HGNC Symbol;Acc:HGNC:16687] |
| ENSG00000185127 | HGLibA_06440 | 33 | -0.820217724 | C6orf120 | chromosome 6 open reading frame 120 [Source:HGNC Symbol;Acc:HGNC:21247] |
| ENSG00000185156 | HGLibA_29110 | 33 | -0.820217724 | MFSD6L | major facilitator superfamily domain containing 6 like [Source:HGNC Symbol;Acc:HGNC:26656] |
| ENSG00000185640 | HGLibA_25548 | 33 | -0.820217724 | KRT79 | keratin 79 [Source:HGNC Symbol;Acc:HGNC:28930] |
| ENSG00000186150 | HGLibA_52709 | 33 | -0.820217724 | UBL4B | ubiquitin like 4B [Source:HGNC Symbol;Acc:HGNC:32309] |
| ENSG00000186660 | HGLibA_55243 | 33 | -0.820217724 | ZFP91 | ZFP91 zinc finger protein [Source:HGNC Symbol;Acc:HGNC:14983] |
| ENSG00000187122 | HGLibA_45506 | 33 | -0.820217724 | SLIT1 | slit guidance ligand 1 [Source:HGNC Symbol;Acc:HGNC:11085] |
| ENSG00000187170 | HGLibA_26145 | 33 | -0.820217724 | LCE4A | late cornified envelope 4A [Source:HGNC Symbol;Acc:HGNC:16613] |
| ENSG00000187581 | HGLibA_10878 | 33 | -0.820217724 | COX8C | cytochrome c oxidase subunit 8C [Source:HGNC Symbol;Acc:HGNC:24382] |
| ENSG00000187720 | HGLibA_49404 | 33 | -0.820217724 | THSD4 | thrombospondin type 1 domain containing 4 [Source:HGNC Symbol;Acc:HGNC:25835] |
| ENSG00000188112 | HGLibA_06444 | 33 | -0.820217724 | C6orf132 | chromosome 6 open reading frame 132 [Source:HGNC Symbol;Acc:HGNC:21288] |
| ENSG00000188352 | HGLibA_17776 | 33 | -0.820217724 | FOCAD | focadhesin [Source:HGNC Symbol;Acc:HGNC:23377] |
| ENSG00000188554 | HGLibA_31249 | 33 | -0.820217724 | NBR1 | autophagy cargo receptor [Source:HGNC Symbol;Acc:HGNC:6746]" |
| ENSG00000189144 | HGLibA_56285 | 33 | -0.820217724 | ZNF573 | zinc finger protein 573 [Source:HGNC Symbol;Acc:HGNC:26420] |
| ENSG00000189253 | HGLibA_51578 | 33 | -0.820217724 | TRIM64B | tripartite motif containing 64B [Source:HGNC Symbol;Acc:HGNC:37147] |
| ENSG00000196242 | HGLibA_33779 | 33 | -0.820217724 | OR2C3 | olfactory receptor family 2 subfamily C member 3 [Source:HGNC Symbol;Acc:HGNC:15005] |
| ENSG00000196431 | HGLibA_11259 | 33 | -0.820217724 | CRYBA4 | crystallin beta A4 [Source:HGNC Symbol;Acc:HGNC:2396] |
| ENSG00000196935 | HGLibA_46966 | 33 | -0.820217724 | SRGAP1 | SLIT-ROBO Rho GTPase activating protein 1 [Source:HGNC Symbol;Acc:HGNC:17382] |
| ENSG00000197140 | HGLibA_00774 | 33 | -0.820217724 | ADAM32 | ADAM metallopeptidase domain 32 [Source:HGNC Symbol;Acc:HGNC:15479] |
| ENSG00000197614 | HGLibA_29055 | 33 | -0.820217724 | MFAP5 | microfibril associated protein 5 [Source:HGNC Symbol;Acc:HGNC:29673] |
| ENSG00000197641 | HGLibA_43545 | 33 | -0.820217724 | SERPINB13 | serpin family B member 13 [Source:HGNC Symbol;Acc:HGNC:8944] |
| ENSG00000197746 | HGLibA_39000 | 33 | -0.820217724 | PSAP | prosaposin [Source:HGNC Symbol;Acc:HGNC:9498] |
| ENSG00000198026 | HGLibA_55824 | 33 | -0.820217724 | ZNF335 | zinc finger protein 335 [Source:HGNC Symbol;Acc:HGNC:15807] |
| ENSG00000198825 | HGLibA_23356 | 33 | -0.820217724 | INPP5F | inositol polyphosphate-5-phosphatase F [Source:HGNC Symbol;Acc:HGNC:17054] |
| ENSG00000198925 | HGLibA_03505 | 33 | -0.820217724 | ATG9A | autophagy related 9A [Source:HGNC Symbol;Acc:HGNC:22408] |
| ENSG00000203661 | HGLibA_33887 | 33 | -0.820217724 | OR2T5 | olfactory receptor family 2 subfamily T member 5 [Source:HGNC Symbol;Acc:HGNC:15017] |
| ENSG00000203756 | HGLibA_50297 | 33 | -0.820217724 | TMEM244 | transmembrane protein 244 [Source:HGNC Symbol;Acc:HGNC:21571] |
| ENSG00000204021 | HGLibA_26625 | 33 | -0.820217724 | LIPK | lipase family member K [Source:HGNC Symbol;Acc:HGNC:23444] |
| ENSG00000204482 | HGLibA_27573 | 33 | -0.820217724 | LST1 | leukocyte specific transcript 1 [Source:HGNC Symbol;Acc:HGNC:14189] |
| ENSG00000204653 | HGLibA_03344 | 33 | -0.820217724 | ASPDH | aspartate dehydrogenase domain containing [Source:HGNC Symbol;Acc:HGNC:33856] |
| ENSG00000204711 | HGLibA_06679 | 33 | -0.820217724 | C9orf135 | chromosome 9 open reading frame 135 [Source:HGNC Symbol;Acc:HGNC:31422] |
| ENSG00000204764 | HGLibA_40197 | 33 | -0.820217724 | RANBP17 | RAN binding protein 17 [Source:HGNC Symbol;Acc:HGNC:14428] |
| ENSG00000205809 | HGLibA_25288 | 33 | -0.820217724 | KLRC2 | killer cell lectin like receptor C2 [Source:HGNC Symbol;Acc:HGNC:6375] |
| ENSG00000205864 | HGLibA_25802 | 33 | -0.820217724 | KRTAP5-6 | keratin associated protein 5-6 [Source:HGNC Symbol;Acc:HGNC:23600] |
| ENSG00000206150 | HGLibA_41399 | 33 | -0.820217724 | RNASE13 | ribonuclease A family member 13 (inactive) [Source:HGNC Symbol;Acc:HGNC:25285] |
| ENSG00000223611 | HGLibA_47812 | 33 | -0.820217724 | SUPT20HL2 | SUPT20H like 2 [Source:HGNC Symbol;Acc:HGNC:31797] |
| ENSG00000224089 | HGLibA_11519 | 33 | -0.820217724 | CT47A10 | member A10 [Source:HGNC Symbol;Acc:HGNC:33291]" |
| ENSG00000224982 | HGLibA_50265 | 33 | -0.820217724 | TMEM233 | transmembrane protein 233 [Source:HGNC Symbol;Acc:HGNC:37219] |
| ENSG00000235268 | HGLibA_24518 | 33 | -0.820217724 | KDM4E | lysine demethylase 4E [Source:HGNC Symbol;Acc:HGNC:37098] |
| ENSG00000237440 | HGLibA_56656 | 33 | -0.820217724 | ZNF737 | zinc finger protein 737 [Source:HGNC Symbol;Acc:HGNC:32468] |
| ENSG00000237515 | HGLibA_44055 | 33 | -0.820217724 | SHISA9 | shisa family member 9 [Source:HGNC Symbol;Acc:HGNC:37231] |
| ENSG00000242441 | HGLibA_20522 | 33 | -0.820217724 | GTF2A1L | general transcription factor IIA subunit 1 like [Source:HGNC Symbol;Acc:HGNC:30727] |
| ENSG00000242574 | HGLibA_21605 | 33 | -0.820217724 | HLA-DMB | DM beta [Source:HGNC Symbol;Acc:HGNC:4935]" |
| ENSG00000243480 | HGLibA_01850 | 33 | -0.820217724 | AMY2A | alpha 2A (pancreatic) [Source:HGNC Symbol;Acc:HGNC:477]" |
| ENSG00000244754 | HGLibA_30959 | 33 | -0.820217724 | N4BP2L2 | NEDD4 binding protein 2 like 2 [Source:HGNC Symbol;Acc:HGNC:26916] |
| ENSG00000251623 | HGLibA_16171 | 33 | -0.820217724 | FAM153B | - |
| ENSG00000253846 | HGLibA_35521 | 33 | -0.820217724 | PCDHGA10 | 10 [Source:HGNC Symbol;Acc:HGNC:8697]" |
| ENSG00000256087 | HGLibA_55985 | 33 | -0.820217724 | ZNF432 | zinc finger protein 432 [Source:HGNC Symbol;Acc:HGNC:20810] |
| ENSG00000256683 | HGLibA_55859 | 33 | -0.820217724 | ZNF350 | zinc finger protein 350 [Source:HGNC Symbol;Acc:HGNC:16656] |
| ENSG00000259024 | HGLibA_52365 | 33 | -0.820217724 | TVP23C-CDRT4 | TVP23C-CDRT4 readthrough [Source:HGNC Symbol;Acc:HGNC:42961] |
| ENSG00000268738 | HGLibA_22238 | 33 | -0.820217724 | HSFX2 | X-linked 2 [Source:HGNC Symbol;Acc:HGNC:32701]" |
| ENSG00000274274 | HGLibA_18464 | 33 | -0.820217724 | GAGE13 | G antigen 13 [Source:HGNC Symbol;Acc:HGNC:29081] |
| FAM150B | HGLibA_16159 | 33 | -0.820217724 | FAM150B | - |
| FAM212B | HGLibA_16468 | 33 | -0.820217724 | FAM212B | - |
| FAM64A | HGLibA_16666 | 33 | -0.820217724 | FAM64A | - |
| GPR116 | HGLibA_19865 | 33 | -0.820217724 | GPR116 | - |
| HIST1H2AA | HGLibA_21375 | 33 | -0.820217724 | HIST1H2AA | - |
| hsa-let-7a-3 | HGLibA_57104 | 33 | -0.820217724 | hsa-let-7a-3 | - |
| hsa-mir-1303 | HGLibA_57726 | 33 | -0.820217724 | hsa-mir-1303 | - |
| hsa-mir-190b | HGLibA_58040 | 33 | -0.820217724 | hsa-mir-190b | - |
| hsa-mir-1972-2 | HGLibA_58112 | 33 | -0.820217724 | hsa-mir-1972-2 | - |
| hsa-mir-2113 | HGLibA_58225 | 33 | -0.820217724 | hsa-mir-2113 | - |
| hsa-mir-3120 | HGLibA_58532 | 33 | -0.820217724 | hsa-mir-3120 | - |
| hsa-mir-3185 | HGLibA_58839 | 33 | -0.820217724 | hsa-mir-3185 | - |
| hsa-mir-329-1 | HGLibA_58980 | 33 | -0.820217724 | hsa-mir-329-1 | - |
| hsa-mir-3661 | HGLibA_59197 | 33 | -0.820217724 | hsa-mir-3661 | - |
| hsa-mir-3688-1 | HGLibA_59307 | 33 | -0.820217724 | hsa-mir-3688-1 | - |
| hsa-mir-3921 | HGLibA_59559 | 33 | -0.820217724 | hsa-mir-3921 | - |
| hsa-mir-3927 | HGLibA_59583 | 33 | -0.820217724 | hsa-mir-3927 | - |
| hsa-mir-4264 | HGLibA_59762 | 33 | -0.820217724 | hsa-mir-4264 | - |
| hsa-mir-4274 | HGLibA_59801 | 33 | -0.820217724 | hsa-mir-4274 | - |
| hsa-mir-4321 | HGLibA_60006 | 33 | -0.820217724 | hsa-mir-4321 | - |
| hsa-mir-4330 | HGLibA_60048 | 33 | -0.820217724 | hsa-mir-4330 | - |
| hsa-mir-4423 | HGLibA_60081 | 33 | -0.820217724 | hsa-mir-4423 | - |
| hsa-mir-4461 | HGLibA_60251 | 33 | -0.820217724 | hsa-mir-4461 | - |
| hsa-mir-4517 | HGLibA_60512 | 33 | -0.820217724 | hsa-mir-4517 | - |
| hsa-mir-4652 | HGLibA_60714 | 33 | -0.820217724 | hsa-mir-4652 | - |

| | | | | | |
|---|---|---|---|---|---|
| hsa-mir-4728 | HGLibA_61011 | 33 | -0.820217724 | hsa-mir-4728 | - |
| hsa-mir-4767 | HGLibA_61163 | 33 | -0.820217724 | hsa-mir-4767 | - |
| hsa-mir-4777 | HGLibA_61214 | 33 | -0.820217724 | hsa-mir-4777 | - |
| hsa-mir-494 | HGLibA_61375 | 33 | -0.820217724 | hsa-mir-494 | - |
| hsa-mir-518a-1 | HGLibA_61651 | 33 | -0.820217724 | hsa-mir-518a-1 | - |
| hsa-mir-519a-2 | HGLibA_61716 | 33 | -0.820217724 | hsa-mir-519a-2 | - |
| hsa-mir-550b-1 | HGLibA_62111 | 33 | -0.820217724 | hsa-mir-550b-1 | - |
| hsa-mir-567 | HGLibA_62228 | 33 | -0.820217724 | hsa-mir-567 | - |
| hsa-mir-585 | HGLibA_62437 | 33 | -0.820217724 | hsa-mir-585 | - |
| hsa-mir-611 | HGLibA_62636 | 33 | -0.820217724 | hsa-mir-611 | - |
| hsa-mir-642a | HGLibA_62806 | 33 | -0.820217724 | hsa-mir-642a | - |
| hsa-mir-6502 | HGLibA_62859 | 33 | -0.820217724 | hsa-mir-6502 | - |
| hsa-mir-6511a-1 | HGLibA_62899 | 33 | -0.820217724 | hsa-mir-6511a-1 | - |
| hsa-mir-659 | HGLibA_62970 | 33 | -0.820217724 | hsa-mir-659 | - |
| hsa-mir-670 | HGLibA_63011 | 33 | -0.820217724 | hsa-mir-670 | - |
| hsa-mir-6735 | HGLibA_63092 | 33 | -0.820217724 | hsa-mir-6735 | - |
| hsa-mir-6840 | HGLibA_63537 | 33 | -0.820217724 | hsa-mir-6840 | - |
| hsa-mir-6857 | HGLibA_63599 | 33 | -0.820217724 | hsa-mir-6857 | - |
| hsa-mir-769 | HGLibA_63936 | 33 | -0.820217724 | hsa-mir-769 | - |
| KIAA0196 | HGLibA_24603 | 33 | -0.820217724 | KIAA0196 | - |
| KIAA2018 | HGLibA_24842 | 33 | -0.820217724 | KIAA2018 | - |
| LOC100132146 | HGLibA_26803 | 33 | -0.820217724 | LOC100132146 | - |
| LOC100506422 | HGLibA_26860 | 33 | -0.820217724 | LOC100506422 | - |
| PCNXL3 | HGLibA_35650 | 33 | -0.820217724 | PCNXL3 | - |
| PLCXD1_Y | HGLibA_37022 | 33 | -0.820217724 | PLCXD1_Y | - |
| PPAP2A | HGLibA_37817 | 33 | -0.820217724 | PPAP2A | - |
| PPIAL4B | HGLibA_37900 | 33 | -0.820217724 | PPIAL4B | - |
| PRUNE | HGLibA_38986 | 33 | -0.820217724 | PRUNE | - |
| PTPLB | HGLibA_39423 | 33 | -0.820217724 | PTPLB | - |
| TMEM110-MUSTN1 | HGLibA_49877 | 33 | -0.820217724 | TMEM110-MUSTN1 | - |
| WBSCR28 | HGLibA_54001 | 33 | -0.820217724 | WBSCR28 | - |
| XAGE1E | HGLibA_54477 | 33 | -0.820217724 | XAGE1E | - |
| ZCCHC5 | HGLibA_55039 | 33 | -0.820217724 | ZCCHC5 | - |
| AARS | HGLibA_00068 | 32 | -0.830168761 | AARS | - |
| ATP5F1 | HGLibA_03652 | 32 | -0.830168761 | ATP5F1 | - |
| C16orf97 | HGLibA_05514 | 32 | -0.830168761 | C16orf97 | - |
| C19orf70 | HGLibA_05699 | 32 | -0.830168761 | C19orf70 | - |
| C6orf57 | HGLibA_06498 | 32 | -0.830168761 | C6orf57 | - |
| C9orf3 | HGLibA_06723 | 32 | -0.830168761 | C9orf3 | - |
| CCDC101 | HGLibA_07505 | 32 | -0.830168761 | CCDC101 | - |
| CXorf64 | HGLibA_11956 | 32 | -0.830168761 | CXorf64 | - |
| ENSG00000001460 | HGLibA_47557 | 32 | -0.830168761 | STPG1 | sperm tail PG-rich repeat containing 1 [Source:HGNC Symbol;Acc:HGNC:28070] |
| ENSG00000004779 | HGLibA_31478 | 32 | -0.830168761 | NDUFAB1 | NADH:ubiquinone oxidoreductase subunit AB1 [Source:HGNC Symbol;Acc:HGNC:7694] |
| ENSG00000006468 | HGLibA_15648 | 32 | -0.830168761 | ETV1 | ETS variant 1 [Source:HGNC Symbol;Acc:HGNC:3490] |
| ENSG00000006607 | HGLibA_16878 | 32 | -0.830168761 | FARP2 | ARH/RhoGEF and pleckstrin domain protein 2 [Source:HGNC Symbol;Acc:HGNC:16460]" |
| ENSG00000009830 | HGLibA_37672 | 32 | -0.830168761 | POMT2 | protein O-mannosyltransferase 2 [Source:HGNC Symbol;Acc:HGNC:19743] |
| ENSG00000010292 | HGLibA_31263 | 32 | -0.830168761 | NCAPD2 | non-SMC condensin I complex subunit D2 [Source:HGNC Symbol;Acc:HGNC:24305] |
| ENSG00000011083 | HGLibA_45337 | 32 | -0.830168761 | SLC6A7 | solute carrier family 6 member 7 [Source:HGNC Symbol;Acc:HGNC:11054] |
| ENSG00000024526 | HGLibA_12973 | 32 | -0.830168761 | DEPDC1 | DEP domain containing 1 [Source:HGNC Symbol;Acc:HGNC:22949] |
| ENSG00000025423 | HGLibA_22197 | 32 | -0.830168761 | HSD17B6 | hydroxysteroid 17-beta dehydrogenase 6 [Source:HGNC Symbol;Acc:HGNC:23316] |
| ENSG00000034693 | HGLibA_36114 | 32 | -0.830168761 | PEX3 | peroxisomal biogenesis factor 3 [Source:HGNC Symbol;Acc:HGNC:8858] |
| ENSG00000040608 | HGLibA_42413 | 32 | -0.830168761 | RTN4R | reticulon 4 receptor [Source:HGNC Symbol;Acc:HGNC:18601] |
| ENSG00000055813 | HGLibA_07893 | 32 | -0.830168761 | CCDC85A | coiled-coil domain containing 85A [Source:HGNC Symbol;Acc:HGNC:29400] |
| ENSG00000068120 | HGLibA_10459 | 32 | -0.830168761 | COASY | Coenzyme A synthase [Source:HGNC Symbol;Acc:HGNC:29932] |
| ENSG00000070669 | HGLibA_03334 | 32 | -0.830168761 | ASNS | asparagine synthetase (glutamine-hydrolyzing) [Source:HGNC Symbol;Acc:HGNC:753] |
| ENSG00000070778 | HGLibA_39458 | 32 | -0.830168761 | PTPN21 | non-receptor type 21 [Source:HGNC Symbol;Acc:HGNC:9651]" |
| ENSG00000071537 | HGLibA_43276 | 32 | -0.830168761 | SEL1L | ERAD E3 ligase adaptor subunit [Source:HGNC Symbol;Acc:HGNC:10717]" |
| ENSG00000075643 | HGLibA_29608 | 32 | -0.830168761 | MOCOS | molybdenum cofactor sulfurase [Source:HGNC Symbol;Acc:HGNC:18234] |
| ENSG00000079101 | HGLibA_10164 | 32 | -0.830168761 | CLUL1 | clusterin like 1 [Source:HGNC Symbol;Acc:HGNC:2096] |
| ENSG00000081818 | HGLibA_35498 | 32 | -0.830168761 | PCDHB4 | protocadherin beta 4 [Source:HGNC Symbol;Acc:HGNC:8689] |
| ENSG00000083290 | HGLibA_52972 | 32 | -0.830168761 | ULK2 | unc-51 like autophagy activating kinase 2 [Source:HGNC Symbol;Acc:HGNC:13480] |
| ENSG00000087191 | HGLibA_39143 | 32 | -0.830168761 | PSMC5 | ATPase 5 [Source:HGNC Symbol;Acc:HGNC:9552]" |
| ENSG00000089169 | HGLibA_41845 | 32 | -0.830168761 | RPH3A | rabphilin 3A [Source:HGNC Symbol;Acc:HGNC:17056] |
| ENSG00000090686 | HGLibA_53384 | 32 | -0.830168761 | USP48 | ubiquitin specific peptidase 48 [Source:HGNC Symbol;Acc:HGNC:18533] |
| ENSG00000091127 | HGLibA_39603 | 32 | -0.830168761 | PUS7 | pseudouridine synthase 7 [Source:HGNC Symbol;Acc:HGNC:26033] |
| ENSG00000095110 | HGLibA_33150 | 32 | -0.830168761 | NXPE1 | neurexophilin and PC-esterase domain family member 1 [Source:HGNC Symbol;Acc:HGNC:28527] |
| ENSG00000099984 | HGLibA_20500 | 32 | -0.830168761 | GSTT2 | glutathione S-transferase theta 2 (gene/pseudogene) [Source:HGNC Symbol;Acc:HGNC:4642] |
| ENSG00000100403 | HGLibA_54985 | 32 | -0.830168761 | ZC3H7B | zinc finger CCCH-type containing 7B [Source:HGNC Symbol;Acc:HGNC:30869] |
| ENSG00000100987 | HGLibA_53875 | 32 | -0.830168761 | VSX1 | visual system homeobox 1 [Source:HGNC Symbol;Acc:HGNC:12723] |
| ENSG00000101138 | HGLibA_11484 | 32 | -0.830168761 | CSTF1 | cleavage stimulation factor subunit 1 [Source:HGNC Symbol;Acc:HGNC:2483] |
| ENSG00000101290 | HGLibA_08884 | 32 | -0.830168761 | CDS2 | CDP-diacylglycerol synthase 2 [Source:HGNC Symbol;Acc:HGNC:1801] |
| ENSG00000101940 | HGLibA_54032 | 32 | -0.830168761 | WDR13 | WD repeat domain 13 [Source:HGNC Symbol;Acc:HGNC:14352] |
| ENSG00000102057 | HGLibA_24174 | 32 | -0.830168761 | KCND1 | potassium voltage-gated channel subfamily D member 1 [Source:HGNC Symbol;Acc:HGNC:6237] |
| ENSG00000102349 | HGLibA_25085 | 32 | -0.830168761 | KLF8 | Kruppel like factor 8 [Source:HGNC Symbol;Acc:HGNC:6351] |
| ENSG00000103591 | HGLibA_00047 | 32 | -0.830168761 | AAGAB | alpha and gamma adaptin binding protein [Source:HGNC Symbol;Acc:HGNC:25662] |
| ENSG00000104611 | HGLibA_43899 | 32 | -0.830168761 | SH2D4A | SH2 domain containing 4A [Source:HGNC Symbol;Acc:HGNC:26102] |
| ENSG00000105088 | HGLibA_33346 | 32 | -0.830168761 | OLFM2 | olfactomedin 2 [Source:HGNC Symbol;Acc:HGNC:17189] |
| ENSG00000105204 | HGLibA_14273 | 32 | -0.830168761 | DYRK1B | dual specificity tyrosine phosphorylation regulated kinase 1B [Source:HGNC Symbol;Acc:HGNC:3092] |
| ENSG00000105372 | HGLibA_42095 | 32 | -0.830168761 | RPS19 | ribosomal protein S19 [Source:HGNC Symbol;Acc:HGNC:10402] |
| ENSG00000105419 | HGLibA_28876 | 32 | -0.830168761 | MEIS3 | Meis homeobox 3 [Source:HGNC Symbol;Acc:HGNC:29537] |
| ENSG00000105447 | HGLibA_20385 | 32 | -0.830168761 | GRWD1 | glutamate rich WD repeat containing 1 [Source:HGNC Symbol;Acc:HGNC:21270] |
| ENSG00000108946 | HGLibA_38521 | 32 | -0.830168761 | PRKAR1A | protein kinase cAMP-dependent type I regulatory subunit alpha [Source:HGNC Symbol;Acc:HGNC:9388] |
| ENSG00000109475 | HGLibA_41944 | 32 | -0.830168761 | RPL34 | ribosomal protein L34 [Source:HGNC Symbol;Acc:HGNC:10340] |
| ENSG00000109743 | HGLibA_04882 | 32 | -0.830168761 | BST1 | bone marrow stromal cell antigen 1 [Source:HGNC Symbol;Acc:HGNC:1118] |
| ENSG00000110844 | HGLibA_38738 | 32 | -0.830168761 | PRPF40B | pre-mRNA processing factor 40 homolog B [Source:HGNC Symbol;Acc:HGNC:25031] |
| ENSG00000110887 | HGLibA_12310 | 32 | -0.830168761 | DAO | D-amino acid oxidase [Source:HGNC Symbol;Acc:HGNC:2671] |
| ENSG00000111962 | HGLibA_53425 | 32 | -0.830168761 | UST | uronyl 2-sulfotransferase [Source:HGNC Symbol;Acc:HGNC:17223] |
| ENSG00000112303 | HGLibA_53712 | 32 | -0.830168761 | VNN2 | vanin 2 [Source:HGNC Symbol;Acc:HGNC:12706] |
| ENSG00000113739 | HGLibA_47418 | 32 | -0.830168761 | STC2 | stanniocalcin 2 [Source:HGNC Symbol;Acc:HGNC:11374] |
| ENSG00000114423 | HGLibA_07398 | 32 | -0.830168761 | CBLB | Cbl proto-oncogene B [Source:HGNC Symbol;Acc:HGNC:1542] |
| ENSG00000115392 | HGLibA_16857 | 32 | -0.830168761 | FANCL | FA complementation group L [Source:HGNC Symbol;Acc:HGNC:20748] |
| ENSG00000115756 | HGLibA_22018 | 32 | -0.830168761 | HPCAL1 | hippocalcin like 1 [Source:HGNC Symbol;Acc:HGNC:5145] |
| ENSG00000115993 | HGLibA_51278 | 32 | -0.830168761 | TRAK2 | trafficking kinesin protein 2 [Source:HGNC Symbol;Acc:HGNC:13206] |
| ENSG00000118432 | HGLibA_10375 | 32 | -0.830168761 | CNR1 | cannabinoid receptor 1 [Source:HGNC Symbol;Acc:HGNC:2159] |
| ENSG00000119285 | HGLibA_21067 | 32 | -0.830168761 | HEATR1 | HEAT repeat containing 1 [Source:HGNC Symbol;Acc:HGNC:25517] |
| ENSG00000120088 | HGLibA_11140 | 32 | -0.830168761 | CRHR1 | corticotropin releasing hormone receptor 1 [Source:HGNC Symbol;Acc:HGNC:2357] |
| ENSG00000120211 | HGLibA_23386 | 32 | -0.830168761 | INSL4 | insulin like 4 [Source:HGNC Symbol;Acc:HGNC:6087] |
| ENSG00000120314 | HGLibA_54127 | 32 | -0.830168761 | WDR55 | WD repeat domain 55 [Source:HGNC Symbol;Acc:HGNC:25971] |
| ENSG00000120318 | HGLibA_02631 | 32 | -0.830168761 | ARAP3 | ankyrin repeat and PH domain 3 [Source:HGNC Symbol;Acc:HGNC:24097]" |
| ENSG00000120690 | HGLibA_14891 | 32 | -0.830168761 | ELF1 | E74 like ETS transcription factor 1 [Source:HGNC Symbol;Acc:HGNC:3316] |
| ENSG00000123975 | HGLibA_09791 | 32 | -0.830168761 | CKS2 | CDC28 protein kinase regulatory subunit 2 [Source:HGNC Symbol;Acc:HGNC:2000] |
| ENSG00000124613 | HGLibA_55905 | 32 | -0.830168761 | ZNF391 | zinc finger protein 391 [Source:HGNC Symbol;Acc:HGNC:18779] |
| ENSG00000125351 | HGLibA_53066 | 32 | -0.830168761 | UPF3B | regulator of nonsense mediated mRNA decay [Source:HGNC Symbol;Acc:HGNC:20439]" |
| ENSG00000125414 | HGLibA_30721 | 32 | -0.830168761 | MYH2 | myosin heavy chain 2 [Source:HGNC Symbol;Acc:HGNC:7572] |
| ENSG00000125450 | HGLibA_33069 | 32 | -0.830168761 | NUP85 | nucleoporin 85 [Source:HGNC Symbol;Acc:HGNC:8734] |
| ENSG00000125864 | HGLibA_04454 | 32 | -0.830168761 | BFSP1 | beaded filament structural protein 1 [Source:HGNC Symbol;Acc:HGNC:1040] |
| ENSG00000125991 | HGLibA_15473 | 32 | -0.830168761 | ERGIC3 | ERGIC and golgi 3 [Source:HGNC Symbol;Acc:HGNC:15927] |
| ENSG00000126778 | HGLibA_44244 | 32 | -0.830168761 | SIX1 | SIX homeobox 1 [Source:HGNC Symbol;Acc:HGNC:10887] |
| ENSG00000126814 | HGLibA_51692 | 32 | -0.830168761 | TRMT5 | tRNA methyltransferase 5 [Source:HGNC Symbol;Acc:HGNC:23141] |
| ENSG00000127995 | HGLibA_07282 | 32 | -0.830168761 | CASD1 | CAS1 domain containing 1 [Source:HGNC Symbol;Acc:HGNC:16014] |
| ENSG00000128513 | HGLibA_37713 | 32 | -0.830168761 | POT1 | protection of telomeres 1 [Source:HGNC Symbol;Acc:HGNC:17284] |
| ENSG00000128578 | HGLibA_47590 | 32 | -0.830168761 | STRIP2 | striatin interacting protein 2 [Source:HGNC Symbol;Acc:HGNC:22209] |
| ENSG00000129158 | HGLibA_43477 | 32 | -0.830168761 | SERGEF | secretion regulating guanine nucleotide exchange factor [Source:HGNC Symbol;Acc:HGNC:17499] |
| ENSG00000130347 | HGLibA_42411 | 32 | -0.830168761 | RTN4IP1 | reticulon 4 interacting protein 1 [Source:HGNC Symbol;Acc:HGNC:18647] |
| ENSG00000130656 | HGLibA_20943 | 32 | -0.830168761 | HBZ | hemoglobin subunit zeta [Source:HGNC Symbol;Acc:HGNC:4835] |
| ENSG00000130711 | HGLibA_38371 | 32 | -0.830168761 | PRDM12 | PR/SET domain 12 [Source:HGNC Symbol;Acc:HGNC:13997] |
| ENSG00000130768 | HGLibA_45818 | 32 | -0.830168761 | SMPDL3B | sphingomyelin phosphodiesterase acid like 3B [Source:HGNC Symbol;Acc:HGNC:21416] |
| ENSG00000130844 | HGLibA_55816 | 32 | -0.830168761 | ZNF331 | zinc finger protein 331 [Source:HGNC Symbol;Acc:HGNC:15489] |
| ENSG00000131165 | HGLibA_09473 | 32 | -0.830168761 | CHMP1A | charged multivesicular body protein 1A [Source:HGNC Symbol;Acc:HGNC:8740] |
| ENSG00000131469 | HGLibA_41920 | 32 | -0.830168761 | RPL27 | ribosomal protein L27 [Source:HGNC Symbol;Acc:HGNC:10328] |
| ENSG00000131480 | HGLibA_02271 | 32 | -0.830168761 | AOC2 | copper containing 2 [Source:HGNC Symbol;Acc:HGNC:549]" |
| ENSG00000132361 | HGLibA_10160 | 32 | -0.830168761 | CLUH | clustered mitochondria homolog [Source:HGNC Symbol;Acc:HGNC:29094] |
| ENSG00000132446 | HGLibA_18123 | 32 | -0.830168761 | FTHL17 | ferritin heavy chain like 17 [Source:HGNC Symbol;Acc:HGNC:3987] |
| ENSG00000132535 | HGLibA_13333 | 32 | -0.830168761 | DLG4 | discs large MAGUK scaffold protein 4 [Source:HGNC Symbol;Acc:HGNC:2903] |
| ENSG00000132792 | HGLibA_11655 | 32 | -0.830168761 | CTNNBL1 | catenin beta like 1 [Source:HGNC Symbol;Acc:HGNC:15879] |
| ENSG00000132837 | HGLibA_13406 | 32 | -0.830168761 | DMGDH | dimethylglycine dehydrogenase [Source:HGNC Symbol;Acc:HGNC:24475] |
| ENSG00000133858 | HGLibA_55167 | 32 | -0.830168761 | ZFC3H1 | zinc finger C3H1-type containing [Source:HGNC Symbol;Acc:HGNC:28328] |
| ENSG00000134086 | HGLibA_53647 | 32 | -0.830168761 | VHL | von Hippel-Lindau tumor suppressor [Source:HGNC Symbol;Acc:HGNC:12687] |
| ENSG00000134108 | HGLibA_03000 | 32 | -0.830168761 | ARL8B | ADP ribosylation factor like GTPase 8B [Source:HGNC Symbol;Acc:HGNC:25564] |
| ENSG00000134184 | HGLibA_20471 | 32 | -0.830168761 | GSTM1 | glutathione S-transferase mu 1 [Source:HGNC Symbol;Acc:HGNC:4632] |
| ENSG00000135070 | HGLibA_23646 | 32 | -0.830168761 | ISCA1 | iron-sulfur cluster assembly 1 [Source:HGNC Symbol;Acc:HGNC:28660] |
| ENSG00000135903 | HGLibA_35302 | 32 | -0.830168761 | PAX3 | paired box 3 [Source:HGNC Symbol;Acc:HGNC:8617] |
| ENSG00000136449 | HGLibA_30671 | 32 | -0.830168761 | MYCBPAP | MYCBP associated protein [Source:HGNC Symbol;Acc:HGNC:19677] |
| ENSG00000136521 | HGLibA_31522 | 32 | -0.830168761 | NDUFB5 | NADH:ubiquinone oxidoreductase subunit B5 [Source:HGNC Symbol;Acc:HGNC:7700] |
| ENSG00000136695 | HGLibA_23153 | 32 | -0.830168761 | IL36RN | interleukin 36 receptor antagonist [Source:HGNC Symbol;Acc:HGNC:15561] |

| | | | | | |
|---|---|---|---|---|---|
| ENSG00000138029 | HGLibA_20810 | 32 | -0.830168761 | HADHB | hydroxyacyl-CoA dehydrogenase trifunctional multienzyme complex subunit beta [Source:HGNC Symbol;Acc:HGNC:4803] |
| ENSG00000138472 | HGLibA_20639 | 32 | -0.830168761 | GUCA1C | guanylate cyclase activator 1C [Source:HGNC Symbol;Acc:HGNC:4680] |
| ENSG00000138623 | HGLibA_43376 | 32 | -0.830168761 | SEMA7A | semaphorin 7A (John Milton Hagen blood group) [Source:HGNC Symbol;Acc:HGNC:10741] |
| ENSG00000139178 | HGLibA_05971 | 32 | -0.830168761 | C1RL | complement C1r subcomponent like [Source:HGNC Symbol;Acc:HGNC:21265] |
| ENSG00000140279 | HGLibA_14101 | 32 | -0.830168761 | DUOX2 | dual oxidase 2 [Source:HGNC Symbol;Acc:HGNC:13273] |
| ENSG00000140694 | HGLibA_35209 | 32 | -0.830168761 | PARN | poly(A)-specific ribonuclease [Source:HGNC Symbol;Acc:HGNC:8609] |
| ENSG00000142731 | HGLibA_37196 | 32 | -0.830168761 | PLK4 | polo like kinase 4 [Source:HGNC Symbol;Acc:HGNC:11397] |
| ENSG00000143319 | HGLibA_23662 | 32 | -0.830168761 | ISG20L2 | interferon stimulated exonuclease gene 20 like 2 [Source:HGNC Symbol;Acc:HGNC:25745] |
| ENSG00000143575 | HGLibA_20903 | 32 | -0.830168761 | HAX1 | HCLS1 associated protein X-1 [Source:HGNC Symbol;Acc:HGNC:16915] |
| ENSG00000143595 | HGLibA_02580 | 32 | -0.830168761 | AQP10 | aquaporin 10 [Source:HGNC Symbol;Acc:HGNC:16029] |
| ENSG00000143771 | HGLibA_10293 | 32 | -0.830168761 | CNIH4 | cornichon family AMPA receptor auxiliary protein 4 [Source:HGNC Symbol;Acc:HGNC:25013] |
| ENSG00000144647 | HGLibA_37661 | 32 | -0.830168761 | POMGNT2 | 4-) [Source:HGNC Symbol;Acc:HGNC:25902]" |
| ENSG00000145949 | HGLibA_30792 | 32 | -0.830168761 | MYLK4 | myosin light chain kinase family member 4 [Source:HGNC Symbol;Acc:HGNC:27972] |
| ENSG00000146433 | HGLibA_50099 | 32 | -0.830168761 | TMEM181 | transmembrane protein 181 [Source:HGNC Symbol;Acc:HGNC:20958] |
| ENSG00000147130 | HGLibA_55397 | 32 | -0.830168761 | ZMYM3 | zinc finger MYM-type containing 3 [Source:HGNC Symbol;Acc:HGNC:13054] |
| ENSG00000147183 | HGLibA_11032 | 32 | -0.830168761 | CPXCR1 | candidate 1 [Source:HGNC Symbol;Acc:HGNC:2332]" |
| ENSG00000147457 | HGLibA_09502 | 32 | -0.830168761 | CHMP7 | charged multivesicular body protein 7 [Source:HGNC Symbol;Acc:HGNC:28439] |
| ENSG00000149016 | HGLibA_52355 | 32 | -0.830168761 | TUT1 | U6 snRNA-specific [Source:HGNC Symbol;Acc:HGNC:26184]" |
| ENSG00000149201 | HGLibA_07880 | 32 | -0.830168761 | CCDC81 | coiled-coil domain containing 81 [Source:HGNC Symbol;Acc:HGNC:26281] |
| ENSG00000149483 | HGLibA_49954 | 32 | -0.830168761 | TMEM138 | transmembrane protein 138 [Source:HGNC Symbol;Acc:HGNC:26944] |
| ENSG00000149554 | HGLibA_09420 | 32 | -0.830168761 | CHEK1 | checkpoint kinase 1 [Source:HGNC Symbol;Acc:HGNC:1925] |
| ENSG00000150316 | HGLibA_11811 | 32 | -0.830168761 | CWC15 | CWC15 spliceosome associated protein homolog [Source:HGNC Symbol;Acc:HGNC:26939] |
| ENSG00000150753 | HGLibA_08205 | 32 | -0.830168761 | CCT5 | chaperonin containing TCP1 subunit 5 [Source:HGNC Symbol;Acc:HGNC:1618] |
| ENSG00000151012 | HGLibA_45352 | 32 | -0.830168761 | SLC7A11 | solute carrier family 7 member 11 [Source:HGNC Symbol;Acc:HGNC:11059] |
| ENSG00000151164 | HGLibA_40110 | 32 | -0.830168761 | RAD9B | RAD9 checkpoint clamp component B [Source:HGNC Symbol;Acc:HGNC:21700] |
| ENSG00000152595 | HGLibA_28905 | 32 | -0.830168761 | MEPE | matrix extracellular phosphoglycoprotein [Source:HGNC Symbol;Acc:HGNC:13361] |
| ENSG00000153827 | HGLibA_51639 | 32 | -0.830168761 | TRIP12 | thyroid hormone receptor interactor 12 [Source:HGNC Symbol;Acc:HGNC:12306] |
| ENSG00000154719 | HGLibA_29971 | 32 | -0.830168761 | MRPL39 | mitochondrial ribosomal protein L39 [Source:HGNC Symbol;Acc:HGNC:14027] |
| ENSG00000156970 | HGLibA_04991 | 32 | -0.830168761 | BUB1B | BUB1 mitotic checkpoint serine/threonine kinase B [Source:HGNC Symbol;Acc:HGNC:1149] |
| ENSG00000160293 | HGLibA_53551 | 32 | -0.830168761 | VAV2 | vav guanine nucleotide exchange factor 2 [Source:HGNC Symbol;Acc:HGNC:12658] |
| ENSG00000161847 | HGLibA_40401 | 32 | -0.830168761 | RAVER1 | PTB binding 1 [Source:HGNC Symbol;Acc:HGNC:30296]" |
| ENSG00000162298 | HGLibA_48112 | 32 | -0.830168761 | SYVN1 | synoviolin 1 [Source:HGNC Symbol;Acc:HGNC:20738] |
| ENSG00000162434 | HGLibA_23912 | 32 | -0.830168761 | JAK1 | Janus kinase 1 [Source:HGNC Symbol;Acc:HGNC:6190] |
| ENSG00000163462 | HGLibA_51514 | 32 | -0.830168761 | TRIM46 | tripartite motif containing 46 [Source:HGNC Symbol;Acc:HGNC:19019] |
| ENSG00000163467 | HGLibA_51809 | 32 | -0.830168761 | TSACC | TSSK6 activating cochaperone [Source:HGNC Symbol;Acc:HGNC:30636] |
| ENSG00000163519 | HGLibA_51342 | 32 | -0.830168761 | TRAT1 | T cell receptor associated transmembrane adaptor 1 [Source:HGNC Symbol;Acc:HGNC:30698] |
| ENSG00000163554 | HGLibA_46872 | 32 | -0.830168761 | SPTA1 | erythrocytic 1 [Source:HGNC Symbol;Acc:HGNC:11272]" |
| ENSG00000163637 | HGLibA_38474 | 32 | -0.830168761 | PRICKLE2 | prickle planar cell polarity protein 2 [Source:HGNC Symbol;Acc:HGNC:20340] |
| ENSG00000163840 | HGLibA_14087 | 32 | -0.830168761 | DTX3L | deltex E3 ubiquitin ligase 3L [Source:HGNC Symbol;Acc:HGNC:30323] |
| ENSG00000164241 | HGLibA_06418 | 32 | -0.830168761 | C5orf63 | chromosome 5 open reading frame 63 [Source:HGNC Symbol;Acc:HGNC:40051] |
| ENSG00000164256 | HGLibA_38403 | 32 | -0.830168761 | PRDM9 | PR/SET domain 9 [Source:HGNC Symbol;Acc:HGNC:13994] |
| ENSG00000164587 | HGLibA_42074 | 32 | -0.830168761 | RPS14 | ribosomal protein S14 [Source:HGNC Symbol;Acc:HGNC:10387] |
| ENSG00000164683 | HGLibA_21215 | 32 | -0.830168761 | HEY1 | hes related family bHLH transcription factor with YRPW motif 1 [Source:HGNC Symbol;Acc:HGNC:4880] |
| ENSG00000165671 | HGLibA_32746 | 32 | -0.830168761 | NSD1 | nuclear receptor binding SET domain protein 1 [Source:HGNC Symbol;Acc:HGNC:14234] |
| ENSG00000165816 | HGLibA_53899 | 32 | -0.830168761 | VWA2 | von Willebrand factor A domain containing 2 [Source:HGNC Symbol;Acc:HGNC:24709] |
| ENSG00000166173 | HGLibA_26039 | 32 | -0.830168761 | LARP6 | La ribonucleoprotein domain family member 6 [Source:HGNC Symbol;Acc:HGNC:24012] |
| ENSG00000166363 | HGLibA_33471 | 32 | -0.830168761 | OR10A5 | olfactory receptor family 10 subfamily A member 5 [Source:HGNC Symbol;Acc:HGNC:15131] |
| ENSG00000166557 | HGLibA_49808 | 32 | -0.830168761 | TMED3 | transmembrane p24 trafficking protein 3 [Source:HGNC Symbol;Acc:HGNC:28889] |
| ENSG00000166747 | HGLibA_02283 | 32 | -0.830168761 | AP1G1 | adaptor related protein complex 1 subunit gamma 1 [Source:HGNC Symbol;Acc:HGNC:555] |
| ENSG00000166831 | HGLibA_40656 | 32 | -0.830168761 | RBPMS2 | mRNA processing factor 2 [Source:HGNC Symbol;Acc:HGNC:19098]" |
| ENSG00000168002 | HGLibA_37583 | 32 | -0.830168761 | POLR2G | RNA polymerase II subunit G [Source:HGNC Symbol;Acc:HGNC:9194] |
| ENSG00000168269 | HGLibA_17877 | 32 | -0.830168761 | FOXI1 | forkhead box I1 [Source:HGNC Symbol;Acc:HGNC:3815] |
| ENSG00000168488 | HGLibA_03839 | 32 | -0.830168761 | ATXN2L | ataxin 2 like [Source:HGNC Symbol;Acc:HGNC:31326] |
| ENSG00000168995 | HGLibA_44148 | 32 | -0.830168761 | SIGLEC7 | sialic acid binding Ig like lectin 7 [Source:HGNC Symbol;Acc:HGNC:10876] |
| ENSG00000169570 | HGLibA_14075 | 32 | -0.830168761 | DTWD2 | DTW domain containing 2 [Source:HGNC Symbol;Acc:HGNC:19334] |
| ENSG00000170946 | HGLibA_13604 | 32 | -0.830168761 | DNAJC24 | DnaJ heat shock protein family (Hsp40) member C24 [Source:HGNC Symbol;Acc:HGNC:26979] |
| ENSG00000171357 | HGLibA_27631 | 32 | -0.830168761 | LURAP1 | leucine rich adaptor protein 1 [Source:HGNC Symbol;Acc:HGNC:32327] |
| ENSG00000171596 | HGLibA_32213 | 32 | -0.830168761 | NMUR1 | neuromedin U receptor 1 [Source:HGNC Symbol;Acc:HGNC:4518] |
| ENSG00000172339 | HGLibA_01618 | 32 | -0.830168761 | ALG14 | UDP-N-acetylglucosaminyltransferase subunit [Source:HGNC Symbol;Acc:HGNC:28287]" |
| ENSG00000172352 | HGLibA_08908 | 32 | -0.830168761 | CDY1B | chromodomain Y-linked 1B [Source:HGNC Symbol;Acc:HGNC:23920] |
| ENSG00000172379 | HGLibA_03064 | 32 | -0.830168761 | ARNT2 | aryl hydrocarbon receptor nuclear translocator 2 [Source:HGNC Symbol;Acc:HGNC:16876] |
| ENSG00000172748 | HGLibA_56350 | 32 | -0.830168761 | ZNF596 | zinc finger protein 596 [Source:HGNC Symbol;Acc:HGNC:27268] |
| ENSG00000174021 | HGLibA_19529 | 32 | -0.830168761 | GNG5 | G protein subunit gamma 5 [Source:HGNC Symbol;Acc:HGNC:4408] |
| ENSG00000174059 | HGLibA_08357 | 32 | -0.830168761 | CD34 | CD34 molecule [Source:HGNC Symbol;Acc:HGNC:1662] |
| ENSG00000174136 | HGLibA_41017 | 32 | -0.830168761 | RGMB | repulsive guidance molecule BMP co-receptor b [Source:HGNC Symbol;Acc:HGNC:26896] |
| ENSG00000174562 | HGLibA_25250 | 32 | -0.830168761 | KLK15 | kallikrein related peptidase 15 [Source:HGNC Symbol;Acc:HGNC:20453] |
| ENSG00000174963 | HGLibA_55329 | 32 | -0.830168761 | ZIC4 | Zic family member 4 [Source:HGNC Symbol;Acc:HGNC:20393] |
| ENSG00000175387 | HGLibA_45573 | 32 | -0.830168761 | SMAD2 | SMAD family member 2 [Source:HGNC Symbol;Acc:HGNC:6768] |
| ENSG00000175792 | HGLibA_42474 | 32 | -0.830168761 | RUVBL1 | RuvB like AAA ATPase 1 [Source:HGNC Symbol;Acc:HGNC:10474] |
| ENSG00000175899 | HGLibA_00007 | 32 | -0.830168761 | A2M | alpha-2-macroglobulin [Source:HGNC Symbol;Acc:HGNC:7] |
| ENSG00000176619 | HGLibA_26698 | 32 | -0.830168761 | LMNB2 | lamin B2 [Source:HGNC Symbol;Acc:HGNC:6638] |
| ENSG00000176920 | HGLibA_18188 | 32 | -0.830168761 | FUT2 | fucosyltransferase 2 [Source:HGNC Symbol;Acc:HGNC:4013] |
| ENSG00000176971 | HGLibA_17500 | 32 | -0.830168761 | FIBIN | fin bud initiation factor homolog [Source:HGNC Symbol;Acc:HGNC:33747] |
| ENSG00000177239 | HGLibA_28063 | 32 | -0.830168761 | MAN1B1 | mannosidase alpha class 1B member 1 [Source:HGNC Symbol;Acc:HGNC:6823] |
| ENSG00000177485 | HGLibA_54861 | 32 | -0.830168761 | ZBTB33 | zinc finger and BTB domain containing 33 [Source:HGNC Symbol;Acc:HGNC:16682] |
| ENSG00000178038 | HGLibA_01722 | 32 | -0.830168761 | ALS2CL | ALS2 C-terminal like [Source:HGNC Symbol;Acc:HGNC:20605] |
| ENSG00000178381 | HGLibA_55147 | 32 | -0.830168761 | ZFAND2A | zinc finger AN1-type containing 2A [Source:HGNC Symbol;Acc:HGNC:28073] |
| ENSG00000178982 | HGLibA_14794 | 32 | -0.830168761 | EIF3K | eukaryotic translation initiation factor 3 subunit K [Source:HGNC Symbol;Acc:HGNC:24656] |
| ENSG00000179468 | HGLibA_34563 | 32 | -0.830168761 | OR9A2 | olfactory receptor family 9 subfamily A member 2 [Source:HGNC Symbol;Acc:HGNC:15093] |
| ENSG00000179965 | HGLibA_56708 | 32 | -0.830168761 | ZNF771 | zinc finger protein 771 [Source:HGNC Symbol;Acc:HGNC:29653] |
| ENSG00000181789 | HGLibA_10704 | 32 | -0.830168761 | COPG1 | coatomer protein complex subunit gamma 1 [Source:HGNC Symbol;Acc:HGNC:2236] |
| ENSG00000182613 | HGLibA_33898 | 32 | -0.830168761 | OR2V2 | olfactory receptor family 2 subfamily V member 2 [Source:HGNC Symbol;Acc:HGNC:15341] |
| ENSG00000182674 | HGLibA_24159 | 32 | -0.830168761 | KCNB2 | potassium voltage-gated channel subfamily B member 2 [Source:HGNC Symbol;Acc:HGNC:6232] |
| ENSG00000182898 | HGLibA_48806 | 32 | -0.830168761 | TCHHL1 | trichohyalin like 1 [Source:HGNC Symbol;Acc:HGNC:31796] |
| ENSG00000183484 | HGLibA_19887 | 32 | -0.830168761 | GPR132 | G protein-coupled receptor 132 [Source:HGNC Symbol;Acc:HGNC:17482] |
| ENSG00000183640 | HGLibA_25826 | 32 | -0.830168761 | KRTAP8-1 | keratin associated protein 8-1 [Source:HGNC Symbol;Acc:HGNC:18935] |
| ENSG00000184254 | HGLibA_01546 | 32 | -0.830168761 | ALDH1A3 | aldehyde dehydrogenase 1 family member A3 [Source:HGNC Symbol;Acc:HGNC:409] |
| ENSG00000184840 | HGLibA_49829 | 32 | -0.830168761 | TMED9 | transmembrane p24 trafficking protein 9 [Source:HGNC Symbol;Acc:HGNC:24878] |
| ENSG00000185070 | HGLibA_17658 | 32 | -0.830168761 | FLRT2 | fibronectin leucine rich transmembrane protein 2 [Source:HGNC Symbol;Acc:HGNC:3761] |
| ENSG00000185088 | HGLibA_42128 | 32 | -0.830168761 | RPS27L | ribosomal protein S27 like [Source:HGNC Symbol;Acc:HGNC:18476] |
| ENSG00000185437 | HGLibA_43914 | 32 | -0.830168761 | SH3BGR | SH3 domain binding glutamate rich protein [Source:HGNC Symbol;Acc:HGNC:10822] |
| ENSG00000185760 | HGLibA_24378 | 32 | -0.830168761 | KCNQ5 | potassium voltage-gated channel subfamily Q member 5 [Source:HGNC Symbol;Acc:HGNC:6299] |
| ENSG00000185933 | HGLibA_07014 | 32 | -0.830168761 | CALHM1 | calcium homeostasis modulator 1 [Source:HGNC Symbol;Acc:HGNC:23494] |
| ENSG00000185972 | HGLibA_07950 | 32 | -0.830168761 | CCIN | calicin [Source:HGNC Symbol;Acc:HGNC:1568] |
| ENSG00000186174 | HGLibA_04350 | 32 | -0.830168761 | BCL9L | B cell CLL/lymphoma 9 like [Source:HGNC Symbol;Acc:HGNC:23688] |
| ENSG00000186340 | HGLibA_49324 | 32 | -0.830168761 | THBS2 | thrombospondin 2 [Source:HGNC Symbol;Acc:HGNC:11786] |
| ENSG00000186666 | HGLibA_04273 | 32 | -0.830168761 | BCDIN3D | BCDIN3 domain containing RNA methyltransferase [Source:HGNC Symbol;Acc:HGNC:27050] |
| ENSG00000186967 | HGLibA_25671 | 32 | -0.830168761 | KRTAP19-4 | keratin associated protein 19-4 [Source:HGNC Symbol;Acc:HGNC:18939] |
| ENSG00000187857 | HGLibA_34408 | 32 | -0.830168761 | OR6C75 | olfactory receptor family 6 subfamily C member 75 [Source:HGNC Symbol;Acc:HGNC:31304] |
| ENSG00000188152 | HGLibA_33111 | 32 | -0.830168761 | NUTM2G | NUT family member 2G [Source:HGNC Symbol;Acc:HGNC:23449] |
| ENSG00000188266 | HGLibA_22454 | 32 | -0.830168761 | HYKK | hydroxylysine kinase [Source:HGNC Symbol;Acc:HGNC:34403] |
| ENSG00000196156 | HGLibA_25761 | 32 | -0.830168761 | KRTAP4-3 | keratin associated protein 4-3 [Source:HGNC Symbol;Acc:HGNC:18908] |
| ENSG00000196284 | HGLibA_47815 | 32 | -0.830168761 | SUPT3H | SAGA and STAGA complex component [Source:HGNC Symbol;Acc:HGNC:11466]" |
| ENSG00000196535 | HGLibA_30810 | 32 | -0.830168761 | MYO18A | myosin XVIIIA [Source:HGNC Symbol;Acc:HGNC:31104] |
| ENSG00000197265 | HGLibA_20535 | 32 | -0.830168761 | GTF2E2 | general transcription factor IIE subunit 2 [Source:HGNC Symbol;Acc:HGNC:4651] |
| ENSG00000197771 | HGLibA_28632 | 32 | -0.830168761 | MCMBP | minichromosome maintenance complex binding protein [Source:HGNC Symbol;Acc:HGNC:25782] |
| ENSG00000197857 | HGLibA_55999 | 32 | -0.830168761 | ZNF44 | zinc finger protein 44 [Source:HGNC Symbol;Acc:HGNC:13110] |
| ENSG00000198189 | HGLibA_22174 | 32 | -0.830168761 | HSD17B11 | hydroxysteroid 17-beta dehydrogenase 11 [Source:HGNC Symbol;Acc:HGNC:22960] |
| ENSG00000198677 | HGLibA_52126 | 32 | -0.830168761 | TTC37 | tetratricopeptide repeat domain 37 [Source:HGNC Symbol;Acc:HGNC:23639] |
| ENSG00000198805 | HGLibA_37402 | 32 | -0.830168761 | PNP | purine nucleoside phosphorylase [Source:HGNC Symbol;Acc:HGNC:7892] |
| ENSG00000204175 | HGLibA_20142 | 32 | -0.830168761 | GPRIN2 | G protein regulated inducer of neurite outgrowth 2 [Source:HGNC Symbol;Acc:HGNC:23730] |
| ENSG00000204176 | HGLibA_48063 | 32 | -0.830168761 | SYT15 | synaptotagmin 15 [Source:HGNC Symbol;Acc:HGNC:17167] |
| ENSG00000204611 | HGLibA_56396 | 32 | -0.830168761 | ZNF616 | zinc finger protein 616 [Source:HGNC Symbol;Acc:HGNC:28062] |
| ENSG00000205362 | HGLibA_30277 | 32 | -0.830168761 | MT1A | metallothionein 1A [Source:HGNC Symbol;Acc:HGNC:7393] |
| ENSG00000205846 | HGLibA_10004 | 32 | -0.830168761 | CLEC6A | C-type lectin domain containing 6A [Source:HGNC Symbol;Acc:HGNC:14556] |
| ENSG00000213215 | HGLibA_33789 | 32 | -0.830168761 | OR2F1 | olfactory receptor family 2 subfamily F member 1 (gene/pseudogene) [Source:HGNC Symbol;Acc:HGNC:8246] |
| ENSG00000214274 | HGLibA_01891 | 32 | -0.830168761 | ANG | angiogenin [Source:HGNC Symbol;Acc:HGNC:483] |
| ENSG00000214575 | HGLibA_10918 | 32 | -0.830168761 | CPEB1 | cytoplasmic polyadenylation element binding protein 1 [Source:HGNC Symbol;Acc:HGNC:21744] |
| ENSG00000214842 | HGLibA_40085 | 32 | -0.830168761 | RAD51AP2 | RAD51 associated protein 2 [Source:HGNC Symbol;Acc:HGNC:34417] |
| ENSG00000215343 | HGLibA_56593 | 32 | -0.830168761 | ZNF705D | zinc finger protein 705D [Source:HGNC Symbol;Acc:HGNC:33202] |
| ENSG00000221826 | HGLibA_39042 | 32 | -0.830168761 | PSG3 | pregnancy specific beta-1-glycoprotein 3 [Source:HGNC Symbol;Acc:HGNC:9520] |
| ENSG00000221914 | HGLibA_38147 | 32 | -0.830168761 | PPP2R2A | protein phosphatase 2 regulatory subunit Balpha [Source:HGNC Symbol;Acc:HGNC:9304] |
| ENSG00000224420 | HGLibA_00997 | 32 | -0.830168761 | ADM5 | adrenomedullin 5 (putative) [Source:HGNC Symbol;Acc:HGNC:27293] |
| ENSG00000228696 | HGLibA_02954 | 32 | -0.830168761 | ARL17B | ADP ribosylation factor like GTPase 17B [Source:HGNC Symbol;Acc:HGNC:32387] |
| ENSG00000228836 | HGLibA_11511 | 32 | -0.830168761 | CT45A5 | cancer/testis antigen family 45 member A5 [Source:HGNC Symbol;Acc:HGNC:33270] |
| ENSG00000229859 | HGLibA_36187 | 32 | -0.830168761 | PGA3 | group I (pepsinogen A) [Source:HGNC Symbol;Acc:HGNC:8885]" |
| ENSG00000234829 | HGLibA_22672 | 32 | -0.830168761 | IFNA17 | interferon alpha 17 [Source:HGNC Symbol;Acc:HGNC:5422] |
| ENSG00000241106 | HGLibA_21612 | 32 | -0.830168761 | HLA-DOB | DO beta [Source:HGNC Symbol;Acc:HGNC:4937]" |
| ENSG00000253368 | HGLibA_51710 | 32 | -0.830168761 | TRNP1 | TMF1-regulated nuclear protein 1 [Source:HGNC Symbol;Acc:HGNC:34348] |
| ENSG00000257108 | HGLibA_31905 | 32 | -0.830168761 | NHLRC4 | NHL repeat containing 4 [Source:HGNC Symbol;Acc:HGNC:26700] |
| ENSG00000258405 | HGLibA_56298 | 32 | -0.830168761 | ZNF578 | zinc finger protein 578 [Source:HGNC Symbol;Acc:HGNC:26449] |
| ENSG00000260325 | HGLibA_22322 | 32 | -0.830168761 | HSPB9 | heat shock protein family B (small) member 9 [Source:HGNC Symbol;Acc:HGNC:30589] |
| ENSG00000262246 | HGLibA_10790 | 32 | -0.830168761 | CORO7 | coronin 7 [Source:HGNC Symbol;Acc:HGNC:26161] |
| ENSG00000271079 | HGLibA_11570 | 32 | -0.830168761 | CTAGE15 | CTAGE family member 15 [Source:HGNC Symbol;Acc:HGNC:37295] |
| ENSG00000272414 | HGLibA_16621 | 32 | -0.830168761 | FAM47E-STBD1 | FAM47E-STBD1 readthrough [Source:HGNC Symbol;Acc:HGNC:44667] |

| | | | | | |
|---|---|---|---|---|---|
| ENSG00000274391 | HGLibA_51214 | 32 | -0.830168761 | TPTE | transmembrane phosphatase with tensin homology [Source:HGNC Symbol;Acc:HGNC:12023] |
| HIST1H1C | HGLibA_21362 | 32 | -0.830168761 | HIST1H1C | - |
| HIST1H3F | HGLibA_21471 | 32 | -0.830168761 | HIST1H3F | - |
| HIST1H3F | HGLibA_21470 | 32 | -0.830168761 | HIST1H3F | - |
| hsa-mir-10a | HGLibA_57199 | 32 | -0.830168761 | hsa-mir-10a | - |
| hsa-mir-1288 | HGLibA_57617 | 32 | -0.830168761 | hsa-mir-1288 | - |
| hsa-mir-1294 | HGLibA_57652 | 32 | -0.830168761 | hsa-mir-1294 | - |
| hsa-mir-1302-8 | HGLibA_57721 | 32 | -0.830168761 | hsa-mir-1302-8 | - |
| hsa-mir-151b | HGLibA_57899 | 32 | -0.830168761 | hsa-mir-151b | - |
| hsa-mir-1825 | HGLibA_57988 | 32 | -0.830168761 | hsa-mir-1825 | - |
| hsa-mir-3116-1 | HGLibA_58505 | 32 | -0.830168761 | hsa-mir-3116-1 | - |
| hsa-mir-409 | HGLibA_59674 | 32 | -0.830168761 | hsa-mir-409 | - |
| hsa-mir-4287 | HGLibA_59854 | 32 | -0.830168761 | hsa-mir-4287 | - |
| hsa-mir-4291 | HGLibA_59874 | 32 | -0.830168761 | hsa-mir-4291 | - |
| hsa-mir-4490 | HGLibA_60379 | 32 | -0.830168761 | hsa-mir-4490 | - |
| hsa-mir-4656 | HGLibA_60730 | 32 | -0.830168761 | hsa-mir-4656 | - |
| hsa-mir-4731 | HGLibA_61022 | 32 | -0.830168761 | hsa-mir-4731 | - |
| hsa-mir-4738 | HGLibA_61049 | 32 | -0.830168761 | hsa-mir-4738 | - |
| hsa-mir-4749 | HGLibA_61092 | 32 | -0.830168761 | hsa-mir-4749 | - |
| hsa-mir-4756 | HGLibA_61120 | 32 | -0.830168761 | hsa-mir-4756 | - |
| hsa-mir-4788 | HGLibA_61259 | 32 | -0.830168761 | hsa-mir-4788 | - |
| hsa-mir-4794 | HGLibA_61282 | 32 | -0.830168761 | hsa-mir-4794 | - |
| hsa-mir-5191 | HGLibA_61683 | 32 | -0.830168761 | hsa-mir-5191 | - |
| hsa-mir-548f-1 | HGLibA_61972 | 32 | -0.830168761 | hsa-mir-548f-1 | - |
| hsa-mir-549a | HGLibA_62095 | 32 | -0.830168761 | hsa-mir-549a | - |
| hsa-mir-5682 | HGLibA_62247 | 32 | -0.830168761 | hsa-mir-5682 | - |
| hsa-mir-593 | HGLibA_62469 | 32 | -0.830168761 | hsa-mir-593 | - |
| hsa-mir-608 | HGLibA_62575 | 32 | -0.830168761 | hsa-mir-608 | - |
| hsa-mir-621 | HGLibA_62723 | 32 | -0.830168761 | hsa-mir-621 | - |
| hsa-mir-6507 | HGLibA_62877 | 32 | -0.830168761 | hsa-mir-6507 | - |
| hsa-mir-6728 | HGLibA_63065 | 32 | -0.830168761 | hsa-mir-6728 | - |
| hsa-mir-6791 | HGLibA_63343 | 32 | -0.830168761 | hsa-mir-6791 | - |
| hsa-mir-6866 | HGLibA_63649 | 32 | -0.830168761 | hsa-mir-6866 | - |
| hsa-mir-6881 | HGLibA_63710 | 32 | -0.830168761 | hsa-mir-6881 | - |
| hsa-mir-7153 | HGLibA_63839 | 32 | -0.830168761 | hsa-mir-7153 | - |
| hsa-mir-7849 | HGLibA_63984 | 32 | -0.830168761 | hsa-mir-7849 | - |
| hsa-mir-7977 | HGLibA_64037 | 32 | -0.830168761 | hsa-mir-7977 | - |
| hsa-mir-920 | HGLibA_64275 | 32 | -0.830168761 | hsa-mir-920 | - |
| KIAA0247 | HGLibA_24615 | 32 | -0.830168761 | KIAA0247 | - |
| KIAA0947 | HGLibA_24674 | 32 | -0.830168761 | KIAA0947 | - |
| LOC100132247 | HGLibA_26806 | 32 | -0.830168761 | LOC100132247 | - |
| LOC100144595 | HGLibA_26812 | 32 | -0.830168761 | LOC100144595 | - |
| 14-Sep | HGLibA_43405 | 32 | -0.830168761 | 14-Sep | - |
| STRA13 | HGLibA_47564 | 32 | -0.830168761 | STRA13 | - |
| TROVE2 | HGLibA_51721 | 32 | -0.830168761 | TROVE2 | - |
| XAGE1D | HGLibA_54474 | 32 | -0.830168761 | XAGE1D | - |
| BAI3 | HGLibA_04118 | 31 | -0.840119799 | BAI3 | - |
| C7orf63 | HGLibA_06571 | 31 | -0.840119799 | C7orf63 | - |
| CCDC53 | HGLibA_07800 | 31 | -0.840119799 | CCDC53 | - |
| CLLU1OS | HGLibA_10065 | 31 | -0.840119799 | CLLU1OS | - |
| DIEXF | HGLibA_13233 | 31 | -0.840119799 | DIEXF | - |
| ENSG00000004478 | HGLibA_17577 | 31 | -0.840119799 | FKBP4 | FK506 binding protein 4 [Source:HGNC Symbol;Acc:HGNC:3720] |
| ENSG00000004897 | HGLibA_08539 | 31 | -0.840119799 | CDC27 | cell division cycle 27 [Source:HGNC Symbol;Acc:HGNC:1728] |
| ENSG00000006377 | HGLibA_13387 | 31 | -0.840119799 | DLX6 | distal-less homeobox 6 [Source:HGNC Symbol;Acc:HGNC:2919] |
| ENSG00000008323 | HGLibA_37120 | 31 | -0.840119799 | PLEKHG6 | pleckstrin homology and RhoGEF domain containing G6 [Source:HGNC Symbol;Acc:HGNC:25562] |
| ENSG00000014914 | HGLibA_30415 | 31 | -0.840119799 | MTMR11 | myotubularin related protein 11 [Source:HGNC Symbol;Acc:HGNC:24307] |
| ENSG00000022355 | HGLibA_18357 | 31 | -0.840119799 | GABRA1 | gamma-aminobutyric acid type A receptor alpha1 subunit [Source:HGNC Symbol;Acc:HGNC:4075] |
| ENSG00000023228 | HGLibA_31546 | 31 | -0.840119799 | NDUFS1 | NADH:ubiquinone oxidoreductase core subunit S1 [Source:HGNC Symbol;Acc:HGNC:7707] |
| ENSG00000033122 | HGLibA_27412 | 31 | -0.840119799 | LRRC7 | leucine rich repeat containing 7 [Source:HGNC Symbol;Acc:HGNC:18531] |
| ENSG00000047230 | HGLibA_11670 | 31 | -0.840119799 | CTPS2 | CTP synthase 2 [Source:HGNC Symbol;Acc:HGNC:2520] |
| ENSG00000048707 | HGLibA_53741 | 31 | -0.840119799 | VPS13D | vacuolar protein sorting 13 homolog D [Source:HGNC Symbol;Acc:HGNC:23595] |
| ENSG00000050393 | HGLibA_28663 | 31 | -0.840119799 | MCUR1 | mitochondrial calcium uniporter regulator 1 [Source:HGNC Symbol;Acc:HGNC:21097] |
| ENSG00000060762 | HGLibA_29701 | 31 | -0.840119799 | MPC1 | mitochondrial pyruvate carrier 1 [Source:HGNC Symbol;Acc:HGNC:21606] |
| ENSG00000065325 | HGLibA_19294 | 31 | -0.840119799 | GLP2R | glucagon like peptide 2 receptor [Source:HGNC Symbol;Acc:HGNC:4325] |
| ENSG00000066117 | HGLibA_45628 | 31 | -0.840119799 | SMARCD1 | member 1 [Source:HGNC Symbol;Acc:HGNC:11106]" |
| ENSG00000066427 | HGLibA_03844 | 31 | -0.840119799 | ATXN3 | ataxin 3 [Source:HGNC Symbol;Acc:HGNC:7106] |
| ENSG00000068976 | HGLibA_39691 | 31 | -0.840119799 | PYGM | muscle associated [Source:HGNC Symbol;Acc:HGNC:9726]" |
| ENSG00000071655 | HGLibA_28469 | 31 | -0.840119799 | MBD3 | methyl-CpG binding domain protein 3 [Source:HGNC Symbol;Acc:HGNC:6918] |
| ENSG00000074054 | HGLibA_09792 | 31 | -0.840119799 | CLASP1 | cytoplasmic linker associated protein 1 [Source:HGNC Symbol;Acc:HGNC:17088] |
| ENSG00000074201 | HGLibA_10091 | 31 | -0.840119799 | CLNS1A | chloride nucleotide-sensitive channel 1A [Source:HGNC Symbol;Acc:HGNC:2080] |
| ENSG00000076043 | HGLibA_40897 | 31 | -0.840119799 | REXO2 | RNA exonuclease 2 [Source:HGNC Symbol;Acc:HGNC:17851] |
| ENSG00000076351 | HGLibA_45184 | 31 | -0.840119799 | SLC46A1 | solute carrier family 46 member 1 [Source:HGNC Symbol;Acc:HGNC:30521] |
| ENSG00000081248 | HGLibA_06903 | 31 | -0.840119799 | CACNA1S | calcium voltage-gated channel subunit alpha1 S [Source:HGNC Symbol;Acc:HGNC:1397] |
| ENSG00000087995 | HGLibA_29001 | 31 | -0.840119799 | METTL2A | methyltransferase like 2A [Source:HGNC Symbol;Acc:HGNC:25755] |
| ENSG00000089163 | HGLibA_44225 | 31 | -0.840119799 | SIRT4 | sirtuin 4 [Source:HGNC Symbol;Acc:HGNC:14932] |
| ENSG00000090273 | HGLibA_32918 | 31 | -0.840119799 | NUDC | dynein complex regulator [Source:HGNC Symbol;Acc:HGNC:8045]" |
| ENSG00000090565 | HGLibA_39798 | 31 | -0.840119799 | RAB11FIP3 | RAB11 family interacting protein 3 [Source:HGNC Symbol;Acc:HGNC:17224] |
| ENSG00000095574 | HGLibA_22941 | 31 | -0.840119799 | IKZF5 | IKAROS family zinc finger 5 [Source:HGNC Symbol;Acc:HGNC:14283] |
| ENSG00000099769 | HGLibA_22813 | 31 | -0.840119799 | IGFALS | insulin like growth factor binding protein acid labile subunit [Source:HGNC Symbol;Acc:HGNC:5468] |
| ENSG00000100359 | HGLibA_43865 | 31 | -0.840119799 | SGSM3 | small G protein signaling modulator 3 [Source:HGNC Symbol;Acc:HGNC:25228] |
| ENSG00000101452 | HGLibA_13186 | 31 | -0.840119799 | DHX35 | DEAH-box helicase 35 [Source:HGNC Symbol;Acc:HGNC:15861] |
| ENSG00000101746 | HGLibA_32267 | 31 | -0.840119799 | NOL4 | nucleolar protein 4 [Source:HGNC Symbol;Acc:HGNC:7870] |
| ENSG00000101811 | HGLibA_11485 | 31 | -0.840119799 | CSTF2 | cleavage stimulation factor subunit 2 [Source:HGNC Symbol;Acc:HGNC:2484] |
| ENSG00000102384 | HGLibA_09058 | 31 | -0.840119799 | CENPI | centromere protein I [Source:HGNC Symbol;Acc:HGNC:3968] |
| ENSG00000104892 | HGLibA_25034 | 31 | -0.840119799 | KLC3 | kinesin light chain 3 [Source:HGNC Symbol;Acc:HGNC:20717] |
| ENSG00000105679 | HGLibA_18616 | 31 | -0.840119799 | GAPDHS | spermatogenic [Source:HGNC Symbol;Acc:HGNC:24864]" |
| ENSG00000105705 | HGLibA_47718 | 31 | -0.840119799 | SUGP1 | SURP and G-patch domain containing 1 [Source:HGNC Symbol;Acc:HGNC:18643] |
| ENSG00000107949 | HGLibA_04268 | 31 | -0.840119799 | BCCIP | BRCA2 and CDKN1A interacting protein [Source:HGNC Symbol;Acc:HGNC:978] |
| ENSG00000108559 | HGLibA_33072 | 31 | -0.840119799 | NUP88 | nucleoporin 88 [Source:HGNC Symbol;Acc:HGNC:8067] |
| ENSG00000109920 | HGLibA_17741 | 31 | -0.840119799 | FNBP4 | formin binding protein 4 [Source:HGNC Symbol;Acc:HGNC:19752] |
| ENSG00000111348 | HGLibA_02798 | 31 | -0.840119799 | ARHGDIB | Rho GDP dissociation inhibitor beta [Source:HGNC Symbol;Acc:HGNC:679] |
| ENSG00000111783 | HGLibA_40972 | 31 | -0.840119799 | RFX4 | regulatory factor X4 [Source:HGNC Symbol;Acc:HGNC:9985] |
| ENSG00000111817 | HGLibA_14015 | 31 | -0.840119799 | DSE | dermatan sulfate epimerase [Source:HGNC Symbol;Acc:HGNC:21144] |
| ENSG00000112149 | HGLibA_08461 | 31 | -0.840119799 | CD83 | CD83 molecule [Source:HGNC Symbol;Acc:HGNC:1703] |
| ENSG00000112312 | HGLibA_19417 | 31 | -0.840119799 | GMNN | DNA replication inhibitor [Source:HGNC Symbol;Acc:HGNC:17493]" |
| ENSG00000112667 | HGLibA_13714 | 31 | -0.840119799 | DNPH1 | 2'-deoxynucleoside 5'-phosphate N-hydrolase 1 [Source:HGNC Symbol;Acc:HGNC:21218] |
| ENSG00000112782 | HGLibA_10033 | 31 | -0.840119799 | CLIC5 | chloride intracellular channel 5 [Source:HGNC Symbol;Acc:HGNC:13517] |
| ENSG00000113448 | HGLibA_35797 | 31 | -0.840119799 | PDE4D | phosphodiesterase 4D [Source:HGNC Symbol;Acc:HGNC:8783] |
| ENSG00000114503 | HGLibA_31283 | 31 | -0.840119799 | NCBP2 | nuclear cap binding protein subunit 2 [Source:HGNC Symbol;Acc:HGNC:7659] |
| ENSG00000115604 | HGLibA_23028 | 31 | -0.840119799 | IL18R1 | interleukin 18 receptor 1 [Source:HGNC Symbol;Acc:HGNC:5988] |
| ENSG00000116212 | HGLibA_27360 | 31 | -0.840119799 | LRRC42 | leucine rich repeat containing 42 [Source:HGNC Symbol;Acc:HGNC:28792] |
| ENSG00000116717 | HGLibA_18415 | 31 | -0.840119799 | GADD45A | growth arrest and DNA damage inducible alpha [Source:HGNC Symbol;Acc:HGNC:4095] |
| ENSG00000117525 | HGLibA_15873 | 31 | -0.840119799 | F3 | tissue factor [Source:HGNC Symbol;Acc:HGNC:3541]" |
| ENSG00000118137 | HGLibA_02449 | 31 | -0.840119799 | APOA1 | apolipoprotein A1 [Source:HGNC Symbol;Acc:HGNC:600] |
| ENSG00000118245 | HGLibA_50893 | 31 | -0.840119799 | TNP1 | transition protein 1 [Source:HGNC Symbol;Acc:HGNC:11951] |
| ENSG00000118412 | HGLibA_07328 | 31 | -0.840119799 | CASP8AP2 | caspase 8 associated protein 2 [Source:HGNC Symbol;Acc:HGNC:1510] |
| ENSG00000118873 | HGLibA_39922 | 31 | -0.840119799 | RAB3GAP2 | RAB3 GTPase activating non-catalytic protein subunit 2 [Source:HGNC Symbol;Acc:HGNC:17168] |
| ENSG00000119471 | HGLibA_22216 | 31 | -0.840119799 | HSDL2 | hydroxysteroid dehydrogenase like 2 [Source:HGNC Symbol;Acc:HGNC:18572] |
| ENSG00000119608 | HGLibA_38703 | 31 | -0.840119799 | PROX2 | prospero homeobox 2 [Source:HGNC Symbol;Acc:HGNC:26715] |
| ENSG00000119682 | HGLibA_02643 | 31 | -0.840119799 | AREL1 | apoptosis resistant E3 ubiquitin protein ligase 1 [Source:HGNC Symbol;Acc:HGNC:20363] |
| ENSG00000120899 | HGLibA_39383 | 31 | -0.840119799 | PTK2B | protein tyrosine kinase 2 beta [Source:HGNC Symbol;Acc:HGNC:9612] |
| ENSG00000121957 | HGLibA_20157 | 31 | -0.840119799 | GPSM2 | G protein signaling modulator 2 [Source:HGNC Symbol;Acc:HGNC:29501] |
| ENSG00000122122 | HGLibA_42765 | 31 | -0.840119799 | SASH3 | SAM and SH3 domain containing 3 [Source:HGNC Symbol;Acc:HGNC:15975] |
| ENSG00000122194 | HGLibA_37159 | 31 | -0.840119799 | PLG | plasminogen [Source:HGNC Symbol;Acc:HGNC:9071] |
| ENSG00000122863 | HGLibA_09633 | 31 | -0.840119799 | CHST3 | carbohydrate sulfotransferase 3 [Source:HGNC Symbol;Acc:HGNC:1971] |
| ENSG00000124523 | HGLibA_44229 | 31 | -0.840119799 | SIRT5 | sirtuin 5 [Source:HGNC Symbol;Acc:HGNC:14933] |
| ENSG00000124688 | HGLibA_27847 | 31 | -0.840119799 | MAD2L1BP | MAD2L1 binding protein [Source:HGNC Symbol;Acc:HGNC:21059] |
| ENSG00000125812 | HGLibA_20716 | 31 | -0.840119799 | GZF1 | GDNF inducible zinc finger protein 1 [Source:HGNC Symbol;Acc:HGNC:15808] |
| ENSG00000125968 | HGLibA_22524 | 31 | -0.840119799 | ID1 | HLH protein [Source:HGNC Symbol;Acc:HGNC:5360]" |
| ENSG00000126460 | HGLibA_38871 | 31 | -0.840119799 | PRRG2 | proline rich and Gla domain 2 [Source:HGNC Symbol;Acc:HGNC:9470] |
| ENSG00000126705 | HGLibA_01295 | 31 | -0.840119799 | AHDC1 | AT-hook DNA binding motif containing 1 [Source:HGNC Symbol;Acc:HGNC:25230] |
| ENSG00000126767 | HGLibA_14913 | 31 | -0.840119799 | ELK1 | ETS transcription factor [Source:HGNC Symbol;Acc:HGNC:3321]" |
| ENSG00000127663 | HGLibA_24510 | 31 | -0.840119799 | KDM4B | lysine demethylase 4B [Source:HGNC Symbol;Acc:HGNC:29136] |
| ENSG00000127804 | HGLibA_28965 | 31 | -0.840119799 | METTL16 | methyltransferase like 16 [Source:HGNC Symbol;Acc:HGNC:28484] |
| ENSG00000128607 | HGLibA_25094 | 31 | -0.840119799 | KLHDC10 | kelch domain containing 10 [Source:HGNC Symbol;Acc:HGNC:22194] |
| ENSG00000130402 | HGLibA_00645 | 31 | -0.840119799 | ACTN4 | actinin alpha 4 [Source:HGNC Symbol;Acc:HGNC:166] |
| ENSG00000130529 | HGLibA_51762 | 31 | -0.840119799 | TRPM4 | transient receptor potential cation channel subfamily M member 4 [Source:HGNC Symbol;Acc:HGNC:17993] |
| ENSG00000131620 | HGLibA_02171 | 31 | -0.840119799 | ANO1 | anoctamin 1 [Source:HGNC Symbol;Acc:HGNC:21625] |
| ENSG00000131652 | HGLibA_49375 | 31 | -0.840119799 | THOC6 | THO complex 6 [Source:HGNC Symbol;Acc:HGNC:28369] |
| ENSG00000132819 | HGLibA_40540 | 31 | -0.840119799 | RBM38 | RNA binding motif protein 38 [Source:HGNC Symbol;Acc:HGNC:15818] |
| ENSG00000133800 | HGLibA_27779 | 31 | -0.840119799 | LYVE1 | lymphatic vessel endothelial hyaluronan receptor 1 [Source:HGNC Symbol;Acc:HGNC:14687] |
| ENSG00000134240 | HGLibA_21714 | 31 | -0.840119799 | HMGCS2 | 3-hydroxy-3-methylglutaryl-CoA synthase 2 [Source:HGNC Symbol;Acc:HGNC:5008] |
| ENSG00000134321 | HGLibA_42299 | 31 | -0.840119799 | RSAD2 | radical S-adenosyl methionine domain containing 2 [Source:HGNC Symbol;Acc:HGNC:30908] |
| ENSG00000134594 | HGLibA_39880 | 31 | -0.840119799 | RAB33A | member RAS oncogene family [Source:HGNC Symbol;Acc:HGNC:9773]" |
| ENSG00000135317 | HGLibA_46038 | 31 | -0.840119799 | SNX14 | sorting nexin 14 [Source:HGNC Symbol;Acc:HGNC:14977] |

| | | | | | |
|---|---|---|---|---|---|
| ENSG00000135318 | HGLibA_32830 | 31 | -0.840119799 | NT5E | 5'-nucleotidase ecto [Source:HGNC Symbol;Acc:HGNC:8021] |
| ENSG00000135596 | HGLibA_29201 | 31 | -0.840119799 | MICAL1 | calponin and LIM domain containing 1 [Source:HGNC Symbol;Acc:HGNC:20619]" |
| ENSG00000135917 | HGLibA_44505 | 31 | -0.840119799 | SLC19A3 | solute carrier family 19 member 3 [Source:HGNC Symbol;Acc:HGNC:16266] |
| ENSG00000136044 | HGLibA_02570 | 31 | -0.840119799 | APPL2 | phosphotyrosine interacting with PH domain and leucine zipper 2 [Source:HGNC Symbol;Acc:HGNC:18242]" |
| ENSG00000136631 | HGLibA_53793 | 31 | -0.840119799 | VPS45 | vacuolar protein sorting 45 homolog [Source:HGNC Symbol;Acc:HGNC:14579] |
| ENSG00000136859 | HGLibA_01912 | 31 | -0.840119799 | ANGPTL2 | angiopoietin like 2 [Source:HGNC Symbol;Acc:HGNC:490] |
| ENSG00000137033 | HGLibA_23139 | 31 | -0.840119799 | IL33 | interleukin 33 [Source:HGNC Symbol;Acc:HGNC:16028] |
| ENSG00000137462 | HGLibA_49647 | 31 | -0.840119799 | TLR2 | toll like receptor 2 [Source:HGNC Symbol;Acc:HGNC:11848] |
| ENSG00000137563 | HGLibA_18997 | 31 | -0.840119799 | GGH | gamma-glutamyl hydrolase [Source:HGNC Symbol;Acc:HGNC:4248] |
| ENSG00000137672 | HGLibA_51746 | 31 | -0.840119799 | TRPC6 | transient receptor potential cation channel subfamily C member 6 [Source:HGNC Symbol;Acc:HGNC:12338] |
| ENSG00000138379 | HGLibA_30264 | 31 | -0.840119799 | MSTN | myostatin [Source:HGNC Symbol;Acc:HGNC:4223] |
| ENSG00000138395 | HGLibA_08741 | 31 | -0.840119799 | CDK15 | cyclin dependent kinase 15 [Source:HGNC Symbol;Acc:HGNC:14434] |
| ENSG00000139218 | HGLibA_42823 | 31 | -0.840119799 | SCAF11 | SR-related CTD associated factor 11 [Source:HGNC Symbol;Acc:HGNC:10784] |
| ENSG00000139679 | HGLibA_27068 | 31 | -0.840119799 | LPAR6 | lysophosphatidic acid receptor 6 [Source:HGNC Symbol;Acc:HGNC:15520] |
| ENSG00000139687 | HGLibA_40412 | 31 | -0.840119799 | RB1 | RB transcriptional corepressor 1 [Source:HGNC Symbol;Acc:HGNC:9884] |
| ENSG00000140391 | HGLibA_51926 | 31 | -0.840119799 | TSPAN3 | tetraspanin 3 [Source:HGNC Symbol;Acc:HGNC:17752] |
| ENSG00000140396 | HGLibA_31339 | 31 | -0.840119799 | NCOA2 | nuclear receptor coactivator 2 [Source:HGNC Symbol;Acc:HGNC:7669] |
| ENSG00000140798 | HGLibA_00168 | 31 | -0.840119799 | ABCC12 | ATP binding cassette subfamily C member 12 [Source:HGNC Symbol;Acc:HGNC:14640] |
| ENSG00000141499 | HGLibA_54399 | 31 | -0.840119799 | WRAP53 | WD repeat containing antisense to TP53 [Source:HGNC Symbol;Acc:HGNC:25522] |
| ENSG00000141543 | HGLibA_14810 | 31 | -0.840119799 | EIF4A3 | eukaryotic translation initiation factor 4A3 [Source:HGNC Symbol;Acc:HGNC:18683] |
| ENSG00000141933 | HGLibA_51123 | 31 | -0.840119799 | TPGS1 | tubulin polyglutamylase complex subunit 1 [Source:HGNC Symbol;Acc:HGNC:25058] |
| ENSG00000142039 | HGLibA_07939 | 31 | -0.840119799 | CCDC97 | coiled-coil domain containing 97 [Source:HGNC Symbol;Acc:HGNC:28289] |
| ENSG00000143473 | HGLibA_24211 | 31 | -0.840119799 | KCNH1 | potassium voltage-gated channel subfamily H member 1 [Source:HGNC Symbol;Acc:HGNC:6250] |
| ENSG00000145365 | HGLibA_49457 | 31 | -0.840119799 | TIFA | TRAF interacting protein with forkhead associated domain [Source:HGNC Symbol;Acc:HGNC:19075] |
| ENSG00000145919 | HGLibA_04669 | 31 | -0.840119799 | BOD1 | biorientation of chromosomes in cell division 1 [Source:HGNC Symbol;Acc:HGNC:25114] |
| ENSG00000146233 | HGLibA_12151 | 31 | -0.840119799 | CYP39A1 | cytochrome P450 family 39 subfamily A member 1 [Source:HGNC Symbol;Acc:HGNC:17449] |
| ENSG00000147255 | HGLibA_22876 | 31 | -0.840119799 | IGSF1 | immunoglobulin superfamily member 1 [Source:HGNC Symbol;Acc:HGNC:5948] |
| ENSG00000148826 | HGLibA_32058 | 31 | -0.840119799 | NKX6-2 | NK6 homeobox 2 [Source:HGNC Symbol;Acc:HGNC:19321] |
| ENSG00000150394 | HGLibA_08692 | 31 | -0.840119799 | CDH8 | cadherin 8 [Source:HGNC Symbol;Acc:HGNC:1767] |
| ENSG00000150477 | HGLibA_24743 | 31 | -0.840119799 | KIAA1328 | KIAA1328 [Source:HGNC Symbol;Acc:HGNC:29248] |
| ENSG00000151465 | HGLibA_08508 | 31 | -0.840119799 | CDC123 | cell division cycle 123 [Source:HGNC Symbol;Acc:HGNC:16827] |
| ENSG00000152093 | HGLibA_09252 | 31 | -0.840119799 | CFC1B | cryptic family 1B [Source:HGNC Symbol;Acc:HGNC:33983]" |
| ENSG00000152455 | HGLibA_47861 | 31 | -0.840119799 | SUV39H2 | suppressor of variegation 3-9 homolog 2 [Source:HGNC Symbol;Acc:HGNC:17287] |
| ENSG00000153574 | HGLibA_41849 | 31 | -0.840119799 | RPIA | ribose 5-phosphate isomerase A [Source:HGNC Symbol;Acc:HGNC:10297] |
| ENSG00000155249 | HGLibA_34016 | 31 | -0.840119799 | OR4K1 | olfactory receptor family 4 subfamily K member 1 [Source:HGNC Symbol;Acc:HGNC:14726] |
| ENSG00000155957 | HGLibA_49741 | 31 | -0.840119799 | TMBIM4 | transmembrane BAX inhibitor motif containing 4 [Source:HGNC Symbol;Acc:HGNC:24257] |
| ENSG00000156222 | HGLibA_44832 | 31 | -0.840119799 | SLC28A1 | solute carrier family 28 member 1 [Source:HGNC Symbol;Acc:HGNC:11001] |
| ENSG00000156603 | HGLibA_28770 | 31 | -0.840119799 | MED19 | mediator complex subunit 19 [Source:HGNC Symbol;Acc:HGNC:29600] |
| ENSG00000156642 | HGLibA_32529 | 31 | -0.840119799 | NPTN | neuroplastin [Source:HGNC Symbol;Acc:HGNC:17867] |
| ENSG00000156738 | HGLibA_30126 | 31 | -0.840119799 | MS4A1 | membrane spanning 4-domains A1 [Source:HGNC Symbol;Acc:HGNC:7315] |
| ENSG00000158352 | HGLibA_44097 | 31 | -0.840119799 | SHROOM4 | shroom family member 4 [Source:HGNC Symbol;Acc:HGNC:29215] |
| ENSG00000159387 | HGLibA_23644 | 31 | -0.840119799 | IRX6 | iroquois homeobox 6 [Source:HGNC Symbol;Acc:HGNC:14675] |
| ENSG00000159399 | HGLibA_21583 | 31 | -0.840119799 | HK2 | hexokinase 2 [Source:HGNC Symbol;Acc:HGNC:4923] |
| ENSG00000159882 | HGLibA_55636 | 31 | -0.840119799 | ZNF230 | zinc finger protein 230 [Source:HGNC Symbol;Acc:HGNC:13024] |
| ENSG00000159899 | HGLibA_32510 | 31 | -0.840119799 | NPR2 | natriuretic peptide receptor 2 [Source:HGNC Symbol;Acc:HGNC:7944] |
| ENSG00000160014 | HGLibA_07029 | 31 | -0.840119799 | CALM3 | calmodulin 3 [Source:HGNC Symbol;Acc:HGNC:1449] |
| ENSG00000160051 | HGLibA_23494 | 31 | -0.840119799 | IQCC | IQ motif containing C [Source:HGNC Symbol;Acc:HGNC:25545] |
| ENSG00000160789 | HGLibA_26692 | 31 | -0.840119799 | LMNA | lamin A/C [Source:HGNC Symbol;Acc:HGNC:6636] |
| ENSG00000161011 | HGLibA_46919 | 31 | -0.840119799 | SQSTM1 | sequestosome 1 [Source:HGNC Symbol;Acc:HGNC:11280] |
| ENSG00000161921 | HGLibA_11860 | 31 | -0.840119799 | CXCL16 | C-X-C motif chemokine ligand 16 [Source:HGNC Symbol;Acc:HGNC:16642] |
| ENSG00000161929 | HGLibA_42945 | 31 | -0.840119799 | SCIMP | SLP adaptor and CSK interacting membrane protein [Source:HGNC Symbol;Acc:HGNC:33504] |
| ENSG00000162337 | HGLibA_27224 | 31 | -0.840119799 | LRP5 | LDL receptor related protein 5 [Source:HGNC Symbol;Acc:HGNC:6697] |
| ENSG00000162763 | HGLibA_27386 | 31 | -0.840119799 | LRRC52 | leucine rich repeat containing 52 [Source:HGNC Symbol;Acc:HGNC:32156] |
| ENSG00000163328 | HGLibA_19942 | 31 | -0.840119799 | GPR155 | G protein-coupled receptor 155 [Source:HGNC Symbol;Acc:HGNC:22951] |
| ENSG00000163378 | HGLibA_15224 | 31 | -0.840119799 | EOGT | EGF domain specific O-linked N-acetylglucosamine transferase [Source:HGNC Symbol;Acc:HGNC:28526] |
| ENSG00000163914 | HGLibA_41164 | 31 | -0.840119799 | RHO | rhodopsin [Source:HGNC Symbol;Acc:HGNC:10012] |
| ENSG00000164129 | HGLibA_32560 | 31 | -0.840119799 | NPY5R | neuropeptide Y receptor Y5 [Source:HGNC Symbol;Acc:HGNC:7958] |
| ENSG00000164175 | HGLibA_45176 | 31 | -0.840119799 | SLC45A2 | solute carrier family 45 member 2 [Source:HGNC Symbol;Acc:HGNC:16472] |
| ENSG00000164326 | HGLibA_07264 | 31 | -0.840119799 | CARTPT | CART prepropeptide [Source:HGNC Symbol;Acc:HGNC:24323] |
| ENSG00000164512 | HGLibA_02127 | 31 | -0.840119799 | ANKRD55 | ankyrin repeat domain 55 [Source:HGNC Symbol;Acc:HGNC:25681] |
| ENSG00000164543 | HGLibA_47462 | 31 | -0.840119799 | STK17A | serine/threonine kinase 17a [Source:HGNC Symbol;Acc:HGNC:11395] |
| ENSG00000164822 | HGLibA_12806 | 31 | -0.840119799 | DEFA6 | defensin alpha 6 [Source:HGNC Symbol;Acc:HGNC:2765] |
| ENSG00000164893 | HGLibA_45355 | 31 | -0.840119799 | SLC7A13 | solute carrier family 7 member 13 [Source:HGNC Symbol;Acc:HGNC:23092] |
| ENSG00000164941 | HGLibA_23433 | 31 | -0.840119799 | INTS8 | integrator complex subunit 8 [Source:HGNC Symbol;Acc:HGNC:26048] |
| ENSG00000165188 | HGLibA_41588 | 31 | -0.840119799 | RNF183 | ring finger protein 183 [Source:HGNC Symbol;Acc:HGNC:28721] |
| ENSG00000165462 | HGLibA_36450 | 31 | -0.840119799 | PHOX2A | paired like homeobox 2A [Source:HGNC Symbol;Acc:HGNC:691] |
| ENSG00000166126 | HGLibA_01807 | 31 | -0.840119799 | AMN | amnion associated transmembrane protein [Source:HGNC Symbol;Acc:HGNC:14604] |
| ENSG00000166152 | HGLibA_05484 | 31 | -0.840119799 | C16orf78 | chromosome 16 open reading frame 78 [Source:HGNC Symbol;Acc:HGNC:28479] |
| ENSG00000166343 | HGLibA_30251 | 31 | -0.840119799 | MSS51 | MSS51 mitochondrial translational activator [Source:HGNC Symbol;Acc:HGNC:21000] |
| ENSG00000166589 | HGLibA_08646 | 31 | -0.840119799 | CDH16 | cadherin 16 [Source:HGNC Symbol;Acc:HGNC:1755] |
| ENSG00000166845 | HGLibA_05617 | 31 | -0.840119799 | C18orf54 | chromosome 18 open reading frame 54 [Source:HGNC Symbol;Acc:HGNC:13796] |
| ENSG00000167005 | HGLibA_32969 | 31 | -0.840119799 | NUDT21 | nudix hydrolase 21 [Source:HGNC Symbol;Acc:HGNC:13870] |
| ENSG00000167037 | HGLibA_43860 | 31 | -0.840119799 | SGSM1 | small G protein signaling modulator 1 [Source:HGNC Symbol;Acc:HGNC:29410] |
| ENSG00000167470 | HGLibA_29241 | 31 | -0.840119799 | MIDN | midnolin [Source:HGNC Symbol;Acc:HGNC:16298] |
| ENSG00000167552 | HGLibA_52242 | 31 | -0.840119799 | TUBA1A | tubulin alpha 1a [Source:HGNC Symbol;Acc:HGNC:20766] |
| ENSG00000167604 | HGLibA_31828 | 31 | -0.840119799 | NFKBID | NFKB inhibitor delta [Source:HGNC Symbol;Acc:HGNC:15671] |
| ENSG00000167700 | HGLibA_29099 | 31 | -0.840119799 | MFSD3 | major facilitator superfamily domain containing 3 [Source:HGNC Symbol;Acc:HGNC:25157] |
| ENSG00000167775 | HGLibA_08351 | 31 | -0.840119799 | CD320 | CD320 molecule [Source:HGNC Symbol;Acc:HGNC:16692] |
| ENSG00000167994 | HGLibA_39925 | 31 | -0.840119799 | RAB3IL1 | RAB3A interacting protein like 1 [Source:HGNC Symbol;Acc:HGNC:9780] |
| ENSG00000168546 | HGLibA_18967 | 31 | -0.840119799 | GFRA2 | GDNF family receptor alpha 2 [Source:HGNC Symbol;Acc:HGNC:4244] |
| ENSG00000168658 | HGLibA_53906 | 31 | -0.840119799 | VWA3B | von Willebrand factor A domain containing 3B [Source:HGNC Symbol;Acc:HGNC:28385] |
| ENSG00000168970 | HGLibA_23965 | 31 | -0.840119799 | JMJD7-PLA2G4B | JMJD7-PLA2G4B readthrough [Source:HGNC Symbol;Acc:HGNC:34449] |
| ENSG00000168995 | HGLibA_44147 | 31 | -0.840119799 | SIGLEC7 | sialic acid binding Ig like lectin 7 [Source:HGNC Symbol;Acc:HGNC:10876] |
| ENSG00000169220 | HGLibA_41064 | 31 | -0.840119799 | RGS14 | regulator of G protein signaling 14 [Source:HGNC Symbol;Acc:HGNC:9996] |
| ENSG00000169696 | HGLibA_03366 | 31 | -0.840119799 | ASPSCR1 | UBX domain containing tether for SLC2A4 [Source:HGNC Symbol;Acc:HGNC:13825]" |
| ENSG00000170178 | HGLibA_21992 | 31 | -0.840119799 | HOXD12 | homeobox D12 [Source:HGNC Symbol;Acc:HGNC:5135] |
| ENSG00000170464 | HGLibA_13587 | 31 | -0.840119799 | DNAJC18 | DnaJ heat shock protein family (Hsp40) member C18 [Source:HGNC Symbol;Acc:HGNC:28429] |
| ENSG00000170545 | HGLibA_45592 | 31 | -0.840119799 | SMAGP | small cell adhesion glycoprotein [Source:HGNC Symbol;Acc:HGNC:26918] |
| ENSG00000170929 | HGLibA_33702 | 31 | -0.840119799 | OR1M1 | olfactory receptor family 1 subfamily M member 1 [Source:HGNC Symbol;Acc:HGNC:8220] |
| ENSG00000171433 | HGLibA_19288 | 31 | -0.840119799 | GLOD5 | glyoxalase domain containing 5 [Source:HGNC Symbol;Acc:HGNC:33358] |
| ENSG00000171451 | HGLibA_14018 | 31 | -0.840119799 | DSEL | dermatan sulfate epimerase like [Source:HGNC Symbol;Acc:HGNC:18144] |
| ENSG00000171456 | HGLibA_03388 | 31 | -0.840119799 | ASXL1 | ASXL transcriptional regulator 1 [Source:HGNC Symbol;Acc:HGNC:18318] |
| ENSG00000171659 | HGLibA_20027 | 31 | -0.840119799 | GPR34 | G protein-coupled receptor 34 [Source:HGNC Symbol;Acc:HGNC:4490] |
| ENSG00000171865 | HGLibA_41424 | 31 | -0.840119799 | RNASEH1 | ribonuclease H1 [Source:HGNC Symbol;Acc:HGNC:18466] |
| ENSG00000172932 | HGLibA_02008 | 31 | -0.840119799 | ANKRD13D | ankyrin repeat domain 13D [Source:HGNC Symbol;Acc:HGNC:27880] |
| ENSG00000173451 | HGLibA_49295 | 31 | -0.840119799 | THAP2 | THAP domain containing 2 [Source:HGNC Symbol;Acc:HGNC:20854] |
| ENSG00000174099 | HGLibA_30247 | 31 | -0.840119799 | MSRB3 | methionine sulfoxide reductase B3 [Source:HGNC Symbol;Acc:HGNC:27375] |
| ENSG00000174599 | HGLibA_51286 | 31 | -0.840119799 | TRAM1L1 | translocation associated membrane protein 1 like 1 [Source:HGNC Symbol;Acc:HGNC:28371] |
| ENSG00000176715 | HGLibA_00552 | 31 | -0.840119799 | ACSF3 | acyl-CoA synthetase family member 3 [Source:HGNC Symbol;Acc:HGNC:27288] |
| ENSG00000176774 | HGLibA_27941 | 31 | -0.840119799 | MAGEB18 | MAGE family member B18 [Source:HGNC Symbol;Acc:HGNC:28515] |
| ENSG00000177082 | HGLibA_54168 | 31 | -0.840119799 | WDR73 | WD repeat domain 73 [Source:HGNC Symbol;Acc:HGNC:25928] |
| ENSG00000177301 | HGLibA_24129 | 31 | -0.840119799 | KCNA2 | potassium voltage-gated channel subfamily A member 2 [Source:HGNC Symbol;Acc:HGNC:6220] |
| ENSG00000177576 | HGLibA_05613 | 31 | -0.840119799 | C18orf32 | chromosome 18 open reading frame 32 [Source:HGNC Symbol;Acc:HGNC:31690] |
| ENSG00000177693 | HGLibA_34006 | 31 | -0.840119799 | OR4F4 | olfactory receptor family 4 subfamily F member 4 [Source:HGNC Symbol;Acc:HGNC:8301] |
| ENSG00000178233 | HGLibA_50004 | 31 | -0.840119799 | TMEM151B | transmembrane protein 151B [Source:HGNC Symbol;Acc:HGNC:21315] |
| ENSG00000178718 | HGLibA_42030 | 31 | -0.840119799 | RPP25 | ribonuclease P and MRP subunit p25 [Source:HGNC Symbol;Acc:HGNC:30361] |
| ENSG00000179751 | HGLibA_47926 | 31 | -0.840119799 | SYCN | syncollin [Source:HGNC Symbol;Acc:HGNC:18442] |
| ENSG00000180035 | HGLibA_56069 | 31 | -0.840119799 | ZNF48 | zinc finger protein 48 [Source:HGNC Symbol;Acc:HGNC:13114] |
| ENSG00000181690 | HGLibA_36952 | 31 | -0.840119799 | PLAG1 | PLAG1 zinc finger [Source:HGNC Symbol;Acc:HGNC:9045] |
| ENSG00000181958 | HGLibA_33923 | 31 | -0.840119799 | OR4A15 | olfactory receptor family 4 subfamily A member 15 [Source:HGNC Symbol;Acc:HGNC:15152] |
| ENSG00000183048 | HGLibA_44636 | 31 | -0.840119799 | SLC25A10 | solute carrier family 25 member 10 [Source:HGNC Symbol;Acc:HGNC:10980] |
| ENSG00000183072 | HGLibA_32042 | 31 | -0.840119799 | NKX2-5 | NK2 homeobox 5 [Source:HGNC Symbol;Acc:HGNC:2488] |
| ENSG00000183340 | HGLibA_23993 | 31 | -0.840119799 | JRKL | JRK like [Source:HGNC Symbol;Acc:HGNC:6200] |
| ENSG00000183379 | HGLibA_47973 | 31 | -0.840119799 | SYNDIG1L | synapse differentiation inducing 1 like [Source:HGNC Symbol;Acc:HGNC:32388] |
| ENSG00000184108 | HGLibA_51620 | 31 | -0.840119799 | TRIML1 | tripartite motif family like 1 [Source:HGNC Symbol;Acc:HGNC:26698] |
| ENSG00000184709 | HGLibA_27294 | 31 | -0.840119799 | LRRC26 | leucine rich repeat containing 26 [Source:HGNC Symbol;Acc:HGNC:31409] |
| ENSG00000184743 | HGLibA_03522 | 31 | -0.840119799 | ATL3 | atlastin GTPase 3 [Source:HGNC Symbol;Acc:HGNC:24526] |
| ENSG00000185306 | HGLibA_05266 | 31 | -0.840119799 | C12orf56 | chromosome 12 open reading frame 56 [Source:HGNC Symbol;Acc:HGNC:26967] |
| ENSG00000185950 | HGLibA_23622 | 31 | -0.840119799 | IRS2 | insulin receptor substrate 2 [Source:HGNC Symbol;Acc:HGNC:6126] |
| ENSG00000186298 | HGLibA_38010 | 31 | -0.840119799 | PPP1CC | protein phosphatase 1 catalytic subunit gamma [Source:HGNC Symbol;Acc:HGNC:9283] |
| ENSG00000186314 | HGLibA_38431 | 31 | -0.840119799 | PRELID2 | PRELI domain containing 2 [Source:HGNC Symbol;Acc:HGNC:28306] |
| ENSG00000186469 | HGLibA_19521 | 31 | -0.840119799 | GNG2 | G protein subunit gamma 2 [Source:HGNC Symbol;Acc:HGNC:4404] |
| ENSG00000186895 | HGLibA_17404 | 31 | -0.840119799 | FGF3 | fibroblast growth factor 3 [Source:HGNC Symbol;Acc:HGNC:3681] |
| ENSG00000187051 | HGLibA_42098 | 31 | -0.840119799 | RPS19BP1 | ribosomal protein S19 binding protein 1 [Source:HGNC Symbol;Acc:HGNC:28749] |
| ENSG00000187191 | HGLibA_12359 | 31 | -0.840119799 | DAZ3 | deleted in azoospermia 3 [Source:HGNC Symbol;Acc:HGNC:15965] |
| ENSG00000187479 | HGLibA_05221 | 31 | -0.840119799 | C11orf96 | chromosome 11 open reading frame 96 [Source:HGNC Symbol;Acc:HGNC:38675] |
| ENSG00000187607 | HGLibA_55749 | 31 | -0.840119799 | ZNF286A | zinc finger protein 286A [Source:HGNC Symbol;Acc:HGNC:13501] |
| ENSG00000187772 | HGLibA_26560 | 31 | -0.840119799 | LIN28B | lin-28 homolog B [Source:HGNC Symbol;Acc:HGNC:32207] |
| ENSG00000187808 | HGLibA_46221 | 31 | -0.840119799 | SOWAHD | sosondowah ankyrin repeat domain family member D [Source:HGNC Symbol;Acc:HGNC:32960] |
| ENSG00000187987 | HGLibA_57006 | 31 | -0.840119799 | ZSCAN23 | zinc finger and SCAN domain containing 23 [Source:HGNC Symbol;Acc:HGNC:21193] |
| ENSG00000188306 | HGLibA_27462 | 31 | -0.840119799 | LRRIQ4 | leucine rich repeats and IQ motif containing 4 [Source:HGNC Symbol;Acc:HGNC:34298] |
| ENSG00000188313 | HGLibA_37232 | 31 | -0.840119799 | PLSCR1 | phospholipid scramblase 1 [Source:HGNC Symbol;Acc:HGNC:9092] |
| ENSG00000188501 | HGLibA_26208 | 31 | -0.840119799 | LCTL | lactase like [Source:HGNC Symbol;Acc:HGNC:15583] |
| ENSG00000188706 | HGLibA_55133 | 31 | -0.840119799 | ZDHHC9 | zinc finger DHHC-type containing 9 [Source:HGNC Symbol;Acc:HGNC:18475] |
| ENSG00000196091 | HGLibA_30649 | 31 | -0.840119799 | MYBPC1 | slow type [Source:HGNC Symbol;Acc:HGNC:7549]" |

| | | | | | |
|---|---|---|---|---|---|
| ENSG00000196262 | HGLibA_37894 | 31 | -0.840119799 | PPIA | peptidylprolyl isomerase A [Source:HGNC Symbol;Acc:HGNC:9253] |
| ENSG00000196353 | HGLibA_10963 | 31 | -0.840119799 | CPNE4 | copine 4 [Source:HGNC Symbol;Acc:HGNC:2317] |
| ENSG00000196517 | HGLibA_45343 | 31 | -0.840119799 | SLC6A9 | solute carrier family 6 member 9 [Source:HGNC Symbol;Acc:HGNC:11056] |
| ENSG00000196578 | HGLibA_34236 | 31 | -0.840119799 | OR5AC2 | olfactory receptor family 5 subfamily AC member 2 [Source:HGNC Symbol;Acc:HGNC:15431] |
| ENSG00000196591 | HGLibA_21007 | 31 | -0.840119799 | HDAC2 | histone deacetylase 2 [Source:HGNC Symbol;Acc:HGNC:4853] |
| ENSG00000196700 | HGLibA_56134 | 31 | -0.840119799 | ZNF512B | zinc finger protein 512B [Source:HGNC Symbol;Acc:HGNC:29212] |
| ENSG00000197329 | HGLibA_36033 | 31 | -0.840119799 | PELI1 | pellino E3 ubiquitin protein ligase 1 [Source:HGNC Symbol;Acc:HGNC:8827] |
| ENSG00000197467 | HGLibA_10510 | 31 | -0.840119799 | COL13A1 | collagen type XIII alpha 1 chain [Source:HGNC Symbol;Acc:HGNC:2190] |
| ENSG00000197557 | HGLibA_52104 | 31 | -0.840119799 | TTC30A | tetratricopeptide repeat domain 30A [Source:HGNC Symbol;Acc:HGNC:25853] |
| ENSG00000197658 | HGLibA_44575 | 31 | -0.840119799 | SLC22A24 | solute carrier family 22 member 24 [Source:HGNC Symbol;Acc:HGNC:28542] |
| ENSG00000198162 | HGLibA_28060 | 31 | -0.840119799 | MAN1A2 | mannosidase alpha class 1A member 2 [Source:HGNC Symbol;Acc:HGNC:6822] |
| ENSG00000198754 | HGLibA_34816 | 31 | -0.840119799 | OXCT2 | 3-oxoacid CoA-transferase 2 [Source:HGNC Symbol;Acc:HGNC:18606] |
| ENSG00000198918 | HGLibA_41974 | 31 | -0.840119799 | RPL39 | ribosomal protein L39 [Source:HGNC Symbol;Acc:HGNC:10350] |
| ENSG00000204310 | HGLibA_01218 | 31 | -0.840119799 | AGPAT1 | 1-acylglycerol-3-phosphate O-acyltransferase 1 [Source:HGNC Symbol;Acc:HGNC:324] |
| ENSG00000204344 | HGLibA_47468 | 31 | -0.840119799 | STK19 | serine/threonine kinase 19 [Source:HGNC Symbol;Acc:HGNC:11398] |
| ENSG00000204568 | HGLibA_30054 | 31 | -0.840119799 | MRPS18B | mitochondrial ribosomal protein S18B [Source:HGNC Symbol;Acc:HGNC:14516] |
| ENSG00000204710 | HGLibA_46576 | 31 | -0.840119799 | SPDYC | speedy/RINGO cell cycle regulator family member C [Source:HGNC Symbol;Acc:HGNC:32681] |
| ENSG00000205002 | HGLibA_00064 | 31 | -0.840119799 | AARD | alanine and arginine rich domain containing protein [Source:HGNC Symbol;Acc:HGNC:33842] |
| ENSG00000215274 | HGLibA_18434 | 31 | -0.840119799 | GAGE10 | G antigen 10 [Source:HGNC Symbol;Acc:HGNC:30968] |
| ENSG00000216490 | HGLibA_22605 | 31 | -0.840119799 | IFI30 | lysosomal thiol reductase [Source:HGNC Symbol;Acc:HGNC:5398]" |
| ENSG00000225327 | HGLibA_53271 | 31 | -0.840119799 | USP17L3 | ubiquitin specific peptidase 17-like family member 3 [Source:HGNC Symbol;Acc:HGNC:37175] |
| ENSG00000263002 | HGLibA_55645 | 31 | -0.840119799 | ZNF234 | zinc finger protein 234 [Source:HGNC Symbol;Acc:HGNC:13027] |
| ENSG00000266412 | HGLibA_31344 | 31 | -0.840119799 | NCOA4 | nuclear receptor coactivator 4 [Source:HGNC Symbol;Acc:HGNC:7671] |
| ENSG00000266412 | HGLibA_31345 | 31 | -0.840119799 | NCOA4 | nuclear receptor coactivator 4 [Source:HGNC Symbol;Acc:HGNC:7671] |
| ENSG00000267206 | HGLibA_26182 | 31 | -0.840119799 | LCN6 | lipocalin 6 [Source:HGNC Symbol;Acc:HGNC:17337] |
| ENSG00000276409 | HGLibA_07970 | 31 | -0.840119799 | CCL14 | C-C motif chemokine ligand 14 [Source:HGNC Symbol;Acc:HGNC:10612] |
| ENSG00000276644 | HGLibA_12275 | 31 | -0.840119799 | DACH1 | dachshund family transcription factor 1 [Source:HGNC Symbol;Acc:HGNC:2663] |
| ENSG00000279514 | HGLibA_33951 | 31 | -0.840119799 | OR4C16 | olfactory receptor family 4 subfamily C member 16 (gene/pseudogene) [Source:HGNC Symbol;Acc:HGNC:15172] |
| FAM122B | HGLibA_16068 | 31 | -0.840119799 | FAM122B | - |
| FAM132A | HGLibA_16114 | 31 | -0.840119799 | FAM132A | - |
| FAM49B | HGLibA_16627 | 31 | -0.840119799 | FAM49B | - |
| HIST2H2AA3 | HGLibA_21521 | 31 | -0.840119799 | HIST2H2AA3 | - |
| HRSP12 | HGLibA_22113 | 31 | -0.840119799 | HRSP12 | - |
| hsa-mir-1245b | HGLibA_57378 | 31 | -0.840119799 | hsa-mir-1245b | - |
| hsa-mir-195 | HGLibA_58088 | 31 | -0.840119799 | hsa-mir-195 | - |
| hsa-mir-214 | HGLibA_58248 | 31 | -0.840119799 | hsa-mir-214 | - |
| hsa-mir-3150a | HGLibA_58656 | 31 | -0.840119799 | hsa-mir-3150a | - |
| hsa-mir-3614 | HGLibA_59086 | 31 | -0.840119799 | hsa-mir-3614 | - |
| hsa-mir-362 | HGLibA_59110 | 31 | -0.840119799 | hsa-mir-362 | - |
| hsa-mir-3659 | HGLibA_59183 | 31 | -0.840119799 | hsa-mir-3659 | - |
| hsa-mir-4663 | HGLibA_60764 | 31 | -0.840119799 | hsa-mir-4663 | - |
| hsa-mir-4677 | HGLibA_60826 | 31 | -0.840119799 | hsa-mir-4677 | - |
| hsa-mir-4724 | HGLibA_60992 | 31 | -0.840119799 | hsa-mir-4724 | - |
| hsa-mir-5002 | HGLibA_61415 | 31 | -0.840119799 | hsa-mir-5002 | - |
| hsa-mir-5004 | HGLibA_61423 | 31 | -0.840119799 | hsa-mir-5004 | - |
| hsa-mir-501 | HGLibA_61451 | 31 | -0.840119799 | hsa-mir-501 | - |
| hsa-mir-514a-3 | HGLibA_61593 | 31 | -0.840119799 | hsa-mir-514a-3 | - |
| hsa-mir-526a-1 | HGLibA_61791 | 31 | -0.840119799 | hsa-mir-526a-1 | - |
| hsa-mir-604 | HGLibA_62507 | 31 | -0.840119799 | hsa-mir-604 | - |
| hsa-mir-6073 | HGLibA_62543 | 31 | -0.840119799 | hsa-mir-6073 | - |
| hsa-mir-6130 | HGLibA_62673 | 31 | -0.840119799 | hsa-mir-6130 | - |
| hsa-mir-6720 | HGLibA_63042 | 31 | -0.840119799 | hsa-mir-6720 | - |
| hsa-mir-6793 | HGLibA_63348 | 31 | -0.840119799 | hsa-mir-6793 | - |
| hsa-mir-6814 | HGLibA_63432 | 31 | -0.840119799 | hsa-mir-6814 | - |
| hsa-mir-6818 | HGLibA_63451 | 31 | -0.840119799 | hsa-mir-6818 | - |
| hsa-mir-6881 | HGLibA_63708 | 31 | -0.840119799 | hsa-mir-6881 | - |
| hsa-mir-7848 | HGLibA_63983 | 31 | -0.840119799 | hsa-mir-7848 | - |
| hsa-mir-873 | HGLibA_64202 | 31 | -0.840119799 | hsa-mir-873 | - |
| hsa-mir-937 | HGLibA_64323 | 31 | -0.840119799 | hsa-mir-937 | - |
| LOC100144595 | HGLibA_26813 | 31 | -0.840119799 | LOC100144595 | - |
| LOC100288524 | HGLibA_26835 | 31 | -0.840119799 | LOC100288524 | - |
| METTL21B | HGLibA_28978 | 31 | -0.840119799 | METTL21B | - |
| MPP5 | HGLibA_29759 | 31 | -0.840119799 | MPP5 | - |
| SMCR9 | HGLibA_45685 | 31 | -0.840119799 | SMCR9 | - |
| VAMP7_Y | HGLibA_53506 | 31 | -0.840119799 | VAMP7_Y | - |
| WDR63 | HGLibA_54147 | 31 | -0.840119799 | WDR63 | - |
| WDR92 | HGLibA_54221 | 31 | -0.840119799 | WDR92 | - |
| WISP3 | HGLibA_54325 | 31 | -0.840119799 | WISP3 | - |
| ADRBK2 | HGLibA_01067 | 30 | -0.850070836 | ADRBK2 | - |
| AGXT2L2 | HGLibA_01279 | 30 | -0.850070836 | AGXT2L2 | - |
| B3GNT1 | HGLibA_03980 | 30 | -0.850070836 | B3GNT1 | - |
| C10orf129 | HGLibA_05051 | 30 | -0.850070836 | C10orf129 | - |
| C10orf25 | HGLibA_05063 | 30 | -0.850070836 | C10orf25 | - |
| C11orf73 | HGLibA_05181 | 30 | -0.850070836 | C11orf73 | - |
| C22orf26 | HGLibA_06062 | 30 | -0.850070836 | C22orf26 | - |
| CCDC130 | HGLibA_07591 | 30 | -0.850070836 | CCDC130 | - |
| ENSG00000006453 | HGLibA_04125 | 30 | -0.850070836 | BAIAP2L1 | BAI1 associated protein 2 like 1 [Source:HGNC Symbol;Acc:HGNC:21649] |
| ENSG00000006756 | HGLibA_03133 | 30 | -0.850070836 | ARSD | arylsulfatase D [Source:HGNC Symbol;Acc:HGNC:717] |
| ENSG00000007516 | HGLibA_04131 | 30 | -0.850070836 | BAIAP3 | BAI1 associated protein 3 [Source:HGNC Symbol;Acc:HGNC:948] |
| ENSG00000016082 | HGLibA_23664 | 30 | -0.850070836 | ISL1 | ISL LIM homeobox 1 [Source:HGNC Symbol;Acc:HGNC:6132] |
| ENSG00000028528 | HGLibA_46024 | 30 | -0.850070836 | SNX1 | sorting nexin 1 [Source:HGNC Symbol;Acc:HGNC:11172] |
| ENSG00000051620 | HGLibA_21088 | 30 | -0.850070836 | HEBP2 | heme binding protein 2 [Source:HGNC Symbol;Acc:HGNC:15716] |
| ENSG00000065923 | HGLibA_45423 | 30 | -0.850070836 | SLC9A7 | solute carrier family 9 member A7 [Source:HGNC Symbol;Acc:HGNC:17123] |
| ENSG00000066135 | HGLibA_24506 | 30 | -0.850070836 | KDM4A | lysine demethylase 4A [Source:HGNC Symbol;Acc:HGNC:22978] |
| ENSG00000067082 | HGLibA_25078 | 30 | -0.850070836 | KLF6 | Kruppel like factor 6 [Source:HGNC Symbol;Acc:HGNC:2235] |
| ENSG00000070444 | HGLibA_29575 | 30 | -0.850070836 | MNT | MAX network transcriptional repressor [Source:HGNC Symbol;Acc:HGNC:7188] |
| ENSG00000072080 | HGLibA_46772 | 30 | -0.850070836 | SPP2 | secreted phosphoprotein 2 [Source:HGNC Symbol;Acc:HGNC:11256] |
| ENSG00000072778 | HGLibA_00378 | 30 | -0.850070836 | ACADVL | acyl-CoA dehydrogenase very long chain [Source:HGNC Symbol;Acc:HGNC:92] |
| ENSG00000072818 | HGLibA_00382 | 30 | -0.850070836 | ACAP1 | ankyrin repeat and PH domains 1 [Source:HGNC Symbol;Acc:HGNC:16467]" |
| ENSG00000079805 | HGLibA_13690 | 30 | -0.850070836 | DNM2 | dynamin 2 [Source:HGNC Symbol;Acc:HGNC:2974] |
| ENSG00000081052 | HGLibA_10577 | 30 | -0.850070836 | COL4A4 | collagen type IV alpha 4 chain [Source:HGNC Symbol;Acc:HGNC:2206] |
| ENSG00000083123 | HGLibA_04281 | 30 | -0.850070836 | BCKDHB | branched chain keto acid dehydrogenase E1 subunit beta [Source:HGNC Symbol;Acc:HGNC:987] |
| ENSG00000086205 | HGLibA_17781 | 30 | -0.850070836 | FOLH1 | folate hydrolase 1 [Source:HGNC Symbol;Acc:HGNC:3788] |
| ENSG00000088256 | HGLibA_19435 | 30 | -0.850070836 | GNA11 | G protein subunit alpha 11 [Source:HGNC Symbol;Acc:HGNC:4379] |
| ENSG00000088325 | HGLibA_51219 | 30 | -0.850070836 | TPX2 | microtubule nucleation factor [Source:HGNC Symbol;Acc:HGNC:1249]" |
| ENSG00000089195 | HGLibA_51696 | 30 | -0.850070836 | TRMT6 | tRNA methyltransferase 6 [Source:HGNC Symbol;Acc:HGNC:20900] |
| ENSG00000089199 | HGLibA_09434 | 30 | -0.850070836 | CHGB | chromogranin B [Source:HGNC Symbol;Acc:HGNC:1930] |
| ENSG00000089685 | HGLibA_04520 | 30 | -0.850070836 | BIRC5 | baculoviral IAP repeat containing 5 [Source:HGNC Symbol;Acc:HGNC:593] |
| ENSG00000093217 | HGLibA_54615 | 30 | -0.850070836 | XYLB | xylulokinase [Source:HGNC Symbol;Acc:HGNC:12839] |
| ENSG00000099246 | HGLibA_39822 | 30 | -0.850070836 | RAB18 | member RAS oncogene family [Source:HGNC Symbol;Acc:HGNC:14244]" |
| ENSG00000100300 | HGLibA_51959 | 30 | -0.850070836 | TSPO | translocator protein [Source:HGNC Symbol;Acc:HGNC:1158] |
| ENSG00000100330 | HGLibA_30428 | 30 | -0.850070836 | MTMR3 | myotubularin related protein 3 [Source:HGNC Symbol;Acc:HGNC:7451] |
| ENSG00000101236 | HGLibA_41653 | 30 | -0.850070836 | RNF24 | ring finger protein 24 [Source:HGNC Symbol;Acc:HGNC:13779] |
| ENSG00000101413 | HGLibA_42048 | 30 | -0.850070836 | RPRD1B | regulation of nuclear pre-mRNA domain containing 1B [Source:HGNC Symbol;Acc:HGNC:16209] |
| ENSG00000102409 | HGLibA_04447 | 30 | -0.850070836 | BEX4 | brain expressed X-linked 4 [Source:HGNC Symbol;Acc:HGNC:25475] |
| ENSG00000102837 | HGLibA_33352 | 30 | -0.850070836 | OLFM4 | olfactomedin 4 [Source:HGNC Symbol;Acc:HGNC:17190] |
| ENSG00000104290 | HGLibA_18269 | 30 | -0.850070836 | FZD3 | frizzled class receptor 3 [Source:HGNC Symbol;Acc:HGNC:4041] |
| ENSG00000104332 | HGLibA_43735 | 30 | -0.850070836 | SFRP1 | secreted frizzled related protein 1 [Source:HGNC Symbol;Acc:HGNC:10776] |
| ENSG00000104888 | HGLibA_44477 | 30 | -0.850070836 | SLC17A7 | solute carrier family 17 member 7 [Source:HGNC Symbol;Acc:HGNC:16704] |
| ENSG00000105227 | HGLibA_38992 | 30 | -0.850070836 | PRX | periaxin [Source:HGNC Symbol;Acc:HGNC:13797] |
| ENSG00000105357 | HGLibA_30714 | 30 | -0.850070836 | MYH14 | myosin heavy chain 14 [Source:HGNC Symbol;Acc:HGNC:23212] |
| ENSG00000105835 | HGLibA_31086 | 30 | -0.850070836 | NAMPT | nicotinamide phosphoribosyltransferase [Source:HGNC Symbol;Acc:HGNC:30092] |
| ENSG00000106305 | HGLibA_01358 | 30 | -0.850070836 | AIMP2 | aminoacyl tRNA synthetase complex interacting multifunctional protein 2 [Source:HGNC Symbol;Acc:HGNC:20609] |
| ENSG00000106560 | HGLibA_19075 | 30 | -0.850070836 | GIMAP2 | IMAP family member 2 [Source:HGNC Symbol;Acc:HGNC:21789]" |
| ENSG00000107745 | HGLibA_29222 | 30 | -0.850070836 | MICU1 | mitochondrial calcium uptake 1 [Source:HGNC Symbol;Acc:HGNC:1530] |
| ENSG00000111206 | HGLibA_17907 | 30 | -0.850070836 | FOXM1 | forkhead box M1 [Source:HGNC Symbol;Acc:HGNC:3818] |
| ENSG00000111276 | HGLibA_08825 | 30 | -0.850070836 | CDKN1B | cyclin dependent kinase inhibitor 1B [Source:HGNC Symbol;Acc:HGNC:1785] |
| ENSG00000111727 | HGLibA_20963 | 30 | -0.850070836 | HCFC2 | host cell factor C2 [Source:HGNC Symbol;Acc:HGNC:24972] |
| ENSG00000111846 | HGLibA_18812 | 30 | -0.850070836 | GCNT2 | glucosaminyl (N-acetyl) transferase 2 (I blood group) [Source:HGNC Symbol;Acc:HGNC:4204] |
| ENSG00000112118 | HGLibA_28607 | 30 | -0.850070836 | MCM3 | minichromosome maintenance complex component 3 [Source:HGNC Symbol;Acc:HGNC:6945] |
| ENSG00000112335 | HGLibA_46082 | 30 | -0.850070836 | SNX3 | sorting nexin 3 [Source:HGNC Symbol;Acc:HGNC:11174] |
| ENSG00000112902 | HGLibA_43359 | 30 | -0.850070836 | SEMA5A | semaphorin 5A [Source:HGNC Symbol;Acc:HGNC:10736] |
| ENSG00000113328 | HGLibA_08092 | 30 | -0.850070836 | CCNG1 | cyclin G1 [Source:HGNC Symbol;Acc:HGNC:1592] |
| ENSG00000113657 | HGLibA_13929 | 30 | -0.850070836 | DPYSL3 | dihydropyrimidinase like 3 [Source:HGNC Symbol;Acc:HGNC:3015] |
| ENSG00000114670 | HGLibA_31643 | 30 | -0.850070836 | NEK11 | NIMA related kinase 11 [Source:HGNC Symbol;Acc:HGNC:18593] |
| ENSG00000116406 | HGLibA_14460 | 30 | -0.850070836 | EDEM3 | ER degradation enhancing alpha-mannosidase like protein 3 [Source:HGNC Symbol;Acc:HGNC:16787] |
| ENSG00000116731 | HGLibA_38386 | 30 | -0.850070836 | PRDM2 | PR/SET domain 2 [Source:HGNC Symbol;Acc:HGNC:9347] |
| ENSG00000116745 | HGLibA_41826 | 30 | -0.850070836 | RPE65 | retinoid isomerohydrolase [Source:HGNC Symbol;Acc:HGNC:10294]" |
| ENSG00000117222 | HGLibA_40432 | 30 | -0.850070836 | RBBP5 | histone lysine methyltransferase complex subunit [Source:HGNC Symbol;Acc:HGNC:9888]" |
| ENSG00000118620 | HGLibA_55977 | 30 | -0.850070836 | ZNF430 | zinc finger protein 430 [Source:HGNC Symbol;Acc:HGNC:20808] |
| ENSG00000119878 | HGLibA_11163 | 30 | -0.850070836 | CRIPT | CXXC repeat containing interactor of PDZ3 domain [Source:HGNC Symbol;Acc:HGNC:14312] |
| ENSG00000119922 | HGLibA_22630 | 30 | -0.850070836 | IFIT2 | interferon induced protein with tetratricopeptide repeats 2 [Source:HGNC Symbol;Acc:HGNC:5409] |
| ENSG00000121579 | HGLibA_31000 | 30 | -0.850070836 | NAA50 | NatE catalytic subunit [Source:HGNC Symbol;Acc:HGNC:29533]" |
| ENSG00000123552 | HGLibA_53375 | 30 | -0.850070836 | USP45 | ubiquitin specific peptidase 45 [Source:HGNC Symbol;Acc:HGNC:20080] |
| ENSG00000124102 | HGLibA_36490 | 30 | -0.850070836 | PI3 | peptidase inhibitor 3 [Source:HGNC Symbol;Acc:HGNC:8947] |

| | | | | | |
|---|---|---|---|---|---|
| ENSG00000124191 | HGLibA_51039 | 30 | -0.850070836 | TOX2 | TOX high mobility group box family member 2 [Source:HGNC Symbol;Acc:HGNC:16095] |
| ENSG00000124469 | HGLibA_08953 | 30 | -0.850070836 | CEACAM8 | carcinoembryonic antigen related cell adhesion molecule 8 [Source:HGNC Symbol;Acc:HGNC:1820] |
| ENSG00000125449 | HGLibA_03030 | 30 | -0.850070836 | ARMC7 | armadillo repeat containing 7 [Source:HGNC Symbol;Acc:HGNC:26168] |
| ENSG00000125869 | HGLibA_25985 | 30 | -0.850070836 | LAMP5 | lysosomal associated membrane protein family member 5 [Source:HGNC Symbol;Acc:HGNC:16097] |
| ENSG00000125875 | HGLibA_48498 | 30 | -0.850070836 | TBC1D20 | TBC1 domain family member 20 [Source:HGNC Symbol;Acc:HGNC:16133] |
| ENSG00000126010 | HGLibA_20376 | 30 | -0.850070836 | GRPR | gastrin releasing peptide receptor [Source:HGNC Symbol;Acc:HGNC:4609] |
| ENSG00000126351 | HGLibA_49387 | 30 | -0.850070836 | THRA | thyroid hormone receptor alpha [Source:HGNC Symbol;Acc:HGNC:11796] |
| ENSG00000127125 | HGLibA_37862 | 30 | -0.850070836 | PPCS | phosphopantothenoylcysteine synthetase [Source:HGNC Symbol;Acc:HGNC:25686] |
| ENSG00000127561 | HGLibA_47997 | 30 | -0.850070836 | SYNGR3 | synaptogyrin 3 [Source:HGNC Symbol;Acc:HGNC:11501] |
| ENSG00000127774 | HGLibA_15017 | 30 | -0.850070836 | EMC6 | ER membrane protein complex subunit 6 [Source:HGNC Symbol;Acc:HGNC:28430] |
| ENSG00000127928 | HGLibA_19538 | 30 | -0.850070836 | GNGT1 | G protein subunit gamma transducin 1 [Source:HGNC Symbol;Acc:HGNC:4411] |
| ENSG00000127955 | HGLibA_19450 | 30 | -0.850070836 | GNAI1 | G protein subunit alpha i1 [Source:HGNC Symbol;Acc:HGNC:4384] |
| ENSG00000128298 | HGLibA_04127 | 30 | -0.850070836 | BAIAP2L2 | BAI1 associated protein 2 like 2 [Source:HGNC Symbol;Acc:HGNC:26203] |
| ENSG00000128609 | HGLibA_31465 | 30 | -0.850070836 | NDUFA5 | NADH:ubiquinone oxidoreductase subunit A5 [Source:HGNC Symbol;Acc:HGNC:7688] |
| ENSG00000128714 | HGLibA_21995 | 30 | -0.850070836 | HOXD13 | homeobox D13 [Source:HGNC Symbol;Acc:HGNC:5136] |
| ENSG00000129873 | HGLibA_08914 | 30 | -0.850070836 | CDY2B | chromodomain Y-linked 2B [Source:HGNC Symbol;Acc:HGNC:23921] |
| ENSG00000130224 | HGLibA_27140 | 30 | -0.850070836 | LRCH2 | leucine rich repeats and calponin homology domain containing 2 [Source:HGNC Symbol;Acc:HGNC:29292] |
| ENSG00000130255 | HGLibA_41953 | 30 | -0.850070836 | RPL36 | ribosomal protein L36 [Source:HGNC Symbol;Acc:HGNC:13631] |
| ENSG00000130309 | HGLibA_10640 | 30 | -0.850070836 | COLGALT1 | collagen beta(1-O)galactosyltransferase 1 [Source:HGNC Symbol;Acc:HGNC:26182] |
| ENSG00000130772 | HGLibA_28765 | 30 | -0.850070836 | MED18 | mediator complex subunit 18 [Source:HGNC Symbol;Acc:HGNC:25944] |
| ENSG00000131055 | HGLibA_10832 | 30 | -0.850070836 | COX4I2 | cytochrome c oxidase subunit 4I2 [Source:HGNC Symbol;Acc:HGNC:16232] |
| ENSG00000131379 | HGLibA_06217 | 30 | -0.850070836 | C3orf20 | chromosome 3 open reading frame 20 [Source:HGNC Symbol;Acc:HGNC:25320] |
| ENSG00000131508 | HGLibA_52585 | 30 | -0.850070836 | UBE2D2 | ubiquitin conjugating enzyme E2 D2 [Source:HGNC Symbol;Acc:HGNC:12475] |
| ENSG00000132122 | HGLibA_46521 | 30 | -0.850070836 | SPATA6 | spermatogenesis associated 6 [Source:HGNC Symbol;Acc:HGNC:18309] |
| ENSG00000132549 | HGLibA_53735 | 30 | -0.850070836 | VPS13B | vacuolar protein sorting 13 homolog B [Source:HGNC Symbol;Acc:HGNC:2183] |
| ENSG00000132631 | HGLibA_43037 | 30 | -0.850070836 | SCP2D1 | SCP2 sterol binding domain containing 1 [Source:HGNC Symbol;Acc:HGNC:16211] |
| ENSG00000133059 | HGLibA_14050 | 30 | -0.850070836 | DSTYK | dual serine/threonine and tyrosine protein kinase [Source:HGNC Symbol;Acc:HGNC:29043] |
| ENSG00000133112 | HGLibA_51211 | 30 | -0.850070836 | TPT1 | translationally-controlled 1 [Source:HGNC Symbol;Acc:HGNC:12022]" |
| ENSG00000133124 | HGLibA_23625 | 30 | -0.850070836 | IRS4 | insulin receptor substrate 4 [Source:HGNC Symbol;Acc:HGNC:6128] |
| ENSG00000133142 | HGLibA_48713 | 30 | -0.850070836 | TCEAL4 | transcription elongation factor A like 4 [Source:HGNC Symbol;Acc:HGNC:26121] |
| ENSG00000135451 | HGLibA_51718 | 30 | -0.850070836 | TROAP | trophinin associated protein [Source:HGNC Symbol;Acc:HGNC:12327] |
| ENSG00000135502 | HGLibA_44782 | 30 | -0.850070836 | SLC26A10 | solute carrier family 26 member 10 [Source:HGNC Symbol;Acc:HGNC:14470] |
| ENSG00000136451 | HGLibA_53626 | 30 | -0.850070836 | VEZF1 | vascular endothelial zinc finger 1 [Source:HGNC Symbol;Acc:HGNC:12949] |
| ENSG00000136643 | HGLibA_42181 | 30 | -0.850070836 | RPS6KC1 | ribosomal protein S6 kinase C1 [Source:HGNC Symbol;Acc:HGNC:10439] |
| ENSG00000136842 | HGLibA_50594 | 30 | -0.850070836 | TMOD1 | tropomodulin 1 [Source:HGNC Symbol;Acc:HGNC:11871] |
| ENSG00000137142 | HGLibA_22835 | 30 | -0.850070836 | IGFBPL1 | insulin like growth factor binding protein like 1 [Source:HGNC Symbol;Acc:HGNC:20081] |
| ENSG00000137817 | HGLibA_35242 | 30 | -0.850070836 | PARP6 | poly(ADP-ribose) polymerase family member 6 [Source:HGNC Symbol;Acc:HGNC:26921] |
| ENSG00000139874 | HGLibA_47175 | 30 | -0.850070836 | SSTR1 | somatostatin receptor 1 [Source:HGNC Symbol;Acc:HGNC:11330] |
| ENSG00000140262 | HGLibA_48761 | 30 | -0.850070836 | TCF12 | transcription factor 12 [Source:HGNC Symbol;Acc:HGNC:11623] |
| ENSG00000140455 | HGLibA_53325 | 30 | -0.850070836 | USP3 | ubiquitin specific peptidase 3 [Source:HGNC Symbol;Acc:HGNC:12626] |
| ENSG00000140522 | HGLibA_41342 | 30 | -0.850070836 | RLBP1 | retinaldehyde binding protein 1 [Source:HGNC Symbol;Acc:HGNC:10024] |
| ENSG00000141294 | HGLibA_27368 | 30 | -0.850070836 | LRRC46 | leucine rich repeat containing 46 [Source:HGNC Symbol;Acc:HGNC:25047] |
| ENSG00000141404 | HGLibA_19461 | 30 | -0.850070836 | GNAL | G protein subunit alpha L [Source:HGNC Symbol;Acc:HGNC:4388] |
| ENSG00000141738 | HGLibA_20232 | 30 | -0.850070836 | GRB7 | growth factor receptor bound protein 7 [Source:HGNC Symbol;Acc:HGNC:4567] |
| ENSG00000142686 | HGLibA_05832 | 30 | -0.850070836 | C1orf216 | chromosome 1 open reading frame 216 [Source:HGNC Symbol;Acc:HGNC:26800] |
| ENSG00000142856 | HGLibA_23783 | 30 | -0.850070836 | ITGB3BP | integrin subunit beta 3 binding protein [Source:HGNC Symbol;Acc:HGNC:6157] |
| ENSG00000143228 | HGLibA_32996 | 30 | -0.850070836 | NUF2 | NDC80 kinetochore complex component NUF2 [Source:HGNC Symbol;Acc:HGNC:14621] |
| ENSG00000143469 | HGLibA_48060 | 30 | -0.850070836 | SYT14 | synaptotagmin 14 [Source:HGNC Symbol;Acc:HGNC:23143] |
| ENSG00000143786 | HGLibA_10289 | 30 | -0.850070836 | CNIH3 | cornichon family AMPA receptor auxiliary protein 3 [Source:HGNC Symbol;Acc:HGNC:26802] |
| ENSG00000144566 | HGLibA_39962 | 30 | -0.850070836 | RAB5A | member RAS oncogene family [Source:HGNC Symbol;Acc:HGNC:9783]" |
| ENSG00000144802 | HGLibA_31836 | 30 | -0.850070836 | NFKBIZ | NFKB inhibitor zeta [Source:HGNC Symbol;Acc:HGNC:29805] |
| ENSG00000145192 | HGLibA_01318 | 30 | -0.850070836 | AHSG | alpha 2-HS glycoprotein [Source:HGNC Symbol;Acc:HGNC:349] |
| ENSG00000145284 | HGLibA_42887 | 30 | -0.850070836 | SCD5 | stearoyl-CoA desaturase 5 [Source:HGNC Symbol;Acc:HGNC:21088] |
| ENSG00000145681 | HGLibA_20841 | 30 | -0.850070836 | HAPLN1 | hyaluronan and proteoglycan link protein 1 [Source:HGNC Symbol;Acc:HGNC:2380] |
| ENSG00000146007 | HGLibA_55370 | 30 | -0.850070836 | ZMAT2 | zinc finger matrin-type 2 [Source:HGNC Symbol;Acc:HGNC:26433] |
| ENSG00000146457 | HGLibA_54431 | 30 | -0.850070836 | WTAP | WT1 associated protein [Source:HGNC Symbol;Acc:HGNC:16846] |
| ENSG00000146733 | HGLibA_39226 | 30 | -0.850070836 | PSPH | phosphoserine phosphatase [Source:HGNC Symbol;Acc:HGNC:9577] |
| ENSG00000146909 | HGLibA_32286 | 30 | -0.850070836 | NOM1 | nucleolar protein with MIF4G domain 1 [Source:HGNC Symbol;Acc:HGNC:13244] |
| ENSG00000147010 | HGLibA_43962 | 30 | -0.850070836 | SH3KBP1 | SH3 domain containing kinase binding protein 1 [Source:HGNC Symbol;Acc:HGNC:13867] |
| ENSG00000148488 | HGLibA_47304 | 30 | -0.850070836 | ST8SIA6 | 8-sialyltransferase 6 [Source:HGNC Symbol;Acc:HGNC:23317]" |
| ENSG00000148677 | HGLibA_01987 | 30 | -0.850070836 | ANKRD1 | ankyrin repeat domain 1 [Source:HGNC Symbol;Acc:HGNC:15819] |
| ENSG00000149133 | HGLibA_34287 | 30 | -0.850070836 | OR5F1 | olfactory receptor family 5 subfamily F member 1 [Source:HGNC Symbol;Acc:HGNC:8343] |
| ENSG00000149571 | HGLibA_25005 | 30 | -0.850070836 | KIRREL3 | kirre like nephrin family adhesion molecule 3 [Source:HGNC Symbol;Acc:HGNC:23204] |
| ENSG00000149679 | HGLibA_06845 | 30 | -0.850070836 | CABLES2 | Cdk5 and Abl enzyme substrate 2 [Source:HGNC Symbol;Acc:HGNC:16143] |
| ENSG00000150394 | HGLibA_08693 | 30 | -0.850070836 | CDH8 | cadherin 8 [Source:HGNC Symbol;Acc:HGNC:1767] |
| ENSG00000150995 | HGLibA_23852 | 30 | -0.850070836 | ITPR1 | 5-trisphosphate receptor type 1 [Source:HGNC Symbol;Acc:HGNC:6180]" |
| ENSG00000152128 | HGLibA_50031 | 30 | -0.850070836 | TMEM163 | transmembrane protein 163 [Source:HGNC Symbol;Acc:HGNC:25380] |
| ENSG00000152240 | HGLibA_20875 | 30 | -0.850070836 | HAUS1 | HAUS augmin like complex subunit 1 [Source:HGNC Symbol;Acc:HGNC:25174] |
| ENSG00000152556 | HGLibA_36167 | 30 | -0.850070836 | PFKM | muscle [Source:HGNC Symbol;Acc:HGNC:8877]" |
| ENSG00000152782 | HGLibA_35098 | 30 | -0.850070836 | PANK1 | pantothenate kinase 1 [Source:HGNC Symbol;Acc:HGNC:8598] |
| ENSG00000157542 | HGLibA_24286 | 30 | -0.850070836 | KCNJ6 | potassium voltage-gated channel subfamily J member 6 [Source:HGNC Symbol;Acc:HGNC:6267] |
| ENSG00000158716 | HGLibA_14165 | 30 | -0.850070836 | DUSP23 | dual specificity phosphatase 23 [Source:HGNC Symbol;Acc:HGNC:21480] |
| ENSG00000161533 | HGLibA_00496 | 30 | -0.850070836 | ACOX1 | acyl-CoA oxidase 1 [Source:HGNC Symbol;Acc:HGNC:119] |
| ENSG00000161618 | HGLibA_01536 | 30 | -0.850070836 | ALDH16A1 | aldehyde dehydrogenase 16 family member A1 [Source:HGNC Symbol;Acc:HGNC:28114] |
| ENSG00000162241 | HGLibA_44750 | 30 | -0.850070836 | SLC25A45 | solute carrier family 25 member 45 [Source:HGNC Symbol;Acc:HGNC:27442] |
| ENSG00000162241 | HGLibA_44751 | 30 | -0.850070836 | SLC25A45 | solute carrier family 25 member 45 [Source:HGNC Symbol;Acc:HGNC:27442] |
| ENSG00000162521 | HGLibA_40428 | 30 | -0.850070836 | RBBP4 | chromatin remodeling factor [Source:HGNC Symbol;Acc:HGNC:9887]" |
| ENSG00000163216 | HGLibA_46815 | 30 | -0.850070836 | SPRR2D | small proline rich protein 2D [Source:HGNC Symbol;Acc:HGNC:11264] |
| ENSG00000163464 | HGLibA_11882 | 30 | -0.850070836 | CXCR1 | C-X-C motif chemokine receptor 1 [Source:HGNC Symbol;Acc:HGNC:6026] |
| ENSG00000163808 | HGLibA_24864 | 30 | -0.850070836 | KIF15 | kinesin family member 15 [Source:HGNC Symbol;Acc:HGNC:17273] |
| ENSG00000163874 | HGLibA_54953 | 30 | -0.850070836 | ZC3H12A | zinc finger CCCH-type containing 12A [Source:HGNC Symbol;Acc:HGNC:26259] |
| ENSG00000164136 | HGLibA_22979 | 30 | -0.850070836 | IL15 | interleukin 15 [Source:HGNC Symbol;Acc:HGNC:5977] |
| ENSG00000164161 | HGLibA_21262 | 30 | -0.850070836 | HHIP | hedgehog interacting protein [Source:HGNC Symbol;Acc:HGNC:14866] |
| ENSG00000164161 | HGLibA_21261 | 30 | -0.850070836 | HHIP | hedgehog interacting protein [Source:HGNC Symbol;Acc:HGNC:14866] |
| ENSG00000164933 | HGLibA_44707 | 30 | -0.850070836 | SLC25A32 | solute carrier family 25 member 32 [Source:HGNC Symbol;Acc:HGNC:29683] |
| ENSG00000166012 | HGLibA_48223 | 30 | -0.850070836 | TAF1D | RNA polymerase I subunit D [Source:HGNC Symbol;Acc:HGNC:28759]" |
| ENSG00000166225 | HGLibA_18048 | 30 | -0.850070836 | FRS2 | fibroblast growth factor receptor substrate 2 [Source:HGNC Symbol;Acc:HGNC:16971] |
| ENSG00000166707 | HGLibA_55023 | 30 | -0.850070836 | ZCCHC18 | zinc finger CCHC-type containing 18 [Source:HGNC Symbol;Acc:HGNC:32459] |
| ENSG00000166949 | HGLibA_45575 | 30 | -0.850070836 | SMAD3 | SMAD family member 3 [Source:HGNC Symbol;Acc:HGNC:6769] |
| ENSG00000167703 | HGLibA_45152 | 30 | -0.850070836 | SLC43A2 | solute carrier family 43 member 2 [Source:HGNC Symbol;Acc:HGNC:23087] |
| ENSG00000167771 | HGLibA_40712 | 30 | -0.850070836 | RCOR2 | REST corepressor 2 [Source:HGNC Symbol;Acc:HGNC:27455] |
| ENSG00000167797 | HGLibA_08765 | 30 | -0.850070836 | CDK2AP2 | cyclin dependent kinase 2 associated protein 2 [Source:HGNC Symbol;Acc:HGNC:30833] |
| ENSG00000168589 | HGLibA_14262 | 30 | -0.850070836 | DYNLRB2 | dynein light chain roadblock-type 2 [Source:HGNC Symbol;Acc:HGNC:15467] |
| ENSG00000169021 | HGLibA_53120 | 30 | -0.850070836 | UQCRFS1 | Rieske iron-sulfur polypeptide 1 [Source:HGNC Symbol;Acc:HGNC:12587]" |
| ENSG00000169032 | HGLibA_28138 | 30 | -0.850070836 | MAP2K1 | mitogen-activated protein kinase kinase 1 [Source:HGNC Symbol;Acc:HGNC:6840] |
| ENSG00000169064 | HGLibA_54783 | 30 | -0.850070836 | ZBBX | zinc finger B-box domain containing [Source:HGNC Symbol;Acc:HGNC:26245] |
| ENSG00000169118 | HGLibA_11393 | 30 | -0.850070836 | CSNK1G1 | casein kinase 1 gamma 1 [Source:HGNC Symbol;Acc:HGNC:2454] |
| ENSG00000169397 | HGLibA_41405 | 30 | -0.850070836 | RNASE3 | ribonuclease A family member 3 [Source:HGNC Symbol;Acc:HGNC:10046] |
| ENSG00000170100 | HGLibA_56727 | 30 | -0.850070836 | ZNF778 | zinc finger protein 778 [Source:HGNC Symbol;Acc:HGNC:26479] |
| ENSG00000170348 | HGLibA_49802 | 30 | -0.850070836 | TMED10 | transmembrane p24 trafficking protein 10 [Source:HGNC Symbol;Acc:HGNC:16998] |
| ENSG00000170953 | HGLibA_34494 | 30 | -0.850070836 | OR8B12 | olfactory receptor family 8 subfamily B member 12 [Source:HGNC Symbol;Acc:HGNC:15307] |
| ENSG00000171791 | HGLibA_04296 | 30 | -0.850070836 | BCL2 | apoptosis regulator [Source:HGNC Symbol;Acc:HGNC:990]" |
| ENSG00000172005 | HGLibA_28021 | 30 | -0.850070836 | MAL | T cell differentiation protein [Source:HGNC Symbol;Acc:HGNC:6817]" |
| ENSG00000172362 | HGLibA_34256 | 30 | -0.850070836 | OR5B12 | olfactory receptor family 5 subfamily B member 12 [Source:HGNC Symbol;Acc:HGNC:15432] |
| ENSG00000172765 | HGLibA_49773 | 30 | -0.850070836 | TMCC1 | transmembrane and coiled-coil domain family 1 [Source:HGNC Symbol;Acc:HGNC:29116] |
| ENSG00000172782 | HGLibA_15944 | 30 | -0.850070836 | FADS6 | fatty acid desaturase 6 [Source:HGNC Symbol;Acc:HGNC:30459] |
| ENSG00000172927 | HGLibA_30692 | 30 | -0.850070836 | MYEOV | myeloma overexpressed [Source:HGNC Symbol;Acc:HGNC:7563] |
| ENSG00000172932 | HGLibA_02010 | 30 | -0.850070836 | ANKRD13D | ankyrin repeat domain 13D [Source:HGNC Symbol;Acc:HGNC:27880] |
| ENSG00000173258 | HGLibA_56073 | 30 | -0.850070836 | ZNF483 | zinc finger protein 483 [Source:HGNC Symbol;Acc:HGNC:23384] |
| ENSG00000173572 | HGLibA_32108 | 30 | -0.850070836 | NLRP13 | NLR family pyrin domain containing 13 [Source:HGNC Symbol;Acc:HGNC:22937] |
| ENSG00000173715 | HGLibA_05186 | 30 | -0.850070836 | C11orf80 | chromosome 11 open reading frame 80 [Source:HGNC Symbol;Acc:HGNC:26197] |
| ENSG00000173757 | HGLibA_47396 | 30 | -0.850070836 | STAT5B | signal transducer and activator of transcription 5B [Source:HGNC Symbol;Acc:HGNC:11367] |
| ENSG00000174173 | HGLibA_51666 | 30 | -0.850070836 | TRMT10C | mitochondrial RNase P subunit [Source:HGNC Symbol;Acc:HGNC:26022]" |
| ENSG00000174482 | HGLibA_26587 | 30 | -0.850070836 | LINGO2 | leucine rich repeat and Ig domain containing 2 [Source:HGNC Symbol;Acc:HGNC:21207] |
| ENSG00000174695 | HGLibA_50039 | 30 | -0.850070836 | TMEM167A | transmembrane protein 167A [Source:HGNC Symbol;Acc:HGNC:28330] |
| ENSG00000174914 | HGLibA_34569 | 30 | -0.850070836 | OR9G1 | olfactory receptor family 9 subfamily G member 1 [Source:HGNC Symbol;Acc:HGNC:15319] |
| ENSG00000175106 | HGLibA_52362 | 30 | -0.850070836 | TVP23C | trans-golgi network vesicle protein 23 homolog C [Source:HGNC Symbol;Acc:HGNC:30453] |
| ENSG00000175324 | HGLibA_27519 | 30 | -0.850070836 | LSM1 | mRNA degradation associated [Source:HGNC Symbol;Acc:HGNC:20472]" |
| ENSG00000176742 | HGLibA_34137 | 30 | -0.850070836 | OR51V1 | olfactory receptor family 51 subfamily V member 1 [Source:HGNC Symbol;Acc:HGNC:19597] |
| ENSG00000176787 | HGLibA_34157 | 30 | -0.850070836 | OR52E2 | olfactory receptor family 52 subfamily E member 2 [Source:HGNC Symbol;Acc:HGNC:14769] |
| ENSG00000177034 | HGLibA_30518 | 30 | -0.850070836 | MTX3 | metaxin 3 [Source:HGNC Symbol;Acc:HGNC:24812] |
| ENSG00000177354 | HGLibA_05092 | 30 | -0.850070836 | C10orf71 | chromosome 10 open reading frame 71 [Source:HGNC Symbol;Acc:HGNC:26973] |
| ENSG00000177613 | HGLibA_11489 | 30 | -0.850070836 | CSTF2T | cleavage stimulation factor subunit 2 tau variant [Source:HGNC Symbol;Acc:HGNC:17086] |
| ENSG00000177854 | HGLibA_50128 | 30 | -0.850070836 | TMEM187 | transmembrane protein 187 [Source:HGNC Symbol;Acc:HGNC:13705] |
| ENSG00000177984 | HGLibA_26176 | 30 | -0.850070836 | LCN15 | lipocalin 15 [Source:HGNC Symbol;Acc:HGNC:33777] |
| ENSG00000179455 | HGLibA_29354 | 30 | -0.850070836 | MKRN3 | makorin ring finger protein 3 [Source:HGNC Symbol;Acc:HGNC:7114] |
| ENSG00000180346 | HGLibA_49466 | 30 | -0.850070836 | TIGD2 | tigger transposable element derived 2 [Source:HGNC Symbol;Acc:HGNC:18333] |
| ENSG00000180432 | HGLibA_12216 | 30 | -0.850070836 | CYP8B1 | cytochrome P450 family 8 subfamily B member 1 [Source:HGNC Symbol;Acc:HGNC:2653] |
| ENSG00000181617 | HGLibA_17250 | 30 | -0.850070836 | FDCSP | follicular dendritic cell secreted protein [Source:HGNC Symbol;Acc:HGNC:19215] |
| ENSG00000182077 | HGLibA_39280 | 30 | -0.850070836 | PTCHD3 | patched domain containing 3 [Source:HGNC Symbol;Acc:HGNC:24776] |
| ENSG00000182199 | HGLibA_44063 | 30 | -0.850070836 | SHMT2 | serine hydroxymethyltransferase 2 [Source:HGNC Symbol;Acc:HGNC:10852] |
| ENSG00000182447 | HGLibA_34732 | 30 | -0.850070836 | OTOL1 | otolin 1 [Source:HGNC Symbol;Acc:HGNC:34071] |
| ENSG00000183117 | HGLibA_11362 | 30 | -0.850070836 | CSMD1 | CUB and Sushi multiple domains 1 [Source:HGNC Symbol;Acc:HGNC:14026] |
| ENSG00000183401 | HGLibA_07667 | 30 | -0.850070836 | CCDC159 | coiled-coil domain containing 159 [Source:HGNC Symbol;Acc:HGNC:26996] |
| ENSG00000183638 | HGLibA_41790 | 30 | -0.850070836 | RP1L1 | RP1 like 1 [Source:HGNC Symbol;Acc:HGNC:15946] |

| | | | | | |
|---|---|---|---|---|---|
| ENSG00000183831 | HGLibA_02105 | 30 | -0.850070836 | ANKRD45 | ankyrin repeat domain 45 [Source:HGNC Symbol;Acc:HGNC:24786] |
| ENSG00000184148 | HGLibA_46830 | 30 | -0.850070836 | SPRR4 | small proline rich protein 4 [Source:HGNC Symbol;Acc:HGNC:23173] |
| ENSG00000184388 | HGLibA_34930 | 30 | -0.850070836 | PABPC1L2B | poly(A) binding protein cytoplasmic 1 like 2B [Source:HGNC Symbol;Acc:HGNC:31852] |
| ENSG00000184478 | HGLibA_34214 | 30 | -0.850070836 | OR56A3 | olfactory receptor family 56 subfamily A member 3 [Source:HGNC Symbol;Acc:HGNC:14786] |
| ENSG00000184999 | HGLibA_44536 | 30 | -0.850070836 | SLC22A10 | solute carrier family 22 member 10 [Source:HGNC Symbol;Acc:HGNC:18057] |
| ENSG00000185219 | HGLibA_56016 | 30 | -0.850070836 | ZNF445 | zinc finger protein 445 [Source:HGNC Symbol;Acc:HGNC:21018] |
| ENSG00000185269 | HGLibA_32359 | 30 | -0.850070836 | NOTUM | palmitoleoyl-protein carboxylesterase [Source:HGNC Symbol;Acc:HGNC:27106]" |
| ENSG00000186017 | HGLibA_56259 | 30 | -0.850070836 | ZNF566 | zinc finger protein 566 [Source:HGNC Symbol;Acc:HGNC:25919] |
| ENSG00000186086 | HGLibA_31238 | 30 | -0.850070836 | NBPF6 | NBPF member 6 [Source:HGNC Symbol;Acc:HGNC:31988] |
| ENSG00000187902 | HGLibA_44047 | 30 | -0.850070836 | SHISA7 | shisa family member 7 [Source:HGNC Symbol;Acc:HGNC:35409] |
| ENSG00000188735 | HGLibA_49899 | 30 | -0.850070836 | TMEM120B | transmembrane protein 120B [Source:HGNC Symbol;Acc:HGNC:32008] |
| ENSG00000189052 | HGLibA_09309 | 30 | -0.850070836 | CGB5 | chorionic gonadotropin subunit beta 5 [Source:HGNC Symbol;Acc:HGNC:16452] |
| ENSG00000196104 | HGLibA_46753 | 30 | -0.850070836 | SPOCK3 | cwcv and kazal like domains proteoglycan 3 [Source:HGNC Symbol;Acc:HGNC:13565]" |
| ENSG00000196199 | HGLibA_29730 | 30 | -0.850070836 | MPHOSPH8 | M-phase phosphoprotein 8 [Source:HGNC Symbol;Acc:HGNC:29810] |
| ENSG00000196268 | HGLibA_56097 | 30 | -0.850070836 | ZNF493 | zinc finger protein 493 [Source:HGNC Symbol;Acc:HGNC:23708] |
| ENSG00000196337 | HGLibA_09312 | 30 | -0.850070836 | CGB7 | chorionic gonadotropin subunit beta 7 [Source:HGNC Symbol;Acc:HGNC:16451] |
| ENSG00000196352 | HGLibA_08412 | 30 | -0.850070836 | CD55 | CD55 molecule (Cromer blood group) [Source:HGNC Symbol;Acc:HGNC:2665] |
| ENSG00000196468 | HGLibA_17377 | 30 | -0.850070836 | FGF16 | fibroblast growth factor 16 [Source:HGNC Symbol;Acc:HGNC:3672] |
| ENSG00000196562 | HGLibA_47727 | 30 | -0.850070836 | SULF2 | sulfatase 2 [Source:HGNC Symbol;Acc:HGNC:20392] |
| ENSG00000197114 | HGLibA_55305 | 30 | -0.850070836 | ZGPAT | zinc finger CCCH-type and G-patch domain containing [Source:HGNC Symbol;Acc:HGNC:15948] |
| ENSG00000197226 | HGLibA_48576 | 30 | -0.850070836 | TBC1D9B | TBC1 domain family member 9B [Source:HGNC Symbol;Acc:HGNC:29097] |
| ENSG00000197275 | HGLibA_40100 | 30 | -0.850070836 | RAD54B | RAD54 homolog B [Source:HGNC Symbol;Acc:HGNC:17228] |
| ENSG00000197386 | HGLibA_22419 | 30 | -0.850070836 | HTT | huntingtin [Source:HGNC Symbol;Acc:HGNC:4851] |
| ENSG00000197487 | HGLibA_18585 | 30 | -0.850070836 | GALP | galanin like peptide [Source:HGNC Symbol;Acc:HGNC:24840] |
| ENSG00000197561 | HGLibA_14877 | 30 | -0.850070836 | ELANE | neutrophil expressed [Source:HGNC Symbol;Acc:HGNC:3309]" |
| ENSG00000197734 | HGLibA_05345 | 30 | -0.850070836 | C14orf178 | chromosome 14 open reading frame 178 [Source:HGNC Symbol;Acc:HGNC:26385] |
| ENSG00000197841 | HGLibA_55540 | 30 | -0.850070836 | ZNF181 | zinc finger protein 181 [Source:HGNC Symbol;Acc:HGNC:12971] |
| ENSG00000198298 | HGLibA_56080 | 30 | -0.850070836 | ZNF485 | zinc finger protein 485 [Source:HGNC Symbol;Acc:HGNC:23440] |
| ENSG00000198478 | HGLibA_43919 | 30 | -0.850070836 | SH3BGRL2 | SH3 domain binding glutamate rich protein like 2 [Source:HGNC Symbol;Acc:HGNC:15567] |
| ENSG00000198765 | HGLibA_47928 | 30 | -0.850070836 | SYCP1 | synaptonemal complex protein 1 [Source:HGNC Symbol;Acc:HGNC:11487] |
| ENSG00000198797 | HGLibA_04814 | 30 | -0.850070836 | BRINP2 | BMP/retinoic acid inducible neural specific 2 [Source:HGNC Symbol;Acc:HGNC:13746] |
| ENSG00000203737 | HGLibA_20049 | 30 | -0.850070836 | GPR52 | G protein-coupled receptor 52 [Source:HGNC Symbol;Acc:HGNC:4508] |
| ENSG00000203995 | HGLibA_57081 | 30 | -0.850070836 | ZYG11A | cell cycle regulator [Source:HGNC Symbol;Acc:HGNC:32058]" |
| ENSG00000204560 | HGLibA_13170 | 30 | -0.850070836 | DHX16 | DEAH-box helicase 16 [Source:HGNC Symbol;Acc:HGNC:2739] |
| ENSG00000205864 | HGLibA_25804 | 30 | -0.850070836 | KRTAP5-6 | keratin associated protein 5-6 [Source:HGNC Symbol;Acc:HGNC:23600] |
| ENSG00000205922 | HGLibA_33398 | 30 | -0.850070836 | ONECUT3 | one cut homeobox 3 [Source:HGNC Symbol;Acc:HGNC:13399] |
| ENSG00000211445 | HGLibA_20176 | 30 | -0.850070836 | GPX3 | glutathione peroxidase 3 [Source:HGNC Symbol;Acc:HGNC:4555] |
| ENSG00000212993 | HGLibA_37790 | 30 | -0.850070836 | POU5F1B | POU class 5 homeobox 1B [Source:HGNC Symbol;Acc:HGNC:9223] |
| ENSG00000213231 | HGLibA_48818 | 30 | -0.850070836 | TCL1B | T cell leukemia/lymphoma 1B [Source:HGNC Symbol;Acc:HGNC:11649] |
| ENSG00000213719 | HGLibA_10021 | 30 | -0.850070836 | CLIC1 | chloride intracellular channel 1 [Source:HGNC Symbol;Acc:HGNC:2062] |
| ENSG00000214530 | HGLibA_47352 | 30 | -0.850070836 | STARD10 | StAR related lipid transfer domain containing 10 [Source:HGNC Symbol;Acc:HGNC:10666] |
| ENSG00000214575 | HGLibA_10917 | 30 | -0.850070836 | CPEB1 | cytoplasmic polyadenylation element binding protein 1 [Source:HGNC Symbol;Acc:HGNC:21744] |
| ENSG00000215568 | HGLibA_18329 | 30 | -0.850070836 | GAB4 | GRB2 associated binding protein family member 4 [Source:HGNC Symbol;Acc:HGNC:18325] |
| ENSG00000215612 | HGLibA_21759 | 30 | -0.850070836 | HMX1 | H6 family homeobox 1 [Source:HGNC Symbol;Acc:HGNC:5017] |
| ENSG00000215853 | HGLibA_42201 | 30 | -0.850070836 | RPTN | repetin [Source:HGNC Symbol;Acc:HGNC:26809] |
| ENSG00000221829 | HGLibA_16850 | 30 | -0.850070836 | FANCG | FA complementation group G [Source:HGNC Symbol;Acc:HGNC:3588] |
| ENSG00000221977 | HGLibA_33985 | 30 | -0.850070836 | OR4E2 | olfactory receptor family 4 subfamily E member 2 [Source:HGNC Symbol;Acc:HGNC:8297] |
| ENSG00000226763 | HGLibA_47053 | 30 | -0.850070836 | SRRM5 | serine/arginine repetitive matrix 5 [Source:HGNC Symbol;Acc:HGNC:37248] |
| ENSG00000235711 | HGLibA_02081 | 30 | -0.850070836 | ANKRD34C | ankyrin repeat domain 34C [Source:HGNC Symbol;Acc:HGNC:33888] |
| ENSG00000241370 | HGLibA_42026 | 30 | -0.850070836 | RPP21 | ribonuclease P/MRP subunit p21 [Source:HGNC Symbol;Acc:HGNC:21300] |
| ENSG00000241476 | HGLibA_47197 | 30 | -0.850070836 | SSX2 | SSX family member 2 [Source:HGNC Symbol;Acc:HGNC:11336] |
| ENSG00000241973 | HGLibA_36499 | 30 | -0.850070836 | PI4KA | phosphatidylinositol 4-kinase alpha [Source:HGNC Symbol;Acc:HGNC:8983] |
| ENSG00000248405 | HGLibA_38845 | 30 | -0.850070836 | PRR5-ARHGAP8 | PRR5-ARHGAP8 readthrough [Source:HGNC Symbol;Acc:HGNC:34512] |
| ENSG00000248487 | HGLibA_00247 | 30 | -0.850070836 | ABHD14A | abhydrolase domain containing 14A [Source:HGNC Symbol;Acc:HGNC:24538] |
| ENSG00000251369 | HGLibA_56213 | 30 | -0.850070836 | ZNF550 | zinc finger protein 550 [Source:HGNC Symbol;Acc:HGNC:28643] |
| ENSG00000255181 | HGLibA_07672 | 30 | -0.850070836 | CCDC166 | coiled-coil domain containing 166 [Source:HGNC Symbol;Acc:HGNC:41910] |
| ENSG00000258102 | HGLibA_28127 | 30 | -0.850070836 | MAP1LC3B2 | microtubule associated protein 1 light chain 3 beta 2 [Source:HGNC Symbol;Acc:HGNC:34390] |
| ENSG00000263002 | HGLibA_55646 | 30 | -0.850070836 | ZNF234 | zinc finger protein 234 [Source:HGNC Symbol;Acc:HGNC:13027] |
| ENSG00000268940 | HGLibA_11498 | 30 | -0.850070836 | CT45A1 | cancer/testis antigen family 45 member A1 [Source:HGNC Symbol;Acc:HGNC:33267] |
| ENSG00000279012 | HGLibA_34081 | 30 | -0.850070836 | OR51B2 | olfactory receptor family 51 subfamily B member 2 (gene/pseudogene) [Source:HGNC Symbol;Acc:HGNC:14703] |
| FAM188A | HGLibA_16346 | 30 | -0.850070836 | FAM188A | - |
| GSG2 | HGLibA_20426 | 30 | -0.850070836 | GSG2 | - |
| H3F3A | HGLibA_20777 | 30 | -0.850070836 | H3F3A | - |
| hsa-mir-1245b | HGLibA_57377 | 30 | -0.850070836 | hsa-mir-1245b | - |
| hsa-mir-1302-4 | HGLibA_57704 | 30 | -0.850070836 | hsa-mir-1302-4 | - |
| hsa-mir-188 | HGLibA_58014 | 30 | -0.850070836 | hsa-mir-188 | - |
| hsa-mir-18a | HGLibA_58020 | 30 | -0.850070836 | hsa-mir-18a | - |
| hsa-mir-2116 | HGLibA_58236 | 30 | -0.850070836 | hsa-mir-2116 | - |
| hsa-mir-217 | HGLibA_58266 | 30 | -0.850070836 | hsa-mir-217 | - |
| hsa-mir-301b | HGLibA_58433 | 30 | -0.850070836 | hsa-mir-301b | - |
| hsa-mir-3179-1 | HGLibA_58788 | 30 | -0.850070836 | hsa-mir-3179-1 | - |
| hsa-mir-3617 | HGLibA_59098 | 30 | -0.850070836 | hsa-mir-3617 | - |
| hsa-mir-3622b | HGLibA_59124 | 30 | -0.850070836 | hsa-mir-3622b | - |
| hsa-mir-369 | HGLibA_59340 | 30 | -0.850070836 | hsa-mir-369 | - |
| hsa-mir-369 | HGLibA_59343 | 30 | -0.850070836 | hsa-mir-369 | - |
| hsa-mir-376a-2 | HGLibA_59410 | 30 | -0.850070836 | hsa-mir-376a-2 | - |
| hsa-mir-3921 | HGLibA_59562 | 30 | -0.850070836 | hsa-mir-3921 | - |
| hsa-mir-3938 | HGLibA_59612 | 30 | -0.850070836 | hsa-mir-3938 | - |
| hsa-mir-4258 | HGLibA_59738 | 30 | -0.850070836 | hsa-mir-4258 | - |
| hsa-mir-4269 | HGLibA_59785 | 30 | -0.850070836 | hsa-mir-4269 | - |
| hsa-mir-4275 | HGLibA_59802 | 30 | -0.850070836 | hsa-mir-4275 | - |
| hsa-mir-4291 | HGLibA_59876 | 30 | -0.850070836 | hsa-mir-4291 | - |
| hsa-mir-4296 | HGLibA_59897 | 30 | -0.850070836 | hsa-mir-4296 | - |
| hsa-mir-4424 | HGLibA_60083 | 30 | -0.850070836 | hsa-mir-4424 | - |
| hsa-mir-4450 | HGLibA_60207 | 30 | -0.850070836 | hsa-mir-4450 | - |
| hsa-mir-4483 | HGLibA_60350 | 30 | -0.850070836 | hsa-mir-4483 | - |
| hsa-mir-449c | HGLibA_60426 | 30 | -0.850070836 | hsa-mir-449c | - |
| hsa-mir-4502 | HGLibA_60434 | 30 | -0.850070836 | hsa-mir-4502 | - |
| hsa-mir-4658 | HGLibA_60740 | 30 | -0.850070836 | hsa-mir-4658 | - |
| hsa-mir-4667 | HGLibA_60784 | 30 | -0.850070836 | hsa-mir-4667 | - |
| hsa-mir-4723 | HGLibA_60988 | 30 | -0.850070836 | hsa-mir-4723 | - |
| hsa-mir-4736 | HGLibA_61041 | 30 | -0.850070836 | hsa-mir-4736 | - |
| hsa-mir-4753 | HGLibA_61108 | 30 | -0.850070836 | hsa-mir-4753 | - |
| hsa-mir-548as | HGLibA_61920 | 30 | -0.850070836 | hsa-mir-548as | - |
| hsa-mir-548l | HGLibA_62038 | 30 | -0.850070836 | hsa-mir-548l | - |
| hsa-mir-5687 | HGLibA_62265 | 30 | -0.850070836 | hsa-mir-5687 | - |
| hsa-mir-620 | HGLibA_62718 | 30 | -0.850070836 | hsa-mir-620 | - |
| hsa-mir-634 | HGLibA_62777 | 30 | -0.850070836 | hsa-mir-634 | - |
| hsa-mir-654 | HGLibA_62948 | 30 | -0.850070836 | hsa-mir-654 | - |
| hsa-mir-659 | HGLibA_62969 | 30 | -0.850070836 | hsa-mir-659 | - |
| hsa-mir-6763 | HGLibA_63212 | 30 | -0.850070836 | hsa-mir-6763 | - |
| hsa-mir-6764 | HGLibA_63219 | 30 | -0.850070836 | hsa-mir-6764 | - |
| hsa-mir-6834 | HGLibA_63514 | 30 | -0.850070836 | hsa-mir-6834 | - |
| hsa-mir-7152 | HGLibA_63836 | 30 | -0.850070836 | hsa-mir-7152 | - |
| hsa-mir-8070 | HGLibA_64117 | 30 | -0.850070836 | hsa-mir-8070 | - |
| hsa-mir-890 | HGLibA_64237 | 30 | -0.850070836 | hsa-mir-890 | - |
| hsa-mir-891a | HGLibA_64244 | 30 | -0.850070836 | hsa-mir-891a | - |
| IL3RA_Y | HGLibA_23162 | 30 | -0.850070836 | IL3RA_Y | - |
| IL8 | HGLibA_23197 | 30 | -0.850070836 | IL8 | - |
| KDELC1 | HGLibA_24472 | 30 | -0.850070836 | KDELC1 | - |
| LOC730159 | HGLibA_27003 | 30 | -0.850070836 | LOC730159 | - |
| LOC730183 | HGLibA_27005 | 30 | -0.850070836 | LOC730183 | - |
| NOV | HGLibA_32361 | 30 | -0.850070836 | NOV | - |
| NUTMF | HGLibA_33115 | 30 | -0.850070836 | NUTMF | - |
| PDDC1 | HGLibA_35757 | 30 | -0.850070836 | PDDC1 | - |
| PNMA6C | HGLibA_37380 | 30 | -0.850070836 | PNMA6C | - |
| QARS | HGLibA_39717 | 30 | -0.850070836 | QARS | - |
| SMIM11 | HGLibA_45723 | 30 | -0.850070836 | SMIM11 | - |
| TMEM159 | HGLibA_50019 | 30 | -0.850070836 | TMEM159 | - |
| VAMP7_X | HGLibA_53503 | 30 | -0.850070836 | VAMP7_X | - |
| C14orf2 | HGLibA_05356 | 29 | -0.860021873 | C14orf2 | - |
| C1orf177 | HGLibA_05800 | 29 | -0.860021873 | C1orf177 | - |
| C22orf28 | HGLibA_06064 | 29 | -0.860021873 | C22orf28 | - |
| C6orf165 | HGLibA_06458 | 29 | -0.860021873 | C6orf165 | - |
| C7orf71 | HGLibA_06584 | 29 | -0.860021873 | C7orf71 | - |
| ENSG00000003509 | HGLibA_31499 | 29 | -0.860021873 | NDUFAF7 | NADH:ubiquinone oxidoreductase complex assembly factor 7 [Source:HGNC Symbol;Acc:HGNC:28816] |
| ENSG00000005471 | HGLibA_00141 | 29 | -0.860021873 | ABCB4 | ATP binding cassette subfamily B member 4 [Source:HGNC Symbol;Acc:HGNC:45] |
| ENSG00000007341 | HGLibA_47285 | 29 | -0.860021873 | ST7L | suppression of tumorigenicity 7 like [Source:HGNC Symbol;Acc:HGNC:18441] |
| ENSG00000011132 | HGLibA_02375 | 29 | -0.860021873 | APBA3 | amyloid beta precursor protein binding family A member 3 [Source:HGNC Symbol;Acc:HGNC:580] |
| ENSG00000013503 | HGLibA_37611 | 29 | -0.860021873 | POLR3B | RNA polymerase III subunit B [Source:HGNC Symbol;Acc:HGNC:30348] |
| ENSG00000021574 | HGLibA_46423 | 29 | -0.860021873 | SPAST | spastin [Source:HGNC Symbol;Acc:HGNC:11233] |
| ENSG00000029534 | HGLibA_01935 | 29 | -0.860021873 | ANK1 | ankyrin 1 [Source:HGNC Symbol;Acc:HGNC:492] |
| ENSG00000030419 | HGLibA_22932 | 29 | -0.860021873 | IKZF2 | IKAROS family zinc finger 2 [Source:HGNC Symbol;Acc:HGNC:13177] |
| ENSG00000033011 | HGLibA_01602 | 29 | -0.860021873 | ALG1 | chitobiosyldiphosphodolichol beta-mannosyltransferase [Source:HGNC Symbol;Acc:HGNC:18294]" |
| ENSG00000049249 | HGLibA_50794 | 29 | -0.860021873 | TNFRSF9 | TNF receptor superfamily member 9 [Source:HGNC Symbol;Acc:HGNC:11924] |

| | | | | | |
|---|---|---|---|---|---|
| ENSG00000053501 | HGLibA_53163 | 29 | -0.860021873 | USE1 | unconventional SNARE in the ER 1 [Source:HGNC Symbol;Acc:HGNC:30882] |
| ENSG00000059122 | HGLibA_17680 | 29 | -0.860021873 | FLYWCH1 | FLYWCH-type zinc finger 1 [Source:HGNC Symbol;Acc:HGNC:25404] |
| ENSG00000063180 | HGLibA_06791 | 29 | -0.860021873 | CA11 | carbonic anhydrase 11 [Source:HGNC Symbol;Acc:HGNC:1370] |
| ENSG00000064419 | HGLibA_50903 | 29 | -0.860021873 | TNPO3 | transportin 3 [Source:HGNC Symbol;Acc:HGNC:17103] |
| ENSG00000064651 | HGLibA_44360 | 29 | -0.860021873 | SLC12A2 | solute carrier family 12 member 2 [Source:HGNC Symbol;Acc:HGNC:10911] |
| ENSG00000065665 | HGLibA_43249 | 29 | -0.860021873 | SEC61A2 | Sec61 translocon alpha 2 subunit [Source:HGNC Symbol;Acc:HGNC:17702] |
| ENSG00000066455 | HGLibA_19603 | 29 | -0.860021873 | GOLGA5 | golgin A5 [Source:HGNC Symbol;Acc:HGNC:4428] |
| ENSG00000068903 | HGLibA_44218 | 29 | -0.860021873 | SIRT2 | sirtuin 2 [Source:HGNC Symbol;Acc:HGNC:10886] |
| ENSG00000070614 | HGLibA_31425 | 29 | -0.860021873 | NDST1 | N-deacetylase and N-sulfotransferase 1 [Source:HGNC Symbol;Acc:HGNC:7680] |
| ENSG00000071894 | HGLibA_10993 | 29 | -0.860021873 | CPSF1 | cleavage and polyadenylation specific factor 1 [Source:HGNC Symbol;Acc:HGNC:2324] |
| ENSG00000076108 | HGLibA_04192 | 29 | -0.860021873 | BAZ2A | bromodomain adjacent to zinc finger domain 2A [Source:HGNC Symbol;Acc:HGNC:962] |
| ENSG00000079974 | HGLibA_40030 | 29 | -0.860021873 | RABL2B | member of RAS oncogene family like 2B [Source:HGNC Symbol;Acc:HGNC:9800]" |
| ENSG00000080345 | HGLibA_41243 | 29 | -0.860021873 | RIF1 | replication timing regulatory factor 1 [Source:HGNC Symbol;Acc:HGNC:23207] |
| ENSG00000081985 | HGLibA_22969 | 29 | -0.860021873 | IL12RB2 | interleukin 12 receptor subunit beta 2 [Source:HGNC Symbol;Acc:HGNC:5972] |
| ENSG00000082641 | HGLibA_31791 | 29 | -0.860021873 | NFE2L1 | erythroid 2 like 1 [Source:HGNC Symbol;Acc:HGNC:7781]" |
| ENSG00000084764 | HGLibA_28319 | 29 | -0.860021873 | MAPRE3 | microtubule associated protein RP/EB family member 3 [Source:HGNC Symbol;Acc:HGNC:6892] |
| ENSG00000085760 | HGLibA_30396 | 29 | -0.860021873 | MTIF2 | mitochondrial translational initiation factor 2 [Source:HGNC Symbol;Acc:HGNC:7441] |
| ENSG00000087338 | HGLibA_19392 | 29 | -0.860021873 | GMCL1 | spermatogenesis associated 1 [Source:HGNC Symbol;Acc:HGNC:23843]" |
| ENSG00000096395 | HGLibA_29426 | 29 | -0.860021873 | MLN | motilin [Source:HGNC Symbol;Acc:HGNC:7141] |
| ENSG00000096395 | HGLibA_29425 | 29 | -0.860021873 | MLN | motilin [Source:HGNC Symbol;Acc:HGNC:7141] |
| ENSG00000096968 | HGLibA_23915 | 29 | -0.860021873 | JAK2 | Janus kinase 2 [Source:HGNC Symbol;Acc:HGNC:6192] |
| ENSG00000099875 | HGLibA_29344 | 29 | -0.860021873 | MKNK2 | MAP kinase interacting serine/threonine kinase 2 [Source:HGNC Symbol;Acc:HGNC:7111] |
| ENSG00000099901 | HGLibA_40191 | 29 | -0.860021873 | RANBP1 | RAN binding protein 1 [Source:HGNC Symbol;Acc:HGNC:9847] |
| ENSG00000099995 | HGLibA_43691 | 29 | -0.860021873 | SF3A1 | splicing factor 3a subunit 1 [Source:HGNC Symbol;Acc:HGNC:10765] |
| ENSG00000100320 | HGLibA_40459 | 29 | -0.860021873 | RBFOX2 | RNA binding fox-1 homolog 2 [Source:HGNC Symbol;Acc:HGNC:9906] |
| ENSG00000100580 | HGLibA_49825 | 29 | -0.860021873 | TMED8 | transmembrane p24 trafficking protein family member 8 [Source:HGNC Symbol;Acc:HGNC:18633] |
| ENSG00000100811 | HGLibA_54758 | 29 | -0.860021873 | YY1 | YY1 transcription factor [Source:HGNC Symbol;Acc:HGNC:12856] |
| ENSG00000101412 | HGLibA_14306 | 29 | -0.860021873 | E2F1 | E2F transcription factor 1 [Source:HGNC Symbol;Acc:HGNC:3113] |
| ENSG00000102158 | HGLibA_28012 | 29 | -0.860021873 | MAGT1 | magnesium transporter 1 [Source:HGNC Symbol;Acc:HGNC:28880] |
| ENSG00000102924 | HGLibA_07406 | 29 | -0.860021873 | CBLN1 | cerebellin 1 precursor [Source:HGNC Symbol;Acc:HGNC:1543] |
| ENSG00000102935 | HGLibA_55961 | 29 | -0.860021873 | ZNF423 | zinc finger protein 423 [Source:HGNC Symbol;Acc:HGNC:16762] |
| ENSG00000103528 | HGLibA_48068 | 29 | -0.860021873 | SYT17 | synaptotagmin 17 [Source:HGNC Symbol;Acc:HGNC:24119] |
| ENSG00000103723 | HGLibA_02323 | 29 | -0.860021873 | AP3B2 | adaptor related protein complex 3 subunit beta 2 [Source:HGNC Symbol;Acc:HGNC:567] |
| ENSG00000104365 | HGLibA_22920 | 29 | -0.860021873 | IKBKB | inhibitor of nuclear factor kappa B kinase subunit beta [Source:HGNC Symbol;Acc:HGNC:5960] |
| ENSG00000104755 | HGLibA_00748 | 29 | -0.860021873 | ADAM2 | ADAM metallopeptidase domain 2 [Source:HGNC Symbol;Acc:HGNC:198] |
| ENSG00000104848 | HGLibA_24144 | 29 | -0.860021873 | KCNA7 | potassium voltage-gated channel subfamily A member 7 [Source:HGNC Symbol;Acc:HGNC:6226] |
| ENSG00000104903 | HGLibA_27704 | 29 | -0.860021873 | LYL1 | basic helix-loop-helix family member [Source:HGNC Symbol;Acc:HGNC:6734]" |
| ENSG00000105287 | HGLibA_38569 | 29 | -0.860021873 | PRKD2 | protein kinase D2 [Source:HGNC Symbol;Acc:HGNC:17293] |
| ENSG00000105705 | HGLibA_47717 | 29 | -0.860021873 | SUGP1 | SURP and G-patch domain containing 1 [Source:HGNC Symbol;Acc:HGNC:18643] |
| ENSG00000106012 | HGLibA_23498 | 29 | -0.860021873 | IQCE | IQ motif containing E [Source:HGNC Symbol;Acc:HGNC:29171] |
| ENSG00000106384 | HGLibA_29630 | 29 | -0.860021873 | MOGAT3 | monoacylglycerol O-acyltransferase 3 [Source:HGNC Symbol;Acc:HGNC:23249] |
| ENSG00000106404 | HGLibA_09863 | 29 | -0.860021873 | CLDN15 | claudin 15 [Source:HGNC Symbol;Acc:HGNC:2036] |
| ENSG00000106588 | HGLibA_39074 | 29 | -0.860021873 | PSMA2 | proteasome subunit alpha 2 [Source:HGNC Symbol;Acc:HGNC:9531] |
| ENSG00000106624 | HGLibA_01086 | 29 | -0.860021873 | AEBP1 | AE binding protein 1 [Source:HGNC Symbol;Acc:HGNC:303] |
| ENSG00000108406 | HGLibA_13199 | 29 | -0.860021873 | DHX40 | DEAH-box helicase 40 [Source:HGNC Symbol;Acc:HGNC:18018] |
| ENSG00000108788 | HGLibA_29437 | 29 | -0.860021873 | MLX | MAX dimerization protein [Source:HGNC Symbol;Acc:HGNC:11645]" |
| ENSG00000108960 | HGLibA_29461 | 29 | -0.860021873 | MMD | monocyte to macrophage differentiation associated [Source:HGNC Symbol;Acc:HGNC:7153] |
| ENSG00000109927 | HGLibA_48954 | 29 | -0.860021873 | TECTA | tectorin alpha [Source:HGNC Symbol;Acc:HGNC:11720] |
| ENSG00000110148 | HGLibA_07958 | 29 | -0.860021873 | CCKBR | cholecystokinin B receptor [Source:HGNC Symbol;Acc:HGNC:1571] |
| ENSG00000110172 | HGLibA_09513 | 29 | -0.860021873 | CHORDC1 | cysteine and histidine rich domain containing 1 [Source:HGNC Symbol;Acc:HGNC:14525] |
| ENSG00000110651 | HGLibA_08457 | 29 | -0.860021873 | CD81 | CD81 molecule [Source:HGNC Symbol;Acc:HGNC:1701] |
| ENSG00000110900 | HGLibA_51896 | 29 | -0.860021873 | TSPAN11 | tetraspanin 11 [Source:HGNC Symbol;Acc:HGNC:30795] |
| ENSG00000111319 | HGLibA_43012 | 29 | -0.860021873 | SCNN1A | sodium channel epithelial 1 alpha subunit [Source:HGNC Symbol;Acc:HGNC:10599] |
| ENSG00000111667 | HGLibA_53389 | 29 | -0.860021873 | USP5 | ubiquitin specific peptidase 5 [Source:HGNC Symbol;Acc:HGNC:12628] |
| ENSG00000112077 | HGLibA_41121 | 29 | -0.860021873 | RHAG | Rh associated glycoprotein [Source:HGNC Symbol;Acc:HGNC:10006] |
| ENSG00000112739 | HGLibA_38741 | 29 | -0.860021873 | PRPF4B | pre-mRNA processing factor 4B [Source:HGNC Symbol;Acc:HGNC:17346] |
| ENSG00000112877 | HGLibA_09160 | 29 | -0.860021873 | CEP72 | centrosomal protein 72 [Source:HGNC Symbol;Acc:HGNC:25547] |
| ENSG00000113328 | HGLibA_08094 | 29 | -0.860021873 | CCNG1 | cyclin G1 [Source:HGNC Symbol;Acc:HGNC:1592] |
| ENSG00000113657 | HGLibA_13928 | 29 | -0.860021873 | DPYSL3 | dihydropyrimidinase like 3 [Source:HGNC Symbol;Acc:HGNC:3015] |
| ENSG00000113716 | HGLibA_21732 | 29 | -0.860021873 | HMGXB3 | HMG-box containing 3 [Source:HGNC Symbol;Acc:HGNC:28982] |
| ENSG00000115107 | HGLibA_47430 | 29 | -0.860021873 | STEAP3 | STEAP3 metalloreductase [Source:HGNC Symbol;Acc:HGNC:24592] |
| ENSG00000115561 | HGLibA_09483 | 29 | -0.860021873 | CHMP3 | charged multivesicular body protein 3 [Source:HGNC Symbol;Acc:HGNC:29865] |
| ENSG00000115602 | HGLibA_23061 | 29 | -0.860021873 | IL1RL1 | interleukin 1 receptor like 1 [Source:HGNC Symbol;Acc:HGNC:5998] |
| ENSG00000115977 | HGLibA_00049 | 29 | -0.860021873 | AAK1 | AP2 associated kinase 1 [Source:HGNC Symbol;Acc:HGNC:19679] |
| ENSG00000116675 | HGLibA_13633 | 29 | -0.860021873 | DNAJC6 | DnaJ heat shock protein family (Hsp40) member C6 [Source:HGNC Symbol;Acc:HGNC:15469] |
| ENSG00000116852 | HGLibA_24901 | 29 | -0.860021873 | KIF21B | kinesin family member 21B [Source:HGNC Symbol;Acc:HGNC:29442] |
| ENSG00000117143 | HGLibA_52508 | 29 | -0.860021873 | UAP1 | UDP-N-acetylglucosamine pyrophosphorylase 1 [Source:HGNC Symbol;Acc:HGNC:12457] |
| ENSG00000117461 | HGLibA_36653 | 29 | -0.860021873 | PIK3R3 | phosphoinositide-3-kinase regulatory subunit 3 [Source:HGNC Symbol;Acc:HGNC:8981] |
| ENSG00000118997 | HGLibA_13496 | 29 | -0.860021873 | DNAH7 | dynein axonemal heavy chain 7 [Source:HGNC Symbol;Acc:HGNC:18661] |
| ENSG00000119283 | HGLibA_51589 | 29 | -0.860021873 | TRIM67 | tripartite motif containing 67 [Source:HGNC Symbol;Acc:HGNC:31859] |
| ENSG00000119986 | HGLibA_03901 | 29 | -0.860021873 | AVPI1 | arginine vasopressin induced 1 [Source:HGNC Symbol;Acc:HGNC:30898] |
| ENSG00000120008 | HGLibA_54024 | 29 | -0.860021873 | WDR11 | WD repeat domain 11 [Source:HGNC Symbol;Acc:HGNC:13831] |
| ENSG00000120279 | HGLibA_30681 | 29 | -0.860021873 | MYCT1 | MYC target 1 [Source:HGNC Symbol;Acc:HGNC:23172] |
| ENSG00000120688 | HGLibA_53984 | 29 | -0.860021873 | WBP4 | WW domain binding protein 4 [Source:HGNC Symbol;Acc:HGNC:12739] |
| ENSG00000120708 | HGLibA_49221 | 29 | -0.860021873 | TGFBI | transforming growth factor beta induced [Source:HGNC Symbol;Acc:HGNC:11771] |
| ENSG00000120727 | HGLibA_35030 | 29 | -0.860021873 | PAIP2 | poly(A) binding protein interacting protein 2 [Source:HGNC Symbol;Acc:HGNC:17970] |
| ENSG00000122435 | HGLibA_51678 | 29 | -0.860021873 | TRMT13 | tRNA methyltransferase 13 homolog [Source:HGNC Symbol;Acc:HGNC:25502] |
| ENSG00000122643 | HGLibA_32813 | 29 | -0.860021873 | NT5C3A | cytosolic IIIA [Source:HGNC Symbol;Acc:HGNC:17820]" |
| ENSG00000124702 | HGLibA_25099 | 29 | -0.860021873 | KLHDC3 | kelch domain containing 3 [Source:HGNC Symbol;Acc:HGNC:20704] |
| ENSG00000125735 | HGLibA_50815 | 29 | -0.860021873 | TNFSF14 | TNF superfamily member 14 [Source:HGNC Symbol;Acc:HGNC:11930] |
| ENSG00000125846 | HGLibA_55464 | 29 | -0.860021873 | ZNF133 | zinc finger protein 133 [Source:HGNC Symbol;Acc:HGNC:12917] |
| ENSG00000126883 | HGLibA_33044 | 29 | -0.860021873 | NUP214 | nucleoporin 214 [Source:HGNC Symbol;Acc:HGNC:8064] |
| ENSG00000128739 | HGLibA_45995 | 29 | -0.860021873 | SNRPN | small nuclear ribonucleoprotein polypeptide N [Source:HGNC Symbol;Acc:HGNC:11164] |
| ENSG00000130479 | HGLibA_28134 | 29 | -0.860021873 | MAP1S | microtubule associated protein 1S [Source:HGNC Symbol;Acc:HGNC:15715] |
| ENSG00000131686 | HGLibA_06819 | 29 | -0.860021873 | CA6 | carbonic anhydrase 6 [Source:HGNC Symbol;Acc:HGNC:1380] |
| ENSG00000131781 | HGLibA_17713 | 29 | -0.860021873 | FMO5 | flavin containing monooxygenase 5 [Source:HGNC Symbol;Acc:HGNC:3773] |
| ENSG00000131828 | HGLibA_35862 | 29 | -0.860021873 | PDHA1 | pyruvate dehydrogenase E1 alpha 1 subunit [Source:HGNC Symbol;Acc:HGNC:8806] |
| ENSG00000132688 | HGLibA_31697 | 29 | -0.860021873 | NES | nestin [Source:HGNC Symbol;Acc:HGNC:7756] |
| ENSG00000133488 | HGLibA_43198 | 29 | -0.860021873 | SEC14L4 | SEC14 like lipid binding 4 [Source:HGNC Symbol;Acc:HGNC:20627] |
| ENSG00000133835 | HGLibA_22193 | 29 | -0.860021873 | HSD17B4 | hydroxysteroid 17-beta dehydrogenase 4 [Source:HGNC Symbol;Acc:HGNC:5213] |
| ENSG00000134152 | HGLibA_24085 | 29 | -0.860021873 | KATNBL1 | katanin regulatory subunit B1 like 1 [Source:HGNC Symbol;Acc:HGNC:26199] |
| ENSG00000134762 | HGLibA_13994 | 29 | -0.860021873 | DSC3 | desmocollin 3 [Source:HGNC Symbol;Acc:HGNC:3037] |
| ENSG00000134780 | HGLibA_12294 | 29 | -0.860021873 | DAGLA | diacylglycerol lipase alpha [Source:HGNC Symbol;Acc:HGNC:1165] |
| ENSG00000135040 | HGLibA_30992 | 29 | -0.860021873 | NAA35 | NatC auxiliary subunit [Source:HGNC Symbol;Acc:HGNC:24340]" |
| ENSG00000135443 | HGLibA_25571 | 29 | -0.860021873 | KRT85 | keratin 85 [Source:HGNC Symbol;Acc:HGNC:6462] |
| ENSG00000135486 | HGLibA_21793 | 29 | -0.860021873 | HNRNPA1 | heterogeneous nuclear ribonucleoprotein A1 [Source:HGNC Symbol;Acc:HGNC:5031] |
| ENSG00000135720 | HGLibA_14244 | 29 | -0.860021873 | DYNC1LI2 | dynein cytoplasmic 1 light intermediate chain 2 [Source:HGNC Symbol;Acc:HGNC:2966] |
| ENSG00000135828 | HGLibA_41440 | 29 | -0.860021873 | RNASEL | ribonuclease L [Source:HGNC Symbol;Acc:HGNC:10050] |
| ENSG00000136518 | HGLibA_00619 | 29 | -0.860021873 | ACTL6A | actin like 6A [Source:HGNC Symbol;Acc:HGNC:24124] |
| ENSG00000136816 | HGLibA_51023 | 29 | -0.860021873 | TOR1B | torsin family 1 member B [Source:HGNC Symbol;Acc:HGNC:11995] |
| ENSG00000136960 | HGLibA_15170 | 29 | -0.860021873 | ENPP2 | ectonucleotide pyrophosphatase/phosphodiesterase 2 [Source:HGNC Symbol;Acc:HGNC:3357] |
| ENSG00000138400 | HGLibA_28684 | 29 | -0.860021873 | MDH1B | malate dehydrogenase 1B [Source:HGNC Symbol;Acc:HGNC:17836] |
| ENSG00000139211 | HGLibA_01797 | 29 | -0.860021873 | AMIGO2 | adhesion molecule with Ig like domain 2 [Source:HGNC Symbol;Acc:HGNC:24073] |
| ENSG00000139263 | HGLibA_27181 | 29 | -0.860021873 | LRIG3 | leucine rich repeats and immunoglobulin like domains 3 [Source:HGNC Symbol;Acc:HGNC:30991] |
| ENSG00000139684 | HGLibA_15562 | 29 | -0.860021873 | ESD | esterase D [Source:HGNC Symbol;Acc:HGNC:3465] |
| ENSG00000139746 | HGLibA_40522 | 29 | -0.860021873 | RBM26 | RNA binding motif protein 26 [Source:HGNC Symbol;Acc:HGNC:20327] |
| ENSG00000140506 | HGLibA_26659 | 29 | -0.860021873 | LMAN1L | mannose binding 1 like [Source:HGNC Symbol;Acc:HGNC:6632]" |
| ENSG00000143006 | HGLibA_13432 | 29 | -0.860021873 | DMRTB1 | DMRT like family B with proline rich C-terminal 1 [Source:HGNC Symbol;Acc:HGNC:13913] |
| ENSG00000143367 | HGLibA_52327 | 29 | -0.860021873 | TUFT1 | tuftelin 1 [Source:HGNC Symbol;Acc:HGNC:12422] |
| ENSG00000143793 | HGLibA_05854 | 29 | -0.860021873 | C1orf35 | chromosome 1 open reading frame 35 [Source:HGNC Symbol;Acc:HGNC:19032] |
| ENSG00000144231 | HGLibA_37574 | 29 | -0.860021873 | POLR2D | RNA polymerase II subunit D [Source:HGNC Symbol;Acc:HGNC:9191] |
| ENSG00000144355 | HGLibA_13371 | 29 | -0.860021873 | DLX1 | distal-less homeobox 1 [Source:HGNC Symbol;Acc:HGNC:2914] |
| ENSG00000145349 | HGLibA_07073 | 29 | -0.860021873 | CAMK2D | calcium/calmodulin dependent protein kinase II delta [Source:HGNC Symbol;Acc:HGNC:1462] |
| ENSG00000145832 | HGLibA_44759 | 29 | -0.860021873 | SLC25A48 | solute carrier family 25 member 48 [Source:HGNC Symbol;Acc:HGNC:30451] |
| ENSG00000146477 | HGLibA_44583 | 29 | -0.860021873 | SLC22A3 | solute carrier family 22 member 3 [Source:HGNC Symbol;Acc:HGNC:10967] |
| ENSG00000148296 | HGLibA_47840 | 29 | -0.860021873 | SURF6 | surfeit 6 [Source:HGNC Symbol;Acc:HGNC:11478] |
| ENSG00000148690 | HGLibA_17972 | 29 | -0.860021873 | FRA10AC1 | FRA10A associated CGG repeat 1 [Source:HGNC Symbol;Acc:HGNC:1162] |
| ENSG00000149577 | HGLibA_44118 | 29 | -0.860021873 | SIDT2 | SID1 transmembrane family member 2 [Source:HGNC Symbol;Acc:HGNC:24272] |
| ENSG00000149679 | HGLibA_06846 | 29 | -0.860021873 | CABLES2 | Cdk5 and Abl enzyme substrate 2 [Source:HGNC Symbol;Acc:HGNC:16143] |
| ENSG00000150637 | HGLibA_08299 | 29 | -0.860021873 | CD226 | CD226 molecule [Source:HGNC Symbol;Acc:HGNC:16961] |
| ENSG00000152689 | HGLibA_40351 | 29 | -0.860021873 | RASGRP3 | RAS guanyl releasing protein 3 [Source:HGNC Symbol;Acc:HGNC:14545] |
| ENSG00000154639 | HGLibA_11840 | 29 | -0.860021873 | CXADR | Ig-like cell adhesion molecule [Source:HGNC Symbol;Acc:HGNC:2559]" |
| ENSG00000154642 | HGLibA_06050 | 29 | -0.860021873 | C21orf91 | chromosome 21 open reading frame 91 [Source:HGNC Symbol;Acc:HGNC:16459] |
| ENSG00000154764 | HGLibA_54382 | 29 | -0.860021873 | WNT7A | Wnt family member 7A [Source:HGNC Symbol;Acc:HGNC:12786] |
| ENSG00000154781 | HGLibA_07704 | 29 | -0.860021873 | CCDC174 | coiled-coil domain containing 174 [Source:HGNC Symbol;Acc:HGNC:28033] |
| ENSG00000156232 | HGLibA_54290 | 29 | -0.860021873 | WHAMM | golgi membranes and microtubules [Source:HGNC Symbol;Acc:HGNC:30493]" |
| ENSG00000156384 | HGLibA_43733 | 29 | -0.860021873 | SFR1 | SWI5 dependent homologous recombination repair protein 1 [Source:HGNC Symbol;Acc:HGNC:29574] |
| ENSG00000157087 | HGLibA_03616 | 29 | -0.860021873 | ATP2B2 | ATPase plasma membrane Ca2+ transporting 2 [Source:HGNC Symbol;Acc:HGNC:815] |
| ENSG00000157227 | HGLibA_29493 | 29 | -0.860021873 | MMP14 | matrix metallopeptidase 14 [Source:HGNC Symbol;Acc:HGNC:7160] |
| ENSG00000159167 | HGLibA_47414 | 29 | -0.860021873 | STC1 | stanniocalcin 1 [Source:HGNC Symbol;Acc:HGNC:11373] |
| ENSG00000160209 | HGLibA_35961 | 29 | -0.860021873 | PDXK | pyridoxal kinase [Source:HGNC Symbol;Acc:HGNC:8819] |
| ENSG00000161202 | HGLibA_14214 | 29 | -0.860021873 | DVL3 | dishevelled segment polarity protein 3 [Source:HGNC Symbol;Acc:HGNC:3087] |
| ENSG00000161800 | HGLibA_40050 | 29 | -0.860021873 | RACGAP1 | Rac GTPase activating protein 1 [Source:HGNC Symbol;Acc:HGNC:9804] |
| ENSG00000162599 | HGLibA_31799 | 29 | -0.860021873 | NFIA | nuclear factor I A [Source:HGNC Symbol;Acc:HGNC:7784] |
| ENSG00000163633 | HGLibA_06326 | 29 | -0.860021873 | C4orf36 | chromosome 4 open reading frame 36 [Source:HGNC Symbol;Acc:HGNC:28386] |

| | | | | | |
|---|---|---|---|---|---|
| ENSG00000163638 | HGLibA_00845 | 29 | -0.860021873 | ADAMTS9 | ADAM metallopeptidase with thrombospondin type 1 motif 9 [Source:HGNC Symbol;Acc:HGNC:13202] |
| ENSG00000164258 | HGLibA_31553 | 29 | -0.860021873 | NDUFS4 | NADH:ubiquinone oxidoreductase subunit S4 [Source:HGNC Symbol;Acc:HGNC:7711] |
| ENSG00000164713 | HGLibA_04804 | 29 | -0.860021873 | BRI3 | brain protein I3 [Source:HGNC Symbol;Acc:HGNC:1109] |
| ENSG00000164756 | HGLibA_44921 | 29 | -0.860021873 | SLC30A8 | solute carrier family 30 member 8 [Source:HGNC Symbol;Acc:HGNC:20303] |
| ENSG00000165138 | HGLibA_02163 | 29 | -0.860021873 | ANKS6 | ankyrin repeat and sterile alpha motif domain containing 6 [Source:HGNC Symbol;Acc:HGNC:26724] |
| ENSG00000165275 | HGLibA_51664 | 29 | -0.860021873 | TRMT10B | tRNA methyltransferase 10B [Source:HGNC Symbol;Acc:HGNC:26454] |
| ENSG00000165655 | HGLibA_56116 | 29 | -0.860021873 | ZNF503 | zinc finger protein 503 [Source:HGNC Symbol;Acc:HGNC:23589] |
| ENSG00000165699 | HGLibA_51812 | 29 | -0.860021873 | TSC1 | TSC complex subunit 1 [Source:HGNC Symbol;Acc:HGNC:12362] |
| ENSG00000165959 | HGLibA_10068 | 29 | -0.860021873 | CLMN | calmin [Source:HGNC Symbol;Acc:HGNC:19972] |
| ENSG00000166220 | HGLibA_48458 | 29 | -0.860021873 | TBATA | brain and testes associated [Source:HGNC Symbol;Acc:HGNC:23511]" |
| ENSG00000166479 | HGLibA_50694 | 29 | -0.860021873 | TMX3 | thioredoxin related transmembrane protein 3 [Source:HGNC Symbol;Acc:HGNC:24718] |
| ENSG00000166925 | HGLibA_51825 | 29 | -0.860021873 | TSC22D4 | TSC22 domain family member 4 [Source:HGNC Symbol;Acc:HGNC:21696] |
| ENSG00000166984 | HGLibA_48839 | 29 | -0.860021873 | TCP10L2 | t-complex 10 like 2 [Source:HGNC Symbol;Acc:HGNC:21254] |
| ENSG00000167447 | HGLibA_45711 | 29 | -0.860021873 | SMG8 | nonsense mediated mRNA decay factor [Source:HGNC Symbol;Acc:HGNC:25551]" |
| ENSG00000167523 | HGLibA_46509 | 29 | -0.860021873 | SPATA33 | spermatogenesis associated 33 [Source:HGNC Symbol;Acc:HGNC:26463] |
| ENSG00000167656 | HGLibA_27652 | 29 | -0.860021873 | LY6D | lymphocyte antigen 6 family member D [Source:HGNC Symbol;Acc:HGNC:13348] |
| ENSG00000168131 | HGLibA_33766 | 29 | -0.860021873 | OR2B2 | olfactory receptor family 2 subfamily B member 2 [Source:HGNC Symbol;Acc:HGNC:13966] |
| ENSG00000168228 | HGLibA_55037 | 29 | -0.860021873 | ZCCHC4 | zinc finger CCHC-type containing 4 [Source:HGNC Symbol;Acc:HGNC:22917] |
| ENSG00000168653 | HGLibA_31558 | 29 | -0.860021873 | NDUFS5 | NADH:ubiquinone oxidoreductase subunit S5 [Source:HGNC Symbol;Acc:HGNC:7712] |
| ENSG00000168778 | HGLibA_48876 | 29 | -0.860021873 | TCTN2 | tectonic family member 2 [Source:HGNC Symbol;Acc:HGNC:25774] |
| ENSG00000168785 | HGLibA_51939 | 29 | -0.860021873 | TSPAN5 | tetraspanin 5 [Source:HGNC Symbol;Acc:HGNC:17753] |
| ENSG00000169564 | HGLibA_35366 | 29 | -0.860021873 | PCBP1 | poly(rC) binding protein 1 [Source:HGNC Symbol;Acc:HGNC:8647] |
| ENSG00000169783 | HGLibA_26585 | 29 | -0.860021873 | LINGO1 | leucine rich repeat and Ig domain containing 1 [Source:HGNC Symbol;Acc:HGNC:21205] |
| ENSG00000170345 | HGLibA_17796 | 29 | -0.860021873 | FOS | AP-1 transcription factor subunit [Source:HGNC Symbol;Acc:HGNC:3796]" |
| ENSG00000170537 | HGLibA_49766 | 29 | -0.860021873 | TMC7 | transmembrane channel like 7 [Source:HGNC Symbol;Acc:HGNC:23000] |
| ENSG00000170925 | HGLibA_49060 | 29 | -0.860021873 | TEX13B | testis expressed 13B [Source:HGNC Symbol;Acc:HGNC:11736] |
| ENSG00000171604 | HGLibA_11974 | 29 | -0.860021873 | CXXC5 | CXXC finger protein 5 [Source:HGNC Symbol;Acc:HGNC:26943] |
| ENSG00000171877 | HGLibA_18019 | 29 | -0.860021873 | FRMD5 | FERM domain containing 5 [Source:HGNC Symbol;Acc:HGNC:28214] |
| ENSG00000172046 | HGLibA_53292 | 29 | -0.860021873 | USP19 | ubiquitin specific peptidase 19 [Source:HGNC Symbol;Acc:HGNC:12617] |
| ENSG00000172058 | HGLibA_43468 | 29 | -0.860021873 | SERF1A | small EDRK-rich factor 1A [Source:HGNC Symbol;Acc:HGNC:10755] |
| ENSG00000172769 | HGLibA_34268 | 29 | -0.860021873 | OR5B3 | olfactory receptor family 5 subfamily B member 3 [Source:HGNC Symbol;Acc:HGNC:8324] |
| ENSG00000173080 | HGLibA_42502 | 29 | -0.860021873 | RXFP4 | relaxin family peptide/INSL5 receptor 4 [Source:HGNC Symbol;Acc:HGNC:14666] |
| ENSG00000173875 | HGLibA_56766 | 29 | -0.860021873 | ZNF791 | zinc finger protein 791 [Source:HGNC Symbol;Acc:HGNC:26895] |
| ENSG00000175697 | HGLibA_19945 | 29 | -0.860021873 | GPR156 | G protein-coupled receptor 156 [Source:HGNC Symbol;Acc:HGNC:20844] |
| ENSG00000175756 | HGLibA_03875 | 29 | -0.860021873 | AURKAIP1 | aurora kinase A interacting protein 1 [Source:HGNC Symbol;Acc:HGNC:24114] |
| ENSG00000175895 | HGLibA_37097 | 29 | -0.860021873 | PLEKHF2 | pleckstrin homology and FYVE domain containing 2 [Source:HGNC Symbol;Acc:HGNC:20757] |
| ENSG00000176774 | HGLibA_27942 | 29 | -0.860021873 | MAGEB18 | MAGE family member B18 [Source:HGNC Symbol;Acc:HGNC:28515] |
| ENSG00000177409 | HGLibA_42702 | 29 | -0.860021873 | SAMD9L | sterile alpha motif domain containing 9 like [Source:HGNC Symbol;Acc:HGNC:1349] |
| ENSG00000177938 | HGLibA_07210 | 29 | -0.860021873 | CAPZA3 | capping actin protein of muscle Z-line subunit alpha 3 [Source:HGNC Symbol;Acc:HGNC:24205] |
| ENSG00000179331 | HGLibA_39901 | 29 | -0.860021873 | RAB39A | member RAS oncogene family [Source:HGNC Symbol;Acc:HGNC:16521]" |
| ENSG00000180332 | HGLibA_24454 | 29 | -0.860021873 | KCTD4 | potassium channel tetramerization domain containing 4 [Source:HGNC Symbol;Acc:HGNC:23227] |
| ENSG00000181027 | HGLibA_17597 | 29 | -0.860021873 | FKRP | fukutin related protein [Source:HGNC Symbol;Acc:HGNC:17997] |
| ENSG00000181444 | HGLibA_56044 | 29 | -0.860021873 | ZNF467 | zinc finger protein 467 [Source:HGNC Symbol;Acc:HGNC:23154] |
| ENSG00000181634 | HGLibA_50818 | 29 | -0.860021873 | TNFSF15 | TNF superfamily member 15 [Source:HGNC Symbol;Acc:HGNC:11931] |
| ENSG00000181804 | HGLibA_45430 | 29 | -0.860021873 | SLC9A9 | solute carrier family 9 member A9 [Source:HGNC Symbol;Acc:HGNC:20653] |
| ENSG00000182247 | HGLibA_52598 | 29 | -0.860021873 | UBE2E2 | ubiquitin conjugating enzyme E2 E2 [Source:HGNC Symbol;Acc:HGNC:12478] |
| ENSG00000182447 | HGLibA_34733 | 29 | -0.860021873 | OTOL1 | otolin 1 [Source:HGNC Symbol;Acc:HGNC:34071] |
| ENSG00000182732 | HGLibA_41099 | 29 | -0.860021873 | RGS6 | regulator of G protein signaling 6 [Source:HGNC Symbol;Acc:HGNC:10002] |
| ENSG00000183255 | HGLibA_39564 | 29 | -0.860021873 | PTTG1IP | PTTG1 interacting protein [Source:HGNC Symbol;Acc:HGNC:13524] |
| ENSG00000183337 | HGLibA_04363 | 29 | -0.860021873 | BCOR | BCL6 corepressor [Source:HGNC Symbol;Acc:HGNC:20893] |
| ENSG00000183621 | HGLibA_55994 | 29 | -0.860021873 | ZNF438 | zinc finger protein 438 [Source:HGNC Symbol;Acc:HGNC:21029] |
| ENSG00000184221 | HGLibA_33368 | 29 | -0.860021873 | OLIG1 | oligodendrocyte transcription factor 1 [Source:HGNC Symbol;Acc:HGNC:16983] |
| ENSG00000184374 | HGLibA_10633 | 29 | -0.860021873 | COLEC10 | collectin subfamily member 10 [Source:HGNC Symbol;Acc:HGNC:2220] |
| ENSG00000184895 | HGLibA_47100 | 29 | -0.860021873 | SRY | sex determining region Y [Source:HGNC Symbol;Acc:HGNC:11311] |
| ENSG00000184990 | HGLibA_44240 | 29 | -0.860021873 | SIVA1 | SIVA1 apoptosis inducing factor [Source:HGNC Symbol;Acc:HGNC:17712] |
| ENSG00000185013 | HGLibA_32806 | 29 | -0.860021873 | NT5C1B | cytosolic IB [Source:HGNC Symbol;Acc:HGNC:17818]" |
| ENSG00000185052 | HGLibA_44621 | 29 | -0.860021873 | SLC24A3 | solute carrier family 24 member 3 [Source:HGNC Symbol;Acc:HGNC:10977] |
| ENSG00000185361 | HGLibA_50721 | 29 | -0.860021873 | TNFAIP8L1 | TNF alpha induced protein 8 like 1 [Source:HGNC Symbol;Acc:HGNC:28279] |
| ENSG00000185883 | HGLibA_03715 | 29 | -0.860021873 | ATP6V0C | ATPase H+ transporting V0 subunit c [Source:HGNC Symbol;Acc:HGNC:855] |
| ENSG00000185963 | HGLibA_04496 | 29 | -0.860021873 | BICD2 | BICD cargo adaptor 2 [Source:HGNC Symbol;Acc:HGNC:17208] |
| ENSG00000186919 | HGLibA_54766 | 29 | -0.860021873 | ZACN | zinc activated ion channel [Source:HGNC Symbol;Acc:HGNC:29504] |
| ENSG00000186951 | HGLibA_37837 | 29 | -0.860021873 | PPARA | peroxisome proliferator activated receptor alpha [Source:HGNC Symbol;Acc:HGNC:9232] |
| ENSG00000186980 | HGLibA_25723 | 29 | -0.860021873 | KRTAP23-1 | keratin associated protein 23-1 [Source:HGNC Symbol;Acc:HGNC:18928] |
| ENSG00000187555 | HGLibA_53409 | 29 | -0.860021873 | USP7 | ubiquitin specific peptidase 7 [Source:HGNC Symbol;Acc:HGNC:12630] |
| ENSG00000188000 | HGLibA_34473 | 29 | -0.860021873 | OR7D2 | olfactory receptor family 7 subfamily D member 2 [Source:HGNC Symbol;Acc:HGNC:8378] |
| ENSG00000189013 | HGLibA_24987 | 29 | -0.860021873 | KIR2DL4 | two Ig domains and long cytoplasmic tail 4 [Source:HGNC Symbol;Acc:HGNC:6332]" |
| ENSG00000196110 | HGLibA_56566 | 29 | -0.860021873 | ZNF699 | zinc finger protein 699 [Source:HGNC Symbol;Acc:HGNC:24750] |
| ENSG00000196323 | HGLibA_54893 | 29 | -0.860021873 | ZBTB44 | zinc finger and BTB domain containing 44 [Source:HGNC Symbol;Acc:HGNC:25001] |
| ENSG00000196878 | HGLibA_25962 | 29 | -0.860021873 | LAMB3 | laminin subunit beta 3 [Source:HGNC Symbol;Acc:HGNC:6490] |
| ENSG00000197408 | HGLibA_12112 | 29 | -0.860021873 | CYP2B6 | cytochrome P450 family 2 subfamily B member 6 [Source:HGNC Symbol;Acc:HGNC:2615] |
| ENSG00000197483 | HGLibA_56428 | 29 | -0.860021873 | ZNF628 | zinc finger protein 628 [Source:HGNC Symbol;Acc:HGNC:28054] |
| ENSG00000197779 | HGLibA_56800 | 29 | -0.860021873 | ZNF81 | zinc finger protein 81 [Source:HGNC Symbol;Acc:HGNC:13156] |
| ENSG00000198099 | HGLibA_00955 | 29 | -0.860021873 | ADH4 | pi polypeptide [Source:HGNC Symbol;Acc:HGNC:252]" |
| ENSG00000198612 | HGLibA_10733 | 29 | -0.860021873 | COPS8 | COP9 signalosome subunit 8 [Source:HGNC Symbol;Acc:HGNC:24335] |
| ENSG00000198633 | HGLibA_56181 | 29 | -0.860021873 | ZNF534 | zinc finger protein 534 [Source:HGNC Symbol;Acc:HGNC:26337] |
| ENSG00000198879 | HGLibA_43725 | 29 | -0.860021873 | SFMBT2 | Scm like with four mbt domains 2 [Source:HGNC Symbol;Acc:HGNC:20256] |
| ENSG00000204301 | HGLibA_32352 | 29 | -0.860021873 | NOTCH4 | notch 4 [Source:HGNC Symbol;Acc:HGNC:7884] |
| ENSG00000204315 | HGLibA_17595 | 29 | -0.860021873 | FKBPL | FK506 binding protein like [Source:HGNC Symbol;Acc:HGNC:13949] |
| ENSG00000204604 | HGLibA_56047 | 29 | -0.860021873 | ZNF468 | zinc finger protein 468 [Source:HGNC Symbol;Acc:HGNC:33105] |
| ENSG00000204616 | HGLibA_51459 | 29 | -0.860021873 | TRIM31 | tripartite motif containing 31 [Source:HGNC Symbol;Acc:HGNC:16289] |
| ENSG00000205323 | HGLibA_42747 | 29 | -0.860021873 | SARNP | SAP domain containing ribonucleoprotein [Source:HGNC Symbol;Acc:HGNC:24432] |
| ENSG00000206557 | HGLibA_51601 | 29 | -0.860021873 | TRIM71 | tripartite motif containing 71 [Source:HGNC Symbol;Acc:HGNC:32669] |
| ENSG00000211445 | HGLibA_20177 | 29 | -0.860021873 | GPX3 | glutathione peroxidase 3 [Source:HGNC Symbol;Acc:HGNC:4555] |
| ENSG00000213380 | HGLibA_10492 | 29 | -0.860021873 | COG8 | component of oligomeric golgi complex 8 [Source:HGNC Symbol;Acc:HGNC:18623] |
| ENSG00000213512 | HGLibA_18745 | 29 | -0.860021873 | GBP7 | guanylate binding protein 7 [Source:HGNC Symbol;Acc:HGNC:29606] |
| ENSG00000215305 | HGLibA_53743 | 29 | -0.860021873 | VPS16 | CORVET/HOPS core subunit [Source:HGNC Symbol;Acc:HGNC:14584]" |
| ENSG00000215644 | HGLibA_18779 | 29 | -0.860021873 | GCGR | glucagon receptor [Source:HGNC Symbol;Acc:HGNC:4192] |
| ENSG00000216588 | HGLibA_22891 | 29 | -0.860021873 | IGSF23 | immunoglobulin superfamily member 23 [Source:HGNC Symbol;Acc:HGNC:40040] |
| ENSG00000223572 | HGLibA_09777 | 29 | -0.860021873 | CKMT1A | mitochondrial 1A [Source:HGNC Symbol;Acc:HGNC:31736]" |
| ENSG00000226742 | HGLibA_22155 | 29 | -0.860021873 | HSBP1L1 | heat shock factor binding protein 1 like 1 [Source:HGNC Symbol;Acc:HGNC:37243] |
| ENSG00000235194 | HGLibA_38110 | 29 | -0.860021873 | PPP1R3E | protein phosphatase 1 regulatory subunit 3E [Source:HGNC Symbol;Acc:HGNC:14943] |
| ENSG00000237649 | HGLibA_24969 | 29 | -0.860021873 | KIFC1 | kinesin family member C1 [Source:HGNC Symbol;Acc:HGNC:6389] |
| ENSG00000237763 | HGLibA_01840 | 29 | -0.860021873 | AMY1A | alpha 1A (salivary) [Source:HGNC Symbol;Acc:HGNC:474]" |
| ENSG00000241794 | HGLibA_46807 | 29 | -0.860021873 | SPRR2A | small proline rich protein 2A [Source:HGNC Symbol;Acc:HGNC:11261] |
| ENSG00000244694 | HGLibA_39284 | 29 | -0.860021873 | PTCHD4 | patched domain containing 4 [Source:HGNC Symbol;Acc:HGNC:21345] |
| ENSG00000250506 | HGLibA_08768 | 29 | -0.860021873 | CDK3 | cyclin dependent kinase 3 [Source:HGNC Symbol;Acc:HGNC:1772] |
| ENSG00000261587 | HGLibA_50312 | 29 | -0.860021873 | TMEM249 | transmembrane protein 249 [Source:HGNC Symbol;Acc:HGNC:44155] |
| ENSG00000261934 | HGLibA_35548 | 29 | -0.860021873 | PCDHGA9 | 9 [Source:HGNC Symbol;Acc:HGNC:8707]" |
| ENSG00000263465 | HGLibA_47089 | 29 | -0.860021873 | SRSF8 | serine and arginine rich splicing factor 8 [Source:HGNC Symbol;Acc:HGNC:16988] |
| ENSG00000267631 | HGLibA_09303 | 29 | -0.860021873 | CGB1 | chorionic gonadotropin subunit beta 1 [Source:HGNC Symbol;Acc:HGNC:16721] |
| ENSG00000276023 | HGLibA_14143 | 29 | -0.860021873 | DUSP14 | dual specificity phosphatase 14 [Source:HGNC Symbol;Acc:HGNC:17007] |
| ENSG00000278570 | HGLibA_32608 | 29 | -0.860021873 | NR2E3 | nuclear receptor subfamily 2 group E member 3 [Source:HGNC Symbol;Acc:HGNC:7974] |
| ENSG00000285077 | HGLibA_02704 | 29 | -0.860021873 | ARHGAP11B | Rho GTPase activating protein 11B [Source:HGNC Symbol;Acc:HGNC:15782] |
| ENSG00000288547 | HGLibA_06236 | 29 | -0.860021873 | C3orf36 | - |
| EPB49 | HGLibA_15262 | 29 | -0.860021873 | EPB49 | - |
| FAM101A | HGLibA_15973 | 29 | -0.860021873 | FAM101A | - |
| FAM26E | HGLibA_16564 | 29 | -0.860021873 | FAM26E | - |
| H3F3C | HGLibA_20784 | 29 | -0.860021873 | H3F3C | - |
| HIST1H2BM | HGLibA_21446 | 29 | -0.860021873 | HIST1H2BM | - |
| HIST1H4A | HGLibA_21486 | 29 | -0.860021873 | HIST1H4A | - |
| HIST2H2AC | HGLibA_21531 | 29 | -0.860021873 | HIST2H2AC | - |
| hsa-mir-1245a | HGLibA_57373 | 29 | -0.860021873 | hsa-mir-1245a | - |
| hsa-mir-1246 | HGLibA_57381 | 29 | -0.860021873 | hsa-mir-1246 | - |
| hsa-mir-1247 | HGLibA_57386 | 29 | -0.860021873 | hsa-mir-1247 | - |
| hsa-mir-1267 | HGLibA_57491 | 29 | -0.860021873 | hsa-mir-1267 | - |
| hsa-mir-149 | HGLibA_57888 | 29 | -0.860021873 | hsa-mir-149 | - |
| hsa-mir-188 | HGLibA_58015 | 29 | -0.860021873 | hsa-mir-188 | - |
| hsa-mir-219b | HGLibA_58285 | 29 | -0.860021873 | hsa-mir-219b | - |
| hsa-mir-31 | HGLibA_58497 | 29 | -0.860021873 | hsa-mir-31 | - |
| hsa-mir-3119-1 | HGLibA_58523 | 29 | -0.860021873 | hsa-mir-3119-1 | - |
| hsa-mir-3128 | HGLibA_58564 | 29 | -0.860021873 | hsa-mir-3128 | - |
| hsa-mir-3156-1 | HGLibA_58688 | 29 | -0.860021873 | hsa-mir-3156-1 | - |
| hsa-mir-3160-2 | HGLibA_58719 | 29 | -0.860021873 | hsa-mir-3160-2 | - |
| hsa-mir-3160-2 | HGLibA_58721 | 29 | -0.860021873 | hsa-mir-3160-2 | - |
| hsa-mir-3682 | HGLibA_59282 | 29 | -0.860021873 | hsa-mir-3682 | - |
| hsa-mir-376c | HGLibA_59417 | 29 | -0.860021873 | hsa-mir-376c | - |
| hsa-mir-378j | HGLibA_59466 | 29 | -0.860021873 | hsa-mir-378j | - |
| hsa-mir-3929 | HGLibA_59591 | 29 | -0.860021873 | hsa-mir-3929 | - |
| hsa-mir-4252 | HGLibA_59715 | 29 | -0.860021873 | hsa-mir-4252 | - |
| hsa-mir-4308 | HGLibA_59945 | 29 | -0.860021873 | hsa-mir-4308 | - |
| hsa-mir-4441 | HGLibA_60166 | 29 | -0.860021873 | hsa-mir-4441 | - |
| hsa-mir-4482 | HGLibA_60347 | 29 | -0.860021873 | hsa-mir-4482 | - |
| hsa-mir-4656 | HGLibA_60733 | 29 | -0.860021873 | hsa-mir-4656 | - |
| hsa-mir-4662b | HGLibA_60760 | 29 | -0.860021873 | hsa-mir-4662b | - |
| hsa-mir-4694 | HGLibA_60883 | 29 | -0.860021873 | hsa-mir-4694 | - |

| | | | | | |
|---|---|---|---|---|---|
| hsa-mir-4742 | HGLibA_61067 | 29 | -0.860021873 | hsa-mir-4742 | - |
| hsa-mir-4773-1 | HGLibA_61193 | 29 | -0.860021873 | hsa-mir-4773-1 | - |
| hsa-mir-548ar | HGLibA_61918 | 29 | -0.860021873 | hsa-mir-548ar | - |
| hsa-mir-619 | HGLibA_62715 | 29 | -0.860021873 | hsa-mir-619 | - |
| hsa-mir-637 | HGLibA_62787 | 29 | -0.860021873 | hsa-mir-637 | - |
| hsa-mir-641 | HGLibA_62804 | 29 | -0.860021873 | hsa-mir-641 | - |
| hsa-mir-650 | HGLibA_62848 | 29 | -0.860021873 | hsa-mir-650 | - |
| hsa-mir-663b | HGLibA_62991 | 29 | -0.860021873 | hsa-mir-663b | - |
| hsa-mir-6785 | HGLibA_63318 | 29 | -0.860021873 | hsa-mir-6785 | - |
| hsa-mir-6822 | HGLibA_63465 | 29 | -0.860021873 | hsa-mir-6822 | - |
| hsa-mir-6830 | HGLibA_63498 | 29 | -0.860021873 | hsa-mir-6830 | - |
| hsa-mir-7844 | HGLibA_63964 | 29 | -0.860021873 | hsa-mir-7844 | - |
| hsa-mir-877 | HGLibA_64220 | 29 | -0.860021873 | hsa-mir-877 | - |
| hsa-mir-891b | HGLibA_64246 | 29 | -0.860021873 | hsa-mir-891b | - |
| hsa-mir-95 | HGLibA_64367 | 29 | -0.860021873 | hsa-mir-95 | - |
| LOC643355 | HGLibA_26967 | 29 | -0.860021873 | LOC643355 | - |
| MEF2BNB | HGLibA_28833 | 29 | -0.860021873 | MEF2BNB | - |
| PHF16 | HGLibA_36352 | 29 | -0.860021873 | PHF16 | - |
| SPATA31A2 | HGLibA_46470 | 29 | -0.860021873 | SPATA31A2 | - |
| WISP3 | HGLibA_54324 | 29 | -0.860021873 | WISP3 | - |
| BAGE5 | HGLibA_04103 | 28 | -0.869972911 | BAGE5 | - |
| C11orf57 | HGLibA_05160 | 28 | -0.869972911 | C11orf57 | - |
| C9orf3 | HGLibA_06722 | 28 | -0.869972911 | C9orf3 | - |
| CD99_X | HGLibA_08488 | 28 | -0.869972911 | CD99_X | - |
| DHRSX_Y | HGLibA_13161 | 28 | -0.869972911 | DHRSX_Y | - |
| ENSG00000005075 | HGLibA_37592 | 28 | -0.869972911 | POLR2J | RNA polymerase II subunit J [Source:HGNC Symbol;Acc:HGNC:9197] |
| ENSG00000014216 | HGLibA_07142 | 28 | -0.869972911 | CAPN1 | calpain 1 [Source:HGNC Symbol;Acc:HGNC:1476] |
| ENSG00000015676 | HGLibA_32927 | 28 | -0.869972911 | NUDCD3 | NudC domain containing 3 [Source:HGNC Symbol;Acc:HGNC:22208] |
| ENSG00000025796 | HGLibA_43262 | 28 | -0.869972911 | SEC63 | protein translocation regulator [Source:HGNC Symbol;Acc:HGNC:21082]" |
| ENSG00000027075 | HGLibA_38551 | 28 | -0.869972911 | PRKCH | protein kinase C eta [Source:HGNC Symbol;Acc:HGNC:9403] |
| ENSG00000044524 | HGLibA_15292 | 28 | -0.869972911 | EPHA3 | EPH receptor A3 [Source:HGNC Symbol;Acc:HGNC:3387] |
| ENSG00000049541 | HGLibA_40904 | 28 | -0.869972911 | RFC2 | replication factor C subunit 2 [Source:HGNC Symbol;Acc:HGNC:9970] |
| ENSG00000049759 | HGLibA_31606 | 28 | -0.869972911 | NEDD4L | E3 ubiquitin protein ligase [Source:HGNC Symbol;Acc:HGNC:7728]" |
| ENSG00000059769 | HGLibA_13607 | 28 | -0.869972911 | DNAJC25 | DnaJ heat shock protein family (Hsp40) member C25 [Source:HGNC Symbol;Acc:HGNC:34187] |
| ENSG00000063761 | HGLibA_00890 | 28 | -0.869972911 | ADCK1 | aarF domain containing kinase 1 [Source:HGNC Symbol;Acc:HGNC:19038] |
| ENSG00000066827 | HGLibA_55166 | 28 | -0.869972911 | ZFAT | zinc finger and AT-hook domain containing [Source:HGNC Symbol;Acc:HGNC:19899] |
| ENSG00000070404 | HGLibA_18104 | 28 | -0.869972911 | FSTL3 | follistatin like 3 [Source:HGNC Symbol;Acc:HGNC:3973] |
| ENSG00000073754 | HGLibA_08419 | 28 | -0.869972911 | CD5L | CD5 molecule like [Source:HGNC Symbol;Acc:HGNC:1690] |
| ENSG00000076201 | HGLibA_39463 | 28 | -0.869972911 | PTPN23 | non-receptor type 23 [Source:HGNC Symbol;Acc:HGNC:14406]" |
| ENSG00000077522 | HGLibA_00641 | 28 | -0.869972911 | ACTN2 | actinin alpha 2 [Source:HGNC Symbol;Acc:HGNC:164] |
| ENSG00000077984 | HGLibA_11466 | 28 | -0.869972911 | CST7 | cystatin F [Source:HGNC Symbol;Acc:HGNC:2479] |
| ENSG00000078668 | HGLibA_53604 | 28 | -0.869972911 | VDAC3 | voltage dependent anion channel 3 [Source:HGNC Symbol;Acc:HGNC:12674] |
| ENSG00000079335 | HGLibA_08509 | 28 | -0.869972911 | CDC14A | cell division cycle 14A [Source:HGNC Symbol;Acc:HGNC:1718] |
| ENSG00000082196 | HGLibA_05942 | 28 | -0.869972911 | C1QTNF3 | C1q and TNF related 3 [Source:HGNC Symbol;Acc:HGNC:14326] |
| ENSG00000083782 | HGLibA_15394 | 28 | -0.869972911 | EPYC | epiphycan [Source:HGNC Symbol;Acc:HGNC:3053] |
| ENSG00000096006 | HGLibA_11172 | 28 | -0.869972911 | CRISP3 | cysteine rich secretory protein 3 [Source:HGNC Symbol;Acc:HGNC:16904] |
| ENSG00000096395 | HGLibA_29427 | 28 | -0.869972911 | MLN | motilin [Source:HGNC Symbol;Acc:HGNC:7141] |
| ENSG00000100060 | HGLibA_29076 | 28 | -0.869972911 | MFNG | MFNG O-fucosylpeptide 3-beta-N-acetylglucosaminyltransferase [Source:HGNC Symbol;Acc:HGNC:7038] |
| ENSG00000100351 | HGLibA_20216 | 28 | -0.869972911 | GRAP2 | GRB2 related adaptor protein 2 [Source:HGNC Symbol;Acc:HGNC:4563] |
| ENSG00000101161 | HGLibA_38742 | 28 | -0.869972911 | PRPF6 | pre-mRNA processing factor 6 [Source:HGNC Symbol;Acc:HGNC:15860] |
| ENSG00000101981 | HGLibA_15896 | 28 | -0.869972911 | F9 | coagulation factor IX [Source:HGNC Symbol;Acc:HGNC:3551] |
| ENSG00000102172 | HGLibA_45829 | 28 | -0.869972911 | SMS | spermine synthase [Source:HGNC Symbol;Acc:HGNC:11123] |
| ENSG00000102302 | HGLibA_17341 | 28 | -0.869972911 | FGD1 | RhoGEF and PH domain containing 1 [Source:HGNC Symbol;Acc:HGNC:3663]" |
| ENSG00000102804 | HGLibA_51818 | 28 | -0.869972911 | TSC22D1 | TSC22 domain family member 1 [Source:HGNC Symbol;Acc:HGNC:16826] |
| ENSG00000102870 | HGLibA_56432 | 28 | -0.869972911 | ZNF629 | zinc finger protein 629 [Source:HGNC Symbol;Acc:HGNC:29008] |
| ENSG00000104177 | HGLibA_30688 | 28 | -0.869972911 | MYEF2 | myelin expression factor 2 [Source:HGNC Symbol;Acc:HGNC:17940] |
| ENSG00000105171 | HGLibA_37690 | 28 | -0.869972911 | POP4 | ribonuclease P/MRP subunit [Source:HGNC Symbol;Acc:HGNC:30081]" |
| ENSG00000105254 | HGLibA_48581 | 28 | -0.869972911 | TBCB | tubulin folding cofactor B [Source:HGNC Symbol;Acc:HGNC:1989] |
| ENSG00000105669 | HGLibA_10700 | 28 | -0.869972911 | COPE | coatomer protein complex subunit epsilon [Source:HGNC Symbol;Acc:HGNC:2234] |
| ENSG00000105865 | HGLibA_14124 | 28 | -0.869972911 | DUS4L | dihydrouridine synthase 4 like [Source:HGNC Symbol;Acc:HGNC:21517] |
| ENSG00000106605 | HGLibA_04574 | 28 | -0.869972911 | BLVRA | biliverdin reductase A [Source:HGNC Symbol;Acc:HGNC:1062] |
| ENSG00000108175 | HGLibA_55382 | 28 | -0.869972911 | ZMIZ1 | zinc finger MIZ-type containing 1 [Source:HGNC Symbol;Acc:HGNC:16493] |
| ENSG00000108848 | HGLibA_27625 | 28 | -0.869972911 | LUC7L3 | LUC7 like 3 pre-mRNA splicing factor [Source:HGNC Symbol;Acc:HGNC:24309] |
| ENSG00000108854 | HGLibA_45849 | 28 | -0.869972911 | SMURF2 | SMAD specific E3 ubiquitin protein ligase 2 [Source:HGNC Symbol;Acc:HGNC:16809] |
| ENSG00000109182 | HGLibA_11829 | 28 | -0.869972911 | CWH43 | cell wall biogenesis 43 C-terminal homolog [Source:HGNC Symbol;Acc:HGNC:26133] |
| ENSG00000109689 | HGLibA_47444 | 28 | -0.869972911 | STIM2 | stromal interaction molecule 2 [Source:HGNC Symbol;Acc:HGNC:19205] |
| ENSG00000112137 | HGLibA_36296 | 28 | -0.869972911 | PHACTR1 | phosphatase and actin regulator 1 [Source:HGNC Symbol;Acc:HGNC:20990] |
| ENSG00000112175 | HGLibA_04612 | 28 | -0.869972911 | BMP5 | bone morphogenetic protein 5 [Source:HGNC Symbol;Acc:HGNC:1072] |
| ENSG00000112499 | HGLibA_44566 | 28 | -0.869972911 | SLC22A2 | solute carrier family 22 member 2 [Source:HGNC Symbol;Acc:HGNC:10966] |
| ENSG00000113649 | HGLibA_48755 | 28 | -0.869972911 | TCERG1 | transcription elongation regulator 1 [Source:HGNC Symbol;Acc:HGNC:15630] |
| ENSG00000114107 | HGLibA_09157 | 28 | -0.869972911 | CEP70 | centrosomal protein 70 [Source:HGNC Symbol;Acc:HGNC:29972] |
| ENSG00000114520 | HGLibA_46097 | 28 | -0.869972911 | SNX4 | sorting nexin 4 [Source:HGNC Symbol;Acc:HGNC:11175] |
| ENSG00000115053 | HGLibA_31326 | 28 | -0.869972911 | NCL | nucleolin [Source:HGNC Symbol;Acc:HGNC:7667] |
| ENSG00000115112 | HGLibA_49149 | 28 | -0.869972911 | TFCP2L1 | transcription factor CP2 like 1 [Source:HGNC Symbol;Acc:HGNC:17925] |
| ENSG00000116014 | HGLibA_25011 | 28 | -0.869972911 | KISS1R | KISS1 receptor [Source:HGNC Symbol;Acc:HGNC:4510] |
| ENSG00000116750 | HGLibA_52808 | 28 | -0.869972911 | UCHL5 | ubiquitin C-terminal hydrolase L5 [Source:HGNC Symbol;Acc:HGNC:19678] |
| ENSG00000117523 | HGLibA_38866 | 28 | -0.869972911 | PRRC2C | proline rich coiled-coil 2C [Source:HGNC Symbol;Acc:HGNC:24903] |
| ENSG00000119801 | HGLibA_54719 | 28 | -0.869972911 | YPEL5 | yippee like 5 [Source:HGNC Symbol;Acc:HGNC:18329] |
| ENSG00000120669 | HGLibA_46165 | 28 | -0.869972911 | SOHLH2 | spermatogenesis and oogenesis specific basic helix-loop-helix 2 [Source:HGNC Symbol;Acc:HGNC:26026] |
| ENSG00000120837 | HGLibA_31858 | 28 | -0.869972911 | NFYB | nuclear transcription factor Y subunit beta [Source:HGNC Symbol;Acc:HGNC:7805] |
| ENSG00000121390 | HGLibA_39223 | 28 | -0.869972911 | PSPC1 | paraspeckle component 1 [Source:HGNC Symbol;Acc:HGNC:20320] |
| ENSG00000122859 | HGLibA_31751 | 28 | -0.869972911 | NEUROG3 | neurogenin 3 [Source:HGNC Symbol;Acc:HGNC:13806] |
| ENSG00000123219 | HGLibA_09065 | 28 | -0.869972911 | CENPK | centromere protein K [Source:HGNC Symbol;Acc:HGNC:29479] |
| ENSG00000124257 | HGLibA_31727 | 28 | -0.869972911 | NEURL2 | neuralized E3 ubiquitin protein ligase 2 [Source:HGNC Symbol;Acc:HGNC:16156] |
| ENSG00000124789 | HGLibA_33024 | 28 | -0.869972911 | NUP153 | nucleoporin 153 [Source:HGNC Symbol;Acc:HGNC:8062] |
| ENSG00000125633 | HGLibA_07930 | 28 | -0.869972911 | CCDC93 | coiled-coil domain containing 93 [Source:HGNC Symbol;Acc:HGNC:25611] |
| ENSG00000125746 | HGLibA_15058 | 28 | -0.869972911 | EML2 | echinoderm microtubule associated protein like 2 [Source:HGNC Symbol;Acc:HGNC:18035] |
| ENSG00000126432 | HGLibA_38420 | 28 | -0.869972911 | PRDX5 | peroxiredoxin 5 [Source:HGNC Symbol;Acc:HGNC:9355] |
| ENSG00000127328 | HGLibA_39927 | 28 | -0.869972911 | RAB3IP | RAB3A interacting protein [Source:HGNC Symbol;Acc:HGNC:16508] |
| ENSG00000127603 | HGLibA_27832 | 28 | -0.869972911 | MACF1 | microtubule-actin crosslinking factor 1 [Source:HGNC Symbol;Acc:HGNC:13664] |
| ENSG00000127947 | HGLibA_39435 | 28 | -0.869972911 | PTPN12 | non-receptor type 12 [Source:HGNC Symbol;Acc:HGNC:9645]" |
| ENSG00000130283 | HGLibA_18852 | 28 | -0.869972911 | GDF1 | growth differentiation factor 1 [Source:HGNC Symbol;Acc:HGNC:4214] |
| ENSG00000130283 | HGLibA_18850 | 28 | -0.869972911 | GDF1 | growth differentiation factor 1 [Source:HGNC Symbol;Acc:HGNC:4214] |
| ENSG00000130706 | HGLibA_01069 | 28 | -0.869972911 | ADRM1 | adhesion regulating molecule 1 [Source:HGNC Symbol;Acc:HGNC:15759] |
| ENSG00000131013 | HGLibA_37948 | 28 | -0.869972911 | PPIL4 | peptidylprolyl isomerase like 4 [Source:HGNC Symbol;Acc:HGNC:15702] |
| ENSG00000131462 | HGLibA_52303 | 28 | -0.869972911 | TUBG1 | tubulin gamma 1 [Source:HGNC Symbol;Acc:HGNC:12417] |
| ENSG00000131725 | HGLibA_54095 | 28 | -0.869972911 | WDR44 | WD repeat domain 44 [Source:HGNC Symbol;Acc:HGNC:30512] |
| ENSG00000131747 | HGLibA_50993 | 28 | -0.869972911 | TOP2A | DNA topoisomerase II alpha [Source:HGNC Symbol;Acc:HGNC:11989] |
| ENSG00000131981 | HGLibA_26363 | 28 | -0.869972911 | LGALS3 | galectin 3 [Source:HGNC Symbol;Acc:HGNC:6563] |
| ENSG00000132109 | HGLibA_51429 | 28 | -0.869972911 | TRIM21 | tripartite motif containing 21 [Source:HGNC Symbol;Acc:HGNC:11312] |
| ENSG00000132635 | HGLibA_35584 | 28 | -0.869972911 | PCED1A | PC-esterase domain containing 1A [Source:HGNC Symbol;Acc:HGNC:16212] |
| ENSG00000134242 | HGLibA_39461 | 28 | -0.869972911 | PTPN22 | non-receptor type 22 [Source:HGNC Symbol;Acc:HGNC:9652]" |
| ENSG00000135249 | HGLibA_41303 | 28 | -0.869972911 | RINT1 | RAD50 interactor 1 [Source:HGNC Symbol;Acc:HGNC:21876] |
| ENSG00000135913 | HGLibA_53351 | 28 | -0.869972911 | USP37 | ubiquitin specific peptidase 37 [Source:HGNC Symbol;Acc:HGNC:20063] |
| ENSG00000136682 | HGLibA_07433 | 28 | -0.869972911 | CBWD2 | COBW domain containing 2 [Source:HGNC Symbol;Acc:HGNC:17907] |
| ENSG00000136819 | HGLibA_06762 | 28 | -0.869972911 | C9orf78 | chromosome 9 open reading frame 78 [Source:HGNC Symbol;Acc:HGNC:24932] |
| ENSG00000136935 | HGLibA_19593 | 28 | -0.869972911 | GOLGA1 | golgin A1 [Source:HGNC Symbol;Acc:HGNC:4424] |
| ENSG00000137070 | HGLibA_22955 | 28 | -0.869972911 | IL11RA | interleukin 11 receptor subunit alpha [Source:HGNC Symbol;Acc:HGNC:5967] |
| ENSG00000137204 | HGLibA_44596 | 28 | -0.869972911 | SLC22A7 | solute carrier family 22 member 7 [Source:HGNC Symbol;Acc:HGNC:10971] |
| ENSG00000137500 | HGLibA_07921 | 28 | -0.869972911 | CCDC90B | coiled-coil domain containing 90B [Source:HGNC Symbol;Acc:HGNC:28108] |
| ENSG00000138152 | HGLibA_04902 | 28 | -0.869972911 | BTBD16 | BTB domain containing 16 [Source:HGNC Symbol;Acc:HGNC:26340] |
| ENSG00000138160 | HGLibA_24850 | 28 | -0.869972911 | KIF11 | kinesin family member 11 [Source:HGNC Symbol;Acc:HGNC:6388] |
| ENSG00000138400 | HGLibA_28685 | 28 | -0.869972911 | MDH1B | malate dehydrogenase 1B [Source:HGNC Symbol;Acc:HGNC:17836] |
| ENSG00000138653 | HGLibA_31434 | 28 | -0.869972911 | NDST4 | N-deacetylase and N-sulfotransferase 4 [Source:HGNC Symbol;Acc:HGNC:20779] |
| ENSG00000140478 | HGLibA_19615 | 28 | -0.869972911 | GOLGA6D | golgin A6 family member D [Source:HGNC Symbol;Acc:HGNC:32204] |
| ENSG00000141232 | HGLibA_50933 | 28 | -0.869972911 | TOB1 | 1 [Source:HGNC Symbol;Acc:HGNC:11979]" |
| ENSG00000141759 | HGLibA_52438 | 28 | -0.869972911 | TXNL4A | thioredoxin like 4A [Source:HGNC Symbol;Acc:HGNC:30551] |
| ENSG00000141837 | HGLibA_06876 | 28 | -0.869972911 | CACNA1A | calcium voltage-gated channel subunit alpha1 A [Source:HGNC Symbol;Acc:HGNC:1388] |
| ENSG00000142538 | HGLibA_39369 | 28 | -0.869972911 | PTH2 | parathyroid hormone 2 [Source:HGNC Symbol;Acc:HGNC:30828] |
| ENSG00000143367 | HGLibA_52328 | 28 | -0.869972911 | TUFT1 | tuftelin 1 [Source:HGNC Symbol;Acc:HGNC:12422] |
| ENSG00000143971 | HGLibA_15613 | 28 | -0.869972911 | ETAA1 | ATR kinase activator [Source:HGNC Symbol;Acc:HGNC:24648]" |
| ENSG00000146054 | HGLibA_51596 | 28 | -0.869972911 | TRIM7 | tripartite motif containing 7 [Source:HGNC Symbol;Acc:HGNC:16278] |
| ENSG00000146112 | HGLibA_38063 | 28 | -0.869972911 | PPP1R18 | protein phosphatase 1 regulatory subunit 18 [Source:HGNC Symbol;Acc:HGNC:29413] |
| ENSG00000146648 | HGLibA_14635 | 28 | -0.869972911 | EGFR | epidermal growth factor receptor [Source:HGNC Symbol;Acc:HGNC:3236] |
| ENSG00000146833 | HGLibA_51489 | 28 | -0.869972911 | TRIM4 | tripartite motif containing 4 [Source:HGNC Symbol;Acc:HGNC:16275] |
| ENSG00000148303 | HGLibA_41995 | 28 | -0.869972911 | RPL7A | ribosomal protein L7a [Source:HGNC Symbol;Acc:HGNC:10364] |
| ENSG00000148308 | HGLibA_20592 | 28 | -0.869972911 | GTF3C5 | general transcription factor IIIC subunit 5 [Source:HGNC Symbol;Acc:HGNC:4668] |
| ENSG00000148925 | HGLibA_04896 | 28 | -0.869972911 | BTBD10 | BTB domain containing 10 [Source:HGNC Symbol;Acc:HGNC:21445] |
| ENSG00000151148 | HGLibA_52681 | 28 | -0.869972911 | UBE3B | ubiquitin protein ligase E3B [Source:HGNC Symbol;Acc:HGNC:13478] |
| ENSG00000151572 | HGLibA_02179 | 28 | -0.869972911 | ANO4 | anoctamin 4 [Source:HGNC Symbol;Acc:HGNC:23837] |
| ENSG00000151718 | HGLibA_54442 | 28 | -0.869972911 | WWC2 | WW and C2 domain containing 2 [Source:HGNC Symbol;Acc:HGNC:24148] |
| ENSG00000151838 | HGLibA_07706 | 28 | -0.869972911 | CCDC175 | coiled-coil domain containing 175 [Source:HGNC Symbol;Acc:HGNC:19847] |
| ENSG00000153048 | HGLibA_07247 | 28 | -0.869972911 | CARHSP1 | calcium regulated heat stable protein 1 [Source:HGNC Symbol;Acc:HGNC:17150] |
| ENSG00000153201 | HGLibA_40200 | 28 | -0.869972911 | RANBP2 | RAN binding protein 2 [Source:HGNC Symbol;Acc:HGNC:9848] |
| ENSG00000153823 | HGLibA_36529 | 28 | -0.869972911 | PID1 | phosphotyrosine interaction domain containing 1 [Source:HGNC Symbol;Acc:HGNC:26084] |

| | | | | | |
|---|---|---|---|---|---|
| ENSG00000153922 | HGLibA_09384 | 28 | -0.869972911 | CHD1 | chromodomain helicase DNA binding protein 1 [Source:HGNC Symbol;Acc:HGNC:1915] |
| ENSG00000154736 | HGLibA_00832 | 28 | -0.869972911 | ADAMTS5 | ADAM metallopeptidase with thrombospondin type 1 motif 5 [Source:HGNC Symbol;Acc:HGNC:221] |
| ENSG00000154839 | HGLibA_44262 | 28 | -0.869972911 | SKA1 | spindle and kinetochore associated complex subunit 1 [Source:HGNC Symbol;Acc:HGNC:28109] |
| ENSG00000154930 | HGLibA_00587 | 28 | -0.869972911 | ACSS1 | acyl-CoA synthetase short chain family member 1 [Source:HGNC Symbol;Acc:HGNC:16091] |
| ENSG00000155970 | HGLibA_29228 | 28 | -0.869972911 | MICU3 | mitochondrial calcium uptake family member 3 [Source:HGNC Symbol;Acc:HGNC:27820] |
| ENSG00000156395 | HGLibA_46190 | 28 | -0.869972911 | SORCS3 | sortilin related VPS10 domain containing receptor 3 [Source:HGNC Symbol;Acc:HGNC:16699] |
| ENSG00000156466 | HGLibA_18873 | 28 | -0.869972911 | GDF6 | growth differentiation factor 6 [Source:HGNC Symbol;Acc:HGNC:4221] |
| ENSG00000157349 | HGLibA_12666 | 28 | -0.869972911 | DDX19B | DEAD-box helicase 19B [Source:HGNC Symbol;Acc:HGNC:2742] |
| ENSG00000157613 | HGLibA_11093 | 28 | -0.869972911 | CREB3L1 | cAMP responsive element binding protein 3 like 1 [Source:HGNC Symbol;Acc:HGNC:18856] |
| ENSG00000157823 | HGLibA_02338 | 28 | -0.869972911 | AP3S2 | adaptor related protein complex 3 subunit sigma 2 [Source:HGNC Symbol;Acc:HGNC:571] |
| ENSG00000157884 | HGLibA_09690 | 28 | -0.869972911 | CIB4 | calcium and integrin binding family member 4 [Source:HGNC Symbol;Acc:HGNC:33703] |
| ENSG00000158062 | HGLibA_52780 | 28 | -0.869972911 | UBXN11 | UBX domain protein 11 [Source:HGNC Symbol;Acc:HGNC:30600] |
| ENSG00000158163 | HGLibA_14301 | 28 | -0.869972911 | DZIP1L | DAZ interacting zinc finger protein 1 like [Source:HGNC Symbol;Acc:HGNC:26551] |
| ENSG00000158164 | HGLibA_50659 | 28 | -0.869972911 | TMSB15A | thymosin beta 15a [Source:HGNC Symbol;Acc:HGNC:30744] |
| ENSG00000159348 | HGLibA_12000 | 28 | -0.869972911 | CYB5R1 | cytochrome b5 reductase 1 [Source:HGNC Symbol;Acc:HGNC:13397] |
| ENSG00000159915 | HGLibA_55643 | 28 | -0.869972911 | ZNF233 | zinc finger protein 233 [Source:HGNC Symbol;Acc:HGNC:30946] |
| ENSG00000160183 | HGLibA_50638 | 28 | -0.869972911 | TMPRSS3 | transmembrane serine protease 3 [Source:HGNC Symbol;Acc:HGNC:11877] |
| ENSG00000160360 | HGLibA_20155 | 28 | -0.869972911 | GPSM1 | G protein signaling modulator 1 [Source:HGNC Symbol;Acc:HGNC:17858] |
| ENSG00000160688 | HGLibA_17602 | 28 | -0.869972911 | FLAD1 | flavin adenine dinucleotide synthetase 1 [Source:HGNC Symbol;Acc:HGNC:24671] |
| ENSG00000160951 | HGLibA_39314 | 28 | -0.869972911 | PTGER1 | prostaglandin E receptor 1 [Source:HGNC Symbol;Acc:HGNC:9593] |
| ENSG00000161021 | HGLibA_28044 | 28 | -0.869972911 | MAML1 | mastermind like transcriptional coactivator 1 [Source:HGNC Symbol;Acc:HGNC:13632] |
| ENSG00000161057 | HGLibA_39129 | 28 | -0.869972911 | PSMC2 | ATPase 2 [Source:HGNC Symbol;Acc:HGNC:9548]" |
| ENSG00000161328 | HGLibA_27393 | 28 | -0.869972911 | LRRC56 | leucine rich repeat containing 56 [Source:HGNC Symbol;Acc:HGNC:25430] |
| ENSG00000161981 | HGLibA_45944 | 28 | -0.869972911 | SNRNP25 | small nuclear ribonucleoprotein U11/U12 subunit 25 [Source:HGNC Symbol;Acc:HGNC:14161] |
| ENSG00000162139 | HGLibA_31716 | 28 | -0.869972911 | NEU3 | neuraminidase 3 [Source:HGNC Symbol;Acc:HGNC:7760] |
| ENSG00000162267 | HGLibA_23811 | 28 | -0.869972911 | ITIH3 | inter-alpha-trypsin inhibitor heavy chain 3 [Source:HGNC Symbol;Acc:HGNC:6168] |
| ENSG00000162456 | HGLibA_25335 | 28 | -0.869972911 | KNCN | kinocilin [Source:HGNC Symbol;Acc:HGNC:26488] |
| ENSG00000162460 | HGLibA_50523 | 28 | -0.869972911 | TMEM82 | transmembrane protein 82 [Source:HGNC Symbol;Acc:HGNC:32350] |
| ENSG00000162594 | HGLibA_23099 | 28 | -0.869972911 | IL23R | interleukin 23 receptor [Source:HGNC Symbol;Acc:HGNC:19100] |
| ENSG00000162804 | HGLibA_45922 | 28 | -0.869972911 | SNED1 | nidogen and EGF like domains 1 [Source:HGNC Symbol;Acc:HGNC:24696]" |
| ENSG00000163156 | HGLibA_43009 | 28 | -0.869972911 | SCNM1 | sodium channel modifier 1 [Source:HGNC Symbol;Acc:HGNC:23136] |
| ENSG00000163191 | HGLibA_42537 | 28 | -0.869972911 | S100A11 | S100 calcium binding protein A11 [Source:HGNC Symbol;Acc:HGNC:10488] |
| ENSG00000163818 | HGLibA_27800 | 28 | -0.869972911 | LZTFL1 | leucine zipper transcription factor like 1 [Source:HGNC Symbol;Acc:HGNC:6741] |
| ENSG00000163877 | HGLibA_45929 | 28 | -0.869972911 | SNIP1 | Smad nuclear interacting protein 1 [Source:HGNC Symbol;Acc:HGNC:30587] |
| ENSG00000164061 | HGLibA_04869 | 28 | -0.869972911 | BSN | bassoon presynaptic cytomatrix protein [Source:HGNC Symbol;Acc:HGNC:1117] |
| ENSG00000164434 | HGLibA_15928 | 28 | -0.869972911 | FABP7 | fatty acid binding protein 7 [Source:HGNC Symbol;Acc:HGNC:3562] |
| ENSG00000164932 | HGLibA_11630 | 28 | -0.869972911 | CTHRC1 | collagen triple helix repeat containing 1 [Source:HGNC Symbol;Acc:HGNC:18831] |
| ENSG00000165502 | HGLibA_41962 | 28 | -0.869972911 | RPL36AL | ribosomal protein L36a like [Source:HGNC Symbol;Acc:HGNC:10346] |
| ENSG00000165630 | HGLibA_38709 | 28 | -0.869972911 | PRPF18 | pre-mRNA processing factor 18 [Source:HGNC Symbol;Acc:HGNC:17351] |
| ENSG00000165675 | HGLibA_15160 | 28 | -0.869972911 | ENOX2 | ecto-NOX disulfide-thiol exchanger 2 [Source:HGNC Symbol;Acc:HGNC:2259] |
| ENSG00000165802 | HGLibA_32776 | 28 | -0.869972911 | NSMF | NMDA receptor synaptonuclear signaling and neuronal migration factor [Source:HGNC Symbol;Acc:HGNC:29843] |
| ENSG00000165973 | HGLibA_31683 | 28 | -0.869972911 | NELL1 | neural EGFL like 1 [Source:HGNC Symbol;Acc:HGNC:7750] |
| ENSG00000166396 | HGLibA_43562 | 28 | -0.869972911 | SERPINB7 | serpin family B member 7 [Source:HGNC Symbol;Acc:HGNC:13902] |
| ENSG00000167183 | HGLibA_38789 | 28 | -0.869972911 | PRR15L | proline rich 15 like [Source:HGNC Symbol;Acc:HGNC:28149] |
| ENSG00000167588 | HGLibA_19784 | 28 | -0.869972911 | GPD1 | glycerol-3-phosphate dehydrogenase 1 [Source:HGNC Symbol;Acc:HGNC:4455] |
| ENSG00000167751 | HGLibA_25253 | 28 | -0.869972911 | KLK2 | kallikrein related peptidase 2 [Source:HGNC Symbol;Acc:HGNC:6363] |
| ENSG00000167759 | HGLibA_25243 | 28 | -0.869972911 | KLK13 | kallikrein related peptidase 13 [Source:HGNC Symbol;Acc:HGNC:6361] |
| ENSG00000167842 | HGLibA_29303 | 28 | -0.869972911 | MIS12 | kinetochore complex component [Source:HGNC Symbol;Acc:HGNC:24967]" |
| ENSG00000167925 | HGLibA_19035 | 28 | -0.869972911 | GHDC | GH3 domain containing [Source:HGNC Symbol;Acc:HGNC:24438] |
| ENSG00000168234 | HGLibA_52139 | 28 | -0.869972911 | TTC39C | tetratricopeptide repeat domain 39C [Source:HGNC Symbol;Acc:HGNC:26595] |
| ENSG00000168890 | HGLibA_49991 | 28 | -0.869972911 | TMEM150A | transmembrane protein 150A [Source:HGNC Symbol;Acc:HGNC:24677] |
| ENSG00000169181 | HGLibA_20422 | 28 | -0.869972911 | GSG1L | GSG1 like [Source:HGNC Symbol;Acc:HGNC:28283] |
| ENSG00000169188 | HGLibA_02411 | 28 | -0.869972911 | APEX2 | apurinic/apyrimidinic endodeoxyribonuclease 2 [Source:HGNC Symbol;Acc:HGNC:17889] |
| ENSG00000169718 | HGLibA_14111 | 28 | -0.869972911 | DUS1L | dihydrouridine synthase 1 like [Source:HGNC Symbol;Acc:HGNC:30086] |
| ENSG00000169851 | HGLibA_35416 | 28 | -0.869972911 | PCDH7 | protocadherin 7 [Source:HGNC Symbol;Acc:HGNC:8659] |
| ENSG00000170500 | HGLibA_27022 | 28 | -0.869972911 | LONRF2 | LON peptidase N-terminal domain and ring finger 2 [Source:HGNC Symbol;Acc:HGNC:24788] |
| ENSG00000170889 | HGLibA_42195 | 28 | -0.869972911 | RPS9 | ribosomal protein S9 [Source:HGNC Symbol;Acc:HGNC:10442] |
| ENSG00000170921 | HGLibA_48295 | 28 | -0.869972911 | TANC2 | ankyrin repeat and coiled-coil containing 2 [Source:HGNC Symbol;Acc:HGNC:30212]" |
| ENSG00000171045 | HGLibA_51884 | 28 | -0.869972911 | TSNARE1 | t-SNARE domain containing 1 [Source:HGNC Symbol;Acc:HGNC:26437] |
| ENSG00000171217 | HGLibA_09879 | 28 | -0.869972911 | CLDN20 | claudin 20 [Source:HGNC Symbol;Acc:HGNC:2042] |
| ENSG00000171365 | HGLibA_09832 | 28 | -0.869972911 | CLCN5 | chloride voltage-gated channel 5 [Source:HGNC Symbol;Acc:HGNC:2023] |
| ENSG00000171489 | HGLibA_46332 | 28 | -0.869972911 | SPACA5 | sperm acrosome associated 5 [Source:HGNC Symbol;Acc:HGNC:31353] |
| ENSG00000171621 | HGLibA_46858 | 28 | -0.869972911 | SPSB1 | splA/ryanodine receptor domain and SOCS box containing 1 [Source:HGNC Symbol;Acc:HGNC:30628] |
| ENSG00000172175 | HGLibA_28034 | 28 | -0.869972911 | MALT1 | MALT1 paracaspase [Source:HGNC Symbol;Acc:HGNC:6819] |
| ENSG00000172432 | HGLibA_20605 | 28 | -0.869972911 | GTPBP2 | GTP binding protein 2 [Source:HGNC Symbol;Acc:HGNC:4670] |
| ENSG00000173540 | HGLibA_19423 | 28 | -0.869972911 | GMPPB | GDP-mannose pyrophosphorylase B [Source:HGNC Symbol;Acc:HGNC:22932] |
| ENSG00000173679 | HGLibA_33685 | 28 | -0.869972911 | OR1L1 | olfactory receptor family 1 subfamily L member 1 [Source:HGNC Symbol;Acc:HGNC:8213] |
| ENSG00000174276 | HGLibA_56925 | 28 | -0.869972911 | ZNHIT2 | zinc finger HIT-type containing 2 [Source:HGNC Symbol;Acc:HGNC:1177] |
| ENSG00000174348 | HGLibA_37454 | 28 | -0.869972911 | PODN | podocan [Source:HGNC Symbol;Acc:HGNC:23174] |
| ENSG00000175104 | HGLibA_51263 | 28 | -0.869972911 | TRAF6 | TNF receptor associated factor 6 [Source:HGNC Symbol;Acc:HGNC:12036] |
| ENSG00000175398 | HGLibA_33535 | 28 | -0.869972911 | OR10P1 | olfactory receptor family 10 subfamily P member 1 [Source:HGNC Symbol;Acc:HGNC:15378] |
| ENSG00000176049 | HGLibA_23925 | 28 | -0.869972911 | JAKMIP2 | janus kinase and microtubule interacting protein 2 [Source:HGNC Symbol;Acc:HGNC:29067] |
| ENSG00000176198 | HGLibA_33578 | 28 | -0.869972911 | OR11H4 | olfactory receptor family 11 subfamily H member 4 [Source:HGNC Symbol;Acc:HGNC:15347] |
| ENSG00000177398 | HGLibA_52980 | 28 | -0.869972911 | UMODL1 | uromodulin like 1 [Source:HGNC Symbol;Acc:HGNC:12560] |
| ENSG00000179632 | HGLibA_27868 | 28 | -0.869972911 | MAF1 | negative regulator of RNA polymerase III [Source:HGNC Symbol;Acc:HGNC:24966]" |
| ENSG00000179954 | HGLibA_47125 | 28 | -0.869972911 | SSC5D | scavenger receptor cysteine rich family member with 5 domains [Source:HGNC Symbol;Acc:HGNC:26641] |
| ENSG00000180083 | HGLibA_54253 | 28 | -0.869972911 | WFDC11 | WAP four-disulfide core domain 11 [Source:HGNC Symbol;Acc:HGNC:20478] |
| ENSG00000182004 | HGLibA_45984 | 28 | -0.869972911 | SNRPE | small nuclear ribonucleoprotein polypeptide E [Source:HGNC Symbol;Acc:HGNC:11161] |
| ENSG00000182670 | HGLibA_52102 | 28 | -0.869972911 | TTC3 | tetratricopeptide repeat domain 3 [Source:HGNC Symbol;Acc:HGNC:12393] |
| ENSG00000182704 | HGLibA_51874 | 28 | -0.869972911 | TSKU | small leucine rich proteoglycan [Source:HGNC Symbol;Acc:HGNC:28850]" |
| ENSG00000182791 | HGLibA_07905 | 28 | -0.869972911 | CCDC87 | coiled-coil domain containing 87 [Source:HGNC Symbol;Acc:HGNC:25579] |
| ENSG00000183161 | HGLibA_16847 | 28 | -0.869972911 | FANCF | FA complementation group F [Source:HGNC Symbol;Acc:HGNC:3587] |
| ENSG00000185010 | HGLibA_15884 | 28 | -0.869972911 | F8 | coagulation factor VIII [Source:HGNC Symbol;Acc:HGNC:3546] |
| ENSG00000185127 | HGLibA_06439 | 28 | -0.869972911 | C6orf120 | chromosome 6 open reading frame 120 [Source:HGNC Symbol;Acc:HGNC:21247] |
| ENSG00000186431 | HGLibA_17165 | 28 | -0.869972911 | FCAR | Fc fragment of IgA receptor [Source:HGNC Symbol;Acc:HGNC:3608] |
| ENSG00000186652 | HGLibA_38453 | 28 | -0.869972911 | PRG2 | pro eosinophil major basic protein [Source:HGNC Symbol;Acc:HGNC:9362]" |
| ENSG00000188778 | HGLibA_01061 | 28 | -0.869972911 | ADRB3 | adrenoceptor beta 3 [Source:HGNC Symbol;Acc:HGNC:288] |
| ENSG00000189253 | HGLibA_51579 | 28 | -0.869972911 | TRIM64B | tripartite motif containing 64B [Source:HGNC Symbol;Acc:HGNC:37147] |
| ENSG00000196275 | HGLibA_20570 | 28 | -0.869972911 | GTF2IRD2 | GTF2I repeat domain containing 2 [Source:HGNC Symbol;Acc:HGNC:30775] |
| ENSG00000196290 | HGLibA_31929 | 28 | -0.869972911 | NIF3L1 | NGG1 interacting factor 3 like 1 [Source:HGNC Symbol;Acc:HGNC:13390] |
| ENSG00000196408 | HGLibA_32386 | 28 | -0.869972911 | NOXO1 | NADPH oxidase organizer 1 [Source:HGNC Symbol;Acc:HGNC:19404] |
| ENSG00000196415 | HGLibA_38984 | 28 | -0.869972911 | PRTN3 | proteinase 3 [Source:HGNC Symbol;Acc:HGNC:9495] |
| ENSG00000196427 | HGLibA_31236 | 28 | -0.869972911 | NBPF4 | NBPF member 4 [Source:HGNC Symbol;Acc:HGNC:26550] |
| ENSG00000196943 | HGLibA_32319 | 28 | -0.869972911 | NOP9 | NOP9 nucleolar protein [Source:HGNC Symbol;Acc:HGNC:19826] |
| ENSG00000197043 | HGLibA_02247 | 28 | -0.869972911 | ANXA6 | annexin A6 [Source:HGNC Symbol;Acc:HGNC:544] |
| ENSG00000197106 | HGLibA_45310 | 28 | -0.869972911 | SLC6A17 | solute carrier family 6 member 17 [Source:HGNC Symbol;Acc:HGNC:31399] |
| ENSG00000197299 | HGLibA_04548 | 28 | -0.869972911 | BLM | BLM RecQ like helicase [Source:HGNC Symbol;Acc:HGNC:1058] |
| ENSG00000197728 | HGLibA_42120 | 28 | -0.869972911 | RPS26 | ribosomal protein S26 [Source:HGNC Symbol;Acc:HGNC:10414] |
| ENSG00000198053 | HGLibA_44202 | 28 | -0.869972911 | SIRPA | signal regulatory protein alpha [Source:HGNC Symbol;Acc:HGNC:9662] |
| ENSG00000198467 | HGLibA_51143 | 28 | -0.869972911 | TPM2 | tropomyosin 2 [Source:HGNC Symbol;Acc:HGNC:12011] |
| ENSG00000198546 | HGLibA_56128 | 28 | -0.869972911 | ZNF511 | zinc finger protein 511 [Source:HGNC Symbol;Acc:HGNC:28445] |
| ENSG00000198573 | HGLibA_46390 | 28 | -0.869972911 | SPANXC | SPANX family member C [Source:HGNC Symbol;Acc:HGNC:14331] |
| ENSG00000198574 | HGLibA_43886 | 28 | -0.869972911 | SH2D1B | SH2 domain containing 1B [Source:HGNC Symbol;Acc:HGNC:30416] |
| ENSG00000198691 | HGLibA_00112 | 28 | -0.869972911 | ABCA4 | ATP binding cassette subfamily A member 4 [Source:HGNC Symbol;Acc:HGNC:34] |
| ENSG00000198755 | HGLibA_41855 | 28 | -0.869972911 | RPL10A | ribosomal protein L10a [Source:HGNC Symbol;Acc:HGNC:10299] |
| ENSG00000198812 | HGLibA_27248 | 28 | -0.869972911 | LRRC10 | leucine rich repeat containing 10 [Source:HGNC Symbol;Acc:HGNC:20264] |
| ENSG00000203857 | HGLibA_22205 | 28 | -0.869972911 | HSD3B1 | 3 beta- and steroid delta-isomerase 1 [Source:HGNC Symbol;Acc:HGNC:5217]" |
| ENSG00000204022 | HGLibA_26621 | 28 | -0.869972911 | LIPJ | lipase family member J [Source:HGNC Symbol;Acc:HGNC:21773] |
| ENSG00000204287 | HGLibA_21633 | 28 | -0.869972911 | HLA-DRA | DR alpha [Source:HGNC Symbol;Acc:HGNC:4947]" |
| ENSG00000204954 | HGLibA_05291 | 28 | -0.869972911 | C12orf73 | chromosome 12 open reading frame 73 [Source:HGNC Symbol;Acc:HGNC:34450] |
| ENSG00000205495 | HGLibA_34179 | 28 | -0.869972911 | OR52J3 | olfactory receptor family 52 subfamily J member 3 [Source:HGNC Symbol;Acc:HGNC:14799] |
| ENSG00000205572 | HGLibA_43471 | 28 | -0.869972911 | SERF1B | small EDRK-rich factor 1B [Source:HGNC Symbol;Acc:HGNC:10756] |
| ENSG00000205581 | HGLibA_21718 | 28 | -0.869972911 | HMGN1 | high mobility group nucleosome binding domain 1 [Source:HGNC Symbol;Acc:HGNC:4984] |
| ENSG00000205916 | HGLibA_12362 | 28 | -0.869972911 | DAZ4 | deleted in azoospermia 4 [Source:HGNC Symbol;Acc:HGNC:15966] |
| ENSG00000212916 | HGLibA_28112 | 28 | -0.869972911 | MAP10 | microtubule associated protein 10 [Source:HGNC Symbol;Acc:HGNC:29265] |
| ENSG00000213648 | HGLibA_47740 | 28 | -0.869972911 | SULT1A4 | sulfotransferase family 1A member 4 [Source:HGNC Symbol;Acc:HGNC:30004] |
| ENSG00000214216 | HGLibA_23523 | 28 | -0.869972911 | IQCJ | IQ motif containing J [Source:HGNC Symbol;Acc:HGNC:32406] |
| ENSG00000216937 | HGLibA_07846 | 28 | -0.869972911 | CCDC7 | coiled-coil domain containing 7 [Source:HGNC Symbol;Acc:HGNC:26533] |
| ENSG00000217236 | HGLibA_46319 | 28 | -0.869972911 | SP9 | Sp9 transcription factor [Source:HGNC Symbol;Acc:HGNC:30690] |
| ENSG00000219073 | HGLibA_09001 | 28 | -0.869972911 | CELA3B | chymotrypsin like elastase family member 3B [Source:HGNC Symbol;Acc:HGNC:15945] |
| ENSG00000221864 | HGLibA_25634 | 28 | -0.869972911 | KRTAP12-2 | keratin associated protein 12-2 [Source:HGNC Symbol;Acc:HGNC:20530] |
| ENSG00000236637 | HGLibA_22681 | 28 | -0.869972911 | IFNA4 | interferon alpha 4 [Source:HGNC Symbol;Acc:HGNC:5425] |
| ENSG00000239306 | HGLibA_40484 | 28 | -0.869972911 | RBM14 | RNA binding motif protein 14 [Source:HGNC Symbol;Acc:HGNC:14219] |
| ENSG00000240065 | HGLibA_39125 | 28 | -0.869972911 | PSMB9 | proteasome subunit beta 9 [Source:HGNC Symbol;Acc:HGNC:9546] |
| ENSG00000240771 | HGLibA_02838 | 28 | -0.869972911 | ARHGEF25 | Rho guanine nucleotide exchange factor 25 [Source:HGNC Symbol;Acc:HGNC:30275] |
| ENSG00000243811 | HGLibA_02484 | 28 | -0.869972911 | APOBEC3D | apolipoprotein B mRNA editing enzyme catalytic subunit 3D [Source:HGNC Symbol;Acc:HGNC:17354] |
| ENSG00000248712 | HGLibA_07653 | 28 | -0.869972911 | CCDC153 | coiled-coil domain containing 153 [Source:HGNC Symbol;Acc:HGNC:27446] |
| ENSG00000250868 | HGLibA_54540 | 28 | -0.869972911 | XKRY | Y-linked [Source:HGNC Symbol;Acc:HGNC:18571]" |
| ENSG00000251192 | HGLibA_56508 | 28 | -0.869972911 | ZNF674 | zinc finger protein 674 [Source:HGNC Symbol;Acc:HGNC:17625] |
| ENSG00000255221 | HGLibA_07230 | 28 | -0.869972911 | CARD17 | caspase recruitment domain family member 17 [Source:HGNC Symbol;Acc:HGNC:33827] |
| ENSG00000258227 | HGLibA_10001 | 28 | -0.869972911 | CLEC5A | C-type lectin domain containing 5A [Source:HGNC Symbol;Acc:HGNC:2054] |
| ENSG00000260314 | HGLibA_29817 | 28 | -0.869972911 | MRC1 | mannose receptor C-type 1 [Source:HGNC Symbol;Acc:HGNC:7228] |
| ENSG00000263353 | HGLibA_37897 | 28 | -0.869972911 | PPIAL4A | peptidylprolyl isomerase A like 4A [Source:HGNC Symbol;Acc:HGNC:24369] |
| ENSG00000264424 | HGLibA_30727 | 28 | -0.869972911 | MYH4 | myosin heavy chain 4 [Source:HGNC Symbol;Acc:HGNC:7574] |
| ENSG00000265681 | HGLibA_41881 | 28 | -0.869972911 | RPL17 | ribosomal protein L17 [Source:HGNC Symbol;Acc:HGNC:10307] |

| | | | | | |
|---|---|---|---|---|---|
| ENSG00000267206 | HGLibA_26180 | 28 | -0.869972911 | LCN6 | lipocalin 6 [Source:HGNC Symbol;Acc:HGNC:17337] |
| ENSG00000269556 | HGLibA_50120 | 28 | -0.869972911 | TMEM185A | transmembrane protein 185A [Source:HGNC Symbol;Acc:HGNC:17125] |
| ENSG00000271605 | HGLibA_29269 | 28 | -0.869972911 | MILR1 | mast cell immunoglobulin like receptor 1 [Source:HGNC Symbol;Acc:HGNC:27570] |
| ENSG00000277258 | HGLibA_35595 | 28 | -0.869972911 | PCGF2 | polycomb group ring finger 2 [Source:HGNC Symbol;Acc:HGNC:12929] |
| ENSG00000277399 | HGLibA_19978 | 28 | -0.869972911 | GPR179 | G protein-coupled receptor 179 [Source:HGNC Symbol;Acc:HGNC:31371] |
| FAM73A | HGLibA_16715 | 28 | -0.869972911 | FAM73A | - |
| GAGE12I | HGLibA_18458 | 28 | -0.869972911 | GAGE12I | - |
| HIST1H2BB | HGLibA_21413 | 28 | -0.869972911 | HIST1H2BB | - |
| HIST1H3D | HGLibA_21465 | 28 | -0.869972911 | HIST1H3D | - |
| hsa-mir-103b-1 | HGLibA_57170 | 28 | -0.869972911 | hsa-mir-103b-1 | - |
| hsa-mir-106a | HGLibA_57186 | 28 | -0.869972911 | hsa-mir-106a | - |
| hsa-mir-2278 | HGLibA_58316 | 28 | -0.869972911 | hsa-mir-2278 | - |
| hsa-mir-29b-2 | HGLibA_58416 | 28 | -0.869972911 | hsa-mir-29b-2 | - |
| hsa-mir-30a | HGLibA_58470 | 28 | -0.869972911 | hsa-mir-30a | - |
| hsa-mir-30c-2 | HGLibA_58483 | 28 | -0.869972911 | hsa-mir-30c-2 | - |
| hsa-mir-3116-2 | HGLibA_58509 | 28 | -0.869972911 | hsa-mir-3116-2 | - |
| hsa-mir-3137 | HGLibA_58606 | 28 | -0.869972911 | hsa-mir-3137 | - |
| hsa-mir-3180-4 | HGLibA_58813 | 28 | -0.869972911 | hsa-mir-3180-4 | - |
| hsa-mir-3614 | HGLibA_59085 | 28 | -0.869972911 | hsa-mir-3614 | - |
| hsa-mir-3618 | HGLibA_59101 | 28 | -0.869972911 | hsa-mir-3618 | - |
| hsa-mir-3688-2 | HGLibA_59309 | 28 | -0.869972911 | hsa-mir-3688-2 | - |
| hsa-mir-3938 | HGLibA_59611 | 28 | -0.869972911 | hsa-mir-3938 | - |
| hsa-mir-3943 | HGLibA_59633 | 28 | -0.869972911 | hsa-mir-3943 | - |
| hsa-mir-3972 | HGLibA_59648 | 28 | -0.869972911 | hsa-mir-3972 | - |
| hsa-mir-4303 | HGLibA_59924 | 28 | -0.869972911 | hsa-mir-4303 | - |
| hsa-mir-4444-2 | HGLibA_60183 | 28 | -0.869972911 | hsa-mir-4444-2 | - |
| hsa-mir-4494 | HGLibA_60393 | 28 | -0.869972911 | hsa-mir-4494 | - |
| hsa-mir-4522 | HGLibA_60547 | 28 | -0.869972911 | hsa-mir-4522 | - |
| hsa-mir-4676 | HGLibA_60821 | 28 | -0.869972911 | hsa-mir-4676 | - |
| hsa-mir-4683 | HGLibA_60844 | 28 | -0.869972911 | hsa-mir-4683 | - |
| hsa-mir-4724 | HGLibA_60993 | 28 | -0.869972911 | hsa-mir-4724 | - |
| hsa-mir-484 | HGLibA_61328 | 28 | -0.869972911 | hsa-mir-484 | - |
| hsa-mir-493 | HGLibA_61372 | 28 | -0.869972911 | hsa-mir-493 | - |
| hsa-mir-5047 | HGLibA_61477 | 28 | -0.869972911 | hsa-mir-5047 | - |
| hsa-mir-5091 | HGLibA_61524 | 28 | -0.869972911 | hsa-mir-5091 | - |
| hsa-mir-545 | HGLibA_61832 | 28 | -0.869972911 | hsa-mir-545 | - |
| hsa-mir-548ax | HGLibA_61938 | 28 | -0.869972911 | hsa-mir-548ax | - |
| hsa-mir-548h-2 | HGLibA_61997 | 28 | -0.869972911 | hsa-mir-548h-2 | - |
| hsa-mir-548o | HGLibA_62050 | 28 | -0.869972911 | hsa-mir-548o | - |
| hsa-mir-548x-2 | HGLibA_62082 | 28 | -0.869972911 | hsa-mir-548x-2 | - |
| hsa-mir-557 | HGLibA_62143 | 28 | -0.869972911 | hsa-mir-557 | - |
| hsa-mir-603 | HGLibA_62504 | 28 | -0.869972911 | hsa-mir-603 | - |
| hsa-mir-6074 | HGLibA_62547 | 28 | -0.869972911 | hsa-mir-6074 | - |
| hsa-mir-615 | HGLibA_62696 | 28 | -0.869972911 | hsa-mir-615 | - |
| hsa-mir-6797 | HGLibA_63366 | 28 | -0.869972911 | hsa-mir-6797 | - |
| hsa-mir-6867 | HGLibA_63654 | 28 | -0.869972911 | hsa-mir-6867 | - |
| hsa-mir-7108 | HGLibA_63791 | 28 | -0.869972911 | hsa-mir-7108 | - |
| hsa-mir-7109 | HGLibA_63798 | 28 | -0.869972911 | hsa-mir-7109 | - |
| hsa-mir-7153 | HGLibA_63842 | 28 | -0.869972911 | hsa-mir-7153 | - |
| hsa-mir-760 | HGLibA_63902 | 28 | -0.869972911 | hsa-mir-760 | - |
| hsa-mir-877 | HGLibA_64217 | 28 | -0.869972911 | hsa-mir-877 | - |
| hsa-mir-890 | HGLibA_64240 | 28 | -0.869972911 | hsa-mir-890 | - |
| hsa-mir-933 | HGLibA_64307 | 28 | -0.869972911 | hsa-mir-933 | - |
| hsa-mir-99b | HGLibA_64382 | 28 | -0.869972911 | hsa-mir-99b | - |
| KIAA1244 | HGLibA_24728 | 28 | -0.869972911 | KIAA1244 | - |
| KIAA1919 | HGLibA_24826 | 28 | -0.869972911 | KIAA1919 | - |
| LOC402160 | HGLibA_26938 | 28 | -0.869972911 | LOC402160 | - |
| MINA | HGLibA_29274 | 28 | -0.869972911 | MINA | - |
| AGAP8 | HGLibA_01161 | 27 | -0.879923948 | AGAP8 | - |
| ATP5G2 | HGLibA_03657 | 27 | -0.879923948 | ATP5G2 | - |
| C19orf77 | HGLibA_05709 | 27 | -0.879923948 | C19orf77 | - |
| C6orf211 | HGLibA_06472 | 27 | -0.879923948 | C6orf211 | - |
| CCDC53 | HGLibA_07799 | 27 | -0.879923948 | CCDC53 | - |
| CGB | HGLibA_09299 | 27 | -0.879923948 | CGB | - |
| CT45A4 | HGLibA_11506 | 27 | -0.879923948 | CT45A4 | - |
| CXorf23 | HGLibA_11907 | 27 | -0.879923948 | CXorf23 | - |
| CXorf48 | HGLibA_11930 | 27 | -0.879923948 | CXorf48 | - |
| DDX26B | HGLibA_12683 | 27 | -0.879923948 | DDX26B | - |
| ENSG00000006712 | HGLibA_34986 | 27 | -0.879923948 | PAF1 | Paf1/RNA polymerase II complex component [Source:HGNC Symbol;Acc:HGNC:25459]" |
| ENSG00000011114 | HGLibA_04921 | 27 | -0.879923948 | BTBD7 | BTB domain containing 7 [Source:HGNC Symbol;Acc:HGNC:18269] |
| ENSG00000039987 | HGLibA_04422 | 27 | -0.879923948 | BEST2 | bestrophin 2 [Source:HGNC Symbol;Acc:HGNC:17107] |
| ENSG00000042832 | HGLibA_49198 | 27 | -0.879923948 | TG | thyroglobulin [Source:HGNC Symbol;Acc:HGNC:11764] |
| ENSG00000047188 | HGLibA_54728 | 27 | -0.879923948 | YTHDC2 | YTH domain containing 2 [Source:HGNC Symbol;Acc:HGNC:24721] |
| ENSG00000052749 | HGLibA_42268 | 27 | -0.879923948 | RRP12 | ribosomal RNA processing 12 homolog [Source:HGNC Symbol;Acc:HGNC:29100] |
| ENSG00000054654 | HGLibA_47978 | 27 | -0.879923948 | SYNE2 | spectrin repeat containing nuclear envelope protein 2 [Source:HGNC Symbol;Acc:HGNC:17084] |
| ENSG00000057657 | HGLibA_38363 | 27 | -0.879923948 | PRDM1 | PR/SET domain 1 [Source:HGNC Symbol;Acc:HGNC:9346] |
| ENSG00000065491 | HGLibA_48506 | 27 | -0.879923948 | TBC1D22B | TBC1 domain family member 22B [Source:HGNC Symbol;Acc:HGNC:21602] |
| ENSG00000066136 | HGLibA_31860 | 27 | -0.879923948 | NFYC | nuclear transcription factor Y subunit gamma [Source:HGNC Symbol;Acc:HGNC:7806] |
| ENSG00000066926 | HGLibA_17270 | 27 | -0.879923948 | FECH | ferrochelatase [Source:HGNC Symbol;Acc:HGNC:3647] |
| ENSG00000067596 | HGLibA_13209 | 27 | -0.879923948 | DHX8 | DEAH-box helicase 8 [Source:HGNC Symbol;Acc:HGNC:2749] |
| ENSG00000069345 | HGLibA_13514 | 27 | -0.879923948 | DNAJA2 | DnaJ heat shock protein family (Hsp40) member A2 [Source:HGNC Symbol;Acc:HGNC:14884] |
| ENSG00000071967 | HGLibA_12021 | 27 | -0.879923948 | CYBRD1 | cytochrome b reductase 1 [Source:HGNC Symbol;Acc:HGNC:20797] |
| ENSG00000075275 | HGLibA_09023 | 27 | -0.879923948 | CELSR1 | cadherin EGF LAG seven-pass G-type receptor 1 [Source:HGNC Symbol;Acc:HGNC:1850] |
| ENSG00000076321 | HGLibA_25151 | 27 | -0.879923948 | KLHL20 | kelch like family member 20 [Source:HGNC Symbol;Acc:HGNC:25056] |
| ENSG00000077713 | HGLibA_44743 | 27 | -0.879923948 | SLC25A43 | solute carrier family 25 member 43 [Source:HGNC Symbol;Acc:HGNC:30557] |
| ENSG00000079102 | HGLibA_42458 | 27 | -0.879923948 | RUNX1T1 | RUNX1 translocation partner 1 [Source:HGNC Symbol;Acc:HGNC:1535] |
| ENSG00000085719 | HGLibA_10959 | 27 | -0.879923948 | CPNE3 | copine 3 [Source:HGNC Symbol;Acc:HGNC:2316] |
| ENSG00000086506 | HGLibA_20938 | 27 | -0.879923948 | HBQ1 | hemoglobin subunit theta 1 [Source:HGNC Symbol;Acc:HGNC:4833] |
| ENSG00000089048 | HGLibA_15566 | 27 | -0.879923948 | ESF1 | ESF1 nucleolar pre-rRNA processing protein homolog [Source:HGNC Symbol;Acc:HGNC:15898] |
| ENSG00000089876 | HGLibA_13179 | 27 | -0.879923948 | DHX32 | DEAH-box helicase 32 (putative) [Source:HGNC Symbol;Acc:HGNC:16717] |
| ENSG00000090104 | HGLibA_41048 | 27 | -0.879923948 | RGS1 | regulator of G protein signaling 1 [Source:HGNC Symbol;Acc:HGNC:9991] |
| ENSG00000090432 | HGLibA_30562 | 27 | -0.879923948 | MUL1 | mitochondrial E3 ubiquitin protein ligase 1 [Source:HGNC Symbol;Acc:HGNC:25762] |
| ENSG00000090534 | HGLibA_49385 | 27 | -0.879923948 | THPO | thrombopoietin [Source:HGNC Symbol;Acc:HGNC:11795] |
| ENSG00000091947 | HGLibA_49842 | 27 | -0.879923948 | TMEM101 | transmembrane protein 101 [Source:HGNC Symbol;Acc:HGNC:28653] |
| ENSG00000092445 | HGLibA_52469 | 27 | -0.879923948 | TYRO3 | TYRO3 protein tyrosine kinase [Source:HGNC Symbol;Acc:HGNC:12446] |
| ENSG00000100360 | HGLibA_22754 | 27 | -0.879923948 | IFT27 | intraflagellar transport 27 [Source:HGNC Symbol;Acc:HGNC:18626] |
| ENSG00000100461 | HGLibA_40512 | 27 | -0.879923948 | RBM23 | RNA binding motif protein 23 [Source:HGNC Symbol;Acc:HGNC:20155] |
| ENSG00000101280 | HGLibA_01907 | 27 | -0.879923948 | ANGPT4 | angiopoietin 4 [Source:HGNC Symbol;Acc:HGNC:487] |
| ENSG00000104517 | HGLibA_52760 | 27 | -0.879923948 | UBR5 | ubiquitin protein ligase E3 component n-recognin 5 [Source:HGNC Symbol;Acc:HGNC:16806] |
| ENSG00000105135 | HGLibA_23227 | 27 | -0.879923948 | ILVBL | ilvB acetolactate synthase like [Source:HGNC Symbol;Acc:HGNC:6041] |
| ENSG00000106571 | HGLibA_19253 | 27 | -0.879923948 | GLI3 | GLI family zinc finger 3 [Source:HGNC Symbol;Acc:HGNC:4319] |
| ENSG00000107611 | HGLibA_11763 | 27 | -0.879923948 | CUBN | cubilin [Source:HGNC Symbol;Acc:HGNC:2548] |
| ENSG00000108439 | HGLibA_37430 | 27 | -0.879923948 | PNPO | pyridoxamine 5'-phosphate oxidase [Source:HGNC Symbol;Acc:HGNC:30260] |
| ENSG00000113070 | HGLibA_20923 | 27 | -0.879923948 | HBEGF | heparin binding EGF like growth factor [Source:HGNC Symbol;Acc:HGNC:3059] |
| ENSG00000113430 | HGLibA_23637 | 27 | -0.879923948 | IRX4 | iroquois homeobox 4 [Source:HGNC Symbol;Acc:HGNC:6129] |
| ENSG00000114107 | HGLibA_09158 | 27 | -0.879923948 | CEP70 | centrosomal protein 70 [Source:HGNC Symbol;Acc:HGNC:29972] |
| ENSG00000114268 | HGLibA_36162 | 27 | -0.879923948 | PFKFB4 | 6-biphosphatase 4 [Source:HGNC Symbol;Acc:HGNC:8875]" |
| ENSG00000114455 | HGLibA_21269 | 27 | -0.879923948 | HHLA2 | HERV-H LTR-associating 2 [Source:HGNC Symbol;Acc:HGNC:4905] |
| ENSG00000114988 | HGLibA_26667 | 27 | -0.879923948 | LMAN2L | mannose binding 2 like [Source:HGNC Symbol;Acc:HGNC:19263]" |
| ENSG00000115761 | HGLibA_32256 | 27 | -0.879923948 | NOL10 | nucleolar protein 10 [Source:HGNC Symbol;Acc:HGNC:25862] |
| ENSG00000116005 | HGLibA_35693 | 27 | -0.879923948 | PCYOX1 | prenylcysteine oxidase 1 [Source:HGNC Symbol;Acc:HGNC:20588] |
| ENSG00000116711 | HGLibA_36899 | 27 | -0.879923948 | PLA2G4A | phospholipase A2 group IVA [Source:HGNC Symbol;Acc:HGNC:9035] |
| ENSG00000117148 | HGLibA_00632 | 27 | -0.879923948 | ACTL8 | actin like 8 [Source:HGNC Symbol;Acc:HGNC:24018] |
| ENSG00000117222 | HGLibA_40431 | 27 | -0.879923948 | RBBP5 | histone lysine methyltransferase complex subunit [Source:HGNC Symbol;Acc:HGNC:9888]" |
| ENSG00000117448 | HGLibA_01468 | 27 | -0.879923948 | AKR1A1 | aldo-keto reductase family 1 member A1 [Source:HGNC Symbol;Acc:HGNC:380] |
| ENSG00000118263 | HGLibA_25080 | 27 | -0.879923948 | KLF7 | Kruppel like factor 7 [Source:HGNC Symbol;Acc:HGNC:6350] |
| ENSG00000118579 | HGLibA_28796 | 27 | -0.879923948 | MED28 | mediator complex subunit 28 [Source:HGNC Symbol;Acc:HGNC:24628] |
| ENSG00000118946 | HGLibA_35403 | 27 | -0.879923948 | PCDH17 | protocadherin 17 [Source:HGNC Symbol;Acc:HGNC:14267] |
| ENSG00000119616 | HGLibA_17176 | 27 | -0.879923948 | FCF1 | rRNA-processing protein [Source:HGNC Symbol;Acc:HGNC:20220]" |
| ENSG00000120162 | HGLibA_29597 | 27 | -0.879923948 | MOB3B | MOB kinase activator 3B [Source:HGNC Symbol;Acc:HGNC:23825] |
| ENSG00000120833 | HGLibA_46126 | 27 | -0.879923948 | SOCS2 | suppressor of cytokine signaling 2 [Source:HGNC Symbol;Acc:HGNC:19382] |
| ENSG00000121022 | HGLibA_10721 | 27 | -0.879923948 | COPS5 | COP9 signalosome subunit 5 [Source:HGNC Symbol;Acc:HGNC:2240] |
| ENSG00000121764 | HGLibA_20987 | 27 | -0.879923948 | HCRTR1 | hypocretin receptor 1 [Source:HGNC Symbol;Acc:HGNC:4848] |
| ENSG00000122008 | HGLibA_37535 | 27 | -0.879923948 | POLK | DNA polymerase kappa [Source:HGNC Symbol;Acc:HGNC:9183] |
| ENSG00000123191 | HGLibA_03771 | 27 | -0.879923948 | ATP7B | ATPase copper transporting beta [Source:HGNC Symbol;Acc:HGNC:870] |
| ENSG00000123411 | HGLibA_22938 | 27 | -0.879923948 | IKZF4 | IKAROS family zinc finger 4 [Source:HGNC Symbol;Acc:HGNC:13179] |
| ENSG00000124171 | HGLibA_35186 | 27 | -0.879923948 | PARD6B | par-6 family cell polarity regulator beta [Source:HGNC Symbol;Acc:HGNC:16245] |
| ENSG00000124313 | HGLibA_23543 | 27 | -0.879923948 | IQSEC2 | IQ motif and Sec7 domain 2 [Source:HGNC Symbol;Acc:HGNC:29059] |
| ENSG00000125356 | HGLibA_31437 | 27 | -0.879923948 | NDUFA1 | NADH:ubiquinone oxidoreductase subunit A1 [Source:HGNC Symbol;Acc:HGNC:7683] |
| ENSG00000125378 | HGLibA_04608 | 27 | -0.879923948 | BMP4 | bone morphogenetic protein 4 [Source:HGNC Symbol;Acc:HGNC:1071] |
| ENSG00000125457 | HGLibA_29265 | 27 | -0.879923948 | MIF4GD | MIF4G domain containing [Source:HGNC Symbol;Acc:HGNC:24030] |
| ENSG00000125835 | HGLibA_45969 | 27 | -0.879923948 | SNRPB | small nuclear ribonucleoprotein polypeptides B and B1 [Source:HGNC Symbol;Acc:HGNC:11153] |
| ENSG00000125970 | HGLibA_40175 | 27 | -0.879923948 | RALY | RALY heterogeneous nuclear ribonucleoprotein [Source:HGNC Symbol;Acc:HGNC:15921] |
| ENSG00000128591 | HGLibA_17645 | 27 | -0.879923948 | FLNC | filamin C [Source:HGNC Symbol;Acc:HGNC:3756] |
| ENSG00000128595 | HGLibA_07053 | 27 | -0.879923948 | CALU | calumenin [Source:HGNC Symbol;Acc:HGNC:1458] |

| | | | | | |
|---|---|---|---|---|---|
| ENSG00000128683 | HGLibA_18409 | 27 | -0.879923948 | GAD1 | glutamate decarboxylase 1 [Source:HGNC Symbol;Acc:HGNC:4092] |
| ENSG00000129158 | HGLibA_43479 | 27 | -0.879923948 | SERGEF | secretion regulating guanine nucleotide exchange factor [Source:HGNC Symbol;Acc:HGNC:17499] |
| ENSG00000129518 | HGLibA_14341 | 27 | -0.879923948 | EAPP | E2F associated phosphoprotein [Source:HGNC Symbol;Acc:HGNC:19312] |
| ENSG00000130307 | HGLibA_53182 | 27 | -0.879923948 | USHBP1 | USH1 protein network component harmonin binding protein 1 [Source:HGNC Symbol;Acc:HGNC:24058] |
| ENSG00000130522 | HGLibA_24009 | 27 | -0.879923948 | JUND | AP-1 transcription factor subunit [Source:HGNC Symbol;Acc:HGNC:6206]" |
| ENSG00000130810 | HGLibA_37809 | 27 | -0.879923948 | PPAN | peter pan homolog [Source:HGNC Symbol;Acc:HGNC:9227] |
| ENSG00000132676 | HGLibA_12319 | 27 | -0.879923948 | DAP3 | death associated protein 3 [Source:HGNC Symbol;Acc:HGNC:2673] |
| ENSG00000132716 | HGLibA_12458 | 27 | -0.879923948 | DCAF8 | DDB1 and CUL4 associated factor 8 [Source:HGNC Symbol;Acc:HGNC:24891] |
| ENSG00000134253 | HGLibA_51510 | 27 | -0.879923948 | TRIM45 | tripartite motif containing 45 [Source:HGNC Symbol;Acc:HGNC:19018] |
| ENSG00000134698 | HGLibA_01213 | 27 | -0.879923948 | AGO4 | RISC catalytic component [Source:HGNC Symbol;Acc:HGNC:18424]" |
| ENSG00000135093 | HGLibA_53330 | 27 | -0.879923948 | USP30 | ubiquitin specific peptidase 30 [Source:HGNC Symbol;Acc:HGNC:20065] |
| ENSG00000135744 | HGLibA_01257 | 27 | -0.879923948 | AGT | angiotensinogen [Source:HGNC Symbol;Acc:HGNC:333] |
| ENSG00000136206 | HGLibA_46579 | 27 | -0.879923948 | SPDYE1 | speedy/RINGO cell cycle regulator family member E1 [Source:HGNC Symbol;Acc:HGNC:16408] |
| ENSG00000136487 | HGLibA_19031 | 27 | -0.879923948 | GH2 | growth hormone 2 [Source:HGNC Symbol;Acc:HGNC:4262] |
| ENSG00000136699 | HGLibA_45811 | 27 | -0.879923948 | SMPD4 | sphingomyelin phosphodiesterase 4 [Source:HGNC Symbol;Acc:HGNC:32949] |
| ENSG00000136950 | HGLibA_03095 | 27 | -0.879923948 | ARPC5L | actin related protein 2/3 complex subunit 5 like [Source:HGNC Symbol;Acc:HGNC:23366] |
| ENSG00000137312 | HGLibA_17647 | 27 | -0.879923948 | FLOT1 | flotillin 1 [Source:HGNC Symbol;Acc:HGNC:3757] |
| ENSG00000137547 | HGLibA_29905 | 27 | -0.879923948 | MRPL15 | mitochondrial ribosomal protein L15 [Source:HGNC Symbol;Acc:HGNC:14054] |
| ENSG00000138795 | HGLibA_26271 | 27 | -0.879923948 | LEF1 | lymphoid enhancer binding factor 1 [Source:HGNC Symbol;Acc:HGNC:6551] |
| ENSG00000139350 | HGLibA_31598 | 27 | -0.879923948 | NEDD1 | developmentally down-regulated 1 [Source:HGNC Symbol;Acc:HGNC:7723]" |
| ENSG00000139428 | HGLibA_29454 | 27 | -0.879923948 | MMAB | metabolism of cobalamin associated B [Source:HGNC Symbol;Acc:HGNC:19331] |
| ENSG00000139567 | HGLibA_00702 | 27 | -0.879923948 | ACVRL1 | activin A receptor like type 1 [Source:HGNC Symbol;Acc:HGNC:175] |
| ENSG00000140511 | HGLibA_20847 | 27 | -0.879923948 | HAPLN3 | hyaluronan and proteoglycan link protein 3 [Source:HGNC Symbol;Acc:HGNC:21446] |
| ENSG00000141012 | HGLibA_18523 | 27 | -0.879923948 | GALNS | galactosamine (N-acetyl)-6-sulfatase [Source:HGNC Symbol;Acc:HGNC:4122] |
| ENSG00000141294 | HGLibA_27367 | 27 | -0.879923948 | LRRC46 | leucine rich repeat containing 46 [Source:HGNC Symbol;Acc:HGNC:25047] |
| ENSG00000141424 | HGLibA_45119 | 27 | -0.879923948 | SLC39A6 | solute carrier family 39 member 6 [Source:HGNC Symbol;Acc:HGNC:18607] |
| ENSG00000141510 | HGLibA_51048 | 27 | -0.879923948 | TP53 | tumor protein p53 [Source:HGNC Symbol;Acc:HGNC:11998] |
| ENSG00000141748 | HGLibA_02980 | 27 | -0.879923948 | ARL5C | ADP ribosylation factor like GTPase 5C [Source:HGNC Symbol;Acc:HGNC:31111] |
| ENSG00000141759 | HGLibA_52437 | 27 | -0.879923948 | TXNL4A | thioredoxin like 4A [Source:HGNC Symbol;Acc:HGNC:30551] |
| ENSG00000141858 | HGLibA_42660 | 27 | -0.879923948 | SAMD1 | sterile alpha motif domain containing 1 [Source:HGNC Symbol;Acc:HGNC:17958] |
| ENSG00000142039 | HGLibA_07940 | 27 | -0.879923948 | CCDC97 | coiled-coil domain containing 97 [Source:HGNC Symbol;Acc:HGNC:28289] |
| ENSG00000142731 | HGLibA_37197 | 27 | -0.879923948 | PLK4 | polo like kinase 4 [Source:HGNC Symbol;Acc:HGNC:11397] |
| ENSG00000143158 | HGLibA_29708 | 27 | -0.879923948 | MPC2 | mitochondrial pyruvate carrier 2 [Source:HGNC Symbol;Acc:HGNC:24515] |
| ENSG00000143194 | HGLibA_27863 | 27 | -0.879923948 | MAEL | maelstrom spermatogenic transposon silencer [Source:HGNC Symbol;Acc:HGNC:25929] |
| ENSG00000143369 | HGLibA_14411 | 27 | -0.879923948 | ECM1 | extracellular matrix protein 1 [Source:HGNC Symbol;Acc:HGNC:3153] |
| ENSG00000143473 | HGLibA_24209 | 27 | -0.879923948 | KCNH1 | potassium voltage-gated channel subfamily H member 1 [Source:HGNC Symbol;Acc:HGNC:6250] |
| ENSG00000145022 | HGLibA_48854 | 27 | -0.879923948 | TCTA | T cell leukemia translocation altered [Source:HGNC Symbol;Acc:HGNC:11692] |
| ENSG00000145247 | HGLibA_33249 | 27 | -0.879923948 | OCIAD2 | OCIA domain containing 2 [Source:HGNC Symbol;Acc:HGNC:28685] |
| ENSG00000146192 | HGLibA_17345 | 27 | -0.879923948 | FGD2 | RhoGEF and PH domain containing 2 [Source:HGNC Symbol;Acc:HGNC:3664]" |
| ENSG00000146859 | HGLibA_49962 | 27 | -0.879923948 | TMEM140 | transmembrane protein 140 [Source:HGNC Symbol;Acc:HGNC:21870] |
| ENSG00000147044 | HGLibA_07285 | 27 | -0.879923948 | CASK | calcium/calmodulin dependent serine protein kinase [Source:HGNC Symbol;Acc:HGNC:1497] |
| ENSG00000147789 | HGLibA_56568 | 27 | -0.879923948 | ZNF7 | zinc finger protein 7 [Source:HGNC Symbol;Acc:HGNC:13139] |
| ENSG00000148291 | HGLibA_47836 | 27 | -0.879923948 | SURF2 | surfeit 2 [Source:HGNC Symbol;Acc:HGNC:11475] |
| ENSG00000149179 | HGLibA_05148 | 27 | -0.879923948 | C11orf49 | chromosome 11 open reading frame 49 [Source:HGNC Symbol;Acc:HGNC:28720] |
| ENSG00000149809 | HGLibA_49716 | 27 | -0.879923948 | TM7SF2 | transmembrane 7 superfamily member 2 [Source:HGNC Symbol;Acc:HGNC:11863] |
| ENSG00000150244 | HGLibA_51520 | 27 | -0.879923948 | TRIM48 | tripartite motif containing 48 [Source:HGNC Symbol;Acc:HGNC:19021] |
| ENSG00000150636 | HGLibA_07510 | 27 | -0.879923948 | CCDC102B | coiled-coil domain containing 102B [Source:HGNC Symbol;Acc:HGNC:26295] |
| ENSG00000150893 | HGLibA_17986 | 27 | -0.879923948 | FREM2 | FRAS1 related extracellular matrix protein 2 [Source:HGNC Symbol;Acc:HGNC:25396] |
| ENSG00000150961 | HGLibA_43238 | 27 | -0.879923948 | SEC24D | COPII coat complex component [Source:HGNC Symbol;Acc:HGNC:10706]" |
| ENSG00000152475 | HGLibA_56846 | 27 | -0.879923948 | ZNF837 | zinc finger protein 837 [Source:HGNC Symbol;Acc:HGNC:25164] |
| ENSG00000152705 | HGLibA_07362 | 27 | -0.879923948 | CATSPER3 | cation channel sperm associated 3 [Source:HGNC Symbol;Acc:HGNC:20819] |
| ENSG00000153187 | HGLibA_21846 | 27 | -0.879923948 | HNRNPU | heterogeneous nuclear ribonucleoprotein U [Source:HGNC Symbol;Acc:HGNC:5048] |
| ENSG00000154096 | HGLibA_49423 | 27 | -0.879923948 | THY1 | Thy-1 cell surface antigen [Source:HGNC Symbol;Acc:HGNC:11801] |
| ENSG00000154743 | HGLibA_51831 | 27 | -0.879923948 | TSEN2 | tRNA splicing endonuclease subunit 2 [Source:HGNC Symbol;Acc:HGNC:28422] |
| ENSG00000155304 | HGLibA_22264 | 27 | -0.879923948 | HSPA13 | heat shock protein family A (Hsp70) member 13 [Source:HGNC Symbol;Acc:HGNC:11375] |
| ENSG00000155307 | HGLibA_42710 | 27 | -0.879923948 | SAMSN1 | SH3 domain and nuclear localization signals 1 [Source:HGNC Symbol;Acc:HGNC:10528]" |
| ENSG00000155329 | HGLibA_55003 | 27 | -0.879923948 | ZCCHC10 | zinc finger CCHC-type containing 10 [Source:HGNC Symbol;Acc:HGNC:25954] |
| ENSG00000155974 | HGLibA_20321 | 27 | -0.879923948 | GRIP1 | glutamate receptor interacting protein 1 [Source:HGNC Symbol;Acc:HGNC:18708] |
| ENSG00000156671 | HGLibA_42695 | 27 | -0.879923948 | SAMD8 | sterile alpha motif domain containing 8 [Source:HGNC Symbol;Acc:HGNC:26320] |
| ENSG00000156697 | HGLibA_53434 | 27 | -0.879923948 | UTP14A | small subunit processome component [Source:HGNC Symbol;Acc:HGNC:10665]" |
| ENSG00000156928 | HGLibA_28030 | 27 | -0.879923948 | MALSU1 | mitochondrial assembly of ribosomal large subunit 1 [Source:HGNC Symbol;Acc:HGNC:21721] |
| ENSG00000157020 | HGLibA_43187 | 27 | -0.879923948 | SEC13 | nuclear pore and COPII coat complex component [Source:HGNC Symbol;Acc:HGNC:10697]" |
| ENSG00000157869 | HGLibA_39862 | 27 | -0.879923948 | RAB28 | member RAS oncogene family [Source:HGNC Symbol;Acc:HGNC:9768]" |
| ENSG00000158109 | HGLibA_51183 | 27 | -0.879923948 | TPRG1L | tumor protein p63 regulated 1 like [Source:HGNC Symbol;Acc:HGNC:27007] |
| ENSG00000158615 | HGLibA_38051 | 27 | -0.879923948 | PPP1R15B | protein phosphatase 1 regulatory subunit 15B [Source:HGNC Symbol;Acc:HGNC:14951] |
| ENSG00000158623 | HGLibA_10706 | 27 | -0.879923948 | COPG2 | coatomer protein complex subunit gamma 2 [Source:HGNC Symbol;Acc:HGNC:2237] |
| ENSG00000159267 | HGLibA_21652 | 27 | -0.879923948 | HLCS | holocarboxylase synthetase [Source:HGNC Symbol;Acc:HGNC:4976] |
| ENSG00000159322 | HGLibA_01022 | 27 | -0.879923948 | ADPGK | ADP dependent glucokinase [Source:HGNC Symbol;Acc:HGNC:25250] |
| ENSG00000159899 | HGLibA_32512 | 27 | -0.879923948 | NPR2 | natriuretic peptide receptor 2 [Source:HGNC Symbol;Acc:HGNC:7944] |
| ENSG00000160803 | HGLibA_52740 | 27 | -0.879923948 | UBQLN4 | ubiquilin 4 [Source:HGNC Symbol;Acc:HGNC:1237] |
| ENSG00000160949 | HGLibA_50986 | 27 | -0.879923948 | TONSL | DNA repair protein [Source:HGNC Symbol;Acc:HGNC:7801]" |
| ENSG00000161217 | HGLibA_35699 | 27 | -0.879923948 | PCYT1A | alpha [Source:HGNC Symbol;Acc:HGNC:8754]" |
| ENSG00000162727 | HGLibA_33841 | 27 | -0.879923948 | OR2M5 | olfactory receptor family 2 subfamily M member 5 [Source:HGNC Symbol;Acc:HGNC:19576] |
| ENSG00000162971 | HGLibA_52489 | 27 | -0.879923948 | TYW5 | tRNA-yW synthesizing protein 5 [Source:HGNC Symbol;Acc:HGNC:26754] |
| ENSG00000163040 | HGLibA_07862 | 27 | -0.879923948 | CCDC74A | coiled-coil domain containing 74A [Source:HGNC Symbol;Acc:HGNC:25197] |
| ENSG00000163221 | HGLibA_42540 | 27 | -0.879923948 | S100A12 | S100 calcium binding protein A12 [Source:HGNC Symbol;Acc:HGNC:10489] |
| ENSG00000163376 | HGLibA_24116 | 27 | -0.879923948 | KBTBD8 | kelch repeat and BTB domain containing 8 [Source:HGNC Symbol;Acc:HGNC:30691] |
| ENSG00000163576 | HGLibA_14568 | 27 | -0.879923948 | EFHB | EF-hand domain family member B [Source:HGNC Symbol;Acc:HGNC:26330] |
| ENSG00000163607 | HGLibA_20621 | 27 | -0.879923948 | GTPBP8 | GTP binding protein 8 (putative) [Source:HGNC Symbol;Acc:HGNC:25007] |
| ENSG00000163701 | HGLibA_23016 | 27 | -0.879923948 | IL17RE | interleukin 17 receptor E [Source:HGNC Symbol;Acc:HGNC:18439] |
| ENSG00000163798 | HGLibA_45211 | 27 | -0.879923948 | SLC4A1AP | solute carrier family 4 member 1 adaptor protein [Source:HGNC Symbol;Acc:HGNC:13813] |
| ENSG00000164076 | HGLibA_07096 | 27 | -0.879923948 | CAMKV | CaM kinase like vesicle associated [Source:HGNC Symbol;Acc:HGNC:28788] |
| ENSG00000164123 | HGLibA_06333 | 27 | -0.879923948 | C4orf45 | chromosome 4 open reading frame 45 [Source:HGNC Symbol;Acc:HGNC:26342] |
| ENSG00000164172 | HGLibA_29615 | 27 | -0.879923948 | MOCS2 | molybdenum cofactor synthesis 2 [Source:HGNC Symbol;Acc:HGNC:7193] |
| ENSG00000164180 | HGLibA_50028 | 27 | -0.879923948 | TMEM161B | transmembrane protein 161B [Source:HGNC Symbol;Acc:HGNC:28483] |
| ENSG00000164258 | HGLibA_31554 | 27 | -0.879923948 | NDUFS4 | NADH:ubiquinone oxidoreductase subunit S4 [Source:HGNC Symbol;Acc:HGNC:7711] |
| ENSG00000164985 | HGLibA_39061 | 27 | -0.879923948 | PSIP1 | PC4 and SFRS1 interacting protein 1 [Source:HGNC Symbol;Acc:HGNC:9527] |
| ENSG00000165512 | HGLibA_55607 | 27 | -0.879923948 | ZNF22 | zinc finger protein 22 [Source:HGNC Symbol;Acc:HGNC:13012] |
| ENSG00000165799 | HGLibA_41416 | 27 | -0.879923948 | RNASE7 | ribonuclease A family member 7 [Source:HGNC Symbol;Acc:HGNC:19278] |
| ENSG00000165807 | HGLibA_38091 | 27 | -0.879923948 | PPP1R36 | protein phosphatase 1 regulatory subunit 36 [Source:HGNC Symbol;Acc:HGNC:20097] |
| ENSG00000165891 | HGLibA_14324 | 27 | -0.879923948 | E2F7 | E2F transcription factor 7 [Source:HGNC Symbol;Acc:HGNC:23820] |
| ENSG00000166415 | HGLibA_54167 | 27 | -0.879923948 | WDR72 | WD repeat domain 72 [Source:HGNC Symbol;Acc:HGNC:26790] |
| ENSG00000166452 | HGLibA_01455 | 27 | -0.879923948 | AKIP1 | A-kinase interacting protein 1 [Source:HGNC Symbol;Acc:HGNC:1170] |
| ENSG00000166454 | HGLibA_03527 | 27 | -0.879923948 | ATMIN | ATM interactor [Source:HGNC Symbol;Acc:HGNC:29034] |
| ENSG00000167004 | HGLibA_35876 | 27 | -0.879923948 | PDIA3 | protein disulfide isomerase family A member 3 [Source:HGNC Symbol;Acc:HGNC:4606] |
| ENSG00000167074 | HGLibA_48962 | 27 | -0.879923948 | TEF | PAR bZIP transcription factor [Source:HGNC Symbol;Acc:HGNC:11722]" |
| ENSG00000168301 | HGLibA_24460 | 27 | -0.879923948 | KCTD6 | potassium channel tetramerization domain containing 6 [Source:HGNC Symbol;Acc:HGNC:22235] |
| ENSG00000169018 | HGLibA_17277 | 27 | -0.879923948 | FEM1B | fem-1 homolog B [Source:HGNC Symbol;Acc:HGNC:3649] |
| ENSG00000169087 | HGLibA_22326 | 27 | -0.879923948 | HSPBAP1 | HSPB1 associated protein 1 [Source:HGNC Symbol;Acc:HGNC:16389] |
| ENSG00000169291 | HGLibA_44020 | 27 | -0.879923948 | SHE | Src homology 2 domain containing E [Source:HGNC Symbol;Acc:HGNC:27004] |
| ENSG00000170561 | HGLibA_23632 | 27 | -0.879923948 | IRX2 | iroquois homeobox 2 [Source:HGNC Symbol;Acc:HGNC:14359] |
| ENSG00000170873 | HGLibA_30497 | 27 | -0.879923948 | MTSS1 | I-BAR domain containing [Source:HGNC Symbol;Acc:HGNC:20443]" |
| ENSG00000170881 | HGLibA_41517 | 27 | -0.879923948 | RNF139 | ring finger protein 139 [Source:HGNC Symbol;Acc:HGNC:17023] |
| ENSG00000171236 | HGLibA_27168 | 27 | -0.879923948 | LRG1 | leucine rich alpha-2-glycoprotein 1 [Source:HGNC Symbol;Acc:HGNC:29480] |
| ENSG00000171604 | HGLibA_11973 | 27 | -0.879923948 | CXXC5 | CXXC finger protein 5 [Source:HGNC Symbol;Acc:HGNC:26943] |
| ENSG00000171766 | HGLibA_18694 | 27 | -0.879923948 | GATM | glycine amidinotransferase [Source:HGNC Symbol;Acc:HGNC:4175] |
| ENSG00000172059 | HGLibA_25044 | 27 | -0.879923948 | KLF11 | Kruppel like factor 11 [Source:HGNC Symbol;Acc:HGNC:11811] |
| ENSG00000175329 | HGLibA_23692 | 27 | -0.879923948 | ISX | intestine specific homeobox [Source:HGNC Symbol;Acc:HGNC:28084] |
| ENSG00000175619 | HGLibA_33935 | 27 | -0.879923948 | OR4B1 | olfactory receptor family 4 subfamily B member 1 [Source:HGNC Symbol;Acc:HGNC:8290] |
| ENSG00000176105 | HGLibA_54661 | 27 | -0.879923948 | YES1 | Src family tyrosine kinase [Source:HGNC Symbol;Acc:HGNC:12841]" |
| ENSG00000176679 | HGLibA_49251 | 27 | -0.879923948 | TGIF2LY | TGFB induced factor homeobox 2 like Y-linked [Source:HGNC Symbol;Acc:HGNC:18569] |
| ENSG00000176681 | HGLibA_27327 | 27 | -0.879923948 | LRRC37A | leucine rich repeat containing 37A [Source:HGNC Symbol;Acc:HGNC:29069] |
| ENSG00000177151 | HGLibA_33881 | 27 | -0.879923948 | OR2T35 | olfactory receptor family 2 subfamily T member 35 [Source:HGNC Symbol;Acc:HGNC:31257] |
| ENSG00000177182 | HGLibA_10166 | 27 | -0.879923948 | CLVS1 | clavesin 1 [Source:HGNC Symbol;Acc:HGNC:23139] |
| ENSG00000177489 | HGLibA_33794 | 27 | -0.879923948 | OR2G2 | olfactory receptor family 2 subfamily G member 2 [Source:HGNC Symbol;Acc:HGNC:15007] |
| ENSG00000178184 | HGLibA_35189 | 27 | -0.879923948 | PARD6G | par-6 family cell polarity regulator gamma [Source:HGNC Symbol;Acc:HGNC:16076] |
| ENSG00000181315 | HGLibA_55798 | 27 | -0.879923948 | ZNF322 | zinc finger protein 322 [Source:HGNC Symbol;Acc:HGNC:23640] |
| ENSG00000181374 | HGLibA_07968 | 27 | -0.879923948 | CCL13 | C-C motif chemokine ligand 13 [Source:HGNC Symbol;Acc:HGNC:10611] |
| ENSG00000182170 | HGLibA_29844 | 27 | -0.879923948 | MRGPRG | MAS related GPR family member G [Source:HGNC Symbol;Acc:HGNC:24829] |
| ENSG00000182389 | HGLibA_06926 | 27 | -0.879923948 | CACNB4 | calcium voltage-gated channel auxiliary subunit beta 4 [Source:HGNC Symbol;Acc:HGNC:1404] |
| ENSG00000182568 | HGLibA_42777 | 27 | -0.879923948 | SATB1 | SATB homeobox 1 [Source:HGNC Symbol;Acc:HGNC:10541] |
| ENSG00000184650 | HGLibA_33296 | 27 | -0.879923948 | ODF4 | outer dense fiber of sperm tails 4 [Source:HGNC Symbol;Acc:HGNC:19056] |
| ENSG00000184895 | HGLibA_47099 | 27 | -0.879923948 | SRY | sex determining region Y [Source:HGNC Symbol;Acc:HGNC:11311] |
| ENSG00000184923 | HGLibA_33104 | 27 | -0.879923948 | NUTM2A | NUT family member 2A [Source:HGNC Symbol;Acc:HGNC:23438] |
| ENSG00000185013 | HGLibA_32805 | 27 | -0.879923948 | NT5C1B | cytosolic IB [Source:HGNC Symbol;Acc:HGNC:17818]" |
| ENSG00000185339 | HGLibA_48825 | 27 | -0.879923948 | TCN2 | transcobalamin 2 [Source:HGNC Symbol;Acc:HGNC:11653] |
| ENSG00000185532 | HGLibA_38579 | 27 | -0.879923948 | PRKG1 | protein kinase cGMP-dependent 1 [Source:HGNC Symbol;Acc:HGNC:9414] |
| ENSG00000185551 | HGLibA_32613 | 27 | -0.879923948 | NR2F2 | nuclear receptor subfamily 2 group F member 2 [Source:HGNC Symbol;Acc:HGNC:7976] |
| ENSG00000185615 | HGLibA_35873 | 27 | -0.879923948 | PDIA2 | protein disulfide isomerase family A member 2 [Source:HGNC Symbol;Acc:HGNC:14180] |
| ENSG00000185742 | HGLibA_05206 | 27 | -0.879923948 | C11orf87 | chromosome 11 open reading frame 87 [Source:HGNC Symbol;Acc:HGNC:33788] |
| ENSG00000186451 | HGLibA_46426 | 27 | -0.879923948 | SPATA12 | spermatogenesis associated 12 [Source:HGNC Symbol;Acc:HGNC:23221] |
| ENSG00000186493 | HGLibA_06381 | 27 | -0.879923948 | C5orf38 | chromosome 5 open reading frame 38 [Source:HGNC Symbol;Acc:HGNC:24226] |
| ENSG00000186665 | HGLibA_05550 | 27 | -0.879923948 | C17orf58 | chromosome 17 open reading frame 58 [Source:HGNC Symbol;Acc:HGNC:27568] |
| ENSG00000186687 | HGLibA_27757 | 27 | -0.879923948 | LYRM7 | LYR motif containing 7 [Source:HGNC Symbol;Acc:HGNC:28072] |
| ENSG00000187054 | HGLibA_50611 | 27 | -0.879923948 | TMPRSS11A | transmembrane serine protease 11A [Source:HGNC Symbol;Acc:HGNC:27954] |

| | | | | | |
|---|---|---|---|---|---|
| ENSG00000187514 | HGLibA_39392 | 27 | -0.879923948 | PTMA | prothymosin alpha [Source:HGNC Symbol;Acc:HGNC:9623] |
| ENSG00000187624 | HGLibA_05598 | 27 | -0.879923948 | C17orf97 | chromosome 17 open reading frame 97 [Source:HGNC Symbol;Acc:HGNC:33800] |
| ENSG00000187857 | HGLibA_34409 | 27 | -0.879923948 | OR6C75 | olfactory receptor family 6 subfamily C member 75 [Source:HGNC Symbol;Acc:HGNC:31304] |
| ENSG00000189091 | HGLibA_43710 | 27 | -0.879923948 | SF3B3 | splicing factor 3b subunit 3 [Source:HGNC Symbol;Acc:HGNC:10770] |
| ENSG00000196542 | HGLibA_46904 | 27 | -0.879923948 | SPTSSB | serine palmitoyltransferase small subunit B [Source:HGNC Symbol;Acc:HGNC:24045] |
| ENSG00000196562 | HGLibA_47728 | 27 | -0.879923948 | SULF2 | sulfatase 2 [Source:HGNC Symbol;Acc:HGNC:20392] |
| ENSG00000196792 | HGLibA_47597 | 27 | -0.879923948 | STRN3 | striatin 3 [Source:HGNC Symbol;Acc:HGNC:15720] |
| ENSG00000197124 | HGLibA_56530 | 27 | -0.879923948 | ZNF682 | zinc finger protein 682 [Source:HGNC Symbol;Acc:HGNC:28857] |
| ENSG00000197361 | HGLibA_17003 | 27 | -0.879923948 | FBXL22 | F-box and leucine rich repeat protein 22 [Source:HGNC Symbol;Acc:HGNC:27537] |
| ENSG00000197651 | HGLibA_07943 | 27 | -0.879923948 | CCER1 | coiled-coil glutamate rich protein 1 [Source:HGNC Symbol;Acc:HGNC:28373] |
| ENSG00000197889 | HGLibA_28865 | 27 | -0.879923948 | MEIG1 | meiosis/spermiogenesis associated 1 [Source:HGNC Symbol;Acc:HGNC:23429] |
| ENSG00000198755 | HGLibA_41857 | 27 | -0.879923948 | RPL10A | ribosomal protein L10a [Source:HGNC Symbol;Acc:HGNC:10299] |
| ENSG00000198911 | HGLibA_46951 | 27 | -0.879923948 | SREBF2 | sterol regulatory element binding transcription factor 2 [Source:HGNC Symbol;Acc:HGNC:11290] |
| ENSG00000203710 | HGLibA_11046 | 27 | -0.879923948 | CR1 | complement C3b/C4b receptor 1 (Knops blood group) [Source:HGNC Symbol;Acc:HGNC:2334] |
| ENSG00000203923 | HGLibA_46403 | 27 | -0.879923948 | SPANXN1 | SPANX family member N1 [Source:HGNC Symbol;Acc:HGNC:33174] |
| ENSG00000204542 | HGLibA_06451 | 27 | -0.879923948 | C6orf15 | chromosome 6 open reading frame 15 [Source:HGNC Symbol;Acc:HGNC:13927] |
| ENSG00000205277 | HGLibA_30522 | 27 | -0.879923948 | MUC12 | cell surface associated [Source:HGNC Symbol;Acc:HGNC:7510]" |
| ENSG00000205323 | HGLibA_42745 | 27 | -0.879923948 | SARNP | SAP domain containing ribonucleoprotein [Source:HGNC Symbol;Acc:HGNC:24432] |
| ENSG00000205642 | HGLibA_53591 | 27 | -0.879923948 | VCX3B | variable charge X-linked 3B [Source:HGNC Symbol;Acc:HGNC:31838] |
| ENSG00000206559 | HGLibA_55061 | 27 | -0.879923948 | ZCWPW2 | zinc finger CW-type and PWWP domain containing 2 [Source:HGNC Symbol;Acc:HGNC:23574] |
| ENSG00000213903 | HGLibA_27584 | 27 | -0.879923948 | LTB4R | leukotriene B4 receptor [Source:HGNC Symbol;Acc:HGNC:6713] |
| ENSG00000213937 | HGLibA_09912 | 27 | -0.879923948 | CLDN9 | claudin 9 [Source:HGNC Symbol;Acc:HGNC:2051] |
| ENSG00000214160 | HGLibA_01629 | 27 | -0.879923948 | ALG3 | 3- mannosyltransferase [Source:HGNC Symbol;Acc:HGNC:23056]" |
| ENSG00000220008 | HGLibA_26591 | 27 | -0.879923948 | LINGO3 | leucine rich repeat and Ig domain containing 3 [Source:HGNC Symbol;Acc:HGNC:21206] |
| ENSG00000221968 | HGLibA_15939 | 27 | -0.879923948 | FADS3 | fatty acid desaturase 3 [Source:HGNC Symbol;Acc:HGNC:3576] |
| ENSG00000240542 | HGLibA_25830 | 27 | -0.879923948 | KRTAP9-1 | keratin associated protein 9-1 [Source:HGNC Symbol;Acc:HGNC:18912] |
| ENSG00000243207 | HGLibA_37812 | 27 | -0.879923948 | PPAN-P2RY11 | PPAN-P2RY11 readthrough [Source:HGNC Symbol;Acc:HGNC:33526] |
| ENSG00000243284 | HGLibA_53852 | 27 | -0.879923948 | VSIG8 | V-set and immunoglobulin domain containing 8 [Source:HGNC Symbol;Acc:HGNC:32063] |
| ENSG00000249139 | HGLibA_15358 | 27 | -0.879923948 | EPPIN-WFDC6 | EPPIN-WFDC6 readthrough [Source:HGNC Symbol;Acc:HGNC:38825] |
| ENSG00000256812 | HGLibA_07188 | 27 | -0.879923948 | CAPNS2 | calpain small subunit 2 [Source:HGNC Symbol;Acc:HGNC:16371] |
| ENSG00000261221 | HGLibA_56885 | 27 | -0.879923948 | ZNF865 | zinc finger protein 865 [Source:HGNC Symbol;Acc:HGNC:38705] |
| ENSG00000269791 | HGLibA_47213 | 27 | -0.879923948 | SSX4B | SSX family member 4B [Source:HGNC Symbol;Acc:HGNC:16880] |
| ENSG00000272804 | HGLibA_25619 | 27 | -0.879923948 | KRTAP10-7 | keratin associated protein 10-7 [Source:HGNC Symbol;Acc:HGNC:22970] |
| ENSG00000273111 | HGLibA_27723 | 27 | -0.879923948 | LYPD4 | LY6/PLAUR domain containing 4 [Source:HGNC Symbol;Acc:HGNC:28659] |
| ENSG00000277972 | HGLibA_09734 | 27 | -0.879923948 | CISD3 | CDGSH iron sulfur domain 3 [Source:HGNC Symbol;Acc:HGNC:27578] |
| ERBB2IP | HGLibA_15416 | 27 | -0.879923948 | ERBB2IP | - |
| FDX1L | HGLibA_17261 | 27 | -0.879923948 | FDX1L | - |
| HIST1H2BI | HGLibA_21434 | 27 | -0.879923948 | HIST1H2BI | - |
| hsa-mir-1250 | HGLibA_57398 | 27 | -0.879923948 | hsa-mir-1250 | - |
| hsa-mir-1471 | HGLibA_57868 | 27 | -0.879923948 | hsa-mir-1471 | - |
| hsa-mir-1914 | HGLibA_58062 | 27 | -0.879923948 | hsa-mir-1914 | - |
| hsa-mir-196a-2 | HGLibA_58096 | 27 | -0.879923948 | hsa-mir-196a-2 | - |
| hsa-mir-30d | HGLibA_58489 | 27 | -0.879923948 | hsa-mir-30d | - |
| hsa-mir-3136 | HGLibA_58604 | 27 | -0.879923948 | hsa-mir-3136 | - |
| hsa-mir-3202-1 | HGLibA_58916 | 27 | -0.879923948 | hsa-mir-3202-1 | - |
| hsa-mir-345 | HGLibA_59027 | 27 | -0.879923948 | hsa-mir-345 | - |
| hsa-mir-3622b | HGLibA_59125 | 27 | -0.879923948 | hsa-mir-3622b | - |
| hsa-mir-3663 | HGLibA_59202 | 27 | -0.879923948 | hsa-mir-3663 | - |
| hsa-mir-4320 | HGLibA_60005 | 27 | -0.879923948 | hsa-mir-4320 | - |
| hsa-mir-4517 | HGLibA_60511 | 27 | -0.879923948 | hsa-mir-4517 | - |
| hsa-mir-4662a | HGLibA_60756 | 27 | -0.879923948 | hsa-mir-4662a | - |
| hsa-mir-4694 | HGLibA_60885 | 27 | -0.879923948 | hsa-mir-4694 | - |
| hsa-mir-4696 | HGLibA_60893 | 27 | -0.879923948 | hsa-mir-4696 | - |
| hsa-mir-4709 | HGLibA_60938 | 27 | -0.879923948 | hsa-mir-4709 | - |
| hsa-mir-4720 | HGLibA_60977 | 27 | -0.879923948 | hsa-mir-4720 | - |
| hsa-mir-4753 | HGLibA_61110 | 27 | -0.879923948 | hsa-mir-4753 | - |
| hsa-mir-4768 | HGLibA_61168 | 27 | -0.879923948 | hsa-mir-4768 | - |
| hsa-mir-514a-2 | HGLibA_61589 | 27 | -0.879923948 | hsa-mir-514a-2 | - |
| hsa-mir-548ab | HGLibA_61856 | 27 | -0.879923948 | hsa-mir-548ab | - |
| hsa-mir-548ao | HGLibA_61909 | 27 | -0.879923948 | hsa-mir-548ao | - |
| hsa-mir-548as | HGLibA_61919 | 27 | -0.879923948 | hsa-mir-548as | - |
| hsa-mir-548m | HGLibA_62042 | 27 | -0.879923948 | hsa-mir-548m | - |
| hsa-mir-5572 | HGLibA_62150 | 27 | -0.879923948 | hsa-mir-5572 | - |
| hsa-mir-5692c-1 | HGLibA_62300 | 27 | -0.879923948 | hsa-mir-5692c-1 | - |
| hsa-mir-6077-1 | HGLibA_62561 | 27 | -0.879923948 | hsa-mir-6077-1 | - |
| hsa-mir-6131 | HGLibA_62676 | 27 | -0.879923948 | hsa-mir-6131 | - |
| hsa-mir-6505 | HGLibA_62870 | 27 | -0.879923948 | hsa-mir-6505 | - |
| hsa-mir-655 | HGLibA_62955 | 27 | -0.879923948 | hsa-mir-655 | - |
| hsa-mir-7847 | HGLibA_63978 | 27 | -0.879923948 | hsa-mir-7847 | - |
| hsa-mir-7852 | HGLibA_63996 | 27 | -0.879923948 | hsa-mir-7852 | - |
| hsa-mir-8056 | HGLibA_64064 | 27 | -0.879923948 | hsa-mir-8056 | - |
| KIAA0141 | HGLibA_24592 | 27 | -0.879923948 | KIAA0141 | - |
| KIAA2018 | HGLibA_24840 | 27 | -0.879923948 | KIAA2018 | - |
| LOC100287177 | HGLibA_26820 | 27 | -0.879923948 | LOC100287177 | - |
| MYEOV2 | HGLibA_30695 | 27 | -0.879923948 | MYEOV2 | - |
| NGFRAP1 | HGLibA_31878 | 27 | -0.879923948 | NGFRAP1 | - |
| PPAP2B | HGLibA_37820 | 27 | -0.879923948 | PPAP2B | - |
| TCEB1 | HGLibA_48735 | 27 | -0.879923948 | TCEB1 | - |
| TNFAIP8L2-SCNM1 | HGLibA_50726 | 27 | -0.879923948 | TNFAIP8L2-SCNM1 | - |
| C10orf91 | HGLibA_05106 | 26 | -0.889874986 | C10orf91 | - |
| C19orf21 | HGLibA_05636 | 26 | -0.889874986 | C19orf21 | - |
| C1orf123 | HGLibA_05763 | 26 | -0.889874986 | C1orf123 | - |
| CXorf40A | HGLibA_11922 | 26 | -0.889874986 | CXorf40A | - |
| DOM3Z | HGLibA_13794 | 26 | -0.889874986 | DOM3Z | - |
| ENSG00000004660 | HGLibA_07088 | 26 | -0.889874986 | CAMKK1 | calcium/calmodulin dependent protein kinase kinase 1 [Source:HGNC Symbol;Acc:HGNC:1469] |
| ENSG00000010610 | HGLibA_08382 | 26 | -0.889874986 | CD4 | CD4 molecule [Source:HGNC Symbol;Acc:HGNC:1678] |
| ENSG00000012817 | HGLibA_24529 | 26 | -0.889874986 | KDM5D | lysine demethylase 5D [Source:HGNC Symbol;Acc:HGNC:11115] |
| ENSG00000037042 | HGLibA_52307 | 26 | -0.889874986 | TUBG2 | tubulin gamma 2 [Source:HGNC Symbol;Acc:HGNC:12419] |
| ENSG00000038210 | HGLibA_36495 | 26 | -0.889874986 | PI4K2B | phosphatidylinositol 4-kinase type 2 beta [Source:HGNC Symbol;Acc:HGNC:18215] |
| ENSG00000047662 | HGLibA_16329 | 26 | -0.889874986 | FAM184B | family with sequence similarity 184 member B [Source:HGNC Symbol;Acc:HGNC:29235] |
| ENSG00000050426 | HGLibA_26338 | 26 | -0.889874986 | LETMD1 | LETM1 domain containing 1 [Source:HGNC Symbol;Acc:HGNC:24241] |
| ENSG00000050748 | HGLibA_28295 | 26 | -0.889874986 | MAPK9 | mitogen-activated protein kinase 9 [Source:HGNC Symbol;Acc:HGNC:6886] |
| ENSG00000052841 | HGLibA_52061 | 26 | -0.889874986 | TTC17 | tetratricopeptide repeat domain 17 [Source:HGNC Symbol;Acc:HGNC:25596] |
| ENSG00000053501 | HGLibA_53165 | 26 | -0.889874986 | USE1 | unconventional SNARE in the ER 1 [Source:HGNC Symbol;Acc:HGNC:30882] |
| ENSG00000056291 | HGLibA_32443 | 26 | -0.889874986 | NPFFR2 | neuropeptide FF receptor 2 [Source:HGNC Symbol;Acc:HGNC:4525] |
| ENSG00000064787 | HGLibA_04252 | 26 | -0.889874986 | BCAS1 | breast carcinoma amplified sequence 1 [Source:HGNC Symbol;Acc:HGNC:974] |
| ENSG00000064933 | HGLibA_37334 | 26 | -0.889874986 | PMS1 | mismatch repair system component [Source:HGNC Symbol;Acc:HGNC:9121]" |
| ENSG00000065548 | HGLibA_54968 | 26 | -0.889874986 | ZC3H15 | zinc finger CCCH-type containing 15 [Source:HGNC Symbol;Acc:HGNC:29528] |
| ENSG00000072778 | HGLibA_00376 | 26 | -0.889874986 | ACADVL | acyl-CoA dehydrogenase very long chain [Source:HGNC Symbol;Acc:HGNC:92] |
| ENSG00000073067 | HGLibA_12148 | 26 | -0.889874986 | CYP2W1 | cytochrome P450 family 2 subfamily W member 1 [Source:HGNC Symbol;Acc:HGNC:20243] |
| ENSG00000075420 | HGLibA_17750 | 26 | -0.889874986 | FNDC3B | fibronectin type III domain containing 3B [Source:HGNC Symbol;Acc:HGNC:24670] |
| ENSG00000075785 | HGLibA_39978 | 26 | -0.889874986 | RAB7A | member RAS oncogene family [Source:HGNC Symbol;Acc:HGNC:9788]" |
| ENSG00000078295 | HGLibA_00911 | 26 | -0.889874986 | ADCY2 | adenylate cyclase 2 [Source:HGNC Symbol;Acc:HGNC:233] |
| ENSG00000078967 | HGLibA_52591 | 26 | -0.889874986 | UBE2D4 | ubiquitin conjugating enzyme E2 D4 (putative) [Source:HGNC Symbol;Acc:HGNC:21647] |
| ENSG00000079435 | HGLibA_26607 | 26 | -0.889874986 | LIPE | hormone sensitive type [Source:HGNC Symbol;Acc:HGNC:6621]" |
| ENSG00000080986 | HGLibA_31383 | 26 | -0.889874986 | NDC80 | kinetochore complex component [Source:HGNC Symbol;Acc:HGNC:16909]" |
| ENSG00000081014 | HGLibA_02343 | 26 | -0.889874986 | AP4E1 | adaptor related protein complex 4 subunit epsilon 1 [Source:HGNC Symbol;Acc:HGNC:573] |
| ENSG00000081800 | HGLibA_44383 | 26 | -0.889874986 | SLC13A1 | solute carrier family 13 member 1 [Source:HGNC Symbol;Acc:HGNC:10916] |
| ENSG00000083807 | HGLibA_44826 | 26 | -0.889874986 | SLC27A5 | solute carrier family 27 member 5 [Source:HGNC Symbol;Acc:HGNC:10999] |
| ENSG00000083828 | HGLibA_56327 | 26 | -0.889874986 | ZNF586 | zinc finger protein 586 [Source:HGNC Symbol;Acc:HGNC:25949] |
| ENSG00000084090 | HGLibA_47372 | 26 | -0.889874986 | STARD7 | StAR related lipid transfer domain containing 7 [Source:HGNC Symbol;Acc:HGNC:18063] |
| ENSG00000090889 | HGLibA_24943 | 26 | -0.889874986 | KIF4A | kinesin family member 4A [Source:HGNC Symbol;Acc:HGNC:13339] |
| ENSG00000091542 | HGLibA_01657 | 26 | -0.889874986 | ALKBH5 | RNA demethylase [Source:HGNC Symbol;Acc:HGNC:25996]" |
| ENSG00000092148 | HGLibA_21093 | 26 | -0.889874986 | HECTD1 | HECT domain E3 ubiquitin protein ligase 1 [Source:HGNC Symbol;Acc:HGNC:20157] |
| ENSG00000100228 | HGLibA_39893 | 26 | -0.889874986 | RAB36 | member RAS oncogene family [Source:HGNC Symbol;Acc:HGNC:9775]" |
| ENSG00000100345 | HGLibA_30742 | 26 | -0.889874986 | MYH9 | myosin heavy chain 9 [Source:HGNC Symbol;Acc:HGNC:7579] |
| ENSG00000100478 | HGLibA_02351 | 26 | -0.889874986 | AP4S1 | adaptor related protein complex 4 subunit sigma 1 [Source:HGNC Symbol;Acc:HGNC:575] |
| ENSG00000100906 | HGLibA_31822 | 26 | -0.889874986 | NFKBIA | NFKB inhibitor alpha [Source:HGNC Symbol;Acc:HGNC:7797] |
| ENSG00000100991 | HGLibA_51737 | 26 | -0.889874986 | TRPC4AP | transient receptor potential cation channel subfamily C member 4 associated protein [Source:HGNC Symbol;Acc:HGNC:16181] |
| ENSG00000101104 | HGLibA_34923 | 26 | -0.889874986 | PABPC1L | poly(A) binding protein cytoplasmic 1 like [Source:HGNC Symbol;Acc:HGNC:15797] |
| ENSG00000101883 | HGLibA_41214 | 26 | -0.889874986 | RHOXF1 | Rhox homeobox family member 1 [Source:HGNC Symbol;Acc:HGNC:29993] |
| ENSG00000102119 | HGLibA_15031 | 26 | -0.889874986 | EMD | emerin [Source:HGNC Symbol;Acc:HGNC:3331] |
| ENSG00000102189 | HGLibA_14485 | 26 | -0.889874986 | EEA1 | early endosome antigen 1 [Source:HGNC Symbol;Acc:HGNC:3185] |
| ENSG00000102763 | HGLibA_53917 | 26 | -0.889874986 | VWA8 | von Willebrand factor A domain containing 8 [Source:HGNC Symbol;Acc:HGNC:29071] |
| ENSG00000103326 | HGLibA_07159 | 26 | -0.889874986 | CAPN15 | calpain 15 [Source:HGNC Symbol;Acc:HGNC:11182] |
| ENSG00000104164 | HGLibA_04573 | 26 | -0.889874986 | BLOC1S6 | biogenesis of lysosomal organelles complex 1 subunit 6 [Source:HGNC Symbol;Acc:HGNC:8549] |
| ENSG00000104369 | HGLibA_23978 | 26 | -0.889874986 | JPH1 | junctophilin 1 [Source:HGNC Symbol;Acc:HGNC:14201] |
| ENSG00000104691 | HGLibA_52799 | 26 | -0.889874986 | UBXN8 | UBX domain protein 8 [Source:HGNC Symbol;Acc:HGNC:30307] |
| ENSG00000105278 | HGLibA_55260 | 26 | -0.889874986 | ZFR2 | zinc finger RNA binding protein 2 [Source:HGNC Symbol;Acc:HGNC:29189] |
| ENSG00000105519 | HGLibA_07197 | 26 | -0.889874986 | CAPS | calcyphosine [Source:HGNC Symbol;Acc:HGNC:1487] |
| ENSG00000106443 | HGLibA_36346 | 26 | -0.889874986 | PHF14 | PHD finger protein 14 [Source:HGNC Symbol;Acc:HGNC:22203] |
| ENSG00000108468 | HGLibA_07444 | 26 | -0.889874986 | CBX1 | chromobox 1 [Source:HGNC Symbol;Acc:HGNC:1551] |
| ENSG00000108515 | HGLibA_15147 | 26 | -0.889874986 | ENO3 | enolase 3 [Source:HGNC Symbol;Acc:HGNC:3354] |
| ENSG00000108883 | HGLibA_14617 | 26 | -0.889874986 | EFTUD2 | elongation factor Tu GTP binding domain containing 2 [Source:HGNC Symbol;Acc:HGNC:30858] |
| ENSG00000110031 | HGLibA_27129 | 26 | -0.889874986 | LPXN | leupaxin [Source:HGNC Symbol;Acc:HGNC:14061] |

| | | | | | |
|---|---|---|---|---|---|
| ENSG00000110422 | HGLibA_21344 | 26 | -0.889874986 | HIPK3 | homeodomain interacting protein kinase 3 [Source:HGNC Symbol;Acc:HGNC:4915] |
| ENSG00000110492 | HGLibA_28690 | 26 | -0.889874986 | MDK | midkine [Source:HGNC Symbol;Acc:HGNC:6972] |
| ENSG00000111196 | HGLibA_28010 | 26 | -0.889874986 | MAGOHB | exon junction complex subunit [Source:HGNC Symbol;Acc:HGNC:25504]" |
| ENSG00000111837 | HGLibA_28016 | 26 | -0.889874986 | MAK | male germ cell associated kinase [Source:HGNC Symbol;Acc:HGNC:6816] |
| ENSG00000112214 | HGLibA_17489 | 26 | -0.889874986 | FHL5 | four and a half LIM domains 5 [Source:HGNC Symbol;Acc:HGNC:17371] |
| ENSG00000112339 | HGLibA_20939 | 26 | -0.889874986 | HBS1L | HBS1 like translational GTPase [Source:HGNC Symbol;Acc:HGNC:4834] |
| ENSG00000112818 | HGLibA_28896 | 26 | -0.889874986 | MEP1A | meprin A subunit alpha [Source:HGNC Symbol;Acc:HGNC:7015] |
| ENSG00000112996 | HGLibA_30087 | 26 | -0.889874986 | MRPS30 | mitochondrial ribosomal protein S30 [Source:HGNC Symbol;Acc:HGNC:8769] |
| ENSG00000113360 | HGLibA_13984 | 26 | -0.889874986 | DROSHA | drosha ribonuclease III [Source:HGNC Symbol;Acc:HGNC:17904] |
| ENSG00000113645 | HGLibA_54438 | 26 | -0.889874986 | WWC1 | WW and C2 domain containing 1 [Source:HGNC Symbol;Acc:HGNC:29435] |
| ENSG00000114650 | HGLibA_42858 | 26 | -0.889874986 | SCAP | SREBF chaperone [Source:HGNC Symbol;Acc:HGNC:30634] |
| ENSG00000115009 | HGLibA_07990 | 26 | -0.889874986 | CCL20 | C-C motif chemokine ligand 20 [Source:HGNC Symbol;Acc:HGNC:10619] |
| ENSG00000115091 | HGLibA_00660 | 26 | -0.889874986 | ACTR3 | ARP3 actin related protein 3 homolog [Source:HGNC Symbol;Acc:HGNC:170] |
| ENSG00000115317 | HGLibA_22411 | 26 | -0.889874986 | HTRA2 | HtrA serine peptidase 2 [Source:HGNC Symbol;Acc:HGNC:14348] |
| ENSG00000115484 | HGLibA_08201 | 26 | -0.889874986 | CCT4 | chaperonin containing TCP1 subunit 4 [Source:HGNC Symbol;Acc:HGNC:1617] |
| ENSG00000115738 | HGLibA_22528 | 26 | -0.889874986 | ID2 | inhibitor of DNA binding 2 [Source:HGNC Symbol;Acc:HGNC:5361] |
| ENSG00000115884 | HGLibA_43096 | 26 | -0.889874986 | SDC1 | syndecan 1 [Source:HGNC Symbol;Acc:HGNC:10658] |
| ENSG00000117153 | HGLibA_25129 | 26 | -0.889874986 | KLHL12 | kelch like family member 12 [Source:HGNC Symbol;Acc:HGNC:19360] |
| ENSG00000117394 | HGLibA_44852 | 26 | -0.889874986 | SLC2A1 | solute carrier family 2 member 1 [Source:HGNC Symbol;Acc:HGNC:11005] |
| ENSG00000117481 | HGLibA_32787 | 26 | -0.889874986 | NSUN4 | NOP2/Sun RNA methyltransferase family member 4 [Source:HGNC Symbol;Acc:HGNC:31802] |
| ENSG00000118514 | HGLibA_01586 | 26 | -0.889874986 | ALDH8A1 | aldehyde dehydrogenase 8 family member A1 [Source:HGNC Symbol;Acc:HGNC:15471] |
| ENSG00000119559 | HGLibA_05642 | 26 | -0.889874986 | C19orf25 | chromosome 19 open reading frame 25 [Source:HGNC Symbol;Acc:HGNC:26711] |
| ENSG00000120693 | HGLibA_45589 | 26 | -0.889874986 | SMAD9 | SMAD family member 9 [Source:HGNC Symbol;Acc:HGNC:6774] |
| ENSG00000121552 | HGLibA_11477 | 26 | -0.889874986 | CSTA | cystatin A [Source:HGNC Symbol;Acc:HGNC:2481] |
| ENSG00000121691 | HGLibA_07353 | 26 | -0.889874986 | CAT | catalase [Source:HGNC Symbol;Acc:HGNC:1516] |
| ENSG00000122176 | HGLibA_17716 | 26 | -0.889874986 | FMOD | fibromodulin [Source:HGNC Symbol;Acc:HGNC:3774] |
| ENSG00000122481 | HGLibA_42489 | 26 | -0.889874986 | RWDD3 | RWD domain containing 3 [Source:HGNC Symbol;Acc:HGNC:21393] |
| ENSG00000123131 | HGLibA_38416 | 26 | -0.889874986 | PRDX4 | peroxiredoxin 4 [Source:HGNC Symbol;Acc:HGNC:17169] |
| ENSG00000123374 | HGLibA_08756 | 26 | -0.889874986 | CDK2 | cyclin dependent kinase 2 [Source:HGNC Symbol;Acc:HGNC:1771] |
| ENSG00000123411 | HGLibA_22937 | 26 | -0.889874986 | IKZF4 | IKAROS family zinc finger 4 [Source:HGNC Symbol;Acc:HGNC:13179] |
| ENSG00000124422 | HGLibA_53306 | 26 | -0.889874986 | USP22 | ubiquitin specific peptidase 22 [Source:HGNC Symbol;Acc:HGNC:12621] |
| ENSG00000125861 | HGLibA_18973 | 26 | -0.889874986 | GFRA4 | GDNF family receptor alpha 4 [Source:HGNC Symbol;Acc:HGNC:13821] |
| ENSG00000125872 | HGLibA_27478 | 26 | -0.889874986 | LRRN4 | leucine rich repeat neuronal 4 [Source:HGNC Symbol;Acc:HGNC:16208] |
| ENSG00000126603 | HGLibA_19274 | 26 | -0.889874986 | GLIS2 | GLIS family zinc finger 2 [Source:HGNC Symbol;Acc:HGNC:29450] |
| ENSG00000127954 | HGLibA_47431 | 26 | -0.889874986 | STEAP4 | STEAP4 metalloreductase [Source:HGNC Symbol;Acc:HGNC:21923] |
| ENSG00000128250 | HGLibA_40929 | 26 | -0.889874986 | RFPL1 | ret finger protein like 1 [Source:HGNC Symbol;Acc:HGNC:9977] |
| ENSG00000128524 | HGLibA_03753 | 26 | -0.889874986 | ATP6V1F | ATPase H+ transporting V1 subunit F [Source:HGNC Symbol;Acc:HGNC:16832] |
| ENSG00000129226 | HGLibA_08429 | 26 | -0.889874986 | CD68 | CD68 molecule [Source:HGNC Symbol;Acc:HGNC:1693] |
| ENSG00000130305 | HGLibA_32791 | 26 | -0.889874986 | NSUN5 | NOP2/Sun RNA methyltransferase family member 5 [Source:HGNC Symbol;Acc:HGNC:16385] |
| ENSG00000130312 | HGLibA_29956 | 26 | -0.889874986 | MRPL34 | mitochondrial ribosomal protein L34 [Source:HGNC Symbol;Acc:HGNC:14488] |
| ENSG00000131669 | HGLibA_31939 | 26 | -0.889874986 | NINJ1 | ninjurin 1 [Source:HGNC Symbol;Acc:HGNC:7824] |
| ENSG00000131788 | HGLibA_36514 | 26 | -0.889874986 | PIAS3 | protein inhibitor of activated STAT 3 [Source:HGNC Symbol;Acc:HGNC:16861] |
| ENSG00000132003 | HGLibA_57045 | 26 | -0.889874986 | ZSWIM4 | zinc finger SWIM-type containing 4 [Source:HGNC Symbol;Acc:HGNC:25704] |
| ENSG00000132681 | HGLibA_03590 | 26 | -0.889874986 | ATP1A4 | ATPase Na+/K+ transporting subunit alpha 4 [Source:HGNC Symbol;Acc:HGNC:14073] |
| ENSG00000132840 | HGLibA_04487 | 26 | -0.889874986 | BHMT2 | betaine--homocysteine S-methyltransferase 2 [Source:HGNC Symbol;Acc:HGNC:1048] |
| ENSG00000133110 | HGLibA_37710 | 26 | -0.889874986 | POSTN | periostin [Source:HGNC Symbol;Acc:HGNC:16953] |
| ENSG00000133636 | HGLibA_32883 | 26 | -0.889874986 | NTS | neurotensin [Source:HGNC Symbol;Acc:HGNC:8038] |
| ENSG00000134042 | HGLibA_29865 | 26 | -0.889874986 | MRO | maestro [Source:HGNC Symbol;Acc:HGNC:24121] |
| ENSG00000134077 | HGLibA_49422 | 26 | -0.889874986 | THUMPD3 | THUMP domain containing 3 [Source:HGNC Symbol;Acc:HGNC:24493] |
| ENSG00000134490 | HGLibA_50288 | 26 | -0.889874986 | TMEM241 | transmembrane protein 241 [Source:HGNC Symbol;Acc:HGNC:31723] |
| ENSG00000135333 | HGLibA_15304 | 26 | -0.889874986 | EPHA7 | EPH receptor A7 [Source:HGNC Symbol;Acc:HGNC:3390] |
| ENSG00000136448 | HGLibA_32205 | 26 | -0.889874986 | NMT1 | N-myristoyltransferase 1 [Source:HGNC Symbol;Acc:HGNC:7857] |
| ENSG00000136960 | HGLibA_15169 | 26 | -0.889874986 | ENPP2 | ectonucleotide pyrophosphatase/phosphodiesterase 2 [Source:HGNC Symbol;Acc:HGNC:3357] |
| ENSG00000137642 | HGLibA_46195 | 26 | -0.889874986 | SORL1 | sortilin related receptor 1 [Source:HGNC Symbol;Acc:HGNC:11185] |
| ENSG00000137871 | HGLibA_55733 | 26 | -0.889874986 | ZNF280D | zinc finger protein 280D [Source:HGNC Symbol;Acc:HGNC:25953] |
| ENSG00000137975 | HGLibA_09808 | 26 | -0.889874986 | CLCA2 | chloride channel accessory 2 [Source:HGNC Symbol;Acc:HGNC:2016] |
| ENSG00000138378 | HGLibA_47391 | 26 | -0.889874986 | STAT4 | signal transducer and activator of transcription 4 [Source:HGNC Symbol;Acc:HGNC:11365] |
| ENSG00000138399 | HGLibA_16900 | 26 | -0.889874986 | FASTKD1 | FAST kinase domains 1 [Source:HGNC Symbol;Acc:HGNC:26150] |
| ENSG00000141504 | HGLibA_42773 | 26 | -0.889874986 | SAT2 | spermidine/spermine N1-acetyltransferase family member 2 [Source:HGNC Symbol;Acc:HGNC:23160] |
| ENSG00000141756 | HGLibA_17551 | 26 | -0.889874986 | FKBP10 | FK506 binding protein 10 [Source:HGNC Symbol;Acc:HGNC:18169] |
| ENSG00000142186 | HGLibA_43086 | 26 | -0.889874986 | SCYL1 | SCY1 like pseudokinase 1 [Source:HGNC Symbol;Acc:HGNC:14372] |
| ENSG00000143434 | HGLibA_43369 | 26 | -0.889874986 | SEMA6C | semaphorin 6C [Source:HGNC Symbol;Acc:HGNC:10740] |
| ENSG00000144848 | HGLibA_03486 | 26 | -0.889874986 | ATG3 | autophagy related 3 [Source:HGNC Symbol;Acc:HGNC:20962] |
| ENSG00000145029 | HGLibA_31919 | 26 | -0.889874986 | NICN1 | nicolin 1 [Source:HGNC Symbol;Acc:HGNC:18317] |
| ENSG00000145536 | HGLibA_00810 | 26 | -0.889874986 | ADAMTS16 | ADAM metallopeptidase with thrombospondin type 1 motif 16 [Source:HGNC Symbol;Acc:HGNC:17108] |
| ENSG00000145819 | HGLibA_02740 | 26 | -0.889874986 | ARHGAP26 | Rho GTPase activating protein 26 [Source:HGNC Symbol;Acc:HGNC:17073] |
| ENSG00000146063 | HGLibA_51494 | 26 | -0.889874986 | TRIM41 | tripartite motif containing 41 [Source:HGNC Symbol;Acc:HGNC:19013] |
| ENSG00000146233 | HGLibA_12150 | 26 | -0.889874986 | CYP39A1 | cytochrome P450 family 39 subfamily A member 1 [Source:HGNC Symbol;Acc:HGNC:17449] |
| ENSG00000146555 | HGLibA_43152 | 26 | -0.889874986 | SDK1 | sidekick cell adhesion molecule 1 [Source:HGNC Symbol;Acc:HGNC:19307] |
| ENSG00000146731 | HGLibA_08208 | 26 | -0.889874986 | CCT6A | chaperonin containing TCP1 subunit 6A [Source:HGNC Symbol;Acc:HGNC:1620] |
| ENSG00000148584 | HGLibA_00005 | 26 | -0.889874986 | A1CF | APOBEC1 complementation factor [Source:HGNC Symbol;Acc:HGNC:24086] |
| ENSG00000154553 | HGLibA_35913 | 26 | -0.889874986 | PDLIM3 | PDZ and LIM domain 3 [Source:HGNC Symbol;Acc:HGNC:20767] |
| ENSG00000155189 | HGLibA_01228 | 26 | -0.889874986 | AGPAT5 | 1-acylglycerol-3-phosphate O-acyltransferase 5 [Source:HGNC Symbol;Acc:HGNC:20886] |
| ENSG00000155380 | HGLibA_44416 | 26 | -0.889874986 | SLC16A1 | solute carrier family 16 member 1 [Source:HGNC Symbol;Acc:HGNC:10922] |
| ENSG00000156282 | HGLibA_09868 | 26 | -0.889874986 | CLDN17 | claudin 17 [Source:HGNC Symbol;Acc:HGNC:2038] |
| ENSG00000158092 | HGLibA_31305 | 26 | -0.889874986 | NCK1 | NCK adaptor protein 1 [Source:HGNC Symbol;Acc:HGNC:7664] |
| ENSG00000158402 | HGLibA_08532 | 26 | -0.889874986 | CDC25C | cell division cycle 25C [Source:HGNC Symbol;Acc:HGNC:1727] |
| ENSG00000159214 | HGLibA_07737 | 26 | -0.889874986 | CCDC24 | coiled-coil domain containing 24 [Source:HGNC Symbol;Acc:HGNC:28688] |
| ENSG00000160202 | HGLibA_11246 | 26 | -0.889874986 | CRYAA | crystallin alpha A [Source:HGNC Symbol;Acc:HGNC:2388] |
| ENSG00000160678 | HGLibA_42530 | 26 | -0.889874986 | S100A1 | S100 calcium binding protein A1 [Source:HGNC Symbol;Acc:HGNC:10486] |
| ENSG00000160753 | HGLibA_42466 | 26 | -0.889874986 | RUSC1 | RUN and SH3 domain containing 1 [Source:HGNC Symbol;Acc:HGNC:17153] |
| ENSG00000162728 | HGLibA_24291 | 26 | -0.889874986 | KCNJ9 | potassium voltage-gated channel subfamily J member 9 [Source:HGNC Symbol;Acc:HGNC:6270] |
| ENSG00000163217 | HGLibA_04592 | 26 | -0.889874986 | BMP10 | bone morphogenetic protein 10 [Source:HGNC Symbol;Acc:HGNC:20869] |
| ENSG00000163219 | HGLibA_02738 | 26 | -0.889874986 | ARHGAP25 | Rho GTPase activating protein 25 [Source:HGNC Symbol;Acc:HGNC:28951] |
| ENSG00000163235 | HGLibA_49204 | 26 | -0.889874986 | TGFA | transforming growth factor alpha [Source:HGNC Symbol;Acc:HGNC:11765] |
| ENSG00000163257 | HGLibA_12433 | 26 | -0.889874986 | DCAF16 | DDB1 and CUL4 associated factor 16 [Source:HGNC Symbol;Acc:HGNC:25987] |
| ENSG00000163319 | HGLibA_30057 | 26 | -0.889874986 | MRPS18C | mitochondrial ribosomal protein S18C [Source:HGNC Symbol;Acc:HGNC:16633] |
| ENSG00000163516 | HGLibA_02164 | 26 | -0.889874986 | ANKZF1 | ankyrin repeat and zinc finger domain containing 1 [Source:HGNC Symbol;Acc:HGNC:25527] |
| ENSG00000163631 | HGLibA_01530 | 26 | -0.889874986 | ALB | albumin [Source:HGNC Symbol;Acc:HGNC:399] |
| ENSG00000163702 | HGLibA_23011 | 26 | -0.889874986 | IL17RC | interleukin 17 receptor C [Source:HGNC Symbol;Acc:HGNC:18358] |
| ENSG00000163762 | HGLibA_49695 | 26 | -0.889874986 | TM4SF18 | transmembrane 4 L six family member 18 [Source:HGNC Symbol;Acc:HGNC:25181] |
| ENSG00000163794 | HGLibA_52821 | 26 | -0.889874986 | UCN | urocortin [Source:HGNC Symbol;Acc:HGNC:12516] |
| ENSG00000163914 | HGLibA_41163 | 26 | -0.889874986 | RHO | rhodopsin [Source:HGNC Symbol;Acc:HGNC:10012] |
| ENSG00000164124 | HGLibA_49969 | 26 | -0.889874986 | TMEM144 | transmembrane protein 144 [Source:HGNC Symbol;Acc:HGNC:25633] |
| ENSG00000164296 | HGLibA_49478 | 26 | -0.889874986 | TIGD6 | tigger transposable element derived 6 [Source:HGNC Symbol;Acc:HGNC:18332] |
| ENSG00000164347 | HGLibA_18949 | 26 | -0.889874986 | GFM2 | G elongation factor mitochondrial 2 [Source:HGNC Symbol;Acc:HGNC:29682] |
| ENSG00000164466 | HGLibA_43781 | 26 | -0.889874986 | SFXN1 | sideroflexin 1 [Source:HGNC Symbol;Acc:HGNC:16085] |
| ENSG00000164532 | HGLibA_48659 | 26 | -0.889874986 | TBX20 | T-box 20 [Source:HGNC Symbol;Acc:HGNC:11598] |
| ENSG00000164713 | HGLibA_04802 | 26 | -0.889874986 | BRI3 | brain protein I3 [Source:HGNC Symbol;Acc:HGNC:1109] |
| ENSG00000166037 | HGLibA_09146 | 26 | -0.889874986 | CEP57 | centrosomal protein 57 [Source:HGNC Symbol;Acc:HGNC:30794] |
| ENSG00000166106 | HGLibA_00805 | 26 | -0.889874986 | ADAMTS15 | ADAM metallopeptidase with thrombospondin type 1 motif 15 [Source:HGNC Symbol;Acc:HGNC:16305] |
| ENSG00000166575 | HGLibA_49949 | 26 | -0.889874986 | TMEM135 | transmembrane protein 135 [Source:HGNC Symbol;Acc:HGNC:26167] |
| ENSG00000166689 | HGLibA_37081 | 26 | -0.889874986 | PLEKHA7 | pleckstrin homology domain containing A7 [Source:HGNC Symbol;Acc:HGNC:27049] |
| ENSG00000166747 | HGLibA_02284 | 26 | -0.889874986 | AP1G1 | adaptor related protein complex 1 subunit gamma 1 [Source:HGNC Symbol;Acc:HGNC:555] |
| ENSG00000166747 | HGLibA_02285 | 26 | -0.889874986 | AP1G1 | adaptor related protein complex 1 subunit gamma 1 [Source:HGNC Symbol;Acc:HGNC:555] |
| ENSG00000166847 | HGLibA_12556 | 26 | -0.889874986 | DCTN5 | dynactin subunit 5 [Source:HGNC Symbol;Acc:HGNC:24594] |
| ENSG00000166938 | HGLibA_13275 | 26 | -0.889874986 | DIS3L | DIS3 like exosome 3'-5' exoribonuclease [Source:HGNC Symbol;Acc:HGNC:28698] |
| ENSG00000167098 | HGLibA_47798 | 26 | -0.889874986 | SUN5 | Sad1 and UNC84 domain containing 5 [Source:HGNC Symbol;Acc:HGNC:16252] |
| ENSG00000167182 | HGLibA_46298 | 26 | -0.889874986 | SP2 | Sp2 transcription factor [Source:HGNC Symbol;Acc:HGNC:11207] |
| ENSG00000167323 | HGLibA_47440 | 26 | -0.889874986 | STIM1 | stromal interaction molecule 1 [Source:HGNC Symbol;Acc:HGNC:11386] |
| ENSG00000167615 | HGLibA_26300 | 26 | -0.889874986 | LENG8 | leukocyte receptor cluster member 8 [Source:HGNC Symbol;Acc:HGNC:15500] |
| ENSG00000167792 | HGLibA_31570 | 26 | -0.889874986 | NDUFV1 | NADH:ubiquinone oxidoreductase core subunit V1 [Source:HGNC Symbol;Acc:HGNC:7716] |
| ENSG00000168066 | HGLibA_43689 | 26 | -0.889874986 | SF1 | splicing factor 1 [Source:HGNC Symbol;Acc:HGNC:12950] |
| ENSG00000169621 | HGLibA_02432 | 26 | -0.889874986 | APLF | aprataxin and PNKP like factor [Source:HGNC Symbol;Acc:HGNC:28724] |
| ENSG00000170074 | HGLibA_16169 | 26 | -0.889874986 | FAM153A | family with sequence similarity 153 member A [Source:HGNC Symbol;Acc:HGNC:29940] |
| ENSG00000170100 | HGLibA_56728 | 26 | -0.889874986 | ZNF778 | zinc finger protein 778 [Source:HGNC Symbol;Acc:HGNC:26479] |
| ENSG00000170367 | HGLibA_11460 | 26 | -0.889874986 | CST5 | cystatin D [Source:HGNC Symbol;Acc:HGNC:2477] |
| ENSG00000171992 | HGLibA_48017 | 26 | -0.889874986 | SYNPO | synaptopodin [Source:HGNC Symbol;Acc:HGNC:30672] |
| ENSG00000172457 | HGLibA_34572 | 26 | -0.889874986 | OR9G4 | olfactory receptor family 9 subfamily G member 4 [Source:HGNC Symbol;Acc:HGNC:15322] |
| ENSG00000172568 | HGLibA_17763 | 26 | -0.889874986 | FNDC9 | fibronectin type III domain containing 9 [Source:HGNC Symbol;Acc:HGNC:33547] |
| ENSG00000172575 | HGLibA_40344 | 26 | -0.889874986 | RASGRP1 | RAS guanyl releasing protein 1 [Source:HGNC Symbol;Acc:HGNC:9878] |
| ENSG00000172922 | HGLibA_41434 | 26 | -0.889874986 | RNASEH2C | ribonuclease H2 subunit C [Source:HGNC Symbol;Acc:HGNC:24116] |
| ENSG00000174013 | HGLibA_17108 | 26 | -0.889874986 | FBXO45 | F-box protein 45 [Source:HGNC Symbol;Acc:HGNC:29148] |
| ENSG00000175110 | HGLibA_30064 | 26 | -0.889874986 | MRPS22 | mitochondrial ribosomal protein S22 [Source:HGNC Symbol;Acc:HGNC:14508] |
| ENSG00000175311 | HGLibA_02160 | 26 | -0.889874986 | ANKS4B | ankyrin repeat and sterile alpha motif domain containing 4B [Source:HGNC Symbol;Acc:HGNC:26795] |
| ENSG00000175544 | HGLibA_06854 | 26 | -0.889874986 | CABP4 | calcium binding protein 4 [Source:HGNC Symbol;Acc:HGNC:1386] |
| ENSG00000175575 | HGLibA_34917 | 26 | -0.889874986 | PAAF1 | proteasomal ATPase associated factor 1 [Source:HGNC Symbol;Acc:HGNC:25687] |
| ENSG00000176219 | HGLibA_33582 | 26 | -0.889874986 | OR11H6 | olfactory receptor family 11 subfamily H member 6 [Source:HGNC Symbol;Acc:HGNC:15349] |
| ENSG00000176986 | HGLibA_43236 | 26 | -0.889874986 | SEC24C | COPII coat complex component [Source:HGNC Symbol;Acc:HGNC:10705]" |
| ENSG00000177693 | HGLibA_34007 | 26 | -0.889874986 | OR4F4 | olfactory receptor family 4 subfamily F member 4 [Source:HGNC Symbol;Acc:HGNC:8301] |
| ENSG00000178104 | HGLibA_35799 | 26 | -0.889874986 | PDE4DIP | phosphodiesterase 4D interacting protein [Source:HGNC Symbol;Acc:HGNC:15580] |
| ENSG00000178175 | HGLibA_55880 | 26 | -0.889874986 | ZNF366 | zinc finger protein 366 [Source:HGNC Symbol;Acc:HGNC:18316] |
| ENSG00000178821 | HGLibA_50432 | 26 | -0.889874986 | TMEM52 | transmembrane protein 52 [Source:HGNC Symbol;Acc:HGNC:27916] |
| ENSG00000180190 | HGLibA_48924 | 26 | -0.889874986 | TDRP | testis development related protein [Source:HGNC Symbol;Acc:HGNC:26951] |
| ENSG00000180776 | HGLibA_55100 | 26 | -0.889874986 | ZDHHC20 | zinc finger DHHC-type containing 20 [Source:HGNC Symbol;Acc:HGNC:20749] |

| | | | | | |
|---|---|---|---|---|---|
| ENSG00000182687 | HGLibA_18590 | 26 | -0.889874986 | GALR2 | galanin receptor 2 [Source:HGNC Symbol;Acc:HGNC:4133] |
| ENSG00000182742 | HGLibA_21939 | 26 | -0.889874986 | HOXB4 | homeobox B4 [Source:HGNC Symbol;Acc:HGNC:5115] |
| ENSG00000183111 | HGLibA_02855 | 26 | -0.889874986 | ARHGEF37 | Rho guanine nucleotide exchange factor 37 [Source:HGNC Symbol;Acc:HGNC:34430] |
| ENSG00000183734 | HGLibA_03270 | 26 | -0.889874986 | ASCL2 | achaete-scute family bHLH transcription factor 2 [Source:HGNC Symbol;Acc:HGNC:739] |
| ENSG00000183844 | HGLibA_16579 | 26 | -0.889874986 | FAM3B | family with sequence similarity 3 member B [Source:HGNC Symbol;Acc:HGNC:1253] |
| ENSG00000183908 | HGLibA_27388 | 26 | -0.889874986 | LRRC55 | leucine rich repeat containing 55 [Source:HGNC Symbol;Acc:HGNC:32324] |
| ENSG00000184672 | HGLibA_40176 | 26 | -0.889874986 | RALYL | RALY RNA binding protein like [Source:HGNC Symbol;Acc:HGNC:27036] |
| ENSG00000185052 | HGLibA_44620 | 26 | -0.889874986 | SLC24A3 | solute carrier family 24 member 3 [Source:HGNC Symbol;Acc:HGNC:10977] |
| ENSG00000185238 | HGLibA_38627 | 26 | -0.889874986 | PRMT3 | protein arginine methyltransferase 3 [Source:HGNC Symbol;Acc:HGNC:30163] |
| ENSG00000185361 | HGLibA_50722 | 26 | -0.889874986 | TNFAIP8L1 | TNF alpha induced protein 8 like 1 [Source:HGNC Symbol;Acc:HGNC:28279] |
| ENSG00000186198 | HGLibA_45240 | 26 | -0.889874986 | SLC51B | solute carrier family 51 beta subunit [Source:HGNC Symbol;Acc:HGNC:29956] |
| ENSG00000186532 | HGLibA_45859 | 26 | -0.889874986 | SMYD4 | SET and MYND domain containing 4 [Source:HGNC Symbol;Acc:HGNC:21067] |
| ENSG00000187024 | HGLibA_39550 | 26 | -0.889874986 | PTRH1 | peptidyl-tRNA hydrolase 1 homolog [Source:HGNC Symbol;Acc:HGNC:27039] |
| ENSG00000187048 | HGLibA_12173 | 26 | -0.889874986 | CYP4A11 | cytochrome P450 family 4 subfamily A member 11 [Source:HGNC Symbol;Acc:HGNC:2642] |
| ENSG00000187553 | HGLibA_12090 | 26 | -0.889874986 | CYP26C1 | cytochrome P450 family 26 subfamily C member 1 [Source:HGNC Symbol;Acc:HGNC:20577] |
| ENSG00000187713 | HGLibA_50186 | 26 | -0.889874986 | TMEM203 | transmembrane protein 203 [Source:HGNC Symbol;Acc:HGNC:28217] |
| ENSG00000187714 | HGLibA_44492 | 26 | -0.889874986 | SLC18A3 | solute carrier family 18 member A3 [Source:HGNC Symbol;Acc:HGNC:10936] |
| ENSG00000187733 | HGLibA_01848 | 26 | -0.889874986 | AMY1C | alpha 1C (salivary) [Source:HGNC Symbol;Acc:HGNC:476]" |
| ENSG00000188176 | HGLibA_45837 | 26 | -0.889874986 | SMTNL2 | smoothelin like 2 [Source:HGNC Symbol;Acc:HGNC:24764] |
| ENSG00000188786 | HGLibA_30348 | 26 | -0.889874986 | MTF1 | metal regulatory transcription factor 1 [Source:HGNC Symbol;Acc:HGNC:7428] |
| ENSG00000188803 | HGLibA_44044 | 26 | -0.889874986 | SHISA6 | shisa family member 6 [Source:HGNC Symbol;Acc:HGNC:34491] |
| ENSG00000189043 | HGLibA_31458 | 26 | -0.889874986 | NDUFA4 | mitochondrial complex associated [Source:HGNC Symbol;Acc:HGNC:7687]" |
| ENSG00000189320 | HGLibA_16311 | 26 | -0.889874986 | FAM180A | family with sequence similarity 180 member A [Source:HGNC Symbol;Acc:HGNC:33773] |
| ENSG00000189339 | HGLibA_44992 | 26 | -0.889874986 | SLC35E2B | solute carrier family 35 member E2B [Source:HGNC Symbol;Acc:HGNC:33941] |
| ENSG00000196109 | HGLibA_56515 | 26 | -0.889874986 | ZNF676 | zinc finger protein 676 [Source:HGNC Symbol;Acc:HGNC:20429] |
| ENSG00000196475 | HGLibA_19198 | 26 | -0.889874986 | GK2 | glycerol kinase 2 [Source:HGNC Symbol;Acc:HGNC:4291] |
| ENSG00000196503 | HGLibA_03002 | 26 | -0.889874986 | ARL9 | ADP ribosylation factor like GTPase 9 [Source:HGNC Symbol;Acc:HGNC:23592] |
| ENSG00000196834 | HGLibA_37742 | 26 | -0.889874986 | POTEI | POTE ankyrin domain family member I [Source:HGNC Symbol;Acc:HGNC:37093] |
| ENSG00000196981 | HGLibA_54132 | 26 | -0.889874986 | WDR5B | WD repeat domain 5B [Source:HGNC Symbol;Acc:HGNC:17826] |
| ENSG00000197405 | HGLibA_06354 | 26 | -0.889874986 | C5AR1 | complement C5a receptor 1 [Source:HGNC Symbol;Acc:HGNC:1338] |
| ENSG00000197786 | HGLibA_34260 | 26 | -0.889874986 | OR5B17 | olfactory receptor family 5 subfamily B member 17 [Source:HGNC Symbol;Acc:HGNC:15267] |
| ENSG00000197980 | HGLibA_26279 | 26 | -0.889874986 | LEKR1 | glutamate and lysine rich 1 [Source:HGNC Symbol;Acc:HGNC:33765]" |
| ENSG00000198246 | HGLibA_44845 | 26 | -0.889874986 | SLC29A3 | solute carrier family 29 member 3 [Source:HGNC Symbol;Acc:HGNC:23096] |
| ENSG00000198625 | HGLibA_28701 | 26 | -0.889874986 | MDM4 | p53 regulator [Source:HGNC Symbol;Acc:HGNC:6974]" |
| ENSG00000198736 | HGLibA_30241 | 26 | -0.889874986 | MSRB1 | methionine sulfoxide reductase B1 [Source:HGNC Symbol;Acc:HGNC:14133] |
| ENSG00000198783 | HGLibA_56833 | 26 | -0.889874986 | ZNF830 | zinc finger protein 830 [Source:HGNC Symbol;Acc:HGNC:28291] |
| ENSG00000203872 | HGLibA_06454 | 26 | -0.889874986 | C6orf163 | chromosome 6 open reading frame 163 [Source:HGNC Symbol;Acc:HGNC:21403] |
| ENSG00000203972 | HGLibA_19379 | 26 | -0.889874986 | GLYATL3 | glycine-N-acyltransferase like 3 [Source:HGNC Symbol;Acc:HGNC:21349] |
| ENSG00000204237 | HGLibA_34824 | 26 | -0.889874986 | OXLD1 | oxidoreductase like domain containing 1 [Source:HGNC Symbol;Acc:HGNC:27901] |
| ENSG00000204657 | HGLibA_33806 | 26 | -0.889874986 | OR2H2 | olfactory receptor family 2 subfamily H member 2 [Source:HGNC Symbol;Acc:HGNC:8253] |
| ENSG00000206013 | HGLibA_22650 | 26 | -0.889874986 | IFITM5 | interferon induced transmembrane protein 5 [Source:HGNC Symbol;Acc:HGNC:16644] |
| ENSG00000214102 | HGLibA_54240 | 26 | -0.889874986 | WEE2 | WEE1 homolog 2 [Source:HGNC Symbol;Acc:HGNC:19684] |
| ENSG00000214652 | HGLibA_56634 | 26 | -0.889874986 | ZNF727 | zinc finger protein 727 [Source:HGNC Symbol;Acc:HGNC:22785] |
| ENSG00000222046 | HGLibA_12486 | 26 | -0.889874986 | DCDC2B | doublecortin domain containing 2B [Source:HGNC Symbol;Acc:HGNC:32576] |
| ENSG00000228716 | HGLibA_13104 | 26 | -0.889874986 | DHFR | dihydrofolate reductase [Source:HGNC Symbol;Acc:HGNC:2861] |
| ENSG00000234545 | HGLibA_16119 | 26 | -0.889874986 | FAM133B | family with sequence similarity 133 member B [Source:HGNC Symbol;Acc:HGNC:28629] |
| ENSG00000237412 | HGLibA_38966 | 26 | -0.889874986 | PRSS56 | serine protease 56 [Source:HGNC Symbol;Acc:HGNC:39433] |
| ENSG00000239382 | HGLibA_01660 | 26 | -0.889874986 | ALKBH6 | alkB homolog 6 [Source:HGNC Symbol;Acc:HGNC:28243] |
| ENSG00000242220 | HGLibA_48835 | 26 | -0.889874986 | TCP10L | t-complex 10 like [Source:HGNC Symbol;Acc:HGNC:11657] |
| ENSG00000243207 | HGLibA_37813 | 26 | -0.889874986 | PPAN-P2RY11 | PPAN-P2RY11 readthrough [Source:HGNC Symbol;Acc:HGNC:33526] |
| ENSG00000243509 | HGLibA_50787 | 26 | -0.889874986 | TNFRSF6B | TNF receptor superfamily member 6b [Source:HGNC Symbol;Acc:HGNC:11921] |
| ENSG00000255012 | HGLibA_34329 | 26 | -0.889874986 | OR5M1 | olfactory receptor family 5 subfamily M member 1 [Source:HGNC Symbol;Acc:HGNC:8352] |
| ENSG00000259224 | HGLibA_45027 | 26 | -0.889874986 | SLC35G6 | solute carrier family 35 member G6 [Source:HGNC Symbol;Acc:HGNC:31351] |
| ENSG00000261652 | HGLibA_05435 | 26 | -0.889874986 | C15orf65 | chromosome 15 open reading frame 65 [Source:HGNC Symbol;Acc:HGNC:44654] |
| ENSG00000261787 | HGLibA_48779 | 26 | -0.889874986 | TCF24 | transcription factor 24 [Source:HGNC Symbol;Acc:HGNC:32275] |
| ENSG00000264230 | HGLibA_02255 | 26 | -0.889874986 | ANXA8L1 | annexin A8 like 1 [Source:HGNC Symbol;Acc:HGNC:23334] |
| ENSG00000269502 | HGLibA_13436 | 26 | -0.889874986 | DMRTC1 | DMRT like family C1 [Source:HGNC Symbol;Acc:HGNC:13910] |
| ENSG00000270647 | HGLibA_48209 | 26 | -0.889874986 | TAF15 | TATA-box binding protein associated factor 15 [Source:HGNC Symbol;Acc:HGNC:11547] |
| ENSG00000284680 | HGLibA_34495 | 26 | -0.889874986 | OR8B2 | olfactory receptor family 8 subfamily B member 2 [Source:HGNC Symbol;Acc:HGNC:8471] |
| FTSJD1 | HGLibA_18144 | 26 | -0.889874986 | FTSJD1 | - |
| H2AFY2 | HGLibA_20766 | 26 | -0.889874986 | H2AFY2 | - |
| HIST1H3J | HGLibA_21484 | 26 | -0.889874986 | HIST1H3J | - |
| HIST1H4K | HGLibA_21515 | 26 | -0.889874986 | HIST1H4K | - |
| hsa-let-7a-1 | HGLibA_57098 | 26 | -0.889874986 | hsa-let-7a-1 | - |
| hsa-let-7g | HGLibA_57132 | 26 | -0.889874986 | hsa-let-7g | - |
| hsa-mir-1256 | HGLibA_57433 | 26 | -0.889874986 | hsa-mir-1256 | - |
| hsa-mir-1257 | HGLibA_57437 | 26 | -0.889874986 | hsa-mir-1257 | - |
| hsa-mir-1297 | HGLibA_57667 | 26 | -0.889874986 | hsa-mir-1297 | - |
| hsa-mir-1302-7 | HGLibA_57717 | 26 | -0.889874986 | hsa-mir-1302-7 | - |
| hsa-mir-135a-2 | HGLibA_57796 | 26 | -0.889874986 | hsa-mir-135a-2 | - |
| hsa-mir-146b | HGLibA_57862 | 26 | -0.889874986 | hsa-mir-146b | - |
| hsa-mir-203a | HGLibA_58166 | 26 | -0.889874986 | hsa-mir-203a | - |
| hsa-mir-2054 | HGLibA_58187 | 26 | -0.889874986 | hsa-mir-2054 | - |
| hsa-mir-2054 | HGLibA_58188 | 26 | -0.889874986 | hsa-mir-2054 | - |
| hsa-mir-296 | HGLibA_58396 | 26 | -0.889874986 | hsa-mir-296 | - |
| hsa-mir-3118-4 | HGLibA_58518 | 26 | -0.889874986 | hsa-mir-3118-4 | - |
| hsa-mir-3118-6 | HGLibA_58522 | 26 | -0.889874986 | hsa-mir-3118-6 | - |
| hsa-mir-3150b | HGLibA_58660 | 26 | -0.889874986 | hsa-mir-3150b | - |
| hsa-mir-3176 | HGLibA_58777 | 26 | -0.889874986 | hsa-mir-3176 | - |
| hsa-mir-320b-2 | HGLibA_58933 | 26 | -0.889874986 | hsa-mir-320b-2 | - |
| hsa-mir-320c-1 | HGLibA_58937 | 26 | -0.889874986 | hsa-mir-320c-1 | - |
| hsa-mir-3611 | HGLibA_59075 | 26 | -0.889874986 | hsa-mir-3611 | - |
| hsa-mir-378a | HGLibA_59427 | 26 | -0.889874986 | hsa-mir-378a | - |
| hsa-mir-4432 | HGLibA_60116 | 26 | -0.889874986 | hsa-mir-4432 | - |
| hsa-mir-4528 | HGLibA_60572 | 26 | -0.889874986 | hsa-mir-4528 | - |
| hsa-mir-4532 | HGLibA_60587 | 26 | -0.889874986 | hsa-mir-4532 | - |
| hsa-mir-499b | HGLibA_61405 | 26 | -0.889874986 | hsa-mir-499b | - |
| hsa-mir-545 | HGLibA_61833 | 26 | -0.889874986 | hsa-mir-545 | - |
| hsa-mir-569 | HGLibA_62277 | 26 | -0.889874986 | hsa-mir-569 | - |
| hsa-mir-6077-2 | HGLibA_62565 | 26 | -0.889874986 | hsa-mir-6077-2 | - |
| hsa-mir-6133 | HGLibA_62685 | 26 | -0.889874986 | hsa-mir-6133 | - |
| hsa-mir-616 | HGLibA_62698 | 26 | -0.889874986 | hsa-mir-616 | - |
| hsa-mir-642b | HGLibA_62811 | 26 | -0.889874986 | hsa-mir-642b | - |
| hsa-mir-6499 | HGLibA_62843 | 26 | -0.889874986 | hsa-mir-6499 | - |
| hsa-mir-6743 | HGLibA_63127 | 26 | -0.889874986 | hsa-mir-6743 | - |
| hsa-mir-6760 | HGLibA_63203 | 26 | -0.889874986 | hsa-mir-6760 | - |
| hsa-mir-6829 | HGLibA_63495 | 26 | -0.889874986 | hsa-mir-6829 | - |
| hsa-mir-6877 | HGLibA_63691 | 26 | -0.889874986 | hsa-mir-6877 | - |
| hsa-mir-8052 | HGLibA_64046 | 26 | -0.889874986 | hsa-mir-8052 | - |
| KIAA1199 | HGLibA_24710 | 26 | -0.889874986 | KIAA1199 | - |
| LOC643802 | HGLibA_26973 | 26 | -0.889874986 | LOC643802 | - |
| MRP63 | HGLibA_29886 | 26 | -0.889874986 | MRP63 | - |
| NDNL2 | HGLibA_31403 | 26 | -0.889874986 | NDNL2 | - |
| PTPLA | HGLibA_39414 | 26 | -0.889874986 | PTPLA | - |
| PTPLB | HGLibA_39425 | 26 | -0.889874986 | PTPLB | - |
| PVRL3 | HGLibA_39627 | 26 | -0.889874986 | PVRL3 | - |
| SCAND3 | HGLibA_42855 | 26 | -0.889874986 | SCAND3 | - |
| TMEM183B | HGLibA_50107 | 26 | -0.889874986 | TMEM183B | - |
| XAGE1C | HGLibA_54472 | 26 | -0.889874986 | XAGE1C | - |
| ADC | HGLibA_00886 | 25 | -0.899826023 | ADC | - |
| ANKRD20A4 | HGLibA_02036 | 25 | -0.899826023 | ANKRD20A4 | - |
| CCDC114 | HGLibA_07550 | 25 | -0.899826023 | CCDC114 | - |
| CCDC58 | HGLibA_07805 | 25 | -0.899826023 | CCDC58 | - |
| CCRL1 | HGLibA_08174 | 25 | -0.899826023 | CCRL1 | - |
| ENSG00000007541 | HGLibA_36580 | 25 | -0.899826023 | PIGQ | phosphatidylinositol glycan anchor biosynthesis class Q [Source:HGNC Symbol;Acc:HGNC:14135] |
| ENSG00000010244 | HGLibA_55580 | 25 | -0.899826023 | ZNF207 | zinc finger protein 207 [Source:HGNC Symbol;Acc:HGNC:12998] |
| ENSG00000010803 | HGLibA_42956 | 25 | -0.899826023 | SCMH1 | Scm polycomb group protein homolog 1 [Source:HGNC Symbol;Acc:HGNC:19003] |
| ENSG00000012779 | HGLibA_01689 | 25 | -0.899826023 | ALOX5 | arachidonate 5-lipoxygenase [Source:HGNC Symbol;Acc:HGNC:435] |
| ENSG00000022267 | HGLibA_17479 | 25 | -0.899826023 | FHL1 | four and a half LIM domains 1 [Source:HGNC Symbol;Acc:HGNC:3702] |
| ENSG00000022355 | HGLibA_18355 | 25 | -0.899826023 | GABRA1 | gamma-aminobutyric acid type A receptor alpha1 subunit [Source:HGNC Symbol;Acc:HGNC:4075] |
| ENSG00000042980 | HGLibA_00764 | 25 | -0.899826023 | ADAM28 | ADAM metallopeptidase domain 28 [Source:HGNC Symbol;Acc:HGNC:206] |
| ENSG00000047648 | HGLibA_02786 | 25 | -0.899826023 | ARHGAP6 | Rho GTPase activating protein 6 [Source:HGNC Symbol;Acc:HGNC:676] |
| ENSG00000048991 | HGLibA_39772 | 25 | -0.899826023 | R3HDM1 | R3H domain containing 1 [Source:HGNC Symbol;Acc:HGNC:9757] |
| ENSG00000058799 | HGLibA_54670 | 25 | -0.899826023 | YIPF1 | Yip1 domain family member 1 [Source:HGNC Symbol;Acc:HGNC:25231] |
| ENSG00000060642 | HGLibA_36593 | 25 | -0.899826023 | PIGV | phosphatidylinositol glycan anchor biosynthesis class V [Source:HGNC Symbol;Acc:HGNC:26031] |
| ENSG00000064225 | HGLibA_47251 | 25 | -0.899826023 | ST3GAL6 | 3-sialyltransferase 6 [Source:HGNC Symbol;Acc:HGNC:18080]" |
| ENSG00000067704 | HGLibA_22475 | 25 | -0.899826023 | IARS2 | mitochondrial [Source:HGNC Symbol;Acc:HGNC:29685]" |
| ENSG00000070729 | HGLibA_10274 | 25 | -0.899826023 | CNGB1 | cyclic nucleotide gated channel beta 1 [Source:HGNC Symbol;Acc:HGNC:2151] |
| ENSG00000073331 | HGLibA_01701 | 25 | -0.899826023 | ALPK1 | alpha kinase 1 [Source:HGNC Symbol;Acc:HGNC:20917] |
| ENSG00000073598 | HGLibA_17758 | 25 | -0.899826023 | FNDC8 | fibronectin type III domain containing 8 [Source:HGNC Symbol;Acc:HGNC:25286] |
| ENSG00000075711 | HGLibA_13324 | 25 | -0.899826023 | DLG1 | discs large MAGUK scaffold protein 1 [Source:HGNC Symbol;Acc:HGNC:2900] |
| ENSG00000075826 | HGLibA_43243 | 25 | -0.899826023 | SEC31B | COPII coat complex component [Source:HGNC Symbol;Acc:HGNC:23197]" |
| ENSG00000075945 | HGLibA_24968 | 25 | -0.899826023 | KIFAP3 | kinesin associated protein 3 [Source:HGNC Symbol;Acc:HGNC:17060] |
| ENSG00000085265 | HGLibA_17216 | 25 | -0.899826023 | FCN1 | ficolin 1 [Source:HGNC Symbol;Acc:HGNC:3623] |
| ENSG00000092330 | HGLibA_49554 | 25 | -0.899826023 | TINF2 | TERF1 interacting nuclear factor 2 [Source:HGNC Symbol;Acc:HGNC:11824] |

| | | | | | |
|---|---|---|---|---|---|
| ENSG00000095627 | HGLibA_48895 | 25 | -0.899826023 | TDRD1 | tudor domain containing 1 [Source:HGNC Symbol;Acc:HGNC:11712] |
| ENSG00000099810 | HGLibA_30318 | 25 | -0.899826023 | MTAP | methylthioadenosine phosphorylase [Source:HGNC Symbol;Acc:HGNC:7413] |
| ENSG00000099812 | HGLibA_29313 | 25 | -0.899826023 | MISP | mitotic spindle positioning [Source:HGNC Symbol;Acc:HGNC:27000] |
| ENSG00000100490 | HGLibA_08806 | 25 | -0.899826023 | CDKL1 | cyclin dependent kinase like 1 [Source:HGNC Symbol;Acc:HGNC:1781] |
| ENSG00000101350 | HGLibA_24938 | 25 | -0.899826023 | KIF3B | kinesin family member 3B [Source:HGNC Symbol;Acc:HGNC:6320] |
| ENSG00000101695 | HGLibA_41492 | 25 | -0.899826023 | RNF125 | ring finger protein 125 [Source:HGNC Symbol;Acc:HGNC:21150] |
| ENSG00000101782 | HGLibA_41314 | 25 | -0.899826023 | RIOK3 | RIO kinase 3 [Source:HGNC Symbol;Acc:HGNC:11451] |
| ENSG00000101883 | HGLibA_41213 | 25 | -0.899826023 | RHOXF1 | Rhox homeobox family member 1 [Source:HGNC Symbol;Acc:HGNC:29993] |
| ENSG00000101928 | HGLibA_29684 | 25 | -0.899826023 | MOSPD1 | motile sperm domain containing 1 [Source:HGNC Symbol;Acc:HGNC:25235] |
| ENSG00000102053 | HGLibA_54956 | 25 | -0.899826023 | ZC3H12B | zinc finger CCCH-type containing 12B [Source:HGNC Symbol;Acc:HGNC:17407] |
| ENSG00000102385 | HGLibA_13986 | 25 | -0.899826023 | DRP2 | dystrophin related protein 2 [Source:HGNC Symbol;Acc:HGNC:3032] |
| ENSG00000103154 | HGLibA_31588 | 25 | -0.899826023 | NECAB2 | N-terminal EF-hand calcium binding protein 2 [Source:HGNC Symbol;Acc:HGNC:23746] |
| ENSG00000104047 | HGLibA_14074 | 25 | -0.899826023 | DTWD1 | DTW domain containing 1 [Source:HGNC Symbol;Acc:HGNC:30926] |
| ENSG00000104205 | HGLibA_43832 | 25 | -0.899826023 | SGK3 | serum/glucocorticoid regulated kinase family member 3 [Source:HGNC Symbol;Acc:HGNC:10812] |
| ENSG00000104613 | HGLibA_23409 | 25 | -0.899826023 | INTS10 | integrator complex subunit 10 [Source:HGNC Symbol;Acc:HGNC:25548] |
| ENSG00000105063 | HGLibA_38220 | 25 | -0.899826023 | PPP6R1 | protein phosphatase 6 regulatory subunit 1 [Source:HGNC Symbol;Acc:HGNC:29195] |
| ENSG00000105669 | HGLibA_10702 | 25 | -0.899826023 | COPE | coatomer protein complex subunit epsilon [Source:HGNC Symbol;Acc:HGNC:2234] |
| ENSG00000106080 | HGLibA_17559 | 25 | -0.899826023 | FKBP14 | FK506 binding protein 14 [Source:HGNC Symbol;Acc:HGNC:18625] |
| ENSG00000106780 | HGLibA_28858 | 25 | -0.899826023 | MEGF9 | multiple EGF like domains 9 [Source:HGNC Symbol;Acc:HGNC:3234] |
| ENSG00000107951 | HGLibA_30456 | 25 | -0.899826023 | MTPAP | mitochondrial poly(A) polymerase [Source:HGNC Symbol;Acc:HGNC:25532] |
| ENSG00000108588 | HGLibA_07789 | 25 | -0.899826023 | CCDC47 | coiled-coil domain containing 47 [Source:HGNC Symbol;Acc:HGNC:24856] |
| ENSG00000109436 | HGLibA_48572 | 25 | -0.899826023 | TBC1D9 | TBC1 domain family member 9 [Source:HGNC Symbol;Acc:HGNC:21710] |
| ENSG00000110435 | HGLibA_35870 | 25 | -0.899826023 | PDHX | pyruvate dehydrogenase complex component X [Source:HGNC Symbol;Acc:HGNC:21350] |
| ENSG00000111678 | HGLibA_05271 | 25 | -0.899826023 | C12orf57 | chromosome 12 open reading frame 57 [Source:HGNC Symbol;Acc:HGNC:29521] |
| ENSG00000112096 | HGLibA_46147 | 25 | -0.899826023 | SOD2 | superoxide dismutase 2 [Source:HGNC Symbol;Acc:HGNC:11180] |
| ENSG00000112667 | HGLibA_13713 | 25 | -0.899826023 | DNPH1 | 2'-deoxynucleoside 5'-phosphate N-hydrolase 1 [Source:HGNC Symbol;Acc:HGNC:21218] |
| ENSG00000114030 | HGLibA_25354 | 25 | -0.899826023 | KPNA1 | karyopherin subunit alpha 1 [Source:HGNC Symbol;Acc:HGNC:6394] |
| ENSG00000114735 | HGLibA_21131 | 25 | -0.899826023 | HEMK1 | HemK methyltransferase family member 1 [Source:HGNC Symbol;Acc:HGNC:24923] |
| ENSG00000114982 | HGLibA_24044 | 25 | -0.899826023 | KANSL3 | KAT8 regulatory NSL complex subunit 3 [Source:HGNC Symbol;Acc:HGNC:25473] |
| ENSG00000115084 | HGLibA_45010 | 25 | -0.899826023 | SLC35F5 | solute carrier family 35 member F5 [Source:HGNC Symbol;Acc:HGNC:23617] |
| ENSG00000115137 | HGLibA_13610 | 25 | -0.899826023 | DNAJC27 | DnaJ heat shock protein family (Hsp40) member C27 [Source:HGNC Symbol;Acc:HGNC:30290] |
| ENSG00000115368 | HGLibA_54174 | 25 | -0.899826023 | WDR75 | WD repeat domain 75 [Source:HGNC Symbol;Acc:HGNC:25725] |
| ENSG00000115459 | HGLibA_14944 | 25 | -0.899826023 | ELMOD3 | ELMO domain containing 3 [Source:HGNC Symbol;Acc:HGNC:26158] |
| ENSG00000115648 | HGLibA_29433 | 25 | -0.899826023 | MLPH | melanophilin [Source:HGNC Symbol;Acc:HGNC:29643] |
| ENSG00000115657 | HGLibA_00146 | 25 | -0.899826023 | ABCB6 | ATP binding cassette subfamily B member 6 (Langereis blood group) [Source:HGNC Symbol;Acc:HGNC:47] |
| ENSG00000115825 | HGLibA_38572 | 25 | -0.899826023 | PRKD3 | protein kinase D3 [Source:HGNC Symbol;Acc:HGNC:9408] |
| ENSG00000116670 | HGLibA_27850 | 25 | -0.899826023 | MAD2L2 | mitotic arrest deficient 2 like 2 [Source:HGNC Symbol;Acc:HGNC:6764] |
| ENSG00000116690 | HGLibA_38458 | 25 | -0.899826023 | PRG4 | proteoglycan 4 [Source:HGNC Symbol;Acc:HGNC:9364] |
| ENSG00000116701 | HGLibA_31300 | 25 | -0.899826023 | NCF2 | neutrophil cytosolic factor 2 [Source:HGNC Symbol;Acc:HGNC:7661] |
| ENSG00000118193 | HGLibA_24861 | 25 | -0.899826023 | KIF14 | kinesin family member 14 [Source:HGNC Symbol;Acc:HGNC:19181] |
| ENSG00000119321 | HGLibA_17562 | 25 | -0.899826023 | FKBP15 | FK506 binding protein 15 [Source:HGNC Symbol;Acc:HGNC:23397] |
| ENSG00000119471 | HGLibA_22218 | 25 | -0.899826023 | HSDL2 | hydroxysteroid dehydrogenase like 2 [Source:HGNC Symbol;Acc:HGNC:18572] |
| ENSG00000119718 | HGLibA_14739 | 25 | -0.899826023 | EIF2B2 | eukaryotic translation initiation factor 2B subunit beta [Source:HGNC Symbol;Acc:HGNC:3258] |
| ENSG00000121905 | HGLibA_22017 | 25 | -0.899826023 | HPCA | hippocalcin [Source:HGNC Symbol;Acc:HGNC:5144] |
| ENSG00000122026 | HGLibA_41894 | 25 | -0.899826023 | RPL21 | ribosomal protein L21 [Source:HGNC Symbol;Acc:HGNC:10313] |
| ENSG00000122965 | HGLibA_40502 | 25 | -0.899826023 | RBM19 | RNA binding motif protein 19 [Source:HGNC Symbol;Acc:HGNC:29098] |
| ENSG00000123096 | HGLibA_47147 | 25 | -0.899826023 | SSPN | sarcospan [Source:HGNC Symbol;Acc:HGNC:11322] |
| ENSG00000123143 | HGLibA_36836 | 25 | -0.899826023 | PKN1 | protein kinase N1 [Source:HGNC Symbol;Acc:HGNC:9405] |
| ENSG00000123154 | HGLibA_54192 | 25 | -0.899826023 | WDR83 | WD repeat domain 83 [Source:HGNC Symbol;Acc:HGNC:32672] |
| ENSG00000123200 | HGLibA_54962 | 25 | -0.899826023 | ZC3H13 | zinc finger CCCH-type containing 13 [Source:HGNC Symbol;Acc:HGNC:20368] |
| ENSG00000123561 | HGLibA_43528 | 25 | -0.899826023 | SERPINA7 | serpin family A member 7 [Source:HGNC Symbol;Acc:HGNC:11583] |
| ENSG00000123892 | HGLibA_39897 | 25 | -0.899826023 | RAB38 | member RAS oncogene family [Source:HGNC Symbol;Acc:HGNC:9776]" |
| ENSG00000125285 | HGLibA_46256 | 25 | -0.899826023 | SOX21 | SRY-box 21 [Source:HGNC Symbol;Acc:HGNC:11197] |
| ENSG00000125482 | HGLibA_52169 | 25 | -0.899826023 | TTF1 | transcription termination factor 1 [Source:HGNC Symbol;Acc:HGNC:12397] |
| ENSG00000125826 | HGLibA_40450 | 25 | -0.899826023 | RBCK1 | RANBP2-type and C3HC4-type zinc finger containing 1 [Source:HGNC Symbol;Acc:HGNC:15864] |
| ENSG00000125995 | HGLibA_41758 | 25 | -0.899826023 | ROMO1 | reactive oxygen species modulator 1 [Source:HGNC Symbol;Acc:HGNC:16185] |
| ENSG00000126070 | HGLibA_01211 | 25 | -0.899826023 | AGO3 | RISC catalytic component [Source:HGNC Symbol;Acc:HGNC:18421]" |
| ENSG00000126581 | HGLibA_04394 | 25 | -0.899826023 | BECN1 | beclin 1 [Source:HGNC Symbol;Acc:HGNC:1034] |
| ENSG00000127452 | HGLibA_16973 | 25 | -0.899826023 | FBXL12 | F-box and leucine rich repeat protein 12 [Source:HGNC Symbol;Acc:HGNC:13611] |
| ENSG00000128040 | HGLibA_46692 | 25 | -0.899826023 | SPINK2 | Kazal type 2 [Source:HGNC Symbol;Acc:HGNC:11245]" |
| ENSG00000128683 | HGLibA_18411 | 25 | -0.899826023 | GAD1 | glutamate decarboxylase 1 [Source:HGNC Symbol;Acc:HGNC:4092] |
| ENSG00000128908 | HGLibA_23319 | 25 | -0.899826023 | INO80 | INO80 complex subunit [Source:HGNC Symbol;Acc:HGNC:26956] |
| ENSG00000130810 | HGLibA_37810 | 25 | -0.899826023 | PPAN | peter pan homolog [Source:HGNC Symbol;Acc:HGNC:9227] |
| ENSG00000132963 | HGLibA_37667 | 25 | -0.899826023 | POMP | proteasome maturation protein [Source:HGNC Symbol;Acc:HGNC:20330] |
| ENSG00000134308 | HGLibA_54751 | 25 | -0.899826023 | YWHAQ | tyrosine 3-monooxygenase/tryptophan 5-monooxygenase activation protein theta [Source:HGNC Symbol;Acc:HGNC:12854] |
| ENSG00000134905 | HGLibA_07262 | 25 | -0.899826023 | CARS2 | mitochondrial [Source:HGNC Symbol;Acc:HGNC:25695]" |
| ENSG00000135269 | HGLibA_49021 | 25 | -0.899826023 | TES | testin LIM domain protein [Source:HGNC Symbol;Acc:HGNC:14620] |
| ENSG00000135655 | HGLibA_53210 | 25 | -0.899826023 | USP15 | ubiquitin specific peptidase 15 [Source:HGNC Symbol;Acc:HGNC:12613] |
| ENSG00000135722 | HGLibA_17021 | 25 | -0.899826023 | FBXL8 | F-box and leucine rich repeat protein 8 [Source:HGNC Symbol;Acc:HGNC:17875] |
| ENSG00000135919 | HGLibA_43579 | 25 | -0.899826023 | SERPINE2 | serpin family E member 2 [Source:HGNC Symbol;Acc:HGNC:8951] |
| ENSG00000135925 | HGLibA_54347 | 25 | -0.899826023 | WNT10A | Wnt family member 10A [Source:HGNC Symbol;Acc:HGNC:13829] |
| ENSG00000136108 | HGLibA_09754 | 25 | -0.899826023 | CKAP2 | cytoskeleton associated protein 2 [Source:HGNC Symbol;Acc:HGNC:1990] |
| ENSG00000136108 | HGLibA_09753 | 25 | -0.899826023 | CKAP2 | cytoskeleton associated protein 2 [Source:HGNC Symbol;Acc:HGNC:1990] |
| ENSG00000136444 | HGLibA_42297 | 25 | -0.899826023 | RSAD1 | radical S-adenosyl methionine domain containing 1 [Source:HGNC Symbol;Acc:HGNC:25634] |
| ENSG00000136488 | HGLibA_11352 | 25 | -0.899826023 | CSH1 | chorionic somatomammotropin hormone 1 [Source:HGNC Symbol;Acc:HGNC:2440] |
| ENSG00000137409 | HGLibA_30324 | 25 | -0.899826023 | MTCH1 | mitochondrial carrier 1 [Source:HGNC Symbol;Acc:HGNC:17586] |
| ENSG00000137496 | HGLibA_23026 | 25 | -0.899826023 | IL18BP | interleukin 18 binding protein [Source:HGNC Symbol;Acc:HGNC:5987] |
| ENSG00000138496 | HGLibA_35246 | 25 | -0.899826023 | PARP9 | poly(ADP-ribose) polymerase family member 9 [Source:HGNC Symbol;Acc:HGNC:24118] |
| ENSG00000139780 | HGLibA_28981 | 25 | -0.899826023 | METTL21C | methyltransferase like 21C [Source:HGNC Symbol;Acc:HGNC:33717] |
| ENSG00000141524 | HGLibA_49764 | 25 | -0.899826023 | TMC6 | transmembrane channel like 6 [Source:HGNC Symbol;Acc:HGNC:18021] |
| ENSG00000143458 | HGLibA_18352 | 25 | -0.899826023 | GABPB2 | GA binding protein transcription factor subunit beta 2 [Source:HGNC Symbol;Acc:HGNC:28441] |
| ENSG00000143816 | HGLibA_54393 | 25 | -0.899826023 | WNT9A | Wnt family member 9A [Source:HGNC Symbol;Acc:HGNC:12778] |
| ENSG00000144791 | HGLibA_26531 | 25 | -0.899826023 | LIMD1 | LIM domains containing 1 [Source:HGNC Symbol;Acc:HGNC:6612] |
| ENSG00000144893 | HGLibA_28745 | 25 | -0.899826023 | MED12L | mediator complex subunit 12 like [Source:HGNC Symbol;Acc:HGNC:16050] |
| ENSG00000145723 | HGLibA_19097 | 25 | -0.899826023 | GIN1 | gypsy retrotransposon integrase 1 [Source:HGNC Symbol;Acc:HGNC:25959] |
| ENSG00000147099 | HGLibA_21024 | 25 | -0.899826023 | HDAC8 | histone deacetylase 8 [Source:HGNC Symbol;Acc:HGNC:13315] |
| ENSG00000147180 | HGLibA_56615 | 25 | -0.899826023 | ZNF711 | zinc finger protein 711 [Source:HGNC Symbol;Acc:HGNC:13128] |
| ENSG00000147262 | HGLibA_19866 | 25 | -0.899826023 | GPR119 | G protein-coupled receptor 119 [Source:HGNC Symbol;Acc:HGNC:19060] |
| ENSG00000147885 | HGLibA_22669 | 25 | -0.899826023 | IFNA16 | interferon alpha 16 [Source:HGNC Symbol;Acc:HGNC:5421] |
| ENSG00000147905 | HGLibA_55046 | 25 | -0.899826023 | ZCCHC7 | zinc finger CCHC-type containing 7 [Source:HGNC Symbol;Acc:HGNC:26209] |
| ENSG00000148426 | HGLibA_38698 | 25 | -0.899826023 | PROSER2 | proline and serine rich 2 [Source:HGNC Symbol;Acc:HGNC:23728] |
| ENSG00000148513 | HGLibA_02061 | 25 | -0.899826023 | ANKRD30A | ankyrin repeat domain 30A [Source:HGNC Symbol;Acc:HGNC:17234] |
| ENSG00000148513 | HGLibA_02059 | 25 | -0.899826023 | ANKRD30A | ankyrin repeat domain 30A [Source:HGNC Symbol;Acc:HGNC:17234] |
| ENSG00000148660 | HGLibA_07076 | 25 | -0.899826023 | CAMK2G | calcium/calmodulin dependent protein kinase II gamma [Source:HGNC Symbol;Acc:HGNC:1463] |
| ENSG00000149021 | HGLibA_42907 | 25 | -0.899826023 | SCGB1A1 | secretoglobin family 1A member 1 [Source:HGNC Symbol;Acc:HGNC:12523] |
| ENSG00000149547 | HGLibA_14689 | 25 | -0.899826023 | EI24 | autophagy associated transmembrane protein [Source:HGNC Symbol;Acc:HGNC:13276]" |
| ENSG00000151474 | HGLibA_18015 | 25 | -0.899826023 | FRMD4A | FERM domain containing 4A [Source:HGNC Symbol;Acc:HGNC:25491] |
| ENSG00000151876 | HGLibA_17089 | 25 | -0.899826023 | FBXO4 | F-box protein 4 [Source:HGNC Symbol;Acc:HGNC:13583] |
| ENSG00000152315 | HGLibA_24303 | 25 | -0.899826023 | KCNK13 | potassium two pore domain channel subfamily K member 13 [Source:HGNC Symbol;Acc:HGNC:6275] |
| ENSG00000152779 | HGLibA_44426 | 25 | -0.899826023 | SLC16A12 | solute carrier family 16 member 12 [Source:HGNC Symbol;Acc:HGNC:23094] |
| ENSG00000153930 | HGLibA_01954 | 25 | -0.899826023 | ANKFN1 | ankyrin repeat and fibronectin type III domain containing 1 [Source:HGNC Symbol;Acc:HGNC:26766] |
| ENSG00000155269 | HGLibA_20085 | 25 | -0.899826023 | GPR78 | G protein-coupled receptor 78 [Source:HGNC Symbol;Acc:HGNC:4528] |
| ENSG00000155622 | HGLibA_54481 | 25 | -0.899826023 | XAGE2 | X antigen family member 2 [Source:HGNC Symbol;Acc:HGNC:4112] |
| ENSG00000155636 | HGLibA_40559 | 25 | -0.899826023 | RBM45 | RNA binding motif protein 45 [Source:HGNC Symbol;Acc:HGNC:24468] |
| ENSG00000156050 | HGLibA_16220 | 25 | -0.899826023 | FAM161B | centrosomal protein [Source:HGNC Symbol;Acc:HGNC:19854]" |
| ENSG00000156482 | HGLibA_41935 | 25 | -0.899826023 | RPL30 | ribosomal protein L30 [Source:HGNC Symbol;Acc:HGNC:10333] |
| ENSG00000156711 | HGLibA_28258 | 25 | -0.899826023 | MAPK13 | mitogen-activated protein kinase 13 [Source:HGNC Symbol;Acc:HGNC:6875] |
| ENSG00000158411 | HGLibA_29315 | 25 | -0.899826023 | MITD1 | microtubule interacting and trafficking domain containing 1 [Source:HGNC Symbol;Acc:HGNC:25207] |
| ENSG00000159231 | HGLibA_07421 | 25 | -0.899826023 | CBR3 | carbonyl reductase 3 [Source:HGNC Symbol;Acc:HGNC:1549] |
| ENSG00000160213 | HGLibA_11481 | 25 | -0.899826023 | CSTB | cystatin B [Source:HGNC Symbol;Acc:HGNC:2482] |
| ENSG00000161849 | HGLibA_25566 | 25 | -0.899826023 | KRT84 | keratin 84 [Source:HGNC Symbol;Acc:HGNC:6461] |
| ENSG00000162438 | HGLibA_11683 | 25 | -0.899826023 | CTRC | chymotrypsin C [Source:HGNC Symbol;Acc:HGNC:2523] |
| ENSG00000162645 | HGLibA_18730 | 25 | -0.899826023 | GBP2 | guanylate binding protein 2 [Source:HGNC Symbol;Acc:HGNC:4183] |
| ENSG00000162736 | HGLibA_31377 | 25 | -0.899826023 | NCSTN | nicastrin [Source:HGNC Symbol;Acc:HGNC:17091] |
| ENSG00000162761 | HGLibA_26731 | 25 | -0.899826023 | LMX1A | LIM homeobox transcription factor 1 alpha [Source:HGNC Symbol;Acc:HGNC:6653] |
| ENSG00000162944 | HGLibA_40952 | 25 | -0.899826023 | RFTN2 | raftlin family member 2 [Source:HGNC Symbol;Acc:HGNC:26402] |
| ENSG00000163106 | HGLibA_22034 | 25 | -0.899826023 | HPGDS | hematopoietic prostaglandin D synthase [Source:HGNC Symbol;Acc:HGNC:17890] |
| ENSG00000163254 | HGLibA_11276 | 25 | -0.899826023 | CRYGC | crystallin gamma C [Source:HGNC Symbol;Acc:HGNC:2410] |
| ENSG00000163444 | HGLibA_50104 | 25 | -0.899826023 | TMEM183A | transmembrane protein 183A [Source:HGNC Symbol;Acc:HGNC:20173] |
| ENSG00000164283 | HGLibA_15570 | 25 | -0.899826023 | ESM1 | endothelial cell specific molecule 1 [Source:HGNC Symbol;Acc:HGNC:3466] |
| ENSG00000164342 | HGLibA_49650 | 25 | -0.899826023 | TLR3 | toll like receptor 3 [Source:HGNC Symbol;Acc:HGNC:11849] |
| ENSG00000164764 | HGLibA_42815 | 25 | -0.899826023 | SBSPON | somatomedin B and thrombospondin type 1 domain containing [Source:HGNC Symbol;Acc:HGNC:30362] |
| ENSG00000165264 | HGLibA_31523 | 25 | -0.899826023 | NDUFB6 | NADH:ubiquinone oxidoreductase subunit B6 [Source:HGNC Symbol;Acc:HGNC:7701] |
| ENSG00000165462 | HGLibA_36449 | 25 | -0.899826023 | PHOX2A | paired like homeobox 2A [Source:HGNC Symbol;Acc:HGNC:691] |
| ENSG00000165583 | HGLibA_47216 | 25 | -0.899826023 | SSX5 | SSX family member 5 [Source:HGNC Symbol;Acc:HGNC:11339] |
| ENSG00000165609 | HGLibA_32983 | 25 | -0.899826023 | NUDT5 | nudix hydrolase 5 [Source:HGNC Symbol;Acc:HGNC:8052] |
| ENSG00000166927 | HGLibA_30164 | 25 | -0.899826023 | MS4A7 | membrane spanning 4-domains A7 [Source:HGNC Symbol;Acc:HGNC:13378] |
| ENSG00000167528 | HGLibA_56444 | 25 | -0.899826023 | ZNF641 | zinc finger protein 641 [Source:HGNC Symbol;Acc:HGNC:31834] |
| ENSG00000167633 | HGLibA_24991 | 25 | -0.899826023 | KIR3DL1 | three Ig domains and long cytoplasmic tail 1 [Source:HGNC Symbol;Acc:HGNC:6338]" |
| ENSG00000167720 | HGLibA_47039 | 25 | -0.899826023 | SRR | serine racemase [Source:HGNC Symbol;Acc:HGNC:14398] |
| ENSG00000168014 | HGLibA_06093 | 25 | -0.899826023 | C2CD3 | C2 calcium dependent domain containing 3 [Source:HGNC Symbol;Acc:HGNC:24564] |
| ENSG00000168234 | HGLibA_52137 | 25 | -0.899826023 | TTC39C | tetratricopeptide repeat domain 39C [Source:HGNC Symbol;Acc:HGNC:26595] |
| ENSG00000168702 | HGLibA_27209 | 25 | -0.899826023 | LRP1B | LDL receptor related protein 1B [Source:HGNC Symbol;Acc:HGNC:6693] |
| ENSG00000169297 | HGLibA_32569 | 25 | -0.899826023 | NR0B1 | nuclear receptor subfamily 0 group B member 1 [Source:HGNC Symbol;Acc:HGNC:7960] |
| ENSG00000169439 | HGLibA_43099 | 25 | -0.899826023 | SDC2 | syndecan 2 [Source:HGNC Symbol;Acc:HGNC:10659] |

| | | | | | |
|---|---|---|---|---|---|
| ENSG00000169692 | HGLibA_01219 | 25 | -0.899826023 | AGPAT2 | 1-acylglycerol-3-phosphate O-acyltransferase 2 [Source:HGNC Symbol;Acc:HGNC:325] |
| ENSG00000169814 | HGLibA_04931 | 25 | -0.899826023 | BTD | biotinidase [Source:HGNC Symbol;Acc:HGNC:1122] |
| ENSG00000170260 | HGLibA_55589 | 25 | -0.899826023 | ZNF212 | zinc finger protein 212 [Source:HGNC Symbol;Acc:HGNC:13004] |
| ENSG00000170345 | HGLibA_17794 | 25 | -0.899826023 | FOS | AP-1 transcription factor subunit [Source:HGNC Symbol;Acc:HGNC:3796]" |
| ENSG00000171148 | HGLibA_48191 | 25 | -0.899826023 | TADA3 | transcriptional adaptor 3 [Source:HGNC Symbol;Acc:HGNC:19422] |
| ENSG00000171302 | HGLibA_07126 | 25 | -0.899826023 | CANT1 | calcium activated nucleotidase 1 [Source:HGNC Symbol;Acc:HGNC:19721] |
| ENSG00000171604 | HGLibA_11975 | 25 | -0.899826023 | CXXC5 | CXXC finger protein 5 [Source:HGNC Symbol;Acc:HGNC:26943] |
| ENSG00000172995 | HGLibA_03101 | 25 | -0.899826023 | ARPP21 | cAMP regulated phosphoprotein 21 [Source:HGNC Symbol;Acc:HGNC:16968] |
| ENSG00000173706 | HGLibA_21107 | 25 | -0.899826023 | HEG1 | heart development protein with EGF like domains 1 [Source:HGNC Symbol;Acc:HGNC:29227] |
| ENSG00000174586 | HGLibA_56105 | 25 | -0.899826023 | ZNF497 | zinc finger protein 497 [Source:HGNC Symbol;Acc:HGNC:23714] |
| ENSG00000174775 | HGLibA_22072 | 25 | -0.899826023 | HRAS | GTPase [Source:HGNC Symbol;Acc:HGNC:5173]" |
| ENSG00000175274 | HGLibA_51059 | 25 | -0.899826023 | TP53I11 | tumor protein p53 inducible protein 11 [Source:HGNC Symbol;Acc:HGNC:16842] |
| ENSG00000175567 | HGLibA_52834 | 25 | -0.899826023 | UCP2 | uncoupling protein 2 [Source:HGNC Symbol;Acc:HGNC:12518] |
| ENSG00000175787 | HGLibA_55518 | 25 | -0.899826023 | ZNF169 | zinc finger protein 169 [Source:HGNC Symbol;Acc:HGNC:12957] |
| ENSG00000176746 | HGLibA_27955 | 25 | -0.899826023 | MAGEB6 | MAGE family member B6 [Source:HGNC Symbol;Acc:HGNC:23796] |
| ENSG00000178460 | HGLibA_28634 | 25 | -0.899826023 | MCMDC2 | minichromosome maintenance domain containing 2 [Source:HGNC Symbol;Acc:HGNC:26368] |
| ENSG00000178764 | HGLibA_55316 | 25 | -0.899826023 | ZHX2 | zinc fingers and homeoboxes 2 [Source:HGNC Symbol;Acc:HGNC:18513] |
| ENSG00000178826 | HGLibA_49959 | 25 | -0.899826023 | TMEM139 | transmembrane protein 139 [Source:HGNC Symbol;Acc:HGNC:22058] |
| ENSG00000179168 | HGLibA_19000 | 25 | -0.899826023 | GGN | gametogenetin [Source:HGNC Symbol;Acc:HGNC:18869] |
| ENSG00000179915 | HGLibA_32733 | 25 | -0.899826023 | NRXN1 | neurexin 1 [Source:HGNC Symbol;Acc:HGNC:8008] |
| ENSG00000180900 | HGLibA_43046 | 25 | -0.899826023 | SCRIB | scribbled planar cell polarity protein [Source:HGNC Symbol;Acc:HGNC:30377] |
| ENSG00000181467 | HGLibA_40238 | 25 | -0.899826023 | RAP2B | member of RAS oncogene family [Source:HGNC Symbol;Acc:HGNC:9862]" |
| ENSG00000181924 | HGLibA_10449 | 25 | -0.899826023 | COA4 | cytochrome c oxidase assembly factor 4 homolog [Source:HGNC Symbol;Acc:HGNC:24604] |
| ENSG00000181924 | HGLibA_10450 | 25 | -0.899826023 | COA4 | cytochrome c oxidase assembly factor 4 homolog [Source:HGNC Symbol;Acc:HGNC:24604] |
| ENSG00000182195 | HGLibA_26255 | 25 | -0.899826023 | LDOC1 | regulator of NFKB signaling [Source:HGNC Symbol;Acc:HGNC:6548]" |
| ENSG00000182240 | HGLibA_04062 | 25 | -0.899826023 | BACE2 | beta-secretase 2 [Source:HGNC Symbol;Acc:HGNC:934] |
| ENSG00000182858 | HGLibA_01612 | 25 | -0.899826023 | ALG12 | 6-mannosyltransferase [Source:HGNC Symbol;Acc:HGNC:19358]" |
| ENSG00000182944 | HGLibA_15712 | 25 | -0.899826023 | EWSR1 | EWS RNA binding protein 1 [Source:HGNC Symbol;Acc:HGNC:3508] |
| ENSG00000183379 | HGLibA_47974 | 25 | -0.899826023 | SYNDIG1L | synapse differentiation inducing 1 like [Source:HGNC Symbol;Acc:HGNC:32388] |
| ENSG00000183617 | HGLibA_30020 | 25 | -0.899826023 | MRPL54 | mitochondrial ribosomal protein L54 [Source:HGNC Symbol;Acc:HGNC:16685] |
| ENSG00000184194 | HGLibA_19970 | 25 | -0.899826023 | GPR173 | G protein-coupled receptor 173 [Source:HGNC Symbol;Acc:HGNC:18186] |
| ENSG00000184481 | HGLibA_17926 | 25 | -0.899826023 | FOXO4 | forkhead box O4 [Source:HGNC Symbol;Acc:HGNC:7139] |
| ENSG00000184677 | HGLibA_54880 | 25 | -0.899826023 | ZBTB40 | zinc finger and BTB domain containing 40 [Source:HGNC Symbol;Acc:HGNC:29045] |
| ENSG00000185565 | HGLibA_27512 | 25 | -0.899826023 | LSAMP | limbic system associated membrane protein [Source:HGNC Symbol;Acc:HGNC:6705] |
| ENSG00000185745 | HGLibA_22623 | 25 | -0.899826023 | IFIT1 | interferon induced protein with tetratricopeptide repeats 1 [Source:HGNC Symbol;Acc:HGNC:5407] |
| ENSG00000185838 | HGLibA_19486 | 25 | -0.899826023 | GNB1L | G protein subunit beta 1 like [Source:HGNC Symbol;Acc:HGNC:4397] |
| ENSG00000186288 | HGLibA_34926 | 25 | -0.899826023 | PABPC1L2A | poly(A) binding protein cytoplasmic 1 like 2A [Source:HGNC Symbol;Acc:HGNC:27989] |
| ENSG00000186971 | HGLibA_25650 | 25 | -0.899826023 | KRTAP13-4 | keratin associated protein 13-4 [Source:HGNC Symbol;Acc:HGNC:18926] |
| ENSG00000186994 | HGLibA_24031 | 25 | -0.899826023 | KANK3 | KN motif and ankyrin repeat domains 3 [Source:HGNC Symbol;Acc:HGNC:24796] |
| ENSG00000187272 | HGLibA_25848 | 25 | -0.899826023 | KRTAP9-8 | keratin associated protein 9-8 [Source:HGNC Symbol;Acc:HGNC:17231] |
| ENSG00000187720 | HGLibA_49403 | 25 | -0.899826023 | THSD4 | thrombospondin type 1 domain containing 4 [Source:HGNC Symbol;Acc:HGNC:25835] |
| ENSG00000188517 | HGLibA_10551 | 25 | -0.899826023 | COL25A1 | collagen type XXV alpha 1 chain [Source:HGNC Symbol;Acc:HGNC:18603] |
| ENSG00000188641 | HGLibA_13921 | 25 | -0.899826023 | DPYD | dihydropyrimidine dehydrogenase [Source:HGNC Symbol;Acc:HGNC:3012] |
| ENSG00000188643 | HGLibA_42549 | 25 | -0.899826023 | S100A16 | S100 calcium binding protein A16 [Source:HGNC Symbol;Acc:HGNC:20441] |
| ENSG00000189050 | HGLibA_41704 | 25 | -0.899826023 | RNFT1 | transmembrane 1 [Source:HGNC Symbol;Acc:HGNC:30206]" |
| ENSG00000196099 | HGLibA_34428 | 25 | -0.899826023 | OR6M1 | olfactory receptor family 6 subfamily M member 1 [Source:HGNC Symbol;Acc:HGNC:14711] |
| ENSG00000196345 | HGLibA_55360 | 25 | -0.899826023 | ZKSCAN7 | zinc finger with KRAB and SCAN domains 7 [Source:HGNC Symbol;Acc:HGNC:12955] |
| ENSG00000196369 | HGLibA_46974 | 25 | -0.899826023 | SRGAP2B | SLIT-ROBO Rho GTPase activating protein 2B [Source:HGNC Symbol;Acc:HGNC:35237] |
| ENSG00000197070 | HGLibA_03113 | 25 | -0.899826023 | ARRDC1 | arrestin domain containing 1 [Source:HGNC Symbol;Acc:HGNC:28633] |
| ENSG00000197150 | HGLibA_00153 | 25 | -0.899826023 | ABCB8 | ATP binding cassette subfamily B member 8 [Source:HGNC Symbol;Acc:HGNC:49] |
| ENSG00000197372 | HGLibA_56513 | 25 | -0.899826023 | ZNF675 | zinc finger protein 675 [Source:HGNC Symbol;Acc:HGNC:30768] |
| ENSG00000197694 | HGLibA_46873 | 25 | -0.899826023 | SPTAN1 | non-erythrocytic 1 [Source:HGNC Symbol;Acc:HGNC:11273]" |
| ENSG00000197746 | HGLibA_39001 | 25 | -0.899826023 | PSAP | prosaposin [Source:HGNC Symbol;Acc:HGNC:9498] |
| ENSG00000197757 | HGLibA_21975 | 25 | -0.899826023 | HOXC6 | homeobox C6 [Source:HGNC Symbol;Acc:HGNC:5128] |
| ENSG00000198185 | HGLibA_55822 | 25 | -0.899826023 | ZNF334 | zinc finger protein 334 [Source:HGNC Symbol;Acc:HGNC:15806] |
| ENSG00000198265 | HGLibA_21124 | 25 | -0.899826023 | HELZ | helicase with zinc finger [Source:HGNC Symbol;Acc:HGNC:16878] |
| ENSG00000198331 | HGLibA_22457 | 25 | -0.899826023 | HYLS1 | centriolar and ciliogenesis associated [Source:HGNC Symbol;Acc:HGNC:26558]" |
| ENSG00000198722 | HGLibA_52995 | 25 | -0.899826023 | UNC13B | unc-13 homolog B [Source:HGNC Symbol;Acc:HGNC:12566] |
| ENSG00000198795 | HGLibA_56159 | 25 | -0.899826023 | ZNF521 | zinc finger protein 521 [Source:HGNC Symbol;Acc:HGNC:24605] |
| ENSG00000198865 | HGLibA_07650 | 25 | -0.899826023 | CCDC152 | coiled-coil domain containing 152 [Source:HGNC Symbol;Acc:HGNC:34438] |
| ENSG00000198873 | HGLibA_20331 | 25 | -0.899826023 | GRK5 | G protein-coupled receptor kinase 5 [Source:HGNC Symbol;Acc:HGNC:4544] |
| ENSG00000203737 | HGLibA_20050 | 25 | -0.899826023 | GPR52 | G protein-coupled receptor 52 [Source:HGNC Symbol;Acc:HGNC:4508] |
| ENSG00000203870 | HGLibA_45773 | 25 | -0.899826023 | SMIM9 | small integral membrane protein 9 [Source:HGNC Symbol;Acc:HGNC:41915] |
| ENSG00000203952 | HGLibA_07670 | 25 | -0.899826023 | CCDC160 | coiled-coil domain containing 160 [Source:HGNC Symbol;Acc:HGNC:37286] |
| ENSG00000204614 | HGLibA_51492 | 25 | -0.899826023 | TRIM40 | tripartite motif containing 40 [Source:HGNC Symbol;Acc:HGNC:18736] |
| ENSG00000204694 | HGLibA_33562 | 25 | -0.899826023 | OR11A1 | olfactory receptor family 11 subfamily A member 1 [Source:HGNC Symbol;Acc:HGNC:8176] |
| ENSG00000205629 | HGLibA_26159 | 25 | -0.899826023 | LCMT1 | leucine carboxyl methyltransferase 1 [Source:HGNC Symbol;Acc:HGNC:17557] |
| ENSG00000205863 | HGLibA_05962 | 25 | -0.899826023 | C1QTNF9B | C1q and TNF related 9B [Source:HGNC Symbol;Acc:HGNC:34072] |
| ENSG00000206418 | HGLibA_39807 | 25 | -0.899826023 | RAB12 | member RAS oncogene family [Source:HGNC Symbol;Acc:HGNC:31332]" |
| ENSG00000212916 | HGLibA_28113 | 25 | -0.899826023 | MAP10 | microtubule associated protein 10 [Source:HGNC Symbol;Acc:HGNC:29265] |
| ENSG00000213024 | HGLibA_33064 | 25 | -0.899826023 | NUP62 | nucleoporin 62 [Source:HGNC Symbol;Acc:HGNC:8066] |
| ENSG00000213638 | HGLibA_00884 | 25 | -0.899826023 | ADAT3 | tRNA specific 3 [Source:HGNC Symbol;Acc:HGNC:25151]" |
| ENSG00000214681 | HGLibA_23512 | 25 | -0.899826023 | IQCF5 | IQ motif containing F5 [Source:HGNC Symbol;Acc:HGNC:35159] |
| ENSG00000214694 | HGLibA_02850 | 25 | -0.899826023 | ARHGEF33 | Rho guanine nucleotide exchange factor 33 [Source:HGNC Symbol;Acc:HGNC:37252] |
| ENSG00000217442 | HGLibA_47923 | 25 | -0.899826023 | SYCE3 | synaptonemal complex central element protein 3 [Source:HGNC Symbol;Acc:HGNC:35245] |
| ENSG00000224420 | HGLibA_00999 | 25 | -0.899826023 | ADM5 | adrenomedullin 5 (putative) [Source:HGNC Symbol;Acc:HGNC:27293] |
| ENSG00000230031 | HGLibA_37720 | 25 | -0.899826023 | POTEB2 | POTE ankyrin domain family member B2 [Source:HGNC Symbol;Acc:HGNC:48327] |
| ENSG00000233917 | HGLibA_37717 | 25 | -0.899826023 | POTEB | POTE ankyrin domain family member B [Source:HGNC Symbol;Acc:HGNC:33734] |
| ENSG00000234444 | HGLibA_56654 | 25 | -0.899826023 | ZNF736 | zinc finger protein 736 [Source:HGNC Symbol;Acc:HGNC:32467] |
| ENSG00000240021 | HGLibA_49099 | 25 | -0.899826023 | TEX35 | testis expressed 35 [Source:HGNC Symbol;Acc:HGNC:25366] |
| ENSG00000241598 | HGLibA_25797 | 25 | -0.899826023 | KRTAP5-4 | keratin associated protein 5-4 [Source:HGNC Symbol;Acc:HGNC:23599] |
| ENSG00000244646 | HGLibA_54543 | 25 | -0.899826023 | XKRY2 | Y-linked 2 [Source:HGNC Symbol;Acc:HGNC:23922]" |
| ENSG00000253276 | HGLibA_07856 | 25 | -0.899826023 | CCDC71L | coiled-coil domain containing 71 like [Source:HGNC Symbol;Acc:HGNC:26685] |
| ENSG00000258453 | HGLibA_33574 | 25 | -0.899826023 | OR11H2 | olfactory receptor family 11 subfamily H member 2 [Source:HGNC Symbol;Acc:HGNC:14716] |
| ENSG00000260238 | HGLibA_37306 | 25 | -0.899826023 | PMF1-BGLAP | PMF1-BGLAP readthrough [Source:HGNC Symbol;Acc:HGNC:42953] |
| ENSG00000263639 | HGLibA_30224 | 25 | -0.899826023 | MSMB | microseminoprotein beta [Source:HGNC Symbol;Acc:HGNC:7372] |
| ENSG00000269096 | HGLibA_11503 | 25 | -0.899826023 | CT45A3 | cancer/testis antigen family 45 member A3 [Source:HGNC Symbol;Acc:HGNC:33268] |
| FAM65A | HGLibA_16669 | 25 | -0.899826023 | FAM65A | - |
| FAM69C | HGLibA_16684 | 25 | -0.899826023 | FAM69C | - |
| FAM84A | HGLibA_16763 | 25 | -0.899826023 | FAM84A | - |
| HDHD1 | HGLibA_21051 | 25 | -0.899826023 | HDHD1 | - |
| hsa-mir-1247 | HGLibA_57387 | 25 | -0.899826023 | hsa-mir-1247 | - |
| hsa-mir-190b | HGLibA_58039 | 25 | -0.899826023 | hsa-mir-190b | - |
| hsa-mir-211 | HGLibA_58217 | 25 | -0.899826023 | hsa-mir-211 | - |
| hsa-mir-2276 | HGLibA_58308 | 25 | -0.899826023 | hsa-mir-2276 | - |
| hsa-mir-25 | HGLibA_58354 | 25 | -0.899826023 | hsa-mir-25 | - |
| hsa-mir-298 | HGLibA_58400 | 25 | -0.899826023 | hsa-mir-298 | - |
| hsa-mir-3171 | HGLibA_58762 | 25 | -0.899826023 | hsa-mir-3171 | - |
| hsa-mir-328 | HGLibA_58974 | 25 | -0.899826023 | hsa-mir-328 | - |
| hsa-mir-329-2 | HGLibA_58984 | 25 | -0.899826023 | hsa-mir-329-2 | - |
| hsa-mir-367 | HGLibA_59230 | 25 | -0.899826023 | hsa-mir-367 | - |
| hsa-mir-377 | HGLibA_59421 | 25 | -0.899826023 | hsa-mir-377 | - |
| hsa-mir-3909 | HGLibA_59500 | 25 | -0.899826023 | hsa-mir-3909 | - |
| hsa-mir-449a | HGLibA_60415 | 25 | -0.899826023 | hsa-mir-449a | - |
| hsa-mir-4535 | HGLibA_60599 | 25 | -0.899826023 | hsa-mir-4535 | - |
| hsa-mir-4640 | HGLibA_60665 | 25 | -0.899826023 | hsa-mir-4640 | - |
| hsa-mir-4718 | HGLibA_60974 | 25 | -0.899826023 | hsa-mir-4718 | - |
| hsa-mir-4728 | HGLibA_61009 | 25 | -0.899826023 | hsa-mir-4728 | - |
| hsa-mir-4776-2 | HGLibA_61210 | 25 | -0.899826023 | hsa-mir-4776-2 | - |
| hsa-mir-516b-2 | HGLibA_61622 | 25 | -0.899826023 | hsa-mir-516b-2 | - |
| hsa-mir-5186 | HGLibA_61634 | 25 | -0.899826023 | hsa-mir-5186 | - |
| hsa-mir-518b | HGLibA_61661 | 25 | -0.899826023 | hsa-mir-518b | - |
| hsa-mir-5692b | HGLibA_62296 | 25 | -0.899826023 | hsa-mir-5692b | - |
| hsa-mir-591 | HGLibA_62460 | 25 | -0.899826023 | hsa-mir-591 | - |
| hsa-mir-636 | HGLibA_62784 | 25 | -0.899826023 | hsa-mir-636 | - |
| hsa-mir-6764 | HGLibA_63218 | 25 | -0.899826023 | hsa-mir-6764 | - |
| hsa-mir-6780a | HGLibA_63295 | 25 | -0.899826023 | hsa-mir-6780a | - |
| hsa-mir-6825 | HGLibA_63476 | 25 | -0.899826023 | hsa-mir-6825 | - |
| hsa-mir-6885 | HGLibA_63723 | 25 | -0.899826023 | hsa-mir-6885 | - |
| hsa-mir-7848 | HGLibA_63982 | 25 | -0.899826023 | hsa-mir-7848 | - |
| hsa-mir-802 | HGLibA_64043 | 25 | -0.899826023 | hsa-mir-802 | - |
| hsa-mir-8081 | HGLibA_64166 | 25 | -0.899826023 | hsa-mir-8081 | - |
| hsa-mir-95 | HGLibA_64364 | 25 | -0.899826023 | hsa-mir-95 | - |
| KIAA0195 | HGLibA_24599 | 25 | -0.899826023 | KIAA0195 | - |
| KIAA1804 | HGLibA_24821 | 25 | -0.899826023 | KIAA1804 | - |
| LOC100287036 | HGLibA_26816 | 25 | -0.899826023 | LOC100287036 | - |
| LOC100653515 | HGLibA_26875 | 25 | -0.899826023 | LOC100653515 | - |
| LOC100653515 | HGLibA_26877 | 25 | -0.899826023 | LOC100653515 | - |
| LOC339862 | HGLibA_26920 | 25 | -0.899826023 | LOC339862 | - |
| PARK2 | HGLibA_35196 | 25 | -0.899826023 | PARK2 | - |
| UFD1L | HGLibA_52847 | 25 | -0.899826023 | UFD1L | - |
| 9-Mar | HGLibA_28351 | 24 | -0.909777061 | 9-Mar | - |
| ANKRD20A3 | HGLibA_02032 | 24 | -0.909777061 | ANKRD20A3 | - |
| C16orf55 | HGLibA_05459 | 24 | -0.909777061 | C16orf55 | - |
| C20orf166 | HGLibA_05992 | 24 | -0.909777061 | C20orf166 | - |

| | | | | | |
|---|---|---|---|---|---|
| C22orf43 | HGLibA_06081 | 24 | -0.909777061 | C22orf43 | - |
| C6orf48 | HGLibA_06491 | 24 | -0.909777061 | C6orf48 | - |
| DHFRL1 | HGLibA_13105 | 24 | -0.909777061 | DHFRL1 | - |
| ENSG00000000457 | HGLibA_43091 | 24 | -0.909777061 | SCYL3 | SCY1 like pseudokinase 3 [Source:HGNC Symbol;Acc:HGNC:19285] |
| ENSG00000005022 | HGLibA_44761 | 24 | -0.909777061 | SLC25A5 | solute carrier family 25 member 5 [Source:HGNC Symbol;Acc:HGNC:10991] |
| ENSG00000006555 | HGLibA_52075 | 24 | -0.909777061 | TTC22 | tetratricopeptide repeat domain 22 [Source:HGNC Symbol;Acc:HGNC:26067] |
| ENSG00000007264 | HGLibA_28425 | 24 | -0.909777061 | MATK | megakaryocyte-associated tyrosine kinase [Source:HGNC Symbol;Acc:HGNC:6906] |
| ENSG00000007516 | HGLibA_04130 | 24 | -0.909777061 | BAIAP3 | BAI1 associated protein 3 [Source:HGNC Symbol;Acc:HGNC:948] |
| ENSG00000008196 | HGLibA_49124 | 24 | -0.909777061 | TFAP2B | transcription factor AP-2 beta [Source:HGNC Symbol;Acc:HGNC:11743] |
| ENSG00000009335 | HGLibA_52684 | 24 | -0.909777061 | UBE3C | ubiquitin protein ligase E3C [Source:HGNC Symbol;Acc:HGNC:16803] |
| ENSG00000011304 | HGLibA_39250 | 24 | -0.909777061 | PTBP1 | polypyrimidine tract binding protein 1 [Source:HGNC Symbol;Acc:HGNC:9583] |
| ENSG00000012174 | HGLibA_28539 | 24 | -0.909777061 | MBTPS2 | site 2 [Source:HGNC Symbol;Acc:HGNC:15455]" |
| ENSG00000018869 | HGLibA_56310 | 24 | -0.909777061 | ZNF582 | zinc finger protein 582 [Source:HGNC Symbol;Acc:HGNC:26421] |
| ENSG00000023839 | HGLibA_00170 | 24 | -0.909777061 | ABCC2 | ATP binding cassette subfamily C member 2 [Source:HGNC Symbol;Acc:HGNC:53] |
| ENSG00000036054 | HGLibA_48509 | 24 | -0.909777061 | TBC1D23 | TBC1 domain family member 23 [Source:HGNC Symbol;Acc:HGNC:25622] |
| ENSG00000036828 | HGLibA_07341 | 24 | -0.909777061 | CASR | calcium sensing receptor [Source:HGNC Symbol;Acc:HGNC:1514] |
| ENSG00000038295 | HGLibA_49627 | 24 | -0.909777061 | TLL1 | tolloid like 1 [Source:HGNC Symbol;Acc:HGNC:11843] |
| ENSG00000052749 | HGLibA_42270 | 24 | -0.909777061 | RRP12 | ribosomal RNA processing 12 homolog [Source:HGNC Symbol;Acc:HGNC:29100] |
| ENSG00000057704 | HGLibA_49779 | 24 | -0.909777061 | TMCC3 | transmembrane and coiled-coil domain family 3 [Source:HGNC Symbol;Acc:HGNC:29199] |
| ENSG00000064309 | HGLibA_08858 | 24 | -0.909777061 | CDON | oncogene regulated [Source:HGNC Symbol;Acc:HGNC:17104]" |
| ENSG00000067596 | HGLibA_13207 | 24 | -0.909777061 | DHX8 | DEAH-box helicase 8 [Source:HGNC Symbol;Acc:HGNC:2749] |
| ENSG00000073464 | HGLibA_09830 | 24 | -0.909777061 | CLCN4 | chloride voltage-gated channel 4 [Source:HGNC Symbol;Acc:HGNC:2022] |
| ENSG00000073734 | HGLibA_00137 | 24 | -0.909777061 | ABCB11 | ATP binding cassette subfamily B member 11 [Source:HGNC Symbol;Acc:HGNC:42] |
| ENSG00000075239 | HGLibA_00392 | 24 | -0.909777061 | ACAT1 | acetyl-CoA acetyltransferase 1 [Source:HGNC Symbol;Acc:HGNC:93] |
| ENSG00000077009 | HGLibA_32198 | 24 | -0.909777061 | NMRK2 | nicotinamide riboside kinase 2 [Source:HGNC Symbol;Acc:HGNC:17871] |
| ENSG00000078808 | HGLibA_43131 | 24 | -0.909777061 | SDF4 | stromal cell derived factor 4 [Source:HGNC Symbol;Acc:HGNC:24188] |
| ENSG00000081051 | HGLibA_01128 | 24 | -0.909777061 | AFP | alpha fetoprotein [Source:HGNC Symbol;Acc:HGNC:317] |
| ENSG00000083642 | HGLibA_35946 | 24 | -0.909777061 | PDS5B | PDS5 cohesin associated factor B [Source:HGNC Symbol;Acc:HGNC:20418] |
| ENSG00000091583 | HGLibA_02524 | 24 | -0.909777061 | APOH | apolipoprotein H [Source:HGNC Symbol;Acc:HGNC:616] |
| ENSG00000091732 | HGLibA_54998 | 24 | -0.909777061 | ZC3HC1 | zinc finger C3HC-type containing 1 [Source:HGNC Symbol;Acc:HGNC:29913] |
| ENSG00000100211 | HGLibA_07470 | 24 | -0.909777061 | CBY1 | beta catenin antagonist [Source:HGNC Symbol;Acc:HGNC:1307]" |
| ENSG00000100221 | HGLibA_23972 | 24 | -0.909777061 | JOSD1 | Josephin domain containing 1 [Source:HGNC Symbol;Acc:HGNC:28953] |
| ENSG00000100304 | HGLibA_52195 | 24 | -0.909777061 | TTLL12 | tubulin tyrosine ligase like 12 [Source:HGNC Symbol;Acc:HGNC:28974] |
| ENSG00000100926 | HGLibA_49721 | 24 | -0.909777061 | TM9SF1 | transmembrane 9 superfamily member 1 [Source:HGNC Symbol;Acc:HGNC:11864] |
| ENSG00000101974 | HGLibA_03560 | 24 | -0.909777061 | ATP11C | ATPase phospholipid transporting 11C [Source:HGNC Symbol;Acc:HGNC:13554] |
| ENSG00000104133 | HGLibA_46629 | 24 | -0.909777061 | SPG11 | spatacsin vesicle trafficking associated [Source:HGNC Symbol;Acc:HGNC:11226]" |
| ENSG00000104499 | HGLibA_19412 | 24 | -0.909777061 | GML | glycosylphosphatidylinositol anchored molecule like [Source:HGNC Symbol;Acc:HGNC:4375] |
| ENSG00000104901 | HGLibA_13306 | 24 | -0.909777061 | DKKL1 | dickkopf like acrosomal protein 1 [Source:HGNC Symbol;Acc:HGNC:16528] |
| ENSG00000105290 | HGLibA_02439 | 24 | -0.909777061 | APLP1 | amyloid beta precursor like protein 1 [Source:HGNC Symbol;Acc:HGNC:597] |
| ENSG00000106631 | HGLibA_30776 | 24 | -0.909777061 | MYL7 | myosin light chain 7 [Source:HGNC Symbol;Acc:HGNC:21719] |
| ENSG00000106948 | HGLibA_01462 | 24 | -0.909777061 | AKNA | AT-hook transcription factor [Source:HGNC Symbol;Acc:HGNC:24108] |
| ENSG00000107295 | HGLibA_43950 | 24 | -0.909777061 | SH3GL2 | endophilin A1 [Source:HGNC Symbol;Acc:HGNC:10831]" |
| ENSG00000107372 | HGLibA_55160 | 24 | -0.909777061 | ZFAND5 | zinc finger AN1-type containing 5 [Source:HGNC Symbol;Acc:HGNC:13008] |
| ENSG00000107859 | HGLibA_36769 | 24 | -0.909777061 | PITX3 | paired like homeodomain 3 [Source:HGNC Symbol;Acc:HGNC:9006] |
| ENSG00000109220 | HGLibA_09447 | 24 | -0.909777061 | CHIC2 | cysteine rich hydrophobic domain 2 [Source:HGNC Symbol;Acc:HGNC:1935] |
| ENSG00000110693 | HGLibA_46269 | 24 | -0.909777061 | SOX6 | SRY-box 6 [Source:HGNC Symbol;Acc:HGNC:16421] |
| ENSG00000111046 | HGLibA_30699 | 24 | -0.909777061 | MYF6 | myogenic factor 6 [Source:HGNC Symbol;Acc:HGNC:7566] |
| ENSG00000111530 | HGLibA_07121 | 24 | -0.909777061 | CAND1 | cullin associated and neddylation dissociated 1 [Source:HGNC Symbol;Acc:HGNC:30688] |
| ENSG00000112706 | HGLibA_23262 | 24 | -0.909777061 | IMPG1 | interphotoreceptor matrix proteoglycan 1 [Source:HGNC Symbol;Acc:HGNC:6055] |
| ENSG00000114062 | HGLibA_52677 | 24 | -0.909777061 | UBE3A | ubiquitin protein ligase E3A [Source:HGNC Symbol;Acc:HGNC:12496] |
| ENSG00000114982 | HGLibA_24046 | 24 | -0.909777061 | KANSL3 | KAT8 regulatory NSL complex subunit 3 [Source:HGNC Symbol;Acc:HGNC:25473] |
| ENSG00000116117 | HGLibA_35180 | 24 | -0.909777061 | PARD3B | par-3 family cell polarity regulator beta [Source:HGNC Symbol;Acc:HGNC:14446] |
| ENSG00000116128 | HGLibA_04348 | 24 | -0.909777061 | BCL9 | B cell CLL/lymphoma 9 [Source:HGNC Symbol;Acc:HGNC:1008] |
| ENSG00000116824 | HGLibA_08279 | 24 | -0.909777061 | CD2 | CD2 molecule [Source:HGNC Symbol;Acc:HGNC:1639] |
| ENSG00000117010 | HGLibA_56535 | 24 | -0.909777061 | ZNF684 | zinc finger protein 684 [Source:HGNC Symbol;Acc:HGNC:28418] |
| ENSG00000117151 | HGLibA_11590 | 24 | -0.909777061 | CTBS | chitobiase [Source:HGNC Symbol;Acc:HGNC:2496] |
| ENSG00000117262 | HGLibA_20108 | 24 | -0.909777061 | GPR89A | G protein-coupled receptor 89A [Source:HGNC Symbol;Acc:HGNC:31984] |
| ENSG00000117399 | HGLibA_08519 | 24 | -0.909777061 | CDC20 | cell division cycle 20 [Source:HGNC Symbol;Acc:HGNC:1723] |
| ENSG00000118503 | HGLibA_50711 | 24 | -0.909777061 | TNFAIP3 | TNF alpha induced protein 3 [Source:HGNC Symbol;Acc:HGNC:11896] |
| ENSG00000118564 | HGLibA_17011 | 24 | -0.909777061 | FBXL5 | F-box and leucine rich repeat protein 5 [Source:HGNC Symbol;Acc:HGNC:13602] |
| ENSG00000119900 | HGLibA_33319 | 24 | -0.909777061 | OGFRL1 | opioid growth factor receptor like 1 [Source:HGNC Symbol;Acc:HGNC:21378] |
| ENSG00000119950 | HGLibA_30618 | 24 | -0.909777061 | MXI1 | dimerization protein [Source:HGNC Symbol;Acc:HGNC:7534]" |
| ENSG00000120686 | HGLibA_52851 | 24 | -0.909777061 | UFM1 | ubiquitin fold modifier 1 [Source:HGNC Symbol;Acc:HGNC:20597] |
| ENSG00000121361 | HGLibA_24289 | 24 | -0.909777061 | KCNJ8 | potassium voltage-gated channel subfamily J member 8 [Source:HGNC Symbol;Acc:HGNC:6269] |
| ENSG00000121440 | HGLibA_35996 | 24 | -0.909777061 | PDZRN3 | PDZ domain containing ring finger 3 [Source:HGNC Symbol;Acc:HGNC:17704] |
| ENSG00000121481 | HGLibA_41607 | 24 | -0.909777061 | RNF2 | ring finger protein 2 [Source:HGNC Symbol;Acc:HGNC:10061] |
| ENSG00000122971 | HGLibA_00371 | 24 | -0.909777061 | ACADS | acyl-CoA dehydrogenase short chain [Source:HGNC Symbol;Acc:HGNC:90] |
| ENSG00000124222 | HGLibA_47624 | 24 | -0.909777061 | STX16 | syntaxin 16 [Source:HGNC Symbol;Acc:HGNC:11431] |
| ENSG00000124228 | HGLibA_12688 | 24 | -0.909777061 | DDX27 | DEAD-box helicase 27 [Source:HGNC Symbol;Acc:HGNC:15837] |
| ENSG00000125458 | HGLibA_32798 | 24 | -0.909777061 | NT5C | cytosolic [Source:HGNC Symbol;Acc:HGNC:17144]" |
| ENSG00000125798 | HGLibA_17809 | 24 | -0.909777061 | FOXA2 | forkhead box A2 [Source:HGNC Symbol;Acc:HGNC:5022] |
| ENSG00000127585 | HGLibA_16985 | 24 | -0.909777061 | FBXL16 | F-box and leucine rich repeat protein 16 [Source:HGNC Symbol;Acc:HGNC:14150] |
| ENSG00000128242 | HGLibA_18496 | 24 | -0.909777061 | GAL3ST1 | galactose-3-O-sulfotransferase 1 [Source:HGNC Symbol;Acc:HGNC:24240] |
| ENSG00000129116 | HGLibA_35064 | 24 | -0.909777061 | PALLD | cytoskeletal associated protein [Source:HGNC Symbol;Acc:HGNC:17068]" |
| ENSG00000130584 | HGLibA_54897 | 24 | -0.909777061 | ZBTB46 | zinc finger and BTB domain containing 46 [Source:HGNC Symbol;Acc:HGNC:16094] |
| ENSG00000130589 | HGLibA_21125 | 24 | -0.909777061 | HELZ2 | helicase with zinc finger 2 [Source:HGNC Symbol;Acc:HGNC:30021] |
| ENSG00000132549 | HGLibA_53733 | 24 | -0.909777061 | VPS13B | vacuolar protein sorting 13 homolog B [Source:HGNC Symbol;Acc:HGNC:2183] |
| ENSG00000133460 | HGLibA_44857 | 24 | -0.909777061 | SLC2A11 | solute carrier family 2 member 11 [Source:HGNC Symbol;Acc:HGNC:14239] |
| ENSG00000133961 | HGLibA_33013 | 24 | -0.909777061 | NUMB | endocytic adaptor protein [Source:HGNC Symbol;Acc:HGNC:8060]" |
| ENSG00000135119 | HGLibA_41707 | 24 | -0.909777061 | RNFT2 | transmembrane 2 [Source:HGNC Symbol;Acc:HGNC:25905]" |
| ENSG00000135241 | HGLibA_37427 | 24 | -0.909777061 | PNPLA8 | patatin like phospholipase domain containing 8 [Source:HGNC Symbol;Acc:HGNC:28900] |
| ENSG00000135862 | HGLibA_25967 | 24 | -0.909777061 | LAMC1 | laminin subunit gamma 1 [Source:HGNC Symbol;Acc:HGNC:6492] |
| ENSG00000136141 | HGLibA_27137 | 24 | -0.909777061 | LRCH1 | leucine rich repeats and calponin homology domain containing 1 [Source:HGNC Symbol;Acc:HGNC:20309] |
| ENSG00000136634 | HGLibA_22945 | 24 | -0.909777061 | IL10 | interleukin 10 [Source:HGNC Symbol;Acc:HGNC:5962] |
| ENSG00000137261 | HGLibA_24620 | 24 | -0.909777061 | KIAA0319 | KIAA0319 [Source:HGNC Symbol;Acc:HGNC:21580] |
| ENSG00000137434 | HGLibA_06494 | 24 | -0.909777061 | C6orf52 | chromosome 6 open reading frame 52 [Source:HGNC Symbol;Acc:HGNC:20881] |
| ENSG00000137574 | HGLibA_49277 | 24 | -0.909777061 | TGS1 | trimethylguanosine synthase 1 [Source:HGNC Symbol;Acc:HGNC:17843] |
| ENSG00000137822 | HGLibA_52316 | 24 | -0.909777061 | TUBGCP4 | tubulin gamma complex associated protein 4 [Source:HGNC Symbol;Acc:HGNC:16691] |
| ENSG00000137947 | HGLibA_20529 | 24 | -0.909777061 | GTF2B | general transcription factor IIB [Source:HGNC Symbol;Acc:HGNC:4648] |
| ENSG00000137955 | HGLibA_40022 | 24 | -0.909777061 | RABGGTB | Rab geranylgeranyltransferase subunit beta [Source:HGNC Symbol;Acc:HGNC:9796] |
| ENSG00000138131 | HGLibA_27047 | 24 | -0.909777061 | LOXL4 | lysyl oxidase like 4 [Source:HGNC Symbol;Acc:HGNC:17171] |
| ENSG00000138375 | HGLibA_45617 | 24 | -0.909777061 | SMARCAL1 | subfamily a like 1 [Source:HGNC Symbol;Acc:HGNC:11102]" |
| ENSG00000138376 | HGLibA_04156 | 24 | -0.909777061 | BARD1 | BRCA1 associated RING domain 1 [Source:HGNC Symbol;Acc:HGNC:952] |
| ENSG00000139645 | HGLibA_02118 | 24 | -0.909777061 | ANKRD52 | ankyrin repeat domain 52 [Source:HGNC Symbol;Acc:HGNC:26614] |
| ENSG00000140044 | HGLibA_23942 | 24 | -0.909777061 | JDP2 | Jun dimerization protein 2 [Source:HGNC Symbol;Acc:HGNC:17546] |
| ENSG00000140263 | HGLibA_46192 | 24 | -0.909777061 | SORD | sorbitol dehydrogenase [Source:HGNC Symbol;Acc:HGNC:11184] |
| ENSG00000140320 | HGLibA_04111 | 24 | -0.909777061 | BAHD1 | bromo adjacent homology domain containing 1 [Source:HGNC Symbol;Acc:HGNC:29153] |
| ENSG00000140905 | HGLibA_18834 | 24 | -0.909777061 | GCSH | glycine cleavage system protein H [Source:HGNC Symbol;Acc:HGNC:4208] |
| ENSG00000140932 | HGLibA_10212 | 24 | -0.909777061 | CMTM2 | CKLF like MARVEL transmembrane domain containing 2 [Source:HGNC Symbol;Acc:HGNC:19173] |
| ENSG00000141293 | HGLibA_44269 | 24 | -0.909777061 | SKAP1 | src kinase associated phosphoprotein 1 [Source:HGNC Symbol;Acc:HGNC:15605] |
| ENSG00000141367 | HGLibA_10149 | 24 | -0.909777061 | CLTC | clathrin heavy chain [Source:HGNC Symbol;Acc:HGNC:2092] |
| ENSG00000142556 | HGLibA_56390 | 24 | -0.909777061 | ZNF614 | zinc finger protein 614 [Source:HGNC Symbol;Acc:HGNC:24722] |
| ENSG00000144218 | HGLibA_01113 | 24 | -0.909777061 | AFF3 | AF4/FMR2 family member 3 [Source:HGNC Symbol;Acc:HGNC:6473] |
| ENSG00000145216 | HGLibA_17533 | 24 | -0.909777061 | FIP1L1 | factor interacting with PAPOLA and CPSF1 [Source:HGNC Symbol;Acc:HGNC:19124] |
| ENSG00000145248 | HGLibA_44338 | 24 | -0.909777061 | SLC10A4 | solute carrier family 10 member 4 [Source:HGNC Symbol;Acc:HGNC:22980] |
| ENSG00000145715 | HGLibA_40296 | 24 | -0.909777061 | RASA1 | RAS p21 protein activator 1 [Source:HGNC Symbol;Acc:HGNC:9871] |
| ENSG00000145916 | HGLibA_41386 | 24 | -0.909777061 | RMND5B | required for meiotic nuclear division 5 homolog B [Source:HGNC Symbol;Acc:HGNC:26181] |
| ENSG00000146592 | HGLibA_11105 | 24 | -0.909777061 | CREB5 | cAMP responsive element binding protein 5 [Source:HGNC Symbol;Acc:HGNC:16844] |
| ENSG00000146950 | HGLibA_44090 | 24 | -0.909777061 | SHROOM2 | shroom family member 2 [Source:HGNC Symbol;Acc:HGNC:630] |
| ENSG00000147138 | HGLibA_19972 | 24 | -0.909777061 | GPR174 | G protein-coupled receptor 174 [Source:HGNC Symbol;Acc:HGNC:30245] |
| ENSG00000147533 | HGLibA_19634 | 24 | -0.909777061 | GOLGA7 | golgin A7 [Source:HGNC Symbol;Acc:HGNC:24876] |
| ENSG00000149273 | HGLibA_42136 | 24 | -0.909777061 | RPS3 | ribosomal protein S3 [Source:HGNC Symbol;Acc:HGNC:10420] |
| ENSG00000150244 | HGLibA_51519 | 24 | -0.909777061 | TRIM48 | tripartite motif containing 48 [Source:HGNC Symbol;Acc:HGNC:19021] |
| ENSG00000151650 | HGLibA_53620 | 24 | -0.909777061 | VENTX | VENT homeobox [Source:HGNC Symbol;Acc:HGNC:13639] |
| ENSG00000152256 | HGLibA_35896 | 24 | -0.909777061 | PDK1 | pyruvate dehydrogenase kinase 1 [Source:HGNC Symbol;Acc:HGNC:8809] |
| ENSG00000152804 | HGLibA_21258 | 24 | -0.909777061 | HHEX | hematopoietically expressed homeobox [Source:HGNC Symbol;Acc:HGNC:4901] |
| ENSG00000154319 | HGLibA_16240 | 24 | -0.909777061 | FAM167A | family with sequence similarity 167 member A [Source:HGNC Symbol;Acc:HGNC:15549] |
| ENSG00000154529 | HGLibA_10428 | 24 | -0.909777061 | CNTNAP3B | contactin associated protein like 3B [Source:HGNC Symbol;Acc:HGNC:32035] |
| ENSG00000155463 | HGLibA_34811 | 24 | -0.909777061 | OXA1L | mitochondrial inner membrane protein [Source:HGNC Symbol;Acc:HGNC:8526]" |
| ENSG00000155827 | HGLibA_41609 | 24 | -0.909777061 | RNF20 | ring finger protein 20 [Source:HGNC Symbol;Acc:HGNC:10062] |
| ENSG00000156886 | HGLibA_23751 | 24 | -0.909777061 | ITGAD | integrin subunit alpha D [Source:HGNC Symbol;Acc:HGNC:6146] |
| ENSG00000157483 | HGLibA_30833 | 24 | -0.909777061 | MYO1E | myosin IE [Source:HGNC Symbol;Acc:HGNC:7599] |
| ENSG00000160818 | HGLibA_19746 | 24 | -0.909777061 | GPATCH4 | G-patch domain containing 4 [Source:HGNC Symbol;Acc:HGNC:25982] |
| ENSG00000161681 | HGLibA_43988 | 24 | -0.909777061 | SHANK1 | SH3 and multiple ankyrin repeat domains 1 [Source:HGNC Symbol;Acc:HGNC:15474] |
| ENSG00000162601 | HGLibA_30923 | 24 | -0.909777061 | MYSM1 | SWIRM and MPN domains 1 [Source:HGNC Symbol;Acc:HGNC:29401]" |
| ENSG00000162706 | HGLibA_06970 | 24 | -0.909777061 | CADM3 | cell adhesion molecule 3 [Source:HGNC Symbol;Acc:HGNC:17601] |
| ENSG00000163527 | HGLibA_47608 | 24 | -0.909777061 | STT3B | catalytic subunit of the oligosaccharyltransferase complex [Source:HGNC Symbol;Acc:HGNC:30611]" |
| ENSG00000163659 | HGLibA_49556 | 24 | -0.909777061 | TIPARP | TCDD inducible poly(ADP-ribose) polymerase [Source:HGNC Symbol;Acc:HGNC:23696] |
| ENSG00000163902 | HGLibA_42017 | 24 | -0.909777061 | RPN1 | ribophorin I [Source:HGNC Symbol;Acc:HGNC:10381] |
| ENSG00000163961 | HGLibA_41565 | 24 | -0.909777061 | RNF168 | ring finger protein 168 [Source:HGNC Symbol;Acc:HGNC:26661] |
| ENSG00000164520 | HGLibA_40121 | 24 | -0.909777061 | RAET1E | retinoic acid early transcript 1E [Source:HGNC Symbol;Acc:HGNC:16793] |
| ENSG00000164690 | HGLibA_44029 | 24 | -0.909777061 | SHH | sonic hedgehog [Source:HGNC Symbol;Acc:HGNC:10848] |
| ENSG00000165055 | HGLibA_29002 | 24 | -0.909777061 | METTL2B | methyltransferase like 2B [Source:HGNC Symbol;Acc:HGNC:18272] |
| ENSG00000165487 | HGLibA_29224 | 24 | -0.909777061 | MICU2 | mitochondrial calcium uptake 2 [Source:HGNC Symbol;Acc:HGNC:31830] |
| ENSG00000165678 | HGLibA_19037 | 24 | -0.909777061 | GHITM | growth hormone inducible transmembrane protein [Source:HGNC Symbol;Acc:HGNC:17281] |

| | | | | | |
|---|---|---|---|---|---|
| ENSG00000166477 | HGLibA_26305 | 24 | -0.909777061 | LEO1 | Paf1/RNA polymerase II complex component [Source:HGNC Symbol;Acc:HGNC:30401]" |
| ENSG00000166508 | HGLibA_28623 | 24 | -0.909777061 | MCM7 | minichromosome maintenance complex component 7 [Source:HGNC Symbol;Acc:HGNC:6950] |
| ENSG00000166839 | HGLibA_01946 | 24 | -0.909777061 | ANKDD1A | ankyrin repeat and death domain containing 1A [Source:HGNC Symbol;Acc:HGNC:28002] |
| ENSG00000166913 | HGLibA_54740 | 24 | -0.909777061 | YWHAB | tyrosine 3-monooxygenase/tryptophan 5-monooxygenase activation protein beta [Source:HGNC Symbol;Acc:HGNC:12849] |
| ENSG00000166965 | HGLibA_40689 | 24 | -0.909777061 | RCCD1 | RCC1 domain containing 1 [Source:HGNC Symbol;Acc:HGNC:30457] |
| ENSG00000167315 | HGLibA_00345 | 24 | -0.909777061 | ACAA2 | acetyl-CoA acyltransferase 2 [Source:HGNC Symbol;Acc:HGNC:83] |
| ENSG00000167419 | HGLibA_27107 | 24 | -0.909777061 | LPO | lactoperoxidase [Source:HGNC Symbol;Acc:HGNC:6678] |
| ENSG00000167978 | HGLibA_47048 | 24 | -0.909777061 | SRRM2 | serine/arginine repetitive matrix 2 [Source:HGNC Symbol;Acc:HGNC:16639] |
| ENSG00000168283 | HGLibA_04587 | 24 | -0.909777061 | BMI1 | polycomb ring finger [Source:HGNC Symbol;Acc:HGNC:1066]" |
| ENSG00000168765 | HGLibA_20480 | 24 | -0.909777061 | GSTM4 | glutathione S-transferase mu 4 [Source:HGNC Symbol;Acc:HGNC:4636] |
| ENSG00000169062 | HGLibA_53062 | 24 | -0.909777061 | UPF3A | regulator of nonsense mediated mRNA decay [Source:HGNC Symbol;Acc:HGNC:20332]" |
| ENSG00000169230 | HGLibA_38427 | 24 | -0.909777061 | PRELID1 | PRELI domain containing 1 [Source:HGNC Symbol;Acc:HGNC:30255] |
| ENSG00000169248 | HGLibA_11848 | 24 | -0.909777061 | CXCL11 | C-X-C motif chemokine ligand 11 [Source:HGNC Symbol;Acc:HGNC:10638] |
| ENSG00000169410 | HGLibA_39481 | 24 | -0.909777061 | PTPN9 | non-receptor type 9 [Source:HGNC Symbol;Acc:HGNC:9661]" |
| ENSG00000170264 | HGLibA_16215 | 24 | -0.909777061 | FAM161A | centrosomal protein [Source:HGNC Symbol;Acc:HGNC:25808]" |
| ENSG00000171402 | HGLibA_54485 | 24 | -0.909777061 | XAGE3 | X antigen family member 3 [Source:HGNC Symbol;Acc:HGNC:14618] |
| ENSG00000171612 | HGLibA_44710 | 24 | -0.909777061 | SLC25A33 | solute carrier family 25 member 33 [Source:HGNC Symbol;Acc:HGNC:29681] |
| ENSG00000172331 | HGLibA_04702 | 24 | -0.909777061 | BPGM | bisphosphoglycerate mutase [Source:HGNC Symbol;Acc:HGNC:1093] |
| ENSG00000172350 | HGLibA_00221 | 24 | -0.909777061 | ABCG4 | ATP binding cassette subfamily G member 4 [Source:HGNC Symbol;Acc:HGNC:13884] |
| ENSG00000172785 | HGLibA_07429 | 24 | -0.909777061 | CBWD1 | COBW domain containing 1 [Source:HGNC Symbol;Acc:HGNC:17134] |
| ENSG00000173264 | HGLibA_19897 | 24 | -0.909777061 | GPR137 | G protein-coupled receptor 137 [Source:HGNC Symbol;Acc:HGNC:24300] |
| ENSG00000173451 | HGLibA_49296 | 24 | -0.909777061 | THAP2 | THAP domain containing 2 [Source:HGNC Symbol;Acc:HGNC:20854] |
| ENSG00000173930 | HGLibA_45472 | 24 | -0.909777061 | SLCO4C1 | solute carrier organic anion transporter family member 4C1 [Source:HGNC Symbol;Acc:HGNC:23612] |
| ENSG00000174197 | HGLibA_29122 | 24 | -0.909777061 | MGA | MAX dimerization protein [Source:HGNC Symbol;Acc:HGNC:14010]" |
| ENSG00000174231 | HGLibA_38745 | 24 | -0.909777061 | PRPF8 | pre-mRNA processing factor 8 [Source:HGNC Symbol;Acc:HGNC:17340] |
| ENSG00000174672 | HGLibA_04851 | 24 | -0.909777061 | BRSK2 | BR serine/threonine kinase 2 [Source:HGNC Symbol;Acc:HGNC:11405] |
| ENSG00000175931 | HGLibA_52642 | 24 | -0.909777061 | UBE2O | ubiquitin conjugating enzyme E2 O [Source:HGNC Symbol;Acc:HGNC:29554] |
| ENSG00000176198 | HGLibA_33577 | 24 | -0.909777061 | OR11H4 | olfactory receptor family 11 subfamily H member 4 [Source:HGNC Symbol;Acc:HGNC:15347] |
| ENSG00000176871 | HGLibA_54418 | 24 | -0.909777061 | WSB2 | WD repeat and SOCS box containing 2 [Source:HGNC Symbol;Acc:HGNC:19222] |
| ENSG00000177352 | HGLibA_07852 | 24 | -0.909777061 | CCDC71 | coiled-coil domain containing 71 [Source:HGNC Symbol;Acc:HGNC:25760] |
| ENSG00000177354 | HGLibA_05091 | 24 | -0.909777061 | C10orf71 | chromosome 10 open reading frame 71 [Source:HGNC Symbol;Acc:HGNC:26973] |
| ENSG00000177414 | HGLibA_52663 | 24 | -0.909777061 | UBE2U | ubiquitin conjugating enzyme E2 U [Source:HGNC Symbol;Acc:HGNC:28559] |
| ENSG00000177565 | HGLibA_48612 | 24 | -0.909777061 | TBL1XR1 | transducin beta like 1 X-linked receptor 1 [Source:HGNC Symbol;Acc:HGNC:29529] |
| ENSG00000177692 | HGLibA_13613 | 24 | -0.909777061 | DNAJC28 | DnaJ heat shock protein family (Hsp40) member C28 [Source:HGNC Symbol;Acc:HGNC:1297] |
| ENSG00000178538 | HGLibA_06825 | 24 | -0.909777061 | CA8 | carbonic anhydrase 8 [Source:HGNC Symbol;Acc:HGNC:1382] |
| ENSG00000178951 | HGLibA_54916 | 24 | -0.909777061 | ZBTB7A | zinc finger and BTB domain containing 7A [Source:HGNC Symbol;Acc:HGNC:18078] |
| ENSG00000179097 | HGLibA_22369 | 24 | -0.909777061 | HTR1F | 5-hydroxytryptamine receptor 1F [Source:HGNC Symbol;Acc:HGNC:5292] |
| ENSG00000179151 | HGLibA_14441 | 24 | -0.909777061 | EDC3 | enhancer of mRNA decapping 3 [Source:HGNC Symbol;Acc:HGNC:26114] |
| ENSG00000181767 | HGLibA_34528 | 24 | -0.909777061 | OR8H2 | olfactory receptor family 8 subfamily H member 2 [Source:HGNC Symbol;Acc:HGNC:15308] |
| ENSG00000183134 | HGLibA_39308 | 24 | -0.909777061 | PTGDR2 | prostaglandin D2 receptor 2 [Source:HGNC Symbol;Acc:HGNC:4502] |
| ENSG00000183495 | HGLibA_15232 | 24 | -0.909777061 | EP400 | E1A binding protein p400 [Source:HGNC Symbol;Acc:HGNC:11958] |
| ENSG00000184478 | HGLibA_34215 | 24 | -0.909777061 | OR56A3 | olfactory receptor family 56 subfamily A member 3 [Source:HGNC Symbol;Acc:HGNC:14786] |
| ENSG00000185022 | HGLibA_27879 | 24 | -0.909777061 | MAFF | MAF bZIP transcription factor F [Source:HGNC Symbol;Acc:HGNC:6780] |
| ENSG00000185049 | HGLibA_31672 | 24 | -0.909777061 | NELFA | negative elongation factor complex member A [Source:HGNC Symbol;Acc:HGNC:12768] |
| ENSG00000185467 | HGLibA_25371 | 24 | -0.909777061 | KPNA7 | karyopherin subunit alpha 7 [Source:HGNC Symbol;Acc:HGNC:21839] |
| ENSG00000186118 | HGLibA_49109 | 24 | -0.909777061 | TEX38 | testis expressed 38 [Source:HGNC Symbol;Acc:HGNC:29589] |
| ENSG00000186572 | HGLibA_12836 | 24 | -0.909777061 | DEFB107A | defensin beta 107A [Source:HGNC Symbol;Acc:HGNC:18086] |
| ENSG00000186844 | HGLibA_26100 | 24 | -0.909777061 | LCE1A | late cornified envelope 1A [Source:HGNC Symbol;Acc:HGNC:29459] |
| ENSG00000188155 | HGLibA_25614 | 24 | -0.909777061 | KRTAP10-6 | keratin associated protein 10-6 [Source:HGNC Symbol;Acc:HGNC:20523] |
| ENSG00000188505 | HGLibA_31287 | 24 | -0.909777061 | NCCRP1 | non-specific cytotoxic cell receptor protein 1 homolog (zebrafish) [Source:HGNC Symbol;Acc:HGNC:33739] |
| ENSG00000196653 | HGLibA_56113 | 24 | -0.909777061 | ZNF502 | zinc finger protein 502 [Source:HGNC Symbol;Acc:HGNC:23718] |
| ENSG00000196659 | HGLibA_52107 | 24 | -0.909777061 | TTC30B | tetratricopeptide repeat domain 30B [Source:HGNC Symbol;Acc:HGNC:26425] |
| ENSG00000196666 | HGLibA_16314 | 24 | -0.909777061 | FAM180B | family with sequence similarity 180 member B [Source:HGNC Symbol;Acc:HGNC:34451] |
| ENSG00000196975 | HGLibA_02241 | 24 | -0.909777061 | ANXA4 | annexin A4 [Source:HGNC Symbol;Acc:HGNC:542] |
| ENSG00000197430 | HGLibA_33411 | 24 | -0.909777061 | OPALIN | oligodendrocytic myelin paranodal and inner loop protein [Source:HGNC Symbol;Acc:HGNC:20707] |
| ENSG00000197798 | HGLibA_16050 | 24 | -0.909777061 | FAM118B | family with sequence similarity 118 member B [Source:HGNC Symbol;Acc:HGNC:26110] |
| ENSG00000197912 | HGLibA_46638 | 24 | -0.909777061 | SPG7 | paraplegin matrix AAA peptidase subunit [Source:HGNC Symbol;Acc:HGNC:11237]" |
| ENSG00000198471 | HGLibA_42426 | 24 | -0.909777061 | RTP2 | receptor transporter protein 2 [Source:HGNC Symbol;Acc:HGNC:32486] |
| ENSG00000198960 | HGLibA_03055 | 24 | -0.909777061 | ARMCX6 | armadillo repeat containing X-linked 6 [Source:HGNC Symbol;Acc:HGNC:26094] |
| ENSG00000203907 | HGLibA_33401 | 24 | -0.909777061 | OOEP | oocyte expressed protein [Source:HGNC Symbol;Acc:HGNC:21382] |
| ENSG00000203926 | HGLibA_46381 | 24 | -0.909777061 | SPANXA2 | SPANX family member A2 [Source:HGNC Symbol;Acc:HGNC:14328] |
| ENSG00000204287 | HGLibA_21632 | 24 | -0.909777061 | HLA-DRA | DR alpha [Source:HGNC Symbol;Acc:HGNC:4947]" |
| ENSG00000204361 | HGLibA_33153 | 24 | -0.909777061 | NXPE2 | neurexophilin and PC-esterase domain family member 2 [Source:HGNC Symbol;Acc:HGNC:26331] |
| ENSG00000204681 | HGLibA_18340 | 24 | -0.909777061 | GABBR1 | gamma-aminobutyric acid type B receptor subunit 1 [Source:HGNC Symbol;Acc:HGNC:4070] |
| ENSG00000204873 | HGLibA_25837 | 24 | -0.909777061 | KRTAP9-3 | keratin associated protein 9-3 [Source:HGNC Symbol;Acc:HGNC:16927] |
| ENSG00000204946 | HGLibA_56743 | 24 | -0.909777061 | ZNF783 | zinc finger family member 783 [Source:HGNC Symbol;Acc:HGNC:27222] |
| ENSG00000205754 | HGLibA_45455 | 24 | -0.909777061 | SLCO1B7 | solute carrier organic anion transporter family member 1B7 (putative) [Source:HGNC Symbol;Acc:HGNC:32934] |
| ENSG00000213030 | HGLibA_09314 | 24 | -0.909777061 | CGB8 | chorionic gonadotropin subunit beta 8 [Source:HGNC Symbol;Acc:HGNC:16453] |
| ENSG00000214415 | HGLibA_19478 | 24 | -0.909777061 | GNAT3 | G protein subunit alpha transducin 3 [Source:HGNC Symbol;Acc:HGNC:22800] |
| ENSG00000215126 | HGLibA_07441 | 24 | -0.909777061 | CBWD6 | COBW domain containing 6 [Source:HGNC Symbol;Acc:HGNC:31978] |
| ENSG00000221968 | HGLibA_15941 | 24 | -0.909777061 | FADS3 | fatty acid desaturase 3 [Source:HGNC Symbol;Acc:HGNC:3576] |
| ENSG00000225327 | HGLibA_53273 | 24 | -0.909777061 | USP17L3 | ubiquitin specific peptidase 17-like family member 3 [Source:HGNC Symbol;Acc:HGNC:37175] |
| ENSG00000232382 | HGLibA_34309 | 24 | -0.909777061 | OR5K1 | olfactory receptor family 5 subfamily K member 1 [Source:HGNC Symbol;Acc:HGNC:8349] |
| ENSG00000237038 | HGLibA_53288 | 24 | -0.909777061 | USP17L8 | ubiquitin specific peptidase 17-like family member 8 [Source:HGNC Symbol;Acc:HGNC:37181] |
| ENSG00000237289 | HGLibA_09781 | 24 | -0.909777061 | CKMT1B | mitochondrial 1B [Source:HGNC Symbol;Acc:HGNC:1995]" |
| ENSG00000241598 | HGLibA_25798 | 24 | -0.909777061 | KRTAP5-4 | keratin associated protein 5-4 [Source:HGNC Symbol;Acc:HGNC:23599] |
| ENSG00000242852 | HGLibA_56605 | 24 | -0.909777061 | ZNF709 | zinc finger protein 709 [Source:HGNC Symbol;Acc:HGNC:20629] |
| ENSG00000251655 | HGLibA_38338 | 24 | -0.909777061 | PRB1 | proline rich protein BstNI subfamily 1 [Source:HGNC Symbol;Acc:HGNC:9337] |
| ENSG00000257365 | HGLibA_17773 | 24 | -0.909777061 | FNTB | beta [Source:HGNC Symbol;Acc:HGNC:3785]" |
| ENSG00000258890 | HGLibA_09180 | 24 | -0.909777061 | CEP95 | centrosomal protein 95 [Source:HGNC Symbol;Acc:HGNC:25141] |
| ENSG00000260001 | HGLibA_49231 | 24 | -0.909777061 | TGFBR3L | transforming growth factor beta receptor 3 like [Source:HGNC Symbol;Acc:HGNC:44152] |
| ENSG00000262814 | HGLibA_29897 | 24 | -0.909777061 | MRPL12 | mitochondrial ribosomal protein L12 [Source:HGNC Symbol;Acc:HGNC:10378] |
| ENSG00000268651 | HGLibA_11558 | 24 | -0.909777061 | CTAG1A | cancer/testis antigen 1A [Source:HGNC Symbol;Acc:HGNC:24198] |
| ENSG00000273706 | HGLibA_26447 | 24 | -0.909777061 | LHX1 | LIM homeobox 1 [Source:HGNC Symbol;Acc:HGNC:6593] |
| ENSG00000275489 | HGLibA_05601 | 24 | -0.909777061 | C17orf98 | chromosome 17 open reading frame 98 [Source:HGNC Symbol;Acc:HGNC:34492] |
| ENSG00000280109 | HGLibA_36938 | 24 | -0.909777061 | PLAC4 | placenta specific 4 [Source:HGNC Symbol;Acc:HGNC:14616] |
| GBAS | HGLibA_18715 | 24 | -0.909777061 | GBAS | - |
| GPR110 | HGLibA_19850 | 24 | -0.909777061 | GPR110 | - |
| GPR110 | HGLibA_19849 | 24 | -0.909777061 | GPR110 | - |
| HIST1H2AA | HGLibA_21374 | 24 | -0.909777061 | HIST1H2AA | - |
| HIST1H2AJ | HGLibA_21399 | 24 | -0.909777061 | HIST1H2AJ | - |
| HIST1H2BH | HGLibA_21432 | 24 | -0.909777061 | HIST1H2BH | - |
| HIST2H2AB | HGLibA_21528 | 24 | -0.909777061 | HIST2H2AB | - |
| hsa-mir-1238 | HGLibA_57343 | 24 | -0.909777061 | hsa-mir-1238 | - |
| hsa-mir-1258 | HGLibA_57441 | 24 | -0.909777061 | hsa-mir-1258 | - |
| hsa-mir-1277 | HGLibA_57565 | 24 | -0.909777061 | hsa-mir-1277 | - |
| hsa-mir-128-1 | HGLibA_57574 | 24 | -0.909777061 | hsa-mir-128-1 | - |
| hsa-mir-183 | HGLibA_57996 | 24 | -0.909777061 | hsa-mir-183 | - |
| hsa-mir-186 | HGLibA_58005 | 24 | -0.909777061 | hsa-mir-186 | - |
| hsa-mir-24-2 | HGLibA_58344 | 24 | -0.909777061 | hsa-mir-24-2 | - |
| hsa-mir-30e | HGLibA_58491 | 24 | -0.909777061 | hsa-mir-30e | - |
| hsa-mir-3119-2 | HGLibA_58527 | 24 | -0.909777061 | hsa-mir-3119-2 | - |
| hsa-mir-3142 | HGLibA_58628 | 24 | -0.909777061 | hsa-mir-3142 | - |
| hsa-mir-3160-1 | HGLibA_58715 | 24 | -0.909777061 | hsa-mir-3160-1 | - |
| hsa-mir-3164 | HGLibA_58736 | 24 | -0.909777061 | hsa-mir-3164 | - |
| hsa-mir-3176 | HGLibA_58778 | 24 | -0.909777061 | hsa-mir-3176 | - |
| hsa-mir-3198-2 | HGLibA_58895 | 24 | -0.909777061 | hsa-mir-3198-2 | - |
| hsa-mir-3199-2 | HGLibA_58901 | 24 | -0.909777061 | hsa-mir-3199-2 | - |
| hsa-mir-3202-1 | HGLibA_58914 | 24 | -0.909777061 | hsa-mir-3202-1 | - |
| hsa-mir-3673 | HGLibA_59245 | 24 | -0.909777061 | hsa-mir-3673 | - |
| hsa-mir-3685 | HGLibA_59294 | 24 | -0.909777061 | hsa-mir-3685 | - |
| hsa-mir-3909 | HGLibA_59501 | 24 | -0.909777061 | hsa-mir-3909 | - |
| hsa-mir-3975 | HGLibA_59661 | 24 | -0.909777061 | hsa-mir-3975 | - |
| hsa-mir-4261 | HGLibA_59750 | 24 | -0.909777061 | hsa-mir-4261 | - |
| hsa-mir-4279 | HGLibA_59820 | 24 | -0.909777061 | hsa-mir-4279 | - |
| hsa-mir-4320 | HGLibA_60002 | 24 | -0.909777061 | hsa-mir-4320 | - |
| hsa-mir-4488 | HGLibA_60371 | 24 | -0.909777061 | hsa-mir-4488 | - |
| hsa-mir-4662b | HGLibA_60761 | 24 | -0.909777061 | hsa-mir-4662b | - |
| hsa-mir-4667 | HGLibA_60787 | 24 | -0.909777061 | hsa-mir-4667 | - |
| hsa-mir-5096 | HGLibA_61541 | 24 | -0.909777061 | hsa-mir-5096 | - |
| hsa-mir-514a-1 | HGLibA_61585 | 24 | -0.909777061 | hsa-mir-514a-1 | - |
| hsa-mir-518f | HGLibA_61675 | 24 | -0.909777061 | hsa-mir-518f | - |
| hsa-mir-541 | HGLibA_61816 | 24 | -0.909777061 | hsa-mir-541 | - |
| hsa-mir-5690 | HGLibA_62278 | 24 | -0.909777061 | hsa-mir-5690 | - |
| hsa-mir-570 | HGLibA_62333 | 24 | -0.909777061 | hsa-mir-570 | - |
| hsa-mir-575 | HGLibA_62394 | 24 | -0.909777061 | hsa-mir-575 | - |
| hsa-mir-6080 | HGLibA_62581 | 24 | -0.909777061 | hsa-mir-6080 | - |
| hsa-mir-6090 | HGLibA_62629 | 24 | -0.909777061 | hsa-mir-6090 | - |
| hsa-mir-6126 | HGLibA_62652 | 24 | -0.909777061 | hsa-mir-6126 | - |
| hsa-mir-618 | HGLibA_62710 | 24 | -0.909777061 | hsa-mir-618 | - |
| hsa-mir-633 | HGLibA_62770 | 24 | -0.909777061 | hsa-mir-633 | - |
| hsa-mir-635 | HGLibA_62778 | 24 | -0.909777061 | hsa-mir-635 | - |
| hsa-mir-6511a-4 | HGLibA_62911 | 24 | -0.909777061 | hsa-mir-6511a-4 | - |
| hsa-mir-6722 | HGLibA_63051 | 24 | -0.909777061 | hsa-mir-6722 | - |
| KIAA1279 | HGLibA_24732 | 24 | -0.909777061 | KIAA1279 | - |

| | | | | | |
|---|---|---|---|---|---|
| MPP5 | HGLibA_29760 | 24 | -0.909777061 | MPP5 | - |
| NUPL1 | HGLibA_33082 | 24 | -0.909777061 | NUPL1 | - |
| PCNX | HGLibA_35644 | 24 | -0.909777061 | PCNX | - |
| SF3B14 | HGLibA_43704 | 24 | -0.909777061 | SF3B14 | - |
| TGIF2-C20orf24 | HGLibA_49243 | 24 | -0.909777061 | TGIF2-C20orf24 | - |
| ALS2CR11 | HGLibA_01725 | 23 | -0.919728098 | ALS2CR11 | - |
| BAGE3 | HGLibA_04097 | 23 | -0.919728098 | BAGE3 | - |
| C15orf56 | HGLibA_05416 | 23 | -0.919728098 | C15orf56 | - |
| C3orf30 | HGLibA_06228 | 23 | -0.919728098 | C3orf30 | - |
| C7orf34 | HGLibA_06538 | 23 | -0.919728098 | C7orf34 | - |
| ENSG00000007168 | HGLibA_34990 | 23 | -0.919728098 | PAFAH1B1 | platelet activating factor acetylhydrolase 1b regulatory subunit 1 [Source:HGNC Symbol;Acc:HGNC:8574] |
| ENSG00000010310 | HGLibA_19128 | 23 | -0.919728098 | GIPR | gastric inhibitory polypeptide receptor [Source:HGNC Symbol;Acc:HGNC:4271] |
| ENSG00000010327 | HGLibA_47306 | 23 | -0.919728098 | STAB1 | stabilin 1 [Source:HGNC Symbol;Acc:HGNC:18628] |
| ENSG00000013561 | HGLibA_41519 | 23 | -0.919728098 | RNF14 | ring finger protein 14 [Source:HGNC Symbol;Acc:HGNC:10058] |
| ENSG00000018699 | HGLibA_52093 | 23 | -0.919728098 | TTC27 | tetratricopeptide repeat domain 27 [Source:HGNC Symbol;Acc:HGNC:25986] |
| ENSG00000046647 | HGLibA_18926 | 23 | -0.919728098 | GEMIN8 | gem nuclear organelle associated protein 8 [Source:HGNC Symbol;Acc:HGNC:26044] |
| ENSG00000059728 | HGLibA_30610 | 23 | -0.919728098 | MXD1 | MAX dimerization protein 1 [Source:HGNC Symbol;Acc:HGNC:6761] |
| ENSG00000065328 | HGLibA_28601 | 23 | -0.919728098 | MCM10 | minichromosome maintenance 10 replication initiation factor [Source:HGNC Symbol;Acc:HGNC:18043] |
| ENSG00000066654 | HGLibA_49414 | 23 | -0.919728098 | THUMPD1 | THUMP domain containing 1 [Source:HGNC Symbol;Acc:HGNC:23807] |
| ENSG00000069424 | HGLibA_24149 | 23 | -0.919728098 | KCNAB2 | potassium voltage-gated channel subfamily A regulatory beta subunit 2 [Source:HGNC Symbol;Acc:HGNC:6229] |
| ENSG00000069966 | HGLibA_19501 | 23 | -0.919728098 | GNB5 | G protein subunit beta 5 [Source:HGNC Symbol;Acc:HGNC:4401] |
| ENSG00000071655 | HGLibA_28468 | 23 | -0.919728098 | MBD3 | methyl-CpG binding domain protein 3 [Source:HGNC Symbol;Acc:HGNC:6918] |
| ENSG00000073008 | HGLibA_39615 | 23 | -0.919728098 | PVR | poliovirus receptor [Source:HGNC Symbol;Acc:HGNC:9705] |
| ENSG00000073584 | HGLibA_45639 | 23 | -0.919728098 | SMARCE1 | member 1 [Source:HGNC Symbol;Acc:HGNC:11109]" |
| ENSG00000073792 | HGLibA_22804 | 23 | -0.919728098 | IGF2BP2 | insulin like growth factor 2 mRNA binding protein 2 [Source:HGNC Symbol;Acc:HGNC:28867] |
| ENSG00000075292 | HGLibA_56437 | 23 | -0.919728098 | ZNF638 | zinc finger protein 638 [Source:HGNC Symbol;Acc:HGNC:17894] |
| ENSG00000075461 | HGLibA_06938 | 23 | -0.919728098 | CACNG4 | calcium voltage-gated channel auxiliary subunit gamma 4 [Source:HGNC Symbol;Acc:HGNC:1408] |
| ENSG00000076984 | HGLibA_28158 | 23 | -0.919728098 | MAP2K7 | mitogen-activated protein kinase kinase 7 [Source:HGNC Symbol;Acc:HGNC:6847] |
| ENSG00000077935 | HGLibA_45643 | 23 | -0.919728098 | SMC1B | structural maintenance of chromosomes 1B [Source:HGNC Symbol;Acc:HGNC:11112] |
| ENSG00000078687 | HGLibA_50920 | 23 | -0.919728098 | TNRC6C | trinucleotide repeat containing 6C [Source:HGNC Symbol;Acc:HGNC:29318] |
| ENSG00000080007 | HGLibA_12718 | 23 | -0.919728098 | DDX43 | DEAD-box helicase 43 [Source:HGNC Symbol;Acc:HGNC:18677] |
| ENSG00000080371 | HGLibA_39837 | 23 | -0.919728098 | RAB21 | member RAS oncogene family [Source:HGNC Symbol;Acc:HGNC:18263]" |
| ENSG00000080822 | HGLibA_09917 | 23 | -0.919728098 | CLDND1 | claudin domain containing 1 [Source:HGNC Symbol;Acc:HGNC:1322] |
| ENSG00000081189 | HGLibA_28839 | 23 | -0.919728098 | MEF2C | myocyte enhancer factor 2C [Source:HGNC Symbol;Acc:HGNC:6996] |
| ENSG00000082212 | HGLibA_28711 | 23 | -0.919728098 | ME2 | malic enzyme 2 [Source:HGNC Symbol;Acc:HGNC:6984] |
| ENSG00000083844 | HGLibA_55696 | 23 | -0.919728098 | ZNF264 | zinc finger protein 264 [Source:HGNC Symbol;Acc:HGNC:13057] |
| ENSG00000087510 | HGLibA_49126 | 23 | -0.919728098 | TFAP2C | transcription factor AP-2 gamma [Source:HGNC Symbol;Acc:HGNC:11744] |
| ENSG00000088888 | HGLibA_28444 | 23 | -0.919728098 | MAVS | mitochondrial antiviral signaling protein [Source:HGNC Symbol;Acc:HGNC:29233] |
| ENSG00000090621 | HGLibA_34937 | 23 | -0.919728098 | PABPC4 | poly(A) binding protein cytoplasmic 4 [Source:HGNC Symbol;Acc:HGNC:8557] |
| ENSG00000091262 | HGLibA_00181 | 23 | -0.919728098 | ABCC6 | ATP binding cassette subfamily C member 6 [Source:HGNC Symbol;Acc:HGNC:57] |
| ENSG00000096401 | HGLibA_08590 | 23 | -0.919728098 | CDC5L | cell division cycle 5 like [Source:HGNC Symbol;Acc:HGNC:1743] |
| ENSG00000096746 | HGLibA_21827 | 23 | -0.919728098 | HNRNPH3 | heterogeneous nuclear ribonucleoprotein H3 [Source:HGNC Symbol;Acc:HGNC:5043] |
| ENSG00000099622 | HGLibA_09722 | 23 | -0.919728098 | CIRBP | cold inducible RNA binding protein [Source:HGNC Symbol;Acc:HGNC:1982] |
| ENSG00000099942 | HGLibA_11183 | 23 | -0.919728098 | CRKL | adaptor protein [Source:HGNC Symbol;Acc:HGNC:2363]" |
| ENSG00000099991 | HGLibA_06838 | 23 | -0.919728098 | CABIN1 | calcineurin binding protein 1 [Source:HGNC Symbol;Acc:HGNC:24187] |
| ENSG00000100038 | HGLibA_51004 | 23 | -0.919728098 | TOP3B | DNA topoisomerase III beta [Source:HGNC Symbol;Acc:HGNC:11993] |
| ENSG00000100312 | HGLibA_00531 | 23 | -0.919728098 | ACR | acrosin [Source:HGNC Symbol;Acc:HGNC:126] |
| ENSG00000100425 | HGLibA_04770 | 23 | -0.919728098 | BRD1 | bromodomain containing 1 [Source:HGNC Symbol;Acc:HGNC:1102] |
| ENSG00000100605 | HGLibA_23840 | 23 | -0.919728098 | ITPK1 | inositol-tetrakisphosphate 1-kinase [Source:HGNC Symbol;Acc:HGNC:6177] |
| ENSG00000100650 | HGLibA_47080 | 23 | -0.919728098 | SRSF5 | serine and arginine rich splicing factor 5 [Source:HGNC Symbol;Acc:HGNC:10787] |
| ENSG00000101019 | HGLibA_53097 | 23 | -0.919728098 | UQCC1 | ubiquinol-cytochrome c reductase complex assembly factor 1 [Source:HGNC Symbol;Acc:HGNC:15891] |
| ENSG00000101074 | HGLibA_39780 | 23 | -0.919728098 | R3HDML | R3H domain containing like [Source:HGNC Symbol;Acc:HGNC:16249] |
| ENSG00000101096 | HGLibA_31776 | 23 | -0.919728098 | NFATC2 | nuclear factor of activated T cells 2 [Source:HGNC Symbol;Acc:HGNC:7776] |
| ENSG00000101146 | HGLibA_40116 | 23 | -0.919728098 | RAE1 | ribonucleic acid export 1 [Source:HGNC Symbol;Acc:HGNC:9828] |
| ENSG00000101347 | HGLibA_42705 | 23 | -0.919728098 | SAMHD1 | SAM and HD domain containing deoxynucleoside triphosphate triphosphohydrolase 1 [Source:HGNC Symbol;Acc:HGNC:15925] |
| ENSG00000102683 | HGLibA_43808 | 23 | -0.919728098 | SGCG | sarcoglycan gamma [Source:HGNC Symbol;Acc:HGNC:10809] |
| ENSG00000102974 | HGLibA_11597 | 23 | -0.919728098 | CTCF | CCCTC-binding factor [Source:HGNC Symbol;Acc:HGNC:13723] |
| ENSG00000103121 | HGLibA_10187 | 23 | -0.919728098 | CMC2 | C-X9-C motif containing 2 [Source:HGNC Symbol;Acc:HGNC:24447] |
| ENSG00000104823 | HGLibA_14392 | 23 | -0.919728098 | ECH1 | enoyl-CoA hydratase 1 [Source:HGNC Symbol;Acc:HGNC:3149] |
| ENSG00000104872 | HGLibA_36609 | 23 | -0.919728098 | PIH1D1 | PIH1 domain containing 1 [Source:HGNC Symbol;Acc:HGNC:26075] |
| ENSG00000106804 | HGLibA_06351 | 23 | -0.919728098 | C5 | complement C5 [Source:HGNC Symbol;Acc:HGNC:1331] |
| ENSG00000106976 | HGLibA_13684 | 23 | -0.919728098 | DNM1 | dynamin 1 [Source:HGNC Symbol;Acc:HGNC:2972] |
| ENSG00000107341 | HGLibA_52655 | 23 | -0.919728098 | UBE2R2 | ubiquitin conjugating enzyme E2 R2 [Source:HGNC Symbol;Acc:HGNC:19907] |
| ENSG00000107959 | HGLibA_36758 | 23 | -0.919728098 | PITRM1 | pitrilysin metallopeptidase 1 [Source:HGNC Symbol;Acc:HGNC:17663] |
| ENSG00000108312 | HGLibA_52772 | 23 | -0.919728098 | UBTF | upstream binding transcription factor [Source:HGNC Symbol;Acc:HGNC:12511] |
| ENSG00000109107 | HGLibA_01599 | 23 | -0.919728098 | ALDOC | fructose-bisphosphate C [Source:HGNC Symbol;Acc:HGNC:418]" |
| ENSG00000110422 | HGLibA_21346 | 23 | -0.919728098 | HIPK3 | homeodomain interacting protein kinase 3 [Source:HGNC Symbol;Acc:HGNC:4915] |
| ENSG00000111331 | HGLibA_33205 | 23 | -0.919728098 | OAS3 | 2'-5'-oligoadenylate synthetase 3 [Source:HGNC Symbol;Acc:HGNC:8088] |
| ENSG00000112312 | HGLibA_19419 | 23 | -0.919728098 | GMNN | DNA replication inhibitor [Source:HGNC Symbol;Acc:HGNC:17493]" |
| ENSG00000112787 | HGLibA_16969 | 23 | -0.919728098 | FBRSL1 | fibrosin like 1 [Source:HGNC Symbol;Acc:HGNC:29308] |
| ENSG00000113312 | HGLibA_52045 | 23 | -0.919728098 | TTC1 | tetratricopeptide repeat domain 1 [Source:HGNC Symbol;Acc:HGNC:12391] |
| ENSG00000114378 | HGLibA_22438 | 23 | -0.919728098 | HYAL1 | hyaluronidase 1 [Source:HGNC Symbol;Acc:HGNC:5320] |
| ENSG00000115226 | HGLibA_17752 | 23 | -0.919728098 | FNDC4 | fibronectin type III domain containing 4 [Source:HGNC Symbol;Acc:HGNC:20239] |
| ENSG00000115271 | HGLibA_18759 | 23 | -0.919728098 | GCA | grancalcin [Source:HGNC Symbol;Acc:HGNC:15990] |
| ENSG00000115421 | HGLibA_35141 | 23 | -0.919728098 | PAPOLG | poly(A) polymerase gamma [Source:HGNC Symbol;Acc:HGNC:14982] |
| ENSG00000116774 | HGLibA_33365 | 23 | -0.919728098 | OLFML3 | olfactomedin like 3 [Source:HGNC Symbol;Acc:HGNC:24956] |
| ENSG00000118946 | HGLibA_35402 | 23 | -0.919728098 | PCDH17 | protocadherin 17 [Source:HGNC Symbol;Acc:HGNC:14267] |
| ENSG00000119913 | HGLibA_48956 | 23 | -0.919728098 | TECTB | tectorin beta [Source:HGNC Symbol;Acc:HGNC:11721] |
| ENSG00000120158 | HGLibA_40698 | 23 | -0.919728098 | RCL1 | RNA terminal phosphate cyclase like 1 [Source:HGNC Symbol;Acc:HGNC:17687] |
| ENSG00000120215 | HGLibA_29362 | 23 | -0.919728098 | MLANA | melan-A [Source:HGNC Symbol;Acc:HGNC:7124] |
| ENSG00000122912 | HGLibA_44655 | 23 | -0.919728098 | SLC25A16 | solute carrier family 25 member 16 [Source:HGNC Symbol;Acc:HGNC:10986] |
| ENSG00000123297 | HGLibA_51842 | 23 | -0.919728098 | TSFM | mitochondrial [Source:HGNC Symbol;Acc:HGNC:12367]" |
| ENSG00000124193 | HGLibA_47085 | 23 | -0.919728098 | SRSF6 | serine and arginine rich splicing factor 6 [Source:HGNC Symbol;Acc:HGNC:10788] |
| ENSG00000124257 | HGLibA_31728 | 23 | -0.919728098 | NEURL2 | neuralized E3 ubiquitin protein ligase 2 [Source:HGNC Symbol;Acc:HGNC:16156] |
| ENSG00000124459 | HGLibA_56027 | 23 | -0.919728098 | ZNF45 | zinc finger protein 45 [Source:HGNC Symbol;Acc:HGNC:13111] |
| ENSG00000124508 | HGLibA_04965 | 23 | -0.919728098 | BTN2A2 | butyrophilin subfamily 2 member A2 [Source:HGNC Symbol;Acc:HGNC:1137] |
| ENSG00000125775 | HGLibA_43111 | 23 | -0.919728098 | SDCBP2 | syndecan binding protein 2 [Source:HGNC Symbol;Acc:HGNC:15756] |
| ENSG00000126934 | HGLibA_28143 | 23 | -0.919728098 | MAP2K2 | mitogen-activated protein kinase kinase 2 [Source:HGNC Symbol;Acc:HGNC:6842] |
| ENSG00000128228 | HGLibA_43128 | 23 | -0.919728098 | SDF2L1 | stromal cell derived factor 2 like 1 [Source:HGNC Symbol;Acc:HGNC:10676] |
| ENSG00000128731 | HGLibA_21155 | 23 | -0.919728098 | HERC2 | HECT and RLD domain containing E3 ubiquitin protein ligase 2 [Source:HGNC Symbol;Acc:HGNC:4868] |
| ENSG00000129451 | HGLibA_25235 | 23 | -0.919728098 | KLK10 | kallikrein related peptidase 10 [Source:HGNC Symbol;Acc:HGNC:6358] |
| ENSG00000130414 | HGLibA_31440 | 23 | -0.919728098 | NDUFA10 | NADH:ubiquinone oxidoreductase subunit A10 [Source:HGNC Symbol;Acc:HGNC:7684] |
| ENSG00000131652 | HGLibA_49376 | 23 | -0.919728098 | THOC6 | THO complex 6 [Source:HGNC Symbol;Acc:HGNC:28369] |
| ENSG00000132846 | HGLibA_54797 | 23 | -0.919728098 | ZBED3 | zinc finger BED-type containing 3 [Source:HGNC Symbol;Acc:HGNC:20711] |
| ENSG00000134001 | HGLibA_14753 | 23 | -0.919728098 | EIF2S1 | eukaryotic translation initiation factor 2 subunit alpha [Source:HGNC Symbol;Acc:HGNC:3265] |
| ENSG00000134698 | HGLibA_01215 | 23 | -0.919728098 | AGO4 | RISC catalytic component [Source:HGNC Symbol;Acc:HGNC:18424]" |
| ENSG00000134809 | HGLibA_49493 | 23 | -0.919728098 | TIMM10 | translocase of inner mitochondrial membrane 10 [Source:HGNC Symbol;Acc:HGNC:11814] |
| ENSG00000134996 | HGLibA_34712 | 23 | -0.919728098 | OSTF1 | osteoclast stimulating factor 1 [Source:HGNC Symbol;Acc:HGNC:8510] |
| ENSG00000135338 | HGLibA_26090 | 23 | -0.919728098 | LCA5 | lebercilin [Source:HGNC Symbol;Acc:HGNC:31923]" |
| ENSG00000135413 | HGLibA_25913 | 23 | -0.919728098 | LACRT | lacritin [Source:HGNC Symbol;Acc:HGNC:16430] |
| ENSG00000135823 | HGLibA_47654 | 23 | -0.919728098 | STX6 | syntaxin 6 [Source:HGNC Symbol;Acc:HGNC:11441] |
| ENSG00000136026 | HGLibA_09761 | 23 | -0.919728098 | CKAP4 | cytoskeleton associated protein 4 [Source:HGNC Symbol;Acc:HGNC:16991] |
| ENSG00000136546 | HGLibA_43002 | 23 | -0.919728098 | SCN7A | sodium voltage-gated channel alpha subunit 7 [Source:HGNC Symbol;Acc:HGNC:10594] |
| ENSG00000136826 | HGLibA_25073 | 23 | -0.919728098 | KLF4 | Kruppel like factor 4 [Source:HGNC Symbol;Acc:HGNC:6348] |
| ENSG00000136861 | HGLibA_08786 | 23 | -0.919728098 | CDK5RAP2 | CDK5 regulatory subunit associated protein 2 [Source:HGNC Symbol;Acc:HGNC:18672] |
| ENSG00000137449 | HGLibA_10920 | 23 | -0.919728098 | CPEB2 | cytoplasmic polyadenylation element binding protein 2 [Source:HGNC Symbol;Acc:HGNC:21745] |
| ENSG00000137573 | HGLibA_47726 | 23 | -0.919728098 | SULF1 | sulfatase 1 [Source:HGNC Symbol;Acc:HGNC:20391] |
| ENSG00000138303 | HGLibA_03256 | 23 | -0.919728098 | ASCC1 | activating signal cointegrator 1 complex subunit 1 [Source:HGNC Symbol;Acc:HGNC:24268] |
| ENSG00000138315 | HGLibA_33336 | 23 | -0.919728098 | OIT3 | oncoprotein induced transcript 3 [Source:HGNC Symbol;Acc:HGNC:29953] |
| ENSG00000138772 | HGLibA_02236 | 23 | -0.919728098 | ANXA3 | annexin A3 [Source:HGNC Symbol;Acc:HGNC:541] |
| ENSG00000139351 | HGLibA_47936 | 23 | -0.919728098 | SYCP3 | synaptonemal complex protein 3 [Source:HGNC Symbol;Acc:HGNC:18130] |
| ENSG00000139370 | HGLibA_44414 | 23 | -0.919728098 | SLC15A4 | solute carrier family 15 member 4 [Source:HGNC Symbol;Acc:HGNC:23090] |
| ENSG00000140044 | HGLibA_23943 | 23 | -0.919728098 | JDP2 | Jun dimerization protein 2 [Source:HGNC Symbol;Acc:HGNC:17546] |
| ENSG00000140299 | HGLibA_04652 | 23 | -0.919728098 | BNIP2 | BCL2 interacting protein 2 [Source:HGNC Symbol;Acc:HGNC:1083] |
| ENSG00000140319 | HGLibA_46999 | 23 | -0.919728098 | SRP14 | signal recognition particle 14 [Source:HGNC Symbol;Acc:HGNC:11299] |
| ENSG00000141642 | HGLibA_14869 | 23 | -0.919728098 | ELAC1 | elaC ribonuclease Z 1 [Source:HGNC Symbol;Acc:HGNC:14197] |
| ENSG00000141644 | HGLibA_28463 | 23 | -0.919728098 | MBD1 | methyl-CpG binding domain protein 1 [Source:HGNC Symbol;Acc:HGNC:6916] |
| ENSG00000141753 | HGLibA_22824 | 23 | -0.919728098 | IGFBP4 | insulin like growth factor binding protein 4 [Source:HGNC Symbol;Acc:HGNC:5473] |
| ENSG00000142698 | HGLibA_05907 | 23 | -0.919728098 | C1orf94 | chromosome 1 open reading frame 94 [Source:HGNC Symbol;Acc:HGNC:28250] |
| ENSG00000143079 | HGLibA_11746 | 23 | -0.919728098 | CTTNBP2NL | CTTNBP2 N-terminal like [Source:HGNC Symbol;Acc:HGNC:25330] |
| ENSG00000143469 | HGLibA_48061 | 23 | -0.919728098 | SYT14 | synaptotagmin 14 [Source:HGNC Symbol;Acc:HGNC:23143] |
| ENSG00000144045 | HGLibA_13938 | 23 | -0.919728098 | DQX1 | DEAQ-box RNA dependent ATPase 1 [Source:HGNC Symbol;Acc:HGNC:20410] |
| ENSG00000144161 | HGLibA_54988 | 23 | -0.919728098 | ZC3H8 | zinc finger CCCH-type containing 8 [Source:HGNC Symbol;Acc:HGNC:30941] |
| ENSG00000144285 | HGLibA_42973 | 23 | -0.919728098 | SCN1A | sodium voltage-gated channel alpha subunit 1 [Source:HGNC Symbol;Acc:HGNC:10585] |
| ENSG00000144648 | HGLibA_00443 | 23 | -0.919728098 | ACKR2 | atypical chemokine receptor 2 [Source:HGNC Symbol;Acc:HGNC:1565] |
| ENSG00000145626 | HGLibA_52932 | 23 | -0.919728098 | UGT3A1 | UDP glycosyltransferase family 3 member A1 [Source:HGNC Symbol;Acc:HGNC:26625] |
| ENSG00000145912 | HGLibA_31907 | 23 | -0.919728098 | NHP2 | NHP2 ribonucleoprotein [Source:HGNC Symbol;Acc:HGNC:14377] |
| ENSG00000146373 | HGLibA_41634 | 23 | -0.919728098 | RNF217 | ring finger protein 217 [Source:HGNC Symbol;Acc:HGNC:21487] |
| ENSG00000146540 | HGLibA_06552 | 23 | -0.919728098 | C7orf50 | chromosome 7 open reading frame 50 [Source:HGNC Symbol;Acc:HGNC:22421] |
| ENSG00000146926 | HGLibA_03206 | 23 | -0.919728098 | ASB10 | ankyrin repeat and SOCS box containing 10 [Source:HGNC Symbol;Acc:HGNC:17185] |
| ENSG00000147118 | HGLibA_55543 | 23 | -0.919728098 | ZNF182 | zinc finger protein 182 [Source:HGNC Symbol;Acc:HGNC:13001] |
| ENSG00000147274 | HGLibA_40596 | 23 | -0.919728098 | RBMX | RNA binding motif protein X-linked [Source:HGNC Symbol;Acc:HGNC:9910] |
| ENSG00000147533 | HGLibA_19633 | 23 | -0.919728098 | GOLGA7 | golgin A7 [Source:HGNC Symbol;Acc:HGNC:24876] |
| ENSG00000149823 | HGLibA_53802 | 23 | -0.919728098 | VPS51 | GARP complex subunit [Source:HGNC Symbol;Acc:HGNC:1172]" |
| ENSG00000150630 | HGLibA_53618 | 23 | -0.919728098 | VEGFC | vascular endothelial growth factor C [Source:HGNC Symbol;Acc:HGNC:12682] |

| | | | | | |
|---|---|---|---|---|---|
| ENSG00000151360 | HGLibA_01669 | 23 | -0.919728098 | ALLC | allantoicase [Source:HGNC Symbol;Acc:HGNC:17377] |
| ENSG00000153012 | HGLibA_26396 | 23 | -0.919728098 | LGI2 | leucine rich repeat LGI family member 2 [Source:HGNC Symbol;Acc:HGNC:18710] |
| ENSG00000153551 | HGLibA_10228 | 23 | -0.919728098 | CMTM7 | CKLF like MARVEL transmembrane domain containing 7 [Source:HGNC Symbol;Acc:HGNC:19178] |
| ENSG00000154118 | HGLibA_23984 | 23 | -0.919728098 | JPH3 | junctophilin 3 [Source:HGNC Symbol;Acc:HGNC:14203] |
| ENSG00000154144 | HGLibA_48635 | 23 | -0.919728098 | TBRG1 | transforming growth factor beta regulator 1 [Source:HGNC Symbol;Acc:HGNC:29551] |
| ENSG00000155428 | HGLibA_51610 | 23 | -0.919728098 | TRIM74 | tripartite motif containing 74 [Source:HGNC Symbol;Acc:HGNC:17453] |
| ENSG00000156564 | HGLibA_27156 | 23 | -0.919728098 | LRFN2 | leucine rich repeat and fibronectin type III domain containing 2 [Source:HGNC Symbol;Acc:HGNC:21226] |
| ENSG00000158516 | HGLibA_10888 | 23 | -0.919728098 | CPA2 | carboxypeptidase A2 [Source:HGNC Symbol;Acc:HGNC:2297] |
| ENSG00000158864 | HGLibA_31548 | 23 | -0.919728098 | NDUFS2 | NADH:ubiquinone oxidoreductase core subunit S2 [Source:HGNC Symbol;Acc:HGNC:7708] |
| ENSG00000159228 | HGLibA_07417 | 23 | -0.919728098 | CBR1 | carbonyl reductase 1 [Source:HGNC Symbol;Acc:HGNC:1548] |
| ENSG00000160224 | HGLibA_01366 | 23 | -0.919728098 | AIRE | autoimmune regulator [Source:HGNC Symbol;Acc:HGNC:360] |
| ENSG00000161016 | HGLibA_42001 | 23 | -0.919728098 | RPL8 | ribosomal protein L8 [Source:HGNC Symbol;Acc:HGNC:10368] |
| ENSG00000162068 | HGLibA_32855 | 23 | -0.919728098 | NTN3 | netrin 3 [Source:HGNC Symbol;Acc:HGNC:8030] |
| ENSG00000162882 | HGLibA_20791 | 23 | -0.919728098 | HAAO | 4-dioxygenase [Source:HGNC Symbol;Acc:HGNC:4796]" |
| ENSG00000163060 | HGLibA_48980 | 23 | -0.919728098 | TEKT4 | tektin 4 [Source:HGNC Symbol;Acc:HGNC:31012] |
| ENSG00000163945 | HGLibA_53476 | 23 | -0.919728098 | UVSSA | UV stimulated scaffold protein A [Source:HGNC Symbol;Acc:HGNC:29304] |
| ENSG00000163960 | HGLibA_52796 | 23 | -0.919728098 | UBXN7 | UBX domain protein 7 [Source:HGNC Symbol;Acc:HGNC:29119] |
| ENSG00000165118 | HGLibA_06750 | 23 | -0.919728098 | C9orf64 | chromosome 9 open reading frame 64 [Source:HGNC Symbol;Acc:HGNC:28144] |
| ENSG00000165409 | HGLibA_51859 | 23 | -0.919728098 | TSHR | thyroid stimulating hormone receptor [Source:HGNC Symbol;Acc:HGNC:12373] |
| ENSG00000166579 | HGLibA_31390 | 23 | -0.919728098 | NDEL1 | nudE neurodevelopment protein 1 like 1 [Source:HGNC Symbol;Acc:HGNC:17620] |
| ENSG00000166922 | HGLibA_42905 | 23 | -0.919728098 | SCG5 | secretogranin V [Source:HGNC Symbol;Acc:HGNC:10816] |
| ENSG00000166925 | HGLibA_51826 | 23 | -0.919728098 | TSC22D4 | TSC22 domain family member 4 [Source:HGNC Symbol;Acc:HGNC:21696] |
| ENSG00000167004 | HGLibA_35877 | 23 | -0.919728098 | PDIA3 | protein disulfide isomerase family A member 3 [Source:HGNC Symbol;Acc:HGNC:4606] |
| ENSG00000167580 | HGLibA_02591 | 23 | -0.919728098 | AQP2 | aquaporin 2 [Source:HGNC Symbol;Acc:HGNC:634] |
| ENSG00000167770 | HGLibA_34753 | 23 | -0.919728098 | OTUB1 | ubiquitin aldehyde binding 1 [Source:HGNC Symbol;Acc:HGNC:23077]" |
| ENSG00000168216 | HGLibA_26676 | 23 | -0.919728098 | LMBRD1 | LMBR1 domain containing 1 [Source:HGNC Symbol;Acc:HGNC:23038] |
| ENSG00000168589 | HGLibA_14261 | 23 | -0.919728098 | DYNLRB2 | dynein light chain roadblock-type 2 [Source:HGNC Symbol;Acc:HGNC:15467] |
| ENSG00000168701 | HGLibA_50200 | 23 | -0.919728098 | TMEM208 | transmembrane protein 208 [Source:HGNC Symbol;Acc:HGNC:25015] |
| ENSG00000169432 | HGLibA_43007 | 23 | -0.919728098 | SCN9A | sodium voltage-gated channel alpha subunit 9 [Source:HGNC Symbol;Acc:HGNC:10597] |
| ENSG00000170525 | HGLibA_36159 | 23 | -0.919728098 | PFKFB3 | 6-biphosphatase 3 [Source:HGNC Symbol;Acc:HGNC:8874]" |
| ENSG00000171094 | HGLibA_01644 | 23 | -0.919728098 | ALK | ALK receptor tyrosine kinase [Source:HGNC Symbol;Acc:HGNC:427] |
| ENSG00000171815 | HGLibA_35469 | 23 | -0.919728098 | PCDHB1 | protocadherin beta 1 [Source:HGNC Symbol;Acc:HGNC:8680] |
| ENSG00000172459 | HGLibA_34247 | 23 | -0.919728098 | OR5AR1 | olfactory receptor family 5 subfamily AR member 1 (gene/pseudogene) [Source:HGNC Symbol;Acc:HGNC:15260] |
| ENSG00000172738 | HGLibA_50225 | 23 | -0.919728098 | TMEM217 | transmembrane protein 217 [Source:HGNC Symbol;Acc:HGNC:21238] |
| ENSG00000173198 | HGLibA_12227 | 23 | -0.919728098 | CYSLTR1 | cysteinyl leukotriene receptor 1 [Source:HGNC Symbol;Acc:HGNC:17451] |
| ENSG00000175215 | HGLibA_11612 | 23 | -0.919728098 | CTDSP2 | CTD small phosphatase 2 [Source:HGNC Symbol;Acc:HGNC:17077] |
| ENSG00000175581 | HGLibA_30001 | 23 | -0.919728098 | MRPL48 | mitochondrial ribosomal protein L48 [Source:HGNC Symbol;Acc:HGNC:16653] |
| ENSG00000176246 | HGLibA_34037 | 23 | -0.919728098 | OR4L1 | olfactory receptor family 4 subfamily L member 1 [Source:HGNC Symbol;Acc:HGNC:15356] |
| ENSG00000176695 | HGLibA_33996 | 23 | -0.919728098 | OR4F17 | olfactory receptor family 4 subfamily F member 17 [Source:HGNC Symbol;Acc:HGNC:15381] |
| ENSG00000176887 | HGLibA_46231 | 23 | -0.919728098 | SOX11 | SRY-box 11 [Source:HGNC Symbol;Acc:HGNC:11191] |
| ENSG00000177200 | HGLibA_09410 | 23 | -0.919728098 | CHD9 | chromodomain helicase DNA binding protein 9 [Source:HGNC Symbol;Acc:HGNC:25701] |
| ENSG00000177752 | HGLibA_54687 | 23 | -0.919728098 | YIPF7 | Yip1 domain family member 7 [Source:HGNC Symbol;Acc:HGNC:26825] |
| ENSG00000177889 | HGLibA_52635 | 23 | -0.919728098 | UBE2N | ubiquitin conjugating enzyme E2 N [Source:HGNC Symbol;Acc:HGNC:12492] |
| ENSG00000178150 | HGLibA_55444 | 23 | -0.919728098 | ZNF114 | zinc finger protein 114 [Source:HGNC Symbol;Acc:HGNC:12894] |
| ENSG00000179387 | HGLibA_14943 | 23 | -0.919728098 | ELMOD2 | ELMO domain containing 2 [Source:HGNC Symbol;Acc:HGNC:28111] |
| ENSG00000179841 | HGLibA_01436 | 23 | -0.919728098 | AKAP5 | A-kinase anchoring protein 5 [Source:HGNC Symbol;Acc:HGNC:375] |
| ENSG00000180340 | HGLibA_18265 | 23 | -0.919728098 | FZD2 | frizzled class receptor 2 [Source:HGNC Symbol;Acc:HGNC:4040] |
| ENSG00000180370 | HGLibA_35043 | 23 | -0.919728098 | PAK2 | p21 (RAC1) activated kinase 2 [Source:HGNC Symbol;Acc:HGNC:8591] |
| ENSG00000181163 | HGLibA_32486 | 23 | -0.919728098 | NPM1 | nucleophosmin 1 [Source:HGNC Symbol;Acc:HGNC:7910] |
| ENSG00000182264 | HGLibA_23892 | 23 | -0.919728098 | IZUMO1 | izumo sperm-egg fusion 1 [Source:HGNC Symbol;Acc:HGNC:28539] |
| ENSG00000183313 | HGLibA_34186 | 23 | -0.919728098 | OR52L1 | olfactory receptor family 52 subfamily L member 1 [Source:HGNC Symbol;Acc:HGNC:14785] |
| ENSG00000183454 | HGLibA_20299 | 23 | -0.919728098 | GRIN2A | glutamate ionotropic receptor NMDA type subunit 2A [Source:HGNC Symbol;Acc:HGNC:4585] |
| ENSG00000184076 | HGLibA_53103 | 23 | -0.919728098 | UQCR10 | complex III subunit X [Source:HGNC Symbol;Acc:HGNC:30863]" |
| ENSG00000184508 | HGLibA_21035 | 23 | -0.919728098 | HDDC3 | HD domain containing 3 [Source:HGNC Symbol;Acc:HGNC:30522] |
| ENSG00000185236 | HGLibA_39790 | 23 | -0.919728098 | RAB11B | member RAS oncogene family [Source:HGNC Symbol;Acc:HGNC:9761]" |
| ENSG00000185372 | HGLibA_33896 | 23 | -0.919728098 | OR2V1 | olfactory receptor family 2 subfamily V member 1 [Source:HGNC Symbol;Acc:HGNC:8280] |
| ENSG00000185658 | HGLibA_04853 | 23 | -0.919728098 | BRWD1 | bromodomain and WD repeat domain containing 1 [Source:HGNC Symbol;Acc:HGNC:12760] |
| ENSG00000185829 | HGLibA_02952 | 23 | -0.919728098 | ARL17A | ADP ribosylation factor like GTPase 17A [Source:HGNC Symbol;Acc:HGNC:24096] |
| ENSG00000185974 | HGLibA_20326 | 23 | -0.919728098 | GRK1 | G protein-coupled receptor kinase 1 [Source:HGNC Symbol;Acc:HGNC:10013] |
| ENSG00000186377 | HGLibA_12202 | 23 | -0.919728098 | CYP4X1 | cytochrome P450 family 4 subfamily X member 1 [Source:HGNC Symbol;Acc:HGNC:20244] |
| ENSG00000187097 | HGLibA_15208 | 23 | -0.919728098 | ENTPD5 | ectonucleoside triphosphate diphosphohydrolase 5 [Source:HGNC Symbol;Acc:HGNC:3367] |
| ENSG00000188487 | HGLibA_23373 | 23 | -0.919728098 | INSC | spindle orientation adaptor protein [Source:HGNC Symbol;Acc:HGNC:33116]" |
| ENSG00000189042 | HGLibA_56262 | 23 | -0.919728098 | ZNF567 | zinc finger protein 567 [Source:HGNC Symbol;Acc:HGNC:28696] |
| ENSG00000189233 | HGLibA_33005 | 23 | -0.919728098 | NUGGC | germinal center associated [Source:HGNC Symbol;Acc:HGNC:33550]" |
| ENSG00000196365 | HGLibA_27014 | 23 | -0.919728098 | LONP1 | mitochondrial [Source:HGNC Symbol;Acc:HGNC:9479]" |
| ENSG00000196406 | HGLibA_46394 | 23 | -0.919728098 | SPANXD | SPANX family member D [Source:HGNC Symbol;Acc:HGNC:14332] |
| ENSG00000196705 | HGLibA_55982 | 23 | -0.919728098 | ZNF431 | zinc finger protein 431 [Source:HGNC Symbol;Acc:HGNC:20809] |
| ENSG00000196734 | HGLibA_26103 | 23 | -0.919728098 | LCE1B | late cornified envelope 1B [Source:HGNC Symbol;Acc:HGNC:16611] |
| ENSG00000196772 | HGLibA_33630 | 23 | -0.919728098 | OR14A16 | olfactory receptor family 14 subfamily A member 16 [Source:HGNC Symbol;Acc:HGNC:15022] |
| ENSG00000197147 | HGLibA_27430 | 23 | -0.919728098 | LRRC8B | leucine rich repeat containing 8 VRAC subunit B [Source:HGNC Symbol;Acc:HGNC:30692] |
| ENSG00000197345 | HGLibA_29928 | 23 | -0.919728098 | MRPL21 | mitochondrial ribosomal protein L21 [Source:HGNC Symbol;Acc:HGNC:14479] |
| ENSG00000197653 | HGLibA_13472 | 23 | -0.919728098 | DNAH10 | dynein axonemal heavy chain 10 [Source:HGNC Symbol;Acc:HGNC:2941] |
| ENSG00000198128 | HGLibA_33823 | 23 | -0.919728098 | OR2L3 | olfactory receptor family 2 subfamily L member 3 [Source:HGNC Symbol;Acc:HGNC:15009] |
| ENSG00000198522 | HGLibA_19825 | 23 | -0.919728098 | GPN1 | GPN-loop GTPase 1 [Source:HGNC Symbol;Acc:HGNC:17030] |
| ENSG00000198570 | HGLibA_40725 | 23 | -0.919728098 | RD3 | GUCY2D regulator [Source:HGNC Symbol;Acc:HGNC:19689]" |
| ENSG00000203786 | HGLibA_25379 | 23 | -0.919728098 | KPRP | keratinocyte proline rich protein [Source:HGNC Symbol;Acc:HGNC:31823] |
| ENSG00000203877 | HGLibA_41332 | 23 | -0.919728098 | RIPPLY2 | ripply transcriptional repressor 2 [Source:HGNC Symbol;Acc:HGNC:21390] |
| ENSG00000204176 | HGLibA_48062 | 23 | -0.919728098 | SYT15 | synaptotagmin 15 [Source:HGNC Symbol;Acc:HGNC:17167] |
| ENSG00000204379 | HGLibA_54465 | 23 | -0.919728098 | XAGE1A | X antigen family member 1A [Source:HGNC Symbol;Acc:HGNC:4111] |
| ENSG00000204397 | HGLibA_07226 | 23 | -0.919728098 | CARD16 | caspase recruitment domain family member 16 [Source:HGNC Symbol;Acc:HGNC:33701] |
| ENSG00000204536 | HGLibA_07947 | 23 | -0.919728098 | CCHCR1 | coiled-coil alpha-helical rod protein 1 [Source:HGNC Symbol;Acc:HGNC:13930] |
| ENSG00000215717 | HGLibA_50042 | 23 | -0.919728098 | TMEM167B | transmembrane protein 167B [Source:HGNC Symbol;Acc:HGNC:30187] |
| ENSG00000221858 | HGLibA_33721 | 23 | -0.919728098 | OR2A12 | olfactory receptor family 2 subfamily A member 12 [Source:HGNC Symbol;Acc:HGNC:15082] |
| ENSG00000225899 | HGLibA_18000 | 23 | -0.919728098 | FRG2B | FSHD region gene 2 family member B [Source:HGNC Symbol;Acc:HGNC:33518] |
| ENSG00000228278 | HGLibA_34623 | 23 | -0.919728098 | ORM2 | orosomucoid 2 [Source:HGNC Symbol;Acc:HGNC:8499] |
| ENSG00000230062 | HGLibA_02140 | 23 | -0.919728098 | ANKRD66 | ankyrin repeat domain 66 [Source:HGNC Symbol;Acc:HGNC:44669] |
| ENSG00000232810 | HGLibA_50704 | 23 | -0.919728098 | TNF | tumor necrosis factor [Source:HGNC Symbol;Acc:HGNC:11892] |
| ENSG00000236279 | HGLibA_09971 | 23 | -0.919728098 | CLEC2L | C-type lectin domain family 2 member L [Source:HGNC Symbol;Acc:HGNC:21969] |
| ENSG00000243543 | HGLibA_54271 | 23 | -0.919728098 | WFDC6 | WAP four-disulfide core domain 6 [Source:HGNC Symbol;Acc:HGNC:16164] |
| ENSG00000249861 | HGLibA_26358 | 23 | -0.919728098 | LGALS16 | galectin 16 [Source:HGNC Symbol;Acc:HGNC:40039] |
| ENSG00000250799 | HGLibA_38662 | 23 | -0.919728098 | PRODH2 | proline dehydrogenase 2 [Source:HGNC Symbol;Acc:HGNC:17325] |
| ENSG00000256294 | HGLibA_55620 | 23 | -0.919728098 | ZNF225 | zinc finger protein 225 [Source:HGNC Symbol;Acc:HGNC:13018] |
| ENSG00000269713 | HGLibA_31246 | 23 | -0.919728098 | NBPF9 | NBPF member 9 [Source:HGNC Symbol;Acc:HGNC:31991] |
| ENSG00000269897 | HGLibA_10662 | 23 | -0.919728098 | COMMD3-BMI1 | COMMD3-BMI1 readthrough [Source:HGNC Symbol;Acc:HGNC:48326] |
| ENSG00000274102 | HGLibA_34044 | 23 | -0.919728098 | OR4M2 | olfactory receptor family 4 subfamily M member 2 [Source:HGNC Symbol;Acc:HGNC:15373] |
| ENSG00000275152 | HGLibA_07977 | 23 | -0.919728098 | CCL16 | C-C motif chemokine ligand 16 [Source:HGNC Symbol;Acc:HGNC:10614] |
| FUK | HGLibA_18161 | 23 | -0.919728098 | FUK | - |
| GPER | HGLibA_19793 | 23 | -0.919728098 | GPER | - |
| GPR133 | HGLibA_19890 | 23 | -0.919728098 | GPR133 | - |
| H2AFX | HGLibA_20761 | 23 | -0.919728098 | H2AFX | - |
| H3F3C | HGLibA_20783 | 23 | -0.919728098 | H3F3C | - |
| HIST2H2AB | HGLibA_21527 | 23 | -0.919728098 | HIST2H2AB | - |
| hsa-mir-103a-2 | HGLibA_57165 | 23 | -0.919728098 | hsa-mir-103a-2 | - |
| hsa-mir-107 | HGLibA_57192 | 23 | -0.919728098 | hsa-mir-107 | - |
| hsa-mir-10a | HGLibA_57197 | 23 | -0.919728098 | hsa-mir-10a | - |
| hsa-mir-1245a | HGLibA_57374 | 23 | -0.919728098 | hsa-mir-1245a | - |
| hsa-mir-1257 | HGLibA_57435 | 23 | -0.919728098 | hsa-mir-1257 | - |
| hsa-mir-1268b | HGLibA_57499 | 23 | -0.919728098 | hsa-mir-1268b | - |
| hsa-mir-192 | HGLibA_58069 | 23 | -0.919728098 | hsa-mir-192 | - |
| hsa-mir-2053 | HGLibA_58183 | 23 | -0.919728098 | hsa-mir-2053 | - |
| hsa-mir-29c | HGLibA_58421 | 23 | -0.919728098 | hsa-mir-29c | - |
| hsa-mir-3115 | HGLibA_58500 | 23 | -0.919728098 | hsa-mir-3115 | - |
| hsa-mir-3193 | HGLibA_58871 | 23 | -0.919728098 | hsa-mir-3193 | - |
| hsa-mir-4451 | HGLibA_60209 | 23 | -0.919728098 | hsa-mir-4451 | - |
| hsa-mir-4508 | HGLibA_60458 | 23 | -0.919728098 | hsa-mir-4508 | - |
| hsa-mir-452 | HGLibA_60531 | 23 | -0.919728098 | hsa-mir-452 | - |
| hsa-mir-4639 | HGLibA_60661 | 23 | -0.919728098 | hsa-mir-4639 | - |
| hsa-mir-5010 | HGLibA_61455 | 23 | -0.919728098 | hsa-mir-5010 | - |
| hsa-mir-502 | HGLibA_61462 | 23 | -0.919728098 | hsa-mir-502 | - |
| hsa-mir-503 | HGLibA_61469 | 23 | -0.919728098 | hsa-mir-503 | - |
| hsa-mir-548q | HGLibA_62059 | 23 | -0.919728098 | hsa-mir-548q | - |
| hsa-mir-564 | HGLibA_62221 | 23 | -0.919728098 | hsa-mir-564 | - |
| hsa-mir-645 | HGLibA_62823 | 23 | -0.919728098 | hsa-mir-645 | - |
| hsa-mir-657 | HGLibA_62960 | 23 | -0.919728098 | hsa-mir-657 | - |
| hsa-mir-6741 | HGLibA_63117 | 23 | -0.919728098 | hsa-mir-6741 | - |
| hsa-mir-6759 | HGLibA_63192 | 23 | -0.919728098 | hsa-mir-6759 | - |
| hsa-mir-6780a | HGLibA_63294 | 23 | -0.919728098 | hsa-mir-6780a | - |
| hsa-mir-6886 | HGLibA_63727 | 23 | -0.919728098 | hsa-mir-6886 | - |
| hsa-mir-7973-2 | HGLibA_64021 | 23 | -0.919728098 | hsa-mir-7973-2 | - |
| hsa-mir-8053 | HGLibA_64052 | 23 | -0.919728098 | hsa-mir-8053 | - |
| ICK | HGLibA_22509 | 23 | -0.919728098 | ICK | - |
| SSPO | HGLibA_47151 | 23 | -0.919728098 | SSPO | - |
| TMEM99 | HGLibA_50574 | 23 | -0.919728098 | TMEM99 | - |
| WISP1 | HGLibA_54318 | 23 | -0.919728098 | WISP1 | - |
| ADCK4 | HGLibA_00899 | 22 | -0.929679135 | ADCK4 | - |

| | | | | | |
|---|---|---|---|---|---|
| ATP5F1 | HGLibA_03651 | 22 | -0.929679135 | ATP5F1 | - |
| C10orf54 | HGLibA_05077 | 22 | -0.929679135 | C10orf54 | - |
| C11orf83 | HGLibA_05193 | 22 | -0.929679135 | C11orf83 | - |
| C12orf5 | HGLibA_05257 | 22 | -0.929679135 | C12orf5 | - |
| C16orf55 | HGLibA_05458 | 22 | -0.929679135 | C16orf55 | - |
| ENSG00000005421 | HGLibA_37679 | 22 | -0.929679135 | PON1 | paraoxonase 1 [Source:HGNC Symbol;Acc:HGNC:9204] |
| ENSG00000006652 | HGLibA_22738 | 22 | -0.929679135 | IFRD1 | interferon related developmental regulator 1 [Source:HGNC Symbol;Acc:HGNC:5456] |
| ENSG00000007908 | HGLibA_43285 | 22 | -0.929679135 | SELE | selectin E [Source:HGNC Symbol;Acc:HGNC:10718] |
| ENSG00000014914 | HGLibA_30416 | 22 | -0.929679135 | MTMR11 | myotubularin related protein 11 [Source:HGNC Symbol;Acc:HGNC:24307] |
| ENSG00000019169 | HGLibA_28361 | 22 | -0.929679135 | MARCO | macrophage receptor with collagenous structure [Source:HGNC Symbol;Acc:HGNC:6895] |
| ENSG00000020426 | HGLibA_29560 | 22 | -0.929679135 | MNAT1 | CDK activating kinase assembly factor [Source:HGNC Symbol;Acc:HGNC:7181]" |
| ENSG00000029363 | HGLibA_04354 | 22 | -0.929679135 | BCLAF1 | BCL2 associated transcription factor 1 [Source:HGNC Symbol;Acc:HGNC:16863] |
| ENSG00000040199 | HGLibA_36438 | 22 | -0.929679135 | PHLPP2 | PH domain and leucine rich repeat protein phosphatase 2 [Source:HGNC Symbol;Acc:HGNC:29149] |
| ENSG00000047936 | HGLibA_41783 | 22 | -0.929679135 | ROS1 | receptor tyrosine kinase [Source:HGNC Symbol;Acc:HGNC:10261]" |
| ENSG00000052802 | HGLibA_30226 | 22 | -0.929679135 | MSMO1 | methylsterol monooxygenase 1 [Source:HGNC Symbol;Acc:HGNC:10545] |
| ENSG00000059804 | HGLibA_44872 | 22 | -0.929679135 | SLC2A3 | solute carrier family 2 member 3 [Source:HGNC Symbol;Acc:HGNC:11007] |
| ENSG00000064313 | HGLibA_48228 | 22 | -0.929679135 | TAF2 | TATA-box binding protein associated factor 2 [Source:HGNC Symbol;Acc:HGNC:11536] |
| ENSG00000064726 | HGLibA_04894 | 22 | -0.929679135 | BTBD1 | BTB domain containing 1 [Source:HGNC Symbol;Acc:HGNC:1120] |
| ENSG00000064763 | HGLibA_16871 | 22 | -0.929679135 | FAR2 | fatty acyl-CoA reductase 2 [Source:HGNC Symbol;Acc:HGNC:25531] |
| ENSG00000069275 | HGLibA_32917 | 22 | -0.929679135 | NUCKS1 | nuclear casein kinase and cyclin dependent kinase substrate 1 [Source:HGNC Symbol;Acc:HGNC:29923] |
| ENSG00000069345 | HGLibA_13512 | 22 | -0.929679135 | DNAJA2 | DnaJ heat shock protein family (Hsp40) member A2 [Source:HGNC Symbol;Acc:HGNC:14884] |
| ENSG00000074590 | HGLibA_32891 | 22 | -0.929679135 | NUAK1 | NUAK family kinase 1 [Source:HGNC Symbol;Acc:HGNC:14311] |
| ENSG00000074964 | HGLibA_02809 | 22 | -0.929679135 | ARHGEF10L | Rho guanine nucleotide exchange factor 10 like [Source:HGNC Symbol;Acc:HGNC:25540] |
| ENSG00000078098 | HGLibA_16866 | 22 | -0.929679135 | FAP | fibroblast activation protein alpha [Source:HGNC Symbol;Acc:HGNC:3590] |
| ENSG00000079308 | HGLibA_50922 | 22 | -0.929679135 | TNS1 | tensin 1 [Source:HGNC Symbol;Acc:HGNC:11973] |
| ENSG00000081019 | HGLibA_42302 | 22 | -0.929679135 | RSBN1 | round spermatid basic protein 1 [Source:HGNC Symbol;Acc:HGNC:25642] |
| ENSG00000081087 | HGLibA_34715 | 22 | -0.929679135 | OSTM1 | osteoclastogenesis associated transmembrane protein 1 [Source:HGNC Symbol;Acc:HGNC:21652] |
| ENSG00000085231 | HGLibA_01390 | 22 | -0.929679135 | AK6 | adenylate kinase 6 [Source:HGNC Symbol;Acc:HGNC:49151] |
| ENSG00000085733 | HGLibA_11740 | 22 | -0.929679135 | CTTN | cortactin [Source:HGNC Symbol;Acc:HGNC:3338] |
| ENSG00000086475 | HGLibA_43413 | 22 | -0.929679135 | SEPHS1 | selenophosphate synthetase 1 [Source:HGNC Symbol;Acc:HGNC:19685] |
| ENSG00000087128 | HGLibA_50620 | 22 | -0.929679135 | TMPRSS11E | transmembrane serine protease 11E [Source:HGNC Symbol;Acc:HGNC:24465] |
| ENSG00000089775 | HGLibA_54851 | 22 | -0.929679135 | ZBTB25 | zinc finger and BTB domain containing 25 [Source:HGNC Symbol;Acc:HGNC:13112] |
| ENSG00000094963 | HGLibA_17704 | 22 | -0.929679135 | FMO2 | flavin containing monooxygenase 2 [Source:HGNC Symbol;Acc:HGNC:3770] |
| ENSG00000099904 | HGLibA_55130 | 22 | -0.929679135 | ZDHHC8 | zinc finger DHHC-type containing 8 [Source:HGNC Symbol;Acc:HGNC:18474] |
| ENSG00000100075 | HGLibA_44632 | 22 | -0.929679135 | SLC25A1 | solute carrier family 25 member 1 [Source:HGNC Symbol;Acc:HGNC:10979] |
| ENSG00000100196 | HGLibA_24484 | 22 | -0.929679135 | KDELR3 | KDEL endoplasmic reticulum protein retention receptor 3 [Source:HGNC Symbol;Acc:HGNC:6306] |
| ENSG00000100298 | HGLibA_02495 | 22 | -0.929679135 | APOBEC3H | apolipoprotein B mRNA editing enzyme catalytic subunit 3H [Source:HGNC Symbol;Acc:HGNC:24100] |
| ENSG00000100577 | HGLibA_20505 | 22 | -0.929679135 | GSTZ1 | glutathione S-transferase zeta 1 [Source:HGNC Symbol;Acc:HGNC:4643] |
| ENSG00000100603 | HGLibA_46021 | 22 | -0.929679135 | SNW1 | SNW domain containing 1 [Source:HGNC Symbol;Acc:HGNC:16696] |
| ENSG00000100815 | HGLibA_51635 | 22 | -0.929679135 | TRIP11 | thyroid hormone receptor interactor 11 [Source:HGNC Symbol;Acc:HGNC:12305] |
| ENSG00000102699 | HGLibA_35238 | 22 | -0.929679135 | PARP4 | poly(ADP-ribose) polymerase family member 4 [Source:HGNC Symbol;Acc:HGNC:271] |
| ENSG00000102780 | HGLibA_13073 | 22 | -0.929679135 | DGKH | diacylglycerol kinase eta [Source:HGNC Symbol;Acc:HGNC:2854] |
| ENSG00000102974 | HGLibA_11596 | 22 | -0.929679135 | CTCF | CCCTC-binding factor [Source:HGNC Symbol;Acc:HGNC:13723] |
| ENSG00000103034 | HGLibA_31422 | 22 | -0.929679135 | NDRG4 | NDRG family member 4 [Source:HGNC Symbol;Acc:HGNC:14466] |
| ENSG00000103769 | HGLibA_39786 | 22 | -0.929679135 | RAB11A | member RAS oncogene family [Source:HGNC Symbol;Acc:HGNC:9760]" |
| ENSG00000104237 | HGLibA_41786 | 22 | -0.929679135 | RP1 | axonemal microtubule associated [Source:HGNC Symbol;Acc:HGNC:10263]" |
| ENSG00000104756 | HGLibA_24469 | 22 | -0.929679135 | KCTD9 | potassium channel tetramerization domain containing 9 [Source:HGNC Symbol;Acc:HGNC:22401] |
| ENSG00000104853 | HGLibA_10113 | 22 | -0.929679135 | CLPTM1 | transmembrane protein [Source:HGNC Symbol;Acc:HGNC:2087]" |
| ENSG00000105289 | HGLibA_49576 | 22 | -0.929679135 | TJP3 | tight junction protein 3 [Source:HGNC Symbol;Acc:HGNC:11829] |
| ENSG00000105369 | HGLibA_08446 | 22 | -0.929679135 | CD79A | CD79a molecule [Source:HGNC Symbol;Acc:HGNC:1698] |
| ENSG00000105383 | HGLibA_08354 | 22 | -0.929679135 | CD33 | CD33 molecule [Source:HGNC Symbol;Acc:HGNC:1659] |
| ENSG00000105556 | HGLibA_29254 | 22 | -0.929679135 | MIER2 | MIER family member 2 [Source:HGNC Symbol;Acc:HGNC:29210] |
| ENSG00000105675 | HGLibA_03631 | 22 | -0.929679135 | ATP4A | ATPase H+/K+ transporting subunit alpha [Source:HGNC Symbol;Acc:HGNC:819] |
| ENSG00000106976 | HGLibA_13682 | 22 | -0.929679135 | DNM1 | dynamin 1 [Source:HGNC Symbol;Acc:HGNC:2972] |
| ENSG00000108064 | HGLibA_49117 | 22 | -0.929679135 | TFAM | mitochondrial [Source:HGNC Symbol;Acc:HGNC:11741]" |
| ENSG00000108179 | HGLibA_37931 | 22 | -0.929679135 | PPIF | peptidylprolyl isomerase F [Source:HGNC Symbol;Acc:HGNC:9259] |
| ENSG00000109911 | HGLibA_14984 | 22 | -0.929679135 | ELP4 | elongator acetyltransferase complex subunit 4 [Source:HGNC Symbol;Acc:HGNC:1171] |
| ENSG00000110169 | HGLibA_22068 | 22 | -0.929679135 | HPX | hemopexin [Source:HGNC Symbol;Acc:HGNC:5171] |
| ENSG00000110367 | HGLibA_12758 | 22 | -0.929679135 | DDX6 | DEAD-box helicase 6 [Source:HGNC Symbol;Acc:HGNC:2747] |
| ENSG00000110583 | HGLibA_30998 | 22 | -0.929679135 | NAA40 | NatD catalytic subunit [Source:HGNC Symbol;Acc:HGNC:25845]" |
| ENSG00000111877 | HGLibA_28629 | 22 | -0.929679135 | MCM9 | minichromosome maintenance 9 homologous recombination repair factor [Source:HGNC Symbol;Acc:HGNC:21484] |
| ENSG00000112425 | HGLibA_15335 | 22 | -0.929679135 | EPM2A | laforin glucan phosphatase [Source:HGNC Symbol;Acc:HGNC:3413]" |
| ENSG00000113303 | HGLibA_04980 | 22 | -0.929679135 | BTNL8 | butyrophilin like 8 [Source:HGNC Symbol;Acc:HGNC:26131] |
| ENSG00000114023 | HGLibA_16221 | 22 | -0.929679135 | FAM162A | family with sequence similarity 162 member A [Source:HGNC Symbol;Acc:HGNC:17865] |
| ENSG00000114120 | HGLibA_44721 | 22 | -0.929679135 | SLC25A36 | solute carrier family 25 member 36 [Source:HGNC Symbol;Acc:HGNC:25554] |
| ENSG00000114346 | HGLibA_14423 | 22 | -0.929679135 | ECT2 | epithelial cell transforming 2 [Source:HGNC Symbol;Acc:HGNC:3155] |
| ENSG00000115241 | HGLibA_37977 | 22 | -0.929679135 | PPM1G | Mg2+/Mn2+ dependent 1G [Source:HGNC Symbol;Acc:HGNC:9278]" |
| ENSG00000115252 | HGLibA_35768 | 22 | -0.929679135 | PDE1A | phosphodiesterase 1A [Source:HGNC Symbol;Acc:HGNC:8774] |
| ENSG00000116194 | HGLibA_01909 | 22 | -0.929679135 | ANGPTL1 | angiopoietin like 1 [Source:HGNC Symbol;Acc:HGNC:489] |
| ENSG00000116688 | HGLibA_29073 | 22 | -0.929679135 | MFN2 | mitofusin 2 [Source:HGNC Symbol;Acc:HGNC:16877] |
| ENSG00000117228 | HGLibA_18727 | 22 | -0.929679135 | GBP1 | guanylate binding protein 1 [Source:HGNC Symbol;Acc:HGNC:4182] |
| ENSG00000117461 | HGLibA_36655 | 22 | -0.929679135 | PIK3R3 | phosphoinositide-3-kinase regulatory subunit 3 [Source:HGNC Symbol;Acc:HGNC:8981] |
| ENSG00000117594 | HGLibA_22161 | 22 | -0.929679135 | HSD11B1 | hydroxysteroid 11-beta dehydrogenase 1 [Source:HGNC Symbol;Acc:HGNC:5208] |
| ENSG00000117643 | HGLibA_28066 | 22 | -0.929679135 | MAN1C1 | mannosidase alpha class 1C member 1 [Source:HGNC Symbol;Acc:HGNC:19080] |
| ENSG00000118271 | HGLibA_52227 | 22 | -0.929679135 | TTR | transthyretin [Source:HGNC Symbol;Acc:HGNC:12405] |
| ENSG00000120071 | HGLibA_24037 | 22 | -0.929679135 | KANSL1 | KAT8 regulatory NSL complex subunit 1 [Source:HGNC Symbol;Acc:HGNC:24565] |
| ENSG00000121361 | HGLibA_24288 | 22 | -0.929679135 | KCNJ8 | potassium voltage-gated channel subfamily J member 8 [Source:HGNC Symbol;Acc:HGNC:6269] |
| ENSG00000121406 | HGLibA_56209 | 22 | -0.929679135 | ZNF549 | zinc finger protein 549 [Source:HGNC Symbol;Acc:HGNC:26632] |
| ENSG00000124614 | HGLibA_42060 | 22 | -0.929679135 | RPS10 | ribosomal protein S10 [Source:HGNC Symbol;Acc:HGNC:10383] |
| ENSG00000125255 | HGLibA_44332 | 22 | -0.929679135 | SLC10A2 | solute carrier family 10 member 2 [Source:HGNC Symbol;Acc:HGNC:10906] |
| ENSG00000125337 | HGLibA_24913 | 22 | -0.929679135 | KIF25 | kinesin family member 25 [Source:HGNC Symbol;Acc:HGNC:6390] |
| ENSG00000125827 | HGLibA_50697 | 22 | -0.929679135 | TMX4 | thioredoxin related transmembrane protein 4 [Source:HGNC Symbol;Acc:HGNC:25237] |
| ENSG00000125848 | HGLibA_17660 | 22 | -0.929679135 | FLRT3 | fibronectin leucine rich transmembrane protein 3 [Source:HGNC Symbol;Acc:HGNC:3762] |
| ENSG00000127472 | HGLibA_36919 | 22 | -0.929679135 | PLA2G5 | phospholipase A2 group V [Source:HGNC Symbol;Acc:HGNC:9038] |
| ENSG00000128849 | HGLibA_09324 | 22 | -0.929679135 | CGNL1 | cingulin like 1 [Source:HGNC Symbol;Acc:HGNC:25931] |
| ENSG00000129636 | HGLibA_23706 | 22 | -0.929679135 | ITFG1 | integrin alpha FG-GAP repeat containing 1 [Source:HGNC Symbol;Acc:HGNC:30697] |
| ENSG00000129675 | HGLibA_02874 | 22 | -0.929679135 | ARHGEF6 | Rac/Cdc42 guanine nucleotide exchange factor 6 [Source:HGNC Symbol;Acc:HGNC:685] |
| ENSG00000129757 | HGLibA_08827 | 22 | -0.929679135 | CDKN1C | cyclin dependent kinase inhibitor 1C [Source:HGNC Symbol;Acc:HGNC:1786] |
| ENSG00000130165 | HGLibA_14954 | 22 | -0.929679135 | ELOF1 | elongation factor 1 homolog [Source:HGNC Symbol;Acc:HGNC:28691] |
| ENSG00000130176 | HGLibA_10304 | 22 | -0.929679135 | CNN1 | calponin 1 [Source:HGNC Symbol;Acc:HGNC:2155] |
| ENSG00000130749 | HGLibA_54977 | 22 | -0.929679135 | ZC3H4 | zinc finger CCCH-type containing 4 [Source:HGNC Symbol;Acc:HGNC:17808] |
| ENSG00000131067 | HGLibA_19020 | 22 | -0.929679135 | GGT7 | gamma-glutamyltransferase 7 [Source:HGNC Symbol;Acc:HGNC:4259] |
| ENSG00000131183 | HGLibA_44938 | 22 | -0.929679135 | SLC34A1 | solute carrier family 34 member 1 [Source:HGNC Symbol;Acc:HGNC:11019] |
| ENSG00000132424 | HGLibA_37344 | 22 | -0.929679135 | PNISR | PNN interacting serine and arginine rich protein [Source:HGNC Symbol;Acc:HGNC:21222] |
| ENSG00000134216 | HGLibA_09441 | 22 | -0.929679135 | CHIA | acidic [Source:HGNC Symbol;Acc:HGNC:17432]" |
| ENSG00000134531 | HGLibA_15074 | 22 | -0.929679135 | EMP1 | epithelial membrane protein 1 [Source:HGNC Symbol;Acc:HGNC:3333] |
| ENSG00000134853 | HGLibA_35854 | 22 | -0.929679135 | PDGFRA | platelet derived growth factor receptor alpha [Source:HGNC Symbol;Acc:HGNC:8803] |
| ENSG00000135930 | HGLibA_14821 | 22 | -0.929679135 | EIF4E2 | eukaryotic translation initiation factor 4E family member 2 [Source:HGNC Symbol;Acc:HGNC:3293] |
| ENSG00000136352 | HGLibA_32031 | 22 | -0.929679135 | NKX2-1 | NK2 homeobox 1 [Source:HGNC Symbol;Acc:HGNC:11825] |
| ENSG00000136379 | HGLibA_00268 | 22 | -0.929679135 | ABHD17C | abhydrolase domain containing 17C [Source:HGNC Symbol;Acc:HGNC:26925] |
| ENSG00000137106 | HGLibA_20256 | 22 | -0.929679135 | GRHPR | glyoxylate and hydroxypyruvate reductase [Source:HGNC Symbol;Acc:HGNC:4570] |
| ENSG00000137509 | HGLibA_38360 | 22 | -0.929679135 | PRCP | prolylcarboxypeptidase [Source:HGNC Symbol;Acc:HGNC:9344] |
| ENSG00000138180 | HGLibA_09142 | 22 | -0.929679135 | CEP55 | centrosomal protein 55 [Source:HGNC Symbol;Acc:HGNC:1161] |
| ENSG00000139364 | HGLibA_49933 | 22 | -0.929679135 | TMEM132B | transmembrane protein 132B [Source:HGNC Symbol;Acc:HGNC:29397] |
| ENSG00000140285 | HGLibA_17416 | 22 | -0.929679135 | FGF7 | fibroblast growth factor 7 [Source:HGNC Symbol;Acc:HGNC:3685] |
| ENSG00000140740 | HGLibA_53115 | 22 | -0.929679135 | UQCRC2 | ubiquinol-cytochrome c reductase core protein 2 [Source:HGNC Symbol;Acc:HGNC:12586] |
| ENSG00000140948 | HGLibA_55015 | 22 | -0.929679135 | ZCCHC14 | zinc finger CCHC-type containing 14 [Source:HGNC Symbol;Acc:HGNC:24134] |
| ENSG00000141551 | HGLibA_11386 | 22 | -0.929679135 | CSNK1D | casein kinase 1 delta [Source:HGNC Symbol;Acc:HGNC:2452] |
| ENSG00000142347 | HGLibA_30835 | 22 | -0.929679135 | MYO1F | myosin IF [Source:HGNC Symbol;Acc:HGNC:7600] |
| ENSG00000142611 | HGLibA_38384 | 22 | -0.929679135 | PRDM16 | PR/SET domain 16 [Source:HGNC Symbol;Acc:HGNC:14000] |
| ENSG00000143032 | HGLibA_04160 | 22 | -0.929679135 | BARHL2 | BarH like homeobox 2 [Source:HGNC Symbol;Acc:HGNC:954] |
| ENSG00000144306 | HGLibA_43056 | 22 | -0.929679135 | SCRN3 | secernin 3 [Source:HGNC Symbol;Acc:HGNC:30382] |
| ENSG00000145107 | HGLibA_49696 | 22 | -0.929679135 | TM4SF19 | transmembrane 4 L six family member 19 [Source:HGNC Symbol;Acc:HGNC:25167] |
| ENSG00000145623 | HGLibA_34696 | 22 | -0.929679135 | OSMR | oncostatin M receptor [Source:HGNC Symbol;Acc:HGNC:8507] |
| ENSG00000145649 | HGLibA_20719 | 22 | -0.929679135 | GZMA | granzyme A [Source:HGNC Symbol;Acc:HGNC:4708] |
| ENSG00000146282 | HGLibA_40292 | 22 | -0.929679135 | RARS2 | mitochondrial [Source:HGNC Symbol;Acc:HGNC:21406]" |
| ENSG00000148346 | HGLibA_26177 | 22 | -0.929679135 | LCN2 | lipocalin 2 [Source:HGNC Symbol;Acc:HGNC:6526] |
| ENSG00000149292 | HGLibA_52047 | 22 | -0.929679135 | TTC12 | tetratricopeptide repeat domain 12 [Source:HGNC Symbol;Acc:HGNC:23700] |
| ENSG00000149516 | HGLibA_30149 | 22 | -0.929679135 | MS4A3 | membrane spanning 4-domains A3 [Source:HGNC Symbol;Acc:HGNC:7317] |
| ENSG00000151338 | HGLibA_29296 | 22 | -0.929679135 | MIPOL1 | mirror-image polydactyly 1 [Source:HGNC Symbol;Acc:HGNC:21460] |
| ENSG00000151365 | HGLibA_49396 | 22 | -0.929679135 | THRSP | thyroid hormone responsive [Source:HGNC Symbol;Acc:HGNC:11800] |
| ENSG00000152291 | HGLibA_49273 | 22 | -0.929679135 | TGOLN2 | trans-golgi network protein 2 [Source:HGNC Symbol;Acc:HGNC:15450] |
| ENSG00000152413 | HGLibA_21864 | 22 | -0.929679135 | HOMER1 | homer scaffold protein 1 [Source:HGNC Symbol;Acc:HGNC:17512] |
| ENSG00000152818 | HGLibA_53457 | 22 | -0.929679135 | UTRN | utrophin [Source:HGNC Symbol;Acc:HGNC:12635] |
| ENSG00000153157 | HGLibA_47935 | 22 | -0.929679135 | SYCP2L | synaptonemal complex protein 2 like [Source:HGNC Symbol;Acc:HGNC:21537] |
| ENSG00000153395 | HGLibA_27071 | 22 | -0.929679135 | LPCAT1 | lysophosphatidylcholine acyltransferase 1 [Source:HGNC Symbol;Acc:HGNC:25718] |
| ENSG00000154370 | HGLibA_51405 | 22 | -0.929679135 | TRIM11 | tripartite motif containing 11 [Source:HGNC Symbol;Acc:HGNC:16281] |
| ENSG00000156521 | HGLibA_52477 | 22 | -0.929679135 | TYSND1 | trypsin domain containing 1 [Source:HGNC Symbol;Acc:HGNC:28531] |
| ENSG00000156711 | HGLibA_28259 | 22 | -0.929679135 | MAPK13 | mitogen-activated protein kinase 13 [Source:HGNC Symbol;Acc:HGNC:6875] |
| ENSG00000160908 | HGLibA_55909 | 22 | -0.929679135 | ZNF394 | zinc finger protein 394 [Source:HGNC Symbol;Acc:HGNC:18832] |
| ENSG00000160973 | HGLibA_17874 | 22 | -0.929679135 | FOXH1 | forkhead box H1 [Source:HGNC Symbol;Acc:HGNC:3814] |
| ENSG00000161013 | HGLibA_29144 | 22 | -0.929679135 | MGAT4B | 3-mannosyl-glycoprotein 4-beta-N-acetylglucosaminyltransferase B [Source:HGNC Symbol;Acc:HGNC:7048]" |
| ENSG00000161654 | HGLibA_27526 | 22 | -0.929679135 | LSM12 | LSM12 homolog [Source:HGNC Symbol;Acc:HGNC:26407] |
| ENSG00000162368 | HGLibA_10199 | 22 | -0.929679135 | CMPK1 | cytidine/uridine monophosphate kinase 1 [Source:HGNC Symbol;Acc:HGNC:18170] |
| ENSG00000162438 | HGLibA_11682 | 22 | -0.929679135 | CTRC | chymotrypsin C [Source:HGNC Symbol;Acc:HGNC:2523] |

| | | | | | |
|---|---|---|---|---|---|
| ENSG00000162620 | HGLibA_27459 | 22 | -0.929679135 | LRRIQ3 | leucine rich repeats and IQ motif containing 3 [Source:HGNC Symbol;Acc:HGNC:28318] |
| ENSG00000163521 | HGLibA_19220 | 22 | -0.929679135 | GLB1L | galactosidase beta 1 like [Source:HGNC Symbol;Acc:HGNC:28129] |
| ENSG00000163534 | HGLibA_17226 | 22 | -0.929679135 | FCRL1 | Fc receptor like 1 [Source:HGNC Symbol;Acc:HGNC:18509] |
| ENSG00000163563 | HGLibA_29568 | 22 | -0.929679135 | MNDA | myeloid cell nuclear differentiation antigen [Source:HGNC Symbol;Acc:HGNC:7183] |
| ENSG00000163600 | HGLibA_22515 | 22 | -0.929679135 | ICOS | inducible T cell costimulator [Source:HGNC Symbol;Acc:HGNC:5351] |
| ENSG00000164100 | HGLibA_31431 | 22 | -0.929679135 | NDST3 | N-deacetylase and N-sulfotransferase 3 [Source:HGNC Symbol;Acc:HGNC:7682] |
| ENSG00000164117 | HGLibA_17130 | 22 | -0.929679135 | FBXO8 | F-box protein 8 [Source:HGNC Symbol;Acc:HGNC:13587] |
| ENSG00000164733 | HGLibA_11692 | 22 | -0.929679135 | CTSB | cathepsin B [Source:HGNC Symbol;Acc:HGNC:2527] |
| ENSG00000164938 | HGLibA_51068 | 22 | -0.929679135 | TP53INP1 | tumor protein p53 inducible nuclear protein 1 [Source:HGNC Symbol;Acc:HGNC:18022] |
| ENSG00000165118 | HGLibA_06749 | 22 | -0.929679135 | C9orf64 | chromosome 9 open reading frame 64 [Source:HGNC Symbol;Acc:HGNC:28144] |
| ENSG00000165282 | HGLibA_36574 | 22 | -0.929679135 | PIGO | phosphatidylinositol glycan anchor biosynthesis class O [Source:HGNC Symbol;Acc:HGNC:23215] |
| ENSG00000165349 | HGLibA_45365 | 22 | -0.929679135 | SLC7A3 | solute carrier family 7 member 3 [Source:HGNC Symbol;Acc:HGNC:11061] |
| ENSG00000165584 | HGLibA_47208 | 22 | -0.929679135 | SSX3 | SSX family member 3 [Source:HGNC Symbol;Acc:HGNC:11337] |
| ENSG00000166123 | HGLibA_20167 | 22 | -0.929679135 | GPT2 | glutamic--pyruvic transaminase 2 [Source:HGNC Symbol;Acc:HGNC:18062] |
| ENSG00000166169 | HGLibA_37536 | 22 | -0.929679135 | POLL | DNA polymerase lambda [Source:HGNC Symbol;Acc:HGNC:9184] |
| ENSG00000166426 | HGLibA_11053 | 22 | -0.929679135 | CRABP1 | cellular retinoic acid binding protein 1 [Source:HGNC Symbol;Acc:HGNC:2338] |
| ENSG00000167131 | HGLibA_07514 | 22 | -0.929679135 | CCDC103 | coiled-coil domain containing 103 [Source:HGNC Symbol;Acc:HGNC:32700] |
| ENSG00000167487 | HGLibA_25168 | 22 | -0.929679135 | KLHL26 | kelch like family member 26 [Source:HGNC Symbol;Acc:HGNC:25623] |
| ENSG00000167531 | HGLibA_25937 | 22 | -0.929679135 | LALBA | lactalbumin alpha [Source:HGNC Symbol;Acc:HGNC:6480] |
| ENSG00000167543 | HGLibA_51062 | 22 | -0.929679135 | TP53I13 | tumor protein p53 inducible protein 13 [Source:HGNC Symbol;Acc:HGNC:25102] |
| ENSG00000167646 | HGLibA_13464 | 22 | -0.929679135 | DNAAF3 | dynein axonemal assembly factor 3 [Source:HGNC Symbol;Acc:HGNC:30492] |
| ENSG00000167721 | HGLibA_51994 | 22 | -0.929679135 | TSR1 | ribosome maturation factor [Source:HGNC Symbol;Acc:HGNC:25542]" |
| ENSG00000167825 | HGLibA_34304 | 22 | -0.929679135 | OR5I1 | olfactory receptor family 5 subfamily I member 1 [Source:HGNC Symbol;Acc:HGNC:8347] |
| ENSG00000168062 | HGLibA_04176 | 22 | -0.929679135 | BATF2 | basic leucine zipper ATF-like transcription factor 2 [Source:HGNC Symbol;Acc:HGNC:25163] |
| ENSG00000168175 | HGLibA_28267 | 22 | -0.929679135 | MAPK1IP1L | mitogen-activated protein kinase 1 interacting protein 1 like [Source:HGNC Symbol;Acc:HGNC:19840] |
| ENSG00000168412 | HGLibA_30446 | 22 | -0.929679135 | MTNR1A | melatonin receptor 1A [Source:HGNC Symbol;Acc:HGNC:7463] |
| ENSG00000168564 | HGLibA_08832 | 22 | -0.929679135 | CDKN2AIP | CDKN2A interacting protein [Source:HGNC Symbol;Acc:HGNC:24325] |
| ENSG00000168811 | HGLibA_22960 | 22 | -0.929679135 | IL12A | interleukin 12A [Source:HGNC Symbol;Acc:HGNC:5969] |
| ENSG00000168917 | HGLibA_45018 | 22 | -0.929679135 | SLC35G2 | solute carrier family 35 member G2 [Source:HGNC Symbol;Acc:HGNC:28480] |
| ENSG00000170807 | HGLibA_26721 | 22 | -0.929679135 | LMOD2 | leiomodin 2 [Source:HGNC Symbol;Acc:HGNC:6648] |
| ENSG00000171236 | HGLibA_27166 | 22 | -0.929679135 | LRG1 | leucine rich alpha-2-glycoprotein 1 [Source:HGNC Symbol;Acc:HGNC:29480] |
| ENSG00000171606 | HGLibA_55711 | 22 | -0.929679135 | ZNF274 | zinc finger protein 274 [Source:HGNC Symbol;Acc:HGNC:13068] |
| ENSG00000171773 | HGLibA_33143 | 22 | -0.929679135 | NXNL1 | nucleoredoxin like 1 [Source:HGNC Symbol;Acc:HGNC:25179] |
| ENSG00000172273 | HGLibA_21320 | 22 | -0.929679135 | HINFP | histone H4 transcription factor [Source:HGNC Symbol;Acc:HGNC:17850] |
| ENSG00000172785 | HGLibA_07431 | 22 | -0.929679135 | CBWD1 | COBW domain containing 1 [Source:HGNC Symbol;Acc:HGNC:17134] |
| ENSG00000173812 | HGLibA_14706 | 22 | -0.929679135 | EIF1 | eukaryotic translation initiation factor 1 [Source:HGNC Symbol;Acc:HGNC:3249] |
| ENSG00000175048 | HGLibA_55077 | 22 | -0.929679135 | ZDHHC14 | zinc finger DHHC-type containing 14 [Source:HGNC Symbol;Acc:HGNC:20341] |
| ENSG00000175806 | HGLibA_30239 | 22 | -0.929679135 | MSRA | methionine sulfoxide reductase A [Source:HGNC Symbol;Acc:HGNC:7377] |
| ENSG00000175832 | HGLibA_15659 | 22 | -0.929679135 | ETV4 | ETS variant 4 [Source:HGNC Symbol;Acc:HGNC:3493] |
| ENSG00000176293 | HGLibA_55472 | 22 | -0.929679135 | ZNF135 | zinc finger protein 135 [Source:HGNC Symbol;Acc:HGNC:12919] |
| ENSG00000176974 | HGLibA_44060 | 22 | -0.929679135 | SHMT1 | serine hydroxymethyltransferase 1 [Source:HGNC Symbol;Acc:HGNC:10850] |
| ENSG00000177025 | HGLibA_05634 | 22 | -0.929679135 | C19orf18 | chromosome 19 open reading frame 18 [Source:HGNC Symbol;Acc:HGNC:28642] |
| ENSG00000177700 | HGLibA_37604 | 22 | -0.929679135 | POLR2L | RNA polymerase II subunit L [Source:HGNC Symbol;Acc:HGNC:9199] |
| ENSG00000177873 | HGLibA_56402 | 22 | -0.929679135 | ZNF619 | zinc finger protein 619 [Source:HGNC Symbol;Acc:HGNC:26910] |
| ENSG00000180357 | HGLibA_56377 | 22 | -0.929679135 | ZNF609 | zinc finger protein 609 [Source:HGNC Symbol;Acc:HGNC:29003] |
| ENSG00000181163 | HGLibA_32488 | 22 | -0.929679135 | NPM1 | nucleophosmin 1 [Source:HGNC Symbol;Acc:HGNC:7910] |
| ENSG00000181894 | HGLibA_55809 | 22 | -0.929679135 | ZNF329 | zinc finger protein 329 [Source:HGNC Symbol;Acc:HGNC:14209] |
| ENSG00000182185 | HGLibA_40087 | 22 | -0.929679135 | RAD51B | RAD51 paralog B [Source:HGNC Symbol;Acc:HGNC:9822] |
| ENSG00000182256 | HGLibA_18394 | 22 | -0.929679135 | GABRG3 | gamma-aminobutyric acid type A receptor gamma3 subunit [Source:HGNC Symbol;Acc:HGNC:4088] |
| ENSG00000182261 | HGLibA_32099 | 22 | -0.929679135 | NLRP10 | NLR family pyrin domain containing 10 [Source:HGNC Symbol;Acc:HGNC:21464] |
| ENSG00000182606 | HGLibA_51275 | 22 | -0.929679135 | TRAK1 | trafficking kinesin protein 1 [Source:HGNC Symbol;Acc:HGNC:29947] |
| ENSG00000183035 | HGLibA_12042 | 22 | -0.929679135 | CYLC1 | cylicin 1 [Source:HGNC Symbol;Acc:HGNC:2582] |
| ENSG00000183643 | HGLibA_05385 | 22 | -0.929679135 | C15orf32 | chromosome 15 open reading frame 32 [Source:HGNC Symbol;Acc:HGNC:26549] |
| ENSG00000183643 | HGLibA_05383 | 22 | -0.929679135 | C15orf32 | chromosome 15 open reading frame 32 [Source:HGNC Symbol;Acc:HGNC:26549] |
| ENSG00000183668 | HGLibA_39058 | 22 | -0.929679135 | PSG9 | pregnancy specific beta-1-glycoprotein 9 [Source:HGNC Symbol;Acc:HGNC:9526] |
| ENSG00000184226 | HGLibA_35421 | 22 | -0.929679135 | PCDH9 | protocadherin 9 [Source:HGNC Symbol;Acc:HGNC:8661] |
| ENSG00000184867 | HGLibA_03040 | 22 | -0.929679135 | ARMCX2 | armadillo repeat containing X-linked 2 [Source:HGNC Symbol;Acc:HGNC:16869] |
| ENSG00000184956 | HGLibA_30554 | 22 | -0.929679135 | MUC6 | oligomeric mucus/gel-forming [Source:HGNC Symbol;Acc:HGNC:7517]" |
| ENSG00000185088 | HGLibA_42127 | 22 | -0.929679135 | RPS27L | ribosomal protein S27 like [Source:HGNC Symbol;Acc:HGNC:18476] |
| ENSG00000185344 | HGLibA_03706 | 22 | -0.929679135 | ATP6V0A2 | ATPase H+ transporting V0 subunit a2 [Source:HGNC Symbol;Acc:HGNC:18481] |
| ENSG00000186416 | HGLibA_32022 | 22 | -0.929679135 | NKRF | NFKB repressing factor [Source:HGNC Symbol;Acc:HGNC:19374] |
| ENSG00000186638 | HGLibA_24910 | 22 | -0.929679135 | KIF24 | kinesin family member 24 [Source:HGNC Symbol;Acc:HGNC:19916] |
| ENSG00000186638 | HGLibA_24911 | 22 | -0.929679135 | KIF24 | kinesin family member 24 [Source:HGNC Symbol;Acc:HGNC:19916] |
| ENSG00000187173 | HGLibA_26118 | 22 | -0.929679135 | LCE2A | late cornified envelope 2A [Source:HGNC Symbol;Acc:HGNC:29469] |
| ENSG00000187608 | HGLibA_23655 | 22 | -0.929679135 | ISG15 | ISG15 ubiquitin-like modifier [Source:HGNC Symbol;Acc:HGNC:4053] |
| ENSG00000187862 | HGLibA_52085 | 22 | -0.929679135 | TTC24 | tetratricopeptide repeat domain 24 [Source:HGNC Symbol;Acc:HGNC:32348] |
| ENSG00000188215 | HGLibA_12571 | 22 | -0.929679135 | DCUN1D3 | defective in cullin neddylation 1 domain containing 3 [Source:HGNC Symbol;Acc:HGNC:28734] |
| ENSG00000188883 | HGLibA_25311 | 22 | -0.929679135 | KLRG2 | killer cell lectin like receptor G2 [Source:HGNC Symbol;Acc:HGNC:24778] |
| ENSG00000189308 | HGLibA_26570 | 22 | -0.929679135 | LIN54 | lin-54 DREAM MuvB core complex component [Source:HGNC Symbol;Acc:HGNC:25397] |
| ENSG00000196236 | HGLibA_54560 | 22 | -0.929679135 | XPNPEP3 | X-prolyl aminopeptidase 3 [Source:HGNC Symbol;Acc:HGNC:28052] |
| ENSG00000196440 | HGLibA_03048 | 22 | -0.929679135 | ARMCX4 | armadillo repeat containing X-linked 4 [Source:HGNC Symbol;Acc:HGNC:28615] |
| ENSG00000196659 | HGLibA_52108 | 22 | -0.929679135 | TTC30B | tetratricopeptide repeat domain 30B [Source:HGNC Symbol;Acc:HGNC:26425] |
| ENSG00000197429 | HGLibA_23481 | 22 | -0.929679135 | IPP | intracisternal A particle-promoted polypeptide [Source:HGNC Symbol;Acc:HGNC:6108] |
| ENSG00000197540 | HGLibA_20730 | 22 | -0.929679135 | GZMM | granzyme M [Source:HGNC Symbol;Acc:HGNC:4712] |
| ENSG00000197935 | HGLibA_55780 | 22 | -0.929679135 | ZNF311 | zinc finger protein 311 [Source:HGNC Symbol;Acc:HGNC:13847] |
| ENSG00000198104 | HGLibA_33890 | 22 | -0.929679135 | OR2T6 | olfactory receptor family 2 subfamily T member 6 [Source:HGNC Symbol;Acc:HGNC:15018] |
| ENSG00000198862 | HGLibA_27613 | 22 | -0.929679135 | LTN1 | listerin E3 ubiquitin protein ligase 1 [Source:HGNC Symbol;Acc:HGNC:13082] |
| ENSG00000198937 | HGLibA_07675 | 22 | -0.929679135 | CCDC167 | coiled-coil domain containing 167 [Source:HGNC Symbol;Acc:HGNC:21239] |
| ENSG00000204371 | HGLibA_14687 | 22 | -0.929679135 | EHMT2 | euchromatic histone lysine methyltransferase 2 [Source:HGNC Symbol;Acc:HGNC:14129] |
| ENSG00000214530 | HGLibA_47351 | 22 | -0.929679135 | STARD10 | StAR related lipid transfer domain containing 10 [Source:HGNC Symbol;Acc:HGNC:10666] |
| ENSG00000221880 | HGLibA_25581 | 22 | -0.929679135 | KRTAP1-3 | keratin associated protein 1-3 [Source:HGNC Symbol;Acc:HGNC:16771] |
| ENSG00000231852 | HGLibA_12079 | 22 | -0.929679135 | CYP21A2 | cytochrome P450 family 21 subfamily A member 2 [Source:HGNC Symbol;Acc:HGNC:2600] |
| ENSG00000236446 | HGLibA_11551 | 22 | -0.929679135 | CT47B1 | member B1 [Source:HGNC Symbol;Acc:HGNC:33293]" |
| ENSG00000237649 | HGLibA_24971 | 22 | -0.929679135 | KIFC1 | kinesin family member C1 [Source:HGNC Symbol;Acc:HGNC:6389] |
| ENSG00000239810 | HGLibA_38283 | 22 | -0.929679135 | PRAMEF11 | PRAME family member 11 [Source:HGNC Symbol;Acc:HGNC:14086] |
| ENSG00000250423 | HGLibA_24713 | 22 | -0.929679135 | KIAA1210 | KIAA1210 [Source:HGNC Symbol;Acc:HGNC:29218] |
| ENSG00000254858 | HGLibA_29789 | 22 | -0.929679135 | MPV17L2 | MPV17 mitochondrial inner membrane protein like 2 [Source:HGNC Symbol;Acc:HGNC:28177] |
| ENSG00000255823 | HGLibA_30490 | 22 | -0.929679135 | MTRNR2L8 | MT-RNR2 like 8 [Source:HGNC Symbol;Acc:HGNC:37165] |
| ENSG00000262664 | HGLibA_34788 | 22 | -0.929679135 | OVCA2 | serine hydrolase domain containing [Source:HGNC Symbol;Acc:HGNC:24203]" |
| ENSG00000264230 | HGLibA_02256 | 22 | -0.929679135 | ANXA8L1 | annexin A8 like 1 [Source:HGNC Symbol;Acc:HGNC:23334] |
| ENSG00000265203 | HGLibA_40635 | 22 | -0.929679135 | RBP3 | retinol binding protein 3 [Source:HGNC Symbol;Acc:HGNC:9921] |
| ENSG00000265817 | HGLibA_18063 | 22 | -0.929679135 | FSBP | fibrinogen silencer binding protein [Source:HGNC Symbol;Acc:HGNC:43653] |
| ENSG00000275718 | HGLibA_07974 | 22 | -0.929679135 | CCL15 | C-C motif chemokine ligand 15 [Source:HGNC Symbol;Acc:HGNC:10613] |
| ENSG00000283154 | HGLibA_23527 | 22 | -0.929679135 | IQCJ-SCHIP1 | IQCJ-SCHIP1 readthrough [Source:HGNC Symbol;Acc:HGNC:38842] |
| FAM154B | HGLibA_16176 | 22 | -0.929679135 | FAM154B | - |
| HIAT1 | HGLibA_21276 | 22 | -0.929679135 | HIAT1 | - |
| HIST1H4I | HGLibA_21511 | 22 | -0.929679135 | HIST1H4I | - |
| HN1L | HGLibA_21770 | 22 | -0.929679135 | HN1L | - |
| hsa-mir-103a-1 | HGLibA_57163 | 22 | -0.929679135 | hsa-mir-103a-1 | - |
| hsa-mir-1180 | HGLibA_57215 | 22 | -0.929679135 | hsa-mir-1180 | - |
| hsa-mir-1234 | HGLibA_57332 | 22 | -0.929679135 | hsa-mir-1234 | - |
| hsa-mir-1255b-2 | HGLibA_57426 | 22 | -0.929679135 | hsa-mir-1255b-2 | - |
| hsa-mir-125a | HGLibA_57443 | 22 | -0.929679135 | hsa-mir-125a | - |
| hsa-mir-1283-2 | HGLibA_57591 | 22 | -0.929679135 | hsa-mir-1283-2 | - |
| hsa-mir-1286 | HGLibA_57609 | 22 | -0.929679135 | hsa-mir-1286 | - |
| hsa-mir-181b-1 | HGLibA_57965 | 22 | -0.929679135 | hsa-mir-181b-1 | - |
| hsa-mir-1972-1 | HGLibA_58108 | 22 | -0.929679135 | hsa-mir-1972-1 | - |
| hsa-mir-204 | HGLibA_58176 | 22 | -0.929679135 | hsa-mir-204 | - |
| hsa-mir-3133 | HGLibA_58587 | 22 | -0.929679135 | hsa-mir-3133 | - |
| hsa-mir-3135b | HGLibA_58599 | 22 | -0.929679135 | hsa-mir-3135b | - |
| hsa-mir-3161 | HGLibA_58725 | 22 | -0.929679135 | hsa-mir-3161 | - |
| hsa-mir-3180-1 | HGLibA_58801 | 22 | -0.929679135 | hsa-mir-3180-1 | - |
| hsa-mir-320b-1 | HGLibA_58928 | 22 | -0.929679135 | hsa-mir-320b-1 | - |
| hsa-mir-320b-2 | HGLibA_58930 | 22 | -0.929679135 | hsa-mir-320b-2 | - |
| hsa-mir-320c-1 | HGLibA_58935 | 22 | -0.929679135 | hsa-mir-320c-1 | - |
| hsa-mir-326 | HGLibA_58970 | 22 | -0.929679135 | hsa-mir-326 | - |
| hsa-mir-3688-1 | HGLibA_59306 | 22 | -0.929679135 | hsa-mir-3688-1 | - |
| hsa-mir-4281 | HGLibA_59826 | 22 | -0.929679135 | hsa-mir-4281 | - |
| hsa-mir-4287 | HGLibA_59855 | 22 | -0.929679135 | hsa-mir-4287 | - |
| hsa-mir-4444-1 | HGLibA_60179 | 22 | -0.929679135 | hsa-mir-4444-1 | - |
| hsa-mir-4525 | HGLibA_60562 | 22 | -0.929679135 | hsa-mir-4525 | - |
| hsa-mir-4655 | HGLibA_60726 | 22 | -0.929679135 | hsa-mir-4655 | - |
| hsa-mir-4678 | HGLibA_60830 | 22 | -0.929679135 | hsa-mir-4678 | - |
| hsa-mir-4723 | HGLibA_60991 | 22 | -0.929679135 | hsa-mir-4723 | - |
| hsa-mir-4746 | HGLibA_61082 | 22 | -0.929679135 | hsa-mir-4746 | - |
| hsa-mir-4803 | HGLibA_61316 | 22 | -0.929679135 | hsa-mir-4803 | - |
| hsa-mir-5004 | HGLibA_61424 | 22 | -0.929679135 | hsa-mir-5004 | - |
| hsa-mir-519a-1 | HGLibA_61709 | 22 | -0.929679135 | hsa-mir-519a-1 | - |
| hsa-mir-548at | HGLibA_61923 | 22 | -0.929679135 | hsa-mir-548at | - |
| hsa-mir-548x-2 | HGLibA_62081 | 22 | -0.929679135 | hsa-mir-548x-2 | - |
| hsa-mir-550a-2 | HGLibA_62101 | 22 | -0.929679135 | hsa-mir-550a-2 | - |
| hsa-mir-5585 | HGLibA_62180 | 22 | -0.929679135 | hsa-mir-5585 | - |
| hsa-mir-6131 | HGLibA_62674 | 22 | -0.929679135 | hsa-mir-6131 | - |
| hsa-mir-6845 | HGLibA_63553 | 22 | -0.929679135 | hsa-mir-6845 | - |
| hsa-mir-6855 | HGLibA_63592 | 22 | -0.929679135 | hsa-mir-6855 | - |

| | | | | | |
|---|---|---|---|---|---|
| hsa-mir-6865 | HGLibA_63646 | 22 | -0.929679135 | hsa-mir-6865 | - |
| KIAA1257 | HGLibA_24731 | 22 | -0.929679135 | KIAA1257 | - |
| LOC100129520 | HGLibA_26765 | 22 | -0.929679135 | LOC100129520 | - |
| OR9G9 | HGLibA_34575 | 22 | -0.929679135 | OR9G9 | - |
| PIH1D3 | HGLibA_36616 | 22 | -0.929679135 | PIH1D3 | - |
| REXO1L1 | HGLibA_40894 | 22 | -0.929679135 | REXO1L1 | - |
| RQCD1 | HGLibA_42219 | 22 | -0.929679135 | RQCD1 | - |
| AGAP7 | HGLibA_01158 | 21 | -0.939630173 | AGAP7 | - |
| ASMTL_X | HGLibA_03325 | 21 | -0.939630173 | ASMTL_X | - |
| ATP5C1 | HGLibA_03645 | 21 | -0.939630173 | ATP5C1 | - |
| C12orf65 | HGLibA_05279 | 21 | -0.939630173 | C12orf65 | - |
| C13orf45 | HGLibA_05313 | 21 | -0.939630173 | C13orf45 | - |
| C4orf21 | HGLibA_06301 | 21 | -0.939630173 | C4orf21 | - |
| C7orf41 | HGLibA_06542 | 21 | -0.939630173 | C7orf41 | - |
| CCDC64 | HGLibA_07826 | 21 | -0.939630173 | CCDC64 | - |
| ENSG00000004939 | HGLibA_45201 | 21 | -0.939630173 | SLC4A1 | solute carrier family 4 member 1 (Diego blood group) [Source:HGNC Symbol;Acc:HGNC:11027] |
| ENSG00000005007 | HGLibA_53056 | 21 | -0.939630173 | UPF1 | RNA helicase and ATPase [Source:HGNC Symbol;Acc:HGNC:9962]" |
| ENSG00000007306 | HGLibA_08949 | 21 | -0.939630173 | CEACAM7 | carcinoembryonic antigen related cell adhesion molecule 7 [Source:HGNC Symbol;Acc:HGNC:1819] |
| ENSG00000007866 | HGLibA_48931 | 21 | -0.939630173 | TEAD3 | TEA domain transcription factor 3 [Source:HGNC Symbol;Acc:HGNC:11716] |
| ENSG00000010244 | HGLibA_55578 | 21 | -0.939630173 | ZNF207 | zinc finger protein 207 [Source:HGNC Symbol;Acc:HGNC:12998] |
| ENSG00000042317 | HGLibA_46528 | 21 | -0.939630173 | SPATA7 | spermatogenesis associated 7 [Source:HGNC Symbol;Acc:HGNC:20423] |
| ENSG00000047849 | HGLibA_28207 | 21 | -0.939630173 | MAP4 | microtubule associated protein 4 [Source:HGNC Symbol;Acc:HGNC:6862] |
| ENSG00000060709 | HGLibA_41258 | 21 | -0.939630173 | RIMBP2 | RIMS binding protein 2 [Source:HGNC Symbol;Acc:HGNC:30339] |
| ENSG00000075388 | HGLibA_17408 | 21 | -0.939630173 | FGF4 | fibroblast growth factor 4 [Source:HGNC Symbol;Acc:HGNC:3682] |
| ENSG00000077254 | HGLibA_53337 | 21 | -0.939630173 | USP33 | ubiquitin specific peptidase 33 [Source:HGNC Symbol;Acc:HGNC:20059] |
| ENSG00000078053 | HGLibA_01833 | 21 | -0.939630173 | AMPH | amphiphysin [Source:HGNC Symbol;Acc:HGNC:471] |
| ENSG00000078804 | HGLibA_51071 | 21 | -0.939630173 | TP53INP2 | tumor protein p53 inducible nuclear protein 2 [Source:HGNC Symbol;Acc:HGNC:16104] |
| ENSG00000080511 | HGLibA_40752 | 21 | -0.939630173 | RDH8 | retinol dehydrogenase 8 [Source:HGNC Symbol;Acc:HGNC:14423] |
| ENSG00000085274 | HGLibA_30799 | 21 | -0.939630173 | MYNN | myoneurin [Source:HGNC Symbol;Acc:HGNC:14955] |
| ENSG00000085365 | HGLibA_42835 | 21 | -0.939630173 | SCAMP1 | secretory carrier membrane protein 1 [Source:HGNC Symbol;Acc:HGNC:10563] |
| ENSG00000088280 | HGLibA_03200 | 21 | -0.939630173 | ASAP3 | ankyrin repeat and PH domain 3 [Source:HGNC Symbol;Acc:HGNC:14987]" |
| ENSG00000090565 | HGLibA_39799 | 21 | -0.939630173 | RAB11FIP3 | RAB11 family interacting protein 3 [Source:HGNC Symbol;Acc:HGNC:17224] |
| ENSG00000091536 | HGLibA_30804 | 21 | -0.939630173 | MYO15A | myosin XVA [Source:HGNC Symbol;Acc:HGNC:7594] |
| ENSG00000095015 | HGLibA_28160 | 21 | -0.939630173 | MAP3K1 | mitogen-activated protein kinase kinase kinase 1 [Source:HGNC Symbol;Acc:HGNC:6848] |
| ENSG00000099246 | HGLibA_39823 | 21 | -0.939630173 | RAB18 | member RAS oncogene family [Source:HGNC Symbol;Acc:HGNC:14244]" |
| ENSG00000100426 | HGLibA_54798 | 21 | -0.939630173 | ZBED4 | zinc finger BED-type containing 4 [Source:HGNC Symbol;Acc:HGNC:20721] |
| ENSG00000100490 | HGLibA_08805 | 21 | -0.939630173 | CDKL1 | cyclin dependent kinase like 1 [Source:HGNC Symbol;Acc:HGNC:1781] |
| ENSG00000100764 | HGLibA_39128 | 21 | -0.939630173 | PSMC1 | ATPase 1 [Source:HGNC Symbol;Acc:HGNC:9547]" |
| ENSG00000102054 | HGLibA_40437 | 21 | -0.939630173 | RBBP7 | chromatin remodeling factor [Source:HGNC Symbol;Acc:HGNC:9890]" |
| ENSG00000103510 | HGLibA_24070 | 21 | -0.939630173 | KAT8 | lysine acetyltransferase 8 [Source:HGNC Symbol;Acc:HGNC:17933] |
| ENSG00000104142 | HGLibA_53746 | 21 | -0.939630173 | VPS18 | CORVET/HOPS core subunit [Source:HGNC Symbol;Acc:HGNC:15972]" |
| ENSG00000104687 | HGLibA_20444 | 21 | -0.939630173 | GSR | glutathione-disulfide reductase [Source:HGNC Symbol;Acc:HGNC:4623] |
| ENSG00000104888 | HGLibA_44476 | 21 | -0.939630173 | SLC17A7 | solute carrier family 17 member 7 [Source:HGNC Symbol;Acc:HGNC:16704] |
| ENSG00000105048 | HGLibA_50883 | 21 | -0.939630173 | TNNT1 | slow skeletal type [Source:HGNC Symbol;Acc:HGNC:11948]" |
| ENSG00000105518 | HGLibA_50191 | 21 | -0.939630173 | TMEM205 | transmembrane protein 205 [Source:HGNC Symbol;Acc:HGNC:29631] |
| ENSG00000106367 | HGLibA_02296 | 21 | -0.939630173 | AP1S1 | adaptor related protein complex 1 subunit sigma 1 [Source:HGNC Symbol;Acc:HGNC:559] |
| ENSG00000106415 | HGLibA_19229 | 21 | -0.939630173 | GLCCI1 | glucocorticoid induced 1 [Source:HGNC Symbol;Acc:HGNC:18713] |
| ENSG00000107140 | HGLibA_49027 | 21 | -0.939630173 | TESK1 | testis associated actin remodelling kinase 1 [Source:HGNC Symbol;Acc:HGNC:11731] |
| ENSG00000107164 | HGLibA_18151 | 21 | -0.939630173 | FUBP3 | far upstream element binding protein 3 [Source:HGNC Symbol;Acc:HGNC:4005] |
| ENSG00000107438 | HGLibA_35908 | 21 | -0.939630173 | PDLIM1 | PDZ and LIM domain 1 [Source:HGNC Symbol;Acc:HGNC:2067] |
| ENSG00000107672 | HGLibA_32772 | 21 | -0.939630173 | NSMCE4A | SMC5-SMC6 complex component [Source:HGNC Symbol;Acc:HGNC:25935]" |
| ENSG00000108788 | HGLibA_29438 | 21 | -0.939630173 | MLX | MAX dimerization protein [Source:HGNC Symbol;Acc:HGNC:11645]" |
| ENSG00000108797 | HGLibA_10419 | 21 | -0.939630173 | CNTNAP1 | contactin associated protein 1 [Source:HGNC Symbol;Acc:HGNC:8011] |
| ENSG00000108813 | HGLibA_13382 | 21 | -0.939630173 | DLX4 | distal-less homeobox 4 [Source:HGNC Symbol;Acc:HGNC:2917] |
| ENSG00000109270 | HGLibA_25994 | 21 | -0.939630173 | LAMTOR3 | MAPK and MTOR activator 3 [Source:HGNC Symbol;Acc:HGNC:15606]" |
| ENSG00000110375 | HGLibA_53074 | 21 | -0.939630173 | UPK2 | uroplakin 2 [Source:HGNC Symbol;Acc:HGNC:12579] |
| ENSG00000111834 | HGLibA_42336 | 21 | -0.939630173 | RSPH4A | radial spoke head component 4A [Source:HGNC Symbol;Acc:HGNC:21558] |
| ENSG00000112320 | HGLibA_46119 | 21 | -0.939630173 | SOBP | sine oculis binding protein homolog [Source:HGNC Symbol;Acc:HGNC:29256] |
| ENSG00000112685 | HGLibA_15735 | 21 | -0.939630173 | EXOC2 | exocyst complex component 2 [Source:HGNC Symbol;Acc:HGNC:24968] |
| ENSG00000113262 | HGLibA_20357 | 21 | -0.939630173 | GRM6 | glutamate metabotropic receptor 6 [Source:HGNC Symbol;Acc:HGNC:4598] |
| ENSG00000113272 | HGLibA_49354 | 21 | -0.939630173 | THG1L | tRNA-histidine guanylyltransferase 1 like [Source:HGNC Symbol;Acc:HGNC:26053] |
| ENSG00000113448 | HGLibA_35796 | 21 | -0.939630173 | PDE4D | phosphodiesterase 4D [Source:HGNC Symbol;Acc:HGNC:8783] |
| ENSG00000113615 | HGLibA_43229 | 21 | -0.939630173 | SEC24A | COPII coat complex component [Source:HGNC Symbol;Acc:HGNC:10703]" |
| ENSG00000113763 | HGLibA_53015 | 21 | -0.939630173 | UNC5A | unc-5 netrin receptor A [Source:HGNC Symbol;Acc:HGNC:12567] |
| ENSG00000115661 | HGLibA_47459 | 21 | -0.939630173 | STK16 | serine/threonine kinase 16 [Source:HGNC Symbol;Acc:HGNC:11394] |
| ENSG00000116560 | HGLibA_43731 | 21 | -0.939630173 | SFPQ | splicing factor proline and glutamine rich [Source:HGNC Symbol;Acc:HGNC:10774] |
| ENSG00000117174 | HGLibA_56931 | 21 | -0.939630173 | ZNHIT6 | zinc finger HIT-type containing 6 [Source:HGNC Symbol;Acc:HGNC:26089] |
| ENSG00000118518 | HGLibA_41534 | 21 | -0.939630173 | RNF146 | ring finger protein 146 [Source:HGNC Symbol;Acc:HGNC:21336] |
| ENSG00000118900 | HGLibA_52719 | 21 | -0.939630173 | UBN1 | ubinuclein 1 [Source:HGNC Symbol;Acc:HGNC:12506] |
| ENSG00000120709 | HGLibA_16641 | 21 | -0.939630173 | FAM53C | family with sequence similarity 53 member C [Source:HGNC Symbol;Acc:HGNC:1336] |
| ENSG00000121335 | HGLibA_38342 | 21 | -0.939630173 | PRB2 | proline rich protein BstNI subfamily 2 [Source:HGNC Symbol;Acc:HGNC:9338] |
| ENSG00000121864 | HGLibA_56440 | 21 | -0.939630173 | ZNF639 | zinc finger protein 639 [Source:HGNC Symbol;Acc:HGNC:30950] |
| ENSG00000122692 | HGLibA_45838 | 21 | -0.939630173 | SMU1 | DNA replication regulator and spliceosomal factor [Source:HGNC Symbol;Acc:HGNC:18247]" |
| ENSG00000123444 | HGLibA_24108 | 21 | -0.939630173 | KBTBD4 | kelch repeat and BTB domain containing 4 [Source:HGNC Symbol;Acc:HGNC:23761] |
| ENSG00000124067 | HGLibA_44367 | 21 | -0.939630173 | SLC12A4 | solute carrier family 12 member 4 [Source:HGNC Symbol;Acc:HGNC:10913] |
| ENSG00000124260 | HGLibA_27892 | 21 | -0.939630173 | MAGEA10 | MAGE family member A10 [Source:HGNC Symbol;Acc:HGNC:6797] |
| ENSG00000124613 | HGLibA_55907 | 21 | -0.939630173 | ZNF391 | zinc finger protein 391 [Source:HGNC Symbol;Acc:HGNC:18779] |
| ENSG00000125388 | HGLibA_20330 | 21 | -0.939630173 | GRK4 | G protein-coupled receptor kinase 4 [Source:HGNC Symbol;Acc:HGNC:4543] |
| ENSG00000125731 | HGLibA_43893 | 21 | -0.939630173 | SH2D3A | SH2 domain containing 3A [Source:HGNC Symbol;Acc:HGNC:16885] |
| ENSG00000126267 | HGLibA_10849 | 21 | -0.939630173 | COX6B1 | cytochrome c oxidase subunit 6B1 [Source:HGNC Symbol;Acc:HGNC:2280] |
| ENSG00000126550 | HGLibA_22352 | 21 | -0.939630173 | HTN1 | histatin 1 [Source:HGNC Symbol;Acc:HGNC:5283] |
| ENSG00000126861 | HGLibA_33387 | 21 | -0.939630173 | OMG | oligodendrocyte myelin glycoprotein [Source:HGNC Symbol;Acc:HGNC:8135] |
| ENSG00000127080 | HGLibA_23485 | 21 | -0.939630173 | IPPK | inositol-pentakisphosphate 2-kinase [Source:HGNC Symbol;Acc:HGNC:14645] |
| ENSG00000128513 | HGLibA_37715 | 21 | -0.939630173 | POT1 | protection of telomeres 1 [Source:HGNC Symbol;Acc:HGNC:17284] |
| ENSG00000128965 | HGLibA_09334 | 21 | -0.939630173 | CHAC1 | ChaC glutathione specific gamma-glutamylcyclotransferase 1 [Source:HGNC Symbol;Acc:HGNC:28680] |
| ENSG00000129197 | HGLibA_41812 | 21 | -0.939630173 | RPAIN | RPA interacting protein [Source:HGNC Symbol;Acc:HGNC:28641] |
| ENSG00000129990 | HGLibA_48082 | 21 | -0.939630173 | SYT5 | synaptotagmin 5 [Source:HGNC Symbol;Acc:HGNC:11513] |
| ENSG00000130433 | HGLibA_06945 | 21 | -0.939630173 | CACNG6 | calcium voltage-gated channel auxiliary subunit gamma 6 [Source:HGNC Symbol;Acc:HGNC:13625] |
| ENSG00000130433 | HGLibA_06943 | 21 | -0.939630173 | CACNG6 | calcium voltage-gated channel auxiliary subunit gamma 6 [Source:HGNC Symbol;Acc:HGNC:13625] |
| ENSG00000130669 | HGLibA_35050 | 21 | -0.939630173 | PAK4 | p21 (RAC1) activated kinase 4 [Source:HGNC Symbol;Acc:HGNC:16059] |
| ENSG00000130935 | HGLibA_32260 | 21 | -0.939630173 | NOL11 | nucleolar protein 11 [Source:HGNC Symbol;Acc:HGNC:24557] |
| ENSG00000131738 | HGLibA_25482 | 21 | -0.939630173 | KRT33B | keratin 33B [Source:HGNC Symbol;Acc:HGNC:6451] |
| ENSG00000132207 | HGLibA_45556 | 21 | -0.939630173 | SLX1A | structure-specific endonuclease subunit [Source:HGNC Symbol;Acc:HGNC:20922]" |
| ENSG00000132256 | HGLibA_51535 | 21 | -0.939630173 | TRIM5 | tripartite motif containing 5 [Source:HGNC Symbol;Acc:HGNC:16276] |
| ENSG00000132692 | HGLibA_04235 | 21 | -0.939630173 | BCAN | brevican [Source:HGNC Symbol;Acc:HGNC:23059] |
| ENSG00000133657 | HGLibA_03572 | 21 | -0.939630173 | ATP13A3 | ATPase 13A3 [Source:HGNC Symbol;Acc:HGNC:24113] |
| ENSG00000134809 | HGLibA_49492 | 21 | -0.939630173 | TIMM10 | translocase of inner mitochondrial membrane 10 [Source:HGNC Symbol;Acc:HGNC:11814] |
| ENSG00000136141 | HGLibA_27138 | 21 | -0.939630173 | LRCH1 | leucine rich repeats and calponin homology domain containing 1 [Source:HGNC Symbol;Acc:HGNC:20309] |
| ENSG00000136436 | HGLibA_07002 | 21 | -0.939630173 | CALCOCO2 | calcium binding and coiled-coil domain 2 [Source:HGNC Symbol;Acc:HGNC:29912] |
| ENSG00000136891 | HGLibA_49046 | 21 | -0.939630173 | TEX10 | testis expressed 10 [Source:HGNC Symbol;Acc:HGNC:25988] |
| ENSG00000137033 | HGLibA_23138 | 21 | -0.939630173 | IL33 | interleukin 33 [Source:HGNC Symbol;Acc:HGNC:16028] |
| ENSG00000137135 | HGLibA_02862 | 21 | -0.939630173 | ARHGEF39 | Rho guanine nucleotide exchange factor 39 [Source:HGNC Symbol;Acc:HGNC:25909] |
| ENSG00000137145 | HGLibA_12956 | 21 | -0.939630173 | DENND4C | DENN domain containing 4C [Source:HGNC Symbol;Acc:HGNC:26079] |
| ENSG00000137601 | HGLibA_31637 | 21 | -0.939630173 | NEK1 | NIMA related kinase 1 [Source:HGNC Symbol;Acc:HGNC:7744] |
| ENSG00000137976 | HGLibA_13667 | 21 | -0.939630173 | DNASE2B | deoxyribonuclease 2 beta [Source:HGNC Symbol;Acc:HGNC:28875] |
| ENSG00000139200 | HGLibA_36503 | 21 | -0.939630173 | PIANP | PILR alpha associated neural protein [Source:HGNC Symbol;Acc:HGNC:25338] |
| ENSG00000139574 | HGLibA_32435 | 21 | -0.939630173 | NPFF | neuropeptide FF-amide peptide precursor [Source:HGNC Symbol;Acc:HGNC:7901] |
| ENSG00000140986 | HGLibA_41978 | 21 | -0.939630173 | RPL3L | ribosomal protein L3 like [Source:HGNC Symbol;Acc:HGNC:10351] |
| ENSG00000141376 | HGLibA_04258 | 21 | -0.939630173 | BCAS3 | microtubule associated cell migration factor [Source:HGNC Symbol;Acc:HGNC:14347]" |
| ENSG00000141505 | HGLibA_03287 | 21 | -0.939630173 | ASGR1 | asialoglycoprotein receptor 1 [Source:HGNC Symbol;Acc:HGNC:742] |
| ENSG00000142273 | HGLibA_07400 | 21 | -0.939630173 | CBLC | Cbl proto-oncogene C [Source:HGNC Symbol;Acc:HGNC:15961] |
| ENSG00000143013 | HGLibA_26711 | 21 | -0.939630173 | LMO4 | LIM domain only 4 [Source:HGNC Symbol;Acc:HGNC:6644] |
| ENSG00000143147 | HGLibA_19956 | 21 | -0.939630173 | GPR161 | G protein-coupled receptor 161 [Source:HGNC Symbol;Acc:HGNC:23694] |
| ENSG00000143167 | HGLibA_19718 | 21 | -0.939630173 | GPA33 | glycoprotein A33 [Source:HGNC Symbol;Acc:HGNC:4445] |
| ENSG00000143178 | HGLibA_48653 | 21 | -0.939630173 | TBX19 | T-box 19 [Source:HGNC Symbol;Acc:HGNC:11596] |
| ENSG00000143919 | HGLibA_07095 | 21 | -0.939630173 | CAMKMT | calmodulin-lysine N-methyltransferase [Source:HGNC Symbol;Acc:HGNC:26276] |
| ENSG00000144406 | HGLibA_53031 | 21 | -0.939630173 | UNC80 | NALCN channel complex subunit [Source:HGNC Symbol;Acc:HGNC:26582]" |
| ENSG00000144908 | HGLibA_01552 | 21 | -0.939630173 | ALDH1L1 | aldehyde dehydrogenase 1 family member L1 [Source:HGNC Symbol;Acc:HGNC:3978] |
| ENSG00000144909 | HGLibA_34647 | 21 | -0.939630173 | OSBPL11 | oxysterol binding protein like 11 [Source:HGNC Symbol;Acc:HGNC:16397] |
| ENSG00000145147 | HGLibA_45507 | 21 | -0.939630173 | SLIT2 | slit guidance ligand 2 [Source:HGNC Symbol;Acc:HGNC:11086] |
| ENSG00000145354 | HGLibA_09729 | 21 | -0.939630173 | CISD2 | CDGSH iron sulfur domain 2 [Source:HGNC Symbol;Acc:HGNC:24212] |
| ENSG00000147654 | HGLibA_14345 | 21 | -0.939630173 | EBAG9 | 9 [Source:HGNC Symbol;Acc:HGNC:3123]" |
| ENSG00000150687 | HGLibA_38916 | 21 | -0.939630173 | PRSS23 | serine protease 23 [Source:HGNC Symbol;Acc:HGNC:14370] |
| ENSG00000150750 | HGLibA_05155 | 21 | -0.939630173 | C11orf53 | chromosome 11 open reading frame 53 [Source:HGNC Symbol;Acc:HGNC:30527] |
| ENSG00000151012 | HGLibA_45353 | 21 | -0.939630173 | SLC7A11 | solute carrier family 7 member 11 [Source:HGNC Symbol;Acc:HGNC:11059] |
| ENSG00000151117 | HGLibA_50524 | 21 | -0.939630173 | TMEM86A | transmembrane protein 86A [Source:HGNC Symbol;Acc:HGNC:26890] |
| ENSG00000151208 | HGLibA_13337 | 21 | -0.939630173 | DLG5 | discs large MAGUK scaffold protein 5 [Source:HGNC Symbol;Acc:HGNC:2904] |
| ENSG00000152291 | HGLibA_49275 | 21 | -0.939630173 | TGOLN2 | trans-golgi network protein 2 [Source:HGNC Symbol;Acc:HGNC:15450] |
| ENSG00000152377 | HGLibA_46747 | 21 | -0.939630173 | SPOCK1 | cwcv and kazal like domains proteoglycan 1 [Source:HGNC Symbol;Acc:HGNC:11251]" |
| ENSG00000153779 | HGLibA_49246 | 21 | -0.939630173 | TGIF2LX | TGFB induced factor homeobox 2 like X-linked [Source:HGNC Symbol;Acc:HGNC:18570] |
| ENSG00000153814 | HGLibA_23940 | 21 | -0.939630173 | JAZF1 | JAZF zinc finger 1 [Source:HGNC Symbol;Acc:HGNC:28917] |
| ENSG00000153930 | HGLibA_01955 | 21 | -0.939630173 | ANKFN1 | ankyrin repeat and fibronectin type III domain containing 1 [Source:HGNC Symbol;Acc:HGNC:26766] |
| ENSG00000154143 | HGLibA_35115 | 21 | -0.939630173 | PANX3 | pannexin 3 [Source:HGNC Symbol;Acc:HGNC:20573] |
| ENSG00000156076 | HGLibA_54301 | 21 | -0.939630173 | WIF1 | WNT inhibitory factor 1 [Source:HGNC Symbol;Acc:HGNC:18081] |
| ENSG00000157426 | HGLibA_00077 | 21 | -0.939630173 | AASDH | aminoadipate-semialdehyde dehydrogenase [Source:HGNC Symbol;Acc:HGNC:23993] |

| | | | | | |
|---|---|---|---|---|---|
| ENSG00000157764 | HGLibA_04752 | 21 | -0.939630173 | BRAF | serine/threonine kinase [Source:HGNC Symbol;Acc:HGNC:1097]" |
| ENSG00000158528 | HGLibA_38127 | 21 | -0.939630173 | PPP1R9A | protein phosphatase 1 regulatory subunit 9A [Source:HGNC Symbol;Acc:HGNC:14946] |
| ENSG00000160191 | HGLibA_35834 | 21 | -0.939630173 | PDE9A | phosphodiesterase 9A [Source:HGNC Symbol;Acc:HGNC:8795] |
| ENSG00000160703 | HGLibA_32138 | 21 | -0.939630173 | NLRX1 | NLR family member X1 [Source:HGNC Symbol;Acc:HGNC:29890] |
| ENSG00000161328 | HGLibA_27392 | 21 | -0.939630173 | LRRC56 | leucine rich repeat containing 56 [Source:HGNC Symbol;Acc:HGNC:25430] |
| ENSG00000161509 | HGLibA_20306 | 21 | -0.939630173 | GRIN2C | glutamate ionotropic receptor NMDA type subunit 2C [Source:HGNC Symbol;Acc:HGNC:4587] |
| ENSG00000162174 | HGLibA_03367 | 21 | -0.939630173 | ASRGL1 | asparaginase like 1 [Source:HGNC Symbol;Acc:HGNC:16448] |
| ENSG00000162441 | HGLibA_27798 | 21 | -0.939630173 | LZIC | leucine zipper and CTNNBIP1 domain containing [Source:HGNC Symbol;Acc:HGNC:17497] |
| ENSG00000162571 | HGLibA_52189 | 21 | -0.939630173 | TTLL10 | tubulin tyrosine ligase like 10 [Source:HGNC Symbol;Acc:HGNC:26693] |
| ENSG00000162747 | HGLibA_17198 | 21 | -0.939630173 | FCGR3B | Fc fragment of IgG receptor IIIb [Source:HGNC Symbol;Acc:HGNC:3620] |
| ENSG00000162813 | HGLibA_04737 | 21 | -0.939630173 | BPNT1 | 5'-bisphosphate nucleotidase 1 [Source:HGNC Symbol;Acc:HGNC:1096]" |
| ENSG00000162819 | HGLibA_04837 | 21 | -0.939630173 | BROX | BRO1 domain and CAAX motif containing [Source:HGNC Symbol;Acc:HGNC:26512] |
| ENSG00000163239 | HGLibA_48900 | 21 | -0.939630173 | TDRD10 | tudor domain containing 10 [Source:HGNC Symbol;Acc:HGNC:25316] |
| ENSG00000163584 | HGLibA_41901 | 21 | -0.939630173 | RPL22L1 | ribosomal protein L22 like 1 [Source:HGNC Symbol;Acc:HGNC:27610] |
| ENSG00000163751 | HGLibA_10889 | 21 | -0.939630173 | CPA3 | carboxypeptidase A3 [Source:HGNC Symbol;Acc:HGNC:2298] |
| ENSG00000163803 | HGLibA_36968 | 21 | -0.939630173 | PLB1 | phospholipase B1 [Source:HGNC Symbol;Acc:HGNC:30041] |
| ENSG00000164294 | HGLibA_20190 | 21 | -0.939630173 | GPX8 | glutathione peroxidase 8 (putative) [Source:HGNC Symbol;Acc:HGNC:33100] |
| ENSG00000164307 | HGLibA_15403 | 21 | -0.939630173 | ERAP1 | endoplasmic reticulum aminopeptidase 1 [Source:HGNC Symbol;Acc:HGNC:18173] |
| ENSG00000164694 | HGLibA_17743 | 21 | -0.939630173 | FNDC1 | fibronectin type III domain containing 1 [Source:HGNC Symbol;Acc:HGNC:21184] |
| ENSG00000165113 | HGLibA_19206 | 21 | -0.939630173 | GKAP1 | G kinase anchoring protein 1 [Source:HGNC Symbol;Acc:HGNC:17496] |
| ENSG00000165995 | HGLibA_06920 | 21 | -0.939630173 | CACNB2 | calcium voltage-gated channel auxiliary subunit beta 2 [Source:HGNC Symbol;Acc:HGNC:1402] |
| ENSG00000166135 | HGLibA_21301 | 21 | -0.939630173 | HIF1AN | hypoxia inducible factor 1 subunit alpha inhibitor [Source:HGNC Symbol;Acc:HGNC:17113] |
| ENSG00000166220 | HGLibA_48457 | 21 | -0.939630173 | TBATA | brain and testes associated [Source:HGNC Symbol;Acc:HGNC:23511]" |
| ENSG00000166446 | HGLibA_08920 | 21 | -0.939630173 | CDYL2 | chromodomain Y like 2 [Source:HGNC Symbol;Acc:HGNC:23030] |
| ENSG00000166527 | HGLibA_09985 | 21 | -0.939630173 | CLEC4D | C-type lectin domain family 4 member D [Source:HGNC Symbol;Acc:HGNC:14554] |
| ENSG00000167578 | HGLibA_39957 | 21 | -0.939630173 | RAB4B | member RAS oncogene family [Source:HGNC Symbol;Acc:HGNC:9782]" |
| ENSG00000167614 | HGLibA_52232 | 21 | -0.939630173 | TTYH1 | tweety family member 1 [Source:HGNC Symbol;Acc:HGNC:13476] |
| ENSG00000168101 | HGLibA_32954 | 21 | -0.939630173 | NUDT16L1 | nudix hydrolase 16 like 1 [Source:HGNC Symbol;Acc:HGNC:28154] |
| ENSG00000168530 | HGLibA_30746 | 21 | -0.939630173 | MYL1 | myosin light chain 1 [Source:HGNC Symbol;Acc:HGNC:7582] |
| ENSG00000168671 | HGLibA_52937 | 21 | -0.939630173 | UGT3A2 | UDP glycosyltransferase family 3 member A2 [Source:HGNC Symbol;Acc:HGNC:27266] |
| ENSG00000168802 | HGLibA_09664 | 21 | -0.939630173 | CHTF8 | chromosome transmission fidelity factor 8 [Source:HGNC Symbol;Acc:HGNC:24353] |
| ENSG00000168818 | HGLibA_47629 | 21 | -0.939630173 | STX18 | syntaxin 18 [Source:HGNC Symbol;Acc:HGNC:15942] |
| ENSG00000169714 | HGLibA_10247 | 21 | -0.939630173 | CNBP | CCHC-type zinc finger nucleic acid binding protein [Source:HGNC Symbol;Acc:HGNC:13164] |
| ENSG00000170577 | HGLibA_44246 | 21 | -0.939630173 | SIX2 | SIX homeobox 2 [Source:HGNC Symbol;Acc:HGNC:10888] |
| ENSG00000170906 | HGLibA_31455 | 21 | -0.939630173 | NDUFA3 | NADH:ubiquinone oxidoreductase subunit A3 [Source:HGNC Symbol;Acc:HGNC:7686] |
| ENSG00000171241 | HGLibA_44012 | 21 | -0.939630173 | SHCBP1 | SHC binding and spindle associated 1 [Source:HGNC Symbol;Acc:HGNC:29547] |
| ENSG00000171360 | HGLibA_25498 | 21 | -0.939630173 | KRT38 | keratin 38 [Source:HGNC Symbol;Acc:HGNC:6456] |
| ENSG00000171533 | HGLibA_28225 | 21 | -0.939630173 | MAP6 | microtubule associated protein 6 [Source:HGNC Symbol;Acc:HGNC:6868] |
| ENSG00000171943 | HGLibA_46977 | 21 | -0.939630173 | SRGAP2C | SLIT-ROBO Rho GTPase activating protein 2C [Source:HGNC Symbol;Acc:HGNC:30584] |
| ENSG00000171953 | HGLibA_03801 | 21 | -0.939630173 | ATPAF2 | ATP synthase mitochondrial F1 complex assembly factor 2 [Source:HGNC Symbol;Acc:HGNC:18802] |
| ENSG00000172000 | HGLibA_56227 | 21 | -0.939630173 | ZNF556 | zinc finger protein 556 [Source:HGNC Symbol;Acc:HGNC:25669] |
| ENSG00000172602 | HGLibA_41445 | 21 | -0.939630173 | RND1 | Rho family GTPase 1 [Source:HGNC Symbol;Acc:HGNC:18314] |
| ENSG00000172785 | HGLibA_07430 | 21 | -0.939630173 | CBWD1 | COBW domain containing 1 [Source:HGNC Symbol;Acc:HGNC:17134] |
| ENSG00000173093 | HGLibA_07823 | 21 | -0.939630173 | CCDC63 | coiled-coil domain containing 63 [Source:HGNC Symbol;Acc:HGNC:26669] |
| ENSG00000173285 | HGLibA_33531 | 21 | -0.939630173 | OR10K1 | olfactory receptor family 10 subfamily K member 1 [Source:HGNC Symbol;Acc:HGNC:14693] |
| ENSG00000173531 | HGLibA_30254 | 21 | -0.939630173 | MST1 | macrophage stimulating 1 [Source:HGNC Symbol;Acc:HGNC:7380] |
| ENSG00000174177 | HGLibA_11751 | 21 | -0.939630173 | CTU2 | cytosolic thiouridylase subunit 2 [Source:HGNC Symbol;Acc:HGNC:28005] |
| ENSG00000174243 | HGLibA_12674 | 21 | -0.939630173 | DDX23 | DEAD-box helicase 23 [Source:HGNC Symbol;Acc:HGNC:17347] |
| ENSG00000175535 | HGLibA_37354 | 21 | -0.939630173 | PNLIP | pancreatic lipase [Source:HGNC Symbol;Acc:HGNC:9155] |
| ENSG00000176253 | HGLibA_34018 | 21 | -0.939630173 | OR4K13 | olfactory receptor family 4 subfamily K member 13 [Source:HGNC Symbol;Acc:HGNC:15351] |
| ENSG00000176256 | HGLibA_21698 | 21 | -0.939630173 | HMGB4 | high mobility group box 4 [Source:HGNC Symbol;Acc:HGNC:24954] |
| ENSG00000176299 | HGLibA_34041 | 21 | -0.939630173 | OR4M1 | olfactory receptor family 4 subfamily M member 1 [Source:HGNC Symbol;Acc:HGNC:14735] |
| ENSG00000177034 | HGLibA_30517 | 21 | -0.939630173 | MTX3 | metaxin 3 [Source:HGNC Symbol;Acc:HGNC:24812] |
| ENSG00000178057 | HGLibA_31489 | 21 | -0.939630173 | NDUFAF3 | NADH:ubiquinone oxidoreductase complex assembly factor 3 [Source:HGNC Symbol;Acc:HGNC:29918] |
| ENSG00000178645 | HGLibA_05073 | 21 | -0.939630173 | C10orf53 | chromosome 10 open reading frame 53 [Source:HGNC Symbol;Acc:HGNC:27421] |
| ENSG00000179051 | HGLibA_40687 | 21 | -0.939630173 | RCC2 | regulator of chromosome condensation 2 [Source:HGNC Symbol;Acc:HGNC:30297] |
| ENSG00000179407 | HGLibA_13556 | 21 | -0.939630173 | DNAJB8 | DnaJ heat shock protein family (Hsp40) member B8 [Source:HGNC Symbol;Acc:HGNC:23699] |
| ENSG00000179454 | HGLibA_25172 | 21 | -0.939630173 | KLHL28 | kelch like family member 28 [Source:HGNC Symbol;Acc:HGNC:19741] |
| ENSG00000179674 | HGLibA_02935 | 21 | -0.939630173 | ARL14 | ADP ribosylation factor like GTPase 14 [Source:HGNC Symbol;Acc:HGNC:22974] |
| ENSG00000179965 | HGLibA_56707 | 21 | -0.939630173 | ZNF771 | zinc finger protein 771 [Source:HGNC Symbol;Acc:HGNC:29653] |
| ENSG00000182870 | HGLibA_18576 | 21 | -0.939630173 | GALNT9 | polypeptide N-acetylgalactosaminyltransferase 9 [Source:HGNC Symbol;Acc:HGNC:4131] |
| ENSG00000182903 | HGLibA_56629 | 21 | -0.939630173 | ZNF721 | zinc finger protein 721 [Source:HGNC Symbol;Acc:HGNC:29425] |
| ENSG00000183474 | HGLibA_20550 | 21 | -0.939630173 | GTF2H2C | GTF2H2 family member C [Source:HGNC Symbol;Acc:HGNC:31394] |
| ENSG00000184178 | HGLibA_42896 | 21 | -0.939630173 | SCFD2 | sec1 family domain containing 2 [Source:HGNC Symbol;Acc:HGNC:30676] |
| ENSG00000185294 | HGLibA_46782 | 21 | -0.939630173 | SPPL2C | signal peptide peptidase like 2C [Source:HGNC Symbol;Acc:HGNC:28902] |
| ENSG00000185303 | HGLibA_43770 | 21 | -0.939630173 | SFTPA2 | surfactant protein A2 [Source:HGNC Symbol;Acc:HGNC:10799] |
| ENSG00000185803 | HGLibA_45248 | 21 | -0.939630173 | SLC52A2 | solute carrier family 52 member 2 [Source:HGNC Symbol;Acc:HGNC:30224] |
| ENSG00000186480 | HGLibA_23376 | 21 | -0.939630173 | INSIG1 | insulin induced gene 1 [Source:HGNC Symbol;Acc:HGNC:6083] |
| ENSG00000187134 | HGLibA_01480 | 21 | -0.939630173 | AKR1C1 | aldo-keto reductase family 1 member C1 [Source:HGNC Symbol;Acc:HGNC:384] |
| ENSG00000187510 | HGLibA_37123 | 21 | -0.939630173 | PLEKHG7 | pleckstrin homology and RhoGEF domain containing G7 [Source:HGNC Symbol;Acc:HGNC:33829] |
| ENSG00000187741 | HGLibA_16830 | 21 | -0.939630173 | FANCA | FA complementation group A [Source:HGNC Symbol;Acc:HGNC:3582] |
| ENSG00000187848 | HGLibA_34849 | 21 | -0.939630173 | P2RX2 | purinergic receptor P2X 2 [Source:HGNC Symbol;Acc:HGNC:15459] |
| ENSG00000188120 | HGLibA_12353 | 21 | -0.939630173 | DAZ1 | deleted in azoospermia 1 [Source:HGNC Symbol;Acc:HGNC:2682] |
| ENSG00000188153 | HGLibA_10581 | 21 | -0.939630173 | COL4A5 | collagen type IV alpha 5 chain [Source:HGNC Symbol;Acc:HGNC:2207] |
| ENSG00000188818 | HGLibA_55068 | 21 | -0.939630173 | ZDHHC11 | zinc finger DHHC-type containing 11 [Source:HGNC Symbol;Acc:HGNC:19158] |
| ENSG00000189157 | HGLibA_16618 | 21 | -0.939630173 | FAM47E | family with sequence similarity 47 member E [Source:HGNC Symbol;Acc:HGNC:34343] |
| ENSG00000196911 | HGLibA_25365 | 21 | -0.939630173 | KPNA5 | karyopherin subunit alpha 5 [Source:HGNC Symbol;Acc:HGNC:6398] |
| ENSG00000197046 | HGLibA_44137 | 21 | -0.939630173 | SIGLEC15 | sialic acid binding Ig like lectin 15 [Source:HGNC Symbol;Acc:HGNC:27596] |
| ENSG00000197753 | HGLibA_26442 | 21 | -0.939630173 | LHFPL5 | LHFPL tetraspan subfamily member 5 [Source:HGNC Symbol;Acc:HGNC:21253] |
| ENSG00000198373 | HGLibA_54455 | 21 | -0.939630173 | WWP2 | WW domain containing E3 ubiquitin protein ligase 2 [Source:HGNC Symbol;Acc:HGNC:16804] |
| ENSG00000198521 | HGLibA_55974 | 21 | -0.939630173 | ZNF43 | zinc finger protein 43 [Source:HGNC Symbol;Acc:HGNC:13109] |
| ENSG00000198650 | HGLibA_48437 | 21 | -0.939630173 | TAT | tyrosine aminotransferase [Source:HGNC Symbol;Acc:HGNC:11573] |
| ENSG00000203727 | HGLibA_42689 | 21 | -0.939630173 | SAMD5 | sterile alpha motif domain containing 5 [Source:HGNC Symbol;Acc:HGNC:21180] |
| ENSG00000203734 | HGLibA_14427 | 21 | -0.939630173 | ECT2L | epithelial cell transforming 2 like [Source:HGNC Symbol;Acc:HGNC:21118] |
| ENSG00000204305 | HGLibA_01182 | 21 | -0.939630173 | AGER | advanced glycosylation end-product specific receptor [Source:HGNC Symbol;Acc:HGNC:320] |
| ENSG00000204542 | HGLibA_06453 | 21 | -0.939630173 | C6orf15 | chromosome 6 open reading frame 15 [Source:HGNC Symbol;Acc:HGNC:13927] |
| ENSG00000204700 | HGLibA_33809 | 21 | -0.939630173 | OR2J2 | olfactory receptor family 2 subfamily J member 2 [Source:HGNC Symbol;Acc:HGNC:8260] |
| ENSG00000204710 | HGLibA_46575 | 21 | -0.939630173 | SPDYC | speedy/RINGO cell cycle regulator family member C [Source:HGNC Symbol;Acc:HGNC:32681] |
| ENSG00000205496 | HGLibA_34072 | 21 | -0.939630173 | OR51A2 | olfactory receptor family 51 subfamily A member 2 [Source:HGNC Symbol;Acc:HGNC:14764] |
| ENSG00000205944 | HGLibA_12356 | 21 | -0.939630173 | DAZ2 | deleted in azoospermia 2 [Source:HGNC Symbol;Acc:HGNC:15964] |
| ENSG00000214860 | HGLibA_15703 | 21 | -0.939630173 | EVPLL | envoplakin like [Source:HGNC Symbol;Acc:HGNC:35236] |
| ENSG00000215440 | HGLibA_32431 | 21 | -0.939630173 | NPEPL1 | aminopeptidase like 1 [Source:HGNC Symbol;Acc:HGNC:16244] |
| ENSG00000221947 | HGLibA_54536 | 21 | -0.939630173 | XKR9 | XK related 9 [Source:HGNC Symbol;Acc:HGNC:20937] |
| ENSG00000221978 | HGLibA_08120 | 21 | -0.939630173 | CCNL2 | cyclin L2 [Source:HGNC Symbol;Acc:HGNC:20570] |
| ENSG00000228727 | HGLibA_42732 | 21 | -0.939630173 | SAPCD1 | suppressor APC domain containing 1 [Source:HGNC Symbol;Acc:HGNC:13938] |
| ENSG00000230301 | HGLibA_34300 | 21 | -0.939630173 | OR5H6 | olfactory receptor family 5 subfamily H member 6 (gene/pseudogene) [Source:HGNC Symbol;Acc:HGNC:14767] |
| ENSG00000234224 | HGLibA_50250 | 21 | -0.939630173 | TMEM229A | transmembrane protein 229A [Source:HGNC Symbol;Acc:HGNC:37279] |
| ENSG00000235961 | HGLibA_37377 | 21 | -0.939630173 | PNMA6A | PNMA family member 6A [Source:HGNC Symbol;Acc:HGNC:28248] |
| ENSG00000236981 | HGLibA_33502 | 21 | -0.939630173 | OR10G9 | olfactory receptor family 10 subfamily G member 9 [Source:HGNC Symbol;Acc:HGNC:15129] |
| ENSG00000237441 | HGLibA_41005 | 21 | -0.939630173 | RGL2 | ral guanine nucleotide dissociation stimulator like 2 [Source:HGNC Symbol;Acc:HGNC:9769] |
| ENSG00000240247 | HGLibA_12794 | 21 | -0.939630173 | DEFA1B | defensin alpha 1B [Source:HGNC Symbol;Acc:HGNC:33596] |
| ENSG00000242574 | HGLibA_21607 | 21 | -0.939630173 | HLA-DMB | DM beta [Source:HGNC Symbol;Acc:HGNC:4935]" |
| ENSG00000242612 | HGLibA_12776 | 21 | -0.939630173 | DECR2 | 4-dienoyl-CoA reductase 2 [Source:HGNC Symbol;Acc:HGNC:2754]" |
| ENSG00000246922 | HGLibA_52548 | 21 | -0.939630173 | UBAP1L | ubiquitin associated protein 1 like [Source:HGNC Symbol;Acc:HGNC:40028] |
| ENSG00000247596 | HGLibA_52373 | 21 | -0.939630173 | TWF2 | twinfilin actin binding protein 2 [Source:HGNC Symbol;Acc:HGNC:9621] |
| ENSG00000253719 | HGLibA_03862 | 21 | -0.939630173 | ATXN7L3B | ataxin 7 like 3B [Source:HGNC Symbol;Acc:HGNC:37931] |
| ENSG00000253729 | HGLibA_38576 | 21 | -0.939630173 | PRKDC | catalytic subunit [Source:HGNC Symbol;Acc:HGNC:9413]" |
| ENSG00000255837 | HGLibA_48381 | 21 | -0.939630173 | TAS2R20 | taste 2 receptor member 20 [Source:HGNC Symbol;Acc:HGNC:19109] |
| ENSG00000266200 | HGLibA_37360 | 21 | -0.939630173 | PNLIPRP2 | pancreatic lipase related protein 2 (gene/pseudogene) [Source:HGNC Symbol;Acc:HGNC:9157] |
| ENSG00000271449 | HGLibA_11500 | 21 | -0.939630173 | CT45A2 | cancer/testis antigen family 45 member A2 [Source:HGNC Symbol;Acc:HGNC:28400] |
| FAM160B2 | HGLibA_16213 | 21 | -0.939630173 | FAM160B2 | - |
| FBXO18 | HGLibA_17039 | 21 | -0.939630173 | FBXO18 | - |
| FLJ27352 | HGLibA_17626 | 21 | -0.939630173 | FLJ27352 | - |
| hsa-let-7a-3 | HGLibA_57106 | 21 | -0.939630173 | hsa-let-7a-3 | - |
| hsa-mir-1250 | HGLibA_57399 | 21 | -0.939630173 | hsa-mir-1250 | - |
| hsa-mir-1287 | HGLibA_57614 | 21 | -0.939630173 | hsa-mir-1287 | - |
| hsa-mir-1292 | HGLibA_57643 | 21 | -0.939630173 | hsa-mir-1292 | - |
| hsa-mir-135a-2 | HGLibA_57798 | 21 | -0.939630173 | hsa-mir-135a-2 | - |
| hsa-mir-147b | HGLibA_57875 | 21 | -0.939630173 | hsa-mir-147b | - |
| hsa-mir-215 | HGLibA_58252 | 21 | -0.939630173 | hsa-mir-215 | - |
| hsa-mir-29b-2 | HGLibA_58417 | 21 | -0.939630173 | hsa-mir-29b-2 | - |
| hsa-mir-3125 | HGLibA_58553 | 21 | -0.939630173 | hsa-mir-3125 | - |
| hsa-mir-3180-2 | HGLibA_58804 | 21 | -0.939630173 | hsa-mir-3180-2 | - |
| hsa-mir-340 | HGLibA_59020 | 21 | -0.939630173 | hsa-mir-340 | - |
| hsa-mir-3666 | HGLibA_59216 | 21 | -0.939630173 | hsa-mir-3666 | - |
| hsa-mir-3670-2 | HGLibA_59237 | 21 | -0.939630173 | hsa-mir-3670-2 | - |
| hsa-mir-3928 | HGLibA_59587 | 21 | -0.939630173 | hsa-mir-3928 | - |
| hsa-mir-412 | HGLibA_59689 | 21 | -0.939630173 | hsa-mir-412 | - |
| hsa-mir-4270 | HGLibA_59787 | 21 | -0.939630173 | hsa-mir-4270 | - |
| hsa-mir-4419b | HGLibA_60064 | 21 | -0.939630173 | hsa-mir-4419b | - |
| hsa-mir-4432 | HGLibA_60114 | 21 | -0.939630173 | hsa-mir-4432 | - |
| hsa-mir-4451 | HGLibA_60212 | 21 | -0.939630173 | hsa-mir-4451 | - |
| hsa-mir-4670 | HGLibA_60799 | 21 | -0.939630173 | hsa-mir-4670 | - |
| hsa-mir-4726 | HGLibA_61003 | 21 | -0.939630173 | hsa-mir-4726 | - |
| hsa-mir-4727 | HGLibA_61005 | 21 | -0.939630173 | hsa-mir-4727 | - |

| | | | | | |
|---|---|---|---|---|---|
| hsa-mir-4774 | HGLibA_61200 | 21 | -0.939630173 | hsa-mir-4774 | - |
| hsa-mir-4783 | HGLibA_61239 | 21 | -0.939630173 | hsa-mir-4783 | - |
| hsa-mir-516a-2 | HGLibA_61612 | 21 | -0.939630173 | hsa-mir-516a-2 | - |
| hsa-mir-526a-2 | HGLibA_61794 | 21 | -0.939630173 | hsa-mir-526a-2 | - |
| hsa-mir-5681b | HGLibA_62243 | 21 | -0.939630173 | hsa-mir-5681b | - |
| hsa-mir-5692c-2 | HGLibA_62301 | 21 | -0.939630173 | hsa-mir-5692c-2 | - |
| hsa-mir-6727 | HGLibA_63060 | 21 | -0.939630173 | hsa-mir-6727 | - |
| hsa-mir-6828 | HGLibA_63491 | 21 | -0.939630173 | hsa-mir-6828 | - |
| hsa-mir-6862-2 | HGLibA_63632 | 21 | -0.939630173 | hsa-mir-6862-2 | - |
| hsa-mir-767 | HGLibA_63932 | 21 | -0.939630173 | hsa-mir-767 | - |
| hsa-mir-7973-1 | HGLibA_64017 | 21 | -0.939630173 | hsa-mir-7973-1 | - |
| hsa-mir-8071-1 | HGLibA_64121 | 21 | -0.939630173 | hsa-mir-8071-1 | - |
| hsa-mir-92a-1 | HGLibA_64290 | 21 | -0.939630173 | hsa-mir-92a-1 | - |
| ICT1 | HGLibA_22520 | 21 | -0.939630173 | ICT1 | - |
| IMPAD1 | HGLibA_23254 | 21 | -0.939630173 | IMPAD1 | - |
| LOC100127983 | HGLibA_26750 | 21 | -0.939630173 | LOC100127983 | - |
| LOC100287534 | HGLibA_26824 | 21 | -0.939630173 | LOC100287534 | - |
| LOC653486 | HGLibA_26986 | 21 | -0.939630173 | LOC653486 | - |
| OR8U8 | HGLibA_34559 | 21 | -0.939630173 | OR8U8 | - |
| PHOSPHO2-KLHL23 | HGLibA_36446 | 21 | -0.939630173 | PHOSPHO2-KLHL23 | - |
| C10orf111 | HGLibA_05031 | 20 | -0.94958121 | C10orf111 | - |
| C12orf44 | HGLibA_05247 | 20 | -0.94958121 | C12orf44 | - |
| C19orf77 | HGLibA_05708 | 20 | -0.94958121 | C19orf77 | - |
| C20orf111 | HGLibA_05980 | 20 | -0.94958121 | C20orf111 | - |
| C7orf62 | HGLibA_06570 | 20 | -0.94958121 | C7orf62 | - |
| ENSG00000001461 | HGLibA_31962 | 20 | -0.94958121 | NIPAL3 | NIPA like domain containing 3 [Source:HGNC Symbol;Acc:HGNC:25233] |
| ENSG00000005102 | HGLibA_28889 | 20 | -0.94958121 | MEOX1 | mesenchyme homeobox 1 [Source:HGNC Symbol;Acc:HGNC:7013] |
| ENSG00000007384 | HGLibA_41132 | 20 | -0.94958121 | RHBDF1 | rhomboid 5 homolog 1 [Source:HGNC Symbol;Acc:HGNC:20561] |
| ENSG00000008988 | HGLibA_42105 | 20 | -0.94958121 | RPS20 | ribosomal protein S20 [Source:HGNC Symbol;Acc:HGNC:10405] |
| ENSG00000013306 | HGLibA_44729 | 20 | -0.94958121 | SLC25A39 | solute carrier family 25 member 39 [Source:HGNC Symbol;Acc:HGNC:24279] |
| ENSG00000021645 | HGLibA_32738 | 20 | -0.94958121 | NRXN3 | neurexin 3 [Source:HGNC Symbol;Acc:HGNC:8010] |
| ENSG00000036549 | HGLibA_57094 | 20 | -0.94958121 | ZZZ3 | zinc finger ZZ-type containing 3 [Source:NCBI gene;Acc:26009] |
| ENSG00000038358 | HGLibA_14444 | 20 | -0.94958121 | EDC4 | enhancer of mRNA decapping 4 [Source:HGNC Symbol;Acc:HGNC:17157] |
| ENSG00000049246 | HGLibA_36059 | 20 | -0.94958121 | PER3 | period circadian regulator 3 [Source:HGNC Symbol;Acc:HGNC:8847] |
| ENSG00000055813 | HGLibA_07892 | 20 | -0.94958121 | CCDC85A | coiled-coil domain containing 85A [Source:HGNC Symbol;Acc:HGNC:29400] |
| ENSG00000058804 | HGLibA_31379 | 20 | -0.94958121 | NDC1 | NDC1 transmembrane nucleoporin [Source:HGNC Symbol;Acc:HGNC:25525] |
| ENSG00000060558 | HGLibA_19447 | 20 | -0.94958121 | GNA15 | G protein subunit alpha 15 [Source:HGNC Symbol;Acc:HGNC:4383] |
| ENSG00000061794 | HGLibA_30099 | 20 | -0.94958121 | MRPS35 | mitochondrial ribosomal protein S35 [Source:HGNC Symbol;Acc:HGNC:16635] |
| ENSG00000064961 | HGLibA_21680 | 20 | -0.94958121 | HMG20B | high mobility group 20B [Source:HGNC Symbol;Acc:HGNC:5002] |
| ENSG00000065457 | HGLibA_00878 | 20 | -0.94958121 | ADAT1 | tRNA specific 1 [Source:HGNC Symbol;Acc:HGNC:228]" |
| ENSG00000066422 | HGLibA_54817 | 20 | -0.94958121 | ZBTB11 | zinc finger and BTB domain containing 11 [Source:HGNC Symbol;Acc:HGNC:16740] |
| ENSG00000067560 | HGLibA_41166 | 20 | -0.94958121 | RHOA | ras homolog family member A [Source:HGNC Symbol;Acc:HGNC:667] |
| ENSG00000068885 | HGLibA_22772 | 20 | -0.94958121 | IFT80 | intraflagellar transport 80 [Source:HGNC Symbol;Acc:HGNC:29262] |
| ENSG00000071205 | HGLibA_02700 | 20 | -0.94958121 | ARHGAP10 | Rho GTPase activating protein 10 [Source:HGNC Symbol;Acc:HGNC:26099] |
| ENSG00000072778 | HGLibA_00377 | 20 | -0.94958121 | ACADVL | acyl-CoA dehydrogenase very long chain [Source:HGNC Symbol;Acc:HGNC:92] |
| ENSG00000081870 | HGLibA_22305 | 20 | -0.94958121 | HSPB11 | heat shock protein family B (small) member 11 [Source:HGNC Symbol;Acc:HGNC:25019] |
| ENSG00000081985 | HGLibA_22967 | 20 | -0.94958121 | IL12RB2 | interleukin 12 receptor subunit beta 2 [Source:HGNC Symbol;Acc:HGNC:5972] |
| ENSG00000083807 | HGLibA_44825 | 20 | -0.94958121 | SLC27A5 | solute carrier family 27 member 5 [Source:HGNC Symbol;Acc:HGNC:10999] |
| ENSG00000084636 | HGLibA_10518 | 20 | -0.94958121 | COL16A1 | collagen type XVI alpha 1 chain [Source:HGNC Symbol;Acc:HGNC:2193] |
| ENSG00000087111 | HGLibA_36585 | 20 | -0.94958121 | PIGS | phosphatidylinositol glycan anchor biosynthesis class S [Source:HGNC Symbol;Acc:HGNC:14937] |
| ENSG00000090776 | HGLibA_14596 | 20 | -0.94958121 | EFNB1 | ephrin B1 [Source:HGNC Symbol;Acc:HGNC:3226] |
| ENSG00000090863 | HGLibA_19243 | 20 | -0.94958121 | GLG1 | golgi glycoprotein 1 [Source:HGNC Symbol;Acc:HGNC:4316] |
| ENSG00000096384 | HGLibA_22254 | 20 | -0.94958121 | HSP90AB1 | heat shock protein 90 alpha family class B member 1 [Source:HGNC Symbol;Acc:HGNC:5258] |
| ENSG00000099330 | HGLibA_33244 | 20 | -0.94958121 | OCEL1 | occludin/ELL domain containing 1 [Source:HGNC Symbol;Acc:HGNC:26221] |
| ENSG00000099800 | HGLibA_49499 | 20 | -0.94958121 | TIMM13 | translocase of inner mitochondrial membrane 13 [Source:HGNC Symbol;Acc:HGNC:11816] |
| ENSG00000099821 | HGLibA_37639 | 20 | -0.94958121 | POLRMT | RNA polymerase mitochondrial [Source:HGNC Symbol;Acc:HGNC:9200] |
| ENSG00000101204 | HGLibA_09573 | 20 | -0.94958121 | CHRNA4 | cholinergic receptor nicotinic alpha 4 subunit [Source:HGNC Symbol;Acc:HGNC:1958] |
| ENSG00000101306 | HGLibA_30787 | 20 | -0.94958121 | MYLK2 | myosin light chain kinase 2 [Source:HGNC Symbol;Acc:HGNC:16243] |
| ENSG00000103018 | HGLibA_11991 | 20 | -0.94958121 | CYB5B | cytochrome b5 type B [Source:HGNC Symbol;Acc:HGNC:24374] |
| ENSG00000103540 | HGLibA_08137 | 20 | -0.94958121 | CCP110 | centriolar coiled-coil protein 110 [Source:HGNC Symbol;Acc:HGNC:24342] |
| ENSG00000103995 | HGLibA_09110 | 20 | -0.94958121 | CEP152 | centrosomal protein 152 [Source:HGNC Symbol;Acc:HGNC:29298] |
| ENSG00000104064 | HGLibA_18350 | 20 | -0.94958121 | GABPB1 | GA binding protein transcription factor subunit beta 1 [Source:HGNC Symbol;Acc:HGNC:4074] |
| ENSG00000104081 | HGLibA_04584 | 20 | -0.94958121 | BMF | Bcl2 modifying factor [Source:HGNC Symbol;Acc:HGNC:24132] |
| ENSG00000104884 | HGLibA_15434 | 20 | -0.94958121 | ERCC2 | TFIIH core complex helicase subunit [Source:HGNC Symbol;Acc:HGNC:3434]" |
| ENSG00000105063 | HGLibA_38221 | 20 | -0.94958121 | PPP6R1 | protein phosphatase 6 regulatory subunit 1 [Source:HGNC Symbol;Acc:HGNC:29195] |
| ENSG00000105695 | HGLibA_27886 | 20 | -0.94958121 | MAG | myelin associated glycoprotein [Source:HGNC Symbol;Acc:HGNC:6783] |
| ENSG00000106565 | HGLibA_50074 | 20 | -0.94958121 | TMEM176B | transmembrane protein 176B [Source:HGNC Symbol;Acc:HGNC:29596] |
| ENSG00000106591 | HGLibA_29952 | 20 | -0.94958121 | MRPL32 | mitochondrial ribosomal protein L32 [Source:HGNC Symbol;Acc:HGNC:14035] |
| ENSG00000107951 | HGLibA_30457 | 20 | -0.94958121 | MTPAP | mitochondrial poly(A) polymerase [Source:HGNC Symbol;Acc:HGNC:25532] |
| ENSG00000109586 | HGLibA_18570 | 20 | -0.94958121 | GALNT7 | polypeptide N-acetylgalactosaminyltransferase 7 [Source:HGNC Symbol;Acc:HGNC:4129] |
| ENSG00000109771 | HGLibA_27214 | 20 | -0.94958121 | LRP2BP | LRP2 binding protein [Source:HGNC Symbol;Acc:HGNC:25434] |
| ENSG00000110104 | HGLibA_07902 | 20 | -0.94958121 | CCDC86 | coiled-coil domain containing 86 [Source:HGNC Symbol;Acc:HGNC:28359] |
| ENSG00000110245 | HGLibA_02509 | 20 | -0.94958121 | APOC3 | apolipoprotein C3 [Source:HGNC Symbol;Acc:HGNC:610] |
| ENSG00000110429 | HGLibA_17064 | 20 | -0.94958121 | FBXO3 | F-box protein 3 [Source:HGNC Symbol;Acc:HGNC:13582] |
| ENSG00000110651 | HGLibA_08456 | 20 | -0.94958121 | CD81 | CD81 molecule [Source:HGNC Symbol;Acc:HGNC:1701] |
| ENSG00000110711 | HGLibA_01361 | 20 | -0.94958121 | AIP | aryl hydrocarbon receptor interacting protein [Source:HGNC Symbol;Acc:HGNC:358] |
| ENSG00000112308 | HGLibA_06504 | 20 | -0.94958121 | C6orf62 | chromosome 6 open reading frame 62 [Source:HGNC Symbol;Acc:HGNC:20998] |
| ENSG00000112365 | HGLibA_54847 | 20 | -0.94958121 | ZBTB24 | zinc finger and BTB domain containing 24 [Source:HGNC Symbol;Acc:HGNC:21143] |
| ENSG00000112561 | HGLibA_49162 | 20 | -0.94958121 | TFEB | transcription factor EB [Source:HGNC Symbol;Acc:HGNC:11753] |
| ENSG00000112981 | HGLibA_32165 | 20 | -0.94958121 | NME5 | NME/NM23 family member 5 [Source:HGNC Symbol;Acc:HGNC:7853] |
| ENSG00000113272 | HGLibA_49356 | 20 | -0.94958121 | THG1L | tRNA-histidine guanylyltransferase 1 like [Source:HGNC Symbol;Acc:HGNC:26053] |
| ENSG00000114209 | HGLibA_35718 | 20 | -0.94958121 | PDCD10 | programmed cell death 10 [Source:HGNC Symbol;Acc:HGNC:8761] |
| ENSG00000115252 | HGLibA_35769 | 20 | -0.94958121 | PDE1A | phosphodiesterase 1A [Source:HGNC Symbol;Acc:HGNC:8774] |
| ENSG00000115257 | HGLibA_35676 | 20 | -0.94958121 | PCSK4 | proprotein convertase subtilisin/kexin type 4 [Source:HGNC Symbol;Acc:HGNC:8746] |
| ENSG00000115267 | HGLibA_22621 | 20 | -0.94958121 | IFIH1 | interferon induced with helicase C domain 1 [Source:HGNC Symbol;Acc:HGNC:18873] |
| ENSG00000115386 | HGLibA_40794 | 20 | -0.94958121 | REG1A | regenerating family member 1 alpha [Source:HGNC Symbol;Acc:HGNC:9951] |
| ENSG00000115488 | HGLibA_31712 | 20 | -0.94958121 | NEU2 | neuraminidase 2 [Source:HGNC Symbol;Acc:HGNC:7759] |
| ENSG00000115652 | HGLibA_53479 | 20 | -0.94958121 | UXS1 | UDP-glucuronate decarboxylase 1 [Source:HGNC Symbol;Acc:HGNC:17729] |
| ENSG00000117013 | HGLibA_24375 | 20 | -0.94958121 | KCNQ4 | potassium voltage-gated channel subfamily Q member 4 [Source:HGNC Symbol;Acc:HGNC:6298] |
| ENSG00000117174 | HGLibA_56932 | 20 | -0.94958121 | ZNHIT6 | zinc finger HIT-type containing 6 [Source:HGNC Symbol;Acc:HGNC:26089] |
| ENSG00000117266 | HGLibA_08749 | 20 | -0.94958121 | CDK18 | cyclin dependent kinase 18 [Source:HGNC Symbol;Acc:HGNC:8751] |
| ENSG00000118777 | HGLibA_00218 | 20 | -0.94958121 | ABCG2 | ATP binding cassette subfamily G member 2 (Junior blood group) [Source:HGNC Symbol;Acc:HGNC:74] |
| ENSG00000120137 | HGLibA_35102 | 20 | -0.94958121 | PANK3 | pantothenate kinase 3 [Source:HGNC Symbol;Acc:HGNC:19365] |
| ENSG00000120903 | HGLibA_09569 | 20 | -0.94958121 | CHRNA2 | cholinergic receptor nicotinic alpha 2 subunit [Source:HGNC Symbol;Acc:HGNC:1956] |
| ENSG00000121152 | HGLibA_31276 | 20 | -0.94958121 | NCAPH | non-SMC condensin I complex subunit H [Source:HGNC Symbol;Acc:HGNC:1112] |
| ENSG00000122042 | HGLibA_52703 | 20 | -0.94958121 | UBL3 | ubiquitin like 3 [Source:HGNC Symbol;Acc:HGNC:12504] |
| ENSG00000122367 | HGLibA_26218 | 20 | -0.94958121 | LDB3 | LIM domain binding 3 [Source:HGNC Symbol;Acc:HGNC:15710] |
| ENSG00000122641 | HGLibA_23301 | 20 | -0.94958121 | INHBA | inhibin subunit beta A [Source:HGNC Symbol;Acc:HGNC:6066] |
| ENSG00000125895 | HGLibA_50514 | 20 | -0.94958121 | TMEM74B | transmembrane protein 74B [Source:HGNC Symbol;Acc:HGNC:15893] |
| ENSG00000126545 | HGLibA_11372 | 20 | -0.94958121 | CSN1S1 | casein alpha s1 [Source:HGNC Symbol;Acc:HGNC:2445] |
| ENSG00000127515 | HGLibA_34456 | 20 | -0.94958121 | OR7A10 | olfactory receptor family 7 subfamily A member 10 [Source:HGNC Symbol;Acc:HGNC:8356] |
| ENSG00000128394 | HGLibA_02487 | 20 | -0.94958121 | APOBEC3F | apolipoprotein B mRNA editing enzyme catalytic subunit 3F [Source:HGNC Symbol;Acc:HGNC:17356] |
| ENSG00000128519 | HGLibA_48375 | 20 | -0.94958121 | TAS2R16 | taste 2 receptor member 16 [Source:HGNC Symbol;Acc:HGNC:14921] |
| ENSG00000129991 | HGLibA_50877 | 20 | -0.94958121 | TNNI3 | cardiac type [Source:HGNC Symbol;Acc:HGNC:11947]" |
| ENSG00000130529 | HGLibA_51761 | 20 | -0.94958121 | TRPM4 | transient receptor potential cation channel subfamily M member 4 [Source:HGNC Symbol;Acc:HGNC:17993] |
| ENSG00000131477 | HGLibA_40183 | 20 | -0.94958121 | RAMP2 | receptor activity modifying protein 2 [Source:HGNC Symbol;Acc:HGNC:9844] |
| ENSG00000134352 | HGLibA_23187 | 20 | -0.94958121 | IL6ST | interleukin 6 signal transducer [Source:HGNC Symbol;Acc:HGNC:6021] |
| ENSG00000134531 | HGLibA_15073 | 20 | -0.94958121 | EMP1 | epithelial membrane protein 1 [Source:HGNC Symbol;Acc:HGNC:3333] |
| ENSG00000136048 | HGLibA_13944 | 20 | -0.94958121 | DRAM1 | DNA damage regulated autophagy modulator 1 [Source:HGNC Symbol;Acc:HGNC:25645] |
| ENSG00000137877 | HGLibA_46888 | 20 | -0.94958121 | SPTBN5 | non-erythrocytic 5 [Source:HGNC Symbol;Acc:HGNC:15680]" |
| ENSG00000138382 | HGLibA_29011 | 20 | -0.94958121 | METTL5 | methyltransferase like 5 [Source:HGNC Symbol;Acc:HGNC:25006] |
| ENSG00000139974 | HGLibA_45076 | 20 | -0.94958121 | SLC38A6 | solute carrier family 38 member 6 [Source:HGNC Symbol;Acc:HGNC:19863] |
| ENSG00000140265 | HGLibA_57013 | 20 | -0.94958121 | ZSCAN29 | zinc finger and SCAN domain containing 29 [Source:HGNC Symbol;Acc:HGNC:26673] |
| ENSG00000140386 | HGLibA_42860 | 20 | -0.94958121 | SCAPER | S-phase cyclin A associated protein in the ER [Source:HGNC Symbol;Acc:HGNC:13081] |
| ENSG00000140832 | HGLibA_28385 | 20 | -0.94958121 | MARVELD3 | MARVEL domain containing 3 [Source:HGNC Symbol;Acc:HGNC:30525] |
| ENSG00000141622 | HGLibA_41556 | 20 | -0.94958121 | RNF165 | ring finger protein 165 [Source:HGNC Symbol;Acc:HGNC:31696] |
| ENSG00000142623 | HGLibA_34968 | 20 | -0.94958121 | PADI1 | peptidyl arginine deiminase 1 [Source:HGNC Symbol;Acc:HGNC:18367] |
| ENSG00000143155 | HGLibA_49561 | 20 | -0.94958121 | TIPRL | TOR signaling pathway regulator [Source:HGNC Symbol;Acc:HGNC:30231] |
| ENSG00000143185 | HGLibA_54497 | 20 | -0.94958121 | XCL2 | X-C motif chemokine ligand 2 [Source:HGNC Symbol;Acc:HGNC:10646] |
| ENSG00000143797 | HGLibA_28521 | 20 | -0.94958121 | MBOAT2 | membrane bound O-acyltransferase domain containing 2 [Source:HGNC Symbol;Acc:HGNC:25193] |
| ENSG00000144410 | HGLibA_10980 | 20 | -0.94958121 | CPO | carboxypeptidase O [Source:HGNC Symbol;Acc:HGNC:21011] |
| ENSG00000144847 | HGLibA_22882 | 20 | -0.94958121 | IGSF11 | immunoglobulin superfamily member 11 [Source:HGNC Symbol;Acc:HGNC:16669] |
| ENSG00000144959 | HGLibA_31293 | 20 | -0.94958121 | NCEH1 | neutral cholesterol ester hydrolase 1 [Source:HGNC Symbol;Acc:HGNC:29260] |
| ENSG00000145388 | HGLibA_28959 | 20 | -0.94958121 | METTL14 | methyltransferase like 14 [Source:HGNC Symbol;Acc:HGNC:29330] |
| ENSG00000146250 | HGLibA_38928 | 20 | -0.94958121 | PRSS35 | serine protease 35 [Source:HGNC Symbol;Acc:HGNC:21387] |
| ENSG00000147256 | HGLibA_02767 | 20 | -0.94958121 | ARHGAP36 | Rho GTPase activating protein 36 [Source:HGNC Symbol;Acc:HGNC:26388] |
| ENSG00000147316 | HGLibA_28646 | 20 | -0.94958121 | MCPH1 | microcephalin 1 [Source:HGNC Symbol;Acc:HGNC:6954] |
| ENSG00000147416 | HGLibA_03737 | 20 | -0.94958121 | ATP6V1B2 | ATPase H+ transporting V1 subunit B2 [Source:HGNC Symbol;Acc:HGNC:854] |
| ENSG00000147419 | HGLibA_07739 | 20 | -0.94958121 | CCDC25 | coiled-coil domain containing 25 [Source:HGNC Symbol;Acc:HGNC:25591] |
| ENSG00000147654 | HGLibA_14347 | 20 | -0.94958121 | EBAG9 | 9 [Source:HGNC Symbol;Acc:HGNC:3123]" |
| ENSG00000147996 | HGLibA_07438 | 20 | -0.94958121 | CBWD5 | COBW domain containing 5 [Source:HGNC Symbol;Acc:HGNC:24584] |
| ENSG00000148965 | HGLibA_42617 | 20 | -0.94958121 | SAA4 | constitutive [Source:HGNC Symbol;Acc:HGNC:10516]" |
| ENSG00000149308 | HGLibA_32407 | 20 | -0.94958121 | NPAT | coactivator of histone transcription [Source:HGNC Symbol;Acc:HGNC:7896]" |
| ENSG00000150045 | HGLibA_25302 | 20 | -0.94958121 | KLRF1 | killer cell lectin like receptor F1 [Source:HGNC Symbol;Acc:HGNC:13342] |
| ENSG00000150764 | HGLibA_13288 | 20 | -0.94958121 | DIXDC1 | DIX domain containing 1 [Source:HGNC Symbol;Acc:HGNC:23695] |

| | | | | | |
|---|---|---|---|---|---|
| ENSG00000150907 | HGLibA_17920 | 20 | -0.94958121 | FOXO1 | forkhead box O1 [Source:HGNC Symbol;Acc:HGNC:3819] |
| ENSG00000151239 | HGLibA_52370 | 20 | -0.94958121 | TWF1 | twinfilin actin binding protein 1 [Source:HGNC Symbol;Acc:HGNC:9620] |
| ENSG00000151422 | HGLibA_17285 | 20 | -0.94958121 | FER | FER tyrosine kinase [Source:HGNC Symbol;Acc:HGNC:3655] |
| ENSG00000153048 | HGLibA_07248 | 20 | -0.94958121 | CARHSP1 | calcium regulated heat stable protein 1 [Source:HGNC Symbol;Acc:HGNC:17150] |
| ENSG00000154227 | HGLibA_09205 | 20 | -0.94958121 | CERS3 | ceramide synthase 3 [Source:HGNC Symbol;Acc:HGNC:23752] |
| ENSG00000154767 | HGLibA_54551 | 20 | -0.94958121 | XPC | DNA damage recognition and repair factor [Source:HGNC Symbol;Acc:HGNC:12816]" |
| ENSG00000155511 | HGLibA_20259 | 20 | -0.94958121 | GRIA1 | glutamate ionotropic receptor AMPA type subunit 1 [Source:HGNC Symbol;Acc:HGNC:4571] |
| ENSG00000155961 | HGLibA_39905 | 20 | -0.94958121 | RAB39B | member RAS oncogene family [Source:HGNC Symbol;Acc:HGNC:16499]" |
| ENSG00000156026 | HGLibA_28661 | 20 | -0.94958121 | MCU | mitochondrial calcium uniporter [Source:HGNC Symbol;Acc:HGNC:23526] |
| ENSG00000156253 | HGLibA_42484 | 20 | -0.94958121 | RWDD2B | RWD domain containing 2B [Source:HGNC Symbol;Acc:HGNC:1302] |
| ENSG00000156968 | HGLibA_29786 | 20 | -0.94958121 | MPV17L | MPV17 mitochondrial inner membrane protein like [Source:HGNC Symbol;Acc:HGNC:26827] |
| ENSG00000157856 | HGLibA_13957 | 20 | -0.94958121 | DRC1 | dynein regulatory complex subunit 1 [Source:HGNC Symbol;Acc:HGNC:24245] |
| ENSG00000158109 | HGLibA_51184 | 20 | -0.94958121 | TPRG1L | tumor protein p63 regulated 1 like [Source:HGNC Symbol;Acc:HGNC:27007] |
| ENSG00000158435 | HGLibA_10332 | 20 | -0.94958121 | CNOT11 | CCR4-NOT transcription complex subunit 11 [Source:HGNC Symbol;Acc:HGNC:25217] |
| ENSG00000159455 | HGLibA_26122 | 20 | -0.94958121 | LCE2B | late cornified envelope 2B [Source:HGNC Symbol;Acc:HGNC:16610] |
| ENSG00000159650 | HGLibA_53153 | 20 | -0.94958121 | UROC1 | urocanate hydratase 1 [Source:HGNC Symbol;Acc:HGNC:26444] |
| ENSG00000160094 | HGLibA_55872 | 20 | -0.94958121 | ZNF362 | zinc finger protein 362 [Source:HGNC Symbol;Acc:HGNC:18079] |
| ENSG00000160908 | HGLibA_55910 | 20 | -0.94958121 | ZNF394 | zinc finger protein 394 [Source:HGNC Symbol;Acc:HGNC:18832] |
| ENSG00000161558 | HGLibA_49968 | 20 | -0.94958121 | TMEM143 | transmembrane protein 143 [Source:HGNC Symbol;Acc:HGNC:25603] |
| ENSG00000162591 | HGLibA_28853 | 20 | -0.94958121 | MEGF6 | multiple EGF like domains 6 [Source:HGNC Symbol;Acc:HGNC:3232] |
| ENSG00000163320 | HGLibA_09318 | 20 | -0.94958121 | CGGBP1 | CGG triplet repeat binding protein 1 [Source:HGNC Symbol;Acc:HGNC:1888] |
| ENSG00000163462 | HGLibA_51513 | 20 | -0.94958121 | TRIM46 | tripartite motif containing 46 [Source:HGNC Symbol;Acc:HGNC:19019] |
| ENSG00000163497 | HGLibA_17308 | 20 | -0.94958121 | FEV | ETS transcription factor [Source:HGNC Symbol;Acc:HGNC:18562]" |
| ENSG00000163590 | HGLibA_37989 | 20 | -0.94958121 | PPM1L | Mg2+/Mn2+ dependent 1L [Source:HGNC Symbol;Acc:HGNC:16381]" |
| ENSG00000163600 | HGLibA_22514 | 20 | -0.94958121 | ICOS | inducible T cell costimulator [Source:HGNC Symbol;Acc:HGNC:5351] |
| ENSG00000163655 | HGLibA_19434 | 20 | -0.94958121 | GMPS | guanine monophosphate synthase [Source:HGNC Symbol;Acc:HGNC:4378] |
| ENSG00000163697 | HGLibA_02384 | 20 | -0.94958121 | APBB2 | amyloid beta precursor protein binding family B member 2 [Source:HGNC Symbol;Acc:HGNC:582] |
| ENSG00000164061 | HGLibA_04870 | 20 | -0.94958121 | BSN | bassoon presynaptic cytomatrix protein [Source:HGNC Symbol;Acc:HGNC:1117] |
| ENSG00000164953 | HGLibA_50491 | 20 | -0.94958121 | TMEM67 | transmembrane protein 67 [Source:HGNC Symbol;Acc:HGNC:28396] |
| ENSG00000165115 | HGLibA_24923 | 20 | -0.94958121 | KIF27 | kinesin family member 27 [Source:HGNC Symbol;Acc:HGNC:18632] |
| ENSG00000165392 | HGLibA_54409 | 20 | -0.94958121 | WRN | Werner syndrome RecQ like helicase [Source:HGNC Symbol;Acc:HGNC:12791] |
| ENSG00000165457 | HGLibA_17787 | 20 | -0.94958121 | FOLR2 | folate receptor beta [Source:HGNC Symbol;Acc:HGNC:3793] |
| ENSG00000165633 | HGLibA_53869 | 20 | -0.94958121 | VSTM4 | V-set and transmembrane domain containing 4 [Source:HGNC Symbol;Acc:HGNC:26470] |
| ENSG00000166343 | HGLibA_30249 | 20 | -0.94958121 | MSS51 | MSS51 mitochondrial translational activator [Source:HGNC Symbol;Acc:HGNC:21000] |
| ENSG00000166707 | HGLibA_55025 | 20 | -0.94958121 | ZCCHC18 | zinc finger CCHC-type containing 18 [Source:HGNC Symbol;Acc:HGNC:32459] |
| ENSG00000166987 | HGLibA_28492 | 20 | -0.94958121 | MBD6 | methyl-CpG binding domain protein 6 [Source:HGNC Symbol;Acc:HGNC:20445] |
| ENSG00000167646 | HGLibA_13466 | 20 | -0.94958121 | DNAAF3 | dynein axonemal assembly factor 3 [Source:HGNC Symbol;Acc:HGNC:30492] |
| ENSG00000168101 | HGLibA_32955 | 20 | -0.94958121 | NUDT16L1 | nudix hydrolase 16 like 1 [Source:HGNC Symbol;Acc:HGNC:28154] |
| ENSG00000168303 | HGLibA_29738 | 20 | -0.94958121 | MPLKIP | M-phase specific PLK1 interacting protein [Source:HGNC Symbol;Acc:HGNC:16002] |
| ENSG00000168404 | HGLibA_29390 | 20 | -0.94958121 | MLKL | mixed lineage kinase domain like pseudokinase [Source:HGNC Symbol;Acc:HGNC:26617] |
| ENSG00000169251 | HGLibA_32149 | 20 | -0.94958121 | NMD3 | NMD3 ribosome export adaptor [Source:HGNC Symbol;Acc:HGNC:24250] |
| ENSG00000170190 | HGLibA_44444 | 20 | -0.94958121 | SLC16A5 | solute carrier family 16 member 5 [Source:HGNC Symbol;Acc:HGNC:10926] |
| ENSG00000170782 | HGLibA_33467 | 20 | -0.94958121 | OR10A4 | olfactory receptor family 10 subfamily A member 4 [Source:HGNC Symbol;Acc:HGNC:15130] |
| ENSG00000171291 | HGLibA_55997 | 20 | -0.94958121 | ZNF439 | zinc finger protein 439 [Source:HGNC Symbol;Acc:HGNC:20873] |
| ENSG00000171487 | HGLibA_32123 | 20 | -0.94958121 | NLRP5 | NLR family pyrin domain containing 5 [Source:HGNC Symbol;Acc:HGNC:21269] |
| ENSG00000171490 | HGLibA_42316 | 20 | -0.94958121 | RSL1D1 | ribosomal L1 domain containing 1 [Source:HGNC Symbol;Acc:HGNC:24534] |
| ENSG00000171596 | HGLibA_32215 | 20 | -0.94958121 | NMUR1 | neuromedin U receptor 1 [Source:HGNC Symbol;Acc:HGNC:4518] |
| ENSG00000171786 | HGLibA_31890 | 20 | -0.94958121 | NHLH1 | nescient helix-loop-helix 1 [Source:HGNC Symbol;Acc:HGNC:7817] |
| ENSG00000171790 | HGLibA_45499 | 20 | -0.94958121 | SLFNL1 | schlafen like 1 [Source:HGNC Symbol;Acc:HGNC:26313] |
| ENSG00000171804 | HGLibA_54205 | 20 | -0.94958121 | WDR87 | WD repeat domain 87 [Source:HGNC Symbol;Acc:HGNC:29934] |
| ENSG00000172146 | HGLibA_33641 | 20 | -0.94958121 | OR1A1 | olfactory receptor family 1 subfamily A member 1 [Source:HGNC Symbol;Acc:HGNC:8179] |
| ENSG00000172270 | HGLibA_04865 | 20 | -0.94958121 | BSG | basigin (Ok blood group) [Source:HGNC Symbol;Acc:HGNC:1116] |
| ENSG00000172315 | HGLibA_51074 | 20 | -0.94958121 | TP53RK | TP53 regulating kinase [Source:HGNC Symbol;Acc:HGNC:16197] |
| ENSG00000173467 | HGLibA_01246 | 20 | -0.94958121 | AGR3 | protein disulphide isomerase family member [Source:HGNC Symbol;Acc:HGNC:24167]" |
| ENSG00000173535 | HGLibA_50739 | 20 | -0.94958121 | TNFRSF10C | TNF receptor superfamily member 10c [Source:HGNC Symbol;Acc:HGNC:11906] |
| ENSG00000175018 | HGLibA_49104 | 20 | -0.94958121 | TEX36 | testis expressed 36 [Source:HGNC Symbol;Acc:HGNC:31653] |
| ENSG00000175221 | HGLibA_28760 | 20 | -0.94958121 | MED16 | mediator complex subunit 16 [Source:HGNC Symbol;Acc:HGNC:17556] |
| ENSG00000178015 | HGLibA_19929 | 20 | -0.94958121 | GPR150 | G protein-coupled receptor 150 [Source:HGNC Symbol;Acc:HGNC:23628] |
| ENSG00000178342 | HGLibA_24202 | 20 | -0.94958121 | KCNG2 | potassium voltage-gated channel modifier subfamily G member 2 [Source:HGNC Symbol;Acc:HGNC:6249] |
| ENSG00000178719 | HGLibA_20318 | 20 | -0.94958121 | GRINA | glutamate ionotropic receptor NMDA type subunit associated protein 1 [Source:HGNC Symbol;Acc:HGNC:4589] |
| ENSG00000179604 | HGLibA_08574 | 20 | -0.94958121 | CDC42EP4 | CDC42 effector protein 4 [Source:HGNC Symbol;Acc:HGNC:17147] |
| ENSG00000179636 | HGLibA_51167 | 20 | -0.94958121 | TPPP2 | tubulin polymerization promoting protein family member 2 [Source:HGNC Symbol;Acc:HGNC:19293] |
| ENSG00000179776 | HGLibA_08684 | 20 | -0.94958121 | CDH5 | cadherin 5 [Source:HGNC Symbol;Acc:HGNC:1764] |
| ENSG00000180104 | HGLibA_15738 | 20 | -0.94958121 | EXOC3 | exocyst complex component 3 [Source:HGNC Symbol;Acc:HGNC:30378] |
| ENSG00000180872 | HGLibA_12848 | 20 | -0.94958121 | DEFB112 | defensin beta 112 [Source:HGNC Symbol;Acc:HGNC:18093] |
| ENSG00000181698 | HGLibA_34355 | 20 | -0.94958121 | OR5T1 | olfactory receptor family 5 subfamily T member 1 [Source:HGNC Symbol;Acc:HGNC:14821] |
| ENSG00000182986 | HGLibA_55796 | 20 | -0.94958121 | ZNF320 | zinc finger protein 320 [Source:HGNC Symbol;Acc:HGNC:13842] |
| ENSG00000183024 | HGLibA_33669 | 20 | -0.94958121 | OR1G1 | olfactory receptor family 1 subfamily G member 1 [Source:HGNC Symbol;Acc:HGNC:8204] |
| ENSG00000183735 | HGLibA_48602 | 20 | -0.94958121 | TBK1 | TANK binding kinase 1 [Source:HGNC Symbol;Acc:HGNC:11584] |
| ENSG00000184634 | HGLibA_28743 | 20 | -0.94958121 | MED12 | mediator complex subunit 12 [Source:HGNC Symbol;Acc:HGNC:11957] |
| ENSG00000184845 | HGLibA_13959 | 20 | -0.94958121 | DRD1 | dopamine receptor D1 [Source:HGNC Symbol;Acc:HGNC:3020] |
| ENSG00000185650 | HGLibA_55207 | 20 | -0.94958121 | ZFP36L1 | ZFP36 ring finger protein like 1 [Source:HGNC Symbol;Acc:HGNC:1107] |
| ENSG00000185823 | HGLibA_32391 | 20 | -0.94958121 | NPAP1 | nuclear pore associated protein 1 [Source:HGNC Symbol;Acc:HGNC:1190] |
| ENSG00000185885 | HGLibA_22637 | 20 | -0.94958121 | IFITM1 | interferon induced transmembrane protein 1 [Source:HGNC Symbol;Acc:HGNC:5412] |
| ENSG00000185909 | HGLibA_25113 | 20 | -0.94958121 | KLHDC8B | kelch domain containing 8B [Source:HGNC Symbol;Acc:HGNC:28557] |
| ENSG00000186009 | HGLibA_03635 | 20 | -0.94958121 | ATP4B | ATPase H+/K+ transporting subunit beta [Source:HGNC Symbol;Acc:HGNC:820] |
| ENSG00000186281 | HGLibA_19733 | 20 | -0.94958121 | GPAT2 | mitochondrial [Source:HGNC Symbol;Acc:HGNC:27168]" |
| ENSG00000186468 | HGLibA_42111 | 20 | -0.94958121 | RPS23 | ribosomal protein S23 [Source:HGNC Symbol;Acc:HGNC:10410] |
| ENSG00000186716 | HGLibA_04368 | 20 | -0.94958121 | BCR | RhoGEF and GTPase activating protein [Source:HGNC Symbol;Acc:HGNC:1014]" |
| ENSG00000186868 | HGLibA_28322 | 20 | -0.94958121 | MAPT | microtubule associated protein tau [Source:HGNC Symbol;Acc:HGNC:6893] |
| ENSG00000187554 | HGLibA_49654 | 20 | -0.94958121 | TLR5 | toll like receptor 5 [Source:HGNC Symbol;Acc:HGNC:11851] |
| ENSG00000188234 | HGLibA_01149 | 20 | -0.94958121 | AGAP4 | ankyrin repeat and PH domain 4 [Source:HGNC Symbol;Acc:HGNC:23459]" |
| ENSG00000188266 | HGLibA_22456 | 20 | -0.94958121 | HYKK | hydroxylysine kinase [Source:HGNC Symbol;Acc:HGNC:34403] |
| ENSG00000188725 | HGLibA_45734 | 20 | -0.94958121 | SMIM15 | small integral membrane protein 15 [Source:HGNC Symbol;Acc:HGNC:33861] |
| ENSG00000189050 | HGLibA_41703 | 20 | -0.94958121 | RNFT1 | transmembrane 1 [Source:HGNC Symbol;Acc:HGNC:30206]" |
| ENSG00000189057 | HGLibA_16027 | 20 | -0.94958121 | FAM111B | family with sequence similarity 111 member B [Source:HGNC Symbol;Acc:HGNC:24200] |
| ENSG00000196663 | HGLibA_48944 | 20 | -0.94958121 | TECPR2 | tectonin beta-propeller repeat containing 2 [Source:HGNC Symbol;Acc:HGNC:19957] |
| ENSG00000196954 | HGLibA_07314 | 20 | -0.94958121 | CASP4 | caspase 4 [Source:HGNC Symbol;Acc:HGNC:1505] |
| ENSG00000197093 | HGLibA_18506 | 20 | -0.94958121 | GAL3ST4 | galactose-3-O-sulfotransferase 4 [Source:HGNC Symbol;Acc:HGNC:24145] |
| ENSG00000197594 | HGLibA_15167 | 20 | -0.94958121 | ENPP1 | ectonucleotide pyrophosphatase/phosphodiesterase 1 [Source:HGNC Symbol;Acc:HGNC:3356] |
| ENSG00000197863 | HGLibA_56765 | 20 | -0.94958121 | ZNF790 | zinc finger protein 790 [Source:HGNC Symbol;Acc:HGNC:33114] |
| ENSG00000198453 | HGLibA_56266 | 20 | -0.94958121 | ZNF568 | zinc finger protein 568 [Source:HGNC Symbol;Acc:HGNC:25392] |
| ENSG00000198601 | HGLibA_33832 | 20 | -0.94958121 | OR2M2 | olfactory receptor family 2 subfamily M member 2 [Source:HGNC Symbol;Acc:HGNC:8268] |
| ENSG00000198822 | HGLibA_20347 | 20 | -0.94958121 | GRM3 | glutamate metabotropic receptor 3 [Source:HGNC Symbol;Acc:HGNC:4595] |
| ENSG00000203757 | HGLibA_34422 | 20 | -0.94958121 | OR6K3 | olfactory receptor family 6 subfamily K member 3 [Source:HGNC Symbol;Acc:HGNC:15030] |
| ENSG00000203880 | HGLibA_35630 | 20 | -0.94958121 | PCMTD2 | protein-L-isoaspartate (D-aspartate) O-methyltransferase domain containing 2 [Source:HGNC Symbol;Acc:HGNC:15882] |
| ENSG00000204687 | HGLibA_28392 | 20 | -0.94958121 | MAS1L | G protein-coupled receptor [Source:HGNC Symbol;Acc:HGNC:13961]" |
| ENSG00000206047 | HGLibA_12793 | 20 | -0.94958121 | DEFA1 | defensin alpha 1 [Source:HGNC Symbol;Acc:HGNC:2761] |
| ENSG00000206104 | HGLibA_25704 | 20 | -0.94958121 | KRTAP20-3 | keratin associated protein 20-3 [Source:HGNC Symbol;Acc:HGNC:34001] |
| ENSG00000206422 | HGLibA_27309 | 20 | -0.94958121 | LRRC30 | leucine rich repeat containing 30 [Source:HGNC Symbol;Acc:HGNC:30219] |
| ENSG00000206561 | HGLibA_10647 | 20 | -0.94958121 | COLQ | collagen like tail subunit of asymmetric acetylcholinesterase [Source:HGNC Symbol;Acc:HGNC:2226] |
| ENSG00000213658 | HGLibA_26058 | 20 | -0.94958121 | LAT | linker for activation of T cells [Source:HGNC Symbol;Acc:HGNC:18874] |
| ENSG00000215784 | HGLibA_16713 | 20 | -0.94958121 | FAM72D | family with sequence similarity 72 member D [Source:HGNC Symbol;Acc:HGNC:33593] |
| ENSG00000237765 | HGLibA_16415 | 20 | -0.94958121 | FAM200B | family with sequence similarity 200 member B [Source:HGNC Symbol;Acc:HGNC:27740] |
| ENSG00000239839 | HGLibA_12799 | 20 | -0.94958121 | DEFA3 | defensin alpha 3 [Source:HGNC Symbol;Acc:HGNC:2762] |
| ENSG00000241935 | HGLibA_21860 | 20 | -0.94958121 | HOGA1 | 4-hydroxy-2-oxoglutarate aldolase 1 [Source:HGNC Symbol;Acc:HGNC:25155] |
| ENSG00000244617 | HGLibA_03361 | 20 | -0.94958121 | ASPRV1 | aspartic peptidase retroviral like 1 [Source:HGNC Symbol;Acc:HGNC:26321] |
| ENSG00000248672 | HGLibA_27684 | 20 | -0.94958121 | LY75-CD302 | LY75-CD302 readthrough [Source:HGNC Symbol;Acc:HGNC:38828] |
| ENSG00000249861 | HGLibA_26356 | 20 | -0.94958121 | LGALS16 | galectin 16 [Source:HGNC Symbol;Acc:HGNC:40039] |
| ENSG00000256713 | HGLibA_36193 | 20 | -0.94958121 | PGA5 | group I (pepsinogen A) [Source:HGNC Symbol;Acc:HGNC:8887]" |
| ENSG00000261052 | HGLibA_47737 | 20 | -0.94958121 | SULT1A3 | sulfotransferase family 1A member 3 [Source:HGNC Symbol;Acc:HGNC:11455] |
| ENSG00000276581 | HGLibA_46481 | 20 | -0.94958121 | SPATA31A5 | SPATA31 subfamily A member 5 [Source:HGNC Symbol;Acc:HGNC:32005] |
| ENSG00000283706 | HGLibA_38952 | 20 | -0.94958121 | PRSS50 | serine protease 50 [Source:NCBI gene;Acc:29122] |
| ENSG00000285437 | HGLibA_37596 | 20 | -0.94958121 | POLR2J3 | RNA polymerase II subunit J3 [Source:HGNC Symbol;Acc:HGNC:33853] |
| FAM188B | HGLibA_16348 | 20 | -0.94958121 | FAM188B | - |
| FAM206A | HGLibA_16427 | 20 | -0.94958121 | FAM206A | - |
| GTPBP6_X | HGLibA_20616 | 20 | -0.94958121 | GTPBP6_X | - |
| HIST1H3H | HGLibA_21478 | 20 | -0.94958121 | HIST1H3H | - |
| HIST2H2AA4 | HGLibA_21525 | 20 | -0.94958121 | HIST2H2AA4 | - |
| hsa-mir-125b-2 | HGLibA_57452 | 20 | -0.94958121 | hsa-mir-125b-2 | - |
| hsa-mir-1290 | HGLibA_57637 | 20 | -0.94958121 | hsa-mir-1290 | - |
| hsa-mir-1468 | HGLibA_57849 | 20 | -0.94958121 | hsa-mir-1468 | - |
| hsa-mir-199b | HGLibA_58135 | 20 | -0.94958121 | hsa-mir-199b | - |
| hsa-mir-218-2 | HGLibA_58274 | 20 | -0.94958121 | hsa-mir-218-2 | - |
| hsa-mir-298 | HGLibA_58401 | 20 | -0.94958121 | hsa-mir-298 | - |
| hsa-mir-29c | HGLibA_58423 | 20 | -0.94958121 | hsa-mir-29c | - |
| hsa-mir-3127 | HGLibA_58559 | 20 | -0.94958121 | hsa-mir-3127 | - |
| hsa-mir-3179-3 | HGLibA_58797 | 20 | -0.94958121 | hsa-mir-3179-3 | - |
| hsa-mir-3192 | HGLibA_58864 | 20 | -0.94958121 | hsa-mir-3192 | - |
| hsa-mir-3665 | HGLibA_59213 | 20 | -0.94958121 | hsa-mir-3665 | - |
| hsa-mir-3671 | HGLibA_59241 | 20 | -0.94958121 | hsa-mir-3671 | - |
| hsa-mir-3688-2 | HGLibA_59310 | 20 | -0.94958121 | hsa-mir-3688-2 | - |
| hsa-mir-3911 | HGLibA_59512 | 20 | -0.94958121 | hsa-mir-3911 | - |
| hsa-mir-4264 | HGLibA_59765 | 20 | -0.94958121 | hsa-mir-4264 | - |
| hsa-mir-4290 | HGLibA_59872 | 20 | -0.94958121 | hsa-mir-4290 | - |

| | | | | | |
|---|---|---|---|---|---|
| hsa-mir-4443 | HGLibA_60176 | 20 | -0.94958121 | hsa-mir-4443 | - |
| hsa-mir-4469 | HGLibA_60284 | 20 | -0.94958121 | hsa-mir-4469 | - |
| hsa-mir-4650-1 | HGLibA_60704 | 20 | -0.94958121 | hsa-mir-4650-1 | - |
| hsa-mir-4770 | HGLibA_61177 | 20 | -0.94958121 | hsa-mir-4770 | - |
| hsa-mir-487b | HGLibA_61349 | 20 | -0.94958121 | hsa-mir-487b | - |
| hsa-mir-5006 | HGLibA_61427 | 20 | -0.94958121 | hsa-mir-5006 | - |
| hsa-mir-509-1 | HGLibA_61505 | 20 | -0.94958121 | hsa-mir-509-1 | - |
| hsa-mir-516b-1 | HGLibA_61618 | 20 | -0.94958121 | hsa-mir-516b-1 | - |
| hsa-mir-519a-1 | HGLibA_61712 | 20 | -0.94958121 | hsa-mir-519a-1 | - |
| hsa-mir-550b-2 | HGLibA_62113 | 20 | -0.94958121 | hsa-mir-550b-2 | - |
| hsa-mir-5687 | HGLibA_62263 | 20 | -0.94958121 | hsa-mir-5687 | - |
| hsa-mir-5692a-1 | HGLibA_62288 | 20 | -0.94958121 | hsa-mir-5692a-1 | - |
| hsa-mir-577 | HGLibA_62401 | 20 | -0.94958121 | hsa-mir-577 | - |
| hsa-mir-582 | HGLibA_62428 | 20 | -0.94958121 | hsa-mir-582 | - |
| hsa-mir-6737 | HGLibA_63101 | 20 | -0.94958121 | hsa-mir-6737 | - |
| hsa-mir-6755 | HGLibA_63179 | 20 | -0.94958121 | hsa-mir-6755 | - |
| hsa-mir-6767 | HGLibA_63231 | 20 | -0.94958121 | hsa-mir-6767 | - |
| hsa-mir-6779 | HGLibA_63289 | 20 | -0.94958121 | hsa-mir-6779 | - |
| hsa-mir-6822 | HGLibA_63466 | 20 | -0.94958121 | hsa-mir-6822 | - |
| hsa-mir-761 | HGLibA_63905 | 20 | -0.94958121 | hsa-mir-761 | - |
| hsa-mir-770 | HGLibA_63939 | 20 | -0.94958121 | hsa-mir-770 | - |
| hsa-mir-7706 | HGLibA_63957 | 20 | -0.94958121 | hsa-mir-7706 | - |
| JHDM1D | HGLibA_23946 | 20 | -0.94958121 | JHDM1D | - |
| LACE1 | HGLibA_25912 | 20 | -0.94958121 | LACE1 | - |
| LACE1 | HGLibA_25911 | 20 | -0.94958121 | LACE1 | - |
| LOC100289187 | HGLibA_26840 | 20 | -0.94958121 | LOC100289187 | - |
| LOR | HGLibA_27029 | 20 | -0.94958121 | LOR | - |
| MGEA5 | HGLibA_29155 | 20 | -0.94958121 | MGEA5 | - |
| PPP2R4 | HGLibA_38168 | 20 | -0.94958121 | PPP2R4 | - |
| PPP5D1 | HGLibA_38214 | 20 | -0.94958121 | PPP5D1 | - |
| SMEK1 | HGLibA_45692 | 20 | -0.94958121 | SMEK1 | - |
| TAZ | HGLibA_48456 | 20 | -0.94958121 | TAZ | - |
| TCTEX1D2 | HGLibA_48867 | 20 | -0.94958121 | TCTEX1D2 | - |
| TCTEX1D2 | HGLibA_48865 | 20 | -0.94958121 | TCTEX1D2 | - |
| TMEM48 | HGLibA_50417 | 20 | -0.94958121 | TMEM48 | - |
| ASMTL_Y | HGLibA_03328 | 19 | -0.959532248 | ASMTL_Y | - |
| ATP5G3 | HGLibA_03662 | 19 | -0.959532248 | ATP5G3 | - |
| ATP5H | HGLibA_03665 | 19 | -0.959532248 | ATP5H | - |
| C1orf95 | HGLibA_05910 | 19 | -0.959532248 | C1orf95 | - |
| C2orf71 | HGLibA_06174 | 19 | -0.959532248 | C2orf71 | - |
| C3orf43 | HGLibA_06244 | 19 | -0.959532248 | C3orf43 | - |
| C5orf20 | HGLibA_06361 | 19 | -0.959532248 | C5orf20 | - |
| C9orf37 | HGLibA_06726 | 19 | -0.959532248 | C9orf37 | - |
| DGCR14 | HGLibA_13044 | 19 | -0.959532248 | DGCR14 | - |
| ENSG00000007202 | HGLibA_24588 | 19 | -0.959532248 | KIAA0100 | KIAA0100 [Source:HGNC Symbol;Acc:HGNC:28960] |
| ENSG00000033800 | HGLibA_36506 | 19 | -0.959532248 | PIAS1 | protein inhibitor of activated STAT 1 [Source:HGNC Symbol;Acc:HGNC:2752] |
| ENSG00000036828 | HGLibA_07339 | 19 | -0.959532248 | CASR | calcium sensing receptor [Source:HGNC Symbol;Acc:HGNC:1514] |
| ENSG00000042088 | HGLibA_48890 | 19 | -0.959532248 | TDP1 | tyrosyl-DNA phosphodiesterase 1 [Source:HGNC Symbol;Acc:HGNC:18884] |
| ENSG00000049192 | HGLibA_00837 | 19 | -0.959532248 | ADAMTS6 | ADAM metallopeptidase with thrombospondin type 1 motif 6 [Source:HGNC Symbol;Acc:HGNC:222] |
| ENSG00000049249 | HGLibA_50792 | 19 | -0.959532248 | TNFRSF9 | TNF receptor superfamily member 9 [Source:HGNC Symbol;Acc:HGNC:11924] |
| ENSG00000051180 | HGLibA_40077 | 19 | -0.959532248 | RAD51 | RAD51 recombinase [Source:HGNC Symbol;Acc:HGNC:9817] |
| ENSG00000052723 | HGLibA_44169 | 19 | -0.959532248 | SIKE1 | suppressor of IKBKE 1 [Source:HGNC Symbol;Acc:HGNC:26119] |
| ENSG00000053770 | HGLibA_02357 | 19 | -0.959532248 | AP5M1 | adaptor related protein complex 5 subunit mu 1 [Source:HGNC Symbol;Acc:HGNC:20192] |
| ENSG00000054356 | HGLibA_39521 | 19 | -0.959532248 | PTPRN | receptor type N [Source:HGNC Symbol;Acc:HGNC:9676]" |
| ENSG00000061938 | HGLibA_50847 | 19 | -0.959532248 | TNK2 | tyrosine kinase non receptor 2 [Source:HGNC Symbol;Acc:HGNC:19297] |
| ENSG00000063438 | HGLibA_01311 | 19 | -0.959532248 | AHRR | aryl-hydrocarbon receptor repressor [Source:HGNC Symbol;Acc:HGNC:346] |
| ENSG00000065485 | HGLibA_35883 | 19 | -0.959532248 | PDIA5 | protein disulfide isomerase family A member 5 [Source:HGNC Symbol;Acc:HGNC:24811] |
| ENSG00000071794 | HGLibA_21657 | 19 | -0.959532248 | HLTF | helicase like transcription factor [Source:HGNC Symbol;Acc:HGNC:11099] |
| ENSG00000072195 | HGLibA_46616 | 19 | -0.959532248 | SPEG | SPEG complex locus [Source:HGNC Symbol;Acc:HGNC:16901] |
| ENSG00000073712 | HGLibA_17298 | 19 | -0.959532248 | FERMT2 | fermitin family member 2 [Source:HGNC Symbol;Acc:HGNC:15767] |
| ENSG00000075223 | HGLibA_43325 | 19 | -0.959532248 | SEMA3C | semaphorin 3C [Source:HGNC Symbol;Acc:HGNC:10725] |
| ENSG00000077274 | HGLibA_07171 | 19 | -0.959532248 | CAPN6 | calpain 6 [Source:HGNC Symbol;Acc:HGNC:1483] |
| ENSG00000078043 | HGLibA_36510 | 19 | -0.959532248 | PIAS2 | protein inhibitor of activated STAT 2 [Source:HGNC Symbol;Acc:HGNC:17311] |
| ENSG00000078403 | HGLibA_29410 | 19 | -0.959532248 | MLLT10 | histone lysine methyltransferase DOT1L cofactor [Source:HGNC Symbol;Acc:HGNC:16063]" |
| ENSG00000080166 | HGLibA_12537 | 19 | -0.959532248 | DCT | dopachrome tautomerase [Source:HGNC Symbol;Acc:HGNC:2709] |
| ENSG00000082213 | HGLibA_06365 | 19 | -0.959532248 | C5orf22 | chromosome 5 open reading frame 22 [Source:HGNC Symbol;Acc:HGNC:25639] |
| ENSG00000086189 | HGLibA_13234 | 19 | -0.959532248 | DIMT1 | DIM1 dimethyladenosine transferase 1 homolog [Source:HGNC Symbol;Acc:HGNC:30217] |
| ENSG00000086848 | HGLibA_01640 | 19 | -0.959532248 | ALG9 | 2-mannosyltransferase [Source:HGNC Symbol;Acc:HGNC:15672]" |
| ENSG00000087299 | HGLibA_25889 | 19 | -0.959532248 | L2HGDH | L-2-hydroxyglutarate dehydrogenase [Source:HGNC Symbol;Acc:HGNC:20499] |
| ENSG00000092929 | HGLibA_53003 | 19 | -0.959532248 | UNC13D | unc-13 homolog D [Source:HGNC Symbol;Acc:HGNC:23147] |
| ENSG00000099399 | HGLibA_27945 | 19 | -0.959532248 | MAGEB2 | MAGE family member B2 [Source:HGNC Symbol;Acc:HGNC:6809] |
| ENSG00000099814 | HGLibA_09119 | 19 | -0.959532248 | CEP170B | centrosomal protein 170B [Source:HGNC Symbol;Acc:HGNC:20362] |
| ENSG00000099957 | HGLibA_34862 | 19 | -0.959532248 | P2RX6 | purinergic receptor P2X 6 [Source:HGNC Symbol;Acc:HGNC:8538] |
| ENSG00000101000 | HGLibA_38657 | 19 | -0.959532248 | PROCR | protein C receptor [Source:HGNC Symbol;Acc:HGNC:9452] |
| ENSG00000101040 | HGLibA_55427 | 19 | -0.959532248 | ZMYND8 | zinc finger MYND-type containing 8 [Source:HGNC Symbol;Acc:HGNC:9397] |
| ENSG00000101220 | HGLibA_06023 | 19 | -0.959532248 | C20orf27 | chromosome 20 open reading frame 27 [Source:HGNC Symbol;Acc:HGNC:15873] |
| ENSG00000101413 | HGLibA_42047 | 19 | -0.959532248 | RPRD1B | regulation of nuclear pre-mRNA domain containing 1B [Source:HGNC Symbol;Acc:HGNC:16209] |
| ENSG00000101849 | HGLibA_48608 | 19 | -0.959532248 | TBL1X | transducin beta like 1 X-linked [Source:HGNC Symbol;Acc:HGNC:11585] |
| ENSG00000102934 | HGLibA_37203 | 19 | -0.959532248 | PLLP | plasmolipin [Source:HGNC Symbol;Acc:HGNC:18553] |
| ENSG00000103056 | HGLibA_45808 | 19 | -0.959532248 | SMPD3 | sphingomyelin phosphodiesterase 3 [Source:HGNC Symbol;Acc:HGNC:14240] |
| ENSG00000103479 | HGLibA_40471 | 19 | -0.959532248 | RBL2 | RB transcriptional corepressor like 2 [Source:HGNC Symbol;Acc:HGNC:9894] |
| ENSG00000104213 | HGLibA_35859 | 19 | -0.959532248 | PDGFRL | platelet derived growth factor receptor like [Source:HGNC Symbol;Acc:HGNC:8805] |
| ENSG00000104219 | HGLibA_55096 | 19 | -0.959532248 | ZDHHC2 | zinc finger DHHC-type containing 2 [Source:HGNC Symbol;Acc:HGNC:18469] |
| ENSG00000104812 | HGLibA_20708 | 19 | -0.959532248 | GYS1 | glycogen synthase 1 [Source:HGNC Symbol;Acc:HGNC:4706] |
| ENSG00000104960 | HGLibA_39401 | 19 | -0.959532248 | PTOV1 | prostate tumor overexpressed 1 [Source:HGNC Symbol;Acc:HGNC:9632] |
| ENSG00000105193 | HGLibA_42082 | 19 | -0.959532248 | RPS16 | ribosomal protein S16 [Source:HGNC Symbol;Acc:HGNC:10396] |
| ENSG00000105519 | HGLibA_07195 | 19 | -0.959532248 | CAPS | calcyphosine [Source:HGNC Symbol;Acc:HGNC:1487] |
| ENSG00000105640 | HGLibA_41888 | 19 | -0.959532248 | RPL18A | ribosomal protein L18a [Source:HGNC Symbol;Acc:HGNC:10311] |
| ENSG00000109670 | HGLibA_17154 | 19 | -0.959532248 | FBXW7 | F-box and WD repeat domain containing 7 [Source:HGNC Symbol;Acc:HGNC:16712] |
| ENSG00000109854 | HGLibA_22345 | 19 | -0.959532248 | HTATIP2 | HIV-1 Tat interactive protein 2 [Source:HGNC Symbol;Acc:HGNC:16637] |
| ENSG00000111224 | HGLibA_35217 | 19 | -0.959532248 | PARP11 | poly(ADP-ribose) polymerase family member 11 [Source:HGNC Symbol;Acc:HGNC:1186] |
| ENSG00000112038 | HGLibA_33451 | 19 | -0.959532248 | OPRM1 | opioid receptor mu 1 [Source:HGNC Symbol;Acc:HGNC:8156] |
| ENSG00000113384 | HGLibA_19657 | 19 | -0.959532248 | GOLPH3 | golgi phosphoprotein 3 [Source:HGNC Symbol;Acc:HGNC:15452] |
| ENSG00000114767 | HGLibA_42286 | 19 | -0.959532248 | RRP9 | U3 small nucleolar RNA binding protein [Source:HGNC Symbol;Acc:HGNC:16829]" |
| ENSG00000114770 | HGLibA_00179 | 19 | -0.959532248 | ABCC5 | ATP binding cassette subfamily C member 5 [Source:HGNC Symbol;Acc:HGNC:56] |
| ENSG00000115107 | HGLibA_47428 | 19 | -0.959532248 | STEAP3 | STEAP3 metalloreductase [Source:HGNC Symbol;Acc:HGNC:24592] |
| ENSG00000115163 | HGLibA_09037 | 19 | -0.959532248 | CENPA | centromere protein A [Source:HGNC Symbol;Acc:HGNC:1851] |
| ENSG00000115423 | HGLibA_13491 | 19 | -0.959532248 | DNAH6 | dynein axonemal heavy chain 6 [Source:HGNC Symbol;Acc:HGNC:2951] |
| ENSG00000115598 | HGLibA_23064 | 19 | -0.959532248 | IL1RL2 | interleukin 1 receptor like 2 [Source:HGNC Symbol;Acc:HGNC:5999] |
| ENSG00000116771 | HGLibA_01198 | 19 | -0.959532248 | AGMAT | agmatinase [Source:HGNC Symbol;Acc:HGNC:18407] |
| ENSG00000117362 | HGLibA_02412 | 19 | -0.959532248 | APH1A | gamma-secretase subunit [Source:HGNC Symbol;Acc:HGNC:29509]" |
| ENSG00000118473 | HGLibA_43813 | 19 | -0.959532248 | SGIP1 | SH3 domain GRB2 like endophilin interacting protein 1 [Source:HGNC Symbol;Acc:HGNC:25412] |
| ENSG00000118515 | HGLibA_43818 | 19 | -0.959532248 | SGK1 | serum/glucocorticoid regulated kinase 1 [Source:HGNC Symbol;Acc:HGNC:10810] |
| ENSG00000120053 | HGLibA_19690 | 19 | -0.959532248 | GOT1 | glutamic-oxaloacetic transaminase 1 [Source:HGNC Symbol;Acc:HGNC:4432] |
| ENSG00000120159 | HGLibA_06830 | 19 | -0.959532248 | CAAP1 | caspase activity and apoptosis inhibitor 1 [Source:HGNC Symbol;Acc:HGNC:25834] |
| ENSG00000120289 | HGLibA_27949 | 19 | -0.959532248 | MAGEB4 | MAGE family member B4 [Source:HGNC Symbol;Acc:HGNC:6811] |
| ENSG00000121579 | HGLibA_30999 | 19 | -0.959532248 | NAA50 | NatE catalytic subunit [Source:HGNC Symbol;Acc:HGNC:29533]" |
| ENSG00000122481 | HGLibA_42487 | 19 | -0.959532248 | RWDD3 | RWD domain containing 3 [Source:HGNC Symbol;Acc:HGNC:21393] |
| ENSG00000122870 | HGLibA_04492 | 19 | -0.959532248 | BICC1 | BicC family RNA binding protein 1 [Source:HGNC Symbol;Acc:HGNC:19351] |
| ENSG00000123191 | HGLibA_03772 | 19 | -0.959532248 | ATP7B | ATPase copper transporting beta [Source:HGNC Symbol;Acc:HGNC:870] |
| ENSG00000123472 | HGLibA_03799 | 19 | -0.959532248 | ATPAF1 | ATP synthase mitochondrial F1 complex assembly factor 1 [Source:HGNC Symbol;Acc:HGNC:18803] |
| ENSG00000124429 | HGLibA_37465 | 19 | -0.959532248 | POF1B | actin binding protein [Source:HGNC Symbol;Acc:HGNC:13711]" |
| ENSG00000124721 | HGLibA_13497 | 19 | -0.959532248 | DNAH8 | dynein axonemal heavy chain 8 [Source:HGNC Symbol;Acc:HGNC:2952] |
| ENSG00000124783 | HGLibA_47154 | 19 | -0.959532248 | SSR1 | signal sequence receptor subunit 1 [Source:HGNC Symbol;Acc:HGNC:11323] |
| ENSG00000125409 | HGLibA_48978 | 19 | -0.959532248 | TEKT3 | tektin 3 [Source:HGNC Symbol;Acc:HGNC:14293] |
| ENSG00000125430 | HGLibA_22130 | 19 | -0.959532248 | HS3ST3B1 | heparan sulfate-glucosamine 3-sulfotransferase 3B1 [Source:HGNC Symbol;Acc:HGNC:5198] |
| ENSG00000125952 | HGLibA_28448 | 19 | -0.959532248 | MAX | MYC associated factor X [Source:HGNC Symbol;Acc:HGNC:6913] |
| ENSG00000127580 | HGLibA_54049 | 19 | -0.959532248 | WDR24 | WD repeat domain 24 [Source:HGNC Symbol;Acc:HGNC:20852] |
| ENSG00000129654 | HGLibA_17884 | 19 | -0.959532248 | FOXJ1 | forkhead box J1 [Source:HGNC Symbol;Acc:HGNC:3816] |
| ENSG00000131055 | HGLibA_10834 | 19 | -0.959532248 | COX4I2 | cytochrome c oxidase subunit 4I2 [Source:HGNC Symbol;Acc:HGNC:16232] |
| ENSG00000131459 | HGLibA_18961 | 19 | -0.959532248 | GFPT2 | glutamine-fructose-6-phosphate transaminase 2 [Source:HGNC Symbol;Acc:HGNC:4242] |
| ENSG00000131788 | HGLibA_36512 | 19 | -0.959532248 | PIAS3 | protein inhibitor of activated STAT 3 [Source:HGNC Symbol;Acc:HGNC:16861] |
| ENSG00000132128 | HGLibA_27357 | 19 | -0.959532248 | LRRC41 | leucine rich repeat containing 41 [Source:HGNC Symbol;Acc:HGNC:16917] |
| ENSG00000132300 | HGLibA_39264 | 19 | -0.959532248 | PTCD3 | pentatricopeptide repeat domain 3 [Source:HGNC Symbol;Acc:HGNC:24717] |
| ENSG00000132382 | HGLibA_30641 | 19 | -0.959532248 | MYBBP1A | MYB binding protein 1a [Source:HGNC Symbol;Acc:HGNC:7546] |
| ENSG00000132801 | HGLibA_57042 | 19 | -0.959532248 | ZSWIM3 | zinc finger SWIM-type containing 3 [Source:HGNC Symbol;Acc:HGNC:16157] |
| ENSG00000133243 | HGLibA_04910 | 19 | -0.959532248 | BTBD2 | BTB domain containing 2 [Source:HGNC Symbol;Acc:HGNC:15504] |
| ENSG00000134538 | HGLibA_45447 | 19 | -0.959532248 | SLCO1B1 | solute carrier organic anion transporter family member 1B1 [Source:HGNC Symbol;Acc:HGNC:10959] |
| ENSG00000135845 | HGLibA_36549 | 19 | -0.959532248 | PIGC | phosphatidylinositol glycan anchor biosynthesis class C [Source:HGNC Symbol;Acc:HGNC:8960] |
| ENSG00000135900 | HGLibA_29990 | 19 | -0.959532248 | MRPL44 | mitochondrial ribosomal protein L44 [Source:HGNC Symbol;Acc:HGNC:16650] |
| ENSG00000135903 | HGLibA_35300 | 19 | -0.959532248 | PAX3 | paired box 3 [Source:HGNC Symbol;Acc:HGNC:8617] |
| ENSG00000136059 | HGLibA_53652 | 19 | -0.959532248 | VILL | villin like [Source:HGNC Symbol;Acc:HGNC:30906] |
| ENSG00000136848 | HGLibA_12270 | 19 | -0.959532248 | DAB2IP | DAB2 interacting protein [Source:HGNC Symbol;Acc:HGNC:17294] |
| ENSG00000137831 | HGLibA_52505 | 19 | -0.959532248 | UACA | uveal autoantigen with coiled-coil domains and ankyrin repeats [Source:HGNC Symbol;Acc:HGNC:15947] |
| ENSG00000137965 | HGLibA_22610 | 19 | -0.959532248 | IFI44 | interferon induced protein 44 [Source:HGNC Symbol;Acc:HGNC:16938] |

| | | | | | |
|---|---|---|---|---|---|
| ENSG00000138823 | HGLibA_30502 | 19 | -0.959532248 | MTTP | microsomal triglyceride transfer protein [Source:HGNC Symbol;Acc:HGNC:7467] |
| ENSG00000139629 | HGLibA_18566 | 19 | -0.959532248 | GALNT6 | polypeptide N-acetylgalactosaminyltransferase 6 [Source:HGNC Symbol;Acc:HGNC:4128] |
| ENSG00000140691 | HGLibA_03024 | 19 | -0.959532248 | ARMC5 | armadillo repeat containing 5 [Source:HGNC Symbol;Acc:HGNC:25781] |
| ENSG00000141485 | HGLibA_44397 | 19 | -0.959532248 | SLC13A5 | solute carrier family 13 member 5 [Source:HGNC Symbol;Acc:HGNC:23089] |
| ENSG00000141736 | HGLibA_15414 | 19 | -0.959532248 | ERBB2 | erb-b2 receptor tyrosine kinase 2 [Source:HGNC Symbol;Acc:HGNC:3430] |
| ENSG00000143401 | HGLibA_02207 | 19 | -0.959532248 | ANP32E | acidic nuclear phosphoprotein 32 family member E [Source:HGNC Symbol;Acc:HGNC:16673] |
| ENSG00000144010 | HGLibA_51504 | 19 | -0.959532248 | TRIM43B | tripartite motif containing 43B [Source:HGNC Symbol;Acc:HGNC:37146] |
| ENSG00000144115 | HGLibA_49361 | 19 | -0.959532248 | THNSL2 | threonine synthase like 2 [Source:HGNC Symbol;Acc:HGNC:25602] |
| ENSG00000146587 | HGLibA_40418 | 19 | -0.959532248 | RBAK | RB associated KRAB zinc finger [Source:HGNC Symbol;Acc:HGNC:17680] |
| ENSG00000148396 | HGLibA_43208 | 19 | -0.959532248 | SEC16A | endoplasmic reticulum export factor [Source:HGNC Symbol;Acc:HGNC:29006]" |
| ENSG00000149311 | HGLibA_03524 | 19 | -0.959532248 | ATM | ATM serine/threonine kinase [Source:HGNC Symbol;Acc:HGNC:795] |
| ENSG00000149633 | HGLibA_24816 | 19 | -0.959532248 | KIAA1755 | KIAA1755 [Source:HGNC Symbol;Acc:HGNC:29372] |
| ENSG00000150347 | HGLibA_02910 | 19 | -0.959532248 | ARID5B | AT-rich interaction domain 5B [Source:HGNC Symbol;Acc:HGNC:17362] |
| ENSG00000150687 | HGLibA_38918 | 19 | -0.959532248 | PRSS23 | serine protease 23 [Source:HGNC Symbol;Acc:HGNC:14370] |
| ENSG00000150753 | HGLibA_08204 | 19 | -0.959532248 | CCT5 | chaperonin containing TCP1 subunit 5 [Source:HGNC Symbol;Acc:HGNC:1618] |
| ENSG00000151208 | HGLibA_13336 | 19 | -0.959532248 | DLG5 | discs large MAGUK scaffold protein 5 [Source:HGNC Symbol;Acc:HGNC:2904] |
| ENSG00000151379 | HGLibA_30186 | 19 | -0.959532248 | MSGN1 | mesogenin 1 [Source:HGNC Symbol;Acc:HGNC:14907] |
| ENSG00000152332 | HGLibA_52943 | 19 | -0.959532248 | UHMK1 | U2AF homology motif kinase 1 [Source:HGNC Symbol;Acc:HGNC:19683] |
| ENSG00000152430 | HGLibA_04691 | 19 | -0.959532248 | BOLL | RNA binding protein [Source:HGNC Symbol;Acc:HGNC:14273]" |
| ENSG00000152601 | HGLibA_28509 | 19 | -0.959532248 | MBNL1 | muscleblind like splicing regulator 1 [Source:HGNC Symbol;Acc:HGNC:6923] |
| ENSG00000152953 | HGLibA_47486 | 19 | -0.959532248 | STK32B | serine/threonine kinase 32B [Source:HGNC Symbol;Acc:HGNC:14217] |
| ENSG00000153093 | HGLibA_00507 | 19 | -0.959532248 | ACOXL | acyl-CoA oxidase like [Source:HGNC Symbol;Acc:HGNC:25621] |
| ENSG00000153234 | HGLibA_32627 | 19 | -0.959532248 | NR4A2 | nuclear receptor subfamily 4 group A member 2 [Source:HGNC Symbol;Acc:HGNC:7981] |
| ENSG00000153815 | HGLibA_10193 | 19 | -0.959532248 | CMIP | c-Maf inducing protein [Source:HGNC Symbol;Acc:HGNC:24319] |
| ENSG00000154380 | HGLibA_15103 | 19 | -0.959532248 | ENAH | actin regulator [Source:HGNC Symbol;Acc:HGNC:18271]" |
| ENSG00000154727 | HGLibA_18348 | 19 | -0.959532248 | GABPA | GA binding protein transcription factor subunit alpha [Source:HGNC Symbol;Acc:HGNC:4071] |
| ENSG00000154928 | HGLibA_15310 | 19 | -0.959532248 | EPHB1 | EPH receptor B1 [Source:HGNC Symbol;Acc:HGNC:3392] |
| ENSG00000154945 | HGLibA_02095 | 19 | -0.959532248 | ANKRD40 | ankyrin repeat domain 40 [Source:HGNC Symbol;Acc:HGNC:28233] |
| ENSG00000156253 | HGLibA_42485 | 19 | -0.959532248 | RWDD2B | RWD domain containing 2B [Source:HGNC Symbol;Acc:HGNC:1302] |
| ENSG00000157741 | HGLibA_52723 | 19 | -0.959532248 | UBN2 | ubinuclein 2 [Source:HGNC Symbol;Acc:HGNC:21931] |
| ENSG00000157911 | HGLibA_36080 | 19 | -0.959532248 | PEX10 | peroxisomal biogenesis factor 10 [Source:HGNC Symbol;Acc:HGNC:8851] |
| ENSG00000158290 | HGLibA_11784 | 19 | -0.959532248 | CUL4B | cullin 4B [Source:HGNC Symbol;Acc:HGNC:2555] |
| ENSG00000158769 | HGLibA_15847 | 19 | -0.959532248 | F11R | F11 receptor [Source:HGNC Symbol;Acc:HGNC:14685] |
| ENSG00000158773 | HGLibA_53166 | 19 | -0.959532248 | USF1 | upstream transcription factor 1 [Source:HGNC Symbol;Acc:HGNC:12593] |
| ENSG00000158786 | HGLibA_36893 | 19 | -0.959532248 | PLA2G2F | phospholipase A2 group IIF [Source:HGNC Symbol;Acc:HGNC:30040] |
| ENSG00000159069 | HGLibA_17151 | 19 | -0.959532248 | FBXW5 | F-box and WD repeat domain containing 5 [Source:HGNC Symbol;Acc:HGNC:13613] |
| ENSG00000160014 | HGLibA_07027 | 19 | -0.959532248 | CALM3 | calmodulin 3 [Source:HGNC Symbol;Acc:HGNC:1449] |
| ENSG00000160191 | HGLibA_35835 | 19 | -0.959532248 | PDE9A | phosphodiesterase 9A [Source:HGNC Symbol;Acc:HGNC:8795] |
| ENSG00000160224 | HGLibA_01368 | 19 | -0.959532248 | AIRE | autoimmune regulator [Source:HGNC Symbol;Acc:HGNC:360] |
| ENSG00000160714 | HGLibA_52644 | 19 | -0.959532248 | UBE2Q1 | ubiquitin conjugating enzyme E2 Q1 [Source:HGNC Symbol;Acc:HGNC:15698] |
| ENSG00000161671 | HGLibA_15005 | 19 | -0.959532248 | EMC10 | ER membrane protein complex subunit 10 [Source:HGNC Symbol;Acc:HGNC:27609] |
| ENSG00000161904 | HGLibA_26290 | 19 | -0.959532248 | LEMD2 | LEM domain containing 2 [Source:HGNC Symbol;Acc:HGNC:21244] |
| ENSG00000162409 | HGLibA_38494 | 19 | -0.959532248 | PRKAA2 | protein kinase AMP-activated catalytic subunit alpha 2 [Source:HGNC Symbol;Acc:HGNC:9377] |
| ENSG00000162928 | HGLibA_36095 | 19 | -0.959532248 | PEX13 | peroxisomal biogenesis factor 13 [Source:HGNC Symbol;Acc:HGNC:8855] |
| ENSG00000162980 | HGLibA_02975 | 19 | -0.959532248 | ARL5A | ADP ribosylation factor like GTPase 5A [Source:HGNC Symbol;Acc:HGNC:696] |
| ENSG00000163040 | HGLibA_07863 | 19 | -0.959532248 | CCDC74A | coiled-coil domain containing 74A [Source:HGNC Symbol;Acc:HGNC:25197] |
| ENSG00000163157 | HGLibA_50602 | 19 | -0.959532248 | TMOD4 | tropomodulin 4 [Source:HGNC Symbol;Acc:HGNC:11874] |
| ENSG00000163263 | HGLibA_05811 | 19 | -0.959532248 | C1orf189 | chromosome 1 open reading frame 189 [Source:HGNC Symbol;Acc:HGNC:32305] |
| ENSG00000164197 | HGLibA_41581 | 19 | -0.959532248 | RNF180 | ring finger protein 180 [Source:HGNC Symbol;Acc:HGNC:27752] |
| ENSG00000164209 | HGLibA_44752 | 19 | -0.959532248 | SLC25A46 | solute carrier family 25 member 46 [Source:HGNC Symbol;Acc:HGNC:25198] |
| ENSG00000165304 | HGLibA_28879 | 19 | -0.959532248 | MELK | maternal embryonic leucine zipper kinase [Source:HGNC Symbol;Acc:HGNC:16870] |
| ENSG00000165685 | HGLibA_50436 | 19 | -0.959532248 | TMEM52B | transmembrane protein 52B [Source:HGNC Symbol;Acc:HGNC:26438] |
| ENSG00000165916 | HGLibA_39132 | 19 | -0.959532248 | PSMC3 | ATPase 3 [Source:HGNC Symbol;Acc:HGNC:9549]" |
| ENSG00000166676 | HGLibA_52358 | 19 | -0.959532248 | TVP23A | trans-golgi network vesicle protein 23 homolog A [Source:HGNC Symbol;Acc:HGNC:20398] |
| ENSG00000166920 | HGLibA_05404 | 19 | -0.959532248 | C15orf48 | chromosome 15 open reading frame 48 [Source:HGNC Symbol;Acc:HGNC:29898] |
| ENSG00000167104 | HGLibA_04730 | 19 | -0.959532248 | BPIFB6 | BPI fold containing family B member 6 [Source:HGNC Symbol;Acc:HGNC:16504] |
| ENSG00000167112 | HGLibA_51804 | 19 | -0.959532248 | TRUB2 | TruB pseudouridine synthase family member 2 [Source:HGNC Symbol;Acc:HGNC:17170] |
| ENSG00000167333 | HGLibA_51591 | 19 | -0.959532248 | TRIM68 | tripartite motif containing 68 [Source:HGNC Symbol;Acc:HGNC:21161] |
| ENSG00000167419 | HGLibA_27106 | 19 | -0.959532248 | LPO | lactoperoxidase [Source:HGNC Symbol;Acc:HGNC:6678] |
| ENSG00000167470 | HGLibA_29240 | 19 | -0.959532248 | MIDN | midnolin [Source:HGNC Symbol;Acc:HGNC:16298] |
| ENSG00000167676 | HGLibA_37178 | 19 | -0.959532248 | PLIN4 | perilipin 4 [Source:HGNC Symbol;Acc:HGNC:29393] |
| ENSG00000168288 | HGLibA_29458 | 19 | -0.959532248 | MMADHC | metabolism of cobalamin associated D [Source:HGNC Symbol;Acc:HGNC:25221] |
| ENSG00000169692 | HGLibA_01221 | 19 | -0.959532248 | AGPAT2 | 1-acylglycerol-3-phosphate O-acyltransferase 2 [Source:HGNC Symbol;Acc:HGNC:325] |
| ENSG00000170456 | HGLibA_12961 | 19 | -0.959532248 | DENND5B | DENN domain containing 5B [Source:HGNC Symbol;Acc:HGNC:28338] |
| ENSG00000170873 | HGLibA_30495 | 19 | -0.959532248 | MTSS1 | I-BAR domain containing [Source:HGNC Symbol;Acc:HGNC:20443]" |
| ENSG00000171848 | HGLibA_42255 | 19 | -0.959532248 | RRM2 | ribonucleotide reductase regulatory subunit M2 [Source:HGNC Symbol;Acc:HGNC:10452] |
| ENSG00000171928 | HGLibA_52360 | 19 | -0.959532248 | TVP23B | trans-golgi network vesicle protein 23 homolog B [Source:HGNC Symbol;Acc:HGNC:20399] |
| ENSG00000172208 | HGLibA_34071 | 19 | -0.959532248 | OR4X2 | olfactory receptor family 4 subfamily X member 2 (gene/pseudogene) [Source:HGNC Symbol;Acc:HGNC:15184] |
| ENSG00000172680 | HGLibA_29680 | 19 | -0.959532248 | MOS | serine/threonine kinase [Source:HGNC Symbol;Acc:HGNC:7199]" |
| ENSG00000172890 | HGLibA_31054 | 19 | -0.959532248 | NADSYN1 | NAD synthetase 1 [Source:HGNC Symbol;Acc:HGNC:29832] |
| ENSG00000173960 | HGLibA_52783 | 19 | -0.959532248 | UBXN2A | UBX domain protein 2A [Source:HGNC Symbol;Acc:HGNC:27265] |
| ENSG00000174010 | HGLibA_25138 | 19 | -0.959532248 | KLHL15 | kelch like family member 15 [Source:HGNC Symbol;Acc:HGNC:29347] |
| ENSG00000174788 | HGLibA_35658 | 19 | -0.959532248 | PCP2 | Purkinje cell protein 2 [Source:HGNC Symbol;Acc:HGNC:30209] |
| ENSG00000174982 | HGLibA_34064 | 19 | -0.959532248 | OR4S2 | olfactory receptor family 4 subfamily S member 2 [Source:HGNC Symbol;Acc:HGNC:15183] |
| ENSG00000175879 | HGLibA_22005 | 19 | -0.959532248 | HOXD8 | homeobox D8 [Source:HGNC Symbol;Acc:HGNC:5139] |
| ENSG00000175893 | HGLibA_55103 | 19 | -0.959532248 | ZDHHC21 | zinc finger DHHC-type containing 21 [Source:HGNC Symbol;Acc:HGNC:20750] |
| ENSG00000176438 | HGLibA_47983 | 19 | -0.959532248 | SYNE3 | spectrin repeat containing nuclear envelope family member 3 [Source:HGNC Symbol;Acc:HGNC:19861] |
| ENSG00000176842 | HGLibA_23639 | 19 | -0.959532248 | IRX5 | iroquois homeobox 5 [Source:HGNC Symbol;Acc:HGNC:14361] |
| ENSG00000176978 | HGLibA_13879 | 19 | -0.959532248 | DPP7 | dipeptidyl peptidase 7 [Source:HGNC Symbol;Acc:HGNC:14892] |
| ENSG00000177370 | HGLibA_49512 | 19 | -0.959532248 | TIMM22 | translocase of inner mitochondrial membrane 22 [Source:HGNC Symbol;Acc:HGNC:17317] |
| ENSG00000177548 | HGLibA_40002 | 19 | -0.959532248 | RABEP2 | RAB GTPase binding effector protein 2 [Source:HGNC Symbol;Acc:HGNC:24817]" |
| ENSG00000179715 | HGLibA_35585 | 19 | -0.959532248 | PCED1B | PC-esterase domain containing 1B [Source:HGNC Symbol;Acc:HGNC:28255] |
| ENSG00000180432 | HGLibA_12218 | 19 | -0.959532248 | CYP8B1 | cytochrome P450 family 8 subfamily B member 1 [Source:HGNC Symbol;Acc:HGNC:2653] |
| ENSG00000180979 | HGLibA_27394 | 19 | -0.959532248 | LRRC57 | leucine rich repeat containing 57 [Source:HGNC Symbol;Acc:HGNC:26719] |
| ENSG00000182107 | HGLibA_50363 | 19 | -0.959532248 | TMEM30B | transmembrane protein 30B [Source:HGNC Symbol;Acc:HGNC:27254] |
| ENSG00000182718 | HGLibA_02231 | 19 | -0.959532248 | ANXA2 | annexin A2 [Source:HGNC Symbol;Acc:HGNC:537] |
| ENSG00000183542 | HGLibA_25295 | 19 | -0.959532248 | KLRC4 | killer cell lectin like receptor C4 [Source:HGNC Symbol;Acc:HGNC:6377] |
| ENSG00000183690 | HGLibA_14572 | 19 | -0.959532248 | EFHC2 | EF-hand domain containing 2 [Source:HGNC Symbol;Acc:HGNC:26233] |
| ENSG00000184060 | HGLibA_00865 | 19 | -0.959532248 | ADAP2 | ArfGAP with dual PH domains 2 [Source:HGNC Symbol;Acc:HGNC:16487] |
| ENSG00000184408 | HGLibA_24177 | 19 | -0.959532248 | KCND2 | potassium voltage-gated channel subfamily D member 2 [Source:HGNC Symbol;Acc:HGNC:6238] |
| ENSG00000184659 | HGLibA_17848 | 19 | -0.959532248 | FOXD4L4 | forkhead box D4 like 4 [Source:HGNC Symbol;Acc:HGNC:23762] |
| ENSG00000184828 | HGLibA_54921 | 19 | -0.959532248 | ZBTB7C | zinc finger and BTB domain containing 7C [Source:HGNC Symbol;Acc:HGNC:31700] |
| ENSG00000184922 | HGLibA_17693 | 19 | -0.959532248 | FMNL1 | formin like 1 [Source:HGNC Symbol;Acc:HGNC:1212] |
| ENSG00000185250 | HGLibA_37952 | 19 | -0.959532248 | PPIL6 | peptidylprolyl isomerase like 6 [Source:HGNC Symbol;Acc:HGNC:21557] |
| ENSG00000185988 | HGLibA_37200 | 19 | -0.959532248 | PLK5 | polo like kinase 5 [Source:HGNC Symbol;Acc:HGNC:27001] |
| ENSG00000186439 | HGLibA_51348 | 19 | -0.959532248 | TRDN | triadin [Source:HGNC Symbol;Acc:HGNC:12261] |
| ENSG00000188001 | HGLibA_51179 | 19 | -0.959532248 | TPRG1 | tumor protein p63 regulated 1 [Source:HGNC Symbol;Acc:HGNC:24759] |
| ENSG00000188517 | HGLibA_10552 | 19 | -0.959532248 | COL25A1 | collagen type XXV alpha 1 chain [Source:HGNC Symbol;Acc:HGNC:18603] |
| ENSG00000189366 | HGLibA_01623 | 19 | -0.959532248 | ALG1L | chitobiosyldiphosphodolichol beta-mannosyltransferase like [Source:HGNC Symbol;Acc:HGNC:33721]" |
| ENSG00000196090 | HGLibA_39538 | 19 | -0.959532248 | PTPRT | receptor type T [Source:HGNC Symbol;Acc:HGNC:9682]" |
| ENSG00000196171 | HGLibA_34418 | 19 | -0.959532248 | OR6K2 | olfactory receptor family 6 subfamily K member 2 [Source:HGNC Symbol;Acc:HGNC:15029] |
| ENSG00000196365 | HGLibA_27013 | 19 | -0.959532248 | LONP1 | mitochondrial [Source:HGNC Symbol;Acc:HGNC:9479]" |
| ENSG00000196664 | HGLibA_49661 | 19 | -0.959532248 | TLR7 | toll like receptor 7 [Source:HGNC Symbol;Acc:HGNC:15631] |
| ENSG00000196684 | HGLibA_22247 | 19 | -0.959532248 | HSH2D | hematopoietic SH2 domain containing [Source:HGNC Symbol;Acc:HGNC:24920] |
| ENSG00000197454 | HGLibA_33826 | 19 | -0.959532248 | OR2L5 | olfactory receptor family 2 subfamily L member 5 [Source:HGNC Symbol;Acc:HGNC:15011] |
| ENSG00000197683 | HGLibA_25730 | 19 | -0.959532248 | KRTAP26-1 | keratin associated protein 26-1 [Source:HGNC Symbol;Acc:HGNC:33760] |
| ENSG00000197977 | HGLibA_14960 | 19 | -0.959532248 | ELOVL2 | ELOVL fatty acid elongase 2 [Source:HGNC Symbol;Acc:HGNC:14416] |
| ENSG00000198001 | HGLibA_23566 | 19 | -0.959532248 | IRAK4 | interleukin 1 receptor associated kinase 4 [Source:HGNC Symbol;Acc:HGNC:17967] |
| ENSG00000198108 | HGLibA_09658 | 19 | -0.959532248 | CHSY3 | chondroitin sulfate synthase 3 [Source:HGNC Symbol;Acc:HGNC:24293] |
| ENSG00000198610 | HGLibA_01491 | 19 | -0.959532248 | AKR1C4 | aldo-keto reductase family 1 member C4 [Source:HGNC Symbol;Acc:HGNC:387] |
| ENSG00000198783 | HGLibA_56834 | 19 | -0.959532248 | ZNF830 | zinc finger protein 830 [Source:HGNC Symbol;Acc:HGNC:28291] |
| ENSG00000198924 | HGLibA_12507 | 19 | -0.959532248 | DCLRE1A | DNA cross-link repair 1A [Source:HGNC Symbol;Acc:HGNC:17660] |
| ENSG00000203666 | HGLibA_14536 | 19 | -0.959532248 | EFCAB2 | EF-hand calcium binding domain 2 [Source:HGNC Symbol;Acc:HGNC:28166] |
| ENSG00000203786 | HGLibA_25377 | 19 | -0.959532248 | KPRP | keratinocyte proline rich protein [Source:HGNC Symbol;Acc:HGNC:31823] |
| ENSG00000204304 | HGLibA_35345 | 19 | -0.959532248 | PBX2 | PBX homeobox 2 [Source:HGNC Symbol;Acc:HGNC:8633] |
| ENSG00000204899 | HGLibA_30941 | 19 | -0.959532248 | MZT1 | mitotic spindle organizing protein 1 [Source:HGNC Symbol;Acc:HGNC:33830] |
| ENSG00000205571 | HGLibA_45785 | 19 | -0.959532248 | SMN2 | centromeric [Source:HGNC Symbol;Acc:HGNC:11118]" |
| ENSG00000206536 | HGLibA_34315 | 19 | -0.959532248 | OR5K3 | olfactory receptor family 5 subfamily K member 3 [Source:HGNC Symbol;Acc:HGNC:31290] |
| ENSG00000211455 | HGLibA_47505 | 19 | -0.959532248 | STK38L | serine/threonine kinase 38 like [Source:HGNC Symbol;Acc:HGNC:17848] |
| ENSG00000213160 | HGLibA_25158 | 19 | -0.959532248 | KLHL23 | kelch like family member 23 [Source:HGNC Symbol;Acc:HGNC:27506] |
| ENSG00000213762 | HGLibA_55469 | 19 | -0.959532248 | ZNF134 | zinc finger protein 134 [Source:HGNC Symbol;Acc:HGNC:12918] |
| ENSG00000214107 | HGLibA_27928 | 19 | -0.959532248 | MAGEB1 | MAGE family member B1 [Source:HGNC Symbol;Acc:HGNC:6808] |
| ENSG00000221836 | HGLibA_33739 | 19 | -0.959532248 | OR2A5 | olfactory receptor family 2 subfamily A member 5 [Source:HGNC Symbol;Acc:HGNC:8232] |
| ENSG00000223614 | HGLibA_56649 | 19 | -0.959532248 | ZNF735 | zinc finger protein 735 [Source:HGNC Symbol;Acc:HGNC:32466] |
| ENSG00000226430 | HGLibA_53283 | 19 | -0.959532248 | USP17L7 | ubiquitin specific peptidase 17-like family member 7 [Source:HGNC Symbol;Acc:HGNC:37180] |
| ENSG00000243789 | HGLibA_23962 | 19 | -0.959532248 | JMJD7 | jumonji domain containing 7 [Source:HGNC Symbol;Acc:HGNC:34397] |
| ENSG00000249853 | HGLibA_22136 | 19 | -0.959532248 | HS3ST5 | heparan sulfate-glucosamine 3-sulfotransferase 5 [Source:HGNC Symbol;Acc:HGNC:19419] |
| ENSG00000250254 | HGLibA_39568 | 19 | -0.959532248 | PTTG2 | pituitary tumor-transforming 2 [Source:HGNC Symbol;Acc:HGNC:9691] |
| ENSG00000251258 | HGLibA_40944 | 19 | -0.959532248 | RFPL4B | ret finger protein like 4B [Source:HGNC Symbol;Acc:HGNC:33264] |
| ENSG00000253313 | HGLibA_05829 | 19 | -0.959532248 | C1orf210 | chromosome 1 open reading frame 210 [Source:HGNC Symbol;Acc:HGNC:28755] |
| ENSG00000257529 | HGLibA_41959 | 19 | -0.959532248 | RPL36A-HNRNPH2 | RPL36A-HNRNPH2 readthrough [Source:HGNC Symbol;Acc:HGNC:48349] |
| ENSG00000258436 | HGLibA_41396 | 19 | -0.959532248 | RNASE12 | ribonuclease A family member 12 (inactive) [Source:HGNC Symbol;Acc:HGNC:24211] |
| ENSG00000266028 | HGLibA_46971 | 19 | -0.959532248 | SRGAP2 | SLIT-ROBO Rho GTPase activating protein 2 [Source:HGNC Symbol;Acc:HGNC:19751] |
| ENSG00000268447 | HGLibA_47200 | 19 | -0.959532248 | SSX2B | SSX family member 2B [Source:HGNC Symbol;Acc:HGNC:22263] |
| ENSG00000270800 | HGLibA_42062 | 19 | -0.959532248 | RPS10-NUDT3 | RPS10-NUDT3 readthrough [Source:HGNC Symbol;Acc:HGNC:49181] |

| | | | | | |
|---|---|---|---|---|---|
| ENSG00000275066 | HGLibA_48029 | 19 | -0.959532248 | SYNRG | synergin gamma [Source:HGNC Symbol;Acc:HGNC:557] |
| ENSG00000278289 | HGLibA_11512 | 19 | -0.959532248 | CT45A6 | cancer/testis antigen family 45 member A6 [Source:HGNC Symbol;Acc:HGNC:33271] |
| FAM65B | HGLibA_16670 | 19 | -0.959532248 | FAM65B | - |
| GAGE2D | HGLibA_18476 | 19 | -0.959532248 | GAGE2D | - |
| HIST1H2AG | HGLibA_21391 | 19 | -0.959532248 | HIST1H2AG | - |
| HIST1H2BG | HGLibA_21430 | 19 | -0.959532248 | HIST1H2BG | - |
| hsa-mir-100 | HGLibA_57151 | 19 | -0.959532248 | hsa-mir-100 | - |
| hsa-mir-1248 | HGLibA_57392 | 19 | -0.959532248 | hsa-mir-1248 | - |
| hsa-mir-1272 | HGLibA_57529 | 19 | -0.959532248 | hsa-mir-1272 | - |
| hsa-mir-1302-3 | HGLibA_57701 | 19 | -0.959532248 | hsa-mir-1302-3 | - |
| hsa-mir-132 | HGLibA_57756 | 19 | -0.959532248 | hsa-mir-132 | - |
| hsa-mir-143 | HGLibA_57835 | 19 | -0.959532248 | hsa-mir-143 | - |
| hsa-mir-3115 | HGLibA_58499 | 19 | -0.959532248 | hsa-mir-3115 | - |
| hsa-mir-3199-1 | HGLibA_58897 | 19 | -0.959532248 | hsa-mir-3199-1 | - |
| hsa-mir-329-1 | HGLibA_58979 | 19 | -0.959532248 | hsa-mir-329-1 | - |
| hsa-mir-3672 | HGLibA_59243 | 19 | -0.959532248 | hsa-mir-3672 | - |
| hsa-mir-3923 | HGLibA_59569 | 19 | -0.959532248 | hsa-mir-3923 | - |
| hsa-mir-4519 | HGLibA_60520 | 19 | -0.959532248 | hsa-mir-4519 | - |
| hsa-mir-4726 | HGLibA_61000 | 19 | -0.959532248 | hsa-mir-4726 | - |
| hsa-mir-4798 | HGLibA_61296 | 19 | -0.959532248 | hsa-mir-4798 | - |
| hsa-mir-495 | HGLibA_61382 | 19 | -0.959532248 | hsa-mir-495 | - |
| hsa-mir-517a | HGLibA_61625 | 19 | -0.959532248 | hsa-mir-517a | - |
| hsa-mir-548i-1 | HGLibA_62014 | 19 | -0.959532248 | hsa-mir-548i-1 | - |
| hsa-mir-5692a-2 | HGLibA_62291 | 19 | -0.959532248 | hsa-mir-5692a-2 | - |
| hsa-mir-595 | HGLibA_62473 | 19 | -0.959532248 | hsa-mir-595 | - |
| hsa-mir-6758 | HGLibA_63190 | 19 | -0.959532248 | hsa-mir-6758 | - |
| hsa-mir-6783 | HGLibA_63311 | 19 | -0.959532248 | hsa-mir-6783 | - |
| hsa-mir-875 | HGLibA_64209 | 19 | -0.959532248 | hsa-mir-875 | - |
| KIAA1984 | HGLibA_24835 | 19 | -0.959532248 | KIAA1984 | - |
| LOC100130348 | HGLibA_26777 | 19 | -0.959532248 | LOC100130348 | - |
| LOC728392 | HGLibA_26991 | 19 | -0.959532248 | LOC728392 | - |
| PPP5D1 | HGLibA_38215 | 19 | -0.959532248 | PPP5D1 | - |
| RNMTL1 | HGLibA_41721 | 19 | -0.959532248 | RNMTL1 | - |
| TMEM56-RWDD3 | HGLibA_50454 | 19 | -0.959532248 | TMEM56-RWDD3 | - |
| WDYHV1 | HGLibA_54234 | 19 | -0.959532248 | WDYHV1 | - |
| ATP5G2 | HGLibA_03659 | 18 | -0.969483285 | ATP5G2 | - |
| C10orf107 | HGLibA_05025 | 18 | -0.969483285 | C10orf107 | - |
| C10orf76 | HGLibA_05093 | 18 | -0.969483285 | C10orf76 | - |
| C14orf169 | HGLibA_05339 | 18 | -0.969483285 | C14orf169 | - |
| C16orf70 | HGLibA_05470 | 18 | -0.969483285 | C16orf70 | - |
| CCBP2 | HGLibA_07502 | 18 | -0.969483285 | CCBP2 | - |
| ENSG00000005812 | HGLibA_17007 | 18 | -0.969483285 | FBXL3 | F-box and leucine rich repeat protein 3 [Source:HGNC Symbol;Acc:HGNC:13599] |
| ENSG00000006606 | HGLibA_08008 | 18 | -0.969483285 | CCL26 | C-C motif chemokine ligand 26 [Source:HGNC Symbol;Acc:HGNC:10625] |
| ENSG00000007968 | HGLibA_14310 | 18 | -0.969483285 | E2F2 | E2F transcription factor 2 [Source:HGNC Symbol;Acc:HGNC:3114] |
| ENSG00000010256 | HGLibA_53113 | 18 | -0.969483285 | UQCRC1 | ubiquinol-cytochrome c reductase core protein 1 [Source:HGNC Symbol;Acc:HGNC:12585] |
| ENSG00000012983 | HGLibA_28224 | 18 | -0.969483285 | MAP4K5 | mitogen-activated protein kinase kinase kinase kinase 5 [Source:HGNC Symbol;Acc:HGNC:6867] |
| ENSG00000023318 | HGLibA_15530 | 18 | -0.969483285 | ERP44 | endoplasmic reticulum protein 44 [Source:HGNC Symbol;Acc:HGNC:18311] |
| ENSG00000038002 | HGLibA_01132 | 18 | -0.969483285 | AGA | aspartylglucosaminidase [Source:HGNC Symbol;Acc:HGNC:318] |
| ENSG00000042429 | HGLibA_28763 | 18 | -0.969483285 | MED17 | mediator complex subunit 17 [Source:HGNC Symbol;Acc:HGNC:2375] |
| ENSG00000064195 | HGLibA_13378 | 18 | -0.969483285 | DLX3 | distal-less homeobox 3 [Source:HGNC Symbol;Acc:HGNC:2916] |
| ENSG00000076351 | HGLibA_45185 | 18 | -0.969483285 | SLC46A1 | solute carrier family 46 member 1 [Source:HGNC Symbol;Acc:HGNC:30521] |
| ENSG00000076826 | HGLibA_07112 | 18 | -0.969483285 | CAMSAP3 | calmodulin regulated spectrin associated protein family member 3 [Source:HGNC Symbol;Acc:HGNC:29307] |
| ENSG00000076984 | HGLibA_28157 | 18 | -0.969483285 | MAP2K7 | mitogen-activated protein kinase kinase 7 [Source:HGNC Symbol;Acc:HGNC:6847] |
| ENSG00000085871 | HGLibA_29176 | 18 | -0.969483285 | MGST2 | microsomal glutathione S-transferase 2 [Source:HGNC Symbol;Acc:HGNC:7063] |
| ENSG00000086848 | HGLibA_01641 | 18 | -0.969483285 | ALG9 | 2-mannosyltransferase [Source:HGNC Symbol;Acc:HGNC:15672]" |
| ENSG00000088247 | HGLibA_24582 | 18 | -0.969483285 | KHSRP | KH-type splicing regulatory protein [Source:HGNC Symbol;Acc:HGNC:6316] |
| ENSG00000089356 | HGLibA_18229 | 18 | -0.969483285 | FXYD3 | FXYD domain containing ion transport regulator 3 [Source:HGNC Symbol;Acc:HGNC:4027] |
| ENSG00000089820 | HGLibA_02775 | 18 | -0.969483285 | ARHGAP4 | Rho GTPase activating protein 4 [Source:HGNC Symbol;Acc:HGNC:674] |
| ENSG00000089902 | HGLibA_40710 | 18 | -0.969483285 | RCOR1 | REST corepressor 1 [Source:HGNC Symbol;Acc:HGNC:17441] |
| ENSG00000092470 | HGLibA_54178 | 18 | -0.969483285 | WDR76 | WD repeat domain 76 [Source:HGNC Symbol;Acc:HGNC:25773] |
| ENSG00000092929 | HGLibA_53002 | 18 | -0.969483285 | UNC13D | unc-13 homolog D [Source:HGNC Symbol;Acc:HGNC:23147] |
| ENSG00000093000 | HGLibA_33056 | 18 | -0.969483285 | NUP50 | nucleoporin 50 [Source:HGNC Symbol;Acc:HGNC:8065] |
| ENSG00000094916 | HGLibA_07456 | 18 | -0.969483285 | CBX5 | chromobox 5 [Source:HGNC Symbol;Acc:HGNC:1555] |
| ENSG00000099139 | HGLibA_35678 | 18 | -0.969483285 | PCSK5 | proprotein convertase subtilisin/kexin type 5 [Source:HGNC Symbol;Acc:HGNC:8747] |
| ENSG00000100012 | HGLibA_43197 | 18 | -0.969483285 | SEC14L3 | SEC14 like lipid binding 3 [Source:HGNC Symbol;Acc:HGNC:18655] |
| ENSG00000100104 | HGLibA_47041 | 18 | -0.969483285 | SRRD | SRR1 domain containing [Source:HGNC Symbol;Acc:HGNC:33910] |
| ENSG00000100321 | HGLibA_47991 | 18 | -0.969483285 | SYNGR1 | synaptogyrin 1 [Source:HGNC Symbol;Acc:HGNC:11498] |
| ENSG00000100422 | HGLibA_09193 | 18 | -0.969483285 | CERK | ceramide kinase [Source:HGNC Symbol;Acc:HGNC:19256] |
| ENSG00000100815 | HGLibA_51637 | 18 | -0.969483285 | TRIP11 | thyroid hormone receptor interactor 11 [Source:HGNC Symbol;Acc:HGNC:12305] |
| ENSG00000101134 | HGLibA_13779 | 18 | -0.969483285 | DOK5 | docking protein 5 [Source:HGNC Symbol;Acc:HGNC:16173] |
| ENSG00000101868 | HGLibA_37484 | 18 | -0.969483285 | POLA1 | catalytic subunit [Source:HGNC Symbol;Acc:HGNC:9173]" |
| ENSG00000102908 | HGLibA_31771 | 18 | -0.969483285 | NFAT5 | nuclear factor of activated T cells 5 [Source:HGNC Symbol;Acc:HGNC:7774] |
| ENSG00000104835 | HGLibA_42752 | 18 | -0.969483285 | SARS2 | mitochondrial [Source:HGNC Symbol;Acc:HGNC:17697]" |
| ENSG00000105072 | HGLibA_05664 | 18 | -0.969483285 | C19orf44 | chromosome 19 open reading frame 44 [Source:HGNC Symbol;Acc:HGNC:26141] |
| ENSG00000105701 | HGLibA_17589 | 18 | -0.969483285 | FKBP8 | FK506 binding protein 8 [Source:HGNC Symbol;Acc:HGNC:3724] |
| ENSG00000105793 | HGLibA_20601 | 18 | -0.969483285 | GTPBP10 | GTP binding protein 10 [Source:HGNC Symbol;Acc:HGNC:25106] |
| ENSG00000106089 | HGLibA_47637 | 18 | -0.969483285 | STX1A | syntaxin 1A [Source:HGNC Symbol;Acc:HGNC:11433] |
| ENSG00000106617 | HGLibA_38514 | 18 | -0.969483285 | PRKAG2 | protein kinase AMP-activated non-catalytic subunit gamma 2 [Source:HGNC Symbol;Acc:HGNC:9386] |
| ENSG00000108306 | HGLibA_16999 | 18 | -0.969483285 | FBXL20 | F-box and leucine rich repeat protein 20 [Source:HGNC Symbol;Acc:HGNC:24679] |
| ENSG00000108469 | HGLibA_40772 | 18 | -0.969483285 | RECQL5 | RecQ like helicase 5 [Source:HGNC Symbol;Acc:HGNC:9950] |
| ENSG00000108510 | HGLibA_28749 | 18 | -0.969483285 | MED13 | mediator complex subunit 13 [Source:HGNC Symbol;Acc:HGNC:22474] |
| ENSG00000109572 | HGLibA_09827 | 18 | -0.969483285 | CLCN3 | chloride voltage-gated channel 3 [Source:HGNC Symbol;Acc:HGNC:2021] |
| ENSG00000110536 | HGLibA_39426 | 18 | -0.969483285 | PTPMT1 | mitochondrial 1 [Source:HGNC Symbol;Acc:HGNC:26965]" |
| ENSG00000110958 | HGLibA_39332 | 18 | -0.969483285 | PTGES3 | prostaglandin E synthase 3 [Source:HGNC Symbol;Acc:HGNC:16049] |
| ENSG00000110975 | HGLibA_48048 | 18 | -0.969483285 | SYT10 | synaptotagmin 10 [Source:HGNC Symbol;Acc:HGNC:19266] |
| ENSG00000111348 | HGLibA_02799 | 18 | -0.969483285 | ARHGDIB | Rho GDP dissociation inhibitor beta [Source:HGNC Symbol;Acc:HGNC:679] |
| ENSG00000112110 | HGLibA_29914 | 18 | -0.969483285 | MRPL18 | mitochondrial ribosomal protein L18 [Source:HGNC Symbol;Acc:HGNC:14477] |
| ENSG00000112149 | HGLibA_08463 | 18 | -0.969483285 | CD83 | CD83 molecule [Source:HGNC Symbol;Acc:HGNC:1703] |
| ENSG00000112333 | HGLibA_32604 | 18 | -0.969483285 | NR2E1 | nuclear receptor subfamily 2 group E member 1 [Source:HGNC Symbol;Acc:HGNC:7973] |
| ENSG00000112679 | HGLibA_14163 | 18 | -0.969483285 | DUSP22 | dual specificity phosphatase 22 [Source:HGNC Symbol;Acc:HGNC:16077] |
| ENSG00000112763 | HGLibA_04963 | 18 | -0.969483285 | BTN2A1 | butyrophilin subfamily 2 member A1 [Source:HGNC Symbol;Acc:HGNC:1136] |
| ENSG00000113068 | HGLibA_36137 | 18 | -0.969483285 | PFDN1 | prefoldin subunit 1 [Source:HGNC Symbol;Acc:HGNC:8866] |
| ENSG00000114251 | HGLibA_54374 | 18 | -0.969483285 | WNT5A | Wnt family member 5A [Source:HGNC Symbol;Acc:HGNC:12784] |
| ENSG00000115239 | HGLibA_03237 | 18 | -0.969483285 | ASB3 | ankyrin repeat and SOCS box containing 3 [Source:HGNC Symbol;Acc:HGNC:16013] |
| ENSG00000115306 | HGLibA_46881 | 18 | -0.969483285 | SPTBN1 | non-erythrocytic 1 [Source:HGNC Symbol;Acc:HGNC:11275]" |
| ENSG00000115361 | HGLibA_00365 | 18 | -0.969483285 | ACADL | acyl-CoA dehydrogenase long chain [Source:HGNC Symbol;Acc:HGNC:88] |
| ENSG00000115556 | HGLibA_36996 | 18 | -0.969483285 | PLCD4 | phospholipase C delta 4 [Source:HGNC Symbol;Acc:HGNC:9062] |
| ENSG00000115607 | HGLibA_23032 | 18 | -0.969483285 | IL18RAP | interleukin 18 receptor accessory protein [Source:HGNC Symbol;Acc:HGNC:5989] |
| ENSG00000117477 | HGLibA_07723 | 18 | -0.969483285 | CCDC181 | coiled-coil domain containing 181 [Source:HGNC Symbol;Acc:HGNC:28051] |
| ENSG00000117477 | HGLibA_07725 | 18 | -0.969483285 | CCDC181 | coiled-coil domain containing 181 [Source:HGNC Symbol;Acc:HGNC:28051] |
| ENSG00000117560 | HGLibA_16892 | 18 | -0.969483285 | FASLG | Fas ligand [Source:HGNC Symbol;Acc:HGNC:11936] |
| ENSG00000117593 | HGLibA_12343 | 18 | -0.969483285 | DARS2 | mitochondrial [Source:HGNC Symbol;Acc:HGNC:25538]" |
| ENSG00000118004 | HGLibA_10635 | 18 | -0.969483285 | COLEC11 | collectin subfamily member 11 [Source:HGNC Symbol;Acc:HGNC:17213] |
| ENSG00000118046 | HGLibA_47452 | 18 | -0.969483285 | STK11 | serine/threonine kinase 11 [Source:HGNC Symbol;Acc:HGNC:11389] |
| ENSG00000120324 | HGLibA_35473 | 18 | -0.969483285 | PCDHB10 | protocadherin beta 10 [Source:HGNC Symbol;Acc:HGNC:8681] |
| ENSG00000120942 | HGLibA_52698 | 18 | -0.969483285 | UBIAD1 | UbiA prenyltransferase domain containing 1 [Source:HGNC Symbol;Acc:HGNC:30791] |
| ENSG00000121988 | HGLibA_56976 | 18 | -0.969483285 | ZRANB3 | zinc finger RANBP2-type containing 3 [Source:HGNC Symbol;Acc:HGNC:25249] |
| ENSG00000122176 | HGLibA_17718 | 18 | -0.969483285 | FMOD | fibromodulin [Source:HGNC Symbol;Acc:HGNC:3774] |
| ENSG00000122304 | HGLibA_38612 | 18 | -0.969483285 | PRM2 | protamine 2 [Source:HGNC Symbol;Acc:HGNC:9448] |
| ENSG00000123609 | HGLibA_32180 | 18 | -0.969483285 | NMI | N-myc and STAT interactor [Source:HGNC Symbol;Acc:HGNC:7854] |
| ENSG00000125731 | HGLibA_43891 | 18 | -0.969483285 | SH2D3A | SH2 domain containing 3A [Source:HGNC Symbol;Acc:HGNC:16885] |
| ENSG00000125815 | HGLibA_11467 | 18 | -0.969483285 | CST8 | cystatin 8 [Source:HGNC Symbol;Acc:HGNC:2480] |
| ENSG00000127445 | HGLibA_36684 | 18 | -0.969483285 | PIN1 | NIMA-interacting 1 [Source:HGNC Symbol;Acc:HGNC:8988]" |
| ENSG00000127804 | HGLibA_28963 | 18 | -0.969483285 | METTL16 | methyltransferase like 16 [Source:HGNC Symbol;Acc:HGNC:28484] |
| ENSG00000127870 | HGLibA_41694 | 18 | -0.969483285 | RNF6 | ring finger protein 6 [Source:HGNC Symbol;Acc:HGNC:10069] |
| ENSG00000128294 | HGLibA_51208 | 18 | -0.969483285 | TPST2 | tyrosylprotein sulfotransferase 2 [Source:HGNC Symbol;Acc:HGNC:12021] |
| ENSG00000128298 | HGLibA_04128 | 18 | -0.969483285 | BAIAP2L2 | BAI1 associated protein 2 like 2 [Source:HGNC Symbol;Acc:HGNC:26203] |
| ENSG00000128510 | HGLibA_10893 | 18 | -0.969483285 | CPA4 | carboxypeptidase A4 [Source:HGNC Symbol;Acc:HGNC:15740] |
| ENSG00000129862 | HGLibA_53597 | 18 | -0.969483285 | VCY1B | variable charge Y-linked 1B [Source:HGNC Symbol;Acc:HGNC:31751] |
| ENSG00000130475 | HGLibA_17203 | 18 | -0.969483285 | FCHO1 | FCH domain only 1 [Source:HGNC Symbol;Acc:HGNC:29002] |
| ENSG00000130775 | HGLibA_49353 | 18 | -0.969483285 | THEMIS2 | thymocyte selection associated family member 2 [Source:HGNC Symbol;Acc:HGNC:16839] |
| ENSG00000133121 | HGLibA_47354 | 18 | -0.969483285 | STARD13 | StAR related lipid transfer domain containing 13 [Source:HGNC Symbol;Acc:HGNC:19164] |
| ENSG00000133739 | HGLibA_27444 | 18 | -0.969483285 | LRRCC1 | leucine rich repeat and coiled-coil centrosomal protein 1 [Source:HGNC Symbol;Acc:HGNC:29373] |
| ENSG00000135945 | HGLibA_40885 | 18 | -0.969483285 | REV1 | DNA directed polymerase [Source:HGNC Symbol;Acc:HGNC:14060]" |
| ENSG00000136273 | HGLibA_22425 | 18 | -0.969483285 | HUS1 | HUS1 checkpoint clamp component [Source:HGNC Symbol;Acc:HGNC:5309] |
| ENSG00000136542 | HGLibA_18564 | 18 | -0.969483285 | GALNT5 | polypeptide N-acetylgalactosaminyltransferase 5 [Source:HGNC Symbol;Acc:HGNC:4127] |
| ENSG00000136930 | HGLibA_39119 | 18 | -0.969483285 | PSMB7 | proteasome subunit beta 7 [Source:HGNC Symbol;Acc:HGNC:9544] |
| ENSG00000136938 | HGLibA_02200 | 18 | -0.969483285 | ANP32B | acidic nuclear phosphoprotein 32 family member B [Source:HGNC Symbol;Acc:HGNC:16677] |
| ENSG00000137073 | HGLibA_52552 | 18 | -0.969483285 | UBAP2 | ubiquitin associated protein 2 [Source:HGNC Symbol;Acc:HGNC:14185] |
| ENSG00000137106 | HGLibA_20257 | 18 | -0.969483285 | GRHPR | glyoxylate and hydroxypyruvate reductase [Source:HGNC Symbol;Acc:HGNC:4570] |
| ENSG00000137414 | HGLibA_16789 | 18 | -0.969483285 | FAM8A1 | family with sequence similarity 8 member A1 [Source:HGNC Symbol;Acc:HGNC:16372] |
| ENSG00000137462 | HGLibA_49645 | 18 | -0.969483285 | TLR2 | toll like receptor 2 [Source:HGNC Symbol;Acc:HGNC:11848] |
| ENSG00000139053 | HGLibA_35821 | 18 | -0.969483285 | PDE6H | phosphodiesterase 6H [Source:HGNC Symbol;Acc:HGNC:8790] |
| ENSG00000139613 | HGLibA_45627 | 18 | -0.969483285 | SMARCC2 | actin dependent regulator of chromatin subfamily c member 2 [Source:HGNC Symbol;Acc:HGNC:11105]" |
| ENSG00000140350 | HGLibA_02198 | 18 | -0.969483285 | ANP32A | acidic nuclear phosphoprotein 32 family member A [Source:HGNC Symbol;Acc:HGNC:13233] |

| | | | | | |
|---|---|---|---|---|---|
| ENSG00000141665 | HGLibA_17030 | 18 | -0.969483285 | FBXO15 | F-box protein 15 [Source:HGNC Symbol;Acc:HGNC:13617] |
| ENSG00000142512 | HGLibA_44125 | 18 | -0.969483285 | SIGLEC10 | sialic acid binding Ig like lectin 10 [Source:HGNC Symbol;Acc:HGNC:15620] |
| ENSG00000142611 | HGLibA_38383 | 18 | -0.969483285 | PRDM16 | PR/SET domain 16 [Source:HGNC Symbol;Acc:HGNC:14000] |
| ENSG00000143198 | HGLibA_29181 | 18 | -0.969483285 | MGST3 | microsomal glutathione S-transferase 3 [Source:HGNC Symbol;Acc:HGNC:7064] |
| ENSG00000143590 | HGLibA_14589 | 18 | -0.969483285 | EFNA3 | ephrin A3 [Source:HGNC Symbol;Acc:HGNC:3223] |
| ENSG00000144228 | HGLibA_46767 | 18 | -0.969483285 | SPOPL | speckle type BTB/POZ protein like [Source:HGNC Symbol;Acc:HGNC:27934] |
| ENSG00000144909 | HGLibA_34646 | 18 | -0.969483285 | OSBPL11 | oxysterol binding protein like 11 [Source:HGNC Symbol;Acc:HGNC:16397] |
| ENSG00000145088 | HGLibA_14336 | 18 | -0.969483285 | EAF2 | ELL associated factor 2 [Source:HGNC Symbol;Acc:HGNC:23115] |
| ENSG00000145740 | HGLibA_44911 | 18 | -0.969483285 | SLC30A5 | solute carrier family 30 member 5 [Source:HGNC Symbol;Acc:HGNC:19089] |
| ENSG00000146433 | HGLibA_50098 | 18 | -0.969483285 | TMEM181 | transmembrane protein 181 [Source:HGNC Symbol;Acc:HGNC:20958] |
| ENSG00000147614 | HGLibA_03721 | 18 | -0.969483285 | ATP6V0D2 | ATPase H+ transporting V0 subunit d2 [Source:HGNC Symbol;Acc:HGNC:18266] |
| ENSG00000148053 | HGLibA_32878 | 18 | -0.969483285 | NTRK2 | neurotrophic receptor tyrosine kinase 2 [Source:HGNC Symbol;Acc:HGNC:8032] |
| ENSG00000148426 | HGLibA_38697 | 18 | -0.969483285 | PROSER2 | proline and serine rich 2 [Source:HGNC Symbol;Acc:HGNC:23728] |
| ENSG00000151148 | HGLibA_52680 | 18 | -0.969483285 | UBE3B | ubiquitin protein ligase E3B [Source:HGNC Symbol;Acc:HGNC:13478] |
| ENSG00000152413 | HGLibA_21865 | 18 | -0.969483285 | HOMER1 | homer scaffold protein 1 [Source:HGNC Symbol;Acc:HGNC:17512] |
| ENSG00000152591 | HGLibA_14039 | 18 | -0.969483285 | DSPP | dentin sialophosphoprotein [Source:HGNC Symbol;Acc:HGNC:3054] |
| ENSG00000152767 | HGLibA_16874 | 18 | -0.969483285 | FARP1 | ARH/RhoGEF and pleckstrin domain protein 1 [Source:HGNC Symbol;Acc:HGNC:3591]" |
| ENSG00000153060 | HGLibA_48982 | 18 | -0.969483285 | TEKT5 | tektin 5 [Source:HGNC Symbol;Acc:HGNC:26554] |
| ENSG00000153485 | HGLibA_50319 | 18 | -0.969483285 | TMEM251 | transmembrane protein 251 [Source:HGNC Symbol;Acc:HGNC:20218] |
| ENSG00000153922 | HGLibA_09383 | 18 | -0.969483285 | CHD1 | chromodomain helicase DNA binding protein 1 [Source:HGNC Symbol;Acc:HGNC:1915] |
| ENSG00000154227 | HGLibA_09207 | 18 | -0.969483285 | CERS3 | ceramide synthase 3 [Source:HGNC Symbol;Acc:HGNC:23752] |
| ENSG00000155622 | HGLibA_54482 | 18 | -0.969483285 | XAGE2 | X antigen family member 2 [Source:HGNC Symbol;Acc:HGNC:4112] |
| ENSG00000155980 | HGLibA_24948 | 18 | -0.969483285 | KIF5A | kinesin family member 5A [Source:HGNC Symbol;Acc:HGNC:6323] |
| ENSG00000156535 | HGLibA_08232 | 18 | -0.969483285 | CD109 | CD109 molecule [Source:HGNC Symbol;Acc:HGNC:21685] |
| ENSG00000156853 | HGLibA_56545 | 18 | -0.969483285 | ZNF689 | zinc finger protein 689 [Source:HGNC Symbol;Acc:HGNC:25173] |
| ENSG00000157014 | HGLibA_48443 | 18 | -0.969483285 | TATDN2 | TatD DNase domain containing 2 [Source:HGNC Symbol;Acc:HGNC:28988] |
| ENSG00000157601 | HGLibA_30604 | 18 | -0.969483285 | MX1 | MX dynamin like GTPase 1 [Source:HGNC Symbol;Acc:HGNC:7532] |
| ENSG00000158402 | HGLibA_08534 | 18 | -0.969483285 | CDC25C | cell division cycle 25C [Source:HGNC Symbol;Acc:HGNC:1727] |
| ENSG00000158411 | HGLibA_29314 | 18 | -0.969483285 | MITD1 | microtubule interacting and trafficking domain containing 1 [Source:HGNC Symbol;Acc:HGNC:25207] |
| ENSG00000159713 | HGLibA_51170 | 18 | -0.969483285 | TPPP3 | tubulin polymerization promoting protein family member 3 [Source:HGNC Symbol;Acc:HGNC:24162] |
| ENSG00000160131 | HGLibA_53687 | 18 | -0.969483285 | VMA21 | vacuolar ATPase assembly factor [Source:HGNC Symbol;Acc:HGNC:22082]" |
| ENSG00000160325 | HGLibA_06869 | 18 | -0.969483285 | CACFD1 | calcium channel flower domain containing 1 [Source:HGNC Symbol;Acc:HGNC:1365] |
| ENSG00000160917 | HGLibA_11005 | 18 | -0.969483285 | CPSF4 | cleavage and polyadenylation specific factor 4 [Source:HGNC Symbol;Acc:HGNC:2327] |
| ENSG00000160972 | HGLibA_38052 | 18 | -0.969483285 | PPP1R16A | protein phosphatase 1 regulatory subunit 16A [Source:HGNC Symbol;Acc:HGNC:14941] |
| ENSG00000162419 | HGLibA_19398 | 18 | -0.969483285 | GMEB1 | glucocorticoid modulatory element binding protein 1 [Source:HGNC Symbol;Acc:HGNC:4370] |
| ENSG00000163710 | HGLibA_35656 | 18 | -0.969483285 | PCOLCE2 | procollagen C-endopeptidase enhancer 2 [Source:HGNC Symbol;Acc:HGNC:8739] |
| ENSG00000163714 | HGLibA_52500 | 18 | -0.969483285 | U2SURP | U2 snRNP associated SURP domain containing [Source:HGNC Symbol;Acc:HGNC:30855] |
| ENSG00000163875 | HGLibA_28720 | 18 | -0.969483285 | MEAF6 | MYST/Esa1 associated factor 6 [Source:HGNC Symbol;Acc:HGNC:25674] |
| ENSG00000164463 | HGLibA_11113 | 18 | -0.969483285 | CREBRF | CREB3 regulatory factor [Source:HGNC Symbol;Acc:HGNC:24050] |
| ENSG00000164897 | HGLibA_50683 | 18 | -0.969483285 | TMUB1 | transmembrane and ubiquitin like domain containing 1 [Source:HGNC Symbol;Acc:HGNC:21709] |
| ENSG00000164919 | HGLibA_10854 | 18 | -0.969483285 | COX6C | cytochrome c oxidase subunit 6C [Source:HGNC Symbol;Acc:HGNC:2285] |
| ENSG00000165072 | HGLibA_28037 | 18 | -0.969483285 | MAMDC2 | MAM domain containing 2 [Source:HGNC Symbol;Acc:HGNC:23673] |
| ENSG00000166105 | HGLibA_19226 | 18 | -0.969483285 | GLB1L3 | galactosidase beta 1 like 3 [Source:HGNC Symbol;Acc:HGNC:25147] |
| ENSG00000166860 | HGLibA_54873 | 18 | -0.969483285 | ZBTB39 | zinc finger and BTB domain containing 39 [Source:HGNC Symbol;Acc:HGNC:29014] |
| ENSG00000168476 | HGLibA_40786 | 18 | -0.969483285 | REEP4 | receptor accessory protein 4 [Source:HGNC Symbol;Acc:HGNC:26176] |
| ENSG00000168566 | HGLibA_45957 | 18 | -0.969483285 | SNRNP48 | small nuclear ribonucleoprotein U11/U12 subunit 48 [Source:HGNC Symbol;Acc:HGNC:21368] |
| ENSG00000168781 | HGLibA_37955 | 18 | -0.969483285 | PPIP5K1 | diphosphoinositol pentakisphosphate kinase 1 [Source:HGNC Symbol;Acc:HGNC:29023] |
| ENSG00000169327 | HGLibA_34252 | 18 | -0.969483285 | OR5AU1 | olfactory receptor family 5 subfamily AU member 1 [Source:HGNC Symbol;Acc:HGNC:15362] |
| ENSG00000169379 | HGLibA_02934 | 18 | -0.969483285 | ARL13B | ADP ribosylation factor like GTPase 13B [Source:HGNC Symbol;Acc:HGNC:25419] |
| ENSG00000170832 | HGLibA_53334 | 18 | -0.969483285 | USP32 | ubiquitin specific peptidase 32 [Source:HGNC Symbol;Acc:HGNC:19143] |
| ENSG00000170906 | HGLibA_31456 | 18 | -0.969483285 | NDUFA3 | NADH:ubiquinone oxidoreductase subunit A3 [Source:HGNC Symbol;Acc:HGNC:7686] |
| ENSG00000170920 | HGLibA_34487 | 18 | -0.969483285 | OR7G3 | olfactory receptor family 7 subfamily G member 3 [Source:HGNC Symbol;Acc:HGNC:8467] |
| ENSG00000171428 | HGLibA_31163 | 18 | -0.969483285 | NAT1 | N-acetyltransferase 1 [Source:HGNC Symbol;Acc:HGNC:7645] |
| ENSG00000171858 | HGLibA_42108 | 18 | -0.969483285 | RPS21 | ribosomal protein S21 [Source:HGNC Symbol;Acc:HGNC:10409] |
| ENSG00000171873 | HGLibA_01044 | 18 | -0.969483285 | ADRA1D | adrenoceptor alpha 1D [Source:HGNC Symbol;Acc:HGNC:280] |
| ENSG00000172803 | HGLibA_46089 | 18 | -0.969483285 | SNX32 | sorting nexin 32 [Source:HGNC Symbol;Acc:HGNC:26423] |
| ENSG00000173517 | HGLibA_36005 | 18 | -0.969483285 | PEAK1 | pseudopodium enriched atypical kinase 1 [Source:HGNC Symbol;Acc:HGNC:29431] |
| ENSG00000174059 | HGLibA_08356 | 18 | -0.969483285 | CD34 | CD34 molecule [Source:HGNC Symbol;Acc:HGNC:1662] |
| ENSG00000174547 | HGLibA_29895 | 18 | -0.969483285 | MRPL11 | mitochondrial ribosomal protein L11 [Source:HGNC Symbol;Acc:HGNC:14042] |
| ENSG00000174937 | HGLibA_34337 | 18 | -0.969483285 | OR5M3 | olfactory receptor family 5 subfamily M member 3 [Source:HGNC Symbol;Acc:HGNC:14806] |
| ENSG00000174982 | HGLibA_34065 | 18 | -0.969483285 | OR4S2 | olfactory receptor family 4 subfamily S member 2 [Source:HGNC Symbol;Acc:HGNC:15183] |
| ENSG00000175595 | HGLibA_15439 | 18 | -0.969483285 | ERCC4 | endonuclease catalytic subunit [Source:HGNC Symbol;Acc:HGNC:3436]" |
| ENSG00000175857 | HGLibA_18619 | 18 | -0.969483285 | GAPT | transmembrane [Source:HGNC Symbol;Acc:HGNC:26588]" |
| ENSG00000176281 | HGLibA_34035 | 18 | -0.969483285 | OR4K5 | olfactory receptor family 4 subfamily K member 5 [Source:HGNC Symbol;Acc:HGNC:14745] |
| ENSG00000176953 | HGLibA_31779 | 18 | -0.969483285 | NFATC2IP | nuclear factor of activated T cells 2 interacting protein [Source:HGNC Symbol;Acc:HGNC:25906] |
| ENSG00000177272 | HGLibA_24131 | 18 | -0.969483285 | KCNA3 | potassium voltage-gated channel subfamily A member 3 [Source:HGNC Symbol;Acc:HGNC:6221] |
| ENSG00000177302 | HGLibA_50999 | 18 | -0.969483285 | TOP3A | DNA topoisomerase III alpha [Source:HGNC Symbol;Acc:HGNC:11992] |
| ENSG00000177425 | HGLibA_35292 | 18 | -0.969483285 | PAWR | pro-apoptotic WT1 regulator [Source:HGNC Symbol;Acc:HGNC:8614] |
| ENSG00000177842 | HGLibA_56404 | 18 | -0.969483285 | ZNF620 | zinc finger protein 620 [Source:HGNC Symbol;Acc:HGNC:28742] |
| ENSG00000178538 | HGLibA_06824 | 18 | -0.969483285 | CA8 | carbonic anhydrase 8 [Source:HGNC Symbol;Acc:HGNC:1382] |
| ENSG00000180817 | HGLibA_37805 | 18 | -0.969483285 | PPA1 | pyrophosphatase (inorganic) 1 [Source:HGNC Symbol;Acc:HGNC:9226] |
| ENSG00000181625 | HGLibA_45559 | 18 | -0.969483285 | SLX1B | structure-specific endonuclease subunit [Source:HGNC Symbol;Acc:HGNC:28748]" |
| ENSG00000181638 | HGLibA_55215 | 18 | -0.969483285 | ZFP41 | ZFP41 zinc finger protein [Source:HGNC Symbol;Acc:HGNC:26786] |
| ENSG00000182132 | HGLibA_24235 | 18 | -0.969483285 | KCNIP1 | potassium voltage-gated channel interacting protein 1 [Source:HGNC Symbol;Acc:HGNC:15521] |
| ENSG00000182583 | HGLibA_53581 | 18 | -0.969483285 | VCX | variable charge X-linked [Source:HGNC Symbol;Acc:HGNC:12667] |
| ENSG00000184056 | HGLibA_53767 | 18 | -0.969483285 | VPS33B | late endosome and lysosome associated [Source:HGNC Symbol;Acc:HGNC:12712]" |
| ENSG00000184752 | HGLibA_31447 | 18 | -0.969483285 | NDUFA12 | NADH:ubiquinone oxidoreductase subunit A12 [Source:HGNC Symbol;Acc:HGNC:23987] |
| ENSG00000185262 | HGLibA_52543 | 18 | -0.969483285 | UBALD2 | UBA like domain containing 2 [Source:HGNC Symbol;Acc:HGNC:28438] |
| ENSG00000185437 | HGLibA_43912 | 18 | -0.969483285 | SH3BGR | SH3 domain binding glutamate rich protein [Source:HGNC Symbol;Acc:HGNC:10822] |
| ENSG00000185515 | HGLibA_04767 | 18 | -0.969483285 | BRCC3 | BRCA1/BRCA2-containing complex subunit 3 [Source:HGNC Symbol;Acc:HGNC:24185] |
| ENSG00000186432 | HGLibA_25362 | 18 | -0.969483285 | KPNA4 | karyopherin subunit alpha 4 [Source:HGNC Symbol;Acc:HGNC:6397] |
| ENSG00000186523 | HGLibA_16774 | 18 | -0.969483285 | FAM86B1 | family with sequence similarity 86 member B1 [Source:HGNC Symbol;Acc:HGNC:28268] |
| ENSG00000187045 | HGLibA_50647 | 18 | -0.969483285 | TMPRSS6 | transmembrane serine protease 6 [Source:HGNC Symbol;Acc:HGNC:16517] |
| ENSG00000187123 | HGLibA_27727 | 18 | -0.969483285 | LYPD6 | LY6/PLAUR domain containing 6 [Source:HGNC Symbol;Acc:HGNC:28751] |
| ENSG00000187559 | HGLibA_17847 | 18 | -0.969483285 | FOXD4L3 | forkhead box D4 like 3 [Source:HGNC Symbol;Acc:HGNC:18523] |
| ENSG00000188909 | HGLibA_04888 | 18 | -0.969483285 | BSX | brain specific homeobox [Source:HGNC Symbol;Acc:HGNC:20450] |
| ENSG00000189067 | HGLibA_26640 | 18 | -0.969483285 | LITAF | lipopolysaccharide induced TNF factor [Source:HGNC Symbol;Acc:HGNC:16841] |
| ENSG00000196368 | HGLibA_32936 | 18 | -0.969483285 | NUDT11 | nudix hydrolase 11 [Source:HGNC Symbol;Acc:HGNC:18011] |
| ENSG00000196381 | HGLibA_56736 | 18 | -0.969483285 | ZNF781 | zinc finger protein 781 [Source:HGNC Symbol;Acc:HGNC:26745] |
| ENSG00000196539 | HGLibA_33871 | 18 | -0.969483285 | OR2T3 | olfactory receptor family 2 subfamily T member 3 [Source:HGNC Symbol;Acc:HGNC:14727] |
| ENSG00000196550 | HGLibA_16706 | 18 | -0.969483285 | FAM72A | family with sequence similarity 72 member A [Source:HGNC Symbol;Acc:HGNC:24044] |
| ENSG00000197157 | HGLibA_45919 | 18 | -0.969483285 | SND1 | staphylococcal nuclease and tudor domain containing 1 [Source:HGNC Symbol;Acc:HGNC:30646] |
| ENSG00000197168 | HGLibA_31656 | 18 | -0.969483285 | NEK5 | NIMA related kinase 5 [Source:HGNC Symbol;Acc:HGNC:7748] |
| ENSG00000197226 | HGLibA_48575 | 18 | -0.969483285 | TBC1D9B | TBC1 domain family member 9B [Source:HGNC Symbol;Acc:HGNC:29097] |
| ENSG00000197272 | HGLibA_23111 | 18 | -0.969483285 | IL27 | interleukin 27 [Source:HGNC Symbol;Acc:HGNC:19157] |
| ENSG00000197275 | HGLibA_40098 | 18 | -0.969483285 | RAD54B | RAD54 homolog B [Source:HGNC Symbol;Acc:HGNC:17228] |
| ENSG00000197430 | HGLibA_33409 | 18 | -0.969483285 | OPALIN | oligodendrocytic myelin paranodal and inner loop protein [Source:HGNC Symbol;Acc:HGNC:20707] |
| ENSG00000197467 | HGLibA_10509 | 18 | -0.969483285 | COL13A1 | collagen type XIII alpha 1 chain [Source:HGNC Symbol;Acc:HGNC:2190] |
| ENSG00000197608 | HGLibA_56853 | 18 | -0.969483285 | ZNF841 | zinc finger protein 841 [Source:HGNC Symbol;Acc:HGNC:27611] |
| ENSG00000197705 | HGLibA_25135 | 18 | -0.969483285 | KLHL14 | kelch like family member 14 [Source:HGNC Symbol;Acc:HGNC:29266] |
| ENSG00000198021 | HGLibA_46377 | 18 | -0.969483285 | SPANXA1 | family member A1 [Source:HGNC Symbol;Acc:HGNC:11218]" |
| ENSG00000198429 | HGLibA_56548 | 18 | -0.969483285 | ZNF69 | zinc finger protein 69 [Source:HGNC Symbol;Acc:HGNC:13138] |
| ENSG00000198464 | HGLibA_56070 | 18 | -0.969483285 | ZNF480 | zinc finger protein 480 [Source:HGNC Symbol;Acc:HGNC:23305] |
| ENSG00000198576 | HGLibA_02634 | 18 | -0.969483285 | ARC | activity regulated cytoskeleton associated protein [Source:HGNC Symbol;Acc:HGNC:648] |
| ENSG00000198858 | HGLibA_39778 | 18 | -0.969483285 | R3HDM4 | R3H domain containing 4 [Source:HGNC Symbol;Acc:HGNC:28270] |
| ENSG00000203747 | HGLibA_17196 | 18 | -0.969483285 | FCGR3A | Fc fragment of IgG receptor IIIa [Source:HGNC Symbol;Acc:HGNC:3619] |
| ENSG00000205143 | HGLibA_02897 | 18 | -0.969483285 | ARID3C | AT-rich interaction domain 3C [Source:HGNC Symbol;Acc:HGNC:21209] |
| ENSG00000205502 | HGLibA_06099 | 18 | -0.969483285 | C2CD4B | C2 calcium dependent domain containing 4B [Source:HGNC Symbol;Acc:HGNC:33628] |
| ENSG00000206026 | HGLibA_45750 | 18 | -0.969483285 | SMIM21 | small integral membrane protein 21 [Source:HGNC Symbol;Acc:HGNC:27598] |
| ENSG00000206047 | HGLibA_12791 | 18 | -0.969483285 | DEFA1 | defensin alpha 1 [Source:HGNC Symbol;Acc:HGNC:2761] |
| ENSG00000212721 | HGLibA_25752 | 18 | -0.969483285 | KRTAP4-11 | keratin associated protein 4-11 [Source:HGNC Symbol;Acc:HGNC:18911] |
| ENSG00000214367 | HGLibA_20880 | 18 | -0.969483285 | HAUS3 | HAUS augmin like complex subunit 3 [Source:HGNC Symbol;Acc:HGNC:28719] |
| ENSG00000214513 | HGLibA_32354 | 18 | -0.969483285 | NOTO | notochord homeobox [Source:HGNC Symbol;Acc:HGNC:31839] |
| ENSG00000214944 | HGLibA_02844 | 18 | -0.969483285 | ARHGEF28 | Rho guanine nucleotide exchange factor 28 [Source:HGNC Symbol;Acc:HGNC:30322] |
| ENSG00000227507 | HGLibA_27581 | 18 | -0.969483285 | LTB | lymphotoxin beta [Source:HGNC Symbol;Acc:HGNC:6711] |
| ENSG00000240682 | HGLibA_23695 | 18 | -0.969483285 | ISY1 | ISY1 splicing factor homolog [Source:HGNC Symbol;Acc:HGNC:29201] |
| ENSG00000243716 | HGLibA_32474 | 18 | -0.969483285 | NPIPB5 | nuclear pore complex interacting protein family member B5 [Source:HGNC Symbol;Acc:HGNC:37233] |
| ENSG00000244624 | HGLibA_25698 | 18 | -0.969483285 | KRTAP20-1 | keratin associated protein 20-1 [Source:HGNC Symbol;Acc:HGNC:18943] |
| ENSG00000253831 | HGLibA_15657 | 18 | -0.969483285 | ETV3L | ETS variant 3 like [Source:HGNC Symbol;Acc:HGNC:33834] |
| ENSG00000254709 | HGLibA_22869 | 18 | -0.969483285 | IGLL5 | immunoglobulin lambda like polypeptide 5 [Source:HGNC Symbol;Acc:HGNC:38476] |
| ENSG00000259207 | HGLibA_23781 | 18 | -0.969483285 | ITGB3 | integrin subunit beta 3 [Source:HGNC Symbol;Acc:HGNC:6156] |
| ENSG00000261308 | HGLibA_17525 | 18 | -0.969483285 | FIGNL2 | fidgetin like 2 [Source:HGNC Symbol;Acc:HGNC:13287] |
| ENSG00000271858 | HGLibA_11985 | 18 | -0.969483285 | CYB561D2 | cytochrome b561 family member D2 [Source:NCBI gene;Acc:11068] |
| ENSG00000273706 | HGLibA_26448 | 18 | -0.969483285 | LHX1 | LIM homeobox 1 [Source:HGNC Symbol;Acc:HGNC:6593] |
| FAM134C | HGLibA_16128 | 18 | -0.969483285 | FAM134C | - |
| FAM92A1 | HGLibA_16797 | 18 | -0.969483285 | FAM92A1 | - |
| GPR126 | HGLibA_19883 | 18 | -0.969483285 | GPR126 | - |
| GUCY1A3 | HGLibA_20656 | 18 | -0.969483285 | GUCY1A3 | - |
| hsa-mir-1244-2 | HGLibA_57365 | 18 | -0.969483285 | hsa-mir-1244-2 | - |
| hsa-mir-1244-3 | HGLibA_57369 | 18 | -0.969483285 | hsa-mir-1244-3 | - |
| hsa-mir-1255a | HGLibA_57423 | 18 | -0.969483285 | hsa-mir-1255a | - |
| hsa-mir-1255b-1 | HGLibA_57424 | 18 | -0.969483285 | hsa-mir-1255b-1 | - |
| hsa-mir-1273g | HGLibA_57549 | 18 | -0.969483285 | hsa-mir-1273g | - |
| hsa-mir-1293 | HGLibA_57647 | 18 | -0.969483285 | hsa-mir-1293 | - |
| hsa-mir-135a-1 | HGLibA_57794 | 18 | -0.969483285 | hsa-mir-135a-1 | - |

| | | | | | |
|---|---|---|---|---|---|
| hsa-mir-16-2 | HGLibA_57949 | 18 | -0.969483285 | hsa-mir-16-2 | - |
| hsa-mir-181c | HGLibA_57974 | 18 | -0.969483285 | hsa-mir-181c | - |
| hsa-mir-208a | HGLibA_58195 | 18 | -0.969483285 | hsa-mir-208a | - |
| hsa-mir-2116 | HGLibA_58238 | 18 | -0.969483285 | hsa-mir-2116 | - |
| hsa-mir-24-1 | HGLibA_58339 | 18 | -0.969483285 | hsa-mir-24-1 | - |
| hsa-mir-302a | HGLibA_58437 | 18 | -0.969483285 | hsa-mir-302a | - |
| hsa-mir-3158-1 | HGLibA_58703 | 18 | -0.969483285 | hsa-mir-3158-1 | - |
| hsa-mir-3177 | HGLibA_58780 | 18 | -0.969483285 | hsa-mir-3177 | - |
| hsa-mir-320d-2 | HGLibA_58946 | 18 | -0.969483285 | hsa-mir-320d-2 | - |
| hsa-mir-3529 | HGLibA_59049 | 18 | -0.969483285 | hsa-mir-3529 | - |
| hsa-mir-3622a | HGLibA_59120 | 18 | -0.969483285 | hsa-mir-3622a | - |
| hsa-mir-3670-1 | HGLibA_59233 | 18 | -0.969483285 | hsa-mir-3670-1 | - |
| hsa-mir-4421 | HGLibA_60072 | 18 | -0.969483285 | hsa-mir-4421 | - |
| hsa-mir-4429 | HGLibA_60103 | 18 | -0.969483285 | hsa-mir-4429 | - |
| hsa-mir-4429 | HGLibA_60104 | 18 | -0.969483285 | hsa-mir-4429 | - |
| hsa-mir-4431 | HGLibA_60112 | 18 | -0.969483285 | hsa-mir-4431 | - |
| hsa-mir-4437 | HGLibA_60151 | 18 | -0.969483285 | hsa-mir-4437 | - |
| hsa-mir-4445 | HGLibA_60186 | 18 | -0.969483285 | hsa-mir-4445 | - |
| hsa-mir-4477b | HGLibA_60322 | 18 | -0.969483285 | hsa-mir-4477b | - |
| hsa-mir-4517 | HGLibA_60513 | 18 | -0.969483285 | hsa-mir-4517 | - |
| hsa-mir-4521 | HGLibA_60541 | 18 | -0.969483285 | hsa-mir-4521 | - |
| hsa-mir-4660 | HGLibA_60751 | 18 | -0.969483285 | hsa-mir-4660 | - |
| hsa-mir-516a-1 | HGLibA_61608 | 18 | -0.969483285 | hsa-mir-516a-1 | - |
| hsa-mir-542 | HGLibA_61818 | 18 | -0.969483285 | hsa-mir-542 | - |
| hsa-mir-548a-1 | HGLibA_61835 | 18 | -0.969483285 | hsa-mir-548a-1 | - |
| hsa-mir-624 | HGLibA_62737 | 18 | -0.969483285 | hsa-mir-624 | - |
| hsa-mir-642a | HGLibA_62808 | 18 | -0.969483285 | hsa-mir-642a | - |
| hsa-mir-6512 | HGLibA_62921 | 18 | -0.969483285 | hsa-mir-6512 | - |
| hsa-mir-6721 | HGLibA_63047 | 18 | -0.969483285 | hsa-mir-6721 | - |
| hsa-mir-6726 | HGLibA_63058 | 18 | -0.969483285 | hsa-mir-6726 | - |
| hsa-mir-6834 | HGLibA_63513 | 18 | -0.969483285 | hsa-mir-6834 | - |
| hsa-mir-6870 | HGLibA_63664 | 18 | -0.969483285 | hsa-mir-6870 | - |
| hsa-mir-8061 | HGLibA_64084 | 18 | -0.969483285 | hsa-mir-8061 | - |
| hsa-mir-8071-2 | HGLibA_64125 | 18 | -0.969483285 | hsa-mir-8071-2 | - |
| hsa-mir-892b | HGLibA_64255 | 18 | -0.969483285 | hsa-mir-892b | - |
| KIAA1841 | HGLibA_24823 | 18 | -0.969483285 | KIAA1841 | - |
| LOC100287399 | HGLibA_26822 | 18 | -0.969483285 | LOC100287399 | - |
| LOC100996331 | HGLibA_26882 | 18 | -0.969483285 | LOC100996331 | - |
| LPPR2 | HGLibA_27117 | 18 | -0.969483285 | LPPR2 | - |
| METTL21D | HGLibA_28984 | 18 | -0.969483285 | METTL21D | - |
| NOTCH2NL | HGLibA_32347 | 18 | -0.969483285 | NOTCH2NL | - |
| PRMT10 | HGLibA_38621 | 18 | -0.969483285 | PRMT10 | - |
| SLMO1 | HGLibA_45537 | 18 | -0.969483285 | SLMO1 | - |
| TMEM56 | HGLibA_50451 | 18 | -0.969483285 | TMEM56 | - |
| WDR66 | HGLibA_54153 | 18 | -0.969483285 | WDR66 | - |
| ADRBK1 | HGLibA_01063 | 17 | -0.979434323 | ADRBK1 | - |
| ANKRD20A2 | HGLibA_02030 | 17 | -0.979434323 | ANKRD20A2 | - |
| APOA1BP | HGLibA_02453 | 17 | -0.979434323 | APOA1BP | - |
| ATP5G2 | HGLibA_03658 | 17 | -0.979434323 | ATP5G2 | - |
| C16orf3 | HGLibA_05445 | 17 | -0.979434323 | C16orf3 | - |
| C9orf47 | HGLibA_06737 | 17 | -0.979434323 | C9orf47 | - |
| CASC1 | HGLibA_07268 | 17 | -0.979434323 | CASC1 | - |
| ENSG00000029559 | HGLibA_22483 | 17 | -0.979434323 | IBSP | integrin binding sialoprotein [Source:HGNC Symbol;Acc:HGNC:5341] |
| ENSG00000033867 | HGLibA_45225 | 17 | -0.979434323 | SLC4A7 | solute carrier family 4 member 7 [Source:HGNC Symbol;Acc:HGNC:11033] |
| ENSG00000046774 | HGLibA_27962 | 17 | -0.979434323 | MAGEC2 | MAGE family member C2 [Source:HGNC Symbol;Acc:HGNC:13574] |
| ENSG00000055332 | HGLibA_14725 | 17 | -0.979434323 | EIF2AK2 | eukaryotic translation initiation factor 2 alpha kinase 2 [Source:HGNC Symbol;Acc:HGNC:9437] |
| ENSG00000065371 | HGLibA_41759 | 17 | -0.979434323 | ROPN1 | rhophilin associated tail protein 1 [Source:HGNC Symbol;Acc:HGNC:17692] |
| ENSG00000067177 | HGLibA_36401 | 17 | -0.979434323 | PHKA1 | phosphorylase kinase regulatory subunit alpha 1 [Source:HGNC Symbol;Acc:HGNC:8925] |
| ENSG00000068971 | HGLibA_38174 | 17 | -0.979434323 | PPP2R5B | protein phosphatase 2 regulatory subunit B'beta [Source:HGNC Symbol;Acc:HGNC:9310] |
| ENSG00000070601 | HGLibA_18032 | 17 | -0.979434323 | FRMPD1 | FERM and PDZ domain containing 1 [Source:HGNC Symbol;Acc:HGNC:29159] |
| ENSG00000072518 | HGLibA_28367 | 17 | -0.979434323 | MARK2 | microtubule affinity regulating kinase 2 [Source:HGNC Symbol;Acc:HGNC:3332] |
| ENSG00000072682 | HGLibA_34904 | 17 | -0.979434323 | P4HA2 | prolyl 4-hydroxylase subunit alpha 2 [Source:HGNC Symbol;Acc:HGNC:8547] |
| ENSG00000076351 | HGLibA_45183 | 17 | -0.979434323 | SLC46A1 | solute carrier family 46 member 1 [Source:HGNC Symbol;Acc:HGNC:30521] |
| ENSG00000082556 | HGLibA_33446 | 17 | -0.979434323 | OPRK1 | opioid receptor kappa 1 [Source:HGNC Symbol;Acc:HGNC:8154] |
| ENSG00000085552 | HGLibA_22904 | 17 | -0.979434323 | IGSF9 | immunoglobulin superfamily member 9 [Source:HGNC Symbol;Acc:HGNC:18132] |
| ENSG00000086015 | HGLibA_28403 | 17 | -0.979434323 | MAST2 | microtubule associated serine/threonine kinase 2 [Source:HGNC Symbol;Acc:HGNC:19035] |
| ENSG00000087258 | HGLibA_19464 | 17 | -0.979434323 | GNAO1 | G protein subunit alpha o1 [Source:HGNC Symbol;Acc:HGNC:4389] |
| ENSG00000088881 | HGLibA_14358 | 17 | -0.979434323 | EBF4 | EBF family member 4 [Source:HGNC Symbol;Acc:HGNC:29278] |
| ENSG00000089195 | HGLibA_51694 | 17 | -0.979434323 | TRMT6 | tRNA methyltransferase 6 [Source:HGNC Symbol;Acc:HGNC:20900] |
| ENSG00000089486 | HGLibA_08712 | 17 | -0.979434323 | CDIP1 | cell death inducing p53 target 1 [Source:HGNC Symbol;Acc:HGNC:13234] |
| ENSG00000090520 | HGLibA_13525 | 17 | -0.979434323 | DNAJB11 | DnaJ heat shock protein family (Hsp40) member B11 [Source:HGNC Symbol;Acc:HGNC:14889] |
| ENSG00000091129 | HGLibA_32662 | 17 | -0.979434323 | NRCAM | neuronal cell adhesion molecule [Source:HGNC Symbol;Acc:HGNC:7994] |
| ENSG00000091482 | HGLibA_45822 | 17 | -0.979434323 | SMPX | small muscle protein X-linked [Source:HGNC Symbol;Acc:HGNC:11122] |
| ENSG00000091831 | HGLibA_15581 | 17 | -0.979434323 | ESR1 | estrogen receptor 1 [Source:HGNC Symbol;Acc:HGNC:3467] |
| ENSG00000099139 | HGLibA_35680 | 17 | -0.979434323 | PCSK5 | proprotein convertase subtilisin/kexin type 5 [Source:HGNC Symbol;Acc:HGNC:8747] |
| ENSG00000100599 | HGLibA_41295 | 17 | -0.979434323 | RIN3 | Ras and Rab interactor 3 [Source:HGNC Symbol;Acc:HGNC:18751] |
| ENSG00000100814 | HGLibA_08061 | 17 | -0.979434323 | CCNB1IP1 | cyclin B1 interacting protein 1 [Source:HGNC Symbol;Acc:HGNC:19437] |
| ENSG00000101019 | HGLibA_53098 | 17 | -0.979434323 | UQCC1 | ubiquinol-cytochrome c reductase complex assembly factor 1 [Source:HGNC Symbol;Acc:HGNC:15891] |
| ENSG00000101138 | HGLibA_11482 | 17 | -0.979434323 | CSTF1 | cleavage stimulation factor subunit 1 [Source:HGNC Symbol;Acc:HGNC:2483] |
| ENSG00000101558 | HGLibA_53517 | 17 | -0.979434323 | VAPA | VAMP associated protein A [Source:HGNC Symbol;Acc:HGNC:12648] |
| ENSG00000102081 | HGLibA_17721 | 17 | -0.979434323 | FMR1 | fragile X mental retardation 1 [Source:HGNC Symbol;Acc:HGNC:3775] |
| ENSG00000102271 | HGLibA_25204 | 17 | -0.979434323 | KLHL4 | kelch like family member 4 [Source:HGNC Symbol;Acc:HGNC:6355] |
| ENSG00000103111 | HGLibA_29642 | 17 | -0.979434323 | MON1B | secretory trafficking associated [Source:HGNC Symbol;Acc:HGNC:25020]" |
| ENSG00000104611 | HGLibA_43898 | 17 | -0.979434323 | SH2D4A | SH2 domain containing 4A [Source:HGNC Symbol;Acc:HGNC:26102] |
| ENSG00000104808 | HGLibA_13099 | 17 | -0.979434323 | DHDH | dihydrodiol dehydrogenase [Source:HGNC Symbol;Acc:HGNC:17887] |
| ENSG00000105146 | HGLibA_03881 | 17 | -0.979434323 | AURKC | aurora kinase C [Source:HGNC Symbol;Acc:HGNC:11391] |
| ENSG00000105467 | HGLibA_48001 | 17 | -0.979434323 | SYNGR4 | synaptogyrin 4 [Source:HGNC Symbol;Acc:HGNC:11502] |
| ENSG00000105655 | HGLibA_23700 | 17 | -0.979434323 | ISYNA1 | inositol-3-phosphate synthase 1 [Source:HGNC Symbol;Acc:HGNC:29821] |
| ENSG00000107021 | HGLibA_48477 | 17 | -0.979434323 | TBC1D13 | TBC1 domain family member 13 [Source:HGNC Symbol;Acc:HGNC:25571] |
| ENSG00000107859 | HGLibA_36767 | 17 | -0.979434323 | PITX3 | paired like homeodomain 3 [Source:HGNC Symbol;Acc:HGNC:9006] |
| ENSG00000107897 | HGLibA_00403 | 17 | -0.979434323 | ACBD5 | acyl-CoA binding domain containing 5 [Source:HGNC Symbol;Acc:HGNC:23338] |
| ENSG00000108187 | HGLibA_35335 | 17 | -0.979434323 | PBLD | phenazine biosynthesis like protein domain containing [Source:HGNC Symbol;Acc:HGNC:23301] |
| ENSG00000108406 | HGLibA_13198 | 17 | -0.979434323 | DHX40 | DEAH-box helicase 40 [Source:HGNC Symbol;Acc:HGNC:18018] |
| ENSG00000108651 | HGLibA_53454 | 17 | -0.979434323 | UTP6 | small subunit processome component [Source:HGNC Symbol;Acc:HGNC:18279]" |
| ENSG00000109332 | HGLibA_52587 | 17 | -0.979434323 | UBE2D3 | ubiquitin conjugating enzyme E2 D3 [Source:HGNC Symbol;Acc:HGNC:12476] |
| ENSG00000109452 | HGLibA_23340 | 17 | -0.979434323 | INPP4B | inositol polyphosphate-4-phosphatase type II B [Source:HGNC Symbol;Acc:HGNC:6075] |
| ENSG00000109736 | HGLibA_29083 | 17 | -0.979434323 | MFSD10 | major facilitator superfamily domain containing 10 [Source:HGNC Symbol;Acc:HGNC:16894] |
| ENSG00000110841 | HGLibA_37886 | 17 | -0.979434323 | PPFIBP1 | PPFIA binding protein 1 [Source:HGNC Symbol;Acc:HGNC:9249] |
| ENSG00000112893 | HGLibA_28069 | 17 | -0.979434323 | MAN2A1 | mannosidase alpha class 2A member 1 [Source:HGNC Symbol;Acc:HGNC:6824] |
| ENSG00000113356 | HGLibA_37628 | 17 | -0.979434323 | POLR3G | RNA polymerase III subunit G [Source:HGNC Symbol;Acc:HGNC:30075] |
| ENSG00000113621 | HGLibA_52412 | 17 | -0.979434323 | TXNDC15 | thioredoxin domain containing 15 [Source:HGNC Symbol;Acc:HGNC:20652] |
| ENSG00000114125 | HGLibA_41697 | 17 | -0.979434323 | RNF7 | ring finger protein 7 [Source:HGNC Symbol;Acc:HGNC:10070] |
| ENSG00000114993 | HGLibA_42388 | 17 | -0.979434323 | RTKN | rhotekin [Source:HGNC Symbol;Acc:HGNC:10466] |
| ENSG00000115289 | HGLibA_35591 | 17 | -0.979434323 | PCGF1 | polycomb group ring finger 1 [Source:HGNC Symbol;Acc:HGNC:17615] |
| ENSG00000115486 | HGLibA_18996 | 17 | -0.979434323 | GGCX | gamma-glutamyl carboxylase [Source:HGNC Symbol;Acc:HGNC:4247] |
| ENSG00000116138 | HGLibA_13583 | 17 | -0.979434323 | DNAJC16 | DnaJ heat shock protein family (Hsp40) member C16 [Source:HGNC Symbol;Acc:HGNC:29157] |
| ENSG00000118513 | HGLibA_30636 | 17 | -0.979434323 | MYB | transcription factor [Source:HGNC Symbol;Acc:HGNC:7545]" |
| ENSG00000118707 | HGLibA_49240 | 17 | -0.979434323 | TGIF2 | TGFB induced factor homeobox 2 [Source:HGNC Symbol;Acc:HGNC:15764] |
| ENSG00000118855 | HGLibA_29081 | 17 | -0.979434323 | MFSD1 | major facilitator superfamily domain containing 1 [Source:HGNC Symbol;Acc:HGNC:25874] |
| ENSG00000119655 | HGLibA_32424 | 17 | -0.979434323 | NPC2 | NPC intracellular cholesterol transporter 2 [Source:HGNC Symbol;Acc:HGNC:14537] |
| ENSG00000119669 | HGLibA_23583 | 17 | -0.979434323 | IRF2BPL | interferon regulatory factor 2 binding protein like [Source:HGNC Symbol;Acc:HGNC:14282] |
| ENSG00000119862 | HGLibA_26390 | 17 | -0.979434323 | LGALSL | galectin like [Source:HGNC Symbol;Acc:HGNC:25012] |
| ENSG00000119953 | HGLibA_45789 | 17 | -0.979434323 | SMNDC1 | survival motor neuron domain containing 1 [Source:HGNC Symbol;Acc:HGNC:16900] |
| ENSG00000120075 | HGLibA_21942 | 17 | -0.979434323 | HOXB5 | homeobox B5 [Source:HGNC Symbol;Acc:HGNC:5116] |
| ENSG00000120341 | HGLibA_43212 | 17 | -0.979434323 | SEC16B | endoplasmic reticulum export factor [Source:HGNC Symbol;Acc:HGNC:30301]" |
| ENSG00000120437 | HGLibA_00394 | 17 | -0.979434323 | ACAT2 | acetyl-CoA acetyltransferase 2 [Source:HGNC Symbol;Acc:HGNC:94] |
| ENSG00000121022 | HGLibA_10723 | 17 | -0.979434323 | COPS5 | COP9 signalosome subunit 5 [Source:HGNC Symbol;Acc:HGNC:2240] |
| ENSG00000121316 | HGLibA_36971 | 17 | -0.979434323 | PLBD1 | phospholipase B domain containing 1 [Source:HGNC Symbol;Acc:HGNC:26215] |
| ENSG00000122420 | HGLibA_39341 | 17 | -0.979434323 | PTGFR | prostaglandin F receptor [Source:HGNC Symbol;Acc:HGNC:9600] |
| ENSG00000122674 | HGLibA_08222 | 17 | -0.979434323 | CCZ1 | vacuolar protein trafficking and biogenesis associated [Source:HGNC Symbol;Acc:HGNC:21691]" |
| ENSG00000122870 | HGLibA_04491 | 17 | -0.979434323 | BICC1 | BicC family RNA binding protein 1 [Source:HGNC Symbol;Acc:HGNC:19351] |
| ENSG00000123094 | HGLibA_40394 | 17 | -0.979434323 | RASSF8 | Ras association domain family member 8 [Source:HGNC Symbol;Acc:HGNC:13232] |
| ENSG00000123349 | HGLibA_36148 | 17 | -0.979434323 | PFDN5 | prefoldin subunit 5 [Source:HGNC Symbol;Acc:HGNC:8869] |
| ENSG00000124657 | HGLibA_33774 | 17 | -0.979434323 | OR2B6 | olfactory receptor family 2 subfamily B member 6 [Source:HGNC Symbol;Acc:HGNC:8241] |
| ENSG00000124787 | HGLibA_42041 | 17 | -0.979434323 | RPP40 | ribonuclease P/MRP subunit p40 [Source:HGNC Symbol;Acc:HGNC:20992] |
| ENSG00000125246 | HGLibA_10174 | 17 | -0.979434323 | CLYBL | citrate lyase beta like [Source:HGNC Symbol;Acc:HGNC:18355] |
| ENSG00000125733 | HGLibA_51633 | 17 | -0.979434323 | TRIP10 | thyroid hormone receptor interactor 10 [Source:HGNC Symbol;Acc:HGNC:12304] |
| ENSG00000125835 | HGLibA_45968 | 17 | -0.979434323 | SNRPB | small nuclear ribonucleoprotein polypeptides B and B1 [Source:HGNC Symbol;Acc:HGNC:11153] |
| ENSG00000126214 | HGLibA_25027 | 17 | -0.979434323 | KLC1 | kinesin light chain 1 [Source:HGNC Symbol;Acc:HGNC:6387] |
| ENSG00000126787 | HGLibA_13351 | 17 | -0.979434323 | DLGAP5 | DLG associated protein 5 [Source:HGNC Symbol;Acc:HGNC:16864] |
| ENSG00000126970 | HGLibA_55001 | 17 | -0.979434323 | ZC4H2 | zinc finger C4H2-type containing [Source:HGNC Symbol;Acc:HGNC:24931] |
| ENSG00000127220 | HGLibA_00287 | 17 | -0.979434323 | ABHD8 | abhydrolase domain containing 8 [Source:HGNC Symbol;Acc:HGNC:23759] |
| ENSG00000128849 | HGLibA_09323 | 17 | -0.979434323 | CGNL1 | cingulin like 1 [Source:HGNC Symbol;Acc:HGNC:25931] |
| ENSG00000129159 | HGLibA_24161 | 17 | -0.979434323 | KCNC1 | potassium voltage-gated channel subfamily C member 1 [Source:HGNC Symbol;Acc:HGNC:6233] |
| ENSG00000129691 | HGLibA_03296 | 17 | -0.979434323 | ASH2L | histone lysine methyltransferase complex subunit [Source:HGNC Symbol;Acc:HGNC:744]" |

| | | | | | |
|---|---|---|---|---|---|
| ENSG00000130045 | HGLibA_33148 | 17 | -0.979434323 | NXNL2 | nucleoredoxin like 2 [Source:HGNC Symbol;Acc:HGNC:30482] |
| ENSG00000130175 | HGLibA_38560 | 17 | -0.979434323 | PRKCSH | protein kinase C substrate 80K-H [Source:HGNC Symbol;Acc:HGNC:9411] |
| ENSG00000130226 | HGLibA_13874 | 17 | -0.979434323 | DPP6 | dipeptidyl peptidase like 6 [Source:HGNC Symbol;Acc:HGNC:3010] |
| ENSG00000130783 | HGLibA_07821 | 17 | -0.979434323 | CCDC62 | coiled-coil domain containing 62 [Source:HGNC Symbol;Acc:HGNC:30723] |
| ENSG00000131059 | HGLibA_04716 | 17 | -0.979434323 | BPIFA3 | BPI fold containing family A member 3 [Source:HGNC Symbol;Acc:HGNC:16204] |
| ENSG00000131979 | HGLibA_18783 | 17 | -0.979434323 | GCH1 | GTP cyclohydrolase 1 [Source:HGNC Symbol;Acc:HGNC:4193] |
| ENSG00000132437 | HGLibA_12600 | 17 | -0.979434323 | DDC | dopa decarboxylase [Source:HGNC Symbol;Acc:HGNC:2719] |
| ENSG00000133026 | HGLibA_30706 | 17 | -0.979434323 | MYH10 | myosin heavy chain 10 [Source:HGNC Symbol;Acc:HGNC:7568] |
| ENSG00000133627 | HGLibA_00663 | 17 | -0.979434323 | ACTR3B | ARP3 actin related protein 3 homolog B [Source:HGNC Symbol;Acc:HGNC:17256] |
| ENSG00000134516 | HGLibA_13741 | 17 | -0.979434323 | DOCK2 | dedicator of cytokinesis 2 [Source:HGNC Symbol;Acc:HGNC:2988] |
| ENSG00000134759 | HGLibA_14979 | 17 | -0.979434323 | ELP2 | elongator acetyltransferase complex subunit 2 [Source:HGNC Symbol;Acc:HGNC:18248] |
| ENSG00000135063 | HGLibA_16355 | 17 | -0.979434323 | FAM189A2 | family with sequence similarity 189 member A2 [Source:HGNC Symbol;Acc:HGNC:24820] |
| ENSG00000135517 | HGLibA_29291 | 17 | -0.979434323 | MIP | major intrinsic protein of lens fiber [Source:HGNC Symbol;Acc:HGNC:7103] |
| ENSG00000136826 | HGLibA_25072 | 17 | -0.979434323 | KLF4 | Kruppel like factor 4 [Source:HGNC Symbol;Acc:HGNC:6348] |
| ENSG00000136940 | HGLibA_35747 | 17 | -0.979434323 | PDCL | phosducin like [Source:HGNC Symbol;Acc:HGNC:8770] |
| ENSG00000137656 | HGLibA_04997 | 17 | -0.979434323 | BUD13 | BUD13 homolog [Source:HGNC Symbol;Acc:HGNC:28199] |
| ENSG00000137819 | HGLibA_35163 | 17 | -0.979434323 | PAQR5 | progestin and adipoQ receptor family member 5 [Source:HGNC Symbol;Acc:HGNC:29645] |
| ENSG00000138413 | HGLibA_22538 | 17 | -0.979434323 | IDH1 | cytosolic [Source:HGNC Symbol;Acc:HGNC:5382]" |
| ENSG00000138459 | HGLibA_44959 | 17 | -0.979434323 | SLC35A5 | solute carrier family 35 member A5 [Source:HGNC Symbol;Acc:HGNC:20792] |
| ENSG00000138764 | HGLibA_08095 | 17 | -0.979434323 | CCNG2 | cyclin G2 [Source:HGNC Symbol;Acc:HGNC:1593] |
| ENSG00000139372 | HGLibA_48882 | 17 | -0.979434323 | TDG | thymine DNA glycosylase [Source:HGNC Symbol;Acc:HGNC:11700] |
| ENSG00000141570 | HGLibA_07465 | 17 | -0.979434323 | CBX8 | chromobox 8 [Source:HGNC Symbol;Acc:HGNC:15962] |
| ENSG00000142453 | HGLibA_07254 | 17 | -0.979434323 | CARM1 | coactivator associated arginine methyltransferase 1 [Source:HGNC Symbol;Acc:HGNC:23393] |
| ENSG00000143641 | HGLibA_18555 | 17 | -0.979434323 | GALNT2 | polypeptide N-acetylgalactosaminyltransferase 2 [Source:HGNC Symbol;Acc:HGNC:4124] |
| ENSG00000143669 | HGLibA_27777 | 17 | -0.979434323 | LYST | lysosomal trafficking regulator [Source:HGNC Symbol;Acc:HGNC:1968] |
| ENSG00000145708 | HGLibA_11139 | 17 | -0.979434323 | CRHBP | corticotropin releasing hormone binding protein [Source:HGNC Symbol;Acc:HGNC:2356] |
| ENSG00000145817 | HGLibA_54682 | 17 | -0.979434323 | YIPF5 | Yip1 domain family member 5 [Source:HGNC Symbol;Acc:HGNC:24877] |
| ENSG00000145949 | HGLibA_30793 | 17 | -0.979434323 | MYLK4 | myosin light chain kinase family member 4 [Source:HGNC Symbol;Acc:HGNC:27972] |
| ENSG00000146221 | HGLibA_48857 | 17 | -0.979434323 | TCTE1 | t-complex-associated-testis-expressed 1 [Source:HGNC Symbol;Acc:HGNC:11693] |
| ENSG00000146250 | HGLibA_38929 | 17 | -0.979434323 | PRSS35 | serine protease 35 [Source:HGNC Symbol;Acc:HGNC:21387] |
| ENSG00000146587 | HGLibA_40419 | 17 | -0.979434323 | RBAK | RB associated KRAB zinc finger [Source:HGNC Symbol;Acc:HGNC:17680] |
| ENSG00000146904 | HGLibA_15283 | 17 | -0.979434323 | EPHA1 | EPH receptor A1 [Source:HGNC Symbol;Acc:HGNC:3385] |
| ENSG00000148688 | HGLibA_42036 | 17 | -0.979434323 | RPP30 | ribonuclease P/MRP subunit p30 [Source:HGNC Symbol;Acc:HGNC:17688] |
| ENSG00000149091 | HGLibA_13085 | 17 | -0.979434323 | DGKZ | diacylglycerol kinase zeta [Source:HGNC Symbol;Acc:HGNC:2857] |
| ENSG00000149260 | HGLibA_07170 | 17 | -0.979434323 | CAPN5 | calpain 5 [Source:HGNC Symbol;Acc:HGNC:1482] |
| ENSG00000149534 | HGLibA_30145 | 17 | -0.979434323 | MS4A2 | membrane spanning 4-domains A2 [Source:HGNC Symbol;Acc:HGNC:7316] |
| ENSG00000149923 | HGLibA_38201 | 17 | -0.979434323 | PPP4C | protein phosphatase 4 catalytic subunit [Source:HGNC Symbol;Acc:HGNC:9319] |
| ENSG00000150760 | HGLibA_13732 | 17 | -0.979434323 | DOCK1 | dedicator of cytokinesis 1 [Source:HGNC Symbol;Acc:HGNC:2987] |
| ENSG00000151151 | HGLibA_23457 | 17 | -0.979434323 | IPMK | inositol polyphosphate multikinase [Source:HGNC Symbol;Acc:HGNC:20739] |
| ENSG00000152804 | HGLibA_21257 | 17 | -0.979434323 | HHEX | hematopoietically expressed homeobox [Source:HGNC Symbol;Acc:HGNC:4901] |
| ENSG00000153029 | HGLibA_29805 | 17 | -0.979434323 | MR1 | class I-related [Source:HGNC Symbol;Acc:HGNC:4975]" |
| ENSG00000153561 | HGLibA_41381 | 17 | -0.979434323 | RMND5A | required for meiotic nuclear division 5 homolog A [Source:HGNC Symbol;Acc:HGNC:25850] |
| ENSG00000156671 | HGLibA_42694 | 17 | -0.979434323 | SAMD8 | sterile alpha motif domain containing 8 [Source:HGNC Symbol;Acc:HGNC:26320] |
| ENSG00000157617 | HGLibA_06086 | 17 | -0.979434323 | C2CD2 | C2 calcium dependent domain containing 2 [Source:HGNC Symbol;Acc:HGNC:1266] |
| ENSG00000157764 | HGLibA_04751 | 17 | -0.979434323 | BRAF | serine/threonine kinase [Source:HGNC Symbol;Acc:HGNC:1097]" |
| ENSG00000158006 | HGLibA_34998 | 17 | -0.979434323 | PAFAH2 | platelet activating factor acetylhydrolase 2 [Source:HGNC Symbol;Acc:HGNC:8579] |
| ENSG00000158089 | HGLibA_18542 | 17 | -0.979434323 | GALNT14 | polypeptide N-acetylgalactosaminyltransferase 14 [Source:HGNC Symbol;Acc:HGNC:22946] |
| ENSG00000159251 | HGLibA_00607 | 17 | -0.979434323 | ACTC1 | cardiac muscle 1 [Source:HGNC Symbol;Acc:HGNC:143]" |
| ENSG00000159267 | HGLibA_21650 | 17 | -0.979434323 | HLCS | holocarboxylase synthetase [Source:HGNC Symbol;Acc:HGNC:4976] |
| ENSG00000159479 | HGLibA_28817 | 17 | -0.979434323 | MED8 | mediator complex subunit 8 [Source:HGNC Symbol;Acc:HGNC:19971] |
| ENSG00000160883 | HGLibA_21586 | 17 | -0.979434323 | HK3 | hexokinase 3 [Source:HGNC Symbol;Acc:HGNC:4925] |
| ENSG00000160959 | HGLibA_27254 | 17 | -0.979434323 | LRRC14 | leucine rich repeat containing 14 [Source:HGNC Symbol;Acc:HGNC:20419] |
| ENSG00000161298 | HGLibA_55889 | 17 | -0.979434323 | ZNF382 | zinc finger protein 382 [Source:HGNC Symbol;Acc:HGNC:17409] |
| ENSG00000161677 | HGLibA_23976 | 17 | -0.979434323 | JOSD2 | Josephin domain containing 2 [Source:HGNC Symbol;Acc:HGNC:28853] |
| ENSG00000162004 | HGLibA_07872 | 17 | -0.979434323 | CCDC78 | coiled-coil domain containing 78 [Source:HGNC Symbol;Acc:HGNC:14153] |
| ENSG00000162367 | HGLibA_48278 | 17 | -0.979434323 | TAL1 | erythroid differentiation factor [Source:HGNC Symbol;Acc:HGNC:11556]" |
| ENSG00000162654 | HGLibA_18738 | 17 | -0.979434323 | GBP4 | guanylate binding protein 4 [Source:HGNC Symbol;Acc:HGNC:20480] |
| ENSG00000162819 | HGLibA_04835 | 17 | -0.979434323 | BROX | BRO1 domain and CAAX motif containing [Source:HGNC Symbol;Acc:HGNC:26512] |
| ENSG00000163156 | HGLibA_43010 | 17 | -0.979434323 | SCNM1 | sodium channel modifier 1 [Source:HGNC Symbol;Acc:HGNC:23136] |
| ENSG00000163378 | HGLibA_15225 | 17 | -0.979434323 | EOGT | EGF domain specific O-linked N-acetylglucosamine transferase [Source:HGNC Symbol;Acc:HGNC:28526] |
| ENSG00000163428 | HGLibA_27398 | 17 | -0.979434323 | LRRC58 | leucine rich repeat containing 58 [Source:HGNC Symbol;Acc:HGNC:26968] |
| ENSG00000163605 | HGLibA_38207 | 17 | -0.979434323 | PPP4R2 | protein phosphatase 4 regulatory subunit 2 [Source:HGNC Symbol;Acc:HGNC:18296] |
| ENSG00000164241 | HGLibA_06419 | 17 | -0.979434323 | C5orf63 | chromosome 5 open reading frame 63 [Source:HGNC Symbol;Acc:HGNC:40051] |
| ENSG00000164318 | HGLibA_14632 | 17 | -0.979434323 | EGFLAM | fibronectin type III and laminin G domains [Source:HGNC Symbol;Acc:HGNC:26810]" |
| ENSG00000164438 | HGLibA_49680 | 17 | -0.979434323 | TLX3 | T cell leukemia homeobox 3 [Source:HGNC Symbol;Acc:HGNC:13532] |
| ENSG00000164850 | HGLibA_19795 | 17 | -0.979434323 | GPER1 | G protein-coupled estrogen receptor 1 [Source:HGNC Symbol;Acc:HGNC:4485] |
| ENSG00000165115 | HGLibA_24922 | 17 | -0.979434323 | KIF27 | kinesin family member 27 [Source:HGNC Symbol;Acc:HGNC:18632] |
| ENSG00000165478 | HGLibA_21139 | 17 | -0.979434323 | HEPACAM | hepatic and glial cell adhesion molecule [Source:HGNC Symbol;Acc:HGNC:26361] |
| ENSG00000165548 | HGLibA_50479 | 17 | -0.979434323 | TMEM63C | transmembrane protein 63C [Source:HGNC Symbol;Acc:HGNC:23787] |
| ENSG00000165762 | HGLibA_34031 | 17 | -0.979434323 | OR4K2 | olfactory receptor family 4 subfamily K member 2 [Source:HGNC Symbol;Acc:HGNC:14728] |
| ENSG00000165795 | HGLibA_31415 | 17 | -0.979434323 | NDRG2 | NDRG family member 2 [Source:HGNC Symbol;Acc:HGNC:14460] |
| ENSG00000166228 | HGLibA_35362 | 17 | -0.979434323 | PCBD1 | pterin-4 alpha-carbinolamine dehydratase 1 [Source:HGNC Symbol;Acc:HGNC:8646] |
| ENSG00000166886 | HGLibA_31022 | 17 | -0.979434323 | NAB2 | NGFI-A binding protein 2 [Source:HGNC Symbol;Acc:HGNC:7627] |
| ENSG00000166887 | HGLibA_53789 | 17 | -0.979434323 | VPS39 | HOPS complex subunit [Source:HGNC Symbol;Acc:HGNC:20593]" |
| ENSG00000167114 | HGLibA_44823 | 17 | -0.979434323 | SLC27A4 | solute carrier family 27 member 4 [Source:HGNC Symbol;Acc:HGNC:10998] |
| ENSG00000167118 | HGLibA_53150 | 17 | -0.979434323 | URM1 | ubiquitin related modifier 1 [Source:HGNC Symbol;Acc:HGNC:28378] |
| ENSG00000167394 | HGLibA_56495 | 17 | -0.979434323 | ZNF668 | zinc finger protein 668 [Source:HGNC Symbol;Acc:HGNC:25821] |
| ENSG00000167553 | HGLibA_52250 | 17 | -0.979434323 | TUBA1C | tubulin alpha 1c [Source:HGNC Symbol;Acc:HGNC:20768] |
| ENSG00000167680 | HGLibA_43366 | 17 | -0.979434323 | SEMA6B | semaphorin 6B [Source:HGNC Symbol;Acc:HGNC:10739] |
| ENSG00000168569 | HGLibA_50244 | 17 | -0.979434323 | TMEM223 | transmembrane protein 223 [Source:HGNC Symbol;Acc:HGNC:28464] |
| ENSG00000169554 | HGLibA_55138 | 17 | -0.979434323 | ZEB2 | zinc finger E-box binding homeobox 2 [Source:HGNC Symbol;Acc:HGNC:14881] |
| ENSG00000169609 | HGLibA_05395 | 17 | -0.979434323 | C15orf40 | chromosome 15 open reading frame 40 [Source:HGNC Symbol;Acc:HGNC:28443] |
| ENSG00000169760 | HGLibA_32068 | 17 | -0.979434323 | NLGN1 | neuroligin 1 [Source:HGNC Symbol;Acc:HGNC:14291] |
| ENSG00000169900 | HGLibA_39681 | 17 | -0.979434323 | PYDC1 | pyrin domain containing 1 [Source:HGNC Symbol;Acc:HGNC:30261] |
| ENSG00000170322 | HGLibA_31839 | 17 | -0.979434323 | NFRKB | nuclear factor related to kappaB binding protein [Source:HGNC Symbol;Acc:HGNC:7802] |
| ENSG00000171481 | HGLibA_33689 | 17 | -0.979434323 | OR1L3 | olfactory receptor family 1 subfamily L member 3 [Source:HGNC Symbol;Acc:HGNC:8215] |
| ENSG00000171824 | HGLibA_15773 | 17 | -0.979434323 | EXOSC10 | exosome component 10 [Source:HGNC Symbol;Acc:HGNC:9138] |
| ENSG00000172058 | HGLibA_43470 | 17 | -0.979434323 | SERF1A | small EDRK-rich factor 1A [Source:HGNC Symbol;Acc:HGNC:10755] |
| ENSG00000172199 | HGLibA_34556 | 17 | -0.979434323 | OR8U1 | olfactory receptor family 8 subfamily U member 1 [Source:HGNC Symbol;Acc:HGNC:19611] |
| ENSG00000172809 | HGLibA_41970 | 17 | -0.979434323 | RPL38 | ribosomal protein L38 [Source:HGNC Symbol;Acc:HGNC:10349] |
| ENSG00000173933 | HGLibA_40546 | 17 | -0.979434323 | RBM4 | RNA binding motif protein 4 [Source:HGNC Symbol;Acc:HGNC:9901] |
| ENSG00000174928 | HGLibA_06229 | 17 | -0.979434323 | C3orf33 | chromosome 3 open reading frame 33 [Source:HGNC Symbol;Acc:HGNC:26434] |
| ENSG00000175105 | HGLibA_56468 | 17 | -0.979434323 | ZNF654 | zinc finger protein 654 [Source:HGNC Symbol;Acc:HGNC:25612] |
| ENSG00000175329 | HGLibA_23690 | 17 | -0.979434323 | ISX | intestine specific homeobox [Source:HGNC Symbol;Acc:HGNC:28084] |
| ENSG00000176422 | HGLibA_46854 | 17 | -0.979434323 | SPRYD4 | SPRY domain containing 4 [Source:HGNC Symbol;Acc:HGNC:27468] |
| ENSG00000177045 | HGLibA_44256 | 17 | -0.979434323 | SIX5 | SIX homeobox 5 [Source:HGNC Symbol;Acc:HGNC:10891] |
| ENSG00000177380 | HGLibA_37879 | 17 | -0.979434323 | PPFIA3 | PTPRF interacting protein alpha 3 [Source:HGNC Symbol;Acc:HGNC:9247] |
| ENSG00000178175 | HGLibA_55879 | 17 | -0.979434323 | ZNF366 | zinc finger protein 366 [Source:HGNC Symbol;Acc:HGNC:18316] |
| ENSG00000181038 | HGLibA_28991 | 17 | -0.979434323 | METTL23 | methyltransferase like 23 [Source:HGNC Symbol;Acc:HGNC:26988] |
| ENSG00000181619 | HGLibA_19894 | 17 | -0.979434323 | GPR135 | G protein-coupled receptor 135 [Source:HGNC Symbol;Acc:HGNC:19991] |
| ENSG00000181754 | HGLibA_01794 | 17 | -0.979434323 | AMIGO1 | adhesion molecule with Ig like domain 1 [Source:HGNC Symbol;Acc:HGNC:20824] |
| ENSG00000182508 | HGLibA_26429 | 17 | -0.979434323 | LHFPL1 | LHFPL tetraspan subfamily member 1 [Source:HGNC Symbol;Acc:HGNC:6587] |
| ENSG00000183695 | HGLibA_29848 | 17 | -0.979434323 | MRGPRX2 | MAS related GPR family member X2 [Source:HGNC Symbol;Acc:HGNC:17983] |
| ENSG00000183741 | HGLibA_07461 | 17 | -0.979434323 | CBX6 | chromobox 6 [Source:HGNC Symbol;Acc:HGNC:1556] |
| ENSG00000184363 | HGLibA_36857 | 17 | -0.979434323 | PKP3 | plakophilin 3 [Source:HGNC Symbol;Acc:HGNC:9025] |
| ENSG00000184709 | HGLibA_27292 | 17 | -0.979434323 | LRRC26 | leucine rich repeat containing 26 [Source:HGNC Symbol;Acc:HGNC:31409] |
| ENSG00000184724 | HGLibA_25815 | 17 | -0.979434323 | KRTAP6-1 | keratin associated protein 6-1 [Source:HGNC Symbol;Acc:HGNC:18931] |
| ENSG00000184925 | HGLibA_26171 | 17 | -0.979434323 | LCN12 | lipocalin 12 [Source:HGNC Symbol;Acc:HGNC:28733] |
| ENSG00000184967 | HGLibA_32240 | 17 | -0.979434323 | NOC4L | nucleolar complex associated 4 homolog [Source:HGNC Symbol;Acc:HGNC:28461] |
| ENSG00000184999 | HGLibA_44537 | 17 | -0.979434323 | SLC22A10 | solute carrier family 22 member 10 [Source:HGNC Symbol;Acc:HGNC:18057] |
| ENSG00000185594 | HGLibA_46530 | 17 | -0.979434323 | SPATA8 | spermatogenesis associated 8 [Source:HGNC Symbol;Acc:HGNC:28676] |
| ENSG00000185594 | HGLibA_46531 | 17 | -0.979434323 | SPATA8 | spermatogenesis associated 8 [Source:HGNC Symbol;Acc:HGNC:28676] |
| ENSG00000185608 | HGLibA_29978 | 17 | -0.979434323 | MRPL40 | mitochondrial ribosomal protein L40 [Source:HGNC Symbol;Acc:HGNC:14491] |
| ENSG00000185615 | HGLibA_35875 | 17 | -0.979434323 | PDIA2 | protein disulfide isomerase family A member 2 [Source:HGNC Symbol;Acc:HGNC:14180] |
| ENSG00000186188 | HGLibA_17334 | 17 | -0.979434323 | FFAR4 | free fatty acid receptor 4 [Source:HGNC Symbol;Acc:HGNC:19061] |
| ENSG00000186188 | HGLibA_17332 | 17 | -0.979434323 | FFAR4 | free fatty acid receptor 4 [Source:HGNC Symbol;Acc:HGNC:19061] |
| ENSG00000188001 | HGLibA_51180 | 17 | -0.979434323 | TPRG1 | tumor protein p63 regulated 1 [Source:HGNC Symbol;Acc:HGNC:24759] |
| ENSG00000188124 | HGLibA_33751 | 17 | -0.979434323 | OR2AG2 | olfactory receptor family 2 subfamily AG member 2 [Source:HGNC Symbol;Acc:HGNC:15143] |
| ENSG00000188517 | HGLibA_10550 | 17 | -0.979434323 | COL25A1 | collagen type XXV alpha 1 chain [Source:HGNC Symbol;Acc:HGNC:18603] |
| ENSG00000188612 | HGLibA_47780 | 17 | -0.979434323 | SUMO2 | small ubiquitin-like modifier 2 [Source:HGNC Symbol;Acc:HGNC:11125] |
| ENSG00000189129 | HGLibA_36947 | 17 | -0.979434323 | PLAC9 | placenta specific 9 [Source:HGNC Symbol;Acc:HGNC:19255] |
| ENSG00000189227 | HGLibA_05429 | 17 | -0.979434323 | C15orf61 | chromosome 15 open reading frame 61 [Source:HGNC Symbol;Acc:HGNC:34453] |
| ENSG00000196126 | HGLibA_21635 | 17 | -0.979434323 | HLA-DRB1 | DR beta 1 [Source:HGNC Symbol;Acc:HGNC:4948]" |
| ENSG00000196459 | HGLibA_51311 | 17 | -0.979434323 | TRAPPC2 | trafficking protein particle complex 2 [Source:HGNC Symbol;Acc:HGNC:23068] |
| ENSG00000196591 | HGLibA_21005 | 17 | -0.979434323 | HDAC2 | histone deacetylase 2 [Source:HGNC Symbol;Acc:HGNC:4853] |
| ENSG00000196666 | HGLibA_16315 | 17 | -0.979434323 | FAM180B | family with sequence similarity 180 member B [Source:HGNC Symbol;Acc:HGNC:34451] |
| ENSG00000196954 | HGLibA_07312 | 17 | -0.979434323 | CASP4 | caspase 4 [Source:HGNC Symbol;Acc:HGNC:1505] |
| ENSG00000197302 | HGLibA_56625 | 17 | -0.979434323 | ZNF720 | zinc finger protein 720 [Source:HGNC Symbol;Acc:HGNC:26987] |
| ENSG00000197579 | HGLibA_51012 | 17 | -0.979434323 | TOPORS | TOP1 binding arginine/serine rich protein [Source:HGNC Symbol;Acc:HGNC:21653] |
| ENSG00000198001 | HGLibA_23564 | 17 | -0.979434323 | IRAK4 | interleukin 1 receptor associated kinase 4 [Source:HGNC Symbol;Acc:HGNC:17967] |
| ENSG00000198093 | HGLibA_56459 | 17 | -0.979434323 | ZNF649 | zinc finger protein 649 [Source:HGNC Symbol;Acc:HGNC:25741] |
| ENSG00000198176 | HGLibA_49150 | 17 | -0.979434323 | TFDP1 | transcription factor Dp-1 [Source:HGNC Symbol;Acc:HGNC:11749] |
| ENSG00000198753 | HGLibA_37277 | 17 | -0.979434323 | PLXNB3 | plexin B3 [Source:HGNC Symbol;Acc:HGNC:9105] |
| ENSG00000203837 | HGLibA_37364 | 17 | -0.979434323 | PNLIPRP3 | pancreatic lipase related protein 3 [Source:HGNC Symbol;Acc:HGNC:23492] |
| ENSG00000203910 | HGLibA_05773 | 17 | -0.979434323 | C1orf146 | chromosome 1 open reading frame 146 [Source:HGNC Symbol;Acc:HGNC:24032] |

| | | | | | |
|---|---|---|---|---|---|
| ENSG00000204227 | HGLibA_41297 | 17 | -0.979434323 | RING1 | ring finger protein 1 [Source:HGNC Symbol;Acc:HGNC:10018] |
| ENSG00000204610 | HGLibA_51415 | 17 | -0.979434323 | TRIM15 | tripartite motif containing 15 [Source:HGNC Symbol;Acc:HGNC:16284] |
| ENSG00000204839 | HGLibA_29874 | 17 | -0.979434323 | MROH6 | maestro heat like repeat family member 6 [Source:HGNC Symbol;Acc:HGNC:27814] |
| ENSG00000205730 | HGLibA_23867 | 17 | -0.979434323 | ITPRIPL2 | ITPRIP like 2 [Source:HGNC Symbol;Acc:HGNC:27257] |
| ENSG00000214021 | HGLibA_52202 | 17 | -0.979434323 | TTLL3 | tubulin tyrosine ligase like 3 [Source:HGNC Symbol;Acc:HGNC:24483] |
| ENSG00000215126 | HGLibA_07443 | 17 | -0.979434323 | CBWD6 | COBW domain containing 6 [Source:HGNC Symbol;Acc:HGNC:31978] |
| ENSG00000215440 | HGLibA_32430 | 17 | -0.979434323 | NPEPL1 | aminopeptidase like 1 [Source:HGNC Symbol;Acc:HGNC:16244] |
| ENSG00000221813 | HGLibA_34372 | 17 | -0.979434323 | OR6B1 | olfactory receptor family 6 subfamily B member 1 [Source:HGNC Symbol;Acc:HGNC:8354] |
| ENSG00000221994 | HGLibA_56433 | 17 | -0.979434323 | ZNF630 | zinc finger protein 630 [Source:HGNC Symbol;Acc:HGNC:28855] |
| ENSG00000231396 | HGLibA_53216 | 17 | -0.979434323 | USP17L10 | ubiquitin specific peptidase 17-like family member 10 [Source:HGNC Symbol;Acc:HGNC:44438] |
| ENSG00000234545 | HGLibA_16121 | 17 | -0.979434323 | FAM133B | family with sequence similarity 133 member B [Source:HGNC Symbol;Acc:HGNC:28629] |
| ENSG00000236125 | HGLibA_53278 | 17 | -0.979434323 | USP17L4 | ubiquitin specific peptidase 17-like family member 4 [Source:HGNC Symbol;Acc:HGNC:37176] |
| ENSG00000240247 | HGLibA_12796 | 17 | -0.979434323 | DEFA1B | defensin alpha 1B [Source:HGNC Symbol;Acc:HGNC:33596] |
| ENSG00000241697 | HGLibA_49831 | 17 | -0.979434323 | TMEFF1 | transmembrane protein with EGF like and two follistatin like domains 1 [Source:HGNC Symbol;Acc:HGNC:11866] |
| ENSG00000243955 | HGLibA_20452 | 17 | -0.979434323 | GSTA1 | glutathione S-transferase alpha 1 [Source:HGNC Symbol;Acc:HGNC:4626] |
| ENSG00000244687 | HGLibA_52666 | 17 | -0.979434323 | UBE2V1 | ubiquitin conjugating enzyme E2 V1 [Source:HGNC Symbol;Acc:HGNC:12494] |
| ENSG00000253368 | HGLibA_51709 | 17 | -0.979434323 | TRNP1 | TMF1-regulated nuclear protein 1 [Source:HGNC Symbol;Acc:HGNC:34348] |
| ENSG00000258102 | HGLibA_28128 | 17 | -0.979434323 | MAP1LC3B2 | microtubule associated protein 1 light chain 3 beta 2 [Source:HGNC Symbol;Acc:HGNC:34390] |
| ENSG00000269058 | HGLibA_07048 | 17 | -0.979434323 | CALR3 | calreticulin 3 [Source:HGNC Symbol;Acc:HGNC:20407] |
| ENSG00000272333 | HGLibA_25323 | 17 | -0.979434323 | KMT2B | lysine methyltransferase 2B [Source:HGNC Symbol;Acc:HGNC:15840] |
| ENSG00000272395 | HGLibA_22729 | 17 | -0.979434323 | IFNL4 | interferon lambda 4 (gene/pseudogene) [Source:HGNC Symbol;Acc:HGNC:44480] |
| ENSG00000274286 | HGLibA_01049 | 17 | -0.979434323 | ADRA2B | adrenoceptor alpha 2B [Source:HGNC Symbol;Acc:HGNC:282] |
| ENSG00000279116 | HGLibA_34510 | 17 | -0.979434323 | OR8D2 | olfactory receptor family 8 subfamily D member 2 (gene/pseudogene) [Source:HGNC Symbol;Acc:HGNC:8482] |
| FAM198B | HGLibA_16389 | 17 | -0.979434323 | FAM198B | - |
| FLJ44635 | HGLibA_17634 | 17 | -0.979434323 | FLJ44635 | - |
| FOXD4L2 | HGLibA_17843 | 17 | -0.979434323 | FOXD4L2 | - |
| HIST1H2BB | HGLibA_21415 | 17 | -0.979434323 | HIST1H2BB | - |
| hsa-mir-1193 | HGLibA_57249 | 17 | -0.979434323 | hsa-mir-1193 | - |
| hsa-mir-1290 | HGLibA_57636 | 17 | -0.979434323 | hsa-mir-1290 | - |
| hsa-mir-151a | HGLibA_57897 | 17 | -0.979434323 | hsa-mir-151a | - |
| hsa-mir-200a | HGLibA_58149 | 17 | -0.979434323 | hsa-mir-200a | - |
| hsa-mir-211 | HGLibA_58216 | 17 | -0.979434323 | hsa-mir-211 | - |
| hsa-mir-3658 | HGLibA_59178 | 17 | -0.979434323 | hsa-mir-3658 | - |
| hsa-mir-3937 | HGLibA_59607 | 17 | -0.979434323 | hsa-mir-3937 | - |
| hsa-mir-4266 | HGLibA_59773 | 17 | -0.979434323 | hsa-mir-4266 | - |
| hsa-mir-4421 | HGLibA_60073 | 17 | -0.979434323 | hsa-mir-4421 | - |
| hsa-mir-4424 | HGLibA_60084 | 17 | -0.979434323 | hsa-mir-4424 | - |
| hsa-mir-4449 | HGLibA_60203 | 17 | -0.979434323 | hsa-mir-4449 | - |
| hsa-mir-4497 | HGLibA_60403 | 17 | -0.979434323 | hsa-mir-4497 | - |
| hsa-mir-4692 | HGLibA_60878 | 17 | -0.979434323 | hsa-mir-4692 | - |
| hsa-mir-4769 | HGLibA_61173 | 17 | -0.979434323 | hsa-mir-4769 | - |
| hsa-mir-4789 | HGLibA_61262 | 17 | -0.979434323 | hsa-mir-4789 | - |
| hsa-mir-4802 | HGLibA_61314 | 17 | -0.979434323 | hsa-mir-4802 | - |
| hsa-mir-485 | HGLibA_61333 | 17 | -0.979434323 | hsa-mir-485 | - |
| hsa-mir-517b | HGLibA_61628 | 17 | -0.979434323 | hsa-mir-517b | - |
| hsa-mir-518f | HGLibA_61676 | 17 | -0.979434323 | hsa-mir-518f | - |
| hsa-mir-520c | HGLibA_61741 | 17 | -0.979434323 | hsa-mir-520c | - |
| hsa-mir-548aw | HGLibA_61934 | 17 | -0.979434323 | hsa-mir-548aw | - |
| hsa-mir-5582 | HGLibA_62167 | 17 | -0.979434323 | hsa-mir-5582 | - |
| hsa-mir-563 | HGLibA_62217 | 17 | -0.979434323 | hsa-mir-563 | - |
| hsa-mir-5689 | HGLibA_62272 | 17 | -0.979434323 | hsa-mir-5689 | - |
| hsa-mir-5692c-2 | HGLibA_62303 | 17 | -0.979434323 | hsa-mir-5692c-2 | - |
| hsa-mir-6086 | HGLibA_62603 | 17 | -0.979434323 | hsa-mir-6086 | - |
| hsa-mir-635 | HGLibA_62780 | 17 | -0.979434323 | hsa-mir-635 | - |
| hsa-mir-6719 | HGLibA_63038 | 17 | -0.979434323 | hsa-mir-6719 | - |
| hsa-mir-6734 | HGLibA_63089 | 17 | -0.979434323 | hsa-mir-6734 | - |
| hsa-mir-6801 | HGLibA_63380 | 17 | -0.979434323 | hsa-mir-6801 | - |
| hsa-mir-6806 | HGLibA_63400 | 17 | -0.979434323 | hsa-mir-6806 | - |
| hsa-mir-6819 | HGLibA_63455 | 17 | -0.979434323 | hsa-mir-6819 | - |
| hsa-mir-6859-3 | HGLibA_63616 | 17 | -0.979434323 | hsa-mir-6859-3 | - |
| hsa-mir-759 | HGLibA_63897 | 17 | -0.979434323 | hsa-mir-759 | - |
| KIAA0368 | HGLibA_24628 | 17 | -0.979434323 | KIAA0368 | - |
| KIAA1704 | HGLibA_24805 | 17 | -0.979434323 | KIAA1704 | - |
| LPPR5 | HGLibA_27124 | 17 | -0.979434323 | LPPR5 | - |
| MICALCL | HGLibA_29210 | 17 | -0.979434323 | MICALCL | - |
| MTL5 | HGLibA_30403 | 17 | -0.979434323 | MTL5 | - |
| NOV | HGLibA_32362 | 17 | -0.979434323 | NOV | - |
| RSG1 | HGLibA_42315 | 17 | -0.979434323 | RSG1 | - |
| SARS | HGLibA_42750 | 17 | -0.979434323 | SARS | - |
| TXNRD3NB | HGLibA_52454 | 17 | -0.979434323 | TXNRD3NB | - |
| BAGE4 | HGLibA_04100 | 16 | -0.98938536 | BAGE4 | - |
| C10orf131 | HGLibA_05056 | 16 | -0.98938536 | C10orf131 | - |
| C15orf52 | HGLibA_05408 | 16 | -0.98938536 | C15orf52 | - |
| C16orf3 | HGLibA_05444 | 16 | -0.98938536 | C16orf3 | - |
| C3orf67 | HGLibA_06260 | 16 | -0.98938536 | C3orf67 | - |
| C9orf114 | HGLibA_06662 | 16 | -0.98938536 | C9orf114 | - |
| CXorf23 | HGLibA_11908 | 16 | -0.98938536 | CXorf23 | - |
| DSCR6 | HGLibA_14013 | 16 | -0.98938536 | DSCR6 | - |
| ENSG00000005020 | HGLibA_44272 | 16 | -0.98938536 | SKAP2 | src kinase associated phosphoprotein 2 [Source:HGNC Symbol;Acc:HGNC:15687] |
| ENSG00000010810 | HGLibA_18255 | 16 | -0.98938536 | FYN | Src family tyrosine kinase [Source:HGNC Symbol;Acc:HGNC:4037]" |
| ENSG00000039068 | HGLibA_08628 | 16 | -0.98938536 | CDH1 | cadherin 1 [Source:HGNC Symbol;Acc:HGNC:1748] |
| ENSG00000047056 | HGLibA_54078 | 16 | -0.98938536 | WDR37 | WD repeat domain 37 [Source:HGNC Symbol;Acc:HGNC:31406] |
| ENSG00000049246 | HGLibA_36060 | 16 | -0.98938536 | PER3 | period circadian regulator 3 [Source:HGNC Symbol;Acc:HGNC:8847] |
| ENSG00000058085 | HGLibA_25971 | 16 | -0.98938536 | LAMC2 | laminin subunit gamma 2 [Source:HGNC Symbol;Acc:HGNC:6493] |
| ENSG00000074266 | HGLibA_14487 | 16 | -0.98938536 | EED | embryonic ectoderm development [Source:HGNC Symbol;Acc:HGNC:3188] |
| ENSG00000079156 | HGLibA_34662 | 16 | -0.98938536 | OSBPL6 | oxysterol binding protein like 6 [Source:HGNC Symbol;Acc:HGNC:16388] |
| ENSG00000079974 | HGLibA_40029 | 16 | -0.98938536 | RABL2B | member of RAS oncogene family like 2B [Source:HGNC Symbol;Acc:HGNC:9800]" |
| ENSG00000081803 | HGLibA_06980 | 16 | -0.98938536 | CADPS2 | calcium dependent secretion activator 2 [Source:HGNC Symbol;Acc:HGNC:16018] |
| ENSG00000088543 | HGLibA_06215 | 16 | -0.98938536 | C3orf18 | chromosome 3 open reading frame 18 [Source:HGNC Symbol;Acc:HGNC:24837] |
| ENSG00000092009 | HGLibA_10175 | 16 | -0.98938536 | CMA1 | chymase 1 [Source:HGNC Symbol;Acc:HGNC:2097] |
| ENSG00000092820 | HGLibA_15838 | 16 | -0.98938536 | EZR | ezrin [Source:HGNC Symbol;Acc:HGNC:12691] |
| ENSG00000099139 | HGLibA_35679 | 16 | -0.98938536 | PCSK5 | proprotein convertase subtilisin/kexin type 5 [Source:HGNC Symbol;Acc:HGNC:8747] |
| ENSG00000099338 | HGLibA_07374 | 16 | -0.98938536 | CATSPERG | cation channel sperm associated auxiliary subunit gamma [Source:HGNC Symbol;Acc:HGNC:25243] |
| ENSG00000100228 | HGLibA_39891 | 16 | -0.98938536 | RAB36 | member RAS oncogene family [Source:HGNC Symbol;Acc:HGNC:9775]" |
| ENSG00000100403 | HGLibA_54984 | 16 | -0.98938536 | ZC3H7B | zinc finger CCCH-type containing 7B [Source:HGNC Symbol;Acc:HGNC:30869] |
| ENSG00000100983 | HGLibA_20448 | 16 | -0.98938536 | GSS | glutathione synthetase [Source:HGNC Symbol;Acc:HGNC:4624] |
| ENSG00000100994 | HGLibA_39686 | 16 | -0.98938536 | PYGB | glycogen phosphorylase B [Source:HGNC Symbol;Acc:HGNC:9723] |
| ENSG00000101346 | HGLibA_37468 | 16 | -0.98938536 | POFUT1 | protein O-fucosyltransferase 1 [Source:HGNC Symbol;Acc:HGNC:14988] |
| ENSG00000101446 | HGLibA_46719 | 16 | -0.98938536 | SPINT3 | Kunitz type 3 [Source:HGNC Symbol;Acc:HGNC:11248]" |
| ENSG00000102078 | HGLibA_44648 | 16 | -0.98938536 | SLC25A14 | solute carrier family 25 member 14 [Source:HGNC Symbol;Acc:HGNC:10984] |
| ENSG00000102309 | HGLibA_36686 | 16 | -0.98938536 | PIN4 | NIMA-interacting 4 [Source:HGNC Symbol;Acc:HGNC:8992]" |
| ENSG00000102854 | HGLibA_30219 | 16 | -0.98938536 | MSLN | mesothelin [Source:HGNC Symbol;Acc:HGNC:7371] |
| ENSG00000104883 | HGLibA_36090 | 16 | -0.98938536 | PEX11G | peroxisomal biogenesis factor 11 gamma [Source:HGNC Symbol;Acc:HGNC:20208] |
| ENSG00000105696 | HGLibA_50461 | 16 | -0.98938536 | TMEM59L | transmembrane protein 59 like [Source:HGNC Symbol;Acc:HGNC:13237] |
| ENSG00000107014 | HGLibA_41355 | 16 | -0.98938536 | RLN2 | relaxin 2 [Source:HGNC Symbol;Acc:HGNC:10027] |
| ENSG00000107614 | HGLibA_51346 | 16 | -0.98938536 | TRDMT1 | tRNA aspartic acid methyltransferase 1 [Source:HGNC Symbol;Acc:HGNC:2977] |
| ENSG00000107779 | HGLibA_04628 | 16 | -0.98938536 | BMPR1A | bone morphogenetic protein receptor type 1A [Source:HGNC Symbol;Acc:HGNC:1076] |
| ENSG00000107789 | HGLibA_29282 | 16 | -0.98938536 | MINPP1 | multiple inositol-polyphosphate phosphatase 1 [Source:HGNC Symbol;Acc:HGNC:7102] |
| ENSG00000108771 | HGLibA_13204 | 16 | -0.98938536 | DHX58 | DExH-box helicase 58 [Source:HGNC Symbol;Acc:HGNC:29517] |
| ENSG00000109089 | HGLibA_08869 | 16 | -0.98938536 | CDR2L | cerebellar degeneration related protein 2 like [Source:HGNC Symbol;Acc:HGNC:29999] |
| ENSG00000109111 | HGLibA_47822 | 16 | -0.98938536 | SUPT6H | histone chaperone [Source:HGNC Symbol;Acc:HGNC:11470]" |
| ENSG00000109738 | HGLibA_19309 | 16 | -0.98938536 | GLRB | glycine receptor beta [Source:HGNC Symbol;Acc:HGNC:4329] |
| ENSG00000110871 | HGLibA_10757 | 16 | -0.98938536 | COQ5 | methyltransferase [Source:HGNC Symbol;Acc:HGNC:28722]" |
| ENSG00000111713 | HGLibA_20713 | 16 | -0.98938536 | GYS2 | glycogen synthase 2 [Source:HGNC Symbol;Acc:HGNC:4707] |
| ENSG00000112357 | HGLibA_36126 | 16 | -0.98938536 | PEX7 | peroxisomal biogenesis factor 7 [Source:HGNC Symbol;Acc:HGNC:8860] |
| ENSG00000112499 | HGLibA_44568 | 16 | -0.98938536 | SLC22A2 | solute carrier family 22 member 2 [Source:HGNC Symbol;Acc:HGNC:10966] |
| ENSG00000112759 | HGLibA_44839 | 16 | -0.98938536 | SLC29A1 | solute carrier family 29 member 1 (Augustine blood group) [Source:HGNC Symbol;Acc:HGNC:11003] |
| ENSG00000114349 | HGLibA_19472 | 16 | -0.98938536 | GNAT1 | G protein subunit alpha transducin 1 [Source:HGNC Symbol;Acc:HGNC:4393] |
| ENSG00000115137 | HGLibA_13608 | 16 | -0.98938536 | DNAJC27 | DnaJ heat shock protein family (Hsp40) member C27 [Source:HGNC Symbol;Acc:HGNC:30290] |
| ENSG00000115350 | HGLibA_37518 | 16 | -0.98938536 | POLE4 | accessory subunit [Source:HGNC Symbol;Acc:HGNC:18755]" |
| ENSG00000116016 | HGLibA_15237 | 16 | -0.98938536 | EPAS1 | endothelial PAS domain protein 1 [Source:HGNC Symbol;Acc:HGNC:3374] |
| ENSG00000116251 | HGLibA_41898 | 16 | -0.98938536 | RPL22 | ribosomal protein L22 [Source:HGNC Symbol;Acc:HGNC:10315] |
| ENSG00000119969 | HGLibA_21114 | 16 | -0.98938536 | HELLS | lymphoid specific [Source:HGNC Symbol;Acc:HGNC:4861]" |
| ENSG00000119969 | HGLibA_21115 | 16 | -0.98938536 | HELLS | lymphoid specific [Source:HGNC Symbol;Acc:HGNC:4861]" |
| ENSG00000120334 | HGLibA_09069 | 16 | -0.98938536 | CENPL | centromere protein L [Source:HGNC Symbol;Acc:HGNC:17879] |
| ENSG00000120533 | HGLibA_15222 | 16 | -0.98938536 | ENY2 | transcription and export complex 2 subunit [Source:HGNC Symbol;Acc:HGNC:24449]" |
| ENSG00000121417 | HGLibA_55585 | 16 | -0.98938536 | ZNF211 | zinc finger protein 211 [Source:HGNC Symbol;Acc:HGNC:13003] |
| ENSG00000121957 | HGLibA_20159 | 16 | -0.98938536 | GPSM2 | G protein signaling modulator 2 [Source:HGNC Symbol;Acc:HGNC:29501] |
| ENSG00000122025 | HGLibA_17666 | 16 | -0.98938536 | FLT3 | fms related tyrosine kinase 3 [Source:HGNC Symbol;Acc:HGNC:3765] |
| ENSG00000122224 | HGLibA_27688 | 16 | -0.98938536 | LY9 | lymphocyte antigen 9 [Source:HGNC Symbol;Acc:HGNC:6730] |
| ENSG00000123240 | HGLibA_33457 | 16 | -0.98938536 | OPTN | optineurin [Source:HGNC Symbol;Acc:HGNC:17142] |
| ENSG00000123411 | HGLibA_22939 | 16 | -0.98938536 | IKZF4 | IKAROS family zinc finger 4 [Source:HGNC Symbol;Acc:HGNC:13179] |
| ENSG00000123612 | HGLibA_00692 | 16 | -0.98938536 | ACVR1C | activin A receptor type 1C [Source:HGNC Symbol;Acc:HGNC:18123] |
| ENSG00000124193 | HGLibA_47084 | 16 | -0.98938536 | SRSF6 | serine and arginine rich splicing factor 6 [Source:HGNC Symbol;Acc:HGNC:10788] |
| ENSG00000124237 | HGLibA_06030 | 16 | -0.98938536 | C20orf85 | chromosome 20 open reading frame 85 [Source:HGNC Symbol;Acc:HGNC:16216] |

| | | | | | |
|---|---|---|---|---|---|
| ENSG00000124802 | HGLibA_14503 | 16 | -0.98938536 | EEF1E1 | eukaryotic translation elongation factor 1 epsilon 1 [Source:HGNC Symbol;Acc:HGNC:3212] |
| ENSG00000124812 | HGLibA_11165 | 16 | -0.98938536 | CRISP1 | cysteine rich secretory protein 1 [Source:HGNC Symbol;Acc:HGNC:304] |
| ENSG00000125046 | HGLibA_47192 | 16 | -0.98938536 | SSUH2 | ssu-2 homolog (C. elegans) [Source:HGNC Symbol;Acc:HGNC:24809] |
| ENSG00000126012 | HGLibA_24527 | 16 | -0.98938536 | KDM5C | lysine demethylase 5C [Source:HGNC Symbol;Acc:HGNC:11114] |
| ENSG00000127314 | HGLibA_40223 | 16 | -0.98938536 | RAP1B | member of RAS oncogene family [Source:HGNC Symbol;Acc:HGNC:9857]" |
| ENSG00000128652 | HGLibA_21999 | 16 | -0.98938536 | HOXD3 | homeobox D3 [Source:HGNC Symbol;Acc:HGNC:5137] |
| ENSG00000128699 | HGLibA_34626 | 16 | -0.98938536 | ORMDL1 | ORMDL sphingolipid biosynthesis regulator 1 [Source:HGNC Symbol;Acc:HGNC:16036] |
| ENSG00000130561 | HGLibA_42642 | 16 | -0.98938536 | SAG | S-antigen visual arrestin [Source:HGNC Symbol;Acc:HGNC:10521] |
| ENSG00000130695 | HGLibA_09170 | 16 | -0.98938536 | CEP85 | centrosomal protein 85 [Source:HGNC Symbol;Acc:HGNC:25309] |
| ENSG00000132024 | HGLibA_07476 | 16 | -0.98938536 | CC2D1A | coiled-coil and C2 domain containing 1A [Source:HGNC Symbol;Acc:HGNC:30237] |
| ENSG00000132313 | HGLibA_29961 | 16 | -0.98938536 | MRPL35 | mitochondrial ribosomal protein L35 [Source:HGNC Symbol;Acc:HGNC:14489] |
| ENSG00000132530 | HGLibA_54464 | 16 | -0.98938536 | XAF1 | XIAP associated factor 1 [Source:HGNC Symbol;Acc:HGNC:30932] |
| ENSG00000132664 | HGLibA_37624 | 16 | -0.98938536 | POLR3F | RNA polymerase III subunit F [Source:HGNC Symbol;Acc:HGNC:15763] |
| ENSG00000133106 | HGLibA_15385 | 16 | -0.98938536 | EPSTI1 | epithelial stromal interaction 1 [Source:HGNC Symbol;Acc:HGNC:16465] |
| ENSG00000133316 | HGLibA_54171 | 16 | -0.98938536 | WDR74 | WD repeat domain 74 [Source:HGNC Symbol;Acc:HGNC:25529] |
| ENSG00000134121 | HGLibA_09463 | 16 | -0.98938536 | CHL1 | cell adhesion molecule L1 like [Source:HGNC Symbol;Acc:HGNC:1939] |
| ENSG00000134709 | HGLibA_21875 | 16 | -0.98938536 | HOOK1 | hook microtubule tethering protein 1 [Source:HGNC Symbol;Acc:HGNC:19884] |
| ENSG00000136450 | HGLibA_47060 | 16 | -0.98938536 | SRSF1 | serine and arginine rich splicing factor 1 [Source:HGNC Symbol;Acc:HGNC:10780] |
| ENSG00000136688 | HGLibA_23151 | 16 | -0.98938536 | IL36G | interleukin 36 gamma [Source:HGNC Symbol;Acc:HGNC:15741] |
| ENSG00000138768 | HGLibA_53188 | 16 | -0.98938536 | USO1 | USO1 vesicle transport factor [Source:HGNC Symbol;Acc:HGNC:30904] |
| ENSG00000141052 | HGLibA_30877 | 16 | -0.98938536 | MYOCD | myocardin [Source:HGNC Symbol;Acc:HGNC:16067] |
| ENSG00000141255 | HGLibA_46452 | 16 | -0.98938536 | SPATA22 | spermatogenesis associated 22 [Source:HGNC Symbol;Acc:HGNC:30705] |
| ENSG00000142453 | HGLibA_07252 | 16 | -0.98938536 | CARM1 | coactivator associated arginine methyltransferase 1 [Source:HGNC Symbol;Acc:HGNC:23393] |
| ENSG00000143222 | HGLibA_52843 | 16 | -0.98938536 | UFC1 | ubiquitin-fold modifier conjugating enzyme 1 [Source:HGNC Symbol;Acc:HGNC:26941] |
| ENSG00000143257 | HGLibA_32593 | 16 | -0.98938536 | NR1I3 | nuclear receptor subfamily 1 group I member 3 [Source:HGNC Symbol;Acc:HGNC:7969] |
| ENSG00000143612 | HGLibA_05858 | 16 | -0.98938536 | C1orf43 | chromosome 1 open reading frame 43 [Source:HGNC Symbol;Acc:HGNC:29876] |
| ENSG00000143772 | HGLibA_23846 | 16 | -0.98938536 | ITPKB | inositol-trisphosphate 3-kinase B [Source:HGNC Symbol;Acc:HGNC:6179] |
| ENSG00000144366 | HGLibA_20677 | 16 | -0.98938536 | GULP1 | GULP PTB domain containing engulfment adaptor 1 [Source:HGNC Symbol;Acc:HGNC:18649] |
| ENSG00000144460 | HGLibA_33184 | 16 | -0.98938536 | NYAP2 | neuronal tyrosine-phosphorylated phosphoinositide-3-kinase adaptor 2 [Source:HGNC Symbol;Acc:HGNC:29291] |
| ENSG00000144792 | HGLibA_56477 | 16 | -0.98938536 | ZNF660 | zinc finger protein 660 [Source:HGNC Symbol;Acc:HGNC:26720] |
| ENSG00000146039 | HGLibA_44469 | 16 | -0.98938536 | SLC17A4 | solute carrier family 17 member 4 [Source:HGNC Symbol;Acc:HGNC:10932] |
| ENSG00000147010 | HGLibA_43961 | 16 | -0.98938536 | SH3KBP1 | SH3 domain containing kinase binding protein 1 [Source:HGNC Symbol;Acc:HGNC:13867] |
| ENSG00000147121 | HGLibA_25395 | 16 | -0.98938536 | KRBOX4 | KRAB box domain containing 4 [Source:HGNC Symbol;Acc:HGNC:26007] |
| ENSG00000149929 | HGLibA_21354 | 16 | -0.98938536 | HIRIP3 | HIRA interacting protein 3 [Source:HGNC Symbol;Acc:HGNC:4917] |
| ENSG00000149948 | HGLibA_21686 | 16 | -0.98938536 | HMGA2 | high mobility group AT-hook 2 [Source:HGNC Symbol;Acc:HGNC:5009] |
| ENSG00000150337 | HGLibA_17182 | 16 | -0.98938536 | FCGR1A | Fc fragment of IgG receptor Ia [Source:HGNC Symbol;Acc:HGNC:3613] |
| ENSG00000152219 | HGLibA_02939 | 16 | -0.98938536 | ARL14EP | ADP ribosylation factor like GTPase 14 effector protein [Source:HGNC Symbol;Acc:HGNC:26798] |
| ENSG00000152284 | HGLibA_48794 | 16 | -0.98938536 | TCF7L1 | transcription factor 7 like 1 [Source:HGNC Symbol;Acc:HGNC:11640] |
| ENSG00000152292 | HGLibA_43908 | 16 | -0.98938536 | SH2D6 | SH2 domain containing 6 [Source:HGNC Symbol;Acc:HGNC:30439] |
| ENSG00000152464 | HGLibA_42039 | 16 | -0.98938536 | RPP38 | ribonuclease P/MRP subunit p38 [Source:HGNC Symbol;Acc:HGNC:30329] |
| ENSG00000152611 | HGLibA_07201 | 16 | -0.98938536 | CAPSL | calcyphosine like [Source:HGNC Symbol;Acc:HGNC:28375] |
| ENSG00000152782 | HGLibA_35097 | 16 | -0.98938536 | PANK1 | pantothenate kinase 1 [Source:HGNC Symbol;Acc:HGNC:8598] |
| ENSG00000153207 | HGLibA_01284 | 16 | -0.98938536 | AHCTF1 | AT-hook containing transcription factor 1 [Source:HGNC Symbol;Acc:HGNC:24618] |
| ENSG00000154222 | HGLibA_07477 | 16 | -0.98938536 | CC2D1B | coiled-coil and C2 domain containing 1B [Source:HGNC Symbol;Acc:HGNC:29386] |
| ENSG00000155330 | HGLibA_05493 | 16 | -0.98938536 | C16orf87 | chromosome 16 open reading frame 87 [Source:HGNC Symbol;Acc:HGNC:33754] |
| ENSG00000157404 | HGLibA_25016 | 16 | -0.98938536 | KIT | KIT proto-oncogene receptor tyrosine kinase [Source:HGNC Symbol;Acc:HGNC:6342] |
| ENSG00000158683 | HGLibA_36793 | 16 | -0.98938536 | PKD1L1 | transient receptor potential channel interacting [Source:HGNC Symbol;Acc:HGNC:18053]" |
| ENSG00000159069 | HGLibA_17149 | 16 | -0.98938536 | FBXW5 | F-box and WD repeat domain containing 5 [Source:HGNC Symbol;Acc:HGNC:13613] |
| ENSG00000159314 | HGLibA_02745 | 16 | -0.98938536 | ARHGAP27 | Rho GTPase activating protein 27 [Source:HGNC Symbol;Acc:HGNC:31813] |
| ENSG00000159337 | HGLibA_36908 | 16 | -0.98938536 | PLA2G4D | phospholipase A2 group IVD [Source:HGNC Symbol;Acc:HGNC:30038] |
| ENSG00000159592 | HGLibA_19759 | 16 | -0.98938536 | GPBP1L1 | GC-rich promoter binding protein 1 like 1 [Source:HGNC Symbol;Acc:HGNC:28843] |
| ENSG00000159685 | HGLibA_09378 | 16 | -0.98938536 | CHCHD6 | coiled-coil-helix-coiled-coil-helix domain containing 6 [Source:HGNC Symbol;Acc:HGNC:28184] |
| ENSG00000160325 | HGLibA_06870 | 16 | -0.98938536 | CACFD1 | calcium channel flower domain containing 1 [Source:HGNC Symbol;Acc:HGNC:1365] |
| ENSG00000161594 | HGLibA_25124 | 16 | -0.98938536 | KLHL10 | kelch like family member 10 [Source:HGNC Symbol;Acc:HGNC:18829] |
| ENSG00000161791 | HGLibA_17700 | 16 | -0.98938536 | FMNL3 | formin like 3 [Source:HGNC Symbol;Acc:HGNC:23698] |
| ENSG00000162704 | HGLibA_03091 | 16 | -0.98938536 | ARPC5 | actin related protein 2/3 complex subunit 5 [Source:HGNC Symbol;Acc:HGNC:708] |
| ENSG00000162869 | HGLibA_38078 | 16 | -0.98938536 | PPP1R21 | protein phosphatase 1 regulatory subunit 21 [Source:HGNC Symbol;Acc:HGNC:30595] |
| ENSG00000162909 | HGLibA_07164 | 16 | -0.98938536 | CAPN2 | calpain 2 [Source:HGNC Symbol;Acc:HGNC:1479] |
| ENSG00000163032 | HGLibA_53855 | 16 | -0.98938536 | VSNL1 | visinin like 1 [Source:HGNC Symbol;Acc:HGNC:12722] |
| ENSG00000163162 | HGLibA_41540 | 16 | -0.98938536 | RNF149 | ring finger protein 149 [Source:HGNC Symbol;Acc:HGNC:23137] |
| ENSG00000163467 | HGLibA_51808 | 16 | -0.98938536 | TSACC | TSSK6 activating cochaperone [Source:HGNC Symbol;Acc:HGNC:30636] |
| ENSG00000163833 | HGLibA_17093 | 16 | -0.98938536 | FBXO40 | F-box protein 40 [Source:HGNC Symbol;Acc:HGNC:29816] |
| ENSG00000164053 | HGLibA_03816 | 16 | -0.98938536 | ATRIP | ATR interacting protein [Source:HGNC Symbol;Acc:HGNC:33499] |
| ENSG00000164326 | HGLibA_07266 | 16 | -0.98938536 | CARTPT | CART prepropeptide [Source:HGNC Symbol;Acc:HGNC:24323] |
| ENSG00000164406 | HGLibA_26261 | 16 | -0.98938536 | LEAP2 | liver enriched antimicrobial peptide 2 [Source:HGNC Symbol;Acc:HGNC:29571] |
| ENSG00000165548 | HGLibA_50480 | 16 | -0.98938536 | TMEM63C | transmembrane protein 63C [Source:HGNC Symbol;Acc:HGNC:23787] |
| ENSG00000165694 | HGLibA_18026 | 16 | -0.98938536 | FRMD7 | FERM domain containing 7 [Source:HGNC Symbol;Acc:HGNC:8079] |
| ENSG00000166123 | HGLibA_20168 | 16 | -0.98938536 | GPT2 | glutamic--pyruvic transaminase 2 [Source:HGNC Symbol;Acc:HGNC:18062] |
| ENSG00000166233 | HGLibA_02913 | 16 | -0.98938536 | ARIH1 | ariadne RBR E3 ubiquitin protein ligase 1 [Source:HGNC Symbol;Acc:HGNC:689] |
| ENSG00000166289 | HGLibA_37094 | 16 | -0.98938536 | PLEKHF1 | pleckstrin homology and FYVE domain containing 1 [Source:HGNC Symbol;Acc:HGNC:20764] |
| ENSG00000166401 | HGLibA_43565 | 16 | -0.98938536 | SERPINB8 | serpin family B member 8 [Source:HGNC Symbol;Acc:HGNC:8952] |
| ENSG00000167916 | HGLibA_25457 | 16 | -0.98938536 | KRT24 | keratin 24 [Source:HGNC Symbol;Acc:HGNC:18527] |
| ENSG00000167978 | HGLibA_47047 | 16 | -0.98938536 | SRRM2 | serine/arginine repetitive matrix 2 [Source:HGNC Symbol;Acc:HGNC:16639] |
| ENSG00000168131 | HGLibA_33767 | 16 | -0.98938536 | OR2B2 | olfactory receptor family 2 subfamily B member 2 [Source:HGNC Symbol;Acc:HGNC:13966] |
| ENSG00000168175 | HGLibA_28269 | 16 | -0.98938536 | MAPK1IP1L | mitogen-activated protein kinase 1 interacting protein 1 like [Source:HGNC Symbol;Acc:HGNC:19840] |
| ENSG00000168487 | HGLibA_04591 | 16 | -0.98938536 | BMP1 | bone morphogenetic protein 1 [Source:HGNC Symbol;Acc:HGNC:1067] |
| ENSG00000169371 | HGLibA_46016 | 16 | -0.98938536 | SNUPN | snurportin 1 [Source:HGNC Symbol;Acc:HGNC:14245] |
| ENSG00000169607 | HGLibA_09756 | 16 | -0.98938536 | CKAP2L | cytoskeleton associated protein 2 like [Source:HGNC Symbol;Acc:HGNC:26877] |
| ENSG00000169684 | HGLibA_09578 | 16 | -0.98938536 | CHRNA5 | cholinergic receptor nicotinic alpha 5 subunit [Source:HGNC Symbol;Acc:HGNC:1959] |
| ENSG00000170837 | HGLibA_20012 | 16 | -0.98938536 | GPR27 | G protein-coupled receptor 27 [Source:HGNC Symbol;Acc:HGNC:4482] |
| ENSG00000171133 | HGLibA_33814 | 16 | -0.98938536 | OR2K2 | olfactory receptor family 2 subfamily K member 2 [Source:HGNC Symbol;Acc:HGNC:8264] |
| ENSG00000171766 | HGLibA_18696 | 16 | -0.98938536 | GATM | glycine amidinotransferase [Source:HGNC Symbol;Acc:HGNC:4175] |
| ENSG00000171815 | HGLibA_35468 | 16 | -0.98938536 | PCDHB1 | protocadherin beta 1 [Source:HGNC Symbol;Acc:HGNC:8680] |
| ENSG00000172113 | HGLibA_32170 | 16 | -0.98938536 | NME6 | NME/NM23 nucleoside diphosphate kinase 6 [Source:HGNC Symbol;Acc:HGNC:20567] |
| ENSG00000172155 | HGLibA_26109 | 16 | -0.98938536 | LCE1D | late cornified envelope 1D [Source:HGNC Symbol;Acc:HGNC:29465] |
| ENSG00000172554 | HGLibA_46009 | 16 | -0.98938536 | SNTG2 | syntrophin gamma 2 [Source:HGNC Symbol;Acc:HGNC:13741] |
| ENSG00000172809 | HGLibA_41969 | 16 | -0.98938536 | RPL38 | ribosomal protein L38 [Source:HGNC Symbol;Acc:HGNC:10349] |
| ENSG00000173250 | HGLibA_19934 | 16 | -0.98938536 | GPR151 | G protein-coupled receptor 151 [Source:HGNC Symbol;Acc:HGNC:23624] |
| ENSG00000173585 | HGLibA_08170 | 16 | -0.98938536 | CCR9 | C-C motif chemokine receptor 9 [Source:HGNC Symbol;Acc:HGNC:1610] |
| ENSG00000175063 | HGLibA_52578 | 16 | -0.98938536 | UBE2C | ubiquitin conjugating enzyme E2 C [Source:HGNC Symbol;Acc:HGNC:15937] |
| ENSG00000175928 | HGLibA_27470 | 16 | -0.98938536 | LRRN1 | leucine rich repeat neuronal 1 [Source:HGNC Symbol;Acc:HGNC:20980] |
| ENSG00000176555 | HGLibA_34062 | 16 | -0.98938536 | OR4S1 | olfactory receptor family 4 subfamily S member 1 [Source:HGNC Symbol;Acc:HGNC:14705] |
| ENSG00000177201 | HGLibA_33860 | 16 | -0.98938536 | OR2T12 | olfactory receptor family 2 subfamily T member 12 [Source:HGNC Symbol;Acc:HGNC:19592] |
| ENSG00000177465 | HGLibA_00481 | 16 | -0.98938536 | ACOT4 | acyl-CoA thioesterase 4 [Source:HGNC Symbol;Acc:HGNC:19748] |
| ENSG00000177606 | HGLibA_24003 | 16 | -0.98938536 | JUN | AP-1 transcription factor subunit [Source:HGNC Symbol;Acc:HGNC:6204]" |
| ENSG00000179455 | HGLibA_29355 | 16 | -0.98938536 | MKRN3 | makorin ring finger protein 3 [Source:HGNC Symbol;Acc:HGNC:7114] |
| ENSG00000180304 | HGLibA_33216 | 16 | -0.98938536 | OAZ2 | ornithine decarboxylase antizyme 2 [Source:HGNC Symbol;Acc:HGNC:8096] |
| ENSG00000180901 | HGLibA_24445 | 16 | -0.98938536 | KCTD2 | potassium channel tetramerization domain containing 2 [Source:HGNC Symbol;Acc:HGNC:21294] |
| ENSG00000180998 | HGLibA_19903 | 16 | -0.98938536 | GPR137C | G protein-coupled receptor 137C [Source:HGNC Symbol;Acc:HGNC:25445] |
| ENSG00000181038 | HGLibA_28992 | 16 | -0.98938536 | METTL23 | methyltransferase like 23 [Source:HGNC Symbol;Acc:HGNC:26988] |
| ENSG00000181693 | HGLibA_34527 | 16 | -0.98938536 | OR8H1 | olfactory receptor family 8 subfamily H member 1 [Source:HGNC Symbol;Acc:HGNC:14824] |
| ENSG00000182667 | HGLibA_32848 | 16 | -0.98938536 | NTM | neurotrimin [Source:HGNC Symbol;Acc:HGNC:17941] |
| ENSG00000183114 | HGLibA_16589 | 16 | -0.98938536 | FAM43B | family with sequence similarity 43 member B [Source:HGNC Symbol;Acc:HGNC:31791] |
| ENSG00000183463 | HGLibA_53130 | 16 | -0.98938536 | URAD | ureidoimidazoline (2-oxo-4-hydroxy-4-carboxy-5-) decarboxylase [Source:HGNC Symbol;Acc:HGNC:17785] |
| ENSG00000184058 | HGLibA_48642 | 16 | -0.98938536 | TBX1 | T-box 1 [Source:HGNC Symbol;Acc:HGNC:11592] |
| ENSG00000184261 | HGLibA_24301 | 16 | -0.98938536 | KCNK12 | potassium two pore domain channel subfamily K member 12 [Source:HGNC Symbol;Acc:HGNC:6274] |
| ENSG00000185053 | HGLibA_43811 | 16 | -0.98938536 | SGCZ | sarcoglycan zeta [Source:HGNC Symbol;Acc:HGNC:14075] |
| ENSG00000185245 | HGLibA_19699 | 16 | -0.98938536 | GP1BA | glycoprotein Ib platelet subunit alpha [Source:HGNC Symbol;Acc:HGNC:4439] |
| ENSG00000185313 | HGLibA_42967 | 16 | -0.98938536 | SCN10A | sodium voltage-gated channel alpha subunit 10 [Source:HGNC Symbol;Acc:HGNC:10582] |
| ENSG00000185627 | HGLibA_39161 | 16 | -0.98938536 | PSMD13 | non-ATPase 13 [Source:HGNC Symbol;Acc:HGNC:9558]" |
| ENSG00000186094 | HGLibA_01176 | 16 | -0.98938536 | AGBL4 | ATP/GTP binding protein like 4 [Source:HGNC Symbol;Acc:HGNC:25892] |
| ENSG00000186231 | HGLibA_25187 | 16 | -0.98938536 | KLHL32 | kelch like family member 32 [Source:HGNC Symbol;Acc:HGNC:21221] |
| ENSG00000186472 | HGLibA_35618 | 16 | -0.98938536 | PCLO | piccolo presynaptic cytomatrix protein [Source:HGNC Symbol;Acc:HGNC:13406] |
| ENSG00000186562 | HGLibA_12826 | 16 | -0.98938536 | DEFB105A | defensin beta 105A [Source:HGNC Symbol;Acc:HGNC:18087] |
| ENSG00000187957 | HGLibA_13675 | 16 | -0.98938536 | DNER | delta/notch like EGF repeat containing [Source:HGNC Symbol;Acc:HGNC:24456] |
| ENSG00000188647 | HGLibA_39248 | 16 | -0.98938536 | PTAR1 | protein prenyltransferase alpha subunit repeat containing 1 [Source:HGNC Symbol;Acc:HGNC:30449] |
| ENSG00000188672 | HGLibA_41144 | 16 | -0.98938536 | RHCE | Rh blood group CcEe antigens [Source:HGNC Symbol;Acc:HGNC:10008] |
| ENSG00000189152 | HGLibA_20217 | 16 | -0.98938536 | GRAPL | GRB2 related adaptor protein like [Source:HGNC Symbol;Acc:HGNC:37240] |
| ENSG00000189266 | HGLibA_37439 | 16 | -0.98938536 | PNRC2 | proline rich nuclear receptor coactivator 2 [Source:HGNC Symbol;Acc:HGNC:23158] |
| ENSG00000197063 | HGLibA_27881 | 16 | -0.98938536 | MAFG | MAF bZIP transcription factor G [Source:HGNC Symbol;Acc:HGNC:6781] |
| ENSG00000197110 | HGLibA_22725 | 16 | -0.98938536 | IFNL3 | interferon lambda 3 [Source:HGNC Symbol;Acc:HGNC:18365] |
| ENSG00000197119 | HGLibA_44697 | 16 | -0.98938536 | SLC25A29 | solute carrier family 25 member 29 [Source:HGNC Symbol;Acc:HGNC:20116] |
| ENSG00000197233 | HGLibA_33678 | 16 | -0.98938536 | OR1J2 | olfactory receptor family 1 subfamily J member 2 [Source:HGNC Symbol;Acc:HGNC:8209] |
| ENSG00000197889 | HGLibA_28864 | 16 | -0.98938536 | MEIG1 | meiosis/spermiogenesis associated 1 [Source:HGNC Symbol;Acc:HGNC:23429] |
| ENSG00000197993 | HGLibA_24551 | 16 | -0.98938536 | KEL | Kell metallo-endopeptidase (Kell blood group) [Source:HGNC Symbol;Acc:HGNC:6308] |
| ENSG00000198346 | HGLibA_56806 | 16 | -0.98938536 | ZNF813 | zinc finger protein 813 [Source:HGNC Symbol;Acc:HGNC:33257] |
| ENSG00000198626 | HGLibA_42523 | 16 | -0.98938536 | RYR2 | ryanodine receptor 2 [Source:HGNC Symbol;Acc:HGNC:10484] |
| ENSG00000198932 | HGLibA_20120 | 16 | -0.98938536 | GPRASP1 | G protein-coupled receptor associated sorting protein 1 [Source:HGNC Symbol;Acc:HGNC:24834] |
| ENSG00000203724 | HGLibA_05871 | 16 | -0.98938536 | C1orf53 | chromosome 1 open reading frame 53 [Source:HGNC Symbol;Acc:HGNC:30003] |
| ENSG00000204195 | HGLibA_03911 | 16 | -0.98938536 | AWAT1 | acyl-CoA wax alcohol acyltransferase 1 [Source:HGNC Symbol;Acc:HGNC:23252] |
| ENSG00000204231 | HGLibA_42508 | 16 | -0.98938536 | RXRB | retinoid X receptor beta [Source:HGNC Symbol;Acc:HGNC:10478] |
| ENSG00000204655 | HGLibA_29621 | 16 | -0.98938536 | MOG | myelin oligodendrocyte glycoprotein [Source:HGNC Symbol;Acc:HGNC:7197] |
| ENSG00000206536 | HGLibA_34316 | 16 | -0.98938536 | OR5K3 | olfactory receptor family 5 subfamily K member 3 [Source:HGNC Symbol;Acc:HGNC:31290] |
| ENSG00000213625 | HGLibA_26327 | 16 | -0.98938536 | LEPROT | leptin receptor overlapping transcript [Source:HGNC Symbol;Acc:HGNC:29477] |
| ENSG00000214107 | HGLibA_27929 | 16 | -0.98938536 | MAGEB1 | MAGE family member B1 [Source:HGNC Symbol;Acc:HGNC:6808] |

| | | | | | |
|---|---|---|---|---|---|
| ENSG00000214212 | HGLibA_05655 | 16 | -0.98938536 | C19orf38 | chromosome 19 open reading frame 38 [Source:HGNC Symbol;Acc:HGNC:34073] |
| ENSG00000214643 | HGLibA_12902 | 16 | -0.98938536 | DEFB133 | defensin beta 133 [Source:HGNC Symbol;Acc:HGNC:31331] |
| ENSG00000229549 | HGLibA_51975 | 16 | -0.98938536 | TSPY8 | testis specific protein Y-linked 8 [Source:HGNC Symbol;Acc:HGNC:37471] |
| ENSG00000232838 | HGLibA_36075 | 16 | -0.98938536 | PET117 | PET117 homolog [Source:HGNC Symbol;Acc:HGNC:40045] |
| ENSG00000244509 | HGLibA_02483 | 16 | -0.98938536 | APOBEC3C | apolipoprotein B mRNA editing enzyme catalytic subunit 3C [Source:HGNC Symbol;Acc:HGNC:17353] |
| ENSG00000251258 | HGLibA_40945 | 16 | -0.98938536 | RFPL4B | ret finger protein like 4B [Source:HGNC Symbol;Acc:HGNC:33264] |
| ENSG00000255221 | HGLibA_07229 | 16 | -0.98938536 | CARD17 | caspase recruitment domain family member 17 [Source:HGNC Symbol;Acc:HGNC:33827] |
| ENSG00000256618 | HGLibA_30472 | 16 | -0.98938536 | MTRNR2L1 | MT-RNR2 like 1 [Source:HGNC Symbol;Acc:HGNC:37155] |
| ENSG00000256618 | HGLibA_30471 | 16 | -0.98938536 | MTRNR2L1 | MT-RNR2 like 1 [Source:HGNC Symbol;Acc:HGNC:37155] |
| ENSG00000272398 | HGLibA_08302 | 16 | -0.98938536 | CD24 | CD24 molecule [Source:HGNC Symbol;Acc:HGNC:1645] |
| ENSG00000273514 | HGLibA_17854 | 16 | -0.98938536 | FOXD4L6 | forkhead box D4 like 6 [Source:HGNC Symbol;Acc:HGNC:31986] |
| ENSG00000278845 | HGLibA_29992 | 16 | -0.98938536 | MRPL45 | mitochondrial ribosomal protein L45 [Source:HGNC Symbol;Acc:HGNC:16651] |
| ERO1L | HGLibA_15519 | 16 | -0.98938536 | ERO1L | - |
| FAM57A | HGLibA_16643 | 16 | -0.98938536 | FAM57A | - |
| hsa-mir-1185-1 | HGLibA_57240 | 16 | -0.98938536 | hsa-mir-1185-1 | - |
| hsa-mir-1263 | HGLibA_57476 | 16 | -0.98938536 | hsa-mir-1263 | - |
| hsa-mir-1282 | HGLibA_57584 | 16 | -0.98938536 | hsa-mir-1282 | - |
| hsa-mir-16-2 | HGLibA_57952 | 16 | -0.98938536 | hsa-mir-16-2 | - |
| hsa-mir-200b | HGLibA_58155 | 16 | -0.98938536 | hsa-mir-200b | - |
| hsa-mir-23a | HGLibA_58329 | 16 | -0.98938536 | hsa-mir-23a | - |
| hsa-mir-3140 | HGLibA_58617 | 16 | -0.98938536 | hsa-mir-3140 | - |
| hsa-mir-3619 | HGLibA_59107 | 16 | -0.98938536 | hsa-mir-3619 | - |
| hsa-mir-3655 | HGLibA_59167 | 16 | -0.98938536 | hsa-mir-3655 | - |
| hsa-mir-367 | HGLibA_59231 | 16 | -0.98938536 | hsa-mir-367 | - |
| hsa-mir-3673 | HGLibA_59247 | 16 | -0.98938536 | hsa-mir-3673 | - |
| hsa-mir-3673 | HGLibA_59246 | 16 | -0.98938536 | hsa-mir-3673 | - |
| hsa-mir-4311 | HGLibA_59958 | 16 | -0.98938536 | hsa-mir-4311 | - |
| hsa-mir-4427 | HGLibA_60094 | 16 | -0.98938536 | hsa-mir-4427 | - |
| hsa-mir-4477a | HGLibA_60317 | 16 | -0.98938536 | hsa-mir-4477a | - |
| hsa-mir-4496 | HGLibA_60400 | 16 | -0.98938536 | hsa-mir-4496 | - |
| hsa-mir-4674 | HGLibA_60813 | 16 | -0.98938536 | hsa-mir-4674 | - |
| hsa-mir-4699 | HGLibA_60905 | 16 | -0.98938536 | hsa-mir-4699 | - |
| hsa-mir-4723 | HGLibA_60989 | 16 | -0.98938536 | hsa-mir-4723 | - |
| hsa-mir-4724 | HGLibA_60995 | 16 | -0.98938536 | hsa-mir-4724 | - |
| hsa-mir-4765 | HGLibA_61155 | 16 | -0.98938536 | hsa-mir-4765 | - |
| hsa-mir-4766 | HGLibA_61162 | 16 | -0.98938536 | hsa-mir-4766 | - |
| hsa-mir-4791 | HGLibA_61271 | 16 | -0.98938536 | hsa-mir-4791 | - |
| hsa-mir-5008 | HGLibA_61434 | 16 | -0.98938536 | hsa-mir-5008 | - |
| hsa-mir-509-3 | HGLibA_61513 | 16 | -0.98938536 | hsa-mir-509-3 | - |
| hsa-mir-517c | HGLibA_61632 | 16 | -0.98938536 | hsa-mir-517c | - |
| hsa-mir-518f | HGLibA_61674 | 16 | -0.98938536 | hsa-mir-518f | - |
| hsa-mir-523 | HGLibA_61777 | 16 | -0.98938536 | hsa-mir-523 | - |
| hsa-mir-548t | HGLibA_62065 | 16 | -0.98938536 | hsa-mir-548t | - |
| hsa-mir-569 | HGLibA_62275 | 16 | -0.98938536 | hsa-mir-569 | - |
| hsa-mir-605 | HGLibA_62511 | 16 | -0.98938536 | hsa-mir-605 | - |
| hsa-mir-609 | HGLibA_62622 | 16 | -0.98938536 | hsa-mir-609 | - |
| hsa-mir-6749 | HGLibA_63148 | 16 | -0.98938536 | hsa-mir-6749 | - |
| hsa-mir-6806 | HGLibA_63403 | 16 | -0.98938536 | hsa-mir-6806 | - |
| hsa-mir-6817 | HGLibA_63444 | 16 | -0.98938536 | hsa-mir-6817 | - |
| hsa-mir-6888 | HGLibA_63738 | 16 | -0.98938536 | hsa-mir-6888 | - |
| hsa-mir-764 | HGLibA_63911 | 16 | -0.98938536 | hsa-mir-764 | - |
| hsa-mir-8053 | HGLibA_64051 | 16 | -0.98938536 | hsa-mir-8053 | - |
| KIAA1524 | HGLibA_24775 | 16 | -0.98938536 | KIAA1524 | - |
| LOC100288255 | HGLibA_26829 | 16 | -0.98938536 | LOC100288255 | - |
| LSMD1 | HGLibA_27555 | 16 | -0.98938536 | LSMD1 | - |
| TCEB3C | HGLibA_48746 | 16 | -0.98938536 | TCEB3C | - |
| TWISTNB | HGLibA_52382 | 16 | -0.98938536 | TWISTNB | - |
| UQCC | HGLibA_53095 | 16 | -0.98938536 | UQCC | - |
| C11orf85 | HGLibA_05199 | 15 | -0.999336397 | C11orf85 | - |
| C17orf77 | HGLibA_05577 | 15 | -0.999336397 | C17orf77 | - |
| C6orf222 | HGLibA_06476 | 15 | -0.999336397 | C6orf222 | - |
| C6orf48 | HGLibA_06492 | 15 | -0.999336397 | C6orf48 | - |
| C9orf142 | HGLibA_06687 | 15 | -0.999336397 | C9orf142 | - |
| CCDC23 | HGLibA_07733 | 15 | -0.999336397 | CCDC23 | - |
| ENSG00000000005 | HGLibA_50858 | 15 | -0.999336397 | TNMD | tenomodulin [Source:HGNC Symbol;Acc:HGNC:17757] |
| ENSG00000008197 | HGLibA_49130 | 15 | -0.999336397 | TFAP2D | transcription factor AP-2 delta [Source:HGNC Symbol;Acc:HGNC:15581] |
| ENSG00000010017 | HGLibA_40212 | 15 | -0.999336397 | RANBP9 | RAN binding protein 9 [Source:HGNC Symbol;Acc:HGNC:13727] |
| ENSG00000023191 | HGLibA_41713 | 15 | -0.999336397 | RNH1 | ribonuclease/angiogenin inhibitor 1 [Source:HGNC Symbol;Acc:HGNC:10074] |
| ENSG00000032742 | HGLibA_22778 | 15 | -0.999336397 | IFT88 | intraflagellar transport 88 [Source:HGNC Symbol;Acc:HGNC:20606] |
| ENSG00000033178 | HGLibA_52529 | 15 | -0.999336397 | UBA6 | ubiquitin like modifier activating enzyme 6 [Source:HGNC Symbol;Acc:HGNC:25581] |
| ENSG00000037474 | HGLibA_32780 | 15 | -0.999336397 | NSUN2 | NOP2/Sun RNA methyltransferase family member 2 [Source:HGNC Symbol;Acc:HGNC:25994] |
| ENSG00000039560 | HGLibA_40141 | 15 | -0.999336397 | RAI14 | retinoic acid induced 14 [Source:HGNC Symbol;Acc:HGNC:14873] |
| ENSG00000044012 | HGLibA_20647 | 15 | -0.999336397 | GUCA2B | guanylate cyclase activator 2B [Source:HGNC Symbol;Acc:HGNC:4683] |
| ENSG00000054983 | HGLibA_18510 | 15 | -0.999336397 | GALC | galactosylceramidase [Source:HGNC Symbol;Acc:HGNC:4115] |
| ENSG00000057935 | HGLibA_30317 | 15 | -0.999336397 | MTA3 | metastasis associated 1 family member 3 [Source:HGNC Symbol;Acc:HGNC:23784] |
| ENSG00000070770 | HGLibA_11405 | 15 | -0.999336397 | CSNK2A2 | casein kinase 2 alpha 2 [Source:HGNC Symbol;Acc:HGNC:2459] |
| ENSG00000080815 | HGLibA_39024 | 15 | -0.999336397 | PSEN1 | presenilin 1 [Source:HGNC Symbol;Acc:HGNC:9508] |
| ENSG00000082438 | HGLibA_10464 | 15 | -0.999336397 | COBLL1 | cordon-bleu WH2 repeat protein like 1 [Source:HGNC Symbol;Acc:HGNC:23571] |
| ENSG00000087448 | HGLibA_25214 | 15 | -0.999336397 | KLHL42 | kelch like family member 42 [Source:HGNC Symbol;Acc:HGNC:29252] |
| ENSG00000089597 | HGLibA_18606 | 15 | -0.999336397 | GANAB | glucosidase II alpha subunit [Source:HGNC Symbol;Acc:HGNC:4138] |
| ENSG00000095203 | HGLibA_15255 | 15 | -0.999336397 | EPB41L4B | erythrocyte membrane protein band 4.1 like 4B [Source:HGNC Symbol;Acc:HGNC:19818] |
| ENSG00000100626 | HGLibA_18548 | 15 | -0.999336397 | GALNT16 | polypeptide N-acetylgalactosaminyltransferase 16 [Source:HGNC Symbol;Acc:HGNC:23233] |
| ENSG00000101255 | HGLibA_51396 | 15 | -0.999336397 | TRIB3 | tribbles pseudokinase 3 [Source:HGNC Symbol;Acc:HGNC:16228] |
| ENSG00000101639 | HGLibA_09126 | 15 | -0.999336397 | CEP192 | centrosomal protein 192 [Source:HGNC Symbol;Acc:HGNC:25515] |
| ENSG00000102878 | HGLibA_22230 | 15 | -0.999336397 | HSF4 | heat shock transcription factor 4 [Source:HGNC Symbol;Acc:HGNC:5227] |
| ENSG00000102897 | HGLibA_27747 | 15 | -0.999336397 | LYRM1 | LYR motif containing 1 [Source:HGNC Symbol;Acc:HGNC:25074] |
| ENSG00000102901 | HGLibA_09086 | 15 | -0.999336397 | CENPT | centromere protein T [Source:HGNC Symbol;Acc:HGNC:25787] |
| ENSG00000103035 | HGLibA_39181 | 15 | -0.999336397 | PSMD7 | non-ATPase 7 [Source:HGNC Symbol;Acc:HGNC:9565]" |
| ENSG00000103249 | HGLibA_09838 | 15 | -0.999336397 | CLCN7 | chloride voltage-gated channel 7 [Source:HGNC Symbol;Acc:HGNC:2025] |
| ENSG00000107018 | HGLibA_41353 | 15 | -0.999336397 | RLN1 | relaxin 1 [Source:HGNC Symbol;Acc:HGNC:10026] |
| ENSG00000107566 | HGLibA_15494 | 15 | -0.999336397 | ERLIN1 | ER lipid raft associated 1 [Source:HGNC Symbol;Acc:HGNC:16947] |
| ENSG00000107736 | HGLibA_08668 | 15 | -0.999336397 | CDH23 | cadherin related 23 [Source:HGNC Symbol;Acc:HGNC:13733] |
| ENSG00000107862 | HGLibA_18722 | 15 | -0.999336397 | GBF1 | golgi brefeldin A resistant guanine nucleotide exchange factor 1 [Source:HGNC Symbol;Acc:HGNC:4181] |
| ENSG00000108312 | HGLibA_52770 | 15 | -0.999336397 | UBTF | upstream binding transcription factor [Source:HGNC Symbol;Acc:HGNC:12511] |
| ENSG00000109321 | HGLibA_02641 | 15 | -0.999336397 | AREG | amphiregulin [Source:HGNC Symbol;Acc:HGNC:651] |
| ENSG00000109534 | HGLibA_18624 | 15 | -0.999336397 | GAR1 | GAR1 ribonucleoprotein [Source:HGNC Symbol;Acc:HGNC:14264] |
| ENSG00000109736 | HGLibA_29084 | 15 | -0.999336397 | MFSD10 | major facilitator superfamily domain containing 10 [Source:HGNC Symbol;Acc:HGNC:16894] |
| ENSG00000111640 | HGLibA_18614 | 15 | -0.999336397 | GAPDH | glyceraldehyde-3-phosphate dehydrogenase [Source:HGNC Symbol;Acc:HGNC:4141] |
| ENSG00000111783 | HGLibA_40970 | 15 | -0.999336397 | RFX4 | regulatory factor X4 [Source:HGNC Symbol;Acc:HGNC:9985] |
| ENSG00000112299 | HGLibA_53711 | 15 | -0.999336397 | VNN1 | vanin 1 [Source:HGNC Symbol;Acc:HGNC:12705] |
| ENSG00000112473 | HGLibA_45120 | 15 | -0.999336397 | SLC39A7 | solute carrier family 39 member 7 [Source:HGNC Symbol;Acc:HGNC:4927] |
| ENSG00000113119 | HGLibA_49797 | 15 | -0.999336397 | TMCO6 | transmembrane and coiled-coil domains 6 [Source:HGNC Symbol;Acc:HGNC:28814] |
| ENSG00000113456 | HGLibA_40055 | 15 | -0.999336397 | RAD1 | RAD1 checkpoint DNA exonuclease [Source:HGNC Symbol;Acc:HGNC:9806] |
| ENSG00000114107 | HGLibA_09159 | 15 | -0.999336397 | CEP70 | centrosomal protein 70 [Source:HGNC Symbol;Acc:HGNC:29972] |
| ENSG00000116176 | HGLibA_51202 | 15 | -0.999336397 | TPSG1 | tryptase gamma 1 [Source:HGNC Symbol;Acc:HGNC:14134] |
| ENSG00000116285 | HGLibA_15534 | 15 | -0.999336397 | ERRFI1 | ERBB receptor feedback inhibitor 1 [Source:HGNC Symbol;Acc:HGNC:18185] |
| ENSG00000116285 | HGLibA_15532 | 15 | -0.999336397 | ERRFI1 | ERBB receptor feedback inhibitor 1 [Source:HGNC Symbol;Acc:HGNC:18185] |
| ENSG00000116679 | HGLibA_23884 | 15 | -0.999336397 | IVNS1ABP | influenza virus NS1A binding protein [Source:HGNC Symbol;Acc:HGNC:16951] |
| ENSG00000116711 | HGLibA_36900 | 15 | -0.999336397 | PLA2G4A | phospholipase A2 group IVA [Source:HGNC Symbol;Acc:HGNC:9035] |
| ENSG00000117133 | HGLibA_41829 | 15 | -0.999336397 | RPF1 | ribosome production factor 1 homolog [Source:HGNC Symbol;Acc:HGNC:30350] |
| ENSG00000119318 | HGLibA_40072 | 15 | -0.999336397 | RAD23B | nucleotide excision repair protein [Source:HGNC Symbol;Acc:HGNC:9813]" |
| ENSG00000119866 | HGLibA_04289 | 15 | -0.999336397 | BCL11A | B cell CLL/lymphoma 11A [Source:HGNC Symbol;Acc:HGNC:13221] |
| ENSG00000119973 | HGLibA_38602 | 15 | -0.999336397 | PRLHR | prolactin releasing hormone receptor [Source:HGNC Symbol;Acc:HGNC:4464] |
| ENSG00000121039 | HGLibA_40731 | 15 | -0.999336397 | RDH10 | retinol dehydrogenase 10 [Source:HGNC Symbol;Acc:HGNC:19975] |
| ENSG00000122642 | HGLibA_17591 | 15 | -0.999336397 | FKBP9 | FK506 binding protein 9 [Source:HGNC Symbol;Acc:HGNC:3725] |
| ENSG00000122644 | HGLibA_02965 | 15 | -0.999336397 | ARL4A | ADP ribosylation factor like GTPase 4A [Source:HGNC Symbol;Acc:HGNC:695] |
| ENSG00000122707 | HGLibA_40765 | 15 | -0.999336397 | RECK | reversion inducing cysteine rich protein with kazal motifs [Source:HGNC Symbol;Acc:HGNC:11345] |
| ENSG00000122877 | HGLibA_14652 | 15 | -0.999336397 | EGR2 | early growth response 2 [Source:HGNC Symbol;Acc:HGNC:3239] |
| ENSG00000123124 | HGLibA_54451 | 15 | -0.999336397 | WWP1 | WW domain containing E3 ubiquitin protein ligase 1 [Source:HGNC Symbol;Acc:HGNC:17004] |
| ENSG00000124535 | HGLibA_54412 | 15 | -0.999336397 | WRNIP1 | Werner helicase interacting protein 1 [Source:HGNC Symbol;Acc:HGNC:20876] |
| ENSG00000125246 | HGLibA_10172 | 15 | -0.999336397 | CLYBL | citrate lyase beta like [Source:HGNC Symbol;Acc:HGNC:18355] |
| ENSG00000125861 | HGLibA_18974 | 15 | -0.999336397 | GFRA4 | GDNF family receptor alpha 4 [Source:HGNC Symbol;Acc:HGNC:13821] |
| ENSG00000126067 | HGLibA_39104 | 15 | -0.999336397 | PSMB2 | proteasome subunit beta 2 [Source:HGNC Symbol;Acc:HGNC:9539] |
| ENSG00000126353 | HGLibA_08165 | 15 | -0.999336397 | CCR7 | C-C motif chemokine receptor 7 [Source:HGNC Symbol;Acc:HGNC:1608] |
| ENSG00000126524 | HGLibA_42792 | 15 | -0.999336397 | SBDS | ribosome maturation factor [Source:HGNC Symbol;Acc:HGNC:19440]" |
| ENSG00000128000 | HGLibA_56735 | 15 | -0.999336397 | ZNF780B | zinc finger protein 780B [Source:HGNC Symbol;Acc:HGNC:33109] |
| ENSG00000128595 | HGLibA_07051 | 15 | -0.999336397 | CALU | calumenin [Source:HGNC Symbol;Acc:HGNC:1458] |
| ENSG00000128989 | HGLibA_03097 | 15 | -0.999336397 | ARPP19 | cAMP regulated phosphoprotein 19 [Source:HGNC Symbol;Acc:HGNC:16967] |
| ENSG00000129354 | HGLibA_02292 | 15 | -0.999336397 | AP1M2 | adaptor related protein complex 1 subunit mu 2 [Source:HGNC Symbol;Acc:HGNC:558] |
| ENSG00000130340 | HGLibA_46112 | 15 | -0.999336397 | SNX9 | sorting nexin 9 [Source:HGNC Symbol;Acc:HGNC:14973] |
| ENSG00000131153 | HGLibA_19107 | 15 | -0.999336397 | GINS2 | GINS complex subunit 2 [Source:HGNC Symbol;Acc:HGNC:24575] |
| ENSG00000131462 | HGLibA_52302 | 15 | -0.999336397 | TUBG1 | tubulin gamma 1 [Source:HGNC Symbol;Acc:HGNC:12417] |
| ENSG00000132485 | HGLibA_56975 | 15 | -0.999336397 | ZRANB2 | zinc finger RANBP2-type containing 2 [Source:HGNC Symbol;Acc:HGNC:13058] |
| ENSG00000134215 | HGLibA_53553 | 15 | -0.999336397 | VAV3 | vav guanine nucleotide exchange factor 3 [Source:HGNC Symbol;Acc:HGNC:12659] |
| ENSG00000134265 | HGLibA_31135 | 15 | -0.999336397 | NAPG | NSF attachment protein gamma [Source:HGNC Symbol;Acc:HGNC:7642] |

| | | | | | |
|---|---|---|---|---|---|
| ENSG00000135750 | HGLibA_24293 | 15 | -0.999336397 | KCNK1 | potassium two pore domain channel subfamily K member 1 [Source:HGNC Symbol;Acc:HGNC:6272] |
| ENSG00000135766 | HGLibA_14639 | 15 | -0.999336397 | EGLN1 | egl-9 family hypoxia inducible factor 1 [Source:HGNC Symbol;Acc:HGNC:1232] |
| ENSG00000136169 | HGLibA_43670 | 15 | -0.999336397 | SETDB2 | SET domain bifurcated 2 [Source:HGNC Symbol;Acc:HGNC:20263] |
| ENSG00000136682 | HGLibA_07434 | 15 | -0.999336397 | CBWD2 | COBW domain containing 2 [Source:HGNC Symbol;Acc:HGNC:17907] |
| ENSG00000136738 | HGLibA_47330 | 15 | -0.999336397 | STAM | signal transducing adaptor molecule [Source:HGNC Symbol;Acc:HGNC:11357] |
| ENSG00000137078 | HGLibA_44238 | 15 | -0.999336397 | SIT1 | signaling threshold regulating transmembrane adaptor 1 [Source:HGNC Symbol;Acc:HGNC:17710] |
| ENSG00000137747 | HGLibA_50630 | 15 | -0.999336397 | TMPRSS13 | transmembrane serine protease 13 [Source:HGNC Symbol;Acc:HGNC:29808] |
| ENSG00000138075 | HGLibA_00225 | 15 | -0.999336397 | ABCG5 | ATP binding cassette subfamily G member 5 [Source:HGNC Symbol;Acc:HGNC:13886] |
| ENSG00000138668 | HGLibA_21812 | 15 | -0.999336397 | HNRNPD | heterogeneous nuclear ribonucleoprotein D [Source:HGNC Symbol;Acc:HGNC:5036] |
| ENSG00000139168 | HGLibA_55055 | 15 | -0.999336397 | ZCRB1 | zinc finger CCHC-type and RNA binding motif containing 1 [Source:HGNC Symbol;Acc:HGNC:29620] |
| ENSG00000139180 | HGLibA_31476 | 15 | -0.999336397 | NDUFA9 | NADH:ubiquinone oxidoreductase subunit A9 [Source:HGNC Symbol;Acc:HGNC:7693] |
| ENSG00000139445 | HGLibA_17918 | 15 | -0.999336397 | FOXN4 | forkhead box N4 [Source:HGNC Symbol;Acc:HGNC:21399] |
| ENSG00000139549 | HGLibA_13110 | 15 | -0.999336397 | DHH | desert hedgehog [Source:HGNC Symbol;Acc:HGNC:2865] |
| ENSG00000143125 | HGLibA_38664 | 15 | -0.999336397 | PROK1 | prokineticin 1 [Source:HGNC Symbol;Acc:HGNC:18454] |
| ENSG00000143257 | HGLibA_32591 | 15 | -0.999336397 | NR1I3 | nuclear receptor subfamily 1 group I member 3 [Source:HGNC Symbol;Acc:HGNC:7969] |
| ENSG00000143799 | HGLibA_35212 | 15 | -0.999336397 | PARP1 | poly(ADP-ribose) polymerase 1 [Source:HGNC Symbol;Acc:HGNC:270] |
| ENSG00000144118 | HGLibA_40150 | 15 | -0.999336397 | RALB | RAS like proto-oncogene B [Source:HGNC Symbol;Acc:HGNC:9840] |
| ENSG00000144554 | HGLibA_16838 | 15 | -0.999336397 | FANCD2 | FA complementation group D2 [Source:HGNC Symbol;Acc:HGNC:3585] |
| ENSG00000145354 | HGLibA_09730 | 15 | -0.999336397 | CISD2 | CDGSH iron sulfur domain 2 [Source:HGNC Symbol;Acc:HGNC:24212] |
| ENSG00000145414 | HGLibA_31059 | 15 | -0.999336397 | NAF1 | nuclear assembly factor 1 ribonucleoprotein [Source:HGNC Symbol;Acc:HGNC:25126] |
| ENSG00000145476 | HGLibA_12199 | 15 | -0.999336397 | CYP4V2 | cytochrome P450 family 4 subfamily V member 2 [Source:HGNC Symbol;Acc:HGNC:23198] |
| ENSG00000146263 | HGLibA_29555 | 15 | -0.999336397 | MMS22L | DNA repair protein [Source:HGNC Symbol;Acc:HGNC:21475]" |
| ENSG00000146457 | HGLibA_54430 | 15 | -0.999336397 | WTAP | WT1 associated protein [Source:HGNC Symbol;Acc:HGNC:16846] |
| ENSG00000146469 | HGLibA_53662 | 15 | -0.999336397 | VIP | vasoactive intestinal peptide [Source:HGNC Symbol;Acc:HGNC:12693] |
| ENSG00000146776 | HGLibA_03853 | 15 | -0.999336397 | ATXN7L1 | ataxin 7 like 1 [Source:HGNC Symbol;Acc:HGNC:22210] |
| ENSG00000147036 | HGLibA_26009 | 15 | -0.999336397 | LANCL3 | LanC like 3 [Source:HGNC Symbol;Acc:HGNC:24767] |
| ENSG00000148384 | HGLibA_23352 | 15 | -0.999336397 | INPP5E | inositol polyphosphate-5-phosphatase E [Source:HGNC Symbol;Acc:HGNC:21474] |
| ENSG00000148444 | HGLibA_10660 | 15 | -0.999336397 | COMMD3 | COMM domain containing 3 [Source:HGNC Symbol;Acc:HGNC:23332] |
| ENSG00000148814 | HGLibA_27296 | 15 | -0.999336397 | LRRC27 | leucine rich repeat containing 27 [Source:HGNC Symbol;Acc:HGNC:29346] |
| ENSG00000149269 | HGLibA_35038 | 15 | -0.999336397 | PAK1 | p21 (RAC1) activated kinase 1 [Source:HGNC Symbol;Acc:HGNC:8590] |
| ENSG00000150457 | HGLibA_26067 | 15 | -0.999336397 | LATS2 | large tumor suppressor kinase 2 [Source:HGNC Symbol;Acc:HGNC:6515] |
| ENSG00000150540 | HGLibA_21787 | 15 | -0.999336397 | HNMT | histamine N-methyltransferase [Source:HGNC Symbol;Acc:HGNC:5028] |
| ENSG00000150712 | HGLibA_30419 | 15 | -0.999336397 | MTMR12 | myotubularin related protein 12 [Source:HGNC Symbol;Acc:HGNC:18191] |
| ENSG00000152380 | HGLibA_16165 | 15 | -0.999336397 | FAM151B | family with sequence similarity 151 member B [Source:HGNC Symbol;Acc:HGNC:33716] |
| ENSG00000153179 | HGLibA_40380 | 15 | -0.999336397 | RASSF3 | Ras association domain family member 3 [Source:HGNC Symbol;Acc:HGNC:14271] |
| ENSG00000153561 | HGLibA_41383 | 15 | -0.999336397 | RMND5A | required for meiotic nuclear division 5 homolog A [Source:HGNC Symbol;Acc:HGNC:25850] |
| ENSG00000154380 | HGLibA_15105 | 15 | -0.999336397 | ENAH | actin regulator [Source:HGNC Symbol;Acc:HGNC:18271]" |
| ENSG00000156966 | HGLibA_03998 | 15 | -0.999336397 | B3GNT7 | 3-N-acetylglucosaminyltransferase 7 [Source:HGNC Symbol;Acc:HGNC:18811]" |
| ENSG00000160058 | HGLibA_04863 | 15 | -0.999336397 | BSDC1 | BSD domain containing 1 [Source:HGNC Symbol;Acc:HGNC:25501] |
| ENSG00000162746 | HGLibA_17246 | 15 | -0.999336397 | FCRLB | Fc receptor like B [Source:HGNC Symbol;Acc:HGNC:26431] |
| ENSG00000163577 | HGLibA_14857 | 15 | -0.999336397 | EIF5A2 | eukaryotic translation initiation factor 5A2 [Source:HGNC Symbol;Acc:HGNC:3301] |
| ENSG00000163625 | HGLibA_54009 | 15 | -0.999336397 | WDFY3 | WD repeat and FYVE domain containing 3 [Source:HGNC Symbol;Acc:HGNC:20751] |
| ENSG00000164053 | HGLibA_03817 | 15 | -0.999336397 | ATRIP | ATR interacting protein [Source:HGNC Symbol;Acc:HGNC:33499] |
| ENSG00000164406 | HGLibA_26262 | 15 | -0.999336397 | LEAP2 | liver enriched antimicrobial peptide 2 [Source:HGNC Symbol;Acc:HGNC:29571] |
| ENSG00000164620 | HGLibA_40820 | 15 | -0.999336397 | RELL2 | RELT like 2 [Source:HGNC Symbol;Acc:HGNC:26902] |
| ENSG00000165475 | HGLibA_11289 | 15 | -0.999336397 | CRYL1 | crystallin lambda 1 [Source:HGNC Symbol;Acc:HGNC:18246] |
| ENSG00000165672 | HGLibA_38412 | 15 | -0.999336397 | PRDX3 | peroxiredoxin 3 [Source:HGNC Symbol;Acc:HGNC:9354] |
| ENSG00000166090 | HGLibA_23107 | 15 | -0.999336397 | IL25 | interleukin 25 [Source:HGNC Symbol;Acc:HGNC:13765] |
| ENSG00000166128 | HGLibA_39988 | 15 | -0.999336397 | RAB8B | member RAS oncogene family [Source:HGNC Symbol;Acc:HGNC:30273]" |
| ENSG00000166526 | HGLibA_55765 | 15 | -0.999336397 | ZNF3 | zinc finger protein 3 [Source:HGNC Symbol;Acc:HGNC:13089] |
| ENSG00000166562 | HGLibA_43183 | 15 | -0.999336397 | SEC11C | signal peptidase complex subunit [Source:HGNC Symbol;Acc:HGNC:23400]" |
| ENSG00000167186 | HGLibA_10765 | 15 | -0.999336397 | COQ7 | hydroxylase [Source:HGNC Symbol;Acc:HGNC:2244]" |
| ENSG00000167244 | HGLibA_22797 | 15 | -0.999336397 | IGF2 | insulin like growth factor 2 [Source:HGNC Symbol;Acc:HGNC:5466] |
| ENSG00000167531 | HGLibA_25938 | 15 | -0.999336397 | LALBA | lactalbumin alpha [Source:HGNC Symbol;Acc:HGNC:6480] |
| ENSG00000167565 | HGLibA_43610 | 15 | -0.999336397 | SERTAD3 | SERTA domain containing 3 [Source:HGNC Symbol;Acc:HGNC:17931] |
| ENSG00000167925 | HGLibA_19033 | 15 | -0.999336397 | GHDC | GH3 domain containing [Source:HGNC Symbol;Acc:HGNC:24438] |
| ENSG00000168246 | HGLibA_52769 | 15 | -0.999336397 | UBTD2 | ubiquitin domain containing 2 [Source:HGNC Symbol;Acc:HGNC:24463] |
| ENSG00000168476 | HGLibA_40785 | 15 | -0.999336397 | REEP4 | receptor accessory protein 4 [Source:HGNC Symbol;Acc:HGNC:26176] |
| ENSG00000168679 | HGLibA_44440 | 15 | -0.999336397 | SLC16A4 | solute carrier family 16 member 4 [Source:HGNC Symbol;Acc:HGNC:10925] |
| ENSG00000169155 | HGLibA_54888 | 15 | -0.999336397 | ZBTB43 | zinc finger and BTB domain containing 43 [Source:HGNC Symbol;Acc:HGNC:17908] |
| ENSG00000169857 | HGLibA_03889 | 15 | -0.999336397 | AVEN | apoptosis and caspase activation inhibitor [Source:HGNC Symbol;Acc:HGNC:13509] |
| ENSG00000170340 | HGLibA_03985 | 15 | -0.999336397 | B3GNT2 | 3-N-acetylglucosaminyltransferase 2 [Source:HGNC Symbol;Acc:HGNC:15629]" |
| ENSG00000170484 | HGLibA_25535 | 15 | -0.999336397 | KRT74 | keratin 74 [Source:HGNC Symbol;Acc:HGNC:28929] |
| ENSG00000171488 | HGLibA_27433 | 15 | -0.999336397 | LRRC8C | leucine rich repeat containing 8 VRAC subunit C [Source:HGNC Symbol;Acc:HGNC:25075] |
| ENSG00000172318 | HGLibA_03955 | 15 | -0.999336397 | B3GALT1 | 3-galactosyltransferase 1 [Source:HGNC Symbol;Acc:HGNC:916]" |
| ENSG00000172336 | HGLibA_37695 | 15 | -0.999336397 | POP7 | ribonuclease P/MRP subunit [Source:HGNC Symbol;Acc:HGNC:19949]" |
| ENSG00000172340 | HGLibA_47701 | 15 | -0.999336397 | SUCLG2 | succinate-CoA ligase GDP-forming beta subunit [Source:HGNC Symbol;Acc:HGNC:11450] |
| ENSG00000172586 | HGLibA_09360 | 15 | -0.999336397 | CHCHD1 | coiled-coil-helix-coiled-coil-helix domain containing 1 [Source:HGNC Symbol;Acc:HGNC:23518] |
| ENSG00000172638 | HGLibA_14563 | 15 | -0.999336397 | EFEMP2 | EGF containing fibulin extracellular matrix protein 2 [Source:HGNC Symbol;Acc:HGNC:3219] |
| ENSG00000172893 | HGLibA_13093 | 15 | -0.999336397 | DHCR7 | 7-dehydrocholesterol reductase [Source:HGNC Symbol;Acc:HGNC:2860] |
| ENSG00000173349 | HGLibA_43758 | 15 | -0.999336397 | SFT2D3 | SFT2 domain containing 3 [Source:HGNC Symbol;Acc:HGNC:28767] |
| ENSG00000173678 | HGLibA_46586 | 15 | -0.999336397 | SPDYE2B | speedy/RINGO cell cycle regulator family member E2B [Source:HGNC Symbol;Acc:HGNC:48334] |
| ENSG00000174137 | HGLibA_16636 | 15 | -0.999336397 | FAM53A | family with sequence similarity 53 member A [Source:HGNC Symbol;Acc:HGNC:31860] |
| ENSG00000175513 | HGLibA_51850 | 15 | -0.999336397 | TSGA10IP | testis specific 10 interacting protein [Source:HGNC Symbol;Acc:HGNC:26555] |
| ENSG00000175592 | HGLibA_17802 | 15 | -0.999336397 | FOSL1 | AP-1 transcription factor subunit [Source:HGNC Symbol;Acc:HGNC:13718]" |
| ENSG00000175894 | HGLibA_51954 | 15 | -0.999336397 | TSPEAR | thrombospondin type laminin G domain and EAR repeats [Source:HGNC Symbol;Acc:HGNC:1268] |
| ENSG00000176148 | HGLibA_48844 | 15 | -0.999336397 | TCP11L1 | t-complex 11 like 1 [Source:HGNC Symbol;Acc:HGNC:25655] |
| ENSG00000176246 | HGLibA_34038 | 15 | -0.999336397 | OR4L1 | olfactory receptor family 4 subfamily L member 1 [Source:HGNC Symbol;Acc:HGNC:15356] |
| ENSG00000176261 | HGLibA_54930 | 15 | -0.999336397 | ZBTB8OS | zinc finger and BTB domain containing 8 opposite strand [Source:HGNC Symbol;Acc:HGNC:24094] |
| ENSG00000176299 | HGLibA_34040 | 15 | -0.999336397 | OR4M1 | olfactory receptor family 4 subfamily M member 1 [Source:HGNC Symbol;Acc:HGNC:14735] |
| ENSG00000177181 | HGLibA_41271 | 15 | -0.999336397 | RIMKLA | ribosomal modification protein rimK like family member A [Source:HGNC Symbol;Acc:HGNC:28725] |
| ENSG00000177272 | HGLibA_24132 | 15 | -0.999336397 | KCNA3 | potassium voltage-gated channel subfamily A member 3 [Source:HGNC Symbol;Acc:HGNC:6221] |
| ENSG00000177432 | HGLibA_31122 | 15 | -0.999336397 | NAP1L5 | nucleosome assembly protein 1 like 5 [Source:HGNC Symbol;Acc:HGNC:19968] |
| ENSG00000177830 | HGLibA_09452 | 15 | -0.999336397 | CHID1 | chitinase domain containing 1 [Source:HGNC Symbol;Acc:HGNC:28474] |
| ENSG00000177889 | HGLibA_52637 | 15 | -0.999336397 | UBE2N | ubiquitin conjugating enzyme E2 N [Source:HGNC Symbol;Acc:HGNC:12492] |
| ENSG00000178279 | HGLibA_50896 | 15 | -0.999336397 | TNP2 | transition protein 2 [Source:HGNC Symbol;Acc:HGNC:11952] |
| ENSG00000179817 | HGLibA_29854 | 15 | -0.999336397 | MRGPRX4 | MAS related GPR family member X4 [Source:HGNC Symbol;Acc:HGNC:17617] |
| ENSG00000180694 | HGLibA_50483 | 15 | -0.999336397 | TMEM64 | transmembrane protein 64 [Source:HGNC Symbol;Acc:HGNC:25441] |
| ENSG00000182827 | HGLibA_00397 | 15 | -0.999336397 | ACBD3 | acyl-CoA binding domain containing 3 [Source:HGNC Symbol;Acc:HGNC:15453] |
| ENSG00000183160 | HGLibA_49892 | 15 | -0.999336397 | TMEM119 | transmembrane protein 119 [Source:HGNC Symbol;Acc:HGNC:27884] |
| ENSG00000183615 | HGLibA_16243 | 15 | -0.999336397 | FAM167B | family with sequence similarity 167 member B [Source:HGNC Symbol;Acc:HGNC:28133] |
| ENSG00000184898 | HGLibA_40553 | 15 | -0.999336397 | RBM43 | RNA binding motif protein 43 [Source:HGNC Symbol;Acc:HGNC:24790] |
| ENSG00000185261 | HGLibA_24655 | 15 | -0.999336397 | KIAA0825 | KIAA0825 [Source:HGNC Symbol;Acc:HGNC:28532] |
| ENSG00000185869 | HGLibA_56826 | 15 | -0.999336397 | ZNF829 | zinc finger protein 829 [Source:HGNC Symbol;Acc:HGNC:34032] |
| ENSG00000186352 | HGLibA_02089 | 15 | -0.999336397 | ANKRD37 | ankyrin repeat domain 37 [Source:HGNC Symbol;Acc:HGNC:29593] |
| ENSG00000186487 | HGLibA_30929 | 15 | -0.999336397 | MYT1L | myelin transcription factor 1 like [Source:HGNC Symbol;Acc:HGNC:7623] |
| ENSG00000187144 | HGLibA_46449 | 15 | -0.999336397 | SPATA21 | spermatogenesis associated 21 [Source:HGNC Symbol;Acc:HGNC:28026] |
| ENSG00000187527 | HGLibA_03577 | 15 | -0.999336397 | ATP13A5 | ATPase 13A5 [Source:HGNC Symbol;Acc:HGNC:31789] |
| ENSG00000188175 | HGLibA_21140 | 15 | -0.999336397 | HEPACAM2 | HEPACAM family member 2 [Source:HGNC Symbol;Acc:HGNC:27364] |
| ENSG00000188763 | HGLibA_18288 | 15 | -0.999336397 | FZD9 | frizzled class receptor 9 [Source:HGNC Symbol;Acc:HGNC:4047] |
| ENSG00000188859 | HGLibA_16732 | 15 | -0.999336397 | FAM78B | family with sequence similarity 78 member B [Source:HGNC Symbol;Acc:HGNC:13495] |
| ENSG00000189280 | HGLibA_19168 | 15 | -0.999336397 | GJB5 | gap junction protein beta 5 [Source:HGNC Symbol;Acc:HGNC:4287] |
| ENSG00000196126 | HGLibA_21636 | 15 | -0.999336397 | HLA-DRB1 | DR beta 1 [Source:HGNC Symbol;Acc:HGNC:4948]" |
| ENSG00000196411 | HGLibA_15320 | 15 | -0.999336397 | EPHB4 | EPH receptor B4 [Source:HGNC Symbol;Acc:HGNC:3395] |
| ENSG00000196805 | HGLibA_46810 | 15 | -0.999336397 | SPRR2B | small proline rich protein 2B [Source:HGNC Symbol;Acc:HGNC:11262] |
| ENSG00000197044 | HGLibA_56006 | 15 | -0.999336397 | ZNF441 | zinc finger protein 441 [Source:HGNC Symbol;Acc:HGNC:20875] |
| ENSG00000197498 | HGLibA_41833 | 15 | -0.999336397 | RPF2 | ribosome production factor 2 homolog [Source:HGNC Symbol;Acc:HGNC:20870] |
| ENSG00000197622 | HGLibA_08581 | 15 | -0.999336397 | CDC42SE1 | CDC42 small effector 1 [Source:HGNC Symbol;Acc:HGNC:17719] |
| ENSG00000198492 | HGLibA_54732 | 15 | -0.999336397 | YTHDF2 | YTH N6-methyladenosine RNA binding protein 2 [Source:HGNC Symbol;Acc:HGNC:31675] |
| ENSG00000198554 | HGLibA_54016 | 15 | -0.999336397 | WDHD1 | WD repeat and HMG-box DNA binding protein 1 [Source:HGNC Symbol;Acc:HGNC:23170] |
| ENSG00000198648 | HGLibA_47508 | 15 | -0.999336397 | STK39 | serine/threonine kinase 39 [Source:HGNC Symbol;Acc:HGNC:17717] |
| ENSG00000198721 | HGLibA_14408 | 15 | -0.999336397 | ECI2 | enoyl-CoA delta isomerase 2 [Source:HGNC Symbol;Acc:HGNC:14601] |
| ENSG00000205097 | HGLibA_17997 | 15 | -0.999336397 | FRG2 | FSHD region gene 2 [Source:HGNC Symbol;Acc:HGNC:19136] |
| ENSG00000212124 | HGLibA_48379 | 15 | -0.999336397 | TAS2R19 | taste 2 receptor member 19 [Source:HGNC Symbol;Acc:HGNC:19108] |
| ENSG00000213741 | HGLibA_42135 | 15 | -0.999336397 | RPS29 | ribosomal protein S29 [Source:HGNC Symbol;Acc:HGNC:10419] |
| ENSG00000213853 | HGLibA_15077 | 15 | -0.999336397 | EMP2 | epithelial membrane protein 2 [Source:HGNC Symbol;Acc:HGNC:3334] |
| ENSG00000214193 | HGLibA_43944 | 15 | -0.999336397 | SH3D21 | SH3 domain containing 21 [Source:HGNC Symbol;Acc:HGNC:26236] |
| ENSG00000221837 | HGLibA_25623 | 15 | -0.999336397 | KRTAP10-9 | keratin associated protein 10-9 [Source:HGNC Symbol;Acc:HGNC:22971] |
| ENSG00000239839 | HGLibA_12797 | 15 | -0.999336397 | DEFA3 | defensin alpha 3 [Source:HGNC Symbol;Acc:HGNC:2762] |
| ENSG00000241935 | HGLibA_21861 | 15 | -0.999336397 | HOGA1 | 4-hydroxy-2-oxoglutarate aldolase 1 [Source:HGNC Symbol;Acc:HGNC:25155] |
| ENSG00000242019 | HGLibA_24997 | 15 | -0.999336397 | KIR3DL3 | three Ig domains and long cytoplasmic tail 3 [Source:HGNC Symbol;Acc:HGNC:16312]" |
| ENSG00000247077 | HGLibA_36205 | 15 | -0.999336397 | PGAM5 | mitochondrial serine/threonine protein phosphatase [Source:HGNC Symbol;Acc:HGNC:28763]" |
| ENSG00000256870 | HGLibA_45283 | 15 | -0.999336397 | SLC5A8 | solute carrier family 5 member 8 [Source:HGNC Symbol;Acc:HGNC:19119] |
| ENSG00000258405 | HGLibA_56300 | 15 | -0.999336397 | ZNF578 | zinc finger protein 578 [Source:HGNC Symbol;Acc:HGNC:26449] |
| ENSG00000260001 | HGLibA_49233 | 15 | -0.999336397 | TGFBR3L | transforming growth factor beta receptor 3 like [Source:HGNC Symbol;Acc:HGNC:44152] |
| ENSG00000269190 | HGLibA_17035 | 15 | -0.999336397 | FBXO17 | F-box protein 17 [Source:HGNC Symbol;Acc:HGNC:18754] |
| ENSG00000276040 | HGLibA_46486 | 15 | -0.999336397 | SPATA31A7 | SPATA31 subfamily A member 7 [Source:HGNC Symbol;Acc:HGNC:32007] |
| ENSG00000288722 | HGLibA_15885 | 15 | -0.999336397 | F8A1 | - |
| FAM196A | HGLibA_16380 | 15 | -0.999336397 | FAM196A | - |
| HEXDC | HGLibA_21207 | 15 | -0.999336397 | HEXDC | - |
| HFE2 | HGLibA_21229 | 15 | -0.999336397 | HFE2 | - |
| HIST1H2AH | HGLibA_21393 | 15 | -0.999336397 | HIST1H2AH | - |
| HIST1H2BA | HGLibA_21410 | 15 | -0.999336397 | HIST1H2BA | - |
| HIST1H2BE | HGLibA_21422 | 15 | -0.999336397 | HIST1H2BE | - |
| HIST1H3H | HGLibA_21476 | 15 | -0.999336397 | HIST1H3H | - |

| | | | | | |
|---|---|---|---|---|---|
| HIST1H4G | HGLibA_21505 | 15 | -0.999336397 | HIST1H4G | - |
| hsa-mir-1182 | HGLibA_57220 | 15 | -0.999336397 | hsa-mir-1182 | - |
| hsa-mir-140 | HGLibA_57825 | 15 | -0.999336397 | hsa-mir-140 | - |
| hsa-mir-2054 | HGLibA_58189 | 15 | -0.999336397 | hsa-mir-2054 | - |
| hsa-mir-3157 | HGLibA_58700 | 15 | -0.999336397 | hsa-mir-3157 | - |
| hsa-mir-3165 | HGLibA_58739 | 15 | -0.999336397 | hsa-mir-3165 | - |
| hsa-mir-3188 | HGLibA_58849 | 15 | -0.999336397 | hsa-mir-3188 | - |
| hsa-mir-3197 | HGLibA_58884 | 15 | -0.999336397 | hsa-mir-3197 | - |
| hsa-mir-3675 | HGLibA_59254 | 15 | -0.999336397 | hsa-mir-3675 | - |
| hsa-mir-384 | HGLibA_59488 | 15 | -0.999336397 | hsa-mir-384 | - |
| hsa-mir-3916 | HGLibA_59542 | 15 | -0.999336397 | hsa-mir-3916 | - |
| hsa-mir-3975 | HGLibA_59658 | 15 | -0.999336397 | hsa-mir-3975 | - |
| hsa-mir-4311 | HGLibA_59960 | 15 | -0.999336397 | hsa-mir-4311 | - |
| hsa-mir-4318 | HGLibA_59990 | 15 | -0.999336397 | hsa-mir-4318 | - |
| hsa-mir-4329 | HGLibA_60038 | 15 | -0.999336397 | hsa-mir-4329 | - |
| hsa-mir-4460 | HGLibA_60246 | 15 | -0.999336397 | hsa-mir-4460 | - |
| hsa-mir-449c | HGLibA_60423 | 15 | -0.999336397 | hsa-mir-449c | - |
| hsa-mir-4637 | HGLibA_60654 | 15 | -0.999336397 | hsa-mir-4637 | - |
| hsa-mir-4708 | HGLibA_60932 | 15 | -0.999336397 | hsa-mir-4708 | - |
| hsa-mir-4714 | HGLibA_60956 | 15 | -0.999336397 | hsa-mir-4714 | - |
| hsa-mir-513a-2 | HGLibA_61576 | 15 | -0.999336397 | hsa-mir-513a-2 | - |
| hsa-mir-514b | HGLibA_61600 | 15 | -0.999336397 | hsa-mir-514b | - |
| hsa-mir-516b-2 | HGLibA_61621 | 15 | -0.999336397 | hsa-mir-516b-2 | - |
| hsa-mir-519e | HGLibA_61731 | 15 | -0.999336397 | hsa-mir-519e | - |
| hsa-mir-548l | HGLibA_62035 | 15 | -0.999336397 | hsa-mir-548l | - |
| hsa-mir-597 | HGLibA_62479 | 15 | -0.999336397 | hsa-mir-597 | - |
| hsa-mir-617 | HGLibA_62706 | 15 | -0.999336397 | hsa-mir-617 | - |
| hsa-mir-6862-1 | HGLibA_63628 | 15 | -0.999336397 | hsa-mir-6862-1 | - |
| hsa-mir-6884 | HGLibA_63720 | 15 | -0.999336397 | hsa-mir-6884 | - |
| PRKRIR | HGLibA_38590 | 15 | -0.999336397 | PRKRIR | - |
| TMEM257 | HGLibA_50339 | 15 | -0.999336397 | TMEM257 | - |
| UQCC | HGLibA_53094 | 15 | -0.999336397 | UQCC | - |
| WDR16 | HGLibA_54034 | 15 | -0.999336397 | WDR16 | - |
| ADSS | HGLibA_01075 | 14 | -1.009287435 | ADSS | - |
| AIM1 | HGLibA_01349 | 14 | -1.009287435 | AIM1 | - |
| C15orf59 | HGLibA_05422 | 14 | -1.009287435 | C15orf59 | - |
| C15orf60 | HGLibA_05427 | 14 | -1.009287435 | C15orf60 | - |
| C17orf85 | HGLibA_05589 | 14 | -1.009287435 | C17orf85 | - |
| CTAGE5 | HGLibA_11576 | 14 | -1.009287435 | CTAGE5 | - |
| DHRSX_X | HGLibA_13158 | 14 | -1.009287435 | DHRSX_X | - |
| ENSG00000003756 | HGLibA_40576 | 14 | -1.009287435 | RBM5 | RNA binding motif protein 5 [Source:HGNC Symbol;Acc:HGNC:9902] |
| ENSG00000008197 | HGLibA_49129 | 14 | -1.009287435 | TFAP2D | transcription factor AP-2 delta [Source:HGNC Symbol;Acc:HGNC:15581] |
| ENSG00000008838 | HGLibA_28785 | 14 | -1.009287435 | MED24 | mediator complex subunit 24 [Source:HGNC Symbol;Acc:HGNC:22963] |
| ENSG00000011052 | HGLibA_32153 | 14 | -1.009287435 | NME1-NME2 | NME1-NME2 readthrough [Source:HGNC Symbol;Acc:HGNC:33531] |
| ENSG00000011590 | HGLibA_54860 | 14 | -1.009287435 | ZBTB32 | zinc finger and BTB domain containing 32 [Source:HGNC Symbol;Acc:HGNC:16763] |
| ENSG00000013288 | HGLibA_28079 | 14 | -1.009287435 | MAN2B2 | mannosidase alpha class 2B member 2 [Source:HGNC Symbol;Acc:HGNC:29623] |
| ENSG00000029534 | HGLibA_01934 | 14 | -1.009287435 | ANK1 | ankyrin 1 [Source:HGNC Symbol;Acc:HGNC:492] |
| ENSG00000029639 | HGLibA_49139 | 14 | -1.009287435 | TFB1M | mitochondrial [Source:HGNC Symbol;Acc:HGNC:17037]" |
| ENSG00000031823 | HGLibA_40203 | 14 | -1.009287435 | RANBP3 | RAN binding protein 3 [Source:HGNC Symbol;Acc:HGNC:9850] |
| ENSG00000033100 | HGLibA_09527 | 14 | -1.009287435 | CHPF2 | chondroitin polymerizing factor 2 [Source:HGNC Symbol;Acc:HGNC:29270] |
| ENSG00000052344 | HGLibA_38975 | 14 | -1.009287435 | PRSS8 | serine protease 8 [Source:HGNC Symbol;Acc:HGNC:9491] |
| ENSG00000065491 | HGLibA_48505 | 14 | -1.009287435 | TBC1D22B | TBC1 domain family member 22B [Source:HGNC Symbol;Acc:HGNC:21602] |
| ENSG00000075218 | HGLibA_20625 | 14 | -1.009287435 | GTSE1 | G2 and S-phase expressed 1 [Source:HGNC Symbol;Acc:HGNC:13698] |
| ENSG00000077264 | HGLibA_35046 | 14 | -1.009287435 | PAK3 | p21 (RAC1) activated kinase 3 [Source:HGNC Symbol;Acc:HGNC:8592] |
| ENSG00000078043 | HGLibA_36511 | 14 | -1.009287435 | PIAS2 | protein inhibitor of activated STAT 2 [Source:HGNC Symbol;Acc:HGNC:17311] |
| ENSG00000079616 | HGLibA_24905 | 14 | -1.009287435 | KIF22 | kinesin family member 22 [Source:HGNC Symbol;Acc:HGNC:6391] |
| ENSG00000090006 | HGLibA_27599 | 14 | -1.009287435 | LTBP4 | latent transforming growth factor beta binding protein 4 [Source:HGNC Symbol;Acc:HGNC:6717] |
| ENSG00000090905 | HGLibA_50913 | 14 | -1.009287435 | TNRC6A | trinucleotide repeat containing 6A [Source:HGNC Symbol;Acc:HGNC:11969] |
| ENSG00000091039 | HGLibA_34666 | 14 | -1.009287435 | OSBPL8 | oxysterol binding protein like 8 [Source:HGNC Symbol;Acc:HGNC:16396] |
| ENSG00000092199 | HGLibA_21807 | 14 | -1.009287435 | HNRNPC | heterogeneous nuclear ribonucleoprotein C (C1/C2) [Source:HGNC Symbol;Acc:HGNC:5035] |
| ENSG00000100226 | HGLibA_20598 | 14 | -1.009287435 | GTPBP1 | GTP binding protein 1 [Source:HGNC Symbol;Acc:HGNC:4669] |
| ENSG00000100263 | HGLibA_41129 | 14 | -1.009287435 | RHBDD3 | rhomboid domain containing 3 [Source:HGNC Symbol;Acc:HGNC:1308] |
| ENSG00000100307 | HGLibA_07464 | 14 | -1.009287435 | CBX7 | chromobox 7 [Source:HGNC Symbol;Acc:HGNC:1557] |
| ENSG00000100379 | HGLibA_24436 | 14 | -1.009287435 | KCTD17 | potassium channel tetramerization domain containing 17 [Source:HGNC Symbol;Acc:HGNC:25705] |
| ENSG00000101282 | HGLibA_42352 | 14 | -1.009287435 | RSPO4 | R-spondin 4 [Source:HGNC Symbol;Acc:HGNC:16175] |
| ENSG00000102145 | HGLibA_18665 | 14 | -1.009287435 | GATA1 | GATA binding protein 1 [Source:HGNC Symbol;Acc:HGNC:4170] |
| ENSG00000102606 | HGLibA_02875 | 14 | -1.009287435 | ARHGEF7 | Rho guanine nucleotide exchange factor 7 [Source:HGNC Symbol;Acc:HGNC:15607] |
| ENSG00000103037 | HGLibA_43655 | 14 | -1.009287435 | SETD6 | SET domain containing 6 [Source:HGNC Symbol;Acc:HGNC:26116] |
| ENSG00000103091 | HGLibA_54129 | 14 | -1.009287435 | WDR59 | WD repeat domain 59 [Source:HGNC Symbol;Acc:HGNC:25706] |
| ENSG00000103253 | HGLibA_20818 | 14 | -1.009287435 | HAGHL | hydroxyacylglutathione hydrolase like [Source:HGNC Symbol;Acc:HGNC:14177] |
| ENSG00000104341 | HGLibA_26019 | 14 | -1.009287435 | LAPTM4B | lysosomal protein transmembrane 4 beta [Source:HGNC Symbol;Acc:HGNC:13646] |
| ENSG00000105085 | HGLibA_28791 | 14 | -1.009287435 | MED26 | mediator complex subunit 26 [Source:HGNC Symbol;Acc:HGNC:2376] |
| ENSG00000105321 | HGLibA_07920 | 14 | -1.009287435 | CCDC9 | coiled-coil domain containing 9 [Source:HGNC Symbol;Acc:HGNC:24560] |
| ENSG00000105443 | HGLibA_12239 | 14 | -1.009287435 | CYTH2 | cytohesin 2 [Source:HGNC Symbol;Acc:HGNC:9502] |
| ENSG00000105695 | HGLibA_27888 | 14 | -1.009287435 | MAG | myelin associated glycoprotein [Source:HGNC Symbol;Acc:HGNC:6783] |
| ENSG00000105954 | HGLibA_32541 | 14 | -1.009287435 | NPVF | neuropeptide VF precursor [Source:HGNC Symbol;Acc:HGNC:13782] |
| ENSG00000106049 | HGLibA_21283 | 14 | -1.009287435 | HIBADH | 3-hydroxyisobutyrate dehydrogenase [Source:HGNC Symbol;Acc:HGNC:4907] |
| ENSG00000107959 | HGLibA_36759 | 14 | -1.009287435 | PITRM1 | pitrilysin metallopeptidase 1 [Source:HGNC Symbol;Acc:HGNC:17663] |
| ENSG00000108389 | HGLibA_30429 | 14 | -1.009287435 | MTMR4 | myotubularin related protein 4 [Source:HGNC Symbol;Acc:HGNC:7452] |
| ENSG00000108823 | HGLibA_43796 | 14 | -1.009287435 | SGCA | sarcoglycan alpha [Source:HGNC Symbol;Acc:HGNC:10805] |
| ENSG00000109103 | HGLibA_52987 | 14 | -1.009287435 | UNC119 | unc-119 lipid binding chaperone [Source:HGNC Symbol;Acc:HGNC:12565] |
| ENSG00000109111 | HGLibA_47823 | 14 | -1.009287435 | SUPT6H | histone chaperone [Source:HGNC Symbol;Acc:HGNC:11470]" |
| ENSG00000109475 | HGLibA_41942 | 14 | -1.009287435 | RPL34 | ribosomal protein L34 [Source:HGNC Symbol;Acc:HGNC:10340] |
| ENSG00000111252 | HGLibA_43879 | 14 | -1.009287435 | SH2B3 | SH2B adaptor protein 3 [Source:HGNC Symbol;Acc:HGNC:29605] |
| ENSG00000112936 | HGLibA_06518 | 14 | -1.009287435 | C7 | complement C7 [Source:HGNC Symbol;Acc:HGNC:1346] |
| ENSG00000113140 | HGLibA_46417 | 14 | -1.009287435 | SPARC | secreted protein acidic and cysteine rich [Source:HGNC Symbol;Acc:HGNC:11219] |
| ENSG00000114200 | HGLibA_04275 | 14 | -1.009287435 | BCHE | butyrylcholinesterase [Source:HGNC Symbol;Acc:HGNC:983] |
| ENSG00000114248 | HGLibA_27310 | 14 | -1.009287435 | LRRC31 | leucine rich repeat containing 31 [Source:HGNC Symbol;Acc:HGNC:26261] |
| ENSG00000114473 | HGLibA_23517 | 14 | -1.009287435 | IQCG | IQ motif containing G [Source:HGNC Symbol;Acc:HGNC:25251] |
| ENSG00000114933 | HGLibA_23329 | 14 | -1.009287435 | INO80D | INO80 complex subunit D [Source:HGNC Symbol;Acc:HGNC:25997] |
| ENSG00000115266 | HGLibA_02393 | 14 | -1.009287435 | APC2 | WNT signaling pathway regulator [Source:HGNC Symbol;Acc:HGNC:24036]" |
| ENSG00000116147 | HGLibA_50908 | 14 | -1.009287435 | TNR | tenascin R [Source:HGNC Symbol;Acc:HGNC:11953] |
| ENSG00000116213 | HGLibA_54402 | 14 | -1.009287435 | WRAP73 | antisense to TP73 [Source:HGNC Symbol;Acc:HGNC:12759]" |
| ENSG00000117868 | HGLibA_15607 | 14 | -1.009287435 | ESYT2 | extended synaptotagmin 2 [Source:HGNC Symbol;Acc:HGNC:22211] |
| ENSG00000119760 | HGLibA_47827 | 14 | -1.009287435 | SUPT7L | STAGA complex gamma subunit [Source:HGNC Symbol;Acc:HGNC:30632]" |
| ENSG00000120697 | HGLibA_01631 | 14 | -1.009287435 | ALG5 | dolichyl-phosphate beta-glucosyltransferase [Source:HGNC Symbol;Acc:HGNC:20266]" |
| ENSG00000122643 | HGLibA_32814 | 14 | -1.009287435 | NT5C3A | cytosolic IIIA [Source:HGNC Symbol;Acc:HGNC:17820]" |
| ENSG00000123352 | HGLibA_46546 | 14 | -1.009287435 | SPATS2 | spermatogenesis associated serine rich 2 [Source:HGNC Symbol;Acc:HGNC:18650] |
| ENSG00000124216 | HGLibA_45866 | 14 | -1.009287435 | SNAI1 | snail family transcriptional repressor 1 [Source:HGNC Symbol;Acc:HGNC:11128] |
| ENSG00000124596 | HGLibA_33194 | 14 | -1.009287435 | OARD1 | O-acyl-ADP-ribose deacylase 1 [Source:HGNC Symbol;Acc:HGNC:21257] |
| ENSG00000124664 | HGLibA_46566 | 14 | -1.009287435 | SPDEF | SAM pointed domain containing ETS transcription factor [Source:HGNC Symbol;Acc:HGNC:17257] |
| ENSG00000125378 | HGLibA_04607 | 14 | -1.009287435 | BMP4 | bone morphogenetic protein 4 [Source:HGNC Symbol;Acc:HGNC:1071] |
| ENSG00000126217 | HGLibA_28581 | 14 | -1.009287435 | MCF2L | MCF.2 cell line derived transforming sequence like [Source:HGNC Symbol;Acc:HGNC:14576] |
| ENSG00000126267 | HGLibA_10847 | 14 | -1.009287435 | COX6B1 | cytochrome c oxidase subunit 6B1 [Source:HGNC Symbol;Acc:HGNC:2280] |
| ENSG00000129158 | HGLibA_43478 | 14 | -1.009287435 | SERGEF | secretion regulating guanine nucleotide exchange factor [Source:HGNC Symbol;Acc:HGNC:17499] |
| ENSG00000130707 | HGLibA_03370 | 14 | -1.009287435 | ASS1 | argininosuccinate synthase 1 [Source:HGNC Symbol;Acc:HGNC:758] |
| ENSG00000130749 | HGLibA_54976 | 14 | -1.009287435 | ZC3H4 | zinc finger CCCH-type containing 4 [Source:HGNC Symbol;Acc:HGNC:17808] |
| ENSG00000131043 | HGLibA_00061 | 14 | -1.009287435 | AAR2 | AAR2 splicing factor homolog [Source:HGNC Symbol;Acc:HGNC:15886] |
| ENSG00000131467 | HGLibA_39197 | 14 | -1.009287435 | PSME3 | proteasome activator subunit 3 [Source:HGNC Symbol;Acc:HGNC:9570] |
| ENSG00000132436 | HGLibA_17522 | 14 | -1.009287435 | FIGNL1 | fidgetin like 1 [Source:HGNC Symbol;Acc:HGNC:13286] |
| ENSG00000132669 | HGLibA_41293 | 14 | -1.009287435 | RIN2 | Ras and Rab interactor 2 [Source:HGNC Symbol;Acc:HGNC:18750] |
| ENSG00000133055 | HGLibA_30659 | 14 | -1.009287435 | MYBPH | myosin binding protein H [Source:HGNC Symbol;Acc:HGNC:7552] |
| ENSG00000133398 | HGLibA_28737 | 14 | -1.009287435 | MED10 | mediator complex subunit 10 [Source:HGNC Symbol;Acc:HGNC:28760] |
| ENSG00000134375 | HGLibA_49501 | 14 | -1.009287435 | TIMM17A | translocase of inner mitochondrial membrane 17A [Source:HGNC Symbol;Acc:HGNC:17315] |
| ENSG00000134954 | HGLibA_15640 | 14 | -1.009287435 | ETS1 | transcription factor [Source:HGNC Symbol;Acc:HGNC:3488]" |
| ENSG00000135972 | HGLibA_30113 | 14 | -1.009287435 | MRPS9 | mitochondrial ribosomal protein S9 [Source:HGNC Symbol;Acc:HGNC:14501] |
| ENSG00000136152 | HGLibA_10477 | 14 | -1.009287435 | COG3 | component of oligomeric golgi complex 3 [Source:HGNC Symbol;Acc:HGNC:18619] |
| ENSG00000136158 | HGLibA_46837 | 14 | -1.009287435 | SPRY2 | sprouty RTK signaling antagonist 2 [Source:HGNC Symbol;Acc:HGNC:11270] |
| ENSG00000136770 | HGLibA_13561 | 14 | -1.009287435 | DNAJC1 | DnaJ heat shock protein family (Hsp40) member C1 [Source:HGNC Symbol;Acc:HGNC:20090] |
| ENSG00000136842 | HGLibA_50593 | 14 | -1.009287435 | TMOD1 | tropomodulin 1 [Source:HGNC Symbol;Acc:HGNC:11871] |
| ENSG00000136877 | HGLibA_17959 | 14 | -1.009287435 | FPGS | folylpolyglutamate synthase [Source:HGNC Symbol;Acc:HGNC:3824] |
| ENSG00000138074 | HGLibA_45276 | 14 | -1.009287435 | SLC5A6 | solute carrier family 5 member 6 [Source:HGNC Symbol;Acc:HGNC:11041] |
| ENSG00000138315 | HGLibA_33335 | 14 | -1.009287435 | OIT3 | oncoprotein induced transcript 3 [Source:HGNC Symbol;Acc:HGNC:29953] |
| ENSG00000138363 | HGLibA_03512 | 14 | -1.009287435 | ATIC | 5-aminoimidazole-4-carboxamide ribonucleotide formyltransferase/IMP cyclohydrolase [Source:HGNC Symbol;Acc:HGNC:794] |
| ENSG00000139354 | HGLibA_18649 | 14 | -1.009287435 | GAS2L3 | growth arrest specific 2 like 3 [Source:HGNC Symbol;Acc:HGNC:27475] |
| ENSG00000143393 | HGLibA_36502 | 14 | -1.009287435 | PI4KB | phosphatidylinositol 4-kinase beta [Source:HGNC Symbol;Acc:HGNC:8984] |
| ENSG00000143520 | HGLibA_17613 | 14 | -1.009287435 | FLG2 | filaggrin family member 2 [Source:HGNC Symbol;Acc:HGNC:33276] |
| ENSG00000143630 | HGLibA_20978 | 14 | -1.009287435 | HCN3 | hyperpolarization activated cyclic nucleotide gated potassium channel 3 [Source:HGNC Symbol;Acc:HGNC:19183] |
| ENSG00000143819 | HGLibA_15324 | 14 | -1.009287435 | EPHX1 | epoxide hydrolase 1 [Source:HGNC Symbol;Acc:HGNC:3401] |
| ENSG00000144481 | HGLibA_51773 | 14 | -1.009287435 | TRPM8 | transient receptor potential cation channel subfamily M member 8 [Source:HGNC Symbol;Acc:HGNC:17961] |
| ENSG00000146166 | HGLibA_26416 | 14 | -1.009287435 | LGSN | lens protein with glutamine synthetase domain [Source:HGNC Symbol;Acc:HGNC:21016]" |
| ENSG00000146386 | HGLibA_00330 | 14 | -1.009287435 | ABRACL | ABRA C-terminal like [Source:HGNC Symbol;Acc:HGNC:21230] |
| ENSG00000147459 | HGLibA_13750 | 14 | -1.009287435 | DOCK5 | dedicator of cytokinesis 5 [Source:HGNC Symbol;Acc:HGNC:23476] |
| ENSG00000147874 | HGLibA_20889 | 14 | -1.009287435 | HAUS6 | HAUS augmin like complex subunit 6 [Source:HGNC Symbol;Acc:HGNC:25948] |
| ENSG00000150594 | HGLibA_01046 | 14 | -1.009287435 | ADRA2A | adrenoceptor alpha 2A [Source:HGNC Symbol;Acc:HGNC:281] |

| | | | | | |
|---|---|---|---|---|---|
| ENSG00000151025 | HGLibA_19950 | 14 | -1.009287435 | GPR158 | G protein-coupled receptor 158 [Source:HGNC Symbol;Acc:HGNC:23689] |
| ENSG00000151532 | HGLibA_53886 | 14 | -1.009287435 | VTI1A | vesicle transport through interaction with t-SNAREs 1A [Source:HGNC Symbol;Acc:HGNC:17792] |
| ENSG00000152620 | HGLibA_31047 | 14 | -1.009287435 | NADK2 | mitochondrial [Source:HGNC Symbol;Acc:HGNC:26404]" |
| ENSG00000153060 | HGLibA_48984 | 14 | -1.009287435 | TEKT5 | tektin 5 [Source:HGNC Symbol;Acc:HGNC:26554] |
| ENSG00000154479 | HGLibA_07701 | 14 | -1.009287435 | CCDC173 | coiled-coil domain containing 173 [Source:HGNC Symbol;Acc:HGNC:25064] |
| ENSG00000155111 | HGLibA_08753 | 14 | -1.009287435 | CDK19 | cyclin dependent kinase 19 [Source:HGNC Symbol;Acc:HGNC:19338] |
| ENSG00000155926 | HGLibA_44301 | 14 | -1.009287435 | SLA | Src like adaptor [Source:HGNC Symbol;Acc:HGNC:10902] |
| ENSG00000156521 | HGLibA_52478 | 14 | -1.009287435 | TYSND1 | trypsin domain containing 1 [Source:HGNC Symbol;Acc:HGNC:28531] |
| ENSG00000156858 | HGLibA_38778 | 14 | -1.009287435 | PRR14 | proline rich 14 [Source:HGNC Symbol;Acc:HGNC:28458] |
| ENSG00000159650 | HGLibA_53151 | 14 | -1.009287435 | UROC1 | urocanate hydratase 1 [Source:HGNC Symbol;Acc:HGNC:26444] |
| ENSG00000162614 | HGLibA_31755 | 14 | -1.009287435 | NEXN | nexilin F-actin binding protein [Source:HGNC Symbol;Acc:HGNC:29557] |
| ENSG00000162889 | HGLibA_28302 | 14 | -1.009287435 | MAPKAPK2 | mitogen-activated protein kinase-activated protein kinase 2 [Source:HGNC Symbol;Acc:HGNC:6887] |
| ENSG00000163092 | HGLibA_54514 | 14 | -1.009287435 | XIRP2 | xin actin binding repeat containing 2 [Source:HGNC Symbol;Acc:HGNC:14303] |
| ENSG00000163170 | HGLibA_04689 | 14 | -1.009287435 | BOLA3 | bolA family member 3 [Source:HGNC Symbol;Acc:HGNC:24415] |
| ENSG00000163354 | HGLibA_12532 | 14 | -1.009287435 | DCST2 | DC-STAMP domain containing 2 [Source:HGNC Symbol;Acc:HGNC:26562] |
| ENSG00000163536 | HGLibA_43598 | 14 | -1.009287435 | SERPINI1 | serpin family I member 1 [Source:HGNC Symbol;Acc:HGNC:8943] |
| ENSG00000163811 | HGLibA_54091 | 14 | -1.009287435 | WDR43 | WD repeat domain 43 [Source:HGNC Symbol;Acc:HGNC:28945] |
| ENSG00000164022 | HGLibA_01354 | 14 | -1.009287435 | AIMP1 | aminoacyl tRNA synthetase complex interacting multifunctional protein 1 [Source:HGNC Symbol;Acc:HGNC:10648] |
| ENSG00000164338 | HGLibA_53439 | 14 | -1.009287435 | UTP15 | small subunit processome component [Source:HGNC Symbol;Acc:HGNC:25758]" |
| ENSG00000164576 | HGLibA_42729 | 14 | -1.009287435 | SAP30L | SAP30 like [Source:HGNC Symbol;Acc:HGNC:25663] |
| ENSG00000164691 | HGLibA_48266 | 14 | -1.009287435 | TAGAP | T cell activation RhoGTPase activating protein [Source:HGNC Symbol;Acc:HGNC:15669] |
| ENSG00000165887 | HGLibA_02024 | 14 | -1.009287435 | ANKRD2 | ankyrin repeat domain 2 [Source:HGNC Symbol;Acc:HGNC:495] |
| ENSG00000166250 | HGLibA_10072 | 14 | -1.009287435 | CLMP | CXADR like membrane protein [Source:HGNC Symbol;Acc:HGNC:24039] |
| ENSG00000166930 | HGLibA_30155 | 14 | -1.009287435 | MS4A5 | membrane spanning 4-domains A5 [Source:HGNC Symbol;Acc:HGNC:13374] |
| ENSG00000167555 | HGLibA_56169 | 14 | -1.009287435 | ZNF528 | zinc finger protein 528 [Source:HGNC Symbol;Acc:HGNC:29384] |
| ENSG00000168016 | HGLibA_51291 | 14 | -1.009287435 | TRANK1 | tetratricopeptide repeat and ankyrin repeat containing 1 [Source:HGNC Symbol;Acc:HGNC:29011] |
| ENSG00000168903 | HGLibA_04978 | 14 | -1.009287435 | BTNL3 | butyrophilin like 3 [Source:HGNC Symbol;Acc:HGNC:1143] |
| ENSG00000169006 | HGLibA_32888 | 14 | -1.009287435 | NTSR2 | neurotensin receptor 2 [Source:HGNC Symbol;Acc:HGNC:8040] |
| ENSG00000169067 | HGLibA_00604 | 14 | -1.009287435 | ACTBL2 | beta like 2 [Source:HGNC Symbol;Acc:HGNC:17780]" |
| ENSG00000169248 | HGLibA_11849 | 14 | -1.009287435 | CXCL11 | C-X-C motif chemokine ligand 11 [Source:HGNC Symbol;Acc:HGNC:10638] |
| ENSG00000169402 | HGLibA_42330 | 14 | -1.009287435 | RSPH10B2 | radial spoke head 10 homolog B2 [Source:HGNC Symbol;Acc:HGNC:34385] |
| ENSG00000170561 | HGLibA_23630 | 14 | -1.009287435 | IRX2 | iroquois homeobox 2 [Source:HGNC Symbol;Acc:HGNC:14359] |
| ENSG00000170954 | HGLibA_55943 | 14 | -1.009287435 | ZNF415 | zinc finger protein 415 [Source:HGNC Symbol;Acc:HGNC:20636] |
| ENSG00000171163 | HGLibA_56553 | 14 | -1.009287435 | ZNF692 | zinc finger protein 692 [Source:HGNC Symbol;Acc:HGNC:26049] |
| ENSG00000171314 | HGLibA_36195 | 14 | -1.009287435 | PGAM1 | phosphoglycerate mutase 1 [Source:HGNC Symbol;Acc:HGNC:8888] |
| ENSG00000171823 | HGLibA_16979 | 14 | -1.009287435 | FBXL14 | F-box and leucine rich repeat protein 14 [Source:HGNC Symbol;Acc:HGNC:28624] |
| ENSG00000171956 | HGLibA_17817 | 14 | -1.009287435 | FOXB1 | forkhead box B1 [Source:HGNC Symbol;Acc:HGNC:3799] |
| ENSG00000173175 | HGLibA_00919 | 14 | -1.009287435 | ADCY5 | adenylate cyclase 5 [Source:HGNC Symbol;Acc:HGNC:236] |
| ENSG00000173917 | HGLibA_21932 | 14 | -1.009287435 | HOXB2 | homeobox B2 [Source:HGNC Symbol;Acc:HGNC:5113] |
| ENSG00000174326 | HGLibA_44423 | 14 | -1.009287435 | SLC16A11 | solute carrier family 16 member 11 [Source:HGNC Symbol;Acc:HGNC:23093] |
| ENSG00000174775 | HGLibA_22074 | 14 | -1.009287435 | HRAS | GTPase [Source:HGNC Symbol;Acc:HGNC:5173]" |
| ENSG00000174827 | HGLibA_35992 | 14 | -1.009287435 | PDZK1 | PDZ domain containing 1 [Source:HGNC Symbol;Acc:HGNC:8821] |
| ENSG00000175105 | HGLibA_56466 | 14 | -1.009287435 | ZNF654 | zinc finger protein 654 [Source:HGNC Symbol;Acc:HGNC:25612] |
| ENSG00000175471 | HGLibA_28651 | 14 | -1.009287435 | MCTP1 | multiple C2 and transmembrane domain containing 1 [Source:HGNC Symbol;Acc:HGNC:26183] |
| ENSG00000176601 | HGLibA_28177 | 14 | -1.009287435 | MAP3K19 | mitogen-activated protein kinase kinase kinase 19 [Source:HGNC Symbol;Acc:HGNC:26249] |
| ENSG00000176601 | HGLibA_28179 | 14 | -1.009287435 | MAP3K19 | mitogen-activated protein kinase kinase kinase 19 [Source:HGNC Symbol;Acc:HGNC:26249] |
| ENSG00000177994 | HGLibA_06180 | 14 | -1.009287435 | C2orf73 | chromosome 2 open reading frame 73 [Source:HGNC Symbol;Acc:HGNC:26861] |
| ENSG00000178149 | HGLibA_12304 | 14 | -1.009287435 | DALRD3 | DALR anticodon binding domain containing 3 [Source:HGNC Symbol;Acc:HGNC:25536] |
| ENSG00000178974 | HGLibA_17077 | 14 | -1.009287435 | FBXO34 | F-box protein 34 [Source:HGNC Symbol;Acc:HGNC:20201] |
| ENSG00000179364 | HGLibA_34957 | 14 | -1.009287435 | PACS2 | phosphofurin acidic cluster sorting protein 2 [Source:HGNC Symbol;Acc:HGNC:23794] |
| ENSG00000179603 | HGLibA_20362 | 14 | -1.009287435 | GRM8 | glutamate metabotropic receptor 8 [Source:HGNC Symbol;Acc:HGNC:4600] |
| ENSG00000180386 | HGLibA_25846 | 14 | -1.009287435 | KRTAP9-7 | keratin associated protein 9-7 [Source:HGNC Symbol;Acc:HGNC:18915] |
| ENSG00000182511 | HGLibA_17302 | 14 | -1.009287435 | FES | tyrosine kinase [Source:HGNC Symbol;Acc:HGNC:3657]" |
| ENSG00000183318 | HGLibA_46594 | 14 | -1.009287435 | SPDYE4 | speedy/RINGO cell cycle regulator family member E4 [Source:HGNC Symbol;Acc:HGNC:35463] |
| ENSG00000184785 | HGLibA_45718 | 14 | -1.009287435 | SMIM10 | small integral membrane protein 10 [Source:HGNC Symbol;Acc:HGNC:41913] |
| ENSG00000185650 | HGLibA_55208 | 14 | -1.009287435 | ZFP36L1 | ZFP36 ring finger protein like 1 [Source:HGNC Symbol;Acc:HGNC:1107] |
| ENSG00000186075 | HGLibA_56964 | 14 | -1.009287435 | ZPBP2 | zona pellucida binding protein 2 [Source:HGNC Symbol;Acc:HGNC:20678] |
| ENSG00000186967 | HGLibA_25673 | 14 | -1.009287435 | KRTAP19-4 | keratin associated protein 19-4 [Source:HGNC Symbol;Acc:HGNC:18939] |
| ENSG00000186976 | HGLibA_14549 | 14 | -1.009287435 | EFCAB6 | EF-hand calcium binding domain 6 [Source:HGNC Symbol;Acc:HGNC:24204] |
| ENSG00000187531 | HGLibA_44233 | 14 | -1.009287435 | SIRT7 | sirtuin 7 [Source:HGNC Symbol;Acc:HGNC:14935] |
| ENSG00000188051 | HGLibA_50237 | 14 | -1.009287435 | TMEM221 | transmembrane protein 221 [Source:HGNC Symbol;Acc:HGNC:21943] |
| ENSG00000198042 | HGLibA_28020 | 14 | -1.009287435 | MAK16 | MAK16 homolog [Source:HGNC Symbol;Acc:HGNC:13703] |
| ENSG00000198522 | HGLibA_19826 | 14 | -1.009287435 | GPN1 | GPN-loop GTPase 1 [Source:HGNC Symbol;Acc:HGNC:17030] |
| ENSG00000198947 | HGLibA_13403 | 14 | -1.009287435 | DMD | dystrophin [Source:HGNC Symbol;Acc:HGNC:2928] |
| ENSG00000204655 | HGLibA_29620 | 14 | -1.009287435 | MOG | myelin oligodendrocyte glycoprotein [Source:HGNC Symbol;Acc:HGNC:7197] |
| ENSG00000205221 | HGLibA_53675 | 14 | -1.009287435 | VIT | vitrin [Source:HGNC Symbol;Acc:HGNC:12697] |
| ENSG00000205809 | HGLibA_25289 | 14 | -1.009287435 | KLRC2 | killer cell lectin like receptor C2 [Source:HGNC Symbol;Acc:HGNC:6375] |
| ENSG00000213015 | HGLibA_56305 | 14 | -1.009287435 | ZNF580 | zinc finger protein 580 [Source:HGNC Symbol;Acc:HGNC:29473] |
| ENSG00000213341 | HGLibA_09671 | 14 | -1.009287435 | CHUK | conserved helix-loop-helix ubiquitous kinase [Source:HGNC Symbol;Acc:HGNC:1974] |
| ENSG00000213424 | HGLibA_25451 | 14 | -1.009287435 | KRT222 | keratin 222 [Source:HGNC Symbol;Acc:HGNC:28695] |
| ENSG00000213676 | HGLibA_03449 | 14 | -1.009287435 | ATF6B | activating transcription factor 6 beta [Source:HGNC Symbol;Acc:HGNC:2349] |
| ENSG00000214300 | HGLibA_46591 | 14 | -1.009287435 | SPDYE3 | speedy/RINGO cell cycle regulator family member E3 [Source:HGNC Symbol;Acc:HGNC:35462] |
| ENSG00000217930 | HGLibA_35085 | 14 | -1.009287435 | PAM16 | presequence translocase associated motor 16 [Source:HGNC Symbol;Acc:HGNC:29679] |
| ENSG00000219073 | HGLibA_09002 | 14 | -1.009287435 | CELA3B | chymotrypsin like elastase family member 3B [Source:HGNC Symbol;Acc:HGNC:15945] |
| ENSG00000222011 | HGLibA_16333 | 14 | -1.009287435 | FAM185A | family with sequence similarity 185 member A [Source:HGNC Symbol;Acc:HGNC:22412] |
| ENSG00000226650 | HGLibA_24946 | 14 | -1.009287435 | KIF4B | kinesin family member 4B [Source:HGNC Symbol;Acc:HGNC:6322] |
| ENSG00000233041 | HGLibA_36395 | 14 | -1.009287435 | PHGR1 | histidine and glycine rich 1 [Source:HGNC Symbol;Acc:HGNC:37226]" |
| ENSG00000234965 | HGLibA_44050 | 14 | -1.009287435 | SHISA8 | shisa family member 8 [Source:HGNC Symbol;Acc:HGNC:18351] |
| ENSG00000237172 | HGLibA_04004 | 14 | -1.009287435 | B3GNT9 | 3-N-acetylglucosaminyltransferase 9 [Source:HGNC Symbol;Acc:HGNC:28714]" |
| ENSG00000237412 | HGLibA_38965 | 14 | -1.009287435 | PRSS56 | serine protease 56 [Source:HGNC Symbol;Acc:HGNC:39433] |
| ENSG00000241233 | HGLibA_25810 | 14 | -1.009287435 | KRTAP5-8 | keratin associated protein 5-8 [Source:HGNC Symbol;Acc:HGNC:23603] |
| ENSG00000242852 | HGLibA_56606 | 14 | -1.009287435 | ZNF709 | zinc finger protein 709 [Source:HGNC Symbol;Acc:HGNC:20629] |
| ENSG00000249860 | HGLibA_30484 | 14 | -1.009287435 | MTRNR2L5 | MT-RNR2 like 5 [Source:HGNC Symbol;Acc:HGNC:37162] |
| ENSG00000258453 | HGLibA_33575 | 14 | -1.009287435 | OR11H2 | olfactory receptor family 11 subfamily H member 2 [Source:HGNC Symbol;Acc:HGNC:14716] |
| ENSG00000268009 | HGLibA_47210 | 14 | -1.009287435 | SSX4 | SSX family member 4 [Source:HGNC Symbol;Acc:HGNC:11338] |
| ENSG00000269897 | HGLibA_10663 | 14 | -1.009287435 | COMMD3-BMI1 | COMMD3-BMI1 readthrough [Source:HGNC Symbol;Acc:HGNC:48326] |
| ENSG00000270800 | HGLibA_42061 | 14 | -1.009287435 | RPS10-NUDT3 | RPS10-NUDT3 readthrough [Source:HGNC Symbol;Acc:HGNC:49181] |
| ENSG00000270872 | HGLibA_46980 | 14 | -1.009287435 | SRGAP2D | SLIT-ROBO Rho GTPase activating protein 2D (pseudogene) [Source:HGNC Symbol;Acc:HGNC:43932] |
| ENSG00000273749 | HGLibA_12030 | 14 | -1.009287435 | CYFIP1 | cytoplasmic FMR1 interacting protein 1 [Source:HGNC Symbol;Acc:HGNC:13759] |
| ENSG00000277150 | HGLibA_15892 | 14 | -1.009287435 | F8A3 | coagulation factor VIII associated 3 [Source:HGNC Symbol;Acc:HGNC:31850] |
| ENSG00000278053 | HGLibA_12737 | 14 | -1.009287435 | DDX52 | DExD-box helicase 52 [Source:HGNC Symbol;Acc:HGNC:20038] |
| FAM63A | HGLibA_16658 | 14 | -1.009287435 | FAM63A | - |
| hsa-mir-106b | HGLibA_57189 | 14 | -1.009287435 | hsa-mir-106b | - |
| hsa-mir-1-2 | HGLibA_57146 | 14 | -1.009287435 | hsa-mir-1-2 | - |
| hsa-mir-1264 | HGLibA_57481 | 14 | -1.009287435 | hsa-mir-1264 | - |
| hsa-mir-1273c | HGLibA_57537 | 14 | -1.009287435 | hsa-mir-1273c | - |
| hsa-mir-130a | HGLibA_57748 | 14 | -1.009287435 | hsa-mir-130a | - |
| hsa-mir-153-1 | HGLibA_57907 | 14 | -1.009287435 | hsa-mir-153-1 | - |
| hsa-mir-218-2 | HGLibA_58271 | 14 | -1.009287435 | hsa-mir-218-2 | - |
| hsa-mir-302c | HGLibA_58444 | 14 | -1.009287435 | hsa-mir-302c | - |
| hsa-mir-3074 | HGLibA_58466 | 14 | -1.009287435 | hsa-mir-3074 | - |
| hsa-mir-3074 | HGLibA_58469 | 14 | -1.009287435 | hsa-mir-3074 | - |
| hsa-mir-3133 | HGLibA_58586 | 14 | -1.009287435 | hsa-mir-3133 | - |
| hsa-mir-3142 | HGLibA_58626 | 14 | -1.009287435 | hsa-mir-3142 | - |
| hsa-mir-3158-2 | HGLibA_58707 | 14 | -1.009287435 | hsa-mir-3158-2 | - |
| hsa-mir-3161 | HGLibA_58723 | 14 | -1.009287435 | hsa-mir-3161 | - |
| hsa-mir-3163 | HGLibA_58733 | 14 | -1.009287435 | hsa-mir-3163 | - |
| hsa-mir-3189 | HGLibA_58852 | 14 | -1.009287435 | hsa-mir-3189 | - |
| hsa-mir-3202-2 | HGLibA_58919 | 14 | -1.009287435 | hsa-mir-3202-2 | - |
| hsa-mir-329-2 | HGLibA_58983 | 14 | -1.009287435 | hsa-mir-329-2 | - |
| hsa-mir-33a | HGLibA_59010 | 14 | -1.009287435 | hsa-mir-33a | - |
| hsa-mir-3667 | HGLibA_59220 | 14 | -1.009287435 | hsa-mir-3667 | - |
| hsa-mir-3914-1 | HGLibA_59528 | 14 | -1.009287435 | hsa-mir-3914-1 | - |
| hsa-mir-4262 | HGLibA_59755 | 14 | -1.009287435 | hsa-mir-4262 | - |
| hsa-mir-4477a | HGLibA_60318 | 14 | -1.009287435 | hsa-mir-4477a | - |
| hsa-mir-4495 | HGLibA_60397 | 14 | -1.009287435 | hsa-mir-4495 | - |
| hsa-mir-4696 | HGLibA_60892 | 14 | -1.009287435 | hsa-mir-4696 | - |
| hsa-mir-4739 | HGLibA_61052 | 14 | -1.009287435 | hsa-mir-4739 | - |
| hsa-mir-4764 | HGLibA_61153 | 14 | -1.009287435 | hsa-mir-4764 | - |
| hsa-mir-4782 | HGLibA_61236 | 14 | -1.009287435 | hsa-mir-4782 | - |
| hsa-mir-5000 | HGLibA_61407 | 14 | -1.009287435 | hsa-mir-5000 | - |
| hsa-mir-513a-1 | HGLibA_61570 | 14 | -1.009287435 | hsa-mir-513a-1 | - |
| hsa-mir-517b | HGLibA_61629 | 14 | -1.009287435 | hsa-mir-517b | - |
| hsa-mir-517b | HGLibA_61627 | 14 | -1.009287435 | hsa-mir-517b | - |
| hsa-mir-518e | HGLibA_61673 | 14 | -1.009287435 | hsa-mir-518e | - |
| hsa-mir-548f-5 | HGLibA_61986 | 14 | -1.009287435 | hsa-mir-548f-5 | - |
| hsa-mir-548o | HGLibA_62049 | 14 | -1.009287435 | hsa-mir-548o | - |
| hsa-mir-550a-1 | HGLibA_62097 | 14 | -1.009287435 | hsa-mir-550a-1 | - |
| hsa-mir-550b-1 | HGLibA_62109 | 14 | -1.009287435 | hsa-mir-550b-1 | - |
| hsa-mir-5692a-1 | HGLibA_62287 | 14 | -1.009287435 | hsa-mir-5692a-1 | - |
| hsa-mir-6077-1 | HGLibA_62559 | 14 | -1.009287435 | hsa-mir-6077-1 | - |
| hsa-mir-6077-2 | HGLibA_62563 | 14 | -1.009287435 | hsa-mir-6077-2 | - |
| hsa-mir-637 | HGLibA_62786 | 14 | -1.009287435 | hsa-mir-637 | - |
| hsa-mir-653 | HGLibA_62945 | 14 | -1.009287435 | hsa-mir-653 | - |
| hsa-mir-6768 | HGLibA_63233 | 14 | -1.009287435 | hsa-mir-6768 | - |

| | | | | | |
|---|---|---|---|---|---|
| hsa-mir-6849 | HGLibA_63570 | 14 | -1.009287435 | hsa-mir-6849 | - |
| hsa-mir-6889 | HGLibA_63741 | 14 | -1.009287435 | hsa-mir-6889 | - |
| hsa-mir-7845 | HGLibA_63970 | 14 | -1.009287435 | hsa-mir-7845 | - |
| hsa-mir-8057 | HGLibA_64066 | 14 | -1.009287435 | hsa-mir-8057 | - |
| hsa-mir-8060 | HGLibA_64080 | 14 | -1.009287435 | hsa-mir-8060 | - |
| LDOC1L | HGLibA_26259 | 14 | -1.009287435 | LDOC1L | - |
| LOC152586 | HGLibA_26893 | 14 | -1.009287435 | LOC152586 | - |
| LOC643802 | HGLibA_26972 | 14 | -1.009287435 | LOC643802 | - |
| PCNXL2 | HGLibA_35645 | 14 | -1.009287435 | PCNXL2 | - |
| SDCCAG3 | HGLibA_43116 | 14 | -1.009287435 | SDCCAG3 | - |
| ATP5SL | HGLibA_03690 | 13 | -1.019238472 | ATP5SL | - |
| B3GALTL | HGLibA_03970 | 13 | -1.019238472 | B3GALTL | - |
| C19orf52 | HGLibA_05674 | 13 | -1.019238472 | C19orf52 | - |
| C1orf177 | HGLibA_05802 | 13 | -1.019238472 | C1orf177 | - |
| C1orf228 | HGLibA_05840 | 13 | -1.019238472 | C1orf228 | - |
| C1orf229 | HGLibA_05842 | 13 | -1.019238472 | C1orf229 | - |
| C3orf72 | HGLibA_06265 | 13 | -1.019238472 | C3orf72 | - |
| C9orf9 | HGLibA_06772 | 13 | -1.019238472 | C9orf9 | - |
| CNTD2 | HGLibA_10390 | 13 | -1.019238472 | CNTD2 | - |
| EMR3 | HGLibA_15089 | 13 | -1.019238472 | EMR3 | - |
| ENSG00000000460 | HGLibA_05750 | 13 | -1.019238472 | C1orf112 | chromosome 1 open reading frame 112 [Source:HGNC Symbol;Acc:HGNC:25565] |
| ENSG00000005249 | HGLibA_38531 | 13 | -1.019238472 | PRKAR2B | protein kinase cAMP-dependent type II regulatory subunit beta [Source:HGNC Symbol;Acc:HGNC:9392] |
| ENSG00000010438 | HGLibA_38923 | 13 | -1.019238472 | PRSS3 | serine protease 3 [Source:HGNC Symbol;Acc:HGNC:9486] |
| ENSG00000019991 | HGLibA_21241 | 13 | -1.019238472 | HGF | hepatocyte growth factor [Source:HGNC Symbol;Acc:HGNC:4893] |
| ENSG00000020426 | HGLibA_29561 | 13 | -1.019238472 | MNAT1 | CDK activating kinase assembly factor [Source:HGNC Symbol;Acc:HGNC:7181]" |
| ENSG00000023892 | HGLibA_12785 | 13 | -1.019238472 | DEF6 | guanine nucleotide exchange factor [Source:HGNC Symbol;Acc:HGNC:2760]" |
| ENSG00000030066 | HGLibA_33031 | 13 | -1.019238472 | NUP160 | nucleoporin 160 [Source:HGNC Symbol;Acc:HGNC:18017] |
| ENSG00000034152 | HGLibA_28146 | 13 | -1.019238472 | MAP2K3 | mitogen-activated protein kinase kinase 3 [Source:HGNC Symbol;Acc:HGNC:6843] |
| ENSG00000053918 | HGLibA_24367 | 13 | -1.019238472 | KCNQ1 | potassium voltage-gated channel subfamily Q member 1 [Source:HGNC Symbol;Acc:HGNC:6294] |
| ENSG00000058804 | HGLibA_31380 | 13 | -1.019238472 | NDC1 | NDC1 transmembrane nucleoporin [Source:HGNC Symbol;Acc:HGNC:25525] |
| ENSG00000065978 | HGLibA_54642 | 13 | -1.019238472 | YBX1 | Y-box binding protein 1 [Source:HGNC Symbol;Acc:HGNC:8014] |
| ENSG00000067208 | HGLibA_15691 | 13 | -1.019238472 | EVI5 | ecotropic viral integration site 5 [Source:HGNC Symbol;Acc:HGNC:3501] |
| ENSG00000069018 | HGLibA_51750 | 13 | -1.019238472 | TRPC7 | transient receptor potential cation channel subfamily C member 7 [Source:HGNC Symbol;Acc:HGNC:20754] |
| ENSG00000070756 | HGLibA_34922 | 13 | -1.019238472 | PABPC1 | poly(A) binding protein cytoplasmic 1 [Source:HGNC Symbol;Acc:HGNC:8554] |
| ENSG00000071203 | HGLibA_30132 | 13 | -1.019238472 | MS4A12 | membrane spanning 4-domains A12 [Source:HGNC Symbol;Acc:HGNC:13370] |
| ENSG00000072818 | HGLibA_00383 | 13 | -1.019238472 | ACAP1 | ankyrin repeat and PH domains 1 [Source:HGNC Symbol;Acc:HGNC:16467]" |
| ENSG00000078399 | HGLibA_21924 | 13 | -1.019238472 | HOXA9 | homeobox A9 [Source:HGNC Symbol;Acc:HGNC:5109] |
| ENSG00000079156 | HGLibA_34661 | 13 | -1.019238472 | OSBPL6 | oxysterol binding protein like 6 [Source:HGNC Symbol;Acc:HGNC:16388] |
| ENSG00000080298 | HGLibA_40967 | 13 | -1.019238472 | RFX3 | regulatory factor X3 [Source:HGNC Symbol;Acc:HGNC:9984] |
| ENSG00000083168 | HGLibA_24060 | 13 | -1.019238472 | KAT6A | lysine acetyltransferase 6A [Source:HGNC Symbol;Acc:HGNC:13013] |
| ENSG00000084731 | HGLibA_24941 | 13 | -1.019238472 | KIF3C | kinesin family member 3C [Source:HGNC Symbol;Acc:HGNC:6321] |
| ENSG00000084754 | HGLibA_20808 | 13 | -1.019238472 | HADHA | hydroxyacyl-CoA dehydrogenase trifunctional multienzyme complex subunit alpha [Source:HGNC Symbol;Acc:HGNC:4801] |
| ENSG00000087053 | HGLibA_30423 | 13 | -1.019238472 | MTMR2 | myotubularin related protein 2 [Source:HGNC Symbol;Acc:HGNC:7450] |
| ENSG00000089775 | HGLibA_54850 | 13 | -1.019238472 | ZBTB25 | zinc finger and BTB domain containing 25 [Source:HGNC Symbol;Acc:HGNC:13112] |
| ENSG00000092054 | HGLibA_30733 | 13 | -1.019238472 | MYH7 | myosin heavy chain 7 [Source:HGNC Symbol;Acc:HGNC:7577] |
| ENSG00000096088 | HGLibA_36229 | 13 | -1.019238472 | PGC | progastricsin [Source:HGNC Symbol;Acc:HGNC:8890] |
| ENSG00000100246 | HGLibA_13647 | 13 | -1.019238472 | DNAL4 | dynein axonemal light chain 4 [Source:HGNC Symbol;Acc:HGNC:2955] |
| ENSG00000100412 | HGLibA_00465 | 13 | -1.019238472 | ACO2 | aconitase 2 [Source:HGNC Symbol;Acc:HGNC:118] |
| ENSG00000100596 | HGLibA_46894 | 13 | -1.019238472 | SPTLC2 | serine palmitoyltransferase long chain base subunit 2 [Source:HGNC Symbol;Acc:HGNC:11278] |
| ENSG00000102144 | HGLibA_36240 | 13 | -1.019238472 | PGK1 | phosphoglycerate kinase 1 [Source:HGNC Symbol;Acc:HGNC:8896] |
| ENSG00000103226 | HGLibA_32296 | 13 | -1.019238472 | NOMO3 | NODAL modulator 3 [Source:HGNC Symbol;Acc:HGNC:25242] |
| ENSG00000103310 | HGLibA_56954 | 13 | -1.019238472 | ZP2 | zona pellucida glycoprotein 2 [Source:HGNC Symbol;Acc:HGNC:13188] |
| ENSG00000104324 | HGLibA_10988 | 13 | -1.019238472 | CPQ | carboxypeptidase Q [Source:HGNC Symbol;Acc:HGNC:16910] |
| ENSG00000104804 | HGLibA_52334 | 13 | -1.019238472 | TULP2 | tubby like protein 2 [Source:HGNC Symbol;Acc:HGNC:12424] |
| ENSG00000104804 | HGLibA_52332 | 13 | -1.019238472 | TULP2 | tubby like protein 2 [Source:HGNC Symbol;Acc:HGNC:12424] |
| ENSG00000105887 | HGLibA_30461 | 13 | -1.019238472 | MTPN | myotrophin [Source:HGNC Symbol;Acc:HGNC:15667] |
| ENSG00000106070 | HGLibA_20224 | 13 | -1.019238472 | GRB10 | growth factor receptor bound protein 10 [Source:HGNC Symbol;Acc:HGNC:4564] |
| ENSG00000107140 | HGLibA_49029 | 13 | -1.019238472 | TESK1 | testis associated actin remodelling kinase 1 [Source:HGNC Symbol;Acc:HGNC:11731] |
| ENSG00000108861 | HGLibA_14177 | 13 | -1.019238472 | DUSP3 | dual specificity phosphatase 3 [Source:HGNC Symbol;Acc:HGNC:3069] |
| ENSG00000108953 | HGLibA_54743 | 13 | -1.019238472 | YWHAE | tyrosine 3-monooxygenase/tryptophan 5-monooxygenase activation protein epsilon [Source:HGNC Symbol;Acc:HGNC:12851] |
| ENSG00000109182 | HGLibA_11831 | 13 | -1.019238472 | CWH43 | cell wall biogenesis 43 C-terminal homolog [Source:HGNC Symbol;Acc:HGNC:26133] |
| ENSG00000109193 | HGLibA_47755 | 13 | -1.019238472 | SULT1E1 | sulfotransferase family 1E member 1 [Source:HGNC Symbol;Acc:HGNC:11377] |
| ENSG00000110841 | HGLibA_37884 | 13 | -1.019238472 | PPFIBP1 | PPFIA binding protein 1 [Source:HGNC Symbol;Acc:HGNC:9249] |
| ENSG00000110921 | HGLibA_30597 | 13 | -1.019238472 | MVK | mevalonate kinase [Source:HGNC Symbol;Acc:HGNC:7530] |
| ENSG00000111640 | HGLibA_18613 | 13 | -1.019238472 | GAPDH | glyceraldehyde-3-phosphate dehydrogenase [Source:HGNC Symbol;Acc:HGNC:4141] |
| ENSG00000112578 | HGLibA_05007 | 13 | -1.019238472 | BYSL | bystin like [Source:HGNC Symbol;Acc:HGNC:1157] |
| ENSG00000113141 | HGLibA_22910 | 13 | -1.019238472 | IK | IK cytokine [Source:HGNC Symbol;Acc:HGNC:5958] |
| ENSG00000113296 | HGLibA_49332 | 13 | -1.019238472 | THBS4 | thrombospondin 4 [Source:HGNC Symbol;Acc:HGNC:11788] |
| ENSG00000114302 | HGLibA_38528 | 13 | -1.019238472 | PRKAR2A | protein kinase cAMP-dependent type II regulatory subunit alpha [Source:HGNC Symbol;Acc:HGNC:9391] |
| ENSG00000114784 | HGLibA_14718 | 13 | -1.019238472 | EIF1B | eukaryotic translation initiation factor 1B [Source:HGNC Symbol;Acc:HGNC:30792] |
| ENSG00000115677 | HGLibA_21060 | 13 | -1.019238472 | HDLBP | high density lipoprotein binding protein [Source:HGNC Symbol;Acc:HGNC:4857] |
| ENSG00000116273 | HGLibA_36342 | 13 | -1.019238472 | PHF13 | PHD finger protein 13 [Source:HGNC Symbol;Acc:HGNC:22983] |
| ENSG00000116750 | HGLibA_52807 | 13 | -1.019238472 | UCHL5 | ubiquitin C-terminal hydrolase L5 [Source:HGNC Symbol;Acc:HGNC:19678] |
| ENSG00000117090 | HGLibA_44312 | 13 | -1.019238472 | SLAMF1 | signaling lymphocytic activation molecule family member 1 [Source:HGNC Symbol;Acc:HGNC:10903] |
| ENSG00000117394 | HGLibA_44851 | 13 | -1.019238472 | SLC2A1 | solute carrier family 2 member 1 [Source:HGNC Symbol;Acc:HGNC:11005] |
| ENSG00000118733 | HGLibA_33350 | 13 | -1.019238472 | OLFM3 | olfactomedin 3 [Source:HGNC Symbol;Acc:HGNC:17990] |
| ENSG00000118762 | HGLibA_36797 | 13 | -1.019238472 | PKD2 | transient receptor potential cation channel [Source:HGNC Symbol;Acc:HGNC:9009]" |
| ENSG00000118785 | HGLibA_46770 | 13 | -1.019238472 | SPP1 | secreted phosphoprotein 1 [Source:HGNC Symbol;Acc:HGNC:11255] |
| ENSG00000119650 | HGLibA_22758 | 13 | -1.019238472 | IFT43 | intraflagellar transport 43 [Source:HGNC Symbol;Acc:HGNC:29669] |
| ENSG00000120158 | HGLibA_40697 | 13 | -1.019238472 | RCL1 | RNA terminal phosphate cyclase like 1 [Source:HGNC Symbol;Acc:HGNC:17687] |
| ENSG00000120519 | HGLibA_44349 | 13 | -1.019238472 | SLC10A7 | solute carrier family 10 member 7 [Source:HGNC Symbol;Acc:HGNC:23088] |
| ENSG00000120685 | HGLibA_38696 | 13 | -1.019238472 | PROSER1 | proline and serine rich 1 [Source:HGNC Symbol;Acc:HGNC:20291] |
| ENSG00000120802 | HGLibA_50606 | 13 | -1.019238472 | TMPO | thymopoietin [Source:HGNC Symbol;Acc:HGNC:11875] |
| ENSG00000121057 | HGLibA_01404 | 13 | -1.019238472 | AKAP1 | A-kinase anchoring protein 1 [Source:HGNC Symbol;Acc:HGNC:367] |
| ENSG00000123307 | HGLibA_31740 | 13 | -1.019238472 | NEUROD4 | neuronal differentiation 4 [Source:HGNC Symbol;Acc:HGNC:13802] |
| ENSG00000125434 | HGLibA_44717 | 13 | -1.019238472 | SLC25A35 | solute carrier family 25 member 35 [Source:HGNC Symbol;Acc:HGNC:31921] |
| ENSG00000125812 | HGLibA_20714 | 13 | -1.019238472 | GZF1 | GDNF inducible zinc finger protein 1 [Source:HGNC Symbol;Acc:HGNC:15808] |
| ENSG00000127084 | HGLibA_17349 | 13 | -1.019238472 | FGD3 | RhoGEF and PH domain containing 3 [Source:HGNC Symbol;Acc:HGNC:16027]" |
| ENSG00000127952 | HGLibA_47690 | 13 | -1.019238472 | STYXL1 | serine/threonine/tyrosine interacting like 1 [Source:HGNC Symbol;Acc:HGNC:18165] |
| ENSG00000128886 | HGLibA_14926 | 13 | -1.019238472 | ELL3 | elongation factor for RNA polymerase II 3 [Source:HGNC Symbol;Acc:HGNC:23113] |
| ENSG00000130479 | HGLibA_28133 | 13 | -1.019238472 | MAP1S | microtubule associated protein 1S [Source:HGNC Symbol;Acc:HGNC:15715] |
| ENSG00000133243 | HGLibA_04912 | 13 | -1.019238472 | BTBD2 | BTB domain containing 2 [Source:HGNC Symbol;Acc:HGNC:15504] |
| ENSG00000133835 | HGLibA_22194 | 13 | -1.019238472 | HSD17B4 | hydroxysteroid 17-beta dehydrogenase 4 [Source:HGNC Symbol;Acc:HGNC:5213] |
| ENSG00000134419 | HGLibA_42081 | 13 | -1.019238472 | RPS15A | ribosomal protein S15a [Source:HGNC Symbol;Acc:HGNC:10389] |
| ENSG00000134508 | HGLibA_06843 | 13 | -1.019238472 | CABLES1 | Cdk5 and Abl enzyme substrate 1 [Source:HGNC Symbol;Acc:HGNC:25097] |
| ENSG00000135097 | HGLibA_30204 | 13 | -1.019238472 | MSI1 | musashi RNA binding protein 1 [Source:HGNC Symbol;Acc:HGNC:7330] |
| ENSG00000135686 | HGLibA_25199 | 13 | -1.019238472 | KLHL36 | kelch like family member 36 [Source:HGNC Symbol;Acc:HGNC:17844] |
| ENSG00000136381 | HGLibA_23568 | 13 | -1.019238472 | IREB2 | iron responsive element binding protein 2 [Source:HGNC Symbol;Acc:HGNC:6115] |
| ENSG00000136694 | HGLibA_23144 | 13 | -1.019238472 | IL36A | interleukin 36 alpha [Source:HGNC Symbol;Acc:HGNC:15562] |
| ENSG00000136715 | HGLibA_42714 | 13 | -1.019238472 | SAP130 | Sin3A associated protein 130 [Source:HGNC Symbol;Acc:HGNC:29813] |
| ENSG00000136870 | HGLibA_55551 | 13 | -1.019238472 | ZNF189 | zinc finger protein 189 [Source:HGNC Symbol;Acc:HGNC:12980] |
| ENSG00000137207 | HGLibA_54676 | 13 | -1.019238472 | YIPF3 | Yip1 domain family member 3 [Source:HGNC Symbol;Acc:HGNC:21023] |
| ENSG00000137766 | HGLibA_52999 | 13 | -1.019238472 | UNC13C | unc-13 homolog C [Source:HGNC Symbol;Acc:HGNC:23149] |
| ENSG00000139531 | HGLibA_47799 | 13 | -1.019238472 | SUOX | sulfite oxidase [Source:HGNC Symbol;Acc:HGNC:11460] |
| ENSG00000140265 | HGLibA_57012 | 13 | -1.019238472 | ZSCAN29 | zinc finger and SCAN domain containing 29 [Source:HGNC Symbol;Acc:HGNC:26673] |
| ENSG00000140718 | HGLibA_18131 | 13 | -1.019238472 | FTO | alpha-ketoglutarate dependent dioxygenase [Source:HGNC Symbol;Acc:HGNC:24678]" |
| ENSG00000140839 | HGLibA_09946 | 13 | -1.019238472 | CLEC18B | C-type lectin domain family 18 member B [Source:HGNC Symbol;Acc:HGNC:33849] |
| ENSG00000141027 | HGLibA_31357 | 13 | -1.019238472 | NCOR1 | nuclear receptor corepressor 1 [Source:HGNC Symbol;Acc:HGNC:7672] |
| ENSG00000141258 | HGLibA_43862 | 13 | -1.019238472 | SGSM2 | small G protein signaling modulator 2 [Source:HGNC Symbol;Acc:HGNC:29026] |
| ENSG00000142178 | HGLibA_44163 | 13 | -1.019238472 | SIK1 | salt inducible kinase 1 [Source:HGNC Symbol;Acc:HGNC:11142] |
| ENSG00000143028 | HGLibA_48038 | 13 | -1.019238472 | SYPL2 | synaptophysin like 2 [Source:HGNC Symbol;Acc:HGNC:27638] |
| ENSG00000144028 | HGLibA_45940 | 13 | -1.019238472 | SNRNP200 | small nuclear ribonucleoprotein U5 subunit 200 [Source:HGNC Symbol;Acc:HGNC:30859] |
| ENSG00000144395 | HGLibA_07642 | 13 | -1.019238472 | CCDC150 | coiled-coil domain containing 150 [Source:HGNC Symbol;Acc:HGNC:26834] |
| ENSG00000147854 | HGLibA_52955 | 13 | -1.019238472 | UHRF2 | ubiquitin like with PHD and ring finger domains 2 [Source:HGNC Symbol;Acc:HGNC:12557] |
| ENSG00000148339 | HGLibA_44685 | 13 | -1.019238472 | SLC25A25 | solute carrier family 25 member 25 [Source:HGNC Symbol;Acc:HGNC:20663] |
| ENSG00000148356 | HGLibA_27498 | 13 | -1.019238472 | LRSAM1 | leucine rich repeat and sterile alpha motif containing 1 [Source:HGNC Symbol;Acc:HGNC:25135] |
| ENSG00000148444 | HGLibA_10659 | 13 | -1.019238472 | COMMD3 | COMM domain containing 3 [Source:HGNC Symbol;Acc:HGNC:23332] |
| ENSG00000149187 | HGLibA_09003 | 13 | -1.019238472 | CELF1 | CUGBP Elav-like family member 1 [Source:HGNC Symbol;Acc:HGNC:2549] |
| ENSG00000150281 | HGLibA_11620 | 13 | -1.019238472 | CTF1 | cardiotrophin 1 [Source:HGNC Symbol;Acc:HGNC:2499] |
| ENSG00000150625 | HGLibA_19818 | 13 | -1.019238472 | GPM6A | glycoprotein M6A [Source:HGNC Symbol;Acc:HGNC:4460] |
| ENSG00000151065 | HGLibA_12519 | 13 | -1.019238472 | DCP1B | decapping mRNA 1B [Source:HGNC Symbol;Acc:HGNC:24451] |
| ENSG00000152520 | HGLibA_35095 | 13 | -1.019238472 | PAN3 | poly(A) specific ribonuclease subunit PAN3 [Source:HGNC Symbol;Acc:HGNC:29991] |
| ENSG00000152669 | HGLibA_08124 | 13 | -1.019238472 | CCNO | cyclin O [Source:HGNC Symbol;Acc:HGNC:18576] |
| ENSG00000154447 | HGLibA_43971 | 13 | -1.019238472 | SH3RF1 | SH3 domain containing ring finger 1 [Source:HGNC Symbol;Acc:HGNC:17650] |
| ENSG00000157150 | HGLibA_49543 | 13 | -1.019238472 | TIMP4 | TIMP metallopeptidase inhibitor 4 [Source:HGNC Symbol;Acc:HGNC:11823] |
| ENSG00000157890 | HGLibA_28849 | 13 | -1.019238472 | MEGF11 | multiple EGF like domains 11 [Source:HGNC Symbol;Acc:HGNC:29635] |
| ENSG00000158042 | HGLibA_29913 | 13 | -1.019238472 | MRPL17 | mitochondrial ribosomal protein L17 [Source:HGNC Symbol;Acc:HGNC:14053] |
| ENSG00000158615 | HGLibA_38049 | 13 | -1.019238472 | PPP1R15B | protein phosphatase 1 regulatory subunit 15B [Source:HGNC Symbol;Acc:HGNC:14951] |
| ENSG00000158748 | HGLibA_22400 | 13 | -1.019238472 | HTR6 | 5-hydroxytryptamine receptor 6 [Source:HGNC Symbol;Acc:HGNC:5301] |
| ENSG00000158825 | HGLibA_08498 | 13 | -1.019238472 | CDA | cytidine deaminase [Source:HGNC Symbol;Acc:HGNC:1712] |
| ENSG00000159086 | HGLibA_35321 | 13 | -1.019238472 | PAXBP1 | PAX3 and PAX7 binding protein 1 [Source:HGNC Symbol;Acc:HGNC:13579] |
| ENSG00000159289 | HGLibA_19606 | 13 | -1.019238472 | GOLGA6A | golgin A6 family member A [Source:HGNC Symbol;Acc:HGNC:13567] |
| ENSG00000160209 | HGLibA_35960 | 13 | -1.019238472 | PDXK | pyridoxal kinase [Source:HGNC Symbol;Acc:HGNC:8819] |
| ENSG00000160396 | HGLibA_21348 | 13 | -1.019238472 | HIPK4 | homeodomain interacting protein kinase 4 [Source:HGNC Symbol;Acc:HGNC:19007] |
| ENSG00000160867 | HGLibA_17450 | 13 | -1.019238472 | FGFR4 | fibroblast growth factor receptor 4 [Source:HGNC Symbol;Acc:HGNC:3691] |

| | | | | | |
|---|---|---|---|---|---|
| ENSG00000161888 | HGLibA_46551 | 13 | -1.019238472 | SPC24 | NDC80 kinetochore complex component [Source:HGNC Symbol;Acc:HGNC:26913]" |
| ENSG00000162426 | HGLibA_45173 | 13 | -1.019238472 | SLC45A1 | solute carrier family 45 member 1 [Source:HGNC Symbol;Acc:HGNC:17939] |
| ENSG00000162543 | HGLibA_52776 | 13 | -1.019238472 | UBXN10 | UBX domain protein 10 [Source:HGNC Symbol;Acc:HGNC:26354] |
| ENSG00000163702 | HGLibA_23010 | 13 | -1.019238472 | IL17RC | interleukin 17 receptor C [Source:HGNC Symbol;Acc:HGNC:18358] |
| ENSG00000163902 | HGLibA_42016 | 13 | -1.019238472 | RPN1 | ribophorin I [Source:HGNC Symbol;Acc:HGNC:10381] |
| ENSG00000164040 | HGLibA_36292 | 13 | -1.019238472 | PGRMC2 | progesterone receptor membrane component 2 [Source:HGNC Symbol;Acc:HGNC:16089] |
| ENSG00000164088 | HGLibA_37993 | 13 | -1.019238472 | PPM1M | Mg2+/Mn2+ dependent 1M [Source:HGNC Symbol;Acc:HGNC:26506]" |
| ENSG00000165572 | HGLibA_24111 | 13 | -1.019238472 | KBTBD6 | kelch repeat and BTB domain containing 6 [Source:HGNC Symbol;Acc:HGNC:25340] |
| ENSG00000165637 | HGLibA_53603 | 13 | -1.019238472 | VDAC2 | voltage dependent anion channel 2 [Source:HGNC Symbol;Acc:HGNC:12672] |
| ENSG00000165732 | HGLibA_12671 | 13 | -1.019238472 | DDX21 | DExD-box helicase 21 [Source:HGNC Symbol;Acc:HGNC:2744] |
| ENSG00000165966 | HGLibA_36000 | 13 | -1.019238472 | PDZRN4 | PDZ domain containing ring finger 4 [Source:HGNC Symbol;Acc:HGNC:30552] |
| ENSG00000166435 | HGLibA_54611 | 13 | -1.019238472 | XRRA1 | X-ray radiation resistance associated 1 [Source:HGNC Symbol;Acc:HGNC:18868] |
| ENSG00000166450 | HGLibA_38981 | 13 | -1.019238472 | PRTG | protogenin [Source:HGNC Symbol;Acc:HGNC:26373] |
| ENSG00000166507 | HGLibA_31427 | 13 | -1.019238472 | NDST2 | N-deacetylase and N-sulfotransferase 2 [Source:HGNC Symbol;Acc:HGNC:7681] |
| ENSG00000166598 | HGLibA_22255 | 13 | -1.019238472 | HSP90B1 | heat shock protein 90 beta family member 1 [Source:HGNC Symbol;Acc:HGNC:12028] |
| ENSG00000166813 | HGLibA_24961 | 13 | -1.019238472 | KIF7 | kinesin family member 7 [Source:HGNC Symbol;Acc:HGNC:30497] |
| ENSG00000167612 | HGLibA_02069 | 13 | -1.019238472 | ANKRD33 | ankyrin repeat domain 33 [Source:HGNC Symbol;Acc:HGNC:13788] |
| ENSG00000167676 | HGLibA_37180 | 13 | -1.019238472 | PLIN4 | perilipin 4 [Source:HGNC Symbol;Acc:HGNC:29393] |
| ENSG00000167965 | HGLibA_29434 | 13 | -1.019238472 | MLST8 | LST8 homolog [Source:HGNC Symbol;Acc:HGNC:24825]" |
| ENSG00000168071 | HGLibA_07909 | 13 | -1.019238472 | CCDC88B | coiled-coil domain containing 88B [Source:HGNC Symbol;Acc:HGNC:26757] |
| ENSG00000168301 | HGLibA_24461 | 13 | -1.019238472 | KCTD6 | potassium channel tetramerization domain containing 6 [Source:HGNC Symbol;Acc:HGNC:22235] |
| ENSG00000169946 | HGLibA_55254 | 13 | -1.019238472 | ZFPM2 | FOG family member 2 [Source:HGNC Symbol;Acc:HGNC:16700]" |
| ENSG00000170088 | HGLibA_50151 | 13 | -1.019238472 | TMEM192 | transmembrane protein 192 [Source:HGNC Symbol;Acc:HGNC:26775] |
| ENSG00000170745 | HGLibA_24389 | 13 | -1.019238472 | KCNS3 | potassium voltage-gated channel modifier subfamily S member 3 [Source:HGNC Symbol;Acc:HGNC:6302] |
| ENSG00000170820 | HGLibA_18089 | 13 | -1.019238472 | FSHR | follicle stimulating hormone receptor [Source:HGNC Symbol;Acc:HGNC:3969] |
| ENSG00000170889 | HGLibA_42194 | 13 | -1.019238472 | RPS9 | ribosomal protein S9 [Source:HGNC Symbol;Acc:HGNC:10442] |
| ENSG00000171109 | HGLibA_29070 | 13 | -1.019238472 | MFN1 | mitofusin 1 [Source:HGNC Symbol;Acc:HGNC:18262] |
| ENSG00000171155 | HGLibA_05725 | 13 | -1.019238472 | C1GALT1C1 | C1GALT1 specific chaperone 1 [Source:HGNC Symbol;Acc:HGNC:24338] |
| ENSG00000171295 | HGLibA_56002 | 13 | -1.019238472 | ZNF440 | zinc finger protein 440 [Source:HGNC Symbol;Acc:HGNC:20874] |
| ENSG00000171564 | HGLibA_17338 | 13 | -1.019238472 | FGB | fibrinogen beta chain [Source:HGNC Symbol;Acc:HGNC:3662] |
| ENSG00000171954 | HGLibA_12191 | 13 | -1.019238472 | CYP4F22 | cytochrome P450 family 4 subfamily F member 22 [Source:HGNC Symbol;Acc:HGNC:26820] |
| ENSG00000172058 | HGLibA_43469 | 13 | -1.019238472 | SERF1A | small EDRK-rich factor 1A [Source:HGNC Symbol;Acc:HGNC:10755] |
| ENSG00000172264 | HGLibA_27838 | 13 | -1.019238472 | MACROD2 | MACRO domain containing 2 [Source:HGNC Symbol;Acc:HGNC:16126] |
| ENSG00000172575 | HGLibA_40345 | 13 | -1.019238472 | RASGRP1 | RAS guanyl releasing protein 1 [Source:HGNC Symbol;Acc:HGNC:9878] |
| ENSG00000172831 | HGLibA_09220 | 13 | -1.019238472 | CES2 | carboxylesterase 2 [Source:HGNC Symbol;Acc:HGNC:1864] |
| ENSG00000174332 | HGLibA_19272 | 13 | -1.019238472 | GLIS1 | GLIS family zinc finger 1 [Source:HGNC Symbol;Acc:HGNC:29525] |
| ENSG00000174498 | HGLibA_22786 | 13 | -1.019238472 | IGDCC3 | immunoglobulin superfamily DCC subclass member 3 [Source:HGNC Symbol;Acc:HGNC:9700] |
| ENSG00000176853 | HGLibA_16794 | 13 | -1.019238472 | FAM91A1 | family with sequence similarity 91 member A1 [Source:HGNC Symbol;Acc:HGNC:26306] |
| ENSG00000177675 | HGLibA_08245 | 13 | -1.019238472 | CD163L1 | CD163 molecule like 1 [Source:HGNC Symbol;Acc:HGNC:30375] |
| ENSG00000180205 | HGLibA_54277 | 13 | -1.019238472 | WFDC9 | WAP four-disulfide core domain 9 [Source:HGNC Symbol;Acc:HGNC:20380] |
| ENSG00000180708 | HGLibA_33534 | 13 | -1.019238472 | OR10K2 | olfactory receptor family 10 subfamily K member 2 [Source:HGNC Symbol;Acc:HGNC:14826] |
| ENSG00000180921 | HGLibA_16762 | 13 | -1.019238472 | FAM83H | family with sequence similarity 83 member H [Source:HGNC Symbol;Acc:HGNC:24797] |
| ENSG00000182150 | HGLibA_15451 | 13 | -1.019238472 | ERCC6L2 | ERCC excision repair 6 like 2 [Source:HGNC Symbol;Acc:HGNC:26922] |
| ENSG00000182400 | HGLibA_51334 | 13 | -1.019238472 | TRAPPC6B | trafficking protein particle complex 6B [Source:HGNC Symbol;Acc:HGNC:23066] |
| ENSG00000182591 | HGLibA_25626 | 13 | -1.019238472 | KRTAP11-1 | keratin associated protein 11-1 [Source:HGNC Symbol;Acc:HGNC:18922] |
| ENSG00000182747 | HGLibA_44984 | 13 | -1.019238472 | SLC35D3 | solute carrier family 35 member D3 [Source:HGNC Symbol;Acc:HGNC:15621] |
| ENSG00000182963 | HGLibA_19177 | 13 | -1.019238472 | GJC1 | gap junction protein gamma 1 [Source:HGNC Symbol;Acc:HGNC:4280] |
| ENSG00000182986 | HGLibA_55795 | 13 | -1.019238472 | ZNF320 | zinc finger protein 320 [Source:HGNC Symbol;Acc:HGNC:13842] |
| ENSG00000184164 | HGLibA_11130 | 13 | -1.019238472 | CRELD2 | cysteine rich with EGF like domains 2 [Source:HGNC Symbol;Acc:HGNC:28150] |
| ENSG00000184983 | HGLibA_31467 | 13 | -1.019238472 | NDUFA6 | NADH:ubiquinone oxidoreductase subunit A6 [Source:HGNC Symbol;Acc:HGNC:7690] |
| ENSG00000185046 | HGLibA_02153 | 13 | -1.019238472 | ANKS1B | ankyrin repeat and sterile alpha motif domain containing 1B [Source:HGNC Symbol;Acc:HGNC:24600] |
| ENSG00000185164 | HGLibA_32292 | 13 | -1.019238472 | NOMO2 | NODAL modulator 2 [Source:HGNC Symbol;Acc:HGNC:22652] |
| ENSG00000185499 | HGLibA_30521 | 13 | -1.019238472 | MUC1 | cell surface associated [Source:HGNC Symbol;Acc:HGNC:7508]" |
| ENSG00000186532 | HGLibA_45860 | 13 | -1.019238472 | SMYD4 | SET and MYND domain containing 4 [Source:HGNC Symbol;Acc:HGNC:21067] |
| ENSG00000186660 | HGLibA_55244 | 13 | -1.019238472 | ZFP91 | ZFP91 zinc finger protein [Source:HGNC Symbol;Acc:HGNC:14983] |
| ENSG00000186834 | HGLibA_21211 | 13 | -1.019238472 | HEXIM1 | hexamethylene bisacetamide inducible 1 [Source:HGNC Symbol;Acc:HGNC:24953] |
| ENSG00000186854 | HGLibA_51232 | 13 | -1.019238472 | TRABD2A | TraB domain containing 2A [Source:HGNC Symbol;Acc:HGNC:27013] |
| ENSG00000187266 | HGLibA_15352 | 13 | -1.019238472 | EPOR | erythropoietin receptor [Source:HGNC Symbol;Acc:HGNC:3416] |
| ENSG00000188056 | HGLibA_51367 | 13 | -1.019238472 | TREML4 | triggering receptor expressed on myeloid cells like 4 [Source:HGNC Symbol;Acc:HGNC:30807] |
| ENSG00000188811 | HGLibA_31902 | 13 | -1.019238472 | NHLRC3 | NHL repeat containing 3 [Source:HGNC Symbol;Acc:HGNC:33751] |
| ENSG00000196620 | HGLibA_52919 | 13 | -1.019238472 | UGT2B15 | UDP glucuronosyltransferase family 2 member B15 [Source:HGNC Symbol;Acc:HGNC:12546] |
| ENSG00000196646 | HGLibA_55475 | 13 | -1.019238472 | ZNF136 | zinc finger protein 136 [Source:HGNC Symbol;Acc:HGNC:12920] |
| ENSG00000196873 | HGLibA_07435 | 13 | -1.019238472 | CBWD3 | COBW domain containing 3 [Source:HGNC Symbol;Acc:HGNC:18519] |
| ENSG00000198129 | HGLibA_12839 | 13 | -1.019238472 | DEFB107B | defensin beta 107B [Source:HGNC Symbol;Acc:HGNC:31918] |
| ENSG00000198774 | HGLibA_40398 | 13 | -1.019238472 | RASSF9 | Ras association domain family member 9 [Source:HGNC Symbol;Acc:HGNC:15739] |
| ENSG00000198797 | HGLibA_04816 | 13 | -1.019238472 | BRINP2 | BMP/retinoic acid inducible neural specific 2 [Source:HGNC Symbol;Acc:HGNC:13746] |
| ENSG00000203910 | HGLibA_05774 | 13 | -1.019238472 | C1orf146 | chromosome 1 open reading frame 146 [Source:HGNC Symbol;Acc:HGNC:24032] |
| ENSG00000203965 | HGLibA_14552 | 13 | -1.019238472 | EFCAB7 | EF-hand calcium binding domain 7 [Source:HGNC Symbol;Acc:HGNC:29379] |
| ENSG00000204700 | HGLibA_33810 | 13 | -1.019238472 | OR2J2 | olfactory receptor family 2 subfamily J member 2 [Source:HGNC Symbol;Acc:HGNC:8260] |
| ENSG00000205238 | HGLibA_46583 | 13 | -1.019238472 | SPDYE2 | speedy/RINGO cell cycle regulator family member E2 [Source:HGNC Symbol;Acc:HGNC:33841] |
| ENSG00000205309 | HGLibA_32832 | 13 | -1.019238472 | NT5M | mitochondrial [Source:HGNC Symbol;Acc:HGNC:15769]" |
| ENSG00000205339 | HGLibA_23472 | 13 | -1.019238472 | IPO7 | importin 7 [Source:HGNC Symbol;Acc:HGNC:9852] |
| ENSG00000205359 | HGLibA_45478 | 13 | -1.019238472 | SLCO6A1 | solute carrier organic anion transporter family member 6A1 [Source:HGNC Symbol;Acc:HGNC:23613] |
| ENSG00000205572 | HGLibA_43472 | 13 | -1.019238472 | SERF1B | small EDRK-rich factor 1B [Source:HGNC Symbol;Acc:HGNC:10756] |
| ENSG00000206127 | HGLibA_19646 | 13 | -1.019238472 | GOLGA8O | golgin A8 family member O [Source:HGNC Symbol;Acc:HGNC:44406] |
| ENSG00000213221 | HGLibA_13680 | 13 | -1.019238472 | DNLZ | DNL-type zinc finger [Source:HGNC Symbol;Acc:HGNC:33879] |
| ENSG00000213445 | HGLibA_44189 | 13 | -1.019238472 | SIPA1 | signal-induced proliferation-associated 1 [Source:HGNC Symbol;Acc:HGNC:10885] |
| ENSG00000214026 | HGLibA_29933 | 13 | -1.019238472 | MRPL23 | mitochondrial ribosomal protein L23 [Source:HGNC Symbol;Acc:HGNC:10322] |
| ENSG00000214212 | HGLibA_05653 | 13 | -1.019238472 | C19orf38 | chromosome 19 open reading frame 38 [Source:HGNC Symbol;Acc:HGNC:34073] |
| ENSG00000221890 | HGLibA_32539 | 13 | -1.019238472 | NPTXR | neuronal pentraxin receptor [Source:HGNC Symbol;Acc:HGNC:7954] |
| ENSG00000221944 | HGLibA_49464 | 13 | -1.019238472 | TIGD1 | tigger transposable element derived 1 [Source:HGNC Symbol;Acc:HGNC:14523] |
| ENSG00000223510 | HGLibA_08874 | 13 | -1.019238472 | CDRT15 | CMT1A duplicated region transcript 15 [Source:HGNC Symbol;Acc:HGNC:14395] |
| ENSG00000231861 | HGLibA_34314 | 13 | -1.019238472 | OR5K2 | olfactory receptor family 5 subfamily K member 2 [Source:HGNC Symbol;Acc:HGNC:14774] |
| ENSG00000234127 | HGLibA_51444 | 13 | -1.019238472 | TRIM26 | tripartite motif containing 26 [Source:HGNC Symbol;Acc:HGNC:12962] |
| ENSG00000235098 | HGLibA_02137 | 13 | -1.019238472 | ANKRD65 | ankyrin repeat domain 65 [Source:HGNC Symbol;Acc:HGNC:42950] |
| ENSG00000235711 | HGLibA_02082 | 13 | -1.019238472 | ANKRD34C | ankyrin repeat domain 34C [Source:HGNC Symbol;Acc:HGNC:33888] |
| ENSG00000241484 | HGLibA_02790 | 13 | -1.019238472 | ARHGAP8 | Rho GTPase activating protein 8 [Source:HGNC Symbol;Acc:HGNC:677] |
| ENSG00000242114 | HGLibA_30357 | 13 | -1.019238472 | MTFP1 | mitochondrial fission process 1 [Source:HGNC Symbol;Acc:HGNC:26945] |
| ENSG00000244038 | HGLibA_12629 | 13 | -1.019238472 | DDOST | dolichyl-diphosphooligosaccharide--protein glycosyltransferase non-catalytic subunit [Source:HGNC Symbol;Acc:HGNC:2728] |
| ENSG00000250510 | HGLibA_19959 | 13 | -1.019238472 | GPR162 | G protein-coupled receptor 162 [Source:HGNC Symbol;Acc:HGNC:16693] |
| ENSG00000253309 | HGLibA_43584 | 13 | -1.019238472 | SERPINE3 | serpin family E member 3 [Source:HGNC Symbol;Acc:HGNC:24774] |
| ENSG00000255423 | HGLibA_14366 | 13 | -1.019238472 | EBLN2 | endogenous Bornavirus like nucleoprotein 2 [Source:HGNC Symbol;Acc:HGNC:25493] |
| ENSG00000259112 | HGLibA_31541 | 13 | -1.019238472 | NDUFC2-KCTD14 | NDUFC2-KCTD14 readthrough [Source:HGNC Symbol;Acc:HGNC:42956] |
| ENSG00000261796 | HGLibA_23698 | 13 | -1.019238472 | ISY1-RAB43 | ISY1-RAB43 readthrough [Source:HGNC Symbol;Acc:HGNC:42969] |
| ENSG00000266200 | HGLibA_37359 | 13 | -1.019238472 | PNLIPRP2 | pancreatic lipase related protein 2 (gene/pseudogene) [Source:HGNC Symbol;Acc:HGNC:9157] |
| ENSG00000273079 | HGLibA_20302 | 13 | -1.019238472 | GRIN2B | glutamate ionotropic receptor NMDA type subunit 2B [Source:HGNC Symbol;Acc:HGNC:4586] |
| ENSG00000274749 | HGLibA_25823 | 13 | -1.019238472 | KRTAP7-1 | keratin associated protein 7-1 (gene/pseudogene) [Source:HGNC Symbol;Acc:HGNC:18934] |
| ENSG00000275718 | HGLibA_07973 | 13 | -1.019238472 | CCL15 | C-C motif chemokine ligand 15 [Source:HGNC Symbol;Acc:HGNC:10613] |
| FAM69C | HGLibA_16683 | 13 | -1.019238472 | FAM69C | - |
| HEATR2 | HGLibA_21070 | 13 | -1.019238472 | HEATR2 | - |
| hsa-mir-1273g | HGLibA_57548 | 13 | -1.019238472 | hsa-mir-1273g | - |
| hsa-mir-1323 | HGLibA_57763 | 13 | -1.019238472 | hsa-mir-1323 | - |
| hsa-mir-2053 | HGLibA_58184 | 13 | -1.019238472 | hsa-mir-2053 | - |
| hsa-mir-218-1 | HGLibA_58268 | 13 | -1.019238472 | hsa-mir-218-1 | - |
| hsa-mir-3186 | HGLibA_58842 | 13 | -1.019238472 | hsa-mir-3186 | - |
| hsa-mir-3911 | HGLibA_59514 | 13 | -1.019238472 | hsa-mir-3911 | - |
| hsa-mir-3914-2 | HGLibA_59532 | 13 | -1.019238472 | hsa-mir-3914-2 | - |
| hsa-mir-4259 | HGLibA_59744 | 13 | -1.019238472 | hsa-mir-4259 | - |
| hsa-mir-4292 | HGLibA_59880 | 13 | -1.019238472 | hsa-mir-4292 | - |
| hsa-mir-4433 | HGLibA_60120 | 13 | -1.019238472 | hsa-mir-4433 | - |
| hsa-mir-4433b | HGLibA_60124 | 13 | -1.019238472 | hsa-mir-4433b | - |
| hsa-mir-4445 | HGLibA_60187 | 13 | -1.019238472 | hsa-mir-4445 | - |
| hsa-mir-4468 | HGLibA_60277 | 13 | -1.019238472 | hsa-mir-4468 | - |
| hsa-mir-4470 | HGLibA_60287 | 13 | -1.019238472 | hsa-mir-4470 | - |
| hsa-mir-4636 | HGLibA_60648 | 13 | -1.019238472 | hsa-mir-4636 | - |
| hsa-mir-4759 | HGLibA_61133 | 13 | -1.019238472 | hsa-mir-4759 | - |
| hsa-mir-548b | HGLibA_61950 | 13 | -1.019238472 | hsa-mir-548b | - |
| hsa-mir-551a | HGLibA_62117 | 13 | -1.019238472 | hsa-mir-551a | - |
| hsa-mir-5584 | HGLibA_62177 | 13 | -1.019238472 | hsa-mir-5584 | - |
| hsa-mir-562 | HGLibA_62212 | 13 | -1.019238472 | hsa-mir-562 | - |
| hsa-mir-633 | HGLibA_62771 | 13 | -1.019238472 | hsa-mir-633 | - |
| hsa-mir-6782 | HGLibA_63306 | 13 | -1.019238472 | hsa-mir-6782 | - |
| hsa-mir-6884 | HGLibA_63719 | 13 | -1.019238472 | hsa-mir-6884 | - |
| hsa-mir-8056 | HGLibA_64061 | 13 | -1.019238472 | hsa-mir-8056 | - |
| LOC440335 | HGLibA_26945 | 13 | -1.019238472 | LOC440335 | - |
| PRHOXNB | HGLibA_38466 | 13 | -1.019238472 | PRHOXNB | - |
| SF3B14 | HGLibA_43702 | 13 | -1.019238472 | SF3B14 | - |
| ALS2CR12 | HGLibA_01727 | 12 | -1.02918951 | ALS2CR12 | - |
| CD3EAP | HGLibA_08374 | 12 | -1.02918951 | CD3EAP | - |
| ENSG00000005448 | HGLibA_54125 | 12 | -1.02918951 | WDR54 | WD repeat domain 54 [Source:HGNC Symbol;Acc:HGNC:25770] |
| ENSG00000012232 | HGLibA_15815 | 12 | -1.02918951 | EXTL3 | exostosin like glycosyltransferase 3 [Source:HGNC Symbol;Acc:HGNC:3518] |
| ENSG00000012232 | HGLibA_15814 | 12 | -1.02918951 | EXTL3 | exostosin like glycosyltransferase 3 [Source:HGNC Symbol;Acc:HGNC:3518] |
| ENSG00000020577 | HGLibA_42682 | 12 | -1.02918951 | SAMD4A | sterile alpha motif domain containing 4A [Source:HGNC Symbol;Acc:HGNC:23023] |
| ENSG00000025708 | HGLibA_52459 | 12 | -1.02918951 | TYMP | thymidine phosphorylase [Source:HGNC Symbol;Acc:HGNC:3148] |
| ENSG00000035403 | HGLibA_53573 | 12 | -1.02918951 | VCL | vinculin [Source:HGNC Symbol;Acc:HGNC:12665] |

| | | | | | |
|---|---|---|---|---|---|
| ENSG00000042493 | HGLibA_07138 | 12 | -1.02918951 | CAPG | gelsolin like [Source:HGNC Symbol;Acc:HGNC:1474]" |
| ENSG00000050327 | HGLibA_02869 | 12 | -1.02918951 | ARHGEF5 | Rho guanine nucleotide exchange factor 5 [Source:HGNC Symbol;Acc:HGNC:13209] |
| ENSG00000062096 | HGLibA_03140 | 12 | -1.02918951 | ARSF | arylsulfatase F [Source:HGNC Symbol;Acc:HGNC:721] |
| ENSG00000063978 | HGLibA_41675 | 12 | -1.02918951 | RNF4 | ring finger protein 4 [Source:HGNC Symbol;Acc:HGNC:10067] |
| ENSG00000066382 | HGLibA_29774 | 12 | -1.02918951 | MPPED2 | metallophosphoesterase domain containing 2 [Source:HGNC Symbol;Acc:HGNC:1180] |
| ENSG00000067955 | HGLibA_07391 | 12 | -1.02918951 | CBFB | core-binding factor subunit beta [Source:HGNC Symbol;Acc:HGNC:1539] |
| ENSG00000069535 | HGLibA_28109 | 12 | -1.02918951 | MAOB | monoamine oxidase B [Source:HGNC Symbol;Acc:HGNC:6834] |
| ENSG00000073756 | HGLibA_39361 | 12 | -1.02918951 | PTGS2 | prostaglandin-endoperoxide synthase 2 [Source:HGNC Symbol;Acc:HGNC:9605] |
| ENSG00000075218 | HGLibA_20624 | 12 | -1.02918951 | GTSE1 | G2 and S-phase expressed 1 [Source:HGNC Symbol;Acc:HGNC:13698] |
| ENSG00000075234 | HGLibA_52130 | 12 | -1.02918951 | TTC38 | tetratricopeptide repeat domain 38 [Source:HGNC Symbol;Acc:HGNC:26082] |
| ENSG00000075914 | HGLibA_15789 | 12 | -1.02918951 | EXOSC7 | exosome component 7 [Source:HGNC Symbol;Acc:HGNC:28112] |
| ENSG00000079393 | HGLibA_14140 | 12 | -1.02918951 | DUSP13 | dual specificity phosphatase 13 [Source:HGNC Symbol;Acc:HGNC:19681] |
| ENSG00000081377 | HGLibA_08513 | 12 | -1.02918951 | CDC14B | cell division cycle 14B [Source:HGNC Symbol;Acc:HGNC:1719] |
| ENSG00000083444 | HGLibA_37207 | 12 | -1.02918951 | PLOD1 | 2-oxoglutarate 5-dioxygenase 1 [Source:HGNC Symbol;Acc:HGNC:9081]" |
| ENSG00000085999 | HGLibA_40101 | 12 | -1.02918951 | RAD54L | RAD54 like [Source:HGNC Symbol;Acc:HGNC:9826] |
| ENSG00000091583 | HGLibA_02523 | 12 | -1.02918951 | APOH | apolipoprotein H [Source:HGNC Symbol;Acc:HGNC:616] |
| ENSG00000091640 | HGLibA_46365 | 12 | -1.02918951 | SPAG7 | sperm associated antigen 7 [Source:HGNC Symbol;Acc:HGNC:11216] |
| ENSG00000091831 | HGLibA_15582 | 12 | -1.02918951 | ESR1 | estrogen receptor 1 [Source:HGNC Symbol;Acc:HGNC:3467] |
| ENSG00000092345 | HGLibA_12370 | 12 | -1.02918951 | DAZL | deleted in azoospermia like [Source:HGNC Symbol;Acc:HGNC:2685] |
| ENSG00000093167 | HGLibA_27452 | 12 | -1.02918951 | LRRFIP2 | LRR binding FLII interacting protein 2 [Source:HGNC Symbol;Acc:HGNC:6703] |
| ENSG00000099326 | HGLibA_30936 | 12 | -1.02918951 | MZF1 | myeloid zinc finger 1 [Source:HGNC Symbol;Acc:HGNC:13108] |
| ENSG00000100053 | HGLibA_11266 | 12 | -1.02918951 | CRYBB3 | crystallin beta B3 [Source:HGNC Symbol;Acc:HGNC:2400] |
| ENSG00000101951 | HGLibA_35013 | 12 | -1.02918951 | PAGE4 | PAGE family member 4 [Source:HGNC Symbol;Acc:HGNC:4108] |
| ENSG00000102362 | HGLibA_48105 | 12 | -1.02918951 | SYTL4 | synaptotagmin like 4 [Source:HGNC Symbol;Acc:HGNC:15588] |
| ENSG00000104228 | HGLibA_51471 | 12 | -1.02918951 | TRIM35 | tripartite motif containing 35 [Source:HGNC Symbol;Acc:HGNC:16285] |
| ENSG00000105146 | HGLibA_03882 | 12 | -1.02918951 | AURKC | aurora kinase C [Source:HGNC Symbol;Acc:HGNC:11391] |
| ENSG00000105472 | HGLibA_09924 | 12 | -1.02918951 | CLEC11A | C-type lectin domain containing 11A [Source:HGNC Symbol;Acc:HGNC:10576] |
| ENSG00000105993 | HGLibA_13548 | 12 | -1.02918951 | DNAJB6 | DnaJ heat shock protein family (Hsp40) member B6 [Source:HGNC Symbol;Acc:HGNC:14888] |
| ENSG00000106330 | HGLibA_29691 | 12 | -1.02918951 | MOSPD3 | motile sperm domain containing 3 [Source:HGNC Symbol;Acc:HGNC:25078] |
| ENSG00000108602 | HGLibA_01563 | 12 | -1.02918951 | ALDH3A1 | aldehyde dehydrogenase 3 family member A1 [Source:HGNC Symbol;Acc:HGNC:405] |
| ENSG00000108651 | HGLibA_53455 | 12 | -1.02918951 | UTP6 | small subunit processome component [Source:HGNC Symbol;Acc:HGNC:18279]" |
| ENSG00000108823 | HGLibA_43795 | 12 | -1.02918951 | SGCA | sarcoglycan alpha [Source:HGNC Symbol;Acc:HGNC:10805] |
| ENSG00000110429 | HGLibA_17062 | 12 | -1.02918951 | FBXO3 | F-box protein 3 [Source:HGNC Symbol;Acc:HGNC:13582] |
| ENSG00000110876 | HGLibA_43307 | 12 | -1.02918951 | SELPLG | selectin P ligand [Source:HGNC Symbol;Acc:HGNC:10722] |
| ENSG00000113356 | HGLibA_37627 | 12 | -1.02918951 | POLR3G | RNA polymerase III subunit G [Source:HGNC Symbol;Acc:HGNC:30075] |
| ENSG00000113716 | HGLibA_21733 | 12 | -1.02918951 | HMGXB3 | HMG-box containing 3 [Source:HGNC Symbol;Acc:HGNC:28982] |
| ENSG00000114767 | HGLibA_42287 | 12 | -1.02918951 | RRP9 | U3 small nucleolar RNA binding protein [Source:HGNC Symbol;Acc:HGNC:16829]" |
| ENSG00000115286 | HGLibA_31563 | 12 | -1.02918951 | NDUFS7 | NADH:ubiquinone oxidoreductase core subunit S7 [Source:HGNC Symbol;Acc:HGNC:7714] |
| ENSG00000116039 | HGLibA_03732 | 12 | -1.02918951 | ATP6V1B1 | ATPase H+ transporting V1 subunit B1 [Source:HGNC Symbol;Acc:HGNC:853] |
| ENSG00000116205 | HGLibA_48730 | 12 | -1.02918951 | TCEANC2 | transcription elongation factor A N-terminal and central domain containing 2 [Source:HGNC Symbol;Acc:HGNC:26494] |
| ENSG00000117054 | HGLibA_00369 | 12 | -1.02918951 | ACADM | acyl-CoA dehydrogenase medium chain [Source:HGNC Symbol;Acc:HGNC:89] |
| ENSG00000117152 | HGLibA_41093 | 12 | -1.02918951 | RGS4 | regulator of G protein signaling 4 [Source:HGNC Symbol;Acc:HGNC:10000] |
| ENSG00000119326 | HGLibA_11647 | 12 | -1.02918951 | CTNNAL1 | catenin alpha like 1 [Source:HGNC Symbol;Acc:HGNC:2512] |
| ENSG00000124469 | HGLibA_08954 | 12 | -1.02918951 | CEACAM8 | carcinoembryonic antigen related cell adhesion molecule 8 [Source:HGNC Symbol;Acc:HGNC:1820] |
| ENSG00000125746 | HGLibA_15060 | 12 | -1.02918951 | EML2 | echinoderm microtubule associated protein like 2 [Source:HGNC Symbol;Acc:HGNC:18035] |
| ENSG00000126545 | HGLibA_11371 | 12 | -1.02918951 | CSN1S1 | casein alpha s1 [Source:HGNC Symbol;Acc:HGNC:2445] |
| ENSG00000127511 | HGLibA_44187 | 12 | -1.02918951 | SIN3B | SIN3 transcription regulator family member B [Source:HGNC Symbol;Acc:HGNC:19354] |
| ENSG00000129353 | HGLibA_45160 | 12 | -1.02918951 | SLC44A2 | solute carrier family 44 member 2 [Source:HGNC Symbol;Acc:HGNC:17292] |
| ENSG00000130935 | HGLibA_32258 | 12 | -1.02918951 | NOL11 | nucleolar protein 11 [Source:HGNC Symbol;Acc:HGNC:24557] |
| ENSG00000131149 | HGLibA_20417 | 12 | -1.02918951 | GSE1 | Gse1 coiled-coil protein [Source:HGNC Symbol;Acc:HGNC:28979] |
| ENSG00000131370 | HGLibA_43934 | 12 | -1.02918951 | SH3BP5 | SH3 domain binding protein 5 [Source:HGNC Symbol;Acc:HGNC:10827] |
| ENSG00000132600 | HGLibA_38636 | 12 | -1.02918951 | PRMT7 | protein arginine methyltransferase 7 [Source:HGNC Symbol;Acc:HGNC:25557] |
| ENSG00000133135 | HGLibA_41499 | 12 | -1.02918951 | RNF128 | E3 ubiquitin protein ligase [Source:HGNC Symbol;Acc:HGNC:21153]" |
| ENSG00000133142 | HGLibA_48712 | 12 | -1.02918951 | TCEAL4 | transcription elongation factor A like 4 [Source:HGNC Symbol;Acc:HGNC:26121] |
| ENSG00000134046 | HGLibA_28465 | 12 | -1.02918951 | MBD2 | methyl-CpG binding domain protein 2 [Source:HGNC Symbol;Acc:HGNC:6917] |
| ENSG00000134717 | HGLibA_04938 | 12 | -1.02918951 | BTF3L4 | basic transcription factor 3 like 4 [Source:HGNC Symbol;Acc:HGNC:30547] |
| ENSG00000135097 | HGLibA_30206 | 12 | -1.02918951 | MSI1 | musashi RNA binding protein 1 [Source:HGNC Symbol;Acc:HGNC:7330] |
| ENSG00000135596 | HGLibA_29202 | 12 | -1.02918951 | MICAL1 | calponin and LIM domain containing 1 [Source:HGNC Symbol;Acc:HGNC:20619]" |
| ENSG00000135744 | HGLibA_01255 | 12 | -1.02918951 | AGT | angiotensinogen [Source:HGNC Symbol;Acc:HGNC:333] |
| ENSG00000136040 | HGLibA_37280 | 12 | -1.02918951 | PLXNC1 | plexin C1 [Source:HGNC Symbol;Acc:HGNC:9106] |
| ENSG00000136104 | HGLibA_41431 | 12 | -1.02918951 | RNASEH2B | ribonuclease H2 subunit B [Source:HGNC Symbol;Acc:HGNC:25671] |
| ENSG00000136878 | HGLibA_53300 | 12 | -1.02918951 | USP20 | ubiquitin specific peptidase 20 [Source:HGNC Symbol;Acc:HGNC:12619] |
| ENSG00000137101 | HGLibA_08440 | 12 | -1.02918951 | CD72 | CD72 molecule [Source:HGNC Symbol;Acc:HGNC:1696] |
| ENSG00000138028 | HGLibA_09325 | 12 | -1.02918951 | CGREF1 | cell growth regulator with EF-hand domain 1 [Source:HGNC Symbol;Acc:HGNC:16962] |
| ENSG00000139324 | HGLibA_50675 | 12 | -1.02918951 | TMTC3 | transmembrane and tetratricopeptide repeat containing 3 [Source:HGNC Symbol;Acc:HGNC:26899] |
| ENSG00000139540 | HGLibA_45114 | 12 | -1.02918951 | SLC39A5 | solute carrier family 39 member 5 [Source:HGNC Symbol;Acc:HGNC:20502] |
| ENSG00000139625 | HGLibA_28168 | 12 | -1.02918951 | MAP3K12 | mitogen-activated protein kinase kinase kinase 12 [Source:HGNC Symbol;Acc:HGNC:6851] |
| ENSG00000141040 | HGLibA_55755 | 12 | -1.02918951 | ZNF287 | zinc finger protein 287 [Source:HGNC Symbol;Acc:HGNC:13502] |
| ENSG00000141480 | HGLibA_03110 | 12 | -1.02918951 | ARRB2 | arrestin beta 2 [Source:HGNC Symbol;Acc:HGNC:712] |
| ENSG00000142208 | HGLibA_01504 | 12 | -1.02918951 | AKT1 | AKT serine/threonine kinase 1 [Source:HGNC Symbol;Acc:HGNC:391] |
| ENSG00000142208 | HGLibA_01506 | 12 | -1.02918951 | AKT1 | AKT serine/threonine kinase 1 [Source:HGNC Symbol;Acc:HGNC:391] |
| ENSG00000143393 | HGLibA_36500 | 12 | -1.02918951 | PI4KB | phosphatidylinositol 4-kinase beta [Source:HGNC Symbol;Acc:HGNC:8984] |
| ENSG00000143398 | HGLibA_36714 | 12 | -1.02918951 | PIP5K1A | phosphatidylinositol-4-phosphate 5-kinase type 1 alpha [Source:HGNC Symbol;Acc:HGNC:8994] |
| ENSG00000143878 | HGLibA_41170 | 12 | -1.02918951 | RHOB | ras homolog family member B [Source:HGNC Symbol;Acc:HGNC:668] |
| ENSG00000144057 | HGLibA_47261 | 12 | -1.02918951 | ST6GAL2 | 6-sialyltransferase 2 [Source:HGNC Symbol;Acc:HGNC:10861]" |
| ENSG00000144677 | HGLibA_11613 | 12 | -1.02918951 | CTDSPL | CTD small phosphatase like [Source:HGNC Symbol;Acc:HGNC:16890] |
| ENSG00000144744 | HGLibA_52519 | 12 | -1.02918951 | UBA3 | ubiquitin like modifier activating enzyme 3 [Source:HGNC Symbol;Acc:HGNC:12470] |
| ENSG00000144935 | HGLibA_51727 | 12 | -1.02918951 | TRPC1 | transient receptor potential cation channel subfamily C member 1 [Source:HGNC Symbol;Acc:HGNC:12333] |
| ENSG00000145365 | HGLibA_49458 | 12 | -1.02918951 | TIFA | TRAF interacting protein with forkhead associated domain [Source:HGNC Symbol;Acc:HGNC:19075] |
| ENSG00000145990 | HGLibA_18953 | 12 | -1.02918951 | GFOD1 | glucose-fructose oxidoreductase domain containing 1 [Source:HGNC Symbol;Acc:HGNC:21096] |
| ENSG00000146678 | HGLibA_22815 | 12 | -1.02918951 | IGFBP1 | insulin like growth factor binding protein 1 [Source:HGNC Symbol;Acc:HGNC:5469] |
| ENSG00000146963 | HGLibA_27622 | 12 | -1.02918951 | LUC7L2 | pre-mRNA splicing factor [Source:HGNC Symbol;Acc:HGNC:21608]" |
| ENSG00000147050 | HGLibA_24534 | 12 | -1.02918951 | KDM6A | lysine demethylase 6A [Source:HGNC Symbol;Acc:HGNC:12637] |
| ENSG00000147316 | HGLibA_28647 | 12 | -1.02918951 | MCPH1 | microcephalin 1 [Source:HGNC Symbol;Acc:HGNC:6954] |
| ENSG00000148541 | HGLibA_16148 | 12 | -1.02918951 | FAM13C | family with sequence similarity 13 member C [Source:HGNC Symbol;Acc:HGNC:19371] |
| ENSG00000149177 | HGLibA_39512 | 12 | -1.02918951 | PTPRJ | receptor type J [Source:HGNC Symbol;Acc:HGNC:9673]" |
| ENSG00000149243 | HGLibA_25196 | 12 | -1.02918951 | KLHL35 | kelch like family member 35 [Source:HGNC Symbol;Acc:HGNC:26597] |
| ENSG00000149548 | HGLibA_07641 | 12 | -1.02918951 | CCDC15 | coiled-coil domain containing 15 [Source:HGNC Symbol;Acc:HGNC:25798] |
| ENSG00000150093 | HGLibA_23769 | 12 | -1.02918951 | ITGB1 | integrin subunit beta 1 [Source:HGNC Symbol;Acc:HGNC:6153] |
| ENSG00000151748 | HGLibA_42784 | 12 | -1.02918951 | SAV1 | salvador family WW domain containing protein 1 [Source:HGNC Symbol;Acc:HGNC:17795] |
| ENSG00000152520 | HGLibA_35093 | 12 | -1.02918951 | PAN3 | poly(A) specific ribonuclease subunit PAN3 [Source:HGNC Symbol;Acc:HGNC:29991] |
| ENSG00000152785 | HGLibA_04606 | 12 | -1.02918951 | BMP3 | bone morphogenetic protein 3 [Source:HGNC Symbol;Acc:HGNC:1070] |
| ENSG00000153443 | HGLibA_52541 | 12 | -1.02918951 | UBALD1 | UBA like domain containing 1 [Source:HGNC Symbol;Acc:HGNC:29576] |
| ENSG00000156006 | HGLibA_31177 | 12 | -1.02918951 | NAT2 | N-acetyltransferase 2 [Source:HGNC Symbol;Acc:HGNC:7646] |
| ENSG00000158234 | HGLibA_15965 | 12 | -1.02918951 | FAIM | Fas apoptotic inhibitory molecule [Source:HGNC Symbol;Acc:HGNC:18703] |
| ENSG00000160716 | HGLibA_09591 | 12 | -1.02918951 | CHRNB2 | cholinergic receptor nicotinic beta 2 subunit [Source:HGNC Symbol;Acc:HGNC:1962] |
| ENSG00000161981 | HGLibA_45945 | 12 | -1.02918951 | SNRNP25 | small nuclear ribonucleoprotein U11/U12 subunit 25 [Source:HGNC Symbol;Acc:HGNC:14161] |
| ENSG00000163191 | HGLibA_42536 | 12 | -1.02918951 | S100A11 | S100 calcium binding protein A11 [Source:HGNC Symbol;Acc:HGNC:10488] |
| ENSG00000163207 | HGLibA_23880 | 12 | -1.02918951 | IVL | involucrin [Source:HGNC Symbol;Acc:HGNC:6187] |
| ENSG00000164587 | HGLibA_42075 | 12 | -1.02918951 | RPS14 | ribosomal protein S14 [Source:HGNC Symbol;Acc:HGNC:10387] |
| ENSG00000164816 | HGLibA_12805 | 12 | -1.02918951 | DEFA5 | defensin alpha 5 [Source:HGNC Symbol;Acc:HGNC:2764] |
| ENSG00000165879 | HGLibA_17977 | 12 | -1.02918951 | FRAT1 | WNT signaling pathway regulator [Source:HGNC Symbol;Acc:HGNC:3944]" |
| ENSG00000166479 | HGLibA_50695 | 12 | -1.02918951 | TMX3 | thioredoxin related transmembrane protein 3 [Source:HGNC Symbol;Acc:HGNC:24718] |
| ENSG00000167103 | HGLibA_36722 | 12 | -1.02918951 | PIP5KL1 | phosphatidylinositol-4-phosphate 5-kinase like 1 [Source:HGNC Symbol;Acc:HGNC:28711] |
| ENSG00000167625 | HGLibA_56164 | 12 | -1.02918951 | ZNF526 | zinc finger protein 526 [Source:HGNC Symbol;Acc:HGNC:29415] |
| ENSG00000168411 | HGLibA_40959 | 12 | -1.02918951 | RFWD3 | ring finger and WD repeat domain 3 [Source:HGNC Symbol;Acc:HGNC:25539] |
| ENSG00000168828 | HGLibA_33627 | 12 | -1.02918951 | OR13J1 | olfactory receptor family 13 subfamily J member 1 [Source:HGNC Symbol;Acc:HGNC:15108] |
| ENSG00000169777 | HGLibA_48363 | 12 | -1.02918951 | TAS2R1 | taste 2 receptor member 1 [Source:HGNC Symbol;Acc:HGNC:14909] |
| ENSG00000170370 | HGLibA_15094 | 12 | -1.02918951 | EMX2 | empty spiracles homeobox 2 [Source:HGNC Symbol;Acc:HGNC:3341] |
| ENSG00000171509 | HGLibA_42495 | 12 | -1.02918951 | RXFP1 | relaxin family peptide receptor 1 [Source:HGNC Symbol;Acc:HGNC:19718] |
| ENSG00000171790 | HGLibA_45500 | 12 | -1.02918951 | SLFNL1 | schlafen like 1 [Source:HGNC Symbol;Acc:HGNC:26313] |
| ENSG00000171867 | HGLibA_38645 | 12 | -1.02918951 | PRNP | prion protein [Source:HGNC Symbol;Acc:HGNC:9449] |
| ENSG00000172007 | HGLibA_39883 | 12 | -1.02918951 | RAB33B | member RAS oncogene family [Source:HGNC Symbol;Acc:HGNC:16075]" |
| ENSG00000172377 | HGLibA_34578 | 12 | -1.02918951 | OR9I1 | olfactory receptor family 9 subfamily I member 1 [Source:HGNC Symbol;Acc:HGNC:14718] |
| ENSG00000174177 | HGLibA_11752 | 12 | -1.02918951 | CTU2 | cytosolic thiouridylase subunit 2 [Source:HGNC Symbol;Acc:HGNC:28005] |
| ENSG00000174444 | HGLibA_41983 | 12 | -1.02918951 | RPL4 | ribosomal protein L4 [Source:HGNC Symbol;Acc:HGNC:10353] |
| ENSG00000174844 | HGLibA_13476 | 12 | -1.02918951 | DNAH12 | dynein axonemal heavy chain 12 [Source:HGNC Symbol;Acc:HGNC:2943] |
| ENSG00000175305 | HGLibA_08086 | 12 | -1.02918951 | CCNE2 | cyclin E2 [Source:HGNC Symbol;Acc:HGNC:1590] |
| ENSG00000175344 | HGLibA_09582 | 12 | -1.02918951 | CHRNA7 | cholinergic receptor nicotinic alpha 7 subunit [Source:HGNC Symbol;Acc:HGNC:1960] |
| ENSG00000175449 | HGLibA_40917 | 12 | -1.02918951 | RFESD | Rieske Fe-S domain containing [Source:HGNC Symbol;Acc:HGNC:29587] |
| ENSG00000176915 | HGLibA_01975 | 12 | -1.02918951 | ANKLE2 | ankyrin repeat and LEM domain containing 2 [Source:HGNC Symbol;Acc:HGNC:29101] |
| ENSG00000179097 | HGLibA_22368 | 12 | -1.02918951 | HTR1F | 5-hydroxytryptamine receptor 1F [Source:HGNC Symbol;Acc:HGNC:5292] |
| ENSG00000179133 | HGLibA_05086 | 12 | -1.02918951 | C10orf67 | chromosome 10 open reading frame 67 [Source:HGNC Symbol;Acc:HGNC:28716] |
| ENSG00000180855 | HGLibA_56011 | 12 | -1.02918951 | ZNF443 | zinc finger protein 443 [Source:HGNC Symbol;Acc:HGNC:20878] |
| ENSG00000181927 | HGLibA_34054 | 12 | -1.02918951 | OR4P4 | olfactory receptor family 4 subfamily P member 4 [Source:HGNC Symbol;Acc:HGNC:15180] |
| ENSG00000182568 | HGLibA_42776 | 12 | -1.02918951 | SATB1 | SATB homeobox 1 [Source:HGNC Symbol;Acc:HGNC:10541] |
| ENSG00000182774 | HGLibA_42086 | 12 | -1.02918951 | RPS17 | ribosomal protein S17 [Source:HGNC Symbol;Acc:HGNC:10397] |
| ENSG00000182923 | HGLibA_09152 | 12 | -1.02918951 | CEP63 | centrosomal protein 63 [Source:HGNC Symbol;Acc:HGNC:25815] |
| ENSG00000183166 | HGLibA_07044 | 12 | -1.02918951 | CALN1 | calneuron 1 [Source:HGNC Symbol;Acc:HGNC:13248] |
| ENSG00000183763 | HGLibA_51274 | 12 | -1.02918951 | TRAIP | TRAF interacting protein [Source:HGNC Symbol;Acc:HGNC:30764] |
| ENSG00000184007 | HGLibA_39405 | 12 | -1.02918951 | PTP4A2 | member 2 [Source:HGNC Symbol;Acc:HGNC:9635]" |
| ENSG00000184033 | HGLibA_11560 | 12 | -1.02918951 | CTAG1B | cancer/testis antigen 1B [Source:HGNC Symbol;Acc:HGNC:2491] |
| ENSG00000184194 | HGLibA_19969 | 12 | -1.02918951 | GPR173 | G protein-coupled receptor 173 [Source:HGNC Symbol;Acc:HGNC:18186] |
| ENSG00000184454 | HGLibA_31332 | 12 | -1.02918951 | NCMAP | non-compact myelin associated protein [Source:HGNC Symbol;Acc:HGNC:29332] |
| ENSG00000185002 | HGLibA_40976 | 12 | -1.02918951 | RFX6 | regulatory factor X6 [Source:HGNC Symbol;Acc:HGNC:21478] |

| | | | | | |
|---|---|---|---|---|---|
| ENSG00000186074 | HGLibA_08342 | 12 | -1.02918951 | CD300LF | CD300 molecule like family member f [Source:HGNC Symbol;Acc:HGNC:29883] |
| ENSG00000186844 | HGLibA_26101 | 12 | -1.02918951 | LCE1A | late cornified envelope 1A [Source:HGNC Symbol;Acc:HGNC:29459] |
| ENSG00000187147 | HGLibA_41640 | 12 | -1.02918951 | RNF220 | ring finger protein 220 [Source:HGNC Symbol;Acc:HGNC:25552] |
| ENSG00000188610 | HGLibA_16711 | 12 | -1.02918951 | FAM72B | family with sequence similarity 72 member B [Source:HGNC Symbol;Acc:HGNC:24805] |
| ENSG00000196453 | HGLibA_56724 | 12 | -1.02918951 | ZNF777 | zinc finger protein 777 [Source:HGNC Symbol;Acc:HGNC:22213] |
| ENSG00000196850 | HGLibA_38238 | 12 | -1.02918951 | PPTC7 | PTC7 protein phosphatase homolog [Source:HGNC Symbol;Acc:HGNC:30695] |
| ENSG00000197157 | HGLibA_45920 | 12 | -1.02918951 | SND1 | staphylococcal nuclease and tudor domain containing 1 [Source:HGNC Symbol;Acc:HGNC:30646] |
| ENSG00000197632 | HGLibA_43546 | 12 | -1.02918951 | SERPINB2 | serpin family B member 2 [Source:HGNC Symbol;Acc:HGNC:8584] |
| ENSG00000197785 | HGLibA_03409 | 12 | -1.02918951 | ATAD3A | AAA domain containing 3A [Source:HGNC Symbol;Acc:HGNC:25567]" |
| ENSG00000197808 | HGLibA_56038 | 12 | -1.02918951 | ZNF461 | zinc finger protein 461 [Source:HGNC Symbol;Acc:HGNC:21629] |
| ENSG00000198369 | HGLibA_46793 | 12 | -1.02918951 | SPRED2 | sprouty related EVH1 domain containing 2 [Source:HGNC Symbol;Acc:HGNC:17722] |
| ENSG00000198836 | HGLibA_33405 | 12 | -1.02918951 | OPA1 | mitochondrial dynamin like GTPase [Source:HGNC Symbol;Acc:HGNC:8140]" |
| ENSG00000198858 | HGLibA_39777 | 12 | -1.02918951 | R3HDM4 | R3H domain containing 4 [Source:HGNC Symbol;Acc:HGNC:28270] |
| ENSG00000198934 | HGLibA_27981 | 12 | -1.02918951 | MAGEE1 | MAGE family member E1 [Source:HGNC Symbol;Acc:HGNC:24934] |
| ENSG00000204186 | HGLibA_55063 | 12 | -1.02918951 | ZDBF2 | zinc finger DBF-type containing 2 [Source:HGNC Symbol;Acc:HGNC:29313] |
| ENSG00000204612 | HGLibA_17818 | 12 | -1.02918951 | FOXB2 | forkhead box B2 [Source:HGNC Symbol;Acc:HGNC:23315] |
| ENSG00000204928 | HGLibA_20393 | 12 | -1.02918951 | GRXCR2 | glutaredoxin and cysteine rich domain containing 2 [Source:HGNC Symbol;Acc:HGNC:33862] |
| ENSG00000205155 | HGLibA_39030 | 12 | -1.02918951 | PSENEN | gamma-secretase subunit [Source:HGNC Symbol;Acc:HGNC:30100]" |
| ENSG00000205642 | HGLibA_53589 | 12 | -1.02918951 | VCX3B | variable charge X-linked 3B [Source:HGNC Symbol;Acc:HGNC:31838] |
| ENSG00000206531 | HGLibA_08287 | 12 | -1.02918951 | CD200R1L | CD200 receptor 1 like [Source:HGNC Symbol;Acc:HGNC:24665] |
| ENSG00000213339 | HGLibA_39759 | 12 | -1.02918951 | QTRT1 | queuine tRNA-ribosyltransferase catalytic subunit 1 [Source:HGNC Symbol;Acc:HGNC:23797] |
| ENSG00000213585 | HGLibA_53599 | 12 | -1.02918951 | VDAC1 | voltage dependent anion channel 1 [Source:HGNC Symbol;Acc:HGNC:12669] |
| ENSG00000214128 | HGLibA_50214 | 12 | -1.02918951 | TMEM213 | transmembrane protein 213 [Source:HGNC Symbol;Acc:HGNC:27220] |
| ENSG00000214491 | HGLibA_43206 | 12 | -1.02918951 | SEC14L6 | SEC14 like lipid binding 6 [Source:HGNC Symbol;Acc:HGNC:40047] |
| ENSG00000214711 | HGLibA_07157 | 12 | -1.02918951 | CAPN14 | calpain 14 [Source:HGNC Symbol;Acc:HGNC:16664] |
| ENSG00000215547 | HGLibA_12859 | 12 | -1.02918951 | DEFB115 | defensin beta 115 [Source:HGNC Symbol;Acc:HGNC:18096] |
| ENSG00000221818 | HGLibA_14352 | 12 | -1.02918951 | EBF2 | EBF transcription factor 2 [Source:HGNC Symbol;Acc:HGNC:19090] |
| ENSG00000224389 | HGLibA_06285 | 12 | -1.02918951 | C4B | complement C4B (Chido blood group) [Source:HGNC Symbol;Acc:HGNC:1324] |
| ENSG00000227802 | HGLibA_13539 | 12 | -1.02918951 | DNAJB3 | DnaJ heat shock protein family (Hsp40) member B3 [Source:HGNC Symbol;Acc:HGNC:32397] |
| ENSG00000228049 | HGLibA_37594 | 12 | -1.02918951 | POLR2J2 | RNA polymerase II subunit J2 [Source:HGNC Symbol;Acc:HGNC:23208] |
| ENSG00000228696 | HGLibA_02955 | 12 | -1.02918951 | ARL17B | ADP ribosylation factor like GTPase 17B [Source:HGNC Symbol;Acc:HGNC:32387] |
| ENSG00000240230 | HGLibA_10825 | 12 | -1.02918951 | COX19 | cytochrome c oxidase assembly factor COX19 [Source:HGNC Symbol;Acc:HGNC:28074] |
| ENSG00000245848 | HGLibA_08955 | 12 | -1.02918951 | CEBPA | CCAAT enhancer binding protein alpha [Source:HGNC Symbol;Acc:HGNC:1833] |
| ENSG00000254772 | HGLibA_14505 | 12 | -1.02918951 | EEF1G | eukaryotic translation elongation factor 1 gamma [Source:HGNC Symbol;Acc:HGNC:3213] |
| ENSG00000256574 | HGLibA_33593 | 12 | -1.02918951 | OR13A1 | olfactory receptor family 13 subfamily A member 1 [Source:HGNC Symbol;Acc:HGNC:14772] |
| ENSG00000266412 | HGLibA_31343 | 12 | -1.02918951 | NCOA4 | nuclear receptor coactivator 4 [Source:HGNC Symbol;Acc:HGNC:7671] |
| ENSG00000268629 | HGLibA_49059 | 12 | -1.02918951 | TEX13A | testis expressed 13A [Source:HGNC Symbol;Acc:HGNC:11735] |
| ENSG00000270765 | HGLibA_18646 | 12 | -1.02918951 | GAS2L2 | growth arrest specific 2 like 2 [Source:HGNC Symbol;Acc:HGNC:24846] |
| ENSG00000272414 | HGLibA_16620 | 12 | -1.02918951 | FAM47E-STBD1 | FAM47E-STBD1 readthrough [Source:HGNC Symbol;Acc:HGNC:44667] |
| ENSG00000272602 | HGLibA_56347 | 12 | -1.02918951 | ZNF595 | zinc finger protein 595 [Source:HGNC Symbol;Acc:HGNC:27196] |
| ENSG00000277972 | HGLibA_09733 | 12 | -1.02918951 | CISD3 | CDGSH iron sulfur domain 3 [Source:HGNC Symbol;Acc:HGNC:27578] |
| FAM175A | HGLibA_16289 | 12 | -1.02918951 | FAM175A | - |
| FAM19A1 | HGLibA_16395 | 12 | -1.02918951 | FAM19A1 | - |
| GTPBP5 | HGLibA_20612 | 12 | -1.02918951 | GTPBP5 | - |
| hsa-mir-1185-2 | HGLibA_57244 | 12 | -1.02918951 | hsa-mir-1185-2 | - |
| hsa-mir-15a | HGLibA_57938 | 12 | -1.02918951 | hsa-mir-15a | - |
| hsa-mir-299 | HGLibA_58406 | 12 | -1.02918951 | hsa-mir-299 | - |
| hsa-mir-29a | HGLibA_58409 | 12 | -1.02918951 | hsa-mir-29a | - |
| hsa-mir-3130-1 | HGLibA_58573 | 12 | -1.02918951 | hsa-mir-3130-1 | - |
| hsa-mir-3149 | HGLibA_58652 | 12 | -1.02918951 | hsa-mir-3149 | - |
| hsa-mir-3179-3 | HGLibA_58799 | 12 | -1.02918951 | hsa-mir-3179-3 | - |
| hsa-mir-320d-1 | HGLibA_58942 | 12 | -1.02918951 | hsa-mir-320d-1 | - |
| hsa-mir-3661 | HGLibA_59195 | 12 | -1.02918951 | hsa-mir-3661 | - |
| hsa-mir-3673 | HGLibA_59244 | 12 | -1.02918951 | hsa-mir-3673 | - |
| hsa-mir-3914-1 | HGLibA_59530 | 12 | -1.02918951 | hsa-mir-3914-1 | - |
| hsa-mir-3914-2 | HGLibA_59534 | 12 | -1.02918951 | hsa-mir-3914-2 | - |
| hsa-mir-425 | HGLibA_59707 | 12 | -1.02918951 | hsa-mir-425 | - |
| hsa-mir-4319 | HGLibA_59994 | 12 | -1.02918951 | hsa-mir-4319 | - |
| hsa-mir-4417 | HGLibA_60053 | 12 | -1.02918951 | hsa-mir-4417 | - |
| hsa-mir-448 | HGLibA_60333 | 12 | -1.02918951 | hsa-mir-448 | - |
| hsa-mir-4480 | HGLibA_60338 | 12 | -1.02918951 | hsa-mir-4480 | - |
| hsa-mir-4681 | HGLibA_60834 | 12 | -1.02918951 | hsa-mir-4681 | - |
| hsa-mir-5007 | HGLibA_61430 | 12 | -1.02918951 | hsa-mir-5007 | - |
| hsa-mir-516b-1 | HGLibA_61617 | 12 | -1.02918951 | hsa-mir-516b-1 | - |
| hsa-mir-519a-1 | HGLibA_61710 | 12 | -1.02918951 | hsa-mir-519a-1 | - |
| hsa-mir-6077-1 | HGLibA_62562 | 12 | -1.02918951 | hsa-mir-6077-1 | - |
| hsa-mir-6077-2 | HGLibA_62566 | 12 | -1.02918951 | hsa-mir-6077-2 | - |
| hsa-mir-6800 | HGLibA_63378 | 12 | -1.02918951 | hsa-mir-6800 | - |
| hsa-mir-7-1 | HGLibA_63767 | 12 | -1.02918951 | hsa-mir-7-1 | - |
| KIAA1524 | HGLibA_24776 | 12 | -1.02918951 | KIAA1524 | - |
| LOC100288524 | HGLibA_26834 | 12 | -1.02918951 | LOC100288524 | - |
| LOC646498 | HGLibA_26974 | 12 | -1.02918951 | LOC646498 | - |
| MRVI1 | HGLibA_30125 | 12 | -1.02918951 | MRVI1 | - |
| PRSS45 | HGLibA_38944 | 12 | -1.02918951 | PRSS45 | - |
| TNFAIP8L2-SCNM1 | HGLibA_50727 | 12 | -1.02918951 | TNFAIP8L2-SCNM1 | - |
| C11orf93 | HGLibA_05214 | 11 | -1.039140547 | C11orf93 | - |
| C17orf59 | HGLibA_05552 | 11 | -1.039140547 | C17orf59 | - |
| C1orf61 | HGLibA_05878 | 11 | -1.039140547 | C1orf61 | - |
| C5orf42 | HGLibA_06382 | 11 | -1.039140547 | C5orf42 | - |
| CCDC23 | HGLibA_07734 | 11 | -1.039140547 | CCDC23 | - |
| CXorf30 | HGLibA_11913 | 11 | -1.039140547 | CXorf30 | - |
| ENSG00000005302 | HGLibA_30216 | 11 | -1.039140547 | MSL3 | MSL complex subunit 3 [Source:HGNC Symbol;Acc:HGNC:7370] |
| ENSG00000006606 | HGLibA_08010 | 11 | -1.039140547 | CCL26 | C-C motif chemokine ligand 26 [Source:HGNC Symbol;Acc:HGNC:10625] |
| ENSG00000008277 | HGLibA_00758 | 11 | -1.039140547 | ADAM22 | ADAM metallopeptidase domain 22 [Source:HGNC Symbol;Acc:HGNC:201] |
| ENSG00000013523 | HGLibA_01894 | 11 | -1.039140547 | ANGEL1 | angel homolog 1 [Source:HGNC Symbol;Acc:HGNC:19961] |
| ENSG00000014919 | HGLibA_10813 | 11 | -1.039140547 | COX15 | cytochrome c oxidase assembly homolog COX15 [Source:HGNC Symbol;Acc:HGNC:2263] |
| ENSG00000015171 | HGLibA_55415 | 11 | -1.039140547 | ZMYND11 | zinc finger MYND-type containing 11 [Source:HGNC Symbol;Acc:HGNC:16966] |
| ENSG00000025800 | HGLibA_25370 | 11 | -1.039140547 | KPNA6 | karyopherin subunit alpha 6 [Source:HGNC Symbol;Acc:HGNC:6399] |
| ENSG00000035115 | HGLibA_43985 | 11 | -1.039140547 | SH3YL1 | SH3 and SYLF domain containing 1 [Source:HGNC Symbol;Acc:HGNC:29546] |
| ENSG00000056291 | HGLibA_32442 | 11 | -1.039140547 | NPFFR2 | neuropeptide FF receptor 2 [Source:HGNC Symbol;Acc:HGNC:4525] |
| ENSG00000064835 | HGLibA_37749 | 11 | -1.039140547 | POU1F1 | POU class 1 homeobox 1 [Source:HGNC Symbol;Acc:HGNC:9210] |
| ENSG00000067248 | HGLibA_13172 | 11 | -1.039140547 | DHX29 | DExH-box helicase 29 [Source:HGNC Symbol;Acc:HGNC:15815] |
| ENSG00000071246 | HGLibA_53530 | 11 | -1.039140547 | VASH1 | vasohibin 1 [Source:HGNC Symbol;Acc:HGNC:19964] |
| ENSG00000071282 | HGLibA_26680 | 11 | -1.039140547 | LMCD1 | LIM and cysteine rich domains 1 [Source:HGNC Symbol;Acc:HGNC:6633] |
| ENSG00000072694 | HGLibA_17191 | 11 | -1.039140547 | FCGR2B | Fc fragment of IgG receptor IIb [Source:HGNC Symbol;Acc:HGNC:3618] |
| ENSG00000076242 | HGLibA_29381 | 11 | -1.039140547 | MLH1 | mutL homolog 1 [Source:HGNC Symbol;Acc:HGNC:7127] |
| ENSG00000080293 | HGLibA_43066 | 11 | -1.039140547 | SCTR | secretin receptor [Source:HGNC Symbol;Acc:HGNC:10608] |
| ENSG00000081237 | HGLibA_39491 | 11 | -1.039140547 | PTPRC | receptor type C [Source:HGNC Symbol;Acc:HGNC:9666]" |
| ENSG00000084093 | HGLibA_40868 | 11 | -1.039140547 | REST | RE1 silencing transcription factor [Source:HGNC Symbol;Acc:HGNC:9966] |
| ENSG00000085832 | HGLibA_15369 | 11 | -1.039140547 | EPS15 | epidermal growth factor receptor pathway substrate 15 [Source:HGNC Symbol;Acc:HGNC:3419] |
| ENSG00000086288 | HGLibA_32175 | 11 | -1.039140547 | NME8 | NME/NM23 family member 8 [Source:HGNC Symbol;Acc:HGNC:16473] |
| ENSG00000087237 | HGLibA_09242 | 11 | -1.039140547 | CETP | cholesteryl ester transfer protein [Source:HGNC Symbol;Acc:HGNC:1869] |
| ENSG00000087253 | HGLibA_27073 | 11 | -1.039140547 | LPCAT2 | lysophosphatidylcholine acyltransferase 2 [Source:HGNC Symbol;Acc:HGNC:26032] |
| ENSG00000087301 | HGLibA_52414 | 11 | -1.039140547 | TXNDC16 | thioredoxin domain containing 16 [Source:HGNC Symbol;Acc:HGNC:19965] |
| ENSG00000089094 | HGLibA_24497 | 11 | -1.039140547 | KDM2B | lysine demethylase 2B [Source:HGNC Symbol;Acc:HGNC:13610] |
| ENSG00000091622 | HGLibA_36756 | 11 | -1.039140547 | PITPNM3 | PITPNM family member 3 [Source:HGNC Symbol;Acc:HGNC:21043] |
| ENSG00000099822 | HGLibA_20976 | 11 | -1.039140547 | HCN2 | hyperpolarization activated cyclic nucleotide gated potassium and sodium channel 2 [Source:HGNC Symbol;Acc:HGNC:4846] |
| ENSG00000101057 | HGLibA_30645 | 11 | -1.039140547 | MYBL2 | MYB proto-oncogene like 2 [Source:HGNC Symbol;Acc:HGNC:7548] |
| ENSG00000101181 | HGLibA_30372 | 11 | -1.039140547 | MTG2 | mitochondrial ribosome associated GTPase 2 [Source:HGNC Symbol;Acc:HGNC:16239] |
| ENSG00000101654 | HGLibA_41719 | 11 | -1.039140547 | RNMT | RNA guanine-7 methyltransferase [Source:HGNC Symbol;Acc:HGNC:10075] |
| ENSG00000101892 | HGLibA_03603 | 11 | -1.039140547 | ATP1B4 | ATPase Na+/K+ transporting family member beta 4 [Source:HGNC Symbol;Acc:HGNC:808] |
| ENSG00000102078 | HGLibA_44647 | 11 | -1.039140547 | SLC25A14 | solute carrier family 25 member 14 [Source:HGNC Symbol;Acc:HGNC:10984] |
| ENSG00000103723 | HGLibA_02322 | 11 | -1.039140547 | AP3B2 | adaptor related protein complex 3 subunit beta 2 [Source:HGNC Symbol;Acc:HGNC:567] |
| ENSG00000104447 | HGLibA_51777 | 11 | -1.039140547 | TRPS1 | transcriptional repressor GATA binding 1 [Source:HGNC Symbol;Acc:HGNC:12340] |
| ENSG00000104856 | HGLibA_40815 | 11 | -1.039140547 | RELB | NF-kB subunit [Source:HGNC Symbol;Acc:HGNC:9956]" |
| ENSG00000106689 | HGLibA_26451 | 11 | -1.039140547 | LHX2 | LIM homeobox 2 [Source:HGNC Symbol;Acc:HGNC:6594] |
| ENSG00000108666 | HGLibA_05572 | 11 | -1.039140547 | C17orf75 | chromosome 17 open reading frame 75 [Source:HGNC Symbol;Acc:HGNC:30173] |
| ENSG00000110076 | HGLibA_32736 | 11 | -1.039140547 | NRXN2 | neurexin 2 [Source:HGNC Symbol;Acc:HGNC:8009] |
| ENSG00000110237 | HGLibA_02825 | 11 | -1.039140547 | ARHGEF17 | Rho guanine nucleotide exchange factor 17 [Source:HGNC Symbol;Acc:HGNC:21726] |
| ENSG00000110328 | HGLibA_18551 | 11 | -1.039140547 | GALNT18 | polypeptide N-acetylgalactosaminyltransferase 18 [Source:HGNC Symbol;Acc:HGNC:30488] |
| ENSG00000111358 | HGLibA_20556 | 11 | -1.039140547 | GTF2H3 | general transcription factor IIH subunit 3 [Source:HGNC Symbol;Acc:HGNC:4657] |
| ENSG00000111850 | HGLibA_45769 | 11 | -1.039140547 | SMIM8 | small integral membrane protein 8 [Source:HGNC Symbol;Acc:HGNC:21401] |
| ENSG00000113249 | HGLibA_20898 | 11 | -1.039140547 | HAVCR1 | hepatitis A virus cellular receptor 1 [Source:HGNC Symbol;Acc:HGNC:17866] |
| ENSG00000113761 | HGLibA_55848 | 11 | -1.039140547 | ZNF346 | zinc finger protein 346 [Source:HGNC Symbol;Acc:HGNC:16403] |
| ENSG00000114771 | HGLibA_00029 | 11 | -1.039140547 | AADAC | arylacetamide deacetylase [Source:HGNC Symbol;Acc:HGNC:17] |
| ENSG00000115363 | HGLibA_15671 | 11 | -1.039140547 | EVA1A | regulator of programmed cell death [Source:HGNC Symbol;Acc:HGNC:25816]" |
| ENSG00000115464 | HGLibA_53340 | 11 | -1.039140547 | USP34 | ubiquitin specific peptidase 34 [Source:HGNC Symbol;Acc:HGNC:20066] |
| ENSG00000115904 | HGLibA_46200 | 11 | -1.039140547 | SOS1 | SOS Ras/Rac guanine nucleotide exchange factor 1 [Source:HGNC Symbol;Acc:HGNC:11187] |
| ENSG00000116183 | HGLibA_35149 | 11 | -1.039140547 | PAPPA2 | pappalysin 2 [Source:HGNC Symbol;Acc:HGNC:14615] |
| ENSG00000116525 | HGLibA_51569 | 11 | -1.039140547 | TRIM62 | tripartite motif containing 62 [Source:HGNC Symbol;Acc:HGNC:25574] |
| ENSG00000116574 | HGLibA_41207 | 11 | -1.039140547 | RHOU | ras homolog family member U [Source:HGNC Symbol;Acc:HGNC:17794] |
| ENSG00000116903 | HGLibA_15763 | 11 | -1.039140547 | EXOC8 | exocyst complex component 8 [Source:HGNC Symbol;Acc:HGNC:24659] |
| ENSG00000116985 | HGLibA_04624 | 11 | -1.039140547 | BMP8B | bone morphogenetic protein 8b [Source:HGNC Symbol;Acc:HGNC:1075] |
| ENSG00000119314 | HGLibA_39256 | 11 | -1.039140547 | PTBP3 | polypyrimidine tract binding protein 3 [Source:HGNC Symbol;Acc:HGNC:10253] |
| ENSG00000121152 | HGLibA_31275 | 11 | -1.039140547 | NCAPH | non-SMC condensin I complex subunit H [Source:HGNC Symbol;Acc:HGNC:1112] |

| | | | | | |
|---|---|---|---|---|---|
| ENSG00000122068 | HGLibA_18258 | 11 | -1.039140547 | FYTTD1 | forty-two-three domain containing 1 [Source:HGNC Symbol;Acc:HGNC:25407] |
| ENSG00000123636 | HGLibA_04194 | 11 | -1.039140547 | BAZ2B | bromodomain adjacent to zinc finger domain 2B [Source:HGNC Symbol;Acc:HGNC:963] |
| ENSG00000124279 | HGLibA_16907 | 11 | -1.039140547 | FASTKD3 | FAST kinase domains 3 [Source:HGNC Symbol;Acc:HGNC:28758] |
| ENSG00000124602 | HGLibA_53022 | 11 | -1.039140547 | UNC5CL | unc-5 family C-terminal like [Source:HGNC Symbol;Acc:HGNC:21203] |
| ENSG00000125870 | HGLibA_45970 | 11 | -1.039140547 | SNRPB2 | small nuclear ribonucleoprotein polypeptide B2 [Source:HGNC Symbol;Acc:HGNC:11155] |
| ENSG00000126767 | HGLibA_14912 | 11 | -1.039140547 | ELK1 | ETS transcription factor [Source:HGNC Symbol;Acc:HGNC:3321]" |
| ENSG00000126822 | HGLibA_37106 | 11 | -1.039140547 | PLEKHG3 | pleckstrin homology and RhoGEF domain containing G3 [Source:HGNC Symbol;Acc:HGNC:20364] |
| ENSG00000127780 | HGLibA_33661 | 11 | -1.039140547 | OR1E2 | olfactory receptor family 1 subfamily E member 2 [Source:HGNC Symbol;Acc:HGNC:8190] |
| ENSG00000128050 | HGLibA_35024 | 11 | -1.039140547 | PAICS | phosphoribosylaminoimidazole carboxylase and phosphoribosylaminoimidazolesuccinocarboxamide synthase [Source:HGNC Symbol;Acc:HGNC:8587] |
| ENSG00000129534 | HGLibA_29308 | 11 | -1.039140547 | MIS18BP1 | MIS18 binding protein 1 [Source:HGNC Symbol;Acc:HGNC:20190] |
| ENSG00000130338 | HGLibA_52339 | 11 | -1.039140547 | TULP4 | tubby like protein 4 [Source:HGNC Symbol;Acc:HGNC:15530] |
| ENSG00000131037 | HGLibA_15378 | 11 | -1.039140547 | EPS8L1 | EPS8 like 1 [Source:HGNC Symbol;Acc:HGNC:21295] |
| ENSG00000131100 | HGLibA_03748 | 11 | -1.039140547 | ATP6V1E1 | ATPase H+ transporting V1 subunit E1 [Source:HGNC Symbol;Acc:HGNC:857] |
| ENSG00000132507 | HGLibA_14855 | 11 | -1.039140547 | EIF5A | eukaryotic translation initiation factor 5A [Source:HGNC Symbol;Acc:HGNC:3300] |
| ENSG00000132842 | HGLibA_02320 | 11 | -1.039140547 | AP3B1 | adaptor related protein complex 3 subunit beta 1 [Source:HGNC Symbol;Acc:HGNC:566] |
| ENSG00000133055 | HGLibA_30658 | 11 | -1.039140547 | MYBPH | myosin binding protein H [Source:HGNC Symbol;Acc:HGNC:7552] |
| ENSG00000133107 | HGLibA_51734 | 11 | -1.039140547 | TRPC4 | transient receptor potential cation channel subfamily C member 4 [Source:HGNC Symbol;Acc:HGNC:12336] |
| ENSG00000133460 | HGLibA_44858 | 11 | -1.039140547 | SLC2A11 | solute carrier family 2 member 11 [Source:HGNC Symbol;Acc:HGNC:14239] |
| ENSG00000135316 | HGLibA_47966 | 11 | -1.039140547 | SYNCRIP | synaptotagmin binding cytoplasmic RNA interacting protein [Source:HGNC Symbol;Acc:HGNC:16918] |
| ENSG00000136021 | HGLibA_43087 | 11 | -1.039140547 | SCYL2 | SCY1 like pseudokinase 2 [Source:HGNC Symbol;Acc:HGNC:19286] |
| ENSG00000136457 | HGLibA_09342 | 11 | -1.039140547 | CHAD | chondroadherin [Source:HGNC Symbol;Acc:HGNC:1909] |
| ENSG00000137100 | HGLibA_12549 | 11 | -1.039140547 | DCTN3 | dynactin subunit 3 [Source:HGNC Symbol;Acc:HGNC:2713] |
| ENSG00000137700 | HGLibA_45052 | 11 | -1.039140547 | SLC37A4 | solute carrier family 37 member 4 [Source:HGNC Symbol;Acc:HGNC:4061] |
| ENSG00000137726 | HGLibA_18240 | 11 | -1.039140547 | FXYD6 | FXYD domain containing ion transport regulator 6 [Source:HGNC Symbol;Acc:HGNC:4030] |
| ENSG00000138363 | HGLibA_03513 | 11 | -1.039140547 | ATIC | 5-aminoimidazole-4-carboxamide ribonucleotide formyltransferase/IMP cyclohydrolase [Source:HGNC Symbol;Acc:HGNC:794] |
| ENSG00000138459 | HGLibA_44958 | 11 | -1.039140547 | SLC35A5 | solute carrier family 35 member A5 [Source:HGNC Symbol;Acc:HGNC:20792] |
| ENSG00000138604 | HGLibA_19233 | 11 | -1.039140547 | GLCE | glucuronic acid epimerase [Source:HGNC Symbol;Acc:HGNC:17855] |
| ENSG00000138642 | HGLibA_21169 | 11 | -1.039140547 | HERC6 | HECT and RLD domain containing E3 ubiquitin protein ligase family member 6 [Source:HGNC Symbol;Acc:HGNC:26072] |
| ENSG00000139835 | HGLibA_20383 | 11 | -1.039140547 | GRTP1 | growth hormone regulated TBC protein 1 [Source:HGNC Symbol;Acc:HGNC:20310] |
| ENSG00000140199 | HGLibA_44373 | 11 | -1.039140547 | SLC12A6 | solute carrier family 12 member 6 [Source:HGNC Symbol;Acc:HGNC:10914] |
| ENSG00000142319 | HGLibA_45326 | 11 | -1.039140547 | SLC6A3 | solute carrier family 6 member 3 [Source:HGNC Symbol;Acc:HGNC:11049] |
| ENSG00000142408 | HGLibA_06950 | 11 | -1.039140547 | CACNG8 | calcium voltage-gated channel auxiliary subunit gamma 8 [Source:HGNC Symbol;Acc:HGNC:13628] |
| ENSG00000142698 | HGLibA_05906 | 11 | -1.039140547 | C1orf94 | chromosome 1 open reading frame 94 [Source:HGNC Symbol;Acc:HGNC:28250] |
| ENSG00000143179 | HGLibA_52813 | 11 | -1.039140547 | UCK2 | uridine-cytidine kinase 2 [Source:HGNC Symbol;Acc:HGNC:12562] |
| ENSG00000143952 | HGLibA_53813 | 11 | -1.039140547 | VPS54 | GARP complex subunit [Source:HGNC Symbol;Acc:HGNC:18652]" |
| ENSG00000144278 | HGLibA_18538 | 11 | -1.039140547 | GALNT13 | polypeptide N-acetylgalactosaminyltransferase 13 [Source:HGNC Symbol;Acc:HGNC:23242] |
| ENSG00000144355 | HGLibA_13372 | 11 | -1.039140547 | DLX1 | distal-less homeobox 1 [Source:HGNC Symbol;Acc:HGNC:2914] |
| ENSG00000144452 | HGLibA_00100 | 11 | -1.039140547 | ABCA12 | ATP binding cassette subfamily A member 12 [Source:HGNC Symbol;Acc:HGNC:14637] |
| ENSG00000144711 | HGLibA_23540 | 11 | -1.039140547 | IQSEC1 | IQ motif and Sec7 domain 1 [Source:HGNC Symbol;Acc:HGNC:29112] |
| ENSG00000144893 | HGLibA_28746 | 11 | -1.039140547 | MED12L | mediator complex subunit 12 like [Source:HGNC Symbol;Acc:HGNC:16050] |
| ENSG00000145526 | HGLibA_08654 | 11 | -1.039140547 | CDH18 | cadherin 18 [Source:HGNC Symbol;Acc:HGNC:1757] |
| ENSG00000148153 | HGLibA_23314 | 11 | -1.039140547 | INIP | INTS3 and NABP interacting protein [Source:HGNC Symbol;Acc:HGNC:24994] |
| ENSG00000149115 | HGLibA_50854 | 11 | -1.039140547 | TNKS1BP1 | tankyrase 1 binding protein 1 [Source:HGNC Symbol;Acc:HGNC:19081] |
| ENSG00000150269 | HGLibA_34343 | 11 | -1.039140547 | OR5M9 | olfactory receptor family 5 subfamily M member 9 [Source:HGNC Symbol;Acc:HGNC:15294] |
| ENSG00000151718 | HGLibA_54443 | 11 | -1.039140547 | WWC2 | WW and C2 domain containing 2 [Source:HGNC Symbol;Acc:HGNC:24148] |
| ENSG00000154920 | HGLibA_15035 | 11 | -1.039140547 | EME1 | essential meiotic structure-specific endonuclease 1 [Source:HGNC Symbol;Acc:HGNC:24965] |
| ENSG00000156219 | HGLibA_03161 | 11 | -1.039140547 | ART3 | ADP-ribosyltransferase 3 [Source:HGNC Symbol;Acc:HGNC:725] |
| ENSG00000156381 | HGLibA_02148 | 11 | -1.039140547 | ANKRD9 | ankyrin repeat domain 9 [Source:HGNC Symbol;Acc:HGNC:20096] |
| ENSG00000156384 | HGLibA_43734 | 11 | -1.039140547 | SFR1 | SWI5 dependent homologous recombination repair protein 1 [Source:HGNC Symbol;Acc:HGNC:29574] |
| ENSG00000156804 | HGLibA_17072 | 11 | -1.039140547 | FBXO32 | F-box protein 32 [Source:HGNC Symbol;Acc:HGNC:16731] |
| ENSG00000157014 | HGLibA_48442 | 11 | -1.039140547 | TATDN2 | TatD DNase domain containing 2 [Source:HGNC Symbol;Acc:HGNC:28988] |
| ENSG00000157193 | HGLibA_27233 | 11 | -1.039140547 | LRP8 | LDL receptor related protein 8 [Source:HGNC Symbol;Acc:HGNC:6700] |
| ENSG00000157335 | HGLibA_09948 | 11 | -1.039140547 | CLEC18C | C-type lectin domain family 18 member C [Source:HGNC Symbol;Acc:HGNC:28538] |
| ENSG00000158104 | HGLibA_22025 | 11 | -1.039140547 | HPD | 4-hydroxyphenylpyruvate dioxygenase [Source:HGNC Symbol;Acc:HGNC:5147] |
| ENSG00000160111 | HGLibA_10902 | 11 | -1.039140547 | CPAMD8 | alpha-2-macroglobulin domain containing 8 [Source:HGNC Symbol;Acc:HGNC:23228]" |
| ENSG00000160882 | HGLibA_12055 | 11 | -1.039140547 | CYP11B1 | cytochrome P450 family 11 subfamily B member 1 [Source:HGNC Symbol;Acc:HGNC:2591] |
| ENSG00000161888 | HGLibA_46553 | 11 | -1.039140547 | SPC24 | NDC80 kinetochore complex component [Source:HGNC Symbol;Acc:HGNC:26913]" |
| ENSG00000162543 | HGLibA_52778 | 11 | -1.039140547 | UBXN10 | UBX domain protein 10 [Source:HGNC Symbol;Acc:HGNC:26354] |
| ENSG00000162771 | HGLibA_16687 | 11 | -1.039140547 | FAM71A | family with sequence similarity 71 member A [Source:HGNC Symbol;Acc:HGNC:26541] |
| ENSG00000163219 | HGLibA_02739 | 11 | -1.039140547 | ARHGAP25 | Rho GTPase activating protein 25 [Source:HGNC Symbol;Acc:HGNC:28951] |
| ENSG00000163344 | HGLibA_37340 | 11 | -1.039140547 | PMVK | phosphomevalonate kinase [Source:HGNC Symbol;Acc:HGNC:9141] |
| ENSG00000163684 | HGLibA_42023 | 11 | -1.039140547 | RPP14 | ribonuclease P/MRP subunit p14 [Source:HGNC Symbol;Acc:HGNC:30327] |
| ENSG00000163738 | HGLibA_30386 | 11 | -1.039140547 | MTHFD2L | methylenetetrahydrofolate dehydrogenase (NADP+ dependent) 2 like [Source:HGNC Symbol;Acc:HGNC:31865] |
| ENSG00000163785 | HGLibA_42517 | 11 | -1.039140547 | RYK | receptor-like tyrosine kinase [Source:HGNC Symbol;Acc:HGNC:10481] |
| ENSG00000163812 | HGLibA_55113 | 11 | -1.039140547 | ZDHHC3 | zinc finger DHHC-type containing 3 [Source:HGNC Symbol;Acc:HGNC:18470] |
| ENSG00000163935 | HGLibA_43721 | 11 | -1.039140547 | SFMBT1 | Scm like with four mbt domains 1 [Source:HGNC Symbol;Acc:HGNC:20255] |
| ENSG00000164327 | HGLibA_41242 | 11 | -1.039140547 | RICTOR | RPTOR independent companion of MTOR complex 2 [Source:HGNC Symbol;Acc:HGNC:28611] |
| ENSG00000164344 | HGLibA_25275 | 11 | -1.039140547 | KLKB1 | kallikrein B1 [Source:HGNC Symbol;Acc:HGNC:6371] |
| ENSG00000164535 | HGLibA_12297 | 11 | -1.039140547 | DAGLB | diacylglycerol lipase beta [Source:HGNC Symbol;Acc:HGNC:28923] |
| ENSG00000164778 | HGLibA_15100 | 11 | -1.039140547 | EN2 | engrailed homeobox 2 [Source:HGNC Symbol;Acc:HGNC:3343] |
| ENSG00000166592 | HGLibA_42222 | 11 | -1.039140547 | RRAD | Ras related glycolysis inhibitor and calcium channel regulator [Source:HGNC Symbol;Acc:HGNC:10446]" |
| ENSG00000166682 | HGLibA_50646 | 11 | -1.039140547 | TMPRSS5 | transmembrane serine protease 5 [Source:HGNC Symbol;Acc:HGNC:14908] |
| ENSG00000166833 | HGLibA_31197 | 11 | -1.039140547 | NAV2 | neuron navigator 2 [Source:HGNC Symbol;Acc:HGNC:15997] |
| ENSG00000166839 | HGLibA_01947 | 11 | -1.039140547 | ANKDD1A | ankyrin repeat and death domain containing 1A [Source:HGNC Symbol;Acc:HGNC:28002] |
| ENSG00000166924 | HGLibA_33179 | 11 | -1.039140547 | NYAP1 | neuronal tyrosine phosphorylated phosphoinositide-3-kinase adaptor 1 [Source:HGNC Symbol;Acc:HGNC:22009] |
| ENSG00000167173 | HGLibA_05394 | 11 | -1.039140547 | C15orf39 | chromosome 15 open reading frame 39 [Source:HGNC Symbol;Acc:HGNC:24497] |
| ENSG00000167207 | HGLibA_32247 | 11 | -1.039140547 | NOD2 | nucleotide binding oligomerization domain containing 2 [Source:HGNC Symbol;Acc:HGNC:5331] |
| ENSG00000167325 | HGLibA_42252 | 11 | -1.039140547 | RRM1 | ribonucleotide reductase catalytic subunit M1 [Source:HGNC Symbol;Acc:HGNC:10451] |
| ENSG00000167359 | HGLibA_34114 | 11 | -1.039140547 | OR51I1 | olfactory receptor family 51 subfamily I member 1 [Source:HGNC Symbol;Acc:HGNC:15200] |
| ENSG00000168092 | HGLibA_34991 | 11 | -1.039140547 | PAFAH1B2 | platelet activating factor acetylhydrolase 1b catalytic subunit 2 [Source:HGNC Symbol;Acc:HGNC:8575] |
| ENSG00000168393 | HGLibA_14093 | 11 | -1.039140547 | DTYMK | deoxythymidylate kinase [Source:HGNC Symbol;Acc:HGNC:3061] |
| ENSG00000168539 | HGLibA_09547 | 11 | -1.039140547 | CHRM1 | cholinergic receptor muscarinic 1 [Source:HGNC Symbol;Acc:HGNC:1950] |
| ENSG00000168734 | HGLibA_36824 | 11 | -1.039140547 | PKIG | cAMP-dependent protein kinase inhibitor gamma [Source:HGNC Symbol;Acc:HGNC:9019] |
| ENSG00000169223 | HGLibA_26662 | 11 | -1.039140547 | LMAN2 | mannose binding 2 [Source:HGNC Symbol;Acc:HGNC:16986]" |
| ENSG00000169641 | HGLibA_27637 | 11 | -1.039140547 | LUZP1 | leucine zipper protein 1 [Source:HGNC Symbol;Acc:HGNC:14985] |
| ENSG00000169981 | HGLibA_55855 | 11 | -1.039140547 | ZNF35 | zinc finger protein 35 [Source:HGNC Symbol;Acc:HGNC:13099] |
| ENSG00000170448 | HGLibA_31852 | 11 | -1.039140547 | NFXL1 | X-box binding like 1 [Source:HGNC Symbol;Acc:HGNC:18726]" |
| ENSG00000171014 | HGLibA_33978 | 11 | -1.039140547 | OR4D5 | olfactory receptor family 4 subfamily D member 5 [Source:HGNC Symbol;Acc:HGNC:14852] |
| ENSG00000171100 | HGLibA_30406 | 11 | -1.039140547 | MTM1 | myotubularin 1 [Source:HGNC Symbol;Acc:HGNC:7448] |
| ENSG00000171132 | HGLibA_38546 | 11 | -1.039140547 | PRKCE | protein kinase C epsilon [Source:HGNC Symbol;Acc:HGNC:9401] |
| ENSG00000171135 | HGLibA_23911 | 11 | -1.039140547 | JAGN1 | jagunal homolog 1 [Source:HGNC Symbol;Acc:HGNC:26926] |
| ENSG00000171466 | HGLibA_56247 | 11 | -1.039140547 | ZNF562 | zinc finger protein 562 [Source:HGNC Symbol;Acc:HGNC:25950] |
| ENSG00000171497 | HGLibA_37925 | 11 | -1.039140547 | PPID | peptidylprolyl isomerase D [Source:HGNC Symbol;Acc:HGNC:9257] |
| ENSG00000171819 | HGLibA_01927 | 11 | -1.039140547 | ANGPTL7 | angiopoietin like 7 [Source:HGNC Symbol;Acc:HGNC:24078] |
| ENSG00000172164 | HGLibA_46001 | 11 | -1.039140547 | SNTB1 | syntrophin beta 1 [Source:HGNC Symbol;Acc:HGNC:11168] |
| ENSG00000172199 | HGLibA_34555 | 11 | -1.039140547 | OR8U1 | olfactory receptor family 8 subfamily U member 1 [Source:HGNC Symbol;Acc:HGNC:19611] |
| ENSG00000172817 | HGLibA_12215 | 11 | -1.039140547 | CYP7B1 | cytochrome P450 family 7 subfamily B member 1 [Source:HGNC Symbol;Acc:HGNC:2652] |
| ENSG00000173638 | HGLibA_44498 | 11 | -1.039140547 | SLC19A1 | solute carrier family 19 member 1 [Source:HGNC Symbol;Acc:HGNC:10937] |
| ENSG00000174567 | HGLibA_19662 | 11 | -1.039140547 | GOLT1A | golgi transport 1A [Source:HGNC Symbol;Acc:HGNC:24766] |
| ENSG00000174667 | HGLibA_34474 | 11 | -1.039140547 | OR7D4 | olfactory receptor family 7 subfamily D member 4 [Source:HGNC Symbol;Acc:HGNC:8380] |
| ENSG00000174827 | HGLibA_35990 | 11 | -1.039140547 | PDZK1 | PDZ domain containing 1 [Source:HGNC Symbol;Acc:HGNC:8821] |
| ENSG00000174898 | HGLibA_07370 | 11 | -1.039140547 | CATSPERD | cation channel sperm associated auxiliary subunit delta [Source:HGNC Symbol;Acc:HGNC:28598] |
| ENSG00000175224 | HGLibA_03467 | 11 | -1.039140547 | ATG13 | autophagy related 13 [Source:HGNC Symbol;Acc:HGNC:29091] |
| ENSG00000175634 | HGLibA_42180 | 11 | -1.039140547 | RPS6KB2 | ribosomal protein S6 kinase B2 [Source:HGNC Symbol;Acc:HGNC:10437] |
| ENSG00000176165 | HGLibA_17871 | 11 | -1.039140547 | FOXG1 | forkhead box G1 [Source:HGNC Symbol;Acc:HGNC:3811] |
| ENSG00000177181 | HGLibA_41272 | 11 | -1.039140547 | RIMKLA | ribosomal modification protein rimK like family member A [Source:HGNC Symbol;Acc:HGNC:28725] |
| ENSG00000178776 | HGLibA_06389 | 11 | -1.039140547 | C5orf46 | chromosome 5 open reading frame 46 [Source:HGNC Symbol;Acc:HGNC:33768] |
| ENSG00000179364 | HGLibA_34958 | 11 | -1.039140547 | PACS2 | phosphofurin acidic cluster sorting protein 2 [Source:HGNC Symbol;Acc:HGNC:23794] |
| ENSG00000180104 | HGLibA_15736 | 11 | -1.039140547 | EXOC3 | exocyst complex component 3 [Source:HGNC Symbol;Acc:HGNC:30378] |
| ENSG00000180113 | HGLibA_48911 | 11 | -1.039140547 | TDRD6 | tudor domain containing 6 [Source:HGNC Symbol;Acc:HGNC:21339] |
| ENSG00000180660 | HGLibA_27821 | 11 | -1.039140547 | MAB21L1 | mab-21 like 1 [Source:HGNC Symbol;Acc:HGNC:6757] |
| ENSG00000181649 | HGLibA_36421 | 11 | -1.039140547 | PHLDA2 | pleckstrin homology like domain family A member 2 [Source:HGNC Symbol;Acc:HGNC:12385] |
| ENSG00000182108 | HGLibA_13015 | 11 | -1.039140547 | DEXI | Dexi homolog [Source:HGNC Symbol;Acc:HGNC:13267] |
| ENSG00000182173 | HGLibA_51838 | 11 | -1.039140547 | TSEN54 | tRNA splicing endonuclease subunit 54 [Source:HGNC Symbol;Acc:HGNC:27561] |
| ENSG00000182870 | HGLibA_18575 | 11 | -1.039140547 | GALNT9 | polypeptide N-acetylgalactosaminyltransferase 9 [Source:HGNC Symbol;Acc:HGNC:4131] |
| ENSG00000183091 | HGLibA_31577 | 11 | -1.039140547 | NEB | nebulin [Source:HGNC Symbol;Acc:HGNC:7720] |
| ENSG00000184216 | HGLibA_23554 | 11 | -1.039140547 | IRAK1 | interleukin 1 receptor associated kinase 1 [Source:HGNC Symbol;Acc:HGNC:6112] |
| ENSG00000184220 | HGLibA_10205 | 11 | -1.039140547 | CMSS1 | cms1 ribosomal small subunit homolog [Source:HGNC Symbol;Acc:HGNC:28666] |
| ENSG00000184388 | HGLibA_34929 | 11 | -1.039140547 | PABPC1L2B | poly(A) binding protein cytoplasmic 1 like 2B [Source:HGNC Symbol;Acc:HGNC:31852] |
| ENSG00000184489 | HGLibA_39408 | 11 | -1.039140547 | PTP4A3 | member 3 [Source:HGNC Symbol;Acc:HGNC:9636]" |
| ENSG00000184497 | HGLibA_50332 | 11 | -1.039140547 | TMEM255B | transmembrane protein 255B [Source:HGNC Symbol;Acc:HGNC:28297] |
| ENSG00000184557 | HGLibA_46128 | 11 | -1.039140547 | SOCS3 | suppressor of cytokine signaling 3 [Source:HGNC Symbol;Acc:HGNC:19391] |
| ENSG00000185610 | HGLibA_12408 | 11 | -1.039140547 | DBX2 | developing brain homeobox 2 [Source:HGNC Symbol;Acc:HGNC:33186] |
| ENSG00000185728 | HGLibA_54736 | 11 | -1.039140547 | YTHDF3 | YTH N6-methyladenosine RNA binding protein 3 [Source:HGNC Symbol;Acc:HGNC:26465] |
| ENSG00000187658 | HGLibA_06404 | 11 | -1.039140547 | C5orf52 | chromosome 5 open reading frame 52 [Source:HGNC Symbol;Acc:HGNC:35121] |
| ENSG00000189221 | HGLibA_28107 | 11 | -1.039140547 | MAOA | monoamine oxidase A [Source:HGNC Symbol;Acc:HGNC:6833] |
| ENSG00000197045 | HGLibA_19402 | 11 | -1.039140547 | GMFB | glia maturation factor beta [Source:HGNC Symbol;Acc:HGNC:4373] |
| ENSG00000197054 | HGLibA_56687 | 11 | -1.039140547 | ZNF763 | zinc finger protein 763 [Source:HGNC Symbol;Acc:HGNC:27614] |
| ENSG00000197586 | HGLibA_15211 | 11 | -1.039140547 | ENTPD6 | ectonucleoside triphosphate diphosphohydrolase 6 (putative) [Source:HGNC Symbol;Acc:HGNC:3368] |
| ENSG00000197935 | HGLibA_55779 | 11 | -1.039140547 | ZNF311 | zinc finger protein 311 [Source:HGNC Symbol;Acc:HGNC:13847] |
| ENSG00000198643 | HGLibA_16583 | 11 | -1.039140547 | FAM3D | family with sequence similarity 3 member D [Source:HGNC Symbol;Acc:HGNC:18665] |
| ENSG00000203722 | HGLibA_40122 | 11 | -1.039140547 | RAET1G | retinoic acid early transcript 1G [Source:HGNC Symbol;Acc:HGNC:16795] |
| ENSG00000203760 | HGLibA_09091 | 11 | -1.039140547 | CENPW | centromere protein W [Source:HGNC Symbol;Acc:HGNC:21488] |
| ENSG00000203780 | HGLibA_16864 | 11 | -1.039140547 | FANK1 | fibronectin type III and ankyrin repeat domains 1 [Source:HGNC Symbol;Acc:HGNC:23527] |
| ENSG00000204356 | HGLibA_31679 | 11 | -1.039140547 | NELFE | negative elongation factor complex member E [Source:HGNC Symbol;Acc:HGNC:13974] |

| | | | | | |
|---|---|---|---|---|---|
| ENSG00000204574 | HGLibA_00205 | 11 | -1.039140547 | ABCF1 | ATP binding cassette subfamily F member 1 [Source:HGNC Symbol;Acc:HGNC:70] |
| ENSG00000205758 | HGLibA_11296 | 11 | -1.039140547 | CRYZL1 | crystallin zeta like 1 [Source:HGNC Symbol;Acc:HGNC:2420] |
| ENSG00000213512 | HGLibA_18747 | 11 | -1.039140547 | GBP7 | guanylate binding protein 7 [Source:HGNC Symbol;Acc:HGNC:29606] |
| ENSG00000213782 | HGLibA_12723 | 11 | -1.039140547 | DDX47 | DEAD-box helicase 47 [Source:HGNC Symbol;Acc:HGNC:18682] |
| ENSG00000214050 | HGLibA_17033 | 11 | -1.039140547 | FBXO16 | F-box protein 16 [Source:HGNC Symbol;Acc:HGNC:13618] |
| ENSG00000228727 | HGLibA_42730 | 11 | -1.039140547 | SAPCD1 | suppressor APC domain containing 1 [Source:HGNC Symbol;Acc:HGNC:13938] |
| ENSG00000232237 | HGLibA_03278 | 11 | -1.039140547 | ASCL5 | achaete-scute family bHLH transcription factor 5 [Source:HGNC Symbol;Acc:HGNC:33169] |
| ENSG00000232263 | HGLibA_25727 | 11 | -1.039140547 | KRTAP25-1 | keratin associated protein 25-1 [Source:HGNC Symbol;Acc:HGNC:34003] |
| ENSG00000239672 | HGLibA_32152 | 11 | -1.039140547 | NME1 | NME/NM23 nucleoside diphosphate kinase 1 [Source:HGNC Symbol;Acc:HGNC:7849] |
| ENSG00000250479 | HGLibA_09362 | 11 | -1.039140547 | CHCHD10 | coiled-coil-helix-coiled-coil-helix domain containing 10 [Source:HGNC Symbol;Acc:HGNC:15559] |
| ENSG00000256977 | HGLibA_26551 | 11 | -1.039140547 | LIMS3 | LIM zinc finger domain containing 3 [Source:HGNC Symbol;Acc:HGNC:30047] |
| ENSG00000272391 | HGLibA_37645 | 11 | -1.039140547 | POM121C | POM121 transmembrane nucleoporin C [Source:HGNC Symbol;Acc:HGNC:34005] |
| ENSG00000277893 | HGLibA_46944 | 11 | -1.039140547 | SRD5A2 | steroid 5 alpha-reductase 2 [Source:HGNC Symbol;Acc:HGNC:11285] |
| ENSG00000280094 | HGLibA_33646 | 11 | -1.039140547 | OR1B1 | olfactory receptor family 1 subfamily B member 1 (gene/pseudogene) [Source:HGNC Symbol;Acc:HGNC:8181] |
| ENSG00000288611 | HGLibA_32411 | 11 | -1.039140547 | NPBWR1 | - |
| ENSG00000288709 | HGLibA_15888 | 11 | -1.039140547 | F8A2 | - |
| HIST1H2AK | HGLibA_21403 | 11 | -1.039140547 | HIST1H2AK | - |
| hsa-let-7a-1 | HGLibA_57097 | 11 | -1.039140547 | hsa-let-7a-1 | - |
| hsa-mir-1262 | HGLibA_57471 | 11 | -1.039140547 | hsa-mir-1262 | - |
| hsa-mir-1306 | HGLibA_57738 | 11 | -1.039140547 | hsa-mir-1306 | - |
| hsa-mir-2114 | HGLibA_58227 | 11 | -1.039140547 | hsa-mir-2114 | - |
| hsa-mir-3202-1 | HGLibA_58915 | 11 | -1.039140547 | hsa-mir-3202-1 | - |
| hsa-mir-3202-2 | HGLibA_58920 | 11 | -1.039140547 | hsa-mir-3202-2 | - |
| hsa-mir-329-2 | HGLibA_58982 | 11 | -1.039140547 | hsa-mir-329-2 | - |
| hsa-mir-3661 | HGLibA_59198 | 11 | -1.039140547 | hsa-mir-3661 | - |
| hsa-mir-3670-2 | HGLibA_59239 | 11 | -1.039140547 | hsa-mir-3670-2 | - |
| hsa-mir-4285 | HGLibA_59848 | 11 | -1.039140547 | hsa-mir-4285 | - |
| hsa-mir-4458 | HGLibA_60240 | 11 | -1.039140547 | hsa-mir-4458 | - |
| hsa-mir-4633 | HGLibA_60638 | 11 | -1.039140547 | hsa-mir-4633 | - |
| hsa-mir-4730 | HGLibA_61019 | 11 | -1.039140547 | hsa-mir-4730 | - |
| hsa-mir-4751 | HGLibA_61102 | 11 | -1.039140547 | hsa-mir-4751 | - |
| hsa-mir-4767 | HGLibA_61165 | 11 | -1.039140547 | hsa-mir-4767 | - |
| hsa-mir-503 | HGLibA_61467 | 11 | -1.039140547 | hsa-mir-503 | - |
| hsa-mir-508 | HGLibA_61490 | 11 | -1.039140547 | hsa-mir-508 | - |
| hsa-mir-519a-2 | HGLibA_61714 | 11 | -1.039140547 | hsa-mir-519a-2 | - |
| hsa-mir-548f-4 | HGLibA_61985 | 11 | -1.039140547 | hsa-mir-548f-4 | - |
| hsa-mir-623 | HGLibA_62731 | 11 | -1.039140547 | hsa-mir-623 | - |
| hsa-mir-6514 | HGLibA_62931 | 11 | -1.039140547 | hsa-mir-6514 | - |
| hsa-mir-6759 | HGLibA_63195 | 11 | -1.039140547 | hsa-mir-6759 | - |
| hsa-mir-6859-1 | HGLibA_63608 | 11 | -1.039140547 | hsa-mir-6859-1 | - |
| hsa-mir-7641-2 | HGLibA_63919 | 11 | -1.039140547 | hsa-mir-7641-2 | - |
| hsa-mir-8064 | HGLibA_64096 | 11 | -1.039140547 | hsa-mir-8064 | - |
| hsa-mir-940 | HGLibA_64334 | 11 | -1.039140547 | hsa-mir-940 | - |
| KIAA0391 | HGLibA_24632 | 11 | -1.039140547 | KIAA0391 | - |
| LIMS3L | HGLibA_26555 | 11 | -1.039140547 | LIMS3L | - |
| LOC100287177 | HGLibA_26819 | 11 | -1.039140547 | LOC100287177 | - |
| LOC100652824 | HGLibA_26874 | 11 | -1.039140547 | LOC100652824 | - |
| OR8U8 | HGLibA_34558 | 11 | -1.039140547 | OR8U8 | - |
| RARRES3 | HGLibA_40288 | 11 | -1.039140547 | RARRES3 | - |
| 3-Sep | HGLibA_43440 | 11 | -1.039140547 | 3-Sep | - |
| WBSCR17 | HGLibA_53993 | 11 | -1.039140547 | WBSCR17 | - |
| WDR16 | HGLibA_54033 | 11 | -1.039140547 | WDR16 | - |
| ATP5J | HGLibA_03669 | 10 | -1.049091584 | ATP5J | - |
| C12orf10 | HGLibA_05223 | 10 | -1.049091584 | C12orf10 | - |
| C20orf195 | HGLibA_06001 | 10 | -1.049091584 | C20orf195 | - |
| C4orf6 | HGLibA_06348 | 10 | -1.049091584 | C4orf6 | - |
| C7orf55-LUC7L2 | HGLibA_06556 | 10 | -1.049091584 | C7orf55-LUC7L2 | - |
| ENSG00000001036 | HGLibA_18157 | 10 | -1.049091584 | FUCA2 | alpha-L-fucosidase 2 [Source:HGNC Symbol;Acc:HGNC:4008] |
| ENSG00000006530 | HGLibA_01194 | 10 | -1.049091584 | AGK | acylglycerol kinase [Source:HGNC Symbol;Acc:HGNC:21869] |
| ENSG00000007255 | HGLibA_51329 | 10 | -1.049091584 | TRAPPC6A | trafficking protein particle complex 6A [Source:HGNC Symbol;Acc:HGNC:23069] |
| ENSG00000013588 | HGLibA_20124 | 10 | -1.049091584 | GPRC5A | G protein-coupled receptor class C group 5 member A [Source:HGNC Symbol;Acc:HGNC:9836] |
| ENSG00000025293 | HGLibA_36363 | 10 | -1.049091584 | PHF20 | PHD finger protein 20 [Source:HGNC Symbol;Acc:HGNC:16098] |
| ENSG00000026508 | HGLibA_08391 | 10 | -1.049091584 | CD44 | CD44 molecule (Indian blood group) [Source:HGNC Symbol;Acc:HGNC:1681] |
| ENSG00000032219 | HGLibA_02900 | 10 | -1.049091584 | ARID4A | AT-rich interaction domain 4A [Source:HGNC Symbol;Acc:HGNC:9885] |
| ENSG00000047644 | HGLibA_54444 | 10 | -1.049091584 | WWC3 | WWC family member 3 [Source:HGNC Symbol;Acc:HGNC:29237] |
| ENSG00000049192 | HGLibA_00835 | 10 | -1.049091584 | ADAMTS6 | ADAM metallopeptidase with thrombospondin type 1 motif 6 [Source:HGNC Symbol;Acc:HGNC:222] |
| ENSG00000065609 | HGLibA_45887 | 10 | -1.049091584 | SNAP91 | synaptosome associated protein 91 [Source:HGNC Symbol;Acc:HGNC:14986] |
| ENSG00000066056 | HGLibA_49454 | 10 | -1.049091584 | TIE1 | tyrosine kinase with immunoglobulin like and EGF like domains 1 [Source:HGNC Symbol;Acc:HGNC:11809] |
| ENSG00000069345 | HGLibA_13513 | 10 | -1.049091584 | DNAJA2 | DnaJ heat shock protein family (Hsp40) member A2 [Source:HGNC Symbol;Acc:HGNC:14884] |
| ENSG00000070423 | HGLibA_41496 | 10 | -1.049091584 | RNF126 | ring finger protein 126 [Source:HGNC Symbol;Acc:HGNC:21151] |
| ENSG00000071859 | HGLibA_16629 | 10 | -1.049091584 | FAM50A | family with sequence similarity 50 member A [Source:HGNC Symbol;Acc:HGNC:18786] |
| ENSG00000072364 | HGLibA_01114 | 10 | -1.049091584 | AFF4 | AF4/FMR2 family member 4 [Source:HGNC Symbol;Acc:HGNC:17869] |
| ENSG00000073536 | HGLibA_32063 | 10 | -1.049091584 | NLE1 | notchless homolog 1 [Source:HGNC Symbol;Acc:HGNC:19889] |
| ENSG00000075407 | HGLibA_55884 | 10 | -1.049091584 | ZNF37A | zinc finger protein 37A [Source:HGNC Symbol;Acc:HGNC:13102] |
| ENSG00000077232 | HGLibA_13563 | 10 | -1.049091584 | DNAJC10 | DnaJ heat shock protein family (Hsp40) member C10 [Source:HGNC Symbol;Acc:HGNC:24637] |
| ENSG00000079156 | HGLibA_34660 | 10 | -1.049091584 | OSBPL6 | oxysterol binding protein like 6 [Source:HGNC Symbol;Acc:HGNC:16388] |
| ENSG00000082212 | HGLibA_28713 | 10 | -1.049091584 | ME2 | malic enzyme 2 [Source:HGNC Symbol;Acc:HGNC:6984] |
| ENSG00000085415 | HGLibA_43273 | 10 | -1.049091584 | SEH1L | SEH1 like nucleoporin [Source:HGNC Symbol;Acc:HGNC:30379] |
| ENSG00000088812 | HGLibA_03819 | 10 | -1.049091584 | ATRN | attractin [Source:HGNC Symbol;Acc:HGNC:885] |
| ENSG00000089053 | HGLibA_01885 | 10 | -1.049091584 | ANAPC5 | anaphase promoting complex subunit 5 [Source:HGNC Symbol;Acc:HGNC:15713] |
| ENSG00000099797 | HGLibA_48946 | 10 | -1.049091584 | TECR | 3-enoyl-CoA reductase [Source:HGNC Symbol;Acc:HGNC:4551]" |
| ENSG00000099866 | HGLibA_27853 | 10 | -1.049091584 | MADCAM1 | mucosal vascular addressin cell adhesion molecule 1 [Source:HGNC Symbol;Acc:HGNC:6765] |
| ENSG00000099984 | HGLibA_20499 | 10 | -1.049091584 | GSTT2 | glutathione S-transferase theta 2 (gene/pseudogene) [Source:HGNC Symbol;Acc:HGNC:4642] |
| ENSG00000100116 | HGLibA_18761 | 10 | -1.049091584 | GCAT | glycine C-acetyltransferase [Source:HGNC Symbol;Acc:HGNC:4188] |
| ENSG00000100348 | HGLibA_52402 | 10 | -1.049091584 | TXN2 | thioredoxin 2 [Source:HGNC Symbol;Acc:HGNC:17772] |
| ENSG00000100365 | HGLibA_31302 | 10 | -1.049091584 | NCF4 | neutrophil cytosolic factor 4 [Source:HGNC Symbol;Acc:HGNC:7662] |
| ENSG00000100802 | HGLibA_05376 | 10 | -1.049091584 | C14orf93 | chromosome 14 open reading frame 93 [Source:HGNC Symbol;Acc:HGNC:20162] |
| ENSG00000101746 | HGLibA_32269 | 10 | -1.049091584 | NOL4 | nucleolar protein 4 [Source:HGNC Symbol;Acc:HGNC:7870] |
| ENSG00000102218 | HGLibA_41794 | 10 | -1.049091584 | RP2 | ARL3 GTPase activating protein [Source:HGNC Symbol;Acc:HGNC:10274]" |
| ENSG00000103005 | HGLibA_53161 | 10 | -1.049091584 | USB1 | U6 snRNA biogenesis phosphodiesterase 1 [Source:HGNC Symbol;Acc:HGNC:25792] |
| ENSG00000103175 | HGLibA_54245 | 10 | -1.049091584 | WFDC1 | WAP four-disulfide core domain 1 [Source:HGNC Symbol;Acc:HGNC:15466] |
| ENSG00000103351 | HGLibA_10158 | 10 | -1.049091584 | CLUAP1 | clusterin associated protein 1 [Source:HGNC Symbol;Acc:HGNC:19009] |
| ENSG00000104064 | HGLibA_18351 | 10 | -1.049091584 | GABPB1 | GA binding protein transcription factor subunit beta 1 [Source:HGNC Symbol;Acc:HGNC:4074] |
| ENSG00000104980 | HGLibA_49516 | 10 | -1.049091584 | TIMM44 | translocase of inner mitochondrial membrane 44 [Source:HGNC Symbol;Acc:HGNC:17316] |
| ENSG00000105251 | HGLibA_44019 | 10 | -1.049091584 | SHD | Src homology 2 domain containing transforming protein D [Source:HGNC Symbol;Acc:HGNC:30633] |
| ENSG00000105388 | HGLibA_08943 | 10 | -1.049091584 | CEACAM5 | carcinoembryonic antigen related cell adhesion molecule 5 [Source:HGNC Symbol;Acc:HGNC:1817] |
| ENSG00000106460 | HGLibA_49855 | 10 | -1.049091584 | TMEM106B | transmembrane protein 106B [Source:HGNC Symbol;Acc:HGNC:22407] |
| ENSG00000106772 | HGLibA_38988 | 10 | -1.049091584 | PRUNE2 | prune homolog 2 [Source:HGNC Symbol;Acc:HGNC:25209] |
| ENSG00000106823 | HGLibA_14415 | 10 | -1.049091584 | ECM2 | extracellular matrix protein 2 [Source:HGNC Symbol;Acc:HGNC:3154] |
| ENSG00000107643 | HGLibA_28283 | 10 | -1.049091584 | MAPK8 | mitogen-activated protein kinase 8 [Source:HGNC Symbol;Acc:HGNC:6881] |
| ENSG00000108417 | HGLibA_25496 | 10 | -1.049091584 | KRT37 | keratin 37 [Source:HGNC Symbol;Acc:HGNC:6455] |
| ENSG00000109511 | HGLibA_02222 | 10 | -1.049091584 | ANXA10 | annexin A10 [Source:HGNC Symbol;Acc:HGNC:534] |
| ENSG00000109576 | HGLibA_00040 | 10 | -1.049091584 | AADAT | aminoadipate aminotransferase [Source:HGNC Symbol;Acc:HGNC:17929] |
| ENSG00000109805 | HGLibA_31270 | 10 | -1.049091584 | NCAPG | non-SMC condensin I complex subunit G [Source:HGNC Symbol;Acc:HGNC:24304] |
| ENSG00000111432 | HGLibA_18263 | 10 | -1.049091584 | FZD10 | frizzled class receptor 10 [Source:HGNC Symbol;Acc:HGNC:4039] |
| ENSG00000111912 | HGLibA_31352 | 10 | -1.049091584 | NCOA7 | nuclear receptor coactivator 7 [Source:HGNC Symbol;Acc:HGNC:21081] |
| ENSG00000113575 | HGLibA_38134 | 10 | -1.049091584 | PPP2CA | protein phosphatase 2 catalytic subunit alpha [Source:HGNC Symbol;Acc:HGNC:9299] |
| ENSG00000114948 | HGLibA_00760 | 10 | -1.049091584 | ADAM23 | ADAM metallopeptidase domain 23 [Source:HGNC Symbol;Acc:HGNC:202] |
| ENSG00000115461 | HGLibA_22827 | 10 | -1.049091584 | IGFBP5 | insulin like growth factor binding protein 5 [Source:HGNC Symbol;Acc:HGNC:5474] |
| ENSG00000115685 | HGLibA_38122 | 10 | -1.049091584 | PPP1R7 | protein phosphatase 1 regulatory subunit 7 [Source:HGNC Symbol;Acc:HGNC:9295] |
| ENSG00000117308 | HGLibA_18512 | 10 | -1.049091584 | GALE | UDP-galactose-4-epimerase [Source:HGNC Symbol;Acc:HGNC:4116] |
| ENSG00000118246 | HGLibA_16904 | 10 | -1.049091584 | FASTKD2 | FAST kinase domains 2 [Source:HGNC Symbol;Acc:HGNC:29160] |
| ENSG00000118322 | HGLibA_03547 | 10 | -1.049091584 | ATP10B | ATPase phospholipid transporting 10B (putative) [Source:HGNC Symbol;Acc:HGNC:13543] |
| ENSG00000118495 | HGLibA_36954 | 10 | -1.049091584 | PLAGL1 | PLAG1 like zinc finger 1 [Source:HGNC Symbol;Acc:HGNC:9046] |
| ENSG00000118508 | HGLibA_39876 | 10 | -1.049091584 | RAB32 | member RAS oncogene family [Source:HGNC Symbol;Acc:HGNC:9772]" |
| ENSG00000119138 | HGLibA_25087 | 10 | -1.049091584 | KLF9 | Kruppel like factor 9 [Source:HGNC Symbol;Acc:HGNC:1123] |
| ENSG00000119655 | HGLibA_32423 | 10 | -1.049091584 | NPC2 | NPC intracellular cholesterol transporter 2 [Source:HGNC Symbol;Acc:HGNC:14537] |
| ENSG00000119715 | HGLibA_15596 | 10 | -1.049091584 | ESRRB | estrogen related receptor beta [Source:HGNC Symbol;Acc:HGNC:3473] |
| ENSG00000120498 | HGLibA_49052 | 10 | -1.049091584 | TEX11 | testis expressed 11 [Source:HGNC Symbol;Acc:HGNC:11733] |
| ENSG00000120733 | HGLibA_24502 | 10 | -1.049091584 | KDM3B | lysine demethylase 3B [Source:HGNC Symbol;Acc:HGNC:1337] |
| ENSG00000121211 | HGLibA_29565 | 10 | -1.049091584 | MND1 | meiotic nuclear divisions 1 [Source:HGNC Symbol;Acc:HGNC:24839] |
| ENSG00000121481 | HGLibA_41608 | 10 | -1.049091584 | RNF2 | ring finger protein 2 [Source:HGNC Symbol;Acc:HGNC:10061] |
| ENSG00000121644 | HGLibA_13011 | 10 | -1.049091584 | DESI2 | desumoylating isopeptidase 2 [Source:HGNC Symbol;Acc:HGNC:24264] |
| ENSG00000122557 | HGLibA_21174 | 10 | -1.049091584 | HERPUD2 | HERPUD family member 2 [Source:HGNC Symbol;Acc:HGNC:21915] |
| ENSG00000124225 | HGLibA_37301 | 10 | -1.049091584 | PMEPA1 | androgen induced 1 [Source:HGNC Symbol;Acc:HGNC:14107]" |
| ENSG00000124786 | HGLibA_44965 | 10 | -1.049091584 | SLC35B3 | solute carrier family 35 member B3 [Source:HGNC Symbol;Acc:HGNC:21601] |
| ENSG00000125384 | HGLibA_39317 | 10 | -1.049091584 | PTGER2 | prostaglandin E receptor 2 [Source:HGNC Symbol;Acc:HGNC:9594] |
| ENSG00000126261 | HGLibA_52517 | 10 | -1.049091584 | UBA2 | ubiquitin like modifier activating enzyme 2 [Source:HGNC Symbol;Acc:HGNC:30661] |
| ENSG00000127399 | HGLibA_27406 | 10 | -1.049091584 | LRRC61 | leucine rich repeat containing 61 [Source:HGNC Symbol;Acc:HGNC:21704] |
| ENSG00000132639 | HGLibA_45878 | 10 | -1.049091584 | SNAP25 | synaptosome associated protein 25 [Source:HGNC Symbol;Acc:HGNC:11132] |
| ENSG00000133597 | HGLibA_00894 | 10 | -1.049091584 | ADCK2 | aarF domain containing kinase 2 [Source:HGNC Symbol;Acc:HGNC:19039] |
| ENSG00000133665 | HGLibA_14223 | 10 | -1.049091584 | DYDC2 | DPY30 domain containing 2 [Source:HGNC Symbol;Acc:HGNC:23468] |
| ENSG00000133980 | HGLibA_53832 | 10 | -1.049091584 | VRTN | vertebrae development associated [Source:HGNC Symbol;Acc:HGNC:20223] |
| ENSG00000134020 | HGLibA_36014 | 10 | -1.049091584 | PEBP4 | phosphatidylethanolamine binding protein 4 [Source:HGNC Symbol;Acc:HGNC:28319] |
| ENSG00000134874 | HGLibA_14297 | 10 | -1.049091584 | DZIP1 | DAZ interacting zinc finger protein 1 [Source:HGNC Symbol;Acc:HGNC:20908] |

| | | | | | |
|---|---|---|---|---|---|
| ENSG00000135407 | HGLibA_03890 | 10 | -1.049091584 | AVIL | advillin [Source:HGNC Symbol;Acc:HGNC:14188] |
| ENSG00000135638 | HGLibA_15091 | 10 | -1.049091584 | EMX1 | empty spiracles homeobox 1 [Source:HGNC Symbol;Acc:HGNC:3340] |
| ENSG00000136574 | HGLibA_18675 | 10 | -1.049091584 | GATA4 | GATA binding protein 4 [Source:HGNC Symbol;Acc:HGNC:4173] |
| ENSG00000138411 | HGLibA_21105 | 10 | -1.049091584 | HECW2 | C2 and WW domain containing E3 ubiquitin protein ligase 2 [Source:HGNC Symbol;Acc:HGNC:29853]" |
| ENSG00000138472 | HGLibA_20640 | 10 | -1.049091584 | GUCA1C | guanylate cyclase activator 1C [Source:HGNC Symbol;Acc:HGNC:4680] |
| ENSG00000138621 | HGLibA_37857 | 10 | -1.049091584 | PPCDC | phosphopantothenoylcysteine decarboxylase [Source:HGNC Symbol;Acc:HGNC:28107] |
| ENSG00000138785 | HGLibA_23412 | 10 | -1.049091584 | INTS12 | integrator complex subunit 12 [Source:HGNC Symbol;Acc:HGNC:25067] |
| ENSG00000139946 | HGLibA_36035 | 10 | -1.049091584 | PELI2 | pellino E3 ubiquitin protein ligase family member 2 [Source:HGNC Symbol;Acc:HGNC:8828] |
| ENSG00000140548 | HGLibA_56611 | 10 | -1.049091584 | ZNF710 | zinc finger protein 710 [Source:HGNC Symbol;Acc:HGNC:25352] |
| ENSG00000140995 | HGLibA_12788 | 10 | -1.049091584 | DEF8 | differentially expressed in FDCP 8 homolog [Source:HGNC Symbol;Acc:HGNC:25969] |
| ENSG00000141252 | HGLibA_53810 | 10 | -1.049091584 | VPS53 | GARP complex subunit [Source:HGNC Symbol;Acc:HGNC:25608]" |
| ENSG00000141384 | HGLibA_48236 | 10 | -1.049091584 | TAF4B | TATA-box binding protein associated factor 4b [Source:HGNC Symbol;Acc:HGNC:11538] |
| ENSG00000144028 | HGLibA_45942 | 10 | -1.049091584 | SNRNP200 | small nuclear ribonucleoprotein U5 subunit 200 [Source:HGNC Symbol;Acc:HGNC:30859] |
| ENSG00000144524 | HGLibA_10730 | 10 | -1.049091584 | COPS7B | COP9 signalosome subunit 7B [Source:HGNC Symbol;Acc:HGNC:16760] |
| ENSG00000144681 | HGLibA_47313 | 10 | -1.049091584 | STAC | SH3 and cysteine rich domain [Source:HGNC Symbol;Acc:HGNC:11353] |
| ENSG00000144747 | HGLibA_50579 | 10 | -1.049091584 | TMF1 | TATA element modulatory factor 1 [Source:HGNC Symbol;Acc:HGNC:11870] |
| ENSG00000146385 | HGLibA_48135 | 10 | -1.049091584 | TAAR8 | trace amine associated receptor 8 [Source:HGNC Symbol;Acc:HGNC:14964] |
| ENSG00000147465 | HGLibA_47347 | 10 | -1.049091584 | STAR | steroidogenic acute regulatory protein [Source:HGNC Symbol;Acc:HGNC:11359] |
| ENSG00000147601 | HGLibA_49014 | 10 | -1.049091584 | TERF1 | telomeric repeat binding factor 1 [Source:HGNC Symbol;Acc:HGNC:11728] |
| ENSG00000148225 | HGLibA_54065 | 10 | -1.049091584 | WDR31 | WD repeat domain 31 [Source:HGNC Symbol;Acc:HGNC:21421] |
| ENSG00000148386 | HGLibA_26188 | 10 | -1.049091584 | LCN9 | lipocalin 9 [Source:HGNC Symbol;Acc:HGNC:17442] |
| ENSG00000148734 | HGLibA_32438 | 10 | -1.049091584 | NPFFR1 | neuropeptide FF receptor 1 [Source:HGNC Symbol;Acc:HGNC:17425] |
| ENSG00000148848 | HGLibA_00733 | 10 | -1.049091584 | ADAM12 | ADAM metallopeptidase domain 12 [Source:HGNC Symbol;Acc:HGNC:190] |
| ENSG00000151117 | HGLibA_50525 | 10 | -1.049091584 | TMEM86A | transmembrane protein 86A [Source:HGNC Symbol;Acc:HGNC:26890] |
| ENSG00000152582 | HGLibA_46613 | 10 | -1.049091584 | SPEF2 | sperm flagellar 2 [Source:HGNC Symbol;Acc:HGNC:26293] |
| ENSG00000152767 | HGLibA_16875 | 10 | -1.049091584 | FARP1 | ARH/RhoGEF and pleckstrin domain protein 1 [Source:HGNC Symbol;Acc:HGNC:3591]" |
| ENSG00000153815 | HGLibA_10194 | 10 | -1.049091584 | CMIP | c-Maf inducing protein [Source:HGNC Symbol;Acc:HGNC:24319] |
| ENSG00000153989 | HGLibA_33092 | 10 | -1.049091584 | NUS1 | dehydrodolichyl diphosphate synthase subunit [Source:HGNC Symbol;Acc:HGNC:21042]" |
| ENSG00000154928 | HGLibA_15311 | 10 | -1.049091584 | EPHB1 | EPH receptor B1 [Source:HGNC Symbol;Acc:HGNC:3392] |
| ENSG00000157216 | HGLibA_47119 | 10 | -1.049091584 | SSBP3 | single stranded DNA binding protein 3 [Source:HGNC Symbol;Acc:HGNC:15674] |
| ENSG00000157703 | HGLibA_47895 | 10 | -1.049091584 | SVOPL | SVOP like [Source:HGNC Symbol;Acc:HGNC:27034] |
| ENSG00000158485 | HGLibA_08267 | 10 | -1.049091584 | CD1B | CD1b molecule [Source:HGNC Symbol;Acc:HGNC:1635] |
| ENSG00000158486 | HGLibA_13487 | 10 | -1.049091584 | DNAH3 | dynein axonemal heavy chain 3 [Source:HGNC Symbol;Acc:HGNC:2949] |
| ENSG00000158941 | HGLibA_07489 | 10 | -1.049091584 | CCAR2 | cell cycle and apoptosis regulator 2 [Source:HGNC Symbol;Acc:HGNC:23360] |
| ENSG00000161265 | HGLibA_52495 | 10 | -1.049091584 | U2AF1L4 | U2 small nuclear RNA auxiliary factor 1 like 4 [Source:HGNC Symbol;Acc:HGNC:23020] |
| ENSG00000162526 | HGLibA_52014 | 10 | -1.049091584 | TSSK3 | testis specific serine kinase 3 [Source:HGNC Symbol;Acc:HGNC:15473] |
| ENSG00000163518 | HGLibA_17235 | 10 | -1.049091584 | FCRL4 | Fc receptor like 4 [Source:HGNC Symbol;Acc:HGNC:18507] |
| ENSG00000163684 | HGLibA_42024 | 10 | -1.049091584 | RPP14 | ribonuclease P/MRP subunit p14 [Source:HGNC Symbol;Acc:HGNC:30327] |
| ENSG00000164050 | HGLibA_37271 | 10 | -1.049091584 | PLXNB1 | plexin B1 [Source:HGNC Symbol;Acc:HGNC:9103] |
| ENSG00000164113 | HGLibA_00719 | 10 | -1.049091584 | ADAD1 | adenosine deaminase domain containing 1 [Source:HGNC Symbol;Acc:HGNC:30713] |
| ENSG00000164134 | HGLibA_30976 | 10 | -1.049091584 | NAA15 | NatA auxiliary subunit [Source:HGNC Symbol;Acc:HGNC:30782]" |
| ENSG00000164604 | HGLibA_20098 | 10 | -1.049091584 | GPR85 | G protein-coupled receptor 85 [Source:HGNC Symbol;Acc:HGNC:4536] |
| ENSG00000166866 | HGLibA_30821 | 10 | -1.049091584 | MYO1A | myosin IA [Source:HGNC Symbol;Acc:HGNC:7595] |
| ENSG00000166912 | HGLibA_30413 | 10 | -1.049091584 | MTMR10 | myotubularin related protein 10 [Source:HGNC Symbol;Acc:HGNC:25999] |
| ENSG00000167105 | HGLibA_50560 | 10 | -1.049091584 | TMEM92 | transmembrane protein 92 [Source:HGNC Symbol;Acc:HGNC:26579] |
| ENSG00000167414 | HGLibA_19536 | 10 | -1.049091584 | GNG8 | G protein subunit gamma 8 [Source:HGNC Symbol;Acc:HGNC:19664] |
| ENSG00000168916 | HGLibA_56373 | 10 | -1.049091584 | ZNF608 | zinc finger protein 608 [Source:HGNC Symbol;Acc:HGNC:29238] |
| ENSG00000169575 | HGLibA_53721 | 10 | -1.049091584 | VPREB1 | V-set pre-B cell surrogate light chain 1 [Source:HGNC Symbol;Acc:HGNC:12709] |
| ENSG00000169641 | HGLibA_27638 | 10 | -1.049091584 | LUZP1 | leucine zipper protein 1 [Source:HGNC Symbol;Acc:HGNC:14985] |
| ENSG00000169738 | HGLibA_12582 | 10 | -1.049091584 | DCXR | dicarbonyl and L-xylulose reductase [Source:HGNC Symbol;Acc:HGNC:18985] |
| ENSG00000169764 | HGLibA_52873 | 10 | -1.049091584 | UGP2 | UDP-glucose pyrophosphorylase 2 [Source:HGNC Symbol;Acc:HGNC:12527] |
| ENSG00000170498 | HGLibA_25009 | 10 | -1.049091584 | KISS1 | KiSS-1 metastasis suppressor [Source:HGNC Symbol;Acc:HGNC:6341] |
| ENSG00000170759 | HGLibA_24953 | 10 | -1.049091584 | KIF5B | kinesin family member 5B [Source:HGNC Symbol;Acc:HGNC:6324] |
| ENSG00000171790 | HGLibA_45498 | 10 | -1.049091584 | SLFNL1 | schlafen like 1 [Source:HGNC Symbol;Acc:HGNC:26313] |
| ENSG00000173040 | HGLibA_15682 | 10 | -1.049091584 | EVC2 | EvC ciliary complex subunit 2 [Source:HGNC Symbol;Acc:HGNC:19747] |
| ENSG00000173641 | HGLibA_22315 | 10 | -1.049091584 | HSPB7 | heat shock protein family B (small) member 7 [Source:HGNC Symbol;Acc:HGNC:5249] |
| ENSG00000173809 | HGLibA_48901 | 10 | -1.049091584 | TDRD12 | tudor domain containing 12 [Source:HGNC Symbol;Acc:HGNC:25044] |
| ENSG00000175455 | HGLibA_07613 | 10 | -1.049091584 | CCDC14 | coiled-coil domain containing 14 [Source:HGNC Symbol;Acc:HGNC:25766] |
| ENSG00000175745 | HGLibA_32609 | 10 | -1.049091584 | NR2F1 | nuclear receptor subfamily 2 group F member 1 [Source:HGNC Symbol;Acc:HGNC:7975] |
| ENSG00000176473 | HGLibA_54051 | 10 | -1.049091584 | WDR25 | WD repeat domain 25 [Source:HGNC Symbol;Acc:HGNC:21064] |
| ENSG00000176495 | HGLibA_34241 | 10 | -1.049091584 | OR5AN1 | olfactory receptor family 5 subfamily AN member 1 [Source:HGNC Symbol;Acc:HGNC:15255] |
| ENSG00000176571 | HGLibA_10242 | 10 | -1.049091584 | CNBD1 | cyclic nucleotide binding domain containing 1 [Source:HGNC Symbol;Acc:HGNC:26663] |
| ENSG00000176658 | HGLibA_30829 | 10 | -1.049091584 | MYO1D | myosin ID [Source:HGNC Symbol;Acc:HGNC:7598] |
| ENSG00000177182 | HGLibA_10167 | 10 | -1.049091584 | CLVS1 | clavesin 1 [Source:HGNC Symbol;Acc:HGNC:23139] |
| ENSG00000177508 | HGLibA_23635 | 10 | -1.049091584 | IRX3 | iroquois homeobox 3 [Source:HGNC Symbol;Acc:HGNC:14360] |
| ENSG00000177576 | HGLibA_05611 | 10 | -1.049091584 | C18orf32 | chromosome 18 open reading frame 32 [Source:HGNC Symbol;Acc:HGNC:31690] |
| ENSG00000177791 | HGLibA_30901 | 10 | -1.049091584 | MYOZ1 | myozenin 1 [Source:HGNC Symbol;Acc:HGNC:13752] |
| ENSG00000177971 | HGLibA_23238 | 10 | -1.049091584 | IMP3 | U3 small nucleolar ribonucleoprotein [Source:HGNC Symbol;Acc:HGNC:14497]" |
| ENSG00000178125 | HGLibA_38120 | 10 | -1.049091584 | PPP1R42 | protein phosphatase 1 regulatory subunit 42 [Source:HGNC Symbol;Acc:HGNC:33732] |
| ENSG00000178695 | HGLibA_24419 | 10 | -1.049091584 | KCTD12 | potassium channel tetramerization domain containing 12 [Source:HGNC Symbol;Acc:HGNC:14678] |
| ENSG00000181029 | HGLibA_51326 | 10 | -1.049091584 | TRAPPC5 | trafficking protein particle complex 5 [Source:HGNC Symbol;Acc:HGNC:23067] |
| ENSG00000181222 | HGLibA_37564 | 10 | -1.049091584 | POLR2A | RNA polymerase II subunit A [Source:HGNC Symbol;Acc:HGNC:9187] |
| ENSG00000181333 | HGLibA_21146 | 10 | -1.049091584 | HEPHL1 | hephaestin like 1 [Source:HGNC Symbol;Acc:HGNC:30477] |
| ENSG00000181616 | HGLibA_34168 | 10 | -1.049091584 | OR52H1 | olfactory receptor family 52 subfamily H member 1 [Source:HGNC Symbol;Acc:HGNC:15218] |
| ENSG00000183549 | HGLibA_00583 | 10 | -1.049091584 | ACSM5 | acyl-CoA synthetase medium chain family member 5 [Source:HGNC Symbol;Acc:HGNC:26060] |
| ENSG00000183571 | HGLibA_36283 | 10 | -1.049091584 | PGPEP1L | pyroglutamyl-peptidase I like [Source:HGNC Symbol;Acc:HGNC:27080] |
| ENSG00000183765 | HGLibA_09423 | 10 | -1.049091584 | CHEK2 | checkpoint kinase 2 [Source:HGNC Symbol;Acc:HGNC:16627] |
| ENSG00000184205 | HGLibA_51983 | 10 | -1.049091584 | TSPYL2 | TSPY like 2 [Source:HGNC Symbol;Acc:HGNC:24358] |
| ENSG00000184619 | HGLibA_25391 | 10 | -1.049091584 | KRBA2 | KRAB-A domain containing 2 [Source:HGNC Symbol;Acc:HGNC:26989] |
| ENSG00000185352 | HGLibA_22149 | 10 | -1.049091584 | HS6ST3 | heparan sulfate 6-O-sulfotransferase 3 [Source:HGNC Symbol;Acc:HGNC:19134] |
| ENSG00000186051 | HGLibA_48283 | 10 | -1.049091584 | TAL2 | TAL bHLH transcription factor 2 [Source:HGNC Symbol;Acc:HGNC:11557] |
| ENSG00000186074 | HGLibA_08343 | 10 | -1.049091584 | CD300LF | CD300 molecule like family member f [Source:HGNC Symbol;Acc:HGNC:29883] |
| ENSG00000186092 | HGLibA_34010 | 10 | -1.049091584 | OR4F5 | olfactory receptor family 4 subfamily F member 5 [Source:HGNC Symbol;Acc:HGNC:14825] |
| ENSG00000186479 | HGLibA_41106 | 10 | -1.049091584 | RGS7BP | regulator of G protein signaling 7 binding protein [Source:HGNC Symbol;Acc:HGNC:23271] |
| ENSG00000186603 | HGLibA_22028 | 10 | -1.049091584 | HPDL | 4-hydroxyphenylpyruvate dioxygenase like [Source:HGNC Symbol;Acc:HGNC:28242] |
| ENSG00000186827 | HGLibA_50784 | 10 | -1.049091584 | TNFRSF4 | TNF receptor superfamily member 4 [Source:HGNC Symbol;Acc:HGNC:11918] |
| ENSG00000187955 | HGLibA_10512 | 10 | -1.049091584 | COL14A1 | collagen type XIV alpha 1 chain [Source:HGNC Symbol;Acc:HGNC:2191] |
| ENSG00000188340 | HGLibA_34433 | 10 | -1.049091584 | OR6N2 | olfactory receptor family 6 subfamily N member 2 [Source:HGNC Symbol;Acc:HGNC:15035] |
| ENSG00000189190 | HGLibA_56362 | 10 | -1.049091584 | ZNF600 | zinc finger protein 600 [Source:HGNC Symbol;Acc:HGNC:30951] |
| ENSG00000196150 | HGLibA_55665 | 10 | -1.049091584 | ZNF250 | zinc finger protein 250 [Source:HGNC Symbol;Acc:HGNC:13044] |
| ENSG00000196526 | HGLibA_01097 | 10 | -1.049091584 | AFAP1 | actin filament associated protein 1 [Source:HGNC Symbol;Acc:HGNC:24017] |
| ENSG00000196597 | HGLibA_56739 | 10 | -1.049091584 | ZNF782 | zinc finger protein 782 [Source:HGNC Symbol;Acc:HGNC:33110] |
| ENSG00000197283 | HGLibA_47987 | 10 | -1.049091584 | SYNGAP1 | synaptic Ras GTPase activating protein 1 [Source:HGNC Symbol;Acc:HGNC:11497] |
| ENSG00000197771 | HGLibA_28631 | 10 | -1.049091584 | MCMBP | minichromosome maintenance complex binding protein [Source:HGNC Symbol;Acc:HGNC:25782] |
| ENSG00000197798 | HGLibA_16052 | 10 | -1.049091584 | FAM118B | family with sequence similarity 118 member B [Source:HGNC Symbol;Acc:HGNC:26110] |
| ENSG00000198075 | HGLibA_47751 | 10 | -1.049091584 | SULT1C4 | sulfotransferase family 1C member 4 [Source:HGNC Symbol;Acc:HGNC:11457] |
| ENSG00000198563 | HGLibA_12698 | 10 | -1.049091584 | DDX39B | DExD-box helicase 39B [Source:HGNC Symbol;Acc:HGNC:13917] |
| ENSG00000198625 | HGLibA_28699 | 10 | -1.049091584 | MDM4 | p53 regulator [Source:HGNC Symbol;Acc:HGNC:6974]" |
| ENSG00000198754 | HGLibA_34817 | 10 | -1.049091584 | OXCT2 | 3-oxoacid CoA-transferase 2 [Source:HGNC Symbol;Acc:HGNC:18606] |
| ENSG00000198815 | HGLibA_17890 | 10 | -1.049091584 | FOXJ3 | forkhead box J3 [Source:HGNC Symbol;Acc:HGNC:29178] |
| ENSG00000205212 | HGLibA_07624 | 10 | -1.049091584 | CCDC144NL | N-terminal like [Source:HGNC Symbol;Acc:HGNC:33735]" |
| ENSG00000205302 | HGLibA_46056 | 10 | -1.049091584 | SNX2 | sorting nexin 2 [Source:HGNC Symbol;Acc:HGNC:11173] |
| ENSG00000205517 | HGLibA_41006 | 10 | -1.049091584 | RGL3 | ral guanine nucleotide dissociation stimulator like 3 [Source:HGNC Symbol;Acc:HGNC:30282] |
| ENSG00000205572 | HGLibA_43473 | 10 | -1.049091584 | SERF1B | small EDRK-rich factor 1B [Source:HGNC Symbol;Acc:HGNC:10756] |
| ENSG00000205693 | HGLibA_28104 | 10 | -1.049091584 | MANSC4 | MANSC domain containing 4 [Source:HGNC Symbol;Acc:HGNC:40023] |
| ENSG00000205937 | HGLibA_41731 | 10 | -1.049091584 | RNPS1 | RNA binding protein with serine rich domain 1 [Source:HGNC Symbol;Acc:HGNC:10080] |
| ENSG00000213886 | HGLibA_52569 | 10 | -1.049091584 | UBD | ubiquitin D [Source:HGNC Symbol;Acc:HGNC:18795] |
| ENSG00000213937 | HGLibA_09914 | 10 | -1.049091584 | CLDN9 | claudin 9 [Source:HGNC Symbol;Acc:HGNC:2051] |
| ENSG00000215912 | HGLibA_52120 | 10 | -1.049091584 | TTC34 | tetratricopeptide repeat domain 34 [Source:HGNC Symbol;Acc:HGNC:34297] |
| ENSG00000233701 | HGLibA_38826 | 10 | -1.049091584 | PRR23C | proline rich 23C [Source:HGNC Symbol;Acc:HGNC:37173] |
| ENSG00000234616 | HGLibA_23990 | 10 | -1.049091584 | JRK | Jrk helix-turn-helix protein [Source:HGNC Symbol;Acc:HGNC:6199] |
| ENSG00000247315 | HGLibA_55033 | 10 | -1.049091584 | ZCCHC3 | zinc finger CCHC-type containing 3 [Source:HGNC Symbol;Acc:HGNC:16230] |
| ENSG00000249104 | HGLibA_53230 | 10 | -1.049091584 | USP17L17 | ubiquitin specific peptidase 17-like family member 17 [Source:HGNC Symbol;Acc:HGNC:44445] |
| ENSG00000250571 | HGLibA_19257 | 10 | -1.049091584 | GLI4 | GLI family zinc finger 4 [Source:HGNC Symbol;Acc:HGNC:4320] |
| ENSG00000250745 | HGLibA_53245 | 10 | -1.049091584 | USP17L20 | ubiquitin specific peptidase 17-like family member 20 [Source:HGNC Symbol;Acc:HGNC:44448] |
| ENSG00000250844 | HGLibA_53233 | 10 | -1.049091584 | USP17L18 | ubiquitin specific peptidase 17-like family member 18 [Source:HGNC Symbol;Acc:HGNC:44446] |
| ENSG00000251623 | HGLibA_16172 | 10 | -1.049091584 | FAM153B | - |
| ENSG00000258223 | HGLibA_38972 | 10 | -1.049091584 | PRSS58 | serine protease 58 [Source:HGNC Symbol;Acc:HGNC:39125] |
| ENSG00000275793 | HGLibA_41263 | 10 | -1.049091584 | RIMBP3 | RIMS binding protein 3 [Source:HGNC Symbol;Acc:HGNC:29344] |
| ENSG00000284194 | HGLibA_43028 | 10 | -1.049091584 | SCO2 | cytochrome c oxidase assembly protein [Source:NCBI gene;Acc:9997]" |
| ENSG00000285053 | HGLibA_48592 | 10 | -1.049091584 | TBCE | tubulin folding cofactor E [Source:NCBI gene;Acc:6905] |
| FAM105A | HGLibA_15993 | 10 | -1.049091584 | FAM105A | - |
| FAM105B | HGLibA_15997 | 10 | -1.049091584 | FAM105B | - |
| FAM5B | HGLibA_16651 | 10 | -1.049091584 | FAM5B | - |
| GPR125 | HGLibA_19879 | 10 | -1.049091584 | GPR125 | - |
| HIST1H2BL | HGLibA_21443 | 10 | -1.049091584 | HIST1H2BL | - |
| hsa-mir-124-2 | HGLibA_57351 | 10 | -1.049091584 | hsa-mir-124-2 | - |
| hsa-mir-1293 | HGLibA_57649 | 10 | -1.049091584 | hsa-mir-1293 | - |
| hsa-mir-153-2 | HGLibA_57911 | 10 | -1.049091584 | hsa-mir-153-2 | - |
| hsa-mir-199b | HGLibA_58136 | 10 | -1.049091584 | hsa-mir-199b | - |
| hsa-mir-2110 | HGLibA_58220 | 10 | -1.049091584 | hsa-mir-2110 | - |
| hsa-mir-302c | HGLibA_58446 | 10 | -1.049091584 | hsa-mir-302c | - |
| hsa-mir-31 | HGLibA_58495 | 10 | -1.049091584 | hsa-mir-31 | - |
| hsa-mir-3117 | HGLibA_58513 | 10 | -1.049091584 | hsa-mir-3117 | - |
| hsa-mir-3153 | HGLibA_58670 | 10 | -1.049091584 | hsa-mir-3153 | - |

| | | | | | |
|---|---|---|---|---|---|
| hsa-mir-3180-1 | HGLibA_58802 | 10 | -1.049091584 | hsa-mir-3180-1 | - |
| hsa-mir-374c | HGLibA_59398 | 10 | -1.049091584 | hsa-mir-374c | - |
| hsa-mir-380 | HGLibA_59472 | 10 | -1.049091584 | hsa-mir-380 | - |
| hsa-mir-4262 | HGLibA_59757 | 10 | -1.049091584 | hsa-mir-4262 | - |
| hsa-mir-4307 | HGLibA_59940 | 10 | -1.049091584 | hsa-mir-4307 | - |
| hsa-mir-4418 | HGLibA_60054 | 10 | -1.049091584 | hsa-mir-4418 | - |
| hsa-mir-4475 | HGLibA_60312 | 10 | -1.049091584 | hsa-mir-4475 | - |
| hsa-mir-4527 | HGLibA_60569 | 10 | -1.049091584 | hsa-mir-4527 | - |
| hsa-mir-4765 | HGLibA_61157 | 10 | -1.049091584 | hsa-mir-4765 | - |
| hsa-mir-491 | HGLibA_61366 | 10 | -1.049091584 | hsa-mir-491 | - |
| hsa-mir-509-2 | HGLibA_61509 | 10 | -1.049091584 | hsa-mir-509-2 | - |
| hsa-mir-548ad | HGLibA_61863 | 10 | -1.049091584 | hsa-mir-548ad | - |
| hsa-mir-548d-2 | HGLibA_61966 | 10 | -1.049091584 | hsa-mir-548d-2 | - |
| hsa-mir-570 | HGLibA_62334 | 10 | -1.049091584 | hsa-mir-570 | - |
| hsa-mir-610 | HGLibA_62631 | 10 | -1.049091584 | hsa-mir-610 | - |
| hsa-mir-6124 | HGLibA_62643 | 10 | -1.049091584 | hsa-mir-6124 | - |
| hsa-mir-6128 | HGLibA_62661 | 10 | -1.049091584 | hsa-mir-6128 | - |
| hsa-mir-642a | HGLibA_62807 | 10 | -1.049091584 | hsa-mir-642a | - |
| hsa-mir-642b | HGLibA_62810 | 10 | -1.049091584 | hsa-mir-642b | - |
| hsa-mir-6507 | HGLibA_62879 | 10 | -1.049091584 | hsa-mir-6507 | - |
| hsa-mir-6728 | HGLibA_63066 | 10 | -1.049091584 | hsa-mir-6728 | - |
| hsa-mir-6728 | HGLibA_63067 | 10 | -1.049091584 | hsa-mir-6728 | - |
| hsa-mir-6800 | HGLibA_63377 | 10 | -1.049091584 | hsa-mir-6800 | - |
| hsa-mir-6837 | HGLibA_63524 | 10 | -1.049091584 | hsa-mir-6837 | - |
| hsa-mir-7155 | HGLibA_63850 | 10 | -1.049091584 | hsa-mir-7155 | - |
| hsa-mir-7159 | HGLibA_63866 | 10 | -1.049091584 | hsa-mir-7159 | - |
| hsa-mir-934 | HGLibA_64310 | 10 | -1.049091584 | hsa-mir-934 | - |
| KIAA0247 | HGLibA_24614 | 10 | -1.049091584 | KIAA0247 | - |
| LOC440243 | HGLibA_26942 | 10 | -1.049091584 | LOC440243 | - |
| LRRC48 | HGLibA_27375 | 10 | -1.049091584 | LRRC48 | - |
| 12-Sep | HGLibA_43432 | 10 | -1.049091584 | 12-Sep | - |
| BAGE3 | HGLibA_04098 | 9 | -1.059042622 | BAGE3 | - |
| C16orf45 | HGLibA_05446 | 9 | -1.059042622 | C16orf45 | - |
| C1orf233 | HGLibA_05847 | 9 | -1.059042622 | C1orf233 | - |
| C9orf41 | HGLibA_06730 | 9 | -1.059042622 | C9orf41 | - |
| CARKD | HGLibA_07249 | 9 | -1.059042622 | CARKD | - |
| CXorf27 | HGLibA_11912 | 9 | -1.059042622 | CXorf27 | - |
| ENSG00000013725 | HGLibA_08422 | 9 | -1.059042622 | CD6 | CD6 molecule [Source:HGNC Symbol;Acc:HGNC:1691] |
| ENSG00000042781 | HGLibA_53180 | 9 | -1.059042622 | USH2A | usherin [Source:HGNC Symbol;Acc:HGNC:12601] |
| ENSG00000047410 | HGLibA_51175 | 9 | -1.059042622 | TPR | nuclear basket protein [Source:HGNC Symbol;Acc:HGNC:12017]" |
| ENSG00000055917 | HGLibA_39583 | 9 | -1.059042622 | PUM2 | pumilio RNA binding family member 2 [Source:HGNC Symbol;Acc:HGNC:14958] |
| ENSG00000058091 | HGLibA_08738 | 9 | -1.059042622 | CDK14 | cyclin dependent kinase 14 [Source:HGNC Symbol;Acc:HGNC:8883] |
| ENSG00000063601 | HGLibA_30409 | 9 | -1.059042622 | MTMR1 | myotubularin related protein 1 [Source:HGNC Symbol;Acc:HGNC:7449] |
| ENSG00000064703 | HGLibA_12670 | 9 | -1.059042622 | DDX20 | DEAD-box helicase 20 [Source:HGNC Symbol;Acc:HGNC:2743] |
| ENSG00000065361 | HGLibA_15419 | 9 | -1.059042622 | ERBB3 | erb-b2 receptor tyrosine kinase 3 [Source:HGNC Symbol;Acc:HGNC:3431] |
| ENSG00000065613 | HGLibA_45530 | 9 | -1.059042622 | SLK | STE20 like kinase [Source:HGNC Symbol;Acc:HGNC:11088] |
| ENSG00000066135 | HGLibA_24505 | 9 | -1.059042622 | KDM4A | lysine demethylase 4A [Source:HGNC Symbol;Acc:HGNC:22978] |
| ENSG00000072501 | HGLibA_45641 | 9 | -1.059042622 | SMC1A | structural maintenance of chromosomes 1A [Source:HGNC Symbol;Acc:HGNC:11111] |
| ENSG00000081760 | HGLibA_00027 | 9 | -1.059042622 | AACS | acetoacetyl-CoA synthetase [Source:HGNC Symbol;Acc:HGNC:21298] |
| ENSG00000083812 | HGLibA_55800 | 9 | -1.059042622 | ZNF324 | zinc finger protein 324 [Source:HGNC Symbol;Acc:HGNC:14096] |
| ENSG00000087087 | HGLibA_47057 | 9 | -1.059042622 | SRRT | RNA effector molecule [Source:HGNC Symbol;Acc:HGNC:24101]" |
| ENSG00000087095 | HGLibA_32083 | 9 | -1.059042622 | NLK | nemo like kinase [Source:HGNC Symbol;Acc:HGNC:29858] |
| ENSG00000096093 | HGLibA_14571 | 9 | -1.059042622 | EFHC1 | EF-hand domain containing 1 [Source:HGNC Symbol;Acc:HGNC:16406] |
| ENSG00000100033 | HGLibA_38658 | 9 | -1.059042622 | PRODH | proline dehydrogenase 1 [Source:HGNC Symbol;Acc:HGNC:9453] |
| ENSG00000100726 | HGLibA_48985 | 9 | -1.059042622 | TELO2 | telomere maintenance 2 [Source:HGNC Symbol;Acc:HGNC:29099] |
| ENSG00000101199 | HGLibA_02662 | 9 | -1.059042622 | ARFGAP1 | ADP ribosylation factor GTPase activating protein 1 [Source:HGNC Symbol;Acc:HGNC:15852] |
| ENSG00000101460 | HGLibA_28122 | 9 | -1.059042622 | MAP1LC3A | microtubule associated protein 1 light chain 3 alpha [Source:HGNC Symbol;Acc:HGNC:6838] |
| ENSG00000102452 | HGLibA_31083 | 9 | -1.059042622 | NALCN | non-selective [Source:HGNC Symbol;Acc:HGNC:19082]" |
| ENSG00000103415 | HGLibA_21750 | 9 | -1.059042622 | HMOX2 | heme oxygenase 2 [Source:HGNC Symbol;Acc:HGNC:5014] |
| ENSG00000106327 | HGLibA_49192 | 9 | -1.059042622 | TFR2 | transferrin receptor 2 [Source:HGNC Symbol;Acc:HGNC:11762] |
| ENSG00000108578 | HGLibA_04552 | 9 | -1.059042622 | BLMH | bleomycin hydrolase [Source:HGNC Symbol;Acc:HGNC:1059] |
| ENSG00000108669 | HGLibA_12234 | 9 | -1.059042622 | CYTH1 | cytohesin 1 [Source:HGNC Symbol;Acc:HGNC:9501] |
| ENSG00000108854 | HGLibA_45847 | 9 | -1.059042622 | SMURF2 | SMAD specific E3 ubiquitin protein ligase 2 [Source:HGNC Symbol;Acc:HGNC:16809] |
| ENSG00000110218 | HGLibA_35109 | 9 | -1.059042622 | PANX1 | pannexin 1 [Source:HGNC Symbol;Acc:HGNC:8599] |
| ENSG00000111247 | HGLibA_40082 | 9 | -1.059042622 | RAD51AP1 | RAD51 associated protein 1 [Source:HGNC Symbol;Acc:HGNC:16956] |
| ENSG00000111644 | HGLibA_00534 | 9 | -1.059042622 | ACRBP | acrosin binding protein [Source:HGNC Symbol;Acc:HGNC:17195] |
| ENSG00000111737 | HGLibA_39890 | 9 | -1.059042622 | RAB35 | member RAS oncogene family [Source:HGNC Symbol;Acc:HGNC:9774]" |
| ENSG00000111790 | HGLibA_17441 | 9 | -1.059042622 | FGFR1OP2 | FGFR1 oncogene partner 2 [Source:HGNC Symbol;Acc:HGNC:23098] |
| ENSG00000112462 | HGLibA_33589 | 9 | -1.059042622 | OR12D3 | olfactory receptor family 12 subfamily D member 3 [Source:HGNC Symbol;Acc:HGNC:13963] |
| ENSG00000112559 | HGLibA_28669 | 9 | -1.059042622 | MDFI | MyoD family inhibitor [Source:HGNC Symbol;Acc:HGNC:6967] |
| ENSG00000114383 | HGLibA_52344 | 9 | -1.059042622 | TUSC2 | mitochondrial calcium regulator [Source:HGNC Symbol;Acc:HGNC:17034]" |
| ENSG00000115042 | HGLibA_15957 | 9 | -1.059042622 | FAHD2A | fumarylacetoacetate hydrolase domain containing 2A [Source:HGNC Symbol;Acc:HGNC:24252] |
| ENSG00000116874 | HGLibA_53947 | 9 | -1.059042622 | WARS2 | mitochondrial [Source:HGNC Symbol;Acc:HGNC:12730]" |
| ENSG00000120992 | HGLibA_27736 | 9 | -1.059042622 | LYPLA1 | lysophospholipase I [Source:HGNC Symbol;Acc:HGNC:6737] |
| ENSG00000121454 | HGLibA_26455 | 9 | -1.059042622 | LHX4 | LIM homeobox 4 [Source:HGNC Symbol;Acc:HGNC:21734] |
| ENSG00000122484 | HGLibA_41818 | 9 | -1.059042622 | RPAP2 | RNA polymerase II associated protein 2 [Source:HGNC Symbol;Acc:HGNC:25791] |
| ENSG00000122692 | HGLibA_45839 | 9 | -1.059042622 | SMU1 | DNA replication regulator and spliceosomal factor [Source:HGNC Symbol;Acc:HGNC:18247]" |
| ENSG00000122779 | HGLibA_51438 | 9 | -1.059042622 | TRIM24 | tripartite motif containing 24 [Source:HGNC Symbol;Acc:HGNC:11812] |
| ENSG00000123179 | HGLibA_14375 | 9 | -1.059042622 | EBPL | EBP like [Source:HGNC Symbol;Acc:HGNC:18061] |
| ENSG00000124614 | HGLibA_42058 | 9 | -1.059042622 | RPS10 | ribosomal protein S10 [Source:HGNC Symbol;Acc:HGNC:10383] |
| ENSG00000124839 | HGLibA_39820 | 9 | -1.059042622 | RAB17 | member RAS oncogene family [Source:HGNC Symbol;Acc:HGNC:16523]" |
| ENSG00000125851 | HGLibA_35674 | 9 | -1.059042622 | PCSK2 | proprotein convertase subtilisin/kexin type 2 [Source:HGNC Symbol;Acc:HGNC:8744] |
| ENSG00000125931 | HGLibA_09741 | 9 | -1.059042622 | CITED1 | Cbp/p300 interacting transactivator with Glu/Asp rich carboxy-terminal domain 1 [Source:HGNC Symbol;Acc:HGNC:1986] |
| ENSG00000127554 | HGLibA_18937 | 9 | -1.059042622 | GFER | augmenter of liver regeneration [Source:HGNC Symbol;Acc:HGNC:4236]" |
| ENSG00000127780 | HGLibA_33662 | 9 | -1.059042622 | OR1E2 | olfactory receptor family 1 subfamily E member 2 [Source:HGNC Symbol;Acc:HGNC:8190] |
| ENSG00000130598 | HGLibA_50874 | 9 | -1.059042622 | TNNI2 | fast skeletal type [Source:HGNC Symbol;Acc:HGNC:11946]" |
| ENSG00000130764 | HGLibA_27371 | 9 | -1.059042622 | LRRC47 | leucine rich repeat containing 47 [Source:HGNC Symbol;Acc:HGNC:29207] |
| ENSG00000131050 | HGLibA_04712 | 9 | -1.059042622 | BPIFA2 | BPI fold containing family A member 2 [Source:HGNC Symbol;Acc:HGNC:16203] |
| ENSG00000132000 | HGLibA_37456 | 9 | -1.059042622 | PODNL1 | podocan like 1 [Source:HGNC Symbol;Acc:HGNC:26275] |
| ENSG00000132254 | HGLibA_02679 | 9 | -1.059042622 | ARFIP2 | ADP ribosylation factor interacting protein 2 [Source:HGNC Symbol;Acc:HGNC:17160] |
| ENSG00000132259 | HGLibA_10273 | 9 | -1.059042622 | CNGA4 | cyclic nucleotide gated channel alpha 4 [Source:HGNC Symbol;Acc:HGNC:2152] |
| ENSG00000132554 | HGLibA_41089 | 9 | -1.059042622 | RGS22 | regulator of G protein signaling 22 [Source:HGNC Symbol;Acc:HGNC:24499] |
| ENSG00000132561 | HGLibA_28431 | 9 | -1.059042622 | MATN2 | matrilin 2 [Source:HGNC Symbol;Acc:HGNC:6908] |
| ENSG00000132635 | HGLibA_35582 | 9 | -1.059042622 | PCED1A | PC-esterase domain containing 1A [Source:HGNC Symbol;Acc:HGNC:16212] |
| ENSG00000132879 | HGLibA_17104 | 9 | -1.059042622 | FBXO44 | F-box protein 44 [Source:HGNC Symbol;Acc:HGNC:24847] |
| ENSG00000133318 | HGLibA_42405 | 9 | -1.059042622 | RTN3 | reticulon 3 [Source:HGNC Symbol;Acc:HGNC:10469] |
| ENSG00000135052 | HGLibA_19654 | 9 | -1.059042622 | GOLM1 | golgi membrane protein 1 [Source:HGNC Symbol;Acc:HGNC:15451] |
| ENSG00000137807 | HGLibA_24908 | 9 | -1.059042622 | KIF23 | kinesin family member 23 [Source:HGNC Symbol;Acc:HGNC:6392] |
| ENSG00000138375 | HGLibA_45618 | 9 | -1.059042622 | SMARCAL1 | subfamily a like 1 [Source:HGNC Symbol;Acc:HGNC:11102]" |
| ENSG00000139445 | HGLibA_17919 | 9 | -1.059042622 | FOXN4 | forkhead box N4 [Source:HGNC Symbol;Acc:HGNC:21399] |
| ENSG00000139572 | HGLibA_20094 | 9 | -1.059042622 | GPR84 | G protein-coupled receptor 84 [Source:HGNC Symbol;Acc:HGNC:4535] |
| ENSG00000139746 | HGLibA_40520 | 9 | -1.059042622 | RBM26 | RNA binding motif protein 26 [Source:HGNC Symbol;Acc:HGNC:20327] |
| ENSG00000140459 | HGLibA_12053 | 9 | -1.059042622 | CYP11A1 | cytochrome P450 family 11 subfamily A member 1 [Source:HGNC Symbol;Acc:HGNC:2590] |
| ENSG00000141759 | HGLibA_52439 | 9 | -1.059042622 | TXNL4A | thioredoxin like 4A [Source:HGNC Symbol;Acc:HGNC:30551] |
| ENSG00000141873 | HGLibA_45109 | 9 | -1.059042622 | SLC39A3 | solute carrier family 39 member 3 [Source:HGNC Symbol;Acc:HGNC:17128] |
| ENSG00000142515 | HGLibA_25255 | 9 | -1.059042622 | KLK3 | kallikrein related peptidase 3 [Source:HGNC Symbol;Acc:HGNC:6364] |
| ENSG00000142751 | HGLibA_19828 | 9 | -1.059042622 | GPN2 | GPN-loop GTPase 2 [Source:HGNC Symbol;Acc:HGNC:25513] |
| ENSG00000143353 | HGLibA_27742 | 9 | -1.059042622 | LYPLAL1 | lysophospholipase like 1 [Source:HGNC Symbol;Acc:HGNC:20440] |
| ENSG00000143498 | HGLibA_48213 | 9 | -1.059042622 | TAF1A | RNA polymerase I subunit A [Source:HGNC Symbol;Acc:HGNC:11532]" |
| ENSG00000144026 | HGLibA_56140 | 9 | -1.059042622 | ZNF514 | zinc finger protein 514 [Source:HGNC Symbol;Acc:HGNC:25894] |
| ENSG00000145623 | HGLibA_34695 | 9 | -1.059042622 | OSMR | oncostatin M receptor [Source:HGNC Symbol;Acc:HGNC:8507] |
| ENSG00000146083 | HGLibA_41689 | 9 | -1.059042622 | RNF44 | ring finger protein 44 [Source:HGNC Symbol;Acc:HGNC:19180] |
| ENSG00000146828 | HGLibA_44380 | 9 | -1.059042622 | SLC12A9 | solute carrier family 12 member 9 [Source:HGNC Symbol;Acc:HGNC:17435] |
| ENSG00000150991 | HGLibA_52567 | 9 | -1.059042622 | UBC | ubiquitin C [Source:HGNC Symbol;Acc:HGNC:12468] |
| ENSG00000151743 | HGLibA_01812 | 9 | -1.059042622 | AMN1 | antagonist of mitotic exit network 1 homolog [Source:HGNC Symbol;Acc:HGNC:27281] |
| ENSG00000151746 | HGLibA_04494 | 9 | -1.059042622 | BICD1 | BICD cargo adaptor 1 [Source:HGNC Symbol;Acc:HGNC:1049] |
| ENSG00000152056 | HGLibA_02302 | 9 | -1.059042622 | AP1S3 | adaptor related protein complex 1 subunit sigma 3 [Source:HGNC Symbol;Acc:HGNC:18971] |
| ENSG00000152253 | HGLibA_46556 | 9 | -1.059042622 | SPC25 | NDC80 kinetochore complex component [Source:HGNC Symbol;Acc:HGNC:24031]" |
| ENSG00000152359 | HGLibA_37451 | 9 | -1.059042622 | POC5 | POC5 centriolar protein [Source:HGNC Symbol;Acc:HGNC:26658] |
| ENSG00000153896 | HGLibA_56360 | 9 | -1.059042622 | ZNF599 | zinc finger protein 599 [Source:HGNC Symbol;Acc:HGNC:26408] |
| ENSG00000154144 | HGLibA_48634 | 9 | -1.059042622 | TBRG1 | transforming growth factor beta regulator 1 [Source:HGNC Symbol;Acc:HGNC:29551] |
| ENSG00000155755 | HGLibA_50277 | 9 | -1.059042622 | TMEM237 | transmembrane protein 237 [Source:HGNC Symbol;Acc:HGNC:14432] |
| ENSG00000156170 | HGLibA_31498 | 9 | -1.059042622 | NDUFAF6 | NADH:ubiquinone oxidoreductase complex assembly factor 6 [Source:HGNC Symbol;Acc:HGNC:28625] |
| ENSG00000156687 | HGLibA_53027 | 9 | -1.059042622 | UNC5D | unc-5 netrin receptor D [Source:HGNC Symbol;Acc:HGNC:18634] |
| ENSG00000157227 | HGLibA_29492 | 9 | -1.059042622 | MMP14 | matrix metallopeptidase 14 [Source:HGNC Symbol;Acc:HGNC:7160] |
| ENSG00000157322 | HGLibA_09942 | 9 | -1.059042622 | CLEC18A | C-type lectin domain family 18 member A [Source:HGNC Symbol;Acc:HGNC:30388] |
| ENSG00000157600 | HGLibA_50032 | 9 | -1.059042622 | TMEM164 | transmembrane protein 164 [Source:HGNC Symbol;Acc:HGNC:26217] |
| ENSG00000158941 | HGLibA_07491 | 9 | -1.059042622 | CCAR2 | cell cycle and apoptosis regulator 2 [Source:HGNC Symbol;Acc:HGNC:23360] |
| ENSG00000159720 | HGLibA_03718 | 9 | -1.059042622 | ATP6V0D1 | ATPase H+ transporting V0 subunit d1 [Source:HGNC Symbol;Acc:HGNC:13724] |
| ENSG00000160255 | HGLibA_23779 | 9 | -1.059042622 | ITGB2 | integrin subunit beta 2 [Source:HGNC Symbol;Acc:HGNC:6155] |
| ENSG00000160799 | HGLibA_07561 | 9 | -1.059042622 | CCDC12 | coiled-coil domain containing 12 [Source:HGNC Symbol;Acc:HGNC:28332] |
| ENSG00000161243 | HGLibA_17057 | 9 | -1.059042622 | FBXO27 | F-box protein 27 [Source:HGNC Symbol;Acc:HGNC:18753] |
| ENSG00000161542 | HGLibA_38764 | 9 | -1.059042622 | PRPSAP1 | phosphoribosyl pyrophosphate synthetase associated protein 1 [Source:HGNC Symbol;Acc:HGNC:9466] |
| ENSG00000162385 | HGLibA_28007 | 9 | -1.059042622 | MAGOH | exon junction complex subunit [Source:HGNC Symbol;Acc:HGNC:6815]" |
| ENSG00000163060 | HGLibA_48981 | 9 | -1.059042622 | TEKT4 | tektin 4 [Source:HGNC Symbol;Acc:HGNC:31012] |

| | | | | | |
|---|---|---|---|---|---|
| ENSG00000163539 | HGLibA_09797 | 9 | -1.059042622 | CLASP2 | cytoplasmic linker associated protein 2 [Source:HGNC Symbol;Acc:HGNC:17078] |
| ENSG00000164077 | HGLibA_29640 | 9 | -1.059042622 | MON1A | secretory trafficking associated [Source:HGNC Symbol;Acc:HGNC:28207]" |
| ENSG00000164600 | HGLibA_31743 | 9 | -1.059042622 | NEUROD6 | neuronal differentiation 6 [Source:HGNC Symbol;Acc:HGNC:13804] |
| ENSG00000164647 | HGLibA_47420 | 9 | -1.059042622 | STEAP1 | STEAP family member 1 [Source:HGNC Symbol;Acc:HGNC:11378] |
| ENSG00000165186 | HGLibA_39273 | 9 | -1.059042622 | PTCHD1 | patched domain containing 1 [Source:HGNC Symbol;Acc:HGNC:26392] |
| ENSG00000165280 | HGLibA_53576 | 9 | -1.059042622 | VCP | valosin containing protein [Source:HGNC Symbol;Acc:HGNC:12666] |
| ENSG00000165355 | HGLibA_17075 | 9 | -1.059042622 | FBXO33 | F-box protein 33 [Source:HGNC Symbol;Acc:HGNC:19833] |
| ENSG00000166974 | HGLibA_28317 | 9 | -1.059042622 | MAPRE2 | microtubule associated protein RP/EB family member 2 [Source:HGNC Symbol;Acc:HGNC:6891] |
| ENSG00000167186 | HGLibA_10763 | 9 | -1.059042622 | COQ7 | hydroxylase [Source:HGNC Symbol;Acc:HGNC:2244]" |
| ENSG00000167550 | HGLibA_41156 | 9 | -1.059042622 | RHEBL1 | RHEB like 1 [Source:HGNC Symbol;Acc:HGNC:21166] |
| ENSG00000167785 | HGLibA_56234 | 9 | -1.059042622 | ZNF558 | zinc finger protein 558 [Source:HGNC Symbol;Acc:HGNC:26422] |
| ENSG00000168014 | HGLibA_06091 | 9 | -1.059042622 | C2CD3 | C2 calcium dependent domain containing 3 [Source:HGNC Symbol;Acc:HGNC:24564] |
| ENSG00000168495 | HGLibA_37617 | 9 | -1.059042622 | POLR3D | RNA polymerase III subunit D [Source:HGNC Symbol;Acc:HGNC:1080] |
| ENSG00000168924 | HGLibA_26333 | 9 | -1.059042622 | LETM1 | leucine zipper and EF-hand containing transmembrane protein 1 [Source:HGNC Symbol;Acc:HGNC:6556] |
| ENSG00000168994 | HGLibA_39645 | 9 | -1.059042622 | PXDC1 | PX domain containing 1 [Source:HGNC Symbol;Acc:HGNC:21361] |
| ENSG00000169989 | HGLibA_49473 | 9 | -1.059042622 | TIGD4 | tigger transposable element derived 4 [Source:HGNC Symbol;Acc:HGNC:18335] |
| ENSG00000170074 | HGLibA_16167 | 9 | -1.059042622 | FAM153A | family with sequence similarity 153 member A [Source:HGNC Symbol;Acc:HGNC:29940] |
| ENSG00000170271 | HGLibA_16930 | 9 | -1.059042622 | FAXDC2 | fatty acid hydroxylase domain containing 2 [Source:HGNC Symbol;Acc:HGNC:1334] |
| ENSG00000170832 | HGLibA_53335 | 9 | -1.059042622 | USP32 | ubiquitin specific peptidase 32 [Source:HGNC Symbol;Acc:HGNC:19143] |
| ENSG00000171044 | HGLibA_54526 | 9 | -1.059042622 | XKR6 | XK related 6 [Source:HGNC Symbol;Acc:HGNC:27806] |
| ENSG00000171885 | HGLibA_02595 | 9 | -1.059042622 | AQP4 | aquaporin 4 [Source:HGNC Symbol;Acc:HGNC:637] |
| ENSG00000172164 | HGLibA_45999 | 9 | -1.059042622 | SNTB1 | syntrophin beta 1 [Source:HGNC Symbol;Acc:HGNC:11168] |
| ENSG00000172772 | HGLibA_33554 | 9 | -1.059042622 | OR10W1 | olfactory receptor family 10 subfamily W member 1 [Source:HGNC Symbol;Acc:HGNC:15139] |
| ENSG00000172818 | HGLibA_34801 | 9 | -1.059042622 | OVOL1 | ovo like transcriptional repressor 1 [Source:HGNC Symbol;Acc:HGNC:8525] |
| ENSG00000173093 | HGLibA_07822 | 9 | -1.059042622 | CCDC63 | coiled-coil domain containing 63 [Source:HGNC Symbol;Acc:HGNC:26669] |
| ENSG00000173692 | HGLibA_39147 | 9 | -1.059042622 | PSMD1 | non-ATPase 1 [Source:HGNC Symbol;Acc:HGNC:9554]" |
| ENSG00000173848 | HGLibA_31702 | 9 | -1.059042622 | NET1 | neuroepithelial cell transforming 1 [Source:HGNC Symbol;Acc:HGNC:14592] |
| ENSG00000174607 | HGLibA_52938 | 9 | -1.059042622 | UGT8 | UDP glycosyltransferase 8 [Source:HGNC Symbol;Acc:HGNC:12555] |
| ENSG00000174640 | HGLibA_45459 | 9 | -1.059042622 | SLCO2A1 | solute carrier organic anion transporter family member 2A1 [Source:HGNC Symbol;Acc:HGNC:10955] |
| ENSG00000175602 | HGLibA_07895 | 9 | -1.059042622 | CCDC85B | coiled-coil domain containing 85B [Source:HGNC Symbol;Acc:HGNC:24926] |
| ENSG00000176435 | HGLibA_09934 | 9 | -1.059042622 | CLEC14A | C-type lectin domain containing 14A [Source:HGNC Symbol;Acc:HGNC:19832] |
| ENSG00000176563 | HGLibA_10385 | 9 | -1.059042622 | CNTD1 | cyclin N-terminal domain containing 1 [Source:HGNC Symbol;Acc:HGNC:26847] |
| ENSG00000178966 | HGLibA_41374 | 9 | -1.059042622 | RMI1 | RecQ mediated genome instability 1 [Source:HGNC Symbol;Acc:HGNC:25764] |
| ENSG00000179913 | HGLibA_03987 | 9 | -1.059042622 | B3GNT3 | 3-N-acetylglucosaminyltransferase 3 [Source:HGNC Symbol;Acc:HGNC:13528]" |
| ENSG00000180138 | HGLibA_11385 | 9 | -1.059042622 | CSNK1A1L | casein kinase 1 alpha 1 like [Source:HGNC Symbol;Acc:HGNC:20289] |
| ENSG00000180269 | HGLibA_19907 | 9 | -1.059042622 | GPR139 | G protein-coupled receptor 139 [Source:HGNC Symbol;Acc:HGNC:19995] |
| ENSG00000180616 | HGLibA_47176 | 9 | -1.059042622 | SSTR2 | somatostatin receptor 2 [Source:HGNC Symbol;Acc:HGNC:11331] |
| ENSG00000181072 | HGLibA_09550 | 9 | -1.059042622 | CHRM2 | cholinergic receptor muscarinic 2 [Source:HGNC Symbol;Acc:HGNC:1951] |
| ENSG00000181371 | HGLibA_34341 | 9 | -1.059042622 | OR5M8 | olfactory receptor family 5 subfamily M member 8 [Source:HGNC Symbol;Acc:HGNC:14846] |
| ENSG00000181458 | HGLibA_50408 | 9 | -1.059042622 | TMEM45A | transmembrane protein 45A [Source:HGNC Symbol;Acc:HGNC:25480] |
| ENSG00000182187 | HGLibA_11272 | 9 | -1.059042622 | CRYGB | crystallin gamma B [Source:HGNC Symbol;Acc:HGNC:2409] |
| ENSG00000182253 | HGLibA_48014 | 9 | -1.059042622 | SYNM | synemin [Source:HGNC Symbol;Acc:HGNC:24466] |
| ENSG00000183137 | HGLibA_09148 | 9 | -1.059042622 | CEP57L1 | centrosomal protein 57 like 1 [Source:HGNC Symbol;Acc:HGNC:21561] |
| ENSG00000183347 | HGLibA_18742 | 9 | -1.059042622 | GBP6 | guanylate binding protein family member 6 [Source:HGNC Symbol;Acc:HGNC:25395] |
| ENSG00000184029 | HGLibA_14011 | 9 | -1.059042622 | DSCR4 | Down syndrome critical region 4 [Source:HGNC Symbol;Acc:HGNC:3045] |
| ENSG00000184465 | HGLibA_54057 | 9 | -1.059042622 | WDR27 | WD repeat domain 27 [Source:HGNC Symbol;Acc:HGNC:21248] |
| ENSG00000184956 | HGLibA_30552 | 9 | -1.059042622 | MUC6 | oligomeric mucus/gel-forming [Source:HGNC Symbol;Acc:HGNC:7517]" |
| ENSG00000186115 | HGLibA_12188 | 9 | -1.059042622 | CYP4F2 | cytochrome P450 family 4 subfamily F member 2 [Source:HGNC Symbol;Acc:HGNC:2645] |
| ENSG00000186265 | HGLibA_04955 | 9 | -1.059042622 | BTLA | B and T lymphocyte associated [Source:HGNC Symbol;Acc:HGNC:21087] |
| ENSG00000186496 | HGLibA_55914 | 9 | -1.059042622 | ZNF396 | zinc finger protein 396 [Source:HGNC Symbol;Acc:HGNC:18824] |
| ENSG00000186566 | HGLibA_19749 | 9 | -1.059042622 | GPATCH8 | G-patch domain containing 8 [Source:HGNC Symbol;Acc:HGNC:29066] |
| ENSG00000187815 | HGLibA_55230 | 9 | -1.059042622 | ZFP69 | ZFP69 zinc finger protein [Source:HGNC Symbol;Acc:HGNC:24708] |
| ENSG00000188694 | HGLibA_25725 | 9 | -1.059042622 | KRTAP24-1 | keratin associated protein 24-1 [Source:HGNC Symbol;Acc:HGNC:33902] |
| ENSG00000189052 | HGLibA_09307 | 9 | -1.059042622 | CGB5 | chorionic gonadotropin subunit beta 5 [Source:HGNC Symbol;Acc:HGNC:16452] |
| ENSG00000197208 | HGLibA_44588 | 9 | -1.059042622 | SLC22A4 | solute carrier family 22 member 4 [Source:HGNC Symbol;Acc:HGNC:10968] |
| ENSG00000197608 | HGLibA_56854 | 9 | -1.059042622 | ZNF841 | zinc finger protein 841 [Source:HGNC Symbol;Acc:HGNC:27611] |
| ENSG00000197712 | HGLibA_16031 | 9 | -1.059042622 | FAM114A1 | family with sequence similarity 114 member A1 [Source:HGNC Symbol;Acc:HGNC:25087] |
| ENSG00000197753 | HGLibA_26440 | 9 | -1.059042622 | LHFPL5 | LHFPL tetraspan subfamily member 5 [Source:HGNC Symbol;Acc:HGNC:21253] |
| ENSG00000198171 | HGLibA_12638 | 9 | -1.059042622 | DDRGK1 | DDRGK domain containing 1 [Source:HGNC Symbol;Acc:HGNC:16110] |
| ENSG00000198918 | HGLibA_41973 | 9 | -1.059042622 | RPL39 | ribosomal protein L39 [Source:HGNC Symbol;Acc:HGNC:10350] |
| ENSG00000203985 | HGLibA_26240 | 9 | -1.059042622 | LDLRAD1 | low density lipoprotein receptor class A domain containing 1 [Source:HGNC Symbol;Acc:HGNC:32069] |
| ENSG00000204540 | HGLibA_39216 | 9 | -1.059042622 | PSORS1C1 | psoriasis susceptibility 1 candidate 1 [Source:HGNC Symbol;Acc:HGNC:17202] |
| ENSG00000205330 | HGLibA_34383 | 9 | -1.059042622 | OR6C1 | olfactory receptor family 6 subfamily C member 1 [Source:HGNC Symbol;Acc:HGNC:8355] |
| ENSG00000213047 | HGLibA_12931 | 9 | -1.059042622 | DENND1B | DENN domain containing 1B [Source:HGNC Symbol;Acc:HGNC:28404] |
| ENSG00000213654 | HGLibA_20162 | 9 | -1.059042622 | GPSM3 | G protein signaling modulator 3 [Source:HGNC Symbol;Acc:HGNC:13945] |
| ENSG00000214518 | HGLibA_25691 | 9 | -1.059042622 | KRTAP2-2 | keratin associated protein 2-2 [Source:HGNC Symbol;Acc:HGNC:18905] |
| ENSG00000221916 | HGLibA_05705 | 9 | -1.059042622 | C19orf73 | chromosome 19 open reading frame 73 [Source:HGNC Symbol;Acc:HGNC:25534] |
| ENSG00000239672 | HGLibA_32151 | 9 | -1.059042622 | NME1 | NME/NM23 nucleoside diphosphate kinase 1 [Source:HGNC Symbol;Acc:HGNC:7849] |
| ENSG00000241258 | HGLibA_11081 | 9 | -1.059042622 | CRCP | CGRP receptor component [Source:HGNC Symbol;Acc:HGNC:17888] |
| ENSG00000244038 | HGLibA_12631 | 9 | -1.059042622 | DDOST | dolichyl-diphosphooligosaccharide--protein glycosyltransferase non-catalytic subunit [Source:HGNC Symbol;Acc:HGNC:2728] |
| ENSG00000249437 | HGLibA_31080 | 9 | -1.059042622 | NAIP | NLR family apoptosis inhibitory protein [Source:HGNC Symbol;Acc:HGNC:7634] |
| ENSG00000253910 | HGLibA_35552 | 9 | -1.059042622 | PCDHGB2 | 2 [Source:HGNC Symbol;Acc:HGNC:8709]" |
| ENSG00000270231 | HGLibA_31243 | 9 | -1.059042622 | NBPF8 | NBPF member 8 [Source:HGNC Symbol;Acc:HGNC:31990] |
| ENSG00000273976 | HGLibA_19620 | 9 | -1.059042622 | GOLGA6L1 | golgin A6 family-like 1 [Source:HGNC Symbol;Acc:HGNC:37444] |
| ENSG00000279782 | HGLibA_37913 | 9 | -1.059042622 | PPIAL4F | peptidylprolyl isomerase A like 4F [Source:HGNC Symbol;Acc:HGNC:33999] |
| FAM134B | HGLibA_16125 | 9 | -1.059042622 | FAM134B | - |
| GAGE2B | HGLibA_18471 | 9 | -1.059042622 | GAGE2B | - |
| hsa-mir-10b | HGLibA_57200 | 9 | -1.059042622 | hsa-mir-10b | - |
| hsa-mir-1250 | HGLibA_57397 | 9 | -1.059042622 | hsa-mir-1250 | - |
| hsa-mir-1307 | HGLibA_57743 | 9 | -1.059042622 | hsa-mir-1307 | - |
| hsa-mir-146b | HGLibA_57861 | 9 | -1.059042622 | hsa-mir-146b | - |
| hsa-mir-1973 | HGLibA_58118 | 9 | -1.059042622 | hsa-mir-1973 | - |
| hsa-mir-29b-2 | HGLibA_58419 | 9 | -1.059042622 | hsa-mir-29b-2 | - |
| hsa-mir-3121 | HGLibA_58535 | 9 | -1.059042622 | hsa-mir-3121 | - |
| hsa-mir-3621 | HGLibA_59116 | 9 | -1.059042622 | hsa-mir-3621 | - |
| hsa-mir-3685 | HGLibA_59295 | 9 | -1.059042622 | hsa-mir-3685 | - |
| hsa-mir-411 | HGLibA_59682 | 9 | -1.059042622 | hsa-mir-411 | - |
| hsa-mir-4255 | HGLibA_59727 | 9 | -1.059042622 | hsa-mir-4255 | - |
| hsa-mir-4437 | HGLibA_60149 | 9 | -1.059042622 | hsa-mir-4437 | - |
| hsa-mir-4477b | HGLibA_60321 | 9 | -1.059042622 | hsa-mir-4477b | - |
| hsa-mir-449c | HGLibA_60424 | 9 | -1.059042622 | hsa-mir-449c | - |
| hsa-mir-4508 | HGLibA_60459 | 9 | -1.059042622 | hsa-mir-4508 | - |
| hsa-mir-4538 | HGLibA_60614 | 9 | -1.059042622 | hsa-mir-4538 | - |
| hsa-mir-4708 | HGLibA_60934 | 9 | -1.059042622 | hsa-mir-4708 | - |
| hsa-mir-4755 | HGLibA_61117 | 9 | -1.059042622 | hsa-mir-4755 | - |
| hsa-mir-4756 | HGLibA_61121 | 9 | -1.059042622 | hsa-mir-4756 | - |
| hsa-mir-486-2 | HGLibA_61340 | 9 | -1.059042622 | hsa-mir-486-2 | - |
| hsa-mir-517a | HGLibA_61624 | 9 | -1.059042622 | hsa-mir-517a | - |
| hsa-mir-548aa-2 | HGLibA_61854 | 9 | -1.059042622 | hsa-mir-548aa-2 | - |
| hsa-mir-550b-2 | HGLibA_62114 | 9 | -1.059042622 | hsa-mir-550b-2 | - |
| hsa-mir-5584 | HGLibA_62179 | 9 | -1.059042622 | hsa-mir-5584 | - |
| hsa-mir-5695 | HGLibA_62313 | 9 | -1.059042622 | hsa-mir-5695 | - |
| hsa-mir-5700 | HGLibA_62338 | 9 | -1.059042622 | hsa-mir-5700 | - |
| hsa-mir-579 | HGLibA_62416 | 9 | -1.059042622 | hsa-mir-579 | - |
| hsa-mir-6846 | HGLibA_63558 | 9 | -1.059042622 | hsa-mir-6846 | - |
| hsa-mir-8079 | HGLibA_64157 | 9 | -1.059042622 | hsa-mir-8079 | - |
| hsa-mir-8079 | HGLibA_64158 | 9 | -1.059042622 | hsa-mir-8079 | - |
| LRRC16B | HGLibA_27265 | 9 | -1.059042622 | LRRC16B | - |
| MLLT4 | HGLibA_29419 | 9 | -1.059042622 | MLLT4 | - |
| PROSC | HGLibA_38692 | 9 | -1.059042622 | PROSC | - |
| SHFM1 | HGLibA_44028 | 9 | -1.059042622 | SHFM1 | - |
| TCTEX1D4 | HGLibA_48870 | 9 | -1.059042622 | TCTEX1D4 | - |
| ZNRD1 | HGLibA_56934 | 9 | -1.059042622 | ZNRD1 | - |
| C10orf129 | HGLibA_05052 | 8 | -1.068993659 | C10orf129 | - |
| C20orf201 | HGLibA_06012 | 8 | -1.068993659 | C20orf201 | - |
| C6orf70 | HGLibA_06510 | 8 | -1.068993659 | C6orf70 | - |
| CCBL1 | HGLibA_07497 | 8 | -1.068993659 | CCBL1 | - |
| CCDC104 | HGLibA_07516 | 8 | -1.068993659 | CCDC104 | - |
| ENSG00000008128 | HGLibA_08724 | 8 | -1.068993659 | CDK11A | cyclin dependent kinase 11A [Source:HGNC Symbol;Acc:HGNC:1730] |
| ENSG00000011243 | HGLibA_01447 | 8 | -1.068993659 | AKAP8L | A-kinase anchoring protein 8 like [Source:HGNC Symbol;Acc:HGNC:29857] |
| ENSG00000012660 | HGLibA_14969 | 8 | -1.068993659 | ELOVL5 | ELOVL fatty acid elongase 5 [Source:HGNC Symbol;Acc:HGNC:21308] |
| ENSG00000034533 | HGLibA_03375 | 8 | -1.068993659 | ASTE1 | asteroid homolog 1 [Source:HGNC Symbol;Acc:HGNC:25021] |
| ENSG00000052749 | HGLibA_42269 | 8 | -1.068993659 | RRP12 | ribosomal RNA processing 12 homolog [Source:HGNC Symbol;Acc:HGNC:29100] |
| ENSG00000064692 | HGLibA_45910 | 8 | -1.068993659 | SNCAIP | synuclein alpha interacting protein [Source:HGNC Symbol;Acc:HGNC:11139] |
| ENSG00000066651 | HGLibA_51670 | 8 | -1.068993659 | TRMT11 | tRNA methyltransferase 11 homolog [Source:HGNC Symbol;Acc:HGNC:21080] |
| ENSG00000073614 | HGLibA_24520 | 8 | -1.068993659 | KDM5A | lysine demethylase 5A [Source:HGNC Symbol;Acc:HGNC:9886] |
| ENSG00000074855 | HGLibA_02191 | 8 | -1.068993659 | ANO8 | anoctamin 8 [Source:HGNC Symbol;Acc:HGNC:29329] |
| ENSG00000075388 | HGLibA_17407 | 8 | -1.068993659 | FGF4 | fibroblast growth factor 4 [Source:HGNC Symbol;Acc:HGNC:3682] |
| ENSG00000081041 | HGLibA_11865 | 8 | -1.068993659 | CXCL2 | C-X-C motif chemokine ligand 2 [Source:HGNC Symbol;Acc:HGNC:4603] |
| ENSG00000081059 | HGLibA_48790 | 8 | -1.068993659 | TCF7 | transcription factor 7 [Source:HGNC Symbol;Acc:HGNC:11639] |
| ENSG00000081692 | HGLibA_23955 | 8 | -1.068993659 | JMJD4 | jumonji domain containing 4 [Source:HGNC Symbol;Acc:HGNC:25724] |
| ENSG00000084652 | HGLibA_52390 | 8 | -1.068993659 | TXLNA | taxilin alpha [Source:HGNC Symbol;Acc:HGNC:30685] |
| ENSG00000087087 | HGLibA_47058 | 8 | -1.068993659 | SRRT | RNA effector molecule [Source:HGNC Symbol;Acc:HGNC:24101]" |
| ENSG00000088298 | HGLibA_14458 | 8 | -1.068993659 | EDEM2 | ER degradation enhancing alpha-mannosidase like protein 2 [Source:HGNC Symbol;Acc:HGNC:15877] |
| ENSG00000092020 | HGLibA_38163 | 8 | -1.068993659 | PPP2R3C | protein phosphatase 2 regulatory subunit B''gamma [Source:HGNC Symbol;Acc:HGNC:17485] |

| | | | | | |
|---|---|---|---|---|---|
| ENSG00000092330 | HGLibA_49552 | 8 | -1.068993659 | TINF2 | TERF1 interacting nuclear factor 2 [Source:HGNC Symbol;Acc:HGNC:11824] |
| ENSG00000093000 | HGLibA_33058 | 8 | -1.068993659 | NUP50 | nucleoporin 50 [Source:HGNC Symbol;Acc:HGNC:8065] |
| ENSG00000100156 | HGLibA_44452 | 8 | -1.068993659 | SLC16A8 | solute carrier family 16 member 8 [Source:HGNC Symbol;Acc:HGNC:16270] |
| ENSG00000100216 | HGLibA_50961 | 8 | -1.068993659 | TOMM22 | translocase of outer mitochondrial membrane 22 [Source:HGNC Symbol;Acc:HGNC:18002] |
| ENSG00000100234 | HGLibA_49542 | 8 | -1.068993659 | TIMP3 | TIMP metallopeptidase inhibitor 3 [Source:HGNC Symbol;Acc:HGNC:11822] |
| ENSG00000100401 | HGLibA_40215 | 8 | -1.068993659 | RANGAP1 | Ran GTPase activating protein 1 [Source:HGNC Symbol;Acc:HGNC:9854] |
| ENSG00000100665 | HGLibA_43519 | 8 | -1.068993659 | SERPINA4 | serpin family A member 4 [Source:HGNC Symbol;Acc:HGNC:8948] |
| ENSG00000100916 | HGLibA_04834 | 8 | -1.068993659 | BRMS1L | BRMS1 like transcriptional repressor [Source:HGNC Symbol;Acc:HGNC:20512] |
| ENSG00000101361 | HGLibA_32312 | 8 | -1.068993659 | NOP56 | NOP56 ribonucleoprotein [Source:HGNC Symbol;Acc:HGNC:15911] |
| ENSG00000101460 | HGLibA_28121 | 8 | -1.068993659 | MAP1LC3A | microtubule associated protein 1 light chain 3 alpha [Source:HGNC Symbol;Acc:HGNC:6838] |
| ENSG00000101871 | HGLibA_29231 | 8 | -1.068993659 | MID1 | midline 1 [Source:HGNC Symbol;Acc:HGNC:7095] |
| ENSG00000101981 | HGLibA_15895 | 8 | -1.068993659 | F9 | coagulation factor IX [Source:HGNC Symbol;Acc:HGNC:3551] |
| ENSG00000103266 | HGLibA_47612 | 8 | -1.068993659 | STUB1 | STIP1 homology and U-box containing protein 1 [Source:HGNC Symbol;Acc:HGNC:11427] |
| ENSG00000103496 | HGLibA_47647 | 8 | -1.068993659 | STX4 | syntaxin 4 [Source:HGNC Symbol;Acc:HGNC:11439] |
| ENSG00000103855 | HGLibA_08320 | 8 | -1.068993659 | CD276 | CD276 molecule [Source:HGNC Symbol;Acc:HGNC:19137] |
| ENSG00000104848 | HGLibA_24145 | 8 | -1.068993659 | KCNA7 | potassium voltage-gated channel subfamily A member 7 [Source:HGNC Symbol;Acc:HGNC:6226] |
| ENSG00000105127 | HGLibA_01445 | 8 | -1.068993659 | AKAP8 | A-kinase anchoring protein 8 [Source:HGNC Symbol;Acc:HGNC:378] |
| ENSG00000105866 | HGLibA_46303 | 8 | -1.068993659 | SP4 | Sp4 transcription factor [Source:HGNC Symbol;Acc:HGNC:11209] |
| ENSG00000106609 | HGLibA_50309 | 8 | -1.068993659 | TMEM248 | transmembrane protein 248 [Source:HGNC Symbol;Acc:HGNC:25476] |
| ENSG00000108091 | HGLibA_07810 | 8 | -1.068993659 | CCDC6 | coiled-coil domain containing 6 [Source:HGNC Symbol;Acc:HGNC:18782] |
| ENSG00000108370 | HGLibA_41112 | 8 | -1.068993659 | RGS9 | regulator of G protein signaling 9 [Source:HGNC Symbol;Acc:HGNC:10004] |
| ENSG00000109929 | HGLibA_42817 | 8 | -1.068993659 | SC5D | sterol-C5-desaturase [Source:HGNC Symbol;Acc:HGNC:10547] |
| ENSG00000110696 | HGLibA_05164 | 8 | -1.068993659 | C11orf58 | chromosome 11 open reading frame 58 [Source:HGNC Symbol;Acc:HGNC:16990] |
| ENSG00000110876 | HGLibA_43306 | 8 | -1.068993659 | SELPLG | selectin P ligand [Source:HGNC Symbol;Acc:HGNC:10722] |
| ENSG00000110934 | HGLibA_04509 | 8 | -1.068993659 | BIN2 | bridging integrator 2 [Source:HGNC Symbol;Acc:HGNC:1053] |
| ENSG00000111218 | HGLibA_38639 | 8 | -1.068993659 | PRMT8 | protein arginine methyltransferase 8 [Source:HGNC Symbol;Acc:HGNC:5188] |
| ENSG00000112619 | HGLibA_38753 | 8 | -1.068993659 | PRPH2 | peripherin 2 [Source:HGNC Symbol;Acc:HGNC:9942] |
| ENSG00000112992 | HGLibA_32227 | 8 | -1.068993659 | NNT | nicotinamide nucleotide transhydrogenase [Source:HGNC Symbol;Acc:HGNC:7863] |
| ENSG00000113583 | HGLibA_06360 | 8 | -1.068993659 | C5orf15 | chromosome 5 open reading frame 15 [Source:HGNC Symbol;Acc:HGNC:20656] |
| ENSG00000114251 | HGLibA_54373 | 8 | -1.068993659 | WNT5A | Wnt family member 5A [Source:HGNC Symbol;Acc:HGNC:12784] |
| ENSG00000115750 | HGLibA_48217 | 8 | -1.068993659 | TAF1B | RNA polymerase I subunit B [Source:HGNC Symbol;Acc:HGNC:11533]" |
| ENSG00000116830 | HGLibA_52172 | 8 | -1.068993659 | TTF2 | transcription termination factor 2 [Source:HGNC Symbol;Acc:HGNC:12398] |
| ENSG00000117906 | HGLibA_40703 | 8 | -1.068993659 | RCN2 | reticulocalbin 2 [Source:HGNC Symbol;Acc:HGNC:9935] |
| ENSG00000118058 | HGLibA_25322 | 8 | -1.068993659 | KMT2A | lysine methyltransferase 2A [Source:HGNC Symbol;Acc:HGNC:7132] |
| ENSG00000120094 | HGLibA_21926 | 8 | -1.068993659 | HOXB1 | homeobox B1 [Source:HGNC Symbol;Acc:HGNC:5111] |
| ENSG00000120160 | HGLibA_15397 | 8 | -1.068993659 | EQTN | equatorin [Source:HGNC Symbol;Acc:HGNC:1359] |
| ENSG00000120327 | HGLibA_35483 | 8 | -1.068993659 | PCDHB14 | protocadherin beta 14 [Source:HGNC Symbol;Acc:HGNC:8685] |
| ENSG00000122126 | HGLibA_33262 | 8 | -1.068993659 | OCRL | inositol polyphosphate-5-phosphatase [Source:HGNC Symbol;Acc:HGNC:8108]" |
| ENSG00000123500 | HGLibA_10496 | 8 | -1.068993659 | COL10A1 | collagen type X alpha 1 chain [Source:HGNC Symbol;Acc:HGNC:2185] |
| ENSG00000125823 | HGLibA_11495 | 8 | -1.068993659 | CSTL1 | cystatin like 1 [Source:HGNC Symbol;Acc:HGNC:15958] |
| ENSG00000126088 | HGLibA_53154 | 8 | -1.068993659 | UROD | uroporphyrinogen decarboxylase [Source:HGNC Symbol;Acc:HGNC:12591] |
| ENSG00000126953 | HGLibA_49524 | 8 | -1.068993659 | TIMM8A | translocase of inner mitochondrial membrane 8A [Source:HGNC Symbol;Acc:HGNC:11817] |
| ENSG00000129194 | HGLibA_46242 | 8 | -1.068993659 | SOX15 | SRY-box 15 [Source:HGNC Symbol;Acc:HGNC:11196] |
| ENSG00000130024 | HGLibA_36334 | 8 | -1.068993659 | PHF10 | PHD finger protein 10 [Source:HGNC Symbol;Acc:HGNC:18250] |
| ENSG00000130304 | HGLibA_44813 | 8 | -1.068993659 | SLC27A1 | solute carrier family 27 member 1 [Source:HGNC Symbol;Acc:HGNC:10995] |
| ENSG00000130368 | HGLibA_28388 | 8 | -1.068993659 | MAS1 | G protein-coupled receptor [Source:HGNC Symbol;Acc:HGNC:6899]" |
| ENSG00000130956 | HGLibA_20796 | 8 | -1.068993659 | HABP4 | hyaluronan binding protein 4 [Source:HGNC Symbol;Acc:HGNC:17062] |
| ENSG00000132199 | HGLibA_15156 | 8 | -1.068993659 | ENOSF1 | enolase superfamily member 1 [Source:HGNC Symbol;Acc:HGNC:30365] |
| ENSG00000132670 | HGLibA_39485 | 8 | -1.068993659 | PTPRA | receptor type A [Source:HGNC Symbol;Acc:HGNC:9664]" |
| ENSG00000132854 | HGLibA_24033 | 8 | -1.068993659 | KANK4 | KN motif and ankyrin repeat domains 4 [Source:HGNC Symbol;Acc:HGNC:27263] |
| ENSG00000133422 | HGLibA_29651 | 8 | -1.068993659 | MORC2 | MORC family CW-type zinc finger 2 [Source:HGNC Symbol;Acc:HGNC:23573] |
| ENSG00000134817 | HGLibA_02438 | 8 | -1.068993659 | APLNR | apelin receptor [Source:HGNC Symbol;Acc:HGNC:339] |
| ENSG00000134982 | HGLibA_02389 | 8 | -1.068993659 | APC | WNT signaling pathway regulator [Source:HGNC Symbol;Acc:HGNC:583]" |
| ENSG00000138385 | HGLibA_47112 | 8 | -1.068993659 | SSB | Sjogren syndrome antigen B [Source:HGNC Symbol;Acc:HGNC:11316] |
| ENSG00000139505 | HGLibA_30434 | 8 | -1.068993659 | MTMR6 | myotubularin related protein 6 [Source:HGNC Symbol;Acc:HGNC:7453] |
| ENSG00000139631 | HGLibA_11303 | 8 | -1.068993659 | CSAD | cysteine sulfinic acid decarboxylase [Source:HGNC Symbol;Acc:HGNC:18966] |
| ENSG00000142207 | HGLibA_53134 | 8 | -1.068993659 | URB1 | URB1 ribosome biogenesis homolog [Source:HGNC Symbol;Acc:HGNC:17344] |
| ENSG00000143494 | HGLibA_53534 | 8 | -1.068993659 | VASH2 | vasohibin 2 [Source:HGNC Symbol;Acc:HGNC:25723] |
| ENSG00000143569 | HGLibA_52556 | 8 | -1.068993659 | UBAP2L | ubiquitin associated protein 2 like [Source:HGNC Symbol;Acc:HGNC:29877] |
| ENSG00000143578 | HGLibA_11102 | 8 | -1.068993659 | CREB3L4 | cAMP responsive element binding protein 3 like 4 [Source:HGNC Symbol;Acc:HGNC:18854] |
| ENSG00000145832 | HGLibA_44760 | 8 | -1.068993659 | SLC25A48 | solute carrier family 25 member 48 [Source:HGNC Symbol;Acc:HGNC:30451] |
| ENSG00000146918 | HGLibA_31271 | 8 | -1.068993659 | NCAPG2 | non-SMC condensin II complex subunit G2 [Source:HGNC Symbol;Acc:HGNC:21904] |
| ENSG00000147647 | HGLibA_13923 | 8 | -1.068993659 | DPYS | dihydropyrimidinase [Source:HGNC Symbol;Acc:HGNC:3013] |
| ENSG00000147799 | HGLibA_02772 | 8 | -1.068993659 | ARHGAP39 | Rho GTPase activating protein 39 [Source:HGNC Symbol;Acc:HGNC:29351] |
| ENSG00000148019 | HGLibA_09166 | 8 | -1.068993659 | CEP78 | centrosomal protein 78 [Source:HGNC Symbol;Acc:HGNC:25740] |
| ENSG00000148053 | HGLibA_32876 | 8 | -1.068993659 | NTRK2 | neurotrophic receptor tyrosine kinase 2 [Source:HGNC Symbol;Acc:HGNC:8032] |
| ENSG00000148344 | HGLibA_39326 | 8 | -1.068993659 | PTGES | prostaglandin E synthase [Source:HGNC Symbol;Acc:HGNC:9599] |
| ENSG00000148848 | HGLibA_00735 | 8 | -1.068993659 | ADAM12 | ADAM metallopeptidase domain 12 [Source:HGNC Symbol;Acc:HGNC:190] |
| ENSG00000149735 | HGLibA_19799 | 8 | -1.068993659 | GPHA2 | glycoprotein hormone alpha 2 [Source:HGNC Symbol;Acc:HGNC:18054] |
| ENSG00000151233 | HGLibA_20682 | 8 | -1.068993659 | GXYLT1 | glucoside xylosyltransferase 1 [Source:HGNC Symbol;Acc:HGNC:27482] |
| ENSG00000153391 | HGLibA_23324 | 8 | -1.068993659 | INO80C | INO80 complex subunit C [Source:HGNC Symbol;Acc:HGNC:26994] |
| ENSG00000153495 | HGLibA_49092 | 8 | -1.068993659 | TEX29 | testis expressed 29 [Source:HGNC Symbol;Acc:HGNC:20370] |
| ENSG00000153531 | HGLibA_01027 | 8 | -1.068993659 | ADPRHL1 | ADP-ribosylhydrolase like 1 [Source:HGNC Symbol;Acc:HGNC:21303] |
| ENSG00000154144 | HGLibA_48636 | 8 | -1.068993659 | TBRG1 | transforming growth factor beta regulator 1 [Source:HGNC Symbol;Acc:HGNC:29551] |
| ENSG00000154814 | HGLibA_34828 | 8 | -1.068993659 | OXNAD1 | oxidoreductase NAD binding domain containing 1 [Source:HGNC Symbol;Acc:HGNC:25128] |
| ENSG00000155903 | HGLibA_40299 | 8 | -1.068993659 | RASA2 | RAS p21 protein activator 2 [Source:HGNC Symbol;Acc:HGNC:9872] |
| ENSG00000157869 | HGLibA_39861 | 8 | -1.068993659 | RAB28 | member RAS oncogene family [Source:HGNC Symbol;Acc:HGNC:9768]" |
| ENSG00000158006 | HGLibA_34997 | 8 | -1.068993659 | PAFAH2 | platelet activating factor acetylhydrolase 2 [Source:HGNC Symbol;Acc:HGNC:8579] |
| ENSG00000158008 | HGLibA_15808 | 8 | -1.068993659 | EXTL1 | exostosin like glycosyltransferase 1 [Source:HGNC Symbol;Acc:HGNC:3515] |
| ENSG00000159055 | HGLibA_29306 | 8 | -1.068993659 | MIS18A | MIS18 kinetochore protein A [Source:HGNC Symbol;Acc:HGNC:1286] |
| ENSG00000159166 | HGLibA_25922 | 8 | -1.068993659 | LAD1 | ladinin 1 [Source:HGNC Symbol;Acc:HGNC:6472] |
| ENSG00000159197 | HGLibA_24188 | 8 | -1.068993659 | KCNE2 | potassium voltage-gated channel subfamily E regulatory subunit 2 [Source:HGNC Symbol;Acc:HGNC:6242] |
| ENSG00000160948 | HGLibA_53759 | 8 | -1.068993659 | VPS28 | ESCRT-I subunit [Source:HGNC Symbol;Acc:HGNC:18178]" |
| ENSG00000162073 | HGLibA_35159 | 8 | -1.068993659 | PAQR4 | progestin and adipoQ receptor family member 4 [Source:HGNC Symbol;Acc:HGNC:26386] |
| ENSG00000162631 | HGLibA_32866 | 8 | -1.068993659 | NTNG1 | netrin G1 [Source:HGNC Symbol;Acc:HGNC:23319] |
| ENSG00000163285 | HGLibA_18390 | 8 | -1.068993659 | GABRG1 | gamma-aminobutyric acid type A receptor gamma1 subunit [Source:HGNC Symbol;Acc:HGNC:4086] |
| ENSG00000163395 | HGLibA_22853 | 8 | -1.068993659 | IGFN1 | immunoglobulin-like and fibronectin type III domain containing 1 [Source:HGNC Symbol;Acc:HGNC:24607] |
| ENSG00000163602 | HGLibA_42516 | 8 | -1.068993659 | RYBP | RING1 and YY1 binding protein [Source:HGNC Symbol;Acc:HGNC:10480] |
| ENSG00000163626 | HGLibA_10820 | 8 | -1.068993659 | COX18 | cytochrome c oxidase assembly factor COX18 [Source:HGNC Symbol;Acc:HGNC:26801] |
| ENSG00000163754 | HGLibA_20687 | 8 | -1.068993659 | GYG1 | glycogenin 1 [Source:HGNC Symbol;Acc:HGNC:4699] |
| ENSG00000164163 | HGLibA_00203 | 8 | -1.068993659 | ABCE1 | ATP binding cassette subfamily E member 1 [Source:HGNC Symbol;Acc:HGNC:69] |
| ENSG00000164265 | HGLibA_42934 | 8 | -1.068993659 | SCGB3A2 | secretoglobin family 3A member 2 [Source:HGNC Symbol;Acc:HGNC:18391] |
| ENSG00000164729 | HGLibA_45023 | 8 | -1.068993659 | SLC35G3 | solute carrier family 35 member G3 [Source:HGNC Symbol;Acc:HGNC:26848] |
| ENSG00000164880 | HGLibA_23405 | 8 | -1.068993659 | INTS1 | integrator complex subunit 1 [Source:HGNC Symbol;Acc:HGNC:24555] |
| ENSG00000165030 | HGLibA_31809 | 8 | -1.068993659 | NFIL3 | interleukin 3 regulated [Source:HGNC Symbol;Acc:HGNC:7787]" |
| ENSG00000165119 | HGLibA_21831 | 8 | -1.068993659 | HNRNPK | heterogeneous nuclear ribonucleoprotein K [Source:HGNC Symbol;Acc:HGNC:5044] |
| ENSG00000165272 | HGLibA_02593 | 8 | -1.068993659 | AQP3 | aquaporin 3 (Gill blood group) [Source:HGNC Symbol;Acc:HGNC:636] |
| ENSG00000165495 | HGLibA_36850 | 8 | -1.068993659 | PKNOX2 | PBX/knotted 1 homeobox 2 [Source:HGNC Symbol;Acc:HGNC:16714] |
| ENSG00000165923 | HGLibA_01168 | 8 | -1.068993659 | AGBL2 | ATP/GTP binding protein like 2 [Source:HGNC Symbol;Acc:HGNC:26296] |
| ENSG00000165934 | HGLibA_10997 | 8 | -1.068993659 | CPSF2 | cleavage and polyadenylation specific factor 2 [Source:HGNC Symbol;Acc:HGNC:2325] |
| ENSG00000166435 | HGLibA_54610 | 8 | -1.068993659 | XRRA1 | X-ray radiation resistance associated 1 [Source:HGNC Symbol;Acc:HGNC:18868] |
| ENSG00000166483 | HGLibA_54238 | 8 | -1.068993659 | WEE1 | WEE1 G2 checkpoint kinase [Source:HGNC Symbol;Acc:HGNC:12761] |
| ENSG00000166819 | HGLibA_37171 | 8 | -1.068993659 | PLIN1 | perilipin 1 [Source:HGNC Symbol;Acc:HGNC:9076] |
| ENSG00000166884 | HGLibA_33979 | 8 | -1.068993659 | OR4D6 | olfactory receptor family 4 subfamily D member 6 [Source:HGNC Symbol;Acc:HGNC:15175] |
| ENSG00000167964 | HGLibA_39852 | 8 | -1.068993659 | RAB26 | member RAS oncogene family [Source:HGNC Symbol;Acc:HGNC:14259]" |
| ENSG00000167971 | HGLibA_07290 | 8 | -1.068993659 | CASKIN1 | CASK interacting protein 1 [Source:HGNC Symbol;Acc:HGNC:20879] |
| ENSG00000168032 | HGLibA_15204 | 8 | -1.068993659 | ENTPD3 | ectonucleoside triphosphate diphosphohydrolase 3 [Source:HGNC Symbol;Acc:HGNC:3365] |
| ENSG00000168172 | HGLibA_21882 | 8 | -1.068993659 | HOOK3 | hook microtubule tethering protein 3 [Source:HGNC Symbol;Acc:HGNC:23576] |
| ENSG00000168350 | HGLibA_12924 | 8 | -1.068993659 | DEGS2 | sphingolipid 2 [Source:HGNC Symbol;Acc:HGNC:20113]" |
| ENSG00000168564 | HGLibA_08834 | 8 | -1.068993659 | CDKN2AIP | CDKN2A interacting protein [Source:HGNC Symbol;Acc:HGNC:24325] |
| ENSG00000169783 | HGLibA_26586 | 8 | -1.068993659 | LINGO1 | leucine rich repeat and Ig domain containing 1 [Source:HGNC Symbol;Acc:HGNC:21205] |
| ENSG00000170004 | HGLibA_09391 | 8 | -1.068993659 | CHD3 | chromodomain helicase DNA binding protein 3 [Source:HGNC Symbol;Acc:HGNC:1918] |
| ENSG00000170035 | HGLibA_52600 | 8 | -1.068993659 | UBE2E3 | ubiquitin conjugating enzyme E2 E3 [Source:HGNC Symbol;Acc:HGNC:12479] |
| ENSG00000170092 | HGLibA_46596 | 8 | -1.068993659 | SPDYE5 | speedy/RINGO cell cycle regulator family member E5 [Source:HGNC Symbol;Acc:HGNC:35464] |
| ENSG00000170961 | HGLibA_20866 | 8 | -1.068993659 | HAS2 | hyaluronan synthase 2 [Source:HGNC Symbol;Acc:HGNC:4819] |
| ENSG00000171560 | HGLibA_17336 | 8 | -1.068993659 | FGA | fibrinogen alpha chain [Source:HGNC Symbol;Acc:HGNC:3661] |
| ENSG00000172717 | HGLibA_16695 | 8 | -1.068993659 | FAM71D | family with sequence similarity 71 member D [Source:HGNC Symbol;Acc:HGNC:20101] |
| ENSG00000174600 | HGLibA_10198 | 8 | -1.068993659 | CMKLR1 | chemerin chemokine-like receptor 1 [Source:HGNC Symbol;Acc:HGNC:2121] |
| ENSG00000174939 | HGLibA_03352 | 8 | -1.068993659 | ASPHD1 | aspartate beta-hydroxylase domain containing 1 [Source:HGNC Symbol;Acc:HGNC:27380] |
| ENSG00000175220 | HGLibA_02696 | 8 | -1.068993659 | ARHGAP1 | Rho GTPase activating protein 1 [Source:HGNC Symbol;Acc:HGNC:673] |
| ENSG00000175416 | HGLibA_10146 | 8 | -1.068993659 | CLTB | clathrin light chain B [Source:HGNC Symbol;Acc:HGNC:2091] |
| ENSG00000177363 | HGLibA_27482 | 8 | -1.068993659 | LRRN4CL | LRRN4 C-terminal like [Source:HGNC Symbol;Acc:HGNC:33724] |
| ENSG00000177570 | HGLibA_42668 | 8 | -1.068993659 | SAMD12 | sterile alpha motif domain containing 12 [Source:HGNC Symbol;Acc:HGNC:31750] |
| ENSG00000179820 | HGLibA_30630 | 8 | -1.068993659 | MYADM | myeloid associated differentiation marker [Source:HGNC Symbol;Acc:HGNC:7544] |
| ENSG00000180697 | HGLibA_06221 | 8 | -1.068993659 | C3orf22 | chromosome 3 open reading frame 22 [Source:HGNC Symbol;Acc:HGNC:28534] |
| ENSG00000182580 | HGLibA_15317 | 8 | -1.068993659 | EPHB3 | EPH receptor B3 [Source:HGNC Symbol;Acc:HGNC:3394] |
| ENSG00000183054 | HGLibA_41039 | 8 | -1.068993659 | RGPD6 | RANBP2-like and GRIP domain containing 6 [Source:HGNC Symbol;Acc:HGNC:32419] |
| ENSG00000183735 | HGLibA_48603 | 8 | -1.068993659 | TBK1 | TANK binding kinase 1 [Source:HGNC Symbol;Acc:HGNC:11584] |
| ENSG00000184735 | HGLibA_12741 | 8 | -1.068993659 | DDX53 | DEAD-box helicase 53 [Source:HGNC Symbol;Acc:HGNC:20083] |
| ENSG00000186474 | HGLibA_25240 | 8 | -1.068993659 | KLK12 | kallikrein related peptidase 12 [Source:HGNC Symbol;Acc:HGNC:6360] |
| ENSG00000186496 | HGLibA_55916 | 8 | -1.068993659 | ZNF396 | zinc finger protein 396 [Source:HGNC Symbol;Acc:HGNC:18824] |
| ENSG00000196208 | HGLibA_20236 | 8 | -1.068993659 | GREB1 | growth regulating estrogen receptor binding 1 [Source:HGNC Symbol;Acc:HGNC:24885] |
| ENSG00000196284 | HGLibA_47814 | 8 | -1.068993659 | SUPT3H | SAGA and STAGA complex component [Source:HGNC Symbol;Acc:HGNC:11466]" |
| ENSG00000197780 | HGLibA_48208 | 8 | -1.068993659 | TAF13 | TATA-box binding protein associated factor 13 [Source:HGNC Symbol;Acc:HGNC:11546] |
| ENSG00000204344 | HGLibA_47469 | 8 | -1.068993659 | STK19 | serine/threonine kinase 19 [Source:HGNC Symbol;Acc:HGNC:11398] |

| | | | | | |
|---|---|---|---|---|---|
| ENSG00000204713 | HGLibA_51446 | 8 | -1.068993659 | TRIM27 | tripartite motif containing 27 [Source:HGNC Symbol;Acc:HGNC:9975] |
| ENSG00000204977 | HGLibA_51407 | 8 | -1.068993659 | TRIM13 | tripartite motif containing 13 [Source:HGNC Symbol;Acc:HGNC:9976] |
| ENSG00000205238 | HGLibA_46581 | 8 | -1.068993659 | SPDYE2 | speedy/RINGO cell cycle regulator family member E2 [Source:HGNC Symbol;Acc:HGNC:33841] |
| ENSG00000205581 | HGLibA_21716 | 8 | -1.068993659 | HMGN1 | high mobility group nucleosome binding domain 1 [Source:HGNC Symbol;Acc:HGNC:4984] |
| ENSG00000213030 | HGLibA_09313 | 8 | -1.068993659 | CGB8 | chorionic gonadotropin subunit beta 8 [Source:HGNC Symbol;Acc:HGNC:16453] |
| ENSG00000216649 | HGLibA_18447 | 8 | -1.068993659 | GAGE12E | G antigen 12E [Source:HGNC Symbol;Acc:HGNC:31905] |
| ENSG00000233136 | HGLibA_53218 | 8 | -1.068993659 | USP17L11 | ubiquitin specific peptidase 17-like family member 11 [Source:HGNC Symbol;Acc:HGNC:44439] |
| ENSG00000239697 | HGLibA_50801 | 8 | -1.068993659 | TNFSF12 | TNF superfamily member 12 [Source:HGNC Symbol;Acc:HGNC:11927] |
| ENSG00000242247 | HGLibA_02666 | 8 | -1.068993659 | ARFGAP3 | ADP ribosylation factor GTPase activating protein 3 [Source:HGNC Symbol;Acc:HGNC:661] |
| ENSG00000243649 | HGLibA_09244 | 8 | -1.068993659 | CFB | complement factor B [Source:HGNC Symbol;Acc:HGNC:1037] |
| ENSG00000244187 | HGLibA_49963 | 8 | -1.068993659 | TMEM141 | transmembrane protein 141 [Source:HGNC Symbol;Acc:HGNC:28211] |
| ENSG00000272047 | HGLibA_20561 | 8 | -1.068993659 | GTF2H5 | general transcription factor IIH subunit 5 [Source:HGNC Symbol;Acc:HGNC:21157] |
| ENSG00000272333 | HGLibA_25324 | 8 | -1.068993659 | KMT2B | lysine methyltransferase 2B [Source:HGNC Symbol;Acc:HGNC:15840] |
| ENSG00000275004 | HGLibA_55727 | 8 | -1.068993659 | ZNF280B | zinc finger protein 280B [Source:HGNC Symbol;Acc:HGNC:23022] |
| ENSG00000276040 | HGLibA_46485 | 8 | -1.068993659 | SPATA31A7 | SPATA31 subfamily A member 7 [Source:HGNC Symbol;Acc:HGNC:32007] |
| FAM5B | HGLibA_16649 | 8 | -1.068993659 | FAM5B | - |
| GIF | HGLibA_19060 | 8 | -1.068993659 | GIF | - |
| hsa-mir-1185-1 | HGLibA_57241 | 8 | -1.068993659 | hsa-mir-1185-1 | - |
| hsa-mir-124-3 | HGLibA_57355 | 8 | -1.068993659 | hsa-mir-124-3 | - |
| hsa-mir-138-1 | HGLibA_57813 | 8 | -1.068993659 | hsa-mir-138-1 | - |
| hsa-mir-139 | HGLibA_57819 | 8 | -1.068993659 | hsa-mir-139 | - |
| hsa-mir-1470 | HGLibA_57864 | 8 | -1.068993659 | hsa-mir-1470 | - |
| hsa-mir-27b | HGLibA_58380 | 8 | -1.068993659 | hsa-mir-27b | - |
| hsa-mir-29a | HGLibA_58410 | 8 | -1.068993659 | hsa-mir-29a | - |
| hsa-mir-3137 | HGLibA_58608 | 8 | -1.068993659 | hsa-mir-3137 | - |
| hsa-mir-3148 | HGLibA_58649 | 8 | -1.068993659 | hsa-mir-3148 | - |
| hsa-mir-3169 | HGLibA_58757 | 8 | -1.068993659 | hsa-mir-3169 | - |
| hsa-mir-365b | HGLibA_59191 | 8 | -1.068993659 | hsa-mir-365b | - |
| hsa-mir-3678 | HGLibA_59263 | 8 | -1.068993659 | hsa-mir-3678 | - |
| hsa-mir-378d-2 | HGLibA_59440 | 8 | -1.068993659 | hsa-mir-378d-2 | - |
| hsa-mir-4445 | HGLibA_60185 | 8 | -1.068993659 | hsa-mir-4445 | - |
| hsa-mir-4477a | HGLibA_60319 | 8 | -1.068993659 | hsa-mir-4477a | - |
| hsa-mir-4503 | HGLibA_60439 | 8 | -1.068993659 | hsa-mir-4503 | - |
| hsa-mir-4741 | HGLibA_61061 | 8 | -1.068993659 | hsa-mir-4741 | - |
| hsa-mir-4999 | HGLibA_61398 | 8 | -1.068993659 | hsa-mir-4999 | - |
| hsa-mir-5192 | HGLibA_61688 | 8 | -1.068993659 | hsa-mir-5192 | - |
| hsa-mir-520b | HGLibA_61738 | 8 | -1.068993659 | hsa-mir-520b | - |
| hsa-mir-520d | HGLibA_61747 | 8 | -1.068993659 | hsa-mir-520d | - |
| hsa-mir-554 | HGLibA_62131 | 8 | -1.068993659 | hsa-mir-554 | - |
| hsa-mir-5581 | HGLibA_62163 | 8 | -1.068993659 | hsa-mir-5581 | - |
| hsa-mir-5697 | HGLibA_62322 | 8 | -1.068993659 | hsa-mir-5697 | - |
| hsa-mir-6076 | HGLibA_62555 | 8 | -1.068993659 | hsa-mir-6076 | - |
| hsa-mir-6081 | HGLibA_62583 | 8 | -1.068993659 | hsa-mir-6081 | - |
| hsa-mir-6083 | HGLibA_62592 | 8 | -1.068993659 | hsa-mir-6083 | - |
| hsa-mir-642b | HGLibA_62812 | 8 | -1.068993659 | hsa-mir-642b | - |
| hsa-mir-6516 | HGLibA_62939 | 8 | -1.068993659 | hsa-mir-6516 | - |
| hsa-mir-6716 | HGLibA_63027 | 8 | -1.068993659 | hsa-mir-6716 | - |
| hsa-mir-6834 | HGLibA_63515 | 8 | -1.068993659 | hsa-mir-6834 | - |
| hsa-mir-6853 | HGLibA_63586 | 8 | -1.068993659 | hsa-mir-6853 | - |
| KIAA1683 | HGLibA_24801 | 8 | -1.068993659 | KIAA1683 | - |
| LOC100129216 | HGLibA_26757 | 8 | -1.068993659 | LOC100129216 | - |
| MYCL1 | HGLibA_30675 | 8 | -1.068993659 | MYCL1 | - |
| NARFL | HGLibA_31145 | 8 | -1.068993659 | NARFL | - |
| RABL5 | HGLibA_40035 | 8 | -1.068993659 | RABL5 | - |
| ST5 | HGLibA_47256 | 8 | -1.068993659 | ST5 | - |
| WDR67 | HGLibA_54157 | 8 | -1.068993659 | WDR67 | - |
| BAGE5 | HGLibA_04104 | 7 | -1.078944697 | BAGE5 | - |
| C11orf57 | HGLibA_05159 | 7 | -1.078944697 | C11orf57 | - |
| C1orf27 | HGLibA_05853 | 7 | -1.078944697 | C1orf27 | - |
| C4B_2 | HGLibA_06287 | 7 | -1.078944697 | C4B_2 | - |
| ENSG00000001631 | HGLibA_25409 | 7 | -1.078944697 | KRIT1 | ankyrin repeat containing [Source:HGNC Symbol;Acc:HGNC:1573]" |
| ENSG00000005961 | HGLibA_23728 | 7 | -1.078944697 | ITGA2B | integrin subunit alpha 2b [Source:HGNC Symbol;Acc:HGNC:6138] |
| ENSG00000007350 | HGLibA_49588 | 7 | -1.078944697 | TKTL1 | transketolase like 1 [Source:HGNC Symbol;Acc:HGNC:11835] |
| ENSG00000012061 | HGLibA_15431 | 7 | -1.078944697 | ERCC1 | endonuclease non-catalytic subunit [Source:HGNC Symbol;Acc:HGNC:3433]" |
| ENSG00000021300 | HGLibA_37087 | 7 | -1.078944697 | PLEKHB1 | pleckstrin homology domain containing B1 [Source:HGNC Symbol;Acc:HGNC:19079] |
| ENSG00000023041 | HGLibA_55124 | 7 | -1.078944697 | ZDHHC6 | zinc finger DHHC-type containing 6 [Source:HGNC Symbol;Acc:HGNC:19160] |
| ENSG00000023572 | HGLibA_19317 | 7 | -1.078944697 | GLRX2 | glutaredoxin 2 [Source:HGNC Symbol;Acc:HGNC:16065] |
| ENSG00000036448 | HGLibA_30891 | 7 | -1.078944697 | MYOM2 | myomesin 2 [Source:HGNC Symbol;Acc:HGNC:7614] |
| ENSG00000041802 | HGLibA_27514 | 7 | -1.078944697 | LSG1 | large 60S subunit nuclear export GTPase 1 [Source:HGNC Symbol;Acc:HGNC:25652] |
| ENSG00000054116 | HGLibA_51319 | 7 | -1.078944697 | TRAPPC3 | trafficking protein particle complex 3 [Source:HGNC Symbol;Acc:HGNC:19942] |
| ENSG00000064787 | HGLibA_04250 | 7 | -1.078944697 | BCAS1 | breast carcinoma amplified sequence 1 [Source:HGNC Symbol;Acc:HGNC:974] |
| ENSG00000066336 | HGLibA_46653 | 7 | -1.078944697 | SPI1 | Spi-1 proto-oncogene [Source:HGNC Symbol;Acc:HGNC:11241] |
| ENSG00000068724 | HGLibA_52150 | 7 | -1.078944697 | TTC7A | tetratricopeptide repeat domain 7A [Source:HGNC Symbol;Acc:HGNC:19750] |
| ENSG00000073803 | HGLibA_28172 | 7 | -1.078944697 | MAP3K13 | mitogen-activated protein kinase kinase kinase 13 [Source:HGNC Symbol;Acc:HGNC:6852] |
| ENSG00000074755 | HGLibA_57092 | 7 | -1.078944697 | ZZEF1 | zinc finger ZZ-type and EF-hand domain containing 1 [Source:HGNC Symbol;Acc:HGNC:29027] |
| ENSG00000074855 | HGLibA_02192 | 7 | -1.078944697 | ANO8 | anoctamin 8 [Source:HGNC Symbol;Acc:HGNC:29329] |
| ENSG00000076555 | HGLibA_00350 | 7 | -1.078944697 | ACACB | acetyl-CoA carboxylase beta [Source:HGNC Symbol;Acc:HGNC:85] |
| ENSG00000076604 | HGLibA_51259 | 7 | -1.078944697 | TRAF4 | TNF receptor associated factor 4 [Source:HGNC Symbol;Acc:HGNC:12034] |
| ENSG00000077942 | HGLibA_16942 | 7 | -1.078944697 | FBLN1 | fibulin 1 [Source:HGNC Symbol;Acc:HGNC:3600] |
| ENSG00000080007 | HGLibA_12717 | 7 | -1.078944697 | DDX43 | DEAD-box helicase 43 [Source:HGNC Symbol;Acc:HGNC:18677] |
| ENSG00000084234 | HGLibA_02442 | 7 | -1.078944697 | APLP2 | amyloid beta precursor like protein 2 [Source:HGNC Symbol;Acc:HGNC:598] |
| ENSG00000085465 | HGLibA_34799 | 7 | -1.078944697 | OVGP1 | oviductal glycoprotein 1 [Source:HGNC Symbol;Acc:HGNC:8524] |
| ENSG00000090661 | HGLibA_09208 | 7 | -1.078944697 | CERS4 | ceramide synthase 4 [Source:HGNC Symbol;Acc:HGNC:23747] |
| ENSG00000099974 | HGLibA_12644 | 7 | -1.078944697 | DDTL | D-dopachrome tautomerase like [Source:HGNC Symbol;Acc:HGNC:33446] |
| ENSG00000101132 | HGLibA_36143 | 7 | -1.078944697 | PFDN4 | prefoldin subunit 4 [Source:HGNC Symbol;Acc:HGNC:8868] |
| ENSG00000102317 | HGLibA_40530 | 7 | -1.078944697 | RBM3 | RNA binding motif protein 3 [Source:HGNC Symbol;Acc:HGNC:9900] |
| ENSG00000102910 | HGLibA_27017 | 7 | -1.078944697 | LONP2 | peroxisomal [Source:HGNC Symbol;Acc:HGNC:20598]" |
| ENSG00000104889 | HGLibA_41426 | 7 | -1.078944697 | RNASEH2A | ribonuclease H2 subunit A [Source:HGNC Symbol;Acc:HGNC:18518] |
| ENSG00000106615 | HGLibA_41153 | 7 | -1.078944697 | RHEB | mTORC1 binding [Source:HGNC Symbol;Acc:HGNC:10011]" |
| ENSG00000108591 | HGLibA_13976 | 7 | -1.078944697 | DRG2 | developmentally regulated GTP binding protein 2 [Source:HGNC Symbol;Acc:HGNC:3030] |
| ENSG00000108799 | HGLibA_15833 | 7 | -1.078944697 | EZH1 | enhancer of zeste 1 polycomb repressive complex 2 subunit [Source:HGNC Symbol;Acc:HGNC:3526] |
| ENSG00000109787 | HGLibA_25068 | 7 | -1.078944697 | KLF3 | Kruppel like factor 3 [Source:HGNC Symbol;Acc:HGNC:16516] |
| ENSG00000110925 | HGLibA_11426 | 7 | -1.078944697 | CSRNP2 | cysteine and serine rich nuclear protein 2 [Source:HGNC Symbol;Acc:HGNC:16006] |
| ENSG00000111011 | HGLibA_42361 | 7 | -1.078944697 | RSRC2 | arginine and serine rich coiled-coil 2 [Source:HGNC Symbol;Acc:HGNC:30559] |
| ENSG00000115009 | HGLibA_07992 | 7 | -1.078944697 | CCL20 | C-C motif chemokine ligand 20 [Source:HGNC Symbol;Acc:HGNC:10619] |
| ENSG00000115041 | HGLibA_24240 | 7 | -1.078944697 | KCNIP3 | potassium voltage-gated channel interacting protein 3 [Source:HGNC Symbol;Acc:HGNC:15523] |
| ENSG00000115884 | HGLibA_43097 | 7 | -1.078944697 | SDC1 | syndecan 1 [Source:HGNC Symbol;Acc:HGNC:10658] |
| ENSG00000116337 | HGLibA_01826 | 7 | -1.078944697 | AMPD2 | adenosine monophosphate deaminase 2 [Source:HGNC Symbol;Acc:HGNC:469] |
| ENSG00000118004 | HGLibA_10636 | 7 | -1.078944697 | COLEC11 | collectin subfamily member 11 [Source:HGNC Symbol;Acc:HGNC:17213] |
| ENSG00000118363 | HGLibA_46562 | 7 | -1.078944697 | SPCS2 | signal peptidase complex subunit 2 [Source:HGNC Symbol;Acc:HGNC:28962] |
| ENSG00000118503 | HGLibA_50712 | 7 | -1.078944697 | TNFAIP3 | TNF alpha induced protein 3 [Source:HGNC Symbol;Acc:HGNC:11896] |
| ENSG00000118922 | HGLibA_25048 | 7 | -1.078944697 | KLF12 | Kruppel like factor 12 [Source:HGNC Symbol;Acc:HGNC:6346] |
| ENSG00000119471 | HGLibA_22217 | 7 | -1.078944697 | HSDL2 | hydroxysteroid dehydrogenase like 2 [Source:HGNC Symbol;Acc:HGNC:18572] |
| ENSG00000119661 | HGLibA_13644 | 7 | -1.078944697 | DNAL1 | dynein axonemal light chain 1 [Source:HGNC Symbol;Acc:HGNC:23247] |
| ENSG00000122257 | HGLibA_40435 | 7 | -1.078944697 | RBBP6 | ubiquitin ligase [Source:HGNC Symbol;Acc:HGNC:9889]" |
| ENSG00000122958 | HGLibA_53753 | 7 | -1.078944697 | VPS26A | retromer complex component A [Source:HGNC Symbol;Acc:HGNC:12711]" |
| ENSG00000122986 | HGLibA_22434 | 7 | -1.078944697 | HVCN1 | hydrogen voltage gated channel 1 [Source:HGNC Symbol;Acc:HGNC:28240] |
| ENSG00000124003 | HGLibA_29625 | 7 | -1.078944697 | MOGAT1 | monoacylglycerol O-acyltransferase 1 [Source:HGNC Symbol;Acc:HGNC:18210] |
| ENSG00000124160 | HGLibA_31348 | 7 | -1.078944697 | NCOA5 | nuclear receptor coactivator 5 [Source:HGNC Symbol;Acc:HGNC:15909] |
| ENSG00000125571 | HGLibA_23157 | 7 | -1.078944697 | IL37 | interleukin 37 [Source:HGNC Symbol;Acc:HGNC:15563] |
| ENSG00000125733 | HGLibA_51634 | 7 | -1.078944697 | TRIP10 | thyroid hormone receptor interactor 10 [Source:HGNC Symbol;Acc:HGNC:12304] |
| ENSG00000125912 | HGLibA_31328 | 7 | -1.078944697 | NCLN | nicalin [Source:HGNC Symbol;Acc:HGNC:26923] |
| ENSG00000126524 | HGLibA_42791 | 7 | -1.078944697 | SBDS | ribosome maturation factor [Source:HGNC Symbol;Acc:HGNC:19440]" |
| ENSG00000127362 | HGLibA_48383 | 7 | -1.078944697 | TAS2R3 | taste 2 receptor member 3 [Source:HGNC Symbol;Acc:HGNC:14910] |
| ENSG00000129315 | HGLibA_08127 | 7 | -1.078944697 | CCNT1 | cyclin T1 [Source:HGNC Symbol;Acc:HGNC:1599] |
| ENSG00000132388 | HGLibA_52607 | 7 | -1.078944697 | UBE2G1 | ubiquitin conjugating enzyme E2 G1 [Source:HGNC Symbol;Acc:HGNC:12482] |
| ENSG00000132938 | HGLibA_30509 | 7 | -1.078944697 | MTUS2 | microtubule associated scaffold protein 2 [Source:HGNC Symbol;Acc:HGNC:20595] |
| ENSG00000133028 | HGLibA_43024 | 7 | -1.078944697 | SCO1 | cytochrome c oxidase assembly protein [Source:HGNC Symbol;Acc:HGNC:10603]" |
| ENSG00000133256 | HGLibA_35809 | 7 | -1.078944697 | PDE6B | phosphodiesterase 6B [Source:HGNC Symbol;Acc:HGNC:8786] |
| ENSG00000134283 | HGLibA_37892 | 7 | -1.078944697 | PPHLN1 | periphilin 1 [Source:HGNC Symbol;Acc:HGNC:19369] |
| ENSG00000134480 | HGLibA_08099 | 7 | -1.078944697 | CCNH | cyclin H [Source:HGNC Symbol;Acc:HGNC:1594] |
| ENSG00000135775 | HGLibA_10474 | 7 | -1.078944697 | COG2 | component of oligomeric golgi complex 2 [Source:HGNC Symbol;Acc:HGNC:6546] |
| ENSG00000137776 | HGLibA_45549 | 7 | -1.078944697 | SLTM | SAFB like transcription modulator [Source:HGNC Symbol;Acc:HGNC:20709] |
| ENSG00000137801 | HGLibA_49323 | 7 | -1.078944697 | THBS1 | thrombospondin 1 [Source:HGNC Symbol;Acc:HGNC:11785] |
| ENSG00000138311 | HGLibA_55876 | 7 | -1.078944697 | ZNF365 | zinc finger protein 365 [Source:HGNC Symbol;Acc:HGNC:18194] |
| ENSG00000138380 | HGLibA_07245 | 7 | -1.078944697 | CARF | calcium responsive transcription factor [Source:HGNC Symbol;Acc:HGNC:14435] |
| ENSG00000139187 | HGLibA_25310 | 7 | -1.078944697 | KLRG1 | killer cell lectin like receptor G1 [Source:HGNC Symbol;Acc:HGNC:6380] |
| ENSG00000140264 | HGLibA_43475 | 7 | -1.078944697 | SERF2 | small EDRK-rich factor 2 [Source:HGNC Symbol;Acc:HGNC:10757] |
| ENSG00000140854 | HGLibA_24080 | 7 | -1.078944697 | KATNB1 | katanin regulatory subunit B1 [Source:HGNC Symbol;Acc:HGNC:6217] |
| ENSG00000142511 | HGLibA_20021 | 7 | -1.078944697 | GPR32 | G protein-coupled receptor 32 [Source:HGNC Symbol;Acc:HGNC:4487] |
| ENSG00000142515 | HGLibA_25256 | 7 | -1.078944697 | KLK3 | kallikrein related peptidase 3 [Source:HGNC Symbol;Acc:HGNC:6364] |
| ENSG00000143401 | HGLibA_02206 | 7 | -1.078944697 | ANP32E | acidic nuclear phosphoprotein 32 family member E [Source:HGNC Symbol;Acc:HGNC:16673] |
| ENSG00000144591 | HGLibA_19422 | 7 | -1.078944697 | GMPPA | GDP-mannose pyrophosphorylase A [Source:HGNC Symbol;Acc:HGNC:22923] |
| ENSG00000147059 | HGLibA_46671 | 7 | -1.078944697 | SPIN2A | spindlin family member 2A [Source:HGNC Symbol;Acc:HGNC:20694] |
| ENSG00000147223 | HGLibA_41329 | 7 | -1.078944697 | RIPPLY1 | ripply transcriptional repressor 1 [Source:HGNC Symbol;Acc:HGNC:25117] |

| | | | | | |
|---|---|---|---|---|---|
| ENSG00000147394 | HGLibA_55549 | 7 | -1.078944697 | ZNF185 | zinc finger protein 185 with LIM domain [Source:HGNC Symbol;Acc:HGNC:12976] |
| ENSG00000149929 | HGLibA_21355 | 7 | -1.078944697 | HIRIP3 | HIRA interacting protein 3 [Source:HGNC Symbol;Acc:HGNC:4917] |
| ENSG00000152454 | HGLibA_55677 | 7 | -1.078944697 | ZNF256 | zinc finger protein 256 [Source:HGNC Symbol;Acc:HGNC:13049] |
| ENSG00000156304 | HGLibA_42826 | 7 | -1.078944697 | SCAF4 | SR-related CTD associated factor 4 [Source:HGNC Symbol;Acc:HGNC:19304] |
| ENSG00000158488 | HGLibA_08277 | 7 | -1.078944697 | CD1E | CD1e molecule [Source:HGNC Symbol;Acc:HGNC:1638] |
| ENSG00000159761 | HGLibA_05489 | 7 | -1.078944697 | C16orf86 | chromosome 16 open reading frame 86 [Source:HGNC Symbol;Acc:HGNC:33755] |
| ENSG00000159784 | HGLibA_16109 | 7 | -1.078944697 | FAM131B | family with sequence similarity 131 member B [Source:HGNC Symbol;Acc:HGNC:22202] |
| ENSG00000160339 | HGLibA_17220 | 7 | -1.078944697 | FCN2 | ficolin 2 [Source:HGNC Symbol;Acc:HGNC:3624] |
| ENSG00000160695 | HGLibA_53729 | 7 | -1.078944697 | VPS11 | CORVET/HOPS core subunit [Source:HGNC Symbol;Acc:HGNC:14583]" |
| ENSG00000160785 | HGLibA_44746 | 7 | -1.078944697 | SLC25A44 | solute carrier family 25 member 44 [Source:HGNC Symbol;Acc:HGNC:29036] |
| ENSG00000161904 | HGLibA_26289 | 7 | -1.078944697 | LEMD2 | LEM domain containing 2 [Source:HGNC Symbol;Acc:HGNC:21244] |
| ENSG00000162736 | HGLibA_31378 | 7 | -1.078944697 | NCSTN | nicastrin [Source:HGNC Symbol;Acc:HGNC:17091] |
| ENSG00000163607 | HGLibA_20623 | 7 | -1.078944697 | GTPBP8 | GTP binding protein 8 (putative) [Source:HGNC Symbol;Acc:HGNC:25007] |
| ENSG00000163806 | HGLibA_46574 | 7 | -1.078944697 | SPDYA | speedy/RINGO cell cycle regulator family member A [Source:HGNC Symbol;Acc:HGNC:30613] |
| ENSG00000164129 | HGLibA_32559 | 7 | -1.078944697 | NPY5R | neuropeptide Y receptor Y5 [Source:HGNC Symbol;Acc:HGNC:7958] |
| ENSG00000164287 | HGLibA_08521 | 7 | -1.078944697 | CDC20B | cell division cycle 20B [Source:HGNC Symbol;Acc:HGNC:24222] |
| ENSG00000164880 | HGLibA_23406 | 7 | -1.078944697 | INTS1 | integrator complex subunit 1 [Source:HGNC Symbol;Acc:HGNC:24555] |
| ENSG00000165934 | HGLibA_10996 | 7 | -1.078944697 | CPSF2 | cleavage and polyadenylation specific factor 2 [Source:HGNC Symbol;Acc:HGNC:2325] |
| ENSG00000165943 | HGLibA_29581 | 7 | -1.078944697 | MOAP1 | modulator of apoptosis 1 [Source:HGNC Symbol;Acc:HGNC:16658] |
| ENSG00000166091 | HGLibA_10222 | 7 | -1.078944697 | CMTM5 | CKLF like MARVEL transmembrane domain containing 5 [Source:HGNC Symbol;Acc:HGNC:19176] |
| ENSG00000167578 | HGLibA_39959 | 7 | -1.078944697 | RAB4B | member RAS oncogene family [Source:HGNC Symbol;Acc:HGNC:9782]" |
| ENSG00000167615 | HGLibA_26301 | 7 | -1.078944697 | LENG8 | leukocyte receptor cluster member 8 [Source:HGNC Symbol;Acc:HGNC:15500] |
| ENSG00000167664 | HGLibA_50588 | 7 | -1.078944697 | TMIGD2 | transmembrane and immunoglobulin domain containing 2 [Source:HGNC Symbol;Acc:HGNC:28324] |
| ENSG00000168404 | HGLibA_29391 | 7 | -1.078944697 | MLKL | mixed lineage kinase domain like pseudokinase [Source:HGNC Symbol;Acc:HGNC:26617] |
| ENSG00000168961 | HGLibA_26381 | 7 | -1.078944697 | LGALS9 | galectin 9 [Source:HGNC Symbol;Acc:HGNC:6570] |
| ENSG00000169231 | HGLibA_49328 | 7 | -1.078944697 | THBS3 | thrombospondin 3 [Source:HGNC Symbol;Acc:HGNC:11787] |
| ENSG00000169403 | HGLibA_39244 | 7 | -1.078944697 | PTAFR | platelet activating factor receptor [Source:HGNC Symbol;Acc:HGNC:9582] |
| ENSG00000171631 | HGLibA_34890 | 7 | -1.078944697 | P2RY6 | pyrimidinergic receptor P2Y6 [Source:HGNC Symbol;Acc:HGNC:8543] |
| ENSG00000171970 | HGLibA_56273 | 7 | -1.078944697 | ZNF57 | zinc finger protein 57 [Source:HGNC Symbol;Acc:HGNC:13125] |
| ENSG00000172350 | HGLibA_00220 | 7 | -1.078944697 | ABCG4 | ATP binding cassette subfamily G member 4 [Source:HGNC Symbol;Acc:HGNC:13884] |
| ENSG00000173120 | HGLibA_24495 | 7 | -1.078944697 | KDM2A | lysine demethylase 2A [Source:HGNC Symbol;Acc:HGNC:13606] |
| ENSG00000173674 | HGLibA_14711 | 7 | -1.078944697 | EIF1AX | eukaryotic translation initiation factor 1A X-linked [Source:HGNC Symbol;Acc:HGNC:3250] |
| ENSG00000173917 | HGLibA_21931 | 7 | -1.078944697 | HOXB2 | homeobox B2 [Source:HGNC Symbol;Acc:HGNC:5113] |
| ENSG00000174231 | HGLibA_38746 | 7 | -1.078944697 | PRPF8 | pre-mRNA processing factor 8 [Source:HGNC Symbol;Acc:HGNC:17340] |
| ENSG00000174628 | HGLibA_23529 | 7 | -1.078944697 | IQCK | IQ motif containing K [Source:HGNC Symbol;Acc:HGNC:28556] |
| ENSG00000174791 | HGLibA_41290 | 7 | -1.078944697 | RIN1 | Ras and Rab interactor 1 [Source:HGNC Symbol;Acc:HGNC:18749] |
| ENSG00000175054 | HGLibA_03811 | 7 | -1.078944697 | ATR | ATR serine/threonine kinase [Source:HGNC Symbol;Acc:HGNC:882] |
| ENSG00000175634 | HGLibA_42178 | 7 | -1.078944697 | RPS6KB2 | ribosomal protein S6 kinase B2 [Source:HGNC Symbol;Acc:HGNC:10437] |
| ENSG00000176219 | HGLibA_33580 | 7 | -1.078944697 | OR11H6 | olfactory receptor family 11 subfamily H member 6 [Source:HGNC Symbol;Acc:HGNC:15349] |
| ENSG00000176454 | HGLibA_27081 | 7 | -1.078944697 | LPCAT4 | lysophosphatidylcholine acyltransferase 4 [Source:HGNC Symbol;Acc:HGNC:30059] |
| ENSG00000176490 | HGLibA_13257 | 7 | -1.078944697 | DIRAS1 | DIRAS family GTPase 1 [Source:HGNC Symbol;Acc:HGNC:19127] |
| ENSG00000177990 | HGLibA_13908 | 7 | -1.078944697 | DPY19L2 | dpy-19 like 2 [Source:HGNC Symbol;Acc:HGNC:19414] |
| ENSG00000178935 | HGLibA_56217 | 7 | -1.078944697 | ZNF552 | zinc finger protein 552 [Source:HGNC Symbol;Acc:HGNC:26135] |
| ENSG00000181092 | HGLibA_00976 | 7 | -1.078944697 | ADIPOQ | C1Q and collagen domain containing [Source:HGNC Symbol;Acc:HGNC:13633]" |
| ENSG00000182944 | HGLibA_15714 | 7 | -1.078944697 | EWSR1 | EWS RNA binding protein 1 [Source:HGNC Symbol;Acc:HGNC:3508] |
| ENSG00000183655 | HGLibA_25165 | 7 | -1.078944697 | KLHL25 | kelch like family member 25 [Source:HGNC Symbol;Acc:HGNC:25732] |
| ENSG00000185053 | HGLibA_43812 | 7 | -1.078944697 | SGCZ | sarcoglycan zeta [Source:HGNC Symbol;Acc:HGNC:14075] |
| ENSG00000185352 | HGLibA_22147 | 7 | -1.078944697 | HS6ST3 | heparan sulfate 6-O-sulfotransferase 3 [Source:HGNC Symbol;Acc:HGNC:19134] |
| ENSG00000185838 | HGLibA_19487 | 7 | -1.078944697 | GNB1L | G protein subunit beta 1 like [Source:HGNC Symbol;Acc:HGNC:4397] |
| ENSG00000186924 | HGLibA_25715 | 7 | -1.078944697 | KRTAP22-1 | keratin associated protein 22-1 [Source:HGNC Symbol;Acc:HGNC:18947] |
| ENSG00000188620 | HGLibA_21764 | 7 | -1.078944697 | HMX3 | H6 family homeobox 3 [Source:HGNC Symbol;Acc:HGNC:5019] |
| ENSG00000196083 | HGLibA_23052 | 7 | -1.078944697 | IL1RAP | interleukin 1 receptor accessory protein [Source:HGNC Symbol;Acc:HGNC:5995] |
| ENSG00000196214 | HGLibA_56695 | 7 | -1.078944697 | ZNF766 | zinc finger protein 766 [Source:HGNC Symbol;Acc:HGNC:28063] |
| ENSG00000196337 | HGLibA_09311 | 7 | -1.078944697 | CGB7 | chorionic gonadotropin subunit beta 7 [Source:HGNC Symbol;Acc:HGNC:16451] |
| ENSG00000197016 | HGLibA_56054 | 7 | -1.078944697 | ZNF470 | zinc finger protein 470 [Source:HGNC Symbol;Acc:HGNC:22220] |
| ENSG00000197451 | HGLibA_21805 | 7 | -1.078944697 | HNRNPAB | heterogeneous nuclear ribonucleoprotein A/B [Source:HGNC Symbol;Acc:HGNC:5034] |
| ENSG00000197617 | HGLibA_53706 | 7 | -1.078944697 | VN1R5 | vomeronasal 1 receptor 5 (gene/pseudogene) [Source:HGNC Symbol;Acc:HGNC:19870] |
| ENSG00000198083 | HGLibA_25852 | 7 | -1.078944697 | KRTAP9-9 | keratin associated protein 9-9 [Source:HGNC Symbol;Acc:HGNC:16773] |
| ENSG00000198796 | HGLibA_01703 | 7 | -1.078944697 | ALPK2 | alpha kinase 2 [Source:HGNC Symbol;Acc:HGNC:20565] |
| ENSG00000198814 | HGLibA_19195 | 7 | -1.078944697 | GK | glycerol kinase [Source:HGNC Symbol;Acc:HGNC:4289] |
| ENSG00000204065 | HGLibA_48715 | 7 | -1.078944697 | TCEAL5 | transcription elongation factor A like 5 [Source:HGNC Symbol;Acc:HGNC:22282] |
| ENSG00000204071 | HGLibA_48718 | 7 | -1.078944697 | TCEAL6 | transcription elongation factor A like 6 [Source:HGNC Symbol;Acc:HGNC:24553] |
| ENSG00000204574 | HGLibA_00207 | 7 | -1.078944697 | ABCF1 | ATP binding cassette subfamily F member 1 [Source:HGNC Symbol;Acc:HGNC:70] |
| ENSG00000204669 | HGLibA_06742 | 7 | -1.078944697 | C9orf57 | chromosome 9 open reading frame 57 [Source:HGNC Symbol;Acc:HGNC:27037] |
| ENSG00000204928 | HGLibA_20391 | 7 | -1.078944697 | GRXCR2 | glutaredoxin and cysteine rich domain containing 2 [Source:HGNC Symbol;Acc:HGNC:33862] |
| ENSG00000204970 | HGLibA_35425 | 7 | -1.078944697 | PCDHA1 | protocadherin alpha 1 [Source:HGNC Symbol;Acc:HGNC:8663] |
| ENSG00000206013 | HGLibA_22649 | 7 | -1.078944697 | IFITM5 | interferon induced transmembrane protein 5 [Source:HGNC Symbol;Acc:HGNC:16644] |
| ENSG00000213015 | HGLibA_56304 | 7 | -1.078944697 | ZNF580 | zinc finger protein 580 [Source:HGNC Symbol;Acc:HGNC:29473] |
| ENSG00000213171 | HGLibA_26595 | 7 | -1.078944697 | LINGO4 | leucine rich repeat and Ig domain containing 4 [Source:HGNC Symbol;Acc:HGNC:31814] |
| ENSG00000227729 | HGLibA_40729 | 7 | -1.078944697 | RD3L | retinal degeneration 3 like [Source:HGNC Symbol;Acc:HGNC:40912] |
| ENSG00000235034 | HGLibA_05713 | 7 | -1.078944697 | C19orf81 | chromosome 19 open reading frame 81 [Source:HGNC Symbol;Acc:HGNC:40041] |
| ENSG00000237671 | HGLibA_18441 | 7 | -1.078944697 | GAGE12C | G antigen 12C [Source:HGNC Symbol;Acc:HGNC:28402] |
| ENSG00000243811 | HGLibA_02486 | 7 | -1.078944697 | APOBEC3D | apolipoprotein B mRNA editing enzyme catalytic subunit 3D [Source:HGNC Symbol;Acc:HGNC:17354] |
| ENSG00000244731 | HGLibA_06282 | 7 | -1.078944697 | C4A | complement C4A (Rodgers blood group) [Source:HGNC Symbol;Acc:HGNC:1323] |
| ENSG00000248920 | HGLibA_53235 | 7 | -1.078944697 | USP17L19 | ubiquitin specific peptidase 17-like family member 19 [Source:HGNC Symbol;Acc:HGNC:44447] |
| ENSG00000249884 | HGLibA_41460 | 7 | -1.078944697 | RNF103-CHMP3 | RNF103-CHMP3 readthrough [Source:HGNC Symbol;Acc:HGNC:38847] |
| ENSG00000253276 | HGLibA_07855 | 7 | -1.078944697 | CCDC71L | coiled-coil domain containing 71 like [Source:HGNC Symbol;Acc:HGNC:26685] |
| ENSG00000253953 | HGLibA_35559 | 7 | -1.078944697 | PCDHGB4 | 4 [Source:HGNC Symbol;Acc:HGNC:8711]" |
| ENSG00000254505 | HGLibA_09486 | 7 | -1.078944697 | CHMP4A | charged multivesicular body protein 4A [Source:HGNC Symbol;Acc:HGNC:20274] |
| ENSG00000266919 | HGLibA_59700 | 7 | -1.078944697 | hsa-mir-423 | microRNA 3184 |
| ENSG00000268221 | HGLibA_33426 | 7 | -1.078944697 | OPN1MW | medium wave sensitive [Source:HGNC Symbol;Acc:HGNC:4206]" |
| ENSG00000274274 | HGLibA_18465 | 7 | -1.078944697 | GAGE13 | G antigen 13 [Source:HGNC Symbol;Acc:HGNC:29081] |
| ENSG00000278129 | HGLibA_56779 | 7 | -1.078944697 | ZNF8 | zinc finger protein 8 [Source:HGNC Symbol;Acc:HGNC:13154] |
| FAM211A | HGLibA_16459 | 7 | -1.078944697 | FAM211A | - |
| hsa-mir-1262 | HGLibA_57470 | 7 | -1.078944697 | hsa-mir-1262 | - |
| hsa-mir-1303 | HGLibA_57728 | 7 | -1.078944697 | hsa-mir-1303 | - |
| hsa-mir-24-1 | HGLibA_58342 | 7 | -1.078944697 | hsa-mir-24-1 | - |
| hsa-mir-3180-2 | HGLibA_58806 | 7 | -1.078944697 | hsa-mir-3180-2 | - |
| hsa-mir-3180-2 | HGLibA_58805 | 7 | -1.078944697 | hsa-mir-3180-2 | - |
| hsa-mir-3195 | HGLibA_58876 | 7 | -1.078944697 | hsa-mir-3195 | - |
| hsa-mir-3920 | HGLibA_59556 | 7 | -1.078944697 | hsa-mir-3920 | - |
| hsa-mir-4273 | HGLibA_59794 | 7 | -1.078944697 | hsa-mir-4273 | - |
| hsa-mir-4316 | HGLibA_59985 | 7 | -1.078944697 | hsa-mir-4316 | - |
| hsa-mir-4432 | HGLibA_60115 | 7 | -1.078944697 | hsa-mir-4432 | - |
| hsa-mir-4435-2 | HGLibA_60133 | 7 | -1.078944697 | hsa-mir-4435-2 | - |
| hsa-mir-4446 | HGLibA_60190 | 7 | -1.078944697 | hsa-mir-4446 | - |
| hsa-mir-4477b | HGLibA_60323 | 7 | -1.078944697 | hsa-mir-4477b | - |
| hsa-mir-4659a | HGLibA_60743 | 7 | -1.078944697 | hsa-mir-4659a | - |
| hsa-mir-4697 | HGLibA_60895 | 7 | -1.078944697 | hsa-mir-4697 | - |
| hsa-mir-4722 | HGLibA_60986 | 7 | -1.078944697 | hsa-mir-4722 | - |
| hsa-mir-517c | HGLibA_61633 | 7 | -1.078944697 | hsa-mir-517c | - |
| hsa-mir-548ag-2 | HGLibA_61878 | 7 | -1.078944697 | hsa-mir-548ag-2 | - |
| hsa-mir-548i-1 | HGLibA_62012 | 7 | -1.078944697 | hsa-mir-548i-1 | - |
| hsa-mir-550a-1 | HGLibA_62098 | 7 | -1.078944697 | hsa-mir-550a-1 | - |
| hsa-mir-589 | HGLibA_62454 | 7 | -1.078944697 | hsa-mir-589 | - |
| hsa-mir-607 | HGLibA_62530 | 7 | -1.078944697 | hsa-mir-607 | - |
| hsa-mir-622 | HGLibA_62726 | 7 | -1.078944697 | hsa-mir-622 | - |
| hsa-mir-645 | HGLibA_62825 | 7 | -1.078944697 | hsa-mir-645 | - |
| hsa-mir-663b | HGLibA_62990 | 7 | -1.078944697 | hsa-mir-663b | - |
| hsa-mir-8075 | HGLibA_64144 | 7 | -1.078944697 | hsa-mir-8075 | - |
| LOC642799 | HGLibA_26961 | 7 | -1.078944697 | LOC642799 | - |
| PPAPDC3 | HGLibA_37835 | 7 | -1.078944697 | PPAPDC3 | - |
| USP17L1P | HGLibA_53238 | 7 | -1.078944697 | USP17L1P | - |
| VIMP | HGLibA_53658 | 7 | -1.078944697 | VIMP | - |
| ZNF259 | HGLibA_55685 | 7 | -1.078944697 | ZNF259 | - |
| C15orf59 | HGLibA_05423 | 6 | -1.088895734 | C15orf59 | - |
| CGB | HGLibA_09298 | 6 | -1.088895734 | CGB | - |
| COL4A3BP | HGLibA_10574 | 6 | -1.088895734 | COL4A3BP | - |
| ELTD1 | HGLibA_14995 | 6 | -1.088895734 | ELTD1 | - |
| ENSG00000003400 | HGLibA_07299 | 6 | -1.088895734 | CASP10 | caspase 10 [Source:HGNC Symbol;Acc:HGNC:1500] |
| ENSG00000007520 | HGLibA_51999 | 6 | -1.088895734 | TSR3 | acp transferase ribosome maturation factor [Source:HGNC Symbol;Acc:HGNC:14175]" |
| ENSG00000015285 | HGLibA_53951 | 6 | -1.088895734 | WAS | Wiskott-Aldrich syndrome [Source:HGNC Symbol;Acc:HGNC:12731] |
| ENSG00000022976 | HGLibA_56848 | 6 | -1.088895734 | ZNF839 | zinc finger protein 839 [Source:HGNC Symbol;Acc:HGNC:20345] |
| ENSG00000042286 | HGLibA_01341 | 6 | -1.088895734 | AIFM2 | mitochondria associated 2 [Source:HGNC Symbol;Acc:HGNC:21411]" |
| ENSG00000049618 | HGLibA_02886 | 6 | -1.088895734 | ARID1B | AT-rich interaction domain 1B [Source:HGNC Symbol;Acc:HGNC:18040] |
| ENSG00000054598 | HGLibA_17822 | 6 | -1.088895734 | FOXC1 | forkhead box C1 [Source:HGNC Symbol;Acc:HGNC:3800] |
| ENSG00000055813 | HGLibA_07891 | 6 | -1.088895734 | CCDC85A | coiled-coil domain containing 85A [Source:HGNC Symbol;Acc:HGNC:29400] |
| ENSG00000068650 | HGLibA_03555 | 6 | -1.088895734 | ATP11A | ATPase phospholipid transporting 11A [Source:HGNC Symbol;Acc:HGNC:13552] |
| ENSG00000074319 | HGLibA_51845 | 6 | -1.088895734 | TSG101 | tumor susceptibility 101 [Source:HGNC Symbol;Acc:HGNC:15971] |
| ENSG00000074803 | HGLibA_44358 | 6 | -1.088895734 | SLC12A1 | solute carrier family 12 member 1 [Source:HGNC Symbol;Acc:HGNC:10910] |
| ENSG00000075539 | HGLibA_18057 | 6 | -1.088895734 | FRYL | FRY like transcription coactivator [Source:HGNC Symbol;Acc:HGNC:29127] |
| ENSG00000075790 | HGLibA_04239 | 6 | -1.088895734 | BCAP29 | B cell receptor associated protein 29 [Source:HGNC Symbol;Acc:HGNC:24131] |
| ENSG00000082196 | HGLibA_05943 | 6 | -1.088895734 | C1QTNF3 | C1q and TNF related 3 [Source:HGNC Symbol;Acc:HGNC:14326] |
| ENSG00000084234 | HGLibA_02443 | 6 | -1.088895734 | APLP2 | amyloid beta precursor like protein 2 [Source:HGNC Symbol;Acc:HGNC:598] |
| ENSG00000088782 | HGLibA_12885 | 6 | -1.088895734 | DEFB127 | defensin beta 127 [Source:HGNC Symbol;Acc:HGNC:16206] |

| | | | | | |
|---|---|---|---|---|---|
| ENSG00000089597 | HGLibA_18605 | 6 | -1.088895734 | GANAB | glucosidase II alpha subunit [Source:HGNC Symbol;Acc:HGNC:4138] |
| ENSG00000090266 | HGLibA_31513 | 6 | -1.088895734 | NDUFB2 | NADH:ubiquinone oxidoreductase subunit B2 [Source:HGNC Symbol;Acc:HGNC:7697] |
| ENSG00000095485 | HGLibA_11824 | 6 | -1.088895734 | CWF19L1 | CWF19 like cell cycle control factor 1 [Source:HGNC Symbol;Acc:HGNC:25613] |
| ENSG00000096264 | HGLibA_31364 | 6 | -1.088895734 | NCR2 | natural cytotoxicity triggering receptor 2 [Source:HGNC Symbol;Acc:HGNC:6732] |
| ENSG00000099942 | HGLibA_11184 | 6 | -1.088895734 | CRKL | adaptor protein [Source:HGNC Symbol;Acc:HGNC:2363]" |
| ENSG00000099949 | HGLibA_27803 | 6 | -1.088895734 | LZTR1 | leucine zipper like transcription regulator 1 [Source:HGNC Symbol;Acc:HGNC:6742] |
| ENSG00000100353 | HGLibA_14773 | 6 | -1.088895734 | EIF3D | eukaryotic translation initiation factor 3 subunit D [Source:HGNC Symbol;Acc:HGNC:3278] |
| ENSG00000101311 | HGLibA_17293 | 6 | -1.088895734 | FERMT1 | fermitin family member 1 [Source:HGNC Symbol;Acc:HGNC:15889] |
| ENSG00000103023 | HGLibA_38959 | 6 | -1.088895734 | PRSS54 | serine protease 54 [Source:HGNC Symbol;Acc:HGNC:26336] |
| ENSG00000103876 | HGLibA_15951 | 6 | -1.088895734 | FAH | fumarylacetoacetate hydrolase [Source:HGNC Symbol;Acc:HGNC:3579] |
| ENSG00000105835 | HGLibA_31088 | 6 | -1.088895734 | NAMPT | nicotinamide phosphoribosyltransferase [Source:HGNC Symbol;Acc:HGNC:30092] |
| ENSG00000106113 | HGLibA_11144 | 6 | -1.088895734 | CRHR2 | corticotropin releasing hormone receptor 2 [Source:HGNC Symbol;Acc:HGNC:2358] |
| ENSG00000106462 | HGLibA_15835 | 6 | -1.088895734 | EZH2 | enhancer of zeste 2 polycomb repressive complex 2 subunit [Source:HGNC Symbol;Acc:HGNC:3527] |
| ENSG00000106809 | HGLibA_33325 | 6 | -1.088895734 | OGN | osteoglycin [Source:HGNC Symbol;Acc:HGNC:8126] |
| ENSG00000107362 | HGLibA_00265 | 6 | -1.088895734 | ABHD17B | abhydrolase domain containing 17B [Source:HGNC Symbol;Acc:HGNC:24278] |
| ENSG00000107611 | HGLibA_11765 | 6 | -1.088895734 | CUBN | cubilin [Source:HGNC Symbol;Acc:HGNC:2548] |
| ENSG00000109390 | HGLibA_31535 | 6 | -1.088895734 | NDUFC1 | NADH:ubiquinone oxidoreductase subunit C1 [Source:HGNC Symbol;Acc:HGNC:7705] |
| ENSG00000109610 | HGLibA_46150 | 6 | -1.088895734 | SOD3 | superoxide dismutase 3 [Source:HGNC Symbol;Acc:HGNC:11181] |
| ENSG00000110076 | HGLibA_32737 | 6 | -1.088895734 | NRXN2 | neurexin 2 [Source:HGNC Symbol;Acc:HGNC:8009] |
| ENSG00000110583 | HGLibA_30997 | 6 | -1.088895734 | NAA40 | NatD catalytic subunit [Source:HGNC Symbol;Acc:HGNC:25845]" |
| ENSG00000110852 | HGLibA_09964 | 6 | -1.088895734 | CLEC2B | C-type lectin domain family 2 member B [Source:HGNC Symbol;Acc:HGNC:2053] |
| ENSG00000110880 | HGLibA_10779 | 6 | -1.088895734 | CORO1C | coronin 1C [Source:HGNC Symbol;Acc:HGNC:2254] |
| ENSG00000111058 | HGLibA_00592 | 6 | -1.088895734 | ACSS3 | acyl-CoA synthetase short chain family member 3 [Source:HGNC Symbol;Acc:HGNC:24723] |
| ENSG00000111670 | HGLibA_19574 | 6 | -1.088895734 | GNPTAB | N-acetylglucosamine-1-phosphate transferase subunits alpha and beta [Source:HGNC Symbol;Acc:HGNC:29670] |
| ENSG00000111802 | HGLibA_48893 | 6 | -1.088895734 | TDP2 | tyrosyl-DNA phosphodiesterase 2 [Source:HGNC Symbol;Acc:HGNC:17768] |
| ENSG00000115310 | HGLibA_42406 | 6 | -1.088895734 | RTN4 | reticulon 4 [Source:HGNC Symbol;Acc:HGNC:14085] |
| ENSG00000116641 | HGLibA_13755 | 6 | -1.088895734 | DOCK7 | dedicator of cytokinesis 7 [Source:HGNC Symbol;Acc:HGNC:19190] |
| ENSG00000119138 | HGLibA_25088 | 6 | -1.088895734 | KLF9 | Kruppel like factor 9 [Source:HGNC Symbol;Acc:HGNC:1123] |
| ENSG00000120337 | HGLibA_50819 | 6 | -1.088895734 | TNFSF18 | TNF superfamily member 18 [Source:HGNC Symbol;Acc:HGNC:11932] |
| ENSG00000120690 | HGLibA_14890 | 6 | -1.088895734 | ELF1 | E74 like ETS transcription factor 1 [Source:HGNC Symbol;Acc:HGNC:3316] |
| ENSG00000124935 | HGLibA_42916 | 6 | -1.088895734 | SCGB1D2 | secretoglobin family 1D member 2 [Source:HGNC Symbol;Acc:HGNC:18396] |
| ENSG00000125831 | HGLibA_11448 | 6 | -1.088895734 | CST11 | cystatin 11 [Source:HGNC Symbol;Acc:HGNC:15959] |
| ENSG00000126822 | HGLibA_37108 | 6 | -1.088895734 | PLEKHG3 | pleckstrin homology and RhoGEF domain containing G3 [Source:HGNC Symbol;Acc:HGNC:20364] |
| ENSG00000127580 | HGLibA_54048 | 6 | -1.088895734 | WDR24 | WD repeat domain 24 [Source:HGNC Symbol;Acc:HGNC:20852] |
| ENSG00000127914 | HGLibA_01452 | 6 | -1.088895734 | AKAP9 | A-kinase anchoring protein 9 [Source:HGNC Symbol;Acc:HGNC:379] |
| ENSG00000128829 | HGLibA_14731 | 6 | -1.088895734 | EIF2AK4 | eukaryotic translation initiation factor 2 alpha kinase 4 [Source:HGNC Symbol;Acc:HGNC:19687] |
| ENSG00000130176 | HGLibA_10305 | 6 | -1.088895734 | CNN1 | calponin 1 [Source:HGNC Symbol;Acc:HGNC:2155] |
| ENSG00000130595 | HGLibA_50889 | 6 | -1.088895734 | TNNT3 | fast skeletal type [Source:HGNC Symbol;Acc:HGNC:11950]" |
| ENSG00000131233 | HGLibA_19153 | 6 | -1.088895734 | GJA9 | gap junction protein alpha 9 [Source:HGNC Symbol;Acc:HGNC:19155] |
| ENSG00000132744 | HGLibA_00706 | 6 | -1.088895734 | ACY3 | aminoacylase 3 [Source:HGNC Symbol;Acc:HGNC:24104] |
| ENSG00000133318 | HGLibA_42404 | 6 | -1.088895734 | RTN3 | reticulon 3 [Source:HGNC Symbol;Acc:HGNC:10469] |
| ENSG00000134072 | HGLibA_07059 | 6 | -1.088895734 | CAMK1 | calcium/calmodulin dependent protein kinase I [Source:HGNC Symbol;Acc:HGNC:1459] |
| ENSG00000134533 | HGLibA_40859 | 6 | -1.088895734 | RERG | RAS like estrogen regulated growth inhibitor [Source:HGNC Symbol;Acc:HGNC:15980] |
| ENSG00000135924 | HGLibA_13538 | 6 | -1.088895734 | DNAJB2 | DnaJ heat shock protein family (Hsp40) member B2 [Source:HGNC Symbol;Acc:HGNC:5228] |
| ENSG00000136197 | HGLibA_06524 | 6 | -1.088895734 | C7orf25 | chromosome 7 open reading frame 25 [Source:HGNC Symbol;Acc:HGNC:21703] |
| ENSG00000136942 | HGLibA_41945 | 6 | -1.088895734 | RPL35 | ribosomal protein L35 [Source:HGNC Symbol;Acc:HGNC:10344] |
| ENSG00000137338 | HGLibA_36216 | 6 | -1.088895734 | PGBD1 | piggyBac transposable element derived 1 [Source:HGNC Symbol;Acc:HGNC:19398] |
| ENSG00000137343 | HGLibA_03423 | 6 | -1.088895734 | ATAT1 | alpha tubulin acetyltransferase 1 [Source:HGNC Symbol;Acc:HGNC:21186] |
| ENSG00000137364 | HGLibA_51154 | 6 | -1.088895734 | TPMT | thiopurine S-methyltransferase [Source:HGNC Symbol;Acc:HGNC:12014] |
| ENSG00000137642 | HGLibA_46194 | 6 | -1.088895734 | SORL1 | sortilin related receptor 1 [Source:HGNC Symbol;Acc:HGNC:11185] |
| ENSG00000137968 | HGLibA_45168 | 6 | -1.088895734 | SLC44A5 | solute carrier family 44 member 5 [Source:HGNC Symbol;Acc:HGNC:28524] |
| ENSG00000138698 | HGLibA_40233 | 6 | -1.088895734 | RAP1GDS1 | Rap1 GTPase-GDP dissociation stimulator 1 [Source:HGNC Symbol;Acc:HGNC:9859] |
| ENSG00000138759 | HGLibA_17976 | 6 | -1.088895734 | FRAS1 | Fraser extracellular matrix complex subunit 1 [Source:HGNC Symbol;Acc:HGNC:19185] |
| ENSG00000139546 | HGLibA_48334 | 6 | -1.088895734 | TARBP2 | RISC loading complex RNA binding subunit [Source:HGNC Symbol;Acc:HGNC:11569]" |
| ENSG00000139629 | HGLibA_18565 | 6 | -1.088895734 | GALNT6 | polypeptide N-acetylgalactosaminyltransferase 6 [Source:HGNC Symbol;Acc:HGNC:4128] |
| ENSG00000139800 | HGLibA_55333 | 6 | -1.088895734 | ZIC5 | Zic family member 5 [Source:HGNC Symbol;Acc:HGNC:20322] |
| ENSG00000140943 | HGLibA_28535 | 6 | -1.088895734 | MBTPS1 | site 1 [Source:HGNC Symbol;Acc:HGNC:15456]" |
| ENSG00000143344 | HGLibA_41000 | 6 | -1.088895734 | RGL1 | ral guanine nucleotide dissociation stimulator like 1 [Source:HGNC Symbol;Acc:HGNC:30281] |
| ENSG00000143457 | HGLibA_19659 | 6 | -1.088895734 | GOLPH3L | golgi phosphoprotein 3 like [Source:HGNC Symbol;Acc:HGNC:24882] |
| ENSG00000143727 | HGLibA_00510 | 6 | -1.088895734 | ACP1 | acid phosphatase 1 [Source:HGNC Symbol;Acc:HGNC:122] |
| ENSG00000144040 | HGLibA_43794 | 6 | -1.088895734 | SFXN5 | sideroflexin 5 [Source:HGNC Symbol;Acc:HGNC:16073] |
| ENSG00000145375 | HGLibA_46515 | 6 | -1.088895734 | SPATA5 | spermatogenesis associated 5 [Source:HGNC Symbol;Acc:HGNC:18119] |
| ENSG00000146151 | HGLibA_21705 | 6 | -1.088895734 | HMGCLL1 | 3-hydroxymethyl-3-methylglutaryl-CoA lyase like 1 [Source:HGNC Symbol;Acc:HGNC:21359] |
| ENSG00000147130 | HGLibA_55396 | 6 | -1.088895734 | ZMYM3 | zinc finger MYM-type containing 3 [Source:HGNC Symbol;Acc:HGNC:13054] |
| ENSG00000147434 | HGLibA_09581 | 6 | -1.088895734 | CHRNA6 | cholinergic receptor nicotinic alpha 6 subunit [Source:HGNC Symbol;Acc:HGNC:15963] |
| ENSG00000147601 | HGLibA_49013 | 6 | -1.088895734 | TERF1 | telomeric repeat binding factor 1 [Source:HGNC Symbol;Acc:HGNC:11728] |
| ENSG00000148468 | HGLibA_16265 | 6 | -1.088895734 | FAM171A1 | family with sequence similarity 171 member A1 [Source:HGNC Symbol;Acc:HGNC:23522] |
| ENSG00000148832 | HGLibA_35118 | 6 | -1.088895734 | PAOX | polyamine oxidase [Source:HGNC Symbol;Acc:HGNC:20837] |
| ENSG00000151136 | HGLibA_04900 | 6 | -1.088895734 | BTBD11 | BTB domain containing 11 [Source:HGNC Symbol;Acc:HGNC:23844] |
| ENSG00000151715 | HGLibA_50412 | 6 | -1.088895734 | TMEM45B | transmembrane protein 45B [Source:HGNC Symbol;Acc:HGNC:25194] |
| ENSG00000152939 | HGLibA_28383 | 6 | -1.088895734 | MARVELD2 | MARVEL domain containing 2 [Source:HGNC Symbol;Acc:HGNC:26401] |
| ENSG00000154080 | HGLibA_09651 | 6 | -1.088895734 | CHST9 | carbohydrate sulfotransferase 9 [Source:HGNC Symbol;Acc:HGNC:19898] |
| ENSG00000154217 | HGLibA_36746 | 6 | -1.088895734 | PITPNC1 | phosphatidylinositol transfer protein cytoplasmic 1 [Source:HGNC Symbol;Acc:HGNC:21045] |
| ENSG00000154856 | HGLibA_02395 | 6 | -1.088895734 | APCDD1 | APC down-regulated 1 [Source:HGNC Symbol;Acc:HGNC:15718] |
| ENSG00000155066 | HGLibA_38683 | 6 | -1.088895734 | PROM2 | prominin 2 [Source:HGNC Symbol;Acc:HGNC:20685] |
| ENSG00000155508 | HGLibA_10353 | 6 | -1.088895734 | CNOT8 | CCR4-NOT transcription complex subunit 8 [Source:HGNC Symbol;Acc:HGNC:9207] |
| ENSG00000157500 | HGLibA_02567 | 6 | -1.088895734 | APPL1 | phosphotyrosine interacting with PH domain and leucine zipper 1 [Source:HGNC Symbol;Acc:HGNC:24035]" |
| ENSG00000157613 | HGLibA_11092 | 6 | -1.088895734 | CREB3L1 | cAMP responsive element binding protein 3 like 1 [Source:HGNC Symbol;Acc:HGNC:18856] |
| ENSG00000157778 | HGLibA_39210 | 6 | -1.088895734 | PSMG3 | proteasome assembly chaperone 3 [Source:HGNC Symbol;Acc:HGNC:22420] |
| ENSG00000162777 | HGLibA_12943 | 6 | -1.088895734 | DENND2D | DENN domain containing 2D [Source:HGNC Symbol;Acc:HGNC:26192] |
| ENSG00000163565 | HGLibA_22592 | 6 | -1.088895734 | IFI16 | interferon gamma inducible protein 16 [Source:HGNC Symbol;Acc:HGNC:5395] |
| ENSG00000164093 | HGLibA_36766 | 6 | -1.088895734 | PITX2 | paired like homeodomain 2 [Source:HGNC Symbol;Acc:HGNC:9005] |
| ENSG00000164411 | HGLibA_19176 | 6 | -1.088895734 | GJB7 | gap junction protein beta 7 [Source:HGNC Symbol;Acc:HGNC:16690] |
| ENSG00000164483 | HGLibA_42680 | 6 | -1.088895734 | SAMD3 | sterile alpha motif domain containing 3 [Source:HGNC Symbol;Acc:HGNC:21574] |
| ENSG00000165084 | HGLibA_06610 | 6 | -1.088895734 | C8orf34 | chromosome 8 open reading frame 34 [Source:HGNC Symbol;Acc:HGNC:30905] |
| ENSG00000165202 | HGLibA_33711 | 6 | -1.088895734 | OR1Q1 | olfactory receptor family 1 subfamily Q member 1 [Source:HGNC Symbol;Acc:HGNC:8223] |
| ENSG00000166855 | HGLibA_10120 | 6 | -1.088895734 | CLPX | caseinolytic mitochondrial matrix peptidase chaperone subunit [Source:HGNC Symbol;Acc:HGNC:2088] |
| ENSG00000166862 | HGLibA_06932 | 6 | -1.088895734 | CACNG2 | calcium voltage-gated channel auxiliary subunit gamma 2 [Source:HGNC Symbol;Acc:HGNC:1406] |
| ENSG00000166863 | HGLibA_48152 | 6 | -1.088895734 | TAC3 | tachykinin 3 [Source:HGNC Symbol;Acc:HGNC:11521] |
| ENSG00000168268 | HGLibA_32822 | 6 | -1.088895734 | NT5DC2 | 5'-nucleotidase domain containing 2 [Source:HGNC Symbol;Acc:HGNC:25717] |
| ENSG00000169221 | HGLibA_48468 | 6 | -1.088895734 | TBC1D10B | TBC1 domain family member 10B [Source:HGNC Symbol;Acc:HGNC:24510] |
| ENSG00000169515 | HGLibA_07874 | 6 | -1.088895734 | CCDC8 | coiled-coil domain containing 8 [Source:HGNC Symbol;Acc:HGNC:25367] |
| ENSG00000169599 | HGLibA_31845 | 6 | -1.088895734 | NFU1 | NFU1 iron-sulfur cluster scaffold [Source:HGNC Symbol;Acc:HGNC:16287] |
| ENSG00000170967 | HGLibA_12608 | 6 | -1.088895734 | DDI1 | DNA damage inducible 1 homolog 1 [Source:HGNC Symbol;Acc:HGNC:18961] |
| ENSG00000172985 | HGLibA_43977 | 6 | -1.088895734 | SH3RF3 | SH3 domain containing ring finger 3 [Source:HGNC Symbol;Acc:HGNC:24699] |
| ENSG00000173402 | HGLibA_12292 | 6 | -1.088895734 | DAG1 | dystroglycan 1 [Source:HGNC Symbol;Acc:HGNC:2666] |
| ENSG00000173467 | HGLibA_01248 | 6 | -1.088895734 | AGR3 | protein disulphide isomerase family member [Source:HGNC Symbol;Acc:HGNC:24167]" |
| ENSG00000173809 | HGLibA_48903 | 6 | -1.088895734 | TDRD12 | tudor domain containing 12 [Source:HGNC Symbol;Acc:HGNC:25044] |
| ENSG00000173914 | HGLibA_40573 | 6 | -1.088895734 | RBM4B | RNA binding motif protein 4B [Source:HGNC Symbol;Acc:HGNC:28842] |
| ENSG00000174780 | HGLibA_47011 | 6 | -1.088895734 | SRP72 | signal recognition particle 72 [Source:HGNC Symbol;Acc:HGNC:11303] |
| ENSG00000176014 | HGLibA_52292 | 6 | -1.088895734 | TUBB6 | tubulin beta 6 class V [Source:HGNC Symbol;Acc:HGNC:20776] |
| ENSG00000176269 | HGLibA_33998 | 6 | -1.088895734 | OR4F21 | olfactory receptor family 4 subfamily F member 21 [Source:HGNC Symbol;Acc:HGNC:19583] |
| ENSG00000177150 | HGLibA_16453 | 6 | -1.088895734 | FAM210A | family with sequence similarity 210 member A [Source:HGNC Symbol;Acc:HGNC:28346] |
| ENSG00000178568 | HGLibA_15422 | 6 | -1.088895734 | ERBB4 | erb-b2 receptor tyrosine kinase 4 [Source:HGNC Symbol;Acc:HGNC:3432] |
| ENSG00000180176 | HGLibA_49279 | 6 | -1.088895734 | TH | tyrosine hydroxylase [Source:HGNC Symbol;Acc:HGNC:11782] |
| ENSG00000181617 | HGLibA_17248 | 6 | -1.088895734 | FDCSP | follicular dendritic cell secreted protein [Source:HGNC Symbol;Acc:HGNC:19215] |
| ENSG00000182674 | HGLibA_24158 | 6 | -1.088895734 | KCNB2 | potassium voltage-gated channel subfamily B member 2 [Source:HGNC Symbol;Acc:HGNC:6232] |
| ENSG00000182831 | HGLibA_05477 | 6 | -1.088895734 | C16orf72 | chromosome 16 open reading frame 72 [Source:HGNC Symbol;Acc:HGNC:30103] |
| ENSG00000183273 | HGLibA_07814 | 6 | -1.088895734 | CCDC60 | coiled-coil domain containing 60 [Source:HGNC Symbol;Acc:HGNC:28610] |
| ENSG00000183684 | HGLibA_01741 | 6 | -1.088895734 | ALYREF | Aly/REF export factor [Source:HGNC Symbol;Acc:HGNC:19071] |
| ENSG00000184185 | HGLibA_24254 | 6 | -1.088895734 | KCNJ12 | potassium voltage-gated channel subfamily J member 12 [Source:HGNC Symbol;Acc:HGNC:6258] |
| ENSG00000184344 | HGLibA_18865 | 6 | -1.088895734 | GDF3 | growth differentiation factor 3 [Source:HGNC Symbol;Acc:HGNC:4218] |
| ENSG00000185250 | HGLibA_37951 | 6 | -1.088895734 | PPIL6 | peptidylprolyl isomerase like 6 [Source:HGNC Symbol;Acc:HGNC:21557] |
| ENSG00000185359 | HGLibA_21245 | 6 | -1.088895734 | HGS | hepatocyte growth factor-regulated tyrosine kinase substrate [Source:HGNC Symbol;Acc:HGNC:4897] |
| ENSG00000186119 | HGLibA_34284 | 6 | -1.088895734 | OR5D18 | olfactory receptor family 5 subfamily D member 18 [Source:HGNC Symbol;Acc:HGNC:15285] |
| ENSG00000186288 | HGLibA_34927 | 6 | -1.088895734 | PABPC1L2A | poly(A) binding protein cytoplasmic 1 like 2A [Source:HGNC Symbol;Acc:HGNC:27989] |
| ENSG00000187754 | HGLibA_47219 | 6 | -1.088895734 | SSX7 | SSX family member 7 [Source:HGNC Symbol;Acc:HGNC:19653] |
| ENSG00000187806 | HGLibA_50183 | 6 | -1.088895734 | TMEM202 | transmembrane protein 202 [Source:HGNC Symbol;Acc:HGNC:33733] |
| ENSG00000188828 | HGLibA_19308 | 6 | -1.088895734 | GLRA4 | glycine receptor alpha 4 [Source:HGNC Symbol;Acc:HGNC:31715] |
| ENSG00000189169 | HGLibA_25600 | 6 | -1.088895734 | KRTAP10-12 | keratin associated protein 10-12 [Source:HGNC Symbol;Acc:HGNC:20533] |
| ENSG00000189252 | HGLibA_46407 | 6 | -1.088895734 | SPANXN3 | SPANX family member N3 [Source:HGNC Symbol;Acc:HGNC:33176] |
| ENSG00000189266 | HGLibA_37438 | 6 | -1.088895734 | PNRC2 | proline rich nuclear receptor coactivator 2 [Source:HGNC Symbol;Acc:HGNC:23158] |
| ENSG00000196465 | HGLibA_30771 | 6 | -1.088895734 | MYL6B | myosin light chain 6B [Source:HGNC Symbol;Acc:HGNC:29823] |
| ENSG00000197170 | HGLibA_39157 | 6 | -1.088895734 | PSMD12 | non-ATPase 12 [Source:HGNC Symbol;Acc:HGNC:9557]" |
| ENSG00000197766 | HGLibA_09255 | 6 | -1.088895734 | CFD | complement factor D [Source:HGNC Symbol;Acc:HGNC:2771] |
| ENSG00000198077 | HGLibA_12110 | 6 | -1.088895734 | CYP2A7 | cytochrome P450 family 2 subfamily A member 7 [Source:HGNC Symbol;Acc:HGNC:2611] |
| ENSG00000204427 | HGLibA_00257 | 6 | -1.088895734 | ABHD16A | abhydrolase domain containing 16A [Source:HGNC Symbol;Acc:HGNC:13921] |
| ENSG00000206127 | HGLibA_19647 | 6 | -1.088895734 | GOLGA8O | golgin A8 family member O [Source:HGNC Symbol;Acc:HGNC:44406] |
| ENSG00000213516 | HGLibA_40602 | 6 | -1.088895734 | RBMXL1 | RBMX like 1 [Source:HGNC Symbol;Acc:HGNC:25073] |
| ENSG00000213906 | HGLibA_27588 | 6 | -1.088895734 | LTB4R2 | leukotriene B4 receptor 2 [Source:HGNC Symbol;Acc:HGNC:19260] |
| ENSG00000214160 | HGLibA_01627 | 6 | -1.088895734 | ALG3 | 3- mannosyltransferase [Source:HGNC Symbol;Acc:HGNC:23056]" |
| ENSG00000215114 | HGLibA_52786 | 6 | -1.088895734 | UBXN2B | UBX domain protein 2B [Source:HGNC Symbol;Acc:HGNC:27035] |
| ENSG00000236362 | HGLibA_18450 | 6 | -1.088895734 | GAGE12F | G antigen 12F [Source:HGNC Symbol;Acc:HGNC:31906] |
| ENSG00000236980 | HGLibA_06277 | 6 | -1.088895734 | C3orf84 | chromosome 3 open reading frame 84 [Source:HGNC Symbol;Acc:HGNC:44666] |
| ENSG00000250745 | HGLibA_53244 | 6 | -1.088895734 | USP17L20 | ubiquitin specific peptidase 17-like family member 20 [Source:HGNC Symbol;Acc:HGNC:44448] |

| | | | | | |
|---|---|---|---|---|---|
| ENSG00000255072 | HGLibA_36604 | 6 | -1.088895734 | PIGY | phosphatidylinositol glycan anchor biosynthesis class Y [Source:HGNC Symbol;Acc:HGNC:28213] |
| ENSG00000255302 | HGLibA_14694 | 6 | -1.088895734 | EID1 | EP300 interacting inhibitor of differentiation 1 [Source:HGNC Symbol;Acc:HGNC:1191] |
| ENSG00000256892 | HGLibA_30489 | 6 | -1.088895734 | MTRNR2L7 | MT-RNR2 like 7 [Source:HGNC Symbol;Acc:HGNC:37164] |
| ENSG00000256892 | HGLibA_30488 | 6 | -1.088895734 | MTRNR2L7 | MT-RNR2 like 7 [Source:HGNC Symbol;Acc:HGNC:37164] |
| ENSG00000260458 | HGLibA_24269 | 6 | -1.088895734 | KCNJ18 | potassium voltage-gated channel subfamily J member 18 [Source:HGNC Symbol;Acc:HGNC:39080] |
| ENSG00000260811 | HGLibA_33955 | 6 | -1.088895734 | OR4C45 | olfactory receptor family 4 subfamily C member 45 (gene/pseudogene) [Source:HGNC Symbol;Acc:HGNC:31270] |
| ENSG00000270011 | HGLibA_56239 | 6 | -1.088895734 | ZNF559-ZNF177 | ZNF559-ZNF177 readthrough [Source:HGNC Symbol;Acc:HGNC:42964] |
| ENSG00000284922 | HGLibA_27505 | 6 | -1.088895734 | LRTOMT | novel protein |
| GAGE6 | HGLibA_18486 | 6 | -1.088895734 | GAGE6 | - |
| GAGE7 | HGLibA_18489 | 6 | -1.088895734 | GAGE7 | - |
| GPR98 | HGLibA_20115 | 6 | -1.088895734 | GPR98 | - |
| H3F3A | HGLibA_20778 | 6 | -1.088895734 | H3F3A | - |
| HIST2H2AC | HGLibA_21530 | 6 | -1.088895734 | HIST2H2AC | - |
| hsa-mir-1181 | HGLibA_57219 | 6 | -1.088895734 | hsa-mir-1181 | - |
| hsa-mir-1185-2 | HGLibA_57245 | 6 | -1.088895734 | hsa-mir-1185-2 | - |
| hsa-mir-1302-9 | HGLibA_57725 | 6 | -1.088895734 | hsa-mir-1302-9 | - |
| hsa-mir-144 | HGLibA_57839 | 6 | -1.088895734 | hsa-mir-144 | - |
| hsa-mir-199a-2 | HGLibA_58134 | 6 | -1.088895734 | hsa-mir-199a-2 | - |
| hsa-mir-3184 | HGLibA_58833 | 6 | -1.088895734 | hsa-mir-3184 | - |
| hsa-mir-342 | HGLibA_59023 | 6 | -1.088895734 | hsa-mir-342 | - |
| hsa-mir-345 | HGLibA_59028 | 6 | -1.088895734 | hsa-mir-345 | - |
| hsa-mir-4466 | HGLibA_60270 | 6 | -1.088895734 | hsa-mir-4466 | - |
| hsa-mir-4485 | HGLibA_60357 | 6 | -1.088895734 | hsa-mir-4485 | - |
| hsa-mir-4644 | HGLibA_60680 | 6 | -1.088895734 | hsa-mir-4644 | - |
| hsa-mir-4755 | HGLibA_61116 | 6 | -1.088895734 | hsa-mir-4755 | - |
| hsa-mir-486 | HGLibA_61337 | 6 | -1.088895734 | hsa-mir-486 | - |
| hsa-mir-519a-2 | HGLibA_61715 | 6 | -1.088895734 | hsa-mir-519a-2 | - |
| hsa-mir-520h | HGLibA_61764 | 6 | -1.088895734 | hsa-mir-520h | - |
| hsa-mir-548k | HGLibA_62031 | 6 | -1.088895734 | hsa-mir-548k | - |
| hsa-mir-548w | HGLibA_62080 | 6 | -1.088895734 | hsa-mir-548w | - |
| hsa-mir-551a | HGLibA_62119 | 6 | -1.088895734 | hsa-mir-551a | - |
| hsa-mir-606 | HGLibA_62515 | 6 | -1.088895734 | hsa-mir-606 | - |
| hsa-mir-6071 | HGLibA_62536 | 6 | -1.088895734 | hsa-mir-6071 | - |
| hsa-mir-617 | HGLibA_62708 | 6 | -1.088895734 | hsa-mir-617 | - |
| hsa-mir-6509 | HGLibA_62886 | 6 | -1.088895734 | hsa-mir-6509 | - |
| hsa-mir-6718 | HGLibA_63034 | 6 | -1.088895734 | hsa-mir-6718 | - |
| hsa-mir-6832 | HGLibA_63505 | 6 | -1.088895734 | hsa-mir-6832 | - |
| hsa-mir-7161 | HGLibA_63874 | 6 | -1.088895734 | hsa-mir-7161 | - |
| hsa-mir-7703 | HGLibA_63947 | 6 | -1.088895734 | hsa-mir-7703 | - |
| hsa-mir-8075 | HGLibA_64142 | 6 | -1.088895734 | hsa-mir-8075 | - |
| hsa-mir-875 | HGLibA_64211 | 6 | -1.088895734 | hsa-mir-875 | - |
| KIAA1033 | HGLibA_24684 | 6 | -1.088895734 | KIAA1033 | - |
| MESDC1 | HGLibA_28911 | 6 | -1.088895734 | MESDC1 | - |
| MLL | HGLibA_29392 | 6 | -1.088895734 | MLL | - |
| MLL4 | HGLibA_29403 | 6 | -1.088895734 | MLL4 | - |
| MNF1 | HGLibA_29571 | 6 | -1.088895734 | MNF1 | - |
| PAPD7 | HGLibA_35126 | 6 | -1.088895734 | PAPD7 | - |
| SARS | HGLibA_42748 | 6 | -1.088895734 | SARS | - |
| SDPR | HGLibA_43157 | 6 | -1.088895734 | SDPR | - |
| SMCR7L | HGLibA_45680 | 6 | -1.088895734 | SMCR7L | - |
| ZNF812 | HGLibA_56803 | 6 | -1.088895734 | ZNF812 | - |
| 5-Mar | HGLibA_28340 | 5 | -1.098846772 | 5-Mar | - |
| C15orf41 | HGLibA_05398 | 5 | -1.098846772 | C15orf41 | - |
| C22orf26 | HGLibA_06061 | 5 | -1.098846772 | C22orf26 | - |
| C4orf27 | HGLibA_06312 | 5 | -1.098846772 | C4orf27 | - |
| C7orf55-LUC7L2 | HGLibA_06558 | 5 | -1.098846772 | C7orf55-LUC7L2 | - |
| CASC1 | HGLibA_07269 | 5 | -1.098846772 | CASC1 | - |
| CSF2RA_Y | HGLibA_11332 | 5 | -1.098846772 | CSF2RA_Y | - |
| CXorf57 | HGLibA_11947 | 5 | -1.098846772 | CXorf57 | - |
| ENSG00000006282 | HGLibA_46447 | 5 | -1.098846772 | SPATA20 | spermatogenesis associated 20 [Source:HGNC Symbol;Acc:HGNC:26125] |
| ENSG00000028310 | HGLibA_04788 | 5 | -1.098846772 | BRD9 | bromodomain containing 9 [Source:HGNC Symbol;Acc:HGNC:25818] |
| ENSG00000067334 | HGLibA_13723 | 5 | -1.098846772 | DNTTIP2 | deoxynucleotidyltransferase terminal interacting protein 2 [Source:HGNC Symbol;Acc:HGNC:24013] |
| ENSG00000067533 | HGLibA_42272 | 5 | -1.098846772 | RRP15 | ribosomal RNA processing 15 homolog [Source:HGNC Symbol;Acc:HGNC:24255] |
| ENSG00000068024 | HGLibA_21013 | 5 | -1.098846772 | HDAC4 | histone deacetylase 4 [Source:HGNC Symbol;Acc:HGNC:14063] |
| ENSG00000068985 | HGLibA_35004 | 5 | -1.098846772 | PAGE1 | PAGE family member 1 [Source:HGNC Symbol;Acc:HGNC:4107] |
| ENSG00000077463 | HGLibA_44231 | 5 | -1.098846772 | SIRT6 | sirtuin 6 [Source:HGNC Symbol;Acc:HGNC:14934] |
| ENSG00000079819 | HGLibA_15246 | 5 | -1.098846772 | EPB41L2 | erythrocyte membrane protein band 4.1 like 2 [Source:HGNC Symbol;Acc:HGNC:3379] |
| ENSG00000079841 | HGLibA_41277 | 5 | -1.098846772 | RIMS1 | regulating synaptic membrane exocytosis 1 [Source:HGNC Symbol;Acc:HGNC:17282] |
| ENSG00000081760 | HGLibA_00025 | 5 | -1.098846772 | AACS | acetoacetyl-CoA synthetase [Source:HGNC Symbol;Acc:HGNC:21298] |
| ENSG00000086696 | HGLibA_22188 | 5 | -1.098846772 | HSD17B2 | hydroxysteroid 17-beta dehydrogenase 2 [Source:HGNC Symbol;Acc:HGNC:5211] |
| ENSG00000087152 | HGLibA_03858 | 5 | -1.098846772 | ATXN7L3 | ataxin 7 like 3 [Source:HGNC Symbol;Acc:HGNC:25416] |
| ENSG00000088448 | HGLibA_01991 | 5 | -1.098846772 | ANKRD10 | ankyrin repeat domain 10 [Source:HGNC Symbol;Acc:HGNC:20265] |
| ENSG00000088836 | HGLibA_45207 | 5 | -1.098846772 | SLC4A11 | solute carrier family 4 member 11 [Source:HGNC Symbol;Acc:HGNC:16438] |
| ENSG00000089876 | HGLibA_13178 | 5 | -1.098846772 | DHX32 | DEAH-box helicase 32 (putative) [Source:HGNC Symbol;Acc:HGNC:16717] |
| ENSG00000090273 | HGLibA_32919 | 5 | -1.098846772 | NUDC | dynein complex regulator [Source:HGNC Symbol;Acc:HGNC:8045]" |
| ENSG00000091106 | HGLibA_32092 | 5 | -1.098846772 | NLRC4 | NLR family CARD domain containing 4 [Source:HGNC Symbol;Acc:HGNC:16412] |
| ENSG00000100146 | HGLibA_46228 | 5 | -1.098846772 | SOX10 | SRY-box 10 [Source:HGNC Symbol;Acc:HGNC:11190] |
| ENSG00000100348 | HGLibA_52403 | 5 | -1.098846772 | TXN2 | thioredoxin 2 [Source:HGNC Symbol;Acc:HGNC:17772] |
| ENSG00000100721 | HGLibA_48814 | 5 | -1.098846772 | TCL1A | T cell leukemia/lymphoma 1A [Source:HGNC Symbol;Acc:HGNC:11648] |
| ENSG00000101200 | HGLibA_03898 | 5 | -1.098846772 | AVP | arginine vasopressin [Source:HGNC Symbol;Acc:HGNC:894] |
| ENSG00000101276 | HGLibA_45250 | 5 | -1.098846772 | SLC52A3 | solute carrier family 52 member 3 [Source:HGNC Symbol;Acc:HGNC:16187] |
| ENSG00000101444 | HGLibA_01287 | 5 | -1.098846772 | AHCY | adenosylhomocysteinase [Source:HGNC Symbol;Acc:HGNC:343] |
| ENSG00000101638 | HGLibA_47300 | 5 | -1.098846772 | ST8SIA5 | 8-sialyltransferase 5 [Source:HGNC Symbol;Acc:HGNC:17827]" |
| ENSG00000101844 | HGLibA_03487 | 5 | -1.098846772 | ATG4A | autophagy related 4A cysteine peptidase [Source:HGNC Symbol;Acc:HGNC:16489] |
| ENSG00000102572 | HGLibA_47472 | 5 | -1.098846772 | STK24 | serine/threonine kinase 24 [Source:HGNC Symbol;Acc:HGNC:11403] |
| ENSG00000102967 | HGLibA_13111 | 5 | -1.098846772 | DHODH | dihydroorotate dehydrogenase (quinone) [Source:HGNC Symbol;Acc:HGNC:2867] |
| ENSG00000103512 | HGLibA_32288 | 5 | -1.098846772 | NOMO1 | NODAL modulator 1 [Source:HGNC Symbol;Acc:HGNC:30060] |
| ENSG00000103549 | HGLibA_41679 | 5 | -1.098846772 | RNF40 | ring finger protein 40 [Source:HGNC Symbol;Acc:HGNC:16867] |
| ENSG00000104427 | HGLibA_54936 | 5 | -1.098846772 | ZC2HC1A | zinc finger C2HC-type containing 1A [Source:HGNC Symbol;Acc:HGNC:24277] |
| ENSG00000105656 | HGLibA_14920 | 5 | -1.098846772 | ELL | elongation factor for RNA polymerase II [Source:HGNC Symbol;Acc:HGNC:23114] |
| ENSG00000106524 | HGLibA_01981 | 5 | -1.098846772 | ANKMY2 | ankyrin repeat and MYND domain containing 2 [Source:HGNC Symbol;Acc:HGNC:25370] |
| ENSG00000106683 | HGLibA_26541 | 5 | -1.098846772 | LIMK1 | LIM domain kinase 1 [Source:HGNC Symbol;Acc:HGNC:6613] |
| ENSG00000111664 | HGLibA_19497 | 5 | -1.098846772 | GNB3 | G protein subunit beta 3 [Source:HGNC Symbol;Acc:HGNC:4400] |
| ENSG00000111845 | HGLibA_35039 | 5 | -1.098846772 | PAK1IP1 | PAK1 interacting protein 1 [Source:HGNC Symbol;Acc:HGNC:20882] |
| ENSG00000112210 | HGLibA_39845 | 5 | -1.098846772 | RAB23 | member RAS oncogene family [Source:HGNC Symbol;Acc:HGNC:14263]" |
| ENSG00000112511 | HGLibA_36330 | 5 | -1.098846772 | PHF1 | PHD finger protein 1 [Source:HGNC Symbol;Acc:HGNC:8919] |
| ENSG00000112787 | HGLibA_16971 | 5 | -1.098846772 | FBRSL1 | fibrosin like 1 [Source:HGNC Symbol;Acc:HGNC:29308] |
| ENSG00000112799 | HGLibA_27687 | 5 | -1.098846772 | LY86 | lymphocyte antigen 86 [Source:HGNC Symbol;Acc:HGNC:16837] |
| ENSG00000113141 | HGLibA_22912 | 5 | -1.098846772 | IK | IK cytokine [Source:HGNC Symbol;Acc:HGNC:5958] |
| ENSG00000116874 | HGLibA_53946 | 5 | -1.098846772 | WARS2 | mitochondrial [Source:HGNC Symbol;Acc:HGNC:12730]" |
| ENSG00000117305 | HGLibA_21703 | 5 | -1.098846772 | HMGCL | 3-hydroxy-3-methylglutaryl-CoA lyase [Source:HGNC Symbol;Acc:HGNC:5005] |
| ENSG00000117543 | HGLibA_13849 | 5 | -1.098846772 | DPH5 | diphthamide biosynthesis 5 [Source:HGNC Symbol;Acc:HGNC:24270] |
| ENSG00000118017 | HGLibA_00021 | 5 | -1.098846772 | A4GNT | 4-N-acetylglucosaminyltransferase [Source:HGNC Symbol;Acc:HGNC:17968]" |
| ENSG00000118434 | HGLibA_46323 | 5 | -1.098846772 | SPACA1 | sperm acrosome associated 1 [Source:HGNC Symbol;Acc:HGNC:14967] |
| ENSG00000118514 | HGLibA_01585 | 5 | -1.098846772 | ALDH8A1 | aldehyde dehydrogenase 8 family member A1 [Source:HGNC Symbol;Acc:HGNC:15471] |
| ENSG00000118557 | HGLibA_37308 | 5 | -1.098846772 | PMFBP1 | polyamine modulated factor 1 binding protein 1 [Source:HGNC Symbol;Acc:HGNC:17728] |
| ENSG00000122591 | HGLibA_16080 | 5 | -1.098846772 | FAM126A | family with sequence similarity 126 member A [Source:HGNC Symbol;Acc:HGNC:24587] |
| ENSG00000124370 | HGLibA_28573 | 5 | -1.098846772 | MCEE | methylmalonyl-CoA epimerase [Source:HGNC Symbol;Acc:HGNC:16732] |
| ENSG00000124939 | HGLibA_42924 | 5 | -1.098846772 | SCGB2A1 | secretoglobin family 2A member 1 [Source:HGNC Symbol;Acc:HGNC:7051] |
| ENSG00000127337 | HGLibA_54659 | 5 | -1.098846772 | YEATS4 | YEATS domain containing 4 [Source:HGNC Symbol;Acc:HGNC:24859] |
| ENSG00000127540 | HGLibA_53108 | 5 | -1.098846772 | UQCR11 | complex III subunit XI [Source:HGNC Symbol;Acc:HGNC:30862]" |
| ENSG00000129910 | HGLibA_08645 | 5 | -1.098846772 | CDH15 | cadherin 15 [Source:HGNC Symbol;Acc:HGNC:1754] |
| ENSG00000130826 | HGLibA_13291 | 5 | -1.098846772 | DKC1 | dyskerin pseudouridine synthase 1 [Source:HGNC Symbol;Acc:HGNC:2890] |
| ENSG00000131471 | HGLibA_02272 | 5 | -1.098846772 | AOC3 | copper containing 3 [Source:HGNC Symbol;Acc:HGNC:550]" |
| ENSG00000132323 | HGLibA_23223 | 5 | -1.098846772 | ILKAP | ILK associated serine/threonine phosphatase [Source:HGNC Symbol;Acc:HGNC:15566] |
| ENSG00000132938 | HGLibA_30507 | 5 | -1.098846772 | MTUS2 | microtubule associated scaffold protein 2 [Source:HGNC Symbol;Acc:HGNC:20595] |
| ENSG00000133027 | HGLibA_36046 | 5 | -1.098846772 | PEMT | phosphatidylethanolamine N-methyltransferase [Source:HGNC Symbol;Acc:HGNC:8830] |
| ENSG00000134153 | HGLibA_15021 | 5 | -1.098846772 | EMC7 | ER membrane protein complex subunit 7 [Source:HGNC Symbol;Acc:HGNC:24301] |
| ENSG00000134258 | HGLibA_53884 | 5 | -1.098846772 | VTCN1 | V-set domain containing T cell activation inhibitor 1 [Source:HGNC Symbol;Acc:HGNC:28873] |
| ENSG00000135047 | HGLibA_11716 | 5 | -1.098846772 | CTSL | cathepsin L [Source:HGNC Symbol;Acc:HGNC:2537] |
| ENSG00000135069 | HGLibA_39007 | 5 | -1.098846772 | PSAT1 | phosphoserine aminotransferase 1 [Source:HGNC Symbol;Acc:HGNC:19129] |
| ENSG00000135624 | HGLibA_08214 | 5 | -1.098846772 | CCT7 | chaperonin containing TCP1 subunit 7 [Source:HGNC Symbol;Acc:HGNC:1622] |
| ENSG00000137656 | HGLibA_04998 | 5 | -1.098846772 | BUD13 | BUD13 homolog [Source:HGNC Symbol;Acc:HGNC:28199] |
| ENSG00000137845 | HGLibA_00728 | 5 | -1.098846772 | ADAM10 | ADAM metallopeptidase domain 10 [Source:HGNC Symbol;Acc:HGNC:188] |
| ENSG00000138744 | HGLibA_31005 | 5 | -1.098846772 | NAAA | N-acylethanolamine acid amidase [Source:HGNC Symbol;Acc:HGNC:736] |
| ENSG00000139083 | HGLibA_15664 | 5 | -1.098846772 | ETV6 | ETS variant 6 [Source:HGNC Symbol;Acc:HGNC:3495] |
| ENSG00000140543 | HGLibA_13013 | 5 | -1.098846772 | DET1 | COP1 ubiquitin ligase partner [Source:HGNC Symbol;Acc:HGNC:25477]" |
| ENSG00000141404 | HGLibA_19460 | 5 | -1.098846772 | GNAL | G protein subunit alpha L [Source:HGNC Symbol;Acc:HGNC:4388] |
| ENSG00000143028 | HGLibA_48040 | 5 | -1.098846772 | SYPL2 | synaptophysin like 2 [Source:HGNC Symbol;Acc:HGNC:27638] |
| ENSG00000143355 | HGLibA_26468 | 5 | -1.098846772 | LHX9 | LIM homeobox 9 [Source:HGNC Symbol;Acc:HGNC:14222] |
| ENSG00000143768 | HGLibA_26276 | 5 | -1.098846772 | LEFTY2 | left-right determination factor 2 [Source:HGNC Symbol;Acc:HGNC:3122] |
| ENSG00000144867 | HGLibA_47029 | 5 | -1.098846772 | SRPRB | SRP receptor subunit beta [Source:HGNC Symbol;Acc:HGNC:24085] |
| ENSG00000145391 | HGLibA_43657 | 5 | -1.098846772 | SETD7 | SET domain containing lysine methyltransferase 7 [Source:HGNC Symbol;Acc:HGNC:30412] |
| ENSG00000147324 | HGLibA_29064 | 5 | -1.098846772 | MFHAS1 | malignant fibrous histiocytoma amplified sequence 1 [Source:HGNC Symbol;Acc:HGNC:16982] |
| ENSG00000147421 | HGLibA_21665 | 5 | -1.098846772 | HMBOX1 | homeobox containing 1 [Source:HGNC Symbol;Acc:HGNC:26137] |

| | | | | | |
|---|---|---|---|---|---|
| ENSG00000147571 | HGLibA_11135 | 5 | -1.098846772 | CRH | corticotropin releasing hormone [Source:HGNC Symbol;Acc:HGNC:2355] |
| ENSG00000148136 | HGLibA_33602 | 5 | -1.098846772 | OR13C4 | olfactory receptor family 13 subfamily C member 4 [Source:HGNC Symbol;Acc:HGNC:14722] |
| ENSG00000148200 | HGLibA_32639 | 5 | -1.098846772 | NR6A1 | nuclear receptor subfamily 6 group A member 1 [Source:HGNC Symbol;Acc:HGNC:7985] |
| ENSG00000149480 | HGLibA_30312 | 5 | -1.098846772 | MTA2 | metastasis associated 1 family member 2 [Source:HGNC Symbol;Acc:HGNC:7411] |
| ENSG00000149557 | HGLibA_17312 | 5 | -1.098846772 | FEZ1 | fasciculation and elongation protein zeta 1 [Source:HGNC Symbol;Acc:HGNC:3659] |
| ENSG00000149927 | HGLibA_13724 | 5 | -1.098846772 | DOC2A | double C2 domain alpha [Source:HGNC Symbol;Acc:HGNC:2985] |
| ENSG00000150627 | HGLibA_54038 | 5 | -1.098846772 | WDR17 | WD repeat domain 17 [Source:HGNC Symbol;Acc:HGNC:16661] |
| ENSG00000151276 | HGLibA_27996 | 5 | -1.098846772 | MAGI1 | WW and PDZ domain containing 1 [Source:HGNC Symbol;Acc:HGNC:946]" |
| ENSG00000151366 | HGLibA_31539 | 5 | -1.098846772 | NDUFC2 | NADH:ubiquinone oxidoreductase subunit C2 [Source:HGNC Symbol;Acc:HGNC:7706] |
| ENSG00000151715 | HGLibA_50410 | 5 | -1.098846772 | TMEM45B | transmembrane protein 45B [Source:HGNC Symbol;Acc:HGNC:25194] |
| ENSG00000157017 | HGLibA_19048 | 5 | -1.098846772 | GHRL | ghrelin and obestatin prepropeptide [Source:HGNC Symbol;Acc:HGNC:18129] |
| ENSG00000158234 | HGLibA_15964 | 5 | -1.098846772 | FAIM | Fas apoptotic inhibitory molecule [Source:HGNC Symbol;Acc:HGNC:18703] |
| ENSG00000158806 | HGLibA_32491 | 5 | -1.098846772 | NPM2 | nucleophosmin/nucleoplasmin 2 [Source:HGNC Symbol;Acc:HGNC:7930] |
| ENSG00000159335 | HGLibA_39394 | 5 | -1.098846772 | PTMS | parathymosin [Source:HGNC Symbol;Acc:HGNC:9629] |
| ENSG00000160691 | HGLibA_44001 | 5 | -1.098846772 | SHC1 | SHC adaptor protein 1 [Source:HGNC Symbol;Acc:HGNC:10840] |
| ENSG00000160991 | HGLibA_34592 | 5 | -1.098846772 | ORAI2 | ORAI calcium release-activated calcium modulator 2 [Source:HGNC Symbol;Acc:HGNC:21667] |
| ENSG00000161217 | HGLibA_35701 | 5 | -1.098846772 | PCYT1A | alpha [Source:HGNC Symbol;Acc:HGNC:8754]" |
| ENSG00000161328 | HGLibA_27391 | 5 | -1.098846772 | LRRC56 | leucine rich repeat containing 56 [Source:HGNC Symbol;Acc:HGNC:25430] |
| ENSG00000162961 | HGLibA_13916 | 5 | -1.098846772 | DPY30 | histone methyltransferase complex regulatory subunit [Source:HGNC Symbol;Acc:HGNC:24590]" |
| ENSG00000163485 | HGLibA_01009 | 5 | -1.098846772 | ADORA1 | adenosine A1 receptor [Source:HGNC Symbol;Acc:HGNC:262] |
| ENSG00000163636 | HGLibA_39177 | 5 | -1.098846772 | PSMD6 | non-ATPase 6 [Source:HGNC Symbol;Acc:HGNC:9564]" |
| ENSG00000163666 | HGLibA_21199 | 5 | -1.098846772 | HESX1 | HESX homeobox 1 [Source:HGNC Symbol;Acc:HGNC:4877] |
| ENSG00000165118 | HGLibA_06748 | 5 | -1.098846772 | C9orf64 | chromosome 9 open reading frame 64 [Source:HGNC Symbol;Acc:HGNC:28144] |
| ENSG00000165449 | HGLibA_44455 | 5 | -1.098846772 | SLC16A9 | solute carrier family 16 member 9 [Source:HGNC Symbol;Acc:HGNC:23520] |
| ENSG00000165807 | HGLibA_38093 | 5 | -1.098846772 | PPP1R36 | protein phosphatase 1 regulatory subunit 36 [Source:HGNC Symbol;Acc:HGNC:20097] |
| ENSG00000165943 | HGLibA_29583 | 5 | -1.098846772 | MOAP1 | modulator of apoptosis 1 [Source:HGNC Symbol;Acc:HGNC:16658] |
| ENSG00000166160 | HGLibA_33429 | 5 | -1.098846772 | OPN1MW2 | medium wave sensitive 2 [Source:HGNC Symbol;Acc:HGNC:26952]" |
| ENSG00000167791 | HGLibA_06850 | 5 | -1.098846772 | CABP2 | calcium binding protein 2 [Source:HGNC Symbol;Acc:HGNC:1385] |
| ENSG00000167851 | HGLibA_08333 | 5 | -1.098846772 | CD300A | CD300a molecule [Source:HGNC Symbol;Acc:HGNC:19319] |
| ENSG00000168228 | HGLibA_55036 | 5 | -1.098846772 | ZCCHC4 | zinc finger CCHC-type containing 4 [Source:HGNC Symbol;Acc:HGNC:22917] |
| ENSG00000168300 | HGLibA_35628 | 5 | -1.098846772 | PCMTD1 | protein-L-isoaspartate (D-aspartate) O-methyltransferase domain containing 1 [Source:HGNC Symbol;Acc:HGNC:30483] |
| ENSG00000168389 | HGLibA_29093 | 5 | -1.098846772 | MFSD2A | major facilitator superfamily domain containing 2A [Source:HGNC Symbol;Acc:HGNC:25897] |
| ENSG00000168803 | HGLibA_00726 | 5 | -1.098846772 | ADAL | adenosine deaminase like [Source:HGNC Symbol;Acc:HGNC:31853] |
| ENSG00000169393 | HGLibA_14992 | 5 | -1.098846772 | ELSPBP1 | epididymal sperm binding protein 1 [Source:HGNC Symbol;Acc:HGNC:14417] |
| ENSG00000169684 | HGLibA_09576 | 5 | -1.098846772 | CHRNA5 | cholinergic receptor nicotinic alpha 5 subunit [Source:HGNC Symbol;Acc:HGNC:1959] |
| ENSG00000169692 | HGLibA_01220 | 5 | -1.098846772 | AGPAT2 | 1-acylglycerol-3-phosphate O-acyltransferase 2 [Source:HGNC Symbol;Acc:HGNC:325] |
| ENSG00000169860 | HGLibA_34866 | 5 | -1.098846772 | P2RY1 | purinergic receptor P2Y1 [Source:HGNC Symbol;Acc:HGNC:8539] |
| ENSG00000169992 | HGLibA_32070 | 5 | -1.098846772 | NLGN2 | neuroligin 2 [Source:HGNC Symbol;Acc:HGNC:14290] |
| ENSG00000170144 | HGLibA_21800 | 5 | -1.098846772 | HNRNPA3 | heterogeneous nuclear ribonucleoprotein A3 [Source:HGNC Symbol;Acc:HGNC:24941] |
| ENSG00000170234 | HGLibA_39641 | 5 | -1.098846772 | PWWP2A | PWWP domain containing 2A [Source:HGNC Symbol;Acc:HGNC:29406] |
| ENSG00000170683 | HGLibA_33464 | 5 | -1.098846772 | OR10A3 | olfactory receptor family 10 subfamily A member 3 [Source:HGNC Symbol;Acc:HGNC:8162] |
| ENSG00000170892 | HGLibA_51834 | 5 | -1.098846772 | TSEN34 | tRNA splicing endonuclease subunit 34 [Source:HGNC Symbol;Acc:HGNC:15506] |
| ENSG00000170915 | HGLibA_35172 | 5 | -1.098846772 | PAQR8 | progestin and adipoQ receptor family member 8 [Source:HGNC Symbol;Acc:HGNC:15708] |
| ENSG00000171056 | HGLibA_46273 | 5 | -1.098846772 | SOX7 | SRY-box 7 [Source:HGNC Symbol;Acc:HGNC:18196] |
| ENSG00000171160 | HGLibA_29676 | 5 | -1.098846772 | MORN4 | MORN repeat containing 4 [Source:HGNC Symbol;Acc:HGNC:24001] |
| ENSG00000171792 | HGLibA_41159 | 5 | -1.098846772 | RHNO1 | RAD9-HUS1-RAD1 interacting nuclear orphan 1 [Source:HGNC Symbol;Acc:HGNC:28206] |
| ENSG00000171863 | HGLibA_42187 | 5 | -1.098846772 | RPS7 | ribosomal protein S7 [Source:HGNC Symbol;Acc:HGNC:10440] |
| ENSG00000172020 | HGLibA_18610 | 5 | -1.098846772 | GAP43 | growth associated protein 43 [Source:HGNC Symbol;Acc:HGNC:4140] |
| ENSG00000173678 | HGLibA_46584 | 5 | -1.098846772 | SPDYE2B | speedy/RINGO cell cycle regulator family member E2B [Source:HGNC Symbol;Acc:HGNC:48334] |
| ENSG00000174695 | HGLibA_50038 | 5 | -1.098846772 | TMEM167A | transmembrane protein 167A [Source:HGNC Symbol;Acc:HGNC:28330] |
| ENSG00000175203 | HGLibA_12548 | 5 | -1.098846772 | DCTN2 | dynactin subunit 2 [Source:HGNC Symbol;Acc:HGNC:2712] |
| ENSG00000176087 | HGLibA_44957 | 5 | -1.098846772 | SLC35A4 | solute carrier family 35 member A4 [Source:HGNC Symbol;Acc:HGNC:20753] |
| ENSG00000176165 | HGLibA_17870 | 5 | -1.098846772 | FOXG1 | forkhead box G1 [Source:HGNC Symbol;Acc:HGNC:3811] |
| ENSG00000177045 | HGLibA_44254 | 5 | -1.098846772 | SIX5 | SIX homeobox 5 [Source:HGNC Symbol;Acc:HGNC:10891] |
| ENSG00000177455 | HGLibA_08262 | 5 | -1.098846772 | CD19 | CD19 molecule [Source:HGNC Symbol;Acc:HGNC:1633] |
| ENSG00000178031 | HGLibA_00849 | 5 | -1.098846772 | ADAMTSL1 | ADAMTS like 1 [Source:HGNC Symbol;Acc:HGNC:14632] |
| ENSG00000178217 | HGLibA_43902 | 5 | -1.098846772 | SH2D4B | SH2 domain containing 4B [Source:HGNC Symbol;Acc:HGNC:31440] |
| ENSG00000178234 | HGLibA_18534 | 5 | -1.098846772 | GALNT11 | polypeptide N-acetylgalactosaminyltransferase 11 [Source:HGNC Symbol;Acc:HGNC:19875] |
| ENSG00000179695 | HGLibA_34385 | 5 | -1.098846772 | OR6C2 | olfactory receptor family 6 subfamily C member 2 [Source:HGNC Symbol;Acc:HGNC:15436] |
| ENSG00000181617 | HGLibA_17249 | 5 | -1.098846772 | FDCSP | follicular dendritic cell secreted protein [Source:HGNC Symbol;Acc:HGNC:19215] |
| ENSG00000182173 | HGLibA_51837 | 5 | -1.098846772 | TSEN54 | tRNA splicing endonuclease subunit 54 [Source:HGNC Symbol;Acc:HGNC:27561] |
| ENSG00000183091 | HGLibA_31578 | 5 | -1.098846772 | NEB | nebulin [Source:HGNC Symbol;Acc:HGNC:7720] |
| ENSG00000183856 | HGLibA_23538 | 5 | -1.098846772 | IQGAP3 | IQ motif containing GTPase activating protein 3 [Source:HGNC Symbol;Acc:HGNC:20669] |
| ENSG00000184923 | HGLibA_33106 | 5 | -1.098846772 | NUTM2A | NUT family member 2A [Source:HGNC Symbol;Acc:HGNC:23438] |
| ENSG00000185164 | HGLibA_32291 | 5 | -1.098846772 | NOMO2 | NODAL modulator 2 [Source:HGNC Symbol;Acc:HGNC:22652] |
| ENSG00000185306 | HGLibA_05267 | 5 | -1.098846772 | C12orf56 | chromosome 12 open reading frame 56 [Source:HGNC Symbol;Acc:HGNC:26967] |
| ENSG00000185940 | HGLibA_25801 | 5 | -1.098846772 | KRTAP5-5 | keratin associated protein 5-5 [Source:HGNC Symbol;Acc:HGNC:23601] |
| ENSG00000187187 | HGLibA_56199 | 5 | -1.098846772 | ZNF546 | zinc finger protein 546 [Source:HGNC Symbol;Acc:HGNC:28671] |
| ENSG00000189283 | HGLibA_17478 | 5 | -1.098846772 | FHIT | fragile histidine triad [Source:HGNC Symbol;Acc:HGNC:3701] |
| ENSG00000196109 | HGLibA_56514 | 5 | -1.098846772 | ZNF676 | zinc finger protein 676 [Source:HGNC Symbol;Acc:HGNC:20429] |
| ENSG00000196748 | HGLibA_10109 | 5 | -1.098846772 | CLPSL2 | colipase like 2 [Source:HGNC Symbol;Acc:HGNC:21250] |
| ENSG00000197757 | HGLibA_21974 | 5 | -1.098846772 | HOXC6 | homeobox C6 [Source:HGNC Symbol;Acc:HGNC:5128] |
| ENSG00000198860 | HGLibA_51829 | 5 | -1.098846772 | TSEN15 | tRNA splicing endonuclease subunit 15 [Source:HGNC Symbol;Acc:HGNC:16791] |
| ENSG00000198873 | HGLibA_20332 | 5 | -1.098846772 | GRK5 | G protein-coupled receptor kinase 5 [Source:HGNC Symbol;Acc:HGNC:4544] |
| ENSG00000204020 | HGLibA_26629 | 5 | -1.098846772 | LIPN | lipase family member N [Source:HGNC Symbol;Acc:HGNC:23452] |
| ENSG00000204920 | HGLibA_55505 | 5 | -1.098846772 | ZNF155 | zinc finger protein 155 [Source:HGNC Symbol;Acc:HGNC:12940] |
| ENSG00000205869 | HGLibA_25781 | 5 | -1.098846772 | KRTAP5-1 | keratin associated protein 5-1 [Source:HGNC Symbol;Acc:HGNC:23596] |
| ENSG00000214652 | HGLibA_56635 | 5 | -1.098846772 | ZNF727 | zinc finger protein 727 [Source:HGNC Symbol;Acc:HGNC:22785] |
| ENSG00000224940 | HGLibA_38889 | 5 | -1.098846772 | PRRT4 | proline rich transmembrane protein 4 [Source:HGNC Symbol;Acc:HGNC:37280] |
| ENSG00000229579 | HGLibA_53259 | 5 | -1.098846772 | USP17L26 | ubiquitin specific peptidase 17-like family member 26 [Source:HGNC Symbol;Acc:HGNC:44454] |
| ENSG00000230301 | HGLibA_34302 | 5 | -1.098846772 | OR5H6 | olfactory receptor family 5 subfamily H member 6 (gene/pseudogene) [Source:HGNC Symbol;Acc:HGNC:14767] |
| ENSG00000235780 | HGLibA_53262 | 5 | -1.098846772 | USP17L27 | ubiquitin specific peptidase 17-like family member 27 [Source:HGNC Symbol;Acc:HGNC:44455] |
| ENSG00000237190 | HGLibA_08836 | 5 | -1.098846772 | CDKN2AIPNL | CDKN2A interacting protein N-terminal like [Source:HGNC Symbol;Acc:HGNC:30545] |
| ENSG00000243896 | HGLibA_33744 | 5 | -1.098846772 | OR2A7 | olfactory receptor family 2 subfamily A member 7 [Source:HGNC Symbol;Acc:HGNC:8234] |
| ENSG00000248933 | HGLibA_53250 | 5 | -1.098846772 | USP17L22 | ubiquitin specific peptidase 17-like family member 22 [Source:HGNC Symbol;Acc:HGNC:44450] |
| ENSG00000249884 | HGLibA_41461 | 5 | -1.098846772 | RNF103-CHMP3 | RNF103-CHMP3 readthrough [Source:HGNC Symbol;Acc:HGNC:38847] |
| ENSG00000250844 | HGLibA_53232 | 5 | -1.098846772 | USP17L18 | ubiquitin specific peptidase 17-like family member 18 [Source:HGNC Symbol;Acc:HGNC:44446] |
| ENSG00000253117 | HGLibA_33236 | 5 | -1.098846772 | OC90 | otoconin 90 [Source:HGNC Symbol;Acc:HGNC:8100] |
| ENSG00000256870 | HGLibA_45282 | 5 | -1.098846772 | SLC5A8 | solute carrier family 5 member 8 [Source:HGNC Symbol;Acc:HGNC:19119] |
| ENSG00000260097 | HGLibA_46600 | 5 | -1.098846772 | SPDYE6 | speedy/RINGO cell cycle regulator family member E6 [Source:HGNC Symbol;Acc:HGNC:35465] |
| ENSG00000265190 | HGLibA_02253 | 5 | -1.098846772 | ANXA8 | annexin A8 [Source:HGNC Symbol;Acc:HGNC:546] |
| ENSG00000266472 | HGLibA_30062 | 5 | -1.098846772 | MRPS21 | mitochondrial ribosomal protein S21 [Source:HGNC Symbol;Acc:HGNC:14046] |
| ENSG00000266919 | HGLibA_59699 | 5 | -1.098846772 | hsa-mir-423 | microRNA 3184 |
| ENSG00000285437 | HGLibA_37598 | 5 | -1.098846772 | POLR2J3 | RNA polymerase II subunit J3 [Source:HGNC Symbol;Acc:HGNC:33853] |
| GAGE2C | HGLibA_18474 | 5 | -1.098846772 | GAGE2C | - |
| GAGE2D | HGLibA_18477 | 5 | -1.098846772 | GAGE2D | - |
| HIST1H2BB | HGLibA_21414 | 5 | -1.098846772 | HIST1H2BB | - |
| HIST1H2BC | HGLibA_21418 | 5 | -1.098846772 | HIST1H2BC | - |
| hsa-mir-1260b | HGLibA_57463 | 5 | -1.098846772 | hsa-mir-1260b | - |
| hsa-mir-1273d | HGLibA_57538 | 5 | -1.098846772 | hsa-mir-1273d | - |
| hsa-mir-1304 | HGLibA_57730 | 5 | -1.098846772 | hsa-mir-1304 | - |
| hsa-mir-185 | HGLibA_58001 | 5 | -1.098846772 | hsa-mir-185 | - |
| hsa-mir-219a-2 | HGLibA_58281 | 5 | -1.098846772 | hsa-mir-219a-2 | - |
| hsa-mir-335 | HGLibA_58994 | 5 | -1.098846772 | hsa-mir-335 | - |
| hsa-mir-3651 | HGLibA_59151 | 5 | -1.098846772 | hsa-mir-3651 | - |
| hsa-mir-3667 | HGLibA_59221 | 5 | -1.098846772 | hsa-mir-3667 | - |
| hsa-mir-374b | HGLibA_59394 | 5 | -1.098846772 | hsa-mir-374b | - |
| hsa-mir-3914-2 | HGLibA_59533 | 5 | -1.098846772 | hsa-mir-3914-2 | - |
| hsa-mir-4417 | HGLibA_60050 | 5 | -1.098846772 | hsa-mir-4417 | - |
| hsa-mir-4699 | HGLibA_60903 | 5 | -1.098846772 | hsa-mir-4699 | - |
| hsa-mir-4718 | HGLibA_60972 | 5 | -1.098846772 | hsa-mir-4718 | - |
| hsa-mir-4765 | HGLibA_61156 | 5 | -1.098846772 | hsa-mir-4765 | - |
| hsa-mir-483 | HGLibA_61325 | 5 | -1.098846772 | hsa-mir-483 | - |
| hsa-mir-5090 | HGLibA_61517 | 5 | -1.098846772 | hsa-mir-5090 | - |
| hsa-mir-554 | HGLibA_62134 | 5 | -1.098846772 | hsa-mir-554 | - |
| hsa-mir-605 | HGLibA_62512 | 5 | -1.098846772 | hsa-mir-605 | - |
| hsa-mir-642a | HGLibA_62809 | 5 | -1.098846772 | hsa-mir-642a | - |
| hsa-mir-671 | HGLibA_63014 | 5 | -1.098846772 | hsa-mir-671 | - |
| hsa-mir-6719 | HGLibA_63039 | 5 | -1.098846772 | hsa-mir-6719 | - |
| hsa-mir-6739 | HGLibA_63108 | 5 | -1.098846772 | hsa-mir-6739 | - |
| hsa-mir-6796 | HGLibA_63360 | 5 | -1.098846772 | hsa-mir-6796 | - |
| hsa-mir-6810 | HGLibA_63418 | 5 | -1.098846772 | hsa-mir-6810 | - |
| hsa-mir-6877 | HGLibA_63693 | 5 | -1.098846772 | hsa-mir-6877 | - |
| LOC100132247 | HGLibA_26807 | 5 | -1.098846772 | LOC100132247 | - |
| LOC440243 | HGLibA_26943 | 5 | -1.098846772 | LOC440243 | - |
| NPIP | HGLibA_32460 | 5 | -1.098846772 | NPIP | - |
| NPIPL3 | HGLibA_32479 | 5 | -1.098846772 | NPIPL3 | - |
| PAPL | HGLibA_35129 | 5 | -1.098846772 | PAPL | - |
| PRR21 | HGLibA_38815 | 5 | -1.098846772 | PRR21 | - |
| REXO1L1 | HGLibA_40893 | 5 | -1.098846772 | REXO1L1 | - |
| SGOL1 | HGLibA_43841 | 5 | -1.098846772 | SGOL1 | - |
| WISP3 | HGLibA_54326 | 5 | -1.098846772 | WISP3 | - |
| AHSA2 | HGLibA_01317 | 4 | -1.108797809 | AHSA2 | - |
| AQPEP | HGLibA_02613 | 4 | -1.108797809 | AQPEP | - |
| C1orf110 | HGLibA_05744 | 4 | -1.108797809 | C1orf110 | - |

| | | | | | |
|---|---|---|---|---|---|
| C1orf63 | HGLibA_05881 | 4 | -1.108797809 | C1orf63 | - |
| CTSL1 | HGLibA_11718 | 4 | -1.108797809 | CTSL1 | - |
| ENSG00000011052 | HGLibA_32154 | 4 | -1.108797809 | NME1-NME2 | NME1-NME2 readthrough [Source:HGNC Symbol;Acc:HGNC:33531] |
| ENSG00000011478 | HGLibA_39730 | 4 | -1.108797809 | QPCTL | glutaminyl-peptide cyclotransferase like [Source:HGNC Symbol;Acc:HGNC:25952] |
| ENSG00000047634 | HGLibA_42958 | 4 | -1.108797809 | SCML1 | Scm polycomb group protein like 1 [Source:HGNC Symbol;Acc:HGNC:10580] |
| ENSG00000048028 | HGLibA_53321 | 4 | -1.108797809 | USP28 | ubiquitin specific peptidase 28 [Source:HGNC Symbol;Acc:HGNC:12625] |
| ENSG00000049540 | HGLibA_14950 | 4 | -1.108797809 | ELN | elastin [Source:HGNC Symbol;Acc:HGNC:3327] |
| ENSG00000056998 | HGLibA_20690 | 4 | -1.108797809 | GYG2 | glycogenin 2 [Source:HGNC Symbol;Acc:HGNC:4700] |
| ENSG00000057704 | HGLibA_49778 | 4 | -1.108797809 | TMCC3 | transmembrane and coiled-coil domain family 3 [Source:HGNC Symbol;Acc:HGNC:29199] |
| ENSG00000059377 | HGLibA_48683 | 4 | -1.108797809 | TBXAS1 | thromboxane A synthase 1 [Source:HGNC Symbol;Acc:HGNC:11609] |
| ENSG00000059804 | HGLibA_44874 | 4 | -1.108797809 | SLC2A3 | solute carrier family 2 member 3 [Source:HGNC Symbol;Acc:HGNC:11007] |
| ENSG00000063046 | HGLibA_14814 | 4 | -1.108797809 | EIF4B | eukaryotic translation initiation factor 4B [Source:HGNC Symbol;Acc:HGNC:3285] |
| ENSG00000067167 | HGLibA_51281 | 4 | -1.108797809 | TRAM1 | translocation associated membrane protein 1 [Source:HGNC Symbol;Acc:HGNC:20568] |
| ENSG00000075290 | HGLibA_54391 | 4 | -1.108797809 | WNT8B | Wnt family member 8B [Source:HGNC Symbol;Acc:HGNC:12789] |
| ENSG00000076003 | HGLibA_28620 | 4 | -1.108797809 | MCM6 | minichromosome maintenance complex component 6 [Source:HGNC Symbol;Acc:HGNC:6949] |
| ENSG00000078140 | HGLibA_52624 | 4 | -1.108797809 | UBE2K | ubiquitin conjugating enzyme E2 K [Source:HGNC Symbol;Acc:HGNC:4914] |
| ENSG00000078369 | HGLibA_19483 | 4 | -1.108797809 | GNB1 | G protein subunit beta 1 [Source:HGNC Symbol;Acc:HGNC:4396] |
| ENSG00000078401 | HGLibA_14468 | 4 | -1.108797809 | EDN1 | endothelin 1 [Source:HGNC Symbol;Acc:HGNC:3176] |
| ENSG00000078699 | HGLibA_07385 | 4 | -1.108797809 | CBFA2T2 | CBFA2/RUNX1 translocation partner 2 [Source:HGNC Symbol;Acc:HGNC:1536] |
| ENSG00000082515 | HGLibA_29930 | 4 | -1.108797809 | MRPL22 | mitochondrial ribosomal protein L22 [Source:HGNC Symbol;Acc:HGNC:14480] |
| ENSG00000088356 | HGLibA_35939 | 4 | -1.108797809 | PDRG1 | p53 and DNA damage regulated 1 [Source:HGNC Symbol;Acc:HGNC:16119] |
| ENSG00000089723 | HGLibA_34755 | 4 | -1.108797809 | OTUB2 | ubiquitin aldehyde binding 2 [Source:HGNC Symbol;Acc:HGNC:20351]" |
| ENSG00000091527 | HGLibA_08894 | 4 | -1.108797809 | CDV3 | CDV3 homolog [Source:HGNC Symbol;Acc:HGNC:26928] |
| ENSG00000100014 | HGLibA_46606 | 4 | -1.108797809 | SPECC1L | sperm antigen with calponin homology and coiled-coil domains 1 like [Source:HGNC Symbol;Acc:HGNC:29022] |
| ENSG00000100448 | HGLibA_11708 | 4 | -1.108797809 | CTSG | cathepsin G [Source:HGNC Symbol;Acc:HGNC:2532] |
| ENSG00000100941 | HGLibA_37394 | 4 | -1.108797809 | PNN | desmosome associated protein [Source:HGNC Symbol;Acc:HGNC:9162]" |
| ENSG00000101152 | HGLibA_13624 | 4 | -1.108797809 | DNAJC5 | DnaJ heat shock protein family (Hsp40) member C5 [Source:HGNC Symbol;Acc:HGNC:16235] |
| ENSG00000101384 | HGLibA_23903 | 4 | -1.108797809 | JAG1 | jagged 1 [Source:HGNC Symbol;Acc:HGNC:6188] |
| ENSG00000101544 | HGLibA_01005 | 4 | -1.108797809 | ADNP2 | ADNP homeobox 2 [Source:HGNC Symbol;Acc:HGNC:23803] |
| ENSG00000101558 | HGLibA_53519 | 4 | -1.108797809 | VAPA | VAMP associated protein A [Source:HGNC Symbol;Acc:HGNC:12648] |
| ENSG00000103335 | HGLibA_36535 | 4 | -1.108797809 | PIEZO1 | piezo type mechanosensitive ion channel component 1 [Source:HGNC Symbol;Acc:HGNC:28993] |
| ENSG00000103512 | HGLibA_32289 | 4 | -1.108797809 | NOMO1 | NODAL modulator 1 [Source:HGNC Symbol;Acc:HGNC:30060] |
| ENSG00000104763 | HGLibA_03186 | 4 | -1.108797809 | ASAH1 | N-acylsphingosine amidohydrolase 1 [Source:HGNC Symbol;Acc:HGNC:735] |
| ENSG00000104824 | HGLibA_21834 | 4 | -1.108797809 | HNRNPL | heterogeneous nuclear ribonucleoprotein L [Source:HGNC Symbol;Acc:HGNC:5045] |
| ENSG00000105072 | HGLibA_05663 | 4 | -1.108797809 | C19orf44 | chromosome 19 open reading frame 44 [Source:HGNC Symbol;Acc:HGNC:26141] |
| ENSG00000106803 | HGLibA_43254 | 4 | -1.108797809 | SEC61B | Sec61 translocon beta subunit [Source:HGNC Symbol;Acc:HGNC:16993] |
| ENSG00000108010 | HGLibA_19318 | 4 | -1.108797809 | GLRX3 | glutaredoxin 3 [Source:HGNC Symbol;Acc:HGNC:15987] |
| ENSG00000112541 | HGLibA_35761 | 4 | -1.108797809 | PDE10A | phosphodiesterase 10A [Source:HGNC Symbol;Acc:HGNC:8772] |
| ENSG00000114113 | HGLibA_40632 | 4 | -1.108797809 | RBP2 | retinol binding protein 2 [Source:HGNC Symbol;Acc:HGNC:9920] |
| ENSG00000116985 | HGLibA_04622 | 4 | -1.108797809 | BMP8B | bone morphogenetic protein 8b [Source:HGNC Symbol;Acc:HGNC:1075] |
| ENSG00000116990 | HGLibA_30673 | 4 | -1.108797809 | MYCL | bHLH transcription factor [Source:HGNC Symbol;Acc:HGNC:7555]" |
| ENSG00000117601 | HGLibA_43571 | 4 | -1.108797809 | SERPINC1 | serpin family C member 1 [Source:HGNC Symbol;Acc:HGNC:775] |
| ENSG00000118412 | HGLibA_07329 | 4 | -1.108797809 | CASP8AP2 | caspase 8 associated protein 2 [Source:HGNC Symbol;Acc:HGNC:1510] |
| ENSG00000120539 | HGLibA_28411 | 4 | -1.108797809 | MASTL | microtubule associated serine/threonine kinase like [Source:HGNC Symbol;Acc:HGNC:19042] |
| ENSG00000121005 | HGLibA_11173 | 4 | -1.108797809 | CRISPLD1 | cysteine rich secretory protein LCCL domain containing 1 [Source:HGNC Symbol;Acc:HGNC:18206] |
| ENSG00000121903 | HGLibA_56998 | 4 | -1.108797809 | ZSCAN20 | zinc finger and SCAN domain containing 20 [Source:HGNC Symbol;Acc:HGNC:13093] |
| ENSG00000122678 | HGLibA_37539 | 4 | -1.108797809 | POLM | DNA polymerase mu [Source:HGNC Symbol;Acc:HGNC:9185] |
| ENSG00000123080 | HGLibA_08841 | 4 | -1.108797809 | CDKN2C | cyclin dependent kinase inhibitor 2C [Source:HGNC Symbol;Acc:HGNC:1789] |
| ENSG00000124939 | HGLibA_42923 | 4 | -1.108797809 | SCGB2A1 | secretoglobin family 2A member 1 [Source:HGNC Symbol;Acc:HGNC:7051] |
| ENSG00000127249 | HGLibA_03576 | 4 | -1.108797809 | ATP13A4 | ATPase 13A4 [Source:HGNC Symbol;Acc:HGNC:25422] |
| ENSG00000128245 | HGLibA_54747 | 4 | -1.108797809 | YWHAH | tyrosine 3-monooxygenase/tryptophan 5-monooxygenase activation protein eta [Source:HGNC Symbol;Acc:HGNC:12853] |
| ENSG00000128283 | HGLibA_08565 | 4 | -1.108797809 | CDC42EP1 | CDC42 effector protein 1 [Source:HGNC Symbol;Acc:HGNC:17014] |
| ENSG00000128654 | HGLibA_30513 | 4 | -1.108797809 | MTX2 | metaxin 2 [Source:HGNC Symbol;Acc:HGNC:7506] |
| ENSG00000129128 | HGLibA_46563 | 4 | -1.108797809 | SPCS3 | signal peptidase complex subunit 3 [Source:HGNC Symbol;Acc:HGNC:26212] |
| ENSG00000129946 | HGLibA_44003 | 4 | -1.108797809 | SHC2 | SHC adaptor protein 2 [Source:HGNC Symbol;Acc:HGNC:29869] |
| ENSG00000131584 | HGLibA_00390 | 4 | -1.108797809 | ACAP3 | ankyrin repeat and PH domains 3 [Source:HGNC Symbol;Acc:HGNC:16754]" |
| ENSG00000136931 | HGLibA_32635 | 4 | -1.108797809 | NR5A1 | nuclear receptor subfamily 5 group A member 1 [Source:HGNC Symbol;Acc:HGNC:7983] |
| ENSG00000137288 | HGLibA_53100 | 4 | -1.108797809 | UQCC2 | ubiquinol-cytochrome c reductase complex assembly factor 2 [Source:HGNC Symbol;Acc:HGNC:21237] |
| ENSG00000137804 | HGLibA_33096 | 4 | -1.108797809 | NUSAP1 | nucleolar and spindle associated protein 1 [Source:HGNC Symbol;Acc:HGNC:18538] |
| ENSG00000138138 | HGLibA_03402 | 4 | -1.108797809 | ATAD1 | AAA domain containing 1 [Source:HGNC Symbol;Acc:HGNC:25903]" |
| ENSG00000138794 | HGLibA_07319 | 4 | -1.108797809 | CASP6 | caspase 6 [Source:HGNC Symbol;Acc:HGNC:1507] |
| ENSG00000138867 | HGLibA_20650 | 4 | -1.108797809 | GUCD1 | guanylyl cyclase domain containing 1 [Source:HGNC Symbol;Acc:HGNC:14237] |
| ENSG00000139187 | HGLibA_25308 | 4 | -1.108797809 | KLRG1 | killer cell lectin like receptor G1 [Source:HGNC Symbol;Acc:HGNC:6380] |
| ENSG00000139719 | HGLibA_53764 | 4 | -1.108797809 | VPS33A | CORVET/HOPS core subunit [Source:HGNC Symbol;Acc:HGNC:18179]" |
| ENSG00000141401 | HGLibA_23248 | 4 | -1.108797809 | IMPA2 | inositol monophosphatase 2 [Source:HGNC Symbol;Acc:HGNC:6051] |
| ENSG00000141568 | HGLibA_17898 | 4 | -1.108797809 | FOXK2 | forkhead box K2 [Source:HGNC Symbol;Acc:HGNC:6036] |
| ENSG00000142538 | HGLibA_39370 | 4 | -1.108797809 | PTH2 | parathyroid hormone 2 [Source:HGNC Symbol;Acc:HGNC:30828] |
| ENSG00000142546 | HGLibA_32335 | 4 | -1.108797809 | NOSIP | nitric oxide synthase interacting protein [Source:HGNC Symbol;Acc:HGNC:17946] |
| ENSG00000142867 | HGLibA_04288 | 4 | -1.108797809 | BCL10 | B cell CLL/lymphoma 10 [Source:HGNC Symbol;Acc:HGNC:989] |
| ENSG00000144401 | HGLibA_28977 | 4 | -1.108797809 | METTL21A | methyltransferase like 21A [Source:HGNC Symbol;Acc:HGNC:30476] |
| ENSG00000144659 | HGLibA_44725 | 4 | -1.108797809 | SLC25A38 | solute carrier family 25 member 38 [Source:HGNC Symbol;Acc:HGNC:26054] |
| ENSG00000145431 | HGLibA_35847 | 4 | -1.108797809 | PDGFC | platelet derived growth factor C [Source:HGNC Symbol;Acc:HGNC:8801] |
| ENSG00000145736 | HGLibA_20547 | 4 | -1.108797809 | GTF2H2 | general transcription factor IIH subunit 2 [Source:HGNC Symbol;Acc:HGNC:4656] |
| ENSG00000145777 | HGLibA_51877 | 4 | -1.108797809 | TSLP | thymic stromal lymphopoietin [Source:HGNC Symbol;Acc:HGNC:30743] |
| ENSG00000146701 | HGLibA_28689 | 4 | -1.108797809 | MDH2 | malate dehydrogenase 2 [Source:HGNC Symbol;Acc:HGNC:6971] |
| ENSG00000147684 | HGLibA_31533 | 4 | -1.108797809 | NDUFB9 | NADH:ubiquinone oxidoreductase subunit B9 [Source:HGNC Symbol;Acc:HGNC:7704] |
| ENSG00000148773 | HGLibA_29325 | 4 | -1.108797809 | MKI67 | marker of proliferation Ki-67 [Source:HGNC Symbol;Acc:HGNC:7107] |
| ENSG00000149131 | HGLibA_43593 | 4 | -1.108797809 | SERPING1 | serpin family G member 1 [Source:HGNC Symbol;Acc:HGNC:1228] |
| ENSG00000150750 | HGLibA_05153 | 4 | -1.108797809 | C11orf53 | chromosome 11 open reading frame 53 [Source:HGNC Symbol;Acc:HGNC:30527] |
| ENSG00000150938 | HGLibA_11146 | 4 | -1.108797809 | CRIM1 | cysteine rich transmembrane BMP regulator 1 [Source:HGNC Symbol;Acc:HGNC:2359] |
| ENSG00000150991 | HGLibA_52568 | 4 | -1.108797809 | UBC | ubiquitin C [Source:HGNC Symbol;Acc:HGNC:12468] |
| ENSG00000151176 | HGLibA_36976 | 4 | -1.108797809 | PLBD2 | phospholipase B domain containing 2 [Source:HGNC Symbol;Acc:HGNC:27283] |
| ENSG00000151651 | HGLibA_00781 | 4 | -1.108797809 | ADAM8 | ADAM metallopeptidase domain 8 [Source:HGNC Symbol;Acc:HGNC:215] |
| ENSG00000151689 | HGLibA_23335 | 4 | -1.108797809 | INPP1 | inositol polyphosphate-1-phosphatase [Source:HGNC Symbol;Acc:HGNC:6071] |
| ENSG00000152580 | HGLibA_22877 | 4 | -1.108797809 | IGSF10 | immunoglobulin superfamily member 10 [Source:HGNC Symbol;Acc:HGNC:26384] |
| ENSG00000153165 | HGLibA_41030 | 4 | -1.108797809 | RGPD3 | RANBP2-like and GRIP domain containing 3 [Source:HGNC Symbol;Acc:HGNC:32416] |
| ENSG00000153291 | HGLibA_44690 | 4 | -1.108797809 | SLC25A27 | solute carrier family 25 member 27 [Source:HGNC Symbol;Acc:HGNC:21065] |
| ENSG00000153498 | HGLibA_46340 | 4 | -1.108797809 | SPACA7 | sperm acrosome associated 7 [Source:HGNC Symbol;Acc:HGNC:29575] |
| ENSG00000154065 | HGLibA_02057 | 4 | -1.108797809 | ANKRD29 | ankyrin repeat domain 29 [Source:HGNC Symbol;Acc:HGNC:27110] |
| ENSG00000154813 | HGLibA_13842 | 4 | -1.108797809 | DPH3 | diphthamide biosynthesis 3 [Source:HGNC Symbol;Acc:HGNC:27717] |
| ENSG00000155096 | HGLibA_03940 | 4 | -1.108797809 | AZIN1 | antizyme inhibitor 1 [Source:HGNC Symbol;Acc:HGNC:16432] |
| ENSG00000157020 | HGLibA_43186 | 4 | -1.108797809 | SEC13 | nuclear pore and COPII coat complex component [Source:HGNC Symbol;Acc:HGNC:10697]" |
| ENSG00000157107 | HGLibA_17206 | 4 | -1.108797809 | FCHO2 | FCH domain only 2 [Source:HGNC Symbol;Acc:HGNC:25180] |
| ENSG00000157110 | HGLibA_40653 | 4 | -1.108797809 | RBPMS | mRNA processing factor [Source:HGNC Symbol;Acc:HGNC:19097]" |
| ENSG00000157150 | HGLibA_49545 | 4 | -1.108797809 | TIMP4 | TIMP metallopeptidase inhibitor 4 [Source:HGNC Symbol;Acc:HGNC:11823] |
| ENSG00000157168 | HGLibA_32677 | 4 | -1.108797809 | NRG1 | neuregulin 1 [Source:HGNC Symbol;Acc:HGNC:7997] |
| ENSG00000157510 | HGLibA_01101 | 4 | -1.108797809 | AFAP1L1 | actin filament associated protein 1 like 1 [Source:HGNC Symbol;Acc:HGNC:26714] |
| ENSG00000160410 | HGLibA_44058 | 4 | -1.108797809 | SHKBP1 | SH3KBP1 binding protein 1 [Source:HGNC Symbol;Acc:HGNC:19214] |
| ENSG00000162409 | HGLibA_38495 | 4 | -1.108797809 | PRKAA2 | protein kinase AMP-activated catalytic subunit alpha 2 [Source:HGNC Symbol;Acc:HGNC:9377] |
| ENSG00000162669 | HGLibA_21231 | 4 | -1.108797809 | HFM1 | ATP dependent DNA helicase homolog [Source:HGNC Symbol;Acc:HGNC:20193]" |
| ENSG00000163898 | HGLibA_26616 | 4 | -1.108797809 | LIPH | lipase H [Source:HGNC Symbol;Acc:HGNC:18483] |
| ENSG00000164932 | HGLibA_11628 | 4 | -1.108797809 | CTHRC1 | collagen triple helix repeat containing 1 [Source:HGNC Symbol;Acc:HGNC:18831] |
| ENSG00000164949 | HGLibA_18908 | 4 | -1.108797809 | GEM | GTP binding protein overexpressed in skeletal muscle [Source:HGNC Symbol;Acc:HGNC:4234] |
| ENSG00000165684 | HGLibA_45900 | 4 | -1.108797809 | SNAPC4 | small nuclear RNA activating complex polypeptide 4 [Source:HGNC Symbol;Acc:HGNC:11137] |
| ENSG00000166262 | HGLibA_16530 | 4 | -1.108797809 | FAM227B | family with sequence similarity 227 member B [Source:HGNC Symbol;Acc:HGNC:26543] |
| ENSG00000166546 | HGLibA_04391 | 4 | -1.108797809 | BEAN1 | brain expressed associated with NEDD4 1 [Source:HGNC Symbol;Acc:HGNC:24160] |
| ENSG00000166710 | HGLibA_03945 | 4 | -1.108797809 | B2M | beta-2-microglobulin [Source:HGNC Symbol;Acc:HGNC:914] |
| ENSG00000167670 | HGLibA_09346 | 4 | -1.108797809 | CHAF1A | chromatin assembly factor 1 subunit A [Source:HGNC Symbol;Acc:HGNC:1910] |
| ENSG00000168081 | HGLibA_37399 | 4 | -1.108797809 | PNOC | prepronociceptin [Source:HGNC Symbol;Acc:HGNC:9163] |
| ENSG00000168661 | HGLibA_55767 | 4 | -1.108797809 | ZNF30 | zinc finger protein 30 [Source:HGNC Symbol;Acc:HGNC:13090] |
| ENSG00000168890 | HGLibA_49992 | 4 | -1.108797809 | TMEM150A | transmembrane protein 150A [Source:HGNC Symbol;Acc:HGNC:24677] |
| ENSG00000169228 | HGLibA_39847 | 4 | -1.108797809 | RAB24 | member RAS oncogene family [Source:HGNC Symbol;Acc:HGNC:9765]" |
| ENSG00000172247 | HGLibA_05946 | 4 | -1.108797809 | C1QTNF4 | C1q and TNF related 4 [Source:HGNC Symbol;Acc:HGNC:14346] |
| ENSG00000172461 | HGLibA_18210 | 4 | -1.108797809 | FUT9 | fucosyltransferase 9 [Source:HGNC Symbol;Acc:HGNC:4020] |
| ENSG00000173210 | HGLibA_00313 | 4 | -1.108797809 | ABLIM3 | actin binding LIM protein family member 3 [Source:HGNC Symbol;Acc:HGNC:29132] |
| ENSG00000174808 | HGLibA_04930 | 4 | -1.108797809 | BTC | betacellulin [Source:HGNC Symbol;Acc:HGNC:1121] |
| ENSG00000175354 | HGLibA_39449 | 4 | -1.108797809 | PTPN2 | non-receptor type 2 [Source:HGNC Symbol;Acc:HGNC:9650]" |
| ENSG00000176049 | HGLibA_23926 | 4 | -1.108797809 | JAKMIP2 | janus kinase and microtubule interacting protein 2 [Source:HGNC Symbol;Acc:HGNC:29067] |
| ENSG00000179010 | HGLibA_29828 | 4 | -1.108797809 | MRFAP1 | Morf4 family associated protein 1 [Source:HGNC Symbol;Acc:HGNC:24549] |
| ENSG00000180155 | HGLibA_27711 | 4 | -1.108797809 | LYNX1 | Ly6/neurotoxin 1 [Source:HGNC Symbol;Acc:HGNC:29604] |
| ENSG00000180658 | HGLibA_33735 | 4 | -1.108797809 | OR2A4 | olfactory receptor family 2 subfamily A member 4 [Source:HGNC Symbol;Acc:HGNC:14729] |
| ENSG00000180758 | HGLibA_19947 | 4 | -1.108797809 | GPR157 | G protein-coupled receptor 157 [Source:HGNC Symbol;Acc:HGNC:23687] |
| ENSG00000180878 | HGLibA_05140 | 4 | -1.108797809 | C11orf42 | chromosome 11 open reading frame 42 [Source:HGNC Symbol;Acc:HGNC:28541] |
| ENSG00000182261 | HGLibA_32100 | 4 | -1.108797809 | NLRP10 | NLR family pyrin domain containing 10 [Source:HGNC Symbol;Acc:HGNC:21464] |
| ENSG00000183426 | HGLibA_32464 | 4 | -1.108797809 | NPIPA1 | nuclear pore complex interacting protein family member A1 [Source:HGNC Symbol;Acc:HGNC:7909] |
| ENSG00000184207 | HGLibA_36276 | 4 | -1.108797809 | PGP | phosphoglycolate phosphatase [Source:HGNC Symbol;Acc:HGNC:8909] |
| ENSG00000186480 | HGLibA_23377 | 4 | -1.108797809 | INSIG1 | insulin induced gene 1 [Source:HGNC Symbol;Acc:HGNC:6083] |
| ENSG00000186564 | HGLibA_17830 | 4 | -1.108797809 | FOXD2 | forkhead box D2 [Source:HGNC Symbol;Acc:HGNC:3803] |
| ENSG00000187838 | HGLibA_37236 | 4 | -1.108797809 | PLSCR3 | phospholipid scramblase 3 [Source:HGNC Symbol;Acc:HGNC:16495] |
| ENSG00000187922 | HGLibA_26168 | 4 | -1.108797809 | LCN10 | lipocalin 10 [Source:HGNC Symbol;Acc:HGNC:20892] |
| ENSG00000188042 | HGLibA_02969 | 4 | -1.108797809 | ARL4C | ADP ribosylation factor like GTPase 4C [Source:HGNC Symbol;Acc:HGNC:698] |
| ENSG00000196228 | HGLibA_47749 | 4 | -1.108797809 | SULT1C3 | sulfotransferase family 1C member 3 [Source:HGNC Symbol;Acc:HGNC:33543] |
| ENSG00000197019 | HGLibA_43604 | 4 | -1.108797809 | SERTAD1 | SERTA domain containing 1 [Source:HGNC Symbol;Acc:HGNC:17932] |
| ENSG00000197454 | HGLibA_33828 | 4 | -1.108797809 | OR2L5 | olfactory receptor family 2 subfamily L member 5 [Source:HGNC Symbol;Acc:HGNC:15011] |
| ENSG00000197535 | HGLibA_30851 | 4 | -1.108797809 | MYO5A | myosin VA [Source:HGNC Symbol;Acc:HGNC:7602] |

| | | | | | |
|---|---|---|---|---|---|
| ENSG00000197562 | HGLibA_39941 | 4 | -1.108797809 | RAB40C | member RAS oncogene family [Source:HGNC Symbol;Acc:HGNC:18285]" |
| ENSG00000197951 | HGLibA_56607 | 4 | -1.108797809 | ZNF71 | zinc finger protein 71 [Source:HGNC Symbol;Acc:HGNC:13141] |
| ENSG00000198216 | HGLibA_06887 | 4 | -1.108797809 | CACNA1E | calcium voltage-gated channel subunit alpha1 E [Source:HGNC Symbol;Acc:HGNC:1392] |
| ENSG00000204936 | HGLibA_08255 | 4 | -1.108797809 | CD177 | CD177 molecule [Source:HGNC Symbol;Acc:HGNC:30072] |
| ENSG00000205777 | HGLibA_18432 | 4 | -1.108797809 | GAGE1 | G antigen 1 [Source:HGNC Symbol;Acc:HGNC:4098] |
| ENSG00000213215 | HGLibA_33788 | 4 | -1.108797809 | OR2F1 | olfactory receptor family 2 subfamily F member 1 (gene/pseudogene) [Source:HGNC Symbol;Acc:HGNC:8246] |
| ENSG00000223953 | HGLibA_05949 | 4 | -1.108797809 | C1QTNF5 | C1q and TNF related 5 [Source:HGNC Symbol;Acc:HGNC:14344] |
| ENSG00000224659 | HGLibA_18462 | 4 | -1.108797809 | GAGE12J | G antigen 12J [Source:HGNC Symbol;Acc:HGNC:17778] |
| ENSG00000227345 | HGLibA_35193 | 4 | -1.108797809 | PARG | poly(ADP-ribose) glycohydrolase [Source:HGNC Symbol;Acc:HGNC:8605] |
| ENSG00000231051 | HGLibA_53265 | 4 | -1.108797809 | USP17L28 | ubiquitin specific peptidase 17-like family member 28 [Source:HGNC Symbol;Acc:HGNC:44456] |
| ENSG00000231637 | HGLibA_53268 | 4 | -1.108797809 | USP17L29 | ubiquitin specific peptidase 17-like family member 29 [Source:HGNC Symbol;Acc:HGNC:44457] |
| ENSG00000233136 | HGLibA_53217 | 4 | -1.108797809 | USP17L11 | ubiquitin specific peptidase 17-like family member 11 [Source:HGNC Symbol;Acc:HGNC:44439] |
| ENSG00000236125 | HGLibA_53277 | 4 | -1.108797809 | USP17L4 | ubiquitin specific peptidase 17-like family member 4 [Source:HGNC Symbol;Acc:HGNC:37176] |
| ENSG00000249811 | HGLibA_53247 | 4 | -1.108797809 | USP17L21 | ubiquitin specific peptidase 17-like family member 21 [Source:HGNC Symbol;Acc:HGNC:44449] |
| ENSG00000254585 | HGLibA_27992 | 4 | -1.108797809 | MAGEL2 | MAGE family member L2 [Source:HGNC Symbol;Acc:HGNC:6814] |
| ENSG00000256660 | HGLibA_09932 | 4 | -1.108797809 | CLEC12B | C-type lectin domain family 12 member B [Source:HGNC Symbol;Acc:HGNC:31966] |
| ENSG00000260097 | HGLibA_46601 | 4 | -1.108797809 | SPDYE6 | speedy/RINGO cell cycle regulator family member E6 [Source:HGNC Symbol;Acc:HGNC:35465] |
| ENSG00000275113 | HGLibA_18480 | 4 | -1.108797809 | GAGE2E | G antigen 2E [Source:HGNC Symbol;Acc:HGNC:31960] |
| ENSG00000278558 | HGLibA_50144 | 4 | -1.108797809 | TMEM191B | transmembrane protein 191B [Source:HGNC Symbol;Acc:HGNC:33600] |
| ENSG00000284733 | HGLibA_34000 | 4 | -1.108797809 | OR4F29 | olfactory receptor family 4 subfamily F member 29 [Source:HGNC Symbol;Acc:HGNC:31275] |
| FAM173B | HGLibA_16278 | 4 | -1.108797809 | FAM173B | - |
| FAM19A2 | HGLibA_16400 | 4 | -1.108797809 | FAM19A2 | - |
| FAM84B | HGLibA_16766 | 4 | -1.108797809 | FAM84B | - |
| GAGE12I | HGLibA_18459 | 4 | -1.108797809 | GAGE12I | - |
| GPR128 | HGLibA_19886 | 4 | -1.108797809 | GPR128 | - |
| HRASLS5 | HGLibA_22081 | 4 | -1.108797809 | HRASLS5 | - |
| hsa-mir-135b | HGLibA_57802 | 4 | -1.108797809 | hsa-mir-135b | - |
| hsa-mir-152 | HGLibA_57905 | 4 | -1.108797809 | hsa-mir-152 | - |
| hsa-mir-24-1 | HGLibA_58341 | 4 | -1.108797809 | hsa-mir-24-1 | - |
| hsa-mir-30a | HGLibA_58471 | 4 | -1.108797809 | hsa-mir-30a | - |
| hsa-mir-3119-1 | HGLibA_58524 | 4 | -1.108797809 | hsa-mir-3119-1 | - |
| hsa-mir-3123 | HGLibA_58544 | 4 | -1.108797809 | hsa-mir-3123 | - |
| hsa-mir-3179-3 | HGLibA_58796 | 4 | -1.108797809 | hsa-mir-3179-3 | - |
| hsa-mir-3180-1 | HGLibA_58803 | 4 | -1.108797809 | hsa-mir-3180-1 | - |
| hsa-mir-3184 | HGLibA_58832 | 4 | -1.108797809 | hsa-mir-3184 | - |
| hsa-mir-3670-1 | HGLibA_59235 | 4 | -1.108797809 | hsa-mir-3670-1 | - |
| hsa-mir-3689d-1 | HGLibA_59326 | 4 | -1.108797809 | hsa-mir-3689d-1 | - |
| hsa-mir-3918 | HGLibA_59549 | 4 | -1.108797809 | hsa-mir-3918 | - |
| hsa-mir-412 | HGLibA_59688 | 4 | -1.108797809 | hsa-mir-412 | - |
| hsa-mir-4262 | HGLibA_59754 | 4 | -1.108797809 | hsa-mir-4262 | - |
| hsa-mir-4305 | HGLibA_59932 | 4 | -1.108797809 | hsa-mir-4305 | - |
| hsa-mir-4509-1 | HGLibA_60462 | 4 | -1.108797809 | hsa-mir-4509-1 | - |
| hsa-mir-4659b | HGLibA_60746 | 4 | -1.108797809 | hsa-mir-4659b | - |
| hsa-mir-4712 | HGLibA_60949 | 4 | -1.108797809 | hsa-mir-4712 | - |
| hsa-mir-486 | HGLibA_61336 | 4 | -1.108797809 | hsa-mir-486 | - |
| hsa-mir-520g | HGLibA_61759 | 4 | -1.108797809 | hsa-mir-520g | - |
| hsa-mir-527 | HGLibA_61802 | 4 | -1.108797809 | hsa-mir-527 | - |
| hsa-mir-548ac | HGLibA_61861 | 4 | -1.108797809 | hsa-mir-548ac | - |
| hsa-mir-548h-5 | HGLibA_62008 | 4 | -1.108797809 | hsa-mir-548h-5 | - |
| hsa-mir-5587 | HGLibA_62190 | 4 | -1.108797809 | hsa-mir-5587 | - |
| hsa-mir-566 | HGLibA_62222 | 4 | -1.108797809 | hsa-mir-566 | - |
| hsa-mir-580 | HGLibA_62420 | 4 | -1.108797809 | hsa-mir-580 | - |
| hsa-mir-614 | HGLibA_62693 | 4 | -1.108797809 | hsa-mir-614 | - |
| hsa-mir-639 | HGLibA_62797 | 4 | -1.108797809 | hsa-mir-639 | - |
| hsa-mir-652 | HGLibA_62943 | 4 | -1.108797809 | hsa-mir-652 | - |
| hsa-mir-6718 | HGLibA_63035 | 4 | -1.108797809 | hsa-mir-6718 | - |
| hsa-mir-6741 | HGLibA_63118 | 4 | -1.108797809 | hsa-mir-6741 | - |
| hsa-mir-6820 | HGLibA_63458 | 4 | -1.108797809 | hsa-mir-6820 | - |
| hsa-mir-6864 | HGLibA_63639 | 4 | -1.108797809 | hsa-mir-6864 | - |
| hsa-mir-7-1 | HGLibA_63769 | 4 | -1.108797809 | hsa-mir-7-1 | - |
| hsa-mir-7704 | HGLibA_63950 | 4 | -1.108797809 | hsa-mir-7704 | - |
| hsa-mir-8054 | HGLibA_64056 | 4 | -1.108797809 | hsa-mir-8054 | - |
| LOC100288332 | HGLibA_26830 | 4 | -1.108797809 | LOC100288332 | - |
| LOC642778 | HGLibA_26957 | 4 | -1.108797809 | LOC642778 | - |
| NGFRAP1 | HGLibA_31879 | 4 | -1.108797809 | NGFRAP1 | - |
| QARS | HGLibA_39719 | 4 | -1.108797809 | QARS | - |
| RPS17L | HGLibA_42089 | 4 | -1.108797809 | RPS17L | - |
| ZHX1-C8ORF76 | HGLibA_55312 | 4 | -1.108797809 | ZHX1-C8ORF76 | - |
| 8-Mar | HGLibA_28350 | 3 | -1.118748846 | 8-Mar | - |
| C9orf156 | HGLibA_06696 | 3 | -1.118748846 | C9orf156 | - |
| CCBL2 | HGLibA_07500 | 3 | -1.118748846 | CCBL2 | - |
| CCDC23 | HGLibA_07732 | 3 | -1.118748846 | CCDC23 | - |
| CSF2RA_X | HGLibA_11329 | 3 | -1.118748846 | CSF2RA_X | - |
| ENSG00000004059 | HGLibA_02656 | 3 | -1.118748846 | ARF5 | ADP ribosylation factor 5 [Source:HGNC Symbol;Acc:HGNC:658] |
| ENSG00000009694 | HGLibA_48996 | 3 | -1.118748846 | TENM1 | teneurin transmembrane protein 1 [Source:HGNC Symbol;Acc:HGNC:8117] |
| ENSG00000010539 | HGLibA_55570 | 3 | -1.118748846 | ZNF200 | zinc finger protein 200 [Source:HGNC Symbol;Acc:HGNC:12993] |
| ENSG00000013374 | HGLibA_32899 | 3 | -1.118748846 | NUB1 | negative regulator of ubiquitin like proteins 1 [Source:HGNC Symbol;Acc:HGNC:17623] |
| ENSG00000015568 | HGLibA_41037 | 3 | -1.118748846 | RGPD5 | RANBP2-like and GRIP domain containing 5 [Source:HGNC Symbol;Acc:HGNC:32418] |
| ENSG00000018510 | HGLibA_01240 | 3 | -1.118748846 | AGPS | alkylglycerone phosphate synthase [Source:HGNC Symbol;Acc:HGNC:327] |
| ENSG00000028116 | HGLibA_53827 | 3 | -1.118748846 | VRK2 | VRK serine/threonine kinase 2 [Source:HGNC Symbol;Acc:HGNC:12719] |
| ENSG00000034239 | HGLibA_14521 | 3 | -1.118748846 | EFCAB1 | EF-hand calcium binding domain 1 [Source:HGNC Symbol;Acc:HGNC:25678] |
| ENSG00000036565 | HGLibA_44485 | 3 | -1.118748846 | SLC18A1 | solute carrier family 18 member A1 [Source:HGNC Symbol;Acc:HGNC:10934] |
| ENSG00000055955 | HGLibA_23813 | 3 | -1.118748846 | ITIH4 | inter-alpha-trypsin inhibitor heavy chain family member 4 [Source:HGNC Symbol;Acc:HGNC:6169] |
| ENSG00000063015 | HGLibA_43679 | 3 | -1.118748846 | SEZ6 | seizure related 6 homolog [Source:HGNC Symbol;Acc:HGNC:15955] |
| ENSG00000064490 | HGLibA_40986 | 3 | -1.118748846 | RFXANK | regulatory factor X associated ankyrin containing protein [Source:HGNC Symbol;Acc:HGNC:9987] |
| ENSG00000065518 | HGLibA_31519 | 3 | -1.118748846 | NDUFB4 | NADH:ubiquinone oxidoreductase subunit B4 [Source:HGNC Symbol;Acc:HGNC:7699] |
| ENSG00000068796 | HGLibA_24926 | 3 | -1.118748846 | KIF2A | kinesin family member 2A [Source:HGNC Symbol;Acc:HGNC:6318] |
| ENSG00000069943 | HGLibA_36546 | 3 | -1.118748846 | PIGB | phosphatidylinositol glycan anchor biosynthesis class B [Source:HGNC Symbol;Acc:HGNC:8959] |
| ENSG00000073734 | HGLibA_00138 | 3 | -1.118748846 | ABCB11 | ATP binding cassette subfamily B member 11 [Source:HGNC Symbol;Acc:HGNC:42] |
| ENSG00000076716 | HGLibA_19771 | 3 | -1.118748846 | GPC4 | glypican 4 [Source:HGNC Symbol;Acc:HGNC:4452] |
| ENSG00000077044 | HGLibA_13064 | 3 | -1.118748846 | DGKD | diacylglycerol kinase delta [Source:HGNC Symbol;Acc:HGNC:2851] |
| ENSG00000077327 | HGLibA_46362 | 3 | -1.118748846 | SPAG6 | sperm associated antigen 6 [Source:HGNC Symbol;Acc:HGNC:11215] |
| ENSG00000079432 | HGLibA_09694 | 3 | -1.118748846 | CIC | capicua transcriptional repressor [Source:HGNC Symbol;Acc:HGNC:14214] |
| ENSG00000081479 | HGLibA_27213 | 3 | -1.118748846 | LRP2 | LDL receptor related protein 2 [Source:HGNC Symbol;Acc:HGNC:6694] |
| ENSG00000085491 | HGLibA_44680 | 3 | -1.118748846 | SLC25A24 | solute carrier family 25 member 24 [Source:HGNC Symbol;Acc:HGNC:20662] |
| ENSG00000085832 | HGLibA_15367 | 3 | -1.118748846 | EPS15 | epidermal growth factor receptor pathway substrate 15 [Source:HGNC Symbol;Acc:HGNC:3419] |
| ENSG00000087157 | HGLibA_36294 | 3 | -1.118748846 | PGS1 | phosphatidylglycerophosphate synthase 1 [Source:HGNC Symbol;Acc:HGNC:30029] |
| ENSG00000089692 | HGLibA_25927 | 3 | -1.118748846 | LAG3 | lymphocyte activating 3 [Source:HGNC Symbol;Acc:HGNC:6476] |
| ENSG00000091128 | HGLibA_25964 | 3 | -1.118748846 | LAMB4 | laminin subunit beta 4 [Source:HGNC Symbol;Acc:HGNC:6491] |
| ENSG00000100122 | HGLibA_11261 | 3 | -1.118748846 | CRYBB1 | crystallin beta B1 [Source:HGNC Symbol;Acc:HGNC:2397] |
| ENSG00000100605 | HGLibA_23841 | 3 | -1.118748846 | ITPK1 | inositol-tetrakisphosphate 1-kinase [Source:HGNC Symbol;Acc:HGNC:6177] |
| ENSG00000101161 | HGLibA_38743 | 3 | -1.118748846 | PRPF6 | pre-mRNA processing factor 6 [Source:HGNC Symbol;Acc:HGNC:15860] |
| ENSG00000101216 | HGLibA_19400 | 3 | -1.118748846 | GMEB2 | glucocorticoid modulatory element binding protein 2 [Source:HGNC Symbol;Acc:HGNC:4371] |
| ENSG00000101773 | HGLibA_40440 | 3 | -1.118748846 | RBBP8 | endonuclease [Source:HGNC Symbol;Acc:HGNC:9891]" |
| ENSG00000101974 | HGLibA_03561 | 3 | -1.118748846 | ATP11C | ATPase phospholipid transporting 11C [Source:HGNC Symbol;Acc:HGNC:13554] |
| ENSG00000102104 | HGLibA_42293 | 3 | -1.118748846 | RS1 | retinoschisin 1 [Source:HGNC Symbol;Acc:HGNC:10457] |
| ENSG00000104369 | HGLibA_23979 | 3 | -1.118748846 | JPH1 | junctophilin 1 [Source:HGNC Symbol;Acc:HGNC:14201] |
| ENSG00000107562 | HGLibA_11851 | 3 | -1.118748846 | CXCL12 | C-X-C motif chemokine ligand 12 [Source:HGNC Symbol;Acc:HGNC:10672] |
| ENSG00000107874 | HGLibA_11770 | 3 | -1.118748846 | CUEDC2 | CUE domain containing 2 [Source:HGNC Symbol;Acc:HGNC:28352] |
| ENSG00000108587 | HGLibA_19685 | 3 | -1.118748846 | GOSR1 | golgi SNAP receptor complex member 1 [Source:HGNC Symbol;Acc:HGNC:4430] |
| ENSG00000108771 | HGLibA_13206 | 3 | -1.118748846 | DHX58 | DExH-box helicase 58 [Source:HGNC Symbol;Acc:HGNC:29517] |
| ENSG00000110429 | HGLibA_17063 | 3 | -1.118748846 | FBXO3 | F-box protein 3 [Source:HGNC Symbol;Acc:HGNC:13582] |
| ENSG00000111670 | HGLibA_19575 | 3 | -1.118748846 | GNPTAB | N-acetylglucosamine-1-phosphate transferase subunits alpha and beta [Source:HGNC Symbol;Acc:HGNC:29670] |
| ENSG00000111783 | HGLibA_40971 | 3 | -1.118748846 | RFX4 | regulatory factor X4 [Source:HGNC Symbol;Acc:HGNC:9985] |
| ENSG00000112592 | HGLibA_48622 | 3 | -1.118748846 | TBP | TATA-box binding protein [Source:HGNC Symbol;Acc:HGNC:11588] |
| ENSG00000113916 | HGLibA_04331 | 3 | -1.118748846 | BCL6 | B cell CLL/lymphoma 6 [Source:HGNC Symbol;Acc:HGNC:1001] |
| ENSG00000114204 | HGLibA_43600 | 3 | -1.118748846 | SERPINI2 | serpin family I member 2 [Source:HGNC Symbol;Acc:HGNC:8945] |
| ENSG00000116497 | HGLibA_42586 | 3 | -1.118748846 | S100PBP | S100P binding protein [Source:HGNC Symbol;Acc:HGNC:25768] |
| ENSG00000116833 | HGLibA_32637 | 3 | -1.118748846 | NR5A2 | nuclear receptor subfamily 5 group A member 2 [Source:HGNC Symbol;Acc:HGNC:7984] |
| ENSG00000117479 | HGLibA_44502 | 3 | -1.118748846 | SLC19A2 | solute carrier family 19 member 2 [Source:HGNC Symbol;Acc:HGNC:10938] |
| ENSG00000119508 | HGLibA_32630 | 3 | -1.118748846 | NR4A3 | nuclear receptor subfamily 4 group A member 3 [Source:HGNC Symbol;Acc:HGNC:7982] |
| ENSG00000119614 | HGLibA_53879 | 3 | -1.118748846 | VSX2 | visual system homeobox 2 [Source:HGNC Symbol;Acc:HGNC:1975] |
| ENSG00000119650 | HGLibA_22759 | 3 | -1.118748846 | IFT43 | intraflagellar transport 43 [Source:HGNC Symbol;Acc:HGNC:29669] |
| ENSG00000120868 | HGLibA_02365 | 3 | -1.118748846 | APAF1 | apoptotic peptidase activating factor 1 [Source:HGNC Symbol;Acc:HGNC:576] |
| ENSG00000123338 | HGLibA_31315 | 3 | -1.118748846 | NCKAP1L | NCK associated protein 1 like [Source:HGNC Symbol;Acc:HGNC:4862] |
| ENSG00000124201 | HGLibA_56919 | 3 | -1.118748846 | ZNFX1 | zinc finger NFX1-type containing 1 [Source:HGNC Symbol;Acc:HGNC:29271] |
| ENSG00000124215 | HGLibA_08674 | 3 | -1.118748846 | CDH26 | cadherin 26 [Source:HGNC Symbol;Acc:HGNC:15902] |
| ENSG00000124785 | HGLibA_32709 | 3 | -1.118748846 | NRN1 | neuritin 1 [Source:HGNC Symbol;Acc:HGNC:17972] |
| ENSG00000125534 | HGLibA_37865 | 3 | -1.118748846 | PPDPF | pancreatic progenitor cell differentiation and proliferation factor [Source:HGNC Symbol;Acc:HGNC:16142] |
| ENSG00000125868 | HGLibA_14047 | 3 | -1.118748846 | DSTN | actin depolymerizing factor [Source:HGNC Symbol;Acc:HGNC:15750]" |
| ENSG00000126549 | HGLibA_47402 | 3 | -1.118748846 | STATH | statherin [Source:HGNC Symbol;Acc:HGNC:11369] |
| ENSG00000127220 | HGLibA_00286 | 3 | -1.118748846 | ABHD8 | abhydrolase domain containing 8 [Source:HGNC Symbol;Acc:HGNC:23759] |
| ENSG00000128000 | HGLibA_56733 | 3 | -1.118748846 | ZNF780B | zinc finger protein 780B [Source:HGNC Symbol;Acc:HGNC:33109] |
| ENSG00000130023 | HGLibA_15504 | 3 | -1.118748846 | ERMARD | ER membrane associated RNA degradation [Source:HGNC Symbol;Acc:HGNC:21056] |
| ENSG00000130766 | HGLibA_43623 | 3 | -1.118748846 | SESN2 | sestrin 2 [Source:HGNC Symbol;Acc:HGNC:20746] |

| | | | | | |
|---|---|---|---|---|---|
| ENSG00000131650 | HGLibA_25405 | 3 | -1.118748846 | KREMEN2 | kringle containing transmembrane protein 2 [Source:HGNC Symbol;Acc:HGNC:18797] |
| ENSG00000132376 | HGLibA_23362 | 3 | -1.118748846 | INPP5K | inositol polyphosphate-5-phosphatase K [Source:HGNC Symbol;Acc:HGNC:33882] |
| ENSG00000132671 | HGLibA_47182 | 3 | -1.118748846 | SSTR4 | somatostatin receptor 4 [Source:HGNC Symbol;Acc:HGNC:11333] |
| ENSG00000133055 | HGLibA_30657 | 3 | -1.118748846 | MYBPH | myosin binding protein H [Source:HGNC Symbol;Acc:HGNC:7552] |
| ENSG00000133169 | HGLibA_04440 | 3 | -1.118748846 | BEX1 | brain expressed X-linked 1 [Source:HGNC Symbol;Acc:HGNC:1036] |
| ENSG00000134201 | HGLibA_20485 | 3 | -1.118748846 | GSTM5 | glutathione S-transferase mu 5 [Source:HGNC Symbol;Acc:HGNC:4637] |
| ENSG00000134574 | HGLibA_12598 | 3 | -1.118748846 | DDB2 | damage specific DNA binding protein 2 [Source:HGNC Symbol;Acc:HGNC:2718] |
| ENSG00000135116 | HGLibA_22106 | 3 | -1.118748846 | HRK | BCL2 interacting protein [Source:HGNC Symbol;Acc:HGNC:5185]" |
| ENSG00000135750 | HGLibA_24294 | 3 | -1.118748846 | KCNK1 | potassium two pore domain channel subfamily K member 1 [Source:HGNC Symbol;Acc:HGNC:6272] |
| ENSG00000135973 | HGLibA_20044 | 3 | -1.118748846 | GPR45 | G protein-coupled receptor 45 [Source:HGNC Symbol;Acc:HGNC:4503] |
| ENSG00000136205 | HGLibA_50925 | 3 | -1.118748846 | TNS3 | tensin 3 [Source:HGNC Symbol;Acc:HGNC:21616] |
| ENSG00000136463 | HGLibA_48169 | 3 | -1.118748846 | TACO1 | translational activator of cytochrome c oxidase I [Source:HGNC Symbol;Acc:HGNC:24316] |
| ENSG00000136492 | HGLibA_04822 | 3 | -1.118748846 | BRIP1 | BRCA1 interacting protein C-terminal helicase 1 [Source:HGNC Symbol;Acc:HGNC:20473] |
| ENSG00000136697 | HGLibA_23044 | 3 | -1.118748846 | IL1F10 | interleukin 1 family member 10 [Source:HGNC Symbol;Acc:HGNC:15552] |
| ENSG00000137094 | HGLibA_13545 | 3 | -1.118748846 | DNAJB5 | DnaJ heat shock protein family (Hsp40) member B5 [Source:HGNC Symbol;Acc:HGNC:14887] |
| ENSG00000137857 | HGLibA_14097 | 3 | -1.118748846 | DUOX1 | dual oxidase 1 [Source:HGNC Symbol;Acc:HGNC:3062] |
| ENSG00000138663 | HGLibA_10718 | 3 | -1.118748846 | COPS4 | COP9 signalosome subunit 4 [Source:HGNC Symbol;Acc:HGNC:16702] |
| ENSG00000140718 | HGLibA_18132 | 3 | -1.118748846 | FTO | alpha-ketoglutarate dependent dioxygenase [Source:HGNC Symbol;Acc:HGNC:24678]" |
| ENSG00000141298 | HGLibA_47136 | 3 | -1.118748846 | SSH2 | slingshot protein phosphatase 2 [Source:HGNC Symbol;Acc:HGNC:30580] |
| ENSG00000141579 | HGLibA_56673 | 3 | -1.118748846 | ZNF750 | zinc finger protein 750 [Source:HGNC Symbol;Acc:HGNC:25843] |
| ENSG00000141642 | HGLibA_14871 | 3 | -1.118748846 | ELAC1 | elaC ribonuclease Z 1 [Source:HGNC Symbol;Acc:HGNC:14197] |
| ENSG00000143954 | HGLibA_40804 | 3 | -1.118748846 | REG3G | regenerating family member 3 gamma [Source:HGNC Symbol;Acc:HGNC:29595] |
| ENSG00000144029 | HGLibA_30104 | 3 | -1.118748846 | MRPS5 | mitochondrial ribosomal protein S5 [Source:HGNC Symbol;Acc:HGNC:14498] |
| ENSG00000145934 | HGLibA_48997 | 3 | -1.118748846 | TENM2 | teneurin transmembrane protein 2 [Source:HGNC Symbol;Acc:HGNC:29943] |
| ENSG00000146576 | HGLibA_06527 | 3 | -1.118748846 | C7orf26 | chromosome 7 open reading frame 26 [Source:HGNC Symbol;Acc:HGNC:21702] |
| ENSG00000146592 | HGLibA_11104 | 3 | -1.118748846 | CREB5 | cAMP responsive element binding protein 5 [Source:HGNC Symbol;Acc:HGNC:16844] |
| ENSG00000146618 | HGLibA_17290 | 3 | -1.118748846 | FERD3L | Fer3 like bHLH transcription factor [Source:HGNC Symbol;Acc:HGNC:16660] |
| ENSG00000147604 | HGLibA_41992 | 3 | -1.118748846 | RPL7 | ribosomal protein L7 [Source:HGNC Symbol;Acc:HGNC:10363] |
| ENSG00000148143 | HGLibA_56041 | 3 | -1.118748846 | ZNF462 | zinc finger protein 462 [Source:HGNC Symbol;Acc:HGNC:21684] |
| ENSG00000148175 | HGLibA_47530 | 3 | -1.118748846 | STOM | stomatin [Source:HGNC Symbol;Acc:HGNC:3383] |
| ENSG00000151090 | HGLibA_49394 | 3 | -1.118748846 | THRB | thyroid hormone receptor beta [Source:HGNC Symbol;Acc:HGNC:11799] |
| ENSG00000151948 | HGLibA_19330 | 3 | -1.118748846 | GLT1D1 | glycosyltransferase 1 domain containing 1 [Source:HGNC Symbol;Acc:HGNC:26483] |
| ENSG00000152266 | HGLibA_39365 | 3 | -1.118748846 | PTH | parathyroid hormone [Source:HGNC Symbol;Acc:HGNC:9606] |
| ENSG00000153162 | HGLibA_04613 | 3 | -1.118748846 | BMP6 | bone morphogenetic protein 6 [Source:HGNC Symbol;Acc:HGNC:1073] |
| ENSG00000155249 | HGLibA_34017 | 3 | -1.118748846 | OR4K1 | olfactory receptor family 4 subfamily K member 1 [Source:HGNC Symbol;Acc:HGNC:14726] |
| ENSG00000155363 | HGLibA_29694 | 3 | -1.118748846 | MOV10 | Mov10 RISC complex RNA helicase [Source:HGNC Symbol;Acc:HGNC:7200] |
| ENSG00000156256 | HGLibA_53211 | 3 | -1.118748846 | USP16 | ubiquitin specific peptidase 16 [Source:HGNC Symbol;Acc:HGNC:12614] |
| ENSG00000156453 | HGLibA_35385 | 3 | -1.118748846 | PCDH1 | protocadherin 1 [Source:HGNC Symbol;Acc:HGNC:8655] |
| ENSG00000156976 | HGLibA_14806 | 3 | -1.118748846 | EIF4A2 | eukaryotic translation initiation factor 4A2 [Source:HGNC Symbol;Acc:HGNC:3284] |
| ENSG00000157368 | HGLibA_23142 | 3 | -1.118748846 | IL34 | interleukin 34 [Source:HGNC Symbol;Acc:HGNC:28529] |
| ENSG00000158055 | HGLibA_20253 | 3 | -1.118748846 | GRHL3 | grainyhead like transcription factor 3 [Source:HGNC Symbol;Acc:HGNC:25839] |
| ENSG00000158560 | HGLibA_14236 | 3 | -1.118748846 | DYNC1I1 | dynein cytoplasmic 1 intermediate chain 1 [Source:HGNC Symbol;Acc:HGNC:2963] |
| ENSG00000158716 | HGLibA_14166 | 3 | -1.118748846 | DUSP23 | dual specificity phosphatase 23 [Source:HGNC Symbol;Acc:HGNC:21480] |
| ENSG00000162039 | HGLibA_28867 | 3 | -1.118748846 | MEIOB | meiosis specific with OB domains [Source:HGNC Symbol;Acc:HGNC:28569] |
| ENSG00000162227 | HGLibA_48248 | 3 | -1.118748846 | TAF6L | TATA-box binding protein associated factor 6 like [Source:HGNC Symbol;Acc:HGNC:17305] |
| ENSG00000162772 | HGLibA_03437 | 3 | -1.118748846 | ATF3 | activating transcription factor 3 [Source:HGNC Symbol;Acc:HGNC:785] |
| ENSG00000163515 | HGLibA_40878 | 3 | -1.118748846 | RETNLB | resistin like beta [Source:HGNC Symbol;Acc:HGNC:20388] |
| ENSG00000163661 | HGLibA_39570 | 3 | -1.118748846 | PTX3 | pentraxin 3 [Source:HGNC Symbol;Acc:HGNC:9692] |
| ENSG00000163738 | HGLibA_30384 | 3 | -1.118748846 | MTHFD2L | methylenetetrahydrofolate dehydrogenase (NADP+ dependent) 2 like [Source:HGNC Symbol;Acc:HGNC:31865] |
| ENSG00000163781 | HGLibA_51010 | 3 | -1.118748846 | TOPBP1 | DNA topoisomerase II binding protein 1 [Source:HGNC Symbol;Acc:HGNC:17008] |
| ENSG00000163900 | HGLibA_50393 | 3 | -1.118748846 | TMEM41A | transmembrane protein 41A [Source:HGNC Symbol;Acc:HGNC:30544] |
| ENSG00000164096 | HGLibA_06316 | 3 | -1.118748846 | C4orf3 | chromosome 4 open reading frame 3 [Source:HGNC Symbol;Acc:HGNC:19225] |
| ENSG00000165076 | HGLibA_38936 | 3 | -1.118748846 | PRSS37 | serine protease 37 [Source:HGNC Symbol;Acc:HGNC:29211] |
| ENSG00000166200 | HGLibA_10713 | 3 | -1.118748846 | COPS2 | COP9 signalosome subunit 2 [Source:HGNC Symbol;Acc:HGNC:30747] |
| ENSG00000166289 | HGLibA_37096 | 3 | -1.118748846 | PLEKHF1 | pleckstrin homology and FYVE domain containing 1 [Source:HGNC Symbol;Acc:HGNC:20764] |
| ENSG00000167074 | HGLibA_48963 | 3 | -1.118748846 | TEF | PAR bZIP transcription factor [Source:HGNC Symbol;Acc:HGNC:11722]" |
| ENSG00000167914 | HGLibA_20404 | 3 | -1.118748846 | GSDMA | gasdermin A [Source:HGNC Symbol;Acc:HGNC:13311] |
| ENSG00000169398 | HGLibA_39379 | 3 | -1.118748846 | PTK2 | protein tyrosine kinase 2 [Source:HGNC Symbol;Acc:HGNC:9611] |
| ENSG00000169474 | HGLibA_46801 | 3 | -1.118748846 | SPRR1A | small proline rich protein 1A [Source:HGNC Symbol;Acc:HGNC:11259] |
| ENSG00000169629 | HGLibA_41042 | 3 | -1.118748846 | RGPD8 | RANBP2-like and GRIP domain containing 8 [Source:HGNC Symbol;Acc:HGNC:9849] |
| ENSG00000170166 | HGLibA_22001 | 3 | -1.118748846 | HOXD4 | homeobox D4 [Source:HGNC Symbol;Acc:HGNC:5138] |
| ENSG00000170382 | HGLibA_27473 | 3 | -1.118748846 | LRRN2 | leucine rich repeat neuronal 2 [Source:HGNC Symbol;Acc:HGNC:16914] |
| ENSG00000170634 | HGLibA_00714 | 3 | -1.118748846 | ACYP2 | acylphosphatase 2 [Source:HGNC Symbol;Acc:HGNC:180] |
| ENSG00000170734 | HGLibA_37527 | 3 | -1.118748846 | POLH | DNA polymerase eta [Source:HGNC Symbol;Acc:HGNC:9181] |
| ENSG00000170779 | HGLibA_08609 | 3 | -1.118748846 | CDCA4 | cell division cycle associated 4 [Source:HGNC Symbol;Acc:HGNC:14625] |
| ENSG00000170921 | HGLibA_48293 | 3 | -1.118748846 | TANC2 | ankyrin repeat and coiled-coil containing 2 [Source:HGNC Symbol;Acc:HGNC:30212]" |
| ENSG00000171014 | HGLibA_33977 | 3 | -1.118748846 | OR4D5 | olfactory receptor family 4 subfamily D member 5 [Source:HGNC Symbol;Acc:HGNC:14852] |
| ENSG00000171435 | HGLibA_25867 | 3 | -1.118748846 | KSR2 | kinase suppressor of ras 2 [Source:HGNC Symbol;Acc:HGNC:18610] |
| ENSG00000171612 | HGLibA_44711 | 3 | -1.118748846 | SLC25A33 | solute carrier family 25 member 33 [Source:HGNC Symbol;Acc:HGNC:29681] |
| ENSG00000171827 | HGLibA_56274 | 3 | -1.118748846 | ZNF570 | zinc finger protein 570 [Source:HGNC Symbol;Acc:HGNC:26416] |
| ENSG00000173678 | HGLibA_46585 | 3 | -1.118748846 | SPDYE2B | speedy/RINGO cell cycle regulator family member E2B [Source:HGNC Symbol;Acc:HGNC:48334] |
| ENSG00000173875 | HGLibA_56768 | 3 | -1.118748846 | ZNF791 | zinc finger protein 791 [Source:HGNC Symbol;Acc:HGNC:26895] |
| ENSG00000174827 | HGLibA_35991 | 3 | -1.118748846 | PDZK1 | PDZ domain containing 1 [Source:HGNC Symbol;Acc:HGNC:8821] |
| ENSG00000176208 | HGLibA_03419 | 3 | -1.118748846 | ATAD5 | AAA domain containing 5 [Source:HGNC Symbol;Acc:HGNC:25752]" |
| ENSG00000176222 | HGLibA_55924 | 3 | -1.118748846 | ZNF404 | zinc finger protein 404 [Source:HGNC Symbol;Acc:HGNC:19417] |
| ENSG00000176893 | HGLibA_34111 | 3 | -1.118748846 | OR51G2 | olfactory receptor family 51 subfamily G member 2 [Source:HGNC Symbol;Acc:HGNC:15198] |
| ENSG00000177119 | HGLibA_02185 | 3 | -1.118748846 | ANO6 | anoctamin 6 [Source:HGNC Symbol;Acc:HGNC:25240] |
| ENSG00000177283 | HGLibA_18285 | 3 | -1.118748846 | FZD8 | frizzled class receptor 8 [Source:HGNC Symbol;Acc:HGNC:4046] |
| ENSG00000177535 | HGLibA_33764 | 3 | -1.118748846 | OR2B11 | olfactory receptor family 2 subfamily B member 11 [Source:HGNC Symbol;Acc:HGNC:31249] |
| ENSG00000179051 | HGLibA_40685 | 3 | -1.118748846 | RCC2 | regulator of chromosome condensation 2 [Source:HGNC Symbol;Acc:HGNC:30297] |
| ENSG00000179241 | HGLibA_26248 | 3 | -1.118748846 | LDLRAD3 | low density lipoprotein receptor class A domain containing 3 [Source:HGNC Symbol;Acc:HGNC:27046] |
| ENSG00000179335 | HGLibA_10059 | 3 | -1.118748846 | CLK3 | CDC like kinase 3 [Source:HGNC Symbol;Acc:HGNC:2071] |
| ENSG00000179409 | HGLibA_18914 | 3 | -1.118748846 | GEMIN4 | gem nuclear organelle associated protein 4 [Source:HGNC Symbol;Acc:HGNC:15717] |
| ENSG00000180787 | HGLibA_55198 | 3 | -1.118748846 | ZFP3 | ZFP3 zinc finger protein [Source:HGNC Symbol;Acc:HGNC:12861] |
| ENSG00000181026 | HGLibA_01092 | 3 | -1.118748846 | AEN | apoptosis enhancing nuclease [Source:HGNC Symbol;Acc:HGNC:25722] |
| ENSG00000182134 | HGLibA_48921 | 3 | -1.118748846 | TDRKH | tudor and KH domain containing [Source:HGNC Symbol;Acc:HGNC:11713] |
| ENSG00000182149 | HGLibA_23689 | 3 | -1.118748846 | IST1 | ESCRT-III associated factor [Source:HGNC Symbol;Acc:HGNC:28977]" |
| ENSG00000182521 | HGLibA_48629 | 3 | -1.118748846 | TBPL2 | TATA-box binding protein like 2 [Source:HGNC Symbol;Acc:HGNC:19841] |
| ENSG00000182634 | HGLibA_33498 | 3 | -1.118748846 | OR10G7 | olfactory receptor family 10 subfamily G member 7 [Source:HGNC Symbol;Acc:HGNC:14842] |
| ENSG00000182902 | HGLibA_44660 | 3 | -1.118748846 | SLC25A18 | solute carrier family 25 member 18 [Source:HGNC Symbol;Acc:HGNC:10988] |
| ENSG00000183426 | HGLibA_32463 | 3 | -1.118748846 | NPIPA1 | nuclear pore complex interacting protein family member A1 [Source:HGNC Symbol;Acc:HGNC:7909] |
| ENSG00000183570 | HGLibA_35373 | 3 | -1.118748846 | PCBP3 | poly(rC) binding protein 3 [Source:HGNC Symbol;Acc:HGNC:8651] |
| ENSG00000183793 | HGLibA_32472 | 3 | -1.118748846 | NPIPA5 | nuclear pore complex interacting protein family member A5 [Source:HGNC Symbol;Acc:HGNC:41980] |
| ENSG00000184166 | HGLibA_33654 | 3 | -1.118748846 | OR1D2 | olfactory receptor family 1 subfamily D member 2 [Source:HGNC Symbol;Acc:HGNC:8183] |
| ENSG00000184925 | HGLibA_26173 | 3 | -1.118748846 | LCN12 | lipocalin 12 [Source:HGNC Symbol;Acc:HGNC:28733] |
| ENSG00000185651 | HGLibA_52626 | 3 | -1.118748846 | UBE2L3 | ubiquitin conjugating enzyme E2 L3 [Source:HGNC Symbol;Acc:HGNC:12488] |
| ENSG00000186160 | HGLibA_12206 | 3 | -1.118748846 | CYP4Z1 | cytochrome P450 family 4 subfamily Z member 1 [Source:HGNC Symbol;Acc:HGNC:20583] |
| ENSG00000186453 | HGLibA_16534 | 3 | -1.118748846 | FAM228A | family with sequence similarity 228 member A [Source:HGNC Symbol;Acc:HGNC:34418] |
| ENSG00000186496 | HGLibA_55915 | 3 | -1.118748846 | ZNF396 | zinc finger protein 396 [Source:HGNC Symbol;Acc:HGNC:18824] |
| ENSG00000186951 | HGLibA_37836 | 3 | -1.118748846 | PPARA | peroxisome proliferator activated receptor alpha [Source:HGNC Symbol;Acc:HGNC:9232] |
| ENSG00000187068 | HGLibA_06262 | 3 | -1.118748846 | C3orf70 | chromosome 3 open reading frame 70 [Source:HGNC Symbol;Acc:HGNC:33731] |
| ENSG00000187323 | HGLibA_12475 | 3 | -1.118748846 | DCC | DCC netrin 1 receptor [Source:HGNC Symbol;Acc:HGNC:2701] |
| ENSG00000187918 | HGLibA_34117 | 3 | -1.118748846 | OR51I2 | olfactory receptor family 51 subfamily I member 2 [Source:HGNC Symbol;Acc:HGNC:15201] |
| ENSG00000188779 | HGLibA_44292 | 3 | -1.118748846 | SKOR1 | SKI family transcriptional corepressor 1 [Source:HGNC Symbol;Acc:HGNC:21326] |
| ENSG00000189132 | HGLibA_16612 | 3 | -1.118748846 | FAM47B | family with sequence similarity 47 member B [Source:HGNC Symbol;Acc:HGNC:26659] |
| ENSG00000189376 | HGLibA_06647 | 3 | -1.118748846 | C8orf76 | chromosome 8 open reading frame 76 [Source:HGNC Symbol;Acc:HGNC:25924] |
| ENSG00000196378 | HGLibA_55837 | 3 | -1.118748846 | ZNF34 | zinc finger protein 34 [Source:HGNC Symbol;Acc:HGNC:13098] |
| ENSG00000196628 | HGLibA_48789 | 3 | -1.118748846 | TCF4 | transcription factor 4 [Source:HGNC Symbol;Acc:HGNC:11634] |
| ENSG00000196684 | HGLibA_22246 | 3 | -1.118748846 | HSH2D | hematopoietic SH2 domain containing [Source:HGNC Symbol;Acc:HGNC:24920] |
| ENSG00000197919 | HGLibA_22655 | 3 | -1.118748846 | IFNA1 | interferon alpha 1 [Source:HGNC Symbol;Acc:HGNC:5417] |
| ENSG00000198034 | HGLibA_42142 | 3 | -1.118748846 | RPS4X | ribosomal protein S4 X-linked [Source:HGNC Symbol;Acc:HGNC:10424] |
| ENSG00000198315 | HGLibA_55363 | 3 | -1.118748846 | ZKSCAN8 | zinc finger with KRAB and SCAN domains 8 [Source:HGNC Symbol;Acc:HGNC:12983] |
| ENSG00000198429 | HGLibA_56547 | 3 | -1.118748846 | ZNF69 | zinc finger protein 69 [Source:HGNC Symbol;Acc:HGNC:13138] |
| ENSG00000198892 | HGLibA_44040 | 3 | -1.118748846 | SHISA4 | shisa family member 4 [Source:HGNC Symbol;Acc:HGNC:27139] |
| ENSG00000203757 | HGLibA_34420 | 3 | -1.118748846 | OR6K3 | olfactory receptor family 6 subfamily K member 3 [Source:HGNC Symbol;Acc:HGNC:15030] |
| ENSG00000203965 | HGLibA_14553 | 3 | -1.118748846 | EFCAB7 | EF-hand calcium binding domain 7 [Source:HGNC Symbol;Acc:HGNC:29379] |
| ENSG00000204086 | HGLibA_41809 | 3 | -1.118748846 | RPA4 | replication protein A4 [Source:HGNC Symbol;Acc:HGNC:30305] |
| ENSG00000204278 | HGLibA_50271 | 3 | -1.118748846 | TMEM235 | transmembrane protein 235 [Source:HGNC Symbol;Acc:HGNC:27563] |
| ENSG00000205111 | HGLibA_08816 | 3 | -1.118748846 | CDKL4 | cyclin dependent kinase like 4 [Source:HGNC Symbol;Acc:HGNC:19287] |
| ENSG00000205436 | HGLibA_15747 | 3 | -1.118748846 | EXOC3L4 | exocyst complex component 3 like 4 [Source:HGNC Symbol;Acc:HGNC:20120] |
| ENSG00000212722 | HGLibA_25779 | 3 | -1.118748846 | KRTAP4-9 | keratin associated protein 4-9 [Source:HGNC Symbol;Acc:HGNC:18910] |
| ENSG00000214655 | HGLibA_57059 | 3 | -1.118748846 | ZSWIM8 | zinc finger SWIM-type containing 8 [Source:HGNC Symbol;Acc:HGNC:23528] |
| ENSG00000218823 | HGLibA_35139 | 3 | -1.118748846 | PAPOLB | poly(A) polymerase beta [Source:HGNC Symbol;Acc:HGNC:15970] |
| ENSG00000227488 | HGLibA_18444 | 3 | -1.118748846 | GAGE12D | G antigen 12D [Source:HGNC Symbol;Acc:HGNC:31904] |
| ENSG00000227551 | HGLibA_53220 | 3 | -1.118748846 | USP17L12 | ubiquitin specific peptidase 17-like family member 12 [Source:HGNC Symbol;Acc:HGNC:44440] |
| ENSG00000229972 | HGLibA_23508 | 3 | -1.118748846 | IQCF3 | IQ motif containing F3 [Source:HGNC Symbol;Acc:HGNC:31816] |
| ENSG00000232264 | HGLibA_53253 | 3 | -1.118748846 | USP17L24 | ubiquitin specific peptidase 17-like family member 24 [Source:HGNC Symbol;Acc:HGNC:44453] |
| ENSG00000232629 | HGLibA_21630 | 3 | -1.118748846 | HLA-DQB2 | DQ beta 2 [Source:HGNC Symbol;Acc:HGNC:4945]" |
| ENSG00000233670 | HGLibA_36731 | 3 | -1.118748846 | PIRT | phosphoinositide interacting regulator of transient receptor potential channels [Source:HGNC Symbol;Acc:HGNC:37239] |
| ENSG00000236737 | HGLibA_18438 | 3 | -1.118748846 | GAGE12B | G antigen 12B [Source:HGNC Symbol;Acc:HGNC:26779] |
| ENSG00000239474 | HGLibA_25211 | 3 | -1.118748846 | KLHL41 | kelch like family member 41 [Source:HGNC Symbol;Acc:HGNC:16905] |
| ENSG00000241343 | HGLibA_41954 | 3 | -1.118748846 | RPL36A | ribosomal protein L36a [Source:HGNC Symbol;Acc:HGNC:10359] |
| ENSG00000254852 | HGLibA_32467 | 3 | -1.118748846 | NPIPA2 | nuclear pore complex interacting protein family member A2 [Source:HGNC Symbol;Acc:HGNC:41979] |
| ENSG00000254852 | HGLibA_32466 | 3 | -1.118748846 | NPIPA2 | nuclear pore complex interacting protein family member A2 [Source:HGNC Symbol;Acc:HGNC:41979] |
| ENSG00000258643 | HGLibA_04327 | 3 | -1.118748846 | BCL2L2-PABPN1 | BCL2L2-PABPN1 readthrough [Source:HGNC Symbol;Acc:HGNC:42959] |

| | | | | | |
|---|---|---|---|---|---|
| ENSG00000263465 | HGLibA_47090 | 3 | -1.118748846 | SRSF8 | serine and arginine rich splicing factor 8 [Source:HGNC Symbol;Acc:HGNC:16988] |
| ENSG00000265972 | HGLibA_52433 | 3 | -1.118748846 | TXNIP | thioredoxin interacting protein [Source:HGNC Symbol;Acc:HGNC:16952] |
| ENSG00000271043 | HGLibA_30475 | 3 | -1.118748846 | MTRNR2L2 | MT-RNR2 like 2 [Source:HGNC Symbol;Acc:HGNC:37156] |
| H2AFZ | HGLibA_20770 | 3 | -1.118748846 | H2AFZ | - |
| HIST3H3 | HGLibA_21562 | 3 | -1.118748846 | HIST3H3 | - |
| hsa-mir-135b | HGLibA_57799 | 3 | -1.118748846 | hsa-mir-135b | - |
| hsa-mir-1912 | HGLibA_58053 | 3 | -1.118748846 | hsa-mir-1912 | - |
| hsa-mir-1913 | HGLibA_58057 | 3 | -1.118748846 | hsa-mir-1913 | - |
| hsa-mir-302d | HGLibA_58450 | 3 | -1.118748846 | hsa-mir-302d | - |
| hsa-mir-3119-2 | HGLibA_58528 | 3 | -1.118748846 | hsa-mir-3119-2 | - |
| hsa-mir-3130-2 | HGLibA_58577 | 3 | -1.118748846 | hsa-mir-3130-2 | - |
| hsa-mir-3155a | HGLibA_58679 | 3 | -1.118748846 | hsa-mir-3155a | - |
| hsa-mir-3189 | HGLibA_58855 | 3 | -1.118748846 | hsa-mir-3189 | - |
| hsa-mir-3665 | HGLibA_59212 | 3 | -1.118748846 | hsa-mir-3665 | - |
| hsa-mir-4277 | HGLibA_59810 | 3 | -1.118748846 | hsa-mir-4277 | - |
| hsa-mir-4427 | HGLibA_60095 | 3 | -1.118748846 | hsa-mir-4427 | - |
| hsa-mir-4473 | HGLibA_60301 | 3 | -1.118748846 | hsa-mir-4473 | - |
| hsa-mir-4660 | HGLibA_60749 | 3 | -1.118748846 | hsa-mir-4660 | - |
| hsa-mir-4728 | HGLibA_61008 | 3 | -1.118748846 | hsa-mir-4728 | - |
| hsa-mir-4732 | HGLibA_61027 | 3 | -1.118748846 | hsa-mir-4732 | - |
| hsa-mir-486-2 | HGLibA_61341 | 3 | -1.118748846 | hsa-mir-486-2 | - |
| hsa-mir-487b | HGLibA_61347 | 3 | -1.118748846 | hsa-mir-487b | - |
| hsa-mir-518b | HGLibA_61659 | 3 | -1.118748846 | hsa-mir-518b | - |
| hsa-mir-526a-2 | HGLibA_61793 | 3 | -1.118748846 | hsa-mir-526a-2 | - |
| hsa-mir-548s | HGLibA_62064 | 3 | -1.118748846 | hsa-mir-548s | - |
| hsa-mir-550a-2 | HGLibA_62102 | 3 | -1.118748846 | hsa-mir-550a-2 | - |
| hsa-mir-5583-2 | HGLibA_62172 | 3 | -1.118748846 | hsa-mir-5583-2 | - |
| hsa-mir-5695 | HGLibA_62314 | 3 | -1.118748846 | hsa-mir-5695 | - |
| hsa-mir-600 | HGLibA_62494 | 3 | -1.118748846 | hsa-mir-600 | - |
| hsa-mir-646 | HGLibA_62826 | 3 | -1.118748846 | hsa-mir-646 | - |
| hsa-mir-6751 | HGLibA_63160 | 3 | -1.118748846 | hsa-mir-6751 | - |
| hsa-mir-6881 | HGLibA_63709 | 3 | -1.118748846 | hsa-mir-6881 | - |
| hsa-mir-744 | HGLibA_63886 | 3 | -1.118748846 | hsa-mir-744 | - |
| hsa-mir-8076 | HGLibA_64147 | 3 | -1.118748846 | hsa-mir-8076 | - |
| LOC100127983 | HGLibA_26749 | 3 | -1.118748846 | LOC100127983 | - |
| LOC100132247 | HGLibA_26808 | 3 | -1.118748846 | LOC100132247 | - |
| LOC100996331 | HGLibA_26883 | 3 | -1.118748846 | LOC100996331 | - |
| LOC649330 | HGLibA_26981 | 3 | -1.118748846 | LOC649330 | - |
| NPIP | HGLibA_32461 | 3 | -1.118748846 | NPIP | - |
| RARRES3 | HGLibA_40286 | 3 | -1.118748846 | RARRES3 | - |
| SPDYE2L | HGLibA_46589 | 3 | -1.118748846 | SPDYE2L | - |
| ALPPL2 | HGLibA_01716 | 2 | -1.128699884 | ALPPL2 | - |
| BAGE4 | HGLibA_04101 | 2 | -1.128699884 | BAGE4 | - |
| C14orf2 | HGLibA_05357 | 2 | -1.128699884 | C14orf2 | - |
| C16orf52 | HGLibA_05453 | 2 | -1.128699884 | C16orf52 | - |
| C17orf72 | HGLibA_05567 | 2 | -1.128699884 | C17orf72 | - |
| C2orf71 | HGLibA_06172 | 2 | -1.128699884 | C2orf71 | - |
| C7orf41 | HGLibA_06543 | 2 | -1.128699884 | C7orf41 | - |
| C9orf62 | HGLibA_06746 | 2 | -1.128699884 | C9orf62 | - |
| C9orf69 | HGLibA_06755 | 2 | -1.128699884 | C9orf69 | - |
| CD97 | HGLibA_08487 | 2 | -1.128699884 | CD97 | - |
| EFCAB4A | HGLibA_14542 | 2 | -1.128699884 | EFCAB4A | - |
| ENSG00000004700 | HGLibA_40766 | 2 | -1.128699884 | RECQL | RecQ like helicase [Source:HGNC Symbol;Acc:HGNC:9948] |
| ENSG00000006042 | HGLibA_50571 | 2 | -1.128699884 | TMEM98 | transmembrane protein 98 [Source:HGNC Symbol;Acc:HGNC:24529] |
| ENSG00000008294 | HGLibA_46372 | 2 | -1.128699884 | SPAG9 | sperm associated antigen 9 [Source:HGNC Symbol;Acc:HGNC:14524] |
| ENSG00000008311 | HGLibA_00084 | 2 | -1.128699884 | AASS | aminoadipate-semialdehyde synthase [Source:HGNC Symbol;Acc:HGNC:17366] |
| ENSG00000010319 | HGLibA_43337 | 2 | -1.128699884 | SEMA3G | semaphorin 3G [Source:HGNC Symbol;Acc:HGNC:30400] |
| ENSG00000010361 | HGLibA_18212 | 2 | -1.128699884 | FUZ | fuzzy planar cell polarity protein [Source:HGNC Symbol;Acc:HGNC:26219] |
| ENSG00000010704 | HGLibA_21224 | 2 | -1.128699884 | HFE | homeostatic iron regulator [Source:HGNC Symbol;Acc:HGNC:4886] |
| ENSG00000011295 | HGLibA_52067 | 2 | -1.128699884 | TTC19 | tetratricopeptide repeat domain 19 [Source:HGNC Symbol;Acc:HGNC:26006] |
| ENSG00000012963 | HGLibA_52761 | 2 | -1.128699884 | UBR7 | ubiquitin protein ligase E3 component n-recognin 7 (putative) [Source:HGNC Symbol;Acc:HGNC:20344] |
| ENSG00000015592 | HGLibA_47525 | 2 | -1.128699884 | STMN4 | stathmin 4 [Source:HGNC Symbol;Acc:HGNC:16078] |
| ENSG00000016391 | HGLibA_09415 | 2 | -1.128699884 | CHDH | choline dehydrogenase [Source:HGNC Symbol;Acc:HGNC:24288] |
| ENSG00000019169 | HGLibA_28362 | 2 | -1.128699884 | MARCO | macrophage receptor with collagenous structure [Source:HGNC Symbol;Acc:HGNC:6895] |
| ENSG00000019995 | HGLibA_56971 | 2 | -1.128699884 | ZRANB1 | zinc finger RANBP2-type containing 1 [Source:HGNC Symbol;Acc:HGNC:18224] |
| ENSG00000020129 | HGLibA_31289 | 2 | -1.128699884 | NCDN | neurochondrin [Source:HGNC Symbol;Acc:HGNC:17597] |
| ENSG00000023697 | HGLibA_12991 | 2 | -1.128699884 | DERA | deoxyribose-phosphate aldolase [Source:HGNC Symbol;Acc:HGNC:24269] |
| ENSG00000034971 | HGLibA_30874 | 2 | -1.128699884 | MYOC | myocilin [Source:HGNC Symbol;Acc:HGNC:7610] |
| ENSG00000037280 | HGLibA_17673 | 2 | -1.128699884 | FLT4 | fms related tyrosine kinase 4 [Source:HGNC Symbol;Acc:HGNC:3767] |
| ENSG00000038382 | HGLibA_51627 | 2 | -1.128699884 | TRIO | trio Rho guanine nucleotide exchange factor [Source:HGNC Symbol;Acc:HGNC:12303] |
| ENSG00000039600 | HGLibA_46260 | 2 | -1.128699884 | SOX30 | SRY-box 30 [Source:HGNC Symbol;Acc:HGNC:30635] |
| ENSG00000043355 | HGLibA_55324 | 2 | -1.128699884 | ZIC2 | Zic family member 2 [Source:HGNC Symbol;Acc:HGNC:12873] |
| ENSG00000049759 | HGLibA_31604 | 2 | -1.128699884 | NEDD4L | E3 ubiquitin protein ligase [Source:HGNC Symbol;Acc:HGNC:7728]" |
| ENSG00000049860 | HGLibA_21203 | 2 | -1.128699884 | HEXB | hexosaminidase subunit beta [Source:HGNC Symbol;Acc:HGNC:4879] |
| ENSG00000051523 | HGLibA_12016 | 2 | -1.128699884 | CYBA | cytochrome b-245 alpha chain [Source:HGNC Symbol;Acc:HGNC:2577] |
| ENSG00000054277 | HGLibA_33433 | 2 | -1.128699884 | OPN3 | opsin 3 [Source:HGNC Symbol;Acc:HGNC:14007] |
| ENSG00000057593 | HGLibA_15880 | 2 | -1.128699884 | F7 | coagulation factor VII [Source:HGNC Symbol;Acc:HGNC:3544] |
| ENSG00000065029 | HGLibA_56684 | 2 | -1.128699884 | ZNF76 | zinc finger protein 76 [Source:HGNC Symbol;Acc:HGNC:13149] |
| ENSG00000067646 | HGLibA_55266 | 2 | -1.128699884 | ZFY | zinc finger protein Y-linked [Source:HGNC Symbol;Acc:HGNC:12870] |
| ENSG00000068078 | HGLibA_17448 | 2 | -1.128699884 | FGFR3 | fibroblast growth factor receptor 3 [Source:HGNC Symbol;Acc:HGNC:3690] |
| ENSG00000072401 | HGLibA_52582 | 2 | -1.128699884 | UBE2D1 | ubiquitin conjugating enzyme E2 D1 [Source:HGNC Symbol;Acc:HGNC:12474] |
| ENSG00000076770 | HGLibA_28513 | 2 | -1.128699884 | MBNL3 | muscleblind like splicing regulator 3 [Source:HGNC Symbol;Acc:HGNC:20564] |
| ENSG00000077235 | HGLibA_20581 | 2 | -1.128699884 | GTF3C1 | general transcription factor IIIC subunit 1 [Source:HGNC Symbol;Acc:HGNC:4664] |
| ENSG00000077235 | HGLibA_20579 | 2 | -1.128699884 | GTF3C1 | general transcription factor IIIC subunit 1 [Source:HGNC Symbol;Acc:HGNC:4664] |
| ENSG00000078795 | HGLibA_36805 | 2 | -1.128699884 | PKD2L2 | transient receptor potential cation channel [Source:HGNC Symbol;Acc:HGNC:9012]" |
| ENSG00000079246 | HGLibA_54596 | 2 | -1.128699884 | XRCC5 | X-ray repair cross complementing 5 [Source:HGNC Symbol;Acc:HGNC:12833] |
| ENSG00000079308 | HGLibA_50921 | 2 | -1.128699884 | TNS1 | tensin 1 [Source:HGNC Symbol;Acc:HGNC:11973] |
| ENSG00000082805 | HGLibA_15425 | 2 | -1.128699884 | ERC1 | ELKS/RAB6-interacting/CAST family member 1 [Source:HGNC Symbol;Acc:HGNC:17072] |
| ENSG00000085117 | HGLibA_08460 | 2 | -1.128699884 | CD82 | CD82 molecule [Source:HGNC Symbol;Acc:HGNC:6210] |
| ENSG00000087274 | HGLibA_00940 | 2 | -1.128699884 | ADD1 | adducin 1 [Source:HGNC Symbol;Acc:HGNC:243] |
| ENSG00000089060 | HGLibA_45397 | 2 | -1.128699884 | SLC8B1 | solute carrier family 8 member B1 [Source:HGNC Symbol;Acc:HGNC:26175] |
| ENSG00000089159 | HGLibA_39663 | 2 | -1.128699884 | PXN | paxillin [Source:HGNC Symbol;Acc:HGNC:9718] |
| ENSG00000090238 | HGLibA_54712 | 2 | -1.128699884 | YPEL3 | yippee like 3 [Source:HGNC Symbol;Acc:HGNC:18327] |
| ENSG00000090924 | HGLibA_37103 | 2 | -1.128699884 | PLEKHG2 | pleckstrin homology and RhoGEF domain containing G2 [Source:HGNC Symbol;Acc:HGNC:29515] |
| ENSG00000095752 | HGLibA_22954 | 2 | -1.128699884 | IL11 | interleukin 11 [Source:HGNC Symbol;Acc:HGNC:5966] |
| ENSG00000096063 | HGLibA_47018 | 2 | -1.128699884 | SRPK1 | SRSF protein kinase 1 [Source:HGNC Symbol;Acc:HGNC:11305] |
| ENSG00000096088 | HGLibA_36227 | 2 | -1.128699884 | PGC | progastricsin [Source:HGNC Symbol;Acc:HGNC:8890] |
| ENSG00000099795 | HGLibA_31526 | 2 | -1.128699884 | NDUFB7 | NADH:ubiquinone oxidoreductase subunit B7 [Source:HGNC Symbol;Acc:HGNC:7702] |
| ENSG00000099889 | HGLibA_03175 | 2 | -1.128699884 | ARVCF | delta catenin family member [Source:HGNC Symbol;Acc:HGNC:728]" |
| ENSG00000100197 | HGLibA_12128 | 2 | -1.128699884 | CYP2D6 | cytochrome P450 family 2 subfamily D member 6 [Source:HGNC Symbol;Acc:HGNC:2625] |
| ENSG00000100479 | HGLibA_37513 | 2 | -1.128699884 | POLE2 | accessory subunit [Source:HGNC Symbol;Acc:HGNC:9178]" |
| ENSG00000100650 | HGLibA_47081 | 2 | -1.128699884 | SRSF5 | serine and arginine rich splicing factor 5 [Source:HGNC Symbol;Acc:HGNC:10787] |
| ENSG00000100836 | HGLibA_34943 | 2 | -1.128699884 | PABPN1 | poly(A) binding protein nuclear 1 [Source:HGNC Symbol;Acc:HGNC:8565] |
| ENSG00000100991 | HGLibA_51736 | 2 | -1.128699884 | TRPC4AP | transient receptor potential cation channel subfamily C member 4 associated protein [Source:HGNC Symbol;Acc:HGNC:16181] |
| ENSG00000101265 | HGLibA_40376 | 2 | -1.128699884 | RASSF2 | Ras association domain family member 2 [Source:HGNC Symbol;Acc:HGNC:9883] |
| ENSG00000101871 | HGLibA_29232 | 2 | -1.128699884 | MID1 | midline 1 [Source:HGNC Symbol;Acc:HGNC:7095] |
| ENSG00000102316 | HGLibA_27972 | 2 | -1.128699884 | MAGED2 | MAGE family member D2 [Source:HGNC Symbol;Acc:HGNC:16353] |
| ENSG00000102921 | HGLibA_30950 | 2 | -1.128699884 | N4BP1 | NEDD4 binding protein 1 [Source:HGNC Symbol;Acc:HGNC:29850] |
| ENSG00000103546 | HGLibA_45320 | 2 | -1.128699884 | SLC6A2 | solute carrier family 6 member 2 [Source:HGNC Symbol;Acc:HGNC:11048] |
| ENSG00000103740 | HGLibA_00541 | 2 | -1.128699884 | ACSBG1 | acyl-CoA synthetase bubblegum family member 1 [Source:HGNC Symbol;Acc:HGNC:29567] |
| ENSG00000104765 | HGLibA_04660 | 2 | -1.128699884 | BNIP3L | BCL2 interacting protein 3 like [Source:HGNC Symbol;Acc:HGNC:1085] |
| ENSG00000104808 | HGLibA_13100 | 2 | -1.128699884 | DHDH | dihydrodiol dehydrogenase [Source:HGNC Symbol;Acc:HGNC:17887] |
| ENSG00000104870 | HGLibA_17200 | 2 | -1.128699884 | FCGRT | Fc fragment of IgG receptor and transporter [Source:HGNC Symbol;Acc:HGNC:3621] |
| ENSG00000104915 | HGLibA_47615 | 2 | -1.128699884 | STX10 | syntaxin 10 [Source:HGNC Symbol;Acc:HGNC:11428] |
| ENSG00000104967 | HGLibA_32366 | 2 | -1.128699884 | NOVA2 | NOVA alternative splicing regulator 2 [Source:HGNC Symbol;Acc:HGNC:7887] |
| ENSG00000105137 | HGLibA_47939 | 2 | -1.128699884 | SYDE1 | synapse defective Rho GTPase homolog 1 [Source:HGNC Symbol;Acc:HGNC:25824] |
| ENSG00000106031 | HGLibA_21901 | 2 | -1.128699884 | HOXA13 | homeobox A13 [Source:HGNC Symbol;Acc:HGNC:5102] |
| ENSG00000107020 | HGLibA_37166 | 2 | -1.128699884 | PLGRKT | plasminogen receptor with a C-terminal lysine [Source:HGNC Symbol;Acc:HGNC:23633] |
| ENSG00000107611 | HGLibA_11764 | 2 | -1.128699884 | CUBN | cubilin [Source:HGNC Symbol;Acc:HGNC:2548] |
| ENSG00000107745 | HGLibA_29221 | 2 | -1.128699884 | MICU1 | mitochondrial calcium uptake 1 [Source:HGNC Symbol;Acc:HGNC:1530] |
| ENSG00000107959 | HGLibA_36760 | 2 | -1.128699884 | PITRM1 | pitrilysin metallopeptidase 1 [Source:HGNC Symbol;Acc:HGNC:17663] |
| ENSG00000108219 | HGLibA_51905 | 2 | -1.128699884 | TSPAN14 | tetraspanin 14 [Source:HGNC Symbol;Acc:HGNC:23303] |
| ENSG00000108528 | HGLibA_44638 | 2 | -1.128699884 | SLC25A11 | solute carrier family 25 member 11 [Source:HGNC Symbol;Acc:HGNC:10981] |
| ENSG00000108591 | HGLibA_13977 | 2 | -1.128699884 | DRG2 | developmentally regulated GTP binding protein 2 [Source:HGNC Symbol;Acc:HGNC:3030] |
| ENSG00000108759 | HGLibA_25478 | 2 | -1.128699884 | KRT32 | keratin 32 [Source:HGNC Symbol;Acc:HGNC:6449] |
| ENSG00000108828 | HGLibA_53541 | 2 | -1.128699884 | VAT1 | vesicle amine transport 1 [Source:HGNC Symbol;Acc:HGNC:16919] |
| ENSG00000110713 | HGLibA_33078 | 2 | -1.128699884 | NUP98 | nucleoporin 98 [Source:HGNC Symbol;Acc:HGNC:8068] |
| ENSG00000110852 | HGLibA_09963 | 2 | -1.128699884 | CLEC2B | C-type lectin domain family 2 member B [Source:HGNC Symbol;Acc:HGNC:2053] |
| ENSG00000111711 | HGLibA_19667 | 2 | -1.128699884 | GOLT1B | golgi transport 1B [Source:HGNC Symbol;Acc:HGNC:20175] |
| ENSG00000111752 | HGLibA_36317 | 2 | -1.128699884 | PHC1 | polyhomeotic homolog 1 [Source:HGNC Symbol;Acc:HGNC:3182] |
| ENSG00000112183 | HGLibA_40515 | 2 | -1.128699884 | RBM24 | RNA binding motif protein 24 [Source:HGNC Symbol;Acc:HGNC:21539] |
| ENSG00000112210 | HGLibA_39843 | 2 | -1.128699884 | RAB23 | member RAS oncogene family [Source:HGNC Symbol;Acc:HGNC:14263]" |
| ENSG00000112249 | HGLibA_03262 | 2 | -1.128699884 | ASCC3 | activating signal cointegrator 1 complex subunit 3 [Source:HGNC Symbol;Acc:HGNC:18697] |
| ENSG00000112685 | HGLibA_15734 | 2 | -1.128699884 | EXOC2 | exocyst complex component 2 [Source:HGNC Symbol;Acc:HGNC:24968] |
| ENSG00000113638 | HGLibA_52116 | 2 | -1.128699884 | TTC33 | tetratricopeptide repeat domain 33 [Source:HGNC Symbol;Acc:HGNC:29959] |

| | | | | | |
|---|---|---|---|---|---|
| ENSG00000114405 | HGLibA_06210 | 2 | -1.128699884 | C3orf14 | chromosome 3 open reading frame 14 [Source:HGNC Symbol;Acc:HGNC:25024] |
| ENSG00000114554 | HGLibA_37258 | 2 | -1.128699884 | PLXNA1 | plexin A1 [Source:HGNC Symbol;Acc:HGNC:9099] |
| ENSG00000114737 | HGLibA_09735 | 2 | -1.128699884 | CISH | cytokine inducible SH2 containing protein [Source:HGNC Symbol;Acc:HGNC:1984] |
| ENSG00000114923 | HGLibA_45216 | 2 | -1.128699884 | SLC4A3 | solute carrier family 4 member 3 [Source:HGNC Symbol;Acc:HGNC:11029] |
| ENSG00000115263 | HGLibA_18776 | 2 | -1.128699884 | GCG | glucagon [Source:HGNC Symbol;Acc:HGNC:4191] |
| ENSG00000115310 | HGLibA_42408 | 2 | -1.128699884 | RTN4 | reticulon 4 [Source:HGNC Symbol;Acc:HGNC:14085] |
| ENSG00000115414 | HGLibA_17727 | 2 | -1.128699884 | FN1 | fibronectin 1 [Source:HGNC Symbol;Acc:HGNC:3778] |
| ENSG00000115568 | HGLibA_55490 | 2 | -1.128699884 | ZNF142 | zinc finger protein 142 [Source:HGNC Symbol;Acc:HGNC:12927] |
| ENSG00000115665 | HGLibA_45281 | 2 | -1.128699884 | SLC5A7 | solute carrier family 5 member 7 [Source:HGNC Symbol;Acc:HGNC:14025] |
| ENSG00000116176 | HGLibA_51200 | 2 | -1.128699884 | TPSG1 | tryptase gamma 1 [Source:HGNC Symbol;Acc:HGNC:14134] |
| ENSG00000116194 | HGLibA_01911 | 2 | -1.128699884 | ANGPTL1 | angiopoietin like 1 [Source:HGNC Symbol;Acc:HGNC:489] |
| ENSG00000116285 | HGLibA_15533 | 2 | -1.128699884 | ERRFI1 | ERBB receptor feedback inhibitor 1 [Source:HGNC Symbol;Acc:HGNC:18185] |
| ENSG00000117676 | HGLibA_42158 | 2 | -1.128699884 | RPS6KA1 | ribosomal protein S6 kinase A1 [Source:HGNC Symbol;Acc:HGNC:10430] |
| ENSG00000119042 | HGLibA_42780 | 2 | -1.128699884 | SATB2 | SATB homeobox 2 [Source:HGNC Symbol;Acc:HGNC:21637] |
| ENSG00000119408 | HGLibA_31659 | 2 | -1.128699884 | NEK6 | NIMA related kinase 6 [Source:HGNC Symbol;Acc:HGNC:7749] |
| ENSG00000119411 | HGLibA_04879 | 2 | -1.128699884 | BSPRY | B-box and SPRY domain containing [Source:HGNC Symbol;Acc:HGNC:18232] |
| ENSG00000120341 | HGLibA_43211 | 2 | -1.128699884 | SEC16B | endoplasmic reticulum export factor [Source:HGNC Symbol;Acc:HGNC:30301]" |
| ENSG00000120438 | HGLibA_48830 | 2 | -1.128699884 | TCP1 | t-complex 1 [Source:HGNC Symbol;Acc:HGNC:11655] |
| ENSG00000120616 | HGLibA_15266 | 2 | -1.128699884 | EPC1 | enhancer of polycomb homolog 1 [Source:HGNC Symbol;Acc:HGNC:19876] |
| ENSG00000120686 | HGLibA_52853 | 2 | -1.128699884 | UFM1 | ubiquitin fold modifier 1 [Source:HGNC Symbol;Acc:HGNC:20597] |
| ENSG00000120903 | HGLibA_09567 | 2 | -1.128699884 | CHRNA2 | cholinergic receptor nicotinic alpha 2 subunit [Source:HGNC Symbol;Acc:HGNC:1956] |
| ENSG00000121377 | HGLibA_48427 | 2 | -1.128699884 | TAS2R7 | taste 2 receptor member 7 [Source:HGNC Symbol;Acc:HGNC:14913] |
| ENSG00000122203 | HGLibA_24706 | 2 | -1.128699884 | KIAA1191 | KIAA1191 [Source:HGNC Symbol;Acc:HGNC:29209] |
| ENSG00000122679 | HGLibA_40185 | 2 | -1.128699884 | RAMP3 | receptor activity modifying protein 3 [Source:HGNC Symbol;Acc:HGNC:9845] |
| ENSG00000123454 | HGLibA_12381 | 2 | -1.128699884 | DBH | dopamine beta-hydroxylase [Source:HGNC Symbol;Acc:HGNC:2689] |
| ENSG00000124216 | HGLibA_45867 | 2 | -1.128699884 | SNAI1 | snail family transcriptional repressor 1 [Source:HGNC Symbol;Acc:HGNC:11128] |
| ENSG00000124249 | HGLibA_24306 | 2 | -1.128699884 | KCNK15 | potassium two pore domain channel subfamily K member 15 [Source:HGNC Symbol;Acc:HGNC:13814] |
| ENSG00000124827 | HGLibA_18804 | 2 | -1.128699884 | GCM2 | glial cells missing homolog 2 [Source:HGNC Symbol;Acc:HGNC:4198] |
| ENSG00000125485 | HGLibA_12694 | 2 | -1.128699884 | DDX31 | DEAD-box helicase 31 [Source:HGNC Symbol;Acc:HGNC:16715] |
| ENSG00000125538 | HGLibA_23040 | 2 | -1.128699884 | IL1B | interleukin 1 beta [Source:HGNC Symbol;Acc:HGNC:5992] |
| ENSG00000126351 | HGLibA_49388 | 2 | -1.128699884 | THRA | thyroid hormone receptor alpha [Source:HGNC Symbol;Acc:HGNC:11796] |
| ENSG00000126775 | HGLibA_03469 | 2 | -1.128699884 | ATG14 | autophagy related 14 [Source:HGNC Symbol;Acc:HGNC:19962] |
| ENSG00000126821 | HGLibA_43850 | 2 | -1.128699884 | SGPP1 | sphingosine-1-phosphate phosphatase 1 [Source:HGNC Symbol;Acc:HGNC:17720] |
| ENSG00000127152 | HGLibA_04292 | 2 | -1.128699884 | BCL11B | B cell CLL/lymphoma 11B [Source:HGNC Symbol;Acc:HGNC:13222] |
| ENSG00000128294 | HGLibA_51206 | 2 | -1.128699884 | TPST2 | tyrosylprotein sulfotransferase 2 [Source:HGNC Symbol;Acc:HGNC:12021] |
| ENSG00000128513 | HGLibA_37714 | 2 | -1.128699884 | POT1 | protection of telomeres 1 [Source:HGNC Symbol;Acc:HGNC:17284] |
| ENSG00000128591 | HGLibA_17646 | 2 | -1.128699884 | FLNC | filamin C [Source:HGNC Symbol;Acc:HGNC:3756] |
| ENSG00000129521 | HGLibA_14646 | 2 | -1.128699884 | EGLN3 | egl-9 family hypoxia inducible factor 3 [Source:HGNC Symbol;Acc:HGNC:14661] |
| ENSG00000130208 | HGLibA_02504 | 2 | -1.128699884 | APOC1 | apolipoprotein C1 [Source:HGNC Symbol;Acc:HGNC:607] |
| ENSG00000130427 | HGLibA_15351 | 2 | -1.128699884 | EPO | erythropoietin [Source:HGNC Symbol;Acc:HGNC:3415] |
| ENSG00000130517 | HGLibA_36278 | 2 | -1.128699884 | PGPEP1 | pyroglutamyl-peptidase I [Source:HGNC Symbol;Acc:HGNC:13568] |
| ENSG00000130751 | HGLibA_32395 | 2 | -1.128699884 | NPAS1 | neuronal PAS domain protein 1 [Source:HGNC Symbol;Acc:HGNC:7894] |
| ENSG00000130779 | HGLibA_10042 | 2 | -1.128699884 | CLIP1 | CAP-Gly domain containing linker protein 1 [Source:HGNC Symbol;Acc:HGNC:10461] |
| ENSG00000130881 | HGLibA_27217 | 2 | -1.128699884 | LRP3 | LDL receptor related protein 3 [Source:HGNC Symbol;Acc:HGNC:6695] |
| ENSG00000131899 | HGLibA_26649 | 2 | -1.128699884 | LLGL1 | scribble cell polarity complex component [Source:HGNC Symbol;Acc:HGNC:6628]" |
| ENSG00000131910 | HGLibA_32571 | 2 | -1.128699884 | NR0B2 | nuclear receptor subfamily 0 group B member 2 [Source:HGNC Symbol;Acc:HGNC:7961] |
| ENSG00000132274 | HGLibA_51431 | 2 | -1.128699884 | TRIM22 | tripartite motif containing 22 [Source:HGNC Symbol;Acc:HGNC:16379] |
| ENSG00000132329 | HGLibA_40179 | 2 | -1.128699884 | RAMP1 | receptor activity modifying protein 1 [Source:HGNC Symbol;Acc:HGNC:9843] |
| ENSG00000132640 | HGLibA_04913 | 2 | -1.128699884 | BTBD3 | BTB domain containing 3 [Source:HGNC Symbol;Acc:HGNC:15854] |
| ENSG00000133065 | HGLibA_45139 | 2 | -1.128699884 | SLC41A1 | solute carrier family 41 member 1 [Source:HGNC Symbol;Acc:HGNC:19429] |
| ENSG00000133606 | HGLibA_29349 | 2 | -1.128699884 | MKRN1 | makorin ring finger protein 1 [Source:HGNC Symbol;Acc:HGNC:7112] |
| ENSG00000134058 | HGLibA_08795 | 2 | -1.128699884 | CDK7 | cyclin dependent kinase 7 [Source:HGNC Symbol;Acc:HGNC:1778] |
| ENSG00000134115 | HGLibA_10417 | 2 | -1.128699884 | CNTN6 | contactin 6 [Source:HGNC Symbol;Acc:HGNC:2176] |
| ENSG00000134644 | HGLibA_39580 | 2 | -1.128699884 | PUM1 | pumilio RNA binding family member 1 [Source:HGNC Symbol;Acc:HGNC:14957] |
| ENSG00000135049 | HGLibA_01260 | 2 | -1.128699884 | AGTPBP1 | ATP/GTP binding protein 1 [Source:HGNC Symbol;Acc:HGNC:17258] |
| ENSG00000135525 | HGLibA_28231 | 2 | -1.128699884 | MAP7 | microtubule associated protein 7 [Source:HGNC Symbol;Acc:HGNC:6869] |
| ENSG00000135845 | HGLibA_36548 | 2 | -1.128699884 | PIGC | phosphatidylinositol glycan anchor biosynthesis class C [Source:HGNC Symbol;Acc:HGNC:8960] |
| ENSG00000136280 | HGLibA_08046 | 2 | -1.128699884 | CCM2 | CCM2 scaffold protein [Source:HGNC Symbol;Acc:HGNC:21708] |
| ENSG00000137033 | HGLibA_23140 | 2 | -1.128699884 | IL33 | interleukin 33 [Source:HGNC Symbol;Acc:HGNC:16028] |
| ENSG00000137216 | HGLibA_50478 | 2 | -1.128699884 | TMEM63B | transmembrane protein 63B [Source:HGNC Symbol;Acc:HGNC:17735] |
| ENSG00000137473 | HGLibA_52099 | 2 | -1.128699884 | TTC29 | tetratricopeptide repeat domain 29 [Source:HGNC Symbol;Acc:HGNC:29936] |
| ENSG00000137507 | HGLibA_27313 | 2 | -1.128699884 | LRRC32 | leucine rich repeat containing 32 [Source:HGNC Symbol;Acc:HGNC:4161] |
| ENSG00000137693 | HGLibA_54632 | 2 | -1.128699884 | YAP1 | Yes associated protein 1 [Source:HGNC Symbol;Acc:HGNC:16262] |
| ENSG00000137868 | HGLibA_47568 | 2 | -1.128699884 | STRA6 | stimulated by retinoic acid 6 [Source:HGNC Symbol;Acc:HGNC:30650] |
| ENSG00000137965 | HGLibA_22612 | 2 | -1.128699884 | IFI44 | interferon induced protein 44 [Source:HGNC Symbol;Acc:HGNC:16938] |
| ENSG00000137976 | HGLibA_13668 | 2 | -1.128699884 | DNASE2B | deoxyribonuclease 2 beta [Source:HGNC Symbol;Acc:HGNC:28875] |
| ENSG00000138101 | HGLibA_14066 | 2 | -1.128699884 | DTNB | dystrobrevin beta [Source:HGNC Symbol;Acc:HGNC:3058] |
| ENSG00000138669 | HGLibA_38581 | 2 | -1.128699884 | PRKG2 | protein kinase cGMP-dependent 2 [Source:HGNC Symbol;Acc:HGNC:9416] |
| ENSG00000138696 | HGLibA_04632 | 2 | -1.128699884 | BMPR1B | bone morphogenetic protein receptor type 1B [Source:HGNC Symbol;Acc:HGNC:1077] |
| ENSG00000140854 | HGLibA_24081 | 2 | -1.128699884 | KATNB1 | katanin regulatory subunit B1 [Source:HGNC Symbol;Acc:HGNC:6217] |
| ENSG00000141026 | HGLibA_28820 | 2 | -1.128699884 | MED9 | mediator complex subunit 9 [Source:HGNC Symbol;Acc:HGNC:25487] |
| ENSG00000141127 | HGLibA_38768 | 2 | -1.128699884 | PRPSAP2 | phosphoribosyl pyrophosphate synthetase associated protein 2 [Source:HGNC Symbol;Acc:HGNC:9467] |
| ENSG00000142684 | HGLibA_56340 | 2 | -1.128699884 | ZNF593 | zinc finger protein 593 [Source:HGNC Symbol;Acc:HGNC:30943] |
| ENSG00000142748 | HGLibA_17222 | 2 | -1.128699884 | FCN3 | ficolin 3 [Source:HGNC Symbol;Acc:HGNC:3625] |
| ENSG00000144476 | HGLibA_00446 | 2 | -1.128699884 | ACKR3 | atypical chemokine receptor 3 [Source:HGNC Symbol;Acc:HGNC:23692] |
| ENSG00000145198 | HGLibA_53911 | 2 | -1.128699884 | VWA5B2 | von Willebrand factor A domain containing 5B2 [Source:HGNC Symbol;Acc:HGNC:25144] |
| ENSG00000145331 | HGLibA_51659 | 2 | -1.128699884 | TRMT10A | tRNA methyltransferase 10A [Source:HGNC Symbol;Acc:HGNC:28403] |
| ENSG00000145555 | HGLibA_30801 | 2 | -1.128699884 | MYO10 | myosin X [Source:HGNC Symbol;Acc:HGNC:7593] |
| ENSG00000145675 | HGLibA_36648 | 2 | -1.128699884 | PIK3R1 | phosphoinositide-3-kinase regulatory subunit 1 [Source:HGNC Symbol;Acc:HGNC:8979] |
| ENSG00000145703 | HGLibA_23536 | 2 | -1.128699884 | IQGAP2 | IQ motif containing GTPase activating protein 2 [Source:HGNC Symbol;Acc:HGNC:6111] |
| ENSG00000145945 | HGLibA_16632 | 2 | -1.128699884 | FAM50B | family with sequence similarity 50 member B [Source:HGNC Symbol;Acc:HGNC:18789] |
| ENSG00000146416 | HGLibA_01345 | 2 | -1.128699884 | AIG1 | androgen induced 1 [Source:HGNC Symbol;Acc:HGNC:21607] |
| ENSG00000146707 | HGLibA_37674 | 2 | -1.128699884 | POMZP3 | POM121 and ZP3 fusion [Source:HGNC Symbol;Acc:HGNC:9203] |
| ENSG00000147118 | HGLibA_55544 | 2 | -1.128699884 | ZNF182 | zinc finger protein 182 [Source:HGNC Symbol;Acc:HGNC:13001] |
| ENSG00000147613 | HGLibA_39066 | 2 | -1.128699884 | PSKH2 | protein serine kinase H2 [Source:HGNC Symbol;Acc:HGNC:18997] |
| ENSG00000148136 | HGLibA_33603 | 2 | -1.128699884 | OR13C4 | olfactory receptor family 13 subfamily C member 4 [Source:HGNC Symbol;Acc:HGNC:14722] |
| ENSG00000148468 | HGLibA_16263 | 2 | -1.128699884 | FAM171A1 | family with sequence similarity 171 member A1 [Source:HGNC Symbol;Acc:HGNC:23522] |
| ENSG00000148737 | HGLibA_48797 | 2 | -1.128699884 | TCF7L2 | transcription factor 7 like 2 [Source:HGNC Symbol;Acc:HGNC:11641] |
| ENSG00000149295 | HGLibA_13963 | 2 | -1.128699884 | DRD2 | dopamine receptor D2 [Source:HGNC Symbol;Acc:HGNC:3023] |
| ENSG00000149489 | HGLibA_41753 | 2 | -1.128699884 | ROM1 | retinal outer segment membrane protein 1 [Source:HGNC Symbol;Acc:HGNC:10254] |
| ENSG00000149573 | HGLibA_29797 | 2 | -1.128699884 | MPZL2 | myelin protein zero like 2 [Source:HGNC Symbol;Acc:HGNC:3496] |
| ENSG00000149792 | HGLibA_30005 | 2 | -1.128699884 | MRPL49 | mitochondrial ribosomal protein L49 [Source:HGNC Symbol;Acc:HGNC:1176] |
| ENSG00000151006 | HGLibA_38955 | 2 | -1.128699884 | PRSS53 | serine protease 53 [Source:HGNC Symbol;Acc:HGNC:34407] |
| ENSG00000151422 | HGLibA_17284 | 2 | -1.128699884 | FER | FER tyrosine kinase [Source:HGNC Symbol;Acc:HGNC:3655] |
| ENSG00000151650 | HGLibA_53621 | 2 | -1.128699884 | VENTX | VENT homeobox [Source:HGNC Symbol;Acc:HGNC:13639] |
| ENSG00000152818 | HGLibA_53458 | 2 | -1.128699884 | UTRN | utrophin [Source:HGNC Symbol;Acc:HGNC:12635] |
| ENSG00000153485 | HGLibA_50318 | 2 | -1.128699884 | TMEM251 | transmembrane protein 251 [Source:HGNC Symbol;Acc:HGNC:20218] |
| ENSG00000153495 | HGLibA_49091 | 2 | -1.128699884 | TEX29 | testis expressed 29 [Source:HGNC Symbol;Acc:HGNC:20370] |
| ENSG00000153936 | HGLibA_22117 | 2 | -1.128699884 | HS2ST1 | heparan sulfate 2-O-sulfotransferase 1 [Source:HGNC Symbol;Acc:HGNC:5193] |
| ENSG00000154188 | HGLibA_01901 | 2 | -1.128699884 | ANGPT1 | angiopoietin 1 [Source:HGNC Symbol;Acc:HGNC:484] |
| ENSG00000155093 | HGLibA_39523 | 2 | -1.128699884 | PTPRN2 | receptor type N2 [Source:HGNC Symbol;Acc:HGNC:9677]" |
| ENSG00000155393 | HGLibA_21073 | 2 | -1.128699884 | HEATR3 | HEAT repeat containing 3 [Source:HGNC Symbol;Acc:HGNC:26087] |
| ENSG00000155530 | HGLibA_27169 | 2 | -1.128699884 | LRGUK | leucine rich repeats and guanylate kinase domain containing [Source:HGNC Symbol;Acc:HGNC:21964] |
| ENSG00000155621 | HGLibA_06767 | 2 | -1.128699884 | C9orf85 | chromosome 9 open reading frame 85 [Source:HGNC Symbol;Acc:HGNC:28784] |
| ENSG00000155970 | HGLibA_29229 | 2 | -1.128699884 | MICU3 | mitochondrial calcium uptake family member 3 [Source:HGNC Symbol;Acc:HGNC:27820] |
| ENSG00000156052 | HGLibA_19466 | 2 | -1.128699884 | GNAQ | G protein subunit alpha q [Source:HGNC Symbol;Acc:HGNC:4390] |
| ENSG00000156162 | HGLibA_13913 | 2 | -1.128699884 | DPY19L4 | dpy-19 like 4 [Source:HGNC Symbol;Acc:HGNC:27829] |
| ENSG00000156650 | HGLibA_24064 | 2 | -1.128699884 | KAT6B | lysine acetyltransferase 6B [Source:HGNC Symbol;Acc:HGNC:17582] |
| ENSG00000157303 | HGLibA_47851 | 2 | -1.128699884 | SUSD3 | sushi domain containing 3 [Source:HGNC Symbol;Acc:HGNC:28391] |
| ENSG00000157388 | HGLibA_06883 | 2 | -1.128699884 | CACNA1D | calcium voltage-gated channel subunit alpha1 D [Source:HGNC Symbol;Acc:HGNC:1391] |
| ENSG00000158856 | HGLibA_13446 | 2 | -1.128699884 | DMTN | dematin actin binding protein [Source:HGNC Symbol;Acc:HGNC:3382] |
| ENSG00000159147 | HGLibA_13796 | 2 | -1.128699884 | DONSON | downstream neighbor of SON [Source:HGNC Symbol;Acc:HGNC:2993] |
| ENSG00000159792 | HGLibA_39063 | 2 | -1.128699884 | PSKH1 | protein serine kinase H1 [Source:HGNC Symbol;Acc:HGNC:9529] |
| ENSG00000159873 | HGLibA_07560 | 2 | -1.128699884 | CCDC117 | coiled-coil domain containing 117 [Source:HGNC Symbol;Acc:HGNC:26599] |
| ENSG00000161040 | HGLibA_16976 | 2 | -1.128699884 | FBXL13 | F-box and leucine rich repeat protein 13 [Source:HGNC Symbol;Acc:HGNC:21658] |
| ENSG00000161911 | HGLibA_51361 | 2 | -1.128699884 | TREML1 | triggering receptor expressed on myeloid cells like 1 [Source:HGNC Symbol;Acc:HGNC:20434] |
| ENSG00000161958 | HGLibA_17366 | 2 | -1.128699884 | FGF11 | fibroblast growth factor 11 [Source:HGNC Symbol;Acc:HGNC:3667] |
| ENSG00000162373 | HGLibA_04411 | 2 | -1.128699884 | BEND5 | BEN domain containing 5 [Source:HGNC Symbol;Acc:HGNC:25668] |
| ENSG00000162437 | HGLibA_40404 | 2 | -1.128699884 | RAVER2 | PTB binding 2 [Source:HGNC Symbol;Acc:HGNC:25577]" |
| ENSG00000162624 | HGLibA_26464 | 2 | -1.128699884 | LHX8 | LIM homeobox 8 [Source:HGNC Symbol;Acc:HGNC:28838] |
| ENSG00000163071 | HGLibA_46437 | 2 | -1.128699884 | SPATA18 | spermatogenesis associated 18 [Source:HGNC Symbol;Acc:HGNC:29579] |
| ENSG00000163191 | HGLibA_42535 | 2 | -1.128699884 | S100A11 | S100 calcium binding protein A11 [Source:HGNC Symbol;Acc:HGNC:10488] |
| ENSG00000163374 | HGLibA_54760 | 2 | -1.128699884 | YY1AP1 | YY1 associated protein 1 [Source:HGNC Symbol;Acc:HGNC:30935] |
| ENSG00000163637 | HGLibA_38472 | 2 | -1.128699884 | PRICKLE2 | prickle planar cell polarity protein 2 [Source:HGNC Symbol;Acc:HGNC:20340] |
| ENSG00000163638 | HGLibA_00844 | 2 | -1.128699884 | ADAMTS9 | ADAM metallopeptidase with thrombospondin type 1 motif 9 [Source:HGNC Symbol;Acc:HGNC:13202] |
| ENSG00000163646 | HGLibA_10123 | 2 | -1.128699884 | CLRN1 | clarin 1 [Source:HGNC Symbol;Acc:HGNC:12605] |
| ENSG00000163832 | HGLibA_14991 | 2 | -1.128699884 | ELP6 | elongator acetyltransferase complex subunit 6 [Source:HGNC Symbol;Acc:HGNC:25976] |
| ENSG00000164049 | HGLibA_17141 | 2 | -1.128699884 | FBXW12 | F-box and WD repeat domain containing 12 [Source:HGNC Symbol;Acc:HGNC:20729] |
| ENSG00000164283 | HGLibA_15568 | 2 | -1.128699884 | ESM1 | endothelial cell specific molecule 1 [Source:HGNC Symbol;Acc:HGNC:3466] |
| ENSG00000164291 | HGLibA_03155 | 2 | -1.128699884 | ARSK | arylsulfatase family member K [Source:HGNC Symbol;Acc:HGNC:25239] |
| ENSG00000164403 | HGLibA_44086 | 2 | -1.128699884 | SHROOM1 | shroom family member 1 [Source:HGNC Symbol;Acc:HGNC:24084] |
| ENSG00000164543 | HGLibA_47461 | 2 | -1.128699884 | STK17A | serine/threonine kinase 17a [Source:HGNC Symbol;Acc:HGNC:11395] |
| ENSG00000164715 | HGLibA_26725 | 2 | -1.128699884 | LMTK2 | lemur tyrosine kinase 2 [Source:HGNC Symbol;Acc:HGNC:17880] |
| ENSG00000164855 | HGLibA_50112 | 2 | -1.128699884 | TMEM184A | transmembrane protein 184A [Source:HGNC Symbol;Acc:HGNC:28797] |
| ENSG00000164929 | HGLibA_04050 | 2 | -1.128699884 | BAALC | MAP3K1 and KLF4 binding [Source:HGNC Symbol;Acc:HGNC:14333]" |

| | | | | | |
|---|---|---|---|---|---|
| ENSG00000165186 | HGLibA_39275 | 2 | -1.128699884 | PTCHD1 | patched domain containing 1 [Source:HGNC Symbol;Acc:HGNC:26392] |
| ENSG00000165512 | HGLibA_55606 | 2 | -1.128699884 | ZNF22 | zinc finger protein 22 [Source:HGNC Symbol;Acc:HGNC:13012] |
| ENSG00000165935 | HGLibA_45666 | 2 | -1.128699884 | SMCO2 | single-pass membrane protein with coiled-coil domains 2 [Source:HGNC Symbol;Acc:HGNC:34448] |
| ENSG00000166130 | HGLibA_22913 | 2 | -1.128699884 | IKBIP | IKBKB interacting protein [Source:HGNC Symbol;Acc:HGNC:26430] |
| ENSG00000166402 | HGLibA_52241 | 2 | -1.128699884 | TUB | tubby bipartite transcription factor [Source:HGNC Symbol;Acc:HGNC:12406] |
| ENSG00000166402 | HGLibA_52239 | 2 | -1.128699884 | TUB | tubby bipartite transcription factor [Source:HGNC Symbol;Acc:HGNC:12406] |
| ENSG00000166428 | HGLibA_37045 | 2 | -1.128699884 | PLD4 | phospholipase D family member 4 [Source:HGNC Symbol;Acc:HGNC:23792] |
| ENSG00000167011 | HGLibA_31172 | 2 | -1.128699884 | NAT16 | N-acetyltransferase 16 (putative) [Source:HGNC Symbol;Acc:HGNC:22030] |
| ENSG00000167107 | HGLibA_00547 | 2 | -1.128699884 | ACSF2 | acyl-CoA synthetase family member 2 [Source:HGNC Symbol;Acc:HGNC:26101] |
| ENSG00000167191 | HGLibA_20129 | 2 | -1.128699884 | GPRC5B | G protein-coupled receptor class C group 5 member B [Source:HGNC Symbol;Acc:HGNC:13308] |
| ENSG00000167371 | HGLibA_38884 | 2 | -1.128699884 | PRRT2 | proline rich transmembrane protein 2 [Source:HGNC Symbol;Acc:HGNC:30500] |
| ENSG00000167434 | HGLibA_06808 | 2 | -1.128699884 | CA4 | carbonic anhydrase 4 [Source:HGNC Symbol;Acc:HGNC:1375] |
| ENSG00000167645 | HGLibA_54667 | 2 | -1.128699884 | YIF1B | membrane trafficking protein [Source:HGNC Symbol;Acc:HGNC:30511]" |
| ENSG00000167749 | HGLibA_25258 | 2 | -1.128699884 | KLK4 | kallikrein related peptidase 4 [Source:HGNC Symbol;Acc:HGNC:6365] |
| ENSG00000167778 | HGLibA_46851 | 2 | -1.128699884 | SPRYD3 | SPRY domain containing 3 [Source:HGNC Symbol;Acc:HGNC:25920] |
| ENSG00000167889 | HGLibA_29152 | 2 | -1.128699884 | MGAT5B | 6-mannosylglycoprotein 6-beta-N-acetylglucosaminyltransferase B [Source:HGNC Symbol;Acc:HGNC:24140]" |
| ENSG00000167968 | HGLibA_13660 | 2 | -1.128699884 | DNASE1L2 | deoxyribonuclease 1 like 2 [Source:HGNC Symbol;Acc:HGNC:2958] |
| ENSG00000168079 | HGLibA_42866 | 2 | -1.128699884 | SCARA5 | scavenger receptor class A member 5 [Source:HGNC Symbol;Acc:HGNC:28701] |
| ENSG00000168116 | HGLibA_24786 | 2 | -1.128699884 | KIAA1586 | KIAA1586 [Source:HGNC Symbol;Acc:HGNC:21360] |
| ENSG00000168118 | HGLibA_39954 | 2 | -1.128699884 | RAB4A | member RAS oncogene family [Source:HGNC Symbol;Acc:HGNC:9781]" |
| ENSG00000168490 | HGLibA_36473 | 2 | -1.128699884 | PHYHIP | phytanoyl-CoA 2-hydroxylase interacting protein [Source:HGNC Symbol;Acc:HGNC:16865] |
| ENSG00000168634 | HGLibA_54259 | 2 | -1.128699884 | WFDC13 | WAP four-disulfide core domain 13 [Source:HGNC Symbol;Acc:HGNC:16131] |
| ENSG00000168765 | HGLibA_20481 | 2 | -1.128699884 | GSTM4 | glutathione S-transferase mu 4 [Source:HGNC Symbol;Acc:HGNC:4636] |
| ENSG00000169047 | HGLibA_23619 | 2 | -1.128699884 | IRS1 | insulin receptor substrate 1 [Source:HGNC Symbol;Acc:HGNC:6125] |
| ENSG00000169067 | HGLibA_00605 | 2 | -1.128699884 | ACTBL2 | beta like 2 [Source:HGNC Symbol;Acc:HGNC:17780]" |
| ENSG00000169509 | HGLibA_11083 | 2 | -1.128699884 | CRCT1 | cysteine rich C-terminal 1 [Source:HGNC Symbol;Acc:HGNC:29875] |
| ENSG00000169607 | HGLibA_09758 | 2 | -1.128699884 | CKAP2L | cytoskeleton associated protein 2 like [Source:HGNC Symbol;Acc:HGNC:26877] |
| ENSG00000170017 | HGLibA_01531 | 2 | -1.128699884 | ALCAM | activated leukocyte cell adhesion molecule [Source:HGNC Symbol;Acc:HGNC:400] |
| ENSG00000170185 | HGLibA_53354 | 2 | -1.128699884 | USP38 | ubiquitin specific peptidase 38 [Source:HGNC Symbol;Acc:HGNC:20067] |
| ENSG00000170367 | HGLibA_11459 | 2 | -1.128699884 | CST5 | cystatin D [Source:HGNC Symbol;Acc:HGNC:2477] |
| ENSG00000170430 | HGLibA_29165 | 2 | -1.128699884 | MGMT | O-6-methylguanine-DNA methyltransferase [Source:HGNC Symbol;Acc:HGNC:7059] |
| ENSG00000170889 | HGLibA_42193 | 2 | -1.128699884 | RPS9 | ribosomal protein S9 [Source:HGNC Symbol;Acc:HGNC:10442] |
| ENSG00000170967 | HGLibA_12609 | 2 | -1.128699884 | DDI1 | DNA damage inducible 1 homolog 1 [Source:HGNC Symbol;Acc:HGNC:18961] |
| ENSG00000171004 | HGLibA_22145 | 2 | -1.128699884 | HS6ST2 | heparan sulfate 6-O-sulfotransferase 2 [Source:HGNC Symbol;Acc:HGNC:19133] |
| ENSG00000171053 | HGLibA_35270 | 2 | -1.128699884 | PATE1 | prostate and testis expressed 1 [Source:HGNC Symbol;Acc:HGNC:24664] |
| ENSG00000171180 | HGLibA_33838 | 2 | -1.128699884 | OR2M4 | olfactory receptor family 2 subfamily M member 4 [Source:HGNC Symbol;Acc:HGNC:8270] |
| ENSG00000171421 | HGLibA_29963 | 2 | -1.128699884 | MRPL36 | mitochondrial ribosomal protein L36 [Source:HGNC Symbol;Acc:HGNC:14490] |
| ENSG00000171488 | HGLibA_27435 | 2 | -1.128699884 | LRRC8C | leucine rich repeat containing 8 VRAC subunit C [Source:HGNC Symbol;Acc:HGNC:25075] |
| ENSG00000172159 | HGLibA_18011 | 2 | -1.128699884 | FRMD3 | FERM domain containing 3 [Source:HGNC Symbol;Acc:HGNC:24125] |
| ENSG00000172183 | HGLibA_23657 | 2 | -1.128699884 | ISG20 | interferon stimulated exonuclease gene 20 [Source:HGNC Symbol;Acc:HGNC:6130] |
| ENSG00000172236 | HGLibA_51194 | 2 | -1.128699884 | TPSAB1 | tryptase alpha/beta 1 [Source:HGNC Symbol;Acc:HGNC:12019] |
| ENSG00000172375 | HGLibA_06090 | 2 | -1.128699884 | C2CD2L | C2CD2 like [Source:HGNC Symbol;Acc:HGNC:29000] |
| ENSG00000172731 | HGLibA_27280 | 2 | -1.128699884 | LRRC20 | leucine rich repeat containing 20 [Source:HGNC Symbol;Acc:HGNC:23421] |
| ENSG00000173175 | HGLibA_00920 | 2 | -1.128699884 | ADCY5 | adenylate cyclase 5 [Source:HGNC Symbol;Acc:HGNC:236] |
| ENSG00000173673 | HGLibA_21182 | 2 | -1.128699884 | HES3 | hes family bHLH transcription factor 3 [Source:HGNC Symbol;Acc:HGNC:26226] |
| ENSG00000173762 | HGLibA_08435 | 2 | -1.128699884 | CD7 | CD7 molecule [Source:HGNC Symbol;Acc:HGNC:1695] |
| ENSG00000174177 | HGLibA_11753 | 2 | -1.128699884 | CTU2 | cytosolic thiouridylase subunit 2 [Source:HGNC Symbol;Acc:HGNC:28005] |
| ENSG00000174325 | HGLibA_13264 | 2 | -1.128699884 | DIRC1 | disrupted in renal carcinoma 1 [Source:HGNC Symbol;Acc:HGNC:15760] |
| ENSG00000175115 | HGLibA_34953 | 2 | -1.128699884 | PACS1 | phosphofurin acidic cluster sorting protein 1 [Source:HGNC Symbol;Acc:HGNC:30032] |
| ENSG00000175216 | HGLibA_09762 | 2 | -1.128699884 | CKAP5 | cytoskeleton associated protein 5 [Source:HGNC Symbol;Acc:HGNC:28959] |
| ENSG00000176371 | HGLibA_56996 | 2 | -1.128699884 | ZSCAN2 | zinc finger and SCAN domain containing 2 [Source:HGNC Symbol;Acc:HGNC:20994] |
| ENSG00000176788 | HGLibA_04171 | 2 | -1.128699884 | BASP1 | brain abundant membrane attached signal protein 1 [Source:HGNC Symbol;Acc:HGNC:957] |
| ENSG00000177108 | HGLibA_55106 | 2 | -1.128699884 | ZDHHC22 | zinc finger DHHC-type containing 22 [Source:HGNC Symbol;Acc:HGNC:20106] |
| ENSG00000177119 | HGLibA_02187 | 2 | -1.128699884 | ANO6 | anoctamin 6 [Source:HGNC Symbol;Acc:HGNC:25240] |
| ENSG00000177628 | HGLibA_18710 | 2 | -1.128699884 | GBA | glucosylceramidase beta [Source:HGNC Symbol;Acc:HGNC:4177] |
| ENSG00000177710 | HGLibA_45025 | 2 | -1.128699884 | SLC35G5 | solute carrier family 35 member G5 [Source:HGNC Symbol;Acc:HGNC:15546] |
| ENSG00000178234 | HGLibA_18533 | 2 | -1.128699884 | GALNT11 | polypeptide N-acetylgalactosaminyltransferase 11 [Source:HGNC Symbol;Acc:HGNC:19875] |
| ENSG00000178498 | HGLibA_14086 | 2 | -1.128699884 | DTX3 | deltex E3 ubiquitin ligase 3 [Source:HGNC Symbol;Acc:HGNC:24457] |
| ENSG00000178665 | HGLibA_56618 | 2 | -1.128699884 | ZNF713 | zinc finger protein 713 [Source:HGNC Symbol;Acc:HGNC:22043] |
| ENSG00000179172 | HGLibA_21810 | 2 | -1.128699884 | HNRNPCL1 | heterogeneous nuclear ribonucleoprotein C-like 1 [Source:HGNC Symbol;Acc:HGNC:29295] |
| ENSG00000181045 | HGLibA_44785 | 2 | -1.128699884 | SLC26A11 | solute carrier family 26 member 11 [Source:HGNC Symbol;Acc:HGNC:14471] |
| ENSG00000181143 | HGLibA_30528 | 2 | -1.128699884 | MUC16 | cell surface associated [Source:HGNC Symbol;Acc:HGNC:15582]" |
| ENSG00000181513 | HGLibA_00400 | 2 | -1.128699884 | ACBD4 | acyl-CoA binding domain containing 4 [Source:HGNC Symbol;Acc:HGNC:23337] |
| ENSG00000182271 | HGLibA_50586 | 2 | -1.128699884 | TMIGD1 | transmembrane and immunoglobulin domain containing 1 [Source:HGNC Symbol;Acc:HGNC:32431] |
| ENSG00000182631 | HGLibA_42499 | 2 | -1.128699884 | RXFP3 | relaxin family peptide receptor 3 [Source:HGNC Symbol;Acc:HGNC:24883] |
| ENSG00000182872 | HGLibA_40472 | 2 | -1.128699884 | RBM10 | RNA binding motif protein 10 [Source:HGNC Symbol;Acc:HGNC:9896] |
| ENSG00000183111 | HGLibA_02854 | 2 | -1.128699884 | ARHGEF37 | Rho guanine nucleotide exchange factor 37 [Source:HGNC Symbol;Acc:HGNC:34430] |
| ENSG00000183578 | HGLibA_50731 | 2 | -1.128699884 | TNFAIP8L3 | TNF alpha induced protein 8 like 3 [Source:HGNC Symbol;Acc:HGNC:20620] |
| ENSG00000183621 | HGLibA_55993 | 2 | -1.128699884 | ZNF438 | zinc finger protein 438 [Source:HGNC Symbol;Acc:HGNC:21029] |
| ENSG00000183655 | HGLibA_25164 | 2 | -1.128699884 | KLHL25 | kelch like family member 25 [Source:HGNC Symbol;Acc:HGNC:25732] |
| ENSG00000184497 | HGLibA_50333 | 2 | -1.128699884 | TMEM255B | transmembrane protein 255B [Source:HGNC Symbol;Acc:HGNC:28297] |
| ENSG00000184544 | HGLibA_13150 | 2 | -1.128699884 | DHRS7C | dehydrogenase/reductase 7C [Source:HGNC Symbol;Acc:HGNC:32423] |
| ENSG00000184574 | HGLibA_27066 | 2 | -1.128699884 | LPAR5 | lysophosphatidic acid receptor 5 [Source:HGNC Symbol;Acc:HGNC:13307] |
| ENSG00000184752 | HGLibA_31446 | 2 | -1.128699884 | NDUFA12 | NADH:ubiquinone oxidoreductase subunit A12 [Source:HGNC Symbol;Acc:HGNC:23987] |
| ENSG00000184995 | HGLibA_22703 | 2 | -1.128699884 | IFNE | interferon epsilon [Source:HGNC Symbol;Acc:HGNC:18163] |
| ENSG00000185305 | HGLibA_02945 | 2 | -1.128699884 | ARL15 | ADP ribosylation factor like GTPase 15 [Source:HGNC Symbol;Acc:HGNC:25945] |
| ENSG00000185515 | HGLibA_04766 | 2 | -1.128699884 | BRCC3 | BRCA1/BRCA2-containing complex subunit 3 [Source:HGNC Symbol;Acc:HGNC:24185] |
| ENSG00000185753 | HGLibA_11921 | 2 | -1.128699884 | CXorf38 | chromosome X open reading frame 38 [Source:HGNC Symbol;Acc:HGNC:28589] |
| ENSG00000186113 | HGLibA_34278 | 2 | -1.128699884 | OR5D14 | olfactory receptor family 5 subfamily D member 14 [Source:HGNC Symbol;Acc:HGNC:15281] |
| ENSG00000186300 | HGLibA_56223 | 2 | -1.128699884 | ZNF555 | zinc finger protein 555 [Source:HGNC Symbol;Acc:HGNC:28382] |
| ENSG00000186310 | HGLibA_31118 | 2 | -1.128699884 | NAP1L3 | nucleosome assembly protein 1 like 3 [Source:HGNC Symbol;Acc:HGNC:7639] |
| ENSG00000186453 | HGLibA_16533 | 2 | -1.128699884 | FAM228A | family with sequence similarity 228 member A [Source:HGNC Symbol;Acc:HGNC:34418] |
| ENSG00000187240 | HGLibA_14247 | 2 | -1.128699884 | DYNC2H1 | dynein cytoplasmic 2 heavy chain 1 [Source:HGNC Symbol;Acc:HGNC:2962] |
| ENSG00000188100 | HGLibA_16553 | 2 | -1.128699884 | FAM25A | family with sequence similarity 25 member A [Source:HGNC Symbol;Acc:HGNC:23436] |
| ENSG00000188186 | HGLibA_25999 | 2 | -1.128699884 | LAMTOR4 | MAPK and MTOR activator 4 [Source:HGNC Symbol;Acc:HGNC:33772]" |
| ENSG00000188312 | HGLibA_09079 | 2 | -1.128699884 | CENPP | centromere protein P [Source:HGNC Symbol;Acc:HGNC:32933] |
| ENSG00000189120 | HGLibA_46310 | 2 | -1.128699884 | SP6 | Sp6 transcription factor [Source:HGNC Symbol;Acc:HGNC:14530] |
| ENSG00000189182 | HGLibA_25542 | 2 | -1.128699884 | KRT77 | keratin 77 [Source:HGNC Symbol;Acc:HGNC:20411] |
| ENSG00000196549 | HGLibA_29468 | 2 | -1.128699884 | MME | membrane metalloendopeptidase [Source:HGNC Symbol;Acc:HGNC:7154] |
| ENSG00000196754 | HGLibA_42552 | 2 | -1.128699884 | S100A2 | S100 calcium binding protein A2 [Source:HGNC Symbol;Acc:HGNC:10492] |
| ENSG00000197172 | HGLibA_27918 | 2 | -1.128699884 | MAGEA6 | MAGE family member A6 [Source:HGNC Symbol;Acc:HGNC:6804] |
| ENSG00000197324 | HGLibA_27200 | 2 | -1.128699884 | LRP10 | LDL receptor related protein 10 [Source:HGNC Symbol;Acc:HGNC:14553] |
| ENSG00000197417 | HGLibA_44079 | 2 | -1.128699884 | SHPK | sedoheptulokinase [Source:HGNC Symbol;Acc:HGNC:1492] |
| ENSG00000197565 | HGLibA_10585 | 2 | -1.128699884 | COL4A6 | collagen type IV alpha 6 chain [Source:HGNC Symbol;Acc:HGNC:2208] |
| ENSG00000197892 | HGLibA_24859 | 2 | -1.128699884 | KIF13B | kinesin family member 13B [Source:HGNC Symbol;Acc:HGNC:14405] |
| ENSG00000198033 | HGLibA_52252 | 2 | -1.128699884 | TUBA3C | tubulin alpha 3c [Source:HGNC Symbol;Acc:HGNC:12408] |
| ENSG00000198062 | HGLibA_37737 | 2 | -1.128699884 | POTEH | POTE ankyrin domain family member H [Source:HGNC Symbol;Acc:HGNC:133] |
| ENSG00000198643 | HGLibA_16584 | 2 | -1.128699884 | FAM3D | family with sequence similarity 3 member D [Source:HGNC Symbol;Acc:HGNC:18665] |
| ENSG00000198704 | HGLibA_20184 | 2 | -1.128699884 | GPX6 | glutathione peroxidase 6 [Source:HGNC Symbol;Acc:HGNC:4558] |
| ENSG00000198853 | HGLibA_42470 | 2 | -1.128699884 | RUSC2 | RUN and SH3 domain containing 2 [Source:HGNC Symbol;Acc:HGNC:23625] |
| ENSG00000198865 | HGLibA_07648 | 2 | -1.128699884 | CCDC152 | coiled-coil domain containing 152 [Source:HGNC Symbol;Acc:HGNC:34438] |
| ENSG00000204025 | HGLibA_51744 | 2 | -1.128699884 | TRPC5OS | TRPC5 opposite strand [Source:HGNC Symbol;Acc:HGNC:40593] |
| ENSG00000204852 | HGLibA_48872 | 2 | -1.128699884 | TCTN1 | tectonic family member 1 [Source:HGNC Symbol;Acc:HGNC:26113] |
| ENSG00000205358 | HGLibA_30292 | 2 | -1.128699884 | MT1H | metallothionein 1H [Source:HGNC Symbol;Acc:HGNC:7400] |
| ENSG00000205944 | HGLibA_12354 | 2 | -1.128699884 | DAZ2 | deleted in azoospermia 2 [Source:HGNC Symbol;Acc:HGNC:15964] |
| ENSG00000206557 | HGLibA_51599 | 2 | -1.128699884 | TRIM71 | tripartite motif containing 71 [Source:HGNC Symbol;Acc:HGNC:32669] |
| ENSG00000214595 | HGLibA_15071 | 2 | -1.128699884 | EML6 | echinoderm microtubule associated protein like 6 [Source:HGNC Symbol;Acc:HGNC:35412] |
| ENSG00000214827 | HGLibA_30331 | 2 | -1.128699884 | MTCP1 | mature T cell proliferation 1 [Source:HGNC Symbol;Acc:HGNC:7423] |
| ENSG00000215045 | HGLibA_20278 | 2 | -1.128699884 | GRID2IP | Grid2 interacting protein [Source:HGNC Symbol;Acc:HGNC:18464] |
| ENSG00000215305 | HGLibA_53744 | 2 | -1.128699884 | VPS16 | CORVET/HOPS core subunit [Source:HGNC Symbol;Acc:HGNC:14584]" |
| ENSG00000221874 | HGLibA_56814 | 2 | -1.128699884 | ZNF816-ZNF321P | ZNF816-ZNF321P readthrough [Source:HGNC Symbol;Acc:HGNC:38879] |
| ENSG00000221883 | HGLibA_02918 | 2 | -1.128699884 | ARIH2OS | ariadne RBR E3 ubiquitin protein ligase 2 opposite strand [Source:HGNC Symbol;Acc:HGNC:34425] |
| ENSG00000223731 | HGLibA_47808 | 2 | -1.128699884 | SUPT20HL1 | SUPT20H like 1 [Source:HGNC Symbol;Acc:HGNC:30773] |
| ENSG00000224712 | HGLibA_32469 | 2 | -1.128699884 | NPIPA3 | nuclear pore complex interacting protein family member A3 [Source:HGNC Symbol;Acc:HGNC:41978] |
| ENSG00000226887 | HGLibA_15542 | 2 | -1.128699884 | ERVMER34-1 | envelope [Source:HGNC Symbol;Acc:HGNC:42970]" |
| ENSG00000228856 | HGLibA_53274 | 2 | -1.128699884 | USP17L30 | ubiquitin specific peptidase 17-like family member 30 [Source:HGNC Symbol;Acc:HGNC:44458] |
| ENSG00000230430 | HGLibA_53256 | 2 | -1.128699884 | USP17L25 | ubiquitin specific peptidase 17-like family member 25 [Source:HGNC Symbol;Acc:HGNC:44452] |
| ENSG00000232399 | HGLibA_53224 | 2 | -1.128699884 | USP17L13 | ubiquitin specific peptidase 17-like family member 13 [Source:HGNC Symbol;Acc:HGNC:44441] |
| ENSG00000233917 | HGLibA_37718 | 2 | -1.128699884 | POTEB | POTE ankyrin domain family member B [Source:HGNC Symbol;Acc:HGNC:33734] |
| ENSG00000237521 | HGLibA_34477 | 2 | -1.128699884 | OR7E24 | olfactory receptor family 7 subfamily E member 24 [Source:HGNC Symbol;Acc:HGNC:8396] |
| ENSG00000243056 | HGLibA_14833 | 2 | -1.128699884 | EIF4EBP3 | eukaryotic translation initiation factor 4E binding protein 3 [Source:HGNC Symbol;Acc:HGNC:3290] |
| ENSG00000243716 | HGLibA_32476 | 2 | -1.128699884 | NPIPB5 | nuclear pore complex interacting protein family member B5 [Source:HGNC Symbol;Acc:HGNC:37233] |
| ENSG00000243716 | HGLibA_32475 | 2 | -1.128699884 | NPIPB5 | nuclear pore complex interacting protein family member B5 [Source:HGNC Symbol;Acc:HGNC:37233] |
| ENSG00000244537 | HGLibA_25758 | 2 | -1.128699884 | KRTAP4-2 | keratin associated protein 4-2 [Source:HGNC Symbol;Acc:HGNC:18900] |
| ENSG00000248933 | HGLibA_53251 | 2 | -1.128699884 | USP17L22 | ubiquitin specific peptidase 17-like family member 22 [Source:HGNC Symbol;Acc:HGNC:44450] |
| ENSG00000249481 | HGLibA_46544 | 2 | -1.128699884 | SPATS1 | spermatogenesis associated serine rich 1 [Source:HGNC Symbol;Acc:HGNC:22957] |
| ENSG00000253117 | HGLibA_33237 | 2 | -1.128699884 | OC90 | otoconin 90 [Source:HGNC Symbol;Acc:HGNC:8100] |
| ENSG00000254004 | HGLibA_55690 | 2 | -1.128699884 | ZNF260 | zinc finger protein 260 [Source:HGNC Symbol;Acc:HGNC:13499] |
| ENSG00000254402 | HGLibA_27286 | 2 | -1.128699884 | LRRC24 | leucine rich repeat containing 24 [Source:HGNC Symbol;Acc:HGNC:28947] |
| ENSG00000254726 | HGLibA_29027 | 2 | -1.128699884 | MEX3A | mex-3 RNA binding family member A [Source:HGNC Symbol;Acc:HGNC:33482] |
| ENSG00000255298 | HGLibA_34524 | 2 | -1.128699884 | OR8G5 | olfactory receptor family 8 subfamily G member 5 [Source:HGNC Symbol;Acc:HGNC:19622] |
| ENSG00000256436 | HGLibA_48389 | 2 | -1.128699884 | TAS2R31 | taste 2 receptor member 31 [Source:HGNC Symbol;Acc:HGNC:19113] |
| ENSG00000260230 | HGLibA_18045 | 2 | -1.128699884 | FRRS1L | ferric chelate reductase 1 like [Source:HGNC Symbol;Acc:HGNC:1362] |
| ENSG00000271567 | HGLibA_37910 | 2 | -1.128699884 | PPIAL4E | peptidylprolyl isomerase A like 4E [Source:HGNC Symbol;Acc:HGNC:33997] |
| ENSG00000276043 | HGLibA_52944 | 2 | -1.128699884 | UHRF1 | ubiquitin like with PHD and ring finger domains 1 [Source:HGNC Symbol;Acc:HGNC:12556] |

| | | | | | |
|---|---|---|---|---|---|
| ENSG00000280109 | HGLibA_36939 | 2 | -1.128699884 | PLAC4 | placenta specific 4 [Source:HGNC Symbol;Acc:HGNC:14616] |
| ENSG00000281887 | HGLibA_19074 | 2 | -1.128699884 | GIMAP1-GIMAP5 | GIMAP1-GIMAP5 readthrough [Source:HGNC Symbol;Acc:HGNC:51257] |
| ENSG00000283632 | HGLibA_15744 | 2 | -1.128699884 | EXOC3L2 | exocyst complex component 3 like 2 [Source:HGNC Symbol;Acc:HGNC:30162] |
| ENSG00000284662 | HGLibA_33992 | 2 | -1.128699884 | OR4F16 | olfactory receptor family 4 subfamily F member 16 [Source:HGNC Symbol;Acc:HGNC:15079] |
| ENSG00000288701 | HGLibA_38862 | 2 | -1.128699884 | PRRC2B | - |
| FAM134A | HGLibA_16124 | 2 | -1.128699884 | FAM134A | - |
| GAGE5 | HGLibA_18483 | 2 | -1.128699884 | GAGE5 | - |
| GPR123 | HGLibA_19874 | 2 | -1.128699884 | GPR123 | - |
| GSTT1 | HGLibA_20495 | 2 | -1.128699884 | GSTT1 | - |
| HIST1H2BF | HGLibA_21426 | 2 | -1.128699884 | HIST1H2BF | - |
| HIST1H2BJ | HGLibA_21438 | 2 | -1.128699884 | HIST1H2BJ | - |
| HIST1H3B | HGLibA_21459 | 2 | -1.128699884 | HIST1H3B | - |
| HMP19 | HGLibA_21753 | 2 | -1.128699884 | HMP19 | - |
| hsa-mir-1289-1 | HGLibA_57621 | 2 | -1.128699884 | hsa-mir-1289-1 | - |
| hsa-mir-181b-1 | HGLibA_57967 | 2 | -1.128699884 | hsa-mir-181b-1 | - |
| hsa-mir-211 | HGLibA_58215 | 2 | -1.128699884 | hsa-mir-211 | - |
| hsa-mir-3074 | HGLibA_58468 | 2 | -1.128699884 | hsa-mir-3074 | - |
| hsa-mir-3155b | HGLibA_58683 | 2 | -1.128699884 | hsa-mir-3155b | - |
| hsa-mir-3164 | HGLibA_58737 | 2 | -1.128699884 | hsa-mir-3164 | - |
| hsa-mir-3166 | HGLibA_58745 | 2 | -1.128699884 | hsa-mir-3166 | - |
| hsa-mir-34b | HGLibA_59039 | 2 | -1.128699884 | hsa-mir-34b | - |
| hsa-mir-3613 | HGLibA_59082 | 2 | -1.128699884 | hsa-mir-3613 | - |
| hsa-mir-3621 | HGLibA_59117 | 2 | -1.128699884 | hsa-mir-3621 | - |
| hsa-mir-3650 | HGLibA_59144 | 2 | -1.128699884 | hsa-mir-3650 | - |
| hsa-mir-384 | HGLibA_59490 | 2 | -1.128699884 | hsa-mir-384 | - |
| hsa-mir-4263 | HGLibA_59761 | 2 | -1.128699884 | hsa-mir-4263 | - |
| hsa-mir-4291 | HGLibA_59875 | 2 | -1.128699884 | hsa-mir-4291 | - |
| hsa-mir-4330 | HGLibA_60046 | 2 | -1.128699884 | hsa-mir-4330 | - |
| hsa-mir-4479 | HGLibA_60330 | 2 | -1.128699884 | hsa-mir-4479 | - |
| hsa-mir-4489 | HGLibA_60374 | 2 | -1.128699884 | hsa-mir-4489 | - |
| hsa-mir-4501 | HGLibA_60432 | 2 | -1.128699884 | hsa-mir-4501 | - |
| hsa-mir-4508 | HGLibA_60457 | 2 | -1.128699884 | hsa-mir-4508 | - |
| hsa-mir-4509-2 | HGLibA_60465 | 2 | -1.128699884 | hsa-mir-4509-2 | - |
| hsa-mir-4518 | HGLibA_60516 | 2 | -1.128699884 | hsa-mir-4518 | - |
| hsa-mir-4533 | HGLibA_60592 | 2 | -1.128699884 | hsa-mir-4533 | - |
| hsa-mir-4645 | HGLibA_60684 | 2 | -1.128699884 | hsa-mir-4645 | - |
| hsa-mir-4717 | HGLibA_60970 | 2 | -1.128699884 | hsa-mir-4717 | - |
| hsa-mir-4773-2 | HGLibA_61198 | 2 | -1.128699884 | hsa-mir-4773-2 | - |
| hsa-mir-518d | HGLibA_61667 | 2 | -1.128699884 | hsa-mir-518d | - |
| hsa-mir-519e | HGLibA_61729 | 2 | -1.128699884 | hsa-mir-519e | - |
| hsa-mir-548ag-2 | HGLibA_61880 | 2 | -1.128699884 | hsa-mir-548ag-2 | - |
| hsa-mir-548aj-2 | HGLibA_61890 | 2 | -1.128699884 | hsa-mir-548aj-2 | - |
| hsa-mir-548o-2 | HGLibA_62052 | 2 | -1.128699884 | hsa-mir-548o-2 | - |
| hsa-mir-550b-1 | HGLibA_62110 | 2 | -1.128699884 | hsa-mir-550b-1 | - |
| hsa-mir-5583-1 | HGLibA_62168 | 2 | -1.128699884 | hsa-mir-5583-1 | - |
| hsa-mir-5585 | HGLibA_62182 | 2 | -1.128699884 | hsa-mir-5585 | - |
| hsa-mir-5681b | HGLibA_62245 | 2 | -1.128699884 | hsa-mir-5681b | - |
| hsa-mir-5702 | HGLibA_62350 | 2 | -1.128699884 | hsa-mir-5702 | - |
| hsa-mir-6078 | HGLibA_62569 | 2 | -1.128699884 | hsa-mir-6078 | - |
| hsa-mir-618 | HGLibA_62713 | 2 | -1.128699884 | hsa-mir-618 | - |
| hsa-mir-622 | HGLibA_62729 | 2 | -1.128699884 | hsa-mir-622 | - |
| hsa-mir-623 | HGLibA_62732 | 2 | -1.128699884 | hsa-mir-623 | - |
| hsa-mir-625 | HGLibA_62741 | 2 | -1.128699884 | hsa-mir-625 | - |
| hsa-mir-6717 | HGLibA_63028 | 2 | -1.128699884 | hsa-mir-6717 | - |
| hsa-mir-6799 | HGLibA_63375 | 2 | -1.128699884 | hsa-mir-6799 | - |
| hsa-mir-6845 | HGLibA_63554 | 2 | -1.128699884 | hsa-mir-6845 | - |
| hsa-mir-6879 | HGLibA_63700 | 2 | -1.128699884 | hsa-mir-6879 | - |
| hsa-mir-6890 | HGLibA_63744 | 2 | -1.128699884 | hsa-mir-6890 | - |
| hsa-mir-7162 | HGLibA_63876 | 2 | -1.128699884 | hsa-mir-7162 | - |
| hsa-mir-7641-1 | HGLibA_63914 | 2 | -1.128699884 | hsa-mir-7641-1 | - |
| hsa-mir-8058 | HGLibA_64070 | 2 | -1.128699884 | hsa-mir-8058 | - |
| hsa-mir-8059 | HGLibA_64074 | 2 | -1.128699884 | hsa-mir-8059 | - |
| IRG1 | HGLibA_23608 | 2 | -1.128699884 | IRG1 | - |
| KIAA1161 | HGLibA_24702 | 2 | -1.128699884 | KIAA1161 | - |
| KIAA1429 | HGLibA_24751 | 2 | -1.128699884 | KIAA1429 | - |
| LOC402160 | HGLibA_26940 | 2 | -1.128699884 | LOC402160 | - |
| LOC642799 | HGLibA_26960 | 2 | -1.128699884 | LOC642799 | - |
| LRRC6 | HGLibA_27405 | 2 | -1.128699884 | LRRC6 | - |
| LUZP6 | HGLibA_27647 | 2 | -1.128699884 | LUZP6 | - |
| PPAPDC1B | HGLibA_37828 | 2 | -1.128699884 | PPAPDC1B | - |
| PRAMEF21 | HGLibA_38311 | 2 | -1.128699884 | PRAMEF21 | - |
| RTDR1 | HGLibA_42377 | 2 | -1.128699884 | RTDR1 | - |
| STRA13 | HGLibA_47565 | 2 | -1.128699884 | STRA13 | - |
| T | HGLibA_48119 | 2 | -1.128699884 | T | - |
| ZUFSP | HGLibA_57060 | 2 | -1.128699884 | ZUFSP | - |
| ACPL2 | HGLibA_00520 | 1 | -1.138650921 | ACPL2 | - |
| C14orf80 | HGLibA_05371 | 1 | -1.138650921 | C14orf80 | - |
| C15orf60 | HGLibA_05426 | 1 | -1.138650921 | C15orf60 | - |
| C16orf58 | HGLibA_05462 | 1 | -1.138650921 | C16orf58 | - |
| C17orf105 | HGLibA_05525 | 1 | -1.138650921 | C17orf105 | - |
| C17orf53 | HGLibA_05547 | 1 | -1.138650921 | C17orf53 | - |
| C17orf77 | HGLibA_05575 | 1 | -1.138650921 | C17orf77 | - |
| C20orf111 | HGLibA_05981 | 1 | -1.138650921 | C20orf111 | - |
| C20orf194 | HGLibA_06000 | 1 | -1.138650921 | C20orf194 | - |
| C21orf33 | HGLibA_06037 | 1 | -1.138650921 | C21orf33 | - |
| C2orf57 | HGLibA_06152 | 1 | -1.138650921 | C2orf57 | - |
| C2orf82 | HGLibA_06192 | 1 | -1.138650921 | C2orf82 | - |
| C3orf83 | HGLibA_06275 | 1 | -1.138650921 | C3orf83 | - |
| C5orf28 | HGLibA_06372 | 1 | -1.138650921 | C5orf28 | - |
| C6orf99 | HGLibA_06515 | 1 | -1.138650921 | C6orf99 | - |
| C7orf66 | HGLibA_06577 | 1 | -1.138650921 | C7orf66 | - |
| C8orf46 | HGLibA_06628 | 1 | -1.138650921 | C8orf46 | - |
| C9orf139 | HGLibA_06683 | 1 | -1.138650921 | C9orf139 | - |
| C9orf96 | HGLibA_06783 | 1 | -1.138650921 | C9orf96 | - |
| DEFB131 | HGLibA_12898 | 1 | -1.138650921 | DEFB131 | - |
| DFNB31 | HGLibA_13027 | 1 | -1.138650921 | DFNB31 | - |
| ENSG00000000460 | HGLibA_05751 | 1 | -1.138650921 | C1orf112 | chromosome 1 open reading frame 112 [Source:HGNC Symbol;Acc:HGNC:25565] |
| ENSG00000001460 | HGLibA_47559 | 1 | -1.138650921 | STPG1 | sperm tail PG-rich repeat containing 1 [Source:HGNC Symbol;Acc:HGNC:28070] |
| ENSG00000001631 | HGLibA_25407 | 1 | -1.138650921 | KRIT1 | ankyrin repeat containing [Source:HGNC Symbol;Acc:HGNC:1573]" |
| ENSG00000003137 | HGLibA_12089 | 1 | -1.138650921 | CYP26B1 | cytochrome P450 family 26 subfamily B member 1 [Source:HGNC Symbol;Acc:HGNC:20581] |
| ENSG00000003756 | HGLibA_40574 | 1 | -1.138650921 | RBM5 | RNA binding motif protein 5 [Source:HGNC Symbol;Acc:HGNC:9902] |
| ENSG00000004487 | HGLibA_24489 | 1 | -1.138650921 | KDM1A | lysine demethylase 1A [Source:HGNC Symbol;Acc:HGNC:29079] |
| ENSG00000004777 | HGLibA_02761 | 1 | -1.138650921 | ARHGAP33 | Rho GTPase activating protein 33 [Source:HGNC Symbol;Acc:HGNC:23085] |
| ENSG00000005471 | HGLibA_00139 | 1 | -1.138650921 | ABCB4 | ATP binding cassette subfamily B member 4 [Source:HGNC Symbol;Acc:HGNC:45] |
| ENSG00000005882 | HGLibA_35897 | 1 | -1.138650921 | PDK2 | pyruvate dehydrogenase kinase 2 [Source:HGNC Symbol;Acc:HGNC:8810] |
| ENSG00000005981 | HGLibA_03240 | 1 | -1.138650921 | ASB4 | ankyrin repeat and SOCS box containing 4 [Source:HGNC Symbol;Acc:HGNC:16009] |
| ENSG00000006047 | HGLibA_54645 | 1 | -1.138650921 | YBX2 | Y-box binding protein 2 [Source:HGNC Symbol;Acc:HGNC:17948] |
| ENSG00000006116 | HGLibA_06936 | 1 | -1.138650921 | CACNG3 | calcium voltage-gated channel auxiliary subunit gamma 3 [Source:HGNC Symbol;Acc:HGNC:1407] |
| ENSG00000006118 | HGLibA_49931 | 1 | -1.138650921 | TMEM132A | transmembrane protein 132A [Source:HGNC Symbol;Acc:HGNC:31092] |
| ENSG00000006576 | HGLibA_36465 | 1 | -1.138650921 | PHTF2 | putative homeodomain transcription factor 2 [Source:HGNC Symbol;Acc:HGNC:13411] |
| ENSG00000008086 | HGLibA_08818 | 1 | -1.138650921 | CDKL5 | cyclin dependent kinase like 5 [Source:HGNC Symbol;Acc:HGNC:11411] |
| ENSG00000009950 | HGLibA_29444 | 1 | -1.138650921 | MLXIPL | MLX interacting protein like [Source:HGNC Symbol;Acc:HGNC:12744] |
| ENSG00000011198 | HGLibA_00282 | 1 | -1.138650921 | ABHD5 | abhydrolase domain containing 5 [Source:HGNC Symbol;Acc:HGNC:21396] |
| ENSG00000011260 | HGLibA_53442 | 1 | -1.138650921 | UTP18 | small subunit processome component [Source:HGNC Symbol;Acc:HGNC:24274]" |
| ENSG00000016402 | HGLibA_23076 | 1 | -1.138650921 | IL20RA | interleukin 20 receptor subunit alpha [Source:HGNC Symbol;Acc:HGNC:6003] |
| ENSG00000018236 | HGLibA_10402 | 1 | -1.138650921 | CNTN1 | contactin 1 [Source:HGNC Symbol;Acc:HGNC:2171] |
| ENSG00000021300 | HGLibA_37086 | 1 | -1.138650921 | PLEKHB1 | pleckstrin homology domain containing B1 [Source:HGNC Symbol;Acc:HGNC:19079] |
| ENSG00000025156 | HGLibA_22224 | 1 | -1.138650921 | HSF2 | heat shock transcription factor 2 [Source:HGNC Symbol;Acc:HGNC:5225] |
| ENSG00000025800 | HGLibA_25369 | 1 | -1.138650921 | KPNA6 | karyopherin subunit alpha 6 [Source:HGNC Symbol;Acc:HGNC:6399] |
| ENSG00000026652 | HGLibA_01225 | 1 | -1.138650921 | AGPAT4 | 1-acylglycerol-3-phosphate O-acyltransferase 4 [Source:HGNC Symbol;Acc:HGNC:20885] |
| ENSG00000026751 | HGLibA_44319 | 1 | -1.138650921 | SLAMF7 | SLAM family member 7 [Source:HGNC Symbol;Acc:HGNC:21394] |
| ENSG00000033100 | HGLibA_09526 | 1 | -1.138650921 | CHPF2 | chondroitin polymerizing factor 2 [Source:HGNC Symbol;Acc:HGNC:29270] |
| ENSG00000038002 | HGLibA_01133 | 1 | -1.138650921 | AGA | aspartylglucosaminidase [Source:HGNC Symbol;Acc:HGNC:318] |
| ENSG00000043093 | HGLibA_12566 | 1 | -1.138650921 | DCUN1D1 | defective in cullin neddylation 1 domain containing 1 [Source:HGNC Symbol;Acc:HGNC:18184] |
| ENSG00000047365 | HGLibA_02628 | 1 | -1.138650921 | ARAP2 | ankyrin repeat and PH domain 2 [Source:HGNC Symbol;Acc:HGNC:16924]" |
| ENSG00000049239 | HGLibA_20788 | 1 | -1.138650921 | H6PD | hexose-6-phosphate dehydrogenase/glucose 1-dehydrogenase [Source:HGNC Symbol;Acc:HGNC:4795] |
| ENSG00000049768 | HGLibA_17937 | 1 | -1.138650921 | FOXP3 | forkhead box P3 [Source:HGNC Symbol;Acc:HGNC:6106] |
| ENSG00000049883 | HGLibA_39262 | 1 | -1.138650921 | PTCD2 | pentatricopeptide repeat domain 2 [Source:HGNC Symbol;Acc:HGNC:25734] |
| ENSG00000050405 | HGLibA_26526 | 1 | -1.138650921 | LIMA1 | LIM domain and actin binding 1 [Source:HGNC Symbol;Acc:HGNC:24636] |
| ENSG00000052802 | HGLibA_30225 | 1 | -1.138650921 | MSMO1 | methylsterol monooxygenase 1 [Source:HGNC Symbol;Acc:HGNC:10545] |
| ENSG00000052850 | HGLibA_01739 | 1 | -1.138650921 | ALX4 | ALX homeobox 4 [Source:HGNC Symbol;Acc:HGNC:450] |
| ENSG00000053770 | HGLibA_02355 | 1 | -1.138650921 | AP5M1 | adaptor related protein complex 5 subunit mu 1 [Source:HGNC Symbol;Acc:HGNC:20192] |
| ENSG00000054967 | HGLibA_40827 | 1 | -1.138650921 | RELT | TNF receptor [Source:HGNC Symbol;Acc:HGNC:13764]" |
| ENSG00000058085 | HGLibA_25972 | 1 | -1.138650921 | LAMC2 | laminin subunit gamma 2 [Source:HGNC Symbol;Acc:HGNC:6493] |
| ENSG00000059758 | HGLibA_08745 | 1 | -1.138650921 | CDK17 | cyclin dependent kinase 17 [Source:HGNC Symbol;Acc:HGNC:8750] |

| | | | | | |
|---|---|---|---|---|---|
| ENSG00000064042 | HGLibA_26527 | 1 | -1.138650921 | LIMCH1 | LIM and calponin homology domains 1 [Source:HGNC Symbol;Acc:HGNC:29191] |
| ENSG00000064218 | HGLibA_13424 | 1 | -1.138650921 | DMRT3 | doublesex and mab-3 related transcription factor 3 [Source:HGNC Symbol;Acc:HGNC:13909] |
| ENSG00000064545 | HGLibA_50024 | 1 | -1.138650921 | TMEM161A | transmembrane protein 161A [Source:HGNC Symbol;Acc:HGNC:26020] |
| ENSG00000065882 | HGLibA_48461 | 1 | -1.138650921 | TBC1D1 | TBC1 domain family member 1 [Source:HGNC Symbol;Acc:HGNC:11578] |
| ENSG00000069011 | HGLibA_36762 | 1 | -1.138650921 | PITX1 | paired like homeodomain 1 [Source:HGNC Symbol;Acc:HGNC:9004] |
| ENSG00000070193 | HGLibA_17363 | 1 | -1.138650921 | FGF10 | fibroblast growth factor 10 [Source:HGNC Symbol;Acc:HGNC:3666] |
| ENSG00000070371 | HGLibA_10151 | 1 | -1.138650921 | CLTCL1 | clathrin heavy chain like 1 [Source:HGNC Symbol;Acc:HGNC:2093] |
| ENSG00000070540 | HGLibA_54312 | 1 | -1.138650921 | WIPI1 | phosphoinositide interacting 1 [Source:HGNC Symbol;Acc:HGNC:25471]" |
| ENSG00000070756 | HGLibA_34920 | 1 | -1.138650921 | PABPC1 | poly(A) binding protein cytoplasmic 1 [Source:HGNC Symbol;Acc:HGNC:8554] |
| ENSG00000071189 | HGLibA_46036 | 1 | -1.138650921 | SNX13 | sorting nexin 13 [Source:HGNC Symbol;Acc:HGNC:21335] |
| ENSG00000072041 | HGLibA_45304 | 1 | -1.138650921 | SLC6A15 | solute carrier family 6 member 15 [Source:HGNC Symbol;Acc:HGNC:13621] |
| ENSG00000072134 | HGLibA_15345 | 1 | -1.138650921 | EPN2 | epsin 2 [Source:HGNC Symbol;Acc:HGNC:18639] |
| ENSG00000072501 | HGLibA_45640 | 1 | -1.138650921 | SMC1A | structural maintenance of chromosomes 1A [Source:HGNC Symbol;Acc:HGNC:11111] |
| ENSG00000073536 | HGLibA_32065 | 1 | -1.138650921 | NLE1 | notchless homolog 1 [Source:HGNC Symbol;Acc:HGNC:19889] |
| ENSG00000073756 | HGLibA_39360 | 1 | -1.138650921 | PTGS2 | prostaglandin-endoperoxide synthase 2 [Source:HGNC Symbol;Acc:HGNC:9605] |
| ENSG00000074657 | HGLibA_56180 | 1 | -1.138650921 | ZNF532 | zinc finger protein 532 [Source:HGNC Symbol;Acc:HGNC:30940] |
| ENSG00000074657 | HGLibA_56178 | 1 | -1.138650921 | ZNF532 | zinc finger protein 532 [Source:HGNC Symbol;Acc:HGNC:30940] |
| ENSG00000075275 | HGLibA_09021 | 1 | -1.138650921 | CELSR1 | cadherin EGF LAG seven-pass G-type receptor 1 [Source:HGNC Symbol;Acc:HGNC:1850] |
| ENSG00000075399 | HGLibA_53821 | 1 | -1.138650921 | VPS9D1 | VPS9 domain containing 1 [Source:HGNC Symbol;Acc:HGNC:13526] |
| ENSG00000075407 | HGLibA_55885 | 1 | -1.138650921 | ZNF37A | zinc finger protein 37A [Source:HGNC Symbol;Acc:HGNC:13102] |
| ENSG00000076382 | HGLibA_46360 | 1 | -1.138650921 | SPAG5 | sperm associated antigen 5 [Source:HGNC Symbol;Acc:HGNC:13452] |
| ENSG00000077458 | HGLibA_16725 | 1 | -1.138650921 | FAM76B | family with sequence similarity 76 member B [Source:HGNC Symbol;Acc:HGNC:28492] |
| ENSG00000077549 | HGLibA_07214 | 1 | -1.138650921 | CAPZB | capping actin protein of muscle Z-line subunit beta [Source:HGNC Symbol;Acc:HGNC:1491] |
| ENSG00000078018 | HGLibA_28137 | 1 | -1.138650921 | MAP2 | microtubule associated protein 2 [Source:HGNC Symbol;Acc:HGNC:6839] |
| ENSG00000078142 | HGLibA_36629 | 1 | -1.138650921 | PIK3C3 | phosphatidylinositol 3-kinase catalytic subunit type 3 [Source:HGNC Symbol;Acc:HGNC:8974] |
| ENSG00000078304 | HGLibA_38176 | 1 | -1.138650921 | PPP2R5C | protein phosphatase 2 regulatory subunit B'gamma [Source:HGNC Symbol;Acc:HGNC:9311] |
| ENSG00000078369 | HGLibA_19484 | 1 | -1.138650921 | GNB1 | G protein subunit beta 1 [Source:HGNC Symbol;Acc:HGNC:4396] |
| ENSG00000078487 | HGLibA_55057 | 1 | -1.138650921 | ZCWPW1 | zinc finger CW-type and PWWP domain containing 1 [Source:HGNC Symbol;Acc:HGNC:23486] |
| ENSG00000079263 | HGLibA_46292 | 1 | -1.138650921 | SP140 | SP140 nuclear body protein [Source:HGNC Symbol;Acc:HGNC:17133] |
| ENSG00000080293 | HGLibA_43068 | 1 | -1.138650921 | SCTR | secretin receptor [Source:HGNC Symbol;Acc:HGNC:10608] |
| ENSG00000080503 | HGLibA_45606 | 1 | -1.138650921 | SMARCA2 | member 2 [Source:HGNC Symbol;Acc:HGNC:11098]" |
| ENSG00000080546 | HGLibA_43620 | 1 | -1.138650921 | SESN1 | sestrin 1 [Source:HGNC Symbol;Acc:HGNC:21595] |
| ENSG00000080573 | HGLibA_10593 | 1 | -1.138650921 | COL5A3 | collagen type V alpha 3 chain [Source:HGNC Symbol;Acc:HGNC:14864] |
| ENSG00000080603 | HGLibA_46932 | 1 | -1.138650921 | SRCAP | Snf2 related CREBBP activator protein [Source:HGNC Symbol;Acc:HGNC:16974] |
| ENSG00000080603 | HGLibA_46931 | 1 | -1.138650921 | SRCAP | Snf2 related CREBBP activator protein [Source:HGNC Symbol;Acc:HGNC:16974] |
| ENSG00000080802 | HGLibA_10342 | 1 | -1.138650921 | CNOT4 | CCR4-NOT transcription complex subunit 4 [Source:HGNC Symbol;Acc:HGNC:7880] |
| ENSG00000081014 | HGLibA_02345 | 1 | -1.138650921 | AP4E1 | adaptor related protein complex 4 subunit epsilon 1 [Source:HGNC Symbol;Acc:HGNC:573] |
| ENSG00000081059 | HGLibA_48792 | 1 | -1.138650921 | TCF7 | transcription factor 7 [Source:HGNC Symbol;Acc:HGNC:11639] |
| ENSG00000081307 | HGLibA_52521 | 1 | -1.138650921 | UBA5 | ubiquitin like modifier activating enzyme 5 [Source:HGNC Symbol;Acc:HGNC:23230] |
| ENSG00000082293 | HGLibA_10526 | 1 | -1.138650921 | COL19A1 | collagen type XIX alpha 1 chain [Source:HGNC Symbol;Acc:HGNC:2196] |
| ENSG00000082556 | HGLibA_33445 | 1 | -1.138650921 | OPRK1 | opioid receptor kappa 1 [Source:HGNC Symbol;Acc:HGNC:8154] |
| ENSG00000084710 | HGLibA_14608 | 1 | -1.138650921 | EFR3B | EFR3 homolog B [Source:HGNC Symbol;Acc:HGNC:29155] |
| ENSG00000085224 | HGLibA_03824 | 1 | -1.138650921 | ATRX | chromatin remodeler [Source:HGNC Symbol;Acc:HGNC:886]" |
| ENSG00000085274 | HGLibA_30798 | 1 | -1.138650921 | MYNN | myoneurin [Source:HGNC Symbol;Acc:HGNC:14955] |
| ENSG00000087008 | HGLibA_00504 | 1 | -1.138650921 | ACOX3 | pristanoyl [Source:HGNC Symbol;Acc:HGNC:121]" |
| ENSG00000087253 | HGLibA_27075 | 1 | -1.138650921 | LPCAT2 | lysophosphatidylcholine acyltransferase 2 [Source:HGNC Symbol;Acc:HGNC:26032] |
| ENSG00000088035 | HGLibA_01633 | 1 | -1.138650921 | ALG6 | 3-glucosyltransferase [Source:HGNC Symbol;Acc:HGNC:23157]" |
| ENSG00000088766 | HGLibA_11197 | 1 | -1.138650921 | CRLS1 | cardiolipin synthase 1 [Source:HGNC Symbol;Acc:HGNC:16148] |
| ENSG00000089127 | HGLibA_33197 | 1 | -1.138650921 | OAS1 | 2'-5'-oligoadenylate synthetase 1 [Source:HGNC Symbol;Acc:HGNC:8086] |
| ENSG00000089225 | HGLibA_48674 | 1 | -1.138650921 | TBX5 | T-box 5 [Source:HGNC Symbol;Acc:HGNC:11604] |
| ENSG00000090316 | HGLibA_27861 | 1 | -1.138650921 | MAEA | macrophage erythroblast attacher [Source:HGNC Symbol;Acc:HGNC:13731] |
| ENSG00000090971 | HGLibA_31169 | 1 | -1.138650921 | NAT14 | N-acetyltransferase 14 (putative) [Source:HGNC Symbol;Acc:HGNC:28918] |
| ENSG00000091972 | HGLibA_08281 | 1 | -1.138650921 | CD200 | CD200 molecule [Source:HGNC Symbol;Acc:HGNC:7203] |
| ENSG00000092067 | HGLibA_08964 | 1 | -1.138650921 | CEBPE | CCAAT enhancer binding protein epsilon [Source:HGNC Symbol;Acc:HGNC:1836] |
| ENSG00000092140 | HGLibA_18297 | 1 | -1.138650921 | G2E3 | G2/M-phase specific E3 ubiquitin protein ligase [Source:HGNC Symbol;Acc:HGNC:20338] |
| ENSG00000093010 | HGLibA_10686 | 1 | -1.138650921 | COMT | catechol-O-methyltransferase [Source:HGNC Symbol;Acc:HGNC:2228] |
| ENSG00000095383 | HGLibA_48494 | 1 | -1.138650921 | TBC1D2 | TBC1 domain family member 2 [Source:HGNC Symbol;Acc:HGNC:18026] |
| ENSG00000096060 | HGLibA_17579 | 1 | -1.138650921 | FKBP5 | FK506 binding protein 5 [Source:HGNC Symbol;Acc:HGNC:3721] |
| ENSG00000096872 | HGLibA_22769 | 1 | -1.138650921 | IFT74 | intraflagellar transport 74 [Source:HGNC Symbol;Acc:HGNC:21424] |
| ENSG00000099968 | HGLibA_04314 | 1 | -1.138650921 | BCL2L13 | BCL2 like 13 [Source:HGNC Symbol;Acc:HGNC:17164] |
| ENSG00000100027 | HGLibA_54706 | 1 | -1.138650921 | YPEL1 | yippee like 1 [Source:HGNC Symbol;Acc:HGNC:12845] |
| ENSG00000100170 | HGLibA_45253 | 1 | -1.138650921 | SLC5A1 | solute carrier family 5 member 1 [Source:HGNC Symbol;Acc:HGNC:11036] |
| ENSG00000100241 | HGLibA_42795 | 1 | -1.138650921 | SBF1 | SET binding factor 1 [Source:HGNC Symbol;Acc:HGNC:10542] |
| ENSG00000100249 | HGLibA_06070 | 1 | -1.138650921 | C22orf31 | chromosome 22 open reading frame 31 [Source:HGNC Symbol;Acc:HGNC:26931] |
| ENSG00000100307 | HGLibA_07462 | 1 | -1.138650921 | CBX7 | chromobox 7 [Source:HGNC Symbol;Acc:HGNC:1557] |
| ENSG00000100311 | HGLibA_35843 | 1 | -1.138650921 | PDGFB | platelet derived growth factor subunit B [Source:HGNC Symbol;Acc:HGNC:8800] |
| ENSG00000100321 | HGLibA_47992 | 1 | -1.138650921 | SYNGR1 | synaptogyrin 1 [Source:HGNC Symbol;Acc:HGNC:11498] |
| ENSG00000100342 | HGLibA_02528 | 1 | -1.138650921 | APOL1 | apolipoprotein L1 [Source:HGNC Symbol;Acc:HGNC:618] |
| ENSG00000100364 | HGLibA_24670 | 1 | -1.138650921 | KIAA0930 | KIAA0930 [Source:HGNC Symbol;Acc:HGNC:1314] |
| ENSG00000100372 | HGLibA_44658 | 1 | -1.138650921 | SLC25A17 | solute carrier family 25 member 17 [Source:HGNC Symbol;Acc:HGNC:10987] |
| ENSG00000100612 | HGLibA_13146 | 1 | -1.138650921 | DHRS7 | dehydrogenase/reductase 7 [Source:HGNC Symbol;Acc:HGNC:21524] |
| ENSG00000100814 | HGLibA_08060 | 1 | -1.138650921 | CCNB1IP1 | cyclin B1 interacting protein 1 [Source:HGNC Symbol;Acc:HGNC:19437] |
| ENSG00000100889 | HGLibA_35615 | 1 | -1.138650921 | PCK2 | mitochondrial [Source:HGNC Symbol;Acc:HGNC:8725]" |
| ENSG00000100916 | HGLibA_04832 | 1 | -1.138650921 | BRMS1L | BRMS1 like transcriptional repressor [Source:HGNC Symbol;Acc:HGNC:20512] |
| ENSG00000100938 | HGLibA_19430 | 1 | -1.138650921 | GMPR2 | guanosine monophosphate reductase 2 [Source:HGNC Symbol;Acc:HGNC:4377] |
| ENSG00000100985 | HGLibA_29544 | 1 | -1.138650921 | MMP9 | matrix metallopeptidase 9 [Source:HGNC Symbol;Acc:HGNC:7176] |
| ENSG00000101052 | HGLibA_22763 | 1 | -1.138650921 | IFT52 | intraflagellar transport 52 [Source:HGNC Symbol;Acc:HGNC:15901] |
| ENSG00000101079 | HGLibA_31419 | 1 | -1.138650921 | NDRG3 | NDRG family member 3 [Source:HGNC Symbol;Acc:HGNC:14462] |
| ENSG00000101146 | HGLibA_40118 | 1 | -1.138650921 | RAE1 | ribonucleic acid export 1 [Source:HGNC Symbol;Acc:HGNC:9828] |
| ENSG00000101198 | HGLibA_31998 | 1 | -1.138650921 | NKAIN4 | sodium/potassium transporting ATPase interacting 4 [Source:HGNC Symbol;Acc:HGNC:16191] |
| ENSG00000101446 | HGLibA_46717 | 1 | -1.138650921 | SPINT3 | Kunitz type 3 [Source:HGNC Symbol;Acc:HGNC:11248]" |
| ENSG00000101452 | HGLibA_13187 | 1 | -1.138650921 | DHX35 | DEAH-box helicase 35 [Source:HGNC Symbol;Acc:HGNC:15861] |
| ENSG00000101825 | HGLibA_30623 | 1 | -1.138650921 | MXRA5 | matrix remodeling associated 5 [Source:HGNC Symbol;Acc:HGNC:7539] |
| ENSG00000101958 | HGLibA_19302 | 1 | -1.138650921 | GLRA2 | glycine receptor alpha 2 [Source:HGNC Symbol;Acc:HGNC:4327] |
| ENSG00000101997 | HGLibA_07729 | 1 | -1.138650921 | CCDC22 | coiled-coil domain containing 22 [Source:HGNC Symbol;Acc:HGNC:28909] |
| ENSG00000102096 | HGLibA_36679 | 1 | -1.138650921 | PIM2 | serine/threonine kinase [Source:HGNC Symbol;Acc:HGNC:8987]" |
| ENSG00000102359 | HGLibA_47035 | 1 | -1.138650921 | SRPX2 | sushi repeat containing protein X-linked 2 [Source:HGNC Symbol;Acc:HGNC:30668] |
| ENSG00000102409 | HGLibA_04445 | 1 | -1.138650921 | BEX4 | brain expressed X-linked 4 [Source:HGNC Symbol;Acc:HGNC:25475] |
| ENSG00000102891 | HGLibA_30307 | 1 | -1.138650921 | MT4 | metallothionein 4 [Source:HGNC Symbol;Acc:HGNC:18705] |
| ENSG00000102981 | HGLibA_35184 | 1 | -1.138650921 | PARD6A | par-6 family cell polarity regulator alpha [Source:HGNC Symbol;Acc:HGNC:15943] |
| ENSG00000103852 | HGLibA_52077 | 1 | -1.138650921 | TTC23 | tetratricopeptide repeat domain 23 [Source:HGNC Symbol;Acc:HGNC:25730] |
| ENSG00000103942 | HGLibA_21867 | 1 | -1.138650921 | HOMER2 | homer scaffold protein 2 [Source:HGNC Symbol;Acc:HGNC:17513] |
| ENSG00000103942 | HGLibA_21866 | 1 | -1.138650921 | HOMER2 | homer scaffold protein 2 [Source:HGNC Symbol;Acc:HGNC:17513] |
| ENSG00000104371 | HGLibA_13304 | 1 | -1.138650921 | DKK4 | dickkopf WNT signaling pathway inhibitor 4 [Source:HGNC Symbol;Acc:HGNC:2894] |
| ENSG00000104738 | HGLibA_28613 | 1 | -1.138650921 | MCM4 | minichromosome maintenance complex component 4 [Source:HGNC Symbol;Acc:HGNC:6947] |
| ENSG00000104826 | HGLibA_26421 | 1 | -1.138650921 | LHB | luteinizing hormone beta polypeptide [Source:HGNC Symbol;Acc:HGNC:6584] |
| ENSG00000104863 | HGLibA_26575 | 1 | -1.138650921 | LIN7B | crumbs cell polarity complex component [Source:HGNC Symbol;Acc:HGNC:17788]" |
| ENSG00000104918 | HGLibA_40876 | 1 | -1.138650921 | RETN | resistin [Source:HGNC Symbol;Acc:HGNC:20389] |
| ENSG00000105251 | HGLibA_44017 | 1 | -1.138650921 | SHD | Src homology 2 domain containing transforming protein D [Source:HGNC Symbol;Acc:HGNC:30633] |
| ENSG00000105538 | HGLibA_40356 | 1 | -1.138650921 | RASIP1 | Ras interacting protein 1 [Source:HGNC Symbol;Acc:HGNC:24716] |
| ENSG00000105549 | HGLibA_49334 | 1 | -1.138650921 | THEG | theg spermatid protein [Source:HGNC Symbol;Acc:HGNC:13706] |
| ENSG00000105609 | HGLibA_26518 | 1 | -1.138650921 | LILRB5 | leukocyte immunoglobulin like receptor B5 [Source:HGNC Symbol;Acc:HGNC:6609] |
| ENSG00000105619 | HGLibA_49191 | 1 | -1.138650921 | TFPT | TCF3 fusion partner [Source:HGNC Symbol;Acc:HGNC:13630] |
| ENSG00000105967 | HGLibA_49167 | 1 | -1.138650921 | TFEC | transcription factor EC [Source:HGNC Symbol;Acc:HGNC:11754] |
| ENSG00000106038 | HGLibA_15708 | 1 | -1.138650921 | EVX1 | even-skipped homeobox 1 [Source:HGNC Symbol;Acc:HGNC:3506] |
| ENSG00000106631 | HGLibA_30775 | 1 | -1.138650921 | MYL7 | myosin light chain 7 [Source:HGNC Symbol;Acc:HGNC:21719] |
| ENSG00000106723 | HGLibA_46666 | 1 | -1.138650921 | SPIN1 | spindlin 1 [Source:HGNC Symbol;Acc:HGNC:11243] |
| ENSG00000106952 | HGLibA_50825 | 1 | -1.138650921 | TNFSF8 | TNF superfamily member 8 [Source:HGNC Symbol;Acc:HGNC:11938] |
| ENSG00000107147 | HGLibA_24394 | 1 | -1.138650921 | KCNT1 | potassium sodium-activated channel subfamily T member 1 [Source:HGNC Symbol;Acc:HGNC:18865] |
| ENSG00000107164 | HGLibA_18152 | 1 | -1.138650921 | FUBP3 | far upstream element binding protein 3 [Source:HGNC Symbol;Acc:HGNC:4005] |
| ENSG00000107186 | HGLibA_29713 | 1 | -1.138650921 | MPDZ | multiple PDZ domain crumbs cell polarity complex component [Source:HGNC Symbol;Acc:HGNC:7208] |
| ENSG00000108106 | HGLibA_52657 | 1 | -1.138650921 | UBE2S | ubiquitin conjugating enzyme E2 S [Source:HGNC Symbol;Acc:HGNC:17895] |
| ENSG00000108175 | HGLibA_55381 | 1 | -1.138650921 | ZMIZ1 | zinc finger MIZ-type containing 1 [Source:HGNC Symbol;Acc:HGNC:16493] |
| ENSG00000108309 | HGLibA_42449 | 1 | -1.138650921 | RUNDC3A | RUN domain containing 3A [Source:HGNC Symbol;Acc:HGNC:16984] |
| ENSG00000108604 | HGLibA_45632 | 1 | -1.138650921 | SMARCD2 | member 2 [Source:HGNC Symbol;Acc:HGNC:11107]" |
| ENSG00000108830 | HGLibA_41448 | 1 | -1.138650921 | RND2 | Rho family GTPase 2 [Source:HGNC Symbol;Acc:HGNC:18315] |
| ENSG00000109083 | HGLibA_22752 | 1 | -1.138650921 | IFT20 | intraflagellar transport 20 [Source:HGNC Symbol;Acc:HGNC:30989] |
| ENSG00000109132 | HGLibA_36454 | 1 | -1.138650921 | PHOX2B | paired like homeobox 2B [Source:HGNC Symbol;Acc:HGNC:9143] |
| ENSG00000109171 | HGLibA_44309 | 1 | -1.138650921 | SLAIN2 | SLAIN motif family member 2 [Source:HGNC Symbol;Acc:HGNC:29282] |
| ENSG00000109205 | HGLibA_33268 | 1 | -1.138650921 | ODAM | ameloblast associated [Source:HGNC Symbol;Acc:HGNC:26043]" |
| ENSG00000109458 | HGLibA_18320 | 1 | -1.138650921 | GAB1 | GRB2 associated binding protein 1 [Source:HGNC Symbol;Acc:HGNC:4066] |
| ENSG00000109536 | HGLibA_17992 | 1 | -1.138650921 | FRG1 | FSHD region gene 1 [Source:HGNC Symbol;Acc:HGNC:3954] |
| ENSG00000109606 | HGLibA_13167 | 1 | -1.138650921 | DHX15 | DEAH-box helicase 15 [Source:HGNC Symbol;Acc:HGNC:2738] |
| ENSG00000109705 | HGLibA_32052 | 1 | -1.138650921 | NKX3-2 | NK3 homeobox 2 [Source:HGNC Symbol;Acc:HGNC:951] |
| ENSG00000110013 | HGLibA_44103 | 1 | -1.138650921 | SIAE | sialic acid acetylesterase [Source:HGNC Symbol;Acc:HGNC:18187] |
| ENSG00000110057 | HGLibA_53037 | 1 | -1.138650921 | UNC93B1 | TLR signaling regulator [Source:HGNC Symbol;Acc:HGNC:13481]" |
| ENSG00000110104 | HGLibA_07901 | 1 | -1.138650921 | CCDC86 | coiled-coil domain containing 86 [Source:HGNC Symbol;Acc:HGNC:28359] |
| ENSG00000110104 | HGLibA_07900 | 1 | -1.138650921 | CCDC86 | coiled-coil domain containing 86 [Source:HGNC Symbol;Acc:HGNC:28359] |
| ENSG00000110148 | HGLibA_07957 | 1 | -1.138650921 | CCKBR | cholecystokinin B receptor [Source:HGNC Symbol;Acc:HGNC:1571] |
| ENSG00000110169 | HGLibA_22066 | 1 | -1.138650921 | HPX | hemopexin [Source:HGNC Symbol;Acc:HGNC:5171] |
| ENSG00000110315 | HGLibA_41523 | 1 | -1.138650921 | RNF141 | ring finger protein 141 [Source:HGNC Symbol;Acc:HGNC:21159] |
| ENSG00000110344 | HGLibA_52689 | 1 | -1.138650921 | UBE4A | ubiquitination factor E4A [Source:HGNC Symbol;Acc:HGNC:12499] |
| ENSG00000110446 | HGLibA_44412 | 1 | -1.138650921 | SLC15A3 | solute carrier family 15 member 3 [Source:HGNC Symbol;Acc:HGNC:18068] |
| ENSG00000110786 | HGLibA_39471 | 1 | -1.138650921 | PTPN5 | non-receptor type 5 [Source:HGNC Symbol;Acc:HGNC:9657]" |
| ENSG00000110888 | HGLibA_07192 | 1 | -1.138650921 | CAPRIN2 | caprin family member 2 [Source:HGNC Symbol;Acc:HGNC:21259] |

| | | | | | |
|---|---|---|---|---|---|
| ENSG00000111049 | HGLibA_30698 | 1 | -1.138650921 | MYF5 | myogenic factor 5 [Source:HGNC Symbol;Acc:HGNC:7565] |
| ENSG00000111145 | HGLibA_14915 | 1 | -1.138650921 | ELK3 | ETS transcription factor [Source:HGNC Symbol;Acc:HGNC:3325]" |
| ENSG00000111231 | HGLibA_19831 | 1 | -1.138650921 | GPN3 | GPN-loop GTPase 3 [Source:HGNC Symbol;Acc:HGNC:30186] |
| ENSG00000111341 | HGLibA_29169 | 1 | -1.138650921 | MGP | matrix Gla protein [Source:HGNC Symbol;Acc:HGNC:7060] |
| ENSG00000111445 | HGLibA_40913 | 1 | -1.138650921 | RFC5 | replication factor C subunit 5 [Source:HGNC Symbol;Acc:HGNC:9973] |
| ENSG00000111490 | HGLibA_48533 | 1 | -1.138650921 | TBC1D30 | TBC1 domain family member 30 [Source:HGNC Symbol;Acc:HGNC:29164] |
| ENSG00000112167 | HGLibA_42788 | 1 | -1.138650921 | SAYSD1 | SAYSVFN motif domain containing 1 [Source:HGNC Symbol;Acc:HGNC:21025] |
| ENSG00000112282 | HGLibA_28781 | 1 | -1.138650921 | MED23 | mediator complex subunit 23 [Source:HGNC Symbol;Acc:HGNC:2372] |
| ENSG00000112293 | HGLibA_19817 | 1 | -1.138650921 | GPLD1 | glycosylphosphatidylinositol specific phospholipase D1 [Source:HGNC Symbol;Acc:HGNC:4459] |
| ENSG00000112406 | HGLibA_21089 | 1 | -1.138650921 | HECA | cell cycle regulator [Source:HGNC Symbol;Acc:HGNC:21041]" |
| ENSG00000112514 | HGLibA_11796 | 1 | -1.138650921 | CUTA | cutA divalent cation tolerance homolog [Source:HGNC Symbol;Acc:HGNC:21101] |
| ENSG00000112584 | HGLibA_16060 | 1 | -1.138650921 | FAM120B | family with sequence similarity 120B [Source:HGNC Symbol;Acc:HGNC:21109] |
| ENSG00000112659 | HGLibA_11793 | 1 | -1.138650921 | CUL9 | cullin 9 [Source:HGNC Symbol;Acc:HGNC:15982] |
| ENSG00000113231 | HGLibA_35833 | 1 | -1.138650921 | PDE8B | phosphodiesterase 8B [Source:HGNC Symbol;Acc:HGNC:8794] |
| ENSG00000113318 | HGLibA_30194 | 1 | -1.138650921 | MSH3 | mutS homolog 3 [Source:HGNC Symbol;Acc:HGNC:7326] |
| ENSG00000113593 | HGLibA_38243 | 1 | -1.138650921 | PPWD1 | peptidylprolyl isomerase domain and WD repeat containing 1 [Source:HGNC Symbol;Acc:HGNC:28954] |
| ENSG00000113593 | HGLibA_38242 | 1 | -1.138650921 | PPWD1 | peptidylprolyl isomerase domain and WD repeat containing 1 [Source:HGNC Symbol;Acc:HGNC:28954] |
| ENSG00000113621 | HGLibA_52411 | 1 | -1.138650921 | TXNDC15 | thioredoxin domain containing 15 [Source:HGNC Symbol;Acc:HGNC:20652] |
| ENSG00000113712 | HGLibA_11381 | 1 | -1.138650921 | CSNK1A1 | casein kinase 1 alpha 1 [Source:HGNC Symbol;Acc:HGNC:2451] |
| ENSG00000113761 | HGLibA_55849 | 1 | -1.138650921 | ZNF346 | zinc finger protein 346 [Source:HGNC Symbol;Acc:HGNC:16403] |
| ENSG00000114270 | HGLibA_10612 | 1 | -1.138650921 | COL7A1 | collagen type VII alpha 1 chain [Source:HGNC Symbol;Acc:HGNC:2214] |
| ENSG00000114735 | HGLibA_21133 | 1 | -1.138650921 | HEMK1 | HemK methyltransferase family member 1 [Source:HGNC Symbol;Acc:HGNC:24923] |
| ENSG00000115107 | HGLibA_47429 | 1 | -1.138650921 | STEAP3 | STEAP3 metalloreductase [Source:HGNC Symbol;Acc:HGNC:24592] |
| ENSG00000115170 | HGLibA_00687 | 1 | -1.138650921 | ACVR1 | activin A receptor type 1 [Source:HGNC Symbol;Acc:HGNC:171] |
| ENSG00000115170 | HGLibA_00686 | 1 | -1.138650921 | ACVR1 | activin A receptor type 1 [Source:HGNC Symbol;Acc:HGNC:171] |
| ENSG00000115318 | HGLibA_27043 | 1 | -1.138650921 | LOXL3 | lysyl oxidase like 3 [Source:HGNC Symbol;Acc:HGNC:13869] |
| ENSG00000115526 | HGLibA_09614 | 1 | -1.138650921 | CHST10 | carbohydrate sulfotransferase 10 [Source:HGNC Symbol;Acc:HGNC:19650] |
| ENSG00000115539 | HGLibA_35754 | 1 | -1.138650921 | PDCL3 | phosducin like 3 [Source:HGNC Symbol;Acc:HGNC:28860] |
| ENSG00000115641 | HGLibA_17483 | 1 | -1.138650921 | FHL2 | four and a half LIM domains 2 [Source:HGNC Symbol;Acc:HGNC:3703] |
| ENSG00000115970 | HGLibA_49284 | 1 | -1.138650921 | THADA | armadillo repeat containing [Source:HGNC Symbol;Acc:HGNC:19217]" |
| ENSG00000116161 | HGLibA_06959 | 1 | -1.138650921 | CACYBP | calcyclin binding protein [Source:HGNC Symbol;Acc:HGNC:30423] |
| ENSG00000116661 | HGLibA_17041 | 1 | -1.138650921 | FBXO2 | F-box protein 2 [Source:HGNC Symbol;Acc:HGNC:13581] |
| ENSG00000116819 | HGLibA_49133 | 1 | -1.138650921 | TFAP2E | transcription factor AP-2 epsilon [Source:HGNC Symbol;Acc:HGNC:30774] |
| ENSG00000116962 | HGLibA_31924 | 1 | -1.138650921 | NID1 | nidogen 1 [Source:HGNC Symbol;Acc:HGNC:7821] |
| ENSG00000117122 | HGLibA_29042 | 1 | -1.138650921 | MFAP2 | microfibril associated protein 2 [Source:HGNC Symbol;Acc:HGNC:7033] |
| ENSG00000117262 | HGLibA_20106 | 1 | -1.138650921 | GPR89A | G protein-coupled receptor 89A [Source:HGNC Symbol;Acc:HGNC:31984] |
| ENSG00000117407 | HGLibA_03169 | 1 | -1.138650921 | ARTN | artemin [Source:HGNC Symbol;Acc:HGNC:727] |
| ENSG00000117625 | HGLibA_40715 | 1 | -1.138650921 | RCOR3 | REST corepressor 3 [Source:HGNC Symbol;Acc:HGNC:25594] |
| ENSG00000118363 | HGLibA_46560 | 1 | -1.138650921 | SPCS2 | signal peptidase complex subunit 2 [Source:HGNC Symbol;Acc:HGNC:28962] |
| ENSG00000118496 | HGLibA_17066 | 1 | -1.138650921 | FBXO30 | F-box protein 30 [Source:HGNC Symbol;Acc:HGNC:15600] |
| ENSG00000118507 | HGLibA_01442 | 1 | -1.138650921 | AKAP7 | A-kinase anchoring protein 7 [Source:HGNC Symbol;Acc:HGNC:377] |
| ENSG00000118518 | HGLibA_41535 | 1 | -1.138650921 | RNF146 | ring finger protein 146 [Source:HGNC Symbol;Acc:HGNC:21336] |
| ENSG00000118922 | HGLibA_25049 | 1 | -1.138650921 | KLF12 | Kruppel like factor 12 [Source:HGNC Symbol;Acc:HGNC:6346] |
| ENSG00000119042 | HGLibA_42778 | 1 | -1.138650921 | SATB2 | SATB homeobox 2 [Source:HGNC Symbol;Acc:HGNC:21637] |
| ENSG00000119227 | HGLibA_36607 | 1 | -1.138650921 | PIGZ | phosphatidylinositol glycan anchor biosynthesis class Z [Source:HGNC Symbol;Acc:HGNC:30596] |
| ENSG00000119457 | HGLibA_45187 | 1 | -1.138650921 | SLC46A2 | solute carrier family 46 member 2 [Source:HGNC Symbol;Acc:HGNC:16055] |
| ENSG00000119729 | HGLibA_41200 | 1 | -1.138650921 | RHOQ | ras homolog family member Q [Source:HGNC Symbol;Acc:HGNC:17736] |
| ENSG00000119915 | HGLibA_14964 | 1 | -1.138650921 | ELOVL3 | ELOVL fatty acid elongase 3 [Source:HGNC Symbol;Acc:HGNC:18047] |
| ENSG00000119950 | HGLibA_30619 | 1 | -1.138650921 | MXI1 | dimerization protein [Source:HGNC Symbol;Acc:HGNC:7534]" |
| ENSG00000119965 | HGLibA_05099 | 1 | -1.138650921 | C10orf88 | chromosome 10 open reading frame 88 [Source:HGNC Symbol;Acc:HGNC:25822] |
| ENSG00000120054 | HGLibA_10947 | 1 | -1.138650921 | CPN1 | carboxypeptidase N subunit 1 [Source:HGNC Symbol;Acc:HGNC:2312] |
| ENSG00000120217 | HGLibA_08318 | 1 | -1.138650921 | CD274 | CD274 molecule [Source:HGNC Symbol;Acc:HGNC:17635] |
| ENSG00000120327 | HGLibA_35485 | 1 | -1.138650921 | PCDHB14 | protocadherin beta 14 [Source:HGNC Symbol;Acc:HGNC:8685] |
| ENSG00000120334 | HGLibA_09067 | 1 | -1.138650921 | CENPL | centromere protein L [Source:HGNC Symbol;Acc:HGNC:17879] |
| ENSG00000120685 | HGLibA_38695 | 1 | -1.138650921 | PROSER1 | proline and serine rich 1 [Source:HGNC Symbol;Acc:HGNC:20291] |
| ENSG00000120694 | HGLibA_22344 | 1 | -1.138650921 | HSPH1 | heat shock protein family H (Hsp110) member 1 [Source:HGNC Symbol;Acc:HGNC:16969] |
| ENSG00000121039 | HGLibA_40732 | 1 | -1.138650921 | RDH10 | retinol dehydrogenase 10 [Source:HGNC Symbol;Acc:HGNC:19975] |
| ENSG00000121057 | HGLibA_01403 | 1 | -1.138650921 | AKAP1 | A-kinase anchoring protein 1 [Source:HGNC Symbol;Acc:HGNC:367] |
| ENSG00000121068 | HGLibA_48655 | 1 | -1.138650921 | TBX2 | T-box 2 [Source:HGNC Symbol;Acc:HGNC:11597] |
| ENSG00000121289 | HGLibA_09176 | 1 | -1.138650921 | CEP89 | centrosomal protein 89 [Source:HGNC Symbol;Acc:HGNC:25907] |
| ENSG00000121314 | HGLibA_48429 | 1 | -1.138650921 | TAS2R8 | taste 2 receptor member 8 [Source:HGNC Symbol;Acc:HGNC:14915] |
| ENSG00000121653 | HGLibA_28286 | 1 | -1.138650921 | MAPK8IP1 | mitogen-activated protein kinase 8 interacting protein 1 [Source:HGNC Symbol;Acc:HGNC:6882] |
| ENSG00000121853 | HGLibA_19051 | 1 | -1.138650921 | GHSR | growth hormone secretagogue receptor [Source:HGNC Symbol;Acc:HGNC:4267] |
| ENSG00000122012 | HGLibA_47879 | 1 | -1.138650921 | SV2C | synaptic vesicle glycoprotein 2C [Source:HGNC Symbol;Acc:HGNC:30670] |
| ENSG00000122034 | HGLibA_20576 | 1 | -1.138650921 | GTF3A | general transcription factor IIIA [Source:HGNC Symbol;Acc:HGNC:4662] |
| ENSG00000122194 | HGLibA_37158 | 1 | -1.138650921 | PLG | plasminogen [Source:HGNC Symbol;Acc:HGNC:9071] |
| ENSG00000122512 | HGLibA_37336 | 1 | -1.138650921 | PMS2 | mismatch repair system component [Source:HGNC Symbol;Acc:HGNC:9122]" |
| ENSG00000122735 | HGLibA_13505 | 1 | -1.138650921 | DNAI1 | dynein axonemal intermediate chain 1 [Source:HGNC Symbol;Acc:HGNC:2954] |
| ENSG00000123080 | HGLibA_08843 | 1 | -1.138650921 | CDKN2C | cyclin dependent kinase inhibitor 2C [Source:HGNC Symbol;Acc:HGNC:1789] |
| ENSG00000123609 | HGLibA_32181 | 1 | -1.138650921 | NMI | N-myc and STAT interactor [Source:HGNC Symbol;Acc:HGNC:7854] |
| ENSG00000123610 | HGLibA_50715 | 1 | -1.138650921 | TNFAIP6 | TNF alpha induced protein 6 [Source:HGNC Symbol;Acc:HGNC:11898] |
| ENSG00000123684 | HGLibA_27084 | 1 | -1.138650921 | LPGAT1 | lysophosphatidylglycerol acyltransferase 1 [Source:HGNC Symbol;Acc:HGNC:28985] |
| ENSG00000123689 | HGLibA_18293 | 1 | -1.138650921 | G0S2 | G0/G1 switch 2 [Source:HGNC Symbol;Acc:HGNC:30229] |
| ENSG00000123739 | HGLibA_36870 | 1 | -1.138650921 | PLA2G12A | phospholipase A2 group XIIA [Source:HGNC Symbol;Acc:HGNC:18554] |
| ENSG00000123836 | HGLibA_36155 | 1 | -1.138650921 | PFKFB2 | 6-biphosphatase 2 [Source:HGNC Symbol;Acc:HGNC:8873]" |
| ENSG00000123975 | HGLibA_09790 | 1 | -1.138650921 | CKS2 | CDC28 protein kinase regulatory subunit 2 [Source:HGNC Symbol;Acc:HGNC:2000] |
| ENSG00000124120 | HGLibA_52224 | 1 | -1.138650921 | TTPAL | alpha tocopherol transfer protein like [Source:HGNC Symbol;Acc:HGNC:16114] |
| ENSG00000124214 | HGLibA_47404 | 1 | -1.138650921 | STAU1 | staufen double-stranded RNA binding protein 1 [Source:HGNC Symbol;Acc:HGNC:11370] |
| ENSG00000124253 | HGLibA_35613 | 1 | -1.138650921 | PCK1 | phosphoenolpyruvate carboxykinase 1 [Source:HGNC Symbol;Acc:HGNC:8724] |
| ENSG00000124370 | HGLibA_28574 | 1 | -1.138650921 | MCEE | methylmalonyl-CoA epimerase [Source:HGNC Symbol;Acc:HGNC:16732] |
| ENSG00000124795 | HGLibA_12926 | 1 | -1.138650921 | DEK | DEK proto-oncogene [Source:HGNC Symbol;Acc:HGNC:2768] |
| ENSG00000125510 | HGLibA_33449 | 1 | -1.138650921 | OPRL1 | opioid related nociceptin receptor 1 [Source:HGNC Symbol;Acc:HGNC:8155] |
| ENSG00000125726 | HGLibA_08439 | 1 | -1.138650921 | CD70 | CD70 molecule [Source:HGNC Symbol;Acc:HGNC:11937] |
| ENSG00000125734 | HGLibA_19845 | 1 | -1.138650921 | GPR108 | G protein-coupled receptor 108 [Source:HGNC Symbol;Acc:HGNC:17829] |
| ENSG00000125779 | HGLibA_35100 | 1 | -1.138650921 | PANK2 | pantothenate kinase 2 [Source:HGNC Symbol;Acc:HGNC:15894] |
| ENSG00000125844 | HGLibA_42243 | 1 | -1.138650921 | RRBP1 | ribosome binding protein 1 [Source:HGNC Symbol;Acc:HGNC:10448] |
| ENSG00000125872 | HGLibA_27480 | 1 | -1.138650921 | LRRN4 | leucine rich repeat neuronal 4 [Source:HGNC Symbol;Acc:HGNC:16208] |
| ENSG00000125965 | HGLibA_18868 | 1 | -1.138650921 | GDF5 | growth differentiation factor 5 [Source:HGNC Symbol;Acc:HGNC:4220] |
| ENSG00000126003 | HGLibA_36958 | 1 | -1.138650921 | PLAGL2 | PLAG1 like zinc finger 2 [Source:HGNC Symbol;Acc:HGNC:9047] |
| ENSG00000126458 | HGLibA_42236 | 1 | -1.138650921 | RRAS | RAS related [Source:HGNC Symbol;Acc:HGNC:10447] |
| ENSG00000126756 | HGLibA_53482 | 1 | -1.138650921 | UXT | ubiquitously expressed prefoldin like chaperone [Source:HGNC Symbol;Acc:HGNC:12641] |
| ENSG00000126860 | HGLibA_15687 | 1 | -1.138650921 | EVI2A | ecotropic viral integration site 2A [Source:HGNC Symbol;Acc:HGNC:3499] |
| ENSG00000126945 | HGLibA_21824 | 1 | -1.138650921 | HNRNPH2 | heterogeneous nuclear ribonucleoprotein H2 [Source:HGNC Symbol;Acc:HGNC:5042] |
| ENSG00000127325 | HGLibA_04424 | 1 | -1.138650921 | BEST3 | bestrophin 3 [Source:HGNC Symbol;Acc:HGNC:17105] |
| ENSG00000127445 | HGLibA_36685 | 1 | -1.138650921 | PIN1 | NIMA-interacting 1 [Source:HGNC Symbol;Acc:HGNC:8988]" |
| ENSG00000127452 | HGLibA_16972 | 1 | -1.138650921 | FBXL12 | F-box and leucine rich repeat protein 12 [Source:HGNC Symbol;Acc:HGNC:13611] |
| ENSG00000127472 | HGLibA_36918 | 1 | -1.138650921 | PLA2G5 | phospholipase A2 group V [Source:HGNC Symbol;Acc:HGNC:9038] |
| ENSG00000127863 | HGLibA_50768 | 1 | -1.138650921 | TNFRSF19 | TNF receptor superfamily member 19 [Source:HGNC Symbol;Acc:HGNC:11915] |
| ENSG00000127995 | HGLibA_07284 | 1 | -1.138650921 | CASD1 | CAS1 domain containing 1 [Source:HGNC Symbol;Acc:HGNC:16014] |
| ENSG00000128253 | HGLibA_40933 | 1 | -1.138650921 | RFPL2 | ret finger protein like 2 [Source:HGNC Symbol;Acc:HGNC:9979] |
| ENSG00000128346 | HGLibA_06056 | 1 | -1.138650921 | C22orf23 | chromosome 22 open reading frame 23 [Source:HGNC Symbol;Acc:HGNC:18589] |
| ENSG00000128917 | HGLibA_13367 | 1 | -1.138650921 | DLL4 | delta like canonical Notch ligand 4 [Source:HGNC Symbol;Acc:HGNC:2910] |
| ENSG00000128928 | HGLibA_23876 | 1 | -1.138650921 | IVD | isovaleryl-CoA dehydrogenase [Source:HGNC Symbol;Acc:HGNC:6186] |
| ENSG00000129007 | HGLibA_07035 | 1 | -1.138650921 | CALML4 | calmodulin like 4 [Source:HGNC Symbol;Acc:HGNC:18445] |
| ENSG00000129083 | HGLibA_10696 | 1 | -1.138650921 | COPB1 | coatomer protein complex subunit beta 1 [Source:HGNC Symbol;Acc:HGNC:2231] |
| ENSG00000129221 | HGLibA_01364 | 1 | -1.138650921 | AIPL1 | aryl hydrocarbon receptor interacting protein like 1 [Source:HGNC Symbol;Acc:HGNC:359] |
| ENSG00000129226 | HGLibA_08428 | 1 | -1.138650921 | CD68 | CD68 molecule [Source:HGNC Symbol;Acc:HGNC:1693] |
| ENSG00000129315 | HGLibA_08126 | 1 | -1.138650921 | CCNT1 | cyclin T1 [Source:HGNC Symbol;Acc:HGNC:1599] |
| ENSG00000129472 | HGLibA_39867 | 1 | -1.138650921 | RAB2B | member RAS oncogene family [Source:HGNC Symbol;Acc:HGNC:20246]" |
| ENSG00000129484 | HGLibA_35232 | 1 | -1.138650921 | PARP2 | poly(ADP-ribose) polymerase 2 [Source:HGNC Symbol;Acc:HGNC:272] |
| ENSG00000129596 | HGLibA_08853 | 1 | -1.138650921 | CDO1 | cysteine dioxygenase type 1 [Source:HGNC Symbol;Acc:HGNC:1795] |
| ENSG00000129933 | HGLibA_28441 | 1 | -1.138650921 | MAU2 | MAU2 sister chromatid cohesion factor [Source:HGNC Symbol;Acc:HGNC:29140] |
| ENSG00000130182 | HGLibA_56986 | 1 | -1.138650921 | ZSCAN10 | zinc finger and SCAN domain containing 10 [Source:HGNC Symbol;Acc:HGNC:12997] |
| ENSG00000130208 | HGLibA_02503 | 1 | -1.138650921 | APOC1 | apolipoprotein C1 [Source:HGNC Symbol;Acc:HGNC:607] |
| ENSG00000130429 | HGLibA_03077 | 1 | -1.138650921 | ARPC1B | actin related protein 2/3 complex subunit 1B [Source:HGNC Symbol;Acc:HGNC:704] |
| ENSG00000130511 | HGLibA_47122 | 1 | -1.138650921 | SSBP4 | single stranded DNA binding protein 4 [Source:HGNC Symbol;Acc:HGNC:15676] |
| ENSG00000130545 | HGLibA_11074 | 1 | -1.138650921 | CRB3 | cell polarity complex component [Source:HGNC Symbol;Acc:HGNC:20237]" |
| ENSG00000130595 | HGLibA_50888 | 1 | -1.138650921 | TNNT3 | fast skeletal type [Source:HGNC Symbol;Acc:HGNC:11950]" |
| ENSG00000130595 | HGLibA_50890 | 1 | -1.138650921 | TNNT3 | fast skeletal type [Source:HGNC Symbol;Acc:HGNC:11950]" |
| ENSG00000130822 | HGLibA_37343 | 1 | -1.138650921 | PNCK | pregnancy up-regulated nonubiquitous CaM kinase [Source:HGNC Symbol;Acc:HGNC:13415] |
| ENSG00000130827 | HGLibA_37263 | 1 | -1.138650921 | PLXNA3 | plexin A3 [Source:HGNC Symbol;Acc:HGNC:9101] |
| ENSG00000131037 | HGLibA_15377 | 1 | -1.138650921 | EPS8L1 | EPS8 like 1 [Source:HGNC Symbol;Acc:HGNC:21295] |
| ENSG00000131174 | HGLibA_10867 | 1 | -1.138650921 | COX7B | cytochrome c oxidase subunit 7B [Source:HGNC Symbol;Acc:HGNC:2291] |
| ENSG00000131188 | HGLibA_38851 | 1 | -1.138650921 | PRR7 | synaptic [Source:HGNC Symbol;Acc:HGNC:28130]" |
| ENSG00000131379 | HGLibA_06219 | 1 | -1.138650921 | C3orf20 | chromosome 3 open reading frame 20 [Source:HGNC Symbol;Acc:HGNC:25320] |
| ENSG00000131620 | HGLibA_02170 | 1 | -1.138650921 | ANO1 | anoctamin 1 [Source:HGNC Symbol;Acc:HGNC:21625] |
| ENSG00000131730 | HGLibA_09785 | 1 | -1.138650921 | CKMT2 | mitochondrial 2 [Source:HGNC Symbol;Acc:HGNC:1996]" |
| ENSG00000131773 | HGLibA_24572 | 1 | -1.138650921 | KHDRBS3 | signal transduction associated 3 [Source:HGNC Symbol;Acc:HGNC:18117]" |
| ENSG00000131831 | HGLibA_40145 | 1 | -1.138650921 | RAI2 | retinoic acid induced 2 [Source:HGNC Symbol;Acc:HGNC:9835] |
| ENSG00000132002 | HGLibA_13521 | 1 | -1.138650921 | DNAJB1 | DnaJ heat shock protein family (Hsp40) member B1 [Source:HGNC Symbol;Acc:HGNC:5270] |
| ENSG00000132329 | HGLibA_40181 | 1 | -1.138650921 | RAMP1 | receptor activity modifying protein 1 [Source:HGNC Symbol;Acc:HGNC:9843] |
| ENSG00000132478 | HGLibA_53047 | 1 | -1.138650921 | UNK | unkempt family zinc finger [Source:HGNC Symbol;Acc:HGNC:29369] |
| ENSG00000132481 | HGLibA_51517 | 1 | -1.138650921 | TRIM47 | tripartite motif containing 47 [Source:HGNC Symbol;Acc:HGNC:19020] |
| ENSG00000132570 | HGLibA_35364 | 1 | -1.138650921 | PCBD2 | pterin-4 alpha-carbinolamine dehydratase 2 [Source:HGNC Symbol;Acc:HGNC:24474] |
| ENSG00000132570 | HGLibA_35365 | 1 | -1.138650921 | PCBD2 | pterin-4 alpha-carbinolamine dehydratase 2 [Source:HGNC Symbol;Acc:HGNC:24474] |

| | | | | | |
|---|---|---|---|---|---|
| ENSG00000132716 | HGLibA_12456 | 1 | -1.138650921 | DCAF8 | DDB1 and CUL4 associated factor 8 [Source:HGNC Symbol;Acc:HGNC:24891] |
| ENSG00000132740 | HGLibA_22858 | 1 | -1.138650921 | IGHMBP2 | immunoglobulin mu DNA binding protein 2 [Source:HGNC Symbol;Acc:HGNC:5542] |
| ENSG00000132911 | HGLibA_32216 | 1 | -1.138650921 | NMUR2 | neuromedin U receptor 2 [Source:HGNC Symbol;Acc:HGNC:16454] |
| ENSG00000133477 | HGLibA_16756 | 1 | -1.138650921 | FAM83F | family with sequence similarity 83 member F [Source:HGNC Symbol;Acc:HGNC:25148] |
| ENSG00000133704 | HGLibA_23475 | 1 | -1.138650921 | IPO8 | importin 8 [Source:HGNC Symbol;Acc:HGNC:9853] |
| ENSG00000134058 | HGLibA_08793 | 1 | -1.138650921 | CDK7 | cyclin dependent kinase 7 [Source:HGNC Symbol;Acc:HGNC:1778] |
| ENSG00000134061 | HGLibA_08257 | 1 | -1.138650921 | CD180 | CD180 molecule [Source:HGNC Symbol;Acc:HGNC:6726] |
| ENSG00000134108 | HGLibA_02998 | 1 | -1.138650921 | ARL8B | ADP ribosylation factor like GTPase 8B [Source:HGNC Symbol;Acc:HGNC:25564] |
| ENSG00000134186 | HGLibA_38725 | 1 | -1.138650921 | PRPF38B | pre-mRNA processing factor 38B [Source:HGNC Symbol;Acc:HGNC:25512] |
| ENSG00000134291 | HGLibA_49858 | 1 | -1.138650921 | TMEM106C | transmembrane protein 106C [Source:HGNC Symbol;Acc:HGNC:28775] |
| ENSG00000134333 | HGLibA_26219 | 1 | -1.138650921 | LDHA | lactate dehydrogenase A [Source:HGNC Symbol;Acc:HGNC:6535] |
| ENSG00000134709 | HGLibA_21876 | 1 | -1.138650921 | HOOK1 | hook microtubule tethering protein 1 [Source:HGNC Symbol;Acc:HGNC:19884] |
| ENSG00000134874 | HGLibA_14299 | 1 | -1.138650921 | DZIP1 | DAZ interacting zinc finger protein 1 [Source:HGNC Symbol;Acc:HGNC:20908] |
| ENSG00000134962 | HGLibA_25025 | 1 | -1.138650921 | KLB | klotho beta [Source:HGNC Symbol;Acc:HGNC:15527] |
| ENSG00000135355 | HGLibA_19139 | 1 | -1.138650921 | GJA10 | gap junction protein alpha 10 [Source:HGNC Symbol;Acc:HGNC:16995] |
| ENSG00000135446 | HGLibA_08770 | 1 | -1.138650921 | CDK4 | cyclin dependent kinase 4 [Source:HGNC Symbol;Acc:HGNC:1773] |
| ENSG00000135517 | HGLibA_29292 | 1 | -1.138650921 | MIP | major intrinsic protein of lens fiber [Source:HGNC Symbol;Acc:HGNC:7103] |
| ENSG00000135605 | HGLibA_48939 | 1 | -1.138650921 | TEC | tec protein tyrosine kinase [Source:HGNC Symbol;Acc:HGNC:11719] |
| ENSG00000135625 | HGLibA_14658 | 1 | -1.138650921 | EGR4 | early growth response 4 [Source:HGNC Symbol;Acc:HGNC:3241] |
| ENSG00000135801 | HGLibA_48243 | 1 | -1.138650921 | TAF5L | TATA-box binding protein associated factor 5 like [Source:HGNC Symbol;Acc:HGNC:17304] |
| ENSG00000136235 | HGLibA_19835 | 1 | -1.138650921 | GPNMB | glycoprotein nmb [Source:HGNC Symbol;Acc:HGNC:4462] |
| ENSG00000136379 | HGLibA_00269 | 1 | -1.138650921 | ABHD17C | abhydrolase domain containing 17C [Source:HGNC Symbol;Acc:HGNC:26925] |
| ENSG00000136634 | HGLibA_22944 | 1 | -1.138650921 | IL10 | interleukin 10 [Source:HGNC Symbol;Acc:HGNC:5962] |
| ENSG00000136870 | HGLibA_55553 | 1 | -1.138650921 | ZNF189 | zinc finger protein 189 [Source:HGNC Symbol;Acc:HGNC:12980] |
| ENSG00000136944 | HGLibA_26734 | 1 | -1.138650921 | LMX1B | LIM homeobox transcription factor 1 beta [Source:HGNC Symbol;Acc:HGNC:6654] |
| ENSG00000137040 | HGLibA_40208 | 1 | -1.138650921 | RANBP6 | RAN binding protein 6 [Source:HGNC Symbol;Acc:HGNC:9851] |
| ENSG00000137103 | HGLibA_50549 | 1 | -1.138650921 | TMEM8B | transmembrane protein 8B [Source:HGNC Symbol;Acc:HGNC:21427] |
| ENSG00000137203 | HGLibA_49121 | 1 | -1.138650921 | TFAP2A | transcription factor AP-2 alpha [Source:HGNC Symbol;Acc:HGNC:11742] |
| ENSG00000137216 | HGLibA_50476 | 1 | -1.138650921 | TMEM63B | transmembrane protein 63B [Source:HGNC Symbol;Acc:HGNC:17735] |
| ENSG00000137571 | HGLibA_45475 | 1 | -1.138650921 | SLCO5A1 | solute carrier organic anion transporter family member 5A1 [Source:HGNC Symbol;Acc:HGNC:19046] |
| ENSG00000137574 | HGLibA_49278 | 1 | -1.138650921 | TGS1 | trimethylguanosine synthase 1 [Source:HGNC Symbol;Acc:HGNC:17843] |
| ENSG00000137675 | HGLibA_29527 | 1 | -1.138650921 | MMP27 | matrix metallopeptidase 27 [Source:HGNC Symbol;Acc:HGNC:14250] |
| ENSG00000137809 | HGLibA_23722 | 1 | -1.138650921 | ITGA11 | integrin subunit alpha 11 [Source:HGNC Symbol;Acc:HGNC:6136] |
| ENSG00000137843 | HGLibA_35052 | 1 | -1.138650921 | PAK6 | p21 (RAC1) activated kinase 6 [Source:HGNC Symbol;Acc:HGNC:16061] |
| ENSG00000137878 | HGLibA_18823 | 1 | -1.138650921 | GCOM1 | GRINL1A complex locus 1 [Source:HGNC Symbol;Acc:HGNC:26424] |
| ENSG00000137948 | HGLibA_04790 | 1 | -1.138650921 | BRDT | bromodomain testis associated [Source:HGNC Symbol;Acc:HGNC:1105] |
| ENSG00000138107 | HGLibA_00650 | 1 | -1.138650921 | ACTR1A | ARP1 actin related protein 1 homolog A [Source:HGNC Symbol;Acc:HGNC:167] |
| ENSG00000138115 | HGLibA_12120 | 1 | -1.138650921 | CYP2C8 | cytochrome P450 family 2 subfamily C member 8 [Source:HGNC Symbol;Acc:HGNC:2622] |
| ENSG00000138398 | HGLibA_37932 | 1 | -1.138650921 | PPIG | peptidylprolyl isomerase G [Source:HGNC Symbol;Acc:HGNC:14650] |
| ENSG00000138629 | HGLibA_52715 | 1 | -1.138650921 | UBL7 | ubiquitin like 7 [Source:HGNC Symbol;Acc:HGNC:28221] |
| ENSG00000138646 | HGLibA_21166 | 1 | -1.138650921 | HERC5 | HECT and RLD domain containing E3 ubiquitin protein ligase 5 [Source:HGNC Symbol;Acc:HGNC:24368] |
| ENSG00000138738 | HGLibA_38393 | 1 | -1.138650921 | PRDM5 | PR/SET domain 5 [Source:HGNC Symbol;Acc:HGNC:9349] |
| ENSG00000138755 | HGLibA_11877 | 1 | -1.138650921 | CXCL9 | C-X-C motif chemokine ligand 9 [Source:HGNC Symbol;Acc:HGNC:7098] |
| ENSG00000138771 | HGLibA_44094 | 1 | -1.138650921 | SHROOM3 | shroom family member 3 [Source:HGNC Symbol;Acc:HGNC:30422] |
| ENSG00000139410 | HGLibA_43176 | 1 | -1.138650921 | SDSL | serine dehydratase like [Source:HGNC Symbol;Acc:HGNC:30404] |
| ENSG00000139722 | HGLibA_53780 | 1 | -1.138650921 | VPS37B | ESCRT-I subunit [Source:HGNC Symbol;Acc:HGNC:25754]" |
| ENSG00000139890 | HGLibA_40834 | 1 | -1.138650921 | REM2 | RRAD and GEM like GTPase 2 [Source:HGNC Symbol;Acc:HGNC:20248] |
| ENSG00000139910 | HGLibA_32363 | 1 | -1.138650921 | NOVA1 | NOVA alternative splicing regulator 1 [Source:HGNC Symbol;Acc:HGNC:7886] |
| ENSG00000140044 | HGLibA_23944 | 1 | -1.138650921 | JDP2 | Jun dimerization protein 2 [Source:HGNC Symbol;Acc:HGNC:17546] |
| ENSG00000140107 | HGLibA_44757 | 1 | -1.138650921 | SLC25A47 | solute carrier family 25 member 47 [Source:HGNC Symbol;Acc:HGNC:20115] |
| ENSG00000140538 | HGLibA_32881 | 1 | -1.138650921 | NTRK3 | neurotrophic receptor tyrosine kinase 3 [Source:HGNC Symbol;Acc:HGNC:8033] |
| ENSG00000140563 | HGLibA_28656 | 1 | -1.138650921 | MCTP2 | multiple C2 and transmembrane domain containing 2 [Source:HGNC Symbol;Acc:HGNC:25636] |
| ENSG00000140945 | HGLibA_08640 | 1 | -1.138650921 | CDH13 | cadherin 13 [Source:HGNC Symbol;Acc:HGNC:1753] |
| ENSG00000141084 | HGLibA_40195 | 1 | -1.138650921 | RANBP10 | RAN binding protein 10 [Source:HGNC Symbol;Acc:HGNC:29285] |
| ENSG00000141316 | HGLibA_46327 | 1 | -1.138650921 | SPACA3 | sperm acrosome associated 3 [Source:HGNC Symbol;Acc:HGNC:16260] |
| ENSG00000141428 | HGLibA_05607 | 1 | -1.138650921 | C18orf21 | chromosome 18 open reading frame 21 [Source:HGNC Symbol;Acc:HGNC:28802] |
| ENSG00000141433 | HGLibA_00936 | 1 | -1.138650921 | ADCYAP1 | adenylate cyclase activating polypeptide 1 [Source:HGNC Symbol;Acc:HGNC:241] |
| ENSG00000141434 | HGLibA_28898 | 1 | -1.138650921 | MEP1B | meprin A subunit beta [Source:HGNC Symbol;Acc:HGNC:7020] |
| ENSG00000141504 | HGLibA_42772 | 1 | -1.138650921 | SAT2 | spermidine/spermine N1-acetyltransferase family member 2 [Source:HGNC Symbol;Acc:HGNC:23160] |
| ENSG00000141506 | HGLibA_36661 | 1 | -1.138650921 | PIK3R5 | phosphoinositide-3-kinase regulatory subunit 5 [Source:HGNC Symbol;Acc:HGNC:30035] |
| ENSG00000141560 | HGLibA_17731 | 1 | -1.138650921 | FN3KRP | fructosamine 3 kinase related protein [Source:HGNC Symbol;Acc:HGNC:25700] |
| ENSG00000141564 | HGLibA_42204 | 1 | -1.138650921 | RPTOR | regulatory associated protein of MTOR complex 1 [Source:HGNC Symbol;Acc:HGNC:30287] |
| ENSG00000141867 | HGLibA_04779 | 1 | -1.138650921 | BRD4 | bromodomain containing 4 [Source:HGNC Symbol;Acc:HGNC:13575] |
| ENSG00000141873 | HGLibA_45110 | 1 | -1.138650921 | SLC39A3 | solute carrier family 39 member 3 [Source:HGNC Symbol;Acc:HGNC:17128] |
| ENSG00000141971 | HGLibA_30588 | 1 | -1.138650921 | MVB12A | multivesicular body subunit 12A [Source:HGNC Symbol;Acc:HGNC:25153] |
| ENSG00000142082 | HGLibA_44222 | 1 | -1.138650921 | SIRT3 | sirtuin 3 [Source:HGNC Symbol;Acc:HGNC:14931] |
| ENSG00000142166 | HGLibA_22695 | 1 | -1.138650921 | IFNAR1 | interferon alpha and beta receptor subunit 1 [Source:HGNC Symbol;Acc:HGNC:5432] |
| ENSG00000142449 | HGLibA_16961 | 1 | -1.138650921 | FBN3 | fibrillin 3 [Source:HGNC Symbol;Acc:HGNC:18794] |
| ENSG00000142556 | HGLibA_56389 | 1 | -1.138650921 | ZNF614 | zinc finger protein 614 [Source:HGNC Symbol;Acc:HGNC:24722] |
| ENSG00000142687 | HGLibA_24623 | 1 | -1.138650921 | KIAA0319L | KIAA0319 like [Source:HGNC Symbol;Acc:HGNC:30071] |
| ENSG00000142765 | HGLibA_48096 | 1 | -1.138650921 | SYTL1 | synaptotagmin like 1 [Source:HGNC Symbol;Acc:HGNC:15584] |
| ENSG00000142789 | HGLibA_08998 | 1 | -1.138650921 | CELA3A | chymotrypsin like elastase family member 3A [Source:HGNC Symbol;Acc:HGNC:15944] |
| ENSG00000142892 | HGLibA_36560 | 1 | -1.138650921 | PIGK | phosphatidylinositol glycan anchor biosynthesis class K [Source:HGNC Symbol;Acc:HGNC:8965] |
| ENSG00000143107 | HGLibA_17757 | 1 | -1.138650921 | FNDC7 | fibronectin type III domain containing 7 [Source:HGNC Symbol;Acc:HGNC:26668] |
| ENSG00000143147 | HGLibA_19957 | 1 | -1.138650921 | GPR161 | G protein-coupled receptor 161 [Source:HGNC Symbol;Acc:HGNC:23694] |
| ENSG00000143157 | HGLibA_37473 | 1 | -1.138650921 | POGK | pogo transposable element derived with KRAB domain [Source:HGNC Symbol;Acc:HGNC:18800] |
| ENSG00000143321 | HGLibA_21038 | 1 | -1.138650921 | HDGF | heparin binding growth factor [Source:HGNC Symbol;Acc:HGNC:4856] |
| ENSG00000143373 | HGLibA_56539 | 1 | -1.138650921 | ZNF687 | zinc finger protein 687 [Source:HGNC Symbol;Acc:HGNC:29277] |
| ENSG00000143536 | HGLibA_11206 | 1 | -1.138650921 | CRNN | cornulin [Source:HGNC Symbol;Acc:HGNC:1230] |
| ENSG00000143653 | HGLibA_42881 | 1 | -1.138650921 | SCCPDH | saccharopine dehydrogenase (putative) [Source:HGNC Symbol;Acc:HGNC:24275] |
| ENSG00000143768 | HGLibA_26277 | 1 | -1.138650921 | LEFTY2 | left-right determination factor 2 [Source:HGNC Symbol;Acc:HGNC:3122] |
| ENSG00000143858 | HGLibA_48072 | 1 | -1.138650921 | SYT2 | synaptotagmin 2 [Source:HGNC Symbol;Acc:HGNC:11510] |
| ENSG00000143882 | HGLibA_03742 | 1 | -1.138650921 | ATP6V1C2 | ATPase H+ transporting V1 subunit C2 [Source:HGNC Symbol;Acc:HGNC:18264] |
| ENSG00000144120 | HGLibA_50078 | 1 | -1.138650921 | TMEM177 | transmembrane protein 177 [Source:HGNC Symbol;Acc:HGNC:28143] |
| ENSG00000144488 | HGLibA_15579 | 1 | -1.138650921 | ESPNL | espin like [Source:HGNC Symbol;Acc:HGNC:27937] |
| ENSG00000144674 | HGLibA_19601 | 1 | -1.138650921 | GOLGA4 | golgin A4 [Source:HGNC Symbol;Acc:HGNC:4427] |
| ENSG00000144711 | HGLibA_23541 | 1 | -1.138650921 | IQSEC1 | IQ motif and Sec7 domain 1 [Source:HGNC Symbol;Acc:HGNC:29112] |
| ENSG00000144741 | HGLibA_44687 | 1 | -1.138650921 | SLC25A26 | solute carrier family 25 member 26 [Source:HGNC Symbol;Acc:HGNC:20661] |
| ENSG00000144840 | HGLibA_40032 | 1 | -1.138650921 | RABL3 | member of RAS oncogene family like 3 [Source:HGNC Symbol;Acc:HGNC:18072]" |
| ENSG00000145348 | HGLibA_48600 | 1 | -1.138650921 | TBCK | TBC1 domain containing kinase [Source:HGNC Symbol;Acc:HGNC:28261] |
| ENSG00000145390 | HGLibA_53399 | 1 | -1.138650921 | USP53 | ubiquitin specific peptidase 53 [Source:HGNC Symbol;Acc:HGNC:29255] |
| ENSG00000145777 | HGLibA_51878 | 1 | -1.138650921 | TSLP | thymic stromal lymphopoietin [Source:HGNC Symbol;Acc:HGNC:30743] |
| ENSG00000145888 | HGLibA_19297 | 1 | -1.138650921 | GLRA1 | glycine receptor alpha 1 [Source:HGNC Symbol;Acc:HGNC:4326] |
| ENSG00000145996 | HGLibA_08803 | 1 | -1.138650921 | CDKAL1 | CDK5 regulatory subunit associated protein 1 like 1 [Source:HGNC Symbol;Acc:HGNC:21050] |
| ENSG00000146151 | HGLibA_21706 | 1 | -1.138650921 | HMGCLL1 | 3-hydroxymethyl-3-methylglutaryl-CoA lyase like 1 [Source:HGNC Symbol;Acc:HGNC:21359] |
| ENSG00000146276 | HGLibA_18403 | 1 | -1.138650921 | GABRR1 | gamma-aminobutyric acid type A receptor rho1 subunit [Source:HGNC Symbol;Acc:HGNC:4090] |
| ENSG00000146399 | HGLibA_48122 | 1 | -1.138650921 | TAAR1 | trace amine associated receptor 1 [Source:HGNC Symbol;Acc:HGNC:17734] |
| ENSG00000146678 | HGLibA_22814 | 1 | -1.138650921 | IGFBP1 | insulin like growth factor binding protein 1 [Source:HGNC Symbol;Acc:HGNC:5469] |
| ENSG00000146731 | HGLibA_08206 | 1 | -1.138650921 | CCT6A | chaperonin containing TCP1 subunit 6A [Source:HGNC Symbol;Acc:HGNC:1620] |
| ENSG00000146966 | HGLibA_12937 | 1 | -1.138650921 | DENND2A | DENN domain containing 2A [Source:HGNC Symbol;Acc:HGNC:22212] |
| ENSG00000147133 | HGLibA_48194 | 1 | -1.138650921 | TAF1 | TATA-box binding protein associated factor 1 [Source:HGNC Symbol;Acc:HGNC:11535] |
| ENSG00000147183 | HGLibA_11033 | 1 | -1.138650921 | CPXCR1 | candidate 1 [Source:HGNC Symbol;Acc:HGNC:2332]" |
| ENSG00000147246 | HGLibA_22377 | 1 | -1.138650921 | HTR2C | 5-hydroxytryptamine receptor 2C [Source:HGNC Symbol;Acc:HGNC:5295] |
| ENSG00000147485 | HGLibA_39653 | 1 | -1.138650921 | PXDNL | peroxidasin like [Source:HGNC Symbol;Acc:HGNC:26359] |
| ENSG00000147606 | HGLibA_44804 | 1 | -1.138650921 | SLC26A7 | solute carrier family 26 member 7 [Source:HGNC Symbol;Acc:HGNC:14467] |
| ENSG00000147655 | HGLibA_42346 | 1 | -1.138650921 | RSPO2 | R-spondin 2 [Source:HGNC Symbol;Acc:HGNC:28583] |
| ENSG00000147676 | HGLibA_28024 | 1 | -1.138650921 | MAL2 | T cell differentiation protein 2 (gene/pseudogene) [Source:HGNC Symbol;Acc:HGNC:13634]" |
| ENSG00000147677 | HGLibA_14786 | 1 | -1.138650921 | EIF3H | eukaryotic translation initiation factor 3 subunit H [Source:HGNC Symbol;Acc:HGNC:3273] |
| ENSG00000148331 | HGLibA_03244 | 1 | -1.138650921 | ASB6 | ankyrin repeat and SOCS box containing 6 [Source:HGNC Symbol;Acc:HGNC:17181] |
| ENSG00000148358 | HGLibA_19842 | 1 | -1.138650921 | GPR107 | G protein-coupled receptor 107 [Source:HGNC Symbol;Acc:HGNC:17830] |
| ENSG00000148672 | HGLibA_19360 | 1 | -1.138650921 | GLUD1 | glutamate dehydrogenase 1 [Source:HGNC Symbol;Acc:HGNC:4335] |
| ENSG00000148702 | HGLibA_20792 | 1 | -1.138650921 | HABP2 | hyaluronan binding protein 2 [Source:HGNC Symbol;Acc:HGNC:4798] |
| ENSG00000148834 | HGLibA_20488 | 1 | -1.138650921 | GSTO1 | glutathione S-transferase omega 1 [Source:HGNC Symbol;Acc:HGNC:13312] |
| ENSG00000148908 | HGLibA_41053 | 1 | -1.138650921 | RGS10 | regulator of G protein signaling 10 [Source:HGNC Symbol;Acc:HGNC:9992] |
| ENSG00000149089 | HGLibA_02423 | 1 | -1.138650921 | APIP | APAF1 interacting protein [Source:HGNC Symbol;Acc:HGNC:17581] |
| ENSG00000149294 | HGLibA_31253 | 1 | -1.138650921 | NCAM1 | neural cell adhesion molecule 1 [Source:HGNC Symbol;Acc:HGNC:7656] |
| ENSG00000149418 | HGLibA_47226 | 1 | -1.138650921 | ST14 | suppression of tumorigenicity 14 [Source:HGNC Symbol;Acc:HGNC:11344] |
| ENSG00000149418 | HGLibA_47225 | 1 | -1.138650921 | ST14 | suppression of tumorigenicity 14 [Source:HGNC Symbol;Acc:HGNC:11344] |
| ENSG00000149485 | HGLibA_15935 | 1 | -1.138650921 | FADS1 | fatty acid desaturase 1 [Source:HGNC Symbol;Acc:HGNC:3574] |
| ENSG00000149499 | HGLibA_15062 | 1 | -1.138650921 | EML3 | echinoderm microtubule associated protein like 3 [Source:HGNC Symbol;Acc:HGNC:26666] |
| ENSG00000149527 | HGLibA_37010 | 1 | -1.138650921 | PLCH2 | phospholipase C eta 2 [Source:HGNC Symbol;Acc:HGNC:29037] |
| ENSG00000149798 | HGLibA_08568 | 1 | -1.138650921 | CDC42EP2 | CDC42 effector protein 2 [Source:HGNC Symbol;Acc:HGNC:16263] |
| ENSG00000149798 | HGLibA_08569 | 1 | -1.138650921 | CDC42EP2 | CDC42 effector protein 2 [Source:HGNC Symbol;Acc:HGNC:16263] |
| ENSG00000149972 | HGLibA_10414 | 1 | -1.138650921 | CNTN5 | contactin 5 [Source:HGNC Symbol;Acc:HGNC:2175] |
| ENSG00000150361 | HGLibA_25119 | 1 | -1.138650921 | KLHL1 | kelch like family member 1 [Source:HGNC Symbol;Acc:HGNC:6352] |
| ENSG00000150625 | HGLibA_19819 | 1 | -1.138650921 | GPM6A | glycoprotein M6A [Source:HGNC Symbol;Acc:HGNC:4460] |
| ENSG00000151322 | HGLibA_32400 | 1 | -1.138650921 | NPAS3 | neuronal PAS domain protein 3 [Source:HGNC Symbol;Acc:HGNC:19311] |
| ENSG00000151327 | HGLibA_16293 | 1 | -1.138650921 | FAM177A1 | family with sequence similarity 177 member A1 [Source:HGNC Symbol;Acc:HGNC:19829] |
| ENSG00000151650 | HGLibA_53619 | 1 | -1.138650921 | VENTX | VENT homeobox [Source:HGNC Symbol;Acc:HGNC:13639] |
| ENSG00000151773 | HGLibA_07572 | 1 | -1.138650921 | CCDC122 | coiled-coil domain containing 122 [Source:HGNC Symbol;Acc:HGNC:26478] |
| ENSG00000152402 | HGLibA_20651 | 1 | -1.138650921 | GUCY1A2 | guanylate cyclase 1 soluble subunit alpha 2 [Source:HGNC Symbol;Acc:HGNC:4684] |
| ENSG00000152422 | HGLibA_54591 | 1 | -1.138650921 | XRCC4 | X-ray repair cross complementing 4 [Source:HGNC Symbol;Acc:HGNC:12831] |
| ENSG00000152492 | HGLibA_07792 | 1 | -1.138650921 | CCDC50 | coiled-coil domain containing 50 [Source:HGNC Symbol;Acc:HGNC:18111] |
| ENSG00000152779 | HGLibA_44425 | 1 | -1.138650921 | SLC16A12 | solute carrier family 16 member 12 [Source:HGNC Symbol;Acc:HGNC:23094] |
| ENSG00000152953 | HGLibA_47487 | 1 | -1.138650921 | STK32B | serine/threonine kinase 32B [Source:HGNC Symbol;Acc:HGNC:14217] |

| | | | | | |
|---|---|---|---|---|---|
| ENSG00000153071 | HGLibA_12269 | 1 | -1.138650921 | DAB2 | clathrin adaptor protein [Source:HGNC Symbol;Acc:HGNC:2662]" |
| ENSG00000153714 | HGLibA_27636 | 1 | -1.138650921 | LURAP1L | leucine rich adaptor protein 1 like [Source:HGNC Symbol;Acc:HGNC:31452] |
| ENSG00000154025 | HGLibA_45255 | 1 | -1.138650921 | SLC5A10 | solute carrier family 5 member 10 [Source:HGNC Symbol;Acc:HGNC:23155] |
| ENSG00000154127 | HGLibA_52560 | 1 | -1.138650921 | UBASH3B | ubiquitin associated and SH3 domain containing B [Source:HGNC Symbol;Acc:HGNC:29884] |
| ENSG00000154127 | HGLibA_52562 | 1 | -1.138650921 | UBASH3B | ubiquitin associated and SH3 domain containing B [Source:HGNC Symbol;Acc:HGNC:29884] |
| ENSG00000154175 | HGLibA_00299 | 1 | -1.138650921 | ABI3BP | ABI family member 3 binding protein [Source:HGNC Symbol;Acc:HGNC:17265] |
| ENSG00000154274 | HGLibA_06300 | 1 | -1.138650921 | C4orf19 | chromosome 4 open reading frame 19 [Source:HGNC Symbol;Acc:HGNC:25618] |
| ENSG00000154342 | HGLibA_54367 | 1 | -1.138650921 | WNT3A | Wnt family member 3A [Source:HGNC Symbol;Acc:HGNC:15983] |
| ENSG00000154342 | HGLibA_54368 | 1 | -1.138650921 | WNT3A | Wnt family member 3A [Source:HGNC Symbol;Acc:HGNC:15983] |
| ENSG00000154429 | HGLibA_08185 | 1 | -1.138650921 | CCSAP | cilia and spindle associated protein [Source:HGNC Symbol;Acc:HGNC:29578]" |
| ENSG00000154529 | HGLibA_10427 | 1 | -1.138650921 | CNTNAP3B | contactin associated protein like 3B [Source:HGNC Symbol;Acc:HGNC:32035] |
| ENSG00000154832 | HGLibA_11965 | 1 | -1.138650921 | CXXC1 | CXXC finger protein 1 [Source:HGNC Symbol;Acc:HGNC:24343] |
| ENSG00000154914 | HGLibA_53367 | 1 | -1.138650921 | USP43 | ubiquitin specific peptidase 43 [Source:HGNC Symbol;Acc:HGNC:20072] |
| ENSG00000155115 | HGLibA_20596 | 1 | -1.138650921 | GTF3C6 | general transcription factor IIIC subunit 6 [Source:HGNC Symbol;Acc:HGNC:20872] |
| ENSG00000155189 | HGLibA_01229 | 1 | -1.138650921 | AGPAT5 | 1-acylglycerol-3-phosphate O-acyltransferase 5 [Source:HGNC Symbol;Acc:HGNC:20886] |
| ENSG00000155269 | HGLibA_20087 | 1 | -1.138650921 | GPR78 | G protein-coupled receptor 78 [Source:HGNC Symbol;Acc:HGNC:4528] |
| ENSG00000155846 | HGLibA_37850 | 1 | -1.138650921 | PPARGC1B | PPARG coactivator 1 beta [Source:HGNC Symbol;Acc:HGNC:30022] |
| ENSG00000155868 | HGLibA_28813 | 1 | -1.138650921 | MED7 | mediator complex subunit 7 [Source:HGNC Symbol;Acc:HGNC:2378] |
| ENSG00000155903 | HGLibA_40298 | 1 | -1.138650921 | RASA2 | RAS p21 protein activator 2 [Source:HGNC Symbol;Acc:HGNC:9872] |
| ENSG00000156049 | HGLibA_19446 | 1 | -1.138650921 | GNA14 | G protein subunit alpha 14 [Source:HGNC Symbol;Acc:HGNC:4382] |
| ENSG00000156486 | HGLibA_24388 | 1 | -1.138650921 | KCNS2 | potassium voltage-gated channel modifier subfamily S member 2 [Source:HGNC Symbol;Acc:HGNC:6301] |
| ENSG00000157601 | HGLibA_30605 | 1 | -1.138650921 | MX1 | MX dynamin like GTPase 1 [Source:HGNC Symbol;Acc:HGNC:7532] |
| ENSG00000157625 | HGLibA_48146 | 1 | -1.138650921 | TAB3 | TGF-beta activated kinase 1 (MAP3K7) binding protein 3 [Source:HGNC Symbol;Acc:HGNC:30681] |
| ENSG00000157765 | HGLibA_44942 | 1 | -1.138650921 | SLC34A2 | solute carrier family 34 member 2 [Source:HGNC Symbol;Acc:HGNC:11020] |
| ENSG00000158106 | HGLibA_41222 | 1 | -1.138650921 | RHPN1 | rhophilin Rho GTPase binding protein 1 [Source:HGNC Symbol;Acc:HGNC:19973] |
| ENSG00000158113 | HGLibA_27362 | 1 | -1.138650921 | LRRC43 | leucine rich repeat containing 43 [Source:HGNC Symbol;Acc:HGNC:28562] |
| ENSG00000158301 | HGLibA_20123 | 1 | -1.138650921 | GPRASP2 | G protein-coupled receptor associated sorting protein 2 [Source:HGNC Symbol;Acc:HGNC:25169] |
| ENSG00000158555 | HGLibA_18901 | 1 | -1.138650921 | GDPD5 | glycerophosphodiester phosphodiesterase domain containing 5 [Source:HGNC Symbol;Acc:HGNC:28804] |
| ENSG00000158813 | HGLibA_14430 | 1 | -1.138650921 | EDA | ectodysplasin A [Source:HGNC Symbol;Acc:HGNC:3157] |
| ENSG00000159023 | HGLibA_15239 | 1 | -1.138650921 | EPB41 | erythrocyte membrane protein band 4.1 [Source:HGNC Symbol;Acc:HGNC:3377] |
| ENSG00000159063 | HGLibA_01636 | 1 | -1.138650921 | ALG8 | 3-glucosyltransferase [Source:HGNC Symbol;Acc:HGNC:23161]" |
| ENSG00000159224 | HGLibA_19116 | 1 | -1.138650921 | GIP | gastric inhibitory polypeptide [Source:HGNC Symbol;Acc:HGNC:4270] |
| ENSG00000159228 | HGLibA_07418 | 1 | -1.138650921 | CBR1 | carbonyl reductase 1 [Source:HGNC Symbol;Acc:HGNC:1548] |
| ENSG00000159377 | HGLibA_39110 | 1 | -1.138650921 | PSMB4 | proteasome subunit beta 4 [Source:HGNC Symbol;Acc:HGNC:9541] |
| ENSG00000159761 | HGLibA_05490 | 1 | -1.138650921 | C16orf86 | chromosome 16 open reading frame 86 [Source:HGNC Symbol;Acc:HGNC:33755] |
| ENSG00000159917 | HGLibA_55648 | 1 | -1.138650921 | ZNF235 | zinc finger protein 235 [Source:HGNC Symbol;Acc:HGNC:12866] |
| ENSG00000160190 | HGLibA_45044 | 1 | -1.138650921 | SLC37A1 | solute carrier family 37 member 1 [Source:HGNC Symbol;Acc:HGNC:11024] |
| ENSG00000160207 | HGLibA_22226 | 1 | -1.138650921 | HSF2BP | heat shock transcription factor 2 binding protein [Source:HGNC Symbol;Acc:HGNC:5226] |
| ENSG00000160284 | HGLibA_46541 | 1 | -1.138650921 | SPATC1L | spermatogenesis and centriole associated 1 like [Source:HGNC Symbol;Acc:HGNC:1298] |
| ENSG00000160326 | HGLibA_44886 | 1 | -1.138650921 | SLC2A6 | solute carrier family 2 member 6 [Source:HGNC Symbol;Acc:HGNC:11011] |
| ENSG00000160801 | HGLibA_39368 | 1 | -1.138650921 | PTH1R | parathyroid hormone 1 receptor [Source:HGNC Symbol;Acc:HGNC:9608] |
| ENSG00000160948 | HGLibA_53757 | 1 | -1.138650921 | VPS28 | ESCRT-I subunit [Source:HGNC Symbol;Acc:HGNC:18178]" |
| ENSG00000161082 | HGLibA_09016 | 1 | -1.138650921 | CELF5 | CUGBP Elav-like family member 5 [Source:HGNC Symbol;Acc:HGNC:14058] |
| ENSG00000161664 | HGLibA_03225 | 1 | -1.138650921 | ASB16 | ankyrin repeat and SOCS box containing 16 [Source:HGNC Symbol;Acc:HGNC:19768] |
| ENSG00000161905 | HGLibA_01683 | 1 | -1.138650921 | ALOX15 | arachidonate 15-lipoxygenase [Source:HGNC Symbol;Acc:HGNC:433] |
| ENSG00000161999 | HGLibA_23966 | 1 | -1.138650921 | JMJD8 | jumonji domain containing 8 [Source:HGNC Symbol;Acc:HGNC:14148] |
| ENSG00000162004 | HGLibA_07871 | 1 | -1.138650921 | CCDC78 | coiled-coil domain containing 78 [Source:HGNC Symbol;Acc:HGNC:14153] |
| ENSG00000162086 | HGLibA_56676 | 1 | -1.138650921 | ZNF75A | zinc finger protein 75a [Source:HGNC Symbol;Acc:HGNC:13146] |
| ENSG00000162408 | HGLibA_32280 | 1 | -1.138650921 | NOL9 | nucleolar protein 9 [Source:HGNC Symbol;Acc:HGNC:26265] |
| ENSG00000162496 | HGLibA_13134 | 1 | -1.138650921 | DHRS3 | dehydrogenase/reductase 3 [Source:HGNC Symbol;Acc:HGNC:17693] |
| ENSG00000162614 | HGLibA_31754 | 1 | -1.138650921 | NEXN | nexilin F-actin binding protein [Source:HGNC Symbol;Acc:HGNC:29557] |
| ENSG00000162645 | HGLibA_18731 | 1 | -1.138650921 | GBP2 | guanylate binding protein 2 [Source:HGNC Symbol;Acc:HGNC:4183] |
| ENSG00000162670 | HGLibA_04819 | 1 | -1.138650921 | BRINP3 | BMP/retinoic acid inducible neural specific 3 [Source:HGNC Symbol;Acc:HGNC:22393] |
| ENSG00000162695 | HGLibA_44916 | 1 | -1.138650921 | SLC30A7 | solute carrier family 30 member 7 [Source:HGNC Symbol;Acc:HGNC:19306] |
| ENSG00000162910 | HGLibA_30024 | 1 | -1.138650921 | MRPL55 | mitochondrial ribosomal protein L55 [Source:HGNC Symbol;Acc:HGNC:16686] |
| ENSG00000162951 | HGLibA_27486 | 1 | -1.138650921 | LRRTM1 | leucine rich repeat transmembrane neuronal 1 [Source:HGNC Symbol;Acc:HGNC:19408] |
| ENSG00000163053 | HGLibA_44433 | 1 | -1.138650921 | SLC16A14 | solute carrier family 16 member 14 [Source:HGNC Symbol;Acc:HGNC:26417] |
| ENSG00000163053 | HGLibA_44431 | 1 | -1.138650921 | SLC16A14 | solute carrier family 16 member 14 [Source:HGNC Symbol;Acc:HGNC:26417] |
| ENSG00000163081 | HGLibA_07615 | 1 | -1.138650921 | CCDC140 | coiled-coil domain containing 140 [Source:HGNC Symbol;Acc:HGNC:26514] |
| ENSG00000163170 | HGLibA_04690 | 1 | -1.138650921 | BOLA3 | bolA family member 3 [Source:HGNC Symbol;Acc:HGNC:24415] |
| ENSG00000163209 | HGLibA_46826 | 1 | -1.138650921 | SPRR3 | small proline rich protein 3 [Source:HGNC Symbol;Acc:HGNC:11268] |
| ENSG00000163239 | HGLibA_48899 | 1 | -1.138650921 | TDRD10 | tudor domain containing 10 [Source:HGNC Symbol;Acc:HGNC:25316] |
| ENSG00000163344 | HGLibA_37339 | 1 | -1.138650921 | PMVK | phosphomevalonate kinase [Source:HGNC Symbol;Acc:HGNC:9141] |
| ENSG00000163539 | HGLibA_09796 | 1 | -1.138650921 | CLASP2 | cytoplasmic linker associated protein 2 [Source:HGNC Symbol;Acc:HGNC:17078] |
| ENSG00000163541 | HGLibA_47699 | 1 | -1.138650921 | SUCLG1 | succinate-CoA ligase alpha subunit [Source:HGNC Symbol;Acc:HGNC:11449] |
| ENSG00000163576 | HGLibA_14566 | 1 | -1.138650921 | EFHB | EF-hand domain family member B [Source:HGNC Symbol;Acc:HGNC:26330] |
| ENSG00000163781 | HGLibA_51009 | 1 | -1.138650921 | TOPBP1 | DNA topoisomerase II binding protein 1 [Source:HGNC Symbol;Acc:HGNC:17008] |
| ENSG00000163808 | HGLibA_24865 | 1 | -1.138650921 | KIF15 | kinesin family member 15 [Source:HGNC Symbol;Acc:HGNC:17273] |
| ENSG00000163817 | HGLibA_45323 | 1 | -1.138650921 | SLC6A20 | solute carrier family 6 member 20 [Source:HGNC Symbol;Acc:HGNC:30927] |
| ENSG00000163840 | HGLibA_14089 | 1 | -1.138650921 | DTX3L | deltex E3 ubiquitin ligase 3L [Source:HGNC Symbol;Acc:HGNC:30323] |
| ENSG00000163882 | HGLibA_37584 | 1 | -1.138650921 | POLR2H | RNA polymerase II subunit H [Source:HGNC Symbol;Acc:HGNC:9195] |
| ENSG00000163931 | HGLibA_49587 | 1 | -1.138650921 | TKT | transketolase [Source:HGNC Symbol;Acc:HGNC:11834] |
| ENSG00000163933 | HGLibA_40947 | 1 | -1.138650921 | RFT1 | RFT1 homolog [Source:HGNC Symbol;Acc:HGNC:30220] |
| ENSG00000163939 | HGLibA_35341 | 1 | -1.138650921 | PBRM1 | polybromo 1 [Source:HGNC Symbol;Acc:HGNC:30064] |
| ENSG00000163961 | HGLibA_41566 | 1 | -1.138650921 | RNF168 | ring finger protein 168 [Source:HGNC Symbol;Acc:HGNC:26661] |
| ENSG00000164048 | HGLibA_56335 | 1 | -1.138650921 | ZNF589 | zinc finger protein 589 [Source:HGNC Symbol;Acc:HGNC:16747] |
| ENSG00000164300 | HGLibA_43497 | 1 | -1.138650921 | SERINC5 | serine incorporator 5 [Source:HGNC Symbol;Acc:HGNC:18825] |
| ENSG00000164438 | HGLibA_49679 | 1 | -1.138650921 | TLX3 | T cell leukemia homeobox 3 [Source:HGNC Symbol;Acc:HGNC:13532] |
| ENSG00000164506 | HGLibA_47674 | 1 | -1.138650921 | STXBP5 | syntaxin binding protein 5 [Source:HGNC Symbol;Acc:HGNC:19665] |
| ENSG00000164574 | HGLibA_18531 | 1 | -1.138650921 | GALNT10 | polypeptide N-acetylgalactosaminyltransferase 10 [Source:HGNC Symbol;Acc:HGNC:19873] |
| ENSG00000164880 | HGLibA_23407 | 1 | -1.138650921 | INTS1 | integrator complex subunit 1 [Source:HGNC Symbol;Acc:HGNC:24555] |
| ENSG00000164924 | HGLibA_54754 | 1 | -1.138650921 | YWHAZ | tyrosine 3-monooxygenase/tryptophan 5-monooxygenase activation protein zeta [Source:HGNC Symbol;Acc:HGNC:12855] |
| ENSG00000164935 | HGLibA_12536 | 1 | -1.138650921 | DCSTAMP | dendrocyte expressed seven transmembrane protein [Source:HGNC Symbol;Acc:HGNC:18549] |
| ENSG00000164989 | HGLibA_07693 | 1 | -1.138650921 | CCDC171 | coiled-coil domain containing 171 [Source:HGNC Symbol;Acc:HGNC:29828] |
| ENSG00000165006 | HGLibA_52545 | 1 | -1.138650921 | UBAP1 | ubiquitin associated protein 1 [Source:HGNC Symbol;Acc:HGNC:12461] |
| ENSG00000165105 | HGLibA_40325 | 1 | -1.138650921 | RASEF | RAS and EF-hand domain containing [Source:HGNC Symbol;Acc:HGNC:26464] |
| ENSG00000165156 | HGLibA_55309 | 1 | -1.138650921 | ZHX1 | zinc fingers and homeoboxes 1 [Source:HGNC Symbol;Acc:HGNC:12871] |
| ENSG00000165186 | HGLibA_39274 | 1 | -1.138650921 | PTCHD1 | patched domain containing 1 [Source:HGNC Symbol;Acc:HGNC:26392] |
| ENSG00000165244 | HGLibA_55883 | 1 | -1.138650921 | ZNF367 | zinc finger protein 367 [Source:HGNC Symbol;Acc:HGNC:18320] |
| ENSG00000165246 | HGLibA_32078 | 1 | -1.138650921 | NLGN4Y | neuroligin 4 Y-linked [Source:HGNC Symbol;Acc:HGNC:15529] |
| ENSG00000165264 | HGLibA_31525 | 1 | -1.138650921 | NDUFB6 | NADH:ubiquinone oxidoreductase subunit B6 [Source:HGNC Symbol;Acc:HGNC:7701] |
| ENSG00000165322 | HGLibA_02709 | 1 | -1.138650921 | ARHGAP12 | Rho GTPase activating protein 12 [Source:HGNC Symbol;Acc:HGNC:16348] |
| ENSG00000165338 | HGLibA_21097 | 1 | -1.138650921 | HECTD2 | HECT domain E3 ubiquitin protein ligase 2 [Source:HGNC Symbol;Acc:HGNC:26736] |
| ENSG00000165476 | HGLibA_40781 | 1 | -1.138650921 | REEP3 | receptor accessory protein 3 [Source:HGNC Symbol;Acc:HGNC:23711] |
| ENSG00000165533 | HGLibA_52156 | 1 | -1.138650921 | TTC8 | tetratricopeptide repeat domain 8 [Source:HGNC Symbol;Acc:HGNC:20087] |
| ENSG00000165623 | HGLibA_52818 | 1 | -1.138650921 | UCMA | upper zone of growth plate and cartilage matrix associated [Source:HGNC Symbol;Acc:HGNC:25205] |
| ENSG00000165633 | HGLibA_53868 | 1 | -1.138650921 | VSTM4 | V-set and transmembrane domain containing 4 [Source:HGNC Symbol;Acc:HGNC:26470] |
| ENSG00000165633 | HGLibA_53870 | 1 | -1.138650921 | VSTM4 | V-set and transmembrane domain containing 4 [Source:HGNC Symbol;Acc:HGNC:26470] |
| ENSG00000165685 | HGLibA_50435 | 1 | -1.138650921 | TMEM52B | transmembrane protein 52B [Source:HGNC Symbol;Acc:HGNC:26438] |
| ENSG00000165732 | HGLibA_12672 | 1 | -1.138650921 | DDX21 | DExD-box helicase 21 [Source:HGNC Symbol;Acc:HGNC:2744] |
| ENSG00000165917 | HGLibA_40270 | 1 | -1.138650921 | RAPSN | receptor associated protein of the synapse [Source:HGNC Symbol;Acc:HGNC:9863] |
| ENSG00000165948 | HGLibA_22599 | 1 | -1.138650921 | IFI27L1 | interferon alpha inducible protein 27 like 1 [Source:HGNC Symbol;Acc:HGNC:19754] |
| ENSG00000166037 | HGLibA_09145 | 1 | -1.138650921 | CEP57 | centrosomal protein 57 [Source:HGNC Symbol;Acc:HGNC:30794] |
| ENSG00000166073 | HGLibA_19976 | 1 | -1.138650921 | GPR176 | G protein-coupled receptor 176 [Source:HGNC Symbol;Acc:HGNC:32370] |
| ENSG00000166153 | HGLibA_12980 | 1 | -1.138650921 | DEPDC4 | DEP domain containing 4 [Source:HGNC Symbol;Acc:HGNC:22952] |
| ENSG00000166189 | HGLibA_22058 | 1 | -1.138650921 | HPS6 | biogenesis of lysosomal organelles complex 2 subunit 3 [Source:HGNC Symbol;Acc:HGNC:18817]" |
| ENSG00000166206 | HGLibA_18381 | 1 | -1.138650921 | GABRB3 | gamma-aminobutyric acid type A receptor beta3 subunit [Source:HGNC Symbol;Acc:HGNC:4083] |
| ENSG00000166295 | HGLibA_01878 | 1 | -1.138650921 | ANAPC16 | anaphase promoting complex subunit 16 [Source:HGNC Symbol;Acc:HGNC:26976] |
| ENSG00000166337 | HGLibA_48199 | 1 | -1.138650921 | TAF10 | TATA-box binding protein associated factor 10 [Source:HGNC Symbol;Acc:HGNC:11543] |
| ENSG00000166377 | HGLibA_03794 | 1 | -1.138650921 | ATP9B | ATPase phospholipid transporting 9B (putative) [Source:HGNC Symbol;Acc:HGNC:13541] |
| ENSG00000166387 | HGLibA_37888 | 1 | -1.138650921 | PPFIBP2 | PPFIA binding protein 2 [Source:HGNC Symbol;Acc:HGNC:9250] |
| ENSG00000166411 | HGLibA_22546 | 1 | -1.138650921 | IDH3A | isocitrate dehydrogenase 3 (NAD(+)) alpha [Source:HGNC Symbol;Acc:HGNC:5384] |
| ENSG00000166448 | HGLibA_49925 | 1 | -1.138650921 | TMEM130 | transmembrane protein 130 [Source:HGNC Symbol;Acc:HGNC:25429] |
| ENSG00000166569 | HGLibA_10941 | 1 | -1.138650921 | CPLX4 | complexin 4 [Source:HGNC Symbol;Acc:HGNC:24330] |
| ENSG00000166743 | HGLibA_00570 | 1 | -1.138650921 | ACSM1 | acyl-CoA synthetase medium chain family member 1 [Source:HGNC Symbol;Acc:HGNC:18049] |
| ENSG00000166762 | HGLibA_07358 | 1 | -1.138650921 | CATSPER2 | cation channel sperm associated 2 [Source:HGNC Symbol;Acc:HGNC:18810] |
| ENSG00000166793 | HGLibA_54714 | 1 | -1.138650921 | YPEL4 | yippee like 4 [Source:HGNC Symbol;Acc:HGNC:18328] |
| ENSG00000166796 | HGLibA_26233 | 1 | -1.138650921 | LDHC | lactate dehydrogenase C [Source:HGNC Symbol;Acc:HGNC:6544] |
| ENSG00000166855 | HGLibA_10119 | 1 | -1.138650921 | CLPX | caseinolytic mitochondrial matrix peptidase chaperone subunit [Source:HGNC Symbol;Acc:HGNC:2088] |
| ENSG00000166856 | HGLibA_19987 | 1 | -1.138650921 | GPR182 | G protein-coupled receptor 182 [Source:HGNC Symbol;Acc:HGNC:13708] |
| ENSG00000166926 | HGLibA_30161 | 1 | -1.138650921 | MS4A6E | membrane spanning 4-domains A6E [Source:HGNC Symbol;Acc:HGNC:14285] |
| ENSG00000166959 | HGLibA_30165 | 1 | -1.138650921 | MS4A8 | membrane spanning 4-domains A8 [Source:HGNC Symbol;Acc:HGNC:13380] |
| ENSG00000166997 | HGLibA_10371 | 1 | -1.138650921 | CNPY4 | canopy FGF signaling regulator 4 [Source:HGNC Symbol;Acc:HGNC:28631] |
| ENSG00000167005 | HGLibA_32970 | 1 | -1.138650921 | NUDT21 | nudix hydrolase 21 [Source:HGNC Symbol;Acc:HGNC:13870] |
| ENSG00000167037 | HGLibA_43859 | 1 | -1.138650921 | SGSM1 | small G protein signaling modulator 1 [Source:HGNC Symbol;Acc:HGNC:29410] |
| ENSG00000167113 | HGLibA_10754 | 1 | -1.138650921 | COQ4 | coenzyme Q4 [Source:HGNC Symbol;Acc:HGNC:19693] |
| ENSG00000167118 | HGLibA_53149 | 1 | -1.138650921 | URM1 | ubiquitin related modifier 1 [Source:HGNC Symbol;Acc:HGNC:28378] |
| ENSG00000167136 | HGLibA_15117 | 1 | -1.138650921 | ENDOG | endonuclease G [Source:HGNC Symbol;Acc:HGNC:3346] |
| ENSG00000167397 | HGLibA_53677 | 1 | -1.138650921 | VKORC1 | vitamin K epoxide reductase complex subunit 1 [Source:HGNC Symbol;Acc:HGNC:23663] |
| ENSG00000167600 | HGLibA_12142 | 1 | -1.138650921 | CYP2S1 | cytochrome P450 family 2 subfamily S member 1 [Source:HGNC Symbol;Acc:HGNC:15654] |
| ENSG00000167645 | HGLibA_54666 | 1 | -1.138650921 | YIF1B | membrane trafficking protein [Source:HGNC Symbol;Acc:HGNC:30511]" |
| ENSG00000167840 | HGLibA_55639 | 1 | -1.138650921 | ZNF232 | zinc finger protein 232 [Source:HGNC Symbol;Acc:HGNC:13026] |
| ENSG00000167962 | HGLibA_56356 | 1 | -1.138650921 | ZNF598 | zinc finger protein 598 [Source:HGNC Symbol;Acc:HGNC:28079] |
| ENSG00000168036 | HGLibA_11649 | 1 | -1.138650921 | CTNNB1 | catenin beta 1 [Source:HGNC Symbol;Acc:HGNC:2514] |
| ENSG00000168286 | HGLibA_49293 | 1 | -1.138650921 | THAP11 | THAP domain containing 11 [Source:HGNC Symbol;Acc:HGNC:23194] |

| | | | | | |
|---|---|---|---|---|---|
| ENSG00000168303 | HGLibA_29739 | 1 | -1.138650921 | MPLKIP | M-phase specific PLK1 interacting protein [Source:HGNC Symbol;Acc:HGNC:16002] |
| ENSG00000168309 | HGLibA_16000 | 1 | -1.138650921 | FAM107A | family with sequence similarity 107 member A [Source:HGNC Symbol;Acc:HGNC:30827] |
| ENSG00000168496 | HGLibA_17281 | 1 | -1.138650921 | FEN1 | flap structure-specific endonuclease 1 [Source:HGNC Symbol;Acc:HGNC:3650] |
| ENSG00000168505 | HGLibA_18751 | 1 | -1.138650921 | GBX2 | gastrulation brain homeobox 2 [Source:HGNC Symbol;Acc:HGNC:4186] |
| ENSG00000168582 | HGLibA_11269 | 1 | -1.138650921 | CRYGA | crystallin gamma A [Source:HGNC Symbol;Acc:HGNC:2408] |
| ENSG00000168758 | HGLibA_43346 | 1 | -1.138650921 | SEMA4C | semaphorin 4C [Source:HGNC Symbol;Acc:HGNC:10731] |
| ENSG00000168887 | HGLibA_06164 | 1 | -1.138650921 | C2orf68 | chromosome 2 open reading frame 68 [Source:HGNC Symbol;Acc:HGNC:34353] |
| ENSG00000168887 | HGLibA_06163 | 1 | -1.138650921 | C2orf68 | chromosome 2 open reading frame 68 [Source:HGNC Symbol;Acc:HGNC:34353] |
| ENSG00000168899 | HGLibA_53501 | 1 | -1.138650921 | VAMP5 | vesicle associated membrane protein 5 [Source:HGNC Symbol;Acc:HGNC:12646] |
| ENSG00000168907 | HGLibA_36915 | 1 | -1.138650921 | PLA2G4F | phospholipase A2 group IVF [Source:HGNC Symbol;Acc:HGNC:27396] |
| ENSG00000168907 | HGLibA_36916 | 1 | -1.138650921 | PLA2G4F | phospholipase A2 group IVF [Source:HGNC Symbol;Acc:HGNC:27396] |
| ENSG00000168924 | HGLibA_26332 | 1 | -1.138650921 | LETM1 | leucine zipper and EF-hand containing transmembrane protein 1 [Source:HGNC Symbol;Acc:HGNC:6556] |
| ENSG00000169019 | HGLibA_10676 | 1 | -1.138650921 | COMMD8 | COMM domain containing 8 [Source:HGNC Symbol;Acc:HGNC:26036] |
| ENSG00000169181 | HGLibA_20423 | 1 | -1.138650921 | GSG1L | GSG1 like [Source:HGNC Symbol;Acc:HGNC:28283] |
| ENSG00000169194 | HGLibA_22970 | 1 | -1.138650921 | IL13 | interleukin 13 [Source:HGNC Symbol;Acc:HGNC:5973] |
| ENSG00000169297 | HGLibA_32567 | 1 | -1.138650921 | NR0B1 | nuclear receptor subfamily 0 group B member 1 [Source:HGNC Symbol;Acc:HGNC:7960] |
| ENSG00000169519 | HGLibA_28961 | 1 | -1.138650921 | METTL15 | methyltransferase like 15 [Source:HGNC Symbol;Acc:HGNC:26606] |
| ENSG00000169564 | HGLibA_35367 | 1 | -1.138650921 | PCBP1 | poly(rC) binding protein 1 [Source:HGNC Symbol;Acc:HGNC:8647] |
| ENSG00000169925 | HGLibA_04776 | 1 | -1.138650921 | BRD3 | bromodomain containing 3 [Source:HGNC Symbol;Acc:HGNC:1104] |
| ENSG00000170085 | HGLibA_44181 | 1 | -1.138650921 | SIMC1 | SUMO interacting motifs containing 1 [Source:HGNC Symbol;Acc:HGNC:24779] |
| ENSG00000170214 | HGLibA_01040 | 1 | -1.138650921 | ADRA1B | adrenoceptor alpha 1B [Source:HGNC Symbol;Acc:HGNC:278] |
| ENSG00000170214 | HGLibA_01039 | 1 | -1.138650921 | ADRA1B | adrenoceptor alpha 1B [Source:HGNC Symbol;Acc:HGNC:278] |
| ENSG00000170275 | HGLibA_11226 | 1 | -1.138650921 | CRTAP | cartilage associated protein [Source:HGNC Symbol;Acc:HGNC:2379] |
| ENSG00000170348 | HGLibA_49803 | 1 | -1.138650921 | TMED10 | transmembrane p24 trafficking protein 10 [Source:HGNC Symbol;Acc:HGNC:16998] |
| ENSG00000170448 | HGLibA_31851 | 1 | -1.138650921 | NFXL1 | X-box binding like 1 [Source:HGNC Symbol;Acc:HGNC:18726]" |
| ENSG00000170464 | HGLibA_13589 | 1 | -1.138650921 | DNAJC18 | DnaJ heat shock protein family (Hsp40) member C18 [Source:HGNC Symbol;Acc:HGNC:28429] |
| ENSG00000170476 | HGLibA_30933 | 1 | -1.138650921 | MZB1 | marginal zone B and B1 cell specific protein [Source:HGNC Symbol;Acc:HGNC:30125] |
| ENSG00000170522 | HGLibA_14972 | 1 | -1.138650921 | ELOVL6 | ELOVL fatty acid elongase 6 [Source:HGNC Symbol;Acc:HGNC:15829] |
| ENSG00000170820 | HGLibA_18090 | 1 | -1.138650921 | FSHR | follicle stimulating hormone receptor [Source:HGNC Symbol;Acc:HGNC:3969] |
| ENSG00000170871 | HGLibA_24610 | 1 | -1.138650921 | KIAA0232 | KIAA0232 [Source:HGNC Symbol;Acc:HGNC:28992] |
| ENSG00000170956 | HGLibA_08939 | 1 | -1.138650921 | CEACAM3 | carcinoembryonic antigen related cell adhesion molecule 3 [Source:HGNC Symbol;Acc:HGNC:1815] |
| ENSG00000171401 | HGLibA_25424 | 1 | -1.138650921 | KRT13 | keratin 13 [Source:HGNC Symbol;Acc:HGNC:6415] |
| ENSG00000171446 | HGLibA_25464 | 1 | -1.138650921 | KRT27 | keratin 27 [Source:HGNC Symbol;Acc:HGNC:30841] |
| ENSG00000171566 | HGLibA_37223 | 1 | -1.138650921 | PLRG1 | pleiotropic regulator 1 [Source:HGNC Symbol;Acc:HGNC:9089] |
| ENSG00000171574 | HGLibA_56316 | 1 | -1.138650921 | ZNF584 | zinc finger protein 584 [Source:HGNC Symbol;Acc:HGNC:27318] |
| ENSG00000171864 | HGLibA_38642 | 1 | -1.138650921 | PRND | prion like protein doppel [Source:HGNC Symbol;Acc:HGNC:15748] |
| ENSG00000171931 | HGLibA_17136 | 1 | -1.138650921 | FBXW10 | F-box and WD repeat domain containing 10 [Source:HGNC Symbol;Acc:HGNC:1211] |
| ENSG00000172324 | HGLibA_34233 | 1 | -1.138650921 | OR5A2 | olfactory receptor family 5 subfamily A member 2 [Source:HGNC Symbol;Acc:HGNC:15249] |
| ENSG00000172365 | HGLibA_34261 | 1 | -1.138650921 | OR5B2 | olfactory receptor family 5 subfamily B member 2 [Source:HGNC Symbol;Acc:HGNC:8323] |
| ENSG00000172410 | HGLibA_23388 | 1 | -1.138650921 | INSL5 | insulin like 5 [Source:HGNC Symbol;Acc:HGNC:6088] |
| ENSG00000172425 | HGLibA_52124 | 1 | -1.138650921 | TTC36 | tetratricopeptide repeat domain 36 [Source:HGNC Symbol;Acc:HGNC:33708] |
| ENSG00000172461 | HGLibA_18208 | 1 | -1.138650921 | FUT9 | fucosyltransferase 9 [Source:HGNC Symbol;Acc:HGNC:4020] |
| ENSG00000172543 | HGLibA_11733 | 1 | -1.138650921 | CTSW | cathepsin W [Source:HGNC Symbol;Acc:HGNC:2546] |
| ENSG00000172548 | HGLibA_31965 | 1 | -1.138650921 | NIPAL4 | NIPA like domain containing 4 [Source:HGNC Symbol;Acc:HGNC:28018] |
| ENSG00000172738 | HGLibA_50224 | 1 | -1.138650921 | TMEM217 | transmembrane protein 217 [Source:HGNC Symbol;Acc:HGNC:21238] |
| ENSG00000172765 | HGLibA_49771 | 1 | -1.138650921 | TMCC1 | transmembrane and coiled-coil domain family 1 [Source:HGNC Symbol;Acc:HGNC:29116] |
| ENSG00000172772 | HGLibA_33553 | 1 | -1.138650921 | OR10W1 | olfactory receptor family 10 subfamily W member 1 [Source:HGNC Symbol;Acc:HGNC:15139] |
| ENSG00000172818 | HGLibA_34800 | 1 | -1.138650921 | OVOL1 | ovo like transcriptional repressor 1 [Source:HGNC Symbol;Acc:HGNC:8525] |
| ENSG00000172824 | HGLibA_09228 | 1 | -1.138650921 | CES4A | carboxylesterase 4A [Source:HGNC Symbol;Acc:HGNC:26741] |
| ENSG00000173113 | HGLibA_51672 | 1 | -1.138650921 | TRMT112 | tRNA methyltransferase subunit 11-2 [Source:HGNC Symbol;Acc:HGNC:26940] |
| ENSG00000173193 | HGLibA_35222 | 1 | -1.138650921 | PARP14 | poly(ADP-ribose) polymerase family member 14 [Source:HGNC Symbol;Acc:HGNC:29232] |
| ENSG00000173200 | HGLibA_35226 | 1 | -1.138650921 | PARP15 | poly(ADP-ribose) polymerase family member 15 [Source:HGNC Symbol;Acc:HGNC:26876] |
| ENSG00000173227 | HGLibA_48055 | 1 | -1.138650921 | SYT12 | synaptotagmin 12 [Source:HGNC Symbol;Acc:HGNC:18381] |
| ENSG00000173334 | HGLibA_51389 | 1 | -1.138650921 | TRIB1 | tribbles pseudokinase 1 [Source:HGNC Symbol;Acc:HGNC:16891] |
| ENSG00000173406 | HGLibA_12264 | 1 | -1.138650921 | DAB1 | reelin adaptor protein [Source:HGNC Symbol;Acc:HGNC:2661]" |
| ENSG00000173409 | HGLibA_03173 | 1 | -1.138650921 | ARV1 | fatty acid homeostasis modulator [Source:HGNC Symbol;Acc:HGNC:29561]" |
| ENSG00000173581 | HGLibA_07523 | 1 | -1.138650921 | CCDC106 | coiled-coil domain containing 106 [Source:HGNC Symbol;Acc:HGNC:30181] |
| ENSG00000173599 | HGLibA_35358 | 1 | -1.138650921 | PC | pyruvate carboxylase [Source:HGNC Symbol;Acc:HGNC:8636] |
| ENSG00000173611 | HGLibA_42833 | 1 | -1.138650921 | SCAI | suppressor of cancer cell invasion [Source:HGNC Symbol;Acc:HGNC:26709] |
| ENSG00000173653 | HGLibA_40692 | 1 | -1.138650921 | RCE1 | Ras converting CAAX endopeptidase 1 [Source:HGNC Symbol;Acc:HGNC:13721] |
| ENSG00000173662 | HGLibA_48353 | 1 | -1.138650921 | TAS1R1 | taste 1 receptor member 1 [Source:HGNC Symbol;Acc:HGNC:14448] |
| ENSG00000173728 | HGLibA_05728 | 1 | -1.138650921 | C1orf100 | chromosome 1 open reading frame 100 [Source:HGNC Symbol;Acc:HGNC:30435] |
| ENSG00000173805 | HGLibA_20838 | 1 | -1.138650921 | HAP1 | huntingtin associated protein 1 [Source:HGNC Symbol;Acc:HGNC:4812] |
| ENSG00000174307 | HGLibA_36423 | 1 | -1.138650921 | PHLDA3 | pleckstrin homology like domain family A member 3 [Source:HGNC Symbol;Acc:HGNC:8934] |
| ENSG00000174332 | HGLibA_19270 | 1 | -1.138650921 | GLIS1 | GLIS family zinc finger 1 [Source:HGNC Symbol;Acc:HGNC:29525] |
| ENSG00000174348 | HGLibA_37452 | 1 | -1.138650921 | PODN | podocan [Source:HGNC Symbol;Acc:HGNC:23174] |
| ENSG00000174579 | HGLibA_30213 | 1 | -1.138650921 | MSL2 | MSL complex subunit 2 [Source:HGNC Symbol;Acc:HGNC:25544] |
| ENSG00000174672 | HGLibA_04850 | 1 | -1.138650921 | BRSK2 | BR serine/threonine kinase 2 [Source:HGNC Symbol;Acc:HGNC:11405] |
| ENSG00000174720 | HGLibA_26044 | 1 | -1.138650921 | LARP7 | La ribonucleoprotein domain family member 7 [Source:HGNC Symbol;Acc:HGNC:24912] |
| ENSG00000174744 | HGLibA_04830 | 1 | -1.138650921 | BRMS1 | transcriptional repressor and anoikis regulator [Source:HGNC Symbol;Acc:HGNC:17262]" |
| ENSG00000174944 | HGLibA_34882 | 1 | -1.138650921 | P2RY14 | purinergic receptor P2Y14 [Source:HGNC Symbol;Acc:HGNC:16442] |
| ENSG00000174957 | HGLibA_34306 | 1 | -1.138650921 | OR5J2 | olfactory receptor family 5 subfamily J member 2 [Source:HGNC Symbol;Acc:HGNC:19612] |
| ENSG00000175077 | HGLibA_42422 | 1 | -1.138650921 | RTP1 | receptor transporter protein 1 [Source:HGNC Symbol;Acc:HGNC:28580] |
| ENSG00000175084 | HGLibA_13003 | 1 | -1.138650921 | DES | desmin [Source:HGNC Symbol;Acc:HGNC:2770] |
| ENSG00000175093 | HGLibA_46869 | 1 | -1.138650921 | SPSB4 | splA/ryanodine receptor domain and SOCS box containing 4 [Source:HGNC Symbol;Acc:HGNC:30630] |
| ENSG00000175137 | HGLibA_43937 | 1 | -1.138650921 | SH3BP5L | SH3 binding domain protein 5 like [Source:HGNC Symbol;Acc:HGNC:29360] |
| ENSG00000175264 | HGLibA_09609 | 1 | -1.138650921 | CHST1 | carbohydrate sulfotransferase 1 [Source:HGNC Symbol;Acc:HGNC:1969] |
| ENSG00000175691 | HGLibA_56701 | 1 | -1.138650921 | ZNF77 | zinc finger protein 77 [Source:HGNC Symbol;Acc:HGNC:13150] |
| ENSG00000175766 | HGLibA_14820 | 1 | -1.138650921 | EIF4E1B | eukaryotic translation initiation factor 4E family member 1B [Source:HGNC Symbol;Acc:HGNC:33179] |
| ENSG00000175970 | HGLibA_52991 | 1 | -1.138650921 | UNC119B | unc-119 lipid binding chaperone B [Source:HGNC Symbol;Acc:HGNC:16488] |
| ENSG00000176049 | HGLibA_23924 | 1 | -1.138650921 | JAKMIP2 | janus kinase and microtubule interacting protein 2 [Source:HGNC Symbol;Acc:HGNC:29067] |
| ENSG00000176153 | HGLibA_20174 | 1 | -1.138650921 | GPX2 | glutathione peroxidase 2 [Source:HGNC Symbol;Acc:HGNC:4554] |
| ENSG00000176198 | HGLibA_33579 | 1 | -1.138650921 | OR11H4 | olfactory receptor family 11 subfamily H member 4 [Source:HGNC Symbol;Acc:HGNC:15347] |
| ENSG00000176299 | HGLibA_34039 | 1 | -1.138650921 | OR4M1 | olfactory receptor family 4 subfamily M member 1 [Source:HGNC Symbol;Acc:HGNC:14735] |
| ENSG00000176302 | HGLibA_17946 | 1 | -1.138650921 | FOXR1 | forkhead box R1 [Source:HGNC Symbol;Acc:HGNC:29980] |
| ENSG00000176381 | HGLibA_38795 | 1 | -1.138650921 | PRR18 | proline rich 18 [Source:HGNC Symbol;Acc:HGNC:28574] |
| ENSG00000176472 | HGLibA_56290 | 1 | -1.138650921 | ZNF575 | zinc finger protein 575 [Source:HGNC Symbol;Acc:HGNC:27606] |
| ENSG00000176563 | HGLibA_10386 | 1 | -1.138650921 | CNTD1 | cyclin N-terminal domain containing 1 [Source:HGNC Symbol;Acc:HGNC:26847] |
| ENSG00000176571 | HGLibA_10241 | 1 | -1.138650921 | CNBD1 | cyclic nucleotide binding domain containing 1 [Source:HGNC Symbol;Acc:HGNC:26663] |
| ENSG00000176678 | HGLibA_17899 | 1 | -1.138650921 | FOXL1 | forkhead box L1 [Source:HGNC Symbol;Acc:HGNC:3817] |
| ENSG00000176715 | HGLibA_00550 | 1 | -1.138650921 | ACSF3 | acyl-CoA synthetase family member 3 [Source:HGNC Symbol;Acc:HGNC:27288] |
| ENSG00000176732 | HGLibA_36182 | 1 | -1.138650921 | PFN4 | profilin family member 4 [Source:HGNC Symbol;Acc:HGNC:31103] |
| ENSG00000176974 | HGLibA_44061 | 1 | -1.138650921 | SHMT1 | serine hydroxymethyltransferase 1 [Source:HGNC Symbol;Acc:HGNC:10850] |
| ENSG00000177084 | HGLibA_37509 | 1 | -1.138650921 | POLE | catalytic subunit [Source:HGNC Symbol;Acc:HGNC:9177]" |
| ENSG00000177294 | HGLibA_17088 | 1 | -1.138650921 | FBXO39 | F-box protein 39 [Source:HGNC Symbol;Acc:HGNC:28565] |
| ENSG00000177311 | HGLibA_54870 | 1 | -1.138650921 | ZBTB38 | zinc finger and BTB domain containing 38 [Source:HGNC Symbol;Acc:HGNC:26636] |
| ENSG00000177511 | HGLibA_47295 | 1 | -1.138650921 | ST8SIA3 | 8-sialyltransferase 3 [Source:HGNC Symbol;Acc:HGNC:14269]" |
| ENSG00000177731 | HGLibA_17617 | 1 | -1.138650921 | FLII | actin remodeling protein [Source:HGNC Symbol;Acc:HGNC:3750]" |
| ENSG00000178093 | HGLibA_52021 | 1 | -1.138650921 | TSSK6 | testis specific serine kinase 6 [Source:HGNC Symbol;Acc:HGNC:30410] |
| ENSG00000178096 | HGLibA_04681 | 1 | -1.138650921 | BOLA1 | bolA family member 1 [Source:HGNC Symbol;Acc:HGNC:24263] |
| ENSG00000178149 | HGLibA_12303 | 1 | -1.138650921 | DALRD3 | DALR anticodon binding domain containing 3 [Source:HGNC Symbol;Acc:HGNC:25536] |
| ENSG00000178342 | HGLibA_24201 | 1 | -1.138650921 | KCNG2 | potassium voltage-gated channel modifier subfamily G member 2 [Source:HGNC Symbol;Acc:HGNC:6249] |
| ENSG00000178761 | HGLibA_16502 | 1 | -1.138650921 | FAM219B | family with sequence similarity 219 member B [Source:HGNC Symbol;Acc:HGNC:24695] |
| ENSG00000178772 | HGLibA_10951 | 1 | -1.138650921 | CPN2 | carboxypeptidase N subunit 2 [Source:HGNC Symbol;Acc:HGNC:2313] |
| ENSG00000178878 | HGLibA_02546 | 1 | -1.138650921 | APOLD1 | apolipoprotein L domain containing 1 [Source:HGNC Symbol;Acc:HGNC:25268] |
| ENSG00000179046 | HGLibA_51624 | 1 | -1.138650921 | TRIML2 | tripartite motif family like 2 [Source:HGNC Symbol;Acc:HGNC:26378] |
| ENSG00000179144 | HGLibA_19088 | 1 | -1.138650921 | GIMAP7 | IMAP family member 7 [Source:HGNC Symbol;Acc:HGNC:22404]" |
| ENSG00000179399 | HGLibA_19774 | 1 | -1.138650921 | GPC5 | glypican 5 [Source:HGNC Symbol;Acc:HGNC:4453] |
| ENSG00000179520 | HGLibA_44479 | 1 | -1.138650921 | SLC17A8 | solute carrier family 17 member 8 [Source:HGNC Symbol;Acc:HGNC:20151] |
| ENSG00000179580 | HGLibA_41547 | 1 | -1.138650921 | RNF151 | ring finger protein 151 [Source:HGNC Symbol;Acc:HGNC:23235] |
| ENSG00000179796 | HGLibA_27343 | 1 | -1.138650921 | LRRC3B | leucine rich repeat containing 3B [Source:HGNC Symbol;Acc:HGNC:28105] |
| ENSG00000179813 | HGLibA_16487 | 1 | -1.138650921 | FAM216B | family with sequence similarity 216 member B [Source:HGNC Symbol;Acc:HGNC:26883] |
| ENSG00000179913 | HGLibA_03986 | 1 | -1.138650921 | B3GNT3 | 3-N-acetylglucosaminyltransferase 3 [Source:HGNC Symbol;Acc:HGNC:13528]" |
| ENSG00000179934 | HGLibA_08167 | 1 | -1.138650921 | CCR8 | C-C motif chemokine receptor 8 [Source:HGNC Symbol;Acc:HGNC:1609] |
| ENSG00000180113 | HGLibA_48910 | 1 | -1.138650921 | TDRD6 | tudor domain containing 6 [Source:HGNC Symbol;Acc:HGNC:21339] |
| ENSG00000180233 | HGLibA_56942 | 1 | -1.138650921 | ZNRF2 | zinc and ring finger 2 [Source:HGNC Symbol;Acc:HGNC:22316] |
| ENSG00000180357 | HGLibA_56376 | 1 | -1.138650921 | ZNF609 | zinc finger protein 609 [Source:HGNC Symbol;Acc:HGNC:29003] |
| ENSG00000180425 | HGLibA_05178 | 1 | -1.138650921 | C11orf71 | chromosome 11 open reading frame 71 [Source:HGNC Symbol;Acc:HGNC:25937] |
| ENSG00000180447 | HGLibA_18640 | 1 | -1.138650921 | GAS1 | growth arrest specific 1 [Source:HGNC Symbol;Acc:HGNC:4165] |
| ENSG00000180475 | HGLibA_33540 | 1 | -1.138650921 | OR10Q1 | olfactory receptor family 10 subfamily Q member 1 [Source:HGNC Symbol;Acc:HGNC:15134] |
| ENSG00000180871 | HGLibA_11883 | 1 | -1.138650921 | CXCR2 | C-X-C motif chemokine receptor 2 [Source:HGNC Symbol;Acc:HGNC:6027] |
| ENSG00000181036 | HGLibA_17241 | 1 | -1.138650921 | FCRL6 | Fc receptor like 6 [Source:HGNC Symbol;Acc:HGNC:31910] |
| ENSG00000181826 | HGLibA_40818 | 1 | -1.138650921 | RELL1 | RELT like 1 [Source:HGNC Symbol;Acc:HGNC:27379] |
| ENSG00000181965 | HGLibA_31745 | 1 | -1.138650921 | NEUROG1 | neurogenin 1 [Source:HGNC Symbol;Acc:HGNC:7764] |
| ENSG00000182263 | HGLibA_17520 | 1 | -1.138650921 | FIGN | microtubule severing factor [Source:HGNC Symbol;Acc:HGNC:13285]" |
| ENSG00000182315 | HGLibA_28478 | 1 | -1.138650921 | MBD3L3 | methyl-CpG binding domain protein 3 like 3 [Source:HGNC Symbol;Acc:HGNC:37205] |
| ENSG00000182591 | HGLibA_25628 | 1 | -1.138650921 | KRTAP11-1 | keratin associated protein 11-1 [Source:HGNC Symbol;Acc:HGNC:18922] |
| ENSG00000182634 | HGLibA_33496 | 1 | -1.138650921 | OR10G7 | olfactory receptor family 10 subfamily G member 7 [Source:HGNC Symbol;Acc:HGNC:14842] |
| ENSG00000182676 | HGLibA_38084 | 1 | -1.138650921 | PPP1R27 | protein phosphatase 1 regulatory subunit 27 [Source:HGNC Symbol;Acc:HGNC:16813] |
| ENSG00000182700 | HGLibA_22860 | 1 | -1.138650921 | IGIP | IgA inducing protein [Source:HGNC Symbol;Acc:HGNC:33847] |
| ENSG00000182712 | HGLibA_10191 | 1 | -1.138650921 | CMC4 | C-X9-C motif containing 4 [Source:HGNC Symbol;Acc:HGNC:35428] |
| ENSG00000182902 | HGLibA_44661 | 1 | -1.138650921 | SLC25A18 | solute carrier family 25 member 18 [Source:HGNC Symbol;Acc:HGNC:10988] |
| ENSG00000183098 | HGLibA_19778 | 1 | -1.138650921 | GPC6 | glypican 6 [Source:HGNC Symbol;Acc:HGNC:4454] |
| ENSG00000183255 | HGLibA_39566 | 1 | -1.138650921 | PTTG1IP | PTTG1 interacting protein [Source:HGNC Symbol;Acc:HGNC:13524] |
| ENSG00000183281 | HGLibA_37161 | 1 | -1.138650921 | PLGLB1 | plasminogen-like B1 [Source:HGNC Symbol;Acc:HGNC:9072] |

| | | | | | |
|---|---|---|---|---|---|
| ENSG00000183304 | HGLibA_16819 | 1 | -1.138650921 | FAM9A | family with sequence similarity 9 member A [Source:HGNC Symbol;Acc:HGNC:18403] |
| ENSG00000183379 | HGLibA_47972 | 1 | -1.138650921 | SYNDIG1L | synapse differentiation inducing 1 like [Source:HGNC Symbol;Acc:HGNC:32388] |
| ENSG00000183605 | HGLibA_43791 | 1 | -1.138650921 | SFXN4 | sideroflexin 4 [Source:HGNC Symbol;Acc:HGNC:16088] |
| ENSG00000183648 | HGLibA_31504 | 1 | -1.138650921 | NDUFB1 | NADH:ubiquinone oxidoreductase subunit B1 [Source:HGNC Symbol;Acc:HGNC:7695] |
| ENSG00000183691 | HGLibA_32254 | 1 | -1.138650921 | NOG | noggin [Source:HGNC Symbol;Acc:HGNC:7866] |
| ENSG00000183726 | HGLibA_50423 | 1 | -1.138650921 | TMEM50A | transmembrane protein 50A [Source:HGNC Symbol;Acc:HGNC:30590] |
| ENSG00000183773 | HGLibA_01342 | 1 | -1.138650921 | AIFM3 | mitochondria associated 3 [Source:HGNC Symbol;Acc:HGNC:26398]" |
| ENSG00000183783 | HGLibA_24468 | 1 | -1.138650921 | KCTD8 | potassium channel tetramerization domain containing 8 [Source:HGNC Symbol;Acc:HGNC:22394] |
| ENSG00000183798 | HGLibA_15052 | 1 | -1.138650921 | EMILIN3 | elastin microfibril interfacer 3 [Source:HGNC Symbol;Acc:HGNC:16123] |
| ENSG00000183801 | HGLibA_33355 | 1 | -1.138650921 | OLFML1 | olfactomedin like 1 [Source:HGNC Symbol;Acc:HGNC:24473] |
| ENSG00000183807 | HGLibA_16226 | 1 | -1.138650921 | FAM162B | family with sequence similarity 162 member B [Source:HGNC Symbol;Acc:HGNC:21549] |
| ENSG00000184160 | HGLibA_01052 | 1 | -1.138650921 | ADRA2C | adrenoceptor alpha 2C [Source:HGNC Symbol;Acc:HGNC:283] |
| ENSG00000184220 | HGLibA_10207 | 1 | -1.138650921 | CMSS1 | cms1 ribosomal small subunit homolog [Source:HGNC Symbol;Acc:HGNC:28666] |
| ENSG00000184227 | HGLibA_00468 | 1 | -1.138650921 | ACOT1 | acyl-CoA thioesterase 1 [Source:HGNC Symbol;Acc:HGNC:33128] |
| ENSG00000184277 | HGLibA_49688 | 1 | -1.138650921 | TM2D3 | TM2 domain containing 3 [Source:HGNC Symbol;Acc:HGNC:24128] |
| ENSG00000184708 | HGLibA_14836 | 1 | -1.138650921 | EIF4ENIF1 | eukaryotic translation initiation factor 4E nuclear import factor 1 [Source:HGNC Symbol;Acc:HGNC:16687] |
| ENSG00000185052 | HGLibA_44622 | 1 | -1.138650921 | SLC24A3 | solute carrier family 24 member 3 [Source:HGNC Symbol;Acc:HGNC:10977] |
| ENSG00000185176 | HGLibA_02587 | 1 | -1.138650921 | AQP12B | aquaporin 12B [Source:HGNC Symbol;Acc:HGNC:6096] |
| ENSG00000185507 | HGLibA_23598 | 1 | -1.138650921 | IRF7 | interferon regulatory factor 7 [Source:HGNC Symbol;Acc:HGNC:6122] |
| ENSG00000186086 | HGLibA_31239 | 1 | -1.138650921 | NBPF6 | NBPF member 6 [Source:HGNC Symbol;Acc:HGNC:31988] |
| ENSG00000186212 | HGLibA_46217 | 1 | -1.138650921 | SOWAHB | sosondowah ankyrin repeat domain family member B [Source:HGNC Symbol;Acc:HGNC:32958] |
| ENSG00000186446 | HGLibA_56109 | 1 | -1.138650921 | ZNF501 | zinc finger protein 501 [Source:HGNC Symbol;Acc:HGNC:23717] |
| ENSG00000186523 | HGLibA_16772 | 1 | -1.138650921 | FAM86B1 | family with sequence similarity 86 member B1 [Source:HGNC Symbol;Acc:HGNC:28268] |
| ENSG00000186583 | HGLibA_46537 | 1 | -1.138650921 | SPATC1 | spermatogenesis and centriole associated 1 [Source:HGNC Symbol;Acc:HGNC:30510] |
| ENSG00000186716 | HGLibA_04369 | 1 | -1.138650921 | BCR | RhoGEF and GTPase activating protein [Source:HGNC Symbol;Acc:HGNC:1014]" |
| ENSG00000186766 | HGLibA_17879 | 1 | -1.138650921 | FOXI2 | forkhead box I2 [Source:HGNC Symbol;Acc:HGNC:32448] |
| ENSG00000186787 | HGLibA_46674 | 1 | -1.138650921 | SPIN2B | spindlin family member 2B [Source:HGNC Symbol;Acc:HGNC:33147] |
| ENSG00000186844 | HGLibA_26099 | 1 | -1.138650921 | LCE1A | late cornified envelope 1A [Source:HGNC Symbol;Acc:HGNC:29459] |
| ENSG00000186868 | HGLibA_28323 | 1 | -1.138650921 | MAPT | microtubule associated protein tau [Source:HGNC Symbol;Acc:HGNC:6893] |
| ENSG00000186907 | HGLibA_42420 | 1 | -1.138650921 | RTN4RL2 | reticulon 4 receptor like 2 [Source:HGNC Symbol;Acc:HGNC:23053] |
| ENSG00000186973 | HGLibA_16323 | 1 | -1.138650921 | FAM183A | family with sequence similarity 183 member A [Source:HGNC Symbol;Acc:HGNC:34347] |
| ENSG00000186977 | HGLibA_25675 | 1 | -1.138650921 | KRTAP19-5 | keratin associated protein 19-5 [Source:HGNC Symbol;Acc:HGNC:18940] |
| ENSG00000187135 | HGLibA_53862 | 1 | -1.138650921 | VSTM2B | V-set and transmembrane domain containing 2B [Source:HGNC Symbol;Acc:HGNC:33595] |
| ENSG00000187193 | HGLibA_30299 | 1 | -1.138650921 | MT1X | metallothionein 1X [Source:HGNC Symbol;Acc:HGNC:7405] |
| ENSG00000187272 | HGLibA_25847 | 1 | -1.138650921 | KRTAP9-8 | keratin associated protein 9-8 [Source:HGNC Symbol;Acc:HGNC:17231] |
| ENSG00000187474 | HGLibA_17969 | 1 | -1.138650921 | FPR3 | formyl peptide receptor 3 [Source:HGNC Symbol;Acc:HGNC:3828] |
| ENSG00000187479 | HGLibA_05220 | 1 | -1.138650921 | C11orf96 | chromosome 11 open reading frame 96 [Source:HGNC Symbol;Acc:HGNC:38675] |
| ENSG00000187664 | HGLibA_20851 | 1 | -1.138650921 | HAPLN4 | hyaluronan and proteoglycan link protein 4 [Source:HGNC Symbol;Acc:HGNC:31357] |
| ENSG00000187701 | HGLibA_33867 | 1 | -1.138650921 | OR2T27 | olfactory receptor family 2 subfamily T member 27 [Source:HGNC Symbol;Acc:HGNC:31252] |
| ENSG00000187766 | HGLibA_25620 | 1 | -1.138650921 | KRTAP10-8 | keratin associated protein 10-8 [Source:HGNC Symbol;Acc:HGNC:20525] |
| ENSG00000187808 | HGLibA_46222 | 1 | -1.138650921 | SOWAHD | sosondowah ankyrin repeat domain family member D [Source:HGNC Symbol;Acc:HGNC:32960] |
| ENSG00000188038 | HGLibA_32713 | 1 | -1.138650921 | NRN1L | neuritin 1 like [Source:HGNC Symbol;Acc:HGNC:29811] |
| ENSG00000188112 | HGLibA_06442 | 1 | -1.138650921 | C6orf132 | chromosome 6 open reading frame 132 [Source:HGNC Symbol;Acc:HGNC:21288] |
| ENSG00000188130 | HGLibA_28257 | 1 | -1.138650921 | MAPK12 | mitogen-activated protein kinase 12 [Source:HGNC Symbol;Acc:HGNC:6874] |
| ENSG00000188158 | HGLibA_31915 | 1 | -1.138650921 | NHS | NHS actin remodeling regulator [Source:HGNC Symbol;Acc:HGNC:7820] |
| ENSG00000188163 | HGLibA_16234 | 1 | -1.138650921 | FAM166A | family with sequence similarity 166 member A [Source:HGNC Symbol;Acc:HGNC:33818] |
| ENSG00000188269 | HGLibA_34462 | 1 | -1.138650921 | OR7A5 | olfactory receptor family 7 subfamily A member 5 [Source:HGNC Symbol;Acc:HGNC:8368] |
| ENSG00000188649 | HGLibA_07484 | 1 | -1.138650921 | CC2D2B | coiled-coil and C2 domain containing 2B [Source:HGNC Symbol;Acc:HGNC:31666] |
| ENSG00000188761 | HGLibA_04320 | 1 | -1.138650921 | BCL2L15 | BCL2 like 15 [Source:HGNC Symbol;Acc:HGNC:33624] |
| ENSG00000188803 | HGLibA_44046 | 1 | -1.138650921 | SHISA6 | shisa family member 6 [Source:HGNC Symbol;Acc:HGNC:34491] |
| ENSG00000189139 | HGLibA_18064 | 1 | -1.138650921 | FSCB | fibrous sheath CABYR binding protein [Source:HGNC Symbol;Acc:HGNC:20494] |
| ENSG00000189252 | HGLibA_46409 | 1 | -1.138650921 | SPANXN3 | SPANX family member N3 [Source:HGNC Symbol;Acc:HGNC:33176] |
| ENSG00000189320 | HGLibA_16312 | 1 | -1.138650921 | FAM180A | family with sequence similarity 180 member A [Source:HGNC Symbol;Acc:HGNC:33773] |
| ENSG00000196091 | HGLibA_30648 | 1 | -1.138650921 | MYBPC1 | slow type [Source:HGNC Symbol;Acc:HGNC:7549]" |
| ENSG00000196154 | HGLibA_42558 | 1 | -1.138650921 | S100A4 | S100 calcium binding protein A4 [Source:HGNC Symbol;Acc:HGNC:10494] |
| ENSG00000196169 | HGLibA_24879 | 1 | -1.138650921 | KIF19 | kinesin family member 19 [Source:HGNC Symbol;Acc:HGNC:26735] |
| ENSG00000196235 | HGLibA_47821 | 1 | -1.138650921 | SUPT5H | DSIF elongation factor subunit [Source:HGNC Symbol;Acc:HGNC:11469]" |
| ENSG00000196353 | HGLibA_10962 | 1 | -1.138650921 | CPNE4 | copine 4 [Source:HGNC Symbol;Acc:HGNC:2317] |
| ENSG00000196358 | HGLibA_32869 | 1 | -1.138650921 | NTNG2 | netrin G2 [Source:HGNC Symbol;Acc:HGNC:14288] |
| ENSG00000196372 | HGLibA_03214 | 1 | -1.138650921 | ASB13 | ankyrin repeat and SOCS box containing 13 [Source:HGNC Symbol;Acc:HGNC:19765] |
| ENSG00000196660 | HGLibA_44899 | 1 | -1.138650921 | SLC30A10 | solute carrier family 30 member 10 [Source:HGNC Symbol;Acc:HGNC:25355] |
| ENSG00000196757 | HGLibA_56575 | 1 | -1.138650921 | ZNF700 | zinc finger protein 700 [Source:HGNC Symbol;Acc:HGNC:25292] |
| ENSG00000196812 | HGLibA_56990 | 1 | -1.138650921 | ZSCAN16 | zinc finger and SCAN domain containing 16 [Source:HGNC Symbol;Acc:HGNC:20813] |
| ENSG00000196937 | HGLibA_16582 | 1 | -1.138650921 | FAM3C | family with sequence similarity 3 member C [Source:HGNC Symbol;Acc:HGNC:18664] |
| ENSG00000196950 | HGLibA_45090 | 1 | -1.138650921 | SLC39A10 | solute carrier family 39 member 10 [Source:HGNC Symbol;Acc:HGNC:20861] |
| ENSG00000197046 | HGLibA_44139 | 1 | -1.138650921 | SIGLEC15 | sialic acid binding Ig like lectin 15 [Source:HGNC Symbol;Acc:HGNC:27596] |
| ENSG00000197142 | HGLibA_00564 | 1 | -1.138650921 | ACSL5 | acyl-CoA synthetase long chain family member 5 [Source:HGNC Symbol;Acc:HGNC:16526] |
| ENSG00000197162 | HGLibA_56750 | 1 | -1.138650921 | ZNF785 | zinc finger protein 785 [Source:HGNC Symbol;Acc:HGNC:26496] |
| ENSG00000197275 | HGLibA_40099 | 1 | -1.138650921 | RAD54B | RAD54 homolog B [Source:HGNC Symbol;Acc:HGNC:17228] |
| ENSG00000197353 | HGLibA_27716 | 1 | -1.138650921 | LYPD2 | LY6/PLAUR domain containing 2 [Source:HGNC Symbol;Acc:HGNC:25215] |
| ENSG00000197405 | HGLibA_06352 | 1 | -1.138650921 | C5AR1 | complement C5a receptor 1 [Source:HGNC Symbol;Acc:HGNC:1338] |
| ENSG00000197901 | HGLibA_44593 | 1 | -1.138650921 | SLC22A6 | solute carrier family 22 member 6 [Source:HGNC Symbol;Acc:HGNC:10970] |
| ENSG00000197948 | HGLibA_17209 | 1 | -1.138650921 | FCHSD1 | FCH and double SH3 domains 1 [Source:HGNC Symbol;Acc:HGNC:25463] |
| ENSG00000198039 | HGLibA_55708 | 1 | -1.138650921 | ZNF273 | zinc finger protein 273 [Source:HGNC Symbol;Acc:HGNC:13067] |
| ENSG00000198056 | HGLibA_38481 | 1 | -1.138650921 | PRIM1 | DNA primase subunit 1 [Source:HGNC Symbol;Acc:HGNC:9369] |
| ENSG00000198092 | HGLibA_50623 | 1 | -1.138650921 | TMPRSS11F | transmembrane serine protease 11F [Source:HGNC Symbol;Acc:HGNC:29994] |
| ENSG00000198104 | HGLibA_33891 | 1 | -1.138650921 | OR2T6 | olfactory receptor family 2 subfamily T member 6 [Source:HGNC Symbol;Acc:HGNC:15018] |
| ENSG00000198131 | HGLibA_56198 | 1 | -1.138650921 | ZNF544 | zinc finger protein 544 [Source:HGNC Symbol;Acc:HGNC:16759] |
| ENSG00000198168 | HGLibA_47888 | 1 | -1.138650921 | SVIP | small VCP interacting protein [Source:HGNC Symbol;Acc:HGNC:25238] |
| ENSG00000198270 | HGLibA_49887 | 1 | -1.138650921 | TMEM116 | transmembrane protein 116 [Source:HGNC Symbol;Acc:HGNC:25084] |
| ENSG00000198443 | HGLibA_25749 | 1 | -1.138650921 | KRTAP4-1 | keratin associated protein 4-1 [Source:HGNC Symbol;Acc:HGNC:18907] |
| ENSG00000198553 | HGLibA_24380 | 1 | -1.138650921 | KCNRG | potassium channel regulator [Source:HGNC Symbol;Acc:HGNC:18893] |
| ENSG00000198680 | HGLibA_52341 | 1 | -1.138650921 | TUSC1 | tumor suppressor candidate 1 [Source:HGNC Symbol;Acc:HGNC:31010] |
| ENSG00000198780 | HGLibA_16252 | 1 | -1.138650921 | FAM169A | family with sequence similarity 169 member A [Source:HGNC Symbol;Acc:HGNC:29138] |
| ENSG00000198821 | HGLibA_08308 | 1 | -1.138650921 | CD247 | CD247 molecule [Source:HGNC Symbol;Acc:HGNC:1677] |
| ENSG00000198829 | HGLibA_47706 | 1 | -1.138650921 | SUCNR1 | succinate receptor 1 [Source:HGNC Symbol;Acc:HGNC:4542] |
| ENSG00000198912 | HGLibA_05799 | 1 | -1.138650921 | C1orf174 | chromosome 1 open reading frame 174 [Source:HGNC Symbol;Acc:HGNC:27915] |
| ENSG00000198965 | HGLibA_33542 | 1 | -1.138650921 | OR10R2 | olfactory receptor family 10 subfamily R member 2 [Source:HGNC Symbol;Acc:HGNC:14820] |
| ENSG00000203785 | HGLibA_46818 | 1 | -1.138650921 | SPRR2E | small proline rich protein 2E [Source:HGNC Symbol;Acc:HGNC:11265] |
| ENSG00000203795 | HGLibA_16545 | 1 | -1.138650921 | FAM24A | family with sequence similarity 24 member A [Source:HGNC Symbol;Acc:HGNC:23470] |
| ENSG00000203933 | HGLibA_11962 | 1 | -1.138650921 | CXorf66 | chromosome X open reading frame 66 [Source:HGNC Symbol;Acc:HGNC:33743] |
| ENSG00000204070 | HGLibA_48042 | 1 | -1.138650921 | SYS1 | golgi trafficking protein [Source:HGNC Symbol;Acc:HGNC:16162]" |
| ENSG00000204228 | HGLibA_22202 | 1 | -1.138650921 | HSD17B8 | hydroxysteroid 17-beta dehydrogenase 8 [Source:HGNC Symbol;Acc:HGNC:3554] |
| ENSG00000204278 | HGLibA_50269 | 1 | -1.138650921 | TMEM235 | transmembrane protein 235 [Source:HGNC Symbol;Acc:HGNC:27563] |
| ENSG00000204301 | HGLibA_32351 | 1 | -1.138650921 | NOTCH4 | notch 4 [Source:HGNC Symbol;Acc:HGNC:7884] |
| ENSG00000204308 | HGLibA_41690 | 1 | -1.138650921 | RNF5 | ring finger protein 5 [Source:HGNC Symbol;Acc:HGNC:10068] |
| ENSG00000204389 | HGLibA_22270 | 1 | -1.138650921 | HSPA1A | heat shock protein family A (Hsp70) member 1A [Source:HGNC Symbol;Acc:HGNC:5232] |
| ENSG00000204435 | HGLibA_11412 | 1 | -1.138650921 | CSNK2B | casein kinase 2 beta [Source:HGNC Symbol;Acc:HGNC:2460] |
| ENSG00000204469 | HGLibA_38860 | 1 | -1.138650921 | PRRC2A | proline rich coiled-coil 2A [Source:HGNC Symbol;Acc:HGNC:13918] |
| ENSG00000204475 | HGLibA_31369 | 1 | -1.138650921 | NCR3 | natural cytotoxicity triggering receptor 3 [Source:HGNC Symbol;Acc:HGNC:19077] |
| ENSG00000204713 | HGLibA_51448 | 1 | -1.138650921 | TRIM27 | tripartite motif containing 27 [Source:HGNC Symbol;Acc:HGNC:9975] |
| ENSG00000204950 | HGLibA_27250 | 1 | -1.138650921 | LRRC10B | leucine rich repeat containing 10B [Source:HGNC Symbol;Acc:HGNC:37215] |
| ENSG00000205238 | HGLibA_46582 | 1 | -1.138650921 | SPDYE2 | speedy/RINGO cell cycle regulator family member E2 [Source:HGNC Symbol;Acc:HGNC:33841] |
| ENSG00000205309 | HGLibA_32833 | 1 | -1.138650921 | NT5M | mitochondrial [Source:HGNC Symbol;Acc:HGNC:15769]" |
| ENSG00000205323 | HGLibA_42746 | 1 | -1.138650921 | SARNP | SAP domain containing ribonucleoprotein [Source:HGNC Symbol;Acc:HGNC:24432] |
| ENSG00000205457 | HGLibA_51083 | 1 | -1.138650921 | TP53TG3C | TP53 target 3C [Source:HGNC Symbol;Acc:HGNC:42962] |
| ENSG00000205629 | HGLibA_26160 | 1 | -1.138650921 | LCMT1 | leucine carboxyl methyltransferase 1 [Source:HGNC Symbol;Acc:HGNC:17557] |
| ENSG00000205649 | HGLibA_22353 | 1 | -1.138650921 | HTN3 | histatin 3 [Source:HGNC Symbol;Acc:HGNC:5284] |
| ENSG00000205678 | HGLibA_48950 | 1 | -1.138650921 | TECRL | 3-enoyl-CoA reductase like [Source:HGNC Symbol;Acc:HGNC:27365]" |
| ENSG00000205683 | HGLibA_13834 | 1 | -1.138650921 | DPF3 | double PHD fingers 3 [Source:HGNC Symbol;Acc:HGNC:17427] |
| ENSG00000205765 | HGLibA_06401 | 1 | -1.138650921 | C5orf51 | chromosome 5 open reading frame 51 [Source:HGNC Symbol;Acc:HGNC:27750] |
| ENSG00000205916 | HGLibA_12360 | 1 | -1.138650921 | DAZ4 | deleted in azoospermia 4 [Source:HGNC Symbol;Acc:HGNC:15966] |
| ENSG00000206026 | HGLibA_45749 | 1 | -1.138650921 | SMIM21 | small integral membrane protein 21 [Source:HGNC Symbol;Acc:HGNC:27598] |
| ENSG00000206384 | HGLibA_10608 | 1 | -1.138650921 | COL6A6 | collagen type VI alpha 6 chain [Source:HGNC Symbol;Acc:HGNC:27023] |
| ENSG00000211448 | HGLibA_13240 | 1 | -1.138650921 | DIO2 | iodothyronine deiodinase 2 [Source:HGNC Symbol;Acc:HGNC:2884] |
| ENSG00000213347 | HGLibA_30614 | 1 | -1.138650921 | MXD3 | MAX dimerization protein 3 [Source:HGNC Symbol;Acc:HGNC:14008] |
| ENSG00000213639 | HGLibA_38009 | 1 | -1.138650921 | PPP1CB | protein phosphatase 1 catalytic subunit beta [Source:HGNC Symbol;Acc:HGNC:9282] |
| ENSG00000213759 | HGLibA_52915 | 1 | -1.138650921 | UGT2B11 | UDP glucuronosyltransferase family 2 member B11 [Source:HGNC Symbol;Acc:HGNC:12545] |
| ENSG00000213965 | HGLibA_32964 | 1 | -1.138650921 | NUDT19 | nudix hydrolase 19 [Source:HGNC Symbol;Acc:HGNC:32036] |
| ENSG00000214491 | HGLibA_43204 | 1 | -1.138650921 | SEC14L6 | SEC14 like lipid binding 6 [Source:HGNC Symbol;Acc:HGNC:40047] |
| ENSG00000214595 | HGLibA_15072 | 1 | -1.138650921 | EML6 | echinoderm microtubule associated protein like 6 [Source:HGNC Symbol;Acc:HGNC:35412] |
| ENSG00000214860 | HGLibA_15705 | 1 | -1.138650921 | EVPLL | envoplakin like [Source:HGNC Symbol;Acc:HGNC:35236] |
| ENSG00000221864 | HGLibA_25632 | 1 | -1.138650921 | KRTAP12-2 | keratin associated protein 12-2 [Source:HGNC Symbol;Acc:HGNC:20530] |
| ENSG00000221890 | HGLibA_32538 | 1 | -1.138650921 | NPTXR | neuronal pentraxin receptor [Source:HGNC Symbol;Acc:HGNC:7954] |
| ENSG00000222036 | HGLibA_37748 | 1 | -1.138650921 | POTEM | POTE ankyrin domain family member M [Source:HGNC Symbol;Acc:HGNC:37096] |
| ENSG00000224470 | HGLibA_03833 | 1 | -1.138650921 | ATXN1L | ataxin 1 like [Source:HGNC Symbol;Acc:HGNC:33279] |
| ENSG00000224712 | HGLibA_32470 | 1 | -1.138650921 | NPIPA3 | nuclear pore complex interacting protein family member A3 [Source:HGNC Symbol;Acc:HGNC:41978] |
| ENSG00000224902 | HGLibA_18455 | 1 | -1.138650921 | GAGE12H | G antigen 12H [Source:HGNC Symbol;Acc:HGNC:31908] |
| ENSG00000225556 | HGLibA_06105 | 1 | -1.138650921 | C2CD4D | C2 calcium dependent domain containing 4D [Source:HGNC Symbol;Acc:HGNC:37210] |
| ENSG00000225614 | HGLibA_56051 | 1 | -1.138650921 | ZNF469 | zinc finger protein 469 [Source:HGNC Symbol;Acc:HGNC:23216] |
| ENSG00000226784 | HGLibA_36202 | 1 | -1.138650921 | PGAM4 | phosphoglycerate mutase family member 4 [Source:HGNC Symbol;Acc:HGNC:21731] |
| ENSG00000226941 | HGLibA_40625 | 1 | -1.138650921 | RBMY1J | member J [Source:HGNC Symbol;Acc:HGNC:23917]" |
| ENSG00000227059 | HGLibA_01932 | 1 | -1.138650921 | ANHX | anomalous homeobox [Source:HGNC Symbol;Acc:HGNC:40024] |
| ENSG00000228517 | HGLibA_11544 | 1 | -1.138650921 | CT47A7 | member A7 [Source:HGNC Symbol;Acc:HGNC:33288]" |
| ENSG00000228696 | HGLibA_02953 | 1 | -1.138650921 | ARL17B | ADP ribosylation factor like GTPase 17B [Source:HGNC Symbol;Acc:HGNC:32387] |

| | | | | | |
|---|---|---|---|---|---|
| ENSG00000230031 | HGLibA_37721 | 1 | -1.138650921 | POTEB2 | POTE ankyrin domain family member B2 [Source:HGNC Symbol;Acc:HGNC:48327] |
| ENSG00000230124 | HGLibA_00406 | 1 | -1.138650921 | ACBD6 | acyl-CoA binding domain containing 6 [Source:HGNC Symbol;Acc:HGNC:23339] |
| ENSG00000230178 | HGLibA_34003 | 1 | -1.138650921 | OR4F3 | olfactory receptor family 4 subfamily F member 3 [Source:HGNC Symbol;Acc:HGNC:8300] |
| ENSG00000230797 | HGLibA_54764 | 1 | -1.138650921 | YY2 | YY2 transcription factor [Source:HGNC Symbol;Acc:HGNC:31684] |
| ENSG00000231389 | HGLibA_21616 | 1 | -1.138650921 | HLA-DPA1 | DP alpha 1 [Source:HGNC Symbol;Acc:HGNC:4938]" |
| ENSG00000232629 | HGLibA_21629 | 1 | -1.138650921 | HLA-DQB2 | DQ beta 2 [Source:HGNC Symbol;Acc:HGNC:4945]" |
| ENSG00000233816 | HGLibA_22662 | 1 | -1.138650921 | IFNA13 | interferon alpha 13 [Source:HGNC Symbol;Acc:HGNC:5419] |
| ENSG00000235961 | HGLibA_37378 | 1 | -1.138650921 | PNMA6A | PNMA family member 6A [Source:HGNC Symbol;Acc:HGNC:28248] |
| ENSG00000236126 | HGLibA_11532 | 1 | -1.138650921 | CT47A3 | member A3 [Source:HGNC Symbol;Acc:HGNC:33284]" |
| ENSG00000236438 | HGLibA_16191 | 1 | -1.138650921 | FAM157A | family with sequence similarity 157 member A [Source:HGNC Symbol;Acc:HGNC:34079] |
| ENSG00000237110 | HGLibA_48139 | 1 | -1.138650921 | TAAR9 | trace amine associated receptor 9 (gene/pseudogene) [Source:HGNC Symbol;Acc:HGNC:20977] |
| ENSG00000237353 | HGLibA_35281 | 1 | -1.138650921 | PATE4 | prostate and testis expressed 4 [Source:HGNC Symbol;Acc:HGNC:35427] |
| ENSG00000239305 | HGLibA_41457 | 1 | -1.138650921 | RNF103 | ring finger protein 103 [Source:HGNC Symbol;Acc:HGNC:12859] |
| ENSG00000240403 | HGLibA_24995 | 1 | -1.138650921 | KIR3DL2 | three Ig domains and long cytoplasmic tail 2 [Source:HGNC Symbol;Acc:HGNC:6339]" |
| ENSG00000240694 | HGLibA_37368 | 1 | -1.138650921 | PNMA2 | PNMA family member 2 [Source:HGNC Symbol;Acc:HGNC:9159] |
| ENSG00000241644 | HGLibA_23316 | 1 | -1.138650921 | INMT | indolethylamine N-methyltransferase [Source:HGNC Symbol;Acc:HGNC:6069] |
| ENSG00000241697 | HGLibA_49832 | 1 | -1.138650921 | TMEFF1 | transmembrane protein with EGF like and two follistatin like domains 1 [Source:HGNC Symbol;Acc:HGNC:11866] |
| ENSG00000242852 | HGLibA_56604 | 1 | -1.138650921 | ZNF709 | zinc finger protein 709 [Source:HGNC Symbol;Acc:HGNC:20629] |
| ENSG00000243480 | HGLibA_01849 | 1 | -1.138650921 | AMY2A | alpha 2A (pancreatic) [Source:HGNC Symbol;Acc:HGNC:477]" |
| ENSG00000243772 | HGLibA_24984 | 1 | -1.138650921 | KIR2DL3 | two Ig domains and long cytoplasmic tail 3 [Source:HGNC Symbol;Acc:HGNC:6331]" |
| ENSG00000244607 | HGLibA_07590 | 1 | -1.138650921 | CCDC13 | coiled-coil domain containing 13 [Source:HGNC Symbol;Acc:HGNC:26358] |
| ENSG00000244754 | HGLibA_30957 | 1 | -1.138650921 | N4BP2L2 | NEDD4 binding protein 2 like 2 [Source:HGNC Symbol;Acc:HGNC:26916] |
| ENSG00000249104 | HGLibA_53229 | 1 | -1.138650921 | USP17L17 | ubiquitin specific peptidase 17-like family member 17 [Source:HGNC Symbol;Acc:HGNC:44445] |
| ENSG00000250317 | HGLibA_45746 | 1 | -1.138650921 | SMIM20 | small integral membrane protein 20 [Source:HGNC Symbol;Acc:HGNC:37260] |
| ENSG00000254466 | HGLibA_33967 | 1 | -1.138650921 | OR4D10 | olfactory receptor family 4 subfamily D member 10 [Source:HGNC Symbol;Acc:HGNC:15173] |
| ENSG00000254997 | HGLibA_25812 | 1 | -1.138650921 | KRTAP5-9 | keratin associated protein 5-9 [Source:HGNC Symbol;Acc:HGNC:23604] |
| ENSG00000255071 | HGLibA_42614 | 1 | -1.138650921 | SAA2-SAA4 | SAA2-SAA4 readthrough [Source:HGNC Symbol;Acc:HGNC:39550] |
| ENSG00000255245 | HGLibA_18241 | 1 | -1.138650921 | FXYD6-FXYD2 | FXYD6-FXYD2 readthrough [Source:HGNC Symbol;Acc:HGNC:39978] |
| ENSG00000255974 | HGLibA_12106 | 1 | -1.138650921 | CYP2A6 | cytochrome P450 family 2 subfamily A member 6 [Source:HGNC Symbol;Acc:HGNC:2610] |
| ENSG00000256806 | HGLibA_05515 | 1 | -1.138650921 | C17orf100 | chromosome 17 open reading frame 100 [Source:HGNC Symbol;Acc:HGNC:34494] |
| ENSG00000256806 | HGLibA_05516 | 1 | -1.138650921 | C17orf100 | chromosome 17 open reading frame 100 [Source:HGNC Symbol;Acc:HGNC:34494] |
| ENSG00000258083 | HGLibA_34564 | 1 | -1.138650921 | OR9A4 | olfactory receptor family 9 subfamily A member 4 [Source:HGNC Symbol;Acc:HGNC:15095] |
| ENSG00000261115 | HGLibA_50085 | 1 | -1.138650921 | TMEM178B | transmembrane protein 178B [Source:HGNC Symbol;Acc:HGNC:44112] |
| ENSG00000261236 | HGLibA_04694 | 1 | -1.138650921 | BOP1 | block of proliferation 1 [Source:HGNC Symbol;Acc:HGNC:15519] |
| ENSG00000261587 | HGLibA_50313 | 1 | -1.138650921 | TMEM249 | transmembrane protein 249 [Source:HGNC Symbol;Acc:HGNC:44155] |
| ENSG00000261609 | HGLibA_18602 | 1 | -1.138650921 | GAN | gigaxonin [Source:HGNC Symbol;Acc:HGNC:4137] |
| ENSG00000262628 | HGLibA_33656 | 1 | -1.138650921 | OR1D5 | olfactory receptor family 1 subfamily D member 5 [Source:HGNC Symbol;Acc:HGNC:8186] |
| ENSG00000266028 | HGLibA_46969 | 1 | -1.138650921 | SRGAP2 | SLIT-ROBO Rho GTPase activating protein 2 [Source:HGNC Symbol;Acc:HGNC:19751] |
| ENSG00000266173 | HGLibA_47572 | 1 | -1.138650921 | STRADA | STE20-related kinase adaptor alpha [Source:HGNC Symbol;Acc:HGNC:30172] |
| ENSG00000269058 | HGLibA_07050 | 1 | -1.138650921 | CALR3 | calreticulin 3 [Source:HGNC Symbol;Acc:HGNC:20407] |
| ENSG00000274211 | HGLibA_46140 | 1 | -1.138650921 | SOCS7 | suppressor of cytokine signaling 7 [Source:HGNC Symbol;Acc:HGNC:29846] |
| ENSG00000274226 | HGLibA_48555 | 1 | -1.138650921 | TBC1D3H | TBC1 domain family member 3H [Source:HGNC Symbol;Acc:HGNC:30708] |
| ENSG00000274588 | HGLibA_13079 | 1 | -1.138650921 | DGKK | diacylglycerol kinase kappa [Source:HGNC Symbol;Acc:HGNC:32395] |
| ENSG00000274808 | HGLibA_48543 | 1 | -1.138650921 | TBC1D3B | TBC1 domain family member 3B [Source:HGNC Symbol;Acc:HGNC:27011] |
| ENSG00000275793 | HGLibA_41261 | 1 | -1.138650921 | RIMBP3 | RIMS binding protein 3 [Source:HGNC Symbol;Acc:HGNC:29344] |
| ENSG00000275896 | HGLibA_38907 | 1 | -1.138650921 | PRSS2 | serine protease 2 [Source:HGNC Symbol;Acc:HGNC:9483] |
| ENSG00000275969 | HGLibA_46473 | 1 | -1.138650921 | SPATA31A3 | SPATA31 subfamily A member 3 [Source:HGNC Symbol;Acc:HGNC:32003] |
| ENSG00000276043 | HGLibA_52945 | 1 | -1.138650921 | UHRF1 | ubiquitin like with PHD and ring finger domains 1 [Source:HGNC Symbol;Acc:HGNC:12556] |
| ENSG00000277443 | HGLibA_28354 | 1 | -1.138650921 | MARCKS | myristoylated alanine rich protein kinase C substrate [Source:HGNC Symbol;Acc:HGNC:6759] |
| ENSG00000277462 | HGLibA_56500 | 1 | -1.138650921 | ZNF670 | zinc finger protein 670 [Source:HGNC Symbol;Acc:HGNC:28167] |
| ENSG00000277494 | HGLibA_19809 | 1 | -1.138650921 | GPIHBP1 | glycosylphosphatidylinositol anchored high density lipoprotein binding protein 1 [Source:HGNC Symbol;Acc:HGNC:24945] |
| ENSG00000278053 | HGLibA_12738 | 1 | -1.138650921 | DDX52 | DExD-box helicase 52 [Source:HGNC Symbol;Acc:HGNC:20038] |
| ENSG00000278299 | HGLibA_48544 | 1 | -1.138650921 | TBC1D3C | TBC1 domain family member 3C [Source:HGNC Symbol;Acc:HGNC:24889] |
| ENSG00000278870 | HGLibA_34109 | 1 | -1.138650921 | OR51G1 | olfactory receptor family 51 subfamily G member 1 (gene/pseudogene) [Source:HGNC Symbol;Acc:HGNC:14738] |
| ENSG00000279263 | HGLibA_33830 | 1 | -1.138650921 | OR2L8 | olfactory receptor family 2 subfamily L member 8 (gene/pseudogene) [Source:HGNC Symbol;Acc:HGNC:15014] |
| ENSG00000280094 | HGLibA_33648 | 1 | -1.138650921 | OR1B1 | olfactory receptor family 1 subfamily B member 1 (gene/pseudogene) [Source:HGNC Symbol;Acc:HGNC:8181] |
| ENSG00000289549 | HGLibA_37906 | 1 | -1.138650921 | PPIAL4D | - |
| FAM105B | HGLibA_15998 | 1 | -1.138650921 | FAM105B | - |
| FAM132A | HGLibA_16115 | 1 | -1.138650921 | FAM132A | - |
| FAM192A | HGLibA_16360 | 1 | -1.138650921 | FAM192A | - |
| FAM195B | HGLibA_16377 | 1 | -1.138650921 | FAM195B | - |
| FAM198A | HGLibA_16386 | 1 | -1.138650921 | FAM198A | - |
| FAM19A4 | HGLibA_16404 | 1 | -1.138650921 | FAM19A4 | - |
| FAM69A | HGLibA_16677 | 1 | -1.138650921 | FAM69A | - |
| FAM73B | HGLibA_16720 | 1 | -1.138650921 | FAM73B | - |
| GLTSCR2 | HGLibA_19359 | 1 | -1.138650921 | GLTSCR2 | - |
| HIST1H1T | HGLibA_21371 | 1 | -1.138650921 | HIST1H1T | - |
| HIST1H2AI | HGLibA_21395 | 1 | -1.138650921 | HIST1H2AI | - |
| HIST1H2BH | HGLibA_21433 | 1 | -1.138650921 | HIST1H2BH | - |
| HKR1 | HGLibA_21590 | 1 | -1.138650921 | HKR1 | - |
| HKR1 | HGLibA_21591 | 1 | -1.138650921 | HKR1 | - |
| HMHA1 | HGLibA_21737 | 1 | -1.138650921 | HMHA1 | - |
| hsa-let-7c | HGLibA_57112 | 1 | -1.138650921 | hsa-let-7c | - |
| hsa-let-7i | HGLibA_57136 | 1 | -1.138650921 | hsa-let-7i | - |
| hsa-mir-105-1 | HGLibA_57176 | 1 | -1.138650921 | hsa-mir-105-1 | - |
| hsa-mir-1199 | HGLibA_57256 | 1 | -1.138650921 | hsa-mir-1199 | - |
| hsa-mir-1207 | HGLibA_57283 | 1 | -1.138650921 | hsa-mir-1207 | - |
| hsa-mir-122 | HGLibA_57291 | 1 | -1.138650921 | hsa-mir-122 | - |
| hsa-mir-1229 | HGLibA_57316 | 1 | -1.138650921 | hsa-mir-1229 | - |
| hsa-mir-1233-1 | HGLibA_57322 | 1 | -1.138650921 | hsa-mir-1233-1 | - |
| hsa-mir-1233-2 | HGLibA_57326 | 1 | -1.138650921 | hsa-mir-1233-2 | - |
| hsa-mir-124-1 | HGLibA_57346 | 1 | -1.138650921 | hsa-mir-124-1 | - |
| hsa-mir-1248 | HGLibA_57390 | 1 | -1.138650921 | hsa-mir-1248 | - |
| hsa-mir-1278 | HGLibA_57568 | 1 | -1.138650921 | hsa-mir-1278 | - |
| hsa-mir-1279 | HGLibA_57569 | 1 | -1.138650921 | hsa-mir-1279 | - |
| hsa-mir-1323 | HGLibA_57766 | 1 | -1.138650921 | hsa-mir-1323 | - |
| hsa-mir-154 | HGLibA_57925 | 1 | -1.138650921 | hsa-mir-154 | - |
| hsa-mir-192 | HGLibA_58070 | 1 | -1.138650921 | hsa-mir-192 | - |
| hsa-mir-194-2 | HGLibA_58084 | 1 | -1.138650921 | hsa-mir-194-2 | - |
| hsa-mir-1976 | HGLibA_58122 | 1 | -1.138650921 | hsa-mir-1976 | - |
| hsa-mir-19a | HGLibA_58141 | 1 | -1.138650921 | hsa-mir-19a | - |
| hsa-mir-210 | HGLibA_58214 | 1 | -1.138650921 | hsa-mir-210 | - |
| hsa-mir-221 | HGLibA_58294 | 1 | -1.138650921 | hsa-mir-221 | - |
| hsa-mir-2909 | HGLibA_58393 | 1 | -1.138650921 | hsa-mir-2909 | - |
| hsa-mir-3125 | HGLibA_58552 | 1 | -1.138650921 | hsa-mir-3125 | - |
| hsa-mir-3138 | HGLibA_58611 | 1 | -1.138650921 | hsa-mir-3138 | - |
| hsa-mir-3141 | HGLibA_58621 | 1 | -1.138650921 | hsa-mir-3141 | - |
| hsa-mir-3155b | HGLibA_58685 | 1 | -1.138650921 | hsa-mir-3155b | - |
| hsa-mir-3170 | HGLibA_58760 | 1 | -1.138650921 | hsa-mir-3170 | - |
| hsa-mir-3185 | HGLibA_58838 | 1 | -1.138650921 | hsa-mir-3185 | - |
| hsa-mir-3186 | HGLibA_58840 | 1 | -1.138650921 | hsa-mir-3186 | - |
| hsa-mir-3201 | HGLibA_58911 | 1 | -1.138650921 | hsa-mir-3201 | - |
| hsa-mir-320b-1 | HGLibA_58926 | 1 | -1.138650921 | hsa-mir-320b-1 | - |
| hsa-mir-324 | HGLibA_58964 | 1 | -1.138650921 | hsa-mir-324 | - |
| hsa-mir-342 | HGLibA_59025 | 1 | -1.138650921 | hsa-mir-342 | - |
| hsa-mir-346 | HGLibA_59031 | 1 | -1.138650921 | hsa-mir-346 | - |
| hsa-mir-3656 | HGLibA_59170 | 1 | -1.138650921 | hsa-mir-3656 | - |
| hsa-mir-3661 | HGLibA_59196 | 1 | -1.138650921 | hsa-mir-3661 | - |
| hsa-mir-3662 | HGLibA_59199 | 1 | -1.138650921 | hsa-mir-3662 | - |
| hsa-mir-3689a | HGLibA_59313 | 1 | -1.138650921 | hsa-mir-3689a | - |
| hsa-mir-3689f | HGLibA_59337 | 1 | -1.138650921 | hsa-mir-3689f | - |
| hsa-mir-380 | HGLibA_59475 | 1 | -1.138650921 | hsa-mir-380 | - |
| hsa-mir-383 | HGLibA_59484 | 1 | -1.138650921 | hsa-mir-383 | - |
| hsa-mir-3914-2 | HGLibA_59531 | 1 | -1.138650921 | hsa-mir-3914-2 | - |
| hsa-mir-3917 | HGLibA_59546 | 1 | -1.138650921 | hsa-mir-3917 | - |
| hsa-mir-3943 | HGLibA_59630 | 1 | -1.138650921 | hsa-mir-3943 | - |
| hsa-mir-3944 | HGLibA_59635 | 1 | -1.138650921 | hsa-mir-3944 | - |
| hsa-mir-4257 | HGLibA_59734 | 1 | -1.138650921 | hsa-mir-4257 | - |
| hsa-mir-4258 | HGLibA_59741 | 1 | -1.138650921 | hsa-mir-4258 | - |
| hsa-mir-4267 | HGLibA_59775 | 1 | -1.138650921 | hsa-mir-4267 | - |
| hsa-mir-4278 | HGLibA_59815 | 1 | -1.138650921 | hsa-mir-4278 | - |
| hsa-mir-4312 | HGLibA_59963 | 1 | -1.138650921 | hsa-mir-4312 | - |
| hsa-mir-4313 | HGLibA_59967 | 1 | -1.138650921 | hsa-mir-4313 | - |
| hsa-mir-4324 | HGLibA_60021 | 1 | -1.138650921 | hsa-mir-4324 | - |
| hsa-mir-4326 | HGLibA_60029 | 1 | -1.138650921 | hsa-mir-4326 | - |
| hsa-mir-4439 | HGLibA_60160 | 1 | -1.138650921 | hsa-mir-4439 | - |
| hsa-mir-4451 | HGLibA_60210 | 1 | -1.138650921 | hsa-mir-4451 | - |
| hsa-mir-4451 | HGLibA_60211 | 1 | -1.138650921 | hsa-mir-4451 | - |
| hsa-mir-4462 | HGLibA_60256 | 1 | -1.138650921 | hsa-mir-4462 | - |
| hsa-mir-4478 | HGLibA_60325 | 1 | -1.138650921 | hsa-mir-4478 | - |
| hsa-mir-4481 | HGLibA_60342 | 1 | -1.138650921 | hsa-mir-4481 | - |
| hsa-mir-4485 | HGLibA_60358 | 1 | -1.138650921 | hsa-mir-4485 | - |
| hsa-mir-4509-3 | HGLibA_60472 | 1 | -1.138650921 | hsa-mir-4509-3 | - |
| hsa-mir-4509-3 | HGLibA_60470 | 1 | -1.138650921 | hsa-mir-4509-3 | - |
| hsa-mir-4509-3 | HGLibA_60469 | 1 | -1.138650921 | hsa-mir-4509-3 | - |

| | | | | | |
|---|---|---|---|---|---|
| hsa-mir-4535 | HGLibA_60598 | 1 | -1.138650921 | hsa-mir-4535 | - |
| hsa-mir-4537 | HGLibA_60608 | 1 | -1.138650921 | hsa-mir-4537 | - |
| hsa-mir-4642 | HGLibA_60672 | 1 | -1.138650921 | hsa-mir-4642 | - |
| hsa-mir-4648 | HGLibA_60695 | 1 | -1.138650921 | hsa-mir-4648 | - |
| hsa-mir-4656 | HGLibA_60731 | 1 | -1.138650921 | hsa-mir-4656 | - |
| hsa-mir-4732 | HGLibA_61025 | 1 | -1.138650921 | hsa-mir-4732 | - |
| hsa-mir-4734 | HGLibA_61033 | 1 | -1.138650921 | hsa-mir-4734 | - |
| hsa-mir-4739 | HGLibA_61054 | 1 | -1.138650921 | hsa-mir-4739 | - |
| hsa-mir-4748 | HGLibA_61088 | 1 | -1.138650921 | hsa-mir-4748 | - |
| hsa-mir-4771-2 | HGLibA_61183 | 1 | -1.138650921 | hsa-mir-4771-2 | - |
| hsa-mir-4773-1 | HGLibA_61192 | 1 | -1.138650921 | hsa-mir-4773-1 | - |
| hsa-mir-4776-1 | HGLibA_61208 | 1 | -1.138650921 | hsa-mir-4776-1 | - |
| hsa-mir-4786 | HGLibA_61251 | 1 | -1.138650921 | hsa-mir-4786 | - |
| hsa-mir-4791 | HGLibA_61272 | 1 | -1.138650921 | hsa-mir-4791 | - |
| hsa-mir-498 | HGLibA_61392 | 1 | -1.138650921 | hsa-mir-498 | - |
| hsa-mir-5003 | HGLibA_61421 | 1 | -1.138650921 | hsa-mir-5003 | - |
| hsa-mir-513b | HGLibA_61577 | 1 | -1.138650921 | hsa-mir-513b | - |
| hsa-mir-513c | HGLibA_61584 | 1 | -1.138650921 | hsa-mir-513c | - |
| hsa-mir-515-1 | HGLibA_61603 | 1 | -1.138650921 | hsa-mir-515-1 | - |
| hsa-mir-518d | HGLibA_61668 | 1 | -1.138650921 | hsa-mir-518d | - |
| hsa-mir-519d | HGLibA_61725 | 1 | -1.138650921 | hsa-mir-519d | - |
| hsa-mir-522 | HGLibA_61773 | 1 | -1.138650921 | hsa-mir-522 | - |
| hsa-mir-544a | HGLibA_61823 | 1 | -1.138650921 | hsa-mir-544a | - |
| hsa-mir-548f-1 | HGLibA_61974 | 1 | -1.138650921 | hsa-mir-548f-1 | - |
| hsa-mir-548f-3 | HGLibA_61981 | 1 | -1.138650921 | hsa-mir-548f-3 | - |
| hsa-mir-548f-5 | HGLibA_61989 | 1 | -1.138650921 | hsa-mir-548f-5 | - |
| hsa-mir-548h-2 | HGLibA_61999 | 1 | -1.138650921 | hsa-mir-548h-2 | - |
| hsa-mir-548i-3 | HGLibA_62022 | 1 | -1.138650921 | hsa-mir-548i-3 | - |
| hsa-mir-548o | HGLibA_62047 | 1 | -1.138650921 | hsa-mir-548o | - |
| hsa-mir-5582 | HGLibA_62166 | 1 | -1.138650921 | hsa-mir-5582 | - |
| hsa-mir-559 | HGLibA_62199 | 1 | -1.138650921 | hsa-mir-559 | - |
| hsa-mir-567 | HGLibA_62229 | 1 | -1.138650921 | hsa-mir-567 | - |
| hsa-mir-5681a | HGLibA_62241 | 1 | -1.138650921 | hsa-mir-5681a | - |
| hsa-mir-5688 | HGLibA_62269 | 1 | -1.138650921 | hsa-mir-5688 | - |
| hsa-mir-5691 | HGLibA_62284 | 1 | -1.138650921 | hsa-mir-5691 | - |
| hsa-mir-5696 | HGLibA_62317 | 1 | -1.138650921 | hsa-mir-5696 | - |
| hsa-mir-5697 | HGLibA_62321 | 1 | -1.138650921 | hsa-mir-5697 | - |
| hsa-mir-572 | HGLibA_62379 | 1 | -1.138650921 | hsa-mir-572 | - |
| hsa-mir-582 | HGLibA_62427 | 1 | -1.138650921 | hsa-mir-582 | - |
| hsa-mir-603 | HGLibA_62503 | 1 | -1.138650921 | hsa-mir-603 | - |
| hsa-mir-6073 | HGLibA_62544 | 1 | -1.138650921 | hsa-mir-6073 | - |
| hsa-mir-6089-2 | HGLibA_62620 | 1 | -1.138650921 | hsa-mir-6089-2 | - |
| hsa-mir-610 | HGLibA_62633 | 1 | -1.138650921 | hsa-mir-610 | - |
| hsa-mir-6125 | HGLibA_62647 | 1 | -1.138650921 | hsa-mir-6125 | - |
| hsa-mir-631 | HGLibA_62763 | 1 | -1.138650921 | hsa-mir-631 | - |
| hsa-mir-631 | HGLibA_62765 | 1 | -1.138650921 | hsa-mir-631 | - |
| hsa-mir-6508 | HGLibA_62883 | 1 | -1.138650921 | hsa-mir-6508 | - |
| hsa-mir-6511a-4 | HGLibA_62909 | 1 | -1.138650921 | hsa-mir-6511a-4 | - |
| hsa-mir-658 | HGLibA_62966 | 1 | -1.138650921 | hsa-mir-658 | - |
| hsa-mir-6721 | HGLibA_63046 | 1 | -1.138650921 | hsa-mir-6721 | - |
| hsa-mir-6760 | HGLibA_63201 | 1 | -1.138650921 | hsa-mir-6760 | - |
| hsa-mir-6764 | HGLibA_63217 | 1 | -1.138650921 | hsa-mir-6764 | - |
| hsa-mir-6765 | HGLibA_63220 | 1 | -1.138650921 | hsa-mir-6765 | - |
| hsa-mir-6780b | HGLibA_63297 | 1 | -1.138650921 | hsa-mir-6780b | - |
| hsa-mir-6787 | HGLibA_63327 | 1 | -1.138650921 | hsa-mir-6787 | - |
| hsa-mir-6801 | HGLibA_63381 | 1 | -1.138650921 | hsa-mir-6801 | - |
| hsa-mir-6812 | HGLibA_63425 | 1 | -1.138650921 | hsa-mir-6812 | - |
| hsa-mir-6827 | HGLibA_63486 | 1 | -1.138650921 | hsa-mir-6827 | - |
| hsa-mir-6867 | HGLibA_63652 | 1 | -1.138650921 | hsa-mir-6867 | - |
| hsa-mir-7112-1 | HGLibA_63814 | 1 | -1.138650921 | hsa-mir-7112-1 | - |
| hsa-mir-7-3 | HGLibA_63775 | 1 | -1.138650921 | hsa-mir-7-3 | - |
| hsa-mir-744 | HGLibA_63883 | 1 | -1.138650921 | hsa-mir-744 | - |
| hsa-mir-759 | HGLibA_63898 | 1 | -1.138650921 | hsa-mir-759 | - |
| hsa-mir-766 | HGLibA_63929 | 1 | -1.138650921 | hsa-mir-766 | - |
| hsa-mir-7845 | HGLibA_63968 | 1 | -1.138650921 | hsa-mir-7845 | - |
| hsa-mir-7853 | HGLibA_64002 | 1 | -1.138650921 | hsa-mir-7853 | - |
| hsa-mir-8057 | HGLibA_64067 | 1 | -1.138650921 | hsa-mir-8057 | - |
| hsa-mir-8059 | HGLibA_64076 | 1 | -1.138650921 | hsa-mir-8059 | - |
| hsa-mir-8064 | HGLibA_64095 | 1 | -1.138650921 | hsa-mir-8064 | - |
| hsa-mir-8083 | HGLibA_64175 | 1 | -1.138650921 | hsa-mir-8083 | - |
| hsa-mir-936 | HGLibA_64320 | 1 | -1.138650921 | hsa-mir-936 | - |
| KIAA0101 | HGLibA_24590 | 1 | -1.138650921 | KIAA0101 | - |
| KIAA0430 | HGLibA_24637 | 1 | -1.138650921 | KIAA0430 | - |
| LOC100129480 | HGLibA_26762 | 1 | -1.138650921 | LOC100129480 | - |
| LOC100130705 | HGLibA_26796 | 1 | -1.138650921 | LOC100130705 | - |
| LOC100289561 | HGLibA_26843 | 1 | -1.138650921 | LOC100289561 | - |
| LOC440563 | HGLibA_26948 | 1 | -1.138650921 | LOC440563 | - |
| LOC642778 | HGLibA_26958 | 1 | -1.138650921 | LOC642778 | - |
| LOC643037 | HGLibA_26963 | 1 | -1.138650921 | LOC643037 | - |
| LOC729020 | HGLibA_26997 | 1 | -1.138650921 | LOC729020 | - |
| LPPR2 | HGLibA_27115 | 1 | -1.138650921 | LPPR2 | - |
| LRRC48 | HGLibA_27373 | 1 | -1.138650921 | LRRC48 | - |
| METTL20 | HGLibA_28973 | 1 | -1.138650921 | METTL20 | - |
| NADKD1 | HGLibA_31050 | 1 | -1.138650921 | NADKD1 | - |
| PAPD4 | HGLibA_35121 | 1 | -1.138650921 | PAPD4 | - |
| PNMA6C | HGLibA_37381 | 1 | -1.138650921 | PNMA6C | - |
| PRAMEF16 | HGLibA_38296 | 1 | -1.138650921 | PRAMEF16 | - |
| PRR25 | HGLibA_38833 | 1 | -1.138650921 | PRR25 | - |
| RGAG1 | HGLibA_40993 | 1 | -1.138650921 | RGAG1 | - |
| RQCD1 | HGLibA_42218 | 1 | -1.138650921 | RQCD1 | - |
| SELO | HGLibA_43300 | 1 | -1.138650921 | SELO | - |
| 4-Sep | HGLibA_43442 | 1 | -1.138650921 | 4-Sep | - |
| 7-Sep | HGLibA_43451 | 1 | -1.138650921 | 7-Sep | - |
| 8-Sep | HGLibA_43455 | 1 | -1.138650921 | 8-Sep | - |
| TENC1 | HGLibA_48991 | 1 | -1.138650921 | TENC1 | - |
| TMEM159 | HGLibA_50018 | 1 | -1.138650921 | TMEM159 | - |
| TMEM57 | HGLibA_50455 | 1 | -1.138650921 | TMEM57 | - |
| WARS | HGLibA_53943 | 1 | -1.138650921 | WARS | - |
| WISP2 | HGLibA_54322 | 1 | -1.138650921 | WISP2 | - |

**Table S1 SgRNAs enriched in the sorted RED-negative cells (2nd)**

| gene | unique.id | count | Zscore | name | Description |
|---|---|---|---|---|---|
| ENSG00000131828 | HGLibA_35861 | 2263 | 19.3349175 | PDHA1 | pyruvate dehydrogenase E1 alpha 1 subunit [Source:HGNC Symbol;Acc:HGNC:8806] |
| ENSG00000172292 | HGLibA_09216 | 1709 | 14.32379753 | CERS6 | ceramide synthase 6 [Source:HGNC Symbol;Acc:HGNC:23826] |
| ENSG00000040933 | HGLibA_23337 | 1305 | 10.669479 | INPP4A | inositol polyphosphate-4-phosphatase type I A [Source:HGNC Symbol;Acc:HGNC:6074] |
| ELTD1 | HGLibA_14995 | 1089 | 8.715684933 | ELTD1 | - |
| ENSG00000116717 | HGLibA_18416 | 1054 | 8.399097931 | GADD45A | growth arrest and DNA damage inducible alpha [Source:HGNC Symbol;Acc:HGNC:4095] |
| ENSG00000090686 | HGLibA_53382 | 1037 | 8.245327102 | USP48 | ubiquitin specific peptidase 48 [Source:HGNC Symbol;Acc:HGNC:18533] |
| ENSG00000168772 | HGLibA_11971 | 931 | 7.286520754 | CXXC4 | CXXC finger protein 4 [Source:HGNC Symbol;Acc:HGNC:24593] |
| ENSG00000183638 | HGLibA_41791 | 911 | 7.105613896 | RP1L1 | RP1 like 1 [Source:HGNC Symbol;Acc:HGNC:15946] |
| ENSG00000127831 | HGLibA_53649 | 910 | 7.096568553 | VIL1 | villin 1 [Source:HGNC Symbol;Acc:HGNC:12690] |
| ENSG00000100503 | HGLibA_31934 | 892 | 6.933752381 | NIN | ninein [Source:HGNC Symbol;Acc:HGNC:14906] |
| ENSG00000262246 | HGLibA_10791 | 888 | 6.897571009 | CORO7 | coronin 7 [Source:HGNC Symbol;Acc:HGNC:26161] |
| ENSG00000136867 | HGLibA_44929 | 875 | 6.779981551 | SLC31A2 | solute carrier family 31 member 2 [Source:HGNC Symbol;Acc:HGNC:11017] |
| ENSG00000146281 | HGLibA_37287 | 866 | 6.698573465 | PM20D2 | peptidase M20 domain containing 2 [Source:HGNC Symbol;Acc:HGNC:21408] |
| ENSG00000172292 | HGLibA_09214 | 849 | 6.544802636 | CERS6 | ceramide synthase 6 [Source:HGNC Symbol;Acc:HGNC:23826] |
| ENSG00000121351 | HGLibA_22469 | 834 | 6.409122492 | IAPP | islet amyloid polypeptide [Source:HGNC Symbol;Acc:HGNC:5329] |
| ENSG00000171368 | HGLibA_51164 | 833 | 6.400077149 | TPPP | tubulin polymerization promoting protein [Source:HGNC Symbol;Acc:HGNC:24164] |
| ENSG00000135503 | HGLibA_00688 | 829 | 6.363895778 | ACVR1B | activin A receptor type 1B [Source:HGNC Symbol;Acc:HGNC:172] |
| hsa-mir-941-1 | HGLibA_64338 | 829 | 6.363895778 | hsa-mir-941-1 | - |
| ENSG00000198178 | HGLibA_09982 | 824 | 6.318669063 | CLEC4C | C-type lectin domain family 4 member C [Source:HGNC Symbol;Acc:HGNC:13258] |
| ENSG00000172292 | HGLibA_09215 | 821 | 6.291533034 | CERS6 | ceramide synthase 6 [Source:HGNC Symbol;Acc:HGNC:23826] |
| ENSG00000132031 | HGLibA_28434 | 817 | 6.255351663 | MATN3 | matrilin 3 [Source:HGNC Symbol;Acc:HGNC:6909] |
| ENSG00000204103 | HGLibA_27874 | 817 | 6.255351663 | MAFB | MAF bZIP transcription factor B [Source:HGNC Symbol;Acc:HGNC:6408] |
| ENSG00000060762 | HGLibA_29703 | 813 | 6.219170291 | MPC1 | mitochondrial pyruvate carrier 1 [Source:HGNC Symbol;Acc:HGNC:21606] |
| ENSG00000196154 | HGLibA_42556 | 809 | 6.182988919 | S100A4 | S100 calcium binding protein A4 [Source:HGNC Symbol;Acc:HGNC:10494] |
| ENSG00000168772 | HGLibA_11970 | 803 | 6.128716862 | CXXC4 | CXXC finger protein 4 [Source:HGNC Symbol;Acc:HGNC:24593] |
| ENSG00000087494 | HGLibA_39376 | 792 | 6.02921809 | PTHLH | parathyroid hormone like hormone [Source:HGNC Symbol;Acc:HGNC:9607] |
| ENSG00000127586 | HGLibA_09660 | 789 | 6.002082061 | CHTF18 | chromosome transmission fidelity factor 18 [Source:HGNC Symbol;Acc:HGNC:18435] |
| ENSG00000189280 | HGLibA_19169 | 784 | 5.956855347 | GJB5 | gap junction protein beta 5 [Source:HGNC Symbol;Acc:HGNC:4287] |
| ENSG00000172955 | HGLibA_00962 | 779 | 5.911628632 | ADH6 | alcohol dehydrogenase 6 (class V) [Source:HGNC Symbol;Acc:HGNC:255] |
| ENSG00000151914 | HGLibA_14042 | 775 | 5.875447261 | DST | dystonin [Source:HGNC Symbol;Acc:HGNC:1090] |
| ENSG00000163754 | HGLibA_20688 | 773 | 5.857356575 | GYG1 | glycogenin 1 [Source:HGNC Symbol;Acc:HGNC:4699] |
| ENSG00000121039 | HGLibA_40730 | 767 | 5.803084518 | RDH10 | retinol dehydrogenase 10 [Source:HGNC Symbol;Acc:HGNC:19975] |
| ENSG00000204644 | HGLibA_55223 | 762 | 5.757857803 | ZFP57 | ZFP57 zinc finger protein [Source:HGNC Symbol;Acc:HGNC:18791] |
| ENSG00000179314 | HGLibA_54420 | 759 | 5.730721774 | WSCD1 | WSC domain containing 1 [Source:HGNC Symbol;Acc:HGNC:29060] |

| | | | | | |
|---|---|---|---|---|---|
| ENSG00000165175 | HGLibA_29235 | 750 | 5.649313688 | MID1IP1 | MID1 interacting protein 1 [Source:HGNC Symbol;Acc:HGNC:20715] |
| ENSG00000140564 | HGLibA_18172 | 747 | 5.622177659 | FURIN | paired basic amino acid cleaving enzyme [Source:HGNC Symbol;Acc:HGNC:8568]" |
| ENSG00000162009 | HGLibA_47187 | 743 | 5.585996288 | SSTR5 | somatostatin receptor 5 [Source:HGNC Symbol;Acc:HGNC:11334] |
| ENSG00000159228 | HGLibA_07419 | 741 | 5.567905602 | CBR1 | carbonyl reductase 1 [Source:HGNC Symbol;Acc:HGNC:1548] |
| C4orf6 | HGLibA_06346 | 740 | 5.558860259 | C4orf6 | - |
| ENSG00000218891 | HGLibA_56303 | 738 | 5.540769573 | ZNF579 | zinc finger protein 579 [Source:HGNC Symbol;Acc:HGNC:26646] |
| ENSG00000174373 | HGLibA_40155 | 733 | 5.495542859 | RALGAPA1 | Ral GTPase activating protein catalytic alpha subunit 1 [Source:HGNC Symbol;Acc:HGNC:17770] |
| ENSG00000121410 | HGLibA_00003 | 732 | 5.486497516 | A1BG | alpha-1-B glycoprotein [Source:HGNC Symbol;Acc:HGNC:5] |
| ENSG00000053501 | HGLibA_53164 | 728 | 5.450316144 | USE1 | unconventional SNARE in the ER 1 [Source:HGNC Symbol;Acc:HGNC:30882] |
| hsa-mir-7106 | HGLibA_63785 | 728 | 5.450316144 | hsa-mir-7106 | - |
| ENSG00000182508 | HGLibA_26430 | 719 | 5.368908058 | LHFPL1 | LHFPL tetraspan subfamily member 1 [Source:HGNC Symbol;Acc:HGNC:6587] |
| hsa-mir-5682 | HGLibA_62249 | 719 | 5.368908058 | hsa-mir-5682 | - |
| ENSG00000162891 | HGLibA_23072 | 717 | 5.350817372 | IL20 | interleukin 20 [Source:HGNC Symbol;Acc:HGNC:6002] |
| ENSG00000184227 | HGLibA_00466 | 717 | 5.350817372 | ACOT1 | acyl-CoA thioesterase 1 [Source:HGNC Symbol;Acc:HGNC:33128] |
| ENSG00000131398 | HGLibA_24167 | 716 | 5.341772029 | KCNC3 | potassium voltage-gated channel subfamily C member 3 [Source:HGNC Symbol;Acc:HGNC:6235] |
| ENSG00000115919 | HGLibA_25880 | 713 | 5.314636001 | KYNU | kynureninase [Source:HGNC Symbol;Acc:HGNC:6469] |
| ENSG00000162897 | HGLibA_17163 | 713 | 5.314636001 | FCAMR | Fc fragment of IgA and IgM receptor [Source:HGNC Symbol;Acc:HGNC:24692] |
| hsa-mir-7848 | HGLibA_63980 | 711 | 5.296545315 | hsa-mir-7848 | - |
| ENSG00000099977 | HGLibA_12643 | 707 | 5.260363943 | DDT | D-dopachrome tautomerase [Source:HGNC Symbol;Acc:HGNC:2732] |
| ENSG00000111716 | HGLibA_26230 | 699 | 5.1880012 | LDHB | lactate dehydrogenase B [Source:HGNC Symbol;Acc:HGNC:6541] |
| ENSG00000119922 | HGLibA_22630 | 698 | 5.178955857 | IFIT2 | interferon induced protein with tetratricopeptide repeats 2 [Source:HGNC Symbol;Acc:HGNC:5409] |
| ENSG00000149599 | HGLibA_14146 | 698 | 5.178955857 | DUSP15 | dual specificity phosphatase 15 [Source:HGNC Symbol;Acc:HGNC:16236] |
| ENSG00000128872 | HGLibA_50598 | 692 | 5.1246838 | TMOD2 | tropomodulin 2 [Source:HGNC Symbol;Acc:HGNC:11872] |
| ENSG00000169313 | HGLibA_34875 | 691 | 5.115638457 | P2RY12 | purinergic receptor P2Y12 [Source:HGNC Symbol;Acc:HGNC:18124] |
| METTL12 | HGLibA_28952 | 691 | 5.115638457 | METTL12 | - |
| ENSG00000179869 | HGLibA_00103 | 689 | 5.097547771 | ABCA13 | ATP binding cassette subfamily A member 13 [Source:HGNC Symbol;Acc:HGNC:14638] |
| ENSG00000101945 | HGLibA_47859 | 687 | 5.079457085 | SUV39H1 | suppressor of variegation 3-9 homolog 1 [Source:HGNC Symbol;Acc:HGNC:11479] |
| ENSG00000089685 | HGLibA_04521 | 686 | 5.070411742 | BIRC5 | baculoviral IAP repeat containing 5 [Source:HGNC Symbol;Acc:HGNC:593] |
| ENSG00000133980 | HGLibA_53833 | 686 | 5.070411742 | VRTN | vertebrae development associated [Source:HGNC Symbol;Acc:HGNC:20223] |
| ENSG00000149968 | HGLibA_29534 | 686 | 5.070411742 | MMP3 | matrix metallopeptidase 3 [Source:HGNC Symbol;Acc:HGNC:7173] |
| ENSG00000196152 | HGLibA_56760 | 685 | 5.061366399 | ZNF79 | zinc finger protein 79 [Source:HGNC Symbol;Acc:HGNC:13153] |
| ENSG00000179456 | HGLibA_54832 | 684 | 5.052321057 | ZBTB18 | zinc finger and BTB domain containing 18 [Source:HGNC Symbol;Acc:HGNC:13030] |
| ENSG00000055208 | HGLibA_48143 | 683 | 5.043275714 | TAB2 | TGF-beta activated kinase 1 (MAP3K7) binding protein 2 [Source:HGNC Symbol;Acc:HGNC:17075] |
| ENSG00000101850 | HGLibA_19915 | 683 | 5.043275714 | GPR143 | G protein-coupled receptor 143 [Source:HGNC Symbol;Acc:HGNC:20145] |
| ENSG00000153406 | HGLibA_32193 | 681 | 5.025185028 | NMRAL1 | NmrA like redox sensor 1 [Source:HGNC Symbol;Acc:HGNC:24987] |
| ENSG00000198633 | HGLibA_56183 | 680 | 5.016139685 | ZNF534 | zinc finger protein 534 [Source:HGNC Symbol;Acc:HGNC:26337] |
| ENSG00000136247 | HGLibA_55117 | 676 | 4.979958313 | ZDHHC4 | zinc finger DHHC-type containing 4 [Source:HGNC Symbol;Acc:HGNC:18471] |
| ENSG00000161179 | HGLibA_54653 | 676 | 4.979958313 | YDJC | YdjC chitooligosaccharide deacetylase homolog [Source:HGNC Symbol;Acc:HGNC:27158] |
| ENSG00000129152 | HGLibA_30880 | 675 | 4.97091297 | MYOD1 | myogenic differentiation 1 [Source:HGNC Symbol;Acc:HGNC:7611] |
| ENSG00000179055 | HGLibA_33613 | 672 | 4.943776942 | OR13D1 | olfactory receptor family 13 subfamily D member 1 [Source:HGNC Symbol;Acc:HGNC:14695] |
| ENSG00000185105 | HGLibA_30633 | 672 | 4.943776942 | MYADML2 | myeloid associated differentiation marker like 2 [Source:HGNC Symbol;Acc:HGNC:34548] |
| hsa-mir-6872 | HGLibA_63673 | 672 | 4.943776942 | hsa-mir-6872 | - |
| ENSG00000187672 | HGLibA_15427 | 670 | 4.925686256 | ERC2 | ELKS/RAB6-interacting/CAST family member 2 [Source:HGNC Symbol;Acc:HGNC:31922] |
| ENSG00000106304 | HGLibA_46376 | 667 | 4.898550227 | SPAM1 | sperm adhesion molecule 1 [Source:HGNC Symbol;Acc:HGNC:11217] |
| ENSG00000114841 | HGLibA_13468 | 667 | 4.898550227 | DNAH1 | dynein axonemal heavy chain 1 [Source:HGNC Symbol;Acc:HGNC:2940] |
| ENSG00000103150 | HGLibA_29446 | 666 | 4.889504884 | MLYCD | malonyl-CoA decarboxylase [Source:HGNC Symbol;Acc:HGNC:7150] |
| ENSG00000138867 | HGLibA_20648 | 666 | 4.889504884 | GUCD1 | guanylyl cyclase domain containing 1 [Source:HGNC Symbol;Acc:HGNC:14237] |
| ENSG00000085511 | HGLibA_28187 | 664 | 4.871414198 | MAP3K4 | mitogen-activated protein kinase kinase kinase 4 [Source:HGNC Symbol;Acc:HGNC:6856] |
| ENSG00000181722 | HGLibA_54838 | 664 | 4.871414198 | ZBTB20 | zinc finger and BTB domain containing 20 [Source:HGNC Symbol;Acc:HGNC:13503] |
| ENSG00000185201 | HGLibA_22645 | 664 | 4.871414198 | IFITM2 | interferon induced transmembrane protein 2 [Source:HGNC Symbol;Acc:HGNC:5413] |
| ENSG00000165606 | HGLibA_13981 | 663 | 4.862368856 | DRGX | dorsal root ganglia homeobox [Source:HGNC Symbol;Acc:HGNC:21536] |
| ENSG00000089060 | HGLibA_45398 | 662 | 4.853323513 | SLC8B1 | solute carrier family 8 member B1 [Source:HGNC Symbol;Acc:HGNC:26175] |
| hsa-mir-31 | HGLibA_58496 | 662 | 4.853323513 | hsa-mir-31 | - |
| ENSG00000151552 | HGLibA_39720 | 661 | 4.84427817 | QDPR | quinoid dihydropteridine reductase [Source:HGNC Symbol;Acc:HGNC:9752] |
| ENSG00000169717 | HGLibA_00681 | 661 | 4.84427817 | ACTRT2 | actin related protein T2 [Source:HGNC Symbol;Acc:HGNC:24026] |
| ENSG00000101292 | HGLibA_38675 | 660 | 4.835232827 | PROKR2 | prokineticin receptor 2 [Source:HGNC Symbol;Acc:HGNC:15836] |
| ENSG00000165806 | HGLibA_07321 | 655 | 4.790006112 | CASP7 | caspase 7 [Source:HGNC Symbol;Acc:HGNC:1508] |
| ENSG00000108342 | HGLibA_11338 | 653 | 4.771915426 | CSF3 | colony stimulating factor 3 [Source:HGNC Symbol;Acc:HGNC:2438] |
| ENSG00000116016 | HGLibA_15236 | 653 | 4.771915426 | EPAS1 | endothelial PAS domain protein 1 [Source:HGNC Symbol;Acc:HGNC:3374] |
| ENSG00000238205 | HGLibA_29704 | 653 | 4.771915426 | MPC1L | mitochondrial pyruvate carrier 1 like [Source:HGNC Symbol;Acc:HGNC:44205] |
| ENSG00000016864 | HGLibA_19336 | 652 | 4.762870084 | GLT8D1 | glycosyltransferase 8 domain containing 1 [Source:HGNC Symbol;Acc:HGNC:24870] |
| ENSG00000100519 | HGLibA_39144 | 652 | 4.762870084 | PSMC6 | ATPase 6 [Source:HGNC Symbol;Acc:HGNC:9553]" |
| ENSG00000173542 | HGLibA_29587 | 652 | 4.762870084 | MOB1B | MOB kinase activator 1B [Source:HGNC Symbol;Acc:HGNC:29801] |
| ENSG00000171169 | HGLibA_31077 | 650 | 4.744779398 | NAIF1 | nuclear apoptosis inducing factor 1 [Source:HGNC Symbol;Acc:HGNC:25446] |
| ENSG00000136485 | HGLibA_12455 | 649 | 4.735734055 | DCAF7 | DDB1 and CUL4 associated factor 7 [Source:HGNC Symbol;Acc:HGNC:30915] |
| ENSG00000058866 | HGLibA_13069 | 647 | 4.717643369 | DGKG | diacylglycerol kinase gamma [Source:HGNC Symbol;Acc:HGNC:2853] |
| FAM109A | HGLibA_16005 | 647 | 4.717643369 | FAM109A | - |
| PTPLAD2 | HGLibA_39421 | 647 | 4.717643369 | PTPLAD2 | - |
| ENSG00000132334 | HGLibA_39498 | 646 | 4.708598026 | PTPRE | receptor type E [Source:HGNC Symbol;Acc:HGNC:9669]" |
| ENSG00000244411 | HGLibA_25807 | 646 | 4.708598026 | KRTAP5-7 | keratin associated protein 5-7 [Source:HGNC Symbol;Acc:HGNC:23602] |
| ENSG00000156345 | HGLibA_08758 | 645 | 4.699552683 | CDK20 | cyclin dependent kinase 20 [Source:HGNC Symbol;Acc:HGNC:21420] |
| ENSG00000179163 | HGLibA_18154 | 645 | 4.699552683 | FUCA1 | alpha-L-fucosidase 1 [Source:HGNC Symbol;Acc:HGNC:4006] |
| ENSG00000125246 | HGLibA_10174 | 644 | 4.69050734 | CLYBL | citrate lyase beta like [Source:HGNC Symbol;Acc:HGNC:18355] |
| ENSG00000130045 | HGLibA_33146 | 644 | 4.69050734 | NXNL2 | nucleoredoxin like 2 [Source:HGNC Symbol;Acc:HGNC:30482] |
| ENSG00000156453 | HGLibA_35386 | 644 | 4.69050734 | PCDH1 | protocadherin 1 [Source:HGNC Symbol;Acc:HGNC:8655] |
| ENSG00000166342 | HGLibA_31704 | 644 | 4.69050734 | NETO1 | neuropilin and tolloid like 1 [Source:HGNC Symbol;Acc:HGNC:13823] |
| ENSG00000066294 | HGLibA_08465 | 643 | 4.681461997 | CD84 | CD84 molecule [Source:HGNC Symbol;Acc:HGNC:1704] |
| ENSG00000181830 | HGLibA_44971 | 643 | 4.681461997 | SLC35C1 | solute carrier family 35 member C1 [Source:HGNC Symbol;Acc:HGNC:20197] |
| ENSG00000269335 | HGLibA_22925 | 643 | 4.681461997 | IKBKG | inhibitor of nuclear factor kappa B kinase subunit gamma [Source:HGNC Symbol;Acc:HGNC:5961] |
| ENSG00000008083 | HGLibA_23936 | 642 | 4.672416655 | JARID2 | jumonji and AT-rich interaction domain containing 2 [Source:HGNC Symbol;Acc:HGNC:6196] |
| ENSG00000170949 | HGLibA_55514 | 642 | 4.672416655 | ZNF160 | zinc finger protein 160 [Source:HGNC Symbol;Acc:HGNC:12948] |
| ENSG00000065559 | HGLibA_28147 | 641 | 4.663371312 | MAP2K4 | mitogen-activated protein kinase kinase 4 [Source:HGNC Symbol;Acc:HGNC:6844] |
| ENSG00000095261 | HGLibA_39174 | 639 | 4.645280626 | PSMD5 | non-ATPase 5 [Source:HGNC Symbol;Acc:HGNC:9563]" |
| ENSG00000182022 | HGLibA_09628 | 639 | 4.645280626 | CHST15 | carbohydrate sulfotransferase 15 [Source:HGNC Symbol;Acc:HGNC:18137] |
| hsa-mir-3198-2 | HGLibA_58893 | 639 | 4.645280626 | hsa-mir-3198-2 | - |
| ENSG00000115828 | HGLibA_39728 | 638 | 4.636235283 | QPCT | glutaminyl-peptide cyclotransferase [Source:HGNC Symbol;Acc:HGNC:9753] |
| ENSG00000169740 | HGLibA_55793 | 638 | 4.636235283 | ZNF32 | zinc finger protein 32 [Source:HGNC Symbol;Acc:HGNC:13095] |
| CTSL2 | HGLibA_11722 | 637 | 4.62718994 | CTSL2 | - |
| ENSG00000143632 | HGLibA_00595 | 637 | 4.62718994 | ACTA1 | skeletal muscle [Source:HGNC Symbol;Acc:HGNC:129]" |
| ENSG00000006555 | HGLibA_52074 | 636 | 4.618144597 | TTC22 | tetratricopeptide repeat domain 22 [Source:HGNC Symbol;Acc:HGNC:26067] |
| ENSG00000214042 | HGLibA_22689 | 635 | 4.609099254 | IFNA7 | interferon alpha 7 [Source:HGNC Symbol;Acc:HGNC:5428] |
| hsa-mir-6825 | HGLibA_63478 | 634 | 4.600053911 | hsa-mir-6825 | - |
| ENSG00000178996 | HGLibA_46050 | 632 | 4.581963226 | SNX18 | sorting nexin 18 [Source:HGNC Symbol;Acc:HGNC:19245] |
| FAM115A | HGLibA_16035 | 631 | 4.572917883 | FAM115A | - |
| ENSG00000042813 | HGLibA_56963 | 629 | 4.554827197 | ZPBP | zona pellucida binding protein [Source:HGNC Symbol;Acc:HGNC:15662] |
| ENSG00000075891 | HGLibA_35299 | 628 | 4.545781854 | PAX2 | paired box 2 [Source:HGNC Symbol;Acc:HGNC:8616] |
| ENSG00000137142 | HGLibA_22836 | 628 | 4.545781854 | IGFBPL1 | insulin like growth factor binding protein like 1 [Source:HGNC Symbol;Acc:HGNC:20081] |
| ENSG00000143595 | HGLibA_02581 | 627 | 4.536736511 | AQP10 | aquaporin 10 [Source:HGNC Symbol;Acc:HGNC:16029] |
| ENSG00000131828 | HGLibA_35863 | 625 | 4.518645825 | PDHA1 | pyruvate dehydrogenase E1 alpha 1 subunit [Source:HGNC Symbol;Acc:HGNC:8806] |
| ENSG00000176170 | HGLibA_46643 | 625 | 4.518645825 | SPHK1 | sphingosine kinase 1 [Source:HGNC Symbol;Acc:HGNC:11240] |
| KIAA1462 | HGLibA_24762 | 625 | 4.518645825 | KIAA1462 | - |
| ENSG00000140368 | HGLibA_39238 | 623 | 4.500555139 | PSTPIP1 | proline-serine-threonine phosphatase interacting protein 1 [Source:HGNC Symbol;Acc:HGNC:9580] |
| ENSG00000173153 | HGLibA_15594 | 623 | 4.500555139 | ESRRA | estrogen related receptor alpha [Source:HGNC Symbol;Acc:HGNC:3471] |
| ENSG00000164379 | HGLibA_17942 | 622 | 4.491509796 | FOXQ1 | forkhead box Q1 [Source:HGNC Symbol;Acc:HGNC:20951] |
| ENSG00000105750 | HGLibA_56870 | 621 | 4.482464454 | ZNF85 | zinc finger protein 85 [Source:HGNC Symbol;Acc:HGNC:13160] |
| hsa-mir-4515 | HGLibA_60503 | 621 | 4.482464454 | hsa-mir-4515 | - |
| ENSG00000171872 | HGLibA_25064 | 620 | 4.473419111 | KLF17 | Kruppel like factor 17 [Source:HGNC Symbol;Acc:HGNC:18830] |
| ENSG00000078900 | HGLibA_51095 | 619 | 4.464373768 | TP73 | tumor protein p73 [Source:HGNC Symbol;Acc:HGNC:12003] |
| ENSG00000160111 | HGLibA_10903 | 619 | 4.464373768 | CPAMD8 | alpha-2-macroglobulin domain containing 8 [Source:HGNC Symbol;Acc:HGNC:23228]" |
| ENSG00000106028 | HGLibA_47115 | 618 | 4.455328425 | SSBP1 | single stranded DNA binding protein 1 [Source:HGNC Symbol;Acc:HGNC:11317] |
| KIAA1683 | HGLibA_24803 | 617 | 4.446283082 | KIAA1683 | - |
| ENSG00000078081 | HGLibA_25983 | 616 | 4.437237739 | LAMP3 | lysosomal associated membrane protein 3 [Source:HGNC Symbol;Acc:HGNC:14582] |
| ENSG00000085563 | HGLibA_00130 | 616 | 4.437237739 | ABCB1 | ATP binding cassette subfamily B member 1 [Source:HGNC Symbol;Acc:HGNC:40] |
| ENSG00000125498 | HGLibA_24981 | 616 | 4.437237739 | KIR2DL1 | two Ig domains and long cytoplasmic tail 1 [Source:HGNC Symbol;Acc:HGNC:6329]" |
| ENSG00000123636 | HGLibA_04194 | 614 | 4.419147053 | BAZ2B | bromodomain adjacent to zinc finger domain 2B [Source:HGNC Symbol;Acc:HGNC:963] |
| ENSG00000131668 | HGLibA_04165 | 614 | 4.419147053 | BARX1 | BARX homeobox 1 [Source:HGNC Symbol;Acc:HGNC:955] |
| ENSG00000165495 | HGLibA_36848 | 614 | 4.419147053 | PKNOX2 | PBX/knotted 1 homeobox 2 [Source:HGNC Symbol;Acc:HGNC:16714] |
| ENSG00000188976 | HGLibA_32234 | 612 | 4.401056367 | NOC2L | NOC2 like nucleolar associated transcriptional repressor [Source:HGNC Symbol;Acc:HGNC:24517] |
| ENSG00000136928 | HGLibA_18344 | 611 | 4.392011025 | GABBR2 | gamma-aminobutyric acid type B receptor subunit 2 [Source:HGNC Symbol;Acc:HGNC:4507] |
| ENSG00000182578 | HGLibA_11324 | 608 | 4.364874996 | CSF1R | colony stimulating factor 1 receptor [Source:HGNC Symbol;Acc:HGNC:2433] |
| ENSG00000197696 | HGLibA_32143 | 608 | 4.364874996 | NMB | neuromedin B [Source:HGNC Symbol;Acc:HGNC:7842] |
| ENSG00000182685 | HGLibA_04808 | 607 | 4.355829653 | BRICD5 | BRICHOS domain containing 5 [Source:HGNC Symbol;Acc:HGNC:28309] |
| ENSG00000204695 | HGLibA_33637 | 607 | 4.355829653 | OR14J1 | olfactory receptor family 14 subfamily J member 1 [Source:HGNC Symbol;Acc:HGNC:13971] |
| hsa-mir-6125 | HGLibA_62649 | 607 | 4.355829653 | hsa-mir-6125 | - |
| ENSG00000138439 | HGLibA_16044 | 606 | 4.34678431 | FAM117B | family with sequence similarity 117 member B [Source:HGNC Symbol;Acc:HGNC:14440] |
| ENSG00000159363 | HGLibA_03570 | 606 | 4.34678431 | ATP13A2 | ATPase cation transporting 13A2 [Source:HGNC Symbol;Acc:HGNC:30213] |
| ENSG00000224659 | HGLibA_18460 | 606 | 4.34678431 | GAGE12J | G antigen 12J [Source:HGNC Symbol;Acc:HGNC:17778] |
| ENSG00000237353 | HGLibA_35280 | 606 | 4.34678431 | PATE4 | prostate and testis expressed 4 [Source:HGNC Symbol;Acc:HGNC:35427] |
| ENSG00000029364 | HGLibA_45128 | 605 | 4.337738967 | SLC39A9 | solute carrier family 39 member 9 [Source:HGNC Symbol;Acc:HGNC:20182] |
| ENSG00000115657 | HGLibA_00145 | 604 | 4.328693624 | ABCB6 | ATP binding cassette subfamily B member 6 (Langereis blood group) [Source:HGNC Symbol;Acc:HGNC:47] |
| ENSG00000166152 | HGLibA_05482 | 604 | 4.328693624 | C16orf78 | chromosome 16 open reading frame 78 [Source:HGNC Symbol;Acc:HGNC:28479] |
| ENSG00000175066 | HGLibA_19201 | 604 | 4.328693624 | GK5 | glycerol kinase 5 [Source:HGNC Symbol;Acc:HGNC:28635] |
| ENSG00000145020 | HGLibA_01836 | 603 | 4.319648281 | AMT | aminomethyltransferase [Source:HGNC Symbol;Acc:HGNC:473] |
| ENSG00000169715 | HGLibA_30282 | 603 | 4.319648281 | MT1E | metallothionein 1E [Source:HGNC Symbol;Acc:HGNC:7397] |
| ENSG00000074706 | HGLibA_23453 | 602 | 4.310602938 | IPCEF1 | interaction protein for cytohesin exchange factors 1 [Source:HGNC Symbol;Acc:HGNC:21204] |

| | | | | | |
|---|---|---|---|---|---|
| ENSG00000158411 | HGLibA_29316 | 602 | 4.310602938 | MITD1 | microtubule interacting and trafficking domain containing 1 [Source:HGNC Symbol;Acc:HGNC:25207] |
| ENSG00000144677 | HGLibA_11614 | 601 | 4.301557595 | CTDSPL | CTD small phosphatase like [Source:HGNC Symbol;Acc:HGNC:16890] |
| ENSG00000145781 | HGLibA_10653 | 601 | 4.301557595 | COMMD10 | COMM domain containing 10 [Source:HGNC Symbol;Acc:HGNC:30201] |
| ENSG00000175602 | HGLibA_07896 | 600 | 4.292512253 | CCDC85B | coiled-coil domain containing 85B [Source:HGNC Symbol;Acc:HGNC:24926] |
| hsa-mir-5094 | HGLibA_61533 | 599 | 4.28346691 | hsa-mir-5094 | - |
| ENSG00000127081 | HGLibA_56078 | 597 | 4.265376224 | ZNF484 | zinc finger protein 484 [Source:HGNC Symbol;Acc:HGNC:23385] |
| hsa-mir-5197 | HGLibA_61708 | 597 | 4.265376224 | hsa-mir-5197 | - |
| ENSG00000040608 | HGLibA_42412 | 596 | 4.256330881 | RTN4R | reticulon 4 receptor [Source:HGNC Symbol;Acc:HGNC:18601] |
| LDOC1L | HGLibA_26260 | 596 | 4.256330881 | LDOC1L | - |
| ENSG00000163510 | HGLibA_11815 | 595 | 4.247285538 | CWC22 | CWC22 spliceosome associated protein homolog [Source:HGNC Symbol;Acc:HGNC:29322] |
| PQLC2 | HGLibA_38253 | 595 | 4.247285538 | PQLC2 | - |
| CECR1 | HGLibA_08975 | 594 | 4.238240195 | CECR1 | - |
| ENSG00000135205 | HGLibA_07628 | 594 | 4.238240195 | CCDC146 | coiled-coil domain containing 146 [Source:HGNC Symbol;Acc:HGNC:29296] |
| hsa-mir-3974 | HGLibA_59656 | 594 | 4.238240195 | hsa-mir-3974 | - |
| ENSG00000172016 | HGLibA_40801 | 593 | 4.229194852 | REG3A | regenerating family member 3 alpha [Source:HGNC Symbol;Acc:HGNC:8601] |
| ADC | HGLibA_00888 | 592 | 4.220149509 | ADC | - |
| ENSG00000109065 | HGLibA_31190 | 592 | 4.220149509 | NAT9 | N-acetyltransferase 9 (putative) [Source:HGNC Symbol;Acc:HGNC:23133] |
| ENSG00000128928 | HGLibA_23877 | 592 | 4.220149509 | IVD | isovaleryl-CoA dehydrogenase [Source:HGNC Symbol;Acc:HGNC:6186] |
| ENSG00000172116 | HGLibA_08473 | 592 | 4.220149509 | CD8B | CD8b molecule [Source:HGNC Symbol;Acc:HGNC:1707] |
| ENSG00000183421 | HGLibA_41325 | 592 | 4.220149509 | RIPK4 | receptor interacting serine/threonine kinase 4 [Source:HGNC Symbol;Acc:HGNC:496] |
| ENSG00000184445 | HGLibA_25350 | 592 | 4.220149509 | KNTC1 | kinetochore associated 1 [Source:HGNC Symbol;Acc:HGNC:17255] |
| ENSG00000187094 | HGLibA_07952 | 592 | 4.220149509 | CCK | cholecystokinin [Source:HGNC Symbol;Acc:HGNC:1569] |
| hsa-mir-6848 | HGLibA_63566 | 592 | 4.220149509 | hsa-mir-6848 | - |
| ENSG00000112763 | HGLibA_04962 | 591 | 4.211104166 | BTN2A1 | butyrophilin subfamily 2 member A1 [Source:HGNC Symbol;Acc:HGNC:1136] |
| ENSG00000100591 | HGLibA_01312 | 590 | 4.202058824 | AHSA1 | activator of HSP90 ATPase activity 1 [Source:HGNC Symbol;Acc:HGNC:1189] |
| ENSG00000166598 | HGLibA_22257 | 590 | 4.202058824 | HSP90B1 | heat shock protein 90 beta family member 1 [Source:HGNC Symbol;Acc:HGNC:12028] |
| ENSG00000110013 | HGLibA_44102 | 589 | 4.193013481 | SIAE | sialic acid acetylesterase [Source:HGNC Symbol;Acc:HGNC:18187] |
| ENSG00000152137 | HGLibA_22318 | 589 | 4.193013481 | HSPB8 | heat shock protein family B (small) member 8 [Source:HGNC Symbol;Acc:HGNC:30171] |
| hsa-mir-1266 | HGLibA_57487 | 589 | 4.193013481 | hsa-mir-1266 | - |
| ENSG00000173110 | HGLibA_22292 | 588 | 4.183968138 | HSPA6 | heat shock protein family A (Hsp70) member 6 [Source:HGNC Symbol;Acc:HGNC:5239] |
| ENSG00000169704 | HGLibA_19715 | 587 | 4.174922795 | GP9 | glycoprotein IX platelet [Source:HGNC Symbol;Acc:HGNC:4444] |
| ENSG00000171773 | HGLibA_33144 | 587 | 4.174922795 | NXNL1 | nucleoredoxin like 1 [Source:HGNC Symbol;Acc:HGNC:25179] |
| hsa-mir-4693 | HGLibA_60882 | 586 | 4.165877452 | hsa-mir-4693 | - |
| ENSG00000005187 | HGLibA_00577 | 584 | 4.147786766 | ACSM3 | acyl-CoA synthetase medium chain family member 3 [Source:HGNC Symbol;Acc:HGNC:10522] |
| H2AFB3 | HGLibA_20752 | 584 | 4.147786766 | H2AFB3 | - |
| ENSG00000183044 | HGLibA_00092 | 583 | 4.138741423 | ABAT | 4-aminobutyrate aminotransferase [Source:HGNC Symbol;Acc:HGNC:23] |
| ENSG00000188171 | HGLibA_56421 | 583 | 4.138741423 | ZNF626 | zinc finger protein 626 [Source:HGNC Symbol;Acc:HGNC:30461] |
| hsa-mir-4699 | HGLibA_60906 | 583 | 4.138741423 | hsa-mir-4699 | - |
| ENSG00000005108 | HGLibA_49406 | 582 | 4.12969608 | THSD7A | thrombospondin type 1 domain containing 7A [Source:HGNC Symbol;Acc:HGNC:22207] |
| ENSG00000168515 | HGLibA_42915 | 582 | 4.12969608 | SCGB1D1 | secretoglobin family 1D member 1 [Source:HGNC Symbol;Acc:HGNC:18395] |
| ENSG00000196878 | HGLibA_25961 | 582 | 4.12969608 | LAMB3 | laminin subunit beta 3 [Source:HGNC Symbol;Acc:HGNC:6490] |
| ENSG00000078070 | HGLibA_28566 | 581 | 4.120650737 | MCCC1 | methylcrotonoyl-CoA carboxylase 1 [Source:HGNC Symbol;Acc:HGNC:6936] |
| ENSG00000175325 | HGLibA_38686 | 581 | 4.120650737 | PROP1 | PROP paired-like homeobox 1 [Source:HGNC Symbol;Acc:HGNC:9455] |
| ENSG00000185231 | HGLibA_28543 | 581 | 4.120650737 | MC2R | melanocortin 2 receptor [Source:HGNC Symbol;Acc:HGNC:6930] |
| ENSG00000275385 | HGLibA_07982 | 581 | 4.120650737 | CCL18 | C-C motif chemokine ligand 18 [Source:HGNC Symbol;Acc:HGNC:10616] |
| ENSG00000116711 | HGLibA_36901 | 580 | 4.111605395 | PLA2G4A | phospholipase A2 group IVA [Source:HGNC Symbol;Acc:HGNC:9035] |
| ENSG00000102218 | HGLibA_41792 | 579 | 4.102560052 | RP2 | ARL3 GTPase activating protein [Source:HGNC Symbol;Acc:HGNC:10274]" |
| ENSG00000125522 | HGLibA_32416 | 579 | 4.102560052 | NPBWR2 | neuropeptides B and W receptor 2 [Source:HGNC Symbol;Acc:HGNC:4530] |
| ENSG00000162729 | HGLibA_22903 | 579 | 4.102560052 | IGSF8 | immunoglobulin superfamily member 8 [Source:HGNC Symbol;Acc:HGNC:17813] |
| ENSG00000132259 | HGLibA_10272 | 578 | 4.093514709 | CNGA4 | cyclic nucleotide gated channel alpha 4 [Source:HGNC Symbol;Acc:HGNC:2152] |
| ENSG00000144339 | HGLibA_49836 | 578 | 4.093514709 | TMEFF2 | transmembrane protein with EGF like and two follistatin like domains 2 [Source:HGNC Symbol;Acc:HGNC:11867] |
| ENSG00000264364 | HGLibA_14256 | 578 | 4.093514709 | DYNLL2 | dynein light chain LC8-type 2 [Source:HGNC Symbol;Acc:HGNC:24596] |
| ENSG00000108798 | HGLibA_00295 | 577 | 4.084469366 | ABI3 | ABI family member 3 [Source:HGNC Symbol;Acc:HGNC:29859] |
| ENSG00000101365 | HGLibA_22548 | 576 | 4.075424023 | IDH3B | isocitrate dehydrogenase 3 (NAD(+)) beta [Source:HGNC Symbol;Acc:HGNC:5385] |
| ENSG00000175894 | HGLibA_51956 | 576 | 4.075424023 | TSPEAR | thrombospondin type laminin G domain and EAR repeats [Source:HGNC Symbol;Acc:HGNC:1268] |
| ENSG00000184954 | HGLibA_34403 | 576 | 4.075424023 | OR6C70 | olfactory receptor family 6 subfamily C member 70 [Source:HGNC Symbol;Acc:HGNC:31299] |
| hsa-mir-922 | HGLibA_64281 | 576 | 4.075424023 | hsa-mir-922 | - |
| ENSG00000078403 | HGLibA_29411 | 575 | 4.06637868 | MLLT10 | histone lysine methyltransferase DOT1L cofactor [Source:HGNC Symbol;Acc:HGNC:16063]" |
| RGAG4 | HGLibA_40996 | 575 | 4.06637868 | RGAG4 | - |
| ENSG00000104921 | HGLibA_17175 | 574 | 4.057333337 | FCER2 | Fc fragment of IgE receptor II [Source:HGNC Symbol;Acc:HGNC:3612] |
| ENSG00000149294 | HGLibA_31255 | 574 | 4.057333337 | NCAM1 | neural cell adhesion molecule 1 [Source:HGNC Symbol;Acc:HGNC:7656] |
| ENSG00000166569 | HGLibA_10942 | 574 | 4.057333337 | CPLX4 | complexin 4 [Source:HGNC Symbol;Acc:HGNC:24330] |
| ENSG00000081148 | HGLibA_23265 | 573 | 4.048287994 | IMPG2 | interphotoreceptor matrix proteoglycan 2 [Source:HGNC Symbol;Acc:HGNC:18362] |
| ENSG00000090615 | HGLibA_19597 | 573 | 4.048287994 | GOLGA3 | golgin A3 [Source:HGNC Symbol;Acc:HGNC:4426] |
| ENSG00000167384 | HGLibA_55536 | 573 | 4.048287994 | ZNF180 | zinc finger protein 180 [Source:HGNC Symbol;Acc:HGNC:12970] |
| ENSG00000257591 | HGLibA_56419 | 573 | 4.048287994 | ZNF625 | zinc finger protein 625 [Source:HGNC Symbol;Acc:HGNC:30571] |
| hsa-mir-1236 | HGLibA_57336 | 573 | 4.048287994 | hsa-mir-1236 | - |
| ENSG00000065989 | HGLibA_35788 | 572 | 4.039242651 | PDE4A | phosphodiesterase 4A [Source:HGNC Symbol;Acc:HGNC:8780] |
| ENSG00000122694 | HGLibA_19269 | 572 | 4.039242651 | GLIPR2 | GLI pathogenesis related 2 [Source:HGNC Symbol;Acc:HGNC:18007] |
| ENSG00000149600 | HGLibA_10673 | 572 | 4.039242651 | COMMD7 | COMM domain containing 7 [Source:HGNC Symbol;Acc:HGNC:16223] |
| ENSG00000229937 | HGLibA_38759 | 572 | 4.039242651 | PRPS1L1 | phosphoribosyl pyrophosphate synthetase 1-like 1 [Source:HGNC Symbol;Acc:HGNC:9463] |
| hsa-mir-6827 | HGLibA_63485 | 572 | 4.039242651 | hsa-mir-6827 | - |
| ENSG00000198939 | HGLibA_55191 | 571 | 4.030197308 | ZFP2 | ZFP2 zinc finger protein [Source:HGNC Symbol;Acc:HGNC:26138] |
| ENSG00000204140 | HGLibA_10106 | 571 | 4.030197308 | CLPSL1 | colipase like 1 [Source:HGNC Symbol;Acc:HGNC:21251] |
| ENSG00000123576 | HGLibA_15601 | 570 | 4.021151965 | ESX1 | ESX homeobox 1 [Source:HGNC Symbol;Acc:HGNC:14865] |
| ENSG00000141959 | HGLibA_36166 | 570 | 4.021151965 | PFKL | liver type [Source:HGNC Symbol;Acc:HGNC:8876]" |
| ENSG00000152402 | HGLibA_20653 | 570 | 4.021151965 | GUCY1A2 | guanylate cyclase 1 soluble subunit alpha 2 [Source:HGNC Symbol;Acc:HGNC:4684] |
| ENSG00000189420 | HGLibA_55246 | 570 | 4.021151965 | ZFP92 | ZFP92 zinc finger protein [Source:HGNC Symbol;Acc:HGNC:12865] |
| PGBD3 | HGLibA_36223 | 570 | 4.021151965 | PGBD3 | - |
| ENSG00000010030 | HGLibA_15668 | 569 | 4.012106623 | ETV7 | ETS variant 7 [Source:HGNC Symbol;Acc:HGNC:18160] |
| ENSG00000158639 | HGLibA_35015 | 569 | 4.012106623 | PAGE5 | PAGE family member 5 [Source:HGNC Symbol;Acc:HGNC:29992] |
| ENSG00000161955 | HGLibA_50807 | 569 | 4.012106623 | TNFSF13 | TNF superfamily member 13 [Source:HGNC Symbol;Acc:HGNC:11928] |
| ENSG00000205309 | HGLibA_32831 | 569 | 4.012106623 | NT5M | mitochondrial [Source:HGNC Symbol;Acc:HGNC:15769]" |
| ENSG00000100445 | HGLibA_43163 | 568 | 4.00306128 | SDR39U1 | short chain dehydrogenase/reductase family 39U member 1 [Source:HGNC Symbol;Acc:HGNC:20275] |
| ENSG00000102128 | HGLibA_39934 | 568 | 4.00306128 | RAB40AL | RAB40A like [Source:HGNC Symbol;Acc:HGNC:25410] |
| ENSG00000167414 | HGLibA_19536 | 568 | 4.00306128 | GNG8 | G protein subunit gamma 8 [Source:HGNC Symbol;Acc:HGNC:19664] |
| hsa-mir-7704 | HGLibA_63953 | 568 | 4.00306128 | hsa-mir-7704 | - |
| ENSG00000105699 | HGLibA_27567 | 567 | 3.994015937 | LSR | lipolysis stimulated lipoprotein receptor [Source:HGNC Symbol;Acc:HGNC:29572] |
| ENSG00000182747 | HGLibA_44982 | 567 | 3.994015937 | SLC35D3 | solute carrier family 35 member D3 [Source:HGNC Symbol;Acc:HGNC:15621] |
| ENSG00000006625 | HGLibA_18992 | 566 | 3.984970594 | GGCT | gamma-glutamylcyclotransferase [Source:HGNC Symbol;Acc:HGNC:21705] |
| ENSG00000171174 | HGLibA_40465 | 566 | 3.984970594 | RBKS | ribokinase [Source:HGNC Symbol;Acc:HGNC:30325] |
| ENSG00000184486 | HGLibA_37767 | 566 | 3.984970594 | POU3F2 | POU class 3 homeobox 2 [Source:HGNC Symbol;Acc:HGNC:9215] |
| ENSG00000163982 | HGLibA_34736 | 565 | 3.975925251 | OTOP1 | otopetrin 1 [Source:HGNC Symbol;Acc:HGNC:19656] |
| ENSG00000109705 | HGLibA_32051 | 564 | 3.966879908 | NKX3-2 | NK3 homeobox 2 [Source:HGNC Symbol;Acc:HGNC:951] |
| ENSG00000198680 | HGLibA_52342 | 564 | 3.966879908 | TUSC1 | tumor suppressor candidate 1 [Source:HGNC Symbol;Acc:HGNC:31010] |
| ENSG00000215009 | HGLibA_00580 | 564 | 3.966879908 | ACSM4 | acyl-CoA synthetase medium chain family member 4 [Source:HGNC Symbol;Acc:HGNC:32016] |
| ENSG00000100246 | HGLibA_13646 | 563 | 3.957834565 | DNAL4 | dynein axonemal light chain 4 [Source:HGNC Symbol;Acc:HGNC:2955] |
| ENSG00000157985 | HGLibA_01137 | 563 | 3.957834565 | AGAP1 | ankyrin repeat and PH domain 1 [Source:HGNC Symbol;Acc:HGNC:16922]" |
| ENSG00000159459 | HGLibA_52746 | 563 | 3.957834565 | UBR1 | ubiquitin protein ligase E3 component n-recognin 1 [Source:HGNC Symbol;Acc:HGNC:16808] |
| ENSG00000119321 | HGLibA_17561 | 562 | 3.948789222 | FKBP15 | FK506 binding protein 15 [Source:HGNC Symbol;Acc:HGNC:23397] |
| ENSG00000138796 | HGLibA_20804 | 562 | 3.948789222 | HADH | hydroxyacyl-CoA dehydrogenase [Source:HGNC Symbol;Acc:HGNC:4799] |
| ENSG00000149090 | HGLibA_35088 | 562 | 3.948789222 | PAMR1 | peptidase domain containing associated with muscle regeneration 1 [Source:HGNC Symbol;Acc:HGNC:24554] |
| 3-Sep | HGLibA_43438 | 562 | 3.948789222 | 3-Sep | - |
| 5-Sep | HGLibA_43445 | 562 | 3.948789222 | 5-Sep | - |
| ENSG00000094975 | HGLibA_47707 | 561 | 3.939743879 | SUCO | SUN domain containing ossification factor [Source:HGNC Symbol;Acc:HGNC:1240] |
| hsa-mir-4303 | HGLibA_59924 | 561 | 3.939743879 | hsa-mir-4303 | - |
| ENSG00000159958 | HGLibA_50757 | 560 | 3.930698536 | TNFRSF13C | TNF receptor superfamily member 13C [Source:HGNC Symbol;Acc:HGNC:17755] |
| ENSG00000172752 | HGLibA_10604 | 560 | 3.930698536 | COL6A5 | collagen type VI alpha 5 chain [Source:HGNC Symbol;Acc:HGNC:26674] |
| ENSG00000196570 | HGLibA_36180 | 560 | 3.930698536 | PFN3 | profilin 3 [Source:HGNC Symbol;Acc:HGNC:18627] |
| C14orf182 | HGLibA_05350 | 559 | 3.921653194 | C14orf182 | - |
| ENSG00000115486 | HGLibA_18996 | 559 | 3.921653194 | GGCX | gamma-glutamyl carboxylase [Source:HGNC Symbol;Acc:HGNC:4247] |
| ENSG00000123096 | HGLibA_47146 | 559 | 3.921653194 | SSPN | sarcospan [Source:HGNC Symbol;Acc:HGNC:11322] |
| ENSG00000140284 | HGLibA_44815 | 559 | 3.921653194 | SLC27A2 | solute carrier family 27 member 2 [Source:HGNC Symbol;Acc:HGNC:10996] |
| ENSG00000183304 | HGLibA_16818 | 558 | 3.912607851 | FAM9A | family with sequence similarity 9 member A [Source:HGNC Symbol;Acc:HGNC:18403] |
| ENSG00000188522 | HGLibA_16759 | 558 | 3.912607851 | FAM83G | family with sequence similarity 83 member G [Source:HGNC Symbol;Acc:HGNC:32554] |
| ENSG00000154803 | HGLibA_17606 | 557 | 3.903562508 | FLCN | folliculin [Source:HGNC Symbol;Acc:HGNC:27310] |
| ENSG00000164530 | HGLibA_36485 | 557 | 3.903562508 | PI16 | peptidase inhibitor 16 [Source:HGNC Symbol;Acc:HGNC:21245] |
| ENSG00000132359 | HGLibA_40231 | 556 | 3.894517165 | RAP1GAP2 | RAP1 GTPase activating protein 2 [Source:HGNC Symbol;Acc:HGNC:29176] |
| ENSG00000135205 | HGLibA_07627 | 556 | 3.894517165 | CCDC146 | coiled-coil domain containing 146 [Source:HGNC Symbol;Acc:HGNC:29296] |
| ENSG00000171735 | HGLibA_07115 | 556 | 3.894517165 | CAMTA1 | calmodulin binding transcription activator 1 [Source:HGNC Symbol;Acc:HGNC:18806] |
| ENSG00000135443 | HGLibA_25569 | 554 | 3.876426479 | KRT85 | keratin 85 [Source:HGNC Symbol;Acc:HGNC:6462] |
| ENSG00000181191 | HGLibA_36782 | 554 | 3.876426479 | PJA1 | praja ring finger ubiquitin ligase 1 [Source:HGNC Symbol;Acc:HGNC:16648] |
| ENSG00000182575 | HGLibA_33168 | 554 | 3.876426479 | NXPH3 | neurexophilin 3 [Source:HGNC Symbol;Acc:HGNC:8077] |
| ENSG00000244623 | HGLibA_33746 | 553 | 3.867381136 | OR2AE1 | olfactory receptor family 2 subfamily AE member 1 [Source:HGNC Symbol;Acc:HGNC:15087] |
| ENSG00000109610 | HGLibA_46151 | 552 | 3.858335793 | SOD3 | superoxide dismutase 3 [Source:HGNC Symbol;Acc:HGNC:11181] |
| ENSG00000163281 | HGLibA_19568 | 552 | 3.858335793 | GNPDA2 | glucosamine-6-phosphate deaminase 2 [Source:HGNC Symbol;Acc:HGNC:21526] |
| ENSG00000187021 | HGLibA_37357 | 552 | 3.858335793 | PNLIPRP1 | pancreatic lipase related protein 1 [Source:HGNC Symbol;Acc:HGNC:9156] |
| LOC100505841 | HGLibA_26855 | 552 | 3.858335793 | LOC100505841 | - |
| LPPR3 | HGLibA_27119 | 552 | 3.858335793 | LPPR3 | - |
| ENSG00000090339 | HGLibA_22493 | 551 | 3.84929045 | ICAM1 | intercellular adhesion molecule 1 [Source:HGNC Symbol;Acc:HGNC:5344] |
| ENSG00000124523 | HGLibA_44228 | 551 | 3.84929045 | SIRT5 | sirtuin 5 [Source:HGNC Symbol;Acc:HGNC:14933] |
| ENSG00000153832 | HGLibA_17082 | 551 | 3.84929045 | FBXO36 | F-box protein 36 [Source:HGNC Symbol;Acc:HGNC:27020] |
| ENSG00000161921 | HGLibA_11859 | 551 | 3.84929045 | CXCL16 | C-X-C motif chemokine ligand 16 [Source:HGNC Symbol;Acc:HGNC:16642] |
| hsa-mir-485 | HGLibA_61334 | 551 | 3.84929045 | hsa-mir-485 | - |
| ENSG00000087589 | HGLibA_07344 | 550 | 3.840245107 | CASS4 | Cas scaffold protein family member 4 [Source:HGNC Symbol;Acc:HGNC:15878] |

| | | | | | |
|---|---|---|---|---|---|
| ENSG00000137474 | HGLibA_30861 | 550 | 3.840245107 | MYO7A | myosin VIIA [Source:HGNC Symbol;Acc:HGNC:7606] |
| ENSG00000006757 | HGLibA_37415 | 549 | 3.831199764 | PNPLA4 | patatin like phospholipase domain containing 4 [Source:HGNC Symbol;Acc:HGNC:24887] |
| ENSG00000049759 | HGLibA_31605 | 549 | 3.831199764 | NEDD4L | E3 ubiquitin protein ligase [Source:HGNC Symbol;Acc:HGNC:7728]" |
| ENSG00000135838 | HGLibA_32481 | 549 | 3.831199764 | NPL | N-acetylneuraminate pyruvate lyase [Source:HGNC Symbol;Acc:HGNC:16781] |
| ENSG00000144642 | HGLibA_40594 | 549 | 3.831199764 | RBMS3 | RNA binding motif single stranded interacting protein 3 [Source:HGNC Symbol;Acc:HGNC:13427] |
| ENSG00000144648 | HGLibA_00444 | 549 | 3.831199764 | ACKR2 | atypical chemokine receptor 2 [Source:HGNC Symbol;Acc:HGNC:1565] |
| ENSG00000146281 | HGLibA_37288 | 549 | 3.831199764 | PM20D2 | peptidase M20 domain containing 2 [Source:HGNC Symbol;Acc:HGNC:21408] |
| ENSG00000183798 | HGLibA_15054 | 549 | 3.831199764 | EMILIN3 | elastin microfibril interfacer 3 [Source:HGNC Symbol;Acc:HGNC:16123] |
| hsa-mir-4327 | HGLibA_60032 | 549 | 3.831199764 | hsa-mir-4327 | - |
| ENSG00000135862 | HGLibA_25968 | 548 | 3.822154422 | LAMC1 | laminin subunit gamma 1 [Source:HGNC Symbol;Acc:HGNC:6492] |
| ENSG00000155093 | HGLibA_39524 | 548 | 3.822154422 | PTPRN2 | receptor type N2 [Source:HGNC Symbol;Acc:HGNC:9677]" |
| ENSG00000173821 | HGLibA_41622 | 548 | 3.822154422 | RNF213 | ring finger protein 213 [Source:HGNC Symbol;Acc:HGNC:14539] |
| ENSG00000188026 | HGLibA_41254 | 548 | 3.822154422 | RILPL1 | Rab interacting lysosomal protein like 1 [Source:HGNC Symbol;Acc:HGNC:26814] |
| PLAC1L | HGLibA_36935 | 547 | 3.813109079 | PLAC1L | - |
| ENSG00000115504 | HGLibA_14660 | 546 | 3.804063736 | EHBP1 | EH domain binding protein 1 [Source:HGNC Symbol;Acc:HGNC:29144] |
| ENSG00000119655 | HGLibA_32425 | 546 | 3.804063736 | NPC2 | NPC intracellular cholesterol transporter 2 [Source:HGNC Symbol;Acc:HGNC:14537] |
| ENSG00000132122 | HGLibA_46522 | 546 | 3.804063736 | SPATA6 | spermatogenesis associated 6 [Source:HGNC Symbol;Acc:HGNC:18309] |
| ENSG00000156413 | HGLibA_18201 | 546 | 3.804063736 | FUT6 | fucosyltransferase 6 [Source:HGNC Symbol;Acc:HGNC:4017] |
| ENSG00000172938 | HGLibA_29838 | 546 | 3.804063736 | MRGPRD | MAS related GPR family member D [Source:HGNC Symbol;Acc:HGNC:29626] |
| ENSG00000091409 | HGLibA_23738 | 545 | 3.795018393 | ITGA6 | integrin subunit alpha 6 [Source:HGNC Symbol;Acc:HGNC:6142] |
| ENSG00000187010 | HGLibA_41151 | 545 | 3.795018393 | RHD | Rh blood group D antigen [Source:HGNC Symbol;Acc:HGNC:10009] |
| ENSG00000183695 | HGLibA_29850 | 544 | 3.78597305 | MRGPRX2 | MAS related GPR family member X2 [Source:HGNC Symbol;Acc:HGNC:17983] |
| ENSG00000005884 | HGLibA_23731 | 543 | 3.776927707 | ITGA3 | integrin subunit alpha 3 [Source:HGNC Symbol;Acc:HGNC:6139] |
| ENSG00000070886 | HGLibA_15307 | 543 | 3.776927707 | EPHA8 | EPH receptor A8 [Source:HGNC Symbol;Acc:HGNC:3391] |
| ENSG00000083454 | HGLibA_34857 | 543 | 3.776927707 | P2RX5 | purinergic receptor P2X 5 [Source:HGNC Symbol;Acc:HGNC:8536] |
| ENSG00000123454 | HGLibA_12383 | 543 | 3.776927707 | DBH | dopamine beta-hydroxylase [Source:HGNC Symbol;Acc:HGNC:2689] |
| ENSG00000133056 | HGLibA_36625 | 543 | 3.776927707 | PIK3C2B | phosphatidylinositol-4-phosphate 3-kinase catalytic subunit type 2 beta [Source:HGNC Symbol;Acc:HGNC:8972] |
| ENSG00000204869 | HGLibA_22847 | 543 | 3.776927707 | IGFL4 | IGF like family member 4 [Source:HGNC Symbol;Acc:HGNC:32931] |
| ENSG00000112365 | HGLibA_54848 | 542 | 3.767882364 | ZBTB24 | zinc finger and BTB domain containing 24 [Source:HGNC Symbol;Acc:HGNC:21143] |
| ENSG00000113389 | HGLibA_32513 | 542 | 3.767882364 | NPR3 | natriuretic peptide receptor 3 [Source:HGNC Symbol;Acc:HGNC:7945] |
| ENSG00000183034 | HGLibA_34737 | 542 | 3.767882364 | OTOP2 | otopetrin 2 [Source:HGNC Symbol;Acc:HGNC:19657] |
| ENSG00000049618 | HGLibA_02885 | 540 | 3.749791678 | ARID1B | AT-rich interaction domain 1B [Source:HGNC Symbol;Acc:HGNC:18040] |
| ENSG00000151948 | HGLibA_19331 | 540 | 3.749791678 | GLT1D1 | glycosyltransferase 1 domain containing 1 [Source:HGNC Symbol;Acc:HGNC:26483] |
| ENSG00000204424 | HGLibA_27671 | 540 | 3.749791678 | LY6G6F | lymphocyte antigen 6 family member G6F [Source:HGNC Symbol;Acc:HGNC:13933] |
| TCEB3 | HGLibA_48740 | 540 | 3.749791678 | TCEB3 | - |
| ENSG00000183145 | HGLibA_41334 | 539 | 3.740746335 | RIPPLY3 | ripply transcriptional repressor 3 [Source:HGNC Symbol;Acc:HGNC:3047] |
| hsa-mir-492 | HGLibA_61367 | 539 | 3.740746335 | hsa-mir-492 | - |
| ENSG00000115226 | HGLibA_17753 | 538 | 3.731700993 | FNDC4 | fibronectin type III domain containing 4 [Source:HGNC Symbol;Acc:HGNC:20239] |
| ENSG00000162415 | HGLibA_57050 | 538 | 3.731700993 | ZSWIM5 | zinc finger SWIM-type containing 5 [Source:HGNC Symbol;Acc:HGNC:29299] |
| ENSG00000162496 | HGLibA_13133 | 538 | 3.731700993 | DHRS3 | dehydrogenase/reductase 3 [Source:HGNC Symbol;Acc:HGNC:17693] |
| ENSG00000171700 | HGLibA_41077 | 538 | 3.731700993 | RGS19 | regulator of G protein signaling 19 [Source:HGNC Symbol;Acc:HGNC:13735] |
| ENSG00000224940 | HGLibA_38891 | 538 | 3.731700993 | PRRT4 | proline rich transmembrane protein 4 [Source:HGNC Symbol;Acc:HGNC:37280] |
| ENSG00000100591 | HGLibA_01314 | 537 | 3.72265565 | AHSA1 | activator of HSP90 ATPase activity 1 [Source:HGNC Symbol;Acc:HGNC:1189] |
| ENSG00000157927 | HGLibA_40114 | 537 | 3.72265565 | RADIL | Rap associating with DIL domain [Source:HGNC Symbol;Acc:HGNC:22226] |
| hsa-mir-542 | HGLibA_61820 | 537 | 3.72265565 | hsa-mir-542 | - |
| ENSG00000204116 | HGLibA_09444 | 536 | 3.713610307 | CHIC1 | cysteine rich hydrophobic domain 1 [Source:HGNC Symbol;Acc:HGNC:1934] |
| ENSG00000224586 | HGLibA_20182 | 536 | 3.713610307 | GPX5 | glutathione peroxidase 5 [Source:HGNC Symbol;Acc:HGNC:4557] |
| ENSG00000241973 | HGLibA_36498 | 536 | 3.713610307 | PI4KA | phosphatidylinositol 4-kinase alpha [Source:HGNC Symbol;Acc:HGNC:8983] |
| C14orf177 | HGLibA_05342 | 535 | 3.704564964 | C14orf177 | - |
| ENSG00000126549 | HGLibA_47403 | 535 | 3.704564964 | STATH | statherin [Source:HGNC Symbol;Acc:HGNC:11369] |
| ENSG00000164488 | HGLibA_12283 | 534 | 3.695519621 | DACT2 | dishevelled binding antagonist of beta catenin 2 [Source:HGNC Symbol;Acc:HGNC:21231] |
| ENSG00000166359 | HGLibA_54209 | 534 | 3.695519621 | WDR88 | WD repeat domain 88 [Source:HGNC Symbol;Acc:HGNC:26999] |
| ENSG00000151090 | HGLibA_49393 | 533 | 3.686474278 | THRB | thyroid hormone receptor beta [Source:HGNC Symbol;Acc:HGNC:11799] |
| ENSG00000179299 | HGLibA_32797 | 533 | 3.686474278 | NSUN7 | NOP2/Sun RNA methyltransferase family member 7 [Source:HGNC Symbol;Acc:HGNC:25857] |
| ENSG00000203883 | HGLibA_46249 | 533 | 3.686474278 | SOX18 | SRY-box 18 [Source:HGNC Symbol;Acc:HGNC:11194] |
| ENSG00000121053 | HGLibA_15391 | 532 | 3.677428935 | EPX | eosinophil peroxidase [Source:HGNC Symbol;Acc:HGNC:3423] |
| ENSG00000141905 | HGLibA_31807 | 532 | 3.677428935 | NFIC | nuclear factor I C [Source:HGNC Symbol;Acc:HGNC:7786] |
| ENSG00000155307 | HGLibA_42711 | 532 | 3.677428935 | SAMSN1 | SH3 domain and nuclear localization signals 1 [Source:HGNC Symbol;Acc:HGNC:10528]" |
| ENSG00000133661 | HGLibA_43778 | 531 | 3.668383592 | SFTPD | surfactant protein D [Source:HGNC Symbol;Acc:HGNC:10803] |
| ENSG00000135597 | HGLibA_40847 | 531 | 3.668383592 | REPS1 | RALBP1 associated Eps domain containing 1 [Source:HGNC Symbol;Acc:HGNC:15578] |
| hsa-mir-711 | HGLibA_63799 | 531 | 3.668383592 | hsa-mir-711 | - |
| ENSG00000146147 | HGLibA_29386 | 530 | 3.659338249 | MLIP | muscular LMNA interacting protein [Source:HGNC Symbol;Acc:HGNC:21355] |
| ENSG00000151806 | HGLibA_20669 | 530 | 3.659338249 | GUF1 | GTPase [Source:HGNC Symbol;Acc:HGNC:25799]" |
| ENSG00000244067 | HGLibA_20453 | 530 | 3.659338249 | GSTA2 | glutathione S-transferase alpha 2 [Source:HGNC Symbol;Acc:HGNC:4627] |
| ENSG00000155846 | HGLibA_37848 | 529 | 3.650292906 | PPARGC1B | PPARG coactivator 1 beta [Source:HGNC Symbol;Acc:HGNC:30022] |
| ENSG00000175084 | HGLibA_13005 | 529 | 3.650292906 | DES | desmin [Source:HGNC Symbol;Acc:HGNC:2770] |
| ENSG00000100744 | HGLibA_20433 | 528 | 3.641247564 | GSKIP | GSK3B interacting protein [Source:HGNC Symbol;Acc:HGNC:20343] |
| ENSG00000104371 | HGLibA_13303 | 528 | 3.641247564 | DKK4 | dickkopf WNT signaling pathway inhibitor 4 [Source:HGNC Symbol;Acc:HGNC:2894] |
| ENSG00000136279 | HGLibA_12396 | 528 | 3.641247564 | DBNL | drebrin like [Source:HGNC Symbol;Acc:HGNC:2696] |
| ENSG00000137076 | HGLibA_49634 | 528 | 3.641247564 | TLN1 | talin 1 [Source:HGNC Symbol;Acc:HGNC:11845] |
| ENSG00000175535 | HGLibA_37353 | 528 | 3.641247564 | PNLIP | pancreatic lipase [Source:HGNC Symbol;Acc:HGNC:9155] |
| ENSG00000108641 | HGLibA_04043 | 527 | 3.632202221 | B9D1 | B9 domain containing 1 [Source:HGNC Symbol;Acc:HGNC:24123] |
| ENSG00000124749 | HGLibA_10539 | 527 | 3.632202221 | COL21A1 | collagen type XXI alpha 1 chain [Source:HGNC Symbol;Acc:HGNC:17025] |
| ENSG00000138756 | HGLibA_04602 | 527 | 3.632202221 | BMP2K | BMP2 inducible kinase [Source:HGNC Symbol;Acc:HGNC:18041] |
| ENSG00000154237 | HGLibA_27463 | 527 | 3.632202221 | LRRK1 | leucine rich repeat kinase 1 [Source:HGNC Symbol;Acc:HGNC:18608] |
| ENSG00000173641 | HGLibA_22317 | 527 | 3.632202221 | HSPB7 | heat shock protein family B (small) member 7 [Source:HGNC Symbol;Acc:HGNC:5249] |
| ENSG00000175077 | HGLibA_42421 | 527 | 3.632202221 | RTP1 | receptor transporter protein 1 [Source:HGNC Symbol;Acc:HGNC:28580] |
| ENSG00000198542 | HGLibA_23802 | 527 | 3.632202221 | ITGBL1 | integrin subunit beta like 1 [Source:HGNC Symbol;Acc:HGNC:6164] |
| ENSG00000205268 | HGLibA_35822 | 527 | 3.632202221 | PDE7A | phosphodiesterase 7A [Source:HGNC Symbol;Acc:HGNC:8791] |
| ENSG00000067798 | HGLibA_31199 | 526 | 3.623156878 | NAV3 | neuron navigator 3 [Source:HGNC Symbol;Acc:HGNC:15998] |
| ENSG00000080845 | HGLibA_13349 | 526 | 3.623156878 | DLGAP4 | DLG associated protein 4 [Source:HGNC Symbol;Acc:HGNC:24476] |
| ENSG00000184719 | HGLibA_41716 | 526 | 3.623156878 | RNLS | FAD dependent amine oxidase [Source:HGNC Symbol;Acc:HGNC:25641]" |
| hsa-mir-4533 | HGLibA_60593 | 526 | 3.623156878 | hsa-mir-4533 | - |
| ENSG00000167900 | HGLibA_49579 | 525 | 3.614111535 | TK1 | thymidine kinase 1 [Source:HGNC Symbol;Acc:HGNC:11830] |
| ENSG00000173457 | HGLibA_38039 | 525 | 3.614111535 | PPP1R14B | protein phosphatase 1 regulatory inhibitor subunit 14B [Source:HGNC Symbol;Acc:HGNC:9057] |
| ENSG00000007933 | HGLibA_17708 | 524 | 3.605066192 | FMO3 | flavin containing monooxygenase 3 [Source:HGNC Symbol;Acc:HGNC:3771] |
| ENSG00000091127 | HGLibA_39605 | 524 | 3.605066192 | PUS7 | pseudouridine synthase 7 [Source:HGNC Symbol;Acc:HGNC:26033] |
| ENSG00000126070 | HGLibA_01212 | 524 | 3.605066192 | AGO3 | RISC catalytic component [Source:HGNC Symbol;Acc:HGNC:18421]" |
| ENSG00000181894 | HGLibA_55810 | 524 | 3.605066192 | ZNF329 | zinc finger protein 329 [Source:HGNC Symbol;Acc:HGNC:14209] |
| HIST1H1A | HGLibA_21357 | 524 | 3.605066192 | HIST1H1A | - |
| hsa-mir-3176 | HGLibA_58776 | 524 | 3.605066192 | hsa-mir-3176 | - |
| ENSG00000101049 | HGLibA_43826 | 523 | 3.596020849 | SGK2 | serine/threonine kinase 2 [Source:HGNC Symbol;Acc:HGNC:13900]" |
| ENSG00000142675 | HGLibA_10296 | 523 | 3.596020849 | CNKSR1 | connector enhancer of kinase suppressor of Ras 1 [Source:HGNC Symbol;Acc:HGNC:19700] |
| ENSG00000162065 | HGLibA_48512 | 523 | 3.596020849 | TBC1D24 | TBC1 domain family member 24 [Source:HGNC Symbol;Acc:HGNC:29203] |
| ENSG00000267855 | HGLibA_31470 | 523 | 3.596020849 | NDUFA7 | NADH:ubiquinone oxidoreductase subunit A7 [Source:HGNC Symbol;Acc:HGNC:7691] |
| ENSG00000283632 | HGLibA_15743 | 523 | 3.596020849 | EXOC3L2 | exocyst complex component 3 like 2 [Source:HGNC Symbol;Acc:HGNC:30162] |
| hsa-mir-1307 | HGLibA_57742 | 523 | 3.596020849 | hsa-mir-1307 | - |
| hsa-mir-181d | HGLibA_57977 | 522 | 3.586975506 | hsa-mir-181d | - |
| hsa-mir-4506 | HGLibA_60450 | 521 | 3.577930163 | hsa-mir-4506 | - |
| ENSG00000100307 | HGLibA_07463 | 520 | 3.56888482 | CBX7 | chromobox 7 [Source:HGNC Symbol;Acc:HGNC:1557] |
| ENSG00000154252 | HGLibA_18501 | 520 | 3.56888482 | GAL3ST2 | galactose-3-O-sulfotransferase 2 [Source:HGNC Symbol;Acc:HGNC:24869] |
| ENSG00000187175 | HGLibA_25629 | 520 | 3.56888482 | KRTAP12-1 | keratin associated protein 12-1 [Source:HGNC Symbol;Acc:HGNC:20529] |
| ENSG00000213672 | HGLibA_31322 | 520 | 3.56888482 | NCKIPSD | NCK interacting protein with SH3 domain [Source:HGNC Symbol;Acc:HGNC:15486] |
| ATHL1 | HGLibA_03508 | 519 | 3.559839477 | ATHL1 | - |
| ENSG00000153498 | HGLibA_46338 | 519 | 3.559839477 | SPACA7 | sperm acrosome associated 7 [Source:HGNC Symbol;Acc:HGNC:29575] |
| ENSG00000064115 | HGLibA_49717 | 518 | 3.550794134 | TM7SF3 | transmembrane 7 superfamily member 3 [Source:HGNC Symbol;Acc:HGNC:23049] |
| ENSG00000142279 | HGLibA_54435 | 518 | 3.550794134 | WTIP | WT1 interacting protein [Source:HGNC Symbol;Acc:HGNC:20964] |
| ENSG00000169957 | HGLibA_56697 | 518 | 3.550794134 | ZNF768 | zinc finger protein 768 [Source:HGNC Symbol;Acc:HGNC:26273] |
| ENSG00000170545 | HGLibA_45594 | 518 | 3.550794134 | SMAGP | small cell adhesion glycoprotein [Source:HGNC Symbol;Acc:HGNC:26918] |
| ENSG00000196208 | HGLibA_20235 | 518 | 3.550794134 | GREB1 | growth regulating estrogen receptor binding 1 [Source:HGNC Symbol;Acc:HGNC:24885] |
| ENSG00000242689 | HGLibA_10391 | 518 | 3.550794134 | CNTF | ciliary neurotrophic factor [Source:HGNC Symbol;Acc:HGNC:2169] |
| ENSG00000050165 | HGLibA_13299 | 517 | 3.541748792 | DKK3 | dickkopf WNT signaling pathway inhibitor 3 [Source:HGNC Symbol;Acc:HGNC:2893] |
| ENSG00000152669 | HGLibA_08122 | 517 | 3.541748792 | CCNO | cyclin O [Source:HGNC Symbol;Acc:HGNC:18576] |
| ENSG00000107263 | HGLibA_40246 | 516 | 3.532703449 | RAPGEF1 | Rap guanine nucleotide exchange factor 1 [Source:HGNC Symbol;Acc:HGNC:4568] |
| ENSG00000120690 | HGLibA_14891 | 516 | 3.532703449 | ELF1 | E74 like ETS transcription factor 1 [Source:HGNC Symbol;Acc:HGNC:3316] |
| ENSG00000123643 | HGLibA_45030 | 516 | 3.532703449 | SLC36A1 | solute carrier family 36 member 1 [Source:HGNC Symbol;Acc:HGNC:18761] |
| ENSG00000165879 | HGLibA_17979 | 516 | 3.532703449 | FRAT1 | WNT signaling pathway regulator [Source:HGNC Symbol;Acc:HGNC:3944]" |
| ZCCHC6 | HGLibA_55043 | 516 | 3.532703449 | ZCCHC6 | - |
| ENSG00000004809 | HGLibA_44555 | 515 | 3.523658106 | SLC22A16 | solute carrier family 22 member 16 [Source:HGNC Symbol;Acc:HGNC:20302] |
| ENSG00000049247 | HGLibA_53462 | 515 | 3.523658106 | UTS2 | urotensin 2 [Source:HGNC Symbol;Acc:HGNC:12636] |
| ENSG00000100142 | HGLibA_37579 | 515 | 3.523658106 | POLR2F | RNA polymerase II subunit F [Source:HGNC Symbol;Acc:HGNC:9193] |
| ENSG00000142303 | HGLibA_00793 | 515 | 3.523658106 | ADAMTS10 | ADAM metallopeptidase with thrombospondin type 1 motif 10 [Source:HGNC Symbol;Acc:HGNC:13201] |
| ENSG00000196652 | HGLibA_55358 | 515 | 3.523658106 | ZKSCAN5 | zinc finger with KRAB and SCAN domains 5 [Source:HGNC Symbol;Acc:HGNC:12867] |
| ENSG00000197555 | HGLibA_44193 | 515 | 3.523658106 | SIPA1L1 | signal induced proliferation associated 1 like 1 [Source:HGNC Symbol;Acc:HGNC:20284] |
| WHSC1 | HGLibA_54293 | 515 | 3.523658106 | WHSC1 | - |
| ENSG00000221970 | HGLibA_33718 | 514 | 3.514612763 | OR2A1 | olfactory receptor family 2 subfamily A member 1 [Source:HGNC Symbol;Acc:HGNC:8229] |
| hsa-mir-1178 | HGLibA_57205 | 514 | 3.514612763 | hsa-mir-1178 | - |
| ENSG00000130720 | HGLibA_17499 | 513 | 3.50556742 | FIBCD1 | fibrinogen C domain containing 1 [Source:HGNC Symbol;Acc:HGNC:25922] |
| ENSG00000178537 | HGLibA_44670 | 513 | 3.50556742 | SLC25A20 | solute carrier family 25 member 20 [Source:HGNC Symbol;Acc:HGNC:1421] |
| ENSG00000101940 | HGLibA_54031 | 512 | 3.496522077 | WDR13 | WD repeat domain 13 [Source:HGNC Symbol;Acc:HGNC:14352] |
| ENSG00000106066 | HGLibA_11029 | 512 | 3.496522077 | CPVL | vitellogenic like [Source:HGNC Symbol;Acc:HGNC:14399]" |
| ENSG00000139055 | HGLibA_15523 | 512 | 3.496522077 | ERP27 | endoplasmic reticulum protein 27 [Source:HGNC Symbol;Acc:HGNC:26495] |
| ENSG00000141639 | HGLibA_28273 | 512 | 3.496522077 | MAPK4 | mitogen-activated protein kinase 4 [Source:HGNC Symbol;Acc:HGNC:6878] |
| ENSG00000143512 | HGLibA_21268 | 512 | 3.496522077 | HHIPL2 | HHIP like 2 [Source:HGNC Symbol;Acc:HGNC:25842] |
| ENSG00000167984 | HGLibA_32089 | 512 | 3.496522077 | NLRC3 | NLR family CARD domain containing 3 [Source:HGNC Symbol;Acc:HGNC:29889] |

| | | | | | |
|---|---|---|---|---|---|
| ENSG00000174473 | HGLibA_18581 | 512 | 3.496522077 | GALNTL6 | polypeptide N-acetylgalactosaminyltransferase like 6 [Source:HGNC Symbol;Acc:HGNC:33844] |
| ENSG00000175520 | HGLibA_52739 | 512 | 3.496522077 | UBQLN3 | ubiquilin 3 [Source:HGNC Symbol;Acc:HGNC:12510] |
| ENSG00000183605 | HGLibA_43790 | 512 | 3.496522077 | SFXN4 | sideroflexin 4 [Source:HGNC Symbol;Acc:HGNC:16088] |
| H1FNT | HGLibA_20737 | 512 | 3.496522077 | H1FNT | - |
| ENSG00000155393 | HGLibA_21072 | 511 | 3.487476734 | HEATR3 | HEAT repeat containing 3 [Source:HGNC Symbol;Acc:HGNC:26087] |
| ENSG00000213760 | HGLibA_03759 | 511 | 3.487476734 | ATP6V1G2 | ATPase H+ transporting V1 subunit G2 [Source:HGNC Symbol;Acc:HGNC:862] |
| ENSG00000070985 | HGLibA_51764 | 510 | 3.478431391 | TRPM5 | transient receptor potential cation channel subfamily M member 5 [Source:HGNC Symbol;Acc:HGNC:14323] |
| ENSG00000107821 | HGLibA_24088 | 510 | 3.478431391 | KAZALD1 | Kazal type serine peptidase inhibitor domain 1 [Source:HGNC Symbol;Acc:HGNC:25460] |
| ENSG00000157657 | HGLibA_56399 | 510 | 3.478431391 | ZNF618 | zinc finger protein 618 [Source:HGNC Symbol;Acc:HGNC:29416] |
| ENSG00000161055 | HGLibA_42933 | 510 | 3.478431391 | SCGB3A1 | secretoglobin family 3A member 1 [Source:HGNC Symbol;Acc:HGNC:18384] |
| ENSG00000175348 | HGLibA_50576 | 510 | 3.478431391 | TMEM9B | TMEM9 domain family member B [Source:HGNC Symbol;Acc:HGNC:1168] |
| ENSG00000259207 | HGLibA_23780 | 510 | 3.478431391 | ITGB3 | integrin subunit beta 3 [Source:HGNC Symbol;Acc:HGNC:6156] |
| IMPAD1 | HGLibA_23252 | 510 | 3.478431391 | IMPAD1 | - |
| ENSG00000100439 | HGLibA_00278 | 509 | 3.469386048 | ABHD4 | abhydrolase domain containing 4 [Source:HGNC Symbol;Acc:HGNC:20154] |
| ENSG00000101222 | HGLibA_46608 | 509 | 3.469386048 | SPEF1 | sperm flagellar 1 [Source:HGNC Symbol;Acc:HGNC:15874] |
| ENSG00000130540 | HGLibA_47764 | 509 | 3.469386048 | SULT4A1 | sulfotransferase family 4A member 1 [Source:HGNC Symbol;Acc:HGNC:14903] |
| ENSG00000144048 | HGLibA_14134 | 509 | 3.469386048 | DUSP11 | dual specificity phosphatase 11 [Source:HGNC Symbol;Acc:HGNC:3066] |
| ENSG00000155897 | HGLibA_00928 | 509 | 3.469386048 | ADCY8 | adenylate cyclase 8 [Source:HGNC Symbol;Acc:HGNC:239] |
| ENSG00000188257 | HGLibA_36884 | 509 | 3.469386048 | PLA2G2A | phospholipase A2 group IIA [Source:HGNC Symbol;Acc:HGNC:9031] |
| ENSG00000197415 | HGLibA_53624 | 509 | 3.469386048 | VEPH1 | ventricular zone expressed PH domain containing 1 [Source:HGNC Symbol;Acc:HGNC:25735] |
| ENSG00000204475 | HGLibA_31368 | 509 | 3.469386048 | NCR3 | natural cytotoxicity triggering receptor 3 [Source:HGNC Symbol;Acc:HGNC:19077] |
| ENSG00000087095 | HGLibA_32081 | 508 | 3.460340705 | NLK | nemo like kinase [Source:HGNC Symbol;Acc:HGNC:29858] |
| ENSG00000115290 | HGLibA_20226 | 508 | 3.460340705 | GRB14 | growth factor receptor bound protein 14 [Source:HGNC Symbol;Acc:HGNC:4565] |
| ENSG00000147145 | HGLibA_27061 | 508 | 3.460340705 | LPAR4 | lysophosphatidic acid receptor 4 [Source:HGNC Symbol;Acc:HGNC:4478] |
| ENSG00000158201 | HGLibA_00274 | 508 | 3.460340705 | ABHD3 | abhydrolase domain containing 3 [Source:HGNC Symbol;Acc:HGNC:18718] |
| FAM64A | HGLibA_16665 | 508 | 3.460340705 | FAM64A | - |
| ENSG00000068976 | HGLibA_39690 | 507 | 3.451295363 | PYGM | muscle associated [Source:HGNC Symbol;Acc:HGNC:9726]" |
| ENSG00000095066 | HGLibA_21878 | 507 | 3.451295363 | HOOK2 | hook microtubule tethering protein 2 [Source:HGNC Symbol;Acc:HGNC:19885] |
| ENSG00000130830 | HGLibA_29746 | 507 | 3.451295363 | MPP1 | membrane palmitoylated protein 1 [Source:HGNC Symbol;Acc:HGNC:7219] |
| ENSG00000163751 | HGLibA_10890 | 507 | 3.451295363 | CPA3 | carboxypeptidase A3 [Source:HGNC Symbol;Acc:HGNC:2298] |
| ENSG00000164081 | HGLibA_49084 | 507 | 3.451295363 | TEX264 | testis expressed 264 [Source:HGNC Symbol;Acc:HGNC:30247] |
| ENSG00000188677 | HGLibA_35259 | 507 | 3.451295363 | PARVB | parvin beta [Source:HGNC Symbol;Acc:HGNC:14653] |
| ENSG00000236125 | HGLibA_53279 | 507 | 3.451295363 | USP17L4 | ubiquitin specific peptidase 17-like family member 4 [Source:HGNC Symbol;Acc:HGNC:37176] |
| ENSG00000256223 | HGLibA_55428 | 507 | 3.451295363 | ZNF10 | zinc finger protein 10 [Source:HGNC Symbol;Acc:HGNC:12879] |
| ENSG00000151418 | HGLibA_03762 | 506 | 3.44225002 | ATP6V1G3 | ATPase H+ transporting V1 subunit G3 [Source:HGNC Symbol;Acc:HGNC:18265] |
| ENSG00000179088 | HGLibA_05240 | 506 | 3.44225002 | C12orf42 | chromosome 12 open reading frame 42 [Source:HGNC Symbol;Acc:HGNC:24729] |
| ENSG00000163512 | HGLibA_03937 | 505 | 3.433204677 | AZI2 | 5-azacytidine induced 2 [Source:HGNC Symbol;Acc:HGNC:24002] |
| KIAA1024 | HGLibA_24679 | 505 | 3.433204677 | KIAA1024 | - |
| ENSG00000126016 | HGLibA_01813 | 504 | 3.424159334 | AMOT | angiomotin [Source:HGNC Symbol;Acc:HGNC:17810] |
| ENSG00000129682 | HGLibA_17371 | 504 | 3.424159334 | FGF13 | fibroblast growth factor 13 [Source:HGNC Symbol;Acc:HGNC:3670] |
| ENSG00000130517 | HGLibA_36280 | 504 | 3.424159334 | PGPEP1 | pyroglutamyl-peptidase I [Source:HGNC Symbol;Acc:HGNC:13568] |
| ENSG00000138867 | HGLibA_20649 | 504 | 3.424159334 | GUCD1 | guanylyl cyclase domain containing 1 [Source:HGNC Symbol;Acc:HGNC:14237] |
| ENSG00000164663 | HGLibA_53386 | 504 | 3.424159334 | USP49 | ubiquitin specific peptidase 49 [Source:HGNC Symbol;Acc:HGNC:20078] |
| TMEM194A | HGLibA_50152 | 504 | 3.424159334 | TMEM194A | - |
| ENSG00000175029 | HGLibA_11588 | 503 | 3.415113991 | CTBP2 | C-terminal binding protein 2 [Source:HGNC Symbol;Acc:HGNC:2495] |
| ENSG00000183378 | HGLibA_34795 | 502 | 3.406068648 | OVCH2 | ovochymase 2 (gene/pseudogene) [Source:HGNC Symbol;Acc:HGNC:29970] |
| ENSG00000184788 | HGLibA_42781 | 502 | 3.406068648 | SATL1 | spermidine/spermine N1-acetyl transferase like 1 [Source:HGNC Symbol;Acc:HGNC:27992] |
| ENSG00000155026 | HGLibA_42326 | 501 | 3.397023305 | RSPH10B | radial spoke head 10 homolog B [Source:HGNC Symbol;Acc:HGNC:27362] |
| ENSG00000157064 | HGLibA_32188 | 501 | 3.397023305 | NMNAT2 | nicotinamide nucleotide adenylyltransferase 2 [Source:HGNC Symbol;Acc:HGNC:16789] |
| ENSG00000166455 | HGLibA_05449 | 501 | 3.397023305 | C16orf46 | chromosome 16 open reading frame 46 [Source:HGNC Symbol;Acc:HGNC:26525] |
| ENSG00000196361 | HGLibA_14884 | 501 | 3.397023305 | ELAVL3 | ELAV like RNA binding protein 3 [Source:HGNC Symbol;Acc:HGNC:3314] |
| ENSG00000115488 | HGLibA_31714 | 500 | 3.387977962 | NEU2 | neuraminidase 2 [Source:HGNC Symbol;Acc:HGNC:7759] |
| ENSG00000174915 | HGLibA_39291 | 500 | 3.387977962 | PTDSS2 | phosphatidylserine synthase 2 [Source:HGNC Symbol;Acc:HGNC:15463] |
| ENSG00000182749 | HGLibA_35169 | 500 | 3.387977962 | PAQR7 | progestin and adipoQ receptor family member 7 [Source:HGNC Symbol;Acc:HGNC:23146] |
| ENSG00000198369 | HGLibA_46792 | 500 | 3.387977962 | SPRED2 | sprouty related EVH1 domain containing 2 [Source:HGNC Symbol;Acc:HGNC:17722] |
| ENSG00000180016 | HGLibA_33660 | 499 | 3.378932619 | OR1E1 | olfactory receptor family 1 subfamily E member 1 [Source:HGNC Symbol;Acc:HGNC:8189] |
| ENSG00000214686 | HGLibA_23513 | 499 | 3.378932619 | IQCF6 | IQ motif containing F6 [Source:HGNC Symbol;Acc:HGNC:35158] |
| hsa-let-7b | HGLibA_57110 | 499 | 3.378932619 | hsa-let-7b | - |
| ENSG00000072201 | HGLibA_26744 | 498 | 3.369887276 | LNX1 | ligand of numb-protein X 1 [Source:HGNC Symbol;Acc:HGNC:6657] |
| ENSG00000116337 | HGLibA_01827 | 498 | 3.369887276 | AMPD2 | adenosine monophosphate deaminase 2 [Source:HGNC Symbol;Acc:HGNC:469] |
| ENSG00000138686 | HGLibA_04224 | 498 | 3.369887276 | BBS7 | Bardet-Biedl syndrome 7 [Source:HGNC Symbol;Acc:HGNC:18758] |
| ENSG00000151498 | HGLibA_00360 | 498 | 3.369887276 | ACAD8 | acyl-CoA dehydrogenase family member 8 [Source:HGNC Symbol;Acc:HGNC:87] |
| hsa-mir-6808 | HGLibA_63409 | 498 | 3.369887276 | hsa-mir-6808 | - |
| ENSG00000087842 | HGLibA_36730 | 497 | 3.360841933 | PIR | pirin [Source:HGNC Symbol;Acc:HGNC:30048] |
| ENSG00000137802 | HGLibA_28309 | 497 | 3.360841933 | MAPKBP1 | mitogen-activated protein kinase binding protein 1 [Source:HGNC Symbol;Acc:HGNC:29536] |
| ENSG00000148156 | HGLibA_00630 | 497 | 3.360841933 | ACTL7B | actin like 7B [Source:HGNC Symbol;Acc:HGNC:162] |
| ENSG00000204539 | HGLibA_08886 | 497 | 3.360841933 | CDSN | corneodesmosin [Source:HGNC Symbol;Acc:HGNC:1802] |
| ENSG00000106633 | HGLibA_18789 | 496 | 3.351796591 | GCK | glucokinase [Source:HGNC Symbol;Acc:HGNC:4195] |
| ENSG00000152229 | HGLibA_39241 | 496 | 3.351796591 | PSTPIP2 | proline-serine-threonine phosphatase interacting protein 2 [Source:HGNC Symbol;Acc:HGNC:9581] |
| ENSG00000153066 | HGLibA_52406 | 496 | 3.351796591 | TXNDC11 | thioredoxin domain containing 11 [Source:HGNC Symbol;Acc:HGNC:28030] |
| ENSG00000155592 | HGLibA_55348 | 496 | 3.351796591 | ZKSCAN2 | zinc finger with KRAB and SCAN domains 2 [Source:HGNC Symbol;Acc:HGNC:25677] |
| ENSG00000166211 | HGLibA_46657 | 496 | 3.351796591 | SPIC | Spi-C transcription factor [Source:HGNC Symbol;Acc:HGNC:29549] |
| ENSG00000180901 | HGLibA_24444 | 496 | 3.351796591 | KCTD2 | potassium channel tetramerization domain containing 2 [Source:HGNC Symbol;Acc:HGNC:21294] |
| hsa-mir-370 | HGLibA_59360 | 496 | 3.351796591 | hsa-mir-370 | - |
| ENSG00000165066 | HGLibA_32061 | 495 | 3.342751248 | NKX6-3 | NK6 homeobox 3 [Source:HGNC Symbol;Acc:HGNC:26328] |
| ENSG00000187569 | HGLibA_13889 | 495 | 3.342751248 | DPPA3 | developmental pluripotency associated 3 [Source:HGNC Symbol;Acc:HGNC:19199] |
| ENSG00000075035 | HGLibA_54423 | 494 | 3.333705905 | WSCD2 | WSC domain containing 2 [Source:HGNC Symbol;Acc:HGNC:29117] |
| ENSG00000102007 | HGLibA_37218 | 494 | 3.333705905 | PLP2 | proteolipid protein 2 [Source:HGNC Symbol;Acc:HGNC:9087] |
| ENSG00000160678 | HGLibA_42529 | 494 | 3.333705905 | S100A1 | S100 calcium binding protein A1 [Source:HGNC Symbol;Acc:HGNC:10486] |
| ENSG00000179083 | HGLibA_16117 | 494 | 3.333705905 | FAM133A | family with sequence similarity 133 member A [Source:HGNC Symbol;Acc:HGNC:26748] |
| ENSG00000241635 | HGLibA_52877 | 494 | 3.333705905 | UGT1A1 | UDP glucuronosyltransferase family 1 member A1 [Source:HGNC Symbol;Acc:HGNC:12530] |
| ENSG00000070748 | HGLibA_09357 | 493 | 3.324660562 | CHAT | choline O-acetyltransferase [Source:HGNC Symbol;Acc:HGNC:1912] |
| ENSG00000181752 | HGLibA_34549 | 493 | 3.324660562 | OR8K5 | olfactory receptor family 8 subfamily K member 5 [Source:HGNC Symbol;Acc:HGNC:15315] |
| ENSG00000232070 | HGLibA_50325 | 493 | 3.324660562 | TMEM253 | transmembrane protein 253 [Source:HGNC Symbol;Acc:HGNC:32545] |
| hsa-mir-519d | HGLibA_61725 | 493 | 3.324660562 | hsa-mir-519d | - |
| LOC256021 | HGLibA_26905 | 493 | 3.324660562 | LOC256021 | - |
| ENSG00000100994 | HGLibA_39684 | 492 | 3.315615219 | PYGB | glycogen phosphorylase B [Source:HGNC Symbol;Acc:HGNC:9723] |
| ENSG00000109832 | HGLibA_12681 | 492 | 3.315615219 | DDX25 | DEAD-box helicase 25 [Source:HGNC Symbol;Acc:HGNC:18698] |
| ENSG00000126062 | HGLibA_49883 | 492 | 3.315615219 | TMEM115 | transmembrane protein 115 [Source:HGNC Symbol;Acc:HGNC:30055] |
| ENSG00000134107 | HGLibA_04479 | 492 | 3.315615219 | BHLHE40 | basic helix-loop-helix family member e40 [Source:HGNC Symbol;Acc:HGNC:1046] |
| ENSG00000142798 | HGLibA_22339 | 492 | 3.315615219 | HSPG2 | heparan sulfate proteoglycan 2 [Source:HGNC Symbol;Acc:HGNC:5273] |
| ENSG00000174807 | HGLibA_08313 | 492 | 3.315615219 | CD248 | CD248 molecule [Source:HGNC Symbol;Acc:HGNC:18219] |
| ENSG00000185176 | HGLibA_02586 | 492 | 3.315615219 | AQP12B | aquaporin 12B [Source:HGNC Symbol;Acc:HGNC:6096] |
| ENSG00000080293 | HGLibA_43067 | 491 | 3.306569876 | SCTR | secretin receptor [Source:HGNC Symbol;Acc:HGNC:10608] |
| ENSG00000127824 | HGLibA_52261 | 491 | 3.306569876 | TUBA4A | tubulin alpha 4a [Source:HGNC Symbol;Acc:HGNC:12407] |
| ENSG00000136327 | HGLibA_32046 | 491 | 3.306569876 | NKX2-8 | NK2 homeobox 8 [Source:HGNC Symbol;Acc:HGNC:16364] |
| ENSG00000196539 | HGLibA_33872 | 491 | 3.306569876 | OR2T3 | olfactory receptor family 2 subfamily T member 3 [Source:HGNC Symbol;Acc:HGNC:14727] |
| hsa-mir-604 | HGLibA_62509 | 491 | 3.306569876 | hsa-mir-604 | - |
| hsa-mir-6869 | HGLibA_63659 | 491 | 3.306569876 | hsa-mir-6869 | - |
| ENSG00000172771 | HGLibA_14527 | 490 | 3.297524533 | EFCAB12 | EF-hand calcium binding domain 12 [Source:HGNC Symbol;Acc:HGNC:28061] |
| ENSG00000074966 | HGLibA_52387 | 489 | 3.28847919 | TXK | TXK tyrosine kinase [Source:HGNC Symbol;Acc:HGNC:12434] |
| ENSG00000099250 | HGLibA_32715 | 489 | 3.28847919 | NRP1 | neuropilin 1 [Source:HGNC Symbol;Acc:HGNC:8004] |
| ENSG00000054219 | HGLibA_27681 | 488 | 3.279433847 | LY75 | lymphocyte antigen 75 [Source:HGNC Symbol;Acc:HGNC:6729] |
| ENSG00000095370 | HGLibA_43894 | 488 | 3.279433847 | SH2D3C | SH2 domain containing 3C [Source:HGNC Symbol;Acc:HGNC:16884] |
| ENSG00000134955 | HGLibA_45046 | 488 | 3.279433847 | SLC37A2 | solute carrier family 37 member 2 [Source:HGNC Symbol;Acc:HGNC:20644] |
| ENSG00000168899 | HGLibA_53499 | 488 | 3.279433847 | VAMP5 | vesicle associated membrane protein 5 [Source:HGNC Symbol;Acc:HGNC:12646] |
| ENSG00000172201 | HGLibA_22533 | 488 | 3.279433847 | ID4 | HLH protein [Source:HGNC Symbol;Acc:HGNC:5363]" |
| FAM198A | HGLibA_16388 | 488 | 3.279433847 | FAM198A | - |
| ENSG00000130477 | HGLibA_52992 | 487 | 3.270388504 | UNC13A | unc-13 homolog A [Source:HGNC Symbol;Acc:HGNC:23150] |
| ENSG00000136514 | HGLibA_42430 | 487 | 3.270388504 | RTP4 | receptor transporter protein 4 [Source:HGNC Symbol;Acc:HGNC:23992] |
| ENSG00000160191 | HGLibA_35836 | 487 | 3.270388504 | PDE9A | phosphodiesterase 9A [Source:HGNC Symbol;Acc:HGNC:8795] |
| ENSG00000177182 | HGLibA_10168 | 487 | 3.270388504 | CLVS1 | clavesin 1 [Source:HGNC Symbol;Acc:HGNC:23139] |
| ENSG00000189184 | HGLibA_35406 | 487 | 3.270388504 | PCDH18 | protocadherin 18 [Source:HGNC Symbol;Acc:HGNC:14268] |
| ENSG00000141485 | HGLibA_44396 | 486 | 3.261343162 | SLC13A5 | solute carrier family 13 member 5 [Source:HGNC Symbol;Acc:HGNC:23089] |
| ENSG00000143753 | HGLibA_12921 | 486 | 3.261343162 | DEGS1 | sphingolipid 1 [Source:HGNC Symbol;Acc:HGNC:13709]" |
| ENSG00000171119 | HGLibA_32730 | 486 | 3.261343162 | NRTN | neurturin [Source:HGNC Symbol;Acc:HGNC:8007] |
| ENSG00000171819 | HGLibA_01928 | 486 | 3.261343162 | ANGPTL7 | angiopoietin like 7 [Source:HGNC Symbol;Acc:HGNC:24078] |
| ENSG00000180535 | HGLibA_04468 | 486 | 3.261343162 | BHLHA15 | basic helix-loop-helix family member a15 [Source:HGNC Symbol;Acc:HGNC:22265] |
| ENSG00000184330 | HGLibA_42568 | 486 | 3.261343162 | S100A7A | S100 calcium binding protein A7A [Source:HGNC Symbol;Acc:HGNC:21657] |
| ENSG00000196531 | HGLibA_31030 | 486 | 3.261343162 | NACA | nascent polypeptide associated complex subunit alpha [Source:HGNC Symbol;Acc:HGNC:7629] |
| ENSG00000213809 | HGLibA_25314 | 486 | 3.261343162 | KLRK1 | killer cell lectin like receptor K1 [Source:HGNC Symbol;Acc:HGNC:18788] |
| ENSG00000223638 | HGLibA_40937 | 486 | 3.261343162 | RFPL4A | ret finger protein like 4A [Source:HGNC Symbol;Acc:HGNC:16449] |
| TMEM5 | HGLibA_50419 | 486 | 3.261343162 | TMEM5 | - |
| ENSG00000064999 | HGLibA_02150 | 485 | 3.252297819 | ANKS1A | ankyrin repeat and sterile alpha motif domain containing 1A [Source:HGNC Symbol;Acc:HGNC:20961] |
| ENSG00000158525 | HGLibA_10897 | 485 | 3.252297819 | CPA5 | carboxypeptidase A5 [Source:HGNC Symbol;Acc:HGNC:15722] |
| ENSG00000182687 | HGLibA_18590 | 485 | 3.252297819 | GALR2 | galanin receptor 2 [Source:HGNC Symbol;Acc:HGNC:4133] |
| ENSG00000188322 | HGLibA_42800 | 485 | 3.252297819 | SBK1 | SH3 domain binding kinase 1 [Source:HGNC Symbol;Acc:HGNC:17699] |
| ENSG00000205426 | HGLibA_25559 | 485 | 3.252297819 | KRT81 | keratin 81 [Source:HGNC Symbol;Acc:HGNC:6458] |
| ENSG00000040933 | HGLibA_23336 | 484 | 3.243252476 | INPP4A | inositol polyphosphate-4-phosphatase type I A [Source:HGNC Symbol;Acc:HGNC:6074] |
| ENSG00000071564 | HGLibA_48785 | 484 | 3.243252476 | TCF3 | transcription factor 3 [Source:HGNC Symbol;Acc:HGNC:11633] |
| ENSG00000116857 | HGLibA_50554 | 484 | 3.243252476 | TMEM9 | transmembrane protein 9 [Source:HGNC Symbol;Acc:HGNC:18823] |
| ENSG00000125895 | HGLibA_50512 | 484 | 3.243252476 | TMEM74B | transmembrane protein 74B [Source:HGNC Symbol;Acc:HGNC:15893] |
| ENSG00000142661 | HGLibA_30894 | 484 | 3.243252476 | MYOM3 | myomesin 3 [Source:HGNC Symbol;Acc:HGNC:26679] |
| ENSG00000166887 | HGLibA_53787 | 484 | 3.243252476 | VPS39 | HOPS complex subunit [Source:HGNC Symbol;Acc:HGNC:20593]" |
| ENSG00000182372 | HGLibA_10084 | 484 | 3.243252476 | CLN8 | transmembrane ER and ERGIC protein [Source:HGNC Symbol;Acc:HGNC:2079]" |

| | | | | | |
|---|---|---|---|---|---|
| ENSG00000184164 | HGLibA_11128 | 484 | 3.243252476 | CRELD2 | cysteine rich with EGF like domains 2 [Source:HGNC Symbol;Acc:HGNC:28150] |
| ENSG00000197142 | HGLibA_00563 | 484 | 3.243252476 | ACSL5 | acyl-CoA synthetase long chain family member 5 [Source:HGNC Symbol;Acc:HGNC:16526] |
| ENSG00000197213 | HGLibA_57031 | 484 | 3.243252476 | ZSCAN5B | zinc finger and SCAN domain containing 5B [Source:HGNC Symbol;Acc:HGNC:34246] |
| ENSG00000244734 | HGLibA_20912 | 484 | 3.243252476 | HBB | hemoglobin subunit beta [Source:HGNC Symbol;Acc:HGNC:4827] |
| ENSG00000105605 | HGLibA_06948 | 483 | 3.234207133 | CACNG7 | calcium voltage-gated channel auxiliary subunit gamma 7 [Source:HGNC Symbol;Acc:HGNC:13626] |
| ENSG00000129968 | HGLibA_00262 | 483 | 3.234207133 | ABHD17A | abhydrolase domain containing 17A [Source:HGNC Symbol;Acc:HGNC:28756] |
| ENSG00000134504 | HGLibA_24412 | 483 | 3.234207133 | KCTD1 | potassium channel tetramerization domain containing 1 [Source:HGNC Symbol;Acc:HGNC:18249] |
| ENSG00000144730 | HGLibA_23013 | 483 | 3.234207133 | IL17RD | interleukin 17 receptor D [Source:HGNC Symbol;Acc:HGNC:17616] |
| ENSG00000156076 | HGLibA_54300 | 483 | 3.234207133 | WIF1 | WNT inhibitory factor 1 [Source:HGNC Symbol;Acc:HGNC:18081] |
| ENSG00000187715 | HGLibA_24097 | 483 | 3.234207133 | KBTBD12 | kelch repeat and BTB domain containing 12 [Source:HGNC Symbol;Acc:HGNC:25731] |
| ENSG00000204548 | HGLibA_12871 | 483 | 3.234207133 | DEFB121 | defensin beta 121 [Source:HGNC Symbol;Acc:HGNC:18101] |
| hsa-mir-125a | HGLibA_57444 | 483 | 3.234207133 | hsa-mir-125a | - |
| hsa-mir-2113 | HGLibA_58224 | 483 | 3.234207133 | hsa-mir-2113 | - |
| ENSG00000077092 | HGLibA_40275 | 482 | 3.22516179 | RARB | retinoic acid receptor beta [Source:HGNC Symbol;Acc:HGNC:9865] |
| ENSG00000147100 | HGLibA_44435 | 482 | 3.22516179 | SLC16A2 | solute carrier family 16 member 2 [Source:HGNC Symbol;Acc:HGNC:10923] |
| ENSG00000157680 | HGLibA_13075 | 482 | 3.22516179 | DGKI | diacylglycerol kinase iota [Source:HGNC Symbol;Acc:HGNC:2855] |
| ENSG00000188051 | HGLibA_50236 | 482 | 3.22516179 | TMEM221 | transmembrane protein 221 [Source:HGNC Symbol;Acc:HGNC:21943] |
| hsa-mir-378e | HGLibA_59445 | 482 | 3.22516179 | hsa-mir-378e | - |
| hsa-mir-6085 | HGLibA_62599 | 482 | 3.22516179 | hsa-mir-6085 | - |
| ENSG00000070501 | HGLibA_37489 | 481 | 3.216116447 | POLB | DNA polymerase beta [Source:HGNC Symbol;Acc:HGNC:9174] |
| ENSG00000142552 | HGLibA_40707 | 481 | 3.216116447 | RCN3 | reticulocalbin 3 [Source:HGNC Symbol;Acc:HGNC:21145] |
| ENSG00000206073 | HGLibA_43554 | 481 | 3.216116447 | SERPINB4 | serpin family B member 4 [Source:HGNC Symbol;Acc:HGNC:10570] |
| ENSG00000006704 | HGLibA_20569 | 480 | 3.207071104 | GTF2IRD1 | GTF2I repeat domain containing 1 [Source:HGNC Symbol;Acc:HGNC:4661] |
| ENSG00000111962 | HGLibA_53424 | 480 | 3.207071104 | UST | uronyl 2-sulfotransferase [Source:HGNC Symbol;Acc:HGNC:17223] |
| ENSG00000136156 | HGLibA_23831 | 480 | 3.207071104 | ITM2B | integral membrane protein 2B [Source:HGNC Symbol;Acc:HGNC:6174] |
| ENSG00000136250 | HGLibA_02263 | 480 | 3.207071104 | AOAH | acyloxyacyl hydrolase [Source:HGNC Symbol;Acc:HGNC:548] |
| ENSG00000141349 | HGLibA_18312 | 480 | 3.207071104 | G6PC3 | glucose-6-phosphatase catalytic subunit 3 [Source:HGNC Symbol;Acc:HGNC:24861] |
| ENSG00000158092 | HGLibA_31304 | 480 | 3.207071104 | NCK1 | NCK adaptor protein 1 [Source:HGNC Symbol;Acc:HGNC:7664] |
| ENSG00000164363 | HGLibA_45314 | 480 | 3.207071104 | SLC6A18 | solute carrier family 6 member 18 [Source:HGNC Symbol;Acc:HGNC:26441] |
| ENSG00000028839 | HGLibA_48627 | 479 | 3.198025761 | TBPL1 | TATA-box binding protein like 1 [Source:HGNC Symbol;Acc:HGNC:11589] |
| ENSG00000102468 | HGLibA_22371 | 479 | 3.198025761 | HTR2A | 5-hydroxytryptamine receptor 2A [Source:HGNC Symbol;Acc:HGNC:5293] |
| ENSG00000118292 | HGLibA_05873 | 479 | 3.198025761 | C1orf54 | chromosome 1 open reading frame 54 [Source:HGNC Symbol;Acc:HGNC:26258] |
| ENSG00000177627 | HGLibA_05265 | 479 | 3.198025761 | C12orf54 | chromosome 12 open reading frame 54 [Source:HGNC Symbol;Acc:HGNC:28553] |
| ENSG00000188089 | HGLibA_36911 | 479 | 3.198025761 | PLA2G4E | phospholipase A2 group IVE [Source:HGNC Symbol;Acc:HGNC:24791] |
| hsa-mir-516b-2 | HGLibA_61619 | 479 | 3.198025761 | hsa-mir-516b-2 | - |
| hsa-mir-7854 | HGLibA_64004 | 479 | 3.198025761 | hsa-mir-7854 | - |
| C22orf29 | HGLibA_06068 | 478 | 3.188980418 | C22orf29 | - |
| ENSG00000050820 | HGLibA_04244 | 478 | 3.188980418 | BCAR1 | Cas family scaffold protein [Source:HGNC Symbol;Acc:HGNC:971]" |
| ENSG00000068078 | HGLibA_17446 | 478 | 3.188980418 | FGFR3 | fibroblast growth factor receptor 3 [Source:HGNC Symbol;Acc:HGNC:3690] |
| ENSG00000101435 | HGLibA_11475 | 478 | 3.188980418 | CST9L | cystatin 9 like [Source:HGNC Symbol;Acc:HGNC:16233] |
| ENSG00000143891 | HGLibA_18520 | 478 | 3.188980418 | GALM | galactose mutarotase [Source:HGNC Symbol;Acc:HGNC:24063] |
| ENSG00000171298 | HGLibA_18317 | 478 | 3.188980418 | GAA | acid [Source:HGNC Symbol;Acc:HGNC:4065]" |
| ENSG00000197980 | HGLibA_26280 | 478 | 3.188980418 | LEKR1 | glutamate and lysine rich 1 [Source:HGNC Symbol;Acc:HGNC:33765]" |
| hsa-mir-223 | HGLibA_58301 | 478 | 3.188980418 | hsa-mir-223 | - |
| KIAA1324 | HGLibA_24737 | 478 | 3.188980418 | KIAA1324 | - |
| ENSG00000012211 | HGLibA_38477 | 477 | 3.179935075 | PRICKLE3 | prickle planar cell polarity protein 3 [Source:HGNC Symbol;Acc:HGNC:6645] |
| ENSG00000135899 | HGLibA_46288 | 477 | 3.179935075 | SP110 | SP110 nuclear body protein [Source:HGNC Symbol;Acc:HGNC:5401] |
| ENSG00000158517 | HGLibA_31296 | 477 | 3.179935075 | NCF1 | neutrophil cytosolic factor 1 [Source:HGNC Symbol;Acc:HGNC:7660] |
| ENSG00000163866 | HGLibA_45725 | 477 | 3.179935075 | SMIM12 | small integral membrane protein 12 [Source:HGNC Symbol;Acc:HGNC:25154] |
| ENSG00000168872 | HGLibA_12663 | 477 | 3.179935075 | DDX19A | DEAD-box helicase 19A [Source:HGNC Symbol;Acc:HGNC:25628] |
| ENSG00000169967 | HGLibA_28181 | 477 | 3.179935075 | MAP3K2 | mitogen-activated protein kinase kinase kinase 2 [Source:HGNC Symbol;Acc:HGNC:6854] |
| ENSG00000172728 | HGLibA_18181 | 477 | 3.179935075 | FUT10 | fucosyltransferase 10 [Source:HGNC Symbol;Acc:HGNC:19234] |
| ENSG00000016864 | HGLibA_19337 | 476 | 3.170889733 | GLT8D1 | glycosyltransferase 8 domain containing 1 [Source:HGNC Symbol;Acc:HGNC:24870] |
| ENSG00000116954 | HGLibA_42229 | 476 | 3.170889733 | RRAGC | Ras related GTP binding C [Source:HGNC Symbol;Acc:HGNC:19902] |
| ENSG00000125817 | HGLibA_09040 | 476 | 3.170889733 | CENPB | centromere protein B [Source:HGNC Symbol;Acc:HGNC:1852] |
| ENSG00000132965 | HGLibA_01690 | 476 | 3.170889733 | ALOX5AP | arachidonate 5-lipoxygenase activating protein [Source:HGNC Symbol;Acc:HGNC:436] |
| ENSG00000138002 | HGLibA_22749 | 476 | 3.170889733 | IFT172 | intraflagellar transport 172 [Source:HGNC Symbol;Acc:HGNC:30391] |
| ENSG00000162951 | HGLibA_27485 | 476 | 3.170889733 | LRRTM1 | leucine rich repeat transmembrane neuronal 1 [Source:HGNC Symbol;Acc:HGNC:19408] |
| ENSG00000184571 | HGLibA_36777 | 476 | 3.170889733 | PIWIL3 | piwi like RNA-mediated gene silencing 3 [Source:HGNC Symbol;Acc:HGNC:18443] |
| ENSG00000033327 | HGLibA_18323 | 475 | 3.16184439 | GAB2 | GRB2 associated binding protein 2 [Source:HGNC Symbol;Acc:HGNC:14458] |
| ENSG00000123933 | HGLibA_30617 | 475 | 3.16184439 | MXD4 | MAX dimerization protein 4 [Source:HGNC Symbol;Acc:HGNC:13906] |
| ENSG00000143476 | HGLibA_14060 | 475 | 3.16184439 | DTL | denticleless E3 ubiquitin protein ligase homolog [Source:HGNC Symbol;Acc:HGNC:30288] |
| ENSG00000141424 | HGLibA_45119 | 474 | 3.152799047 | SLC39A6 | solute carrier family 39 member 6 [Source:HGNC Symbol;Acc:HGNC:18607] |
| ENSG00000148204 | HGLibA_11071 | 474 | 3.152799047 | CRB2 | cell polarity complex component [Source:HGNC Symbol;Acc:HGNC:18688]" |
| ENSG00000157168 | HGLibA_32676 | 474 | 3.152799047 | NRG1 | neuregulin 1 [Source:HGNC Symbol;Acc:HGNC:7997] |
| ENSG00000167123 | HGLibA_09191 | 474 | 3.152799047 | CERCAM | cerebral endothelial cell adhesion molecule [Source:HGNC Symbol;Acc:HGNC:23723] |
| ENSG00000175938 | HGLibA_34594 | 474 | 3.152799047 | ORAI3 | ORAI calcium release-activated calcium modulator 3 [Source:HGNC Symbol;Acc:HGNC:28185] |
| ENSG00000188580 | HGLibA_31992 | 474 | 3.152799047 | NKAIN2 | sodium/potassium transporting ATPase interacting 2 [Source:HGNC Symbol;Acc:HGNC:16443] |
| ENSG00000196730 | HGLibA_12321 | 474 | 3.152799047 | DAPK1 | death associated protein kinase 1 [Source:HGNC Symbol;Acc:HGNC:2674] |
| ENSG00000111801 | HGLibA_04974 | 473 | 3.143753704 | BTN3A3 | butyrophilin subfamily 3 member A3 [Source:HGNC Symbol;Acc:HGNC:1140] |
| ENSG00000145040 | HGLibA_52826 | 473 | 3.143753704 | UCN2 | urocortin 2 [Source:HGNC Symbol;Acc:HGNC:18414] |
| ENSG00000145107 | HGLibA_49698 | 473 | 3.143753704 | TM4SF19 | transmembrane 4 L six family member 19 [Source:HGNC Symbol;Acc:HGNC:25167] |
| ENSG00000163534 | HGLibA_17225 | 473 | 3.143753704 | FCRL1 | Fc receptor like 1 [Source:HGNC Symbol;Acc:HGNC:18509] |
| ENSG00000197249 | HGLibA_43504 | 473 | 3.143753704 | SERPINA1 | serpin family A member 1 [Source:HGNC Symbol;Acc:HGNC:8941] |
| ENSG00000273706 | HGLibA_26446 | 473 | 3.143753704 | LHX1 | LIM homeobox 1 [Source:HGNC Symbol;Acc:HGNC:6593] |
| H2AFV | HGLibA_20758 | 473 | 3.143753704 | H2AFV | - |
| MFI2 | HGLibA_29066 | 473 | 3.143753704 | MFI2 | - |
| ENSG00000169919 | HGLibA_20680 | 472 | 3.134708361 | GUSB | glucuronidase beta [Source:HGNC Symbol;Acc:HGNC:4696] |
| ENSG00000173409 | HGLibA_03174 | 472 | 3.134708361 | ARV1 | fatty acid homeostasis modulator [Source:HGNC Symbol;Acc:HGNC:29561]" |
| ENSG00000204021 | HGLibA_26624 | 472 | 3.134708361 | LIPK | lipase family member K [Source:HGNC Symbol;Acc:HGNC:23444] |
| hsa-mir-6870 | HGLibA_63664 | 472 | 3.134708361 | hsa-mir-6870 | - |
| ENSG00000088256 | HGLibA_19437 | 471 | 3.125663018 | GNA11 | G protein subunit alpha 11 [Source:HGNC Symbol;Acc:HGNC:4379] |
| ENSG00000108244 | HGLibA_25454 | 471 | 3.125663018 | KRT23 | keratin 23 [Source:HGNC Symbol;Acc:HGNC:6438] |
| ENSG00000132024 | HGLibA_07475 | 471 | 3.125663018 | CC2D1A | coiled-coil and C2 domain containing 1A [Source:HGNC Symbol;Acc:HGNC:30237] |
| ENSG00000136630 | HGLibA_21659 | 471 | 3.125663018 | HLX | H2.0 like homeobox [Source:HGNC Symbol;Acc:HGNC:4978] |
| ENSG00000155158 | HGLibA_52136 | 471 | 3.125663018 | TTC39B | tetratricopeptide repeat domain 39B [Source:HGNC Symbol;Acc:HGNC:23704] |
| ENSG00000183230 | HGLibA_11644 | 471 | 3.125663018 | CTNNA3 | catenin alpha 3 [Source:HGNC Symbol;Acc:HGNC:2511] |
| ENSG00000234745 | HGLibA_21597 | 471 | 3.125663018 | HLA-B | B [Source:HGNC Symbol;Acc:HGNC:4932]" |
| ENSG00000274736 | HGLibA_07999 | 471 | 3.125663018 | CCL23 | C-C motif chemokine ligand 23 [Source:HGNC Symbol;Acc:HGNC:10622] |
| ENSG00000111801 | HGLibA_04973 | 470 | 3.116617675 | BTN3A3 | butyrophilin subfamily 3 member A3 [Source:HGNC Symbol;Acc:HGNC:1140] |
| ENSG00000120798 | HGLibA_32596 | 470 | 3.116617675 | NR2C1 | nuclear receptor subfamily 2 group C member 1 [Source:HGNC Symbol;Acc:HGNC:7971] |
| ENSG00000148702 | HGLibA_20793 | 470 | 3.116617675 | HABP2 | hyaluronan binding protein 2 [Source:HGNC Symbol;Acc:HGNC:4798] |
| ENSG00000162763 | HGLibA_27385 | 470 | 3.116617675 | LRRC52 | leucine rich repeat containing 52 [Source:HGNC Symbol;Acc:HGNC:32156] |
| ENSG00000186792 | HGLibA_22442 | 470 | 3.116617675 | HYAL3 | hyaluronidase 3 [Source:HGNC Symbol;Acc:HGNC:5322] |
| ENSG00000223572 | HGLibA_09777 | 470 | 3.116617675 | CKMT1A | mitochondrial 1A [Source:HGNC Symbol;Acc:HGNC:31736]" |
| ENSG00000061938 | HGLibA_50848 | 469 | 3.107572332 | TNK2 | tyrosine kinase non receptor 2 [Source:HGNC Symbol;Acc:HGNC:19297] |
| ENSG00000092841 | HGLibA_30768 | 469 | 3.107572332 | MYL6 | myosin light chain 6 [Source:HGNC Symbol;Acc:HGNC:7587] |
| ENSG00000113742 | HGLibA_10926 | 469 | 3.107572332 | CPEB4 | cytoplasmic polyadenylation element binding protein 4 [Source:HGNC Symbol;Acc:HGNC:21747] |
| ENSG00000168763 | HGLibA_10320 | 469 | 3.107572332 | CNNM3 | cyclin and CBS domain divalent metal cation transport mediator 3 [Source:HGNC Symbol;Acc:HGNC:104] |
| hsa-mir-936 | HGLibA_64317 | 469 | 3.107572332 | hsa-mir-936 | - |
| ENSG00000166682 | HGLibA_50644 | 468 | 3.098526989 | TMPRSS5 | transmembrane serine protease 5 [Source:HGNC Symbol;Acc:HGNC:14908] |
| ENSG00000168124 | HGLibA_33665 | 468 | 3.098526989 | OR1F1 | olfactory receptor family 1 subfamily F member 1 [Source:HGNC Symbol;Acc:HGNC:8194] |
| ENSG00000175093 | HGLibA_46867 | 468 | 3.098526989 | SPSB4 | splA/ryanodine receptor domain and SOCS box containing 4 [Source:HGNC Symbol;Acc:HGNC:30630] |
| ENSG00000182518 | HGLibA_15990 | 468 | 3.098526989 | FAM104B | family with sequence similarity 104 member B [Source:HGNC Symbol;Acc:HGNC:25085] |
| ENSG00000197140 | HGLibA_00773 | 468 | 3.098526989 | ADAM32 | ADAM metallopeptidase domain 32 [Source:HGNC Symbol;Acc:HGNC:15479] |
| ENSG00000271321 | HGLibA_11578 | 468 | 3.098526989 | CTAGE6 | CTAGE family member 6 [Source:HGNC Symbol;Acc:HGNC:28644] |
| C1orf110 | HGLibA_05745 | 467 | 3.089481646 | C1orf110 | - |
| ENSG00000089050 | HGLibA_40447 | 467 | 3.089481646 | RBBP9 | serine hydrolase [Source:HGNC Symbol;Acc:HGNC:9892]" |
| ENSG00000127329 | HGLibA_39486 | 467 | 3.089481646 | PTPRB | receptor type B [Source:HGNC Symbol;Acc:HGNC:9665]" |
| ENSG00000159261 | HGLibA_09860 | 467 | 3.089481646 | CLDN14 | claudin 14 [Source:HGNC Symbol;Acc:HGNC:2035] |
| ENSG00000183775 | HGLibA_24432 | 467 | 3.089481646 | KCTD16 | potassium channel tetramerization domain containing 16 [Source:HGNC Symbol;Acc:HGNC:29244] |
| ENSG00000224940 | HGLibA_38890 | 467 | 3.089481646 | PRRT4 | proline rich transmembrane protein 4 [Source:HGNC Symbol;Acc:HGNC:37280] |
| hsa-mir-676 | HGLibA_63199 | 467 | 3.089481646 | hsa-mir-676 | - |
| ENSG00000087274 | HGLibA_00941 | 466 | 3.080436303 | ADD1 | adducin 1 [Source:HGNC Symbol;Acc:HGNC:243] |
| ENSG00000111305 | HGLibA_20420 | 466 | 3.080436303 | GSG1 | germ cell associated 1 [Source:HGNC Symbol;Acc:HGNC:19716] |
| ENSG00000154803 | HGLibA_17607 | 466 | 3.080436303 | FLCN | folliculin [Source:HGNC Symbol;Acc:HGNC:27310] |
| ENSG00000167360 | HGLibA_34126 | 466 | 3.080436303 | OR51Q1 | olfactory receptor family 51 subfamily Q member 1 (gene/pseudogene) [Source:HGNC Symbol;Acc:HGNC:14851] |
| ENSG00000167657 | HGLibA_12327 | 466 | 3.080436303 | DAPK3 | death associated protein kinase 3 [Source:HGNC Symbol;Acc:HGNC:2676] |
| ENSG00000168077 | HGLibA_42863 | 466 | 3.080436303 | SCARA3 | scavenger receptor class A member 3 [Source:HGNC Symbol;Acc:HGNC:19000] |
| ENSG00000095303 | HGLibA_39358 | 465 | 3.071390961 | PTGS1 | prostaglandin-endoperoxide synthase 1 [Source:HGNC Symbol;Acc:HGNC:9604] |
| ENSG00000146755 | HGLibA_51538 | 465 | 3.071390961 | TRIM50 | tripartite motif containing 50 [Source:HGNC Symbol;Acc:HGNC:19017] |
| ENSG00000150676 | HGLibA_07887 | 465 | 3.071390961 | CCDC83 | coiled-coil domain containing 83 [Source:HGNC Symbol;Acc:HGNC:28535] |
| ENSG00000221826 | HGLibA_39044 | 465 | 3.071390961 | PSG3 | pregnancy specific beta-1-glycoprotein 3 [Source:HGNC Symbol;Acc:HGNC:9520] |
| ENSG00000242247 | HGLibA_02667 | 465 | 3.071390961 | ARFGAP3 | ADP ribosylation factor GTPase activating protein 3 [Source:HGNC Symbol;Acc:HGNC:661] |
| ENSG00000012660 | HGLibA_14968 | 464 | 3.062345618 | ELOVL5 | ELOVL fatty acid elongase 5 [Source:HGNC Symbol;Acc:HGNC:21308] |
| ENSG00000112367 | HGLibA_17509 | 464 | 3.062345618 | FIG4 | FIG4 phosphoinositide 5-phosphatase [Source:HGNC Symbol;Acc:HGNC:16873] |
| hsa-mir-548ao | HGLibA_61910 | 464 | 3.062345618 | hsa-mir-548ao | - |
| ENSG00000081181 | HGLibA_02688 | 463 | 3.053300275 | ARG2 | arginase 2 [Source:HGNC Symbol;Acc:HGNC:664] |
| ENSG00000100196 | HGLibA_24485 | 463 | 3.053300275 | KDELR3 | KDEL endoplasmic reticulum protein retention receptor 3 [Source:HGNC Symbol;Acc:HGNC:6306] |
| ENSG00000107833 | HGLibA_32493 | 463 | 3.053300275 | NPM3 | nucleophosmin/nucleoplasmin 3 [Source:HGNC Symbol;Acc:HGNC:7931] |
| ENSG00000137699 | HGLibA_51454 | 463 | 3.053300275 | TRIM29 | tripartite motif containing 29 [Source:HGNC Symbol;Acc:HGNC:17274] |
| ENSG00000181722 | HGLibA_54839 | 463 | 3.053300275 | ZBTB20 | zinc finger and BTB domain containing 20 [Source:HGNC Symbol;Acc:HGNC:13503] |
| ENSG00000119888 | HGLibA_15271 | 462 | 3.044254932 | EPCAM | epithelial cell adhesion molecule [Source:HGNC Symbol;Acc:HGNC:11529] |
| ENSG00000132698 | HGLibA_39849 | 462 | 3.044254932 | RAB25 | member RAS oncogene family [Source:HGNC Symbol;Acc:HGNC:18238]" |
| ENSG00000150782 | HGLibA_23021 | 462 | 3.044254932 | IL18 | interleukin 18 [Source:HGNC Symbol;Acc:HGNC:5986] |

| | | | | | |
|---|---|---|---|---|---|
| ENSG00000152092 | HGLibA_03379 | 462 | 3.044254932 | ASTN1 | astrotactin 1 [Source:HGNC Symbol;Acc:HGNC:773] |
| ENSG00000166262 | HGLibA_16531 | 462 | 3.044254932 | FAM227B | family with sequence similarity 227 member B [Source:HGNC Symbol;Acc:HGNC:26543] |
| ENSG00000182771 | HGLibA_20273 | 462 | 3.044254932 | GRID1 | glutamate ionotropic receptor delta type subunit 1 [Source:HGNC Symbol;Acc:HGNC:4575] |
| ENSG00000186458 | HGLibA_12901 | 462 | 3.044254932 | DEFB132 | defensin beta 132 [Source:HGNC Symbol;Acc:HGNC:33806] |
| ENSG00000180423 | HGLibA_20852 | 461 | 3.035209589 | HARBI1 | harbinger transposase derived 1 [Source:HGNC Symbol;Acc:HGNC:26522] |
| ENSG00000187122 | HGLibA_45505 | 461 | 3.035209589 | SLIT1 | slit guidance ligand 1 [Source:HGNC Symbol;Acc:HGNC:11085] |
| ENSG00000254402 | HGLibA_27287 | 461 | 3.035209589 | LRRC24 | leucine rich repeat containing 24 [Source:HGNC Symbol;Acc:HGNC:28947] |
| ENSG00000254636 | HGLibA_03059 | 461 | 3.035209589 | ARMS2 | age-related maculopathy susceptibility 2 [Source:HGNC Symbol;Acc:HGNC:32685] |
| LOC100130451 | HGLibA_26787 | 461 | 3.035209589 | LOC100130451 | - |
| ENSG00000134343 | HGLibA_02177 | 460 | 3.026164246 | ANO3 | anoctamin 3 [Source:HGNC Symbol;Acc:HGNC:14004] |
| ENSG00000141756 | HGLibA_17553 | 460 | 3.026164246 | FKBP10 | FK506 binding protein 10 [Source:HGNC Symbol;Acc:HGNC:18169] |
| ENSG00000165471 | HGLibA_28500 | 460 | 3.026164246 | MBL2 | mannose binding lectin 2 [Source:HGNC Symbol;Acc:HGNC:6922] |
| ENSG00000166033 | HGLibA_22407 | 460 | 3.026164246 | HTRA1 | HtrA serine peptidase 1 [Source:HGNC Symbol;Acc:HGNC:9476] |
| ENSG00000178690 | HGLibA_14228 | 460 | 3.026164246 | DYNAP | dynactin associated protein [Source:HGNC Symbol;Acc:HGNC:26808] |
| ENSG00000180921 | HGLibA_16761 | 460 | 3.026164246 | FAM83H | family with sequence similarity 83 member H [Source:HGNC Symbol;Acc:HGNC:24797] |
| ENSG00000184007 | HGLibA_39405 | 460 | 3.026164246 | PTP4A2 | member 2 [Source:HGNC Symbol;Acc:HGNC:9635]" |
| ENSG00000185813 | HGLibA_35706 | 460 | 3.026164246 | PCYT2 | ethanolamine [Source:HGNC Symbol;Acc:HGNC:8756]" |
| ENSG00000187608 | HGLibA_23654 | 460 | 3.026164246 | ISG15 | ISG15 ubiquitin-like modifier [Source:HGNC Symbol;Acc:HGNC:4053] |
| ENSG00000198046 | HGLibA_56491 | 460 | 3.026164246 | ZNF667 | zinc finger protein 667 [Source:HGNC Symbol;Acc:HGNC:28854] |
| ENSG00000243335 | HGLibA_24465 | 460 | 3.026164246 | KCTD7 | potassium channel tetramerization domain containing 7 [Source:HGNC Symbol;Acc:HGNC:21957] |
| hsa-mir-4756 | HGLibA_61122 | 460 | 3.026164246 | hsa-mir-4756 | - |
| ENSG00000092067 | HGLibA_08965 | 459 | 3.017118903 | CEBPE | CCAAT enhancer binding protein epsilon [Source:HGNC Symbol;Acc:HGNC:1836] |
| ENSG00000126822 | HGLibA_37107 | 459 | 3.017118903 | PLEKHG3 | pleckstrin homology and RhoGEF domain containing G3 [Source:HGNC Symbol;Acc:HGNC:20364] |
| ENSG00000128699 | HGLibA_34625 | 459 | 3.017118903 | ORMDL1 | ORMDL sphingolipid biosynthesis regulator 1 [Source:HGNC Symbol;Acc:HGNC:16036] |
| ENSG00000145839 | HGLibA_23198 | 459 | 3.017118903 | IL9 | interleukin 9 [Source:HGNC Symbol;Acc:HGNC:6029] |
| ENSG00000157240 | HGLibA_18261 | 459 | 3.017118903 | FZD1 | frizzled class receptor 1 [Source:HGNC Symbol;Acc:HGNC:4038] |
| ENSG00000171045 | HGLibA_51882 | 459 | 3.017118903 | TSNARE1 | t-SNARE domain containing 1 [Source:HGNC Symbol;Acc:HGNC:26437] |
| ENSG00000171174 | HGLibA_40463 | 459 | 3.017118903 | RBKS | ribokinase [Source:HGNC Symbol;Acc:HGNC:30325] |
| ENSG00000188064 | HGLibA_54384 | 459 | 3.017118903 | WNT7B | Wnt family member 7B [Source:HGNC Symbol;Acc:HGNC:12787] |
| KIAA0556 | HGLibA_24643 | 459 | 3.017118903 | KIAA0556 | - |
| ENSG00000140320 | HGLibA_04109 | 458 | 3.00807356 | BAHD1 | bromo adjacent homology domain containing 1 [Source:HGNC Symbol;Acc:HGNC:29153] |
| ENSG00000151892 | HGLibA_18966 | 458 | 3.00807356 | GFRA1 | GDNF family receptor alpha 1 [Source:HGNC Symbol;Acc:HGNC:4243] |
| ENSG00000160695 | HGLibA_53728 | 458 | 3.00807356 | VPS11 | CORVET/HOPS core subunit [Source:HGNC Symbol;Acc:HGNC:14583]" |
| ENSG00000172403 | HGLibA_48021 | 458 | 3.00807356 | SYNPO2 | synaptopodin 2 [Source:HGNC Symbol;Acc:HGNC:17732] |
| ENSG00000179981 | HGLibA_51861 | 458 | 3.00807356 | TSHZ1 | teashirt zinc finger homeobox 1 [Source:HGNC Symbol;Acc:HGNC:10669] |
| ENSG00000187609 | HGLibA_15723 | 458 | 3.00807356 | EXD3 | exonuclease 3'-5' domain containing 3 [Source:HGNC Symbol;Acc:HGNC:26023] |
| ENSG00000221946 | HGLibA_18245 | 458 | 3.00807356 | FXYD7 | FXYD domain containing ion transport regulator 7 [Source:HGNC Symbol;Acc:HGNC:4034] |
| hsa-mir-3713 | HGLibA_59365 | 458 | 3.00807356 | hsa-mir-3713 | - |
| hsa-mir-6795 | HGLibA_63359 | 458 | 3.00807356 | hsa-mir-6795 | - |
| ENSG00000083444 | HGLibA_37208 | 457 | 2.999028217 | PLOD1 | 2-oxoglutarate 5-dioxygenase 1 [Source:HGNC Symbol;Acc:HGNC:9081]" |
| ENSG00000103126 | HGLibA_03922 | 457 | 2.999028217 | AXIN1 | axin 1 [Source:HGNC Symbol;Acc:HGNC:903] |
| ENSG00000126814 | HGLibA_51691 | 457 | 2.999028217 | TRMT5 | tRNA methyltransferase 5 [Source:HGNC Symbol;Acc:HGNC:23141] |
| ENSG00000134897 | HGLibA_04534 | 457 | 2.999028217 | BIVM | immunoglobulin-like variable motif containing [Source:HGNC Symbol;Acc:HGNC:16034]" |
| ENSG00000184545 | HGLibA_14192 | 457 | 2.999028217 | DUSP8 | dual specificity phosphatase 8 [Source:HGNC Symbol;Acc:HGNC:3074] |
| ENSG00000205927 | HGLibA_33370 | 457 | 2.999028217 | OLIG2 | oligodendrocyte transcription factor 2 [Source:HGNC Symbol;Acc:HGNC:9398] |
| FAM46D | HGLibA_16605 | 457 | 2.999028217 | FAM46D | - |
| ENSG00000100034 | HGLibA_37974 | 456 | 2.989982874 | PPM1F | Mg2+/Mn2+ dependent 1F [Source:HGNC Symbol;Acc:HGNC:19388]" |
| ENSG00000136732 | HGLibA_20704 | 456 | 2.989982874 | GYPC | glycophorin C (Gerbich blood group) [Source:HGNC Symbol;Acc:HGNC:4704] |
| ENSG00000147459 | HGLibA_13748 | 456 | 2.989982874 | DOCK5 | dedicator of cytokinesis 5 [Source:HGNC Symbol;Acc:HGNC:23476] |
| ENSG00000149300 | HGLibA_05150 | 456 | 2.989982874 | C11orf52 | chromosome 11 open reading frame 52 [Source:HGNC Symbol;Acc:HGNC:30531] |
| ENSG00000166147 | HGLibA_16954 | 456 | 2.989982874 | FBN1 | fibrillin 1 [Source:HGNC Symbol;Acc:HGNC:3603] |
| ENSG00000167770 | HGLibA_34752 | 456 | 2.989982874 | OTUB1 | ubiquitin aldehyde binding 1 [Source:HGNC Symbol;Acc:HGNC:23077]" |
| ENSG00000174032 | HGLibA_44703 | 456 | 2.989982874 | SLC25A30 | solute carrier family 25 member 30 [Source:HGNC Symbol;Acc:HGNC:27371] |
| ENSG00000184730 | HGLibA_02499 | 456 | 2.989982874 | APOBR | apolipoprotein B receptor [Source:HGNC Symbol;Acc:HGNC:24087] |
| ENSG00000197580 | HGLibA_04359 | 456 | 2.989982874 | BCO2 | beta-carotene oxygenase 2 [Source:HGNC Symbol;Acc:HGNC:18503] |
| ENSG00000112576 | HGLibA_08078 | 455 | 2.980937532 | CCND3 | cyclin D3 [Source:HGNC Symbol;Acc:HGNC:1585] |
| ENSG00000167747 | HGLibA_05672 | 455 | 2.980937532 | C19orf48 | chromosome 19 open reading frame 48 [Source:HGNC Symbol;Acc:HGNC:29667] |
| ENSG00000173769 | HGLibA_51007 | 455 | 2.980937532 | TOPAZ1 | testis and ovary specific PAZ domain containing 1 [Source:HGNC Symbol;Acc:HGNC:24746] |
| ENSG00000184731 | HGLibA_16017 | 455 | 2.980937532 | FAM110C | family with sequence similarity 110 member C [Source:HGNC Symbol;Acc:HGNC:33340] |
| ENSG00000241404 | HGLibA_14631 | 455 | 2.980937532 | EGFL8 | EGF like domain multiple 8 [Source:HGNC Symbol;Acc:HGNC:13944] |
| ENSG00000247077 | HGLibA_36204 | 455 | 2.980937532 | PGAM5 | mitochondrial serine/threonine protein phosphatase [Source:HGNC Symbol;Acc:HGNC:28763]" |
| ENSG00000254788 | HGLibA_09771 | 455 | 2.980937532 | CKLF-CMTM1 | CKLF-CMTM1 readthrough [Source:HGNC Symbol;Acc:HGNC:39977] |
| ENSG00000256660 | HGLibA_09931 | 455 | 2.980937532 | CLEC12B | C-type lectin domain family 12 member B [Source:HGNC Symbol;Acc:HGNC:31966] |
| SUV420H1 | HGLibA_47865 | 455 | 2.980937532 | SUV420H1 | - |
| ENSG00000070018 | HGLibA_27229 | 454 | 2.971892189 | LRP6 | LDL receptor related protein 6 [Source:HGNC Symbol;Acc:HGNC:6698] |
| ENSG00000106537 | HGLibA_51900 | 454 | 2.971892189 | TSPAN13 | tetraspanin 13 [Source:HGNC Symbol;Acc:HGNC:21643] |
| ENSG00000130811 | HGLibA_14783 | 454 | 2.971892189 | EIF3G | eukaryotic translation initiation factor 3 subunit G [Source:HGNC Symbol;Acc:HGNC:3274] |
| ENSG00000213023 | HGLibA_48074 | 454 | 2.971892189 | SYT3 | synaptotagmin 3 [Source:HGNC Symbol;Acc:HGNC:11511] |
| ENSG00000123570 | HGLibA_39993 | 453 | 2.962846846 | RAB9B | member RAS oncogene family [Source:HGNC Symbol;Acc:HGNC:14090]" |
| ENSG00000147257 | HGLibA_19767 | 453 | 2.962846846 | GPC3 | glypican 3 [Source:HGNC Symbol;Acc:HGNC:4451] |
| ENSG00000161647 | HGLibA_29753 | 453 | 2.962846846 | MPP3 | membrane palmitoylated protein 3 [Source:HGNC Symbol;Acc:HGNC:7221] |
| ENSG00000185386 | HGLibA_28252 | 453 | 2.962846846 | MAPK11 | mitogen-activated protein kinase 11 [Source:HGNC Symbol;Acc:HGNC:6873] |
| ENSG00000196739 | HGLibA_10554 | 453 | 2.962846846 | COL27A1 | collagen type XXVII alpha 1 chain [Source:HGNC Symbol;Acc:HGNC:22986] |
| ENSG00000269404 | HGLibA_46655 | 453 | 2.962846846 | SPIB | Spi-B transcription factor [Source:HGNC Symbol;Acc:HGNC:11242] |
| ENSG00000114904 | HGLibA_31652 | 452 | 2.953801503 | NEK4 | NIMA related kinase 4 [Source:HGNC Symbol;Acc:HGNC:11399] |
| ENSG00000136271 | HGLibA_12750 | 452 | 2.953801503 | DDX56 | DEAD-box helicase 56 [Source:HGNC Symbol;Acc:HGNC:18193] |
| ENSG00000272804 | HGLibA_25618 | 452 | 2.953801503 | KRTAP10-7 | keratin associated protein 10-7 [Source:HGNC Symbol;Acc:HGNC:22970] |
| HIST1H4A | HGLibA_21485 | 452 | 2.953801503 | HIST1H4A | - |
| hsa-mir-23c | HGLibA_58338 | 452 | 2.953801503 | hsa-mir-23c | - |
| hsa-mir-614 | HGLibA_62691 | 452 | 2.953801503 | hsa-mir-614 | - |
| ENSG00000105643 | HGLibA_03115 | 451 | 2.94475616 | ARRDC2 | arrestin domain containing 2 [Source:HGNC Symbol;Acc:HGNC:25225] |
| ENSG00000118245 | HGLibA_50891 | 451 | 2.94475616 | TNP1 | transition protein 1 [Source:HGNC Symbol;Acc:HGNC:11951] |
| hsa-mir-500b | HGLibA_61446 | 451 | 2.94475616 | hsa-mir-500b | - |
| hsa-mir-6784 | HGLibA_63313 | 451 | 2.94475616 | hsa-mir-6784 | - |
| ENSG00000025708 | HGLibA_52460 | 450 | 2.935710817 | TYMP | thymidine phosphorylase [Source:HGNC Symbol;Acc:HGNC:3148] |
| ENSG00000101017 | HGLibA_08383 | 450 | 2.935710817 | CD40 | CD40 molecule [Source:HGNC Symbol;Acc:HGNC:11919] |
| ENSG00000169228 | HGLibA_39848 | 450 | 2.935710817 | RAB24 | member RAS oncogene family [Source:HGNC Symbol;Acc:HGNC:9765]" |
| ENSG00000173456 | HGLibA_41658 | 450 | 2.935710817 | RNF26 | ring finger protein 26 [Source:HGNC Symbol;Acc:HGNC:14646] |
| ENSG00000197774 | HGLibA_15037 | 450 | 2.935710817 | EME2 | essential meiotic structure-specific endonuclease subunit 2 [Source:HGNC Symbol;Acc:HGNC:27289] |
| hsa-mir-1911 | HGLibA_58050 | 450 | 2.935710817 | hsa-mir-1911 | - |
| ENSG00000067082 | HGLibA_25077 | 449 | 2.926665474 | KLF6 | Kruppel like factor 6 [Source:HGNC Symbol;Acc:HGNC:2235] |
| ENSG00000069509 | HGLibA_18163 | 449 | 2.926665474 | FUNDC1 | FUN14 domain containing 1 [Source:HGNC Symbol;Acc:HGNC:28746] |
| ENSG00000137948 | HGLibA_04792 | 449 | 2.926665474 | BRDT | bromodomain testis associated [Source:HGNC Symbol;Acc:HGNC:1105] |
| ENSG00000138741 | HGLibA_51731 | 449 | 2.926665474 | TRPC3 | transient receptor potential cation channel subfamily C member 3 [Source:HGNC Symbol;Acc:HGNC:12335] |
| ENSG00000176029 | HGLibA_05119 | 449 | 2.926665474 | C11orf16 | chromosome 11 open reading frame 16 [Source:HGNC Symbol;Acc:HGNC:1169] |
| ENSG00000179954 | HGLibA_47126 | 449 | 2.926665474 | SSC5D | scavenger receptor cysteine rich family member with 5 domains [Source:HGNC Symbol;Acc:HGNC:26641] |
| ENSG00000184999 | HGLibA_44538 | 449 | 2.926665474 | SLC22A10 | solute carrier family 22 member 10 [Source:HGNC Symbol;Acc:HGNC:18057] |
| ENSG00000112655 | HGLibA_39389 | 448 | 2.917620131 | PTK7 | protein tyrosine kinase 7 (inactive) [Source:HGNC Symbol;Acc:HGNC:9618] |
| ENSG00000124104 | HGLibA_46063 | 448 | 2.917620131 | SNX21 | sorting nexin family member 21 [Source:HGNC Symbol;Acc:HGNC:16154] |
| ENSG00000141753 | HGLibA_22825 | 448 | 2.917620131 | IGFBP4 | insulin like growth factor binding protein 4 [Source:HGNC Symbol;Acc:HGNC:5473] |
| ENSG00000188095 | HGLibA_28920 | 448 | 2.917620131 | MESP2 | mesoderm posterior bHLH transcription factor 2 [Source:HGNC Symbol;Acc:HGNC:29659] |
| ENSG00000064225 | HGLibA_47252 | 447 | 2.908574788 | ST3GAL6 | 3-sialyltransferase 6 [Source:HGNC Symbol;Acc:HGNC:18080]" |
| ENSG00000105278 | HGLibA_55261 | 447 | 2.908574788 | ZFR2 | zinc finger RNA binding protein 2 [Source:HGNC Symbol;Acc:HGNC:29189] |
| ENSG00000113269 | HGLibA_41506 | 447 | 2.908574788 | RNF130 | ring finger protein 130 [Source:HGNC Symbol;Acc:HGNC:18280] |
| ENSG00000116539 | HGLibA_03293 | 447 | 2.908574788 | ASH1L | ASH1 like histone lysine methyltransferase [Source:HGNC Symbol;Acc:HGNC:19088] |
| ENSG00000121064 | HGLibA_43039 | 447 | 2.908574788 | SCPEP1 | serine carboxypeptidase 1 [Source:HGNC Symbol;Acc:HGNC:29507] |
| ENSG00000150627 | HGLibA_54037 | 447 | 2.908574788 | WDR17 | WD repeat domain 17 [Source:HGNC Symbol;Acc:HGNC:16661] |
| ENSG00000158715 | HGLibA_45178 | 447 | 2.908574788 | SLC45A3 | solute carrier family 45 member 3 [Source:HGNC Symbol;Acc:HGNC:8642] |
| ENSG00000166823 | HGLibA_28917 | 447 | 2.908574788 | MESP1 | mesoderm posterior bHLH transcription factor 1 [Source:HGNC Symbol;Acc:HGNC:29658] |
| ENSG00000196811 | HGLibA_09608 | 447 | 2.908574788 | CHRNG | cholinergic receptor nicotinic gamma subunit [Source:HGNC Symbol;Acc:HGNC:1967] |
| ENSG00000092201 | HGLibA_47804 | 446 | 2.899529445 | SUPT16H | facilitates chromatin remodeling subunit [Source:HGNC Symbol;Acc:HGNC:11465]" |
| ENSG00000112592 | HGLibA_48624 | 446 | 2.899529445 | TBP | TATA-box binding protein [Source:HGNC Symbol;Acc:HGNC:11588] |
| ENSG00000126353 | HGLibA_08164 | 446 | 2.899529445 | CCR7 | C-C motif chemokine receptor 7 [Source:HGNC Symbol;Acc:HGNC:1608] |
| ENSG00000157992 | HGLibA_25856 | 446 | 2.899529445 | KRTCAP3 | keratinocyte associated protein 3 [Source:HGNC Symbol;Acc:HGNC:28943] |
| ENSG00000175175 | HGLibA_37972 | 446 | 2.899529445 | PPM1E | Mg2+/Mn2+ dependent 1E [Source:HGNC Symbol;Acc:HGNC:19322]" |
| ENSG00000180329 | HGLibA_07786 | 446 | 2.899529445 | CCDC43 | coiled-coil domain containing 43 [Source:HGNC Symbol;Acc:HGNC:26472] |
| ENSG00000198513 | HGLibA_03516 | 446 | 2.899529445 | ATL1 | atlastin GTPase 1 [Source:HGNC Symbol;Acc:HGNC:11231] |
| AMICA1 | HGLibA_01791 | 445 | 2.890484102 | AMICA1 | - |
| BAI2 | HGLibA_04116 | 445 | 2.890484102 | BAI2 | - |
| ENSG00000105738 | HGLibA_44197 | 445 | 2.890484102 | SIPA1L3 | signal induced proliferation associated 1 like 3 [Source:HGNC Symbol;Acc:HGNC:23801] |
| ENSG00000116649 | HGLibA_46993 | 445 | 2.890484102 | SRM | spermidine synthase [Source:HGNC Symbol;Acc:HGNC:11296] |
| ENSG00000118160 | HGLibA_45392 | 445 | 2.890484102 | SLC8A2 | solute carrier family 8 member A2 [Source:HGNC Symbol;Acc:HGNC:11069] |
| ENSG00000125740 | HGLibA_17798 | 445 | 2.890484102 | FOSB | AP-1 transcription factor subunit [Source:HGNC Symbol;Acc:HGNC:3797]" |
| ENSG00000132386 | HGLibA_43587 | 445 | 2.890484102 | SERPINF1 | serpin family F member 1 [Source:HGNC Symbol;Acc:HGNC:8824] |
| ENSG00000171942 | HGLibA_33510 | 445 | 2.890484102 | OR10H2 | olfactory receptor family 10 subfamily H member 2 [Source:HGNC Symbol;Acc:HGNC:8173] |
| ENSG00000184349 | HGLibA_14593 | 445 | 2.890484102 | EFNA5 | ephrin A5 [Source:HGNC Symbol;Acc:HGNC:3225] |
| ENSG00000225968 | HGLibA_14905 | 445 | 2.890484102 | ELFN1 | extracellular leucine rich repeat and fibronectin type III domain containing 1 [Source:HGNC Symbol;Acc:HGNC:33154] |
| ENSG00000268089 | HGLibA_18401 | 445 | 2.890484102 | GABRQ | gamma-aminobutyric acid type A receptor theta subunit [Source:HGNC Symbol;Acc:HGNC:14454] |
| ENSG00000073008 | HGLibA_39616 | 444 | 2.88143876 | PVR | poliovirus receptor [Source:HGNC Symbol;Acc:HGNC:9705] |
| ENSG00000101405 | HGLibA_34840 | 444 | 2.88143876 | OXT | oxytocin/neurophysin I prepropeptide [Source:HGNC Symbol;Acc:HGNC:8528] |
| ENSG00000112425 | HGLibA_15334 | 444 | 2.88143876 | EPM2A | laforin glucan phosphatase [Source:HGNC Symbol;Acc:HGNC:3413]" |
| ENSG00000115694 | HGLibA_47473 | 444 | 2.88143876 | STK25 | serine/threonine kinase 25 [Source:HGNC Symbol;Acc:HGNC:11404] |
| ENSG00000116191 | HGLibA_40172 | 444 | 2.88143876 | RALGPS2 | Ral GEF with PH domain and SH3 binding motif 2 [Source:HGNC Symbol;Acc:HGNC:30279] |
| ENSG00000153094 | HGLibA_04308 | 444 | 2.88143876 | BCL2L11 | BCL2 like 11 [Source:HGNC Symbol;Acc:HGNC:994] |
| ENSG00000158286 | HGLibA_41614 | 444 | 2.88143876 | RNF207 | ring finger protein 207 [Source:HGNC Symbol;Acc:HGNC:32947] |

| | | | | | |
|---|---|---|---|---|---|
| ENSG00000188315 | HGLibA_06257 | 444 | 2.88143876 | C3orf62 | chromosome 3 open reading frame 62 [Source:HGNC Symbol;Acc:HGNC:24771] |
| C19orf57 | HGLibA_05684 | 443 | 2.872393417 | C19orf57 | - |
| ENSG00000088386 | HGLibA_44404 | 443 | 2.872393417 | SLC15A1 | solute carrier family 15 member 1 [Source:HGNC Symbol;Acc:HGNC:10920] |
| ENSG00000113083 | HGLibA_27033 | 443 | 2.872393417 | LOX | lysyl oxidase [Source:HGNC Symbol;Acc:HGNC:6664] |
| ENSG00000126777 | HGLibA_25872 | 443 | 2.872393417 | KTN1 | kinectin 1 [Source:HGNC Symbol;Acc:HGNC:6467] |
| ENSG00000137700 | HGLibA_45051 | 443 | 2.872393417 | SLC37A4 | solute carrier family 37 member 4 [Source:HGNC Symbol;Acc:HGNC:4061] |
| ENSG00000177186 | HGLibA_33846 | 443 | 2.872393417 | OR2M7 | olfactory receptor family 2 subfamily M member 7 [Source:HGNC Symbol;Acc:HGNC:19594] |
| ENSG00000179431 | HGLibA_17550 | 443 | 2.872393417 | FJX1 | four-jointed box kinase 1 [Source:HGNC Symbol;Acc:HGNC:17166] |
| FAM46A | HGLibA_16596 | 443 | 2.872393417 | FAM46A | - |
| GLTSCR1 | HGLibA_19352 | 443 | 2.872393417 | GLTSCR1 | - |
| hsa-mir-6841 | HGLibA_63539 | 443 | 2.872393417 | hsa-mir-6841 | - |
| ENSG00000005884 | HGLibA_23730 | 442 | 2.863348074 | ITGA3 | integrin subunit alpha 3 [Source:HGNC Symbol;Acc:HGNC:6139] |
| ENSG00000086712 | HGLibA_52395 | 442 | 2.863348074 | TXLNG | taxilin gamma [Source:HGNC Symbol;Acc:HGNC:18578] |
| ENSG00000127952 | HGLibA_47689 | 442 | 2.863348074 | STYXL1 | serine/threonine/tyrosine interacting like 1 [Source:HGNC Symbol;Acc:HGNC:18165] |
| ENSG00000168237 | HGLibA_19381 | 442 | 2.863348074 | GLYCTK | glycerate kinase [Source:HGNC Symbol;Acc:HGNC:24247] |
| ENSG00000178187 | HGLibA_56031 | 442 | 2.863348074 | ZNF454 | zinc finger protein 454 [Source:HGNC Symbol;Acc:HGNC:21200] |
| ENSG00000215421 | HGLibA_55926 | 442 | 2.863348074 | ZNF407 | zinc finger protein 407 [Source:HGNC Symbol;Acc:HGNC:19904] |
| ENSG00000128274 | HGLibA_00018 | 441 | 2.854302731 | A4GALT | 4-galactosyltransferase (P blood group) [Source:HGNC Symbol;Acc:HGNC:18149]" |
| ENSG00000142188 | HGLibA_50425 | 441 | 2.854302731 | TMEM50B | transmembrane protein 50B [Source:HGNC Symbol;Acc:HGNC:1280] |
| ENSG00000163251 | HGLibA_18274 | 441 | 2.854302731 | FZD5 | frizzled class receptor 5 [Source:HGNC Symbol;Acc:HGNC:4043] |
| ENSG00000170899 | HGLibA_20459 | 441 | 2.854302731 | GSTA4 | glutathione S-transferase alpha 4 [Source:HGNC Symbol;Acc:HGNC:4629] |
| ENSG00000185052 | HGLibA_44621 | 441 | 2.854302731 | SLC24A3 | solute carrier family 24 member 3 [Source:HGNC Symbol;Acc:HGNC:10977] |
| ENSG00000213523 | HGLibA_46921 | 441 | 2.854302731 | SRA1 | steroid receptor RNA activator 1 [Source:HGNC Symbol;Acc:HGNC:11281] |
| ENSG00000240303 | HGLibA_00355 | 441 | 2.854302731 | ACAD11 | acyl-CoA dehydrogenase family member 11 [Source:HGNC Symbol;Acc:HGNC:30211] |
| ENSG00000007171 | HGLibA_32329 | 440 | 2.845257388 | NOS2 | nitric oxide synthase 2 [Source:HGNC Symbol;Acc:HGNC:7873] |
| ENSG00000016391 | HGLibA_09417 | 440 | 2.845257388 | CHDH | choline dehydrogenase [Source:HGNC Symbol;Acc:HGNC:24288] |
| ENSG00000121764 | HGLibA_20989 | 440 | 2.845257388 | HCRTR1 | hypocretin receptor 1 [Source:HGNC Symbol;Acc:HGNC:4848] |
| ENSG00000139433 | HGLibA_19344 | 440 | 2.845257388 | GLTP | glycolipid transfer protein [Source:HGNC Symbol;Acc:HGNC:24867] |
| ENSG00000165816 | HGLibA_53900 | 440 | 2.845257388 | VWA2 | von Willebrand factor A domain containing 2 [Source:HGNC Symbol;Acc:HGNC:24709] |
| ENSG00000168256 | HGLibA_32018 | 440 | 2.845257388 | NKIRAS2 | NFKB inhibitor interacting Ras like 2 [Source:HGNC Symbol;Acc:HGNC:17898] |
| ENSG00000170502 | HGLibA_32993 | 440 | 2.845257388 | NUDT9 | nudix hydrolase 9 [Source:HGNC Symbol;Acc:HGNC:8056] |
| ENSG00000186635 | HGLibA_02624 | 440 | 2.845257388 | ARAP1 | ankyrin repeat and PH domain 1 [Source:HGNC Symbol;Acc:HGNC:16925]" |
| ENSG00000188816 | HGLibA_21762 | 440 | 2.845257388 | HMX2 | H6 family homeobox 2 [Source:HGNC Symbol;Acc:HGNC:5018] |
| hsa-mir-658 | HGLibA_62965 | 440 | 2.845257388 | hsa-mir-658 | - |
| ENSG00000074755 | HGLibA_57091 | 439 | 2.836212045 | ZZEF1 | zinc finger ZZ-type and EF-hand domain containing 1 [Source:HGNC Symbol;Acc:HGNC:29027] |
| ENSG00000090470 | HGLibA_35744 | 439 | 2.836212045 | PDCD7 | programmed cell death 7 [Source:HGNC Symbol;Acc:HGNC:8767] |
| ENSG00000100441 | HGLibA_24577 | 439 | 2.836212045 | KHNYN | KH and NYN domain containing [Source:HGNC Symbol;Acc:HGNC:20166] |
| ENSG00000110987 | HGLibA_04339 | 439 | 2.836212045 | BCL7A | BCL tumor suppressor 7A [Source:HGNC Symbol;Acc:HGNC:1004] |
| ENSG00000111144 | HGLibA_27578 | 439 | 2.836212045 | LTA4H | leukotriene A4 hydrolase [Source:HGNC Symbol;Acc:HGNC:6710] |
| ENSG00000112742 | HGLibA_52179 | 439 | 2.836212045 | TTK | TTK protein kinase [Source:HGNC Symbol;Acc:HGNC:12401] |
| ENSG00000114487 | HGLibA_29648 | 439 | 2.836212045 | MORC1 | MORC family CW-type zinc finger 1 [Source:HGNC Symbol;Acc:HGNC:7198] |
| ENSG00000125869 | HGLibA_25986 | 439 | 2.836212045 | LAMP5 | lysosomal associated membrane protein family member 5 [Source:HGNC Symbol;Acc:HGNC:16097] |
| ENSG00000129194 | HGLibA_46243 | 439 | 2.836212045 | SOX15 | SRY-box 15 [Source:HGNC Symbol;Acc:HGNC:11196] |
| ENSG00000166333 | HGLibA_23220 | 439 | 2.836212045 | ILK | integrin linked kinase [Source:HGNC Symbol;Acc:HGNC:6040] |
| ENSG00000168267 | HGLibA_39301 | 439 | 2.836212045 | PTF1A | pancreas associated transcription factor 1a [Source:HGNC Symbol;Acc:HGNC:23734] |
| ENSG00000173261 | HGLibA_36944 | 439 | 2.836212045 | PLAC8L1 | PLAC8 like 1 [Source:HGNC Symbol;Acc:HGNC:31746] |
| ENSG00000176893 | HGLibA_34112 | 439 | 2.836212045 | OR51G2 | olfactory receptor family 51 subfamily G member 2 [Source:HGNC Symbol;Acc:HGNC:15198] |
| ENSG00000186119 | HGLibA_34284 | 439 | 2.836212045 | OR5D18 | olfactory receptor family 5 subfamily D member 18 [Source:HGNC Symbol;Acc:HGNC:15285] |
| ENSG00000187244 | HGLibA_04232 | 439 | 2.836212045 | BCAM | basal cell adhesion molecule (Lutheran blood group) [Source:HGNC Symbol;Acc:HGNC:6722] |
| ENSG00000248099 | HGLibA_23382 | 439 | 2.836212045 | INSL3 | insulin like 3 [Source:HGNC Symbol;Acc:HGNC:6086] |
| ENSG00000258713 | HGLibA_05986 | 439 | 2.836212045 | C20orf141 | chromosome 20 open reading frame 141 [Source:HGNC Symbol;Acc:HGNC:16134] |
| ENSG00000269343 | HGLibA_56332 | 439 | 2.836212045 | ZNF587B | zinc finger protein 587B [Source:HGNC Symbol;Acc:HGNC:37142] |
| ENSG00000026559 | HGLibA_24197 | 438 | 2.827166702 | KCNG1 | potassium voltage-gated channel modifier subfamily G member 1 [Source:HGNC Symbol;Acc:HGNC:6248] |
| ENSG00000125356 | HGLibA_31438 | 438 | 2.827166702 | NDUFA1 | NADH:ubiquinone oxidoreductase subunit A1 [Source:HGNC Symbol;Acc:HGNC:7683] |
| ENSG00000137752 | HGLibA_07295 | 438 | 2.827166702 | CASP1 | caspase 1 [Source:HGNC Symbol;Acc:HGNC:1499] |
| ENSG00000143748 | HGLibA_33120 | 438 | 2.827166702 | NVL | nuclear VCP-like [Source:HGNC Symbol;Acc:HGNC:8070] |
| ENSG00000172927 | HGLibA_30690 | 438 | 2.827166702 | MYEOV | myeloma overexpressed [Source:HGNC Symbol;Acc:HGNC:7563] |
| ENSG00000250317 | HGLibA_45747 | 438 | 2.827166702 | SMIM20 | small integral membrane protein 20 [Source:HGNC Symbol;Acc:HGNC:37260] |
| LOC286238 | HGLibA_26919 | 438 | 2.827166702 | LOC286238 | - |
| ENSG00000069974 | HGLibA_39857 | 437 | 2.818121359 | RAB27A | member RAS oncogene family [Source:HGNC Symbol;Acc:HGNC:9766]" |
| ENSG00000083750 | HGLibA_42227 | 437 | 2.818121359 | RRAGB | Ras related GTP binding B [Source:HGNC Symbol;Acc:HGNC:19901] |
| ENSG00000086730 | HGLibA_26061 | 437 | 2.818121359 | LAT2 | linker for activation of T cells family member 2 [Source:HGNC Symbol;Acc:HGNC:12749] |
| ENSG00000088002 | HGLibA_47762 | 437 | 2.818121359 | SULT2B1 | sulfotransferase family 2B member 1 [Source:HGNC Symbol;Acc:HGNC:11459] |
| ENSG00000124091 | HGLibA_18821 | 437 | 2.818121359 | GCNT7 | glucosaminyl (N-acetyl) transferase family member 7 [Source:HGNC Symbol;Acc:HGNC:16099] |
| ENSG00000125089 | HGLibA_43979 | 437 | 2.818121359 | SH3TC1 | SH3 domain and tetratricopeptide repeats 1 [Source:HGNC Symbol;Acc:HGNC:26009] |
| ENSG00000147606 | HGLibA_44805 | 437 | 2.818121359 | SLC26A7 | solute carrier family 26 member 7 [Source:HGNC Symbol;Acc:HGNC:14467] |
| ENSG00000148908 | HGLibA_41052 | 437 | 2.818121359 | RGS10 | regulator of G protein signaling 10 [Source:HGNC Symbol;Acc:HGNC:9992] |
| ENSG00000167769 | HGLibA_00427 | 437 | 2.818121359 | ACER1 | alkaline ceramidase 1 [Source:HGNC Symbol;Acc:HGNC:18356] |
| ENSG00000178988 | HGLibA_29830 | 437 | 2.818121359 | MRFAP1L1 | Morf4 family associated protein 1 like 1 [Source:HGNC Symbol;Acc:HGNC:28796] |
| ENSG00000180914 | HGLibA_34842 | 437 | 2.818121359 | OXTR | oxytocin receptor [Source:HGNC Symbol;Acc:HGNC:8529] |
| ENSG00000203877 | HGLibA_41330 | 437 | 2.818121359 | RIPPLY2 | ripply transcriptional repressor 2 [Source:HGNC Symbol;Acc:HGNC:21390] |
| ENSG00000005812 | HGLibA_17005 | 436 | 2.809076016 | FBXL3 | F-box and leucine rich repeat protein 3 [Source:HGNC Symbol;Acc:HGNC:13599] |
| ENSG00000077264 | HGLibA_35045 | 436 | 2.809076016 | PAK3 | p21 (RAC1) activated kinase 3 [Source:HGNC Symbol;Acc:HGNC:8592] |
| ENSG00000080189 | HGLibA_44973 | 436 | 2.809076016 | SLC35C2 | solute carrier family 35 member C2 [Source:HGNC Symbol;Acc:HGNC:17117] |
| ENSG00000099385 | HGLibA_04345 | 436 | 2.809076016 | BCL7C | BCL tumor suppressor 7C [Source:HGNC Symbol;Acc:HGNC:1006] |
| ENSG00000104154 | HGLibA_44909 | 436 | 2.809076016 | SLC30A4 | solute carrier family 30 member 4 [Source:HGNC Symbol;Acc:HGNC:11015] |
| ENSG00000126775 | HGLibA_03470 | 436 | 2.809076016 | ATG14 | autophagy related 14 [Source:HGNC Symbol;Acc:HGNC:19962] |
| ENSG00000139044 | HGLibA_04016 | 436 | 2.809076016 | B4GALNT3 | 4-N-acetyl-galactosaminyltransferase 3 [Source:HGNC Symbol;Acc:HGNC:24137]" |
| ENSG00000152223 | HGLibA_15277 | 436 | 2.809076016 | EPG5 | ectopic P-granules autophagy protein 5 homolog [Source:HGNC Symbol;Acc:HGNC:29331] |
| ENSG00000153140 | HGLibA_09240 | 436 | 2.809076016 | CETN3 | centrin 3 [Source:HGNC Symbol;Acc:HGNC:1868] |
| ENSG00000161558 | HGLibA_49966 | 436 | 2.809076016 | TMEM143 | transmembrane protein 143 [Source:HGNC Symbol;Acc:HGNC:25603] |
| ENSG00000176340 | HGLibA_10875 | 436 | 2.809076016 | COX8A | cytochrome c oxidase subunit 8A [Source:HGNC Symbol;Acc:HGNC:2294] |
| ENSG00000189319 | HGLibA_16639 | 436 | 2.809076016 | FAM53B | family with sequence similarity 53 member B [Source:HGNC Symbol;Acc:HGNC:28968] |
| ENSG00000198704 | HGLibA_20185 | 436 | 2.809076016 | GPX6 | glutathione peroxidase 6 [Source:HGNC Symbol;Acc:HGNC:4558] |
| ENSG00000103199 | HGLibA_56107 | 435 | 2.800030673 | ZNF500 | zinc finger protein 500 [Source:HGNC Symbol;Acc:HGNC:23716] |
| ENSG00000108448 | HGLibA_51419 | 435 | 2.800030673 | TRIM16L | tripartite motif containing 16 like [Source:HGNC Symbol;Acc:HGNC:32670] |
| ENSG00000114248 | HGLibA_27312 | 435 | 2.800030673 | LRRC31 | leucine rich repeat containing 31 [Source:HGNC Symbol;Acc:HGNC:26261] |
| ENSG00000155542 | HGLibA_43665 | 435 | 2.800030673 | SETD9 | SET domain containing 9 [Source:HGNC Symbol;Acc:HGNC:28508] |
| ENSG00000173805 | HGLibA_20837 | 435 | 2.800030673 | HAP1 | huntingtin associated protein 1 [Source:HGNC Symbol;Acc:HGNC:4812] |
| ENSG00000196693 | HGLibA_55835 | 435 | 2.800030673 | ZNF33B | zinc finger protein 33B [Source:HGNC Symbol;Acc:HGNC:13097] |
| hsa-mir-1227 | HGLibA_57308 | 435 | 2.800030673 | hsa-mir-1227 | - |
| hsa-mir-6072 | HGLibA_62541 | 435 | 2.800030673 | hsa-mir-6072 | - |
| SCAND3 | HGLibA_42854 | 435 | 2.800030673 | SCAND3 | - |
| ENSG00000085365 | HGLibA_42837 | 434 | 2.790985331 | SCAMP1 | secretory carrier membrane protein 1 [Source:HGNC Symbol;Acc:HGNC:10563] |
| ENSG00000184814 | HGLibA_38825 | 434 | 2.790985331 | PRR23B | proline rich 23B [Source:HGNC Symbol;Acc:HGNC:33764] |
| ENSG00000198538 | HGLibA_55720 | 434 | 2.790985331 | ZNF28 | zinc finger protein 28 [Source:HGNC Symbol;Acc:HGNC:13073] |
| ENSG00000198951 | HGLibA_31064 | 434 | 2.790985331 | NAGA | alpha-N-acetylgalactosaminidase [Source:HGNC Symbol;Acc:HGNC:7631] |
| ENSG00000215274 | HGLibA_18433 | 434 | 2.790985331 | GAGE10 | G antigen 10 [Source:HGNC Symbol;Acc:HGNC:30968] |
| ENSG00000228198 | HGLibA_33835 | 434 | 2.790985331 | OR2M3 | olfactory receptor family 2 subfamily M member 3 [Source:HGNC Symbol;Acc:HGNC:8269] |
| ENSG00000101557 | HGLibA_53205 | 433 | 2.781939988 | USP14 | ubiquitin specific peptidase 14 [Source:HGNC Symbol;Acc:HGNC:12612] |
| ENSG00000128276 | HGLibA_40936 | 433 | 2.781939988 | RFPL3 | ret finger protein like 3 [Source:HGNC Symbol;Acc:HGNC:9980] |
| ENSG00000142224 | HGLibA_23035 | 433 | 2.781939988 | IL19 | interleukin 19 [Source:HGNC Symbol;Acc:HGNC:5990] |
| ENSG00000196628 | HGLibA_48788 | 433 | 2.781939988 | TCF4 | transcription factor 4 [Source:HGNC Symbol;Acc:HGNC:11634] |
| ENSG00000225828 | HGLibA_16541 | 433 | 2.781939988 | FAM229A | family with sequence similarity 229 member A [Source:HGNC Symbol;Acc:HGNC:44652] |
| ENSG00000104979 | HGLibA_05678 | 432 | 2.772894645 | C19orf53 | chromosome 19 open reading frame 53 [Source:HGNC Symbol;Acc:HGNC:24991] |
| ENSG00000112679 | HGLibA_14162 | 432 | 2.772894645 | DUSP22 | dual specificity phosphatase 22 [Source:HGNC Symbol;Acc:HGNC:16077] |
| ENSG00000162460 | HGLibA_50521 | 432 | 2.772894645 | TMEM82 | transmembrane protein 82 [Source:HGNC Symbol;Acc:HGNC:32350] |
| ENSG00000173267 | HGLibA_45918 | 432 | 2.772894645 | SNCG | synuclein gamma [Source:HGNC Symbol;Acc:HGNC:11141] |
| ENSG00000182492 | HGLibA_04465 | 432 | 2.772894645 | BGN | biglycan [Source:HGNC Symbol;Acc:HGNC:1044] |
| ENSG00000196455 | HGLibA_36657 | 432 | 2.772894645 | PIK3R4 | phosphoinositide-3-kinase regulatory subunit 4 [Source:HGNC Symbol;Acc:HGNC:8982] |
| ENSG00000197870 | HGLibA_38344 | 432 | 2.772894645 | PRB3 | proline rich protein BstNI subfamily 3 [Source:HGNC Symbol;Acc:HGNC:9339] |
| ENSG00000242220 | HGLibA_48837 | 432 | 2.772894645 | TCP10L | t-complex 10 like [Source:HGNC Symbol;Acc:HGNC:11657] |
| ENSG00000069424 | HGLibA_24150 | 431 | 2.763849302 | KCNAB2 | potassium voltage-gated channel subfamily A regulatory beta subunit 2 [Source:HGNC Symbol;Acc:HGNC:6229] |
| ENSG00000081818 | HGLibA_35499 | 431 | 2.763849302 | PCDHB4 | protocadherin beta 4 [Source:HGNC Symbol;Acc:HGNC:8689] |
| ENSG00000136541 | HGLibA_15507 | 431 | 2.763849302 | ERMN | ermin [Source:HGNC Symbol;Acc:HGNC:29208] |
| ENSG00000175970 | HGLibA_52989 | 431 | 2.763849302 | UNC119B | unc-119 lipid binding chaperone B [Source:HGNC Symbol;Acc:HGNC:16488] |
| ENSG00000180287 | HGLibA_37046 | 431 | 2.763849302 | PLD5 | phospholipase D family member 5 [Source:HGNC Symbol;Acc:HGNC:26879] |
| ENSG00000188393 | HGLibA_09960 | 431 | 2.763849302 | CLEC2A | C-type lectin domain family 2 member A [Source:HGNC Symbol;Acc:HGNC:24191] |
| hsa-mir-3917 | HGLibA_59543 | 431 | 2.763849302 | hsa-mir-3917 | - |
| ENSG00000112855 | HGLibA_20859 | 430 | 2.754803959 | HARS2 | mitochondrial [Source:HGNC Symbol;Acc:HGNC:4817]" |
| ENSG00000134644 | HGLibA_39579 | 430 | 2.754803959 | PUM1 | pumilio RNA binding family member 1 [Source:HGNC Symbol;Acc:HGNC:14957] |
| ENSG00000139687 | HGLibA_40413 | 430 | 2.754803959 | RB1 | RB transcriptional corepressor 1 [Source:HGNC Symbol;Acc:HGNC:9884] |
| ENSG00000167395 | HGLibA_56453 | 430 | 2.754803959 | ZNF646 | zinc finger protein 646 [Source:HGNC Symbol;Acc:HGNC:29004] |
| ENSG00000183723 | HGLibA_10219 | 430 | 2.754803959 | CMTM4 | CKLF like MARVEL transmembrane domain containing 4 [Source:HGNC Symbol;Acc:HGNC:19175] |
| hsa-mir-598 | HGLibA_62485 | 430 | 2.754803959 | hsa-mir-598 | - |
| ENSG00000124422 | HGLibA_53304 | 429 | 2.745758616 | USP22 | ubiquitin specific peptidase 22 [Source:HGNC Symbol;Acc:HGNC:12621] |
| ENSG00000129455 | HGLibA_25269 | 429 | 2.745758616 | KLK8 | kallikrein related peptidase 8 [Source:HGNC Symbol;Acc:HGNC:6369] |
| ENSG00000134460 | HGLibA_23119 | 429 | 2.745758616 | IL2RA | interleukin 2 receptor subunit alpha [Source:HGNC Symbol;Acc:HGNC:6008] |
| ENSG00000135406 | HGLibA_38749 | 429 | 2.745758616 | PRPH | peripherin [Source:HGNC Symbol;Acc:HGNC:9461] |
| ENSG00000163508 | HGLibA_15228 | 429 | 2.745758616 | EOMES | eomesodermin [Source:HGNC Symbol;Acc:HGNC:3372] |
| hsa-mir-4727 | HGLibA_61004 | 429 | 2.745758616 | hsa-mir-4727 | - |
| C15orf26 | HGLibA_05379 | 428 | 2.736713273 | C15orf26 | - |
| ENSG00000103310 | HGLibA_56953 | 428 | 2.736713273 | ZP2 | zona pellucida glycoprotein 2 [Source:HGNC Symbol;Acc:HGNC:13188] |
| ENSG00000143740 | HGLibA_45884 | 428 | 2.736713273 | SNAP47 | synaptosome associated protein 47 [Source:HGNC Symbol;Acc:HGNC:30669] |
| ENSG00000145623 | HGLibA_34696 | 428 | 2.736713273 | OSMR | oncostatin M receptor [Source:HGNC Symbol;Acc:HGNC:8507] |

| | | | | | |
|---|---|---|---|---|---|
| ENSG00000146955 | HGLibA_39826 | 428 | 2.736713273 | RAB19 | member RAS oncogene family [Source:HGNC Symbol;Acc:HGNC:19982]" |
| ENSG00000147592 | HGLibA_25920 | 428 | 2.736713273 | LACTB2 | lactamase beta 2 [Source:HGNC Symbol;Acc:HGNC:18512] |
| ENSG00000172270 | HGLibA_04866 | 428 | 2.736713273 | BSG | basigin (Ok blood group) [Source:HGNC Symbol;Acc:HGNC:1116] |
| ENSG00000176532 | HGLibA_38786 | 428 | 2.736713273 | PRR15 | proline rich 15 [Source:HGNC Symbol;Acc:HGNC:22310] |
| ENSG00000182557 | HGLibA_46739 | 428 | 2.736713273 | SPNS3 | sphingolipid transporter 3 (putative) [Source:HGNC Symbol;Acc:HGNC:28433] |
| ENSG00000184232 | HGLibA_33193 | 428 | 2.736713273 | OAF | out at first homolog [Source:HGNC Symbol;Acc:HGNC:28752] |
| ENSG00000196704 | HGLibA_01859 | 428 | 2.736713273 | AMZ2 | archaelysin family metallopeptidase 2 [Source:HGNC Symbol;Acc:HGNC:28041] |
| ENSG00000197780 | HGLibA_48206 | 428 | 2.736713273 | TAF13 | TATA-box binding protein associated factor 13 [Source:HGNC Symbol;Acc:HGNC:11546] |
| ENSG00000272031 | HGLibA_02076 | 428 | 2.736713273 | ANKRD34A | ankyrin repeat domain 34A [Source:HGNC Symbol;Acc:HGNC:27639] |
| ENSG00000019102 | HGLibA_53845 | 427 | 2.72766793 | VSIG2 | V-set and immunoglobulin domain containing 2 [Source:HGNC Symbol;Acc:HGNC:17149] |
| ENSG00000031003 | HGLibA_16143 | 427 | 2.72766793 | FAM13B | family with sequence similarity 13 member B [Source:HGNC Symbol;Acc:HGNC:1335] |
| ENSG00000083782 | HGLibA_15396 | 427 | 2.72766793 | EPYC | epiphycan [Source:HGNC Symbol;Acc:HGNC:3053] |
| ENSG00000100601 | HGLibA_01647 | 427 | 2.72766793 | ALKBH1 | histone H2A dioxygenase [Source:HGNC Symbol;Acc:HGNC:17911]" |
| ENSG00000101412 | HGLibA_14307 | 427 | 2.72766793 | E2F1 | E2F transcription factor 1 [Source:HGNC Symbol;Acc:HGNC:3113] |
| ENSG00000115183 | HGLibA_48291 | 427 | 2.72766793 | TANC1 | ankyrin repeat and coiled-coil containing 1 [Source:HGNC Symbol;Acc:HGNC:29364]" |
| ENSG00000115468 | HGLibA_14576 | 427 | 2.72766793 | EFHD1 | EF-hand domain family member D1 [Source:HGNC Symbol;Acc:HGNC:29556] |
| ENSG00000148671 | HGLibA_00985 | 427 | 2.72766793 | ADIRF | adipogenesis regulatory factor [Source:HGNC Symbol;Acc:HGNC:24043] |
| ENSG00000161896 | HGLibA_23450 | 427 | 2.72766793 | IP6K3 | inositol hexakisphosphate kinase 3 [Source:HGNC Symbol;Acc:HGNC:17269] |
| ENSG00000175356 | HGLibA_43074 | 427 | 2.72766793 | SCUBE2 | CUB domain and EGF like domain containing 2 [Source:HGNC Symbol;Acc:HGNC:30425]" |
| ENSG00000178093 | HGLibA_52022 | 427 | 2.72766793 | TSSK6 | testis specific serine kinase 6 [Source:HGNC Symbol;Acc:HGNC:30410] |
| ENSG00000181638 | HGLibA_55217 | 427 | 2.72766793 | ZFP41 | ZFP41 zinc finger protein [Source:HGNC Symbol;Acc:HGNC:26786] |
| ENSG00000206384 | HGLibA_10607 | 427 | 2.72766793 | COL6A6 | collagen type VI alpha 6 chain [Source:HGNC Symbol;Acc:HGNC:27023] |
| hsa-mir-4754 | HGLibA_61112 | 427 | 2.72766793 | hsa-mir-4754 | - |
| ENSG00000090006 | HGLibA_27598 | 426 | 2.718622587 | LTBP4 | latent transforming growth factor beta binding protein 4 [Source:HGNC Symbol;Acc:HGNC:6717] |
| ENSG00000109861 | HGLibA_11695 | 426 | 2.718622587 | CTSC | cathepsin C [Source:HGNC Symbol;Acc:HGNC:2528] |
| ENSG00000127588 | HGLibA_19518 | 426 | 2.718622587 | GNG13 | G protein subunit gamma 13 [Source:HGNC Symbol;Acc:HGNC:14131] |
| ENSG00000132746 | HGLibA_01572 | 426 | 2.718622587 | ALDH3B2 | aldehyde dehydrogenase 3 family member B2 [Source:HGNC Symbol;Acc:HGNC:411] |
| ENSG00000140481 | HGLibA_07756 | 426 | 2.718622587 | CCDC33 | coiled-coil domain containing 33 [Source:HGNC Symbol;Acc:HGNC:26552] |
| ENSG00000140955 | HGLibA_00723 | 426 | 2.718622587 | ADAD2 | adenosine deaminase domain containing 2 [Source:HGNC Symbol;Acc:HGNC:30714] |
| ENSG00000141469 | HGLibA_44398 | 426 | 2.718622587 | SLC14A1 | solute carrier family 14 member 1 (Kidd blood group) [Source:HGNC Symbol;Acc:HGNC:10918] |
| ENSG00000176900 | HGLibA_34132 | 426 | 2.718622587 | OR51T1 | olfactory receptor family 51 subfamily T member 1 [Source:HGNC Symbol;Acc:HGNC:15205] |
| ENSG00000242252 | HGLibA_04462 | 426 | 2.718622587 | BGLAP | bone gamma-carboxyglutamate protein [Source:HGNC Symbol;Acc:HGNC:1043] |
| hsa-mir-7703 | HGLibA_63948 | 426 | 2.718622587 | hsa-mir-7703 | - |
| ENSG00000102178 | HGLibA_52704 | 425 | 2.709577244 | UBL4A | ubiquitin like 4A [Source:HGNC Symbol;Acc:HGNC:12505] |
| ENSG00000119715 | HGLibA_15596 | 425 | 2.709577244 | ESRRB | estrogen related receptor beta [Source:HGNC Symbol;Acc:HGNC:3473] |
| ENSG00000119922 | HGLibA_22628 | 425 | 2.709577244 | IFIT2 | interferon induced protein with tetratricopeptide repeats 2 [Source:HGNC Symbol;Acc:HGNC:5409] |
| ENSG00000133401 | HGLibA_35974 | 425 | 2.709577244 | PDZD2 | PDZ domain containing 2 [Source:HGNC Symbol;Acc:HGNC:18486] |
| ENSG00000143954 | HGLibA_40803 | 425 | 2.709577244 | REG3G | regenerating family member 3 gamma [Source:HGNC Symbol;Acc:HGNC:29595] |
| ENSG00000168014 | HGLibA_06093 | 425 | 2.709577244 | C2CD3 | C2 calcium dependent domain containing 3 [Source:HGNC Symbol;Acc:HGNC:24564] |
| ENSG00000169856 | HGLibA_33393 | 425 | 2.709577244 | ONECUT1 | one cut homeobox 1 [Source:HGNC Symbol;Acc:HGNC:8138] |
| ENSG00000176165 | HGLibA_17869 | 425 | 2.709577244 | FOXG1 | forkhead box G1 [Source:HGNC Symbol;Acc:HGNC:3811] |
| ENSG00000178502 | HGLibA_25127 | 425 | 2.709577244 | KLHL11 | kelch like family member 11 [Source:HGNC Symbol;Acc:HGNC:19008] |
| C9orf62 | HGLibA_06745 | 424 | 2.700531902 | C9orf62 | - |
| ENSG00000073584 | HGLibA_45637 | 424 | 2.700531902 | SMARCE1 | member 1 [Source:HGNC Symbol;Acc:HGNC:11109]" |
| ENSG00000084090 | HGLibA_47373 | 424 | 2.700531902 | STARD7 | StAR related lipid transfer domain containing 7 [Source:HGNC Symbol;Acc:HGNC:18063] |
| ENSG00000103995 | HGLibA_09109 | 424 | 2.700531902 | CEP152 | centrosomal protein 152 [Source:HGNC Symbol;Acc:HGNC:29298] |
| ENSG00000112246 | HGLibA_44174 | 424 | 2.700531902 | SIM1 | SIM bHLH transcription factor 1 [Source:HGNC Symbol;Acc:HGNC:10882] |
| ENSG00000125629 | HGLibA_23378 | 424 | 2.700531902 | INSIG2 | insulin induced gene 2 [Source:HGNC Symbol;Acc:HGNC:20452] |
| ENSG00000133106 | HGLibA_15387 | 424 | 2.700531902 | EPSTI1 | epithelial stromal interaction 1 [Source:HGNC Symbol;Acc:HGNC:16465] |
| ENSG00000133789 | HGLibA_47898 | 424 | 2.700531902 | SWAP70 | switching B cell complex subunit SWAP70 [Source:HGNC Symbol;Acc:HGNC:17070] |
| ENSG00000167554 | HGLibA_56381 | 424 | 2.700531902 | ZNF610 | zinc finger protein 610 [Source:HGNC Symbol;Acc:HGNC:26687] |
| ENSG00000169436 | HGLibA_10541 | 424 | 2.700531902 | COL22A1 | collagen type XXII alpha 1 chain [Source:HGNC Symbol;Acc:HGNC:22989] |
| ENSG00000172350 | HGLibA_00222 | 424 | 2.700531902 | ABCG4 | ATP binding cassette subfamily G member 4 [Source:HGNC Symbol;Acc:HGNC:13884] |
| ENSG00000174225 | HGLibA_02929 | 424 | 2.700531902 | ARL13A | ADP ribosylation factor like GTPase 13A [Source:HGNC Symbol;Acc:HGNC:31709] |
| ENSG00000176246 | HGLibA_34036 | 424 | 2.700531902 | OR4L1 | olfactory receptor family 4 subfamily L member 1 [Source:HGNC Symbol;Acc:HGNC:15356] |
| ENSG00000197125 | HGLibA_34505 | 424 | 2.700531902 | OR8B8 | olfactory receptor family 8 subfamily B member 8 [Source:HGNC Symbol;Acc:HGNC:8477] |
| hsa-mir-3944 | HGLibA_59637 | 424 | 2.700531902 | hsa-mir-3944 | - |
| hsa-mir-4284 | HGLibA_59842 | 424 | 2.700531902 | hsa-mir-4284 | - |
| ENSG00000108423 | HGLibA_52296 | 423 | 2.691486559 | TUBD1 | tubulin delta 1 [Source:HGNC Symbol;Acc:HGNC:16811] |
| ENSG00000145703 | HGLibA_23535 | 423 | 2.691486559 | IQGAP2 | IQ motif containing GTPase activating protein 2 [Source:HGNC Symbol;Acc:HGNC:6111] |
| ENSG00000148158 | HGLibA_46084 | 423 | 2.691486559 | SNX30 | sorting nexin family member 30 [Source:HGNC Symbol;Acc:HGNC:23685] |
| ENSG00000148634 | HGLibA_21163 | 423 | 2.691486559 | HERC4 | HECT and RLD domain containing E3 ubiquitin protein ligase 4 [Source:HGNC Symbol;Acc:HGNC:24521] |
| ENSG00000163482 | HGLibA_47499 | 423 | 2.691486559 | STK36 | serine/threonine kinase 36 [Source:HGNC Symbol;Acc:HGNC:17209] |
| ENSG00000169155 | HGLibA_54890 | 423 | 2.691486559 | ZBTB43 | zinc finger and BTB domain containing 43 [Source:HGNC Symbol;Acc:HGNC:17908] |
| ENSG00000178974 | HGLibA_17078 | 423 | 2.691486559 | FBXO34 | F-box protein 34 [Source:HGNC Symbol;Acc:HGNC:20201] |
| ENSG00000181896 | HGLibA_55435 | 423 | 2.691486559 | ZNF101 | zinc finger protein 101 [Source:HGNC Symbol;Acc:HGNC:12881] |
| ENSG00000187824 | HGLibA_50233 | 423 | 2.691486559 | TMEM220 | transmembrane protein 220 [Source:HGNC Symbol;Acc:HGNC:33757] |
| ENSG00000206422 | HGLibA_27308 | 423 | 2.691486559 | LRRC30 | leucine rich repeat containing 30 [Source:HGNC Symbol;Acc:HGNC:30219] |
| ENSG00000240891 | HGLibA_37025 | 423 | 2.691486559 | PLCXD2 | phosphatidylinositol specific phospholipase C X domain containing 2 [Source:HGNC Symbol;Acc:HGNC:26462] |
| hsa-mir-4259 | HGLibA_59742 | 423 | 2.691486559 | hsa-mir-4259 | - |
| ENSG00000062485 | HGLibA_11301 | 422 | 2.682441216 | CS | citrate synthase [Source:HGNC Symbol;Acc:HGNC:2422] |
| ENSG00000102882 | HGLibA_28271 | 422 | 2.682441216 | MAPK3 | mitogen-activated protein kinase 3 [Source:HGNC Symbol;Acc:HGNC:6877] |
| ENSG00000123352 | HGLibA_46545 | 422 | 2.682441216 | SPATS2 | spermatogenesis associated serine rich 2 [Source:HGNC Symbol;Acc:HGNC:18650] |
| ENSG00000124459 | HGLibA_56025 | 422 | 2.682441216 | ZNF45 | zinc finger protein 45 [Source:HGNC Symbol;Acc:HGNC:13111] |
| ENSG00000142319 | HGLibA_45324 | 422 | 2.682441216 | SLC6A3 | solute carrier family 6 member 3 [Source:HGNC Symbol;Acc:HGNC:11049] |
| ENSG00000144057 | HGLibA_47262 | 422 | 2.682441216 | ST6GAL2 | 6-sialyltransferase 2 [Source:HGNC Symbol;Acc:HGNC:10861]" |
| ENSG00000153303 | HGLibA_18008 | 422 | 2.682441216 | FRMD1 | FERM domain containing 1 [Source:HGNC Symbol;Acc:HGNC:21240] |
| ENSG00000176986 | HGLibA_43235 | 422 | 2.682441216 | SEC24C | COPII coat complex component [Source:HGNC Symbol;Acc:HGNC:10705]" |
| ENSG00000186280 | HGLibA_24515 | 422 | 2.682441216 | KDM4D | lysine demethylase 4D [Source:HGNC Symbol;Acc:HGNC:25498] |
| ENSG00000197993 | HGLibA_24550 | 422 | 2.682441216 | KEL | Kell metallo-endopeptidase (Kell blood group) [Source:HGNC Symbol;Acc:HGNC:6308] |
| ENSG00000260314 | HGLibA_29816 | 422 | 2.682441216 | MRC1 | mannose receptor C-type 1 [Source:HGNC Symbol;Acc:HGNC:7228] |
| ENSG00000006071 | HGLibA_00186 | 421 | 2.673395873 | ABCC8 | ATP binding cassette subfamily C member 8 [Source:HGNC Symbol;Acc:HGNC:59] |
| ENSG00000068028 | HGLibA_40373 | 421 | 2.673395873 | RASSF1 | Ras association domain family member 1 [Source:HGNC Symbol;Acc:HGNC:9882] |
| ENSG00000100239 | HGLibA_38223 | 421 | 2.673395873 | PPP6R2 | protein phosphatase 6 regulatory subunit 2 [Source:HGNC Symbol;Acc:HGNC:19253] |
| ENSG00000110436 | HGLibA_44510 | 421 | 2.673395873 | SLC1A2 | solute carrier family 1 member 2 [Source:HGNC Symbol;Acc:HGNC:10940] |
| ENSG00000119335 | HGLibA_43630 | 421 | 2.673395873 | SET | SET nuclear proto-oncogene [Source:HGNC Symbol;Acc:HGNC:10760] |
| ENSG00000121297 | HGLibA_51869 | 421 | 2.673395873 | TSHZ3 | teashirt zinc finger homeobox 3 [Source:HGNC Symbol;Acc:HGNC:30700] |
| ENSG00000127083 | HGLibA_33384 | 421 | 2.673395873 | OMD | osteomodulin [Source:HGNC Symbol;Acc:HGNC:8134] |
| ENSG00000128274 | HGLibA_00016 | 421 | 2.673395873 | A4GALT | 4-galactosyltransferase (P blood group) [Source:HGNC Symbol;Acc:HGNC:18149]" |
| ENSG00000132671 | HGLibA_47184 | 421 | 2.673395873 | SSTR4 | somatostatin receptor 4 [Source:HGNC Symbol;Acc:HGNC:11333] |
| ENSG00000132840 | HGLibA_04489 | 421 | 2.673395873 | BHMT2 | betaine--homocysteine S-methyltransferase 2 [Source:HGNC Symbol;Acc:HGNC:1048] |
| ENSG00000141682 | HGLibA_37292 | 421 | 2.673395873 | PMAIP1 | phorbol-12-myristate-13-acetate-induced protein 1 [Source:HGNC Symbol;Acc:HGNC:9108] |
| ENSG00000147576 | HGLibA_00969 | 421 | 2.673395873 | ADHFE1 | iron containing 1 [Source:HGNC Symbol;Acc:HGNC:16354]" |
| ENSG00000166963 | HGLibA_28114 | 421 | 2.673395873 | MAP1A | microtubule associated protein 1A [Source:HGNC Symbol;Acc:HGNC:6835] |
| ENSG00000011332 | HGLibA_13828 | 420 | 2.66435053 | DPF1 | double PHD fingers 1 [Source:HGNC Symbol;Acc:HGNC:20225] |
| ENSG00000067141 | HGLibA_31695 | 420 | 2.66435053 | NEO1 | neogenin 1 [Source:HGNC Symbol;Acc:HGNC:7754] |
| ENSG00000088682 | HGLibA_10767 | 420 | 2.66435053 | COQ9 | coenzyme Q9 [Source:HGNC Symbol;Acc:HGNC:25302] |
| ENSG00000104518 | HGLibA_20414 | 420 | 2.66435053 | GSDMD | gasdermin D [Source:HGNC Symbol;Acc:HGNC:25697] |
| ENSG00000124155 | HGLibA_36588 | 420 | 2.66435053 | PIGT | phosphatidylinositol glycan anchor biosynthesis class T [Source:HGNC Symbol;Acc:HGNC:14938] |
| ENSG00000126264 | HGLibA_20995 | 420 | 2.66435053 | HCST | hematopoietic cell signal transducer [Source:HGNC Symbol;Acc:HGNC:16977] |
| ENSG00000132740 | HGLibA_22857 | 420 | 2.66435053 | IGHMBP2 | immunoglobulin mu DNA binding protein 2 [Source:HGNC Symbol;Acc:HGNC:5542] |
| ENSG00000137692 | HGLibA_12578 | 420 | 2.66435053 | DCUN1D5 | defective in cullin neddylation 1 domain containing 5 [Source:HGNC Symbol;Acc:HGNC:28409] |
| ENSG00000158485 | HGLibA_08268 | 420 | 2.66435053 | CD1B | CD1b molecule [Source:HGNC Symbol;Acc:HGNC:1635] |
| ENSG00000163520 | HGLibA_16946 | 420 | 2.66435053 | FBLN2 | fibulin 2 [Source:HGNC Symbol;Acc:HGNC:3601] |
| ENSG00000163793 | HGLibA_13631 | 420 | 2.66435053 | DNAJC5G | DnaJ heat shock protein family (Hsp40) member C5 gamma [Source:HGNC Symbol;Acc:HGNC:24844] |
| ENSG00000178222 | HGLibA_41618 | 420 | 2.66435053 | RNF212 | ring finger protein 212 [Source:HGNC Symbol;Acc:HGNC:27729] |
| ENSG00000197619 | HGLibA_56392 | 420 | 2.66435053 | ZNF615 | zinc finger protein 615 [Source:HGNC Symbol;Acc:HGNC:24740] |
| ENSG00000213424 | HGLibA_25449 | 420 | 2.66435053 | KRT222 | keratin 222 [Source:HGNC Symbol;Acc:HGNC:28695] |
| ENSG00000256162 | HGLibA_45780 | 420 | 2.66435053 | SMLR1 | small leucine rich protein 1 [Source:HGNC Symbol;Acc:HGNC:44670] |
| ENSG00000099958 | HGLibA_13002 | 419 | 2.655305187 | DERL3 | derlin 3 [Source:HGNC Symbol;Acc:HGNC:14236] |
| ENSG00000115875 | HGLibA_47088 | 419 | 2.655305187 | SRSF7 | serine and arginine rich splicing factor 7 [Source:HGNC Symbol;Acc:HGNC:10789] |
| ENSG00000128652 | HGLibA_21997 | 419 | 2.655305187 | HOXD3 | homeobox D3 [Source:HGNC Symbol;Acc:HGNC:5137] |
| ENSG00000135253 | HGLibA_24407 | 419 | 2.655305187 | KCP | kielin cysteine rich BMP regulator [Source:HGNC Symbol;Acc:HGNC:17585] |
| ENSG00000164746 | HGLibA_06560 | 419 | 2.655305187 | C7orf57 | chromosome 7 open reading frame 57 [Source:HGNC Symbol;Acc:HGNC:22247] |
| ENSG00000166171 | HGLibA_13813 | 419 | 2.655305187 | DPCD | deleted in primary ciliary dyskinesia homolog (mouse) [Source:HGNC Symbol;Acc:HGNC:24542] |
| ENSG00000166762 | HGLibA_07359 | 419 | 2.655305187 | CATSPER2 | cation channel sperm associated 2 [Source:HGNC Symbol;Acc:HGNC:18810] |
| ENSG00000167644 | HGLibA_05648 | 419 | 2.655305187 | C19orf33 | chromosome 19 open reading frame 33 [Source:HGNC Symbol;Acc:HGNC:16668] |
| ENSG00000175582 | HGLibA_39970 | 419 | 2.655305187 | RAB6A | member RAS oncogene family [Source:HGNC Symbol;Acc:HGNC:9786]" |
| ENSG00000182111 | HGLibA_56624 | 419 | 2.655305187 | ZNF716 | zinc finger protein 716 [Source:HGNC Symbol;Acc:HGNC:32458] |
| ENSG00000197128 | HGLibA_56710 | 419 | 2.655305187 | ZNF772 | zinc finger protein 772 [Source:HGNC Symbol;Acc:HGNC:33106] |
| ENSG00000213445 | HGLibA_44190 | 419 | 2.655305187 | SIPA1 | signal-induced proliferation-associated 1 [Source:HGNC Symbol;Acc:HGNC:10885] |
| ERO1L | HGLibA_15518 | 419 | 2.655305187 | ERO1L | - |
| hsa-mir-6765 | HGLibA_63222 | 419 | 2.655305187 | hsa-mir-6765 | - |
| C15orf26 | HGLibA_05378 | 418 | 2.646259844 | C15orf26 | - |
| ENSG00000110711 | HGLibA_01362 | 418 | 2.646259844 | AIP | aryl hydrocarbon receptor interacting protein [Source:HGNC Symbol;Acc:HGNC:358] |
| ENSG00000114030 | HGLibA_25353 | 418 | 2.646259844 | KPNA1 | karyopherin subunit alpha 1 [Source:HGNC Symbol;Acc:HGNC:6394] |
| ENSG00000138303 | HGLibA_03257 | 418 | 2.646259844 | ASCC1 | activating signal cointegrator 1 complex subunit 1 [Source:HGNC Symbol;Acc:HGNC:24268] |
| ENSG00000147454 | HGLibA_44723 | 418 | 2.646259844 | SLC25A37 | solute carrier family 25 member 37 [Source:HGNC Symbol;Acc:HGNC:29786] |
| ENSG00000169118 | HGLibA_11392 | 418 | 2.646259844 | CSNK1G1 | casein kinase 1 gamma 1 [Source:HGNC Symbol;Acc:HGNC:2454] |
| hsa-mir-141 | HGLibA_57827 | 418 | 2.646259844 | hsa-mir-141 | - |
| hsa-mir-4514 | HGLibA_60498 | 418 | 2.646259844 | hsa-mir-4514 | - |
| ENSG00000011451 | HGLibA_54328 | 417 | 2.637214501 | WIZ | widely interspaced zinc finger motifs [Source:HGNC Symbol;Acc:HGNC:30917] |
| ENSG00000068001 | HGLibA_22441 | 417 | 2.637214501 | HYAL2 | hyaluronidase 2 [Source:HGNC Symbol;Acc:HGNC:5321] |
| ENSG00000085788 | HGLibA_12606 | 417 | 2.637214501 | DDHD2 | DDHD domain containing 2 [Source:HGNC Symbol;Acc:HGNC:29106] |
| ENSG00000095596 | HGLibA_12086 | 417 | 2.637214501 | CYP26A1 | cytochrome P450 family 26 subfamily A member 1 [Source:HGNC Symbol;Acc:HGNC:2603] |

| | | | | | |
|---|---|---|---|---|---|
| ENSG00000109180 | HGLibA_33247 | 417 | 2.637214501 | OCIAD1 | OCIA domain containing 1 [Source:HGNC Symbol;Acc:HGNC:16074] |
| ENSG00000150054 | HGLibA_29766 | 417 | 2.637214501 | MPP7 | membrane palmitoylated protein 7 [Source:HGNC Symbol;Acc:HGNC:26542] |
| ENSG00000155428 | HGLibA_51608 | 417 | 2.637214501 | TRIM74 | tripartite motif containing 74 [Source:HGNC Symbol;Acc:HGNC:17453] |
| ENSG00000165102 | HGLibA_21248 | 417 | 2.637214501 | HGSNAT | heparan-alpha-glucosaminide N-acetyltransferase [Source:HGNC Symbol;Acc:HGNC:26527] |
| ENSG00000169594 | HGLibA_04644 | 417 | 2.637214501 | BNC1 | basonuclin 1 [Source:HGNC Symbol;Acc:HGNC:1081] |
| ENSG00000180720 | HGLibA_09555 | 417 | 2.637214501 | CHRM4 | cholinergic receptor muscarinic 4 [Source:HGNC Symbol;Acc:HGNC:1953] |
| ENSG00000197938 | HGLibA_34298 | 417 | 2.637214501 | OR5H2 | olfactory receptor family 5 subfamily H member 2 [Source:HGNC Symbol;Acc:HGNC:14752] |
| ENSG00000008283 | HGLibA_11978 | 416 | 2.628169158 | CYB561 | cytochrome b561 [Source:HGNC Symbol;Acc:HGNC:2571] |
| ENSG00000099203 | HGLibA_49800 | 416 | 2.628169158 | TMED1 | transmembrane p24 trafficking protein 1 [Source:HGNC Symbol;Acc:HGNC:17291] |
| ENSG00000113905 | HGLibA_22090 | 416 | 2.628169158 | HRG | histidine rich glycoprotein [Source:HGNC Symbol;Acc:HGNC:5181] |
| ENSG00000129991 | HGLibA_50878 | 416 | 2.628169158 | TNNI3 | cardiac type [Source:HGNC Symbol;Acc:HGNC:11947]" |
| ENSG00000154639 | HGLibA_11838 | 416 | 2.628169158 | CXADR | Ig-like cell adhesion molecule [Source:HGNC Symbol;Acc:HGNC:2559]" |
| ENSG00000162148 | HGLibA_38086 | 416 | 2.628169158 | PPP1R32 | protein phosphatase 1 regulatory subunit 32 [Source:HGNC Symbol;Acc:HGNC:28869] |
| ENSG00000163328 | HGLibA_19943 | 416 | 2.628169158 | GPR155 | G protein-coupled receptor 155 [Source:HGNC Symbol;Acc:HGNC:22951] |
| ENSG00000163346 | HGLibA_35356 | 416 | 2.628169158 | PBXIP1 | PBX homeobox interacting protein 1 [Source:HGNC Symbol;Acc:HGNC:21199] |
| ENSG00000164692 | HGLibA_10534 | 416 | 2.628169158 | COL1A2 | collagen type I alpha 2 chain [Source:HGNC Symbol;Acc:HGNC:2198] |
| ENSG00000166368 | HGLibA_33782 | 416 | 2.628169158 | OR2D2 | olfactory receptor family 2 subfamily D member 2 [Source:HGNC Symbol;Acc:HGNC:8244] |
| ENSG00000189143 | HGLibA_09899 | 416 | 2.628169158 | CLDN4 | claudin 4 [Source:HGNC Symbol;Acc:HGNC:2046] |
| hsa-mir-19b-2 | HGLibA_58146 | 416 | 2.628169158 | hsa-mir-19b-2 | - |
| ENSG00000010030 | HGLibA_15669 | 415 | 2.619123815 | ETV7 | ETS variant 7 [Source:HGNC Symbol;Acc:HGNC:18160] |
| ENSG00000114744 | HGLibA_10657 | 415 | 2.619123815 | COMMD2 | COMM domain containing 2 [Source:HGNC Symbol;Acc:HGNC:24993] |
| ENSG00000120756 | HGLibA_37224 | 415 | 2.619123815 | PLS1 | plastin 1 [Source:HGNC Symbol;Acc:HGNC:9090] |
| ENSG00000142149 | HGLibA_22423 | 415 | 2.619123815 | HUNK | hormonally up-regulated Neu-associated kinase [Source:HGNC Symbol;Acc:HGNC:13326] |
| ENSG00000167900 | HGLibA_49580 | 415 | 2.619123815 | TK1 | thymidine kinase 1 [Source:HGNC Symbol;Acc:HGNC:11830] |
| ENSG00000184949 | HGLibA_16528 | 415 | 2.619123815 | FAM227A | family with sequence similarity 227 member A [Source:HGNC Symbol;Acc:HGNC:44197] |
| ENSG00000206190 | HGLibA_03545 | 415 | 2.619123815 | ATP10A | ATPase phospholipid transporting 10A (putative) [Source:HGNC Symbol;Acc:HGNC:13542] |
| ENSG00000215545 | HGLibA_12862 | 415 | 2.619123815 | DEFB116 | defensin beta 116 [Source:HGNC Symbol;Acc:HGNC:18097] |
| hsa-mir-1539 | HGLibA_57923 | 415 | 2.619123815 | hsa-mir-1539 | - |
| hsa-mir-4669 | HGLibA_60792 | 415 | 2.619123815 | hsa-mir-4669 | - |
| hsa-mir-6841 | HGLibA_63538 | 415 | 2.619123815 | hsa-mir-6841 | - |
| ENSG00000035141 | HGLibA_16137 | 414 | 2.610078472 | FAM136A | family with sequence similarity 136 member A [Source:HGNC Symbol;Acc:HGNC:25911] |
| ENSG00000079101 | HGLibA_10163 | 414 | 2.610078472 | CLUL1 | clusterin like 1 [Source:HGNC Symbol;Acc:HGNC:2096] |
| ENSG00000110934 | HGLibA_04508 | 414 | 2.610078472 | BIN2 | bridging integrator 2 [Source:HGNC Symbol;Acc:HGNC:1053] |
| ENSG00000120949 | HGLibA_50790 | 414 | 2.610078472 | TNFRSF8 | TNF receptor superfamily member 8 [Source:HGNC Symbol;Acc:HGNC:11923] |
| ENSG00000125257 | HGLibA_00177 | 414 | 2.610078472 | ABCC4 | ATP binding cassette subfamily C member 4 [Source:HGNC Symbol;Acc:HGNC:55] |
| ENSG00000130363 | HGLibA_42332 | 414 | 2.610078472 | RSPH3 | radial spoke head 3 homolog [Source:HGNC Symbol;Acc:HGNC:21054] |
| ENSG00000130803 | HGLibA_55782 | 414 | 2.610078472 | ZNF317 | zinc finger protein 317 [Source:HGNC Symbol;Acc:HGNC:13507] |
| ENSG00000134824 | HGLibA_15936 | 414 | 2.610078472 | FADS2 | fatty acid desaturase 2 [Source:HGNC Symbol;Acc:HGNC:3575] |
| ENSG00000143512 | HGLibA_21266 | 414 | 2.610078472 | HHIPL2 | HHIP like 2 [Source:HGNC Symbol;Acc:HGNC:25842] |
| ENSG00000158901 | HGLibA_54273 | 414 | 2.610078472 | WFDC8 | WAP four-disulfide core domain 8 [Source:HGNC Symbol;Acc:HGNC:16163] |
| ENSG00000165682 | HGLibA_09959 | 414 | 2.610078472 | CLEC1B | C-type lectin domain family 1 member B [Source:HGNC Symbol;Acc:HGNC:24356] |
| ENSG00000171243 | HGLibA_46209 | 414 | 2.610078472 | SOSTDC1 | sclerostin domain containing 1 [Source:HGNC Symbol;Acc:HGNC:21748] |
| ENSG00000196227 | HGLibA_16491 | 414 | 2.610078472 | FAM217B | family with sequence similarity 217 member B [Source:HGNC Symbol;Acc:HGNC:16170] |
| ENSG00000075234 | HGLibA_52129 | 413 | 2.60103313 | TTC38 | tetratricopeptide repeat domain 38 [Source:HGNC Symbol;Acc:HGNC:26082] |
| ENSG00000088992 | HGLibA_49025 | 413 | 2.60103313 | TESC | tescalcin [Source:HGNC Symbol;Acc:HGNC:26065] |
| ENSG00000089505 | HGLibA_10210 | 413 | 2.60103313 | CMTM1 | CKLF like MARVEL transmembrane domain containing 1 [Source:HGNC Symbol;Acc:HGNC:19172] |
| ENSG00000138802 | HGLibA_43232 | 413 | 2.60103313 | SEC24B | COPII coat complex component [Source:HGNC Symbol;Acc:HGNC:10704]" |
| ENSG00000173889 | HGLibA_36323 | 413 | 2.60103313 | PHC3 | polyhomeotic homolog 3 [Source:HGNC Symbol;Acc:HGNC:15682] |
| ENSG00000250067 | HGLibA_54690 | 413 | 2.60103313 | YJEFN3 | YjeF N-terminal domain containing 3 [Source:HGNC Symbol;Acc:HGNC:24785] |
| ENSG00000254245 | HGLibA_35531 | 413 | 2.60103313 | PCDHGA3 | 3 [Source:HGNC Symbol;Acc:HGNC:8701]" |
| HIST3H2A | HGLibA_21555 | 413 | 2.60103313 | HIST3H2A | - |
| hsa-mir-517b | HGLibA_61629 | 413 | 2.60103313 | hsa-mir-517b | - |
| ENSG00000100304 | HGLibA_52195 | 412 | 2.591987787 | TTLL12 | tubulin tyrosine ligase like 12 [Source:HGNC Symbol;Acc:HGNC:28974] |
| ENSG00000101439 | HGLibA_11453 | 412 | 2.591987787 | CST3 | cystatin C [Source:HGNC Symbol;Acc:HGNC:2475] |
| ENSG00000107242 | HGLibA_36716 | 412 | 2.591987787 | PIP5K1B | phosphatidylinositol-4-phosphate 5-kinase type 1 beta [Source:HGNC Symbol;Acc:HGNC:8995] |
| ENSG00000114861 | HGLibA_17931 | 412 | 2.591987787 | FOXP1 | forkhead box P1 [Source:HGNC Symbol;Acc:HGNC:3823] |
| ENSG00000115828 | HGLibA_39726 | 412 | 2.591987787 | QPCT | glutaminyl-peptide cyclotransferase [Source:HGNC Symbol;Acc:HGNC:9753] |
| ENSG00000117226 | HGLibA_18733 | 412 | 2.591987787 | GBP3 | guanylate binding protein 3 [Source:HGNC Symbol;Acc:HGNC:4184] |
| ENSG00000131400 | HGLibA_31139 | 412 | 2.591987787 | NAPSA | napsin A aspartic peptidase [Source:HGNC Symbol;Acc:HGNC:13395] |
| ENSG00000165626 | HGLibA_04415 | 412 | 2.591987787 | BEND7 | BEN domain containing 7 [Source:HGNC Symbol;Acc:HGNC:23514] |
| ENSG00000166557 | HGLibA_49807 | 412 | 2.591987787 | TMED3 | transmembrane p24 trafficking protein 3 [Source:HGNC Symbol;Acc:HGNC:28889] |
| ENSG00000167291 | HGLibA_48484 | 412 | 2.591987787 | TBC1D16 | TBC1 domain family member 16 [Source:HGNC Symbol;Acc:HGNC:28356] |
| ENSG00000175718 | HGLibA_40607 | 412 | 2.591987787 | RBMXL3 | RBMX like 3 [Source:HGNC Symbol;Acc:HGNC:26859] |
| ENSG00000215704 | HGLibA_08996 | 412 | 2.591987787 | CELA2B | chymotrypsin like elastase family member 2B [Source:HGNC Symbol;Acc:HGNC:29995] |
| ENSG00000234438 | HGLibA_24100 | 412 | 2.591987787 | KBTBD13 | kelch repeat and BTB domain containing 13 [Source:HGNC Symbol;Acc:HGNC:37227] |
| ENSG00000234560 | HGLibA_33500 | 412 | 2.591987787 | OR10G8 | olfactory receptor family 10 subfamily G member 8 [Source:HGNC Symbol;Acc:HGNC:14845] |
| hsa-mir-4292 | HGLibA_59879 | 412 | 2.591987787 | hsa-mir-4292 | - |
| CECR5 | HGLibA_08981 | 411 | 2.582942444 | CECR5 | - |
| ENSG00000064932 | HGLibA_42808 | 411 | 2.582942444 | SBNO2 | strawberry notch homolog 2 [Source:HGNC Symbol;Acc:HGNC:29158] |
| ENSG00000075275 | HGLibA_09022 | 411 | 2.582942444 | CELSR1 | cadherin EGF LAG seven-pass G-type receptor 1 [Source:HGNC Symbol;Acc:HGNC:1850] |
| ENSG00000112378 | HGLibA_36064 | 411 | 2.582942444 | PERP | TP53 apoptosis effector [Source:HGNC Symbol;Acc:HGNC:17637]" |
| ENSG00000135148 | HGLibA_51271 | 411 | 2.582942444 | TRAFD1 | TRAF-type zinc finger domain containing 1 [Source:HGNC Symbol;Acc:HGNC:24808] |
| ENSG00000168769 | HGLibA_49039 | 411 | 2.582942444 | TET2 | tet methylcytosine dioxygenase 2 [Source:HGNC Symbol;Acc:HGNC:25941] |
| ENSG00000177946 | HGLibA_09043 | 411 | 2.582942444 | CENPBD1 | CENPB DNA-binding domain containing 1 [Source:HGNC Symbol;Acc:HGNC:28272] |
| ENSG00000179965 | HGLibA_56706 | 411 | 2.582942444 | ZNF771 | zinc finger protein 771 [Source:HGNC Symbol;Acc:HGNC:29653] |
| ENSG00000180305 | HGLibA_54248 | 411 | 2.582942444 | WFDC10A | WAP four-disulfide core domain 10A [Source:HGNC Symbol;Acc:HGNC:16139] |
| ENSG00000183153 | HGLibA_19190 | 411 | 2.582942444 | GJD3 | gap junction protein delta 3 [Source:HGNC Symbol;Acc:HGNC:19147] |
| ENSG00000187612 | HGLibA_34367 | 411 | 2.582942444 | OR5W2 | olfactory receptor family 5 subfamily W member 2 [Source:HGNC Symbol;Acc:HGNC:15299] |
| ENSG00000198090 | HGLibA_25769 | 411 | 2.582942444 | KRTAP4-6 | keratin associated protein 4-6 [Source:HGNC Symbol;Acc:HGNC:18909] |
| ENSG00000198208 | HGLibA_42186 | 411 | 2.582942444 | RPS6KL1 | ribosomal protein S6 kinase like 1 [Source:HGNC Symbol;Acc:HGNC:20222] |
| ENSG00000009830 | HGLibA_37671 | 410 | 2.573897101 | POMT2 | protein O-mannosyltransferase 2 [Source:HGNC Symbol;Acc:HGNC:19743] |
| ENSG00000134463 | HGLibA_14399 | 410 | 2.573897101 | ECHDC3 | enoyl-CoA hydratase domain containing 3 [Source:HGNC Symbol;Acc:HGNC:23489] |
| ENSG00000138767 | HGLibA_10347 | 410 | 2.573897101 | CNOT6L | CCR4-NOT transcription complex subunit 6 like [Source:HGNC Symbol;Acc:HGNC:18042] |
| ENSG00000147606 | HGLibA_44803 | 410 | 2.573897101 | SLC26A7 | solute carrier family 26 member 7 [Source:HGNC Symbol;Acc:HGNC:14467] |
| ENSG00000160223 | HGLibA_22519 | 410 | 2.573897101 | ICOSLG | inducible T cell costimulator ligand [Source:HGNC Symbol;Acc:HGNC:17087] |
| hsa-mir-3619 | HGLibA_59106 | 410 | 2.573897101 | hsa-mir-3619 | - |
| ENSG00000028277 | HGLibA_37758 | 409 | 2.564851758 | POU2F2 | POU class 2 homeobox 2 [Source:HGNC Symbol;Acc:HGNC:9213] |
| ENSG00000073737 | HGLibA_13154 | 409 | 2.564851758 | DHRS9 | dehydrogenase/reductase 9 [Source:HGNC Symbol;Acc:HGNC:16888] |
| ENSG00000101577 | HGLibA_27099 | 409 | 2.564851758 | LPIN2 | lipin 2 [Source:HGNC Symbol;Acc:HGNC:14450] |
| ENSG00000124701 | HGLibA_02469 | 409 | 2.564851758 | APOBEC2 | apolipoprotein B mRNA editing enzyme catalytic subunit 2 [Source:HGNC Symbol;Acc:HGNC:605] |
| ENSG00000139428 | HGLibA_29452 | 409 | 2.564851758 | MMAB | metabolism of cobalamin associated B [Source:HGNC Symbol;Acc:HGNC:19331] |
| ENSG00000143631 | HGLibA_17610 | 409 | 2.564851758 | FLG | filaggrin [Source:HGNC Symbol;Acc:HGNC:3748] |
| ENSG00000160307 | HGLibA_42578 | 409 | 2.564851758 | S100B | S100 calcium binding protein B [Source:HGNC Symbol;Acc:HGNC:10500] |
| ENSG00000172840 | HGLibA_35929 | 409 | 2.564851758 | PDP2 | pyruvate dehyrogenase phosphatase catalytic subunit 2 [Source:HGNC Symbol;Acc:HGNC:30263] |
| ENSG00000175485 | HGLibA_34207 | 409 | 2.564851758 | OR52W1 | olfactory receptor family 52 subfamily W member 1 [Source:HGNC Symbol;Acc:HGNC:15239] |
| ENSG00000176273 | HGLibA_45015 | 409 | 2.564851758 | SLC35G1 | solute carrier family 35 member G1 [Source:HGNC Symbol;Acc:HGNC:26607] |
| hsa-mir-378d-2 | HGLibA_59441 | 409 | 2.564851758 | hsa-mir-378d-2 | - |
| hsa-mir-492 | HGLibA_61370 | 409 | 2.564851758 | hsa-mir-492 | - |
| C11orf84 | HGLibA_05197 | 408 | 2.555806415 | C11orf84 | - |
| ENSG00000044446 | HGLibA_36404 | 408 | 2.555806415 | PHKA2 | phosphorylase kinase regulatory subunit alpha 2 [Source:HGNC Symbol;Acc:HGNC:8926] |
| ENSG00000078814 | HGLibA_30735 | 408 | 2.555806415 | MYH7B | myosin heavy chain 7B [Source:HGNC Symbol;Acc:HGNC:15906] |
| ENSG00000099337 | HGLibA_24331 | 408 | 2.555806415 | KCNK6 | potassium two pore domain channel subfamily K member 6 [Source:HGNC Symbol;Acc:HGNC:6281] |
| ENSG00000100124 | HGLibA_02124 | 408 | 2.555806415 | ANKRD54 | ankyrin repeat domain 54 [Source:HGNC Symbol;Acc:HGNC:25185] |
| ENSG00000102032 | HGLibA_40840 | 408 | 2.555806415 | RENBP | renin binding protein [Source:HGNC Symbol;Acc:HGNC:9959] |
| ENSG00000112303 | HGLibA_53713 | 408 | 2.555806415 | VNN2 | vanin 2 [Source:HGNC Symbol;Acc:HGNC:12706] |
| ENSG00000114487 | HGLibA_29649 | 408 | 2.555806415 | MORC1 | MORC family CW-type zinc finger 1 [Source:HGNC Symbol;Acc:HGNC:7198] |
| ENSG00000143801 | HGLibA_39029 | 408 | 2.555806415 | PSEN2 | presenilin 2 [Source:HGNC Symbol;Acc:HGNC:9509] |
| ENSG00000179774 | HGLibA_03537 | 408 | 2.555806415 | ATOH7 | atonal bHLH transcription factor 7 [Source:HGNC Symbol;Acc:HGNC:13907] |
| ENSG00000198597 | HGLibA_56185 | 408 | 2.555806415 | ZNF536 | zinc finger protein 536 [Source:HGNC Symbol;Acc:HGNC:29025] |
| ENSG00000243902 | HGLibA_14908 | 408 | 2.555806415 | ELFN2 | extracellular leucine rich repeat and fibronectin type III domain containing 2 [Source:NCBI gene;Acc:114794] |
| ENSG00000274588 | HGLibA_13078 | 408 | 2.555806415 | DGKK | diacylglycerol kinase kappa [Source:HGNC Symbol;Acc:HGNC:32395] |
| hsa-mir-6744 | HGLibA_63129 | 408 | 2.555806415 | hsa-mir-6744 | - |
| hsa-mir-8073 | HGLibA_64136 | 408 | 2.555806415 | hsa-mir-8073 | - |
| hsa-mir-921 | HGLibA_64280 | 408 | 2.555806415 | hsa-mir-921 | - |
| ENSG00000105289 | HGLibA_49577 | 407 | 2.546761072 | TJP3 | tight junction protein 3 [Source:HGNC Symbol;Acc:HGNC:11829] |
| ENSG00000114648 | HGLibA_25144 | 407 | 2.546761072 | KLHL18 | kelch like family member 18 [Source:HGNC Symbol;Acc:HGNC:29120] |
| ENSG00000129194 | HGLibA_46244 | 407 | 2.546761072 | SOX15 | SRY-box 15 [Source:HGNC Symbol;Acc:HGNC:11196] |
| ENSG00000143257 | HGLibA_32592 | 407 | 2.546761072 | NR1I3 | nuclear receptor subfamily 1 group I member 3 [Source:HGNC Symbol;Acc:HGNC:7969] |
| ENSG00000160014 | HGLibA_07027 | 407 | 2.546761072 | CALM3 | calmodulin 3 [Source:HGNC Symbol;Acc:HGNC:1449] |
| ENSG00000163785 | HGLibA_42518 | 407 | 2.546761072 | RYK | receptor-like tyrosine kinase [Source:HGNC Symbol;Acc:HGNC:10481] |
| ENSG00000172742 | HGLibA_33984 | 407 | 2.546761072 | OR4D9 | olfactory receptor family 4 subfamily D member 9 [Source:HGNC Symbol;Acc:HGNC:15178] |
| ENSG00000181903 | HGLibA_33963 | 407 | 2.546761072 | OR4C6 | olfactory receptor family 4 subfamily C member 6 [Source:HGNC Symbol;Acc:HGNC:14743] |
| ENSG00000182459 | HGLibA_49069 | 407 | 2.546761072 | TEX19 | testis expressed 19 [Source:HGNC Symbol;Acc:HGNC:33802] |
| ENSG00000187860 | HGLibA_07660 | 407 | 2.546761072 | CCDC157 | coiled-coil domain containing 157 [Source:HGNC Symbol;Acc:HGNC:33854] |
| ENSG00000214700 | HGLibA_05287 | 407 | 2.546761072 | C12orf71 | chromosome 12 open reading frame 71 [Source:HGNC Symbol;Acc:HGNC:34452] |
| ENSG00000255974 | HGLibA_12107 | 407 | 2.546761072 | CYP2A6 | cytochrome P450 family 2 subfamily A member 6 [Source:HGNC Symbol;Acc:HGNC:2610] |
| hsa-mir-1976 | HGLibA_58119 | 407 | 2.546761072 | hsa-mir-1976 | - |
| hsa-mir-573 | HGLibA_62382 | 407 | 2.546761072 | hsa-mir-573 | - |
| ENSG00000055070 | HGLibA_48115 | 406 | 2.537715729 | SZRD1 | SUZ RNA binding domain containing 1 [Source:HGNC Symbol;Acc:HGNC:30232] |
| ENSG00000068976 | HGLibA_39692 | 406 | 2.537715729 | PYGM | muscle associated [Source:HGNC Symbol;Acc:HGNC:9726]" |
| ENSG00000086288 | HGLibA_32175 | 406 | 2.537715729 | NME8 | NME/NM23 family member 8 [Source:HGNC Symbol;Acc:HGNC:16473] |
| ENSG00000113296 | HGLibA_49331 | 406 | 2.537715729 | THBS4 | thrombospondin 4 [Source:HGNC Symbol;Acc:HGNC:11788] |
| ENSG00000126947 | HGLibA_03037 | 406 | 2.537715729 | ARMCX1 | armadillo repeat containing X-linked 1 [Source:HGNC Symbol;Acc:HGNC:18073] |
| ENSG00000133561 | HGLibA_19084 | 406 | 2.537715729 | GIMAP6 | IMAP family member 6 [Source:HGNC Symbol;Acc:HGNC:21918]" |
| ENSG00000135973 | HGLibA_20045 | 406 | 2.537715729 | GPR45 | G protein-coupled receptor 45 [Source:HGNC Symbol;Acc:HGNC:4503] |

| | | | | | |
|---|---|---|---|---|---|
| ENSG00000142798 | HGLibA_22340 | 406 | 2.537715729 | HSPG2 | heparan sulfate proteoglycan 2 [Source:HGNC Symbol;Acc:HGNC:5273] |
| ENSG00000161091 | HGLibA_29090 | 406 | 2.537715729 | MFSD12 | major facilitator superfamily domain containing 12 [Source:HGNC Symbol;Acc:HGNC:28299] |
| ENSG00000165521 | HGLibA_15067 | 406 | 2.537715729 | EML5 | echinoderm microtubule associated protein like 5 [Source:HGNC Symbol;Acc:HGNC:18197] |
| ENSG00000185247 | HGLibA_27898 | 406 | 2.537715729 | MAGEA11 | MAGE family member A11 [Source:HGNC Symbol;Acc:HGNC:6798] |
| ENSG00000204366 | HGLibA_54821 | 406 | 2.537715729 | ZBTB12 | zinc finger and BTB domain containing 12 [Source:HGNC Symbol;Acc:HGNC:19066] |
| hsa-mir-4298 | HGLibA_59904 | 406 | 2.537715729 | hsa-mir-4298 | - |
| hsa-mir-7846 | HGLibA_63972 | 406 | 2.537715729 | hsa-mir-7846 | - |
| ENSG00000125787 | HGLibA_19582 | 405 | 2.528670386 | GNRH2 | gonadotropin releasing hormone 2 [Source:HGNC Symbol;Acc:HGNC:4420] |
| ENSG00000125971 | HGLibA_14260 | 405 | 2.528670386 | DYNLRB1 | dynein light chain roadblock-type 1 [Source:HGNC Symbol;Acc:HGNC:15468] |
| ENSG00000134108 | HGLibA_02999 | 405 | 2.528670386 | ARL8B | ADP ribosylation factor like GTPase 8B [Source:HGNC Symbol;Acc:HGNC:25564] |
| ENSG00000163873 | HGLibA_20288 | 405 | 2.528670386 | GRIK3 | glutamate ionotropic receptor kainate type subunit 3 [Source:HGNC Symbol;Acc:HGNC:4581] |
| ENSG00000165762 | HGLibA_34030 | 405 | 2.528670386 | OR4K2 | olfactory receptor family 4 subfamily K member 2 [Source:HGNC Symbol;Acc:HGNC:14728] |
| ENSG00000172731 | HGLibA_27282 | 405 | 2.528670386 | LRRC20 | leucine rich repeat containing 20 [Source:HGNC Symbol;Acc:HGNC:23421] |
| ENSG00000197776 | HGLibA_25090 | 405 | 2.528670386 | KLHDC1 | kelch domain containing 1 [Source:HGNC Symbol;Acc:HGNC:19836] |
| ENSG00000203985 | HGLibA_26242 | 405 | 2.528670386 | LDLRAD1 | low density lipoprotein receptor class A domain containing 1 [Source:HGNC Symbol;Acc:HGNC:32069] |
| ENSG00000277363 | HGLibA_46935 | 405 | 2.528670386 | SRCIN1 | SRC kinase signaling inhibitor 1 [Source:HGNC Symbol;Acc:HGNC:29506] |
| hsa-mir-4529 | HGLibA_60576 | 405 | 2.528670386 | hsa-mir-4529 | - |
| hsa-mir-590 | HGLibA_62457 | 405 | 2.528670386 | hsa-mir-590 | - |
| ENSG00000070182 | HGLibA_46877 | 404 | 2.519625043 | SPTB | erythrocytic [Source:HGNC Symbol;Acc:HGNC:11274]" |
| ENSG00000104133 | HGLibA_46631 | 404 | 2.519625043 | SPG11 | spatacsin vesicle trafficking associated [Source:HGNC Symbol;Acc:HGNC:11226]" |
| ENSG00000113739 | HGLibA_47418 | 404 | 2.519625043 | STC2 | stanniocalcin 2 [Source:HGNC Symbol;Acc:HGNC:11374] |
| ENSG00000125384 | HGLibA_39317 | 404 | 2.519625043 | PTGER2 | prostaglandin E receptor 2 [Source:HGNC Symbol;Acc:HGNC:9594] |
| ENSG00000129351 | HGLibA_23216 | 404 | 2.519625043 | ILF3 | interleukin enhancer binding factor 3 [Source:HGNC Symbol;Acc:HGNC:6038] |
| ENSG00000138316 | HGLibA_00802 | 404 | 2.519625043 | ADAMTS14 | ADAM metallopeptidase with thrombospondin type 1 motif 14 [Source:HGNC Symbol;Acc:HGNC:14899] |
| ENSG00000152242 | HGLibA_05610 | 404 | 2.519625043 | C18orf25 | chromosome 18 open reading frame 25 [Source:HGNC Symbol;Acc:HGNC:28172] |
| ENSG00000160932 | HGLibA_27657 | 404 | 2.519625043 | LY6E | lymphocyte antigen 6 family member E [Source:HGNC Symbol;Acc:HGNC:6727] |
| ENSG00000176177 | HGLibA_15192 | 404 | 2.519625043 | ENTHD1 | ENTH domain containing 1 [Source:HGNC Symbol;Acc:HGNC:26352] |
| ENSG00000188783 | HGLibA_38433 | 404 | 2.519625043 | PRELP | proline and arginine rich end leucine rich repeat protein [Source:HGNC Symbol;Acc:HGNC:9357] |
| ENSG00000197992 | HGLibA_10009 | 404 | 2.519625043 | CLEC9A | C-type lectin domain containing 9A [Source:HGNC Symbol;Acc:HGNC:26705] |
| H2AFB2 | HGLibA_20749 | 404 | 2.519625043 | H2AFB2 | - |
| ENSG00000075711 | HGLibA_13324 | 403 | 2.510579701 | DLG1 | discs large MAGUK scaffold protein 1 [Source:HGNC Symbol;Acc:HGNC:2900] |
| ENSG00000089123 | HGLibA_48434 | 403 | 2.510579701 | TASP1 | taspase 1 [Source:HGNC Symbol;Acc:HGNC:15859] |
| ENSG00000100154 | HGLibA_52096 | 403 | 2.510579701 | TTC28 | tetratricopeptide repeat domain 28 [Source:HGNC Symbol;Acc:HGNC:29179] |
| ENSG00000100418 | HGLibA_13008 | 403 | 2.510579701 | DESI1 | desumoylating isopeptidase 1 [Source:HGNC Symbol;Acc:HGNC:24577] |
| ENSG00000104447 | HGLibA_51775 | 403 | 2.510579701 | TRPS1 | transcriptional repressor GATA binding 1 [Source:HGNC Symbol;Acc:HGNC:12340] |
| ENSG00000138449 | HGLibA_45136 | 403 | 2.510579701 | SLC40A1 | solute carrier family 40 member 1 [Source:HGNC Symbol;Acc:HGNC:10909] |
| ENSG00000140009 | HGLibA_15584 | 403 | 2.510579701 | ESR2 | estrogen receptor 2 [Source:HGNC Symbol;Acc:HGNC:3468] |
| ENSG00000150527 | HGLibA_29186 | 403 | 2.510579701 | MIA2 | MIA SH3 domain ER export factor 2 [Source:HGNC Symbol;Acc:HGNC:18432] |
| ENSG00000152495 | HGLibA_07084 | 403 | 2.510579701 | CAMK4 | calcium/calmodulin dependent protein kinase IV [Source:HGNC Symbol;Acc:HGNC:1464] |
| ENSG00000169213 | HGLibA_39910 | 403 | 2.510579701 | RAB3B | member RAS oncogene family [Source:HGNC Symbol;Acc:HGNC:9778]" |
| ENSG00000173237 | HGLibA_05201 | 403 | 2.510579701 | C11orf86 | chromosome 11 open reading frame 86 [Source:HGNC Symbol;Acc:HGNC:34442] |
| ENSG00000278259 | HGLibA_30818 | 403 | 2.510579701 | MYO19 | myosin XIX [Source:HGNC Symbol;Acc:HGNC:26234] |
| FAM65B | HGLibA_16671 | 403 | 2.510579701 | FAM65B | - |
| ENSG00000076641 | HGLibA_35002 | 402 | 2.501534358 | PAG1 | phosphoprotein membrane anchor with glycosphingolipid microdomains 1 [Source:HGNC Symbol;Acc:HGNC:30043] |
| ENSG00000117009 | HGLibA_25318 | 402 | 2.501534358 | KMO | kynurenine 3-monooxygenase [Source:HGNC Symbol;Acc:HGNC:6381] |
| ENSG00000135404 | HGLibA_08425 | 402 | 2.501534358 | CD63 | CD63 molecule [Source:HGNC Symbol;Acc:HGNC:1692] |
| ENSG00000153936 | HGLibA_22119 | 402 | 2.501534358 | HS2ST1 | heparan sulfate 2-O-sulfotransferase 1 [Source:HGNC Symbol;Acc:HGNC:5193] |
| ENSG00000263002 | HGLibA_55644 | 402 | 2.501534358 | ZNF234 | zinc finger protein 234 [Source:HGNC Symbol;Acc:HGNC:13027] |
| hsa-mir-431 | HGLibA_59952 | 402 | 2.501534358 | hsa-mir-431 | - |
| PRAC | HGLibA_38259 | 402 | 2.501534358 | PRAC | - |
| C20orf166 | HGLibA_05993 | 401 | 2.492489015 | C20orf166 | - |
| ENSG00000070540 | HGLibA_54313 | 401 | 2.492489015 | WIPI1 | phosphoinositide interacting 1 [Source:HGNC Symbol;Acc:HGNC:25471]" |
| ENSG00000075426 | HGLibA_17803 | 401 | 2.492489015 | FOSL2 | AP-1 transcription factor subunit [Source:HGNC Symbol;Acc:HGNC:3798]" |
| ENSG00000101493 | HGLibA_56144 | 401 | 2.492489015 | ZNF516 | zinc finger protein 516 [Source:HGNC Symbol;Acc:HGNC:28990] |
| ENSG00000144481 | HGLibA_51772 | 401 | 2.492489015 | TRPM8 | transient receptor potential cation channel subfamily M member 8 [Source:HGNC Symbol;Acc:HGNC:17961] |
| ENSG00000149452 | HGLibA_44601 | 401 | 2.492489015 | SLC22A8 | solute carrier family 22 member 8 [Source:HGNC Symbol;Acc:HGNC:10972] |
| ENSG00000149488 | HGLibA_49751 | 401 | 2.492489015 | TMC2 | transmembrane channel like 2 [Source:HGNC Symbol;Acc:HGNC:16527] |
| ENSG00000261115 | HGLibA_50084 | 401 | 2.492489015 | TMEM178B | transmembrane protein 178B [Source:HGNC Symbol;Acc:HGNC:44112] |
| hsa-mir-532 | HGLibA_61807 | 401 | 2.492489015 | hsa-mir-532 | - |
| ENSG00000115507 | HGLibA_34782 | 400 | 2.483443672 | OTX1 | orthodenticle homeobox 1 [Source:HGNC Symbol;Acc:HGNC:8521] |
| ENSG00000124145 | HGLibA_43107 | 400 | 2.483443672 | SDC4 | syndecan 4 [Source:HGNC Symbol;Acc:HGNC:10661] |
| ENSG00000131849 | HGLibA_55461 | 400 | 2.483443672 | ZNF132 | zinc finger protein 132 [Source:HGNC Symbol;Acc:HGNC:12916] |
| ENSG00000169214 | HGLibA_34415 | 400 | 2.483443672 | OR6F1 | olfactory receptor family 6 subfamily F member 1 [Source:HGNC Symbol;Acc:HGNC:15027] |
| ENSG00000171757 | HGLibA_27319 | 400 | 2.483443672 | LRRC34 | leucine rich repeat containing 34 [Source:HGNC Symbol;Acc:HGNC:28408] |
| ENSG00000175785 | HGLibA_38487 | 400 | 2.483443672 | PRIMA1 | proline rich membrane anchor 1 [Source:HGNC Symbol;Acc:HGNC:18319] |
| ENSG00000176204 | HGLibA_27494 | 400 | 2.483443672 | LRRTM4 | leucine rich repeat transmembrane neuronal 4 [Source:HGNC Symbol;Acc:HGNC:19411] |
| ENSG00000185561 | HGLibA_49599 | 400 | 2.483443672 | TLCD2 | TLC domain containing 2 [Source:HGNC Symbol;Acc:HGNC:33522] |
| ENSG00000204176 | HGLibA_48062 | 400 | 2.483443672 | SYT15 | synaptotagmin 15 [Source:HGNC Symbol;Acc:HGNC:17167] |
| ENSG00000214435 | HGLibA_03182 | 400 | 2.483443672 | AS3MT | arsenite methyltransferase [Source:HGNC Symbol;Acc:HGNC:17452] |
| ENSG00000233013 | HGLibA_16196 | 400 | 2.483443672 | FAM157B | family with sequence similarity 157 member B [Source:HGNC Symbol;Acc:HGNC:34080] |
| hsa-mir-125b-1 | HGLibA_57448 | 400 | 2.483443672 | hsa-mir-125b-1 | - |
| hsa-mir-1468 | HGLibA_57847 | 400 | 2.483443672 | hsa-mir-1468 | - |
| ENSG00000072858 | HGLibA_44113 | 399 | 2.474398329 | SIDT1 | SID1 transmembrane family member 1 [Source:HGNC Symbol;Acc:HGNC:25967] |
| ENSG00000120896 | HGLibA_46179 | 399 | 2.474398329 | SORBS3 | sorbin and SH3 domain containing 3 [Source:HGNC Symbol;Acc:HGNC:30907] |
| ENSG00000134779 | HGLibA_51127 | 399 | 2.474398329 | TPGS2 | tubulin polyglutamylase complex subunit 2 [Source:HGNC Symbol;Acc:HGNC:24561] |
| ENSG00000166682 | HGLibA_50646 | 399 | 2.474398329 | TMPRSS5 | transmembrane serine protease 5 [Source:HGNC Symbol;Acc:HGNC:14908] |
| ENSG00000180386 | HGLibA_25845 | 399 | 2.474398329 | KRTAP9-7 | keratin associated protein 9-7 [Source:HGNC Symbol;Acc:HGNC:18915] |
| ENSG00000180902 | HGLibA_12256 | 399 | 2.474398329 | D2HGDH | D-2-hydroxyglutarate dehydrogenase [Source:HGNC Symbol;Acc:HGNC:28358] |
| ENSG00000182752 | HGLibA_35145 | 399 | 2.474398329 | PAPPA | pappalysin 1 [Source:HGNC Symbol;Acc:HGNC:8602] |
| hsa-mir-4529 | HGLibA_60577 | 399 | 2.474398329 | hsa-mir-4529 | - |
| hsa-mir-492 | HGLibA_61368 | 399 | 2.474398329 | hsa-mir-492 | - |
| PGBD3 | HGLibA_36222 | 399 | 2.474398329 | PGBD3 | - |
| ENSG00000100368 | HGLibA_11337 | 398 | 2.465352986 | CSF2RB | colony stimulating factor 2 receptor beta common subunit [Source:HGNC Symbol;Acc:HGNC:2436] |
| ENSG00000112599 | HGLibA_20636 | 398 | 2.465352986 | GUCA1B | guanylate cyclase activator 1B [Source:HGNC Symbol;Acc:HGNC:4679] |
| ENSG00000115507 | HGLibA_34784 | 398 | 2.465352986 | OTX1 | orthodenticle homeobox 1 [Source:HGNC Symbol;Acc:HGNC:8521] |
| ENSG00000124391 | HGLibA_22995 | 398 | 2.465352986 | IL17C | interleukin 17C [Source:HGNC Symbol;Acc:HGNC:5983] |
| ENSG00000125753 | HGLibA_53538 | 398 | 2.465352986 | VASP | vasodilator stimulated phosphoprotein [Source:HGNC Symbol;Acc:HGNC:12652] |
| ENSG00000130383 | HGLibA_18197 | 398 | 2.465352986 | FUT5 | fucosyltransferase 5 [Source:HGNC Symbol;Acc:HGNC:4016] |
| ENSG00000132274 | HGLibA_51433 | 398 | 2.465352986 | TRIM22 | tripartite motif containing 22 [Source:HGNC Symbol;Acc:HGNC:16379] |
| ENSG00000137204 | HGLibA_44597 | 398 | 2.465352986 | SLC22A7 | solute carrier family 22 member 7 [Source:HGNC Symbol;Acc:HGNC:10971] |
| ENSG00000142494 | HGLibA_45193 | 398 | 2.465352986 | SLC47A1 | solute carrier family 47 member 1 [Source:HGNC Symbol;Acc:HGNC:25588] |
| ENSG00000143344 | HGLibA_41001 | 398 | 2.465352986 | RGL1 | ral guanine nucleotide dissociation stimulator like 1 [Source:HGNC Symbol;Acc:HGNC:30281] |
| ENSG00000151176 | HGLibA_36974 | 398 | 2.465352986 | PLBD2 | phospholipase B domain containing 2 [Source:HGNC Symbol;Acc:HGNC:27283] |
| ENSG00000154545 | HGLibA_27975 | 398 | 2.465352986 | MAGED4 | MAGE family member D4 [Source:HGNC Symbol;Acc:HGNC:23793] |
| ENSG00000196335 | HGLibA_47479 | 398 | 2.465352986 | STK31 | serine/threonine kinase 31 [Source:HGNC Symbol;Acc:HGNC:11407] |
| ENSG00000237038 | HGLibA_53287 | 398 | 2.465352986 | USP17L8 | ubiquitin specific peptidase 17-like family member 8 [Source:HGNC Symbol;Acc:HGNC:37181] |
| ENSG00000288642 | HGLibA_08864 | 398 | 2.465352986 | CDR1 | - |
| FAM208B | HGLibA_16435 | 398 | 2.465352986 | FAM208B | - |
| hsa-mir-6836 | HGLibA_63521 | 398 | 2.465352986 | hsa-mir-6836 | - |
| LOC100130880 | HGLibA_26799 | 398 | 2.465352986 | LOC100130880 | - |
| ENSG00000095383 | HGLibA_48493 | 397 | 2.456307643 | TBC1D2 | TBC1 domain family member 2 [Source:HGNC Symbol;Acc:HGNC:18026] |
| ENSG00000152056 | HGLibA_02303 | 397 | 2.456307643 | AP1S3 | adaptor related protein complex 1 subunit sigma 3 [Source:HGNC Symbol;Acc:HGNC:18971] |
| ENSG00000158022 | HGLibA_51574 | 397 | 2.456307643 | TRIM63 | tripartite motif containing 63 [Source:HGNC Symbol;Acc:HGNC:16007] |
| ENSG00000181433 | HGLibA_42645 | 397 | 2.456307643 | SAGE1 | sarcoma antigen 1 [Source:HGNC Symbol;Acc:HGNC:30369] |
| FAM46A | HGLibA_16595 | 397 | 2.456307643 | FAM46A | - |
| hsa-mir-1538 | HGLibA_57918 | 397 | 2.456307643 | hsa-mir-1538 | - |
| ENSG00000004809 | HGLibA_44556 | 396 | 2.4472623 | SLC22A16 | solute carrier family 22 member 16 [Source:HGNC Symbol;Acc:HGNC:20302] |
| ENSG00000078070 | HGLibA_28565 | 396 | 2.4472623 | MCCC1 | methylcrotonoyl-CoA carboxylase 1 [Source:HGNC Symbol;Acc:HGNC:6936] |
| ENSG00000102053 | HGLibA_54955 | 396 | 2.4472623 | ZC3H12B | zinc finger CCCH-type containing 12B [Source:HGNC Symbol;Acc:HGNC:17407] |
| ENSG00000110244 | HGLibA_02459 | 396 | 2.4472623 | APOA4 | apolipoprotein A4 [Source:HGNC Symbol;Acc:HGNC:602] |
| ENSG00000134531 | HGLibA_15073 | 396 | 2.4472623 | EMP1 | epithelial membrane protein 1 [Source:HGNC Symbol;Acc:HGNC:3333] |
| ENSG00000144045 | HGLibA_13937 | 396 | 2.4472623 | DQX1 | DEAQ-box RNA dependent ATPase 1 [Source:HGNC Symbol;Acc:HGNC:20410] |
| ENSG00000182749 | HGLibA_35170 | 396 | 2.4472623 | PAQR7 | progestin and adipoQ receptor family member 7 [Source:HGNC Symbol;Acc:HGNC:23146] |
| ENSG00000187323 | HGLibA_12474 | 396 | 2.4472623 | DCC | DCC netrin 1 receptor [Source:HGNC Symbol;Acc:HGNC:2701] |
| ENSG00000198874 | HGLibA_52479 | 396 | 2.4472623 | TYW1 | tRNA-yW synthesizing protein 1 homolog [Source:HGNC Symbol;Acc:HGNC:25598] |
| ENSG00000225190 | HGLibA_37136 | 396 | 2.4472623 | PLEKHM1 | pleckstrin homology and RUN domain containing M1 [Source:HGNC Symbol;Acc:HGNC:29017] |
| ENSG00000234602 | HGLibA_28594 | 396 | 2.4472623 | MCIDAS | multiciliate differentiation and DNA synthesis associated cell cycle protein [Source:HGNC Symbol;Acc:HGNC:40050] |
| ENSG00000242173 | HGLibA_02800 | 396 | 2.4472623 | ARHGDIG | Rho GDP dissociation inhibitor gamma [Source:HGNC Symbol;Acc:HGNC:680] |
| ENSG00000278570 | HGLibA_32606 | 396 | 2.4472623 | NR2E3 | nuclear receptor subfamily 2 group E member 3 [Source:HGNC Symbol;Acc:HGNC:7974] |
| hsa-mir-372 | HGLibA_59383 | 396 | 2.4472623 | hsa-mir-372 | - |
| hsa-mir-6128 | HGLibA_62659 | 396 | 2.4472623 | hsa-mir-6128 | - |
| TLDC1 | HGLibA_49600 | 396 | 2.4472623 | TLDC1 | - |
| ENSG00000099769 | HGLibA_22811 | 395 | 2.438216957 | IGFALS | insulin like growth factor binding protein acid labile subunit [Source:HGNC Symbol;Acc:HGNC:5468] |
| ENSG00000100568 | HGLibA_53890 | 395 | 2.438216957 | VTI1B | vesicle transport through interaction with t-SNAREs 1B [Source:HGNC Symbol;Acc:HGNC:17793] |
| ENSG00000102226 | HGLibA_53198 | 395 | 2.438216957 | USP11 | ubiquitin specific peptidase 11 [Source:HGNC Symbol;Acc:HGNC:12609] |
| ENSG00000105204 | HGLibA_14275 | 395 | 2.438216957 | DYRK1B | dual specificity tyrosine phosphorylation regulated kinase 1B [Source:HGNC Symbol;Acc:HGNC:3092] |
| ENSG00000121316 | HGLibA_36973 | 395 | 2.438216957 | PLBD1 | phospholipase B domain containing 1 [Source:HGNC Symbol;Acc:HGNC:26215] |
| ENSG00000130429 | HGLibA_03076 | 395 | 2.438216957 | ARPC1B | actin related protein 2/3 complex subunit 1B [Source:HGNC Symbol;Acc:HGNC:704] |
| ENSG00000135446 | HGLibA_08771 | 395 | 2.438216957 | CDK4 | cyclin dependent kinase 4 [Source:HGNC Symbol;Acc:HGNC:1773] |
| ENSG00000140612 | HGLibA_43181 | 395 | 2.438216957 | SEC11A | signal peptidase complex subunit [Source:HGNC Symbol;Acc:HGNC:17718]" |
| ENSG00000153162 | HGLibA_04615 | 395 | 2.438216957 | BMP6 | bone morphogenetic protein 6 [Source:HGNC Symbol;Acc:HGNC:1073] |
| ENSG00000156535 | HGLibA_08231 | 395 | 2.438216957 | CD109 | CD109 molecule [Source:HGNC Symbol;Acc:HGNC:21685] |
| ENSG00000158014 | HGLibA_44902 | 395 | 2.438216957 | SLC30A2 | solute carrier family 30 member 2 [Source:HGNC Symbol;Acc:HGNC:11013] |
| ENSG00000162627 | HGLibA_46105 | 395 | 2.438216957 | SNX7 | sorting nexin 7 [Source:HGNC Symbol;Acc:HGNC:14971] |
| ENSG00000179588 | HGLibA_55251 | 395 | 2.438216957 | ZFPM1 | FOG family member 1 [Source:HGNC Symbol;Acc:HGNC:19762]" |

| | | | | | |
|---|---|---|---|---|---|
| ENSG00000180739 | HGLibA_42606 | 395 | 2.438216957 | S1PR5 | sphingosine-1-phosphate receptor 5 [Source:HGNC Symbol;Acc:HGNC:14299] |
| ENSG00000257315 | HGLibA_54801 | 395 | 2.438216957 | ZBED6 | zinc finger BED-type containing 6 [Source:HGNC Symbol;Acc:HGNC:33273] |
| hsa-mir-3192 | HGLibA_58864 | 395 | 2.438216957 | hsa-mir-3192 | - |
| DEFB131 | HGLibA_12897 | 394 | 2.429171614 | DEFB131 | - |
| ENSG00000003056 | HGLibA_27815 | 394 | 2.429171614 | M6PR | cation dependent [Source:HGNC Symbol;Acc:HGNC:6752]" |
| ENSG00000099998 | HGLibA_19013 | 394 | 2.429171614 | GGT5 | gamma-glutamyltransferase 5 [Source:HGNC Symbol;Acc:HGNC:4260] |
| ENSG00000136933 | HGLibA_40005 | 394 | 2.429171614 | RABEPK | Rab9 effector protein with kelch motifs [Source:HGNC Symbol;Acc:HGNC:16896] |
| ENSG00000147394 | HGLibA_55550 | 394 | 2.429171614 | ZNF185 | zinc finger protein 185 with LIM domain [Source:HGNC Symbol;Acc:HGNC:12976] |
| ENSG00000151006 | HGLibA_38957 | 394 | 2.429171614 | PRSS53 | serine protease 53 [Source:HGNC Symbol;Acc:HGNC:34407] |
| ENSG00000173699 | HGLibA_46464 | 394 | 2.429171614 | SPATA3 | spermatogenesis associated 3 [Source:HGNC Symbol;Acc:HGNC:17884] |
| ENSG00000175048 | HGLibA_55079 | 394 | 2.429171614 | ZDHHC14 | zinc finger DHHC-type containing 14 [Source:HGNC Symbol;Acc:HGNC:20341] |
| hsa-mir-409 | HGLibA_59674 | 394 | 2.429171614 | hsa-mir-409 | - |
| hsa-mir-4293 | HGLibA_59885 | 394 | 2.429171614 | hsa-mir-4293 | - |
| C15orf56 | HGLibA_05418 | 393 | 2.420126271 | C15orf56 | - |
| ENSG00000068079 | HGLibA_22609 | 393 | 2.420126271 | IFI35 | interferon induced protein 35 [Source:HGNC Symbol;Acc:HGNC:5399] |
| ENSG00000089335 | HGLibA_55775 | 393 | 2.420126271 | ZNF302 | zinc finger protein 302 [Source:HGNC Symbol;Acc:HGNC:13848] |
| ENSG00000103507 | HGLibA_04285 | 393 | 2.420126271 | BCKDK | branched chain ketoacid dehydrogenase kinase [Source:HGNC Symbol;Acc:HGNC:16902] |
| ENSG00000116031 | HGLibA_08292 | 393 | 2.420126271 | CD207 | CD207 molecule [Source:HGNC Symbol;Acc:HGNC:17935] |
| ENSG00000121621 | HGLibA_24875 | 393 | 2.420126271 | KIF18A | kinesin family member 18A [Source:HGNC Symbol;Acc:HGNC:29441] |
| ENSG00000125457 | HGLibA_29263 | 393 | 2.420126271 | MIF4GD | MIF4G domain containing [Source:HGNC Symbol;Acc:HGNC:24030] |
| ENSG00000125903 | HGLibA_12892 | 393 | 2.420126271 | DEFB129 | defensin beta 129 [Source:HGNC Symbol;Acc:HGNC:16218] |
| ENSG00000140497 | HGLibA_42839 | 393 | 2.420126271 | SCAMP2 | secretory carrier membrane protein 2 [Source:HGNC Symbol;Acc:HGNC:10564] |
| ENSG00000146809 | HGLibA_03221 | 393 | 2.420126271 | ASB15 | ankyrin repeat and SOCS box containing 15 [Source:HGNC Symbol;Acc:HGNC:19767] |
| ENSG00000146872 | HGLibA_49626 | 393 | 2.420126271 | TLK2 | tousled like kinase 2 [Source:HGNC Symbol;Acc:HGNC:11842] |
| ENSG00000156787 | HGLibA_48535 | 393 | 2.420126271 | TBC1D31 | TBC1 domain family member 31 [Source:HGNC Symbol;Acc:HGNC:30888] |
| ENSG00000162391 | HGLibA_16162 | 393 | 2.420126271 | FAM151A | family with sequence similarity 151 member A [Source:HGNC Symbol;Acc:HGNC:25032] |
| ENSG00000168491 | HGLibA_07539 | 393 | 2.420126271 | CCDC110 | coiled-coil domain containing 110 [Source:HGNC Symbol;Acc:HGNC:28504] |
| ENSG00000172464 | HGLibA_34243 | 393 | 2.420126271 | OR5AP2 | olfactory receptor family 5 subfamily AP member 2 [Source:HGNC Symbol;Acc:HGNC:15258] |
| ENSG00000183208 | HGLibA_18905 | 393 | 2.420126271 | GDPGP1 | GDP-D-glucose phosphorylase 1 [Source:HGNC Symbol;Acc:HGNC:34360] |
| ENSG00000188313 | HGLibA_37231 | 393 | 2.420126271 | PLSCR1 | phospholipid scramblase 1 [Source:HGNC Symbol;Acc:HGNC:9092] |
| hsa-mir-491 | HGLibA_61365 | 393 | 2.420126271 | hsa-mir-491 | - |
| hsa-mir-6852 | HGLibA_63581 | 393 | 2.420126271 | hsa-mir-6852 | - |
| hsa-mir-92b | HGLibA_64299 | 393 | 2.420126271 | hsa-mir-92b | - |
| ENSG00000105607 | HGLibA_18770 | 392 | 2.411080929 | GCDH | glutaryl-CoA dehydrogenase [Source:HGNC Symbol;Acc:HGNC:4189] |
| ENSG00000107104 | HGLibA_24023 | 392 | 2.411080929 | KANK1 | KN motif and ankyrin repeat domains 1 [Source:HGNC Symbol;Acc:HGNC:19309] |
| ENSG00000108798 | HGLibA_00296 | 392 | 2.411080929 | ABI3 | ABI family member 3 [Source:HGNC Symbol;Acc:HGNC:29859] |
| ENSG00000116514 | HGLibA_41603 | 392 | 2.411080929 | RNF19B | ring finger protein 19B [Source:HGNC Symbol;Acc:HGNC:26886] |
| ENSG00000118620 | HGLibA_55977 | 392 | 2.411080929 | ZNF430 | zinc finger protein 430 [Source:HGNC Symbol;Acc:HGNC:20808] |
| ENSG00000169884 | HGLibA_54350 | 392 | 2.411080929 | WNT10B | Wnt family member 10B [Source:HGNC Symbol;Acc:HGNC:12775] |
| ENSG00000183260 | HGLibA_00260 | 392 | 2.411080929 | ABHD16B | abhydrolase domain containing 16B [Source:HGNC Symbol;Acc:HGNC:16128] |
| ENSG00000187097 | HGLibA_15210 | 392 | 2.411080929 | ENTPD5 | ectonucleoside triphosphate diphosphohydrolase 5 [Source:HGNC Symbol;Acc:HGNC:3367] |
| ENSG00000189430 | HGLibA_31361 | 392 | 2.411080929 | NCR1 | natural cytotoxicity triggering receptor 1 [Source:HGNC Symbol;Acc:HGNC:6731] |
| ENSG00000196357 | HGLibA_56257 | 392 | 2.411080929 | ZNF565 | zinc finger protein 565 [Source:HGNC Symbol;Acc:HGNC:26726] |
| ENSG00000197921 | HGLibA_21190 | 392 | 2.411080929 | HES5 | hes family bHLH transcription factor 5 [Source:HGNC Symbol;Acc:HGNC:19764] |
| ENSG00000198952 | HGLibA_45700 | 392 | 2.411080929 | SMG5 | nonsense mediated mRNA decay factor [Source:HGNC Symbol;Acc:HGNC:24644]" |
| hsa-mir-1307 | HGLibA_57745 | 392 | 2.411080929 | hsa-mir-1307 | - |
| hsa-mir-7157 | HGLibA_63857 | 392 | 2.411080929 | hsa-mir-7157 | - |
| C7orf73 | HGLibA_06591 | 391 | 2.402035586 | C7orf73 | - |
| ENSG00000018408 | HGLibA_54458 | 391 | 2.402035586 | WWTR1 | WW domain containing transcription regulator 1 [Source:HGNC Symbol;Acc:HGNC:24042] |
| ENSG00000122122 | HGLibA_42764 | 391 | 2.402035586 | SASH3 | SAM and SH3 domain containing 3 [Source:HGNC Symbol;Acc:HGNC:15975] |
| ENSG00000122254 | HGLibA_22124 | 391 | 2.402035586 | HS3ST2 | heparan sulfate-glucosamine 3-sulfotransferase 2 [Source:HGNC Symbol;Acc:HGNC:5195] |
| ENSG00000132434 | HGLibA_26007 | 391 | 2.402035586 | LANCL2 | LanC like 2 [Source:HGNC Symbol;Acc:HGNC:6509] |
| ENSG00000141279 | HGLibA_32434 | 391 | 2.402035586 | NPEPPS | aminopeptidase puromycin sensitive [Source:HGNC Symbol;Acc:HGNC:7900] |
| ENSG00000144410 | HGLibA_10978 | 391 | 2.402035586 | CPO | carboxypeptidase O [Source:HGNC Symbol;Acc:HGNC:21011] |
| ENSG00000150455 | HGLibA_49565 | 391 | 2.402035586 | TIRAP | TIR domain containing adaptor protein [Source:HGNC Symbol;Acc:HGNC:17192] |
| ENSG00000156265 | HGLibA_28200 | 391 | 2.402035586 | MAP3K7CL | MAP3K7 C-terminal like [Source:HGNC Symbol;Acc:HGNC:16457] |
| ENSG00000165948 | HGLibA_22598 | 391 | 2.402035586 | IFI27L1 | interferon alpha inducible protein 27 like 1 [Source:HGNC Symbol;Acc:HGNC:19754] |
| ENSG00000183559 | HGLibA_05046 | 391 | 2.402035586 | C10orf120 | chromosome 10 open reading frame 120 [Source:HGNC Symbol;Acc:HGNC:25707] |
| ENSG00000197614 | HGLibA_29054 | 391 | 2.402035586 | MFAP5 | microfibril associated protein 5 [Source:HGNC Symbol;Acc:HGNC:29673] |
| hsa-mir-7-2 | HGLibA_63774 | 391 | 2.402035586 | hsa-mir-7-2 | - |
| LOC100130357 | HGLibA_26781 | 391 | 2.402035586 | LOC100130357 | - |
| ENSG00000043462 | HGLibA_26203 | 390 | 2.392990243 | LCP2 | lymphocyte cytosolic protein 2 [Source:HGNC Symbol;Acc:HGNC:6529] |
| ENSG00000081377 | HGLibA_08512 | 390 | 2.392990243 | CDC14B | cell division cycle 14B [Source:HGNC Symbol;Acc:HGNC:1719] |
| ENSG00000105576 | HGLibA_50900 | 390 | 2.392990243 | TNPO2 | transportin 2 [Source:HGNC Symbol;Acc:HGNC:19998] |
| ENSG00000153814 | HGLibA_23941 | 390 | 2.392990243 | JAZF1 | JAZF zinc finger 1 [Source:HGNC Symbol;Acc:HGNC:28917] |
| ENSG00000160746 | HGLibA_02175 | 390 | 2.392990243 | ANO10 | anoctamin 10 [Source:HGNC Symbol;Acc:HGNC:25519] |
| ENSG00000161714 | HGLibA_36992 | 390 | 2.392990243 | PLCD3 | phospholipase C delta 3 [Source:HGNC Symbol;Acc:HGNC:9061] |
| ENSG00000166546 | HGLibA_04393 | 390 | 2.392990243 | BEAN1 | brain expressed associated with NEDD4 1 [Source:HGNC Symbol;Acc:HGNC:24160] |
| ENSG00000189157 | HGLibA_16617 | 390 | 2.392990243 | FAM47E | family with sequence similarity 47 member E [Source:HGNC Symbol;Acc:HGNC:34343] |
| ENSG00000212126 | HGLibA_48419 | 390 | 2.392990243 | TAS2R50 | taste 2 receptor member 50 [Source:HGNC Symbol;Acc:HGNC:18882] |
| ENSG00000216490 | HGLibA_22606 | 390 | 2.392990243 | IFI30 | lysosomal thiol reductase [Source:HGNC Symbol;Acc:HGNC:5398]" |
| PQLC1 | HGLibA_38252 | 390 | 2.392990243 | PQLC1 | - |
| TARSL2 | HGLibA_48350 | 390 | 2.392990243 | TARSL2 | - |
| TMEM110 | HGLibA_49873 | 390 | 2.392990243 | TMEM110 | - |
| ENSG00000005206 | HGLibA_46779 | 389 | 2.3839449 | SPPL2B | signal peptide peptidase like 2B [Source:HGNC Symbol;Acc:HGNC:30627] |
| ENSG00000090971 | HGLibA_31171 | 389 | 2.3839449 | NAT14 | N-acetyltransferase 14 (putative) [Source:HGNC Symbol;Acc:HGNC:28918] |
| ENSG00000105270 | HGLibA_10049 | 389 | 2.3839449 | CLIP3 | CAP-Gly domain containing linker protein 3 [Source:HGNC Symbol;Acc:HGNC:24314] |
| ENSG00000123983 | HGLibA_00556 | 389 | 2.3839449 | ACSL3 | acyl-CoA synthetase long chain family member 3 [Source:HGNC Symbol;Acc:HGNC:3570] |
| ENSG00000159335 | HGLibA_39394 | 389 | 2.3839449 | PTMS | parathymosin [Source:HGNC Symbol;Acc:HGNC:9629] |
| ENSG00000166923 | HGLibA_20241 | 389 | 2.3839449 | GREM1 | DAN family BMP antagonist [Source:HGNC Symbol;Acc:HGNC:2001]" |
| ENSG00000171989 | HGLibA_26227 | 389 | 2.3839449 | LDHAL6B | lactate dehydrogenase A like 6B [Source:HGNC Symbol;Acc:HGNC:21481] |
| ENSG00000173404 | HGLibA_23393 | 389 | 2.3839449 | INSM1 | INSM transcriptional repressor 1 [Source:HGNC Symbol;Acc:HGNC:6090] |
| ENSG00000175324 | HGLibA_27518 | 389 | 2.3839449 | LSM1 | mRNA degradation associated [Source:HGNC Symbol;Acc:HGNC:20472]" |
| ENSG00000176788 | HGLibA_04169 | 389 | 2.3839449 | BASP1 | brain abundant membrane attached signal protein 1 [Source:HGNC Symbol;Acc:HGNC:957] |
| ENSG00000176809 | HGLibA_27331 | 389 | 2.3839449 | LRRC37A3 | leucine rich repeat containing 37 member A3 [Source:HGNC Symbol;Acc:HGNC:32427] |
| ENSG00000182636 | HGLibA_31398 | 389 | 2.3839449 | NDN | MAGE family member [Source:HGNC Symbol;Acc:HGNC:7675]" |
| ENSG00000184508 | HGLibA_21037 | 389 | 2.3839449 | HDDC3 | HD domain containing 3 [Source:HGNC Symbol;Acc:HGNC:30522] |
| ENSG00000197706 | HGLibA_34406 | 389 | 2.3839449 | OR6C74 | olfactory receptor family 6 subfamily C member 74 [Source:HGNC Symbol;Acc:HGNC:31303] |
| ENSG00000203737 | HGLibA_20051 | 389 | 2.3839449 | GPR52 | G protein-coupled receptor 52 [Source:HGNC Symbol;Acc:HGNC:4508] |
| hsa-mir-4796 | HGLibA_61289 | 389 | 2.3839449 | hsa-mir-4796 | - |
| CXorf48 | HGLibA_11929 | 388 | 2.374899557 | CXorf48 | - |
| ENSG00000005421 | HGLibA_37679 | 388 | 2.374899557 | PON1 | paraoxonase 1 [Source:HGNC Symbol;Acc:HGNC:9204] |
| ENSG00000087074 | HGLibA_38048 | 388 | 2.374899557 | PPP1R15A | protein phosphatase 1 regulatory subunit 15A [Source:HGNC Symbol;Acc:HGNC:14375] |
| ENSG00000108839 | HGLibA_01677 | 388 | 2.374899557 | ALOX12 | 12S type [Source:HGNC Symbol;Acc:HGNC:429]" |
| ENSG00000136003 | HGLibA_23651 | 388 | 2.374899557 | ISCU | iron-sulfur cluster assembly enzyme [Source:HGNC Symbol;Acc:HGNC:29882] |
| ENSG00000141179 | HGLibA_35690 | 388 | 2.374899557 | PCTP | phosphatidylcholine transfer protein [Source:HGNC Symbol;Acc:HGNC:8752] |
| ENSG00000147804 | HGLibA_45111 | 388 | 2.374899557 | SLC39A4 | solute carrier family 39 member 4 [Source:HGNC Symbol;Acc:HGNC:17129] |
| ENSG00000150667 | HGLibA_18091 | 388 | 2.374899557 | FSIP1 | fibrous sheath interacting protein 1 [Source:HGNC Symbol;Acc:HGNC:21674] |
| ENSG00000167110 | HGLibA_19595 | 388 | 2.374899557 | GOLGA2 | golgin A2 [Source:HGNC Symbol;Acc:HGNC:4425] |
| ENSG00000176845 | HGLibA_28941 | 388 | 2.374899557 | METRNL | glial cell differentiation regulator [Source:HGNC Symbol;Acc:HGNC:27584]" |
| ENSG00000188729 | HGLibA_34718 | 388 | 2.374899557 | OSTN | osteocrin [Source:HGNC Symbol;Acc:HGNC:29961] |
| ENSG00000188833 | HGLibA_15219 | 388 | 2.374899557 | ENTPD8 | ectonucleoside triphosphate diphosphohydrolase 8 [Source:HGNC Symbol;Acc:HGNC:24860] |
| hsa-mir-10b | HGLibA_57201 | 388 | 2.374899557 | hsa-mir-10b | - |
| AGPAT9 | HGLibA_01234 | 387 | 2.365854214 | AGPAT9 | - |
| ENSG00000009724 | HGLibA_28397 | 387 | 2.365854214 | MASP2 | mannan binding lectin serine peptidase 2 [Source:HGNC Symbol;Acc:HGNC:6902] |
| ENSG00000109743 | HGLibA_04880 | 387 | 2.365854214 | BST1 | bone marrow stromal cell antigen 1 [Source:HGNC Symbol;Acc:HGNC:1118] |
| ENSG00000114554 | HGLibA_37259 | 387 | 2.365854214 | PLXNA1 | plexin A1 [Source:HGNC Symbol;Acc:HGNC:9099] |
| ENSG00000118454 | HGLibA_02005 | 387 | 2.365854214 | ANKRD13C | ankyrin repeat domain 13C [Source:HGNC Symbol;Acc:HGNC:25374] |
| ENSG00000120725 | HGLibA_44170 | 387 | 2.365854214 | SIL1 | SIL1 nucleotide exchange factor [Source:HGNC Symbol;Acc:HGNC:24624] |
| ENSG00000123104 | HGLibA_23857 | 387 | 2.365854214 | ITPR2 | 5-trisphosphate receptor type 2 [Source:HGNC Symbol;Acc:HGNC:6181]" |
| ENSG00000125378 | HGLibA_04608 | 387 | 2.365854214 | BMP4 | bone morphogenetic protein 4 [Source:HGNC Symbol;Acc:HGNC:1071] |
| ENSG00000130529 | HGLibA_51760 | 387 | 2.365854214 | TRPM4 | transient receptor potential cation channel subfamily M member 4 [Source:HGNC Symbol;Acc:HGNC:17993] |
| ENSG00000146966 | HGLibA_12939 | 387 | 2.365854214 | DENND2A | DENN domain containing 2A [Source:HGNC Symbol;Acc:HGNC:22212] |
| ENSG00000167685 | HGLibA_56015 | 387 | 2.365854214 | ZNF444 | zinc finger protein 444 [Source:HGNC Symbol;Acc:HGNC:16052] |
| ENSG00000169174 | HGLibA_35687 | 387 | 2.365854214 | PCSK9 | proprotein convertase subtilisin/kexin type 9 [Source:HGNC Symbol;Acc:HGNC:20001] |
| ENSG00000171195 | HGLibA_30556 | 387 | 2.365854214 | MUC7 | secreted [Source:HGNC Symbol;Acc:HGNC:7518]" |
| ENSG00000174567 | HGLibA_19662 | 387 | 2.365854214 | GOLT1A | golgi transport 1A [Source:HGNC Symbol;Acc:HGNC:24766] |
| ENSG00000189337 | HGLibA_24090 | 387 | 2.365854214 | KAZN | periplakin interacting protein [Source:HGNC Symbol;Acc:HGNC:29173]" |
| ENSG00000237521 | HGLibA_34479 | 387 | 2.365854214 | OR7E24 | olfactory receptor family 7 subfamily E member 24 [Source:HGNC Symbol;Acc:HGNC:8396] |
| NEURL | HGLibA_31722 | 387 | 2.365854214 | NEURL | - |
| ENSG00000011275 | HGLibA_41632 | 386 | 2.356808871 | RNF216 | ring finger protein 216 [Source:HGNC Symbol;Acc:HGNC:21698] |
| ENSG00000039537 | HGLibA_06425 | 386 | 2.356808871 | C6 | complement C6 [Source:HGNC Symbol;Acc:HGNC:1339] |
| ENSG00000100196 | HGLibA_24486 | 386 | 2.356808871 | KDELR3 | KDEL endoplasmic reticulum protein retention receptor 3 [Source:HGNC Symbol;Acc:HGNC:6306] |
| ENSG00000120289 | HGLibA_27950 | 386 | 2.356808871 | MAGEB4 | MAGE family member B4 [Source:HGNC Symbol;Acc:HGNC:6811] |
| ENSG00000171298 | HGLibA_18316 | 386 | 2.356808871 | GAA | acid [Source:HGNC Symbol;Acc:HGNC:4065]" |
| ENSG00000171346 | HGLibA_25429 | 386 | 2.356808871 | KRT15 | keratin 15 [Source:HGNC Symbol;Acc:HGNC:6421] |
| ENSG00000187605 | HGLibA_49044 | 386 | 2.356808871 | TET3 | tet methylcytosine dioxygenase 3 [Source:HGNC Symbol;Acc:HGNC:28313] |
| ENSG00000198889 | HGLibA_12422 | 386 | 2.356808871 | DCAF12L1 | DDB1 and CUL4 associated factor 12 like 1 [Source:HGNC Symbol;Acc:HGNC:29395] |
| ENSG00000109738 | HGLibA_19311 | 385 | 2.347763528 | GLRB | glycine receptor beta [Source:HGNC Symbol;Acc:HGNC:4329] |
| ENSG00000116668 | HGLibA_47903 | 385 | 2.347763528 | SWT1 | RNA endoribonuclease homolog [Source:HGNC Symbol;Acc:HGNC:16785]" |
| ENSG00000116685 | HGLibA_24838 | 385 | 2.347763528 | KIAA2013 | KIAA2013 [Source:HGNC Symbol;Acc:HGNC:28513] |
| ENSG00000132874 | HGLibA_44401 | 385 | 2.347763528 | SLC14A2 | solute carrier family 14 member 2 [Source:HGNC Symbol;Acc:HGNC:10919] |
| ENSG00000137273 | HGLibA_17866 | 385 | 2.347763528 | FOXF2 | forkhead box F2 [Source:HGNC Symbol;Acc:HGNC:3810] |
| ENSG00000138030 | HGLibA_24574 | 385 | 2.347763528 | KHK | ketohexokinase [Source:HGNC Symbol;Acc:HGNC:6315] |
| ENSG00000141293 | HGLibA_44270 | 385 | 2.347763528 | SKAP1 | src kinase associated phosphoprotein 1 [Source:HGNC Symbol;Acc:HGNC:15605] |

| | | | | | |
|---|---|---|---|---|---|
| ENSG00000152969 | HGLibA_23923 | 385 | 2.347763528 | JAKMIP1 | janus kinase and microtubule interacting protein 1 [Source:HGNC Symbol;Acc:HGNC:26460] |
| ENSG00000165553 | HGLibA_31864 | 385 | 2.347763528 | NGB | neuroglobin [Source:HGNC Symbol;Acc:HGNC:14077] |
| ENSG00000167004 | HGLibA_35878 | 385 | 2.347763528 | PDIA3 | protein disulfide isomerase family A member 3 [Source:HGNC Symbol;Acc:HGNC:4606] |
| ENSG00000167637 | HGLibA_55740 | 385 | 2.347763528 | ZNF283 | zinc finger protein 283 [Source:HGNC Symbol;Acc:HGNC:13077] |
| ENSG00000172954 | HGLibA_26157 | 385 | 2.347763528 | LCLAT1 | lysocardiolipin acyltransferase 1 [Source:HGNC Symbol;Acc:HGNC:26756] |
| ENSG00000181761 | HGLibA_34531 | 385 | 2.347763528 | OR8H3 | olfactory receptor family 8 subfamily H member 3 [Source:HGNC Symbol;Acc:HGNC:15309] |
| ENSG00000182783 | HGLibA_33869 | 385 | 2.347763528 | OR2T29 | olfactory receptor family 2 subfamily T member 29 [Source:HGNC Symbol;Acc:HGNC:31253] |
| ENSG00000196083 | HGLibA_23052 | 385 | 2.347763528 | IL1RAP | interleukin 1 receptor accessory protein [Source:HGNC Symbol;Acc:HGNC:5995] |
| ENSG00000239264 | HGLibA_52424 | 385 | 2.347763528 | TXNDC5 | thioredoxin domain containing 5 [Source:HGNC Symbol;Acc:HGNC:21073] |
| ENSG00000266967 | HGLibA_00074 | 385 | 2.347763528 | AARSD1 | alanyl-tRNA synthetase domain containing 1 [Source:HGNC Symbol;Acc:HGNC:28417] |
| hsa-mir-611 | HGLibA_62635 | 385 | 2.347763528 | hsa-mir-611 | - |
| C16orf58 | HGLibA_05462 | 384 | 2.338718185 | C16orf58 | - |
| ENSG00000082701 | HGLibA_20431 | 384 | 2.338718185 | GSK3B | glycogen synthase kinase 3 beta [Source:HGNC Symbol;Acc:HGNC:4617] |
| ENSG00000105369 | HGLibA_08448 | 384 | 2.338718185 | CD79A | CD79a molecule [Source:HGNC Symbol;Acc:HGNC:1698] |
| ENSG00000105464 | HGLibA_20307 | 384 | 2.338718185 | GRIN2D | glutamate ionotropic receptor NMDA type subunit 2D [Source:HGNC Symbol;Acc:HGNC:4588] |
| ENSG00000110848 | HGLibA_08433 | 384 | 2.338718185 | CD69 | CD69 molecule [Source:HGNC Symbol;Acc:HGNC:1694] |
| ENSG00000116815 | HGLibA_08415 | 384 | 2.338718185 | CD58 | CD58 molecule [Source:HGNC Symbol;Acc:HGNC:1688] |
| ENSG00000128311 | HGLibA_52023 | 384 | 2.338718185 | TST | thiosulfate sulfurtransferase [Source:HGNC Symbol;Acc:HGNC:12388] |
| ENSG00000130762 | HGLibA_02822 | 384 | 2.338718185 | ARHGEF16 | Rho guanine nucleotide exchange factor 16 [Source:HGNC Symbol;Acc:HGNC:15515] |
| ENSG00000134504 | HGLibA_24410 | 384 | 2.338718185 | KCTD1 | potassium channel tetramerization domain containing 1 [Source:HGNC Symbol;Acc:HGNC:18249] |
| ENSG00000154217 | HGLibA_36746 | 384 | 2.338718185 | PITPNC1 | phosphatidylinositol transfer protein cytoplasmic 1 [Source:HGNC Symbol;Acc:HGNC:21045] |
| ENSG00000163534 | HGLibA_17224 | 384 | 2.338718185 | FCRL1 | Fc receptor like 1 [Source:HGNC Symbol;Acc:HGNC:18509] |
| ENSG00000164588 | HGLibA_20972 | 384 | 2.338718185 | HCN1 | hyperpolarization activated cyclic nucleotide gated potassium channel 1 [Source:HGNC Symbol;Acc:HGNC:4845] |
| ENSG00000165917 | HGLibA_40269 | 384 | 2.338718185 | RAPSN | receptor associated protein of the synapse [Source:HGNC Symbol;Acc:HGNC:9863] |
| ENSG00000166326 | HGLibA_51506 | 384 | 2.338718185 | TRIM44 | tripartite motif containing 44 [Source:HGNC Symbol;Acc:HGNC:19016] |
| ENSG00000172845 | HGLibA_46300 | 384 | 2.338718185 | SP3 | Sp3 transcription factor [Source:HGNC Symbol;Acc:HGNC:11208] |
| ENSG00000197870 | HGLibA_38345 | 384 | 2.338718185 | PRB3 | proline rich protein BstNI subfamily 3 [Source:HGNC Symbol;Acc:HGNC:9339] |
| ENSG00000198624 | HGLibA_07845 | 384 | 2.338718185 | CCDC69 | coiled-coil domain containing 69 [Source:HGNC Symbol;Acc:HGNC:24487] |
| hsa-mir-302b | HGLibA_58440 | 384 | 2.338718185 | hsa-mir-302b | - |
| hsa-mir-3120 | HGLibA_58534 | 384 | 2.338718185 | hsa-mir-3120 | - |
| hsa-mir-4269 | HGLibA_59782 | 384 | 2.338718185 | hsa-mir-4269 | - |
| ENSG00000060656 | HGLibA_39541 | 383 | 2.329672842 | PTPRU | receptor type U [Source:HGNC Symbol;Acc:HGNC:9683]" |
| ENSG00000096696 | HGLibA_14037 | 383 | 2.329672842 | DSP | desmoplakin [Source:HGNC Symbol;Acc:HGNC:3052] |
| ENSG00000110851 | HGLibA_38388 | 383 | 2.329672842 | PRDM4 | PR/SET domain 4 [Source:HGNC Symbol;Acc:HGNC:9348] |
| ENSG00000112852 | HGLibA_35492 | 383 | 2.329672842 | PCDHB2 | protocadherin beta 2 [Source:HGNC Symbol;Acc:HGNC:8687] |
| ENSG00000117154 | HGLibA_22884 | 383 | 2.329672842 | IGSF21 | immunoglobin superfamily member 21 [Source:HGNC Symbol;Acc:HGNC:28246] |
| ENSG00000125753 | HGLibA_53539 | 383 | 2.329672842 | VASP | vasodilator stimulated phosphoprotein [Source:HGNC Symbol;Acc:HGNC:12652] |
| ENSG00000132740 | HGLibA_22856 | 383 | 2.329672842 | IGHMBP2 | immunoglobulin mu DNA binding protein 2 [Source:HGNC Symbol;Acc:HGNC:5542] |
| ENSG00000136811 | HGLibA_33278 | 383 | 2.329672842 | ODF2 | outer dense fiber of sperm tails 2 [Source:HGNC Symbol;Acc:HGNC:8114] |
| ENSG00000138363 | HGLibA_03511 | 383 | 2.329672842 | ATIC | 5-aminoimidazole-4-carboxamide ribonucleotide formyltransferase/IMP cyclohydrolase [Source:HGNC Symbol;Acc:HGNC:794] |
| ENSG00000145526 | HGLibA_08652 | 383 | 2.329672842 | CDH18 | cadherin 18 [Source:HGNC Symbol;Acc:HGNC:1757] |
| ENSG00000163781 | HGLibA_51010 | 383 | 2.329672842 | TOPBP1 | DNA topoisomerase II binding protein 1 [Source:HGNC Symbol;Acc:HGNC:17008] |
| ENSG00000197081 | HGLibA_22808 | 383 | 2.329672842 | IGF2R | insulin like growth factor 2 receptor [Source:HGNC Symbol;Acc:HGNC:5467] |
| hsa-mir-4534 | HGLibA_60597 | 383 | 2.329672842 | hsa-mir-4534 | - |
| hsa-mir-7108 | HGLibA_63793 | 383 | 2.329672842 | hsa-mir-7108 | - |
| ENSG00000102401 | HGLibA_03044 | 382 | 2.3206275 | ARMCX3 | armadillo repeat containing X-linked 3 [Source:HGNC Symbol;Acc:HGNC:24065] |
| ENSG00000105676 | HGLibA_03025 | 382 | 2.3206275 | ARMC6 | armadillo repeat containing 6 [Source:HGNC Symbol;Acc:HGNC:25049] |
| ENSG00000126890 | HGLibA_11564 | 382 | 2.3206275 | CTAG2 | cancer/testis antigen 2 [Source:HGNC Symbol;Acc:HGNC:2492] |
| ENSG00000143297 | HGLibA_17237 | 382 | 2.3206275 | FCRL5 | Fc receptor like 5 [Source:HGNC Symbol;Acc:HGNC:18508] |
| ENSG00000163746 | HGLibA_37235 | 382 | 2.3206275 | PLSCR2 | phospholipid scramblase 2 [Source:HGNC Symbol;Acc:HGNC:16494] |
| ENSG00000165060 | HGLibA_18216 | 382 | 2.3206275 | FXN | frataxin [Source:HGNC Symbol;Acc:HGNC:3951] |
| ENSG00000169710 | HGLibA_16894 | 382 | 2.3206275 | FASN | fatty acid synthase [Source:HGNC Symbol;Acc:HGNC:3594] |
| ENSG00000175066 | HGLibA_19202 | 382 | 2.3206275 | GK5 | glycerol kinase 5 [Source:HGNC Symbol;Acc:HGNC:28635] |
| ENSG00000176396 | HGLibA_14697 | 382 | 2.3206275 | EID2 | EP300 interacting inhibitor of differentiation 2 [Source:HGNC Symbol;Acc:HGNC:28292] |
| ENSG00000182255 | HGLibA_24136 | 382 | 2.3206275 | KCNA4 | potassium voltage-gated channel subfamily A member 4 [Source:HGNC Symbol;Acc:HGNC:6222] |
| ENSG00000205867 | HGLibA_25791 | 382 | 2.3206275 | KRTAP5-2 | keratin associated protein 5-2 [Source:HGNC Symbol;Acc:HGNC:23597] |
| ENSG00000276430 | HGLibA_16554 | 382 | 2.3206275 | FAM25C | family with sequence similarity 25 member C [Source:HGNC Symbol;Acc:HGNC:23586] |
| FAM26D | HGLibA_16562 | 382 | 2.3206275 | FAM26D | - |
| hsa-mir-6759 | HGLibA_63193 | 382 | 2.3206275 | hsa-mir-6759 | - |
| ENSG00000066735 | HGLibA_24916 | 381 | 2.311582157 | KIF26A | kinesin family member 26A [Source:HGNC Symbol;Acc:HGNC:20226] |
| ENSG00000070413 | HGLibA_13046 | 381 | 2.311582157 | DGCR2 | DiGeorge syndrome critical region gene 2 [Source:HGNC Symbol;Acc:HGNC:2845] |
| ENSG00000102393 | HGLibA_19215 | 381 | 2.311582157 | GLA | galactosidase alpha [Source:HGNC Symbol;Acc:HGNC:4296] |
| ENSG00000110395 | HGLibA_07395 | 381 | 2.311582157 | CBL | Cbl proto-oncogene [Source:HGNC Symbol;Acc:HGNC:1541] |
| ENSG00000120563 | HGLibA_27785 | 381 | 2.311582157 | LYZL1 | lysozyme like 1 [Source:HGNC Symbol;Acc:HGNC:30502] |
| ENSG00000120659 | HGLibA_50798 | 381 | 2.311582157 | TNFSF11 | TNF superfamily member 11 [Source:HGNC Symbol;Acc:HGNC:11926] |
| ENSG00000136950 | HGLibA_03096 | 381 | 2.311582157 | ARPC5L | actin related protein 2/3 complex subunit 5 like [Source:HGNC Symbol;Acc:HGNC:23366] |
| ENSG00000154485 | HGLibA_29514 | 381 | 2.311582157 | MMP21 | matrix metallopeptidase 21 [Source:HGNC Symbol;Acc:HGNC:14357] |
| ENSG00000154645 | HGLibA_09511 | 381 | 2.311582157 | CHODL | chondrolectin [Source:HGNC Symbol;Acc:HGNC:17807] |
| ENSG00000162747 | HGLibA_17199 | 381 | 2.311582157 | FCGR3B | Fc fragment of IgG receptor IIIb [Source:HGNC Symbol;Acc:HGNC:3620] |
| ENSG00000168060 | HGLibA_31013 | 381 | 2.311582157 | NAALADL1 | N-acetylated alpha-linked acidic dipeptidase like 1 [Source:HGNC Symbol;Acc:HGNC:23536] |
| ENSG00000168135 | HGLibA_24279 | 381 | 2.311582157 | KCNJ4 | potassium voltage-gated channel subfamily J member 4 [Source:HGNC Symbol;Acc:HGNC:6265] |
| ENSG00000169228 | HGLibA_39847 | 381 | 2.311582157 | RAB24 | member RAS oncogene family [Source:HGNC Symbol;Acc:HGNC:9765]" |
| ENSG00000169435 | HGLibA_40389 | 381 | 2.311582157 | RASSF6 | Ras association domain family member 6 [Source:HGNC Symbol;Acc:HGNC:20796] |
| ENSG00000172992 | HGLibA_12467 | 381 | 2.311582157 | DCAKD | dephospho-CoA kinase domain containing [Source:HGNC Symbol;Acc:HGNC:26238] |
| ENSG00000173013 | HGLibA_07937 | 381 | 2.311582157 | CCDC96 | coiled-coil domain containing 96 [Source:HGNC Symbol;Acc:HGNC:26900] |
| ENSG00000186111 | HGLibA_36720 | 381 | 2.311582157 | PIP5K1C | phosphatidylinositol-4-phosphate 5-kinase type 1 gamma [Source:HGNC Symbol;Acc:HGNC:8996] |
| ENSG00000196405 | HGLibA_15697 | 381 | 2.311582157 | EVL | Enah/Vasp-like [Source:HGNC Symbol;Acc:HGNC:20234] |
| ENSG00000213923 | HGLibA_11391 | 381 | 2.311582157 | CSNK1E | casein kinase 1 epsilon [Source:HGNC Symbol;Acc:HGNC:2453] |
| ENSG00000273899 | HGLibA_32263 | 381 | 2.311582157 | NOL12 | nucleolar protein 12 [Source:HGNC Symbol;Acc:HGNC:28585] |
| hsa-mir-4757 | HGLibA_61127 | 381 | 2.311582157 | hsa-mir-4757 | - |
| ENSG00000060491 | HGLibA_33317 | 380 | 2.302536814 | OGFR | opioid growth factor receptor [Source:HGNC Symbol;Acc:HGNC:15768] |
| ENSG00000106689 | HGLibA_26449 | 380 | 2.302536814 | LHX2 | LIM homeobox 2 [Source:HGNC Symbol;Acc:HGNC:6594] |
| ENSG00000118113 | HGLibA_29540 | 380 | 2.302536814 | MMP8 | matrix metallopeptidase 8 [Source:HGNC Symbol;Acc:HGNC:7175] |
| ENSG00000118418 | HGLibA_21724 | 380 | 2.302536814 | HMGN3 | high mobility group nucleosomal binding domain 3 [Source:HGNC Symbol;Acc:HGNC:12312] |
| ENSG00000139364 | HGLibA_49934 | 380 | 2.302536814 | TMEM132B | transmembrane protein 132B [Source:HGNC Symbol;Acc:HGNC:29397] |
| ENSG00000142875 | HGLibA_38506 | 380 | 2.302536814 | PRKACB | protein kinase cAMP-activated catalytic subunit beta [Source:HGNC Symbol;Acc:HGNC:9381] |
| ENSG00000154025 | HGLibA_45256 | 380 | 2.302536814 | SLC5A10 | solute carrier family 5 member 10 [Source:HGNC Symbol;Acc:HGNC:23155] |
| ENSG00000167757 | HGLibA_25237 | 380 | 2.302536814 | KLK11 | kallikrein related peptidase 11 [Source:HGNC Symbol;Acc:HGNC:6359] |
| ENSG00000172782 | HGLibA_15942 | 380 | 2.302536814 | FADS6 | fatty acid desaturase 6 [Source:HGNC Symbol;Acc:HGNC:30459] |
| ENSG00000176566 | HGLibA_12444 | 380 | 2.302536814 | DCAF4L2 | DDB1 and CUL4 associated factor 4 like 2 [Source:HGNC Symbol;Acc:HGNC:26657] |
| ENSG00000188937 | HGLibA_33189 | 380 | 2.302536814 | NYX | nyctalopin [Source:HGNC Symbol;Acc:HGNC:8082] |
| ENSG00000269858 | HGLibA_14641 | 380 | 2.302536814 | EGLN2 | egl-9 family hypoxia inducible factor 2 [Source:HGNC Symbol;Acc:HGNC:14660] |
| hsa-mir-451a | HGLibA_60524 | 380 | 2.302536814 | hsa-mir-451a | - |
| KIAA0355 | HGLibA_24627 | 380 | 2.302536814 | KIAA0355 | - |
| ENSG00000065989 | HGLibA_35787 | 379 | 2.293491471 | PDE4A | phosphodiesterase 4A [Source:HGNC Symbol;Acc:HGNC:8780] |
| ENSG00000108239 | HGLibA_48474 | 379 | 2.293491471 | TBC1D12 | TBC1 domain family member 12 [Source:HGNC Symbol;Acc:HGNC:29082] |
| ENSG00000125454 | HGLibA_44662 | 379 | 2.293491471 | SLC25A19 | solute carrier family 25 member 19 [Source:HGNC Symbol;Acc:HGNC:14409] |
| ENSG00000134504 | HGLibA_24411 | 379 | 2.293491471 | KCTD1 | potassium channel tetramerization domain containing 1 [Source:HGNC Symbol;Acc:HGNC:18249] |
| ENSG00000134769 | HGLibA_14063 | 379 | 2.293491471 | DTNA | dystrobrevin alpha [Source:HGNC Symbol;Acc:HGNC:3057] |
| ENSG00000136942 | HGLibA_41947 | 379 | 2.293491471 | RPL35 | ribosomal protein L35 [Source:HGNC Symbol;Acc:HGNC:10344] |
| ENSG00000143375 | HGLibA_09320 | 379 | 2.293491471 | CGN | cingulin [Source:HGNC Symbol;Acc:HGNC:17429] |
| ENSG00000145088 | HGLibA_14337 | 379 | 2.293491471 | EAF2 | ELL associated factor 2 [Source:HGNC Symbol;Acc:HGNC:23115] |
| ENSG00000146733 | HGLibA_39227 | 379 | 2.293491471 | PSPH | phosphoserine phosphatase [Source:HGNC Symbol;Acc:HGNC:9577] |
| ENSG00000155511 | HGLibA_20261 | 379 | 2.293491471 | GRIA1 | glutamate ionotropic receptor AMPA type subunit 1 [Source:HGNC Symbol;Acc:HGNC:4571] |
| ENSG00000162992 | HGLibA_31734 | 379 | 2.293491471 | NEUROD1 | neuronal differentiation 1 [Source:HGNC Symbol;Acc:HGNC:7762] |
| ENSG00000166016 | HGLibA_00337 | 379 | 2.293491471 | ABTB2 | ankyrin repeat and BTB domain containing 2 [Source:HGNC Symbol;Acc:HGNC:23842] |
| ENSG00000166341 | HGLibA_12489 | 379 | 2.293491471 | DCHS1 | dachsous cadherin-related 1 [Source:HGNC Symbol;Acc:HGNC:13681] |
| ENSG00000167207 | HGLibA_32247 | 379 | 2.293491471 | NOD2 | nucleotide binding oligomerization domain containing 2 [Source:HGNC Symbol;Acc:HGNC:5331] |
| ENSG00000169750 | HGLibA_40049 | 379 | 2.293491471 | RAC3 | Rac family small GTPase 3 [Source:HGNC Symbol;Acc:HGNC:9803] |
| ENSG00000171103 | HGLibA_51700 | 379 | 2.293491471 | TRMT61B | tRNA methyltransferase 61B [Source:HGNC Symbol;Acc:HGNC:26070] |
| ENSG00000173239 | HGLibA_26628 | 379 | 2.293491471 | LIPM | lipase family member M [Source:HGNC Symbol;Acc:HGNC:23455] |
| ENSG00000182179 | HGLibA_52532 | 379 | 2.293491471 | UBA7 | ubiquitin like modifier activating enzyme 7 [Source:HGNC Symbol;Acc:HGNC:12471] |
| ENSG00000198920 | HGLibA_24648 | 379 | 2.293491471 | KIAA0753 | KIAA0753 [Source:HGNC Symbol;Acc:HGNC:29110] |
| ENSG00000227729 | HGLibA_40727 | 379 | 2.293491471 | RD3L | retinal degeneration 3 like [Source:HGNC Symbol;Acc:HGNC:40912] |
| ENSG00000005022 | HGLibA_44761 | 378 | 2.284446128 | SLC25A5 | solute carrier family 25 member 5 [Source:HGNC Symbol;Acc:HGNC:10991] |
| ENSG00000006607 | HGLibA_16876 | 378 | 2.284446128 | FARP2 | ARH/RhoGEF and pleckstrin domain protein 2 [Source:HGNC Symbol;Acc:HGNC:16460]" |
| ENSG00000084693 | HGLibA_01178 | 378 | 2.284446128 | AGBL5 | ATP/GTP binding protein like 5 [Source:HGNC Symbol;Acc:HGNC:26147] |
| ENSG00000089163 | HGLibA_44224 | 378 | 2.284446128 | SIRT4 | sirtuin 4 [Source:HGNC Symbol;Acc:HGNC:14932] |
| ENSG00000101115 | HGLibA_42655 | 378 | 2.284446128 | SALL4 | spalt like transcription factor 4 [Source:HGNC Symbol;Acc:HGNC:15924] |
| ENSG00000106366 | HGLibA_43576 | 378 | 2.284446128 | SERPINE1 | serpin family E member 1 [Source:HGNC Symbol;Acc:HGNC:8583] |
| ENSG00000111206 | HGLibA_17905 | 378 | 2.284446128 | FOXM1 | forkhead box M1 [Source:HGNC Symbol;Acc:HGNC:3818] |
| ENSG00000113389 | HGLibA_32514 | 378 | 2.284446128 | NPR3 | natriuretic peptide receptor 3 [Source:HGNC Symbol;Acc:HGNC:7945] |
| ENSG00000114023 | HGLibA_16223 | 378 | 2.284446128 | FAM162A | family with sequence similarity 162 member A [Source:HGNC Symbol;Acc:HGNC:17865] |
| ENSG00000142698 | HGLibA_05905 | 378 | 2.284446128 | C1orf94 | chromosome 1 open reading frame 94 [Source:HGNC Symbol;Acc:HGNC:28250] |
| ENSG00000152208 | HGLibA_20275 | 378 | 2.284446128 | GRID2 | glutamate ionotropic receptor delta type subunit 2 [Source:HGNC Symbol;Acc:HGNC:4576] |
| ENSG00000153214 | HGLibA_50533 | 378 | 2.284446128 | TMEM87B | transmembrane protein 87B [Source:HGNC Symbol;Acc:HGNC:25913] |
| ENSG00000160299 | HGLibA_35639 | 378 | 2.284446128 | PCNT | pericentrin [Source:HGNC Symbol;Acc:HGNC:16068] |
| ENSG00000166920 | HGLibA_05405 | 378 | 2.284446128 | C15orf48 | chromosome 15 open reading frame 48 [Source:HGNC Symbol;Acc:HGNC:29898] |
| ENSG00000169184 | HGLibA_29557 | 378 | 2.284446128 | MN1 | transcriptional regulator [Source:HGNC Symbol;Acc:HGNC:7180]" |
| ENSG00000172146 | HGLibA_33640 | 378 | 2.284446128 | OR1A1 | olfactory receptor family 1 subfamily A member 1 [Source:HGNC Symbol;Acc:HGNC:8179] |
| ENSG00000188917 | HGLibA_51687 | 378 | 2.284446128 | TRMT2B | tRNA methyltransferase 2 homolog B [Source:HGNC Symbol;Acc:HGNC:25748] |
| ENSG00000205307 | HGLibA_42719 | 378 | 2.284446128 | SAP25 | Sin3A associated protein 25 [Source:HGNC Symbol;Acc:HGNC:41908] |
| ENSG00000230246 | HGLibA_46489 | 378 | 2.284446128 | SPATA31C1 | SPATA31 subfamily C member 1 [Source:HGNC Symbol;Acc:HGNC:27846] |
| hsa-mir-1285-1 | HGLibA_57600 | 378 | 2.284446128 | hsa-mir-1285-1 | - |
| hsa-mir-6777 | HGLibA_63283 | 378 | 2.284446128 | hsa-mir-6777 | - |

| | | | | | |
|---|---|---|---|---|---|
| PQLC3 | HGLibA_38256 | 378 | 2.284446128 | PQLC3 | - |
| ENSG00000002330 | HGLibA_04071 | 377 | 2.275400785 | BAD | BCL2 associated agonist of cell death [Source:HGNC Symbol;Acc:HGNC:936] |
| ENSG00000070371 | HGLibA_10153 | 377 | 2.275400785 | CLTCL1 | clathrin heavy chain like 1 [Source:HGNC Symbol;Acc:HGNC:2093] |
| ENSG00000073756 | HGLibA_39362 | 377 | 2.275400785 | PTGS2 | prostaglandin-endoperoxide synthase 2 [Source:HGNC Symbol;Acc:HGNC:9605] |
| ENSG00000074266 | HGLibA_14486 | 377 | 2.275400785 | EED | embryonic ectoderm development [Source:HGNC Symbol;Acc:HGNC:3188] |
| ENSG00000106477 | HGLibA_09137 | 377 | 2.275400785 | CEP41 | centrosomal protein 41 [Source:HGNC Symbol;Acc:HGNC:12370] |
| ENSG00000112852 | HGLibA_35493 | 377 | 2.275400785 | PCDHB2 | protocadherin beta 2 [Source:HGNC Symbol;Acc:HGNC:8687] |
| ENSG00000124713 | HGLibA_19558 | 377 | 2.275400785 | GNMT | glycine N-methyltransferase [Source:HGNC Symbol;Acc:HGNC:4415] |
| ENSG00000127334 | HGLibA_14277 | 377 | 2.275400785 | DYRK2 | dual specificity tyrosine phosphorylation regulated kinase 2 [Source:HGNC Symbol;Acc:HGNC:3093] |
| ENSG00000130684 | HGLibA_55829 | 377 | 2.275400785 | ZNF337 | zinc finger protein 337 [Source:HGNC Symbol;Acc:HGNC:15809] |
| ENSG00000141564 | HGLibA_42202 | 377 | 2.275400785 | RPTOR | regulatory associated protein of MTOR complex 1 [Source:HGNC Symbol;Acc:HGNC:30287] |
| ENSG00000155252 | HGLibA_36492 | 377 | 2.275400785 | PI4K2A | phosphatidylinositol 4-kinase type 2 alpha [Source:HGNC Symbol;Acc:HGNC:30031] |
| ENSG00000162378 | HGLibA_57086 | 377 | 2.275400785 | ZYG11B | cell cycle regulator [Source:HGNC Symbol;Acc:HGNC:25820]" |
| ENSG00000164889 | HGLibA_45213 | 377 | 2.275400785 | SLC4A2 | solute carrier family 4 member 2 [Source:HGNC Symbol;Acc:HGNC:11028] |
| ENSG00000170289 | HGLibA_10277 | 377 | 2.275400785 | CNGB3 | cyclic nucleotide gated channel beta 3 [Source:HGNC Symbol;Acc:HGNC:2153] |
| ENSG00000176256 | HGLibA_21699 | 377 | 2.275400785 | HMGB4 | high mobility group box 4 [Source:HGNC Symbol;Acc:HGNC:24954] |
| ENSG00000176567 | HGLibA_34067 | 377 | 2.275400785 | OR4X1 | olfactory receptor family 4 subfamily X member 1 (gene/pseudogene) [Source:HGNC Symbol;Acc:HGNC:14854] |
| ENSG00000176720 | HGLibA_04677 | 377 | 2.275400785 | BOK | BCL2 family apoptosis regulator BOK [Source:HGNC Symbol;Acc:HGNC:1087] |
| ENSG00000188624 | HGLibA_22846 | 377 | 2.275400785 | IGFL3 | IGF like family member 3 [Source:HGNC Symbol;Acc:HGNC:32930] |
| ENSG00000196184 | HGLibA_33520 | 377 | 2.275400785 | OR10J1 | olfactory receptor family 10 subfamily J member 1 [Source:HGNC Symbol;Acc:HGNC:8175] |
| ENSG00000196581 | HGLibA_01370 | 377 | 2.275400785 | AJAP1 | adherens junctions associated protein 1 [Source:HGNC Symbol;Acc:HGNC:30801] |
| ENSG00000204314 | HGLibA_38881 | 377 | 2.275400785 | PRRT1 | proline rich transmembrane protein 1 [Source:HGNC Symbol;Acc:HGNC:13943] |
| hsa-mir-4695 | HGLibA_60890 | 377 | 2.275400785 | hsa-mir-4695 | - |
| ENSG00000047597 | HGLibA_54518 | 376 | 2.266355442 | XK | X-linked Kx blood group [Source:HGNC Symbol;Acc:HGNC:12811] |
| ENSG00000116521 | HGLibA_42841 | 376 | 2.266355442 | SCAMP3 | secretory carrier membrane protein 3 [Source:HGNC Symbol;Acc:HGNC:10565] |
| ENSG00000116771 | HGLibA_01199 | 376 | 2.266355442 | AGMAT | agmatinase [Source:HGNC Symbol;Acc:HGNC:18407] |
| ENSG00000143819 | HGLibA_15323 | 376 | 2.266355442 | EPHX1 | epoxide hydrolase 1 [Source:HGNC Symbol;Acc:HGNC:3401] |
| ENSG00000158286 | HGLibA_41612 | 376 | 2.266355442 | RNF207 | ring finger protein 207 [Source:HGNC Symbol;Acc:HGNC:32947] |
| ENSG00000159733 | HGLibA_55292 | 376 | 2.266355442 | ZFYVE28 | zinc finger FYVE-type containing 28 [Source:HGNC Symbol;Acc:HGNC:29334] |
| ENSG00000160094 | HGLibA_55873 | 376 | 2.266355442 | ZNF362 | zinc finger protein 362 [Source:HGNC Symbol;Acc:HGNC:18079] |
| ENSG00000164076 | HGLibA_07098 | 376 | 2.266355442 | CAMKV | CaM kinase like vesicle associated [Source:HGNC Symbol;Acc:HGNC:28788] |
| ENSG00000171489 | HGLibA_46333 | 376 | 2.266355442 | SPACA5 | sperm acrosome associated 5 [Source:HGNC Symbol;Acc:HGNC:31353] |
| ENSG00000178467 | HGLibA_34912 | 376 | 2.266355442 | P4HTM | transmembrane [Source:HGNC Symbol;Acc:HGNC:28858]" |
| ENSG00000180902 | HGLibA_12257 | 376 | 2.266355442 | D2HGDH | D-2-hydroxyglutarate dehydrogenase [Source:HGNC Symbol;Acc:HGNC:28358] |
| ENSG00000181856 | HGLibA_44875 | 376 | 2.266355442 | SLC2A4 | solute carrier family 2 member 4 [Source:HGNC Symbol;Acc:HGNC:11009] |
| ENSG00000181963 | HGLibA_34184 | 376 | 2.266355442 | OR52K2 | olfactory receptor family 52 subfamily K member 2 [Source:HGNC Symbol;Acc:HGNC:15223] |
| ENSG00000189037 | HGLibA_14159 | 376 | 2.266355442 | DUSP21 | dual specificity phosphatase 21 [Source:HGNC Symbol;Acc:HGNC:20476] |
| ENSG00000197601 | HGLibA_16867 | 376 | 2.266355442 | FAR1 | fatty acyl-CoA reductase 1 [Source:HGNC Symbol;Acc:HGNC:26222] |
| ENSG00000234068 | HGLibA_35007 | 376 | 2.266355442 | PAGE2 | PAGE family member 2 [Source:HGNC Symbol;Acc:HGNC:31804] |
| PQLC1 | HGLibA_38250 | 376 | 2.266355442 | PQLC1 | - |
| ENSG00000086570 | HGLibA_16917 | 375 | 2.257310099 | FAT2 | FAT atypical cadherin 2 [Source:HGNC Symbol;Acc:HGNC:3596] |
| ENSG00000094841 | HGLibA_53091 | 375 | 2.257310099 | UPRT | uracil phosphoribosyltransferase homolog [Source:HGNC Symbol;Acc:HGNC:28334] |
| ENSG00000113721 | HGLibA_35855 | 375 | 2.257310099 | PDGFRB | platelet derived growth factor receptor beta [Source:HGNC Symbol;Acc:HGNC:8804] |
| ENSG00000126549 | HGLibA_47402 | 375 | 2.257310099 | STATH | statherin [Source:HGNC Symbol;Acc:HGNC:11369] |
| ENSG00000128310 | HGLibA_18592 | 375 | 2.257310099 | GALR3 | galanin receptor 3 [Source:HGNC Symbol;Acc:HGNC:4134] |
| ENSG00000138207 | HGLibA_40637 | 375 | 2.257310099 | RBP4 | retinol binding protein 4 [Source:HGNC Symbol;Acc:HGNC:9922] |
| ENSG00000140450 | HGLibA_03123 | 375 | 2.257310099 | ARRDC4 | arrestin domain containing 4 [Source:HGNC Symbol;Acc:HGNC:28087] |
| ENSG00000155158 | HGLibA_52134 | 375 | 2.257310099 | TTC39B | tetratricopeptide repeat domain 39B [Source:HGNC Symbol;Acc:HGNC:23704] |
| ENSG00000160349 | HGLibA_26167 | 375 | 2.257310099 | LCN1 | lipocalin 1 [Source:HGNC Symbol;Acc:HGNC:6525] |
| ENSG00000196240 | HGLibA_33862 | 375 | 2.257310099 | OR2T2 | olfactory receptor family 2 subfamily T member 2 [Source:HGNC Symbol;Acc:HGNC:14725] |
| ENSG00000204595 | HGLibA_13900 | 375 | 2.257310099 | DPRX | divergent-paired related homeobox [Source:HGNC Symbol;Acc:HGNC:32166] |
| ENSG00000217128 | HGLibA_17765 | 375 | 2.257310099 | FNIP1 | folliculin interacting protein 1 [Source:HGNC Symbol;Acc:HGNC:29418] |
| ENSG00000241973 | HGLibA_36497 | 375 | 2.257310099 | PI4KA | phosphatidylinositol 4-kinase alpha [Source:HGNC Symbol;Acc:HGNC:8983] |
| hsa-mir-4499 | HGLibA_60413 | 375 | 2.257310099 | hsa-mir-4499 | - |
| ENSG00000085276 | HGLibA_28723 | 374 | 2.248264756 | MECOM | MDS1 and EVI1 complex locus [Source:HGNC Symbol;Acc:HGNC:3498] |
| ENSG00000099985 | HGLibA_34692 | 374 | 2.248264756 | OSM | oncostatin M [Source:HGNC Symbol;Acc:HGNC:8506] |
| ENSG00000105058 | HGLibA_16571 | 374 | 2.248264756 | FAM32A | family with sequence similarity 32 member A [Source:HGNC Symbol;Acc:HGNC:24563] |
| ENSG00000139083 | HGLibA_15666 | 374 | 2.248264756 | ETV6 | ETS variant 6 [Source:HGNC Symbol;Acc:HGNC:3495] |
| ENSG00000146083 | HGLibA_41687 | 374 | 2.248264756 | RNF44 | ring finger protein 44 [Source:HGNC Symbol;Acc:HGNC:19180] |
| ENSG00000146926 | HGLibA_03205 | 374 | 2.248264756 | ASB10 | ankyrin repeat and SOCS box containing 10 [Source:HGNC Symbol;Acc:HGNC:17185] |
| ENSG00000150551 | HGLibA_27712 | 374 | 2.248264756 | LYPD1 | LY6/PLAUR domain containing 1 [Source:HGNC Symbol;Acc:HGNC:28431] |
| ENSG00000163214 | HGLibA_13202 | 374 | 2.248264756 | DHX57 | DExH-box helicase 57 [Source:HGNC Symbol;Acc:HGNC:20086] |
| ENSG00000163873 | HGLibA_20286 | 374 | 2.248264756 | GRIK3 | glutamate ionotropic receptor kainate type subunit 3 [Source:HGNC Symbol;Acc:HGNC:4581] |
| ENSG00000172349 | HGLibA_22986 | 374 | 2.248264756 | IL16 | interleukin 16 [Source:HGNC Symbol;Acc:HGNC:5980] |
| ENSG00000187024 | HGLibA_39551 | 374 | 2.248264756 | PTRH1 | peptidyl-tRNA hydrolase 1 homolog [Source:HGNC Symbol;Acc:HGNC:27039] |
| ENSG00000188886 | HGLibA_03378 | 374 | 2.248264756 | ASTL | astacin like metalloendopeptidase [Source:HGNC Symbol;Acc:HGNC:31704] |
| ENSG00000189090 | HGLibA_16558 | 374 | 2.248264756 | FAM25G | family with sequence similarity 25 member G [Source:HGNC Symbol;Acc:HGNC:23590] |
| ENSG00000288596 | HGLibA_06624 | 374 | 2.248264756 | C8orf44 | - |
| FAM155A | HGLibA_16181 | 374 | 2.248264756 | FAM155A | - |
| hsa-mir-802 | HGLibA_64042 | 374 | 2.248264756 | hsa-mir-802 | - |
| ENSG00000096433 | HGLibA_23859 | 373 | 2.239219413 | ITPR3 | 5-trisphosphate receptor type 3 [Source:HGNC Symbol;Acc:HGNC:6182]" |
| ENSG00000100739 | HGLibA_04380 | 373 | 2.239219413 | BDKRB1 | bradykinin receptor B1 [Source:HGNC Symbol;Acc:HGNC:1029] |
| ENSG00000112787 | HGLibA_16970 | 373 | 2.239219413 | FBRSL1 | fibrosin like 1 [Source:HGNC Symbol;Acc:HGNC:29308] |
| ENSG00000118965 | HGLibA_54072 | 373 | 2.239219413 | WDR35 | WD repeat domain 35 [Source:HGNC Symbol;Acc:HGNC:29250] |
| ENSG00000121104 | HGLibA_16041 | 373 | 2.239219413 | FAM117A | family with sequence similarity 117 member A [Source:HGNC Symbol;Acc:HGNC:24179] |
| ENSG00000126368 | HGLibA_32574 | 373 | 2.239219413 | NR1D1 | nuclear receptor subfamily 1 group D member 1 [Source:HGNC Symbol;Acc:HGNC:7962] |
| ENSG00000129009 | HGLibA_23671 | 373 | 2.239219413 | ISLR | immunoglobulin superfamily containing leucine rich repeat [Source:HGNC Symbol;Acc:HGNC:6133] |
| ENSG00000148377 | HGLibA_22557 | 373 | 2.239219413 | IDI2 | isopentenyl-diphosphate delta isomerase 2 [Source:HGNC Symbol;Acc:HGNC:23487] |
| ENSG00000156009 | HGLibA_27920 | 373 | 2.239219413 | MAGEA8 | MAGE family member A8 [Source:HGNC Symbol;Acc:HGNC:6806] |
| ENSG00000167515 | HGLibA_51315 | 373 | 2.239219413 | TRAPPC2L | trafficking protein particle complex 2 like [Source:HGNC Symbol;Acc:HGNC:30887] |
| ENSG00000168818 | HGLibA_47630 | 373 | 2.239219413 | STX18 | syntaxin 18 [Source:HGNC Symbol;Acc:HGNC:15942] |
| ENSG00000168878 | HGLibA_43771 | 373 | 2.239219413 | SFTPB | surfactant protein B [Source:HGNC Symbol;Acc:HGNC:10801] |
| ENSG00000168955 | HGLibA_49700 | 373 | 2.239219413 | TM4SF20 | transmembrane 4 L six family member 20 [Source:HGNC Symbol;Acc:HGNC:26230] |
| ENSG00000180210 | HGLibA_15859 | 373 | 2.239219413 | F2 | thrombin [Source:HGNC Symbol;Acc:HGNC:3535]" |
| ENSG00000198892 | HGLibA_44040 | 373 | 2.239219413 | SHISA4 | shisa family member 4 [Source:HGNC Symbol;Acc:HGNC:27139] |
| ENSG00000259120 | HGLibA_45763 | 373 | 2.239219413 | SMIM6 | small integral membrane protein 6 [Source:HGNC Symbol;Acc:HGNC:40032] |
| hsa-mir-218-1 | HGLibA_58270 | 373 | 2.239219413 | hsa-mir-218-1 | - |
| hsa-mir-4697 | HGLibA_60898 | 373 | 2.239219413 | hsa-mir-4697 | - |
| ENSG00000101856 | HGLibA_36287 | 372 | 2.230174071 | PGRMC1 | progesterone receptor membrane component 1 [Source:HGNC Symbol;Acc:HGNC:16090] |
| ENSG00000104859 | HGLibA_09800 | 372 | 2.230174071 | CLASRP | CLK4 associating serine/arginine rich protein [Source:HGNC Symbol;Acc:HGNC:17731] |
| ENSG00000127081 | HGLibA_56077 | 372 | 2.230174071 | ZNF484 | zinc finger protein 484 [Source:HGNC Symbol;Acc:HGNC:23385] |
| ENSG00000135569 | HGLibA_48129 | 372 | 2.230174071 | TAAR5 | trace amine associated receptor 5 [Source:HGNC Symbol;Acc:HGNC:30236] |
| ENSG00000151224 | HGLibA_28415 | 372 | 2.230174071 | MAT1A | methionine adenosyltransferase 1A [Source:HGNC Symbol;Acc:HGNC:6903] |
| ENSG00000155249 | HGLibA_34015 | 372 | 2.230174071 | OR4K1 | olfactory receptor family 4 subfamily K member 1 [Source:HGNC Symbol;Acc:HGNC:14726] |
| ENSG00000159217 | HGLibA_22799 | 372 | 2.230174071 | IGF2BP1 | insulin like growth factor 2 mRNA binding protein 1 [Source:HGNC Symbol;Acc:HGNC:28866] |
| ENSG00000163935 | HGLibA_43721 | 372 | 2.230174071 | SFMBT1 | Scm like with four mbt domains 1 [Source:HGNC Symbol;Acc:HGNC:20255] |
| ENSG00000164935 | HGLibA_12535 | 372 | 2.230174071 | DCSTAMP | dendrocyte expressed seven transmembrane protein [Source:HGNC Symbol;Acc:HGNC:18549] |
| ENSG00000166432 | HGLibA_55367 | 372 | 2.230174071 | ZMAT1 | zinc finger matrin-type 1 [Source:HGNC Symbol;Acc:HGNC:29377] |
| ENSG00000172164 | HGLibA_46000 | 372 | 2.230174071 | SNTB1 | syntrophin beta 1 [Source:HGNC Symbol;Acc:HGNC:11168] |
| ENSG00000175029 | HGLibA_11587 | 372 | 2.230174071 | CTBP2 | C-terminal binding protein 2 [Source:HGNC Symbol;Acc:HGNC:2495] |
| ENSG00000176018 | HGLibA_27770 | 372 | 2.230174071 | LYSMD3 | LysM domain containing 3 [Source:HGNC Symbol;Acc:HGNC:26969] |
| ENSG00000177688 | HGLibA_47784 | 372 | 2.230174071 | SUMO4 | small ubiquitin-like modifier 4 [Source:HGNC Symbol;Acc:HGNC:21181] |
| ENSG00000182318 | HGLibA_57005 | 372 | 2.230174071 | ZSCAN22 | zinc finger and SCAN domain containing 22 [Source:HGNC Symbol;Acc:HGNC:4929] |
| ENSG00000182330 | HGLibA_38329 | 372 | 2.230174071 | PRAMEF8 | PRAME family member 8 [Source:HGNC Symbol;Acc:HGNC:24074] |
| ENSG00000185989 | HGLibA_40302 | 372 | 2.230174071 | RASA3 | RAS p21 protein activator 3 [Source:HGNC Symbol;Acc:HGNC:20331] |
| ENSG00000196570 | HGLibA_36179 | 372 | 2.230174071 | PFN3 | profilin 3 [Source:HGNC Symbol;Acc:HGNC:18627] |
| ENSG00000197241 | HGLibA_44888 | 372 | 2.230174071 | SLC2A7 | solute carrier family 2 member 7 [Source:HGNC Symbol;Acc:HGNC:13445] |
| ENSG00000197893 | HGLibA_32644 | 372 | 2.230174071 | NRAP | nebulin related anchoring protein [Source:HGNC Symbol;Acc:HGNC:7988] |
| ENSG00000198429 | HGLibA_56549 | 372 | 2.230174071 | ZNF69 | zinc finger protein 69 [Source:HGNC Symbol;Acc:HGNC:13138] |
| ENSG00000198574 | HGLibA_43885 | 372 | 2.230174071 | SH2D1B | SH2 domain containing 1B [Source:HGNC Symbol;Acc:HGNC:30416] |
| ENSG00000253731 | HGLibA_35539 | 372 | 2.230174071 | PCDHGA6 | 6 [Source:HGNC Symbol;Acc:HGNC:8704]" |
| hsa-mir-639 | HGLibA_62795 | 372 | 2.230174071 | hsa-mir-639 | - |
| 4-Sep | HGLibA_43443 | 372 | 2.230174071 | 4-Sep | - |
| BAI3 | HGLibA_04119 | 371 | 2.221128728 | BAI3 | - |
| ENSG00000057657 | HGLibA_38363 | 371 | 2.221128728 | PRDM1 | PR/SET domain 1 [Source:HGNC Symbol;Acc:HGNC:9346] |
| ENSG00000106366 | HGLibA_43577 | 371 | 2.221128728 | SERPINE1 | serpin family E member 1 [Source:HGNC Symbol;Acc:HGNC:8583] |
| ENSG00000124788 | HGLibA_03827 | 371 | 2.221128728 | ATXN1 | ataxin 1 [Source:HGNC Symbol;Acc:HGNC:10548] |
| ENSG00000137393 | HGLibA_41528 | 371 | 2.221128728 | RNF144B | ring finger protein 144B [Source:HGNC Symbol;Acc:HGNC:21578] |
| ENSG00000143653 | HGLibA_42880 | 371 | 2.221128728 | SCCPDH | saccharopine dehydrogenase (putative) [Source:HGNC Symbol;Acc:HGNC:24275] |
| ENSG00000147059 | HGLibA_46670 | 371 | 2.221128728 | SPIN2A | spindlin family member 2A [Source:HGNC Symbol;Acc:HGNC:20694] |
| ENSG00000164938 | HGLibA_51070 | 371 | 2.221128728 | TP53INP1 | tumor protein p53 inducible nuclear protein 1 [Source:HGNC Symbol;Acc:HGNC:18022] |
| ENSG00000166971 | HGLibA_01516 | 371 | 2.221128728 | AKTIP | AKT interacting protein [Source:HGNC Symbol;Acc:HGNC:16710] |
| ENSG00000169740 | HGLibA_55791 | 371 | 2.221128728 | ZNF32 | zinc finger protein 32 [Source:HGNC Symbol;Acc:HGNC:13095] |
| ENSG00000175105 | HGLibA_56467 | 371 | 2.221128728 | ZNF654 | zinc finger protein 654 [Source:HGNC Symbol;Acc:HGNC:25612] |
| ENSG00000180638 | HGLibA_45196 | 371 | 2.221128728 | SLC47A2 | solute carrier family 47 member 2 [Source:HGNC Symbol;Acc:HGNC:26439] |
| ENSG00000185519 | HGLibA_16112 | 371 | 2.221128728 | FAM131C | family with sequence similarity 131 member C [Source:HGNC Symbol;Acc:HGNC:26717] |
| ENSG00000198604 | HGLibA_04186 | 371 | 2.221128728 | BAZ1A | bromodomain adjacent to zinc finger domain 1A [Source:HGNC Symbol;Acc:HGNC:960] |
| ENSG00000204634 | HGLibA_48566 | 371 | 2.221128728 | TBC1D8 | TBC1 domain family member 8 [Source:HGNC Symbol;Acc:HGNC:17791] |
| ENSG00000205457 | HGLibA_51085 | 371 | 2.221128728 | TP53TG3C | TP53 target 3C [Source:HGNC Symbol;Acc:HGNC:42962] |
| ENSG00000205517 | HGLibA_41007 | 371 | 2.221128728 | RGL3 | ral guanine nucleotide dissociation stimulator like 3 [Source:HGNC Symbol;Acc:HGNC:30282] |
| ENSG00000257335 | HGLibA_29125 | 371 | 2.221128728 | MGAM | maltase-glucoamylase [Source:HGNC Symbol;Acc:HGNC:7043] |
| hsa-mir-3681 | HGLibA_59279 | 371 | 2.221128728 | hsa-mir-3681 | - |
| hsa-mir-3691 | HGLibA_59352 | 371 | 2.221128728 | hsa-mir-3691 | - |
| hsa-mir-4510 | HGLibA_60485 | 371 | 2.221128728 | hsa-mir-4510 | - |
| hsa-mir-6824 | HGLibA_63473 | 371 | 2.221128728 | hsa-mir-6824 | - |

| | | | | | |
|---|---|---|---|---|---|
| ENSG00000100154 | HGLibA_52095 | 370 | 2.212083385 | TTC28 | tetratricopeptide repeat domain 28 [Source:HGNC Symbol;Acc:HGNC:29179] |
| ENSG00000122420 | HGLibA_39339 | 370 | 2.212083385 | PTGFR | prostaglandin F receptor [Source:HGNC Symbol;Acc:HGNC:9600] |
| ENSG00000125735 | HGLibA_50813 | 370 | 2.212083385 | TNFSF14 | TNF superfamily member 14 [Source:HGNC Symbol;Acc:HGNC:11930] |
| ENSG00000130598 | HGLibA_50875 | 370 | 2.212083385 | TNNI2 | fast skeletal type [Source:HGNC Symbol;Acc:HGNC:11946]" |
| ENSG00000131398 | HGLibA_24169 | 370 | 2.212083385 | KCNC3 | potassium voltage-gated channel subfamily C member 3 [Source:HGNC Symbol;Acc:HGNC:6235] |
| ENSG00000136108 | HGLibA_09753 | 370 | 2.212083385 | CKAP2 | cytoskeleton associated protein 2 [Source:HGNC Symbol;Acc:HGNC:1990] |
| ENSG00000147885 | HGLibA_22667 | 370 | 2.212083385 | IFNA16 | interferon alpha 16 [Source:HGNC Symbol;Acc:HGNC:5421] |
| ENSG00000168334 | HGLibA_54511 | 370 | 2.212083385 | XIRP1 | xin actin binding repeat containing 1 [Source:HGNC Symbol;Acc:HGNC:14301] |
| ENSG00000168615 | HGLibA_00784 | 370 | 2.212083385 | ADAM9 | ADAM metallopeptidase domain 9 [Source:HGNC Symbol;Acc:HGNC:216] |
| ENSG00000174130 | HGLibA_49657 | 370 | 2.212083385 | TLR6 | toll like receptor 6 [Source:HGNC Symbol;Acc:HGNC:16711] |
| ENSG00000177432 | HGLibA_31124 | 370 | 2.212083385 | NAP1L5 | nucleosome assembly protein 1 like 5 [Source:HGNC Symbol;Acc:HGNC:19968] |
| ENSG00000108556 | HGLibA_09603 | 369 | 2.203038042 | CHRNE | cholinergic receptor nicotinic epsilon subunit [Source:HGNC Symbol;Acc:HGNC:1966] |
| ENSG00000124532 | HGLibA_30118 | 369 | 2.203038042 | MRS2 | magnesium transporter MRS2 [Source:HGNC Symbol;Acc:HGNC:13785] |
| ENSG00000136002 | HGLibA_02864 | 369 | 2.203038042 | ARHGEF4 | Rho guanine nucleotide exchange factor 4 [Source:HGNC Symbol;Acc:HGNC:684] |
| ENSG00000140538 | HGLibA_32880 | 369 | 2.203038042 | NTRK3 | neurotrophic receptor tyrosine kinase 3 [Source:HGNC Symbol;Acc:HGNC:8033] |
| ENSG00000156587 | HGLibA_52631 | 369 | 2.203038042 | UBE2L6 | ubiquitin conjugating enzyme E2 L6 [Source:HGNC Symbol;Acc:HGNC:12490] |
| ENSG00000168398 | HGLibA_04382 | 369 | 2.203038042 | BDKRB2 | bradykinin receptor B2 [Source:HGNC Symbol;Acc:HGNC:1030] |
| ENSG00000172354 | HGLibA_19489 | 369 | 2.203038042 | GNB2 | G protein subunit beta 2 [Source:HGNC Symbol;Acc:HGNC:4398] |
| ENSG00000182334 | HGLibA_34348 | 369 | 2.203038042 | OR5P3 | olfactory receptor family 5 subfamily P member 3 [Source:HGNC Symbol;Acc:HGNC:14784] |
| ENSG00000187961 | HGLibA_25142 | 369 | 2.203038042 | KLHL17 | kelch like family member 17 [Source:HGNC Symbol;Acc:HGNC:24023] |
| ENSG00000204406 | HGLibA_28491 | 369 | 2.203038042 | MBD5 | methyl-CpG binding domain protein 5 [Source:HGNC Symbol;Acc:HGNC:20444] |
| ENSG00000230937 | HGLibA_29301 | 369 | 2.203038042 | MIR205HG | MIR205 host gene [Source:HGNC Symbol;Acc:HGNC:43562] |
| ENSG00000254470 | HGLibA_02354 | 369 | 2.203038042 | AP5B1 | adaptor related protein complex 5 subunit beta 1 [Source:HGNC Symbol;Acc:HGNC:25104] |
| ENSG00000258873 | HGLibA_14205 | 369 | 2.203038042 | DUXA | double homeobox A [Source:HGNC Symbol;Acc:HGNC:32179] |
| hsa-mir-4259 | HGLibA_59744 | 369 | 2.203038042 | hsa-mir-4259 | - |
| hsa-mir-429 | HGLibA_59868 | 369 | 2.203038042 | hsa-mir-429 | - |
| hsa-mir-891b | HGLibA_64248 | 369 | 2.203038042 | hsa-mir-891b | - |
| MLL3 | HGLibA_29399 | 369 | 2.203038042 | MLL3 | - |
| ENSG00000032444 | HGLibA_37420 | 368 | 2.193992699 | PNPLA6 | patatin like phospholipase domain containing 6 [Source:HGNC Symbol;Acc:HGNC:16268] |
| ENSG00000073464 | HGLibA_09829 | 368 | 2.193992699 | CLCN4 | chloride voltage-gated channel 4 [Source:HGNC Symbol;Acc:HGNC:2022] |
| ENSG00000087074 | HGLibA_38046 | 368 | 2.193992699 | PPP1R15A | protein phosphatase 1 regulatory subunit 15A [Source:HGNC Symbol;Acc:HGNC:14375] |
| ENSG00000092098 | HGLibA_41660 | 368 | 2.193992699 | RNF31 | ring finger protein 31 [Source:HGNC Symbol;Acc:HGNC:16031] |
| ENSG00000101096 | HGLibA_31775 | 368 | 2.193992699 | NFATC2 | nuclear factor of activated T cells 2 [Source:HGNC Symbol;Acc:HGNC:7776] |
| ENSG00000102265 | HGLibA_49536 | 368 | 2.193992699 | TIMP1 | TIMP metallopeptidase inhibitor 1 [Source:HGNC Symbol;Acc:HGNC:11820] |
| ENSG00000103769 | HGLibA_39788 | 368 | 2.193992699 | RAB11A | member RAS oncogene family [Source:HGNC Symbol;Acc:HGNC:9760]" |
| ENSG00000112658 | HGLibA_46962 | 368 | 2.193992699 | SRF | serum response factor [Source:HGNC Symbol;Acc:HGNC:11291] |
| ENSG00000116731 | HGLibA_38386 | 368 | 2.193992699 | PRDM2 | PR/SET domain 2 [Source:HGNC Symbol;Acc:HGNC:9347] |
| ENSG00000130589 | HGLibA_21126 | 368 | 2.193992699 | HELZ2 | helicase with zinc finger 2 [Source:HGNC Symbol;Acc:HGNC:30021] |
| ENSG00000151726 | HGLibA_00553 | 368 | 2.193992699 | ACSL1 | acyl-CoA synthetase long chain family member 1 [Source:HGNC Symbol;Acc:HGNC:3569] |
| ENSG00000158516 | HGLibA_10886 | 368 | 2.193992699 | CPA2 | carboxypeptidase A2 [Source:HGNC Symbol;Acc:HGNC:2297] |
| ENSG00000165046 | HGLibA_26336 | 368 | 2.193992699 | LETM2 | leucine zipper and EF-hand containing transmembrane protein 2 [Source:HGNC Symbol;Acc:HGNC:14648] |
| ENSG00000182132 | HGLibA_24233 | 368 | 2.193992699 | KCNIP1 | potassium voltage-gated channel interacting protein 1 [Source:HGNC Symbol;Acc:HGNC:15521] |
| ENSG00000182798 | HGLibA_27937 | 368 | 2.193992699 | MAGEB17 | MAGE family member B17 [Source:HGNC Symbol;Acc:HGNC:17418] |
| ENSG00000196944 | HGLibA_33883 | 368 | 2.193992699 | OR2T4 | olfactory receptor family 2 subfamily T member 4 [Source:HGNC Symbol;Acc:HGNC:15016] |
| ENSG00000198108 | HGLibA_09658 | 368 | 2.193992699 | CHSY3 | chondroitin sulfate synthase 3 [Source:HGNC Symbol;Acc:HGNC:24293] |
| ENSG00000213654 | HGLibA_20161 | 368 | 2.193992699 | GPSM3 | G protein signaling modulator 3 [Source:HGNC Symbol;Acc:HGNC:13945] |
| hsa-mir-103a-2 | HGLibA_57164 | 368 | 2.193992699 | hsa-mir-103a-2 | - |
| C9orf142 | HGLibA_06685 | 367 | 2.184947356 | C9orf142 | - |
| ENSG00000056277 | HGLibA_55728 | 367 | 2.184947356 | ZNF280C | zinc finger protein 280C [Source:HGNC Symbol;Acc:HGNC:25955] |
| ENSG00000084676 | HGLibA_31336 | 367 | 2.184947356 | NCOA1 | nuclear receptor coactivator 1 [Source:HGNC Symbol;Acc:HGNC:7668] |
| ENSG00000100302 | HGLibA_40322 | 367 | 2.184947356 | RASD2 | RASD family member 2 [Source:HGNC Symbol;Acc:HGNC:18229] |
| ENSG00000103642 | HGLibA_25917 | 367 | 2.184947356 | LACTB | lactamase beta [Source:HGNC Symbol;Acc:HGNC:16468] |
| ENSG00000110675 | HGLibA_14938 | 367 | 2.184947356 | ELMOD1 | ELMO domain containing 1 [Source:HGNC Symbol;Acc:HGNC:25334] |
| ENSG00000121454 | HGLibA_26456 | 367 | 2.184947356 | LHX4 | LIM homeobox 4 [Source:HGNC Symbol;Acc:HGNC:21734] |
| ENSG00000162066 | HGLibA_01762 | 367 | 2.184947356 | AMDHD2 | amidohydrolase domain containing 2 [Source:HGNC Symbol;Acc:HGNC:24262] |
| ENSG00000171813 | HGLibA_39643 | 367 | 2.184947356 | PWWP2B | PWWP domain containing 2B [Source:HGNC Symbol;Acc:HGNC:25150] |
| ENSG00000180044 | HGLibA_06273 | 367 | 2.184947356 | C3orf80 | chromosome 3 open reading frame 80 [Source:HGNC Symbol;Acc:HGNC:40048] |
| ENSG00000182676 | HGLibA_38083 | 367 | 2.184947356 | PPP1R27 | protein phosphatase 1 regulatory subunit 27 [Source:HGNC Symbol;Acc:HGNC:16813] |
| ENSG00000196549 | HGLibA_29469 | 367 | 2.184947356 | MME | membrane metalloendopeptidase [Source:HGNC Symbol;Acc:HGNC:7154] |
| ENSG00000204882 | HGLibA_19997 | 367 | 2.184947356 | GPR20 | G protein-coupled receptor 20 [Source:HGNC Symbol;Acc:HGNC:4475] |
| ENSG00000205864 | HGLibA_25803 | 367 | 2.184947356 | KRTAP5-6 | keratin associated protein 5-6 [Source:HGNC Symbol;Acc:HGNC:23600] |
| TARSL2 | HGLibA_48351 | 367 | 2.184947356 | TARSL2 | - |
| TMEM180 | HGLibA_50096 | 367 | 2.184947356 | TMEM180 | - |
| ENSG00000046604 | HGLibA_14024 | 366 | 2.175902013 | DSG2 | desmoglein 2 [Source:HGNC Symbol;Acc:HGNC:3049] |
| ENSG00000099866 | HGLibA_27854 | 366 | 2.175902013 | MADCAM1 | mucosal vascular addressin cell adhesion molecule 1 [Source:HGNC Symbol;Acc:HGNC:6765] |
| ENSG00000113742 | HGLibA_10927 | 366 | 2.175902013 | CPEB4 | cytoplasmic polyadenylation element binding protein 4 [Source:HGNC Symbol;Acc:HGNC:21747] |
| ENSG00000115183 | HGLibA_48290 | 366 | 2.175902013 | TANC1 | ankyrin repeat and coiled-coil containing 1 [Source:HGNC Symbol;Acc:HGNC:29364]" |
| ENSG00000130427 | HGLibA_15351 | 366 | 2.175902013 | EPO | erythropoietin [Source:HGNC Symbol;Acc:HGNC:3415] |
| ENSG00000132965 | HGLibA_01691 | 366 | 2.175902013 | ALOX5AP | arachidonate 5-lipoxygenase activating protein [Source:HGNC Symbol;Acc:HGNC:436] |
| ENSG00000134321 | HGLibA_42299 | 366 | 2.175902013 | RSAD2 | radical S-adenosyl methionine domain containing 2 [Source:HGNC Symbol;Acc:HGNC:30908] |
| ENSG00000137473 | HGLibA_52100 | 366 | 2.175902013 | TTC29 | tetratricopeptide repeat domain 29 [Source:HGNC Symbol;Acc:HGNC:29936] |
| ENSG00000139433 | HGLibA_19342 | 366 | 2.175902013 | GLTP | glycolipid transfer protein [Source:HGNC Symbol;Acc:HGNC:24867] |
| ENSG00000144746 | HGLibA_02989 | 366 | 2.175902013 | ARL6IP5 | ADP ribosylation factor like GTPase 6 interacting protein 5 [Source:HGNC Symbol;Acc:HGNC:16937] |
| ENSG00000147394 | HGLibA_55548 | 366 | 2.175902013 | ZNF185 | zinc finger protein 185 with LIM domain [Source:HGNC Symbol;Acc:HGNC:12976] |
| ENSG00000151577 | HGLibA_13964 | 366 | 2.175902013 | DRD3 | dopamine receptor D3 [Source:HGNC Symbol;Acc:HGNC:3024] |
| ENSG00000154734 | HGLibA_00792 | 366 | 2.175902013 | ADAMTS1 | ADAM metallopeptidase with thrombospondin type 1 motif 1 [Source:HGNC Symbol;Acc:HGNC:217] |
| ENSG00000155256 | HGLibA_55288 | 366 | 2.175902013 | ZFYVE27 | zinc finger FYVE-type containing 27 [Source:HGNC Symbol;Acc:HGNC:26559] |
| ENSG00000162623 | HGLibA_52486 | 366 | 2.175902013 | TYW3 | tRNA-yW synthesizing protein 3 homolog [Source:HGNC Symbol;Acc:HGNC:24757] |
| ENSG00000171757 | HGLibA_27321 | 366 | 2.175902013 | LRRC34 | leucine rich repeat containing 34 [Source:HGNC Symbol;Acc:HGNC:28408] |
| ENSG00000175352 | HGLibA_32697 | 366 | 2.175902013 | NRIP3 | nuclear receptor interacting protein 3 [Source:HGNC Symbol;Acc:HGNC:1167] |
| ENSG00000196260 | HGLibA_43760 | 366 | 2.175902013 | SFTA2 | surfactant associated 2 [Source:HGNC Symbol;Acc:HGNC:18386] |
| ENSG00000197245 | HGLibA_16022 | 366 | 2.175902013 | FAM110D | family with sequence similarity 110 member D [Source:HGNC Symbol;Acc:HGNC:25860] |
| ENSG00000213022 | HGLibA_25274 | 366 | 2.175902013 | KLK9 | kallikrein related peptidase 9 [Source:HGNC Symbol;Acc:HGNC:6370] |
| HEATR2 | HGLibA_21069 | 366 | 2.175902013 | HEATR2 | - |
| hsa-mir-4317 | HGLibA_59988 | 366 | 2.175902013 | hsa-mir-4317 | - |
| hsa-mir-8065 | HGLibA_64099 | 366 | 2.175902013 | hsa-mir-8065 | - |
| ENSG00000074047 | HGLibA_19249 | 365 | 2.16685667 | GLI2 | GLI family zinc finger 2 [Source:HGNC Symbol;Acc:HGNC:4318] |
| ENSG00000106144 | HGLibA_07306 | 365 | 2.16685667 | CASP2 | caspase 2 [Source:HGNC Symbol;Acc:HGNC:1503] |
| ENSG00000107521 | HGLibA_22046 | 365 | 2.16685667 | HPS1 | biogenesis of lysosomal organelles complex 3 subunit 1 [Source:HGNC Symbol;Acc:HGNC:5163]" |
| ENSG00000118557 | HGLibA_37309 | 365 | 2.16685667 | PMFBP1 | polyamine modulated factor 1 binding protein 1 [Source:HGNC Symbol;Acc:HGNC:17728] |
| ENSG00000131236 | HGLibA_07132 | 365 | 2.16685667 | CAP1 | cyclase associated actin cytoskeleton regulatory protein 1 [Source:HGNC Symbol;Acc:HGNC:20040] |
| ENSG00000139190 | HGLibA_53488 | 365 | 2.16685667 | VAMP1 | vesicle associated membrane protein 1 [Source:HGNC Symbol;Acc:HGNC:12642] |
| ENSG00000139323 | HGLibA_37444 | 365 | 2.16685667 | POC1B | POC1 centriolar protein B [Source:HGNC Symbol;Acc:HGNC:30836] |
| ENSG00000160679 | HGLibA_09667 | 365 | 2.16685667 | CHTOP | chromatin target of PRMT1 [Source:HGNC Symbol;Acc:HGNC:24511] |
| ENSG00000164266 | HGLibA_46681 | 365 | 2.16685667 | SPINK1 | Kazal type 1 [Source:HGNC Symbol;Acc:HGNC:11244]" |
| ENSG00000166188 | HGLibA_55788 | 365 | 2.16685667 | ZNF319 | zinc finger protein 319 [Source:HGNC Symbol;Acc:HGNC:13644] |
| ENSG00000167077 | HGLibA_28861 | 365 | 2.16685667 | MEI1 | meiotic double-stranded break formation protein 1 [Source:HGNC Symbol;Acc:HGNC:28613] |
| ENSG00000168828 | HGLibA_33626 | 365 | 2.16685667 | OR13J1 | olfactory receptor family 13 subfamily J member 1 [Source:HGNC Symbol;Acc:HGNC:15108] |
| ENSG00000169567 | HGLibA_21323 | 365 | 2.16685667 | HINT1 | histidine triad nucleotide binding protein 1 [Source:HGNC Symbol;Acc:HGNC:4912] |
| ENSG00000175866 | HGLibA_04121 | 365 | 2.16685667 | BAIAP2 | BAI1 associated protein 2 [Source:HGNC Symbol;Acc:HGNC:947] |
| ENSG00000178229 | HGLibA_56195 | 365 | 2.16685667 | ZNF543 | zinc finger protein 543 [Source:HGNC Symbol;Acc:HGNC:25281] |
| ENSG00000181552 | HGLibA_14451 | 365 | 2.16685667 | EDDM3B | epididymal protein 3B [Source:HGNC Symbol;Acc:HGNC:19223] |
| ENSG00000198821 | HGLibA_08309 | 365 | 2.16685667 | CD247 | CD247 molecule [Source:HGNC Symbol;Acc:HGNC:1677] |
| FAM46D | HGLibA_16606 | 365 | 2.16685667 | FAM46D | - |
| hsa-mir-1252 | HGLibA_57405 | 365 | 2.16685667 | hsa-mir-1252 | - |
| hsa-mir-497 | HGLibA_61390 | 365 | 2.16685667 | hsa-mir-497 | - |
| hsa-mir-6755 | HGLibA_63176 | 365 | 2.16685667 | hsa-mir-6755 | - |
| ENSG00000067798 | HGLibA_31200 | 364 | 2.157811327 | NAV3 | neuron navigator 3 [Source:HGNC Symbol;Acc:HGNC:15998] |
| ENSG00000099256 | HGLibA_38976 | 364 | 2.157811327 | PRTFDC1 | phosphoribosyl transferase domain containing 1 [Source:HGNC Symbol;Acc:HGNC:23333] |
| ENSG00000100226 | HGLibA_20599 | 364 | 2.157811327 | GTPBP1 | GTP binding protein 1 [Source:HGNC Symbol;Acc:HGNC:4669] |
| ENSG00000100934 | HGLibA_43221 | 364 | 2.157811327 | SEC23A | coat complex II component [Source:HGNC Symbol;Acc:HGNC:10701]" |
| ENSG00000105327 | HGLibA_04196 | 364 | 2.157811327 | BBC3 | BCL2 binding component 3 [Source:HGNC Symbol;Acc:HGNC:17868] |
| ENSG00000105865 | HGLibA_14124 | 364 | 2.157811327 | DUS4L | dihydrouridine synthase 4 like [Source:HGNC Symbol;Acc:HGNC:21517] |
| ENSG00000106268 | HGLibA_32931 | 364 | 2.157811327 | NUDT1 | nudix hydrolase 1 [Source:HGNC Symbol;Acc:HGNC:8048] |
| ENSG00000121898 | HGLibA_11039 | 364 | 2.157811327 | CPXM2 | M14 family member 2 [Source:HGNC Symbol;Acc:HGNC:26977]" |
| ENSG00000125975 | HGLibA_05997 | 364 | 2.157811327 | C20orf173 | chromosome 20 open reading frame 173 [Source:HGNC Symbol;Acc:HGNC:16166] |
| ENSG00000131068 | HGLibA_12864 | 364 | 2.157811327 | DEFB118 | defensin beta 118 [Source:HGNC Symbol;Acc:HGNC:16196] |
| ENSG00000140519 | HGLibA_41149 | 364 | 2.157811327 | RHCG | Rh family C glycoprotein [Source:HGNC Symbol;Acc:HGNC:18140] |
| ENSG00000171502 | HGLibA_10549 | 364 | 2.157811327 | COL24A1 | collagen type XXIV alpha 1 chain [Source:HGNC Symbol;Acc:HGNC:20821] |
| ENSG00000184601 | HGLibA_05349 | 364 | 2.157811327 | C14orf180 | chromosome 14 open reading frame 180 [Source:HGNC Symbol;Acc:HGNC:33795] |
| ENSG00000186715 | HGLibA_30255 | 364 | 2.157811327 | MST1L | macrophage stimulating 1 like [Source:HGNC Symbol;Acc:HGNC:7390] |
| GPR1 | HGLibA_19838 | 364 | 2.157811327 | GPR1 | - |
| hsa-mir-655 | HGLibA_62952 | 364 | 2.157811327 | hsa-mir-655 | - |
| PPAPDC3 | HGLibA_37835 | 364 | 2.157811327 | PPAPDC3 | - |
| ENSG00000023171 | HGLibA_20198 | 363 | 2.148765984 | GRAMD1B | GRAM domain containing 1B [Source:HGNC Symbol;Acc:HGNC:29214] |
| ENSG00000053918 | HGLibA_24365 | 363 | 2.148765984 | KCNQ1 | potassium voltage-gated channel subfamily Q member 1 [Source:HGNC Symbol;Acc:HGNC:6294] |
| ENSG00000079819 | HGLibA_15245 | 363 | 2.148765984 | EPB41L2 | erythrocyte membrane protein band 4.1 like 2 [Source:HGNC Symbol;Acc:HGNC:3379] |
| ENSG00000100034 | HGLibA_37975 | 363 | 2.148765984 | PPM1F | Mg2+/Mn2+ dependent 1F [Source:HGNC Symbol;Acc:HGNC:19388]" |
| ENSG00000106070 | HGLibA_20223 | 363 | 2.148765984 | GRB10 | growth factor receptor bound protein 10 [Source:HGNC Symbol;Acc:HGNC:4564] |
| ENSG00000110171 | HGLibA_51455 | 363 | 2.148765984 | TRIM3 | tripartite motif containing 3 [Source:HGNC Symbol;Acc:HGNC:10064] |
| ENSG00000114124 | HGLibA_20338 | 363 | 2.148765984 | GRK7 | G protein-coupled receptor kinase 7 [Source:HGNC Symbol;Acc:HGNC:17031] |
| ENSG00000115750 | HGLibA_48217 | 363 | 2.148765984 | TAF1B | RNA polymerase I subunit B [Source:HGNC Symbol;Acc:HGNC:11533]" |
| ENSG00000116791 | HGLibA_11294 | 363 | 2.148765984 | CRYZ | crystallin zeta [Source:HGNC Symbol;Acc:HGNC:2419] |
| ENSG00000116830 | HGLibA_52171 | 363 | 2.148765984 | TTF2 | transcription termination factor 2 [Source:HGNC Symbol;Acc:HGNC:12398] |
| ENSG00000131015 | HGLibA_52964 | 363 | 2.148765984 | ULBP2 | UL16 binding protein 2 [Source:HGNC Symbol;Acc:HGNC:14894] |

| | | | | | |
|---|---|---|---|---|---|
| ENSG00000134107 | HGLibA_04478 | 363 | 2.148765984 | BHLHE40 | basic helix-loop-helix family member e40 [Source:HGNC Symbol;Acc:HGNC:1046] |
| ENSG00000135324 | HGLibA_29811 | 363 | 2.148765984 | MRAP2 | melanocortin 2 receptor accessory protein 2 [Source:HGNC Symbol;Acc:HGNC:21232] |
| ENSG00000153048 | HGLibA_07246 | 363 | 2.148765984 | CARHSP1 | calcium regulated heat stable protein 1 [Source:HGNC Symbol;Acc:HGNC:17150] |
| ENSG00000154274 | HGLibA_06299 | 363 | 2.148765984 | C4orf19 | chromosome 4 open reading frame 19 [Source:HGNC Symbol;Acc:HGNC:25618] |
| ENSG00000161813 | HGLibA_26035 | 363 | 2.148765984 | LARP4 | La ribonucleoprotein domain family member 4 [Source:HGNC Symbol;Acc:HGNC:24320] |
| ENSG00000166869 | HGLibA_09519 | 363 | 2.148765984 | CHP2 | calcineurin like EF-hand protein 2 [Source:HGNC Symbol;Acc:HGNC:24927] |
| ENSG00000168411 | HGLibA_40958 | 363 | 2.148765984 | RFWD3 | ring finger and WD repeat domain 3 [Source:HGNC Symbol;Acc:HGNC:25539] |
| ENSG00000169855 | HGLibA_41734 | 363 | 2.148765984 | ROBO1 | roundabout guidance receptor 1 [Source:HGNC Symbol;Acc:HGNC:10249] |
| ENSG00000173917 | HGLibA_21933 | 363 | 2.148765984 | HOXB2 | homeobox B2 [Source:HGNC Symbol;Acc:HGNC:5113] |
| ENSG00000177519 | HGLibA_42053 | 363 | 2.148765984 | RPRM | TP53 dependent G2 arrest mediator homolog [Source:HGNC Symbol;Acc:HGNC:24201]" |
| ENSG00000179148 | HGLibA_01694 | 363 | 2.148765984 | ALOXE3 | arachidonate lipoxygenase 3 [Source:HGNC Symbol;Acc:HGNC:13743] |
| ENSG00000187243 | HGLibA_27977 | 363 | 2.148765984 | MAGED4B | MAGE family member D4B [Source:HGNC Symbol;Acc:HGNC:22880] |
| ENSG00000197712 | HGLibA_16029 | 363 | 2.148765984 | FAM114A1 | family with sequence similarity 114 member A1 [Source:HGNC Symbol;Acc:HGNC:25087] |
| ENSG00000204219 | HGLibA_48702 | 363 | 2.148765984 | TCEA3 | transcription elongation factor A3 [Source:HGNC Symbol;Acc:HGNC:11615] |
| hsa-mir-372 | HGLibA_59381 | 363 | 2.148765984 | hsa-mir-372 | - |
| C1QTNF9B-AS1 | HGLibA_05965 | 362 | 2.139720641 | C1QTNF9B-AS1 | - |
| ENSG00000004776 | HGLibA_22314 | 362 | 2.139720641 | HSPB6 | heat shock protein family B (small) member 6 [Source:HGNC Symbol;Acc:HGNC:26511] |
| ENSG00000063180 | HGLibA_06792 | 362 | 2.139720641 | CA11 | carbonic anhydrase 11 [Source:HGNC Symbol;Acc:HGNC:1370] |
| ENSG00000099940 | HGLibA_45880 | 362 | 2.139720641 | SNAP29 | synaptosome associated protein 29 [Source:HGNC Symbol;Acc:HGNC:11133] |
| ENSG00000114654 | HGLibA_14557 | 362 | 2.139720641 | EFCC1 | EF-hand and coiled-coil domain containing 1 [Source:HGNC Symbol;Acc:HGNC:25692] |
| ENSG00000120690 | HGLibA_14892 | 362 | 2.139720641 | ELF1 | E74 like ETS transcription factor 1 [Source:HGNC Symbol;Acc:HGNC:3316] |
| ENSG00000125249 | HGLibA_40235 | 362 | 2.139720641 | RAP2A | member of RAS oncogene family [Source:HGNC Symbol;Acc:HGNC:9861]" |
| ENSG00000125831 | HGLibA_11448 | 362 | 2.139720641 | CST11 | cystatin 11 [Source:HGNC Symbol;Acc:HGNC:15959] |
| ENSG00000138073 | HGLibA_38425 | 362 | 2.139720641 | PREB | prolactin regulatory element binding [Source:HGNC Symbol;Acc:HGNC:9356] |
| ENSG00000151327 | HGLibA_16294 | 362 | 2.139720641 | FAM177A1 | family with sequence similarity 177 member A1 [Source:HGNC Symbol;Acc:HGNC:19829] |
| ENSG00000161055 | HGLibA_42931 | 362 | 2.139720641 | SCGB3A1 | secretoglobin family 3A member 1 [Source:HGNC Symbol;Acc:HGNC:18384] |
| ENSG00000164520 | HGLibA_40119 | 362 | 2.139720641 | RAET1E | retinoic acid early transcript 1E [Source:HGNC Symbol;Acc:HGNC:16793] |
| ENSG00000166349 | HGLibA_40132 | 362 | 2.139720641 | RAG1 | recombination activating 1 [Source:HGNC Symbol;Acc:HGNC:9831] |
| ENSG00000167755 | HGLibA_25265 | 362 | 2.139720641 | KLK6 | kallikrein related peptidase 6 [Source:HGNC Symbol;Acc:HGNC:6367] |
| ENSG00000168661 | HGLibA_55769 | 362 | 2.139720641 | ZNF30 | zinc finger protein 30 [Source:HGNC Symbol;Acc:HGNC:13090] |
| ENSG00000171155 | HGLibA_05727 | 362 | 2.139720641 | C1GALT1C1 | C1GALT1 specific chaperone 1 [Source:HGNC Symbol;Acc:HGNC:24338] |
| ENSG00000176209 | HGLibA_45743 | 362 | 2.139720641 | SMIM19 | small integral membrane protein 19 [Source:HGNC Symbol;Acc:HGNC:25166] |
| ENSG00000176922 | HGLibA_34130 | 362 | 2.139720641 | OR51S1 | olfactory receptor family 51 subfamily S member 1 [Source:HGNC Symbol;Acc:HGNC:15204] |
| ENSG00000186090 | HGLibA_22389 | 362 | 2.139720641 | HTR3D | 5-hydroxytryptamine receptor 3D [Source:HGNC Symbol;Acc:HGNC:24004] |
| ENSG00000186815 | HGLibA_51105 | 362 | 2.139720641 | TPCN1 | two pore segment channel 1 [Source:HGNC Symbol;Acc:HGNC:18182] |
| hsa-mir-429 | HGLibA_59866 | 362 | 2.139720641 | hsa-mir-429 | - |
| hsa-mir-549a | HGLibA_62093 | 362 | 2.139720641 | hsa-mir-549a | - |
| hsa-mir-6881 | HGLibA_63707 | 362 | 2.139720641 | hsa-mir-6881 | - |
| ENSG00000111981 | HGLibA_52961 | 361 | 2.130675299 | ULBP1 | UL16 binding protein 1 [Source:HGNC Symbol;Acc:HGNC:14893] |
| ENSG00000115592 | HGLibA_38517 | 361 | 2.130675299 | PRKAG3 | protein kinase AMP-activated non-catalytic subunit gamma 3 [Source:HGNC Symbol;Acc:HGNC:9387] |
| ENSG00000119227 | HGLibA_36605 | 361 | 2.130675299 | PIGZ | phosphatidylinositol glycan anchor biosynthesis class Z [Source:HGNC Symbol;Acc:HGNC:30596] |
| ENSG00000121900 | HGLibA_50441 | 361 | 2.130675299 | TMEM54 | transmembrane protein 54 [Source:HGNC Symbol;Acc:HGNC:24143] |
| ENSG00000139974 | HGLibA_45075 | 361 | 2.130675299 | SLC38A6 | solute carrier family 38 member 6 [Source:HGNC Symbol;Acc:HGNC:19863] |
| ENSG00000140470 | HGLibA_00813 | 361 | 2.130675299 | ADAMTS17 | ADAM metallopeptidase with thrombospondin type 1 motif 17 [Source:HGNC Symbol;Acc:HGNC:17109] |
| ENSG00000166086 | HGLibA_23933 | 361 | 2.130675299 | JAM3 | junctional adhesion molecule 3 [Source:HGNC Symbol;Acc:HGNC:15532] |
| ENSG00000167685 | HGLibA_56013 | 361 | 2.130675299 | ZNF444 | zinc finger protein 444 [Source:HGNC Symbol;Acc:HGNC:16052] |
| ENSG00000173511 | HGLibA_53614 | 361 | 2.130675299 | VEGFB | vascular endothelial growth factor B [Source:HGNC Symbol;Acc:HGNC:12681] |
| ENSG00000205356 | HGLibA_48941 | 361 | 2.130675299 | TECPR1 | tectonin beta-propeller repeat containing 1 [Source:HGNC Symbol;Acc:HGNC:22214] |
| ENSG00000205667 | HGLibA_03145 | 361 | 2.130675299 | ARSH | arylsulfatase family member H [Source:HGNC Symbol;Acc:HGNC:32488] |
| hsa-mir-195 | HGLibA_58087 | 361 | 2.130675299 | hsa-mir-195 | - |
| hsa-mir-4655 | HGLibA_60729 | 361 | 2.130675299 | hsa-mir-4655 | - |
| ENSG00000101624 | HGLibA_09165 | 360 | 2.121629956 | CEP76 | centrosomal protein 76 [Source:HGNC Symbol;Acc:HGNC:25727] |
| ENSG00000105617 | HGLibA_26296 | 360 | 2.121629956 | LENG1 | leukocyte receptor cluster member 1 [Source:HGNC Symbol;Acc:HGNC:15502] |
| ENSG00000135269 | HGLibA_49022 | 360 | 2.121629956 | TES | testin LIM domain protein [Source:HGNC Symbol;Acc:HGNC:14620] |
| ENSG00000139344 | HGLibA_01759 | 360 | 2.121629956 | AMDHD1 | amidohydrolase domain containing 1 [Source:HGNC Symbol;Acc:HGNC:28577] |
| ENSG00000149084 | HGLibA_22178 | 360 | 2.121629956 | HSD17B12 | hydroxysteroid 17-beta dehydrogenase 12 [Source:HGNC Symbol;Acc:HGNC:18646] |
| ENSG00000149534 | HGLibA_30145 | 360 | 2.121629956 | MS4A2 | membrane spanning 4-domains A2 [Source:HGNC Symbol;Acc:HGNC:7316] |
| ENSG00000153107 | HGLibA_01862 | 360 | 2.121629956 | ANAPC1 | anaphase promoting complex subunit 1 [Source:HGNC Symbol;Acc:HGNC:19988] |
| ENSG00000158062 | HGLibA_52779 | 360 | 2.121629956 | UBXN11 | UBX domain protein 11 [Source:HGNC Symbol;Acc:HGNC:30600] |
| ENSG00000162782 | HGLibA_48908 | 360 | 2.121629956 | TDRD5 | tudor domain containing 5 [Source:HGNC Symbol;Acc:HGNC:20614] |
| ENSG00000164047 | HGLibA_07102 | 360 | 2.121629956 | CAMP | cathelicidin antimicrobial peptide [Source:HGNC Symbol;Acc:HGNC:1472] |
| ENSG00000176239 | HGLibA_34090 | 360 | 2.121629956 | OR51B6 | olfactory receptor family 51 subfamily B member 6 [Source:HGNC Symbol;Acc:HGNC:19600] |
| ENSG00000205884 | HGLibA_12912 | 360 | 2.121629956 | DEFB136 | defensin beta 136 [Source:HGNC Symbol;Acc:HGNC:34433] |
| ENSG00000213694 | HGLibA_42600 | 360 | 2.121629956 | S1PR3 | sphingosine-1-phosphate receptor 3 [Source:HGNC Symbol;Acc:HGNC:3167] |
| ENSG00000221938 | HGLibA_33724 | 360 | 2.121629956 | OR2A14 | olfactory receptor family 2 subfamily A member 14 [Source:HGNC Symbol;Acc:HGNC:15084] |
| hsa-mir-6791 | HGLibA_63341 | 360 | 2.121629956 | hsa-mir-6791 | - |
| hsa-mir-6815 | HGLibA_63439 | 360 | 2.121629956 | hsa-mir-6815 | - |
| hsa-mir-6837 | HGLibA_63522 | 360 | 2.121629956 | hsa-mir-6837 | - |
| ENSG00000081853 | HGLibA_35528 | 359 | 2.112584613 | PCDHGA2 | 2 [Source:HGNC Symbol;Acc:HGNC:8700]" |
| ENSG00000124143 | HGLibA_02777 | 359 | 2.112584613 | ARHGAP40 | Rho GTPase activating protein 40 [Source:HGNC Symbol;Acc:HGNC:16226] |
| ENSG00000135175 | HGLibA_33261 | 359 | 2.112584613 | OCM2 | oncomodulin 2 [Source:HGNC Symbol;Acc:HGNC:34396] |
| ENSG00000139767 | HGLibA_47050 | 359 | 2.112584613 | SRRM4 | serine/arginine repetitive matrix 4 [Source:HGNC Symbol;Acc:HGNC:29389] |
| ENSG00000139890 | HGLibA_40833 | 359 | 2.112584613 | REM2 | RRAD and GEM like GTPase 2 [Source:HGNC Symbol;Acc:HGNC:20248] |
| ENSG00000145220 | HGLibA_27695 | 359 | 2.112584613 | LYAR | Ly1 antibody reactive [Source:HGNC Symbol;Acc:HGNC:26021] |
| ENSG00000145734 | HGLibA_04389 | 359 | 2.112584613 | BDP1 | subunit of RNA polymerase III transcription initiation factor IIIB [Source:HGNC Symbol;Acc:HGNC:13652]" |
| ENSG00000151332 | HGLibA_28497 | 359 | 2.112584613 | MBIP | MAP3K12 binding inhibitory protein 1 [Source:HGNC Symbol;Acc:HGNC:20427] |
| ENSG00000155016 | HGLibA_12145 | 359 | 2.112584613 | CYP2U1 | cytochrome P450 family 2 subfamily U member 1 [Source:HGNC Symbol;Acc:HGNC:20582] |
| ENSG00000162366 | HGLibA_35993 | 359 | 2.112584613 | PDZK1IP1 | PDZK1 interacting protein 1 [Source:HGNC Symbol;Acc:HGNC:16887] |
| ENSG00000163704 | HGLibA_38888 | 359 | 2.112584613 | PRRT3 | proline rich transmembrane protein 3 [Source:HGNC Symbol;Acc:HGNC:26591] |
| ENSG00000167654 | HGLibA_03424 | 359 | 2.112584613 | ATCAY | caytaxin [Source:HGNC Symbol;Acc:HGNC:779]" |
| ENSG00000168913 | HGLibA_15132 | 359 | 2.112584613 | ENHO | energy homeostasis associated [Source:HGNC Symbol;Acc:HGNC:24838] |
| ENSG00000175003 | HGLibA_44533 | 359 | 2.112584613 | SLC22A1 | solute carrier family 22 member 1 [Source:HGNC Symbol;Acc:HGNC:10963] |
| ENSG00000175267 | HGLibA_53903 | 359 | 2.112584613 | VWA3A | von Willebrand factor A domain containing 3A [Source:HGNC Symbol;Acc:HGNC:27088] |
| ENSG00000182853 | HGLibA_53691 | 359 | 2.112584613 | VMO1 | vitelline membrane outer layer 1 homolog [Source:HGNC Symbol;Acc:HGNC:30387] |
| ENSG00000186971 | HGLibA_25652 | 359 | 2.112584613 | KRTAP13-4 | keratin associated protein 13-4 [Source:HGNC Symbol;Acc:HGNC:18926] |
| HIST1H4L | HGLibA_21518 | 359 | 2.112584613 | HIST1H4L | - |
| ENSG00000033050 | HGLibA_00208 | 358 | 2.10353927 | ABCF2 | ATP binding cassette subfamily F member 2 [Source:HGNC Symbol;Acc:HGNC:71] |
| ENSG00000073803 | HGLibA_28173 | 358 | 2.10353927 | MAP3K13 | mitogen-activated protein kinase kinase kinase 13 [Source:HGNC Symbol;Acc:HGNC:6852] |
| ENSG00000089775 | HGLibA_54850 | 358 | 2.10353927 | ZBTB25 | zinc finger and BTB domain containing 25 [Source:HGNC Symbol;Acc:HGNC:13112] |
| ENSG00000106682 | HGLibA_14848 | 358 | 2.10353927 | EIF4H | eukaryotic translation initiation factor 4H [Source:HGNC Symbol;Acc:HGNC:12741] |
| ENSG00000110108 | HGLibA_49867 | 358 | 2.10353927 | TMEM109 | transmembrane protein 109 [Source:HGNC Symbol;Acc:HGNC:28771] |
| ENSG00000120903 | HGLibA_09569 | 358 | 2.10353927 | CHRNA2 | cholinergic receptor nicotinic alpha 2 subunit [Source:HGNC Symbol;Acc:HGNC:1956] |
| ENSG00000125656 | HGLibA_10101 | 358 | 2.10353927 | CLPP | caseinolytic mitochondrial matrix peptidase proteolytic subunit [Source:HGNC Symbol;Acc:HGNC:2084] |
| ENSG00000136881 | HGLibA_04053 | 358 | 2.10353927 | BAAT | bile acid-CoA:amino acid N-acyltransferase [Source:HGNC Symbol;Acc:HGNC:932] |
| ENSG00000137745 | HGLibA_29489 | 358 | 2.10353927 | MMP13 | matrix metallopeptidase 13 [Source:HGNC Symbol;Acc:HGNC:7159] |
| ENSG00000140995 | HGLibA_12790 | 358 | 2.10353927 | DEF8 | differentially expressed in FDCP 8 homolog [Source:HGNC Symbol;Acc:HGNC:25969] |
| ENSG00000146109 | HGLibA_00331 | 358 | 2.10353927 | ABT1 | activator of basal transcription 1 [Source:HGNC Symbol;Acc:HGNC:17369] |
| ENSG00000156564 | HGLibA_27155 | 358 | 2.10353927 | LRFN2 | leucine rich repeat and fibronectin type III domain containing 2 [Source:HGNC Symbol;Acc:HGNC:21226] |
| ENSG00000169594 | HGLibA_04643 | 358 | 2.10353927 | BNC1 | basonuclin 1 [Source:HGNC Symbol;Acc:HGNC:1081] |
| ENSG00000170275 | HGLibA_11224 | 358 | 2.10353927 | CRTAP | cartilage associated protein [Source:HGNC Symbol;Acc:HGNC:2379] |
| ENSG00000188153 | HGLibA_10580 | 358 | 2.10353927 | COL4A5 | collagen type IV alpha 5 chain [Source:HGNC Symbol;Acc:HGNC:2207] |
| ENSG00000189164 | HGLibA_56166 | 358 | 2.10353927 | ZNF527 | zinc finger protein 527 [Source:HGNC Symbol;Acc:HGNC:29385] |
| ENSG00000198862 | HGLibA_27614 | 358 | 2.10353927 | LTN1 | listerin E3 ubiquitin protein ligase 1 [Source:HGNC Symbol;Acc:HGNC:13082] |
| ENSG00000258947 | HGLibA_52282 | 358 | 2.10353927 | TUBB3 | tubulin beta 3 class III [Source:HGNC Symbol;Acc:HGNC:20772] |
| ENSG00000279804 | HGLibA_38303 | 358 | 2.10353927 | PRAMEF18 | PRAME family member 18 [Source:HGNC Symbol;Acc:HGNC:30693] |
| hsa-mir-188 | HGLibA_58016 | 358 | 2.10353927 | hsa-mir-188 | - |
| hsa-mir-574 | HGLibA_62391 | 358 | 2.10353927 | hsa-mir-574 | - |
| hsa-mir-6890 | HGLibA_63746 | 358 | 2.10353927 | hsa-mir-6890 | - |
| ENSG00000004399 | HGLibA_37283 | 357 | 2.094493927 | PLXND1 | plexin D1 [Source:HGNC Symbol;Acc:HGNC:9107] |
| ENSG00000004838 | HGLibA_55410 | 357 | 2.094493927 | ZMYND10 | zinc finger MYND-type containing 10 [Source:HGNC Symbol;Acc:HGNC:19412] |
| ENSG00000008196 | HGLibA_49124 | 357 | 2.094493927 | TFAP2B | transcription factor AP-2 beta [Source:HGNC Symbol;Acc:HGNC:11743] |
| ENSG00000026508 | HGLibA_08391 | 357 | 2.094493927 | CD44 | CD44 molecule (Indian blood group) [Source:HGNC Symbol;Acc:HGNC:1681] |
| ENSG00000063015 | HGLibA_43680 | 357 | 2.094493927 | SEZ6 | seizure related 6 homolog [Source:HGNC Symbol;Acc:HGNC:15955] |
| ENSG00000066427 | HGLibA_03842 | 357 | 2.094493927 | ATXN3 | ataxin 3 [Source:HGNC Symbol;Acc:HGNC:7106] |
| ENSG00000109107 | HGLibA_01598 | 357 | 2.094493927 | ALDOC | fructose-bisphosphate C [Source:HGNC Symbol;Acc:HGNC:418]" |
| ENSG00000110047 | HGLibA_14666 | 357 | 2.094493927 | EHD1 | EH domain containing 1 [Source:HGNC Symbol;Acc:HGNC:3242] |
| ENSG00000111344 | HGLibA_40310 | 357 | 2.094493927 | RASAL1 | RAS protein activator like 1 [Source:HGNC Symbol;Acc:HGNC:9873] |
| ENSG00000119408 | HGLibA_31658 | 357 | 2.094493927 | NEK6 | NIMA related kinase 6 [Source:HGNC Symbol;Acc:HGNC:7749] |
| ENSG00000122584 | HGLibA_33163 | 357 | 2.094493927 | NXPH1 | neurexophilin 1 [Source:HGNC Symbol;Acc:HGNC:20693] |
| ENSG00000125414 | HGLibA_30720 | 357 | 2.094493927 | MYH2 | myosin heavy chain 2 [Source:HGNC Symbol;Acc:HGNC:7572] |
| ENSG00000136603 | HGLibA_44281 | 357 | 2.094493927 | SKIL | SKI like proto-oncogene [Source:HGNC Symbol;Acc:HGNC:10897] |
| ENSG00000139112 | HGLibA_18334 | 357 | 2.094493927 | GABARAPL1 | GABA type A receptor associated protein like 1 [Source:HGNC Symbol;Acc:HGNC:4068] |
| ENSG00000141738 | HGLibA_20234 | 357 | 2.094493927 | GRB7 | growth factor receptor bound protein 7 [Source:HGNC Symbol;Acc:HGNC:4567] |
| ENSG00000152583 | HGLibA_46421 | 357 | 2.094493927 | SPARCL1 | SPARC like 1 [Source:HGNC Symbol;Acc:HGNC:11220] |
| ENSG00000169871 | HGLibA_51548 | 357 | 2.094493927 | TRIM56 | tripartite motif containing 56 [Source:HGNC Symbol;Acc:HGNC:19028] |
| ENSG00000169914 | HGLibA_34761 | 357 | 2.094493927 | OTUD3 | OTU deubiquitinase 3 [Source:HGNC Symbol;Acc:HGNC:29038] |
| ENSG00000176024 | HGLibA_56387 | 357 | 2.094493927 | ZNF613 | zinc finger protein 613 [Source:HGNC Symbol;Acc:HGNC:25827] |
| ENSG00000182858 | HGLibA_01612 | 357 | 2.094493927 | ALG12 | 6-mannosyltransferase [Source:HGNC Symbol;Acc:HGNC:19358]" |
| ENSG00000184730 | HGLibA_02500 | 357 | 2.094493927 | APOBR | apolipoprotein B receptor [Source:HGNC Symbol;Acc:HGNC:24087] |
| ENSG00000204421 | HGLibA_27665 | 357 | 2.094493927 | LY6G6C | lymphocyte antigen 6 family member G6C [Source:HGNC Symbol;Acc:HGNC:13936] |
| hsa-mir-4504 | HGLibA_60444 | 357 | 2.094493927 | hsa-mir-4504 | - |
| LRRC48 | HGLibA_27374 | 357 | 2.094493927 | LRRC48 | - |
| ENSG00000002746 | HGLibA_21103 | 356 | 2.085448584 | HECW1 | C2 and WW domain containing E3 ubiquitin protein ligase 1 [Source:HGNC Symbol;Acc:HGNC:22195]" |
| ENSG00000070031 | HGLibA_43065 | 356 | 2.085448584 | SCT | secretin [Source:HGNC Symbol;Acc:HGNC:10607] |
| ENSG00000083535 | HGLibA_36518 | 356 | 2.085448584 | PIBF1 | progesterone immunomodulatory binding factor 1 [Source:HGNC Symbol;Acc:HGNC:23352] |

| | | | | | |
|---|---|---|---|---|---|
| ENSG00000102385 | HGLibA_13986 | 356 | 2.085448584 | DRP2 | dystrophin related protein 2 [Source:HGNC Symbol;Acc:HGNC:3032] |
| ENSG00000109743 | HGLibA_04881 | 356 | 2.085448584 | BST1 | bone marrow stromal cell antigen 1 [Source:HGNC Symbol;Acc:HGNC:1118] |
| ENSG00000118271 | HGLibA_52227 | 356 | 2.085448584 | TTR | transthyretin [Source:HGNC Symbol;Acc:HGNC:12405] |
| ENSG00000119927 | HGLibA_19725 | 356 | 2.085448584 | GPAM | mitochondrial [Source:HGNC Symbol;Acc:HGNC:24865]" |
| ENSG00000120054 | HGLibA_10946 | 356 | 2.085448584 | CPN1 | carboxypeptidase N subunit 1 [Source:HGNC Symbol;Acc:HGNC:2312] |
| ENSG00000134470 | HGLibA_22983 | 356 | 2.085448584 | IL15RA | interleukin 15 receptor subunit alpha [Source:HGNC Symbol;Acc:HGNC:5978] |
| ENSG00000142408 | HGLibA_06951 | 356 | 2.085448584 | CACNG8 | calcium voltage-gated channel auxiliary subunit gamma 8 [Source:HGNC Symbol;Acc:HGNC:13628] |
| ENSG00000150637 | HGLibA_08299 | 356 | 2.085448584 | CD226 | CD226 molecule [Source:HGNC Symbol;Acc:HGNC:16961] |
| ENSG00000152217 | HGLibA_43634 | 356 | 2.085448584 | SETBP1 | SET binding protein 1 [Source:HGNC Symbol;Acc:HGNC:15573] |
| ENSG00000166415 | HGLibA_54166 | 356 | 2.085448584 | WDR72 | WD repeat domain 72 [Source:HGNC Symbol;Acc:HGNC:26790] |
| ENSG00000167183 | HGLibA_38788 | 356 | 2.085448584 | PRR15L | proline rich 15 like [Source:HGNC Symbol;Acc:HGNC:28149] |
| ENSG00000170236 | HGLibA_53392 | 356 | 2.085448584 | USP50 | ubiquitin specific peptidase 50 [Source:HGNC Symbol;Acc:HGNC:20079] |
| ENSG00000170469 | HGLibA_46455 | 356 | 2.085448584 | SPATA24 | spermatogenesis associated 24 [Source:HGNC Symbol;Acc:HGNC:27322] |
| ENSG00000171227 | HGLibA_50374 | 356 | 2.085448584 | TMEM37 | transmembrane protein 37 [Source:HGNC Symbol;Acc:HGNC:18216] |
| ENSG00000172146 | HGLibA_33642 | 356 | 2.085448584 | OR1A1 | olfactory receptor family 1 subfamily A member 1 [Source:HGNC Symbol;Acc:HGNC:8179] |
| ENSG00000177108 | HGLibA_55105 | 356 | 2.085448584 | ZDHHC22 | zinc finger DHHC-type containing 22 [Source:HGNC Symbol;Acc:HGNC:20106] |
| ENSG00000181803 | HGLibA_34442 | 356 | 2.085448584 | OR6S1 | olfactory receptor family 6 subfamily S member 1 [Source:HGNC Symbol;Acc:HGNC:15363] |
| ENSG00000183150 | HGLibA_19993 | 356 | 2.085448584 | GPR19 | G protein-coupled receptor 19 [Source:HGNC Symbol;Acc:HGNC:4473] |
| ENSG00000196917 | HGLibA_20947 | 356 | 2.085448584 | HCAR1 | hydroxycarboxylic acid receptor 1 [Source:HGNC Symbol;Acc:HGNC:4532] |
| ENSG00000241360 | HGLibA_35965 | 356 | 2.085448584 | PDXP | pyridoxal phosphatase [Source:HGNC Symbol;Acc:HGNC:30259] |
| hsa-mir-885 | HGLibA_64222 | 356 | 2.085448584 | hsa-mir-885 | - |
| 12-Sep | HGLibA_43433 | 356 | 2.085448584 | 12-Sep | - |
| ENSG00000003137 | HGLibA_12087 | 355 | 2.076403241 | CYP26B1 | cytochrome P450 family 26 subfamily B member 1 [Source:HGNC Symbol;Acc:HGNC:20581] |
| ENSG00000050426 | HGLibA_26339 | 355 | 2.076403241 | LETMD1 | LETM1 domain containing 1 [Source:HGNC Symbol;Acc:HGNC:24241] |
| ENSG00000099977 | HGLibA_12641 | 355 | 2.076403241 | DDT | D-dopachrome tautomerase [Source:HGNC Symbol;Acc:HGNC:2732] |
| ENSG00000100744 | HGLibA_20435 | 355 | 2.076403241 | GSKIP | GSK3B interacting protein [Source:HGNC Symbol;Acc:HGNC:20343] |
| ENSG00000129152 | HGLibA_30881 | 355 | 2.076403241 | MYOD1 | myogenic differentiation 1 [Source:HGNC Symbol;Acc:HGNC:7611] |
| ENSG00000168397 | HGLibA_03492 | 355 | 2.076403241 | ATG4B | autophagy related 4B cysteine peptidase [Source:HGNC Symbol;Acc:HGNC:20790] |
| ENSG00000179057 | HGLibA_22887 | 355 | 2.076403241 | IGSF22 | immunoglobulin superfamily member 22 [Source:HGNC Symbol;Acc:HGNC:26750] |
| ENSG00000180720 | HGLibA_09557 | 355 | 2.076403241 | CHRM4 | cholinergic receptor muscarinic 4 [Source:HGNC Symbol;Acc:HGNC:1953] |
| ENSG00000204482 | HGLibA_27572 | 355 | 2.076403241 | LST1 | leukocyte specific transcript 1 [Source:HGNC Symbol;Acc:HGNC:14189] |
| ENSG00000234745 | HGLibA_21596 | 355 | 2.076403241 | HLA-B | B [Source:HGNC Symbol;Acc:HGNC:4932]" |
| ENSG00000236699 | HGLibA_02859 | 355 | 2.076403241 | ARHGEF38 | Rho guanine nucleotide exchange factor 38 [Source:HGNC Symbol;Acc:HGNC:25968] |
| ENSG00000259431 | HGLibA_49412 | 355 | 2.076403241 | THTPA | thiamine triphosphatase [Source:HGNC Symbol;Acc:HGNC:18987] |
| hsa-mir-4725 | HGLibA_60996 | 355 | 2.076403241 | hsa-mir-4725 | - |
| hsa-mir-520e | HGLibA_61749 | 355 | 2.076403241 | hsa-mir-520e | - |
| ENSG00000078018 | HGLibA_28135 | 354 | 2.067357898 | MAP2 | microtubule associated protein 2 [Source:HGNC Symbol;Acc:HGNC:6839] |
| ENSG00000078401 | HGLibA_14470 | 354 | 2.067357898 | EDN1 | endothelin 1 [Source:HGNC Symbol;Acc:HGNC:3176] |
| ENSG00000080511 | HGLibA_40753 | 354 | 2.067357898 | RDH8 | retinol dehydrogenase 8 [Source:HGNC Symbol;Acc:HGNC:14423] |
| ENSG00000080573 | HGLibA_10594 | 354 | 2.067357898 | COL5A3 | collagen type V alpha 3 chain [Source:HGNC Symbol;Acc:HGNC:14864] |
| ENSG00000113525 | HGLibA_23174 | 354 | 2.067357898 | IL5 | interleukin 5 [Source:HGNC Symbol;Acc:HGNC:6016] |
| ENSG00000114021 | HGLibA_31985 | 354 | 2.067357898 | NIT2 | nitrilase family member 2 [Source:HGNC Symbol;Acc:HGNC:29878] |
| ENSG00000114739 | HGLibA_00698 | 354 | 2.067357898 | ACVR2B | activin A receptor type 2B [Source:HGNC Symbol;Acc:HGNC:174] |
| ENSG00000119392 | HGLibA_19240 | 354 | 2.067357898 | GLE1 | RNA export mediator [Source:HGNC Symbol;Acc:HGNC:4315]" |
| ENSG00000119919 | HGLibA_32038 | 354 | 2.067357898 | NKX2-3 | NK2 homeobox 3 [Source:HGNC Symbol;Acc:HGNC:7836] |
| ENSG00000127415 | HGLibA_22569 | 354 | 2.067357898 | IDUA | alpha-L- [Source:HGNC Symbol;Acc:HGNC:5391]" |
| ENSG00000134571 | HGLibA_30656 | 354 | 2.067357898 | MYBPC3 | cardiac [Source:HGNC Symbol;Acc:HGNC:7551]" |
| ENSG00000138074 | HGLibA_45278 | 354 | 2.067357898 | SLC5A6 | solute carrier family 5 member 6 [Source:HGNC Symbol;Acc:HGNC:11041] |
| ENSG00000138109 | HGLibA_12123 | 354 | 2.067357898 | CYP2C9 | cytochrome P450 family 2 subfamily C member 9 [Source:HGNC Symbol;Acc:HGNC:2623] |
| ENSG00000138650 | HGLibA_35387 | 354 | 2.067357898 | PCDH10 | protocadherin 10 [Source:HGNC Symbol;Acc:HGNC:13404] |
| ENSG00000145198 | HGLibA_53912 | 354 | 2.067357898 | VWA5B2 | von Willebrand factor A domain containing 5B2 [Source:HGNC Symbol;Acc:HGNC:25144] |
| ENSG00000146021 | HGLibA_25176 | 354 | 2.067357898 | KLHL3 | kelch like family member 3 [Source:HGNC Symbol;Acc:HGNC:6354] |
| ENSG00000147526 | HGLibA_48160 | 354 | 2.067357898 | TACC1 | transforming acidic coiled-coil containing protein 1 [Source:HGNC Symbol;Acc:HGNC:11522] |
| ENSG00000156639 | HGLibA_55153 | 354 | 2.067357898 | ZFAND3 | zinc finger AN1-type containing 3 [Source:HGNC Symbol;Acc:HGNC:18019] |
| ENSG00000160410 | HGLibA_44056 | 354 | 2.067357898 | SHKBP1 | SH3KBP1 binding protein 1 [Source:HGNC Symbol;Acc:HGNC:19214] |
| ENSG00000164244 | HGLibA_38858 | 354 | 2.067357898 | PRRC1 | proline rich coiled-coil 1 [Source:HGNC Symbol;Acc:HGNC:28164] |
| ENSG00000173875 | HGLibA_56767 | 354 | 2.067357898 | ZNF791 | zinc finger protein 791 [Source:HGNC Symbol;Acc:HGNC:26895] |
| ENSG00000175806 | HGLibA_30238 | 354 | 2.067357898 | MSRA | methionine sulfoxide reductase A [Source:HGNC Symbol;Acc:HGNC:7377] |
| ENSG00000185238 | HGLibA_38626 | 354 | 2.067357898 | PRMT3 | protein arginine methyltransferase 3 [Source:HGNC Symbol;Acc:HGNC:30163] |
| ENSG00000205497 | HGLibA_34077 | 354 | 2.067357898 | OR51A4 | olfactory receptor family 51 subfamily A member 4 [Source:HGNC Symbol;Acc:HGNC:14795] |
| hsa-mir-1910 | HGLibA_58045 | 354 | 2.067357898 | hsa-mir-1910 | - |
| ENSG00000035664 | HGLibA_12324 | 353 | 2.058312555 | DAPK2 | death associated protein kinase 2 [Source:HGNC Symbol;Acc:HGNC:2675] |
| ENSG00000101204 | HGLibA_09575 | 353 | 2.058312555 | CHRNA4 | cholinergic receptor nicotinic alpha 4 subunit [Source:HGNC Symbol;Acc:HGNC:1958] |
| ENSG00000103042 | HGLibA_45080 | 353 | 2.058312555 | SLC38A7 | solute carrier family 38 member 7 [Source:HGNC Symbol;Acc:HGNC:25582] |
| ENSG00000106701 | HGLibA_18081 | 353 | 2.058312555 | FSD1L | fibronectin type III and SPRY domain containing 1 like [Source:HGNC Symbol;Acc:HGNC:13753] |
| ENSG00000113196 | HGLibA_20826 | 353 | 2.058312555 | HAND1 | heart and neural crest derivatives expressed 1 [Source:HGNC Symbol;Acc:HGNC:4807] |
| ENSG00000116251 | HGLibA_41898 | 353 | 2.058312555 | RPL22 | ribosomal protein L22 [Source:HGNC Symbol;Acc:HGNC:10315] |
| ENSG00000126861 | HGLibA_33386 | 353 | 2.058312555 | OMG | oligodendrocyte myelin glycoprotein [Source:HGNC Symbol;Acc:HGNC:8135] |
| ENSG00000139291 | HGLibA_50137 | 353 | 2.058312555 | TMEM19 | transmembrane protein 19 [Source:HGNC Symbol;Acc:HGNC:25605] |
| ENSG00000142279 | HGLibA_54436 | 353 | 2.058312555 | WTIP | WT1 interacting protein [Source:HGNC Symbol;Acc:HGNC:20964] |
| ENSG00000162433 | HGLibA_01384 | 353 | 2.058312555 | AK4 | adenylate kinase 4 [Source:HGNC Symbol;Acc:HGNC:363] |
| ENSG00000162949 | HGLibA_07153 | 353 | 2.058312555 | CAPN13 | calpain 13 [Source:HGNC Symbol;Acc:HGNC:16663] |
| ENSG00000163093 | HGLibA_04220 | 353 | 2.058312555 | BBS5 | Bardet-Biedl syndrome 5 [Source:HGNC Symbol;Acc:HGNC:970] |
| ENSG00000163734 | HGLibA_11869 | 353 | 2.058312555 | CXCL3 | C-X-C motif chemokine ligand 3 [Source:HGNC Symbol;Acc:HGNC:4604] |
| ENSG00000164265 | HGLibA_42935 | 353 | 2.058312555 | SCGB3A2 | secretoglobin family 3A member 2 [Source:HGNC Symbol;Acc:HGNC:18391] |
| ENSG00000166415 | HGLibA_54165 | 353 | 2.058312555 | WDR72 | WD repeat domain 72 [Source:HGNC Symbol;Acc:HGNC:26790] |
| ENSG00000167543 | HGLibA_51063 | 353 | 2.058312555 | TP53I13 | tumor protein p53 inducible protein 13 [Source:HGNC Symbol;Acc:HGNC:25102] |
| ENSG00000167895 | HGLibA_49768 | 353 | 2.058312555 | TMC8 | transmembrane channel like 8 [Source:HGNC Symbol;Acc:HGNC:20474] |
| ENSG00000167972 | HGLibA_00111 | 353 | 2.058312555 | ABCA3 | ATP binding cassette subfamily A member 3 [Source:HGNC Symbol;Acc:HGNC:33] |
| ENSG00000168569 | HGLibA_50242 | 353 | 2.058312555 | TMEM223 | transmembrane protein 223 [Source:HGNC Symbol;Acc:HGNC:28464] |
| ENSG00000172830 | HGLibA_47138 | 353 | 2.058312555 | SSH3 | slingshot protein phosphatase 3 [Source:HGNC Symbol;Acc:HGNC:30581] |
| ENSG00000177984 | HGLibA_26176 | 353 | 2.058312555 | LCN15 | lipocalin 15 [Source:HGNC Symbol;Acc:HGNC:33777] |
| ENSG00000204590 | HGLibA_19545 | 353 | 2.058312555 | GNL1 | G protein nucleolar 1 (putative) [Source:HGNC Symbol;Acc:HGNC:4413] |
| ENSG00000215183 | HGLibA_30228 | 353 | 2.058312555 | MSMP | prostate associated [Source:HGNC Symbol;Acc:HGNC:29663]" |
| ENSG00000227268 | HGLibA_25279 | 353 | 2.058312555 | KLLN | p53 regulated DNA replication inhibitor [Source:HGNC Symbol;Acc:HGNC:37212]" |
| ENSG00000274736 | HGLibA_08001 | 353 | 2.058312555 | CCL23 | C-C motif chemokine ligand 23 [Source:HGNC Symbol;Acc:HGNC:10622] |
| hsa-mir-4485 | HGLibA_60359 | 353 | 2.058312555 | hsa-mir-4485 | - |
| ENSG00000063854 | HGLibA_20815 | 352 | 2.049267212 | HAGH | hydroxyacylglutathione hydrolase [Source:HGNC Symbol;Acc:HGNC:4805] |
| ENSG00000068697 | HGLibA_26017 | 352 | 2.049267212 | LAPTM4A | lysosomal protein transmembrane 4 alpha [Source:HGNC Symbol;Acc:HGNC:6924] |
| ENSG00000100814 | HGLibA_08059 | 352 | 2.049267212 | CCNB1IP1 | cyclin B1 interacting protein 1 [Source:HGNC Symbol;Acc:HGNC:19437] |
| ENSG00000101353 | HGLibA_29878 | 352 | 2.049267212 | MROH8 | maestro heat like repeat family member 8 [Source:HGNC Symbol;Acc:HGNC:16125] |
| ENSG00000104142 | HGLibA_53747 | 352 | 2.049267212 | VPS18 | CORVET/HOPS core subunit [Source:HGNC Symbol;Acc:HGNC:15972]" |
| ENSG00000112139 | HGLibA_28677 | 352 | 2.049267212 | MDGA1 | MAM domain containing glycosylphosphatidylinositol anchor 1 [Source:HGNC Symbol;Acc:HGNC:19267] |
| ENSG00000114956 | HGLibA_13087 | 352 | 2.049267212 | DGUOK | deoxyguanosine kinase [Source:HGNC Symbol;Acc:HGNC:2858] |
| ENSG00000123505 | HGLibA_01758 | 352 | 2.049267212 | AMD1 | adenosylmethionine decarboxylase 1 [Source:HGNC Symbol;Acc:HGNC:457] |
| ENSG00000124357 | HGLibA_31067 | 352 | 2.049267212 | NAGK | N-acetylglucosamine kinase [Source:HGNC Symbol;Acc:HGNC:17174] |
| ENSG00000124882 | HGLibA_15458 | 352 | 2.049267212 | EREG | epiregulin [Source:HGNC Symbol;Acc:HGNC:3443] |
| ENSG00000129159 | HGLibA_24162 | 352 | 2.049267212 | KCNC1 | potassium voltage-gated channel subfamily C member 1 [Source:HGNC Symbol;Acc:HGNC:6233] |
| ENSG00000133313 | HGLibA_10253 | 352 | 2.049267212 | CNDP2 | carnosine dipeptidase 2 [Source:HGNC Symbol;Acc:HGNC:24437] |
| ENSG00000149600 | HGLibA_10675 | 352 | 2.049267212 | COMMD7 | COMM domain containing 7 [Source:HGNC Symbol;Acc:HGNC:16223] |
| ENSG00000153993 | HGLibA_43328 | 352 | 2.049267212 | SEMA3D | semaphorin 3D [Source:HGNC Symbol;Acc:HGNC:10726] |
| ENSG00000157093 | HGLibA_27791 | 352 | 2.049267212 | LYZL4 | lysozyme like 4 [Source:HGNC Symbol;Acc:HGNC:28387] |
| ENSG00000164081 | HGLibA_49085 | 352 | 2.049267212 | TEX264 | testis expressed 264 [Source:HGNC Symbol;Acc:HGNC:30247] |
| ENSG00000168010 | HGLibA_03477 | 352 | 2.049267212 | ATG16L2 | autophagy related 16 like 2 [Source:HGNC Symbol;Acc:HGNC:25464] |
| ENSG00000171421 | HGLibA_29964 | 352 | 2.049267212 | MRPL36 | mitochondrial ribosomal protein L36 [Source:HGNC Symbol;Acc:HGNC:14490] |
| ENSG00000172346 | HGLibA_11313 | 352 | 2.049267212 | CSDC2 | cold shock domain containing C2 [Source:HGNC Symbol;Acc:HGNC:30359] |
| ENSG00000187513 | HGLibA_19145 | 352 | 2.049267212 | GJA4 | gap junction protein alpha 4 [Source:HGNC Symbol;Acc:HGNC:4278] |
| ENSG00000188706 | HGLibA_55131 | 352 | 2.049267212 | ZDHHC9 | zinc finger DHHC-type containing 9 [Source:HGNC Symbol;Acc:HGNC:18475] |
| ENSG00000197774 | HGLibA_15038 | 352 | 2.049267212 | EME2 | essential meiotic structure-specific endonuclease subunit 2 [Source:HGNC Symbol;Acc:HGNC:27289] |
| ENSG00000240204 | HGLibA_45775 | 352 | 2.049267212 | SMKR1 | small lysine rich protein 1 [Source:HGNC Symbol;Acc:HGNC:43561] |
| ENSG00000007237 | HGLibA_18656 | 351 | 2.04022187 | GAS7 | growth arrest specific 7 [Source:HGNC Symbol;Acc:HGNC:4169] |
| ENSG00000086159 | HGLibA_02603 | 351 | 2.04022187 | AQP6 | aquaporin 6 [Source:HGNC Symbol;Acc:HGNC:639] |
| ENSG00000086730 | HGLibA_26062 | 351 | 2.04022187 | LAT2 | linker for activation of T cells family member 2 [Source:HGNC Symbol;Acc:HGNC:12749] |
| ENSG00000102302 | HGLibA_17343 | 351 | 2.04022187 | FGD1 | RhoGEF and PH domain containing 1 [Source:HGNC Symbol;Acc:HGNC:3663]" |
| ENSG00000104825 | HGLibA_31823 | 351 | 2.04022187 | NFKBIB | NFKB inhibitor beta [Source:HGNC Symbol;Acc:HGNC:7798] |
| ENSG00000112200 | HGLibA_56029 | 351 | 2.04022187 | ZNF451 | zinc finger protein 451 [Source:HGNC Symbol;Acc:HGNC:21091] |
| ENSG00000120068 | HGLibA_21951 | 351 | 2.04022187 | HOXB8 | homeobox B8 [Source:HGNC Symbol;Acc:HGNC:5119] |
| ENSG00000124780 | HGLibA_24312 | 351 | 2.04022187 | KCNK17 | potassium two pore domain channel subfamily K member 17 [Source:HGNC Symbol;Acc:HGNC:14465] |
| ENSG00000130402 | HGLibA_00643 | 351 | 2.04022187 | ACTN4 | actinin alpha 4 [Source:HGNC Symbol;Acc:HGNC:166] |
| ENSG00000139154 | HGLibA_01089 | 351 | 2.04022187 | AEBP2 | AE binding protein 2 [Source:HGNC Symbol;Acc:HGNC:24051] |
| ENSG00000148356 | HGLibA_27496 | 351 | 2.04022187 | LRSAM1 | leucine rich repeat and sterile alpha motif containing 1 [Source:HGNC Symbol;Acc:HGNC:25135] |
| ENSG00000154099 | HGLibA_13458 | 351 | 2.04022187 | DNAAF1 | dynein axonemal assembly factor 1 [Source:HGNC Symbol;Acc:HGNC:30539] |
| ENSG00000162390 | HGLibA_00469 | 351 | 2.04022187 | ACOT11 | acyl-CoA thioesterase 11 [Source:HGNC Symbol;Acc:HGNC:18156] |
| ENSG00000166866 | HGLibA_30820 | 351 | 2.04022187 | MYO1A | myosin IA [Source:HGNC Symbol;Acc:HGNC:7595] |
| ENSG00000177151 | HGLibA_33880 | 351 | 2.04022187 | OR2T35 | olfactory receptor family 2 subfamily T member 35 [Source:HGNC Symbol;Acc:HGNC:31257] |
| ENSG00000182993 | HGLibA_05274 | 351 | 2.04022187 | C12orf60 | chromosome 12 open reading frame 60 [Source:HGNC Symbol;Acc:HGNC:28726] |
| ENSG00000185758 | HGLibA_09889 | 351 | 2.04022187 | CLDN24 | claudin 24 [Source:HGNC Symbol;Acc:HGNC:37200] |
| ENSG00000196678 | HGLibA_15482 | 351 | 2.04022187 | ERI2 | ERI1 exoribonuclease family member 2 [Source:HGNC Symbol;Acc:HGNC:30541] |
| ENSG00000198934 | HGLibA_27980 | 351 | 2.04022187 | MAGEE1 | MAGE family member E1 [Source:HGNC Symbol;Acc:HGNC:24934] |
| ENSG00000274736 | HGLibA_08000 | 351 | 2.04022187 | CCL23 | C-C motif chemokine ligand 23 [Source:HGNC Symbol;Acc:HGNC:10622] |
| hsa-mir-4685 | HGLibA_60851 | 351 | 2.04022187 | hsa-mir-4685 | - |
| hsa-mir-8066 | HGLibA_64101 | 351 | 2.04022187 | hsa-mir-8066 | - |
| CHDC2 | HGLibA_09413 | 350 | 2.031176527 | CHDC2 | - |
| ENSG00000008382 | HGLibA_29742 | 350 | 2.031176527 | MPND | MPN domain containing [Source:HGNC Symbol;Acc:HGNC:25934] |
| ENSG00000011478 | HGLibA_39730 | 350 | 2.031176527 | QPCTL | glutaminyl-peptide cyclotransferase like [Source:HGNC Symbol;Acc:HGNC:25952] |

| | | | | | |
|---|---|---|---|---|---|
| ENSG00000047634 | HGLibA_42959 | 350 | 2.031176527 | SCML1 | Scm polycomb group protein like 1 [Source:HGNC Symbol;Acc:HGNC:10580] |
| ENSG00000048140 | HGLibA_51914 | 350 | 2.031176527 | TSPAN17 | tetraspanin 17 [Source:HGNC Symbol;Acc:HGNC:13594] |
| ENSG00000100884 | HGLibA_10969 | 350 | 2.031176527 | CPNE6 | copine 6 [Source:HGNC Symbol;Acc:HGNC:2319] |
| ENSG00000101134 | HGLibA_13780 | 350 | 2.031176527 | DOK5 | docking protein 5 [Source:HGNC Symbol;Acc:HGNC:16173] |
| ENSG00000101190 | HGLibA_48799 | 350 | 2.031176527 | TCFL5 | transcription factor like 5 [Source:HGNC Symbol;Acc:HGNC:11646] |
| ENSG00000106397 | HGLibA_37213 | 350 | 2.031176527 | PLOD3 | 2-oxoglutarate 5-dioxygenase 3 [Source:HGNC Symbol;Acc:HGNC:9083]" |
| ENSG00000112280 | HGLibA_10619 | 350 | 2.031176527 | COL9A1 | collagen type IX alpha 1 chain [Source:HGNC Symbol;Acc:HGNC:2217] |
| ENSG00000115677 | HGLibA_21059 | 350 | 2.031176527 | HDLBP | high density lipoprotein binding protein [Source:HGNC Symbol;Acc:HGNC:4857] |
| ENSG00000115902 | HGLibA_44515 | 350 | 2.031176527 | SLC1A4 | solute carrier family 1 member 4 [Source:HGNC Symbol;Acc:HGNC:10942] |
| ENSG00000124209 | HGLibA_39842 | 350 | 2.031176527 | RAB22A | member RAS oncogene family [Source:HGNC Symbol;Acc:HGNC:9764]" |
| ENSG00000131508 | HGLibA_52584 | 350 | 2.031176527 | UBE2D2 | ubiquitin conjugating enzyme E2 D2 [Source:HGNC Symbol;Acc:HGNC:12475] |
| ENSG00000135111 | HGLibA_48668 | 350 | 2.031176527 | TBX3 | T-box 3 [Source:HGNC Symbol;Acc:HGNC:11602] |
| ENSG00000171942 | HGLibA_33508 | 350 | 2.031176527 | OR10H2 | olfactory receptor family 10 subfamily H member 2 [Source:HGNC Symbol;Acc:HGNC:8173] |
| ENSG00000173237 | HGLibA_05203 | 350 | 2.031176527 | C11orf86 | chromosome 11 open reading frame 86 [Source:HGNC Symbol;Acc:HGNC:34442] |
| ENSG00000180694 | HGLibA_50483 | 350 | 2.031176527 | TMEM64 | transmembrane protein 64 [Source:HGNC Symbol;Acc:HGNC:25441] |
| ENSG00000186924 | HGLibA_25717 | 350 | 2.031176527 | KRTAP22-1 | keratin associated protein 22-1 [Source:HGNC Symbol;Acc:HGNC:18947] |
| ENSG00000187714 | HGLibA_44493 | 350 | 2.031176527 | SLC18A3 | solute carrier family 18 member A3 [Source:HGNC Symbol;Acc:HGNC:10936] |
| ENSG00000197563 | HGLibA_36570 | 350 | 2.031176527 | PIGN | phosphatidylinositol glycan anchor biosynthesis class N [Source:HGNC Symbol;Acc:HGNC:8967] |
| ENSG00000261594 | HGLibA_51102 | 350 | 2.031176527 | TPBGL | trophoblast glycoprotein like [Source:HGNC Symbol;Acc:HGNC:44159] |
| hsa-mir-4268 | HGLibA_59781 | 350 | 2.031176527 | hsa-mir-4268 | - |
| hsa-mir-7161 | HGLibA_63872 | 350 | 2.031176527 | hsa-mir-7161 | - |
| ENSG00000069702 | HGLibA_49228 | 349 | 2.022131184 | TGFBR3 | transforming growth factor beta receptor 3 [Source:HGNC Symbol;Acc:HGNC:11774] |
| ENSG00000081842 | HGLibA_35450 | 349 | 2.022131184 | PCDHA6 | protocadherin alpha 6 [Source:HGNC Symbol;Acc:HGNC:8672] |
| ENSG00000100227 | HGLibA_37508 | 349 | 2.022131184 | POLDIP3 | DNA polymerase delta interacting protein 3 [Source:HGNC Symbol;Acc:HGNC:23782] |
| ENSG00000100813 | HGLibA_00440 | 349 | 2.022131184 | ACIN1 | apoptotic chromatin condensation inducer 1 [Source:HGNC Symbol;Acc:HGNC:17066] |
| ENSG00000101276 | HGLibA_45249 | 349 | 2.022131184 | SLC52A3 | solute carrier family 52 member 3 [Source:HGNC Symbol;Acc:HGNC:16187] |
| ENSG00000138802 | HGLibA_43233 | 349 | 2.022131184 | SEC24B | COPII coat complex component [Source:HGNC Symbol;Acc:HGNC:10704]" |
| ENSG00000140835 | HGLibA_09638 | 349 | 2.022131184 | CHST4 | carbohydrate sulfotransferase 4 [Source:HGNC Symbol;Acc:HGNC:1972] |
| ENSG00000142182 | HGLibA_13708 | 349 | 2.022131184 | DNMT3L | DNA methyltransferase 3 like [Source:HGNC Symbol;Acc:HGNC:2980] |
| ENSG00000144560 | HGLibA_53645 | 349 | 2.022131184 | VGLL4 | vestigial like family member 4 [Source:HGNC Symbol;Acc:HGNC:28966] |
| ENSG00000147256 | HGLibA_02768 | 349 | 2.022131184 | ARHGAP36 | Rho GTPase activating protein 36 [Source:HGNC Symbol;Acc:HGNC:26388] |
| ENSG00000156140 | HGLibA_00827 | 349 | 2.022131184 | ADAMTS3 | ADAM metallopeptidase with thrombospondin type 1 motif 3 [Source:HGNC Symbol;Acc:HGNC:219] |
| ENSG00000165023 | HGLibA_13258 | 349 | 2.022131184 | DIRAS2 | DIRAS family GTPase 2 [Source:HGNC Symbol;Acc:HGNC:19323] |
| ENSG00000168334 | HGLibA_54510 | 349 | 2.022131184 | XIRP1 | xin actin binding repeat containing 1 [Source:HGNC Symbol;Acc:HGNC:14301] |
| ENSG00000171102 | HGLibA_33228 | 349 | 2.022131184 | OBP2B | odorant binding protein 2B [Source:HGNC Symbol;Acc:HGNC:23381] |
| ENSG00000172197 | HGLibA_28516 | 349 | 2.022131184 | MBOAT1 | membrane bound O-acyltransferase domain containing 1 [Source:HGNC Symbol;Acc:HGNC:21579] |
| ENSG00000174839 | HGLibA_12964 | 349 | 2.022131184 | DENND6A | DENN domain containing 6A [Source:HGNC Symbol;Acc:HGNC:26635] |
| ENSG00000175183 | HGLibA_11434 | 349 | 2.022131184 | CSRP2 | cysteine and glycine rich protein 2 [Source:HGNC Symbol;Acc:HGNC:2470] |
| ENSG00000187140 | HGLibA_17834 | 349 | 2.022131184 | FOXD3 | forkhead box D3 [Source:HGNC Symbol;Acc:HGNC:3804] |
| ENSG00000187550 | HGLibA_42802 | 349 | 2.022131184 | SBK2 | SH3 domain binding kinase family member 2 [Source:HGNC Symbol;Acc:HGNC:34416] |
| ENSG00000204300 | HGLibA_50245 | 349 | 2.022131184 | TMEM225 | transmembrane protein 225 [Source:HGNC Symbol;Acc:HGNC:32390] |
| ENSG00000204970 | HGLibA_35423 | 349 | 2.022131184 | PCDHA1 | protocadherin alpha 1 [Source:HGNC Symbol;Acc:HGNC:8663] |
| ENSG00000221996 | HGLibA_34148 | 349 | 2.022131184 | OR52B4 | olfactory receptor family 52 subfamily B member 4 (gene/pseudogene) [Source:HGNC Symbol;Acc:HGNC:15209] |
| hsa-mir-6802 | HGLibA_63386 | 349 | 2.022131184 | hsa-mir-6802 | - |
| hsa-mir-6805 | HGLibA_63397 | 349 | 2.022131184 | hsa-mir-6805 | - |
| ENSG00000017427 | HGLibA_22792 | 348 | 2.013085841 | IGF1 | insulin like growth factor 1 [Source:HGNC Symbol;Acc:HGNC:5464] |
| ENSG00000021645 | HGLibA_32738 | 348 | 2.013085841 | NRXN3 | neurexin 3 [Source:HGNC Symbol;Acc:HGNC:8010] |
| ENSG00000078328 | HGLibA_40454 | 348 | 2.013085841 | RBFOX1 | RNA binding fox-1 homolog 1 [Source:HGNC Symbol;Acc:HGNC:18222] |
| ENSG00000101448 | HGLibA_15355 | 348 | 2.013085841 | EPPIN | epididymal peptidase inhibitor [Source:HGNC Symbol;Acc:HGNC:15932] |
| ENSG00000112195 | HGLibA_51362 | 348 | 2.013085841 | TREML2 | triggering receptor expressed on myeloid cells like 2 [Source:HGNC Symbol;Acc:HGNC:21092] |
| ENSG00000120656 | HGLibA_48203 | 348 | 2.013085841 | TAF12 | TATA-box binding protein associated factor 12 [Source:HGNC Symbol;Acc:HGNC:11545] |
| ENSG00000127884 | HGLibA_14402 | 348 | 2.013085841 | ECHS1 | short chain 1 [Source:HGNC Symbol;Acc:HGNC:3151]" |
| ENSG00000137834 | HGLibA_45583 | 348 | 2.013085841 | SMAD6 | SMAD family member 6 [Source:HGNC Symbol;Acc:HGNC:6772] |
| ENSG00000139540 | HGLibA_45116 | 348 | 2.013085841 | SLC39A5 | solute carrier family 39 member 5 [Source:HGNC Symbol;Acc:HGNC:20502] |
| ENSG00000147160 | HGLibA_03916 | 348 | 2.013085841 | AWAT2 | acyl-CoA wax alcohol acyltransferase 2 [Source:HGNC Symbol;Acc:HGNC:23251] |
| ENSG00000147596 | HGLibA_38376 | 348 | 2.013085841 | PRDM14 | PR/SET domain 14 [Source:HGNC Symbol;Acc:HGNC:14001] |
| ENSG00000149635 | HGLibA_33265 | 348 | 2.013085841 | OCSTAMP | osteoclast stimulatory transmembrane protein [Source:HGNC Symbol;Acc:HGNC:16116] |
| ENSG00000159905 | HGLibA_55608 | 348 | 2.013085841 | ZNF221 | zinc finger protein 221 [Source:HGNC Symbol;Acc:HGNC:13014] |
| ENSG00000171206 | HGLibA_51616 | 348 | 2.013085841 | TRIM8 | tripartite motif containing 8 [Source:HGNC Symbol;Acc:HGNC:15579] |
| ENSG00000178401 | HGLibA_13601 | 348 | 2.013085841 | DNAJC22 | DnaJ heat shock protein family (Hsp40) member C22 [Source:HGNC Symbol;Acc:HGNC:25802] |
| ENSG00000182326 | HGLibA_05976 | 348 | 2.013085841 | C1S | complement C1s [Source:HGNC Symbol;Acc:HGNC:1247] |
| ENSG00000183134 | HGLibA_39307 | 348 | 2.013085841 | PTGDR2 | prostaglandin D2 receptor 2 [Source:HGNC Symbol;Acc:HGNC:4502] |
| ENSG00000197446 | HGLibA_12133 | 348 | 2.013085841 | CYP2F1 | cytochrome P450 family 2 subfamily F member 1 [Source:HGNC Symbol;Acc:HGNC:2632] |
| ENSG00000204514 | HGLibA_56809 | 348 | 2.013085841 | ZNF814 | zinc finger protein 814 [Source:HGNC Symbol;Acc:HGNC:33258] |
| ENSG00000204694 | HGLibA_33564 | 348 | 2.013085841 | OR11A1 | olfactory receptor family 11 subfamily A member 1 [Source:HGNC Symbol;Acc:HGNC:8176] |
| ENSG00000241839 | HGLibA_37151 | 348 | 2.013085841 | PLEKHO2 | pleckstrin homology domain containing O2 [Source:HGNC Symbol;Acc:HGNC:30026] |
| FIGF | HGLibA_17512 | 348 | 2.013085841 | FIGF | - |
| hsa-mir-490 | HGLibA_61362 | 348 | 2.013085841 | hsa-mir-490 | - |
| MFSD4 | HGLibA_29101 | 348 | 2.013085841 | MFSD4 | - |
| ENSG00000009830 | HGLibA_37673 | 347 | 2.004040498 | POMT2 | protein O-mannosyltransferase 2 [Source:HGNC Symbol;Acc:HGNC:19743] |
| ENSG00000078269 | HGLibA_48005 | 347 | 2.004040498 | SYNJ2 | synaptojanin 2 [Source:HGNC Symbol;Acc:HGNC:11504] |
| ENSG00000101846 | HGLibA_47604 | 347 | 2.004040498 | STS | steroid sulfatase [Source:HGNC Symbol;Acc:HGNC:11425] |
| ENSG00000121281 | HGLibA_00927 | 347 | 2.004040498 | ADCY7 | adenylate cyclase 7 [Source:HGNC Symbol;Acc:HGNC:238] |
| ENSG00000135722 | HGLibA_17020 | 347 | 2.004040498 | FBXL8 | F-box and leucine rich repeat protein 8 [Source:HGNC Symbol;Acc:HGNC:17875] |
| ENSG00000136099 | HGLibA_35417 | 347 | 2.004040498 | PCDH8 | protocadherin 8 [Source:HGNC Symbol;Acc:HGNC:8660] |
| ENSG00000136261 | HGLibA_05016 | 347 | 2.004040498 | BZW2 | basic leucine zipper and W2 domains 2 [Source:HGNC Symbol;Acc:HGNC:18808] |
| ENSG00000137824 | HGLibA_41370 | 347 | 2.004040498 | RMDN3 | regulator of microtubule dynamics 3 [Source:HGNC Symbol;Acc:HGNC:25550] |
| ENSG00000139219 | HGLibA_10559 | 347 | 2.004040498 | COL2A1 | collagen type II alpha 1 chain [Source:HGNC Symbol;Acc:HGNC:2200] |
| ENSG00000148926 | HGLibA_00993 | 347 | 2.004040498 | ADM | adrenomedullin [Source:HGNC Symbol;Acc:HGNC:259] |
| ENSG00000155970 | HGLibA_29227 | 347 | 2.004040498 | MICU3 | mitochondrial calcium uptake family member 3 [Source:HGNC Symbol;Acc:HGNC:27820] |
| ENSG00000157036 | HGLibA_15766 | 347 | 2.004040498 | EXOG | exo/endonuclease G [Source:HGNC Symbol;Acc:HGNC:3347] |
| ENSG00000167113 | HGLibA_10755 | 347 | 2.004040498 | COQ4 | coenzyme Q4 [Source:HGNC Symbol;Acc:HGNC:19693] |
| ENSG00000168938 | HGLibA_37922 | 347 | 2.004040498 | PPIC | peptidylprolyl isomerase C [Source:HGNC Symbol;Acc:HGNC:9256] |
| ENSG00000172081 | HGLibA_29595 | 347 | 2.004040498 | MOB3A | MOB kinase activator 3A [Source:HGNC Symbol;Acc:HGNC:29802] |
| ENSG00000175497 | HGLibA_13866 | 347 | 2.004040498 | DPP10 | dipeptidyl peptidase like 10 [Source:HGNC Symbol;Acc:HGNC:20823] |
| ENSG00000179477 | HGLibA_01680 | 347 | 2.004040498 | ALOX12B | 12R type [Source:HGNC Symbol;Acc:HGNC:430]" |
| ENSG00000183607 | HGLibA_19210 | 347 | 2.004040498 | GKN2 | gastrokine 2 [Source:HGNC Symbol;Acc:HGNC:24588] |
| ENSG00000183783 | HGLibA_24467 | 347 | 2.004040498 | KCTD8 | potassium channel tetramerization domain containing 8 [Source:HGNC Symbol;Acc:HGNC:22394] |
| ENSG00000189114 | HGLibA_04562 | 347 | 2.004040498 | BLOC1S3 | biogenesis of lysosomal organelles complex 1 subunit 3 [Source:HGNC Symbol;Acc:HGNC:20914] |
| ENSG00000198286 | HGLibA_07220 | 347 | 2.004040498 | CARD11 | caspase recruitment domain family member 11 [Source:HGNC Symbol;Acc:HGNC:16393] |
| ENSG00000198300 | HGLibA_36030 | 347 | 2.004040498 | PEG3 | paternally expressed 3 [Source:HGNC Symbol;Acc:HGNC:8826] |
| ENSG00000248383 | HGLibA_35463 | 347 | 2.004040498 | PCDHAC1 | 1 [Source:HGNC Symbol;Acc:HGNC:8676]" |
| GRASP | HGLibA_20220 | 347 | 2.004040498 | GRASP | - |
| WIBG | HGLibA_54299 | 347 | 2.004040498 | WIBG | - |
| C12orf39 | HGLibA_05232 | 346 | 1.994995155 | C12orf39 | - |
| C5orf54 | HGLibA_06408 | 346 | 1.994995155 | C5orf54 | - |
| ENSG00000080298 | HGLibA_40969 | 346 | 1.994995155 | RFX3 | regulatory factor X3 [Source:HGNC Symbol;Acc:HGNC:9984] |
| ENSG00000100385 | HGLibA_23120 | 346 | 1.994995155 | IL2RB | interleukin 2 receptor subunit beta [Source:HGNC Symbol;Acc:HGNC:6009] |
| ENSG00000100422 | HGLibA_09195 | 346 | 1.994995155 | CERK | ceramide kinase [Source:HGNC Symbol;Acc:HGNC:19256] |
| ENSG00000106682 | HGLibA_14849 | 346 | 1.994995155 | EIF4H | eukaryotic translation initiation factor 4H [Source:HGNC Symbol;Acc:HGNC:12741] |
| ENSG00000114115 | HGLibA_40628 | 346 | 1.994995155 | RBP1 | retinol binding protein 1 [Source:HGNC Symbol;Acc:HGNC:9919] |
| ENSG00000124783 | HGLibA_47153 | 346 | 1.994995155 | SSR1 | signal sequence receptor subunit 1 [Source:HGNC Symbol;Acc:HGNC:11323] |
| ENSG00000127083 | HGLibA_33382 | 346 | 1.994995155 | OMD | osteomodulin [Source:HGNC Symbol;Acc:HGNC:8134] |
| ENSG00000135318 | HGLibA_32829 | 346 | 1.994995155 | NT5E | 5'-nucleotidase ecto [Source:HGNC Symbol;Acc:HGNC:8021] |
| ENSG00000141401 | HGLibA_23246 | 346 | 1.994995155 | IMPA2 | inositol monophosphatase 2 [Source:HGNC Symbol;Acc:HGNC:6051] |
| ENSG00000144749 | HGLibA_27177 | 346 | 1.994995155 | LRIG1 | leucine rich repeats and immunoglobulin like domains 1 [Source:HGNC Symbol;Acc:HGNC:17360] |
| ENSG00000157131 | HGLibA_06593 | 346 | 1.994995155 | C8A | complement C8 alpha chain [Source:HGNC Symbol;Acc:HGNC:1352] |
| ENSG00000159200 | HGLibA_40667 | 346 | 1.994995155 | RCAN1 | regulator of calcineurin 1 [Source:HGNC Symbol;Acc:HGNC:3040] |
| ENSG00000175711 | HGLibA_04008 | 346 | 1.994995155 | B3GNTL1 | 3-N-acetylglucosaminyltransferase like 1 [Source:HGNC Symbol;Acc:HGNC:21727]" |
| ENSG00000176903 | HGLibA_37365 | 346 | 1.994995155 | PNMA1 | PNMA family member 1 [Source:HGNC Symbol;Acc:HGNC:9158] |
| ENSG00000185432 | HGLibA_29019 | 346 | 1.994995155 | METTL7A | methyltransferase like 7A [Source:HGNC Symbol;Acc:HGNC:24550] |
| ENSG00000204128 | HGLibA_06175 | 346 | 1.994995155 | C2orf72 | chromosome 2 open reading frame 72 [Source:HGNC Symbol;Acc:HGNC:27418] |
| ENSG00000204564 | HGLibA_06446 | 346 | 1.994995155 | C6orf136 | chromosome 6 open reading frame 136 [Source:HGNC Symbol;Acc:HGNC:21301] |
| hsa-mir-5698 | HGLibA_62326 | 346 | 1.994995155 | hsa-mir-5698 | - |
| hsa-mir-5699 | HGLibA_62330 | 346 | 1.994995155 | hsa-mir-5699 | - |
| CASC4 | HGLibA_07276 | 345 | 1.985949812 | CASC4 | - |
| DFNB31 | HGLibA_13029 | 345 | 1.985949812 | DFNB31 | - |
| ENSG00000054803 | HGLibA_07415 | 345 | 1.985949812 | CBLN4 | cerebellin 4 precursor [Source:HGNC Symbol;Acc:HGNC:16231] |
| ENSG00000075391 | HGLibA_40315 | 345 | 1.985949812 | RASAL2 | RAS protein activator like 2 [Source:HGNC Symbol;Acc:HGNC:9874] |
| ENSG00000103021 | HGLibA_07546 | 345 | 1.985949812 | CCDC113 | coiled-coil domain containing 113 [Source:HGNC Symbol;Acc:HGNC:25002] |
| ENSG00000110324 | HGLibA_22947 | 345 | 1.985949812 | IL10RA | interleukin 10 receptor subunit alpha [Source:HGNC Symbol;Acc:HGNC:5964] |
| ENSG00000128652 | HGLibA_21999 | 345 | 1.985949812 | HOXD3 | homeobox D3 [Source:HGNC Symbol;Acc:HGNC:5137] |
| ENSG00000130037 | HGLibA_24137 | 345 | 1.985949812 | KCNA5 | potassium voltage-gated channel subfamily A member 5 [Source:HGNC Symbol;Acc:HGNC:6224] |
| ENSG00000134020 | HGLibA_36016 | 345 | 1.985949812 | PEBP4 | phosphatidylethanolamine binding protein 4 [Source:HGNC Symbol;Acc:HGNC:28319] |
| ENSG00000140395 | HGLibA_54141 | 345 | 1.985949812 | WDR61 | WD repeat domain 61 [Source:HGNC Symbol;Acc:HGNC:30300] |
| ENSG00000146072 | HGLibA_50779 | 345 | 1.985949812 | TNFRSF21 | TNF receptor superfamily member 21 [Source:HGNC Symbol;Acc:HGNC:13469] |
| ENSG00000151458 | HGLibA_02115 | 345 | 1.985949812 | ANKRD50 | ankyrin repeat domain 50 [Source:HGNC Symbol;Acc:HGNC:29223] |
| ENSG00000158050 | HGLibA_14157 | 345 | 1.985949812 | DUSP2 | dual specificity phosphatase 2 [Source:HGNC Symbol;Acc:HGNC:3068] |
| ENSG00000158220 | HGLibA_15611 | 345 | 1.985949812 | ESYT3 | extended synaptotagmin 3 [Source:HGNC Symbol;Acc:HGNC:24295] |
| ENSG00000159899 | HGLibA_32512 | 345 | 1.985949812 | NPR2 | natriuretic peptide receptor 2 [Source:HGNC Symbol;Acc:HGNC:7944] |
| ENSG00000162971 | HGLibA_52490 | 345 | 1.985949812 | TYW5 | tRNA-yW synthesizing protein 5 [Source:HGNC Symbol;Acc:HGNC:26754] |
| ENSG00000168875 | HGLibA_46241 | 345 | 1.985949812 | SOX14 | SRY-box 14 [Source:HGNC Symbol;Acc:HGNC:11193] |
| ENSG00000178662 | HGLibA_11429 | 345 | 1.985949812 | CSRNP3 | cysteine and serine rich nuclear protein 3 [Source:HGNC Symbol;Acc:HGNC:30729] |
| ENSG00000178772 | HGLibA_10949 | 345 | 1.985949812 | CPN2 | carboxypeptidase N subunit 2 [Source:HGNC Symbol;Acc:HGNC:2313] |
| ENSG00000181449 | HGLibA_46253 | 345 | 1.985949812 | SOX2 | SRY-box 2 [Source:HGNC Symbol;Acc:HGNC:11195] |

| | | | | | |
|---|---|---|---|---|---|
| ENSG00000187650 | HGLibA_53690 | 345 | 1.985949812 | VMAC | vimentin type intermediate filament associated coiled-coil protein [Source:HGNC Symbol;Acc:HGNC:33803] |
| GPR133 | HGLibA_19891 | 345 | 1.985949812 | GPR133 | - |
| hsa-mir-3126 | HGLibA_58555 | 345 | 1.985949812 | hsa-mir-3126 | - |
| hsa-mir-4784 | HGLibA_61243 | 345 | 1.985949812 | hsa-mir-4784 | - |
| hsa-mir-6763 | HGLibA_63215 | 345 | 1.985949812 | hsa-mir-6763 | - |
| C20orf111 | HGLibA_05982 | 344 | 1.976904469 | C20orf111 | - |
| CCDC176 | HGLibA_07708 | 344 | 1.976904469 | CCDC176 | - |
| ENSG00000012223 | HGLibA_27609 | 344 | 1.976904469 | LTF | lactotransferrin [Source:HGNC Symbol;Acc:HGNC:6720] |
| ENSG00000021461 | HGLibA_12156 | 344 | 1.976904469 | CYP3A43 | cytochrome P450 family 3 subfamily A member 43 [Source:HGNC Symbol;Acc:HGNC:17450] |
| ENSG00000106483 | HGLibA_43742 | 344 | 1.976904469 | SFRP4 | secreted frizzled related protein 4 [Source:HGNC Symbol;Acc:HGNC:10778] |
| ENSG00000111906 | HGLibA_21034 | 344 | 1.976904469 | HDDC2 | HD domain containing 2 [Source:HGNC Symbol;Acc:HGNC:21078] |
| ENSG00000118960 | HGLibA_22115 | 344 | 1.976904469 | HS1BP3 | HCLS1 binding protein 3 [Source:HGNC Symbol;Acc:HGNC:24979] |
| ENSG00000132004 | HGLibA_17159 | 344 | 1.976904469 | FBXW9 | F-box and WD repeat domain containing 9 [Source:HGNC Symbol;Acc:HGNC:28136] |
| ENSG00000138606 | HGLibA_44023 | 344 | 1.976904469 | SHF | Src homology 2 domain containing F [Source:HGNC Symbol;Acc:HGNC:25116] |
| ENSG00000142765 | HGLibA_48095 | 344 | 1.976904469 | SYTL1 | synaptotagmin like 1 [Source:HGNC Symbol;Acc:HGNC:15584] |
| ENSG00000157601 | HGLibA_30603 | 344 | 1.976904469 | MX1 | MX dynamin like GTPase 1 [Source:HGNC Symbol;Acc:HGNC:7532] |
| ENSG00000163518 | HGLibA_17233 | 344 | 1.976904469 | FCRL4 | Fc receptor like 4 [Source:HGNC Symbol;Acc:HGNC:18507] |
| ENSG00000163945 | HGLibA_53475 | 344 | 1.976904469 | UVSSA | UV stimulated scaffold protein A [Source:HGNC Symbol;Acc:HGNC:29304] |
| ENSG00000166704 | HGLibA_56367 | 344 | 1.976904469 | ZNF606 | zinc finger protein 606 [Source:HGNC Symbol;Acc:HGNC:25879] |
| ENSG00000171540 | HGLibA_34749 | 344 | 1.976904469 | OTP | orthopedia homeobox [Source:HGNC Symbol;Acc:HGNC:8518] |
| ENSG00000176340 | HGLibA_10876 | 344 | 1.976904469 | COX8A | cytochrome c oxidase subunit 8A [Source:HGNC Symbol;Acc:HGNC:2294] |
| ENSG00000182771 | HGLibA_20271 | 344 | 1.976904469 | GRID1 | glutamate ionotropic receptor delta type subunit 1 [Source:HGNC Symbol;Acc:HGNC:4575] |
| ENSG00000185002 | HGLibA_40976 | 344 | 1.976904469 | RFX6 | regulatory factor X6 [Source:HGNC Symbol;Acc:HGNC:21478] |
| ENSG00000185028 | HGLibA_27257 | 344 | 1.976904469 | LRRC14B | leucine rich repeat containing 14B [Source:HGNC Symbol;Acc:HGNC:37268] |
| ENSG00000185252 | HGLibA_56659 | 344 | 1.976904469 | ZNF74 | zinc finger protein 74 [Source:HGNC Symbol;Acc:HGNC:13144] |
| ENSG00000188959 | HGLibA_06689 | 344 | 1.976904469 | C9orf152 | chromosome 9 open reading frame 152 [Source:HGNC Symbol;Acc:HGNC:31455] |
| ENSG00000197114 | HGLibA_55307 | 344 | 1.976904469 | ZGPAT | zinc finger CCCH-type and G-patch domain containing [Source:HGNC Symbol;Acc:HGNC:15948] |
| ENSG00000205784 | HGLibA_03126 | 344 | 1.976904469 | ARRDC5 | arrestin domain containing 5 [Source:HGNC Symbol;Acc:HGNC:31407] |
| hsa-mir-595 | HGLibA_62473 | 344 | 1.976904469 | hsa-mir-595 | - |
| hsa-mir-644a | HGLibA_62821 | 344 | 1.976904469 | hsa-mir-644a | - |
| ENSG00000004142 | HGLibA_37505 | 343 | 1.967859126 | POLDIP2 | DNA polymerase delta interacting protein 2 [Source:HGNC Symbol;Acc:HGNC:23781] |
| ENSG00000055957 | HGLibA_23804 | 343 | 1.967859126 | ITIH1 | inter-alpha-trypsin inhibitor heavy chain 1 [Source:HGNC Symbol;Acc:HGNC:6166] |
| ENSG00000064490 | HGLibA_40985 | 343 | 1.967859126 | RFXANK | regulatory factor X associated ankyrin containing protein [Source:HGNC Symbol;Acc:HGNC:9987] |
| ENSG00000082153 | HGLibA_05013 | 343 | 1.967859126 | BZW1 | basic leucine zipper and W2 domains 1 [Source:HGNC Symbol;Acc:HGNC:18380] |
| ENSG00000089876 | HGLibA_13177 | 343 | 1.967859126 | DHX32 | DEAH-box helicase 32 (putative) [Source:HGNC Symbol;Acc:HGNC:16717] |
| ENSG00000106689 | HGLibA_26450 | 343 | 1.967859126 | LHX2 | LIM homeobox 2 [Source:HGNC Symbol;Acc:HGNC:6594] |
| ENSG00000124568 | HGLibA_44460 | 343 | 1.967859126 | SLC17A1 | solute carrier family 17 member 1 [Source:HGNC Symbol;Acc:HGNC:10929] |
| ENSG00000133895 | HGLibA_28886 | 343 | 1.967859126 | MEN1 | menin 1 [Source:HGNC Symbol;Acc:HGNC:7010] |
| ENSG00000135374 | HGLibA_14903 | 343 | 1.967859126 | ELF5 | E74 like ETS transcription factor 5 [Source:HGNC Symbol;Acc:HGNC:3320] |
| ENSG00000140067 | HGLibA_16317 | 343 | 1.967859126 | FAM181A | family with sequence similarity 181 member A [Source:HGNC Symbol;Acc:HGNC:20491] |
| ENSG00000143870 | HGLibA_35885 | 343 | 1.967859126 | PDIA6 | protein disulfide isomerase family A member 6 [Source:HGNC Symbol;Acc:HGNC:30168] |
| ENSG00000154639 | HGLibA_11840 | 343 | 1.967859126 | CXADR | Ig-like cell adhesion molecule [Source:HGNC Symbol;Acc:HGNC:2559]" |
| ENSG00000163235 | HGLibA_49206 | 343 | 1.967859126 | TGFA | transforming growth factor alpha [Source:HGNC Symbol;Acc:HGNC:11765] |
| ENSG00000186352 | HGLibA_02090 | 343 | 1.967859126 | ANKRD37 | ankyrin repeat domain 37 [Source:HGNC Symbol;Acc:HGNC:29593] |
| ENSG00000186973 | HGLibA_16323 | 343 | 1.967859126 | FAM183A | family with sequence similarity 183 member A [Source:HGNC Symbol;Acc:HGNC:34347] |
| ENSG00000198910 | HGLibA_25884 | 343 | 1.967859126 | L1CAM | L1 cell adhesion molecule [Source:HGNC Symbol;Acc:HGNC:6470] |
| ENSG00000225327 | HGLibA_53271 | 343 | 1.967859126 | USP17L3 | ubiquitin specific peptidase 17-like family member 3 [Source:HGNC Symbol;Acc:HGNC:37175] |
| ENSG00000251349 | HGLibA_30177 | 343 | 1.967859126 | MSANTD3-TMEFF1 | MSANTD3-TMEFF1 readthrough [Source:HGNC Symbol;Acc:HGNC:38838] |
| hsa-let-7g | HGLibA_57132 | 343 | 1.967859126 | hsa-let-7g | - |
| hsa-mir-3686 | HGLibA_59297 | 343 | 1.967859126 | hsa-mir-3686 | - |
| ARSE | HGLibA_03137 | 342 | 1.958813783 | ARSE | - |
| ENSG00000100644 | HGLibA_21297 | 342 | 1.958813783 | HIF1A | hypoxia inducible factor 1 subunit alpha [Source:HGNC Symbol;Acc:HGNC:4910] |
| ENSG00000104823 | HGLibA_14391 | 342 | 1.958813783 | ECH1 | enoyl-CoA hydratase 1 [Source:HGNC Symbol;Acc:HGNC:3149] |
| ENSG00000137757 | HGLibA_07316 | 342 | 1.958813783 | CASP5 | caspase 5 [Source:HGNC Symbol;Acc:HGNC:1506] |
| ENSG00000152056 | HGLibA_02301 | 342 | 1.958813783 | AP1S3 | adaptor related protein complex 1 subunit sigma 3 [Source:HGNC Symbol;Acc:HGNC:18971] |
| ENSG00000153558 | HGLibA_16996 | 342 | 1.958813783 | FBXL2 | F-box and leucine rich repeat protein 2 [Source:HGNC Symbol;Acc:HGNC:13598] |
| ENSG00000162873 | HGLibA_25110 | 342 | 1.958813783 | KLHDC8A | kelch domain containing 8A [Source:HGNC Symbol;Acc:HGNC:25573] |
| ENSG00000165997 | HGLibA_02979 | 342 | 1.958813783 | ARL5B | ADP ribosylation factor like GTPase 5B [Source:HGNC Symbol;Acc:HGNC:23052] |
| ENSG00000169738 | HGLibA_12583 | 342 | 1.958813783 | DCXR | dicarbonyl and L-xylulose reductase [Source:HGNC Symbol;Acc:HGNC:18985] |
| ENSG00000170615 | HGLibA_44797 | 342 | 1.958813783 | SLC26A5 | solute carrier family 26 member 5 [Source:HGNC Symbol;Acc:HGNC:9359] |
| ENSG00000181001 | HGLibA_34192 | 342 | 1.958813783 | OR52N1 | olfactory receptor family 52 subfamily N member 1 [Source:HGNC Symbol;Acc:HGNC:14853] |
| ENSG00000188283 | HGLibA_55892 | 342 | 1.958813783 | ZNF383 | zinc finger protein 383 [Source:HGNC Symbol;Acc:HGNC:18609] |
| ENSG00000204897 | HGLibA_25458 | 342 | 1.958813783 | KRT25 | keratin 25 [Source:HGNC Symbol;Acc:HGNC:30839] |
| ENSG00000257108 | HGLibA_31905 | 342 | 1.958813783 | NHLRC4 | NHL repeat containing 4 [Source:HGNC Symbol;Acc:HGNC:26700] |
| hsa-mir-1179 | HGLibA_57211 | 342 | 1.958813783 | hsa-mir-1179 | - |
| MICALCL | HGLibA_29209 | 342 | 1.958813783 | MICALCL | - |
| SPRY3_X | HGLibA_46842 | 342 | 1.958813783 | SPRY3_X | - |
| ENSG00000074276 | HGLibA_08702 | 341 | 1.94976844 | CDHR2 | cadherin related family member 2 [Source:HGNC Symbol;Acc:HGNC:18231] |
| ENSG00000078081 | HGLibA_25982 | 341 | 1.94976844 | LAMP3 | lysosomal associated membrane protein 3 [Source:HGNC Symbol;Acc:HGNC:14582] |
| ENSG00000082684 | HGLibA_43362 | 341 | 1.94976844 | SEMA5B | semaphorin 5B [Source:HGNC Symbol;Acc:HGNC:10737] |
| ENSG00000091482 | HGLibA_45820 | 341 | 1.94976844 | SMPX | small muscle protein X-linked [Source:HGNC Symbol;Acc:HGNC:11122] |
| ENSG00000100105 | HGLibA_35290 | 341 | 1.94976844 | PATZ1 | POZ/BTB and AT hook containing zinc finger 1 [Source:HGNC Symbol;Acc:HGNC:13071] |
| ENSG00000111331 | HGLibA_33204 | 341 | 1.94976844 | OAS3 | 2'-5'-oligoadenylate synthetase 3 [Source:HGNC Symbol;Acc:HGNC:8088] |
| ENSG00000140848 | HGLibA_10955 | 341 | 1.94976844 | CPNE2 | copine 2 [Source:HGNC Symbol;Acc:HGNC:2315] |
| ENSG00000145414 | HGLibA_31059 | 341 | 1.94976844 | NAF1 | nuclear assembly factor 1 ribonucleoprotein [Source:HGNC Symbol;Acc:HGNC:25126] |
| ENSG00000151379 | HGLibA_30188 | 341 | 1.94976844 | MSGN1 | mesogenin 1 [Source:HGNC Symbol;Acc:HGNC:14907] |
| ENSG00000154359 | HGLibA_27019 | 341 | 1.94976844 | LONRF1 | LON peptidase N-terminal domain and ring finger 1 [Source:HGNC Symbol;Acc:HGNC:26302] |
| ENSG00000165188 | HGLibA_41590 | 341 | 1.94976844 | RNF183 | ring finger protein 183 [Source:HGNC Symbol;Acc:HGNC:28721] |
| ENSG00000165806 | HGLibA_07322 | 341 | 1.94976844 | CASP7 | caspase 7 [Source:HGNC Symbol;Acc:HGNC:1508] |
| ENSG00000168062 | HGLibA_04177 | 341 | 1.94976844 | BATF2 | basic leucine zipper ATF-like transcription factor 2 [Source:HGNC Symbol;Acc:HGNC:25163] |
| ENSG00000170315 | HGLibA_52565 | 341 | 1.94976844 | UBB | ubiquitin B [Source:HGNC Symbol;Acc:HGNC:12463] |
| ENSG00000174990 | HGLibA_06812 | 341 | 1.94976844 | CA5A | carbonic anhydrase 5A [Source:HGNC Symbol;Acc:HGNC:1377] |
| ENSG00000177479 | HGLibA_02916 | 341 | 1.94976844 | ARIH2 | ariadne RBR E3 ubiquitin protein ligase 2 [Source:HGNC Symbol;Acc:HGNC:690] |
| ENSG00000184911 | HGLibA_13438 | 341 | 1.94976844 | DMRTC1B | DMRT like family C1B [Source:HGNC Symbol;Acc:HGNC:31686] |
| ENSG00000215788 | HGLibA_50782 | 341 | 1.94976844 | TNFRSF25 | TNF receptor superfamily member 25 [Source:HGNC Symbol;Acc:HGNC:11910] |
| ENSG00000221988 | HGLibA_38236 | 341 | 1.94976844 | PPT2 | palmitoyl-protein thioesterase 2 [Source:HGNC Symbol;Acc:HGNC:9326] |
| ENSG00000241852 | HGLibA_06637 | 341 | 1.94976844 | C8orf58 | chromosome 8 open reading frame 58 [Source:HGNC Symbol;Acc:HGNC:32233] |
| hsa-mir-127 | HGLibA_57510 | 341 | 1.94976844 | hsa-mir-127 | - |
| hsa-mir-451b | HGLibA_60528 | 341 | 1.94976844 | hsa-mir-451b | - |
| hsa-mir-4661 | HGLibA_60753 | 341 | 1.94976844 | hsa-mir-4661 | - |
| hsa-mir-4701 | HGLibA_60914 | 341 | 1.94976844 | hsa-mir-4701 | - |
| hsa-mir-943 | HGLibA_64360 | 341 | 1.94976844 | hsa-mir-943 | - |
| ENSG00000018625 | HGLibA_03585 | 340 | 1.940723098 | ATP1A2 | ATPase Na+/K+ transporting subunit alpha 2 [Source:HGNC Symbol;Acc:HGNC:800] |
| ENSG00000086967 | HGLibA_30652 | 340 | 1.940723098 | MYBPC2 | fast type [Source:HGNC Symbol;Acc:HGNC:7550]" |
| ENSG00000095739 | HGLibA_04137 | 340 | 1.940723098 | BAMBI | BMP and activin membrane bound inhibitor [Source:HGNC Symbol;Acc:HGNC:30251] |
| ENSG00000103502 | HGLibA_08717 | 340 | 1.940723098 | CDIPT | CDP-diacylglycerol--inositol 3-phosphatidyltransferase [Source:HGNC Symbol;Acc:HGNC:1769] |
| ENSG00000119698 | HGLibA_38208 | 340 | 1.940723098 | PPP4R4 | protein phosphatase 4 regulatory subunit 4 [Source:HGNC Symbol;Acc:HGNC:23788] |
| ENSG00000122507 | HGLibA_04227 | 340 | 1.940723098 | BBS9 | Bardet-Biedl syndrome 9 [Source:HGNC Symbol;Acc:HGNC:30000] |
| ENSG00000125821 | HGLibA_14051 | 340 | 1.940723098 | DTD1 | D-tyrosyl-tRNA deacylase 1 [Source:HGNC Symbol;Acc:HGNC:16219] |
| ENSG00000125834 | HGLibA_47494 | 340 | 1.940723098 | STK35 | serine/threonine kinase 35 [Source:HGNC Symbol;Acc:HGNC:16254] |
| ENSG00000135272 | HGLibA_28672 | 340 | 1.940723098 | MDFIC | MyoD family inhibitor domain containing [Source:HGNC Symbol;Acc:HGNC:28870] |
| ENSG00000136514 | HGLibA_42432 | 340 | 1.940723098 | RTP4 | receptor transporter protein 4 [Source:HGNC Symbol;Acc:HGNC:23992] |
| ENSG00000137831 | HGLibA_52504 | 340 | 1.940723098 | UACA | uveal autoantigen with coiled-coil domains and ankyrin repeats [Source:HGNC Symbol;Acc:HGNC:15947] |
| ENSG00000153094 | HGLibA_04309 | 340 | 1.940723098 | BCL2L11 | BCL2 like 11 [Source:HGNC Symbol;Acc:HGNC:994] |
| ENSG00000164853 | HGLibA_53042 | 340 | 1.940723098 | UNCX | UNC homeobox [Source:HGNC Symbol;Acc:HGNC:33194] |
| ENSG00000167914 | HGLibA_20403 | 340 | 1.940723098 | GSDMA | gasdermin A [Source:HGNC Symbol;Acc:HGNC:13311] |
| ENSG00000179915 | HGLibA_32732 | 340 | 1.940723098 | NRXN1 | neurexin 1 [Source:HGNC Symbol;Acc:HGNC:8008] |
| ENSG00000181315 | HGLibA_55797 | 340 | 1.940723098 | ZNF322 | zinc finger protein 322 [Source:HGNC Symbol;Acc:HGNC:23640] |
| ENSG00000185155 | HGLibA_29320 | 340 | 1.940723098 | MIXL1 | Mix paired-like homeobox [Source:HGNC Symbol;Acc:HGNC:13363] |
| ENSG00000187607 | HGLibA_55751 | 340 | 1.940723098 | ZNF286A | zinc finger protein 286A [Source:HGNC Symbol;Acc:HGNC:13501] |
| ENSG00000196381 | HGLibA_56737 | 340 | 1.940723098 | ZNF781 | zinc finger protein 781 [Source:HGNC Symbol;Acc:HGNC:26745] |
| ENSG00000217442 | HGLibA_47922 | 340 | 1.940723098 | SYCE3 | synaptonemal complex central element protein 3 [Source:HGNC Symbol;Acc:HGNC:35245] |
| hsa-mir-107 | HGLibA_57194 | 340 | 1.940723098 | hsa-mir-107 | - |
| hsa-mir-3922 | HGLibA_59563 | 340 | 1.940723098 | hsa-mir-3922 | - |
| hsa-mir-6767 | HGLibA_63230 | 340 | 1.940723098 | hsa-mir-6767 | - |
| hsa-mir-6840 | HGLibA_63534 | 340 | 1.940723098 | hsa-mir-6840 | - |
| ENSG00000103855 | HGLibA_08322 | 339 | 1.931677755 | CD276 | CD276 molecule [Source:HGNC Symbol;Acc:HGNC:19137] |
| ENSG00000132623 | HGLibA_01953 | 339 | 1.931677755 | ANKEF1 | ankyrin repeat and EF-hand domain containing 1 [Source:HGNC Symbol;Acc:HGNC:15803] |
| ENSG00000134287 | HGLibA_02650 | 339 | 1.931677755 | ARF3 | ADP ribosylation factor 3 [Source:HGNC Symbol;Acc:HGNC:654] |
| ENSG00000136159 | HGLibA_32950 | 339 | 1.931677755 | NUDT15 | nudix hydrolase 15 [Source:HGNC Symbol;Acc:HGNC:23063] |
| ENSG00000137440 | HGLibA_17426 | 339 | 1.931677755 | FGFBP1 | fibroblast growth factor binding protein 1 [Source:HGNC Symbol;Acc:HGNC:19695] |
| ENSG00000137441 | HGLibA_17430 | 339 | 1.931677755 | FGFBP2 | fibroblast growth factor binding protein 2 [Source:HGNC Symbol;Acc:HGNC:29451] |
| ENSG00000143514 | HGLibA_51057 | 339 | 1.931677755 | TP53BP2 | tumor protein p53 binding protein 2 [Source:HGNC Symbol;Acc:HGNC:12000] |
| ENSG00000144136 | HGLibA_44529 | 339 | 1.931677755 | SLC20A1 | solute carrier family 20 member 1 [Source:HGNC Symbol;Acc:HGNC:10946] |
| ENSG00000144460 | HGLibA_33184 | 339 | 1.931677755 | NYAP2 | neuronal tyrosine-phosphorylated phosphoinositide-3-kinase adaptor 2 [Source:HGNC Symbol;Acc:HGNC:29291] |
| ENSG00000145919 | HGLibA_04669 | 339 | 1.931677755 | BOD1 | biorientation of chromosomes in cell division 1 [Source:HGNC Symbol;Acc:HGNC:25114] |
| ENSG00000163749 | HGLibA_07665 | 339 | 1.931677755 | CCDC158 | coiled-coil domain containing 158 [Source:HGNC Symbol;Acc:HGNC:26374] |
| ENSG00000163810 | HGLibA_49262 | 339 | 1.931677755 | TGM4 | transglutaminase 4 [Source:HGNC Symbol;Acc:HGNC:11780] |
| ENSG00000164185 | HGLibA_56062 | 339 | 1.931677755 | ZNF474 | zinc finger protein 474 [Source:HGNC Symbol;Acc:HGNC:23245] |
| ENSG00000166086 | HGLibA_23935 | 339 | 1.931677755 | JAM3 | junctional adhesion molecule 3 [Source:HGNC Symbol;Acc:HGNC:15532] |
| ENSG00000167264 | HGLibA_14114 | 339 | 1.931677755 | DUS2 | dihydrouridine synthase 2 [Source:HGNC Symbol;Acc:HGNC:26014] |
| ENSG00000168038 | HGLibA_52975 | 339 | 1.931677755 | ULK4 | unc-51 like kinase 4 [Source:HGNC Symbol;Acc:HGNC:15784] |
| ENSG00000170099 | HGLibA_43526 | 339 | 1.931677755 | SERPINA6 | serpin family A member 6 [Source:HGNC Symbol;Acc:HGNC:1540] |
| ENSG00000171478 | HGLibA_46336 | 339 | 1.931677755 | SPACA5B | sperm acrosome associated 5B [Source:HGNC Symbol;Acc:HGNC:19142] |
| ENSG00000177000 | HGLibA_30387 | 339 | 1.931677755 | MTHFR | methylenetetrahydrofolate reductase [Source:HGNC Symbol;Acc:HGNC:7436] |

| | | | | | |
|---|---|---|---|---|---|
| ENSG00000185823 | HGLibA_32390 | 339 | 1.931677755 | NPAP1 | nuclear pore associated protein 1 [Source:HGNC Symbol;Acc:HGNC:1190] |
| ENSG00000189337 | HGLibA_24091 | 339 | 1.931677755 | KAZN | periplakin interacting protein [Source:HGNC Symbol;Acc:HGNC:29173]" |
| ENSG00000196743 | HGLibA_19389 | 339 | 1.931677755 | GM2A | GM2 ganglioside activator [Source:HGNC Symbol;Acc:HGNC:4367] |
| ENSG00000206107 | HGLibA_25734 | 339 | 1.931677755 | KRTAP27-1 | keratin associated protein 27-1 [Source:HGNC Symbol;Acc:HGNC:33864] |
| ENSG00000213563 | HGLibA_06649 | 339 | 1.931677755 | C8orf82 | chromosome 8 open reading frame 82 [Source:HGNC Symbol;Acc:HGNC:33826] |
| ENSG00000214013 | HGLibA_18609 | 339 | 1.931677755 | GANC | neutral C [Source:HGNC Symbol;Acc:HGNC:4139]" |
| ENSG00000221994 | HGLibA_56434 | 339 | 1.931677755 | ZNF630 | zinc finger protein 630 [Source:HGNC Symbol;Acc:HGNC:28855] |
| ENSG00000231887 | HGLibA_38461 | 339 | 1.931677755 | PRH1 | proline rich protein HaeIII subfamily 1 [Source:HGNC Symbol;Acc:HGNC:9366] |
| ENSG00000231925 | HGLibA_48322 | 339 | 1.931677755 | TAPBP | TAP binding protein [Source:HGNC Symbol;Acc:HGNC:11566] |
| ENSG00000255150 | HGLibA_14701 | 339 | 1.931677755 | EID3 | EP300 interacting inhibitor of differentiation 3 [Source:HGNC Symbol;Acc:HGNC:32961] |
| hsa-mir-6850 | HGLibA_63573 | 339 | 1.931677755 | hsa-mir-6850 | - |
| TCTE3 | HGLibA_48859 | 339 | 1.931677755 | TCTE3 | - |
| C1orf168 | HGLibA_05789 | 338 | 1.922632412 | C1orf168 | - |
| ENSG00000067066 | HGLibA_46286 | 338 | 1.922632412 | SP100 | SP100 nuclear antigen [Source:HGNC Symbol;Acc:HGNC:11206] |
| ENSG00000100124 | HGLibA_02122 | 338 | 1.922632412 | ANKRD54 | ankyrin repeat domain 54 [Source:HGNC Symbol;Acc:HGNC:25185] |
| ENSG00000102539 | HGLibA_29429 | 338 | 1.922632412 | MLNR | motilin receptor [Source:HGNC Symbol;Acc:HGNC:4495] |
| ENSG00000115380 | HGLibA_14560 | 338 | 1.922632412 | EFEMP1 | EGF containing fibulin extracellular matrix protein 1 [Source:HGNC Symbol;Acc:HGNC:3218] |
| ENSG00000119401 | HGLibA_51462 | 338 | 1.922632412 | TRIM32 | tripartite motif containing 32 [Source:HGNC Symbol;Acc:HGNC:16380] |
| ENSG00000119457 | HGLibA_45188 | 338 | 1.922632412 | SLC46A2 | solute carrier family 46 member 2 [Source:HGNC Symbol;Acc:HGNC:16055] |
| ENSG00000125686 | HGLibA_28733 | 338 | 1.922632412 | MED1 | mediator complex subunit 1 [Source:HGNC Symbol;Acc:HGNC:9234] |
| ENSG00000132334 | HGLibA_39500 | 338 | 1.922632412 | PTPRE | receptor type E [Source:HGNC Symbol;Acc:HGNC:9669]" |
| ENSG00000144591 | HGLibA_19420 | 338 | 1.922632412 | GMPPA | GDP-mannose pyrophosphorylase A [Source:HGNC Symbol;Acc:HGNC:22923] |
| ENSG00000162706 | HGLibA_06971 | 338 | 1.922632412 | CADM3 | cell adhesion molecule 3 [Source:HGNC Symbol;Acc:HGNC:17601] |
| ENSG00000166169 | HGLibA_37537 | 338 | 1.922632412 | POLL | DNA polymerase lambda [Source:HGNC Symbol;Acc:HGNC:9184] |
| ENSG00000169609 | HGLibA_05396 | 338 | 1.922632412 | C15orf40 | chromosome 15 open reading frame 40 [Source:HGNC Symbol;Acc:HGNC:28443] |
| ENSG00000172789 | HGLibA_21970 | 338 | 1.922632412 | HOXC5 | homeobox C5 [Source:HGNC Symbol;Acc:HGNC:5127] |
| ENSG00000174059 | HGLibA_08356 | 338 | 1.922632412 | CD34 | CD34 molecule [Source:HGNC Symbol;Acc:HGNC:1662] |
| ENSG00000176293 | HGLibA_55471 | 338 | 1.922632412 | ZNF135 | zinc finger protein 135 [Source:HGNC Symbol;Acc:HGNC:12919] |
| ENSG00000176834 | HGLibA_53839 | 338 | 1.922632412 | VSIG10 | V-set and immunoglobulin domain containing 10 [Source:HGNC Symbol;Acc:HGNC:26078] |
| ENSG00000185271 | HGLibA_25189 | 338 | 1.922632412 | KLHL33 | kelch like family member 33 [Source:HGNC Symbol;Acc:HGNC:31952] |
| ENSG00000186716 | HGLibA_04367 | 338 | 1.922632412 | BCR | RhoGEF and GTPase activating protein [Source:HGNC Symbol;Acc:HGNC:1014]" |
| ENSG00000198759 | HGLibA_14624 | 338 | 1.922632412 | EGFL6 | EGF like domain multiple 6 [Source:HGNC Symbol;Acc:HGNC:3235] |
| ENSG00000206177 | HGLibA_20932 | 338 | 1.922632412 | HBM | hemoglobin subunit mu [Source:HGNC Symbol;Acc:HGNC:4826] |
| ENSG00000241839 | HGLibA_37153 | 338 | 1.922632412 | PLEKHO2 | pleckstrin homology domain containing O2 [Source:HGNC Symbol;Acc:HGNC:30026] |
| hsa-mir-3653 | HGLibA_59157 | 338 | 1.922632412 | hsa-mir-3653 | - |
| hsa-mir-3915 | HGLibA_59535 | 338 | 1.922632412 | hsa-mir-3915 | - |
| hsa-mir-802 | HGLibA_64044 | 338 | 1.922632412 | hsa-mir-802 | - |
| C19orf10 | HGLibA_05628 | 337 | 1.913587069 | C19orf10 | - |
| ENSG00000020256 | HGLibA_55229 | 337 | 1.913587069 | ZFP64 | ZFP64 zinc finger protein [Source:HGNC Symbol;Acc:HGNC:15940] |
| ENSG00000058085 | HGLibA_25970 | 337 | 1.913587069 | LAMC2 | laminin subunit gamma 2 [Source:HGNC Symbol;Acc:HGNC:6493] |
| ENSG00000074410 | HGLibA_06795 | 337 | 1.913587069 | CA12 | carbonic anhydrase 12 [Source:HGNC Symbol;Acc:HGNC:1371] |
| ENSG00000091527 | HGLibA_08892 | 337 | 1.913587069 | CDV3 | CDV3 homolog [Source:HGNC Symbol;Acc:HGNC:26928] |
| ENSG00000101210 | HGLibA_14493 | 337 | 1.913587069 | EEF1A2 | eukaryotic translation elongation factor 1 alpha 2 [Source:HGNC Symbol;Acc:HGNC:3192] |
| ENSG00000102924 | HGLibA_07405 | 337 | 1.913587069 | CBLN1 | cerebellin 1 precursor [Source:HGNC Symbol;Acc:HGNC:1543] |
| ENSG00000111481 | HGLibA_10738 | 337 | 1.913587069 | COPZ1 | coatomer protein complex subunit zeta 1 [Source:HGNC Symbol;Acc:HGNC:2243] |
| ENSG00000111981 | HGLibA_52959 | 337 | 1.913587069 | ULBP1 | UL16 binding protein 1 [Source:HGNC Symbol;Acc:HGNC:14893] |
| ENSG00000112561 | HGLibA_49163 | 337 | 1.913587069 | TFEB | transcription factor EB [Source:HGNC Symbol;Acc:HGNC:11753] |
| ENSG00000115226 | HGLibA_17754 | 337 | 1.913587069 | FNDC4 | fibronectin type III domain containing 4 [Source:HGNC Symbol;Acc:HGNC:20239] |
| ENSG00000122707 | HGLibA_40763 | 337 | 1.913587069 | RECK | reversion inducing cysteine rich protein with kazal motifs [Source:HGNC Symbol;Acc:HGNC:11345] |
| ENSG00000139714 | HGLibA_29672 | 337 | 1.913587069 | MORN3 | MORN repeat containing 3 [Source:HGNC Symbol;Acc:HGNC:29807] |
| ENSG00000140379 | HGLibA_04300 | 337 | 1.913587069 | BCL2A1 | BCL2 related protein A1 [Source:HGNC Symbol;Acc:HGNC:991] |
| ENSG00000142046 | HGLibA_50557 | 337 | 1.913587069 | TMEM91 | transmembrane protein 91 [Source:HGNC Symbol;Acc:HGNC:32393] |
| ENSG00000147378 | HGLibA_16921 | 337 | 1.913587069 | FATE1 | fetal and adult testis expressed 1 [Source:HGNC Symbol;Acc:HGNC:24683] |
| ENSG00000156097 | HGLibA_20062 | 337 | 1.913587069 | GPR61 | G protein-coupled receptor 61 [Source:HGNC Symbol;Acc:HGNC:13300] |
| ENSG00000162174 | HGLibA_03369 | 337 | 1.913587069 | ASRGL1 | asparaginase like 1 [Source:HGNC Symbol;Acc:HGNC:16448] |
| ENSG00000177025 | HGLibA_05632 | 337 | 1.913587069 | C19orf18 | chromosome 19 open reading frame 18 [Source:HGNC Symbol;Acc:HGNC:28642] |
| ENSG00000179083 | HGLibA_16118 | 337 | 1.913587069 | FAM133A | family with sequence similarity 133 member A [Source:HGNC Symbol;Acc:HGNC:26748] |
| ENSG00000197714 | HGLibA_56034 | 337 | 1.913587069 | ZNF460 | zinc finger protein 460 [Source:HGNC Symbol;Acc:HGNC:21628] |
| ENSG00000204227 | HGLibA_41299 | 337 | 1.913587069 | RING1 | ring finger protein 1 [Source:HGNC Symbol;Acc:HGNC:10018] |
| ENSG00000237693 | HGLibA_23613 | 337 | 1.913587069 | IRGM | immunity related GTPase M [Source:HGNC Symbol;Acc:HGNC:29597] |
| ENSG00000239389 | HGLibA_35435 | 337 | 1.913587069 | PCDHA13 | protocadherin alpha 13 [Source:HGNC Symbol;Acc:HGNC:8667] |
| ENSG00000253958 | HGLibA_09885 | 337 | 1.913587069 | CLDN23 | claudin 23 [Source:HGNC Symbol;Acc:HGNC:17591] |
| hsa-mir-769 | HGLibA_63936 | 337 | 1.913587069 | hsa-mir-769 | - |
| ENSG00000008735 | HGLibA_28289 | 336 | 1.904541726 | MAPK8IP2 | mitogen-activated protein kinase 8 interacting protein 2 [Source:HGNC Symbol;Acc:HGNC:6883] |
| ENSG00000057657 | HGLibA_38362 | 336 | 1.904541726 | PRDM1 | PR/SET domain 1 [Source:HGNC Symbol;Acc:HGNC:9346] |
| ENSG00000079337 | HGLibA_40250 | 336 | 1.904541726 | RAPGEF3 | Rap guanine nucleotide exchange factor 3 [Source:HGNC Symbol;Acc:HGNC:16629] |
| ENSG00000087085 | HGLibA_00438 | 336 | 1.904541726 | ACHE | acetylcholinesterase (Cartwright blood group) [Source:HGNC Symbol;Acc:HGNC:108] |
| ENSG00000100711 | HGLibA_55282 | 336 | 1.904541726 | ZFYVE21 | zinc finger FYVE-type containing 21 [Source:HGNC Symbol;Acc:HGNC:20760] |
| ENSG00000103647 | HGLibA_10786 | 336 | 1.904541726 | CORO2B | coronin 2B [Source:HGNC Symbol;Acc:HGNC:2256] |
| ENSG00000106327 | HGLibA_49192 | 336 | 1.904541726 | TFR2 | transferrin receptor 2 [Source:HGNC Symbol;Acc:HGNC:11762] |
| ENSG00000106624 | HGLibA_01084 | 336 | 1.904541726 | AEBP1 | AE binding protein 1 [Source:HGNC Symbol;Acc:HGNC:303] |
| ENSG00000107159 | HGLibA_06828 | 336 | 1.904541726 | CA9 | carbonic anhydrase 9 [Source:HGNC Symbol;Acc:HGNC:1383] |
| ENSG00000111331 | HGLibA_33203 | 336 | 1.904541726 | OAS3 | 2'-5'-oligoadenylate synthetase 3 [Source:HGNC Symbol;Acc:HGNC:8088] |
| ENSG00000114654 | HGLibA_14559 | 336 | 1.904541726 | EFCC1 | EF-hand and coiled-coil domain containing 1 [Source:HGNC Symbol;Acc:HGNC:25692] |
| ENSG00000135097 | HGLibA_30205 | 336 | 1.904541726 | MSI1 | musashi RNA binding protein 1 [Source:HGNC Symbol;Acc:HGNC:7330] |
| ENSG00000163431 | HGLibA_26716 | 336 | 1.904541726 | LMOD1 | leiomodin 1 [Source:HGNC Symbol;Acc:HGNC:6647] |
| ENSG00000163995 | HGLibA_00312 | 336 | 1.904541726 | ABLIM2 | actin binding LIM protein family member 2 [Source:HGNC Symbol;Acc:HGNC:19195] |
| ENSG00000166192 | HGLibA_43404 | 336 | 1.904541726 | SENP8 | NEDD8 specific [Source:HGNC Symbol;Acc:HGNC:22992]" |
| ENSG00000167800 | HGLibA_48645 | 336 | 1.904541726 | TBX10 | T-box 10 [Source:HGNC Symbol;Acc:HGNC:11593] |
| ENSG00000168505 | HGLibA_18753 | 336 | 1.904541726 | GBX2 | gastrulation brain homeobox 2 [Source:HGNC Symbol;Acc:HGNC:4186] |
| ENSG00000168685 | HGLibA_23194 | 336 | 1.904541726 | IL7R | interleukin 7 receptor [Source:HGNC Symbol;Acc:HGNC:6024] |
| ENSG00000171611 | HGLibA_39287 | 336 | 1.904541726 | PTCRA | pre T cell antigen receptor alpha [Source:HGNC Symbol;Acc:HGNC:21290] |
| ENSG00000173890 | HGLibA_19955 | 336 | 1.904541726 | GPR160 | G protein-coupled receptor 160 [Source:HGNC Symbol;Acc:HGNC:23693] |
| ENSG00000181804 | HGLibA_45429 | 336 | 1.904541726 | SLC9A9 | solute carrier family 9 member A9 [Source:HGNC Symbol;Acc:HGNC:20653] |
| ENSG00000182968 | HGLibA_46226 | 336 | 1.904541726 | SOX1 | SRY-box 1 [Source:HGNC Symbol;Acc:HGNC:11189] |
| ENSG00000189326 | HGLibA_46410 | 336 | 1.904541726 | SPANXN4 | SPANX family member N4 [Source:HGNC Symbol;Acc:HGNC:33177] |
| ENSG00000205863 | HGLibA_05963 | 336 | 1.904541726 | C1QTNF9B | C1q and TNF related 9B [Source:HGNC Symbol;Acc:HGNC:34072] |
| ENSG00000239998 | HGLibA_26491 | 336 | 1.904541726 | LILRA2 | leukocyte immunoglobulin like receptor A2 [Source:HGNC Symbol;Acc:HGNC:6603] |
| ENSG00000277363 | HGLibA_46934 | 336 | 1.904541726 | SRCIN1 | SRC kinase signaling inhibitor 1 [Source:HGNC Symbol;Acc:HGNC:29506] |
| hsa-mir-4634 | HGLibA_60640 | 336 | 1.904541726 | hsa-mir-4634 | - |
| PVRL1 | HGLibA_39623 | 336 | 1.904541726 | PVRL1 | - |
| ENSG00000054938 | HGLibA_09542 | 335 | 1.895496383 | CHRDL2 | chordin like 2 [Source:HGNC Symbol;Acc:HGNC:24168] |
| ENSG00000114378 | HGLibA_22437 | 335 | 1.895496383 | HYAL1 | hyaluronidase 1 [Source:HGNC Symbol;Acc:HGNC:5320] |
| ENSG00000118096 | HGLibA_22760 | 335 | 1.895496383 | IFT46 | intraflagellar transport 46 [Source:HGNC Symbol;Acc:HGNC:26146] |
| ENSG00000120656 | HGLibA_48205 | 335 | 1.895496383 | TAF12 | TATA-box binding protein associated factor 12 [Source:HGNC Symbol;Acc:HGNC:11545] |
| ENSG00000134461 | HGLibA_02013 | 335 | 1.895496383 | ANKRD16 | ankyrin repeat domain 16 [Source:HGNC Symbol;Acc:HGNC:23471] |
| ENSG00000151929 | HGLibA_04080 | 335 | 1.895496383 | BAG3 | BCL2 associated athanogene 3 [Source:HGNC Symbol;Acc:HGNC:939] |
| ENSG00000168301 | HGLibA_24460 | 335 | 1.895496383 | KCTD6 | potassium channel tetramerization domain containing 6 [Source:HGNC Symbol;Acc:HGNC:22235] |
| ENSG00000168490 | HGLibA_36475 | 335 | 1.895496383 | PHYHIP | phytanoyl-CoA 2-hydroxylase interacting protein [Source:HGNC Symbol;Acc:HGNC:16865] |
| ENSG00000168795 | HGLibA_54909 | 335 | 1.895496383 | ZBTB5 | zinc finger and BTB domain containing 5 [Source:HGNC Symbol;Acc:HGNC:23836] |
| ENSG00000198089 | HGLibA_43719 | 335 | 1.895496383 | SFI1 | SFI1 centrin binding protein [Source:HGNC Symbol;Acc:HGNC:29064] |
| ENSG00000198185 | HGLibA_55821 | 335 | 1.895496383 | ZNF334 | zinc finger protein 334 [Source:HGNC Symbol;Acc:HGNC:15806] |
| ENSG00000204967 | HGLibA_35444 | 335 | 1.895496383 | PCDHA4 | protocadherin alpha 4 [Source:HGNC Symbol;Acc:HGNC:8670] |
| ENSG00000205476 | HGLibA_07898 | 335 | 1.895496383 | CCDC85C | coiled-coil domain containing 85C [Source:HGNC Symbol;Acc:HGNC:35459] |
| ENSG00000269190 | HGLibA_17036 | 335 | 1.895496383 | FBXO17 | F-box protein 17 [Source:HGNC Symbol;Acc:HGNC:18754] |
| hsa-mir-139 | HGLibA_57821 | 335 | 1.895496383 | hsa-mir-139 | - |
| hsa-mir-670 | HGLibA_63011 | 335 | 1.895496383 | hsa-mir-670 | - |
| hsa-mir-6795 | HGLibA_63357 | 335 | 1.895496383 | hsa-mir-6795 | - |
| C19orf59 | HGLibA_05687 | 334 | 1.88645104 | C19orf59 | - |
| ENSG00000092969 | HGLibA_49214 | 334 | 1.88645104 | TGFB2 | transforming growth factor beta 2 [Source:HGNC Symbol;Acc:HGNC:11768] |
| ENSG00000099250 | HGLibA_32714 | 334 | 1.88645104 | NRP1 | neuropilin 1 [Source:HGNC Symbol;Acc:HGNC:8004] |
| ENSG00000101384 | HGLibA_23904 | 334 | 1.88645104 | JAG1 | jagged 1 [Source:HGNC Symbol;Acc:HGNC:6188] |
| ENSG00000104549 | HGLibA_46912 | 334 | 1.88645104 | SQLE | squalene epoxidase [Source:HGNC Symbol;Acc:HGNC:11279] |
| ENSG00000105393 | HGLibA_04057 | 334 | 1.88645104 | BABAM1 | BRISC and BRCA1 A complex member 1 [Source:HGNC Symbol;Acc:HGNC:25008] |
| ENSG00000106128 | HGLibA_19047 | 334 | 1.88645104 | GHRHR | growth hormone releasing hormone receptor [Source:HGNC Symbol;Acc:HGNC:4266] |
| ENSG00000113732 | HGLibA_03723 | 334 | 1.88645104 | ATP6V0E1 | ATPase H+ transporting V0 subunit e1 [Source:HGNC Symbol;Acc:HGNC:863] |
| ENSG00000113761 | HGLibA_55850 | 334 | 1.88645104 | ZNF346 | zinc finger protein 346 [Source:HGNC Symbol;Acc:HGNC:16403] |
| ENSG00000117595 | HGLibA_23596 | 334 | 1.88645104 | IRF6 | interferon regulatory factor 6 [Source:HGNC Symbol;Acc:HGNC:6121] |
| ENSG00000126453 | HGLibA_04311 | 334 | 1.88645104 | BCL2L12 | BCL2 like 12 [Source:HGNC Symbol;Acc:HGNC:13787] |
| ENSG00000133687 | HGLibA_50670 | 334 | 1.88645104 | TMTC1 | transmembrane and tetratricopeptide repeat containing 1 [Source:HGNC Symbol;Acc:HGNC:24099] |
| ENSG00000145545 | HGLibA_46939 | 334 | 1.88645104 | SRD5A1 | steroid 5 alpha-reductase 1 [Source:HGNC Symbol;Acc:HGNC:11284] |
| ENSG00000148602 | HGLibA_27186 | 334 | 1.88645104 | LRIT1 | Ig-like and transmembrane domains 1 [Source:HGNC Symbol;Acc:HGNC:23404]" |
| ENSG00000150768 | HGLibA_13308 | 334 | 1.88645104 | DLAT | dihydrolipoamide S-acetyltransferase [Source:HGNC Symbol;Acc:HGNC:2896] |
| ENSG00000153714 | HGLibA_27634 | 334 | 1.88645104 | LURAP1L | leucine rich adaptor protein 1 like [Source:HGNC Symbol;Acc:HGNC:31452] |
| ENSG00000161847 | HGLibA_40402 | 334 | 1.88645104 | RAVER1 | PTB binding 1 [Source:HGNC Symbol;Acc:HGNC:30296]" |
| ENSG00000171853 | HGLibA_51306 | 334 | 1.88645104 | TRAPPC12 | trafficking protein particle complex 12 [Source:HGNC Symbol;Acc:HGNC:24284] |
| ENSG00000180016 | HGLibA_33658 | 334 | 1.88645104 | OR1E1 | olfactory receptor family 1 subfamily E member 1 [Source:HGNC Symbol;Acc:HGNC:8189] |
| ENSG00000180697 | HGLibA_06220 | 334 | 1.88645104 | C3orf22 | chromosome 3 open reading frame 22 [Source:HGNC Symbol;Acc:HGNC:28534] |
| ENSG00000196387 | HGLibA_55482 | 334 | 1.88645104 | ZNF140 | zinc finger protein 140 [Source:HGNC Symbol;Acc:HGNC:12925] |
| ENSG00000204536 | HGLibA_07946 | 334 | 1.88645104 | CCHCR1 | coiled-coil alpha-helical rod protein 1 [Source:HGNC Symbol;Acc:HGNC:13930] |
| hsa-mir-6126 | HGLibA_62653 | 334 | 1.88645104 | hsa-mir-6126 | - |
| hsa-mir-624 | HGLibA_62735 | 334 | 1.88645104 | hsa-mir-624 | - |
| ENSG00000087237 | HGLibA_09241 | 333 | 1.877405697 | CETP | cholesteryl ester transfer protein [Source:HGNC Symbol;Acc:HGNC:1869] |
| ENSG00000100473 | HGLibA_10468 | 333 | 1.877405697 | COCH | cochlin [Source:HGNC Symbol;Acc:HGNC:2180] |
| ENSG00000102977 | HGLibA_00420 | 333 | 1.877405697 | ACD | shelterin complex subunit and telomerase recruitment factor [Source:HGNC Symbol;Acc:HGNC:25070]" |

| | | | | | |
|---|---|---|---|---|---|
| ENSG00000108774 | HGLibA_39967 | 333 | 1.877405697 | RAB5C | member RAS oncogene family [Source:HGNC Symbol;Acc:HGNC:9785]" |
| ENSG00000114654 | HGLibA_14558 | 333 | 1.877405697 | EFCC1 | EF-hand and coiled-coil domain containing 1 [Source:HGNC Symbol;Acc:HGNC:25692] |
| ENSG00000117054 | HGLibA_00367 | 333 | 1.877405697 | ACADM | acyl-CoA dehydrogenase medium chain [Source:HGNC Symbol;Acc:HGNC:89] |
| ENSG00000126602 | HGLibA_51294 | 333 | 1.877405697 | TRAP1 | TNF receptor associated protein 1 [Source:HGNC Symbol;Acc:HGNC:16264] |
| ENSG00000129911 | HGLibA_25061 | 333 | 1.877405697 | KLF16 | Kruppel like factor 16 [Source:HGNC Symbol;Acc:HGNC:16857] |
| ENSG00000144445 | HGLibA_24040 | 333 | 1.877405697 | KANSL1L | KAT8 regulatory NSL complex subunit 1 like [Source:HGNC Symbol;Acc:HGNC:26310] |
| ENSG00000152580 | HGLibA_22879 | 333 | 1.877405697 | IGSF10 | immunoglobulin superfamily member 10 [Source:HGNC Symbol;Acc:HGNC:26384] |
| ENSG00000162512 | HGLibA_43103 | 333 | 1.877405697 | SDC3 | syndecan 3 [Source:HGNC Symbol;Acc:HGNC:10660] |
| ENSG00000162641 | HGLibA_01467 | 333 | 1.877405697 | AKNAD1 | AKNA domain containing 1 [Source:HGNC Symbol;Acc:HGNC:28398] |
| ENSG00000169550 | HGLibA_30525 | 333 | 1.877405697 | MUC15 | cell surface associated [Source:HGNC Symbol;Acc:HGNC:14956]" |
| ENSG00000171385 | HGLibA_24179 | 333 | 1.877405697 | KCND3 | potassium voltage-gated channel subfamily D member 3 [Source:HGNC Symbol;Acc:HGNC:6239] |
| ENSG00000182631 | HGLibA_42500 | 333 | 1.877405697 | RXFP3 | relaxin family peptide receptor 3 [Source:HGNC Symbol;Acc:HGNC:24883] |
| ENSG00000186001 | HGLibA_27144 | 333 | 1.877405697 | LRCH3 | leucine rich repeats and calponin homology domain containing 3 [Source:HGNC Symbol;Acc:HGNC:28637] |
| ENSG00000188782 | HGLibA_07365 | 333 | 1.877405697 | CATSPER4 | cation channel sperm associated 4 [Source:HGNC Symbol;Acc:HGNC:23220] |
| ENSG00000197256 | HGLibA_24027 | 333 | 1.877405697 | KANK2 | KN motif and ankyrin repeat domains 2 [Source:HGNC Symbol;Acc:HGNC:29300] |
| ENSG00000214652 | HGLibA_56636 | 333 | 1.877405697 | ZNF727 | zinc finger protein 727 [Source:HGNC Symbol;Acc:HGNC:22785] |
| ENSG00000221838 | HGLibA_02346 | 333 | 1.877405697 | AP4M1 | adaptor related protein complex 4 subunit mu 1 [Source:HGNC Symbol;Acc:HGNC:574] |
| ENSG00000273079 | HGLibA_20301 | 333 | 1.877405697 | GRIN2B | glutamate ionotropic receptor NMDA type subunit 2B [Source:HGNC Symbol;Acc:HGNC:4586] |
| hsa-mir-1343 | HGLibA_57787 | 333 | 1.877405697 | hsa-mir-1343 | - |
| hsa-mir-378f | HGLibA_59448 | 333 | 1.877405697 | hsa-mir-378f | - |
| hsa-mir-4511 | HGLibA_60487 | 333 | 1.877405697 | hsa-mir-4511 | - |
| hsa-mir-526b | HGLibA_61799 | 333 | 1.877405697 | hsa-mir-526b | - |
| hsa-mir-6778 | HGLibA_63286 | 333 | 1.877405697 | hsa-mir-6778 | - |
| hsa-mir-6811 | HGLibA_63421 | 333 | 1.877405697 | hsa-mir-6811 | - |
| 8-Sep | HGLibA_43454 | 333 | 1.877405697 | 8-Sep | - |
| C11orf73 | HGLibA_05180 | 332 | 1.868360354 | C11orf73 | - |
| C9orf16 | HGLibA_06697 | 332 | 1.868360354 | C9orf16 | - |
| ENSG00000100711 | HGLibA_55283 | 332 | 1.868360354 | ZFYVE21 | zinc finger FYVE-type containing 21 [Source:HGNC Symbol;Acc:HGNC:20760] |
| ENSG00000104826 | HGLibA_26419 | 332 | 1.868360354 | LHB | luteinizing hormone beta polypeptide [Source:HGNC Symbol;Acc:HGNC:6584] |
| ENSG00000106799 | HGLibA_49222 | 332 | 1.868360354 | TGFBR1 | transforming growth factor beta receptor 1 [Source:HGNC Symbol;Acc:HGNC:11772] |
| ENSG00000110057 | HGLibA_53038 | 332 | 1.868360354 | UNC93B1 | TLR signaling regulator [Source:HGNC Symbol;Acc:HGNC:13481]" |
| ENSG00000111058 | HGLibA_00592 | 332 | 1.868360354 | ACSS3 | acyl-CoA synthetase short chain family member 3 [Source:HGNC Symbol;Acc:HGNC:24723] |
| ENSG00000112164 | HGLibA_19293 | 332 | 1.868360354 | GLP1R | glucagon like peptide 1 receptor [Source:HGNC Symbol;Acc:HGNC:4324] |
| ENSG00000115257 | HGLibA_35677 | 332 | 1.868360354 | PCSK4 | proprotein convertase subtilisin/kexin type 4 [Source:HGNC Symbol;Acc:HGNC:8746] |
| ENSG00000121743 | HGLibA_19142 | 332 | 1.868360354 | GJA3 | gap junction protein alpha 3 [Source:HGNC Symbol;Acc:HGNC:4277] |
| ENSG00000126264 | HGLibA_20993 | 332 | 1.868360354 | HCST | hematopoietic cell signal transducer [Source:HGNC Symbol;Acc:HGNC:16977] |
| ENSG00000130227 | HGLibA_54574 | 332 | 1.868360354 | XPO7 | exportin 7 [Source:HGNC Symbol;Acc:HGNC:14108] |
| ENSG00000133065 | HGLibA_45140 | 332 | 1.868360354 | SLC41A1 | solute carrier family 41 member 1 [Source:HGNC Symbol;Acc:HGNC:19429] |
| ENSG00000135074 | HGLibA_00745 | 332 | 1.868360354 | ADAM19 | ADAM metallopeptidase domain 19 [Source:HGNC Symbol;Acc:HGNC:197] |
| ENSG00000139209 | HGLibA_45070 | 332 | 1.868360354 | SLC38A4 | solute carrier family 38 member 4 [Source:HGNC Symbol;Acc:HGNC:14679] |
| ENSG00000143891 | HGLibA_18521 | 332 | 1.868360354 | GALM | galactose mutarotase [Source:HGNC Symbol;Acc:HGNC:24063] |
| ENSG00000145936 | HGLibA_24343 | 332 | 1.868360354 | KCNMB1 | potassium calcium-activated channel subfamily M regulatory beta subunit 1 [Source:HGNC Symbol;Acc:HGNC:6285] |
| ENSG00000152954 | HGLibA_32725 | 332 | 1.868360354 | NRSN1 | neurensin 1 [Source:HGNC Symbol;Acc:HGNC:17881] |
| ENSG00000163497 | HGLibA_17309 | 332 | 1.868360354 | FEV | ETS transcription factor [Source:HGNC Symbol;Acc:HGNC:18562]" |
| ENSG00000168282 | HGLibA_29135 | 332 | 1.868360354 | MGAT2 | 2-N-acetylglucosaminyltransferase [Source:HGNC Symbol;Acc:HGNC:7045]" |
| ENSG00000171451 | HGLibA_14019 | 332 | 1.868360354 | DSEL | dermatan sulfate epimerase like [Source:HGNC Symbol;Acc:HGNC:18144] |
| ENSG00000181381 | HGLibA_12766 | 332 | 1.868360354 | DDX60L | DExD/H-box 60 like [Source:HGNC Symbol;Acc:HGNC:26429] |
| ENSG00000181523 | HGLibA_43855 | 332 | 1.868360354 | SGSH | N-sulfoglucosamine sulfohydrolase [Source:HGNC Symbol;Acc:HGNC:10818] |
| ENSG00000198704 | HGLibA_20186 | 332 | 1.868360354 | GPX6 | glutathione peroxidase 6 [Source:HGNC Symbol;Acc:HGNC:4558] |
| ENSG00000198945 | HGLibA_25901 | 332 | 1.868360354 | L3MBTL3 | histone methyl-lysine binding protein [Source:HGNC Symbol;Acc:HGNC:23035]" |
| hsa-mir-3175 | HGLibA_58775 | 332 | 1.868360354 | hsa-mir-3175 | - |
| hsa-mir-4666b | HGLibA_60783 | 332 | 1.868360354 | hsa-mir-4666b | - |
| hsa-mir-517c | HGLibA_61630 | 332 | 1.868360354 | hsa-mir-517c | - |
| hsa-mir-5692a-2 | HGLibA_62293 | 332 | 1.868360354 | hsa-mir-5692a-2 | - |
| PRAMEF16 | HGLibA_38295 | 332 | 1.868360354 | PRAMEF16 | - |
| ENSG00000079112 | HGLibA_08649 | 331 | 1.859315011 | CDH17 | cadherin 17 [Source:HGNC Symbol;Acc:HGNC:1756] |
| ENSG00000105982 | HGLibA_41665 | 331 | 1.859315011 | RNF32 | ring finger protein 32 [Source:HGNC Symbol;Acc:HGNC:17118] |
| ENSG00000115705 | HGLibA_51157 | 331 | 1.859315011 | TPO | thyroid peroxidase [Source:HGNC Symbol;Acc:HGNC:12015] |
| ENSG00000121864 | HGLibA_56441 | 331 | 1.859315011 | ZNF639 | zinc finger protein 639 [Source:HGNC Symbol;Acc:HGNC:30950] |
| ENSG00000140538 | HGLibA_32879 | 331 | 1.859315011 | NTRK3 | neurotrophic receptor tyrosine kinase 3 [Source:HGNC Symbol;Acc:HGNC:8033] |
| ENSG00000159164 | HGLibA_47873 | 331 | 1.859315011 | SV2A | synaptic vesicle glycoprotein 2A [Source:HGNC Symbol;Acc:HGNC:20566] |
| ENSG00000159917 | HGLibA_55648 | 331 | 1.859315011 | ZNF235 | zinc finger protein 235 [Source:HGNC Symbol;Acc:HGNC:12866] |
| ENSG00000161640 | HGLibA_44130 | 331 | 1.859315011 | SIGLEC11 | sialic acid binding Ig like lectin 11 [Source:HGNC Symbol;Acc:HGNC:15622] |
| ENSG00000167139 | HGLibA_48501 | 331 | 1.859315011 | TBC1D21 | TBC1 domain family member 21 [Source:HGNC Symbol;Acc:HGNC:28536] |
| ENSG00000171303 | HGLibA_24322 | 331 | 1.859315011 | KCNK3 | potassium two pore domain channel subfamily K member 3 [Source:HGNC Symbol;Acc:HGNC:6278] |
| ENSG00000171700 | HGLibA_41076 | 331 | 1.859315011 | RGS19 | regulator of G protein signaling 19 [Source:HGNC Symbol;Acc:HGNC:13735] |
| ENSG00000175518 | HGLibA_52743 | 331 | 1.859315011 | UBQLNL | ubiquilin like [Source:HGNC Symbol;Acc:HGNC:28294] |
| ENSG00000176641 | HGLibA_41549 | 331 | 1.859315011 | RNF152 | ring finger protein 152 [Source:HGNC Symbol;Acc:HGNC:26811] |
| ENSG00000182782 | HGLibA_20949 | 331 | 1.859315011 | HCAR2 | hydroxycarboxylic acid receptor 2 [Source:HGNC Symbol;Acc:HGNC:24827] |
| ENSG00000189430 | HGLibA_31363 | 331 | 1.859315011 | NCR1 | natural cytotoxicity triggering receptor 1 [Source:HGNC Symbol;Acc:HGNC:6731] |
| ENSG00000196932 | HGLibA_50348 | 331 | 1.859315011 | TMEM26 | transmembrane protein 26 [Source:HGNC Symbol;Acc:HGNC:28550] |
| hsa-mir-1225 | HGLibA_57298 | 331 | 1.859315011 | hsa-mir-1225 | - |
| hsa-mir-3934 | HGLibA_59595 | 331 | 1.859315011 | hsa-mir-3934 | - |
| hsa-mir-941-3 | HGLibA_64348 | 331 | 1.859315011 | hsa-mir-941-3 | - |
| SMCR9 | HGLibA_45687 | 331 | 1.859315011 | SMCR9 | - |
| C14orf79 | HGLibA_05369 | 330 | 1.850269669 | C14orf79 | - |
| ENSG00000100147 | HGLibA_07597 | 330 | 1.850269669 | CCDC134 | coiled-coil domain containing 134 [Source:HGNC Symbol;Acc:HGNC:26185] |
| ENSG00000102837 | HGLibA_33353 | 330 | 1.850269669 | OLFM4 | olfactomedin 4 [Source:HGNC Symbol;Acc:HGNC:17190] |
| ENSG00000103145 | HGLibA_20960 | 330 | 1.850269669 | HCFC1R1 | host cell factor C1 regulator 1 [Source:HGNC Symbol;Acc:HGNC:21198] |
| ENSG00000104892 | HGLibA_25032 | 330 | 1.850269669 | KLC3 | kinesin light chain 3 [Source:HGNC Symbol;Acc:HGNC:20717] |
| ENSG00000113108 | HGLibA_02386 | 330 | 1.850269669 | APBB3 | amyloid beta precursor protein binding family B member 3 [Source:HGNC Symbol;Acc:HGNC:20708] |
| ENSG00000113595 | HGLibA_51435 | 330 | 1.850269669 | TRIM23 | tripartite motif containing 23 [Source:HGNC Symbol;Acc:HGNC:660] |
| ENSG00000134815 | HGLibA_13185 | 330 | 1.850269669 | DHX34 | DExH-box helicase 34 [Source:HGNC Symbol;Acc:HGNC:16719] |
| ENSG00000137843 | HGLibA_35053 | 330 | 1.850269669 | PAK6 | p21 (RAC1) activated kinase 6 [Source:HGNC Symbol;Acc:HGNC:16061] |
| ENSG00000139899 | HGLibA_07411 | 330 | 1.850269669 | CBLN3 | cerebellin 3 precursor [Source:HGNC Symbol;Acc:HGNC:20146] |
| ENSG00000147677 | HGLibA_14787 | 330 | 1.850269669 | EIF3H | eukaryotic translation initiation factor 3 subunit H [Source:HGNC Symbol;Acc:HGNC:3273] |
| ENSG00000148826 | HGLibA_32058 | 330 | 1.850269669 | NKX6-2 | NK6 homeobox 2 [Source:HGNC Symbol;Acc:HGNC:19321] |
| ENSG00000152382 | HGLibA_48184 | 330 | 1.850269669 | TADA1 | transcriptional adaptor 1 [Source:HGNC Symbol;Acc:HGNC:30631] |
| ENSG00000155974 | HGLibA_20320 | 330 | 1.850269669 | GRIP1 | glutamate receptor interacting protein 1 [Source:HGNC Symbol;Acc:HGNC:18708] |
| ENSG00000162065 | HGLibA_48511 | 330 | 1.850269669 | TBC1D24 | TBC1 domain family member 24 [Source:HGNC Symbol;Acc:HGNC:29203] |
| ENSG00000163378 | HGLibA_15223 | 330 | 1.850269669 | EOGT | EGF domain specific O-linked N-acetylglucosamine transferase [Source:HGNC Symbol;Acc:HGNC:28526] |
| ENSG00000164465 | HGLibA_12469 | 330 | 1.850269669 | DCBLD1 | CUB and LCCL domain containing 1 [Source:HGNC Symbol;Acc:HGNC:21479]" |
| ENSG00000169895 | HGLibA_47908 | 330 | 1.850269669 | SYAP1 | synapse associated protein 1 [Source:HGNC Symbol;Acc:HGNC:16273] |
| ENSG00000173456 | HGLibA_41659 | 330 | 1.850269669 | RNF26 | ring finger protein 26 [Source:HGNC Symbol;Acc:HGNC:14646] |
| ENSG00000176742 | HGLibA_34135 | 330 | 1.850269669 | OR51V1 | olfactory receptor family 51 subfamily V member 1 [Source:HGNC Symbol;Acc:HGNC:19597] |
| ENSG00000180532 | HGLibA_57024 | 330 | 1.850269669 | ZSCAN4 | zinc finger and SCAN domain containing 4 [Source:HGNC Symbol;Acc:HGNC:23709] |
| ENSG00000204548 | HGLibA_12870 | 330 | 1.850269669 | DEFB121 | defensin beta 121 [Source:HGNC Symbol;Acc:HGNC:18101] |
| ENSG00000258986 | HGLibA_50088 | 330 | 1.850269669 | TMEM179 | transmembrane protein 179 [Source:HGNC Symbol;Acc:HGNC:20137] |
| hsa-mir-718 | HGLibA_63882 | 330 | 1.850269669 | hsa-mir-718 | - |
| KIAA1841 | HGLibA_24824 | 330 | 1.850269669 | KIAA1841 | - |
| ENSG00000007237 | HGLibA_18657 | 329 | 1.841224326 | GAS7 | growth arrest specific 7 [Source:HGNC Symbol;Acc:HGNC:4169] |
| ENSG00000050820 | HGLibA_04245 | 329 | 1.841224326 | BCAR1 | Cas family scaffold protein [Source:HGNC Symbol;Acc:HGNC:971]" |
| ENSG00000057935 | HGLibA_30317 | 329 | 1.841224326 | MTA3 | metastasis associated 1 family member 3 [Source:HGNC Symbol;Acc:HGNC:23784] |
| ENSG00000072315 | HGLibA_51739 | 329 | 1.841224326 | TRPC5 | transient receptor potential cation channel subfamily C member 5 [Source:HGNC Symbol;Acc:HGNC:12337] |
| ENSG00000100079 | HGLibA_26361 | 329 | 1.841224326 | LGALS2 | galectin 2 [Source:HGNC Symbol;Acc:HGNC:6562] |
| ENSG00000106853 | HGLibA_39351 | 329 | 1.841224326 | PTGR1 | prostaglandin reductase 1 [Source:HGNC Symbol;Acc:HGNC:18429] |
| ENSG00000111305 | HGLibA_20419 | 329 | 1.841224326 | GSG1 | germ cell associated 1 [Source:HGNC Symbol;Acc:HGNC:19716] |
| ENSG00000137364 | HGLibA_51152 | 329 | 1.841224326 | TPMT | thiopurine S-methyltransferase [Source:HGNC Symbol;Acc:HGNC:12014] |
| ENSG00000141622 | HGLibA_41557 | 329 | 1.841224326 | RNF165 | ring finger protein 165 [Source:HGNC Symbol;Acc:HGNC:31696] |
| ENSG00000156958 | HGLibA_18517 | 329 | 1.841224326 | GALK2 | galactokinase 2 [Source:HGNC Symbol;Acc:HGNC:4119] |
| ENSG00000162882 | HGLibA_20790 | 329 | 1.841224326 | HAAO | 4-dioxygenase [Source:HGNC Symbol;Acc:HGNC:4796]" |
| ENSG00000164296 | HGLibA_49478 | 329 | 1.841224326 | TIGD6 | tigger transposable element derived 6 [Source:HGNC Symbol;Acc:HGNC:18332] |
| ENSG00000169299 | HGLibA_36264 | 329 | 1.841224326 | PGM2 | phosphoglucomutase 2 [Source:HGNC Symbol;Acc:HGNC:8906] |
| ENSG00000173114 | HGLibA_27477 | 329 | 1.841224326 | LRRN3 | leucine rich repeat neuronal 3 [Source:HGNC Symbol;Acc:HGNC:17200] |
| ENSG00000173156 | HGLibA_41185 | 329 | 1.841224326 | RHOD | ras homolog family member D [Source:HGNC Symbol;Acc:HGNC:670] |
| ENSG00000177354 | HGLibA_05091 | 329 | 1.841224326 | C10orf71 | chromosome 10 open reading frame 71 [Source:HGNC Symbol;Acc:HGNC:26973] |
| ENSG00000186579 | HGLibA_12831 | 329 | 1.841224326 | DEFB106A | defensin beta 106A [Source:HGNC Symbol;Acc:HGNC:18088] |
| ENSG00000198055 | HGLibA_20336 | 329 | 1.841224326 | GRK6 | G protein-coupled receptor kinase 6 [Source:HGNC Symbol;Acc:HGNC:4545] |
| ENSG00000224420 | HGLibA_00998 | 329 | 1.841224326 | ADM5 | adrenomedullin 5 (putative) [Source:HGNC Symbol;Acc:HGNC:27293] |
| hsa-mir-498 | HGLibA_61394 | 329 | 1.841224326 | hsa-mir-498 | - |
| hsa-mir-5190 | HGLibA_61680 | 329 | 1.841224326 | hsa-mir-5190 | - |
| LOC100507462 | HGLibA_26870 | 329 | 1.841224326 | LOC100507462 | - |
| ENSG00000100865 | HGLibA_09714 | 328 | 1.832178983 | CINP | cyclin dependent kinase 2 interacting protein [Source:HGNC Symbol;Acc:HGNC:23789] |
| ENSG00000104375 | HGLibA_47477 | 328 | 1.832178983 | STK3 | serine/threonine kinase 3 [Source:HGNC Symbol;Acc:HGNC:11406] |
| ENSG00000104549 | HGLibA_46914 | 328 | 1.832178983 | SQLE | squalene epoxidase [Source:HGNC Symbol;Acc:HGNC:11279] |
| ENSG00000109182 | HGLibA_11830 | 328 | 1.832178983 | CWH43 | cell wall biogenesis 43 C-terminal homolog [Source:HGNC Symbol;Acc:HGNC:26133] |
| ENSG00000122335 | HGLibA_43462 | 328 | 1.832178983 | SERAC1 | serine active site containing 1 [Source:HGNC Symbol;Acc:HGNC:21061] |
| ENSG00000124942 | HGLibA_01300 | 328 | 1.832178983 | AHNAK | AHNAK nucleoprotein [Source:HGNC Symbol;Acc:HGNC:347] |
| ENSG00000128284 | HGLibA_02534 | 328 | 1.832178983 | APOL3 | apolipoprotein L3 [Source:HGNC Symbol;Acc:HGNC:14868] |
| ENSG00000128567 | HGLibA_37459 | 328 | 1.832178983 | PODXL | podocalyxin like [Source:HGNC Symbol;Acc:HGNC:9171] |
| ENSG00000139192 | HGLibA_48324 | 328 | 1.832178983 | TAPBPL | TAP binding protein like [Source:HGNC Symbol;Acc:HGNC:30683] |
| ENSG00000139531 | HGLibA_47801 | 328 | 1.832178983 | SUOX | sulfite oxidase [Source:HGNC Symbol;Acc:HGNC:11460] |
| ENSG00000159399 | HGLibA_21581 | 328 | 1.832178983 | HK2 | hexokinase 2 [Source:HGNC Symbol;Acc:HGNC:4923] |
| ENSG00000161513 | HGLibA_17267 | 328 | 1.832178983 | FDXR | ferredoxin reductase [Source:HGNC Symbol;Acc:HGNC:3642] |
| ENSG00000163914 | HGLibA_41162 | 328 | 1.832178983 | RHO | rhodopsin [Source:HGNC Symbol;Acc:HGNC:10012] |

| | | | | | |
|---|---|---|---|---|---|
| ENSG00000164776 | HGLibA_36410 | 328 | 1.832178983 | PHKG1 | phosphorylase kinase catalytic subunit gamma 1 [Source:HGNC Symbol;Acc:HGNC:8930] |
| ENSG00000167822 | HGLibA_34541 | 328 | 1.832178983 | OR8J3 | olfactory receptor family 8 subfamily J member 3 [Source:HGNC Symbol;Acc:HGNC:15312] |
| ENSG00000171503 | HGLibA_15626 | 328 | 1.832178983 | ETFDH | electron transfer flavoprotein dehydrogenase [Source:HGNC Symbol;Acc:HGNC:3483] |
| ENSG00000182685 | HGLibA_04809 | 328 | 1.832178983 | BRICD5 | BRICHOS domain containing 5 [Source:HGNC Symbol;Acc:HGNC:28309] |
| ENSG00000183048 | HGLibA_44635 | 328 | 1.832178983 | SLC25A10 | solute carrier family 25 member 10 [Source:HGNC Symbol;Acc:HGNC:10980] |
| ENSG00000185519 | HGLibA_16111 | 328 | 1.832178983 | FAM131C | family with sequence similarity 131 member C [Source:HGNC Symbol;Acc:HGNC:26717] |
| ENSG00000197181 | HGLibA_36773 | 328 | 1.832178983 | PIWIL2 | piwi like RNA-mediated gene silencing 2 [Source:HGNC Symbol;Acc:HGNC:17644] |
| ENSG00000204687 | HGLibA_28391 | 328 | 1.832178983 | MAS1L | G protein-coupled receptor [Source:HGNC Symbol;Acc:HGNC:13961]" |
| GPR113 | HGLibA_19854 | 328 | 1.832178983 | GPR113 | - |
| hsa-mir-4497 | HGLibA_60405 | 328 | 1.832178983 | hsa-mir-4497 | - |
| hsa-mir-4721 | HGLibA_60981 | 328 | 1.832178983 | hsa-mir-4721 | - |
| hsa-mir-762 | HGLibA_63909 | 328 | 1.832178983 | hsa-mir-762 | - |
| C14orf183 | HGLibA_05354 | 327 | 1.82313364 | C14orf183 | - |
| ENSG00000075223 | HGLibA_43324 | 327 | 1.82313364 | SEMA3C | semaphorin 3C [Source:HGNC Symbol;Acc:HGNC:10725] |
| ENSG00000100596 | HGLibA_46896 | 327 | 1.82313364 | SPTLC2 | serine palmitoyltransferase long chain base subunit 2 [Source:HGNC Symbol;Acc:HGNC:11278] |
| ENSG00000101825 | HGLibA_30622 | 327 | 1.82313364 | MXRA5 | matrix remodeling associated 5 [Source:HGNC Symbol;Acc:HGNC:7539] |
| ENSG00000103522 | HGLibA_23085 | 327 | 1.82313364 | IL21R | interleukin 21 receptor [Source:HGNC Symbol;Acc:HGNC:6006] |
| ENSG00000105509 | HGLibA_20863 | 327 | 1.82313364 | HAS1 | hyaluronan synthase 1 [Source:HGNC Symbol;Acc:HGNC:4818] |
| ENSG00000112983 | HGLibA_04784 | 327 | 1.82313364 | BRD8 | bromodomain containing 8 [Source:HGNC Symbol;Acc:HGNC:19874] |
| ENSG00000114480 | HGLibA_18718 | 327 | 1.82313364 | GBE1 | 4-alpha-glucan branching enzyme 1 [Source:HGNC Symbol;Acc:HGNC:4180]" |
| ENSG00000115762 | HGLibA_37088 | 327 | 1.82313364 | PLEKHB2 | pleckstrin homology domain containing B2 [Source:HGNC Symbol;Acc:HGNC:19236] |
| ENSG00000120963 | HGLibA_56595 | 327 | 1.82313364 | ZNF706 | zinc finger protein 706 [Source:HGNC Symbol;Acc:HGNC:24992] |
| ENSG00000124429 | HGLibA_37464 | 327 | 1.82313364 | POF1B | actin binding protein [Source:HGNC Symbol;Acc:HGNC:13711]" |
| ENSG00000127328 | HGLibA_39928 | 327 | 1.82313364 | RAB3IP | RAB3A interacting protein [Source:HGNC Symbol;Acc:HGNC:16508] |
| ENSG00000128045 | HGLibA_40368 | 327 | 1.82313364 | RASL11B | RAS like family 11 member B [Source:HGNC Symbol;Acc:HGNC:23804] |
| ENSG00000128609 | HGLibA_31464 | 327 | 1.82313364 | NDUFA5 | NADH:ubiquinone oxidoreductase subunit A5 [Source:HGNC Symbol;Acc:HGNC:7688] |
| ENSG00000135241 | HGLibA_37425 | 327 | 1.82313364 | PNPLA8 | patatin like phospholipase domain containing 8 [Source:HGNC Symbol;Acc:HGNC:28900] |
| ENSG00000135926 | HGLibA_49739 | 327 | 1.82313364 | TMBIM1 | transmembrane BAX inhibitor motif containing 1 [Source:HGNC Symbol;Acc:HGNC:23410] |
| ENSG00000138829 | HGLibA_16959 | 327 | 1.82313364 | FBN2 | fibrillin 2 [Source:HGNC Symbol;Acc:HGNC:3604] |
| ENSG00000140107 | HGLibA_44756 | 327 | 1.82313364 | SLC25A47 | solute carrier family 25 member 47 [Source:HGNC Symbol;Acc:HGNC:20115] |
| ENSG00000142233 | HGLibA_32861 | 327 | 1.82313364 | NTN5 | netrin 5 [Source:HGNC Symbol;Acc:HGNC:25208] |
| ENSG00000150676 | HGLibA_07886 | 327 | 1.82313364 | CCDC83 | coiled-coil domain containing 83 [Source:HGNC Symbol;Acc:HGNC:28535] |
| ENSG00000162434 | HGLibA_23913 | 327 | 1.82313364 | JAK1 | Janus kinase 1 [Source:HGNC Symbol;Acc:HGNC:6190] |
| ENSG00000171552 | HGLibA_04302 | 327 | 1.82313364 | BCL2L1 | BCL2 like 1 [Source:HGNC Symbol;Acc:HGNC:992] |
| ENSG00000177551 | HGLibA_31892 | 327 | 1.82313364 | NHLH2 | nescient helix-loop-helix 2 [Source:HGNC Symbol;Acc:HGNC:7818] |
| ENSG00000179361 | HGLibA_02895 | 327 | 1.82313364 | ARID3B | AT-rich interaction domain 3B [Source:HGNC Symbol;Acc:HGNC:14350] |
| ENSG00000179750 | HGLibA_02478 | 327 | 1.82313364 | APOBEC3B | apolipoprotein B mRNA editing enzyme catalytic subunit 3B [Source:HGNC Symbol;Acc:HGNC:17352] |
| ENSG00000187796 | HGLibA_07241 | 327 | 1.82313364 | CARD9 | caspase recruitment domain family member 9 [Source:HGNC Symbol;Acc:HGNC:16391] |
| ENSG00000187860 | HGLibA_07661 | 327 | 1.82313364 | CCDC157 | coiled-coil domain containing 157 [Source:HGNC Symbol;Acc:HGNC:33854] |
| ENSG00000187959 | HGLibA_11008 | 327 | 1.82313364 | CPSF4L | cleavage and polyadenylation specific factor 4 like [Source:HGNC Symbol;Acc:HGNC:33632] |
| ENSG00000189056 | HGLibA_40824 | 327 | 1.82313364 | RELN | reelin [Source:HGNC Symbol;Acc:HGNC:9957] |
| ENSG00000204478 | HGLibA_38309 | 327 | 1.82313364 | PRAMEF20 | PRAME family member 20 [Source:HGNC Symbol;Acc:HGNC:25224] |
| ENSG00000204616 | HGLibA_51460 | 327 | 1.82313364 | TRIM31 | tripartite motif containing 31 [Source:HGNC Symbol;Acc:HGNC:16289] |
| ENSG00000262209 | HGLibA_35557 | 327 | 1.82313364 | PCDHGB3 | 3 [Source:HGNC Symbol;Acc:HGNC:8710]" |
| ENSG00000033100 | HGLibA_09525 | 326 | 1.814088297 | CHPF2 | chondroitin polymerizing factor 2 [Source:HGNC Symbol;Acc:HGNC:29270] |
| ENSG00000088448 | HGLibA_01992 | 326 | 1.814088297 | ANKRD10 | ankyrin repeat domain 10 [Source:HGNC Symbol;Acc:HGNC:20265] |
| ENSG00000091986 | HGLibA_07876 | 326 | 1.814088297 | CCDC80 | coiled-coil domain containing 80 [Source:HGNC Symbol;Acc:HGNC:30649] |
| ENSG00000094963 | HGLibA_17705 | 326 | 1.814088297 | FMO2 | flavin containing monooxygenase 2 [Source:HGNC Symbol;Acc:HGNC:3770] |
| ENSG00000099860 | HGLibA_18420 | 326 | 1.814088297 | GADD45B | growth arrest and DNA damage inducible beta [Source:HGNC Symbol;Acc:HGNC:4096] |
| ENSG00000100003 | HGLibA_43194 | 326 | 1.814088297 | SEC14L2 | SEC14 like lipid binding 2 [Source:HGNC Symbol;Acc:HGNC:10699] |
| ENSG00000101194 | HGLibA_44484 | 326 | 1.814088297 | SLC17A9 | solute carrier family 17 member 9 [Source:HGNC Symbol;Acc:HGNC:16192] |
| ENSG00000103196 | HGLibA_11177 | 326 | 1.814088297 | CRISPLD2 | cysteine rich secretory protein LCCL domain containing 2 [Source:HGNC Symbol;Acc:HGNC:25248] |
| ENSG00000105376 | HGLibA_22505 | 326 | 1.814088297 | ICAM5 | intercellular adhesion molecule 5 [Source:HGNC Symbol;Acc:HGNC:5348] |
| ENSG00000115756 | HGLibA_22020 | 326 | 1.814088297 | HPCAL1 | hippocalcin like 1 [Source:HGNC Symbol;Acc:HGNC:5145] |
| ENSG00000119685 | HGLibA_52206 | 326 | 1.814088297 | TTLL5 | tubulin tyrosine ligase like 5 [Source:HGNC Symbol;Acc:HGNC:19963] |
| ENSG00000121903 | HGLibA_56998 | 326 | 1.814088297 | ZSCAN20 | zinc finger and SCAN domain containing 20 [Source:HGNC Symbol;Acc:HGNC:13093] |
| ENSG00000123560 | HGLibA_37215 | 326 | 1.814088297 | PLP1 | proteolipid protein 1 [Source:HGNC Symbol;Acc:HGNC:9086] |
| ENSG00000126545 | HGLibA_11373 | 326 | 1.814088297 | CSN1S1 | casein alpha s1 [Source:HGNC Symbol;Acc:HGNC:2445] |
| ENSG00000130300 | HGLibA_37250 | 326 | 1.814088297 | PLVAP | plasmalemma vesicle associated protein [Source:HGNC Symbol;Acc:HGNC:13635] |
| ENSG00000146828 | HGLibA_44382 | 326 | 1.814088297 | SLC12A9 | solute carrier family 12 member 9 [Source:HGNC Symbol;Acc:HGNC:17435] |
| ENSG00000147124 | HGLibA_55934 | 326 | 1.814088297 | ZNF41 | zinc finger protein 41 [Source:HGNC Symbol;Acc:HGNC:13107] |
| ENSG00000147251 | HGLibA_13737 | 326 | 1.814088297 | DOCK11 | dedicator of cytokinesis 11 [Source:HGNC Symbol;Acc:HGNC:23483] |
| ENSG00000149054 | HGLibA_55598 | 326 | 1.814088297 | ZNF215 | zinc finger protein 215 [Source:HGNC Symbol;Acc:HGNC:13007] |
| ENSG00000157800 | HGLibA_45050 | 326 | 1.814088297 | SLC37A3 | solute carrier family 37 member 3 [Source:HGNC Symbol;Acc:HGNC:20651] |
| ENSG00000160293 | HGLibA_53550 | 326 | 1.814088297 | VAV2 | vav guanine nucleotide exchange factor 2 [Source:HGNC Symbol;Acc:HGNC:12658] |
| ENSG00000164096 | HGLibA_06317 | 326 | 1.814088297 | C4orf3 | chromosome 4 open reading frame 3 [Source:HGNC Symbol;Acc:HGNC:19225] |
| ENSG00000170323 | HGLibA_15918 | 326 | 1.814088297 | FABP4 | fatty acid binding protein 4 [Source:HGNC Symbol;Acc:HGNC:3559] |
| ENSG00000177646 | HGLibA_00362 | 326 | 1.814088297 | ACAD9 | acyl-CoA dehydrogenase family member 9 [Source:HGNC Symbol;Acc:HGNC:21497] |
| ENSG00000177963 | HGLibA_41236 | 326 | 1.814088297 | RIC8A | RIC8 guanine nucleotide exchange factor A [Source:HGNC Symbol;Acc:HGNC:29550] |
| ENSG00000179262 | HGLibA_40068 | 326 | 1.814088297 | RAD23A | nucleotide excision repair protein [Source:HGNC Symbol;Acc:HGNC:9812]" |
| ENSG00000214402 | HGLibA_26191 | 326 | 1.814088297 | LCNL1 | lipocalin like 1 [Source:HGNC Symbol;Acc:HGNC:34436] |
| ENSG00000248385 | HGLibA_48338 | 326 | 1.814088297 | TARM1 | activating receptor on myeloid cells 1 [Source:HGNC Symbol;Acc:HGNC:37250]" |
| ENSG00000249853 | HGLibA_22137 | 326 | 1.814088297 | HS3ST5 | heparan sulfate-glucosamine 3-sulfotransferase 5 [Source:HGNC Symbol;Acc:HGNC:19419] |
| ENSG00000257594 | HGLibA_18561 | 326 | 1.814088297 | GALNT4 | polypeptide N-acetylgalactosaminyltransferase 4 [Source:HGNC Symbol;Acc:HGNC:4126] |
| hsa-mir-4450 | HGLibA_60205 | 326 | 1.814088297 | hsa-mir-4450 | - |
| KDELC1 | HGLibA_24473 | 326 | 1.814088297 | KDELC1 | - |
| ENSG00000080031 | HGLibA_39509 | 325 | 1.805042954 | PTPRH | receptor type H [Source:HGNC Symbol;Acc:HGNC:9672]" |
| ENSG00000088298 | HGLibA_14456 | 325 | 1.805042954 | EDEM2 | ER degradation enhancing alpha-mannosidase like protein 2 [Source:HGNC Symbol;Acc:HGNC:15877] |
| ENSG00000100417 | HGLibA_37315 | 325 | 1.805042954 | PMM1 | phosphomannomutase 1 [Source:HGNC Symbol;Acc:HGNC:9114] |
| ENSG00000100427 | HGLibA_29365 | 325 | 1.805042954 | MLC1 | megalencephalic leukoencephalopathy with subcortical cysts 1 [Source:HGNC Symbol;Acc:HGNC:17082] |
| ENSG00000102316 | HGLibA_27970 | 325 | 1.805042954 | MAGED2 | MAGE family member D2 [Source:HGNC Symbol;Acc:HGNC:16353] |
| ENSG00000103647 | HGLibA_10785 | 325 | 1.805042954 | CORO2B | coronin 2B [Source:HGNC Symbol;Acc:HGNC:2256] |
| ENSG00000105258 | HGLibA_37589 | 325 | 1.805042954 | POLR2I | RNA polymerase II subunit I [Source:HGNC Symbol;Acc:HGNC:9196] |
| ENSG00000107518 | HGLibA_03822 | 325 | 1.805042954 | ATRNL1 | attractin like 1 [Source:HGNC Symbol;Acc:HGNC:29063] |
| ENSG00000108823 | HGLibA_43797 | 325 | 1.805042954 | SGCA | sarcoglycan alpha [Source:HGNC Symbol;Acc:HGNC:10805] |
| ENSG00000112936 | HGLibA_06517 | 325 | 1.805042954 | C7 | complement C7 [Source:HGNC Symbol;Acc:HGNC:1346] |
| ENSG00000118849 | HGLibA_40280 | 325 | 1.805042954 | RARRES1 | retinoic acid receptor responder 1 [Source:HGNC Symbol;Acc:HGNC:9867] |
| ENSG00000123607 | HGLibA_52071 | 325 | 1.805042954 | TTC21B | tetratricopeptide repeat domain 21B [Source:HGNC Symbol;Acc:HGNC:25660] |
| ENSG00000132972 | HGLibA_41571 | 325 | 1.805042954 | RNF17 | ring finger protein 17 [Source:HGNC Symbol;Acc:HGNC:10060] |
| ENSG00000141179 | HGLibA_35692 | 325 | 1.805042954 | PCTP | phosphatidylcholine transfer protein [Source:HGNC Symbol;Acc:HGNC:8752] |
| ENSG00000141295 | HGLibA_43051 | 325 | 1.805042954 | SCRN2 | secernin 2 [Source:HGNC Symbol;Acc:HGNC:30381] |
| ENSG00000143632 | HGLibA_00597 | 325 | 1.805042954 | ACTA1 | skeletal muscle [Source:HGNC Symbol;Acc:HGNC:129]" |
| ENSG00000145451 | HGLibA_19303 | 325 | 1.805042954 | GLRA3 | glycine receptor alpha 3 [Source:HGNC Symbol;Acc:HGNC:4328] |
| ENSG00000147419 | HGLibA_07739 | 325 | 1.805042954 | CCDC25 | coiled-coil domain containing 25 [Source:HGNC Symbol;Acc:HGNC:25591] |
| ENSG00000151422 | HGLibA_17286 | 325 | 1.805042954 | FER | FER tyrosine kinase [Source:HGNC Symbol;Acc:HGNC:3655] |
| ENSG00000152578 | HGLibA_20268 | 325 | 1.805042954 | GRIA4 | glutamate ionotropic receptor AMPA type subunit 4 [Source:HGNC Symbol;Acc:HGNC:4574] |
| ENSG00000159409 | HGLibA_09009 | 325 | 1.805042954 | CELF3 | CUGBP Elav-like family member 3 [Source:HGNC Symbol;Acc:HGNC:11967] |
| ENSG00000164841 | HGLibA_50509 | 325 | 1.805042954 | TMEM74 | transmembrane protein 74 [Source:HGNC Symbol;Acc:HGNC:26409] |
| ENSG00000165868 | HGLibA_22259 | 325 | 1.805042954 | HSPA12A | heat shock protein family A (Hsp70) member 12A [Source:HGNC Symbol;Acc:HGNC:19022] |
| ENSG00000174851 | HGLibA_54665 | 325 | 1.805042954 | YIF1A | membrane trafficking protein [Source:HGNC Symbol;Acc:HGNC:16688]" |
| ENSG00000196092 | HGLibA_35307 | 325 | 1.805042954 | PAX5 | paired box 5 [Source:HGNC Symbol;Acc:HGNC:8619] |
| ENSG00000197790 | HGLibA_34190 | 325 | 1.805042954 | OR52M1 | olfactory receptor family 52 subfamily M member 1 [Source:HGNC Symbol;Acc:HGNC:15225] |
| ENSG00000198276 | HGLibA_52815 | 325 | 1.805042954 | UCKL1 | uridine-cytidine kinase 1 like 1 [Source:HGNC Symbol;Acc:HGNC:15938] |
| ENSG00000198604 | HGLibA_04184 | 325 | 1.805042954 | BAZ1A | bromodomain adjacent to zinc finger domain 1A [Source:HGNC Symbol;Acc:HGNC:960] |
| ENSG00000214046 | HGLibA_45768 | 325 | 1.805042954 | SMIM7 | small integral membrane protein 7 [Source:HGNC Symbol;Acc:HGNC:28419] |
| ENSG00000248485 | HGLibA_35664 | 325 | 1.805042954 | PCP4L1 | Purkinje cell protein 4 like 1 [Source:HGNC Symbol;Acc:HGNC:20448] |
| ENSG00000260916 | HGLibA_08142 | 325 | 1.805042954 | CCPG1 | cell cycle progression 1 [Source:HGNC Symbol;Acc:HGNC:24227] |
| hsa-mir-4322 | HGLibA_60012 | 325 | 1.805042954 | hsa-mir-4322 | - |
| ITFG3 | HGLibA_23712 | 325 | 1.805042954 | ITFG3 | - |
| KIAA0195 | HGLibA_24599 | 325 | 1.805042954 | KIAA0195 | - |
| ENSG00000027075 | HGLibA_38552 | 324 | 1.795997611 | PRKCH | protein kinase C eta [Source:HGNC Symbol;Acc:HGNC:9403] |
| ENSG00000038219 | HGLibA_04672 | 324 | 1.795997611 | BOD1L1 | biorientation of chromosomes in cell division 1 like 1 [Source:HGNC Symbol;Acc:HGNC:31792] |
| ENSG00000064651 | HGLibA_44361 | 324 | 1.795997611 | SLC12A2 | solute carrier family 12 member 2 [Source:HGNC Symbol;Acc:HGNC:10911] |
| ENSG00000076248 | HGLibA_53045 | 324 | 1.795997611 | UNG | uracil DNA glycosylase [Source:HGNC Symbol;Acc:HGNC:12572] |
| ENSG00000100429 | HGLibA_21001 | 324 | 1.795997611 | HDAC10 | histone deacetylase 10 [Source:HGNC Symbol;Acc:HGNC:18128] |
| ENSG00000120278 | HGLibA_37100 | 324 | 1.795997611 | PLEKHG1 | pleckstrin homology and RhoGEF domain containing G1 [Source:HGNC Symbol;Acc:HGNC:20884] |
| ENSG00000121211 | HGLibA_29563 | 324 | 1.795997611 | MND1 | meiotic nuclear divisions 1 [Source:HGNC Symbol;Acc:HGNC:24839] |
| ENSG00000124831 | HGLibA_27448 | 324 | 1.795997611 | LRRFIP1 | LRR binding FLII interacting protein 1 [Source:HGNC Symbol;Acc:HGNC:6702] |
| ENSG00000125247 | HGLibA_50678 | 324 | 1.795997611 | TMTC4 | transmembrane and tetratricopeptide repeat containing 4 [Source:HGNC Symbol;Acc:HGNC:25904] |
| ENSG00000144283 | HGLibA_36861 | 324 | 1.795997611 | PKP4 | plakophilin 4 [Source:HGNC Symbol;Acc:HGNC:9026] |
| ENSG00000153064 | HGLibA_04146 | 324 | 1.795997611 | BANK1 | B cell scaffold protein with ankyrin repeats 1 [Source:HGNC Symbol;Acc:HGNC:18233] |
| ENSG00000158985 | HGLibA_08585 | 324 | 1.795997611 | CDC42SE2 | CDC42 small effector 2 [Source:HGNC Symbol;Acc:HGNC:18547] |
| ENSG00000159184 | HGLibA_21929 | 324 | 1.795997611 | HOXB13 | homeobox B13 [Source:HGNC Symbol;Acc:HGNC:5112] |
| ENSG00000164136 | HGLibA_22979 | 324 | 1.795997611 | IL15 | interleukin 15 [Source:HGNC Symbol;Acc:HGNC:5977] |
| ENSG00000171428 | HGLibA_31164 | 324 | 1.795997611 | NAT1 | N-acetyltransferase 1 [Source:HGNC Symbol;Acc:HGNC:7645] |
| ENSG00000173335 | HGLibA_11472 | 324 | 1.795997611 | CST9 | cystatin 9 [Source:HGNC Symbol;Acc:HGNC:13261] |
| ENSG00000184347 | HGLibA_45509 | 324 | 1.795997611 | SLIT3 | slit guidance ligand 3 [Source:HGNC Symbol;Acc:HGNC:11087] |
| ENSG00000196345 | HGLibA_55359 | 324 | 1.795997611 | ZKSCAN7 | zinc finger with KRAB and SCAN domains 7 [Source:HGNC Symbol;Acc:HGNC:12955] |
| ENSG00000204962 | HGLibA_35457 | 324 | 1.795997611 | PCDHA8 | protocadherin alpha 8 [Source:HGNC Symbol;Acc:HGNC:8674] |
| hsa-mir-8082 | HGLibA_64169 | 324 | 1.795997611 | hsa-mir-8082 | - |
| LOC389895 | HGLibA_26930 | 324 | 1.795997611 | LOC389895 | - |
| 14-Sep | HGLibA_43407 | 324 | 1.795997611 | 14-Sep | - |
| ATP5G1 | HGLibA_03654 | 323 | 1.786952268 | ATP5G1 | - |
| CCBL1 | HGLibA_07496 | 323 | 1.786952268 | CCBL1 | - |
| ENSG00000110031 | HGLibA_27127 | 323 | 1.786952268 | LPXN | leupaxin [Source:HGNC Symbol;Acc:HGNC:14061] |

| | | | | | |
|---|---|---|---|---|---|
| ENSG00000118985 | HGLibA_14924 | 323 | 1.786952268 | ELL2 | elongation factor for RNA polymerase II 2 [Source:HGNC Symbol;Acc:HGNC:17064] |
| ENSG00000122133 | HGLibA_34983 | 323 | 1.786952268 | PAEP | progestagen associated endometrial protein [Source:HGNC Symbol;Acc:HGNC:8573] |
| ENSG00000123700 | HGLibA_24273 | 323 | 1.786952268 | KCNJ2 | potassium voltage-gated channel subfamily J member 2 [Source:HGNC Symbol;Acc:HGNC:6263] |
| ENSG00000128311 | HGLibA_52024 | 323 | 1.786952268 | TST | thiosulfate sulfurtransferase [Source:HGNC Symbol;Acc:HGNC:12388] |
| ENSG00000133687 | HGLibA_50671 | 323 | 1.786952268 | TMTC1 | transmembrane and tetratricopeptide repeat containing 1 [Source:HGNC Symbol;Acc:HGNC:24099] |
| ENSG00000138028 | HGLibA_09326 | 323 | 1.786952268 | CGREF1 | cell growth regulator with EF-hand domain 1 [Source:HGNC Symbol;Acc:HGNC:16962] |
| ENSG00000147573 | HGLibA_51547 | 323 | 1.786952268 | TRIM55 | tripartite motif containing 55 [Source:HGNC Symbol;Acc:HGNC:14215] |
| ENSG00000153291 | HGLibA_44689 | 323 | 1.786952268 | SLC25A27 | solute carrier family 25 member 27 [Source:HGNC Symbol;Acc:HGNC:21065] |
| ENSG00000161281 | HGLibA_10858 | 323 | 1.786952268 | COX7A1 | cytochrome c oxidase subunit 7A1 [Source:HGNC Symbol;Acc:HGNC:2287] |
| ENSG00000162641 | HGLibA_01465 | 323 | 1.786952268 | AKNAD1 | AKNA domain containing 1 [Source:HGNC Symbol;Acc:HGNC:28398] |
| ENSG00000163295 | HGLibA_01696 | 323 | 1.786952268 | ALPI | intestinal [Source:HGNC Symbol;Acc:HGNC:437]" |
| ENSG00000174021 | HGLibA_19530 | 323 | 1.786952268 | GNG5 | G protein subunit gamma 5 [Source:HGNC Symbol;Acc:HGNC:4408] |
| ENSG00000180155 | HGLibA_27709 | 323 | 1.786952268 | LYNX1 | Ly6/neurotoxin 1 [Source:HGNC Symbol;Acc:HGNC:29604] |
| ENSG00000196743 | HGLibA_19388 | 323 | 1.786952268 | GM2A | GM2 ganglioside activator [Source:HGNC Symbol;Acc:HGNC:4367] |
| ENSG00000197959 | HGLibA_13692 | 323 | 1.786952268 | DNM3 | dynamin 3 [Source:HGNC Symbol;Acc:HGNC:29125] |
| ENSG00000197982 | HGLibA_05760 | 323 | 1.786952268 | C1orf122 | chromosome 1 open reading frame 122 [Source:HGNC Symbol;Acc:HGNC:24789] |
| ENSG00000198589 | HGLibA_27133 | 323 | 1.786952268 | LRBA | LPS responsive beige-like anchor protein [Source:HGNC Symbol;Acc:HGNC:1742] |
| ENSG00000204525 | HGLibA_21600 | 323 | 1.786952268 | HLA-C | C [Source:HGNC Symbol;Acc:HGNC:4933]" |
| ENSG00000204963 | HGLibA_35453 | 323 | 1.786952268 | PCDHA7 | protocadherin alpha 7 [Source:HGNC Symbol;Acc:HGNC:8673] |
| ENSG00000204965 | HGLibA_35448 | 323 | 1.786952268 | PCDHA5 | protocadherin alpha 5 [Source:HGNC Symbol;Acc:HGNC:8671] |
| ENSG00000205426 | HGLibA_25558 | 323 | 1.786952268 | KRT81 | keratin 81 [Source:HGNC Symbol;Acc:HGNC:6458] |
| ENSG00000214700 | HGLibA_05288 | 323 | 1.786952268 | C12orf71 | chromosome 12 open reading frame 71 [Source:HGNC Symbol;Acc:HGNC:34452] |
| FAM159B | HGLibA_16202 | 323 | 1.786952268 | FAM159B | - |
| hsa-mir-1183 | HGLibA_57227 | 323 | 1.786952268 | hsa-mir-1183 | - |
| hsa-mir-4264 | HGLibA_59763 | 323 | 1.786952268 | hsa-mir-4264 | - |
| hsa-mir-6750 | HGLibA_63159 | 323 | 1.786952268 | hsa-mir-6750 | - |
| LPPR3 | HGLibA_27120 | 323 | 1.786952268 | LPPR3 | - |
| 11-Mar | HGLibA_28331 | 322 | 1.777906925 | 11-Mar | - |
| ENSG00000008441 | HGLibA_31812 | 322 | 1.777906925 | NFIX | nuclear factor I X [Source:HGNC Symbol;Acc:HGNC:7788] |
| ENSG00000067167 | HGLibA_51282 | 322 | 1.777906925 | TRAM1 | translocation associated membrane protein 1 [Source:HGNC Symbol;Acc:HGNC:20568] |
| ENSG00000101298 | HGLibA_45935 | 322 | 1.777906925 | SNPH | syntaphilin [Source:HGNC Symbol;Acc:HGNC:15931] |
| ENSG00000114166 | HGLibA_24054 | 322 | 1.777906925 | KAT2B | lysine acetyltransferase 2B [Source:HGNC Symbol;Acc:HGNC:8638] |
| ENSG00000115935 | HGLibA_54303 | 322 | 1.777906925 | WIPF1 | WAS/WASL interacting protein family member 1 [Source:HGNC Symbol;Acc:HGNC:12736] |
| ENSG00000116288 | HGLibA_35199 | 322 | 1.777906925 | PARK7 | Parkinsonism associated deglycase [Source:HGNC Symbol;Acc:HGNC:16369] |
| ENSG00000138760 | HGLibA_42873 | 322 | 1.777906925 | SCARB2 | scavenger receptor class B member 2 [Source:HGNC Symbol;Acc:HGNC:1665] |
| ENSG00000140795 | HGLibA_30789 | 322 | 1.777906925 | MYLK3 | myosin light chain kinase 3 [Source:HGNC Symbol;Acc:HGNC:29826] |
| ENSG00000144792 | HGLibA_56475 | 322 | 1.777906925 | ZNF660 | zinc finger protein 660 [Source:HGNC Symbol;Acc:HGNC:26720] |
| ENSG00000151276 | HGLibA_27995 | 322 | 1.777906925 | MAGI1 | WW and PDZ domain containing 1 [Source:HGNC Symbol;Acc:HGNC:946]" |
| ENSG00000151364 | HGLibA_24425 | 322 | 1.777906925 | KCTD14 | potassium channel tetramerization domain containing 14 [Source:HGNC Symbol;Acc:HGNC:23295] |
| ENSG00000157423 | HGLibA_22450 | 322 | 1.777906925 | HYDIN | axonemal central pair apparatus protein [Source:HGNC Symbol;Acc:HGNC:19368]" |
| ENSG00000158716 | HGLibA_14165 | 322 | 1.777906925 | DUSP23 | dual specificity phosphatase 23 [Source:HGNC Symbol;Acc:HGNC:21480] |
| ENSG00000162630 | HGLibA_03957 | 322 | 1.777906925 | B3GALT2 | 3-galactosyltransferase 2 [Source:HGNC Symbol;Acc:HGNC:917]" |
| ENSG00000163013 | HGLibA_17095 | 322 | 1.777906925 | FBXO41 | F-box protein 41 [Source:HGNC Symbol;Acc:HGNC:29409] |
| ENSG00000167207 | HGLibA_32246 | 322 | 1.777906925 | NOD2 | nucleotide binding oligomerization domain containing 2 [Source:HGNC Symbol;Acc:HGNC:5331] |
| ENSG00000168785 | HGLibA_51939 | 322 | 1.777906925 | TSPAN5 | tetraspanin 5 [Source:HGNC Symbol;Acc:HGNC:17753] |
| ENSG00000171243 | HGLibA_46211 | 322 | 1.777906925 | SOSTDC1 | sclerostin domain containing 1 [Source:HGNC Symbol;Acc:HGNC:21748] |
| ENSG00000177034 | HGLibA_30517 | 322 | 1.777906925 | MTX3 | metaxin 3 [Source:HGNC Symbol;Acc:HGNC:24812] |
| ENSG00000182175 | HGLibA_41012 | 322 | 1.777906925 | RGMA | repulsive guidance molecule BMP co-receptor a [Source:HGNC Symbol;Acc:HGNC:30308] |
| ENSG00000182264 | HGLibA_23891 | 322 | 1.777906925 | IZUMO1 | izumo sperm-egg fusion 1 [Source:HGNC Symbol;Acc:HGNC:28539] |
| ENSG00000188452 | HGLibA_09196 | 322 | 1.777906925 | CERKL | ceramide kinase like [Source:HGNC Symbol;Acc:HGNC:21699] |
| ENSG00000197629 | HGLibA_29716 | 322 | 1.777906925 | MPEG1 | macrophage expressed 1 [Source:HGNC Symbol;Acc:HGNC:29619] |
| ENSG00000204969 | HGLibA_35438 | 322 | 1.777906925 | PCDHA2 | protocadherin alpha 2 [Source:HGNC Symbol;Acc:HGNC:8668] |
| ENSG00000244274 | HGLibA_12393 | 322 | 1.777906925 | DBNDD2 | dysbindin domain containing 2 [Source:HGNC Symbol;Acc:HGNC:15881] |
| ENSG00000251664 | HGLibA_35432 | 322 | 1.777906925 | PCDHA12 | protocadherin alpha 12 [Source:HGNC Symbol;Acc:HGNC:8666] |
| hsa-mir-3130-1 | HGLibA_58573 | 322 | 1.777906925 | hsa-mir-3130-1 | - |
| hsa-mir-6823 | HGLibA_63469 | 322 | 1.777906925 | hsa-mir-6823 | - |
| hsa-mir-7150 | HGLibA_63830 | 322 | 1.777906925 | hsa-mir-7150 | - |
| hsa-mir-941-1 | HGLibA_64340 | 322 | 1.777906925 | hsa-mir-941-1 | - |
| LOC100288814 | HGLibA_26836 | 322 | 1.777906925 | LOC100288814 | - |
| C20orf78 | HGLibA_06025 | 321 | 1.768861582 | C20orf78 | - |
| C7orf62 | HGLibA_06570 | 321 | 1.768861582 | C7orf62 | - |
| C7orf63 | HGLibA_06571 | 321 | 1.768861582 | C7orf63 | - |
| DIRC2 | HGLibA_13268 | 321 | 1.768861582 | DIRC2 | - |
| ENSG00000033100 | HGLibA_09527 | 321 | 1.768861582 | CHPF2 | chondroitin polymerizing factor 2 [Source:HGNC Symbol;Acc:HGNC:29270] |
| ENSG00000054690 | HGLibA_37126 | 321 | 1.768861582 | PLEKHH1 | MyTH4 and FERM domain containing H1 [Source:HGNC Symbol;Acc:HGNC:17733]" |
| ENSG00000075426 | HGLibA_17805 | 321 | 1.768861582 | FOSL2 | AP-1 transcription factor subunit [Source:HGNC Symbol;Acc:HGNC:3798]" |
| ENSG00000088782 | HGLibA_12884 | 321 | 1.768861582 | DEFB127 | defensin beta 127 [Source:HGNC Symbol;Acc:HGNC:16206] |
| ENSG00000101367 | HGLibA_28312 | 321 | 1.768861582 | MAPRE1 | microtubule associated protein RP/EB family member 1 [Source:HGNC Symbol;Acc:HGNC:6890] |
| ENSG00000104967 | HGLibA_32368 | 321 | 1.768861582 | NOVA2 | NOVA alternative splicing regulator 2 [Source:HGNC Symbol;Acc:HGNC:7887] |
| ENSG00000129355 | HGLibA_08846 | 321 | 1.768861582 | CDKN2D | cyclin dependent kinase inhibitor 2D [Source:HGNC Symbol;Acc:HGNC:1790] |
| ENSG00000135094 | HGLibA_43172 | 321 | 1.768861582 | SDS | serine dehydratase [Source:HGNC Symbol;Acc:HGNC:10691] |
| ENSG00000138641 | HGLibA_21158 | 321 | 1.768861582 | HERC3 | HECT and RLD domain containing E3 ubiquitin protein ligase 3 [Source:HGNC Symbol;Acc:HGNC:4876] |
| ENSG00000143632 | HGLibA_00596 | 321 | 1.768861582 | ACTA1 | skeletal muscle [Source:HGNC Symbol;Acc:HGNC:129]" |
| ENSG00000151615 | HGLibA_37781 | 321 | 1.768861582 | POU4F2 | POU class 4 homeobox 2 [Source:HGNC Symbol;Acc:HGNC:9219] |
| ENSG00000153012 | HGLibA_26397 | 321 | 1.768861582 | LGI2 | leucine rich repeat LGI family member 2 [Source:HGNC Symbol;Acc:HGNC:18710] |
| ENSG00000161996 | HGLibA_54215 | 321 | 1.768861582 | WDR90 | WD repeat domain 90 [Source:HGNC Symbol;Acc:HGNC:26960] |
| ENSG00000162383 | HGLibA_44524 | 321 | 1.768861582 | SLC1A7 | solute carrier family 1 member 7 [Source:HGNC Symbol;Acc:HGNC:10945] |
| ENSG00000162769 | HGLibA_17676 | 321 | 1.768861582 | FLVCR1 | feline leukemia virus subgroup C cellular receptor 1 [Source:HGNC Symbol;Acc:HGNC:24682] |
| ENSG00000162779 | HGLibA_03918 | 321 | 1.768861582 | AXDND1 | axonemal dynein light chain domain containing 1 [Source:HGNC Symbol;Acc:HGNC:26564] |
| ENSG00000163879 | HGLibA_13650 | 321 | 1.768861582 | DNALI1 | dynein axonemal light intermediate chain 1 [Source:HGNC Symbol;Acc:HGNC:14353] |
| ENSG00000164708 | HGLibA_36197 | 321 | 1.768861582 | PGAM2 | phosphoglycerate mutase 2 [Source:HGNC Symbol;Acc:HGNC:8889] |
| ENSG00000171798 | HGLibA_25338 | 321 | 1.768861582 | KNDC1 | kinase non-catalytic C-lobe domain containing 1 [Source:HGNC Symbol;Acc:HGNC:29374] |
| ENSG00000175489 | HGLibA_27289 | 321 | 1.768861582 | LRRC25 | leucine rich repeat containing 25 [Source:HGNC Symbol;Acc:HGNC:29806] |
| ENSG00000177030 | HGLibA_12769 | 321 | 1.768861582 | DEAF1 | DEAF1 transcription factor [Source:HGNC Symbol;Acc:HGNC:14677] |
| ENSG00000182253 | HGLibA_48016 | 321 | 1.768861582 | SYNM | synemin [Source:HGNC Symbol;Acc:HGNC:24466] |
| ENSG00000183049 | HGLibA_07060 | 321 | 1.768861582 | CAMK1D | calcium/calmodulin dependent protein kinase ID [Source:HGNC Symbol;Acc:HGNC:19341] |
| ENSG00000185730 | HGLibA_56560 | 321 | 1.768861582 | ZNF696 | zinc finger protein 696 [Source:HGNC Symbol;Acc:HGNC:25872] |
| ENSG00000186925 | HGLibA_25677 | 321 | 1.768861582 | KRTAP19-6 | keratin associated protein 19-6 [Source:HGNC Symbol;Acc:HGNC:18941] |
| ENSG00000196440 | HGLibA_03046 | 321 | 1.768861582 | ARMCX4 | armadillo repeat containing X-linked 4 [Source:HGNC Symbol;Acc:HGNC:28615] |
| ENSG00000197372 | HGLibA_56512 | 321 | 1.768861582 | ZNF675 | zinc finger protein 675 [Source:HGNC Symbol;Acc:HGNC:30768] |
| ENSG00000204779 | HGLibA_17851 | 321 | 1.768861582 | FOXD4L5 | forkhead box D4 like 5 [Source:HGNC Symbol;Acc:HGNC:18522] |
| ENSG00000204961 | HGLibA_35459 | 321 | 1.768861582 | PCDHA9 | protocadherin alpha 9 [Source:HGNC Symbol;Acc:HGNC:8675] |
| ENSG00000227802 | HGLibA_13540 | 321 | 1.768861582 | DNAJB3 | DnaJ heat shock protein family (Hsp40) member B3 [Source:HGNC Symbol;Acc:HGNC:32397] |
| ENSG00000242802 | HGLibA_02361 | 321 | 1.768861582 | AP5Z1 | adaptor related protein complex 5 subunit zeta 1 [Source:HGNC Symbol;Acc:HGNC:22197] |
| ENSG00000250298 | HGLibA_19093 | 321 | 1.768861582 | GIMD1 | GIMAP family P-loop NTPase domain containing 1 [Source:HGNC Symbol;Acc:HGNC:44141] |
| hsa-mir-493 | HGLibA_61374 | 321 | 1.768861582 | hsa-mir-493 | - |
| hsa-mir-762 | HGLibA_63906 | 321 | 1.768861582 | hsa-mir-762 | - |
| LOC646498 | HGLibA_26976 | 321 | 1.768861582 | LOC646498 | - |
| AARS | HGLibA_00069 | 320 | 1.75981624 | AARS | - |
| C14orf142 | HGLibA_05327 | 320 | 1.75981624 | C14orf142 | - |
| ENSG00000005893 | HGLibA_25979 | 320 | 1.75981624 | LAMP2 | lysosomal associated membrane protein 2 [Source:HGNC Symbol;Acc:HGNC:6501] |
| ENSG00000056277 | HGLibA_55730 | 320 | 1.75981624 | ZNF280C | zinc finger protein 280C [Source:HGNC Symbol;Acc:HGNC:25955] |
| ENSG00000070371 | HGLibA_10152 | 320 | 1.75981624 | CLTCL1 | clathrin heavy chain like 1 [Source:HGNC Symbol;Acc:HGNC:2093] |
| ENSG00000107372 | HGLibA_55160 | 320 | 1.75981624 | ZFAND5 | zinc finger AN1-type containing 5 [Source:HGNC Symbol;Acc:HGNC:13008] |
| ENSG00000133027 | HGLibA_36044 | 320 | 1.75981624 | PEMT | phosphatidylethanolamine N-methyltransferase [Source:HGNC Symbol;Acc:HGNC:8830] |
| ENSG00000134317 | HGLibA_20247 | 320 | 1.75981624 | GRHL1 | grainyhead like transcription factor 1 [Source:HGNC Symbol;Acc:HGNC:17923] |
| ENSG00000135346 | HGLibA_09296 | 320 | 1.75981624 | CGA | alpha polypeptide [Source:HGNC Symbol;Acc:HGNC:1885]" |
| ENSG00000136011 | HGLibA_47310 | 320 | 1.75981624 | STAB2 | stabilin 2 [Source:HGNC Symbol;Acc:HGNC:18629] |
| ENSG00000137672 | HGLibA_51746 | 320 | 1.75981624 | TRPC6 | transient receptor potential cation channel subfamily C member 6 [Source:HGNC Symbol;Acc:HGNC:12338] |
| ENSG00000137996 | HGLibA_42370 | 320 | 1.75981624 | RTCA | RNA 3'-terminal phosphate cyclase [Source:HGNC Symbol;Acc:HGNC:17981] |
| ENSG00000138175 | HGLibA_02963 | 320 | 1.75981624 | ARL3 | ADP ribosylation factor like GTPase 3 [Source:HGNC Symbol;Acc:HGNC:694] |
| ENSG00000142789 | HGLibA_08997 | 320 | 1.75981624 | CELA3A | chymotrypsin like elastase family member 3A [Source:HGNC Symbol;Acc:HGNC:15944] |
| ENSG00000152969 | HGLibA_23922 | 320 | 1.75981624 | JAKMIP1 | janus kinase and microtubule interacting protein 1 [Source:HGNC Symbol;Acc:HGNC:26460] |
| ENSG00000163394 | HGLibA_07955 | 320 | 1.75981624 | CCKAR | cholecystokinin A receptor [Source:HGNC Symbol;Acc:HGNC:1570] |
| ENSG00000167566 | HGLibA_31321 | 320 | 1.75981624 | NCKAP5L | NCK associated protein 5 like [Source:HGNC Symbol;Acc:HGNC:29321] |
| ENSG00000168300 | HGLibA_35629 | 320 | 1.75981624 | PCMTD1 | protein-L-isoaspartate (D-aspartate) O-methyltransferase domain containing 1 [Source:HGNC Symbol;Acc:HGNC:30483] |
| ENSG00000174279 | HGLibA_15711 | 320 | 1.75981624 | EVX2 | even-skipped homeobox 2 [Source:HGNC Symbol;Acc:HGNC:3507] |
| ENSG00000180592 | HGLibA_44279 | 320 | 1.75981624 | SKIDA1 | SKI/DACH domain containing 1 [Source:HGNC Symbol;Acc:HGNC:32697] |
| ENSG00000184903 | HGLibA_23232 | 320 | 1.75981624 | IMMP2L | inner mitochondrial membrane peptidase subunit 2 [Source:HGNC Symbol;Acc:HGNC:14598] |
| ENSG00000185624 | HGLibA_34909 | 320 | 1.75981624 | P4HB | prolyl 4-hydroxylase subunit beta [Source:HGNC Symbol;Acc:HGNC:8548] |
| ENSG00000198780 | HGLibA_16251 | 320 | 1.75981624 | FAM169A | family with sequence similarity 169 member A [Source:HGNC Symbol;Acc:HGNC:29138] |
| ENSG00000204262 | HGLibA_10591 | 320 | 1.75981624 | COL5A2 | collagen type V alpha 2 chain [Source:HGNC Symbol;Acc:HGNC:2210] |
| ENSG00000204928 | HGLibA_20392 | 320 | 1.75981624 | GRXCR2 | glutaredoxin and cysteine rich domain containing 2 [Source:HGNC Symbol;Acc:HGNC:33862] |
| ENSG00000205356 | HGLibA_48942 | 320 | 1.75981624 | TECPR1 | tectonin beta-propeller repeat containing 1 [Source:HGNC Symbol;Acc:HGNC:22214] |
| ENSG00000258083 | HGLibA_34566 | 320 | 1.75981624 | OR9A4 | olfactory receptor family 9 subfamily A member 4 [Source:HGNC Symbol;Acc:HGNC:15095] |
| hsa-mir-4764 | HGLibA_61154 | 320 | 1.75981624 | hsa-mir-4764 | - |
| hsa-mir-548y | HGLibA_62086 | 320 | 1.75981624 | hsa-mir-548y | - |
| LRRC33 | HGLibA_27318 | 320 | 1.75981624 | LRRC33 | - |
| WDR67 | HGLibA_54157 | 320 | 1.75981624 | WDR67 | - |
| ENSG00000004142 | HGLibA_37503 | 319 | 1.750770897 | POLDIP2 | DNA polymerase delta interacting protein 2 [Source:HGNC Symbol;Acc:HGNC:23781] |
| ENSG00000007933 | HGLibA_17709 | 319 | 1.750770897 | FMO3 | flavin containing monooxygenase 3 [Source:HGNC Symbol;Acc:HGNC:3771] |
| ENSG00000084693 | HGLibA_01179 | 319 | 1.750770897 | AGBL5 | ATP/GTP binding protein like 5 [Source:HGNC Symbol;Acc:HGNC:26147] |
| ENSG00000089818 | HGLibA_31592 | 319 | 1.750770897 | NECAP1 | NECAP endocytosis associated 1 [Source:HGNC Symbol;Acc:HGNC:24539] |
| ENSG00000099954 | HGLibA_08978 | 319 | 1.750770897 | CECR2 | histone acetyl-lysine reader [Source:HGNC Symbol;Acc:HGNC:1840]" |
| ENSG00000110448 | HGLibA_08401 | 319 | 1.750770897 | CD5 | CD5 molecule [Source:HGNC Symbol;Acc:HGNC:1685] |

| | | | | | |
|---|---|---|---|---|---|
| ENSG00000117480 | HGLibA_15900 | 319 | 1.750770897 | FAAH | fatty acid amide hydrolase [Source:HGNC Symbol;Acc:HGNC:3553] |
| ENSG00000120088 | HGLibA_11142 | 319 | 1.750770897 | CRHR1 | corticotropin releasing hormone receptor 1 [Source:HGNC Symbol;Acc:HGNC:2357] |
| ENSG00000135424 | HGLibA_23742 | 319 | 1.750770897 | ITGA7 | integrin subunit alpha 7 [Source:HGNC Symbol;Acc:HGNC:6143] |
| ENSG00000135956 | HGLibA_49915 | 319 | 1.750770897 | TMEM127 | transmembrane protein 127 [Source:HGNC Symbol;Acc:HGNC:26038] |
| ENSG00000136542 | HGLibA_18564 | 319 | 1.750770897 | GALNT5 | polypeptide N-acetylgalactosaminyltransferase 5 [Source:HGNC Symbol;Acc:HGNC:4127] |
| ENSG00000147027 | HGLibA_50413 | 319 | 1.750770897 | TMEM47 | transmembrane protein 47 [Source:HGNC Symbol;Acc:HGNC:18515] |
| ENSG00000147649 | HGLibA_30334 | 319 | 1.750770897 | MTDH | metadherin [Source:HGNC Symbol;Acc:HGNC:29608] |
| ENSG00000151025 | HGLibA_19952 | 319 | 1.750770897 | GPR158 | G protein-coupled receptor 158 [Source:HGNC Symbol;Acc:HGNC:23689] |
| ENSG00000161652 | HGLibA_23896 | 319 | 1.750770897 | IZUMO2 | IZUMO family member 2 [Source:HGNC Symbol;Acc:HGNC:28518] |
| ENSG00000162073 | HGLibA_35160 | 319 | 1.750770897 | PAQR4 | progestin and adipoQ receptor family member 4 [Source:HGNC Symbol;Acc:HGNC:26386] |
| ENSG00000168658 | HGLibA_53904 | 319 | 1.750770897 | VWA3B | von Willebrand factor A domain containing 3B [Source:HGNC Symbol;Acc:HGNC:28385] |
| ENSG00000168894 | HGLibA_41584 | 319 | 1.750770897 | RNF181 | ring finger protein 181 [Source:HGNC Symbol;Acc:HGNC:28037] |
| ENSG00000176444 | HGLibA_10057 | 319 | 1.750770897 | CLK2 | CDC like kinase 2 [Source:HGNC Symbol;Acc:HGNC:2069] |
| ENSG00000180432 | HGLibA_12217 | 319 | 1.750770897 | CYP8B1 | cytochrome P450 family 8 subfamily B member 1 [Source:HGNC Symbol;Acc:HGNC:2653] |
| ENSG00000182866 | HGLibA_26155 | 319 | 1.750770897 | LCK | Src family tyrosine kinase [Source:HGNC Symbol;Acc:HGNC:6524]" |
| ENSG00000184208 | HGLibA_06083 | 319 | 1.750770897 | C22orf46 | chromosome 22 open reading frame 46 [Source:HGNC Symbol;Acc:HGNC:26294] |
| ENSG00000188883 | HGLibA_25312 | 319 | 1.750770897 | KLRG2 | killer cell lectin like receptor G2 [Source:HGNC Symbol;Acc:HGNC:24778] |
| ENSG00000196350 | HGLibA_56640 | 319 | 1.750770897 | ZNF729 | zinc finger protein 729 [Source:HGNC Symbol;Acc:HGNC:32464] |
| ENSG00000204439 | HGLibA_06488 | 319 | 1.750770897 | C6orf47 | chromosome 6 open reading frame 47 [Source:HGNC Symbol;Acc:HGNC:19076] |
| ENSG00000213967 | HGLibA_56632 | 319 | 1.750770897 | ZNF726 | zinc finger protein 726 [Source:HGNC Symbol;Acc:HGNC:32462] |
| ENSG00000255398 | HGLibA_20953 | 319 | 1.750770897 | HCAR3 | hydroxycarboxylic acid receptor 3 [Source:HGNC Symbol;Acc:HGNC:16824] |
| ENSG00000260458 | HGLibA_24271 | 319 | 1.750770897 | KCNJ18 | potassium voltage-gated channel subfamily J member 18 [Source:HGNC Symbol;Acc:HGNC:39080] |
| ENSG00000271601 | HGLibA_26645 | 319 | 1.750770897 | LIX1L | limb and CNS expressed 1 like [Source:HGNC Symbol;Acc:HGNC:28715] |
| hsa-mir-3188 | HGLibA_58848 | 319 | 1.750770897 | hsa-mir-3188 | - |
| ENSG00000000003 | HGLibA_51944 | 318 | 1.741725554 | TSPAN6 | tetraspanin 6 [Source:HGNC Symbol;Acc:HGNC:11858] |
| ENSG00000006059 | HGLibA_25479 | 318 | 1.741725554 | KRT33A | keratin 33A [Source:HGNC Symbol;Acc:HGNC:6450] |
| ENSG00000064042 | HGLibA_26528 | 318 | 1.741725554 | LIMCH1 | LIM and calponin homology domains 1 [Source:HGNC Symbol;Acc:HGNC:29191] |
| ENSG00000072786 | HGLibA_47450 | 318 | 1.741725554 | STK10 | serine/threonine kinase 10 [Source:HGNC Symbol;Acc:HGNC:11388] |
| ENSG00000072832 | HGLibA_11200 | 318 | 1.741725554 | CRMP1 | collapsin response mediator protein 1 [Source:HGNC Symbol;Acc:HGNC:2365] |
| ENSG00000076321 | HGLibA_25151 | 318 | 1.741725554 | KLHL20 | kelch like family member 20 [Source:HGNC Symbol;Acc:HGNC:25056] |
| ENSG00000083857 | HGLibA_16912 | 318 | 1.741725554 | FAT1 | FAT atypical cadherin 1 [Source:HGNC Symbol;Acc:HGNC:3595] |
| ENSG00000104219 | HGLibA_55097 | 318 | 1.741725554 | ZDHHC2 | zinc finger DHHC-type containing 2 [Source:HGNC Symbol;Acc:HGNC:18469] |
| ENSG00000108100 | HGLibA_08132 | 318 | 1.741725554 | CCNY | cyclin Y [Source:HGNC Symbol;Acc:HGNC:23354] |
| ENSG00000114757 | HGLibA_36121 | 318 | 1.741725554 | PEX5L | peroxisomal biogenesis factor 5 like [Source:HGNC Symbol;Acc:HGNC:30024] |
| ENSG00000115234 | HGLibA_46048 | 318 | 1.741725554 | SNX17 | sorting nexin 17 [Source:HGNC Symbol;Acc:HGNC:14979] |
| ENSG00000124713 | HGLibA_19560 | 318 | 1.741725554 | GNMT | glycine N-methyltransferase [Source:HGNC Symbol;Acc:HGNC:4415] |
| ENSG00000124780 | HGLibA_24311 | 318 | 1.741725554 | KCNK17 | potassium two pore domain channel subfamily K member 17 [Source:HGNC Symbol;Acc:HGNC:14465] |
| ENSG00000129187 | HGLibA_12540 | 318 | 1.741725554 | DCTD | dCMP deaminase [Source:HGNC Symbol;Acc:HGNC:2710] |
| ENSG00000135541 | HGLibA_01298 | 318 | 1.741725554 | AHI1 | Abelson helper integration site 1 [Source:HGNC Symbol;Acc:HGNC:21575] |
| ENSG00000137494 | HGLibA_02100 | 318 | 1.741725554 | ANKRD42 | ankyrin repeat domain 42 [Source:HGNC Symbol;Acc:HGNC:26752] |
| ENSG00000142453 | HGLibA_07252 | 318 | 1.741725554 | CARM1 | coactivator associated arginine methyltransferase 1 [Source:HGNC Symbol;Acc:HGNC:23393] |
| ENSG00000148826 | HGLibA_32059 | 318 | 1.741725554 | NKX6-2 | NK6 homeobox 2 [Source:HGNC Symbol;Acc:HGNC:19321] |
| ENSG00000149743 | HGLibA_51779 | 318 | 1.741725554 | TRPT1 | tRNA phosphotransferase 1 [Source:HGNC Symbol;Acc:HGNC:20316] |
| ENSG00000149930 | HGLibA_48308 | 318 | 1.741725554 | TAOK2 | TAO kinase 2 [Source:HGNC Symbol;Acc:HGNC:16835] |
| ENSG00000163374 | HGLibA_54761 | 318 | 1.741725554 | YY1AP1 | YY1 associated protein 1 [Source:HGNC Symbol;Acc:HGNC:30935] |
| ENSG00000173801 | HGLibA_24012 | 318 | 1.741725554 | JUP | junction plakoglobin [Source:HGNC Symbol;Acc:HGNC:6207] |
| ENSG00000178226 | HGLibA_38933 | 318 | 1.741725554 | PRSS36 | serine protease 36 [Source:HGNC Symbol;Acc:HGNC:26906] |
| ENSG00000178385 | HGLibA_37143 | 318 | 1.741725554 | PLEKHM3 | pleckstrin homology domain containing M3 [Source:HGNC Symbol;Acc:HGNC:34006] |
| ENSG00000185610 | HGLibA_12410 | 318 | 1.741725554 | DBX2 | developing brain homeobox 2 [Source:HGNC Symbol;Acc:HGNC:33186] |
| ENSG00000186395 | HGLibA_25418 | 318 | 1.741725554 | KRT10 | keratin 10 [Source:HGNC Symbol;Acc:HGNC:6413] |
| ENSG00000197444 | HGLibA_33304 | 318 | 1.741725554 | OGDHL | oxoglutarate dehydrogenase like [Source:HGNC Symbol;Acc:HGNC:25590] |
| ENSG00000204618 | HGLibA_41672 | 318 | 1.741725554 | RNF39 | ring finger protein 39 [Source:HGNC Symbol;Acc:HGNC:18064] |
| ENSG00000236032 | HGLibA_34292 | 318 | 1.741725554 | OR5H14 | olfactory receptor family 5 subfamily H member 14 [Source:HGNC Symbol;Acc:HGNC:31286] |
| ENSG00000275410 | HGLibA_21778 | 318 | 1.741725554 | HNF1B | HNF1 homeobox B [Source:HGNC Symbol;Acc:HGNC:11630] |
| hsa-mir-1228 | HGLibA_57312 | 318 | 1.741725554 | hsa-mir-1228 | - |
| hsa-mir-151b | HGLibA_57901 | 318 | 1.741725554 | hsa-mir-151b | - |
| hsa-mir-3115 | HGLibA_58500 | 318 | 1.741725554 | hsa-mir-3115 | - |
| hsa-mir-3622b | HGLibA_59126 | 318 | 1.741725554 | hsa-mir-3622b | - |
| hsa-mir-575 | HGLibA_62395 | 318 | 1.741725554 | hsa-mir-575 | - |
| hsa-mir-6884 | HGLibA_63722 | 318 | 1.741725554 | hsa-mir-6884 | - |
| C7orf60 | HGLibA_06563 | 317 | 1.732680211 | C7orf60 | - |
| ENSG00000003436 | HGLibA_49185 | 317 | 1.732680211 | TFPI | tissue factor pathway inhibitor [Source:HGNC Symbol;Acc:HGNC:11760] |
| ENSG00000100129 | HGLibA_14799 | 317 | 1.732680211 | EIF3L | eukaryotic translation initiation factor 3 subunit L [Source:HGNC Symbol;Acc:HGNC:18138] |
| ENSG00000116977 | HGLibA_26378 | 317 | 1.732680211 | LGALS8 | galectin 8 [Source:HGNC Symbol;Acc:HGNC:6569] |
| ENSG00000119042 | HGLibA_42779 | 317 | 1.732680211 | SATB2 | SATB homeobox 2 [Source:HGNC Symbol;Acc:HGNC:21637] |
| ENSG00000122035 | HGLibA_40365 | 317 | 1.732680211 | RASL11A | RAS like family 11 member A [Source:HGNC Symbol;Acc:HGNC:23802] |
| ENSG00000129084 | HGLibA_39070 | 317 | 1.732680211 | PSMA1 | proteasome subunit alpha 1 [Source:HGNC Symbol;Acc:HGNC:9530] |
| ENSG00000130948 | HGLibA_22189 | 317 | 1.732680211 | HSD17B3 | hydroxysteroid 17-beta dehydrogenase 3 [Source:HGNC Symbol;Acc:HGNC:5212] |
| ENSG00000134668 | HGLibA_46745 | 317 | 1.732680211 | SPOCD1 | SPOC domain containing 1 [Source:HGNC Symbol;Acc:HGNC:26338] |
| ENSG00000136002 | HGLibA_02863 | 317 | 1.732680211 | ARHGEF4 | Rho guanine nucleotide exchange factor 4 [Source:HGNC Symbol;Acc:HGNC:684] |
| ENSG00000137673 | HGLibA_29536 | 317 | 1.732680211 | MMP7 | matrix metallopeptidase 7 [Source:HGNC Symbol;Acc:HGNC:7174] |
| ENSG00000139445 | HGLibA_17918 | 317 | 1.732680211 | FOXN4 | forkhead box N4 [Source:HGNC Symbol;Acc:HGNC:21399] |
| ENSG00000156968 | HGLibA_29787 | 317 | 1.732680211 | MPV17L | MPV17 mitochondrial inner membrane protein like [Source:HGNC Symbol;Acc:HGNC:26827] |
| ENSG00000167701 | HGLibA_20165 | 317 | 1.732680211 | GPT | glutamic--pyruvic transaminase [Source:HGNC Symbol;Acc:HGNC:4552] |
| ENSG00000169393 | HGLibA_14993 | 317 | 1.732680211 | ELSPBP1 | epididymal sperm binding protein 1 [Source:HGNC Symbol;Acc:HGNC:14417] |
| ENSG00000170633 | HGLibA_41667 | 317 | 1.732680211 | RNF34 | ring finger protein 34 [Source:HGNC Symbol;Acc:HGNC:17297] |
| ENSG00000172236 | HGLibA_51195 | 317 | 1.732680211 | TPSAB1 | tryptase alpha/beta 1 [Source:HGNC Symbol;Acc:HGNC:12019] |
| ENSG00000177853 | HGLibA_56150 | 317 | 1.732680211 | ZNF518A | zinc finger protein 518A [Source:HGNC Symbol;Acc:HGNC:29009] |
| ENSG00000178719 | HGLibA_20317 | 317 | 1.732680211 | GRINA | glutamate ionotropic receptor NMDA type subunit associated protein 1 [Source:HGNC Symbol;Acc:HGNC:4589] |
| ENSG00000179071 | HGLibA_07915 | 317 | 1.732680211 | CCDC89 | coiled-coil domain containing 89 [Source:HGNC Symbol;Acc:HGNC:26762] |
| ENSG00000184117 | HGLibA_31970 | 317 | 1.732680211 | NIPSNAP1 | nipsnap homolog 1 [Source:HGNC Symbol;Acc:HGNC:7827] |
| ENSG00000185238 | HGLibA_38625 | 317 | 1.732680211 | PRMT3 | protein arginine methyltransferase 3 [Source:HGNC Symbol;Acc:HGNC:30163] |
| ENSG00000197181 | HGLibA_36774 | 317 | 1.732680211 | PIWIL2 | piwi like RNA-mediated gene silencing 2 [Source:HGNC Symbol;Acc:HGNC:17644] |
| ENSG00000215186 | HGLibA_19609 | 317 | 1.732680211 | GOLGA6B | golgin A6 family member B [Source:HGNC Symbol;Acc:HGNC:32205] |
| ENSG00000243811 | HGLibA_02486 | 317 | 1.732680211 | APOBEC3D | apolipoprotein B mRNA editing enzyme catalytic subunit 3D [Source:HGNC Symbol;Acc:HGNC:17354] |
| hsa-mir-455 | HGLibA_60630 | 317 | 1.732680211 | hsa-mir-455 | - |
| hsa-mir-7853 | HGLibA_64000 | 317 | 1.732680211 | hsa-mir-7853 | - |
| KIRREL | HGLibA_25001 | 317 | 1.732680211 | KIRREL | - |
| ENSG00000005483 | HGLibA_25333 | 316 | 1.723634868 | KMT2E | lysine methyltransferase 2E [Source:HGNC Symbol;Acc:HGNC:18541] |
| ENSG00000023318 | HGLibA_15530 | 316 | 1.723634868 | ERP44 | endoplasmic reticulum protein 44 [Source:HGNC Symbol;Acc:HGNC:18311] |
| ENSG00000091428 | HGLibA_40253 | 316 | 1.723634868 | RAPGEF4 | Rap guanine nucleotide exchange factor 4 [Source:HGNC Symbol;Acc:HGNC:16626] |
| ENSG00000100354 | HGLibA_50917 | 316 | 1.723634868 | TNRC6B | trinucleotide repeat containing 6B [Source:HGNC Symbol;Acc:HGNC:29190] |
| ENSG00000100360 | HGLibA_22756 | 316 | 1.723634868 | IFT27 | intraflagellar transport 27 [Source:HGNC Symbol;Acc:HGNC:18626] |
| ENSG00000106028 | HGLibA_47113 | 316 | 1.723634868 | SSBP1 | single stranded DNA binding protein 1 [Source:HGNC Symbol;Acc:HGNC:11317] |
| ENSG00000108702 | HGLibA_07962 | 316 | 1.723634868 | CCL1 | C-C motif chemokine ligand 1 [Source:HGNC Symbol;Acc:HGNC:10609] |
| ENSG00000122711 | HGLibA_46693 | 316 | 1.723634868 | SPINK4 | Kazal type 4 [Source:HGNC Symbol;Acc:HGNC:16646]" |
| ENSG00000126882 | HGLibA_16728 | 316 | 1.723634868 | FAM78A | family with sequence similarity 78 member A [Source:HGNC Symbol;Acc:HGNC:25465] |
| ENSG00000129472 | HGLibA_39869 | 316 | 1.723634868 | RAB2B | member RAS oncogene family [Source:HGNC Symbol;Acc:HGNC:20246]" |
| ENSG00000131242 | HGLibA_39802 | 316 | 1.723634868 | RAB11FIP4 | RAB11 family interacting protein 4 [Source:HGNC Symbol;Acc:HGNC:30267] |
| ENSG00000137177 | HGLibA_24855 | 316 | 1.723634868 | KIF13A | kinesin family member 13A [Source:HGNC Symbol;Acc:HGNC:14566] |
| ENSG00000137571 | HGLibA_45476 | 316 | 1.723634868 | SLCO5A1 | solute carrier organic anion transporter family member 5A1 [Source:HGNC Symbol;Acc:HGNC:19046] |
| ENSG00000139436 | HGLibA_19134 | 316 | 1.723634868 | GIT2 | GIT ArfGAP 2 [Source:HGNC Symbol;Acc:HGNC:4273] |
| ENSG00000141642 | HGLibA_14870 | 316 | 1.723634868 | ELAC1 | elaC ribonuclease Z 1 [Source:HGNC Symbol;Acc:HGNC:14197] |
| ENSG00000142661 | HGLibA_30895 | 316 | 1.723634868 | MYOM3 | myomesin 3 [Source:HGNC Symbol;Acc:HGNC:26679] |
| ENSG00000145907 | HGLibA_18299 | 316 | 1.723634868 | G3BP1 | G3BP stress granule assembly factor 1 [Source:HGNC Symbol;Acc:HGNC:30292] |
| ENSG00000147383 | HGLibA_32749 | 316 | 1.723634868 | NSDHL | NAD(P) dependent steroid dehydrogenase-like [Source:HGNC Symbol;Acc:HGNC:13398] |
| ENSG00000148795 | HGLibA_12062 | 316 | 1.723634868 | CYP17A1 | cytochrome P450 family 17 subfamily A member 1 [Source:HGNC Symbol;Acc:HGNC:2593] |
| ENSG00000149300 | HGLibA_05152 | 316 | 1.723634868 | C11orf52 | chromosome 11 open reading frame 52 [Source:HGNC Symbol;Acc:HGNC:30531] |
| ENSG00000152463 | HGLibA_33340 | 316 | 1.723634868 | OLAH | oleoyl-ACP hydrolase [Source:HGNC Symbol;Acc:HGNC:25625] |
| ENSG00000159871 | HGLibA_27726 | 316 | 1.723634868 | LYPD5 | LY6/PLAUR domain containing 5 [Source:HGNC Symbol;Acc:HGNC:26397] |
| ENSG00000160285 | HGLibA_27570 | 316 | 1.723634868 | LSS | lanosterol synthase [Source:HGNC Symbol;Acc:HGNC:6708] |
| ENSG00000160602 | HGLibA_31665 | 316 | 1.723634868 | NEK8 | NIMA related kinase 8 [Source:HGNC Symbol;Acc:HGNC:13387] |
| ENSG00000167791 | HGLibA_06850 | 316 | 1.723634868 | CABP2 | calcium binding protein 2 [Source:HGNC Symbol;Acc:HGNC:1385] |
| ENSG00000169469 | HGLibA_46805 | 316 | 1.723634868 | SPRR1B | small proline rich protein 1B [Source:HGNC Symbol;Acc:HGNC:11260] |
| ENSG00000170558 | HGLibA_08660 | 316 | 1.723634868 | CDH2 | cadherin 2 [Source:HGNC Symbol;Acc:HGNC:1759] |
| ENSG00000180758 | HGLibA_19948 | 316 | 1.723634868 | GPR157 | G protein-coupled receptor 157 [Source:HGNC Symbol;Acc:HGNC:23687] |
| ENSG00000181817 | HGLibA_27522 | 316 | 1.723634868 | LSM10 | U7 small nuclear RNA associated [Source:HGNC Symbol;Acc:HGNC:17562]" |
| ENSG00000204410 | HGLibA_30198 | 316 | 1.723634868 | MSH5 | mutS homolog 5 [Source:HGNC Symbol;Acc:HGNC:7328] |
| ENSG00000214063 | HGLibA_51938 | 316 | 1.723634868 | TSPAN4 | tetraspanin 4 [Source:HGNC Symbol;Acc:HGNC:11859] |
| ENSG00000262246 | HGLibA_10790 | 316 | 1.723634868 | CORO7 | coronin 7 [Source:HGNC Symbol;Acc:HGNC:26161] |
| ENSG00000266094 | HGLibA_40386 | 316 | 1.723634868 | RASSF5 | Ras association domain family member 5 [Source:HGNC Symbol;Acc:HGNC:17609] |
| FAM194B | HGLibA_16372 | 316 | 1.723634868 | FAM194B | - |
| hsa-mir-323a | HGLibA_58954 | 316 | 1.723634868 | hsa-mir-323a | - |
| hsa-mir-625 | HGLibA_62738 | 316 | 1.723634868 | hsa-mir-625 | - |
| DUS2L | HGLibA_14119 | 315 | 1.714589525 | DUS2L | - |
| ENSG00000004399 | HGLibA_37281 | 315 | 1.714589525 | PLXND1 | plexin D1 [Source:HGNC Symbol;Acc:HGNC:9107] |
| ENSG00000076043 | HGLibA_40897 | 315 | 1.714589525 | REXO2 | RNA exonuclease 2 [Source:HGNC Symbol;Acc:HGNC:17851] |
| ENSG00000099795 | HGLibA_31527 | 315 | 1.714589525 | NDUFB7 | NADH:ubiquinone oxidoreductase subunit B7 [Source:HGNC Symbol;Acc:HGNC:7702] |
| ENSG00000106927 | HGLibA_01751 | 315 | 1.714589525 | AMBP | alpha-1-microglobulin/bikunin precursor [Source:HGNC Symbol;Acc:HGNC:453] |
| ENSG00000113924 | HGLibA_21238 | 315 | 1.714589525 | HGD | 2-dioxygenase [Source:HGNC Symbol;Acc:HGNC:4892]" |
| ENSG00000116194 | HGLibA_01910 | 315 | 1.714589525 | ANGPTL1 | angiopoietin like 1 [Source:HGNC Symbol;Acc:HGNC:489] |
| ENSG00000120616 | HGLibA_15267 | 315 | 1.714589525 | EPC1 | enhancer of polycomb homolog 1 [Source:HGNC Symbol;Acc:HGNC:19876] |
| ENSG00000120658 | HGLibA_15158 | 315 | 1.714589525 | ENOX1 | ecto-NOX disulfide-thiol exchanger 1 [Source:HGNC Symbol;Acc:HGNC:25474] |
| ENSG00000132359 | HGLibA_40229 | 315 | 1.714589525 | RAP1GAP2 | RAP1 GTPase activating protein 2 [Source:HGNC Symbol;Acc:HGNC:29176] |
| ENSG00000133256 | HGLibA_35807 | 315 | 1.714589525 | PDE6B | phosphodiesterase 6B [Source:HGNC Symbol;Acc:HGNC:8786] |

| | | | | | |
|---|---|---|---|---|---|
| ENSG00000137168 | HGLibA_37938 | 315 | 1.714589525 | PPIL1 | peptidylprolyl isomerase like 1 [Source:HGNC Symbol;Acc:HGNC:9260] |
| ENSG00000137486 | HGLibA_03107 | 315 | 1.714589525 | ARRB1 | arrestin beta 1 [Source:HGNC Symbol;Acc:HGNC:711] |
| ENSG00000147155 | HGLibA_14372 | 315 | 1.714589525 | EBP | cholestenol delta-isomerase [Source:HGNC Symbol;Acc:HGNC:3133]" |
| ENSG00000161849 | HGLibA_25568 | 315 | 1.714589525 | KRT84 | keratin 84 [Source:HGNC Symbol;Acc:HGNC:6461] |
| ENSG00000162891 | HGLibA_23073 | 315 | 1.714589525 | IL20 | interleukin 20 [Source:HGNC Symbol;Acc:HGNC:6002] |
| ENSG00000163485 | HGLibA_01010 | 315 | 1.714589525 | ADORA1 | adenosine A1 receptor [Source:HGNC Symbol;Acc:HGNC:262] |
| ENSG00000163751 | HGLibA_10891 | 315 | 1.714589525 | CPA3 | carboxypeptidase A3 [Source:HGNC Symbol;Acc:HGNC:2298] |
| ENSG00000163820 | HGLibA_18250 | 315 | 1.714589525 | FYCO1 | FYVE and coiled-coil domain containing 1 [Source:HGNC Symbol;Acc:HGNC:14673] |
| ENSG00000166589 | HGLibA_08647 | 315 | 1.714589525 | CDH16 | cadherin 16 [Source:HGNC Symbol;Acc:HGNC:1755] |
| ENSG00000167791 | HGLibA_06851 | 315 | 1.714589525 | CABP2 | calcium binding protein 2 [Source:HGNC Symbol;Acc:HGNC:1385] |
| ENSG00000169131 | HGLibA_55860 | 315 | 1.714589525 | ZNF354A | zinc finger protein 354A [Source:HGNC Symbol;Acc:HGNC:11628] |
| ENSG00000171316 | HGLibA_09403 | 315 | 1.714589525 | CHD7 | chromodomain helicase DNA binding protein 7 [Source:HGNC Symbol;Acc:HGNC:20626] |
| ENSG00000172476 | HGLibA_39931 | 315 | 1.714589525 | RAB40A | member RAS oncogene family [Source:HGNC Symbol;Acc:HGNC:18283]" |
| ENSG00000180138 | HGLibA_11383 | 315 | 1.714589525 | CSNK1A1L | casein kinase 1 alpha 1 like [Source:HGNC Symbol;Acc:HGNC:20289] |
| ENSG00000184160 | HGLibA_01053 | 315 | 1.714589525 | ADRA2C | adrenoceptor alpha 2C [Source:HGNC Symbol;Acc:HGNC:283] |
| ENSG00000186510 | HGLibA_09841 | 315 | 1.714589525 | CLCNKA | chloride voltage-gated channel Ka [Source:HGNC Symbol;Acc:HGNC:2026] |
| ENSG00000186787 | HGLibA_46673 | 315 | 1.714589525 | SPIN2B | spindlin family member 2B [Source:HGNC Symbol;Acc:HGNC:33147] |
| ENSG00000198417 | HGLibA_30285 | 315 | 1.714589525 | MT1F | metallothionein 1F [Source:HGNC Symbol;Acc:HGNC:7398] |
| ENSG00000215695 | HGLibA_42308 | 315 | 1.714589525 | RSC1A1 | regulator of solute carriers 1 [Source:HGNC Symbol;Acc:HGNC:10458] |
| hsa-mir-941-2 | HGLibA_64344 | 315 | 1.714589525 | hsa-mir-941-2 | - |
| hsa-mir-941-4 | HGLibA_64352 | 315 | 1.714589525 | hsa-mir-941-4 | - |
| PCNX | HGLibA_35644 | 315 | 1.714589525 | PCNX | - |
| ENSG00000070985 | HGLibA_51763 | 314 | 1.705544182 | TRPM5 | transient receptor potential cation channel subfamily M member 5 [Source:HGNC Symbol;Acc:HGNC:14323] |
| ENSG00000095752 | HGLibA_22953 | 314 | 1.705544182 | IL11 | interleukin 11 [Source:HGNC Symbol;Acc:HGNC:5966] |
| ENSG00000099875 | HGLibA_29345 | 314 | 1.705544182 | MKNK2 | MAP kinase interacting serine/threonine kinase 2 [Source:HGNC Symbol;Acc:HGNC:7111] |
| ENSG00000100722 | HGLibA_54964 | 314 | 1.705544182 | ZC3H14 | zinc finger CCCH-type containing 14 [Source:HGNC Symbol;Acc:HGNC:20509] |
| ENSG00000105676 | HGLibA_03026 | 314 | 1.705544182 | ARMC6 | armadillo repeat containing 6 [Source:HGNC Symbol;Acc:HGNC:25049] |
| ENSG00000112812 | HGLibA_38906 | 314 | 1.705544182 | PRSS16 | serine protease 16 [Source:HGNC Symbol;Acc:HGNC:9480] |
| ENSG00000115947 | HGLibA_34610 | 314 | 1.705544182 | ORC4 | origin recognition complex subunit 4 [Source:HGNC Symbol;Acc:HGNC:8490] |
| ENSG00000116095 | HGLibA_37069 | 314 | 1.705544182 | PLEKHA3 | pleckstrin homology domain containing A3 [Source:HGNC Symbol;Acc:HGNC:14338] |
| ENSG00000120907 | HGLibA_01038 | 314 | 1.705544182 | ADRA1A | adrenoceptor alpha 1A [Source:HGNC Symbol;Acc:HGNC:277] |
| ENSG00000129675 | HGLibA_02872 | 314 | 1.705544182 | ARHGEF6 | Rac/Cdc42 guanine nucleotide exchange factor 6 [Source:HGNC Symbol;Acc:HGNC:685] |
| ENSG00000133275 | HGLibA_11396 | 314 | 1.705544182 | CSNK1G2 | casein kinase 1 gamma 2 [Source:HGNC Symbol;Acc:HGNC:2455] |
| ENSG00000137747 | HGLibA_50629 | 314 | 1.705544182 | TMPRSS13 | transmembrane serine protease 13 [Source:HGNC Symbol;Acc:HGNC:29808] |
| ENSG00000151693 | HGLibA_03196 | 314 | 1.705544182 | ASAP2 | ankyrin repeat and PH domain 2 [Source:HGNC Symbol;Acc:HGNC:2721]" |
| ENSG00000153814 | HGLibA_23940 | 314 | 1.705544182 | JAZF1 | JAZF zinc finger 1 [Source:HGNC Symbol;Acc:HGNC:28917] |
| ENSG00000163754 | HGLibA_20689 | 314 | 1.705544182 | GYG1 | glycogenin 1 [Source:HGNC Symbol;Acc:HGNC:4699] |
| ENSG00000166710 | HGLibA_03945 | 314 | 1.705544182 | B2M | beta-2-microglobulin [Source:HGNC Symbol;Acc:HGNC:914] |
| ENSG00000169676 | HGLibA_13972 | 314 | 1.705544182 | DRD5 | dopamine receptor D5 [Source:HGNC Symbol;Acc:HGNC:3026] |
| ENSG00000174652 | HGLibA_55699 | 314 | 1.705544182 | ZNF266 | zinc finger protein 266 [Source:HGNC Symbol;Acc:HGNC:13059] |
| ENSG00000178150 | HGLibA_55444 | 314 | 1.705544182 | ZNF114 | zinc finger protein 114 [Source:HGNC Symbol;Acc:HGNC:12894] |
| ENSG00000179119 | HGLibA_46906 | 314 | 1.705544182 | SPTY2D1 | SPT2 chromatin protein domain containing 1 [Source:HGNC Symbol;Acc:HGNC:26818] |
| ENSG00000187783 | HGLibA_50507 | 314 | 1.705544182 | TMEM72 | transmembrane protein 72 [Source:HGNC Symbol;Acc:HGNC:31658] |
| ENSG00000188283 | HGLibA_55891 | 314 | 1.705544182 | ZNF383 | zinc finger protein 383 [Source:HGNC Symbol;Acc:HGNC:18609] |
| ENSG00000196184 | HGLibA_33521 | 314 | 1.705544182 | OR10J1 | olfactory receptor family 10 subfamily J member 1 [Source:HGNC Symbol;Acc:HGNC:8175] |
| ENSG00000274349 | HGLibA_56473 | 314 | 1.705544182 | ZNF658 | zinc finger protein 658 [Source:HGNC Symbol;Acc:HGNC:25226] |
| hsa-mir-339 | HGLibA_59007 | 314 | 1.705544182 | hsa-mir-339 | - |
| ADSSL1 | HGLibA_01080 | 313 | 1.696498839 | ADSSL1 | - |
| ENSG00000087903 | HGLibA_40966 | 313 | 1.696498839 | RFX2 | regulatory factor X2 [Source:HGNC Symbol;Acc:HGNC:9983] |
| ENSG00000112293 | HGLibA_19815 | 313 | 1.696498839 | GPLD1 | glycosylphosphatidylinositol specific phospholipase D1 [Source:HGNC Symbol;Acc:HGNC:4459] |
| ENSG00000112406 | HGLibA_21090 | 313 | 1.696498839 | HECA | cell cycle regulator [Source:HGNC Symbol;Acc:HGNC:21041]" |
| ENSG00000118217 | HGLibA_03445 | 313 | 1.696498839 | ATF6 | activating transcription factor 6 [Source:HGNC Symbol;Acc:HGNC:791] |
| ENSG00000129159 | HGLibA_24163 | 313 | 1.696498839 | KCNC1 | potassium voltage-gated channel subfamily C member 1 [Source:HGNC Symbol;Acc:HGNC:6233] |
| ENSG00000133812 | HGLibA_42796 | 313 | 1.696498839 | SBF2 | SET binding factor 2 [Source:HGNC Symbol;Acc:HGNC:2135] |
| ENSG00000144061 | HGLibA_32446 | 313 | 1.696498839 | NPHP1 | nephrocystin 1 [Source:HGNC Symbol;Acc:HGNC:7905] |
| ENSG00000150967 | HGLibA_00155 | 313 | 1.696498839 | ABCB9 | ATP binding cassette subfamily B member 9 [Source:HGNC Symbol;Acc:HGNC:50] |
| ENSG00000160310 | HGLibA_38622 | 313 | 1.696498839 | PRMT2 | protein arginine methyltransferase 2 [Source:HGNC Symbol;Acc:HGNC:5186] |
| ENSG00000163806 | HGLibA_46573 | 313 | 1.696498839 | SPDYA | speedy/RINGO cell cycle regulator family member A [Source:HGNC Symbol;Acc:HGNC:30613] |
| ENSG00000164823 | HGLibA_34689 | 313 | 1.696498839 | OSGIN2 | oxidative stress induced growth inhibitor family member 2 [Source:HGNC Symbol;Acc:HGNC:1355] |
| ENSG00000165478 | HGLibA_21139 | 313 | 1.696498839 | HEPACAM | hepatic and glial cell adhesion molecule [Source:HGNC Symbol;Acc:HGNC:26361] |
| ENSG00000168077 | HGLibA_42862 | 313 | 1.696498839 | SCARA3 | scavenger receptor class A member 3 [Source:HGNC Symbol;Acc:HGNC:19000] |
| ENSG00000170927 | HGLibA_36813 | 313 | 1.696498839 | PKHD1 | fibrocystin/polyductin [Source:HGNC Symbol;Acc:HGNC:9016]" |
| ENSG00000181220 | HGLibA_56664 | 313 | 1.696498839 | ZNF746 | zinc finger protein 746 [Source:HGNC Symbol;Acc:HGNC:21948] |
| ENSG00000196371 | HGLibA_18193 | 313 | 1.696498839 | FUT4 | fucosyltransferase 4 [Source:HGNC Symbol;Acc:HGNC:4015] |
| ENSG00000197081 | HGLibA_22810 | 313 | 1.696498839 | IGF2R | insulin like growth factor 2 receptor [Source:HGNC Symbol;Acc:HGNC:5467] |
| ENSG00000214063 | HGLibA_51937 | 313 | 1.696498839 | TSPAN4 | tetraspanin 4 [Source:HGNC Symbol;Acc:HGNC:11859] |
| ENSG00000271447 | HGLibA_29530 | 313 | 1.696498839 | MMP28 | matrix metallopeptidase 28 [Source:HGNC Symbol;Acc:HGNC:14366] |
| hsa-mir-16-2 | HGLibA_57951 | 313 | 1.696498839 | hsa-mir-16-2 | - |
| hsa-mir-27a | HGLibA_58377 | 313 | 1.696498839 | hsa-mir-27a | - |
| hsa-mir-3927 | HGLibA_59586 | 313 | 1.696498839 | hsa-mir-3927 | - |
| PAPL | HGLibA_35129 | 313 | 1.696498839 | PAPL | - |
| C17orf72 | HGLibA_05568 | 312 | 1.687453496 | C17orf72 | - |
| ENSG00000004846 | HGLibA_00142 | 312 | 1.687453496 | ABCB5 | ATP binding cassette subfamily B member 5 [Source:HGNC Symbol;Acc:HGNC:46] |
| ENSG00000034713 | HGLibA_18339 | 312 | 1.687453496 | GABARAPL2 | GABA type A receptor associated protein like 2 [Source:HGNC Symbol;Acc:HGNC:13291] |
| ENSG00000072694 | HGLibA_17192 | 312 | 1.687453496 | FCGR2B | Fc fragment of IgG receptor IIb [Source:HGNC Symbol;Acc:HGNC:3618] |
| ENSG00000109689 | HGLibA_47443 | 312 | 1.687453496 | STIM2 | stromal interaction molecule 2 [Source:HGNC Symbol;Acc:HGNC:19205] |
| ENSG00000112186 | HGLibA_07137 | 312 | 1.687453496 | CAP2 | cyclase associated actin cytoskeleton regulatory protein 2 [Source:HGNC Symbol;Acc:HGNC:20039] |
| ENSG00000133818 | HGLibA_42238 | 312 | 1.687453496 | RRAS2 | RAS related 2 [Source:HGNC Symbol;Acc:HGNC:17271] |
| ENSG00000137819 | HGLibA_35164 | 312 | 1.687453496 | PAQR5 | progestin and adipoQ receptor family member 5 [Source:HGNC Symbol;Acc:HGNC:29645] |
| ENSG00000145335 | HGLibA_45907 | 312 | 1.687453496 | SNCA | synuclein alpha [Source:HGNC Symbol;Acc:HGNC:11138] |
| ENSG00000154319 | HGLibA_16241 | 312 | 1.687453496 | FAM167A | family with sequence similarity 167 member A [Source:HGNC Symbol;Acc:HGNC:15549] |
| ENSG00000157107 | HGLibA_17207 | 312 | 1.687453496 | FCHO2 | FCH domain only 2 [Source:HGNC Symbol;Acc:HGNC:25180] |
| ENSG00000167850 | HGLibA_08336 | 312 | 1.687453496 | CD300C | CD300c molecule [Source:HGNC Symbol;Acc:HGNC:19320] |
| ENSG00000168303 | HGLibA_29738 | 312 | 1.687453496 | MPLKIP | M-phase specific PLK1 interacting protein [Source:HGNC Symbol;Acc:HGNC:16002] |
| ENSG00000260314 | HGLibA_29817 | 312 | 1.687453496 | MRC1 | mannose receptor C-type 1 [Source:HGNC Symbol;Acc:HGNC:7228] |
| hsa-mir-641 | HGLibA_62802 | 312 | 1.687453496 | hsa-mir-641 | - |
| KIAA0922 | HGLibA_24666 | 312 | 1.687453496 | KIAA0922 | - |
| ENSG00000072042 | HGLibA_40735 | 311 | 1.678408153 | RDH11 | retinol dehydrogenase 11 [Source:HGNC Symbol;Acc:HGNC:17964] |
| ENSG00000076604 | HGLibA_51257 | 311 | 1.678408153 | TRAF4 | TNF receptor associated factor 4 [Source:HGNC Symbol;Acc:HGNC:12034] |
| ENSG00000095587 | HGLibA_49630 | 311 | 1.678408153 | TLL2 | tolloid like 2 [Source:HGNC Symbol;Acc:HGNC:11844] |
| ENSG00000104059 | HGLibA_16351 | 311 | 1.678408153 | FAM189A1 | family with sequence similarity 189 member A1 [Source:HGNC Symbol;Acc:HGNC:29075] |
| ENSG00000115355 | HGLibA_07907 | 311 | 1.678408153 | CCDC88A | coiled-coil domain containing 88A [Source:HGNC Symbol;Acc:HGNC:25523] |
| ENSG00000119574 | HGLibA_54894 | 311 | 1.678408153 | ZBTB45 | zinc finger and BTB domain containing 45 [Source:HGNC Symbol;Acc:HGNC:23715] |
| ENSG00000131721 | HGLibA_41218 | 311 | 1.678408153 | RHOXF2 | Rhox homeobox family member 2 [Source:HGNC Symbol;Acc:HGNC:30011] |
| ENSG00000136044 | HGLibA_02570 | 311 | 1.678408153 | APPL2 | phosphotyrosine interacting with PH domain and leucine zipper 2 [Source:HGNC Symbol;Acc:HGNC:18242]" |
| ENSG00000136457 | HGLibA_09341 | 311 | 1.678408153 | CHAD | chondroadherin [Source:HGNC Symbol;Acc:HGNC:1909] |
| ENSG00000137463 | HGLibA_29128 | 311 | 1.678408153 | MGARP | mitochondria localized glutamic acid rich protein [Source:HGNC Symbol;Acc:HGNC:29969] |
| ENSG00000139352 | HGLibA_03267 | 311 | 1.678408153 | ASCL1 | achaete-scute family bHLH transcription factor 1 [Source:HGNC Symbol;Acc:HGNC:738] |
| ENSG00000141198 | HGLibA_50948 | 311 | 1.678408153 | TOM1L1 | target of myb1 like 1 membrane trafficking protein [Source:HGNC Symbol;Acc:HGNC:11983] |
| ENSG00000146250 | HGLibA_38929 | 311 | 1.678408153 | PRSS35 | serine protease 35 [Source:HGNC Symbol;Acc:HGNC:21387] |
| ENSG00000148848 | HGLibA_00733 | 311 | 1.678408153 | ADAM12 | ADAM metallopeptidase domain 12 [Source:HGNC Symbol;Acc:HGNC:190] |
| ENSG00000148943 | HGLibA_26579 | 311 | 1.678408153 | LIN7C | crumbs cell polarity complex component [Source:HGNC Symbol;Acc:HGNC:17789]" |
| ENSG00000156103 | HGLibA_29499 | 311 | 1.678408153 | MMP16 | matrix metallopeptidase 16 [Source:HGNC Symbol;Acc:HGNC:7162] |
| ENSG00000159958 | HGLibA_50758 | 311 | 1.678408153 | TNFRSF13C | TNF receptor superfamily member 13C [Source:HGNC Symbol;Acc:HGNC:17755] |
| ENSG00000160685 | HGLibA_54919 | 311 | 1.678408153 | ZBTB7B | zinc finger and BTB domain containing 7B [Source:HGNC Symbol;Acc:HGNC:18668] |
| ENSG00000169288 | HGLibA_29889 | 311 | 1.678408153 | MRPL1 | mitochondrial ribosomal protein L1 [Source:HGNC Symbol;Acc:HGNC:14275] |
| ENSG00000182197 | HGLibA_15801 | 311 | 1.678408153 | EXT1 | exostosin glycosyltransferase 1 [Source:HGNC Symbol;Acc:HGNC:3512] |
| ENSG00000187210 | HGLibA_18809 | 311 | 1.678408153 | GCNT1 | core 2 [Source:HGNC Symbol;Acc:HGNC:4203]" |
| ENSG00000196990 | HGLibA_16231 | 311 | 1.678408153 | FAM163B | family with sequence similarity 163 member B [Source:HGNC Symbol;Acc:HGNC:33277] |
| ENSG00000196998 | HGLibA_54098 | 311 | 1.678408153 | WDR45 | WD repeat domain 45 [Source:HGNC Symbol;Acc:HGNC:28912] |
| ENSG00000198546 | HGLibA_56129 | 311 | 1.678408153 | ZNF511 | zinc finger protein 511 [Source:HGNC Symbol;Acc:HGNC:28445] |
| ENSG00000203926 | HGLibA_46380 | 311 | 1.678408153 | SPANXA2 | SPANX family member A2 [Source:HGNC Symbol;Acc:HGNC:14328] |
| ENSG00000255408 | HGLibA_35443 | 311 | 1.678408153 | PCDHA3 | protocadherin alpha 3 [Source:HGNC Symbol;Acc:HGNC:8669] |
| ENSG00000261272 | HGLibA_30544 | 311 | 1.678408153 | MUC22 | mucin 22 [Source:HGNC Symbol;Acc:HGNC:39755] |
| hsa-mir-4476 | HGLibA_60316 | 311 | 1.678408153 | hsa-mir-4476 | - |
| LOC401052 | HGLibA_26936 | 311 | 1.678408153 | LOC401052 | - |
| TMEM5 | HGLibA_50420 | 311 | 1.678408153 | TMEM5 | - |
| ENSG00000008382 | HGLibA_29741 | 310 | 1.66936281 | MPND | MPN domain containing [Source:HGNC Symbol;Acc:HGNC:25934] |
| ENSG00000008513 | HGLibA_47238 | 310 | 1.66936281 | ST3GAL1 | 3-sialyltransferase 1 [Source:HGNC Symbol;Acc:HGNC:10862]" |
| ENSG00000072786 | HGLibA_47449 | 310 | 1.66936281 | STK10 | serine/threonine kinase 10 [Source:HGNC Symbol;Acc:HGNC:11388] |
| ENSG00000078061 | HGLibA_02622 | 310 | 1.66936281 | ARAF | serine/threonine kinase [Source:HGNC Symbol;Acc:HGNC:646]" |
| ENSG00000079101 | HGLibA_10165 | 310 | 1.66936281 | CLUL1 | clusterin like 1 [Source:HGNC Symbol;Acc:HGNC:2096] |
| ENSG00000101350 | HGLibA_24937 | 310 | 1.66936281 | KIF3B | kinesin family member 3B [Source:HGNC Symbol;Acc:HGNC:6320] |
| ENSG00000105750 | HGLibA_56868 | 310 | 1.66936281 | ZNF85 | zinc finger protein 85 [Source:HGNC Symbol;Acc:HGNC:13160] |
| ENSG00000108961 | HGLibA_40219 | 310 | 1.66936281 | RANGRF | RAN guanine nucleotide release factor [Source:HGNC Symbol;Acc:HGNC:17679] |
| ENSG00000114956 | HGLibA_13089 | 310 | 1.66936281 | DGUOK | deoxyguanosine kinase [Source:HGNC Symbol;Acc:HGNC:2858] |
| ENSG00000115353 | HGLibA_48170 | 310 | 1.66936281 | TACR1 | tachykinin receptor 1 [Source:HGNC Symbol;Acc:HGNC:11526] |
| ENSG00000137642 | HGLibA_46196 | 310 | 1.66936281 | SORL1 | sortilin related receptor 1 [Source:HGNC Symbol;Acc:HGNC:11185] |
| ENSG00000140382 | HGLibA_21678 | 310 | 1.66936281 | HMG20A | high mobility group 20A [Source:HGNC Symbol;Acc:HGNC:5001] |
| ENSG00000143224 | HGLibA_38001 | 310 | 1.66936281 | PPOX | protoporphyrinogen oxidase [Source:HGNC Symbol;Acc:HGNC:9280] |
| ENSG00000143412 | HGLibA_02262 | 310 | 1.66936281 | ANXA9 | annexin A9 [Source:HGNC Symbol;Acc:HGNC:547] |
| ENSG00000147050 | HGLibA_24532 | 310 | 1.66936281 | KDM6A | lysine demethylase 6A [Source:HGNC Symbol;Acc:HGNC:12637] |
| ENSG00000147889 | HGLibA_08831 | 310 | 1.66936281 | CDKN2A | cyclin dependent kinase inhibitor 2A [Source:HGNC Symbol;Acc:HGNC:1787] |
| ENSG00000151151 | HGLibA_23458 | 310 | 1.66936281 | IPMK | inositol polyphosphate multikinase [Source:HGNC Symbol;Acc:HGNC:20739] |
| ENSG00000157330 | HGLibA_05781 | 310 | 1.66936281 | C1orf158 | chromosome 1 open reading frame 158 [Source:HGNC Symbol;Acc:HGNC:28567] |

| | | | | | |
|---|---|---|---|---|---|
| ENSG00000157379 | HGLibA_13119 | 310 | 1.66936281 | DHRS1 | dehydrogenase/reductase 1 [Source:HGNC Symbol;Acc:HGNC:16445] |
| ENSG00000160190 | HGLibA_45042 | 310 | 1.66936281 | SLC37A1 | solute carrier family 37 member 1 [Source:HGNC Symbol;Acc:HGNC:11024] |
| ENSG00000163945 | HGLibA_53477 | 310 | 1.66936281 | UVSSA | UV stimulated scaffold protein A [Source:HGNC Symbol;Acc:HGNC:29304] |
| ENSG00000164086 | HGLibA_14190 | 310 | 1.66936281 | DUSP7 | dual specificity phosphatase 7 [Source:HGNC Symbol;Acc:HGNC:3073] |
| ENSG00000165084 | HGLibA_06611 | 310 | 1.66936281 | C8orf34 | chromosome 8 open reading frame 34 [Source:HGNC Symbol;Acc:HGNC:30905] |
| ENSG00000173572 | HGLibA_32110 | 310 | 1.66936281 | NLRP13 | NLR family pyrin domain containing 13 [Source:HGNC Symbol;Acc:HGNC:22937] |
| ENSG00000174669 | HGLibA_44844 | 310 | 1.66936281 | SLC29A2 | solute carrier family 29 member 2 [Source:HGNC Symbol;Acc:HGNC:11004] |
| ENSG00000175305 | HGLibA_08088 | 310 | 1.66936281 | CCNE2 | cyclin E2 [Source:HGNC Symbol;Acc:HGNC:1590] |
| ENSG00000179168 | HGLibA_19000 | 310 | 1.66936281 | GGN | gametogenetin [Source:HGNC Symbol;Acc:HGNC:18869] |
| ENSG00000182177 | HGLibA_03231 | 310 | 1.66936281 | ASB18 | ankyrin repeat and SOCS box containing 18 [Source:HGNC Symbol;Acc:HGNC:19770] |
| ENSG00000183018 | HGLibA_46737 | 310 | 1.66936281 | SPNS2 | sphingolipid transporter 2 [Source:HGNC Symbol;Acc:HGNC:26992] |
| ENSG00000186119 | HGLibA_34283 | 310 | 1.66936281 | OR5D18 | olfactory receptor family 5 subfamily D member 18 [Source:HGNC Symbol;Acc:HGNC:15285] |
| ENSG00000189042 | HGLibA_56264 | 310 | 1.66936281 | ZNF567 | zinc finger protein 567 [Source:HGNC Symbol;Acc:HGNC:28696] |
| ENSG00000196296 | HGLibA_03606 | 310 | 1.66936281 | ATP2A1 | ATPase sarcoplasmic/endoplasmic reticulum Ca2+ transporting 1 [Source:HGNC Symbol;Acc:HGNC:811] |
| ENSG00000204352 | HGLibA_06674 | 310 | 1.66936281 | C9orf129 | chromosome 9 open reading frame 129 [Source:HGNC Symbol;Acc:HGNC:31116] |
| ENSG00000214860 | HGLibA_15704 | 310 | 1.66936281 | EVPLL | envoplakin like [Source:HGNC Symbol;Acc:HGNC:35236] |
| ENSG00000243232 | HGLibA_35466 | 310 | 1.66936281 | PCDHAC2 | 2 [Source:HGNC Symbol;Acc:HGNC:8677]" |
| GLTPD1 | HGLibA_19345 | 310 | 1.66936281 | GLTPD1 | - |
| hsa-mir-3945 | HGLibA_59641 | 310 | 1.66936281 | hsa-mir-3945 | - |
| hsa-mir-4308 | HGLibA_59942 | 310 | 1.66936281 | hsa-mir-4308 | - |
| TMEM183B | HGLibA_50108 | 310 | 1.66936281 | TMEM183B | - |
| C4orf27 | HGLibA_06312 | 309 | 1.660317468 | C4orf27 | - |
| C7orf63 | HGLibA_06573 | 309 | 1.660317468 | C7orf63 | - |
| ENSG00000035403 | HGLibA_53572 | 309 | 1.660317468 | VCL | vinculin [Source:HGNC Symbol;Acc:HGNC:12665] |
| ENSG00000048740 | HGLibA_09008 | 309 | 1.660317468 | CELF2 | CUGBP Elav-like family member 2 [Source:HGNC Symbol;Acc:HGNC:2550] |
| ENSG00000101544 | HGLibA_01003 | 309 | 1.660317468 | ADNP2 | ADNP homeobox 2 [Source:HGNC Symbol;Acc:HGNC:23803] |
| ENSG00000106479 | HGLibA_56881 | 309 | 1.660317468 | ZNF862 | zinc finger protein 862 [Source:HGNC Symbol;Acc:HGNC:34519] |
| ENSG00000119535 | HGLibA_11342 | 309 | 1.660317468 | CSF3R | colony stimulating factor 3 receptor [Source:HGNC Symbol;Acc:HGNC:2439] |
| ENSG00000125817 | HGLibA_09041 | 309 | 1.660317468 | CENPB | centromere protein B [Source:HGNC Symbol;Acc:HGNC:1852] |
| ENSG00000127364 | HGLibA_48399 | 309 | 1.660317468 | TAS2R4 | taste 2 receptor member 4 [Source:HGNC Symbol;Acc:HGNC:14911] |
| ENSG00000132004 | HGLibA_17158 | 309 | 1.660317468 | FBXW9 | F-box and WD repeat domain containing 9 [Source:HGNC Symbol;Acc:HGNC:28136] |
| ENSG00000132128 | HGLibA_27357 | 309 | 1.660317468 | LRRC41 | leucine rich repeat containing 41 [Source:HGNC Symbol;Acc:HGNC:16917] |
| ENSG00000137343 | HGLibA_03421 | 309 | 1.660317468 | ATAT1 | alpha tubulin acetyltransferase 1 [Source:HGNC Symbol;Acc:HGNC:21186] |
| ENSG00000137504 | HGLibA_11116 | 309 | 1.660317468 | CREBZF | CREB/ATF bZIP transcription factor [Source:HGNC Symbol;Acc:HGNC:24905] |
| ENSG00000152214 | HGLibA_41339 | 309 | 1.660317468 | RIT2 | Ras like without CAAX 2 [Source:HGNC Symbol;Acc:HGNC:10017] |
| ENSG00000160460 | HGLibA_46886 | 309 | 1.660317468 | SPTBN4 | non-erythrocytic 4 [Source:HGNC Symbol;Acc:HGNC:14896]" |
| ENSG00000172602 | HGLibA_41445 | 309 | 1.660317468 | RND1 | Rho family GTPase 1 [Source:HGNC Symbol;Acc:HGNC:18314] |
| ENSG00000179119 | HGLibA_46908 | 309 | 1.660317468 | SPTY2D1 | SPT2 chromatin protein domain containing 1 [Source:HGNC Symbol;Acc:HGNC:26818] |
| ENSG00000181023 | HGLibA_34224 | 309 | 1.660317468 | OR56B1 | olfactory receptor family 56 subfamily B member 1 [Source:HGNC Symbol;Acc:HGNC:15245] |
| ENSG00000183971 | HGLibA_32545 | 309 | 1.660317468 | NPW | neuropeptide W [Source:HGNC Symbol;Acc:HGNC:30509] |
| ENSG00000198851 | HGLibA_08372 | 309 | 1.660317468 | CD3E | CD3e molecule [Source:HGNC Symbol;Acc:HGNC:1674] |
| hsa-mir-4764 | HGLibA_61153 | 309 | 1.660317468 | hsa-mir-4764 | - |
| hsa-mir-532 | HGLibA_61805 | 309 | 1.660317468 | hsa-mir-532 | - |
| hsa-mir-7110 | HGLibA_63805 | 309 | 1.660317468 | hsa-mir-7110 | - |
| MESDC2 | HGLibA_28913 | 309 | 1.660317468 | MESDC2 | - |
| ENSG00000025423 | HGLibA_22196 | 308 | 1.651272125 | HSD17B6 | hydroxysteroid 17-beta dehydrogenase 6 [Source:HGNC Symbol;Acc:HGNC:23316] |
| ENSG00000044446 | HGLibA_36405 | 308 | 1.651272125 | PHKA2 | phosphorylase kinase regulatory subunit alpha 2 [Source:HGNC Symbol;Acc:HGNC:8926] |
| ENSG00000099381 | HGLibA_43636 | 308 | 1.651272125 | SETD1A | SET domain containing 1A [Source:HGNC Symbol;Acc:HGNC:29010] |
| ENSG00000100312 | HGLibA_00531 | 308 | 1.651272125 | ACR | acrosin [Source:HGNC Symbol;Acc:HGNC:126] |
| ENSG00000102390 | HGLibA_35329 | 308 | 1.651272125 | PBDC1 | polysaccharide biosynthesis domain containing 1 [Source:HGNC Symbol;Acc:HGNC:28790] |
| ENSG00000116882 | HGLibA_20836 | 308 | 1.651272125 | HAO2 | hydroxyacid oxidase 2 [Source:HGNC Symbol;Acc:HGNC:4810] |
| ENSG00000117983 | HGLibA_30551 | 308 | 1.651272125 | MUC5B | oligomeric mucus/gel-forming [Source:HGNC Symbol;Acc:HGNC:7516]" |
| ENSG00000118680 | HGLibA_30755 | 308 | 1.651272125 | MYL12B | myosin light chain 12B [Source:HGNC Symbol;Acc:HGNC:29827] |
| ENSG00000119771 | HGLibA_25174 | 308 | 1.651272125 | KLHL29 | kelch like family member 29 [Source:HGNC Symbol;Acc:HGNC:29404] |
| ENSG00000123307 | HGLibA_31739 | 308 | 1.651272125 | NEUROD4 | neuronal differentiation 4 [Source:HGNC Symbol;Acc:HGNC:13802] |
| ENSG00000126759 | HGLibA_09289 | 308 | 1.651272125 | CFP | complement factor properdin [Source:HGNC Symbol;Acc:HGNC:8864] |
| ENSG00000128886 | HGLibA_14927 | 308 | 1.651272125 | ELL3 | elongation factor for RNA polymerase II 3 [Source:HGNC Symbol;Acc:HGNC:23113] |
| ENSG00000131781 | HGLibA_17715 | 308 | 1.651272125 | FMO5 | flavin containing monooxygenase 5 [Source:HGNC Symbol;Acc:HGNC:3773] |
| ENSG00000133318 | HGLibA_42403 | 308 | 1.651272125 | RTN3 | reticulon 3 [Source:HGNC Symbol;Acc:HGNC:10469] |
| ENSG00000137802 | HGLibA_28311 | 308 | 1.651272125 | MAPKBP1 | mitogen-activated protein kinase binding protein 1 [Source:HGNC Symbol;Acc:HGNC:29536] |
| ENSG00000138100 | HGLibA_51543 | 308 | 1.651272125 | TRIM54 | tripartite motif containing 54 [Source:HGNC Symbol;Acc:HGNC:16008] |
| ENSG00000141968 | HGLibA_53549 | 308 | 1.651272125 | VAV1 | vav guanine nucleotide exchange factor 1 [Source:HGNC Symbol;Acc:HGNC:12657] |
| ENSG00000165185 | HGLibA_24829 | 308 | 1.651272125 | KIAA1958 | KIAA1958 [Source:HGNC Symbol;Acc:HGNC:23427] |
| ENSG00000165887 | HGLibA_02023 | 308 | 1.651272125 | ANKRD2 | ankyrin repeat domain 2 [Source:HGNC Symbol;Acc:HGNC:495] |
| ENSG00000167619 | HGLibA_49974 | 308 | 1.651272125 | TMEM145 | transmembrane protein 145 [Source:HGNC Symbol;Acc:HGNC:26912] |
| ENSG00000174469 | HGLibA_10421 | 308 | 1.651272125 | CNTNAP2 | contactin associated protein like 2 [Source:HGNC Symbol;Acc:HGNC:13830] |
| ENSG00000175806 | HGLibA_30237 | 308 | 1.651272125 | MSRA | methionine sulfoxide reductase A [Source:HGNC Symbol;Acc:HGNC:7377] |
| ENSG00000183864 | HGLibA_50938 | 308 | 1.651272125 | TOB2 | 2 [Source:HGNC Symbol;Acc:HGNC:11980]" |
| ENSG00000184486 | HGLibA_37768 | 308 | 1.651272125 | POU3F2 | POU class 3 homeobox 2 [Source:HGNC Symbol;Acc:HGNC:9215] |
| ENSG00000185532 | HGLibA_38578 | 308 | 1.651272125 | PRKG1 | protein kinase cGMP-dependent 1 [Source:HGNC Symbol;Acc:HGNC:9414] |
| ENSG00000186732 | HGLibA_29770 | 308 | 1.651272125 | MPPED1 | metallophosphoesterase domain containing 1 [Source:HGNC Symbol;Acc:HGNC:1306] |
| ENSG00000204700 | HGLibA_33808 | 308 | 1.651272125 | OR2J2 | olfactory receptor family 2 subfamily J member 2 [Source:HGNC Symbol;Acc:HGNC:8260] |
| ENSG00000205111 | HGLibA_08814 | 308 | 1.651272125 | CDKL4 | cyclin dependent kinase like 4 [Source:HGNC Symbol;Acc:HGNC:19287] |
| ENSG00000221855 | HGLibA_48405 | 308 | 1.651272125 | TAS2R41 | taste 2 receptor member 41 [Source:HGNC Symbol;Acc:HGNC:18883] |
| ENSG00000246922 | HGLibA_52550 | 308 | 1.651272125 | UBAP1L | ubiquitin associated protein 1 like [Source:HGNC Symbol;Acc:HGNC:40028] |
| hsa-mir-1193 | HGLibA_57250 | 308 | 1.651272125 | hsa-mir-1193 | - |
| hsa-mir-137 | HGLibA_57807 | 308 | 1.651272125 | hsa-mir-137 | - |
| hsa-mir-3916 | HGLibA_59541 | 308 | 1.651272125 | hsa-mir-3916 | - |
| hsa-mir-499b | HGLibA_61404 | 308 | 1.651272125 | hsa-mir-499b | - |
| hsa-mir-639 | HGLibA_62797 | 308 | 1.651272125 | hsa-mir-639 | - |
| LOC100129480 | HGLibA_26763 | 308 | 1.651272125 | LOC100129480 | - |
| LRMP | HGLibA_27194 | 308 | 1.651272125 | LRMP | - |
| CARKD | HGLibA_07250 | 307 | 1.642226782 | CARKD | - |
| ENSG00000104774 | HGLibA_28076 | 307 | 1.642226782 | MAN2B1 | mannosidase alpha class 2B member 1 [Source:HGNC Symbol;Acc:HGNC:6826] |
| ENSG00000107736 | HGLibA_08667 | 307 | 1.642226782 | CDH23 | cadherin related 23 [Source:HGNC Symbol;Acc:HGNC:13733] |
| ENSG00000114248 | HGLibA_27310 | 307 | 1.642226782 | LRRC31 | leucine rich repeat containing 31 [Source:HGNC Symbol;Acc:HGNC:26261] |
| ENSG00000125864 | HGLibA_04456 | 307 | 1.642226782 | BFSP1 | beaded filament structural protein 1 [Source:HGNC Symbol;Acc:HGNC:1040] |
| ENSG00000134369 | HGLibA_31195 | 307 | 1.642226782 | NAV1 | neuron navigator 1 [Source:HGNC Symbol;Acc:HGNC:15989] |
| ENSG00000147164 | HGLibA_46034 | 307 | 1.642226782 | SNX12 | sorting nexin 12 [Source:HGNC Symbol;Acc:HGNC:14976] |
| ENSG00000159111 | HGLibA_29892 | 307 | 1.642226782 | MRPL10 | mitochondrial ribosomal protein L10 [Source:HGNC Symbol;Acc:HGNC:14055] |
| ENSG00000160959 | HGLibA_27255 | 307 | 1.642226782 | LRRC14 | leucine rich repeat containing 14 [Source:HGNC Symbol;Acc:HGNC:20419] |
| ENSG00000162144 | HGLibA_11979 | 307 | 1.642226782 | CYB561A3 | cytochrome b561 family member A3 [Source:HGNC Symbol;Acc:HGNC:23014] |
| ENSG00000164466 | HGLibA_43781 | 307 | 1.642226782 | SFXN1 | sideroflexin 1 [Source:HGNC Symbol;Acc:HGNC:16085] |
| ENSG00000164600 | HGLibA_31742 | 307 | 1.642226782 | NEUROD6 | neuronal differentiation 6 [Source:HGNC Symbol;Acc:HGNC:13804] |
| ENSG00000164674 | HGLibA_48103 | 307 | 1.642226782 | SYTL3 | synaptotagmin like 3 [Source:HGNC Symbol;Acc:HGNC:15587] |
| ENSG00000169218 | HGLibA_42345 | 307 | 1.642226782 | RSPO1 | R-spondin 1 [Source:HGNC Symbol;Acc:HGNC:21679] |
| ENSG00000169554 | HGLibA_55138 | 307 | 1.642226782 | ZEB2 | zinc finger E-box binding homeobox 2 [Source:HGNC Symbol;Acc:HGNC:14881] |
| ENSG00000170921 | HGLibA_48294 | 307 | 1.642226782 | TANC2 | ankyrin repeat and coiled-coil containing 2 [Source:HGNC Symbol;Acc:HGNC:30212]" |
| ENSG00000171444 | HGLibA_28562 | 307 | 1.642226782 | MCC | WNT signaling pathway regulator [Source:HGNC Symbol;Acc:HGNC:6935]" |
| ENSG00000174099 | HGLibA_30247 | 307 | 1.642226782 | MSRB3 | methionine sulfoxide reductase B3 [Source:HGNC Symbol;Acc:HGNC:27375] |
| ENSG00000174453 | HGLibA_53927 | 307 | 1.642226782 | VWC2L | von Willebrand factor C domain containing 2 like [Source:HGNC Symbol;Acc:HGNC:37203] |
| ENSG00000175182 | HGLibA_16105 | 307 | 1.642226782 | FAM131A | family with sequence similarity 131 member A [Source:HGNC Symbol;Acc:HGNC:28308] |
| ENSG00000181929 | HGLibA_38511 | 307 | 1.642226782 | PRKAG1 | protein kinase AMP-activated non-catalytic subunit gamma 1 [Source:HGNC Symbol;Acc:HGNC:9385] |
| ENSG00000184454 | HGLibA_31333 | 307 | 1.642226782 | NCMAP | non-compact myelin associated protein [Source:HGNC Symbol;Acc:HGNC:29332] |
| ENSG00000187569 | HGLibA_13890 | 307 | 1.642226782 | DPPA3 | developmental pluripotency associated 3 [Source:HGNC Symbol;Acc:HGNC:19199] |
| ENSG00000196366 | HGLibA_06700 | 307 | 1.642226782 | C9orf163 | chromosome 9 open reading frame 163 [Source:HGNC Symbol;Acc:HGNC:26718] |
| ENSG00000250120 | HGLibA_35427 | 307 | 1.642226782 | PCDHA10 | protocadherin alpha 10 [Source:HGNC Symbol;Acc:HGNC:8664] |
| FOPNL | HGLibA_17791 | 307 | 1.642226782 | FOPNL | - |
| hsa-mir-1268b | HGLibA_57499 | 307 | 1.642226782 | hsa-mir-1268b | - |
| hsa-mir-378h | HGLibA_59459 | 307 | 1.642226782 | hsa-mir-378h | - |
| hsa-mir-431 | HGLibA_59951 | 307 | 1.642226782 | hsa-mir-431 | - |
| hsa-mir-4310 | HGLibA_59955 | 307 | 1.642226782 | hsa-mir-4310 | - |
| hsa-mir-543 | HGLibA_61821 | 307 | 1.642226782 | hsa-mir-543 | - |
| hsa-mir-6826 | HGLibA_63482 | 307 | 1.642226782 | hsa-mir-6826 | - |
| ENSG00000061273 | HGLibA_21020 | 306 | 1.633181439 | HDAC7 | histone deacetylase 7 [Source:HGNC Symbol;Acc:HGNC:14067] |
| ENSG00000064607 | HGLibA_47720 | 306 | 1.633181439 | SUGP2 | SURP and G-patch domain containing 2 [Source:HGNC Symbol;Acc:HGNC:18641] |
| ENSG00000073910 | HGLibA_18054 | 306 | 1.633181439 | FRY | FRY microtubule binding protein [Source:HGNC Symbol;Acc:HGNC:20367] |
| ENSG00000104067 | HGLibA_49572 | 306 | 1.633181439 | TJP1 | tight junction protein 1 [Source:HGNC Symbol;Acc:HGNC:11827] |
| ENSG00000111269 | HGLibA_11110 | 306 | 1.633181439 | CREBL2 | cAMP responsive element binding protein like 2 [Source:HGNC Symbol;Acc:HGNC:2350] |
| ENSG00000118162 | HGLibA_25380 | 306 | 1.633181439 | KPTN | actin binding protein [Source:HGNC Symbol;Acc:HGNC:6404]" |
| ENSG00000122035 | HGLibA_40366 | 306 | 1.633181439 | RASL11A | RAS like family 11 member A [Source:HGNC Symbol;Acc:HGNC:23802] |
| ENSG00000130413 | HGLibA_47491 | 306 | 1.633181439 | STK33 | serine/threonine kinase 33 [Source:HGNC Symbol;Acc:HGNC:14568] |
| ENSG00000134321 | HGLibA_42298 | 306 | 1.633181439 | RSAD2 | radical S-adenosyl methionine domain containing 2 [Source:HGNC Symbol;Acc:HGNC:30908] |
| ENSG00000144668 | HGLibA_23748 | 306 | 1.633181439 | ITGA9 | integrin subunit alpha 9 [Source:HGNC Symbol;Acc:HGNC:6145] |
| ENSG00000161509 | HGLibA_20304 | 306 | 1.633181439 | GRIN2C | glutamate ionotropic receptor NMDA type subunit 2C [Source:HGNC Symbol;Acc:HGNC:4587] |
| ENSG00000170653 | HGLibA_03451 | 306 | 1.633181439 | ATF7 | activating transcription factor 7 [Source:HGNC Symbol;Acc:HGNC:792] |
| ENSG00000171608 | HGLibA_36638 | 306 | 1.633181439 | PIK3CD | 5-bisphosphate 3-kinase catalytic subunit delta [Source:HGNC Symbol;Acc:HGNC:8977]" |
| ENSG00000175287 | HGLibA_36471 | 306 | 1.633181439 | PHYHD1 | phytanoyl-CoA dioxygenase domain containing 1 [Source:HGNC Symbol;Acc:HGNC:23396] |
| ENSG00000176248 | HGLibA_01879 | 306 | 1.633181439 | ANAPC2 | anaphase promoting complex subunit 2 [Source:HGNC Symbol;Acc:HGNC:19989] |
| ENSG00000278619 | HGLibA_29860 | 306 | 1.633181439 | MRM1 | mitochondrial rRNA methyltransferase 1 [Source:HGNC Symbol;Acc:HGNC:26202] |
| hsa-mir-3663 | HGLibA_59205 | 306 | 1.633181439 | hsa-mir-3663 | - |
| hsa-mir-7157 | HGLibA_63856 | 306 | 1.633181439 | hsa-mir-7157 | - |
| ENSG00000007129 | HGLibA_08935 | 305 | 1.624136096 | CEACAM21 | carcinoembryonic antigen related cell adhesion molecule 21 [Source:HGNC Symbol;Acc:HGNC:28834] |
| ENSG00000019485 | HGLibA_38369 | 305 | 1.624136096 | PRDM11 | PR/SET domain 11 [Source:HGNC Symbol;Acc:HGNC:13996] |
| ENSG00000081087 | HGLibA_34714 | 305 | 1.624136096 | OSTM1 | osteoclastogenesis associated transmembrane protein 1 [Source:HGNC Symbol;Acc:HGNC:21652] |

| | | | | | |
|---|---|---|---|---|---|
| ENSG00000085382 | HGLibA_20800 | 305 | 1.624136096 | HACE1 | HECT domain and ankyrin repeat containing E3 ubiquitin protein ligase 1 [Source:HGNC Symbol;Acc:HGNC:21033] |
| ENSG00000087301 | HGLibA_52415 | 305 | 1.624136096 | TXNDC16 | thioredoxin domain containing 16 [Source:HGNC Symbol;Acc:HGNC:19965] |
| ENSG00000100239 | HGLibA_38225 | 305 | 1.624136096 | PPP6R2 | protein phosphatase 6 regulatory subunit 2 [Source:HGNC Symbol;Acc:HGNC:19253] |
| ENSG00000108639 | HGLibA_47994 | 305 | 1.624136096 | SYNGR2 | synaptogyrin 2 [Source:HGNC Symbol;Acc:HGNC:11499] |
| ENSG00000111215 | HGLibA_38838 | 305 | 1.624136096 | PRR4 | proline rich 4 [Source:HGNC Symbol;Acc:HGNC:18020] |
| ENSG00000115593 | HGLibA_45852 | 305 | 1.624136096 | SMYD1 | SET and MYND domain containing 1 [Source:HGNC Symbol;Acc:HGNC:20986] |
| ENSG00000116478 | HGLibA_20996 | 305 | 1.624136096 | HDAC1 | histone deacetylase 1 [Source:HGNC Symbol;Acc:HGNC:4852] |
| ENSG00000117245 | HGLibA_24872 | 305 | 1.624136096 | KIF17 | kinesin family member 17 [Source:HGNC Symbol;Acc:HGNC:19167] |
| ENSG00000122958 | HGLibA_53753 | 305 | 1.624136096 | VPS26A | retromer complex component A [Source:HGNC Symbol;Acc:HGNC:12711]" |
| ENSG00000123570 | HGLibA_39995 | 305 | 1.624136096 | RAB9B | member RAS oncogene family [Source:HGNC Symbol;Acc:HGNC:14090]" |
| ENSG00000125363 | HGLibA_01766 | 305 | 1.624136096 | AMELX | amelogenin X-linked [Source:HGNC Symbol;Acc:HGNC:461] |
| ENSG00000129292 | HGLibA_36367 | 305 | 1.624136096 | PHF20L1 | PHD finger protein 20 like 1 [Source:HGNC Symbol;Acc:HGNC:24280] |
| ENSG00000130385 | HGLibA_04597 | 305 | 1.624136096 | BMP15 | bone morphogenetic protein 15 [Source:HGNC Symbol;Acc:HGNC:1068] |
| ENSG00000136295 | HGLibA_52238 | 305 | 1.624136096 | TTYH3 | tweety family member 3 [Source:HGNC Symbol;Acc:HGNC:22222] |
| ENSG00000137500 | HGLibA_07923 | 305 | 1.624136096 | CCDC90B | coiled-coil domain containing 90B [Source:HGNC Symbol;Acc:HGNC:28108] |
| ENSG00000142192 | HGLibA_02559 | 305 | 1.624136096 | APP | amyloid beta precursor protein [Source:HGNC Symbol;Acc:HGNC:620] |
| ENSG00000148019 | HGLibA_09167 | 305 | 1.624136096 | CEP78 | centrosomal protein 78 [Source:HGNC Symbol;Acc:HGNC:25740] |
| ENSG00000155926 | HGLibA_44300 | 305 | 1.624136096 | SLA | Src like adaptor [Source:HGNC Symbol;Acc:HGNC:10902] |
| ENSG00000156052 | HGLibA_19467 | 305 | 1.624136096 | GNAQ | G protein subunit alpha q [Source:HGNC Symbol;Acc:HGNC:4390] |
| ENSG00000160683 | HGLibA_11892 | 305 | 1.624136096 | CXCR5 | C-X-C motif chemokine receptor 5 [Source:HGNC Symbol;Acc:HGNC:1060] |
| ENSG00000162552 | HGLibA_54371 | 305 | 1.624136096 | WNT4 | Wnt family member 4 [Source:HGNC Symbol;Acc:HGNC:12783] |
| ENSG00000169327 | HGLibA_34254 | 305 | 1.624136096 | OR5AU1 | olfactory receptor family 5 subfamily AU member 1 [Source:HGNC Symbol;Acc:HGNC:15362] |
| ENSG00000170364 | HGLibA_43674 | 305 | 1.624136096 | SETMAR | SET domain and mariner transposase fusion gene [Source:HGNC Symbol;Acc:HGNC:10762] |
| ENSG00000176887 | HGLibA_46230 | 305 | 1.624136096 | SOX11 | SRY-box 11 [Source:HGNC Symbol;Acc:HGNC:11191] |
| ENSG00000179639 | HGLibA_17167 | 305 | 1.624136096 | FCER1A | Fc fragment of IgE receptor Ia [Source:HGNC Symbol;Acc:HGNC:3609] |
| ENSG00000181541 | HGLibA_27824 | 305 | 1.624136096 | MAB21L2 | mab-21 like 2 [Source:HGNC Symbol;Acc:HGNC:6758] |
| ENSG00000181577 | HGLibA_06480 | 305 | 1.624136096 | C6orf223 | chromosome 6 open reading frame 223 [Source:HGNC Symbol;Acc:HGNC:28692] |
| ENSG00000184140 | HGLibA_34013 | 305 | 1.624136096 | OR4F6 | olfactory receptor family 4 subfamily F member 6 [Source:HGNC Symbol;Acc:HGNC:15372] |
| ENSG00000184937 | HGLibA_54426 | 305 | 1.624136096 | WT1 | Wilms tumor 1 [Source:HGNC Symbol;Acc:HGNC:12796] |
| ENSG00000189280 | HGLibA_19170 | 305 | 1.624136096 | GJB5 | gap junction protein beta 5 [Source:HGNC Symbol;Acc:HGNC:4287] |
| ENSG00000197937 | HGLibA_55853 | 305 | 1.624136096 | ZNF347 | zinc finger protein 347 [Source:HGNC Symbol;Acc:HGNC:16447] |
| ENSG00000203952 | HGLibA_07670 | 305 | 1.624136096 | CCDC160 | coiled-coil domain containing 160 [Source:HGNC Symbol;Acc:HGNC:37286] |
| ENSG00000205726 | HGLibA_23870 | 305 | 1.624136096 | ITSN1 | intersectin 1 [Source:HGNC Symbol;Acc:HGNC:6183] |
| ENSG00000205867 | HGLibA_25792 | 305 | 1.624136096 | KRTAP5-2 | keratin associated protein 5-2 [Source:HGNC Symbol;Acc:HGNC:23597] |
| ENSG00000221931 | HGLibA_34452 | 305 | 1.624136096 | OR6X1 | olfactory receptor family 6 subfamily X member 1 [Source:HGNC Symbol;Acc:HGNC:14737] |
| ENSG00000240204 | HGLibA_45777 | 305 | 1.624136096 | SMKR1 | small lysine rich protein 1 [Source:HGNC Symbol;Acc:HGNC:43561] |
| ENSG00000257108 | HGLibA_31904 | 305 | 1.624136096 | NHLRC4 | NHL repeat containing 4 [Source:HGNC Symbol;Acc:HGNC:26700] |
| ENSG00000267041 | HGLibA_56871 | 305 | 1.624136096 | ZNF850 | zinc finger protein 850 [Source:HGNC Symbol;Acc:HGNC:27994] |
| ENSG00000277443 | HGLibA_28356 | 305 | 1.624136096 | MARCKS | myristoylated alanine rich protein kinase C substrate [Source:HGNC Symbol;Acc:HGNC:6759] |
| hsa-mir-4253 | HGLibA_59721 | 305 | 1.624136096 | hsa-mir-4253 | - |
| hsa-mir-6732 | HGLibA_63083 | 305 | 1.624136096 | hsa-mir-6732 | - |
| hsa-mir-6811 | HGLibA_63420 | 305 | 1.624136096 | hsa-mir-6811 | - |
| C9orf117 | HGLibA_06669 | 304 | 1.615090753 | C9orf117 | - |
| DFNB59 | HGLibA_13032 | 304 | 1.615090753 | DFNB59 | - |
| ENSG00000000938 | HGLibA_17468 | 304 | 1.615090753 | FGR | Src family tyrosine kinase [Source:HGNC Symbol;Acc:HGNC:3697]" |
| ENSG00000013293 | HGLibA_45357 | 304 | 1.615090753 | SLC7A14 | solute carrier family 7 member 14 [Source:HGNC Symbol;Acc:HGNC:29326] |
| ENSG00000034713 | HGLibA_18338 | 304 | 1.615090753 | GABARAPL2 | GABA type A receptor associated protein like 2 [Source:HGNC Symbol;Acc:HGNC:13291] |
| ENSG00000066777 | HGLibA_02670 | 304 | 1.615090753 | ARFGEF1 | ADP ribosylation factor guanine nucleotide exchange factor 1 [Source:HGNC Symbol;Acc:HGNC:15772] |
| ENSG00000072849 | HGLibA_12999 | 304 | 1.615090753 | DERL2 | derlin 2 [Source:HGNC Symbol;Acc:HGNC:17943] |
| ENSG00000089006 | HGLibA_46100 | 304 | 1.615090753 | SNX5 | sorting nexin 5 [Source:HGNC Symbol;Acc:HGNC:14969] |
| ENSG00000101916 | HGLibA_49665 | 304 | 1.615090753 | TLR8 | toll like receptor 8 [Source:HGNC Symbol;Acc:HGNC:15632] |
| ENSG00000103175 | HGLibA_54245 | 304 | 1.615090753 | WFDC1 | WAP four-disulfide core domain 1 [Source:HGNC Symbol;Acc:HGNC:15466] |
| ENSG00000105976 | HGLibA_28924 | 304 | 1.615090753 | MET | receptor tyrosine kinase [Source:HGNC Symbol;Acc:HGNC:7029]" |
| ENSG00000106927 | HGLibA_01750 | 304 | 1.615090753 | AMBP | alpha-1-microglobulin/bikunin precursor [Source:HGNC Symbol;Acc:HGNC:453] |
| ENSG00000107742 | HGLibA_46751 | 304 | 1.615090753 | SPOCK2 | cwcv and kazal like domains proteoglycan 2 [Source:HGNC Symbol;Acc:HGNC:13564]" |
| ENSG00000108963 | HGLibA_13837 | 304 | 1.615090753 | DPH1 | diphthamide biosynthesis 1 [Source:HGNC Symbol;Acc:HGNC:3003] |
| ENSG00000112706 | HGLibA_23263 | 304 | 1.615090753 | IMPG1 | interphotoreceptor matrix proteoglycan 1 [Source:HGNC Symbol;Acc:HGNC:6055] |
| ENSG00000116096 | HGLibA_46788 | 304 | 1.615090753 | SPR | sepiapterin reductase [Source:HGNC Symbol;Acc:HGNC:11257] |
| ENSG00000117020 | HGLibA_01513 | 304 | 1.615090753 | AKT3 | AKT serine/threonine kinase 3 [Source:HGNC Symbol;Acc:HGNC:393] |
| ENSG00000117308 | HGLibA_18513 | 304 | 1.615090753 | GALE | UDP-galactose-4-epimerase [Source:HGNC Symbol;Acc:HGNC:4116] |
| ENSG00000129474 | HGLibA_01372 | 304 | 1.615090753 | AJUBA | ajuba LIM protein [Source:HGNC Symbol;Acc:HGNC:20250] |
| ENSG00000130561 | HGLibA_42640 | 304 | 1.615090753 | SAG | S-antigen visual arrestin [Source:HGNC Symbol;Acc:HGNC:10521] |
| ENSG00000136492 | HGLibA_04820 | 304 | 1.615090753 | BRIP1 | BRCA1 interacting protein C-terminal helicase 1 [Source:HGNC Symbol;Acc:HGNC:20473] |
| ENSG00000140416 | HGLibA_51141 | 304 | 1.615090753 | TPM1 | tropomyosin 1 [Source:HGNC Symbol;Acc:HGNC:12010] |
| ENSG00000143164 | HGLibA_12452 | 304 | 1.615090753 | DCAF6 | DDB1 and CUL4 associated factor 6 [Source:HGNC Symbol;Acc:HGNC:30002] |
| ENSG00000144589 | HGLibA_47457 | 304 | 1.615090753 | STK11IP | serine/threonine kinase 11 interacting protein [Source:HGNC Symbol;Acc:HGNC:19184] |
| ENSG00000151327 | HGLibA_16295 | 304 | 1.615090753 | FAM177A1 | family with sequence similarity 177 member A1 [Source:HGNC Symbol;Acc:HGNC:19829] |
| ENSG00000153237 | HGLibA_07634 | 304 | 1.615090753 | CCDC148 | coiled-coil domain containing 148 [Source:HGNC Symbol;Acc:HGNC:25191] |
| ENSG00000153976 | HGLibA_22126 | 304 | 1.615090753 | HS3ST3A1 | heparan sulfate-glucosamine 3-sulfotransferase 3A1 [Source:HGNC Symbol;Acc:HGNC:5196] |
| ENSG00000163348 | HGLibA_39696 | 304 | 1.615090753 | PYGO2 | pygopus family PHD finger 2 [Source:HGNC Symbol;Acc:HGNC:30257] |
| ENSG00000166261 | HGLibA_55573 | 304 | 1.615090753 | ZNF202 | zinc finger protein 202 [Source:HGNC Symbol;Acc:HGNC:12994] |
| ENSG00000166341 | HGLibA_12490 | 304 | 1.615090753 | DCHS1 | dachsous cadherin-related 1 [Source:HGNC Symbol;Acc:HGNC:13681] |
| ENSG00000170264 | HGLibA_16217 | 304 | 1.615090753 | FAM161A | centrosomal protein [Source:HGNC Symbol;Acc:HGNC:25808]" |
| ENSG00000175121 | HGLibA_54268 | 304 | 1.615090753 | WFDC5 | WAP four-disulfide core domain 5 [Source:HGNC Symbol;Acc:HGNC:20477] |
| ENSG00000178385 | HGLibA_37142 | 304 | 1.615090753 | PLEKHM3 | pleckstrin homology domain containing M3 [Source:HGNC Symbol;Acc:HGNC:34006] |
| ENSG00000183862 | HGLibA_10266 | 304 | 1.615090753 | CNGA2 | cyclic nucleotide gated channel alpha 2 [Source:HGNC Symbol;Acc:HGNC:2149] |
| ENSG00000186493 | HGLibA_06380 | 304 | 1.615090753 | C5orf38 | chromosome 5 open reading frame 38 [Source:HGNC Symbol;Acc:HGNC:24226] |
| ENSG00000196526 | HGLibA_01097 | 304 | 1.615090753 | AFAP1 | actin filament associated protein 1 [Source:HGNC Symbol;Acc:HGNC:24017] |
| ENSG00000197579 | HGLibA_51013 | 304 | 1.615090753 | TOPORS | TOP1 binding arginine/serine rich protein [Source:HGNC Symbol;Acc:HGNC:21653] |
| ENSG00000198130 | HGLibA_21284 | 304 | 1.615090753 | HIBCH | 3-hydroxyisobutyryl-CoA hydrolase [Source:HGNC Symbol;Acc:HGNC:4908] |
| ENSG00000242221 | HGLibA_39039 | 304 | 1.615090753 | PSG2 | pregnancy specific beta-1-glycoprotein 2 [Source:HGNC Symbol;Acc:HGNC:9519] |
| ENSG00000275410 | HGLibA_21777 | 304 | 1.615090753 | HNF1B | HNF1 homeobox B [Source:HGNC Symbol;Acc:HGNC:11630] |
| ENSG00000280090 | HGLibA_34502 | 304 | 1.615090753 | OR8B4 | olfactory receptor family 8 subfamily B member 4 (gene/pseudogene) [Source:HGNC Symbol;Acc:HGNC:8473] |
| FAM49A | HGLibA_16622 | 304 | 1.615090753 | FAM49A | - |
| hsa-mir-194-1 | HGLibA_58079 | 304 | 1.615090753 | hsa-mir-194-1 | - |
| hsa-mir-3185 | HGLibA_58839 | 304 | 1.615090753 | hsa-mir-3185 | - |
| hsa-mir-4527 | HGLibA_60570 | 304 | 1.615090753 | hsa-mir-4527 | - |
| hsa-mir-5093 | HGLibA_61531 | 304 | 1.615090753 | hsa-mir-5093 | - |
| hsa-mir-6765 | HGLibA_63223 | 304 | 1.615090753 | hsa-mir-6765 | - |
| hsa-mir-7108 | HGLibA_63794 | 304 | 1.615090753 | hsa-mir-7108 | - |
| KIAA0247 | HGLibA_24615 | 304 | 1.615090753 | KIAA0247 | - |
| C9orf173 | HGLibA_06715 | 303 | 1.60604541 | C9orf173 | - |
| ENSG00000010379 | HGLibA_45299 | 303 | 1.60604541 | SLC6A13 | solute carrier family 6 member 13 [Source:HGNC Symbol;Acc:HGNC:11046] |
| ENSG00000055917 | HGLibA_39582 | 303 | 1.60604541 | PUM2 | pumilio RNA binding family member 2 [Source:HGNC Symbol;Acc:HGNC:14958] |
| ENSG00000073734 | HGLibA_00137 | 303 | 1.60604541 | ABCB11 | ATP binding cassette subfamily B member 11 [Source:HGNC Symbol;Acc:HGNC:42] |
| ENSG00000086065 | HGLibA_09497 | 303 | 1.60604541 | CHMP5 | charged multivesicular body protein 5 [Source:HGNC Symbol;Acc:HGNC:26942] |
| ENSG00000089505 | HGLibA_10209 | 303 | 1.60604541 | CMTM1 | CKLF like MARVEL transmembrane domain containing 1 [Source:HGNC Symbol;Acc:HGNC:19172] |
| ENSG00000107447 | HGLibA_13715 | 303 | 1.60604541 | DNTT | DNA nucleotidylexotransferase [Source:HGNC Symbol;Acc:HGNC:2983] |
| ENSG00000107864 | HGLibA_10924 | 303 | 1.60604541 | CPEB3 | cytoplasmic polyadenylation element binding protein 3 [Source:HGNC Symbol;Acc:HGNC:21746] |
| ENSG00000109881 | HGLibA_07761 | 303 | 1.60604541 | CCDC34 | coiled-coil domain containing 34 [Source:HGNC Symbol;Acc:HGNC:25079] |
| ENSG00000113657 | HGLibA_13928 | 303 | 1.60604541 | DPYSL3 | dihydropyrimidinase like 3 [Source:HGNC Symbol;Acc:HGNC:3015] |
| ENSG00000126752 | HGLibA_47195 | 303 | 1.60604541 | SSX1 | SSX family member 1 [Source:HGNC Symbol;Acc:HGNC:11335] |
| ENSG00000131370 | HGLibA_43935 | 303 | 1.60604541 | SH3BP5 | SH3 domain binding protein 5 [Source:HGNC Symbol;Acc:HGNC:10827] |
| ENSG00000131446 | HGLibA_29132 | 303 | 1.60604541 | MGAT1 | 2-N-acetylglucosaminyltransferase [Source:HGNC Symbol;Acc:HGNC:7044]" |
| ENSG00000131759 | HGLibA_40273 | 303 | 1.60604541 | RARA | retinoic acid receptor alpha [Source:HGNC Symbol;Acc:HGNC:9864] |
| ENSG00000131941 | HGLibA_41225 | 303 | 1.60604541 | RHPN2 | rhophilin Rho GTPase binding protein 2 [Source:HGNC Symbol;Acc:HGNC:19974] |
| ENSG00000132965 | HGLibA_01692 | 303 | 1.60604541 | ALOX5AP | arachidonate 5-lipoxygenase activating protein [Source:HGNC Symbol;Acc:HGNC:436] |
| ENSG00000136244 | HGLibA_23180 | 303 | 1.60604541 | IL6 | interleukin 6 [Source:HGNC Symbol;Acc:HGNC:6018] |
| ENSG00000139428 | HGLibA_29453 | 303 | 1.60604541 | MMAB | metabolism of cobalamin associated B [Source:HGNC Symbol;Acc:HGNC:19331] |
| ENSG00000147119 | HGLibA_09645 | 303 | 1.60604541 | CHST7 | carbohydrate sulfotransferase 7 [Source:HGNC Symbol;Acc:HGNC:13817] |
| ENSG00000149657 | HGLibA_27532 | 303 | 1.60604541 | LSM14B | LSM family member 14B [Source:HGNC Symbol;Acc:HGNC:15887] |
| ENSG00000168061 | HGLibA_42624 | 303 | 1.60604541 | SAC3D1 | SAC3 domain containing 1 [Source:HGNC Symbol;Acc:HGNC:30179] |
| ENSG00000169519 | HGLibA_28960 | 303 | 1.60604541 | METTL15 | methyltransferase like 15 [Source:HGNC Symbol;Acc:HGNC:26606] |
| ENSG00000171509 | HGLibA_42493 | 303 | 1.60604541 | RXFP1 | relaxin family peptide receptor 1 [Source:HGNC Symbol;Acc:HGNC:19718] |
| ENSG00000173239 | HGLibA_26627 | 303 | 1.60604541 | LIPM | lipase family member M [Source:HGNC Symbol;Acc:HGNC:23455] |
| ENSG00000179921 | HGLibA_19753 | 303 | 1.60604541 | GPBAR1 | G protein-coupled bile acid receptor 1 [Source:HGNC Symbol;Acc:HGNC:19680] |
| ENSG00000182963 | HGLibA_19179 | 303 | 1.60604541 | GJC1 | gap junction protein gamma 1 [Source:HGNC Symbol;Acc:HGNC:4280] |
| ENSG00000185133 | HGLibA_23358 | 303 | 1.60604541 | INPP5J | inositol polyphosphate-5-phosphatase J [Source:HGNC Symbol;Acc:HGNC:8956] |
| ENSG00000186788 | HGLibA_46499 | 303 | 1.60604541 | SPATA31D3 | SPATA31 subfamily D member 3 [Source:HGNC Symbol;Acc:HGNC:38603] |
| ENSG00000186854 | HGLibA_51230 | 303 | 1.60604541 | TRABD2A | TraB domain containing 2A [Source:HGNC Symbol;Acc:HGNC:27013] |
| ENSG00000188906 | HGLibA_27466 | 303 | 1.60604541 | LRRK2 | leucine rich repeat kinase 2 [Source:HGNC Symbol;Acc:HGNC:18618] |
| ENSG00000196793 | HGLibA_55653 | 303 | 1.60604541 | ZNF239 | zinc finger protein 239 [Source:HGNC Symbol;Acc:HGNC:13031] |
| ENSG00000204410 | HGLibA_30200 | 303 | 1.60604541 | MSH5 | mutS homolog 5 [Source:HGNC Symbol;Acc:HGNC:7328] |
| ENSG00000233041 | HGLibA_36395 | 303 | 1.60604541 | PHGR1 | histidine and glycine rich 1 [Source:HGNC Symbol;Acc:HGNC:37226]" |
| ENSG00000255221 | HGLibA_07230 | 303 | 1.60604541 | CARD17 | caspase recruitment domain family member 17 [Source:HGNC Symbol;Acc:HGNC:33827] |
| hsa-mir-3130-2 | HGLibA_58577 | 303 | 1.60604541 | hsa-mir-3130-2 | - |
| hsa-mir-372 | HGLibA_59382 | 303 | 1.60604541 | hsa-mir-372 | - |
| hsa-mir-4439 | HGLibA_60157 | 303 | 1.60604541 | hsa-mir-4439 | - |
| hsa-mir-4640 | HGLibA_60665 | 303 | 1.60604541 | hsa-mir-4640 | - |
| 8-Mar | HGLibA_28349 | 302 | 1.597000067 | 8-Mar | - |
| C12orf45 | HGLibA_05249 | 302 | 1.597000067 | C12orf45 | - |
| ENSG00000002587 | HGLibA_22120 | 302 | 1.597000067 | HS3ST1 | heparan sulfate-glucosamine 3-sulfotransferase 1 [Source:HGNC Symbol;Acc:HGNC:5194] |
| ENSG00000069206 | HGLibA_00780 | 302 | 1.597000067 | ADAM7 | ADAM metallopeptidase domain 7 [Source:HGNC Symbol;Acc:HGNC:214] |
| ENSG00000076826 | HGLibA_07113 | 302 | 1.597000067 | CAMSAP3 | calmodulin regulated spectrin associated protein family member 3 [Source:HGNC Symbol;Acc:HGNC:29307] |
| ENSG00000101935 | HGLibA_01801 | 302 | 1.597000067 | AMMECR1 | midface hypoplasia and elliptocytosis chromosomal region gene 1 [Source:HGNC Symbol;Acc:HGNC:467]" |

| | | | | | |
|---|---|---|---|---|---|
| ENSG00000108984 | HGLibA_28154 | 302 | 1.597000067 | MAP2K6 | mitogen-activated protein kinase kinase 6 [Source:HGNC Symbol;Acc:HGNC:6846] |
| ENSG00000115170 | HGLibA_00685 | 302 | 1.597000067 | ACVR1 | activin A receptor type 1 [Source:HGNC Symbol;Acc:HGNC:171] |
| ENSG00000115998 | HGLibA_06125 | 302 | 1.597000067 | C2orf42 | chromosome 2 open reading frame 42 [Source:HGNC Symbol;Acc:HGNC:26056] |
| ENSG00000118900 | HGLibA_52721 | 302 | 1.597000067 | UBN1 | ubinuclein 1 [Source:HGNC Symbol;Acc:HGNC:12506] |
| ENSG00000119943 | HGLibA_39707 | 302 | 1.597000067 | PYROXD2 | pyridine nucleotide-disulphide oxidoreductase domain 2 [Source:HGNC Symbol;Acc:HGNC:23517] |
| ENSG00000121634 | HGLibA_19151 | 302 | 1.597000067 | GJA8 | gap junction protein alpha 8 [Source:HGNC Symbol;Acc:HGNC:4281] |
| ENSG00000122986 | HGLibA_22434 | 302 | 1.597000067 | HVCN1 | hydrogen voltage gated channel 1 [Source:HGNC Symbol;Acc:HGNC:28240] |
| ENSG00000123892 | HGLibA_39898 | 302 | 1.597000067 | RAB38 | member RAS oncogene family [Source:HGNC Symbol;Acc:HGNC:9776]" |
| ENSG00000124313 | HGLibA_23544 | 302 | 1.597000067 | IQSEC2 | IQ motif and Sec7 domain 2 [Source:HGNC Symbol;Acc:HGNC:29059] |
| ENSG00000127951 | HGLibA_17466 | 302 | 1.597000067 | FGL2 | fibrinogen like 2 [Source:HGNC Symbol;Acc:HGNC:3696] |
| ENSG00000133863 | HGLibA_49067 | 302 | 1.597000067 | TEX15 | meiosis and synapsis associated [Source:HGNC Symbol;Acc:HGNC:11738]" |
| ENSG00000138085 | HGLibA_03813 | 302 | 1.597000067 | ATRAID | all-trans retinoic acid induced differentiation factor [Source:HGNC Symbol;Acc:HGNC:24090] |
| ENSG00000141985 | HGLibA_43945 | 302 | 1.597000067 | SH3GL1 | endophilin A2 [Source:HGNC Symbol;Acc:HGNC:10830]" |
| ENSG00000144674 | HGLibA_19600 | 302 | 1.597000067 | GOLGA4 | golgin A4 [Source:HGNC Symbol;Acc:HGNC:4427] |
| ENSG00000157191 | HGLibA_31595 | 302 | 1.597000067 | NECAP2 | NECAP endocytosis associated 2 [Source:HGNC Symbol;Acc:HGNC:25528] |
| ENSG00000157193 | HGLibA_27232 | 302 | 1.597000067 | LRP8 | LDL receptor related protein 8 [Source:HGNC Symbol;Acc:HGNC:6700] |
| ENSG00000158806 | HGLibA_32490 | 302 | 1.597000067 | NPM2 | nucleophosmin/nucleoplasmin 2 [Source:HGNC Symbol;Acc:HGNC:7930] |
| ENSG00000160961 | HGLibA_55819 | 302 | 1.597000067 | ZNF333 | zinc finger protein 333 [Source:HGNC Symbol;Acc:HGNC:15624] |
| ENSG00000164929 | HGLibA_04051 | 302 | 1.597000067 | BAALC | MAP3K1 and KLF4 binding [Source:HGNC Symbol;Acc:HGNC:14333]" |
| ENSG00000166140 | HGLibA_55277 | 302 | 1.597000067 | ZFYVE19 | zinc finger FYVE-type containing 19 [Source:HGNC Symbol;Acc:HGNC:20758] |
| ENSG00000167257 | HGLibA_41624 | 302 | 1.597000067 | RNF214 | ring finger protein 214 [Source:HGNC Symbol;Acc:HGNC:25335] |
| ENSG00000168000 | HGLibA_04860 | 302 | 1.597000067 | BSCL2 | seipin lipid droplet biogenesis associated [Source:HGNC Symbol;Acc:HGNC:15832]" |
| ENSG00000173409 | HGLibA_03172 | 302 | 1.597000067 | ARV1 | fatty acid homeostasis modulator [Source:HGNC Symbol;Acc:HGNC:29561]" |
| ENSG00000182983 | HGLibA_56479 | 302 | 1.597000067 | ZNF662 | zinc finger protein 662 [Source:HGNC Symbol;Acc:HGNC:31930] |
| ENSG00000185090 | HGLibA_28095 | 302 | 1.597000067 | MANEAL | mannosidase endo-alpha like [Source:HGNC Symbol;Acc:HGNC:26452] |
| ENSG00000188937 | HGLibA_33188 | 302 | 1.597000067 | NYX | nyctalopin [Source:HGNC Symbol;Acc:HGNC:8082] |
| ENSG00000196268 | HGLibA_56098 | 302 | 1.597000067 | ZNF493 | zinc finger protein 493 [Source:HGNC Symbol;Acc:HGNC:23708] |
| ENSG00000204859 | HGLibA_54904 | 302 | 1.597000067 | ZBTB48 | zinc finger and BTB domain containing 48 [Source:HGNC Symbol;Acc:HGNC:4930] |
| ENSG00000205531 | HGLibA_31120 | 302 | 1.597000067 | NAP1L4 | nucleosome assembly protein 1 like 4 [Source:HGNC Symbol;Acc:HGNC:7640] |
| hsa-mir-4535 | HGLibA_60601 | 302 | 1.597000067 | hsa-mir-4535 | - |
| hsa-mir-4690 | HGLibA_60869 | 302 | 1.597000067 | hsa-mir-4690 | - |
| ENSG00000007516 | HGLibA_04131 | 301 | 1.587954724 | BAIAP3 | BAI1 associated protein 3 [Source:HGNC Symbol;Acc:HGNC:948] |
| ENSG00000018280 | HGLibA_44351 | 301 | 1.587954724 | SLC11A1 | solute carrier family 11 member 1 [Source:HGNC Symbol;Acc:HGNC:10907] |
| ENSG00000038358 | HGLibA_14445 | 301 | 1.587954724 | EDC4 | enhancer of mRNA decapping 4 [Source:HGNC Symbol;Acc:HGNC:17157] |
| ENSG00000066382 | HGLibA_29773 | 301 | 1.587954724 | MPPED2 | metallophosphoesterase domain containing 2 [Source:HGNC Symbol;Acc:HGNC:1180] |
| ENSG00000068615 | HGLibA_40777 | 301 | 1.587954724 | REEP1 | receptor accessory protein 1 [Source:HGNC Symbol;Acc:HGNC:25786] |
| ENSG00000096717 | HGLibA_44217 | 301 | 1.587954724 | SIRT1 | sirtuin 1 [Source:HGNC Symbol;Acc:HGNC:14929] |
| ENSG00000103460 | HGLibA_51041 | 301 | 1.587954724 | TOX3 | TOX high mobility group box family member 3 [Source:HGNC Symbol;Acc:HGNC:11972] |
| ENSG00000106268 | HGLibA_32932 | 301 | 1.587954724 | NUDT1 | nudix hydrolase 1 [Source:HGNC Symbol;Acc:HGNC:8048] |
| ENSG00000109680 | HGLibA_48491 | 301 | 1.587954724 | TBC1D19 | TBC1 domain family member 19 [Source:HGNC Symbol;Acc:HGNC:25624] |
| ENSG00000129083 | HGLibA_10695 | 301 | 1.587954724 | COPB1 | coatomer protein complex subunit beta 1 [Source:HGNC Symbol;Acc:HGNC:2231] |
| ENSG00000134759 | HGLibA_14978 | 301 | 1.587954724 | ELP2 | elongator acetyltransferase complex subunit 2 [Source:HGNC Symbol;Acc:HGNC:18248] |
| ENSG00000138395 | HGLibA_08740 | 301 | 1.587954724 | CDK15 | cyclin dependent kinase 15 [Source:HGNC Symbol;Acc:HGNC:14434] |
| ENSG00000139292 | HGLibA_26411 | 301 | 1.587954724 | LGR5 | leucine rich repeat containing G protein-coupled receptor 5 [Source:HGNC Symbol;Acc:HGNC:4504] |
| ENSG00000140464 | HGLibA_37311 | 301 | 1.587954724 | PML | promyelocytic leukemia [Source:HGNC Symbol;Acc:HGNC:9113] |
| ENSG00000141298 | HGLibA_47136 | 301 | 1.587954724 | SSH2 | slingshot protein phosphatase 2 [Source:HGNC Symbol;Acc:HGNC:30580] |
| ENSG00000147526 | HGLibA_48159 | 301 | 1.587954724 | TACC1 | transforming acidic coiled-coil containing protein 1 [Source:HGNC Symbol;Acc:HGNC:11522] |
| ENSG00000152422 | HGLibA_54592 | 301 | 1.587954724 | XRCC4 | X-ray repair cross complementing 4 [Source:HGNC Symbol;Acc:HGNC:12831] |
| ENSG00000154222 | HGLibA_07477 | 301 | 1.587954724 | CC2D1B | coiled-coil and C2 domain containing 1B [Source:HGNC Symbol;Acc:HGNC:29386] |
| ENSG00000156398 | HGLibA_43784 | 301 | 1.587954724 | SFXN2 | sideroflexin 2 [Source:HGNC Symbol;Acc:HGNC:16086] |
| ENSG00000158793 | HGLibA_31982 | 301 | 1.587954724 | NIT1 | nitrilase 1 [Source:HGNC Symbol;Acc:HGNC:7828] |
| ENSG00000163380 | HGLibA_26723 | 301 | 1.587954724 | LMOD3 | leiomodin 3 [Source:HGNC Symbol;Acc:HGNC:6649] |
| ENSG00000166823 | HGLibA_28915 | 301 | 1.587954724 | MESP1 | mesoderm posterior bHLH transcription factor 1 [Source:HGNC Symbol;Acc:HGNC:29658] |
| ENSG00000170291 | HGLibA_14987 | 301 | 1.587954724 | ELP5 | elongator acetyltransferase complex subunit 5 [Source:HGNC Symbol;Acc:HGNC:30617] |
| ENSG00000176787 | HGLibA_34158 | 301 | 1.587954724 | OR52E2 | olfactory receptor family 52 subfamily E member 2 [Source:HGNC Symbol;Acc:HGNC:14769] |
| ENSG00000177202 | HGLibA_46330 | 301 | 1.587954724 | SPACA4 | sperm acrosome associated 4 [Source:HGNC Symbol;Acc:HGNC:16441] |
| ENSG00000179399 | HGLibA_19775 | 301 | 1.587954724 | GPC5 | glypican 5 [Source:HGNC Symbol;Acc:HGNC:4453] |
| ENSG00000179922 | HGLibA_56745 | 301 | 1.587954724 | ZNF784 | zinc finger protein 784 [Source:HGNC Symbol;Acc:HGNC:33111] |
| ENSG00000179958 | HGLibA_12563 | 301 | 1.587954724 | DCTPP1 | dCTP pyrophosphatase 1 [Source:HGNC Symbol;Acc:HGNC:28777] |
| ENSG00000180176 | HGLibA_49280 | 301 | 1.587954724 | TH | tyrosine hydroxylase [Source:HGNC Symbol;Acc:HGNC:11782] |
| ENSG00000182199 | HGLibA_44064 | 301 | 1.587954724 | SHMT2 | serine hydroxymethyltransferase 2 [Source:HGNC Symbol;Acc:HGNC:10852] |
| ENSG00000182372 | HGLibA_10085 | 301 | 1.587954724 | CLN8 | transmembrane ER and ERGIC protein [Source:HGNC Symbol;Acc:HGNC:2079]" |
| ENSG00000184205 | HGLibA_51981 | 301 | 1.587954724 | TSPYL2 | TSPY like 2 [Source:HGNC Symbol;Acc:HGNC:24358] |
| ENSG00000187531 | HGLibA_44234 | 301 | 1.587954724 | SIRT7 | sirtuin 7 [Source:HGNC Symbol;Acc:HGNC:14935] |
| ENSG00000196475 | HGLibA_19198 | 301 | 1.587954724 | GK2 | glycerol kinase 2 [Source:HGNC Symbol;Acc:HGNC:4291] |
| ENSG00000197753 | HGLibA_26440 | 301 | 1.587954724 | LHFPL5 | LHFPL tetraspan subfamily member 5 [Source:HGNC Symbol;Acc:HGNC:21253] |
| ENSG00000198794 | HGLibA_42848 | 301 | 1.587954724 | SCAMP5 | secretory carrier membrane protein 5 [Source:HGNC Symbol;Acc:HGNC:30386] |
| ENSG00000205358 | HGLibA_30293 | 301 | 1.587954724 | MT1H | metallothionein 1H [Source:HGNC Symbol;Acc:HGNC:7400] |
| hsa-mir-3689f | HGLibA_59336 | 301 | 1.587954724 | hsa-mir-3689f | - |
| hsa-mir-548h-3 | HGLibA_62000 | 301 | 1.587954724 | hsa-mir-548h-3 | - |
| ENSG00000023445 | HGLibA_04518 | 300 | 1.578909381 | BIRC3 | baculoviral IAP repeat containing 3 [Source:HGNC Symbol;Acc:HGNC:591] |
| ENSG00000095261 | HGLibA_39175 | 300 | 1.578909381 | PSMD5 | non-ATPase 5 [Source:HGNC Symbol;Acc:HGNC:9563]" |
| ENSG00000105971 | HGLibA_07379 | 300 | 1.578909381 | CAV2 | caveolin 2 [Source:HGNC Symbol;Acc:HGNC:1528] |
| ENSG00000107872 | HGLibA_16982 | 300 | 1.578909381 | FBXL15 | F-box and leucine rich repeat protein 15 [Source:HGNC Symbol;Acc:HGNC:28155] |
| ENSG00000112031 | HGLibA_30470 | 300 | 1.578909381 | MTRF1L | mitochondrial translational release factor 1 like [Source:HGNC Symbol;Acc:HGNC:21051] |
| ENSG00000115386 | HGLibA_40794 | 300 | 1.578909381 | REG1A | regenerating family member 1 alpha [Source:HGNC Symbol;Acc:HGNC:9951] |
| ENSG00000132003 | HGLibA_57047 | 300 | 1.578909381 | ZSWIM4 | zinc finger SWIM-type containing 4 [Source:HGNC Symbol;Acc:HGNC:25704] |
| ENSG00000145990 | HGLibA_18952 | 300 | 1.578909381 | GFOD1 | glucose-fructose oxidoreductase domain containing 1 [Source:HGNC Symbol;Acc:HGNC:21096] |
| ENSG00000147804 | HGLibA_45112 | 300 | 1.578909381 | SLC39A4 | solute carrier family 39 member 4 [Source:HGNC Symbol;Acc:HGNC:17129] |
| ENSG00000150961 | HGLibA_43239 | 300 | 1.578909381 | SEC24D | COPII coat complex component [Source:HGNC Symbol;Acc:HGNC:10706]" |
| ENSG00000151116 | HGLibA_52839 | 300 | 1.578909381 | UEVLD | UEV and lactate/malate dehyrogenase domains [Source:HGNC Symbol;Acc:HGNC:30866] |
| ENSG00000159263 | HGLibA_44177 | 300 | 1.578909381 | SIM2 | SIM bHLH transcription factor 2 [Source:HGNC Symbol;Acc:HGNC:10883] |
| ENSG00000163082 | HGLibA_43853 | 300 | 1.578909381 | SGPP2 | sphingosine-1-phosphate phosphatase 2 [Source:HGNC Symbol;Acc:HGNC:19953] |
| ENSG00000163530 | HGLibA_13886 | 300 | 1.578909381 | DPPA2 | developmental pluripotency associated 2 [Source:HGNC Symbol;Acc:HGNC:19197] |
| ENSG00000166272 | HGLibA_53974 | 300 | 1.578909381 | WBP1L | WW domain binding protein 1 like [Source:HGNC Symbol;Acc:HGNC:23510] |
| ENSG00000167634 | HGLibA_32130 | 300 | 1.578909381 | NLRP7 | NLR family pyrin domain containing 7 [Source:HGNC Symbol;Acc:HGNC:22947] |
| ENSG00000170430 | HGLibA_29166 | 300 | 1.578909381 | MGMT | O-6-methylguanine-DNA methyltransferase [Source:HGNC Symbol;Acc:HGNC:7059] |
| ENSG00000171860 | HGLibA_06206 | 300 | 1.578909381 | C3AR1 | complement C3a receptor 1 [Source:HGNC Symbol;Acc:HGNC:1319] |
| ENSG00000173068 | HGLibA_04648 | 300 | 1.578909381 | BNC2 | basonuclin 2 [Source:HGNC Symbol;Acc:HGNC:30988] |
| ENSG00000174600 | HGLibA_10196 | 300 | 1.578909381 | CMKLR1 | chemerin chemokine-like receptor 1 [Source:HGNC Symbol;Acc:HGNC:2121] |
| ENSG00000180475 | HGLibA_33539 | 300 | 1.578909381 | OR10Q1 | olfactory receptor family 10 subfamily Q member 1 [Source:HGNC Symbol;Acc:HGNC:15134] |
| ENSG00000183317 | HGLibA_15288 | 300 | 1.578909381 | EPHA10 | EPH receptor A10 [Source:HGNC Symbol;Acc:HGNC:19987] |
| ENSG00000196578 | HGLibA_34235 | 300 | 1.578909381 | OR5AC2 | olfactory receptor family 5 subfamily AC member 2 [Source:HGNC Symbol;Acc:HGNC:15431] |
| ENSG00000197943 | HGLibA_37006 | 300 | 1.578909381 | PLCG2 | phospholipase C gamma 2 [Source:HGNC Symbol;Acc:HGNC:9066] |
| ENSG00000198633 | HGLibA_56182 | 300 | 1.578909381 | ZNF534 | zinc finger protein 534 [Source:HGNC Symbol;Acc:HGNC:26337] |
| ENSG00000259384 | HGLibA_19028 | 300 | 1.578909381 | GH1 | growth hormone 1 [Source:HGNC Symbol;Acc:HGNC:4261] |
| FAM86A | HGLibA_16771 | 300 | 1.578909381 | FAM86A | - |
| H1FX | HGLibA_20743 | 300 | 1.578909381 | H1FX | - |
| hsa-mir-4721 | HGLibA_60980 | 300 | 1.578909381 | hsa-mir-4721 | - |
| hsa-mir-8071-2 | HGLibA_64128 | 300 | 1.578909381 | hsa-mir-8071-2 | - |
| KDELC2 | HGLibA_24475 | 300 | 1.578909381 | KDELC2 | - |
| LOC100507462 | HGLibA_26871 | 300 | 1.578909381 | LOC100507462 | - |
| TMEM8A | HGLibA_50547 | 300 | 1.578909381 | TMEM8A | - |
| ENSG00000003987 | HGLibA_30435 | 299 | 1.569864039 | MTMR7 | myotubularin related protein 7 [Source:HGNC Symbol;Acc:HGNC:7454] |
| ENSG00000009765 | HGLibA_23888 | 299 | 1.569864039 | IYD | iodotyrosine deiodinase [Source:HGNC Symbol;Acc:HGNC:21071] |
| ENSG00000010438 | HGLibA_38922 | 299 | 1.569864039 | PRSS3 | serine protease 3 [Source:HGNC Symbol;Acc:HGNC:9486] |
| ENSG00000011028 | HGLibA_29820 | 299 | 1.569864039 | MRC2 | mannose receptor C type 2 [Source:HGNC Symbol;Acc:HGNC:16875] |
| ENSG00000021355 | HGLibA_43534 | 299 | 1.569864039 | SERPINB1 | serpin family B member 1 [Source:HGNC Symbol;Acc:HGNC:3311] |
| ENSG00000071242 | HGLibA_42160 | 299 | 1.569864039 | RPS6KA2 | ribosomal protein S6 kinase A2 [Source:HGNC Symbol;Acc:HGNC:10431] |
| ENSG00000101104 | HGLibA_34924 | 299 | 1.569864039 | PABPC1L | poly(A) binding protein cytoplasmic 1 like [Source:HGNC Symbol;Acc:HGNC:15797] |
| ENSG00000105697 | HGLibA_20823 | 299 | 1.569864039 | HAMP | hepcidin antimicrobial peptide [Source:HGNC Symbol;Acc:HGNC:15598] |
| ENSG00000131773 | HGLibA_24573 | 299 | 1.569864039 | KHDRBS3 | signal transduction associated 3 [Source:HGNC Symbol;Acc:HGNC:18117]" |
| ENSG00000134762 | HGLibA_13995 | 299 | 1.569864039 | DSC3 | desmocollin 3 [Source:HGNC Symbol;Acc:HGNC:3037] |
| ENSG00000138594 | HGLibA_50599 | 299 | 1.569864039 | TMOD3 | tropomodulin 3 [Source:HGNC Symbol;Acc:HGNC:11873] |
| ENSG00000142405 | HGLibA_32107 | 299 | 1.569864039 | NLRP12 | NLR family pyrin domain containing 12 [Source:HGNC Symbol;Acc:HGNC:22938] |
| ENSG00000146267 | HGLibA_16928 | 299 | 1.569864039 | FAXC | failed axon connections homolog [Source:HGNC Symbol;Acc:HGNC:20742] |
| ENSG00000157992 | HGLibA_25858 | 299 | 1.569864039 | KRTCAP3 | keratinocyte associated protein 3 [Source:HGNC Symbol;Acc:HGNC:28943] |
| ENSG00000158163 | HGLibA_14302 | 299 | 1.569864039 | DZIP1L | DAZ interacting zinc finger protein 1 like [Source:HGNC Symbol;Acc:HGNC:26551] |
| ENSG00000158480 | HGLibA_46445 | 299 | 1.569864039 | SPATA2 | spermatogenesis associated 2 [Source:HGNC Symbol;Acc:HGNC:14681] |
| ENSG00000164056 | HGLibA_46835 | 299 | 1.569864039 | SPRY1 | sprouty RTK signaling antagonist 1 [Source:HGNC Symbol;Acc:HGNC:11269] |
| ENSG00000165863 | HGLibA_05097 | 299 | 1.569864039 | C10orf82 | chromosome 10 open reading frame 82 [Source:HGNC Symbol;Acc:HGNC:28500] |
| ENSG00000166292 | HGLibA_49837 | 299 | 1.569864039 | TMEM100 | transmembrane protein 100 [Source:HGNC Symbol;Acc:HGNC:25607] |
| ENSG00000170683 | HGLibA_33465 | 299 | 1.569864039 | OR10A3 | olfactory receptor family 10 subfamily A member 3 [Source:HGNC Symbol;Acc:HGNC:8162] |
| ENSG00000178343 | HGLibA_44035 | 299 | 1.569864039 | SHISA3 | shisa family member 3 [Source:HGNC Symbol;Acc:HGNC:25159] |
| ENSG00000181007 | HGLibA_55237 | 299 | 1.569864039 | ZFP82 | ZFP82 zinc finger protein [Source:HGNC Symbol;Acc:HGNC:28682] |
| ENSG00000182175 | HGLibA_41013 | 299 | 1.569864039 | RGMA | repulsive guidance molecule BMP co-receptor a [Source:HGNC Symbol;Acc:HGNC:30308] |
| ENSG00000183647 | HGLibA_56175 | 299 | 1.569864039 | ZNF530 | zinc finger protein 530 [Source:HGNC Symbol;Acc:HGNC:29297] |
| ENSG00000188986 | HGLibA_31674 | 299 | 1.569864039 | NELFB | negative elongation factor complex member B [Source:HGNC Symbol;Acc:HGNC:24324] |
| ENSG00000197694 | HGLibA_46873 | 299 | 1.569864039 | SPTAN1 | non-erythrocytic 1 [Source:HGNC Symbol;Acc:HGNC:11273]" |
| ENSG00000198663 | HGLibA_06512 | 299 | 1.569864039 | C6orf89 | chromosome 6 open reading frame 89 [Source:HGNC Symbol;Acc:HGNC:21114] |
| ENSG00000198682 | HGLibA_35154 | 299 | 1.569864039 | PAPSS2 | 3'-phosphoadenosine 5'-phosphosulfate synthase 2 [Source:HGNC Symbol;Acc:HGNC:8604] |
| ENSG00000204592 | HGLibA_21641 | 299 | 1.569864039 | HLA-E | E [Source:HGNC Symbol;Acc:HGNC:4962]" |
| ENSG00000236980 | HGLibA_06279 | 299 | 1.569864039 | C3orf84 | chromosome 3 open reading frame 84 [Source:HGNC Symbol;Acc:HGNC:44666] |
| ENSG00000249158 | HGLibA_35429 | 299 | 1.569864039 | PCDHA11 | protocadherin alpha 11 [Source:HGNC Symbol;Acc:HGNC:8665] |
| ENSG00000250799 | HGLibA_38663 | 299 | 1.569864039 | PRODH2 | proline dehydrogenase 2 [Source:HGNC Symbol;Acc:HGNC:17325] |

| | | | | | |
|---|---|---|---|---|---|
| ENSG00000267467 | HGLibA_02512 | 299 | 1.569864039 | APOC4 | apolipoprotein C4 [Source:HGNC Symbol;Acc:HGNC:611] |
| FAM19A1 | HGLibA_16397 | 299 | 1.569864039 | FAM19A1 | - |
| hsa-mir-135a-1 | HGLibA_57791 | 299 | 1.569864039 | hsa-mir-135a-1 | - |
| hsa-mir-8060 | HGLibA_64079 | 299 | 1.569864039 | hsa-mir-8060 | - |
| RLTPR | HGLibA_41361 | 299 | 1.569864039 | RLTPR | - |
| SEPP1 | HGLibA_43422 | 299 | 1.569864039 | SEPP1 | - |
| ADSSL1 | HGLibA_01079 | 298 | 1.560818696 | ADSSL1 | - |
| C9orf41 | HGLibA_06731 | 298 | 1.560818696 | C9orf41 | - |
| ENSG00000004468 | HGLibA_08366 | 298 | 1.560818696 | CD38 | CD38 molecule [Source:HGNC Symbol;Acc:HGNC:1667] |
| ENSG00000006740 | HGLibA_02781 | 298 | 1.560818696 | ARHGAP44 | Rho GTPase activating protein 44 [Source:HGNC Symbol;Acc:HGNC:29096] |
| ENSG00000014914 | HGLibA_30414 | 298 | 1.560818696 | MTMR11 | myotubularin related protein 11 [Source:HGNC Symbol;Acc:HGNC:24307] |
| ENSG00000048405 | HGLibA_56785 | 298 | 1.560818696 | ZNF800 | zinc finger protein 800 [Source:HGNC Symbol;Acc:HGNC:27267] |
| ENSG00000072182 | HGLibA_03309 | 298 | 1.560818696 | ASIC4 | acid sensing ion channel subunit family member 4 [Source:HGNC Symbol;Acc:HGNC:21263] |
| ENSG00000087495 | HGLibA_36304 | 298 | 1.560818696 | PHACTR3 | phosphatase and actin regulator 3 [Source:HGNC Symbol;Acc:HGNC:15833] |
| ENSG00000099974 | HGLibA_12644 | 298 | 1.560818696 | DDTL | D-dopachrome tautomerase like [Source:HGNC Symbol;Acc:HGNC:33446] |
| ENSG00000100139 | HGLibA_29214 | 298 | 1.560818696 | MICALL1 | MICAL like 1 [Source:HGNC Symbol;Acc:HGNC:29804] |
| ENSG00000105821 | HGLibA_13593 | 298 | 1.560818696 | DNAJC2 | DnaJ heat shock protein family (Hsp40) member C2 [Source:HGNC Symbol;Acc:HGNC:13192] |
| ENSG00000107897 | HGLibA_00404 | 298 | 1.560818696 | ACBD5 | acyl-CoA binding domain containing 5 [Source:HGNC Symbol;Acc:HGNC:23338] |
| ENSG00000112137 | HGLibA_36297 | 298 | 1.560818696 | PHACTR1 | phosphatase and actin regulator 1 [Source:HGNC Symbol;Acc:HGNC:20990] |
| ENSG00000112514 | HGLibA_11798 | 298 | 1.560818696 | CUTA | cutA divalent cation tolerance homolog [Source:HGNC Symbol;Acc:HGNC:21101] |
| ENSG00000120658 | HGLibA_15159 | 298 | 1.560818696 | ENOX1 | ecto-NOX disulfide-thiol exchanger 1 [Source:HGNC Symbol;Acc:HGNC:25474] |
| ENSG00000122643 | HGLibA_32813 | 298 | 1.560818696 | NT5C3A | cytosolic IIIA [Source:HGNC Symbol;Acc:HGNC:17820]" |
| ENSG00000125355 | HGLibA_50330 | 298 | 1.560818696 | TMEM255A | transmembrane protein 255A [Source:HGNC Symbol;Acc:HGNC:26086] |
| ENSG00000128536 | HGLibA_08703 | 298 | 1.560818696 | CDHR3 | cadherin related family member 3 [Source:HGNC Symbol;Acc:HGNC:26308] |
| ENSG00000131263 | HGLibA_41348 | 298 | 1.560818696 | RLIM | LIM domain interacting [Source:HGNC Symbol;Acc:HGNC:13429]" |
| ENSG00000134200 | HGLibA_51856 | 298 | 1.560818696 | TSHB | thyroid stimulating hormone beta [Source:HGNC Symbol;Acc:HGNC:12372] |
| ENSG00000134830 | HGLibA_06357 | 298 | 1.560818696 | C5AR2 | complement component 5a receptor 2 [Source:HGNC Symbol;Acc:HGNC:4527] |
| ENSG00000135083 | HGLibA_08110 | 298 | 1.560818696 | CCNJL | cyclin J like [Source:HGNC Symbol;Acc:HGNC:25876] |
| ENSG00000138443 | HGLibA_00292 | 298 | 1.560818696 | ABI2 | abl interactor 2 [Source:HGNC Symbol;Acc:HGNC:24011] |
| ENSG00000138653 | HGLibA_31434 | 298 | 1.560818696 | NDST4 | N-deacetylase and N-sulfotransferase 4 [Source:HGNC Symbol;Acc:HGNC:20779] |
| ENSG00000141052 | HGLibA_30878 | 298 | 1.560818696 | MYOCD | myocardin [Source:HGNC Symbol;Acc:HGNC:16067] |
| ENSG00000148584 | HGLibA_00004 | 298 | 1.560818696 | A1CF | APOBEC1 complementation factor [Source:HGNC Symbol;Acc:HGNC:24086] |
| ENSG00000151640 | HGLibA_13932 | 298 | 1.560818696 | DPYSL4 | dihydropyrimidinase like 4 [Source:HGNC Symbol;Acc:HGNC:3016] |
| ENSG00000157578 | HGLibA_26094 | 298 | 1.560818696 | LCA5L | lebercilin like [Source:HGNC Symbol;Acc:HGNC:1255]" |
| ENSG00000171431 | HGLibA_25448 | 298 | 1.560818696 | KRT20 | keratin 20 [Source:HGNC Symbol;Acc:HGNC:20412] |
| ENSG00000176014 | HGLibA_52291 | 298 | 1.560818696 | TUBB6 | tubulin beta 6 class V [Source:HGNC Symbol;Acc:HGNC:20776] |
| ENSG00000179583 | HGLibA_09705 | 298 | 1.560818696 | CIITA | class II major histocompatibility complex transactivator [Source:HGNC Symbol;Acc:HGNC:7067] |
| ENSG00000179981 | HGLibA_51862 | 298 | 1.560818696 | TSHZ1 | teashirt zinc finger homeobox 1 [Source:HGNC Symbol;Acc:HGNC:10669] |
| ENSG00000187079 | HGLibA_48927 | 298 | 1.560818696 | TEAD1 | TEA domain transcription factor 1 [Source:HGNC Symbol;Acc:HGNC:11714] |
| ENSG00000188157 | HGLibA_01250 | 298 | 1.560818696 | AGRN | agrin [Source:HGNC Symbol;Acc:HGNC:329] |
| ENSG00000197122 | HGLibA_46927 | 298 | 1.560818696 | SRC | non-receptor tyrosine kinase [Source:HGNC Symbol;Acc:HGNC:11283]" |
| ENSG00000197165 | HGLibA_47735 | 298 | 1.560818696 | SULT1A2 | sulfotransferase family 1A member 2 [Source:HGNC Symbol;Acc:HGNC:11454] |
| ENSG00000198276 | HGLibA_52817 | 298 | 1.560818696 | UCKL1 | uridine-cytidine kinase 1 like 1 [Source:HGNC Symbol;Acc:HGNC:15938] |
| ENSG00000206474 | HGLibA_33484 | 298 | 1.560818696 | OR10C1 | olfactory receptor family 10 subfamily C member 1 (gene/pseudogene) [Source:HGNC Symbol;Acc:HGNC:8165] |
| ENSG00000250151 | HGLibA_03090 | 298 | 1.560818696 | ARPC4-TTLL3 | ARPC4-TTLL3 readthrough [Source:HGNC Symbol;Acc:HGNC:38830] |
| FAM159A | HGLibA_16197 | 298 | 1.560818696 | FAM159A | - |
| hsa-mir-103b-2 | HGLibA_57172 | 298 | 1.560818696 | hsa-mir-103b-2 | - |
| hsa-mir-3125 | HGLibA_58551 | 298 | 1.560818696 | hsa-mir-3125 | - |
| hsa-mir-5090 | HGLibA_61520 | 298 | 1.560818696 | hsa-mir-5090 | - |
| hsa-mir-595 | HGLibA_62471 | 298 | 1.560818696 | hsa-mir-595 | - |
| ENSG00000009950 | HGLibA_29445 | 297 | 1.551773353 | MLXIPL | MLX interacting protein like [Source:HGNC Symbol;Acc:HGNC:12744] |
| ENSG00000081692 | HGLibA_23955 | 297 | 1.551773353 | JMJD4 | jumonji domain containing 4 [Source:HGNC Symbol;Acc:HGNC:25724] |
| ENSG00000088448 | HGLibA_01990 | 297 | 1.551773353 | ANKRD10 | ankyrin repeat domain 10 [Source:HGNC Symbol;Acc:HGNC:20265] |
| ENSG00000107295 | HGLibA_43948 | 297 | 1.551773353 | SH3GL2 | endophilin A1 [Source:HGNC Symbol;Acc:HGNC:10831]" |
| ENSG00000110047 | HGLibA_14665 | 297 | 1.551773353 | EHD1 | EH domain containing 1 [Source:HGNC Symbol;Acc:HGNC:3242] |
| ENSG00000112486 | HGLibA_08161 | 297 | 1.551773353 | CCR6 | C-C motif chemokine receptor 6 [Source:HGNC Symbol;Acc:HGNC:1607] |
| ENSG00000119917 | HGLibA_22631 | 297 | 1.551773353 | IFIT3 | interferon induced protein with tetratricopeptide repeats 3 [Source:HGNC Symbol;Acc:HGNC:5411] |
| ENSG00000120549 | HGLibA_24720 | 297 | 1.551773353 | KIAA1217 | KIAA1217 [Source:HGNC Symbol;Acc:HGNC:25428] |
| ENSG00000124767 | HGLibA_19283 | 297 | 1.551773353 | GLO1 | glyoxalase I [Source:HGNC Symbol;Acc:HGNC:4323] |
| ENSG00000130595 | HGLibA_50889 | 297 | 1.551773353 | TNNT3 | fast skeletal type [Source:HGNC Symbol;Acc:HGNC:11950]" |
| ENSG00000132330 | HGLibA_42954 | 297 | 1.551773353 | SCLY | selenocysteine lyase [Source:HGNC Symbol;Acc:HGNC:18161] |
| ENSG00000138792 | HGLibA_15165 | 297 | 1.551773353 | ENPEP | glutamyl aminopeptidase [Source:HGNC Symbol;Acc:HGNC:3355] |
| ENSG00000157111 | HGLibA_50059 | 297 | 1.551773353 | TMEM171 | transmembrane protein 171 [Source:HGNC Symbol;Acc:HGNC:27031] |
| ENSG00000158079 | HGLibA_39413 | 297 | 1.551773353 | PTPDC1 | protein tyrosine phosphatase domain containing 1 [Source:HGNC Symbol;Acc:HGNC:30184] |
| ENSG00000162714 | HGLibA_56102 | 297 | 1.551773353 | ZNF496 | zinc finger protein 496 [Source:HGNC Symbol;Acc:HGNC:23713] |
| ENSG00000164850 | HGLibA_19796 | 297 | 1.551773353 | GPER1 | G protein-coupled estrogen receptor 1 [Source:HGNC Symbol;Acc:HGNC:4485] |
| ENSG00000167323 | HGLibA_47442 | 297 | 1.551773353 | STIM1 | stromal interaction molecule 1 [Source:HGNC Symbol;Acc:HGNC:11386] |
| ENSG00000169136 | HGLibA_03443 | 297 | 1.551773353 | ATF5 | activating transcription factor 5 [Source:HGNC Symbol;Acc:HGNC:790] |
| ENSG00000169249 | HGLibA_56979 | 297 | 1.551773353 | ZRSR2 | RNA binding motif and serine/arginine rich 2 [Source:HGNC Symbol;Acc:HGNC:23019]" |
| ENSG00000171360 | HGLibA_25497 | 297 | 1.551773353 | KRT38 | keratin 38 [Source:HGNC Symbol;Acc:HGNC:6456] |
| ENSG00000174516 | HGLibA_36039 | 297 | 1.551773353 | PELI3 | pellino E3 ubiquitin protein ligase family member 3 [Source:HGNC Symbol;Acc:HGNC:30010] |
| ENSG00000176463 | HGLibA_45467 | 297 | 1.551773353 | SLCO3A1 | solute carrier organic anion transporter family member 3A1 [Source:HGNC Symbol;Acc:HGNC:10952] |
| ENSG00000177468 | HGLibA_33373 | 297 | 1.551773353 | OLIG3 | oligodendrocyte transcription factor 3 [Source:HGNC Symbol;Acc:HGNC:18003] |
| ENSG00000179603 | HGLibA_20363 | 297 | 1.551773353 | GRM8 | glutamate metabotropic receptor 8 [Source:HGNC Symbol;Acc:HGNC:4600] |
| ENSG00000196418 | HGLibA_55456 | 297 | 1.551773353 | ZNF124 | zinc finger protein 124 [Source:HGNC Symbol;Acc:HGNC:12907] |
| ENSG00000203795 | HGLibA_16546 | 297 | 1.551773353 | FAM24A | family with sequence similarity 24 member A [Source:HGNC Symbol;Acc:HGNC:23470] |
| ENSG00000213347 | HGLibA_30613 | 297 | 1.551773353 | MXD3 | MAX dimerization protein 3 [Source:HGNC Symbol;Acc:HGNC:14008] |
| ENSG00000234776 | HGLibA_05218 | 297 | 1.551773353 | C11orf94 | chromosome 11 open reading frame 94 [Source:HGNC Symbol;Acc:HGNC:37213] |
| ENSG00000236320 | HGLibA_45494 | 297 | 1.551773353 | SLFN14 | schlafen family member 14 [Source:HGNC Symbol;Acc:HGNC:32689] |
| ENSG00000273274 | HGLibA_54927 | 297 | 1.551773353 | ZBTB8B | zinc finger and BTB domain containing 8B [Source:HGNC Symbol;Acc:HGNC:37057] |
| H3F3B | HGLibA_20780 | 297 | 1.551773353 | H3F3B | - |
| hsa-mir-4675 | HGLibA_60816 | 297 | 1.551773353 | hsa-mir-4675 | - |
| KIAA1731 | HGLibA_24811 | 297 | 1.551773353 | KIAA1731 | - |
| WIBG | HGLibA_54297 | 297 | 1.551773353 | WIBG | - |
| ZBED1_X | HGLibA_54787 | 297 | 1.551773353 | ZBED1_X | - |
| C14orf79 | HGLibA_05368 | 296 | 1.54272801 | C14orf79 | - |
| ENSG00000004846 | HGLibA_00144 | 296 | 1.54272801 | ABCB5 | ATP binding cassette subfamily B member 5 [Source:HGNC Symbol;Acc:HGNC:46] |
| ENSG00000102466 | HGLibA_17376 | 296 | 1.54272801 | FGF14 | fibroblast growth factor 14 [Source:HGNC Symbol;Acc:HGNC:3671] |
| ENSG00000105173 | HGLibA_08084 | 296 | 1.54272801 | CCNE1 | cyclin E1 [Source:HGNC Symbol;Acc:HGNC:1589] |
| ENSG00000108700 | HGLibA_08043 | 296 | 1.54272801 | CCL8 | C-C motif chemokine ligand 8 [Source:HGNC Symbol;Acc:HGNC:10635] |
| ENSG00000117751 | HGLibA_38124 | 296 | 1.54272801 | PPP1R8 | protein phosphatase 1 regulatory subunit 8 [Source:HGNC Symbol;Acc:HGNC:9296] |
| ENSG00000137460 | HGLibA_17475 | 296 | 1.54272801 | FHDC1 | FH2 domain containing 1 [Source:HGNC Symbol;Acc:HGNC:29363] |
| ENSG00000139168 | HGLibA_55054 | 296 | 1.54272801 | ZCRB1 | zinc finger CCHC-type and RNA binding motif containing 1 [Source:HGNC Symbol;Acc:HGNC:29620] |
| ENSG00000152705 | HGLibA_07360 | 296 | 1.54272801 | CATSPER3 | cation channel sperm associated 3 [Source:HGNC Symbol;Acc:HGNC:20819] |
| ENSG00000164270 | HGLibA_22394 | 296 | 1.54272801 | HTR4 | 5-hydroxytryptamine receptor 4 [Source:HGNC Symbol;Acc:HGNC:5299] |
| ENSG00000165269 | HGLibA_02604 | 296 | 1.54272801 | AQP7 | aquaporin 7 [Source:HGNC Symbol;Acc:HGNC:640] |
| ENSG00000165730 | HGLibA_47553 | 296 | 1.54272801 | STOX1 | storkhead box 1 [Source:HGNC Symbol;Acc:HGNC:23508] |
| ENSG00000168913 | HGLibA_15131 | 296 | 1.54272801 | ENHO | energy homeostasis associated [Source:HGNC Symbol;Acc:HGNC:24838] |
| ENSG00000169756 | HGLibA_26545 | 296 | 1.54272801 | LIMS1 | LIM zinc finger domain containing 1 [Source:HGNC Symbol;Acc:HGNC:6616] |
| ENSG00000170345 | HGLibA_17795 | 296 | 1.54272801 | FOS | AP-1 transcription factor subunit [Source:HGNC Symbol;Acc:HGNC:3796]" |
| ENSG00000173253 | HGLibA_13419 | 296 | 1.54272801 | DMRT2 | doublesex and mab-3 related transcription factor 2 [Source:HGNC Symbol;Acc:HGNC:2935] |
| ENSG00000173706 | HGLibA_21108 | 296 | 1.54272801 | HEG1 | heart development protein with EGF like domains 1 [Source:HGNC Symbol;Acc:HGNC:29227] |
| ENSG00000173786 | HGLibA_10357 | 296 | 1.54272801 | CNP | 3'-cyclic nucleotide 3' phosphodiesterase [Source:HGNC Symbol;Acc:HGNC:2158]" |
| ENSG00000183283 | HGLibA_12367 | 296 | 1.54272801 | DAZAP2 | DAZ associated protein 2 [Source:HGNC Symbol;Acc:HGNC:2684] |
| ENSG00000186470 | HGLibA_04970 | 296 | 1.54272801 | BTN3A2 | butyrophilin subfamily 3 member A2 [Source:HGNC Symbol;Acc:HGNC:1139] |
| ENSG00000187857 | HGLibA_34408 | 296 | 1.54272801 | OR6C75 | olfactory receptor family 6 subfamily C member 75 [Source:HGNC Symbol;Acc:HGNC:31304] |
| ENSG00000198898 | HGLibA_07207 | 296 | 1.54272801 | CAPZA2 | capping actin protein of muscle Z-line subunit alpha 2 [Source:HGNC Symbol;Acc:HGNC:1490] |
| ENSG00000204764 | HGLibA_40199 | 296 | 1.54272801 | RANBP17 | RAN binding protein 17 [Source:HGNC Symbol;Acc:HGNC:14428] |
| ENSG00000241685 | HGLibA_03074 | 296 | 1.54272801 | ARPC1A | actin related protein 2/3 complex subunit 1A [Source:HGNC Symbol;Acc:HGNC:703] |
| ENSG00000258289 | HGLibA_09674 | 296 | 1.54272801 | CHURC1 | churchill domain containing 1 [Source:HGNC Symbol;Acc:HGNC:20099] |
| hsa-mir-8082 | HGLibA_64170 | 296 | 1.54272801 | hsa-mir-8082 | - |
| C19orf45 | HGLibA_05666 | 295 | 1.533682667 | C19orf45 | - |
| DUS2L | HGLibA_14117 | 295 | 1.533682667 | DUS2L | - |
| ENSG00000047188 | HGLibA_54727 | 295 | 1.533682667 | YTHDC2 | YTH domain containing 2 [Source:HGNC Symbol;Acc:HGNC:24721] |
| ENSG00000082126 | HGLibA_29757 | 295 | 1.533682667 | MPP4 | membrane palmitoylated protein 4 [Source:HGNC Symbol;Acc:HGNC:13680] |
| ENSG00000093217 | HGLibA_54616 | 295 | 1.533682667 | XYLB | xylulokinase [Source:HGNC Symbol;Acc:HGNC:12839] |
| ENSG00000101298 | HGLibA_45936 | 295 | 1.533682667 | SNPH | syntaphilin [Source:HGNC Symbol;Acc:HGNC:15931] |
| ENSG00000109099 | HGLibA_37324 | 295 | 1.533682667 | PMP22 | peripheral myelin protein 22 [Source:HGNC Symbol;Acc:HGNC:9118] |
| ENSG00000109424 | HGLibA_52832 | 295 | 1.533682667 | UCP1 | uncoupling protein 1 [Source:HGNC Symbol;Acc:HGNC:12517] |
| ENSG00000112394 | HGLibA_44419 | 295 | 1.533682667 | SLC16A10 | solute carrier family 16 member 10 [Source:HGNC Symbol;Acc:HGNC:17027] |
| ENSG00000113520 | HGLibA_23166 | 295 | 1.533682667 | IL4 | interleukin 4 [Source:HGNC Symbol;Acc:HGNC:6014] |
| ENSG00000116691 | HGLibA_29267 | 295 | 1.533682667 | MIIP | migration and invasion inhibitory protein [Source:HGNC Symbol;Acc:HGNC:25715] |
| ENSG00000126602 | HGLibA_51293 | 295 | 1.533682667 | TRAP1 | TNF receptor associated protein 1 [Source:HGNC Symbol;Acc:HGNC:16264] |
| ENSG00000131724 | HGLibA_22975 | 295 | 1.533682667 | IL13RA1 | interleukin 13 receptor subunit alpha 1 [Source:HGNC Symbol;Acc:HGNC:5974] |
| ENSG00000133250 | HGLibA_55939 | 295 | 1.533682667 | ZNF414 | zinc finger protein 414 [Source:HGNC Symbol;Acc:HGNC:20630] |
| ENSG00000137267 | HGLibA_52277 | 295 | 1.533682667 | TUBB2A | tubulin beta 2A class IIa [Source:HGNC Symbol;Acc:HGNC:12412] |
| ENSG00000141965 | HGLibA_17274 | 295 | 1.533682667 | FEM1A | fem-1 homolog A [Source:HGNC Symbol;Acc:HGNC:16934] |
| ENSG00000143612 | HGLibA_05857 | 295 | 1.533682667 | C1orf43 | chromosome 1 open reading frame 43 [Source:HGNC Symbol;Acc:HGNC:29876] |
| ENSG00000145283 | HGLibA_44345 | 295 | 1.533682667 | SLC10A6 | solute carrier family 10 member 6 [Source:HGNC Symbol;Acc:HGNC:30603] |
| ENSG00000145425 | HGLibA_42139 | 295 | 1.533682667 | RPS3A | ribosomal protein S3A [Source:HGNC Symbol;Acc:HGNC:10421] |
| ENSG00000152076 | HGLibA_07865 | 295 | 1.533682667 | CCDC74B | coiled-coil domain containing 74B [Source:HGNC Symbol;Acc:HGNC:25267] |
| ENSG00000152932 | HGLibA_39912 | 295 | 1.533682667 | RAB3C | member RAS oncogene family [Source:HGNC Symbol;Acc:HGNC:30269]" |
| ENSG00000162510 | HGLibA_28428 | 295 | 1.533682667 | MATN1 | matrilin 1 [Source:HGNC Symbol;Acc:HGNC:6907] |
| ENSG00000165868 | HGLibA_22260 | 295 | 1.533682667 | HSPA12A | heat shock protein family A (Hsp70) member 12A [Source:HGNC Symbol;Acc:HGNC:19022] |
| ENSG00000196498 | HGLibA_31358 | 295 | 1.533682667 | NCOR2 | nuclear receptor corepressor 2 [Source:HGNC Symbol;Acc:HGNC:7673] |

| | | | | | |
|---|---|---|---|---|---|
| ENSG00000196535 | HGLibA_30812 | 295 | 1.533682667 | MYO18A | myosin XVIIIA [Source:HGNC Symbol;Acc:HGNC:31104] |
| ENSG00000197037 | HGLibA_57009 | 295 | 1.533682667 | ZSCAN25 | zinc finger and SCAN domain containing 25 [Source:HGNC Symbol;Acc:HGNC:21961] |
| FYB | HGLibA_18247 | 295 | 1.533682667 | FYB | - |
| hsa-mir-520b | HGLibA_61737 | 295 | 1.533682667 | hsa-mir-520b | - |
| hsa-mir-548ag-2 | HGLibA_61879 | 295 | 1.533682667 | hsa-mir-548ag-2 | - |
| hsa-mir-5684 | HGLibA_62254 | 295 | 1.533682667 | hsa-mir-5684 | - |
| hsa-mir-6887 | HGLibA_63734 | 295 | 1.533682667 | hsa-mir-6887 | - |
| LOC149373 | HGLibA_26891 | 295 | 1.533682667 | LOC149373 | - |
| ENSG00000003147 | HGLibA_22489 | 294 | 1.524637324 | ICA1 | islet cell autoantigen 1 [Source:HGNC Symbol;Acc:HGNC:5343] |
| ENSG00000011028 | HGLibA_29818 | 294 | 1.524637324 | MRC2 | mannose receptor C type 2 [Source:HGNC Symbol;Acc:HGNC:16875] |
| ENSG00000105514 | HGLibA_39916 | 294 | 1.524637324 | RAB3D | member RAS oncogene family [Source:HGNC Symbol;Acc:HGNC:9779]" |
| ENSG00000105668 | HGLibA_53068 | 294 | 1.524637324 | UPK1A | uroplakin 1A [Source:HGNC Symbol;Acc:HGNC:12577] |
| ENSG00000107140 | HGLibA_49028 | 294 | 1.524637324 | TESK1 | testis associated actin remodelling kinase 1 [Source:HGNC Symbol;Acc:HGNC:11731] |
| ENSG00000111348 | HGLibA_02797 | 294 | 1.524637324 | ARHGDIB | Rho GDP dissociation inhibitor beta [Source:HGNC Symbol;Acc:HGNC:679] |
| ENSG00000115902 | HGLibA_44517 | 294 | 1.524637324 | SLC1A4 | solute carrier family 1 member 4 [Source:HGNC Symbol;Acc:HGNC:10942] |
| ENSG00000128422 | HGLibA_25435 | 294 | 1.524637324 | KRT17 | keratin 17 [Source:HGNC Symbol;Acc:HGNC:6427] |
| ENSG00000135540 | HGLibA_31916 | 294 | 1.524637324 | NHSL1 | NHS like 1 [Source:HGNC Symbol;Acc:HGNC:21021] |
| ENSG00000137193 | HGLibA_36676 | 294 | 1.524637324 | PIM1 | serine/threonine kinase [Source:HGNC Symbol;Acc:HGNC:8986]" |
| ENSG00000137343 | HGLibA_03423 | 294 | 1.524637324 | ATAT1 | alpha tubulin acetyltransferase 1 [Source:HGNC Symbol;Acc:HGNC:21186] |
| ENSG00000143164 | HGLibA_12450 | 294 | 1.524637324 | DCAF6 | DDB1 and CUL4 associated factor 6 [Source:HGNC Symbol;Acc:HGNC:30002] |
| ENSG00000144362 | HGLibA_36445 | 294 | 1.524637324 | PHOSPHO2 | orphan 2 [Source:HGNC Symbol;Acc:HGNC:28316]" |
| ENSG00000145808 | HGLibA_00818 | 294 | 1.524637324 | ADAMTS19 | ADAM metallopeptidase with thrombospondin type 1 motif 19 [Source:HGNC Symbol;Acc:HGNC:17111] |
| ENSG00000154589 | HGLibA_27691 | 294 | 1.524637324 | LY96 | lymphocyte antigen 96 [Source:HGNC Symbol;Acc:HGNC:17156] |
| ENSG00000156675 | HGLibA_39794 | 294 | 1.524637324 | RAB11FIP1 | RAB11 family interacting protein 1 [Source:HGNC Symbol;Acc:HGNC:30265] |
| ENSG00000157796 | HGLibA_54044 | 294 | 1.524637324 | WDR19 | WD repeat domain 19 [Source:HGNC Symbol;Acc:HGNC:18340] |
| ENSG00000157927 | HGLibA_40115 | 294 | 1.524637324 | RADIL | Rap associating with DIL domain [Source:HGNC Symbol;Acc:HGNC:22226] |
| ENSG00000157992 | HGLibA_25857 | 294 | 1.524637324 | KRTCAP3 | keratinocyte associated protein 3 [Source:HGNC Symbol;Acc:HGNC:28943] |
| ENSG00000158467 | HGLibA_01292 | 294 | 1.524637324 | AHCYL2 | adenosylhomocysteinase like 2 [Source:HGNC Symbol;Acc:HGNC:22204] |
| ENSG00000164187 | HGLibA_26677 | 294 | 1.524637324 | LMBRD2 | LMBR1 domain containing 2 [Source:HGNC Symbol;Acc:HGNC:25287] |
| ENSG00000164440 | HGLibA_52394 | 294 | 1.524637324 | TXLNB | taxilin beta [Source:HGNC Symbol;Acc:HGNC:21617] |
| ENSG00000170075 | HGLibA_20035 | 294 | 1.524637324 | GPR37L1 | G protein-coupled receptor 37 like 1 [Source:HGNC Symbol;Acc:HGNC:14923] |
| ENSG00000175866 | HGLibA_04123 | 294 | 1.524637324 | BAIAP2 | BAI1 associated protein 2 [Source:HGNC Symbol;Acc:HGNC:947] |
| ENSG00000176055 | HGLibA_28506 | 294 | 1.524637324 | MBLAC2 | metallo-beta-lactamase domain containing 2 [Source:HGNC Symbol;Acc:HGNC:33711] |
| ENSG00000180269 | HGLibA_19905 | 294 | 1.524637324 | GPR139 | G protein-coupled receptor 139 [Source:HGNC Symbol;Acc:HGNC:19995] |
| ENSG00000188747 | HGLibA_32381 | 294 | 1.524637324 | NOXA1 | NADPH oxidase activator 1 [Source:HGNC Symbol;Acc:HGNC:10668] |
| ENSG00000197646 | HGLibA_35723 | 294 | 1.524637324 | PDCD1LG2 | programmed cell death 1 ligand 2 [Source:HGNC Symbol;Acc:HGNC:18731] |
| ENSG00000197838 | HGLibA_12103 | 294 | 1.524637324 | CYP2A13 | cytochrome P450 family 2 subfamily A member 13 [Source:HGNC Symbol;Acc:HGNC:2608] |
| ENSG00000223572 | HGLibA_09779 | 294 | 1.524637324 | CKMT1A | mitochondrial 1A [Source:HGNC Symbol;Acc:HGNC:31736]" |
| HIST1H2AB | HGLibA_21378 | 294 | 1.524637324 | HIST1H2AB | - |
| hsa-mir-1249 | HGLibA_57395 | 294 | 1.524637324 | hsa-mir-1249 | - |
| hsa-mir-1911 | HGLibA_58052 | 294 | 1.524637324 | hsa-mir-1911 | - |
| hsa-mir-3167 | HGLibA_58746 | 294 | 1.524637324 | hsa-mir-3167 | - |
| C1orf192 | HGLibA_05812 | 293 | 1.515591981 | C1orf192 | - |
| ENSG00000076344 | HGLibA_41056 | 293 | 1.515591981 | RGS11 | regulator of G protein signaling 11 [Source:HGNC Symbol;Acc:HGNC:9993] |
| ENSG00000101443 | HGLibA_54261 | 293 | 1.515591981 | WFDC2 | WAP four-disulfide core domain 2 [Source:HGNC Symbol;Acc:HGNC:15939] |
| ENSG00000103653 | HGLibA_11359 | 293 | 1.515591981 | CSK | C-terminal Src kinase [Source:HGNC Symbol;Acc:HGNC:2444] |
| ENSG00000105662 | HGLibA_11227 | 293 | 1.515591981 | CRTC1 | CREB regulated transcription coactivator 1 [Source:HGNC Symbol;Acc:HGNC:16062] |
| ENSG00000106631 | HGLibA_30776 | 293 | 1.515591981 | MYL7 | myosin light chain 7 [Source:HGNC Symbol;Acc:HGNC:21719] |
| ENSG00000106636 | HGLibA_54694 | 293 | 1.515591981 | YKT6 | YKT6 v-SNARE homolog [Source:HGNC Symbol;Acc:HGNC:16959] |
| ENSG00000111679 | HGLibA_39474 | 293 | 1.515591981 | PTPN6 | non-receptor type 6 [Source:HGNC Symbol;Acc:HGNC:9658]" |
| ENSG00000120549 | HGLibA_24722 | 293 | 1.515591981 | KIAA1217 | KIAA1217 [Source:HGNC Symbol;Acc:HGNC:25428] |
| ENSG00000121101 | HGLibA_49064 | 293 | 1.515591981 | TEX14 | intercellular bridge forming factor [Source:HGNC Symbol;Acc:HGNC:11737]" |
| ENSG00000122145 | HGLibA_48666 | 293 | 1.515591981 | TBX22 | T-box 22 [Source:HGNC Symbol;Acc:HGNC:11600] |
| ENSG00000125999 | HGLibA_04718 | 293 | 1.515591981 | BPIFB1 | BPI fold containing family B member 1 [Source:HGNC Symbol;Acc:HGNC:16108] |
| ENSG00000133195 | HGLibA_45094 | 293 | 1.515591981 | SLC39A11 | solute carrier family 39 member 11 [Source:HGNC Symbol;Acc:HGNC:14463] |
| ENSG00000136816 | HGLibA_51024 | 293 | 1.515591981 | TOR1B | torsin family 1 member B [Source:HGNC Symbol;Acc:HGNC:11995] |
| ENSG00000137434 | HGLibA_06494 | 293 | 1.515591981 | C6orf52 | chromosome 6 open reading frame 52 [Source:HGNC Symbol;Acc:HGNC:20881] |
| ENSG00000141873 | HGLibA_45108 | 293 | 1.515591981 | SLC39A3 | solute carrier family 39 member 3 [Source:HGNC Symbol;Acc:HGNC:17128] |
| ENSG00000145555 | HGLibA_30802 | 293 | 1.515591981 | MYO10 | myosin X [Source:HGNC Symbol;Acc:HGNC:7593] |
| ENSG00000156869 | HGLibA_18041 | 293 | 1.515591981 | FRRS1 | ferric chelate reductase 1 [Source:HGNC Symbol;Acc:HGNC:27622] |
| ENSG00000159216 | HGLibA_42455 | 293 | 1.515591981 | RUNX1 | runt related transcription factor 1 [Source:HGNC Symbol;Acc:HGNC:10471] |
| ENSG00000166819 | HGLibA_37170 | 293 | 1.515591981 | PLIN1 | perilipin 1 [Source:HGNC Symbol;Acc:HGNC:9076] |
| ENSG00000171303 | HGLibA_24320 | 293 | 1.515591981 | KCNK3 | potassium two pore domain channel subfamily K member 3 [Source:HGNC Symbol;Acc:HGNC:6278] |
| ENSG00000176971 | HGLibA_17501 | 293 | 1.515591981 | FIBIN | fin bud initiation factor homolog [Source:HGNC Symbol;Acc:HGNC:33747] |
| ENSG00000184083 | HGLibA_16062 | 293 | 1.515591981 | FAM120C | family with sequence similarity 120C [Source:HGNC Symbol;Acc:HGNC:16949] |
| ENSG00000185250 | HGLibA_37950 | 293 | 1.515591981 | PPIL6 | peptidylprolyl isomerase like 6 [Source:HGNC Symbol;Acc:HGNC:21557] |
| ENSG00000196832 | HGLibA_33565 | 293 | 1.515591981 | OR11G2 | olfactory receptor family 11 subfamily G member 2 [Source:HGNC Symbol;Acc:HGNC:15346] |
| ENSG00000205927 | HGLibA_33372 | 293 | 1.515591981 | OLIG2 | oligodendrocyte transcription factor 2 [Source:HGNC Symbol;Acc:HGNC:9398] |
| ENSG00000255346 | HGLibA_32380 | 293 | 1.515591981 | NOX5 | NADPH oxidase 5 [Source:HGNC Symbol;Acc:HGNC:14874] |
| ENSG00000272573 | HGLibA_30581 | 293 | 1.515591981 | MUSTN1 | embryonic nuclear protein 1 [Source:HGNC Symbol;Acc:HGNC:22144]" |
| hsa-mir-6821 | HGLibA_63463 | 293 | 1.515591981 | hsa-mir-6821 | - |
| hsa-mir-6885 | HGLibA_63724 | 293 | 1.515591981 | hsa-mir-6885 | - |
| C13orf45 | HGLibA_05313 | 292 | 1.506546638 | C13orf45 | - |
| ENSG00000004961 | HGLibA_20956 | 292 | 1.506546638 | HCCS | holocytochrome c synthase [Source:HGNC Symbol;Acc:HGNC:4837] |
| ENSG00000049245 | HGLibA_53494 | 292 | 1.506546638 | VAMP3 | vesicle associated membrane protein 3 [Source:HGNC Symbol;Acc:HGNC:12644] |
| ENSG00000060749 | HGLibA_39750 | 292 | 1.506546638 | QSER1 | glutamine and serine rich 1 [Source:HGNC Symbol;Acc:HGNC:26154] |
| ENSG00000101311 | HGLibA_17293 | 292 | 1.506546638 | FERMT1 | fermitin family member 1 [Source:HGNC Symbol;Acc:HGNC:15889] |
| ENSG00000105675 | HGLibA_03632 | 292 | 1.506546638 | ATP4A | ATPase H+/K+ transporting subunit alpha [Source:HGNC Symbol;Acc:HGNC:819] |
| ENSG00000105974 | HGLibA_07375 | 292 | 1.506546638 | CAV1 | caveolin 1 [Source:HGNC Symbol;Acc:HGNC:1527] |
| ENSG00000108309 | HGLibA_42448 | 292 | 1.506546638 | RUNDC3A | RUN domain containing 3A [Source:HGNC Symbol;Acc:HGNC:16984] |
| ENSG00000123739 | HGLibA_36869 | 292 | 1.506546638 | PLA2G12A | phospholipase A2 group XIIA [Source:HGNC Symbol;Acc:HGNC:18554] |
| ENSG00000128683 | HGLibA_18410 | 292 | 1.506546638 | GAD1 | glutamate decarboxylase 1 [Source:HGNC Symbol;Acc:HGNC:4092] |
| ENSG00000134330 | HGLibA_22466 | 292 | 1.506546638 | IAH1 | isoamyl acetate hydrolyzing esterase 1 (putative) [Source:HGNC Symbol;Acc:HGNC:27696] |
| ENSG00000136463 | HGLibA_48167 | 292 | 1.506546638 | TACO1 | translational activator of cytochrome c oxidase I [Source:HGNC Symbol;Acc:HGNC:24316] |
| ENSG00000144535 | HGLibA_13276 | 292 | 1.506546638 | DIS3L2 | DIS3 like 3'-5' exoribonuclease 2 [Source:HGNC Symbol;Acc:HGNC:28648] |
| ENSG00000147144 | HGLibA_07565 | 292 | 1.506546638 | CCDC120 | coiled-coil domain containing 120 [Source:HGNC Symbol;Acc:HGNC:28910] |
| ENSG00000148948 | HGLibA_27384 | 292 | 1.506546638 | LRRC4C | leucine rich repeat containing 4C [Source:HGNC Symbol;Acc:HGNC:29317] |
| ENSG00000150990 | HGLibA_13193 | 292 | 1.506546638 | DHX37 | DEAH-box helicase 37 [Source:HGNC Symbol;Acc:HGNC:17210] |
| ENSG00000158552 | HGLibA_55150 | 292 | 1.506546638 | ZFAND2B | zinc finger AN1-type containing 2B [Source:HGNC Symbol;Acc:HGNC:25206] |
| ENSG00000163618 | HGLibA_06976 | 292 | 1.506546638 | CADPS | calcium dependent secretion activator [Source:HGNC Symbol;Acc:HGNC:1426] |
| ENSG00000163827 | HGLibA_27278 | 292 | 1.506546638 | LRRC2 | leucine rich repeat containing 2 [Source:HGNC Symbol;Acc:HGNC:14676] |
| ENSG00000169442 | HGLibA_08405 | 292 | 1.506546638 | CD52 | CD52 molecule [Source:HGNC Symbol;Acc:HGNC:1804] |
| ENSG00000170074 | HGLibA_16168 | 292 | 1.506546638 | FAM153A | family with sequence similarity 153 member A [Source:HGNC Symbol;Acc:HGNC:29940] |
| ENSG00000173157 | HGLibA_00823 | 292 | 1.506546638 | ADAMTS20 | ADAM metallopeptidase with thrombospondin type 1 motif 20 [Source:HGNC Symbol;Acc:HGNC:17178] |
| ENSG00000182050 | HGLibA_29146 | 292 | 1.506546638 | MGAT4C | MGAT4 family member C [Source:HGNC Symbol;Acc:HGNC:30871] |
| ENSG00000188060 | HGLibA_39947 | 292 | 1.506546638 | RAB42 | member RAS oncogene family [Source:HGNC Symbol;Acc:HGNC:28702]" |
| ENSG00000196611 | HGLibA_29478 | 292 | 1.506546638 | MMP1 | matrix metallopeptidase 1 [Source:HGNC Symbol;Acc:HGNC:7155] |
| ENSG00000248385 | HGLibA_48339 | 292 | 1.506546638 | TARM1 | activating receptor on myeloid cells 1 [Source:HGNC Symbol;Acc:HGNC:37250]" |
| hsa-mir-23a | HGLibA_58328 | 292 | 1.506546638 | hsa-mir-23a | - |
| hsa-mir-4522 | HGLibA_60544 | 292 | 1.506546638 | hsa-mir-4522 | - |
| hsa-mir-5010 | HGLibA_61457 | 292 | 1.506546638 | hsa-mir-5010 | - |
| hsa-mir-6818 | HGLibA_63449 | 292 | 1.506546638 | hsa-mir-6818 | - |
| ENSG00000004848 | HGLibA_03180 | 291 | 1.497501295 | ARX | aristaless related homeobox [Source:HGNC Symbol;Acc:HGNC:18060] |
| ENSG00000062282 | HGLibA_13036 | 291 | 1.497501295 | DGAT2 | diacylglycerol O-acyltransferase 2 [Source:HGNC Symbol;Acc:HGNC:16940] |
| ENSG00000071051 | HGLibA_31308 | 291 | 1.497501295 | NCK2 | NCK adaptor protein 2 [Source:HGNC Symbol;Acc:HGNC:7665] |
| ENSG00000100453 | HGLibA_20721 | 291 | 1.497501295 | GZMB | granzyme B [Source:HGNC Symbol;Acc:HGNC:4709] |
| ENSG00000103160 | HGLibA_22213 | 291 | 1.497501295 | HSDL1 | hydroxysteroid dehydrogenase like 1 [Source:HGNC Symbol;Acc:HGNC:16475] |
| ENSG00000104133 | HGLibA_46629 | 291 | 1.497501295 | SPG11 | spatacsin vesicle trafficking associated [Source:HGNC Symbol;Acc:HGNC:11226]" |
| ENSG00000110721 | HGLibA_09458 | 291 | 1.497501295 | CHKA | choline kinase alpha [Source:HGNC Symbol;Acc:HGNC:1937] |
| ENSG00000115163 | HGLibA_09037 | 291 | 1.497501295 | CENPA | centromere protein A [Source:HGNC Symbol;Acc:HGNC:1851] |
| ENSG00000124920 | HGLibA_30915 | 291 | 1.497501295 | MYRF | myelin regulatory factor [Source:HGNC Symbol;Acc:HGNC:1181] |
| ENSG00000125779 | HGLibA_35099 | 291 | 1.497501295 | PANK2 | pantothenate kinase 2 [Source:HGNC Symbol;Acc:HGNC:15894] |
| ENSG00000131016 | HGLibA_01412 | 291 | 1.497501295 | AKAP12 | A-kinase anchoring protein 12 [Source:HGNC Symbol;Acc:HGNC:370] |
| ENSG00000131435 | HGLibA_35917 | 291 | 1.497501295 | PDLIM4 | PDZ and LIM domain 4 [Source:HGNC Symbol;Acc:HGNC:16501] |
| ENSG00000140199 | HGLibA_44372 | 291 | 1.497501295 | SLC12A6 | solute carrier family 12 member 6 [Source:HGNC Symbol;Acc:HGNC:10914] |
| ENSG00000140968 | HGLibA_23601 | 291 | 1.497501295 | IRF8 | interferon regulatory factor 8 [Source:HGNC Symbol;Acc:HGNC:5358] |
| ENSG00000142459 | HGLibA_15694 | 291 | 1.497501295 | EVI5L | ecotropic viral integration site 5 like [Source:HGNC Symbol;Acc:HGNC:30464] |
| ENSG00000143127 | HGLibA_23718 | 291 | 1.497501295 | ITGA10 | integrin subunit alpha 10 [Source:HGNC Symbol;Acc:HGNC:6135] |
| ENSG00000145012 | HGLibA_27110 | 291 | 1.497501295 | LPP | LIM domain containing preferred translocation partner in lipoma [Source:HGNC Symbol;Acc:HGNC:6679] |
| ENSG00000146399 | HGLibA_48124 | 291 | 1.497501295 | TAAR1 | trace amine associated receptor 1 [Source:HGNC Symbol;Acc:HGNC:17734] |
| ENSG00000146926 | HGLibA_03207 | 291 | 1.497501295 | ASB10 | ankyrin repeat and SOCS box containing 10 [Source:HGNC Symbol;Acc:HGNC:17185] |
| ENSG00000155393 | HGLibA_21071 | 291 | 1.497501295 | HEATR3 | HEAT repeat containing 3 [Source:HGNC Symbol;Acc:HGNC:26087] |
| ENSG00000158445 | HGLibA_24156 | 291 | 1.497501295 | KCNB1 | potassium voltage-gated channel subfamily B member 1 [Source:HGNC Symbol;Acc:HGNC:6231] |
| ENSG00000162645 | HGLibA_18730 | 291 | 1.497501295 | GBP2 | guanylate binding protein 2 [Source:HGNC Symbol;Acc:HGNC:4183] |
| ENSG00000163218 | HGLibA_36258 | 291 | 1.497501295 | PGLYRP4 | peptidoglycan recognition protein 4 [Source:HGNC Symbol;Acc:HGNC:30015] |
| ENSG00000164904 | HGLibA_01582 | 291 | 1.497501295 | ALDH7A1 | aldehyde dehydrogenase 7 family member A1 [Source:HGNC Symbol;Acc:HGNC:877] |
| ENSG00000166816 | HGLibA_26236 | 291 | 1.497501295 | LDHD | lactate dehydrogenase D [Source:HGNC Symbol;Acc:HGNC:19708] |
| ENSG00000171540 | HGLibA_34751 | 291 | 1.497501295 | OTP | orthopedia homeobox [Source:HGNC Symbol;Acc:HGNC:8518] |
| ENSG00000171617 | HGLibA_15111 | 291 | 1.497501295 | ENC1 | ectodermal-neural cortex 1 [Source:HGNC Symbol;Acc:HGNC:3345] |
| ENSG00000172465 | HGLibA_48704 | 291 | 1.497501295 | TCEAL1 | transcription elongation factor A like 1 [Source:HGNC Symbol;Acc:HGNC:11616] |
| ENSG00000172568 | HGLibA_17761 | 291 | 1.497501295 | FNDC9 | fibronectin type III domain containing 9 [Source:HGNC Symbol;Acc:HGNC:33547] |
| ENSG00000173868 | HGLibA_36440 | 291 | 1.497501295 | PHOSPHO1 | phosphoethanolamine/phosphocholine phosphatase [Source:HGNC Symbol;Acc:HGNC:16815] |
| ENSG00000174939 | HGLibA_03354 | 291 | 1.497501295 | ASPHD1 | aspartate beta-hydroxylase domain containing 1 [Source:HGNC Symbol;Acc:HGNC:27380] |
| ENSG00000175591 | HGLibA_34885 | 291 | 1.497501295 | P2RY2 | purinergic receptor P2Y2 [Source:HGNC Symbol;Acc:HGNC:8541] |
| ENSG00000176714 | HGLibA_07569 | 291 | 1.497501295 | CCDC121 | coiled-coil domain containing 121 [Source:HGNC Symbol;Acc:HGNC:25833] |

| | | | | | |
|---|---|---|---|---|---|
| ENSG00000179292 | HGLibA_50000 | 291 | 1.497501295 | TMEM151A | transmembrane protein 151A [Source:HGNC Symbol;Acc:HGNC:28497] |
| ENSG00000182187 | HGLibA_11274 | 291 | 1.497501295 | CRYGB | crystallin gamma B [Source:HGNC Symbol;Acc:HGNC:2409] |
| ENSG00000182674 | HGLibA_24160 | 291 | 1.497501295 | KCNB2 | potassium voltage-gated channel subfamily B member 2 [Source:HGNC Symbol;Acc:HGNC:6232] |
| ENSG00000183496 | HGLibA_29029 | 291 | 1.497501295 | MEX3B | mex-3 RNA binding family member B [Source:HGNC Symbol;Acc:HGNC:25297] |
| ENSG00000184156 | HGLibA_24371 | 291 | 1.497501295 | KCNQ3 | potassium voltage-gated channel subfamily Q member 3 [Source:HGNC Symbol;Acc:HGNC:6297] |
| ENSG00000184916 | HGLibA_23908 | 291 | 1.497501295 | JAG2 | jagged 2 [Source:HGNC Symbol;Acc:HGNC:6189] |
| ENSG00000186310 | HGLibA_31118 | 291 | 1.497501295 | NAP1L3 | nucleosome assembly protein 1 like 3 [Source:HGNC Symbol;Acc:HGNC:7639] |
| ENSG00000187969 | HGLibA_55013 | 291 | 1.497501295 | ZCCHC13 | zinc finger CCHC-type containing 13 [Source:HGNC Symbol;Acc:HGNC:31749] |
| ENSG00000198455 | HGLibA_57076 | 291 | 1.497501295 | ZXDB | zinc finger X-linked duplicated B [Source:HGNC Symbol;Acc:HGNC:13199] |
| ENSG00000198689 | HGLibA_45420 | 291 | 1.497501295 | SLC9A6 | solute carrier family 9 member A6 [Source:HGNC Symbol;Acc:HGNC:11079] |
| ENSG00000204577 | HGLibA_26514 | 291 | 1.497501295 | LILRB3 | leukocyte immunoglobulin like receptor B3 [Source:HGNC Symbol;Acc:HGNC:6607] |
| ENSG00000237038 | HGLibA_53286 | 291 | 1.497501295 | USP17L8 | ubiquitin specific peptidase 17-like family member 8 [Source:HGNC Symbol;Acc:HGNC:37181] |
| ENSG00000243284 | HGLibA_53852 | 291 | 1.497501295 | VSIG8 | V-set and immunoglobulin domain containing 8 [Source:HGNC Symbol;Acc:HGNC:32063] |
| hsa-mir-548h-4 | HGLibA_62004 | 291 | 1.497501295 | hsa-mir-548h-4 | - |
| KIAA2018 | HGLibA_24840 | 291 | 1.497501295 | KIAA2018 | - |
| AES | HGLibA_01093 | 290 | 1.488455952 | AES | - |
| ENSG00000089250 | HGLibA_32322 | 290 | 1.488455952 | NOS1 | nitric oxide synthase 1 [Source:HGNC Symbol;Acc:HGNC:7872] |
| ENSG00000089327 | HGLibA_18237 | 290 | 1.488455952 | FXYD5 | FXYD domain containing ion transport regulator 5 [Source:HGNC Symbol;Acc:HGNC:4029] |
| ENSG00000100191 | HGLibA_45270 | 290 | 1.488455952 | SLC5A4 | solute carrier family 5 member 4 [Source:HGNC Symbol;Acc:HGNC:11039] |
| ENSG00000100385 | HGLibA_23122 | 290 | 1.488455952 | IL2RB | interleukin 2 receptor subunit beta [Source:HGNC Symbol;Acc:HGNC:6009] |
| ENSG00000101473 | HGLibA_00492 | 290 | 1.488455952 | ACOT8 | acyl-CoA thioesterase 8 [Source:HGNC Symbol;Acc:HGNC:15919] |
| ENSG00000104368 | HGLibA_36959 | 290 | 1.488455952 | PLAT | tissue type [Source:HGNC Symbol;Acc:HGNC:9051]" |
| ENSG00000107669 | HGLibA_03428 | 290 | 1.488455952 | ATE1 | arginyltransferase 1 [Source:HGNC Symbol;Acc:HGNC:782] |
| ENSG00000107789 | HGLibA_29282 | 290 | 1.488455952 | MINPP1 | multiple inositol-polyphosphate phosphatase 1 [Source:HGNC Symbol;Acc:HGNC:7102] |
| ENSG00000110075 | HGLibA_38228 | 290 | 1.488455952 | PPP6R3 | protein phosphatase 6 regulatory subunit 3 [Source:HGNC Symbol;Acc:HGNC:1173] |
| ENSG00000118526 | HGLibA_48772 | 290 | 1.488455952 | TCF21 | transcription factor 21 [Source:HGNC Symbol;Acc:HGNC:11632] |
| ENSG00000122122 | HGLibA_42763 | 290 | 1.488455952 | SASH3 | SAM and SH3 domain containing 3 [Source:HGNC Symbol;Acc:HGNC:15975] |
| ENSG00000123095 | HGLibA_04483 | 290 | 1.488455952 | BHLHE41 | basic helix-loop-helix family member e41 [Source:HGNC Symbol;Acc:HGNC:16617] |
| ENSG00000125730 | HGLibA_06202 | 290 | 1.488455952 | C3 | complement C3 [Source:HGNC Symbol;Acc:HGNC:1318] |
| ENSG00000127249 | HGLibA_03574 | 290 | 1.488455952 | ATP13A4 | ATPase 13A4 [Source:HGNC Symbol;Acc:HGNC:25422] |
| ENSG00000132639 | HGLibA_45877 | 290 | 1.488455952 | SNAP25 | synaptosome associated protein 25 [Source:HGNC Symbol;Acc:HGNC:11132] |
| ENSG00000135541 | HGLibA_01297 | 290 | 1.488455952 | AHI1 | Abelson helper integration site 1 [Source:HGNC Symbol;Acc:HGNC:21575] |
| ENSG00000139233 | HGLibA_26654 | 290 | 1.488455952 | LLPH | long-term synaptic facilitation factor [Source:HGNC Symbol;Acc:HGNC:28229]" |
| ENSG00000139644 | HGLibA_49746 | 290 | 1.488455952 | TMBIM6 | transmembrane BAX inhibitor motif containing 6 [Source:HGNC Symbol;Acc:HGNC:11723] |
| ENSG00000140382 | HGLibA_21677 | 290 | 1.488455952 | HMG20A | high mobility group 20A [Source:HGNC Symbol;Acc:HGNC:5001] |
| ENSG00000147138 | HGLibA_19971 | 290 | 1.488455952 | GPR174 | G protein-coupled receptor 174 [Source:HGNC Symbol;Acc:HGNC:30245] |
| ENSG00000148057 | HGLibA_22560 | 290 | 1.488455952 | IDNK | gluconokinase [Source:HGNC Symbol;Acc:HGNC:31367]" |
| ENSG00000162951 | HGLibA_27484 | 290 | 1.488455952 | LRRTM1 | leucine rich repeat transmembrane neuronal 1 [Source:HGNC Symbol;Acc:HGNC:19408] |
| ENSG00000163638 | HGLibA_00846 | 290 | 1.488455952 | ADAMTS9 | ADAM metallopeptidase with thrombospondin type 1 motif 9 [Source:HGNC Symbol;Acc:HGNC:13202] |
| ENSG00000163950 | HGLibA_44328 | 290 | 1.488455952 | SLBP | stem-loop binding protein [Source:HGNC Symbol;Acc:HGNC:10904] |
| ENSG00000167080 | HGLibA_04013 | 290 | 1.488455952 | B4GALNT2 | 4-N-acetyl-galactosaminyltransferase 2 [Source:HGNC Symbol;Acc:HGNC:24136]" |
| ENSG00000168137 | HGLibA_43653 | 290 | 1.488455952 | SETD5 | SET domain containing 5 [Source:HGNC Symbol;Acc:HGNC:25566] |
| ENSG00000169919 | HGLibA_20678 | 290 | 1.488455952 | GUSB | glucuronidase beta [Source:HGNC Symbol;Acc:HGNC:4696] |
| ENSG00000170486 | HGLibA_25527 | 290 | 1.488455952 | KRT72 | keratin 72 [Source:HGNC Symbol;Acc:HGNC:28932] |
| ENSG00000172716 | HGLibA_45480 | 290 | 1.488455952 | SLFN11 | schlafen family member 11 [Source:HGNC Symbol;Acc:HGNC:26633] |
| ENSG00000178665 | HGLibA_56616 | 290 | 1.488455952 | ZNF713 | zinc finger protein 713 [Source:HGNC Symbol;Acc:HGNC:22043] |
| ENSG00000181896 | HGLibA_55436 | 290 | 1.488455952 | ZNF101 | zinc finger protein 101 [Source:HGNC Symbol;Acc:HGNC:12881] |
| ENSG00000182107 | HGLibA_50362 | 290 | 1.488455952 | TMEM30B | transmembrane protein 30B [Source:HGNC Symbol;Acc:HGNC:27254] |
| ENSG00000183128 | HGLibA_07019 | 290 | 1.488455952 | CALHM3 | calcium homeostasis modulator 3 [Source:HGNC Symbol;Acc:HGNC:23458] |
| ENSG00000184022 | HGLibA_33853 | 290 | 1.488455952 | OR2T10 | olfactory receptor family 2 subfamily T member 10 [Source:HGNC Symbol;Acc:HGNC:19573] |
| ENSG00000185862 | HGLibA_15689 | 290 | 1.488455952 | EVI2B | ecotropic viral integration site 2B [Source:HGNC Symbol;Acc:HGNC:3500] |
| ENSG00000186193 | HGLibA_42735 | 290 | 1.488455952 | SAPCD2 | suppressor APC domain containing 2 [Source:HGNC Symbol;Acc:HGNC:28055] |
| ENSG00000236279 | HGLibA_09970 | 290 | 1.488455952 | CLEC2L | C-type lectin domain family 2 member L [Source:HGNC Symbol;Acc:HGNC:21969] |
| ENSG00000280969 | HGLibA_42150 | 290 | 1.488455952 | RPS4Y2 | ribosomal protein S4 Y-linked 2 [Source:HGNC Symbol;Acc:HGNC:18501] |
| GPR1 | HGLibA_19837 | 290 | 1.488455952 | GPR1 | - |
| hsa-mir-4254 | HGLibA_59725 | 290 | 1.488455952 | hsa-mir-4254 | - |
| hsa-mir-6895 | HGLibA_63764 | 290 | 1.488455952 | hsa-mir-6895 | - |
| TSSC1 | HGLibA_52004 | 290 | 1.488455952 | TSSC1 | - |
| C2orf44 | HGLibA_06132 | 289 | 1.479410609 | C2orf44 | - |
| ENSG00000021852 | HGLibA_06597 | 289 | 1.479410609 | C8B | complement C8 beta chain [Source:HGNC Symbol;Acc:HGNC:1353] |
| ENSG00000068784 | HGLibA_46924 | 289 | 1.479410609 | SRBD1 | S1 RNA binding domain 1 [Source:HGNC Symbol;Acc:HGNC:25521] |
| ENSG00000074319 | HGLibA_51843 | 289 | 1.479410609 | TSG101 | tumor susceptibility 101 [Source:HGNC Symbol;Acc:HGNC:15971] |
| ENSG00000083544 | HGLibA_48906 | 289 | 1.479410609 | TDRD3 | tudor domain containing 3 [Source:HGNC Symbol;Acc:HGNC:20612] |
| ENSG00000092094 | HGLibA_34680 | 289 | 1.479410609 | OSGEP | O-sialoglycoprotein endopeptidase [Source:HGNC Symbol;Acc:HGNC:18028] |
| ENSG00000100097 | HGLibA_26345 | 289 | 1.479410609 | LGALS1 | galectin 1 [Source:HGNC Symbol;Acc:HGNC:6561] |
| ENSG00000103202 | HGLibA_32164 | 289 | 1.479410609 | NME4 | NME/NM23 nucleoside diphosphate kinase 4 [Source:HGNC Symbol;Acc:HGNC:7852] |
| ENSG00000110079 | HGLibA_30152 | 289 | 1.479410609 | MS4A4A | membrane spanning 4-domains A4A [Source:HGNC Symbol;Acc:HGNC:13371] |
| ENSG00000111667 | HGLibA_53389 | 289 | 1.479410609 | USP5 | ubiquitin specific peptidase 5 [Source:HGNC Symbol;Acc:HGNC:12628] |
| ENSG00000116151 | HGLibA_29666 | 289 | 1.479410609 | MORN1 | MORN repeat containing 1 [Source:HGNC Symbol;Acc:HGNC:25852] |
| ENSG00000116750 | HGLibA_52807 | 289 | 1.479410609 | UCHL5 | ubiquitin C-terminal hydrolase L5 [Source:HGNC Symbol;Acc:HGNC:19678] |
| ENSG00000139428 | HGLibA_29454 | 289 | 1.479410609 | MMAB | metabolism of cobalamin associated B [Source:HGNC Symbol;Acc:HGNC:19331] |
| ENSG00000139567 | HGLibA_00700 | 289 | 1.479410609 | ACVRL1 | activin A receptor like type 1 [Source:HGNC Symbol;Acc:HGNC:175] |
| ENSG00000151692 | HGLibA_41526 | 289 | 1.479410609 | RNF144A | ring finger protein 144A [Source:HGNC Symbol;Acc:HGNC:20457] |
| ENSG00000154262 | HGLibA_00119 | 289 | 1.479410609 | ABCA6 | ATP binding cassette subfamily A member 6 [Source:HGNC Symbol;Acc:HGNC:36] |
| ENSG00000162688 | HGLibA_01197 | 289 | 1.479410609 | AGL | 4-alpha-glucanotransferase [Source:HGNC Symbol;Acc:HGNC:321]" |
| ENSG00000162980 | HGLibA_02975 | 289 | 1.479410609 | ARL5A | ADP ribosylation factor like GTPase 5A [Source:HGNC Symbol;Acc:HGNC:696] |
| ENSG00000166558 | HGLibA_45083 | 289 | 1.479410609 | SLC38A8 | solute carrier family 38 member 8 [Source:HGNC Symbol;Acc:HGNC:32434] |
| ENSG00000169116 | HGLibA_35205 | 289 | 1.479410609 | PARM1 | prostate androgen-regulated mucin-like protein 1 [Source:HGNC Symbol;Acc:HGNC:24536] |
| ENSG00000169372 | HGLibA_11059 | 289 | 1.479410609 | CRADD | CASP2 and RIPK1 domain containing adaptor with death domain [Source:HGNC Symbol;Acc:HGNC:2340] |
| ENSG00000169372 | HGLibA_11061 | 289 | 1.479410609 | CRADD | CASP2 and RIPK1 domain containing adaptor with death domain [Source:HGNC Symbol;Acc:HGNC:2340] |
| ENSG00000171433 | HGLibA_19290 | 289 | 1.479410609 | GLOD5 | glyoxalase domain containing 5 [Source:HGNC Symbol;Acc:HGNC:33358] |
| ENSG00000177054 | HGLibA_55074 | 289 | 1.479410609 | ZDHHC13 | zinc finger DHHC-type containing 13 [Source:HGNC Symbol;Acc:HGNC:18413] |
| ENSG00000185177 | HGLibA_56065 | 289 | 1.479410609 | ZNF479 | zinc finger protein 479 [Source:HGNC Symbol;Acc:HGNC:23258] |
| ENSG00000186104 | HGLibA_12139 | 289 | 1.479410609 | CYP2R1 | cytochrome P450 family 2 subfamily R member 1 [Source:HGNC Symbol;Acc:HGNC:20580] |
| ENSG00000196209 | HGLibA_44208 | 289 | 1.479410609 | SIRPB2 | signal regulatory protein beta 2 [Source:HGNC Symbol;Acc:HGNC:16247] |
| ENSG00000196616 | HGLibA_00953 | 289 | 1.479410609 | ADH1B | beta polypeptide [Source:HGNC Symbol;Acc:HGNC:250]" |
| ENSG00000198681 | HGLibA_27891 | 289 | 1.479410609 | MAGEA1 | MAGE family member A1 [Source:HGNC Symbol;Acc:HGNC:6796] |
| ENSG00000204366 | HGLibA_54820 | 289 | 1.479410609 | ZBTB12 | zinc finger and BTB domain containing 12 [Source:HGNC Symbol;Acc:HGNC:19066] |
| ENSG00000260903 | HGLibA_54528 | 289 | 1.479410609 | XKR7 | XK related 7 [Source:HGNC Symbol;Acc:HGNC:23062] |
| FAM86C1 | HGLibA_16780 | 289 | 1.479410609 | FAM86C1 | - |
| hsa-mir-4456 | HGLibA_60229 | 289 | 1.479410609 | hsa-mir-4456 | - |
| hsa-mir-4526 | HGLibA_60567 | 289 | 1.479410609 | hsa-mir-4526 | - |
| hsa-mir-4681 | HGLibA_60833 | 289 | 1.479410609 | hsa-mir-4681 | - |
| hsa-mir-6730 | HGLibA_63074 | 289 | 1.479410609 | hsa-mir-6730 | - |
| C6orf1 | HGLibA_06428 | 288 | 1.470365267 | C6orf1 | - |
| ENSG00000008282 | HGLibA_48037 | 288 | 1.470365267 | SYPL1 | synaptophysin like 1 [Source:HGNC Symbol;Acc:HGNC:11507] |
| ENSG00000032444 | HGLibA_37421 | 288 | 1.470365267 | PNPLA6 | patatin like phospholipase domain containing 6 [Source:HGNC Symbol;Acc:HGNC:16268] |
| ENSG00000078328 | HGLibA_40455 | 288 | 1.470365267 | RBFOX1 | RNA binding fox-1 homolog 1 [Source:HGNC Symbol;Acc:HGNC:18222] |
| ENSG00000082996 | HGLibA_41501 | 288 | 1.470365267 | RNF13 | ring finger protein 13 [Source:HGNC Symbol;Acc:HGNC:10057] |
| ENSG00000102891 | HGLibA_30306 | 288 | 1.470365267 | MT4 | metallothionein 4 [Source:HGNC Symbol;Acc:HGNC:18705] |
| ENSG00000104960 | HGLibA_39400 | 288 | 1.470365267 | PTOV1 | prostate tumor overexpressed 1 [Source:HGNC Symbol;Acc:HGNC:9632] |
| ENSG00000107593 | HGLibA_36802 | 288 | 1.470365267 | PKD2L1 | transient receptor potential cation channel [Source:HGNC Symbol;Acc:HGNC:9011]" |
| ENSG00000116288 | HGLibA_35198 | 288 | 1.470365267 | PARK7 | Parkinsonism associated deglycase [Source:HGNC Symbol;Acc:HGNC:16369] |
| ENSG00000120337 | HGLibA_50820 | 288 | 1.470365267 | TNFSF18 | TNF superfamily member 18 [Source:HGNC Symbol;Acc:HGNC:11932] |
| ENSG00000124588 | HGLibA_32564 | 288 | 1.470365267 | NQO2 | N-ribosyldihydronicotinamide:quinone reductase 2 [Source:HGNC Symbol;Acc:HGNC:7856] |
| ENSG00000124788 | HGLibA_03829 | 288 | 1.470365267 | ATXN1 | ataxin 1 [Source:HGNC Symbol;Acc:HGNC:10548] |
| ENSG00000126778 | HGLibA_44242 | 288 | 1.470365267 | SIX1 | SIX homeobox 1 [Source:HGNC Symbol;Acc:HGNC:10887] |
| ENSG00000132017 | HGLibA_12430 | 288 | 1.470365267 | DCAF15 | DDB1 and CUL4 associated factor 15 [Source:HGNC Symbol;Acc:HGNC:25095] |
| ENSG00000140750 | HGLibA_02713 | 288 | 1.470365267 | ARHGAP17 | Rho GTPase activating protein 17 [Source:HGNC Symbol;Acc:HGNC:18239] |
| ENSG00000142655 | HGLibA_36099 | 288 | 1.470365267 | PEX14 | peroxisomal biogenesis factor 14 [Source:HGNC Symbol;Acc:HGNC:8856] |
| ENSG00000147613 | HGLibA_39067 | 288 | 1.470365267 | PSKH2 | protein serine kinase H2 [Source:HGNC Symbol;Acc:HGNC:18997] |
| ENSG00000153395 | HGLibA_27070 | 288 | 1.470365267 | LPCAT1 | lysophosphatidylcholine acyltransferase 1 [Source:HGNC Symbol;Acc:HGNC:25718] |
| ENSG00000154252 | HGLibA_18500 | 288 | 1.470365267 | GAL3ST2 | galactose-3-O-sulfotransferase 2 [Source:HGNC Symbol;Acc:HGNC:24869] |
| ENSG00000160185 | HGLibA_52559 | 288 | 1.470365267 | UBASH3A | ubiquitin associated and SH3 domain containing A [Source:HGNC Symbol;Acc:HGNC:12462] |
| ENSG00000160207 | HGLibA_22225 | 288 | 1.470365267 | HSF2BP | heat shock transcription factor 2 binding protein [Source:HGNC Symbol;Acc:HGNC:5226] |
| ENSG00000164597 | HGLibA_10482 | 288 | 1.470365267 | COG5 | component of oligomeric golgi complex 5 [Source:HGNC Symbol;Acc:HGNC:14857] |
| ENSG00000169896 | HGLibA_23760 | 288 | 1.470365267 | ITGAM | integrin subunit alpha M [Source:HGNC Symbol;Acc:HGNC:6149] |
| ENSG00000171136 | HGLibA_41358 | 288 | 1.470365267 | RLN3 | relaxin 3 [Source:HGNC Symbol;Acc:HGNC:17135] |
| ENSG00000184454 | HGLibA_31331 | 288 | 1.470365267 | NCMAP | non-compact myelin associated protein [Source:HGNC Symbol;Acc:HGNC:29332] |
| ENSG00000186272 | HGLibA_55523 | 288 | 1.470365267 | ZNF17 | zinc finger protein 17 [Source:HGNC Symbol;Acc:HGNC:12958] |
| ENSG00000186329 | HGLibA_50210 | 288 | 1.470365267 | TMEM212 | transmembrane protein 212 [Source:HGNC Symbol;Acc:HGNC:34295] |
| ENSG00000197465 | HGLibA_20706 | 288 | 1.470365267 | GYPE | glycophorin E (MNS blood group) [Source:HGNC Symbol;Acc:HGNC:4705] |
| ENSG00000198848 | HGLibA_09217 | 288 | 1.470365267 | CES1 | carboxylesterase 1 [Source:HGNC Symbol;Acc:HGNC:1863] |
| ENSG00000204923 | HGLibA_17117 | 288 | 1.470365267 | FBXO48 | F-box protein 48 [Source:HGNC Symbol;Acc:HGNC:33857] |
| ENSG00000215187 | HGLibA_16236 | 288 | 1.470365267 | FAM166B | family with sequence similarity 166 member B [Source:HGNC Symbol;Acc:HGNC:34242] |
| ENSG00000237330 | HGLibA_41647 | 288 | 1.470365267 | RNF223 | ring finger protein 223 [Source:HGNC Symbol;Acc:HGNC:40020] |
| ENSG00000261210 | HGLibA_09951 | 288 | 1.470365267 | CLEC19A | C-type lectin domain containing 19A [Source:HGNC Symbol;Acc:HGNC:34522] |
| hsa-mir-144 | HGLibA_57840 | 288 | 1.470365267 | hsa-mir-144 | - |
| hsa-mir-4504 | HGLibA_60443 | 288 | 1.470365267 | hsa-mir-4504 | - |
| hsa-mir-4780 | HGLibA_61229 | 288 | 1.470365267 | hsa-mir-4780 | - |
| hsa-mir-890 | HGLibA_64238 | 288 | 1.470365267 | hsa-mir-890 | - |
| KIAA1737 | HGLibA_24815 | 288 | 1.470365267 | KIAA1737 | - |
| PVRL4 | HGLibA_39631 | 288 | 1.470365267 | PVRL4 | - |
| SELV | HGLibA_43316 | 288 | 1.470365267 | SELV | - |
| ENSG00000022976 | HGLibA_56849 | 287 | 1.461319924 | ZNF839 | zinc finger protein 839 [Source:HGNC Symbol;Acc:HGNC:20345] |

| | | | | | |
|---|---|---|---|---|---|
| ENSG00000091317 | HGLibA_10225 | 287 | 1.461319924 | CMTM6 | CKLF like MARVEL transmembrane domain containing 6 [Source:HGNC Symbol;Acc:HGNC:19177] |
| ENSG00000092470 | HGLibA_54179 | 287 | 1.461319924 | WDR76 | WD repeat domain 76 [Source:HGNC Symbol;Acc:HGNC:25773] |
| ENSG00000100577 | HGLibA_20504 | 287 | 1.461319924 | GSTZ1 | glutathione S-transferase zeta 1 [Source:HGNC Symbol;Acc:HGNC:4643] |
| ENSG00000101017 | HGLibA_08384 | 287 | 1.461319924 | CD40 | CD40 molecule [Source:HGNC Symbol;Acc:HGNC:11919] |
| ENSG00000107872 | HGLibA_16981 | 287 | 1.461319924 | FBXL15 | F-box and leucine rich repeat protein 15 [Source:HGNC Symbol;Acc:HGNC:28155] |
| ENSG00000108702 | HGLibA_07961 | 287 | 1.461319924 | CCL1 | C-C motif chemokine ligand 1 [Source:HGNC Symbol;Acc:HGNC:10609] |
| ENSG00000116133 | HGLibA_13091 | 287 | 1.461319924 | DHCR24 | 24-dehydrocholesterol reductase [Source:HGNC Symbol;Acc:HGNC:2859] |
| ENSG00000125813 | HGLibA_35296 | 287 | 1.461319924 | PAX1 | paired box 1 [Source:HGNC Symbol;Acc:HGNC:8615] |
| ENSG00000126214 | HGLibA_25028 | 287 | 1.461319924 | KLC1 | kinesin light chain 1 [Source:HGNC Symbol;Acc:HGNC:6387] |
| ENSG00000126804 | HGLibA_54812 | 287 | 1.461319924 | ZBTB1 | zinc finger and BTB domain containing 1 [Source:HGNC Symbol;Acc:HGNC:20259] |
| ENSG00000130538 | HGLibA_33569 | 287 | 1.461319924 | OR11H1 | olfactory receptor family 11 subfamily H member 1 [Source:HGNC Symbol;Acc:HGNC:15404] |
| ENSG00000133028 | HGLibA_43024 | 287 | 1.461319924 | SCO1 | cytochrome c oxidase assembly protein [Source:HGNC Symbol;Acc:HGNC:10603]" |
| ENSG00000148053 | HGLibA_32877 | 287 | 1.461319924 | NTRK2 | neurotrophic receptor tyrosine kinase 2 [Source:HGNC Symbol;Acc:HGNC:8032] |
| ENSG00000148154 | HGLibA_52860 | 287 | 1.461319924 | UGCG | UDP-glucose ceramide glucosyltransferase [Source:HGNC Symbol;Acc:HGNC:12524] |
| ENSG00000149761 | HGLibA_32973 | 287 | 1.461319924 | NUDT22 | nudix hydrolase 22 [Source:HGNC Symbol;Acc:HGNC:28189] |
| ENSG00000155660 | HGLibA_35879 | 287 | 1.461319924 | PDIA4 | protein disulfide isomerase family A member 4 [Source:HGNC Symbol;Acc:HGNC:30167] |
| ENSG00000162849 | HGLibA_24920 | 287 | 1.461319924 | KIF26B | kinesin family member 26B [Source:HGNC Symbol;Acc:HGNC:25484] |
| ENSG00000163866 | HGLibA_45726 | 287 | 1.461319924 | SMIM12 | small integral membrane protein 12 [Source:HGNC Symbol;Acc:HGNC:25154] |
| ENSG00000164253 | HGLibA_54087 | 287 | 1.461319924 | WDR41 | WD repeat domain 41 [Source:HGNC Symbol;Acc:HGNC:25601] |
| ENSG00000164532 | HGLibA_48660 | 287 | 1.461319924 | TBX20 | T-box 20 [Source:HGNC Symbol;Acc:HGNC:11598] |
| ENSG00000166405 | HGLibA_41233 | 287 | 1.461319924 | RIC3 | RIC3 acetylcholine receptor chaperone [Source:HGNC Symbol;Acc:HGNC:30338] |
| ENSG00000166900 | HGLibA_47646 | 287 | 1.461319924 | STX3 | syntaxin 3 [Source:HGNC Symbol;Acc:HGNC:11438] |
| ENSG00000168661 | HGLibA_55768 | 287 | 1.461319924 | ZNF30 | zinc finger protein 30 [Source:HGNC Symbol;Acc:HGNC:13090] |
| ENSG00000168795 | HGLibA_54910 | 287 | 1.461319924 | ZBTB5 | zinc finger and BTB domain containing 5 [Source:HGNC Symbol;Acc:HGNC:23836] |
| ENSG00000175193 | HGLibA_35201 | 287 | 1.461319924 | PARL | presenilin associated rhomboid like [Source:HGNC Symbol;Acc:HGNC:18253] |
| ENSG00000177706 | HGLibA_16449 | 287 | 1.461319924 | FAM20C | golgi associated secretory pathway kinase [Source:HGNC Symbol;Acc:HGNC:22140]" |
| ENSG00000184500 | HGLibA_38688 | 287 | 1.461319924 | PROS1 | protein S [Source:HGNC Symbol;Acc:HGNC:9456] |
| ENSG00000198739 | HGLibA_27492 | 287 | 1.461319924 | LRRTM3 | leucine rich repeat transmembrane neuronal 3 [Source:HGNC Symbol;Acc:HGNC:19410] |
| ENSG00000198848 | HGLibA_09219 | 287 | 1.461319924 | CES1 | carboxylesterase 1 [Source:HGNC Symbol;Acc:HGNC:1863] |
| ENSG00000205143 | HGLibA_02898 | 287 | 1.461319924 | ARID3C | AT-rich interaction domain 3C [Source:HGNC Symbol;Acc:HGNC:21209] |
| ENSG00000212658 | HGLibA_25736 | 287 | 1.461319924 | KRTAP29-1 | keratin associated protein 29-1 [Source:HGNC Symbol;Acc:HGNC:34211] |
| ENSG00000247315 | HGLibA_55034 | 287 | 1.461319924 | ZCCHC3 | zinc finger CCHC-type containing 3 [Source:HGNC Symbol;Acc:HGNC:16230] |
| FAM84A | HGLibA_16765 | 287 | 1.461319924 | FAM84A | - |
| hsa-mir-31 | HGLibA_58494 | 287 | 1.461319924 | hsa-mir-31 | - |
| hsa-mir-5197 | HGLibA_61706 | 287 | 1.461319924 | hsa-mir-5197 | - |
| TMEM110-MUSTN1 | HGLibA_49878 | 287 | 1.461319924 | TMEM110-MUSTN1 | - |
| ZCCHC16 | HGLibA_55018 | 287 | 1.461319924 | ZCCHC16 | - |
| C6orf1 | HGLibA_06429 | 286 | 1.452274581 | C6orf1 | - |
| ENSG00000048342 | HGLibA_07482 | 286 | 1.452274581 | CC2D2A | coiled-coil and C2 domain containing 2A [Source:HGNC Symbol;Acc:HGNC:29253] |
| ENSG00000066084 | HGLibA_13251 | 286 | 1.452274581 | DIP2B | disco interacting protein 2 homolog B [Source:HGNC Symbol;Acc:HGNC:29284] |
| ENSG00000092853 | HGLibA_10132 | 286 | 1.452274581 | CLSPN | claspin [Source:HGNC Symbol;Acc:HGNC:19715] |
| ENSG00000102001 | HGLibA_06890 | 286 | 1.452274581 | CACNA1F | calcium voltage-gated channel subunit alpha1 F [Source:HGNC Symbol;Acc:HGNC:1393] |
| ENSG00000102753 | HGLibA_25359 | 286 | 1.452274581 | KPNA3 | karyopherin subunit alpha 3 [Source:HGNC Symbol;Acc:HGNC:6396] |
| ENSG00000106348 | HGLibA_23255 | 286 | 1.452274581 | IMPDH1 | inosine monophosphate dehydrogenase 1 [Source:HGNC Symbol;Acc:HGNC:6052] |
| ENSG00000109814 | HGLibA_52863 | 286 | 1.452274581 | UGDH | UDP-glucose 6-dehydrogenase [Source:HGNC Symbol;Acc:HGNC:12525] |
| ENSG00000109991 | HGLibA_34851 | 286 | 1.452274581 | P2RX3 | purinergic receptor P2X 3 [Source:HGNC Symbol;Acc:HGNC:8534] |
| ENSG00000110002 | HGLibA_53908 | 286 | 1.452274581 | VWA5A | von Willebrand factor A domain containing 5A [Source:HGNC Symbol;Acc:HGNC:6658] |
| ENSG00000112337 | HGLibA_44462 | 286 | 1.452274581 | SLC17A2 | solute carrier family 17 member 2 [Source:HGNC Symbol;Acc:HGNC:10930] |
| ENSG00000115649 | HGLibA_10359 | 286 | 1.452274581 | CNPPD1 | cyclin Pas1/PHO80 domain containing 1 [Source:HGNC Symbol;Acc:HGNC:25220] |
| ENSG00000115956 | HGLibA_37056 | 286 | 1.452274581 | PLEK | pleckstrin [Source:HGNC Symbol;Acc:HGNC:9070] |
| ENSG00000122550 | HGLibA_25221 | 286 | 1.452274581 | KLHL7 | kelch like family member 7 [Source:HGNC Symbol;Acc:HGNC:15646] |
| ENSG00000132031 | HGLibA_28433 | 286 | 1.452274581 | MATN3 | matrilin 3 [Source:HGNC Symbol;Acc:HGNC:6909] |
| ENSG00000134030 | HGLibA_11633 | 286 | 1.452274581 | CTIF | cap binding complex dependent translation initiation factor [Source:HGNC Symbol;Acc:HGNC:23925] |
| ENSG00000137877 | HGLibA_46888 | 286 | 1.452274581 | SPTBN5 | non-erythrocytic 5 [Source:HGNC Symbol;Acc:HGNC:15680]" |
| ENSG00000139618 | HGLibA_04765 | 286 | 1.452274581 | BRCA2 | DNA repair associated [Source:HGNC Symbol;Acc:HGNC:1101]" |
| ENSG00000141503 | HGLibA_29275 | 286 | 1.452274581 | MINK1 | misshapen like kinase 1 [Source:HGNC Symbol;Acc:HGNC:17565] |
| ENSG00000144655 | HGLibA_11422 | 286 | 1.452274581 | CSRNP1 | cysteine and serine rich nuclear protein 1 [Source:HGNC Symbol;Acc:HGNC:14300] |
| ENSG00000147166 | HGLibA_23776 | 286 | 1.452274581 | ITGB1BP2 | integrin subunit beta 1 binding protein 2 [Source:HGNC Symbol;Acc:HGNC:6154] |
| ENSG00000162598 | HGLibA_05904 | 286 | 1.452274581 | C1orf87 | chromosome 1 open reading frame 87 [Source:HGNC Symbol;Acc:HGNC:28547] |
| ENSG00000167580 | HGLibA_02591 | 286 | 1.452274581 | AQP2 | aquaporin 2 [Source:HGNC Symbol;Acc:HGNC:634] |
| ENSG00000168646 | HGLibA_03923 | 286 | 1.452274581 | AXIN2 | axin 2 [Source:HGNC Symbol;Acc:HGNC:904] |
| ENSG00000169570 | HGLibA_14076 | 286 | 1.452274581 | DTWD2 | DTW domain containing 2 [Source:HGNC Symbol;Acc:HGNC:19334] |
| ENSG00000169727 | HGLibA_20149 | 286 | 1.452274581 | GPS1 | G protein pathway suppressor 1 [Source:HGNC Symbol;Acc:HGNC:4549] |
| ENSG00000171234 | HGLibA_52930 | 286 | 1.452274581 | UGT2B7 | UDP glucuronosyltransferase family 2 member B7 [Source:HGNC Symbol;Acc:HGNC:12554] |
| ENSG00000171388 | HGLibA_02435 | 286 | 1.452274581 | APLN | apelin [Source:HGNC Symbol;Acc:HGNC:16665] |
| ENSG00000171551 | HGLibA_14387 | 286 | 1.452274581 | ECEL1 | endothelin converting enzyme like 1 [Source:HGNC Symbol;Acc:HGNC:3147] |
| ENSG00000177143 | HGLibA_09232 | 286 | 1.452274581 | CETN1 | centrin 1 [Source:HGNC Symbol;Acc:HGNC:1866] |
| ENSG00000180878 | HGLibA_05138 | 286 | 1.452274581 | C11orf42 | chromosome 11 open reading frame 42 [Source:HGNC Symbol;Acc:HGNC:28541] |
| ENSG00000185950 | HGLibA_23623 | 286 | 1.452274581 | IRS2 | insulin receptor substrate 2 [Source:HGNC Symbol;Acc:HGNC:6126] |
| ENSG00000186684 | HGLibA_12099 | 286 | 1.452274581 | CYP27C1 | cytochrome P450 family 27 subfamily C member 1 [Source:HGNC Symbol;Acc:HGNC:33480] |
| ENSG00000187713 | HGLibA_50187 | 286 | 1.452274581 | TMEM203 | transmembrane protein 203 [Source:HGNC Symbol;Acc:HGNC:28217] |
| ENSG00000197557 | HGLibA_52105 | 286 | 1.452274581 | TTC30A | tetratricopeptide repeat domain 30A [Source:HGNC Symbol;Acc:HGNC:25853] |
| ENSG00000198670 | HGLibA_27050 | 286 | 1.452274581 | LPA | lipoprotein(a) [Source:HGNC Symbol;Acc:HGNC:6667] |
| ENSG00000198846 | HGLibA_51036 | 286 | 1.452274581 | TOX | thymocyte selection associated high mobility group box [Source:HGNC Symbol;Acc:HGNC:18988] |
| ENSG00000204941 | HGLibA_39048 | 286 | 1.452274581 | PSG5 | pregnancy specific beta-1-glycoprotein 5 [Source:HGNC Symbol;Acc:HGNC:9522] |
| ENSG00000205899 | HGLibA_04469 | 286 | 1.452274581 | BHLHA9 | basic helix-loop-helix family member a9 [Source:HGNC Symbol;Acc:HGNC:35126] |
| FAM196A | HGLibA_16381 | 286 | 1.452274581 | FAM196A | - |
| hsa-mir-4316 | HGLibA_59982 | 286 | 1.452274581 | hsa-mir-4316 | - |
| hsa-mir-4518 | HGLibA_60517 | 286 | 1.452274581 | hsa-mir-4518 | - |
| hsa-mir-548az | HGLibA_61947 | 286 | 1.452274581 | hsa-mir-548az | - |
| TMEM155 | HGLibA_50010 | 286 | 1.452274581 | TMEM155 | - |
| ENSG00000005889 | HGLibA_55263 | 285 | 1.443229238 | ZFX | zinc finger protein X-linked [Source:HGNC Symbol;Acc:HGNC:12869] |
| ENSG00000047056 | HGLibA_54079 | 285 | 1.443229238 | WDR37 | WD repeat domain 37 [Source:HGNC Symbol;Acc:HGNC:31406] |
| ENSG00000077157 | HGLibA_38024 | 285 | 1.443229238 | PPP1R12B | protein phosphatase 1 regulatory subunit 12B [Source:HGNC Symbol;Acc:HGNC:7619] |
| ENSG00000078269 | HGLibA_48006 | 285 | 1.443229238 | SYNJ2 | synaptojanin 2 [Source:HGNC Symbol;Acc:HGNC:11504] |
| ENSG00000100300 | HGLibA_51957 | 285 | 1.443229238 | TSPO | translocator protein [Source:HGNC Symbol;Acc:HGNC:1158] |
| ENSG00000102886 | HGLibA_18896 | 285 | 1.443229238 | GDPD3 | glycerophosphodiester phosphodiesterase domain containing 3 [Source:HGNC Symbol;Acc:HGNC:28638] |
| ENSG00000103313 | HGLibA_28845 | 285 | 1.443229238 | MEFV | pyrin innate immunity regulator [Source:HGNC Symbol;Acc:HGNC:6998]" |
| ENSG00000105677 | HGLibA_49977 | 285 | 1.443229238 | TMEM147 | transmembrane protein 147 [Source:HGNC Symbol;Acc:HGNC:30414] |
| ENSG00000105855 | HGLibA_23800 | 285 | 1.443229238 | ITGB8 | integrin subunit beta 8 [Source:HGNC Symbol;Acc:HGNC:6163] |
| ENSG00000107959 | HGLibA_36758 | 285 | 1.443229238 | PITRM1 | pitrilysin metallopeptidase 1 [Source:HGNC Symbol;Acc:HGNC:17663] |
| ENSG00000110851 | HGLibA_38389 | 285 | 1.443229238 | PRDM4 | PR/SET domain 4 [Source:HGNC Symbol;Acc:HGNC:9348] |
| ENSG00000112697 | HGLibA_50361 | 285 | 1.443229238 | TMEM30A | transmembrane protein 30A [Source:HGNC Symbol;Acc:HGNC:16667] |
| ENSG00000116717 | HGLibA_18417 | 285 | 1.443229238 | GADD45A | growth arrest and DNA damage inducible alpha [Source:HGNC Symbol;Acc:HGNC:4095] |
| ENSG00000123810 | HGLibA_04048 | 285 | 1.443229238 | B9D2 | B9 domain containing 2 [Source:HGNC Symbol;Acc:HGNC:28636] |
| ENSG00000124232 | HGLibA_40650 | 285 | 1.443229238 | RBPJL | recombination signal binding protein for immunoglobulin kappa J region like [Source:HGNC Symbol;Acc:HGNC:13761] |
| ENSG00000135218 | HGLibA_08359 | 285 | 1.443229238 | CD36 | CD36 molecule [Source:HGNC Symbol;Acc:HGNC:1663] |
| ENSG00000144847 | HGLibA_22881 | 285 | 1.443229238 | IGSF11 | immunoglobulin superfamily member 11 [Source:HGNC Symbol;Acc:HGNC:16669] |
| ENSG00000146376 | HGLibA_02716 | 285 | 1.443229238 | ARHGAP18 | Rho GTPase activating protein 18 [Source:HGNC Symbol;Acc:HGNC:21035] |
| ENSG00000147081 | HGLibA_01432 | 285 | 1.443229238 | AKAP4 | A-kinase anchoring protein 4 [Source:HGNC Symbol;Acc:HGNC:374] |
| ENSG00000155008 | HGLibA_02554 | 285 | 1.443229238 | APOOL | apolipoprotein O like [Source:HGNC Symbol;Acc:HGNC:24009] |
| ENSG00000164649 | HGLibA_08616 | 285 | 1.443229238 | CDCA7L | cell division cycle associated 7 like [Source:HGNC Symbol;Acc:HGNC:30777] |
| ENSG00000164796 | HGLibA_11369 | 285 | 1.443229238 | CSMD3 | CUB and Sushi multiple domains 3 [Source:HGNC Symbol;Acc:HGNC:19291] |
| ENSG00000165272 | HGLibA_02592 | 285 | 1.443229238 | AQP3 | aquaporin 3 (Gill blood group) [Source:HGNC Symbol;Acc:HGNC:636] |
| ENSG00000177201 | HGLibA_33859 | 285 | 1.443229238 | OR2T12 | olfactory receptor family 2 subfamily T member 12 [Source:HGNC Symbol;Acc:HGNC:19592] |
| ENSG00000179041 | HGLibA_42289 | 285 | 1.443229238 | RRS1 | ribosome biogenesis regulator homolog [Source:HGNC Symbol;Acc:HGNC:17083] |
| ENSG00000180332 | HGLibA_24454 | 285 | 1.443229238 | KCTD4 | potassium channel tetramerization domain containing 4 [Source:HGNC Symbol;Acc:HGNC:23227] |
| ENSG00000181552 | HGLibA_14450 | 285 | 1.443229238 | EDDM3B | epididymal protein 3B [Source:HGNC Symbol;Acc:HGNC:19223] |
| ENSG00000182087 | HGLibA_50344 | 285 | 1.443229238 | TMEM259 | transmembrane protein 259 [Source:HGNC Symbol;Acc:HGNC:17039] |
| ENSG00000183153 | HGLibA_19191 | 285 | 1.443229238 | GJD3 | gap junction protein delta 3 [Source:HGNC Symbol;Acc:HGNC:19147] |
| ENSG00000185278 | HGLibA_54867 | 285 | 1.443229238 | ZBTB37 | zinc finger and BTB domain containing 37 [Source:HGNC Symbol;Acc:HGNC:28365] |
| ENSG00000185483 | HGLibA_41770 | 285 | 1.443229238 | ROR1 | receptor tyrosine kinase like orphan receptor 1 [Source:HGNC Symbol;Acc:HGNC:10256] |
| ENSG00000187258 | HGLibA_32527 | 285 | 1.443229238 | NPSR1 | neuropeptide S receptor 1 [Source:HGNC Symbol;Acc:HGNC:23631] |
| ENSG00000189409 | HGLibA_29515 | 285 | 1.443229238 | MMP23B | matrix metallopeptidase 23B [Source:HGNC Symbol;Acc:HGNC:7171] |
| ENSG00000196482 | HGLibA_15600 | 285 | 1.443229238 | ESRRG | estrogen related receptor gamma [Source:HGNC Symbol;Acc:HGNC:3474] |
| ENSG00000231396 | HGLibA_53214 | 285 | 1.443229238 | USP17L10 | ubiquitin specific peptidase 17-like family member 10 [Source:HGNC Symbol;Acc:HGNC:44438] |
| ENSG00000280090 | HGLibA_34501 | 285 | 1.443229238 | OR8B4 | olfactory receptor family 8 subfamily B member 4 (gene/pseudogene) [Source:HGNC Symbol;Acc:HGNC:8473] |
| hsa-mir-4424 | HGLibA_60084 | 285 | 1.443229238 | hsa-mir-4424 | - |
| hsa-mir-4724 | HGLibA_60992 | 285 | 1.443229238 | hsa-mir-4724 | - |
| LOC100129361 | HGLibA_26758 | 285 | 1.443229238 | LOC100129361 | - |
| LOC100130480 | HGLibA_26789 | 285 | 1.443229238 | LOC100130480 | - |
| LPPR4 | HGLibA_27123 | 285 | 1.443229238 | LPPR4 | - |
| MUT | HGLibA_30583 | 285 | 1.443229238 | MUT | - |
| ENSG00000058404 | HGLibA_07070 | 284 | 1.434183895 | CAMK2B | calcium/calmodulin dependent protein kinase II beta [Source:HGNC Symbol;Acc:HGNC:1461] |
| ENSG00000069018 | HGLibA_51748 | 284 | 1.434183895 | TRPC7 | transient receptor potential cation channel subfamily C member 7 [Source:HGNC Symbol;Acc:HGNC:20754] |
| ENSG00000072609 | HGLibA_09427 | 284 | 1.434183895 | CHFR | checkpoint with forkhead and ring finger domains [Source:HGNC Symbol;Acc:HGNC:20455] |
| ENSG00000100450 | HGLibA_20723 | 284 | 1.434183895 | GZMH | granzyme H [Source:HGNC Symbol;Acc:HGNC:4710] |
| ENSG00000100994 | HGLibA_39685 | 284 | 1.434183895 | PYGB | glycogen phosphorylase B [Source:HGNC Symbol;Acc:HGNC:9723] |
| ENSG00000101098 | HGLibA_41286 | 284 | 1.434183895 | RIMS4 | regulating synaptic membrane exocytosis 4 [Source:HGNC Symbol;Acc:HGNC:16183] |
| ENSG00000102076 | HGLibA_33422 | 284 | 1.434183895 | OPN1LW | long wave sensitive [Source:HGNC Symbol;Acc:HGNC:9936]" |
| ENSG00000128310 | HGLibA_18594 | 284 | 1.434183895 | GALR3 | galanin receptor 3 [Source:HGNC Symbol;Acc:HGNC:4134] |
| ENSG00000130338 | HGLibA_52338 | 284 | 1.434183895 | TULP4 | tubby like protein 4 [Source:HGNC Symbol;Acc:HGNC:15530] |
| ENSG00000134369 | HGLibA_31193 | 284 | 1.434183895 | NAV1 | neuron navigator 1 [Source:HGNC Symbol;Acc:HGNC:15989] |
| ENSG00000151079 | HGLibA_24141 | 284 | 1.434183895 | KCNA6 | potassium voltage-gated channel subfamily A member 6 [Source:HGNC Symbol;Acc:HGNC:6225] |
| ENSG00000151500 | HGLibA_49427 | 284 | 1.434183895 | THYN1 | thymocyte nuclear protein 1 [Source:HGNC Symbol;Acc:HGNC:29560] |

| | | | | | |
|---|---|---|---|---|---|
| ENSG00000166928 | HGLibA_30138 | 284 | 1.434183895 | MS4A14 | membrane spanning 4-domains A14 [Source:HGNC Symbol;Acc:HGNC:30706] |
| ENSG00000171723 | HGLibA_19805 | 284 | 1.434183895 | GPHN | gephyrin [Source:HGNC Symbol;Acc:HGNC:15465] |
| ENSG00000172878 | HGLibA_28932 | 284 | 1.434183895 | METAP1D | mitochondrial [Source:HGNC Symbol;Acc:HGNC:32583]" |
| ENSG00000172890 | HGLibA_31053 | 284 | 1.434183895 | NADSYN1 | NAD synthetase 1 [Source:HGNC Symbol;Acc:HGNC:29832] |
| ENSG00000173250 | HGLibA_19932 | 284 | 1.434183895 | GPR151 | G protein-coupled receptor 151 [Source:HGNC Symbol;Acc:HGNC:23624] |
| ENSG00000173626 | HGLibA_51322 | 284 | 1.434183895 | TRAPPC3L | trafficking protein particle complex 3 like [Source:HGNC Symbol;Acc:HGNC:21090] |
| ENSG00000186469 | HGLibA_19521 | 284 | 1.434183895 | GNG2 | G protein subunit gamma 2 [Source:HGNC Symbol;Acc:HGNC:4404] |
| ENSG00000196834 | HGLibA_37740 | 284 | 1.434183895 | POTEI | POTE ankyrin domain family member I [Source:HGNC Symbol;Acc:HGNC:37093] |
| ENSG00000196865 | HGLibA_31899 | 284 | 1.434183895 | NHLRC2 | NHL repeat containing 2 [Source:HGNC Symbol;Acc:HGNC:24731] |
| ENSG00000196911 | HGLibA_25366 | 284 | 1.434183895 | KPNA5 | karyopherin subunit alpha 5 [Source:HGNC Symbol;Acc:HGNC:6398] |
| ENSG00000198816 | HGLibA_55869 | 284 | 1.434183895 | ZNF358 | zinc finger protein 358 [Source:HGNC Symbol;Acc:HGNC:16838] |
| ENSG00000205456 | HGLibA_51086 | 284 | 1.434183895 | TP53TG3D | TP53 target 3D [Source:HGNC Symbol;Acc:HGNC:44657] |
| ENSG00000205476 | HGLibA_07899 | 284 | 1.434183895 | CCDC85C | coiled-coil domain containing 85C [Source:HGNC Symbol;Acc:HGNC:35459] |
| ENSG00000214517 | HGLibA_37999 | 284 | 1.434183895 | PPME1 | protein phosphatase methylesterase 1 [Source:HGNC Symbol;Acc:HGNC:30178] |
| ENSG00000232268 | HGLibA_34171 | 284 | 1.434183895 | OR52I1 | olfactory receptor family 52 subfamily I member 1 [Source:HGNC Symbol;Acc:HGNC:15220] |
| ENSG00000254415 | HGLibA_44134 | 284 | 1.434183895 | SIGLEC14 | sialic acid binding Ig like lectin 14 [Source:HGNC Symbol;Acc:HGNC:32926] |
| ENSG00000277322 | HGLibA_19629 | 284 | 1.434183895 | GOLGA6L6 | golgin A6 family-like 6 [Source:HGNC Symbol;Acc:HGNC:37225] |
| hsa-mir-1289-2 | HGLibA_57625 | 284 | 1.434183895 | hsa-mir-1289-2 | - |
| CXXC11 | HGLibA_11969 | 283 | 1.425138552 | CXXC11 | - |
| ENSG00000069764 | HGLibA_36866 | 283 | 1.425138552 | PLA2G10 | phospholipase A2 group X [Source:HGNC Symbol;Acc:HGNC:9029] |
| ENSG00000070087 | HGLibA_36176 | 283 | 1.425138552 | PFN2 | profilin 2 [Source:HGNC Symbol;Acc:HGNC:8882] |
| ENSG00000084090 | HGLibA_47371 | 283 | 1.425138552 | STARD7 | StAR related lipid transfer domain containing 7 [Source:HGNC Symbol;Acc:HGNC:18063] |
| ENSG00000100053 | HGLibA_11268 | 283 | 1.425138552 | CRYBB3 | crystallin beta B3 [Source:HGNC Symbol;Acc:HGNC:2400] |
| ENSG00000100299 | HGLibA_03129 | 283 | 1.425138552 | ARSA | arylsulfatase A [Source:HGNC Symbol;Acc:HGNC:713] |
| ENSG00000100302 | HGLibA_40324 | 283 | 1.425138552 | RASD2 | RASD family member 2 [Source:HGNC Symbol;Acc:HGNC:18229] |
| ENSG00000103479 | HGLibA_40471 | 283 | 1.425138552 | RBL2 | RB transcriptional corepressor like 2 [Source:HGNC Symbol;Acc:HGNC:9894] |
| ENSG00000104951 | HGLibA_23170 | 283 | 1.425138552 | IL4I1 | interleukin 4 induced 1 [Source:HGNC Symbol;Acc:HGNC:19094] |
| ENSG00000105221 | HGLibA_01511 | 283 | 1.425138552 | AKT2 | AKT serine/threonine kinase 2 [Source:HGNC Symbol;Acc:HGNC:392] |
| ENSG00000105948 | HGLibA_52091 | 283 | 1.425138552 | TTC26 | tetratricopeptide repeat domain 26 [Source:HGNC Symbol;Acc:HGNC:21882] |
| ENSG00000107175 | HGLibA_11090 | 283 | 1.425138552 | CREB3 | cAMP responsive element binding protein 3 [Source:HGNC Symbol;Acc:HGNC:2347] |
| ENSG00000116001 | HGLibA_49430 | 283 | 1.425138552 | TIA1 | TIA1 cytotoxic granule associated RNA binding protein [Source:HGNC Symbol;Acc:HGNC:11802] |
| ENSG00000123240 | HGLibA_33458 | 283 | 1.425138552 | OPTN | optineurin [Source:HGNC Symbol;Acc:HGNC:17142] |
| ENSG00000130997 | HGLibA_37543 | 283 | 1.425138552 | POLN | DNA polymerase nu [Source:HGNC Symbol;Acc:HGNC:18870] |
| ENSG00000132535 | HGLibA_13334 | 283 | 1.425138552 | DLG4 | discs large MAGUK scaffold protein 4 [Source:HGNC Symbol;Acc:HGNC:2903] |
| ENSG00000134873 | HGLibA_09849 | 283 | 1.425138552 | CLDN10 | claudin 10 [Source:HGNC Symbol;Acc:HGNC:2033] |
| ENSG00000138688 | HGLibA_24695 | 283 | 1.425138552 | KIAA1109 | KIAA1109 [Source:HGNC Symbol;Acc:HGNC:26953] |
| ENSG00000140545 | HGLibA_29061 | 283 | 1.425138552 | MFGE8 | milk fat globule-EGF factor 8 protein [Source:HGNC Symbol;Acc:HGNC:7036] |
| ENSG00000141012 | HGLibA_18523 | 283 | 1.425138552 | GALNS | galactosamine (N-acetyl)-6-sulfatase [Source:HGNC Symbol;Acc:HGNC:4122] |
| ENSG00000145321 | HGLibA_18754 | 283 | 1.425138552 | GC | vitamin D binding protein [Source:HGNC Symbol;Acc:HGNC:4187]" |
| ENSG00000157350 | HGLibA_47240 | 283 | 1.425138552 | ST3GAL2 | 3-sialyltransferase 2 [Source:HGNC Symbol;Acc:HGNC:10863]" |
| ENSG00000158186 | HGLibA_29812 | 283 | 1.425138552 | MRAS | muscle RAS oncogene homolog [Source:HGNC Symbol;Acc:HGNC:7227] |
| ENSG00000158604 | HGLibA_49810 | 283 | 1.425138552 | TMED4 | transmembrane p24 trafficking protein 4 [Source:HGNC Symbol;Acc:HGNC:22301] |
| ENSG00000160323 | HGLibA_00799 | 283 | 1.425138552 | ADAMTS13 | ADAM metallopeptidase with thrombospondin type 1 motif 13 [Source:HGNC Symbol;Acc:HGNC:1366] |
| ENSG00000164330 | HGLibA_14349 | 283 | 1.425138552 | EBF1 | EBF transcription factor 1 [Source:HGNC Symbol;Acc:HGNC:3126] |
| ENSG00000167173 | HGLibA_05394 | 283 | 1.425138552 | C15orf39 | chromosome 15 open reading frame 39 [Source:HGNC Symbol;Acc:HGNC:24497] |
| ENSG00000167967 | HGLibA_14330 | 283 | 1.425138552 | E4F1 | E4F transcription factor 1 [Source:HGNC Symbol;Acc:HGNC:3121] |
| ENSG00000168955 | HGLibA_49699 | 283 | 1.425138552 | TM4SF20 | transmembrane 4 L six family member 20 [Source:HGNC Symbol;Acc:HGNC:26230] |
| ENSG00000169184 | HGLibA_29559 | 283 | 1.425138552 | MN1 | transcriptional regulator [Source:HGNC Symbol;Acc:HGNC:7180]" |
| ENSG00000169951 | HGLibA_56690 | 283 | 1.425138552 | ZNF764 | zinc finger protein 764 [Source:HGNC Symbol;Acc:HGNC:28200] |
| ENSG00000185220 | HGLibA_36220 | 283 | 1.425138552 | PGBD2 | piggyBac transposable element derived 2 [Source:HGNC Symbol;Acc:HGNC:19399] |
| ENSG00000198515 | HGLibA_10264 | 283 | 1.425138552 | CNGA1 | cyclic nucleotide gated channel alpha 1 [Source:HGNC Symbol;Acc:HGNC:2148] |
| ENSG00000198719 | HGLibA_13359 | 283 | 1.425138552 | DLL1 | delta like canonical Notch ligand 1 [Source:HGNC Symbol;Acc:HGNC:2908] |
| ENSG00000205445 | HGLibA_25604 | 283 | 1.425138552 | KRTAP10-2 | keratin associated protein 10-2 [Source:HGNC Symbol;Acc:HGNC:22967] |
| ENSG00000237765 | HGLibA_16413 | 283 | 1.425138552 | FAM200B | family with sequence similarity 200 member B [Source:HGNC Symbol;Acc:HGNC:27740] |
| ENSG00000267680 | HGLibA_55619 | 283 | 1.425138552 | ZNF224 | zinc finger protein 224 [Source:HGNC Symbol;Acc:HGNC:13017] |
| hsa-mir-3938 | HGLibA_59612 | 283 | 1.425138552 | hsa-mir-3938 | - |
| hsa-mir-4780 | HGLibA_61227 | 283 | 1.425138552 | hsa-mir-4780 | - |
| hsa-mir-4804 | HGLibA_61322 | 283 | 1.425138552 | hsa-mir-4804 | - |
| hsa-mir-7153 | HGLibA_63842 | 283 | 1.425138552 | hsa-mir-7153 | - |
| IL9R_X | HGLibA_23201 | 283 | 1.425138552 | IL9R_X | - |
| TMEM35 | HGLibA_50371 | 283 | 1.425138552 | TMEM35 | - |
| TNFAIP8L2-SCNM1 | HGLibA_50728 | 283 | 1.425138552 | TNFAIP8L2-SCNM1 | - |
| C20orf194 | HGLibA_05998 | 282 | 1.416093209 | C20orf194 | - |
| ENSG00000010327 | HGLibA_47307 | 282 | 1.416093209 | STAB1 | stabilin 1 [Source:HGNC Symbol;Acc:HGNC:18628] |
| ENSG00000067715 | HGLibA_48045 | 282 | 1.416093209 | SYT1 | synaptotagmin 1 [Source:HGNC Symbol;Acc:HGNC:11509] |
| ENSG00000104093 | HGLibA_13457 | 282 | 1.416093209 | DMXL2 | Dmx like 2 [Source:HGNC Symbol;Acc:HGNC:2938] |
| ENSG00000104472 | HGLibA_09531 | 282 | 1.416093209 | CHRAC1 | chromatin accessibility complex subunit 1 [Source:HGNC Symbol;Acc:HGNC:13544] |
| ENSG00000107099 | HGLibA_13759 | 282 | 1.416093209 | DOCK8 | dedicator of cytokinesis 8 [Source:HGNC Symbol;Acc:HGNC:19191] |
| ENSG00000108984 | HGLibA_28155 | 282 | 1.416093209 | MAP2K6 | mitogen-activated protein kinase kinase 6 [Source:HGNC Symbol;Acc:HGNC:6846] |
| ENSG00000113369 | HGLibA_03118 | 282 | 1.416093209 | ARRDC3 | arrestin domain containing 3 [Source:HGNC Symbol;Acc:HGNC:29263] |
| ENSG00000113615 | HGLibA_43230 | 282 | 1.416093209 | SEC24A | COPII coat complex component [Source:HGNC Symbol;Acc:HGNC:10703]" |
| ENSG00000132254 | HGLibA_02680 | 282 | 1.416093209 | ARFIP2 | ADP ribosylation factor interacting protein 2 [Source:HGNC Symbol;Acc:HGNC:17160] |
| ENSG00000139574 | HGLibA_32436 | 282 | 1.416093209 | NPFF | neuropeptide FF-amide peptide precursor [Source:HGNC Symbol;Acc:HGNC:7901] |
| ENSG00000140464 | HGLibA_37313 | 282 | 1.416093209 | PML | promyelocytic leukemia [Source:HGNC Symbol;Acc:HGNC:9113] |
| ENSG00000145863 | HGLibA_18371 | 282 | 1.416093209 | GABRA6 | gamma-aminobutyric acid type A receptor alpha6 subunit [Source:HGNC Symbol;Acc:HGNC:4080] |
| ENSG00000152454 | HGLibA_55678 | 282 | 1.416093209 | ZNF256 | zinc finger protein 256 [Source:HGNC Symbol;Acc:HGNC:13049] |
| ENSG00000154175 | HGLibA_00298 | 282 | 1.416093209 | ABI3BP | ABI family member 3 binding protein [Source:HGNC Symbol;Acc:HGNC:17265] |
| ENSG00000159905 | HGLibA_55609 | 282 | 1.416093209 | ZNF221 | zinc finger protein 221 [Source:HGNC Symbol;Acc:HGNC:13014] |
| ENSG00000160710 | HGLibA_00870 | 282 | 1.416093209 | ADAR | RNA specific [Source:HGNC Symbol;Acc:HGNC:225]" |
| ENSG00000164619 | HGLibA_04627 | 282 | 1.416093209 | BMPER | BMP binding endothelial regulator [Source:HGNC Symbol;Acc:HGNC:24154] |
| ENSG00000166523 | HGLibA_09989 | 282 | 1.416093209 | CLEC4E | C-type lectin domain family 4 member E [Source:HGNC Symbol;Acc:HGNC:14555] |
| ENSG00000167601 | HGLibA_03926 | 282 | 1.416093209 | AXL | AXL receptor tyrosine kinase [Source:HGNC Symbol;Acc:HGNC:905] |
| ENSG00000168229 | HGLibA_39305 | 282 | 1.416093209 | PTGDR | prostaglandin D2 receptor [Source:HGNC Symbol;Acc:HGNC:9591] |
| ENSG00000168306 | HGLibA_00501 | 282 | 1.416093209 | ACOX2 | acyl-CoA oxidase 2 [Source:HGNC Symbol;Acc:HGNC:120] |
| ENSG00000169688 | HGLibA_30281 | 282 | 1.416093209 | MT1B | metallothionein 1B [Source:HGNC Symbol;Acc:HGNC:7394] |
| ENSG00000170956 | HGLibA_08938 | 282 | 1.416093209 | CEACAM3 | carcinoembryonic antigen related cell adhesion molecule 3 [Source:HGNC Symbol;Acc:HGNC:1815] |
| ENSG00000172243 | HGLibA_10006 | 282 | 1.416093209 | CLEC7A | C-type lectin domain containing 7A [Source:HGNC Symbol;Acc:HGNC:14558] |
| ENSG00000173432 | HGLibA_42609 | 282 | 1.416093209 | SAA1 | serum amyloid A1 [Source:HGNC Symbol;Acc:HGNC:10513] |
| ENSG00000177842 | HGLibA_56403 | 282 | 1.416093209 | ZNF620 | zinc finger protein 620 [Source:HGNC Symbol;Acc:HGNC:28742] |
| ENSG00000179284 | HGLibA_12306 | 282 | 1.416093209 | DAND5 | DAN domain BMP antagonist family member 5 [Source:HGNC Symbol;Acc:HGNC:26780] |
| ENSG00000180340 | HGLibA_18266 | 282 | 1.416093209 | FZD2 | frizzled class receptor 2 [Source:HGNC Symbol;Acc:HGNC:4040] |
| ENSG00000182968 | HGLibA_46225 | 282 | 1.416093209 | SOX1 | SRY-box 1 [Source:HGNC Symbol;Acc:HGNC:11189] |
| ENSG00000183395 | HGLibA_37295 | 282 | 1.416093209 | PMCH | pro-melanin concentrating hormone [Source:HGNC Symbol;Acc:HGNC:9109] |
| ENSG00000184007 | HGLibA_39406 | 282 | 1.416093209 | PTP4A2 | member 2 [Source:HGNC Symbol;Acc:HGNC:9635]" |
| ENSG00000184368 | HGLibA_28238 | 282 | 1.416093209 | MAP7D2 | MAP7 domain containing 2 [Source:HGNC Symbol;Acc:HGNC:25899] |
| ENSG00000186283 | HGLibA_51031 | 282 | 1.416093209 | TOR3A | torsin family 3 member A [Source:HGNC Symbol;Acc:HGNC:11997] |
| ENSG00000187474 | HGLibA_17970 | 282 | 1.416093209 | FPR3 | formyl peptide receptor 3 [Source:HGNC Symbol;Acc:HGNC:3828] |
| ENSG00000188186 | HGLibA_25998 | 282 | 1.416093209 | LAMTOR4 | MAPK and MTOR activator 4 [Source:HGNC Symbol;Acc:HGNC:33772]" |
| ENSG00000205784 | HGLibA_03125 | 282 | 1.416093209 | ARRDC5 | arrestin domain containing 5 [Source:HGNC Symbol;Acc:HGNC:31407] |
| ENSG00000214376 | HGLibA_53871 | 282 | 1.416093209 | VSTM5 | V-set and transmembrane domain containing 5 [Source:HGNC Symbol;Acc:HGNC:34443] |
| ENSG00000232810 | HGLibA_50702 | 282 | 1.416093209 | TNF | tumor necrosis factor [Source:HGNC Symbol;Acc:HGNC:11892] |
| ENSG00000255302 | HGLibA_14693 | 282 | 1.416093209 | EID1 | EP300 interacting inhibitor of differentiation 1 [Source:HGNC Symbol;Acc:HGNC:1191] |
| ENSG00000278870 | HGLibA_34108 | 282 | 1.416093209 | OR51G1 | olfactory receptor family 51 subfamily G member 1 (gene/pseudogene) [Source:HGNC Symbol;Acc:HGNC:14738] |
| ENSG00000283597 | HGLibA_16255 | 282 | 1.416093209 | FAM169B | family with sequence similarity 169 member B [Source:HGNC Symbol;Acc:HGNC:26835] |
| hsa-mir-3677 | HGLibA_59256 | 282 | 1.416093209 | hsa-mir-3677 | - |
| hsa-mir-4308 | HGLibA_59944 | 282 | 1.416093209 | hsa-mir-4308 | - |
| hsa-mir-760 | HGLibA_63902 | 282 | 1.416093209 | hsa-mir-760 | - |
| KDELC1 | HGLibA_24474 | 282 | 1.416093209 | KDELC1 | - |
| CCL3L1 | HGLibA_08021 | 281 | 1.407047866 | CCL3L1 | - |
| ENSG00000057019 | HGLibA_12471 | 281 | 1.407047866 | DCBLD2 | CUB and LCCL domain containing 2 [Source:HGNC Symbol;Acc:HGNC:24627]" |
| ENSG00000074964 | HGLibA_02809 | 281 | 1.407047866 | ARHGEF10L | Rho guanine nucleotide exchange factor 10 like [Source:HGNC Symbol;Acc:HGNC:25540] |
| ENSG00000087086 | HGLibA_18126 | 281 | 1.407047866 | FTL | ferritin light chain [Source:HGNC Symbol;Acc:HGNC:3999] |
| ENSG00000091073 | HGLibA_14081 | 281 | 1.407047866 | DTX2 | deltex E3 ubiquitin ligase 2 [Source:HGNC Symbol;Acc:HGNC:15973] |
| ENSG00000091664 | HGLibA_44474 | 281 | 1.407047866 | SLC17A6 | solute carrier family 17 member 6 [Source:HGNC Symbol;Acc:HGNC:16703] |
| ENSG00000100726 | HGLibA_48987 | 281 | 1.407047866 | TELO2 | telomere maintenance 2 [Source:HGNC Symbol;Acc:HGNC:29099] |
| ENSG00000101425 | HGLibA_04708 | 281 | 1.407047866 | BPI | bactericidal permeability increasing protein [Source:HGNC Symbol;Acc:HGNC:1095] |
| ENSG00000104325 | HGLibA_12773 | 281 | 1.407047866 | DECR1 | 4-dienoyl-CoA reductase 1 [Source:HGNC Symbol;Acc:HGNC:2753]" |
| ENSG00000105954 | HGLibA_32540 | 281 | 1.407047866 | NPVF | neuropeptide VF precursor [Source:HGNC Symbol;Acc:HGNC:13782] |
| ENSG00000108786 | HGLibA_22169 | 281 | 1.407047866 | HSD17B1 | hydroxysteroid 17-beta dehydrogenase 1 [Source:HGNC Symbol;Acc:HGNC:5210] |
| ENSG00000114487 | HGLibA_29647 | 281 | 1.407047866 | MORC1 | MORC family CW-type zinc finger 1 [Source:HGNC Symbol;Acc:HGNC:7198] |
| ENSG00000121594 | HGLibA_08454 | 281 | 1.407047866 | CD80 | CD80 molecule [Source:HGNC Symbol;Acc:HGNC:1700] |
| ENSG00000124374 | HGLibA_35034 | 281 | 1.407047866 | PAIP2B | poly(A) binding protein interacting protein 2B [Source:HGNC Symbol;Acc:HGNC:29200] |
| ENSG00000126218 | HGLibA_15842 | 281 | 1.407047866 | F10 | coagulation factor X [Source:HGNC Symbol;Acc:HGNC:3528] |
| ENSG00000129103 | HGLibA_47772 | 281 | 1.407047866 | SUMF2 | sulfatase modifying factor 2 [Source:HGNC Symbol;Acc:HGNC:20415] |
| ENSG00000130234 | HGLibA_00424 | 281 | 1.407047866 | ACE2 | angiotensin I converting enzyme 2 [Source:HGNC Symbol;Acc:HGNC:13557] |
| ENSG00000130584 | HGLibA_54898 | 281 | 1.407047866 | ZBTB46 | zinc finger and BTB domain containing 46 [Source:HGNC Symbol;Acc:HGNC:16094] |
| ENSG00000133639 | HGLibA_04940 | 281 | 1.407047866 | BTG1 | BTG anti-proliferation factor 1 [Source:HGNC Symbol;Acc:HGNC:1130] |
| ENSG00000135823 | HGLibA_47654 | 281 | 1.407047866 | STX6 | syntaxin 6 [Source:HGNC Symbol;Acc:HGNC:11441] |
| ENSG00000136697 | HGLibA_23042 | 281 | 1.407047866 | IL1F10 | interleukin 1 family member 10 [Source:HGNC Symbol;Acc:HGNC:15552] |
| ENSG00000144724 | HGLibA_39505 | 281 | 1.407047866 | PTPRG | receptor type G [Source:HGNC Symbol;Acc:HGNC:9671]" |
| ENSG00000148688 | HGLibA_42035 | 281 | 1.407047866 | RPP30 | ribonuclease P/MRP subunit p30 [Source:HGNC Symbol;Acc:HGNC:17688] |
| ENSG00000149596 | HGLibA_23981 | 281 | 1.407047866 | JPH2 | junctophilin 2 [Source:HGNC Symbol;Acc:HGNC:14202] |
| ENSG00000153395 | HGLibA_27072 | 281 | 1.407047866 | LPCAT1 | lysophosphatidylcholine acyltransferase 1 [Source:HGNC Symbol;Acc:HGNC:25718] |
| ENSG00000156966 | HGLibA_03999 | 281 | 1.407047866 | B3GNT7 | 3-N-acetylglucosaminyltransferase 7 [Source:HGNC Symbol;Acc:HGNC:18811]" |
| ENSG00000157873 | HGLibA_50761 | 281 | 1.407047866 | TNFRSF14 | TNF receptor superfamily member 14 [Source:HGNC Symbol;Acc:HGNC:11912] |

| | | | | | |
|---|---|---|---|---|---|
| ENSG00000163497 | HGLibA_17310 | 281 | 1.407047866 | FEV | ETS transcription factor [Source:HGNC Symbol;Acc:HGNC:18562]" |
| ENSG00000164342 | HGLibA_49649 | 281 | 1.407047866 | TLR3 | toll like receptor 3 [Source:HGNC Symbol;Acc:HGNC:11849] |
| ENSG00000169083 | HGLibA_02619 | 281 | 1.407047866 | AR | androgen receptor [Source:HGNC Symbol;Acc:HGNC:644] |
| ENSG00000170837 | HGLibA_20010 | 281 | 1.407047866 | GPR27 | G protein-coupled receptor 27 [Source:HGNC Symbol;Acc:HGNC:4482] |
| ENSG00000171611 | HGLibA_39286 | 281 | 1.407047866 | PTCRA | pre T cell antigen receptor alpha [Source:HGNC Symbol;Acc:HGNC:21290] |
| ENSG00000172828 | HGLibA_09225 | 281 | 1.407047866 | CES3 | carboxylesterase 3 [Source:HGNC Symbol;Acc:HGNC:1865] |
| ENSG00000172992 | HGLibA_12465 | 281 | 1.407047866 | DCAKD | dephospho-CoA kinase domain containing [Source:HGNC Symbol;Acc:HGNC:26238] |
| ENSG00000174740 | HGLibA_34938 | 281 | 1.407047866 | PABPC5 | poly(A) binding protein cytoplasmic 5 [Source:HGNC Symbol;Acc:HGNC:13629] |
| ENSG00000178821 | HGLibA_50431 | 281 | 1.407047866 | TMEM52 | transmembrane protein 52 [Source:HGNC Symbol;Acc:HGNC:27916] |
| ENSG00000182983 | HGLibA_56480 | 281 | 1.407047866 | ZNF662 | zinc finger protein 662 [Source:HGNC Symbol;Acc:HGNC:31930] |
| ENSG00000185955 | HGLibA_06565 | 281 | 1.407047866 | C7orf61 | chromosome 7 open reading frame 61 [Source:HGNC Symbol;Acc:HGNC:22135] |
| ENSG00000226288 | HGLibA_34176 | 281 | 1.407047866 | OR52I2 | olfactory receptor family 52 subfamily I member 2 [Source:HGNC Symbol;Acc:HGNC:15221] |
| ENSG00000279051 | HGLibA_34438 | 281 | 1.407047866 | OR6Q1 | olfactory receptor family 6 subfamily Q member 1 (gene/pseudogene) [Source:HGNC Symbol;Acc:HGNC:15302] |
| FAM105A | HGLibA_15994 | 281 | 1.407047866 | FAM105A | - |
| HIST1H2AC | HGLibA_21380 | 281 | 1.407047866 | HIST1H2AC | - |
| hsa-mir-1257 | HGLibA_57435 | 281 | 1.407047866 | hsa-mir-1257 | - |
| hsa-mir-1269a | HGLibA_57504 | 281 | 1.407047866 | hsa-mir-1269a | - |
| hsa-mir-370 | HGLibA_59363 | 281 | 1.407047866 | hsa-mir-370 | - |
| hsa-mir-378d-2 | HGLibA_59440 | 281 | 1.407047866 | hsa-mir-378d-2 | - |
| hsa-mir-4312 | HGLibA_59964 | 281 | 1.407047866 | hsa-mir-4312 | - |
| hsa-mir-4431 | HGLibA_60109 | 281 | 1.407047866 | hsa-mir-4431 | - |
| hsa-mir-4707 | HGLibA_60929 | 281 | 1.407047866 | hsa-mir-4707 | - |
| hsa-mir-6895 | HGLibA_63763 | 281 | 1.407047866 | hsa-mir-6895 | - |
| KIAA1045 | HGLibA_24687 | 281 | 1.407047866 | KIAA1045 | - |
| LOC100130705 | HGLibA_26795 | 281 | 1.407047866 | LOC100130705 | - |
| LOC284385 | HGLibA_26915 | 281 | 1.407047866 | LOC284385 | - |
| SELV | HGLibA_43315 | 281 | 1.407047866 | SELV | - |
| CCDC108 | HGLibA_07528 | 280 | 1.398002523 | CCDC108 | - |
| ENSG00000019485 | HGLibA_38368 | 280 | 1.398002523 | PRDM11 | PR/SET domain 11 [Source:HGNC Symbol;Acc:HGNC:13996] |
| ENSG00000071889 | HGLibA_16575 | 280 | 1.398002523 | FAM3A | family with sequence similarity 3 member A [Source:HGNC Symbol;Acc:HGNC:13749] |
| ENSG00000088727 | HGLibA_24965 | 280 | 1.398002523 | KIF9 | kinesin family member 9 [Source:HGNC Symbol;Acc:HGNC:16666] |
| ENSG00000099992 | HGLibA_48465 | 280 | 1.398002523 | TBC1D10A | TBC1 domain family member 10A [Source:HGNC Symbol;Acc:HGNC:23609] |
| ENSG00000105327 | HGLibA_04197 | 280 | 1.398002523 | BBC3 | BCL2 binding component 3 [Source:HGNC Symbol;Acc:HGNC:17868] |
| ENSG00000111701 | HGLibA_02467 | 280 | 1.398002523 | APOBEC1 | apolipoprotein B mRNA editing enzyme catalytic subunit 1 [Source:HGNC Symbol;Acc:HGNC:604] |
| ENSG00000112186 | HGLibA_07135 | 280 | 1.398002523 | CAP2 | cyclase associated actin cytoskeleton regulatory protein 2 [Source:HGNC Symbol;Acc:HGNC:20039] |
| ENSG00000126903 | HGLibA_44335 | 280 | 1.398002523 | SLC10A3 | solute carrier family 10 member 3 [Source:HGNC Symbol;Acc:HGNC:22979] |
| ENSG00000128000 | HGLibA_56735 | 280 | 1.398002523 | ZNF780B | zinc finger protein 780B [Source:HGNC Symbol;Acc:HGNC:33109] |
| ENSG00000130147 | HGLibA_43930 | 280 | 1.398002523 | SH3BP4 | SH3 domain binding protein 4 [Source:HGNC Symbol;Acc:HGNC:10826] |
| ENSG00000131373 | HGLibA_20801 | 280 | 1.398002523 | HACL1 | 2-hydroxyacyl-CoA lyase 1 [Source:HGNC Symbol;Acc:HGNC:17856] |
| ENSG00000135960 | HGLibA_14437 | 280 | 1.398002523 | EDAR | ectodysplasin A receptor [Source:HGNC Symbol;Acc:HGNC:2895] |
| ENSG00000137819 | HGLibA_35162 | 280 | 1.398002523 | PAQR5 | progestin and adipoQ receptor family member 5 [Source:HGNC Symbol;Acc:HGNC:29645] |
| ENSG00000138029 | HGLibA_20812 | 280 | 1.398002523 | HADHB | hydroxyacyl-CoA dehydrogenase trifunctional multienzyme complex subunit beta [Source:HGNC Symbol;Acc:HGNC:4803] |
| ENSG00000138078 | HGLibA_38441 | 280 | 1.398002523 | PREPL | prolyl endopeptidase like [Source:HGNC Symbol;Acc:HGNC:30228] |
| ENSG00000139737 | HGLibA_44307 | 280 | 1.398002523 | SLAIN1 | SLAIN motif family member 1 [Source:HGNC Symbol;Acc:HGNC:26387] |
| ENSG00000140264 | HGLibA_43475 | 280 | 1.398002523 | SERF2 | small EDRK-rich factor 2 [Source:HGNC Symbol;Acc:HGNC:10757] |
| ENSG00000141448 | HGLibA_18679 | 280 | 1.398002523 | GATA6 | GATA binding protein 6 [Source:HGNC Symbol;Acc:HGNC:4174] |
| ENSG00000142599 | HGLibA_40857 | 280 | 1.398002523 | RERE | arginine-glutamic acid dipeptide repeats [Source:HGNC Symbol;Acc:HGNC:9965] |
| ENSG00000144119 | HGLibA_05926 | 280 | 1.398002523 | C1QL2 | complement C1q like 2 [Source:HGNC Symbol;Acc:HGNC:24181] |
| ENSG00000169031 | HGLibA_10572 | 280 | 1.398002523 | COL4A3 | collagen type IV alpha 3 chain [Source:HGNC Symbol;Acc:HGNC:2204] |
| ENSG00000170790 | HGLibA_33462 | 280 | 1.398002523 | OR10A2 | olfactory receptor family 10 subfamily A member 2 [Source:HGNC Symbol;Acc:HGNC:8161] |
| ENSG00000172156 | HGLibA_07963 | 280 | 1.398002523 | CCL11 | C-C motif chemokine ligand 11 [Source:HGNC Symbol;Acc:HGNC:10610] |
| ENSG00000174456 | HGLibA_05300 | 280 | 1.398002523 | C12orf76 | chromosome 12 open reading frame 76 [Source:HGNC Symbol;Acc:HGNC:33790] |
| ENSG00000174804 | HGLibA_18273 | 280 | 1.398002523 | FZD4 | frizzled class receptor 4 [Source:HGNC Symbol;Acc:HGNC:4042] |
| ENSG00000175104 | HGLibA_51263 | 280 | 1.398002523 | TRAF6 | TNF receptor associated factor 6 [Source:HGNC Symbol;Acc:HGNC:12036] |
| ENSG00000175592 | HGLibA_17800 | 280 | 1.398002523 | FOSL1 | AP-1 transcription factor subunit [Source:HGNC Symbol;Acc:HGNC:13718]" |
| ENSG00000178104 | HGLibA_35799 | 280 | 1.398002523 | PDE4DIP | phosphodiesterase 4D interacting protein [Source:HGNC Symbol;Acc:HGNC:15580] |
| ENSG00000183401 | HGLibA_07668 | 280 | 1.398002523 | CCDC159 | coiled-coil domain containing 159 [Source:HGNC Symbol;Acc:HGNC:26996] |
| ENSG00000186980 | HGLibA_25721 | 280 | 1.398002523 | KRTAP23-1 | keratin associated protein 23-1 [Source:HGNC Symbol;Acc:HGNC:18928] |
| ENSG00000188677 | HGLibA_35258 | 280 | 1.398002523 | PARVB | parvin beta [Source:HGNC Symbol;Acc:HGNC:14653] |
| ENSG00000196482 | HGLibA_15598 | 280 | 1.398002523 | ESRRG | estrogen related receptor gamma [Source:HGNC Symbol;Acc:HGNC:3474] |
| ENSG00000198483 | HGLibA_02083 | 280 | 1.398002523 | ANKRD35 | ankyrin repeat domain 35 [Source:HGNC Symbol;Acc:HGNC:26323] |
| ENSG00000203663 | HGLibA_33822 | 280 | 1.398002523 | OR2L2 | olfactory receptor family 2 subfamily L member 2 [Source:HGNC Symbol;Acc:HGNC:8266] |
| ENSG00000205302 | HGLibA_46057 | 280 | 1.398002523 | SNX2 | sorting nexin 2 [Source:HGNC Symbol;Acc:HGNC:11173] |
| ENSG00000205718 | HGLibA_28482 | 280 | 1.398002523 | MBD3L4 | methyl-CpG binding domain protein 3 like 4 [Source:HGNC Symbol;Acc:HGNC:37206] |
| ENSG00000212807 | HGLibA_33736 | 280 | 1.398002523 | OR2A42 | olfactory receptor family 2 subfamily A member 42 [Source:HGNC Symbol;Acc:HGNC:31230] |
| ENSG00000250067 | HGLibA_54692 | 280 | 1.398002523 | YJEFN3 | YjeF N-terminal domain containing 3 [Source:HGNC Symbol;Acc:HGNC:24785] |
| ENSG00000261509 | HGLibA_51082 | 280 | 1.398002523 | TP53TG3B | TP53 target 3B [Source:HGNC Symbol;Acc:HGNC:37202] |
| ENSG00000262406 | HGLibA_29486 | 280 | 1.398002523 | MMP12 | matrix metallopeptidase 12 [Source:HGNC Symbol;Acc:HGNC:7158] |
| GSTT1 | HGLibA_20497 | 280 | 1.398002523 | GSTT1 | - |
| hsa-mir-194-1 | HGLibA_58082 | 280 | 1.398002523 | hsa-mir-194-1 | - |
| hsa-mir-3679 | HGLibA_59266 | 280 | 1.398002523 | hsa-mir-3679 | - |
| ATP5E | HGLibA_03649 | 279 | 1.38895718 | ATP5E | - |
| ATPIF1 | HGLibA_03806 | 279 | 1.38895718 | ATPIF1 | - |
| ENSG00000050555 | HGLibA_25975 | 279 | 1.38895718 | LAMC3 | laminin subunit gamma 3 [Source:HGNC Symbol;Acc:HGNC:6494] |
| ENSG00000067066 | HGLibA_46285 | 279 | 1.38895718 | SP100 | SP100 nuclear antigen [Source:HGNC Symbol;Acc:HGNC:11206] |
| ENSG00000100307 | HGLibA_07462 | 279 | 1.38895718 | CBX7 | chromobox 7 [Source:HGNC Symbol;Acc:HGNC:1557] |
| ENSG00000100362 | HGLibA_39612 | 279 | 1.38895718 | PVALB | parvalbumin [Source:HGNC Symbol;Acc:HGNC:9704] |
| ENSG00000105325 | HGLibA_18289 | 279 | 1.38895718 | FZR1 | fizzy and cell division cycle 20 related 1 [Source:HGNC Symbol;Acc:HGNC:24824] |
| ENSG00000106829 | HGLibA_49617 | 279 | 1.38895718 | TLE4 | transducin like enhancer of split 4 [Source:HGNC Symbol;Acc:HGNC:11840] |
| ENSG00000109927 | HGLibA_48954 | 279 | 1.38895718 | TECTA | tectorin alpha [Source:HGNC Symbol;Acc:HGNC:11720] |
| ENSG00000112619 | HGLibA_38751 | 279 | 1.38895718 | PRPH2 | peripherin 2 [Source:HGNC Symbol;Acc:HGNC:9942] |
| ENSG00000115641 | HGLibA_17484 | 279 | 1.38895718 | FHL2 | four and a half LIM domains 2 [Source:HGNC Symbol;Acc:HGNC:3703] |
| ENSG00000118620 | HGLibA_55978 | 279 | 1.38895718 | ZNF430 | zinc finger protein 430 [Source:HGNC Symbol;Acc:HGNC:20808] |
| ENSG00000119689 | HGLibA_13370 | 279 | 1.38895718 | DLST | dihydrolipoamide S-succinyltransferase [Source:HGNC Symbol;Acc:HGNC:2911] |
| ENSG00000130701 | HGLibA_40442 | 279 | 1.38895718 | RBBP8NL | RBBP8 N-terminal like [Source:HGNC Symbol;Acc:HGNC:16144] |
| ENSG00000130702 | HGLibA_25952 | 279 | 1.38895718 | LAMA5 | laminin subunit alpha 5 [Source:HGNC Symbol;Acc:HGNC:6485] |
| ENSG00000140090 | HGLibA_44623 | 279 | 1.38895718 | SLC24A4 | solute carrier family 24 member 4 [Source:HGNC Symbol;Acc:HGNC:10978] |
| ENSG00000142303 | HGLibA_00795 | 279 | 1.38895718 | ADAMTS10 | ADAM metallopeptidase with thrombospondin type 1 motif 10 [Source:HGNC Symbol;Acc:HGNC:13201] |
| ENSG00000148143 | HGLibA_56041 | 279 | 1.38895718 | ZNF462 | zinc finger protein 462 [Source:HGNC Symbol;Acc:HGNC:21684] |
| ENSG00000151704 | HGLibA_24245 | 279 | 1.38895718 | KCNJ1 | potassium voltage-gated channel subfamily J member 1 [Source:HGNC Symbol;Acc:HGNC:6255] |
| ENSG00000155974 | HGLibA_20319 | 279 | 1.38895718 | GRIP1 | glutamate receptor interacting protein 1 [Source:HGNC Symbol;Acc:HGNC:18708] |
| ENSG00000160446 | HGLibA_55073 | 279 | 1.38895718 | ZDHHC12 | zinc finger DHHC-type containing 12 [Source:HGNC Symbol;Acc:HGNC:19159] |
| ENSG00000160789 | HGLibA_26692 | 279 | 1.38895718 | LMNA | lamin A/C [Source:HGNC Symbol;Acc:HGNC:6636] |
| ENSG00000163006 | HGLibA_07610 | 279 | 1.38895718 | CCDC138 | coiled-coil domain containing 138 [Source:HGNC Symbol;Acc:HGNC:26531] |
| ENSG00000163104 | HGLibA_45615 | 279 | 1.38895718 | SMARCAD1 | containing DEAD/H box 1 [Source:HGNC Symbol;Acc:HGNC:18398]" |
| ENSG00000166173 | HGLibA_26040 | 279 | 1.38895718 | LARP6 | La ribonucleoprotein domain family member 6 [Source:HGNC Symbol;Acc:HGNC:24012] |
| ENSG00000166206 | HGLibA_18380 | 279 | 1.38895718 | GABRB3 | gamma-aminobutyric acid type A receptor beta3 subunit [Source:HGNC Symbol;Acc:HGNC:4083] |
| ENSG00000167700 | HGLibA_29098 | 279 | 1.38895718 | MFSD3 | major facilitator superfamily domain containing 3 [Source:HGNC Symbol;Acc:HGNC:25157] |
| ENSG00000168394 | HGLibA_48316 | 279 | 1.38895718 | TAP1 | ATP binding cassette subfamily B member [Source:HGNC Symbol;Acc:HGNC:43]" |
| ENSG00000169604 | HGLibA_02212 | 279 | 1.38895718 | ANTXR1 | ANTXR cell adhesion molecule 1 [Source:HGNC Symbol;Acc:HGNC:21014] |
| ENSG00000170310 | HGLibA_47660 | 279 | 1.38895718 | STX8 | syntaxin 8 [Source:HGNC Symbol;Acc:HGNC:11443] |
| ENSG00000184368 | HGLibA_28237 | 279 | 1.38895718 | MAP7D2 | MAP7 domain containing 2 [Source:HGNC Symbol;Acc:HGNC:25899] |
| ENSG00000184716 | HGLibA_43494 | 279 | 1.38895718 | SERINC4 | serine incorporator 4 [Source:HGNC Symbol;Acc:HGNC:32237] |
| ENSG00000184922 | HGLibA_17692 | 279 | 1.38895718 | FMNL1 | formin like 1 [Source:HGNC Symbol;Acc:HGNC:1212] |
| ENSG00000187223 | HGLibA_26127 | 279 | 1.38895718 | LCE2D | late cornified envelope 2D [Source:HGNC Symbol;Acc:HGNC:16518] |
| ENSG00000196812 | HGLibA_56989 | 279 | 1.38895718 | ZSCAN16 | zinc finger and SCAN domain containing 16 [Source:HGNC Symbol;Acc:HGNC:20813] |
| ENSG00000204084 | HGLibA_23346 | 279 | 1.38895718 | INPP5B | inositol polyphosphate-5-phosphatase B [Source:HGNC Symbol;Acc:HGNC:6077] |
| ENSG00000214300 | HGLibA_46590 | 279 | 1.38895718 | SPDYE3 | speedy/RINGO cell cycle regulator family member E3 [Source:HGNC Symbol;Acc:HGNC:35462] |
| ENSG00000215595 | HGLibA_06013 | 279 | 1.38895718 | C20orf202 | chromosome 20 open reading frame 202 [Source:HGNC Symbol;Acc:HGNC:37254] |
| ENSG00000249459 | HGLibA_55752 | 279 | 1.38895718 | ZNF286B | zinc finger protein 286B [Source:HGNC Symbol;Acc:HGNC:33241] |
| hsa-mir-1468 | HGLibA_57848 | 279 | 1.38895718 | hsa-mir-1468 | - |
| hsa-mir-4314 | HGLibA_59972 | 279 | 1.38895718 | hsa-mir-4314 | - |
| hsa-mir-4717 | HGLibA_60969 | 279 | 1.38895718 | hsa-mir-4717 | - |
| C10orf128 | HGLibA_05049 | 278 | 1.379911838 | C10orf128 | - |
| C9orf169 | HGLibA_06705 | 278 | 1.379911838 | C9orf169 | - |
| ENSG00000057593 | HGLibA_15881 | 278 | 1.379911838 | F7 | coagulation factor VII [Source:HGNC Symbol;Acc:HGNC:3544] |
| ENSG00000069696 | HGLibA_13967 | 278 | 1.379911838 | DRD4 | dopamine receptor D4 [Source:HGNC Symbol;Acc:HGNC:3025] |
| ENSG00000082146 | HGLibA_47575 | 278 | 1.379911838 | STRADB | STE20-related kinase adaptor beta [Source:HGNC Symbol;Acc:HGNC:13205] |
| ENSG00000100100 | HGLibA_36644 | 278 | 1.379911838 | PIK3IP1 | phosphoinositide-3-kinase interacting protein 1 [Source:HGNC Symbol;Acc:HGNC:24942] |
| ENSG00000107669 | HGLibA_03427 | 278 | 1.379911838 | ATE1 | arginyltransferase 1 [Source:HGNC Symbol;Acc:HGNC:782] |
| ENSG00000124772 | HGLibA_10965 | 278 | 1.379911838 | CPNE5 | copine 5 [Source:HGNC Symbol;Acc:HGNC:2318] |
| ENSG00000128591 | HGLibA_17645 | 278 | 1.379911838 | FLNC | filamin C [Source:HGNC Symbol;Acc:HGNC:3756] |
| ENSG00000130313 | HGLibA_36245 | 278 | 1.379911838 | PGLS | 6-phosphogluconolactonase [Source:HGNC Symbol;Acc:HGNC:8903] |
| ENSG00000134955 | HGLibA_45047 | 278 | 1.379911838 | SLC37A2 | solute carrier family 37 member 2 [Source:HGNC Symbol;Acc:HGNC:20644] |
| ENSG00000135451 | HGLibA_51719 | 278 | 1.379911838 | TROAP | trophinin associated protein [Source:HGNC Symbol;Acc:HGNC:12327] |
| ENSG00000144810 | HGLibA_10613 | 278 | 1.379911838 | COL8A1 | collagen type VIII alpha 1 chain [Source:HGNC Symbol;Acc:HGNC:2215] |
| ENSG00000146005 | HGLibA_39016 | 278 | 1.379911838 | PSD2 | pleckstrin and Sec7 domain containing 2 [Source:HGNC Symbol;Acc:HGNC:19092] |
| ENSG00000149575 | HGLibA_42982 | 278 | 1.379911838 | SCN2B | sodium voltage-gated channel beta subunit 2 [Source:HGNC Symbol;Acc:HGNC:10589] |
| ENSG00000150594 | HGLibA_01047 | 278 | 1.379911838 | ADRA2A | adrenoceptor alpha 2A [Source:HGNC Symbol;Acc:HGNC:281] |
| ENSG00000151067 | HGLibA_06881 | 278 | 1.379911838 | CACNA1C | calcium voltage-gated channel subunit alpha1 C [Source:HGNC Symbol;Acc:HGNC:1390] |
| ENSG00000152558 | HGLibA_49904 | 278 | 1.379911838 | TMEM123 | transmembrane protein 123 [Source:HGNC Symbol;Acc:HGNC:30138] |
| ENSG00000162032 | HGLibA_46866 | 278 | 1.379911838 | SPSB3 | splA/ryanodine receptor domain and SOCS box containing 3 [Source:HGNC Symbol;Acc:HGNC:30629] |
| ENSG00000164331 | HGLibA_01984 | 278 | 1.379911838 | ANKRA2 | ankyrin repeat family A member 2 [Source:HGNC Symbol;Acc:HGNC:13208] |
| ENSG00000178409 | HGLibA_04405 | 278 | 1.379911838 | BEND3 | BEN domain containing 3 [Source:HGNC Symbol;Acc:HGNC:23040] |
| ENSG00000179057 | HGLibA_22886 | 278 | 1.379911838 | IGSF22 | immunoglobulin superfamily member 22 [Source:HGNC Symbol;Acc:HGNC:26750] |

| | | | | | |
|---|---|---|---|---|---|
| ENSG00000180628 | HGLibA_35600 | 278 | 1.379911838 | PCGF5 | polycomb group ring finger 5 [Source:HGNC Symbol;Acc:HGNC:28264] |
| ENSG00000182628 | HGLibA_44263 | 278 | 1.379911838 | SKA2 | spindle and kinetochore associated complex subunit 2 [Source:HGNC Symbol;Acc:HGNC:28006] |
| ENSG00000185133 | HGLibA_23357 | 278 | 1.379911838 | INPP5J | inositol polyphosphate-5-phosphatase J [Source:HGNC Symbol;Acc:HGNC:8956] |
| ENSG00000186160 | HGLibA_12205 | 278 | 1.379911838 | CYP4Z1 | cytochrome P450 family 4 subfamily Z member 1 [Source:HGNC Symbol;Acc:HGNC:20583] |
| ENSG00000186318 | HGLibA_04058 | 278 | 1.379911838 | BACE1 | beta-secretase 1 [Source:HGNC Symbol;Acc:HGNC:933] |
| ENSG00000187867 | HGLibA_35075 | 278 | 1.379911838 | PALM3 | paralemmin 3 [Source:HGNC Symbol;Acc:HGNC:33274] |
| ENSG00000197465 | HGLibA_20707 | 278 | 1.379911838 | GYPE | glycophorin E (MNS blood group) [Source:HGNC Symbol;Acc:HGNC:4705] |
| ENSG00000203867 | HGLibA_40506 | 278 | 1.379911838 | RBM20 | RNA binding motif protein 20 [Source:HGNC Symbol;Acc:HGNC:27424] |
| ENSG00000204421 | HGLibA_27664 | 278 | 1.379911838 | LY6G6C | lymphocyte antigen 6 family member G6C [Source:HGNC Symbol;Acc:HGNC:13936] |
| ENSG00000244414 | HGLibA_09263 | 278 | 1.379911838 | CFHR1 | complement factor H related 1 [Source:HGNC Symbol;Acc:HGNC:4888] |
| ENSG00000269699 | HGLibA_55339 | 278 | 1.379911838 | ZIM2 | zinc finger imprinted 2 [Source:HGNC Symbol;Acc:HGNC:12875] |
| hsa-mir-1264 | HGLibA_57481 | 278 | 1.379911838 | hsa-mir-1264 | - |
| hsa-mir-193a | HGLibA_58072 | 278 | 1.379911838 | hsa-mir-193a | - |
| hsa-mir-3178 | HGLibA_58787 | 278 | 1.379911838 | hsa-mir-3178 | - |
| hsa-mir-4311 | HGLibA_59959 | 278 | 1.379911838 | hsa-mir-4311 | - |
| hsa-mir-4700 | HGLibA_60907 | 278 | 1.379911838 | hsa-mir-4700 | - |
| hsa-mir-671 | HGLibA_63012 | 278 | 1.379911838 | hsa-mir-671 | - |
| hsa-mir-8089 | HGLibA_64197 | 278 | 1.379911838 | hsa-mir-8089 | - |
| LRRC16A | HGLibA_27264 | 278 | 1.379911838 | LRRC16A | - |
| ENSG00000064545 | HGLibA_50025 | 277 | 1.370866495 | TMEM161A | transmembrane protein 161A [Source:HGNC Symbol;Acc:HGNC:26020] |
| ENSG00000081177 | HGLibA_15720 | 277 | 1.370866495 | EXD2 | exonuclease 3'-5' domain containing 2 [Source:HGNC Symbol;Acc:HGNC:20217] |
| ENSG00000099834 | HGLibA_08711 | 277 | 1.370866495 | CDHR5 | cadherin related family member 5 [Source:HGNC Symbol;Acc:HGNC:7521] |
| ENSG00000103197 | HGLibA_51813 | 277 | 1.370866495 | TSC2 | TSC complex subunit 2 [Source:HGNC Symbol;Acc:HGNC:12363] |
| ENSG00000103249 | HGLibA_09839 | 277 | 1.370866495 | CLCN7 | chloride voltage-gated channel 7 [Source:HGNC Symbol;Acc:HGNC:2025] |
| ENSG00000105048 | HGLibA_50883 | 277 | 1.370866495 | TNNT1 | slow skeletal type [Source:HGNC Symbol;Acc:HGNC:11948]" |
| ENSG00000105198 | HGLibA_26351 | 277 | 1.370866495 | LGALS13 | galectin 13 [Source:HGNC Symbol;Acc:HGNC:15449] |
| ENSG00000105996 | HGLibA_21905 | 277 | 1.370866495 | HOXA2 | homeobox A2 [Source:HGNC Symbol;Acc:HGNC:5103] |
| ENSG00000107263 | HGLibA_40245 | 277 | 1.370866495 | RAPGEF1 | Rap guanine nucleotide exchange factor 1 [Source:HGNC Symbol;Acc:HGNC:4568] |
| ENSG00000110680 | HGLibA_06993 | 277 | 1.370866495 | CALCA | calcitonin related polypeptide alpha [Source:HGNC Symbol;Acc:HGNC:1437] |
| ENSG00000112699 | HGLibA_19394 | 277 | 1.370866495 | GMDS | 6-dehydratase [Source:HGNC Symbol;Acc:HGNC:4369]" |
| ENSG00000115350 | HGLibA_37520 | 277 | 1.370866495 | POLE4 | accessory subunit [Source:HGNC Symbol;Acc:HGNC:18755]" |
| ENSG00000118242 | HGLibA_29824 | 277 | 1.370866495 | MREG | melanoregulin [Source:HGNC Symbol;Acc:HGNC:25478] |
| ENSG00000132879 | HGLibA_17105 | 277 | 1.370866495 | FBXO44 | F-box protein 44 [Source:HGNC Symbol;Acc:HGNC:24847] |
| ENSG00000136867 | HGLibA_44928 | 277 | 1.370866495 | SLC31A2 | solute carrier family 31 member 2 [Source:HGNC Symbol;Acc:HGNC:11017] |
| ENSG00000137080 | HGLibA_22677 | 277 | 1.370866495 | IFNA21 | interferon alpha 21 [Source:HGNC Symbol;Acc:HGNC:5424] |
| ENSG00000138675 | HGLibA_17410 | 277 | 1.370866495 | FGF5 | fibroblast growth factor 5 [Source:HGNC Symbol;Acc:HGNC:3683] |
| ENSG00000149633 | HGLibA_24818 | 277 | 1.370866495 | KIAA1755 | KIAA1755 [Source:HGNC Symbol;Acc:HGNC:29372] |
| ENSG00000155833 | HGLibA_12047 | 277 | 1.370866495 | CYLC2 | cylicin 2 [Source:HGNC Symbol;Acc:HGNC:2583] |
| ENSG00000155961 | HGLibA_39903 | 277 | 1.370866495 | RAB39B | member RAS oncogene family [Source:HGNC Symbol;Acc:HGNC:16499]" |
| ENSG00000163283 | HGLibA_01711 | 277 | 1.370866495 | ALPP | placental [Source:HGNC Symbol;Acc:HGNC:439]" |
| ENSG00000166813 | HGLibA_24962 | 277 | 1.370866495 | KIF7 | kinesin family member 7 [Source:HGNC Symbol;Acc:HGNC:30497] |
| ENSG00000169877 | HGLibA_01323 | 277 | 1.370866495 | AHSP | alpha hemoglobin stabilizing protein [Source:HGNC Symbol;Acc:HGNC:18075] |
| ENSG00000172932 | HGLibA_02010 | 277 | 1.370866495 | ANKRD13D | ankyrin repeat domain 13D [Source:HGNC Symbol;Acc:HGNC:27880] |
| ENSG00000173992 | HGLibA_08184 | 277 | 1.370866495 | CCS | copper chaperone for superoxide dismutase [Source:HGNC Symbol;Acc:HGNC:1613] |
| ENSG00000177989 | HGLibA_33288 | 277 | 1.370866495 | ODF3B | outer dense fiber of sperm tails 3B [Source:HGNC Symbol;Acc:HGNC:34388] |
| ENSG00000180730 | HGLibA_44033 | 277 | 1.370866495 | SHISA2 | shisa family member 2 [Source:HGNC Symbol;Acc:HGNC:20366] |
| ENSG00000184351 | HGLibA_25662 | 277 | 1.370866495 | KRTAP19-1 | keratin associated protein 19-1 [Source:HGNC Symbol;Acc:HGNC:18936] |
| ENSG00000185915 | HGLibA_25192 | 277 | 1.370866495 | KLHL34 | kelch like family member 34 [Source:HGNC Symbol;Acc:HGNC:26634] |
| ENSG00000187492 | HGLibA_08707 | 277 | 1.370866495 | CDHR4 | cadherin related family member 4 [Source:HGNC Symbol;Acc:HGNC:34527] |
| ENSG00000188032 | HGLibA_05696 | 277 | 1.370866495 | C19orf67 | chromosome 19 open reading frame 67 [Source:HGNC Symbol;Acc:HGNC:34354] |
| ENSG00000189357 | HGLibA_46502 | 277 | 1.370866495 | SPATA31D4 | SPATA31 subfamily D member 4 [Source:HGNC Symbol;Acc:HGNC:38601] |
| ENSG00000198271 | HGLibA_25766 | 277 | 1.370866495 | KRTAP4-5 | keratin associated protein 4-5 [Source:HGNC Symbol;Acc:HGNC:18899] |
| ENSG00000204438 | HGLibA_19728 | 277 | 1.370866495 | GPANK1 | G-patch domain and ankyrin repeats 1 [Source:HGNC Symbol;Acc:HGNC:13920] |
| ENSG00000217128 | HGLibA_17766 | 277 | 1.370866495 | FNIP1 | folliculin interacting protein 1 [Source:HGNC Symbol;Acc:HGNC:29418] |
| ENSG00000220008 | HGLibA_26590 | 277 | 1.370866495 | LINGO3 | leucine rich repeat and Ig domain containing 3 [Source:HGNC Symbol;Acc:HGNC:21206] |
| ENSG00000241595 | HGLibA_25839 | 277 | 1.370866495 | KRTAP9-4 | keratin associated protein 9-4 [Source:HGNC Symbol;Acc:HGNC:18902] |
| hsa-mir-326 | HGLibA_58971 | 277 | 1.370866495 | hsa-mir-326 | - |
| hsa-mir-4527 | HGLibA_60571 | 277 | 1.370866495 | hsa-mir-4527 | - |
| hsa-mir-651 | HGLibA_62890 | 277 | 1.370866495 | hsa-mir-651 | - |
| hsa-mir-6895 | HGLibA_63766 | 277 | 1.370866495 | hsa-mir-6895 | - |
| hsa-mir-9-2 | HGLibA_64265 | 277 | 1.370866495 | hsa-mir-9-2 | - |
| RABL5 | HGLibA_40035 | 277 | 1.370866495 | RABL5 | - |
| C4orf32 | HGLibA_06321 | 276 | 1.361821152 | C4orf32 | - |
| ENSG00000010539 | HGLibA_55571 | 276 | 1.361821152 | ZNF200 | zinc finger protein 200 [Source:HGNC Symbol;Acc:HGNC:12993] |
| ENSG00000042493 | HGLibA_07140 | 276 | 1.361821152 | CAPG | gelsolin like [Source:HGNC Symbol;Acc:HGNC:1474]" |
| ENSG00000067177 | HGLibA_36403 | 276 | 1.361821152 | PHKA1 | phosphorylase kinase regulatory subunit alpha 1 [Source:HGNC Symbol;Acc:HGNC:8925] |
| ENSG00000080503 | HGLibA_45605 | 276 | 1.361821152 | SMARCA2 | member 2 [Source:HGNC Symbol;Acc:HGNC:11098]" |
| ENSG00000101224 | HGLibA_08530 | 276 | 1.361821152 | CDC25B | cell division cycle 25B [Source:HGNC Symbol;Acc:HGNC:1726] |
| ENSG00000101323 | HGLibA_20832 | 276 | 1.361821152 | HAO1 | hydroxyacid oxidase 1 [Source:HGNC Symbol;Acc:HGNC:4809] |
| ENSG00000102452 | HGLibA_31085 | 276 | 1.361821152 | NALCN | non-selective [Source:HGNC Symbol;Acc:HGNC:19082]" |
| ENSG00000106006 | HGLibA_21916 | 276 | 1.361821152 | HOXA6 | homeobox A6 [Source:HGNC Symbol;Acc:HGNC:5107] |
| ENSG00000113966 | HGLibA_02985 | 276 | 1.361821152 | ARL6 | ADP ribosylation factor like GTPase 6 [Source:HGNC Symbol;Acc:HGNC:13210] |
| ENSG00000114859 | HGLibA_09824 | 276 | 1.361821152 | CLCN2 | chloride voltage-gated channel 2 [Source:HGNC Symbol;Acc:HGNC:2020] |
| ENSG00000116350 | HGLibA_47077 | 276 | 1.361821152 | SRSF4 | serine and arginine rich splicing factor 4 [Source:HGNC Symbol;Acc:HGNC:10786] |
| ENSG00000121764 | HGLibA_20987 | 276 | 1.361821152 | HCRTR1 | hypocretin receptor 1 [Source:HGNC Symbol;Acc:HGNC:4848] |
| ENSG00000129103 | HGLibA_47774 | 276 | 1.361821152 | SUMF2 | sulfatase modifying factor 2 [Source:HGNC Symbol;Acc:HGNC:20415] |
| ENSG00000135324 | HGLibA_29810 | 276 | 1.361821152 | MRAP2 | melanocortin 2 receptor accessory protein 2 [Source:HGNC Symbol;Acc:HGNC:21232] |
| ENSG00000147459 | HGLibA_13749 | 276 | 1.361821152 | DOCK5 | dedicator of cytokinesis 5 [Source:HGNC Symbol;Acc:HGNC:23476] |
| ENSG00000148356 | HGLibA_27497 | 276 | 1.361821152 | LRSAM1 | leucine rich repeat and sterile alpha motif containing 1 [Source:HGNC Symbol;Acc:HGNC:25135] |
| ENSG00000149798 | HGLibA_08570 | 276 | 1.361821152 | CDC42EP2 | CDC42 effector protein 2 [Source:HGNC Symbol;Acc:HGNC:16263] |
| ENSG00000158486 | HGLibA_13486 | 276 | 1.361821152 | DNAH3 | dynein axonemal heavy chain 3 [Source:HGNC Symbol;Acc:HGNC:2949] |
| ENSG00000158604 | HGLibA_49812 | 276 | 1.361821152 | TMED4 | transmembrane p24 trafficking protein 4 [Source:HGNC Symbol;Acc:HGNC:22301] |
| ENSG00000160447 | HGLibA_36844 | 276 | 1.361821152 | PKN3 | protein kinase N3 [Source:HGNC Symbol;Acc:HGNC:17999] |
| ENSG00000162688 | HGLibA_01195 | 276 | 1.361821152 | AGL | 4-alpha-glucanotransferase [Source:HGNC Symbol;Acc:HGNC:321]" |
| ENSG00000163864 | HGLibA_32191 | 276 | 1.361821152 | NMNAT3 | nicotinamide nucleotide adenylyltransferase 3 [Source:HGNC Symbol;Acc:HGNC:20989] |
| ENSG00000167588 | HGLibA_19782 | 276 | 1.361821152 | GPD1 | glycerol-3-phosphate dehydrogenase 1 [Source:HGNC Symbol;Acc:HGNC:4455] |
| ENSG00000168028 | HGLibA_42197 | 276 | 1.361821152 | RPSA | ribosomal protein SA [Source:HGNC Symbol;Acc:HGNC:6502] |
| ENSG00000170619 | HGLibA_10668 | 276 | 1.361821152 | COMMD5 | COMM domain containing 5 [Source:HGNC Symbol;Acc:HGNC:17902] |
| ENSG00000173705 | HGLibA_47855 | 276 | 1.361821152 | SUSD5 | sushi domain containing 5 [Source:HGNC Symbol;Acc:HGNC:29061] |
| ENSG00000176136 | HGLibA_28553 | 276 | 1.361821152 | MC5R | melanocortin 5 receptor [Source:HGNC Symbol;Acc:HGNC:6933] |
| ENSG00000181631 | HGLibA_34879 | 276 | 1.361821152 | P2RY13 | purinergic receptor P2Y13 [Source:HGNC Symbol;Acc:HGNC:4537] |
| ENSG00000185920 | HGLibA_39267 | 276 | 1.361821152 | PTCH1 | patched 1 [Source:HGNC Symbol;Acc:HGNC:9585] |
| ENSG00000196935 | HGLibA_46968 | 276 | 1.361821152 | SRGAP1 | SLIT-ROBO Rho GTPase activating protein 1 [Source:HGNC Symbol;Acc:HGNC:17382] |
| ENSG00000198876 | HGLibA_12419 | 276 | 1.361821152 | DCAF12 | DDB1 and CUL4 associated factor 12 [Source:HGNC Symbol;Acc:HGNC:19911] |
| ENSG00000198879 | HGLibA_43723 | 276 | 1.361821152 | SFMBT2 | Scm like with four mbt domains 2 [Source:HGNC Symbol;Acc:HGNC:20256] |
| ENSG00000204065 | HGLibA_48716 | 276 | 1.361821152 | TCEAL5 | transcription elongation factor A like 5 [Source:HGNC Symbol;Acc:HGNC:22282] |
| ENSG00000215912 | HGLibA_52119 | 276 | 1.361821152 | TTC34 | tetratricopeptide repeat domain 34 [Source:HGNC Symbol;Acc:HGNC:34297] |
| ENSG00000237172 | HGLibA_04005 | 276 | 1.361821152 | B3GNT9 | 3-N-acetylglucosaminyltransferase 9 [Source:HGNC Symbol;Acc:HGNC:28714]" |
| FAM160B1 | HGLibA_16211 | 276 | 1.361821152 | FAM160B1 | - |
| GPR126 | HGLibA_19881 | 276 | 1.361821152 | GPR126 | - |
| GPR56 | HGLibA_20056 | 276 | 1.361821152 | GPR56 | - |
| hsa-mir-125b-2 | HGLibA_57451 | 276 | 1.361821152 | hsa-mir-125b-2 | - |
| hsa-mir-346 | HGLibA_59030 | 276 | 1.361821152 | hsa-mir-346 | - |
| hsa-mir-5192 | HGLibA_61686 | 276 | 1.361821152 | hsa-mir-5192 | - |
| hsa-mir-7855 | HGLibA_64008 | 276 | 1.361821152 | hsa-mir-7855 | - |
| LOC100652824 | HGLibA_26873 | 276 | 1.361821152 | LOC100652824 | - |
| SPRY3_Y | HGLibA_46845 | 276 | 1.361821152 | SPRY3_Y | - |
| AGPAT6 | HGLibA_01232 | 275 | 1.352775809 | AGPAT6 | - |
| CXorf23 | HGLibA_11909 | 275 | 1.352775809 | CXorf23 | - |
| ENSG00000007952 | HGLibA_32370 | 275 | 1.352775809 | NOX1 | NADPH oxidase 1 [Source:HGNC Symbol;Acc:HGNC:7889] |
| ENSG00000030419 | HGLibA_22931 | 275 | 1.352775809 | IKZF2 | IKAROS family zinc finger 2 [Source:HGNC Symbol;Acc:HGNC:13177] |
| ENSG00000059804 | HGLibA_44874 | 275 | 1.352775809 | SLC2A3 | solute carrier family 2 member 3 [Source:HGNC Symbol;Acc:HGNC:11007] |
| ENSG00000064999 | HGLibA_02151 | 275 | 1.352775809 | ANKS1A | ankyrin repeat and sterile alpha motif domain containing 1A [Source:HGNC Symbol;Acc:HGNC:20961] |
| ENSG00000072694 | HGLibA_17193 | 275 | 1.352775809 | FCGR2B | Fc fragment of IgG receptor IIb [Source:HGNC Symbol;Acc:HGNC:3618] |
| ENSG00000080618 | HGLibA_10907 | 275 | 1.352775809 | CPB2 | carboxypeptidase B2 [Source:HGNC Symbol;Acc:HGNC:2300] |
| ENSG00000103184 | HGLibA_43203 | 275 | 1.352775809 | SEC14L5 | SEC14 like lipid binding 5 [Source:HGNC Symbol;Acc:HGNC:29032] |
| ENSG00000104728 | HGLibA_02807 | 275 | 1.352775809 | ARHGEF10 | Rho guanine nucleotide exchange factor 10 [Source:HGNC Symbol;Acc:HGNC:14103] |
| ENSG00000113790 | HGLibA_14680 | 275 | 1.352775809 | EHHADH | enoyl-CoA hydratase and 3-hydroxyacyl CoA dehydrogenase [Source:HGNC Symbol;Acc:HGNC:3247] |
| ENSG00000114859 | HGLibA_09822 | 275 | 1.352775809 | CLCN2 | chloride voltage-gated channel 2 [Source:HGNC Symbol;Acc:HGNC:2020] |
| ENSG00000115998 | HGLibA_06124 | 275 | 1.352775809 | C2orf42 | chromosome 2 open reading frame 42 [Source:HGNC Symbol;Acc:HGNC:26056] |
| ENSG00000123575 | HGLibA_16394 | 275 | 1.352775809 | FAM199X | X-linked [Source:HGNC Symbol;Acc:HGNC:25195]" |
| ENSG00000126581 | HGLibA_04395 | 275 | 1.352775809 | BECN1 | beclin 1 [Source:HGNC Symbol;Acc:HGNC:1034] |
| ENSG00000127081 | HGLibA_56076 | 275 | 1.352775809 | ZNF484 | zinc finger protein 484 [Source:HGNC Symbol;Acc:HGNC:23385] |
| ENSG00000130208 | HGLibA_02502 | 275 | 1.352775809 | APOC1 | apolipoprotein C1 [Source:HGNC Symbol;Acc:HGNC:607] |
| ENSG00000130876 | HGLibA_45349 | 275 | 1.352775809 | SLC7A10 | solute carrier family 7 member 10 [Source:HGNC Symbol;Acc:HGNC:11058] |
| ENSG00000131845 | HGLibA_55776 | 275 | 1.352775809 | ZNF304 | zinc finger protein 304 [Source:HGNC Symbol;Acc:HGNC:13505] |
| ENSG00000142484 | HGLibA_49707 | 275 | 1.352775809 | TM4SF5 | transmembrane 4 L six family member 5 [Source:HGNC Symbol;Acc:HGNC:11857] |
| ENSG00000142634 | HGLibA_14580 | 275 | 1.352775809 | EFHD2 | EF-hand domain family member D2 [Source:HGNC Symbol;Acc:HGNC:28670] |
| ENSG00000145002 | HGLibA_16777 | 275 | 1.352775809 | FAM86B2 | family with sequence similarity 86 member B2 [Source:HGNC Symbol;Acc:HGNC:32222] |
| ENSG00000145241 | HGLibA_09046 | 275 | 1.352775809 | CENPC | centromere protein C [Source:HGNC Symbol;Acc:HGNC:1854] |
| ENSG00000150457 | HGLibA_26066 | 275 | 1.352775809 | LATS2 | large tumor suppressor kinase 2 [Source:HGNC Symbol;Acc:HGNC:6515] |
| ENSG00000157303 | HGLibA_47850 | 275 | 1.352775809 | SUSD3 | sushi domain containing 3 [Source:HGNC Symbol;Acc:HGNC:28391] |
| ENSG00000157349 | HGLibA_12665 | 275 | 1.352775809 | DDX19B | DEAD-box helicase 19B [Source:HGNC Symbol;Acc:HGNC:2742] |
| ENSG00000157483 | HGLibA_30832 | 275 | 1.352775809 | MYO1E | myosin IE [Source:HGNC Symbol;Acc:HGNC:7599] |
| ENSG00000164418 | HGLibA_20285 | 275 | 1.352775809 | GRIK2 | glutamate ionotropic receptor kainate type subunit 2 [Source:HGNC Symbol;Acc:HGNC:4580] |

| | | | | | |
|---|---|---|---|---|---|
| ENSG00000166482 | HGLibA_29052 | 275 | 1.352775809 | MFAP4 | microfibril associated protein 4 [Source:HGNC Symbol;Acc:HGNC:7035] |
| ENSG00000167261 | HGLibA_13822 | 275 | 1.352775809 | DPEP2 | dipeptidase 2 [Source:HGNC Symbol;Acc:HGNC:23028] |
| ENSG00000168826 | HGLibA_54906 | 275 | 1.352775809 | ZBTB49 | zinc finger and BTB domain containing 49 [Source:HGNC Symbol;Acc:HGNC:19883] |
| ENSG00000171056 | HGLibA_46274 | 275 | 1.352775809 | SOX7 | SRY-box 7 [Source:HGNC Symbol;Acc:HGNC:18196] |
| ENSG00000171502 | HGLibA_10548 | 275 | 1.352775809 | COL24A1 | collagen type XXIV alpha 1 chain [Source:HGNC Symbol;Acc:HGNC:20821] |
| ENSG00000172318 | HGLibA_03954 | 275 | 1.352775809 | B3GALT1 | 3-galactosyltransferase 1 [Source:HGNC Symbol;Acc:HGNC:916]" |
| ENSG00000173302 | HGLibA_19920 | 275 | 1.352775809 | GPR148 | G protein-coupled receptor 148 [Source:HGNC Symbol;Acc:HGNC:23623] |
| ENSG00000174502 | HGLibA_44809 | 275 | 1.352775809 | SLC26A9 | solute carrier family 26 member 9 [Source:HGNC Symbol;Acc:HGNC:14469] |
| ENSG00000175946 | HGLibA_25202 | 275 | 1.352775809 | KLHL38 | kelch like family member 38 [Source:HGNC Symbol;Acc:HGNC:34435] |
| ENSG00000179455 | HGLibA_29354 | 275 | 1.352775809 | MKRN3 | makorin ring finger protein 3 [Source:HGNC Symbol;Acc:HGNC:7114] |
| ENSG00000181481 | HGLibA_41511 | 275 | 1.352775809 | RNF135 | ring finger protein 135 [Source:HGNC Symbol;Acc:HGNC:21158] |
| ENSG00000184471 | HGLibA_05956 | 275 | 1.352775809 | C1QTNF8 | C1q and TNF related 8 [Source:HGNC Symbol;Acc:HGNC:31374] |
| ENSG00000184709 | HGLibA_27293 | 275 | 1.352775809 | LRRC26 | leucine rich repeat containing 26 [Source:HGNC Symbol;Acc:HGNC:31409] |
| ENSG00000185219 | HGLibA_56018 | 275 | 1.352775809 | ZNF445 | zinc finger protein 445 [Source:HGNC Symbol;Acc:HGNC:21018] |
| ENSG00000185900 | HGLibA_37664 | 275 | 1.352775809 | POMK | protein-O-mannose kinase [Source:HGNC Symbol;Acc:HGNC:26267] |
| ENSG00000188816 | HGLibA_21761 | 275 | 1.352775809 | HMX2 | H6 family homeobox 2 [Source:HGNC Symbol;Acc:HGNC:5018] |
| ENSG00000196411 | HGLibA_15319 | 275 | 1.352775809 | EPHB4 | EPH receptor B4 [Source:HGNC Symbol;Acc:HGNC:3395] |
| ENSG00000196642 | HGLibA_40039 | 275 | 1.352775809 | RABL6 | member RAS oncogene family like 6 [Source:HGNC Symbol;Acc:HGNC:24703]" |
| ENSG00000204653 | HGLibA_03345 | 275 | 1.352775809 | ASPDH | aspartate dehydrogenase domain containing [Source:HGNC Symbol;Acc:HGNC:33856] |
| ENSG00000261857 | HGLibA_29184 | 275 | 1.352775809 | MIA | MIA SH3 domain containing [Source:HGNC Symbol;Acc:HGNC:7076] |
| FAM60A | HGLibA_16655 | 275 | 1.352775809 | FAM60A | - |
| hsa-mir-1294 | HGLibA_57651 | 275 | 1.352775809 | hsa-mir-1294 | - |
| hsa-mir-3681 | HGLibA_59278 | 275 | 1.352775809 | hsa-mir-3681 | - |
| hsa-mir-6129 | HGLibA_62664 | 275 | 1.352775809 | hsa-mir-6129 | - |
| OR5R1 | HGLibA_34352 | 275 | 1.352775809 | OR5R1 | - |
| RGAG4 | HGLibA_40995 | 275 | 1.352775809 | RGAG4 | - |
| RSG1 | HGLibA_42315 | 275 | 1.352775809 | RSG1 | - |
| ARMC4 | HGLibA_03021 | 274 | 1.343730466 | ARMC4 | - |
| ENSG00000003393 | HGLibA_01717 | 274 | 1.343730466 | ALS2 | alsin Rho guanine nucleotide exchange factor [Source:HGNC Symbol;Acc:HGNC:443]" |
| ENSG00000021488 | HGLibA_45386 | 274 | 1.343730466 | SLC7A9 | solute carrier family 7 member 9 [Source:HGNC Symbol;Acc:HGNC:11067] |
| ENSG00000055147 | HGLibA_16034 | 274 | 1.343730466 | FAM114A2 | family with sequence similarity 114 member A2 [Source:HGNC Symbol;Acc:HGNC:1333] |
| ENSG00000057704 | HGLibA_49777 | 274 | 1.343730466 | TMCC3 | transmembrane and coiled-coil domain family 3 [Source:HGNC Symbol;Acc:HGNC:29199] |
| ENSG00000101421 | HGLibA_09490 | 274 | 1.343730466 | CHMP4B | charged multivesicular body protein 4B [Source:HGNC Symbol;Acc:HGNC:16171] |
| ENSG00000103023 | HGLibA_38958 | 274 | 1.343730466 | PRSS54 | serine protease 54 [Source:HGNC Symbol;Acc:HGNC:26336] |
| ENSG00000103089 | HGLibA_15897 | 274 | 1.343730466 | FA2H | fatty acid 2-hydroxylase [Source:HGNC Symbol;Acc:HGNC:21197] |
| ENSG00000107281 | HGLibA_32427 | 274 | 1.343730466 | NPDC1 | differentiation and control 1 [Source:HGNC Symbol;Acc:HGNC:7899]" |
| ENSG00000111707 | HGLibA_47711 | 274 | 1.343730466 | SUDS3 | SIN3A corepressor complex component [Source:HGNC Symbol;Acc:HGNC:29545]" |
| ENSG00000111786 | HGLibA_47092 | 274 | 1.343730466 | SRSF9 | serine and arginine rich splicing factor 9 [Source:HGNC Symbol;Acc:HGNC:10791] |
| ENSG00000112290 | HGLibA_53952 | 274 | 1.343730466 | WASF1 | WAS protein family member 1 [Source:HGNC Symbol;Acc:HGNC:12732] |
| ENSG00000124733 | HGLibA_28719 | 274 | 1.343730466 | MEA1 | male-enhanced antigen 1 [Source:HGNC Symbol;Acc:HGNC:6986] |
| ENSG00000125247 | HGLibA_50680 | 274 | 1.343730466 | TMTC4 | transmembrane and tetratricopeptide repeat containing 4 [Source:HGNC Symbol;Acc:HGNC:25904] |
| ENSG00000125910 | HGLibA_42602 | 274 | 1.343730466 | S1PR4 | sphingosine-1-phosphate receptor 4 [Source:HGNC Symbol;Acc:HGNC:3170] |
| ENSG00000130699 | HGLibA_48234 | 274 | 1.343730466 | TAF4 | TATA-box binding protein associated factor 4 [Source:HGNC Symbol;Acc:HGNC:11537] |
| ENSG00000131386 | HGLibA_18544 | 274 | 1.343730466 | GALNT15 | polypeptide N-acetylgalactosaminyltransferase 15 [Source:HGNC Symbol;Acc:HGNC:21531] |
| ENSG00000133055 | HGLibA_30658 | 274 | 1.343730466 | MYBPH | myosin binding protein H [Source:HGNC Symbol;Acc:HGNC:7552] |
| ENSG00000135387 | HGLibA_07190 | 274 | 1.343730466 | CAPRIN1 | cell cycle associated protein 1 [Source:HGNC Symbol;Acc:HGNC:6743] |
| ENSG00000136643 | HGLibA_42183 | 274 | 1.343730466 | RPS6KC1 | ribosomal protein S6 kinase C1 [Source:HGNC Symbol;Acc:HGNC:10439] |
| ENSG00000137648 | HGLibA_50641 | 274 | 1.343730466 | TMPRSS4 | transmembrane serine protease 4 [Source:HGNC Symbol;Acc:HGNC:11878] |
| ENSG00000138622 | HGLibA_20981 | 274 | 1.343730466 | HCN4 | hyperpolarization activated cyclic nucleotide gated potassium channel 4 [Source:HGNC Symbol;Acc:HGNC:16882] |
| ENSG00000141431 | HGLibA_03396 | 274 | 1.343730466 | ASXL3 | ASXL transcriptional regulator 3 [Source:HGNC Symbol;Acc:HGNC:29357] |
| ENSG00000143153 | HGLibA_03592 | 274 | 1.343730466 | ATP1B1 | ATPase Na+/K+ transporting subunit beta 1 [Source:HGNC Symbol;Acc:HGNC:804] |
| ENSG00000145217 | HGLibA_44781 | 274 | 1.343730466 | SLC26A1 | solute carrier family 26 member 1 [Source:HGNC Symbol;Acc:HGNC:10993] |
| ENSG00000151062 | HGLibA_06914 | 274 | 1.343730466 | CACNA2D4 | calcium voltage-gated channel auxiliary subunit alpha2delta 4 [Source:HGNC Symbol;Acc:HGNC:20202] |
| ENSG00000157782 | HGLibA_06849 | 274 | 1.343730466 | CABP1 | calcium binding protein 1 [Source:HGNC Symbol;Acc:HGNC:1384] |
| ENSG00000161692 | HGLibA_12378 | 274 | 1.343730466 | DBF4B | DBF4 zinc finger B [Source:HGNC Symbol;Acc:HGNC:17883] |
| ENSG00000162341 | HGLibA_51107 | 274 | 1.343730466 | TPCN2 | two pore segment channel 2 [Source:HGNC Symbol;Acc:HGNC:20820] |
| ENSG00000163520 | HGLibA_16947 | 274 | 1.343730466 | FBLN2 | fibulin 2 [Source:HGNC Symbol;Acc:HGNC:3601] |
| ENSG00000163577 | HGLibA_14858 | 274 | 1.343730466 | EIF5A2 | eukaryotic translation initiation factor 5A2 [Source:HGNC Symbol;Acc:HGNC:3301] |
| ENSG00000164707 | HGLibA_44392 | 274 | 1.343730466 | SLC13A4 | solute carrier family 13 member 4 [Source:HGNC Symbol;Acc:HGNC:15827] |
| ENSG00000166140 | HGLibA_55275 | 274 | 1.343730466 | ZFYVE19 | zinc finger FYVE-type containing 19 [Source:HGNC Symbol;Acc:HGNC:20758] |
| ENSG00000166272 | HGLibA_53975 | 274 | 1.343730466 | WBP1L | WW domain binding protein 1 like [Source:HGNC Symbol;Acc:HGNC:23510] |
| ENSG00000169006 | HGLibA_32890 | 274 | 1.343730466 | NTSR2 | neurotensin receptor 2 [Source:HGNC Symbol;Acc:HGNC:8040] |
| ENSG00000172725 | HGLibA_10777 | 274 | 1.343730466 | CORO1B | coronin 1B [Source:HGNC Symbol;Acc:HGNC:2253] |
| ENSG00000173875 | HGLibA_56766 | 274 | 1.343730466 | ZNF791 | zinc finger protein 791 [Source:HGNC Symbol;Acc:HGNC:26895] |
| ENSG00000176142 | HGLibA_50385 | 274 | 1.343730466 | TMEM39A | transmembrane protein 39A [Source:HGNC Symbol;Acc:HGNC:25600] |
| ENSG00000177570 | HGLibA_42669 | 274 | 1.343730466 | SAMD12 | sterile alpha motif domain containing 12 [Source:HGNC Symbol;Acc:HGNC:31750] |
| ENSG00000178031 | HGLibA_00847 | 274 | 1.343730466 | ADAMTSL1 | ADAMTS like 1 [Source:HGNC Symbol;Acc:HGNC:14632] |
| ENSG00000179709 | HGLibA_32134 | 274 | 1.343730466 | NLRP8 | NLR family pyrin domain containing 8 [Source:HGNC Symbol;Acc:HGNC:22940] |
| ENSG00000182508 | HGLibA_26429 | 274 | 1.343730466 | LHFPL1 | LHFPL tetraspan subfamily member 1 [Source:HGNC Symbol;Acc:HGNC:6587] |
| ENSG00000185924 | HGLibA_42415 | 274 | 1.343730466 | RTN4RL1 | reticulon 4 receptor like 1 [Source:HGNC Symbol;Acc:HGNC:21329] |
| ENSG00000188505 | HGLibA_31288 | 274 | 1.343730466 | NCCRP1 | non-specific cytotoxic cell receptor protein 1 homolog (zebrafish) [Source:HGNC Symbol;Acc:HGNC:33739] |
| ENSG00000197044 | HGLibA_56004 | 274 | 1.343730466 | ZNF441 | zinc finger protein 441 [Source:HGNC Symbol;Acc:HGNC:20875] |
| ENSG00000222047 | HGLibA_05079 | 274 | 1.343730466 | C10orf55 | chromosome 10 open reading frame 55 [Source:HGNC Symbol;Acc:HGNC:31008] |
| ENSG00000231852 | HGLibA_12078 | 274 | 1.343730466 | CYP21A2 | cytochrome P450 family 21 subfamily A member 2 [Source:HGNC Symbol;Acc:HGNC:2600] |
| ENSG00000238243 | HGLibA_33905 | 274 | 1.343730466 | OR2W3 | olfactory receptor family 2 subfamily W member 3 [Source:HGNC Symbol;Acc:HGNC:15021] |
| FAM129A | HGLibA_16097 | 274 | 1.343730466 | FAM129A | - |
| HNRPLL | HGLibA_21857 | 274 | 1.343730466 | HNRPLL | - |
| hsa-mir-216b | HGLibA_58260 | 274 | 1.343730466 | hsa-mir-216b | - |
| hsa-mir-3609 | HGLibA_59065 | 274 | 1.343730466 | hsa-mir-3609 | - |
| hsa-mir-378e | HGLibA_59444 | 274 | 1.343730466 | hsa-mir-378e | - |
| hsa-mir-5092 | HGLibA_61528 | 274 | 1.343730466 | hsa-mir-5092 | - |
| hsa-mir-8077 | HGLibA_64150 | 274 | 1.343730466 | hsa-mir-8077 | - |
| PPAPDC1A | HGLibA_37826 | 274 | 1.343730466 | PPAPDC1A | - |
| ZAK | HGLibA_54771 | 274 | 1.343730466 | ZAK | - |
| AGXT2L1 | HGLibA_01278 | 273 | 1.334685123 | AGXT2L1 | - |
| C3orf83 | HGLibA_06276 | 273 | 1.334685123 | C3orf83 | - |
| ENSG00000083750 | HGLibA_42228 | 273 | 1.334685123 | RRAGB | Ras related GTP binding B [Source:HGNC Symbol;Acc:HGNC:19901] |
| ENSG00000090539 | HGLibA_09536 | 273 | 1.334685123 | CHRD | chordin [Source:HGNC Symbol;Acc:HGNC:1949] |
| ENSG00000101849 | HGLibA_48607 | 273 | 1.334685123 | TBL1X | transducin beta like 1 X-linked [Source:HGNC Symbol;Acc:HGNC:11585] |
| ENSG00000103653 | HGLibA_11360 | 273 | 1.334685123 | CSK | C-terminal Src kinase [Source:HGNC Symbol;Acc:HGNC:2444] |
| ENSG00000106012 | HGLibA_23500 | 273 | 1.334685123 | IQCE | IQ motif containing E [Source:HGNC Symbol;Acc:HGNC:29171] |
| ENSG00000108106 | HGLibA_52656 | 273 | 1.334685123 | UBE2S | ubiquitin conjugating enzyme E2 S [Source:HGNC Symbol;Acc:HGNC:17895] |
| ENSG00000116014 | HGLibA_25013 | 273 | 1.334685123 | KISS1R | KISS1 receptor [Source:HGNC Symbol;Acc:HGNC:4510] |
| ENSG00000119457 | HGLibA_45186 | 273 | 1.334685123 | SLC46A2 | solute carrier family 46 member 2 [Source:HGNC Symbol;Acc:HGNC:16055] |
| ENSG00000120262 | HGLibA_07690 | 273 | 1.334685123 | CCDC170 | coiled-coil domain containing 170 [Source:HGNC Symbol;Acc:HGNC:21177] |
| ENSG00000123454 | HGLibA_12382 | 273 | 1.334685123 | DBH | dopamine beta-hydroxylase [Source:HGNC Symbol;Acc:HGNC:2689] |
| ENSG00000125656 | HGLibA_10100 | 273 | 1.334685123 | CLPP | caseinolytic mitochondrial matrix peptidase proteolytic subunit [Source:HGNC Symbol;Acc:HGNC:2084] |
| ENSG00000136011 | HGLibA_47309 | 273 | 1.334685123 | STAB2 | stabilin 2 [Source:HGNC Symbol;Acc:HGNC:18629] |
| ENSG00000137834 | HGLibA_45585 | 273 | 1.334685123 | SMAD6 | SMAD family member 6 [Source:HGNC Symbol;Acc:HGNC:6772] |
| ENSG00000138131 | HGLibA_27048 | 273 | 1.334685123 | LOXL4 | lysyl oxidase like 4 [Source:HGNC Symbol;Acc:HGNC:17171] |
| ENSG00000138286 | HGLibA_16152 | 273 | 1.334685123 | FAM149B1 | family with sequence similarity 149 member B1 [Source:HGNC Symbol;Acc:HGNC:29162] |
| ENSG00000151665 | HGLibA_36553 | 273 | 1.334685123 | PIGF | phosphatidylinositol glycan anchor biosynthesis class F [Source:HGNC Symbol;Acc:HGNC:8962] |
| ENSG00000163263 | HGLibA_05811 | 273 | 1.334685123 | C1orf189 | chromosome 1 open reading frame 189 [Source:HGNC Symbol;Acc:HGNC:32305] |
| ENSG00000163958 | HGLibA_55094 | 273 | 1.334685123 | ZDHHC19 | zinc finger DHHC-type containing 19 [Source:HGNC Symbol;Acc:HGNC:20713] |
| ENSG00000166860 | HGLibA_54873 | 273 | 1.334685123 | ZBTB39 | zinc finger and BTB domain containing 39 [Source:HGNC Symbol;Acc:HGNC:29014] |
| ENSG00000167011 | HGLibA_31174 | 273 | 1.334685123 | NAT16 | N-acetyltransferase 16 (putative) [Source:HGNC Symbol;Acc:HGNC:22030] |
| ENSG00000167751 | HGLibA_25251 | 273 | 1.334685123 | KLK2 | kallikrein related peptidase 2 [Source:HGNC Symbol;Acc:HGNC:6363] |
| ENSG00000169895 | HGLibA_47906 | 273 | 1.334685123 | SYAP1 | synapse associated protein 1 [Source:HGNC Symbol;Acc:HGNC:16273] |
| ENSG00000175018 | HGLibA_49103 | 273 | 1.334685123 | TEX36 | testis expressed 36 [Source:HGNC Symbol;Acc:HGNC:31653] |
| ENSG00000184083 | HGLibA_16064 | 273 | 1.334685123 | FAM120C | family with sequence similarity 120C [Source:HGNC Symbol;Acc:HGNC:16949] |
| ENSG00000184434 | HGLibA_27274 | 273 | 1.334685123 | LRRC19 | leucine rich repeat containing 19 [Source:HGNC Symbol;Acc:HGNC:23379] |
| ENSG00000187742 | HGLibA_43266 | 273 | 1.334685123 | SECISBP2 | SECIS binding protein 2 [Source:HGNC Symbol;Acc:HGNC:30972] |
| ENSG00000197279 | HGLibA_55516 | 273 | 1.334685123 | ZNF165 | zinc finger protein 165 [Source:HGNC Symbol;Acc:HGNC:12953] |
| ENSG00000198018 | HGLibA_15215 | 273 | 1.334685123 | ENTPD7 | ectonucleoside triphosphate diphosphohydrolase 7 [Source:HGNC Symbol;Acc:HGNC:19745] |
| ENSG00000206538 | HGLibA_53641 | 273 | 1.334685123 | VGLL3 | vestigial like family member 3 [Source:HGNC Symbol;Acc:HGNC:24327] |
| ENSG00000212657 | HGLibA_25657 | 273 | 1.334685123 | KRTAP16-1 | keratin associated protein 16-1 [Source:HGNC Symbol;Acc:HGNC:18916] |
| ENSG00000212916 | HGLibA_28112 | 273 | 1.334685123 | MAP10 | microtubule associated protein 10 [Source:HGNC Symbol;Acc:HGNC:29265] |
| ENSG00000213402 | HGLibA_39494 | 273 | 1.334685123 | PTPRCAP | receptor type C associated protein [Source:HGNC Symbol;Acc:HGNC:9667]" |
| ENSG00000214357 | HGLibA_31725 | 273 | 1.334685123 | NEURL1B | neuralized E3 ubiquitin protein ligase 1B [Source:HGNC Symbol;Acc:HGNC:35422] |
| ENSG00000215712 | HGLibA_50290 | 273 | 1.334685123 | TMEM242 | transmembrane protein 242 [Source:HGNC Symbol;Acc:HGNC:17206] |
| ENSG00000229972 | HGLibA_23508 | 273 | 1.334685123 | IQCF3 | IQ motif containing F3 [Source:HGNC Symbol;Acc:HGNC:31816] |
| ENSG00000244509 | HGLibA_02481 | 273 | 1.334685123 | APOBEC3C | apolipoprotein B mRNA editing enzyme catalytic subunit 3C [Source:HGNC Symbol;Acc:HGNC:17353] |
| ENSG00000275832 | HGLibA_02733 | 273 | 1.334685123 | ARHGAP23 | Rho GTPase activating protein 23 [Source:HGNC Symbol;Acc:HGNC:29293] |
| FAM122B | HGLibA_16069 | 273 | 1.334685123 | FAM122B | - |
| hsa-mir-106b | HGLibA_57191 | 273 | 1.334685123 | hsa-mir-106b | - |
| hsa-mir-30a | HGLibA_58473 | 273 | 1.334685123 | hsa-mir-30a | - |
| hsa-mir-6792 | HGLibA_63346 | 273 | 1.334685123 | hsa-mir-6792 | - |
| hsa-mir-6824 | HGLibA_63475 | 273 | 1.334685123 | hsa-mir-6824 | - |
| C12orf68 | HGLibA_05285 | 272 | 1.32563978 | C12orf68 | - |
| C1QTNF9B-AS1 | HGLibA_05967 | 272 | 1.32563978 | C1QTNF9B-AS1 | - |
| ENSG00000055163 | HGLibA_12034 | 272 | 1.32563978 | CYFIP2 | cytoplasmic FMR1 interacting protein 2 [Source:HGNC Symbol;Acc:HGNC:13760] |
| ENSG00000076201 | HGLibA_39464 | 272 | 1.32563978 | PTPN23 | non-receptor type 23 [Source:HGNC Symbol;Acc:HGNC:14406]" |
| ENSG00000076928 | HGLibA_02803 | 272 | 1.32563978 | ARHGEF1 | Rho guanine nucleotide exchange factor 1 [Source:HGNC Symbol;Acc:HGNC:681] |
| ENSG00000082512 | HGLibA_51262 | 272 | 1.32563978 | TRAF5 | TNF receptor associated factor 5 [Source:HGNC Symbol;Acc:HGNC:12035] |
| ENSG00000085662 | HGLibA_01472 | 272 | 1.32563978 | AKR1B1 | aldo-keto reductase family 1 member B [Source:HGNC Symbol;Acc:HGNC:381] |

| | | | | | |
|---|---|---|---|---|---|
| ENSG00000092969 | HGLibA_49213 | 272 | 1.32563978 | TGFB2 | transforming growth factor beta 2 [Source:HGNC Symbol;Acc:HGNC:11768] |
| ENSG00000100600 | HGLibA_26406 | 272 | 1.32563978 | LGMN | legumain [Source:HGNC Symbol;Acc:HGNC:9472] |
| ENSG00000101017 | HGLibA_08385 | 272 | 1.32563978 | CD40 | CD40 molecule [Source:HGNC Symbol;Acc:HGNC:11919] |
| ENSG00000103534 | HGLibA_49761 | 272 | 1.32563978 | TMC5 | transmembrane channel like 5 [Source:HGNC Symbol;Acc:HGNC:22999] |
| ENSG00000104783 | HGLibA_24363 | 272 | 1.32563978 | KCNN4 | potassium calcium-activated channel subfamily N member 4 [Source:HGNC Symbol;Acc:HGNC:6293] |
| ENSG00000105122 | HGLibA_40317 | 272 | 1.32563978 | RASAL3 | RAS protein activator like 3 [Source:HGNC Symbol;Acc:HGNC:26129] |
| ENSG00000105556 | HGLibA_29256 | 272 | 1.32563978 | MIER2 | MIER family member 2 [Source:HGNC Symbol;Acc:HGNC:29210] |
| ENSG00000108242 | HGLibA_12114 | 272 | 1.32563978 | CYP2C18 | cytochrome P450 family 2 subfamily C member 18 [Source:HGNC Symbol;Acc:HGNC:2620] |
| ENSG00000108641 | HGLibA_04045 | 272 | 1.32563978 | B9D1 | B9 domain containing 1 [Source:HGNC Symbol;Acc:HGNC:24123] |
| ENSG00000110046 | HGLibA_03478 | 272 | 1.32563978 | ATG2A | autophagy related 2A [Source:HGNC Symbol;Acc:HGNC:29028] |
| ENSG00000111674 | HGLibA_15144 | 272 | 1.32563978 | ENO2 | enolase 2 [Source:HGNC Symbol;Acc:HGNC:3353] |
| ENSG00000115267 | HGLibA_22619 | 272 | 1.32563978 | IFIH1 | interferon induced with helicase C domain 1 [Source:HGNC Symbol;Acc:HGNC:18873] |
| ENSG00000116151 | HGLibA_29667 | 272 | 1.32563978 | MORN1 | MORN repeat containing 1 [Source:HGNC Symbol;Acc:HGNC:25852] |
| ENSG00000136158 | HGLibA_46838 | 272 | 1.32563978 | SPRY2 | sprouty RTK signaling antagonist 2 [Source:HGNC Symbol;Acc:HGNC:11270] |
| ENSG00000141380 | HGLibA_47101 | 272 | 1.32563978 | SS18 | nBAF chromatin remodeling complex subunit [Source:HGNC Symbol;Acc:HGNC:11340]" |
| ENSG00000144119 | HGLibA_05927 | 272 | 1.32563978 | C1QL2 | complement C1q like 2 [Source:HGNC Symbol;Acc:HGNC:24181] |
| ENSG00000148965 | HGLibA_42616 | 272 | 1.32563978 | SAA4 | constitutive [Source:HGNC Symbol;Acc:HGNC:10516]" |
| ENSG00000152382 | HGLibA_48183 | 272 | 1.32563978 | TADA1 | transcriptional adaptor 1 [Source:HGNC Symbol;Acc:HGNC:30631] |
| ENSG00000155833 | HGLibA_12046 | 272 | 1.32563978 | CYLC2 | cylicin 2 [Source:HGNC Symbol;Acc:HGNC:2583] |
| ENSG00000158089 | HGLibA_18541 | 272 | 1.32563978 | GALNT14 | polypeptide N-acetylgalactosaminyltransferase 14 [Source:HGNC Symbol;Acc:HGNC:22946] |
| ENSG00000158764 | HGLibA_23827 | 272 | 1.32563978 | ITLN2 | intelectin 2 [Source:HGNC Symbol;Acc:HGNC:20599] |
| ENSG00000164089 | HGLibA_15638 | 272 | 1.32563978 | ETNPPL | ethanolamine-phosphate phospho-lyase [Source:HGNC Symbol;Acc:HGNC:14404] |
| ENSG00000171346 | HGLibA_25430 | 272 | 1.32563978 | KRT15 | keratin 15 [Source:HGNC Symbol;Acc:HGNC:6421] |
| ENSG00000172818 | HGLibA_34802 | 272 | 1.32563978 | OVOL1 | ovo like transcriptional repressor 1 [Source:HGNC Symbol;Acc:HGNC:8525] |
| ENSG00000173200 | HGLibA_35227 | 272 | 1.32563978 | PARP15 | poly(ADP-ribose) polymerase family member 15 [Source:HGNC Symbol;Acc:HGNC:26876] |
| ENSG00000173208 | HGLibA_00193 | 272 | 1.32563978 | ABCD2 | ATP binding cassette subfamily D member 2 [Source:HGNC Symbol;Acc:HGNC:66] |
| ENSG00000174004 | HGLibA_32721 | 272 | 1.32563978 | NRROS | negative regulator of reactive oxygen species [Source:HGNC Symbol;Acc:HGNC:24613] |
| ENSG00000179304 | HGLibA_16188 | 272 | 1.32563978 | FAM156B | family with sequence similarity 156 member B [Source:HGNC Symbol;Acc:HGNC:31962] |
| ENSG00000180210 | HGLibA_15858 | 272 | 1.32563978 | F2 | thrombin [Source:HGNC Symbol;Acc:HGNC:3535]" |
| ENSG00000184012 | HGLibA_50636 | 272 | 1.32563978 | TMPRSS2 | transmembrane serine protease 2 [Source:HGNC Symbol;Acc:HGNC:11876] |
| ENSG00000198074 | HGLibA_01475 | 272 | 1.32563978 | AKR1B10 | aldo-keto reductase family 1 member B10 [Source:HGNC Symbol;Acc:HGNC:382] |
| ENSG00000204363 | HGLibA_46414 | 272 | 1.32563978 | SPANXN5 | SPANX family member N5 [Source:HGNC Symbol;Acc:HGNC:33178] |
| ENSG00000204519 | HGLibA_56214 | 272 | 1.32563978 | ZNF551 | zinc finger protein 551 [Source:HGNC Symbol;Acc:HGNC:25108] |
| ENSG00000204967 | HGLibA_35446 | 272 | 1.32563978 | PCDHA4 | protocadherin alpha 4 [Source:HGNC Symbol;Acc:HGNC:8670] |
| ENSG00000226742 | HGLibA_22154 | 272 | 1.32563978 | HSBP1L1 | heat shock factor binding protein 1 like 1 [Source:HGNC Symbol;Acc:HGNC:37243] |
| ENSG00000256087 | HGLibA_55985 | 272 | 1.32563978 | ZNF432 | zinc finger protein 432 [Source:HGNC Symbol;Acc:HGNC:20810] |
| ENSG00000267855 | HGLibA_31471 | 272 | 1.32563978 | NDUFA7 | NADH:ubiquinone oxidoreductase subunit A7 [Source:HGNC Symbol;Acc:HGNC:7691] |
| hsa-mir-372 | HGLibA_59380 | 272 | 1.32563978 | hsa-mir-372 | - |
| hsa-mir-4516 | HGLibA_60507 | 272 | 1.32563978 | hsa-mir-4516 | - |
| hsa-mir-492 | HGLibA_61369 | 272 | 1.32563978 | hsa-mir-492 | - |
| hsa-mir-665 | HGLibA_63001 | 272 | 1.32563978 | hsa-mir-665 | - |
| hsa-mir-6778 | HGLibA_63284 | 272 | 1.32563978 | hsa-mir-6778 | - |
| SHOX_X | HGLibA_44068 | 272 | 1.32563978 | SHOX_X | - |
| ENSG00000068383 | HGLibA_23342 | 271 | 1.316594437 | INPP5A | inositol polyphosphate-5-phosphatase A [Source:HGNC Symbol;Acc:HGNC:6076] |
| ENSG00000075239 | HGLibA_00391 | 271 | 1.316594437 | ACAT1 | acetyl-CoA acetyltransferase 1 [Source:HGNC Symbol;Acc:HGNC:93] |
| ENSG00000099949 | HGLibA_27802 | 271 | 1.316594437 | LZTR1 | leucine zipper like transcription regulator 1 [Source:HGNC Symbol;Acc:HGNC:6742] |
| ENSG00000104626 | HGLibA_15479 | 271 | 1.316594437 | ERI1 | exoribonuclease 1 [Source:HGNC Symbol;Acc:HGNC:23994] |
| ENSG00000104695 | HGLibA_38138 | 271 | 1.316594437 | PPP2CB | protein phosphatase 2 catalytic subunit beta [Source:HGNC Symbol;Acc:HGNC:9300] |
| ENSG00000105976 | HGLibA_28925 | 271 | 1.316594437 | MET | receptor tyrosine kinase [Source:HGNC Symbol;Acc:HGNC:7029]" |
| ENSG00000116406 | HGLibA_14461 | 271 | 1.316594437 | EDEM3 | ER degradation enhancing alpha-mannosidase like protein 3 [Source:HGNC Symbol;Acc:HGNC:16787] |
| ENSG00000124762 | HGLibA_08821 | 271 | 1.316594437 | CDKN1A | cyclin dependent kinase inhibitor 1A [Source:HGNC Symbol;Acc:HGNC:1784] |
| ENSG00000125945 | HGLibA_55989 | 271 | 1.316594437 | ZNF436 | zinc finger protein 436 [Source:HGNC Symbol;Acc:HGNC:20814] |
| ENSG00000127084 | HGLibA_17349 | 271 | 1.316594437 | FGD3 | RhoGEF and PH domain containing 3 [Source:HGNC Symbol;Acc:HGNC:16027]" |
| ENSG00000132405 | HGLibA_48479 | 271 | 1.316594437 | TBC1D14 | TBC1 domain family member 14 [Source:HGNC Symbol;Acc:HGNC:29246] |
| ENSG00000138161 | HGLibA_11809 | 271 | 1.316594437 | CUZD1 | CUB and zona pellucida like domains 1 [Source:HGNC Symbol;Acc:HGNC:17937] |
| ENSG00000141428 | HGLibA_05607 | 271 | 1.316594437 | C18orf21 | chromosome 18 open reading frame 21 [Source:HGNC Symbol;Acc:HGNC:28802] |
| ENSG00000141985 | HGLibA_43946 | 271 | 1.316594437 | SH3GL1 | endophilin A2 [Source:HGNC Symbol;Acc:HGNC:10830]" |
| ENSG00000161681 | HGLibA_43989 | 271 | 1.316594437 | SHANK1 | SH3 and multiple ankyrin repeat domains 1 [Source:HGNC Symbol;Acc:HGNC:15474] |
| ENSG00000162458 | HGLibA_16940 | 271 | 1.316594437 | FBLIM1 | filamin binding LIM protein 1 [Source:HGNC Symbol;Acc:HGNC:24686] |
| ENSG00000164305 | HGLibA_07311 | 271 | 1.316594437 | CASP3 | caspase 3 [Source:HGNC Symbol;Acc:HGNC:1504] |
| ENSG00000165006 | HGLibA_52546 | 271 | 1.316594437 | UBAP1 | ubiquitin associated protein 1 [Source:HGNC Symbol;Acc:HGNC:12461] |
| ENSG00000165731 | HGLibA_40871 | 271 | 1.316594437 | RET | ret proto-oncogene [Source:HGNC Symbol;Acc:HGNC:9967] |
| ENSG00000166535 | HGLibA_00012 | 271 | 1.316594437 | A2ML1 | alpha-2-macroglobulin like 1 [Source:HGNC Symbol;Acc:HGNC:23336] |
| ENSG00000166676 | HGLibA_52357 | 271 | 1.316594437 | TVP23A | trans-golgi network vesicle protein 23 homolog A [Source:HGNC Symbol;Acc:HGNC:20398] |
| ENSG00000170954 | HGLibA_55942 | 271 | 1.316594437 | ZNF415 | zinc finger protein 415 [Source:HGNC Symbol;Acc:HGNC:20636] |
| ENSG00000171552 | HGLibA_04301 | 271 | 1.316594437 | BCL2L1 | BCL2 like 1 [Source:HGNC Symbol;Acc:HGNC:992] |
| ENSG00000171700 | HGLibA_41075 | 271 | 1.316594437 | RGS19 | regulator of G protein signaling 19 [Source:HGNC Symbol;Acc:HGNC:13735] |
| ENSG00000174500 | HGLibA_18828 | 271 | 1.316594437 | GCSAM | germinal center associated signaling and motility [Source:HGNC Symbol;Acc:HGNC:20253] |
| ENSG00000183684 | HGLibA_01742 | 271 | 1.316594437 | ALYREF | Aly/REF export factor [Source:HGNC Symbol;Acc:HGNC:19071] |
| ENSG00000184205 | HGLibA_51982 | 271 | 1.316594437 | TSPYL2 | TSPY like 2 [Source:HGNC Symbol;Acc:HGNC:24358] |
| ENSG00000185129 | HGLibA_39585 | 271 | 1.316594437 | PURA | purine rich element binding protein A [Source:HGNC Symbol;Acc:HGNC:9701] |
| ENSG00000186510 | HGLibA_09842 | 271 | 1.316594437 | CLCNKA | chloride voltage-gated channel Ka [Source:HGNC Symbol;Acc:HGNC:2026] |
| ENSG00000187082 | HGLibA_12833 | 271 | 1.316594437 | DEFB106B | defensin beta 106B [Source:HGNC Symbol;Acc:HGNC:28879] |
| ENSG00000189023 | HGLibA_27934 | 271 | 1.316594437 | MAGEB16 | MAGE family member B16 [Source:HGNC Symbol;Acc:HGNC:21188] |
| ENSG00000198033 | HGLibA_52251 | 271 | 1.316594437 | TUBA3C | tubulin alpha 3c [Source:HGNC Symbol;Acc:HGNC:12408] |
| ENSG00000257315 | HGLibA_54803 | 271 | 1.316594437 | ZBED6 | zinc finger BED-type containing 6 [Source:HGNC Symbol;Acc:HGNC:33273] |
| H1FOO | HGLibA_20738 | 271 | 1.316594437 | H1FOO | - |
| hsa-mir-4525 | HGLibA_60562 | 271 | 1.316594437 | hsa-mir-4525 | - |
| hsa-mir-6075 | HGLibA_62551 | 271 | 1.316594437 | hsa-mir-6075 | - |
| hsa-mir-934 | HGLibA_64310 | 271 | 1.316594437 | hsa-mir-934 | - |
| AKAP2 | HGLibA_01427 | 270 | 1.307549094 | AKAP2 | - |
| ENSG00000007038 | HGLibA_38910 | 270 | 1.307549094 | PRSS21 | serine protease 21 [Source:HGNC Symbol;Acc:HGNC:9485] |
| ENSG00000091181 | HGLibA_23179 | 270 | 1.307549094 | IL5RA | interleukin 5 receptor subunit alpha [Source:HGNC Symbol;Acc:HGNC:6017] |
| ENSG00000099804 | HGLibA_08543 | 270 | 1.307549094 | CDC34 | cell division cycle 34 [Source:HGNC Symbol;Acc:HGNC:1734] |
| ENSG00000100276 | HGLibA_40359 | 270 | 1.307549094 | RASL10A | RAS like family 10 member A [Source:HGNC Symbol;Acc:HGNC:16954] |
| ENSG00000102870 | HGLibA_56430 | 270 | 1.307549094 | ZNF629 | zinc finger protein 629 [Source:HGNC Symbol;Acc:HGNC:29008] |
| ENSG00000108587 | HGLibA_19683 | 270 | 1.307549094 | GOSR1 | golgi SNAP receptor complex member 1 [Source:HGNC Symbol;Acc:HGNC:4430] |
| ENSG00000110148 | HGLibA_07959 | 270 | 1.307549094 | CCKBR | cholecystokinin B receptor [Source:HGNC Symbol;Acc:HGNC:1571] |
| ENSG00000111215 | HGLibA_38840 | 270 | 1.307549094 | PRR4 | proline rich 4 [Source:HGNC Symbol;Acc:HGNC:18020] |
| ENSG00000111817 | HGLibA_14017 | 270 | 1.307549094 | DSE | dermatan sulfate epimerase [Source:HGNC Symbol;Acc:HGNC:21144] |
| ENSG00000112246 | HGLibA_44175 | 270 | 1.307549094 | SIM1 | SIM bHLH transcription factor 1 [Source:HGNC Symbol;Acc:HGNC:10882] |
| ENSG00000112486 | HGLibA_08163 | 270 | 1.307549094 | CCR6 | C-C motif chemokine receptor 6 [Source:HGNC Symbol;Acc:HGNC:1607] |
| ENSG00000124449 | HGLibA_23609 | 270 | 1.307549094 | IRGC | immunity related GTPase cinema [Source:HGNC Symbol;Acc:HGNC:28835] |
| ENSG00000130958 | HGLibA_44979 | 270 | 1.307549094 | SLC35D2 | solute carrier family 35 member D2 [Source:HGNC Symbol;Acc:HGNC:20799] |
| ENSG00000131044 | HGLibA_52217 | 270 | 1.307549094 | TTLL9 | tubulin tyrosine ligase like 9 [Source:HGNC Symbol;Acc:HGNC:16118] |
| ENSG00000132471 | HGLibA_53978 | 270 | 1.307549094 | WBP2 | WW domain binding protein 2 [Source:HGNC Symbol;Acc:HGNC:12738] |
| ENSG00000132823 | HGLibA_34677 | 270 | 1.307549094 | OSER1 | oxidative stress responsive serine rich 1 [Source:HGNC Symbol;Acc:HGNC:16105] |
| ENSG00000133710 | HGLibA_46697 | 270 | 1.307549094 | SPINK5 | Kazal type 5 [Source:HGNC Symbol;Acc:HGNC:15464]" |
| ENSG00000135525 | HGLibA_28233 | 270 | 1.307549094 | MAP7 | microtubule associated protein 7 [Source:HGNC Symbol;Acc:HGNC:6869] |
| ENSG00000135835 | HGLibA_24794 | 270 | 1.307549094 | KIAA1614 | KIAA1614 [Source:HGNC Symbol;Acc:HGNC:29327] |
| ENSG00000136928 | HGLibA_18345 | 270 | 1.307549094 | GABBR2 | gamma-aminobutyric acid type B receptor subunit 2 [Source:HGNC Symbol;Acc:HGNC:4507] |
| ENSG00000140557 | HGLibA_47292 | 270 | 1.307549094 | ST8SIA2 | 8-sialyltransferase 2 [Source:HGNC Symbol;Acc:HGNC:10870]" |
| ENSG00000144230 | HGLibA_19963 | 270 | 1.307549094 | GPR17 | G protein-coupled receptor 17 [Source:HGNC Symbol;Acc:HGNC:4471] |
| ENSG00000147262 | HGLibA_19868 | 270 | 1.307549094 | GPR119 | G protein-coupled receptor 119 [Source:HGNC Symbol;Acc:HGNC:19060] |
| ENSG00000147873 | HGLibA_22684 | 270 | 1.307549094 | IFNA5 | interferon alpha 5 [Source:HGNC Symbol;Acc:HGNC:5426] |
| ENSG00000148803 | HGLibA_18170 | 270 | 1.307549094 | FUOM | fucose mutarotase [Source:HGNC Symbol;Acc:HGNC:24733] |
| ENSG00000152284 | HGLibA_48795 | 270 | 1.307549094 | TCF7L1 | transcription factor 7 like 1 [Source:HGNC Symbol;Acc:HGNC:11640] |
| ENSG00000159248 | HGLibA_19186 | 270 | 1.307549094 | GJD2 | gap junction protein delta 2 [Source:HGNC Symbol;Acc:HGNC:19154] |
| ENSG00000160445 | HGLibA_55142 | 270 | 1.307549094 | ZER1 | zyg-11 related cell cycle regulator [Source:HGNC Symbol;Acc:HGNC:30960] |
| ENSG00000163114 | HGLibA_35866 | 270 | 1.307549094 | PDHA2 | pyruvate dehydrogenase E1 alpha 2 subunit [Source:HGNC Symbol;Acc:HGNC:8807] |
| ENSG00000163444 | HGLibA_50105 | 270 | 1.307549094 | TMEM183A | transmembrane protein 183A [Source:HGNC Symbol;Acc:HGNC:20173] |
| ENSG00000165478 | HGLibA_21137 | 270 | 1.307549094 | HEPACAM | hepatic and glial cell adhesion molecule [Source:HGNC Symbol;Acc:HGNC:26361] |
| ENSG00000170456 | HGLibA_12962 | 270 | 1.307549094 | DENND5B | DENN domain containing 5B [Source:HGNC Symbol;Acc:HGNC:28338] |
| ENSG00000170836 | HGLibA_37968 | 270 | 1.307549094 | PPM1D | Mg2+/Mn2+ dependent 1D [Source:HGNC Symbol;Acc:HGNC:9277]" |
| ENSG00000173334 | HGLibA_51390 | 270 | 1.307549094 | TRIB1 | tribbles pseudokinase 1 [Source:HGNC Symbol;Acc:HGNC:16891] |
| ENSG00000178586 | HGLibA_34379 | 270 | 1.307549094 | OR6B3 | olfactory receptor family 6 subfamily B member 3 [Source:HGNC Symbol;Acc:HGNC:15042] |
| ENSG00000184735 | HGLibA_12741 | 270 | 1.307549094 | DDX53 | DEAD-box helicase 53 [Source:HGNC Symbol;Acc:HGNC:20083] |
| ENSG00000187033 | HGLibA_42691 | 270 | 1.307549094 | SAMD7 | sterile alpha motif domain containing 7 [Source:HGNC Symbol;Acc:HGNC:25394] |
| ENSG00000197555 | HGLibA_44191 | 270 | 1.307549094 | SIPA1L1 | signal induced proliferation associated 1 like 1 [Source:HGNC Symbol;Acc:HGNC:20284] |
| ENSG00000197579 | HGLibA_51011 | 270 | 1.307549094 | TOPORS | TOP1 binding arginine/serine rich protein [Source:HGNC Symbol;Acc:HGNC:21653] |
| ENSG00000205856 | HGLibA_06077 | 270 | 1.307549094 | C22orf42 | chromosome 22 open reading frame 42 [Source:HGNC Symbol;Acc:HGNC:27160] |
| ENSG00000213402 | HGLibA_39492 | 270 | 1.307549094 | PTPRCAP | receptor type C associated protein [Source:HGNC Symbol;Acc:HGNC:9667]" |
| ENSG00000233493 | HGLibA_50279 | 270 | 1.307549094 | TMEM238 | transmembrane protein 238 [Source:HGNC Symbol;Acc:HGNC:40042] |
| ENSG00000261308 | HGLibA_17526 | 270 | 1.307549094 | FIGNL2 | fidgetin like 2 [Source:HGNC Symbol;Acc:HGNC:13287] |
| GPR116 | HGLibA_19864 | 270 | 1.307549094 | GPR116 | - |
| hsa-mir-4670 | HGLibA_60797 | 270 | 1.307549094 | hsa-mir-4670 | - |
| C16orf80 | HGLibA_05485 | 269 | 1.298503751 | C16orf80 | - |
| ENSG00000005486 | HGLibA_41126 | 269 | 1.298503751 | RHBDD2 | rhomboid domain containing 2 [Source:HGNC Symbol;Acc:HGNC:23082] |
| ENSG00000006712 | HGLibA_34986 | 269 | 1.298503751 | PAF1 | Paf1/RNA polymerase II complex component [Source:HGNC Symbol;Acc:HGNC:25459]" |
| ENSG00000049323 | HGLibA_27591 | 269 | 1.298503751 | LTBP1 | latent transforming growth factor beta binding protein 1 [Source:HGNC Symbol;Acc:HGNC:6714] |
| ENSG00000089820 | HGLibA_02773 | 269 | 1.298503751 | ARHGAP4 | Rho GTPase activating protein 4 [Source:HGNC Symbol;Acc:HGNC:674] |
| ENSG00000104689 | HGLibA_50733 | 269 | 1.298503751 | TNFRSF10A | TNF receptor superfamily member 10a [Source:HGNC Symbol;Acc:HGNC:11904] |
| ENSG00000105428 | HGLibA_56946 | 269 | 1.298503751 | ZNRF4 | zinc and ring finger 4 [Source:HGNC Symbol;Acc:HGNC:17726] |
| ENSG00000109133 | HGLibA_50369 | 269 | 1.298503751 | TMEM33 | transmembrane protein 33 [Source:HGNC Symbol;Acc:HGNC:25541] |
| ENSG00000122592 | HGLibA_21920 | 269 | 1.298503751 | HOXA7 | homeobox A7 [Source:HGNC Symbol;Acc:HGNC:5108] |

| | | | | | |
|---|---|---|---|---|---|
| ENSG00000123576 | HGLibA_15603 | 269 | 1.298503751 | ESX1 | ESX homeobox 1 [Source:HGNC Symbol;Acc:HGNC:14865] |
| ENSG00000128564 | HGLibA_53633 | 269 | 1.298503751 | VGF | VGF nerve growth factor inducible [Source:HGNC Symbol;Acc:HGNC:12684] |
| ENSG00000132305 | HGLibA_23236 | 269 | 1.298503751 | IMMT | inner membrane mitochondrial protein [Source:HGNC Symbol;Acc:HGNC:6047] |
| ENSG00000134077 | HGLibA_49422 | 269 | 1.298503751 | THUMPD3 | THUMP domain containing 3 [Source:HGNC Symbol;Acc:HGNC:24493] |
| ENSG00000135597 | HGLibA_40849 | 269 | 1.298503751 | REPS1 | RALBP1 associated Eps domain containing 1 [Source:HGNC Symbol;Acc:HGNC:15578] |
| ENSG00000147081 | HGLibA_01434 | 269 | 1.298503751 | AKAP4 | A-kinase anchoring protein 4 [Source:HGNC Symbol;Acc:HGNC:374] |
| ENSG00000156642 | HGLibA_32528 | 269 | 1.298503751 | NPTN | neuroplastin [Source:HGNC Symbol;Acc:HGNC:17867] |
| ENSG00000159871 | HGLibA_27725 | 269 | 1.298503751 | LYPD5 | LY6/PLAUR domain containing 5 [Source:HGNC Symbol;Acc:HGNC:26397] |
| ENSG00000160688 | HGLibA_17604 | 269 | 1.298503751 | FLAD1 | flavin adenine dinucleotide synthetase 1 [Source:HGNC Symbol;Acc:HGNC:24671] |
| ENSG00000160712 | HGLibA_23185 | 269 | 1.298503751 | IL6R | interleukin 6 receptor [Source:HGNC Symbol;Acc:HGNC:6019] |
| ENSG00000163673 | HGLibA_12505 | 269 | 1.298503751 | DCLK3 | doublecortin like kinase 3 [Source:HGNC Symbol;Acc:HGNC:19005] |
| ENSG00000165030 | HGLibA_31808 | 269 | 1.298503751 | NFIL3 | interleukin 3 regulated [Source:HGNC Symbol;Acc:HGNC:7787]" |
| ENSG00000167880 | HGLibA_15700 | 269 | 1.298503751 | EVPL | envoplakin [Source:HGNC Symbol;Acc:HGNC:3503] |
| ENSG00000171444 | HGLibA_28561 | 269 | 1.298503751 | MCC | WNT signaling pathway regulator [Source:HGNC Symbol;Acc:HGNC:6935]" |
| ENSG00000173821 | HGLibA_41623 | 269 | 1.298503751 | RNF213 | ring finger protein 213 [Source:HGNC Symbol;Acc:HGNC:14539] |
| ENSG00000176171 | HGLibA_04657 | 269 | 1.298503751 | BNIP3 | BCL2 interacting protein 3 [Source:HGNC Symbol;Acc:HGNC:1084] |
| ENSG00000178988 | HGLibA_29832 | 269 | 1.298503751 | MRFAP1L1 | Morf4 family associated protein 1 like 1 [Source:HGNC Symbol;Acc:HGNC:28796] |
| ENSG00000183072 | HGLibA_32043 | 269 | 1.298503751 | NKX2-5 | NK2 homeobox 5 [Source:HGNC Symbol;Acc:HGNC:2488] |
| ENSG00000186930 | HGLibA_25818 | 269 | 1.298503751 | KRTAP6-2 | keratin associated protein 6-2 [Source:HGNC Symbol;Acc:HGNC:18932] |
| ENSG00000188536 | HGLibA_20909 | 269 | 1.298503751 | HBA2 | hemoglobin subunit alpha 2 [Source:HGNC Symbol;Acc:HGNC:4824] |
| ENSG00000196428 | HGLibA_51821 | 269 | 1.298503751 | TSC22D2 | TSC22 domain family member 2 [Source:HGNC Symbol;Acc:HGNC:29095] |
| ENSG00000204385 | HGLibA_45167 | 269 | 1.298503751 | SLC44A4 | solute carrier family 44 member 4 [Source:HGNC Symbol;Acc:HGNC:13941] |
| ENSG00000214435 | HGLibA_03181 | 269 | 1.298503751 | AS3MT | arsenite methyltransferase [Source:HGNC Symbol;Acc:HGNC:17452] |
| ENSG00000233198 | HGLibA_41650 | 269 | 1.298503751 | RNF224 | ring finger protein 224 [Source:HGNC Symbol;Acc:HGNC:41912] |
| ENSG00000245680 | HGLibA_56322 | 269 | 1.298503751 | ZNF585B | zinc finger protein 585B [Source:HGNC Symbol;Acc:HGNC:30948] |
| FAM46C | HGLibA_16602 | 269 | 1.298503751 | FAM46C | - |
| FAM46C | HGLibA_16601 | 269 | 1.298503751 | FAM46C | - |
| hsa-mir-1258 | HGLibA_57438 | 269 | 1.298503751 | hsa-mir-1258 | - |
| hsa-mir-1260b | HGLibA_57465 | 269 | 1.298503751 | hsa-mir-1260b | - |
| hsa-mir-4484 | HGLibA_60356 | 269 | 1.298503751 | hsa-mir-4484 | - |
| PTRF | HGLibA_39547 | 269 | 1.298503751 | PTRF | - |
| SLC22A20 | HGLibA_44569 | 269 | 1.298503751 | SLC22A20 | - |
| TTC40 | HGLibA_52145 | 269 | 1.298503751 | TTC40 | - |
| WISP2 | HGLibA_54321 | 269 | 1.298503751 | WISP2 | - |
| CPSF3L | HGLibA_11002 | 268 | 1.289458408 | CPSF3L | - |
| ENSG00000029639 | HGLibA_49140 | 268 | 1.289458408 | TFB1M | mitochondrial [Source:HGNC Symbol;Acc:HGNC:17037]" |
| ENSG00000038219 | HGLibA_04671 | 268 | 1.289458408 | BOD1L1 | biorientation of chromosomes in cell division 1 like 1 [Source:HGNC Symbol;Acc:HGNC:31792] |
| ENSG00000064651 | HGLibA_44359 | 268 | 1.289458408 | SLC12A2 | solute carrier family 12 member 2 [Source:HGNC Symbol;Acc:HGNC:10911] |
| ENSG00000079332 | HGLibA_42737 | 268 | 1.289458408 | SAR1A | secretion associated Ras related GTPase 1A [Source:HGNC Symbol;Acc:HGNC:10534] |
| ENSG00000089225 | HGLibA_48675 | 268 | 1.289458408 | TBX5 | T-box 5 [Source:HGNC Symbol;Acc:HGNC:11604] |
| ENSG00000093167 | HGLibA_27453 | 268 | 1.289458408 | LRRFIP2 | LRR binding FLII interacting protein 2 [Source:HGNC Symbol;Acc:HGNC:6703] |
| ENSG00000105707 | HGLibA_22037 | 268 | 1.289458408 | HPN | hepsin [Source:HGNC Symbol;Acc:HGNC:5155] |
| ENSG00000110047 | HGLibA_14667 | 268 | 1.289458408 | EHD1 | EH domain containing 1 [Source:HGNC Symbol;Acc:HGNC:3242] |
| ENSG00000112167 | HGLibA_42789 | 268 | 1.289458408 | SAYSD1 | SAYSVFN motif domain containing 1 [Source:HGNC Symbol;Acc:HGNC:21025] |
| ENSG00000114349 | HGLibA_19471 | 268 | 1.289458408 | GNAT1 | G protein subunit alpha transducin 1 [Source:HGNC Symbol;Acc:HGNC:4393] |
| ENSG00000117477 | HGLibA_07725 | 268 | 1.289458408 | CCDC181 | coiled-coil domain containing 181 [Source:HGNC Symbol;Acc:HGNC:28051] |
| ENSG00000118194 | HGLibA_50885 | 268 | 1.289458408 | TNNT2 | cardiac type [Source:HGNC Symbol;Acc:HGNC:11949]" |
| ENSG00000127377 | HGLibA_11283 | 268 | 1.289458408 | CRYGN | crystallin gamma N [Source:HGNC Symbol;Acc:HGNC:20458] |
| ENSG00000132357 | HGLibA_07234 | 268 | 1.289458408 | CARD6 | caspase recruitment domain family member 6 [Source:HGNC Symbol;Acc:HGNC:16394] |
| ENSG00000134668 | HGLibA_46746 | 268 | 1.289458408 | SPOCD1 | SPOC domain containing 1 [Source:HGNC Symbol;Acc:HGNC:26338] |
| ENSG00000135324 | HGLibA_29809 | 268 | 1.289458408 | MRAP2 | melanocortin 2 receptor accessory protein 2 [Source:HGNC Symbol;Acc:HGNC:21232] |
| ENSG00000135365 | HGLibA_36370 | 268 | 1.289458408 | PHF21A | PHD finger protein 21A [Source:HGNC Symbol;Acc:HGNC:24156] |
| ENSG00000135480 | HGLibA_25522 | 268 | 1.289458408 | KRT7 | keratin 7 [Source:HGNC Symbol;Acc:HGNC:6445] |
| ENSG00000145743 | HGLibA_16988 | 268 | 1.289458408 | FBXL17 | F-box and leucine rich repeat protein 17 [Source:HGNC Symbol;Acc:HGNC:13615] |
| ENSG00000147421 | HGLibA_21666 | 268 | 1.289458408 | HMBOX1 | homeobox containing 1 [Source:HGNC Symbol;Acc:HGNC:26137] |
| ENSG00000147676 | HGLibA_28024 | 268 | 1.289458408 | MAL2 | T cell differentiation protein 2 (gene/pseudogene) [Source:HGNC Symbol;Acc:HGNC:13634]" |
| ENSG00000149289 | HGLibA_54957 | 268 | 1.289458408 | ZC3H12C | zinc finger CCCH-type containing 12C [Source:HGNC Symbol;Acc:HGNC:29362] |
| ENSG00000151615 | HGLibA_37780 | 268 | 1.289458408 | POU4F2 | POU class 4 homeobox 2 [Source:HGNC Symbol;Acc:HGNC:9219] |
| ENSG00000155714 | HGLibA_35989 | 268 | 1.289458408 | PDZD9 | PDZ domain containing 9 [Source:HGNC Symbol;Acc:HGNC:28740] |
| ENSG00000158806 | HGLibA_32489 | 268 | 1.289458408 | NPM2 | nucleophosmin/nucleoplasmin 2 [Source:HGNC Symbol;Acc:HGNC:7930] |
| ENSG00000164129 | HGLibA_32560 | 268 | 1.289458408 | NPY5R | neuropeptide Y receptor Y5 [Source:HGNC Symbol;Acc:HGNC:7958] |
| ENSG00000164778 | HGLibA_15102 | 268 | 1.289458408 | EN2 | engrailed homeobox 2 [Source:HGNC Symbol;Acc:HGNC:3343] |
| ENSG00000166562 | HGLibA_43184 | 268 | 1.289458408 | SEC11C | signal peptidase complex subunit [Source:HGNC Symbol;Acc:HGNC:23400]" |
| ENSG00000168757 | HGLibA_51966 | 268 | 1.289458408 | TSPY2 | testis specific protein Y-linked 2 [Source:HGNC Symbol;Acc:HGNC:23924] |
| ENSG00000169682 | HGLibA_46732 | 268 | 1.289458408 | SPNS1 | sphingolipid transporter 1 (putative) [Source:HGNC Symbol;Acc:HGNC:30621] |
| ENSG00000170075 | HGLibA_20034 | 268 | 1.289458408 | GPR37L1 | G protein-coupled receptor 37 like 1 [Source:HGNC Symbol;Acc:HGNC:14923] |
| ENSG00000177731 | HGLibA_17619 | 268 | 1.289458408 | FLII | actin remodeling protein [Source:HGNC Symbol;Acc:HGNC:3750]" |
| ENSG00000186723 | HGLibA_33507 | 268 | 1.289458408 | OR10H1 | olfactory receptor family 10 subfamily H member 1 [Source:HGNC Symbol;Acc:HGNC:8172] |
| ENSG00000187969 | HGLibA_55012 | 268 | 1.289458408 | ZCCHC13 | zinc finger CCHC-type containing 13 [Source:HGNC Symbol;Acc:HGNC:31749] |
| ENSG00000226763 | HGLibA_47054 | 268 | 1.289458408 | SRRM5 | serine/arginine repetitive matrix 5 [Source:HGNC Symbol;Acc:HGNC:37248] |
| hsa-mir-3622a | HGLibA_59121 | 268 | 1.289458408 | hsa-mir-3622a | - |
| hsa-mir-676 | HGLibA_63197 | 268 | 1.289458408 | hsa-mir-676 | - |
| hsa-mir-889 | HGLibA_64234 | 268 | 1.289458408 | hsa-mir-889 | - |
| LOC100505549 | HGLibA_26850 | 268 | 1.289458408 | LOC100505549 | - |
| C11orf57 | HGLibA_05161 | 267 | 1.280413066 | C11orf57 | - |
| ENSG00000066697 | HGLibA_30176 | 267 | 1.280413066 | MSANTD3 | Myb/SANT DNA binding domain containing 3 [Source:HGNC Symbol;Acc:HGNC:23370] |
| ENSG00000072858 | HGLibA_44115 | 267 | 1.280413066 | SIDT1 | SID1 transmembrane family member 1 [Source:HGNC Symbol;Acc:HGNC:25967] |
| ENSG00000088756 | HGLibA_02747 | 267 | 1.280413066 | ARHGAP28 | Rho GTPase activating protein 28 [Source:HGNC Symbol;Acc:HGNC:25509] |
| ENSG00000096092 | HGLibA_49980 | 267 | 1.280413066 | TMEM14A | transmembrane protein 14A [Source:HGNC Symbol;Acc:HGNC:21076] |
| ENSG00000100372 | HGLibA_44657 | 267 | 1.280413066 | SLC25A17 | solute carrier family 25 member 17 [Source:HGNC Symbol;Acc:HGNC:10987] |
| ENSG00000100558 | HGLibA_37058 | 267 | 1.280413066 | PLEK2 | pleckstrin 2 [Source:HGNC Symbol;Acc:HGNC:19238] |
| ENSG00000102383 | HGLibA_55081 | 267 | 1.280413066 | ZDHHC15 | zinc finger DHHC-type containing 15 [Source:HGNC Symbol;Acc:HGNC:20342] |
| ENSG00000103066 | HGLibA_36877 | 267 | 1.280413066 | PLA2G15 | phospholipase A2 group XV [Source:HGNC Symbol;Acc:HGNC:17163] |
| ENSG00000103257 | HGLibA_45371 | 267 | 1.280413066 | SLC7A5 | solute carrier family 7 member 5 [Source:HGNC Symbol;Acc:HGNC:11063] |
| ENSG00000103460 | HGLibA_51042 | 267 | 1.280413066 | TOX3 | TOX high mobility group box family member 3 [Source:HGNC Symbol;Acc:HGNC:11972] |
| ENSG00000107554 | HGLibA_13696 | 267 | 1.280413066 | DNMBP | dynamin binding protein [Source:HGNC Symbol;Acc:HGNC:30373] |
| ENSG00000112852 | HGLibA_35494 | 267 | 1.280413066 | PCDHB2 | protocadherin beta 2 [Source:HGNC Symbol;Acc:HGNC:8687] |
| ENSG00000113441 | HGLibA_26740 | 267 | 1.280413066 | LNPEP | leucyl and cystinyl aminopeptidase [Source:HGNC Symbol;Acc:HGNC:6656] |
| ENSG00000113569 | HGLibA_33026 | 267 | 1.280413066 | NUP155 | nucleoporin 155 [Source:HGNC Symbol;Acc:HGNC:8063] |
| ENSG00000114767 | HGLibA_42288 | 267 | 1.280413066 | RRP9 | U3 small nucleolar RNA binding protein [Source:HGNC Symbol;Acc:HGNC:16829]" |
| ENSG00000115464 | HGLibA_53341 | 267 | 1.280413066 | USP34 | ubiquitin specific peptidase 34 [Source:HGNC Symbol;Acc:HGNC:20066] |
| ENSG00000117640 | HGLibA_30363 | 267 | 1.280413066 | MTFR1L | mitochondrial fission regulator 1 like [Source:HGNC Symbol;Acc:HGNC:28836] |
| ENSG00000119703 | HGLibA_54942 | 267 | 1.280413066 | ZC2HC1C | zinc finger C2HC-type containing 1C [Source:HGNC Symbol;Acc:HGNC:20354] |
| ENSG00000119862 | HGLibA_26389 | 267 | 1.280413066 | LGALSL | galectin like [Source:HGNC Symbol;Acc:HGNC:25012] |
| ENSG00000123307 | HGLibA_31741 | 267 | 1.280413066 | NEUROD4 | neuronal differentiation 4 [Source:HGNC Symbol;Acc:HGNC:13802] |
| ENSG00000124103 | HGLibA_16437 | 267 | 1.280413066 | FAM209A | family with sequence similarity 209 member A [Source:HGNC Symbol;Acc:HGNC:16100] |
| ENSG00000134955 | HGLibA_45045 | 267 | 1.280413066 | SLC37A2 | solute carrier family 37 member 2 [Source:HGNC Symbol;Acc:HGNC:20644] |
| ENSG00000141664 | HGLibA_55026 | 267 | 1.280413066 | ZCCHC2 | zinc finger CCHC-type containing 2 [Source:HGNC Symbol;Acc:HGNC:22916] |
| ENSG00000145911 | HGLibA_30962 | 267 | 1.280413066 | N4BP3 | NEDD4 binding protein 3 [Source:HGNC Symbol;Acc:HGNC:29852] |
| ENSG00000162068 | HGLibA_32857 | 267 | 1.280413066 | NTN3 | netrin 3 [Source:HGNC Symbol;Acc:HGNC:8030] |
| ENSG00000167634 | HGLibA_32129 | 267 | 1.280413066 | NLRP7 | NLR family pyrin domain containing 7 [Source:HGNC Symbol;Acc:HGNC:22947] |
| ENSG00000169436 | HGLibA_10542 | 267 | 1.280413066 | COL22A1 | collagen type XXII alpha 1 chain [Source:HGNC Symbol;Acc:HGNC:22989] |
| ENSG00000170465 | HGLibA_25520 | 267 | 1.280413066 | KRT6C | keratin 6C [Source:HGNC Symbol;Acc:HGNC:20406] |
| ENSG00000173338 | HGLibA_24334 | 267 | 1.280413066 | KCNK7 | potassium two pore domain channel subfamily K member 7 [Source:HGNC Symbol;Acc:HGNC:6282] |
| ENSG00000174165 | HGLibA_55110 | 267 | 1.280413066 | ZDHHC24 | zinc finger DHHC-type containing 24 [Source:HGNC Symbol;Acc:HGNC:27387] |
| ENSG00000176472 | HGLibA_56289 | 267 | 1.280413066 | ZNF575 | zinc finger protein 575 [Source:HGNC Symbol;Acc:HGNC:27606] |
| ENSG00000181220 | HGLibA_56666 | 267 | 1.280413066 | ZNF746 | zinc finger protein 746 [Source:HGNC Symbol;Acc:HGNC:21948] |
| ENSG00000185982 | HGLibA_12887 | 267 | 1.280413066 | DEFB128 | defensin beta 128 [Source:HGNC Symbol;Acc:HGNC:18106] |
| ENSG00000187583 | HGLibA_37145 | 267 | 1.280413066 | PLEKHN1 | pleckstrin homology domain containing N1 [Source:HGNC Symbol;Acc:HGNC:25284] |
| ENSG00000196396 | HGLibA_39431 | 267 | 1.280413066 | PTPN1 | non-receptor type 1 [Source:HGNC Symbol;Acc:HGNC:9642]" |
| ENSG00000197728 | HGLibA_42119 | 267 | 1.280413066 | RPS26 | ribosomal protein S26 [Source:HGNC Symbol;Acc:HGNC:10414] |
| ENSG00000197769 | HGLibA_28131 | 267 | 1.280413066 | MAP1LC3C | microtubule associated protein 1 light chain 3 gamma [Source:HGNC Symbol;Acc:HGNC:13353] |
| ENSG00000198157 | HGLibA_21729 | 267 | 1.280413066 | HMGN5 | high mobility group nucleosome binding domain 5 [Source:HGNC Symbol;Acc:HGNC:8013] |
| ENSG00000204099 | HGLibA_31720 | 267 | 1.280413066 | NEU4 | neuraminidase 4 [Source:HGNC Symbol;Acc:HGNC:21328] |
| ENSG00000215187 | HGLibA_16237 | 267 | 1.280413066 | FAM166B | family with sequence similarity 166 member B [Source:HGNC Symbol;Acc:HGNC:34242] |
| ENSG00000221954 | HGLibA_33940 | 267 | 1.280413066 | OR4C12 | olfactory receptor family 4 subfamily C member 12 [Source:HGNC Symbol;Acc:HGNC:15168] |
| ENSG00000240871 | HGLibA_25774 | 267 | 1.280413066 | KRTAP4-7 | keratin associated protein 4-7 [Source:HGNC Symbol;Acc:HGNC:18898] |
| FAM73A | HGLibA_16716 | 267 | 1.280413066 | FAM73A | - |
| GPR115 | HGLibA_19860 | 267 | 1.280413066 | GPR115 | - |
| hsa-mir-1197 | HGLibA_57253 | 267 | 1.280413066 | hsa-mir-1197 | - |
| hsa-mir-1251 | HGLibA_57404 | 267 | 1.280413066 | hsa-mir-1251 | - |
| hsa-mir-1272 | HGLibA_57529 | 267 | 1.280413066 | hsa-mir-1272 | - |
| hsa-mir-134 | HGLibA_57786 | 267 | 1.280413066 | hsa-mir-134 | - |
| hsa-mir-4792 | HGLibA_61274 | 267 | 1.280413066 | hsa-mir-4792 | - |
| hsa-mir-6828 | HGLibA_63490 | 267 | 1.280413066 | hsa-mir-6828 | - |
| hsa-mir-8082 | HGLibA_64171 | 267 | 1.280413066 | hsa-mir-8082 | - |
| LOC643669 | HGLibA_26970 | 267 | 1.280413066 | LOC643669 | - |
| PET112 | HGLibA_36072 | 267 | 1.280413066 | PET112 | - |
| ZNF664-FAM101A | HGLibA_56484 | 267 | 1.280413066 | ZNF664-FAM101A | - |
| ENSG00000011677 | HGLibA_18361 | 266 | 1.271367723 | GABRA3 | gamma-aminobutyric acid type A receptor alpha3 subunit [Source:HGNC Symbol;Acc:HGNC:4077] |
| ENSG00000103316 | HGLibA_11292 | 266 | 1.271367723 | CRYM | crystallin mu [Source:HGNC Symbol;Acc:HGNC:2418] |
| ENSG00000104472 | HGLibA_09532 | 266 | 1.271367723 | CHRAC1 | chromatin accessibility complex subunit 1 [Source:HGNC Symbol;Acc:HGNC:13544] |
| ENSG00000108829 | HGLibA_27400 | 266 | 1.271367723 | LRRC59 | leucine rich repeat containing 59 [Source:HGNC Symbol;Acc:HGNC:28817] |
| ENSG00000109445 | HGLibA_55813 | 266 | 1.271367723 | ZNF330 | zinc finger protein 330 [Source:HGNC Symbol;Acc:HGNC:15462] |
| ENSG00000111790 | HGLibA_17440 | 266 | 1.271367723 | FGFR1OP2 | FGFR1 oncogene partner 2 [Source:HGNC Symbol;Acc:HGNC:23098] |

| | | | | | |
|---|---|---|---|---|---|
| ENSG00000118407 | HGLibA_17528 | 266 | 1.271367723 | FILIP1 | filamin A interacting protein 1 [Source:HGNC Symbol;Acc:HGNC:21015] |
| ENSG00000120659 | HGLibA_50800 | 266 | 1.271367723 | TNFSF11 | TNF superfamily member 11 [Source:HGNC Symbol;Acc:HGNC:11926] |
| ENSG00000122694 | HGLibA_19267 | 266 | 1.271367723 | GLIPR2 | GLI pathogenesis related 2 [Source:HGNC Symbol;Acc:HGNC:18007] |
| ENSG00000124782 | HGLibA_42246 | 266 | 1.271367723 | RREB1 | ras responsive element binding protein 1 [Source:HGNC Symbol;Acc:HGNC:10449] |
| ENSG00000125538 | HGLibA_23041 | 266 | 1.271367723 | IL1B | interleukin 1 beta [Source:HGNC Symbol;Acc:HGNC:5992] |
| ENSG00000125818 | HGLibA_39201 | 266 | 1.271367723 | PSMF1 | proteasome inhibitor subunit 1 [Source:HGNC Symbol;Acc:HGNC:9571] |
| ENSG00000125970 | HGLibA_40174 | 266 | 1.271367723 | RALY | RALY heterogeneous nuclear ribonucleoprotein [Source:HGNC Symbol;Acc:HGNC:15921] |
| ENSG00000127603 | HGLibA_27832 | 266 | 1.271367723 | MACF1 | microtubule-actin crosslinking factor 1 [Source:HGNC Symbol;Acc:HGNC:13664] |
| ENSG00000130638 | HGLibA_03831 | 266 | 1.271367723 | ATXN10 | ataxin 10 [Source:HGNC Symbol;Acc:HGNC:10549] |
| ENSG00000134538 | HGLibA_45449 | 266 | 1.271367723 | SLCO1B1 | solute carrier organic anion transporter family member 1B1 [Source:HGNC Symbol;Acc:HGNC:10959] |
| ENSG00000135040 | HGLibA_30992 | 266 | 1.271367723 | NAA35 | NatC auxiliary subunit [Source:HGNC Symbol;Acc:HGNC:24340]" |
| ENSG00000136167 | HGLibA_26200 | 266 | 1.271367723 | LCP1 | lymphocyte cytosolic protein 1 [Source:HGNC Symbol;Acc:HGNC:6528] |
| ENSG00000138834 | HGLibA_28291 | 266 | 1.271367723 | MAPK8IP3 | mitogen-activated protein kinase 8 interacting protein 3 [Source:HGNC Symbol;Acc:HGNC:6884] |
| ENSG00000139155 | HGLibA_45457 | 266 | 1.271367723 | SLCO1C1 | solute carrier organic anion transporter family member 1C1 [Source:HGNC Symbol;Acc:HGNC:13819] |
| ENSG00000140497 | HGLibA_42840 | 266 | 1.271367723 | SCAMP2 | secretory carrier membrane protein 2 [Source:HGNC Symbol;Acc:HGNC:10564] |
| ENSG00000141431 | HGLibA_03395 | 266 | 1.271367723 | ASXL3 | ASXL transcriptional regulator 3 [Source:HGNC Symbol;Acc:HGNC:29357] |
| ENSG00000154359 | HGLibA_27020 | 266 | 1.271367723 | LONRF1 | LON peptidase N-terminal domain and ring finger 1 [Source:HGNC Symbol;Acc:HGNC:26302] |
| ENSG00000162399 | HGLibA_04873 | 266 | 1.271367723 | BSND | barttin CLCNK type accessory beta subunit [Source:HGNC Symbol;Acc:HGNC:16512] |
| ENSG00000164744 | HGLibA_47793 | 266 | 1.271367723 | SUN3 | Sad1 and UNC84 domain containing 3 [Source:HGNC Symbol;Acc:HGNC:22429] |
| ENSG00000165071 | HGLibA_50505 | 266 | 1.271367723 | TMEM71 | transmembrane protein 71 [Source:HGNC Symbol;Acc:HGNC:26572] |
| ENSG00000165533 | HGLibA_52155 | 266 | 1.271367723 | TTC8 | tetratricopeptide repeat domain 8 [Source:HGNC Symbol;Acc:HGNC:20087] |
| ENSG00000166913 | HGLibA_54739 | 266 | 1.271367723 | YWHAB | tyrosine 3-monooxygenase/tryptophan 5-monooxygenase activation protein beta [Source:HGNC Symbol;Acc:HGNC:12849] |
| ENSG00000167346 | HGLibA_29525 | 266 | 1.271367723 | MMP26 | matrix metallopeptidase 26 [Source:HGNC Symbol;Acc:HGNC:14249] |
| ENSG00000171206 | HGLibA_51614 | 266 | 1.271367723 | TRIM8 | tripartite motif containing 8 [Source:HGNC Symbol;Acc:HGNC:15579] |
| ENSG00000171495 | HGLibA_29869 | 266 | 1.271367723 | MROH2B | maestro heat like repeat family member 2B [Source:HGNC Symbol;Acc:HGNC:26857] |
| ENSG00000177150 | HGLibA_16454 | 266 | 1.271367723 | FAM210A | family with sequence similarity 210 member A [Source:HGNC Symbol;Acc:HGNC:28346] |
| ENSG00000179674 | HGLibA_02936 | 266 | 1.271367723 | ARL14 | ADP ribosylation factor like GTPase 14 [Source:HGNC Symbol;Acc:HGNC:22974] |
| ENSG00000184675 | HGLibA_01771 | 266 | 1.271367723 | AMER1 | APC membrane recruitment protein 1 [Source:HGNC Symbol;Acc:HGNC:26837] |
| ENSG00000185825 | HGLibA_04243 | 266 | 1.271367723 | BCAP31 | B cell receptor associated protein 31 [Source:HGNC Symbol;Acc:HGNC:16695] |
| ENSG00000185875 | HGLibA_49358 | 266 | 1.271367723 | THNSL1 | threonine synthase like 1 [Source:HGNC Symbol;Acc:HGNC:26160] |
| ENSG00000187867 | HGLibA_35076 | 266 | 1.271367723 | PALM3 | paralemmin 3 [Source:HGNC Symbol;Acc:HGNC:33274] |
| ENSG00000196155 | HGLibA_37110 | 266 | 1.271367723 | PLEKHG4 | pleckstrin homology and RhoGEF domain containing G4 [Source:HGNC Symbol;Acc:HGNC:24501] |
| ENSG00000232237 | HGLibA_03277 | 266 | 1.271367723 | ASCL5 | achaete-scute family bHLH transcription factor 5 [Source:HGNC Symbol;Acc:HGNC:33169] |
| ENSG00000243279 | HGLibA_38269 | 266 | 1.271367723 | PRAF2 | PRA1 domain family member 2 [Source:HGNC Symbol;Acc:HGNC:28911] |
| ENSG00000258947 | HGLibA_52283 | 266 | 1.271367723 | TUBB3 | tubulin beta 3 class III [Source:HGNC Symbol;Acc:HGNC:20772] |
| HIST1H3B | HGLibA_21460 | 266 | 1.271367723 | HIST1H3B | - |
| hsa-mir-4254 | HGLibA_59724 | 266 | 1.271367723 | hsa-mir-4254 | - |
| hsa-mir-4293 | HGLibA_59884 | 266 | 1.271367723 | hsa-mir-4293 | - |
| hsa-mir-509-2 | HGLibA_61512 | 266 | 1.271367723 | hsa-mir-509-2 | - |
| hsa-mir-5692a-1 | HGLibA_62289 | 266 | 1.271367723 | hsa-mir-5692a-1 | - |
| hsa-mir-6131 | HGLibA_62674 | 266 | 1.271367723 | hsa-mir-6131 | - |
| hsa-mir-6801 | HGLibA_63381 | 266 | 1.271367723 | hsa-mir-6801 | - |
| C10orf131 | HGLibA_05054 | 265 | 1.26232238 | C10orf131 | - |
| C14orf182 | HGLibA_05352 | 265 | 1.26232238 | C14orf182 | - |
| C3orf55 | HGLibA_06251 | 265 | 1.26232238 | C3orf55 | - |
| DUPD1 | HGLibA_14110 | 265 | 1.26232238 | DUPD1 | - |
| ENSG00000010322 | HGLibA_31980 | 265 | 1.26232238 | NISCH | nischarin [Source:HGNC Symbol;Acc:HGNC:18006] |
| ENSG00000072135 | HGLibA_39446 | 265 | 1.26232238 | PTPN18 | non-receptor type 18 [Source:HGNC Symbol;Acc:HGNC:9649]" |
| ENSG00000074803 | HGLibA_44358 | 265 | 1.26232238 | SLC12A1 | solute carrier family 12 member 1 [Source:HGNC Symbol;Acc:HGNC:10910] |
| ENSG00000088836 | HGLibA_45208 | 265 | 1.26232238 | SLC4A11 | solute carrier family 4 member 11 [Source:HGNC Symbol;Acc:HGNC:16438] |
| ENSG00000092621 | HGLibA_36394 | 265 | 1.26232238 | PHGDH | phosphoglycerate dehydrogenase [Source:HGNC Symbol;Acc:HGNC:8923] |
| ENSG00000096060 | HGLibA_17580 | 265 | 1.26232238 | FKBP5 | FK506 binding protein 5 [Source:HGNC Symbol;Acc:HGNC:3721] |
| ENSG00000101574 | HGLibA_29010 | 265 | 1.26232238 | METTL4 | methyltransferase like 4 [Source:HGNC Symbol;Acc:HGNC:24726] |
| ENSG00000105549 | HGLibA_49334 | 265 | 1.26232238 | THEG | theg spermatid protein [Source:HGNC Symbol;Acc:HGNC:13706] |
| ENSG00000106771 | HGLibA_50301 | 265 | 1.26232238 | TMEM245 | transmembrane protein 245 [Source:HGNC Symbol;Acc:HGNC:1363] |
| ENSG00000110274 | HGLibA_09112 | 265 | 1.26232238 | CEP164 | centrosomal protein 164 [Source:HGNC Symbol;Acc:HGNC:29182] |
| ENSG00000113356 | HGLibA_37626 | 265 | 1.26232238 | POLR3G | RNA polymerase III subunit G [Source:HGNC Symbol;Acc:HGNC:30075] |
| ENSG00000120278 | HGLibA_37102 | 265 | 1.26232238 | PLEKHG1 | pleckstrin homology and RhoGEF domain containing G1 [Source:HGNC Symbol;Acc:HGNC:20884] |
| ENSG00000120937 | HGLibA_32502 | 265 | 1.26232238 | NPPB | natriuretic peptide B [Source:HGNC Symbol;Acc:HGNC:7940] |
| ENSG00000123560 | HGLibA_37217 | 265 | 1.26232238 | PLP1 | proteolipid protein 1 [Source:HGNC Symbol;Acc:HGNC:9086] |
| ENSG00000124006 | HGLibA_33235 | 265 | 1.26232238 | OBSL1 | obscurin like 1 [Source:HGNC Symbol;Acc:HGNC:29092] |
| ENSG00000124006 | HGLibA_33233 | 265 | 1.26232238 | OBSL1 | obscurin like 1 [Source:HGNC Symbol;Acc:HGNC:29092] |
| ENSG00000127324 | HGLibA_51948 | 265 | 1.26232238 | TSPAN8 | tetraspanin 8 [Source:HGNC Symbol;Acc:HGNC:11855] |
| ENSG00000127914 | HGLibA_01450 | 265 | 1.26232238 | AKAP9 | A-kinase anchoring protein 9 [Source:HGNC Symbol;Acc:HGNC:379] |
| ENSG00000130703 | HGLibA_34651 | 265 | 1.26232238 | OSBPL2 | oxysterol binding protein like 2 [Source:HGNC Symbol;Acc:HGNC:15761] |
| ENSG00000132326 | HGLibA_36058 | 265 | 1.26232238 | PER2 | period circadian regulator 2 [Source:HGNC Symbol;Acc:HGNC:8846] |
| ENSG00000132530 | HGLibA_54462 | 265 | 1.26232238 | XAF1 | XIAP associated factor 1 [Source:HGNC Symbol;Acc:HGNC:30932] |
| ENSG00000138722 | HGLibA_29546 | 265 | 1.26232238 | MMRN1 | multimerin 1 [Source:HGNC Symbol;Acc:HGNC:7178] |
| ENSG00000151148 | HGLibA_52681 | 265 | 1.26232238 | UBE3B | ubiquitin protein ligase E3B [Source:HGNC Symbol;Acc:HGNC:13478] |
| ENSG00000152457 | HGLibA_12514 | 265 | 1.26232238 | DCLRE1C | DNA cross-link repair 1C [Source:HGNC Symbol;Acc:HGNC:17642] |
| ENSG00000152977 | HGLibA_55322 | 265 | 1.26232238 | ZIC1 | Zic family member 1 [Source:HGNC Symbol;Acc:HGNC:12872] |
| ENSG00000154760 | HGLibA_45491 | 265 | 1.26232238 | SLFN13 | schlafen family member 13 [Source:HGNC Symbol;Acc:HGNC:26481] |
| ENSG00000158092 | HGLibA_31305 | 265 | 1.26232238 | NCK1 | NCK adaptor protein 1 [Source:HGNC Symbol;Acc:HGNC:7664] |
| ENSG00000160654 | HGLibA_08377 | 265 | 1.26232238 | CD3G | CD3g molecule [Source:HGNC Symbol;Acc:HGNC:1675] |
| ENSG00000162456 | HGLibA_25337 | 265 | 1.26232238 | KNCN | kinocilin [Source:HGNC Symbol;Acc:HGNC:26488] |
| ENSG00000163131 | HGLibA_11727 | 265 | 1.26232238 | CTSS | cathepsin S [Source:HGNC Symbol;Acc:HGNC:2545] |
| ENSG00000163291 | HGLibA_35158 | 265 | 1.26232238 | PAQR3 | progestin and adipoQ receptor family member 3 [Source:HGNC Symbol;Acc:HGNC:30130] |
| ENSG00000164187 | HGLibA_26678 | 265 | 1.26232238 | LMBRD2 | LMBR1 domain containing 2 [Source:HGNC Symbol;Acc:HGNC:25287] |
| ENSG00000164219 | HGLibA_36238 | 265 | 1.26232238 | PGGT1B | protein geranylgeranyltransferase type I subunit beta [Source:HGNC Symbol;Acc:HGNC:8895] |
| ENSG00000166250 | HGLibA_10073 | 265 | 1.26232238 | CLMP | CXADR like membrane protein [Source:HGNC Symbol;Acc:HGNC:24039] |
| ENSG00000167523 | HGLibA_46511 | 265 | 1.26232238 | SPATA33 | spermatogenesis associated 33 [Source:HGNC Symbol;Acc:HGNC:26463] |
| ENSG00000169432 | HGLibA_43008 | 265 | 1.26232238 | SCN9A | sodium voltage-gated channel alpha subunit 9 [Source:HGNC Symbol;Acc:HGNC:10597] |
| ENSG00000170927 | HGLibA_36812 | 265 | 1.26232238 | PKHD1 | fibrocystin/polyductin [Source:HGNC Symbol;Acc:HGNC:9016]" |
| ENSG00000173267 | HGLibA_45917 | 265 | 1.26232238 | SNCG | synuclein gamma [Source:HGNC Symbol;Acc:HGNC:11141] |
| ENSG00000176410 | HGLibA_13617 | 265 | 1.26232238 | DNAJC30 | DnaJ heat shock protein family (Hsp40) member C30 [Source:HGNC Symbol;Acc:HGNC:16410] |
| ENSG00000177519 | HGLibA_42054 | 265 | 1.26232238 | RPRM | TP53 dependent G2 arrest mediator homolog [Source:HGNC Symbol;Acc:HGNC:24201]" |
| ENSG00000178301 | HGLibA_02583 | 265 | 1.26232238 | AQP11 | aquaporin 11 [Source:HGNC Symbol;Acc:HGNC:19940] |
| ENSG00000178498 | HGLibA_14084 | 265 | 1.26232238 | DTX3 | deltex E3 ubiquitin ligase 3 [Source:HGNC Symbol;Acc:HGNC:24457] |
| ENSG00000178919 | HGLibA_17857 | 265 | 1.26232238 | FOXE1 | forkhead box E1 [Source:HGNC Symbol;Acc:HGNC:3806] |
| ENSG00000178996 | HGLibA_46051 | 265 | 1.26232238 | SNX18 | sorting nexin 18 [Source:HGNC Symbol;Acc:HGNC:19245] |
| ENSG00000181939 | HGLibA_33947 | 265 | 1.26232238 | OR4C15 | olfactory receptor family 4 subfamily C member 15 [Source:HGNC Symbol;Acc:HGNC:15171] |
| ENSG00000183186 | HGLibA_06101 | 265 | 1.26232238 | C2CD4C | C2 calcium dependent domain containing 4C [Source:HGNC Symbol;Acc:HGNC:29417] |
| ENSG00000184979 | HGLibA_53291 | 265 | 1.26232238 | USP18 | ubiquitin specific peptidase 18 [Source:HGNC Symbol;Acc:HGNC:12616] |
| ENSG00000197046 | HGLibA_44138 | 265 | 1.26232238 | SIGLEC15 | sialic acid binding Ig like lectin 15 [Source:HGNC Symbol;Acc:HGNC:27596] |
| ENSG00000197734 | HGLibA_05344 | 265 | 1.26232238 | C14orf178 | chromosome 14 open reading frame 178 [Source:HGNC Symbol;Acc:HGNC:26385] |
| ENSG00000198944 | HGLibA_46213 | 265 | 1.26232238 | SOWAHA | sosondowah ankyrin repeat domain family member A [Source:HGNC Symbol;Acc:HGNC:27033] |
| ENSG00000223547 | HGLibA_56861 | 265 | 1.26232238 | ZNF844 | zinc finger protein 844 [Source:HGNC Symbol;Acc:HGNC:25932] |
| hsa-mir-1343 | HGLibA_57790 | 265 | 1.26232238 | hsa-mir-1343 | - |
| hsa-mir-3173 | HGLibA_58765 | 265 | 1.26232238 | hsa-mir-3173 | - |
| hsa-mir-3189 | HGLibA_58852 | 265 | 1.26232238 | hsa-mir-3189 | - |
| hsa-mir-3689d-2 | HGLibA_59331 | 265 | 1.26232238 | hsa-mir-3689d-2 | - |
| hsa-mir-4530 | HGLibA_60578 | 265 | 1.26232238 | hsa-mir-4530 | - |
| hsa-mir-4752 | HGLibA_61106 | 265 | 1.26232238 | hsa-mir-4752 | - |
| hsa-mir-4783 | HGLibA_61240 | 265 | 1.26232238 | hsa-mir-4783 | - |
| hsa-mir-564 | HGLibA_62220 | 265 | 1.26232238 | hsa-mir-564 | - |
| hsa-mir-6858 | HGLibA_63606 | 265 | 1.26232238 | hsa-mir-6858 | - |
| IL9R_Y | HGLibA_23204 | 265 | 1.26232238 | IL9R_Y | - |
| LOC100130451 | HGLibA_26786 | 265 | 1.26232238 | LOC100130451 | - |
| PYCRL | HGLibA_39678 | 265 | 1.26232238 | PYCRL | - |
| ENSG00000010165 | HGLibA_28955 | 264 | 1.253277037 | METTL13 | eEF1A lysine and N-terminal methyltransferase [Source:HGNC Symbol;Acc:HGNC:24248] |
| ENSG00000055118 | HGLibA_24212 | 264 | 1.253277037 | KCNH2 | potassium voltage-gated channel subfamily H member 2 [Source:HGNC Symbol;Acc:HGNC:6251] |
| ENSG00000066697 | HGLibA_30174 | 264 | 1.253277037 | MSANTD3 | Myb/SANT DNA binding domain containing 3 [Source:HGNC Symbol;Acc:HGNC:23370] |
| ENSG00000074416 | HGLibA_29160 | 264 | 1.253277037 | MGLL | monoglyceride lipase [Source:HGNC Symbol;Acc:HGNC:17038] |
| ENSG00000090661 | HGLibA_09210 | 264 | 1.253277037 | CERS4 | ceramide synthase 4 [Source:HGNC Symbol;Acc:HGNC:23747] |
| ENSG00000091009 | HGLibA_40523 | 264 | 1.253277037 | RBM27 | RNA binding motif protein 27 [Source:HGNC Symbol;Acc:HGNC:29243] |
| ENSG00000100426 | HGLibA_54798 | 264 | 1.253277037 | ZBED4 | zinc finger BED-type containing 4 [Source:HGNC Symbol;Acc:HGNC:20721] |
| ENSG00000101883 | HGLibA_41213 | 264 | 1.253277037 | RHOXF1 | Rhox homeobox family member 1 [Source:HGNC Symbol;Acc:HGNC:29993] |
| ENSG00000102057 | HGLibA_24173 | 264 | 1.253277037 | KCND1 | potassium voltage-gated channel subfamily D member 1 [Source:HGNC Symbol;Acc:HGNC:6237] |
| ENSG00000105668 | HGLibA_53069 | 264 | 1.253277037 | UPK1A | uroplakin 1A [Source:HGNC Symbol;Acc:HGNC:12577] |
| ENSG00000109205 | HGLibA_33270 | 264 | 1.253277037 | ODAM | ameloblast associated [Source:HGNC Symbol;Acc:HGNC:26043]" |
| ENSG00000112759 | HGLibA_44841 | 264 | 1.253277037 | SLC29A1 | solute carrier family 29 member 1 (Augustine blood group) [Source:HGNC Symbol;Acc:HGNC:11003] |
| ENSG00000120129 | HGLibA_14128 | 264 | 1.253277037 | DUSP1 | dual specificity phosphatase 1 [Source:HGNC Symbol;Acc:HGNC:3064] |
| ENSG00000122863 | HGLibA_09634 | 264 | 1.253277037 | CHST3 | carbohydrate sulfotransferase 3 [Source:HGNC Symbol;Acc:HGNC:1971] |
| ENSG00000123358 | HGLibA_32624 | 264 | 1.253277037 | NR4A1 | nuclear receptor subfamily 4 group A member 1 [Source:HGNC Symbol;Acc:HGNC:7980] |
| ENSG00000131864 | HGLibA_53323 | 264 | 1.253277037 | USP29 | ubiquitin specific peptidase 29 [Source:HGNC Symbol;Acc:HGNC:18563] |
| ENSG00000137106 | HGLibA_20258 | 264 | 1.253277037 | GRHPR | glyoxylate and hydroxypyruvate reductase [Source:HGNC Symbol;Acc:HGNC:4570] |
| ENSG00000142619 | HGLibA_34974 | 264 | 1.253277037 | PADI3 | peptidyl arginine deiminase 3 [Source:HGNC Symbol;Acc:HGNC:18337] |
| ENSG00000146618 | HGLibA_17291 | 264 | 1.253277037 | FERD3L | Fer3 like bHLH transcription factor [Source:HGNC Symbol;Acc:HGNC:16660] |
| ENSG00000147912 | HGLibA_17023 | 264 | 1.253277037 | FBXO10 | F-box protein 10 [Source:HGNC Symbol;Acc:HGNC:13589] |
| ENSG00000154319 | HGLibA_16239 | 264 | 1.253277037 | FAM167A | family with sequence similarity 167 member A [Source:HGNC Symbol;Acc:HGNC:15549] |
| ENSG00000154451 | HGLibA_18740 | 264 | 1.253277037 | GBP5 | guanylate binding protein 5 [Source:HGNC Symbol;Acc:HGNC:19895] |
| ENSG00000156531 | HGLibA_36384 | 264 | 1.253277037 | PHF6 | PHD finger protein 6 [Source:HGNC Symbol;Acc:HGNC:18145] |
| ENSG00000158985 | HGLibA_08583 | 264 | 1.253277037 | CDC42SE2 | CDC42 small effector 2 [Source:HGNC Symbol;Acc:HGNC:18547] |
| ENSG00000160404 | HGLibA_51028 | 264 | 1.253277037 | TOR2A | torsin family 2 member A [Source:HGNC Symbol;Acc:HGNC:11996] |
| ENSG00000164821 | HGLibA_12800 | 264 | 1.253277037 | DEFA4 | defensin alpha 4 [Source:HGNC Symbol;Acc:HGNC:2763] |

| | | | | | |
|---|---|---|---|---|---|
| ENSG00000167434 | HGLibA_06810 | 264 | 1.253277037 | CA4 | carbonic anhydrase 4 [Source:HGNC Symbol;Acc:HGNC:1375] |
| ENSG00000168356 | HGLibA_42971 | 264 | 1.253277037 | SCN11A | sodium voltage-gated channel alpha subunit 11 [Source:HGNC Symbol;Acc:HGNC:10583] |
| ENSG00000168675 | HGLibA_26250 | 264 | 1.253277037 | LDLRAD4 | low density lipoprotein receptor class A domain containing 4 [Source:HGNC Symbol;Acc:HGNC:1224] |
| ENSG00000169218 | HGLibA_42344 | 264 | 1.253277037 | RSPO1 | R-spondin 1 [Source:HGNC Symbol;Acc:HGNC:21679] |
| ENSG00000172482 | HGLibA_01270 | 264 | 1.253277037 | AGXT | alanine--glyoxylate and serine--pyruvate aminotransferase [Source:HGNC Symbol;Acc:HGNC:341] |
| ENSG00000174371 | HGLibA_15725 | 264 | 1.253277037 | EXO1 | exonuclease 1 [Source:HGNC Symbol;Acc:HGNC:3511] |
| ENSG00000178078 | HGLibA_47344 | 264 | 1.253277037 | STAP2 | signal transducing adaptor family member 2 [Source:HGNC Symbol;Acc:HGNC:30430] |
| ENSG00000178852 | HGLibA_14529 | 264 | 1.253277037 | EFCAB13 | EF-hand calcium binding domain 13 [Source:HGNC Symbol;Acc:HGNC:26864] |
| ENSG00000179542 | HGLibA_45521 | 264 | 1.253277037 | SLITRK4 | SLIT and NTRK like family member 4 [Source:HGNC Symbol;Acc:HGNC:23502] |
| ENSG00000179886 | HGLibA_49474 | 264 | 1.253277037 | TIGD5 | tigger transposable element derived 5 [Source:HGNC Symbol;Acc:HGNC:18336] |
| ENSG00000181027 | HGLibA_17596 | 264 | 1.253277037 | FKRP | fukutin related protein [Source:HGNC Symbol;Acc:HGNC:17997] |
| ENSG00000183313 | HGLibA_34188 | 264 | 1.253277037 | OR52L1 | olfactory receptor family 52 subfamily L member 1 [Source:HGNC Symbol;Acc:HGNC:14785] |
| ENSG00000183918 | HGLibA_43884 | 264 | 1.253277037 | SH2D1A | SH2 domain containing 1A [Source:HGNC Symbol;Acc:HGNC:10820] |
| ENSG00000186625 | HGLibA_24073 | 264 | 1.253277037 | KATNA1 | katanin catalytic subunit A1 [Source:HGNC Symbol;Acc:HGNC:6216] |
| ENSG00000189143 | HGLibA_09898 | 264 | 1.253277037 | CLDN4 | claudin 4 [Source:HGNC Symbol;Acc:HGNC:2046] |
| ENSG00000197951 | HGLibA_56608 | 264 | 1.253277037 | ZNF71 | zinc finger protein 71 [Source:HGNC Symbol;Acc:HGNC:13141] |
| ENSG00000206562 | HGLibA_29014 | 264 | 1.253277037 | METTL6 | methyltransferase like 6 [Source:HGNC Symbol;Acc:HGNC:28343] |
| ENSG00000271303 | HGLibA_47097 | 264 | 1.253277037 | SRXN1 | sulfiredoxin 1 [Source:HGNC Symbol;Acc:HGNC:16132] |
| hsa-mir-4758 | HGLibA_61130 | 264 | 1.253277037 | hsa-mir-4758 | - |
| hsa-mir-555 | HGLibA_62137 | 264 | 1.253277037 | hsa-mir-555 | - |
| ENSG00000002726 | HGLibA_02267 | 263 | 1.244231694 | AOC1 | copper containing 1 [Source:HGNC Symbol;Acc:HGNC:80]" |
| ENSG00000010379 | HGLibA_45298 | 263 | 1.244231694 | SLC6A13 | solute carrier family 6 member 13 [Source:HGNC Symbol;Acc:HGNC:11046] |
| ENSG00000011465 | HGLibA_12517 | 263 | 1.244231694 | DCN | decorin [Source:HGNC Symbol;Acc:HGNC:2705] |
| ENSG00000029364 | HGLibA_45126 | 263 | 1.244231694 | SLC39A9 | solute carrier family 39 member 9 [Source:HGNC Symbol;Acc:HGNC:20182] |
| ENSG00000054938 | HGLibA_09541 | 263 | 1.244231694 | CHRDL2 | chordin like 2 [Source:HGNC Symbol;Acc:HGNC:24168] |
| ENSG00000057608 | HGLibA_18884 | 263 | 1.244231694 | GDI2 | GDP dissociation inhibitor 2 [Source:HGNC Symbol;Acc:HGNC:4227] |
| ENSG00000075461 | HGLibA_06938 | 263 | 1.244231694 | CACNG4 | calcium voltage-gated channel auxiliary subunit gamma 4 [Source:HGNC Symbol;Acc:HGNC:1408] |
| ENSG00000076248 | HGLibA_53043 | 263 | 1.244231694 | UNG | uracil DNA glycosylase [Source:HGNC Symbol;Acc:HGNC:12572] |
| ENSG00000085063 | HGLibA_08417 | 263 | 1.244231694 | CD59 | CD59 molecule (CD59 blood group) [Source:HGNC Symbol;Acc:HGNC:1689] |
| ENSG00000095713 | HGLibA_11219 | 263 | 1.244231694 | CRTAC1 | cartilage acidic protein 1 [Source:HGNC Symbol;Acc:HGNC:14882] |
| ENSG00000100592 | HGLibA_12259 | 263 | 1.244231694 | DAAM1 | dishevelled associated activator of morphogenesis 1 [Source:HGNC Symbol;Acc:HGNC:18142] |
| ENSG00000104529 | HGLibA_14498 | 263 | 1.244231694 | EEF1D | eukaryotic translation elongation factor 1 delta [Source:HGNC Symbol;Acc:HGNC:3211] |
| ENSG00000105127 | HGLibA_01444 | 263 | 1.244231694 | AKAP8 | A-kinase anchoring protein 8 [Source:HGNC Symbol;Acc:HGNC:378] |
| ENSG00000105379 | HGLibA_15624 | 263 | 1.244231694 | ETFB | electron transfer flavoprotein subunit beta [Source:HGNC Symbol;Acc:HGNC:3482] |
| ENSG00000105514 | HGLibA_39915 | 263 | 1.244231694 | RAB3D | member RAS oncogene family [Source:HGNC Symbol;Acc:HGNC:9779]" |
| ENSG00000108953 | HGLibA_54741 | 263 | 1.244231694 | YWHAE | tyrosine 3-monooxygenase/tryptophan 5-monooxygenase activation protein epsilon [Source:HGNC Symbol;Acc:HGNC:12851] |
| ENSG00000109083 | HGLibA_22753 | 263 | 1.244231694 | IFT20 | intraflagellar transport 20 [Source:HGNC Symbol;Acc:HGNC:30989] |
| ENSG00000110756 | HGLibA_22054 | 263 | 1.244231694 | HPS5 | biogenesis of lysosomal organelles complex 2 subunit 2 [Source:HGNC Symbol;Acc:HGNC:17022]" |
| ENSG00000120913 | HGLibA_35911 | 263 | 1.244231694 | PDLIM2 | PDZ and LIM domain 2 [Source:HGNC Symbol;Acc:HGNC:13992] |
| ENSG00000123130 | HGLibA_00493 | 263 | 1.244231694 | ACOT9 | acyl-CoA thioesterase 9 [Source:HGNC Symbol;Acc:HGNC:17152] |
| ENSG00000124613 | HGLibA_55906 | 263 | 1.244231694 | ZNF391 | zinc finger protein 391 [Source:HGNC Symbol;Acc:HGNC:18779] |
| ENSG00000129514 | HGLibA_17806 | 263 | 1.244231694 | FOXA1 | forkhead box A1 [Source:HGNC Symbol;Acc:HGNC:5021] |
| ENSG00000130479 | HGLibA_28133 | 263 | 1.244231694 | MAP1S | microtubule associated protein 1S [Source:HGNC Symbol;Acc:HGNC:15715] |
| ENSG00000138767 | HGLibA_10348 | 263 | 1.244231694 | CNOT6L | CCR4-NOT transcription complex subunit 6 like [Source:HGNC Symbol;Acc:HGNC:18042] |
| ENSG00000146858 | HGLibA_54995 | 263 | 1.244231694 | ZC3HAV1L | antiviral 1 like [Source:HGNC Symbol;Acc:HGNC:22423]" |
| ENSG00000149658 | HGLibA_54730 | 263 | 1.244231694 | YTHDF1 | YTH N6-methyladenosine RNA binding protein 1 [Source:HGNC Symbol;Acc:HGNC:15867] |
| ENSG00000151067 | HGLibA_06882 | 263 | 1.244231694 | CACNA1C | calcium voltage-gated channel subunit alpha1 C [Source:HGNC Symbol;Acc:HGNC:1390] |
| ENSG00000155367 | HGLibA_37983 | 263 | 1.244231694 | PPM1J | Mg2+/Mn2+ dependent 1J [Source:HGNC Symbol;Acc:HGNC:20785]" |
| ENSG00000162591 | HGLibA_28852 | 263 | 1.244231694 | MEGF6 | multiple EGF like domains 6 [Source:HGNC Symbol;Acc:HGNC:3232] |
| ENSG00000163794 | HGLibA_52823 | 263 | 1.244231694 | UCN | urocortin [Source:HGNC Symbol;Acc:HGNC:12516] |
| ENSG00000166886 | HGLibA_31020 | 263 | 1.244231694 | NAB2 | NGFI-A binding protein 2 [Source:HGNC Symbol;Acc:HGNC:7627] |
| ENSG00000167858 | HGLibA_48970 | 263 | 1.244231694 | TEKT1 | tektin 1 [Source:HGNC Symbol;Acc:HGNC:15534] |
| ENSG00000168002 | HGLibA_37583 | 263 | 1.244231694 | POLR2G | RNA polymerase II subunit G [Source:HGNC Symbol;Acc:HGNC:9194] |
| ENSG00000169255 | HGLibA_03948 | 263 | 1.244231694 | B3GALNT1 | 3-N-acetylgalactosaminyltransferase 1 (globoside blood group) [Source:HGNC Symbol;Acc:HGNC:918]" |
| ENSG00000174238 | HGLibA_36742 | 263 | 1.244231694 | PITPNA | phosphatidylinositol transfer protein alpha [Source:HGNC Symbol;Acc:HGNC:9001] |
| ENSG00000178038 | HGLibA_01721 | 263 | 1.244231694 | ALS2CL | ALS2 C-terminal like [Source:HGNC Symbol;Acc:HGNC:20605] |
| ENSG00000179873 | HGLibA_32104 | 263 | 1.244231694 | NLRP11 | NLR family pyrin domain containing 11 [Source:HGNC Symbol;Acc:HGNC:22945] |
| ENSG00000185869 | HGLibA_56828 | 263 | 1.244231694 | ZNF829 | zinc finger protein 829 [Source:HGNC Symbol;Acc:HGNC:34032] |
| ENSG00000196867 | HGLibA_55196 | 263 | 1.244231694 | ZFP28 | ZFP28 zinc finger protein [Source:HGNC Symbol;Acc:HGNC:17801] |
| ENSG00000204388 | HGLibA_22274 | 263 | 1.244231694 | HSPA1B | heat shock protein family A (Hsp70) member 1B [Source:HGNC Symbol;Acc:HGNC:5233] |
| ENSG00000204614 | HGLibA_51491 | 263 | 1.244231694 | TRIM40 | tripartite motif containing 40 [Source:HGNC Symbol;Acc:HGNC:18736] |
| ENSG00000255713 | HGLibA_33973 | 263 | 1.244231694 | OR4D2 | olfactory receptor family 4 subfamily D member 2 [Source:HGNC Symbol;Acc:HGNC:8294] |
| ENSG00000258588 | HGLibA_51561 | 263 | 1.244231694 | TRIM6-TRIM34 | TRIM6-TRIM34 readthrough [Source:HGNC Symbol;Acc:HGNC:33440] |
| ENSG00000261701 | HGLibA_22040 | 263 | 1.244231694 | HPR | haptoglobin-related protein [Source:HGNC Symbol;Acc:HGNC:5156] |
| ENSG00000277586 | HGLibA_31621 | 263 | 1.244231694 | NEFL | neurofilament light [Source:HGNC Symbol;Acc:HGNC:7739] |
| FAM212A | HGLibA_16464 | 263 | 1.244231694 | FAM212A | - |
| GPR115 | HGLibA_19861 | 263 | 1.244231694 | GPR115 | - |
| hsa-mir-147a | HGLibA_57872 | 263 | 1.244231694 | hsa-mir-147a | - |
| hsa-mir-204 | HGLibA_58175 | 263 | 1.244231694 | hsa-mir-204 | - |
| hsa-mir-3130-2 | HGLibA_58576 | 263 | 1.244231694 | hsa-mir-3130-2 | - |
| hsa-mir-4747 | HGLibA_61086 | 263 | 1.244231694 | hsa-mir-4747 | - |
| LOC100129216 | HGLibA_26755 | 263 | 1.244231694 | LOC100129216 | - |
| MLL4 | HGLibA_29401 | 263 | 1.244231694 | MLL4 | - |
| N6AMT2 | HGLibA_30966 | 263 | 1.244231694 | N6AMT2 | - |
| PPP2R3B_X | HGLibA_38158 | 263 | 1.244231694 | PPP2R3B_X | - |
| C10orf11 | HGLibA_05027 | 262 | 1.235186351 | C10orf11 | - |
| C11orf83 | HGLibA_05194 | 262 | 1.235186351 | C11orf83 | - |
| C15orf26 | HGLibA_05377 | 262 | 1.235186351 | C15orf26 | - |
| ENSG00000012779 | HGLibA_01688 | 262 | 1.235186351 | ALOX5 | arachidonate 5-lipoxygenase [Source:HGNC Symbol;Acc:HGNC:435] |
| ENSG00000044459 | HGLibA_10399 | 262 | 1.235186351 | CNTLN | centlein [Source:HGNC Symbol;Acc:HGNC:23432] |
| ENSG00000102890 | HGLibA_14937 | 262 | 1.235186351 | ELMO3 | engulfment and cell motility 3 [Source:HGNC Symbol;Acc:HGNC:17289] |
| ENSG00000104814 | HGLibA_28210 | 262 | 1.235186351 | MAP4K1 | mitogen-activated protein kinase kinase kinase kinase 1 [Source:HGNC Symbol;Acc:HGNC:6863] |
| ENSG00000111832 | HGLibA_42478 | 262 | 1.235186351 | RWDD1 | RWD domain containing 1 [Source:HGNC Symbol;Acc:HGNC:20993] |
| ENSG00000114503 | HGLibA_31285 | 262 | 1.235186351 | NCBP2 | nuclear cap binding protein subunit 2 [Source:HGNC Symbol;Acc:HGNC:7659] |
| ENSG00000124198 | HGLibA_02674 | 262 | 1.235186351 | ARFGEF2 | ADP ribosylation factor guanine nucleotide exchange factor 2 [Source:HGNC Symbol;Acc:HGNC:15853] |
| ENSG00000125845 | HGLibA_04599 | 262 | 1.235186351 | BMP2 | bone morphogenetic protein 2 [Source:HGNC Symbol;Acc:HGNC:1069] |
| ENSG00000126804 | HGLibA_54810 | 262 | 1.235186351 | ZBTB1 | zinc finger and BTB domain containing 1 [Source:HGNC Symbol;Acc:HGNC:20259] |
| ENSG00000133111 | HGLibA_40989 | 262 | 1.235186351 | RFXAP | regulatory factor X associated protein [Source:HGNC Symbol;Acc:HGNC:9988] |
| ENSG00000135617 | HGLibA_38267 | 262 | 1.235186351 | PRADC1 | protease associated domain containing 1 [Source:HGNC Symbol;Acc:HGNC:16047] |
| ENSG00000136694 | HGLibA_23146 | 262 | 1.235186351 | IL36A | interleukin 36 alpha [Source:HGNC Symbol;Acc:HGNC:15562] |
| ENSG00000137077 | HGLibA_07995 | 262 | 1.235186351 | CCL21 | C-C motif chemokine ligand 21 [Source:HGNC Symbol;Acc:HGNC:10620] |
| ENSG00000138061 | HGLibA_12072 | 262 | 1.235186351 | CYP1B1 | cytochrome P450 family 1 subfamily B member 1 [Source:HGNC Symbol;Acc:HGNC:2597] |
| ENSG00000141576 | HGLibA_41554 | 262 | 1.235186351 | RNF157 | ring finger protein 157 [Source:HGNC Symbol;Acc:HGNC:29402] |
| ENSG00000143437 | HGLibA_03063 | 262 | 1.235186351 | ARNT | aryl hydrocarbon receptor nuclear translocator [Source:HGNC Symbol;Acc:HGNC:700] |
| ENSG00000146072 | HGLibA_50778 | 262 | 1.235186351 | TNFRSF21 | TNF receptor superfamily member 21 [Source:HGNC Symbol;Acc:HGNC:13469] |
| ENSG00000146247 | HGLibA_36398 | 262 | 1.235186351 | PHIP | pleckstrin homology domain interacting protein [Source:HGNC Symbol;Acc:HGNC:15673] |
| ENSG00000157873 | HGLibA_50759 | 262 | 1.235186351 | TNFRSF14 | TNF receptor superfamily member 14 [Source:HGNC Symbol;Acc:HGNC:11912] |
| ENSG00000161692 | HGLibA_12379 | 262 | 1.235186351 | DBF4B | DBF4 zinc finger B [Source:HGNC Symbol;Acc:HGNC:17883] |
| ENSG00000162191 | HGLibA_52775 | 262 | 1.235186351 | UBXN1 | UBX domain protein 1 [Source:HGNC Symbol;Acc:HGNC:18402] |
| ENSG00000163421 | HGLibA_38669 | 262 | 1.235186351 | PROK2 | prokineticin 2 [Source:HGNC Symbol;Acc:HGNC:18455] |
| ENSG00000163879 | HGLibA_13649 | 262 | 1.235186351 | DNALI1 | dynein axonemal light intermediate chain 1 [Source:HGNC Symbol;Acc:HGNC:14353] |
| ENSG00000164825 | HGLibA_12810 | 262 | 1.235186351 | DEFB1 | defensin beta 1 [Source:HGNC Symbol;Acc:HGNC:2766] |
| ENSG00000167772 | HGLibA_01918 | 262 | 1.235186351 | ANGPTL4 | angiopoietin like 4 [Source:HGNC Symbol;Acc:HGNC:16039] |
| ENSG00000167889 | HGLibA_29153 | 262 | 1.235186351 | MGAT5B | 6-mannosylglycoprotein 6-beta-N-acetylglucosaminyltransferase B [Source:HGNC Symbol;Acc:HGNC:24140]" |
| ENSG00000168646 | HGLibA_03925 | 262 | 1.235186351 | AXIN2 | axin 2 [Source:HGNC Symbol;Acc:HGNC:904] |
| ENSG00000168724 | HGLibA_13597 | 262 | 1.235186351 | DNAJC21 | DnaJ heat shock protein family (Hsp40) member C21 [Source:HGNC Symbol;Acc:HGNC:27030] |
| ENSG00000171502 | HGLibA_10547 | 262 | 1.235186351 | COL24A1 | collagen type XXIV alpha 1 chain [Source:HGNC Symbol;Acc:HGNC:20821] |
| ENSG00000175398 | HGLibA_33536 | 262 | 1.235186351 | OR10P1 | olfactory receptor family 10 subfamily P member 1 [Source:HGNC Symbol;Acc:HGNC:15378] |
| ENSG00000175485 | HGLibA_34208 | 262 | 1.235186351 | OR52W1 | olfactory receptor family 52 subfamily W member 1 [Source:HGNC Symbol;Acc:HGNC:15239] |
| ENSG00000175575 | HGLibA_34918 | 262 | 1.235186351 | PAAF1 | proteasomal ATPase associated factor 1 [Source:HGNC Symbol;Acc:HGNC:25687] |
| ENSG00000177981 | HGLibA_03252 | 262 | 1.235186351 | ASB8 | ankyrin repeat and SOCS box containing 8 [Source:HGNC Symbol;Acc:HGNC:17183] |
| ENSG00000178343 | HGLibA_44036 | 262 | 1.235186351 | SHISA3 | shisa family member 3 [Source:HGNC Symbol;Acc:HGNC:25159] |
| ENSG00000179071 | HGLibA_07917 | 262 | 1.235186351 | CCDC89 | coiled-coil domain containing 89 [Source:HGNC Symbol;Acc:HGNC:26762] |
| ENSG00000181781 | HGLibA_33292 | 262 | 1.235186351 | ODF3L2 | outer dense fiber of sperm tails 3 like 2 [Source:HGNC Symbol;Acc:HGNC:26841] |
| ENSG00000182931 | HGLibA_54251 | 262 | 1.235186351 | WFDC10B | WAP four-disulfide core domain 10B [Source:HGNC Symbol;Acc:HGNC:20479] |
| ENSG00000184635 | HGLibA_56910 | 262 | 1.235186351 | ZNF93 | zinc finger protein 93 [Source:HGNC Symbol;Acc:HGNC:13169] |
| ENSG00000187134 | HGLibA_01482 | 262 | 1.235186351 | AKR1C1 | aldo-keto reductase family 1 member C1 [Source:HGNC Symbol;Acc:HGNC:384] |
| ENSG00000187664 | HGLibA_20849 | 262 | 1.235186351 | HAPLN4 | hyaluronan and proteoglycan link protein 4 [Source:HGNC Symbol;Acc:HGNC:31357] |
| ENSG00000197124 | HGLibA_56531 | 262 | 1.235186351 | ZNF682 | zinc finger protein 682 [Source:HGNC Symbol;Acc:HGNC:28857] |
| ENSG00000198252 | HGLibA_47687 | 262 | 1.235186351 | STYX | serine/threonine/tyrosine interacting protein [Source:HGNC Symbol;Acc:HGNC:11447] |
| ENSG00000198498 | HGLibA_49732 | 262 | 1.235186351 | TMA16 | translation machinery associated 16 homolog [Source:HGNC Symbol;Acc:HGNC:25638] |
| ENSG00000204611 | HGLibA_56396 | 262 | 1.235186351 | ZNF616 | zinc finger protein 616 [Source:HGNC Symbol;Acc:HGNC:28062] |
| ENSG00000236027 | HGLibA_35276 | 262 | 1.235186351 | PATE3 | prostate and testis expressed 3 [Source:HGNC Symbol;Acc:HGNC:35426] |
| ENSG00000254858 | HGLibA_29788 | 262 | 1.235186351 | MPV17L2 | MPV17 mitochondrial inner membrane protein like 2 [Source:HGNC Symbol;Acc:HGNC:28177] |
| ENSG00000261594 | HGLibA_51103 | 262 | 1.235186351 | TPBGL | trophoblast glycoprotein like [Source:HGNC Symbol;Acc:HGNC:44159] |
| HIST1H2AJ | HGLibA_21399 | 262 | 1.235186351 | HIST1H2AJ | - |
| hsa-mir-3683 | HGLibA_59285 | 262 | 1.235186351 | hsa-mir-3683 | - |
| hsa-mir-4755 | HGLibA_61118 | 262 | 1.235186351 | hsa-mir-4755 | - |
| hsa-mir-6503 | HGLibA_62863 | 262 | 1.235186351 | hsa-mir-6503 | - |
| hsa-mir-7110 | HGLibA_63806 | 262 | 1.235186351 | hsa-mir-7110 | - |
| KIAA1045 | HGLibA_24688 | 262 | 1.235186351 | KIAA1045 | - |
| WDR63 | HGLibA_54149 | 262 | 1.235186351 | WDR63 | - |
| C9orf16 | HGLibA_06699 | 261 | 1.226141008 | C9orf16 | - |
| CCDC67 | HGLibA_07838 | 261 | 1.226141008 | CCDC67 | - |
| ENSG00000000419 | HGLibA_13856 | 261 | 1.226141008 | DPM1 | catalytic [Source:HGNC Symbol;Acc:HGNC:3005]" |

| | | | | | |
|---|---|---|---|---|---|
| ENSG00000002746 | HGLibA_21101 | 261 | 1.226141008 | HECW1 | C2 and WW domain containing E3 ubiquitin protein ligase 1 [Source:HGNC Symbol;Acc:HGNC:22195]" |
| ENSG00000021762 | HGLibA_34658 | 261 | 1.226141008 | OSBPL5 | oxysterol binding protein like 5 [Source:HGNC Symbol;Acc:HGNC:16392] |
| ENSG00000055609 | HGLibA_25326 | 261 | 1.226141008 | KMT2C | lysine methyltransferase 2C [Source:HGNC Symbol;Acc:HGNC:13726] |
| ENSG00000064270 | HGLibA_03629 | 261 | 1.226141008 | ATP2C2 | ATPase secretory pathway Ca2+ transporting 2 [Source:HGNC Symbol;Acc:HGNC:29103] |
| ENSG00000088320 | HGLibA_40830 | 261 | 1.226141008 | REM1 | RRAD and GEM like GTPase 1 [Source:HGNC Symbol;Acc:HGNC:15922] |
| ENSG00000088992 | HGLibA_49026 | 261 | 1.226141008 | TESC | tescalcin [Source:HGNC Symbol;Acc:HGNC:26065] |
| ENSG00000089335 | HGLibA_55773 | 261 | 1.226141008 | ZNF302 | zinc finger protein 302 [Source:HGNC Symbol;Acc:HGNC:13848] |
| ENSG00000090975 | HGLibA_36754 | 261 | 1.226141008 | PITPNM2 | phosphatidylinositol transfer protein membrane associated 2 [Source:HGNC Symbol;Acc:HGNC:21044] |
| ENSG00000091010 | HGLibA_37783 | 261 | 1.226141008 | POU4F3 | POU class 4 homeobox 3 [Source:HGNC Symbol;Acc:HGNC:9220] |
| ENSG00000099999 | HGLibA_41629 | 261 | 1.226141008 | RNF215 | ring finger protein 215 [Source:HGNC Symbol;Acc:HGNC:33434] |
| ENSG00000100433 | HGLibA_24297 | 261 | 1.226141008 | KCNK10 | potassium two pore domain channel subfamily K member 10 [Source:HGNC Symbol;Acc:HGNC:6273] |
| ENSG00000101405 | HGLibA_34841 | 261 | 1.226141008 | OXT | oxytocin/neurophysin I prepropeptide [Source:HGNC Symbol;Acc:HGNC:8528] |
| ENSG00000102243 | HGLibA_53635 | 261 | 1.226141008 | VGLL1 | vestigial like family member 1 [Source:HGNC Symbol;Acc:HGNC:20985] |
| ENSG00000102287 | HGLibA_18386 | 261 | 1.226141008 | GABRE | gamma-aminobutyric acid type A receptor epsilon subunit [Source:HGNC Symbol;Acc:HGNC:4085] |
| ENSG00000105647 | HGLibA_36651 | 261 | 1.226141008 | PIK3R2 | phosphoinositide-3-kinase regulatory subunit 2 [Source:HGNC Symbol;Acc:HGNC:8980] |
| ENSG00000109158 | HGLibA_18365 | 261 | 1.226141008 | GABRA4 | gamma-aminobutyric acid type A receptor alpha4 subunit [Source:HGNC Symbol;Acc:HGNC:4078] |
| ENSG00000111860 | HGLibA_09174 | 261 | 1.226141008 | CEP85L | centrosomal protein 85 like [Source:HGNC Symbol;Acc:HGNC:21638] |
| ENSG00000112137 | HGLibA_36298 | 261 | 1.226141008 | PHACTR1 | phosphatase and actin regulator 1 [Source:HGNC Symbol;Acc:HGNC:20990] |
| ENSG00000119686 | HGLibA_17677 | 261 | 1.226141008 | FLVCR2 | feline leukemia virus subgroup C cellular receptor family member 2 [Source:HGNC Symbol;Acc:HGNC:20105] |
| ENSG00000119965 | HGLibA_05100 | 261 | 1.226141008 | C10orf88 | chromosome 10 open reading frame 88 [Source:HGNC Symbol;Acc:HGNC:25822] |
| ENSG00000120696 | HGLibA_24113 | 261 | 1.226141008 | KBTBD7 | kelch repeat and BTB domain containing 7 [Source:HGNC Symbol;Acc:HGNC:25266] |
| ENSG00000124491 | HGLibA_15852 | 261 | 1.226141008 | F13A1 | coagulation factor XIII A chain [Source:HGNC Symbol;Acc:HGNC:3531] |
| ENSG00000128185 | HGLibA_13051 | 261 | 1.226141008 | DGCR6L | DiGeorge syndrome critical region gene 6 like [Source:HGNC Symbol;Acc:HGNC:18551] |
| ENSG00000132394 | HGLibA_14514 | 261 | 1.226141008 | EEFSEC | selenocysteine-tRNA specific [Source:HGNC Symbol;Acc:HGNC:24614]" |
| ENSG00000135241 | HGLibA_37427 | 261 | 1.226141008 | PNPLA8 | patatin like phospholipase domain containing 8 [Source:HGNC Symbol;Acc:HGNC:28900] |
| ENSG00000137747 | HGLibA_50631 | 261 | 1.226141008 | TMPRSS13 | transmembrane serine protease 13 [Source:HGNC Symbol;Acc:HGNC:29808] |
| ENSG00000142149 | HGLibA_22421 | 261 | 1.226141008 | HUNK | hormonally up-regulated Neu-associated kinase [Source:HGNC Symbol;Acc:HGNC:13326] |
| ENSG00000143126 | HGLibA_09025 | 261 | 1.226141008 | CELSR2 | cadherin EGF LAG seven-pass G-type receptor 2 [Source:HGNC Symbol;Acc:HGNC:3231] |
| ENSG00000144962 | HGLibA_46432 | 261 | 1.226141008 | SPATA16 | spermatogenesis associated 16 [Source:HGNC Symbol;Acc:HGNC:29935] |
| ENSG00000145833 | HGLibA_12719 | 261 | 1.226141008 | DDX46 | DEAD-box helicase 46 [Source:HGNC Symbol;Acc:HGNC:18681] |
| ENSG00000145888 | HGLibA_19299 | 261 | 1.226141008 | GLRA1 | glycine receptor alpha 1 [Source:HGNC Symbol;Acc:HGNC:4326] |
| ENSG00000146383 | HGLibA_48133 | 261 | 1.226141008 | TAAR6 | trace amine associated receptor 6 [Source:HGNC Symbol;Acc:HGNC:20978] |
| ENSG00000149131 | HGLibA_43593 | 261 | 1.226141008 | SERPING1 | serpin family G member 1 [Source:HGNC Symbol;Acc:HGNC:1228] |
| ENSG00000156471 | HGLibA_39288 | 261 | 1.226141008 | PTDSS1 | phosphatidylserine synthase 1 [Source:HGNC Symbol;Acc:HGNC:9587] |
| ENSG00000161243 | HGLibA_17056 | 261 | 1.226141008 | FBXO27 | F-box protein 27 [Source:HGNC Symbol;Acc:HGNC:18753] |
| ENSG00000161956 | HGLibA_43390 | 261 | 1.226141008 | SENP3 | SUMO specific peptidase 3 [Source:HGNC Symbol;Acc:HGNC:17862] |
| ENSG00000162426 | HGLibA_45171 | 261 | 1.226141008 | SLC45A1 | solute carrier family 45 member 1 [Source:HGNC Symbol;Acc:HGNC:17939] |
| ENSG00000164122 | HGLibA_03243 | 261 | 1.226141008 | ASB5 | ankyrin repeat and SOCS box containing 5 [Source:HGNC Symbol;Acc:HGNC:17180] |
| ENSG00000169083 | HGLibA_02618 | 261 | 1.226141008 | AR | androgen receptor [Source:HGNC Symbol;Acc:HGNC:644] |
| ENSG00000172179 | HGLibA_38596 | 261 | 1.226141008 | PRL | prolactin [Source:HGNC Symbol;Acc:HGNC:9445] |
| ENSG00000175764 | HGLibA_52191 | 261 | 1.226141008 | TTLL11 | tubulin tyrosine ligase like 11 [Source:HGNC Symbol;Acc:HGNC:18113] |
| ENSG00000184979 | HGLibA_53290 | 261 | 1.226141008 | USP18 | ubiquitin specific peptidase 18 [Source:HGNC Symbol;Acc:HGNC:12616] |
| ENSG00000188739 | HGLibA_40536 | 261 | 1.226141008 | RBM34 | RNA binding motif protein 34 [Source:HGNC Symbol;Acc:HGNC:28965] |
| ENSG00000189433 | HGLibA_19167 | 261 | 1.226141008 | GJB4 | gap junction protein beta 4 [Source:HGNC Symbol;Acc:HGNC:4286] |
| ENSG00000197496 | HGLibA_44856 | 261 | 1.226141008 | SLC2A10 | solute carrier family 2 member 10 [Source:HGNC Symbol;Acc:HGNC:13444] |
| ENSG00000197706 | HGLibA_34407 | 261 | 1.226141008 | OR6C74 | olfactory receptor family 6 subfamily C member 74 [Source:HGNC Symbol;Acc:HGNC:31303] |
| ENSG00000198021 | HGLibA_46378 | 261 | 1.226141008 | SPANXA1 | family member A1 [Source:HGNC Symbol;Acc:HGNC:11218]" |
| ENSG00000198092 | HGLibA_50624 | 261 | 1.226141008 | TMPRSS11F | transmembrane serine protease 11F [Source:HGNC Symbol;Acc:HGNC:29994] |
| ENSG00000205883 | HGLibA_12908 | 261 | 1.226141008 | DEFB135 | defensin beta 135 [Source:HGNC Symbol;Acc:HGNC:32400] |
| ENSG00000214102 | HGLibA_54242 | 261 | 1.226141008 | WEE2 | WEE1 homolog 2 [Source:HGNC Symbol;Acc:HGNC:19684] |
| HIST1H2BA | HGLibA_21410 | 261 | 1.226141008 | HIST1H2BA | - |
| hsa-mir-215 | HGLibA_58253 | 261 | 1.226141008 | hsa-mir-215 | - |
| hsa-mir-323b | HGLibA_58961 | 261 | 1.226141008 | hsa-mir-323b | - |
| hsa-mir-3689a | HGLibA_59315 | 261 | 1.226141008 | hsa-mir-3689a | - |
| hsa-mir-4652 | HGLibA_60717 | 261 | 1.226141008 | hsa-mir-4652 | - |
| hsa-mir-4704 | HGLibA_60916 | 261 | 1.226141008 | hsa-mir-4704 | - |
| PHF15 | HGLibA_36348 | 261 | 1.226141008 | PHF15 | - |
| CNTD2 | HGLibA_10388 | 260 | 1.217095665 | CNTD2 | - |
| ENSG00000019102 | HGLibA_53844 | 260 | 1.217095665 | VSIG2 | V-set and immunoglobulin domain containing 2 [Source:HGNC Symbol;Acc:HGNC:17149] |
| ENSG00000019485 | HGLibA_38367 | 260 | 1.217095665 | PRDM11 | PR/SET domain 11 [Source:HGNC Symbol;Acc:HGNC:13996] |
| ENSG00000026559 | HGLibA_24198 | 260 | 1.217095665 | KCNG1 | potassium voltage-gated channel modifier subfamily G member 1 [Source:HGNC Symbol;Acc:HGNC:6248] |
| ENSG00000091181 | HGLibA_23177 | 260 | 1.217095665 | IL5RA | interleukin 5 receptor subunit alpha [Source:HGNC Symbol;Acc:HGNC:6017] |
| ENSG00000097007 | HGLibA_00302 | 260 | 1.217095665 | ABL1 | non-receptor tyrosine kinase [Source:HGNC Symbol;Acc:HGNC:76]" |
| ENSG00000102699 | HGLibA_35239 | 260 | 1.217095665 | PARP4 | poly(ADP-ribose) polymerase family member 4 [Source:HGNC Symbol;Acc:HGNC:271] |
| ENSG00000106991 | HGLibA_15125 | 260 | 1.217095665 | ENG | endoglin [Source:HGNC Symbol;Acc:HGNC:3349] |
| ENSG00000111203 | HGLibA_23710 | 260 | 1.217095665 | ITFG2 | integrin alpha FG-GAP repeat containing 2 [Source:HGNC Symbol;Acc:HGNC:30879] |
| ENSG00000113595 | HGLibA_51434 | 260 | 1.217095665 | TRIM23 | tripartite motif containing 23 [Source:HGNC Symbol;Acc:HGNC:660] |
| ENSG00000116761 | HGLibA_11625 | 260 | 1.217095665 | CTH | cystathionine gamma-lyase [Source:HGNC Symbol;Acc:HGNC:2501] |
| ENSG00000125492 | HGLibA_04158 | 260 | 1.217095665 | BARHL1 | BarH like homeobox 1 [Source:HGNC Symbol;Acc:HGNC:953] |
| ENSG00000125788 | HGLibA_12882 | 260 | 1.217095665 | DEFB126 | defensin beta 126 [Source:HGNC Symbol;Acc:HGNC:15900] |
| ENSG00000133657 | HGLibA_03573 | 260 | 1.217095665 | ATP13A3 | ATPase 13A3 [Source:HGNC Symbol;Acc:HGNC:24113] |
| ENSG00000139436 | HGLibA_19133 | 260 | 1.217095665 | GIT2 | GIT ArfGAP 2 [Source:HGNC Symbol;Acc:HGNC:4273] |
| ENSG00000139880 | HGLibA_08670 | 260 | 1.217095665 | CDH24 | cadherin 24 [Source:HGNC Symbol;Acc:HGNC:14265] |
| ENSG00000140365 | HGLibA_10664 | 260 | 1.217095665 | COMMD4 | COMM domain containing 4 [Source:HGNC Symbol;Acc:HGNC:26027] |
| ENSG00000147437 | HGLibA_19579 | 260 | 1.217095665 | GNRH1 | gonadotropin releasing hormone 1 [Source:HGNC Symbol;Acc:HGNC:4419] |
| ENSG00000149782 | HGLibA_36984 | 260 | 1.217095665 | PLCB3 | phospholipase C beta 3 [Source:HGNC Symbol;Acc:HGNC:9056] |
| ENSG00000150455 | HGLibA_49564 | 260 | 1.217095665 | TIRAP | TIR domain containing adaptor protein [Source:HGNC Symbol;Acc:HGNC:17192] |
| ENSG00000151353 | HGLibA_50092 | 260 | 1.217095665 | TMEM18 | transmembrane protein 18 [Source:HGNC Symbol;Acc:HGNC:25257] |
| ENSG00000152359 | HGLibA_37450 | 260 | 1.217095665 | POC5 | POC5 centriolar protein [Source:HGNC Symbol;Acc:HGNC:26658] |
| ENSG00000152465 | HGLibA_32208 | 260 | 1.217095665 | NMT2 | N-myristoyltransferase 2 [Source:HGNC Symbol;Acc:HGNC:7858] |
| ENSG00000153443 | HGLibA_52540 | 260 | 1.217095665 | UBALD1 | UBA like domain containing 1 [Source:HGNC Symbol;Acc:HGNC:29576] |
| ENSG00000159251 | HGLibA_00609 | 260 | 1.217095665 | ACTC1 | cardiac muscle 1 [Source:HGNC Symbol;Acc:HGNC:143]" |
| ENSG00000161649 | HGLibA_08344 | 260 | 1.217095665 | CD300LG | CD300 molecule like family member g [Source:HGNC Symbol;Acc:HGNC:30455] |
| ENSG00000163053 | HGLibA_44432 | 260 | 1.217095665 | SLC16A14 | solute carrier family 16 member 14 [Source:HGNC Symbol;Acc:HGNC:26417] |
| ENSG00000163064 | HGLibA_15099 | 260 | 1.217095665 | EN1 | engrailed homeobox 1 [Source:HGNC Symbol;Acc:HGNC:3342] |
| ENSG00000163156 | HGLibA_43011 | 260 | 1.217095665 | SCNM1 | sodium channel modifier 1 [Source:HGNC Symbol;Acc:HGNC:23136] |
| ENSG00000164483 | HGLibA_42681 | 260 | 1.217095665 | SAMD3 | sterile alpha motif domain containing 3 [Source:HGNC Symbol;Acc:HGNC:21574] |
| ENSG00000168140 | HGLibA_53537 | 260 | 1.217095665 | VASN | vasorin [Source:HGNC Symbol;Acc:HGNC:18517] |
| ENSG00000170162 | HGLibA_53637 | 260 | 1.217095665 | VGLL2 | vestigial like family member 2 [Source:HGNC Symbol;Acc:HGNC:20232] |
| ENSG00000178685 | HGLibA_35215 | 260 | 1.217095665 | PARP10 | poly(ADP-ribose) polymerase family member 10 [Source:HGNC Symbol;Acc:HGNC:25895] |
| ENSG00000179750 | HGLibA_02480 | 260 | 1.217095665 | APOBEC3B | apolipoprotein B mRNA editing enzyme catalytic subunit 3B [Source:HGNC Symbol;Acc:HGNC:17352] |
| ENSG00000181092 | HGLibA_00978 | 260 | 1.217095665 | ADIPOQ | C1Q and collagen domain containing [Source:HGNC Symbol;Acc:HGNC:13633]" |
| ENSG00000196417 | HGLibA_56692 | 260 | 1.217095665 | ZNF765 | zinc finger protein 765 [Source:HGNC Symbol;Acc:HGNC:25092] |
| ENSG00000198739 | HGLibA_27490 | 260 | 1.217095665 | LRRTM3 | leucine rich repeat transmembrane neuronal 3 [Source:HGNC Symbol;Acc:HGNC:19410] |
| ENSG00000203859 | HGLibA_22208 | 260 | 1.217095665 | HSD3B2 | 3 beta- and steroid delta-isomerase 2 [Source:HGNC Symbol;Acc:HGNC:5218]" |
| ENSG00000213859 | HGLibA_24416 | 260 | 1.217095665 | KCTD11 | potassium channel tetramerization domain containing 11 [Source:HGNC Symbol;Acc:HGNC:21302] |
| ENSG00000213886 | HGLibA_52571 | 260 | 1.217095665 | UBD | ubiquitin D [Source:HGNC Symbol;Acc:HGNC:18795] |
| ENSG00000220008 | HGLibA_26592 | 260 | 1.217095665 | LINGO3 | leucine rich repeat and Ig domain containing 3 [Source:HGNC Symbol;Acc:HGNC:21206] |
| ENSG00000234965 | HGLibA_44052 | 260 | 1.217095665 | SHISA8 | shisa family member 8 [Source:HGNC Symbol;Acc:HGNC:18351] |
| ENSG00000253537 | HGLibA_35542 | 260 | 1.217095665 | PCDHGA7 | 7 [Source:HGNC Symbol;Acc:HGNC:8705]" |
| hsa-mir-3911 | HGLibA_59513 | 260 | 1.217095665 | hsa-mir-3911 | - |
| hsa-mir-4447 | HGLibA_60193 | 260 | 1.217095665 | hsa-mir-4447 | - |
| hsa-mir-6126 | HGLibA_62652 | 260 | 1.217095665 | hsa-mir-6126 | - |
| LOC100652824 | HGLibA_26872 | 260 | 1.217095665 | LOC100652824 | - |
| AZI1 | HGLibA_03933 | 259 | 1.208050322 | AZI1 | - |
| CCDC36 | HGLibA_07763 | 259 | 1.208050322 | CCDC36 | - |
| ENSG00000054967 | HGLibA_40828 | 259 | 1.208050322 | RELT | TNF receptor [Source:HGNC Symbol;Acc:HGNC:13764]" |
| ENSG00000079387 | HGLibA_43385 | 259 | 1.208050322 | SENP1 | SUMO specific peptidase 1 [Source:HGNC Symbol;Acc:HGNC:17927] |
| ENSG00000082014 | HGLibA_45634 | 259 | 1.208050322 | SMARCD3 | member 3 [Source:HGNC Symbol;Acc:HGNC:11108]" |
| ENSG00000082175 | HGLibA_36285 | 259 | 1.208050322 | PGR | progesterone receptor [Source:HGNC Symbol;Acc:HGNC:8910] |
| ENSG00000089639 | HGLibA_19408 | 259 | 1.208050322 | GMIP | GEM interacting protein [Source:HGNC Symbol;Acc:HGNC:24852] |
| ENSG00000100122 | HGLibA_11260 | 259 | 1.208050322 | CRYBB1 | crystallin beta B1 [Source:HGNC Symbol;Acc:HGNC:2397] |
| ENSG00000103375 | HGLibA_02607 | 259 | 1.208050322 | AQP8 | aquaporin 8 [Source:HGNC Symbol;Acc:HGNC:642] |
| ENSG00000106392 | HGLibA_05722 | 259 | 1.208050322 | C1GALT1 | glycoprotein-N-acetylgalactosamine 3-beta-galactosyltransferase 1 [Source:HGNC Symbol;Acc:HGNC:24337]" |
| ENSG00000110455 | HGLibA_00413 | 259 | 1.208050322 | ACCS | 1-aminocyclopropane-1-carboxylate synthase homolog (inactive) [Source:HGNC Symbol;Acc:HGNC:23989] |
| ENSG00000113732 | HGLibA_03725 | 259 | 1.208050322 | ATP6V0E1 | ATPase H+ transporting V0 subunit e1 [Source:HGNC Symbol;Acc:HGNC:863] |
| ENSG00000114113 | HGLibA_40632 | 259 | 1.208050322 | RBP2 | retinol binding protein 2 [Source:HGNC Symbol;Acc:HGNC:9920] |
| ENSG00000114120 | HGLibA_44721 | 259 | 1.208050322 | SLC25A36 | solute carrier family 25 member 36 [Source:HGNC Symbol;Acc:HGNC:25554] |
| ENSG00000116882 | HGLibA_20834 | 259 | 1.208050322 | HAO2 | hydroxyacid oxidase 2 [Source:HGNC Symbol;Acc:HGNC:4810] |
| ENSG00000123584 | HGLibA_27923 | 259 | 1.208050322 | MAGEA9 | MAGE family member A9 [Source:HGNC Symbol;Acc:HGNC:6807] |
| ENSG00000124383 | HGLibA_29723 | 259 | 1.208050322 | MPHOSPH10 | M-phase phosphoprotein 10 [Source:HGNC Symbol;Acc:HGNC:7213] |
| ENSG00000131097 | HGLibA_21310 | 259 | 1.208050322 | HIGD1B | HIG1 hypoxia inducible domain family member 1B [Source:HGNC Symbol;Acc:HGNC:24318] |
| ENSG00000131831 | HGLibA_40144 | 259 | 1.208050322 | RAI2 | retinoic acid induced 2 [Source:HGNC Symbol;Acc:HGNC:9835] |
| ENSG00000133983 | HGLibA_10815 | 259 | 1.208050322 | COX16 | cytochrome c oxidase assembly factor COX16 [Source:HGNC Symbol;Acc:HGNC:20213] |
| ENSG00000134250 | HGLibA_32344 | 259 | 1.208050322 | NOTCH2 | notch 2 [Source:HGNC Symbol;Acc:HGNC:7882] |
| ENSG00000138303 | HGLibA_03258 | 259 | 1.208050322 | ASCC1 | activating signal cointegrator 1 complex subunit 1 [Source:HGNC Symbol;Acc:HGNC:24268] |
| ENSG00000156689 | HGLibA_19377 | 259 | 1.208050322 | GLYATL2 | glycine-N-acyltransferase like 2 [Source:HGNC Symbol;Acc:HGNC:24178] |
| ENSG00000162576 | HGLibA_30629 | 259 | 1.208050322 | MXRA8 | matrix remodeling associated 8 [Source:HGNC Symbol;Acc:HGNC:7542] |
| ENSG00000172771 | HGLibA_14526 | 259 | 1.208050322 | EFCAB12 | EF-hand calcium binding domain 12 [Source:HGNC Symbol;Acc:HGNC:28061] |
| ENSG00000172824 | HGLibA_09226 | 259 | 1.208050322 | CES4A | carboxylesterase 4A [Source:HGNC Symbol;Acc:HGNC:26741] |
| ENSG00000173482 | HGLibA_39516 | 259 | 1.208050322 | PTPRM | receptor type M [Source:HGNC Symbol;Acc:HGNC:9675]" |
| ENSG00000174669 | HGLibA_44842 | 259 | 1.208050322 | SLC29A2 | solute carrier family 29 member 2 [Source:HGNC Symbol;Acc:HGNC:11004] |
| ENSG00000181562 | HGLibA_14447 | 259 | 1.208050322 | EDDM3A | epididymal protein 3A [Source:HGNC Symbol;Acc:HGNC:16978] |
| ENSG00000182742 | HGLibA_21938 | 259 | 1.208050322 | HOXB4 | homeobox B4 [Source:HGNC Symbol;Acc:HGNC:5115] |
| ENSG00000188770 | HGLibA_33454 | 259 | 1.208050322 | OPTC | opticin [Source:HGNC Symbol;Acc:HGNC:8158] |

| | | | | | |
|---|---|---|---|---|---|
| ENSG00000198125 | HGLibA_28454 | 259 | 1.208050322 | MB | myoglobin [Source:HGNC Symbol;Acc:HGNC:6915] |
| ENSG00000205328 | HGLibA_34398 | 259 | 1.208050322 | OR6C65 | olfactory receptor family 6 subfamily C member 65 [Source:HGNC Symbol;Acc:HGNC:31295] |
| ENSG00000205362 | HGLibA_30278 | 259 | 1.208050322 | MT1A | metallothionein 1A [Source:HGNC Symbol;Acc:HGNC:7393] |
| ENSG00000223638 | HGLibA_40938 | 259 | 1.208050322 | RFPL4A | ret finger protein like 4A [Source:HGNC Symbol;Acc:HGNC:16449] |
| ENSG00000253910 | HGLibA_35554 | 259 | 1.208050322 | PCDHGB2 | 2 [Source:HGNC Symbol;Acc:HGNC:8709]" |
| ENSG00000258659 | HGLibA_51469 | 259 | 1.208050322 | TRIM34 | tripartite motif containing 34 [Source:HGNC Symbol;Acc:HGNC:10063] |
| ENSG00000288547 | HGLibA_06235 | 259 | 1.208050322 | C3orf36 | - |
| hsa-mir-4792 | HGLibA_61275 | 259 | 1.208050322 | hsa-mir-4792 | - |
| hsa-mir-6861 | HGLibA_63623 | 259 | 1.208050322 | hsa-mir-6861 | - |
| hsa-mir-7641-2 | HGLibA_63918 | 259 | 1.208050322 | hsa-mir-7641-2 | - |
| hsa-mir-8058 | HGLibA_64071 | 259 | 1.208050322 | hsa-mir-8058 | - |
| hsa-mir-8085 | HGLibA_64183 | 259 | 1.208050322 | hsa-mir-8085 | - |
| hsa-mir-938 | HGLibA_64328 | 259 | 1.208050322 | hsa-mir-938 | - |
| KIAA1804 | HGLibA_24819 | 259 | 1.208050322 | KIAA1804 | - |
| RLTPR | HGLibA_41360 | 259 | 1.208050322 | RLTPR | - |
| C10orf12 | HGLibA_05042 | 258 | 1.199004979 | C10orf12 | - |
| ENSG00000005108 | HGLibA_49405 | 258 | 1.199004979 | THSD7A | thrombospondin type 1 domain containing 7A [Source:HGNC Symbol;Acc:HGNC:22207] |
| ENSG00000006016 | HGLibA_11186 | 258 | 1.199004979 | CRLF1 | cytokine receptor like factor 1 [Source:HGNC Symbol;Acc:HGNC:2364] |
| ENSG00000049449 | HGLibA_40700 | 258 | 1.199004979 | RCN1 | reticulocalbin 1 [Source:HGNC Symbol;Acc:HGNC:9934] |
| ENSG00000073712 | HGLibA_17297 | 258 | 1.199004979 | FERMT2 | fermitin family member 2 [Source:HGNC Symbol;Acc:HGNC:15767] |
| ENSG00000075239 | HGLibA_00393 | 258 | 1.199004979 | ACAT1 | acetyl-CoA acetyltransferase 1 [Source:HGNC Symbol;Acc:HGNC:93] |
| ENSG00000078747 | HGLibA_23704 | 258 | 1.199004979 | ITCH | itchy E3 ubiquitin protein ligase [Source:HGNC Symbol;Acc:HGNC:13890] |
| ENSG00000101098 | HGLibA_41285 | 258 | 1.199004979 | RIMS4 | regulating synaptic membrane exocytosis 4 [Source:HGNC Symbol;Acc:HGNC:16183] |
| ENSG00000101353 | HGLibA_29880 | 258 | 1.199004979 | MROH8 | maestro heat like repeat family member 8 [Source:HGNC Symbol;Acc:HGNC:16125] |
| ENSG00000105370 | HGLibA_26521 | 258 | 1.199004979 | LIM2 | lens intrinsic membrane protein 2 [Source:HGNC Symbol;Acc:HGNC:6610] |
| ENSG00000105877 | HGLibA_13474 | 258 | 1.199004979 | DNAH11 | dynein axonemal heavy chain 11 [Source:HGNC Symbol;Acc:HGNC:2942] |
| ENSG00000110442 | HGLibA_10680 | 258 | 1.199004979 | COMMD9 | COMM domain containing 9 [Source:HGNC Symbol;Acc:HGNC:25014] |
| ENSG00000110455 | HGLibA_00412 | 258 | 1.199004979 | ACCS | 1-aminocyclopropane-1-carboxylate synthase homolog (inactive) [Source:HGNC Symbol;Acc:HGNC:23989] |
| ENSG00000123561 | HGLibA_43528 | 258 | 1.199004979 | SERPINA7 | serpin family A member 7 [Source:HGNC Symbol;Acc:HGNC:11583] |
| ENSG00000125648 | HGLibA_44679 | 258 | 1.199004979 | SLC25A23 | solute carrier family 25 member 23 [Source:HGNC Symbol;Acc:HGNC:19375] |
| ENSG00000129467 | HGLibA_00918 | 258 | 1.199004979 | ADCY4 | adenylate cyclase 4 [Source:HGNC Symbol;Acc:HGNC:235] |
| ENSG00000135679 | HGLibA_28696 | 258 | 1.199004979 | MDM2 | MDM2 proto-oncogene [Source:HGNC Symbol;Acc:HGNC:6973] |
| ENSG00000136840 | HGLibA_47273 | 258 | 1.199004979 | ST6GALNAC4 | 6-sialyltransferase 4 [Source:HGNC Symbol;Acc:HGNC:17846]" |
| ENSG00000139437 | HGLibA_48808 | 258 | 1.199004979 | TCHP | trichoplein keratin filament binding [Source:HGNC Symbol;Acc:HGNC:28135] |
| ENSG00000141977 | HGLibA_09688 | 258 | 1.199004979 | CIB3 | calcium and integrin binding family member 3 [Source:HGNC Symbol;Acc:HGNC:24580] |
| ENSG00000143545 | HGLibA_39811 | 258 | 1.199004979 | RAB13 | member RAS oncogene family [Source:HGNC Symbol;Acc:HGNC:9762]" |
| ENSG00000148296 | HGLibA_47842 | 258 | 1.199004979 | SURF6 | surfeit 6 [Source:HGNC Symbol;Acc:HGNC:11478] |
| ENSG00000149115 | HGLibA_50853 | 258 | 1.199004979 | TNKS1BP1 | tankyrase 1 binding protein 1 [Source:HGNC Symbol;Acc:HGNC:19081] |
| ENSG00000155066 | HGLibA_38682 | 258 | 1.199004979 | PROM2 | prominin 2 [Source:HGNC Symbol;Acc:HGNC:20685] |
| ENSG00000165474 | HGLibA_19161 | 258 | 1.199004979 | GJB2 | gap junction protein beta 2 [Source:HGNC Symbol;Acc:HGNC:4284] |
| ENSG00000166128 | HGLibA_39988 | 258 | 1.199004979 | RAB8B | member RAS oncogene family [Source:HGNC Symbol;Acc:HGNC:30273]" |
| ENSG00000166140 | HGLibA_55276 | 258 | 1.199004979 | ZFYVE19 | zinc finger FYVE-type containing 19 [Source:HGNC Symbol;Acc:HGNC:20758] |
| ENSG00000168246 | HGLibA_52768 | 258 | 1.199004979 | UBTD2 | ubiquitin domain containing 2 [Source:HGNC Symbol;Acc:HGNC:24463] |
| ENSG00000169976 | HGLibA_43716 | 258 | 1.199004979 | SF3B5 | splicing factor 3b subunit 5 [Source:HGNC Symbol;Acc:HGNC:21083] |
| ENSG00000170390 | HGLibA_12501 | 258 | 1.199004979 | DCLK2 | doublecortin like kinase 2 [Source:HGNC Symbol;Acc:HGNC:19002] |
| ENSG00000172023 | HGLibA_40797 | 258 | 1.199004979 | REG1B | regenerating family member 1 beta [Source:HGNC Symbol;Acc:HGNC:9952] |
| ENSG00000172216 | HGLibA_08958 | 258 | 1.199004979 | CEBPB | CCAAT enhancer binding protein beta [Source:HGNC Symbol;Acc:HGNC:1834] |
| ENSG00000178860 | HGLibA_30183 | 258 | 1.199004979 | MSC | musculin [Source:HGNC Symbol;Acc:HGNC:7321] |
| ENSG00000181961 | HGLibA_33927 | 258 | 1.199004979 | OR4A16 | olfactory receptor family 4 subfamily A member 16 [Source:HGNC Symbol;Acc:HGNC:15153] |
| ENSG00000184178 | HGLibA_42897 | 258 | 1.199004979 | SCFD2 | sec1 family domain containing 2 [Source:HGNC Symbol;Acc:HGNC:30676] |
| ENSG00000185432 | HGLibA_29017 | 258 | 1.199004979 | METTL7A | methyltransferase like 7A [Source:HGNC Symbol;Acc:HGNC:24550] |
| ENSG00000185499 | HGLibA_30520 | 258 | 1.199004979 | MUC1 | cell surface associated [Source:HGNC Symbol;Acc:HGNC:7508]" |
| ENSG00000185875 | HGLibA_49357 | 258 | 1.199004979 | THNSL1 | threonine synthase like 1 [Source:HGNC Symbol;Acc:HGNC:26160] |
| ENSG00000187754 | HGLibA_47220 | 258 | 1.199004979 | SSX7 | SSX family member 7 [Source:HGNC Symbol;Acc:HGNC:19653] |
| ENSG00000188393 | HGLibA_09962 | 258 | 1.199004979 | CLEC2A | C-type lectin domain family 2 member A [Source:HGNC Symbol;Acc:HGNC:24191] |
| ENSG00000188687 | HGLibA_45224 | 258 | 1.199004979 | SLC4A5 | solute carrier family 4 member 5 [Source:HGNC Symbol;Acc:HGNC:18168] |
| ENSG00000189401 | HGLibA_34770 | 258 | 1.199004979 | OTUD6A | OTU deubiquitinase 6A [Source:HGNC Symbol;Acc:HGNC:32312] |
| ENSG00000197217 | HGLibA_15207 | 258 | 1.199004979 | ENTPD4 | ectonucleoside triphosphate diphosphohydrolase 4 [Source:HGNC Symbol;Acc:HGNC:14573] |
| ENSG00000203970 | HGLibA_12845 | 258 | 1.199004979 | DEFB110 | defensin beta 110 [Source:HGNC Symbol;Acc:HGNC:18091] |
| ENSG00000214050 | HGLibA_17034 | 258 | 1.199004979 | FBXO16 | F-box protein 16 [Source:HGNC Symbol;Acc:HGNC:13618] |
| ENSG00000224470 | HGLibA_03835 | 258 | 1.199004979 | ATXN1L | ataxin 1 like [Source:HGNC Symbol;Acc:HGNC:33279] |
| ENSG00000254415 | HGLibA_44135 | 258 | 1.199004979 | SIGLEC14 | sialic acid binding Ig like lectin 14 [Source:HGNC Symbol;Acc:HGNC:32926] |
| ENSG00000257315 | HGLibA_54802 | 258 | 1.199004979 | ZBED6 | zinc finger BED-type containing 6 [Source:HGNC Symbol;Acc:HGNC:33273] |
| ENSG00000258227 | HGLibA_10001 | 258 | 1.199004979 | CLEC5A | C-type lectin domain containing 5A [Source:HGNC Symbol;Acc:HGNC:2054] |
| ENSG00000260097 | HGLibA_46599 | 258 | 1.199004979 | SPDYE6 | speedy/RINGO cell cycle regulator family member E6 [Source:HGNC Symbol;Acc:HGNC:35465] |
| GBAS | HGLibA_18717 | 258 | 1.199004979 | GBAS | - |
| hsa-mir-3196 | HGLibA_58883 | 258 | 1.199004979 | hsa-mir-3196 | - |
| hsa-mir-4435-2 | HGLibA_60133 | 258 | 1.199004979 | hsa-mir-4435-2 | - |
| hsa-mir-490 | HGLibA_61359 | 258 | 1.199004979 | hsa-mir-490 | - |
| hsa-mir-5189 | HGLibA_61647 | 258 | 1.199004979 | hsa-mir-5189 | - |
| hsa-mir-551b | HGLibA_62121 | 258 | 1.199004979 | hsa-mir-551b | - |
| hsa-mir-6755 | HGLibA_63178 | 258 | 1.199004979 | hsa-mir-6755 | - |
| KIAA0368 | HGLibA_24630 | 258 | 1.199004979 | KIAA0368 | - |
| WHSC1 | HGLibA_54292 | 258 | 1.199004979 | WHSC1 | - |
| BAI1 | HGLibA_04114 | 257 | 1.189959637 | BAI1 | - |
| C2orf82 | HGLibA_06190 | 257 | 1.189959637 | C2orf82 | - |
| C3orf67 | HGLibA_06261 | 257 | 1.189959637 | C3orf67 | - |
| CCDC130 | HGLibA_07593 | 257 | 1.189959637 | CCDC130 | - |
| ENSG00000011600 | HGLibA_52470 | 257 | 1.189959637 | TYROBP | TYRO protein tyrosine kinase binding protein [Source:HGNC Symbol;Acc:HGNC:12449] |
| ENSG00000014641 | HGLibA_28681 | 257 | 1.189959637 | MDH1 | malate dehydrogenase 1 [Source:HGNC Symbol;Acc:HGNC:6970] |
| ENSG00000054598 | HGLibA_17823 | 257 | 1.189959637 | FOXC1 | forkhead box C1 [Source:HGNC Symbol;Acc:HGNC:3800] |
| ENSG00000063438 | HGLibA_01310 | 257 | 1.189959637 | AHRR | aryl-hydrocarbon receptor repressor [Source:HGNC Symbol;Acc:HGNC:346] |
| ENSG00000073969 | HGLibA_32751 | 257 | 1.189959637 | NSF | vesicle fusing ATPase [Source:HGNC Symbol;Acc:HGNC:8016]" |
| ENSG00000100348 | HGLibA_52402 | 257 | 1.189959637 | TXN2 | thioredoxin 2 [Source:HGNC Symbol;Acc:HGNC:17772] |
| ENSG00000111049 | HGLibA_30697 | 257 | 1.189959637 | MYF5 | myogenic factor 5 [Source:HGNC Symbol;Acc:HGNC:7565] |
| ENSG00000128266 | HGLibA_19482 | 257 | 1.189959637 | GNAZ | G protein subunit alpha z [Source:HGNC Symbol;Acc:HGNC:4395] |
| ENSG00000132716 | HGLibA_12457 | 257 | 1.189959637 | DCAF8 | DDB1 and CUL4 associated factor 8 [Source:HGNC Symbol;Acc:HGNC:24891] |
| ENSG00000134256 | HGLibA_08227 | 257 | 1.189959637 | CD101 | CD101 molecule [Source:HGNC Symbol;Acc:HGNC:5949] |
| ENSG00000136867 | HGLibA_44930 | 257 | 1.189959637 | SLC31A2 | solute carrier family 31 member 2 [Source:HGNC Symbol;Acc:HGNC:11017] |
| ENSG00000138347 | HGLibA_30911 | 257 | 1.189959637 | MYPN | myopalladin [Source:HGNC Symbol;Acc:HGNC:23246] |
| ENSG00000142173 | HGLibA_10598 | 257 | 1.189959637 | COL6A2 | collagen type VI alpha 2 chain [Source:HGNC Symbol;Acc:HGNC:2212] |
| ENSG00000147912 | HGLibA_17024 | 257 | 1.189959637 | FBXO10 | F-box protein 10 [Source:HGNC Symbol;Acc:HGNC:13589] |
| ENSG00000157103 | HGLibA_45290 | 257 | 1.189959637 | SLC6A1 | solute carrier family 6 member 1 [Source:HGNC Symbol;Acc:HGNC:11042] |
| ENSG00000157224 | HGLibA_09856 | 257 | 1.189959637 | CLDN12 | claudin 12 [Source:HGNC Symbol;Acc:HGNC:2034] |
| ENSG00000157500 | HGLibA_02566 | 257 | 1.189959637 | APPL1 | phosphotyrosine interacting with PH domain and leucine zipper 1 [Source:HGNC Symbol;Acc:HGNC:24035]" |
| ENSG00000157823 | HGLibA_02339 | 257 | 1.189959637 | AP3S2 | adaptor related protein complex 3 subunit sigma 2 [Source:HGNC Symbol;Acc:HGNC:571] |
| ENSG00000160838 | HGLibA_27420 | 257 | 1.189959637 | LRRC71 | leucine rich repeat containing 71 [Source:HGNC Symbol;Acc:HGNC:26556] |
| ENSG00000162714 | HGLibA_56100 | 257 | 1.189959637 | ZNF496 | zinc finger protein 496 [Source:HGNC Symbol;Acc:HGNC:23713] |
| ENSG00000164164 | HGLibA_34766 | 257 | 1.189959637 | OTUD4 | OTU deubiquitinase 4 [Source:HGNC Symbol;Acc:HGNC:24949] |
| ENSG00000164675 | HGLibA_23550 | 257 | 1.189959637 | IQUB | IQ motif and ubiquitin domain containing [Source:HGNC Symbol;Acc:HGNC:21995] |
| ENSG00000164683 | HGLibA_21215 | 257 | 1.189959637 | HEY1 | hes related family bHLH transcription factor with YRPW motif 1 [Source:HGNC Symbol;Acc:HGNC:4880] |
| ENSG00000167766 | HGLibA_56829 | 257 | 1.189959637 | ZNF83 | zinc finger protein 83 [Source:HGNC Symbol;Acc:HGNC:13158] |
| ENSG00000168427 | HGLibA_25179 | 257 | 1.189959637 | KLHL30 | kelch like family member 30 [Source:HGNC Symbol;Acc:HGNC:24770] |
| ENSG00000169744 | HGLibA_26214 | 257 | 1.189959637 | LDB2 | LIM domain binding 2 [Source:HGNC Symbol;Acc:HGNC:6533] |
| ENSG00000169840 | HGLibA_20508 | 257 | 1.189959637 | GSX1 | GS homeobox 1 [Source:HGNC Symbol;Acc:HGNC:20374] |
| ENSG00000170144 | HGLibA_21801 | 257 | 1.189959637 | HNRNPA3 | heterogeneous nuclear ribonucleoprotein A3 [Source:HGNC Symbol;Acc:HGNC:24941] |
| ENSG00000170748 | HGLibA_40604 | 257 | 1.189959637 | RBMXL2 | RBMX like 2 [Source:HGNC Symbol;Acc:HGNC:17886] |
| ENSG00000171189 | HGLibA_20281 | 257 | 1.189959637 | GRIK1 | glutamate ionotropic receptor kainate type subunit 1 [Source:HGNC Symbol;Acc:HGNC:4579] |
| ENSG00000171703 | HGLibA_48697 | 257 | 1.189959637 | TCEA2 | transcription elongation factor A2 [Source:HGNC Symbol;Acc:HGNC:11614] |
| ENSG00000172215 | HGLibA_11895 | 257 | 1.189959637 | CXCR6 | C-X-C motif chemokine receptor 6 [Source:HGNC Symbol;Acc:HGNC:16647] |
| ENSG00000174473 | HGLibA_18582 | 257 | 1.189959637 | GALNTL6 | polypeptide N-acetylgalactosaminyltransferase like 6 [Source:HGNC Symbol;Acc:HGNC:33844] |
| ENSG00000178187 | HGLibA_56033 | 257 | 1.189959637 | ZNF454 | zinc finger protein 454 [Source:HGNC Symbol;Acc:HGNC:21200] |
| ENSG00000179709 | HGLibA_32132 | 257 | 1.189959637 | NLRP8 | NLR family pyrin domain containing 8 [Source:HGNC Symbol;Acc:HGNC:22940] |
| ENSG00000181562 | HGLibA_14448 | 257 | 1.189959637 | EDDM3A | epididymal protein 3A [Source:HGNC Symbol;Acc:HGNC:16978] |
| ENSG00000184992 | HGLibA_04807 | 257 | 1.189959637 | BRI3BP | BRI3 binding protein [Source:HGNC Symbol;Acc:HGNC:14251] |
| ENSG00000187240 | HGLibA_14246 | 257 | 1.189959637 | DYNC2H1 | dynein cytoplasmic 2 heavy chain 1 [Source:HGNC Symbol;Acc:HGNC:2962] |
| ENSG00000187954 | HGLibA_12039 | 257 | 1.189959637 | CYHR1 | cysteine and histidine rich 1 [Source:HGNC Symbol;Acc:HGNC:17806] |
| ENSG00000188581 | HGLibA_25579 | 257 | 1.189959637 | KRTAP1-1 | keratin associated protein 1-1 [Source:HGNC Symbol;Acc:HGNC:16772] |
| ENSG00000188848 | HGLibA_04406 | 257 | 1.189959637 | BEND4 | BEN domain containing 4 [Source:HGNC Symbol;Acc:HGNC:23815] |
| ENSG00000189077 | HGLibA_49895 | 257 | 1.189959637 | TMEM120A | transmembrane protein 120A [Source:HGNC Symbol;Acc:HGNC:21697] |
| ENSG00000204052 | HGLibA_27426 | 257 | 1.189959637 | LRRC73 | leucine rich repeat containing 73 [Source:HGNC Symbol;Acc:HGNC:21375] |
| ENSG00000206535 | HGLibA_26738 | 257 | 1.189959637 | LNP1 | leukemia NUP98 fusion partner 1 [Source:HGNC Symbol;Acc:HGNC:28014] |
| ENSG00000224531 | HGLibA_45729 | 257 | 1.189959637 | SMIM13 | small integral membrane protein 13 [Source:HGNC Symbol;Acc:HGNC:27356] |
| ENSG00000255374 | HGLibA_48410 | 257 | 1.189959637 | TAS2R43 | taste 2 receptor member 43 [Source:HGNC Symbol;Acc:HGNC:18875] |
| ENSG00000265817 | HGLibA_18062 | 257 | 1.189959637 | FSBP | fibrinogen silencer binding protein [Source:HGNC Symbol;Acc:HGNC:43653] |
| hsa-mir-326 | HGLibA_58972 | 257 | 1.189959637 | hsa-mir-326 | - |
| hsa-mir-5000 | HGLibA_61409 | 257 | 1.189959637 | hsa-mir-5000 | - |
| hsa-mir-508 | HGLibA_61492 | 257 | 1.189959637 | hsa-mir-508 | - |
| hsa-mir-659 | HGLibA_62970 | 257 | 1.189959637 | hsa-mir-659 | - |
| MLL5 | HGLibA_29406 | 257 | 1.189959637 | MLL5 | - |
| PPP2R3B_Y | HGLibA_38160 | 257 | 1.189959637 | PPP2R3B_Y | - |
| SGOL1 | HGLibA_43842 | 257 | 1.189959637 | SGOL1 | - |
| ENSG00000035720 | HGLibA_47342 | 256 | 1.180914294 | STAP1 | signal transducing adaptor family member 1 [Source:HGNC Symbol;Acc:HGNC:24133] |
| ENSG00000038382 | HGLibA_51627 | 256 | 1.180914294 | TRIO | trio Rho guanine nucleotide exchange factor [Source:HGNC Symbol;Acc:HGNC:12303] |
| ENSG00000068781 | HGLibA_47547 | 256 | 1.180914294 | STON1-GTF2A1L | STON1-GTF2A1L readthrough [Source:HGNC Symbol;Acc:HGNC:30651] |

| | | | | | |
|---|---|---|---|---|---|
| ENSG00000072310 | HGLibA_46948 | 256 | 1.180914294 | SREBF1 | sterol regulatory element binding transcription factor 1 [Source:HGNC Symbol;Acc:HGNC:11289] |
| ENSG00000076864 | HGLibA_40226 | 256 | 1.180914294 | RAP1GAP | RAP1 GTPase activating protein [Source:HGNC Symbol;Acc:HGNC:9858] |
| ENSG00000083817 | HGLibA_55944 | 256 | 1.180914294 | ZNF416 | zinc finger protein 416 [Source:HGNC Symbol;Acc:HGNC:20645] |
| ENSG00000085433 | HGLibA_54107 | 256 | 1.180914294 | WDR47 | WD repeat domain 47 [Source:HGNC Symbol;Acc:HGNC:29141] |
| ENSG00000092758 | HGLibA_10626 | 256 | 1.180914294 | COL9A3 | collagen type IX alpha 3 chain [Source:HGNC Symbol;Acc:HGNC:2219] |
| ENSG00000100711 | HGLibA_55281 | 256 | 1.180914294 | ZFYVE21 | zinc finger FYVE-type containing 21 [Source:HGNC Symbol;Acc:HGNC:20760] |
| ENSG00000101916 | HGLibA_49663 | 256 | 1.180914294 | TLR8 | toll like receptor 8 [Source:HGNC Symbol;Acc:HGNC:15632] |
| ENSG00000102265 | HGLibA_49534 | 256 | 1.180914294 | TIMP1 | TIMP metallopeptidase inhibitor 1 [Source:HGNC Symbol;Acc:HGNC:11820] |
| ENSG00000104731 | HGLibA_25103 | 256 | 1.180914294 | KLHDC4 | kelch domain containing 4 [Source:HGNC Symbol;Acc:HGNC:25272] |
| ENSG00000115677 | HGLibA_21060 | 256 | 1.180914294 | HDLBP | high density lipoprotein binding protein [Source:HGNC Symbol;Acc:HGNC:4857] |
| ENSG00000116675 | HGLibA_13634 | 256 | 1.180914294 | DNAJC6 | DnaJ heat shock protein family (Hsp40) member C6 [Source:HGNC Symbol;Acc:HGNC:15469] |
| ENSG00000136848 | HGLibA_12271 | 256 | 1.180914294 | DAB2IP | DAB2 interacting protein [Source:HGNC Symbol;Acc:HGNC:17294] |
| ENSG00000143878 | HGLibA_41169 | 256 | 1.180914294 | RHOB | ras homolog family member B [Source:HGNC Symbol;Acc:HGNC:668] |
| ENSG00000144959 | HGLibA_31293 | 256 | 1.180914294 | NCEH1 | neutral cholesterol ester hydrolase 1 [Source:HGNC Symbol;Acc:HGNC:29260] |
| ENSG00000145794 | HGLibA_28846 | 256 | 1.180914294 | MEGF10 | multiple EGF like domains 10 [Source:HGNC Symbol;Acc:HGNC:29634] |
| ENSG00000147488 | HGLibA_47228 | 256 | 1.180914294 | ST18 | C2H2C-type zinc finger [Source:HGNC Symbol;Acc:HGNC:18695]" |
| ENSG00000150337 | HGLibA_17183 | 256 | 1.180914294 | FCGR1A | Fc fragment of IgG receptor Ia [Source:HGNC Symbol;Acc:HGNC:3613] |
| ENSG00000153885 | HGLibA_24428 | 256 | 1.180914294 | KCTD15 | potassium channel tetramerization domain containing 15 [Source:HGNC Symbol;Acc:HGNC:23297] |
| ENSG00000154646 | HGLibA_50632 | 256 | 1.180914294 | TMPRSS15 | transmembrane serine protease 15 [Source:HGNC Symbol;Acc:HGNC:9490] |
| ENSG00000156482 | HGLibA_41934 | 256 | 1.180914294 | RPL30 | ribosomal protein L30 [Source:HGNC Symbol;Acc:HGNC:10333] |
| ENSG00000161544 | HGLibA_12036 | 256 | 1.180914294 | CYGB | cytoglobin [Source:HGNC Symbol;Acc:HGNC:16505] |
| ENSG00000162777 | HGLibA_12945 | 256 | 1.180914294 | DENND2D | DENN domain containing 2D [Source:HGNC Symbol;Acc:HGNC:26192] |
| ENSG00000164220 | HGLibA_15869 | 256 | 1.180914294 | F2RL2 | coagulation factor II thrombin receptor like 2 [Source:HGNC Symbol;Acc:HGNC:3539] |
| ENSG00000166848 | HGLibA_49016 | 256 | 1.180914294 | TERF2IP | TERF2 interacting protein [Source:HGNC Symbol;Acc:HGNC:19246] |
| ENSG00000171723 | HGLibA_19804 | 256 | 1.180914294 | GPHN | gephyrin [Source:HGNC Symbol;Acc:HGNC:15465] |
| ENSG00000172765 | HGLibA_49773 | 256 | 1.180914294 | TMCC1 | transmembrane and coiled-coil domain family 1 [Source:HGNC Symbol;Acc:HGNC:29116] |
| ENSG00000176256 | HGLibA_21698 | 256 | 1.180914294 | HMGB4 | high mobility group box 4 [Source:HGNC Symbol;Acc:HGNC:24954] |
| ENSG00000176946 | HGLibA_49302 | 256 | 1.180914294 | THAP4 | THAP domain containing 4 [Source:HGNC Symbol;Acc:HGNC:23187] |
| ENSG00000178295 | HGLibA_18930 | 256 | 1.180914294 | GEN1 | Holliday junction 5' flap endonuclease [Source:HGNC Symbol;Acc:HGNC:26881]" |
| ENSG00000179886 | HGLibA_49476 | 256 | 1.180914294 | TIGD5 | tigger transposable element derived 5 [Source:HGNC Symbol;Acc:HGNC:18336] |
| ENSG00000180423 | HGLibA_20854 | 256 | 1.180914294 | HARBI1 | harbinger transposase derived 1 [Source:HGNC Symbol;Acc:HGNC:26522] |
| ENSG00000183862 | HGLibA_10267 | 256 | 1.180914294 | CNGA2 | cyclic nucleotide gated channel alpha 2 [Source:HGNC Symbol;Acc:HGNC:2149] |
| ENSG00000185736 | HGLibA_00875 | 256 | 1.180914294 | ADARB2 | RNA specific B2 (inactive) [Source:HGNC Symbol;Acc:HGNC:227]" |
| ENSG00000187754 | HGLibA_47218 | 256 | 1.180914294 | SSX7 | SSX family member 7 [Source:HGNC Symbol;Acc:HGNC:19653] |
| ENSG00000196371 | HGLibA_18194 | 256 | 1.180914294 | FUT4 | fucosyltransferase 4 [Source:HGNC Symbol;Acc:HGNC:4015] |
| ENSG00000196670 | HGLibA_55224 | 256 | 1.180914294 | ZFP62 | ZFP62 zinc finger protein [Source:HGNC Symbol;Acc:HGNC:23241] |
| ENSG00000198169 | HGLibA_55669 | 256 | 1.180914294 | ZNF251 | zinc finger protein 251 [Source:HGNC Symbol;Acc:HGNC:13045] |
| ENSG00000198597 | HGLibA_56184 | 256 | 1.180914294 | ZNF536 | zinc finger protein 536 [Source:HGNC Symbol;Acc:HGNC:29025] |
| H2AFB3 | HGLibA_20751 | 256 | 1.180914294 | H2AFB3 | - |
| hsa-mir-19a | HGLibA_58139 | 256 | 1.180914294 | hsa-mir-19a | - |
| hsa-mir-302b | HGLibA_58439 | 256 | 1.180914294 | hsa-mir-302b | - |
| hsa-mir-4655 | HGLibA_60727 | 256 | 1.180914294 | hsa-mir-4655 | - |
| hsa-mir-4667 | HGLibA_60785 | 256 | 1.180914294 | hsa-mir-4667 | - |
| hsa-mir-579 | HGLibA_62414 | 256 | 1.180914294 | hsa-mir-579 | - |
| ZNF812 | HGLibA_56804 | 256 | 1.180914294 | ZNF812 | - |
| C9orf9 | HGLibA_06772 | 255 | 1.171868951 | C9orf9 | - |
| ENSG00000011114 | HGLibA_04920 | 255 | 1.171868951 | BTBD7 | BTB domain containing 7 [Source:HGNC Symbol;Acc:HGNC:18269] |
| ENSG00000011114 | HGLibA_04921 | 255 | 1.171868951 | BTBD7 | BTB domain containing 7 [Source:HGNC Symbol;Acc:HGNC:18269] |
| ENSG00000042832 | HGLibA_49200 | 255 | 1.171868951 | TG | thyroglobulin [Source:HGNC Symbol;Acc:HGNC:11764] |
| ENSG00000070159 | HGLibA_39466 | 255 | 1.171868951 | PTPN3 | non-receptor type 3 [Source:HGNC Symbol;Acc:HGNC:9655]" |
| ENSG00000096395 | HGLibA_29427 | 255 | 1.171868951 | MLN | motilin [Source:HGNC Symbol;Acc:HGNC:7141] |
| ENSG00000100614 | HGLibA_37963 | 255 | 1.171868951 | PPM1A | Mg2+/Mn2+ dependent 1A [Source:HGNC Symbol;Acc:HGNC:9275]" |
| ENSG00000108671 | HGLibA_39155 | 255 | 1.171868951 | PSMD11 | non-ATPase 11 [Source:HGNC Symbol;Acc:HGNC:9556]" |
| ENSG00000114942 | HGLibA_14495 | 255 | 1.171868951 | EEF1B2 | eukaryotic translation elongation factor 1 beta 2 [Source:HGNC Symbol;Acc:HGNC:3208] |
| ENSG00000120645 | HGLibA_23546 | 255 | 1.171868951 | IQSEC3 | IQ motif and Sec7 domain 3 [Source:HGNC Symbol;Acc:HGNC:29193] |
| ENSG00000122852 | HGLibA_43766 | 255 | 1.171868951 | SFTPA1 | surfactant protein A1 [Source:HGNC Symbol;Acc:HGNC:10798] |
| ENSG00000124491 | HGLibA_15854 | 255 | 1.171868951 | F13A1 | coagulation factor XIII A chain [Source:HGNC Symbol;Acc:HGNC:3531] |
| ENSG00000130052 | HGLibA_47375 | 255 | 1.171868951 | STARD8 | StAR related lipid transfer domain containing 8 [Source:HGNC Symbol;Acc:HGNC:19161] |
| ENSG00000130513 | HGLibA_18861 | 255 | 1.171868951 | GDF15 | growth differentiation factor 15 [Source:HGNC Symbol;Acc:HGNC:30142] |
| ENSG00000131459 | HGLibA_18963 | 255 | 1.171868951 | GFPT2 | glutamine-fructose-6-phosphate transaminase 2 [Source:HGNC Symbol;Acc:HGNC:4242] |
| ENSG00000131873 | HGLibA_09656 | 255 | 1.171868951 | CHSY1 | chondroitin sulfate synthase 1 [Source:HGNC Symbol;Acc:HGNC:17198] |
| ENSG00000136068 | HGLibA_17641 | 255 | 1.171868951 | FLNB | filamin B [Source:HGNC Symbol;Acc:HGNC:3755] |
| ENSG00000140945 | HGLibA_08642 | 255 | 1.171868951 | CDH13 | cadherin 13 [Source:HGNC Symbol;Acc:HGNC:1753] |
| ENSG00000141086 | HGLibA_11685 | 255 | 1.171868951 | CTRL | chymotrypsin like [Source:HGNC Symbol;Acc:HGNC:2524] |
| ENSG00000151617 | HGLibA_14477 | 255 | 1.171868951 | EDNRA | endothelin receptor type A [Source:HGNC Symbol;Acc:HGNC:3179] |
| ENSG00000154447 | HGLibA_43971 | 255 | 1.171868951 | SH3RF1 | SH3 domain containing ring finger 1 [Source:HGNC Symbol;Acc:HGNC:17650] |
| ENSG00000159403 | HGLibA_05968 | 255 | 1.171868951 | C1R | complement C1r [Source:HGNC Symbol;Acc:HGNC:1246] |
| ENSG00000160685 | HGLibA_54918 | 255 | 1.171868951 | ZBTB7B | zinc finger and BTB domain containing 7B [Source:HGNC Symbol;Acc:HGNC:18668] |
| ENSG00000163904 | HGLibA_43389 | 255 | 1.171868951 | SENP2 | SUMO specific peptidase 2 [Source:HGNC Symbol;Acc:HGNC:23116] |
| ENSG00000164168 | HGLibA_50116 | 255 | 1.171868951 | TMEM184C | transmembrane protein 184C [Source:HGNC Symbol;Acc:HGNC:25587] |
| ENSG00000165300 | HGLibA_45524 | 255 | 1.171868951 | SLITRK5 | SLIT and NTRK like family member 5 [Source:HGNC Symbol;Acc:HGNC:20295] |
| ENSG00000166394 | HGLibA_12003 | 255 | 1.171868951 | CYB5R2 | cytochrome b5 reductase 2 [Source:HGNC Symbol;Acc:HGNC:24376] |
| ENSG00000170049 | HGLibA_24154 | 255 | 1.171868951 | KCNAB3 | potassium voltage-gated channel subfamily A regulatory beta subunit 3 [Source:HGNC Symbol;Acc:HGNC:6230] |
| ENSG00000174136 | HGLibA_41017 | 255 | 1.171868951 | RGMB | repulsive guidance molecule BMP co-receptor b [Source:HGNC Symbol;Acc:HGNC:26896] |
| ENSG00000176922 | HGLibA_34129 | 255 | 1.171868951 | OR51S1 | olfactory receptor family 51 subfamily S member 1 [Source:HGNC Symbol;Acc:HGNC:15204] |
| ENSG00000176986 | HGLibA_43234 | 255 | 1.171868951 | SEC24C | COPII coat complex component [Source:HGNC Symbol;Acc:HGNC:10705]" |
| ENSG00000177181 | HGLibA_41270 | 255 | 1.171868951 | RIMKLA | ribosomal modification protein rimK like family member A [Source:HGNC Symbol;Acc:HGNC:28725] |
| ENSG00000177689 | HGLibA_27932 | 255 | 1.171868951 | MAGEB10 | MAGE family member B10 [Source:HGNC Symbol;Acc:HGNC:25377] |
| ENSG00000180901 | HGLibA_24443 | 255 | 1.171868951 | KCTD2 | potassium channel tetramerization domain containing 2 [Source:HGNC Symbol;Acc:HGNC:21294] |
| ENSG00000185215 | HGLibA_50710 | 255 | 1.171868951 | TNFAIP2 | TNF alpha induced protein 2 [Source:HGNC Symbol;Acc:HGNC:11895] |
| ENSG00000186777 | HGLibA_56648 | 255 | 1.171868951 | ZNF732 | zinc finger protein 732 [Source:HGNC Symbol;Acc:HGNC:37138] |
| ENSG00000188452 | HGLibA_09197 | 255 | 1.171868951 | CERKL | ceramide kinase like [Source:HGNC Symbol;Acc:HGNC:21699] |
| ENSG00000197360 | HGLibA_56913 | 255 | 1.171868951 | ZNF98 | zinc finger protein 98 [Source:HGNC Symbol;Acc:HGNC:13174] |
| ENSG00000243708 | HGLibA_36904 | 255 | 1.171868951 | PLA2G4B | phospholipase A2 group IVB [Source:HGNC Symbol;Acc:HGNC:9036] |
| FAM196B | HGLibA_16383 | 255 | 1.171868951 | FAM196B | - |
| hsa-mir-1291 | HGLibA_57641 | 255 | 1.171868951 | hsa-mir-1291 | - |
| hsa-mir-330 | HGLibA_58988 | 255 | 1.171868951 | hsa-mir-330 | - |
| hsa-mir-4323 | HGLibA_60014 | 255 | 1.171868951 | hsa-mir-4323 | - |
| hsa-mir-4443 | HGLibA_60174 | 255 | 1.171868951 | hsa-mir-4443 | - |
| hsa-mir-6826 | HGLibA_63483 | 255 | 1.171868951 | hsa-mir-6826 | - |
| ENSG00000004478 | HGLibA_17575 | 254 | 1.162823608 | FKBP4 | FK506 binding protein 4 [Source:HGNC Symbol;Acc:HGNC:3720] |
| ENSG00000010244 | HGLibA_55579 | 254 | 1.162823608 | ZNF207 | zinc finger protein 207 [Source:HGNC Symbol;Acc:HGNC:12998] |
| ENSG00000029725 | HGLibA_40001 | 254 | 1.162823608 | RABEP1 | RAB GTPase binding effector protein 1 [Source:HGNC Symbol;Acc:HGNC:17677]" |
| ENSG00000034693 | HGLibA_36115 | 254 | 1.162823608 | PEX3 | peroxisomal biogenesis factor 3 [Source:HGNC Symbol;Acc:HGNC:8858] |
| ENSG00000066230 | HGLibA_45407 | 254 | 1.162823608 | SLC9A3 | solute carrier family 9 member A3 [Source:HGNC Symbol;Acc:HGNC:11073] |
| ENSG00000067842 | HGLibA_03619 | 254 | 1.162823608 | ATP2B3 | ATPase plasma membrane Ca2+ transporting 3 [Source:HGNC Symbol;Acc:HGNC:816] |
| ENSG00000090372 | HGLibA_47600 | 254 | 1.162823608 | STRN4 | striatin 4 [Source:HGNC Symbol;Acc:HGNC:15721] |
| ENSG00000099834 | HGLibA_08709 | 254 | 1.162823608 | CDHR5 | cadherin related family member 5 [Source:HGNC Symbol;Acc:HGNC:7521] |
| ENSG00000099957 | HGLibA_34860 | 254 | 1.162823608 | P2RX6 | purinergic receptor P2X 6 [Source:HGNC Symbol;Acc:HGNC:8538] |
| ENSG00000102098 | HGLibA_42961 | 254 | 1.162823608 | SCML2 | Scm polycomb group protein like 2 [Source:HGNC Symbol;Acc:HGNC:10581] |
| ENSG00000103241 | HGLibA_17864 | 254 | 1.162823608 | FOXF1 | forkhead box F1 [Source:HGNC Symbol;Acc:HGNC:3809] |
| ENSG00000104960 | HGLibA_39399 | 254 | 1.162823608 | PTOV1 | prostate tumor overexpressed 1 [Source:HGNC Symbol;Acc:HGNC:9632] |
| ENSG00000122557 | HGLibA_21173 | 254 | 1.162823608 | HERPUD2 | HERPUD family member 2 [Source:HGNC Symbol;Acc:HGNC:21915] |
| ENSG00000126903 | HGLibA_44336 | 254 | 1.162823608 | SLC10A3 | solute carrier family 10 member 3 [Source:HGNC Symbol;Acc:HGNC:22979] |
| ENSG00000127585 | HGLibA_16986 | 254 | 1.162823608 | FBXL16 | F-box and leucine rich repeat protein 16 [Source:HGNC Symbol;Acc:HGNC:14150] |
| ENSG00000129038 | HGLibA_27037 | 254 | 1.162823608 | LOXL1 | lysyl oxidase like 1 [Source:HGNC Symbol;Acc:HGNC:6665] |
| ENSG00000129484 | HGLibA_35231 | 254 | 1.162823608 | PARP2 | poly(ADP-ribose) polymerase 2 [Source:HGNC Symbol;Acc:HGNC:272] |
| ENSG00000148426 | HGLibA_38699 | 254 | 1.162823608 | PROSER2 | proline and serine rich 2 [Source:HGNC Symbol;Acc:HGNC:23728] |
| ENSG00000157890 | HGLibA_28851 | 254 | 1.162823608 | MEGF11 | multiple EGF like domains 11 [Source:HGNC Symbol;Acc:HGNC:29635] |
| ENSG00000160439 | HGLibA_40739 | 254 | 1.162823608 | RDH13 | retinol dehydrogenase 13 [Source:HGNC Symbol;Acc:HGNC:19978] |
| ENSG00000162620 | HGLibA_27458 | 254 | 1.162823608 | LRRIQ3 | leucine rich repeats and IQ motif containing 3 [Source:HGNC Symbol;Acc:HGNC:28318] |
| ENSG00000164690 | HGLibA_44031 | 254 | 1.162823608 | SHH | sonic hedgehog [Source:HGNC Symbol;Acc:HGNC:10848] |
| ENSG00000164707 | HGLibA_44393 | 254 | 1.162823608 | SLC13A4 | solute carrier family 13 member 4 [Source:HGNC Symbol;Acc:HGNC:15827] |
| ENSG00000165449 | HGLibA_44455 | 254 | 1.162823608 | SLC16A9 | solute carrier family 16 member 9 [Source:HGNC Symbol;Acc:HGNC:23520] |
| ENSG00000166130 | HGLibA_22915 | 254 | 1.162823608 | IKBIP | IKBKB interacting protein [Source:HGNC Symbol;Acc:HGNC:26430] |
| ENSG00000168411 | HGLibA_40960 | 254 | 1.162823608 | RFWD3 | ring finger and WD repeat domain 3 [Source:HGNC Symbol;Acc:HGNC:25539] |
| ENSG00000168575 | HGLibA_44532 | 254 | 1.162823608 | SLC20A2 | solute carrier family 20 member 2 [Source:HGNC Symbol;Acc:HGNC:10947] |
| ENSG00000169885 | HGLibA_07039 | 254 | 1.162823608 | CALML6 | calmodulin like 6 [Source:HGNC Symbol;Acc:HGNC:24193] |
| ENSG00000170128 | HGLibA_20004 | 254 | 1.162823608 | GPR25 | G protein-coupled receptor 25 [Source:HGNC Symbol;Acc:HGNC:4480] |
| ENSG00000171202 | HGLibA_49911 | 254 | 1.162823608 | TMEM126A | transmembrane protein 126A [Source:HGNC Symbol;Acc:HGNC:25382] |
| ENSG00000171903 | HGLibA_12181 | 254 | 1.162823608 | CYP4F11 | cytochrome P450 family 4 subfamily F member 11 [Source:HGNC Symbol;Acc:HGNC:13265] |
| ENSG00000175938 | HGLibA_34595 | 254 | 1.162823608 | ORAI3 | ORAI calcium release-activated calcium modulator 3 [Source:HGNC Symbol;Acc:HGNC:28185] |
| ENSG00000177058 | HGLibA_45085 | 254 | 1.162823608 | SLC38A9 | solute carrier family 38 member 9 [Source:HGNC Symbol;Acc:HGNC:26907] |
| ENSG00000181472 | HGLibA_54834 | 254 | 1.162823608 | ZBTB2 | zinc finger and BTB domain containing 2 [Source:HGNC Symbol;Acc:HGNC:20868] |
| ENSG00000181817 | HGLibA_27520 | 254 | 1.162823608 | LSM10 | U7 small nuclear RNA associated [Source:HGNC Symbol;Acc:HGNC:17562]" |
| ENSG00000182255 | HGLibA_24135 | 254 | 1.162823608 | KCNA4 | potassium voltage-gated channel subfamily A member 4 [Source:HGNC Symbol;Acc:HGNC:6222] |
| ENSG00000183287 | HGLibA_07493 | 254 | 1.162823608 | CCBE1 | collagen and calcium binding EGF domains 1 [Source:HGNC Symbol;Acc:HGNC:29426] |
| ENSG00000183496 | HGLibA_29030 | 254 | 1.162823608 | MEX3B | mex-3 RNA binding family member B [Source:HGNC Symbol;Acc:HGNC:25297] |
| ENSG00000185800 | HGLibA_13449 | 254 | 1.162823608 | DMWD | WD repeat containing [Source:HGNC Symbol;Acc:HGNC:2936]" |
| ENSG00000198816 | HGLibA_55871 | 254 | 1.162823608 | ZNF358 | zinc finger protein 358 [Source:HGNC Symbol;Acc:HGNC:16838] |
| ENSG00000205927 | HGLibA_33371 | 254 | 1.162823608 | OLIG2 | oligodendrocyte transcription factor 2 [Source:HGNC Symbol;Acc:HGNC:9398] |
| ENSG00000213614 | HGLibA_21202 | 254 | 1.162823608 | HEXA | hexosaminidase subunit alpha [Source:HGNC Symbol;Acc:HGNC:4878] |
| ENSG00000215397 | HGLibA_43061 | 254 | 1.162823608 | SCRT2 | scratch family transcriptional repressor 2 [Source:HGNC Symbol;Acc:HGNC:15952] |
| ENSG00000253276 | HGLibA_07856 | 254 | 1.162823608 | CCDC71L | coiled-coil domain containing 71 like [Source:HGNC Symbol;Acc:HGNC:26685] |
| ENSG00000269858 | HGLibA_14642 | 254 | 1.162823608 | EGLN2 | egl-9 family hypoxia inducible factor 2 [Source:HGNC Symbol;Acc:HGNC:14660] |

| | | | | | |
|---|---|---|---|---|---|
| ENSG00000272636 | HGLibA_13728 | 254 | 1.162823608 | DOC2B | double C2 domain beta [Source:HGNC Symbol;Acc:HGNC:2986] |
| hsa-mir-1293 | HGLibA_57648 | 254 | 1.162823608 | hsa-mir-1293 | - |
| hsa-mir-3139 | HGLibA_58615 | 254 | 1.162823608 | hsa-mir-3139 | - |
| hsa-mir-32 | HGLibA_58905 | 254 | 1.162823608 | hsa-mir-32 | - |
| hsa-mir-888 | HGLibA_64232 | 254 | 1.162823608 | hsa-mir-888 | - |
| CNTD2 | HGLibA_10389 | 253 | 1.153778265 | CNTD2 | - |
| ENSG00000011523 | HGLibA_09155 | 253 | 1.153778265 | CEP68 | centrosomal protein 68 [Source:HGNC Symbol;Acc:HGNC:29076] |
| ENSG00000012963 | HGLibA_52761 | 253 | 1.153778265 | UBR7 | ubiquitin protein ligase E3 component n-recognin 7 (putative) [Source:HGNC Symbol;Acc:HGNC:20344] |
| ENSG00000050344 | HGLibA_31798 | 253 | 1.153778265 | NFE2L3 | erythroid 2 like 3 [Source:HGNC Symbol;Acc:HGNC:7783]" |
| ENSG00000073150 | HGLibA_35112 | 253 | 1.153778265 | PANX2 | pannexin 2 [Source:HGNC Symbol;Acc:HGNC:8600] |
| ENSG00000086300 | HGLibA_46027 | 253 | 1.153778265 | SNX10 | sorting nexin 10 [Source:HGNC Symbol;Acc:HGNC:14974] |
| ENSG00000092108 | HGLibA_42892 | 253 | 1.153778265 | SCFD1 | sec1 family domain containing 1 [Source:HGNC Symbol;Acc:HGNC:20726] |
| ENSG00000096070 | HGLibA_04841 | 253 | 1.153778265 | BRPF3 | bromodomain and PHD finger containing 3 [Source:HGNC Symbol;Acc:HGNC:14256] |
| ENSG00000101144 | HGLibA_04616 | 253 | 1.153778265 | BMP7 | bone morphogenetic protein 7 [Source:HGNC Symbol;Acc:HGNC:1074] |
| ENSG00000102076 | HGLibA_33421 | 253 | 1.153778265 | OPN1LW | long wave sensitive [Source:HGNC Symbol;Acc:HGNC:9936]" |
| ENSG00000103257 | HGLibA_45369 | 253 | 1.153778265 | SLC7A5 | solute carrier family 7 member 5 [Source:HGNC Symbol;Acc:HGNC:11063] |
| ENSG00000104413 | HGLibA_15588 | 253 | 1.153778265 | ESRP1 | epithelial splicing regulatory protein 1 [Source:HGNC Symbol;Acc:HGNC:25966] |
| ENSG00000105072 | HGLibA_05664 | 253 | 1.153778265 | C19orf44 | chromosome 19 open reading frame 44 [Source:HGNC Symbol;Acc:HGNC:26141] |
| ENSG00000106554 | HGLibA_09367 | 253 | 1.153778265 | CHCHD3 | coiled-coil-helix-coiled-coil-helix domain containing 3 [Source:HGNC Symbol;Acc:HGNC:21906] |
| ENSG00000112337 | HGLibA_44461 | 253 | 1.153778265 | SLC17A2 | solute carrier family 17 member 2 [Source:HGNC Symbol;Acc:HGNC:10930] |
| ENSG00000117592 | HGLibA_38422 | 253 | 1.153778265 | PRDX6 | peroxiredoxin 6 [Source:HGNC Symbol;Acc:HGNC:16753] |
| ENSG00000119326 | HGLibA_11648 | 253 | 1.153778265 | CTNNAL1 | catenin alpha like 1 [Source:HGNC Symbol;Acc:HGNC:2512] |
| ENSG00000120889 | HGLibA_50736 | 253 | 1.153778265 | TNFRSF10B | TNF receptor superfamily member 10b [Source:HGNC Symbol;Acc:HGNC:11905] |
| ENSG00000125089 | HGLibA_43978 | 253 | 1.153778265 | SH3TC1 | SH3 domain and tetratricopeptide repeats 1 [Source:HGNC Symbol;Acc:HGNC:26009] |
| ENSG00000125888 | HGLibA_04144 | 253 | 1.153778265 | BANF2 | barrier to autointegration factor 2 [Source:HGNC Symbol;Acc:HGNC:16172] |
| ENSG00000127585 | HGLibA_16984 | 253 | 1.153778265 | FBXL16 | F-box and leucine rich repeat protein 16 [Source:HGNC Symbol;Acc:HGNC:14150] |
| ENSG00000129151 | HGLibA_04203 | 253 | 1.153778265 | BBOX1 | gamma-butyrobetaine hydroxylase 1 [Source:HGNC Symbol;Acc:HGNC:964] |
| ENSG00000129214 | HGLibA_43997 | 253 | 1.153778265 | SHBG | sex hormone binding globulin [Source:HGNC Symbol;Acc:HGNC:10839] |
| ENSG00000134640 | HGLibA_30447 | 253 | 1.153778265 | MTNR1B | melatonin receptor 1B [Source:HGNC Symbol;Acc:HGNC:7464] |
| ENSG00000137331 | HGLibA_22575 | 253 | 1.153778265 | IER3 | immediate early response 3 [Source:HGNC Symbol;Acc:HGNC:5392] |
| ENSG00000138031 | HGLibA_00913 | 253 | 1.153778265 | ADCY3 | adenylate cyclase 3 [Source:HGNC Symbol;Acc:HGNC:234] |
| ENSG00000138380 | HGLibA_07244 | 253 | 1.153778265 | CARF | calcium responsive transcription factor [Source:HGNC Symbol;Acc:HGNC:14435] |
| ENSG00000138413 | HGLibA_22539 | 253 | 1.153778265 | IDH1 | cytosolic [Source:HGNC Symbol;Acc:HGNC:5382]" |
| ENSG00000145824 | HGLibA_11858 | 253 | 1.153778265 | CXCL14 | C-X-C motif chemokine ligand 14 [Source:HGNC Symbol;Acc:HGNC:10640] |
| ENSG00000149485 | HGLibA_15933 | 253 | 1.153778265 | FADS1 | fatty acid desaturase 1 [Source:HGNC Symbol;Acc:HGNC:3574] |
| ENSG00000153707 | HGLibA_39495 | 253 | 1.153778265 | PTPRD | receptor type D [Source:HGNC Symbol;Acc:HGNC:9668]" |
| ENSG00000156136 | HGLibA_12497 | 253 | 1.153778265 | DCK | deoxycytidine kinase [Source:HGNC Symbol;Acc:HGNC:2704] |
| ENSG00000157450 | HGLibA_41466 | 253 | 1.153778265 | RNF111 | ring finger protein 111 [Source:HGNC Symbol;Acc:HGNC:17384] |
| ENSG00000160932 | HGLibA_27655 | 253 | 1.153778265 | LY6E | lymphocyte antigen 6 family member E [Source:HGNC Symbol;Acc:HGNC:6727] |
| ENSG00000163006 | HGLibA_07611 | 253 | 1.153778265 | CCDC138 | coiled-coil domain containing 138 [Source:HGNC Symbol;Acc:HGNC:26531] |
| ENSG00000163126 | HGLibA_02042 | 253 | 1.153778265 | ANKRD23 | ankyrin repeat domain 23 [Source:HGNC Symbol;Acc:HGNC:24470] |
| ENSG00000163508 | HGLibA_15227 | 253 | 1.153778265 | EOMES | eomesodermin [Source:HGNC Symbol;Acc:HGNC:3372] |
| ENSG00000164828 | HGLibA_47788 | 253 | 1.153778265 | SUN1 | Sad1 and UNC84 domain containing 1 [Source:HGNC Symbol;Acc:HGNC:18587] |
| ENSG00000167552 | HGLibA_52243 | 253 | 1.153778265 | TUBA1A | tubulin alpha 1a [Source:HGNC Symbol;Acc:HGNC:20766] |
| ENSG00000172831 | HGLibA_09220 | 253 | 1.153778265 | CES2 | carboxylesterase 2 [Source:HGNC Symbol;Acc:HGNC:1864] |
| ENSG00000176160 | HGLibA_22233 | 253 | 1.153778265 | HSF5 | heat shock transcription factor 5 [Source:HGNC Symbol;Acc:HGNC:26862] |
| ENSG00000177556 | HGLibA_03542 | 253 | 1.153778265 | ATOX1 | antioxidant 1 copper chaperone [Source:HGNC Symbol;Acc:HGNC:798] |
| ENSG00000178828 | HGLibA_41595 | 253 | 1.153778265 | RNF186 | ring finger protein 186 [Source:HGNC Symbol;Acc:HGNC:25978] |
| ENSG00000185127 | HGLibA_06441 | 253 | 1.153778265 | C6orf120 | chromosome 6 open reading frame 120 [Source:HGNC Symbol;Acc:HGNC:21247] |
| ENSG00000186395 | HGLibA_25417 | 253 | 1.153778265 | KRT10 | keratin 10 [Source:HGNC Symbol;Acc:HGNC:6413] |
| ENSG00000186458 | HGLibA_12899 | 253 | 1.153778265 | DEFB132 | defensin beta 132 [Source:HGNC Symbol;Acc:HGNC:33806] |
| ENSG00000187790 | HGLibA_16860 | 253 | 1.153778265 | FANCM | FA complementation group M [Source:HGNC Symbol;Acc:HGNC:23168] |
| ENSG00000196734 | HGLibA_26104 | 253 | 1.153778265 | LCE1B | late cornified envelope 1B [Source:HGNC Symbol;Acc:HGNC:16611] |
| ENSG00000197647 | HGLibA_55987 | 253 | 1.153778265 | ZNF433 | zinc finger protein 433 [Source:HGNC Symbol;Acc:HGNC:20811] |
| ENSG00000204421 | HGLibA_27666 | 253 | 1.153778265 | LY6G6C | lymphocyte antigen 6 family member G6C [Source:HGNC Symbol;Acc:HGNC:13936] |
| ENSG00000205189 | HGLibA_54813 | 253 | 1.153778265 | ZBTB10 | zinc finger and BTB domain containing 10 [Source:HGNC Symbol;Acc:HGNC:30953] |
| ENSG00000213931 | HGLibA_20919 | 253 | 1.153778265 | HBE1 | hemoglobin subunit epsilon 1 [Source:HGNC Symbol;Acc:HGNC:4830] |
| ENSG00000221840 | HGLibA_33933 | 253 | 1.153778265 | OR4A5 | olfactory receptor family 4 subfamily A member 5 [Source:HGNC Symbol;Acc:HGNC:15162] |
| ENSG00000237190 | HGLibA_08837 | 253 | 1.153778265 | CDKN2AIPNL | CDKN2A interacting protein N-terminal like [Source:HGNC Symbol;Acc:HGNC:30545] |
| ENSG00000268988 | HGLibA_46404 | 253 | 1.153778265 | SPANXN2 | SPANX family member N2 [Source:HGNC Symbol;Acc:HGNC:33175] |
| GTF2H2D | HGLibA_20552 | 253 | 1.153778265 | GTF2H2D | - |
| H2AFV | HGLibA_20756 | 253 | 1.153778265 | H2AFV | - |
| hsa-mir-147b | HGLibA_57877 | 253 | 1.153778265 | hsa-mir-147b | - |
| hsa-mir-30e | HGLibA_58490 | 253 | 1.153778265 | hsa-mir-30e | - |
| hsa-mir-4649 | HGLibA_60700 | 253 | 1.153778265 | hsa-mir-4649 | - |
| hsa-mir-4694 | HGLibA_60886 | 253 | 1.153778265 | hsa-mir-4694 | - |
| hsa-mir-4745 | HGLibA_61079 | 253 | 1.153778265 | hsa-mir-4745 | - |
| hsa-mir-6863 | HGLibA_63637 | 253 | 1.153778265 | hsa-mir-6863 | - |
| METTL12 | HGLibA_28951 | 253 | 1.153778265 | METTL12 | - |
| TSSC1 | HGLibA_52002 | 253 | 1.153778265 | TSSC1 | - |
| CCDC67 | HGLibA_07837 | 252 | 1.144732922 | CCDC67 | - |
| ENSG00000012822 | HGLibA_06997 | 252 | 1.144732922 | CALCOCO1 | calcium binding and coiled-coil domain 1 [Source:HGNC Symbol;Acc:HGNC:29306] |
| ENSG00000054803 | HGLibA_07414 | 252 | 1.144732922 | CBLN4 | cerebellin 4 precursor [Source:HGNC Symbol;Acc:HGNC:16231] |
| ENSG00000087995 | HGLibA_29000 | 252 | 1.144732922 | METTL2A | methyltransferase like 2A [Source:HGNC Symbol;Acc:HGNC:25755] |
| ENSG00000105202 | HGLibA_16938 | 252 | 1.144732922 | FBL | fibrillarin [Source:HGNC Symbol;Acc:HGNC:3599] |
| ENSG00000105708 | HGLibA_55481 | 252 | 1.144732922 | ZNF14 | zinc finger protein 14 [Source:HGNC Symbol;Acc:HGNC:12924] |
| ENSG00000111679 | HGLibA_39475 | 252 | 1.144732922 | PTPN6 | non-receptor type 6 [Source:HGNC Symbol;Acc:HGNC:9658]" |
| ENSG00000112214 | HGLibA_17490 | 252 | 1.144732922 | FHL5 | four and a half LIM domains 5 [Source:HGNC Symbol;Acc:HGNC:17371] |
| ENSG00000113924 | HGLibA_21236 | 252 | 1.144732922 | HGD | 2-dioxygenase [Source:HGNC Symbol;Acc:HGNC:4892]" |
| ENSG00000116329 | HGLibA_33444 | 252 | 1.144732922 | OPRD1 | opioid receptor delta 1 [Source:HGNC Symbol;Acc:HGNC:8153] |
| ENSG00000117020 | HGLibA_01515 | 252 | 1.144732922 | AKT3 | AKT serine/threonine kinase 3 [Source:HGNC Symbol;Acc:HGNC:393] |
| ENSG00000120053 | HGLibA_19689 | 252 | 1.144732922 | GOT1 | glutamic-oxaloacetic transaminase 1 [Source:HGNC Symbol;Acc:HGNC:4432] |
| ENSG00000124370 | HGLibA_28575 | 252 | 1.144732922 | MCEE | methylmalonyl-CoA epimerase [Source:HGNC Symbol;Acc:HGNC:16732] |
| ENSG00000129244 | HGLibA_03596 | 252 | 1.144732922 | ATP1B2 | ATPase Na+/K+ transporting subunit beta 2 [Source:HGNC Symbol;Acc:HGNC:805] |
| ENSG00000133243 | HGLibA_04911 | 252 | 1.144732922 | BTBD2 | BTB domain containing 2 [Source:HGNC Symbol;Acc:HGNC:15504] |
| ENSG00000139083 | HGLibA_15665 | 252 | 1.144732922 | ETV6 | ETS variant 6 [Source:HGNC Symbol;Acc:HGNC:3495] |
| ENSG00000139540 | HGLibA_45115 | 252 | 1.144732922 | SLC39A5 | solute carrier family 39 member 5 [Source:HGNC Symbol;Acc:HGNC:20502] |
| ENSG00000139549 | HGLibA_13109 | 252 | 1.144732922 | DHH | desert hedgehog [Source:HGNC Symbol;Acc:HGNC:2865] |
| ENSG00000143195 | HGLibA_23210 | 252 | 1.144732922 | ILDR2 | immunoglobulin like domain containing receptor 2 [Source:HGNC Symbol;Acc:HGNC:18131] |
| ENSG00000144283 | HGLibA_36860 | 252 | 1.144732922 | PKP4 | plakophilin 4 [Source:HGNC Symbol;Acc:HGNC:9026] |
| ENSG00000146648 | HGLibA_14635 | 252 | 1.144732922 | EGFR | epidermal growth factor receptor [Source:HGNC Symbol;Acc:HGNC:3236] |
| ENSG00000149489 | HGLibA_41755 | 252 | 1.144732922 | ROM1 | retinal outer segment membrane protein 1 [Source:HGNC Symbol;Acc:HGNC:10254] |
| ENSG00000157540 | HGLibA_14272 | 252 | 1.144732922 | DYRK1A | dual specificity tyrosine phosphorylation regulated kinase 1A [Source:HGNC Symbol;Acc:HGNC:3091] |
| ENSG00000157927 | HGLibA_40113 | 252 | 1.144732922 | RADIL | Rap associating with DIL domain [Source:HGNC Symbol;Acc:HGNC:22226] |
| ENSG00000159374 | HGLibA_27812 | 252 | 1.144732922 | M1AP | meiosis 1 associated protein [Source:HGNC Symbol;Acc:HGNC:25183] |
| ENSG00000164741 | HGLibA_13311 | 252 | 1.144732922 | DLC1 | DLC1 Rho GTPase activating protein [Source:HGNC Symbol;Acc:HGNC:2897] |
| ENSG00000165566 | HGLibA_01776 | 252 | 1.144732922 | AMER2 | APC membrane recruitment protein 2 [Source:HGNC Symbol;Acc:HGNC:26360] |
| ENSG00000166359 | HGLibA_54207 | 252 | 1.144732922 | WDR88 | WD repeat domain 88 [Source:HGNC Symbol;Acc:HGNC:26999] |
| ENSG00000166948 | HGLibA_49267 | 252 | 1.144732922 | TGM6 | transglutaminase 6 [Source:HGNC Symbol;Acc:HGNC:16255] |
| ENSG00000168269 | HGLibA_17876 | 252 | 1.144732922 | FOXI1 | forkhead box I1 [Source:HGNC Symbol;Acc:HGNC:3815] |
| ENSG00000169919 | HGLibA_20679 | 252 | 1.144732922 | GUSB | glucuronidase beta [Source:HGNC Symbol;Acc:HGNC:4696] |
| ENSG00000172354 | HGLibA_19491 | 252 | 1.144732922 | GNB2 | G protein subunit beta 2 [Source:HGNC Symbol;Acc:HGNC:4398] |
| ENSG00000172752 | HGLibA_10606 | 252 | 1.144732922 | COL6A5 | collagen type VI alpha 5 chain [Source:HGNC Symbol;Acc:HGNC:26674] |
| ENSG00000173572 | HGLibA_32109 | 252 | 1.144732922 | NLRP13 | NLR family pyrin domain containing 13 [Source:HGNC Symbol;Acc:HGNC:22937] |
| ENSG00000176900 | HGLibA_34134 | 252 | 1.144732922 | OR51T1 | olfactory receptor family 51 subfamily T member 1 [Source:HGNC Symbol;Acc:HGNC:15205] |
| ENSG00000179965 | HGLibA_56707 | 252 | 1.144732922 | ZNF771 | zinc finger protein 771 [Source:HGNC Symbol;Acc:HGNC:29653] |
| ENSG00000181019 | HGLibA_32562 | 252 | 1.144732922 | NQO1 | NAD(P)H quinone dehydrogenase 1 [Source:HGNC Symbol;Acc:HGNC:2874] |
| ENSG00000182898 | HGLibA_48806 | 252 | 1.144732922 | TCHHL1 | trichohyalin like 1 [Source:HGNC Symbol;Acc:HGNC:31796] |
| ENSG00000183878 | HGLibA_53471 | 252 | 1.144732922 | UTY | Y-linked [Source:HGNC Symbol;Acc:HGNC:12638]" |
| ENSG00000184840 | HGLibA_49829 | 252 | 1.144732922 | TMED9 | transmembrane p24 trafficking protein 9 [Source:HGNC Symbol;Acc:HGNC:24878] |
| ENSG00000186591 | HGLibA_52612 | 252 | 1.144732922 | UBE2H | ubiquitin conjugating enzyme E2 H [Source:HGNC Symbol;Acc:HGNC:12484] |
| ENSG00000187170 | HGLibA_26144 | 252 | 1.144732922 | LCE4A | late cornified envelope 4A [Source:HGNC Symbol;Acc:HGNC:16613] |
| ENSG00000196267 | HGLibA_56842 | 252 | 1.144732922 | ZNF836 | zinc finger protein 836 [Source:HGNC Symbol;Acc:HGNC:34333] |
| ENSG00000198692 | HGLibA_14714 | 252 | 1.144732922 | EIF1AY | eukaryotic translation initiation factor 1A Y-linked [Source:HGNC Symbol;Acc:HGNC:3252] |
| ENSG00000204946 | HGLibA_56744 | 252 | 1.144732922 | ZNF783 | zinc finger family member 783 [Source:HGNC Symbol;Acc:HGNC:27222] |
| ENSG00000205593 | HGLibA_12968 | 252 | 1.144732922 | DENND6B | DENN domain containing 6B [Source:HGNC Symbol;Acc:HGNC:32690] |
| ENSG00000214681 | HGLibA_23510 | 252 | 1.144732922 | IQCF5 | IQ motif containing F5 [Source:HGNC Symbol;Acc:HGNC:35159] |
| ENSG00000251192 | HGLibA_56510 | 252 | 1.144732922 | ZNF674 | zinc finger protein 674 [Source:HGNC Symbol;Acc:HGNC:17625] |
| ENSG00000257017 | HGLibA_22011 | 252 | 1.144732922 | HP | haptoglobin [Source:HGNC Symbol;Acc:HGNC:5141] |
| hsa-mir-18b | HGLibA_58023 | 252 | 1.144732922 | hsa-mir-18b | - |
| hsa-mir-4526 | HGLibA_60566 | 252 | 1.144732922 | hsa-mir-4526 | - |
| hsa-mir-95 | HGLibA_64367 | 252 | 1.144732922 | hsa-mir-95 | - |
| CXorf64 | HGLibA_11956 | 251 | 1.135687579 | CXorf64 | - |
| ENSG00000010318 | HGLibA_36387 | 251 | 1.135687579 | PHF7 | PHD finger protein 7 [Source:HGNC Symbol;Acc:HGNC:18458] |
| ENSG00000012061 | HGLibA_15430 | 251 | 1.135687579 | ERCC1 | endonuclease non-catalytic subunit [Source:HGNC Symbol;Acc:HGNC:3433]" |
| ENSG00000069812 | HGLibA_21179 | 251 | 1.135687579 | HES2 | hes family bHLH transcription factor 2 [Source:HGNC Symbol;Acc:HGNC:16005] |
| ENSG00000078304 | HGLibA_38177 | 251 | 1.135687579 | PPP2R5C | protein phosphatase 2 regulatory subunit B'gamma [Source:HGNC Symbol;Acc:HGNC:9311] |
| ENSG00000090659 | HGLibA_08293 | 251 | 1.135687579 | CD209 | CD209 molecule [Source:HGNC Symbol;Acc:HGNC:1641] |
| ENSG00000093010 | HGLibA_10687 | 251 | 1.135687579 | COMT | catechol-O-methyltransferase [Source:HGNC Symbol;Acc:HGNC:2228] |
| ENSG00000100596 | HGLibA_46894 | 251 | 1.135687579 | SPTLC2 | serine palmitoyltransferase long chain base subunit 2 [Source:HGNC Symbol;Acc:HGNC:11278] |
| ENSG00000102871 | HGLibA_51236 | 251 | 1.135687579 | TRADD | TNFRSF1A associated via death domain [Source:HGNC Symbol;Acc:HGNC:12030] |
| ENSG00000105287 | HGLibA_38570 | 251 | 1.135687579 | PRKD2 | protein kinase D2 [Source:HGNC Symbol;Acc:HGNC:17293] |
| ENSG00000105989 | HGLibA_54359 | 251 | 1.135687579 | WNT2 | Wnt family member 2 [Source:HGNC Symbol;Acc:HGNC:12780] |
| ENSG00000107593 | HGLibA_36801 | 251 | 1.135687579 | PKD2L1 | transient receptor potential cation channel [Source:HGNC Symbol;Acc:HGNC:9011]" |
| ENSG00000107779 | HGLibA_04630 | 251 | 1.135687579 | BMPR1A | bone morphogenetic protein receptor type 1A [Source:HGNC Symbol;Acc:HGNC:1076] |

| | | | | | |
|---|---|---|---|---|---|
| ENSG00000109667 | HGLibA_44894 | 251 | 1.135687579 | SLC2A9 | solute carrier family 2 member 9 [Source:HGNC Symbol;Acc:HGNC:13446] |
| ENSG00000114638 | HGLibA_53071 | 251 | 1.135687579 | UPK1B | uroplakin 1B [Source:HGNC Symbol;Acc:HGNC:12578] |
| ENSG00000120329 | HGLibA_44667 | 251 | 1.135687579 | SLC25A2 | solute carrier family 25 member 2 [Source:HGNC Symbol;Acc:HGNC:22921] |
| ENSG00000130511 | HGLibA_47124 | 251 | 1.135687579 | SSBP4 | single stranded DNA binding protein 4 [Source:HGNC Symbol;Acc:HGNC:15676] |
| ENSG00000130943 | HGLibA_36811 | 251 | 1.135687579 | PKDREJ | polycystin family receptor for egg jelly [Source:HGNC Symbol;Acc:HGNC:9015] |
| ENSG00000133812 | HGLibA_42798 | 251 | 1.135687579 | SBF2 | SET binding factor 2 [Source:HGNC Symbol;Acc:HGNC:2135] |
| ENSG00000137225 | HGLibA_07147 | 251 | 1.135687579 | CAPN11 | calpain 11 [Source:HGNC Symbol;Acc:HGNC:1478] |
| ENSG00000140043 | HGLibA_39355 | 251 | 1.135687579 | PTGR2 | prostaglandin reductase 2 [Source:HGNC Symbol;Acc:HGNC:20149] |
| ENSG00000140391 | HGLibA_51926 | 251 | 1.135687579 | TSPAN3 | tetraspanin 3 [Source:HGNC Symbol;Acc:HGNC:17752] |
| ENSG00000142156 | HGLibA_10596 | 251 | 1.135687579 | COL6A1 | collagen type VI alpha 1 chain [Source:HGNC Symbol;Acc:HGNC:2211] |
| ENSG00000160714 | HGLibA_52646 | 251 | 1.135687579 | UBE2Q1 | ubiquitin conjugating enzyme E2 Q1 [Source:HGNC Symbol;Acc:HGNC:15698] |
| ENSG00000160908 | HGLibA_55910 | 251 | 1.135687579 | ZNF394 | zinc finger protein 394 [Source:HGNC Symbol;Acc:HGNC:18832] |
| ENSG00000162819 | HGLibA_04836 | 251 | 1.135687579 | BROX | BRO1 domain and CAAX motif containing [Source:HGNC Symbol;Acc:HGNC:26512] |
| ENSG00000166532 | HGLibA_41275 | 251 | 1.135687579 | RIMKLB | ribosomal modification protein rimK like family member B [Source:HGNC Symbol;Acc:HGNC:29228] |
| ENSG00000170631 | HGLibA_55509 | 251 | 1.135687579 | ZNF16 | zinc finger protein 16 [Source:HGNC Symbol;Acc:HGNC:12947] |
| ENSG00000171517 | HGLibA_27060 | 251 | 1.135687579 | LPAR3 | lysophosphatidic acid receptor 3 [Source:HGNC Symbol;Acc:HGNC:14298] |
| ENSG00000171940 | HGLibA_55600 | 251 | 1.135687579 | ZNF217 | zinc finger protein 217 [Source:HGNC Symbol;Acc:HGNC:13009] |
| ENSG00000172459 | HGLibA_34247 | 251 | 1.135687579 | OR5AR1 | olfactory receptor family 5 subfamily AR member 1 (gene/pseudogene) [Source:HGNC Symbol;Acc:HGNC:15260] |
| ENSG00000178795 | HGLibA_18899 | 251 | 1.135687579 | GDPD4 | glycerophosphodiester phosphodiesterase domain containing 4 [Source:HGNC Symbol;Acc:HGNC:24849] |
| ENSG00000180483 | HGLibA_12867 | 251 | 1.135687579 | DEFB119 | defensin beta 119 [Source:HGNC Symbol;Acc:HGNC:18099] |
| ENSG00000181634 | HGLibA_50817 | 251 | 1.135687579 | TNFSF15 | TNF superfamily member 15 [Source:HGNC Symbol;Acc:HGNC:11931] |
| ENSG00000182938 | HGLibA_34742 | 251 | 1.135687579 | OTOP3 | otopetrin 3 [Source:HGNC Symbol;Acc:HGNC:19658] |
| ENSG00000183454 | HGLibA_20300 | 251 | 1.135687579 | GRIN2A | glutamate ionotropic receptor NMDA type subunit 2A [Source:HGNC Symbol;Acc:HGNC:4585] |
| ENSG00000196734 | HGLibA_26103 | 251 | 1.135687579 | LCE1B | late cornified envelope 1B [Source:HGNC Symbol;Acc:HGNC:16611] |
| ENSG00000198142 | HGLibA_46220 | 251 | 1.135687579 | SOWAHC | sosondowah ankyrin repeat domain family member C [Source:HGNC Symbol;Acc:HGNC:26149] |
| ENSG00000214944 | HGLibA_02842 | 251 | 1.135687579 | ARHGEF28 | Rho guanine nucleotide exchange factor 28 [Source:HGNC Symbol;Acc:HGNC:30322] |
| ENSG00000254737 | HGLibA_33495 | 251 | 1.135687579 | OR10G4 | olfactory receptor family 10 subfamily G member 4 [Source:HGNC Symbol;Acc:HGNC:14809] |
| ENSG00000255302 | HGLibA_14694 | 251 | 1.135687579 | EID1 | EP300 interacting inhibitor of differentiation 1 [Source:HGNC Symbol;Acc:HGNC:1191] |
| FAM195A | HGLibA_16374 | 251 | 1.135687579 | FAM195A | - |
| hsa-mir-1227 | HGLibA_57305 | 251 | 1.135687579 | hsa-mir-1227 | - |
| hsa-mir-4276 | HGLibA_59806 | 251 | 1.135687579 | hsa-mir-4276 | - |
| hsa-mir-500a | HGLibA_61443 | 251 | 1.135687579 | hsa-mir-500a | - |
| hsa-mir-548f-2 | HGLibA_61975 | 251 | 1.135687579 | hsa-mir-548f-2 | - |
| hsa-mir-6124 | HGLibA_62644 | 251 | 1.135687579 | hsa-mir-6124 | - |
| 11-Sep | HGLibA_43430 | 251 | 1.135687579 | 11-Sep | - |
| SGK196 | HGLibA_43824 | 251 | 1.135687579 | SGK196 | - |
| ZUFSP | HGLibA_57062 | 251 | 1.135687579 | ZUFSP | - |
| ENSG00000008735 | HGLibA_28290 | 250 | 1.126642236 | MAPK8IP2 | mitogen-activated protein kinase 8 interacting protein 2 [Source:HGNC Symbol;Acc:HGNC:6883] |
| ENSG00000015153 | HGLibA_54628 | 250 | 1.126642236 | YAF2 | YY1 associated factor 2 [Source:HGNC Symbol;Acc:HGNC:17363] |
| ENSG00000015532 | HGLibA_54623 | 250 | 1.126642236 | XYLT2 | xylosyltransferase 2 [Source:HGNC Symbol;Acc:HGNC:15517] |
| ENSG00000016602 | HGLibA_09812 | 250 | 1.126642236 | CLCA4 | chloride channel accessory 4 [Source:HGNC Symbol;Acc:HGNC:2018] |
| ENSG00000044090 | HGLibA_11790 | 250 | 1.126642236 | CUL7 | cullin 7 [Source:HGNC Symbol;Acc:HGNC:21024] |
| ENSG00000048140 | HGLibA_51912 | 250 | 1.126642236 | TSPAN17 | tetraspanin 17 [Source:HGNC Symbol;Acc:HGNC:13594] |
| ENSG00000073598 | HGLibA_17759 | 250 | 1.126642236 | FNDC8 | fibronectin type III domain containing 8 [Source:HGNC Symbol;Acc:HGNC:25286] |
| ENSG00000089280 | HGLibA_18176 | 250 | 1.126642236 | FUS | FUS RNA binding protein [Source:HGNC Symbol;Acc:HGNC:4010] |
| ENSG00000091704 | HGLibA_10883 | 250 | 1.126642236 | CPA1 | carboxypeptidase A1 [Source:HGNC Symbol;Acc:HGNC:2296] |
| ENSG00000097096 | HGLibA_47942 | 250 | 1.126642236 | SYDE2 | synapse defective Rho GTPase homolog 2 [Source:HGNC Symbol;Acc:HGNC:25841] |
| ENSG00000100395 | HGLibA_25899 | 250 | 1.126642236 | L3MBTL2 | polycomb repressive complex 1 subunit [Source:HGNC Symbol;Acc:HGNC:18594]" |
| ENSG00000101405 | HGLibA_34839 | 250 | 1.126642236 | OXT | oxytocin/neurophysin I prepropeptide [Source:HGNC Symbol;Acc:HGNC:8528] |
| ENSG00000107731 | HGLibA_53018 | 250 | 1.126642236 | UNC5B | unc-5 netrin receptor B [Source:HGNC Symbol;Acc:HGNC:12568] |
| ENSG00000109854 | HGLibA_22346 | 250 | 1.126642236 | HTATIP2 | HIV-1 Tat interactive protein 2 [Source:HGNC Symbol;Acc:HGNC:16637] |
| ENSG00000110375 | HGLibA_53075 | 250 | 1.126642236 | UPK2 | uroplakin 2 [Source:HGNC Symbol;Acc:HGNC:12579] |
| ENSG00000116198 | HGLibA_09096 | 250 | 1.126642236 | CEP104 | centrosomal protein 104 [Source:HGNC Symbol;Acc:HGNC:24866] |
| ENSG00000117528 | HGLibA_00198 | 250 | 1.126642236 | ABCD3 | ATP binding cassette subfamily D member 3 [Source:HGNC Symbol;Acc:HGNC:67] |
| ENSG00000123610 | HGLibA_50714 | 250 | 1.126642236 | TNFAIP6 | TNF alpha induced protein 6 [Source:HGNC Symbol;Acc:HGNC:11898] |
| ENSG00000129255 | HGLibA_29711 | 250 | 1.126642236 | MPDU1 | mannose-P-dolichol utilization defect 1 [Source:HGNC Symbol;Acc:HGNC:7207] |
| ENSG00000133641 | HGLibA_05228 | 250 | 1.126642236 | C12orf29 | chromosome 12 open reading frame 29 [Source:HGNC Symbol;Acc:HGNC:25322] |
| ENSG00000134323 | HGLibA_30679 | 250 | 1.126642236 | MYCN | bHLH transcription factor [Source:HGNC Symbol;Acc:HGNC:7559]" |
| ENSG00000135108 | HGLibA_17044 | 250 | 1.126642236 | FBXO21 | F-box protein 21 [Source:HGNC Symbol;Acc:HGNC:13592] |
| ENSG00000135406 | HGLibA_38750 | 250 | 1.126642236 | PRPH | peripherin [Source:HGNC Symbol;Acc:HGNC:9461] |
| ENSG00000135540 | HGLibA_31917 | 250 | 1.126642236 | NHSL1 | NHS like 1 [Source:HGNC Symbol;Acc:HGNC:21021] |
| ENSG00000141425 | HGLibA_42044 | 250 | 1.126642236 | RPRD1A | regulation of nuclear pre-mRNA domain containing 1A [Source:HGNC Symbol;Acc:HGNC:25560] |
| ENSG00000149761 | HGLibA_32972 | 250 | 1.126642236 | NUDT22 | nudix hydrolase 22 [Source:HGNC Symbol;Acc:HGNC:28189] |
| ENSG00000161800 | HGLibA_40052 | 250 | 1.126642236 | RACGAP1 | Rac GTPase activating protein 1 [Source:HGNC Symbol;Acc:HGNC:9804] |
| ENSG00000162437 | HGLibA_40403 | 250 | 1.126642236 | RAVER2 | PTB binding 2 [Source:HGNC Symbol;Acc:HGNC:25577]" |
| ENSG00000162814 | HGLibA_46436 | 250 | 1.126642236 | SPATA17 | spermatogenesis associated 17 [Source:HGNC Symbol;Acc:HGNC:25184] |
| ENSG00000165175 | HGLibA_29233 | 250 | 1.126642236 | MID1IP1 | MID1 interacting protein 1 [Source:HGNC Symbol;Acc:HGNC:20715] |
| ENSG00000169756 | HGLibA_26546 | 250 | 1.126642236 | LIMS1 | LIM zinc finger domain containing 1 [Source:HGNC Symbol;Acc:HGNC:6616] |
| ENSG00000170369 | HGLibA_11450 | 250 | 1.126642236 | CST2 | cystatin SA [Source:HGNC Symbol;Acc:HGNC:2474] |
| ENSG00000175581 | HGLibA_30001 | 250 | 1.126642236 | MRPL48 | mitochondrial ribosomal protein L48 [Source:HGNC Symbol;Acc:HGNC:16653] |
| ENSG00000176978 | HGLibA_13879 | 250 | 1.126642236 | DPP7 | dipeptidyl peptidase 7 [Source:HGNC Symbol;Acc:HGNC:14892] |
| ENSG00000187626 | HGLibA_55355 | 250 | 1.126642236 | ZKSCAN4 | zinc finger with KRAB and SCAN domains 4 [Source:HGNC Symbol;Acc:HGNC:13854] |
| ENSG00000188408 | HGLibA_27953 | 250 | 1.126642236 | MAGEB5 | MAGE family member B5 [Source:HGNC Symbol;Acc:HGNC:23795] |
| ENSG00000196542 | HGLibA_46903 | 250 | 1.126642236 | SPTSSB | serine palmitoyltransferase small subunit B [Source:HGNC Symbol;Acc:HGNC:24045] |
| ENSG00000197747 | HGLibA_42532 | 250 | 1.126642236 | S100A10 | S100 calcium binding protein A10 [Source:HGNC Symbol;Acc:HGNC:10487] |
| ENSG00000204516 | HGLibA_29218 | 250 | 1.126642236 | MICB | MHC class I polypeptide-related sequence B [Source:HGNC Symbol;Acc:HGNC:7091] |
| ENSG00000250741 | HGLibA_32809 | 250 | 1.126642236 | NT5C1B-RDH14 | NT5C1B-RDH14 readthrough [Source:HGNC Symbol;Acc:HGNC:38831] |
| HIST1H3J | HGLibA_21482 | 250 | 1.126642236 | HIST1H3J | - |
| hsa-mir-1226 | HGLibA_57304 | 250 | 1.126642236 | hsa-mir-1226 | - |
| hsa-mir-125a | HGLibA_57442 | 250 | 1.126642236 | hsa-mir-125a | - |
| hsa-mir-133b | HGLibA_57781 | 250 | 1.126642236 | hsa-mir-133b | - |
| hsa-mir-182 | HGLibA_57981 | 250 | 1.126642236 | hsa-mir-182 | - |
| hsa-mir-509-1 | HGLibA_61507 | 250 | 1.126642236 | hsa-mir-509-1 | - |
| hsa-mir-8073 | HGLibA_64134 | 250 | 1.126642236 | hsa-mir-8073 | - |
| OR9G9 | HGLibA_34573 | 250 | 1.126642236 | OR9G9 | - |
| C19orf21 | HGLibA_05637 | 249 | 1.117596893 | C19orf21 | - |
| ENSG00000065029 | HGLibA_56683 | 249 | 1.117596893 | ZNF76 | zinc finger protein 76 [Source:HGNC Symbol;Acc:HGNC:13149] |
| ENSG00000070748 | HGLibA_09356 | 249 | 1.117596893 | CHAT | choline O-acetyltransferase [Source:HGNC Symbol;Acc:HGNC:1912] |
| ENSG00000080493 | HGLibA_45220 | 249 | 1.117596893 | SLC4A4 | solute carrier family 4 member 4 [Source:HGNC Symbol;Acc:HGNC:11030] |
| ENSG00000080546 | HGLibA_43618 | 249 | 1.117596893 | SESN1 | sestrin 1 [Source:HGNC Symbol;Acc:HGNC:21595] |
| ENSG00000090975 | HGLibA_36752 | 249 | 1.117596893 | PITPNM2 | phosphatidylinositol transfer protein membrane associated 2 [Source:HGNC Symbol;Acc:HGNC:21044] |
| ENSG00000109790 | HGLibA_25217 | 249 | 1.117596893 | KLHL5 | kelch like family member 5 [Source:HGNC Symbol;Acc:HGNC:6356] |
| ENSG00000111834 | HGLibA_42334 | 249 | 1.117596893 | RSPH4A | radial spoke head component 4A [Source:HGNC Symbol;Acc:HGNC:21558] |
| ENSG00000125124 | HGLibA_04216 | 249 | 1.117596893 | BBS2 | Bardet-Biedl syndrome 2 [Source:HGNC Symbol;Acc:HGNC:967] |
| ENSG00000125888 | HGLibA_04142 | 249 | 1.117596893 | BANF2 | barrier to autointegration factor 2 [Source:HGNC Symbol;Acc:HGNC:16172] |
| ENSG00000127415 | HGLibA_22570 | 249 | 1.117596893 | IDUA | alpha-L- [Source:HGNC Symbol;Acc:HGNC:5391]" |
| ENSG00000129003 | HGLibA_53737 | 249 | 1.117596893 | VPS13C | vacuolar protein sorting 13 homolog C [Source:HGNC Symbol;Acc:HGNC:23594] |
| ENSG00000137760 | HGLibA_01666 | 249 | 1.117596893 | ALKBH8 | tRNA methyltransferase [Source:HGNC Symbol;Acc:HGNC:25189]" |
| ENSG00000140506 | HGLibA_26660 | 249 | 1.117596893 | LMAN1L | mannose binding 1 like [Source:HGNC Symbol;Acc:HGNC:6632]" |
| ENSG00000154813 | HGLibA_13841 | 249 | 1.117596893 | DPH3 | diphthamide biosynthesis 3 [Source:HGNC Symbol;Acc:HGNC:27717] |
| ENSG00000161243 | HGLibA_17057 | 249 | 1.117596893 | FBXO27 | F-box protein 27 [Source:HGNC Symbol;Acc:HGNC:18753] |
| ENSG00000161647 | HGLibA_29754 | 249 | 1.117596893 | MPP3 | membrane palmitoylated protein 3 [Source:HGNC Symbol;Acc:HGNC:7221] |
| ENSG00000161914 | HGLibA_56465 | 249 | 1.117596893 | ZNF653 | zinc finger protein 653 [Source:HGNC Symbol;Acc:HGNC:25196] |
| ENSG00000163637 | HGLibA_38473 | 249 | 1.117596893 | PRICKLE2 | prickle planar cell polarity protein 2 [Source:HGNC Symbol;Acc:HGNC:20340] |
| ENSG00000164761 | HGLibA_50748 | 249 | 1.117596893 | TNFRSF11B | TNF receptor superfamily member 11b [Source:HGNC Symbol;Acc:HGNC:11909] |
| ENSG00000165383 | HGLibA_27273 | 249 | 1.117596893 | LRRC18 | leucine rich repeat containing 18 [Source:HGNC Symbol;Acc:HGNC:23199] |
| ENSG00000165548 | HGLibA_50481 | 249 | 1.117596893 | TMEM63C | transmembrane protein 63C [Source:HGNC Symbol;Acc:HGNC:23787] |
| ENSG00000170367 | HGLibA_11460 | 249 | 1.117596893 | CST5 | cystatin D [Source:HGNC Symbol;Acc:HGNC:2477] |
| ENSG00000170871 | HGLibA_24611 | 249 | 1.117596893 | KIAA0232 | KIAA0232 [Source:HGNC Symbol;Acc:HGNC:28992] |
| ENSG00000171459 | HGLibA_33694 | 249 | 1.117596893 | OR1L6 | olfactory receptor family 1 subfamily L member 6 [Source:HGNC Symbol;Acc:HGNC:8218] |
| ENSG00000171951 | HGLibA_42900 | 249 | 1.117596893 | SCG2 | secretogranin II [Source:HGNC Symbol;Acc:HGNC:10575] |
| ENSG00000172399 | HGLibA_30905 | 249 | 1.117596893 | MYOZ2 | myozenin 2 [Source:HGNC Symbol;Acc:HGNC:1330] |
| ENSG00000172782 | HGLibA_15943 | 249 | 1.117596893 | FADS6 | fatty acid desaturase 6 [Source:HGNC Symbol;Acc:HGNC:30459] |
| ENSG00000176974 | HGLibA_44059 | 249 | 1.117596893 | SHMT1 | serine hydroxymethyltransferase 1 [Source:HGNC Symbol;Acc:HGNC:10850] |
| ENSG00000177576 | HGLibA_05611 | 249 | 1.117596893 | C18orf32 | chromosome 18 open reading frame 32 [Source:HGNC Symbol;Acc:HGNC:31690] |
| ENSG00000179431 | HGLibA_17548 | 249 | 1.117596893 | FJX1 | four-jointed box kinase 1 [Source:HGNC Symbol;Acc:HGNC:17166] |
| ENSG00000181544 | HGLibA_16833 | 249 | 1.117596893 | FANCB | FA complementation group B [Source:HGNC Symbol;Acc:HGNC:3583] |
| ENSG00000183813 | HGLibA_08156 | 249 | 1.117596893 | CCR4 | C-C motif chemokine receptor 4 [Source:HGNC Symbol;Acc:HGNC:1605] |
| ENSG00000184716 | HGLibA_43493 | 249 | 1.117596893 | SERINC4 | serine incorporator 4 [Source:HGNC Symbol;Acc:HGNC:32237] |
| ENSG00000185842 | HGLibA_13479 | 249 | 1.117596893 | DNAH14 | dynein axonemal heavy chain 14 [Source:HGNC Symbol;Acc:HGNC:2945] |
| ENSG00000188766 | HGLibA_46797 | 249 | 1.117596893 | SPRED3 | sprouty related EVH1 domain containing 3 [Source:HGNC Symbol;Acc:HGNC:31041] |
| ENSG00000196372 | HGLibA_03215 | 249 | 1.117596893 | ASB13 | ankyrin repeat and SOCS box containing 13 [Source:HGNC Symbol;Acc:HGNC:19765] |
| ENSG00000233436 | HGLibA_04907 | 249 | 1.117596893 | BTBD18 | BTB domain containing 18 [Source:HGNC Symbol;Acc:HGNC:37214] |
| ENSG00000234906 | HGLibA_02506 | 249 | 1.117596893 | APOC2 | apolipoprotein C2 [Source:HGNC Symbol;Acc:HGNC:609] |
| ENSG00000237190 | HGLibA_08835 | 249 | 1.117596893 | CDKN2AIPNL | CDKN2A interacting protein N-terminal like [Source:HGNC Symbol;Acc:HGNC:30545] |
| ENSG00000237521 | HGLibA_34478 | 249 | 1.117596893 | OR7E24 | olfactory receptor family 7 subfamily E member 24 [Source:HGNC Symbol;Acc:HGNC:8396] |
| ENSG00000240771 | HGLibA_02837 | 249 | 1.117596893 | ARHGEF25 | Rho guanine nucleotide exchange factor 25 [Source:HGNC Symbol;Acc:HGNC:30275] |
| GPR98 | HGLibA_20116 | 249 | 1.117596893 | GPR98 | - |
| hsa-mir-4523 | HGLibA_60551 | 249 | 1.117596893 | hsa-mir-4523 | - |
| hsa-mir-5704 | HGLibA_62357 | 249 | 1.117596893 | hsa-mir-5704 | - |
| hsa-mir-630 | HGLibA_62759 | 249 | 1.117596893 | hsa-mir-630 | - |
| hsa-mir-7702 | HGLibA_63942 | 249 | 1.117596893 | hsa-mir-7702 | - |
| KIAA1919 | HGLibA_24825 | 249 | 1.117596893 | KIAA1919 | - |
| KIAA1967 | HGLibA_24832 | 249 | 1.117596893 | KIAA1967 | - |
| LOC728819 | HGLibA_26992 | 249 | 1.117596893 | LOC728819 | - |

| | | | | | |
|---|---|---|---|---|---|
| C14orf2 | HGLibA_05356 | 248 | 1.10855155 | C14orf2 | - |
| CXorf57 | HGLibA_11946 | 248 | 1.10855155 | CXorf57 | - |
| ENSG00000002745 | HGLibA_54354 | 248 | 1.10855155 | WNT16 | Wnt family member 16 [Source:HGNC Symbol;Acc:HGNC:16267] |
| ENSG00000004534 | HGLibA_40577 | 248 | 1.10855155 | RBM6 | RNA binding motif protein 6 [Source:HGNC Symbol;Acc:HGNC:9903] |
| ENSG00000006432 | HGLibA_28205 | 248 | 1.10855155 | MAP3K9 | mitogen-activated protein kinase kinase kinase 9 [Source:HGNC Symbol;Acc:HGNC:6861] |
| ENSG00000022567 | HGLibA_45181 | 248 | 1.10855155 | SLC45A4 | solute carrier family 45 member 4 [Source:HGNC Symbol;Acc:HGNC:29196] |
| ENSG00000074416 | HGLibA_29158 | 248 | 1.10855155 | MGLL | monoglyceride lipase [Source:HGNC Symbol;Acc:HGNC:17038] |
| ENSG00000101074 | HGLibA_39781 | 248 | 1.10855155 | R3HDML | R3H domain containing like [Source:HGNC Symbol;Acc:HGNC:16249] |
| ENSG00000104848 | HGLibA_24143 | 248 | 1.10855155 | KCNA7 | potassium voltage-gated channel subfamily A member 7 [Source:HGNC Symbol;Acc:HGNC:6226] |
| ENSG00000110375 | HGLibA_53074 | 248 | 1.10855155 | UPK2 | uroplakin 2 [Source:HGNC Symbol;Acc:HGNC:12579] |
| ENSG00000114993 | HGLibA_42389 | 248 | 1.10855155 | RTKN | rhotekin [Source:HGNC Symbol;Acc:HGNC:10466] |
| ENSG00000117335 | HGLibA_08392 | 248 | 1.10855155 | CD46 | CD46 molecule [Source:HGNC Symbol;Acc:HGNC:6953] |
| ENSG00000117543 | HGLibA_13847 | 248 | 1.10855155 | DPH5 | diphthamide biosynthesis 5 [Source:HGNC Symbol;Acc:HGNC:24270] |
| ENSG00000121101 | HGLibA_49063 | 248 | 1.10855155 | TEX14 | intercellular bridge forming factor [Source:HGNC Symbol;Acc:HGNC:11737]" |
| ENSG00000121236 | HGLibA_51559 | 248 | 1.10855155 | TRIM6 | tripartite motif containing 6 [Source:HGNC Symbol;Acc:HGNC:16277] |
| ENSG00000121741 | HGLibA_55394 | 248 | 1.10855155 | ZMYM2 | zinc finger MYM-type containing 2 [Source:HGNC Symbol;Acc:HGNC:12989] |
| ENSG00000124657 | HGLibA_33774 | 248 | 1.10855155 | OR2B6 | olfactory receptor family 2 subfamily B member 6 [Source:HGNC Symbol;Acc:HGNC:8241] |
| ENSG00000133878 | HGLibA_14169 | 248 | 1.10855155 | DUSP26 | dual specificity phosphatase 26 [Source:HGNC Symbol;Acc:HGNC:28161] |
| ENSG00000133937 | HGLibA_20398 | 248 | 1.10855155 | GSC | goosecoid homeobox [Source:HGNC Symbol;Acc:HGNC:4612] |
| ENSG00000134461 | HGLibA_02011 | 248 | 1.10855155 | ANKRD16 | ankyrin repeat domain 16 [Source:HGNC Symbol;Acc:HGNC:23471] |
| ENSG00000136141 | HGLibA_27137 | 248 | 1.10855155 | LRCH1 | leucine rich repeats and calponin homology domain containing 1 [Source:HGNC Symbol;Acc:HGNC:20309] |
| ENSG00000136485 | HGLibA_12454 | 248 | 1.10855155 | DCAF7 | DDB1 and CUL4 associated factor 7 [Source:HGNC Symbol;Acc:HGNC:30915] |
| ENSG00000137101 | HGLibA_08440 | 248 | 1.10855155 | CD72 | CD72 molecule [Source:HGNC Symbol;Acc:HGNC:1696] |
| ENSG00000138166 | HGLibA_14185 | 248 | 1.10855155 | DUSP5 | dual specificity phosphatase 5 [Source:HGNC Symbol;Acc:HGNC:3071] |
| ENSG00000142039 | HGLibA_07939 | 248 | 1.10855155 | CCDC97 | coiled-coil domain containing 97 [Source:HGNC Symbol;Acc:HGNC:28289] |
| ENSG00000145194 | HGLibA_14385 | 248 | 1.10855155 | ECE2 | endothelin converting enzyme 2 [Source:HGNC Symbol;Acc:HGNC:13275] |
| ENSG00000148737 | HGLibA_48796 | 248 | 1.10855155 | TCF7L2 | transcription factor 7 like 2 [Source:HGNC Symbol;Acc:HGNC:11641] |
| ENSG00000154642 | HGLibA_06051 | 248 | 1.10855155 | C21orf91 | chromosome 21 open reading frame 91 [Source:HGNC Symbol;Acc:HGNC:16459] |
| ENSG00000156510 | HGLibA_21588 | 248 | 1.10855155 | HKDC1 | hexokinase domain containing 1 [Source:HGNC Symbol;Acc:HGNC:23302] |
| ENSG00000163431 | HGLibA_26717 | 248 | 1.10855155 | LMOD1 | leiomodin 1 [Source:HGNC Symbol;Acc:HGNC:6647] |
| ENSG00000166886 | HGLibA_31022 | 248 | 1.10855155 | NAB2 | NGFI-A binding protein 2 [Source:HGNC Symbol;Acc:HGNC:7627] |
| ENSG00000167178 | HGLibA_23673 | 248 | 1.10855155 | ISLR2 | immunoglobulin superfamily containing leucine rich repeat 2 [Source:HGNC Symbol;Acc:HGNC:29286] |
| ENSG00000168137 | HGLibA_43651 | 248 | 1.10855155 | SETD5 | SET domain containing 5 [Source:HGNC Symbol;Acc:HGNC:25566] |
| ENSG00000168802 | HGLibA_09665 | 248 | 1.10855155 | CHTF8 | chromosome transmission fidelity factor 8 [Source:HGNC Symbol;Acc:HGNC:24353] |
| ENSG00000172893 | HGLibA_13094 | 248 | 1.10855155 | DHCR7 | 7-dehydrocholesterol reductase [Source:HGNC Symbol;Acc:HGNC:2860] |
| ENSG00000173083 | HGLibA_22062 | 248 | 1.10855155 | HPSE | heparanase [Source:HGNC Symbol;Acc:HGNC:5164] |
| ENSG00000175544 | HGLibA_06853 | 248 | 1.10855155 | CABP4 | calcium binding protein 4 [Source:HGNC Symbol;Acc:HGNC:1386] |
| ENSG00000176927 | HGLibA_14547 | 248 | 1.10855155 | EFCAB5 | EF-hand calcium binding domain 5 [Source:HGNC Symbol;Acc:HGNC:24801] |
| ENSG00000177628 | HGLibA_18709 | 248 | 1.10855155 | GBA | glucosylceramidase beta [Source:HGNC Symbol;Acc:HGNC:4177] |
| ENSG00000178394 | HGLibA_22357 | 248 | 1.10855155 | HTR1A | 5-hydroxytryptamine receptor 1A [Source:HGNC Symbol;Acc:HGNC:5286] |
| ENSG00000178462 | HGLibA_52266 | 248 | 1.10855155 | TUBAL3 | tubulin alpha like 3 [Source:HGNC Symbol;Acc:HGNC:23534] |
| ENSG00000184992 | HGLibA_04806 | 248 | 1.10855155 | BRI3BP | BRI3 binding protein [Source:HGNC Symbol;Acc:HGNC:14251] |
| ENSG00000186487 | HGLibA_30928 | 248 | 1.10855155 | MYT1L | myelin transcription factor 1 like [Source:HGNC Symbol;Acc:HGNC:7623] |
| ENSG00000187840 | HGLibA_14829 | 248 | 1.10855155 | EIF4EBP1 | eukaryotic translation initiation factor 4E binding protein 1 [Source:HGNC Symbol;Acc:HGNC:3288] |
| ENSG00000196453 | HGLibA_56726 | 248 | 1.10855155 | ZNF777 | zinc finger protein 777 [Source:HGNC Symbol;Acc:HGNC:22213] |
| ENSG00000196735 | HGLibA_21621 | 248 | 1.10855155 | HLA-DQA1 | DQ alpha 1 [Source:HGNC Symbol;Acc:HGNC:4942]" |
| ENSG00000197084 | HGLibA_26105 | 248 | 1.10855155 | LCE1C | late cornified envelope 1C [Source:HGNC Symbol;Acc:HGNC:29464] |
| ENSG00000198964 | HGLibA_43836 | 248 | 1.10855155 | SGMS1 | sphingomyelin synthase 1 [Source:HGNC Symbol;Acc:HGNC:29799] |
| ENSG00000214216 | HGLibA_23524 | 248 | 1.10855155 | IQCJ | IQ motif containing J [Source:HGNC Symbol;Acc:HGNC:32406] |
| ENSG00000229544 | HGLibA_32027 | 248 | 1.10855155 | NKX1-2 | NK1 homeobox 2 [Source:HGNC Symbol;Acc:HGNC:31652] |
| ENSG00000241058 | HGLibA_32794 | 248 | 1.10855155 | NSUN6 | NOP2/Sun RNA methyltransferase family member 6 [Source:HGNC Symbol;Acc:HGNC:23529] |
| ENSG00000242715 | HGLibA_07682 | 248 | 1.10855155 | CCDC169 | coiled-coil domain containing 169 [Source:HGNC Symbol;Acc:HGNC:34361] |
| ENSG00000251664 | HGLibA_35434 | 248 | 1.10855155 | PCDHA12 | protocadherin alpha 12 [Source:HGNC Symbol;Acc:HGNC:8666] |
| ENSG00000254521 | HGLibA_44132 | 248 | 1.10855155 | SIGLEC12 | sialic acid binding Ig like lectin 12 (gene/pseudogene) [Source:HGNC Symbol;Acc:HGNC:15482] |
| ENSG00000265763 | HGLibA_56087 | 248 | 1.10855155 | ZNF488 | zinc finger protein 488 [Source:HGNC Symbol;Acc:HGNC:23535] |
| FTSJ2 | HGLibA_18137 | 248 | 1.10855155 | FTSJ2 | - |
| hsa-mir-1269b | HGLibA_57506 | 248 | 1.10855155 | hsa-mir-1269b | - |
| hsa-mir-1273f | HGLibA_57542 | 248 | 1.10855155 | hsa-mir-1273f | - |
| hsa-mir-3157 | HGLibA_58699 | 248 | 1.10855155 | hsa-mir-3157 | - |
| hsa-mir-3171 | HGLibA_58763 | 248 | 1.10855155 | hsa-mir-3171 | - |
| hsa-mir-3177 | HGLibA_58781 | 248 | 1.10855155 | hsa-mir-3177 | - |
| hsa-mir-4526 | HGLibA_60564 | 248 | 1.10855155 | hsa-mir-4526 | - |
| hsa-mir-4787 | HGLibA_61256 | 248 | 1.10855155 | hsa-mir-4787 | - |
| hsa-mir-543 | HGLibA_61822 | 248 | 1.10855155 | hsa-mir-543 | - |
| hsa-mir-6876 | HGLibA_63688 | 248 | 1.10855155 | hsa-mir-6876 | - |
| hsa-mir-6884 | HGLibA_63721 | 248 | 1.10855155 | hsa-mir-6884 | - |
| hsa-mir-941-2 | HGLibA_64342 | 248 | 1.10855155 | hsa-mir-941-2 | - |
| hsa-mir-941-3 | HGLibA_64346 | 248 | 1.10855155 | hsa-mir-941-3 | - |
| WDR92 | HGLibA_54221 | 248 | 1.10855155 | WDR92 | - |
| ENSG00000020633 | HGLibA_42463 | 247 | 1.099506208 | RUNX3 | runt related transcription factor 3 [Source:HGNC Symbol;Acc:HGNC:10473] |
| ENSG00000047597 | HGLibA_54517 | 247 | 1.099506208 | XK | X-linked Kx blood group [Source:HGNC Symbol;Acc:HGNC:12811] |
| ENSG00000048462 | HGLibA_50763 | 247 | 1.099506208 | TNFRSF17 | TNF receptor superfamily member 17 [Source:HGNC Symbol;Acc:HGNC:11913] |
| ENSG00000068024 | HGLibA_21012 | 247 | 1.099506208 | HDAC4 | histone deacetylase 4 [Source:HGNC Symbol;Acc:HGNC:14063] |
| ENSG00000087995 | HGLibA_28999 | 247 | 1.099506208 | METTL2A | methyltransferase like 2A [Source:HGNC Symbol;Acc:HGNC:25755] |
| ENSG00000091073 | HGLibA_14082 | 247 | 1.099506208 | DTX2 | deltex E3 ubiquitin ligase 2 [Source:HGNC Symbol;Acc:HGNC:15973] |
| ENSG00000094661 | HGLibA_33670 | 247 | 1.099506208 | OR1I1 | olfactory receptor family 1 subfamily I member 1 [Source:HGNC Symbol;Acc:HGNC:8207] |
| ENSG00000095002 | HGLibA_30189 | 247 | 1.099506208 | MSH2 | mutS homolog 2 [Source:HGNC Symbol;Acc:HGNC:7325] |
| ENSG00000101200 | HGLibA_03897 | 247 | 1.099506208 | AVP | arginine vasopressin [Source:HGNC Symbol;Acc:HGNC:894] |
| ENSG00000106554 | HGLibA_09368 | 247 | 1.099506208 | CHCHD3 | coiled-coil-helix-coiled-coil-helix domain containing 3 [Source:HGNC Symbol;Acc:HGNC:21906] |
| ENSG00000110492 | HGLibA_28692 | 247 | 1.099506208 | MDK | midkine [Source:HGNC Symbol;Acc:HGNC:6972] |
| ENSG00000114204 | HGLibA_43601 | 247 | 1.099506208 | SERPINI2 | serpin family I member 2 [Source:HGNC Symbol;Acc:HGNC:8945] |
| ENSG00000116793 | HGLibA_36462 | 247 | 1.099506208 | PHTF1 | putative homeodomain transcription factor 1 [Source:HGNC Symbol;Acc:HGNC:8939] |
| ENSG00000120949 | HGLibA_50789 | 247 | 1.099506208 | TNFRSF8 | TNF receptor superfamily member 8 [Source:HGNC Symbol;Acc:HGNC:11923] |
| ENSG00000121570 | HGLibA_13893 | 247 | 1.099506208 | DPPA4 | developmental pluripotency associated 4 [Source:HGNC Symbol;Acc:HGNC:19200] |
| ENSG00000124145 | HGLibA_43105 | 247 | 1.099506208 | SDC4 | syndecan 4 [Source:HGNC Symbol;Acc:HGNC:10661] |
| ENSG00000124588 | HGLibA_32566 | 247 | 1.099506208 | NQO2 | N-ribosyldihydronicotinamide:quinone reductase 2 [Source:HGNC Symbol;Acc:HGNC:7856] |
| ENSG00000124721 | HGLibA_13497 | 247 | 1.099506208 | DNAH8 | dynein axonemal heavy chain 8 [Source:HGNC Symbol;Acc:HGNC:2952] |
| ENSG00000124772 | HGLibA_10966 | 247 | 1.099506208 | CPNE5 | copine 5 [Source:HGNC Symbol;Acc:HGNC:2318] |
| ENSG00000130640 | HGLibA_52308 | 247 | 1.099506208 | TUBGCP2 | tubulin gamma complex associated protein 2 [Source:HGNC Symbol;Acc:HGNC:18599] |
| ENSG00000131507 | HGLibA_31392 | 247 | 1.099506208 | NDFIP1 | Nedd4 family interacting protein 1 [Source:HGNC Symbol;Acc:HGNC:17592] |
| ENSG00000134759 | HGLibA_14979 | 247 | 1.099506208 | ELP2 | elongator acetyltransferase complex subunit 2 [Source:HGNC Symbol;Acc:HGNC:18248] |
| ENSG00000139531 | HGLibA_47800 | 247 | 1.099506208 | SUOX | sulfite oxidase [Source:HGNC Symbol;Acc:HGNC:11460] |
| ENSG00000139618 | HGLibA_04764 | 247 | 1.099506208 | BRCA2 | DNA repair associated [Source:HGNC Symbol;Acc:HGNC:1101]" |
| ENSG00000141519 | HGLibA_07775 | 247 | 1.099506208 | CCDC40 | coiled-coil domain containing 40 [Source:HGNC Symbol;Acc:HGNC:26090] |
| ENSG00000141582 | HGLibA_07455 | 247 | 1.099506208 | CBX4 | chromobox 4 [Source:HGNC Symbol;Acc:HGNC:1554] |
| ENSG00000143199 | HGLibA_00909 | 247 | 1.099506208 | ADCY10 | adenylate cyclase 10 [Source:HGNC Symbol;Acc:HGNC:21285] |
| ENSG00000143416 | HGLibA_43288 | 247 | 1.099506208 | SELENBP1 | selenium binding protein 1 [Source:HGNC Symbol;Acc:HGNC:10719] |
| ENSG00000145384 | HGLibA_15912 | 247 | 1.099506208 | FABP2 | fatty acid binding protein 2 [Source:HGNC Symbol;Acc:HGNC:3556] |
| ENSG00000160695 | HGLibA_53727 | 247 | 1.099506208 | VPS11 | CORVET/HOPS core subunit [Source:HGNC Symbol;Acc:HGNC:14583]" |
| ENSG00000163013 | HGLibA_17097 | 247 | 1.099506208 | FBXO41 | F-box protein 41 [Source:HGNC Symbol;Acc:HGNC:29409] |
| ENSG00000163017 | HGLibA_00614 | 247 | 1.099506208 | ACTG2 | enteric [Source:HGNC Symbol;Acc:HGNC:145]" |
| ENSG00000167460 | HGLibA_51151 | 247 | 1.099506208 | TPM4 | tropomyosin 4 [Source:HGNC Symbol;Acc:HGNC:12013] |
| ENSG00000167699 | HGLibA_19285 | 247 | 1.099506208 | GLOD4 | glyoxalase domain containing 4 [Source:HGNC Symbol;Acc:HGNC:14111] |
| ENSG00000168116 | HGLibA_24788 | 247 | 1.099506208 | KIAA1586 | KIAA1586 [Source:HGNC Symbol;Acc:HGNC:21360] |
| ENSG00000171456 | HGLibA_03388 | 247 | 1.099506208 | ASXL1 | ASXL transcriptional regulator 1 [Source:HGNC Symbol;Acc:HGNC:18318] |
| ENSG00000172179 | HGLibA_38595 | 247 | 1.099506208 | PRL | prolactin [Source:HGNC Symbol;Acc:HGNC:9445] |
| ENSG00000173389 | HGLibA_23501 | 247 | 1.099506208 | IQCF1 | IQ motif containing F1 [Source:HGNC Symbol;Acc:HGNC:28607] |
| ENSG00000176410 | HGLibA_13619 | 247 | 1.099506208 | DNAJC30 | DnaJ heat shock protein family (Hsp40) member C30 [Source:HGNC Symbol;Acc:HGNC:16410] |
| ENSG00000183090 | HGLibA_17991 | 247 | 1.099506208 | FREM3 | FRAS1 related extracellular matrix 3 [Source:HGNC Symbol;Acc:HGNC:25172] |
| ENSG00000184650 | HGLibA_33295 | 247 | 1.099506208 | ODF4 | outer dense fiber of sperm tails 4 [Source:HGNC Symbol;Acc:HGNC:19056] |
| ENSG00000188508 | HGLibA_25859 | 247 | 1.099506208 | KRTDAP | keratinocyte differentiation associated protein [Source:HGNC Symbol;Acc:HGNC:16313] |
| ENSG00000196517 | HGLibA_45344 | 247 | 1.099506208 | SLC6A9 | solute carrier family 6 member 9 [Source:HGNC Symbol;Acc:HGNC:11056] |
| ENSG00000198483 | HGLibA_02084 | 247 | 1.099506208 | ANKRD35 | ankyrin repeat domain 35 [Source:HGNC Symbol;Acc:HGNC:26323] |
| ENSG00000204138 | HGLibA_36305 | 247 | 1.099506208 | PHACTR4 | phosphatase and actin regulator 4 [Source:HGNC Symbol;Acc:HGNC:25793] |
| ENSG00000204267 | HGLibA_48317 | 247 | 1.099506208 | TAP2 | ATP binding cassette subfamily B member [Source:HGNC Symbol;Acc:HGNC:44]" |
| ENSG00000204713 | HGLibA_51446 | 247 | 1.099506208 | TRIM27 | tripartite motif containing 27 [Source:HGNC Symbol;Acc:HGNC:9975] |
| ENSG00000205420 | HGLibA_25514 | 247 | 1.099506208 | KRT6A | keratin 6A [Source:HGNC Symbol;Acc:HGNC:6443] |
| ENSG00000206172 | HGLibA_20906 | 247 | 1.099506208 | HBA1 | hemoglobin subunit alpha 1 [Source:HGNC Symbol;Acc:HGNC:4823] |
| ENSG00000223953 | HGLibA_05948 | 247 | 1.099506208 | C1QTNF5 | C1q and TNF related 5 [Source:HGNC Symbol;Acc:HGNC:14344] |
| ENSG00000253797 | HGLibA_53436 | 247 | 1.099506208 | UTP14C | small subunit processome component [Source:HGNC Symbol;Acc:HGNC:20321]" |
| ENSG00000255582 | HGLibA_33487 | 247 | 1.099506208 | OR10G2 | olfactory receptor family 10 subfamily G member 2 [Source:HGNC Symbol;Acc:HGNC:8170] |
| ENSG00000256294 | HGLibA_55621 | 247 | 1.099506208 | ZNF225 | zinc finger protein 225 [Source:HGNC Symbol;Acc:HGNC:13018] |
| hsa-mir-2110 | HGLibA_58222 | 247 | 1.099506208 | hsa-mir-2110 | - |
| hsa-mir-4435-2 | HGLibA_60136 | 247 | 1.099506208 | hsa-mir-4435-2 | - |
| hsa-mir-6751 | HGLibA_63161 | 247 | 1.099506208 | hsa-mir-6751 | - |
| KAL1 | HGLibA_24017 | 247 | 1.099506208 | KAL1 | - |
| SLMO1 | HGLibA_45538 | 247 | 1.099506208 | SLMO1 | - |
| WBSCR17 | HGLibA_53991 | 247 | 1.099506208 | WBSCR17 | - |
| C7orf29 | HGLibA_06530 | 246 | 1.090460865 | C7orf29 | - |
| CCDC64B | HGLibA_07830 | 246 | 1.090460865 | CCDC64B | - |
| CSB-PGBD3 | HGLibA_11308 | 246 | 1.090460865 | CSB-PGBD3 | - |
| CTGF | HGLibA_11624 | 246 | 1.090460865 | CTGF | - |
| DUSP27 | HGLibA_14171 | 246 | 1.090460865 | DUSP27 | - |
| ENSG00000053702 | HGLibA_32695 | 246 | 1.090460865 | NRIP2 | nuclear receptor interacting protein 2 [Source:HGNC Symbol;Acc:HGNC:23078] |
| ENSG00000065325 | HGLibA_19294 | 246 | 1.090460865 | GLP2R | glucagon like peptide 2 receptor [Source:HGNC Symbol;Acc:HGNC:4325] |

| | | | | | |
|---|---|---|---|---|---|
| ENSG00000087111 | HGLibA_36586 | 246 | 1.090460865 | PIGS | phosphatidylinositol glycan anchor biosynthesis class S [Source:HGNC Symbol;Acc:HGNC:14937] |
| ENSG00000099814 | HGLibA_09118 | 246 | 1.090460865 | CEP170B | centrosomal protein 170B [Source:HGNC Symbol;Acc:HGNC:20362] |
| ENSG00000100226 | HGLibA_20598 | 246 | 1.090460865 | GTPBP1 | GTP binding protein 1 [Source:HGNC Symbol;Acc:HGNC:4669] |
| ENSG00000104228 | HGLibA_51472 | 246 | 1.090460865 | TRIM35 | tripartite motif containing 35 [Source:HGNC Symbol;Acc:HGNC:16285] |
| ENSG00000120322 | HGLibA_35511 | 246 | 1.090460865 | PCDHB8 | protocadherin beta 8 [Source:HGNC Symbol;Acc:HGNC:8693] |
| ENSG00000130997 | HGLibA_37544 | 246 | 1.090460865 | POLN | DNA polymerase nu [Source:HGNC Symbol;Acc:HGNC:18870] |
| ENSG00000135253 | HGLibA_24408 | 246 | 1.090460865 | KCP | kielin cysteine rich BMP regulator [Source:HGNC Symbol;Acc:HGNC:17585] |
| ENSG00000136842 | HGLibA_50595 | 246 | 1.090460865 | TMOD1 | tropomodulin 1 [Source:HGNC Symbol;Acc:HGNC:11871] |
| ENSG00000141524 | HGLibA_49762 | 246 | 1.090460865 | TMC6 | transmembrane channel like 6 [Source:HGNC Symbol;Acc:HGNC:18021] |
| ENSG00000144724 | HGLibA_39504 | 246 | 1.090460865 | PTPRG | receptor type G [Source:HGNC Symbol;Acc:HGNC:9671]" |
| ENSG00000147130 | HGLibA_55395 | 246 | 1.090460865 | ZMYM3 | zinc finger MYM-type containing 3 [Source:HGNC Symbol;Acc:HGNC:13054] |
| ENSG00000149930 | HGLibA_48309 | 246 | 1.090460865 | TAOK2 | TAO kinase 2 [Source:HGNC Symbol;Acc:HGNC:16835] |
| ENSG00000152093 | HGLibA_09252 | 246 | 1.090460865 | CFC1B | cryptic family 1B [Source:HGNC Symbol;Acc:HGNC:33983]" |
| ENSG00000157514 | HGLibA_51824 | 246 | 1.090460865 | TSC22D3 | TSC22 domain family member 3 [Source:HGNC Symbol;Acc:HGNC:3051] |
| ENSG00000159322 | HGLibA_01021 | 246 | 1.090460865 | ADPGK | ADP dependent glucokinase [Source:HGNC Symbol;Acc:HGNC:25250] |
| ENSG00000159692 | HGLibA_11583 | 246 | 1.090460865 | CTBP1 | C-terminal binding protein 1 [Source:HGNC Symbol;Acc:HGNC:2494] |
| ENSG00000162065 | HGLibA_48513 | 246 | 1.090460865 | TBC1D24 | TBC1 domain family member 24 [Source:HGNC Symbol;Acc:HGNC:29203] |
| ENSG00000162545 | HGLibA_07079 | 246 | 1.090460865 | CAMK2N1 | calcium/calmodulin dependent protein kinase II inhibitor 1 [Source:HGNC Symbol;Acc:HGNC:24190] |
| ENSG00000163719 | HGLibA_30422 | 246 | 1.090460865 | MTMR14 | myotubularin related protein 14 [Source:HGNC Symbol;Acc:HGNC:26190] |
| ENSG00000165061 | HGLibA_55375 | 246 | 1.090460865 | ZMAT4 | zinc finger matrin-type 4 [Source:HGNC Symbol;Acc:HGNC:25844] |
| ENSG00000165801 | HGLibA_02866 | 246 | 1.090460865 | ARHGEF40 | Rho guanine nucleotide exchange factor 40 [Source:HGNC Symbol;Acc:HGNC:25516] |
| ENSG00000165886 | HGLibA_52765 | 246 | 1.090460865 | UBTD1 | ubiquitin domain containing 1 [Source:HGNC Symbol;Acc:HGNC:25683] |
| ENSG00000166750 | HGLibA_45496 | 246 | 1.090460865 | SLFN5 | schlafen family member 5 [Source:HGNC Symbol;Acc:HGNC:28286] |
| ENSG00000167964 | HGLibA_39852 | 246 | 1.090460865 | RAB26 | member RAS oncogene family [Source:HGNC Symbol;Acc:HGNC:14259]" |
| ENSG00000168453 | HGLibA_22071 | 246 | 1.090460865 | HR | lysine demethylase and nuclear receptor corepressor [Source:HGNC Symbol;Acc:HGNC:5172]" |
| ENSG00000173369 | HGLibA_05916 | 246 | 1.090460865 | C1QB | complement C1q B chain [Source:HGNC Symbol;Acc:HGNC:1242] |
| ENSG00000174405 | HGLibA_26485 | 246 | 1.090460865 | LIG4 | DNA ligase 4 [Source:HGNC Symbol;Acc:HGNC:6601] |
| ENSG00000177030 | HGLibA_12767 | 246 | 1.090460865 | DEAF1 | DEAF1 transcription factor [Source:HGNC Symbol;Acc:HGNC:14677] |
| ENSG00000185674 | HGLibA_27702 | 246 | 1.090460865 | LYG2 | lysozyme g2 [Source:HGNC Symbol;Acc:HGNC:29615] |
| ENSG00000187607 | HGLibA_55749 | 246 | 1.090460865 | ZNF286A | zinc finger protein 286A [Source:HGNC Symbol;Acc:HGNC:13501] |
| ENSG00000187954 | HGLibA_12041 | 246 | 1.090460865 | CYHR1 | cysteine and histidine rich 1 [Source:HGNC Symbol;Acc:HGNC:17806] |
| ENSG00000198959 | HGLibA_49256 | 246 | 1.090460865 | TGM2 | transglutaminase 2 [Source:HGNC Symbol;Acc:HGNC:11778] |
| ENSG00000231213 | HGLibA_37242 | 246 | 1.090460865 | PLSCR5 | phospholipid scramblase family member 5 [Source:HGNC Symbol;Acc:HGNC:19952] |
| ENSG00000240972 | HGLibA_29260 | 246 | 1.090460865 | MIF | macrophage migration inhibitory factor [Source:HGNC Symbol;Acc:HGNC:7097] |
| ENSG00000241360 | HGLibA_35963 | 246 | 1.090460865 | PDXP | pyridoxal phosphatase [Source:HGNC Symbol;Acc:HGNC:30259] |
| ENSG00000241484 | HGLibA_02789 | 246 | 1.090460865 | ARHGAP8 | Rho GTPase activating protein 8 [Source:HGNC Symbol;Acc:HGNC:677] |
| ENSG00000255501 | HGLibA_07233 | 246 | 1.090460865 | CARD18 | caspase recruitment domain family member 18 [Source:HGNC Symbol;Acc:HGNC:28861] |
| ENSG00000255837 | HGLibA_48380 | 246 | 1.090460865 | TAS2R20 | taste 2 receptor member 20 [Source:HGNC Symbol;Acc:HGNC:19109] |
| ENSG00000280094 | HGLibA_33646 | 246 | 1.090460865 | OR1B1 | olfactory receptor family 1 subfamily B member 1 (gene/pseudogene) [Source:HGNC Symbol;Acc:HGNC:8181] |
| hsa-mir-1283-1 | HGLibA_57589 | 246 | 1.090460865 | hsa-mir-1283-1 | - |
| hsa-mir-15a | HGLibA_57937 | 246 | 1.090460865 | hsa-mir-15a | - |
| hsa-mir-1827 | HGLibA_57991 | 246 | 1.090460865 | hsa-mir-1827 | - |
| hsa-mir-3132 | HGLibA_58582 | 246 | 1.090460865 | hsa-mir-3132 | - |
| hsa-mir-4440 | HGLibA_60161 | 246 | 1.090460865 | hsa-mir-4440 | - |
| hsa-mir-4506 | HGLibA_60452 | 246 | 1.090460865 | hsa-mir-4506 | - |
| hsa-mir-6746 | HGLibA_63138 | 246 | 1.090460865 | hsa-mir-6746 | - |
| hsa-mir-6769a | HGLibA_63239 | 246 | 1.090460865 | hsa-mir-6769a | - |
| MPP5 | HGLibA_29758 | 246 | 1.090460865 | MPP5 | - |
| 10-Sep | HGLibA_43427 | 246 | 1.090460865 | 10-Sep | - |
| ATHL1 | HGLibA_03510 | 245 | 1.081415522 | ATHL1 | - |
| C18orf8 | HGLibA_05624 | 245 | 1.081415522 | C18orf8 | - |
| C4orf32 | HGLibA_06319 | 245 | 1.081415522 | C4orf32 | - |
| ENSG00000040633 | HGLibA_36376 | 245 | 1.081415522 | PHF23 | PHD finger protein 23 [Source:HGNC Symbol;Acc:HGNC:28428] |
| ENSG00000055147 | HGLibA_16033 | 245 | 1.081415522 | FAM114A2 | family with sequence similarity 114 member A2 [Source:HGNC Symbol;Acc:HGNC:1333] |
| ENSG00000090857 | HGLibA_35938 | 245 | 1.081415522 | PDPR | pyruvate dehydrogenase phosphatase regulatory subunit [Source:HGNC Symbol;Acc:HGNC:30264] |
| ENSG00000095066 | HGLibA_21879 | 245 | 1.081415522 | HOOK2 | hook microtubule tethering protein 2 [Source:HGNC Symbol;Acc:HGNC:19885] |
| ENSG00000100014 | HGLibA_46606 | 245 | 1.081415522 | SPECC1L | sperm antigen with calponin homology and coiled-coil domains 1 like [Source:HGNC Symbol;Acc:HGNC:29022] |
| ENSG00000103460 | HGLibA_51043 | 245 | 1.081415522 | TOX3 | TOX high mobility group box family member 3 [Source:HGNC Symbol;Acc:HGNC:11972] |
| ENSG00000106560 | HGLibA_19077 | 245 | 1.081415522 | GIMAP2 | IMAP family member 2 [Source:HGNC Symbol;Acc:HGNC:21789]" |
| ENSG00000109738 | HGLibA_19310 | 245 | 1.081415522 | GLRB | glycine receptor beta [Source:HGNC Symbol;Acc:HGNC:4329] |
| ENSG00000115850 | HGLibA_26205 | 245 | 1.081415522 | LCT | lactase [Source:HGNC Symbol;Acc:HGNC:6530] |
| ENSG00000120057 | HGLibA_43746 | 245 | 1.081415522 | SFRP5 | secreted frizzled related protein 5 [Source:HGNC Symbol;Acc:HGNC:10779] |
| ENSG00000124357 | HGLibA_31065 | 245 | 1.081415522 | NAGK | N-acetylglucosamine kinase [Source:HGNC Symbol;Acc:HGNC:17174] |
| ENSG00000128594 | HGLibA_27350 | 245 | 1.081415522 | LRRC4 | leucine rich repeat containing 4 [Source:HGNC Symbol;Acc:HGNC:15586] |
| ENSG00000130818 | HGLibA_55966 | 245 | 1.081415522 | ZNF426 | zinc finger protein 426 [Source:HGNC Symbol;Acc:HGNC:20725] |
| ENSG00000131477 | HGLibA_40184 | 245 | 1.081415522 | RAMP2 | receptor activity modifying protein 2 [Source:HGNC Symbol;Acc:HGNC:9844] |
| ENSG00000134516 | HGLibA_13739 | 245 | 1.081415522 | DOCK2 | dedicator of cytokinesis 2 [Source:HGNC Symbol;Acc:HGNC:2988] |
| ENSG00000136274 | HGLibA_31037 | 245 | 1.081415522 | NACAD | NAC alpha domain containing [Source:HGNC Symbol;Acc:HGNC:22196] |
| ENSG00000136878 | HGLibA_53299 | 245 | 1.081415522 | USP20 | ubiquitin specific peptidase 20 [Source:HGNC Symbol;Acc:HGNC:12619] |
| ENSG00000142627 | HGLibA_15290 | 245 | 1.081415522 | EPHA2 | EPH receptor A2 [Source:HGNC Symbol;Acc:HGNC:3386] |
| ENSG00000145826 | HGLibA_26268 | 245 | 1.081415522 | LECT2 | leukocyte cell derived chemotaxin 2 [Source:HGNC Symbol;Acc:HGNC:6550] |
| ENSG00000149201 | HGLibA_07879 | 245 | 1.081415522 | CCDC81 | coiled-coil domain containing 81 [Source:HGNC Symbol;Acc:HGNC:26281] |
| ENSG00000149658 | HGLibA_54731 | 245 | 1.081415522 | YTHDF1 | YTH N6-methyladenosine RNA binding protein 1 [Source:HGNC Symbol;Acc:HGNC:15867] |
| ENSG00000151806 | HGLibA_20670 | 245 | 1.081415522 | GUF1 | GTPase [Source:HGNC Symbol;Acc:HGNC:25799]" |
| ENSG00000152595 | HGLibA_28903 | 245 | 1.081415522 | MEPE | matrix extracellular phosphoglycoprotein [Source:HGNC Symbol;Acc:HGNC:13361] |
| ENSG00000153246 | HGLibA_36926 | 245 | 1.081415522 | PLA2R1 | phospholipase A2 receptor 1 [Source:HGNC Symbol;Acc:HGNC:9042] |
| ENSG00000155660 | HGLibA_35881 | 245 | 1.081415522 | PDIA4 | protein disulfide isomerase family A member 4 [Source:HGNC Symbol;Acc:HGNC:30167] |
| ENSG00000156218 | HGLibA_00855 | 245 | 1.081415522 | ADAMTSL3 | ADAMTS like 3 [Source:HGNC Symbol;Acc:HGNC:14633] |
| ENSG00000158856 | HGLibA_13447 | 245 | 1.081415522 | DMTN | dematin actin binding protein [Source:HGNC Symbol;Acc:HGNC:3382] |
| ENSG00000160179 | HGLibA_00214 | 245 | 1.081415522 | ABCG1 | ATP binding cassette subfamily G member 1 [Source:HGNC Symbol;Acc:HGNC:73] |
| ENSG00000163600 | HGLibA_22514 | 245 | 1.081415522 | ICOS | inducible T cell costimulator [Source:HGNC Symbol;Acc:HGNC:5351] |
| ENSG00000165572 | HGLibA_24110 | 245 | 1.081415522 | KBTBD6 | kelch repeat and BTB domain containing 6 [Source:HGNC Symbol;Acc:HGNC:25340] |
| ENSG00000166035 | HGLibA_26602 | 245 | 1.081415522 | LIPC | hepatic type [Source:HGNC Symbol;Acc:HGNC:6619]" |
| ENSG00000167397 | HGLibA_53678 | 245 | 1.081415522 | VKORC1 | vitamin K epoxide reductase complex subunit 1 [Source:HGNC Symbol;Acc:HGNC:23663] |
| ENSG00000167644 | HGLibA_05647 | 245 | 1.081415522 | C19orf33 | chromosome 19 open reading frame 33 [Source:HGNC Symbol;Acc:HGNC:16668] |
| ENSG00000172935 | HGLibA_29839 | 245 | 1.081415522 | MRGPRF | MAS related GPR family member F [Source:HGNC Symbol;Acc:HGNC:24828] |
| ENSG00000174136 | HGLibA_41015 | 245 | 1.081415522 | RGMB | repulsive guidance molecule BMP co-receptor b [Source:HGNC Symbol;Acc:HGNC:26896] |
| ENSG00000175520 | HGLibA_52738 | 245 | 1.081415522 | UBQLN3 | ubiquilin 3 [Source:HGNC Symbol;Acc:HGNC:12510] |
| ENSG00000178597 | HGLibA_39003 | 245 | 1.081415522 | PSAPL1 | prosaposin like 1 (gene/pseudogene) [Source:HGNC Symbol;Acc:HGNC:33131] |
| ENSG00000182759 | HGLibA_27872 | 245 | 1.081415522 | MAFA | MAF bZIP transcription factor A [Source:HGNC Symbol;Acc:HGNC:23145] |
| ENSG00000184313 | HGLibA_29875 | 245 | 1.081415522 | MROH7 | maestro heat like repeat family member 7 [Source:HGNC Symbol;Acc:HGNC:24802] |
| ENSG00000185414 | HGLibA_29947 | 245 | 1.081415522 | MRPL30 | mitochondrial ribosomal protein L30 [Source:HGNC Symbol;Acc:HGNC:14036] |
| ENSG00000185448 | HGLibA_16608 | 245 | 1.081415522 | FAM47A | family with sequence similarity 47 member A [Source:HGNC Symbol;Acc:HGNC:29962] |
| ENSG00000185775 | HGLibA_46483 | 245 | 1.081415522 | SPATA31A6 | SPATA31 subfamily A member 6 [Source:HGNC Symbol;Acc:HGNC:32006] |
| ENSG00000187135 | HGLibA_53863 | 245 | 1.081415522 | VSTM2B | V-set and transmembrane domain containing 2B [Source:HGNC Symbol;Acc:HGNC:33595] |
| ENSG00000187238 | HGLibA_26132 | 245 | 1.081415522 | LCE3B | late cornified envelope 3B [Source:HGNC Symbol;Acc:HGNC:29462] |
| ENSG00000188051 | HGLibA_50238 | 245 | 1.081415522 | TMEM221 | transmembrane protein 221 [Source:HGNC Symbol;Acc:HGNC:21943] |
| ENSG00000196498 | HGLibA_31360 | 245 | 1.081415522 | NCOR2 | nuclear receptor corepressor 2 [Source:HGNC Symbol;Acc:HGNC:7673] |
| ENSG00000196616 | HGLibA_00952 | 245 | 1.081415522 | ADH1B | beta polypeptide [Source:HGNC Symbol;Acc:HGNC:250]" |
| ENSG00000197467 | HGLibA_10510 | 245 | 1.081415522 | COL13A1 | collagen type XIII alpha 1 chain [Source:HGNC Symbol;Acc:HGNC:2190] |
| ENSG00000198053 | HGLibA_44202 | 245 | 1.081415522 | SIRPA | signal regulatory protein alpha [Source:HGNC Symbol;Acc:HGNC:9662] |
| ENSG00000198055 | HGLibA_20334 | 245 | 1.081415522 | GRK6 | G protein-coupled receptor kinase 6 [Source:HGNC Symbol;Acc:HGNC:4545] |
| ENSG00000198157 | HGLibA_21730 | 245 | 1.081415522 | HMGN5 | high mobility group nucleosome binding domain 5 [Source:HGNC Symbol;Acc:HGNC:8013] |
| ENSG00000198523 | HGLibA_37205 | 245 | 1.081415522 | PLN | phospholamban [Source:HGNC Symbol;Acc:HGNC:9080] |
| ENSG00000212124 | HGLibA_48379 | 245 | 1.081415522 | TAS2R19 | taste 2 receptor member 19 [Source:HGNC Symbol;Acc:HGNC:19108] |
| ENSG00000230301 | HGLibA_34300 | 245 | 1.081415522 | OR5H6 | olfactory receptor family 5 subfamily H member 6 (gene/pseudogene) [Source:HGNC Symbol;Acc:HGNC:14767] |
| ENSG00000267206 | HGLibA_26180 | 245 | 1.081415522 | LCN6 | lipocalin 6 [Source:HGNC Symbol;Acc:HGNC:17337] |
| ENSG00000279111 | HGLibA_33557 | 245 | 1.081415522 | OR10X1 | olfactory receptor family 10 subfamily X member 1 (gene/pseudogene) [Source:HGNC Symbol;Acc:HGNC:14995] |
| GPR123 | HGLibA_19872 | 245 | 1.081415522 | GPR123 | - |
| hsa-mir-1281 | HGLibA_57579 | 245 | 1.081415522 | hsa-mir-1281 | - |
| hsa-mir-146a | HGLibA_57856 | 245 | 1.081415522 | hsa-mir-146a | - |
| hsa-mir-2115 | HGLibA_58234 | 245 | 1.081415522 | hsa-mir-2115 | - |
| hsa-mir-31 | HGLibA_58495 | 245 | 1.081415522 | hsa-mir-31 | - |
| hsa-mir-3199-1 | HGLibA_58896 | 245 | 1.081415522 | hsa-mir-3199-1 | - |
| hsa-mir-4489 | HGLibA_60375 | 245 | 1.081415522 | hsa-mir-4489 | - |
| hsa-mir-4726 | HGLibA_61001 | 245 | 1.081415522 | hsa-mir-4726 | - |
| hsa-mir-509-2 | HGLibA_61511 | 245 | 1.081415522 | hsa-mir-509-2 | - |
| KIAA1644 | HGLibA_24797 | 245 | 1.081415522 | KIAA1644 | - |
| C7orf43 | HGLibA_06544 | 244 | 1.072370179 | C7orf43 | - |
| C8orf31 | HGLibA_06606 | 244 | 1.072370179 | C8orf31 | - |
| ENSG00000053108 | HGLibA_18108 | 244 | 1.072370179 | FSTL4 | follistatin like 4 [Source:HGNC Symbol;Acc:HGNC:21389] |
| ENSG00000062598 | HGLibA_14934 | 244 | 1.072370179 | ELMO2 | engulfment and cell motility 2 [Source:HGNC Symbol;Acc:HGNC:17233] |
| ENSG00000068097 | HGLibA_21080 | 244 | 1.072370179 | HEATR6 | HEAT repeat containing 6 [Source:HGNC Symbol;Acc:HGNC:24076] |
| ENSG00000070785 | HGLibA_14740 | 244 | 1.072370179 | EIF2B3 | eukaryotic translation initiation factor 2B subunit gamma [Source:HGNC Symbol;Acc:HGNC:3259] |
| ENSG00000071282 | HGLibA_26682 | 244 | 1.072370179 | LMCD1 | LIM and cysteine rich domains 1 [Source:HGNC Symbol;Acc:HGNC:6633] |
| ENSG00000076067 | HGLibA_40590 | 244 | 1.072370179 | RBMS2 | RNA binding motif single stranded interacting protein 2 [Source:HGNC Symbol;Acc:HGNC:9909] |
| ENSG00000080644 | HGLibA_09570 | 244 | 1.072370179 | CHRNA3 | cholinergic receptor nicotinic alpha 3 subunit [Source:HGNC Symbol;Acc:HGNC:1957] |
| ENSG00000082458 | HGLibA_13331 | 244 | 1.072370179 | DLG3 | discs large MAGUK scaffold protein 3 [Source:HGNC Symbol;Acc:HGNC:2902] |
| ENSG00000085377 | HGLibA_38436 | 244 | 1.072370179 | PREP | prolyl endopeptidase [Source:HGNC Symbol;Acc:HGNC:9358] |
| ENSG00000086544 | HGLibA_23851 | 244 | 1.072370179 | ITPKC | inositol-trisphosphate 3-kinase C [Source:HGNC Symbol;Acc:HGNC:14897] |
| ENSG00000096996 | HGLibA_22964 | 244 | 1.072370179 | IL12RB1 | interleukin 12 receptor subunit beta 1 [Source:HGNC Symbol;Acc:HGNC:5971] |
| ENSG00000100612 | HGLibA_13144 | 244 | 1.072370179 | DHRS7 | dehydrogenase/reductase 7 [Source:HGNC Symbol;Acc:HGNC:21524] |
| ENSG00000105357 | HGLibA_30714 | 244 | 1.072370179 | MYH14 | myosin heavy chain 14 [Source:HGNC Symbol;Acc:HGNC:23212] |
| ENSG00000105507 | HGLibA_06856 | 244 | 1.072370179 | CABP5 | calcium binding protein 5 [Source:HGNC Symbol;Acc:HGNC:13714] |
| ENSG00000107249 | HGLibA_19278 | 244 | 1.072370179 | GLIS3 | GLIS family zinc finger 3 [Source:HGNC Symbol;Acc:HGNC:28510] |

| | | | | | |
|---|---|---|---|---|---|
| ENSG00000113905 | HGLibA_22092 | 244 | 1.072370179 | HRG | histidine rich glycoprotein [Source:HGNC Symbol;Acc:HGNC:5181] |
| ENSG00000114455 | HGLibA_21269 | 244 | 1.072370179 | HHLA2 | HERV-H LTR-associating 2 [Source:HGNC Symbol;Acc:HGNC:4905] |
| ENSG00000115165 | HGLibA_12246 | 244 | 1.072370179 | CYTIP | cytohesin 1 interacting protein [Source:HGNC Symbol;Acc:HGNC:9506] |
| ENSG00000116161 | HGLibA_06958 | 244 | 1.072370179 | CACYBP | calcyclin binding protein [Source:HGNC Symbol;Acc:HGNC:30423] |
| ENSG00000120242 | HGLibA_22691 | 244 | 1.072370179 | IFNA8 | interferon alpha 8 [Source:HGNC Symbol;Acc:HGNC:5429] |
| ENSG00000123612 | HGLibA_00691 | 244 | 1.072370179 | ACVR1C | activin A receptor type 1C [Source:HGNC Symbol;Acc:HGNC:18123] |
| ENSG00000124209 | HGLibA_39840 | 244 | 1.072370179 | RAB22A | member RAS oncogene family [Source:HGNC Symbol;Acc:HGNC:9764]" |
| ENSG00000132622 | HGLibA_22262 | 244 | 1.072370179 | HSPA12B | heat shock protein family A (Hsp70) member 12B [Source:HGNC Symbol;Acc:HGNC:16193] |
| ENSG00000133665 | HGLibA_14222 | 244 | 1.072370179 | DYDC2 | DPY30 domain containing 2 [Source:HGNC Symbol;Acc:HGNC:23468] |
| ENSG00000135535 | HGLibA_08249 | 244 | 1.072370179 | CD164 | CD164 molecule [Source:HGNC Symbol;Acc:HGNC:1632] |
| ENSG00000136918 | HGLibA_54083 | 244 | 1.072370179 | WDR38 | WD repeat domain 38 [Source:HGNC Symbol;Acc:HGNC:23745] |
| ENSG00000138399 | HGLibA_16902 | 244 | 1.072370179 | FASTKD1 | FAST kinase domains 1 [Source:HGNC Symbol;Acc:HGNC:26150] |
| ENSG00000139514 | HGLibA_45345 | 244 | 1.072370179 | SLC7A1 | solute carrier family 7 member 1 [Source:HGNC Symbol;Acc:HGNC:11057] |
| ENSG00000139610 | HGLibA_08990 | 244 | 1.072370179 | CELA1 | chymotrypsin like elastase family member 1 [Source:HGNC Symbol;Acc:HGNC:3308] |
| ENSG00000141639 | HGLibA_28275 | 244 | 1.072370179 | MAPK4 | mitogen-activated protein kinase 4 [Source:HGNC Symbol;Acc:HGNC:6878] |
| ENSG00000143322 | HGLibA_00305 | 244 | 1.072370179 | ABL2 | non-receptor tyrosine kinase [Source:HGNC Symbol;Acc:HGNC:77]" |
| ENSG00000145882 | HGLibA_35697 | 244 | 1.072370179 | PCYOX1L | prenylcysteine oxidase 1 like [Source:HGNC Symbol;Acc:HGNC:28477] |
| ENSG00000145996 | HGLibA_08804 | 244 | 1.072370179 | CDKAL1 | CDK5 regulatory subunit associated protein 1 like 1 [Source:HGNC Symbol;Acc:HGNC:21050] |
| ENSG00000149781 | HGLibA_17299 | 244 | 1.072370179 | FERMT3 | fermitin family member 3 [Source:HGNC Symbol;Acc:HGNC:23151] |
| ENSG00000151065 | HGLibA_12521 | 244 | 1.072370179 | DCP1B | decapping mRNA 1B [Source:HGNC Symbol;Acc:HGNC:24451] |
| ENSG00000151552 | HGLibA_39721 | 244 | 1.072370179 | QDPR | quinoid dihydropteridine reductase [Source:HGNC Symbol;Acc:HGNC:9752] |
| ENSG00000151967 | HGLibA_42941 | 244 | 1.072370179 | SCHIP1 | schwannomin interacting protein 1 [Source:HGNC Symbol;Acc:HGNC:15678] |
| ENSG00000158486 | HGLibA_13485 | 244 | 1.072370179 | DNAH3 | dynein axonemal heavy chain 3 [Source:HGNC Symbol;Acc:HGNC:2949] |
| ENSG00000162009 | HGLibA_47185 | 244 | 1.072370179 | SSTR5 | somatostatin receptor 5 [Source:HGNC Symbol;Acc:HGNC:11334] |
| ENSG00000164080 | HGLibA_40104 | 244 | 1.072370179 | RAD54L2 | RAD54 like 2 [Source:HGNC Symbol;Acc:HGNC:29123] |
| ENSG00000164171 | HGLibA_23724 | 244 | 1.072370179 | ITGA2 | integrin subunit alpha 2 [Source:HGNC Symbol;Acc:HGNC:6137] |
| ENSG00000164287 | HGLibA_08521 | 244 | 1.072370179 | CDC20B | cell division cycle 20B [Source:HGNC Symbol;Acc:HGNC:24222] |
| ENSG00000165568 | HGLibA_01496 | 244 | 1.072370179 | AKR1E2 | aldo-keto reductase family 1 member E2 [Source:HGNC Symbol;Acc:HGNC:23437] |
| ENSG00000165752 | HGLibA_47490 | 244 | 1.072370179 | STK32C | serine/threonine kinase 32C [Source:HGNC Symbol;Acc:HGNC:21332] |
| ENSG00000166963 | HGLibA_28115 | 244 | 1.072370179 | MAP1A | microtubule associated protein 1A [Source:HGNC Symbol;Acc:HGNC:6835] |
| ENSG00000167182 | HGLibA_46298 | 244 | 1.072370179 | SP2 | Sp2 transcription factor [Source:HGNC Symbol;Acc:HGNC:11207] |
| ENSG00000167767 | HGLibA_25556 | 244 | 1.072370179 | KRT80 | keratin 80 [Source:HGNC Symbol;Acc:HGNC:27056] |
| ENSG00000168878 | HGLibA_43773 | 244 | 1.072370179 | SFTPB | surfactant protein B [Source:HGNC Symbol;Acc:HGNC:10801] |
| ENSG00000169548 | HGLibA_55724 | 244 | 1.072370179 | ZNF280A | zinc finger protein 280A [Source:HGNC Symbol;Acc:HGNC:18597] |
| ENSG00000170011 | HGLibA_30918 | 244 | 1.072370179 | MYRIP | myosin VIIA and Rab interacting protein [Source:HGNC Symbol;Acc:HGNC:19156] |
| ENSG00000170385 | HGLibA_44897 | 244 | 1.072370179 | SLC30A1 | solute carrier family 30 member 1 [Source:HGNC Symbol;Acc:HGNC:11012] |
| ENSG00000173431 | HGLibA_41417 | 244 | 1.072370179 | RNASE8 | ribonuclease A family member 8 [Source:HGNC Symbol;Acc:HGNC:19277] |
| ENSG00000175756 | HGLibA_03875 | 244 | 1.072370179 | AURKAIP1 | aurora kinase A interacting protein 1 [Source:HGNC Symbol;Acc:HGNC:24114] |
| ENSG00000175832 | HGLibA_15658 | 244 | 1.072370179 | ETV4 | ETS variant 4 [Source:HGNC Symbol;Acc:HGNC:3493] |
| ENSG00000177565 | HGLibA_48611 | 244 | 1.072370179 | TBL1XR1 | transducin beta like 1 X-linked receptor 1 [Source:HGNC Symbol;Acc:HGNC:29529] |
| ENSG00000179919 | HGLibA_33476 | 244 | 1.072370179 | OR10A7 | olfactory receptor family 10 subfamily A member 7 [Source:HGNC Symbol;Acc:HGNC:15329] |
| ENSG00000184647 | HGLibA_38963 | 244 | 1.072370179 | PRSS55 | serine protease 55 [Source:HGNC Symbol;Acc:HGNC:30824] |
| ENSG00000196071 | HGLibA_33818 | 244 | 1.072370179 | OR2L13 | olfactory receptor family 2 subfamily L member 13 [Source:HGNC Symbol;Acc:HGNC:19578] |
| ENSG00000196358 | HGLibA_32867 | 244 | 1.072370179 | NTNG2 | netrin G2 [Source:HGNC Symbol;Acc:HGNC:14288] |
| ENSG00000220201 | HGLibA_55302 | 244 | 1.072370179 | ZGLP1 | GATA-like protein 1 [Source:HGNC Symbol;Acc:HGNC:37245]" |
| ENSG00000233412 | HGLibA_34296 | 244 | 1.072370179 | OR5H15 | olfactory receptor family 5 subfamily H member 15 [Source:HGNC Symbol;Acc:HGNC:31287] |
| ENSG00000236446 | HGLibA_11551 | 244 | 1.072370179 | CT47B1 | member B1 [Source:HGNC Symbol;Acc:HGNC:33293]" |
| ENSG00000249992 | HGLibA_50015 | 244 | 1.072370179 | TMEM158 | transmembrane protein 158 (gene/pseudogene) [Source:HGNC Symbol;Acc:HGNC:30293] |
| ENSG00000255837 | HGLibA_48381 | 244 | 1.072370179 | TAS2R20 | taste 2 receptor member 20 [Source:HGNC Symbol;Acc:HGNC:19109] |
| ENSG00000256188 | HGLibA_48386 | 244 | 1.072370179 | TAS2R30 | taste 2 receptor member 30 [Source:HGNC Symbol;Acc:HGNC:19112] |
| ENSG00000256980 | HGLibA_24560 | 244 | 1.072370179 | KHDC1L | KH domain containing 1 like [Source:HGNC Symbol;Acc:HGNC:37274] |
| hsa-mir-1224 | HGLibA_57293 | 244 | 1.072370179 | hsa-mir-1224 | - |
| hsa-mir-339 | HGLibA_59009 | 244 | 1.072370179 | hsa-mir-339 | - |
| hsa-mir-563 | HGLibA_62216 | 244 | 1.072370179 | hsa-mir-563 | - |
| hsa-mir-6133 | HGLibA_62684 | 244 | 1.072370179 | hsa-mir-6133 | - |
| hsa-mir-6894 | HGLibA_63761 | 244 | 1.072370179 | hsa-mir-6894 | - |
| hsa-mir-767 | HGLibA_63933 | 244 | 1.072370179 | hsa-mir-767 | - |
| ENSG00000003402 | HGLibA_09288 | 243 | 1.063324836 | CFLAR | CASP8 and FADD like apoptosis regulator [Source:HGNC Symbol;Acc:HGNC:1876] |
| ENSG00000007376 | HGLibA_42206 | 243 | 1.063324836 | RPUSD1 | RNA pseudouridine synthase domain containing 1 [Source:HGNC Symbol;Acc:HGNC:14173] |
| ENSG00000077935 | HGLibA_45644 | 243 | 1.063324836 | SMC1B | structural maintenance of chromosomes 1B [Source:HGNC Symbol;Acc:HGNC:11112] |
| ENSG00000089012 | HGLibA_44212 | 243 | 1.063324836 | SIRPG | signal regulatory protein gamma [Source:HGNC Symbol;Acc:HGNC:15757] |
| ENSG00000091140 | HGLibA_13316 | 243 | 1.063324836 | DLD | dihydrolipoamide dehydrogenase [Source:HGNC Symbol;Acc:HGNC:2898] |
| ENSG00000100234 | HGLibA_49542 | 243 | 1.063324836 | TIMP3 | TIMP metallopeptidase inhibitor 3 [Source:HGNC Symbol;Acc:HGNC:11822] |
| ENSG00000104442 | HGLibA_03006 | 243 | 1.063324836 | ARMC1 | armadillo repeat containing 1 [Source:HGNC Symbol;Acc:HGNC:17684] |
| ENSG00000105523 | HGLibA_16751 | 243 | 1.063324836 | FAM83E | family with sequence similarity 83 member E [Source:HGNC Symbol;Acc:HGNC:25972] |
| ENSG00000106571 | HGLibA_19254 | 243 | 1.063324836 | GLI3 | GLI family zinc finger 3 [Source:HGNC Symbol;Acc:HGNC:4319] |
| ENSG00000111481 | HGLibA_10737 | 243 | 1.063324836 | COPZ1 | coatomer protein complex subunit zeta 1 [Source:HGNC Symbol;Acc:HGNC:2243] |
| ENSG00000116685 | HGLibA_24839 | 243 | 1.063324836 | KIAA2013 | KIAA2013 [Source:HGNC Symbol;Acc:HGNC:28513] |
| ENSG00000120659 | HGLibA_50799 | 243 | 1.063324836 | TNFSF11 | TNF superfamily member 11 [Source:HGNC Symbol;Acc:HGNC:11926] |
| ENSG00000122543 | HGLibA_33258 | 243 | 1.063324836 | OCM | oncomodulin [Source:HGNC Symbol;Acc:HGNC:8105] |
| ENSG00000123384 | HGLibA_27198 | 243 | 1.063324836 | LRP1 | LDL receptor related protein 1 [Source:HGNC Symbol;Acc:HGNC:6692] |
| ENSG00000124279 | HGLibA_16908 | 243 | 1.063324836 | FASTKD3 | FAST kinase domains 3 [Source:HGNC Symbol;Acc:HGNC:28758] |
| ENSG00000124780 | HGLibA_24313 | 243 | 1.063324836 | KCNK17 | potassium two pore domain channel subfamily K member 17 [Source:HGNC Symbol;Acc:HGNC:14465] |
| ENSG00000126243 | HGLibA_27157 | 243 | 1.063324836 | LRFN3 | leucine rich repeat and fibronectin type III domain containing 3 [Source:HGNC Symbol;Acc:HGNC:28370] |
| ENSG00000137497 | HGLibA_33009 | 243 | 1.063324836 | NUMA1 | nuclear mitotic apparatus protein 1 [Source:HGNC Symbol;Acc:HGNC:8059] |
| ENSG00000139116 | HGLibA_24897 | 243 | 1.063324836 | KIF21A | kinesin family member 21A [Source:HGNC Symbol;Acc:HGNC:19349] |
| ENSG00000139168 | HGLibA_55053 | 243 | 1.063324836 | ZCRB1 | zinc finger CCHC-type and RNA binding motif containing 1 [Source:HGNC Symbol;Acc:HGNC:29620] |
| ENSG00000139574 | HGLibA_32435 | 243 | 1.063324836 | NPFF | neuropeptide FF-amide peptide precursor [Source:HGNC Symbol;Acc:HGNC:7901] |
| ENSG00000145687 | HGLibA_47116 | 243 | 1.063324836 | SSBP2 | single stranded DNA binding protein 2 [Source:HGNC Symbol;Acc:HGNC:15831] |
| ENSG00000146147 | HGLibA_29388 | 243 | 1.063324836 | MLIP | muscular LMNA interacting protein [Source:HGNC Symbol;Acc:HGNC:21355] |
| ENSG00000152082 | HGLibA_30947 | 243 | 1.063324836 | MZT2B | mitotic spindle organizing protein 2B [Source:HGNC Symbol;Acc:HGNC:25886] |
| ENSG00000159363 | HGLibA_03568 | 243 | 1.063324836 | ATP13A2 | ATPase cation transporting 13A2 [Source:HGNC Symbol;Acc:HGNC:30213] |
| ENSG00000164007 | HGLibA_09875 | 243 | 1.063324836 | CLDN19 | claudin 19 [Source:HGNC Symbol;Acc:HGNC:2040] |
| ENSG00000166105 | HGLibA_19227 | 243 | 1.063324836 | GLB1L3 | galactosidase beta 1 like 3 [Source:HGNC Symbol;Acc:HGNC:25147] |
| ENSG00000167641 | HGLibA_38035 | 243 | 1.063324836 | PPP1R14A | protein phosphatase 1 regulatory inhibitor subunit 14A [Source:HGNC Symbol;Acc:HGNC:14871] |
| ENSG00000168685 | HGLibA_23192 | 243 | 1.063324836 | IL7R | interleukin 7 receptor [Source:HGNC Symbol;Acc:HGNC:6024] |
| ENSG00000169174 | HGLibA_35688 | 243 | 1.063324836 | PCSK9 | proprotein convertase subtilisin/kexin type 9 [Source:HGNC Symbol;Acc:HGNC:20001] |
| ENSG00000169288 | HGLibA_29888 | 243 | 1.063324836 | MRPL1 | mitochondrial ribosomal protein L1 [Source:HGNC Symbol;Acc:HGNC:14275] |
| ENSG00000169989 | HGLibA_49471 | 243 | 1.063324836 | TIGD4 | tigger transposable element derived 4 [Source:HGNC Symbol;Acc:HGNC:18335] |
| ENSG00000172059 | HGLibA_25045 | 243 | 1.063324836 | KLF11 | Kruppel like factor 11 [Source:HGNC Symbol;Acc:HGNC:11811] |
| ENSG00000172201 | HGLibA_22532 | 243 | 1.063324836 | ID4 | HLH protein [Source:HGNC Symbol;Acc:HGNC:5363]" |
| ENSG00000175398 | HGLibA_33537 | 243 | 1.063324836 | OR10P1 | olfactory receptor family 10 subfamily P member 1 [Source:HGNC Symbol;Acc:HGNC:15378] |
| ENSG00000178343 | HGLibA_44037 | 243 | 1.063324836 | SHISA3 | shisa family member 3 [Source:HGNC Symbol;Acc:HGNC:25159] |
| ENSG00000180914 | HGLibA_34844 | 243 | 1.063324836 | OXTR | oxytocin receptor [Source:HGNC Symbol;Acc:HGNC:8529] |
| ENSG00000181718 | HGLibA_34358 | 243 | 1.063324836 | OR5T2 | olfactory receptor family 5 subfamily T member 2 [Source:HGNC Symbol;Acc:HGNC:15296] |
| ENSG00000183281 | HGLibA_37162 | 243 | 1.063324836 | PLGLB1 | plasminogen-like B1 [Source:HGNC Symbol;Acc:HGNC:9072] |
| ENSG00000183979 | HGLibA_32408 | 243 | 1.063324836 | NPB | neuropeptide B [Source:HGNC Symbol;Acc:HGNC:30099] |
| ENSG00000197461 | HGLibA_35842 | 243 | 1.063324836 | PDGFA | platelet derived growth factor subunit A [Source:HGNC Symbol;Acc:HGNC:8799] |
| ENSG00000198824 | HGLibA_09354 | 243 | 1.063324836 | CHAMP1 | chromosome alignment maintaining phosphoprotein 1 [Source:HGNC Symbol;Acc:HGNC:20311] |
| ENSG00000203730 | HGLibA_48959 | 243 | 1.063324836 | TEDDM1 | transmembrane epididymal protein 1 [Source:HGNC Symbol;Acc:HGNC:30233] |
| ENSG00000206203 | HGLibA_52013 | 243 | 1.063324836 | TSSK2 | testis specific serine kinase 2 [Source:HGNC Symbol;Acc:HGNC:11401] |
| ENSG00000213145 | HGLibA_11151 | 243 | 1.063324836 | CRIP1 | cysteine rich protein 1 [Source:HGNC Symbol;Acc:HGNC:2360] |
| ENSG00000221986 | HGLibA_30661 | 243 | 1.063324836 | MYBPHL | myosin binding protein H like [Source:HGNC Symbol;Acc:HGNC:30434] |
| ENSG00000244355 | HGLibA_27667 | 243 | 1.063324836 | LY6G6D | lymphocyte antigen 6 family member G6D [Source:HGNC Symbol;Acc:HGNC:13935] |
| ENSG00000244355 | HGLibA_27669 | 243 | 1.063324836 | LY6G6D | lymphocyte antigen 6 family member G6D [Source:HGNC Symbol;Acc:HGNC:13935] |
| ENSG00000254122 | HGLibA_35568 | 243 | 1.063324836 | PCDHGB7 | 7 [Source:HGNC Symbol;Acc:HGNC:8714]" |
| ENSG00000270806 | HGLibA_05541 | 243 | 1.063324836 | C17orf50 | chromosome 17 open reading frame 50 [Source:HGNC Symbol;Acc:HGNC:29581] |
| ENSG00000279301 | HGLibA_33856 | 243 | 1.063324836 | OR2T11 | olfactory receptor family 2 subfamily T member 11 (gene/pseudogene) [Source:HGNC Symbol;Acc:HGNC:19574] |
| hsa-mir-1269b | HGLibA_57507 | 243 | 1.063324836 | hsa-mir-1269b | - |
| hsa-mir-155 | HGLibA_57929 | 243 | 1.063324836 | hsa-mir-155 | - |
| hsa-mir-3148 | HGLibA_58648 | 243 | 1.063324836 | hsa-mir-3148 | - |
| hsa-mir-421 | HGLibA_59691 | 243 | 1.063324836 | hsa-mir-421 | - |
| hsa-mir-4292 | HGLibA_59878 | 243 | 1.063324836 | hsa-mir-4292 | - |
| hsa-mir-548ax | HGLibA_61939 | 243 | 1.063324836 | hsa-mir-548ax | - |
| hsa-mir-567 | HGLibA_62228 | 243 | 1.063324836 | hsa-mir-567 | - |
| hsa-mir-941-4 | HGLibA_64350 | 243 | 1.063324836 | hsa-mir-941-4 | - |
| LOC100506388 | HGLibA_26858 | 243 | 1.063324836 | LOC100506388 | - |
| PRAMEF21 | HGLibA_38312 | 243 | 1.063324836 | PRAMEF21 | - |
| ENSG00000003756 | HGLibA_40575 | 242 | 1.054279493 | RBM5 | RNA binding motif protein 5 [Source:HGNC Symbol;Acc:HGNC:9902] |
| ENSG00000006327 | HGLibA_50752 | 242 | 1.054279493 | TNFRSF12A | TNF receptor superfamily member 12A [Source:HGNC Symbol;Acc:HGNC:18152] |
| ENSG00000006715 | HGLibA_53792 | 242 | 1.054279493 | VPS41 | HOPS complex subunit [Source:HGNC Symbol;Acc:HGNC:12713]" |
| ENSG00000011105 | HGLibA_51951 | 242 | 1.054279493 | TSPAN9 | tetraspanin 9 [Source:HGNC Symbol;Acc:HGNC:21640] |
| ENSG00000031691 | HGLibA_09082 | 242 | 1.054279493 | CENPQ | centromere protein Q [Source:HGNC Symbol;Acc:HGNC:21347] |
| ENSG00000064490 | HGLibA_40986 | 242 | 1.054279493 | RFXANK | regulatory factor X associated ankyrin containing protein [Source:HGNC Symbol;Acc:HGNC:9987] |
| ENSG00000100949 | HGLibA_40017 | 242 | 1.054279493 | RABGGTA | Rab geranylgeranyltransferase subunit alpha [Source:HGNC Symbol;Acc:HGNC:9795] |
| ENSG00000101842 | HGLibA_53837 | 242 | 1.054279493 | VSIG1 | V-set and immunoglobulin domain containing 1 [Source:HGNC Symbol;Acc:HGNC:28675] |
| ENSG00000104290 | HGLibA_18270 | 242 | 1.054279493 | FZD3 | frizzled class receptor 3 [Source:HGNC Symbol;Acc:HGNC:4041] |
| ENSG00000104833 | HGLibA_52285 | 242 | 1.054279493 | TUBB4A | tubulin beta 4A class IVa [Source:HGNC Symbol;Acc:HGNC:20774] |
| ENSG00000105088 | HGLibA_33348 | 242 | 1.054279493 | OLFM2 | olfactomedin 2 [Source:HGNC Symbol;Acc:HGNC:17189] |
| ENSG00000106341 | HGLibA_38059 | 242 | 1.054279493 | PPP1R17 | protein phosphatase 1 regulatory subunit 17 [Source:HGNC Symbol;Acc:HGNC:16973] |
| ENSG00000109738 | HGLibA_19309 | 242 | 1.054279493 | GLRB | glycine receptor beta [Source:HGNC Symbol;Acc:HGNC:4329] |
| ENSG00000110048 | HGLibA_34638 | 242 | 1.054279493 | OSBP | oxysterol binding protein [Source:HGNC Symbol;Acc:HGNC:8503] |
| ENSG00000110801 | HGLibA_39187 | 242 | 1.054279493 | PSMD9 | non-ATPase 9 [Source:HGNC Symbol;Acc:HGNC:9567]" |
| ENSG00000114021 | HGLibA_31986 | 242 | 1.054279493 | NIT2 | nitrilase family member 2 [Source:HGNC Symbol;Acc:HGNC:29878] |
| ENSG00000115457 | HGLibA_22819 | 242 | 1.054279493 | IGFBP2 | insulin like growth factor binding protein 2 [Source:HGNC Symbol;Acc:HGNC:5471] |

| | | | | | |
|---|---|---|---|---|---|
| ENSG00000117425 | HGLibA_39271 | 242 | 1.054279493 | PTCH2 | patched 2 [Source:HGNC Symbol;Acc:HGNC:9586] |
| ENSG00000118965 | HGLibA_54073 | 242 | 1.054279493 | WDR35 | WD repeat domain 35 [Source:HGNC Symbol;Acc:HGNC:29250] |
| ENSG00000125611 | HGLibA_09374 | 242 | 1.054279493 | CHCHD5 | coiled-coil-helix-coiled-coil-helix domain containing 5 [Source:HGNC Symbol;Acc:HGNC:17840] |
| ENSG00000128564 | HGLibA_53631 | 242 | 1.054279493 | VGF | VGF nerve growth factor inducible [Source:HGNC Symbol;Acc:HGNC:12684] |
| ENSG00000135250 | HGLibA_47022 | 242 | 1.054279493 | SRPK2 | SRSF protein kinase 2 [Source:HGNC Symbol;Acc:HGNC:11306] |
| ENSG00000136531 | HGLibA_42981 | 242 | 1.054279493 | SCN2A | sodium voltage-gated channel alpha subunit 2 [Source:HGNC Symbol;Acc:HGNC:10588] |
| ENSG00000137941 | HGLibA_52213 | 242 | 1.054279493 | TTLL7 | tubulin tyrosine ligase like 7 [Source:HGNC Symbol;Acc:HGNC:26242] |
| ENSG00000139220 | HGLibA_37875 | 242 | 1.054279493 | PPFIA2 | PTPRF interacting protein alpha 2 [Source:HGNC Symbol;Acc:HGNC:9246] |
| ENSG00000143333 | HGLibA_41067 | 242 | 1.054279493 | RGS16 | regulator of G protein signaling 16 [Source:HGNC Symbol;Acc:HGNC:9997] |
| ENSG00000152779 | HGLibA_44427 | 242 | 1.054279493 | SLC16A12 | solute carrier family 16 member 12 [Source:HGNC Symbol;Acc:HGNC:23094] |
| ENSG00000155026 | HGLibA_42325 | 242 | 1.054279493 | RSPH10B | radial spoke head 10 homolog B [Source:HGNC Symbol;Acc:HGNC:27362] |
| ENSG00000157869 | HGLibA_39863 | 242 | 1.054279493 | RAB28 | member RAS oncogene family [Source:HGNC Symbol;Acc:HGNC:9768]" |
| ENSG00000162039 | HGLibA_28869 | 242 | 1.054279493 | MEIOB | meiosis specific with OB domains [Source:HGNC Symbol;Acc:HGNC:28569] |
| ENSG00000164296 | HGLibA_49477 | 242 | 1.054279493 | TIGD6 | tigger transposable element derived 6 [Source:HGNC Symbol;Acc:HGNC:18332] |
| ENSG00000165091 | HGLibA_49748 | 242 | 1.054279493 | TMC1 | transmembrane channel like 1 [Source:HGNC Symbol;Acc:HGNC:16513] |
| ENSG00000166377 | HGLibA_03795 | 242 | 1.054279493 | ATP9B | ATPase phospholipid transporting 9B (putative) [Source:HGNC Symbol;Acc:HGNC:13541] |
| ENSG00000166407 | HGLibA_26703 | 242 | 1.054279493 | LMO1 | LIM domain only 1 [Source:HGNC Symbol;Acc:HGNC:6641] |
| ENSG00000168280 | HGLibA_24954 | 242 | 1.054279493 | KIF5C | kinesin family member 5C [Source:HGNC Symbol;Acc:HGNC:6325] |
| ENSG00000171794 | HGLibA_53427 | 242 | 1.054279493 | UTF1 | undifferentiated embryonic cell transcription factor 1 [Source:HGNC Symbol;Acc:HGNC:12634] |
| ENSG00000179364 | HGLibA_34957 | 242 | 1.054279493 | PACS2 | phosphofurin acidic cluster sorting protein 2 [Source:HGNC Symbol;Acc:HGNC:23794] |
| ENSG00000179750 | HGLibA_02479 | 242 | 1.054279493 | APOBEC3B | apolipoprotein B mRNA editing enzyme catalytic subunit 3B [Source:HGNC Symbol;Acc:HGNC:17352] |
| ENSG00000181222 | HGLibA_37565 | 242 | 1.054279493 | POLR2A | RNA polymerase II subunit A [Source:HGNC Symbol;Acc:HGNC:9187] |
| ENSG00000182450 | HGLibA_24325 | 242 | 1.054279493 | KCNK4 | potassium two pore domain channel subfamily K member 4 [Source:HGNC Symbol;Acc:HGNC:6279] |
| ENSG00000183114 | HGLibA_16591 | 242 | 1.054279493 | FAM43B | family with sequence similarity 43 member B [Source:HGNC Symbol;Acc:HGNC:31791] |
| ENSG00000183742 | HGLibA_27830 | 242 | 1.054279493 | MACC1 | MET transcriptional regulator MACC1 [Source:HGNC Symbol;Acc:HGNC:30215] |
| ENSG00000185201 | HGLibA_22644 | 242 | 1.054279493 | IFITM2 | interferon induced transmembrane protein 2 [Source:HGNC Symbol;Acc:HGNC:5413] |
| ENSG00000186638 | HGLibA_24910 | 242 | 1.054279493 | KIF24 | kinesin family member 24 [Source:HGNC Symbol;Acc:HGNC:19916] |
| ENSG00000188425 | HGLibA_31100 | 242 | 1.054279493 | NANOS2 | nanos C2HC-type zinc finger 2 [Source:HGNC Symbol;Acc:HGNC:23292] |
| ENSG00000189241 | HGLibA_51978 | 242 | 1.054279493 | TSPYL1 | TSPY like 1 [Source:HGNC Symbol;Acc:HGNC:12382] |
| ENSG00000198453 | HGLibA_56267 | 242 | 1.054279493 | ZNF568 | zinc finger protein 568 [Source:HGNC Symbol;Acc:HGNC:25392] |
| ENSG00000198646 | HGLibA_31351 | 242 | 1.054279493 | NCOA6 | nuclear receptor coactivator 6 [Source:HGNC Symbol;Acc:HGNC:15936] |
| ENSG00000204424 | HGLibA_27672 | 242 | 1.054279493 | LY6G6F | lymphocyte antigen 6 family member G6F [Source:HGNC Symbol;Acc:HGNC:13933] |
| ENSG00000236981 | HGLibA_33504 | 242 | 1.054279493 | OR10G9 | olfactory receptor family 10 subfamily G member 9 [Source:HGNC Symbol;Acc:HGNC:15129] |
| ENSG00000256463 | HGLibA_42654 | 242 | 1.054279493 | SALL3 | spalt like transcription factor 3 [Source:HGNC Symbol;Acc:HGNC:10527] |
| ENSG00000260691 | HGLibA_02026 | 242 | 1.054279493 | ANKRD20A1 | ankyrin repeat domain 20 family member A1 [Source:HGNC Symbol;Acc:HGNC:23665] |
| GNB2L1 | HGLibA_19493 | 242 | 1.054279493 | GNB2L1 | - |
| GPR116 | HGLibA_19865 | 242 | 1.054279493 | GPR116 | - |
| hsa-mir-6084 | HGLibA_62598 | 242 | 1.054279493 | hsa-mir-6084 | - |
| hsa-mir-652 | HGLibA_62943 | 242 | 1.054279493 | hsa-mir-652 | - |
| TMEM5 | HGLibA_50421 | 242 | 1.054279493 | TMEM5 | - |
| AGPAT9 | HGLibA_01235 | 241 | 1.04523415 | AGPAT9 | - |
| BCMO1 | HGLibA_04355 | 241 | 1.04523415 | BCMO1 | - |
| CARKD | HGLibA_07251 | 241 | 1.04523415 | CARKD | - |
| ENSG00000015475 | HGLibA_04500 | 241 | 1.04523415 | BID | BH3 interacting domain death agonist [Source:HGNC Symbol;Acc:HGNC:1050] |
| ENSG00000069702 | HGLibA_49229 | 241 | 1.04523415 | TGFBR3 | transforming growth factor beta receptor 3 [Source:HGNC Symbol;Acc:HGNC:11774] |
| ENSG00000073060 | HGLibA_42869 | 241 | 1.04523415 | SCARB1 | scavenger receptor class B member 1 [Source:HGNC Symbol;Acc:HGNC:1664] |
| ENSG00000077092 | HGLibA_40274 | 241 | 1.04523415 | RARB | retinoic acid receptor beta [Source:HGNC Symbol;Acc:HGNC:9865] |
| ENSG00000100968 | HGLibA_31786 | 241 | 1.04523415 | NFATC4 | nuclear factor of activated T cells 4 [Source:HGNC Symbol;Acc:HGNC:7778] |
| ENSG00000112053 | HGLibA_44807 | 241 | 1.04523415 | SLC26A8 | solute carrier family 26 member 8 [Source:HGNC Symbol;Acc:HGNC:14468] |
| ENSG00000112175 | HGLibA_04610 | 241 | 1.04523415 | BMP5 | bone morphogenetic protein 5 [Source:HGNC Symbol;Acc:HGNC:1072] |
| ENSG00000116852 | HGLibA_24902 | 241 | 1.04523415 | KIF21B | kinesin family member 21B [Source:HGNC Symbol;Acc:HGNC:29442] |
| ENSG00000117115 | HGLibA_34972 | 241 | 1.04523415 | PADI2 | peptidyl arginine deiminase 2 [Source:HGNC Symbol;Acc:HGNC:18341] |
| ENSG00000119888 | HGLibA_15273 | 241 | 1.04523415 | EPCAM | epithelial cell adhesion molecule [Source:HGNC Symbol;Acc:HGNC:11529] |
| ENSG00000123219 | HGLibA_09065 | 241 | 1.04523415 | CENPK | centromere protein K [Source:HGNC Symbol;Acc:HGNC:29479] |
| ENSG00000123360 | HGLibA_35773 | 241 | 1.04523415 | PDE1B | phosphodiesterase 1B [Source:HGNC Symbol;Acc:HGNC:8775] |
| ENSG00000125731 | HGLibA_43891 | 241 | 1.04523415 | SH2D3A | SH2 domain containing 3A [Source:HGNC Symbol;Acc:HGNC:16885] |
| ENSG00000129152 | HGLibA_30879 | 241 | 1.04523415 | MYOD1 | myogenic differentiation 1 [Source:HGNC Symbol;Acc:HGNC:7611] |
| ENSG00000129354 | HGLibA_02294 | 241 | 1.04523415 | AP1M2 | adaptor related protein complex 1 subunit mu 2 [Source:HGNC Symbol;Acc:HGNC:558] |
| ENSG00000132694 | HGLibA_02814 | 241 | 1.04523415 | ARHGEF11 | Rho guanine nucleotide exchange factor 11 [Source:HGNC Symbol;Acc:HGNC:14580] |
| ENSG00000133789 | HGLibA_47897 | 241 | 1.04523415 | SWAP70 | switching B cell complex subunit SWAP70 [Source:HGNC Symbol;Acc:HGNC:17070] |
| ENSG00000133962 | HGLibA_07367 | 241 | 1.04523415 | CATSPERB | cation channel sperm associated auxiliary subunit beta [Source:HGNC Symbol;Acc:HGNC:20500] |
| ENSG00000137463 | HGLibA_29130 | 241 | 1.04523415 | MGARP | mitochondria localized glutamic acid rich protein [Source:HGNC Symbol;Acc:HGNC:29969] |
| ENSG00000138685 | HGLibA_17390 | 241 | 1.04523415 | FGF2 | fibroblast growth factor 2 [Source:HGNC Symbol;Acc:HGNC:3676] |
| ENSG00000139269 | HGLibA_23311 | 241 | 1.04523415 | INHBE | inhibin subunit beta E [Source:HGNC Symbol;Acc:HGNC:24029] |
| ENSG00000139842 | HGLibA_11781 | 241 | 1.04523415 | CUL4A | cullin 4A [Source:HGNC Symbol;Acc:HGNC:2554] |
| ENSG00000140807 | HGLibA_32006 | 241 | 1.04523415 | NKD1 | naked cuticle homolog 1 [Source:HGNC Symbol;Acc:HGNC:17045] |
| ENSG00000141994 | HGLibA_14122 | 241 | 1.04523415 | DUS3L | dihydrouridine synthase 3 like [Source:HGNC Symbol;Acc:HGNC:26920] |
| ENSG00000144635 | HGLibA_14241 | 241 | 1.04523415 | DYNC1LI1 | dynein cytoplasmic 1 light intermediate chain 1 [Source:HGNC Symbol;Acc:HGNC:18745] |
| ENSG00000147724 | HGLibA_16136 | 241 | 1.04523415 | FAM135B | family with sequence similarity 135 member B [Source:HGNC Symbol;Acc:HGNC:28029] |
| ENSG00000148400 | HGLibA_32340 | 241 | 1.04523415 | NOTCH1 | notch 1 [Source:HGNC Symbol;Acc:HGNC:7881] |
| ENSG00000150995 | HGLibA_23853 | 241 | 1.04523415 | ITPR1 | 5-trisphosphate receptor type 1 [Source:HGNC Symbol;Acc:HGNC:6180]" |
| ENSG00000163541 | HGLibA_47698 | 241 | 1.04523415 | SUCLG1 | succinate-CoA ligase alpha subunit [Source:HGNC Symbol;Acc:HGNC:11449] |
| ENSG00000163694 | HGLibA_40565 | 241 | 1.04523415 | RBM47 | RNA binding motif protein 47 [Source:HGNC Symbol;Acc:HGNC:30358] |
| ENSG00000164761 | HGLibA_50747 | 241 | 1.04523415 | TNFRSF11B | TNF receptor superfamily member 11b [Source:HGNC Symbol;Acc:HGNC:11909] |
| ENSG00000167083 | HGLibA_19540 | 241 | 1.04523415 | GNGT2 | G protein subunit gamma transducin 2 [Source:HGNC Symbol;Acc:HGNC:4412] |
| ENSG00000167702 | HGLibA_24973 | 241 | 1.04523415 | KIFC2 | kinesin family member C2 [Source:HGNC Symbol;Acc:HGNC:29530] |
| ENSG00000168256 | HGLibA_32020 | 241 | 1.04523415 | NKIRAS2 | NFKB inhibitor interacting Ras like 2 [Source:HGNC Symbol;Acc:HGNC:17898] |
| ENSG00000169908 | HGLibA_49692 | 241 | 1.04523415 | TM4SF1 | transmembrane 4 L six family member 1 [Source:HGNC Symbol;Acc:HGNC:11853] |
| ENSG00000171860 | HGLibA_06205 | 241 | 1.04523415 | C3AR1 | complement C3a receptor 1 [Source:HGNC Symbol;Acc:HGNC:1319] |
| ENSG00000172464 | HGLibA_34244 | 241 | 1.04523415 | OR5AP2 | olfactory receptor family 5 subfamily AP member 2 [Source:HGNC Symbol;Acc:HGNC:15258] |
| ENSG00000173662 | HGLibA_48354 | 241 | 1.04523415 | TAS1R1 | taste 1 receptor member 1 [Source:HGNC Symbol;Acc:HGNC:14448] |
| ENSG00000173894 | HGLibA_07448 | 241 | 1.04523415 | CBX2 | chromobox 2 [Source:HGNC Symbol;Acc:HGNC:1552] |
| ENSG00000175832 | HGLibA_15660 | 241 | 1.04523415 | ETV4 | ETS variant 4 [Source:HGNC Symbol;Acc:HGNC:3493] |
| ENSG00000176204 | HGLibA_27493 | 241 | 1.04523415 | LRRTM4 | leucine rich repeat transmembrane neuronal 4 [Source:HGNC Symbol;Acc:HGNC:19411] |
| ENSG00000176903 | HGLibA_37366 | 241 | 1.04523415 | PNMA1 | PNMA family member 1 [Source:HGNC Symbol;Acc:HGNC:9158] |
| ENSG00000180921 | HGLibA_16760 | 241 | 1.04523415 | FAM83H | family with sequence similarity 83 member H [Source:HGNC Symbol;Acc:HGNC:24797] |
| ENSG00000184743 | HGLibA_03522 | 241 | 1.04523415 | ATL3 | atlastin GTPase 3 [Source:HGNC Symbol;Acc:HGNC:24526] |
| ENSG00000184831 | HGLibA_02551 | 241 | 1.04523415 | APOO | apolipoprotein O [Source:HGNC Symbol;Acc:HGNC:28727] |
| ENSG00000184916 | HGLibA_23906 | 241 | 1.04523415 | JAG2 | jagged 2 [Source:HGNC Symbol;Acc:HGNC:6189] |
| ENSG00000185385 | HGLibA_34460 | 241 | 1.04523415 | OR7A17 | olfactory receptor family 7 subfamily A member 17 [Source:HGNC Symbol;Acc:HGNC:8363] |
| ENSG00000196565 | HGLibA_20927 | 241 | 1.04523415 | HBG2 | hemoglobin subunit gamma 2 [Source:HGNC Symbol;Acc:HGNC:4832] |
| ENSG00000196730 | HGLibA_12323 | 241 | 1.04523415 | DAPK1 | death associated protein kinase 1 [Source:HGNC Symbol;Acc:HGNC:2674] |
| ENSG00000197757 | HGLibA_21973 | 241 | 1.04523415 | HOXC6 | homeobox C6 [Source:HGNC Symbol;Acc:HGNC:5128] |
| ENSG00000204542 | HGLibA_06451 | 241 | 1.04523415 | C6orf15 | chromosome 6 open reading frame 15 [Source:HGNC Symbol;Acc:HGNC:13927] |
| ENSG00000223510 | HGLibA_08875 | 241 | 1.04523415 | CDRT15 | CMT1A duplicated region transcript 15 [Source:HGNC Symbol;Acc:HGNC:14395] |
| ENSG00000224940 | HGLibA_38889 | 241 | 1.04523415 | PRRT4 | proline rich transmembrane protein 4 [Source:HGNC Symbol;Acc:HGNC:37280] |
| ENSG00000273976 | HGLibA_19618 | 241 | 1.04523415 | GOLGA6L1 | golgin A6 family-like 1 [Source:HGNC Symbol;Acc:HGNC:37444] |
| GLTPD1 | HGLibA_19347 | 241 | 1.04523415 | GLTPD1 | - |
| hsa-mir-1288 | HGLibA_57618 | 241 | 1.04523415 | hsa-mir-1288 | - |
| hsa-mir-187 | HGLibA_58009 | 241 | 1.04523415 | hsa-mir-187 | - |
| hsa-mir-190a | HGLibA_58034 | 241 | 1.04523415 | hsa-mir-190a | - |
| hsa-mir-2110 | HGLibA_58220 | 241 | 1.04523415 | hsa-mir-2110 | - |
| hsa-mir-4255 | HGLibA_59726 | 241 | 1.04523415 | hsa-mir-4255 | - |
| hsa-mir-4257 | HGLibA_59736 | 241 | 1.04523415 | hsa-mir-4257 | - |
| hsa-mir-6770-2 | HGLibA_63249 | 241 | 1.04523415 | hsa-mir-6770-2 | - |
| hsa-mir-6808 | HGLibA_63410 | 241 | 1.04523415 | hsa-mir-6808 | - |
| hsa-mir-8074 | HGLibA_64139 | 241 | 1.04523415 | hsa-mir-8074 | - |
| hsa-mir-99b | HGLibA_64383 | 241 | 1.04523415 | hsa-mir-99b | - |
| ENSG00000004660 | HGLibA_07089 | 240 | 1.036188807 | CAMKK1 | calcium/calmodulin dependent protein kinase kinase 1 [Source:HGNC Symbol;Acc:HGNC:1469] |
| ENSG00000043039 | HGLibA_04166 | 240 | 1.036188807 | BARX2 | BARX homeobox 2 [Source:HGNC Symbol;Acc:HGNC:956] |
| ENSG00000054793 | HGLibA_03791 | 240 | 1.036188807 | ATP9A | ATPase phospholipid transporting 9A (putative) [Source:HGNC Symbol;Acc:HGNC:13540] |
| ENSG00000078549 | HGLibA_00939 | 240 | 1.036188807 | ADCYAP1R1 | ADCYAP receptor type I [Source:HGNC Symbol;Acc:HGNC:242] |
| ENSG00000096746 | HGLibA_21829 | 240 | 1.036188807 | HNRNPH3 | heterogeneous nuclear ribonucleoprotein H3 [Source:HGNC Symbol;Acc:HGNC:5043] |
| ENSG00000105939 | HGLibA_54991 | 240 | 1.036188807 | ZC3HAV1 | antiviral 1 [Source:HGNC Symbol;Acc:HGNC:23721]" |
| ENSG00000105954 | HGLibA_32542 | 240 | 1.036188807 | NPVF | neuropeptide VF precursor [Source:HGNC Symbol;Acc:HGNC:13782] |
| ENSG00000109861 | HGLibA_11694 | 240 | 1.036188807 | CTSC | cathepsin C [Source:HGNC Symbol;Acc:HGNC:2528] |
| ENSG00000111252 | HGLibA_43880 | 240 | 1.036188807 | SH2B3 | SH2B adaptor protein 3 [Source:HGNC Symbol;Acc:HGNC:29605] |
| ENSG00000115963 | HGLibA_41450 | 240 | 1.036188807 | RND3 | Rho family GTPase 3 [Source:HGNC Symbol;Acc:HGNC:671] |
| ENSG00000116991 | HGLibA_44194 | 240 | 1.036188807 | SIPA1L2 | signal induced proliferation associated 1 like 2 [Source:HGNC Symbol;Acc:HGNC:23800] |
| ENSG00000123219 | HGLibA_09066 | 240 | 1.036188807 | CENPK | centromere protein K [Source:HGNC Symbol;Acc:HGNC:29479] |
| ENSG00000132522 | HGLibA_20152 | 240 | 1.036188807 | GPS2 | G protein pathway suppressor 2 [Source:HGNC Symbol;Acc:HGNC:4550] |
| ENSG00000134962 | HGLibA_25023 | 240 | 1.036188807 | KLB | klotho beta [Source:HGNC Symbol;Acc:HGNC:15527] |
| ENSG00000140015 | HGLibA_24223 | 240 | 1.036188807 | KCNH5 | potassium voltage-gated channel subfamily H member 5 [Source:HGNC Symbol;Acc:HGNC:6254] |
| ENSG00000140941 | HGLibA_28124 | 240 | 1.036188807 | MAP1LC3B | microtubule associated protein 1 light chain 3 beta [Source:HGNC Symbol;Acc:HGNC:13352] |
| ENSG00000146966 | HGLibA_12938 | 240 | 1.036188807 | DENND2A | DENN domain containing 2A [Source:HGNC Symbol;Acc:HGNC:22212] |
| ENSG00000149600 | HGLibA_10674 | 240 | 1.036188807 | COMMD7 | COMM domain containing 7 [Source:HGNC Symbol;Acc:HGNC:16223] |
| ENSG00000150093 | HGLibA_23770 | 240 | 1.036188807 | ITGB1 | integrin subunit beta 1 [Source:HGNC Symbol;Acc:HGNC:6153] |
| ENSG00000151475 | HGLibA_44706 | 240 | 1.036188807 | SLC25A31 | solute carrier family 25 member 31 [Source:HGNC Symbol;Acc:HGNC:25319] |
| ENSG00000155275 | HGLibA_51690 | 240 | 1.036188807 | TRMT44 | tRNA methyltransferase 44 homolog [Source:HGNC Symbol;Acc:HGNC:26653] |
| ENSG00000165046 | HGLibA_26337 | 240 | 1.036188807 | LETM2 | leucine zipper and EF-hand containing transmembrane protein 2 [Source:HGNC Symbol;Acc:HGNC:14648] |
| ENSG00000170542 | HGLibA_43567 | 240 | 1.036188807 | SERPINB9 | serpin family B member 9 [Source:HGNC Symbol;Acc:HGNC:8955] |
| ENSG00000171119 | HGLibA_32729 | 240 | 1.036188807 | NRTN | neurturin [Source:HGNC Symbol;Acc:HGNC:8007] |
| ENSG00000172497 | HGLibA_00474 | 240 | 1.036188807 | ACOT12 | acyl-CoA thioesterase 12 [Source:HGNC Symbol;Acc:HGNC:24436] |
| ENSG00000175175 | HGLibA_37973 | 240 | 1.036188807 | PPM1E | Mg2+/Mn2+ dependent 1E [Source:HGNC Symbol;Acc:HGNC:19322]" |
| ENSG00000175782 | HGLibA_44994 | 240 | 1.036188807 | SLC35E3 | solute carrier family 35 member E3 [Source:HGNC Symbol;Acc:HGNC:20864] |

| | | | | | |
|---|---|---|---|---|---|
| ENSG00000176083 | HGLibA_56533 | 240 | 1.036188807 | ZNF683 | zinc finger protein 683 [Source:HGNC Symbol;Acc:HGNC:28495] |
| ENSG00000176723 | HGLibA_56858 | 240 | 1.036188807 | ZNF843 | zinc finger protein 843 [Source:HGNC Symbol;Acc:HGNC:28710] |
| ENSG00000177189 | HGLibA_42165 | 240 | 1.036188807 | RPS6KA3 | ribosomal protein S6 kinase A3 [Source:HGNC Symbol;Acc:HGNC:10432] |
| ENSG00000177663 | HGLibA_23004 | 240 | 1.036188807 | IL17RA | interleukin 17 receptor A [Source:HGNC Symbol;Acc:HGNC:5985] |
| ENSG00000178597 | HGLibA_39004 | 240 | 1.036188807 | PSAPL1 | prosaposin like 1 (gene/pseudogene) [Source:HGNC Symbol;Acc:HGNC:33131] |
| ENSG00000180543 | HGLibA_51987 | 240 | 1.036188807 | TSPYL5 | TSPY like 5 [Source:HGNC Symbol;Acc:HGNC:29367] |
| ENSG00000184661 | HGLibA_08601 | 240 | 1.036188807 | CDCA2 | cell division cycle associated 2 [Source:HGNC Symbol;Acc:HGNC:14623] |
| ENSG00000185163 | HGLibA_12734 | 240 | 1.036188807 | DDX51 | DEAD-box helicase 51 [Source:HGNC Symbol;Acc:HGNC:20082] |
| ENSG00000185304 | HGLibA_41027 | 240 | 1.036188807 | RGPD2 | RANBP2-like and GRIP domain containing 2 [Source:HGNC Symbol;Acc:HGNC:32415] |
| ENSG00000185869 | HGLibA_56827 | 240 | 1.036188807 | ZNF829 | zinc finger protein 829 [Source:HGNC Symbol;Acc:HGNC:34032] |
| ENSG00000186925 | HGLibA_25678 | 240 | 1.036188807 | KRTAP19-6 | keratin associated protein 19-6 [Source:HGNC Symbol;Acc:HGNC:18941] |
| ENSG00000187658 | HGLibA_06404 | 240 | 1.036188807 | C5orf52 | chromosome 5 open reading frame 52 [Source:HGNC Symbol;Acc:HGNC:35121] |
| ENSG00000188368 | HGLibA_38798 | 240 | 1.036188807 | PRR19 | proline rich 19 [Source:HGNC Symbol;Acc:HGNC:33728] |
| ENSG00000196126 | HGLibA_21637 | 240 | 1.036188807 | HLA-DRB1 | DR beta 1 [Source:HGNC Symbol;Acc:HGNC:4948]" |
| ENSG00000263155 | HGLibA_30931 | 240 | 1.036188807 | MYZAP | myocardial zonula adherens protein [Source:HGNC Symbol;Acc:HGNC:43444] |
| hsa-mir-3130-1 | HGLibA_58572 | 240 | 1.036188807 | hsa-mir-3130-1 | - |
| hsa-mir-3159 | HGLibA_58712 | 240 | 1.036188807 | hsa-mir-3159 | - |
| hsa-mir-4330 | HGLibA_60046 | 240 | 1.036188807 | hsa-mir-4330 | - |
| hsa-mir-4712 | HGLibA_60948 | 240 | 1.036188807 | hsa-mir-4712 | - |
| hsa-mir-6125 | HGLibA_62646 | 240 | 1.036188807 | hsa-mir-6125 | - |
| hsa-mir-646 | HGLibA_62826 | 240 | 1.036188807 | hsa-mir-646 | - |
| hsa-mir-6770-1 | HGLibA_63245 | 240 | 1.036188807 | hsa-mir-6770-1 | - |
| LOC100507462 | HGLibA_26869 | 240 | 1.036188807 | LOC100507462 | - |
| SGK223 | HGLibA_43828 | 240 | 1.036188807 | SGK223 | - |
| AZI1 | HGLibA_03932 | 239 | 1.027143464 | AZI1 | - |
| B3GNT1 | HGLibA_03980 | 239 | 1.027143464 | B3GNT1 | - |
| CCBP2 | HGLibA_07501 | 239 | 1.027143464 | CCBP2 | - |
| ENSG00000005801 | HGLibA_55558 | 239 | 1.027143464 | ZNF195 | zinc finger protein 195 [Source:HGNC Symbol;Acc:HGNC:12986] |
| ENSG00000008086 | HGLibA_08817 | 239 | 1.027143464 | CDKL5 | cyclin dependent kinase like 5 [Source:HGNC Symbol;Acc:HGNC:11411] |
| ENSG00000026508 | HGLibA_08390 | 239 | 1.027143464 | CD44 | CD44 molecule (Indian blood group) [Source:HGNC Symbol;Acc:HGNC:1681] |
| ENSG00000063438 | HGLibA_01309 | 239 | 1.027143464 | AHRR | aryl-hydrocarbon receptor repressor [Source:HGNC Symbol;Acc:HGNC:346] |
| ENSG00000071553 | HGLibA_03693 | 239 | 1.027143464 | ATP6AP1 | ATPase H+ transporting accessory protein 1 [Source:HGNC Symbol;Acc:HGNC:868] |
| ENSG00000091831 | HGLibA_15580 | 239 | 1.027143464 | ESR1 | estrogen receptor 1 [Source:HGNC Symbol;Acc:HGNC:3467] |
| ENSG00000102760 | HGLibA_40998 | 239 | 1.027143464 | RGCC | regulator of cell cycle [Source:HGNC Symbol;Acc:HGNC:20369] |
| ENSG00000102886 | HGLibA_18897 | 239 | 1.027143464 | GDPD3 | glycerophosphodiester phosphodiesterase domain containing 3 [Source:HGNC Symbol;Acc:HGNC:28638] |
| ENSG00000103994 | HGLibA_55438 | 239 | 1.027143464 | ZNF106 | zinc finger protein 106 [Source:HGNC Symbol;Acc:HGNC:12886] |
| ENSG00000104412 | HGLibA_15007 | 239 | 1.027143464 | EMC2 | ER membrane protein complex subunit 2 [Source:HGNC Symbol;Acc:HGNC:28963] |
| ENSG00000105141 | HGLibA_07305 | 239 | 1.027143464 | CASP14 | caspase 14 [Source:HGNC Symbol;Acc:HGNC:1502] |
| ENSG00000109501 | HGLibA_54287 | 239 | 1.027143464 | WFS1 | wolframin ER transmembrane glycoprotein [Source:HGNC Symbol;Acc:HGNC:12762] |
| ENSG00000111897 | HGLibA_43485 | 239 | 1.027143464 | SERINC1 | serine incorporator 1 [Source:HGNC Symbol;Acc:HGNC:13464] |
| ENSG00000114030 | HGLibA_25355 | 239 | 1.027143464 | KPNA1 | karyopherin subunit alpha 1 [Source:HGNC Symbol;Acc:HGNC:6394] |
| ENSG00000114544 | HGLibA_45144 | 239 | 1.027143464 | SLC41A3 | solute carrier family 41 member 3 [Source:HGNC Symbol;Acc:HGNC:31046] |
| ENSG00000115145 | HGLibA_47332 | 239 | 1.027143464 | STAM2 | signal transducing adaptor molecule 2 [Source:HGNC Symbol;Acc:HGNC:11358] |
| ENSG00000126231 | HGLibA_38708 | 239 | 1.027143464 | PROZ | vitamin K dependent plasma glycoprotein [Source:HGNC Symbol;Acc:HGNC:9460]" |
| ENSG00000135426 | HGLibA_49034 | 239 | 1.027143464 | TESPA1 | positive selection associated 1 [Source:HGNC Symbol;Acc:HGNC:29109]" |
| ENSG00000138036 | HGLibA_14250 | 239 | 1.027143464 | DYNC2LI1 | dynein cytoplasmic 2 light intermediate chain 1 [Source:HGNC Symbol;Acc:HGNC:24595] |
| ENSG00000140876 | HGLibA_32987 | 239 | 1.027143464 | NUDT7 | nudix hydrolase 7 [Source:HGNC Symbol;Acc:HGNC:8054] |
| ENSG00000141040 | HGLibA_55756 | 239 | 1.027143464 | ZNF287 | zinc finger protein 287 [Source:HGNC Symbol;Acc:HGNC:13502] |
| ENSG00000141756 | HGLibA_17552 | 239 | 1.027143464 | FKBP10 | FK506 binding protein 10 [Source:HGNC Symbol;Acc:HGNC:18169] |
| ENSG00000141946 | HGLibA_55342 | 239 | 1.027143464 | ZIM3 | zinc finger imprinted 3 [Source:HGNC Symbol;Acc:HGNC:16366] |
| ENSG00000142655 | HGLibA_36098 | 239 | 1.027143464 | PEX14 | peroxisomal biogenesis factor 14 [Source:HGNC Symbol;Acc:HGNC:8856] |
| ENSG00000143333 | HGLibA_41066 | 239 | 1.027143464 | RGS16 | regulator of G protein signaling 16 [Source:HGNC Symbol;Acc:HGNC:9997] |
| ENSG00000147010 | HGLibA_43962 | 239 | 1.027143464 | SH3KBP1 | SH3 domain containing kinase binding protein 1 [Source:HGNC Symbol;Acc:HGNC:13867] |
| ENSG00000147202 | HGLibA_13219 | 239 | 1.027143464 | DIAPH2 | diaphanous related formin 2 [Source:HGNC Symbol;Acc:HGNC:2877] |
| ENSG00000155463 | HGLibA_34811 | 239 | 1.027143464 | OXA1L | mitochondrial inner membrane protein [Source:HGNC Symbol;Acc:HGNC:8526]" |
| ENSG00000162391 | HGLibA_16163 | 239 | 1.027143464 | FAM151A | family with sequence similarity 151 member A [Source:HGNC Symbol;Acc:HGNC:25032] |
| ENSG00000163528 | HGLibA_09371 | 239 | 1.027143464 | CHCHD4 | coiled-coil-helix-coiled-coil-helix domain containing 4 [Source:HGNC Symbol;Acc:HGNC:26467] |
| ENSG00000164251 | HGLibA_15866 | 239 | 1.027143464 | F2RL1 | F2R like trypsin receptor 1 [Source:HGNC Symbol;Acc:HGNC:3538] |
| ENSG00000166257 | HGLibA_42988 | 239 | 1.027143464 | SCN3B | sodium voltage-gated channel beta subunit 3 [Source:HGNC Symbol;Acc:HGNC:20665] |
| ENSG00000166368 | HGLibA_33781 | 239 | 1.027143464 | OR2D2 | olfactory receptor family 2 subfamily D member 2 [Source:HGNC Symbol;Acc:HGNC:8244] |
| ENSG00000166839 | HGLibA_01947 | 239 | 1.027143464 | ANKDD1A | ankyrin repeat and death domain containing 1A [Source:HGNC Symbol;Acc:HGNC:28002] |
| ENSG00000171234 | HGLibA_52929 | 239 | 1.027143464 | UGT2B7 | UDP glucuronosyltransferase family 2 member B7 [Source:HGNC Symbol;Acc:HGNC:12554] |
| ENSG00000172915 | HGLibA_31207 | 239 | 1.027143464 | NBEA | neurobeachin [Source:HGNC Symbol;Acc:HGNC:7648] |
| ENSG00000174332 | HGLibA_19272 | 239 | 1.027143464 | GLIS1 | GLIS family zinc finger 1 [Source:HGNC Symbol;Acc:HGNC:29525] |
| ENSG00000183196 | HGLibA_09642 | 239 | 1.027143464 | CHST6 | carbohydrate sulfotransferase 6 [Source:HGNC Symbol;Acc:HGNC:6938] |
| ENSG00000183778 | HGLibA_03962 | 239 | 1.027143464 | B3GALT5 | 3-galactosyltransferase 5 [Source:HGNC Symbol;Acc:HGNC:920]" |
| ENSG00000184304 | HGLibA_38567 | 239 | 1.027143464 | PRKD1 | protein kinase D1 [Source:HGNC Symbol;Acc:HGNC:9407] |
| ENSG00000196562 | HGLibA_47727 | 239 | 1.027143464 | SULF2 | sulfatase 2 [Source:HGNC Symbol;Acc:HGNC:20392] |
| ENSG00000196666 | HGLibA_16315 | 239 | 1.027143464 | FAM180B | family with sequence similarity 180 member B [Source:HGNC Symbol;Acc:HGNC:34451] |
| ENSG00000198398 | HGLibA_50199 | 239 | 1.027143464 | TMEM207 | transmembrane protein 207 [Source:HGNC Symbol;Acc:HGNC:33705] |
| ENSG00000204176 | HGLibA_48064 | 239 | 1.027143464 | SYT15 | synaptotagmin 15 [Source:HGNC Symbol;Acc:HGNC:17167] |
| ENSG00000204252 | HGLibA_21609 | 239 | 1.027143464 | HLA-DOA | DO alpha [Source:HGNC Symbol;Acc:HGNC:4936]" |
| ENSG00000212938 | HGLibA_25820 | 239 | 1.027143464 | KRTAP6-3 | keratin associated protein 6-3 [Source:HGNC Symbol;Acc:HGNC:18933] |
| ENSG00000213160 | HGLibA_25159 | 239 | 1.027143464 | KLHL23 | kelch like family member 23 [Source:HGNC Symbol;Acc:HGNC:27506] |
| ENSG00000226372 | HGLibA_12460 | 239 | 1.027143464 | DCAF8L1 | DDB1 and CUL4 associated factor 8 like 1 [Source:HGNC Symbol;Acc:HGNC:31810] |
| ENSG00000243335 | HGLibA_24463 | 239 | 1.027143464 | KCTD7 | potassium channel tetramerization domain containing 7 [Source:HGNC Symbol;Acc:HGNC:21957] |
| ENSG00000243543 | HGLibA_54270 | 239 | 1.027143464 | WFDC6 | WAP four-disulfide core domain 6 [Source:HGNC Symbol;Acc:HGNC:16164] |
| ENSG00000243708 | HGLibA_36902 | 239 | 1.027143464 | PLA2G4B | phospholipase A2 group IVB [Source:HGNC Symbol;Acc:HGNC:9036] |
| ENSG00000269190 | HGLibA_17037 | 239 | 1.027143464 | FBXO17 | F-box protein 17 [Source:HGNC Symbol;Acc:HGNC:18754] |
| FAM179A | HGLibA_16307 | 239 | 1.027143464 | FAM179A | - |
| HIST1H3F | HGLibA_21470 | 239 | 1.027143464 | HIST1H3F | - |
| HIST1H4G | HGLibA_21503 | 239 | 1.027143464 | HIST1H4G | - |
| hsa-mir-185 | HGLibA_58004 | 239 | 1.027143464 | hsa-mir-185 | - |
| hsa-mir-6769a | HGLibA_63238 | 239 | 1.027143464 | hsa-mir-6769a | - |
| hsa-mir-6831 | HGLibA_63503 | 239 | 1.027143464 | hsa-mir-6831 | - |
| hsa-mir-935 | HGLibA_64313 | 239 | 1.027143464 | hsa-mir-935 | - |
| METTL10 | HGLibA_28947 | 239 | 1.027143464 | METTL10 | - |
| EMR1 | HGLibA_15084 | 238 | 1.018098121 | EMR1 | - |
| ENSG00000006042 | HGLibA_50570 | 238 | 1.018098121 | TMEM98 | transmembrane protein 98 [Source:HGNC Symbol;Acc:HGNC:24529] |
| ENSG00000070601 | HGLibA_18033 | 238 | 1.018098121 | FRMPD1 | FERM and PDZ domain containing 1 [Source:HGNC Symbol;Acc:HGNC:29159] |
| ENSG00000081320 | HGLibA_47466 | 238 | 1.018098121 | STK17B | serine/threonine kinase 17b [Source:HGNC Symbol;Acc:HGNC:11396] |
| ENSG00000099331 | HGLibA_30871 | 238 | 1.018098121 | MYO9B | myosin IXB [Source:HGNC Symbol;Acc:HGNC:7609] |
| ENSG00000100221 | HGLibA_23972 | 238 | 1.018098121 | JOSD1 | Josephin domain containing 1 [Source:HGNC Symbol;Acc:HGNC:28953] |
| ENSG00000100346 | HGLibA_06898 | 238 | 1.018098121 | CACNA1I | calcium voltage-gated channel subunit alpha1 I [Source:HGNC Symbol;Acc:HGNC:1396] |
| ENSG00000101307 | HGLibA_44205 | 238 | 1.018098121 | SIRPB1 | signal regulatory protein beta 1 [Source:HGNC Symbol;Acc:HGNC:15928] |
| ENSG00000103024 | HGLibA_32159 | 238 | 1.018098121 | NME3 | NME/NM23 nucleoside diphosphate kinase 3 [Source:HGNC Symbol;Acc:HGNC:7851] |
| ENSG00000105053 | HGLibA_53830 | 238 | 1.018098121 | VRK3 | VRK serine/threonine kinase 3 [Source:HGNC Symbol;Acc:HGNC:18996] |
| ENSG00000116977 | HGLibA_26377 | 238 | 1.018098121 | LGALS8 | galectin 8 [Source:HGNC Symbol;Acc:HGNC:6569] |
| ENSG00000121904 | HGLibA_11365 | 238 | 1.018098121 | CSMD2 | CUB and Sushi multiple domains 2 [Source:HGNC Symbol;Acc:HGNC:19290] |
| ENSG00000122122 | HGLibA_42765 | 238 | 1.018098121 | SASH3 | SAM and SH3 domain containing 3 [Source:HGNC Symbol;Acc:HGNC:15975] |
| ENSG00000123576 | HGLibA_15602 | 238 | 1.018098121 | ESX1 | ESX homeobox 1 [Source:HGNC Symbol;Acc:HGNC:14865] |
| ENSG00000126010 | HGLibA_20377 | 238 | 1.018098121 | GRPR | gastrin releasing peptide receptor [Source:HGNC Symbol;Acc:HGNC:4609] |
| ENSG00000128833 | HGLibA_30857 | 238 | 1.018098121 | MYO5C | myosin VC [Source:HGNC Symbol;Acc:HGNC:7604] |
| ENSG00000131746 | HGLibA_50929 | 238 | 1.018098121 | TNS4 | tensin 4 [Source:HGNC Symbol;Acc:HGNC:24352] |
| ENSG00000135709 | HGLibA_24642 | 238 | 1.018098121 | KIAA0513 | KIAA0513 [Source:HGNC Symbol;Acc:HGNC:29058] |
| ENSG00000139990 | HGLibA_12447 | 238 | 1.018098121 | DCAF5 | DDB1 and CUL4 associated factor 5 [Source:HGNC Symbol;Acc:HGNC:20224] |
| ENSG00000141540 | HGLibA_52235 | 238 | 1.018098121 | TTYH2 | tweety family member 2 [Source:HGNC Symbol;Acc:HGNC:13877] |
| ENSG00000142677 | HGLibA_23090 | 238 | 1.018098121 | IL22RA1 | interleukin 22 receptor subunit alpha 1 [Source:HGNC Symbol;Acc:HGNC:13700] |
| ENSG00000143036 | HGLibA_45164 | 238 | 1.018098121 | SLC44A3 | solute carrier family 44 member 3 [Source:HGNC Symbol;Acc:HGNC:28689] |
| ENSG00000143458 | HGLibA_18353 | 238 | 1.018098121 | GABPB2 | GA binding protein transcription factor subunit beta 2 [Source:HGNC Symbol;Acc:HGNC:28441] |
| ENSG00000145912 | HGLibA_31909 | 238 | 1.018098121 | NHP2 | NHP2 ribonucleoprotein [Source:HGNC Symbol;Acc:HGNC:14377] |
| ENSG00000148730 | HGLibA_14831 | 238 | 1.018098121 | EIF4EBP2 | eukaryotic translation initiation factor 4E binding protein 2 [Source:HGNC Symbol;Acc:HGNC:3289] |
| ENSG00000149651 | HGLibA_46722 | 238 | 1.018098121 | SPINT4 | Kunitz type 4 [Source:HGNC Symbol;Acc:HGNC:16130]" |
| ENSG00000154889 | HGLibA_29769 | 238 | 1.018098121 | MPPE1 | metallophosphoesterase 1 [Source:HGNC Symbol;Acc:HGNC:15988] |
| ENSG00000163083 | HGLibA_23303 | 238 | 1.018098121 | INHBB | inhibin subunit beta B [Source:HGNC Symbol;Acc:HGNC:6067] |
| ENSG00000163932 | HGLibA_38539 | 238 | 1.018098121 | PRKCD | protein kinase C delta [Source:HGNC Symbol;Acc:HGNC:9399] |
| ENSG00000165650 | HGLibA_35986 | 238 | 1.018098121 | PDZD8 | PDZ domain containing 8 [Source:HGNC Symbol;Acc:HGNC:26974] |
| ENSG00000166311 | HGLibA_45803 | 238 | 1.018098121 | SMPD1 | sphingomyelin phosphodiesterase 1 [Source:HGNC Symbol;Acc:HGNC:11120] |
| ENSG00000167106 | HGLibA_15978 | 238 | 1.018098121 | FAM102A | family with sequence similarity 102 member A [Source:HGNC Symbol;Acc:HGNC:31419] |
| ENSG00000167306 | HGLibA_30853 | 238 | 1.018098121 | MYO5B | myosin VB [Source:HGNC Symbol;Acc:HGNC:7603] |
| ENSG00000172007 | HGLibA_39882 | 238 | 1.018098121 | RAB33B | member RAS oncogene family [Source:HGNC Symbol;Acc:HGNC:16075]" |
| ENSG00000172171 | HGLibA_48966 | 238 | 1.018098121 | TEFM | mitochondrial [Source:HGNC Symbol;Acc:HGNC:26223]" |
| ENSG00000172765 | HGLibA_49772 | 238 | 1.018098121 | TMCC1 | transmembrane and coiled-coil domain family 1 [Source:HGNC Symbol;Acc:HGNC:29116] |
| ENSG00000172939 | HGLibA_34836 | 238 | 1.018098121 | OXSR1 | oxidative stress responsive 1 [Source:HGNC Symbol;Acc:HGNC:8508] |
| ENSG00000173546 | HGLibA_11414 | 238 | 1.018098121 | CSPG4 | chondroitin sulfate proteoglycan 4 [Source:HGNC Symbol;Acc:HGNC:2466] |
| ENSG00000173599 | HGLibA_35359 | 238 | 1.018098121 | PC | pyruvate carboxylase [Source:HGNC Symbol;Acc:HGNC:8636] |
| ENSG00000173714 | HGLibA_54283 | 238 | 1.018098121 | WFIKKN2 | kunitz and netrin domain containing 2 [Source:HGNC Symbol;Acc:HGNC:30916]" |
| ENSG00000174279 | HGLibA_15709 | 238 | 1.018098121 | EVX2 | even-skipped homeobox 2 [Source:HGNC Symbol;Acc:HGNC:3507] |
| ENSG00000175336 | HGLibA_02521 | 238 | 1.018098121 | APOF | apolipoprotein F [Source:HGNC Symbol;Acc:HGNC:615] |
| ENSG00000175395 | HGLibA_55664 | 238 | 1.018098121 | ZNF25 | zinc finger protein 25 [Source:HGNC Symbol;Acc:HGNC:13043] |
| ENSG00000175489 | HGLibA_27291 | 238 | 1.018098121 | LRRC25 | leucine rich repeat containing 25 [Source:HGNC Symbol;Acc:HGNC:29806] |
| ENSG00000177300 | HGLibA_09884 | 238 | 1.018098121 | CLDN22 | claudin 22 [Source:HGNC Symbol;Acc:HGNC:2044] |
| ENSG00000177646 | HGLibA_00361 | 238 | 1.018098121 | ACAD9 | acyl-CoA dehydrogenase family member 9 [Source:HGNC Symbol;Acc:HGNC:21497] |
| ENSG00000181035 | HGLibA_44742 | 238 | 1.018098121 | SLC25A42 | solute carrier family 25 member 42 [Source:HGNC Symbol;Acc:HGNC:28380] |
| ENSG00000183196 | HGLibA_09644 | 238 | 1.018098121 | CHST6 | carbohydrate sulfotransferase 6 [Source:HGNC Symbol;Acc:HGNC:6938] |

| | | | | | |
|---|---|---|---|---|---|
| ENSG00000184428 | HGLibA_50990 | 238 | 1.018098121 | TOP1MT | DNA topoisomerase I mitochondrial [Source:HGNC Symbol;Acc:HGNC:29787] |
| ENSG00000185156 | HGLibA_29111 | 238 | 1.018098121 | MFSD6L | major facilitator superfamily domain containing 6 like [Source:HGNC Symbol;Acc:HGNC:26656] |
| ENSG00000185681 | HGLibA_29677 | 238 | 1.018098121 | MORN5 | MORN repeat containing 5 [Source:HGNC Symbol;Acc:HGNC:17841] |
| ENSG00000186529 | HGLibA_12192 | 238 | 1.018098121 | CYP4F3 | cytochrome P450 family 4 subfamily F member 3 [Source:HGNC Symbol;Acc:HGNC:2646] |
| ENSG00000186603 | HGLibA_22029 | 238 | 1.018098121 | HPDL | 4-hydroxyphenylpyruvate dioxygenase like [Source:HGNC Symbol;Acc:HGNC:28242] |
| ENSG00000188379 | HGLibA_22673 | 238 | 1.018098121 | IFNA2 | interferon alpha 2 [Source:HGNC Symbol;Acc:HGNC:5423] |
| ENSG00000188508 | HGLibA_25861 | 238 | 1.018098121 | KRTDAP | keratinocyte differentiation associated protein [Source:HGNC Symbol;Acc:HGNC:16313] |
| ENSG00000196188 | HGLibA_11700 | 238 | 1.018098121 | CTSE | cathepsin E [Source:HGNC Symbol;Acc:HGNC:2530] |
| ENSG00000196352 | HGLibA_08411 | 238 | 1.018098121 | CD55 | CD55 molecule (Cromer blood group) [Source:HGNC Symbol;Acc:HGNC:2665] |
| ENSG00000197774 | HGLibA_15039 | 238 | 1.018098121 | EME2 | essential meiotic structure-specific endonuclease subunit 2 [Source:HGNC Symbol;Acc:HGNC:27289] |
| ENSG00000206073 | HGLibA_43552 | 238 | 1.018098121 | SERPINB4 | serpin family B member 4 [Source:HGNC Symbol;Acc:HGNC:10570] |
| ENSG00000213023 | HGLibA_48076 | 238 | 1.018098121 | SYT3 | synaptotagmin 3 [Source:HGNC Symbol;Acc:HGNC:11511] |
| ENSG00000215271 | HGLibA_21872 | 238 | 1.018098121 | HOMEZ | homeobox and leucine zipper encoding [Source:HGNC Symbol;Acc:HGNC:20164] |
| ENSG00000227059 | HGLibA_01930 | 238 | 1.018098121 | ANHX | anomalous homeobox [Source:HGNC Symbol;Acc:HGNC:40024] |
| ENSG00000237541 | HGLibA_21625 | 238 | 1.018098121 | HLA-DQA2 | DQ alpha 2 [Source:HGNC Symbol;Acc:HGNC:4943]" |
| hsa-mir-1283-2 | HGLibA_57592 | 238 | 1.018098121 | hsa-mir-1283-2 | - |
| hsa-mir-4738 | HGLibA_61048 | 238 | 1.018098121 | hsa-mir-4738 | - |
| hsa-mir-502 | HGLibA_61465 | 238 | 1.018098121 | hsa-mir-502 | - |
| hsa-mir-548i-3 | HGLibA_62021 | 238 | 1.018098121 | hsa-mir-548i-3 | - |
| PPP5D1 | HGLibA_38215 | 238 | 1.018098121 | PPP5D1 | - |
| SHOX_Y | HGLibA_44071 | 238 | 1.018098121 | SHOX_Y | - |
| TCTEX1D4 | HGLibA_48868 | 238 | 1.018098121 | TCTEX1D4 | - |
| TMEM261 | HGLibA_50353 | 238 | 1.018098121 | TMEM261 | - |
| VWA9 | HGLibA_53921 | 238 | 1.018098121 | VWA9 | - |
| DARC | HGLibA_12338 | 237 | 1.009052778 | DARC | - |
| ENSG00000010539 | HGLibA_55569 | 237 | 1.009052778 | ZNF200 | zinc finger protein 200 [Source:HGNC Symbol;Acc:HGNC:12993] |
| ENSG00000023445 | HGLibA_04517 | 237 | 1.009052778 | BIRC3 | baculoviral IAP repeat containing 3 [Source:HGNC Symbol;Acc:HGNC:591] |
| ENSG00000026103 | HGLibA_16889 | 237 | 1.009052778 | FAS | Fas cell surface death receptor [Source:HGNC Symbol;Acc:HGNC:11920] |
| ENSG00000069020 | HGLibA_28408 | 237 | 1.009052778 | MAST4 | microtubule associated serine/threonine kinase family member 4 [Source:HGNC Symbol;Acc:HGNC:19037] |
| ENSG00000087266 | HGLibA_43929 | 237 | 1.009052778 | SH3BP2 | SH3 domain binding protein 2 [Source:HGNC Symbol;Acc:HGNC:10825] |
| ENSG00000099721 | HGLibA_01770 | 237 | 1.009052778 | AMELY | amelogenin Y-linked [Source:HGNC Symbol;Acc:HGNC:462] |
| ENSG00000103160 | HGLibA_22215 | 237 | 1.009052778 | HSDL1 | hydroxysteroid dehydrogenase like 1 [Source:HGNC Symbol;Acc:HGNC:16475] |
| ENSG00000105492 | HGLibA_44145 | 237 | 1.009052778 | SIGLEC6 | sialic acid binding Ig like lectin 6 [Source:HGNC Symbol;Acc:HGNC:10875] |
| ENSG00000107897 | HGLibA_00403 | 237 | 1.009052778 | ACBD5 | acyl-CoA binding domain containing 5 [Source:HGNC Symbol;Acc:HGNC:23338] |
| ENSG00000109472 | HGLibA_10915 | 237 | 1.009052778 | CPE | carboxypeptidase E [Source:HGNC Symbol;Acc:HGNC:2303] |
| ENSG00000109911 | HGLibA_14984 | 237 | 1.009052778 | ELP4 | elongator acetyltransferase complex subunit 4 [Source:HGNC Symbol;Acc:HGNC:1171] |
| ENSG00000115850 | HGLibA_26206 | 237 | 1.009052778 | LCT | lactase [Source:HGNC Symbol;Acc:HGNC:6530] |
| ENSG00000116147 | HGLibA_50906 | 237 | 1.009052778 | TNR | tenascin R [Source:HGNC Symbol;Acc:HGNC:11953] |
| ENSG00000116544 | HGLibA_13344 | 237 | 1.009052778 | DLGAP3 | DLG associated protein 3 [Source:HGNC Symbol;Acc:HGNC:30368] |
| ENSG00000116691 | HGLibA_29268 | 237 | 1.009052778 | MIIP | migration and invasion inhibitory protein [Source:HGNC Symbol;Acc:HGNC:25715] |
| ENSG00000117069 | HGLibA_47275 | 237 | 1.009052778 | ST6GALNAC5 | 6-sialyltransferase 5 [Source:HGNC Symbol;Acc:HGNC:19342]" |
| ENSG00000122691 | HGLibA_52375 | 237 | 1.009052778 | TWIST1 | twist family bHLH transcription factor 1 [Source:HGNC Symbol;Acc:HGNC:12428] |
| ENSG00000122852 | HGLibA_43767 | 237 | 1.009052778 | SFTPA1 | surfactant protein A1 [Source:HGNC Symbol;Acc:HGNC:10798] |
| ENSG00000123178 | HGLibA_46855 | 237 | 1.009052778 | SPRYD7 | SPRY domain containing 7 [Source:HGNC Symbol;Acc:HGNC:14297] |
| ENSG00000126467 | HGLibA_51872 | 237 | 1.009052778 | TSKS | testis specific serine kinase substrate [Source:HGNC Symbol;Acc:HGNC:30719] |
| ENSG00000129355 | HGLibA_08844 | 237 | 1.009052778 | CDKN2D | cyclin dependent kinase inhibitor 2D [Source:HGNC Symbol;Acc:HGNC:1790] |
| ENSG00000132256 | HGLibA_51533 | 237 | 1.009052778 | TRIM5 | tripartite motif containing 5 [Source:HGNC Symbol;Acc:HGNC:16276] |
| ENSG00000132953 | HGLibA_54564 | 237 | 1.009052778 | XPO4 | exportin 4 [Source:HGNC Symbol;Acc:HGNC:17796] |
| ENSG00000134201 | HGLibA_20483 | 237 | 1.009052778 | GSTM5 | glutathione S-transferase mu 5 [Source:HGNC Symbol;Acc:HGNC:4637] |
| ENSG00000135373 | HGLibA_14679 | 237 | 1.009052778 | EHF | ETS homologous factor [Source:HGNC Symbol;Acc:HGNC:3246] |
| ENSG00000136939 | HGLibA_33692 | 237 | 1.009052778 | OR1L4 | olfactory receptor family 1 subfamily L member 4 [Source:HGNC Symbol;Acc:HGNC:8216] |
| ENSG00000137040 | HGLibA_40210 | 237 | 1.009052778 | RANBP6 | RAN binding protein 6 [Source:HGNC Symbol;Acc:HGNC:9851] |
| ENSG00000146242 | HGLibA_51099 | 237 | 1.009052778 | TPBG | trophoblast glycoprotein [Source:HGNC Symbol;Acc:HGNC:12004] |
| ENSG00000153132 | HGLibA_10015 | 237 | 1.009052778 | CLGN | calmegin [Source:HGNC Symbol;Acc:HGNC:2060] |
| ENSG00000154222 | HGLibA_07479 | 237 | 1.009052778 | CC2D1B | coiled-coil and C2 domain containing 1B [Source:HGNC Symbol;Acc:HGNC:29386] |
| ENSG00000155657 | HGLibA_52220 | 237 | 1.009052778 | TTN | titin [Source:HGNC Symbol;Acc:HGNC:12403] |
| ENSG00000157131 | HGLibA_06592 | 237 | 1.009052778 | C8A | complement C8 alpha chain [Source:HGNC Symbol;Acc:HGNC:1352] |
| ENSG00000157211 | HGLibA_08627 | 237 | 1.009052778 | CDCP2 | CUB domain containing protein 2 [Source:HGNC Symbol;Acc:HGNC:27297] |
| ENSG00000160318 | HGLibA_09919 | 237 | 1.009052778 | CLDND2 | claudin domain containing 2 [Source:HGNC Symbol;Acc:HGNC:28511] |
| ENSG00000161664 | HGLibA_03223 | 237 | 1.009052778 | ASB16 | ankyrin repeat and SOCS box containing 16 [Source:HGNC Symbol;Acc:HGNC:19768] |
| ENSG00000163464 | HGLibA_11880 | 237 | 1.009052778 | CXCR1 | C-X-C motif chemokine receptor 1 [Source:HGNC Symbol;Acc:HGNC:6026] |
| ENSG00000163734 | HGLibA_11870 | 237 | 1.009052778 | CXCL3 | C-X-C motif chemokine ligand 3 [Source:HGNC Symbol;Acc:HGNC:4604] |
| ENSG00000163982 | HGLibA_34735 | 237 | 1.009052778 | OTOP1 | otopetrin 1 [Source:HGNC Symbol;Acc:HGNC:19656] |
| ENSG00000164733 | HGLibA_11693 | 237 | 1.009052778 | CTSB | cathepsin B [Source:HGNC Symbol;Acc:HGNC:2527] |
| ENSG00000165475 | HGLibA_11287 | 237 | 1.009052778 | CRYL1 | crystallin lambda 1 [Source:HGNC Symbol;Acc:HGNC:18246] |
| ENSG00000165556 | HGLibA_08898 | 237 | 1.009052778 | CDX2 | caudal type homeobox 2 [Source:HGNC Symbol;Acc:HGNC:1806] |
| ENSG00000169896 | HGLibA_23761 | 237 | 1.009052778 | ITGAM | integrin subunit alpha M [Source:HGNC Symbol;Acc:HGNC:6149] |
| ENSG00000172497 | HGLibA_00472 | 237 | 1.009052778 | ACOT12 | acyl-CoA thioesterase 12 [Source:HGNC Symbol;Acc:HGNC:24436] |
| ENSG00000173208 | HGLibA_00194 | 237 | 1.009052778 | ABCD2 | ATP binding cassette subfamily D member 2 [Source:HGNC Symbol;Acc:HGNC:66] |
| ENSG00000174567 | HGLibA_19664 | 237 | 1.009052778 | GOLT1A | golgi transport 1A [Source:HGNC Symbol;Acc:HGNC:24766] |
| ENSG00000181192 | HGLibA_13164 | 237 | 1.009052778 | DHTKD1 | dehydrogenase E1 and transketolase domain containing 1 [Source:HGNC Symbol;Acc:HGNC:23537] |
| ENSG00000181827 | HGLibA_40980 | 237 | 1.009052778 | RFX7 | regulatory factor X7 [Source:HGNC Symbol;Acc:HGNC:25777] |
| ENSG00000182557 | HGLibA_46738 | 237 | 1.009052778 | SPNS3 | sphingolipid transporter 3 (putative) [Source:HGNC Symbol;Acc:HGNC:28433] |
| ENSG00000185594 | HGLibA_46530 | 237 | 1.009052778 | SPATA8 | spermatogenesis associated 8 [Source:HGNC Symbol;Acc:HGNC:28676] |
| ENSG00000186513 | HGLibA_34587 | 237 | 1.009052778 | OR9Q2 | olfactory receptor family 9 subfamily Q member 2 [Source:HGNC Symbol;Acc:HGNC:15328] |
| ENSG00000188386 | HGLibA_38196 | 237 | 1.009052778 | PPP3R2 | beta [Source:HGNC Symbol;Acc:HGNC:9318]" |
| ENSG00000196166 | HGLibA_06652 | 237 | 1.009052778 | C8orf86 | chromosome 8 open reading frame 86 [Source:HGNC Symbol;Acc:HGNC:33774] |
| ENSG00000198090 | HGLibA_25770 | 237 | 1.009052778 | KRTAP4-6 | keratin associated protein 4-6 [Source:HGNC Symbol;Acc:HGNC:18909] |
| ENSG00000198488 | HGLibA_03997 | 237 | 1.009052778 | B3GNT6 | 3-N-acetylglucosaminyltransferase 6 [Source:HGNC Symbol;Acc:HGNC:24141]" |
| ENSG00000198590 | HGLibA_06232 | 237 | 1.009052778 | C3orf35 | chromosome 3 open reading frame 35 [Source:HGNC Symbol;Acc:HGNC:24082] |
| ENSG00000212123 | HGLibA_38819 | 237 | 1.009052778 | PRR22 | proline rich 22 [Source:HGNC Symbol;Acc:HGNC:28354] |
| hsa-mir-3973 | HGLibA_59650 | 237 | 1.009052778 | hsa-mir-3973 | - |
| hsa-mir-4701 | HGLibA_60911 | 237 | 1.009052778 | hsa-mir-4701 | - |
| hsa-mir-660 | HGLibA_62974 | 237 | 1.009052778 | hsa-mir-660 | - |
| PPAPDC3 | HGLibA_37833 | 237 | 1.009052778 | PPAPDC3 | - |
| ENSG00000006744 | HGLibA_14874 | 236 | 1.000007436 | ELAC2 | elaC ribonuclease Z 2 [Source:HGNC Symbol;Acc:HGNC:14198] |
| ENSG00000014216 | HGLibA_07142 | 236 | 1.000007436 | CAPN1 | calpain 1 [Source:HGNC Symbol;Acc:HGNC:1476] |
| ENSG00000047410 | HGLibA_51175 | 236 | 1.000007436 | TPR | nuclear basket protein [Source:HGNC Symbol;Acc:HGNC:12017]" |
| ENSG00000084676 | HGLibA_31334 | 236 | 1.000007436 | NCOA1 | nuclear receptor coactivator 1 [Source:HGNC Symbol;Acc:HGNC:7668] |
| ENSG00000089169 | HGLibA_41844 | 236 | 1.000007436 | RPH3A | rabphilin 3A [Source:HGNC Symbol;Acc:HGNC:17056] |
| ENSG00000090612 | HGLibA_55705 | 236 | 1.000007436 | ZNF268 | zinc finger protein 268 [Source:HGNC Symbol;Acc:HGNC:13061] |
| ENSG00000096996 | HGLibA_22965 | 236 | 1.000007436 | IL12RB1 | interleukin 12 receptor subunit beta 1 [Source:HGNC Symbol;Acc:HGNC:5971] |
| ENSG00000103353 | HGLibA_52695 | 236 | 1.000007436 | UBFD1 | ubiquitin family domain containing 1 [Source:HGNC Symbol;Acc:HGNC:30565] |
| ENSG00000107643 | HGLibA_28282 | 236 | 1.000007436 | MAPK8 | mitogen-activated protein kinase 8 [Source:HGNC Symbol;Acc:HGNC:6881] |
| ENSG00000108591 | HGLibA_13976 | 236 | 1.000007436 | DRG2 | developmentally regulated GTP binding protein 2 [Source:HGNC Symbol;Acc:HGNC:3030] |
| ENSG00000110801 | HGLibA_39188 | 236 | 1.000007436 | PSMD9 | non-ATPase 9 [Source:HGNC Symbol;Acc:HGNC:9567]" |
| ENSG00000112218 | HGLibA_20068 | 236 | 1.000007436 | GPR63 | G protein-coupled receptor 63 [Source:HGNC Symbol;Acc:HGNC:13302] |
| ENSG00000115159 | HGLibA_19790 | 236 | 1.000007436 | GPD2 | glycerol-3-phosphate dehydrogenase 2 [Source:HGNC Symbol;Acc:HGNC:4456] |
| ENSG00000115839 | HGLibA_39919 | 236 | 1.000007436 | RAB3GAP1 | RAB3 GTPase activating protein catalytic subunit 1 [Source:HGNC Symbol;Acc:HGNC:17063] |
| ENSG00000124205 | HGLibA_14476 | 236 | 1.000007436 | EDN3 | endothelin 3 [Source:HGNC Symbol;Acc:HGNC:3178] |
| ENSG00000125998 | HGLibA_16745 | 236 | 1.000007436 | FAM83C | family with sequence similarity 83 member C [Source:HGNC Symbol;Acc:HGNC:16121] |
| ENSG00000129451 | HGLibA_25233 | 236 | 1.000007436 | KLK10 | kallikrein related peptidase 10 [Source:HGNC Symbol;Acc:HGNC:6358] |
| ENSG00000134259 | HGLibA_31872 | 236 | 1.000007436 | NGF | nerve growth factor [Source:HGNC Symbol;Acc:HGNC:7808] |
| ENSG00000135144 | HGLibA_14080 | 236 | 1.000007436 | DTX1 | deltex E3 ubiquitin ligase 1 [Source:HGNC Symbol;Acc:HGNC:3060] |
| ENSG00000137494 | HGLibA_02098 | 236 | 1.000007436 | ANKRD42 | ankyrin repeat domain 42 [Source:HGNC Symbol;Acc:HGNC:26752] |
| ENSG00000138279 | HGLibA_02248 | 236 | 1.000007436 | ANXA7 | annexin A7 [Source:HGNC Symbol;Acc:HGNC:545] |
| ENSG00000139517 | HGLibA_26746 | 236 | 1.000007436 | LNX2 | ligand of numb-protein X 2 [Source:HGNC Symbol;Acc:HGNC:20421] |
| ENSG00000141639 | HGLibA_28274 | 236 | 1.000007436 | MAPK4 | mitogen-activated protein kinase 4 [Source:HGNC Symbol;Acc:HGNC:6878] |
| ENSG00000143549 | HGLibA_51146 | 236 | 1.000007436 | TPM3 | tropomyosin 3 [Source:HGNC Symbol;Acc:HGNC:12012] |
| ENSG00000143631 | HGLibA_17609 | 236 | 1.000007436 | FLG | filaggrin [Source:HGNC Symbol;Acc:HGNC:3748] |
| ENSG00000144488 | HGLibA_15577 | 236 | 1.000007436 | ESPNL | espin like [Source:HGNC Symbol;Acc:HGNC:27937] |
| ENSG00000146830 | HGLibA_19063 | 236 | 1.000007436 | GIGYF1 | GRB10 interacting GYF protein 1 [Source:HGNC Symbol;Acc:HGNC:9126] |
| ENSG00000148702 | HGLibA_20794 | 236 | 1.000007436 | HABP2 | hyaluronan binding protein 2 [Source:HGNC Symbol;Acc:HGNC:4798] |
| ENSG00000151287 | HGLibA_49095 | 236 | 1.000007436 | TEX30 | testis expressed 30 [Source:HGNC Symbol;Acc:HGNC:25188] |
| ENSG00000152127 | HGLibA_29150 | 236 | 1.000007436 | MGAT5 | 6-mannosylglycoprotein 6-beta-N-acetylglucosaminyltransferase [Source:HGNC Symbol;Acc:HGNC:7049]" |
| ENSG00000153012 | HGLibA_26395 | 236 | 1.000007436 | LGI2 | leucine rich repeat LGI family member 2 [Source:HGNC Symbol;Acc:HGNC:18710] |
| ENSG00000159792 | HGLibA_39065 | 236 | 1.000007436 | PSKH1 | protein serine kinase H1 [Source:HGNC Symbol;Acc:HGNC:9529] |
| ENSG00000163155 | HGLibA_27764 | 236 | 1.000007436 | LYSMD1 | LysM domain containing 1 [Source:HGNC Symbol;Acc:HGNC:32070] |
| ENSG00000164100 | HGLibA_31431 | 236 | 1.000007436 | NDST3 | N-deacetylase and N-sulfotransferase 3 [Source:HGNC Symbol;Acc:HGNC:7682] |
| ENSG00000164105 | HGLibA_42723 | 236 | 1.000007436 | SAP30 | Sin3A associated protein 30 [Source:HGNC Symbol;Acc:HGNC:10532] |
| ENSG00000167554 | HGLibA_56379 | 236 | 1.000007436 | ZNF610 | zinc finger protein 610 [Source:HGNC Symbol;Acc:HGNC:26687] |
| ENSG00000169174 | HGLibA_35689 | 236 | 1.000007436 | PCSK9 | proprotein convertase subtilisin/kexin type 9 [Source:HGNC Symbol;Acc:HGNC:20001] |
| ENSG00000172349 | HGLibA_22987 | 236 | 1.000007436 | IL16 | interleukin 16 [Source:HGNC Symbol;Acc:HGNC:5980] |
| ENSG00000173218 | HGLibA_53512 | 236 | 1.000007436 | VANGL1 | VANGL planar cell polarity protein 1 [Source:HGNC Symbol;Acc:HGNC:15512] |
| ENSG00000174970 | HGLibA_33483 | 236 | 1.000007436 | OR10AG1 | olfactory receptor family 10 subfamily AG member 1 [Source:HGNC Symbol;Acc:HGNC:19607] |
| ENSG00000175130 | HGLibA_28359 | 236 | 1.000007436 | MARCKSL1 | MARCKS like 1 [Source:HGNC Symbol;Acc:HGNC:7142] |
| ENSG00000175193 | HGLibA_35202 | 236 | 1.000007436 | PARL | presenilin associated rhomboid like [Source:HGNC Symbol;Acc:HGNC:18253] |
| ENSG00000175538 | HGLibA_24193 | 236 | 1.000007436 | KCNE3 | potassium voltage-gated channel subfamily E regulatory subunit 3 [Source:HGNC Symbol;Acc:HGNC:6243] |
| ENSG00000179097 | HGLibA_22368 | 236 | 1.000007436 | HTR1F | 5-hydroxytryptamine receptor 1F [Source:HGNC Symbol;Acc:HGNC:5292] |
| ENSG00000180353 | HGLibA_20970 | 236 | 1.000007436 | HCLS1 | hematopoietic cell-specific Lyn substrate 1 [Source:HGNC Symbol;Acc:HGNC:4844] |
| ENSG00000181016 | HGLibA_27557 | 236 | 1.000007436 | LSMEM1 | leucine rich single-pass membrane protein 1 [Source:HGNC Symbol;Acc:HGNC:22036] |
| ENSG00000188010 | HGLibA_29669 | 236 | 1.000007436 | MORN2 | MORN repeat containing 2 [Source:HGNC Symbol;Acc:HGNC:30166] |
| ENSG00000188312 | HGLibA_09079 | 236 | 1.000007436 | CENPP | centromere protein P [Source:HGNC Symbol;Acc:HGNC:32933] |
| ENSG00000196417 | HGLibA_56691 | 236 | 1.000007436 | ZNF765 | zinc finger protein 765 [Source:HGNC Symbol;Acc:HGNC:25092] |
| ENSG00000196862 | HGLibA_41033 | 236 | 1.000007436 | RGPD4 | RANBP2-like and GRIP domain containing 4 [Source:HGNC Symbol;Acc:HGNC:32417] |

| | | | | | |
|---|---|---|---|---|---|
| ENSG00000197324 | HGLibA_27199 | 236 | 1.000007436 | LRP10 | LDL receptor related protein 10 [Source:HGNC Symbol;Acc:HGNC:14553] |
| ENSG00000197461 | HGLibA_35841 | 236 | 1.000007436 | PDGFA | platelet derived growth factor subunit A [Source:HGNC Symbol;Acc:HGNC:8799] |
| ENSG00000204140 | HGLibA_10107 | 236 | 1.000007436 | CLPSL1 | colipase like 1 [Source:HGNC Symbol;Acc:HGNC:21251] |
| ENSG00000225828 | HGLibA_16540 | 236 | 1.000007436 | FAM229A | family with sequence similarity 229 member A [Source:HGNC Symbol;Acc:HGNC:44652] |
| ENSG00000231887 | HGLibA_38462 | 236 | 1.000007436 | PRH1 | proline rich protein HaeIII subfamily 1 [Source:HGNC Symbol;Acc:HGNC:9366] |
| ENSG00000239388 | HGLibA_03217 | 236 | 1.000007436 | ASB14 | ankyrin repeat and SOCS box containing 14 [Source:HGNC Symbol;Acc:HGNC:19766] |
| ENSG00000243989 | HGLibA_00705 | 236 | 1.000007436 | ACY1 | aminoacylase 1 [Source:HGNC Symbol;Acc:HGNC:177] |
| ENSG00000258227 | HGLibA_09999 | 236 | 1.000007436 | CLEC5A | C-type lectin domain containing 5A [Source:HGNC Symbol;Acc:HGNC:2054] |
| FAM19A4 | HGLibA_16405 | 236 | 1.000007436 | FAM19A4 | - |
| FAM63A | HGLibA_16659 | 236 | 1.000007436 | FAM63A | - |
| hsa-mir-1294 | HGLibA_57654 | 236 | 1.000007436 | hsa-mir-1294 | - |
| hsa-mir-4305 | HGLibA_59933 | 236 | 1.000007436 | hsa-mir-4305 | - |
| hsa-mir-4658 | HGLibA_60739 | 236 | 1.000007436 | hsa-mir-4658 | - |
| hsa-mir-4800 | HGLibA_61306 | 236 | 1.000007436 | hsa-mir-4800 | - |
| hsa-mir-6134 | HGLibA_62689 | 236 | 1.000007436 | hsa-mir-6134 | - |
| hsa-mir-659 | HGLibA_62971 | 236 | 1.000007436 | hsa-mir-659 | - |
| hsa-mir-6852 | HGLibA_63579 | 236 | 1.000007436 | hsa-mir-6852 | - |
| hsa-mir-6864 | HGLibA_63642 | 236 | 1.000007436 | hsa-mir-6864 | - |
| hsa-mir-7112-1 | HGLibA_63813 | 236 | 1.000007436 | hsa-mir-7112-1 | - |
| KIAA0430 | HGLibA_24639 | 236 | 1.000007436 | KIAA0430 | - |
| SELM | HGLibA_43297 | 236 | 1.000007436 | SELM | - |
| DHFRL1 | HGLibA_13107 | 235 | 0.990962093 | DHFRL1 | - |
| ENSG00000002330 | HGLibA_04072 | 235 | 0.990962093 | BAD | BCL2 associated agonist of cell death [Source:HGNC Symbol;Acc:HGNC:936] |
| ENSG00000039319 | HGLibA_55274 | 235 | 0.990962093 | ZFYVE16 | zinc finger FYVE-type containing 16 [Source:HGNC Symbol;Acc:HGNC:20756] |
| ENSG00000046651 | HGLibA_33299 | 235 | 0.990962093 | OFD1 | centriole and centriolar satellite protein [Source:HGNC Symbol;Acc:HGNC:2567]" |
| ENSG00000054118 | HGLibA_49390 | 235 | 0.990962093 | THRAP3 | thyroid hormone receptor associated protein 3 [Source:HGNC Symbol;Acc:HGNC:22964] |
| ENSG00000065057 | HGLibA_32843 | 235 | 0.990962093 | NTHL1 | nth like DNA glycosylase 1 [Source:HGNC Symbol;Acc:HGNC:8028] |
| ENSG00000065665 | HGLibA_43250 | 235 | 0.990962093 | SEC61A2 | Sec61 translocon alpha 2 subunit [Source:HGNC Symbol;Acc:HGNC:17702] |
| ENSG00000083817 | HGLibA_55945 | 235 | 0.990962093 | ZNF416 | zinc finger protein 416 [Source:HGNC Symbol;Acc:HGNC:20645] |
| ENSG00000089248 | HGLibA_15526 | 235 | 0.990962093 | ERP29 | endoplasmic reticulum protein 29 [Source:HGNC Symbol;Acc:HGNC:13799] |
| ENSG00000090020 | HGLibA_45399 | 235 | 0.990962093 | SLC9A1 | solute carrier family 9 member A1 [Source:HGNC Symbol;Acc:HGNC:11071] |
| ENSG00000097021 | HGLibA_00487 | 235 | 0.990962093 | ACOT7 | acyl-CoA thioesterase 7 [Source:HGNC Symbol;Acc:HGNC:24157] |
| ENSG00000100201 | HGLibA_12656 | 235 | 0.990962093 | DDX17 | DEAD-box helicase 17 [Source:HGNC Symbol;Acc:HGNC:2740] |
| ENSG00000102128 | HGLibA_39933 | 235 | 0.990962093 | RAB40AL | RAB40A like [Source:HGNC Symbol;Acc:HGNC:25410] |
| ENSG00000104660 | HGLibA_26329 | 235 | 0.990962093 | LEPROTL1 | leptin receptor overlapping transcript like 1 [Source:HGNC Symbol;Acc:HGNC:6555] |
| ENSG00000109272 | HGLibA_36131 | 235 | 0.990962093 | PF4V1 | platelet factor 4 variant 1 [Source:HGNC Symbol;Acc:HGNC:8862] |
| ENSG00000111605 | HGLibA_11011 | 235 | 0.990962093 | CPSF6 | cleavage and polyadenylation specific factor 6 [Source:HGNC Symbol;Acc:HGNC:13871] |
| ENSG00000125084 | HGLibA_54342 | 235 | 0.990962093 | WNT1 | Wnt family member 1 [Source:HGNC Symbol;Acc:HGNC:12774] |
| ENSG00000125508 | HGLibA_46998 | 235 | 0.990962093 | SRMS | src-related kinase lacking C-terminal regulatory tyrosine and N-terminal myristylation sites [Source:HGNC Symbol;Acc:HGNC:11298] |
| ENSG00000127663 | HGLibA_24509 | 235 | 0.990962093 | KDM4B | lysine demethylase 4B [Source:HGNC Symbol;Acc:HGNC:29136] |
| ENSG00000128602 | HGLibA_45791 | 235 | 0.990962093 | SMO | frizzled class receptor [Source:HGNC Symbol;Acc:HGNC:11119]" |
| ENSG00000128710 | HGLibA_21987 | 235 | 0.990962093 | HOXD10 | homeobox D10 [Source:HGNC Symbol;Acc:HGNC:5133] |
| ENSG00000130766 | HGLibA_43621 | 235 | 0.990962093 | SESN2 | sestrin 2 [Source:HGNC Symbol;Acc:HGNC:20746] |
| ENSG00000131591 | HGLibA_05784 | 235 | 0.990962093 | C1orf159 | chromosome 1 open reading frame 159 [Source:HGNC Symbol;Acc:HGNC:26062] |
| ENSG00000134438 | HGLibA_40407 | 235 | 0.990962093 | RAX | retina and anterior neural fold homeobox [Source:HGNC Symbol;Acc:HGNC:18662] |
| ENSG00000135363 | HGLibA_26705 | 235 | 0.990962093 | LMO2 | LIM domain only 2 [Source:HGNC Symbol;Acc:HGNC:6642] |
| ENSG00000138795 | HGLibA_26272 | 235 | 0.990962093 | LEF1 | lymphoid enhancer binding factor 1 [Source:HGNC Symbol;Acc:HGNC:6551] |
| ENSG00000151746 | HGLibA_04493 | 235 | 0.990962093 | BICD1 | BICD cargo adaptor 1 [Source:HGNC Symbol;Acc:HGNC:1049] |
| ENSG00000153936 | HGLibA_22118 | 235 | 0.990962093 | HS2ST1 | heparan sulfate 2-O-sulfotransferase 1 [Source:HGNC Symbol;Acc:HGNC:5193] |
| ENSG00000155465 | HGLibA_45380 | 235 | 0.990962093 | SLC7A7 | solute carrier family 7 member 7 [Source:HGNC Symbol;Acc:HGNC:11065] |
| ENSG00000156575 | HGLibA_38454 | 235 | 0.990962093 | PRG3 | pro eosinophil major basic protein 2 [Source:HGNC Symbol;Acc:HGNC:9363]" |
| ENSG00000161021 | HGLibA_28043 | 235 | 0.990962093 | MAML1 | mastermind like transcriptional coactivator 1 [Source:HGNC Symbol;Acc:HGNC:13632] |
| ENSG00000162298 | HGLibA_48112 | 235 | 0.990962093 | SYVN1 | synoviolin 1 [Source:HGNC Symbol;Acc:HGNC:20738] |
| ENSG00000167491 | HGLibA_18686 | 235 | 0.990962093 | GATAD2A | GATA zinc finger domain containing 2A [Source:HGNC Symbol;Acc:HGNC:29989] |
| ENSG00000168495 | HGLibA_37618 | 235 | 0.990962093 | POLR3D | RNA polymerase III subunit D [Source:HGNC Symbol;Acc:HGNC:1080] |
| ENSG00000170891 | HGLibA_12251 | 235 | 0.990962093 | CYTL1 | cytokine like 1 [Source:HGNC Symbol;Acc:HGNC:24435] |
| ENSG00000172031 | HGLibA_15331 | 235 | 0.990962093 | EPHX4 | epoxide hydrolase 4 [Source:HGNC Symbol;Acc:HGNC:23758] |
| ENSG00000173302 | HGLibA_19922 | 235 | 0.990962093 | GPR148 | G protein-coupled receptor 148 [Source:HGNC Symbol;Acc:HGNC:23623] |
| ENSG00000175664 | HGLibA_49079 | 235 | 0.990962093 | TEX26 | testis expressed 26 [Source:HGNC Symbol;Acc:HGNC:28622] |
| ENSG00000182768 | HGLibA_31884 | 235 | 0.990962093 | NGRN | neurite outgrowth associated [Source:HGNC Symbol;Acc:HGNC:18077]" |
| ENSG00000183632 | HGLibA_51079 | 235 | 0.990962093 | TP53TG3 | TP53 target 3 [Source:HGNC Symbol;Acc:HGNC:30759] |
| ENSG00000185201 | HGLibA_22643 | 235 | 0.990962093 | IFITM2 | interferon induced transmembrane protein 2 [Source:HGNC Symbol;Acc:HGNC:5413] |
| ENSG00000188691 | HGLibA_34219 | 235 | 0.990962093 | OR56A5 | olfactory receptor family 56 subfamily A member 5 [Source:HGNC Symbol;Acc:HGNC:14792] |
| ENSG00000196151 | HGLibA_54230 | 235 | 0.990962093 | WDSUB1 | sterile alpha motif and U-box domain containing 1 [Source:HGNC Symbol;Acc:HGNC:26697]" |
| ENSG00000196482 | HGLibA_15599 | 235 | 0.990962093 | ESRRG | estrogen related receptor gamma [Source:HGNC Symbol;Acc:HGNC:3474] |
| ENSG00000196867 | HGLibA_55194 | 235 | 0.990962093 | ZFP28 | ZFP28 zinc finger protein [Source:HGNC Symbol;Acc:HGNC:17801] |
| ENSG00000198435 | HGLibA_32645 | 235 | 0.990962093 | NRARP | NOTCH regulated ankyrin repeat protein [Source:HGNC Symbol;Acc:HGNC:33843] |
| ENSG00000204220 | HGLibA_36149 | 235 | 0.990962093 | PFDN6 | prefoldin subunit 6 [Source:HGNC Symbol;Acc:HGNC:4926] |
| ENSG00000204463 | HGLibA_04090 | 235 | 0.990962093 | BAG6 | BCL2 associated athanogene 6 [Source:HGNC Symbol;Acc:HGNC:13919] |
| ENSG00000223614 | HGLibA_56651 | 235 | 0.990962093 | ZNF735 | zinc finger protein 735 [Source:HGNC Symbol;Acc:HGNC:32466] |
| ENSG00000231192 | HGLibA_34290 | 235 | 0.990962093 | OR5H1 | olfactory receptor family 5 subfamily H member 1 [Source:HGNC Symbol;Acc:HGNC:8346] |
| ENSG00000243896 | HGLibA_33743 | 235 | 0.990962093 | OR2A7 | olfactory receptor family 2 subfamily A member 7 [Source:HGNC Symbol;Acc:HGNC:8234] |
| ENSG00000268350 | HGLibA_16186 | 235 | 0.990962093 | FAM156A | family with sequence similarity 156 member A [Source:HGNC Symbol;Acc:HGNC:30114] |
| ENSG00000272920 | HGLibA_57408 | 235 | 0.990962093 | hsa-mir-1253 | hsa-mir-1253 [Source:miRBase;Acc:MI0006387] |
| GTDC2 | HGLibA_20518 | 235 | 0.990962093 | GTDC2 | - |
| hsa-mir-1272 | HGLibA_57526 | 235 | 0.990962093 | hsa-mir-1272 | - |
| hsa-mir-130a | HGLibA_57749 | 235 | 0.990962093 | hsa-mir-130a | - |
| hsa-mir-4268 | HGLibA_59778 | 235 | 0.990962093 | hsa-mir-4268 | - |
| hsa-mir-770 | HGLibA_63938 | 235 | 0.990962093 | hsa-mir-770 | - |
| hsa-mir-8071-1 | HGLibA_64124 | 235 | 0.990962093 | hsa-mir-8071-1 | - |
| hsa-mir-8074 | HGLibA_64137 | 235 | 0.990962093 | hsa-mir-8074 | - |
| LOC643669 | HGLibA_26968 | 235 | 0.990962093 | LOC643669 | - |
| ENSG00000014641 | HGLibA_28683 | 234 | 0.98191675 | MDH1 | malate dehydrogenase 1 [Source:HGNC Symbol;Acc:HGNC:6970] |
| ENSG00000081985 | HGLibA_22968 | 234 | 0.98191675 | IL12RB2 | interleukin 12 receptor subunit beta 2 [Source:HGNC Symbol;Acc:HGNC:5972] |
| ENSG00000089248 | HGLibA_15528 | 234 | 0.98191675 | ERP29 | endoplasmic reticulum protein 29 [Source:HGNC Symbol;Acc:HGNC:13799] |
| ENSG00000101335 | HGLibA_30777 | 234 | 0.98191675 | MYL9 | myosin light chain 9 [Source:HGNC Symbol;Acc:HGNC:15754] |
| ENSG00000104369 | HGLibA_23978 | 234 | 0.98191675 | JPH1 | junctophilin 1 [Source:HGNC Symbol;Acc:HGNC:14201] |
| ENSG00000107949 | HGLibA_04268 | 234 | 0.98191675 | BCCIP | BRCA2 and CDKN1A interacting protein [Source:HGNC Symbol;Acc:HGNC:978] |
| ENSG00000109943 | HGLibA_11222 | 234 | 0.98191675 | CRTAM | cytotoxic and regulatory T cell molecule [Source:HGNC Symbol;Acc:HGNC:24313] |
| ENSG00000110046 | HGLibA_03479 | 234 | 0.98191675 | ATG2A | autophagy related 2A [Source:HGNC Symbol;Acc:HGNC:29028] |
| ENSG00000110442 | HGLibA_10679 | 234 | 0.98191675 | COMMD9 | COMM domain containing 9 [Source:HGNC Symbol;Acc:HGNC:25014] |
| ENSG00000111012 | HGLibA_12096 | 234 | 0.98191675 | CYP27B1 | cytochrome P450 family 27 subfamily B member 1 [Source:HGNC Symbol;Acc:HGNC:2606] |
| ENSG00000113263 | HGLibA_23819 | 234 | 0.98191675 | ITK | IL2 inducible T cell kinase [Source:HGNC Symbol;Acc:HGNC:6171] |
| ENSG00000113552 | HGLibA_19565 | 234 | 0.98191675 | GNPDA1 | glucosamine-6-phosphate deaminase 1 [Source:HGNC Symbol;Acc:HGNC:4417] |
| ENSG00000120068 | HGLibA_21950 | 234 | 0.98191675 | HOXB8 | homeobox B8 [Source:HGNC Symbol;Acc:HGNC:5119] |
| ENSG00000120436 | HGLibA_20016 | 234 | 0.98191675 | GPR31 | G protein-coupled receptor 31 [Source:HGNC Symbol;Acc:HGNC:4486] |
| ENSG00000121680 | HGLibA_36101 | 234 | 0.98191675 | PEX16 | peroxisomal biogenesis factor 16 [Source:HGNC Symbol;Acc:HGNC:8857] |
| ENSG00000127328 | HGLibA_39927 | 234 | 0.98191675 | RAB3IP | RAB3A interacting protein [Source:HGNC Symbol;Acc:HGNC:16508] |
| ENSG00000132535 | HGLibA_13332 | 234 | 0.98191675 | DLG4 | discs large MAGUK scaffold protein 4 [Source:HGNC Symbol;Acc:HGNC:2903] |
| ENSG00000134198 | HGLibA_51922 | 234 | 0.98191675 | TSPAN2 | tetraspanin 2 [Source:HGNC Symbol;Acc:HGNC:20659] |
| ENSG00000134330 | HGLibA_22468 | 234 | 0.98191675 | IAH1 | isoamyl acetate hydrolyzing esterase 1 (putative) [Source:HGNC Symbol;Acc:HGNC:27696] |
| ENSG00000138615 | HGLibA_09708 | 234 | 0.98191675 | CILP | cartilage intermediate layer protein [Source:HGNC Symbol;Acc:HGNC:1980] |
| ENSG00000139187 | HGLibA_25309 | 234 | 0.98191675 | KLRG1 | killer cell lectin like receptor G1 [Source:HGNC Symbol;Acc:HGNC:6380] |
| ENSG00000139880 | HGLibA_08672 | 234 | 0.98191675 | CDH24 | cadherin 24 [Source:HGNC Symbol;Acc:HGNC:14265] |
| ENSG00000141527 | HGLibA_07223 | 234 | 0.98191675 | CARD14 | caspase recruitment domain family member 14 [Source:HGNC Symbol;Acc:HGNC:16446] |
| ENSG00000142748 | HGLibA_17221 | 234 | 0.98191675 | FCN3 | ficolin 3 [Source:HGNC Symbol;Acc:HGNC:3625] |
| ENSG00000146021 | HGLibA_25177 | 234 | 0.98191675 | KLHL3 | kelch like family member 3 [Source:HGNC Symbol;Acc:HGNC:6354] |
| ENSG00000147044 | HGLibA_07286 | 234 | 0.98191675 | CASK | calcium/calmodulin dependent serine protein kinase [Source:HGNC Symbol;Acc:HGNC:1497] |
| ENSG00000156453 | HGLibA_35384 | 234 | 0.98191675 | PCDH1 | protocadherin 1 [Source:HGNC Symbol;Acc:HGNC:8655] |
| ENSG00000157637 | HGLibA_45057 | 234 | 0.98191675 | SLC38A10 | solute carrier family 38 member 10 [Source:HGNC Symbol;Acc:HGNC:28237] |
| ENSG00000160051 | HGLibA_23493 | 234 | 0.98191675 | IQCC | IQ motif containing C [Source:HGNC Symbol;Acc:HGNC:25545] |
| ENSG00000161671 | HGLibA_15005 | 234 | 0.98191675 | EMC10 | ER membrane protein complex subunit 10 [Source:HGNC Symbol;Acc:HGNC:27609] |
| ENSG00000163257 | HGLibA_12433 | 234 | 0.98191675 | DCAF16 | DDB1 and CUL4 associated factor 16 [Source:HGNC Symbol;Acc:HGNC:25987] |
| ENSG00000166888 | HGLibA_47400 | 234 | 0.98191675 | STAT6 | signal transducer and activator of transcription 6 [Source:HGNC Symbol;Acc:HGNC:11368] |
| ENSG00000169071 | HGLibA_41773 | 234 | 0.98191675 | ROR2 | receptor tyrosine kinase like orphan receptor 2 [Source:HGNC Symbol;Acc:HGNC:10257] |
| ENSG00000170759 | HGLibA_24953 | 234 | 0.98191675 | KIF5B | kinesin family member 5B [Source:HGNC Symbol;Acc:HGNC:6324] |
| ENSG00000176435 | HGLibA_09935 | 234 | 0.98191675 | CLEC14A | C-type lectin domain containing 14A [Source:HGNC Symbol;Acc:HGNC:19832] |
| ENSG00000177058 | HGLibA_45086 | 234 | 0.98191675 | SLC38A9 | solute carrier family 38 member 9 [Source:HGNC Symbol;Acc:HGNC:26907] |
| ENSG00000182566 | HGLibA_09995 | 234 | 0.98191675 | CLEC4G | C-type lectin domain family 4 member G [Source:HGNC Symbol;Acc:HGNC:24591] |
| ENSG00000183733 | HGLibA_17517 | 234 | 0.98191675 | FIGLA | folliculogenesis specific bHLH transcription factor [Source:HGNC Symbol;Acc:HGNC:24669] |
| ENSG00000186049 | HGLibA_25530 | 234 | 0.98191675 | KRT73 | keratin 73 [Source:HGNC Symbol;Acc:HGNC:28928] |
| ENSG00000186895 | HGLibA_17405 | 234 | 0.98191675 | FGF3 | fibroblast growth factor 3 [Source:HGNC Symbol;Acc:HGNC:3681] |
| ENSG00000187091 | HGLibA_36991 | 234 | 0.98191675 | PLCD1 | phospholipase C delta 1 [Source:HGNC Symbol;Acc:HGNC:9060] |
| ENSG00000189042 | HGLibA_56263 | 234 | 0.98191675 | ZNF567 | zinc finger protein 567 [Source:HGNC Symbol;Acc:HGNC:28696] |
| ENSG00000197961 | HGLibA_55453 | 234 | 0.98191675 | ZNF121 | zinc finger protein 121 [Source:HGNC Symbol;Acc:HGNC:12904] |
| ENSG00000197977 | HGLibA_14959 | 234 | 0.98191675 | ELOVL2 | ELOVL fatty acid elongase 2 [Source:HGNC Symbol;Acc:HGNC:14416] |
| ENSG00000198932 | HGLibA_20119 | 234 | 0.98191675 | GPRASP1 | G protein-coupled receptor associated sorting protein 1 [Source:HGNC Symbol;Acc:HGNC:24834] |
| ENSG00000203942 | HGLibA_05081 | 234 | 0.98191675 | C10orf62 | chromosome 10 open reading frame 62 [Source:HGNC Symbol;Acc:HGNC:23294] |
| ENSG00000204140 | HGLibA_10108 | 234 | 0.98191675 | CLPSL1 | colipase like 1 [Source:HGNC Symbol;Acc:HGNC:21251] |
| ENSG00000204991 | HGLibA_46728 | 234 | 0.98191675 | SPIRE2 | spire type actin nucleation factor 2 [Source:HGNC Symbol;Acc:HGNC:30623] |
| ENSG00000212864 | HGLibA_41616 | 234 | 0.98191675 | RNF208 | ring finger protein 208 [Source:HGNC Symbol;Acc:HGNC:25420] |
| ENSG00000214694 | HGLibA_02850 | 234 | 0.98191675 | ARHGEF33 | Rho guanine nucleotide exchange factor 33 [Source:HGNC Symbol;Acc:HGNC:37252] |
| ENSG00000219626 | HGLibA_16536 | 234 | 0.98191675 | FAM228B | family with sequence similarity 228 member B [Source:HGNC Symbol;Acc:HGNC:24736] |
| ENSG00000237388 | HGLibA_33930 | 234 | 0.98191675 | OR4A47 | olfactory receptor family 4 subfamily A member 47 [Source:HGNC Symbol;Acc:HGNC:31266] |
| ENSG00000241484 | HGLibA_02790 | 234 | 0.98191675 | ARHGAP8 | Rho GTPase activating protein 8 [Source:HGNC Symbol;Acc:HGNC:677] |

| | | | | | |
|---|---|---|---|---|---|
| hsa-mir-3182 | HGLibA_58827 | 234 | 0.98191675 | hsa-mir-3182 | - |
| hsa-mir-3960 | HGLibA_59643 | 234 | 0.98191675 | hsa-mir-3960 | - |
| hsa-mir-422a | HGLibA_59695 | 234 | 0.98191675 | hsa-mir-422a | - |
| hsa-mir-4421 | HGLibA_60070 | 234 | 0.98191675 | hsa-mir-4421 | - |
| hsa-mir-4447 | HGLibA_60195 | 234 | 0.98191675 | hsa-mir-4447 | - |
| hsa-mir-891b | HGLibA_64247 | 234 | 0.98191675 | hsa-mir-891b | - |
| WDR60 | HGLibA_54140 | 234 | 0.98191675 | WDR60 | - |
| ZFP112 | HGLibA_55186 | 234 | 0.98191675 | ZFP112 | - |
| ATP5G1 | HGLibA_03655 | 233 | 0.972871407 | ATP5G1 | - |
| C7orf71 | HGLibA_06583 | 233 | 0.972871407 | C7orf71 | - |
| ENSG00000012779 | HGLibA_01687 | 233 | 0.972871407 | ALOX5 | arachidonate 5-lipoxygenase [Source:HGNC Symbol;Acc:HGNC:435] |
| ENSG00000013563 | HGLibA_13657 | 233 | 0.972871407 | DNASE1L1 | deoxyribonuclease 1 like 1 [Source:HGNC Symbol;Acc:HGNC:2957] |
| ENSG00000067992 | HGLibA_35901 | 233 | 0.972871407 | PDK3 | pyruvate dehydrogenase kinase 3 [Source:HGNC Symbol;Acc:HGNC:8811] |
| ENSG00000070047 | HGLibA_36458 | 233 | 0.972871407 | PHRF1 | PHD and ring finger domains 1 [Source:HGNC Symbol;Acc:HGNC:24351] |
| ENSG00000078808 | HGLibA_43131 | 233 | 0.972871407 | SDF4 | stromal cell derived factor 4 [Source:HGNC Symbol;Acc:HGNC:24188] |
| ENSG00000100403 | HGLibA_54985 | 233 | 0.972871407 | ZC3H7B | zinc finger CCCH-type containing 7B [Source:HGNC Symbol;Acc:HGNC:30869] |
| ENSG00000110871 | HGLibA_10757 | 233 | 0.972871407 | COQ5 | methyltransferase [Source:HGNC Symbol;Acc:HGNC:28722]" |
| ENSG00000110880 | HGLibA_10778 | 233 | 0.972871407 | CORO1C | coronin 1C [Source:HGNC Symbol;Acc:HGNC:2254] |
| ENSG00000111701 | HGLibA_02466 | 233 | 0.972871407 | APOBEC1 | apolipoprotein B mRNA editing enzyme catalytic subunit 1 [Source:HGNC Symbol;Acc:HGNC:604] |
| ENSG00000121858 | HGLibA_50796 | 233 | 0.972871407 | TNFSF10 | TNF superfamily member 10 [Source:HGNC Symbol;Acc:HGNC:11925] |
| ENSG00000123159 | HGLibA_19117 | 233 | 0.972871407 | GIPC1 | GIPC PDZ domain containing family member 1 [Source:HGNC Symbol;Acc:HGNC:1226] |
| ENSG00000126709 | HGLibA_22616 | 233 | 0.972871407 | IFI6 | interferon alpha inducible protein 6 [Source:HGNC Symbol;Acc:HGNC:4054] |
| ENSG00000127884 | HGLibA_14403 | 233 | 0.972871407 | ECHS1 | short chain 1 [Source:HGNC Symbol;Acc:HGNC:3151]" |
| ENSG00000139547 | HGLibA_40745 | 233 | 0.972871407 | RDH16 | retinol dehydrogenase 16 [Source:HGNC Symbol;Acc:HGNC:29674] |
| ENSG00000140750 | HGLibA_02714 | 233 | 0.972871407 | ARHGAP17 | Rho GTPase activating protein 17 [Source:HGNC Symbol;Acc:HGNC:18239] |
| ENSG00000143851 | HGLibA_39479 | 233 | 0.972871407 | PTPN7 | non-receptor type 7 [Source:HGNC Symbol;Acc:HGNC:9659]" |
| ENSG00000148219 | HGLibA_03384 | 233 | 0.972871407 | ASTN2 | astrotactin 2 [Source:HGNC Symbol;Acc:HGNC:17021] |
| ENSG00000148848 | HGLibA_00734 | 233 | 0.972871407 | ADAM12 | ADAM metallopeptidase domain 12 [Source:HGNC Symbol;Acc:HGNC:190] |
| ENSG00000154945 | HGLibA_02096 | 233 | 0.972871407 | ANKRD40 | ankyrin repeat domain 40 [Source:HGNC Symbol;Acc:HGNC:28233] |
| ENSG00000155918 | HGLibA_40126 | 233 | 0.972871407 | RAET1L | retinoic acid early transcript 1L [Source:HGNC Symbol;Acc:HGNC:16798] |
| ENSG00000159256 | HGLibA_29653 | 233 | 0.972871407 | MORC3 | MORC family CW-type zinc finger 3 [Source:HGNC Symbol;Acc:HGNC:23572] |
| ENSG00000160439 | HGLibA_40741 | 233 | 0.972871407 | RDH13 | retinol dehydrogenase 13 [Source:HGNC Symbol;Acc:HGNC:19978] |
| ENSG00000161202 | HGLibA_14213 | 233 | 0.972871407 | DVL3 | dishevelled segment polarity protein 3 [Source:HGNC Symbol;Acc:HGNC:3087] |
| ENSG00000161681 | HGLibA_43987 | 233 | 0.972871407 | SHANK1 | SH3 and multiple ankyrin repeat domains 1 [Source:HGNC Symbol;Acc:HGNC:15474] |
| ENSG00000163528 | HGLibA_09370 | 233 | 0.972871407 | CHCHD4 | coiled-coil-helix-coiled-coil-helix domain containing 4 [Source:HGNC Symbol;Acc:HGNC:26467] |
| ENSG00000164241 | HGLibA_06419 | 233 | 0.972871407 | C5orf63 | chromosome 5 open reading frame 63 [Source:HGNC Symbol;Acc:HGNC:40051] |
| ENSG00000167315 | HGLibA_00344 | 233 | 0.972871407 | ACAA2 | acetyl-CoA acyltransferase 2 [Source:HGNC Symbol;Acc:HGNC:83] |
| ENSG00000167771 | HGLibA_40712 | 233 | 0.972871407 | RCOR2 | REST corepressor 2 [Source:HGNC Symbol;Acc:HGNC:27455] |
| ENSG00000167881 | HGLibA_47010 | 233 | 0.972871407 | SRP68 | signal recognition particle 68 [Source:HGNC Symbol;Acc:HGNC:11302] |
| ENSG00000168291 | HGLibA_35869 | 233 | 0.972871407 | PDHB | pyruvate dehydrogenase E1 beta subunit [Source:HGNC Symbol;Acc:HGNC:8808] |
| ENSG00000168300 | HGLibA_35627 | 233 | 0.972871407 | PCMTD1 | protein-L-isoaspartate (D-aspartate) O-methyltransferase domain containing 1 [Source:HGNC Symbol;Acc:HGNC:30483] |
| ENSG00000170423 | HGLibA_25547 | 233 | 0.972871407 | KRT78 | keratin 78 [Source:HGNC Symbol;Acc:HGNC:28926] |
| ENSG00000171794 | HGLibA_53429 | 233 | 0.972871407 | UTF1 | undifferentiated embryonic cell transcription factor 1 [Source:HGNC Symbol;Acc:HGNC:12634] |
| ENSG00000173198 | HGLibA_12225 | 233 | 0.972871407 | CYSLTR1 | cysteinyl leukotriene receptor 1 [Source:HGNC Symbol;Acc:HGNC:17451] |
| ENSG00000175606 | HGLibA_50500 | 233 | 0.972871407 | TMEM70 | transmembrane protein 70 [Source:HGNC Symbol;Acc:HGNC:26050] |
| ENSG00000181518 | HGLibA_34515 | 233 | 0.972871407 | OR8D4 | olfactory receptor family 8 subfamily D member 4 [Source:HGNC Symbol;Acc:HGNC:14840] |
| ENSG00000185361 | HGLibA_50720 | 233 | 0.972871407 | TNFAIP8L1 | TNF alpha induced protein 8 like 1 [Source:HGNC Symbol;Acc:HGNC:28279] |
| ENSG00000186007 | HGLibA_26287 | 233 | 0.972871407 | LEMD1 | LEM domain containing 1 [Source:HGNC Symbol;Acc:HGNC:18725] |
| ENSG00000186136 | HGLibA_48407 | 233 | 0.972871407 | TAS2R42 | taste 2 receptor member 42 [Source:HGNC Symbol;Acc:HGNC:18888] |
| ENSG00000187840 | HGLibA_14828 | 233 | 0.972871407 | EIF4EBP1 | eukaryotic translation initiation factor 4E binding protein 1 [Source:HGNC Symbol;Acc:HGNC:3288] |
| ENSG00000196616 | HGLibA_00954 | 233 | 0.972871407 | ADH1B | beta polypeptide [Source:HGNC Symbol;Acc:HGNC:250]" |
| ENSG00000198000 | HGLibA_32278 | 233 | 0.972871407 | NOL8 | nucleolar protein 8 [Source:HGNC Symbol;Acc:HGNC:23387] |
| ENSG00000204604 | HGLibA_56048 | 233 | 0.972871407 | ZNF468 | zinc finger protein 468 [Source:HGNC Symbol;Acc:HGNC:33105] |
| ENSG00000204897 | HGLibA_25460 | 233 | 0.972871407 | KRT25 | keratin 25 [Source:HGNC Symbol;Acc:HGNC:30839] |
| ENSG00000213316 | HGLibA_27605 | 233 | 0.972871407 | LTC4S | leukotriene C4 synthase [Source:HGNC Symbol;Acc:HGNC:6719] |
| ENSG00000243279 | HGLibA_38268 | 233 | 0.972871407 | PRAF2 | PRA1 domain family member 2 [Source:HGNC Symbol;Acc:HGNC:28911] |
| FAM5C | HGLibA_16653 | 233 | 0.972871407 | FAM5C | - |
| HIST4H4 | HGLibA_21563 | 233 | 0.972871407 | HIST4H4 | - |
| hsa-mir-125b-2 | HGLibA_57450 | 233 | 0.972871407 | hsa-mir-125b-2 | - |
| hsa-mir-21 | HGLibA_58209 | 233 | 0.972871407 | hsa-mir-21 | - |
| hsa-mir-3152 | HGLibA_58667 | 233 | 0.972871407 | hsa-mir-3152 | - |
| hsa-mir-3976 | HGLibA_59663 | 233 | 0.972871407 | hsa-mir-3976 | - |
| hsa-mir-551b | HGLibA_62124 | 233 | 0.972871407 | hsa-mir-551b | - |
| hsa-mir-6081 | HGLibA_62584 | 233 | 0.972871407 | hsa-mir-6081 | - |
| hsa-mir-6085 | HGLibA_62600 | 233 | 0.972871407 | hsa-mir-6085 | - |
| hsa-mir-888 | HGLibA_64231 | 233 | 0.972871407 | hsa-mir-888 | - |
| TMEM8A | HGLibA_50546 | 233 | 0.972871407 | TMEM8A | - |
| ABP1 | HGLibA_00320 | 232 | 0.963826064 | ABP1 | - |
| ADRBK1 | HGLibA_01065 | 232 | 0.963826064 | ADRBK1 | - |
| DDX26B | HGLibA_12684 | 232 | 0.963826064 | DDX26B | - |
| ENSG00000027869 | HGLibA_43890 | 232 | 0.963826064 | SH2D2A | SH2 domain containing 2A [Source:HGNC Symbol;Acc:HGNC:10821] |
| ENSG00000055917 | HGLibA_39584 | 232 | 0.963826064 | PUM2 | pumilio RNA binding family member 2 [Source:HGNC Symbol;Acc:HGNC:14958] |
| ENSG00000065320 | HGLibA_32853 | 232 | 0.963826064 | NTN1 | netrin 1 [Source:HGNC Symbol;Acc:HGNC:8029] |
| ENSG00000078674 | HGLibA_35623 | 232 | 0.963826064 | PCM1 | pericentriolar material 1 [Source:HGNC Symbol;Acc:HGNC:8727] |
| ENSG00000078808 | HGLibA_43130 | 232 | 0.963826064 | SDF4 | stromal cell derived factor 4 [Source:HGNC Symbol;Acc:HGNC:24188] |
| ENSG00000083520 | HGLibA_13270 | 232 | 0.963826064 | DIS3 | exosome endoribonuclease and 3'-5' exoribonuclease [Source:HGNC Symbol;Acc:HGNC:20604]" |
| ENSG00000090776 | HGLibA_14598 | 232 | 0.963826064 | EFNB1 | ephrin B1 [Source:HGNC Symbol;Acc:HGNC:3226] |
| ENSG00000095637 | HGLibA_46173 | 232 | 0.963826064 | SORBS1 | sorbin and SH3 domain containing 1 [Source:HGNC Symbol;Acc:HGNC:14565] |
| ENSG00000101665 | HGLibA_45586 | 232 | 0.963826064 | SMAD7 | SMAD family member 7 [Source:HGNC Symbol;Acc:HGNC:6773] |
| ENSG00000104805 | HGLibA_32911 | 232 | 0.963826064 | NUCB1 | nucleobindin 1 [Source:HGNC Symbol;Acc:HGNC:8043] |
| ENSG00000105261 | HGLibA_34807 | 232 | 0.963826064 | OVOL3 | ovo like zinc finger 3 [Source:HGNC Symbol;Acc:HGNC:14186] |
| ENSG00000105664 | HGLibA_10683 | 232 | 0.963826064 | COMP | cartilage oligomeric matrix protein [Source:HGNC Symbol;Acc:HGNC:2227] |
| ENSG00000108599 | HGLibA_01405 | 232 | 0.963826064 | AKAP10 | A-kinase anchoring protein 10 [Source:HGNC Symbol;Acc:HGNC:368] |
| ENSG00000117620 | HGLibA_44952 | 232 | 0.963826064 | SLC35A3 | solute carrier family 35 member A3 [Source:HGNC Symbol;Acc:HGNC:11023] |
| ENSG00000117758 | HGLibA_47621 | 232 | 0.963826064 | STX12 | syntaxin 12 [Source:HGNC Symbol;Acc:HGNC:11430] |
| ENSG00000119138 | HGLibA_25087 | 232 | 0.963826064 | KLF9 | Kruppel like factor 9 [Source:HGNC Symbol;Acc:HGNC:1123] |
| ENSG00000123191 | HGLibA_03772 | 232 | 0.963826064 | ATP7B | ATPase copper transporting beta [Source:HGNC Symbol;Acc:HGNC:870] |
| ENSG00000125657 | HGLibA_50829 | 232 | 0.963826064 | TNFSF9 | TNF superfamily member 9 [Source:HGNC Symbol;Acc:HGNC:11939] |
| ENSG00000130939 | HGLibA_52693 | 232 | 0.963826064 | UBE4B | ubiquitination factor E4B [Source:HGNC Symbol;Acc:HGNC:12500] |
| ENSG00000135953 | HGLibA_29121 | 232 | 0.963826064 | MFSD9 | major facilitator superfamily domain containing 9 [Source:HGNC Symbol;Acc:HGNC:28158] |
| ENSG00000137142 | HGLibA_22835 | 232 | 0.963826064 | IGFBPL1 | insulin like growth factor binding protein like 1 [Source:HGNC Symbol;Acc:HGNC:20081] |
| ENSG00000137216 | HGLibA_50477 | 232 | 0.963826064 | TMEM63B | transmembrane protein 63B [Source:HGNC Symbol;Acc:HGNC:17735] |
| ENSG00000138835 | HGLibA_41090 | 232 | 0.963826064 | RGS3 | regulator of G protein signaling 3 [Source:HGNC Symbol;Acc:HGNC:9999] |
| ENSG00000142538 | HGLibA_39369 | 232 | 0.963826064 | PTH2 | parathyroid hormone 2 [Source:HGNC Symbol;Acc:HGNC:30828] |
| ENSG00000145721 | HGLibA_26641 | 232 | 0.963826064 | LIX1 | limb and CNS expressed 1 [Source:HGNC Symbol;Acc:HGNC:18581] |
| ENSG00000146469 | HGLibA_53661 | 232 | 0.963826064 | VIP | vasoactive intestinal peptide [Source:HGNC Symbol;Acc:HGNC:12693] |
| ENSG00000146833 | HGLibA_51490 | 232 | 0.963826064 | TRIM4 | tripartite motif containing 4 [Source:HGNC Symbol;Acc:HGNC:16275] |
| ENSG00000149050 | HGLibA_55593 | 232 | 0.963826064 | ZNF214 | zinc finger protein 214 [Source:HGNC Symbol;Acc:HGNC:13006] |
| ENSG00000149257 | HGLibA_43595 | 232 | 0.963826064 | SERPINH1 | serpin family H member 1 [Source:HGNC Symbol;Acc:HGNC:1546] |
| ENSG00000159363 | HGLibA_03569 | 232 | 0.963826064 | ATP13A2 | ATPase cation transporting 13A2 [Source:HGNC Symbol;Acc:HGNC:30213] |
| ENSG00000159792 | HGLibA_39064 | 232 | 0.963826064 | PSKH1 | protein serine kinase H1 [Source:HGNC Symbol;Acc:HGNC:9529] |
| ENSG00000160767 | HGLibA_16358 | 232 | 0.963826064 | FAM189B | family with sequence similarity 189 member B [Source:HGNC Symbol;Acc:HGNC:1233] |
| ENSG00000162039 | HGLibA_28867 | 232 | 0.963826064 | MEIOB | meiosis specific with OB domains [Source:HGNC Symbol;Acc:HGNC:28569] |
| ENSG00000162040 | HGLibA_22139 | 232 | 0.963826064 | HS3ST6 | heparan sulfate-glucosamine 3-sulfotransferase 6 [Source:HGNC Symbol;Acc:HGNC:14178] |
| ENSG00000164305 | HGLibA_07309 | 232 | 0.963826064 | CASP3 | caspase 3 [Source:HGNC Symbol;Acc:HGNC:1504] |
| ENSG00000165349 | HGLibA_45363 | 232 | 0.963826064 | SLC7A3 | solute carrier family 7 member 3 [Source:HGNC Symbol;Acc:HGNC:11061] |
| ENSG00000166863 | HGLibA_48152 | 232 | 0.963826064 | TAC3 | tachykinin 3 [Source:HGNC Symbol;Acc:HGNC:11521] |
| ENSG00000168995 | HGLibA_44147 | 232 | 0.963826064 | SIGLEC7 | sialic acid binding Ig like lectin 7 [Source:HGNC Symbol;Acc:HGNC:10876] |
| ENSG00000169154 | HGLibA_19694 | 232 | 0.963826064 | GOT1L1 | glutamic-oxaloacetic transaminase 1 like 1 [Source:HGNC Symbol;Acc:HGNC:28487] |
| ENSG00000169193 | HGLibA_07581 | 232 | 0.963826064 | CCDC126 | coiled-coil domain containing 126 [Source:HGNC Symbol;Acc:HGNC:22398] |
| ENSG00000170624 | HGLibA_43803 | 232 | 0.963826064 | SGCD | sarcoglycan delta [Source:HGNC Symbol;Acc:HGNC:10807] |
| ENSG00000171792 | HGLibA_41159 | 232 | 0.963826064 | RHNO1 | RAD9-HUS1-RAD1 interacting nuclear orphan 1 [Source:HGNC Symbol;Acc:HGNC:28206] |
| ENSG00000173542 | HGLibA_29589 | 232 | 0.963826064 | MOB1B | MOB kinase activator 1B [Source:HGNC Symbol;Acc:HGNC:29801] |
| ENSG00000173801 | HGLibA_24013 | 232 | 0.963826064 | JUP | junction plakoglobin [Source:HGNC Symbol;Acc:HGNC:6207] |
| ENSG00000174607 | HGLibA_52940 | 232 | 0.963826064 | UGT8 | UDP glycosyltransferase 8 [Source:HGNC Symbol;Acc:HGNC:12555] |
| ENSG00000182578 | HGLibA_11325 | 232 | 0.963826064 | CSF1R | colony stimulating factor 1 receptor [Source:HGNC Symbol;Acc:HGNC:2433] |
| ENSG00000182836 | HGLibA_37028 | 232 | 0.963826064 | PLCXD3 | phosphatidylinositol specific phospholipase C X domain containing 3 [Source:HGNC Symbol;Acc:HGNC:31822] |
| ENSG00000183090 | HGLibA_17990 | 232 | 0.963826064 | FREM3 | FRAS1 related extracellular matrix 3 [Source:HGNC Symbol;Acc:HGNC:25172] |
| ENSG00000183318 | HGLibA_46593 | 232 | 0.963826064 | SPDYE4 | speedy/RINGO cell cycle regulator family member E4 [Source:HGNC Symbol;Acc:HGNC:35463] |
| ENSG00000204873 | HGLibA_25836 | 232 | 0.963826064 | KRTAP9-3 | keratin associated protein 9-3 [Source:HGNC Symbol;Acc:HGNC:16927] |
| ENSG00000204956 | HGLibA_35517 | 232 | 0.963826064 | PCDHGA1 | 1 [Source:HGNC Symbol;Acc:HGNC:8696]" |
| ENSG00000205726 | HGLibA_23872 | 232 | 0.963826064 | ITSN1 | intersectin 1 [Source:HGNC Symbol;Acc:HGNC:6183] |
| ENSG00000213339 | HGLibA_39761 | 232 | 0.963826064 | QTRT1 | queuine tRNA-ribosyltransferase catalytic subunit 1 [Source:HGNC Symbol;Acc:HGNC:23797] |
| ENSG00000234224 | HGLibA_50249 | 232 | 0.963826064 | TMEM229A | transmembrane protein 229A [Source:HGNC Symbol;Acc:HGNC:37279] |
| ENSG00000250423 | HGLibA_24712 | 232 | 0.963826064 | KIAA1210 | KIAA1210 [Source:HGNC Symbol;Acc:HGNC:29218] |
| FAM45A | HGLibA_16594 | 232 | 0.963826064 | FAM45A | - |
| HIST1H2AC | HGLibA_21381 | 232 | 0.963826064 | HIST1H2AC | - |
| hsa-mir-1272 | HGLibA_57527 | 232 | 0.963826064 | hsa-mir-1272 | - |
| hsa-mir-4280 | HGLibA_59823 | 232 | 0.963826064 | hsa-mir-4280 | - |
| hsa-mir-4527 | HGLibA_60568 | 232 | 0.963826064 | hsa-mir-4527 | - |
| hsa-mir-548v | HGLibA_62073 | 232 | 0.963826064 | hsa-mir-548v | - |
| hsa-mir-675 | HGLibA_63155 | 232 | 0.963826064 | hsa-mir-675 | - |
| hsa-mir-6791 | HGLibA_63343 | 232 | 0.963826064 | hsa-mir-6791 | - |
| hsa-mir-8061 | HGLibA_64082 | 232 | 0.963826064 | hsa-mir-8061 | - |
| MURC | HGLibA_30571 | 232 | 0.963826064 | MURC | - |

| | | | | | |
|---|---|---|---|---|---|
| ORAOV1 | HGLibA_34599 | 232 | 0.963826064 | ORAOV1 | - |
| C15orf56 | HGLibA_05416 | 231 | 0.954780721 | C15orf56 | - |
| C1orf172 | HGLibA_05792 | 231 | 0.954780721 | C1orf172 | - |
| ENSG00000003987 | HGLibA_30437 | 231 | 0.954780721 | MTMR7 | myotubularin related protein 7 [Source:HGNC Symbol;Acc:HGNC:7454] |
| ENSG00000008405 | HGLibA_11239 | 231 | 0.954780721 | CRY1 | cryptochrome circadian regulator 1 [Source:HGNC Symbol;Acc:HGNC:2384] |
| ENSG00000069702 | HGLibA_49230 | 231 | 0.954780721 | TGFBR3 | transforming growth factor beta receptor 3 [Source:HGNC Symbol;Acc:HGNC:11774] |
| ENSG00000088280 | HGLibA_03199 | 231 | 0.954780721 | ASAP3 | ankyrin repeat and PH domain 3 [Source:HGNC Symbol;Acc:HGNC:14987]" |
| ENSG00000089094 | HGLibA_24498 | 231 | 0.954780721 | KDM2B | lysine demethylase 2B [Source:HGNC Symbol;Acc:HGNC:13610] |
| ENSG00000090581 | HGLibA_19576 | 231 | 0.954780721 | GNPTG | N-acetylglucosamine-1-phosphate transferase subunit gamma [Source:HGNC Symbol;Acc:HGNC:23026] |
| ENSG00000095261 | HGLibA_39176 | 231 | 0.954780721 | PSMD5 | non-ATPase 5 [Source:HGNC Symbol;Acc:HGNC:9563]" |
| ENSG00000100739 | HGLibA_04381 | 231 | 0.954780721 | BDKRB1 | bradykinin receptor B1 [Source:HGNC Symbol;Acc:HGNC:1029] |
| ENSG00000106588 | HGLibA_39072 | 231 | 0.954780721 | PSMA2 | proteasome subunit alpha 2 [Source:HGNC Symbol;Acc:HGNC:9531] |
| ENSG00000114125 | HGLibA_41696 | 231 | 0.954780721 | RNF7 | ring finger protein 7 [Source:HGNC Symbol;Acc:HGNC:10070] |
| ENSG00000116544 | HGLibA_13345 | 231 | 0.954780721 | DLGAP3 | DLG associated protein 3 [Source:HGNC Symbol;Acc:HGNC:30368] |
| ENSG00000119777 | HGLibA_50217 | 231 | 0.954780721 | TMEM214 | transmembrane protein 214 [Source:HGNC Symbol;Acc:HGNC:25983] |
| ENSG00000120088 | HGLibA_11140 | 231 | 0.954780721 | CRHR1 | corticotropin releasing hormone receptor 1 [Source:HGNC Symbol;Acc:HGNC:2357] |
| ENSG00000120332 | HGLibA_50863 | 231 | 0.954780721 | TNN | tenascin N [Source:HGNC Symbol;Acc:HGNC:22942] |
| ENSG00000124490 | HGLibA_11167 | 231 | 0.954780721 | CRISP2 | cysteine rich secretory protein 2 [Source:HGNC Symbol;Acc:HGNC:12024] |
| ENSG00000130779 | HGLibA_10043 | 231 | 0.954780721 | CLIP1 | CAP-Gly domain containing linker protein 1 [Source:HGNC Symbol;Acc:HGNC:10461] |
| ENSG00000138031 | HGLibA_00914 | 231 | 0.954780721 | ADCY3 | adenylate cyclase 3 [Source:HGNC Symbol;Acc:HGNC:234] |
| ENSG00000138669 | HGLibA_38582 | 231 | 0.954780721 | PRKG2 | protein kinase cGMP-dependent 2 [Source:HGNC Symbol;Acc:HGNC:9416] |
| ENSG00000140632 | HGLibA_19386 | 231 | 0.954780721 | GLYR1 | glyoxylate reductase 1 homolog [Source:HGNC Symbol;Acc:HGNC:24434] |
| ENSG00000141086 | HGLibA_11687 | 231 | 0.954780721 | CTRL | chymotrypsin like [Source:HGNC Symbol;Acc:HGNC:2524] |
| ENSG00000143515 | HGLibA_03784 | 231 | 0.954780721 | ATP8B2 | ATPase phospholipid transporting 8B2 [Source:HGNC Symbol;Acc:HGNC:13534] |
| ENSG00000146070 | HGLibA_36923 | 231 | 0.954780721 | PLA2G7 | phospholipase A2 group VII [Source:HGNC Symbol;Acc:HGNC:9040] |
| ENSG00000152102 | HGLibA_16250 | 231 | 0.954780721 | FAM168B | family with sequence similarity 168 member B [Source:HGNC Symbol;Acc:HGNC:27016] |
| ENSG00000155876 | HGLibA_42225 | 231 | 0.954780721 | RRAGA | Ras related GTP binding A [Source:HGNC Symbol;Acc:HGNC:16963] |
| ENSG00000156738 | HGLibA_30128 | 231 | 0.954780721 | MS4A1 | membrane spanning 4-domains A1 [Source:HGNC Symbol;Acc:HGNC:7315] |
| ENSG00000157017 | HGLibA_19049 | 231 | 0.954780721 | GHRL | ghrelin and obestatin prepropeptide [Source:HGNC Symbol;Acc:HGNC:18129] |
| ENSG00000163312 | HGLibA_21118 | 231 | 0.954780721 | HELQ | POLQ like [Source:HGNC Symbol;Acc:HGNC:18536]" |
| ENSG00000163746 | HGLibA_37234 | 231 | 0.954780721 | PLSCR2 | phospholipid scramblase 2 [Source:HGNC Symbol;Acc:HGNC:16494] |
| ENSG00000165702 | HGLibA_18945 | 231 | 0.954780721 | GFI1B | growth factor independent 1B transcriptional repressor [Source:HGNC Symbol;Acc:HGNC:4238] |
| ENSG00000165949 | HGLibA_22595 | 231 | 0.954780721 | IFI27 | interferon alpha inducible protein 27 [Source:HGNC Symbol;Acc:HGNC:5397] |
| ENSG00000166126 | HGLibA_01808 | 231 | 0.954780721 | AMN | amnion associated transmembrane protein [Source:HGNC Symbol;Acc:HGNC:14604] |
| ENSG00000166821 | HGLibA_36085 | 231 | 0.954780721 | PEX11A | peroxisomal biogenesis factor 11 alpha [Source:HGNC Symbol;Acc:HGNC:8852] |
| ENSG00000168273 | HGLibA_45759 | 231 | 0.954780721 | SMIM4 | small integral membrane protein 4 [Source:HGNC Symbol;Acc:HGNC:37257] |
| ENSG00000169071 | HGLibA_41772 | 231 | 0.954780721 | ROR2 | receptor tyrosine kinase like orphan receptor 2 [Source:HGNC Symbol;Acc:HGNC:10257] |
| ENSG00000171291 | HGLibA_55995 | 231 | 0.954780721 | ZNF439 | zinc finger protein 439 [Source:HGNC Symbol;Acc:HGNC:20873] |
| ENSG00000175040 | HGLibA_09632 | 231 | 0.954780721 | CHST2 | carbohydrate sulfotransferase 2 [Source:HGNC Symbol;Acc:HGNC:1970] |
| ENSG00000177294 | HGLibA_17087 | 231 | 0.954780721 | FBXO39 | F-box protein 39 [Source:HGNC Symbol;Acc:HGNC:28565] |
| ENSG00000179636 | HGLibA_51169 | 231 | 0.954780721 | TPPP2 | tubulin polymerization promoting protein family member 2 [Source:HGNC Symbol;Acc:HGNC:19293] |
| ENSG00000179915 | HGLibA_32734 | 231 | 0.954780721 | NRXN1 | neurexin 1 [Source:HGNC Symbol;Acc:HGNC:8008] |
| ENSG00000180773 | HGLibA_45040 | 231 | 0.954780721 | SLC36A4 | solute carrier family 36 member 4 [Source:HGNC Symbol;Acc:HGNC:19660] |
| ENSG00000183648 | HGLibA_31503 | 231 | 0.954780721 | NDUFB1 | NADH:ubiquinone oxidoreductase subunit B1 [Source:HGNC Symbol;Acc:HGNC:7695] |
| ENSG00000187118 | HGLibA_10185 | 231 | 0.954780721 | CMC1 | C-X9-C motif containing 1 [Source:HGNC Symbol;Acc:HGNC:28783] |
| ENSG00000187193 | HGLibA_30298 | 231 | 0.954780721 | MT1X | metallothionein 1X [Source:HGNC Symbol;Acc:HGNC:7405] |
| ENSG00000187535 | HGLibA_22747 | 231 | 0.954780721 | IFT140 | intraflagellar transport 140 [Source:HGNC Symbol;Acc:HGNC:29077] |
| ENSG00000189221 | HGLibA_28105 | 231 | 0.954780721 | MAOA | monoamine oxidase A [Source:HGNC Symbol;Acc:HGNC:6833] |
| ENSG00000197019 | HGLibA_43605 | 231 | 0.954780721 | SERTAD1 | SERTA domain containing 1 [Source:HGNC Symbol;Acc:HGNC:17932] |
| ENSG00000206557 | HGLibA_51600 | 231 | 0.954780721 | TRIM71 | tripartite motif containing 71 [Source:HGNC Symbol;Acc:HGNC:32669] |
| ENSG00000226979 | HGLibA_27576 | 231 | 0.954780721 | LTA | lymphotoxin alpha [Source:HGNC Symbol;Acc:HGNC:6709] |
| ENSG00000237110 | HGLibA_48137 | 231 | 0.954780721 | TAAR9 | trace amine associated receptor 9 (gene/pseudogene) [Source:HGNC Symbol;Acc:HGNC:20977] |
| ENSG00000243449 | HGLibA_06342 | 231 | 0.954780721 | C4orf48 | chromosome 4 open reading frame 48 [Source:HGNC Symbol;Acc:HGNC:34437] |
| ENSG00000255526 | HGLibA_31612 | 231 | 0.954780721 | NEDD8-MDP1 | NEDD8-MDP1 readthrough [Source:HGNC Symbol;Acc:HGNC:39551] |
| ENSG00000257138 | HGLibA_48392 | 231 | 0.954780721 | TAS2R38 | taste 2 receptor member 38 [Source:HGNC Symbol;Acc:HGNC:9584] |
| ENSG00000279804 | HGLibA_38302 | 231 | 0.954780721 | PRAMEF18 | PRAME family member 18 [Source:HGNC Symbol;Acc:HGNC:30693] |
| FUK | HGLibA_18160 | 231 | 0.954780721 | FUK | - |
| hsa-mir-4322 | HGLibA_60013 | 231 | 0.954780721 | hsa-mir-4322 | - |
| hsa-mir-548n | HGLibA_62046 | 231 | 0.954780721 | hsa-mir-548n | - |
| hsa-mir-603 | HGLibA_62506 | 231 | 0.954780721 | hsa-mir-603 | - |
| hsa-mir-6165 | HGLibA_62705 | 231 | 0.954780721 | hsa-mir-6165 | - |
| hsa-mir-6516 | HGLibA_62939 | 231 | 0.954780721 | hsa-mir-6516 | - |
| hsa-mir-7847 | HGLibA_63979 | 231 | 0.954780721 | hsa-mir-7847 | - |
| LOC100130370 | HGLibA_26782 | 231 | 0.954780721 | LOC100130370 | - |
| NHP2L1 | HGLibA_31911 | 231 | 0.954780721 | NHP2L1 | - |
| ACN9 | HGLibA_00459 | 230 | 0.945735378 | ACN9 | - |
| ENSG00000006062 | HGLibA_28175 | 230 | 0.945735378 | MAP3K14 | mitogen-activated protein kinase kinase kinase 14 [Source:HGNC Symbol;Acc:HGNC:6853] |
| ENSG00000008118 | HGLibA_07065 | 230 | 0.945735378 | CAMK1G | calcium/calmodulin dependent protein kinase IG [Source:HGNC Symbol;Acc:HGNC:14585] |
| ENSG00000031003 | HGLibA_16145 | 230 | 0.945735378 | FAM13B | family with sequence similarity 13 member B [Source:HGNC Symbol;Acc:HGNC:1335] |
| ENSG00000043514 | HGLibA_51655 | 230 | 0.945735378 | TRIT1 | tRNA isopentenyltransferase 1 [Source:HGNC Symbol;Acc:HGNC:20286] |
| ENSG00000048471 | HGLibA_46079 | 230 | 0.945735378 | SNX29 | sorting nexin 29 [Source:HGNC Symbol;Acc:HGNC:30542] |
| ENSG00000049541 | HGLibA_40906 | 230 | 0.945735378 | RFC2 | replication factor C subunit 2 [Source:HGNC Symbol;Acc:HGNC:9970] |
| ENSG00000076555 | HGLibA_00349 | 230 | 0.945735378 | ACACB | acetyl-CoA carboxylase beta [Source:HGNC Symbol;Acc:HGNC:85] |
| ENSG00000081041 | HGLibA_11866 | 230 | 0.945735378 | CXCL2 | C-X-C motif chemokine ligand 2 [Source:HGNC Symbol;Acc:HGNC:4603] |
| ENSG00000085563 | HGLibA_00132 | 230 | 0.945735378 | ABCB1 | ATP binding cassette subfamily B member 1 [Source:HGNC Symbol;Acc:HGNC:40] |
| ENSG00000100092 | HGLibA_43924 | 230 | 0.945735378 | SH3BP1 | SH3 domain binding protein 1 [Source:HGNC Symbol;Acc:HGNC:10824] |
| ENSG00000100296 | HGLibA_49374 | 230 | 0.945735378 | THOC5 | THO complex 5 [Source:HGNC Symbol;Acc:HGNC:19074] |
| ENSG00000100889 | HGLibA_35617 | 230 | 0.945735378 | PCK2 | mitochondrial [Source:HGNC Symbol;Acc:HGNC:8725]" |
| ENSG00000104763 | HGLibA_03184 | 230 | 0.945735378 | ASAH1 | N-acylsphingosine amidohydrolase 1 [Source:HGNC Symbol;Acc:HGNC:735] |
| ENSG00000105642 | HGLibA_24354 | 230 | 0.945735378 | KCNN1 | potassium calcium-activated channel subfamily N member 1 [Source:HGNC Symbol;Acc:HGNC:6290] |
| ENSG00000124574 | HGLibA_00162 | 230 | 0.945735378 | ABCC10 | ATP binding cassette subfamily C member 10 [Source:HGNC Symbol;Acc:HGNC:52] |
| ENSG00000125084 | HGLibA_54344 | 230 | 0.945735378 | WNT1 | Wnt family member 1 [Source:HGNC Symbol;Acc:HGNC:12774] |
| ENSG00000129244 | HGLibA_03595 | 230 | 0.945735378 | ATP1B2 | ATPase Na+/K+ transporting subunit beta 2 [Source:HGNC Symbol;Acc:HGNC:805] |
| ENSG00000132004 | HGLibA_17160 | 230 | 0.945735378 | FBXW9 | F-box and WD repeat domain containing 9 [Source:HGNC Symbol;Acc:HGNC:28136] |
| ENSG00000133030 | HGLibA_29778 | 230 | 0.945735378 | MPRIP | myosin phosphatase Rho interacting protein [Source:HGNC Symbol;Acc:HGNC:30321] |
| ENSG00000134627 | HGLibA_36779 | 230 | 0.945735378 | PIWIL4 | piwi like RNA-mediated gene silencing 4 [Source:HGNC Symbol;Acc:HGNC:18444] |
| ENSG00000135424 | HGLibA_23743 | 230 | 0.945735378 | ITGA7 | integrin subunit alpha 7 [Source:HGNC Symbol;Acc:HGNC:6143] |
| ENSG00000138435 | HGLibA_09563 | 230 | 0.945735378 | CHRNA1 | cholinergic receptor nicotinic alpha 1 subunit [Source:HGNC Symbol;Acc:HGNC:1955] |
| ENSG00000139714 | HGLibA_29673 | 230 | 0.945735378 | MORN3 | MORN repeat containing 3 [Source:HGNC Symbol;Acc:HGNC:29807] |
| ENSG00000140941 | HGLibA_28125 | 230 | 0.945735378 | MAP1LC3B | microtubule associated protein 1 light chain 3 beta [Source:HGNC Symbol;Acc:HGNC:13352] |
| ENSG00000142185 | HGLibA_51754 | 230 | 0.945735378 | TRPM2 | transient receptor potential cation channel subfamily M member 2 [Source:HGNC Symbol;Acc:HGNC:12339] |
| ENSG00000145494 | HGLibA_31560 | 230 | 0.945735378 | NDUFS6 | NADH:ubiquinone oxidoreductase subunit S6 [Source:HGNC Symbol;Acc:HGNC:7713] |
| ENSG00000146386 | HGLibA_00330 | 230 | 0.945735378 | ABRACL | ABRA C-terminal like [Source:HGNC Symbol;Acc:HGNC:21230] |
| ENSG00000154096 | HGLibA_49425 | 230 | 0.945735378 | THY1 | Thy-1 cell surface antigen [Source:HGNC Symbol;Acc:HGNC:11801] |
| ENSG00000156265 | HGLibA_28199 | 230 | 0.945735378 | MAP3K7CL | MAP3K7 C-terminal like [Source:HGNC Symbol;Acc:HGNC:16457] |
| ENSG00000158528 | HGLibA_38129 | 230 | 0.945735378 | PPP1R9A | protein phosphatase 1 regulatory subunit 9A [Source:HGNC Symbol;Acc:HGNC:14946] |
| ENSG00000163517 | HGLibA_21003 | 230 | 0.945735378 | HDAC11 | histone deacetylase 11 [Source:HGNC Symbol;Acc:HGNC:19086] |
| ENSG00000163705 | HGLibA_16842 | 230 | 0.945735378 | FANCD2OS | FANCD2 opposite strand [Source:HGNC Symbol;Acc:HGNC:28623] |
| ENSG00000164318 | HGLibA_14633 | 230 | 0.945735378 | EGFLAM | fibronectin type III and laminin G domains [Source:HGNC Symbol;Acc:HGNC:26810]" |
| ENSG00000164749 | HGLibA_21784 | 230 | 0.945735378 | HNF4G | hepatocyte nuclear factor 4 gamma [Source:HGNC Symbol;Acc:HGNC:5026] |
| ENSG00000165966 | HGLibA_35999 | 230 | 0.945735378 | PDZRN4 | PDZ domain containing ring finger 4 [Source:HGNC Symbol;Acc:HGNC:30552] |
| ENSG00000167346 | HGLibA_29526 | 230 | 0.945735378 | MMP26 | matrix metallopeptidase 26 [Source:HGNC Symbol;Acc:HGNC:14249] |
| ENSG00000167994 | HGLibA_39926 | 230 | 0.945735378 | RAB3IL1 | RAB3A interacting protein like 1 [Source:HGNC Symbol;Acc:HGNC:9780] |
| ENSG00000171219 | HGLibA_08564 | 230 | 0.945735378 | CDC42BPG | CDC42 binding protein kinase gamma [Source:HGNC Symbol;Acc:HGNC:29829] |
| ENSG00000171467 | HGLibA_55785 | 230 | 0.945735378 | ZNF318 | zinc finger protein 318 [Source:HGNC Symbol;Acc:HGNC:13578] |
| ENSG00000172578 | HGLibA_25218 | 230 | 0.945735378 | KLHL6 | kelch like family member 6 [Source:HGNC Symbol;Acc:HGNC:18653] |
| ENSG00000175193 | HGLibA_35203 | 230 | 0.945735378 | PARL | presenilin associated rhomboid like [Source:HGNC Symbol;Acc:HGNC:18253] |
| ENSG00000177138 | HGLibA_16822 | 230 | 0.945735378 | FAM9B | family with sequence similarity 9 member B [Source:HGNC Symbol;Acc:HGNC:18404] |
| ENSG00000179588 | HGLibA_55253 | 230 | 0.945735378 | ZFPM1 | FOG family member 1 [Source:HGNC Symbol;Acc:HGNC:19762]" |
| ENSG00000181965 | HGLibA_31747 | 230 | 0.945735378 | NEUROG1 | neurogenin 1 [Source:HGNC Symbol;Acc:HGNC:7764] |
| ENSG00000183801 | HGLibA_33357 | 230 | 0.945735378 | OLFML1 | olfactomedin like 1 [Source:HGNC Symbol;Acc:HGNC:24473] |
| ENSG00000184029 | HGLibA_14011 | 230 | 0.945735378 | DSCR4 | Down syndrome critical region 4 [Source:HGNC Symbol;Acc:HGNC:3045] |
| ENSG00000184155 | HGLibA_33528 | 230 | 0.945735378 | OR10J5 | olfactory receptor family 10 subfamily J member 5 [Source:HGNC Symbol;Acc:HGNC:14993] |
| ENSG00000186479 | HGLibA_41106 | 230 | 0.945735378 | RGS7BP | regulator of G protein signaling 7 binding protein [Source:HGNC Symbol;Acc:HGNC:23271] |
| ENSG00000187994 | HGLibA_41301 | 230 | 0.945735378 | RINL | Ras and Rab interactor like [Source:HGNC Symbol;Acc:HGNC:24795] |
| ENSG00000188039 | HGLibA_33122 | 230 | 0.945735378 | NWD1 | NACHT and WD repeat domain containing 1 [Source:HGNC Symbol;Acc:HGNC:27619] |
| ENSG00000196475 | HGLibA_19199 | 230 | 0.945735378 | GK2 | glycerol kinase 2 [Source:HGNC Symbol;Acc:HGNC:4291] |
| ENSG00000197016 | HGLibA_56053 | 230 | 0.945735378 | ZNF470 | zinc finger protein 470 [Source:HGNC Symbol;Acc:HGNC:22220] |
| ENSG00000197658 | HGLibA_44576 | 230 | 0.945735378 | SLC22A24 | solute carrier family 22 member 24 [Source:HGNC Symbol;Acc:HGNC:28542] |
| ENSG00000198517 | HGLibA_27884 | 230 | 0.945735378 | MAFK | MAF bZIP transcription factor K [Source:HGNC Symbol;Acc:HGNC:6782] |
| ENSG00000198720 | HGLibA_02002 | 230 | 0.945735378 | ANKRD13B | ankyrin repeat domain 13B [Source:HGNC Symbol;Acc:HGNC:26363] |
| ENSG00000204033 | HGLibA_27188 | 230 | 0.945735378 | LRIT2 | Ig-like and transmembrane domains 2 [Source:HGNC Symbol;Acc:HGNC:23443]" |
| ENSG00000206561 | HGLibA_10646 | 230 | 0.945735378 | COLQ | collagen like tail subunit of asymmetric acetylcholinesterase [Source:HGNC Symbol;Acc:HGNC:2226] |
| ENSG00000213614 | HGLibA_21200 | 230 | 0.945735378 | HEXA | hexosaminidase subunit alpha [Source:HGNC Symbol;Acc:HGNC:4878] |
| ENSG00000221843 | HGLibA_06114 | 230 | 0.945735378 | C2orf16 | chromosome 2 open reading frame 16 [Source:HGNC Symbol;Acc:HGNC:25275] |
| ENSG00000223501 | HGLibA_53805 | 230 | 0.945735378 | VPS52 | GARP complex subunit [Source:HGNC Symbol;Acc:HGNC:10518]" |
| ENSG00000228727 | HGLibA_42732 | 230 | 0.945735378 | SAPCD1 | suppressor APC domain containing 1 [Source:HGNC Symbol;Acc:HGNC:13938] |
| ENSG00000231738 | HGLibA_51918 | 230 | 0.945735378 | TSPAN19 | tetraspanin 19 [Source:HGNC Symbol;Acc:HGNC:31886] |
| ENSG00000232125 | HGLibA_14289 | 230 | 0.945735378 | DYTN | dystrotelin [Source:HGNC Symbol;Acc:HGNC:23279] |
| ENSG00000240694 | HGLibA_37369 | 230 | 0.945735378 | PNMA2 | PNMA family member 2 [Source:HGNC Symbol;Acc:HGNC:9159] |
| ENSG00000243364 | HGLibA_14592 | 230 | 0.945735378 | EFNA4 | ephrin A4 [Source:HGNC Symbol;Acc:HGNC:3224] |
| ENSG00000277443 | HGLibA_28355 | 230 | 0.945735378 | MARCKS | myristoylated alanine rich protein kinase C substrate [Source:HGNC Symbol;Acc:HGNC:6759] |
| ENSG00000279486 | HGLibA_33750 | 230 | 0.945735378 | OR2AG1 | olfactory receptor family 2 subfamily AG member 1 (gene/pseudogene) [Source:HGNC Symbol;Acc:HGNC:15142] |
| HIST1H1E | HGLibA_21370 | 230 | 0.945735378 | HIST1H1E | - |
| hsa-mir-1229 | HGLibA_57315 | 230 | 0.945735378 | hsa-mir-1229 | - |

| | | | | | |
|---|---|---|---|---|---|
| hsa-mir-1909 | HGLibA_58029 | 230 | 0.945735378 | hsa-mir-1909 | - |
| hsa-mir-4681 | HGLibA_60834 | 230 | 0.945735378 | hsa-mir-4681 | - |
| SQRDL | HGLibA_46917 | 230 | 0.945735378 | SQRDL | - |
| WBSCR27 | HGLibA_53997 | 230 | 0.945735378 | WBSCR27 | - |
| ACRC | HGLibA_00536 | 229 | 0.936690035 | ACRC | - |
| C17orf82 | HGLibA_05586 | 229 | 0.936690035 | C17orf82 | - |
| ENSG00000007062 | HGLibA_38680 | 229 | 0.936690035 | PROM1 | prominin 1 [Source:HGNC Symbol;Acc:HGNC:9454] |
| ENSG00000032219 | HGLibA_02900 | 229 | 0.936690035 | ARID4A | AT-rich interaction domain 4A [Source:HGNC Symbol;Acc:HGNC:9885] |
| ENSG00000038427 | HGLibA_53568 | 229 | 0.936690035 | VCAN | versican [Source:HGNC Symbol;Acc:HGNC:2464] |
| ENSG00000060140 | HGLibA_47685 | 229 | 0.936690035 | STYK1 | serine/threonine/tyrosine kinase 1 [Source:HGNC Symbol;Acc:HGNC:18889] |
| ENSG00000060642 | HGLibA_36594 | 229 | 0.936690035 | PIGV | phosphatidylinositol glycan anchor biosynthesis class V [Source:HGNC Symbol;Acc:HGNC:26031] |
| ENSG00000065802 | HGLibA_03204 | 229 | 0.936690035 | ASB1 | ankyrin repeat and SOCS box containing 1 [Source:HGNC Symbol;Acc:HGNC:16011] |
| ENSG00000088367 | HGLibA_15243 | 229 | 0.936690035 | EPB41L1 | erythrocyte membrane protein band 4.1 like 1 [Source:HGNC Symbol;Acc:HGNC:3378] |
| ENSG00000091136 | HGLibA_25957 | 229 | 0.936690035 | LAMB1 | laminin subunit beta 1 [Source:HGNC Symbol;Acc:HGNC:6486] |
| ENSG00000100211 | HGLibA_07470 | 229 | 0.936690035 | CBY1 | beta catenin antagonist [Source:HGNC Symbol;Acc:HGNC:1307]" |
| ENSG00000100399 | HGLibA_09344 | 229 | 0.936690035 | CHADL | chondroadherin like [Source:HGNC Symbol;Acc:HGNC:25165] |
| ENSG00000100478 | HGLibA_02350 | 229 | 0.936690035 | AP4S1 | adaptor related protein complex 4 subunit sigma 1 [Source:HGNC Symbol;Acc:HGNC:575] |
| ENSG00000101441 | HGLibA_11456 | 229 | 0.936690035 | CST4 | cystatin S [Source:HGNC Symbol;Acc:HGNC:2476] |
| ENSG00000102743 | HGLibA_44652 | 229 | 0.936690035 | SLC25A15 | solute carrier family 25 member 15 [Source:HGNC Symbol;Acc:HGNC:10985] |
| ENSG00000103710 | HGLibA_40371 | 229 | 0.936690035 | RASL12 | RAS like family 12 [Source:HGNC Symbol;Acc:HGNC:30289] |
| ENSG00000106009 | HGLibA_04759 | 229 | 0.936690035 | BRAT1 | BRCA1 associated ATM activator 1 [Source:HGNC Symbol;Acc:HGNC:21701] |
| ENSG00000111319 | HGLibA_43014 | 229 | 0.936690035 | SCNN1A | sodium channel epithelial 1 alpha subunit [Source:HGNC Symbol;Acc:HGNC:10599] |
| ENSG00000111424 | HGLibA_53608 | 229 | 0.936690035 | VDR | vitamin D receptor [Source:HGNC Symbol;Acc:HGNC:12679] |
| ENSG00000111652 | HGLibA_10727 | 229 | 0.936690035 | COPS7A | COP9 signalosome subunit 7A [Source:HGNC Symbol;Acc:HGNC:16758] |
| ENSG00000115194 | HGLibA_44906 | 229 | 0.936690035 | SLC30A3 | solute carrier family 30 member 3 [Source:HGNC Symbol;Acc:HGNC:11014] |
| ENSG00000116237 | HGLibA_22513 | 229 | 0.936690035 | ICMT | isoprenylcysteine carboxyl methyltransferase [Source:HGNC Symbol;Acc:HGNC:5350] |
| ENSG00000116649 | HGLibA_46994 | 229 | 0.936690035 | SRM | spermidine synthase [Source:HGNC Symbol;Acc:HGNC:11296] |
| ENSG00000117713 | HGLibA_02881 | 229 | 0.936690035 | ARID1A | AT-rich interaction domain 1A [Source:HGNC Symbol;Acc:HGNC:11110] |
| ENSG00000121570 | HGLibA_13894 | 229 | 0.936690035 | DPPA4 | developmental pluripotency associated 4 [Source:HGNC Symbol;Acc:HGNC:19200] |
| ENSG00000126550 | HGLibA_22352 | 229 | 0.936690035 | HTN1 | histatin 1 [Source:HGNC Symbol;Acc:HGNC:5283] |
| ENSG00000132507 | HGLibA_14856 | 229 | 0.936690035 | EIF5A | eukaryotic translation initiation factor 5A [Source:HGNC Symbol;Acc:HGNC:3300] |
| ENSG00000134443 | HGLibA_20369 | 229 | 0.936690035 | GRP | gastrin releasing peptide [Source:HGNC Symbol;Acc:HGNC:4605] |
| ENSG00000138835 | HGLibA_41091 | 229 | 0.936690035 | RGS3 | regulator of G protein signaling 3 [Source:HGNC Symbol;Acc:HGNC:9999] |
| ENSG00000141977 | HGLibA_09689 | 229 | 0.936690035 | CIB3 | calcium and integrin binding family member 3 [Source:HGNC Symbol;Acc:HGNC:24580] |
| ENSG00000143420 | HGLibA_15188 | 229 | 0.936690035 | ENSA | endosulfine alpha [Source:HGNC Symbol;Acc:HGNC:3360] |
| ENSG00000143543 | HGLibA_23999 | 229 | 0.936690035 | JTB | jumping translocation breakpoint [Source:HGNC Symbol;Acc:HGNC:6201] |
| ENSG00000144824 | HGLibA_36429 | 229 | 0.936690035 | PHLDB2 | pleckstrin homology like domain family B member 2 [Source:HGNC Symbol;Acc:HGNC:29573] |
| ENSG00000144857 | HGLibA_04665 | 229 | 0.936690035 | BOC | oncogene regulated [Source:HGNC Symbol;Acc:HGNC:17173]" |
| ENSG00000150687 | HGLibA_38917 | 229 | 0.936690035 | PRSS23 | serine protease 23 [Source:HGNC Symbol;Acc:HGNC:14370] |
| ENSG00000151229 | HGLibA_44865 | 229 | 0.936690035 | SLC2A13 | solute carrier family 2 member 13 [Source:HGNC Symbol;Acc:HGNC:15956] |
| ENSG00000151704 | HGLibA_24247 | 229 | 0.936690035 | KCNJ1 | potassium voltage-gated channel subfamily J member 1 [Source:HGNC Symbol;Acc:HGNC:6255] |
| ENSG00000152128 | HGLibA_50029 | 229 | 0.936690035 | TMEM163 | transmembrane protein 163 [Source:HGNC Symbol;Acc:HGNC:25380] |
| ENSG00000160326 | HGLibA_44885 | 229 | 0.936690035 | SLC2A6 | solute carrier family 2 member 6 [Source:HGNC Symbol;Acc:HGNC:11011] |
| ENSG00000164300 | HGLibA_43495 | 229 | 0.936690035 | SERINC5 | serine incorporator 5 [Source:HGNC Symbol;Acc:HGNC:18825] |
| ENSG00000164749 | HGLibA_21782 | 229 | 0.936690035 | HNF4G | hepatocyte nuclear factor 4 gamma [Source:HGNC Symbol;Acc:HGNC:5026] |
| ENSG00000165584 | HGLibA_47207 | 229 | 0.936690035 | SSX3 | SSX family member 3 [Source:HGNC Symbol;Acc:HGNC:11337] |
| ENSG00000165861 | HGLibA_55269 | 229 | 0.936690035 | ZFYVE1 | zinc finger FYVE-type containing 1 [Source:HGNC Symbol;Acc:HGNC:13180] |
| ENSG00000166006 | HGLibA_24165 | 229 | 0.936690035 | KCNC2 | potassium voltage-gated channel subfamily C member 2 [Source:HGNC Symbol;Acc:HGNC:6234] |
| ENSG00000169683 | HGLibA_27365 | 229 | 0.936690035 | LRRC45 | leucine rich repeat containing 45 [Source:HGNC Symbol;Acc:HGNC:28302] |
| ENSG00000170100 | HGLibA_56729 | 229 | 0.936690035 | ZNF778 | zinc finger protein 778 [Source:HGNC Symbol;Acc:HGNC:26479] |
| ENSG00000172497 | HGLibA_00473 | 229 | 0.936690035 | ACOT12 | acyl-CoA thioesterase 12 [Source:HGNC Symbol;Acc:HGNC:24436] |
| ENSG00000174851 | HGLibA_54663 | 229 | 0.936690035 | YIF1A | membrane trafficking protein [Source:HGNC Symbol;Acc:HGNC:16688]" |
| ENSG00000175662 | HGLibA_50952 | 229 | 0.936690035 | TOM1L2 | target of myb1 like 2 membrane trafficking protein [Source:HGNC Symbol;Acc:HGNC:11984] |
| ENSG00000180934 | HGLibA_34212 | 229 | 0.936690035 | OR56A1 | olfactory receptor family 56 subfamily A member 1 [Source:HGNC Symbol;Acc:HGNC:14781] |
| ENSG00000181927 | HGLibA_34055 | 229 | 0.936690035 | OR4P4 | olfactory receptor family 4 subfamily P member 4 [Source:HGNC Symbol;Acc:HGNC:15180] |
| ENSG00000183770 | HGLibA_17904 | 229 | 0.936690035 | FOXL2 | forkhead box L2 [Source:HGNC Symbol;Acc:HGNC:1092] |
| ENSG00000186416 | HGLibA_32021 | 229 | 0.936690035 | NKRF | NFKB repressing factor [Source:HGNC Symbol;Acc:HGNC:19374] |
| ENSG00000187605 | HGLibA_49042 | 229 | 0.936690035 | TET3 | tet methylcytosine dioxygenase 3 [Source:HGNC Symbol;Acc:HGNC:28313] |
| ENSG00000188037 | HGLibA_09820 | 229 | 0.936690035 | CLCN1 | chloride voltage-gated channel 1 [Source:HGNC Symbol;Acc:HGNC:2019] |
| ENSG00000196376 | HGLibA_45002 | 229 | 0.936690035 | SLC35F1 | solute carrier family 35 member F1 [Source:HGNC Symbol;Acc:HGNC:21483] |
| ENSG00000196859 | HGLibA_25500 | 229 | 0.936690035 | KRT39 | keratin 39 [Source:HGNC Symbol;Acc:HGNC:32971] |
| ENSG00000198049 | HGLibA_03905 | 229 | 0.936690035 | AVPR1B | arginine vasopressin receptor 1B [Source:HGNC Symbol;Acc:HGNC:896] |
| ENSG00000205045 | HGLibA_45487 | 229 | 0.936690035 | SLFN12L | schlafen family member 12 like [Source:HGNC Symbol;Acc:HGNC:33920] |
| ENSG00000224531 | HGLibA_45728 | 229 | 0.936690035 | SMIM13 | small integral membrane protein 13 [Source:HGNC Symbol;Acc:HGNC:27356] |
| ENSG00000243646 | HGLibA_22951 | 229 | 0.936690035 | IL10RB | interleukin 10 receptor subunit beta [Source:HGNC Symbol;Acc:HGNC:5965] |
| ENSG00000261857 | HGLibA_29182 | 229 | 0.936690035 | MIA | MIA SH3 domain containing [Source:HGNC Symbol;Acc:HGNC:7076] |
| hsa-mir-1324 | HGLibA_57768 | 229 | 0.936690035 | hsa-mir-1324 | - |
| hsa-mir-4531 | HGLibA_60585 | 229 | 0.936690035 | hsa-mir-4531 | - |
| hsa-mir-6080 | HGLibA_62582 | 229 | 0.936690035 | hsa-mir-6080 | - |
| hsa-mir-612 | HGLibA_62638 | 229 | 0.936690035 | hsa-mir-612 | - |
| LOC147646 | HGLibA_26887 | 229 | 0.936690035 | LOC147646 | - |
| METTL20 | HGLibA_28974 | 229 | 0.936690035 | METTL20 | - |
| PRAMEF22 | HGLibA_38313 | 229 | 0.936690035 | PRAMEF22 | - |
| C6orf195 | HGLibA_06463 | 228 | 0.927644692 | C6orf195 | - |
| C9orf139 | HGLibA_06682 | 228 | 0.927644692 | C9orf139 | - |
| ENSG00000000971 | HGLibA_09259 | 228 | 0.927644692 | CFH | complement factor H [Source:HGNC Symbol;Acc:HGNC:4883] |
| ENSG00000001626 | HGLibA_09293 | 228 | 0.927644692 | CFTR | cystic fibrosis transmembrane conductance regulator [Source:HGNC Symbol;Acc:HGNC:1884] |
| ENSG00000007384 | HGLibA_41134 | 228 | 0.927644692 | RHBDF1 | rhomboid 5 homolog 1 [Source:HGNC Symbol;Acc:HGNC:20561] |
| ENSG00000073849 | HGLibA_47259 | 228 | 0.927644692 | ST6GAL1 | 6-sialyltransferase 1 [Source:HGNC Symbol;Acc:HGNC:10860]" |
| ENSG00000075151 | HGLibA_14846 | 228 | 0.927644692 | EIF4G3 | eukaryotic translation initiation factor 4 gamma 3 [Source:HGNC Symbol;Acc:HGNC:3298] |
| ENSG00000075292 | HGLibA_56436 | 228 | 0.927644692 | ZNF638 | zinc finger protein 638 [Source:HGNC Symbol;Acc:HGNC:17894] |
| ENSG00000085511 | HGLibA_28186 | 228 | 0.927644692 | MAP3K4 | mitogen-activated protein kinase kinase kinase 4 [Source:HGNC Symbol;Acc:HGNC:6856] |
| ENSG00000100359 | HGLibA_43866 | 228 | 0.927644692 | SGSM3 | small G protein signaling modulator 3 [Source:HGNC Symbol;Acc:HGNC:25228] |
| ENSG00000101098 | HGLibA_41287 | 228 | 0.927644692 | RIMS4 | regulating synaptic membrane exocytosis 4 [Source:HGNC Symbol;Acc:HGNC:16183] |
| ENSG00000108423 | HGLibA_52298 | 228 | 0.927644692 | TUBD1 | tubulin delta 1 [Source:HGNC Symbol;Acc:HGNC:16811] |
| ENSG00000108759 | HGLibA_25476 | 228 | 0.927644692 | KRT32 | keratin 32 [Source:HGNC Symbol;Acc:HGNC:6449] |
| ENSG00000110628 | HGLibA_44561 | 228 | 0.927644692 | SLC22A18 | solute carrier family 22 member 18 [Source:HGNC Symbol;Acc:HGNC:10964] |
| ENSG00000110723 | HGLibA_15800 | 228 | 0.927644692 | EXPH5 | exophilin 5 [Source:HGNC Symbol;Acc:HGNC:30578] |
| ENSG00000114648 | HGLibA_25143 | 228 | 0.927644692 | KLHL18 | kelch like family member 18 [Source:HGNC Symbol;Acc:HGNC:29120] |
| ENSG00000114805 | HGLibA_37008 | 228 | 0.927644692 | PLCH1 | phospholipase C eta 1 [Source:HGNC Symbol;Acc:HGNC:29185] |
| ENSG00000116035 | HGLibA_53561 | 228 | 0.927644692 | VAX2 | ventral anterior homeobox 2 [Source:HGNC Symbol;Acc:HGNC:12661] |
| ENSG00000123836 | HGLibA_36156 | 228 | 0.927644692 | PFKFB2 | 6-biphosphatase 2 [Source:HGNC Symbol;Acc:HGNC:8873]" |
| ENSG00000124181 | HGLibA_37003 | 228 | 0.927644692 | PLCG1 | phospholipase C gamma 1 [Source:HGNC Symbol;Acc:HGNC:9065] |
| ENSG00000125124 | HGLibA_04215 | 228 | 0.927644692 | BBS2 | Bardet-Biedl syndrome 2 [Source:HGNC Symbol;Acc:HGNC:967] |
| ENSG00000127080 | HGLibA_23484 | 228 | 0.927644692 | IPPK | inositol-pentakisphosphate 2-kinase [Source:HGNC Symbol;Acc:HGNC:14645] |
| ENSG00000128610 | HGLibA_17318 | 228 | 0.927644692 | FEZF1 | FEZ family zinc finger 1 [Source:HGNC Symbol;Acc:HGNC:22788] |
| ENSG00000130958 | HGLibA_44981 | 228 | 0.927644692 | SLC35D2 | solute carrier family 35 member D2 [Source:HGNC Symbol;Acc:HGNC:20799] |
| ENSG00000132963 | HGLibA_37666 | 228 | 0.927644692 | POMP | proteasome maturation protein [Source:HGNC Symbol;Acc:HGNC:20330] |
| ENSG00000135119 | HGLibA_41707 | 228 | 0.927644692 | RNFT2 | transmembrane 2 [Source:HGNC Symbol;Acc:HGNC:25905]" |
| ENSG00000135775 | HGLibA_10474 | 228 | 0.927644692 | COG2 | component of oligomeric golgi complex 2 [Source:HGNC Symbol;Acc:HGNC:6546] |
| ENSG00000141750 | HGLibA_47315 | 228 | 0.927644692 | STAC2 | SH3 and cysteine rich domain 2 [Source:HGNC Symbol;Acc:HGNC:23990] |
| ENSG00000142303 | HGLibA_00794 | 228 | 0.927644692 | ADAMTS10 | ADAM metallopeptidase with thrombospondin type 1 motif 10 [Source:HGNC Symbol;Acc:HGNC:13201] |
| ENSG00000143502 | HGLibA_47853 | 228 | 0.927644692 | SUSD4 | sushi domain containing 4 [Source:HGNC Symbol;Acc:HGNC:25470] |
| ENSG00000147872 | HGLibA_37173 | 228 | 0.927644692 | PLIN2 | perilipin 2 [Source:HGNC Symbol;Acc:HGNC:248] |
| ENSG00000151239 | HGLibA_52368 | 228 | 0.927644692 | TWF1 | twinfilin actin binding protein 1 [Source:HGNC Symbol;Acc:HGNC:9620] |
| ENSG00000152192 | HGLibA_37776 | 228 | 0.927644692 | POU4F1 | POU class 4 homeobox 1 [Source:HGNC Symbol;Acc:HGNC:9218] |
| ENSG00000152932 | HGLibA_39913 | 228 | 0.927644692 | RAB3C | member RAS oncogene family [Source:HGNC Symbol;Acc:HGNC:30269]" |
| ENSG00000153989 | HGLibA_33093 | 228 | 0.927644692 | NUS1 | dehydrodolichyl diphosphate synthase subunit [Source:HGNC Symbol;Acc:HGNC:21042]" |
| ENSG00000159374 | HGLibA_27811 | 228 | 0.927644692 | M1AP | meiosis 1 associated protein [Source:HGNC Symbol;Acc:HGNC:25183] |
| ENSG00000160791 | HGLibA_08160 | 228 | 0.927644692 | CCR5 | C-C motif chemokine receptor 5 (gene/pseudogene) [Source:HGNC Symbol;Acc:HGNC:1606] |
| ENSG00000164794 | HGLibA_24401 | 228 | 0.927644692 | KCNV1 | potassium voltage-gated channel modifier subfamily V member 1 [Source:HGNC Symbol;Acc:HGNC:18861] |
| ENSG00000166484 | HGLibA_28281 | 228 | 0.927644692 | MAPK7 | mitogen-activated protein kinase 7 [Source:HGNC Symbol;Acc:HGNC:6880] |
| ENSG00000166510 | HGLibA_07840 | 228 | 0.927644692 | CCDC68 | coiled-coil domain containing 68 [Source:HGNC Symbol;Acc:HGNC:24350] |
| ENSG00000168539 | HGLibA_09548 | 228 | 0.927644692 | CHRM1 | cholinergic receptor muscarinic 1 [Source:HGNC Symbol;Acc:HGNC:1950] |
| ENSG00000170458 | HGLibA_08233 | 228 | 0.927644692 | CD14 | CD14 molecule [Source:HGNC Symbol;Acc:HGNC:1628] |
| ENSG00000172348 | HGLibA_40670 | 228 | 0.927644692 | RCAN2 | regulator of calcineurin 2 [Source:HGNC Symbol;Acc:HGNC:3041] |
| ENSG00000172985 | HGLibA_43975 | 228 | 0.927644692 | SH3RF3 | SH3 domain containing ring finger 3 [Source:HGNC Symbol;Acc:HGNC:24699] |
| ENSG00000172992 | HGLibA_12466 | 228 | 0.927644692 | DCAKD | dephospho-CoA kinase domain containing [Source:HGNC Symbol;Acc:HGNC:26238] |
| ENSG00000174740 | HGLibA_34940 | 228 | 0.927644692 | PABPC5 | poly(A) binding protein cytoplasmic 5 [Source:HGNC Symbol;Acc:HGNC:13629] |
| ENSG00000174982 | HGLibA_34063 | 228 | 0.927644692 | OR4S2 | olfactory receptor family 4 subfamily S member 2 [Source:HGNC Symbol;Acc:HGNC:15183] |
| ENSG00000175463 | HGLibA_48469 | 228 | 0.927644692 | TBC1D10C | TBC1 domain family member 10C [Source:HGNC Symbol;Acc:HGNC:24702] |
| ENSG00000176925 | HGLibA_34107 | 228 | 0.927644692 | OR51F2 | olfactory receptor family 51 subfamily F member 2 [Source:HGNC Symbol;Acc:HGNC:15197] |
| ENSG00000180332 | HGLibA_24456 | 228 | 0.927644692 | KCTD4 | potassium channel tetramerization domain containing 4 [Source:HGNC Symbol;Acc:HGNC:23227] |
| ENSG00000180530 | HGLibA_32690 | 228 | 0.927644692 | NRIP1 | nuclear receptor interacting protein 1 [Source:HGNC Symbol;Acc:HGNC:8001] |
| ENSG00000181371 | HGLibA_34341 | 228 | 0.927644692 | OR5M8 | olfactory receptor family 5 subfamily M member 8 [Source:HGNC Symbol;Acc:HGNC:14846] |
| ENSG00000181577 | HGLibA_06479 | 228 | 0.927644692 | C6orf223 | chromosome 6 open reading frame 223 [Source:HGNC Symbol;Acc:HGNC:28692] |
| ENSG00000181616 | HGLibA_34170 | 228 | 0.927644692 | OR52H1 | olfactory receptor family 52 subfamily H member 1 [Source:HGNC Symbol;Acc:HGNC:15218] |
| ENSG00000182872 | HGLibA_40474 | 228 | 0.927644692 | RBM10 | RNA binding motif protein 10 [Source:HGNC Symbol;Acc:HGNC:9896] |
| ENSG00000184933 | HGLibA_34370 | 228 | 0.927644692 | OR6A2 | olfactory receptor family 6 subfamily A member 2 [Source:HGNC Symbol;Acc:HGNC:15301] |
| ENSG00000185236 | HGLibA_39791 | 228 | 0.927644692 | RAB11B | member RAS oncogene family [Source:HGNC Symbol;Acc:HGNC:9761]" |
| ENSG00000185664 | HGLibA_37298 | 228 | 0.927644692 | PMEL | premelanosome protein [Source:HGNC Symbol;Acc:HGNC:10880] |
| ENSG00000185792 | HGLibA_32135 | 228 | 0.927644692 | NLRP9 | NLR family pyrin domain containing 9 [Source:HGNC Symbol;Acc:HGNC:22941] |
| ENSG00000188886 | HGLibA_03377 | 228 | 0.927644692 | ASTL | astacin like metalloendopeptidase [Source:HGNC Symbol;Acc:HGNC:31704] |
| ENSG00000188886 | HGLibA_03376 | 228 | 0.927644692 | ASTL | astacin like metalloendopeptidase [Source:HGNC Symbol;Acc:HGNC:31704] |
| ENSG00000197045 | HGLibA_19404 | 228 | 0.927644692 | GMFB | glia maturation factor beta [Source:HGNC Symbol;Acc:HGNC:4373] |

| | | | | | |
|---|---|---|---|---|---|
| ENSG00000198380 | HGLibA_18959 | 228 | 0.927644692 | GFPT1 | glutamine--fructose-6-phosphate transaminase 1 [Source:HGNC Symbol;Acc:HGNC:4241] |
| ENSG00000204583 | HGLibA_27148 | 228 | 0.927644692 | LRCOL1 | leucine rich colipase like 1 [Source:HGNC Symbol;Acc:HGNC:44160] |
| ENSG00000205327 | HGLibA_34399 | 228 | 0.927644692 | OR6C68 | olfactory receptor family 6 subfamily C member 68 [Source:HGNC Symbol;Acc:HGNC:31297] |
| ENSG00000205857 | HGLibA_31093 | 228 | 0.927644692 | NANOGNB | NANOG neighbor homeobox [Source:HGNC Symbol;Acc:HGNC:24958] |
| ENSG00000205869 | HGLibA_25782 | 228 | 0.927644692 | KRTAP5-1 | keratin associated protein 5-1 [Source:HGNC Symbol;Acc:HGNC:23596] |
| ENSG00000253305 | HGLibA_35565 | 228 | 0.927644692 | PCDHGB6 | 6 [Source:HGNC Symbol;Acc:HGNC:8713]" |
| hsa-mir-5579 | HGLibA_62154 | 228 | 0.927644692 | hsa-mir-5579 | - |
| hsa-mir-6502 | HGLibA_62860 | 228 | 0.927644692 | hsa-mir-6502 | - |
| hsa-mir-6504 | HGLibA_62867 | 228 | 0.927644692 | hsa-mir-6504 | - |
| hsa-mir-6858 | HGLibA_63605 | 228 | 0.927644692 | hsa-mir-6858 | - |
| LOC100130880 | HGLibA_26798 | 228 | 0.927644692 | LOC100130880 | - |
| 12-Sep | HGLibA_43434 | 228 | 0.927644692 | 12-Sep | - |
| TMEM8C | HGLibA_50552 | 228 | 0.927644692 | TMEM8C | - |
| AIM1 | HGLibA_01348 | 227 | 0.918599349 | AIM1 | - |
| C10orf128 | HGLibA_05050 | 227 | 0.918599349 | C10orf128 | - |
| CECR5 | HGLibA_08979 | 227 | 0.918599349 | CECR5 | - |
| ENSG00000022355 | HGLibA_18356 | 227 | 0.918599349 | GABRA1 | gamma-aminobutyric acid type A receptor alpha1 subunit [Source:HGNC Symbol;Acc:HGNC:4075] |
| ENSG00000048545 | HGLibA_20633 | 227 | 0.918599349 | GUCA1A | guanylate cyclase activator 1A [Source:HGNC Symbol;Acc:HGNC:4678] |
| ENSG00000052344 | HGLibA_38973 | 227 | 0.918599349 | PRSS8 | serine protease 8 [Source:HGNC Symbol;Acc:HGNC:9491] |
| ENSG00000056558 | HGLibA_51239 | 227 | 0.918599349 | TRAF1 | TNF receptor associated factor 1 [Source:HGNC Symbol;Acc:HGNC:12031] |
| ENSG00000060982 | HGLibA_04264 | 227 | 0.918599349 | BCAT1 | branched chain amino acid transaminase 1 [Source:HGNC Symbol;Acc:HGNC:976] |
| ENSG00000077044 | HGLibA_13063 | 227 | 0.918599349 | DGKD | diacylglycerol kinase delta [Source:HGNC Symbol;Acc:HGNC:2851] |
| ENSG00000077380 | HGLibA_14238 | 227 | 0.918599349 | DYNC1I2 | dynein cytoplasmic 1 intermediate chain 2 [Source:HGNC Symbol;Acc:HGNC:2964] |
| ENSG00000078687 | HGLibA_50918 | 227 | 0.918599349 | TNRC6C | trinucleotide repeat containing 6C [Source:HGNC Symbol;Acc:HGNC:29318] |
| ENSG00000079739 | HGLibA_36262 | 227 | 0.918599349 | PGM1 | phosphoglucomutase 1 [Source:HGNC Symbol;Acc:HGNC:8905] |
| ENSG00000099219 | HGLibA_15509 | 227 | 0.918599349 | ERMP1 | endoplasmic reticulum metallopeptidase 1 [Source:HGNC Symbol;Acc:HGNC:23703] |
| ENSG00000100266 | HGLibA_34962 | 227 | 0.918599349 | PACSIN2 | protein kinase C and casein kinase substrate in neurons 2 [Source:HGNC Symbol;Acc:HGNC:8571] |
| ENSG00000100417 | HGLibA_37316 | 227 | 0.918599349 | PMM1 | phosphomannomutase 1 [Source:HGNC Symbol;Acc:HGNC:9114] |
| ENSG00000103168 | HGLibA_48220 | 227 | 0.918599349 | TAF1C | RNA polymerase I subunit C [Source:HGNC Symbol;Acc:HGNC:11534]" |
| ENSG00000106605 | HGLibA_04574 | 227 | 0.918599349 | BLVRA | biliverdin reductase A [Source:HGNC Symbol;Acc:HGNC:1062] |
| ENSG00000106799 | HGLibA_49224 | 227 | 0.918599349 | TGFBR1 | transforming growth factor beta receptor 1 [Source:HGNC Symbol;Acc:HGNC:11772] |
| ENSG00000107331 | HGLibA_00108 | 227 | 0.918599349 | ABCA2 | ATP binding cassette subfamily A member 2 [Source:HGNC Symbol;Acc:HGNC:32] |
| ENSG00000107611 | HGLibA_11765 | 227 | 0.918599349 | CUBN | cubilin [Source:HGNC Symbol;Acc:HGNC:2548] |
| ENSG00000110925 | HGLibA_11427 | 227 | 0.918599349 | CSRNP2 | cysteine and serine rich nuclear protein 2 [Source:HGNC Symbol;Acc:HGNC:16006] |
| ENSG00000113240 | HGLibA_10062 | 227 | 0.918599349 | CLK4 | CDC like kinase 4 [Source:HGNC Symbol;Acc:HGNC:13659] |
| ENSG00000121361 | HGLibA_24289 | 227 | 0.918599349 | KCNJ8 | potassium voltage-gated channel subfamily J member 8 [Source:HGNC Symbol;Acc:HGNC:6269] |
| ENSG00000126895 | HGLibA_03908 | 227 | 0.918599349 | AVPR2 | arginine vasopressin receptor 2 [Source:HGNC Symbol;Acc:HGNC:897] |
| ENSG00000128694 | HGLibA_34683 | 227 | 0.918599349 | OSGEPL1 | O-sialoglycoprotein endopeptidase like 1 [Source:HGNC Symbol;Acc:HGNC:23075] |
| ENSG00000131437 | HGLibA_24934 | 227 | 0.918599349 | KIF3A | kinesin family member 3A [Source:HGNC Symbol;Acc:HGNC:6319] |
| ENSG00000136167 | HGLibA_26198 | 227 | 0.918599349 | LCP1 | lymphocyte cytosolic protein 1 [Source:HGNC Symbol;Acc:HGNC:6528] |
| ENSG00000138772 | HGLibA_02237 | 227 | 0.918599349 | ANXA3 | annexin A3 [Source:HGNC Symbol;Acc:HGNC:541] |
| ENSG00000139625 | HGLibA_28170 | 227 | 0.918599349 | MAP3K12 | mitogen-activated protein kinase kinase kinase 12 [Source:HGNC Symbol;Acc:HGNC:6851] |
| ENSG00000144837 | HGLibA_36865 | 227 | 0.918599349 | PLA1A | phospholipase A1 member A [Source:HGNC Symbol;Acc:HGNC:17661] |
| ENSG00000147573 | HGLibA_51545 | 227 | 0.918599349 | TRIM55 | tripartite motif containing 55 [Source:HGNC Symbol;Acc:HGNC:14215] |
| ENSG00000149547 | HGLibA_14690 | 227 | 0.918599349 | EI24 | autophagy associated transmembrane protein [Source:HGNC Symbol;Acc:HGNC:13276]" |
| ENSG00000154764 | HGLibA_54383 | 227 | 0.918599349 | WNT7A | Wnt family member 7A [Source:HGNC Symbol;Acc:HGNC:12786] |
| ENSG00000162520 | HGLibA_47965 | 227 | 0.918599349 | SYNC | intermediate filament protein [Source:HGNC Symbol;Acc:HGNC:28897]" |
| ENSG00000163217 | HGLibA_04593 | 227 | 0.918599349 | BMP10 | bone morphogenetic protein 10 [Source:HGNC Symbol;Acc:HGNC:20869] |
| ENSG00000163558 | HGLibA_38555 | 227 | 0.918599349 | PRKCI | protein kinase C iota [Source:HGNC Symbol;Acc:HGNC:9404] |
| ENSG00000163736 | HGLibA_37854 | 227 | 0.918599349 | PPBP | pro-platelet basic protein [Source:HGNC Symbol;Acc:HGNC:9240] |
| ENSG00000163793 | HGLibA_13629 | 227 | 0.918599349 | DNAJC5G | DnaJ heat shock protein family (Hsp40) member C5 gamma [Source:HGNC Symbol;Acc:HGNC:24844] |
| ENSG00000164334 | HGLibA_16257 | 227 | 0.918599349 | FAM170A | family with sequence similarity 170 member A [Source:HGNC Symbol;Acc:HGNC:27963] |
| ENSG00000166024 | HGLibA_39768 | 227 | 0.918599349 | R3HCC1L | R3H domain and coiled-coil containing 1 like [Source:HGNC Symbol;Acc:HGNC:23512] |
| ENSG00000168026 | HGLibA_52068 | 227 | 0.918599349 | TTC21A | tetratricopeptide repeat domain 21A [Source:HGNC Symbol;Acc:HGNC:30761] |
| ENSG00000174165 | HGLibA_55111 | 227 | 0.918599349 | ZDHHC24 | zinc finger DHHC-type containing 24 [Source:HGNC Symbol;Acc:HGNC:27387] |
| ENSG00000179403 | HGLibA_53895 | 227 | 0.918599349 | VWA1 | von Willebrand factor A domain containing 1 [Source:HGNC Symbol;Acc:HGNC:30910] |
| ENSG00000180549 | HGLibA_18202 | 227 | 0.918599349 | FUT7 | fucosyltransferase 7 [Source:HGNC Symbol;Acc:HGNC:4018] |
| ENSG00000182107 | HGLibA_50364 | 227 | 0.918599349 | TMEM30B | transmembrane protein 30B [Source:HGNC Symbol;Acc:HGNC:27254] |
| ENSG00000182327 | HGLibA_19350 | 227 | 0.918599349 | GLTPD2 | glycolipid transfer protein domain containing 2 [Source:HGNC Symbol;Acc:HGNC:33756] |
| ENSG00000182631 | HGLibA_42501 | 227 | 0.918599349 | RXFP3 | relaxin family peptide receptor 3 [Source:HGNC Symbol;Acc:HGNC:24883] |
| ENSG00000183665 | HGLibA_51676 | 227 | 0.918599349 | TRMT12 | tRNA methyltransferase 12 homolog [Source:HGNC Symbol;Acc:HGNC:26091] |
| ENSG00000184544 | HGLibA_13152 | 227 | 0.918599349 | DHRS7C | dehydrogenase/reductase 7C [Source:HGNC Symbol;Acc:HGNC:32423] |
| ENSG00000185899 | HGLibA_48422 | 227 | 0.918599349 | TAS2R60 | taste 2 receptor member 60 [Source:HGNC Symbol;Acc:HGNC:20639] |
| ENSG00000186523 | HGLibA_16773 | 227 | 0.918599349 | FAM86B1 | family with sequence similarity 86 member B1 [Source:HGNC Symbol;Acc:HGNC:28268] |
| ENSG00000187758 | HGLibA_00949 | 227 | 0.918599349 | ADH1A | alpha polypeptide [Source:HGNC Symbol;Acc:HGNC:249]" |
| ENSG00000188483 | HGLibA_22584 | 227 | 0.918599349 | IER5L | immediate early response 5 like [Source:HGNC Symbol;Acc:HGNC:23679] |
| ENSG00000188770 | HGLibA_33455 | 227 | 0.918599349 | OPTC | opticin [Source:HGNC Symbol;Acc:HGNC:8158] |
| ENSG00000196236 | HGLibA_54559 | 227 | 0.918599349 | XPNPEP3 | X-prolyl aminopeptidase 3 [Source:HGNC Symbol;Acc:HGNC:28052] |
| ENSG00000196366 | HGLibA_06701 | 227 | 0.918599349 | C9orf163 | chromosome 9 open reading frame 163 [Source:HGNC Symbol;Acc:HGNC:26718] |
| ENSG00000196860 | HGLibA_50958 | 227 | 0.918599349 | TOMM20L | translocase of outer mitochondrial membrane 20 like [Source:HGNC Symbol;Acc:HGNC:33752] |
| ENSG00000198885 | HGLibA_23865 | 227 | 0.918599349 | ITPRIPL1 | ITPRIP like 1 [Source:HGNC Symbol;Acc:HGNC:29371] |
| ENSG00000204160 | HGLibA_55090 | 227 | 0.918599349 | ZDHHC18 | zinc finger DHHC-type containing 18 [Source:HGNC Symbol;Acc:HGNC:20712] |
| ENSG00000205858 | HGLibA_27422 | 227 | 0.918599349 | LRRC72 | leucine rich repeat containing 72 [Source:HGNC Symbol;Acc:HGNC:42972] |
| ENSG00000236104 | HGLibA_54844 | 227 | 0.918599349 | ZBTB22 | zinc finger and BTB domain containing 22 [Source:HGNC Symbol;Acc:HGNC:13085] |
| ENSG00000239697 | HGLibA_50803 | 227 | 0.918599349 | TNFSF12 | TNF superfamily member 12 [Source:HGNC Symbol;Acc:HGNC:11927] |
| ENSG00000268009 | HGLibA_47209 | 227 | 0.918599349 | SSX4 | SSX family member 4 [Source:HGNC Symbol;Acc:HGNC:11338] |
| ENSG00000274286 | HGLibA_01048 | 227 | 0.918599349 | ADRA2B | adrenoceptor alpha 2B [Source:HGNC Symbol;Acc:HGNC:282] |
| hsa-mir-183 | HGLibA_57995 | 227 | 0.918599349 | hsa-mir-183 | - |
| hsa-mir-2681 | HGLibA_58355 | 227 | 0.918599349 | hsa-mir-2681 | - |
| hsa-mir-4435-1 | HGLibA_60130 | 227 | 0.918599349 | hsa-mir-4435-1 | - |
| hsa-mir-4639 | HGLibA_60660 | 227 | 0.918599349 | hsa-mir-4639 | - |
| hsa-mir-4662a | HGLibA_60756 | 227 | 0.918599349 | hsa-mir-4662a | - |
| hsa-mir-4767 | HGLibA_61164 | 227 | 0.918599349 | hsa-mir-4767 | - |
| hsa-mir-499a | HGLibA_61402 | 227 | 0.918599349 | hsa-mir-499a | - |
| hsa-mir-532 | HGLibA_61808 | 227 | 0.918599349 | hsa-mir-532 | - |
| hsa-mir-5683 | HGLibA_62251 | 227 | 0.918599349 | hsa-mir-5683 | - |
| hsa-mir-6771 | HGLibA_63258 | 227 | 0.918599349 | hsa-mir-6771 | - |
| PRSS46 | HGLibA_38946 | 227 | 0.918599349 | PRSS46 | - |
| PTPLA | HGLibA_39416 | 227 | 0.918599349 | PTPLA | - |
| C19orf57 | HGLibA_05683 | 226 | 0.909554007 | C19orf57 | - |
| CCDC155 | HGLibA_07657 | 226 | 0.909554007 | CCDC155 | - |
| ENSG00000001626 | HGLibA_09294 | 226 | 0.909554007 | CFTR | cystic fibrosis transmembrane conductance regulator [Source:HGNC Symbol;Acc:HGNC:1884] |
| ENSG00000008083 | HGLibA_23938 | 226 | 0.909554007 | JARID2 | jumonji and AT-rich interaction domain containing 2 [Source:HGNC Symbol;Acc:HGNC:6196] |
| ENSG00000011465 | HGLibA_12516 | 226 | 0.909554007 | DCN | decorin [Source:HGNC Symbol;Acc:HGNC:2705] |
| ENSG00000011566 | HGLibA_28218 | 226 | 0.909554007 | MAP4K3 | mitogen-activated protein kinase kinase kinase kinase 3 [Source:HGNC Symbol;Acc:HGNC:6865] |
| ENSG00000019505 | HGLibA_48057 | 226 | 0.909554007 | SYT13 | synaptotagmin 13 [Source:HGNC Symbol;Acc:HGNC:14962] |
| ENSG00000036257 | HGLibA_11778 | 226 | 0.909554007 | CUL3 | cullin 3 [Source:HGNC Symbol;Acc:HGNC:2553] |
| ENSG00000040341 | HGLibA_47409 | 226 | 0.909554007 | STAU2 | staufen double-stranded RNA binding protein 2 [Source:HGNC Symbol;Acc:HGNC:11371] |
| ENSG00000070047 | HGLibA_36459 | 226 | 0.909554007 | PHRF1 | PHD and ring finger domains 1 [Source:HGNC Symbol;Acc:HGNC:24351] |
| ENSG00000075290 | HGLibA_54391 | 226 | 0.909554007 | WNT8B | Wnt family member 8B [Source:HGNC Symbol;Acc:HGNC:12789] |
| ENSG00000079385 | HGLibA_08923 | 226 | 0.909554007 | CEACAM1 | carcinoembryonic antigen related cell adhesion molecule 1 [Source:HGNC Symbol;Acc:HGNC:1814] |
| ENSG00000102359 | HGLibA_47036 | 226 | 0.909554007 | SRPX2 | sushi repeat containing protein X-linked 2 [Source:HGNC Symbol;Acc:HGNC:30668] |
| ENSG00000102804 | HGLibA_51818 | 226 | 0.909554007 | TSC22D1 | TSC22 domain family member 1 [Source:HGNC Symbol;Acc:HGNC:16826] |
| ENSG00000103194 | HGLibA_53195 | 226 | 0.909554007 | USP10 | ubiquitin specific peptidase 10 [Source:HGNC Symbol;Acc:HGNC:12608] |
| ENSG00000105185 | HGLibA_35737 | 226 | 0.909554007 | PDCD5 | programmed cell death 5 [Source:HGNC Symbol;Acc:HGNC:8764] |
| ENSG00000108602 | HGLibA_01562 | 226 | 0.909554007 | ALDH3A1 | aldehyde dehydrogenase 3 family member A1 [Source:HGNC Symbol;Acc:HGNC:405] |
| ENSG00000111275 | HGLibA_01560 | 226 | 0.909554007 | ALDH2 | aldehyde dehydrogenase 2 family member [Source:HGNC Symbol;Acc:HGNC:404] |
| ENSG00000112984 | HGLibA_24892 | 226 | 0.909554007 | KIF20A | kinesin family member 20A [Source:HGNC Symbol;Acc:HGNC:9787] |
| ENSG00000113391 | HGLibA_16272 | 226 | 0.909554007 | FAM172A | family with sequence similarity 172 member A [Source:HGNC Symbol;Acc:HGNC:25365] |
| ENSG00000116729 | HGLibA_54330 | 226 | 0.909554007 | WLS | wntless Wnt ligand secretion mediator [Source:HGNC Symbol;Acc:HGNC:30238] |
| ENSG00000124205 | HGLibA_14475 | 226 | 0.909554007 | EDN3 | endothelin 3 [Source:HGNC Symbol;Acc:HGNC:3178] |
| ENSG00000125656 | HGLibA_10102 | 226 | 0.909554007 | CLPP | caseinolytic mitochondrial matrix peptidase proteolytic subunit [Source:HGNC Symbol;Acc:HGNC:2084] |
| ENSG00000127588 | HGLibA_19517 | 226 | 0.909554007 | GNG13 | G protein subunit gamma 13 [Source:HGNC Symbol;Acc:HGNC:14131] |
| ENSG00000128590 | HGLibA_13557 | 226 | 0.909554007 | DNAJB9 | DnaJ heat shock protein family (Hsp40) member B9 [Source:HGNC Symbol;Acc:HGNC:6968] |
| ENSG00000129518 | HGLibA_14339 | 226 | 0.909554007 | EAPP | E2F associated phosphoprotein [Source:HGNC Symbol;Acc:HGNC:19312] |
| ENSG00000130822 | HGLibA_37342 | 226 | 0.909554007 | PNCK | pregnancy up-regulated nonubiquitous CaM kinase [Source:HGNC Symbol;Acc:HGNC:13415] |
| ENSG00000133703 | HGLibA_25384 | 226 | 0.909554007 | KRAS | GTPase [Source:HGNC Symbol;Acc:HGNC:6407]" |
| ENSG00000134996 | HGLibA_34711 | 226 | 0.909554007 | OSTF1 | osteoclast stimulating factor 1 [Source:HGNC Symbol;Acc:HGNC:8510] |
| ENSG00000136541 | HGLibA_15505 | 226 | 0.909554007 | ERMN | ermin [Source:HGNC Symbol;Acc:HGNC:29208] |
| ENSG00000138356 | HGLibA_02275 | 226 | 0.909554007 | AOX1 | aldehyde oxidase 1 [Source:HGNC Symbol;Acc:HGNC:553] |
| ENSG00000142089 | HGLibA_22647 | 226 | 0.909554007 | IFITM3 | interferon induced transmembrane protein 3 [Source:HGNC Symbol;Acc:HGNC:5414] |
| ENSG00000143494 | HGLibA_53534 | 226 | 0.909554007 | VASH2 | vasohibin 2 [Source:HGNC Symbol;Acc:HGNC:25723] |
| ENSG00000153446 | HGLibA_05494 | 226 | 0.909554007 | C16orf89 | chromosome 16 open reading frame 89 [Source:HGNC Symbol;Acc:HGNC:28687] |
| ENSG00000157542 | HGLibA_24284 | 226 | 0.909554007 | KCNJ6 | potassium voltage-gated channel subfamily J member 6 [Source:HGNC Symbol;Acc:HGNC:6267] |
| ENSG00000157703 | HGLibA_47894 | 226 | 0.909554007 | SVOPL | SVOP like [Source:HGNC Symbol;Acc:HGNC:27034] |
| ENSG00000158887 | HGLibA_29793 | 226 | 0.909554007 | MPZ | myelin protein zero [Source:HGNC Symbol;Acc:HGNC:7225] |
| ENSG00000160752 | HGLibA_17254 | 226 | 0.909554007 | FDPS | farnesyl diphosphate synthase [Source:HGNC Symbol;Acc:HGNC:3631] |
| ENSG00000163393 | HGLibA_44553 | 226 | 0.909554007 | SLC22A15 | solute carrier family 22 member 15 [Source:HGNC Symbol;Acc:HGNC:20301] |
| ENSG00000163482 | HGLibA_47498 | 226 | 0.909554007 | STK36 | serine/threonine kinase 36 [Source:HGNC Symbol;Acc:HGNC:17209] |
| ENSG00000163825 | HGLibA_42429 | 226 | 0.909554007 | RTP3 | receptor transporter protein 3 [Source:HGNC Symbol;Acc:HGNC:15572] |
| ENSG00000165637 | HGLibA_53602 | 226 | 0.909554007 | VDAC2 | voltage dependent anion channel 2 [Source:HGNC Symbol;Acc:HGNC:12672] |
| ENSG00000166439 | HGLibA_41568 | 226 | 0.909554007 | RNF169 | ring finger protein 169 [Source:HGNC Symbol;Acc:HGNC:26961] |
| ENSG00000167210 | HGLibA_27035 | 226 | 0.909554007 | LOXHD1 | lipoxygenase homology domains 1 [Source:HGNC Symbol;Acc:HGNC:26521] |
| ENSG00000169554 | HGLibA_55139 | 226 | 0.909554007 | ZEB2 | zinc finger E-box binding homeobox 2 [Source:HGNC Symbol;Acc:HGNC:14881] |
| ENSG00000171169 | HGLibA_31078 | 226 | 0.909554007 | NAIF1 | nuclear apoptosis inducing factor 1 [Source:HGNC Symbol;Acc:HGNC:25446] |

| | | | | | |
|---|---|---|---|---|---|
| ENSG00000173598 | HGLibA_32979 | 226 | 0.909554007 | NUDT4 | nudix hydrolase 4 [Source:HGNC Symbol;Acc:HGNC:8051] |
| ENSG00000173947 | HGLibA_36541 | 226 | 0.909554007 | PIFO | primary cilia formation [Source:HGNC Symbol;Acc:HGNC:27009] |
| ENSG00000177023 | HGLibA_12823 | 226 | 0.909554007 | DEFB104B | defensin beta 104B [Source:HGNC Symbol;Acc:HGNC:26165] |
| ENSG00000179172 | HGLibA_21809 | 226 | 0.909554007 | HNRNPCL1 | heterogeneous nuclear ribonucleoprotein C-like 1 [Source:HGNC Symbol;Acc:HGNC:29295] |
| ENSG00000181904 | HGLibA_06369 | 226 | 0.909554007 | C5orf24 | chromosome 5 open reading frame 24 [Source:HGNC Symbol;Acc:HGNC:26746] |
| ENSG00000183486 | HGLibA_30606 | 226 | 0.909554007 | MX2 | MX dynamin like GTPase 2 [Source:HGNC Symbol;Acc:HGNC:7533] |
| ENSG00000183876 | HGLibA_03150 | 226 | 0.909554007 | ARSI | arylsulfatase family member I [Source:HGNC Symbol;Acc:HGNC:32521] |
| ENSG00000186007 | HGLibA_26286 | 226 | 0.909554007 | LEMD1 | LEM domain containing 1 [Source:HGNC Symbol;Acc:HGNC:18725] |
| ENSG00000186280 | HGLibA_24516 | 226 | 0.909554007 | KDM4D | lysine demethylase 4D [Source:HGNC Symbol;Acc:HGNC:25498] |
| ENSG00000188316 | HGLibA_15148 | 226 | 0.909554007 | ENO4 | enolase 4 [Source:HGNC Symbol;Acc:HGNC:31670] |
| ENSG00000197632 | HGLibA_43546 | 226 | 0.909554007 | SERPINB2 | serpin family B member 2 [Source:HGNC Symbol;Acc:HGNC:8584] |
| ENSG00000197818 | HGLibA_45427 | 226 | 0.909554007 | SLC9A8 | solute carrier family 9 member A8 [Source:HGNC Symbol;Acc:HGNC:20728] |
| ENSG00000197912 | HGLibA_46639 | 226 | 0.909554007 | SPG7 | paraplegin matrix AAA peptidase subunit [Source:HGNC Symbol;Acc:HGNC:11237]" |
| ENSG00000198822 | HGLibA_20348 | 226 | 0.909554007 | GRM3 | glutamate metabotropic receptor 3 [Source:HGNC Symbol;Acc:HGNC:4595] |
| ENSG00000198846 | HGLibA_51035 | 226 | 0.909554007 | TOX | thymocyte selection associated high mobility group box [Source:HGNC Symbol;Acc:HGNC:18988] |
| ENSG00000241644 | HGLibA_23317 | 226 | 0.909554007 | INMT | indolethylamine N-methyltransferase [Source:HGNC Symbol;Acc:HGNC:6069] |
| ENSG00000260325 | HGLibA_22321 | 226 | 0.909554007 | HSPB9 | heat shock protein family B (small) member 9 [Source:HGNC Symbol;Acc:HGNC:30589] |
| ENSG00000261221 | HGLibA_56884 | 226 | 0.909554007 | ZNF865 | zinc finger protein 865 [Source:HGNC Symbol;Acc:HGNC:38705] |
| hsa-mir-378c | HGLibA_59432 | 226 | 0.909554007 | hsa-mir-378c | - |
| hsa-mir-378i | HGLibA_59462 | 226 | 0.909554007 | hsa-mir-378i | - |
| PAPD7 | HGLibA_35127 | 226 | 0.909554007 | PAPD7 | - |
| ZCCHC11 | HGLibA_55005 | 226 | 0.909554007 | ZCCHC11 | - |
| C14orf166B | HGLibA_05336 | 225 | 0.900508664 | C14orf166B | - |
| C2orf70 | HGLibA_06170 | 225 | 0.900508664 | C2orf70 | - |
| CRIPAK | HGLibA_11160 | 225 | 0.900508664 | CRIPAK | - |
| ENSG00000007402 | HGLibA_06909 | 225 | 0.900508664 | CACNA2D2 | calcium voltage-gated channel auxiliary subunit alpha2delta 2 [Source:HGNC Symbol;Acc:HGNC:1400] |
| ENSG00000036828 | HGLibA_07341 | 225 | 0.900508664 | CASR | calcium sensing receptor [Source:HGNC Symbol;Acc:HGNC:1514] |
| ENSG00000056586 | HGLibA_40665 | 225 | 0.900508664 | RC3H2 | ring finger and CCCH-type domains 2 [Source:HGNC Symbol;Acc:HGNC:21461] |
| ENSG00000058404 | HGLibA_07069 | 225 | 0.900508664 | CAMK2B | calcium/calmodulin dependent protein kinase II beta [Source:HGNC Symbol;Acc:HGNC:1461] |
| ENSG00000072518 | HGLibA_28368 | 225 | 0.900508664 | MARK2 | microtubule affinity regulating kinase 2 [Source:HGNC Symbol;Acc:HGNC:3332] |
| ENSG00000087008 | HGLibA_00502 | 225 | 0.900508664 | ACOX3 | pristanoyl [Source:HGNC Symbol;Acc:HGNC:121]" |
| ENSG00000089159 | HGLibA_39664 | 225 | 0.900508664 | PXN | paxillin [Source:HGNC Symbol;Acc:HGNC:9718] |
| ENSG00000100083 | HGLibA_18980 | 225 | 0.900508664 | GGA1 | ARF binding protein 1 [Source:HGNC Symbol;Acc:HGNC:17842]" |
| ENSG00000100109 | HGLibA_49180 | 225 | 0.900508664 | TFIP11 | tuftelin interacting protein 11 [Source:HGNC Symbol;Acc:HGNC:17165] |
| ENSG00000100330 | HGLibA_30426 | 225 | 0.900508664 | MTMR3 | myotubularin related protein 3 [Source:HGNC Symbol;Acc:HGNC:7451] |
| ENSG00000101955 | HGLibA_47034 | 225 | 0.900508664 | SRPX | sushi repeat containing protein X-linked [Source:HGNC Symbol;Acc:HGNC:11309] |
| ENSG00000106462 | HGLibA_15834 | 225 | 0.900508664 | EZH2 | enhancer of zeste 2 polycomb repressive complex 2 subunit [Source:HGNC Symbol;Acc:HGNC:3527] |
| ENSG00000106571 | HGLibA_19253 | 225 | 0.900508664 | GLI3 | GLI family zinc finger 3 [Source:HGNC Symbol;Acc:HGNC:4319] |
| ENSG00000111405 | HGLibA_15119 | 225 | 0.900508664 | ENDOU | poly(U) specific [Source:HGNC Symbol;Acc:HGNC:14369]" |
| ENSG00000112562 | HGLibA_45798 | 225 | 0.900508664 | SMOC2 | SPARC related modular calcium binding 2 [Source:HGNC Symbol;Acc:HGNC:20323] |
| ENSG00000115129 | HGLibA_51067 | 225 | 0.900508664 | TP53I3 | tumor protein p53 inducible protein 3 [Source:HGNC Symbol;Acc:HGNC:19373] |
| ENSG00000116604 | HGLibA_28840 | 225 | 0.900508664 | MEF2D | myocyte enhancer factor 2D [Source:HGNC Symbol;Acc:HGNC:6997] |
| ENSG00000118804 | HGLibA_47410 | 225 | 0.900508664 | STBD1 | starch binding domain 1 [Source:HGNC Symbol;Acc:HGNC:24854] |
| ENSG00000122477 | HGLibA_27342 | 225 | 0.900508664 | LRRC39 | leucine rich repeat containing 39 [Source:HGNC Symbol;Acc:HGNC:28228] |
| ENSG00000125652 | HGLibA_01664 | 225 | 0.900508664 | ALKBH7 | alkB homolog 7 [Source:HGNC Symbol;Acc:HGNC:21306] |
| ENSG00000130560 | HGLibA_52535 | 225 | 0.900508664 | UBAC1 | UBA domain containing 1 [Source:HGNC Symbol;Acc:HGNC:30221] |
| ENSG00000130748 | HGLibA_50021 | 225 | 0.900508664 | TMEM160 | transmembrane protein 160 [Source:HGNC Symbol;Acc:HGNC:26042] |
| ENSG00000137875 | HGLibA_04306 | 225 | 0.900508664 | BCL2L10 | BCL2 like 10 [Source:HGNC Symbol;Acc:HGNC:993] |
| ENSG00000138039 | HGLibA_26423 | 225 | 0.900508664 | LHCGR | luteinizing hormone/choriogonadotropin receptor [Source:HGNC Symbol;Acc:HGNC:6585] |
| ENSG00000138134 | HGLibA_47340 | 225 | 0.900508664 | STAMBPL1 | STAM binding protein like 1 [Source:HGNC Symbol;Acc:HGNC:24105] |
| ENSG00000147166 | HGLibA_23774 | 225 | 0.900508664 | ITGB1BP2 | integrin subunit beta 1 binding protein 2 [Source:HGNC Symbol;Acc:HGNC:6154] |
| ENSG00000147316 | HGLibA_28647 | 225 | 0.900508664 | MCPH1 | microcephalin 1 [Source:HGNC Symbol;Acc:HGNC:6954] |
| ENSG00000148377 | HGLibA_22556 | 225 | 0.900508664 | IDI2 | isopentenyl-diphosphate delta isomerase 2 [Source:HGNC Symbol;Acc:HGNC:23487] |
| ENSG00000149927 | HGLibA_13724 | 225 | 0.900508664 | DOC2A | double C2 domain alpha [Source:HGNC Symbol;Acc:HGNC:2985] |
| ENSG00000151923 | HGLibA_49435 | 225 | 0.900508664 | TIAL1 | TIA1 cytotoxic granule associated RNA binding protein like 1 [Source:HGNC Symbol;Acc:HGNC:11804] |
| ENSG00000152332 | HGLibA_52941 | 225 | 0.900508664 | UHMK1 | U2AF homology motif kinase 1 [Source:HGNC Symbol;Acc:HGNC:19683] |
| ENSG00000154803 | HGLibA_17605 | 225 | 0.900508664 | FLCN | folliculin [Source:HGNC Symbol;Acc:HGNC:27310] |
| ENSG00000161594 | HGLibA_25124 | 225 | 0.900508664 | KLHL10 | kelch like family member 10 [Source:HGNC Symbol;Acc:HGNC:18829] |
| ENSG00000163686 | HGLibA_00284 | 225 | 0.900508664 | ABHD6 | abhydrolase domain containing 6 [Source:HGNC Symbol;Acc:HGNC:21398] |
| ENSG00000164082 | HGLibA_20345 | 225 | 0.900508664 | GRM2 | glutamate metabotropic receptor 2 [Source:HGNC Symbol;Acc:HGNC:4594] |
| ENSG00000164117 | HGLibA_17129 | 225 | 0.900508664 | FBXO8 | F-box protein 8 [Source:HGNC Symbol;Acc:HGNC:13587] |
| ENSG00000165370 | HGLibA_19841 | 225 | 0.900508664 | GPR101 | G protein-coupled receptor 101 [Source:HGNC Symbol;Acc:HGNC:14963] |
| ENSG00000166670 | HGLibA_29480 | 225 | 0.900508664 | MMP10 | matrix metallopeptidase 10 [Source:HGNC Symbol;Acc:HGNC:7156] |
| ENSG00000166927 | HGLibA_30162 | 225 | 0.900508664 | MS4A7 | membrane spanning 4-domains A7 [Source:HGNC Symbol;Acc:HGNC:13378] |
| ENSG00000168795 | HGLibA_54911 | 225 | 0.900508664 | ZBTB5 | zinc finger and BTB domain containing 5 [Source:HGNC Symbol;Acc:HGNC:23836] |
| ENSG00000170689 | HGLibA_21952 | 225 | 0.900508664 | HOXB9 | homeobox B9 [Source:HGNC Symbol;Acc:HGNC:5120] |
| ENSG00000170948 | HGLibA_28472 | 225 | 0.900508664 | MBD3L1 | methyl-CpG binding domain protein 3 like 1 [Source:HGNC Symbol;Acc:HGNC:15774] |
| ENSG00000172081 | HGLibA_29593 | 225 | 0.900508664 | MOB3A | MOB kinase activator 3A [Source:HGNC Symbol;Acc:HGNC:29802] |
| ENSG00000173662 | HGLibA_48355 | 225 | 0.900508664 | TAS1R1 | taste 1 receptor member 1 [Source:HGNC Symbol;Acc:HGNC:14448] |
| ENSG00000174886 | HGLibA_31444 | 225 | 0.900508664 | NDUFA11 | NADH:ubiquinone oxidoreductase subunit A11 [Source:HGNC Symbol;Acc:HGNC:20371] |
| ENSG00000174914 | HGLibA_34567 | 225 | 0.900508664 | OR9G1 | olfactory receptor family 9 subfamily G member 1 [Source:HGNC Symbol;Acc:HGNC:15319] |
| ENSG00000175868 | HGLibA_06995 | 225 | 0.900508664 | CALCB | calcitonin related polypeptide beta [Source:HGNC Symbol;Acc:HGNC:1438] |
| ENSG00000178473 | HGLibA_52828 | 225 | 0.900508664 | UCN3 | urocortin 3 [Source:HGNC Symbol;Acc:HGNC:17781] |
| ENSG00000180739 | HGLibA_42605 | 225 | 0.900508664 | S1PR5 | sphingosine-1-phosphate receptor 5 [Source:HGNC Symbol;Acc:HGNC:14299] |
| ENSG00000180919 | HGLibA_34225 | 225 | 0.900508664 | OR56B4 | olfactory receptor family 56 subfamily B member 4 [Source:HGNC Symbol;Acc:HGNC:15248] |
| ENSG00000181541 | HGLibA_27823 | 225 | 0.900508664 | MAB21L2 | mab-21 like 2 [Source:HGNC Symbol;Acc:HGNC:6758] |
| ENSG00000183117 | HGLibA_11363 | 225 | 0.900508664 | CSMD1 | CUB and Sushi multiple domains 1 [Source:HGNC Symbol;Acc:HGNC:14026] |
| ENSG00000184210 | HGLibA_13040 | 225 | 0.900508664 | DGAT2L6 | diacylglycerol O-acyltransferase 2 like 6 [Source:HGNC Symbol;Acc:HGNC:23250] |
| ENSG00000184302 | HGLibA_44259 | 225 | 0.900508664 | SIX6 | SIX homeobox 6 [Source:HGNC Symbol;Acc:HGNC:10892] |
| ENSG00000186970 | HGLibA_25655 | 225 | 0.900508664 | KRTAP15-1 | keratin associated protein 15-1 [Source:HGNC Symbol;Acc:HGNC:18927] |
| ENSG00000196224 | HGLibA_25794 | 225 | 0.900508664 | KRTAP5-3 | keratin associated protein 5-3 [Source:HGNC Symbol;Acc:HGNC:23598] |
| ENSG00000196227 | HGLibA_16493 | 225 | 0.900508664 | FAM217B | family with sequence similarity 217 member B [Source:HGNC Symbol;Acc:HGNC:16170] |
| ENSG00000196584 | HGLibA_54587 | 225 | 0.900508664 | XRCC2 | X-ray repair cross complementing 2 [Source:HGNC Symbol;Acc:HGNC:12829] |
| ENSG00000197140 | HGLibA_00772 | 225 | 0.900508664 | ADAM32 | ADAM metallopeptidase domain 32 [Source:HGNC Symbol;Acc:HGNC:15479] |
| ENSG00000197241 | HGLibA_44887 | 225 | 0.900508664 | SLC2A7 | solute carrier family 2 member 7 [Source:HGNC Symbol;Acc:HGNC:13445] |
| ENSG00000197745 | HGLibA_42920 | 225 | 0.900508664 | SCGB1D4 | secretoglobin family 1D member 4 [Source:HGNC Symbol;Acc:HGNC:31748] |
| ENSG00000205269 | HGLibA_50056 | 225 | 0.900508664 | TMEM170B | transmembrane protein 170B [Source:HGNC Symbol;Acc:HGNC:34244] |
| ENSG00000235711 | HGLibA_02082 | 225 | 0.900508664 | ANKRD34C | ankyrin repeat domain 34C [Source:HGNC Symbol;Acc:HGNC:33888] |
| ENSG00000244094 | HGLibA_46820 | 225 | 0.900508664 | SPRR2F | small proline rich protein 2F [Source:HGNC Symbol;Acc:HGNC:11266] |
| FAM134A | HGLibA_16123 | 225 | 0.900508664 | FAM134A | - |
| GPR97 | HGLibA_20114 | 225 | 0.900508664 | GPR97 | - |
| GTDC2 | HGLibA_20517 | 225 | 0.900508664 | GTDC2 | - |
| GUCY1A3 | HGLibA_20655 | 225 | 0.900508664 | GUCY1A3 | - |
| hsa-mir-3659 | HGLibA_59182 | 225 | 0.900508664 | hsa-mir-3659 | - |
| hsa-mir-3680-2 | HGLibA_59272 | 225 | 0.900508664 | hsa-mir-3680-2 | - |
| hsa-mir-380 | HGLibA_59473 | 225 | 0.900508664 | hsa-mir-380 | - |
| hsa-mir-4422 | HGLibA_60077 | 225 | 0.900508664 | hsa-mir-4422 | - |
| hsa-mir-4534 | HGLibA_60596 | 225 | 0.900508664 | hsa-mir-4534 | - |
| hsa-mir-4687 | HGLibA_60860 | 225 | 0.900508664 | hsa-mir-4687 | - |
| hsa-mir-656 | HGLibA_62959 | 225 | 0.900508664 | hsa-mir-656 | - |
| hsa-mir-7155 | HGLibA_63848 | 225 | 0.900508664 | hsa-mir-7155 | - |
| KIAA0101 | HGLibA_24591 | 225 | 0.900508664 | KIAA0101 | - |
| KIAA1244 | HGLibA_24728 | 225 | 0.900508664 | KIAA1244 | - |
| KIAA1737 | HGLibA_24813 | 225 | 0.900508664 | KIAA1737 | - |
| PION | HGLibA_36698 | 225 | 0.900508664 | PION | - |
| ENSG00000006194 | HGLibA_55692 | 224 | 0.891463321 | ZNF263 | zinc finger protein 263 [Source:HGNC Symbol;Acc:HGNC:13056] |
| ENSG00000024422 | HGLibA_14668 | 224 | 0.891463321 | EHD2 | EH domain containing 2 [Source:HGNC Symbol;Acc:HGNC:3243] |
| ENSG00000038295 | HGLibA_49628 | 224 | 0.891463321 | TLL1 | tolloid like 1 [Source:HGNC Symbol;Acc:HGNC:11843] |
| ENSG00000066185 | HGLibA_55418 | 224 | 0.891463321 | ZMYND12 | zinc finger MYND-type containing 12 [Source:HGNC Symbol;Acc:HGNC:21192] |
| ENSG00000081479 | HGLibA_27212 | 224 | 0.891463321 | LRP2 | LDL receptor related protein 2 [Source:HGNC Symbol;Acc:HGNC:6694] |
| ENSG00000086288 | HGLibA_32176 | 224 | 0.891463321 | NME8 | NME/NM23 family member 8 [Source:HGNC Symbol;Acc:HGNC:16473] |
| ENSG00000092377 | HGLibA_48614 | 224 | 0.891463321 | TBL1Y | transducin beta like 1 Y-linked [Source:HGNC Symbol;Acc:HGNC:18502] |
| ENSG00000092621 | HGLibA_36392 | 224 | 0.891463321 | PHGDH | phosphoglycerate dehydrogenase [Source:HGNC Symbol;Acc:HGNC:8923] |
| ENSG00000100281 | HGLibA_21734 | 224 | 0.891463321 | HMGXB4 | HMG-box containing 4 [Source:HGNC Symbol;Acc:HGNC:5003] |
| ENSG00000101337 | HGLibA_49729 | 224 | 0.891463321 | TM9SF4 | transmembrane 9 superfamily member 4 [Source:HGNC Symbol;Acc:HGNC:30797] |
| ENSG00000101489 | HGLibA_09012 | 224 | 0.891463321 | CELF4 | CUGBP Elav-like family member 4 [Source:HGNC Symbol;Acc:HGNC:14015] |
| ENSG00000102760 | HGLibA_40997 | 224 | 0.891463321 | RGCC | regulator of cell cycle [Source:HGNC Symbol;Acc:HGNC:20369] |
| ENSG00000111696 | HGLibA_32826 | 224 | 0.891463321 | NT5DC3 | 5'-nucleotidase domain containing 3 [Source:HGNC Symbol;Acc:HGNC:30826] |
| ENSG00000111725 | HGLibA_38496 | 224 | 0.891463321 | PRKAB1 | protein kinase AMP-activated non-catalytic subunit beta 1 [Source:HGNC Symbol;Acc:HGNC:9378] |
| ENSG00000112182 | HGLibA_04069 | 224 | 0.891463321 | BACH2 | BTB domain and CNC homolog 2 [Source:HGNC Symbol;Acc:HGNC:14078] |
| ENSG00000112212 | HGLibA_51962 | 224 | 0.891463321 | TSPO2 | translocator protein 2 [Source:HGNC Symbol;Acc:HGNC:21256] |
| ENSG00000114423 | HGLibA_07397 | 224 | 0.891463321 | CBLB | Cbl proto-oncogene B [Source:HGNC Symbol;Acc:HGNC:1542] |
| ENSG00000115306 | HGLibA_46880 | 224 | 0.891463321 | SPTBN1 | non-erythrocytic 1 [Source:HGNC Symbol;Acc:HGNC:11275]" |
| ENSG00000115556 | HGLibA_36995 | 224 | 0.891463321 | PLCD4 | phospholipase C delta 4 [Source:HGNC Symbol;Acc:HGNC:9062] |
| ENSG00000118246 | HGLibA_16905 | 224 | 0.891463321 | FASTKD2 | FAST kinase domains 2 [Source:HGNC Symbol;Acc:HGNC:29160] |
| ENSG00000119640 | HGLibA_00709 | 224 | 0.891463321 | ACYP1 | acylphosphatase 1 [Source:HGNC Symbol;Acc:HGNC:179] |
| ENSG00000121270 | HGLibA_00163 | 224 | 0.891463321 | ABCC11 | ATP binding cassette subfamily C member 11 [Source:HGNC Symbol;Acc:HGNC:14639] |
| ENSG00000121440 | HGLibA_35998 | 224 | 0.891463321 | PDZRN3 | PDZ domain containing ring finger 3 [Source:HGNC Symbol;Acc:HGNC:17704] |
| ENSG00000124535 | HGLibA_54413 | 224 | 0.891463321 | WRNIP1 | Werner helicase interacting protein 1 [Source:HGNC Symbol;Acc:HGNC:20876] |
| ENSG00000126460 | HGLibA_38872 | 224 | 0.891463321 | PRRG2 | proline rich and Gla domain 2 [Source:HGNC Symbol;Acc:HGNC:9470] |
| ENSG00000130517 | HGLibA_36279 | 224 | 0.891463321 | PGPEP1 | pyroglutamyl-peptidase I [Source:HGNC Symbol;Acc:HGNC:13568] |
| ENSG00000131089 | HGLibA_02879 | 224 | 0.891463321 | ARHGEF9 | Cdc42 guanine nucleotide exchange factor 9 [Source:HGNC Symbol;Acc:HGNC:14561] |
| ENSG00000131389 | HGLibA_45335 | 224 | 0.891463321 | SLC6A6 | solute carrier family 6 member 6 [Source:HGNC Symbol;Acc:HGNC:11052] |
| ENSG00000132361 | HGLibA_10162 | 224 | 0.891463321 | CLUH | clustered mitochondria homolog [Source:HGNC Symbol;Acc:HGNC:29094] |
| ENSG00000132768 | HGLibA_13839 | 224 | 0.891463321 | DPH2 | DPH2 homolog [Source:HGNC Symbol;Acc:HGNC:3004] |

| | | | | | |
|---|---|---|---|---|---|
| ENSG00000132874 | HGLibA_44403 | 224 | 0.891463321 | SLC14A2 | solute carrier family 14 member 2 [Source:HGNC Symbol;Acc:HGNC:10919] |
| ENSG00000133958 | HGLibA_53030 | 224 | 0.891463321 | UNC79 | NALCN channel complex subunit [Source:HGNC Symbol;Acc:HGNC:19966]" |
| ENSG00000136160 | HGLibA_14480 | 224 | 0.891463321 | EDNRB | endothelin receptor type B [Source:HGNC Symbol;Acc:HGNC:3180] |
| ENSG00000136449 | HGLibA_30670 | 224 | 0.891463321 | MYCBPAP | MYCBP associated protein [Source:HGNC Symbol;Acc:HGNC:19677] |
| ENSG00000136869 | HGLibA_49651 | 224 | 0.891463321 | TLR4 | toll like receptor 4 [Source:HGNC Symbol;Acc:HGNC:11850] |
| ENSG00000137393 | HGLibA_41530 | 224 | 0.891463321 | RNF144B | ring finger protein 144B [Source:HGNC Symbol;Acc:HGNC:21578] |
| ENSG00000137573 | HGLibA_47724 | 224 | 0.891463321 | SULF1 | sulfatase 1 [Source:HGNC Symbol;Acc:HGNC:20391] |
| ENSG00000138068 | HGLibA_47766 | 224 | 0.891463321 | SULT6B1 | sulfotransferase family 6B member 1 [Source:HGNC Symbol;Acc:HGNC:33433] |
| ENSG00000140968 | HGLibA_23600 | 224 | 0.891463321 | IRF8 | interferon regulatory factor 8 [Source:HGNC Symbol;Acc:HGNC:5358] |
| ENSG00000141098 | HGLibA_18957 | 224 | 0.891463321 | GFOD2 | glucose-fructose oxidoreductase domain containing 2 [Source:HGNC Symbol;Acc:HGNC:28159] |
| ENSG00000141378 | HGLibA_39552 | 224 | 0.891463321 | PTRH2 | peptidyl-tRNA hydrolase 2 [Source:HGNC Symbol;Acc:HGNC:24265] |
| ENSG00000143442 | HGLibA_37481 | 224 | 0.891463321 | POGZ | pogo transposable element derived with ZNF domain [Source:HGNC Symbol;Acc:HGNC:18801] |
| ENSG00000144746 | HGLibA_02990 | 224 | 0.891463321 | ARL6IP5 | ADP ribosylation factor like GTPase 6 interacting protein 5 [Source:HGNC Symbol;Acc:HGNC:16937] |
| ENSG00000145687 | HGLibA_47117 | 224 | 0.891463321 | SSBP2 | single stranded DNA binding protein 2 [Source:HGNC Symbol;Acc:HGNC:15831] |
| ENSG00000146247 | HGLibA_36400 | 224 | 0.891463321 | PHIP | pleckstrin homology domain interacting protein [Source:HGNC Symbol;Acc:HGNC:15673] |
| ENSG00000147099 | HGLibA_21024 | 224 | 0.891463321 | HDAC8 | histone deacetylase 8 [Source:HGNC Symbol;Acc:HGNC:13315] |
| ENSG00000147853 | HGLibA_01382 | 224 | 0.891463321 | AK3 | adenylate kinase 3 [Source:HGNC Symbol;Acc:HGNC:17376] |
| ENSG00000149527 | HGLibA_37011 | 224 | 0.891463321 | PLCH2 | phospholipase C eta 2 [Source:HGNC Symbol;Acc:HGNC:29037] |
| ENSG00000158109 | HGLibA_51184 | 224 | 0.891463321 | TPRG1L | tumor protein p63 regulated 1 like [Source:HGNC Symbol;Acc:HGNC:27007] |
| ENSG00000160691 | HGLibA_43999 | 224 | 0.891463321 | SHC1 | SHC adaptor protein 1 [Source:HGNC Symbol;Acc:HGNC:10840] |
| ENSG00000162482 | HGLibA_01501 | 224 | 0.891463321 | AKR7A3 | aldo-keto reductase family 7 member A3 [Source:HGNC Symbol;Acc:HGNC:390] |
| ENSG00000164077 | HGLibA_29638 | 224 | 0.891463321 | MON1A | secretory trafficking associated [Source:HGNC Symbol;Acc:HGNC:28207]" |
| ENSG00000165264 | HGLibA_31524 | 224 | 0.891463321 | NDUFB6 | NADH:ubiquinone oxidoreductase subunit B6 [Source:HGNC Symbol;Acc:HGNC:7701] |
| ENSG00000166501 | HGLibA_38536 | 224 | 0.891463321 | PRKCB | protein kinase C beta [Source:HGNC Symbol;Acc:HGNC:9395] |
| ENSG00000167460 | HGLibA_51150 | 224 | 0.891463321 | TPM4 | tropomyosin 4 [Source:HGNC Symbol;Acc:HGNC:12013] |
| ENSG00000173276 | HGLibA_54842 | 224 | 0.891463321 | ZBTB21 | zinc finger and BTB domain containing 21 [Source:HGNC Symbol;Acc:HGNC:13083] |
| ENSG00000176046 | HGLibA_33086 | 224 | 0.891463321 | NUPR1 | transcriptional regulator [Source:HGNC Symbol;Acc:HGNC:29990]" |
| ENSG00000176723 | HGLibA_56857 | 224 | 0.891463321 | ZNF843 | zinc finger protein 843 [Source:HGNC Symbol;Acc:HGNC:28710] |
| ENSG00000183186 | HGLibA_06100 | 224 | 0.891463321 | C2CD4C | C2 calcium dependent domain containing 4C [Source:HGNC Symbol;Acc:HGNC:29417] |
| ENSG00000183484 | HGLibA_19889 | 224 | 0.891463321 | GPR132 | G protein-coupled receptor 132 [Source:HGNC Symbol;Acc:HGNC:17482] |
| ENSG00000183715 | HGLibA_33413 | 224 | 0.891463321 | OPCML | opioid binding protein/cell adhesion molecule like [Source:HGNC Symbol;Acc:HGNC:8143] |
| ENSG00000183840 | HGLibA_20039 | 224 | 0.891463321 | GPR39 | G protein-coupled receptor 39 [Source:HGNC Symbol;Acc:HGNC:4496] |
| ENSG00000188293 | HGLibA_22838 | 224 | 0.891463321 | IGFL1 | IGF like family member 1 [Source:HGNC Symbol;Acc:HGNC:24093] |
| ENSG00000196141 | HGLibA_46548 | 224 | 0.891463321 | SPATS2L | spermatogenesis associated serine rich 2 like [Source:HGNC Symbol;Acc:HGNC:24574] |
| ENSG00000196262 | HGLibA_37894 | 224 | 0.891463321 | PPIA | peptidylprolyl isomerase A [Source:HGNC Symbol;Acc:HGNC:9253] |
| ENSG00000198074 | HGLibA_01474 | 224 | 0.891463321 | AKR1B10 | aldo-keto reductase family 1 member B10 [Source:HGNC Symbol;Acc:HGNC:382] |
| ENSG00000198121 | HGLibA_27053 | 224 | 0.891463321 | LPAR1 | lysophosphatidic acid receptor 1 [Source:HGNC Symbol;Acc:HGNC:3166] |
| ENSG00000198569 | HGLibA_44944 | 224 | 0.891463321 | SLC34A3 | solute carrier family 34 member 3 [Source:HGNC Symbol;Acc:HGNC:20305] |
| ENSG00000203705 | HGLibA_48445 | 224 | 0.891463321 | TATDN3 | TatD DNase domain containing 3 [Source:HGNC Symbol;Acc:HGNC:27010] |
| ENSG00000203970 | HGLibA_12846 | 224 | 0.891463321 | DEFB110 | defensin beta 110 [Source:HGNC Symbol;Acc:HGNC:18091] |
| ENSG00000204335 | HGLibA_46306 | 224 | 0.891463321 | SP5 | Sp5 transcription factor [Source:HGNC Symbol;Acc:HGNC:14529] |
| ENSG00000213694 | HGLibA_42599 | 224 | 0.891463321 | S1PR3 | sphingosine-1-phosphate receptor 3 [Source:HGNC Symbol;Acc:HGNC:3167] |
| ENSG00000213799 | HGLibA_56862 | 224 | 0.891463321 | ZNF845 | zinc finger protein 845 [Source:HGNC Symbol;Acc:HGNC:25112] |
| ENSG00000214511 | HGLibA_21311 | 224 | 0.891463321 | HIGD1C | HIG1 hypoxia inducible domain family member 1C [Source:HGNC Symbol;Acc:HGNC:28044] |
| ENSG00000227500 | HGLibA_42846 | 224 | 0.891463321 | SCAMP4 | secretory carrier membrane protein 4 [Source:HGNC Symbol;Acc:HGNC:30385] |
| ENSG00000265107 | HGLibA_19149 | 224 | 0.891463321 | GJA5 | gap junction protein alpha 5 [Source:HGNC Symbol;Acc:HGNC:4279] |
| ENSG00000270765 | HGLibA_18647 | 224 | 0.891463321 | GAS2L2 | growth arrest specific 2 like 2 [Source:HGNC Symbol;Acc:HGNC:24846] |
| hsa-mir-3146 | HGLibA_58641 | 224 | 0.891463321 | hsa-mir-3146 | - |
| hsa-mir-3652 | HGLibA_59152 | 224 | 0.891463321 | hsa-mir-3652 | - |
| hsa-mir-4290 | HGLibA_59870 | 224 | 0.891463321 | hsa-mir-4290 | - |
| hsa-mir-4461 | HGLibA_60250 | 224 | 0.891463321 | hsa-mir-4461 | - |
| hsa-mir-6861 | HGLibA_63626 | 224 | 0.891463321 | hsa-mir-6861 | - |
| IL9R_Y | HGLibA_23206 | 224 | 0.891463321 | IL9R_Y | - |
| LPHN2 | HGLibA_27090 | 224 | 0.891463321 | LPHN2 | - |
| PHOSPHO2-KLHL23 | HGLibA_36447 | 224 | 0.891463321 | PHOSPHO2-KLHL23 | - |
| ENSG00000065413 | HGLibA_02102 | 223 | 0.882417978 | ANKRD44 | ankyrin repeat domain 44 [Source:HGNC Symbol;Acc:HGNC:25259] |
| ENSG00000068028 | HGLibA_40375 | 223 | 0.882417978 | RASSF1 | Ras association domain family member 1 [Source:HGNC Symbol;Acc:HGNC:9882] |
| ENSG00000080709 | HGLibA_24357 | 223 | 0.882417978 | KCNN2 | potassium calcium-activated channel subfamily N member 2 [Source:HGNC Symbol;Acc:HGNC:6291] |
| ENSG00000101938 | HGLibA_09538 | 223 | 0.882417978 | CHRDL1 | chordin like 1 [Source:HGNC Symbol;Acc:HGNC:29861] |
| ENSG00000102678 | HGLibA_17423 | 223 | 0.882417978 | FGF9 | fibroblast growth factor 9 [Source:HGNC Symbol;Acc:HGNC:3687] |
| ENSG00000103496 | HGLibA_47649 | 223 | 0.882417978 | STX4 | syntaxin 4 [Source:HGNC Symbol;Acc:HGNC:11439] |
| ENSG00000104901 | HGLibA_13307 | 223 | 0.882417978 | DKKL1 | dickkopf like acrosomal protein 1 [Source:HGNC Symbol;Acc:HGNC:16528] |
| ENSG00000108861 | HGLibA_14178 | 223 | 0.882417978 | DUSP3 | dual specificity phosphatase 3 [Source:HGNC Symbol;Acc:HGNC:3069] |
| ENSG00000109193 | HGLibA_47756 | 223 | 0.882417978 | SULT1E1 | sulfotransferase family 1E member 1 [Source:HGNC Symbol;Acc:HGNC:11377] |
| ENSG00000111536 | HGLibA_23109 | 223 | 0.882417978 | IL26 | interleukin 26 [Source:HGNC Symbol;Acc:HGNC:17119] |
| ENSG00000111704 | HGLibA_31090 | 223 | 0.882417978 | NANOG | Nanog homeobox [Source:HGNC Symbol;Acc:HGNC:20857] |
| ENSG00000112164 | HGLibA_19291 | 223 | 0.882417978 | GLP1R | glucagon like peptide 1 receptor [Source:HGNC Symbol;Acc:HGNC:4324] |
| ENSG00000112273 | HGLibA_21041 | 223 | 0.882417978 | HDGFL1 | HDGF like 1 [Source:HGNC Symbol;Acc:HGNC:21095] |
| ENSG00000116996 | HGLibA_56959 | 223 | 0.882417978 | ZP4 | zona pellucida glycoprotein 4 [Source:HGNC Symbol;Acc:HGNC:15770] |
| ENSG00000119688 | HGLibA_00199 | 223 | 0.882417978 | ABCD4 | ATP binding cassette subfamily D member 4 [Source:HGNC Symbol;Acc:HGNC:68] |
| ENSG00000120328 | HGLibA_35477 | 223 | 0.882417978 | PCDHB12 | protocadherin beta 12 [Source:HGNC Symbol;Acc:HGNC:8683] |
| ENSG00000126583 | HGLibA_38549 | 223 | 0.882417978 | PRKCG | protein kinase C gamma [Source:HGNC Symbol;Acc:HGNC:9402] |
| ENSG00000128342 | HGLibA_26475 | 223 | 0.882417978 | LIF | interleukin 6 family cytokine [Source:HGNC Symbol;Acc:HGNC:6596]" |
| ENSG00000128578 | HGLibA_47592 | 223 | 0.882417978 | STRIP2 | striatin interacting protein 2 [Source:HGNC Symbol;Acc:HGNC:22209] |
| ENSG00000134152 | HGLibA_24084 | 223 | 0.882417978 | KATNBL1 | katanin regulatory subunit B1 like 1 [Source:HGNC Symbol;Acc:HGNC:26199] |
| ENSG00000134398 | HGLibA_15516 | 223 | 0.882417978 | ERN2 | endoplasmic reticulum to nucleus signaling 2 [Source:HGNC Symbol;Acc:HGNC:16942] |
| ENSG00000135436 | HGLibA_16339 | 223 | 0.882417978 | FAM186B | family with sequence similarity 186 member B [Source:HGNC Symbol;Acc:HGNC:25296] |
| ENSG00000135506 | HGLibA_34633 | 223 | 0.882417978 | OS9 | endoplasmic reticulum lectin [Source:HGNC Symbol;Acc:HGNC:16994]" |
| ENSG00000138442 | HGLibA_54029 | 223 | 0.882417978 | WDR12 | WD repeat domain 12 [Source:HGNC Symbol;Acc:HGNC:14098] |
| ENSG00000138640 | HGLibA_16142 | 223 | 0.882417978 | FAM13A | family with sequence similarity 13 member A [Source:HGNC Symbol;Acc:HGNC:19367] |
| ENSG00000138802 | HGLibA_43231 | 223 | 0.882417978 | SEC24B | COPII coat complex component [Source:HGNC Symbol;Acc:HGNC:10704]" |
| ENSG00000142583 | HGLibA_44881 | 223 | 0.882417978 | SLC2A5 | solute carrier family 2 member 5 [Source:HGNC Symbol;Acc:HGNC:11010] |
| ENSG00000145779 | HGLibA_50717 | 223 | 0.882417978 | TNFAIP8 | TNF alpha induced protein 8 [Source:HGNC Symbol;Acc:HGNC:17260] |
| ENSG00000146360 | HGLibA_20060 | 223 | 0.882417978 | GPR6 | G protein-coupled receptor 6 [Source:HGNC Symbol;Acc:HGNC:4515] |
| ENSG00000147896 | HGLibA_22715 | 223 | 0.882417978 | IFNK | interferon kappa [Source:HGNC Symbol;Acc:HGNC:21714] |
| ENSG00000148384 | HGLibA_23353 | 223 | 0.882417978 | INPP5E | inositol polyphosphate-5-phosphatase E [Source:HGNC Symbol;Acc:HGNC:21474] |
| ENSG00000151689 | HGLibA_23335 | 223 | 0.882417978 | INPP1 | inositol polyphosphate-1-phosphatase [Source:HGNC Symbol;Acc:HGNC:6071] |
| ENSG00000153201 | HGLibA_40202 | 223 | 0.882417978 | RANBP2 | RAN binding protein 2 [Source:HGNC Symbol;Acc:HGNC:9848] |
| ENSG00000158270 | HGLibA_10637 | 223 | 0.882417978 | COLEC12 | collectin subfamily member 12 [Source:HGNC Symbol;Acc:HGNC:16016] |
| ENSG00000163359 | HGLibA_10603 | 223 | 0.882417978 | COL6A3 | collagen type VI alpha 3 chain [Source:HGNC Symbol;Acc:HGNC:2213] |
| ENSG00000163435 | HGLibA_14896 | 223 | 0.882417978 | ELF3 | E74 like ETS transcription factor 3 [Source:HGNC Symbol;Acc:HGNC:3318] |
| ENSG00000164047 | HGLibA_07104 | 223 | 0.882417978 | CAMP | cathelicidin antimicrobial peptide [Source:HGNC Symbol;Acc:HGNC:1472] |
| ENSG00000164107 | HGLibA_20829 | 223 | 0.882417978 | HAND2 | heart and neural crest derivatives expressed 2 [Source:HGNC Symbol;Acc:HGNC:4808] |
| ENSG00000167491 | HGLibA_18687 | 223 | 0.882417978 | GATAD2A | GATA zinc finger domain containing 2A [Source:HGNC Symbol;Acc:HGNC:29989] |
| ENSG00000168028 | HGLibA_42198 | 223 | 0.882417978 | RPSA | ribosomal protein SA [Source:HGNC Symbol;Acc:HGNC:6502] |
| ENSG00000170634 | HGLibA_00713 | 223 | 0.882417978 | ACYP2 | acylphosphatase 2 [Source:HGNC Symbol;Acc:HGNC:180] |
| ENSG00000177354 | HGLibA_05092 | 223 | 0.882417978 | C10orf71 | chromosome 10 open reading frame 71 [Source:HGNC Symbol;Acc:HGNC:26973] |
| ENSG00000180185 | HGLibA_15954 | 223 | 0.882417978 | FAHD1 | fumarylacetoacetate hydrolase domain containing 1 [Source:HGNC Symbol;Acc:HGNC:14169] |
| ENSG00000182330 | HGLibA_38330 | 223 | 0.882417978 | PRAMEF8 | PRAME family member 8 [Source:HGNC Symbol;Acc:HGNC:24074] |
| ENSG00000184009 | HGLibA_00611 | 223 | 0.882417978 | ACTG1 | actin gamma 1 [Source:HGNC Symbol;Acc:HGNC:144] |
| ENSG00000187689 | HGLibA_01837 | 223 | 0.882417978 | AMTN | amelotin [Source:HGNC Symbol;Acc:HGNC:33188] |
| ENSG00000189067 | HGLibA_26639 | 223 | 0.882417978 | LITAF | lipopolysaccharide induced TNF factor [Source:HGNC Symbol;Acc:HGNC:16841] |
| ENSG00000189108 | HGLibA_23059 | 223 | 0.882417978 | IL1RAPL2 | interleukin 1 receptor accessory protein like 2 [Source:HGNC Symbol;Acc:HGNC:5997] |
| ENSG00000189319 | HGLibA_16637 | 223 | 0.882417978 | FAM53B | family with sequence similarity 53 member B [Source:HGNC Symbol;Acc:HGNC:28968] |
| ENSG00000198283 | HGLibA_34264 | 223 | 0.882417978 | OR5B21 | olfactory receptor family 5 subfamily B member 21 [Source:HGNC Symbol;Acc:HGNC:19616] |
| ENSG00000198883 | HGLibA_37376 | 223 | 0.882417978 | PNMA5 | PNMA family member 5 [Source:HGNC Symbol;Acc:HGNC:18743] |
| ENSG00000198931 | HGLibA_02573 | 223 | 0.882417978 | APRT | adenine phosphoribosyltransferase [Source:HGNC Symbol;Acc:HGNC:626] |
| ENSG00000205277 | HGLibA_30524 | 223 | 0.882417978 | MUC12 | cell surface associated [Source:HGNC Symbol;Acc:HGNC:7510]" |
| ENSG00000217555 | HGLibA_09768 | 223 | 0.882417978 | CKLF | chemokine like factor [Source:HGNC Symbol;Acc:HGNC:13253] |
| ENSG00000221888 | HGLibA_33651 | 223 | 0.882417978 | OR1C1 | olfactory receptor family 1 subfamily C member 1 [Source:HGNC Symbol;Acc:HGNC:8182] |
| ENSG00000223865 | HGLibA_21617 | 223 | 0.882417978 | HLA-DPB1 | DP beta 1 [Source:HGNC Symbol;Acc:HGNC:4940]" |
| ENSG00000226174 | HGLibA_49075 | 223 | 0.882417978 | TEX22 | testis expressed 22 [Source:HGNC Symbol;Acc:HGNC:40026] |
| ENSG00000228474 | HGLibA_34704 | 223 | 0.882417978 | OST4 | non-catalytic [Source:HGNC Symbol;Acc:HGNC:32483]" |
| ENSG00000232263 | HGLibA_25729 | 223 | 0.882417978 | KRTAP25-1 | keratin associated protein 25-1 [Source:HGNC Symbol;Acc:HGNC:34003] |
| ENSG00000256870 | HGLibA_45283 | 223 | 0.882417978 | SLC5A8 | solute carrier family 5 member 8 [Source:HGNC Symbol;Acc:HGNC:19119] |
| ENSG00000269405 | HGLibA_33130 | 223 | 0.882417978 | NXF2 | nuclear RNA export factor 2 [Source:HGNC Symbol;Acc:HGNC:8072] |
| FAM46B | HGLibA_16600 | 223 | 0.882417978 | FAM46B | - |
| HIST1H4H | HGLibA_21506 | 223 | 0.882417978 | HIST1H4H | - |
| hsa-mir-1208 | HGLibA_57288 | 223 | 0.882417978 | hsa-mir-1208 | - |
| hsa-mir-1234 | HGLibA_57331 | 223 | 0.882417978 | hsa-mir-1234 | - |
| hsa-mir-3064 | HGLibA_58460 | 223 | 0.882417978 | hsa-mir-3064 | - |
| hsa-mir-3116-2 | HGLibA_58507 | 223 | 0.882417978 | hsa-mir-3116-2 | - |
| hsa-mir-3614 | HGLibA_59087 | 223 | 0.882417978 | hsa-mir-3614 | - |
| hsa-mir-3664 | HGLibA_59207 | 223 | 0.882417978 | hsa-mir-3664 | - |
| hsa-mir-5010 | HGLibA_61454 | 223 | 0.882417978 | hsa-mir-5010 | - |
| hsa-mir-548e | HGLibA_61967 | 223 | 0.882417978 | hsa-mir-548e | - |
| hsa-mir-6810 | HGLibA_63416 | 223 | 0.882417978 | hsa-mir-6810 | - |
| ZBED1_Y | HGLibA_54790 | 223 | 0.882417978 | ZBED1_Y | - |
| CCDC104 | HGLibA_07516 | 222 | 0.873372635 | CCDC104 | - |
| ENSG00000006757 | HGLibA_37414 | 222 | 0.873372635 | PNPLA4 | patatin like phospholipase domain containing 4 [Source:HGNC Symbol;Acc:HGNC:24887] |
| ENSG00000047457 | HGLibA_10881 | 222 | 0.873372635 | CP | ceruloplasmin [Source:HGNC Symbol;Acc:HGNC:2295] |
| ENSG00000070601 | HGLibA_18031 | 222 | 0.873372635 | FRMPD1 | FERM and PDZ domain containing 1 [Source:HGNC Symbol;Acc:HGNC:29159] |
| ENSG00000087258 | HGLibA_19463 | 222 | 0.873372635 | GNAO1 | G protein subunit alpha o1 [Source:HGNC Symbol;Acc:HGNC:4389] |
| ENSG00000091490 | HGLibA_43284 | 222 | 0.873372635 | SEL1L3 | SEL1L family member 3 [Source:HGNC Symbol;Acc:HGNC:29108] |
| ENSG00000101463 | HGLibA_47970 | 222 | 0.873372635 | SYNDIG1 | synapse differentiation inducing 1 [Source:HGNC Symbol;Acc:HGNC:15885] |

| | | | | | |
|---|---|---|---|---|---|
| ENSG00000101972 | HGLibA_47325 | 222 | 0.873372635 | STAG2 | stromal antigen 2 [Source:HGNC Symbol;Acc:HGNC:11355] |
| ENSG00000103494 | HGLibA_41840 | 222 | 0.873372635 | RPGRIP1L | RPGRIP1 like [Source:HGNC Symbol;Acc:HGNC:29168] |
| ENSG00000106524 | HGLibA_01983 | 222 | 0.873372635 | ANKMY2 | ankyrin repeat and MYND domain containing 2 [Source:HGNC Symbol;Acc:HGNC:25370] |
| ENSG00000110218 | HGLibA_35108 | 222 | 0.873372635 | PANX1 | pannexin 1 [Source:HGNC Symbol;Acc:HGNC:8599] |
| ENSG00000114023 | HGLibA_16221 | 222 | 0.873372635 | FAM162A | family with sequence similarity 162 member A [Source:HGNC Symbol;Acc:HGNC:17865] |
| ENSG00000116254 | HGLibA_09399 | 222 | 0.873372635 | CHD5 | chromodomain helicase DNA binding protein 5 [Source:HGNC Symbol;Acc:HGNC:16816] |
| ENSG00000117425 | HGLibA_39272 | 222 | 0.873372635 | PTCH2 | patched 2 [Source:HGNC Symbol;Acc:HGNC:9586] |
| ENSG00000122585 | HGLibA_32548 | 222 | 0.873372635 | NPY | neuropeptide Y [Source:HGNC Symbol;Acc:HGNC:7955] |
| ENSG00000122859 | HGLibA_31752 | 222 | 0.873372635 | NEUROG3 | neurogenin 3 [Source:HGNC Symbol;Acc:HGNC:13806] |
| ENSG00000123999 | HGLibA_23297 | 222 | 0.873372635 | INHA | inhibin subunit alpha [Source:HGNC Symbol;Acc:HGNC:6065] |
| ENSG00000125246 | HGLibA_10172 | 222 | 0.873372635 | CLYBL | citrate lyase beta like [Source:HGNC Symbol;Acc:HGNC:18355] |
| ENSG00000125249 | HGLibA_40236 | 222 | 0.873372635 | RAP2A | member of RAS oncogene family [Source:HGNC Symbol;Acc:HGNC:9861]" |
| ENSG00000126882 | HGLibA_16727 | 222 | 0.873372635 | FAM78A | family with sequence similarity 78 member A [Source:HGNC Symbol;Acc:HGNC:25465] |
| ENSG00000133962 | HGLibA_07366 | 222 | 0.873372635 | CATSPERB | cation channel sperm associated auxiliary subunit beta [Source:HGNC Symbol;Acc:HGNC:20500] |
| ENSG00000135018 | HGLibA_52731 | 222 | 0.873372635 | UBQLN1 | ubiquilin 1 [Source:HGNC Symbol;Acc:HGNC:12508] |
| ENSG00000141698 | HGLibA_32818 | 222 | 0.873372635 | NT5C3B | cytosolic IIIB [Source:HGNC Symbol;Acc:HGNC:28300]" |
| ENSG00000143756 | HGLibA_17061 | 222 | 0.873372635 | FBXO28 | F-box protein 28 [Source:HGNC Symbol;Acc:HGNC:29046] |
| ENSG00000144824 | HGLibA_36428 | 222 | 0.873372635 | PHLDB2 | pleckstrin homology like domain family B member 2 [Source:HGNC Symbol;Acc:HGNC:29573] |
| ENSG00000144852 | HGLibA_32589 | 222 | 0.873372635 | NR1I2 | nuclear receptor subfamily 1 group I member 2 [Source:HGNC Symbol;Acc:HGNC:7968] |
| ENSG00000146373 | HGLibA_41635 | 222 | 0.873372635 | RNF217 | ring finger protein 217 [Source:HGNC Symbol;Acc:HGNC:21487] |
| ENSG00000153237 | HGLibA_07633 | 222 | 0.873372635 | CCDC148 | coiled-coil domain containing 148 [Source:HGNC Symbol;Acc:HGNC:25191] |
| ENSG00000157349 | HGLibA_12666 | 222 | 0.873372635 | DDX19B | DEAD-box helicase 19B [Source:HGNC Symbol;Acc:HGNC:2742] |
| ENSG00000162992 | HGLibA_31735 | 222 | 0.873372635 | NEUROD1 | neuronal differentiation 1 [Source:HGNC Symbol;Acc:HGNC:7762] |
| ENSG00000163564 | HGLibA_39699 | 222 | 0.873372635 | PYHIN1 | pyrin and HIN domain family member 1 [Source:HGNC Symbol;Acc:HGNC:28894] |
| ENSG00000164309 | HGLibA_10240 | 222 | 0.873372635 | CMYA5 | cardiomyopathy associated 5 [Source:HGNC Symbol;Acc:HGNC:14305] |
| ENSG00000166407 | HGLibA_26702 | 222 | 0.873372635 | LMO1 | LIM domain only 1 [Source:HGNC Symbol;Acc:HGNC:6641] |
| ENSG00000166441 | HGLibA_41923 | 222 | 0.873372635 | RPL27A | ribosomal protein L27a [Source:HGNC Symbol;Acc:HGNC:10329] |
| ENSG00000168906 | HGLibA_28417 | 222 | 0.873372635 | MAT2A | methionine adenosyltransferase 2A [Source:HGNC Symbol;Acc:HGNC:6904] |
| ENSG00000170209 | HGLibA_01972 | 222 | 0.873372635 | ANKK1 | ankyrin repeat and kinase domain containing 1 [Source:HGNC Symbol;Acc:HGNC:21027] |
| ENSG00000171481 | HGLibA_33688 | 222 | 0.873372635 | OR1L3 | olfactory receptor family 1 subfamily L member 3 [Source:HGNC Symbol;Acc:HGNC:8215] |
| ENSG00000171657 | HGLibA_20090 | 222 | 0.873372635 | GPR82 | G protein-coupled receptor 82 [Source:HGNC Symbol;Acc:HGNC:4533] |
| ENSG00000172009 | HGLibA_49382 | 222 | 0.873372635 | THOP1 | thimet oligopeptidase 1 [Source:HGNC Symbol;Acc:HGNC:11793] |
| ENSG00000174502 | HGLibA_44810 | 222 | 0.873372635 | SLC26A9 | solute carrier family 26 member 9 [Source:HGNC Symbol;Acc:HGNC:14469] |
| ENSG00000174871 | HGLibA_10286 | 222 | 0.873372635 | CNIH2 | cornichon family AMPA receptor auxiliary protein 2 [Source:HGNC Symbol;Acc:HGNC:28744] |
| ENSG00000175985 | HGLibA_37093 | 222 | 0.873372635 | PLEKHD1 | pleckstrin homology and coiled-coil domain containing D1 [Source:HGNC Symbol;Acc:HGNC:20148] |
| ENSG00000177807 | HGLibA_24250 | 222 | 0.873372635 | KCNJ10 | potassium voltage-gated channel subfamily J member 10 [Source:HGNC Symbol;Acc:HGNC:6256] |
| ENSG00000177917 | HGLibA_02994 | 222 | 0.873372635 | ARL6IP6 | ADP ribosylation factor like GTPase 6 interacting protein 6 [Source:HGNC Symbol;Acc:HGNC:24048] |
| ENSG00000178445 | HGLibA_19235 | 222 | 0.873372635 | GLDC | glycine decarboxylase [Source:HGNC Symbol;Acc:HGNC:4313] |
| ENSG00000182795 | HGLibA_05756 | 222 | 0.873372635 | C1orf116 | chromosome 1 open reading frame 116 [Source:HGNC Symbol;Acc:HGNC:28667] |
| ENSG00000182923 | HGLibA_09152 | 222 | 0.873372635 | CEP63 | centrosomal protein 63 [Source:HGNC Symbol;Acc:HGNC:25815] |
| ENSG00000183569 | HGLibA_43482 | 222 | 0.873372635 | SERHL2 | serine hydrolase like 2 [Source:HGNC Symbol;Acc:HGNC:29446] |
| ENSG00000183840 | HGLibA_20038 | 222 | 0.873372635 | GPR39 | G protein-coupled receptor 39 [Source:HGNC Symbol;Acc:HGNC:4496] |
| ENSG00000184293 | HGLibA_10013 | 222 | 0.873372635 | CLECL1 | C-type lectin like 1 [Source:HGNC Symbol;Acc:HGNC:24462] |
| ENSG00000185973 | HGLibA_50590 | 222 | 0.873372635 | TMLHE | epsilon [Source:HGNC Symbol;Acc:HGNC:18308]" |
| ENSG00000186847 | HGLibA_25427 | 222 | 0.873372635 | KRT14 | keratin 14 [Source:HGNC Symbol;Acc:HGNC:6416] |
| ENSG00000187010 | HGLibA_41152 | 222 | 0.873372635 | RHD | Rh blood group D antigen [Source:HGNC Symbol;Acc:HGNC:10009] |
| ENSG00000197077 | HGLibA_24798 | 222 | 0.873372635 | KIAA1671 | KIAA1671 [Source:HGNC Symbol;Acc:HGNC:29345] |
| ENSG00000197891 | HGLibA_44544 | 222 | 0.873372635 | SLC22A12 | solute carrier family 22 member 12 [Source:HGNC Symbol;Acc:HGNC:17989] |
| ENSG00000198168 | HGLibA_47889 | 222 | 0.873372635 | SVIP | small VCP interacting protein [Source:HGNC Symbol;Acc:HGNC:25238] |
| ENSG00000204209 | HGLibA_12349 | 222 | 0.873372635 | DAXX | death domain associated protein [Source:HGNC Symbol;Acc:HGNC:2681] |
| ENSG00000204414 | HGLibA_11357 | 222 | 0.873372635 | CSHL1 | chorionic somatomammotropin hormone like 1 [Source:HGNC Symbol;Acc:HGNC:2442] |
| ENSG00000204450 | HGLibA_51575 | 222 | 0.873372635 | TRIM64 | tripartite motif containing 64 [Source:HGNC Symbol;Acc:HGNC:14663] |
| ENSG00000204538 | HGLibA_39221 | 222 | 0.873372635 | PSORS1C2 | psoriasis susceptibility 1 candidate 2 [Source:HGNC Symbol;Acc:HGNC:17199] |
| ENSG00000204849 | HGLibA_46467 | 222 | 0.873372635 | SPATA31A1 | SPATA31 subfamily A member 1 [Source:HGNC Symbol;Acc:HGNC:23394] |
| ENSG00000205758 | HGLibA_11296 | 222 | 0.873372635 | CRYZL1 | crystallin zeta like 1 [Source:HGNC Symbol;Acc:HGNC:2420] |
| ENSG00000212899 | HGLibA_25747 | 222 | 0.873372635 | KRTAP3-3 | keratin associated protein 3-3 [Source:HGNC Symbol;Acc:HGNC:18890] |
| ENSG00000235034 | HGLibA_05714 | 222 | 0.873372635 | C19orf81 | chromosome 19 open reading frame 81 [Source:HGNC Symbol;Acc:HGNC:40041] |
| ENSG00000244165 | HGLibA_34874 | 222 | 0.873372635 | P2RY11 | purinergic receptor P2Y11 [Source:HGNC Symbol;Acc:HGNC:8540] |
| ENSG00000248483 | HGLibA_37791 | 222 | 0.873372635 | POU5F2 | transcription factor 2 [Source:HGNC Symbol;Acc:HGNC:26367]" |
| ENSG00000249437 | HGLibA_31081 | 222 | 0.873372635 | NAIP | NLR family apoptosis inhibitory protein [Source:HGNC Symbol;Acc:HGNC:7634] |
| H2AFB1 | HGLibA_20745 | 222 | 0.873372635 | H2AFB1 | - |
| hsa-mir-1908 | HGLibA_58025 | 222 | 0.873372635 | hsa-mir-1908 | - |
| hsa-mir-4251 | HGLibA_59711 | 222 | 0.873372635 | hsa-mir-4251 | - |
| hsa-mir-4316 | HGLibA_59983 | 222 | 0.873372635 | hsa-mir-4316 | - |
| hsa-mir-4450 | HGLibA_60208 | 222 | 0.873372635 | hsa-mir-4450 | - |
| hsa-mir-4736 | HGLibA_61042 | 222 | 0.873372635 | hsa-mir-4736 | - |
| hsa-mir-498 | HGLibA_61391 | 222 | 0.873372635 | hsa-mir-498 | - |
| hsa-mir-499b | HGLibA_61403 | 222 | 0.873372635 | hsa-mir-499b | - |
| hsa-mir-5197 | HGLibA_61707 | 222 | 0.873372635 | hsa-mir-5197 | - |
| hsa-mir-542 | HGLibA_61819 | 222 | 0.873372635 | hsa-mir-542 | - |
| hsa-mir-548aa-2 | HGLibA_61853 | 222 | 0.873372635 | hsa-mir-548aa-2 | - |
| hsa-mir-5698 | HGLibA_62325 | 222 | 0.873372635 | hsa-mir-5698 | - |
| hsa-mir-6860 | HGLibA_63619 | 222 | 0.873372635 | hsa-mir-6860 | - |
| hsa-mir-8066 | HGLibA_64102 | 222 | 0.873372635 | hsa-mir-8066 | - |
| PLCXD1_Y | HGLibA_37024 | 222 | 0.873372635 | PLCXD1_Y | - |
| SPANXE | HGLibA_46395 | 222 | 0.873372635 | SPANXE | - |
| C20orf24 | HGLibA_06017 | 221 | 0.864327292 | C20orf24 | - |
| C4orf29 | HGLibA_06313 | 221 | 0.864327292 | C4orf29 | - |
| CASC1 | HGLibA_07269 | 221 | 0.864327292 | CASC1 | - |
| ENSG00000005961 | HGLibA_23728 | 221 | 0.864327292 | ITGA2B | integrin subunit alpha 2b [Source:HGNC Symbol;Acc:HGNC:6138] |
| ENSG00000010404 | HGLibA_22566 | 221 | 0.864327292 | IDS | iduronate 2-sulfatase [Source:HGNC Symbol;Acc:HGNC:5389] |
| ENSG00000011021 | HGLibA_09834 | 221 | 0.864327292 | CLCN6 | chloride voltage-gated channel 6 [Source:HGNC Symbol;Acc:HGNC:2024] |
| ENSG00000019169 | HGLibA_28360 | 221 | 0.864327292 | MARCO | macrophage receptor with collagenous structure [Source:HGNC Symbol;Acc:HGNC:6895] |
| ENSG00000048471 | HGLibA_46078 | 221 | 0.864327292 | SNX29 | sorting nexin 29 [Source:HGNC Symbol;Acc:HGNC:30542] |
| ENSG00000053372 | HGLibA_30121 | 221 | 0.864327292 | MRTO4 | ribosome maturation factor [Source:HGNC Symbol;Acc:HGNC:18477]" |
| ENSG00000068650 | HGLibA_03553 | 221 | 0.864327292 | ATP11A | ATPase phospholipid transporting 11A [Source:HGNC Symbol;Acc:HGNC:13552] |
| ENSG00000068654 | HGLibA_37548 | 221 | 0.864327292 | POLR1A | RNA polymerase I subunit A [Source:HGNC Symbol;Acc:HGNC:17264] |
| ENSG00000070882 | HGLibA_34656 | 221 | 0.864327292 | OSBPL3 | oxysterol binding protein like 3 [Source:HGNC Symbol;Acc:HGNC:16370] |
| ENSG00000078902 | HGLibA_50943 | 221 | 0.864327292 | TOLLIP | toll interacting protein [Source:HGNC Symbol;Acc:HGNC:16476] |
| ENSG00000104332 | HGLibA_43735 | 221 | 0.864327292 | SFRP1 | secreted frizzled related protein 1 [Source:HGNC Symbol;Acc:HGNC:10776] |
| ENSG00000104886 | HGLibA_37134 | 221 | 0.864327292 | PLEKHJ1 | pleckstrin homology domain containing J1 [Source:HGNC Symbol;Acc:HGNC:18211] |
| ENSG00000105887 | HGLibA_30459 | 221 | 0.864327292 | MTPN | myotrophin [Source:HGNC Symbol;Acc:HGNC:15667] |
| ENSG00000106701 | HGLibA_18079 | 221 | 0.864327292 | FSD1L | fibronectin type III and SPRY domain containing 1 like [Source:HGNC Symbol;Acc:HGNC:13753] |
| ENSG00000107719 | HGLibA_35061 | 221 | 0.864327292 | PALD1 | phosphatase domain containing paladin 1 [Source:HGNC Symbol;Acc:HGNC:23530] |
| ENSG00000107863 | HGLibA_02725 | 221 | 0.864327292 | ARHGAP21 | Rho GTPase activating protein 21 [Source:HGNC Symbol;Acc:HGNC:23725] |
| ENSG00000111331 | HGLibA_33205 | 221 | 0.864327292 | OAS3 | 2'-5'-oligoadenylate synthetase 3 [Source:HGNC Symbol;Acc:HGNC:8088] |
| ENSG00000112343 | HGLibA_51480 | 221 | 0.864327292 | TRIM38 | tripartite motif containing 38 [Source:HGNC Symbol;Acc:HGNC:10059] |
| ENSG00000115339 | HGLibA_18558 | 221 | 0.864327292 | GALNT3 | polypeptide N-acetylgalactosaminyltransferase 3 [Source:HGNC Symbol;Acc:HGNC:4125] |
| ENSG00000116017 | HGLibA_02892 | 221 | 0.864327292 | ARID3A | AT-rich interaction domain 3A [Source:HGNC Symbol;Acc:HGNC:3031] |
| ENSG00000117154 | HGLibA_22883 | 221 | 0.864327292 | IGSF21 | immunoglobin superfamily member 21 [Source:HGNC Symbol;Acc:HGNC:28246] |
| ENSG00000124256 | HGLibA_54807 | 221 | 0.864327292 | ZBP1 | Z-DNA binding protein 1 [Source:HGNC Symbol;Acc:HGNC:16176] |
| ENSG00000125844 | HGLibA_42242 | 221 | 0.864327292 | RRBP1 | ribosome binding protein 1 [Source:HGNC Symbol;Acc:HGNC:10448] |
| ENSG00000127954 | HGLibA_47433 | 221 | 0.864327292 | STEAP4 | STEAP4 metalloreductase [Source:HGNC Symbol;Acc:HGNC:21923] |
| ENSG00000130270 | HGLibA_03785 | 221 | 0.864327292 | ATP8B3 | ATPase phospholipid transporting 8B3 [Source:HGNC Symbol;Acc:HGNC:13535] |
| ENSG00000132623 | HGLibA_01951 | 221 | 0.864327292 | ANKEF1 | ankyrin repeat and EF-hand domain containing 1 [Source:HGNC Symbol;Acc:HGNC:15803] |
| ENSG00000132912 | HGLibA_12552 | 221 | 0.864327292 | DCTN4 | dynactin subunit 4 [Source:HGNC Symbol;Acc:HGNC:15518] |
| ENSG00000136379 | HGLibA_00270 | 221 | 0.864327292 | ABHD17C | abhydrolase domain containing 17C [Source:HGNC Symbol;Acc:HGNC:26925] |
| ENSG00000139329 | HGLibA_27628 | 221 | 0.864327292 | LUM | lumican [Source:HGNC Symbol;Acc:HGNC:6724] |
| ENSG00000141096 | HGLibA_13825 | 221 | 0.864327292 | DPEP3 | dipeptidase 3 [Source:HGNC Symbol;Acc:HGNC:23029] |
| ENSG00000142511 | HGLibA_20020 | 221 | 0.864327292 | GPR32 | G protein-coupled receptor 32 [Source:HGNC Symbol;Acc:HGNC:4487] |
| ENSG00000143013 | HGLibA_26712 | 221 | 0.864327292 | LMO4 | LIM domain only 4 [Source:HGNC Symbol;Acc:HGNC:6644] |
| ENSG00000145536 | HGLibA_00809 | 221 | 0.864327292 | ADAMTS16 | ADAM metallopeptidase with thrombospondin type 1 motif 16 [Source:HGNC Symbol;Acc:HGNC:17108] |
| ENSG00000153904 | HGLibA_12589 | 221 | 0.864327292 | DDAH1 | dimethylarginine dimethylaminohydrolase 1 [Source:HGNC Symbol;Acc:HGNC:2715] |
| ENSG00000158186 | HGLibA_29814 | 221 | 0.864327292 | MRAS | muscle RAS oncogene homolog [Source:HGNC Symbol;Acc:HGNC:7227] |
| ENSG00000166710 | HGLibA_03944 | 221 | 0.864327292 | B2M | beta-2-microglobulin [Source:HGNC Symbol;Acc:HGNC:914] |
| ENSG00000170017 | HGLibA_01532 | 221 | 0.864327292 | ALCAM | activated leukocyte cell adhesion molecule [Source:HGNC Symbol;Acc:HGNC:400] |
| ENSG00000170290 | HGLibA_45544 | 221 | 0.864327292 | SLN | sarcolipin [Source:HGNC Symbol;Acc:HGNC:11089] |
| ENSG00000170677 | HGLibA_46137 | 221 | 0.864327292 | SOCS6 | suppressor of cytokine signaling 6 [Source:HGNC Symbol;Acc:HGNC:16833] |
| ENSG00000171786 | HGLibA_31889 | 221 | 0.864327292 | NHLH1 | nescient helix-loop-helix 1 [Source:HGNC Symbol;Acc:HGNC:7817] |
| ENSG00000171860 | HGLibA_06207 | 221 | 0.864327292 | C3AR1 | complement C3a receptor 1 [Source:HGNC Symbol;Acc:HGNC:1319] |
| ENSG00000174885 | HGLibA_32126 | 221 | 0.864327292 | NLRP6 | NLR family pyrin domain containing 6 [Source:HGNC Symbol;Acc:HGNC:22944] |
| ENSG00000175315 | HGLibA_11463 | 221 | 0.864327292 | CST6 | cystatin E/M [Source:HGNC Symbol;Acc:HGNC:2478] |
| ENSG00000178163 | HGLibA_56152 | 221 | 0.864327292 | ZNF518B | zinc finger protein 518B [Source:HGNC Symbol;Acc:HGNC:29365] |
| ENSG00000178372 | HGLibA_07036 | 221 | 0.864327292 | CALML5 | calmodulin like 5 [Source:HGNC Symbol;Acc:HGNC:18180] |
| ENSG00000181240 | HGLibA_44737 | 221 | 0.864327292 | SLC25A41 | solute carrier family 25 member 41 [Source:HGNC Symbol;Acc:HGNC:28533] |
| ENSG00000181896 | HGLibA_55434 | 221 | 0.864327292 | ZNF101 | zinc finger protein 101 [Source:HGNC Symbol;Acc:HGNC:12881] |
| ENSG00000183161 | HGLibA_16847 | 221 | 0.864327292 | FANCF | FA complementation group F [Source:HGNC Symbol;Acc:HGNC:3587] |
| ENSG00000183207 | HGLibA_42477 | 221 | 0.864327292 | RUVBL2 | RuvB like AAA ATPase 2 [Source:HGNC Symbol;Acc:HGNC:10475] |
| ENSG00000184113 | HGLibA_09902 | 221 | 0.864327292 | CLDN5 | claudin 5 [Source:HGNC Symbol;Acc:HGNC:2047] |
| ENSG00000186187 | HGLibA_56938 | 221 | 0.864327292 | ZNRF1 | zinc and ring finger 1 [Source:HGNC Symbol;Acc:HGNC:18452] |
| ENSG00000189046 | HGLibA_01648 | 221 | 0.864327292 | ALKBH2 | alpha-ketoglutarate dependent dioxygenase [Source:HGNC Symbol;Acc:HGNC:32487]" |
| ENSG00000197859 | HGLibA_00852 | 221 | 0.864327292 | ADAMTSL2 | ADAMTS like 2 [Source:HGNC Symbol;Acc:HGNC:14631] |
| ENSG00000198113 | HGLibA_51034 | 221 | 0.864327292 | TOR4A | torsin family 4 member A [Source:HGNC Symbol;Acc:HGNC:25981] |
| ENSG00000198467 | HGLibA_51143 | 221 | 0.864327292 | TPM2 | tropomyosin 2 [Source:HGNC Symbol;Acc:HGNC:12011] |
| ENSG00000198873 | HGLibA_20331 | 221 | 0.864327292 | GRK5 | G protein-coupled receptor kinase 5 [Source:HGNC Symbol;Acc:HGNC:4544] |

| | | | | | |
|---|---|---|---|---|---|
| ENSG00000198937 | HGLibA_07676 | 221 | 0.864327292 | CCDC167 | coiled-coil domain containing 167 [Source:HGNC Symbol;Acc:HGNC:21239] |
| ENSG00000206102 | HGLibA_25683 | 221 | 0.864327292 | KRTAP19-8 | keratin associated protein 19-8 [Source:HGNC Symbol;Acc:HGNC:33898] |
| ENSG00000222046 | HGLibA_12487 | 221 | 0.864327292 | DCDC2B | doublecortin domain containing 2B [Source:HGNC Symbol;Acc:HGNC:32576] |
| ENSG00000239900 | HGLibA_01072 | 221 | 0.864327292 | ADSL | adenylosuccinate lyase [Source:HGNC Symbol;Acc:HGNC:291] |
| ENSG00000258588 | HGLibA_51560 | 221 | 0.864327292 | TRIM6-TRIM34 | TRIM6-TRIM34 readthrough [Source:HGNC Symbol;Acc:HGNC:33440] |
| ENSG00000261701 | HGLibA_22041 | 221 | 0.864327292 | HPR | haptoglobin-related protein [Source:HGNC Symbol;Acc:HGNC:5156] |
| H2AFB2 | HGLibA_20747 | 221 | 0.864327292 | H2AFB2 | - |
| hsa-mir-3138 | HGLibA_58610 | 221 | 0.864327292 | hsa-mir-3138 | - |
| hsa-mir-34a | HGLibA_59034 | 221 | 0.864327292 | hsa-mir-34a | - |
| hsa-mir-3682 | HGLibA_59283 | 221 | 0.864327292 | hsa-mir-3682 | - |
| hsa-mir-4480 | HGLibA_60337 | 221 | 0.864327292 | hsa-mir-4480 | - |
| hsa-mir-6825 | HGLibA_63477 | 221 | 0.864327292 | hsa-mir-6825 | - |
| PTPLAD2 | HGLibA_39422 | 221 | 0.864327292 | PTPLAD2 | - |
| C22orf43 | HGLibA_06080 | 220 | 0.855281949 | C22orf43 | - |
| C5orf48 | HGLibA_06395 | 220 | 0.855281949 | C5orf48 | - |
| ENSG00000004455 | HGLibA_01378 | 220 | 0.855281949 | AK2 | adenylate kinase 2 [Source:HGNC Symbol;Acc:HGNC:362] |
| ENSG00000004864 | HGLibA_44645 | 220 | 0.855281949 | SLC25A13 | solute carrier family 25 member 13 [Source:HGNC Symbol;Acc:HGNC:10983] |
| ENSG00000049246 | HGLibA_36060 | 220 | 0.855281949 | PER3 | period circadian regulator 3 [Source:HGNC Symbol;Acc:HGNC:8847] |
| ENSG00000049656 | HGLibA_10116 | 220 | 0.855281949 | CLPTM1L | CLPTM1 like [Source:HGNC Symbol;Acc:HGNC:24308] |
| ENSG00000051382 | HGLibA_36635 | 220 | 0.855281949 | PIK3CB | 5-bisphosphate 3-kinase catalytic subunit beta [Source:HGNC Symbol;Acc:HGNC:8976]" |
| ENSG00000055211 | HGLibA_19101 | 220 | 0.855281949 | GINM1 | glycoprotein integral membrane 1 [Source:HGNC Symbol;Acc:HGNC:21074] |
| ENSG00000064652 | HGLibA_46070 | 220 | 0.855281949 | SNX24 | sorting nexin 24 [Source:HGNC Symbol;Acc:HGNC:21533] |
| ENSG00000072133 | HGLibA_42173 | 220 | 0.855281949 | RPS6KA6 | ribosomal protein S6 kinase A6 [Source:HGNC Symbol;Acc:HGNC:10435] |
| ENSG00000074590 | HGLibA_32892 | 220 | 0.855281949 | NUAK1 | NUAK family kinase 1 [Source:HGNC Symbol;Acc:HGNC:14311] |
| ENSG00000088448 | HGLibA_01991 | 220 | 0.855281949 | ANKRD10 | ankyrin repeat domain 10 [Source:HGNC Symbol;Acc:HGNC:20265] |
| ENSG00000089050 | HGLibA_40446 | 220 | 0.855281949 | RBBP9 | serine hydrolase [Source:HGNC Symbol;Acc:HGNC:9892]" |
| ENSG00000101182 | HGLibA_39087 | 220 | 0.855281949 | PSMA7 | proteasome subunit alpha 7 [Source:HGNC Symbol;Acc:HGNC:9536] |
| ENSG00000106785 | HGLibA_51412 | 220 | 0.855281949 | TRIM14 | tripartite motif containing 14 [Source:HGNC Symbol;Acc:HGNC:16283] |
| ENSG00000110203 | HGLibA_17790 | 220 | 0.855281949 | FOLR3 | folate receptor 3 [Source:HGNC Symbol;Acc:HGNC:3795] |
| ENSG00000110911 | HGLibA_44355 | 220 | 0.855281949 | SLC11A2 | solute carrier family 11 member 2 [Source:HGNC Symbol;Acc:HGNC:10908] |
| ENSG00000112655 | HGLibA_39388 | 220 | 0.855281949 | PTK7 | protein tyrosine kinase 7 (inactive) [Source:HGNC Symbol;Acc:HGNC:9618] |
| ENSG00000113083 | HGLibA_27031 | 220 | 0.855281949 | LOX | lysyl oxidase [Source:HGNC Symbol;Acc:HGNC:6664] |
| ENSG00000113971 | HGLibA_32447 | 220 | 0.855281949 | NPHP3 | nephrocystin 3 [Source:HGNC Symbol;Acc:HGNC:7907] |
| ENSG00000114738 | HGLibA_28304 | 220 | 0.855281949 | MAPKAPK3 | mitogen-activated protein kinase-activated protein kinase 3 [Source:HGNC Symbol;Acc:HGNC:6888] |
| ENSG00000120820 | HGLibA_19340 | 220 | 0.855281949 | GLT8D2 | glycosyltransferase 8 domain containing 2 [Source:HGNC Symbol;Acc:HGNC:24890] |
| ENSG00000126878 | HGLibA_01335 | 220 | 0.855281949 | AIF1L | allograft inflammatory factor 1 like [Source:HGNC Symbol;Acc:HGNC:28904] |
| ENSG00000129219 | HGLibA_37039 | 220 | 0.855281949 | PLD2 | phospholipase D2 [Source:HGNC Symbol;Acc:HGNC:9068] |
| ENSG00000130119 | HGLibA_19552 | 220 | 0.855281949 | GNL3L | G protein nucleolar 3 like [Source:HGNC Symbol;Acc:HGNC:25553] |
| ENSG00000131042 | HGLibA_26509 | 220 | 0.855281949 | LILRB2 | leukocyte immunoglobulin like receptor B2 [Source:HGNC Symbol;Acc:HGNC:6606] |
| ENSG00000133083 | HGLibA_12499 | 220 | 0.855281949 | DCLK1 | doublecortin like kinase 1 [Source:HGNC Symbol;Acc:HGNC:2700] |
| ENSG00000136068 | HGLibA_17642 | 220 | 0.855281949 | FLNB | filamin B [Source:HGNC Symbol;Acc:HGNC:3755] |
| ENSG00000137161 | HGLibA_10368 | 220 | 0.855281949 | CNPY3 | canopy FGF signaling regulator 3 [Source:HGNC Symbol;Acc:HGNC:11968] |
| ENSG00000137203 | HGLibA_49122 | 220 | 0.855281949 | TFAP2A | transcription factor AP-2 alpha [Source:HGNC Symbol;Acc:HGNC:11742] |
| ENSG00000148156 | HGLibA_00628 | 220 | 0.855281949 | ACTL7B | actin like 7B [Source:HGNC Symbol;Acc:HGNC:162] |
| ENSG00000149256 | HGLibA_49004 | 220 | 0.855281949 | TENM4 | teneurin transmembrane protein 4 [Source:HGNC Symbol;Acc:HGNC:29945] |
| ENSG00000149292 | HGLibA_52047 | 220 | 0.855281949 | TTC12 | tetratricopeptide repeat domain 12 [Source:HGNC Symbol;Acc:HGNC:23700] |
| ENSG00000151117 | HGLibA_50526 | 220 | 0.855281949 | TMEM86A | transmembrane protein 86A [Source:HGNC Symbol;Acc:HGNC:26890] |
| ENSG00000154743 | HGLibA_51832 | 220 | 0.855281949 | TSEN2 | tRNA splicing endonuclease subunit 2 [Source:HGNC Symbol;Acc:HGNC:28422] |
| ENSG00000155189 | HGLibA_01230 | 220 | 0.855281949 | AGPAT5 | 1-acylglycerol-3-phosphate O-acyltransferase 5 [Source:HGNC Symbol;Acc:HGNC:20886] |
| ENSG00000158481 | HGLibA_08271 | 220 | 0.855281949 | CD1C | CD1c molecule [Source:HGNC Symbol;Acc:HGNC:1636] |
| ENSG00000158516 | HGLibA_10888 | 220 | 0.855281949 | CPA2 | carboxypeptidase A2 [Source:HGNC Symbol;Acc:HGNC:2297] |
| ENSG00000159527 | HGLibA_36256 | 220 | 0.855281949 | PGLYRP3 | peptidoglycan recognition protein 3 [Source:HGNC Symbol;Acc:HGNC:30014] |
| ENSG00000163032 | HGLibA_53855 | 220 | 0.855281949 | VSNL1 | visinin like 1 [Source:HGNC Symbol;Acc:HGNC:12722] |
| ENSG00000164188 | HGLibA_40206 | 220 | 0.855281949 | RANBP3L | RAN binding protein 3 like [Source:HGNC Symbol;Acc:HGNC:26353] |
| ENSG00000166821 | HGLibA_36083 | 220 | 0.855281949 | PEX11A | peroxisomal biogenesis factor 11 alpha [Source:HGNC Symbol;Acc:HGNC:8852] |
| ENSG00000168438 | HGLibA_08550 | 220 | 0.855281949 | CDC40 | cell division cycle 40 [Source:HGNC Symbol;Acc:HGNC:17350] |
| ENSG00000169035 | HGLibA_25266 | 220 | 0.855281949 | KLK7 | kallikrein related peptidase 7 [Source:HGNC Symbol;Acc:HGNC:6368] |
| ENSG00000171608 | HGLibA_36640 | 220 | 0.855281949 | PIK3CD | 5-bisphosphate 3-kinase catalytic subunit delta [Source:HGNC Symbol;Acc:HGNC:8977]" |
| ENSG00000171813 | HGLibA_39644 | 220 | 0.855281949 | PWWP2B | PWWP domain containing 2B [Source:HGNC Symbol;Acc:HGNC:25150] |
| ENSG00000171970 | HGLibA_56272 | 220 | 0.855281949 | ZNF57 | zinc finger protein 57 [Source:HGNC Symbol;Acc:HGNC:13125] |
| ENSG00000172349 | HGLibA_22985 | 220 | 0.855281949 | IL16 | interleukin 16 [Source:HGNC Symbol;Acc:HGNC:5980] |
| ENSG00000173039 | HGLibA_40812 | 220 | 0.855281949 | RELA | NF-kB subunit [Source:HGNC Symbol;Acc:HGNC:9955]" |
| ENSG00000173482 | HGLibA_39517 | 220 | 0.855281949 | PTPRM | receptor type M [Source:HGNC Symbol;Acc:HGNC:9675]" |
| ENSG00000175520 | HGLibA_52737 | 220 | 0.855281949 | UBQLN3 | ubiquilin 3 [Source:HGNC Symbol;Acc:HGNC:12510] |
| ENSG00000180116 | HGLibA_05237 | 220 | 0.855281949 | C12orf40 | chromosome 12 open reading frame 40 [Source:HGNC Symbol;Acc:HGNC:26846] |
| ENSG00000180398 | HGLibA_28586 | 220 | 0.855281949 | MCFD2 | multiple coagulation factor deficiency 2 [Source:HGNC Symbol;Acc:HGNC:18451] |
| ENSG00000181031 | HGLibA_41848 | 220 | 0.855281949 | RPH3AL | rabphilin 3A like (without C2 domains) [Source:HGNC Symbol;Acc:HGNC:10296] |
| ENSG00000182853 | HGLibA_53693 | 220 | 0.855281949 | VMO1 | vitelline membrane outer layer 1 homolog [Source:HGNC Symbol;Acc:HGNC:30387] |
| ENSG00000188340 | HGLibA_34434 | 220 | 0.855281949 | OR6N2 | olfactory receptor family 6 subfamily N member 2 [Source:HGNC Symbol;Acc:HGNC:15035] |
| ENSG00000188785 | HGLibA_56206 | 220 | 0.855281949 | ZNF548 | zinc finger protein 548 [Source:HGNC Symbol;Acc:HGNC:26561] |
| ENSG00000196663 | HGLibA_48943 | 220 | 0.855281949 | TECPR2 | tectonin beta-propeller repeat containing 2 [Source:HGNC Symbol;Acc:HGNC:19957] |
| ENSG00000198040 | HGLibA_56851 | 220 | 0.855281949 | ZNF84 | zinc finger protein 84 [Source:HGNC Symbol;Acc:HGNC:13159] |
| ENSG00000205864 | HGLibA_25802 | 220 | 0.855281949 | KRTAP5-6 | keratin associated protein 5-6 [Source:HGNC Symbol;Acc:HGNC:23600] |
| ENSG00000234224 | HGLibA_50248 | 220 | 0.855281949 | TMEM229A | transmembrane protein 229A [Source:HGNC Symbol;Acc:HGNC:37279] |
| ENSG00000242950 | HGLibA_15552 | 220 | 0.855281949 | ERVW-1 | envelope [Source:HGNC Symbol;Acc:HGNC:13525]" |
| ENSG00000244094 | HGLibA_46821 | 220 | 0.855281949 | SPRR2F | small proline rich protein 2F [Source:HGNC Symbol;Acc:HGNC:11266] |
| ENSG00000259956 | HGLibA_40493 | 220 | 0.855281949 | RBM15B | RNA binding motif protein 15B [Source:HGNC Symbol;Acc:HGNC:24303] |
| ENSG00000271601 | HGLibA_26646 | 220 | 0.855281949 | LIX1L | limb and CNS expressed 1 like [Source:HGNC Symbol;Acc:HGNC:28715] |
| ENSG00000276547 | HGLibA_35561 | 220 | 0.855281949 | PCDHGB5 | 5 [Source:HGNC Symbol;Acc:HGNC:8712]" |
| FAM150A | HGLibA_16157 | 220 | 0.855281949 | FAM150A | - |
| FAM73B | HGLibA_16718 | 220 | 0.855281949 | FAM73B | - |
| hsa-mir-1237 | HGLibA_57337 | 220 | 0.855281949 | hsa-mir-1237 | - |
| hsa-mir-3178 | HGLibA_58786 | 220 | 0.855281949 | hsa-mir-3178 | - |
| hsa-mir-4742 | HGLibA_61066 | 220 | 0.855281949 | hsa-mir-4742 | - |
| hsa-mir-5003 | HGLibA_61418 | 220 | 0.855281949 | hsa-mir-5003 | - |
| hsa-mir-5091 | HGLibA_61521 | 220 | 0.855281949 | hsa-mir-5091 | - |
| hsa-mir-588 | HGLibA_62451 | 220 | 0.855281949 | hsa-mir-588 | - |
| hsa-mir-6510 | HGLibA_62895 | 220 | 0.855281949 | hsa-mir-6510 | - |
| hsa-mir-6733 | HGLibA_63084 | 220 | 0.855281949 | hsa-mir-6733 | - |
| hsa-mir-7849 | HGLibA_63987 | 220 | 0.855281949 | hsa-mir-7849 | - |
| hsa-mir-802 | HGLibA_64043 | 220 | 0.855281949 | hsa-mir-802 | - |
| LACE1 | HGLibA_25911 | 220 | 0.855281949 | LACE1 | - |
| N6AMT2 | HGLibA_30968 | 220 | 0.855281949 | N6AMT2 | - |
| PRSS46 | HGLibA_38948 | 220 | 0.855281949 | PRSS46 | - |
| ZNF645 | HGLibA_56448 | 220 | 0.855281949 | ZNF645 | - |
| ZNF645 | HGLibA_56450 | 220 | 0.855281949 | ZNF645 | - |
| C12orf66 | HGLibA_05281 | 219 | 0.846236606 | C12orf66 | - |
| C12orf79 | HGLibA_05307 | 219 | 0.846236606 | C12orf79 | - |
| C14orf142 | HGLibA_05328 | 219 | 0.846236606 | C14orf142 | - |
| ENSG00000006606 | HGLibA_08010 | 219 | 0.846236606 | CCL26 | C-C motif chemokine ligand 26 [Source:HGNC Symbol;Acc:HGNC:10625] |
| ENSG00000007216 | HGLibA_44388 | 219 | 0.846236606 | SLC13A2 | solute carrier family 13 member 2 [Source:HGNC Symbol;Acc:HGNC:10917] |
| ENSG00000056998 | HGLibA_20690 | 219 | 0.846236606 | GYG2 | glycogenin 2 [Source:HGNC Symbol;Acc:HGNC:4700] |
| ENSG00000080815 | HGLibA_39025 | 219 | 0.846236606 | PSEN1 | presenilin 1 [Source:HGNC Symbol;Acc:HGNC:9508] |
| ENSG00000084207 | HGLibA_20494 | 219 | 0.846236606 | GSTP1 | glutathione S-transferase pi 1 [Source:HGNC Symbol;Acc:HGNC:4638] |
| ENSG00000087263 | HGLibA_33308 | 219 | 0.846236606 | OGFOD1 | 2-oxoglutarate and iron dependent oxygenase domain containing 1 [Source:HGNC Symbol;Acc:HGNC:25585] |
| ENSG00000088451 | HGLibA_49202 | 219 | 0.846236606 | TGDS | 6-dehydratase [Source:HGNC Symbol;Acc:HGNC:20324]" |
| ENSG00000088992 | HGLibA_49024 | 219 | 0.846236606 | TESC | tescalcin [Source:HGNC Symbol;Acc:HGNC:26065] |
| ENSG00000092377 | HGLibA_48615 | 219 | 0.846236606 | TBL1Y | transducin beta like 1 Y-linked [Source:HGNC Symbol;Acc:HGNC:18502] |
| ENSG00000094804 | HGLibA_08593 | 219 | 0.846236606 | CDC6 | cell division cycle 6 [Source:HGNC Symbol;Acc:HGNC:1744] |
| ENSG00000102225 | HGLibA_08743 | 219 | 0.846236606 | CDK16 | cyclin dependent kinase 16 [Source:HGNC Symbol;Acc:HGNC:8749] |
| ENSG00000105127 | HGLibA_01445 | 219 | 0.846236606 | AKAP8 | A-kinase anchoring protein 8 [Source:HGNC Symbol;Acc:HGNC:378] |
| ENSG00000105711 | HGLibA_42977 | 219 | 0.846236606 | SCN1B | sodium voltage-gated channel beta subunit 1 [Source:HGNC Symbol;Acc:HGNC:10586] |
| ENSG00000106066 | HGLibA_11030 | 219 | 0.846236606 | CPVL | vitellogenic like [Source:HGNC Symbol;Acc:HGNC:14399]" |
| ENSG00000109736 | HGLibA_29084 | 219 | 0.846236606 | MFSD10 | major facilitator superfamily domain containing 10 [Source:HGNC Symbol;Acc:HGNC:16894] |
| ENSG00000111339 | HGLibA_03163 | 219 | 0.846236606 | ART4 | ADP-ribosyltransferase 4 (Dombrock blood group) [Source:HGNC Symbol;Acc:HGNC:726] |
| ENSG00000111647 | HGLibA_52952 | 219 | 0.846236606 | UHRF1BP1L | UHRF1 binding protein 1 like [Source:HGNC Symbol;Acc:HGNC:29102] |
| ENSG00000114999 | HGLibA_52183 | 219 | 0.846236606 | TTL | tubulin tyrosine ligase [Source:HGNC Symbol;Acc:HGNC:21586] |
| ENSG00000115593 | HGLibA_45850 | 219 | 0.846236606 | SMYD1 | SET and MYND domain containing 1 [Source:HGNC Symbol;Acc:HGNC:20986] |
| ENSG00000118971 | HGLibA_08075 | 219 | 0.846236606 | CCND2 | cyclin D2 [Source:HGNC Symbol;Acc:HGNC:1583] |
| ENSG00000120942 | HGLibA_52698 | 219 | 0.846236606 | UBIAD1 | UbiA prenyltransferase domain containing 1 [Source:HGNC Symbol;Acc:HGNC:30791] |
| ENSG00000123165 | HGLibA_00676 | 219 | 0.846236606 | ACTRT1 | actin related protein T1 [Source:HGNC Symbol;Acc:HGNC:24027] |
| ENSG00000123983 | HGLibA_00558 | 219 | 0.846236606 | ACSL3 | acyl-CoA synthetase long chain family member 3 [Source:HGNC Symbol;Acc:HGNC:3570] |
| ENSG00000126705 | HGLibA_01295 | 219 | 0.846236606 | AHDC1 | AT-hook DNA binding motif containing 1 [Source:HGNC Symbol;Acc:HGNC:25230] |
| ENSG00000128849 | HGLibA_09323 | 219 | 0.846236606 | CGNL1 | cingulin like 1 [Source:HGNC Symbol;Acc:HGNC:25931] |
| ENSG00000133020 | HGLibA_30740 | 219 | 0.846236606 | MYH8 | myosin heavy chain 8 [Source:HGNC Symbol;Acc:HGNC:7578] |
| ENSG00000133142 | HGLibA_48714 | 219 | 0.846236606 | TCEAL4 | transcription elongation factor A like 4 [Source:HGNC Symbol;Acc:HGNC:26121] |
| ENSG00000133739 | HGLibA_27443 | 219 | 0.846236606 | LRRCC1 | leucine rich repeat and coiled-coil centrosomal protein 1 [Source:HGNC Symbol;Acc:HGNC:29373] |
| ENSG00000134864 | HGLibA_18988 | 219 | 0.846236606 | GGACT | gamma-glutamylamine cyclotransferase [Source:HGNC Symbol;Acc:HGNC:25100] |
| ENSG00000135333 | HGLibA_15305 | 219 | 0.846236606 | EPHA7 | EPH receptor A7 [Source:HGNC Symbol;Acc:HGNC:3390] |
| ENSG00000136425 | HGLibA_09685 | 219 | 0.846236606 | CIB2 | calcium and integrin binding family member 2 [Source:HGNC Symbol;Acc:HGNC:24579] |
| ENSG00000137364 | HGLibA_51154 | 219 | 0.846236606 | TPMT | thiopurine S-methyltransferase [Source:HGNC Symbol;Acc:HGNC:12014] |
| ENSG00000140829 | HGLibA_13195 | 219 | 0.846236606 | DHX38 | DEAH-box helicase 38 [Source:HGNC Symbol;Acc:HGNC:17211] |
| ENSG00000142192 | HGLibA_02560 | 219 | 0.846236606 | APP | amyloid beta precursor protein [Source:HGNC Symbol;Acc:HGNC:620] |
| ENSG00000142784 | HGLibA_54231 | 219 | 0.846236606 | WDTC1 | WD and tetratricopeptide repeats 1 [Source:HGNC Symbol;Acc:HGNC:29175] |
| ENSG00000143297 | HGLibA_17236 | 219 | 0.846236606 | FCRL5 | Fc receptor like 5 [Source:HGNC Symbol;Acc:HGNC:18508] |

| | | | | | |
|---|---|---|---|---|---|
| ENSG00000143643 | HGLibA_52050 | 219 | 0.846236606 | TTC13 | tetratricopeptide repeat domain 13 [Source:HGNC Symbol;Acc:HGNC:26204] |
| ENSG00000143793 | HGLibA_05855 | 219 | 0.846236606 | C1orf35 | chromosome 1 open reading frame 35 [Source:HGNC Symbol;Acc:HGNC:19032] |
| ENSG00000144619 | HGLibA_10409 | 219 | 0.846236606 | CNTN4 | contactin 4 [Source:HGNC Symbol;Acc:HGNC:2174] |
| ENSG00000146360 | HGLibA_20058 | 219 | 0.846236606 | GPR6 | G protein-coupled receptor 6 [Source:HGNC Symbol;Acc:HGNC:4515] |
| ENSG00000150760 | HGLibA_13732 | 219 | 0.846236606 | DOCK1 | dedicator of cytokinesis 1 [Source:HGNC Symbol;Acc:HGNC:2987] |
| ENSG00000151655 | HGLibA_23807 | 219 | 0.846236606 | ITIH2 | inter-alpha-trypsin inhibitor heavy chain 2 [Source:HGNC Symbol;Acc:HGNC:6167] |
| ENSG00000151917 | HGLibA_04412 | 219 | 0.846236606 | BEND6 | BEN domain containing 6 [Source:HGNC Symbol;Acc:HGNC:20871] |
| ENSG00000152061 | HGLibA_40012 | 219 | 0.846236606 | RABGAP1L | RAB GTPase activating protein 1 like [Source:HGNC Symbol;Acc:HGNC:24663] |
| ENSG00000153982 | HGLibA_18891 | 219 | 0.846236606 | GDPD1 | glycerophosphodiester phosphodiesterase domain containing 1 [Source:HGNC Symbol;Acc:HGNC:20883] |
| ENSG00000155090 | HGLibA_25041 | 219 | 0.846236606 | KLF10 | Kruppel like factor 10 [Source:HGNC Symbol;Acc:HGNC:11810] |
| ENSG00000157259 | HGLibA_18682 | 219 | 0.846236606 | GATAD1 | GATA zinc finger domain containing 1 [Source:HGNC Symbol;Acc:HGNC:29941] |
| ENSG00000157551 | HGLibA_24263 | 219 | 0.846236606 | KCNJ15 | potassium voltage-gated channel subfamily J member 15 [Source:HGNC Symbol;Acc:HGNC:6261] |
| ENSG00000157766 | HGLibA_00379 | 219 | 0.846236606 | ACAN | aggrecan [Source:HGNC Symbol;Acc:HGNC:319] |
| ENSG00000161996 | HGLibA_54214 | 219 | 0.846236606 | WDR90 | WD repeat domain 90 [Source:HGNC Symbol;Acc:HGNC:26960] |
| ENSG00000163214 | HGLibA_13201 | 219 | 0.846236606 | DHX57 | DExH-box helicase 57 [Source:HGNC Symbol;Acc:HGNC:20086] |
| ENSG00000164219 | HGLibA_36237 | 219 | 0.846236606 | PGGT1B | protein geranylgeranyltransferase type I subunit beta [Source:HGNC Symbol;Acc:HGNC:8895] |
| ENSG00000164600 | HGLibA_31744 | 219 | 0.846236606 | NEUROD6 | neuronal differentiation 6 [Source:HGNC Symbol;Acc:HGNC:13804] |
| ENSG00000165704 | HGLibA_22043 | 219 | 0.846236606 | HPRT1 | hypoxanthine phosphoribosyltransferase 1 [Source:HGNC Symbol;Acc:HGNC:5157] |
| ENSG00000166509 | HGLibA_09972 | 219 | 0.846236606 | CLEC3A | C-type lectin domain family 3 member A [Source:HGNC Symbol;Acc:HGNC:2052] |
| ENSG00000168263 | HGLibA_24405 | 219 | 0.846236606 | KCNV2 | potassium voltage-gated channel modifier subfamily V member 2 [Source:HGNC Symbol;Acc:HGNC:19698] |
| ENSG00000170967 | HGLibA_12608 | 219 | 0.846236606 | DDI1 | DNA damage inducible 1 homolog 1 [Source:HGNC Symbol;Acc:HGNC:18961] |
| ENSG00000172915 | HGLibA_31205 | 219 | 0.846236606 | NBEA | neurobeachin [Source:HGNC Symbol;Acc:HGNC:7648] |
| ENSG00000173011 | HGLibA_48190 | 219 | 0.846236606 | TADA2B | transcriptional adaptor 2B [Source:HGNC Symbol;Acc:HGNC:30781] |
| ENSG00000175093 | HGLibA_46868 | 219 | 0.846236606 | SPSB4 | splA/ryanodine receptor domain and SOCS box containing 4 [Source:HGNC Symbol;Acc:HGNC:30630] |
| ENSG00000177947 | HGLibA_33285 | 219 | 0.846236606 | ODF3 | outer dense fiber of sperm tails 3 [Source:HGNC Symbol;Acc:HGNC:19905] |
| ENSG00000182636 | HGLibA_31399 | 219 | 0.846236606 | NDN | MAGE family member [Source:HGNC Symbol;Acc:HGNC:7675]" |
| ENSG00000185043 | HGLibA_09681 | 219 | 0.846236606 | CIB1 | calcium and integrin binding 1 [Source:HGNC Symbol;Acc:HGNC:16920] |
| ENSG00000188542 | HGLibA_14176 | 219 | 0.846236606 | DUSP28 | dual specificity phosphatase 28 [Source:HGNC Symbol;Acc:HGNC:33237] |
| ENSG00000188868 | HGLibA_56250 | 219 | 0.846236606 | ZNF563 | zinc finger protein 563 [Source:HGNC Symbol;Acc:HGNC:30498] |
| ENSG00000189326 | HGLibA_46412 | 219 | 0.846236606 | SPANXN4 | SPANX family member N4 [Source:HGNC Symbol;Acc:HGNC:33177] |
| ENSG00000196581 | HGLibA_01369 | 219 | 0.846236606 | AJAP1 | adherens junctions associated protein 1 [Source:HGNC Symbol;Acc:HGNC:30801] |
| ENSG00000197457 | HGLibA_47521 | 219 | 0.846236606 | STMN3 | stathmin 3 [Source:HGNC Symbol;Acc:HGNC:15926] |
| ENSG00000197891 | HGLibA_44542 | 219 | 0.846236606 | SLC22A12 | solute carrier family 22 member 12 [Source:HGNC Symbol;Acc:HGNC:17989] |
| ENSG00000198018 | HGLibA_15214 | 219 | 0.846236606 | ENTPD7 | ectonucleoside triphosphate diphosphohydrolase 7 [Source:HGNC Symbol;Acc:HGNC:19745] |
| ENSG00000203784 | HGLibA_26284 | 219 | 0.846236606 | LELP1 | late cornified envelope like proline rich 1 [Source:HGNC Symbol;Acc:HGNC:32046] |
| ENSG00000239900 | HGLibA_01074 | 219 | 0.846236606 | ADSL | adenylosuccinate lyase [Source:HGNC Symbol;Acc:HGNC:291] |
| ENSG00000254415 | HGLibA_44136 | 219 | 0.846236606 | SIGLEC14 | sialic acid binding Ig like lectin 14 [Source:HGNC Symbol;Acc:HGNC:32926] |
| ENSG00000256043 | HGLibA_11724 | 219 | 0.846236606 | CTSO | cathepsin O [Source:HGNC Symbol;Acc:HGNC:2542] |
| FAM213A | HGLibA_16471 | 219 | 0.846236606 | FAM213A | - |
| FAM84B | HGLibA_16768 | 219 | 0.846236606 | FAM84B | - |
| HIST3H3 | HGLibA_21562 | 219 | 0.846236606 | HIST3H3 | - |
| hsa-mir-133b | HGLibA_57779 | 219 | 0.846236606 | hsa-mir-133b | - |
| hsa-mir-4279 | HGLibA_59820 | 219 | 0.846236606 | hsa-mir-4279 | - |
| hsa-mir-4683 | HGLibA_60844 | 219 | 0.846236606 | hsa-mir-4683 | - |
| LOC728392 | HGLibA_26990 | 219 | 0.846236606 | LOC728392 | - |
| LOC729020 | HGLibA_26996 | 219 | 0.846236606 | LOC729020 | - |
| MTERFD2 | HGLibA_30343 | 219 | 0.846236606 | MTERFD2 | - |
| AGXT2L2 | HGLibA_01280 | 218 | 0.837191263 | AGXT2L2 | - |
| EMR2 | HGLibA_15086 | 218 | 0.837191263 | EMR2 | - |
| ENSG00000038002 | HGLibA_01134 | 218 | 0.837191263 | AGA | aspartylglucosaminidase [Source:HGNC Symbol;Acc:HGNC:318] |
| ENSG00000064270 | HGLibA_03628 | 218 | 0.837191263 | ATP2C2 | ATPase secretory pathway Ca2+ transporting 2 [Source:HGNC Symbol;Acc:HGNC:29103] |
| ENSG00000070388 | HGLibA_17400 | 218 | 0.837191263 | FGF22 | fibroblast growth factor 22 [Source:HGNC Symbol;Acc:HGNC:3679] |
| ENSG00000088808 | HGLibA_38028 | 218 | 0.837191263 | PPP1R13B | protein phosphatase 1 regulatory subunit 13B [Source:HGNC Symbol;Acc:HGNC:14950] |
| ENSG00000099341 | HGLibA_39183 | 218 | 0.837191263 | PSMD8 | non-ATPase 8 [Source:HGNC Symbol;Acc:HGNC:9566]" |
| ENSG00000100852 | HGLibA_02782 | 218 | 0.837191263 | ARHGAP5 | Rho GTPase activating protein 5 [Source:HGNC Symbol;Acc:HGNC:675] |
| ENSG00000101421 | HGLibA_09491 | 218 | 0.837191263 | CHMP4B | charged multivesicular body protein 4B [Source:HGNC Symbol;Acc:HGNC:16171] |
| ENSG00000103226 | HGLibA_32294 | 218 | 0.837191263 | NOMO3 | NODAL modulator 3 [Source:HGNC Symbol;Acc:HGNC:25242] |
| ENSG00000106638 | HGLibA_48617 | 218 | 0.837191263 | TBL2 | transducin beta like 2 [Source:HGNC Symbol;Acc:HGNC:11586] |
| ENSG00000110801 | HGLibA_39186 | 218 | 0.837191263 | PSMD9 | non-ATPase 9 [Source:HGNC Symbol;Acc:HGNC:9567]" |
| ENSG00000114547 | HGLibA_41762 | 218 | 0.837191263 | ROPN1B | rhophilin associated tail protein 1B [Source:HGNC Symbol;Acc:HGNC:31927] |
| ENSG00000114988 | HGLibA_26666 | 218 | 0.837191263 | LMAN2L | mannose binding 2 like [Source:HGNC Symbol;Acc:HGNC:19263]" |
| ENSG00000119547 | HGLibA_33396 | 218 | 0.837191263 | ONECUT2 | one cut homeobox 2 [Source:HGNC Symbol;Acc:HGNC:8139] |
| ENSG00000123364 | HGLibA_21965 | 218 | 0.837191263 | HOXC13 | homeobox C13 [Source:HGNC Symbol;Acc:HGNC:5125] |
| ENSG00000124256 | HGLibA_54809 | 218 | 0.837191263 | ZBP1 | Z-DNA binding protein 1 [Source:HGNC Symbol;Acc:HGNC:16176] |
| ENSG00000125650 | HGLibA_39230 | 218 | 0.837191263 | PSPN | persephin [Source:HGNC Symbol;Acc:HGNC:9579] |
| ENSG00000125845 | HGLibA_04598 | 218 | 0.837191263 | BMP2 | bone morphogenetic protein 2 [Source:HGNC Symbol;Acc:HGNC:1069] |
| ENSG00000126351 | HGLibA_49387 | 218 | 0.837191263 | THRA | thyroid hormone receptor alpha [Source:HGNC Symbol;Acc:HGNC:11796] |
| ENSG00000131748 | HGLibA_47356 | 218 | 0.837191263 | STARD3 | StAR related lipid transfer domain containing 3 [Source:HGNC Symbol;Acc:HGNC:17579] |
| ENSG00000132958 | HGLibA_51215 | 218 | 0.837191263 | TPTE2 | transmembrane phosphoinositide 3-phosphatase and tensin homolog 2 [Source:HGNC Symbol;Acc:HGNC:17299] |
| ENSG00000133874 | HGLibA_41488 | 218 | 0.837191263 | RNF122 | ring finger protein 122 [Source:HGNC Symbol;Acc:HGNC:21147] |
| ENSG00000137462 | HGLibA_49646 | 218 | 0.837191263 | TLR2 | toll like receptor 2 [Source:HGNC Symbol;Acc:HGNC:11848] |
| ENSG00000137720 | HGLibA_05116 | 218 | 0.837191263 | C11orf1 | chromosome 11 open reading frame 1 [Source:HGNC Symbol;Acc:HGNC:1163] |
| ENSG00000139842 | HGLibA_11783 | 218 | 0.837191263 | CUL4A | cullin 4A [Source:HGNC Symbol;Acc:HGNC:2554] |
| ENSG00000142698 | HGLibA_05907 | 218 | 0.837191263 | C1orf94 | chromosome 1 open reading frame 94 [Source:HGNC Symbol;Acc:HGNC:28250] |
| ENSG00000143398 | HGLibA_36715 | 218 | 0.837191263 | PIP5K1A | phosphatidylinositol-4-phosphate 5-kinase type 1 alpha [Source:HGNC Symbol;Acc:HGNC:8994] |
| ENSG00000144035 | HGLibA_31182 | 218 | 0.837191263 | NAT8 | N-acetyltransferase 8 (putative) [Source:HGNC Symbol;Acc:HGNC:18069] |
| ENSG00000147724 | HGLibA_16135 | 218 | 0.837191263 | FAM135B | family with sequence similarity 135 member B [Source:HGNC Symbol;Acc:HGNC:28029] |
| ENSG00000148840 | HGLibA_38231 | 218 | 0.837191263 | PPRC1 | coactivator-related 1 [Source:HGNC Symbol;Acc:HGNC:30025]" |
| ENSG00000151715 | HGLibA_50411 | 218 | 0.837191263 | TMEM45B | transmembrane protein 45B [Source:HGNC Symbol;Acc:HGNC:25194] |
| ENSG00000154001 | HGLibA_38181 | 218 | 0.837191263 | PPP2R5E | protein phosphatase 2 regulatory subunit B'epsilon [Source:HGNC Symbol;Acc:HGNC:9313] |
| ENSG00000155368 | HGLibA_12385 | 218 | 0.837191263 | DBI | acyl-CoA binding protein [Source:HGNC Symbol;Acc:HGNC:2690]" |
| ENSG00000157349 | HGLibA_12667 | 218 | 0.837191263 | DDX19B | DEAD-box helicase 19B [Source:HGNC Symbol;Acc:HGNC:2742] |
| ENSG00000163082 | HGLibA_43854 | 218 | 0.837191263 | SGPP2 | sphingosine-1-phosphate phosphatase 2 [Source:HGNC Symbol;Acc:HGNC:19953] |
| ENSG00000165863 | HGLibA_05096 | 218 | 0.837191263 | C10orf82 | chromosome 10 open reading frame 82 [Source:HGNC Symbol;Acc:HGNC:28500] |
| ENSG00000166479 | HGLibA_50694 | 218 | 0.837191263 | TMX3 | thioredoxin related transmembrane protein 3 [Source:HGNC Symbol;Acc:HGNC:24718] |
| ENSG00000166825 | HGLibA_02210 | 218 | 0.837191263 | ANPEP | membrane [Source:HGNC Symbol;Acc:HGNC:500]" |
| ENSG00000169344 | HGLibA_52979 | 218 | 0.837191263 | UMOD | uromodulin [Source:HGNC Symbol;Acc:HGNC:12559] |
| ENSG00000169385 | HGLibA_41403 | 218 | 0.837191263 | RNASE2 | ribonuclease A family member 2 [Source:HGNC Symbol;Acc:HGNC:10045] |
| ENSG00000170881 | HGLibA_41516 | 218 | 0.837191263 | RNF139 | ring finger protein 139 [Source:HGNC Symbol;Acc:HGNC:17023] |
| ENSG00000171466 | HGLibA_56249 | 218 | 0.837191263 | ZNF562 | zinc finger protein 562 [Source:HGNC Symbol;Acc:HGNC:25950] |
| ENSG00000172301 | HGLibA_10709 | 218 | 0.837191263 | COPRS | coordinator of PRMT5 and differentiation stimulator [Source:HGNC Symbol;Acc:HGNC:28848] |
| ENSG00000175040 | HGLibA_09630 | 218 | 0.837191263 | CHST2 | carbohydrate sulfotransferase 2 [Source:HGNC Symbol;Acc:HGNC:1970] |
| ENSG00000175895 | HGLibA_37098 | 218 | 0.837191263 | PLEKHF2 | pleckstrin homology and FYVE domain containing 2 [Source:HGNC Symbol;Acc:HGNC:20757] |
| ENSG00000182389 | HGLibA_06926 | 218 | 0.837191263 | CACNB4 | calcium voltage-gated channel auxiliary subunit beta 4 [Source:HGNC Symbol;Acc:HGNC:1404] |
| ENSG00000182793 | HGLibA_20463 | 218 | 0.837191263 | GSTA5 | glutathione S-transferase alpha 5 [Source:HGNC Symbol;Acc:HGNC:19662] |
| ENSG00000183323 | HGLibA_07578 | 218 | 0.837191263 | CCDC125 | coiled-coil domain containing 125 [Source:HGNC Symbol;Acc:HGNC:28924] |
| ENSG00000184911 | HGLibA_13439 | 218 | 0.837191263 | DMRTC1B | DMRT like family C1B [Source:HGNC Symbol;Acc:HGNC:31686] |
| ENSG00000185721 | HGLibA_13974 | 218 | 0.837191263 | DRG1 | developmentally regulated GTP binding protein 1 [Source:HGNC Symbol;Acc:HGNC:3029] |
| ENSG00000186136 | HGLibA_48409 | 218 | 0.837191263 | TAS2R42 | taste 2 receptor member 42 [Source:HGNC Symbol;Acc:HGNC:18888] |
| ENSG00000186951 | HGLibA_37837 | 218 | 0.837191263 | PPARA | peroxisome proliferator activated receptor alpha [Source:HGNC Symbol;Acc:HGNC:9232] |
| ENSG00000196139 | HGLibA_01487 | 218 | 0.837191263 | AKR1C3 | aldo-keto reductase family 1 member C3 [Source:HGNC Symbol;Acc:HGNC:386] |
| ENSG00000196455 | HGLibA_36658 | 218 | 0.837191263 | PIK3R4 | phosphoinositide-3-kinase regulatory subunit 4 [Source:HGNC Symbol;Acc:HGNC:8982] |
| ENSG00000214686 | HGLibA_23515 | 218 | 0.837191263 | IQCF6 | IQ motif containing F6 [Source:HGNC Symbol;Acc:HGNC:35158] |
| ENSG00000239264 | HGLibA_52423 | 218 | 0.837191263 | TXNDC5 | thioredoxin domain containing 5 [Source:HGNC Symbol;Acc:HGNC:21073] |
| ENSG00000257017 | HGLibA_22009 | 218 | 0.837191263 | HP | haptoglobin [Source:HGNC Symbol;Acc:HGNC:5141] |
| ENSG00000261594 | HGLibA_51101 | 218 | 0.837191263 | TPBGL | trophoblast glycoprotein like [Source:HGNC Symbol;Acc:HGNC:44159] |
| ENSG00000277481 | HGLibA_36794 | 218 | 0.837191263 | PKD1L3 | transient receptor potential channel interacting [Source:HGNC Symbol;Acc:HGNC:21716]" |
| hsa-mir-1248 | HGLibA_57389 | 218 | 0.837191263 | hsa-mir-1248 | - |
| hsa-mir-205 | HGLibA_58177 | 218 | 0.837191263 | hsa-mir-205 | - |
| hsa-mir-3689e | HGLibA_59335 | 218 | 0.837191263 | hsa-mir-3689e | - |
| hsa-mir-4265 | HGLibA_59766 | 218 | 0.837191263 | hsa-mir-4265 | - |
| hsa-mir-532 | HGLibA_61806 | 218 | 0.837191263 | hsa-mir-532 | - |
| hsa-mir-6506 | HGLibA_62873 | 218 | 0.837191263 | hsa-mir-6506 | - |
| hsa-mir-6794 | HGLibA_63353 | 218 | 0.837191263 | hsa-mir-6794 | - |
| hsa-mir-9-2 | HGLibA_64267 | 218 | 0.837191263 | hsa-mir-9-2 | - |
| IRG1 | HGLibA_23607 | 218 | 0.837191263 | IRG1 | - |
| LOC100507003 | HGLibA_26868 | 218 | 0.837191263 | LOC100507003 | - |
| CTAGE5 | HGLibA_11575 | 217 | 0.82814592 | CTAGE5 | - |
| CXorf61 | HGLibA_11952 | 217 | 0.82814592 | CXorf61 | - |
| ENSG00000043355 | HGLibA_55325 | 217 | 0.82814592 | ZIC2 | Zic family member 2 [Source:HGNC Symbol;Acc:HGNC:12873] |
| ENSG00000059378 | HGLibA_35219 | 217 | 0.82814592 | PARP12 | poly(ADP-ribose) polymerase family member 12 [Source:HGNC Symbol;Acc:HGNC:21919] |
| ENSG00000087128 | HGLibA_50620 | 217 | 0.82814592 | TMPRSS11E | transmembrane serine protease 11E [Source:HGNC Symbol;Acc:HGNC:24465] |
| ENSG00000095794 | HGLibA_11133 | 217 | 0.82814592 | CREM | cAMP responsive element modulator [Source:HGNC Symbol;Acc:HGNC:2352] |
| ENSG00000105379 | HGLibA_15622 | 217 | 0.82814592 | ETFB | electron transfer flavoprotein subunit beta [Source:HGNC Symbol;Acc:HGNC:3482] |
| ENSG00000108588 | HGLibA_07789 | 217 | 0.82814592 | CCDC47 | coiled-coil domain containing 47 [Source:HGNC Symbol;Acc:HGNC:24856] |
| ENSG00000112234 | HGLibA_17009 | 217 | 0.82814592 | FBXL4 | F-box and leucine rich repeat protein 4 [Source:HGNC Symbol;Acc:HGNC:13601] |
| ENSG00000114450 | HGLibA_19498 | 217 | 0.82814592 | GNB4 | G protein subunit beta 4 [Source:HGNC Symbol;Acc:HGNC:20731] |
| ENSG00000114541 | HGLibA_18016 | 217 | 0.82814592 | FRMD4B | FERM domain containing 4B [Source:HGNC Symbol;Acc:HGNC:24886] |
| ENSG00000114638 | HGLibA_53072 | 217 | 0.82814592 | UPK1B | uroplakin 1B [Source:HGNC Symbol;Acc:HGNC:12578] |
| ENSG00000114742 | HGLibA_54110 | 217 | 0.82814592 | WDR48 | WD repeat domain 48 [Source:HGNC Symbol;Acc:HGNC:30914] |
| ENSG00000119953 | HGLibA_45788 | 217 | 0.82814592 | SMNDC1 | survival motor neuron domain containing 1 [Source:HGNC Symbol;Acc:HGNC:16900] |
| ENSG00000121058 | HGLibA_10495 | 217 | 0.82814592 | COIL | coilin [Source:HGNC Symbol;Acc:HGNC:2184] |
| ENSG00000125991 | HGLibA_15474 | 217 | 0.82814592 | ERGIC3 | ERGIC and golgi 3 [Source:HGNC Symbol;Acc:HGNC:15927] |
| ENSG00000133256 | HGLibA_35808 | 217 | 0.82814592 | PDE6B | phosphodiesterase 6B [Source:HGNC Symbol;Acc:HGNC:8786] |
| ENSG00000135392 | HGLibA_13576 | 217 | 0.82814592 | DNAJC14 | DnaJ heat shock protein family (Hsp40) member C14 [Source:HGNC Symbol;Acc:HGNC:24581] |

| | | | | | |
|---|---|---|---|---|---|
| ENSG00000139144 | HGLibA_36627 | 217 | 0.82814592 | PIK3C2G | phosphatidylinositol-4-phosphate 3-kinase catalytic subunit type 2 gamma [Source:HGNC Symbol;Acc:HGNC:8973] |
| ENSG00000142910 | HGLibA_49550 | 217 | 0.82814592 | TINAGL1 | tubulointerstitial nephritis antigen like 1 [Source:HGNC Symbol;Acc:HGNC:19168] |
| ENSG00000143850 | HGLibA_37077 | 217 | 0.82814592 | PLEKHA6 | pleckstrin homology domain containing A6 [Source:HGNC Symbol;Acc:HGNC:17053] |
| ENSG00000144228 | HGLibA_46765 | 217 | 0.82814592 | SPOPL | speckle type BTB/POZ protein like [Source:HGNC Symbol;Acc:HGNC:27934] |
| ENSG00000145390 | HGLibA_53397 | 217 | 0.82814592 | USP53 | ubiquitin specific peptidase 53 [Source:HGNC Symbol;Acc:HGNC:29255] |
| ENSG00000148572 | HGLibA_32651 | 217 | 0.82814592 | NRBF2 | nuclear receptor binding factor 2 [Source:HGNC Symbol;Acc:HGNC:19692] |
| ENSG00000154743 | HGLibA_51831 | 217 | 0.82814592 | TSEN2 | tRNA splicing endonuclease subunit 2 [Source:HGNC Symbol;Acc:HGNC:28422] |
| ENSG00000156414 | HGLibA_48917 | 217 | 0.82814592 | TDRD9 | tudor domain containing 9 [Source:HGNC Symbol;Acc:HGNC:20122] |
| ENSG00000160396 | HGLibA_21348 | 217 | 0.82814592 | HIPK4 | homeodomain interacting protein kinase 4 [Source:HGNC Symbol;Acc:HGNC:19007] |
| ENSG00000165025 | HGLibA_47948 | 217 | 0.82814592 | SYK | spleen associated tyrosine kinase [Source:HGNC Symbol;Acc:HGNC:11491] |
| ENSG00000166033 | HGLibA_22406 | 217 | 0.82814592 | HTRA1 | HtrA serine peptidase 1 [Source:HGNC Symbol;Acc:HGNC:9476] |
| ENSG00000168830 | HGLibA_22365 | 217 | 0.82814592 | HTR1E | 5-hydroxytryptamine receptor 1E [Source:HGNC Symbol;Acc:HGNC:5291] |
| ENSG00000168903 | HGLibA_04977 | 217 | 0.82814592 | BTNL3 | butyrophilin like 3 [Source:HGNC Symbol;Acc:HGNC:1143] |
| ENSG00000170454 | HGLibA_25537 | 217 | 0.82814592 | KRT75 | keratin 75 [Source:HGNC Symbol;Acc:HGNC:24431] |
| ENSG00000170962 | HGLibA_35851 | 217 | 0.82814592 | PDGFD | platelet derived growth factor D [Source:HGNC Symbol;Acc:HGNC:30620] |
| ENSG00000171446 | HGLibA_25465 | 217 | 0.82814592 | KRT27 | keratin 27 [Source:HGNC Symbol;Acc:HGNC:30841] |
| ENSG00000173226 | HGLibA_23491 | 217 | 0.82814592 | IQCB1 | IQ motif containing B1 [Source:HGNC Symbol;Acc:HGNC:28949] |
| ENSG00000175505 | HGLibA_09816 | 217 | 0.82814592 | CLCF1 | cardiotrophin like cytokine factor 1 [Source:HGNC Symbol;Acc:HGNC:17412] |
| ENSG00000177663 | HGLibA_23005 | 217 | 0.82814592 | IL17RA | interleukin 17 receptor A [Source:HGNC Symbol;Acc:HGNC:5985] |
| ENSG00000184220 | HGLibA_10205 | 217 | 0.82814592 | CMSS1 | cms1 ribosomal small subunit homolog [Source:HGNC Symbol;Acc:HGNC:28666] |
| ENSG00000184574 | HGLibA_27064 | 217 | 0.82814592 | LPAR5 | lysophosphatidic acid receptor 5 [Source:HGNC Symbol;Acc:HGNC:13307] |
| ENSG00000187003 | HGLibA_00627 | 217 | 0.82814592 | ACTL7A | actin like 7A [Source:HGNC Symbol;Acc:HGNC:161] |
| ENSG00000188778 | HGLibA_01062 | 217 | 0.82814592 | ADRB3 | adrenoceptor beta 3 [Source:HGNC Symbol;Acc:HGNC:288] |
| ENSG00000189252 | HGLibA_46408 | 217 | 0.82814592 | SPANXN3 | SPANX family member N3 [Source:HGNC Symbol;Acc:HGNC:33176] |
| ENSG00000198028 | HGLibA_56242 | 217 | 0.82814592 | ZNF560 | zinc finger protein 560 [Source:HGNC Symbol;Acc:HGNC:26484] |
| ENSG00000198663 | HGLibA_06513 | 217 | 0.82814592 | C6orf89 | chromosome 6 open reading frame 89 [Source:HGNC Symbol;Acc:HGNC:21114] |
| ENSG00000204590 | HGLibA_19543 | 217 | 0.82814592 | GNL1 | G protein nucleolar 1 (putative) [Source:HGNC Symbol;Acc:HGNC:4413] |
| ENSG00000205250 | HGLibA_14315 | 217 | 0.82814592 | E2F4 | E2F transcription factor 4 [Source:HGNC Symbol;Acc:HGNC:3118] |
| ENSG00000205642 | HGLibA_53589 | 217 | 0.82814592 | VCX3B | variable charge X-linked 3B [Source:HGNC Symbol;Acc:HGNC:31838] |
| ENSG00000205857 | HGLibA_31094 | 217 | 0.82814592 | NANOGNB | NANOG neighbor homeobox [Source:HGNC Symbol;Acc:HGNC:24958] |
| ENSG00000213722 | HGLibA_12591 | 217 | 0.82814592 | DDAH2 | dimethylarginine dimethylaminohydrolase 2 [Source:HGNC Symbol;Acc:HGNC:2716] |
| ENSG00000213988 | HGLibA_56903 | 217 | 0.82814592 | ZNF90 | zinc finger protein 90 [Source:HGNC Symbol;Acc:HGNC:13165] |
| ENSG00000241563 | HGLibA_10796 | 217 | 0.82814592 | CORT | cortistatin [Source:HGNC Symbol;Acc:HGNC:2257] |
| ENSG00000272602 | HGLibA_56348 | 217 | 0.82814592 | ZNF595 | zinc finger protein 595 [Source:HGNC Symbol;Acc:HGNC:27196] |
| ENSG00000276234 | HGLibA_48186 | 217 | 0.82814592 | TADA2A | transcriptional adaptor 2A [Source:HGNC Symbol;Acc:HGNC:11531] |
| FAM155B | HGLibA_16182 | 217 | 0.82814592 | FAM155B | - |
| FAM160A1 | HGLibA_16203 | 217 | 0.82814592 | FAM160A1 | - |
| FAM45A | HGLibA_16593 | 217 | 0.82814592 | FAM45A | - |
| hsa-mir-125a | HGLibA_57443 | 217 | 0.82814592 | hsa-mir-125a | - |
| hsa-mir-1292 | HGLibA_57644 | 217 | 0.82814592 | hsa-mir-1292 | - |
| hsa-mir-3141 | HGLibA_58624 | 217 | 0.82814592 | hsa-mir-3141 | - |
| hsa-mir-3200 | HGLibA_58909 | 217 | 0.82814592 | hsa-mir-3200 | - |
| hsa-mir-4429 | HGLibA_60101 | 217 | 0.82814592 | hsa-mir-4429 | - |
| hsa-mir-494 | HGLibA_61377 | 217 | 0.82814592 | hsa-mir-494 | - |
| hsa-mir-5581 | HGLibA_62165 | 217 | 0.82814592 | hsa-mir-5581 | - |
| hsa-mir-6076 | HGLibA_62557 | 217 | 0.82814592 | hsa-mir-6076 | - |
| hsa-mir-6803 | HGLibA_63391 | 217 | 0.82814592 | hsa-mir-6803 | - |
| hsa-mir-8087 | HGLibA_64189 | 217 | 0.82814592 | hsa-mir-8087 | - |
| LOC389895 | HGLibA_26929 | 217 | 0.82814592 | LOC389895 | - |
| MTSS1L | HGLibA_30499 | 217 | 0.82814592 | MTSS1L | - |
| PHF17 | HGLibA_36355 | 217 | 0.82814592 | PHF17 | - |
| SPANXE | HGLibA_46396 | 217 | 0.82814592 | SPANXE | - |
| ADCK3 | HGLibA_00897 | 216 | 0.819100577 | ADCK3 | - |
| CRIPAK | HGLibA_11158 | 216 | 0.819100577 | CRIPAK | - |
| ENSG00000023572 | HGLibA_19315 | 216 | 0.819100577 | GLRX2 | glutaredoxin 2 [Source:HGNC Symbol;Acc:HGNC:16065] |
| ENSG00000048462 | HGLibA_50764 | 216 | 0.819100577 | TNFRSF17 | TNF receptor superfamily member 17 [Source:HGNC Symbol;Acc:HGNC:11913] |
| ENSG00000075035 | HGLibA_54425 | 216 | 0.819100577 | WSCD2 | WSC domain containing 2 [Source:HGNC Symbol;Acc:HGNC:29117] |
| ENSG00000076984 | HGLibA_28156 | 216 | 0.819100577 | MAP2K7 | mitogen-activated protein kinase kinase 7 [Source:HGNC Symbol;Acc:HGNC:6847] |
| ENSG00000092820 | HGLibA_15837 | 216 | 0.819100577 | EZR | ezrin [Source:HGNC Symbol;Acc:HGNC:12691] |
| ENSG00000100344 | HGLibA_37411 | 216 | 0.819100577 | PNPLA3 | patatin like phospholipase domain containing 3 [Source:HGNC Symbol;Acc:HGNC:18590] |
| ENSG00000103121 | HGLibA_10188 | 216 | 0.819100577 | CMC2 | C-X9-C motif containing 2 [Source:HGNC Symbol;Acc:HGNC:24447] |
| ENSG00000104938 | HGLibA_09997 | 216 | 0.819100577 | CLEC4M | C-type lectin domain family 4 member M [Source:HGNC Symbol;Acc:HGNC:13523] |
| ENSG00000105205 | HGLibA_09802 | 216 | 0.819100577 | CLC | Charcot-Leyden crystal galectin [Source:HGNC Symbol;Acc:HGNC:2014] |
| ENSG00000105655 | HGLibA_23700 | 216 | 0.819100577 | ISYNA1 | inositol-3-phosphate synthase 1 [Source:HGNC Symbol;Acc:HGNC:29821] |
| ENSG00000108946 | HGLibA_38522 | 216 | 0.819100577 | PRKAR1A | protein kinase cAMP-dependent type I regulatory subunit alpha [Source:HGNC Symbol;Acc:HGNC:9388] |
| ENSG00000118263 | HGLibA_25081 | 216 | 0.819100577 | KLF7 | Kruppel like factor 7 [Source:HGNC Symbol;Acc:HGNC:6350] |
| ENSG00000120256 | HGLibA_27204 | 216 | 0.819100577 | LRP11 | LDL receptor related protein 11 [Source:HGNC Symbol;Acc:HGNC:16936] |
| ENSG00000121060 | HGLibA_51442 | 216 | 0.819100577 | TRIM25 | tripartite motif containing 25 [Source:HGNC Symbol;Acc:HGNC:12932] |
| ENSG00000123685 | HGLibA_04179 | 216 | 0.819100577 | BATF3 | basic leucine zipper ATF-like transcription factor 3 [Source:HGNC Symbol;Acc:HGNC:28915] |
| ENSG00000124449 | HGLibA_23611 | 216 | 0.819100577 | IRGC | immunity related GTPase cinema [Source:HGNC Symbol;Acc:HGNC:28835] |
| ENSG00000127884 | HGLibA_14404 | 216 | 0.819100577 | ECHS1 | short chain 1 [Source:HGNC Symbol;Acc:HGNC:3151]" |
| ENSG00000130307 | HGLibA_53182 | 216 | 0.819100577 | USHBP1 | USH1 protein network component harmonin binding protein 1 [Source:HGNC Symbol;Acc:HGNC:24058] |
| ENSG00000131873 | HGLibA_09655 | 216 | 0.819100577 | CHSY1 | chondroitin sulfate synthase 1 [Source:HGNC Symbol;Acc:HGNC:17198] |
| ENSG00000132466 | HGLibA_02015 | 216 | 0.819100577 | ANKRD17 | ankyrin repeat domain 17 [Source:HGNC Symbol;Acc:HGNC:23575] |
| ENSG00000135862 | HGLibA_25969 | 216 | 0.819100577 | LAMC1 | laminin subunit gamma 1 [Source:HGNC Symbol;Acc:HGNC:6492] |
| ENSG00000137145 | HGLibA_12955 | 216 | 0.819100577 | DENND4C | DENN domain containing 4C [Source:HGNC Symbol;Acc:HGNC:26079] |
| ENSG00000140798 | HGLibA_00168 | 216 | 0.819100577 | ABCC12 | ATP binding cassette subfamily C member 12 [Source:HGNC Symbol;Acc:HGNC:14640] |
| ENSG00000141510 | HGLibA_51049 | 216 | 0.819100577 | TP53 | tumor protein p53 [Source:HGNC Symbol;Acc:HGNC:11998] |
| ENSG00000141540 | HGLibA_52234 | 216 | 0.819100577 | TTYH2 | tweety family member 2 [Source:HGNC Symbol;Acc:HGNC:13877] |
| ENSG00000147588 | HGLibA_37321 | 216 | 0.819100577 | PMP2 | peripheral myelin protein 2 [Source:HGNC Symbol;Acc:HGNC:9117] |
| ENSG00000147873 | HGLibA_22682 | 216 | 0.819100577 | IFNA5 | interferon alpha 5 [Source:HGNC Symbol;Acc:HGNC:5426] |
| ENSG00000148680 | HGLibA_22404 | 216 | 0.819100577 | HTR7 | 5-hydroxytryptamine receptor 7 [Source:HGNC Symbol;Acc:HGNC:5302] |
| ENSG00000151746 | HGLibA_04495 | 216 | 0.819100577 | BICD1 | BICD cargo adaptor 1 [Source:HGNC Symbol;Acc:HGNC:1049] |
| ENSG00000155016 | HGLibA_12146 | 216 | 0.819100577 | CYP2U1 | cytochrome P450 family 2 subfamily U member 1 [Source:HGNC Symbol;Acc:HGNC:20582] |
| ENSG00000155816 | HGLibA_17689 | 216 | 0.819100577 | FMN2 | formin 2 [Source:HGNC Symbol;Acc:HGNC:14074] |
| ENSG00000158639 | HGLibA_35016 | 216 | 0.819100577 | PAGE5 | PAGE family member 5 [Source:HGNC Symbol;Acc:HGNC:29992] |
| ENSG00000159348 | HGLibA_12000 | 216 | 0.819100577 | CYB5R1 | cytochrome b5 reductase 1 [Source:HGNC Symbol;Acc:HGNC:13397] |
| ENSG00000160202 | HGLibA_11246 | 216 | 0.819100577 | CRYAA | crystallin alpha A [Source:HGNC Symbol;Acc:HGNC:2388] |
| ENSG00000162241 | HGLibA_44749 | 216 | 0.819100577 | SLC25A45 | solute carrier family 25 member 45 [Source:HGNC Symbol;Acc:HGNC:27442] |
| ENSG00000164651 | HGLibA_46314 | 216 | 0.819100577 | SP8 | Sp8 transcription factor [Source:HGNC Symbol;Acc:HGNC:19196] |
| ENSG00000164930 | HGLibA_18278 | 216 | 0.819100577 | FZD6 | frizzled class receptor 6 [Source:HGNC Symbol;Acc:HGNC:4044] |
| ENSG00000166037 | HGLibA_09147 | 216 | 0.819100577 | CEP57 | centrosomal protein 57 [Source:HGNC Symbol;Acc:HGNC:30794] |
| ENSG00000166920 | HGLibA_05406 | 216 | 0.819100577 | C15orf48 | chromosome 15 open reading frame 48 [Source:HGNC Symbol;Acc:HGNC:29898] |
| ENSG00000167220 | HGLibA_21053 | 216 | 0.819100577 | HDHD2 | haloacid dehalogenase like hydrolase domain containing 2 [Source:HGNC Symbol;Acc:HGNC:25364] |
| ENSG00000167601 | HGLibA_03927 | 216 | 0.819100577 | AXL | AXL receptor tyrosine kinase [Source:HGNC Symbol;Acc:HGNC:905] |
| ENSG00000169704 | HGLibA_19713 | 216 | 0.819100577 | GP9 | glycoprotein IX platelet [Source:HGNC Symbol;Acc:HGNC:4444] |
| ENSG00000169957 | HGLibA_56699 | 216 | 0.819100577 | ZNF768 | zinc finger protein 768 [Source:HGNC Symbol;Acc:HGNC:26273] |
| ENSG00000170236 | HGLibA_53391 | 216 | 0.819100577 | USP50 | ubiquitin specific peptidase 50 [Source:HGNC Symbol;Acc:HGNC:20079] |
| ENSG00000172554 | HGLibA_46008 | 216 | 0.819100577 | SNTG2 | syntrophin gamma 2 [Source:HGNC Symbol;Acc:HGNC:13741] |
| ENSG00000173281 | HGLibA_38101 | 216 | 0.819100577 | PPP1R3B | protein phosphatase 1 regulatory subunit 3B [Source:HGNC Symbol;Acc:HGNC:14942] |
| ENSG00000177291 | HGLibA_19192 | 216 | 0.819100577 | GJD4 | gap junction protein delta 4 [Source:HGNC Symbol;Acc:HGNC:23296] |
| ENSG00000181291 | HGLibA_49940 | 216 | 0.819100577 | TMEM132E | transmembrane protein 132E [Source:HGNC Symbol;Acc:HGNC:26991] |
| ENSG00000181656 | HGLibA_20103 | 216 | 0.819100577 | GPR88 | G protein-coupled receptor 88 [Source:HGNC Symbol;Acc:HGNC:4539] |
| ENSG00000182533 | HGLibA_07382 | 216 | 0.819100577 | CAV3 | caveolin 3 [Source:HGNC Symbol;Acc:HGNC:1529] |
| ENSG00000183963 | HGLibA_45833 | 216 | 0.819100577 | SMTN | smoothelin [Source:HGNC Symbol;Acc:HGNC:11126] |
| ENSG00000185252 | HGLibA_56660 | 216 | 0.819100577 | ZNF74 | zinc finger protein 74 [Source:HGNC Symbol;Acc:HGNC:13144] |
| ENSG00000186088 | HGLibA_20395 | 216 | 0.819100577 | GSAP | gamma-secretase activating protein [Source:HGNC Symbol;Acc:HGNC:28042] |
| ENSG00000189152 | HGLibA_20219 | 216 | 0.819100577 | GRAPL | GRB2 related adaptor protein like [Source:HGNC Symbol;Acc:HGNC:37240] |
| ENSG00000189377 | HGLibA_11863 | 216 | 0.819100577 | CXCL17 | C-X-C motif chemokine ligand 17 [Source:HGNC Symbol;Acc:HGNC:19232] |
| ENSG00000198453 | HGLibA_56265 | 216 | 0.819100577 | ZNF568 | zinc finger protein 568 [Source:HGNC Symbol;Acc:HGNC:25392] |
| ENSG00000204544 | HGLibA_30542 | 216 | 0.819100577 | MUC21 | cell surface associated [Source:HGNC Symbol;Acc:HGNC:21661]" |
| ENSG00000274600 | HGLibA_41266 | 216 | 0.819100577 | RIMBP3B | RIMS binding protein 3B [Source:HGNC Symbol;Acc:HGNC:33891] |
| ENSG00000275718 | HGLibA_07974 | 216 | 0.819100577 | CCL15 | C-C motif chemokine ligand 15 [Source:HGNC Symbol;Acc:HGNC:10613] |
| hsa-mir-1249 | HGLibA_57394 | 216 | 0.819100577 | hsa-mir-1249 | - |
| hsa-mir-4280 | HGLibA_59825 | 216 | 0.819100577 | hsa-mir-4280 | - |
| hsa-mir-4314 | HGLibA_59973 | 216 | 0.819100577 | hsa-mir-4314 | - |
| hsa-mir-4654 | HGLibA_60723 | 216 | 0.819100577 | hsa-mir-4654 | - |
| hsa-mir-4662b | HGLibA_60760 | 216 | 0.819100577 | hsa-mir-4662b | - |
| hsa-mir-6070 | HGLibA_62532 | 216 | 0.819100577 | hsa-mir-6070 | - |
| hsa-mir-637 | HGLibA_62786 | 216 | 0.819100577 | hsa-mir-637 | - |
| hsa-mir-6509 | HGLibA_62887 | 216 | 0.819100577 | hsa-mir-6509 | - |
| hsa-mir-6775 | HGLibA_63275 | 216 | 0.819100577 | hsa-mir-6775 | - |
| TTC25 | HGLibA_52087 | 216 | 0.819100577 | TTC25 | - |
| ZCCHC16 | HGLibA_55019 | 216 | 0.819100577 | ZCCHC16 | - |
| C3orf79 | HGLibA_06268 | 215 | 0.810055235 | C3orf79 | - |
| C7orf29 | HGLibA_06531 | 215 | 0.810055235 | C7orf29 | - |
| ENSG00000000457 | HGLibA_43092 | 215 | 0.810055235 | SCYL3 | SCY1 like pseudokinase 3 [Source:HGNC Symbol;Acc:HGNC:19285] |
| ENSG00000034677 | HGLibA_41602 | 215 | 0.810055235 | RNF19A | RBR E3 ubiquitin protein ligase [Source:HGNC Symbol;Acc:HGNC:13432]" |
| ENSG00000074047 | HGLibA_19250 | 215 | 0.810055235 | GLI2 | GLI family zinc finger 2 [Source:HGNC Symbol;Acc:HGNC:4318] |
| ENSG00000077713 | HGLibA_44743 | 215 | 0.810055235 | SLC25A43 | solute carrier family 25 member 43 [Source:HGNC Symbol;Acc:HGNC:30557] |
| ENSG00000078814 | HGLibA_30736 | 215 | 0.810055235 | MYH7B | myosin heavy chain 7B [Source:HGNC Symbol;Acc:HGNC:15906] |
| ENSG00000091157 | HGLibA_54161 | 215 | 0.810055235 | WDR7 | WD repeat domain 7 [Source:HGNC Symbol;Acc:HGNC:13490] |
| ENSG00000100813 | HGLibA_00441 | 215 | 0.810055235 | ACIN1 | apoptotic chromatin condensation inducer 1 [Source:HGNC Symbol;Acc:HGNC:17066] |
| ENSG00000101445 | HGLibA_38055 | 215 | 0.810055235 | PPP1R16B | protein phosphatase 1 regulatory subunit 16B [Source:HGNC Symbol;Acc:HGNC:15850] |
| ENSG00000104341 | HGLibA_26019 | 215 | 0.810055235 | LAPTM4B | lysosomal protein transmembrane 4 beta [Source:HGNC Symbol;Acc:HGNC:13646] |

| | | | | | |
|---|---|---|---|---|---|
| ENSG00000105426 | HGLibA_39535 | 215 | 0.810055235 | PTPRS | receptor type S [Source:HGNC Symbol;Acc:HGNC:9681]" |
| ENSG00000105427 | HGLibA_10261 | 215 | 0.810055235 | CNFN | cornifelin [Source:HGNC Symbol;Acc:HGNC:30183] |
| ENSG00000106355 | HGLibA_27545 | 215 | 0.810055235 | LSM5 | U6 small nuclear RNA and mRNA degradation associated [Source:HGNC Symbol;Acc:HGNC:17162]" |
| ENSG00000109339 | HGLibA_28250 | 215 | 0.810055235 | MAPK10 | mitogen-activated protein kinase 10 [Source:HGNC Symbol;Acc:HGNC:6872] |
| ENSG00000111843 | HGLibA_49984 | 215 | 0.810055235 | TMEM14C | transmembrane protein 14C [Source:HGNC Symbol;Acc:HGNC:20952] |
| ENSG00000116661 | HGLibA_17042 | 215 | 0.810055235 | FBXO2 | F-box protein 2 [Source:HGNC Symbol;Acc:HGNC:13581] |
| ENSG00000117724 | HGLibA_09055 | 215 | 0.810055235 | CENPF | centromere protein F [Source:HGNC Symbol;Acc:HGNC:1857] |
| ENSG00000118972 | HGLibA_17403 | 215 | 0.810055235 | FGF23 | fibroblast growth factor 23 [Source:HGNC Symbol;Acc:HGNC:3680] |
| ENSG00000119681 | HGLibA_27593 | 215 | 0.810055235 | LTBP2 | latent transforming growth factor beta binding protein 2 [Source:HGNC Symbol;Acc:HGNC:6715] |
| ENSG00000119777 | HGLibA_50215 | 215 | 0.810055235 | TMEM214 | transmembrane protein 214 [Source:HGNC Symbol;Acc:HGNC:25983] |
| ENSG00000119946 | HGLibA_10314 | 215 | 0.810055235 | CNNM1 | cyclin and CBS domain divalent metal cation transport mediator 1 [Source:HGNC Symbol;Acc:HGNC:102] |
| ENSG00000120235 | HGLibA_22685 | 215 | 0.810055235 | IFNA6 | interferon alpha 6 [Source:HGNC Symbol;Acc:HGNC:5427] |
| ENSG00000121417 | HGLibA_55584 | 215 | 0.810055235 | ZNF211 | zinc finger protein 211 [Source:HGNC Symbol;Acc:HGNC:13003] |
| ENSG00000121690 | HGLibA_12987 | 215 | 0.810055235 | DEPDC7 | DEP domain containing 7 [Source:HGNC Symbol;Acc:HGNC:29899] |
| ENSG00000123416 | HGLibA_52245 | 215 | 0.810055235 | TUBA1B | tubulin alpha 1b [Source:HGNC Symbol;Acc:HGNC:18809] |
| ENSG00000124279 | HGLibA_16906 | 215 | 0.810055235 | FASTKD3 | FAST kinase domains 3 [Source:HGNC Symbol;Acc:HGNC:28758] |
| ENSG00000124466 | HGLibA_27719 | 215 | 0.810055235 | LYPD3 | LY6/PLAUR domain containing 3 [Source:HGNC Symbol;Acc:HGNC:24880] |
| ENSG00000125878 | HGLibA_48764 | 215 | 0.810055235 | TCF15 | transcription factor 15 [Source:HGNC Symbol;Acc:HGNC:11627] |
| ENSG00000128536 | HGLibA_08704 | 215 | 0.810055235 | CDHR3 | cadherin related family member 3 [Source:HGNC Symbol;Acc:HGNC:26308] |
| ENSG00000131059 | HGLibA_04715 | 215 | 0.810055235 | BPIFA3 | BPI fold containing family A member 3 [Source:HGNC Symbol;Acc:HGNC:16204] |
| ENSG00000132170 | HGLibA_37842 | 215 | 0.810055235 | PPARG | peroxisome proliferator activated receptor gamma [Source:HGNC Symbol;Acc:HGNC:9236] |
| ENSG00000133063 | HGLibA_09455 | 215 | 0.810055235 | CHIT1 | chitinase 1 [Source:HGNC Symbol;Acc:HGNC:1936] |
| ENSG00000134760 | HGLibA_14021 | 215 | 0.810055235 | DSG1 | desmoglein 1 [Source:HGNC Symbol;Acc:HGNC:3048] |
| ENSG00000135272 | HGLibA_28674 | 215 | 0.810055235 | MDFIC | MyoD family inhibitor domain containing [Source:HGNC Symbol;Acc:HGNC:28870] |
| ENSG00000136929 | HGLibA_21130 | 215 | 0.810055235 | HEMGN | hemogen [Source:HGNC Symbol;Acc:HGNC:17509] |
| ENSG00000137094 | HGLibA_13547 | 215 | 0.810055235 | DNAJB5 | DnaJ heat shock protein family (Hsp40) member B5 [Source:HGNC Symbol;Acc:HGNC:14887] |
| ENSG00000137274 | HGLibA_04704 | 215 | 0.810055235 | BPHL | biphenyl hydrolase like [Source:HGNC Symbol;Acc:HGNC:1094] |
| ENSG00000138756 | HGLibA_04601 | 215 | 0.810055235 | BMP2K | BMP2 inducible kinase [Source:HGNC Symbol;Acc:HGNC:18041] |
| ENSG00000139330 | HGLibA_24553 | 215 | 0.810055235 | KERA | keratocan [Source:HGNC Symbol;Acc:HGNC:6309] |
| ENSG00000142173 | HGLibA_10600 | 215 | 0.810055235 | COL6A2 | collagen type VI alpha 2 chain [Source:HGNC Symbol;Acc:HGNC:2212] |
| ENSG00000142330 | HGLibA_07146 | 215 | 0.810055235 | CAPN10 | calpain 10 [Source:HGNC Symbol;Acc:HGNC:1477] |
| ENSG00000142544 | HGLibA_11748 | 215 | 0.810055235 | CTU1 | cytosolic thiouridylase subunit 1 [Source:HGNC Symbol;Acc:HGNC:29590] |
| ENSG00000143556 | HGLibA_42567 | 215 | 0.810055235 | S100A7 | S100 calcium binding protein A7 [Source:HGNC Symbol;Acc:HGNC:10497] |
| ENSG00000146094 | HGLibA_13773 | 215 | 0.810055235 | DOK3 | docking protein 3 [Source:HGNC Symbol;Acc:HGNC:24583] |
| ENSG00000148082 | HGLibA_44005 | 215 | 0.810055235 | SHC3 | SHC adaptor protein 3 [Source:HGNC Symbol;Acc:HGNC:18181] |
| ENSG00000148346 | HGLibA_26177 | 215 | 0.810055235 | LCN2 | lipocalin 2 [Source:HGNC Symbol;Acc:HGNC:6526] |
| ENSG00000149557 | HGLibA_17313 | 215 | 0.810055235 | FEZ1 | fasciculation and elongation protein zeta 1 [Source:HGNC Symbol;Acc:HGNC:3659] |
| ENSG00000154493 | HGLibA_05102 | 215 | 0.810055235 | C10orf90 | chromosome 10 open reading frame 90 [Source:HGNC Symbol;Acc:HGNC:26563] |
| ENSG00000156928 | HGLibA_28032 | 215 | 0.810055235 | MALSU1 | mitochondrial assembly of ribosomal large subunit 1 [Source:HGNC Symbol;Acc:HGNC:21721] |
| ENSG00000159267 | HGLibA_21652 | 215 | 0.810055235 | HLCS | holocarboxylase synthetase [Source:HGNC Symbol;Acc:HGNC:4976] |
| ENSG00000162571 | HGLibA_52189 | 215 | 0.810055235 | TTLL10 | tubulin tyrosine ligase like 10 [Source:HGNC Symbol;Acc:HGNC:26693] |
| ENSG00000163563 | HGLibA_29568 | 215 | 0.810055235 | MNDA | myeloid cell nuclear differentiation antigen [Source:HGNC Symbol;Acc:HGNC:7183] |
| ENSG00000164237 | HGLibA_10183 | 215 | 0.810055235 | CMBL | carboxymethylenebutenolidase homolog [Source:HGNC Symbol;Acc:HGNC:25090] |
| ENSG00000165819 | HGLibA_29006 | 215 | 0.810055235 | METTL3 | methyltransferase like 3 [Source:HGNC Symbol;Acc:HGNC:17563] |
| ENSG00000168779 | HGLibA_44075 | 215 | 0.810055235 | SHOX2 | short stature homeobox 2 [Source:HGNC Symbol;Acc:HGNC:10854] |
| ENSG00000171643 | HGLibA_42589 | 215 | 0.810055235 | S100Z | S100 calcium binding protein Z [Source:HGNC Symbol;Acc:HGNC:30367] |
| ENSG00000173627 | HGLibA_02498 | 215 | 0.810055235 | APOBEC4 | apolipoprotein B mRNA editing enzyme catalytic polypeptide like 4 [Source:HGNC Symbol;Acc:HGNC:32152] |
| ENSG00000175768 | HGLibA_50973 | 215 | 0.810055235 | TOMM5 | translocase of outer mitochondrial membrane 5 [Source:HGNC Symbol;Acc:HGNC:31369] |
| ENSG00000180316 | HGLibA_37405 | 215 | 0.810055235 | PNPLA1 | patatin like phospholipase domain containing 1 [Source:HGNC Symbol;Acc:HGNC:21246] |
| ENSG00000183258 | HGLibA_12711 | 215 | 0.810055235 | DDX41 | DEAD-box helicase 41 [Source:HGNC Symbol;Acc:HGNC:18674] |
| ENSG00000185745 | HGLibA_22622 | 215 | 0.810055235 | IFIT1 | interferon induced protein with tetratricopeptide repeats 1 [Source:HGNC Symbol;Acc:HGNC:5407] |
| ENSG00000186094 | HGLibA_01176 | 215 | 0.810055235 | AGBL4 | ATP/GTP binding protein like 4 [Source:HGNC Symbol;Acc:HGNC:25892] |
| ENSG00000188783 | HGLibA_38434 | 215 | 0.810055235 | PRELP | proline and arginine rich end leucine rich repeat protein [Source:HGNC Symbol;Acc:HGNC:9357] |
| ENSG00000188827 | HGLibA_45564 | 215 | 0.810055235 | SLX4 | SLX4 structure-specific endonuclease subunit [Source:HGNC Symbol;Acc:HGNC:23845] |
| ENSG00000196458 | HGLibA_56364 | 215 | 0.810055235 | ZNF605 | zinc finger protein 605 [Source:HGNC Symbol;Acc:HGNC:28068] |
| ENSG00000196782 | HGLibA_28048 | 215 | 0.810055235 | MAML3 | mastermind like transcriptional coactivator 3 [Source:HGNC Symbol;Acc:HGNC:16272] |
| ENSG00000197790 | HGLibA_34189 | 215 | 0.810055235 | OR52M1 | olfactory receptor family 52 subfamily M member 1 [Source:HGNC Symbol;Acc:HGNC:15225] |
| ENSG00000198863 | HGLibA_42447 | 215 | 0.810055235 | RUNDC1 | RUN domain containing 1 [Source:HGNC Symbol;Acc:HGNC:25418] |
| ENSG00000204913 | HGLibA_27347 | 215 | 0.810055235 | LRRC3C | leucine rich repeat containing 3C [Source:HGNC Symbol;Acc:HGNC:40034] |
| ENSG00000205358 | HGLibA_30291 | 215 | 0.810055235 | MT1H | metallothionein 1H [Source:HGNC Symbol;Acc:HGNC:7400] |
| ENSG00000205445 | HGLibA_25602 | 215 | 0.810055235 | KRTAP10-2 | keratin associated protein 10-2 [Source:HGNC Symbol;Acc:HGNC:22967] |
| ENSG00000213672 | HGLibA_31323 | 215 | 0.810055235 | NCKIPSD | NCK interacting protein with SH3 domain [Source:HGNC Symbol;Acc:HGNC:15486] |
| ENSG00000214050 | HGLibA_17033 | 215 | 0.810055235 | FBXO16 | F-box protein 16 [Source:HGNC Symbol;Acc:HGNC:13618] |
| ENSG00000214511 | HGLibA_21312 | 215 | 0.810055235 | HIGD1C | HIG1 hypoxia inducible domain family member 1C [Source:HGNC Symbol;Acc:HGNC:28044] |
| ENSG00000215041 | HGLibA_31730 | 215 | 0.810055235 | NEURL4 | neuralized E3 ubiquitin protein ligase 4 [Source:HGNC Symbol;Acc:HGNC:34410] |
| ENSG00000232263 | HGLibA_25727 | 215 | 0.810055235 | KRTAP25-1 | keratin associated protein 25-1 [Source:HGNC Symbol;Acc:HGNC:34003] |
| ENSG00000235718 | HGLibA_29077 | 215 | 0.810055235 | MFRP | membrane frizzled-related protein [Source:HGNC Symbol;Acc:HGNC:18121] |
| ENSG00000237515 | HGLibA_44053 | 215 | 0.810055235 | SHISA9 | shisa family member 9 [Source:HGNC Symbol;Acc:HGNC:37231] |
| ENSG00000269526 | HGLibA_15544 | 215 | 0.810055235 | ERVV-1 | envelope [Source:HGNC Symbol;Acc:HGNC:26501]" |
| ENSG00000278570 | HGLibA_32607 | 215 | 0.810055235 | NR2E3 | nuclear receptor subfamily 2 group E member 3 [Source:HGNC Symbol;Acc:HGNC:7974] |
| ENSG00000288784 | HGLibA_11572 | 215 | 0.810055235 | CTAGE4 | - |
| FAM109A | HGLibA_16006 | 215 | 0.810055235 | FAM109A | - |
| FAM115A | HGLibA_16036 | 215 | 0.810055235 | FAM115A | - |
| hsa-mir-3155b | HGLibA_58682 | 215 | 0.810055235 | hsa-mir-3155b | - |
| hsa-mir-3689c | HGLibA_59320 | 215 | 0.810055235 | hsa-mir-3689c | - |
| hsa-mir-4687 | HGLibA_60857 | 215 | 0.810055235 | hsa-mir-4687 | - |
| hsa-mir-548o-2 | HGLibA_62053 | 215 | 0.810055235 | hsa-mir-548o-2 | - |
| hsa-mir-6773 | HGLibA_63264 | 215 | 0.810055235 | hsa-mir-6773 | - |
| hsa-mir-6789 | HGLibA_63332 | 215 | 0.810055235 | hsa-mir-6789 | - |
| PAK7 | HGLibA_35054 | 215 | 0.810055235 | PAK7 | - |
| C2orf40 | HGLibA_06123 | 214 | 0.801009892 | C2orf40 | - |
| ENSG00000048405 | HGLibA_56784 | 214 | 0.801009892 | ZNF800 | zinc finger protein 800 [Source:HGNC Symbol;Acc:HGNC:27267] |
| ENSG00000070214 | HGLibA_45157 | 214 | 0.801009892 | SLC44A1 | solute carrier family 44 member 1 [Source:HGNC Symbol;Acc:HGNC:18798] |
| ENSG00000078142 | HGLibA_36629 | 214 | 0.801009892 | PIK3C3 | phosphatidylinositol 3-kinase catalytic subunit type 3 [Source:HGNC Symbol;Acc:HGNC:8974] |
| ENSG00000082175 | HGLibA_36284 | 214 | 0.801009892 | PGR | progesterone receptor [Source:HGNC Symbol;Acc:HGNC:8910] |
| ENSG00000091137 | HGLibA_44794 | 214 | 0.801009892 | SLC26A4 | solute carrier family 26 member 4 [Source:HGNC Symbol;Acc:HGNC:8818] |
| ENSG00000101138 | HGLibA_11483 | 214 | 0.801009892 | CSTF1 | cleavage stimulation factor subunit 1 [Source:HGNC Symbol;Acc:HGNC:2483] |
| ENSG00000101596 | HGLibA_45662 | 214 | 0.801009892 | SMCHD1 | structural maintenance of chromosomes flexible hinge domain containing 1 [Source:HGNC Symbol;Acc:HGNC:29090] |
| ENSG00000104447 | HGLibA_51777 | 214 | 0.801009892 | TRPS1 | transcriptional repressor GATA binding 1 [Source:HGNC Symbol;Acc:HGNC:12340] |
| ENSG00000105726 | HGLibA_03567 | 214 | 0.801009892 | ATP13A1 | ATPase 13A1 [Source:HGNC Symbol;Acc:HGNC:24215] |
| ENSG00000106003 | HGLibA_26341 | 214 | 0.801009892 | LFNG | LFNG O-fucosylpeptide 3-beta-N-acetylglucosaminyltransferase [Source:HGNC Symbol;Acc:HGNC:6560] |
| ENSG00000106536 | HGLibA_37797 | 214 | 0.801009892 | POU6F2 | POU class 6 homeobox 2 [Source:HGNC Symbol;Acc:HGNC:21694] |
| ENSG00000110841 | HGLibA_37886 | 214 | 0.801009892 | PPFIBP1 | PPFIA binding protein 1 [Source:HGNC Symbol;Acc:HGNC:9249] |
| ENSG00000115263 | HGLibA_18777 | 214 | 0.801009892 | GCG | glucagon [Source:HGNC Symbol;Acc:HGNC:4191] |
| ENSG00000115825 | HGLibA_38571 | 214 | 0.801009892 | PRKD3 | protein kinase D3 [Source:HGNC Symbol;Acc:HGNC:9408] |
| ENSG00000116478 | HGLibA_20997 | 214 | 0.801009892 | HDAC1 | histone deacetylase 1 [Source:HGNC Symbol;Acc:HGNC:4852] |
| ENSG00000116906 | HGLibA_19562 | 214 | 0.801009892 | GNPAT | glyceronephosphate O-acyltransferase [Source:HGNC Symbol;Acc:HGNC:4416] |
| ENSG00000124557 | HGLibA_04958 | 214 | 0.801009892 | BTN1A1 | butyrophilin subfamily 1 member A1 [Source:HGNC Symbol;Acc:HGNC:1135] |
| ENSG00000128268 | HGLibA_29137 | 214 | 0.801009892 | MGAT3 | 4-N-acetylglucosaminyltransferase [Source:HGNC Symbol;Acc:HGNC:7046]" |
| ENSG00000130762 | HGLibA_02823 | 214 | 0.801009892 | ARHGEF16 | Rho guanine nucleotide exchange factor 16 [Source:HGNC Symbol;Acc:HGNC:15515] |
| ENSG00000133466 | HGLibA_05950 | 214 | 0.801009892 | C1QTNF6 | C1q and TNF related 6 [Source:HGNC Symbol;Acc:HGNC:14343] |
| ENSG00000133477 | HGLibA_16754 | 214 | 0.801009892 | FAM83F | family with sequence similarity 83 member F [Source:HGNC Symbol;Acc:HGNC:25148] |
| ENSG00000134900 | HGLibA_51162 | 214 | 0.801009892 | TPP2 | tripeptidyl peptidase 2 [Source:HGNC Symbol;Acc:HGNC:12016] |
| ENSG00000136099 | HGLibA_35419 | 214 | 0.801009892 | PCDH8 | protocadherin 8 [Source:HGNC Symbol;Acc:HGNC:8660] |
| ENSG00000136144 | HGLibA_40676 | 214 | 0.801009892 | RCBTB1 | RCC1 and BTB domain containing protein 1 [Source:HGNC Symbol;Acc:HGNC:18243] |
| ENSG00000137285 | HGLibA_52279 | 214 | 0.801009892 | TUBB2B | tubulin beta 2B class IIb [Source:HGNC Symbol;Acc:HGNC:30829] |
| ENSG00000137574 | HGLibA_49276 | 214 | 0.801009892 | TGS1 | trimethylguanosine synthase 1 [Source:HGNC Symbol;Acc:HGNC:17843] |
| ENSG00000137834 | HGLibA_45584 | 214 | 0.801009892 | SMAD6 | SMAD family member 6 [Source:HGNC Symbol;Acc:HGNC:6772] |
| ENSG00000138668 | HGLibA_21814 | 214 | 0.801009892 | HNRNPD | heterogeneous nuclear ribonucleoprotein D [Source:HGNC Symbol;Acc:HGNC:5036] |
| ENSG00000139287 | HGLibA_51132 | 214 | 0.801009892 | TPH2 | tryptophan hydroxylase 2 [Source:HGNC Symbol;Acc:HGNC:20692] |
| ENSG00000140262 | HGLibA_48760 | 214 | 0.801009892 | TCF12 | transcription factor 12 [Source:HGNC Symbol;Acc:HGNC:11623] |
| ENSG00000143127 | HGLibA_23719 | 214 | 0.801009892 | ITGA10 | integrin subunit alpha 10 [Source:HGNC Symbol;Acc:HGNC:6135] |
| ENSG00000143630 | HGLibA_20978 | 214 | 0.801009892 | HCN3 | hyperpolarization activated cyclic nucleotide gated potassium channel 3 [Source:HGNC Symbol;Acc:HGNC:19183] |
| ENSG00000145113 | HGLibA_30548 | 214 | 0.801009892 | MUC4 | cell surface associated [Source:HGNC Symbol;Acc:HGNC:7514]" |
| ENSG00000151092 | HGLibA_31880 | 214 | 0.801009892 | NGLY1 | N-glycanase 1 [Source:HGNC Symbol;Acc:HGNC:17646] |
| ENSG00000152595 | HGLibA_28904 | 214 | 0.801009892 | MEPE | matrix extracellular phosphoglycoprotein [Source:HGNC Symbol;Acc:HGNC:13361] |
| ENSG00000154978 | HGLibA_53717 | 214 | 0.801009892 | VOPP1 | WBP1/VOPP1 family member [Source:HGNC Symbol;Acc:HGNC:34518]" |
| ENSG00000155629 | HGLibA_36619 | 214 | 0.801009892 | PIK3AP1 | phosphoinositide-3-kinase adaptor protein 1 [Source:HGNC Symbol;Acc:HGNC:30034] |
| ENSG00000158882 | HGLibA_50969 | 214 | 0.801009892 | TOMM40L | translocase of outer mitochondrial membrane 40 like [Source:HGNC Symbol;Acc:HGNC:25756] |
| ENSG00000160469 | HGLibA_04849 | 214 | 0.801009892 | BRSK1 | BR serine/threonine kinase 1 [Source:HGNC Symbol;Acc:HGNC:18994] |
| ENSG00000164326 | HGLibA_07264 | 214 | 0.801009892 | CARTPT | CART prepropeptide [Source:HGNC Symbol;Acc:HGNC:24323] |
| ENSG00000164615 | HGLibA_07099 | 214 | 0.801009892 | CAMLG | calcium modulating ligand [Source:HGNC Symbol;Acc:HGNC:1471] |
| ENSG00000167487 | HGLibA_25167 | 214 | 0.801009892 | KLHL26 | kelch like family member 26 [Source:HGNC Symbol;Acc:HGNC:25623] |
| ENSG00000167642 | HGLibA_46716 | 214 | 0.801009892 | SPINT2 | Kunitz type 2 [Source:HGNC Symbol;Acc:HGNC:11247]" |
| ENSG00000168329 | HGLibA_11837 | 214 | 0.801009892 | CX3CR1 | C-X3-C motif chemokine receptor 1 [Source:HGNC Symbol;Acc:HGNC:2558] |
| ENSG00000168484 | HGLibA_43774 | 214 | 0.801009892 | SFTPC | surfactant protein C [Source:HGNC Symbol;Acc:HGNC:10802] |
| ENSG00000170175 | HGLibA_09589 | 214 | 0.801009892 | CHRNB1 | cholinergic receptor nicotinic beta 1 subunit [Source:HGNC Symbol;Acc:HGNC:1961] |
| ENSG00000170442 | HGLibA_25573 | 214 | 0.801009892 | KRT86 | keratin 86 [Source:HGNC Symbol;Acc:HGNC:6463] |
| ENSG00000170893 | HGLibA_51379 | 214 | 0.801009892 | TRH | thyrotropin releasing hormone [Source:HGNC Symbol;Acc:HGNC:12298] |
| ENSG00000171711 | HGLibA_12913 | 214 | 0.801009892 | DEFB4A | defensin beta 4A [Source:HGNC Symbol;Acc:HGNC:2767] |
| ENSG00000171942 | HGLibA_33509 | 214 | 0.801009892 | OR10H2 | olfactory receptor family 10 subfamily H member 2 [Source:HGNC Symbol;Acc:HGNC:8173] |
| ENSG00000173826 | HGLibA_24225 | 214 | 0.801009892 | KCNH6 | potassium voltage-gated channel subfamily H member 6 [Source:HGNC Symbol;Acc:HGNC:18862] |
| ENSG00000175745 | HGLibA_32610 | 214 | 0.801009892 | NR2F1 | nuclear receptor subfamily 2 group F member 1 [Source:HGNC Symbol;Acc:HGNC:7975] |
| ENSG00000179455 | HGLibA_29355 | 214 | 0.801009892 | MKRN3 | makorin ring finger protein 3 [Source:HGNC Symbol;Acc:HGNC:7114] |
| ENSG00000183828 | HGLibA_32945 | 214 | 0.801009892 | NUDT14 | nudix hydrolase 14 [Source:HGNC Symbol;Acc:HGNC:20141] |

| | | | | | |
|---|---|---|---|---|---|
| ENSG00000184207 | HGLibA_36276 | 214 | 0.801009892 | PGP | phosphoglycolate phosphatase [Source:HGNC Symbol;Acc:HGNC:8909] |
| ENSG00000186806 | HGLibA_53841 | 214 | 0.801009892 | VSIG10L | V-set and immunoglobulin domain containing 10 like [Source:HGNC Symbol;Acc:HGNC:27111] |
| ENSG00000187051 | HGLibA_42098 | 214 | 0.801009892 | RPS19BP1 | ribosomal protein S19 binding protein 1 [Source:HGNC Symbol;Acc:HGNC:28749] |
| ENSG00000187699 | HGLibA_06196 | 214 | 0.801009892 | C2orf88 | chromosome 2 open reading frame 88 [Source:HGNC Symbol;Acc:HGNC:28191] |
| ENSG00000188157 | HGLibA_01251 | 214 | 0.801009892 | AGRN | agrin [Source:HGNC Symbol;Acc:HGNC:329] |
| ENSG00000189181 | HGLibA_33636 | 214 | 0.801009892 | OR14I1 | olfactory receptor family 14 subfamily I member 1 [Source:HGNC Symbol;Acc:HGNC:19575] |
| ENSG00000189375 | HGLibA_48520 | 214 | 0.801009892 | TBC1D28 | TBC1 domain family member 28 [Source:HGNC Symbol;Acc:HGNC:26858] |
| ENSG00000196407 | HGLibA_49342 | 214 | 0.801009892 | THEM5 | thioesterase superfamily member 5 [Source:HGNC Symbol;Acc:HGNC:26755] |
| ENSG00000197599 | HGLibA_07654 | 214 | 0.801009892 | CCDC154 | coiled-coil domain containing 154 [Source:HGNC Symbol;Acc:HGNC:34454] |
| ENSG00000198142 | HGLibA_46218 | 214 | 0.801009892 | SOWAHC | sosondowah ankyrin repeat domain family member C [Source:HGNC Symbol;Acc:HGNC:26149] |
| ENSG00000198646 | HGLibA_31350 | 214 | 0.801009892 | NCOA6 | nuclear receptor coactivator 6 [Source:HGNC Symbol;Acc:HGNC:15936] |
| ENSG00000204576 | HGLibA_38835 | 214 | 0.801009892 | PRR3 | proline rich 3 [Source:HGNC Symbol;Acc:HGNC:21149] |
| ENSG00000214253 | HGLibA_17536 | 214 | 0.801009892 | FIS1 | mitochondrial 1 [Source:HGNC Symbol;Acc:HGNC:21689]" |
| ENSG00000232382 | HGLibA_34310 | 214 | 0.801009892 | OR5K1 | olfactory receptor family 5 subfamily K member 1 [Source:HGNC Symbol;Acc:HGNC:8349] |
| ENSG00000266964 | HGLibA_18225 | 214 | 0.801009892 | FXYD1 | FXYD domain containing ion transport regulator 1 [Source:HGNC Symbol;Acc:HGNC:4025] |
| EPT1 | HGLibA_15390 | 214 | 0.801009892 | EPT1 | - |
| GAREML | HGLibA_18630 | 214 | 0.801009892 | GAREML | - |
| hsa-mir-1538 | HGLibA_57917 | 214 | 0.801009892 | hsa-mir-1538 | - |
| hsa-mir-183 | HGLibA_57994 | 214 | 0.801009892 | hsa-mir-183 | - |
| hsa-mir-4472-1 | HGLibA_60293 | 214 | 0.801009892 | hsa-mir-4472-1 | - |
| hsa-mir-4684 | HGLibA_60846 | 214 | 0.801009892 | hsa-mir-4684 | - |
| ATP5S | HGLibA_03689 | 213 | 0.791964549 | ATP5S | - |
| C19orf21 | HGLibA_05635 | 213 | 0.791964549 | C19orf21 | - |
| C7orf41 | HGLibA_06541 | 213 | 0.791964549 | C7orf41 | - |
| ENSG00000076944 | HGLibA_47667 | 213 | 0.791964549 | STXBP2 | syntaxin binding protein 2 [Source:HGNC Symbol;Acc:HGNC:11445] |
| ENSG00000084636 | HGLibA_10518 | 213 | 0.791964549 | COL16A1 | collagen type XVI alpha 1 chain [Source:HGNC Symbol;Acc:HGNC:2193] |
| ENSG00000089063 | HGLibA_50254 | 213 | 0.791964549 | TMEM230 | transmembrane protein 230 [Source:HGNC Symbol;Acc:HGNC:15876] |
| ENSG00000092098 | HGLibA_41662 | 213 | 0.791964549 | RNF31 | ring finger protein 31 [Source:HGNC Symbol;Acc:HGNC:16031] |
| ENSG00000095951 | HGLibA_21568 | 213 | 0.791964549 | HIVEP1 | human immunodeficiency virus type I enhancer binding protein 1 [Source:HGNC Symbol;Acc:HGNC:4920] |
| ENSG00000100678 | HGLibA_45393 | 213 | 0.791964549 | SLC8A3 | solute carrier family 8 member A3 [Source:HGNC Symbol;Acc:HGNC:11070] |
| ENSG00000100987 | HGLibA_53875 | 213 | 0.791964549 | VSX1 | visual system homeobox 1 [Source:HGNC Symbol;Acc:HGNC:12723] |
| ENSG00000102024 | HGLibA_37229 | 213 | 0.791964549 | PLS3 | plastin 3 [Source:HGNC Symbol;Acc:HGNC:9091] |
| ENSG00000103196 | HGLibA_11176 | 213 | 0.791964549 | CRISPLD2 | cysteine rich secretory protein LCCL domain containing 2 [Source:HGNC Symbol;Acc:HGNC:25248] |
| ENSG00000105392 | HGLibA_11237 | 213 | 0.791964549 | CRX | cone-rod homeobox [Source:HGNC Symbol;Acc:HGNC:2383] |
| ENSG00000105583 | HGLibA_54197 | 213 | 0.791964549 | WDR83OS | WD repeat domain 83 opposite strand [Source:HGNC Symbol;Acc:HGNC:30203] |
| ENSG00000106348 | HGLibA_23256 | 213 | 0.791964549 | IMPDH1 | inosine monophosphate dehydrogenase 1 [Source:HGNC Symbol;Acc:HGNC:6052] |
| ENSG00000107560 | HGLibA_39797 | 213 | 0.791964549 | RAB11FIP2 | RAB11 family interacting protein 2 [Source:HGNC Symbol;Acc:HGNC:29152] |
| ENSG00000108255 | HGLibA_11253 | 213 | 0.791964549 | CRYBA1 | crystallin beta A1 [Source:HGNC Symbol;Acc:HGNC:2394] |
| ENSG00000108349 | HGLibA_07275 | 213 | 0.791964549 | CASC3 | exon junction complex subunit [Source:HGNC Symbol;Acc:HGNC:17040]" |
| ENSG00000108511 | HGLibA_21945 | 213 | 0.791964549 | HOXB6 | homeobox B6 [Source:HGNC Symbol;Acc:HGNC:5117] |
| ENSG00000112293 | HGLibA_19816 | 213 | 0.791964549 | GPLD1 | glycosylphosphatidylinositol specific phospholipase D1 [Source:HGNC Symbol;Acc:HGNC:4459] |
| ENSG00000124089 | HGLibA_28548 | 213 | 0.791964549 | MC3R | melanocortin 3 receptor [Source:HGNC Symbol;Acc:HGNC:6931] |
| ENSG00000125967 | HGLibA_31589 | 213 | 0.791964549 | NECAB3 | N-terminal EF-hand calcium binding protein 3 [Source:HGNC Symbol;Acc:HGNC:15851] |
| ENSG00000127452 | HGLibA_16973 | 213 | 0.791964549 | FBXL12 | F-box and leucine rich repeat protein 12 [Source:HGNC Symbol;Acc:HGNC:13611] |
| ENSG00000132256 | HGLibA_51535 | 213 | 0.791964549 | TRIM5 | tripartite motif containing 5 [Source:HGNC Symbol;Acc:HGNC:16276] |
| ENSG00000134640 | HGLibA_30449 | 213 | 0.791964549 | MTNR1B | melatonin receptor 1B [Source:HGNC Symbol;Acc:HGNC:7464] |
| ENSG00000135698 | HGLibA_29727 | 213 | 0.791964549 | MPHOSPH6 | M-phase phosphoprotein 6 [Source:HGNC Symbol;Acc:HGNC:7214] |
| ENSG00000137094 | HGLibA_13546 | 213 | 0.791964549 | DNAJB5 | DnaJ heat shock protein family (Hsp40) member B5 [Source:HGNC Symbol;Acc:HGNC:14887] |
| ENSG00000138160 | HGLibA_24851 | 213 | 0.791964549 | KIF11 | kinesin family member 11 [Source:HGNC Symbol;Acc:HGNC:6388] |
| ENSG00000140519 | HGLibA_41148 | 213 | 0.791964549 | RHCG | Rh family C glycoprotein [Source:HGNC Symbol;Acc:HGNC:18140] |
| ENSG00000140832 | HGLibA_28386 | 213 | 0.791964549 | MARVELD3 | MARVEL domain containing 3 [Source:HGNC Symbol;Acc:HGNC:30525] |
| ENSG00000144451 | HGLibA_46352 | 213 | 0.791964549 | SPAG16 | sperm associated antigen 16 [Source:HGNC Symbol;Acc:HGNC:23225] |
| ENSG00000144579 | HGLibA_11607 | 213 | 0.791964549 | CTDSP1 | CTD small phosphatase 1 [Source:HGNC Symbol;Acc:HGNC:21614] |
| ENSG00000146757 | HGLibA_56908 | 213 | 0.791964549 | ZNF92 | zinc finger protein 92 [Source:HGNC Symbol;Acc:HGNC:13168] |
| ENSG00000148516 | HGLibA_55134 | 213 | 0.791964549 | ZEB1 | zinc finger E-box binding homeobox 1 [Source:HGNC Symbol;Acc:HGNC:11642] |
| ENSG00000158125 | HGLibA_54501 | 213 | 0.791964549 | XDH | xanthine dehydrogenase [Source:HGNC Symbol;Acc:HGNC:12805] |
| ENSG00000160180 | HGLibA_49175 | 213 | 0.791964549 | TFF3 | trefoil factor 3 [Source:HGNC Symbol;Acc:HGNC:11757] |
| ENSG00000160182 | HGLibA_49168 | 213 | 0.791964549 | TFF1 | trefoil factor 1 [Source:HGNC Symbol;Acc:HGNC:11755] |
| ENSG00000161671 | HGLibA_15006 | 213 | 0.791964549 | EMC10 | ER membrane protein complex subunit 10 [Source:HGNC Symbol;Acc:HGNC:27609] |
| ENSG00000162650 | HGLibA_03855 | 213 | 0.791964549 | ATXN7L2 | ataxin 7 like 2 [Source:HGNC Symbol;Acc:HGNC:28713] |
| ENSG00000163346 | HGLibA_35354 | 213 | 0.791964549 | PBXIP1 | PBX homeobox interacting protein 1 [Source:HGNC Symbol;Acc:HGNC:21199] |
| ENSG00000164106 | HGLibA_43044 | 213 | 0.791964549 | SCRG1 | stimulator of chondrogenesis 1 [Source:HGNC Symbol;Acc:HGNC:17036] |
| ENSG00000164736 | HGLibA_46246 | 213 | 0.791964549 | SOX17 | SRY-box 17 [Source:HGNC Symbol;Acc:HGNC:18122] |
| ENSG00000165215 | HGLibA_09895 | 213 | 0.791964549 | CLDN3 | claudin 3 [Source:HGNC Symbol;Acc:HGNC:2045] |
| ENSG00000168259 | HGLibA_13636 | 213 | 0.791964549 | DNAJC7 | DnaJ heat shock protein family (Hsp40) member C7 [Source:HGNC Symbol;Acc:HGNC:12392] |
| ENSG00000169567 | HGLibA_21325 | 213 | 0.791964549 | HINT1 | histidine triad nucleotide binding protein 1 [Source:HGNC Symbol;Acc:HGNC:4912] |
| ENSG00000171195 | HGLibA_30557 | 213 | 0.791964549 | MUC7 | secreted [Source:HGNC Symbol;Acc:HGNC:7518]" |
| ENSG00000171496 | HGLibA_33699 | 213 | 0.791964549 | OR1L8 | olfactory receptor family 1 subfamily L member 8 [Source:HGNC Symbol;Acc:HGNC:15110] |
| ENSG00000171956 | HGLibA_17817 | 213 | 0.791964549 | FOXB1 | forkhead box B1 [Source:HGNC Symbol;Acc:HGNC:3799] |
| ENSG00000174796 | HGLibA_49308 | 213 | 0.791964549 | THAP6 | THAP domain containing 6 [Source:HGNC Symbol;Acc:HGNC:23189] |
| ENSG00000175221 | HGLibA_28761 | 213 | 0.791964549 | MED16 | mediator complex subunit 16 [Source:HGNC Symbol;Acc:HGNC:17556] |
| ENSG00000175354 | HGLibA_39449 | 213 | 0.791964549 | PTPN2 | non-receptor type 2 [Source:HGNC Symbol;Acc:HGNC:9650]" |
| ENSG00000176194 | HGLibA_09698 | 213 | 0.791964549 | CIDEA | cell death-inducing DFFA-like effector a [Source:HGNC Symbol;Acc:HGNC:1976] |
| ENSG00000177943 | HGLibA_28039 | 213 | 0.791964549 | MAMDC4 | MAM domain containing 4 [Source:HGNC Symbol;Acc:HGNC:24083] |
| ENSG00000178567 | HGLibA_15337 | 213 | 0.791964549 | EPM2AIP1 | EPM2A interacting protein 1 [Source:HGNC Symbol;Acc:HGNC:19735] |
| ENSG00000178802 | HGLibA_29731 | 213 | 0.791964549 | MPI | mannose phosphate isomerase [Source:HGNC Symbol;Acc:HGNC:7216] |
| ENSG00000183695 | HGLibA_29849 | 213 | 0.791964549 | MRGPRX2 | MAS related GPR family member X2 [Source:HGNC Symbol;Acc:HGNC:17983] |
| ENSG00000184378 | HGLibA_00682 | 213 | 0.791964549 | ACTRT3 | actin related protein T3 [Source:HGNC Symbol;Acc:HGNC:24022] |
| ENSG00000185261 | HGLibA_24655 | 213 | 0.791964549 | KIAA0825 | KIAA0825 [Source:HGNC Symbol;Acc:HGNC:28532] |
| ENSG00000187609 | HGLibA_15721 | 213 | 0.791964549 | EXD3 | exonuclease 3'-5' domain containing 3 [Source:HGNC Symbol;Acc:HGNC:26023] |
| ENSG00000189091 | HGLibA_43710 | 213 | 0.791964549 | SF3B3 | splicing factor 3b subunit 3 [Source:HGNC Symbol;Acc:HGNC:10770] |
| ENSG00000196670 | HGLibA_55226 | 213 | 0.791964549 | ZFP62 | ZFP62 zinc finger protein [Source:HGNC Symbol;Acc:HGNC:23241] |
| ENSG00000197406 | HGLibA_13243 | 213 | 0.791964549 | DIO3 | iodothyronine deiodinase 3 [Source:HGNC Symbol;Acc:HGNC:2885] |
| ENSG00000198728 | HGLibA_26211 | 213 | 0.791964549 | LDB1 | LIM domain binding 1 [Source:HGNC Symbol;Acc:HGNC:6532] |
| ENSG00000204642 | HGLibA_21645 | 213 | 0.791964549 | HLA-F | F [Source:HGNC Symbol;Acc:HGNC:4963]" |
| ENSG00000228278 | HGLibA_34621 | 213 | 0.791964549 | ORM2 | orosomucoid 2 [Source:HGNC Symbol;Acc:HGNC:8499] |
| ENSG00000244486 | HGLibA_42879 | 213 | 0.791964549 | SCARF2 | scavenger receptor class F member 2 [Source:HGNC Symbol;Acc:HGNC:19869] |
| ENSG00000257103 | HGLibA_27529 | 213 | 0.791964549 | LSM14A | mRNA processing body assembly factor [Source:HGNC Symbol;Acc:HGNC:24489]" |
| ENSG00000265107 | HGLibA_19147 | 213 | 0.791964549 | GJA5 | gap junction protein alpha 5 [Source:HGNC Symbol;Acc:HGNC:4279] |
| ENSG00000269113 | HGLibA_51233 | 213 | 0.791964549 | TRABD2B | TraB domain containing 2B [Source:HGNC Symbol;Acc:HGNC:44200] |
| ENSG00000276747 | HGLibA_34980 | 213 | 0.791964549 | PADI6 | peptidyl arginine deiminase 6 [Source:HGNC Symbol;Acc:HGNC:20449] |
| hsa-mir-107 | HGLibA_57192 | 213 | 0.791964549 | hsa-mir-107 | - |
| hsa-mir-1265 | HGLibA_57484 | 213 | 0.791964549 | hsa-mir-1265 | - |
| hsa-mir-335 | HGLibA_58997 | 213 | 0.791964549 | hsa-mir-335 | - |
| hsa-mir-3612 | HGLibA_59079 | 213 | 0.791964549 | hsa-mir-3612 | - |
| hsa-mir-4297 | HGLibA_59898 | 213 | 0.791964549 | hsa-mir-4297 | - |
| hsa-mir-4315-2 | HGLibA_59980 | 213 | 0.791964549 | hsa-mir-4315-2 | - |
| hsa-mir-4491 | HGLibA_60384 | 213 | 0.791964549 | hsa-mir-4491 | - |
| hsa-mir-7114 | HGLibA_63826 | 213 | 0.791964549 | hsa-mir-7114 | - |
| hsa-mir-8062 | HGLibA_64088 | 213 | 0.791964549 | hsa-mir-8062 | - |
| LOC339862 | HGLibA_26921 | 213 | 0.791964549 | LOC339862 | - |
| OR8U8 | HGLibA_34560 | 213 | 0.791964549 | OR8U8 | - |
| APITD1-CORT | HGLibA_02429 | 212 | 0.782919206 | APITD1-CORT | - |
| BRE | HGLibA_04795 | 212 | 0.782919206 | BRE | - |
| C12orf77 | HGLibA_05302 | 212 | 0.782919206 | C12orf77 | - |
| C5orf55 | HGLibA_06410 | 212 | 0.782919206 | C5orf55 | - |
| DARS | HGLibA_12340 | 212 | 0.782919206 | DARS | - |
| ENSG00000013583 | HGLibA_21085 | 212 | 0.782919206 | HEBP1 | heme binding protein 1 [Source:HGNC Symbol;Acc:HGNC:17176] |
| ENSG00000014138 | HGLibA_37486 | 212 | 0.782919206 | POLA2 | accessory subunit [Source:HGNC Symbol;Acc:HGNC:30073]" |
| ENSG00000019144 | HGLibA_36427 | 212 | 0.782919206 | PHLDB1 | pleckstrin homology like domain family B member 1 [Source:HGNC Symbol;Acc:HGNC:23697] |
| ENSG00000039068 | HGLibA_08629 | 212 | 0.782919206 | CDH1 | cadherin 1 [Source:HGNC Symbol;Acc:HGNC:1748] |
| ENSG00000042304 | HGLibA_06194 | 212 | 0.782919206 | C2orf83 | chromosome 2 open reading frame 83 [Source:HGNC Symbol;Acc:HGNC:25344] |
| ENSG00000047849 | HGLibA_28208 | 212 | 0.782919206 | MAP4 | microtubule associated protein 4 [Source:HGNC Symbol;Acc:HGNC:6862] |
| ENSG00000068796 | HGLibA_24924 | 212 | 0.782919206 | KIF2A | kinesin family member 2A [Source:HGNC Symbol;Acc:HGNC:6318] |
| ENSG00000082014 | HGLibA_45636 | 212 | 0.782919206 | SMARCD3 | member 3 [Source:HGNC Symbol;Acc:HGNC:11108]" |
| ENSG00000086827 | HGLibA_57065 | 212 | 0.782919206 | ZW10 | zw10 kinetochore protein [Source:HGNC Symbol;Acc:HGNC:13194] |
| ENSG00000088179 | HGLibA_39470 | 212 | 0.782919206 | PTPN4 | non-receptor type 4 [Source:HGNC Symbol;Acc:HGNC:9656]" |
| ENSG00000101474 | HGLibA_02445 | 212 | 0.782919206 | APMAP | adipocyte plasma membrane associated protein [Source:HGNC Symbol;Acc:HGNC:13238] |
| ENSG00000103811 | HGLibA_11710 | 212 | 0.782919206 | CTSH | cathepsin H [Source:HGNC Symbol;Acc:HGNC:2535] |
| ENSG00000107249 | HGLibA_19277 | 212 | 0.782919206 | GLIS3 | GLIS family zinc finger 3 [Source:HGNC Symbol;Acc:HGNC:28510] |
| ENSG00000107758 | HGLibA_38189 | 212 | 0.782919206 | PPP3CB | protein phosphatase 3 catalytic subunit beta [Source:HGNC Symbol;Acc:HGNC:9315] |
| ENSG00000108244 | HGLibA_25452 | 212 | 0.782919206 | KRT23 | keratin 23 [Source:HGNC Symbol;Acc:HGNC:6438] |
| ENSG00000110583 | HGLibA_30996 | 212 | 0.782919206 | NAA40 | NatD catalytic subunit [Source:HGNC Symbol;Acc:HGNC:25845]" |
| ENSG00000111679 | HGLibA_39476 | 212 | 0.782919206 | PTPN6 | non-receptor type 6 [Source:HGNC Symbol;Acc:HGNC:9658]" |
| ENSG00000113328 | HGLibA_08093 | 212 | 0.782919206 | CCNG1 | cyclin G1 [Source:HGNC Symbol;Acc:HGNC:1592] |
| ENSG00000114316 | HGLibA_53359 | 212 | 0.782919206 | USP4 | ubiquitin specific peptidase 4 [Source:HGNC Symbol;Acc:HGNC:12627] |
| ENSG00000116260 | HGLibA_39753 | 212 | 0.782919206 | QSOX1 | quiescin sulfhydryl oxidase 1 [Source:HGNC Symbol;Acc:HGNC:9756] |
| ENSG00000116703 | HGLibA_35711 | 212 | 0.782919206 | PDC | phosducin [Source:HGNC Symbol;Acc:HGNC:8759] |
| ENSG00000119596 | HGLibA_54698 | 212 | 0.782919206 | YLPM1 | YLP motif containing 1 [Source:HGNC Symbol;Acc:HGNC:17798] |
| ENSG00000119714 | HGLibA_20078 | 212 | 0.782919206 | GPR68 | G protein-coupled receptor 68 [Source:HGNC Symbol;Acc:HGNC:4519] |
| ENSG00000120437 | HGLibA_00395 | 212 | 0.782919206 | ACAT2 | acetyl-CoA acetyltransferase 2 [Source:HGNC Symbol;Acc:HGNC:94] |
| ENSG00000124233 | HGLibA_43380 | 212 | 0.782919206 | SEMG1 | semenogelin 1 [Source:HGNC Symbol;Acc:HGNC:10742] |
| ENSG00000127743 | HGLibA_22993 | 212 | 0.782919206 | IL17B | interleukin 17B [Source:HGNC Symbol;Acc:HGNC:5982] |
| ENSG00000130311 | HGLibA_12586 | 212 | 0.782919206 | DDA1 | DET1 and DDB1 associated 1 [Source:HGNC Symbol;Acc:HGNC:28360] |
| ENSG00000136938 | HGLibA_02201 | 212 | 0.782919206 | ANP32B | acidic nuclear phosphoprotein 32 family member B [Source:HGNC Symbol;Acc:HGNC:16677] |

| | | | | | |
|---|---|---|---|---|---|
| ENSG00000140265 | HGLibA_57013 | 212 | 0.782919206 | ZSCAN29 | zinc finger and SCAN domain containing 29 [Source:HGNC Symbol;Acc:HGNC:26673] |
| ENSG00000140678 | HGLibA_23766 | 212 | 0.782919206 | ITGAX | integrin subunit alpha X [Source:HGNC Symbol;Acc:HGNC:6152] |
| ENSG00000145242 | HGLibA_15298 | 212 | 0.782919206 | EPHA5 | EPH receptor A5 [Source:HGNC Symbol;Acc:HGNC:3389] |
| ENSG00000154978 | HGLibA_53715 | 212 | 0.782919206 | VOPP1 | WBP1/VOPP1 family member [Source:HGNC Symbol;Acc:HGNC:34518]" |
| ENSG00000156298 | HGLibA_51945 | 212 | 0.782919206 | TSPAN7 | tetraspanin 7 [Source:HGNC Symbol;Acc:HGNC:11854] |
| ENSG00000156983 | HGLibA_04838 | 212 | 0.782919206 | BRPF1 | bromodomain and PHD finger containing 1 [Source:HGNC Symbol;Acc:HGNC:14255] |
| ENSG00000158552 | HGLibA_55151 | 212 | 0.782919206 | ZFAND2B | zinc finger AN1-type containing 2B [Source:HGNC Symbol;Acc:HGNC:25206] |
| ENSG00000164296 | HGLibA_49479 | 212 | 0.782919206 | TIGD6 | tigger transposable element derived 6 [Source:HGNC Symbol;Acc:HGNC:18332] |
| ENSG00000164434 | HGLibA_15927 | 212 | 0.782919206 | FABP7 | fatty acid binding protein 7 [Source:HGNC Symbol;Acc:HGNC:3562] |
| ENSG00000165113 | HGLibA_19204 | 212 | 0.782919206 | GKAP1 | G kinase anchoring protein 1 [Source:HGNC Symbol;Acc:HGNC:17496] |
| ENSG00000167333 | HGLibA_51592 | 212 | 0.782919206 | TRIM68 | tripartite motif containing 68 [Source:HGNC Symbol;Acc:HGNC:21161] |
| ENSG00000169057 | HGLibA_28728 | 212 | 0.782919206 | MECP2 | methyl-CpG binding protein 2 [Source:HGNC Symbol;Acc:HGNC:6990] |
| ENSG00000175183 | HGLibA_11435 | 212 | 0.782919206 | CSRP2 | cysteine and glycine rich protein 2 [Source:HGNC Symbol;Acc:HGNC:2470] |
| ENSG00000177963 | HGLibA_41235 | 212 | 0.782919206 | RIC8A | RIC8 guanine nucleotide exchange factor A [Source:HGNC Symbol;Acc:HGNC:29550] |
| ENSG00000178163 | HGLibA_56151 | 212 | 0.782919206 | ZNF518B | zinc finger protein 518B [Source:HGNC Symbol;Acc:HGNC:29365] |
| ENSG00000180316 | HGLibA_37404 | 212 | 0.782919206 | PNPLA1 | patatin like phospholipase domain containing 1 [Source:HGNC Symbol;Acc:HGNC:21246] |
| ENSG00000182307 | HGLibA_06607 | 212 | 0.782919206 | C8orf33 | chromosome 8 open reading frame 33 [Source:HGNC Symbol;Acc:HGNC:26104] |
| ENSG00000182771 | HGLibA_20272 | 212 | 0.782919206 | GRID1 | glutamate ionotropic receptor delta type subunit 1 [Source:HGNC Symbol;Acc:HGNC:4575] |
| ENSG00000184602 | HGLibA_45933 | 212 | 0.782919206 | SNN | stannin [Source:HGNC Symbol;Acc:HGNC:11149] |
| ENSG00000185112 | HGLibA_16586 | 212 | 0.782919206 | FAM43A | family with sequence similarity 43 member A [Source:HGNC Symbol;Acc:HGNC:26888] |
| ENSG00000185940 | HGLibA_25799 | 212 | 0.782919206 | KRTAP5-5 | keratin associated protein 5-5 [Source:HGNC Symbol;Acc:HGNC:23601] |
| ENSG00000186090 | HGLibA_22388 | 212 | 0.782919206 | HTR3D | 5-hydroxytryptamine receptor 3D [Source:HGNC Symbol;Acc:HGNC:24004] |
| ENSG00000186335 | HGLibA_45035 | 212 | 0.782919206 | SLC36A2 | solute carrier family 36 member 2 [Source:HGNC Symbol;Acc:HGNC:18762] |
| ENSG00000187116 | HGLibA_26502 | 212 | 0.782919206 | LILRA5 | leukocyte immunoglobulin like receptor A5 [Source:HGNC Symbol;Acc:HGNC:16309] |
| ENSG00000187325 | HGLibA_48264 | 212 | 0.782919206 | TAF9B | TATA-box binding protein associated factor 9b [Source:HGNC Symbol;Acc:HGNC:17306] |
| ENSG00000189367 | HGLibA_24634 | 212 | 0.782919206 | KIAA0408 | KIAA0408 [Source:HGNC Symbol;Acc:HGNC:21636] |
| ENSG00000196151 | HGLibA_54229 | 212 | 0.782919206 | WDSUB1 | sterile alpha motif and U-box domain containing 1 [Source:HGNC Symbol;Acc:HGNC:26697]" |
| ENSG00000198252 | HGLibA_47688 | 212 | 0.782919206 | STYX | serine/threonine/tyrosine interacting protein [Source:HGNC Symbol;Acc:HGNC:11447] |
| ENSG00000198553 | HGLibA_24381 | 212 | 0.782919206 | KCNRG | potassium channel regulator [Source:HGNC Symbol;Acc:HGNC:18893] |
| ENSG00000203760 | HGLibA_09091 | 212 | 0.782919206 | CENPW | centromere protein W [Source:HGNC Symbol;Acc:HGNC:21488] |
| ENSG00000204930 | HGLibA_16520 | 212 | 0.782919206 | FAM221B | family with sequence similarity 221 member B [Source:HGNC Symbol;Acc:HGNC:30762] |
| ENSG00000206075 | HGLibA_43555 | 212 | 0.782919206 | SERPINB5 | serpin family B member 5 [Source:HGNC Symbol;Acc:HGNC:8949] |
| ENSG00000213973 | HGLibA_56918 | 212 | 0.782919206 | ZNF99 | zinc finger protein 99 [Source:HGNC Symbol;Acc:HGNC:13175] |
| ENSG00000215545 | HGLibA_12861 | 212 | 0.782919206 | DEFB116 | defensin beta 116 [Source:HGNC Symbol;Acc:HGNC:18097] |
| ENSG00000225766 | HGLibA_13138 | 212 | 0.782919206 | DHRS4L1 | dehydrogenase/reductase 4 like 1 [Source:HGNC Symbol;Acc:HGNC:19732] |
| ENSG00000234438 | HGLibA_24099 | 212 | 0.782919206 | KBTBD13 | kelch repeat and BTB domain containing 13 [Source:HGNC Symbol;Acc:HGNC:37227] |
| ENSG00000261652 | HGLibA_05434 | 212 | 0.782919206 | C15orf65 | chromosome 15 open reading frame 65 [Source:HGNC Symbol;Acc:HGNC:44654] |
| ENSG00000283706 | HGLibA_38952 | 212 | 0.782919206 | PRSS50 | serine protease 50 [Source:NCBI gene;Acc:29122] |
| FAM122C | HGLibA_16072 | 212 | 0.782919206 | FAM122C | - |
| hsa-mir-2117 | HGLibA_58242 | 212 | 0.782919206 | hsa-mir-2117 | - |
| hsa-mir-4263 | HGLibA_59758 | 212 | 0.782919206 | hsa-mir-4263 | - |
| hsa-mir-4454 | HGLibA_60222 | 212 | 0.782919206 | hsa-mir-4454 | - |
| hsa-mir-449a | HGLibA_60416 | 212 | 0.782919206 | hsa-mir-449a | - |
| hsa-mir-4760 | HGLibA_61135 | 212 | 0.782919206 | hsa-mir-4760 | - |
| hsa-mir-6801 | HGLibA_63380 | 212 | 0.782919206 | hsa-mir-6801 | - |
| KIAA1462 | HGLibA_24763 | 212 | 0.782919206 | KIAA1462 | - |
| C17orf105 | HGLibA_05526 | 211 | 0.773873863 | C17orf105 | - |
| C8orf47 | HGLibA_06632 | 211 | 0.773873863 | C8orf47 | - |
| ENSG00000025772 | HGLibA_50965 | 211 | 0.773873863 | TOMM34 | translocase of outer mitochondrial membrane 34 [Source:HGNC Symbol;Acc:HGNC:15746] |
| ENSG00000040731 | HGLibA_08631 | 211 | 0.773873863 | CDH10 | cadherin 10 [Source:HGNC Symbol;Acc:HGNC:1749] |
| ENSG00000079819 | HGLibA_15246 | 211 | 0.773873863 | EPB41L2 | erythrocyte membrane protein band 4.1 like 2 [Source:HGNC Symbol;Acc:HGNC:3379] |
| ENSG00000081818 | HGLibA_35498 | 211 | 0.773873863 | PCDHB4 | protocadherin beta 4 [Source:HGNC Symbol;Acc:HGNC:8689] |
| ENSG00000091972 | HGLibA_08283 | 211 | 0.773873863 | CD200 | CD200 molecule [Source:HGNC Symbol;Acc:HGNC:7203] |
| ENSG00000095951 | HGLibA_21566 | 211 | 0.773873863 | HIVEP1 | human immunodeficiency virus type I enhancer binding protein 1 [Source:HGNC Symbol;Acc:HGNC:4920] |
| ENSG00000104321 | HGLibA_51725 | 211 | 0.773873863 | TRPA1 | transient receptor potential cation channel subfamily A member 1 [Source:HGNC Symbol;Acc:HGNC:497] |
| ENSG00000105392 | HGLibA_11238 | 211 | 0.773873863 | CRX | cone-rod homeobox [Source:HGNC Symbol;Acc:HGNC:2383] |
| ENSG00000106536 | HGLibA_37799 | 211 | 0.773873863 | POU6F2 | POU class 6 homeobox 2 [Source:HGNC Symbol;Acc:HGNC:21694] |
| ENSG00000109684 | HGLibA_10088 | 211 | 0.773873863 | CLNK | cytokine dependent hematopoietic cell linker [Source:HGNC Symbol;Acc:HGNC:17438] |
| ENSG00000109756 | HGLibA_40248 | 211 | 0.773873863 | RAPGEF2 | Rap guanine nucleotide exchange factor 2 [Source:HGNC Symbol;Acc:HGNC:16854] |
| ENSG00000110881 | HGLibA_03298 | 211 | 0.773873863 | ASIC1 | acid sensing ion channel subunit 1 [Source:HGNC Symbol;Acc:HGNC:100] |
| ENSG00000113240 | HGLibA_10064 | 211 | 0.773873863 | CLK4 | CDC like kinase 4 [Source:HGNC Symbol;Acc:HGNC:13659] |
| ENSG00000117395 | HGLibA_14369 | 211 | 0.773873863 | EBNA1BP2 | EBNA1 binding protein 2 [Source:HGNC Symbol;Acc:HGNC:15531] |
| ENSG00000122390 | HGLibA_31003 | 211 | 0.773873863 | NAA60 | NatF catalytic subunit [Source:HGNC Symbol;Acc:HGNC:25875]" |
| ENSG00000125648 | HGLibA_44678 | 211 | 0.773873863 | SLC25A23 | solute carrier family 25 member 23 [Source:HGNC Symbol;Acc:HGNC:19375] |
| ENSG00000125898 | HGLibA_16012 | 211 | 0.773873863 | FAM110A | family with sequence similarity 110 member A [Source:HGNC Symbol;Acc:HGNC:16188] |
| ENSG00000128298 | HGLibA_04129 | 211 | 0.773873863 | BAIAP2L2 | BAI1 associated protein 2 like 2 [Source:HGNC Symbol;Acc:HGNC:26203] |
| ENSG00000131480 | HGLibA_02271 | 211 | 0.773873863 | AOC2 | copper containing 2 [Source:HGNC Symbol;Acc:HGNC:549]" |
| ENSG00000133134 | HGLibA_04444 | 211 | 0.773873863 | BEX2 | brain expressed X-linked 2 [Source:HGNC Symbol;Acc:HGNC:30933] |
| ENSG00000135077 | HGLibA_20901 | 211 | 0.773873863 | HAVCR2 | hepatitis A virus cellular receptor 2 [Source:HGNC Symbol;Acc:HGNC:18437] |
| ENSG00000135596 | HGLibA_29201 | 211 | 0.773873863 | MICAL1 | calponin and LIM domain containing 1 [Source:HGNC Symbol;Acc:HGNC:20619]" |
| ENSG00000137713 | HGLibA_38143 | 211 | 0.773873863 | PPP2R1B | protein phosphatase 2 scaffold subunit Abeta [Source:HGNC Symbol;Acc:HGNC:9303] |
| ENSG00000140285 | HGLibA_17418 | 211 | 0.773873863 | FGF7 | fibroblast growth factor 7 [Source:HGNC Symbol;Acc:HGNC:3685] |
| ENSG00000143507 | HGLibA_14129 | 211 | 0.773873863 | DUSP10 | dual specificity phosphatase 10 [Source:HGNC Symbol;Acc:HGNC:3065] |
| ENSG00000144381 | HGLibA_22332 | 211 | 0.773873863 | HSPD1 | heat shock protein family D (Hsp60) member 1 [Source:HGNC Symbol;Acc:HGNC:5261] |
| ENSG00000144485 | HGLibA_21191 | 211 | 0.773873863 | HES6 | hes family bHLH transcription factor 6 [Source:HGNC Symbol;Acc:HGNC:18254] |
| ENSG00000146842 | HGLibA_50204 | 211 | 0.773873863 | TMEM209 | transmembrane protein 209 [Source:HGNC Symbol;Acc:HGNC:21898] |
| ENSG00000148985 | HGLibA_36209 | 211 | 0.773873863 | PGAP2 | post-GPI attachment to proteins 2 [Source:HGNC Symbol;Acc:HGNC:17893] |
| ENSG00000152672 | HGLibA_09991 | 211 | 0.773873863 | CLEC4F | C-type lectin domain family 4 member F [Source:HGNC Symbol;Acc:HGNC:25357] |
| ENSG00000154059 | HGLibA_23249 | 211 | 0.773873863 | IMPACT | impact RWD domain protein [Source:HGNC Symbol;Acc:HGNC:20387] |
| ENSG00000154328 | HGLibA_31631 | 211 | 0.773873863 | NEIL2 | nei like DNA glycosylase 2 [Source:HGNC Symbol;Acc:HGNC:18956] |
| ENSG00000159231 | HGLibA_07422 | 211 | 0.773873863 | CBR3 | carbonyl reductase 3 [Source:HGNC Symbol;Acc:HGNC:1549] |
| ENSG00000161558 | HGLibA_49968 | 211 | 0.773873863 | TMEM143 | transmembrane protein 143 [Source:HGNC Symbol;Acc:HGNC:25603] |
| ENSG00000162631 | HGLibA_32865 | 211 | 0.773873863 | NTNG1 | netrin G1 [Source:HGNC Symbol;Acc:HGNC:23319] |
| ENSG00000162729 | HGLibA_22902 | 211 | 0.773873863 | IGSF8 | immunoglobulin superfamily member 8 [Source:HGNC Symbol;Acc:HGNC:17813] |
| ENSG00000163810 | HGLibA_49261 | 211 | 0.773873863 | TGM4 | transglutaminase 4 [Source:HGNC Symbol;Acc:HGNC:11780] |
| ENSG00000164237 | HGLibA_10182 | 211 | 0.773873863 | CMBL | carboxymethylenebutenolidase homolog [Source:HGNC Symbol;Acc:HGNC:25090] |
| ENSG00000164398 | HGLibA_00566 | 211 | 0.773873863 | ACSL6 | acyl-CoA synthetase long chain family member 6 [Source:HGNC Symbol;Acc:HGNC:16496] |
| ENSG00000164414 | HGLibA_44947 | 211 | 0.773873863 | SLC35A1 | solute carrier family 35 member A1 [Source:HGNC Symbol;Acc:HGNC:11021] |
| ENSG00000166368 | HGLibA_33783 | 211 | 0.773873863 | OR2D2 | olfactory receptor family 2 subfamily D member 2 [Source:HGNC Symbol;Acc:HGNC:8244] |
| ENSG00000166548 | HGLibA_49582 | 211 | 0.773873863 | TK2 | mitochondrial [Source:HGNC Symbol;Acc:HGNC:11831]" |
| ENSG00000167232 | HGLibA_56905 | 211 | 0.773873863 | ZNF91 | zinc finger protein 91 [Source:HGNC Symbol;Acc:HGNC:13166] |
| ENSG00000169045 | HGLibA_21822 | 211 | 0.773873863 | HNRNPH1 | heterogeneous nuclear ribonucleoprotein H1 [Source:HGNC Symbol;Acc:HGNC:5041] |
| ENSG00000170801 | HGLibA_22412 | 211 | 0.773873863 | HTRA3 | HtrA serine peptidase 3 [Source:HGNC Symbol;Acc:HGNC:30406] |
| ENSG00000170819 | HGLibA_04457 | 211 | 0.773873863 | BFSP2 | beaded filament structural protein 2 [Source:HGNC Symbol;Acc:HGNC:1041] |
| ENSG00000172057 | HGLibA_34631 | 211 | 0.773873863 | ORMDL3 | ORMDL sphingolipid biosynthesis regulator 3 [Source:HGNC Symbol;Acc:HGNC:16038] |
| ENSG00000173083 | HGLibA_22060 | 211 | 0.773873863 | HPSE | heparanase [Source:HGNC Symbol;Acc:HGNC:5164] |
| ENSG00000175756 | HGLibA_03877 | 211 | 0.773873863 | AURKAIP1 | aurora kinase A interacting protein 1 [Source:HGNC Symbol;Acc:HGNC:24114] |
| ENSG00000182816 | HGLibA_25646 | 211 | 0.773873863 | KRTAP13-2 | keratin associated protein 13-2 [Source:HGNC Symbol;Acc:HGNC:18923] |
| ENSG00000183260 | HGLibA_00261 | 211 | 0.773873863 | ABHD16B | abhydrolase domain containing 16B [Source:HGNC Symbol;Acc:HGNC:16128] |
| ENSG00000183751 | HGLibA_48620 | 211 | 0.773873863 | TBL3 | transducin beta like 3 [Source:HGNC Symbol;Acc:HGNC:11587] |
| ENSG00000184731 | HGLibA_16018 | 211 | 0.773873863 | FAM110C | family with sequence similarity 110 member C [Source:HGNC Symbol;Acc:HGNC:33340] |
| ENSG00000186364 | HGLibA_32959 | 211 | 0.773873863 | NUDT17 | nudix hydrolase 17 [Source:HGNC Symbol;Acc:HGNC:26618] |
| ENSG00000187446 | HGLibA_09516 | 211 | 0.773873863 | CHP1 | calcineurin like EF-hand protein 1 [Source:HGNC Symbol;Acc:HGNC:17433] |
| ENSG00000187824 | HGLibA_50235 | 211 | 0.773873863 | TMEM220 | transmembrane protein 220 [Source:HGNC Symbol;Acc:HGNC:33757] |
| ENSG00000188487 | HGLibA_23372 | 211 | 0.773873863 | INSC | spindle orientation adaptor protein [Source:HGNC Symbol;Acc:HGNC:33116]" |
| ENSG00000196236 | HGLibA_54558 | 211 | 0.773873863 | XPNPEP3 | X-prolyl aminopeptidase 3 [Source:HGNC Symbol;Acc:HGNC:28052] |
| ENSG00000196268 | HGLibA_56097 | 211 | 0.773873863 | ZNF493 | zinc finger protein 493 [Source:HGNC Symbol;Acc:HGNC:23708] |
| ENSG00000197472 | HGLibA_56557 | 211 | 0.773873863 | ZNF695 | zinc finger protein 695 [Source:HGNC Symbol;Acc:HGNC:30954] |
| ENSG00000198736 | HGLibA_30240 | 211 | 0.773873863 | MSRB1 | methionine sulfoxide reductase B1 [Source:HGNC Symbol;Acc:HGNC:14133] |
| ENSG00000215009 | HGLibA_00581 | 211 | 0.773873863 | ACSM4 | acyl-CoA synthetase medium chain family member 4 [Source:HGNC Symbol;Acc:HGNC:32016] |
| ENSG00000255398 | HGLibA_20952 | 211 | 0.773873863 | HCAR3 | hydroxycarboxylic acid receptor 3 [Source:HGNC Symbol;Acc:HGNC:16824] |
| ENSG00000276085 | HGLibA_08024 | 211 | 0.773873863 | CCL3L3 | C-C motif chemokine ligand 3 like 1 [Source:HGNC Symbol;Acc:HGNC:10628] |
| hsa-mir-128-1 | HGLibA_57572 | 211 | 0.773873863 | hsa-mir-128-1 | - |
| hsa-mir-205 | HGLibA_58180 | 211 | 0.773873863 | hsa-mir-205 | - |
| hsa-mir-3156-3 | HGLibA_58694 | 211 | 0.773873863 | hsa-mir-3156-3 | - |
| hsa-mir-6736 | HGLibA_63096 | 211 | 0.773873863 | hsa-mir-6736 | - |
| hsa-mir-6741 | HGLibA_63118 | 211 | 0.773873863 | hsa-mir-6741 | - |
| hsa-mir-6756 | HGLibA_63180 | 211 | 0.773873863 | hsa-mir-6756 | - |
| KIAA1683 | HGLibA_24801 | 211 | 0.773873863 | KIAA1683 | - |
| LRRC16B | HGLibA_27266 | 211 | 0.773873863 | LRRC16B | - |
| MFSD7 | HGLibA_29114 | 211 | 0.773873863 | MFSD7 | - |
| PRAMEF22 | HGLibA_38314 | 211 | 0.773873863 | PRAMEF22 | - |
| C10orf91 | HGLibA_05107 | 210 | 0.76482852 | C10orf91 | - |
| C20orf195 | HGLibA_06002 | 210 | 0.76482852 | C20orf195 | - |
| CECR6 | HGLibA_08983 | 210 | 0.76482852 | CECR6 | - |
| ENSG00000037280 | HGLibA_17671 | 210 | 0.76482852 | FLT4 | fms related tyrosine kinase 4 [Source:HGNC Symbol;Acc:HGNC:3767] |
| ENSG00000059769 | HGLibA_13606 | 210 | 0.76482852 | DNAJC25 | DnaJ heat shock protein family (Hsp40) member C25 [Source:HGNC Symbol;Acc:HGNC:34187] |
| ENSG00000065609 | HGLibA_45888 | 210 | 0.76482852 | SNAP91 | synaptosome associated protein 91 [Source:HGNC Symbol;Acc:HGNC:14986] |
| ENSG00000068354 | HGLibA_48515 | 210 | 0.76482852 | TBC1D25 | TBC1 domain family member 25 [Source:HGNC Symbol;Acc:HGNC:8092] |
| ENSG00000070915 | HGLibA_44364 | 210 | 0.76482852 | SLC12A3 | solute carrier family 12 member 3 [Source:HGNC Symbol;Acc:HGNC:10912] |
| ENSG00000073921 | HGLibA_36521 | 210 | 0.76482852 | PICALM | phosphatidylinositol binding clathrin assembly protein [Source:HGNC Symbol;Acc:HGNC:15514] |
| ENSG00000075461 | HGLibA_06939 | 210 | 0.76482852 | CACNG4 | calcium voltage-gated channel auxiliary subunit gamma 4 [Source:HGNC Symbol;Acc:HGNC:1408] |
| ENSG00000083307 | HGLibA_20250 | 210 | 0.76482852 | GRHL2 | grainyhead like transcription factor 2 [Source:HGNC Symbol;Acc:HGNC:2799] |
| ENSG00000094755 | HGLibA_18398 | 210 | 0.76482852 | GABRP | gamma-aminobutyric acid type A receptor pi subunit [Source:HGNC Symbol;Acc:HGNC:4089] |
| ENSG00000099804 | HGLibA_08541 | 210 | 0.76482852 | CDC34 | cell division cycle 34 [Source:HGNC Symbol;Acc:HGNC:1734] |

| | | | | | |
|---|---|---|---|---|---|
| ENSG00000100578 | HGLibA_24647 | 210 | 0.76482852 | KIAA0586 | KIAA0586 [Source:HGNC Symbol;Acc:HGNC:19960] |
| ENSG00000102359 | HGLibA_47037 | 210 | 0.76482852 | SRPX2 | sushi repeat containing protein X-linked 2 [Source:HGNC Symbol;Acc:HGNC:30668] |
| ENSG00000103591 | HGLibA_00046 | 210 | 0.76482852 | AAGAB | alpha and gamma adaptin binding protein [Source:HGNC Symbol;Acc:HGNC:25662] |
| ENSG00000104689 | HGLibA_50734 | 210 | 0.76482852 | TNFRSF10A | TNF receptor superfamily member 10a [Source:HGNC Symbol;Acc:HGNC:11904] |
| ENSG00000110367 | HGLibA_12758 | 210 | 0.76482852 | DDX6 | DEAD-box helicase 6 [Source:HGNC Symbol;Acc:HGNC:2747] |
| ENSG00000113504 | HGLibA_44374 | 210 | 0.76482852 | SLC12A7 | solute carrier family 12 member 7 [Source:HGNC Symbol;Acc:HGNC:10915] |
| ENSG00000116199 | HGLibA_16448 | 210 | 0.76482852 | FAM20B | glycosaminoglycan xylosylkinase [Source:HGNC Symbol;Acc:HGNC:23017]" |
| ENSG00000116785 | HGLibA_09269 | 210 | 0.76482852 | CFHR3 | complement factor H related 3 [Source:HGNC Symbol;Acc:HGNC:16980] |
| ENSG00000123901 | HGLibA_20093 | 210 | 0.76482852 | GPR83 | G protein-coupled receptor 83 [Source:HGNC Symbol;Acc:HGNC:4523] |
| ENSG00000124523 | HGLibA_44227 | 210 | 0.76482852 | SIRT5 | sirtuin 5 [Source:HGNC Symbol;Acc:HGNC:14933] |
| ENSG00000124733 | HGLibA_28718 | 210 | 0.76482852 | MEA1 | male-enhanced antigen 1 [Source:HGNC Symbol;Acc:HGNC:6986] |
| ENSG00000125503 | HGLibA_38027 | 210 | 0.76482852 | PPP1R12C | protein phosphatase 1 regulatory subunit 12C [Source:HGNC Symbol;Acc:HGNC:14947] |
| ENSG00000134531 | HGLibA_15074 | 210 | 0.76482852 | EMP1 | epithelial membrane protein 1 [Source:HGNC Symbol;Acc:HGNC:3333] |
| ENSG00000137077 | HGLibA_07994 | 210 | 0.76482852 | CCL21 | C-C motif chemokine ligand 21 [Source:HGNC Symbol;Acc:HGNC:10620] |
| ENSG00000137142 | HGLibA_22837 | 210 | 0.76482852 | IGFBPL1 | insulin like growth factor binding protein like 1 [Source:HGNC Symbol;Acc:HGNC:20081] |
| ENSG00000139044 | HGLibA_04017 | 210 | 0.76482852 | B4GALNT3 | 4-N-acetyl-galactosaminyltransferase 3 [Source:HGNC Symbol;Acc:HGNC:24137]" |
| ENSG00000140093 | HGLibA_43507 | 210 | 0.76482852 | SERPINA10 | serpin family A member 10 [Source:HGNC Symbol;Acc:HGNC:15996] |
| ENSG00000141194 | HGLibA_33965 | 210 | 0.76482852 | OR4D1 | olfactory receptor family 4 subfamily D member 1 [Source:HGNC Symbol;Acc:HGNC:8293] |
| ENSG00000144821 | HGLibA_30718 | 210 | 0.76482852 | MYH15 | myosin heavy chain 15 [Source:HGNC Symbol;Acc:HGNC:31073] |
| ENSG00000144843 | HGLibA_01025 | 210 | 0.76482852 | ADPRH | ADP-ribosylarginine hydrolase [Source:HGNC Symbol;Acc:HGNC:269] |
| ENSG00000148798 | HGLibA_23269 | 210 | 0.76482852 | INA | internexin neuronal intermediate filament protein alpha [Source:HGNC Symbol;Acc:HGNC:6057] |
| ENSG00000149743 | HGLibA_51780 | 210 | 0.76482852 | TRPT1 | tRNA phosphotransferase 1 [Source:HGNC Symbol;Acc:HGNC:20316] |
| ENSG00000151116 | HGLibA_52840 | 210 | 0.76482852 | UEVLD | UEV and lactate/malate dehyrogenase domains [Source:HGNC Symbol;Acc:HGNC:30866] |
| ENSG00000151892 | HGLibA_18965 | 210 | 0.76482852 | GFRA1 | GDNF family receptor alpha 1 [Source:HGNC Symbol;Acc:HGNC:4243] |
| ENSG00000152954 | HGLibA_32724 | 210 | 0.76482852 | NRSN1 | neurensin 1 [Source:HGNC Symbol;Acc:HGNC:17881] |
| ENSG00000154258 | HGLibA_00128 | 210 | 0.76482852 | ABCA9 | ATP binding cassette subfamily A member 9 [Source:HGNC Symbol;Acc:HGNC:39] |
| ENSG00000155313 | HGLibA_53311 | 210 | 0.76482852 | USP25 | ubiquitin specific peptidase 25 [Source:HGNC Symbol;Acc:HGNC:12624] |
| ENSG00000157315 | HGLibA_49818 | 210 | 0.76482852 | TMED6 | transmembrane p24 trafficking protein 6 [Source:HGNC Symbol;Acc:HGNC:28331] |
| ENSG00000158715 | HGLibA_45177 | 210 | 0.76482852 | SLC45A3 | solute carrier family 45 member 3 [Source:HGNC Symbol;Acc:HGNC:8642] |
| ENSG00000160447 | HGLibA_36843 | 210 | 0.76482852 | PKN3 | protein kinase N3 [Source:HGNC Symbol;Acc:HGNC:17999] |
| ENSG00000161791 | HGLibA_17700 | 210 | 0.76482852 | FMNL3 | formin like 3 [Source:HGNC Symbol;Acc:HGNC:23698] |
| ENSG00000163576 | HGLibA_14567 | 210 | 0.76482852 | EFHB | EF-hand domain family member B [Source:HGNC Symbol;Acc:HGNC:26330] |
| ENSG00000164729 | HGLibA_45023 | 210 | 0.76482852 | SLC35G3 | solute carrier family 35 member G3 [Source:HGNC Symbol;Acc:HGNC:26848] |
| ENSG00000167656 | HGLibA_27654 | 210 | 0.76482852 | LY6D | lymphocyte antigen 6 family member D [Source:HGNC Symbol;Acc:HGNC:13348] |
| ENSG00000167780 | HGLibA_46118 | 210 | 0.76482852 | SOAT2 | sterol O-acyltransferase 2 [Source:HGNC Symbol;Acc:HGNC:11178] |
| ENSG00000168263 | HGLibA_24404 | 210 | 0.76482852 | KCNV2 | potassium voltage-gated channel modifier subfamily V member 2 [Source:HGNC Symbol;Acc:HGNC:19698] |
| ENSG00000168542 | HGLibA_10563 | 210 | 0.76482852 | COL3A1 | collagen type III alpha 1 chain [Source:HGNC Symbol;Acc:HGNC:2201] |
| ENSG00000168874 | HGLibA_03540 | 210 | 0.76482852 | ATOH8 | atonal bHLH transcription factor 8 [Source:HGNC Symbol;Acc:HGNC:24126] |
| ENSG00000169855 | HGLibA_41732 | 210 | 0.76482852 | ROBO1 | roundabout guidance receptor 1 [Source:HGNC Symbol;Acc:HGNC:10249] |
| ENSG00000169989 | HGLibA_49472 | 210 | 0.76482852 | TIGD4 | tigger transposable element derived 4 [Source:HGNC Symbol;Acc:HGNC:18335] |
| ENSG00000172568 | HGLibA_17762 | 210 | 0.76482852 | FNDC9 | fibronectin type III domain containing 9 [Source:HGNC Symbol;Acc:HGNC:33547] |
| ENSG00000172995 | HGLibA_03100 | 210 | 0.76482852 | ARPP21 | cAMP regulated phosphoprotein 21 [Source:HGNC Symbol;Acc:HGNC:16968] |
| ENSG00000176200 | HGLibA_33971 | 210 | 0.76482852 | OR4D11 | olfactory receptor family 4 subfamily D member 11 [Source:HGNC Symbol;Acc:HGNC:15174] |
| ENSG00000177807 | HGLibA_24248 | 210 | 0.76482852 | KCNJ10 | potassium voltage-gated channel subfamily J member 10 [Source:HGNC Symbol;Acc:HGNC:6256] |
| ENSG00000180806 | HGLibA_21980 | 210 | 0.76482852 | HOXC9 | homeobox C9 [Source:HGNC Symbol;Acc:HGNC:5130] |
| ENSG00000181830 | HGLibA_44970 | 210 | 0.76482852 | SLC35C1 | solute carrier family 35 member C1 [Source:HGNC Symbol;Acc:HGNC:20197] |
| ENSG00000182141 | HGLibA_56601 | 210 | 0.76482852 | ZNF708 | zinc finger protein 708 [Source:HGNC Symbol;Acc:HGNC:12945] |
| ENSG00000183092 | HGLibA_04397 | 210 | 0.76482852 | BEGAIN | brain enriched guanylate kinase associated [Source:HGNC Symbol;Acc:HGNC:24163] |
| ENSG00000185238 | HGLibA_38627 | 210 | 0.76482852 | PRMT3 | protein arginine methyltransferase 3 [Source:HGNC Symbol;Acc:HGNC:30163] |
| ENSG00000188643 | HGLibA_42547 | 210 | 0.76482852 | S100A16 | S100 calcium binding protein A16 [Source:HGNC Symbol;Acc:HGNC:20441] |
| ENSG00000196177 | HGLibA_00374 | 210 | 0.76482852 | ACADSB | acyl-CoA dehydrogenase short/branched chain [Source:HGNC Symbol;Acc:HGNC:91] |
| ENSG00000198771 | HGLibA_40718 | 210 | 0.76482852 | RCSD1 | RCSD domain containing 1 [Source:HGNC Symbol;Acc:HGNC:28310] |
| ENSG00000198807 | HGLibA_35320 | 210 | 0.76482852 | PAX9 | paired box 9 [Source:HGNC Symbol;Acc:HGNC:8623] |
| ENSG00000203879 | HGLibA_18882 | 210 | 0.76482852 | GDI1 | GDP dissociation inhibitor 1 [Source:HGNC Symbol;Acc:HGNC:4226] |
| ENSG00000213341 | HGLibA_09670 | 210 | 0.76482852 | CHUK | conserved helix-loop-helix ubiquitous kinase [Source:HGNC Symbol;Acc:HGNC:1974] |
| ENSG00000213996 | HGLibA_49711 | 210 | 0.76482852 | TM6SF2 | transmembrane 6 superfamily member 2 [Source:HGNC Symbol;Acc:HGNC:11861] |
| ENSG00000239961 | HGLibA_26499 | 210 | 0.76482852 | LILRA4 | leukocyte immunoglobulin like receptor A4 [Source:HGNC Symbol;Acc:HGNC:15503] |
| ENSG00000242715 | HGLibA_07683 | 210 | 0.76482852 | CCDC169 | coiled-coil domain containing 169 [Source:HGNC Symbol;Acc:HGNC:34361] |
| ENSG00000251655 | HGLibA_38337 | 210 | 0.76482852 | PRB1 | proline rich protein BstNI subfamily 1 [Source:HGNC Symbol;Acc:HGNC:9337] |
| ENSG00000268606 | HGLibA_27905 | 210 | 0.76482852 | MAGEA2 | MAGE family member A2 [Source:HGNC Symbol;Acc:HGNC:6800] |
| ENSG00000276293 | HGLibA_36709 | 210 | 0.76482852 | PIP4K2B | phosphatidylinositol-5-phosphate 4-kinase type 2 beta [Source:HGNC Symbol;Acc:HGNC:8998] |
| GLTSCR1L | HGLibA_19356 | 210 | 0.76482852 | GLTSCR1L | - |
| hsa-mir-1261 | HGLibA_57466 | 210 | 0.76482852 | hsa-mir-1261 | - |
| hsa-mir-138-2 | HGLibA_57815 | 210 | 0.76482852 | hsa-mir-138-2 | - |
| hsa-mir-4503 | HGLibA_60439 | 210 | 0.76482852 | hsa-mir-4503 | - |
| hsa-mir-548az | HGLibA_61946 | 210 | 0.76482852 | hsa-mir-548az | - |
| hsa-mir-623 | HGLibA_62733 | 210 | 0.76482852 | hsa-mir-623 | - |
| hsa-mir-7107 | HGLibA_63789 | 210 | 0.76482852 | hsa-mir-7107 | - |
| hsa-mir-7152 | HGLibA_63838 | 210 | 0.76482852 | hsa-mir-7152 | - |
| SOLH | HGLibA_46167 | 210 | 0.76482852 | SOLH | - |
| C8orf46 | HGLibA_06629 | 209 | 0.755783177 | C8orf46 | - |
| ENSG00000002587 | HGLibA_22121 | 209 | 0.755783177 | HS3ST1 | heparan sulfate-glucosamine 3-sulfotransferase 1 [Source:HGNC Symbol;Acc:HGNC:5194] |
| ENSG00000070669 | HGLibA_03334 | 209 | 0.755783177 | ASNS | asparagine synthetase (glutamine-hydrolyzing) [Source:HGNC Symbol;Acc:HGNC:753] |
| ENSG00000081138 | HGLibA_08689 | 209 | 0.755783177 | CDH7 | cadherin 7 [Source:HGNC Symbol;Acc:HGNC:1766] |
| ENSG00000087237 | HGLibA_09243 | 209 | 0.755783177 | CETP | cholesteryl ester transfer protein [Source:HGNC Symbol;Acc:HGNC:1869] |
| ENSG00000100284 | HGLibA_50945 | 209 | 0.755783177 | TOM1 | target of myb1 membrane trafficking protein [Source:HGNC Symbol;Acc:HGNC:11982] |
| ENSG00000102858 | HGLibA_29172 | 209 | 0.755783177 | MGRN1 | mahogunin ring finger 1 [Source:HGNC Symbol;Acc:HGNC:20254] |
| ENSG00000102977 | HGLibA_00419 | 209 | 0.755783177 | ACD | shelterin complex subunit and telomerase recruitment factor [Source:HGNC Symbol;Acc:HGNC:25070]" |
| ENSG00000104938 | HGLibA_09998 | 209 | 0.755783177 | CLEC4M | C-type lectin domain family 4 member M [Source:HGNC Symbol;Acc:HGNC:13523] |
| ENSG00000105223 | HGLibA_37040 | 209 | 0.755783177 | PLD3 | phospholipase D family member 3 [Source:HGNC Symbol;Acc:HGNC:17158] |
| ENSG00000106153 | HGLibA_09366 | 209 | 0.755783177 | CHCHD2 | coiled-coil-helix-coiled-coil-helix domain containing 2 [Source:HGNC Symbol;Acc:HGNC:21645] |
| ENSG00000108244 | HGLibA_25453 | 209 | 0.755783177 | KRT23 | keratin 23 [Source:HGNC Symbol;Acc:HGNC:6438] |
| ENSG00000113645 | HGLibA_54440 | 209 | 0.755783177 | WWC1 | WW and C2 domain containing 1 [Source:HGNC Symbol;Acc:HGNC:29435] |
| ENSG00000114520 | HGLibA_46097 | 209 | 0.755783177 | SNX4 | sorting nexin 4 [Source:HGNC Symbol;Acc:HGNC:11175] |
| ENSG00000115459 | HGLibA_14946 | 209 | 0.755783177 | ELMOD3 | ELMO domain containing 3 [Source:HGNC Symbol;Acc:HGNC:26158] |
| ENSG00000119946 | HGLibA_10315 | 209 | 0.755783177 | CNNM1 | cyclin and CBS domain divalent metal cation transport mediator 1 [Source:HGNC Symbol;Acc:HGNC:102] |
| ENSG00000121851 | HGLibA_37630 | 209 | 0.755783177 | POLR3GL | RNA polymerase III subunit G like [Source:HGNC Symbol;Acc:HGNC:28466] |
| ENSG00000123143 | HGLibA_36837 | 209 | 0.755783177 | PKN1 | protein kinase N1 [Source:HGNC Symbol;Acc:HGNC:9405] |
| ENSG00000123329 | HGLibA_02793 | 209 | 0.755783177 | ARHGAP9 | Rho GTPase activating protein 9 [Source:HGNC Symbol;Acc:HGNC:14130] |
| ENSG00000124098 | HGLibA_16456 | 209 | 0.755783177 | FAM210B | family with sequence similarity 210 member B [Source:HGNC Symbol;Acc:HGNC:16102] |
| ENSG00000126003 | HGLibA_36957 | 209 | 0.755783177 | PLAGL2 | PLAG1 like zinc finger 2 [Source:HGNC Symbol;Acc:HGNC:9047] |
| ENSG00000126952 | HGLibA_33139 | 209 | 0.755783177 | NXF5 | nuclear RNA export factor 5 [Source:HGNC Symbol;Acc:HGNC:8075] |
| ENSG00000128482 | HGLibA_41470 | 209 | 0.755783177 | RNF112 | ring finger protein 112 [Source:HGNC Symbol;Acc:HGNC:12968] |
| ENSG00000130332 | HGLibA_27552 | 209 | 0.755783177 | LSM7 | U6 small nuclear RNA and mRNA degradation associated [Source:HGNC Symbol;Acc:HGNC:20470]" |
| ENSG00000131650 | HGLibA_25405 | 209 | 0.755783177 | KREMEN2 | kringle containing transmembrane protein 2 [Source:HGNC Symbol;Acc:HGNC:18797] |
| ENSG00000133315 | HGLibA_27837 | 209 | 0.755783177 | MACROD1 | MACRO domain containing 1 [Source:HGNC Symbol;Acc:HGNC:29598] |
| ENSG00000133874 | HGLibA_41486 | 209 | 0.755783177 | RNF122 | ring finger protein 122 [Source:HGNC Symbol;Acc:HGNC:21147] |
| ENSG00000134873 | HGLibA_09851 | 209 | 0.755783177 | CLDN10 | claudin 10 [Source:HGNC Symbol;Acc:HGNC:2033] |
| ENSG00000139946 | HGLibA_36036 | 209 | 0.755783177 | PELI2 | pellino E3 ubiquitin protein ligase family member 2 [Source:HGNC Symbol;Acc:HGNC:8828] |
| ENSG00000139973 | HGLibA_48066 | 209 | 0.755783177 | SYT16 | synaptotagmin 16 [Source:HGNC Symbol;Acc:HGNC:23142] |
| ENSG00000142186 | HGLibA_43084 | 209 | 0.755783177 | SCYL1 | SCY1 like pseudokinase 1 [Source:HGNC Symbol;Acc:HGNC:14372] |
| ENSG00000143569 | HGLibA_52554 | 209 | 0.755783177 | UBAP2L | ubiquitin associated protein 2 like [Source:HGNC Symbol;Acc:HGNC:29877] |
| ENSG00000144306 | HGLibA_43055 | 209 | 0.755783177 | SCRN3 | secernin 3 [Source:HGNC Symbol;Acc:HGNC:30382] |
| ENSG00000147155 | HGLibA_14374 | 209 | 0.755783177 | EBP | cholestenol delta-isomerase [Source:HGNC Symbol;Acc:HGNC:3133]" |
| ENSG00000151208 | HGLibA_13336 | 209 | 0.755783177 | DLG5 | discs large MAGUK scaffold protein 5 [Source:HGNC Symbol;Acc:HGNC:2904] |
| ENSG00000152904 | HGLibA_19008 | 209 | 0.755783177 | GGPS1 | geranylgeranyl diphosphate synthase 1 [Source:HGNC Symbol;Acc:HGNC:4249] |
| ENSG00000157211 | HGLibA_08626 | 209 | 0.755783177 | CDCP2 | CUB domain containing protein 2 [Source:HGNC Symbol;Acc:HGNC:27297] |
| ENSG00000158747 | HGLibA_31215 | 209 | 0.755783177 | NBL1 | DAN family BMP antagonist [Source:HGNC Symbol;Acc:HGNC:7650]" |
| ENSG00000161249 | HGLibA_13408 | 209 | 0.755783177 | DMKN | dermokine [Source:HGNC Symbol;Acc:HGNC:25063] |
| ENSG00000164078 | HGLibA_30259 | 209 | 0.755783177 | MST1R | macrophage stimulating 1 receptor [Source:HGNC Symbol;Acc:HGNC:7381] |
| ENSG00000165169 | HGLibA_14268 | 209 | 0.755783177 | DYNLT3 | dynein light chain Tctex-type 3 [Source:HGNC Symbol;Acc:HGNC:11694] |
| ENSG00000165887 | HGLibA_02025 | 209 | 0.755783177 | ANKRD2 | ankyrin repeat domain 2 [Source:HGNC Symbol;Acc:HGNC:495] |
| ENSG00000166884 | HGLibA_33980 | 209 | 0.755783177 | OR4D6 | olfactory receptor family 4 subfamily D member 6 [Source:HGNC Symbol;Acc:HGNC:15175] |
| ENSG00000167851 | HGLibA_08333 | 209 | 0.755783177 | CD300A | CD300a molecule [Source:HGNC Symbol;Acc:HGNC:19319] |
| ENSG00000167916 | HGLibA_25455 | 209 | 0.755783177 | KRT24 | keratin 24 [Source:HGNC Symbol;Acc:HGNC:18527] |
| ENSG00000168288 | HGLibA_29459 | 209 | 0.755783177 | MMADHC | metabolism of cobalamin associated D [Source:HGNC Symbol;Acc:HGNC:25221] |
| ENSG00000169955 | HGLibA_56667 | 209 | 0.755783177 | ZNF747 | zinc finger protein 747 [Source:HGNC Symbol;Acc:HGNC:28350] |
| ENSG00000170638 | HGLibA_51227 | 209 | 0.755783177 | TRABD | TraB domain containing [Source:HGNC Symbol;Acc:HGNC:28805] |
| ENSG00000171992 | HGLibA_48017 | 209 | 0.755783177 | SYNPO | synaptopodin [Source:HGNC Symbol;Acc:HGNC:30672] |
| ENSG00000173578 | HGLibA_54498 | 209 | 0.755783177 | XCR1 | X-C motif chemokine receptor 1 [Source:HGNC Symbol;Acc:HGNC:1625] |
| ENSG00000177189 | HGLibA_42164 | 209 | 0.755783177 | RPS6KA3 | ribosomal protein S6 kinase A3 [Source:HGNC Symbol;Acc:HGNC:10432] |
| ENSG00000178217 | HGLibA_43900 | 209 | 0.755783177 | SH2D4B | SH2 domain containing 4B [Source:HGNC Symbol;Acc:HGNC:31440] |
| ENSG00000180818 | HGLibA_21957 | 209 | 0.755783177 | HOXC10 | homeobox C10 [Source:HGNC Symbol;Acc:HGNC:5122] |
| ENSG00000183111 | HGLibA_02855 | 209 | 0.755783177 | ARHGEF37 | Rho guanine nucleotide exchange factor 37 [Source:HGNC Symbol;Acc:HGNC:34430] |
| ENSG00000184389 | HGLibA_00015 | 209 | 0.755783177 | A3GALT2 | 3-galactosyltransferase 2 [Source:HGNC Symbol;Acc:HGNC:30005]" |
| ENSG00000185475 | HGLibA_50091 | 209 | 0.755783177 | TMEM179B | transmembrane protein 179B [Source:HGNC Symbol;Acc:HGNC:33744] |
| ENSG00000186654 | HGLibA_38843 | 209 | 0.755783177 | PRR5 | proline rich 5 [Source:HGNC Symbol;Acc:HGNC:31682] |
| ENSG00000186994 | HGLibA_24029 | 209 | 0.755783177 | KANK3 | KN motif and ankyrin repeat domains 3 [Source:HGNC Symbol;Acc:HGNC:24796] |
| ENSG00000187193 | HGLibA_30297 | 209 | 0.755783177 | MT1X | metallothionein 1X [Source:HGNC Symbol;Acc:HGNC:7405] |
| ENSG00000188938 | HGLibA_16056 | 209 | 0.755783177 | FAM120AOS | family with sequence similarity 120A opposite strand [Source:HGNC Symbol;Acc:HGNC:23389] |
| ENSG00000196547 | HGLibA_28073 | 209 | 0.755783177 | MAN2A2 | mannosidase alpha class 2A member 2 [Source:HGNC Symbol;Acc:HGNC:6825] |
| ENSG00000197658 | HGLibA_44575 | 209 | 0.755783177 | SLC22A24 | solute carrier family 22 member 24 [Source:HGNC Symbol;Acc:HGNC:28542] |
| ENSG00000197969 | HGLibA_53731 | 209 | 0.755783177 | VPS13A | vacuolar protein sorting 13 homolog A [Source:HGNC Symbol;Acc:HGNC:1908] |
| ENSG00000206260 | HGLibA_38821 | 209 | 0.755783177 | PRR23A | proline rich 23A [Source:HGNC Symbol;Acc:HGNC:37172] |
| ENSG00000217442 | HGLibA_47921 | 209 | 0.755783177 | SYCE3 | synaptonemal complex central element protein 3 [Source:HGNC Symbol;Acc:HGNC:35245] |

| | | | | | |
|---|---|---|---|---|---|
| ENSG00000231887 | HGLibA_38460 | 209 | 0.755783177 | PRH1 | proline rich protein HaeIII subfamily 1 [Source:HGNC Symbol;Acc:HGNC:9366] |
| ENSG00000241123 | HGLibA_25613 | 209 | 0.755783177 | KRTAP10-5 | keratin associated protein 10-5 [Source:HGNC Symbol;Acc:HGNC:22969] |
| ENSG00000250741 | HGLibA_32807 | 209 | 0.755783177 | NT5C1B-RDH14 | NT5C1B-RDH14 readthrough [Source:HGNC Symbol;Acc:HGNC:38831] |
| FLJ45513 | HGLibA_17637 | 209 | 0.755783177 | FLJ45513 | - |
| H1FNT | HGLibA_20735 | 209 | 0.755783177 | H1FNT | - |
| hsa-mir-2117 | HGLibA_58240 | 209 | 0.755783177 | hsa-mir-2117 | - |
| hsa-mir-378b | HGLibA_59428 | 209 | 0.755783177 | hsa-mir-378b | - |
| hsa-mir-4323 | HGLibA_60016 | 209 | 0.755783177 | hsa-mir-4323 | - |
| hsa-mir-657 | HGLibA_62962 | 209 | 0.755783177 | hsa-mir-657 | - |
| hsa-mir-6804 | HGLibA_63393 | 209 | 0.755783177 | hsa-mir-6804 | - |
| hsa-mir-6827 | HGLibA_63487 | 209 | 0.755783177 | hsa-mir-6827 | - |
| hsa-mir-6871 | HGLibA_63669 | 209 | 0.755783177 | hsa-mir-6871 | - |
| IGJ | HGLibA_22863 | 209 | 0.755783177 | IGJ | - |
| WDR16 | HGLibA_54033 | 209 | 0.755783177 | WDR16 | - |
| C11orf57 | HGLibA_05159 | 208 | 0.746737834 | C11orf57 | - |
| C9orf172 | HGLibA_06712 | 208 | 0.746737834 | C9orf172 | - |
| ENSG00000008952 | HGLibA_43260 | 208 | 0.746737834 | SEC62 | preprotein translocation factor [Source:HGNC Symbol;Acc:HGNC:11846]" |
| ENSG00000011105 | HGLibA_51952 | 208 | 0.746737834 | TSPAN9 | tetraspanin 9 [Source:HGNC Symbol;Acc:HGNC:21640] |
| ENSG00000030419 | HGLibA_22932 | 208 | 0.746737834 | IKZF2 | IKAROS family zinc finger 2 [Source:HGNC Symbol;Acc:HGNC:13177] |
| ENSG00000058453 | HGLibA_11209 | 208 | 0.746737834 | CROCC | rootletin [Source:HGNC Symbol;Acc:HGNC:21299]" |
| ENSG00000064601 | HGLibA_11688 | 208 | 0.746737834 | CTSA | cathepsin A [Source:HGNC Symbol;Acc:HGNC:9251] |
| ENSG00000065809 | HGLibA_16002 | 208 | 0.746737834 | FAM107B | family with sequence similarity 107 member B [Source:HGNC Symbol;Acc:HGNC:23726] |
| ENSG00000075142 | HGLibA_46989 | 208 | 0.746737834 | SRI | sorcin [Source:HGNC Symbol;Acc:HGNC:11292] |
| ENSG00000083857 | HGLibA_16913 | 208 | 0.746737834 | FAT1 | FAT atypical cadherin 1 [Source:HGNC Symbol;Acc:HGNC:3595] |
| ENSG00000084710 | HGLibA_14610 | 208 | 0.746737834 | EFR3B | EFR3 homolog B [Source:HGNC Symbol;Acc:HGNC:29155] |
| ENSG00000100425 | HGLibA_04771 | 208 | 0.746737834 | BRD1 | bromodomain containing 1 [Source:HGNC Symbol;Acc:HGNC:1102] |
| ENSG00000104044 | HGLibA_33239 | 208 | 0.746737834 | OCA2 | OCA2 melanosomal transmembrane protein [Source:HGNC Symbol;Acc:HGNC:8101] |
| ENSG00000104365 | HGLibA_22921 | 208 | 0.746737834 | IKBKB | inhibitor of nuclear factor kappa B kinase subunit beta [Source:HGNC Symbol;Acc:HGNC:5960] |
| ENSG00000104808 | HGLibA_13099 | 208 | 0.746737834 | DHDH | dihydrodiol dehydrogenase [Source:HGNC Symbol;Acc:HGNC:17887] |
| ENSG00000108468 | HGLibA_07444 | 208 | 0.746737834 | CBX1 | chromobox 1 [Source:HGNC Symbol;Acc:HGNC:1551] |
| ENSG00000110328 | HGLibA_18550 | 208 | 0.746737834 | GALNT18 | polypeptide N-acetylgalactosaminyltransferase 18 [Source:HGNC Symbol;Acc:HGNC:30488] |
| ENSG00000111843 | HGLibA_49986 | 208 | 0.746737834 | TMEM14C | transmembrane protein 14C [Source:HGNC Symbol;Acc:HGNC:20952] |
| ENSG00000114850 | HGLibA_47158 | 208 | 0.746737834 | SSR3 | signal sequence receptor subunit 3 [Source:HGNC Symbol;Acc:HGNC:11325] |
| ENSG00000117984 | HGLibA_11698 | 208 | 0.746737834 | CTSD | cathepsin D [Source:HGNC Symbol;Acc:HGNC:2529] |
| ENSG00000119640 | HGLibA_00710 | 208 | 0.746737834 | ACYP1 | acylphosphatase 1 [Source:HGNC Symbol;Acc:HGNC:179] |
| ENSG00000119844 | HGLibA_01131 | 208 | 0.746737834 | AFTPH | aftiphilin [Source:HGNC Symbol;Acc:HGNC:25951] |
| ENSG00000124164 | HGLibA_53521 | 208 | 0.746737834 | VAPB | VAMP associated protein B and C [Source:HGNC Symbol;Acc:HGNC:12649] |
| ENSG00000124688 | HGLibA_27849 | 208 | 0.746737834 | MAD2L1BP | MAD2L1 binding protein [Source:HGNC Symbol;Acc:HGNC:21059] |
| ENSG00000125611 | HGLibA_09375 | 208 | 0.746737834 | CHCHD5 | coiled-coil-helix-coiled-coil-helix domain containing 5 [Source:HGNC Symbol;Acc:HGNC:17840] |
| ENSG00000125903 | HGLibA_12891 | 208 | 0.746737834 | DEFB129 | defensin beta 129 [Source:HGNC Symbol;Acc:HGNC:16218] |
| ENSG00000126583 | HGLibA_38547 | 208 | 0.746737834 | PRKCG | protein kinase C gamma [Source:HGNC Symbol;Acc:HGNC:9402] |
| ENSG00000130224 | HGLibA_27139 | 208 | 0.746737834 | LRCH2 | leucine rich repeats and calponin homology domain containing 2 [Source:HGNC Symbol;Acc:HGNC:29292] |
| ENSG00000130653 | HGLibA_37423 | 208 | 0.746737834 | PNPLA7 | patatin like phospholipase domain containing 7 [Source:HGNC Symbol;Acc:HGNC:24768] |
| ENSG00000132356 | HGLibA_38490 | 208 | 0.746737834 | PRKAA1 | protein kinase AMP-activated catalytic subunit alpha 1 [Source:HGNC Symbol;Acc:HGNC:9376] |
| ENSG00000134365 | HGLibA_09272 | 208 | 0.746737834 | CFHR4 | complement factor H related 4 [Source:HGNC Symbol;Acc:HGNC:16979] |
| ENSG00000139631 | HGLibA_11302 | 208 | 0.746737834 | CSAD | cysteine sulfinic acid decarboxylase [Source:HGNC Symbol;Acc:HGNC:18966] |
| ENSG00000140506 | HGLibA_26661 | 208 | 0.746737834 | LMAN1L | mannose binding 1 like [Source:HGNC Symbol;Acc:HGNC:6632]" |
| ENSG00000140961 | HGLibA_34686 | 208 | 0.746737834 | OSGIN1 | oxidative stress induced growth inhibitor 1 [Source:HGNC Symbol;Acc:HGNC:30093] |
| ENSG00000143337 | HGLibA_51018 | 208 | 0.746737834 | TOR1AIP1 | torsin 1A interacting protein 1 [Source:HGNC Symbol;Acc:HGNC:29456] |
| ENSG00000144036 | HGLibA_15758 | 208 | 0.746737834 | EXOC6B | exocyst complex component 6B [Source:HGNC Symbol;Acc:HGNC:17085] |
| ENSG00000148660 | HGLibA_07077 | 208 | 0.746737834 | CAMK2G | calcium/calmodulin dependent protein kinase II gamma [Source:HGNC Symbol;Acc:HGNC:1463] |
| ENSG00000149177 | HGLibA_39511 | 208 | 0.746737834 | PTPRJ | receptor type J [Source:HGNC Symbol;Acc:HGNC:9673]" |
| ENSG00000154975 | HGLibA_06789 | 208 | 0.746737834 | CA10 | carbonic anhydrase 10 [Source:HGNC Symbol;Acc:HGNC:1369] |
| ENSG00000155329 | HGLibA_55004 | 208 | 0.746737834 | ZCCHC10 | zinc finger CCHC-type containing 10 [Source:HGNC Symbol;Acc:HGNC:25954] |
| ENSG00000155495 | HGLibA_27958 | 208 | 0.746737834 | MAGEC1 | MAGE family member C1 [Source:HGNC Symbol;Acc:HGNC:6812] |
| ENSG00000158485 | HGLibA_08266 | 208 | 0.746737834 | CD1B | CD1b molecule [Source:HGNC Symbol;Acc:HGNC:1635] |
| ENSG00000160712 | HGLibA_23183 | 208 | 0.746737834 | IL6R | interleukin 6 receptor [Source:HGNC Symbol;Acc:HGNC:6019] |
| ENSG00000162739 | HGLibA_44314 | 208 | 0.746737834 | SLAMF6 | SLAM family member 6 [Source:HGNC Symbol;Acc:HGNC:21392] |
| ENSG00000164885 | HGLibA_08773 | 208 | 0.746737834 | CDK5 | cyclin dependent kinase 5 [Source:HGNC Symbol;Acc:HGNC:1774] |
| ENSG00000166509 | HGLibA_09974 | 208 | 0.746737834 | CLEC3A | C-type lectin domain family 3 member A [Source:HGNC Symbol;Acc:HGNC:2052] |
| ENSG00000167604 | HGLibA_31828 | 208 | 0.746737834 | NFKBID | NFKB inhibitor delta [Source:HGNC Symbol;Acc:HGNC:15671] |
| ENSG00000167965 | HGLibA_29435 | 208 | 0.746737834 | MLST8 | LST8 homolog [Source:HGNC Symbol;Acc:HGNC:24825]" |
| ENSG00000169154 | HGLibA_19693 | 208 | 0.746737834 | GOT1L1 | glutamic-oxaloacetic transaminase 1 like 1 [Source:HGNC Symbol;Acc:HGNC:28487] |
| ENSG00000169214 | HGLibA_34414 | 208 | 0.746737834 | OR6F1 | olfactory receptor family 6 subfamily F member 1 [Source:HGNC Symbol;Acc:HGNC:15027] |
| ENSG00000169239 | HGLibA_06815 | 208 | 0.746737834 | CA5B | carbonic anhydrase 5B [Source:HGNC Symbol;Acc:HGNC:1378] |
| ENSG00000173611 | HGLibA_42834 | 208 | 0.746737834 | SCAI | suppressor of cancer cell invasion [Source:HGNC Symbol;Acc:HGNC:26709] |
| ENSG00000175874 | HGLibA_11122 | 208 | 0.746737834 | CREG2 | cellular repressor of E1A stimulated genes 2 [Source:HGNC Symbol;Acc:HGNC:14272] |
| ENSG00000176798 | HGLibA_34121 | 208 | 0.746737834 | OR51L1 | olfactory receptor family 51 subfamily L member 1 [Source:HGNC Symbol;Acc:HGNC:14759] |
| ENSG00000178531 | HGLibA_11754 | 208 | 0.746737834 | CTXN1 | cortexin 1 [Source:HGNC Symbol;Acc:HGNC:31108] |
| ENSG00000180992 | HGLibA_29902 | 208 | 0.746737834 | MRPL14 | mitochondrial ribosomal protein L14 [Source:HGNC Symbol;Acc:HGNC:14279] |
| ENSG00000182348 | HGLibA_56790 | 208 | 0.746737834 | ZNF804B | zinc finger protein 804B [Source:HGNC Symbol;Acc:HGNC:21958] |
| ENSG00000182858 | HGLibA_01613 | 208 | 0.746737834 | ALG12 | 6-mannosyltransferase [Source:HGNC Symbol;Acc:HGNC:19358]" |
| ENSG00000182952 | HGLibA_21725 | 208 | 0.746737834 | HMGN4 | high mobility group nucleosomal binding domain 4 [Source:HGNC Symbol;Acc:HGNC:4989] |
| ENSG00000184361 | HGLibA_46507 | 208 | 0.746737834 | SPATA32 | spermatogenesis associated 32 [Source:HGNC Symbol;Acc:HGNC:26349] |
| ENSG00000184497 | HGLibA_50334 | 208 | 0.746737834 | TMEM255B | transmembrane protein 255B [Source:HGNC Symbol;Acc:HGNC:28297] |
| ENSG00000184613 | HGLibA_31686 | 208 | 0.746737834 | NELL2 | neural EGFL like 2 [Source:HGNC Symbol;Acc:HGNC:7751] |
| ENSG00000185432 | HGLibA_29018 | 208 | 0.746737834 | METTL7A | methyltransferase like 7A [Source:HGNC Symbol;Acc:HGNC:24550] |
| ENSG00000186814 | HGLibA_57016 | 208 | 0.746737834 | ZSCAN30 | zinc finger and SCAN domain containing 30 [Source:HGNC Symbol;Acc:HGNC:33517] |
| ENSG00000196196 | HGLibA_22088 | 208 | 0.746737834 | HRCT1 | histidine rich carboxyl terminus 1 [Source:HGNC Symbol;Acc:HGNC:33872] |
| ENSG00000196228 | HGLibA_47748 | 208 | 0.746737834 | SULT1C3 | sulfotransferase family 1C member 3 [Source:HGNC Symbol;Acc:HGNC:33543] |
| ENSG00000197818 | HGLibA_45426 | 208 | 0.746737834 | SLC9A8 | solute carrier family 9 member A8 [Source:HGNC Symbol;Acc:HGNC:20728] |
| ENSG00000213401 | HGLibA_27902 | 208 | 0.746737834 | MAGEA12 | MAGE family member A12 [Source:HGNC Symbol;Acc:HGNC:6799] |
| ENSG00000233608 | HGLibA_52378 | 208 | 0.746737834 | TWIST2 | twist family bHLH transcription factor 2 [Source:HGNC Symbol;Acc:HGNC:20670] |
| ENSG00000236398 | HGLibA_48397 | 208 | 0.746737834 | TAS2R39 | taste 2 receptor member 39 [Source:HGNC Symbol;Acc:HGNC:18886] |
| ENSG00000240065 | HGLibA_39124 | 208 | 0.746737834 | PSMB9 | proteasome subunit beta 9 [Source:HGNC Symbol;Acc:HGNC:9546] |
| ENSG00000242419 | HGLibA_35575 | 208 | 0.746737834 | PCDHGC4 | 4 [Source:HGNC Symbol;Acc:HGNC:8717]" |
| ENSG00000254656 | HGLibA_42396 | 208 | 0.746737834 | RTL1 | retrotransposon Gag like 1 [Source:HGNC Symbol;Acc:HGNC:14665] |
| ENSG00000256463 | HGLibA_42653 | 208 | 0.746737834 | SALL3 | spalt like transcription factor 3 [Source:HGNC Symbol;Acc:HGNC:10527] |
| ENSG00000258227 | HGLibA_10000 | 208 | 0.746737834 | CLEC5A | C-type lectin domain containing 5A [Source:HGNC Symbol;Acc:HGNC:2054] |
| ENSG00000258947 | HGLibA_52281 | 208 | 0.746737834 | TUBB3 | tubulin beta 3 class III [Source:HGNC Symbol;Acc:HGNC:20772] |
| ENSG00000273274 | HGLibA_54929 | 208 | 0.746737834 | ZBTB8B | zinc finger and BTB domain containing 8B [Source:HGNC Symbol;Acc:HGNC:37057] |
| hsa-mir-1252 | HGLibA_57406 | 208 | 0.746737834 | hsa-mir-1252 | - |
| hsa-mir-28 | HGLibA_58385 | 208 | 0.746737834 | hsa-mir-28 | - |
| hsa-mir-376b | HGLibA_59412 | 208 | 0.746737834 | hsa-mir-376b | - |
| hsa-mir-4267 | HGLibA_59776 | 208 | 0.746737834 | hsa-mir-4267 | - |
| hsa-mir-4282 | HGLibA_59833 | 208 | 0.746737834 | hsa-mir-4282 | - |
| hsa-mir-4428 | HGLibA_60098 | 208 | 0.746737834 | hsa-mir-4428 | - |
| hsa-mir-4531 | HGLibA_60582 | 208 | 0.746737834 | hsa-mir-4531 | - |
| hsa-mir-5700 | HGLibA_62338 | 208 | 0.746737834 | hsa-mir-5700 | - |
| hsa-mir-7849 | HGLibA_63985 | 208 | 0.746737834 | hsa-mir-7849 | - |
| LOC649330 | HGLibA_26980 | 208 | 0.746737834 | LOC649330 | - |
| TBC1D29 | HGLibA_48524 | 208 | 0.746737834 | TBC1D29 | - |
| TMEM110-MUSTN1 | HGLibA_49876 | 208 | 0.746737834 | TMEM110-MUSTN1 | - |
| C5orf54 | HGLibA_06406 | 207 | 0.737692491 | C5orf54 | - |
| ENSG00000011485 | HGLibA_38212 | 207 | 0.737692491 | PPP5C | protein phosphatase 5 catalytic subunit [Source:HGNC Symbol;Acc:HGNC:9322] |
| ENSG00000018869 | HGLibA_56312 | 207 | 0.737692491 | ZNF582 | zinc finger protein 582 [Source:HGNC Symbol;Acc:HGNC:26421] |
| ENSG00000034533 | HGLibA_03374 | 207 | 0.737692491 | ASTE1 | asteroid homolog 1 [Source:HGNC Symbol;Acc:HGNC:25021] |
| ENSG00000078399 | HGLibA_21923 | 207 | 0.737692491 | HOXA9 | homeobox A9 [Source:HGNC Symbol;Acc:HGNC:5109] |
| ENSG00000092068 | HGLibA_45383 | 207 | 0.737692491 | SLC7A8 | solute carrier family 7 member 8 [Source:HGNC Symbol;Acc:HGNC:11066] |
| ENSG00000096088 | HGLibA_36228 | 207 | 0.737692491 | PGC | progastricsin [Source:HGNC Symbol;Acc:HGNC:8890] |
| ENSG00000100055 | HGLibA_12245 | 207 | 0.737692491 | CYTH4 | cytohesin 4 [Source:HGNC Symbol;Acc:HGNC:9505] |
| ENSG00000100206 | HGLibA_13400 | 207 | 0.737692491 | DMC1 | DNA meiotic recombinase 1 [Source:HGNC Symbol;Acc:HGNC:2927] |
| ENSG00000101076 | HGLibA_21781 | 207 | 0.737692491 | HNF4A | hepatocyte nuclear factor 4 alpha [Source:HGNC Symbol;Acc:HGNC:5024] |
| ENSG00000103485 | HGLibA_39732 | 207 | 0.737692491 | QPRT | quinolinate phosphoribosyltransferase [Source:HGNC Symbol;Acc:HGNC:9755] |
| ENSG00000104408 | HGLibA_14777 | 207 | 0.737692491 | EIF3E | eukaryotic translation initiation factor 3 subunit E [Source:HGNC Symbol;Acc:HGNC:3277] |
| ENSG00000105270 | HGLibA_10047 | 207 | 0.737692491 | CLIP3 | CAP-Gly domain containing linker protein 3 [Source:HGNC Symbol;Acc:HGNC:24314] |
| ENSG00000108091 | HGLibA_07810 | 207 | 0.737692491 | CCDC6 | coiled-coil domain containing 6 [Source:HGNC Symbol;Acc:HGNC:18782] |
| ENSG00000112561 | HGLibA_49162 | 207 | 0.737692491 | TFEB | transcription factor EB [Source:HGNC Symbol;Acc:HGNC:11753] |
| ENSG00000114742 | HGLibA_54108 | 207 | 0.737692491 | WDR48 | WD repeat domain 48 [Source:HGNC Symbol;Acc:HGNC:30914] |
| ENSG00000117450 | HGLibA_38408 | 207 | 0.737692491 | PRDX1 | peroxiredoxin 1 [Source:HGNC Symbol;Acc:HGNC:9352] |
| ENSG00000117984 | HGLibA_11699 | 207 | 0.737692491 | CTSD | cathepsin D [Source:HGNC Symbol;Acc:HGNC:2529] |
| ENSG00000120306 | HGLibA_12232 | 207 | 0.737692491 | CYSTM1 | cysteine rich transmembrane module containing 1 [Source:HGNC Symbol;Acc:HGNC:30239] |
| ENSG00000124557 | HGLibA_04960 | 207 | 0.737692491 | BTN1A1 | butyrophilin subfamily 1 member A1 [Source:HGNC Symbol;Acc:HGNC:1135] |
| ENSG00000127946 | HGLibA_21334 | 207 | 0.737692491 | HIP1 | huntingtin interacting protein 1 [Source:HGNC Symbol;Acc:HGNC:4913] |
| ENSG00000128045 | HGLibA_40369 | 207 | 0.737692491 | RASL11B | RAS like family 11 member B [Source:HGNC Symbol;Acc:HGNC:23804] |
| ENSG00000130433 | HGLibA_06944 | 207 | 0.737692491 | CACNG6 | calcium voltage-gated channel auxiliary subunit gamma 6 [Source:HGNC Symbol;Acc:HGNC:13625] |
| ENSG00000130684 | HGLibA_55828 | 207 | 0.737692491 | ZNF337 | zinc finger protein 337 [Source:HGNC Symbol;Acc:HGNC:15809] |
| ENSG00000133216 | HGLibA_15314 | 207 | 0.737692491 | EPHB2 | EPH receptor B2 [Source:HGNC Symbol;Acc:HGNC:3393] |
| ENSG00000137501 | HGLibA_48099 | 207 | 0.737692491 | SYTL2 | synaptotagmin like 2 [Source:HGNC Symbol;Acc:HGNC:15585] |
| ENSG00000139304 | HGLibA_39530 | 207 | 0.737692491 | PTPRQ | receptor type Q [Source:HGNC Symbol;Acc:HGNC:9679]" |
| ENSG00000143412 | HGLibA_02260 | 207 | 0.737692491 | ANXA9 | annexin A9 [Source:HGNC Symbol;Acc:HGNC:547] |
| ENSG00000143951 | HGLibA_54020 | 207 | 0.737692491 | WDPCP | WD repeat containing planar cell polarity effector [Source:HGNC Symbol;Acc:HGNC:28027] |
| ENSG00000145293 | HGLibA_15153 | 207 | 0.737692491 | ENOPH1 | enolase-phosphatase 1 [Source:HGNC Symbol;Acc:HGNC:24599] |
| ENSG00000145491 | HGLibA_41766 | 207 | 0.737692491 | ROPN1L | rhophilin associated tail protein 1 like [Source:HGNC Symbol;Acc:HGNC:24060] |
| ENSG00000148482 | HGLibA_45098 | 207 | 0.737692491 | SLC39A12 | solute carrier family 39 member 12 [Source:HGNC Symbol;Acc:HGNC:20860] |

| | | | | | |
|---|---|---|---|---|---|
| ENSG00000152705 | HGLibA_07361 | 207 | 0.737692491 | CATSPER3 | cation channel sperm associated 3 [Source:HGNC Symbol;Acc:HGNC:20819] |
| ENSG00000152749 | HGLibA_19984 | 207 | 0.737692491 | GPR180 | G protein-coupled receptor 180 [Source:HGNC Symbol;Acc:HGNC:28899] |
| ENSG00000154114 | HGLibA_48597 | 207 | 0.737692491 | TBCEL | tubulin folding cofactor E like [Source:HGNC Symbol;Acc:HGNC:28115] |
| ENSG00000154611 | HGLibA_39090 | 207 | 0.737692491 | PSMA8 | proteasome subunit alpha 8 [Source:HGNC Symbol;Acc:HGNC:22985] |
| ENSG00000155085 | HGLibA_01399 | 207 | 0.737692491 | AK9 | adenylate kinase 9 [Source:HGNC Symbol;Acc:HGNC:33814] |
| ENSG00000156009 | HGLibA_27919 | 207 | 0.737692491 | MAGEA8 | MAGE family member A8 [Source:HGNC Symbol;Acc:HGNC:6806] |
| ENSG00000156384 | HGLibA_43732 | 207 | 0.737692491 | SFR1 | SWI5 dependent homologous recombination repair protein 1 [Source:HGNC Symbol;Acc:HGNC:29574] |
| ENSG00000159214 | HGLibA_07736 | 207 | 0.737692491 | CCDC24 | coiled-coil domain containing 24 [Source:HGNC Symbol;Acc:HGNC:28688] |
| ENSG00000161179 | HGLibA_54652 | 207 | 0.737692491 | YDJC | YdjC chitooligosaccharide deacetylase homolog [Source:HGNC Symbol;Acc:HGNC:27158] |
| ENSG00000161277 | HGLibA_49313 | 207 | 0.737692491 | THAP8 | THAP domain containing 8 [Source:HGNC Symbol;Acc:HGNC:23191] |
| ENSG00000164303 | HGLibA_15183 | 207 | 0.737692491 | ENPP6 | ectonucleotide pyrophosphatase/phosphodiesterase 6 [Source:HGNC Symbol;Acc:HGNC:23409] |
| ENSG00000165282 | HGLibA_36573 | 207 | 0.737692491 | PIGO | phosphatidylinositol glycan anchor biosynthesis class O [Source:HGNC Symbol;Acc:HGNC:23215] |
| ENSG00000166224 | HGLibA_43848 | 207 | 0.737692491 | SGPL1 | sphingosine-1-phosphate lyase 1 [Source:HGNC Symbol;Acc:HGNC:10817] |
| ENSG00000166788 | HGLibA_42621 | 207 | 0.737692491 | SAAL1 | serum amyloid A like 1 [Source:HGNC Symbol;Acc:HGNC:25158] |
| ENSG00000167528 | HGLibA_56442 | 207 | 0.737692491 | ZNF641 | zinc finger protein 641 [Source:HGNC Symbol;Acc:HGNC:31834] |
| ENSG00000168404 | HGLibA_29389 | 207 | 0.737692491 | MLKL | mixed lineage kinase domain like pseudokinase [Source:HGNC Symbol;Acc:HGNC:26617] |
| ENSG00000170677 | HGLibA_46138 | 207 | 0.737692491 | SOCS6 | suppressor of cytokine signaling 6 [Source:HGNC Symbol;Acc:HGNC:16833] |
| ENSG00000171817 | HGLibA_56189 | 207 | 0.737692491 | ZNF540 | zinc finger protein 540 [Source:HGNC Symbol;Acc:HGNC:25331] |
| ENSG00000172288 | HGLibA_08906 | 207 | 0.737692491 | CDY1 | chromodomain Y-linked 1 [Source:HGNC Symbol;Acc:HGNC:1809] |
| ENSG00000172493 | HGLibA_01107 | 207 | 0.737692491 | AFF1 | AF4/FMR2 family member 1 [Source:HGNC Symbol;Acc:HGNC:7135] |
| ENSG00000173757 | HGLibA_47396 | 207 | 0.737692491 | STAT5B | signal transducer and activator of transcription 5B [Source:HGNC Symbol;Acc:HGNC:11367] |
| ENSG00000174473 | HGLibA_18580 | 207 | 0.737692491 | GALNTL6 | polypeptide N-acetylgalactosaminyltransferase like 6 [Source:HGNC Symbol;Acc:HGNC:33844] |
| ENSG00000181085 | HGLibA_28265 | 207 | 0.737692491 | MAPK15 | mitogen-activated protein kinase 15 [Source:HGNC Symbol;Acc:HGNC:24667] |
| ENSG00000184887 | HGLibA_04918 | 207 | 0.737692491 | BTBD6 | BTB domain containing 6 [Source:HGNC Symbol;Acc:HGNC:19897] |
| ENSG00000185361 | HGLibA_50721 | 207 | 0.737692491 | TNFAIP8L1 | TNF alpha induced protein 8 like 1 [Source:HGNC Symbol;Acc:HGNC:28279] |
| ENSG00000186318 | HGLibA_04060 | 207 | 0.737692491 | BACE1 | beta-secretase 1 [Source:HGNC Symbol;Acc:HGNC:933] |
| ENSG00000186334 | HGLibA_45036 | 207 | 0.737692491 | SLC36A3 | solute carrier family 36 member 3 [Source:HGNC Symbol;Acc:HGNC:19659] |
| ENSG00000186788 | HGLibA_46498 | 207 | 0.737692491 | SPATA31D3 | SPATA31 subfamily D member 3 [Source:HGNC Symbol;Acc:HGNC:38603] |
| ENSG00000186918 | HGLibA_55911 | 207 | 0.737692491 | ZNF395 | zinc finger protein 395 [Source:HGNC Symbol;Acc:HGNC:18737] |
| ENSG00000186977 | HGLibA_25676 | 207 | 0.737692491 | KRTAP19-5 | keratin associated protein 19-5 [Source:HGNC Symbol;Acc:HGNC:18940] |
| ENSG00000187556 | HGLibA_31101 | 207 | 0.737692491 | NANOS3 | nanos C2HC-type zinc finger 3 [Source:HGNC Symbol;Acc:HGNC:22048] |
| ENSG00000187783 | HGLibA_50506 | 207 | 0.737692491 | TMEM72 | transmembrane protein 72 [Source:HGNC Symbol;Acc:HGNC:31658] |
| ENSG00000188372 | HGLibA_56955 | 207 | 0.737692491 | ZP3 | zona pellucida glycoprotein 3 [Source:HGNC Symbol;Acc:HGNC:13189] |
| ENSG00000188707 | HGLibA_54805 | 207 | 0.737692491 | ZBED6CL | ZBED6 C-terminal like [Source:HGNC Symbol;Acc:HGNC:21720] |
| ENSG00000196123 | HGLibA_24660 | 207 | 0.737692491 | KIAA0895L | KIAA0895 like [Source:HGNC Symbol;Acc:HGNC:34408] |
| ENSG00000204256 | HGLibA_04774 | 207 | 0.737692491 | BRD2 | bromodomain containing 2 [Source:HGNC Symbol;Acc:HGNC:1103] |
| ENSG00000204291 | HGLibA_10516 | 207 | 0.737692491 | COL15A1 | collagen type XV alpha 1 chain [Source:HGNC Symbol;Acc:HGNC:2192] |
| ENSG00000206535 | HGLibA_26737 | 207 | 0.737692491 | LNP1 | leukemia NUP98 fusion partner 1 [Source:HGNC Symbol;Acc:HGNC:28014] |
| ENSG00000211584 | HGLibA_45199 | 207 | 0.737692491 | SLC48A1 | solute carrier family 48 member 1 [Source:HGNC Symbol;Acc:HGNC:26035] |
| ENSG00000213047 | HGLibA_12932 | 207 | 0.737692491 | DENND1B | DENN domain containing 1B [Source:HGNC Symbol;Acc:HGNC:28404] |
| ENSG00000213246 | HGLibA_47817 | 207 | 0.737692491 | SUPT4H1 | DSIF elongation factor subunit [Source:HGNC Symbol;Acc:HGNC:11467]" |
| ENSG00000214736 | HGLibA_50975 | 207 | 0.737692491 | TOMM6 | translocase of outer mitochondrial membrane 6 [Source:HGNC Symbol;Acc:HGNC:34528] |
| ENSG00000221837 | HGLibA_25623 | 207 | 0.737692491 | KRTAP10-9 | keratin associated protein 10-9 [Source:HGNC Symbol;Acc:HGNC:22971] |
| ENSG00000221933 | HGLibA_33732 | 207 | 0.737692491 | OR2A25 | olfactory receptor family 2 subfamily A member 25 [Source:HGNC Symbol;Acc:HGNC:19562] |
| ENSG00000239732 | HGLibA_49667 | 207 | 0.737692491 | TLR9 | toll like receptor 9 [Source:HGNC Symbol;Acc:HGNC:15633] |
| ENSG00000254505 | HGLibA_09486 | 207 | 0.737692491 | CHMP4A | charged multivesicular body protein 4A [Source:HGNC Symbol;Acc:HGNC:20274] |
| ENSG00000258429 | HGLibA_35839 | 207 | 0.737692491 | PDF | mitochondrial [Source:HGNC Symbol;Acc:HGNC:30012]" |
| ENSG00000271503 | HGLibA_08037 | 207 | 0.737692491 | CCL5 | C-C motif chemokine ligand 5 [Source:HGNC Symbol;Acc:HGNC:10632] |
| GAREM | HGLibA_18625 | 207 | 0.737692491 | GAREM | - |
| hsa-mir-1469 | HGLibA_57851 | 207 | 0.737692491 | hsa-mir-1469 | - |
| hsa-mir-198 | HGLibA_58124 | 207 | 0.737692491 | hsa-mir-198 | - |
| hsa-mir-4523 | HGLibA_60550 | 207 | 0.737692491 | hsa-mir-4523 | - |
| hsa-mir-587 | HGLibA_62448 | 207 | 0.737692491 | hsa-mir-587 | - |
| hsa-mir-593 | HGLibA_62468 | 207 | 0.737692491 | hsa-mir-593 | - |
| hsa-mir-6840 | HGLibA_63536 | 207 | 0.737692491 | hsa-mir-6840 | - |
| hsa-mir-7112-1 | HGLibA_63812 | 207 | 0.737692491 | hsa-mir-7112-1 | - |
| hsa-mir-7856 | HGLibA_64014 | 207 | 0.737692491 | hsa-mir-7856 | - |
| hsa-mir-8059 | HGLibA_64074 | 207 | 0.737692491 | hsa-mir-8059 | - |
| KIAA0195 | HGLibA_24600 | 207 | 0.737692491 | KIAA0195 | - |
| TMEM110-MUSTN1 | HGLibA_49877 | 207 | 0.737692491 | TMEM110-MUSTN1 | - |
| VARS | HGLibA_53525 | 207 | 0.737692491 | VARS | - |
| AMICA1 | HGLibA_01790 | 206 | 0.728647148 | AMICA1 | - |
| DAK | HGLibA_12301 | 206 | 0.728647148 | DAK | - |
| ENSG00000003096 | HGLibA_25133 | 206 | 0.728647148 | KLHL13 | kelch like family member 13 [Source:HGNC Symbol;Acc:HGNC:22931] |
| ENSG00000007038 | HGLibA_38911 | 206 | 0.728647148 | PRSS21 | serine protease 21 [Source:HGNC Symbol;Acc:HGNC:9485] |
| ENSG00000012963 | HGLibA_52763 | 206 | 0.728647148 | UBR7 | ubiquitin protein ligase E3 component n-recognin 7 (putative) [Source:HGNC Symbol;Acc:HGNC:20344] |
| ENSG00000015171 | HGLibA_55414 | 206 | 0.728647148 | ZMYND11 | zinc finger MYND-type containing 11 [Source:HGNC Symbol;Acc:HGNC:16966] |
| ENSG00000044459 | HGLibA_10397 | 206 | 0.728647148 | CNTLN | centlein [Source:HGNC Symbol;Acc:HGNC:23432] |
| ENSG00000072135 | HGLibA_39444 | 206 | 0.728647148 | PTPN18 | non-receptor type 18 [Source:HGNC Symbol;Acc:HGNC:9649]" |
| ENSG00000081818 | HGLibA_35500 | 206 | 0.728647148 | PCDHB4 | protocadherin beta 4 [Source:HGNC Symbol;Acc:HGNC:8689] |
| ENSG00000105176 | HGLibA_53146 | 206 | 0.728647148 | URI1 | prefoldin like chaperone [Source:HGNC Symbol;Acc:HGNC:13236]" |
| ENSG00000105426 | HGLibA_39536 | 206 | 0.728647148 | PTPRS | receptor type S [Source:HGNC Symbol;Acc:HGNC:9681]" |
| ENSG00000106031 | HGLibA_21902 | 206 | 0.728647148 | HOXA13 | homeobox A13 [Source:HGNC Symbol;Acc:HGNC:5102] |
| ENSG00000107562 | HGLibA_11852 | 206 | 0.728647148 | CXCL12 | C-X-C motif chemokine ligand 12 [Source:HGNC Symbol;Acc:HGNC:10672] |
| ENSG00000113532 | HGLibA_47297 | 206 | 0.728647148 | ST8SIA4 | 8-sialyltransferase 4 [Source:HGNC Symbol;Acc:HGNC:10871]" |
| ENSG00000115159 | HGLibA_19788 | 206 | 0.728647148 | GPD2 | glycerol-3-phosphate dehydrogenase 2 [Source:HGNC Symbol;Acc:HGNC:4456] |
| ENSG00000116039 | HGLibA_03733 | 206 | 0.728647148 | ATP6V1B1 | ATPase H+ transporting V1 subunit B1 [Source:HGNC Symbol;Acc:HGNC:853] |
| ENSG00000117643 | HGLibA_28067 | 206 | 0.728647148 | MAN1C1 | mannosidase alpha class 1C member 1 [Source:HGNC Symbol;Acc:HGNC:19080] |
| ENSG00000118369 | HGLibA_53343 | 206 | 0.728647148 | USP35 | ubiquitin specific peptidase 35 [Source:HGNC Symbol;Acc:HGNC:20061] |
| ENSG00000119514 | HGLibA_18537 | 206 | 0.728647148 | GALNT12 | polypeptide N-acetylgalactosaminyltransferase 12 [Source:HGNC Symbol;Acc:HGNC:19877] |
| ENSG00000122592 | HGLibA_21919 | 206 | 0.728647148 | HOXA7 | homeobox A7 [Source:HGNC Symbol;Acc:HGNC:5108] |
| ENSG00000125901 | HGLibA_30077 | 206 | 0.728647148 | MRPS26 | mitochondrial ribosomal protein S26 [Source:HGNC Symbol;Acc:HGNC:14045] |
| ENSG00000131016 | HGLibA_01413 | 206 | 0.728647148 | AKAP12 | A-kinase anchoring protein 12 [Source:HGNC Symbol;Acc:HGNC:370] |
| ENSG00000133454 | HGLibA_30815 | 206 | 0.728647148 | MYO18B | myosin XVIIIB [Source:HGNC Symbol;Acc:HGNC:18150] |
| ENSG00000136147 | HGLibA_36336 | 206 | 0.728647148 | PHF11 | PHD finger protein 11 [Source:HGNC Symbol;Acc:HGNC:17024] |
| ENSG00000136451 | HGLibA_53625 | 206 | 0.728647148 | VEZF1 | vascular endothelial zinc finger 1 [Source:HGNC Symbol;Acc:HGNC:12949] |
| ENSG00000137449 | HGLibA_10921 | 206 | 0.728647148 | CPEB2 | cytoplasmic polyadenylation element binding protein 2 [Source:HGNC Symbol;Acc:HGNC:21745] |
| ENSG00000137942 | HGLibA_17738 | 206 | 0.728647148 | FNBP1L | formin binding protein 1 like [Source:HGNC Symbol;Acc:HGNC:20851] |
| ENSG00000139194 | HGLibA_40642 | 206 | 0.728647148 | RBP5 | retinol binding protein 5 [Source:HGNC Symbol;Acc:HGNC:15847] |
| ENSG00000142039 | HGLibA_07941 | 206 | 0.728647148 | CCDC97 | coiled-coil domain containing 97 [Source:HGNC Symbol;Acc:HGNC:28289] |
| ENSG00000142599 | HGLibA_40856 | 206 | 0.728647148 | RERE | arginine-glutamic acid dipeptide repeats [Source:HGNC Symbol;Acc:HGNC:9965] |
| ENSG00000149743 | HGLibA_51778 | 206 | 0.728647148 | TRPT1 | tRNA phosphotransferase 1 [Source:HGNC Symbol;Acc:HGNC:20316] |
| ENSG00000150394 | HGLibA_08692 | 206 | 0.728647148 | CDH8 | cadherin 8 [Source:HGNC Symbol;Acc:HGNC:1767] |
| ENSG00000151835 | HGLibA_42629 | 206 | 0.728647148 | SACS | sacsin molecular chaperone [Source:HGNC Symbol;Acc:HGNC:10519] |
| ENSG00000152926 | HGLibA_55446 | 206 | 0.728647148 | ZNF117 | zinc finger protein 117 [Source:HGNC Symbol;Acc:HGNC:12897] |
| ENSG00000153563 | HGLibA_08470 | 206 | 0.728647148 | CD8A | CD8a molecule [Source:HGNC Symbol;Acc:HGNC:1706] |
| ENSG00000157483 | HGLibA_30831 | 206 | 0.728647148 | MYO1E | myosin IE [Source:HGNC Symbol;Acc:HGNC:7599] |
| ENSG00000162892 | HGLibA_23104 | 206 | 0.728647148 | IL24 | interleukin 24 [Source:HGNC Symbol;Acc:HGNC:11346] |
| ENSG00000162971 | HGLibA_52488 | 206 | 0.728647148 | TYW5 | tRNA-yW synthesizing protein 5 [Source:HGNC Symbol;Acc:HGNC:26754] |
| ENSG00000163295 | HGLibA_01698 | 206 | 0.728647148 | ALPI | intestinal [Source:HGNC Symbol;Acc:HGNC:437]" |
| ENSG00000163631 | HGLibA_01529 | 206 | 0.728647148 | ALB | albumin [Source:HGNC Symbol;Acc:HGNC:399] |
| ENSG00000163874 | HGLibA_54953 | 206 | 0.728647148 | ZC3H12A | zinc finger CCCH-type containing 12A [Source:HGNC Symbol;Acc:HGNC:26259] |
| ENSG00000164167 | HGLibA_27548 | 206 | 0.728647148 | LSM6 | U6 small nuclear RNA and mRNA degradation associated [Source:HGNC Symbol;Acc:HGNC:17017]" |
| ENSG00000164241 | HGLibA_06418 | 206 | 0.728647148 | C5orf63 | chromosome 5 open reading frame 63 [Source:HGNC Symbol;Acc:HGNC:40051] |
| ENSG00000165972 | HGLibA_07768 | 206 | 0.728647148 | CCDC38 | coiled-coil domain containing 38 [Source:HGNC Symbol;Acc:HGNC:26843] |
| ENSG00000166840 | HGLibA_19374 | 206 | 0.728647148 | GLYATL1 | glycine-N-acyltransferase like 1 [Source:HGNC Symbol;Acc:HGNC:30519] |
| ENSG00000167800 | HGLibA_48644 | 206 | 0.728647148 | TBX10 | T-box 10 [Source:HGNC Symbol;Acc:HGNC:11593] |
| ENSG00000170776 | HGLibA_01414 | 206 | 0.728647148 | AKAP13 | A-kinase anchoring protein 13 [Source:HGNC Symbol;Acc:HGNC:371] |
| ENSG00000173273 | HGLibA_50849 | 206 | 0.728647148 | TNKS | tankyrase [Source:HGNC Symbol;Acc:HGNC:11941] |
| ENSG00000182083 | HGLibA_34376 | 206 | 0.728647148 | OR6B2 | olfactory receptor family 6 subfamily B member 2 [Source:HGNC Symbol;Acc:HGNC:15041] |
| ENSG00000182993 | HGLibA_05272 | 206 | 0.728647148 | C12orf60 | chromosome 12 open reading frame 60 [Source:HGNC Symbol;Acc:HGNC:28726] |
| ENSG00000184571 | HGLibA_36778 | 206 | 0.728647148 | PIWIL3 | piwi like RNA-mediated gene silencing 3 [Source:HGNC Symbol;Acc:HGNC:18443] |
| ENSG00000185515 | HGLibA_04768 | 206 | 0.728647148 | BRCC3 | BRCA1/BRCA2-containing complex subunit 3 [Source:HGNC Symbol;Acc:HGNC:24185] |
| ENSG00000185811 | HGLibA_22928 | 206 | 0.728647148 | IKZF1 | IKAROS family zinc finger 1 [Source:HGNC Symbol;Acc:HGNC:13176] |
| ENSG00000186517 | HGLibA_02753 | 206 | 0.728647148 | ARHGAP30 | Rho GTPase activating protein 30 [Source:HGNC Symbol;Acc:HGNC:27414] |
| ENSG00000188981 | HGLibA_30170 | 206 | 0.728647148 | MSANTD1 | Myb/SANT DNA binding domain containing 1 [Source:HGNC Symbol;Acc:HGNC:33741] |
| ENSG00000196646 | HGLibA_55473 | 206 | 0.728647148 | ZNF136 | zinc finger protein 136 [Source:HGNC Symbol;Acc:HGNC:12920] |
| ENSG00000198087 | HGLibA_08327 | 206 | 0.728647148 | CD2AP | CD2 associated protein [Source:HGNC Symbol;Acc:HGNC:14258] |
| ENSG00000198740 | HGLibA_56462 | 206 | 0.728647148 | ZNF652 | zinc finger protein 652 [Source:HGNC Symbol;Acc:HGNC:29147] |
| ENSG00000204671 | HGLibA_23131 | 206 | 0.728647148 | IL31 | interleukin 31 [Source:HGNC Symbol;Acc:HGNC:19372] |
| ENSG00000205352 | HGLibA_38776 | 206 | 0.728647148 | PRR13 | proline rich 13 [Source:HGNC Symbol;Acc:HGNC:24528] |
| ENSG00000212864 | HGLibA_41615 | 206 | 0.728647148 | RNF208 | ring finger protein 208 [Source:HGNC Symbol;Acc:HGNC:25420] |
| ENSG00000221852 | HGLibA_25589 | 206 | 0.728647148 | KRTAP1-5 | keratin associated protein 1-5 [Source:HGNC Symbol;Acc:HGNC:16777] |
| ENSG00000269404 | HGLibA_46654 | 206 | 0.728647148 | SPIB | Spi-B transcription factor [Source:HGNC Symbol;Acc:HGNC:11242] |
| ENSG00000270885 | HGLibA_40362 | 206 | 0.728647148 | RASL10B | RAS like family 10 member B [Source:HGNC Symbol;Acc:HGNC:30295] |
| ENSG00000280021 | HGLibA_34103 | 206 | 0.728647148 | OR51F1 | olfactory receptor family 51 subfamily F member 1 (gene/pseudogene) [Source:HGNC Symbol;Acc:HGNC:15196] |
| FAM21B | HGLibA_16506 | 206 | 0.728647148 | FAM21B | - |
| hsa-mir-128-1 | HGLibA_57571 | 206 | 0.728647148 | hsa-mir-128-1 | - |
| hsa-mir-133a-2 | HGLibA_57775 | 206 | 0.728647148 | hsa-mir-133a-2 | - |
| hsa-mir-145 | HGLibA_57845 | 206 | 0.728647148 | hsa-mir-145 | - |
| hsa-mir-17 | HGLibA_57955 | 206 | 0.728647148 | hsa-mir-17 | - |
| hsa-mir-4506 | HGLibA_60449 | 206 | 0.728647148 | hsa-mir-4506 | - |
| hsa-mir-4652 | HGLibA_60715 | 206 | 0.728647148 | hsa-mir-4652 | - |
| hsa-mir-4712 | HGLibA_60949 | 206 | 0.728647148 | hsa-mir-4712 | - |
| hsa-mir-4761 | HGLibA_61140 | 206 | 0.728647148 | hsa-mir-4761 | - |

| | | | | | |
|---|---|---|---|---|---|
| hsa-mir-5001 | HGLibA_61411 | 206 | 0.728647148 | hsa-mir-5001 | - |
| hsa-mir-6819 | HGLibA_63453 | 206 | 0.728647148 | hsa-mir-6819 | - |
| KIAA1468 | HGLibA_24768 | 206 | 0.728647148 | KIAA1468 | - |
| KIAA1715 | HGLibA_24808 | 206 | 0.728647148 | KIAA1715 | - |
| PTPLAD1 | HGLibA_39418 | 206 | 0.728647148 | PTPLAD1 | - |
| C10orf25 | HGLibA_05063 | 205 | 0.719601806 | C10orf25 | - |
| C17orf112 | HGLibA_05530 | 205 | 0.719601806 | C17orf112 | - |
| ENSG00000007047 | HGLibA_28373 | 205 | 0.719601806 | MARK4 | microtubule affinity regulating kinase 4 [Source:HGNC Symbol;Acc:HGNC:13538] |
| ENSG00000053328 | HGLibA_28994 | 205 | 0.719601806 | METTL24 | methyltransferase like 24 [Source:HGNC Symbol;Acc:HGNC:21566] |
| ENSG00000060642 | HGLibA_36593 | 205 | 0.719601806 | PIGV | phosphatidylinositol glycan anchor biosynthesis class V [Source:HGNC Symbol;Acc:HGNC:26031] |
| ENSG00000064989 | HGLibA_07008 | 205 | 0.719601806 | CALCRL | calcitonin receptor like receptor [Source:HGNC Symbol;Acc:HGNC:16709] |
| ENSG00000069345 | HGLibA_13513 | 205 | 0.719601806 | DNAJA2 | DnaJ heat shock protein family (Hsp40) member A2 [Source:HGNC Symbol;Acc:HGNC:14884] |
| ENSG00000075891 | HGLibA_35298 | 205 | 0.719601806 | PAX2 | paired box 2 [Source:HGNC Symbol;Acc:HGNC:8616] |
| ENSG00000086589 | HGLibA_40510 | 205 | 0.719601806 | RBM22 | RNA binding motif protein 22 [Source:HGNC Symbol;Acc:HGNC:25503] |
| ENSG00000089916 | HGLibA_19742 | 205 | 0.719601806 | GPATCH2L | G-patch domain containing 2 like [Source:HGNC Symbol;Acc:HGNC:20210] |
| ENSG00000096395 | HGLibA_29425 | 205 | 0.719601806 | MLN | motilin [Source:HGNC Symbol;Acc:HGNC:7141] |
| ENSG00000102021 | HGLibA_27644 | 205 | 0.719601806 | LUZP4 | leucine zipper protein 4 [Source:HGNC Symbol;Acc:HGNC:24971] |
| ENSG00000106479 | HGLibA_56880 | 205 | 0.719601806 | ZNF862 | zinc finger protein 862 [Source:HGNC Symbol;Acc:HGNC:34519] |
| ENSG00000112511 | HGLibA_36329 | 205 | 0.719601806 | PHF1 | PHD finger protein 1 [Source:HGNC Symbol;Acc:HGNC:8919] |
| ENSG00000115042 | HGLibA_15958 | 205 | 0.719601806 | FAHD2A | fumarylacetoacetate hydrolase domain containing 2A [Source:HGNC Symbol;Acc:HGNC:24252] |
| ENSG00000117362 | HGLibA_02414 | 205 | 0.719601806 | APH1A | gamma-secretase subunit [Source:HGNC Symbol;Acc:HGNC:29509]" |
| ENSG00000118496 | HGLibA_17065 | 205 | 0.719601806 | FBXO30 | F-box protein 30 [Source:HGNC Symbol;Acc:HGNC:15600] |
| ENSG00000119125 | HGLibA_18836 | 205 | 0.719601806 | GDA | guanine deaminase [Source:HGNC Symbol;Acc:HGNC:4212] |
| ENSG00000121904 | HGLibA_11367 | 205 | 0.719601806 | CSMD2 | CUB and Sushi multiple domains 2 [Source:HGNC Symbol;Acc:HGNC:19290] |
| ENSG00000122862 | HGLibA_46984 | 205 | 0.719601806 | SRGN | serglycin [Source:HGNC Symbol;Acc:HGNC:9361] |
| ENSG00000123700 | HGLibA_24274 | 205 | 0.719601806 | KCNJ2 | potassium voltage-gated channel subfamily J member 2 [Source:HGNC Symbol;Acc:HGNC:6263] |
| ENSG00000126603 | HGLibA_19274 | 205 | 0.719601806 | GLIS2 | GLIS family zinc finger 2 [Source:HGNC Symbol;Acc:HGNC:29450] |
| ENSG00000128710 | HGLibA_21985 | 205 | 0.719601806 | HOXD10 | homeobox D10 [Source:HGNC Symbol;Acc:HGNC:5133] |
| ENSG00000128710 | HGLibA_21986 | 205 | 0.719601806 | HOXD10 | homeobox D10 [Source:HGNC Symbol;Acc:HGNC:5133] |
| ENSG00000130675 | HGLibA_29580 | 205 | 0.719601806 | MNX1 | motor neuron and pancreas homeobox 1 [Source:HGNC Symbol;Acc:HGNC:4979] |
| ENSG00000130803 | HGLibA_55784 | 205 | 0.719601806 | ZNF317 | zinc finger protein 317 [Source:HGNC Symbol;Acc:HGNC:13507] |
| ENSG00000131981 | HGLibA_26364 | 205 | 0.719601806 | LGALS3 | galectin 3 [Source:HGNC Symbol;Acc:HGNC:6563] |
| ENSG00000132357 | HGLibA_07236 | 205 | 0.719601806 | CARD6 | caspase recruitment domain family member 6 [Source:HGNC Symbol;Acc:HGNC:16394] |
| ENSG00000134780 | HGLibA_12296 | 205 | 0.719601806 | DAGLA | diacylglycerol lipase alpha [Source:HGNC Symbol;Acc:HGNC:1165] |
| ENSG00000135604 | HGLibA_47617 | 205 | 0.719601806 | STX11 | syntaxin 11 [Source:HGNC Symbol;Acc:HGNC:11429] |
| ENSG00000137561 | HGLibA_52222 | 205 | 0.719601806 | TTPA | alpha tocopherol transfer protein [Source:HGNC Symbol;Acc:HGNC:12404] |
| ENSG00000139648 | HGLibA_25524 | 205 | 0.719601806 | KRT71 | keratin 71 [Source:HGNC Symbol;Acc:HGNC:28927] |
| ENSG00000142583 | HGLibA_44883 | 205 | 0.719601806 | SLC2A5 | solute carrier family 2 member 5 [Source:HGNC Symbol;Acc:HGNC:11010] |
| ENSG00000143105 | HGLibA_24125 | 205 | 0.719601806 | KCNA10 | potassium voltage-gated channel subfamily A member 10 [Source:HGNC Symbol;Acc:HGNC:6219] |
| ENSG00000143126 | HGLibA_09026 | 205 | 0.719601806 | CELSR2 | cadherin EGF LAG seven-pass G-type receptor 2 [Source:HGNC Symbol;Acc:HGNC:3231] |
| ENSG00000148835 | HGLibA_48239 | 205 | 0.719601806 | TAF5 | TATA-box binding protein associated factor 5 [Source:HGNC Symbol;Acc:HGNC:11539] |
| ENSG00000152380 | HGLibA_16166 | 205 | 0.719601806 | FAM151B | family with sequence similarity 151 member B [Source:HGNC Symbol;Acc:HGNC:33716] |
| ENSG00000152683 | HGLibA_44914 | 205 | 0.719601806 | SLC30A6 | solute carrier family 30 member 6 [Source:HGNC Symbol;Acc:HGNC:19305] |
| ENSG00000154415 | HGLibA_38099 | 205 | 0.719601806 | PPP1R3A | protein phosphatase 1 regulatory subunit 3A [Source:HGNC Symbol;Acc:HGNC:9291] |
| ENSG00000154917 | HGLibA_39972 | 205 | 0.719601806 | RAB6B | member RAS oncogene family [Source:HGNC Symbol;Acc:HGNC:14902]" |
| ENSG00000156466 | HGLibA_18873 | 205 | 0.719601806 | GDF6 | growth differentiation factor 6 [Source:HGNC Symbol;Acc:HGNC:4221] |
| ENSG00000157214 | HGLibA_47426 | 205 | 0.719601806 | STEAP2 | STEAP2 metalloreductase [Source:HGNC Symbol;Acc:HGNC:17885] |
| ENSG00000160194 | HGLibA_31576 | 205 | 0.719601806 | NDUFV3 | NADH:ubiquinone oxidoreductase subunit V3 [Source:HGNC Symbol;Acc:HGNC:7719] |
| ENSG00000160613 | HGLibA_35685 | 205 | 0.719601806 | PCSK7 | proprotein convertase subtilisin/kexin type 7 [Source:HGNC Symbol;Acc:HGNC:8748] |
| ENSG00000162927 | HGLibA_39597 | 205 | 0.719601806 | PUS10 | pseudouridine synthase 10 [Source:HGNC Symbol;Acc:HGNC:26505] |
| ENSG00000163516 | HGLibA_02164 | 205 | 0.719601806 | ANKZF1 | ankyrin repeat and zinc finger domain containing 1 [Source:HGNC Symbol;Acc:HGNC:25527] |
| ENSG00000163634 | HGLibA_49379 | 205 | 0.719601806 | THOC7 | THO complex 7 [Source:HGNC Symbol;Acc:HGNC:29874] |
| ENSG00000163864 | HGLibA_32189 | 205 | 0.719601806 | NMNAT3 | nicotinamide nucleotide adenylyltransferase 3 [Source:HGNC Symbol;Acc:HGNC:20989] |
| ENSG00000164221 | HGLibA_07543 | 205 | 0.719601806 | CCDC112 | coiled-coil domain containing 112 [Source:HGNC Symbol;Acc:HGNC:28599] |
| ENSG00000169347 | HGLibA_19705 | 205 | 0.719601806 | GP2 | glycoprotein 2 [Source:HGNC Symbol;Acc:HGNC:4441] |
| ENSG00000172006 | HGLibA_56222 | 205 | 0.719601806 | ZNF554 | zinc finger protein 554 [Source:HGNC Symbol;Acc:HGNC:26629] |
| ENSG00000173120 | HGLibA_24495 | 205 | 0.719601806 | KDM2A | lysine demethylase 2A [Source:HGNC Symbol;Acc:HGNC:13606] |
| ENSG00000173401 | HGLibA_19262 | 205 | 0.719601806 | GLIPR1L1 | GLIPR1 like 1 [Source:HGNC Symbol;Acc:HGNC:28392] |
| ENSG00000176401 | HGLibA_14698 | 205 | 0.719601806 | EID2B | EP300 interacting inhibitor of differentiation 2B [Source:HGNC Symbol;Acc:HGNC:26796] |
| ENSG00000177303 | HGLibA_07291 | 205 | 0.719601806 | CASKIN2 | CASK interacting protein 2 [Source:HGNC Symbol;Acc:HGNC:18200] |
| ENSG00000177910 | HGLibA_46492 | 205 | 0.719601806 | SPATA31C2 | SPATA31 subfamily C member 2 [Source:HGNC Symbol;Acc:HGNC:24508] |
| ENSG00000178750 | HGLibA_47632 | 205 | 0.719601806 | STX19 | syntaxin 19 [Source:HGNC Symbol;Acc:HGNC:19300] |
| ENSG00000181085 | HGLibA_28264 | 205 | 0.719601806 | MAPK15 | mitogen-activated protein kinase 15 [Source:HGNC Symbol;Acc:HGNC:24667] |
| ENSG00000181396 | HGLibA_33314 | 205 | 0.719601806 | OGFOD3 | 2-oxoglutarate and iron dependent oxygenase domain containing 3 [Source:HGNC Symbol;Acc:HGNC:26174] |
| ENSG00000181408 | HGLibA_53468 | 205 | 0.719601806 | UTS2R | urotensin 2 receptor [Source:HGNC Symbol;Acc:HGNC:4468] |
| ENSG00000183963 | HGLibA_45832 | 205 | 0.719601806 | SMTN | smoothelin [Source:HGNC Symbol;Acc:HGNC:11126] |
| ENSG00000184307 | HGLibA_55108 | 205 | 0.719601806 | ZDHHC23 | zinc finger DHHC-type containing 23 [Source:HGNC Symbol;Acc:HGNC:28654] |
| ENSG00000185640 | HGLibA_25549 | 205 | 0.719601806 | KRT79 | keratin 79 [Source:HGNC Symbol;Acc:HGNC:28930] |
| ENSG00000186081 | HGLibA_25509 | 205 | 0.719601806 | KRT5 | keratin 5 [Source:HGNC Symbol;Acc:HGNC:6442] |
| ENSG00000186871 | HGLibA_15449 | 205 | 0.719601806 | ERCC6L | spindle assembly checkpoint helicase [Source:HGNC Symbol;Acc:HGNC:20794]" |
| ENSG00000188324 | HGLibA_34393 | 205 | 0.719601806 | OR6C6 | olfactory receptor family 6 subfamily C member 6 [Source:HGNC Symbol;Acc:HGNC:31293] |
| ENSG00000197933 | HGLibA_56821 | 205 | 0.719601806 | ZNF823 | zinc finger protein 823 [Source:HGNC Symbol;Acc:HGNC:30936] |
| ENSG00000198805 | HGLibA_37403 | 205 | 0.719601806 | PNP | purine nucleoside phosphorylase [Source:HGNC Symbol;Acc:HGNC:7892] |
| ENSG00000205362 | HGLibA_30276 | 205 | 0.719601806 | MT1A | metallothionein 1A [Source:HGNC Symbol;Acc:HGNC:7393] |
| ENSG00000205809 | HGLibA_25287 | 205 | 0.719601806 | KLRC2 | killer cell lectin like receptor C2 [Source:HGNC Symbol;Acc:HGNC:6375] |
| ENSG00000213465 | HGLibA_02958 | 205 | 0.719601806 | ARL2 | ADP ribosylation factor like GTPase 2 [Source:HGNC Symbol;Acc:HGNC:693] |
| ENSG00000213714 | HGLibA_16440 | 205 | 0.719601806 | FAM209B | family with sequence similarity 209 member B [Source:HGNC Symbol;Acc:HGNC:16101] |
| ENSG00000221888 | HGLibA_33650 | 205 | 0.719601806 | OR1C1 | olfactory receptor family 1 subfamily C member 1 [Source:HGNC Symbol;Acc:HGNC:8182] |
| ENSG00000260001 | HGLibA_49232 | 205 | 0.719601806 | TGFBR3L | transforming growth factor beta receptor 3 like [Source:HGNC Symbol;Acc:HGNC:44152] |
| ENSG00000267978 | HGLibA_27925 | 205 | 0.719601806 | MAGEA9B | MAGE family member A9B [Source:HGNC Symbol;Acc:HGNC:31909] |
| ENSG00000274286 | HGLibA_01049 | 205 | 0.719601806 | ADRA2B | adrenoceptor alpha 2B [Source:HGNC Symbol;Acc:HGNC:282] |
| ENSG00000277632 | HGLibA_08017 | 205 | 0.719601806 | CCL3 | C-C motif chemokine ligand 3 [Source:HGNC Symbol;Acc:HGNC:10627] |
| ENSG00000284609 | HGLibA_34498 | 205 | 0.719601806 | OR8B3 | olfactory receptor family 8 subfamily B member 3 [Source:HGNC Symbol;Acc:HGNC:8472] |
| EPRS | HGLibA_15364 | 205 | 0.719601806 | EPRS | - |
| hsa-mir-323a | HGLibA_58957 | 205 | 0.719601806 | hsa-mir-323a | - |
| hsa-mir-4304 | HGLibA_59927 | 205 | 0.719601806 | hsa-mir-4304 | - |
| hsa-mir-4444-2 | HGLibA_60181 | 205 | 0.719601806 | hsa-mir-4444-2 | - |
| hsa-mir-4799 | HGLibA_61300 | 205 | 0.719601806 | hsa-mir-4799 | - |
| hsa-mir-519c | HGLibA_61723 | 205 | 0.719601806 | hsa-mir-519c | - |
| hsa-mir-6818 | HGLibA_63448 | 205 | 0.719601806 | hsa-mir-6818 | - |
| hsa-mir-6841 | HGLibA_63540 | 205 | 0.719601806 | hsa-mir-6841 | - |
| hsa-mir-6882 | HGLibA_63713 | 205 | 0.719601806 | hsa-mir-6882 | - |
| hsa-mir-7-1 | HGLibA_63770 | 205 | 0.719601806 | hsa-mir-7-1 | - |
| RGAG1 | HGLibA_40992 | 205 | 0.719601806 | RGAG1 | - |
| CCDC151 | HGLibA_07647 | 204 | 0.710556463 | CCDC151 | - |
| ENSG00000007264 | HGLibA_28424 | 204 | 0.710556463 | MATK | megakaryocyte-associated tyrosine kinase [Source:HGNC Symbol;Acc:HGNC:6906] |
| ENSG00000012822 | HGLibA_06998 | 204 | 0.710556463 | CALCOCO1 | calcium binding and coiled-coil domain 1 [Source:HGNC Symbol;Acc:HGNC:29306] |
| ENSG00000017797 | HGLibA_40153 | 204 | 0.710556463 | RALBP1 | ralA binding protein 1 [Source:HGNC Symbol;Acc:HGNC:9841] |
| ENSG00000019186 | HGLibA_12083 | 204 | 0.710556463 | CYP24A1 | cytochrome P450 family 24 subfamily A member 1 [Source:HGNC Symbol;Acc:HGNC:2602] |
| ENSG00000019582 | HGLibA_08444 | 204 | 0.710556463 | CD74 | CD74 molecule [Source:HGNC Symbol;Acc:HGNC:1697] |
| ENSG00000049130 | HGLibA_25018 | 204 | 0.710556463 | KITLG | KIT ligand [Source:HGNC Symbol;Acc:HGNC:6343] |
| ENSG00000075539 | HGLibA_18056 | 204 | 0.710556463 | FRYL | FRY like transcription coactivator [Source:HGNC Symbol;Acc:HGNC:29127] |
| ENSG00000095321 | HGLibA_11066 | 204 | 0.710556463 | CRAT | carnitine O-acetyltransferase [Source:HGNC Symbol;Acc:HGNC:2342] |
| ENSG00000099910 | HGLibA_25155 | 204 | 0.710556463 | KLHL22 | kelch like family member 22 [Source:HGNC Symbol;Acc:HGNC:25888] |
| ENSG00000100568 | HGLibA_53891 | 204 | 0.710556463 | VTI1B | vesicle transport through interaction with t-SNAREs 1B [Source:HGNC Symbol;Acc:HGNC:17793] |
| ENSG00000102781 | HGLibA_24075 | 204 | 0.710556463 | KATNAL1 | katanin catalytic subunit A1 like 1 [Source:HGNC Symbol;Acc:HGNC:28361] |
| ENSG00000105227 | HGLibA_38992 | 204 | 0.710556463 | PRX | periaxin [Source:HGNC Symbol;Acc:HGNC:13797] |
| ENSG00000105472 | HGLibA_09925 | 204 | 0.710556463 | CLEC11A | C-type lectin domain containing 11A [Source:HGNC Symbol;Acc:HGNC:10576] |
| ENSG00000107077 | HGLibA_24511 | 204 | 0.710556463 | KDM4C | lysine demethylase 4C [Source:HGNC Symbol;Acc:HGNC:17071] |
| ENSG00000114251 | HGLibA_54372 | 204 | 0.710556463 | WNT5A | Wnt family member 5A [Source:HGNC Symbol;Acc:HGNC:12784] |
| ENSG00000115163 | HGLibA_09038 | 204 | 0.710556463 | CENPA | centromere protein A [Source:HGNC Symbol;Acc:HGNC:1851] |
| ENSG00000119283 | HGLibA_51587 | 204 | 0.710556463 | TRIM67 | tripartite motif containing 67 [Source:HGNC Symbol;Acc:HGNC:31859] |
| ENSG00000121481 | HGLibA_41607 | 204 | 0.710556463 | RNF2 | ring finger protein 2 [Source:HGNC Symbol;Acc:HGNC:10061] |
| ENSG00000122882 | HGLibA_14380 | 204 | 0.710556463 | ECD | ecdysoneless cell cycle regulator [Source:HGNC Symbol;Acc:HGNC:17029] |
| ENSG00000124383 | HGLibA_29722 | 204 | 0.710556463 | MPHOSPH10 | M-phase phosphoprotein 10 [Source:HGNC Symbol;Acc:HGNC:7213] |
| ENSG00000125775 | HGLibA_43112 | 204 | 0.710556463 | SDCBP2 | syndecan binding protein 2 [Source:HGNC Symbol;Acc:HGNC:15756] |
| ENSG00000127184 | HGLibA_10871 | 204 | 0.710556463 | COX7C | cytochrome c oxidase subunit 7C [Source:HGNC Symbol;Acc:HGNC:2292] |
| ENSG00000131196 | HGLibA_31774 | 204 | 0.710556463 | NFATC1 | nuclear factor of activated T cells 1 [Source:HGNC Symbol;Acc:HGNC:7775] |
| ENSG00000132429 | HGLibA_37701 | 204 | 0.710556463 | POPDC3 | popeye domain containing 3 [Source:HGNC Symbol;Acc:HGNC:17649] |
| ENSG00000135045 | HGLibA_06727 | 204 | 0.710556463 | C9orf40 | chromosome 9 open reading frame 40 [Source:HGNC Symbol;Acc:HGNC:23433] |
| ENSG00000135686 | HGLibA_25197 | 204 | 0.710556463 | KLHL36 | kelch like family member 36 [Source:HGNC Symbol;Acc:HGNC:17844] |
| ENSG00000137075 | HGLibA_41670 | 204 | 0.710556463 | RNF38 | ring finger protein 38 [Source:HGNC Symbol;Acc:HGNC:18052] |
| ENSG00000137166 | HGLibA_17940 | 204 | 0.710556463 | FOXP4 | forkhead box P4 [Source:HGNC Symbol;Acc:HGNC:20842] |
| ENSG00000143537 | HGLibA_00738 | 204 | 0.710556463 | ADAM15 | ADAM metallopeptidase domain 15 [Source:HGNC Symbol;Acc:HGNC:193] |
| ENSG00000143799 | HGLibA_35212 | 204 | 0.710556463 | PARP1 | poly(ADP-ribose) polymerase 1 [Source:HGNC Symbol;Acc:HGNC:270] |
| ENSG00000150779 | HGLibA_49525 | 204 | 0.710556463 | TIMM8B | translocase of inner mitochondrial membrane 8 homolog B [Source:HGNC Symbol;Acc:HGNC:11818] |
| ENSG00000151240 | HGLibA_13252 | 204 | 0.710556463 | DIP2C | disco interacting protein 2 homolog C [Source:HGNC Symbol;Acc:HGNC:29150] |
| ENSG00000152558 | HGLibA_49905 | 204 | 0.710556463 | TMEM123 | transmembrane protein 123 [Source:HGNC Symbol;Acc:HGNC:30138] |
| ENSG00000155380 | HGLibA_44416 | 204 | 0.710556463 | SLC16A1 | solute carrier family 16 member 1 [Source:HGNC Symbol;Acc:HGNC:10922] |
| ENSG00000156232 | HGLibA_54289 | 204 | 0.710556463 | WHAMM | golgi membranes and microtubules [Source:HGNC Symbol;Acc:HGNC:30493]" |
| ENSG00000161714 | HGLibA_36994 | 204 | 0.710556463 | PLCD3 | phospholipase C delta 3 [Source:HGNC Symbol;Acc:HGNC:9061] |
| ENSG00000162344 | HGLibA_17387 | 204 | 0.710556463 | FGF19 | fibroblast growth factor 19 [Source:HGNC Symbol;Acc:HGNC:3675] |
| ENSG00000162771 | HGLibA_16686 | 204 | 0.710556463 | FAM71A | family with sequence similarity 71 member A [Source:HGNC Symbol;Acc:HGNC:26541] |
| ENSG00000163216 | HGLibA_46813 | 204 | 0.710556463 | SPRR2D | small proline rich protein 2D [Source:HGNC Symbol;Acc:HGNC:11264] |
| ENSG00000163516 | HGLibA_02166 | 204 | 0.710556463 | ANKZF1 | ankyrin repeat and zinc finger domain containing 1 [Source:HGNC Symbol;Acc:HGNC:25527] |

| | | | | | |
|---|---|---|---|---|---|
| ENSG00000164916 | HGLibA_17893 | 204 | 0.710556463 | FOXK1 | forkhead box K1 [Source:HGNC Symbol;Acc:HGNC:23480] |
| ENSG00000165424 | HGLibA_55031 | 204 | 0.710556463 | ZCCHC24 | zinc finger CCHC-type containing 24 [Source:HGNC Symbol;Acc:HGNC:26911] |
| ENSG00000170369 | HGLibA_11449 | 204 | 0.710556463 | CST2 | cystatin SA [Source:HGNC Symbol;Acc:HGNC:2474] |
| ENSG00000170946 | HGLibA_13602 | 204 | 0.710556463 | DNAJC24 | DnaJ heat shock protein family (Hsp40) member C24 [Source:HGNC Symbol;Acc:HGNC:26979] |
| ENSG00000171552 | HGLibA_04303 | 204 | 0.710556463 | BCL2L1 | BCL2 like 1 [Source:HGNC Symbol;Acc:HGNC:992] |
| ENSG00000174177 | HGLibA_11752 | 204 | 0.710556463 | CTU2 | cytosolic thiouridylase subunit 2 [Source:HGNC Symbol;Acc:HGNC:28005] |
| ENSG00000175513 | HGLibA_51851 | 204 | 0.710556463 | TSGA10IP | testis specific 10 interacting protein [Source:HGNC Symbol;Acc:HGNC:26555] |
| ENSG00000175606 | HGLibA_50502 | 204 | 0.710556463 | TMEM70 | transmembrane protein 70 [Source:HGNC Symbol;Acc:HGNC:26050] |
| ENSG00000177054 | HGLibA_55075 | 204 | 0.710556463 | ZDHHC13 | zinc finger DHHC-type containing 13 [Source:HGNC Symbol;Acc:HGNC:18413] |
| ENSG00000177200 | HGLibA_09410 | 204 | 0.710556463 | CHD9 | chromodomain helicase DNA binding protein 9 [Source:HGNC Symbol;Acc:HGNC:25701] |
| ENSG00000178105 | HGLibA_12650 | 204 | 0.710556463 | DDX10 | DEAD-box helicase 10 [Source:HGNC Symbol;Acc:HGNC:2735] |
| ENSG00000182810 | HGLibA_12691 | 204 | 0.710556463 | DDX28 | DEAD-box helicase 28 [Source:HGNC Symbol;Acc:HGNC:17330] |
| ENSG00000183114 | HGLibA_16590 | 204 | 0.710556463 | FAM43B | family with sequence similarity 43 member B [Source:HGNC Symbol;Acc:HGNC:31791] |
| ENSG00000183336 | HGLibA_04683 | 204 | 0.710556463 | BOLA2 | bolA family member 2 [Source:HGNC Symbol;Acc:HGNC:29488] |
| ENSG00000183918 | HGLibA_43882 | 204 | 0.710556463 | SH2D1A | SH2 domain containing 1A [Source:HGNC Symbol;Acc:HGNC:10820] |
| ENSG00000184743 | HGLibA_03521 | 204 | 0.710556463 | ATL3 | atlastin GTPase 3 [Source:HGNC Symbol;Acc:HGNC:24526] |
| ENSG00000184985 | HGLibA_46186 | 204 | 0.710556463 | SORCS2 | sortilin related VPS10 domain containing receptor 2 [Source:HGNC Symbol;Acc:HGNC:16698] |
| ENSG00000187244 | HGLibA_04234 | 204 | 0.710556463 | BCAM | basal cell adhesion molecule (Lutheran blood group) [Source:HGNC Symbol;Acc:HGNC:6722] |
| ENSG00000187372 | HGLibA_35482 | 204 | 0.710556463 | PCDHB13 | protocadherin beta 13 [Source:HGNC Symbol;Acc:HGNC:8684] |
| ENSG00000187857 | HGLibA_34409 | 204 | 0.710556463 | OR6C75 | olfactory receptor family 6 subfamily C member 75 [Source:HGNC Symbol;Acc:HGNC:31304] |
| ENSG00000188227 | HGLibA_56773 | 204 | 0.710556463 | ZNF793 | zinc finger protein 793 [Source:HGNC Symbol;Acc:HGNC:33115] |
| ENSG00000189144 | HGLibA_56283 | 204 | 0.710556463 | ZNF573 | zinc finger protein 573 [Source:HGNC Symbol;Acc:HGNC:26420] |
| ENSG00000196260 | HGLibA_43759 | 204 | 0.710556463 | SFTA2 | surfactant associated 2 [Source:HGNC Symbol;Acc:HGNC:18386] |
| ENSG00000196542 | HGLibA_46904 | 204 | 0.710556463 | SPTSSB | serine palmitoyltransferase small subunit B [Source:HGNC Symbol;Acc:HGNC:24045] |
| ENSG00000197702 | HGLibA_35255 | 204 | 0.710556463 | PARVA | parvin alpha [Source:HGNC Symbol;Acc:HGNC:14652] |
| ENSG00000197721 | HGLibA_11048 | 204 | 0.710556463 | CR1L | complement C3b/C4b receptor 1 like [Source:HGNC Symbol;Acc:HGNC:2335] |
| ENSG00000203783 | HGLibA_38854 | 204 | 0.710556463 | PRR9 | proline rich 9 [Source:HGNC Symbol;Acc:HGNC:32057] |
| ENSG00000205220 | HGLibA_39097 | 204 | 0.710556463 | PSMB10 | proteasome subunit beta 10 [Source:HGNC Symbol;Acc:HGNC:9538] |
| ENSG00000221900 | HGLibA_37648 | 204 | 0.710556463 | POM121L12 | POM121 transmembrane nucleoporin like 12 [Source:HGNC Symbol;Acc:HGNC:25369] |
| ENSG00000221955 | HGLibA_44378 | 204 | 0.710556463 | SLC12A8 | solute carrier family 12 member 8 [Source:HGNC Symbol;Acc:HGNC:15595] |
| ENSG00000232112 | HGLibA_49737 | 204 | 0.710556463 | TMA7 | translation machinery associated 7 homolog [Source:HGNC Symbol;Acc:HGNC:26932] |
| ENSG00000259075 | HGLibA_37447 | 204 | 0.710556463 | POC1B-GALNT4 | POC1B-GALNT4 readthrough [Source:HGNC Symbol;Acc:HGNC:42957] |
| ENSG00000262628 | HGLibA_33657 | 204 | 0.710556463 | OR1D5 | olfactory receptor family 1 subfamily D member 5 [Source:HGNC Symbol;Acc:HGNC:8186] |
| hsa-mir-3200 | HGLibA_58907 | 204 | 0.710556463 | hsa-mir-3200 | - |
| hsa-mir-3975 | HGLibA_59659 | 204 | 0.710556463 | hsa-mir-3975 | - |
| hsa-mir-3975 | HGLibA_59661 | 204 | 0.710556463 | hsa-mir-3975 | - |
| hsa-mir-4293 | HGLibA_59882 | 204 | 0.710556463 | hsa-mir-4293 | - |
| hsa-mir-4482 | HGLibA_60346 | 204 | 0.710556463 | hsa-mir-4482 | - |
| hsa-mir-4658 | HGLibA_60738 | 204 | 0.710556463 | hsa-mir-4658 | - |
| hsa-mir-645 | HGLibA_62825 | 204 | 0.710556463 | hsa-mir-645 | - |
| hsa-mir-6763 | HGLibA_63214 | 204 | 0.710556463 | hsa-mir-6763 | - |
| hsa-mir-6781 | HGLibA_63301 | 204 | 0.710556463 | hsa-mir-6781 | - |
| LOC100129636 | HGLibA_26769 | 204 | 0.710556463 | LOC100129636 | - |
| LPPR5 | HGLibA_27126 | 204 | 0.710556463 | LPPR5 | - |
| SPATA31A2 | HGLibA_46471 | 204 | 0.710556463 | SPATA31A2 | - |
| TMEM110 | HGLibA_49875 | 204 | 0.710556463 | TMEM110 | - |
| C13orf45 | HGLibA_05312 | 203 | 0.70151112 | C13orf45 | - |
| C16orf71 | HGLibA_05474 | 203 | 0.70151112 | C16orf71 | - |
| CCDC53 | HGLibA_07798 | 203 | 0.70151112 | CCDC53 | - |
| ENSG00000011083 | HGLibA_45337 | 203 | 0.70151112 | SLC6A7 | solute carrier family 6 member 7 [Source:HGNC Symbol;Acc:HGNC:11054] |
| ENSG00000022556 | HGLibA_32115 | 203 | 0.70151112 | NLRP2 | NLR family pyrin domain containing 2 [Source:HGNC Symbol;Acc:HGNC:22948] |
| ENSG00000049130 | HGLibA_25019 | 203 | 0.70151112 | KITLG | KIT ligand [Source:HGNC Symbol;Acc:HGNC:6343] |
| ENSG00000052126 | HGLibA_37074 | 203 | 0.70151112 | PLEKHA5 | pleckstrin homology domain containing A5 [Source:HGNC Symbol;Acc:HGNC:30036] |
| ENSG00000055044 | HGLibA_32315 | 203 | 0.70151112 | NOP58 | NOP58 ribonucleoprotein [Source:HGNC Symbol;Acc:HGNC:29926] |
| ENSG00000085063 | HGLibA_08416 | 203 | 0.70151112 | CD59 | CD59 molecule (CD59 blood group) [Source:HGNC Symbol;Acc:HGNC:1689] |
| ENSG00000088827 | HGLibA_44123 | 203 | 0.70151112 | SIGLEC1 | sialic acid binding Ig like lectin 1 [Source:HGNC Symbol;Acc:HGNC:11127] |
| ENSG00000091592 | HGLibA_32096 | 203 | 0.70151112 | NLRP1 | NLR family pyrin domain containing 1 [Source:HGNC Symbol;Acc:HGNC:14374] |
| ENSG00000101180 | HGLibA_22101 | 203 | 0.70151112 | HRH3 | histamine receptor H3 [Source:HGNC Symbol;Acc:HGNC:5184] |
| ENSG00000101323 | HGLibA_20833 | 203 | 0.70151112 | HAO1 | hydroxyacid oxidase 1 [Source:HGNC Symbol;Acc:HGNC:4809] |
| ENSG00000104081 | HGLibA_04584 | 203 | 0.70151112 | BMF | Bcl2 modifying factor [Source:HGNC Symbol;Acc:HGNC:24132] |
| ENSG00000105784 | HGLibA_42451 | 203 | 0.70151112 | RUNDC3B | RUN domain containing 3B [Source:HGNC Symbol;Acc:HGNC:30286] |
| ENSG00000106328 | HGLibA_18073 | 203 | 0.70151112 | FSCN3 | fascin actin-bundling protein 3 [Source:HGNC Symbol;Acc:HGNC:3961] |
| ENSG00000108176 | HGLibA_13571 | 203 | 0.70151112 | DNAJC12 | DnaJ heat shock protein family (Hsp40) member C12 [Source:HGNC Symbol;Acc:HGNC:28908] |
| ENSG00000108813 | HGLibA_13382 | 203 | 0.70151112 | DLX4 | distal-less homeobox 4 [Source:HGNC Symbol;Acc:HGNC:2917] |
| ENSG00000108946 | HGLibA_38520 | 203 | 0.70151112 | PRKAR1A | protein kinase cAMP-dependent type I regulatory subunit alpha [Source:HGNC Symbol;Acc:HGNC:9388] |
| ENSG00000112419 | HGLibA_36300 | 203 | 0.70151112 | PHACTR2 | phosphatase and actin regulator 2 [Source:HGNC Symbol;Acc:HGNC:20956] |
| ENSG00000114353 | HGLibA_19453 | 203 | 0.70151112 | GNAI2 | G protein subunit alpha i2 [Source:HGNC Symbol;Acc:HGNC:4385] |
| ENSG00000115310 | HGLibA_42408 | 203 | 0.70151112 | RTN4 | reticulon 4 [Source:HGNC Symbol;Acc:HGNC:14085] |
| ENSG00000117525 | HGLibA_15875 | 203 | 0.70151112 | F3 | tissue factor [Source:HGNC Symbol;Acc:HGNC:3541]" |
| ENSG00000118298 | HGLibA_06799 | 203 | 0.70151112 | CA14 | carbonic anhydrase 14 [Source:HGNC Symbol;Acc:HGNC:1372] |
| ENSG00000120992 | HGLibA_27738 | 203 | 0.70151112 | LYPLA1 | lysophospholipase I [Source:HGNC Symbol;Acc:HGNC:6737] |
| ENSG00000123594 | HGLibA_03845 | 203 | 0.70151112 | ATXN3L | ataxin 3 like [Source:HGNC Symbol;Acc:HGNC:24173] |
| ENSG00000125611 | HGLibA_09373 | 203 | 0.70151112 | CHCHD5 | coiled-coil-helix-coiled-coil-helix domain containing 5 [Source:HGNC Symbol;Acc:HGNC:17840] |
| ENSG00000128917 | HGLibA_13365 | 203 | 0.70151112 | DLL4 | delta like canonical Notch ligand 4 [Source:HGNC Symbol;Acc:HGNC:2910] |
| ENSG00000130812 | HGLibA_01926 | 203 | 0.70151112 | ANGPTL6 | angiopoietin like 6 [Source:HGNC Symbol;Acc:HGNC:23140] |
| ENSG00000132286 | HGLibA_49497 | 203 | 0.70151112 | TIMM10B | translocase of inner mitochondrial membrane 10B [Source:HGNC Symbol;Acc:HGNC:4022] |
| ENSG00000132635 | HGLibA_35584 | 203 | 0.70151112 | PCED1A | PC-esterase domain containing 1A [Source:HGNC Symbol;Acc:HGNC:16212] |
| ENSG00000134058 | HGLibA_08794 | 203 | 0.70151112 | CDK7 | cyclin dependent kinase 7 [Source:HGNC Symbol;Acc:HGNC:1778] |
| ENSG00000134193 | HGLibA_40805 | 203 | 0.70151112 | REG4 | regenerating family member 4 [Source:HGNC Symbol;Acc:HGNC:22977] |
| ENSG00000134365 | HGLibA_09273 | 203 | 0.70151112 | CFHR4 | complement factor H related 4 [Source:HGNC Symbol;Acc:HGNC:16979] |
| ENSG00000135144 | HGLibA_14079 | 203 | 0.70151112 | DTX1 | deltex E3 ubiquitin ligase 1 [Source:HGNC Symbol;Acc:HGNC:3060] |
| ENSG00000137090 | HGLibA_13417 | 203 | 0.70151112 | DMRT1 | doublesex and mab-3 related transcription factor 1 [Source:HGNC Symbol;Acc:HGNC:2934] |
| ENSG00000138182 | HGLibA_24894 | 203 | 0.70151112 | KIF20B | kinesin family member 20B [Source:HGNC Symbol;Acc:HGNC:7212] |
| ENSG00000139350 | HGLibA_31600 | 203 | 0.70151112 | NEDD1 | developmentally down-regulated 1 [Source:HGNC Symbol;Acc:HGNC:7723]" |
| ENSG00000139988 | HGLibA_40738 | 203 | 0.70151112 | RDH12 | retinol dehydrogenase 12 [Source:HGNC Symbol;Acc:HGNC:19977] |
| ENSG00000142227 | HGLibA_15080 | 203 | 0.70151112 | EMP3 | epithelial membrane protein 3 [Source:HGNC Symbol;Acc:HGNC:3335] |
| ENSG00000142507 | HGLibA_39114 | 203 | 0.70151112 | PSMB6 | proteasome subunit beta 6 [Source:HGNC Symbol;Acc:HGNC:9543] |
| ENSG00000142686 | HGLibA_05830 | 203 | 0.70151112 | C1orf216 | chromosome 1 open reading frame 216 [Source:HGNC Symbol;Acc:HGNC:26800] |
| ENSG00000144852 | HGLibA_32588 | 203 | 0.70151112 | NR1I2 | nuclear receptor subfamily 1 group I member 2 [Source:HGNC Symbol;Acc:HGNC:7968] |
| ENSG00000148143 | HGLibA_56042 | 203 | 0.70151112 | ZNF462 | zinc finger protein 462 [Source:HGNC Symbol;Acc:HGNC:21684] |
| ENSG00000148408 | HGLibA_06877 | 203 | 0.70151112 | CACNA1B | calcium voltage-gated channel subunit alpha1 B [Source:HGNC Symbol;Acc:HGNC:1389] |
| ENSG00000149968 | HGLibA_29533 | 203 | 0.70151112 | MMP3 | matrix metallopeptidase 3 [Source:HGNC Symbol;Acc:HGNC:7173] |
| ENSG00000153982 | HGLibA_18890 | 203 | 0.70151112 | GDPD1 | glycerophosphodiester phosphodiesterase domain containing 1 [Source:HGNC Symbol;Acc:HGNC:20883] |
| ENSG00000157778 | HGLibA_39211 | 203 | 0.70151112 | PSMG3 | proteasome assembly chaperone 3 [Source:HGNC Symbol;Acc:HGNC:22420] |
| ENSG00000163596 | HGLibA_22492 | 203 | 0.70151112 | ICA1L | islet cell autoantigen 1 like [Source:HGNC Symbol;Acc:HGNC:14442] |
| ENSG00000164111 | HGLibA_02244 | 203 | 0.70151112 | ANXA5 | annexin A5 [Source:HGNC Symbol;Acc:HGNC:543] |
| ENSG00000164113 | HGLibA_00719 | 203 | 0.70151112 | ADAD1 | adenosine deaminase domain containing 1 [Source:HGNC Symbol;Acc:HGNC:30713] |
| ENSG00000164548 | HGLibA_51221 | 203 | 0.70151112 | TRA2A | transformer 2 alpha homolog [Source:HGNC Symbol;Acc:HGNC:16645] |
| ENSG00000165443 | HGLibA_36477 | 203 | 0.70151112 | PHYHIPL | phytanoyl-CoA 2-hydroxylase interacting protein like [Source:HGNC Symbol;Acc:HGNC:29378] |
| ENSG00000166801 | HGLibA_16023 | 203 | 0.70151112 | FAM111A | family with sequence similarity 111 member A [Source:HGNC Symbol;Acc:HGNC:24725] |
| ENSG00000168229 | HGLibA_39304 | 203 | 0.70151112 | PTGDR | prostaglandin D2 receptor [Source:HGNC Symbol;Acc:HGNC:9591] |
| ENSG00000168872 | HGLibA_12662 | 203 | 0.70151112 | DDX19A | DEAD-box helicase 19A [Source:HGNC Symbol;Acc:HGNC:25628] |
| ENSG00000168936 | HGLibA_49923 | 203 | 0.70151112 | TMEM129 | transmembrane protein 129 [Source:HGNC Symbol;Acc:HGNC:25137] |
| ENSG00000170035 | HGLibA_52600 | 203 | 0.70151112 | UBE2E3 | ubiquitin conjugating enzyme E2 E3 [Source:HGNC Symbol;Acc:HGNC:12479] |
| ENSG00000171223 | HGLibA_24006 | 203 | 0.70151112 | JUNB | AP-1 transcription factor subunit [Source:HGNC Symbol;Acc:HGNC:6205]" |
| ENSG00000171595 | HGLibA_13507 | 203 | 0.70151112 | DNAI2 | dynein axonemal intermediate chain 2 [Source:HGNC Symbol;Acc:HGNC:18744] |
| ENSG00000173113 | HGLibA_51673 | 203 | 0.70151112 | TRMT112 | tRNA methyltransferase subunit 11-2 [Source:HGNC Symbol;Acc:HGNC:26940] |
| ENSG00000176108 | HGLibA_09500 | 203 | 0.70151112 | CHMP6 | charged multivesicular body protein 6 [Source:HGNC Symbol;Acc:HGNC:25675] |
| ENSG00000178772 | HGLibA_10950 | 203 | 0.70151112 | CPN2 | carboxypeptidase N subunit 2 [Source:HGNC Symbol;Acc:HGNC:2313] |
| ENSG00000179950 | HGLibA_39576 | 203 | 0.70151112 | PUF60 | poly(U) binding splicing factor 60 [Source:HGNC Symbol;Acc:HGNC:17042] |
| ENSG00000180900 | HGLibA_43047 | 203 | 0.70151112 | SCRIB | scribbled planar cell polarity protein [Source:HGNC Symbol;Acc:HGNC:30377] |
| ENSG00000180914 | HGLibA_34843 | 203 | 0.70151112 | OXTR | oxytocin receptor [Source:HGNC Symbol;Acc:HGNC:8529] |
| ENSG00000181826 | HGLibA_40819 | 203 | 0.70151112 | RELL1 | RELT like 1 [Source:HGNC Symbol;Acc:HGNC:27379] |
| ENSG00000182325 | HGLibA_17014 | 203 | 0.70151112 | FBXL6 | F-box and leucine rich repeat protein 6 [Source:HGNC Symbol;Acc:HGNC:13603] |
| ENSG00000183024 | HGLibA_33668 | 203 | 0.70151112 | OR1G1 | olfactory receptor family 1 subfamily G member 1 [Source:HGNC Symbol;Acc:HGNC:8204] |
| ENSG00000184394 | HGLibA_34053 | 203 | 0.70151112 | OR4N5 | olfactory receptor family 4 subfamily N member 5 [Source:HGNC Symbol;Acc:HGNC:15358] |
| ENSG00000185163 | HGLibA_12735 | 203 | 0.70151112 | DDX51 | DEAD-box helicase 51 [Source:HGNC Symbol;Acc:HGNC:20082] |
| ENSG00000185900 | HGLibA_37662 | 203 | 0.70151112 | POMK | protein-O-mannose kinase [Source:HGNC Symbol;Acc:HGNC:26267] |
| ENSG00000203618 | HGLibA_19702 | 203 | 0.70151112 | GP1BB | glycoprotein Ib platelet subunit beta [Source:HGNC Symbol;Acc:HGNC:4440] |
| ENSG00000204361 | HGLibA_33152 | 203 | 0.70151112 | NXPE2 | neurexophilin and PC-esterase domain family member 2 [Source:HGNC Symbol;Acc:HGNC:26331] |
| ENSG00000214050 | HGLibA_17032 | 203 | 0.70151112 | FBXO16 | F-box protein 16 [Source:HGNC Symbol;Acc:HGNC:13618] |
| ENSG00000214842 | HGLibA_40084 | 203 | 0.70151112 | RAD51AP2 | RAD51 associated protein 2 [Source:HGNC Symbol;Acc:HGNC:34417] |
| ENSG00000221910 | HGLibA_33790 | 203 | 0.70151112 | OR2F2 | olfactory receptor family 2 subfamily F member 2 [Source:HGNC Symbol;Acc:HGNC:8247] |
| ENSG00000227500 | HGLibA_42844 | 203 | 0.70151112 | SCAMP4 | secretory carrier membrane protein 4 [Source:HGNC Symbol;Acc:HGNC:30385] |
| ENSG00000276023 | HGLibA_14141 | 203 | 0.70151112 | DUSP14 | dual specificity phosphatase 14 [Source:HGNC Symbol;Acc:HGNC:17007] |
| hsa-mir-3916 | HGLibA_59540 | 203 | 0.70151112 | hsa-mir-3916 | - |
| hsa-mir-4310 | HGLibA_59954 | 203 | 0.70151112 | hsa-mir-4310 | - |
| hsa-mir-4803 | HGLibA_61315 | 203 | 0.70151112 | hsa-mir-4803 | - |
| hsa-mir-548ai | HGLibA_61884 | 203 | 0.70151112 | hsa-mir-548ai | - |
| hsa-mir-548ay | HGLibA_61944 | 203 | 0.70151112 | hsa-mir-548ay | - |
| hsa-mir-548p | HGLibA_62056 | 203 | 0.70151112 | hsa-mir-548p | - |
| hsa-mir-609 | HGLibA_62623 | 203 | 0.70151112 | hsa-mir-609 | - |
| hsa-mir-635 | HGLibA_62778 | 203 | 0.70151112 | hsa-mir-635 | - |
| hsa-mir-6749 | HGLibA_63150 | 203 | 0.70151112 | hsa-mir-6749 | - |
| hsa-mir-6814 | HGLibA_63434 | 203 | 0.70151112 | hsa-mir-6814 | - |

| | | | | | |
|---|---|---|---|---|---|
| hsa-mir-7111 | HGLibA_63809 | 203 | 0.70151112 | hsa-mir-7111 | - |
| hsa-mir-7151 | HGLibA_63832 | 203 | 0.70151112 | hsa-mir-7151 | - |
| LEPREL2 | HGLibA_26321 | 203 | 0.70151112 | LEPREL2 | - |
| LPPR2 | HGLibA_27116 | 203 | 0.70151112 | LPPR2 | - |
| C10orf25 | HGLibA_05064 | 202 | 0.692465777 | C10orf25 | - |
| CXorf23 | HGLibA_11908 | 202 | 0.692465777 | CXorf23 | - |
| ENSG00000004864 | HGLibA_44646 | 202 | 0.692465777 | SLC25A13 | solute carrier family 25 member 13 [Source:HGNC Symbol;Acc:HGNC:10983] |
| ENSG00000030110 | HGLibA_04133 | 202 | 0.692465777 | BAK1 | BCL2 antagonist/killer 1 [Source:HGNC Symbol;Acc:HGNC:949] |
| ENSG00000065882 | HGLibA_48460 | 202 | 0.692465777 | TBC1D1 | TBC1 domain family member 1 [Source:HGNC Symbol;Acc:HGNC:11578] |
| ENSG00000079931 | HGLibA_29700 | 202 | 0.692465777 | MOXD1 | monooxygenase DBH like 1 [Source:HGNC Symbol;Acc:HGNC:21063] |
| ENSG00000091490 | HGLibA_43283 | 202 | 0.692465777 | SEL1L3 | SEL1L family member 3 [Source:HGNC Symbol;Acc:HGNC:29108] |
| ENSG00000092200 | HGLibA_41837 | 202 | 0.692465777 | RPGRIP1 | RPGR interacting protein 1 [Source:HGNC Symbol;Acc:HGNC:13436] |
| ENSG00000096696 | HGLibA_14038 | 202 | 0.692465777 | DSP | desmoplakin [Source:HGNC Symbol;Acc:HGNC:3052] |
| ENSG00000099984 | HGLibA_20498 | 202 | 0.692465777 | GSTT2 | glutathione S-transferase theta 2 (gene/pseudogene) [Source:HGNC Symbol;Acc:HGNC:4642] |
| ENSG00000100197 | HGLibA_12126 | 202 | 0.692465777 | CYP2D6 | cytochrome P450 family 2 subfamily D member 6 [Source:HGNC Symbol;Acc:HGNC:2625] |
| ENSG00000101331 | HGLibA_08047 | 202 | 0.692465777 | CCM2L | CCM2 like scaffold protein [Source:HGNC Symbol;Acc:HGNC:16153] |
| ENSG00000101624 | HGLibA_09164 | 202 | 0.692465777 | CEP76 | centrosomal protein 76 [Source:HGNC Symbol;Acc:HGNC:25727] |
| ENSG00000102054 | HGLibA_40436 | 202 | 0.692465777 | RBBP7 | chromatin remodeling factor [Source:HGNC Symbol;Acc:HGNC:9890]" |
| ENSG00000102854 | HGLibA_30221 | 202 | 0.692465777 | MSLN | mesothelin [Source:HGNC Symbol;Acc:HGNC:7371] |
| ENSG00000105146 | HGLibA_03882 | 202 | 0.692465777 | AURKC | aurora kinase C [Source:HGNC Symbol;Acc:HGNC:11391] |
| ENSG00000106683 | HGLibA_26539 | 202 | 0.692465777 | LIMK1 | LIM domain kinase 1 [Source:HGNC Symbol;Acc:HGNC:6613] |
| ENSG00000112238 | HGLibA_38375 | 202 | 0.692465777 | PRDM13 | PR/SET domain 13 [Source:HGNC Symbol;Acc:HGNC:13998] |
| ENSG00000112619 | HGLibA_38752 | 202 | 0.692465777 | PRPH2 | peripherin 2 [Source:HGNC Symbol;Acc:HGNC:9942] |
| ENSG00000115085 | HGLibA_54775 | 202 | 0.692465777 | ZAP70 | zeta chain of T cell receptor associated protein kinase 70 [Source:HGNC Symbol;Acc:HGNC:12858] |
| ENSG00000115155 | HGLibA_34727 | 202 | 0.692465777 | OTOF | otoferlin [Source:HGNC Symbol;Acc:HGNC:8515] |
| ENSG00000115239 | HGLibA_03235 | 202 | 0.692465777 | ASB3 | ankyrin repeat and SOCS box containing 3 [Source:HGNC Symbol;Acc:HGNC:16013] |
| ENSG00000115295 | HGLibA_10052 | 202 | 0.692465777 | CLIP4 | CAP-Gly domain containing linker protein family member 4 [Source:HGNC Symbol;Acc:HGNC:26108] |
| ENSG00000117713 | HGLibA_02883 | 202 | 0.692465777 | ARID1A | AT-rich interaction domain 1A [Source:HGNC Symbol;Acc:HGNC:11110] |
| ENSG00000123572 | HGLibA_32701 | 202 | 0.692465777 | NRK | Nik related kinase [Source:HGNC Symbol;Acc:HGNC:25391] |
| ENSG00000124299 | HGLibA_36051 | 202 | 0.692465777 | PEPD | peptidase D [Source:HGNC Symbol;Acc:HGNC:8840] |
| ENSG00000125834 | HGLibA_47495 | 202 | 0.692465777 | STK35 | serine/threonine kinase 35 [Source:HGNC Symbol;Acc:HGNC:16254] |
| ENSG00000127666 | HGLibA_49446 | 202 | 0.692465777 | TICAM1 | toll like receptor adaptor molecule 1 [Source:HGNC Symbol;Acc:HGNC:18348] |
| ENSG00000131019 | HGLibA_52966 | 202 | 0.692465777 | ULBP3 | UL16 binding protein 3 [Source:HGNC Symbol;Acc:HGNC:14895] |
| ENSG00000132671 | HGLibA_47182 | 202 | 0.692465777 | SSTR4 | somatostatin receptor 4 [Source:HGNC Symbol;Acc:HGNC:11333] |
| ENSG00000132694 | HGLibA_02813 | 202 | 0.692465777 | ARHGEF11 | Rho guanine nucleotide exchange factor 11 [Source:HGNC Symbol;Acc:HGNC:14580] |
| ENSG00000133106 | HGLibA_15386 | 202 | 0.692465777 | EPSTI1 | epithelial stromal interaction 1 [Source:HGNC Symbol;Acc:HGNC:16465] |
| ENSG00000136367 | HGLibA_55172 | 202 | 0.692465777 | ZFHX2 | zinc finger homeobox 2 [Source:HGNC Symbol;Acc:HGNC:20152] |
| ENSG00000136839 | HGLibA_33610 | 202 | 0.692465777 | OR13C9 | olfactory receptor family 13 subfamily C member 9 [Source:HGNC Symbol;Acc:HGNC:15104] |
| ENSG00000143797 | HGLibA_28520 | 202 | 0.692465777 | MBOAT2 | membrane bound O-acyltransferase domain containing 2 [Source:HGNC Symbol;Acc:HGNC:25193] |
| ENSG00000144395 | HGLibA_07643 | 202 | 0.692465777 | CCDC150 | coiled-coil domain containing 150 [Source:HGNC Symbol;Acc:HGNC:26834] |
| ENSG00000145685 | HGLibA_26432 | 202 | 0.692465777 | LHFPL2 | LHFPL tetraspan subfamily member 2 [Source:HGNC Symbol;Acc:HGNC:6588] |
| ENSG00000146555 | HGLibA_43151 | 202 | 0.692465777 | SDK1 | sidekick cell adhesion molecule 1 [Source:HGNC Symbol;Acc:HGNC:19307] |
| ENSG00000146674 | HGLibA_22821 | 202 | 0.692465777 | IGFBP3 | insulin like growth factor binding protein 3 [Source:HGNC Symbol;Acc:HGNC:5472] |
| ENSG00000155897 | HGLibA_00929 | 202 | 0.692465777 | ADCY8 | adenylate cyclase 8 [Source:HGNC Symbol;Acc:HGNC:239] |
| ENSG00000156313 | HGLibA_41834 | 202 | 0.692465777 | RPGR | retinitis pigmentosa GTPase regulator [Source:HGNC Symbol;Acc:HGNC:10295] |
| ENSG00000158806 | HGLibA_32491 | 202 | 0.692465777 | NPM2 | nucleophosmin/nucleoplasmin 2 [Source:HGNC Symbol;Acc:HGNC:7930] |
| ENSG00000160741 | HGLibA_11232 | 202 | 0.692465777 | CRTC2 | CREB regulated transcription coactivator 2 [Source:HGNC Symbol;Acc:HGNC:27301] |
| ENSG00000160746 | HGLibA_02173 | 202 | 0.692465777 | ANO10 | anoctamin 10 [Source:HGNC Symbol;Acc:HGNC:25519] |
| ENSG00000163491 | HGLibA_31640 | 202 | 0.692465777 | NEK10 | NIMA related kinase 10 [Source:HGNC Symbol;Acc:HGNC:18592] |
| ENSG00000163956 | HGLibA_27236 | 202 | 0.692465777 | LRPAP1 | LDL receptor related protein associated protein 1 [Source:HGNC Symbol;Acc:HGNC:6701] |
| ENSG00000165583 | HGLibA_47216 | 202 | 0.692465777 | SSX5 | SSX family member 5 [Source:HGNC Symbol;Acc:HGNC:11339] |
| ENSG00000169271 | HGLibA_22309 | 202 | 0.692465777 | HSPB3 | heat shock protein family B (small) member 3 [Source:HGNC Symbol;Acc:HGNC:5248] |
| ENSG00000169575 | HGLibA_53722 | 202 | 0.692465777 | VPREB1 | V-set pre-B cell surrogate light chain 1 [Source:HGNC Symbol;Acc:HGNC:12709] |
| ENSG00000170037 | HGLibA_10440 | 202 | 0.692465777 | CNTROB | centriole duplication and spindle assembly protein [Source:HGNC Symbol;Acc:HGNC:29616]" |
| ENSG00000170142 | HGLibA_52595 | 202 | 0.692465777 | UBE2E1 | ubiquitin conjugating enzyme E2 E1 [Source:HGNC Symbol;Acc:HGNC:12477] |
| ENSG00000170677 | HGLibA_46139 | 202 | 0.692465777 | SOCS6 | suppressor of cytokine signaling 6 [Source:HGNC Symbol;Acc:HGNC:16833] |
| ENSG00000170689 | HGLibA_21953 | 202 | 0.692465777 | HOXB9 | homeobox B9 [Source:HGNC Symbol;Acc:HGNC:5120] |
| ENSG00000171045 | HGLibA_51883 | 202 | 0.692465777 | TSNARE1 | t-SNARE domain containing 1 [Source:HGNC Symbol;Acc:HGNC:26437] |
| ENSG00000171054 | HGLibA_33624 | 202 | 0.692465777 | OR13H1 | olfactory receptor family 13 subfamily H member 1 [Source:HGNC Symbol;Acc:HGNC:14755] |
| ENSG00000171136 | HGLibA_41359 | 202 | 0.692465777 | RLN3 | relaxin 3 [Source:HGNC Symbol;Acc:HGNC:17135] |
| ENSG00000171621 | HGLibA_46860 | 202 | 0.692465777 | SPSB1 | splA/ryanodine receptor domain and SOCS box containing 1 [Source:HGNC Symbol;Acc:HGNC:30628] |
| ENSG00000171794 | HGLibA_53428 | 202 | 0.692465777 | UTF1 | undifferentiated embryonic cell transcription factor 1 [Source:HGNC Symbol;Acc:HGNC:12634] |
| ENSG00000172530 | HGLibA_04149 | 202 | 0.692465777 | BANP | BTG3 associated nuclear protein [Source:HGNC Symbol;Acc:HGNC:13450] |
| ENSG00000179134 | HGLibA_42686 | 202 | 0.692465777 | SAMD4B | sterile alpha motif domain containing 4B [Source:HGNC Symbol;Acc:HGNC:25492] |
| ENSG00000182979 | HGLibA_30309 | 202 | 0.692465777 | MTA1 | metastasis associated 1 [Source:HGNC Symbol;Acc:HGNC:7410] |
| ENSG00000189045 | HGLibA_01948 | 202 | 0.692465777 | ANKDD1B | ankyrin repeat and death domain containing 1B [Source:HGNC Symbol;Acc:HGNC:32525] |
| ENSG00000196098 | HGLibA_34320 | 202 | 0.692465777 | OR5K4 | olfactory receptor family 5 subfamily K member 4 [Source:HGNC Symbol;Acc:HGNC:31291] |
| ENSG00000197520 | HGLibA_16297 | 202 | 0.692465777 | FAM177B | family with sequence similarity 177 member B [Source:HGNC Symbol;Acc:HGNC:34395] |
| ENSG00000198513 | HGLibA_03514 | 202 | 0.692465777 | ATL1 | atlastin GTPase 1 [Source:HGNC Symbol;Acc:HGNC:11231] |
| ENSG00000204887 | HGLibA_25584 | 202 | 0.692465777 | KRTAP1-4 | keratin associated protein 1-4 [Source:HGNC Symbol;Acc:HGNC:18904] |
| ENSG00000206559 | HGLibA_55060 | 202 | 0.692465777 | ZCWPW2 | zinc finger CW-type and PWWP domain containing 2 [Source:HGNC Symbol;Acc:HGNC:23574] |
| ENSG00000213366 | HGLibA_20475 | 202 | 0.692465777 | GSTM2 | glutathione S-transferase mu 2 [Source:HGNC Symbol;Acc:HGNC:4634] |
| ENSG00000215262 | HGLibA_24400 | 202 | 0.692465777 | KCNU1 | potassium calcium-activated channel subfamily U member 1 [Source:HGNC Symbol;Acc:HGNC:18867] |
| ENSG00000229314 | HGLibA_34620 | 202 | 0.692465777 | ORM1 | orosomucoid 1 [Source:HGNC Symbol;Acc:HGNC:8498] |
| ENSG00000261150 | HGLibA_15362 | 202 | 0.692465777 | EPPK1 | epiplakin 1 [Source:HGNC Symbol;Acc:HGNC:15577] |
| ENSG00000269226 | HGLibA_50662 | 202 | 0.692465777 | TMSB15B | thymosin beta 15B [Source:NCBI gene;Acc:286527] |
| FAM150B | HGLibA_16159 | 202 | 0.692465777 | FAM150B | - |
| FAM194B | HGLibA_16373 | 202 | 0.692465777 | FAM194B | - |
| hsa-mir-1304 | HGLibA_57733 | 202 | 0.692465777 | hsa-mir-1304 | - |
| hsa-mir-1470 | HGLibA_57865 | 202 | 0.692465777 | hsa-mir-1470 | - |
| hsa-mir-320d-2 | HGLibA_58949 | 202 | 0.692465777 | hsa-mir-320d-2 | - |
| hsa-mir-412 | HGLibA_59686 | 202 | 0.692465777 | hsa-mir-412 | - |
| hsa-mir-4276 | HGLibA_59807 | 202 | 0.692465777 | hsa-mir-4276 | - |
| hsa-mir-4297 | HGLibA_59900 | 202 | 0.692465777 | hsa-mir-4297 | - |
| hsa-mir-4674 | HGLibA_60812 | 202 | 0.692465777 | hsa-mir-4674 | - |
| hsa-mir-511 | HGLibA_61559 | 202 | 0.692465777 | hsa-mir-511 | - |
| hsa-mir-6505 | HGLibA_62871 | 202 | 0.692465777 | hsa-mir-6505 | - |
| hsa-mir-877 | HGLibA_64218 | 202 | 0.692465777 | hsa-mir-877 | - |
| hsa-mir-892c | HGLibA_64260 | 202 | 0.692465777 | hsa-mir-892c | - |
| MLL5 | HGLibA_29404 | 202 | 0.692465777 | MLL5 | - |
| PDDC1 | HGLibA_35758 | 202 | 0.692465777 | PDDC1 | - |
| C12orf52 | HGLibA_05263 | 201 | 0.683420434 | C12orf52 | - |
| C5orf30 | HGLibA_06374 | 201 | 0.683420434 | C5orf30 | - |
| CCDC94 | HGLibA_07933 | 201 | 0.683420434 | CCDC94 | - |
| ENSG00000008988 | HGLibA_42103 | 201 | 0.683420434 | RPS20 | ribosomal protein S20 [Source:HGNC Symbol;Acc:HGNC:10405] |
| ENSG00000025293 | HGLibA_36363 | 201 | 0.683420434 | PHF20 | PHD finger protein 20 [Source:HGNC Symbol;Acc:HGNC:16098] |
| ENSG00000064655 | HGLibA_15820 | 201 | 0.683420434 | EYA2 | EYA transcriptional coactivator and phosphatase 2 [Source:HGNC Symbol;Acc:HGNC:3520] |
| ENSG00000074219 | HGLibA_48929 | 201 | 0.683420434 | TEAD2 | TEA domain transcription factor 2 [Source:HGNC Symbol;Acc:HGNC:11715] |
| ENSG00000076685 | HGLibA_32810 | 201 | 0.683420434 | NT5C2 | cytosolic II [Source:HGNC Symbol;Acc:HGNC:8022]" |
| ENSG00000084674 | HGLibA_02463 | 201 | 0.683420434 | APOB | apolipoprotein B [Source:HGNC Symbol;Acc:HGNC:603] |
| ENSG00000086205 | HGLibA_17779 | 201 | 0.683420434 | FOLH1 | folate hydrolase 1 [Source:HGNC Symbol;Acc:HGNC:3788] |
| ENSG00000099849 | HGLibA_40392 | 201 | 0.683420434 | RASSF7 | Ras association domain family member 7 [Source:HGNC Symbol;Acc:HGNC:1166] |
| ENSG00000100429 | HGLibA_20999 | 201 | 0.683420434 | HDAC10 | histone deacetylase 10 [Source:HGNC Symbol;Acc:HGNC:18128] |
| ENSG00000102786 | HGLibA_23426 | 201 | 0.683420434 | INTS6 | integrator complex subunit 6 [Source:HGNC Symbol;Acc:HGNC:14879] |
| ENSG00000104064 | HGLibA_18351 | 201 | 0.683420434 | GABPB1 | GA binding protein transcription factor subunit beta 1 [Source:HGNC Symbol;Acc:HGNC:4074] |
| ENSG00000105428 | HGLibA_56948 | 201 | 0.683420434 | ZNRF4 | zinc and ring finger 4 [Source:HGNC Symbol;Acc:HGNC:17726] |
| ENSG00000108175 | HGLibA_55380 | 201 | 0.683420434 | ZMIZ1 | zinc finger MIZ-type containing 1 [Source:HGNC Symbol;Acc:HGNC:16493] |
| ENSG00000108515 | HGLibA_15146 | 201 | 0.683420434 | ENO3 | enolase 3 [Source:HGNC Symbol;Acc:HGNC:3354] |
| ENSG00000110080 | HGLibA_47247 | 201 | 0.683420434 | ST3GAL4 | 3-sialyltransferase 4 [Source:HGNC Symbol;Acc:HGNC:10864]" |
| ENSG00000113273 | HGLibA_03132 | 201 | 0.683420434 | ARSB | arylsulfatase B [Source:HGNC Symbol;Acc:HGNC:714] |
| ENSG00000115738 | HGLibA_22527 | 201 | 0.683420434 | ID2 | inhibitor of DNA binding 2 [Source:HGNC Symbol;Acc:HGNC:5361] |
| ENSG00000116574 | HGLibA_41208 | 201 | 0.683420434 | RHOU | ras homolog family member U [Source:HGNC Symbol;Acc:HGNC:17794] |
| ENSG00000118257 | HGLibA_32718 | 201 | 0.683420434 | NRP2 | neuropilin 2 [Source:HGNC Symbol;Acc:HGNC:8005] |
| ENSG00000121454 | HGLibA_26457 | 201 | 0.683420434 | LHX4 | LIM homeobox 4 [Source:HGNC Symbol;Acc:HGNC:21734] |
| ENSG00000128203 | HGLibA_03356 | 201 | 0.683420434 | ASPHD2 | aspartate beta-hydroxylase domain containing 2 [Source:HGNC Symbol;Acc:HGNC:30437] |
| ENSG00000131408 | HGLibA_32581 | 201 | 0.683420434 | NR1H2 | nuclear receptor subfamily 1 group H member 2 [Source:HGNC Symbol;Acc:HGNC:7965] |
| ENSG00000133740 | HGLibA_14320 | 201 | 0.683420434 | E2F5 | E2F transcription factor 5 [Source:HGNC Symbol;Acc:HGNC:3119] |
| ENSG00000134594 | HGLibA_39880 | 201 | 0.683420434 | RAB33A | member RAS oncogene family [Source:HGNC Symbol;Acc:HGNC:9773]" |
| ENSG00000135750 | HGLibA_24295 | 201 | 0.683420434 | KCNK1 | potassium two pore domain channel subfamily K member 1 [Source:HGNC Symbol;Acc:HGNC:6272] |
| ENSG00000136436 | HGLibA_07000 | 201 | 0.683420434 | CALCOCO2 | calcium binding and coiled-coil domain 2 [Source:HGNC Symbol;Acc:HGNC:29912] |
| ENSG00000137070 | HGLibA_22957 | 201 | 0.683420434 | IL11RA | interleukin 11 receptor subunit alpha [Source:HGNC Symbol;Acc:HGNC:5967] |
| ENSG00000137504 | HGLibA_11117 | 201 | 0.683420434 | CREBZF | CREB/ATF bZIP transcription factor [Source:HGNC Symbol;Acc:HGNC:24905] |
| ENSG00000137507 | HGLibA_27314 | 201 | 0.683420434 | LRRC32 | leucine rich repeat containing 32 [Source:HGNC Symbol;Acc:HGNC:4161] |
| ENSG00000138152 | HGLibA_04903 | 201 | 0.683420434 | BTBD16 | BTB domain containing 16 [Source:HGNC Symbol;Acc:HGNC:26340] |
| ENSG00000143093 | HGLibA_47589 | 201 | 0.683420434 | STRIP1 | striatin interacting protein 1 [Source:HGNC Symbol;Acc:HGNC:25916] |
| ENSG00000144229 | HGLibA_49408 | 201 | 0.683420434 | THSD7B | thrombospondin type 1 domain containing 7B [Source:HGNC Symbol;Acc:HGNC:29348] |
| ENSG00000144589 | HGLibA_47455 | 201 | 0.683420434 | STK11IP | serine/threonine kinase 11 interacting protein [Source:HGNC Symbol;Acc:HGNC:19184] |
| ENSG00000145384 | HGLibA_15913 | 201 | 0.683420434 | FABP2 | fatty acid binding protein 2 [Source:HGNC Symbol;Acc:HGNC:3556] |
| ENSG00000149599 | HGLibA_14145 | 201 | 0.683420434 | DUSP15 | dual specificity phosphatase 15 [Source:HGNC Symbol;Acc:HGNC:16236] |
| ENSG00000154822 | HGLibA_37017 | 201 | 0.683420434 | PLCL2 | phospholipase C like 2 [Source:HGNC Symbol;Acc:HGNC:9064] |
| ENSG00000157570 | HGLibA_51916 | 201 | 0.683420434 | TSPAN18 | tetraspanin 18 [Source:HGNC Symbol;Acc:HGNC:20660] |
| ENSG00000158555 | HGLibA_18902 | 201 | 0.683420434 | GDPD5 | glycerophosphodiester phosphodiesterase domain containing 5 [Source:HGNC Symbol;Acc:HGNC:28804] |
| ENSG00000159314 | HGLibA_02744 | 201 | 0.683420434 | ARHGAP27 | Rho GTPase activating protein 27 [Source:HGNC Symbol;Acc:HGNC:31813] |
| ENSG00000166313 | HGLibA_02376 | 201 | 0.683420434 | APBB1 | amyloid beta precursor protein binding family B member 1 [Source:HGNC Symbol;Acc:HGNC:581] |
| ENSG00000167588 | HGLibA_19783 | 201 | 0.683420434 | GPD1 | glycerol-3-phosphate dehydrogenase 1 [Source:HGNC Symbol;Acc:HGNC:4455] |

| | | | | | |
|---|---|---|---|---|---|
| ENSG00000169230 | HGLibA_38429 | 201 | 0.683420434 | PRELID1 | PRELI domain containing 1 [Source:HGNC Symbol;Acc:HGNC:30255] |
| ENSG00000172115 | HGLibA_12027 | 201 | 0.683420434 | CYCS | somatic [Source:HGNC Symbol;Acc:HGNC:19986]" |
| ENSG00000177463 | HGLibA_32597 | 201 | 0.683420434 | NR2C2 | nuclear receptor subfamily 2 group C member 2 [Source:HGNC Symbol;Acc:HGNC:7972] |
| ENSG00000178971 | HGLibA_11594 | 201 | 0.683420434 | CTC1 | CST telomere replication complex component 1 [Source:HGNC Symbol;Acc:HGNC:26169] |
| ENSG00000184261 | HGLibA_24299 | 201 | 0.683420434 | KCNK12 | potassium two pore domain channel subfamily K member 12 [Source:HGNC Symbol;Acc:HGNC:6274] |
| ENSG00000184857 | HGLibA_50125 | 201 | 0.683420434 | TMEM186 | transmembrane protein 186 [Source:HGNC Symbol;Acc:HGNC:24530] |
| ENSG00000186806 | HGLibA_53842 | 201 | 0.683420434 | VSIG10L | V-set and immunoglobulin domain containing 10 like [Source:HGNC Symbol;Acc:HGNC:27111] |
| ENSG00000187049 | HGLibA_50223 | 201 | 0.683420434 | TMEM216 | transmembrane protein 216 [Source:HGNC Symbol;Acc:HGNC:25018] |
| ENSG00000187257 | HGLibA_42304 | 201 | 0.683420434 | RSBN1L | round spermatid basic protein 1 like [Source:HGNC Symbol;Acc:HGNC:24765] |
| ENSG00000188542 | HGLibA_14174 | 201 | 0.683420434 | DUSP28 | dual specificity phosphatase 28 [Source:HGNC Symbol;Acc:HGNC:33237] |
| ENSG00000196507 | HGLibA_48711 | 201 | 0.683420434 | TCEAL3 | transcription elongation factor A like 3 [Source:HGNC Symbol;Acc:HGNC:28247] |
| ENSG00000196620 | HGLibA_52919 | 201 | 0.683420434 | UGT2B15 | UDP glucuronosyltransferase family 2 member B15 [Source:HGNC Symbol;Acc:HGNC:12546] |
| ENSG00000197487 | HGLibA_18585 | 201 | 0.683420434 | GALP | galanin like peptide [Source:HGNC Symbol;Acc:HGNC:24840] |
| ENSG00000197535 | HGLibA_30850 | 201 | 0.683420434 | MYO5A | myosin VA [Source:HGNC Symbol;Acc:HGNC:7602] |
| ENSG00000197576 | HGLibA_21911 | 201 | 0.683420434 | HOXA4 | homeobox A4 [Source:HGNC Symbol;Acc:HGNC:5105] |
| ENSG00000198851 | HGLibA_08373 | 201 | 0.683420434 | CD3E | CD3e molecule [Source:HGNC Symbol;Acc:HGNC:1674] |
| ENSG00000205277 | HGLibA_30522 | 201 | 0.683420434 | MUC12 | cell surface associated [Source:HGNC Symbol;Acc:HGNC:7510]" |
| ENSG00000214360 | HGLibA_14554 | 201 | 0.683420434 | EFCAB9 | EF-hand calcium binding domain 9 [Source:HGNC Symbol;Acc:HGNC:34530] |
| ENSG00000232859 | HGLibA_27761 | 201 | 0.683420434 | LYRM9 | LYR motif containing 9 [Source:HGNC Symbol;Acc:HGNC:27314] |
| ENSG00000238269 | HGLibA_35010 | 201 | 0.683420434 | PAGE2B | PAGE family member 2B [Source:HGNC Symbol;Acc:HGNC:31805] |
| ENSG00000254466 | HGLibA_33969 | 201 | 0.683420434 | OR4D10 | olfactory receptor family 4 subfamily D member 10 [Source:HGNC Symbol;Acc:HGNC:15173] |
| ENSG00000263465 | HGLibA_47091 | 201 | 0.683420434 | SRSF8 | serine and arginine rich splicing factor 8 [Source:HGNC Symbol;Acc:HGNC:16988] |
| FAM19A3 | HGLibA_16403 | 201 | 0.683420434 | FAM19A3 | - |
| hsa-let-7c | HGLibA_57115 | 201 | 0.683420434 | hsa-let-7c | - |
| hsa-mir-1197 | HGLibA_57254 | 201 | 0.683420434 | hsa-mir-1197 | - |
| hsa-mir-1263 | HGLibA_57476 | 201 | 0.683420434 | hsa-mir-1263 | - |
| hsa-mir-3649 | HGLibA_59140 | 201 | 0.683420434 | hsa-mir-3649 | - |
| hsa-mir-378d-1 | HGLibA_59439 | 201 | 0.683420434 | hsa-mir-378d-1 | - |
| hsa-mir-4325 | HGLibA_60023 | 201 | 0.683420434 | hsa-mir-4325 | - |
| hsa-mir-4502 | HGLibA_60435 | 201 | 0.683420434 | hsa-mir-4502 | - |
| hsa-mir-571 | HGLibA_62376 | 201 | 0.683420434 | hsa-mir-571 | - |
| IL9R_X | HGLibA_23203 | 201 | 0.683420434 | IL9R_X | - |
| KIAA1211 | HGLibA_24715 | 201 | 0.683420434 | KIAA1211 | - |
| SMCR7 | HGLibA_45678 | 201 | 0.683420434 | SMCR7 | - |
| C10orf128 | HGLibA_05048 | 200 | 0.674375091 | C10orf128 | - |
| C15orf60 | HGLibA_05427 | 200 | 0.674375091 | C15orf60 | - |
| ENSG00000005469 | HGLibA_11213 | 200 | 0.674375091 | CROT | carnitine O-octanoyltransferase [Source:HGNC Symbol;Acc:HGNC:2366] |
| ENSG00000006606 | HGLibA_08009 | 200 | 0.674375091 | CCL26 | C-C motif chemokine ligand 26 [Source:HGNC Symbol;Acc:HGNC:10625] |
| ENSG00000011132 | HGLibA_02373 | 200 | 0.674375091 | APBA3 | amyloid beta precursor protein binding family A member 3 [Source:HGNC Symbol;Acc:HGNC:580] |
| ENSG00000012504 | HGLibA_32585 | 200 | 0.674375091 | NR1H4 | nuclear receptor subfamily 1 group H member 4 [Source:HGNC Symbol;Acc:HGNC:7967] |
| ENSG00000048392 | HGLibA_42258 | 200 | 0.674375091 | RRM2B | ribonucleotide reductase regulatory TP53 inducible subunit M2B [Source:HGNC Symbol;Acc:HGNC:17296] |
| ENSG00000057149 | HGLibA_43550 | 200 | 0.674375091 | SERPINB3 | serpin family B member 3 [Source:HGNC Symbol;Acc:HGNC:10569] |
| ENSG00000065361 | HGLibA_15420 | 200 | 0.674375091 | ERBB3 | erb-b2 receptor tyrosine kinase 3 [Source:HGNC Symbol;Acc:HGNC:3431] |
| ENSG00000072042 | HGLibA_40734 | 200 | 0.674375091 | RDH11 | retinol dehydrogenase 11 [Source:HGNC Symbol;Acc:HGNC:17964] |
| ENSG00000077157 | HGLibA_38022 | 200 | 0.674375091 | PPP1R12B | protein phosphatase 1 regulatory subunit 12B [Source:HGNC Symbol;Acc:HGNC:7619] |
| ENSG00000078900 | HGLibA_51097 | 200 | 0.674375091 | TP73 | tumor protein p73 [Source:HGNC Symbol;Acc:HGNC:12003] |
| ENSG00000086991 | HGLibA_32376 | 200 | 0.674375091 | NOX4 | NADPH oxidase 4 [Source:HGNC Symbol;Acc:HGNC:7891] |
| ENSG00000100197 | HGLibA_12127 | 200 | 0.674375091 | CYP2D6 | cytochrome P450 family 2 subfamily D member 6 [Source:HGNC Symbol;Acc:HGNC:2625] |
| ENSG00000102290 | HGLibA_35390 | 200 | 0.674375091 | PCDH11X | protocadherin 11 X-linked [Source:HGNC Symbol;Acc:HGNC:8656] |
| ENSG00000104804 | HGLibA_52333 | 200 | 0.674375091 | TULP2 | tubby like protein 2 [Source:HGNC Symbol;Acc:HGNC:12424] |
| ENSG00000105974 | HGLibA_07377 | 200 | 0.674375091 | CAV1 | caveolin 1 [Source:HGNC Symbol;Acc:HGNC:1527] |
| ENSG00000106069 | HGLibA_09507 | 200 | 0.674375091 | CHN2 | chimerin 2 [Source:HGNC Symbol;Acc:HGNC:1944] |
| ENSG00000106263 | HGLibA_14765 | 200 | 0.674375091 | EIF3B | eukaryotic translation initiation factor 3 subunit B [Source:HGNC Symbol;Acc:HGNC:3280] |
| ENSG00000106366 | HGLibA_43578 | 200 | 0.674375091 | SERPINE1 | serpin family E member 1 [Source:HGNC Symbol;Acc:HGNC:8583] |
| ENSG00000107833 | HGLibA_32492 | 200 | 0.674375091 | NPM3 | nucleophosmin/nucleoplasmin 3 [Source:HGNC Symbol;Acc:HGNC:7931] |
| ENSG00000107882 | HGLibA_47715 | 200 | 0.674375091 | SUFU | SUFU negative regulator of hedgehog signaling [Source:HGNC Symbol;Acc:HGNC:16466] |
| ENSG00000108379 | HGLibA_54364 | 200 | 0.674375091 | WNT3 | Wnt family member 3 [Source:HGNC Symbol;Acc:HGNC:12782] |
| ENSG00000109919 | HGLibA_30329 | 200 | 0.674375091 | MTCH2 | mitochondrial carrier 2 [Source:HGNC Symbol;Acc:HGNC:17587] |
| ENSG00000110011 | HGLibA_13620 | 200 | 0.674375091 | DNAJC4 | DnaJ heat shock protein family (Hsp40) member C4 [Source:HGNC Symbol;Acc:HGNC:5271] |
| ENSG00000110697 | HGLibA_36750 | 200 | 0.674375091 | PITPNM1 | phosphatidylinositol transfer protein membrane associated 1 [Source:HGNC Symbol;Acc:HGNC:9003] |
| ENSG00000110700 | HGLibA_42071 | 200 | 0.674375091 | RPS13 | ribosomal protein S13 [Source:HGNC Symbol;Acc:HGNC:10386] |
| ENSG00000114745 | HGLibA_19677 | 200 | 0.674375091 | GORASP1 | golgi reassembly stacking protein 1 [Source:HGNC Symbol;Acc:HGNC:16769] |
| ENSG00000116691 | HGLibA_29266 | 200 | 0.674375091 | MIIP | migration and invasion inhibitory protein [Source:HGNC Symbol;Acc:HGNC:25715] |
| ENSG00000118156 | HGLibA_56190 | 200 | 0.674375091 | ZNF541 | zinc finger protein 541 [Source:HGNC Symbol;Acc:HGNC:25294] |
| ENSG00000121716 | HGLibA_36673 | 200 | 0.674375091 | PILRB | paired immunoglobin-like type 2 receptor beta [Source:HGNC Symbol;Acc:HGNC:18297] |
| ENSG00000123407 | HGLibA_21962 | 200 | 0.674375091 | HOXC12 | homeobox C12 [Source:HGNC Symbol;Acc:HGNC:5124] |
| ENSG00000125538 | HGLibA_23039 | 200 | 0.674375091 | IL1B | interleukin 1 beta [Source:HGNC Symbol;Acc:HGNC:5992] |
| ENSG00000126259 | HGLibA_25003 | 200 | 0.674375091 | KIRREL2 | kirre like nephrin family adhesion molecule 2 [Source:HGNC Symbol;Acc:HGNC:18816] |
| ENSG00000130427 | HGLibA_15349 | 200 | 0.674375091 | EPO | erythropoietin [Source:HGNC Symbol;Acc:HGNC:3415] |
| ENSG00000130755 | HGLibA_19407 | 200 | 0.674375091 | GMFG | glia maturation factor gamma [Source:HGNC Symbol;Acc:HGNC:4374] |
| ENSG00000130758 | HGLibA_28163 | 200 | 0.674375091 | MAP3K10 | mitogen-activated protein kinase kinase kinase 10 [Source:HGNC Symbol;Acc:HGNC:6849] |
| ENSG00000131437 | HGLibA_24933 | 200 | 0.674375091 | KIF3A | kinesin family member 3A [Source:HGNC Symbol;Acc:HGNC:6319] |
| ENSG00000132688 | HGLibA_31699 | 200 | 0.674375091 | NES | nestin [Source:HGNC Symbol;Acc:HGNC:7756] |
| ENSG00000136238 | HGLibA_40043 | 200 | 0.674375091 | RAC1 | Rac family small GTPase 1 [Source:HGNC Symbol;Acc:HGNC:9801] |
| ENSG00000139151 | HGLibA_37032 | 200 | 0.674375091 | PLCZ1 | phospholipase C zeta 1 [Source:HGNC Symbol;Acc:HGNC:19218] |
| ENSG00000144711 | HGLibA_23542 | 200 | 0.674375091 | IQSEC1 | IQ motif and Sec7 domain 1 [Source:HGNC Symbol;Acc:HGNC:29112] |
| ENSG00000145113 | HGLibA_30546 | 200 | 0.674375091 | MUC4 | cell surface associated [Source:HGNC Symbol;Acc:HGNC:7514]" |
| ENSG00000149571 | HGLibA_25007 | 200 | 0.674375091 | KIRREL3 | kirre like nephrin family adhesion molecule 3 [Source:HGNC Symbol;Acc:HGNC:23204] |
| ENSG00000152034 | HGLibA_28593 | 200 | 0.674375091 | MCHR2 | melanin concentrating hormone receptor 2 [Source:HGNC Symbol;Acc:HGNC:20867] |
| ENSG00000152457 | HGLibA_12515 | 200 | 0.674375091 | DCLRE1C | DNA cross-link repair 1C [Source:HGNC Symbol;Acc:HGNC:17642] |
| ENSG00000156804 | HGLibA_17073 | 200 | 0.674375091 | FBXO32 | F-box protein 32 [Source:HGNC Symbol;Acc:HGNC:16731] |
| ENSG00000158825 | HGLibA_08499 | 200 | 0.674375091 | CDA | cytidine deaminase [Source:HGNC Symbol;Acc:HGNC:1712] |
| ENSG00000162592 | HGLibA_07742 | 200 | 0.674375091 | CCDC27 | coiled-coil domain containing 27 [Source:HGNC Symbol;Acc:HGNC:26546] |
| ENSG00000162630 | HGLibA_03958 | 200 | 0.674375091 | B3GALT2 | 3-galactosyltransferase 2 [Source:HGNC Symbol;Acc:HGNC:917]" |
| ENSG00000163374 | HGLibA_54759 | 200 | 0.674375091 | YY1AP1 | YY1 associated protein 1 [Source:HGNC Symbol;Acc:HGNC:30935] |
| ENSG00000164978 | HGLibA_32966 | 200 | 0.674375091 | NUDT2 | nudix hydrolase 2 [Source:HGNC Symbol;Acc:HGNC:8049] |
| ENSG00000166086 | HGLibA_23934 | 200 | 0.674375091 | JAM3 | junctional adhesion molecule 3 [Source:HGNC Symbol;Acc:HGNC:15532] |
| ENSG00000166845 | HGLibA_05618 | 200 | 0.674375091 | C18orf54 | chromosome 18 open reading frame 54 [Source:HGNC Symbol;Acc:HGNC:13796] |
| ENSG00000167700 | HGLibA_29100 | 200 | 0.674375091 | MFSD3 | major facilitator superfamily domain containing 3 [Source:HGNC Symbol;Acc:HGNC:25157] |
| ENSG00000169967 | HGLibA_28182 | 200 | 0.674375091 | MAP3K2 | mitogen-activated protein kinase kinase kinase 2 [Source:HGNC Symbol;Acc:HGNC:6854] |
| ENSG00000171951 | HGLibA_42898 | 200 | 0.674375091 | SCG2 | secretogranin II [Source:HGNC Symbol;Acc:HGNC:10575] |
| ENSG00000174428 | HGLibA_20573 | 200 | 0.674375091 | GTF2IRD2B | GTF2I repeat domain containing 2B [Source:HGNC Symbol;Acc:HGNC:33125] |
| ENSG00000174500 | HGLibA_18826 | 200 | 0.674375091 | GCSAM | germinal center associated signaling and motility [Source:HGNC Symbol;Acc:HGNC:20253] |
| ENSG00000180745 | HGLibA_10129 | 200 | 0.674375091 | CLRN3 | clarin 3 [Source:HGNC Symbol;Acc:HGNC:20795] |
| ENSG00000183317 | HGLibA_15286 | 200 | 0.674375091 | EPHA10 | EPH receptor A10 [Source:HGNC Symbol;Acc:HGNC:19987] |
| ENSG00000183856 | HGLibA_23539 | 200 | 0.674375091 | IQGAP3 | IQ motif containing GTPase activating protein 3 [Source:HGNC Symbol;Acc:HGNC:20669] |
| ENSG00000186009 | HGLibA_03635 | 200 | 0.674375091 | ATP4B | ATPase H+/K+ transporting subunit beta [Source:HGNC Symbol;Acc:HGNC:820] |
| ENSG00000187033 | HGLibA_42692 | 200 | 0.674375091 | SAMD7 | sterile alpha motif domain containing 7 [Source:HGNC Symbol;Acc:HGNC:25394] |
| ENSG00000188747 | HGLibA_32382 | 200 | 0.674375091 | NOXA1 | NADPH oxidase activator 1 [Source:HGNC Symbol;Acc:HGNC:10668] |
| ENSG00000197406 | HGLibA_13245 | 200 | 0.674375091 | DIO3 | iodothyronine deiodinase 3 [Source:HGNC Symbol;Acc:HGNC:2885] |
| ENSG00000197779 | HGLibA_56801 | 200 | 0.674375091 | ZNF81 | zinc finger protein 81 [Source:HGNC Symbol;Acc:HGNC:13156] |
| ENSG00000204257 | HGLibA_21604 | 200 | 0.674375091 | HLA-DMA | DM alpha [Source:HGNC Symbol;Acc:HGNC:4934]" |
| ENSG00000253846 | HGLibA_35520 | 200 | 0.674375091 | PCDHGA10 | 10 [Source:HGNC Symbol;Acc:HGNC:8697]" |
| ENSG00000255181 | HGLibA_07672 | 200 | 0.674375091 | CCDC166 | coiled-coil domain containing 166 [Source:HGNC Symbol;Acc:HGNC:41910] |
| ENSG00000267060 | HGLibA_39335 | 200 | 0.674375091 | PTGES3L | prostaglandin E synthase 3 like [Source:HGNC Symbol;Acc:HGNC:43943] |
| FAM26F | HGLibA_16568 | 200 | 0.674375091 | FAM26F | - |
| GUCY1B3 | HGLibA_20658 | 200 | 0.674375091 | GUCY1B3 | - |
| hsa-mir-208a | HGLibA_58195 | 200 | 0.674375091 | hsa-mir-208a | - |
| hsa-mir-378b | HGLibA_59431 | 200 | 0.674375091 | hsa-mir-378b | - |
| hsa-mir-424 | HGLibA_59704 | 200 | 0.674375091 | hsa-mir-424 | - |
| hsa-mir-4302 | HGLibA_59920 | 200 | 0.674375091 | hsa-mir-4302 | - |
| hsa-mir-448 | HGLibA_60336 | 200 | 0.674375091 | hsa-mir-448 | - |
| hsa-mir-4727 | HGLibA_61007 | 200 | 0.674375091 | hsa-mir-4727 | - |
| hsa-mir-4754 | HGLibA_61114 | 200 | 0.674375091 | hsa-mir-4754 | - |
| hsa-mir-509-1 | HGLibA_61508 | 200 | 0.674375091 | hsa-mir-509-1 | - |
| hsa-mir-629 | HGLibA_62755 | 200 | 0.674375091 | hsa-mir-629 | - |
| hsa-mir-6500 | HGLibA_62852 | 200 | 0.674375091 | hsa-mir-6500 | - |
| KIAA0907 | HGLibA_24665 | 200 | 0.674375091 | KIAA0907 | - |
| TSSC1 | HGLibA_52003 | 200 | 0.674375091 | TSSC1 | - |
| TTC18 | HGLibA_52063 | 200 | 0.674375091 | TTC18 | - |
| C17orf70 | HGLibA_05565 | 199 | 0.665329748 | C17orf70 | - |
| CXCR7 | HGLibA_11898 | 199 | 0.665329748 | CXCR7 | - |
| CXorf48 | HGLibA_11930 | 199 | 0.665329748 | CXorf48 | - |
| ENSG00000047346 | HGLibA_16478 | 199 | 0.665329748 | FAM214A | family with sequence similarity 214 member A [Source:HGNC Symbol;Acc:HGNC:25609] |
| ENSG00000074181 | HGLibA_32350 | 199 | 0.665329748 | NOTCH3 | notch 3 [Source:HGNC Symbol;Acc:HGNC:7883] |
| ENSG00000075213 | HGLibA_43319 | 199 | 0.665329748 | SEMA3A | semaphorin 3A [Source:HGNC Symbol;Acc:HGNC:10723] |
| ENSG00000078579 | HGLibA_17394 | 199 | 0.665329748 | FGF20 | fibroblast growth factor 20 [Source:HGNC Symbol;Acc:HGNC:3677] |
| ENSG00000080224 | HGLibA_15301 | 199 | 0.665329748 | EPHA6 | EPH receptor A6 [Source:HGNC Symbol;Acc:HGNC:19296] |
| ENSG00000084072 | HGLibA_37927 | 199 | 0.665329748 | PPIE | peptidylprolyl isomerase E [Source:HGNC Symbol;Acc:HGNC:9258] |
| ENSG00000087448 | HGLibA_25212 | 199 | 0.665329748 | KLHL42 | kelch like family member 42 [Source:HGNC Symbol;Acc:HGNC:29252] |
| ENSG00000088882 | HGLibA_11036 | 199 | 0.665329748 | CPXM1 | M14 family member 1 [Source:HGNC Symbol;Acc:HGNC:15771]" |
| ENSG00000094963 | HGLibA_17706 | 199 | 0.665329748 | FMO2 | flavin containing monooxygenase 2 [Source:HGNC Symbol;Acc:HGNC:3770] |
| ENSG00000101473 | HGLibA_00490 | 199 | 0.665329748 | ACOT8 | acyl-CoA thioesterase 8 [Source:HGNC Symbol;Acc:HGNC:15919] |
| ENSG00000103154 | HGLibA_31587 | 199 | 0.665329748 | NECAB2 | N-terminal EF-hand calcium binding protein 2 [Source:HGNC Symbol;Acc:HGNC:23746] |
| ENSG00000105220 | HGLibA_19806 | 199 | 0.665329748 | GPI | glucose-6-phosphate isomerase [Source:HGNC Symbol;Acc:HGNC:4458] |

| | | | | | |
|---|---|---|---|---|---|
| ENSG00000105371 | HGLibA_22502 | 199 | 0.665329748 | ICAM4 | intercellular adhesion molecule 4 (Landsteiner-Wiener blood group) [Source:HGNC Symbol;Acc:HGNC:5347] |
| ENSG00000106686 | HGLibA_46525 | 199 | 0.665329748 | SPATA6L | spermatogenesis associated 6 like [Source:HGNC Symbol;Acc:HGNC:25472] |
| ENSG00000111863 | HGLibA_01083 | 199 | 0.665329748 | ADTRP | androgen dependent TFPI regulating protein [Source:HGNC Symbol;Acc:HGNC:21214] |
| ENSG00000114520 | HGLibA_46095 | 199 | 0.665329748 | SNX4 | sorting nexin 4 [Source:HGNC Symbol;Acc:HGNC:11175] |
| ENSG00000116106 | HGLibA_15297 | 199 | 0.665329748 | EPHA4 | EPH receptor A4 [Source:HGNC Symbol;Acc:HGNC:3388] |
| ENSG00000117528 | HGLibA_00197 | 199 | 0.665329748 | ABCD3 | ATP binding cassette subfamily D member 3 [Source:HGNC Symbol;Acc:HGNC:67] |
| ENSG00000117707 | HGLibA_38700 | 199 | 0.665329748 | PROX1 | prospero homeobox 1 [Source:HGNC Symbol;Acc:HGNC:9459] |
| ENSG00000118276 | HGLibA_04037 | 199 | 0.665329748 | B4GALT6 | 4-galactosyltransferase 6 [Source:HGNC Symbol;Acc:HGNC:929]" |
| ENSG00000119661 | HGLibA_13645 | 199 | 0.665329748 | DNAL1 | dynein axonemal light chain 1 [Source:HGNC Symbol;Acc:HGNC:23247] |
| ENSG00000121057 | HGLibA_01402 | 199 | 0.665329748 | AKAP1 | A-kinase anchoring protein 1 [Source:HGNC Symbol;Acc:HGNC:367] |
| ENSG00000123989 | HGLibA_09522 | 199 | 0.665329748 | CHPF | chondroitin polymerizing factor [Source:HGNC Symbol;Acc:HGNC:24291] |
| ENSG00000124140 | HGLibA_44370 | 199 | 0.665329748 | SLC12A5 | solute carrier family 12 member 5 [Source:HGNC Symbol;Acc:HGNC:13818] |
| ENSG00000125551 | HGLibA_37164 | 199 | 0.665329748 | PLGLB2 | plasminogen-like B2 [Source:HGNC Symbol;Acc:HGNC:9073] |
| ENSG00000126217 | HGLibA_28579 | 199 | 0.665329748 | MCF2L | MCF.2 cell line derived transforming sequence like [Source:HGNC Symbol;Acc:HGNC:14576] |
| ENSG00000129038 | HGLibA_27038 | 199 | 0.665329748 | LOXL1 | lysyl oxidase like 1 [Source:HGNC Symbol;Acc:HGNC:6665] |
| ENSG00000130988 | HGLibA_41020 | 199 | 0.665329748 | RGN | regucalcin [Source:HGNC Symbol;Acc:HGNC:9989] |
| ENSG00000131069 | HGLibA_00590 | 199 | 0.665329748 | ACSS2 | acyl-CoA synthetase short chain family member 2 [Source:HGNC Symbol;Acc:HGNC:15814] |
| ENSG00000135436 | HGLibA_16338 | 199 | 0.665329748 | FAM186B | family with sequence similarity 186 member B [Source:HGNC Symbol;Acc:HGNC:25296] |
| ENSG00000136010 | HGLibA_01556 | 199 | 0.665329748 | ALDH1L2 | aldehyde dehydrogenase 1 family member L2 [Source:HGNC Symbol;Acc:HGNC:26777] |
| ENSG00000136235 | HGLibA_19833 | 199 | 0.665329748 | GPNMB | glycoprotein nmb [Source:HGNC Symbol;Acc:HGNC:4462] |
| ENSG00000140612 | HGLibA_43180 | 199 | 0.665329748 | SEC11A | signal peptidase complex subunit [Source:HGNC Symbol;Acc:HGNC:17718]" |
| ENSG00000141219 | HGLibA_05583 | 199 | 0.665329748 | C17orf80 | chromosome 17 open reading frame 80 [Source:HGNC Symbol;Acc:HGNC:29601] |
| ENSG00000141338 | HGLibA_00124 | 199 | 0.665329748 | ABCA8 | ATP binding cassette subfamily A member 8 [Source:HGNC Symbol;Acc:HGNC:38] |
| ENSG00000143190 | HGLibA_37755 | 199 | 0.665329748 | POU2F1 | POU class 2 homeobox 1 [Source:HGNC Symbol;Acc:HGNC:9212] |
| ENSG00000145103 | HGLibA_23208 | 199 | 0.665329748 | ILDR1 | immunoglobulin like domain containing receptor 1 [Source:HGNC Symbol;Acc:HGNC:28741] |
| ENSG00000145949 | HGLibA_30792 | 199 | 0.665329748 | MYLK4 | myosin light chain kinase family member 4 [Source:HGNC Symbol;Acc:HGNC:27972] |
| ENSG00000146830 | HGLibA_19065 | 199 | 0.665329748 | GIGYF1 | GRB10 interacting GYF protein 1 [Source:HGNC Symbol;Acc:HGNC:9126] |
| ENSG00000148082 | HGLibA_44007 | 199 | 0.665329748 | SHC3 | SHC adaptor protein 3 [Source:HGNC Symbol;Acc:HGNC:18181] |
| ENSG00000148926 | HGLibA_00991 | 199 | 0.665329748 | ADM | adrenomedullin [Source:HGNC Symbol;Acc:HGNC:259] |
| ENSG00000150681 | HGLibA_41073 | 199 | 0.665329748 | RGS18 | regulator of G protein signaling 18 [Source:HGNC Symbol;Acc:HGNC:14261] |
| ENSG00000152785 | HGLibA_04604 | 199 | 0.665329748 | BMP3 | bone morphogenetic protein 3 [Source:HGNC Symbol;Acc:HGNC:1070] |
| ENSG00000154832 | HGLibA_11964 | 199 | 0.665329748 | CXXC1 | CXXC finger protein 1 [Source:HGNC Symbol;Acc:HGNC:24343] |
| ENSG00000155111 | HGLibA_08752 | 199 | 0.665329748 | CDK19 | cyclin dependent kinase 19 [Source:HGNC Symbol;Acc:HGNC:19338] |
| ENSG00000157856 | HGLibA_13956 | 199 | 0.665329748 | DRC1 | dynein regulatory complex subunit 1 [Source:HGNC Symbol;Acc:HGNC:24245] |
| ENSG00000159251 | HGLibA_00608 | 199 | 0.665329748 | ACTC1 | cardiac muscle 1 [Source:HGNC Symbol;Acc:HGNC:143]" |
| ENSG00000163485 | HGLibA_01011 | 199 | 0.665329748 | ADORA1 | adenosine A1 receptor [Source:HGNC Symbol;Acc:HGNC:262] |
| ENSG00000163739 | HGLibA_11843 | 199 | 0.665329748 | CXCL1 | C-X-C motif chemokine ligand 1 [Source:HGNC Symbol;Acc:HGNC:4602] |
| ENSG00000164509 | HGLibA_23132 | 199 | 0.665329748 | IL31RA | interleukin 31 receptor A [Source:HGNC Symbol;Acc:HGNC:18969] |
| ENSG00000166170 | HGLibA_04086 | 199 | 0.665329748 | BAG5 | BCL2 associated athanogene 5 [Source:HGNC Symbol;Acc:HGNC:941] |
| ENSG00000167748 | HGLibA_25230 | 199 | 0.665329748 | KLK1 | kallikrein 1 [Source:HGNC Symbol;Acc:HGNC:6357] |
| ENSG00000167880 | HGLibA_15702 | 199 | 0.665329748 | EVPL | envoplakin [Source:HGNC Symbol;Acc:HGNC:3503] |
| ENSG00000168594 | HGLibA_00766 | 199 | 0.665329748 | ADAM29 | ADAM metallopeptidase domain 29 [Source:HGNC Symbol;Acc:HGNC:207] |
| ENSG00000168952 | HGLibA_47682 | 199 | 0.665329748 | STXBP6 | syntaxin binding protein 6 [Source:HGNC Symbol;Acc:HGNC:19666] |
| ENSG00000168961 | HGLibA_26382 | 199 | 0.665329748 | LGALS9 | galectin 9 [Source:HGNC Symbol;Acc:HGNC:6570] |
| ENSG00000173080 | HGLibA_42502 | 199 | 0.665329748 | RXFP4 | relaxin family peptide/INSL5 receptor 4 [Source:HGNC Symbol;Acc:HGNC:14666] |
| ENSG00000174982 | HGLibA_34064 | 199 | 0.665329748 | OR4S2 | olfactory receptor family 4 subfamily S member 2 [Source:HGNC Symbol;Acc:HGNC:15183] |
| ENSG00000174992 | HGLibA_55297 | 199 | 0.665329748 | ZG16 | zymogen granule protein 16 [Source:HGNC Symbol;Acc:HGNC:30961] |
| ENSG00000175274 | HGLibA_51060 | 199 | 0.665329748 | TP53I11 | tumor protein p53 inducible protein 11 [Source:HGNC Symbol;Acc:HGNC:16842] |
| ENSG00000175535 | HGLibA_37354 | 199 | 0.665329748 | PNLIP | pancreatic lipase [Source:HGNC Symbol;Acc:HGNC:9155] |
| ENSG00000175985 | HGLibA_37092 | 199 | 0.665329748 | PLEKHD1 | pleckstrin homology and coiled-coil domain containing D1 [Source:HGNC Symbol;Acc:HGNC:20148] |
| ENSG00000178623 | HGLibA_20028 | 199 | 0.665329748 | GPR35 | G protein-coupled receptor 35 [Source:HGNC Symbol;Acc:HGNC:4492] |
| ENSG00000179104 | HGLibA_50672 | 199 | 0.665329748 | TMTC2 | transmembrane and tetratricopeptide repeat containing 2 [Source:HGNC Symbol;Acc:HGNC:25440] |
| ENSG00000179387 | HGLibA_14942 | 199 | 0.665329748 | ELMOD2 | ELMO domain containing 2 [Source:HGNC Symbol;Acc:HGNC:28111] |
| ENSG00000179889 | HGLibA_35959 | 199 | 0.665329748 | PDXDC1 | pyridoxal dependent decarboxylase domain containing 1 [Source:HGNC Symbol;Acc:HGNC:28995] |
| ENSG00000181004 | HGLibA_04213 | 199 | 0.665329748 | BBS12 | Bardet-Biedl syndrome 12 [Source:HGNC Symbol;Acc:HGNC:26648] |
| ENSG00000181856 | HGLibA_44877 | 199 | 0.665329748 | SLC2A4 | solute carrier family 2 member 4 [Source:HGNC Symbol;Acc:HGNC:11009] |
| ENSG00000182263 | HGLibA_17518 | 199 | 0.665329748 | FIGN | microtubule severing factor [Source:HGNC Symbol;Acc:HGNC:13285]" |
| ENSG00000186153 | HGLibA_54448 | 199 | 0.665329748 | WWOX | WW domain containing oxidoreductase [Source:HGNC Symbol;Acc:HGNC:12799] |
| ENSG00000188021 | HGLibA_52736 | 199 | 0.665329748 | UBQLN2 | ubiquilin 2 [Source:HGNC Symbol;Acc:HGNC:12509] |
| ENSG00000188162 | HGLibA_34730 | 199 | 0.665329748 | OTOG | otogelin [Source:HGNC Symbol;Acc:HGNC:8516] |
| ENSG00000197766 | HGLibA_09253 | 199 | 0.665329748 | CFD | complement factor D [Source:HGNC Symbol;Acc:HGNC:2771] |
| ENSG00000198914 | HGLibA_37770 | 199 | 0.665329748 | POU3F3 | POU class 3 homeobox 3 [Source:HGNC Symbol;Acc:HGNC:9216] |
| ENSG00000205846 | HGLibA_10003 | 199 | 0.665329748 | CLEC6A | C-type lectin domain containing 6A [Source:HGNC Symbol;Acc:HGNC:14556] |
| ENSG00000212122 | HGLibA_52010 | 199 | 0.665329748 | TSSK1B | testis specific serine kinase 1B [Source:HGNC Symbol;Acc:HGNC:14968] |
| ENSG00000249915 | HGLibA_35738 | 199 | 0.665329748 | PDCD6 | programmed cell death 6 [Source:HGNC Symbol;Acc:HGNC:8765] |
| ENSG00000258817 | HGLibA_33944 | 199 | 0.665329748 | OR4C13 | olfactory receptor family 4 subfamily C member 13 [Source:HGNC Symbol;Acc:HGNC:15169] |
| hsa-mir-1-2 | HGLibA_57144 | 199 | 0.665329748 | hsa-mir-1-2 | - |
| hsa-mir-23c | HGLibA_58335 | 199 | 0.665329748 | hsa-mir-23c | - |
| hsa-mir-6744 | HGLibA_63128 | 199 | 0.665329748 | hsa-mir-6744 | - |
| hsa-mir-6822 | HGLibA_63465 | 199 | 0.665329748 | hsa-mir-6822 | - |
| hsa-mir-6830 | HGLibA_63497 | 199 | 0.665329748 | hsa-mir-6830 | - |
| hsa-mir-7160 | HGLibA_63868 | 199 | 0.665329748 | hsa-mir-7160 | - |
| KIAA0317 | HGLibA_24618 | 199 | 0.665329748 | KIAA0317 | - |
| PPP2R3B_Y | HGLibA_38161 | 199 | 0.665329748 | PPP2R3B_Y | - |
| QTRTD1 | HGLibA_39764 | 199 | 0.665329748 | QTRTD1 | - |
| TMEM133 | HGLibA_49944 | 199 | 0.665329748 | TMEM133 | - |
| TMEM136 | HGLibA_49952 | 199 | 0.665329748 | TMEM136 | - |
| C12orf23 | HGLibA_05226 | 198 | 0.656284405 | C12orf23 | - |
| C13orf35 | HGLibA_05308 | 198 | 0.656284405 | C13orf35 | - |
| C1orf172 | HGLibA_05791 | 198 | 0.656284405 | C1orf172 | - |
| C6orf170 | HGLibA_06462 | 198 | 0.656284405 | C6orf170 | - |
| C9orf37 | HGLibA_06724 | 198 | 0.656284405 | C9orf37 | - |
| CCDC155 | HGLibA_07659 | 198 | 0.656284405 | CCDC155 | - |
| ENSG00000002822 | HGLibA_27841 | 198 | 0.656284405 | MAD1L1 | mitotic arrest deficient 1 like 1 [Source:HGNC Symbol;Acc:HGNC:6762] |
| ENSG00000013588 | HGLibA_20126 | 198 | 0.656284405 | GPRC5A | G protein-coupled receptor class C group 5 member A [Source:HGNC Symbol;Acc:HGNC:9836] |
| ENSG00000049239 | HGLibA_20787 | 198 | 0.656284405 | H6PD | hexose-6-phosphate dehydrogenase/glucose 1-dehydrogenase [Source:HGNC Symbol;Acc:HGNC:4795] |
| ENSG00000060566 | HGLibA_11098 | 198 | 0.656284405 | CREB3L3 | cAMP responsive element binding protein 3 like 3 [Source:HGNC Symbol;Acc:HGNC:18855] |
| ENSG00000067221 | HGLibA_47535 | 198 | 0.656284405 | STOML1 | stomatin like 1 [Source:HGNC Symbol;Acc:HGNC:14560] |
| ENSG00000075420 | HGLibA_17749 | 198 | 0.656284405 | FNDC3B | fibronectin type III domain containing 3B [Source:HGNC Symbol;Acc:HGNC:24670] |
| ENSG00000100079 | HGLibA_26360 | 198 | 0.656284405 | LGALS2 | galectin 2 [Source:HGNC Symbol;Acc:HGNC:6562] |
| ENSG00000100220 | HGLibA_42375 | 198 | 0.656284405 | RTCB | 3'-cyclic phosphate and 5'-OH ligase [Source:HGNC Symbol;Acc:HGNC:26935]" |
| ENSG00000105808 | HGLibA_40306 | 198 | 0.656284405 | RASA4 | RAS p21 protein activator 4 [Source:HGNC Symbol;Acc:HGNC:23181] |
| ENSG00000105887 | HGLibA_30460 | 198 | 0.656284405 | MTPN | myotrophin [Source:HGNC Symbol;Acc:HGNC:15667] |
| ENSG00000106113 | HGLibA_11145 | 198 | 0.656284405 | CRHR2 | corticotropin releasing hormone receptor 2 [Source:HGNC Symbol;Acc:HGNC:2358] |
| ENSG00000107021 | HGLibA_48477 | 198 | 0.656284405 | TBC1D13 | TBC1 domain family member 13 [Source:HGNC Symbol;Acc:HGNC:25571] |
| ENSG00000107185 | HGLibA_41021 | 198 | 0.656284405 | RGP1 | RAB6A GEF complex partner 1 [Source:HGNC Symbol;Acc:HGNC:21965]" |
| ENSG00000108639 | HGLibA_47993 | 198 | 0.656284405 | SYNGR2 | synaptogyrin 2 [Source:HGNC Symbol;Acc:HGNC:11499] |
| ENSG00000114646 | HGLibA_11418 | 198 | 0.656284405 | CSPG5 | chondroitin sulfate proteoglycan 5 [Source:HGNC Symbol;Acc:HGNC:2467] |
| ENSG00000118620 | HGLibA_55979 | 198 | 0.656284405 | ZNF430 | zinc finger protein 430 [Source:HGNC Symbol;Acc:HGNC:20808] |
| ENSG00000127249 | HGLibA_03575 | 198 | 0.656284405 | ATP13A4 | ATPase 13A4 [Source:HGNC Symbol;Acc:HGNC:25422] |
| ENSG00000127318 | HGLibA_23089 | 198 | 0.656284405 | IL22 | interleukin 22 [Source:HGNC Symbol;Acc:HGNC:14900] |
| ENSG00000127947 | HGLibA_39435 | 198 | 0.656284405 | PTPN12 | non-receptor type 12 [Source:HGNC Symbol;Acc:HGNC:9645]" |
| ENSG00000129946 | HGLibA_44002 | 198 | 0.656284405 | SHC2 | SHC adaptor protein 2 [Source:HGNC Symbol;Acc:HGNC:29869] |
| ENSG00000132000 | HGLibA_37457 | 198 | 0.656284405 | PODNL1 | podocan like 1 [Source:HGNC Symbol;Acc:HGNC:26275] |
| ENSG00000132631 | HGLibA_43037 | 198 | 0.656284405 | SCP2D1 | SCP2 sterol binding domain containing 1 [Source:HGNC Symbol;Acc:HGNC:16211] |
| ENSG00000135451 | HGLibA_51718 | 198 | 0.656284405 | TROAP | trophinin associated protein [Source:HGNC Symbol;Acc:HGNC:12327] |
| ENSG00000135517 | HGLibA_29291 | 198 | 0.656284405 | MIP | major intrinsic protein of lens fiber [Source:HGNC Symbol;Acc:HGNC:7103] |
| ENSG00000135905 | HGLibA_13733 | 198 | 0.656284405 | DOCK10 | dedicator of cytokinesis 10 [Source:HGNC Symbol;Acc:HGNC:23479] |
| ENSG00000137133 | HGLibA_21328 | 198 | 0.656284405 | HINT2 | histidine triad nucleotide binding protein 2 [Source:HGNC Symbol;Acc:HGNC:18344] |
| ENSG00000137364 | HGLibA_51153 | 198 | 0.656284405 | TPMT | thiopurine S-methyltransferase [Source:HGNC Symbol;Acc:HGNC:12014] |
| ENSG00000139364 | HGLibA_49935 | 198 | 0.656284405 | TMEM132B | transmembrane protein 132B [Source:HGNC Symbol;Acc:HGNC:29397] |
| ENSG00000139668 | HGLibA_54008 | 198 | 0.656284405 | WDFY2 | WD repeat and FYVE domain containing 2 [Source:HGNC Symbol;Acc:HGNC:20482] |
| ENSG00000142675 | HGLibA_10295 | 198 | 0.656284405 | CNKSR1 | connector enhancer of kinase suppressor of Ras 1 [Source:HGNC Symbol;Acc:HGNC:19700] |
| ENSG00000143224 | HGLibA_38002 | 198 | 0.656284405 | PPOX | protoporphyrinogen oxidase [Source:HGNC Symbol;Acc:HGNC:9280] |
| ENSG00000147206 | HGLibA_33136 | 198 | 0.656284405 | NXF3 | nuclear RNA export factor 3 [Source:HGNC Symbol;Acc:HGNC:8073] |
| ENSG00000152784 | HGLibA_38402 | 198 | 0.656284405 | PRDM8 | PR/SET domain 8 [Source:HGNC Symbol;Acc:HGNC:13993] |
| ENSG00000153046 | HGLibA_08918 | 198 | 0.656284405 | CDYL | chromodomain Y like [Source:HGNC Symbol;Acc:HGNC:1811] |
| ENSG00000154096 | HGLibA_49423 | 198 | 0.656284405 | THY1 | Thy-1 cell surface antigen [Source:HGNC Symbol;Acc:HGNC:11801] |
| ENSG00000158457 | HGLibA_51935 | 198 | 0.656284405 | TSPAN33 | tetraspanin 33 [Source:HGNC Symbol;Acc:HGNC:28743] |
| ENSG00000158859 | HGLibA_00829 | 198 | 0.656284405 | ADAMTS4 | ADAM metallopeptidase with thrombospondin type 1 motif 4 [Source:HGNC Symbol;Acc:HGNC:220] |
| ENSG00000160862 | HGLibA_03931 | 198 | 0.656284405 | AZGP1 | zinc-binding [Source:HGNC Symbol;Acc:HGNC:910]" |
| ENSG00000161944 | HGLibA_03289 | 198 | 0.656284405 | ASGR2 | asialoglycoprotein receptor 2 [Source:HGNC Symbol;Acc:HGNC:743] |
| ENSG00000162817 | HGLibA_05754 | 198 | 0.656284405 | C1orf115 | chromosome 1 open reading frame 115 [Source:HGNC Symbol;Acc:HGNC:25873] |
| ENSG00000163214 | HGLibA_13203 | 198 | 0.656284405 | DHX57 | DExH-box helicase 57 [Source:HGNC Symbol;Acc:HGNC:20086] |
| ENSG00000163491 | HGLibA_31641 | 198 | 0.656284405 | NEK10 | NIMA related kinase 10 [Source:HGNC Symbol;Acc:HGNC:18592] |
| ENSG00000164463 | HGLibA_11115 | 198 | 0.656284405 | CREBRF | CREB3 regulatory factor [Source:HGNC Symbol;Acc:HGNC:24050] |
| ENSG00000169627 | HGLibA_04686 | 198 | 0.656284405 | BOLA2B | bolA family member 2B [Source:HGNC Symbol;Acc:HGNC:32479] |
| ENSG00000170369 | HGLibA_11451 | 198 | 0.656284405 | CST2 | cystatin SA [Source:HGNC Symbol;Acc:HGNC:2474] |
| ENSG00000171492 | HGLibA_27437 | 198 | 0.656284405 | LRRC8D | leucine rich repeat containing 8 VRAC subunit D [Source:HGNC Symbol;Acc:HGNC:16992] |
| ENSG00000171936 | HGLibA_33513 | 198 | 0.656284405 | OR10H3 | olfactory receptor family 10 subfamily H member 3 [Source:HGNC Symbol;Acc:HGNC:8174] |
| ENSG00000175143 | HGLibA_33852 | 198 | 0.656284405 | OR2T1 | olfactory receptor family 2 subfamily T member 1 [Source:HGNC Symbol;Acc:HGNC:8277] |
| ENSG00000175198 | HGLibA_35378 | 198 | 0.656284405 | PCCA | propionyl-CoA carboxylase subunit alpha [Source:HGNC Symbol;Acc:HGNC:8653] |
| ENSG00000177169 | HGLibA_52968 | 198 | 0.656284405 | ULK1 | unc-51 like autophagy activating kinase 1 [Source:HGNC Symbol;Acc:HGNC:12558] |
| ENSG00000177963 | HGLibA_41234 | 198 | 0.656284405 | RIC8A | RIC8 guanine nucleotide exchange factor A [Source:HGNC Symbol;Acc:HGNC:29550] |
| ENSG00000179627 | HGLibA_54886 | 198 | 0.656284405 | ZBTB42 | zinc finger and BTB domain containing 42 [Source:HGNC Symbol;Acc:HGNC:32550] |

| | | | | | |
|---|---|---|---|---|---|
| ENSG00000179914 | HGLibA_23824 | 198 | 0.656284405 | ITLN1 | intelectin 1 [Source:HGNC Symbol;Acc:HGNC:18259] |
| ENSG00000180185 | HGLibA_15955 | 198 | 0.656284405 | FAHD1 | fumarylacetoacetate hydrolase domain containing 1 [Source:HGNC Symbol;Acc:HGNC:14169] |
| ENSG00000180828 | HGLibA_04476 | 198 | 0.656284405 | BHLHE22 | basic helix-loop-helix family member e22 [Source:HGNC Symbol;Acc:HGNC:11963] |
| ENSG00000183246 | HGLibA_41268 | 198 | 0.656284405 | RIMBP3C | RIMS binding protein 3C [Source:HGNC Symbol;Acc:HGNC:33892] |
| ENSG00000183643 | HGLibA_05384 | 198 | 0.656284405 | C15orf32 | chromosome 15 open reading frame 32 [Source:HGNC Symbol;Acc:HGNC:26549] |
| ENSG00000184708 | HGLibA_14837 | 198 | 0.656284405 | EIF4ENIF1 | eukaryotic translation initiation factor 4E nuclear import factor 1 [Source:HGNC Symbol;Acc:HGNC:16687] |
| ENSG00000187612 | HGLibA_34366 | 198 | 0.656284405 | OR5W2 | olfactory receptor family 5 subfamily W member 2 [Source:HGNC Symbol;Acc:HGNC:15299] |
| ENSG00000188910 | HGLibA_19164 | 198 | 0.656284405 | GJB3 | gap junction protein beta 3 [Source:HGNC Symbol;Acc:HGNC:4285] |
| ENSG00000197448 | HGLibA_20469 | 198 | 0.656284405 | GSTK1 | glutathione S-transferase kappa 1 [Source:HGNC Symbol;Acc:HGNC:16906] |
| ENSG00000198839 | HGLibA_55717 | 198 | 0.656284405 | ZNF277 | zinc finger protein 277 [Source:HGNC Symbol;Acc:HGNC:13070] |
| ENSG00000204501 | HGLibA_38293 | 198 | 0.656284405 | PRAMEF15 | PRAME family member 15 [Source:HGNC Symbol;Acc:HGNC:26764] |
| ENSG00000206579 | HGLibA_54523 | 198 | 0.656284405 | XKR4 | XK related 4 [Source:HGNC Symbol;Acc:HGNC:29394] |
| ENSG00000211455 | HGLibA_47504 | 198 | 0.656284405 | STK38L | serine/threonine kinase 38 like [Source:HGNC Symbol;Acc:HGNC:17848] |
| ENSG00000217236 | HGLibA_46317 | 198 | 0.656284405 | SP9 | Sp9 transcription factor [Source:HGNC Symbol;Acc:HGNC:30690] |
| ENSG00000247596 | HGLibA_52372 | 198 | 0.656284405 | TWF2 | twinfilin actin binding protein 2 [Source:HGNC Symbol;Acc:HGNC:9621] |
| ENSG00000249437 | HGLibA_31082 | 198 | 0.656284405 | NAIP | NLR family apoptosis inhibitory protein [Source:HGNC Symbol;Acc:HGNC:7634] |
| HIST1H2AD | HGLibA_21385 | 198 | 0.656284405 | HIST1H2AD | - |
| hsa-mir-1257 | HGLibA_57437 | 198 | 0.656284405 | hsa-mir-1257 | - |
| hsa-mir-1260a | HGLibA_57458 | 198 | 0.656284405 | hsa-mir-1260a | - |
| hsa-mir-3200 | HGLibA_58908 | 198 | 0.656284405 | hsa-mir-3200 | - |
| hsa-mir-3654 | HGLibA_59160 | 198 | 0.656284405 | hsa-mir-3654 | - |
| hsa-mir-4271 | HGLibA_59790 | 198 | 0.656284405 | hsa-mir-4271 | - |
| hsa-mir-4280 | HGLibA_59824 | 198 | 0.656284405 | hsa-mir-4280 | - |
| hsa-mir-4740 | HGLibA_61059 | 198 | 0.656284405 | hsa-mir-4740 | - |
| hsa-mir-500a | HGLibA_61444 | 198 | 0.656284405 | hsa-mir-500a | - |
| hsa-mir-512-2 | HGLibA_61565 | 198 | 0.656284405 | hsa-mir-512-2 | - |
| hsa-mir-586 | HGLibA_62444 | 198 | 0.656284405 | hsa-mir-586 | - |
| hsa-mir-655 | HGLibA_62955 | 198 | 0.656284405 | hsa-mir-655 | - |
| hsa-mir-6750 | HGLibA_63156 | 198 | 0.656284405 | hsa-mir-6750 | - |
| hsa-mir-6780a | HGLibA_63294 | 198 | 0.656284405 | hsa-mir-6780a | - |
| LOH12CR1 | HGLibA_27011 | 198 | 0.656284405 | LOH12CR1 | - |
| C16orf93 | HGLibA_05503 | 197 | 0.647239062 | C16orf93 | - |
| CCDC37 | HGLibA_07766 | 197 | 0.647239062 | CCDC37 | - |
| ENSG00000020129 | HGLibA_31290 | 197 | 0.647239062 | NCDN | neurochondrin [Source:HGNC Symbol;Acc:HGNC:17597] |
| ENSG00000028277 | HGLibA_37759 | 197 | 0.647239062 | POU2F2 | POU class 2 homeobox 2 [Source:HGNC Symbol;Acc:HGNC:9213] |
| ENSG00000077232 | HGLibA_13564 | 197 | 0.647239062 | DNAJC10 | DnaJ heat shock protein family (Hsp40) member C10 [Source:HGNC Symbol;Acc:HGNC:24637] |
| ENSG00000081320 | HGLibA_47464 | 197 | 0.647239062 | STK17B | serine/threonine kinase 17b [Source:HGNC Symbol;Acc:HGNC:11396] |
| ENSG00000084112 | HGLibA_47131 | 197 | 0.647239062 | SSH1 | slingshot protein phosphatase 1 [Source:HGNC Symbol;Acc:HGNC:30579] |
| ENSG00000088367 | HGLibA_15242 | 197 | 0.647239062 | EPB41L1 | erythrocyte membrane protein band 4.1 like 1 [Source:HGNC Symbol;Acc:HGNC:3378] |
| ENSG00000100319 | HGLibA_55377 | 197 | 0.647239062 | ZMAT5 | zinc finger matrin-type 5 [Source:HGNC Symbol;Acc:HGNC:28046] |
| ENSG00000101335 | HGLibA_30778 | 197 | 0.647239062 | MYL9 | myosin light chain 9 [Source:HGNC Symbol;Acc:HGNC:15754] |
| ENSG00000102010 | HGLibA_04640 | 197 | 0.647239062 | BMX | BMX non-receptor tyrosine kinase [Source:HGNC Symbol;Acc:HGNC:1079] |
| ENSG00000105723 | HGLibA_20428 | 197 | 0.647239062 | GSK3A | glycogen synthase kinase 3 alpha [Source:HGNC Symbol;Acc:HGNC:4616] |
| ENSG00000106603 | HGLibA_10444 | 197 | 0.647239062 | COA1 | cytochrome c oxidase assembly factor 1 homolog [Source:HGNC Symbol;Acc:HGNC:21868] |
| ENSG00000108379 | HGLibA_54365 | 197 | 0.647239062 | WNT3 | Wnt family member 3 [Source:HGNC Symbol;Acc:HGNC:12782] |
| ENSG00000110042 | HGLibA_14091 | 197 | 0.647239062 | DTX4 | deltex E3 ubiquitin ligase 4 [Source:HGNC Symbol;Acc:HGNC:29151] |
| ENSG00000110777 | HGLibA_37754 | 197 | 0.647239062 | POU2AF1 | POU class 2 associating factor 1 [Source:HGNC Symbol;Acc:HGNC:9211] |
| ENSG00000111052 | HGLibA_26573 | 197 | 0.647239062 | LIN7A | crumbs cell polarity complex component [Source:HGNC Symbol;Acc:HGNC:17787]" |
| ENSG00000111732 | HGLibA_01326 | 197 | 0.647239062 | AICDA | activation induced cytidine deaminase [Source:HGNC Symbol;Acc:HGNC:13203] |
| ENSG00000112715 | HGLibA_53611 | 197 | 0.647239062 | VEGFA | vascular endothelial growth factor A [Source:HGNC Symbol;Acc:HGNC:12680] |
| ENSG00000113368 | HGLibA_26695 | 197 | 0.647239062 | LMNB1 | lamin B1 [Source:HGNC Symbol;Acc:HGNC:6637] |
| ENSG00000115275 | HGLibA_29632 | 197 | 0.647239062 | MOGS | mannosyl-oligosaccharide glucosidase [Source:HGNC Symbol;Acc:HGNC:24862] |
| ENSG00000115297 | HGLibA_49677 | 197 | 0.647239062 | TLX2 | T cell leukemia homeobox 2 [Source:HGNC Symbol;Acc:HGNC:5057] |
| ENSG00000118503 | HGLibA_50711 | 197 | 0.647239062 | TNFAIP3 | TNF alpha induced protein 3 [Source:HGNC Symbol;Acc:HGNC:11896] |
| ENSG00000120471 | HGLibA_51050 | 197 | 0.647239062 | TP53AIP1 | tumor protein p53 regulated apoptosis inducing protein 1 [Source:HGNC Symbol;Acc:HGNC:29984] |
| ENSG00000120693 | HGLibA_45590 | 197 | 0.647239062 | SMAD9 | SMAD family member 9 [Source:HGNC Symbol;Acc:HGNC:6774] |
| ENSG00000120910 | HGLibA_38190 | 197 | 0.647239062 | PPP3CC | protein phosphatase 3 catalytic subunit gamma [Source:HGNC Symbol;Acc:HGNC:9316] |
| ENSG00000122136 | HGLibA_33225 | 197 | 0.647239062 | OBP2A | odorant binding protein 2A [Source:HGNC Symbol;Acc:HGNC:23380] |
| ENSG00000122877 | HGLibA_14652 | 197 | 0.647239062 | EGR2 | early growth response 2 [Source:HGNC Symbol;Acc:HGNC:3239] |
| ENSG00000124251 | HGLibA_51091 | 197 | 0.647239062 | TP53TG5 | TP53 target 5 [Source:HGNC Symbol;Acc:HGNC:15856] |
| ENSG00000124613 | HGLibA_55907 | 197 | 0.647239062 | ZNF391 | zinc finger protein 391 [Source:HGNC Symbol;Acc:HGNC:18779] |
| ENSG00000125629 | HGLibA_23380 | 197 | 0.647239062 | INSIG2 | insulin induced gene 2 [Source:HGNC Symbol;Acc:HGNC:20452] |
| ENSG00000128313 | HGLibA_02540 | 197 | 0.647239062 | APOL5 | apolipoprotein L5 [Source:HGNC Symbol;Acc:HGNC:14869] |
| ENSG00000128805 | HGLibA_02730 | 197 | 0.647239062 | ARHGAP22 | Rho GTPase activating protein 22 [Source:HGNC Symbol;Acc:HGNC:30320] |
| ENSG00000129744 | HGLibA_03157 | 197 | 0.647239062 | ART1 | ADP-ribosyltransferase 1 [Source:HGNC Symbol;Acc:HGNC:723] |
| ENSG00000131409 | HGLibA_27380 | 197 | 0.647239062 | LRRC4B | leucine rich repeat containing 4B [Source:HGNC Symbol;Acc:HGNC:25042] |
| ENSG00000134375 | HGLibA_49502 | 197 | 0.647239062 | TIMM17A | translocase of inner mitochondrial membrane 17A [Source:HGNC Symbol;Acc:HGNC:17315] |
| ENSG00000135414 | HGLibA_18857 | 197 | 0.647239062 | GDF11 | growth differentiation factor 11 [Source:HGNC Symbol;Acc:HGNC:4216] |
| ENSG00000136327 | HGLibA_32045 | 197 | 0.647239062 | NKX2-8 | NK2 homeobox 8 [Source:HGNC Symbol;Acc:HGNC:16364] |
| ENSG00000137033 | HGLibA_23138 | 197 | 0.647239062 | IL33 | interleukin 33 [Source:HGNC Symbol;Acc:HGNC:16028] |
| ENSG00000137804 | HGLibA_33097 | 197 | 0.647239062 | NUSAP1 | nucleolar and spindle associated protein 1 [Source:HGNC Symbol;Acc:HGNC:18538] |
| ENSG00000139835 | HGLibA_20383 | 197 | 0.647239062 | GRTP1 | growth hormone regulated TBC protein 1 [Source:HGNC Symbol;Acc:HGNC:20310] |
| ENSG00000139988 | HGLibA_40736 | 197 | 0.647239062 | RDH12 | retinol dehydrogenase 12 [Source:HGNC Symbol;Acc:HGNC:19977] |
| ENSG00000140678 | HGLibA_23765 | 197 | 0.647239062 | ITGAX | integrin subunit alpha X [Source:HGNC Symbol;Acc:HGNC:6152] |
| ENSG00000141349 | HGLibA_18310 | 197 | 0.647239062 | G6PC3 | glucose-6-phosphatase catalytic subunit 3 [Source:HGNC Symbol;Acc:HGNC:24861] |
| ENSG00000145911 | HGLibA_30961 | 197 | 0.647239062 | N4BP3 | NEDD4 binding protein 3 [Source:HGNC Symbol;Acc:HGNC:29852] |
| ENSG00000146938 | HGLibA_32077 | 197 | 0.647239062 | NLGN4X | neuroligin 4 X-linked [Source:HGNC Symbol;Acc:HGNC:14287] |
| ENSG00000148841 | HGLibA_23862 | 197 | 0.647239062 | ITPRIP | 5-trisphosphate receptor interacting protein [Source:HGNC Symbol;Acc:HGNC:29370]" |
| ENSG00000150782 | HGLibA_23023 | 197 | 0.647239062 | IL18 | interleukin 18 [Source:HGNC Symbol;Acc:HGNC:5986] |
| ENSG00000154258 | HGLibA_00129 | 197 | 0.647239062 | ABCA9 | ATP binding cassette subfamily A member 9 [Source:HGNC Symbol;Acc:HGNC:39] |
| ENSG00000155100 | HGLibA_34775 | 197 | 0.647239062 | OTUD6B | OTU domain containing 6B [Source:HGNC Symbol;Acc:HGNC:24281] |
| ENSG00000155307 | HGLibA_42710 | 197 | 0.647239062 | SAMSN1 | SH3 domain and nuclear localization signals 1 [Source:HGNC Symbol;Acc:HGNC:10528]" |
| ENSG00000156256 | HGLibA_53213 | 197 | 0.647239062 | USP16 | ubiquitin specific peptidase 16 [Source:HGNC Symbol;Acc:HGNC:12614] |
| ENSG00000156427 | HGLibA_17384 | 197 | 0.647239062 | FGF18 | fibroblast growth factor 18 [Source:HGNC Symbol;Acc:HGNC:3674] |
| ENSG00000159110 | HGLibA_22698 | 197 | 0.647239062 | IFNAR2 | interferon alpha and beta receptor subunit 2 [Source:HGNC Symbol;Acc:HGNC:5433] |
| ENSG00000162415 | HGLibA_57048 | 197 | 0.647239062 | ZSWIM5 | zinc finger SWIM-type containing 5 [Source:HGNC Symbol;Acc:HGNC:29299] |
| ENSG00000163132 | HGLibA_30270 | 197 | 0.647239062 | MSX1 | msh homeobox 1 [Source:HGNC Symbol;Acc:HGNC:7391] |
| ENSG00000164877 | HGLibA_29215 | 197 | 0.647239062 | MICALL2 | MICAL like 2 [Source:HGNC Symbol;Acc:HGNC:29672] |
| ENSG00000170791 | HGLibA_09381 | 197 | 0.647239062 | CHCHD7 | coiled-coil-helix-coiled-coil-helix domain containing 7 [Source:HGNC Symbol;Acc:HGNC:28314] |
| ENSG00000174951 | HGLibA_18179 | 197 | 0.647239062 | FUT1 | fucosyltransferase 1 (H blood group) [Source:HGNC Symbol;Acc:HGNC:4012] |
| ENSG00000178084 | HGLibA_22385 | 197 | 0.647239062 | HTR3C | 5-hydroxytryptamine receptor 3C [Source:HGNC Symbol;Acc:HGNC:24003] |
| ENSG00000178562 | HGLibA_08323 | 197 | 0.647239062 | CD28 | CD28 molecule [Source:HGNC Symbol;Acc:HGNC:1653] |
| ENSG00000178607 | HGLibA_15511 | 197 | 0.647239062 | ERN1 | endoplasmic reticulum to nucleus signaling 1 [Source:HGNC Symbol;Acc:HGNC:3449] |
| ENSG00000178828 | HGLibA_41596 | 197 | 0.647239062 | RNF186 | ring finger protein 186 [Source:HGNC Symbol;Acc:HGNC:25978] |
| ENSG00000179152 | HGLibA_48690 | 197 | 0.647239062 | TCAIM | mitochondrial [Source:HGNC Symbol;Acc:HGNC:25241]" |
| ENSG00000179598 | HGLibA_37051 | 197 | 0.647239062 | PLD6 | phospholipase D family member 6 [Source:HGNC Symbol;Acc:HGNC:30447] |
| ENSG00000183287 | HGLibA_07492 | 197 | 0.647239062 | CCBE1 | collagen and calcium binding EGF domains 1 [Source:HGNC Symbol;Acc:HGNC:29426] |
| ENSG00000183476 | HGLibA_43911 | 197 | 0.647239062 | SH2D7 | SH2 domain containing 7 [Source:HGNC Symbol;Acc:HGNC:34549] |
| ENSG00000183647 | HGLibA_56177 | 197 | 0.647239062 | ZNF530 | zinc finger protein 530 [Source:HGNC Symbol;Acc:HGNC:29297] |
| ENSG00000183762 | HGLibA_25403 | 197 | 0.647239062 | KREMEN1 | kringle containing transmembrane protein 1 [Source:HGNC Symbol;Acc:HGNC:17550] |
| ENSG00000183873 | HGLibA_42999 | 197 | 0.647239062 | SCN5A | sodium voltage-gated channel alpha subunit 5 [Source:HGNC Symbol;Acc:HGNC:10593] |
| ENSG00000184113 | HGLibA_09900 | 197 | 0.647239062 | CLDN5 | claudin 5 [Source:HGNC Symbol;Acc:HGNC:2047] |
| ENSG00000184517 | HGLibA_55179 | 197 | 0.647239062 | ZFP1 | ZFP1 zinc finger protein [Source:HGNC Symbol;Acc:HGNC:23328] |
| ENSG00000185808 | HGLibA_36576 | 197 | 0.647239062 | PIGP | phosphatidylinositol glycan anchor biosynthesis class P [Source:HGNC Symbol;Acc:HGNC:3046] |
| ENSG00000196220 | HGLibA_46981 | 197 | 0.647239062 | SRGAP3 | SLIT-ROBO Rho GTPase activating protein 3 [Source:HGNC Symbol;Acc:HGNC:19744] |
| ENSG00000196372 | HGLibA_03216 | 197 | 0.647239062 | ASB13 | ankyrin repeat and SOCS box containing 13 [Source:HGNC Symbol;Acc:HGNC:19765] |
| ENSG00000197403 | HGLibA_34429 | 197 | 0.647239062 | OR6N1 | olfactory receptor family 6 subfamily N member 1 [Source:HGNC Symbol;Acc:HGNC:15034] |
| ENSG00000197651 | HGLibA_07942 | 197 | 0.647239062 | CCER1 | coiled-coil glutamate rich protein 1 [Source:HGNC Symbol;Acc:HGNC:28373] |
| ENSG00000198759 | HGLibA_14625 | 197 | 0.647239062 | EGFL6 | EGF like domain multiple 6 [Source:HGNC Symbol;Acc:HGNC:3235] |
| ENSG00000205364 | HGLibA_30295 | 197 | 0.647239062 | MT1M | metallothionein 1M [Source:HGNC Symbol;Acc:HGNC:14296] |
| ENSG00000225830 | HGLibA_15445 | 197 | 0.647239062 | ERCC6 | chromatin remodeling factor [Source:HGNC Symbol;Acc:HGNC:3438]" |
| ENSG00000255501 | HGLibA_07232 | 197 | 0.647239062 | CARD18 | caspase recruitment domain family member 18 [Source:HGNC Symbol;Acc:HGNC:28861] |
| ENSG00000261371 | HGLibA_36018 | 197 | 0.647239062 | PECAM1 | platelet and endothelial cell adhesion molecule 1 [Source:HGNC Symbol;Acc:HGNC:8823] |
| ENSG00000275183 | HGLibA_26303 | 197 | 0.647239062 | LENG9 | leukocyte receptor cluster member 9 [Source:HGNC Symbol;Acc:HGNC:16306] |
| FAM129C | HGLibA_16103 | 197 | 0.647239062 | FAM129C | - |
| FAM160B1 | HGLibA_16209 | 197 | 0.647239062 | FAM160B1 | - |
| FBXO18 | HGLibA_17040 | 197 | 0.647239062 | FBXO18 | - |
| hsa-mir-4284 | HGLibA_59845 | 197 | 0.647239062 | hsa-mir-4284 | - |
| hsa-mir-4291 | HGLibA_59876 | 197 | 0.647239062 | hsa-mir-4291 | - |
| hsa-mir-586 | HGLibA_62441 | 197 | 0.647239062 | hsa-mir-586 | - |
| KIAA1467 | HGLibA_24767 | 197 | 0.647239062 | KIAA1467 | - |
| LOC100506422 | HGLibA_26861 | 197 | 0.647239062 | LOC100506422 | - |
| LOC200726 | HGLibA_26903 | 197 | 0.647239062 | LOC200726 | - |
| LOC728392 | HGLibA_26991 | 197 | 0.647239062 | LOC728392 | - |
| MICALCL | HGLibA_29211 | 197 | 0.647239062 | MICALCL | - |
| SCAND3 | HGLibA_42855 | 197 | 0.647239062 | SCAND3 | - |
| SEPW1 | HGLibA_43460 | 197 | 0.647239062 | SEPW1 | - |
| TMEM155 | HGLibA_50009 | 197 | 0.647239062 | TMEM155 | - |
| ATP5J | HGLibA_03671 | 196 | 0.638193719 | ATP5J | - |
| BAGE | HGLibA_04093 | 196 | 0.638193719 | BAGE | - |
| C9orf37 | HGLibA_06726 | 196 | 0.638193719 | C9orf37 | - |
| EFTUD1 | HGLibA_14615 | 196 | 0.638193719 | EFTUD1 | - |
| ENSG00000005073 | HGLibA_21900 | 196 | 0.638193719 | HOXA11 | homeobox A11 [Source:HGNC Symbol;Acc:HGNC:5101] |
| ENSG00000048649 | HGLibA_42310 | 196 | 0.638193719 | RSF1 | remodeling and spacing factor 1 [Source:HGNC Symbol;Acc:HGNC:18118] |
| ENSG00000058600 | HGLibA_37621 | 196 | 0.638193719 | POLR3E | RNA polymerase III subunit E [Source:HGNC Symbol;Acc:HGNC:30347] |

| | | | | | |
|---|---|---|---|---|---|
| ENSG00000068724 | HGLibA_52149 | 196 | 0.638193719 | TTC7A | tetratricopeptide repeat domain 7A [Source:HGNC Symbol;Acc:HGNC:19750] |
| ENSG00000069943 | HGLibA_36547 | 196 | 0.638193719 | PIGB | phosphatidylinositol glycan anchor biosynthesis class B [Source:HGNC Symbol;Acc:HGNC:8959] |
| ENSG00000071894 | HGLibA_10993 | 196 | 0.638193719 | CPSF1 | cleavage and polyadenylation specific factor 1 [Source:HGNC Symbol;Acc:HGNC:2324] |
| ENSG00000075673 | HGLibA_03564 | 196 | 0.638193719 | ATP12A | ATPase H+/K+ transporting non-gastric alpha2 subunit [Source:HGNC Symbol;Acc:HGNC:13816] |
| ENSG00000076555 | HGLibA_00351 | 196 | 0.638193719 | ACACB | acetyl-CoA carboxylase beta [Source:HGNC Symbol;Acc:HGNC:85] |
| ENSG00000083828 | HGLibA_56325 | 196 | 0.638193719 | ZNF586 | zinc finger protein 586 [Source:HGNC Symbol;Acc:HGNC:25949] |
| ENSG00000095777 | HGLibA_30844 | 196 | 0.638193719 | MYO3A | myosin IIIA [Source:HGNC Symbol;Acc:HGNC:7601] |
| ENSG00000099337 | HGLibA_24329 | 196 | 0.638193719 | KCNK6 | potassium two pore domain channel subfamily K member 6 [Source:HGNC Symbol;Acc:HGNC:6281] |
| ENSG00000100209 | HGLibA_22156 | 196 | 0.638193719 | HSCB | HscB mitochondrial iron-sulfur cluster cochaperone [Source:HGNC Symbol;Acc:HGNC:28913] |
| ENSG00000102904 | HGLibA_51888 | 196 | 0.638193719 | TSNAXIP1 | translin associated factor X interacting protein 1 [Source:HGNC Symbol;Acc:HGNC:18586] |
| ENSG00000103549 | HGLibA_41680 | 196 | 0.638193719 | RNF40 | ring finger protein 40 [Source:HGNC Symbol;Acc:HGNC:16867] |
| ENSG00000108622 | HGLibA_22498 | 196 | 0.638193719 | ICAM2 | intercellular adhesion molecule 2 [Source:HGNC Symbol;Acc:HGNC:5345] |
| ENSG00000114416 | HGLibA_18219 | 196 | 0.638193719 | FXR1 | FMR1 autosomal homolog 1 [Source:HGNC Symbol;Acc:HGNC:4023] |
| ENSG00000122787 | HGLibA_01494 | 196 | 0.638193719 | AKR1D1 | aldo-keto reductase family 1 member D1 [Source:HGNC Symbol;Acc:HGNC:388] |
| ENSG00000124177 | HGLibA_09401 | 196 | 0.638193719 | CHD6 | chromodomain helicase DNA binding protein 6 [Source:HGNC Symbol;Acc:HGNC:19057] |
| ENSG00000125967 | HGLibA_31590 | 196 | 0.638193719 | NECAB3 | N-terminal EF-hand calcium binding protein 3 [Source:HGNC Symbol;Acc:HGNC:15851] |
| ENSG00000128791 | HGLibA_52383 | 196 | 0.638193719 | TWSG1 | twisted gastrulation BMP signaling modulator 1 [Source:HGNC Symbol;Acc:HGNC:12429] |
| ENSG00000131061 | HGLibA_55839 | 196 | 0.638193719 | ZNF341 | zinc finger protein 341 [Source:HGNC Symbol;Acc:HGNC:15992] |
| ENSG00000131737 | HGLibA_25485 | 196 | 0.638193719 | KRT34 | keratin 34 [Source:HGNC Symbol;Acc:HGNC:6452] |
| ENSG00000132964 | HGLibA_08798 | 196 | 0.638193719 | CDK8 | cyclin dependent kinase 8 [Source:HGNC Symbol;Acc:HGNC:1779] |
| ENSG00000137266 | HGLibA_44573 | 196 | 0.638193719 | SLC22A23 | solute carrier family 22 member 23 [Source:HGNC Symbol;Acc:HGNC:21106] |
| ENSG00000138395 | HGLibA_08741 | 196 | 0.638193719 | CDK15 | cyclin dependent kinase 15 [Source:HGNC Symbol;Acc:HGNC:14434] |
| ENSG00000138617 | HGLibA_35229 | 196 | 0.638193719 | PARP16 | poly(ADP-ribose) polymerase family member 16 [Source:HGNC Symbol;Acc:HGNC:26040] |
| ENSG00000140287 | HGLibA_21029 | 196 | 0.638193719 | HDC | histidine decarboxylase [Source:HGNC Symbol;Acc:HGNC:4855] |
| ENSG00000141579 | HGLibA_56674 | 196 | 0.638193719 | ZNF750 | zinc finger protein 750 [Source:HGNC Symbol;Acc:HGNC:25843] |
| ENSG00000142687 | HGLibA_24622 | 196 | 0.638193719 | KIAA0319L | KIAA0319 like [Source:HGNC Symbol;Acc:HGNC:30071] |
| ENSG00000143499 | HGLibA_45854 | 196 | 0.638193719 | SMYD2 | SET and MYND domain containing 2 [Source:HGNC Symbol;Acc:HGNC:20982] |
| ENSG00000145681 | HGLibA_20841 | 196 | 0.638193719 | HAPLN1 | hyaluronan and proteoglycan link protein 1 [Source:HGNC Symbol;Acc:HGNC:2380] |
| ENSG00000146676 | HGLibA_39588 | 196 | 0.638193719 | PURB | purine rich element binding protein B [Source:HGNC Symbol;Acc:HGNC:9702] |
| ENSG00000149929 | HGLibA_21355 | 196 | 0.638193719 | HIRIP3 | HIRA interacting protein 3 [Source:HGNC Symbol;Acc:HGNC:4917] |
| ENSG00000150907 | HGLibA_17922 | 196 | 0.638193719 | FOXO1 | forkhead box O1 [Source:HGNC Symbol;Acc:HGNC:3819] |
| ENSG00000152137 | HGLibA_22320 | 196 | 0.638193719 | HSPB8 | heat shock protein family B (small) member 8 [Source:HGNC Symbol;Acc:HGNC:30171] |
| ENSG00000153443 | HGLibA_52539 | 196 | 0.638193719 | UBALD1 | UBA like domain containing 1 [Source:HGNC Symbol;Acc:HGNC:29576] |
| ENSG00000157657 | HGLibA_56397 | 196 | 0.638193719 | ZNF618 | zinc finger protein 618 [Source:HGNC Symbol;Acc:HGNC:29416] |
| ENSG00000163600 | HGLibA_22516 | 196 | 0.638193719 | ICOS | inducible T cell costimulator [Source:HGNC Symbol;Acc:HGNC:5351] |
| ENSG00000164808 | HGLibA_46663 | 196 | 0.638193719 | SPIDR | scaffold protein involved in DNA repair [Source:HGNC Symbol;Acc:HGNC:28971] |
| ENSG00000167851 | HGLibA_08332 | 196 | 0.638193719 | CD300A | CD300a molecule [Source:HGNC Symbol;Acc:HGNC:19319] |
| ENSG00000167962 | HGLibA_56357 | 196 | 0.638193719 | ZNF598 | zinc finger protein 598 [Source:HGNC Symbol;Acc:HGNC:28079] |
| ENSG00000170209 | HGLibA_01973 | 196 | 0.638193719 | ANKK1 | ankyrin repeat and kinase domain containing 1 [Source:HGNC Symbol;Acc:HGNC:21027] |
| ENSG00000170482 | HGLibA_44605 | 196 | 0.638193719 | SLC23A1 | solute carrier family 23 member 1 [Source:HGNC Symbol;Acc:HGNC:10974] |
| ENSG00000170920 | HGLibA_34488 | 196 | 0.638193719 | OR7G3 | olfactory receptor family 7 subfamily G member 3 [Source:HGNC Symbol;Acc:HGNC:8467] |
| ENSG00000171161 | HGLibA_56506 | 196 | 0.638193719 | ZNF672 | zinc finger protein 672 [Source:HGNC Symbol;Acc:HGNC:26179] |
| ENSG00000171557 | HGLibA_17456 | 196 | 0.638193719 | FGG | fibrinogen gamma chain [Source:HGNC Symbol;Acc:HGNC:3694] |
| ENSG00000172543 | HGLibA_11734 | 196 | 0.638193719 | CTSW | cathepsin W [Source:HGNC Symbol;Acc:HGNC:2546] |
| ENSG00000173991 | HGLibA_48692 | 196 | 0.638193719 | TCAP | titin-cap [Source:HGNC Symbol;Acc:HGNC:11610] |
| ENSG00000174032 | HGLibA_44702 | 196 | 0.638193719 | SLC25A30 | solute carrier family 25 member 30 [Source:HGNC Symbol;Acc:HGNC:27371] |
| ENSG00000175489 | HGLibA_27290 | 196 | 0.638193719 | LRRC25 | leucine rich repeat containing 25 [Source:HGNC Symbol;Acc:HGNC:29806] |
| ENSG00000178209 | HGLibA_37054 | 196 | 0.638193719 | PLEC | plectin [Source:HGNC Symbol;Acc:HGNC:9069] |
| ENSG00000180346 | HGLibA_49466 | 196 | 0.638193719 | TIGD2 | tigger transposable element derived 2 [Source:HGNC Symbol;Acc:HGNC:18333] |
| ENSG00000180957 | HGLibA_36744 | 196 | 0.638193719 | PITPNB | phosphatidylinositol transfer protein beta [Source:HGNC Symbol;Acc:HGNC:9002] |
| ENSG00000181092 | HGLibA_00977 | 196 | 0.638193719 | ADIPOQ | C1Q and collagen domain containing [Source:HGNC Symbol;Acc:HGNC:13633]" |
| ENSG00000182132 | HGLibA_24234 | 196 | 0.638193719 | KCNIP1 | potassium voltage-gated channel interacting protein 1 [Source:HGNC Symbol;Acc:HGNC:15521] |
| ENSG00000182901 | HGLibA_41104 | 196 | 0.638193719 | RGS7 | regulator of G protein signaling 7 [Source:HGNC Symbol;Acc:HGNC:10003] |
| ENSG00000184719 | HGLibA_41714 | 196 | 0.638193719 | RNLS | FAD dependent amine oxidase [Source:HGNC Symbol;Acc:HGNC:25641]" |
| ENSG00000186579 | HGLibA_12830 | 196 | 0.638193719 | DEFB106A | defensin beta 106A [Source:HGNC Symbol;Acc:HGNC:18088] |
| ENSG00000187116 | HGLibA_26501 | 196 | 0.638193719 | LILRA5 | leukocyte immunoglobulin like receptor A5 [Source:HGNC Symbol;Acc:HGNC:16309] |
| ENSG00000188315 | HGLibA_06256 | 196 | 0.638193719 | C3orf62 | chromosome 3 open reading frame 62 [Source:HGNC Symbol;Acc:HGNC:24771] |
| ENSG00000188559 | HGLibA_40160 | 196 | 0.638193719 | RALGAPA2 | Ral GTPase activating protein catalytic alpha subunit 2 [Source:HGNC Symbol;Acc:HGNC:16207] |
| ENSG00000196975 | HGLibA_02239 | 196 | 0.638193719 | ANXA4 | annexin A4 [Source:HGNC Symbol;Acc:HGNC:542] |
| ENSG00000198492 | HGLibA_54734 | 196 | 0.638193719 | YTHDF2 | YTH N6-methyladenosine RNA binding protein 2 [Source:HGNC Symbol;Acc:HGNC:31675] |
| ENSG00000205269 | HGLibA_50057 | 196 | 0.638193719 | TMEM170B | transmembrane protein 170B [Source:HGNC Symbol;Acc:HGNC:34244] |
| ENSG00000213672 | HGLibA_31324 | 196 | 0.638193719 | NCKIPSD | NCK interacting protein with SH3 domain [Source:HGNC Symbol;Acc:HGNC:15486] |
| ENSG00000214706 | HGLibA_22741 | 196 | 0.638193719 | IFRD2 | interferon related developmental regulator 2 [Source:HGNC Symbol;Acc:HGNC:5457] |
| ENSG00000237765 | HGLibA_16414 | 196 | 0.638193719 | FAM200B | family with sequence similarity 200 member B [Source:HGNC Symbol;Acc:HGNC:27740] |
| ENSG00000250423 | HGLibA_24711 | 196 | 0.638193719 | KIAA1210 | KIAA1210 [Source:HGNC Symbol;Acc:HGNC:29218] |
| ENSG00000256870 | HGLibA_45282 | 196 | 0.638193719 | SLC5A8 | solute carrier family 5 member 8 [Source:HGNC Symbol;Acc:HGNC:19119] |
| FAM101B | HGLibA_15976 | 196 | 0.638193719 | FAM101B | - |
| HIST1H2BC | HGLibA_21416 | 196 | 0.638193719 | HIST1H2BC | - |
| hsa-mir-29c | HGLibA_58423 | 196 | 0.638193719 | hsa-mir-29c | - |
| hsa-mir-320d-2 | HGLibA_58948 | 196 | 0.638193719 | hsa-mir-320d-2 | - |
| hsa-mir-3689c | HGLibA_59322 | 196 | 0.638193719 | hsa-mir-3689c | - |
| hsa-mir-3689d-2 | HGLibA_59329 | 196 | 0.638193719 | hsa-mir-3689d-2 | - |
| hsa-mir-4304 | HGLibA_59926 | 196 | 0.638193719 | hsa-mir-4304 | - |
| hsa-mir-448 | HGLibA_60334 | 196 | 0.638193719 | hsa-mir-448 | - |
| hsa-mir-6511a-4 | HGLibA_62910 | 196 | 0.638193719 | hsa-mir-6511a-4 | - |
| LOC100996485 | HGLibA_26885 | 196 | 0.638193719 | LOC100996485 | - |
| QTRTD1 | HGLibA_39762 | 196 | 0.638193719 | QTRTD1 | - |
| 15-Sep | HGLibA_43408 | 195 | 0.629148377 | 15-Sep | - |
| ASMTL_Y | HGLibA_03328 | 195 | 0.629148377 | ASMTL_Y | - |
| BAI1 | HGLibA_04112 | 195 | 0.629148377 | BAI1 | - |
| C1orf168 | HGLibA_05788 | 195 | 0.629148377 | C1orf168 | - |
| C20orf201 | HGLibA_06010 | 195 | 0.629148377 | C20orf201 | - |
| CCDC109B | HGLibA_07532 | 195 | 0.629148377 | CCDC109B | - |
| ENSG00000020577 | HGLibA_42684 | 195 | 0.629148377 | SAMD4A | sterile alpha motif domain containing 4A [Source:HGNC Symbol;Acc:HGNC:23023] |
| ENSG00000027869 | HGLibA_43889 | 195 | 0.629148377 | SH2D2A | SH2 domain containing 2A [Source:HGNC Symbol;Acc:HGNC:10821] |
| ENSG00000054392 | HGLibA_21253 | 195 | 0.629148377 | HHAT | hedgehog acyltransferase [Source:HGNC Symbol;Acc:HGNC:18270] |
| ENSG00000065809 | HGLibA_16003 | 195 | 0.629148377 | FAM107B | family with sequence similarity 107 member B [Source:HGNC Symbol;Acc:HGNC:23726] |
| ENSG00000067596 | HGLibA_13208 | 195 | 0.629148377 | DHX8 | DEAH-box helicase 8 [Source:HGNC Symbol;Acc:HGNC:2749] |
| ENSG00000075043 | HGLibA_24368 | 195 | 0.629148377 | KCNQ2 | potassium voltage-gated channel subfamily Q member 2 [Source:HGNC Symbol;Acc:HGNC:6296] |
| ENSG00000075073 | HGLibA_48173 | 195 | 0.629148377 | TACR2 | tachykinin receptor 2 [Source:HGNC Symbol;Acc:HGNC:11527] |
| ENSG00000077616 | HGLibA_31008 | 195 | 0.629148377 | NAALAD2 | N-acetylated alpha-linked acidic dipeptidase 2 [Source:HGNC Symbol;Acc:HGNC:14526] |
| ENSG00000084628 | HGLibA_31990 | 195 | 0.629148377 | NKAIN1 | sodium/potassium transporting ATPase interacting 1 [Source:HGNC Symbol;Acc:HGNC:25743] |
| ENSG00000084674 | HGLibA_02464 | 195 | 0.629148377 | APOB | apolipoprotein B [Source:HGNC Symbol;Acc:HGNC:603] |
| ENSG00000087085 | HGLibA_00437 | 195 | 0.629148377 | ACHE | acetylcholinesterase (Cartwright blood group) [Source:HGNC Symbol;Acc:HGNC:108] |
| ENSG00000087111 | HGLibA_36585 | 195 | 0.629148377 | PIGS | phosphatidylinositol glycan anchor biosynthesis class S [Source:HGNC Symbol;Acc:HGNC:14937] |
| ENSG00000099812 | HGLibA_29312 | 195 | 0.629148377 | MISP | mitotic spindle positioning [Source:HGNC Symbol;Acc:HGNC:27000] |
| ENSG00000100302 | HGLibA_40323 | 195 | 0.629148377 | RASD2 | RASD family member 2 [Source:HGNC Symbol;Acc:HGNC:18229] |
| ENSG00000102119 | HGLibA_15032 | 195 | 0.629148377 | EMD | emerin [Source:HGNC Symbol;Acc:HGNC:3331] |
| ENSG00000103034 | HGLibA_31421 | 195 | 0.629148377 | NDRG4 | NDRG family member 4 [Source:HGNC Symbol;Acc:HGNC:14466] |
| ENSG00000104870 | HGLibA_17201 | 195 | 0.629148377 | FCGRT | Fc fragment of IgG receptor and transporter [Source:HGNC Symbol;Acc:HGNC:3621] |
| ENSG00000105672 | HGLibA_15649 | 195 | 0.629148377 | ETV2 | ETS variant 2 [Source:HGNC Symbol;Acc:HGNC:3491] |
| ENSG00000105991 | HGLibA_21893 | 195 | 0.629148377 | HOXA1 | homeobox A1 [Source:HGNC Symbol;Acc:HGNC:5099] |
| ENSG00000108557 | HGLibA_40139 | 195 | 0.629148377 | RAI1 | retinoic acid induced 1 [Source:HGNC Symbol;Acc:HGNC:9834] |
| ENSG00000112763 | HGLibA_04961 | 195 | 0.629148377 | BTN2A1 | butyrophilin subfamily 2 member A1 [Source:HGNC Symbol;Acc:HGNC:1136] |
| ENSG00000117594 | HGLibA_22159 | 195 | 0.629148377 | HSD11B1 | hydroxysteroid 11-beta dehydrogenase 1 [Source:HGNC Symbol;Acc:HGNC:5208] |
| ENSG00000118557 | HGLibA_37308 | 195 | 0.629148377 | PMFBP1 | polyamine modulated factor 1 binding protein 1 [Source:HGNC Symbol;Acc:HGNC:17728] |
| ENSG00000118640 | HGLibA_53508 | 195 | 0.629148377 | VAMP8 | vesicle associated membrane protein 8 [Source:HGNC Symbol;Acc:HGNC:12647] |
| ENSG00000118972 | HGLibA_17401 | 195 | 0.629148377 | FGF23 | fibroblast growth factor 23 [Source:HGNC Symbol;Acc:HGNC:3680] |
| ENSG00000120784 | HGLibA_55202 | 195 | 0.629148377 | ZFP30 | ZFP30 zinc finger protein [Source:HGNC Symbol;Acc:HGNC:29555] |
| ENSG00000121774 | HGLibA_24567 | 195 | 0.629148377 | KHDRBS1 | signal transduction associated 1 [Source:HGNC Symbol;Acc:HGNC:18116]" |
| ENSG00000122728 | HGLibA_48225 | 195 | 0.629148377 | TAF1L | TATA-box binding protein associated factor 1 like [Source:HGNC Symbol;Acc:HGNC:18056] |
| ENSG00000124209 | HGLibA_39841 | 195 | 0.629148377 | RAB22A | member RAS oncogene family [Source:HGNC Symbol;Acc:HGNC:9764]" |
| ENSG00000124490 | HGLibA_11169 | 195 | 0.629148377 | CRISP2 | cysteine rich secretory protein 2 [Source:HGNC Symbol;Acc:HGNC:12024] |
| ENSG00000125841 | HGLibA_32728 | 195 | 0.629148377 | NRSN2 | neurensin 2 [Source:HGNC Symbol;Acc:HGNC:16229] |
| ENSG00000127412 | HGLibA_51794 | 195 | 0.629148377 | TRPV5 | transient receptor potential cation channel subfamily V member 5 [Source:HGNC Symbol;Acc:HGNC:3145] |
| ENSG00000134825 | HGLibA_50342 | 195 | 0.629148377 | TMEM258 | transmembrane protein 258 [Source:HGNC Symbol;Acc:HGNC:1164] |
| ENSG00000134987 | HGLibA_54077 | 195 | 0.629148377 | WDR36 | WD repeat domain 36 [Source:HGNC Symbol;Acc:HGNC:30696] |
| ENSG00000135454 | HGLibA_04011 | 195 | 0.629148377 | B4GALNT1 | 4-N-acetyl-galactosaminyltransferase 1 [Source:HGNC Symbol;Acc:HGNC:4117]" |
| ENSG00000136694 | HGLibA_23145 | 195 | 0.629148377 | IL36A | interleukin 36 alpha [Source:HGNC Symbol;Acc:HGNC:15562] |
| ENSG00000139344 | HGLibA_01760 | 195 | 0.629148377 | AMDHD1 | amidohydrolase domain containing 1 [Source:HGNC Symbol;Acc:HGNC:28577] |
| ENSG00000142875 | HGLibA_38507 | 195 | 0.629148377 | PRKACB | protein kinase cAMP-activated catalytic subunit beta [Source:HGNC Symbol;Acc:HGNC:9381] |
| ENSG00000144214 | HGLibA_27699 | 195 | 0.629148377 | LYG1 | lysozyme g1 [Source:HGNC Symbol;Acc:HGNC:27014] |
| ENSG00000144712 | HGLibA_07125 | 195 | 0.629148377 | CAND2 | cullin associated and neddylation dissociated 2 (putative) [Source:HGNC Symbol;Acc:HGNC:30689] |
| ENSG00000145214 | HGLibA_13083 | 195 | 0.629148377 | DGKQ | diacylglycerol kinase theta [Source:HGNC Symbol;Acc:HGNC:2856] |
| ENSG00000145332 | HGLibA_25226 | 195 | 0.629148377 | KLHL8 | kelch like family member 8 [Source:HGNC Symbol;Acc:HGNC:18644] |
| ENSG00000146094 | HGLibA_13772 | 195 | 0.629148377 | DOK3 | docking protein 3 [Source:HGNC Symbol;Acc:HGNC:24583] |
| ENSG00000148225 | HGLibA_54063 | 195 | 0.629148377 | WDR31 | WD repeat domain 31 [Source:HGNC Symbol;Acc:HGNC:21421] |
| ENSG00000154611 | HGLibA_39092 | 195 | 0.629148377 | PSMA8 | proteasome subunit alpha 8 [Source:HGNC Symbol;Acc:HGNC:22985] |
| ENSG00000157219 | HGLibA_22399 | 195 | 0.629148377 | HTR5A | 5-hydroxytryptamine receptor 5A [Source:HGNC Symbol;Acc:HGNC:5300] |
| ENSG00000157227 | HGLibA_29491 | 195 | 0.629148377 | MMP14 | matrix metallopeptidase 14 [Source:HGNC Symbol;Acc:HGNC:7160] |
| ENSG00000158480 | HGLibA_46444 | 195 | 0.629148377 | SPATA2 | spermatogenesis associated 2 [Source:HGNC Symbol;Acc:HGNC:14681] |
| ENSG00000161395 | HGLibA_36212 | 195 | 0.629148377 | PGAP3 | post-GPI attachment to proteins 3 [Source:HGNC Symbol;Acc:HGNC:23719] |
| ENSG00000164040 | HGLibA_36290 | 195 | 0.629148377 | PGRMC2 | progesterone receptor membrane component 2 [Source:HGNC Symbol;Acc:HGNC:16089] |
| ENSG00000164096 | HGLibA_06316 | 195 | 0.629148377 | C4orf3 | chromosome 4 open reading frame 3 [Source:HGNC Symbol;Acc:HGNC:19225] |
| ENSG00000164690 | HGLibA_44030 | 195 | 0.629148377 | SHH | sonic hedgehog [Source:HGNC Symbol;Acc:HGNC:10848] |

| | | | | | |
|---|---|---|---|---|---|
| ENSG00000166292 | HGLibA_49838 | 195 | 0.629148377 | TMEM100 | transmembrane protein 100 [Source:HGNC Symbol;Acc:HGNC:25607] |
| ENSG00000166402 | HGLibA_52240 | 195 | 0.629148377 | TUB | tubby bipartite transcription factor [Source:HGNC Symbol;Acc:HGNC:12406] |
| ENSG00000167333 | HGLibA_51590 | 195 | 0.629148377 | TRIM68 | tripartite motif containing 68 [Source:HGNC Symbol;Acc:HGNC:21161] |
| ENSG00000167377 | HGLibA_55633 | 195 | 0.629148377 | ZNF23 | zinc finger protein 23 [Source:HGNC Symbol;Acc:HGNC:13023] |
| ENSG00000168813 | HGLibA_56121 | 195 | 0.629148377 | ZNF507 | zinc finger protein 507 [Source:HGNC Symbol;Acc:HGNC:23783] |
| ENSG00000171954 | HGLibA_12189 | 195 | 0.629148377 | CYP4F22 | cytochrome P450 family 4 subfamily F member 22 [Source:HGNC Symbol;Acc:HGNC:26820] |
| ENSG00000173120 | HGLibA_24494 | 195 | 0.629148377 | KDM2A | lysine demethylase 2A [Source:HGNC Symbol;Acc:HGNC:13606] |
| ENSG00000173264 | HGLibA_19897 | 195 | 0.629148377 | GPR137 | G protein-coupled receptor 137 [Source:HGNC Symbol;Acc:HGNC:24300] |
| ENSG00000176101 | HGLibA_47143 | 195 | 0.629148377 | SSNA1 | SS nuclear autoantigen 1 [Source:HGNC Symbol;Acc:HGNC:11321] |
| ENSG00000178467 | HGLibA_34911 | 195 | 0.629148377 | P4HTM | transmembrane [Source:HGNC Symbol;Acc:HGNC:28858]" |
| ENSG00000180999 | HGLibA_05736 | 195 | 0.629148377 | C1orf105 | chromosome 1 open reading frame 105 [Source:HGNC Symbol;Acc:HGNC:29591] |
| ENSG00000181894 | HGLibA_55811 | 195 | 0.629148377 | ZNF329 | zinc finger protein 329 [Source:HGNC Symbol;Acc:HGNC:14209] |
| ENSG00000183273 | HGLibA_07815 | 195 | 0.629148377 | CCDC60 | coiled-coil domain containing 60 [Source:HGNC Symbol;Acc:HGNC:28610] |
| ENSG00000203910 | HGLibA_05775 | 195 | 0.629148377 | C1orf146 | chromosome 1 open reading frame 146 [Source:HGNC Symbol;Acc:HGNC:24032] |
| ENSG00000205189 | HGLibA_54815 | 195 | 0.629148377 | ZBTB10 | zinc finger and BTB domain containing 10 [Source:HGNC Symbol;Acc:HGNC:30953] |
| ENSG00000213397 | HGLibA_20891 | 195 | 0.629148377 | HAUS7 | HAUS augmin like complex subunit 7 [Source:HGNC Symbol;Acc:HGNC:32979] |
| ENSG00000214517 | HGLibA_38000 | 195 | 0.629148377 | PPME1 | protein phosphatase methylesterase 1 [Source:HGNC Symbol;Acc:HGNC:30178] |
| ENSG00000229809 | HGLibA_56542 | 195 | 0.629148377 | ZNF688 | zinc finger protein 688 [Source:HGNC Symbol;Acc:HGNC:30489] |
| ENSG00000231861 | HGLibA_34314 | 195 | 0.629148377 | OR5K2 | olfactory receptor family 5 subfamily K member 2 [Source:HGNC Symbol;Acc:HGNC:14774] |
| FAM188A | HGLibA_16344 | 195 | 0.629148377 | FAM188A | - |
| HN1L | HGLibA_21771 | 195 | 0.629148377 | HN1L | - |
| hsa-mir-184 | HGLibA_57999 | 195 | 0.629148377 | hsa-mir-184 | - |
| hsa-mir-3170 | HGLibA_58758 | 195 | 0.629148377 | hsa-mir-3170 | - |
| hsa-mir-412 | HGLibA_59689 | 195 | 0.629148377 | hsa-mir-412 | - |
| hsa-mir-4521 | HGLibA_60542 | 195 | 0.629148377 | hsa-mir-4521 | - |
| hsa-mir-521-2 | HGLibA_61772 | 195 | 0.629148377 | hsa-mir-521-2 | - |
| hsa-mir-548h-4 | HGLibA_62005 | 195 | 0.629148377 | hsa-mir-548h-4 | - |
| hsa-mir-6513 | HGLibA_62924 | 195 | 0.629148377 | hsa-mir-6513 | - |
| hsa-mir-6718 | HGLibA_63033 | 195 | 0.629148377 | hsa-mir-6718 | - |
| KIAA1147 | HGLibA_24700 | 195 | 0.629148377 | KIAA1147 | - |
| 2-Mar | HGLibA_28326 | 195 | 0.629148377 | 2-Mar | - |
| PPAP2C | HGLibA_37821 | 195 | 0.629148377 | PPAP2C | - |
| C1orf204 | HGLibA_05823 | 194 | 0.620103034 | C1orf204 | - |
| C5orf20 | HGLibA_06362 | 194 | 0.620103034 | C5orf20 | - |
| ENSG00000080166 | HGLibA_12538 | 194 | 0.620103034 | DCT | dopachrome tautomerase [Source:HGNC Symbol;Acc:HGNC:2709] |
| ENSG00000082153 | HGLibA_05012 | 194 | 0.620103034 | BZW1 | basic leucine zipper and W2 domains 1 [Source:HGNC Symbol;Acc:HGNC:18380] |
| ENSG00000095002 | HGLibA_30190 | 194 | 0.620103034 | MSH2 | mutS homolog 2 [Source:HGNC Symbol;Acc:HGNC:7325] |
| ENSG00000101187 | HGLibA_45468 | 194 | 0.620103034 | SLCO4A1 | solute carrier organic anion transporter family member 4A1 [Source:HGNC Symbol;Acc:HGNC:10953] |
| ENSG00000102098 | HGLibA_42962 | 194 | 0.620103034 | SCML2 | Scm polycomb group protein like 2 [Source:HGNC Symbol;Acc:HGNC:10581] |
| ENSG00000102970 | HGLibA_07980 | 194 | 0.620103034 | CCL17 | C-C motif chemokine ligand 17 [Source:HGNC Symbol;Acc:HGNC:10615] |
| ENSG00000103449 | HGLibA_42648 | 194 | 0.620103034 | SALL1 | spalt like transcription factor 1 [Source:HGNC Symbol;Acc:HGNC:10524] |
| ENSG00000104756 | HGLibA_24469 | 194 | 0.620103034 | KCTD9 | potassium channel tetramerization domain containing 9 [Source:HGNC Symbol;Acc:HGNC:22401] |
| ENSG00000104899 | HGLibA_01785 | 194 | 0.620103034 | AMH | anti-Mullerian hormone [Source:HGNC Symbol;Acc:HGNC:464] |
| ENSG00000105767 | HGLibA_06973 | 194 | 0.620103034 | CADM4 | cell adhesion molecule 4 [Source:HGNC Symbol;Acc:HGNC:30825] |
| ENSG00000110048 | HGLibA_34637 | 194 | 0.620103034 | OSBP | oxysterol binding protein [Source:HGNC Symbol;Acc:HGNC:8503] |
| ENSG00000111886 | HGLibA_18407 | 194 | 0.620103034 | GABRR2 | gamma-aminobutyric acid type A receptor rho2 subunit [Source:HGNC Symbol;Acc:HGNC:4091] |
| ENSG00000112357 | HGLibA_36127 | 194 | 0.620103034 | PEX7 | peroxisomal biogenesis factor 7 [Source:HGNC Symbol;Acc:HGNC:8860] |
| ENSG00000113649 | HGLibA_48755 | 194 | 0.620103034 | TCERG1 | transcription elongation regulator 1 [Source:HGNC Symbol;Acc:HGNC:15630] |
| ENSG00000115255 | HGLibA_40792 | 194 | 0.620103034 | REEP6 | receptor accessory protein 6 [Source:HGNC Symbol;Acc:HGNC:30078] |
| ENSG00000116117 | HGLibA_35181 | 194 | 0.620103034 | PARD3B | par-3 family cell polarity regulator beta [Source:HGNC Symbol;Acc:HGNC:14446] |
| ENSG00000118096 | HGLibA_22761 | 194 | 0.620103034 | IFT46 | intraflagellar transport 46 [Source:HGNC Symbol;Acc:HGNC:26146] |
| ENSG00000119655 | HGLibA_32423 | 194 | 0.620103034 | NPC2 | NPC intracellular cholesterol transporter 2 [Source:HGNC Symbol;Acc:HGNC:14537] |
| ENSG00000119725 | HGLibA_55937 | 194 | 0.620103034 | ZNF410 | zinc finger protein 410 [Source:HGNC Symbol;Acc:HGNC:20144] |
| ENSG00000119888 | HGLibA_15272 | 194 | 0.620103034 | EPCAM | epithelial cell adhesion molecule [Source:HGNC Symbol;Acc:HGNC:11529] |
| ENSG00000120262 | HGLibA_07691 | 194 | 0.620103034 | CCDC170 | coiled-coil domain containing 170 [Source:HGNC Symbol;Acc:HGNC:21177] |
| ENSG00000124067 | HGLibA_44367 | 194 | 0.620103034 | SLC12A4 | solute carrier family 12 member 4 [Source:HGNC Symbol;Acc:HGNC:10913] |
| ENSG00000124160 | HGLibA_31346 | 194 | 0.620103034 | NCOA5 | nuclear receptor coactivator 5 [Source:HGNC Symbol;Acc:HGNC:15909] |
| ENSG00000124383 | HGLibA_29724 | 194 | 0.620103034 | MPHOSPH10 | M-phase phosphoprotein 10 [Source:HGNC Symbol;Acc:HGNC:7213] |
| ENSG00000126091 | HGLibA_47244 | 194 | 0.620103034 | ST3GAL3 | 3-sialyltransferase 3 [Source:HGNC Symbol;Acc:HGNC:10866]" |
| ENSG00000126522 | HGLibA_03316 | 194 | 0.620103034 | ASL | argininosuccinate lyase [Source:HGNC Symbol;Acc:HGNC:746] |
| ENSG00000130383 | HGLibA_18196 | 194 | 0.620103034 | FUT5 | fucosyltransferase 5 [Source:HGNC Symbol;Acc:HGNC:4016] |
| ENSG00000130733 | HGLibA_54672 | 194 | 0.620103034 | YIPF2 | Yip1 domain family member 2 [Source:HGNC Symbol;Acc:HGNC:28476] |
| ENSG00000130844 | HGLibA_55815 | 194 | 0.620103034 | ZNF331 | zinc finger protein 331 [Source:HGNC Symbol;Acc:HGNC:15489] |
| ENSG00000131732 | HGLibA_55052 | 194 | 0.620103034 | ZCCHC9 | zinc finger CCHC-type containing 9 [Source:HGNC Symbol;Acc:HGNC:25424] |
| ENSG00000132005 | HGLibA_40962 | 194 | 0.620103034 | RFX1 | regulatory factor X1 [Source:HGNC Symbol;Acc:HGNC:9982] |
| ENSG00000134077 | HGLibA_49420 | 194 | 0.620103034 | THUMPD3 | THUMP domain containing 3 [Source:HGNC Symbol;Acc:HGNC:24493] |
| ENSG00000134352 | HGLibA_23186 | 194 | 0.620103034 | IL6ST | interleukin 6 signal transducer [Source:HGNC Symbol;Acc:HGNC:6021] |
| ENSG00000136696 | HGLibA_23148 | 194 | 0.620103034 | IL36B | interleukin 36 beta [Source:HGNC Symbol;Acc:HGNC:15564] |
| ENSG00000137522 | HGLibA_41485 | 194 | 0.620103034 | RNF121 | ring finger protein 121 [Source:HGNC Symbol;Acc:HGNC:21070] |
| ENSG00000138641 | HGLibA_21159 | 194 | 0.620103034 | HERC3 | HECT and RLD domain containing E3 ubiquitin protein ligase 3 [Source:HGNC Symbol;Acc:HGNC:4876] |
| ENSG00000143653 | HGLibA_42882 | 194 | 0.620103034 | SCCPDH | saccharopine dehydrogenase (putative) [Source:HGNC Symbol;Acc:HGNC:24275] |
| ENSG00000144445 | HGLibA_24038 | 194 | 0.620103034 | KANSL1L | KAT8 regulatory NSL complex subunit 1 like [Source:HGNC Symbol;Acc:HGNC:26310] |
| ENSG00000145850 | HGLibA_49486 | 194 | 0.620103034 | TIMD4 | T cell immunoglobulin and mucin domain containing 4 [Source:HGNC Symbol;Acc:HGNC:25132] |
| ENSG00000146386 | HGLibA_00328 | 194 | 0.620103034 | ABRACL | ABRA C-terminal like [Source:HGNC Symbol;Acc:HGNC:21230] |
| ENSG00000146411 | HGLibA_44862 | 194 | 0.620103034 | SLC2A12 | solute carrier family 2 member 12 [Source:HGNC Symbol;Acc:HGNC:18067] |
| ENSG00000152254 | HGLibA_18307 | 194 | 0.620103034 | G6PC2 | glucose-6-phosphatase catalytic subunit 2 [Source:HGNC Symbol;Acc:HGNC:28906] |
| ENSG00000153404 | HGLibA_37113 | 194 | 0.620103034 | PLEKHG4B | pleckstrin homology and RhoGEF domain containing G4B [Source:HGNC Symbol;Acc:HGNC:29399] |
| ENSG00000157106 | HGLibA_45698 | 194 | 0.620103034 | SMG1 | nonsense mediated mRNA decay associated PI3K related kinase [Source:HGNC Symbol;Acc:HGNC:30045]" |
| ENSG00000161381 | HGLibA_37252 | 194 | 0.620103034 | PLXDC1 | plexin domain containing 1 [Source:HGNC Symbol;Acc:HGNC:20945] |
| ENSG00000162522 | HGLibA_24773 | 194 | 0.620103034 | KIAA1522 | KIAA1522 [Source:HGNC Symbol;Acc:HGNC:29301] |
| ENSG00000163171 | HGLibA_08572 | 194 | 0.620103034 | CDC42EP3 | CDC42 effector protein 3 [Source:HGNC Symbol;Acc:HGNC:16943] |
| ENSG00000163814 | HGLibA_08624 | 194 | 0.620103034 | CDCP1 | CUB domain containing protein 1 [Source:HGNC Symbol;Acc:HGNC:24357] |
| ENSG00000163814 | HGLibA_08623 | 194 | 0.620103034 | CDCP1 | CUB domain containing protein 1 [Source:HGNC Symbol;Acc:HGNC:24357] |
| ENSG00000164080 | HGLibA_40106 | 194 | 0.620103034 | RAD54L2 | RAD54 like 2 [Source:HGNC Symbol;Acc:HGNC:29123] |
| ENSG00000165092 | HGLibA_01541 | 194 | 0.620103034 | ALDH1A1 | aldehyde dehydrogenase 1 family member A1 [Source:HGNC Symbol;Acc:HGNC:402] |
| ENSG00000166016 | HGLibA_00339 | 194 | 0.620103034 | ABTB2 | ankyrin repeat and BTB domain containing 2 [Source:HGNC Symbol;Acc:HGNC:23842] |
| ENSG00000168243 | HGLibA_19525 | 194 | 0.620103034 | GNG4 | G protein subunit gamma 4 [Source:HGNC Symbol;Acc:HGNC:4407] |
| ENSG00000169567 | HGLibA_21324 | 194 | 0.620103034 | HINT1 | histidine triad nucleotide binding protein 1 [Source:HGNC Symbol;Acc:HGNC:4912] |
| ENSG00000169683 | HGLibA_27366 | 194 | 0.620103034 | LRRC45 | leucine rich repeat containing 45 [Source:HGNC Symbol;Acc:HGNC:28302] |
| ENSG00000170248 | HGLibA_35742 | 194 | 0.620103034 | PDCD6IP | programmed cell death 6 interacting protein [Source:HGNC Symbol;Acc:HGNC:8766] |
| ENSG00000171402 | HGLibA_54485 | 194 | 0.620103034 | XAGE3 | X antigen family member 3 [Source:HGNC Symbol;Acc:HGNC:14618] |
| ENSG00000172000 | HGLibA_56228 | 194 | 0.620103034 | ZNF556 | zinc finger protein 556 [Source:HGNC Symbol;Acc:HGNC:25669] |
| ENSG00000178394 | HGLibA_22356 | 194 | 0.620103034 | HTR1A | 5-hydroxytryptamine receptor 1A [Source:HGNC Symbol;Acc:HGNC:5286] |
| ENSG00000178537 | HGLibA_44669 | 194 | 0.620103034 | SLC25A20 | solute carrier family 25 member 20 [Source:HGNC Symbol;Acc:HGNC:1421] |
| ENSG00000179388 | HGLibA_14654 | 194 | 0.620103034 | EGR3 | early growth response 3 [Source:HGNC Symbol;Acc:HGNC:3240] |
| ENSG00000180332 | HGLibA_24455 | 194 | 0.620103034 | KCTD4 | potassium channel tetramerization domain containing 4 [Source:HGNC Symbol;Acc:HGNC:23227] |
| ENSG00000182053 | HGLibA_51525 | 194 | 0.620103034 | TRIM49B | tripartite motif containing 49B [Source:HGNC Symbol;Acc:HGNC:42955] |
| ENSG00000182634 | HGLibA_33497 | 194 | 0.620103034 | OR10G7 | olfactory receptor family 10 subfamily G member 7 [Source:HGNC Symbol;Acc:HGNC:14842] |
| ENSG00000183196 | HGLibA_09643 | 194 | 0.620103034 | CHST6 | carbohydrate sulfotransferase 6 [Source:HGNC Symbol;Acc:HGNC:6938] |
| ENSG00000187783 | HGLibA_50508 | 194 | 0.620103034 | TMEM72 | transmembrane protein 72 [Source:HGNC Symbol;Acc:HGNC:31658] |
| ENSG00000189308 | HGLibA_26569 | 194 | 0.620103034 | LIN54 | lin-54 DREAM MuvB core complex component [Source:HGNC Symbol;Acc:HGNC:25397] |
| ENSG00000196090 | HGLibA_39538 | 194 | 0.620103034 | PTPRT | receptor type T [Source:HGNC Symbol;Acc:HGNC:9682]" |
| ENSG00000196214 | HGLibA_56696 | 194 | 0.620103034 | ZNF766 | zinc finger protein 766 [Source:HGNC Symbol;Acc:HGNC:28063] |
| ENSG00000197213 | HGLibA_57030 | 194 | 0.620103034 | ZSCAN5B | zinc finger and SCAN domain containing 5B [Source:HGNC Symbol;Acc:HGNC:34246] |
| ENSG00000197454 | HGLibA_33828 | 194 | 0.620103034 | OR2L5 | olfactory receptor family 2 subfamily L member 5 [Source:HGNC Symbol;Acc:HGNC:15011] |
| ENSG00000198522 | HGLibA_19826 | 194 | 0.620103034 | GPN1 | GPN-loop GTPase 1 [Source:HGNC Symbol;Acc:HGNC:17030] |
| ENSG00000198812 | HGLibA_27247 | 194 | 0.620103034 | LRRC10 | leucine rich repeat containing 10 [Source:HGNC Symbol;Acc:HGNC:20264] |
| ENSG00000205364 | HGLibA_30296 | 194 | 0.620103034 | MT1M | metallothionein 1M [Source:HGNC Symbol;Acc:HGNC:14296] |
| ENSG00000213139 | HGLibA_11285 | 194 | 0.620103034 | CRYGS | crystallin gamma S [Source:HGNC Symbol;Acc:HGNC:2417] |
| ENSG00000213463 | HGLibA_48008 | 194 | 0.620103034 | SYNJ2BP | synaptojanin 2 binding protein [Source:HGNC Symbol;Acc:HGNC:18955] |
| ENSG00000213676 | HGLibA_03450 | 194 | 0.620103034 | ATF6B | activating transcription factor 6 beta [Source:HGNC Symbol;Acc:HGNC:2349] |
| ENSG00000232258 | HGLibA_49879 | 194 | 0.620103034 | TMEM114 | transmembrane protein 114 [Source:HGNC Symbol;Acc:HGNC:33227] |
| ENSG00000251201 | HGLibA_49824 | 194 | 0.620103034 | TMED7-TICAM2 | TMED7-TICAM2 readthrough [Source:HGNC Symbol;Acc:HGNC:33945] |
| ENSG00000256797 | HGLibA_25306 | 194 | 0.620103034 | KLRF2 | killer cell lectin like receptor F2 [Source:HGNC Symbol;Acc:HGNC:37646] |
| ENSG00000260027 | HGLibA_21948 | 194 | 0.620103034 | HOXB7 | homeobox B7 [Source:HGNC Symbol;Acc:HGNC:5118] |
| ENSG00000271858 | HGLibA_11986 | 194 | 0.620103034 | CYB561D2 | cytochrome b561 family member D2 [Source:NCBI gene;Acc:11068] |
| ENSG00000279395 | HGLibA_34323 | 194 | 0.620103034 | OR5L1 | olfactory receptor family 5 subfamily L member 1 (gene/pseudogene) [Source:HGNC Symbol;Acc:HGNC:8350] |
| ENSG00000288649 | HGLibA_00616 | 194 | 0.620103034 | ACTL10 | - |
| HIST1H3G | HGLibA_21475 | 194 | 0.620103034 | HIST1H3G | - |
| hsa-mir-489 | HGLibA_61356 | 194 | 0.620103034 | hsa-mir-489 | - |
| hsa-mir-490 | HGLibA_61361 | 194 | 0.620103034 | hsa-mir-490 | - |
| hsa-mir-512-1 | HGLibA_61561 | 194 | 0.620103034 | hsa-mir-512-1 | - |
| hsa-mir-5706 | HGLibA_62363 | 194 | 0.620103034 | hsa-mir-5706 | - |
| hsa-mir-587 | HGLibA_62445 | 194 | 0.620103034 | hsa-mir-587 | - |
| hsa-mir-6716 | HGLibA_63024 | 194 | 0.620103034 | hsa-mir-6716 | - |
| hsa-mir-6718 | HGLibA_63035 | 194 | 0.620103034 | hsa-mir-6718 | - |
| hsa-mir-6876 | HGLibA_63690 | 194 | 0.620103034 | hsa-mir-6876 | - |
| LILRA3 | HGLibA_26496 | 194 | 0.620103034 | LILRA3 | - |
| PVRL3 | HGLibA_39627 | 194 | 0.620103034 | PVRL3 | - |
| WDR16 | HGLibA_54034 | 194 | 0.620103034 | WDR16 | - |
| BCMO1 | HGLibA_04356 | 193 | 0.611057691 | BCMO1 | - |
| C1orf151-NBL1 | HGLibA_05778 | 193 | 0.611057691 | C1orf151-NBL1 | - |
| CCDC132 | HGLibA_07596 | 193 | 0.611057691 | CCDC132 | - |
| ENSG00000006652 | HGLibA_22737 | 193 | 0.611057691 | IFRD1 | interferon related developmental regulator 1 [Source:HGNC Symbol;Acc:HGNC:5456] |
| ENSG00000023608 | HGLibA_45890 | 193 | 0.611057691 | SNAPC1 | small nuclear RNA activating complex polypeptide 1 [Source:HGNC Symbol;Acc:HGNC:11134] |

| | | | | | |
|---|---|---|---|---|---|
| ENSG00000029153 | HGLibA_03071 | 193 | 0.611057691 | ARNTL2 | aryl hydrocarbon receptor nuclear translocator like 2 [Source:HGNC Symbol;Acc:HGNC:18984] |
| ENSG00000051180 | HGLibA_40078 | 193 | 0.611057691 | RAD51 | RAD51 recombinase [Source:HGNC Symbol;Acc:HGNC:9817] |
| ENSG00000061492 | HGLibA_54387 | 193 | 0.611057691 | WNT8A | Wnt family member 8A [Source:HGNC Symbol;Acc:HGNC:12788] |
| ENSG00000069188 | HGLibA_43155 | 193 | 0.611057691 | SDK2 | sidekick cell adhesion molecule 2 [Source:HGNC Symbol;Acc:HGNC:19308] |
| ENSG00000085265 | HGLibA_17216 | 193 | 0.611057691 | FCN1 | ficolin 1 [Source:HGNC Symbol;Acc:HGNC:3623] |
| ENSG00000099219 | HGLibA_15510 | 193 | 0.611057691 | ERMP1 | endoplasmic reticulum metallopeptidase 1 [Source:HGNC Symbol;Acc:HGNC:23703] |
| ENSG00000100292 | HGLibA_21746 | 193 | 0.611057691 | HMOX1 | heme oxygenase 1 [Source:HGNC Symbol;Acc:HGNC:5013] |
| ENSG00000100314 | HGLibA_06860 | 193 | 0.611057691 | CABP7 | calcium binding protein 7 [Source:HGNC Symbol;Acc:HGNC:20834] |
| ENSG00000100351 | HGLibA_20215 | 193 | 0.611057691 | GRAP2 | GRB2 related adaptor protein 2 [Source:HGNC Symbol;Acc:HGNC:4563] |
| ENSG00000113597 | HGLibA_51310 | 193 | 0.611057691 | TRAPPC13 | trafficking protein particle complex 13 [Source:HGNC Symbol;Acc:HGNC:25828] |
| ENSG00000119844 | HGLibA_01130 | 193 | 0.611057691 | AFTPH | aftiphilin [Source:HGNC Symbol;Acc:HGNC:25951] |
| ENSG00000120332 | HGLibA_50861 | 193 | 0.611057691 | TNN | tenascin N [Source:HGNC Symbol;Acc:HGNC:22942] |
| ENSG00000121486 | HGLibA_51679 | 193 | 0.611057691 | TRMT1L | tRNA methyltransferase 1 like [Source:HGNC Symbol;Acc:HGNC:16782] |
| ENSG00000123685 | HGLibA_04180 | 193 | 0.611057691 | BATF3 | basic leucine zipper ATF-like transcription factor 3 [Source:HGNC Symbol;Acc:HGNC:28915] |
| ENSG00000125850 | HGLibA_34804 | 193 | 0.611057691 | OVOL2 | ovo like zinc finger 2 [Source:HGNC Symbol;Acc:HGNC:15804] |
| ENSG00000128872 | HGLibA_50596 | 193 | 0.611057691 | TMOD2 | tropomodulin 2 [Source:HGNC Symbol;Acc:HGNC:11872] |
| ENSG00000129048 | HGLibA_00450 | 193 | 0.611057691 | ACKR4 | atypical chemokine receptor 4 [Source:HGNC Symbol;Acc:HGNC:1611] |
| ENSG00000132185 | HGLibA_17244 | 193 | 0.611057691 | FCRLA | Fc receptor like A [Source:HGNC Symbol;Acc:HGNC:18504] |
| ENSG00000134248 | HGLibA_26001 | 193 | 0.611057691 | LAMTOR5 | MAPK and MTOR activator 5 [Source:HGNC Symbol;Acc:HGNC:17955]" |
| ENSG00000135838 | HGLibA_32482 | 193 | 0.611057691 | NPL | N-acetylneuraminate pyruvate lyase [Source:HGNC Symbol;Acc:HGNC:16781] |
| ENSG00000135929 | HGLibA_12095 | 193 | 0.611057691 | CYP27A1 | cytochrome P450 family 27 subfamily A member 1 [Source:HGNC Symbol;Acc:HGNC:2605] |
| ENSG00000135976 | HGLibA_02086 | 193 | 0.611057691 | ANKRD36 | ankyrin repeat domain 36 [Source:HGNC Symbol;Acc:HGNC:24079] |
| ENSG00000136197 | HGLibA_06524 | 193 | 0.611057691 | C7orf25 | chromosome 7 open reading frame 25 [Source:HGNC Symbol;Acc:HGNC:21703] |
| ENSG00000136842 | HGLibA_50594 | 193 | 0.611057691 | TMOD1 | tropomodulin 1 [Source:HGNC Symbol;Acc:HGNC:11871] |
| ENSG00000137080 | HGLibA_22676 | 193 | 0.611057691 | IFNA21 | interferon alpha 21 [Source:HGNC Symbol;Acc:HGNC:5424] |
| ENSG00000137634 | HGLibA_33160 | 193 | 0.611057691 | NXPE4 | neurexophilin and PC-esterase domain family member 4 [Source:HGNC Symbol;Acc:HGNC:23117] |
| ENSG00000137731 | HGLibA_18226 | 193 | 0.611057691 | FXYD2 | FXYD domain containing ion transport regulator 2 [Source:HGNC Symbol;Acc:HGNC:4026] |
| ENSG00000139354 | HGLibA_18649 | 193 | 0.611057691 | GAS2L3 | growth arrest specific 2 like 3 [Source:HGNC Symbol;Acc:HGNC:27475] |
| ENSG00000139914 | HGLibA_17541 | 193 | 0.611057691 | FITM1 | fat storage inducing transmembrane protein 1 [Source:HGNC Symbol;Acc:HGNC:33714] |
| ENSG00000145022 | HGLibA_48853 | 193 | 0.611057691 | TCTA | T cell leukemia translocation altered [Source:HGNC Symbol;Acc:HGNC:11692] |
| ENSG00000145192 | HGLibA_01319 | 193 | 0.611057691 | AHSG | alpha 2-HS glycoprotein [Source:HGNC Symbol;Acc:HGNC:349] |
| ENSG00000146802 | HGLibA_50046 | 193 | 0.611057691 | TMEM168 | transmembrane protein 168 [Source:HGNC Symbol;Acc:HGNC:25826] |
| ENSG00000154370 | HGLibA_51406 | 193 | 0.611057691 | TRIM11 | tripartite motif containing 11 [Source:HGNC Symbol;Acc:HGNC:16281] |
| ENSG00000154548 | HGLibA_47069 | 193 | 0.611057691 | SRSF12 | serine and arginine rich splicing factor 12 [Source:HGNC Symbol;Acc:HGNC:21220] |
| ENSG00000154957 | HGLibA_55535 | 193 | 0.611057691 | ZNF18 | zinc finger protein 18 [Source:HGNC Symbol;Acc:HGNC:12969] |
| ENSG00000155719 | HGLibA_34723 | 193 | 0.611057691 | OTOA | otoancorin [Source:HGNC Symbol;Acc:HGNC:16378] |
| ENSG00000157150 | HGLibA_49545 | 193 | 0.611057691 | TIMP4 | TIMP metallopeptidase inhibitor 4 [Source:HGNC Symbol;Acc:HGNC:11823] |
| ENSG00000162419 | HGLibA_19397 | 193 | 0.611057691 | GMEB1 | glucocorticoid modulatory element binding protein 1 [Source:HGNC Symbol;Acc:HGNC:4370] |
| ENSG00000162881 | HGLibA_34820 | 193 | 0.611057691 | OXER1 | oxoeicosanoid receptor 1 [Source:HGNC Symbol;Acc:HGNC:24884] |
| ENSG00000162897 | HGLibA_17162 | 193 | 0.611057691 | FCAMR | Fc fragment of IgA and IgM receptor [Source:HGNC Symbol;Acc:HGNC:24692] |
| ENSG00000165370 | HGLibA_19840 | 193 | 0.611057691 | GPR101 | G protein-coupled receptor 101 [Source:HGNC Symbol;Acc:HGNC:14963] |
| ENSG00000166012 | HGLibA_48221 | 193 | 0.611057691 | TAF1D | RNA polymerase I subunit D [Source:HGNC Symbol;Acc:HGNC:28759]" |
| ENSG00000166483 | HGLibA_54238 | 193 | 0.611057691 | WEE1 | WEE1 G2 checkpoint kinase [Source:HGNC Symbol;Acc:HGNC:12761] |
| ENSG00000167210 | HGLibA_27036 | 193 | 0.611057691 | LOXHD1 | lipoxygenase homology domains 1 [Source:HGNC Symbol;Acc:HGNC:26521] |
| ENSG00000167580 | HGLibA_02589 | 193 | 0.611057691 | AQP2 | aquaporin 2 [Source:HGNC Symbol;Acc:HGNC:634] |
| ENSG00000167670 | HGLibA_09347 | 193 | 0.611057691 | CHAF1A | chromatin assembly factor 1 subunit A [Source:HGNC Symbol;Acc:HGNC:1910] |
| ENSG00000168484 | HGLibA_43776 | 193 | 0.611057691 | SFTPC | surfactant protein C [Source:HGNC Symbol;Acc:HGNC:10802] |
| ENSG00000168653 | HGLibA_31556 | 193 | 0.611057691 | NDUFS5 | NADH:ubiquinone oxidoreductase subunit S5 [Source:HGNC Symbol;Acc:HGNC:7712] |
| ENSG00000168743 | HGLibA_32497 | 193 | 0.611057691 | NPNT | nephronectin [Source:HGNC Symbol;Acc:HGNC:27405] |
| ENSG00000169676 | HGLibA_13970 | 193 | 0.611057691 | DRD5 | dopamine receptor D5 [Source:HGNC Symbol;Acc:HGNC:3026] |
| ENSG00000169862 | HGLibA_11662 | 193 | 0.611057691 | CTNND2 | catenin delta 2 [Source:HGNC Symbol;Acc:HGNC:2516] |
| ENSG00000170049 | HGLibA_24153 | 193 | 0.611057691 | KCNAB3 | potassium voltage-gated channel subfamily A regulatory beta subunit 3 [Source:HGNC Symbol;Acc:HGNC:6230] |
| ENSG00000170537 | HGLibA_49765 | 193 | 0.611057691 | TMC7 | transmembrane channel like 7 [Source:HGNC Symbol;Acc:HGNC:23000] |
| ENSG00000172322 | HGLibA_09928 | 193 | 0.611057691 | CLEC12A | C-type lectin domain family 12 member A [Source:HGNC Symbol;Acc:HGNC:31713] |
| ENSG00000176679 | HGLibA_49250 | 193 | 0.611057691 | TGIF2LY | TGFB induced factor homeobox 2 like Y-linked [Source:HGNC Symbol;Acc:HGNC:18569] |
| ENSG00000178363 | HGLibA_07030 | 193 | 0.611057691 | CALML3 | calmodulin like 3 [Source:HGNC Symbol;Acc:HGNC:1452] |
| ENSG00000178695 | HGLibA_24421 | 193 | 0.611057691 | KCTD12 | potassium channel tetramerization domain containing 12 [Source:HGNC Symbol;Acc:HGNC:14678] |
| ENSG00000179409 | HGLibA_18913 | 193 | 0.611057691 | GEMIN4 | gem nuclear organelle associated protein 4 [Source:HGNC Symbol;Acc:HGNC:15717] |
| ENSG00000179902 | HGLibA_05815 | 193 | 0.611057691 | C1orf194 | chromosome 1 open reading frame 194 [Source:HGNC Symbol;Acc:HGNC:32331] |
| ENSG00000184619 | HGLibA_25391 | 193 | 0.611057691 | KRBA2 | KRAB-A domain containing 2 [Source:HGNC Symbol;Acc:HGNC:26989] |
| ENSG00000185338 | HGLibA_46124 | 193 | 0.611057691 | SOCS1 | suppressor of cytokine signaling 1 [Source:HGNC Symbol;Acc:HGNC:19383] |
| ENSG00000186092 | HGLibA_34009 | 193 | 0.611057691 | OR4F5 | olfactory receptor family 4 subfamily F member 5 [Source:HGNC Symbol;Acc:HGNC:14825] |
| ENSG00000186136 | HGLibA_48408 | 193 | 0.611057691 | TAS2R42 | taste 2 receptor member 42 [Source:HGNC Symbol;Acc:HGNC:18888] |
| ENSG00000186642 | HGLibA_35777 | 193 | 0.611057691 | PDE2A | phosphodiesterase 2A [Source:HGNC Symbol;Acc:HGNC:8777] |
| ENSG00000186652 | HGLibA_38451 | 193 | 0.611057691 | PRG2 | pro eosinophil major basic protein [Source:HGNC Symbol;Acc:HGNC:9362]" |
| ENSG00000188807 | HGLibA_50179 | 193 | 0.611057691 | TMEM201 | transmembrane protein 201 [Source:HGNC Symbol;Acc:HGNC:33719] |
| ENSG00000197838 | HGLibA_12102 | 193 | 0.611057691 | CYP2A13 | cytochrome P450 family 2 subfamily A member 13 [Source:HGNC Symbol;Acc:HGNC:2608] |
| ENSG00000197889 | HGLibA_28864 | 193 | 0.611057691 | MEIG1 | meiosis/spermiogenesis associated 1 [Source:HGNC Symbol;Acc:HGNC:23429] |
| ENSG00000204564 | HGLibA_06447 | 193 | 0.611057691 | C6orf136 | chromosome 6 open reading frame 136 [Source:HGNC Symbol;Acc:HGNC:21301] |
| ENSG00000204572 | HGLibA_25785 | 193 | 0.611057691 | KRTAP5-10 | keratin associated protein 5-10 [Source:HGNC Symbol;Acc:HGNC:23605] |
| ENSG00000214517 | HGLibA_37998 | 193 | 0.611057691 | PPME1 | protein phosphatase methylesterase 1 [Source:HGNC Symbol;Acc:HGNC:30178] |
| ENSG00000226979 | HGLibA_27575 | 193 | 0.611057691 | LTA | lymphotoxin alpha [Source:HGNC Symbol;Acc:HGNC:6709] |
| ENSG00000229314 | HGLibA_34619 | 193 | 0.611057691 | ORM1 | orosomucoid 1 [Source:HGNC Symbol;Acc:HGNC:8498] |
| ENSG00000244731 | HGLibA_06281 | 193 | 0.611057691 | C4A | complement C4A (Rodgers blood group) [Source:HGNC Symbol;Acc:HGNC:1323] |
| ENSG00000255423 | HGLibA_14368 | 193 | 0.611057691 | EBLN2 | endogenous Bornavirus like nucleoprotein 2 [Source:HGNC Symbol;Acc:HGNC:25493] |
| ENSG00000256806 | HGLibA_05517 | 193 | 0.611057691 | C17orf100 | chromosome 17 open reading frame 100 [Source:HGNC Symbol;Acc:HGNC:34494] |
| ENSG00000277149 | HGLibA_52482 | 193 | 0.611057691 | TYW1B | tRNA-yW synthesizing protein 1 homolog B [Source:HGNC Symbol;Acc:HGNC:33908] |
| ENSG00000288602 | HGLibA_06625 | 193 | 0.611057691 | C8orf44-SGK3 | - |
| FAM188B | HGLibA_16349 | 193 | 0.611057691 | FAM188B | - |
| hsa-mir-216b | HGLibA_58262 | 193 | 0.611057691 | hsa-mir-216b | - |
| hsa-mir-378d-2 | HGLibA_59443 | 193 | 0.611057691 | hsa-mir-378d-2 | - |
| hsa-mir-4294 | HGLibA_59889 | 193 | 0.611057691 | hsa-mir-4294 | - |
| hsa-mir-450b | HGLibA_60481 | 193 | 0.611057691 | hsa-mir-450b | - |
| TCP10 | HGLibA_48832 | 193 | 0.611057691 | TCP10 | - |
| C10orf76 | HGLibA_05094 | 192 | 0.602012348 | C10orf76 | - |
| C2orf62 | HGLibA_06157 | 192 | 0.602012348 | C2orf62 | - |
| C2orf71 | HGLibA_06173 | 192 | 0.602012348 | C2orf71 | - |
| C9orf89 | HGLibA_06771 | 192 | 0.602012348 | C9orf89 | - |
| DUPD1 | HGLibA_14109 | 192 | 0.602012348 | DUPD1 | - |
| ENSG00000004838 | HGLibA_55411 | 192 | 0.602012348 | ZMYND10 | zinc finger MYND-type containing 10 [Source:HGNC Symbol;Acc:HGNC:19412] |
| ENSG00000013293 | HGLibA_45359 | 192 | 0.602012348 | SLC7A14 | solute carrier family 7 member 14 [Source:HGNC Symbol;Acc:HGNC:29326] |
| ENSG00000064666 | HGLibA_10308 | 192 | 0.602012348 | CNN2 | calponin 2 [Source:HGNC Symbol;Acc:HGNC:2156] |
| ENSG00000067182 | HGLibA_50773 | 192 | 0.602012348 | TNFRSF1A | TNF receptor superfamily member 1A [Source:HGNC Symbol;Acc:HGNC:11916] |
| ENSG00000069020 | HGLibA_28410 | 192 | 0.602012348 | MAST4 | microtubule associated serine/threonine kinase family member 4 [Source:HGNC Symbol;Acc:HGNC:19037] |
| ENSG00000080561 | HGLibA_29236 | 192 | 0.602012348 | MID2 | midline 2 [Source:HGNC Symbol;Acc:HGNC:7096] |
| ENSG00000091137 | HGLibA_44796 | 192 | 0.602012348 | SLC26A4 | solute carrier family 26 member 4 [Source:HGNC Symbol;Acc:HGNC:8818] |
| ENSG00000102910 | HGLibA_27018 | 192 | 0.602012348 | LONP2 | peroxisomal [Source:HGNC Symbol;Acc:HGNC:20598]" |
| ENSG00000105227 | HGLibA_38991 | 192 | 0.602012348 | PRX | periaxin [Source:HGNC Symbol;Acc:HGNC:13797] |
| ENSG00000107290 | HGLibA_43676 | 192 | 0.602012348 | SETX | senataxin [Source:HGNC Symbol;Acc:HGNC:445] |
| ENSG00000108479 | HGLibA_18514 | 192 | 0.602012348 | GALK1 | galactokinase 1 [Source:HGNC Symbol;Acc:HGNC:4118] |
| ENSG00000108691 | HGLibA_07987 | 192 | 0.602012348 | CCL2 | C-C motif chemokine ligand 2 [Source:HGNC Symbol;Acc:HGNC:10618] |
| ENSG00000108932 | HGLibA_44446 | 192 | 0.602012348 | SLC16A6 | solute carrier family 16 member 6 [Source:HGNC Symbol;Acc:HGNC:10927] |
| ENSG00000110921 | HGLibA_30597 | 192 | 0.602012348 | MVK | mevalonate kinase [Source:HGNC Symbol;Acc:HGNC:7530] |
| ENSG00000111880 | HGLibA_41709 | 192 | 0.602012348 | RNGTT | RNA guanylyltransferase and 5'-phosphatase [Source:HGNC Symbol;Acc:HGNC:10073] |
| ENSG00000112164 | HGLibA_19292 | 192 | 0.602012348 | GLP1R | glucagon like peptide 1 receptor [Source:HGNC Symbol;Acc:HGNC:4324] |
| ENSG00000114354 | HGLibA_49177 | 192 | 0.602012348 | TFG | TRK-fused gene [Source:HGNC Symbol;Acc:HGNC:11758] |
| ENSG00000114790 | HGLibA_02839 | 192 | 0.602012348 | ARHGEF26 | Rho guanine nucleotide exchange factor 26 [Source:HGNC Symbol;Acc:HGNC:24490] |
| ENSG00000116786 | HGLibA_37140 | 192 | 0.602012348 | PLEKHM2 | pleckstrin homology and RUN domain containing M2 [Source:HGNC Symbol;Acc:HGNC:29131] |
| ENSG00000117408 | HGLibA_23463 | 192 | 0.602012348 | IPO13 | importin 13 [Source:HGNC Symbol;Acc:HGNC:16853] |
| ENSG00000117505 | HGLibA_13940 | 192 | 0.602012348 | DR1 | down-regulator of transcription 1 [Source:HGNC Symbol;Acc:HGNC:3017] |
| ENSG00000119938 | HGLibA_38103 | 192 | 0.602012348 | PPP1R3C | protein phosphatase 1 regulatory subunit 3C [Source:HGNC Symbol;Acc:HGNC:9293] |
| ENSG00000120211 | HGLibA_23386 | 192 | 0.602012348 | INSL4 | insulin like 4 [Source:HGNC Symbol;Acc:HGNC:6087] |
| ENSG00000122367 | HGLibA_26217 | 192 | 0.602012348 | LDB3 | LIM domain binding 3 [Source:HGNC Symbol;Acc:HGNC:15710] |
| ENSG00000124496 | HGLibA_51369 | 192 | 0.602012348 | TRERF1 | transcriptional regulating factor 1 [Source:HGNC Symbol;Acc:HGNC:18273] |
| ENSG00000125875 | HGLibA_48497 | 192 | 0.602012348 | TBC1D20 | TBC1 domain family member 20 [Source:HGNC Symbol;Acc:HGNC:16133] |
| ENSG00000126247 | HGLibA_07184 | 192 | 0.602012348 | CAPNS1 | calpain small subunit 1 [Source:HGNC Symbol;Acc:HGNC:1481] |
| ENSG00000127948 | HGLibA_37705 | 192 | 0.602012348 | POR | cytochrome p450 oxidoreductase [Source:HGNC Symbol;Acc:HGNC:9208] |
| ENSG00000128654 | HGLibA_30514 | 192 | 0.602012348 | MTX2 | metaxin 2 [Source:HGNC Symbol;Acc:HGNC:7506] |
| ENSG00000128655 | HGLibA_35764 | 192 | 0.602012348 | PDE11A | phosphodiesterase 11A [Source:HGNC Symbol;Acc:HGNC:8773] |
| ENSG00000129219 | HGLibA_37038 | 192 | 0.602012348 | PLD2 | phospholipase D2 [Source:HGNC Symbol;Acc:HGNC:9068] |
| ENSG00000129744 | HGLibA_03159 | 192 | 0.602012348 | ART1 | ADP-ribosyltransferase 1 [Source:HGNC Symbol;Acc:HGNC:723] |
| ENSG00000131055 | HGLibA_10834 | 192 | 0.602012348 | COX4I2 | cytochrome c oxidase subunit 4I2 [Source:HGNC Symbol;Acc:HGNC:16232] |
| ENSG00000131116 | HGLibA_55970 | 192 | 0.602012348 | ZNF428 | zinc finger protein 428 [Source:HGNC Symbol;Acc:HGNC:20804] |
| ENSG00000131626 | HGLibA_37874 | 192 | 0.602012348 | PPFIA1 | PTPRF interacting protein alpha 1 [Source:HGNC Symbol;Acc:HGNC:9245] |
| ENSG00000134853 | HGLibA_35853 | 192 | 0.602012348 | PDGFRA | platelet derived growth factor receptor alpha [Source:HGNC Symbol;Acc:HGNC:8803] |
| ENSG00000136732 | HGLibA_20702 | 192 | 0.602012348 | GYPC | glycophorin C (Gerbich blood group) [Source:HGNC Symbol;Acc:HGNC:4704] |
| ENSG00000143368 | HGLibA_43713 | 192 | 0.602012348 | SF3B4 | splicing factor 3b subunit 4 [Source:HGNC Symbol;Acc:HGNC:10771] |
| ENSG00000143811 | HGLibA_39676 | 192 | 0.602012348 | PYCR2 | pyrroline-5-carboxylate reductase 2 [Source:HGNC Symbol;Acc:HGNC:30262] |
| ENSG00000144426 | HGLibA_31208 | 192 | 0.602012348 | NBEAL1 | neurobeachin like 1 [Source:HGNC Symbol;Acc:HGNC:20681] |
| ENSG00000145391 | HGLibA_43657 | 192 | 0.602012348 | SETD7 | SET domain containing lysine methyltransferase 7 [Source:HGNC Symbol;Acc:HGNC:30412] |
| ENSG00000150722 | HGLibA_38071 | 192 | 0.602012348 | PPP1R1C | protein phosphatase 1 regulatory inhibitor subunit 1C [Source:HGNC Symbol;Acc:HGNC:14940] |
| ENSG00000154957 | HGLibA_55533 | 192 | 0.602012348 | ZNF18 | zinc finger protein 18 [Source:HGNC Symbol;Acc:HGNC:12969] |
| ENSG00000155463 | HGLibA_34810 | 192 | 0.602012348 | OXA1L | mitochondrial inner membrane protein [Source:HGNC Symbol;Acc:HGNC:8526]" |
| ENSG00000156486 | HGLibA_24386 | 192 | 0.602012348 | KCNS2 | potassium voltage-gated channel modifier subfamily S member 2 [Source:HGNC Symbol;Acc:HGNC:6301] |
| ENSG00000158296 | HGLibA_44391 | 192 | 0.602012348 | SLC13A3 | solute carrier family 13 member 3 [Source:HGNC Symbol;Acc:HGNC:14430] |

| | | | | | |
|---|---|---|---|---|---|
| ENSG00000158710 | HGLibA_48273 | 192 | 0.602012348 | TAGLN2 | transgelin 2 [Source:HGNC Symbol;Acc:HGNC:11554] |
| ENSG00000162688 | HGLibA_01196 | 192 | 0.602012348 | AGL | 4-alpha-glucanotransferase [Source:HGNC Symbol;Acc:HGNC:321]" |
| ENSG00000163257 | HGLibA_12432 | 192 | 0.602012348 | DCAF16 | DDB1 and CUL4 associated factor 16 [Source:HGNC Symbol;Acc:HGNC:25987] |
| ENSG00000163806 | HGLibA_46572 | 192 | 0.602012348 | SPDYA | speedy/RINGO cell cycle regulator family member A [Source:HGNC Symbol;Acc:HGNC:30613] |
| ENSG00000165948 | HGLibA_22600 | 192 | 0.602012348 | IFI27L1 | interferon alpha inducible protein 27 like 1 [Source:HGNC Symbol;Acc:HGNC:19754] |
| ENSG00000166012 | HGLibA_48223 | 192 | 0.602012348 | TAF1D | RNA polymerase I subunit D [Source:HGNC Symbol;Acc:HGNC:28759]" |
| ENSG00000167244 | HGLibA_22797 | 192 | 0.602012348 | IGF2 | insulin like growth factor 2 [Source:HGNC Symbol;Acc:HGNC:5466] |
| ENSG00000168374 | HGLibA_02653 | 192 | 0.602012348 | ARF4 | ADP ribosylation factor 4 [Source:HGNC Symbol;Acc:HGNC:655] |
| ENSG00000168748 | HGLibA_06821 | 192 | 0.602012348 | CA7 | carbonic anhydrase 7 [Source:HGNC Symbol;Acc:HGNC:1381] |
| ENSG00000169018 | HGLibA_17276 | 192 | 0.602012348 | FEM1B | fem-1 homolog B [Source:HGNC Symbol;Acc:HGNC:3649] |
| ENSG00000171132 | HGLibA_38544 | 192 | 0.602012348 | PRKCE | protein kinase C epsilon [Source:HGNC Symbol;Acc:HGNC:9401] |
| ENSG00000172005 | HGLibA_28022 | 192 | 0.602012348 | MAL | T cell differentiation protein [Source:HGNC Symbol;Acc:HGNC:6817]" |
| ENSG00000172840 | HGLibA_35928 | 192 | 0.602012348 | PDP2 | pyruvate dehyrogenase phosphatase catalytic subunit 2 [Source:HGNC Symbol;Acc:HGNC:30263] |
| ENSG00000176679 | HGLibA_49249 | 192 | 0.602012348 | TGIF2LY | TGFB induced factor homeobox 2 like Y-linked [Source:HGNC Symbol;Acc:HGNC:18569] |
| ENSG00000177910 | HGLibA_46493 | 192 | 0.602012348 | SPATA31C2 | SPATA31 subfamily C member 2 [Source:HGNC Symbol;Acc:HGNC:24508] |
| ENSG00000179407 | HGLibA_13555 | 192 | 0.602012348 | DNAJB8 | DnaJ heat shock protein family (Hsp40) member B8 [Source:HGNC Symbol;Acc:HGNC:23699] |
| ENSG00000181752 | HGLibA_34550 | 192 | 0.602012348 | OR8K5 | olfactory receptor family 8 subfamily K member 5 [Source:HGNC Symbol;Acc:HGNC:15315] |
| ENSG00000182405 | HGLibA_36226 | 192 | 0.602012348 | PGBD4 | piggyBac transposable element derived 4 [Source:HGNC Symbol;Acc:HGNC:19401] |
| ENSG00000182919 | HGLibA_05157 | 192 | 0.602012348 | C11orf54 | chromosome 11 open reading frame 54 [Source:HGNC Symbol;Acc:HGNC:30204] |
| ENSG00000183060 | HGLibA_27772 | 192 | 0.602012348 | LYSMD4 | LysM domain containing 4 [Source:HGNC Symbol;Acc:HGNC:26571] |
| ENSG00000186306 | HGLibA_33547 | 192 | 0.602012348 | OR10T2 | olfactory receptor family 10 subfamily T member 2 [Source:HGNC Symbol;Acc:HGNC:14816] |
| ENSG00000186812 | HGLibA_55917 | 192 | 0.602012348 | ZNF397 | zinc finger protein 397 [Source:HGNC Symbol;Acc:HGNC:18818] |
| ENSG00000186994 | HGLibA_24031 | 192 | 0.602012348 | KANK3 | KN motif and ankyrin repeat domains 3 [Source:HGNC Symbol;Acc:HGNC:24796] |
| ENSG00000189045 | HGLibA_01949 | 192 | 0.602012348 | ANKDD1B | ankyrin repeat and death domain containing 1B [Source:HGNC Symbol;Acc:HGNC:32525] |
| ENSG00000197134 | HGLibA_55680 | 192 | 0.602012348 | ZNF257 | zinc finger protein 257 [Source:HGNC Symbol;Acc:HGNC:13498] |
| ENSG00000204172 | HGLibA_01164 | 192 | 0.602012348 | AGAP9 | ankyrin repeat and PH domain 9 [Source:HGNC Symbol;Acc:HGNC:23463]" |
| ENSG00000204237 | HGLibA_34825 | 192 | 0.602012348 | OXLD1 | oxidoreductase like domain containing 1 [Source:HGNC Symbol;Acc:HGNC:27901] |
| ENSG00000205143 | HGLibA_02896 | 192 | 0.602012348 | ARID3C | AT-rich interaction domain 3C [Source:HGNC Symbol;Acc:HGNC:21209] |
| ENSG00000215009 | HGLibA_00582 | 192 | 0.602012348 | ACSM4 | acyl-CoA synthetase medium chain family member 4 [Source:HGNC Symbol;Acc:HGNC:32016] |
| ENSG00000223953 | HGLibA_05947 | 192 | 0.602012348 | C1QTNF5 | C1q and TNF related 5 [Source:HGNC Symbol;Acc:HGNC:14344] |
| ENSG00000261609 | HGLibA_18601 | 192 | 0.602012348 | GAN | gigaxonin [Source:HGNC Symbol;Acc:HGNC:4137] |
| ENSG00000276231 | HGLibA_36664 | 192 | 0.602012348 | PIK3R6 | phosphoinositide-3-kinase regulatory subunit 6 [Source:HGNC Symbol;Acc:HGNC:27101] |
| hsa-mir-371a | HGLibA_59373 | 192 | 0.602012348 | hsa-mir-371a | - |
| hsa-mir-484 | HGLibA_61327 | 192 | 0.602012348 | hsa-mir-484 | - |
| hsa-mir-489 | HGLibA_61355 | 192 | 0.602012348 | hsa-mir-489 | - |
| hsa-mir-638 | HGLibA_62792 | 192 | 0.602012348 | hsa-mir-638 | - |
| hsa-mir-7112-2 | HGLibA_63817 | 192 | 0.602012348 | hsa-mir-7112-2 | - |
| BAGE | HGLibA_04091 | 191 | 0.592967005 | BAGE | - |
| CD97 | HGLibA_08486 | 191 | 0.592967005 | CD97 | - |
| ENSG00000015520 | HGLibA_32422 | 191 | 0.592967005 | NPC1L1 | NPC1 like intracellular cholesterol transporter 1 [Source:HGNC Symbol;Acc:HGNC:7898] |
| ENSG00000018408 | HGLibA_54457 | 191 | 0.592967005 | WWTR1 | WW domain containing transcription regulator 1 [Source:HGNC Symbol;Acc:HGNC:24042] |
| ENSG00000018625 | HGLibA_03584 | 191 | 0.592967005 | ATP1A2 | ATPase Na+/K+ transporting subunit alpha 2 [Source:HGNC Symbol;Acc:HGNC:800] |
| ENSG00000021645 | HGLibA_32740 | 191 | 0.592967005 | NRXN3 | neurexin 3 [Source:HGNC Symbol;Acc:HGNC:8010] |
| ENSG00000028528 | HGLibA_46024 | 191 | 0.592967005 | SNX1 | sorting nexin 1 [Source:HGNC Symbol;Acc:HGNC:11172] |
| ENSG00000055732 | HGLibA_28644 | 191 | 0.592967005 | MCOLN3 | mucolipin 3 [Source:HGNC Symbol;Acc:HGNC:13358] |
| ENSG00000068985 | HGLibA_35005 | 191 | 0.592967005 | PAGE1 | PAGE family member 1 [Source:HGNC Symbol;Acc:HGNC:4107] |
| ENSG00000074590 | HGLibA_32893 | 191 | 0.592967005 | NUAK1 | NUAK family kinase 1 [Source:HGNC Symbol;Acc:HGNC:14311] |
| ENSG00000090382 | HGLibA_27782 | 191 | 0.592967005 | LYZ | lysozyme [Source:HGNC Symbol;Acc:HGNC:6740] |
| ENSG00000093144 | HGLibA_14393 | 191 | 0.592967005 | ECHDC1 | ethylmalonyl-CoA decarboxylase 1 [Source:HGNC Symbol;Acc:HGNC:21489] |
| ENSG00000095110 | HGLibA_33151 | 191 | 0.592967005 | NXPE1 | neurexophilin and PC-esterase domain family member 1 [Source:HGNC Symbol;Acc:HGNC:28527] |
| ENSG00000101224 | HGLibA_08531 | 191 | 0.592967005 | CDC25B | cell division cycle 25B [Source:HGNC Symbol;Acc:HGNC:1726] |
| ENSG00000104055 | HGLibA_49265 | 191 | 0.592967005 | TGM5 | transglutaminase 5 [Source:HGNC Symbol;Acc:HGNC:11781] |
| ENSG00000104267 | HGLibA_06802 | 191 | 0.592967005 | CA2 | carbonic anhydrase 2 [Source:HGNC Symbol;Acc:HGNC:1373] |
| ENSG00000105205 | HGLibA_09801 | 191 | 0.592967005 | CLC | Charcot-Leyden crystal galectin [Source:HGNC Symbol;Acc:HGNC:2014] |
| ENSG00000107147 | HGLibA_24393 | 191 | 0.592967005 | KCNT1 | potassium sodium-activated channel subfamily T member 1 [Source:HGNC Symbol;Acc:HGNC:18865] |
| ENSG00000117461 | HGLibA_36655 | 191 | 0.592967005 | PIK3R3 | phosphoinositide-3-kinase regulatory subunit 3 [Source:HGNC Symbol;Acc:HGNC:8981] |
| ENSG00000118454 | HGLibA_02007 | 191 | 0.592967005 | ANKRD13C | ankyrin repeat domain 13C [Source:HGNC Symbol;Acc:HGNC:25374] |
| ENSG00000118777 | HGLibA_00219 | 191 | 0.592967005 | ABCG2 | ATP binding cassette subfamily G member 2 (Junior blood group) [Source:HGNC Symbol;Acc:HGNC:74] |
| ENSG00000118816 | HGLibA_08101 | 191 | 0.592967005 | CCNI | cyclin I [Source:HGNC Symbol;Acc:HGNC:1595] |
| ENSG00000123595 | HGLibA_39991 | 191 | 0.592967005 | RAB9A | member RAS oncogene family [Source:HGNC Symbol;Acc:HGNC:9792]" |
| ENSG00000124232 | HGLibA_40649 | 191 | 0.592967005 | RBPJL | recombination signal binding protein for immunoglobulin kappa J region like [Source:HGNC Symbol;Acc:HGNC:13761] |
| ENSG00000125652 | HGLibA_01663 | 191 | 0.592967005 | ALKBH7 | alkB homolog 7 [Source:HGNC Symbol;Acc:HGNC:21306] |
| ENSG00000126432 | HGLibA_38418 | 191 | 0.592967005 | PRDX5 | peroxiredoxin 5 [Source:HGNC Symbol;Acc:HGNC:9355] |
| ENSG00000126522 | HGLibA_03317 | 191 | 0.592967005 | ASL | argininosuccinate lyase [Source:HGNC Symbol;Acc:HGNC:746] |
| ENSG00000131446 | HGLibA_29131 | 191 | 0.592967005 | MGAT1 | 2-N-acetylglucosaminyltransferase [Source:HGNC Symbol;Acc:HGNC:7044]" |
| ENSG00000131634 | HGLibA_50189 | 191 | 0.592967005 | TMEM204 | transmembrane protein 204 [Source:HGNC Symbol;Acc:HGNC:14158] |
| ENSG00000133794 | HGLibA_03067 | 191 | 0.592967005 | ARNTL | aryl hydrocarbon receptor nuclear translocator like [Source:HGNC Symbol;Acc:HGNC:701] |
| ENSG00000135090 | HGLibA_48311 | 191 | 0.592967005 | TAOK3 | TAO kinase 3 [Source:HGNC Symbol;Acc:HGNC:18133] |
| ENSG00000137185 | HGLibA_57034 | 191 | 0.592967005 | ZSCAN9 | zinc finger and SCAN domain containing 9 [Source:HGNC Symbol;Acc:HGNC:12984] |
| ENSG00000137269 | HGLibA_27244 | 191 | 0.592967005 | LRRC1 | leucine rich repeat containing 1 [Source:HGNC Symbol;Acc:HGNC:14307] |
| ENSG00000137502 | HGLibA_39871 | 191 | 0.592967005 | RAB30 | member RAS oncogene family [Source:HGNC Symbol;Acc:HGNC:9770]" |
| ENSG00000137802 | HGLibA_28310 | 191 | 0.592967005 | MAPKBP1 | mitogen-activated protein kinase binding protein 1 [Source:HGNC Symbol;Acc:HGNC:29536] |
| ENSG00000138190 | HGLibA_15755 | 191 | 0.592967005 | EXOC6 | exocyst complex component 6 [Source:HGNC Symbol;Acc:HGNC:23196] |
| ENSG00000140323 | HGLibA_13285 | 191 | 0.592967005 | DISP2 | dispatched RND transporter family member 2 [Source:HGNC Symbol;Acc:HGNC:19712] |
| ENSG00000141258 | HGLibA_43861 | 191 | 0.592967005 | SGSM2 | small G protein signaling modulator 2 [Source:HGNC Symbol;Acc:HGNC:29026] |
| ENSG00000141401 | HGLibA_23247 | 191 | 0.592967005 | IMPA2 | inositol monophosphatase 2 [Source:HGNC Symbol;Acc:HGNC:6051] |
| ENSG00000142227 | HGLibA_15079 | 191 | 0.592967005 | EMP3 | epithelial membrane protein 3 [Source:HGNC Symbol;Acc:HGNC:3335] |
| ENSG00000142409 | HGLibA_56755 | 191 | 0.592967005 | ZNF787 | zinc finger protein 787 [Source:HGNC Symbol;Acc:HGNC:26998] |
| ENSG00000143184 | HGLibA_54492 | 191 | 0.592967005 | XCL1 | X-C motif chemokine ligand 1 [Source:HGNC Symbol;Acc:HGNC:10645] |
| ENSG00000143373 | HGLibA_56540 | 191 | 0.592967005 | ZNF687 | zinc finger protein 687 [Source:HGNC Symbol;Acc:HGNC:29277] |
| ENSG00000146535 | HGLibA_19439 | 191 | 0.592967005 | GNA12 | G protein subunit alpha 12 [Source:HGNC Symbol;Acc:HGNC:4380] |
| ENSG00000150782 | HGLibA_23022 | 191 | 0.592967005 | IL18 | interleukin 18 [Source:HGNC Symbol;Acc:HGNC:5986] |
| ENSG00000153779 | HGLibA_49246 | 191 | 0.592967005 | TGIF2LX | TGFB induced factor homeobox 2 like X-linked [Source:HGNC Symbol;Acc:HGNC:18570] |
| ENSG00000159884 | HGLibA_07525 | 191 | 0.592967005 | CCDC107 | coiled-coil domain containing 107 [Source:HGNC Symbol;Acc:HGNC:28465] |
| ENSG00000160050 | HGLibA_07748 | 191 | 0.592967005 | CCDC28B | coiled-coil domain containing 28B [Source:HGNC Symbol;Acc:HGNC:28163] |
| ENSG00000161298 | HGLibA_55888 | 191 | 0.592967005 | ZNF382 | zinc finger protein 382 [Source:HGNC Symbol;Acc:HGNC:17409] |
| ENSG00000162594 | HGLibA_23099 | 191 | 0.592967005 | IL23R | interleukin 23 receptor [Source:HGNC Symbol;Acc:HGNC:19100] |
| ENSG00000162892 | HGLibA_23102 | 191 | 0.592967005 | IL24 | interleukin 24 [Source:HGNC Symbol;Acc:HGNC:11346] |
| ENSG00000163389 | HGLibA_37476 | 191 | 0.592967005 | POGLUT1 | protein O-glucosyltransferase 1 [Source:HGNC Symbol;Acc:HGNC:22954] |
| ENSG00000163703 | HGLibA_11125 | 191 | 0.592967005 | CRELD1 | cysteine rich with EGF like domains 1 [Source:HGNC Symbol;Acc:HGNC:14630] |
| ENSG00000164675 | HGLibA_23549 | 191 | 0.592967005 | IQUB | IQ motif and ubiquitin domain containing [Source:HGNC Symbol;Acc:HGNC:21995] |
| ENSG00000168268 | HGLibA_32823 | 191 | 0.592967005 | NT5DC2 | 5'-nucleotidase domain containing 2 [Source:HGNC Symbol;Acc:HGNC:25717] |
| ENSG00000168421 | HGLibA_41194 | 191 | 0.592967005 | RHOH | ras homolog family member H [Source:HGNC Symbol;Acc:HGNC:686] |
| ENSG00000168569 | HGLibA_50244 | 191 | 0.592967005 | TMEM223 | transmembrane protein 223 [Source:HGNC Symbol;Acc:HGNC:28464] |
| ENSG00000169302 | HGLibA_47484 | 191 | 0.592967005 | STK32A | serine/threonine kinase 32A [Source:HGNC Symbol;Acc:HGNC:28317] |
| ENSG00000169991 | HGLibA_22589 | 191 | 0.592967005 | IFFO2 | intermediate filament family orphan 2 [Source:HGNC Symbol;Acc:HGNC:27006] |
| ENSG00000171587 | HGLibA_13998 | 191 | 0.592967005 | DSCAM | DS cell adhesion molecule [Source:HGNC Symbol;Acc:HGNC:3039] |
| ENSG00000174307 | HGLibA_36422 | 191 | 0.592967005 | PHLDA3 | pleckstrin homology like domain family A member 3 [Source:HGNC Symbol;Acc:HGNC:8934] |
| ENSG00000174950 | HGLibA_08253 | 191 | 0.592967005 | CD164L2 | CD164 molecule like 2 [Source:HGNC Symbol;Acc:HGNC:32043] |
| ENSG00000176695 | HGLibA_33995 | 191 | 0.592967005 | OR4F17 | olfactory receptor family 4 subfamily F member 17 [Source:HGNC Symbol;Acc:HGNC:15381] |
| ENSG00000178795 | HGLibA_18900 | 191 | 0.592967005 | GDPD4 | glycerophosphodiester phosphodiesterase domain containing 4 [Source:HGNC Symbol;Acc:HGNC:24849] |
| ENSG00000178952 | HGLibA_52323 | 191 | 0.592967005 | TUFM | mitochondrial [Source:HGNC Symbol;Acc:HGNC:12420]" |
| ENSG00000180638 | HGLibA_45195 | 191 | 0.592967005 | SLC47A2 | solute carrier family 47 member 2 [Source:HGNC Symbol;Acc:HGNC:26439] |
| ENSG00000181704 | HGLibA_54685 | 191 | 0.592967005 | YIPF6 | Yip1 domain family member 6 [Source:HGNC Symbol;Acc:HGNC:28304] |
| ENSG00000182533 | HGLibA_07381 | 191 | 0.592967005 | CAV3 | caveolin 3 [Source:HGNC Symbol;Acc:HGNC:1529] |
| ENSG00000183908 | HGLibA_27389 | 191 | 0.592967005 | LRRC55 | leucine rich repeat containing 55 [Source:HGNC Symbol;Acc:HGNC:32324] |
| ENSG00000183943 | HGLibA_38592 | 191 | 0.592967005 | PRKX | protein kinase X-linked [Source:HGNC Symbol;Acc:HGNC:9441] |
| ENSG00000185585 | HGLibA_33359 | 191 | 0.592967005 | OLFML2A | olfactomedin like 2A [Source:HGNC Symbol;Acc:HGNC:27270] |
| ENSG00000187243 | HGLibA_27976 | 191 | 0.592967005 | MAGED4B | MAGE family member D4B [Source:HGNC Symbol;Acc:HGNC:22880] |
| ENSG00000187554 | HGLibA_49654 | 191 | 0.592967005 | TLR5 | toll like receptor 5 [Source:HGNC Symbol;Acc:HGNC:11851] |
| ENSG00000187713 | HGLibA_50186 | 191 | 0.592967005 | TMEM203 | transmembrane protein 203 [Source:HGNC Symbol;Acc:HGNC:28217] |
| ENSG00000187715 | HGLibA_24096 | 191 | 0.592967005 | KBTBD12 | kelch repeat and BTB domain containing 12 [Source:HGNC Symbol;Acc:HGNC:25731] |
| ENSG00000188158 | HGLibA_31914 | 191 | 0.592967005 | NHS | NHS actin remodeling regulator [Source:HGNC Symbol;Acc:HGNC:7820] |
| ENSG00000197938 | HGLibA_34299 | 191 | 0.592967005 | OR5H2 | olfactory receptor family 5 subfamily H member 2 [Source:HGNC Symbol;Acc:HGNC:14752] |
| ENSG00000203965 | HGLibA_14551 | 191 | 0.592967005 | EFCAB7 | EF-hand calcium binding domain 7 [Source:HGNC Symbol;Acc:HGNC:29379] |
| ENSG00000204427 | HGLibA_00256 | 191 | 0.592967005 | ABHD16A | abhydrolase domain containing 16A [Source:HGNC Symbol;Acc:HGNC:13921] |
| ENSG00000205754 | HGLibA_45455 | 191 | 0.592967005 | SLCO1B7 | solute carrier organic anion transporter family member 1B7 (putative) [Source:HGNC Symbol;Acc:HGNC:32934] |
| ENSG00000242019 | HGLibA_24996 | 191 | 0.592967005 | KIR3DL3 | three Ig domains and long cytoplasmic tail 3 [Source:HGNC Symbol;Acc:HGNC:16312]" |
| ENSG00000255307 | HGLibA_34144 | 191 | 0.592967005 | OR52B2 | olfactory receptor family 52 subfamily B member 2 [Source:HGNC Symbol;Acc:HGNC:15207] |
| ENSG00000258659 | HGLibA_51468 | 191 | 0.592967005 | TRIM34 | tripartite motif containing 34 [Source:HGNC Symbol;Acc:HGNC:10063] |
| ENSG00000276430 | HGLibA_16555 | 191 | 0.592967005 | FAM25C | family with sequence similarity 25 member C [Source:HGNC Symbol;Acc:HGNC:23586] |
| HEXDC | HGLibA_21208 | 191 | 0.592967005 | HEXDC | - |
| HIST1H3I | HGLibA_21480 | 191 | 0.592967005 | HIST1H3I | - |
| hsa-mir-1911 | HGLibA_58051 | 191 | 0.592967005 | hsa-mir-1911 | - |
| hsa-mir-3116-1 | HGLibA_58503 | 191 | 0.592967005 | hsa-mir-3116-1 | - |
| hsa-mir-3199-2 | HGLibA_58900 | 191 | 0.592967005 | hsa-mir-3199-2 | - |
| hsa-mir-3617 | HGLibA_59096 | 191 | 0.592967005 | hsa-mir-3617 | - |
| hsa-mir-4484 | HGLibA_60355 | 191 | 0.592967005 | hsa-mir-4484 | - |
| hsa-mir-4736 | HGLibA_61040 | 191 | 0.592967005 | hsa-mir-4736 | - |
| hsa-mir-4751 | HGLibA_61103 | 191 | 0.592967005 | hsa-mir-4751 | - |
| hsa-mir-4752 | HGLibA_61107 | 191 | 0.592967005 | hsa-mir-4752 | - |
| hsa-mir-6515 | HGLibA_62934 | 191 | 0.592967005 | hsa-mir-6515 | - |
| hsa-mir-8058 | HGLibA_64070 | 191 | 0.592967005 | hsa-mir-8058 | - |
| USMG5 | HGLibA_53184 | 191 | 0.592967005 | USMG5 | - |
| ZAK | HGLibA_54772 | 191 | 0.592967005 | ZAK | - |

| | | | | | |
|---|---|---|---|---|---|
| ADRBK1 | HGLibA_01064 | 190 | 0.583921662 | ADRBK1 | - |
| ATP5S | HGLibA_03687 | 190 | 0.583921662 | ATP5S | - |
| C19orf66 | HGLibA_05694 | 190 | 0.583921662 | C19orf66 | - |
| C19orf77 | HGLibA_05709 | 190 | 0.583921662 | C19orf77 | - |
| C1orf234 | HGLibA_05848 | 190 | 0.583921662 | C1orf234 | - |
| C3orf55 | HGLibA_06252 | 190 | 0.583921662 | C3orf55 | - |
| ENSG00000010438 | HGLibA_38924 | 190 | 0.583921662 | PRSS3 | serine protease 3 [Source:HGNC Symbol;Acc:HGNC:9486] |
| ENSG00000016602 | HGLibA_09811 | 190 | 0.583921662 | CLCA4 | chloride channel accessory 4 [Source:HGNC Symbol;Acc:HGNC:2018] |
| ENSG00000056998 | HGLibA_20692 | 190 | 0.583921662 | GYG2 | glycogenin 2 [Source:HGNC Symbol;Acc:HGNC:4700] |
| ENSG00000069535 | HGLibA_28109 | 190 | 0.583921662 | MAOB | monoamine oxidase B [Source:HGNC Symbol;Acc:HGNC:6834] |
| ENSG00000075290 | HGLibA_54390 | 190 | 0.583921662 | WNT8B | Wnt family member 8B [Source:HGNC Symbol;Acc:HGNC:12789] |
| ENSG00000078177 | HGLibA_30951 | 190 | 0.583921662 | N4BP2 | NEDD4 binding protein 2 [Source:HGNC Symbol;Acc:HGNC:29851] |
| ENSG00000078725 | HGLibA_04811 | 190 | 0.583921662 | BRINP1 | BMP/retinoic acid inducible neural specific 1 [Source:HGNC Symbol;Acc:HGNC:2687] |
| ENSG00000079335 | HGLibA_08509 | 190 | 0.583921662 | CDC14A | cell division cycle 14A [Source:HGNC Symbol;Acc:HGNC:1718] |
| ENSG00000092094 | HGLibA_34682 | 190 | 0.583921662 | OSGEP | O-sialoglycoprotein endopeptidase [Source:HGNC Symbol;Acc:HGNC:18028] |
| ENSG00000100593 | HGLibA_23678 | 190 | 0.583921662 | ISM2 | isthmin 2 [Source:HGNC Symbol;Acc:HGNC:23176] |
| ENSG00000102984 | HGLibA_56818 | 190 | 0.583921662 | ZNF821 | zinc finger protein 821 [Source:HGNC Symbol;Acc:HGNC:28043] |
| ENSG00000104435 | HGLibA_47519 | 190 | 0.583921662 | STMN2 | stathmin 2 [Source:HGNC Symbol;Acc:HGNC:10577] |
| ENSG00000104679 | HGLibA_39765 | 190 | 0.583921662 | R3HCC1 | R3H domain and coiled-coil containing 1 [Source:HGNC Symbol;Acc:HGNC:27329] |
| ENSG00000106560 | HGLibA_19075 | 190 | 0.583921662 | GIMAP2 | IMAP family member 2 [Source:HGNC Symbol;Acc:HGNC:21789]" |
| ENSG00000110651 | HGLibA_08455 | 190 | 0.583921662 | CD81 | CD81 molecule [Source:HGNC Symbol;Acc:HGNC:1701] |
| ENSG00000111237 | HGLibA_53760 | 190 | 0.583921662 | VPS29 | retromer complex component [Source:HGNC Symbol;Acc:HGNC:14340]" |
| ENSG00000115183 | HGLibA_48292 | 190 | 0.583921662 | TANC1 | ankyrin repeat and coiled-coil containing 1 [Source:HGNC Symbol;Acc:HGNC:29364]" |
| ENSG00000118094 | HGLibA_51352 | 190 | 0.583921662 | TREH | trehalase [Source:HGNC Symbol;Acc:HGNC:12266] |
| ENSG00000118197 | HGLibA_12756 | 190 | 0.583921662 | DDX59 | DEAD-box helicase 59 [Source:HGNC Symbol;Acc:HGNC:25360] |
| ENSG00000119559 | HGLibA_05642 | 190 | 0.583921662 | C19orf25 | chromosome 19 open reading frame 25 [Source:HGNC Symbol;Acc:HGNC:26711] |
| ENSG00000120498 | HGLibA_49053 | 190 | 0.583921662 | TEX11 | testis expressed 11 [Source:HGNC Symbol;Acc:HGNC:11733] |
| ENSG00000122711 | HGLibA_46694 | 190 | 0.583921662 | SPINK4 | Kazal type 4 [Source:HGNC Symbol;Acc:HGNC:16646]" |
| ENSG00000124214 | HGLibA_47406 | 190 | 0.583921662 | STAU1 | staufen double-stranded RNA binding protein 1 [Source:HGNC Symbol;Acc:HGNC:11370] |
| ENSG00000125046 | HGLibA_47191 | 190 | 0.583921662 | SSUH2 | ssu-2 homolog (C. elegans) [Source:HGNC Symbol;Acc:HGNC:24809] |
| ENSG00000126464 | HGLibA_38774 | 190 | 0.583921662 | PRR12 | proline rich 12 [Source:HGNC Symbol;Acc:HGNC:29217] |
| ENSG00000127511 | HGLibA_44185 | 190 | 0.583921662 | SIN3B | SIN3 transcription regulator family member B [Source:HGNC Symbol;Acc:HGNC:19354] |
| ENSG00000129353 | HGLibA_45159 | 190 | 0.583921662 | SLC44A2 | solute carrier family 44 member 2 [Source:HGNC Symbol;Acc:HGNC:17292] |
| ENSG00000130711 | HGLibA_38371 | 190 | 0.583921662 | PRDM12 | PR/SET domain 12 [Source:HGNC Symbol;Acc:HGNC:13997] |
| ENSG00000131844 | HGLibA_28569 | 190 | 0.583921662 | MCCC2 | methylcrotonoyl-CoA carboxylase 2 [Source:HGNC Symbol;Acc:HGNC:6937] |
| ENSG00000132109 | HGLibA_51428 | 190 | 0.583921662 | TRIM21 | tripartite motif containing 21 [Source:HGNC Symbol;Acc:HGNC:11312] |
| ENSG00000132821 | HGLibA_53867 | 190 | 0.583921662 | VSTM2L | V-set and transmembrane domain containing 2 like [Source:HGNC Symbol;Acc:HGNC:16096] |
| ENSG00000135094 | HGLibA_43173 | 190 | 0.583921662 | SDS | serine dehydratase [Source:HGNC Symbol;Acc:HGNC:10691] |
| ENSG00000135720 | HGLibA_14243 | 190 | 0.583921662 | DYNC1LI2 | dynein cytoplasmic 1 light intermediate chain 2 [Source:HGNC Symbol;Acc:HGNC:2966] |
| ENSG00000135925 | HGLibA_54345 | 190 | 0.583921662 | WNT10A | Wnt family member 10A [Source:HGNC Symbol;Acc:HGNC:13829] |
| ENSG00000136931 | HGLibA_32634 | 190 | 0.583921662 | NR5A1 | nuclear receptor subfamily 5 group A member 1 [Source:HGNC Symbol;Acc:HGNC:7983] |
| ENSG00000141437 | HGLibA_44769 | 190 | 0.583921662 | SLC25A52 | solute carrier family 25 member 52 [Source:HGNC Symbol;Acc:HGNC:23324] |
| ENSG00000142319 | HGLibA_45325 | 190 | 0.583921662 | SLC6A3 | solute carrier family 6 member 3 [Source:HGNC Symbol;Acc:HGNC:11049] |
| ENSG00000143614 | HGLibA_18688 | 190 | 0.583921662 | GATAD2B | GATA zinc finger domain containing 2B [Source:HGNC Symbol;Acc:HGNC:30778] |
| ENSG00000144857 | HGLibA_04666 | 190 | 0.583921662 | BOC | oncogene regulated [Source:HGNC Symbol;Acc:HGNC:17173]" |
| ENSG00000145029 | HGLibA_31920 | 190 | 0.583921662 | NICN1 | nicolin 1 [Source:HGNC Symbol;Acc:HGNC:18317] |
| ENSG00000145839 | HGLibA_23200 | 190 | 0.583921662 | IL9 | interleukin 9 [Source:HGNC Symbol;Acc:HGNC:6029] |
| ENSG00000147144 | HGLibA_07564 | 190 | 0.583921662 | CCDC120 | coiled-coil domain containing 120 [Source:HGNC Symbol;Acc:HGNC:28910] |
| ENSG00000147183 | HGLibA_11032 | 190 | 0.583921662 | CPXCR1 | candidate 1 [Source:HGNC Symbol;Acc:HGNC:2332]" |
| ENSG00000147676 | HGLibA_28026 | 190 | 0.583921662 | MAL2 | T cell differentiation protein 2 (gene/pseudogene) [Source:HGNC Symbol;Acc:HGNC:13634]" |
| ENSG00000149305 | HGLibA_22382 | 190 | 0.583921662 | HTR3B | 5-hydroxytryptamine receptor 3B [Source:HGNC Symbol;Acc:HGNC:5298] |
| ENSG00000150625 | HGLibA_19820 | 190 | 0.583921662 | GPM6A | glycoprotein M6A [Source:HGNC Symbol;Acc:HGNC:4460] |
| ENSG00000155034 | HGLibA_16990 | 190 | 0.583921662 | FBXL18 | F-box and leucine rich repeat protein 18 [Source:HGNC Symbol;Acc:HGNC:21874] |
| ENSG00000158201 | HGLibA_00275 | 190 | 0.583921662 | ABHD3 | abhydrolase domain containing 3 [Source:HGNC Symbol;Acc:HGNC:18718] |
| ENSG00000159921 | HGLibA_19504 | 190 | 0.583921662 | GNE | glucosamine (UDP-N-acetyl)-2-epimerase/N-acetylmannosamine kinase [Source:HGNC Symbol;Acc:HGNC:23657] |
| ENSG00000160051 | HGLibA_23492 | 190 | 0.583921662 | IQCC | IQ motif containing C [Source:HGNC Symbol;Acc:HGNC:25545] |
| ENSG00000162946 | HGLibA_13279 | 190 | 0.583921662 | DISC1 | DISC1 scaffold protein [Source:HGNC Symbol;Acc:HGNC:2888] |
| ENSG00000164163 | HGLibA_00202 | 190 | 0.583921662 | ABCE1 | ATP binding cassette subfamily E member 1 [Source:HGNC Symbol;Acc:HGNC:69] |
| ENSG00000165805 | HGLibA_05258 | 190 | 0.583921662 | C12orf50 | chromosome 12 open reading frame 50 [Source:HGNC Symbol;Acc:HGNC:26665] |
| ENSG00000166428 | HGLibA_37044 | 190 | 0.583921662 | PLD4 | phospholipase D family member 4 [Source:HGNC Symbol;Acc:HGNC:23792] |
| ENSG00000166927 | HGLibA_30163 | 190 | 0.583921662 | MS4A7 | membrane spanning 4-domains A7 [Source:HGNC Symbol;Acc:HGNC:13378] |
| ENSG00000167110 | HGLibA_19596 | 190 | 0.583921662 | GOLGA2 | golgin A2 [Source:HGNC Symbol;Acc:HGNC:4425] |
| ENSG00000167178 | HGLibA_23672 | 190 | 0.583921662 | ISLR2 | immunoglobulin superfamily containing leucine rich repeat 2 [Source:HGNC Symbol;Acc:HGNC:29286] |
| ENSG00000168067 | HGLibA_28214 | 190 | 0.583921662 | MAP4K2 | mitogen-activated protein kinase kinase kinase kinase 2 [Source:HGNC Symbol;Acc:HGNC:6864] |
| ENSG00000169488 | HGLibA_34026 | 190 | 0.583921662 | OR4K15 | olfactory receptor family 4 subfamily K member 15 [Source:HGNC Symbol;Acc:HGNC:15353] |
| ENSG00000170209 | HGLibA_01974 | 190 | 0.583921662 | ANKK1 | ankyrin repeat and kinase domain containing 1 [Source:HGNC Symbol;Acc:HGNC:21027] |
| ENSG00000170265 | HGLibA_55739 | 190 | 0.583921662 | ZNF282 | zinc finger protein 282 [Source:HGNC Symbol;Acc:HGNC:13076] |
| ENSG00000172159 | HGLibA_18010 | 190 | 0.583921662 | FRMD3 | FERM domain containing 3 [Source:HGNC Symbol;Acc:HGNC:24125] |
| ENSG00000174586 | HGLibA_56104 | 190 | 0.583921662 | ZNF497 | zinc finger protein 497 [Source:HGNC Symbol;Acc:HGNC:23714] |
| ENSG00000174796 | HGLibA_49307 | 190 | 0.583921662 | THAP6 | THAP domain containing 6 [Source:HGNC Symbol;Acc:HGNC:23189] |
| ENSG00000175305 | HGLibA_08086 | 190 | 0.583921662 | CCNE2 | cyclin E2 [Source:HGNC Symbol;Acc:HGNC:1590] |
| ENSG00000179094 | HGLibA_36054 | 190 | 0.583921662 | PER1 | period circadian regulator 1 [Source:HGNC Symbol;Acc:HGNC:8845] |
| ENSG00000179593 | HGLibA_01686 | 190 | 0.583921662 | ALOX15B | type B [Source:HGNC Symbol;Acc:HGNC:434]" |
| ENSG00000179673 | HGLibA_42056 | 190 | 0.583921662 | RPRML | reprimo like [Source:HGNC Symbol;Acc:HGNC:32422] |
| ENSG00000182179 | HGLibA_52531 | 190 | 0.583921662 | UBA7 | ubiquitin like modifier activating enzyme 7 [Source:HGNC Symbol;Acc:HGNC:12471] |
| ENSG00000183036 | HGLibA_35662 | 190 | 0.583921662 | PCP4 | Purkinje cell protein 4 [Source:HGNC Symbol;Acc:HGNC:8742] |
| ENSG00000183048 | HGLibA_44637 | 190 | 0.583921662 | SLC25A10 | solute carrier family 25 member 10 [Source:HGNC Symbol;Acc:HGNC:10980] |
| ENSG00000186326 | HGLibA_41115 | 190 | 0.583921662 | RGS9BP | regulator of G protein signaling 9 binding protein [Source:HGNC Symbol;Acc:HGNC:30304] |
| ENSG00000186767 | HGLibA_46679 | 190 | 0.583921662 | SPIN4 | spindlin family member 4 [Source:HGNC Symbol;Acc:HGNC:27040] |
| ENSG00000187180 | HGLibA_26123 | 190 | 0.583921662 | LCE2C | late cornified envelope 2C [Source:HGNC Symbol;Acc:HGNC:29460] |
| ENSG00000187650 | HGLibA_53689 | 190 | 0.583921662 | VMAC | vimentin type intermediate filament associated coiled-coil protein [Source:HGNC Symbol;Acc:HGNC:33803] |
| ENSG00000187838 | HGLibA_37237 | 190 | 0.583921662 | PLSCR3 | phospholipid scramblase 3 [Source:HGNC Symbol;Acc:HGNC:16495] |
| ENSG00000188158 | HGLibA_31913 | 190 | 0.583921662 | NHS | NHS actin remodeling regulator [Source:HGNC Symbol;Acc:HGNC:7820] |
| ENSG00000188313 | HGLibA_37232 | 190 | 0.583921662 | PLSCR1 | phospholipid scramblase 1 [Source:HGNC Symbol;Acc:HGNC:9092] |
| ENSG00000188786 | HGLibA_30350 | 190 | 0.583921662 | MTF1 | metal regulatory transcription factor 1 [Source:HGNC Symbol;Acc:HGNC:7428] |
| ENSG00000188895 | HGLibA_30212 | 190 | 0.583921662 | MSL1 | male specific lethal 1 homolog [Source:HGNC Symbol;Acc:HGNC:27905] |
| ENSG00000196209 | HGLibA_44207 | 190 | 0.583921662 | SIRPB2 | signal regulatory protein beta 2 [Source:HGNC Symbol;Acc:HGNC:16247] |
| ENSG00000198108 | HGLibA_09659 | 190 | 0.583921662 | CHSY3 | chondroitin sulfate synthase 3 [Source:HGNC Symbol;Acc:HGNC:24293] |
| ENSG00000198440 | HGLibA_56315 | 190 | 0.583921662 | ZNF583 | zinc finger protein 583 [Source:HGNC Symbol;Acc:HGNC:26427] |
| ENSG00000198798 | HGLibA_27946 | 190 | 0.583921662 | MAGEB3 | MAGE family member B3 [Source:HGNC Symbol;Acc:HGNC:6810] |
| ENSG00000198838 | HGLibA_42528 | 190 | 0.583921662 | RYR3 | ryanodine receptor 3 [Source:HGNC Symbol;Acc:HGNC:10485] |
| ENSG00000198855 | HGLibA_17508 | 190 | 0.583921662 | FICD | FIC domain containing [Source:HGNC Symbol;Acc:HGNC:18416] |
| ENSG00000205581 | HGLibA_21718 | 190 | 0.583921662 | HMGN1 | high mobility group nucleosome binding domain 1 [Source:HGNC Symbol;Acc:HGNC:4984] |
| ENSG00000213931 | HGLibA_20918 | 190 | 0.583921662 | HBE1 | hemoglobin subunit epsilon 1 [Source:HGNC Symbol;Acc:HGNC:4830] |
| ENSG00000244405 | HGLibA_15663 | 190 | 0.583921662 | ETV5 | ETS variant 5 [Source:HGNC Symbol;Acc:HGNC:3494] |
| ENSG00000255529 | HGLibA_37607 | 190 | 0.583921662 | POLR2M | RNA polymerase II subunit M [Source:HGNC Symbol;Acc:HGNC:14862] |
| ENSG00000272395 | HGLibA_22727 | 190 | 0.583921662 | IFNL4 | interferon lambda 4 (gene/pseudogene) [Source:HGNC Symbol;Acc:HGNC:44480] |
| FAM49B | HGLibA_16627 | 190 | 0.583921662 | FAM49B | - |
| hsa-mir-1287 | HGLibA_57611 | 190 | 0.583921662 | hsa-mir-1287 | - |
| hsa-mir-3126 | HGLibA_58556 | 190 | 0.583921662 | hsa-mir-3126 | - |
| hsa-mir-4672 | HGLibA_60806 | 190 | 0.583921662 | hsa-mir-4672 | - |
| hsa-mir-493 | HGLibA_61373 | 190 | 0.583921662 | hsa-mir-493 | - |
| hsa-mir-5189 | HGLibA_61648 | 190 | 0.583921662 | hsa-mir-5189 | - |
| MKL2 | HGLibA_29335 | 190 | 0.583921662 | MKL2 | - |
| MYEOV2 | HGLibA_30694 | 190 | 0.583921662 | MYEOV2 | - |
| PQLC3 | HGLibA_38258 | 190 | 0.583921662 | PQLC3 | - |
| C11orf35 | HGLibA_05132 | 189 | 0.574876319 | C11orf35 | - |
| C1orf106 | HGLibA_05738 | 189 | 0.574876319 | C1orf106 | - |
| ENSG00000070388 | HGLibA_17398 | 189 | 0.574876319 | FGF22 | fibroblast growth factor 22 [Source:HGNC Symbol;Acc:HGNC:3679] |
| ENSG00000070444 | HGLibA_29577 | 189 | 0.574876319 | MNT | MAX network transcriptional repressor [Source:HGNC Symbol;Acc:HGNC:7188] |
| ENSG00000071242 | HGLibA_42161 | 189 | 0.574876319 | RPS6KA2 | ribosomal protein S6 kinase A2 [Source:HGNC Symbol;Acc:HGNC:10431] |
| ENSG00000085999 | HGLibA_40103 | 189 | 0.574876319 | RAD54L | RAD54 like [Source:HGNC Symbol;Acc:HGNC:9826] |
| ENSG00000088179 | HGLibA_39469 | 189 | 0.574876319 | PTPN4 | non-receptor type 4 [Source:HGNC Symbol;Acc:HGNC:9656]" |
| ENSG00000091664 | HGLibA_44473 | 189 | 0.574876319 | SLC17A6 | solute carrier family 17 member 6 [Source:HGNC Symbol;Acc:HGNC:16703] |
| ENSG00000100416 | HGLibA_51703 | 189 | 0.574876319 | TRMU | tRNA 5-methylaminomethyl-2-thiouridylate methyltransferase [Source:HGNC Symbol;Acc:HGNC:25481] |
| ENSG00000101938 | HGLibA_09537 | 189 | 0.574876319 | CHRDL1 | chordin like 1 [Source:HGNC Symbol;Acc:HGNC:29861] |
| ENSG00000102531 | HGLibA_17746 | 189 | 0.574876319 | FNDC3A | fibronectin type III domain containing 3A [Source:HGNC Symbol;Acc:HGNC:20296] |
| ENSG00000103274 | HGLibA_32902 | 189 | 0.574876319 | NUBP1 | nucleotide binding protein 1 [Source:HGNC Symbol;Acc:HGNC:8041] |
| ENSG00000103316 | HGLibA_11291 | 189 | 0.574876319 | CRYM | crystallin mu [Source:HGNC Symbol;Acc:HGNC:2418] |
| ENSG00000104142 | HGLibA_53746 | 189 | 0.574876319 | VPS18 | CORVET/HOPS core subunit [Source:HGNC Symbol;Acc:HGNC:15972]" |
| ENSG00000105401 | HGLibA_08546 | 189 | 0.574876319 | CDC37 | cell division cycle 37 [Source:HGNC Symbol;Acc:HGNC:1735] |
| ENSG00000105696 | HGLibA_50463 | 189 | 0.574876319 | TMEM59L | transmembrane protein 59 like [Source:HGNC Symbol;Acc:HGNC:13237] |
| ENSG00000106336 | HGLibA_17050 | 189 | 0.574876319 | FBXO24 | F-box protein 24 [Source:HGNC Symbol;Acc:HGNC:13595] |
| ENSG00000106635 | HGLibA_04341 | 189 | 0.574876319 | BCL7B | BCL tumor suppressor 7B [Source:HGNC Symbol;Acc:HGNC:1005] |
| ENSG00000108474 | HGLibA_36563 | 189 | 0.574876319 | PIGL | phosphatidylinositol glycan anchor biosynthesis class L [Source:HGNC Symbol;Acc:HGNC:8966] |
| ENSG00000108849 | HGLibA_38244 | 189 | 0.574876319 | PPY | pancreatic polypeptide [Source:HGNC Symbol;Acc:HGNC:9327] |
| ENSG00000110328 | HGLibA_18551 | 189 | 0.574876319 | GALNT18 | polypeptide N-acetylgalactosaminyltransferase 18 [Source:HGNC Symbol;Acc:HGNC:30488] |
| ENSG00000111241 | HGLibA_17415 | 189 | 0.574876319 | FGF6 | fibroblast growth factor 6 [Source:HGNC Symbol;Acc:HGNC:3684] |
| ENSG00000116095 | HGLibA_37068 | 189 | 0.574876319 | PLEKHA3 | pleckstrin homology domain containing A3 [Source:HGNC Symbol;Acc:HGNC:14338] |
| ENSG00000116218 | HGLibA_32456 | 189 | 0.574876319 | NPHS2 | podocin [Source:HGNC Symbol;Acc:HGNC:13394]" |
| ENSG00000119673 | HGLibA_00478 | 189 | 0.574876319 | ACOT2 | acyl-CoA thioesterase 2 [Source:HGNC Symbol;Acc:HGNC:18431] |
| ENSG00000121236 | HGLibA_51557 | 189 | 0.574876319 | TRIM6 | tripartite motif containing 6 [Source:HGNC Symbol;Acc:HGNC:16277] |
| ENSG00000122691 | HGLibA_52374 | 189 | 0.574876319 | TWIST1 | twist family bHLH transcription factor 1 [Source:HGNC Symbol;Acc:HGNC:12428] |
| ENSG00000125505 | HGLibA_28526 | 189 | 0.574876319 | MBOAT7 | membrane bound O-acyltransferase domain containing 7 [Source:HGNC Symbol;Acc:HGNC:15505] |
| ENSG00000130764 | HGLibA_27372 | 189 | 0.574876319 | LRRC47 | leucine rich repeat containing 47 [Source:HGNC Symbol;Acc:HGNC:29207] |

| | | | | | |
|---|---|---|---|---|---|
| ENSG00000131808 | HGLibA_18086 | 189 | 0.574876319 | FSHB | follicle stimulating hormone subunit beta [Source:HGNC Symbol;Acc:HGNC:3964] |
| ENSG00000132334 | HGLibA_39499 | 189 | 0.574876319 | PTPRE | receptor type E [Source:HGNC Symbol;Acc:HGNC:9669]" |
| ENSG00000132958 | HGLibA_51217 | 189 | 0.574876319 | TPTE2 | transmembrane phosphoinositide 3-phosphatase and tensin homolog 2 [Source:HGNC Symbol;Acc:HGNC:17299] |
| ENSG00000137225 | HGLibA_07148 | 189 | 0.574876319 | CAPN11 | calpain 11 [Source:HGNC Symbol;Acc:HGNC:1478] |
| ENSG00000138964 | HGLibA_35263 | 189 | 0.574876319 | PARVG | parvin gamma [Source:HGNC Symbol;Acc:HGNC:14654] |
| ENSG00000140459 | HGLibA_12053 | 189 | 0.574876319 | CYP11A1 | cytochrome P450 family 11 subfamily A member 1 [Source:HGNC Symbol;Acc:HGNC:2590] |
| ENSG00000145309 | HGLibA_06862 | 189 | 0.574876319 | CABS1 | spermatid associated 1 [Source:HGNC Symbol;Acc:HGNC:30710]" |
| ENSG00000147140 | HGLibA_32298 | 189 | 0.574876319 | NONO | non-POU domain containing octamer binding [Source:HGNC Symbol;Acc:HGNC:7871] |
| ENSG00000147255 | HGLibA_22876 | 189 | 0.574876319 | IGSF1 | immunoglobulin superfamily member 1 [Source:HGNC Symbol;Acc:HGNC:5948] |
| ENSG00000150768 | HGLibA_13309 | 189 | 0.574876319 | DLAT | dihydrolipoamide S-acetyltransferase [Source:HGNC Symbol;Acc:HGNC:2896] |
| ENSG00000151846 | HGLibA_34932 | 189 | 0.574876319 | PABPC3 | poly(A) binding protein cytoplasmic 3 [Source:HGNC Symbol;Acc:HGNC:8556] |
| ENSG00000154274 | HGLibA_06298 | 189 | 0.574876319 | C4orf19 | chromosome 4 open reading frame 19 [Source:HGNC Symbol;Acc:HGNC:25618] |
| ENSG00000155463 | HGLibA_34809 | 189 | 0.574876319 | OXA1L | mitochondrial inner membrane protein [Source:HGNC Symbol;Acc:HGNC:8526]" |
| ENSG00000156103 | HGLibA_29497 | 189 | 0.574876319 | MMP16 | matrix metallopeptidase 16 [Source:HGNC Symbol;Acc:HGNC:7162] |
| ENSG00000156735 | HGLibA_04083 | 189 | 0.574876319 | BAG4 | BCL2 associated athanogene 4 [Source:HGNC Symbol;Acc:HGNC:940] |
| ENSG00000159322 | HGLibA_01022 | 189 | 0.574876319 | ADPGK | ADP dependent glucokinase [Source:HGNC Symbol;Acc:HGNC:25250] |
| ENSG00000162144 | HGLibA_11980 | 189 | 0.574876319 | CYB561A3 | cytochrome b561 family member A3 [Source:HGNC Symbol;Acc:HGNC:23014] |
| ENSG00000162897 | HGLibA_17161 | 189 | 0.574876319 | FCAMR | Fc fragment of IgA and IgM receptor [Source:HGNC Symbol;Acc:HGNC:24692] |
| ENSG00000163655 | HGLibA_19432 | 189 | 0.574876319 | GMPS | guanine monophosphate synthase [Source:HGNC Symbol;Acc:HGNC:4378] |
| ENSG00000164035 | HGLibA_15028 | 189 | 0.574876319 | EMCN | endomucin [Source:HGNC Symbol;Acc:HGNC:16041] |
| ENSG00000164880 | HGLibA_23405 | 189 | 0.574876319 | INTS1 | integrator complex subunit 1 [Source:HGNC Symbol;Acc:HGNC:24555] |
| ENSG00000165688 | HGLibA_37326 | 189 | 0.574876319 | PMPCA | mitochondrial processing alpha subunit [Source:HGNC Symbol;Acc:HGNC:18667]" |
| ENSG00000166343 | HGLibA_30251 | 189 | 0.574876319 | MSS51 | MSS51 mitochondrial translational activator [Source:HGNC Symbol;Acc:HGNC:21000] |
| ENSG00000166736 | HGLibA_22381 | 189 | 0.574876319 | HTR3A | 5-hydroxytryptamine receptor 3A [Source:HGNC Symbol;Acc:HGNC:5297] |
| ENSG00000167112 | HGLibA_51806 | 189 | 0.574876319 | TRUB2 | TruB pseudouridine synthase family member 2 [Source:HGNC Symbol;Acc:HGNC:17170] |
| ENSG00000167637 | HGLibA_55741 | 189 | 0.574876319 | ZNF283 | zinc finger protein 283 [Source:HGNC Symbol;Acc:HGNC:13077] |
| ENSG00000167749 | HGLibA_25259 | 189 | 0.574876319 | KLK4 | kallikrein related peptidase 4 [Source:HGNC Symbol;Acc:HGNC:6365] |
| ENSG00000167768 | HGLibA_25415 | 189 | 0.574876319 | KRT1 | keratin 1 [Source:HGNC Symbol;Acc:HGNC:6412] |
| ENSG00000169213 | HGLibA_39909 | 189 | 0.574876319 | RAB3B | member RAS oncogene family [Source:HGNC Symbol;Acc:HGNC:9778]" |
| ENSG00000170178 | HGLibA_21991 | 189 | 0.574876319 | HOXD12 | homeobox D12 [Source:HGNC Symbol;Acc:HGNC:5135] |
| ENSG00000170367 | HGLibA_11459 | 189 | 0.574876319 | CST5 | cystatin D [Source:HGNC Symbol;Acc:HGNC:2477] |
| ENSG00000170500 | HGLibA_27023 | 189 | 0.574876319 | LONRF2 | LON peptidase N-terminal domain and ring finger 2 [Source:HGNC Symbol;Acc:HGNC:24788] |
| ENSG00000171115 | HGLibA_19090 | 189 | 0.574876319 | GIMAP8 | IMAP family member 8 [Source:HGNC Symbol;Acc:HGNC:21792]" |
| ENSG00000171116 | HGLibA_22235 | 189 | 0.574876319 | HSFX1 | X-linked 1 [Source:HGNC Symbol;Acc:HGNC:29603]" |
| ENSG00000171843 | HGLibA_29416 | 189 | 0.574876319 | MLLT3 | super elongation complex subunit [Source:HGNC Symbol;Acc:HGNC:7136]" |
| ENSG00000172663 | HGLibA_49946 | 189 | 0.574876319 | TMEM134 | transmembrane protein 134 [Source:HGNC Symbol;Acc:HGNC:26142] |
| ENSG00000174292 | HGLibA_50843 | 189 | 0.574876319 | TNK1 | tyrosine kinase non receptor 1 [Source:HGNC Symbol;Acc:HGNC:11940] |
| ENSG00000174437 | HGLibA_03607 | 189 | 0.574876319 | ATP2A2 | ATPase sarcoplasmic/endoplasmic reticulum Ca2+ transporting 2 [Source:HGNC Symbol;Acc:HGNC:812] |
| ENSG00000176428 | HGLibA_53786 | 189 | 0.574876319 | VPS37D | ESCRT-I subunit [Source:HGNC Symbol;Acc:HGNC:18287]" |
| ENSG00000177103 | HGLibA_14002 | 189 | 0.574876319 | DSCAML1 | DS cell adhesion molecule like 1 [Source:HGNC Symbol;Acc:HGNC:14656] |
| ENSG00000177374 | HGLibA_21287 | 189 | 0.574876319 | HIC1 | HIC ZBTB transcriptional repressor 1 [Source:HGNC Symbol;Acc:HGNC:4909] |
| ENSG00000180061 | HGLibA_49995 | 189 | 0.574876319 | TMEM150B | transmembrane protein 150B [Source:HGNC Symbol;Acc:HGNC:34415] |
| ENSG00000180089 | HGLibA_50527 | 189 | 0.574876319 | TMEM86B | transmembrane protein 86B [Source:HGNC Symbol;Acc:HGNC:28448] |
| ENSG00000181291 | HGLibA_49939 | 189 | 0.574876319 | TMEM132E | transmembrane protein 132E [Source:HGNC Symbol;Acc:HGNC:26991] |
| ENSG00000182087 | HGLibA_50346 | 189 | 0.574876319 | TMEM259 | transmembrane protein 259 [Source:HGNC Symbol;Acc:HGNC:17039] |
| ENSG00000182492 | HGLibA_04463 | 189 | 0.574876319 | BGN | biglycan [Source:HGNC Symbol;Acc:HGNC:1044] |
| ENSG00000183340 | HGLibA_23994 | 189 | 0.574876319 | JRKL | JRK like [Source:HGNC Symbol;Acc:HGNC:6200] |
| ENSG00000184166 | HGLibA_33652 | 189 | 0.574876319 | OR1D2 | olfactory receptor family 1 subfamily D member 2 [Source:HGNC Symbol;Acc:HGNC:8183] |
| ENSG00000184557 | HGLibA_46129 | 189 | 0.574876319 | SOCS3 | suppressor of cytokine signaling 3 [Source:HGNC Symbol;Acc:HGNC:19391] |
| ENSG00000186925 | HGLibA_25679 | 189 | 0.574876319 | KRTAP19-6 | keratin associated protein 19-6 [Source:HGNC Symbol;Acc:HGNC:18941] |
| ENSG00000188738 | HGLibA_18094 | 189 | 0.574876319 | FSIP2 | fibrous sheath interacting protein 2 [Source:HGNC Symbol;Acc:HGNC:21675] |
| ENSG00000196510 | HGLibA_01888 | 189 | 0.574876319 | ANAPC7 | anaphase promoting complex subunit 7 [Source:HGNC Symbol;Acc:HGNC:17380] |
| ENSG00000198883 | HGLibA_37375 | 189 | 0.574876319 | PNMA5 | PNMA family member 5 [Source:HGNC Symbol;Acc:HGNC:18743] |
| ENSG00000204165 | HGLibA_11958 | 189 | 0.574876319 | CXorf65 | chromosome X open reading frame 65 [Source:HGNC Symbol;Acc:HGNC:33713] |
| ENSG00000205002 | HGLibA_00066 | 189 | 0.574876319 | AARD | alanine and arginine rich domain containing protein [Source:HGNC Symbol;Acc:HGNC:33842] |
| ENSG00000205413 | HGLibA_42699 | 189 | 0.574876319 | SAMD9 | sterile alpha motif domain containing 9 [Source:HGNC Symbol;Acc:HGNC:1348] |
| ENSG00000214029 | HGLibA_56899 | 189 | 0.574876319 | ZNF891 | zinc finger protein 891 [Source:HGNC Symbol;Acc:HGNC:38709] |
| ENSG00000221944 | HGLibA_49464 | 189 | 0.574876319 | TIGD1 | tigger transposable element derived 1 [Source:HGNC Symbol;Acc:HGNC:14523] |
| ENSG00000249581 | HGLibA_10126 | 189 | 0.574876319 | CLRN2 | clarin 2 [Source:HGNC Symbol;Acc:HGNC:33939] |
| ENSG00000254709 | HGLibA_22870 | 189 | 0.574876319 | IGLL5 | immunoglobulin lambda like polypeptide 5 [Source:HGNC Symbol;Acc:HGNC:38476] |
| ENSG00000261210 | HGLibA_09953 | 189 | 0.574876319 | CLEC19A | C-type lectin domain containing 19A [Source:HGNC Symbol;Acc:HGNC:34522] |
| ENSG00000277481 | HGLibA_36796 | 189 | 0.574876319 | PKD1L3 | transient receptor potential channel interacting [Source:HGNC Symbol;Acc:HGNC:21716]" |
| FAM26D | HGLibA_16561 | 189 | 0.574876319 | FAM26D | - |
| GATSL2 | HGLibA_18704 | 189 | 0.574876319 | GATSL2 | - |
| HIST2H3D | HGLibA_21547 | 189 | 0.574876319 | HIST2H3D | - |
| hsa-mir-3137 | HGLibA_58608 | 189 | 0.574876319 | hsa-mir-3137 | - |
| hsa-mir-3198-2 | HGLibA_58892 | 189 | 0.574876319 | hsa-mir-3198-2 | - |
| hsa-mir-3612 | HGLibA_59076 | 189 | 0.574876319 | hsa-mir-3612 | - |
| hsa-mir-3911 | HGLibA_59512 | 189 | 0.574876319 | hsa-mir-3911 | - |
| hsa-mir-4310 | HGLibA_59956 | 189 | 0.574876319 | hsa-mir-4310 | - |
| hsa-mir-4681 | HGLibA_60836 | 189 | 0.574876319 | hsa-mir-4681 | - |
| hsa-mir-5572 | HGLibA_62153 | 189 | 0.574876319 | hsa-mir-5572 | - |
| hsa-mir-6757 | HGLibA_63187 | 189 | 0.574876319 | hsa-mir-6757 | - |
| hsa-mir-8065 | HGLibA_64098 | 189 | 0.574876319 | hsa-mir-8065 | - |
| C1orf85 | HGLibA_05896 | 188 | 0.565830976 | C1orf85 | - |
| CLLU1OS | HGLibA_10065 | 188 | 0.565830976 | CLLU1OS | - |
| ENSG00000000419 | HGLibA_13857 | 188 | 0.565830976 | DPM1 | catalytic [Source:HGNC Symbol;Acc:HGNC:3005]" |
| ENSG00000003249 | HGLibA_12390 | 188 | 0.565830976 | DBNDD1 | dysbindin domain containing 1 [Source:HGNC Symbol;Acc:HGNC:28455] |
| ENSG00000018869 | HGLibA_56311 | 188 | 0.565830976 | ZNF582 | zinc finger protein 582 [Source:HGNC Symbol;Acc:HGNC:26421] |
| ENSG00000023318 | HGLibA_15531 | 188 | 0.565830976 | ERP44 | endoplasmic reticulum protein 44 [Source:HGNC Symbol;Acc:HGNC:18311] |
| ENSG00000023516 | HGLibA_01410 | 188 | 0.565830976 | AKAP11 | A-kinase anchoring protein 11 [Source:HGNC Symbol;Acc:HGNC:369] |
| ENSG00000070190 | HGLibA_12335 | 188 | 0.565830976 | DAPP1 | dual adaptor of phosphotyrosine and 3-phosphoinositides 1 [Source:HGNC Symbol;Acc:HGNC:16500] |
| ENSG00000070501 | HGLibA_37488 | 188 | 0.565830976 | POLB | DNA polymerase beta [Source:HGNC Symbol;Acc:HGNC:9174] |
| ENSG00000075420 | HGLibA_17751 | 188 | 0.565830976 | FNDC3B | fibronectin type III domain containing 3B [Source:HGNC Symbol;Acc:HGNC:24670] |
| ENSG00000076513 | HGLibA_02001 | 188 | 0.565830976 | ANKRD13A | ankyrin repeat domain 13A [Source:HGNC Symbol;Acc:HGNC:21268] |
| ENSG00000086200 | HGLibA_23459 | 188 | 0.565830976 | IPO11 | importin 11 [Source:HGNC Symbol;Acc:HGNC:20628] |
| ENSG00000088888 | HGLibA_28446 | 188 | 0.565830976 | MAVS | mitochondrial antiviral signaling protein [Source:HGNC Symbol;Acc:HGNC:29233] |
| ENSG00000100918 | HGLibA_40760 | 188 | 0.565830976 | REC8 | REC8 meiotic recombination protein [Source:HGNC Symbol;Acc:HGNC:16879] |
| ENSG00000101773 | HGLibA_40439 | 188 | 0.565830976 | RBBP8 | endonuclease [Source:HGNC Symbol;Acc:HGNC:9891]" |
| ENSG00000104381 | HGLibA_18840 | 188 | 0.565830976 | GDAP1 | ganglioside induced differentiation associated protein 1 [Source:HGNC Symbol;Acc:HGNC:15968] |
| ENSG00000104774 | HGLibA_28075 | 188 | 0.565830976 | MAN2B1 | mannosidase alpha class 2B member 1 [Source:HGNC Symbol;Acc:HGNC:6826] |
| ENSG00000106436 | HGLibA_30749 | 188 | 0.565830976 | MYL10 | myosin light chain 10 [Source:HGNC Symbol;Acc:HGNC:29825] |
| ENSG00000107105 | HGLibA_14882 | 188 | 0.565830976 | ELAVL2 | ELAV like RNA binding protein 2 [Source:HGNC Symbol;Acc:HGNC:3313] |
| ENSG00000107485 | HGLibA_18671 | 188 | 0.565830976 | GATA3 | GATA binding protein 3 [Source:HGNC Symbol;Acc:HGNC:4172] |
| ENSG00000108381 | HGLibA_03342 | 188 | 0.565830976 | ASPA | aspartoacylase [Source:HGNC Symbol;Acc:HGNC:756] |
| ENSG00000111269 | HGLibA_11112 | 188 | 0.565830976 | CREBL2 | cAMP responsive element binding protein like 2 [Source:HGNC Symbol;Acc:HGNC:2350] |
| ENSG00000111911 | HGLibA_21329 | 188 | 0.565830976 | HINT3 | histidine triad nucleotide binding protein 3 [Source:HGNC Symbol;Acc:HGNC:18468] |
| ENSG00000112115 | HGLibA_22990 | 188 | 0.565830976 | IL17A | interleukin 17A [Source:HGNC Symbol;Acc:HGNC:5981] |
| ENSG00000115263 | HGLibA_18775 | 188 | 0.565830976 | GCG | glucagon [Source:HGNC Symbol;Acc:HGNC:4191] |
| ENSG00000115274 | HGLibA_23323 | 188 | 0.565830976 | INO80B | INO80 complex subunit B [Source:HGNC Symbol;Acc:HGNC:13324] |
| ENSG00000119913 | HGLibA_48956 | 188 | 0.565830976 | TECTB | tectorin beta [Source:HGNC Symbol;Acc:HGNC:11721] |
| ENSG00000120738 | HGLibA_14647 | 188 | 0.565830976 | EGR1 | early growth response 1 [Source:HGNC Symbol;Acc:HGNC:3238] |
| ENSG00000124003 | HGLibA_29624 | 188 | 0.565830976 | MOGAT1 | monoacylglycerol O-acyltransferase 1 [Source:HGNC Symbol;Acc:HGNC:18210] |
| ENSG00000125510 | HGLibA_33450 | 188 | 0.565830976 | OPRL1 | opioid related nociceptin receptor 1 [Source:HGNC Symbol;Acc:HGNC:8155] |
| ENSG00000125895 | HGLibA_50513 | 188 | 0.565830976 | TMEM74B | transmembrane protein 74B [Source:HGNC Symbol;Acc:HGNC:15893] |
| ENSG00000125931 | HGLibA_09741 | 188 | 0.565830976 | CITED1 | Cbp/p300 interacting transactivator with Glu/Asp rich carboxy-terminal domain 1 [Source:HGNC Symbol;Acc:HGNC:1986] |
| ENSG00000126254 | HGLibA_40551 | 188 | 0.565830976 | RBM42 | RNA binding motif protein 42 [Source:HGNC Symbol;Acc:HGNC:28117] |
| ENSG00000129158 | HGLibA_43479 | 188 | 0.565830976 | SERGEF | secretion regulating guanine nucleotide exchange factor [Source:HGNC Symbol;Acc:HGNC:17499] |
| ENSG00000130304 | HGLibA_44814 | 188 | 0.565830976 | SLC27A1 | solute carrier family 27 member 1 [Source:HGNC Symbol;Acc:HGNC:10995] |
| ENSG00000132386 | HGLibA_43586 | 188 | 0.565830976 | SERPINF1 | serpin family F member 1 [Source:HGNC Symbol;Acc:HGNC:8824] |
| ENSG00000134294 | HGLibA_45065 | 188 | 0.565830976 | SLC38A2 | solute carrier family 38 member 2 [Source:HGNC Symbol;Acc:HGNC:13448] |
| ENSG00000134516 | HGLibA_13740 | 188 | 0.565830976 | DOCK2 | dedicator of cytokinesis 2 [Source:HGNC Symbol;Acc:HGNC:2988] |
| ENSG00000136840 | HGLibA_47274 | 188 | 0.565830976 | ST6GALNAC4 | 6-sialyltransferase 4 [Source:HGNC Symbol;Acc:HGNC:17846]" |
| ENSG00000139684 | HGLibA_15562 | 188 | 0.565830976 | ESD | esterase D [Source:HGNC Symbol;Acc:HGNC:3465] |
| ENSG00000142082 | HGLibA_44223 | 188 | 0.565830976 | SIRT3 | sirtuin 3 [Source:HGNC Symbol;Acc:HGNC:14931] |
| ENSG00000142273 | HGLibA_07400 | 188 | 0.565830976 | CBLC | Cbl proto-oncogene C [Source:HGNC Symbol;Acc:HGNC:15961] |
| ENSG00000143167 | HGLibA_19716 | 188 | 0.565830976 | GPA33 | glycoprotein A33 [Source:HGNC Symbol;Acc:HGNC:4445] |
| ENSG00000143257 | HGLibA_32591 | 188 | 0.565830976 | NR1I3 | nuclear receptor subfamily 1 group I member 3 [Source:HGNC Symbol;Acc:HGNC:7969] |
| ENSG00000144648 | HGLibA_00442 | 188 | 0.565830976 | ACKR2 | atypical chemokine receptor 2 [Source:HGNC Symbol;Acc:HGNC:1565] |
| ENSG00000145284 | HGLibA_42886 | 188 | 0.565830976 | SCD5 | stearoyl-CoA desaturase 5 [Source:HGNC Symbol;Acc:HGNC:21088] |
| ENSG00000148734 | HGLibA_32440 | 188 | 0.565830976 | NPFFR1 | neuropeptide FF receptor 1 [Source:HGNC Symbol;Acc:HGNC:17425] |
| ENSG00000156127 | HGLibA_04172 | 188 | 0.565830976 | BATF | basic leucine zipper ATF-like transcription factor [Source:HGNC Symbol;Acc:HGNC:958] |
| ENSG00000156313 | HGLibA_41835 | 188 | 0.565830976 | RPGR | retinitis pigmentosa GTPase regulator [Source:HGNC Symbol;Acc:HGNC:10295] |
| ENSG00000160055 | HGLibA_50268 | 188 | 0.565830976 | TMEM234 | transmembrane protein 234 [Source:HGNC Symbol;Acc:HGNC:28837] |
| ENSG00000162989 | HGLibA_24277 | 188 | 0.565830976 | KCNJ3 | potassium voltage-gated channel subfamily J member 3 [Source:HGNC Symbol;Acc:HGNC:6264] |
| ENSG00000164830 | HGLibA_34830 | 188 | 0.565830976 | OXR1 | oxidation resistance 1 [Source:HGNC Symbol;Acc:HGNC:15822] |
| ENSG00000165140 | HGLibA_16964 | 188 | 0.565830976 | FBP1 | fructose-bisphosphatase 1 [Source:HGNC Symbol;Acc:HGNC:3606] |
| ENSG00000165566 | HGLibA_01775 | 188 | 0.565830976 | AMER2 | APC membrane recruitment protein 2 [Source:HGNC Symbol;Acc:HGNC:26360] |
| ENSG00000168350 | HGLibA_12923 | 188 | 0.565830976 | DEGS2 | sphingolipid 2 [Source:HGNC Symbol;Acc:HGNC:20113]" |
| ENSG00000170577 | HGLibA_44247 | 188 | 0.565830976 | SIX2 | SIX homeobox 2 [Source:HGNC Symbol;Acc:HGNC:10888] |
| ENSG00000170748 | HGLibA_40606 | 188 | 0.565830976 | RBMXL2 | RBMX like 2 [Source:HGNC Symbol;Acc:HGNC:17886] |
| ENSG00000170876 | HGLibA_50401 | 188 | 0.565830976 | TMEM43 | transmembrane protein 43 [Source:HGNC Symbol;Acc:HGNC:28472] |
| ENSG00000171931 | HGLibA_17135 | 188 | 0.565830976 | FBXW10 | F-box and WD repeat domain containing 10 [Source:HGNC Symbol;Acc:HGNC:1211] |
| ENSG00000173065 | HGLibA_16526 | 188 | 0.565830976 | FAM222B | family with sequence similarity 222 member B [Source:HGNC Symbol;Acc:HGNC:25563] |
| ENSG00000173679 | HGLibA_33686 | 188 | 0.565830976 | OR1L1 | olfactory receptor family 1 subfamily L member 1 [Source:HGNC Symbol;Acc:HGNC:8213] |

| | | | | | |
|---|---|---|---|---|---|
| ENSG00000174562 | HGLibA_25248 | 188 | 0.565830976 | KLK15 | kallikrein related peptidase 15 [Source:HGNC Symbol;Acc:HGNC:20453] |
| ENSG00000176782 | HGLibA_12820 | 188 | 0.565830976 | DEFB104A | defensin beta 104A [Source:HGNC Symbol;Acc:HGNC:18115] |
| ENSG00000178338 | HGLibA_55864 | 188 | 0.565830976 | ZNF354B | zinc finger protein 354B [Source:HGNC Symbol;Acc:HGNC:17197] |
| ENSG00000178814 | HGLibA_33420 | 188 | 0.565830976 | OPLAH | ATP-hydrolysing [Source:HGNC Symbol;Acc:HGNC:8149]" |
| ENSG00000179085 | HGLibA_13862 | 188 | 0.565830976 | DPM3 | dolichyl-phosphate mannosyltransferase subunit 3 [Source:HGNC Symbol;Acc:HGNC:3007] |
| ENSG00000181788 | HGLibA_44108 | 188 | 0.565830976 | SIAH2 | siah E3 ubiquitin protein ligase 2 [Source:HGNC Symbol;Acc:HGNC:10858] |
| ENSG00000182919 | HGLibA_05156 | 188 | 0.565830976 | C11orf54 | chromosome 11 open reading frame 54 [Source:HGNC Symbol;Acc:HGNC:30204] |
| ENSG00000185674 | HGLibA_27700 | 188 | 0.565830976 | LYG2 | lysozyme g2 [Source:HGNC Symbol;Acc:HGNC:29615] |
| ENSG00000186881 | HGLibA_33617 | 188 | 0.565830976 | OR13F1 | olfactory receptor family 13 subfamily F member 1 [Source:HGNC Symbol;Acc:HGNC:14723] |
| ENSG00000196470 | HGLibA_44106 | 188 | 0.565830976 | SIAH1 | siah E3 ubiquitin protein ligase 1 [Source:HGNC Symbol;Acc:HGNC:10857] |
| ENSG00000197746 | HGLibA_39002 | 188 | 0.565830976 | PSAP | prosaposin [Source:HGNC Symbol;Acc:HGNC:9498] |
| ENSG00000198788 | HGLibA_30534 | 188 | 0.565830976 | MUC2 | oligomeric mucus/gel-forming [Source:HGNC Symbol;Acc:HGNC:7512]" |
| ENSG00000234602 | HGLibA_28596 | 188 | 0.565830976 | MCIDAS | multiciliate differentiation and DNA synthesis associated cell cycle protein [Source:HGNC Symbol;Acc:HGNC:40050] |
| ENSG00000238269 | HGLibA_35009 | 188 | 0.565830976 | PAGE2B | PAGE family member 2B [Source:HGNC Symbol;Acc:HGNC:31805] |
| ENSG00000248098 | HGLibA_04278 | 188 | 0.565830976 | BCKDHA | alpha polypeptide [Source:HGNC Symbol;Acc:HGNC:986]" |
| ENSG00000254245 | HGLibA_35533 | 188 | 0.565830976 | PCDHGA3 | 3 [Source:HGNC Symbol;Acc:HGNC:8701]" |
| ENSG00000254656 | HGLibA_42395 | 188 | 0.565830976 | RTL1 | retrotransposon Gag like 1 [Source:HGNC Symbol;Acc:HGNC:14665] |
| ENSG00000255359 | HGLibA_07718 | 188 | 0.565830976 | CCDC179 | coiled-coil domain containing 179 [Source:HGNC Symbol;Acc:HGNC:44653] |
| EPRS | HGLibA_15365 | 188 | 0.565830976 | EPRS | - |
| HIST1H2BF | HGLibA_21427 | 188 | 0.565830976 | HIST1H2BF | - |
| hsa-mir-1207 | HGLibA_57284 | 188 | 0.565830976 | hsa-mir-1207 | - |
| hsa-mir-1273c | HGLibA_57536 | 188 | 0.565830976 | hsa-mir-1273c | - |
| hsa-mir-28 | HGLibA_58383 | 188 | 0.565830976 | hsa-mir-28 | - |
| hsa-mir-3935 | HGLibA_59600 | 188 | 0.565830976 | hsa-mir-3935 | - |
| hsa-mir-4289 | HGLibA_59865 | 188 | 0.565830976 | hsa-mir-4289 | - |
| hsa-mir-4783 | HGLibA_61241 | 188 | 0.565830976 | hsa-mir-4783 | - |
| hsa-mir-4789 | HGLibA_61262 | 188 | 0.565830976 | hsa-mir-4789 | - |
| hsa-mir-597 | HGLibA_62480 | 188 | 0.565830976 | hsa-mir-597 | - |
| hsa-mir-648 | HGLibA_62835 | 188 | 0.565830976 | hsa-mir-648 | - |
| TMEM206 | HGLibA_50195 | 188 | 0.565830976 | TMEM206 | - |
| C16orf71 | HGLibA_05475 | 187 | 0.556785633 | C16orf71 | - |
| ENSG00000042088 | HGLibA_48889 | 187 | 0.556785633 | TDP1 | tyrosyl-DNA phosphodiesterase 1 [Source:HGNC Symbol;Acc:HGNC:18884] |
| ENSG00000048540 | HGLibA_26709 | 187 | 0.556785633 | LMO3 | LIM domain only 3 [Source:HGNC Symbol;Acc:HGNC:6643] |
| ENSG00000064309 | HGLibA_08857 | 187 | 0.556785633 | CDON | oncogene regulated [Source:HGNC Symbol;Acc:HGNC:17104]" |
| ENSG00000076067 | HGLibA_40589 | 187 | 0.556785633 | RBMS2 | RNA binding motif single stranded interacting protein 2 [Source:HGNC Symbol;Acc:HGNC:9909] |
| ENSG00000085982 | HGLibA_53361 | 187 | 0.556785633 | USP40 | ubiquitin specific peptidase 40 [Source:HGNC Symbol;Acc:HGNC:20069] |
| ENSG00000100170 | HGLibA_45252 | 187 | 0.556785633 | SLC5A1 | solute carrier family 5 member 1 [Source:HGNC Symbol;Acc:HGNC:11036] |
| ENSG00000104327 | HGLibA_06987 | 187 | 0.556785633 | CALB1 | calbindin 1 [Source:HGNC Symbol;Acc:HGNC:1434] |
| ENSG00000105516 | HGLibA_12401 | 187 | 0.556785633 | DBP | D-box binding PAR bZIP transcription factor [Source:HGNC Symbol;Acc:HGNC:2697] |
| ENSG00000105778 | HGLibA_03893 | 187 | 0.556785633 | AVL9 | AVL9 cell migration associated [Source:HGNC Symbol;Acc:HGNC:28994] |
| ENSG00000108309 | HGLibA_42450 | 187 | 0.556785633 | RUNDC3A | RUN domain containing 3A [Source:HGNC Symbol;Acc:HGNC:16984] |
| ENSG00000111325 | HGLibA_33311 | 187 | 0.556785633 | OGFOD2 | 2-oxoglutarate and iron dependent oxygenase domain containing 2 [Source:HGNC Symbol;Acc:HGNC:25823] |
| ENSG00000112280 | HGLibA_10621 | 187 | 0.556785633 | COL9A1 | collagen type IX alpha 1 chain [Source:HGNC Symbol;Acc:HGNC:2217] |
| ENSG00000114353 | HGLibA_19454 | 187 | 0.556785633 | GNAI2 | G protein subunit alpha i2 [Source:HGNC Symbol;Acc:HGNC:4385] |
| ENSG00000115648 | HGLibA_29433 | 187 | 0.556785633 | MLPH | melanophilin [Source:HGNC Symbol;Acc:HGNC:29643] |
| ENSG00000116288 | HGLibA_35200 | 187 | 0.556785633 | PARK7 | Parkinsonism associated deglycase [Source:HGNC Symbol;Acc:HGNC:16369] |
| ENSG00000116745 | HGLibA_41826 | 187 | 0.556785633 | RPE65 | retinoid isomerohydrolase [Source:HGNC Symbol;Acc:HGNC:10294]" |
| ENSG00000120907 | HGLibA_01037 | 187 | 0.556785633 | ADRA1A | adrenoceptor alpha 1A [Source:HGNC Symbol;Acc:HGNC:277] |
| ENSG00000121075 | HGLibA_48670 | 187 | 0.556785633 | TBX4 | T-box 4 [Source:HGNC Symbol;Acc:HGNC:11603] |
| ENSG00000121742 | HGLibA_19172 | 187 | 0.556785633 | GJB6 | gap junction protein beta 6 [Source:HGNC Symbol;Acc:HGNC:4288] |
| ENSG00000126233 | HGLibA_45554 | 187 | 0.556785633 | SLURP1 | secreted LY6/PLAUR domain containing 1 [Source:HGNC Symbol;Acc:HGNC:18746] |
| ENSG00000126705 | HGLibA_01296 | 187 | 0.556785633 | AHDC1 | AT-hook DNA binding motif containing 1 [Source:HGNC Symbol;Acc:HGNC:25230] |
| ENSG00000129757 | HGLibA_08827 | 187 | 0.556785633 | CDKN1C | cyclin dependent kinase inhibitor 1C [Source:HGNC Symbol;Acc:HGNC:1786] |
| ENSG00000134668 | HGLibA_46744 | 187 | 0.556785633 | SPOCD1 | SPOC domain containing 1 [Source:HGNC Symbol;Acc:HGNC:26338] |
| ENSG00000134954 | HGLibA_15641 | 187 | 0.556785633 | ETS1 | transcription factor [Source:HGNC Symbol;Acc:HGNC:3488]" |
| ENSG00000136383 | HGLibA_01705 | 187 | 0.556785633 | ALPK3 | alpha kinase 3 [Source:HGNC Symbol;Acc:HGNC:17574] |
| ENSG00000136878 | HGLibA_53298 | 187 | 0.556785633 | USP20 | ubiquitin specific peptidase 20 [Source:HGNC Symbol;Acc:HGNC:12619] |
| ENSG00000136935 | HGLibA_19592 | 187 | 0.556785633 | GOLGA1 | golgin A1 [Source:HGNC Symbol;Acc:HGNC:4424] |
| ENSG00000138032 | HGLibA_37967 | 187 | 0.556785633 | PPM1B | Mg2+/Mn2+ dependent 1B [Source:HGNC Symbol;Acc:HGNC:9276]" |
| ENSG00000138696 | HGLibA_04633 | 187 | 0.556785633 | BMPR1B | bone morphogenetic protein receptor type 1B [Source:HGNC Symbol;Acc:HGNC:1077] |
| ENSG00000139926 | HGLibA_18024 | 187 | 0.556785633 | FRMD6 | FERM domain containing 6 [Source:HGNC Symbol;Acc:HGNC:19839] |
| ENSG00000140022 | HGLibA_47550 | 187 | 0.556785633 | STON2 | stonin 2 [Source:HGNC Symbol;Acc:HGNC:30652] |
| ENSG00000140682 | HGLibA_49211 | 187 | 0.556785633 | TGFB1I1 | transforming growth factor beta 1 induced transcript 1 [Source:HGNC Symbol;Acc:HGNC:11767] |
| ENSG00000142632 | HGLibA_02832 | 187 | 0.556785633 | ARHGEF19 | Rho guanine nucleotide exchange factor 19 [Source:HGNC Symbol;Acc:HGNC:26604] |
| ENSG00000142748 | HGLibA_17223 | 187 | 0.556785633 | FCN3 | ficolin 3 [Source:HGNC Symbol;Acc:HGNC:3625] |
| ENSG00000143355 | HGLibA_26469 | 187 | 0.556785633 | LHX9 | LIM homeobox 9 [Source:HGNC Symbol;Acc:HGNC:14222] |
| ENSG00000143578 | HGLibA_11102 | 187 | 0.556785633 | CREB3L4 | cAMP responsive element binding protein 3 like 4 [Source:HGNC Symbol;Acc:HGNC:18854] |
| ENSG00000143621 | HGLibA_23215 | 187 | 0.556785633 | ILF2 | interleukin enhancer binding factor 2 [Source:HGNC Symbol;Acc:HGNC:6037] |
| ENSG00000143994 | HGLibA_00230 | 187 | 0.556785633 | ABHD1 | abhydrolase domain containing 1 [Source:HGNC Symbol;Acc:HGNC:17553] |
| ENSG00000144306 | HGLibA_43056 | 187 | 0.556785633 | SCRN3 | secernin 3 [Source:HGNC Symbol;Acc:HGNC:30382] |
| ENSG00000149679 | HGLibA_06846 | 187 | 0.556785633 | CABLES2 | Cdk5 and Abl enzyme substrate 2 [Source:HGNC Symbol;Acc:HGNC:16143] |
| ENSG00000152284 | HGLibA_48794 | 187 | 0.556785633 | TCF7L1 | transcription factor 7 like 1 [Source:HGNC Symbol;Acc:HGNC:11640] |
| ENSG00000153802 | HGLibA_50617 | 187 | 0.556785633 | TMPRSS11D | transmembrane serine protease 11D [Source:HGNC Symbol;Acc:HGNC:24059] |
| ENSG00000154783 | HGLibA_17355 | 187 | 0.556785633 | FGD5 | RhoGEF and PH domain containing 5 [Source:HGNC Symbol;Acc:HGNC:19117]" |
| ENSG00000158457 | HGLibA_51934 | 187 | 0.556785633 | TSPAN33 | tetraspanin 33 [Source:HGNC Symbol;Acc:HGNC:28743] |
| ENSG00000161055 | HGLibA_42932 | 187 | 0.556785633 | SCGB3A1 | secretoglobin family 3A member 1 [Source:HGNC Symbol;Acc:HGNC:18384] |
| ENSG00000164749 | HGLibA_21783 | 187 | 0.556785633 | HNF4G | hepatocyte nuclear factor 4 gamma [Source:HGNC Symbol;Acc:HGNC:5026] |
| ENSG00000165886 | HGLibA_52766 | 187 | 0.556785633 | UBTD1 | ubiquitin domain containing 1 [Source:HGNC Symbol;Acc:HGNC:25683] |
| ENSG00000169397 | HGLibA_41406 | 187 | 0.556785633 | RNASE3 | ribonuclease A family member 3 [Source:HGNC Symbol;Acc:HGNC:10046] |
| ENSG00000172568 | HGLibA_17763 | 187 | 0.556785633 | FNDC9 | fibronectin type III domain containing 9 [Source:HGNC Symbol;Acc:HGNC:33547] |
| ENSG00000172935 | HGLibA_29841 | 187 | 0.556785633 | MRGPRF | MAS related GPR family member F [Source:HGNC Symbol;Acc:HGNC:24828] |
| ENSG00000174576 | HGLibA_32404 | 187 | 0.556785633 | NPAS4 | neuronal PAS domain protein 4 [Source:HGNC Symbol;Acc:HGNC:18983] |
| ENSG00000175467 | HGLibA_42756 | 187 | 0.556785633 | SART1 | recruiter of U4/U6.U5 tri-snRNP [Source:HGNC Symbol;Acc:HGNC:10538]" |
| ENSG00000176022 | HGLibA_03966 | 187 | 0.556785633 | B3GALT6 | 3-galactosyltransferase 6 [Source:HGNC Symbol;Acc:HGNC:17978]" |
| ENSG00000177370 | HGLibA_49512 | 187 | 0.556785633 | TIMM22 | translocase of inner mitochondrial membrane 22 [Source:HGNC Symbol;Acc:HGNC:17317] |
| ENSG00000178878 | HGLibA_02544 | 187 | 0.556785633 | APOLD1 | apolipoprotein L domain containing 1 [Source:HGNC Symbol;Acc:HGNC:25268] |
| ENSG00000181617 | HGLibA_17249 | 187 | 0.556785633 | FDCSP | follicular dendritic cell secreted protein [Source:HGNC Symbol;Acc:HGNC:19215] |
| ENSG00000181761 | HGLibA_34533 | 187 | 0.556785633 | OR8H3 | olfactory receptor family 8 subfamily H member 3 [Source:HGNC Symbol;Acc:HGNC:15309] |
| ENSG00000181867 | HGLibA_18129 | 187 | 0.556785633 | FTMT | ferritin mitochondrial [Source:HGNC Symbol;Acc:HGNC:17345] |
| ENSG00000184838 | HGLibA_38790 | 187 | 0.556785633 | PRR16 | proline rich 16 [Source:HGNC Symbol;Acc:HGNC:29654] |
| ENSG00000186063 | HGLibA_01329 | 187 | 0.556785633 | AIDA | dorsalization associated [Source:HGNC Symbol;Acc:HGNC:25761]" |
| ENSG00000187492 | HGLibA_08706 | 187 | 0.556785633 | CDHR4 | cadherin related family member 4 [Source:HGNC Symbol;Acc:HGNC:34527] |
| ENSG00000188984 | HGLibA_00035 | 187 | 0.556785633 | AADACL3 | arylacetamide deacetylase like 3 [Source:HGNC Symbol;Acc:HGNC:32037] |
| ENSG00000189051 | HGLibA_41644 | 187 | 0.556785633 | RNF222 | ring finger protein 222 [Source:HGNC Symbol;Acc:HGNC:34517] |
| ENSG00000196507 | HGLibA_48710 | 187 | 0.556785633 | TCEAL3 | transcription elongation factor A like 3 [Source:HGNC Symbol;Acc:HGNC:28247] |
| ENSG00000198774 | HGLibA_40398 | 187 | 0.556785633 | RASSF9 | Ras association domain family member 9 [Source:HGNC Symbol;Acc:HGNC:15739] |
| ENSG00000203896 | HGLibA_26538 | 187 | 0.556785633 | LIME1 | Lck interacting transmembrane adaptor 1 [Source:HGNC Symbol;Acc:HGNC:26016] |
| ENSG00000204428 | HGLibA_27662 | 187 | 0.556785633 | LY6G5C | lymphocyte antigen 6 family member G5C [Source:HGNC Symbol;Acc:HGNC:13932] |
| ENSG00000205439 | HGLibA_25637 | 187 | 0.556785633 | KRTAP12-3 | keratin associated protein 12-3 [Source:HGNC Symbol;Acc:HGNC:20531] |
| ENSG00000205669 | HGLibA_00484 | 187 | 0.556785633 | ACOT6 | acyl-CoA thioesterase 6 [Source:HGNC Symbol;Acc:HGNC:33159] |
| ENSG00000214063 | HGLibA_51936 | 187 | 0.556785633 | TSPAN4 | tetraspanin 4 [Source:HGNC Symbol;Acc:HGNC:11859] |
| ENSG00000232268 | HGLibA_34172 | 187 | 0.556785633 | OR52I1 | olfactory receptor family 52 subfamily I member 1 [Source:HGNC Symbol;Acc:HGNC:15220] |
| ENSG00000242612 | HGLibA_12778 | 187 | 0.556785633 | DECR2 | 4-dienoyl-CoA reductase 2 [Source:HGNC Symbol;Acc:HGNC:2754]" |
| ENSG00000283597 | HGLibA_16256 | 187 | 0.556785633 | FAM169B | family with sequence similarity 169 member B [Source:HGNC Symbol;Acc:HGNC:26835] |
| HIST1H2AI | HGLibA_21396 | 187 | 0.556785633 | HIST1H2AI | - |
| hsa-mir-1193 | HGLibA_57251 | 187 | 0.556785633 | hsa-mir-1193 | - |
| hsa-mir-1202 | HGLibA_57265 | 187 | 0.556785633 | hsa-mir-1202 | - |
| hsa-mir-1257 | HGLibA_57436 | 187 | 0.556785633 | hsa-mir-1257 | - |
| hsa-mir-135b | HGLibA_57799 | 187 | 0.556785633 | hsa-mir-135b | - |
| hsa-mir-208a | HGLibA_58194 | 187 | 0.556785633 | hsa-mir-208a | - |
| hsa-mir-320d-2 | HGLibA_58947 | 187 | 0.556785633 | hsa-mir-320d-2 | - |
| hsa-mir-3653 | HGLibA_59159 | 187 | 0.556785633 | hsa-mir-3653 | - |
| hsa-mir-3680-2 | HGLibA_59274 | 187 | 0.556785633 | hsa-mir-3680-2 | - |
| hsa-mir-4650-1 | HGLibA_60705 | 187 | 0.556785633 | hsa-mir-4650-1 | - |
| hsa-mir-6072 | HGLibA_62539 | 187 | 0.556785633 | hsa-mir-6072 | - |
| hsa-mir-609 | HGLibA_62624 | 187 | 0.556785633 | hsa-mir-609 | - |
| hsa-mir-6512 | HGLibA_62920 | 187 | 0.556785633 | hsa-mir-6512 | - |
| hsa-mir-654 | HGLibA_62948 | 187 | 0.556785633 | hsa-mir-654 | - |
| hsa-mir-7113 | HGLibA_63820 | 187 | 0.556785633 | hsa-mir-7113 | - |
| hsa-mir-7160 | HGLibA_63870 | 187 | 0.556785633 | hsa-mir-7160 | - |
| KIAA1468 | HGLibA_24769 | 187 | 0.556785633 | KIAA1468 | - |
| LOR | HGLibA_27028 | 187 | 0.556785633 | LOR | - |
| PRR25 | HGLibA_38834 | 187 | 0.556785633 | PRR25 | - |
| ELTD1 | HGLibA_14997 | 186 | 0.54774029 | ELTD1 | - |
| ENSG00000010404 | HGLibA_22567 | 186 | 0.54774029 | IDS | iduronate 2-sulfatase [Source:HGNC Symbol;Acc:HGNC:5389] |
| ENSG00000071246 | HGLibA_53529 | 186 | 0.54774029 | VASH1 | vasohibin 1 [Source:HGNC Symbol;Acc:HGNC:19964] |
| ENSG00000072958 | HGLibA_02291 | 186 | 0.54774029 | AP1M1 | adaptor related protein complex 1 subunit mu 1 [Source:HGNC Symbol;Acc:HGNC:13667] |
| ENSG00000075035 | HGLibA_54424 | 186 | 0.54774029 | WSCD2 | WSC domain containing 2 [Source:HGNC Symbol;Acc:HGNC:29117] |
| ENSG00000076242 | HGLibA_29380 | 186 | 0.54774029 | MLH1 | mutL homolog 1 [Source:HGNC Symbol;Acc:HGNC:7127] |
| ENSG00000102174 | HGLibA_36328 | 186 | 0.54774029 | PHEX | phosphate regulating endopeptidase homolog X-linked [Source:HGNC Symbol;Acc:HGNC:8918] |
| ENSG00000104237 | HGLibA_41786 | 186 | 0.54774029 | RP1 | axonemal microtubule associated [Source:HGNC Symbol;Acc:HGNC:10263]" |
| ENSG00000104901 | HGLibA_13305 | 186 | 0.54774029 | DKKL1 | dickkopf like acrosomal protein 1 [Source:HGNC Symbol;Acc:HGNC:16528] |
| ENSG00000105136 | HGLibA_55953 | 186 | 0.54774029 | ZNF419 | zinc finger protein 419 [Source:HGNC Symbol;Acc:HGNC:20648] |
| ENSG00000109689 | HGLibA_47444 | 186 | 0.54774029 | STIM2 | stromal interaction molecule 2 [Source:HGNC Symbol;Acc:HGNC:19205] |
| ENSG00000111676 | HGLibA_03529 | 186 | 0.54774029 | ATN1 | atrophin 1 [Source:HGNC Symbol;Acc:HGNC:3033] |

| | | | | | |
|---|---|---|---|---|---|
| ENSG00000115107 | HGLibA_47430 | 186 | 0.54774029 | STEAP3 | STEAP3 metalloreductase [Source:HGNC Symbol;Acc:HGNC:24592] |
| ENSG00000115386 | HGLibA_40793 | 186 | 0.54774029 | REG1A | regenerating family member 1 alpha [Source:HGNC Symbol;Acc:HGNC:9951] |
| ENSG00000115652 | HGLibA_53479 | 186 | 0.54774029 | UXS1 | UDP-glucuronate decarboxylase 1 [Source:HGNC Symbol;Acc:HGNC:17729] |
| ENSG00000119523 | HGLibA_01626 | 186 | 0.54774029 | ALG2 | 6-mannosyltransferase [Source:HGNC Symbol;Acc:HGNC:23159]" |
| ENSG00000120685 | HGLibA_38696 | 186 | 0.54774029 | PROSER1 | proline and serine rich 1 [Source:HGNC Symbol;Acc:HGNC:20291] |
| ENSG00000124678 | HGLibA_48842 | 186 | 0.54774029 | TCP11 | t-complex 11 [Source:HGNC Symbol;Acc:HGNC:11658] |
| ENSG00000125170 | HGLibA_13775 | 186 | 0.54774029 | DOK4 | docking protein 4 [Source:HGNC Symbol;Acc:HGNC:19868] |
| ENSG00000126217 | HGLibA_28581 | 186 | 0.54774029 | MCF2L | MCF.2 cell line derived transforming sequence like [Source:HGNC Symbol;Acc:HGNC:14576] |
| ENSG00000132109 | HGLibA_51430 | 186 | 0.54774029 | TRIM21 | tripartite motif containing 21 [Source:HGNC Symbol;Acc:HGNC:11312] |
| ENSG00000132612 | HGLibA_53798 | 186 | 0.54774029 | VPS4A | vacuolar protein sorting 4 homolog A [Source:HGNC Symbol;Acc:HGNC:13488] |
| ENSG00000134438 | HGLibA_40406 | 186 | 0.54774029 | RAX | retina and anterior neural fold homeobox [Source:HGNC Symbol;Acc:HGNC:18662] |
| ENSG00000134539 | HGLibA_25299 | 186 | 0.54774029 | KLRD1 | killer cell lectin like receptor D1 [Source:HGNC Symbol;Acc:HGNC:6378] |
| ENSG00000136750 | HGLibA_18413 | 186 | 0.54774029 | GAD2 | glutamate decarboxylase 2 [Source:HGNC Symbol;Acc:HGNC:4093] |
| ENSG00000137880 | HGLibA_18784 | 186 | 0.54774029 | GCHFR | GTP cyclohydrolase I feedback regulator [Source:HGNC Symbol;Acc:HGNC:4194] |
| ENSG00000138135 | HGLibA_09333 | 186 | 0.54774029 | CH25H | cholesterol 25-hydroxylase [Source:HGNC Symbol;Acc:HGNC:1907] |
| ENSG00000141232 | HGLibA_50933 | 186 | 0.54774029 | TOB1 | 1 [Source:HGNC Symbol;Acc:HGNC:11979]" |
| ENSG00000144278 | HGLibA_18538 | 186 | 0.54774029 | GALNT13 | polypeptide N-acetylgalactosaminyltransferase 13 [Source:HGNC Symbol;Acc:HGNC:23242] |
| ENSG00000144645 | HGLibA_34643 | 186 | 0.54774029 | OSBPL10 | oxysterol binding protein like 10 [Source:HGNC Symbol;Acc:HGNC:16395] |
| ENSG00000144659 | HGLibA_44727 | 186 | 0.54774029 | SLC25A38 | solute carrier family 25 member 38 [Source:HGNC Symbol;Acc:HGNC:26054] |
| ENSG00000145217 | HGLibA_44779 | 186 | 0.54774029 | SLC26A1 | solute carrier family 26 member 1 [Source:HGNC Symbol;Acc:HGNC:10993] |
| ENSG00000146676 | HGLibA_39589 | 186 | 0.54774029 | PURB | purine rich element binding protein B [Source:HGNC Symbol;Acc:HGNC:9702] |
| ENSG00000147650 | HGLibA_27205 | 186 | 0.54774029 | LRP12 | LDL receptor related protein 12 [Source:HGNC Symbol;Acc:HGNC:31708] |
| ENSG00000151726 | HGLibA_00555 | 186 | 0.54774029 | ACSL1 | acyl-CoA synthetase long chain family member 1 [Source:HGNC Symbol;Acc:HGNC:3569] |
| ENSG00000152154 | HGLibA_50080 | 186 | 0.54774029 | TMEM178A | transmembrane protein 178A [Source:HGNC Symbol;Acc:HGNC:28517] |
| ENSG00000153814 | HGLibA_23939 | 186 | 0.54774029 | JAZF1 | JAZF zinc finger 1 [Source:HGNC Symbol;Acc:HGNC:28917] |
| ENSG00000154639 | HGLibA_11839 | 186 | 0.54774029 | CXADR | Ig-like cell adhesion molecule [Source:HGNC Symbol;Acc:HGNC:2559]" |
| ENSG00000155330 | HGLibA_05491 | 186 | 0.54774029 | C16orf87 | chromosome 16 open reading frame 87 [Source:HGNC Symbol;Acc:HGNC:33754] |
| ENSG00000155545 | HGLibA_29258 | 186 | 0.54774029 | MIER3 | MIER family member 3 [Source:HGNC Symbol;Acc:HGNC:26678] |
| ENSG00000158104 | HGLibA_22025 | 186 | 0.54774029 | HPD | 4-hydroxyphenylpyruvate dioxygenase [Source:HGNC Symbol;Acc:HGNC:5147] |
| ENSG00000158639 | HGLibA_35017 | 186 | 0.54774029 | PAGE5 | PAGE family member 5 [Source:HGNC Symbol;Acc:HGNC:29992] |
| ENSG00000158859 | HGLibA_00830 | 186 | 0.54774029 | ADAMTS4 | ADAM metallopeptidase with thrombospondin type 1 motif 4 [Source:HGNC Symbol;Acc:HGNC:220] |
| ENSG00000163673 | HGLibA_12504 | 186 | 0.54774029 | DCLK3 | doublecortin like kinase 3 [Source:HGNC Symbol;Acc:HGNC:19005] |
| ENSG00000163873 | HGLibA_20287 | 186 | 0.54774029 | GRIK3 | glutamate ionotropic receptor kainate type subunit 3 [Source:HGNC Symbol;Acc:HGNC:4581] |
| ENSG00000164124 | HGLibA_49969 | 186 | 0.54774029 | TMEM144 | transmembrane protein 144 [Source:HGNC Symbol;Acc:HGNC:25633] |
| ENSG00000164161 | HGLibA_21260 | 186 | 0.54774029 | HHIP | hedgehog interacting protein [Source:HGNC Symbol;Acc:HGNC:14866] |
| ENSG00000166006 | HGLibA_24166 | 186 | 0.54774029 | KCNC2 | potassium voltage-gated channel subfamily C member 2 [Source:HGNC Symbol;Acc:HGNC:6234] |
| ENSG00000166856 | HGLibA_19986 | 186 | 0.54774029 | GPR182 | G protein-coupled receptor 182 [Source:HGNC Symbol;Acc:HGNC:13708] |
| ENSG00000166979 | HGLibA_15676 | 186 | 0.54774029 | EVA1C | eva-1 homolog C [Source:HGNC Symbol;Acc:HGNC:13239] |
| ENSG00000167110 | HGLibA_19594 | 186 | 0.54774029 | GOLGA2 | golgin A2 [Source:HGNC Symbol;Acc:HGNC:4425] |
| ENSG00000167193 | HGLibA_11181 | 186 | 0.54774029 | CRK | adaptor protein [Source:HGNC Symbol;Acc:HGNC:2362]" |
| ENSG00000168309 | HGLibA_16000 | 186 | 0.54774029 | FAM107A | family with sequence similarity 107 member A [Source:HGNC Symbol;Acc:HGNC:30827] |
| ENSG00000168754 | HGLibA_16303 | 186 | 0.54774029 | FAM178B | family with sequence similarity 178 member B [Source:HGNC Symbol;Acc:HGNC:28036] |
| ENSG00000169194 | HGLibA_22971 | 186 | 0.54774029 | IL13 | interleukin 13 [Source:HGNC Symbol;Acc:HGNC:5973] |
| ENSG00000172016 | HGLibA_40799 | 186 | 0.54774029 | REG3A | regenerating family member 3 alpha [Source:HGNC Symbol;Acc:HGNC:8601] |
| ENSG00000172046 | HGLibA_53294 | 186 | 0.54774029 | USP19 | ubiquitin specific peptidase 19 [Source:HGNC Symbol;Acc:HGNC:12617] |
| ENSG00000173272 | HGLibA_30944 | 186 | 0.54774029 | MZT2A | mitotic spindle organizing protein 2A [Source:HGNC Symbol;Acc:HGNC:33187] |
| ENSG00000174197 | HGLibA_29123 | 186 | 0.54774029 | MGA | MAX dimerization protein [Source:HGNC Symbol;Acc:HGNC:14010]" |
| ENSG00000174442 | HGLibA_57068 | 186 | 0.54774029 | ZWILCH | zwilch kinetochore protein [Source:HGNC Symbol;Acc:HGNC:25468] |
| ENSG00000175182 | HGLibA_16106 | 186 | 0.54774029 | FAM131A | family with sequence similarity 131 member A [Source:HGNC Symbol;Acc:HGNC:28308] |
| ENSG00000176383 | HGLibA_03990 | 186 | 0.54774029 | B3GNT4 | 3-N-acetylglucosaminyltransferase 4 [Source:HGNC Symbol;Acc:HGNC:15683]" |
| ENSG00000176495 | HGLibA_34241 | 186 | 0.54774029 | OR5AN1 | olfactory receptor family 5 subfamily AN member 1 [Source:HGNC Symbol;Acc:HGNC:15255] |
| ENSG00000176994 | HGLibA_45682 | 186 | 0.54774029 | SMCR8 | candidate 8 [Source:HGNC Symbol;Acc:HGNC:17921]" |
| ENSG00000178209 | HGLibA_37052 | 186 | 0.54774029 | PLEC | plectin [Source:HGNC Symbol;Acc:HGNC:9069] |
| ENSG00000183486 | HGLibA_30607 | 186 | 0.54774029 | MX2 | MX dynamin like GTPase 2 [Source:HGNC Symbol;Acc:HGNC:7533] |
| ENSG00000184735 | HGLibA_12740 | 186 | 0.54774029 | DDX53 | DEAD-box helicase 53 [Source:HGNC Symbol;Acc:HGNC:20083] |
| ENSG00000186190 | HGLibA_04726 | 186 | 0.54774029 | BPIFB3 | BPI fold containing family B member 3 [Source:HGNC Symbol;Acc:HGNC:16178] |
| ENSG00000187037 | HGLibA_19910 | 186 | 0.54774029 | GPR141 | G protein-coupled receptor 141 [Source:HGNC Symbol;Acc:HGNC:19997] |
| ENSG00000187862 | HGLibA_52084 | 186 | 0.54774029 | TTC24 | tetratricopeptide repeat domain 24 [Source:HGNC Symbol;Acc:HGNC:32348] |
| ENSG00000196169 | HGLibA_24881 | 186 | 0.54774029 | KIF19 | kinesin family member 19 [Source:HGNC Symbol;Acc:HGNC:26735] |
| ENSG00000196406 | HGLibA_46392 | 186 | 0.54774029 | SPANXD | SPANX family member D [Source:HGNC Symbol;Acc:HGNC:14332] |
| ENSG00000196754 | HGLibA_42550 | 186 | 0.54774029 | S100A2 | S100 calcium binding protein A2 [Source:HGNC Symbol;Acc:HGNC:10492] |
| ENSG00000197591 | HGLibA_33583 | 186 | 0.54774029 | OR11L1 | olfactory receptor family 11 subfamily L member 1 [Source:HGNC Symbol;Acc:HGNC:14998] |
| ENSG00000198841 | HGLibA_25869 | 186 | 0.54774029 | KTI12 | KTI12 chromatin associated homolog [Source:HGNC Symbol;Acc:HGNC:25160] |
| ENSG00000203747 | HGLibA_17196 | 186 | 0.54774029 | FCGR3A | Fc fragment of IgG receptor IIIa [Source:HGNC Symbol;Acc:HGNC:3619] |
| ENSG00000204520 | HGLibA_29199 | 186 | 0.54774029 | MICA | MHC class I polypeptide-related sequence A [Source:HGNC Symbol;Acc:HGNC:7090] |
| ENSG00000204704 | HGLibA_33901 | 186 | 0.54774029 | OR2W1 | olfactory receptor family 2 subfamily W member 1 [Source:HGNC Symbol;Acc:HGNC:8281] |
| ENSG00000212659 | HGLibA_25843 | 186 | 0.54774029 | KRTAP9-6 | keratin associated protein 9-6 [Source:HGNC Symbol;Acc:HGNC:18914] |
| ENSG00000213937 | HGLibA_09912 | 186 | 0.54774029 | CLDN9 | claudin 9 [Source:HGNC Symbol;Acc:HGNC:2051] |
| ENSG00000228083 | HGLibA_22666 | 186 | 0.54774029 | IFNA14 | interferon alpha 14 [Source:HGNC Symbol;Acc:HGNC:5420] |
| ENSG00000228623 | HGLibA_56896 | 186 | 0.54774029 | ZNF883 | zinc finger protein 883 [Source:HGNC Symbol;Acc:HGNC:27271] |
| ENSG00000234965 | HGLibA_44050 | 186 | 0.54774029 | SHISA8 | shisa family member 8 [Source:HGNC Symbol;Acc:HGNC:18351] |
| ENSG00000240871 | HGLibA_25772 | 186 | 0.54774029 | KRTAP4-7 | keratin associated protein 4-7 [Source:HGNC Symbol;Acc:HGNC:18898] |
| ENSG00000254834 | HGLibA_34332 | 186 | 0.54774029 | OR5M10 | olfactory receptor family 5 subfamily M member 10 [Source:HGNC Symbol;Acc:HGNC:15290] |
| ENSG00000256812 | HGLibA_07186 | 186 | 0.54774029 | CAPNS2 | calpain small subunit 2 [Source:HGNC Symbol;Acc:HGNC:16371] |
| ENSG00000257923 | HGLibA_11803 | 186 | 0.54774029 | CUX1 | cut like homeobox 1 [Source:HGNC Symbol;Acc:HGNC:2557] |
| ENSG00000263155 | HGLibA_30932 | 186 | 0.54774029 | MYZAP | myocardial zonula adherens protein [Source:HGNC Symbol;Acc:HGNC:43444] |
| ENSG00000272047 | HGLibA_20562 | 186 | 0.54774029 | GTF2H5 | general transcription factor IIH subunit 5 [Source:HGNC Symbol;Acc:HGNC:21157] |
| ENSG00000275023 | HGLibA_29423 | 186 | 0.54774029 | MLLT6 | PHD finger containing [Source:HGNC Symbol;Acc:HGNC:7138]" |
| ENSG00000276119 | HGLibA_33597 | 186 | 0.54774029 | OR13C2 | olfactory receptor family 13 subfamily C member 2 [Source:HGNC Symbol;Acc:HGNC:14701] |
| ENSG00000283154 | HGLibA_23526 | 186 | 0.54774029 | IQCJ-SCHIP1 | IQCJ-SCHIP1 readthrough [Source:HGNC Symbol;Acc:HGNC:38842] |
| hsa-mir-1915 | HGLibA_58066 | 186 | 0.54774029 | hsa-mir-1915 | - |
| hsa-mir-3654 | HGLibA_59162 | 186 | 0.54774029 | hsa-mir-3654 | - |
| hsa-mir-3944 | HGLibA_59634 | 186 | 0.54774029 | hsa-mir-3944 | - |
| hsa-mir-3978 | HGLibA_59670 | 186 | 0.54774029 | hsa-mir-3978 | - |
| hsa-mir-4439 | HGLibA_60159 | 186 | 0.54774029 | hsa-mir-4439 | - |
| hsa-mir-4697 | HGLibA_60895 | 186 | 0.54774029 | hsa-mir-4697 | - |
| hsa-mir-4799 | HGLibA_61301 | 186 | 0.54774029 | hsa-mir-4799 | - |
| hsa-mir-5684 | HGLibA_62255 | 186 | 0.54774029 | hsa-mir-5684 | - |
| hsa-mir-615 | HGLibA_62694 | 186 | 0.54774029 | hsa-mir-615 | - |
| hsa-mir-6746 | HGLibA_63139 | 186 | 0.54774029 | hsa-mir-6746 | - |
| hsa-mir-6823 | HGLibA_63470 | 186 | 0.54774029 | hsa-mir-6823 | - |
| KIAA1033 | HGLibA_24684 | 186 | 0.54774029 | KIAA1033 | - |
| MFSD4 | HGLibA_29102 | 186 | 0.54774029 | MFSD4 | - |
| CCBL1 | HGLibA_07495 | 185 | 0.538694947 | CCBL1 | - |
| ENSG00000007001 | HGLibA_53090 | 185 | 0.538694947 | UPP2 | uridine phosphorylase 2 [Source:HGNC Symbol;Acc:HGNC:23061] |
| ENSG00000064545 | HGLibA_50023 | 185 | 0.538694947 | TMEM161A | transmembrane protein 161A [Source:HGNC Symbol;Acc:HGNC:26020] |
| ENSG00000066084 | HGLibA_13250 | 185 | 0.538694947 | DIP2B | disco interacting protein 2 homolog B [Source:HGNC Symbol;Acc:HGNC:29284] |
| ENSG00000073711 | HGLibA_38155 | 185 | 0.538694947 | PPP2R3A | protein phosphatase 2 regulatory subunit B''alpha [Source:HGNC Symbol;Acc:HGNC:9307] |
| ENSG00000073849 | HGLibA_47257 | 185 | 0.538694947 | ST6GAL1 | 6-sialyltransferase 1 [Source:HGNC Symbol;Acc:HGNC:10860]" |
| ENSG00000076662 | HGLibA_22499 | 185 | 0.538694947 | ICAM3 | intercellular adhesion molecule 3 [Source:HGNC Symbol;Acc:HGNC:5346] |
| ENSG00000076944 | HGLibA_47665 | 185 | 0.538694947 | STXBP2 | syntaxin binding protein 2 [Source:HGNC Symbol;Acc:HGNC:11445] |
| ENSG00000091317 | HGLibA_10223 | 185 | 0.538694947 | CMTM6 | CKLF like MARVEL transmembrane domain containing 6 [Source:HGNC Symbol;Acc:HGNC:19177] |
| ENSG00000097046 | HGLibA_08596 | 185 | 0.538694947 | CDC7 | cell division cycle 7 [Source:HGNC Symbol;Acc:HGNC:1745] |
| ENSG00000100038 | HGLibA_51002 | 185 | 0.538694947 | TOP3B | DNA topoisomerase III beta [Source:HGNC Symbol;Acc:HGNC:11993] |
| ENSG00000100450 | HGLibA_20725 | 185 | 0.538694947 | GZMH | granzyme H [Source:HGNC Symbol;Acc:HGNC:4710] |
| ENSG00000103522 | HGLibA_23084 | 185 | 0.538694947 | IL21R | interleukin 21 receptor [Source:HGNC Symbol;Acc:HGNC:6006] |
| ENSG00000105497 | HGLibA_55528 | 185 | 0.538694947 | ZNF175 | zinc finger protein 175 [Source:HGNC Symbol;Acc:HGNC:12964] |
| ENSG00000106443 | HGLibA_36344 | 185 | 0.538694947 | PHF14 | PHD finger protein 14 [Source:HGNC Symbol;Acc:HGNC:22203] |
| ENSG00000106992 | HGLibA_01375 | 185 | 0.538694947 | AK1 | adenylate kinase 1 [Source:HGNC Symbol;Acc:HGNC:361] |
| ENSG00000108561 | HGLibA_05919 | 185 | 0.538694947 | C1QBP | complement C1q binding protein [Source:HGNC Symbol;Acc:HGNC:1243] |
| ENSG00000111145 | HGLibA_14916 | 185 | 0.538694947 | ELK3 | ETS transcription factor [Source:HGNC Symbol;Acc:HGNC:3325]" |
| ENSG00000111671 | HGLibA_46863 | 185 | 0.538694947 | SPSB2 | splA/ryanodine receptor domain and SOCS box containing 2 [Source:HGNC Symbol;Acc:HGNC:29522] |
| ENSG00000111737 | HGLibA_39888 | 185 | 0.538694947 | RAB35 | member RAS oncogene family [Source:HGNC Symbol;Acc:HGNC:9774]" |
| ENSG00000111962 | HGLibA_53425 | 185 | 0.538694947 | UST | uronyl 2-sulfotransferase [Source:HGNC Symbol;Acc:HGNC:17223] |
| ENSG00000113749 | HGLibA_22098 | 185 | 0.538694947 | HRH2 | histamine receptor H2 [Source:HGNC Symbol;Acc:HGNC:5183] |
| ENSG00000116221 | HGLibA_29965 | 185 | 0.538694947 | MRPL37 | mitochondrial ribosomal protein L37 [Source:HGNC Symbol;Acc:HGNC:14034] |
| ENSG00000116701 | HGLibA_31299 | 185 | 0.538694947 | NCF2 | neutrophil cytosolic factor 2 [Source:HGNC Symbol;Acc:HGNC:7661] |
| ENSG00000118200 | HGLibA_07110 | 185 | 0.538694947 | CAMSAP2 | calmodulin regulated spectrin associated protein family member 2 [Source:HGNC Symbol;Acc:HGNC:29188] |
| ENSG00000120158 | HGLibA_40698 | 185 | 0.538694947 | RCL1 | RNA terminal phosphate cyclase like 1 [Source:HGNC Symbol;Acc:HGNC:17687] |
| ENSG00000120837 | HGLibA_31857 | 185 | 0.538694947 | NFYB | nuclear transcription factor Y subunit beta [Source:HGNC Symbol;Acc:HGNC:7805] |
| ENSG00000121067 | HGLibA_46763 | 185 | 0.538694947 | SPOP | speckle type BTB/POZ protein [Source:HGNC Symbol;Acc:HGNC:11254] |
| ENSG00000121743 | HGLibA_19143 | 185 | 0.538694947 | GJA3 | gap junction protein alpha 3 [Source:HGNC Symbol;Acc:HGNC:4277] |
| ENSG00000124214 | HGLibA_47405 | 185 | 0.538694947 | STAU1 | staufen double-stranded RNA binding protein 1 [Source:HGNC Symbol;Acc:HGNC:11370] |
| ENSG00000124532 | HGLibA_30119 | 185 | 0.538694947 | MRS2 | magnesium transporter MRS2 [Source:HGNC Symbol;Acc:HGNC:13785] |
| ENSG00000130035 | HGLibA_18572 | 185 | 0.538694947 | GALNT8 | polypeptide N-acetylgalactosaminyltransferase 8 [Source:HGNC Symbol;Acc:HGNC:4130] |
| ENSG00000130775 | HGLibA_49351 | 185 | 0.538694947 | THEMIS2 | thymocyte selection associated family member 2 [Source:HGNC Symbol;Acc:HGNC:16839] |
| ENSG00000131067 | HGLibA_19020 | 185 | 0.538694947 | GGT7 | gamma-glutamyltransferase 7 [Source:HGNC Symbol;Acc:HGNC:4259] |
| ENSG00000131374 | HGLibA_48560 | 185 | 0.538694947 | TBC1D5 | TBC1 domain family member 5 [Source:HGNC Symbol;Acc:HGNC:19166] |
| ENSG00000131584 | HGLibA_00388 | 185 | 0.538694947 | ACAP3 | ankyrin repeat and PH domains 3 [Source:HGNC Symbol;Acc:HGNC:16754]" |
| ENSG00000132517 | HGLibA_45244 | 185 | 0.538694947 | SLC52A1 | solute carrier family 52 member 1 [Source:HGNC Symbol;Acc:HGNC:30225] |
| ENSG00000134339 | HGLibA_42612 | 185 | 0.538694947 | SAA2 | serum amyloid A2 [Source:HGNC Symbol;Acc:HGNC:10514] |
| ENSG00000135341 | HGLibA_28196 | 185 | 0.538694947 | MAP3K7 | mitogen-activated protein kinase kinase kinase 7 [Source:HGNC Symbol;Acc:HGNC:6859] |
| ENSG00000137672 | HGLibA_51747 | 185 | 0.538694947 | TRPC6 | transient receptor potential cation channel subfamily C member 6 [Source:HGNC Symbol;Acc:HGNC:12338] |
| ENSG00000138741 | HGLibA_51732 | 185 | 0.538694947 | TRPC3 | transient receptor potential cation channel subfamily C member 3 [Source:HGNC Symbol;Acc:HGNC:12335] |
| ENSG00000139323 | HGLibA_37445 | 185 | 0.538694947 | POC1B | POC1 centriolar protein B [Source:HGNC Symbol;Acc:HGNC:30836] |

| | | | | | |
|---|---|---|---|---|---|
| ENSG00000139574 | HGLibA_32437 | 185 | 0.538694947 | NPFF | neuropeptide FF-amide peptide precursor [Source:HGNC Symbol;Acc:HGNC:7901] |
| ENSG00000141431 | HGLibA_03394 | 185 | 0.538694947 | ASXL3 | ASXL transcriptional regulator 3 [Source:HGNC Symbol;Acc:HGNC:29357] |
| ENSG00000142615 | HGLibA_08992 | 185 | 0.538694947 | CELA2A | chymotrypsin like elastase family member 2A [Source:HGNC Symbol;Acc:HGNC:24609] |
| ENSG00000143320 | HGLibA_11057 | 185 | 0.538694947 | CRABP2 | cellular retinoic acid binding protein 2 [Source:HGNC Symbol;Acc:HGNC:2339] |
| ENSG00000144199 | HGLibA_15961 | 185 | 0.538694947 | FAHD2B | fumarylacetoacetate hydrolase domain containing 2B [Source:HGNC Symbol;Acc:HGNC:25318] |
| ENSG00000145604 | HGLibA_44296 | 185 | 0.538694947 | SKP2 | S-phase kinase associated protein 2 [Source:HGNC Symbol;Acc:HGNC:10901] |
| ENSG00000145920 | HGLibA_10935 | 185 | 0.538694947 | CPLX2 | complexin 2 [Source:HGNC Symbol;Acc:HGNC:2310] |
| ENSG00000146192 | HGLibA_17344 | 185 | 0.538694947 | FGD2 | RhoGEF and PH domain containing 2 [Source:HGNC Symbol;Acc:HGNC:3664]" |
| ENSG00000147364 | HGLibA_17055 | 185 | 0.538694947 | FBXO25 | F-box protein 25 [Source:HGNC Symbol;Acc:HGNC:13596] |
| ENSG00000149548 | HGLibA_07641 | 185 | 0.538694947 | CCDC15 | coiled-coil domain containing 15 [Source:HGNC Symbol;Acc:HGNC:25798] |
| ENSG00000151468 | HGLibA_07751 | 185 | 0.538694947 | CCDC3 | coiled-coil domain containing 3 [Source:HGNC Symbol;Acc:HGNC:23813] |
| ENSG00000151790 | HGLibA_48888 | 185 | 0.538694947 | TDO2 | 3-dioxygenase [Source:HGNC Symbol;Acc:HGNC:11708]" |
| ENSG00000157637 | HGLibA_45058 | 185 | 0.538694947 | SLC38A10 | solute carrier family 38 member 10 [Source:HGNC Symbol;Acc:HGNC:28237] |
| ENSG00000160113 | HGLibA_32616 | 185 | 0.538694947 | NR2F6 | nuclear receptor subfamily 2 group F member 6 [Source:HGNC Symbol;Acc:HGNC:7977] |
| ENSG00000160194 | HGLibA_31575 | 185 | 0.538694947 | NDUFV3 | NADH:ubiquinone oxidoreductase subunit V3 [Source:HGNC Symbol;Acc:HGNC:7719] |
| ENSG00000160310 | HGLibA_38623 | 185 | 0.538694947 | PRMT2 | protein arginine methyltransferase 2 [Source:HGNC Symbol;Acc:HGNC:5186] |
| ENSG00000160808 | HGLibA_30760 | 185 | 0.538694947 | MYL3 | myosin light chain 3 [Source:HGNC Symbol;Acc:HGNC:7584] |
| ENSG00000162877 | HGLibA_37285 | 185 | 0.538694947 | PM20D1 | peptidase M20 domain containing 1 [Source:HGNC Symbol;Acc:HGNC:26518] |
| ENSG00000163110 | HGLibA_35919 | 185 | 0.538694947 | PDLIM5 | PDZ and LIM domain 5 [Source:HGNC Symbol;Acc:HGNC:17468] |
| ENSG00000164692 | HGLibA_10533 | 185 | 0.538694947 | COL1A2 | collagen type I alpha 2 chain [Source:HGNC Symbol;Acc:HGNC:2198] |
| ENSG00000165271 | HGLibA_32272 | 185 | 0.538694947 | NOL6 | nucleolar protein 6 [Source:HGNC Symbol;Acc:HGNC:19910] |
| ENSG00000166923 | HGLibA_20242 | 185 | 0.538694947 | GREM1 | DAN family BMP antagonist [Source:HGNC Symbol;Acc:HGNC:2001]" |
| ENSG00000167419 | HGLibA_27107 | 185 | 0.538694947 | LPO | lactoperoxidase [Source:HGNC Symbol;Acc:HGNC:6678] |
| ENSG00000167614 | HGLibA_52232 | 185 | 0.538694947 | TTYH1 | tweety family member 1 [Source:HGNC Symbol;Acc:HGNC:13476] |
| ENSG00000168234 | HGLibA_52139 | 185 | 0.538694947 | TTC39C | tetratricopeptide repeat domain 39C [Source:HGNC Symbol;Acc:HGNC:26595] |
| ENSG00000170605 | HGLibA_34581 | 185 | 0.538694947 | OR9K2 | olfactory receptor family 9 subfamily K member 2 [Source:HGNC Symbol;Acc:HGNC:15339] |
| ENSG00000171446 | HGLibA_25466 | 185 | 0.538694947 | KRT27 | keratin 27 [Source:HGNC Symbol;Acc:HGNC:30841] |
| ENSG00000172382 | HGLibA_38919 | 185 | 0.538694947 | PRSS27 | serine protease 27 [Source:HGNC Symbol;Acc:HGNC:15475] |
| ENSG00000174469 | HGLibA_10423 | 185 | 0.538694947 | CNTNAP2 | contactin associated protein like 2 [Source:HGNC Symbol;Acc:HGNC:13830] |
| ENSG00000176601 | HGLibA_28179 | 185 | 0.538694947 | MAP3K19 | mitogen-activated protein kinase kinase kinase 19 [Source:HGNC Symbol;Acc:HGNC:26249] |
| ENSG00000176895 | HGLibA_34078 | 185 | 0.538694947 | OR51A7 | olfactory receptor family 51 subfamily A member 7 [Source:HGNC Symbol;Acc:HGNC:15188] |
| ENSG00000178172 | HGLibA_46699 | 185 | 0.538694947 | SPINK6 | Kazal type 6 [Source:HGNC Symbol;Acc:HGNC:29486]" |
| ENSG00000179532 | HGLibA_13676 | 185 | 0.538694947 | DNHD1 | dynein heavy chain domain 1 [Source:HGNC Symbol;Acc:HGNC:26532] |
| ENSG00000182459 | HGLibA_49070 | 185 | 0.538694947 | TEX19 | testis expressed 19 [Source:HGNC Symbol;Acc:HGNC:33802] |
| ENSG00000184408 | HGLibA_24177 | 185 | 0.538694947 | KCND2 | potassium voltage-gated channel subfamily D member 2 [Source:HGNC Symbol;Acc:HGNC:6238] |
| ENSG00000184735 | HGLibA_12742 | 185 | 0.538694947 | DDX53 | DEAD-box helicase 53 [Source:HGNC Symbol;Acc:HGNC:20083] |
| ENSG00000185437 | HGLibA_43912 | 185 | 0.538694947 | SH3BGR | SH3 domain binding glutamate rich protein [Source:HGNC Symbol;Acc:HGNC:10822] |
| ENSG00000185480 | HGLibA_35251 | 185 | 0.538694947 | PARPBP | PARP1 binding protein [Source:HGNC Symbol;Acc:HGNC:26074] |
| ENSG00000188782 | HGLibA_07363 | 185 | 0.538694947 | CATSPER4 | cation channel sperm associated 4 [Source:HGNC Symbol;Acc:HGNC:23220] |
| ENSG00000189241 | HGLibA_51979 | 185 | 0.538694947 | TSPYL1 | TSPY like 1 [Source:HGNC Symbol;Acc:HGNC:12382] |
| ENSG00000189375 | HGLibA_48522 | 185 | 0.538694947 | TBC1D28 | TBC1 domain family member 28 [Source:HGNC Symbol;Acc:HGNC:26858] |
| ENSG00000197128 | HGLibA_56711 | 185 | 0.538694947 | ZNF772 | zinc finger protein 772 [Source:HGNC Symbol;Acc:HGNC:33106] |
| ENSG00000198854 | HGLibA_05890 | 185 | 0.538694947 | C1orf68 | chromosome 1 open reading frame 68 [Source:HGNC Symbol;Acc:HGNC:29468] |
| ENSG00000204897 | HGLibA_25459 | 185 | 0.538694947 | KRT25 | keratin 25 [Source:HGNC Symbol;Acc:HGNC:30839] |
| ENSG00000213398 | HGLibA_26096 | 185 | 0.538694947 | LCAT | lecithin-cholesterol acyltransferase [Source:HGNC Symbol;Acc:HGNC:6522] |
| ENSG00000250254 | HGLibA_39567 | 185 | 0.538694947 | PTTG2 | pituitary tumor-transforming 2 [Source:HGNC Symbol;Acc:HGNC:9691] |
| ENSG00000255561 | HGLibA_17265 | 185 | 0.538694947 | FDXACB1 | ferredoxin-fold anticodon binding domain containing 1 [Source:HGNC Symbol;Acc:HGNC:25110] |
| ENSG00000260230 | HGLibA_18044 | 185 | 0.538694947 | FRRS1L | ferric chelate reductase 1 like [Source:HGNC Symbol;Acc:HGNC:1362] |
| ENSG00000269313 | HGLibA_28004 | 185 | 0.538694947 | MAGIX | X-linked [Source:HGNC Symbol;Acc:HGNC:30006]" |
| ENSG00000278540 | HGLibA_00347 | 185 | 0.538694947 | ACACA | acetyl-CoA carboxylase alpha [Source:HGNC Symbol;Acc:HGNC:84] |
| GRAMD3 | HGLibA_20206 | 185 | 0.538694947 | GRAMD3 | - |
| HIST1H4A | HGLibA_21487 | 185 | 0.538694947 | HIST1H4A | - |
| hsa-mir-1199 | HGLibA_57258 | 185 | 0.538694947 | hsa-mir-1199 | - |
| hsa-mir-1910 | HGLibA_58047 | 185 | 0.538694947 | hsa-mir-1910 | - |
| hsa-mir-3180-4 | HGLibA_58813 | 185 | 0.538694947 | hsa-mir-3180-4 | - |
| hsa-mir-3689a | HGLibA_59312 | 185 | 0.538694947 | hsa-mir-3689a | - |
| hsa-mir-4737 | HGLibA_61044 | 185 | 0.538694947 | hsa-mir-4737 | - |
| hsa-mir-496 | HGLibA_61383 | 185 | 0.538694947 | hsa-mir-496 | - |
| hsa-mir-6762 | HGLibA_63210 | 185 | 0.538694947 | hsa-mir-6762 | - |
| hsa-mir-6857 | HGLibA_63602 | 185 | 0.538694947 | hsa-mir-6857 | - |
| hsa-mir-7845 | HGLibA_63970 | 185 | 0.538694947 | hsa-mir-7845 | - |
| LOC100130301 | HGLibA_26773 | 185 | 0.538694947 | LOC100130301 | - |
| METTL11B | HGLibA_28949 | 185 | 0.538694947 | METTL11B | - |
| PAPD5 | HGLibA_35124 | 185 | 0.538694947 | PAPD5 | - |
| SDCCAG3 | HGLibA_43115 | 185 | 0.538694947 | SDCCAG3 | - |
| TMEM194A | HGLibA_50153 | 185 | 0.538694947 | TMEM194A | - |
| C10orf118 | HGLibA_05040 | 184 | 0.529649605 | C10orf118 | - |
| ENSG00000001630 | HGLibA_12207 | 184 | 0.529649605 | CYP51A1 | cytochrome P450 family 51 subfamily A member 1 [Source:HGNC Symbol;Acc:HGNC:2649] |
| ENSG00000008196 | HGLibA_49125 | 184 | 0.529649605 | TFAP2B | transcription factor AP-2 beta [Source:HGNC Symbol;Acc:HGNC:11743] |
| ENSG00000010818 | HGLibA_21571 | 184 | 0.529649605 | HIVEP2 | human immunodeficiency virus type I enhancer binding protein 2 [Source:HGNC Symbol;Acc:HGNC:4921] |
| ENSG00000070770 | HGLibA_11404 | 184 | 0.529649605 | CSNK2A2 | casein kinase 2 alpha 2 [Source:HGNC Symbol;Acc:HGNC:2459] |
| ENSG00000073578 | HGLibA_43134 | 184 | 0.529649605 | SDHA | succinate dehydrogenase complex flavoprotein subunit A [Source:HGNC Symbol;Acc:HGNC:10680] |
| ENSG00000077549 | HGLibA_07214 | 184 | 0.529649605 | CAPZB | capping actin protein of muscle Z-line subunit beta [Source:HGNC Symbol;Acc:HGNC:1491] |
| ENSG00000081760 | HGLibA_00027 | 184 | 0.529649605 | AACS | acetoacetyl-CoA synthetase [Source:HGNC Symbol;Acc:HGNC:21298] |
| ENSG00000090674 | HGLibA_28636 | 184 | 0.529649605 | MCOLN1 | mucolipin 1 [Source:HGNC Symbol;Acc:HGNC:13356] |
| ENSG00000092345 | HGLibA_12370 | 184 | 0.529649605 | DAZL | deleted in azoospermia like [Source:HGNC Symbol;Acc:HGNC:2685] |
| ENSG00000099953 | HGLibA_29483 | 184 | 0.529649605 | MMP11 | matrix metallopeptidase 11 [Source:HGNC Symbol;Acc:HGNC:7157] |
| ENSG00000100373 | HGLibA_53076 | 184 | 0.529649605 | UPK3A | uroplakin 3A [Source:HGNC Symbol;Acc:HGNC:12580] |
| ENSG00000102543 | HGLibA_08502 | 184 | 0.529649605 | CDADC1 | cytidine and dCMP deaminase domain containing 1 [Source:HGNC Symbol;Acc:HGNC:20299] |
| ENSG00000102755 | HGLibA_17663 | 184 | 0.529649605 | FLT1 | fms related tyrosine kinase 1 [Source:HGNC Symbol;Acc:HGNC:3763] |
| ENSG00000103375 | HGLibA_02609 | 184 | 0.529649605 | AQP8 | aquaporin 8 [Source:HGNC Symbol;Acc:HGNC:642] |
| ENSG00000103657 | HGLibA_21154 | 184 | 0.529649605 | HERC1 | HECT and RLD domain containing E3 ubiquitin protein ligase family member 1 [Source:HGNC Symbol;Acc:HGNC:4867] |
| ENSG00000105700 | HGLibA_25874 | 184 | 0.529649605 | KXD1 | KxDL motif containing 1 [Source:HGNC Symbol;Acc:HGNC:28420] |
| ENSG00000108018 | HGLibA_46182 | 184 | 0.529649605 | SORCS1 | sortilin related VPS10 domain containing receptor 1 [Source:HGNC Symbol;Acc:HGNC:16697] |
| ENSG00000108417 | HGLibA_25495 | 184 | 0.529649605 | KRT37 | keratin 37 [Source:HGNC Symbol;Acc:HGNC:6455] |
| ENSG00000111775 | HGLibA_10843 | 184 | 0.529649605 | COX6A1 | cytochrome c oxidase subunit 6A1 [Source:HGNC Symbol;Acc:HGNC:2277] |
| ENSG00000117152 | HGLibA_41094 | 184 | 0.529649605 | RGS4 | regulator of G protein signaling 4 [Source:HGNC Symbol;Acc:HGNC:10000] |
| ENSG00000119778 | HGLibA_03407 | 184 | 0.529649605 | ATAD2B | AAA domain containing 2B [Source:HGNC Symbol;Acc:HGNC:29230]" |
| ENSG00000121351 | HGLibA_22471 | 184 | 0.529649605 | IAPP | islet amyloid polypeptide [Source:HGNC Symbol;Acc:HGNC:5329] |
| ENSG00000122367 | HGLibA_26216 | 184 | 0.529649605 | LDB3 | LIM domain binding 3 [Source:HGNC Symbol;Acc:HGNC:15710] |
| ENSG00000123444 | HGLibA_24109 | 184 | 0.529649605 | KBTBD4 | kelch repeat and BTB domain containing 4 [Source:HGNC Symbol;Acc:HGNC:23761] |
| ENSG00000124466 | HGLibA_27718 | 184 | 0.529649605 | LYPD3 | LY6/PLAUR domain containing 3 [Source:HGNC Symbol;Acc:HGNC:24880] |
| ENSG00000130707 | HGLibA_03372 | 184 | 0.529649605 | ASS1 | argininosuccinate synthase 1 [Source:HGNC Symbol;Acc:HGNC:758] |
| ENSG00000131059 | HGLibA_04717 | 184 | 0.529649605 | BPIFA3 | BPI fold containing family A member 3 [Source:HGNC Symbol;Acc:HGNC:16204] |
| ENSG00000131669 | HGLibA_31938 | 184 | 0.529649605 | NINJ1 | ninjurin 1 [Source:HGNC Symbol;Acc:HGNC:7824] |
| ENSG00000133067 | HGLibA_26414 | 184 | 0.529649605 | LGR6 | leucine rich repeat containing G protein-coupled receptor 6 [Source:HGNC Symbol;Acc:HGNC:19719] |
| ENSG00000134240 | HGLibA_21714 | 184 | 0.529649605 | HMGCS2 | 3-hydroxy-3-methylglutaryl-CoA synthase 2 [Source:HGNC Symbol;Acc:HGNC:5008] |
| ENSG00000136490 | HGLibA_26533 | 184 | 0.529649605 | LIMD2 | LIM domain containing 2 [Source:HGNC Symbol;Acc:HGNC:28142] |
| ENSG00000137265 | HGLibA_23588 | 184 | 0.529649605 | IRF4 | interferon regulatory factor 4 [Source:HGNC Symbol;Acc:HGNC:6119] |
| ENSG00000138136 | HGLibA_26085 | 184 | 0.529649605 | LBX1 | ladybird homeobox 1 [Source:HGNC Symbol;Acc:HGNC:16960] |
| ENSG00000138246 | HGLibA_13572 | 184 | 0.529649605 | DNAJC13 | DnaJ heat shock protein family (Hsp40) member C13 [Source:HGNC Symbol;Acc:HGNC:30343] |
| ENSG00000140859 | HGLibA_24977 | 184 | 0.529649605 | KIFC3 | kinesin family member C3 [Source:HGNC Symbol;Acc:HGNC:6326] |
| ENSG00000142273 | HGLibA_07399 | 184 | 0.529649605 | CBLC | Cbl proto-oncogene C [Source:HGNC Symbol;Acc:HGNC:15961] |
| ENSG00000142279 | HGLibA_54437 | 184 | 0.529649605 | WTIP | WT1 interacting protein [Source:HGNC Symbol;Acc:HGNC:20964] |
| ENSG00000143110 | HGLibA_05786 | 184 | 0.529649605 | C1orf162 | chromosome 1 open reading frame 162 [Source:HGNC Symbol;Acc:HGNC:28344] |
| ENSG00000143479 | HGLibA_14280 | 184 | 0.529649605 | DYRK3 | dual specificity tyrosine phosphorylation regulated kinase 3 [Source:HGNC Symbol;Acc:HGNC:3094] |
| ENSG00000146090 | HGLibA_40336 | 184 | 0.529649605 | RASGEF1C | RasGEF domain family member 1C [Source:HGNC Symbol;Acc:HGNC:27400] |
| ENSG00000146857 | HGLibA_47571 | 184 | 0.529649605 | STRA8 | stimulated by retinoic acid 8 [Source:HGNC Symbol;Acc:HGNC:30653] |
| ENSG00000148180 | HGLibA_20436 | 184 | 0.529649605 | GSN | gelsolin [Source:HGNC Symbol;Acc:HGNC:4620] |
| ENSG00000149483 | HGLibA_49955 | 184 | 0.529649605 | TMEM138 | transmembrane protein 138 [Source:HGNC Symbol;Acc:HGNC:26944] |
| ENSG00000152578 | HGLibA_20269 | 184 | 0.529649605 | GRIA4 | glutamate ionotropic receptor AMPA type subunit 4 [Source:HGNC Symbol;Acc:HGNC:4574] |
| ENSG00000152939 | HGLibA_28382 | 184 | 0.529649605 | MARVELD2 | MARVEL domain containing 2 [Source:HGNC Symbol;Acc:HGNC:26401] |
| ENSG00000153956 | HGLibA_06905 | 184 | 0.529649605 | CACNA2D1 | calcium voltage-gated channel auxiliary subunit alpha2delta 1 [Source:HGNC Symbol;Acc:HGNC:1399] |
| ENSG00000155974 | HGLibA_20321 | 184 | 0.529649605 | GRIP1 | glutamate receptor interacting protein 1 [Source:HGNC Symbol;Acc:HGNC:18708] |
| ENSG00000156381 | HGLibA_02146 | 184 | 0.529649605 | ANKRD9 | ankyrin repeat domain 9 [Source:HGNC Symbol;Acc:HGNC:20096] |
| ENSG00000156642 | HGLibA_32529 | 184 | 0.529649605 | NPTN | neuroplastin [Source:HGNC Symbol;Acc:HGNC:17867] |
| ENSG00000159216 | HGLibA_42454 | 184 | 0.529649605 | RUNX1 | runt related transcription factor 1 [Source:HGNC Symbol;Acc:HGNC:10471] |
| ENSG00000160785 | HGLibA_44748 | 184 | 0.529649605 | SLC25A44 | solute carrier family 25 member 44 [Source:HGNC Symbol;Acc:HGNC:29036] |
| ENSG00000162600 | HGLibA_33381 | 184 | 0.529649605 | OMA1 | OMA1 zinc metallopeptidase [Source:HGNC Symbol;Acc:HGNC:29661] |
| ENSG00000164081 | HGLibA_49086 | 184 | 0.529649605 | TEX264 | testis expressed 264 [Source:HGNC Symbol;Acc:HGNC:30247] |
| ENSG00000164114 | HGLibA_28245 | 184 | 0.529649605 | MAP9 | microtubule associated protein 9 [Source:HGNC Symbol;Acc:HGNC:26118] |
| ENSG00000164953 | HGLibA_50491 | 184 | 0.529649605 | TMEM67 | transmembrane protein 67 [Source:HGNC Symbol;Acc:HGNC:28396] |
| ENSG00000165097 | HGLibA_24491 | 184 | 0.529649605 | KDM1B | lysine demethylase 1B [Source:HGNC Symbol;Acc:HGNC:21577] |
| ENSG00000165102 | HGLibA_21249 | 184 | 0.529649605 | HGSNAT | heparan-alpha-glucosaminide N-acetyltransferase [Source:HGNC Symbol;Acc:HGNC:26527] |
| ENSG00000166002 | HGLibA_45671 | 184 | 0.529649605 | SMCO4 | single-pass membrane protein with coiled-coil domains 4 [Source:HGNC Symbol;Acc:HGNC:24810] |
| ENSG00000166165 | HGLibA_09766 | 184 | 0.529649605 | CKB | creatine kinase B [Source:HGNC Symbol;Acc:HGNC:1991] |
| ENSG00000166507 | HGLibA_31427 | 184 | 0.529649605 | NDST2 | N-deacetylase and N-sulfotransferase 2 [Source:HGNC Symbol;Acc:HGNC:7681] |
| ENSG00000168374 | HGLibA_02652 | 184 | 0.529649605 | ARF4 | ADP ribosylation factor 4 [Source:HGNC Symbol;Acc:HGNC:655] |
| ENSG00000168439 | HGLibA_47447 | 184 | 0.529649605 | STIP1 | stress induced phosphoprotein 1 [Source:HGNC Symbol;Acc:HGNC:11387] |
| ENSG00000169379 | HGLibA_02933 | 184 | 0.529649605 | ARL13B | ADP ribosylation factor like GTPase 13B [Source:HGNC Symbol;Acc:HGNC:25419] |
| ENSG00000172071 | HGLibA_14730 | 184 | 0.529649605 | EIF2AK3 | eukaryotic translation initiation factor 2 alpha kinase 3 [Source:HGNC Symbol;Acc:HGNC:3255] |
| ENSG00000172519 | HGLibA_33518 | 184 | 0.529649605 | OR10H5 | olfactory receptor family 10 subfamily H member 5 [Source:HGNC Symbol;Acc:HGNC:15389] |
| ENSG00000175662 | HGLibA_50953 | 184 | 0.529649605 | TOM1L2 | target of myb1 like 2 membrane trafficking protein [Source:HGNC Symbol;Acc:HGNC:11984] |
| ENSG00000176571 | HGLibA_10242 | 184 | 0.529649605 | CNBD1 | cyclic nucleotide binding domain containing 1 [Source:HGNC Symbol;Acc:HGNC:26663] |
| ENSG00000178307 | HGLibA_49871 | 184 | 0.529649605 | TMEM11 | transmembrane protein 11 [Source:HGNC Symbol;Acc:HGNC:16823] |

| | | | | | |
|---|---|---|---|---|---|
| ENSG00000179820 | HGLibA_30631 | 184 | 0.529649605 | MYADM | myeloid associated differentiation marker [Source:HGNC Symbol;Acc:HGNC:7544] |
| ENSG00000181761 | HGLibA_34532 | 184 | 0.529649605 | OR8H3 | olfactory receptor family 8 subfamily H member 3 [Source:HGNC Symbol;Acc:HGNC:15309] |
| ENSG00000183706 | HGLibA_34048 | 184 | 0.529649605 | OR4N4 | olfactory receptor family 4 subfamily N member 4 [Source:HGNC Symbol;Acc:HGNC:15375] |
| ENSG00000184227 | HGLibA_00468 | 184 | 0.529649605 | ACOT1 | acyl-CoA thioesterase 1 [Source:HGNC Symbol;Acc:HGNC:33128] |
| ENSG00000186350 | HGLibA_42505 | 184 | 0.529649605 | RXRA | retinoid X receptor alpha [Source:HGNC Symbol;Acc:HGNC:10477] |
| ENSG00000187583 | HGLibA_37146 | 184 | 0.529649605 | PLEKHN1 | pleckstrin homology domain containing N1 [Source:HGNC Symbol;Acc:HGNC:25284] |
| ENSG00000188263 | HGLibA_23020 | 184 | 0.529649605 | IL17REL | interleukin 17 receptor E like [Source:HGNC Symbol;Acc:HGNC:33808] |
| ENSG00000188277 | HGLibA_05432 | 184 | 0.529649605 | C15orf62 | chromosome 15 open reading frame 62 [Source:HGNC Symbol;Acc:HGNC:34489] |
| ENSG00000189067 | HGLibA_26638 | 184 | 0.529649605 | LITAF | lipopolysaccharide induced TNF factor [Source:HGNC Symbol;Acc:HGNC:16841] |
| ENSG00000196152 | HGLibA_56761 | 184 | 0.529649605 | ZNF79 | zinc finger protein 79 [Source:HGNC Symbol;Acc:HGNC:13153] |
| ENSG00000196344 | HGLibA_00964 | 184 | 0.529649605 | ADH7 | mu or sigma polypeptide [Source:HGNC Symbol;Acc:HGNC:256]" |
| ENSG00000196378 | HGLibA_55836 | 184 | 0.529649605 | ZNF34 | zinc finger protein 34 [Source:HGNC Symbol;Acc:HGNC:13098] |
| ENSG00000197006 | HGLibA_29023 | 184 | 0.529649605 | METTL9 | methyltransferase like 9 [Source:HGNC Symbol;Acc:HGNC:24586] |
| ENSG00000198890 | HGLibA_38633 | 184 | 0.529649605 | PRMT6 | protein arginine methyltransferase 6 [Source:HGNC Symbol;Acc:HGNC:18241] |
| ENSG00000204219 | HGLibA_48700 | 184 | 0.529649605 | TCEA3 | transcription elongation factor A3 [Source:HGNC Symbol;Acc:HGNC:11615] |
| ENSG00000212933 | HGLibA_25640 | 184 | 0.529649605 | KRTAP12-4 | keratin associated protein 12-4 [Source:HGNC Symbol;Acc:HGNC:20532] |
| ENSG00000221947 | HGLibA_54536 | 184 | 0.529649605 | XKR9 | XK related 9 [Source:HGNC Symbol;Acc:HGNC:20937] |
| ENSG00000241697 | HGLibA_49833 | 184 | 0.529649605 | TMEFF1 | transmembrane protein with EGF like and two follistatin like domains 1 [Source:HGNC Symbol;Acc:HGNC:11866] |
| ENSG00000244486 | HGLibA_42878 | 184 | 0.529649605 | SCARF2 | scavenger receptor class F member 2 [Source:HGNC Symbol;Acc:HGNC:19869] |
| ENSG00000269502 | HGLibA_13435 | 184 | 0.529649605 | DMRTC1 | DMRT like family C1 [Source:HGNC Symbol;Acc:HGNC:13910] |
| ENSG00000270672 | HGLibA_30485 | 184 | 0.529649605 | MTRNR2L6 | MT-RNR2 like 6 [Source:HGNC Symbol;Acc:HGNC:37163] |
| ENSG00000278023 | HGLibA_40756 | 184 | 0.529649605 | RDM1 | RAD52 motif containing 1 [Source:HGNC Symbol;Acc:HGNC:19950] |
| FAM63B | HGLibA_16663 | 184 | 0.529649605 | FAM63B | - |
| hsa-mir-212 | HGLibA_58245 | 184 | 0.529649605 | hsa-mir-212 | - |
| hsa-mir-3195 | HGLibA_58877 | 184 | 0.529649605 | hsa-mir-3195 | - |
| hsa-mir-4688 | HGLibA_60862 | 184 | 0.529649605 | hsa-mir-4688 | - |
| hsa-mir-4689 | HGLibA_60865 | 184 | 0.529649605 | hsa-mir-4689 | - |
| hsa-mir-558 | HGLibA_62160 | 184 | 0.529649605 | hsa-mir-558 | - |
| hsa-mir-5707 | HGLibA_62367 | 184 | 0.529649605 | hsa-mir-5707 | - |
| hsa-mir-6743 | HGLibA_63124 | 184 | 0.529649605 | hsa-mir-6743 | - |
| hsa-mir-6852 | HGLibA_63580 | 184 | 0.529649605 | hsa-mir-6852 | - |
| hsa-mir-7159 | HGLibA_63863 | 184 | 0.529649605 | hsa-mir-7159 | - |
| P2RY8_Y | HGLibA_34896 | 184 | 0.529649605 | P2RY8_Y | - |
| 5-Sep | HGLibA_43444 | 184 | 0.529649605 | 5-Sep | - |
| SOGA2 | HGLibA_46155 | 184 | 0.529649605 | SOGA2 | - |
| SSSCA1 | HGLibA_47168 | 184 | 0.529649605 | SSSCA1 | - |
| C7orf55-LUC7L2 | HGLibA_06557 | 183 | 0.520604262 | C7orf55-LUC7L2 | - |
| COL4A3BP | HGLibA_10574 | 183 | 0.520604262 | COL4A3BP | - |
| ENSG00000005893 | HGLibA_25981 | 183 | 0.520604262 | LAMP2 | lysosomal associated membrane protein 2 [Source:HGNC Symbol;Acc:HGNC:6501] |
| ENSG00000015413 | HGLibA_13817 | 183 | 0.520604262 | DPEP1 | dipeptidase 1 [Source:HGNC Symbol;Acc:HGNC:3002] |
| ENSG00000047644 | HGLibA_54444 | 183 | 0.520604262 | WWC3 | WWC family member 3 [Source:HGNC Symbol;Acc:HGNC:29237] |
| ENSG00000053747 | HGLibA_25946 | 183 | 0.520604262 | LAMA3 | laminin subunit alpha 3 [Source:HGNC Symbol;Acc:HGNC:6483] |
| ENSG00000066056 | HGLibA_49454 | 183 | 0.520604262 | TIE1 | tyrosine kinase with immunoglobulin like and EGF like domains 1 [Source:HGNC Symbol;Acc:HGNC:11809] |
| ENSG00000100314 | HGLibA_06861 | 183 | 0.520604262 | CABP7 | calcium binding protein 7 [Source:HGNC Symbol;Acc:HGNC:20834] |
| ENSG00000100330 | HGLibA_30428 | 183 | 0.520604262 | MTMR3 | myotubularin related protein 3 [Source:HGNC Symbol;Acc:HGNC:7451] |
| ENSG00000103037 | HGLibA_43655 | 183 | 0.520604262 | SETD6 | SET domain containing 6 [Source:HGNC Symbol;Acc:HGNC:26116] |
| ENSG00000106333 | HGLibA_35652 | 183 | 0.520604262 | PCOLCE | procollagen C-endopeptidase enhancer [Source:HGNC Symbol;Acc:HGNC:8738] |
| ENSG00000106483 | HGLibA_43741 | 183 | 0.520604262 | SFRP4 | secreted frizzled related protein 4 [Source:HGNC Symbol;Acc:HGNC:10778] |
| ENSG00000108878 | HGLibA_06930 | 183 | 0.520604262 | CACNG1 | calcium voltage-gated channel auxiliary subunit gamma 1 [Source:HGNC Symbol;Acc:HGNC:1405] |
| ENSG00000112782 | HGLibA_10032 | 183 | 0.520604262 | CLIC5 | chloride intracellular channel 5 [Source:HGNC Symbol;Acc:HGNC:13517] |
| ENSG00000114374 | HGLibA_53419 | 183 | 0.520604262 | USP9Y | ubiquitin specific peptidase 9 Y-linked [Source:HGNC Symbol;Acc:HGNC:12633] |
| ENSG00000116001 | HGLibA_49429 | 183 | 0.520604262 | TIA1 | TIA1 cytotoxic granule associated RNA binding protein [Source:HGNC Symbol;Acc:HGNC:11802] |
| ENSG00000116198 | HGLibA_09095 | 183 | 0.520604262 | CEP104 | centrosomal protein 104 [Source:HGNC Symbol;Acc:HGNC:24866] |
| ENSG00000119820 | HGLibA_54679 | 183 | 0.520604262 | YIPF4 | Yip1 domain family member 4 [Source:HGNC Symbol;Acc:HGNC:28145] |
| ENSG00000120162 | HGLibA_29598 | 183 | 0.520604262 | MOB3B | MOB kinase activator 3B [Source:HGNC Symbol;Acc:HGNC:23825] |
| ENSG00000121807 | HGLibA_08151 | 183 | 0.520604262 | CCR2 | C-C motif chemokine receptor 2 [Source:HGNC Symbol;Acc:HGNC:1603] |
| ENSG00000124257 | HGLibA_31727 | 183 | 0.520604262 | NEURL2 | neuralized E3 ubiquitin protein ligase 2 [Source:HGNC Symbol;Acc:HGNC:16156] |
| ENSG00000129636 | HGLibA_23707 | 183 | 0.520604262 | ITFG1 | integrin alpha FG-GAP repeat containing 1 [Source:HGNC Symbol;Acc:HGNC:30697] |
| ENSG00000130283 | HGLibA_18851 | 183 | 0.520604262 | GDF1 | growth differentiation factor 1 [Source:HGNC Symbol;Acc:HGNC:4214] |
| ENSG00000131044 | HGLibA_52215 | 183 | 0.520604262 | TTLL9 | tubulin tyrosine ligase like 9 [Source:HGNC Symbol;Acc:HGNC:16118] |
| ENSG00000131504 | HGLibA_13216 | 183 | 0.520604262 | DIAPH1 | diaphanous related formin 1 [Source:HGNC Symbol;Acc:HGNC:2876] |
| ENSG00000133800 | HGLibA_27778 | 183 | 0.520604262 | LYVE1 | lymphatic vessel endothelial hyaluronan receptor 1 [Source:HGNC Symbol;Acc:HGNC:14687] |
| ENSG00000135299 | HGLibA_02128 | 183 | 0.520604262 | ANKRD6 | ankyrin repeat domain 6 [Source:HGNC Symbol;Acc:HGNC:17280] |
| ENSG00000135312 | HGLibA_22358 | 183 | 0.520604262 | HTR1B | 5-hydroxytryptamine receptor 1B [Source:HGNC Symbol;Acc:HGNC:5287] |
| ENSG00000135414 | HGLibA_18858 | 183 | 0.520604262 | GDF11 | growth differentiation factor 11 [Source:HGNC Symbol;Acc:HGNC:4216] |
| ENSG00000138435 | HGLibA_09561 | 183 | 0.520604262 | CHRNA1 | cholinergic receptor nicotinic alpha 1 subunit [Source:HGNC Symbol;Acc:HGNC:1955] |
| ENSG00000138767 | HGLibA_10346 | 183 | 0.520604262 | CNOT6L | CCR4-NOT transcription complex subunit 6 like [Source:HGNC Symbol;Acc:HGNC:18042] |
| ENSG00000144233 | HGLibA_01804 | 183 | 0.520604262 | AMMECR1L | AMMECR1 like [Source:HGNC Symbol;Acc:HGNC:28658] |
| ENSG00000146267 | HGLibA_16929 | 183 | 0.520604262 | FAXC | failed axon connections homolog [Source:HGNC Symbol;Acc:HGNC:20742] |
| ENSG00000148602 | HGLibA_27184 | 183 | 0.520604262 | LRIT1 | Ig-like and transmembrane domains 1 [Source:HGNC Symbol;Acc:HGNC:23404]" |
| ENSG00000152977 | HGLibA_55321 | 183 | 0.520604262 | ZIC1 | Zic family member 1 [Source:HGNC Symbol;Acc:HGNC:12872] |
| ENSG00000153786 | HGLibA_55125 | 183 | 0.520604262 | ZDHHC7 | zinc finger DHHC-type containing 7 [Source:HGNC Symbol;Acc:HGNC:18459] |
| ENSG00000153820 | HGLibA_46648 | 183 | 0.520604262 | SPHKAP | AKAP domain containing [Source:HGNC Symbol;Acc:HGNC:30619]" |
| ENSG00000155545 | HGLibA_29257 | 183 | 0.520604262 | MIER3 | MIER family member 3 [Source:HGNC Symbol;Acc:HGNC:26678] |
| ENSG00000157219 | HGLibA_22398 | 183 | 0.520604262 | HTR5A | 5-hydroxytryptamine receptor 5A [Source:HGNC Symbol;Acc:HGNC:5300] |
| ENSG00000163220 | HGLibA_42575 | 183 | 0.520604262 | S100A9 | S100 calcium binding protein A9 [Source:HGNC Symbol;Acc:HGNC:10499] |
| ENSG00000163738 | HGLibA_30385 | 183 | 0.520604262 | MTHFD2L | methylenetetrahydrofolate dehydrogenase (NADP+ dependent) 2 like [Source:HGNC Symbol;Acc:HGNC:31865] |
| ENSG00000166106 | HGLibA_00807 | 183 | 0.520604262 | ADAMTS15 | ADAM metallopeptidase with thrombospondin type 1 motif 15 [Source:HGNC Symbol;Acc:HGNC:16305] |
| ENSG00000167768 | HGLibA_25413 | 183 | 0.520604262 | KRT1 | keratin 1 [Source:HGNC Symbol;Acc:HGNC:6412] |
| ENSG00000168234 | HGLibA_52137 | 183 | 0.520604262 | TTC39C | tetratricopeptide repeat domain 39C [Source:HGNC Symbol;Acc:HGNC:26595] |
| ENSG00000168874 | HGLibA_03538 | 183 | 0.520604262 | ATOH8 | atonal bHLH transcription factor 8 [Source:HGNC Symbol;Acc:HGNC:24126] |
| ENSG00000169891 | HGLibA_40852 | 183 | 0.520604262 | REPS2 | RALBP1 associated Eps domain containing 2 [Source:HGNC Symbol;Acc:HGNC:9963] |
| ENSG00000171241 | HGLibA_44013 | 183 | 0.520604262 | SHCBP1 | SHC binding and spindle associated 1 [Source:HGNC Symbol;Acc:HGNC:29547] |
| ENSG00000171320 | HGLibA_15560 | 183 | 0.520604262 | ESCO2 | establishment of sister chromatid cohesion N-acetyltransferase 2 [Source:HGNC Symbol;Acc:HGNC:27230] |
| ENSG00000175229 | HGLibA_18502 | 183 | 0.520604262 | GAL3ST3 | galactose-3-O-sulfotransferase 3 [Source:HGNC Symbol;Acc:HGNC:24144] |
| ENSG00000175463 | HGLibA_48471 | 183 | 0.520604262 | TBC1D10C | TBC1 domain family member 10C [Source:HGNC Symbol;Acc:HGNC:24702] |
| ENSG00000175920 | HGLibA_13786 | 183 | 0.520604262 | DOK7 | docking protein 7 [Source:HGNC Symbol;Acc:HGNC:26594] |
| ENSG00000176783 | HGLibA_42437 | 183 | 0.520604262 | RUFY1 | RUN and FYVE domain containing 1 [Source:HGNC Symbol;Acc:HGNC:19760] |
| ENSG00000177694 | HGLibA_31015 | 183 | 0.520604262 | NAALADL2 | N-acetylated alpha-linked acidic dipeptidase like 2 [Source:HGNC Symbol;Acc:HGNC:23219] |
| ENSG00000177842 | HGLibA_56404 | 183 | 0.520604262 | ZNF620 | zinc finger protein 620 [Source:HGNC Symbol;Acc:HGNC:28742] |
| ENSG00000178381 | HGLibA_55146 | 183 | 0.520604262 | ZFAND2A | zinc finger AN1-type containing 2A [Source:HGNC Symbol;Acc:HGNC:28073] |
| ENSG00000179583 | HGLibA_09706 | 183 | 0.520604262 | CIITA | class II major histocompatibility complex transactivator [Source:HGNC Symbol;Acc:HGNC:7067] |
| ENSG00000180537 | HGLibA_41587 | 183 | 0.520604262 | RNF182 | ring finger protein 182 [Source:HGNC Symbol;Acc:HGNC:28522] |
| ENSG00000181518 | HGLibA_34514 | 183 | 0.520604262 | OR8D4 | olfactory receptor family 8 subfamily D member 4 [Source:HGNC Symbol;Acc:HGNC:14840] |
| ENSG00000182324 | HGLibA_24261 | 183 | 0.520604262 | KCNJ14 | potassium voltage-gated channel subfamily J member 14 [Source:HGNC Symbol;Acc:HGNC:6260] |
| ENSG00000183255 | HGLibA_39565 | 183 | 0.520604262 | PTTG1IP | PTTG1 interacting protein [Source:HGNC Symbol;Acc:HGNC:13524] |
| ENSG00000183576 | HGLibA_43646 | 183 | 0.520604262 | SETD3 | SET domain containing 3 [Source:HGNC Symbol;Acc:HGNC:20493] |
| ENSG00000186462 | HGLibA_31115 | 183 | 0.520604262 | NAP1L2 | nucleosome assembly protein 1 like 2 [Source:HGNC Symbol;Acc:HGNC:7638] |
| ENSG00000189139 | HGLibA_18065 | 183 | 0.520604262 | FSCB | fibrous sheath CABYR binding protein [Source:HGNC Symbol;Acc:HGNC:20494] |
| ENSG00000196152 | HGLibA_56762 | 183 | 0.520604262 | ZNF79 | zinc finger protein 79 [Source:HGNC Symbol;Acc:HGNC:13153] |
| ENSG00000196406 | HGLibA_46393 | 183 | 0.520604262 | SPANXD | SPANX family member D [Source:HGNC Symbol;Acc:HGNC:14332] |
| ENSG00000198399 | HGLibA_23875 | 183 | 0.520604262 | ITSN2 | intersectin 2 [Source:HGNC Symbol;Acc:HGNC:6184] |
| ENSG00000198821 | HGLibA_08310 | 183 | 0.520604262 | CD247 | CD247 molecule [Source:HGNC Symbol;Acc:HGNC:1677] |
| ENSG00000203867 | HGLibA_40507 | 183 | 0.520604262 | RBM20 | RNA binding motif protein 20 [Source:HGNC Symbol;Acc:HGNC:27424] |
| ENSG00000204007 | HGLibA_19333 | 183 | 0.520604262 | GLT6D1 | glycosyltransferase 6 domain containing 1 [Source:HGNC Symbol;Acc:HGNC:23671] |
| ENSG00000205572 | HGLibA_43472 | 183 | 0.520604262 | SERF1B | small EDRK-rich factor 1B [Source:HGNC Symbol;Acc:HGNC:10756] |
| ENSG00000206561 | HGLibA_10647 | 183 | 0.520604262 | COLQ | collagen like tail subunit of asymmetric acetylcholinesterase [Source:HGNC Symbol;Acc:HGNC:2226] |
| ENSG00000213402 | HGLibA_39493 | 183 | 0.520604262 | PTPRCAP | receptor type C associated protein [Source:HGNC Symbol;Acc:HGNC:9667]" |
| ENSG00000215455 | HGLibA_25591 | 183 | 0.520604262 | KRTAP10-1 | keratin associated protein 10-1 [Source:HGNC Symbol;Acc:HGNC:22966] |
| ENSG00000221926 | HGLibA_51417 | 183 | 0.520604262 | TRIM16 | tripartite motif containing 16 [Source:HGNC Symbol;Acc:HGNC:17241] |
| ENSG00000221978 | HGLibA_08121 | 183 | 0.520604262 | CCNL2 | cyclin L2 [Source:HGNC Symbol;Acc:HGNC:20570] |
| ENSG00000254709 | HGLibA_22869 | 183 | 0.520604262 | IGLL5 | immunoglobulin lambda like polypeptide 5 [Source:HGNC Symbol;Acc:HGNC:38476] |
| ENSG00000262246 | HGLibA_10792 | 183 | 0.520604262 | CORO7 | coronin 7 [Source:HGNC Symbol;Acc:HGNC:26161] |
| ENSG00000275722 | HGLibA_27795 | 183 | 0.520604262 | LYZL6 | lysozyme like 6 [Source:HGNC Symbol;Acc:HGNC:29614] |
| FAM203A | HGLibA_16416 | 183 | 0.520604262 | FAM203A | - |
| HIST1H2BM | HGLibA_21448 | 183 | 0.520604262 | HIST1H2BM | - |
| HIST3H3 | HGLibA_21561 | 183 | 0.520604262 | HIST3H3 | - |
| hsa-mir-10b | HGLibA_57200 | 183 | 0.520604262 | hsa-mir-10b | - |
| hsa-mir-153-2 | HGLibA_57912 | 183 | 0.520604262 | hsa-mir-153-2 | - |
| hsa-mir-3671 | HGLibA_59242 | 183 | 0.520604262 | hsa-mir-3671 | - |
| hsa-mir-3686 | HGLibA_59299 | 183 | 0.520604262 | hsa-mir-3686 | - |
| hsa-mir-3940 | HGLibA_59621 | 183 | 0.520604262 | hsa-mir-3940 | - |
| hsa-mir-6132 | HGLibA_62680 | 183 | 0.520604262 | hsa-mir-6132 | - |
| hsa-mir-6499 | HGLibA_62842 | 183 | 0.520604262 | hsa-mir-6499 | - |
| hsa-mir-6503 | HGLibA_62862 | 183 | 0.520604262 | hsa-mir-6503 | - |
| hsa-mir-7705 | HGLibA_63955 | 183 | 0.520604262 | hsa-mir-7705 | - |
| hsa-mir-892a | HGLibA_64249 | 183 | 0.520604262 | hsa-mir-892a | - |
| LOC283403 | HGLibA_26910 | 183 | 0.520604262 | LOC283403 | - |
| TCEB3CL2 | HGLibA_48752 | 183 | 0.520604262 | TCEB3CL2 | - |
| BET3L | HGLibA_04438 | 182 | 0.511558919 | BET3L | - |
| C14orf37 | HGLibA_05364 | 182 | 0.511558919 | C14orf37 | - |
| C15orf38 | HGLibA_05388 | 182 | 0.511558919 | C15orf38 | - |
| ENSG00000005469 | HGLibA_11214 | 182 | 0.511558919 | CROT | carnitine O-octanoyltransferase [Source:HGNC Symbol;Acc:HGNC:2366] |
| ENSG00000006025 | HGLibA_34664 | 182 | 0.511558919 | OSBPL7 | oxysterol binding protein like 7 [Source:HGNC Symbol;Acc:HGNC:16387] |
| ENSG00000007080 | HGLibA_07575 | 182 | 0.511558919 | CCDC124 | coiled-coil domain containing 124 [Source:HGNC Symbol;Acc:HGNC:25171] |

| | | | | | |
|---|---|---|---|---|---|
| ENSG00000008282 | HGLibA_48035 | 182 | 0.511558919 | SYPL1 | synaptophysin like 1 [Source:HGNC Symbol;Acc:HGNC:11507] |
| ENSG00000021852 | HGLibA_06595 | 182 | 0.511558919 | C8B | complement C8 beta chain [Source:HGNC Symbol;Acc:HGNC:1353] |
| ENSG00000047936 | HGLibA_41784 | 182 | 0.511558919 | ROS1 | receptor tyrosine kinase [Source:HGNC Symbol;Acc:HGNC:10261]" |
| ENSG00000049540 | HGLibA_14951 | 182 | 0.511558919 | ELN | elastin [Source:HGNC Symbol;Acc:HGNC:3327] |
| ENSG00000051523 | HGLibA_12015 | 182 | 0.511558919 | CYBA | cytochrome b-245 alpha chain [Source:HGNC Symbol;Acc:HGNC:2577] |
| ENSG00000053328 | HGLibA_28995 | 182 | 0.511558919 | METTL24 | methyltransferase like 24 [Source:HGNC Symbol;Acc:HGNC:21566] |
| ENSG00000064933 | HGLibA_37332 | 182 | 0.511558919 | PMS1 | mismatch repair system component [Source:HGNC Symbol;Acc:HGNC:9121]" |
| ENSG00000064999 | HGLibA_02149 | 182 | 0.511558919 | ANKS1A | ankyrin repeat and sterile alpha motif domain containing 1A [Source:HGNC Symbol;Acc:HGNC:20961] |
| ENSG00000065457 | HGLibA_00878 | 182 | 0.511558919 | ADAT1 | tRNA specific 1 [Source:HGNC Symbol;Acc:HGNC:228]" |
| ENSG00000069812 | HGLibA_21180 | 182 | 0.511558919 | HES2 | hes family bHLH transcription factor 2 [Source:HGNC Symbol;Acc:HGNC:16005] |
| ENSG00000073737 | HGLibA_13153 | 182 | 0.511558919 | DHRS9 | dehydrogenase/reductase 9 [Source:HGNC Symbol;Acc:HGNC:16888] |
| ENSG00000084628 | HGLibA_31989 | 182 | 0.511558919 | NKAIN1 | sodium/potassium transporting ATPase interacting 1 [Source:HGNC Symbol;Acc:HGNC:25743] |
| ENSG00000088833 | HGLibA_32753 | 182 | 0.511558919 | NSFL1C | NSFL1 cofactor [Source:HGNC Symbol;Acc:HGNC:15912] |
| ENSG00000101638 | HGLibA_47301 | 182 | 0.511558919 | ST8SIA5 | 8-sialyltransferase 5 [Source:HGNC Symbol;Acc:HGNC:17827]" |
| ENSG00000103381 | HGLibA_10986 | 182 | 0.511558919 | CPPED1 | calcineurin like phosphoesterase domain containing 1 [Source:HGNC Symbol;Acc:HGNC:25632] |
| ENSG00000105989 | HGLibA_54358 | 182 | 0.511558919 | WNT2 | Wnt family member 2 [Source:HGNC Symbol;Acc:HGNC:12780] |
| ENSG00000106038 | HGLibA_15706 | 182 | 0.511558919 | EVX1 | even-skipped homeobox 1 [Source:HGNC Symbol;Acc:HGNC:3506] |
| ENSG00000106244 | HGLibA_35709 | 182 | 0.511558919 | PDAP1 | PDGFA associated protein 1 [Source:HGNC Symbol;Acc:HGNC:14634] |
| ENSG00000106459 | HGLibA_32673 | 182 | 0.511558919 | NRF1 | nuclear respiratory factor 1 [Source:HGNC Symbol;Acc:HGNC:7996] |
| ENSG00000115211 | HGLibA_14743 | 182 | 0.511558919 | EIF2B4 | eukaryotic translation initiation factor 2B subunit delta [Source:HGNC Symbol;Acc:HGNC:3260] |
| ENSG00000118564 | HGLibA_17011 | 182 | 0.511558919 | FBXL5 | F-box and leucine rich repeat protein 5 [Source:HGNC Symbol;Acc:HGNC:13602] |
| ENSG00000124203 | HGLibA_56837 | 182 | 0.511558919 | ZNF831 | zinc finger protein 831 [Source:HGNC Symbol;Acc:HGNC:16167] |
| ENSG00000125347 | HGLibA_23572 | 182 | 0.511558919 | IRF1 | interferon regulatory factor 1 [Source:HGNC Symbol;Acc:HGNC:6116] |
| ENSG00000126953 | HGLibA_49523 | 182 | 0.511558919 | TIMM8A | translocase of inner mitochondrial membrane 8A [Source:HGNC Symbol;Acc:HGNC:11817] |
| ENSG00000130733 | HGLibA_54674 | 182 | 0.511558919 | YIPF2 | Yip1 domain family member 2 [Source:HGNC Symbol;Acc:HGNC:28476] |
| ENSG00000134775 | HGLibA_17496 | 182 | 0.511558919 | FHOD3 | formin homology 2 domain containing 3 [Source:HGNC Symbol;Acc:HGNC:26178] |
| ENSG00000136487 | HGLibA_19031 | 182 | 0.511558919 | GH2 | growth hormone 2 [Source:HGNC Symbol;Acc:HGNC:4262] |
| ENSG00000136936 | HGLibA_54546 | 182 | 0.511558919 | XPA | DNA damage recognition and repair factor [Source:HGNC Symbol;Acc:HGNC:12814]" |
| ENSG00000137878 | HGLibA_18824 | 182 | 0.511558919 | GCOM1 | GRINL1A complex locus 1 [Source:HGNC Symbol;Acc:HGNC:26424] |
| ENSG00000138279 | HGLibA_02249 | 182 | 0.511558919 | ANXA7 | annexin A7 [Source:HGNC Symbol;Acc:HGNC:545] |
| ENSG00000140941 | HGLibA_28123 | 182 | 0.511558919 | MAP1LC3B | microtubule associated protein 1 light chain 3 beta [Source:HGNC Symbol;Acc:HGNC:13352] |
| ENSG00000142892 | HGLibA_36562 | 182 | 0.511558919 | PIGK | phosphatidylinositol glycan anchor biosynthesis class K [Source:HGNC Symbol;Acc:HGNC:8965] |
| ENSG00000143257 | HGLibA_32593 | 182 | 0.511558919 | NR1I3 | nuclear receptor subfamily 1 group I member 3 [Source:HGNC Symbol;Acc:HGNC:7969] |
| ENSG00000144063 | HGLibA_28027 | 182 | 0.511558919 | MALL | T cell differentiation protein like [Source:HGNC Symbol;Acc:HGNC:6818]" |
| ENSG00000145287 | HGLibA_36941 | 182 | 0.511558919 | PLAC8 | placenta specific 8 [Source:HGNC Symbol;Acc:HGNC:19254] |
| ENSG00000150594 | HGLibA_01045 | 182 | 0.511558919 | ADRA2A | adrenoceptor alpha 2A [Source:HGNC Symbol;Acc:HGNC:281] |
| ENSG00000154240 | HGLibA_09097 | 182 | 0.511558919 | CEP112 | centrosomal protein 112 [Source:HGNC Symbol;Acc:HGNC:28514] |
| ENSG00000154277 | HGLibA_52801 | 182 | 0.511558919 | UCHL1 | ubiquitin C-terminal hydrolase L1 [Source:HGNC Symbol;Acc:HGNC:12513] |
| ENSG00000155980 | HGLibA_24949 | 182 | 0.511558919 | KIF5A | kinesin family member 5A [Source:HGNC Symbol;Acc:HGNC:6323] |
| ENSG00000156299 | HGLibA_49439 | 182 | 0.511558919 | TIAM1 | T cell lymphoma invasion and metastasis 1 [Source:HGNC Symbol;Acc:HGNC:11805] |
| ENSG00000163694 | HGLibA_40567 | 182 | 0.511558919 | RBM47 | RNA binding motif protein 47 [Source:HGNC Symbol;Acc:HGNC:30358] |
| ENSG00000163993 | HGLibA_42583 | 182 | 0.511558919 | S100P | S100 calcium binding protein P [Source:HGNC Symbol;Acc:HGNC:10504] |
| ENSG00000164631 | HGLibA_55451 | 182 | 0.511558919 | ZNF12 | zinc finger protein 12 [Source:HGNC Symbol;Acc:HGNC:12902] |
| ENSG00000165417 | HGLibA_20521 | 182 | 0.511558919 | GTF2A1 | general transcription factor IIA subunit 1 [Source:HGNC Symbol;Acc:HGNC:4646] |
| ENSG00000165794 | HGLibA_45105 | 182 | 0.511558919 | SLC39A2 | solute carrier family 39 member 2 [Source:HGNC Symbol;Acc:HGNC:17127] |
| ENSG00000166265 | HGLibA_12254 | 182 | 0.511558919 | CYYR1 | cysteine and tyrosine rich 1 [Source:HGNC Symbol;Acc:HGNC:16274] |
| ENSG00000166321 | HGLibA_32942 | 182 | 0.511558919 | NUDT13 | nudix hydrolase 13 [Source:HGNC Symbol;Acc:HGNC:18827] |
| ENSG00000166669 | HGLibA_03458 | 182 | 0.511558919 | ATF7IP2 | activating transcription factor 7 interacting protein 2 [Source:HGNC Symbol;Acc:HGNC:20397] |
| ENSG00000167355 | HGLibA_34087 | 182 | 0.511558919 | OR51B5 | olfactory receptor family 51 subfamily B member 5 [Source:HGNC Symbol;Acc:HGNC:19599] |
| ENSG00000167604 | HGLibA_31827 | 182 | 0.511558919 | NFKBID | NFKB inhibitor delta [Source:HGNC Symbol;Acc:HGNC:15671] |
| ENSG00000167910 | HGLibA_12211 | 182 | 0.511558919 | CYP7A1 | cytochrome P450 family 7 subfamily A member 1 [Source:HGNC Symbol;Acc:HGNC:2651] |
| ENSG00000170613 | HGLibA_16689 | 182 | 0.511558919 | FAM71B | family with sequence similarity 71 member B [Source:HGNC Symbol;Acc:HGNC:28397] |
| ENSG00000172244 | HGLibA_06376 | 182 | 0.511558919 | C5orf34 | chromosome 5 open reading frame 34 [Source:HGNC Symbol;Acc:HGNC:24738] |
| ENSG00000172748 | HGLibA_56351 | 182 | 0.511558919 | ZNF596 | zinc finger protein 596 [Source:HGNC Symbol;Acc:HGNC:27268] |
| ENSG00000175745 | HGLibA_32611 | 182 | 0.511558919 | NR2F1 | nuclear receptor subfamily 2 group F member 1 [Source:HGNC Symbol;Acc:HGNC:7975] |
| ENSG00000176771 | HGLibA_31317 | 182 | 0.511558919 | NCKAP5 | NCK associated protein 5 [Source:HGNC Symbol;Acc:HGNC:29847] |
| ENSG00000177938 | HGLibA_07210 | 182 | 0.511558919 | CAPZA3 | capping actin protein of muscle Z-line subunit alpha 3 [Source:HGNC Symbol;Acc:HGNC:24205] |
| ENSG00000179051 | HGLibA_40686 | 182 | 0.511558919 | RCC2 | regulator of chromosome condensation 2 [Source:HGNC Symbol;Acc:HGNC:30297] |
| ENSG00000179817 | HGLibA_29855 | 182 | 0.511558919 | MRGPRX4 | MAS related GPR family member X4 [Source:HGNC Symbol;Acc:HGNC:17617] |
| ENSG00000180432 | HGLibA_12216 | 182 | 0.511558919 | CYP8B1 | cytochrome P450 family 8 subfamily B member 1 [Source:HGNC Symbol;Acc:HGNC:2653] |
| ENSG00000182348 | HGLibA_56791 | 182 | 0.511558919 | ZNF804B | zinc finger protein 804B [Source:HGNC Symbol;Acc:HGNC:21958] |
| ENSG00000184345 | HGLibA_23506 | 182 | 0.511558919 | IQCF2 | IQ motif containing F2 [Source:HGNC Symbol;Acc:HGNC:31815] |
| ENSG00000185594 | HGLibA_46532 | 182 | 0.511558919 | SPATA8 | spermatogenesis associated 8 [Source:HGNC Symbol;Acc:HGNC:28676] |
| ENSG00000185760 | HGLibA_24377 | 182 | 0.511558919 | KCNQ5 | potassium voltage-gated channel subfamily Q member 5 [Source:HGNC Symbol;Acc:HGNC:6299] |
| ENSG00000186231 | HGLibA_25185 | 182 | 0.511558919 | KLHL32 | kelch like family member 32 [Source:HGNC Symbol;Acc:HGNC:21221] |
| ENSG00000186409 | HGLibA_07754 | 182 | 0.511558919 | CCDC30 | coiled-coil domain containing 30 [Source:HGNC Symbol;Acc:HGNC:26103] |
| ENSG00000186517 | HGLibA_02752 | 182 | 0.511558919 | ARHGAP30 | Rho GTPase activating protein 30 [Source:HGNC Symbol;Acc:HGNC:27414] |
| ENSG00000186532 | HGLibA_45861 | 182 | 0.511558919 | SMYD4 | SET and MYND domain containing 4 [Source:HGNC Symbol;Acc:HGNC:21067] |
| ENSG00000187961 | HGLibA_25141 | 182 | 0.511558919 | KLHL17 | kelch like family member 17 [Source:HGNC Symbol;Acc:HGNC:24023] |
| ENSG00000188295 | HGLibA_56496 | 182 | 0.511558919 | ZNF669 | zinc finger protein 669 [Source:HGNC Symbol;Acc:HGNC:25736] |
| ENSG00000188707 | HGLibA_54806 | 182 | 0.511558919 | ZBED6CL | ZBED6 C-terminal like [Source:HGNC Symbol;Acc:HGNC:21720] |
| ENSG00000188778 | HGLibA_01061 | 182 | 0.511558919 | ADRB3 | adrenoceptor beta 3 [Source:HGNC Symbol;Acc:HGNC:288] |
| ENSG00000197265 | HGLibA_20536 | 182 | 0.511558919 | GTF2E2 | general transcription factor IIE subunit 2 [Source:HGNC Symbol;Acc:HGNC:4651] |
| ENSG00000197769 | HGLibA_28129 | 182 | 0.511558919 | MAP1LC3C | microtubule associated protein 1 light chain 3 gamma [Source:HGNC Symbol;Acc:HGNC:13353] |
| ENSG00000198892 | HGLibA_44039 | 182 | 0.511558919 | SHISA4 | shisa family member 4 [Source:HGNC Symbol;Acc:HGNC:27139] |
| ENSG00000204632 | HGLibA_21648 | 182 | 0.511558919 | HLA-G | G [Source:HGNC Symbol;Acc:HGNC:4964]" |
| ENSG00000205090 | HGLibA_50284 | 182 | 0.511558919 | TMEM240 | transmembrane protein 240 [Source:HGNC Symbol;Acc:HGNC:25186] |
| ENSG00000212128 | HGLibA_48370 | 182 | 0.511558919 | TAS2R13 | taste 2 receptor member 13 [Source:HGNC Symbol;Acc:HGNC:14919] |
| ENSG00000213047 | HGLibA_12933 | 182 | 0.511558919 | DENND1B | DENN domain containing 1B [Source:HGNC Symbol;Acc:HGNC:28404] |
| ENSG00000213462 | HGLibA_15537 | 182 | 0.511558919 | ERV3-1 | envelope [Source:HGNC Symbol;Acc:HGNC:3454]" |
| ENSG00000213934 | HGLibA_20925 | 182 | 0.511558919 | HBG1 | hemoglobin subunit gamma 1 [Source:HGNC Symbol;Acc:HGNC:4831] |
| ENSG00000221821 | HGLibA_06483 | 182 | 0.511558919 | C6orf226 | chromosome 6 open reading frame 226 [Source:HGNC Symbol;Acc:HGNC:34431] |
| ENSG00000226761 | HGLibA_48413 | 182 | 0.511558919 | TAS2R46 | taste 2 receptor member 46 [Source:HGNC Symbol;Acc:HGNC:18877] |
| ENSG00000230246 | HGLibA_46490 | 182 | 0.511558919 | SPATA31C1 | SPATA31 subfamily C member 1 [Source:HGNC Symbol;Acc:HGNC:27846] |
| ENSG00000239264 | HGLibA_52422 | 182 | 0.511558919 | TXNDC5 | thioredoxin domain containing 5 [Source:HGNC Symbol;Acc:HGNC:21073] |
| ENSG00000255181 | HGLibA_07674 | 182 | 0.511558919 | CCDC166 | coiled-coil domain containing 166 [Source:HGNC Symbol;Acc:HGNC:41910] |
| ENSG00000278889 | HGLibA_33849 | 182 | 0.511558919 | OR2S2 | olfactory receptor family 2 subfamily S member 2 (gene/pseudogene) [Source:HGNC Symbol;Acc:HGNC:8276] |
| GAREM | HGLibA_18626 | 182 | 0.511558919 | GAREM | - |
| GTF2H2D | HGLibA_20554 | 182 | 0.511558919 | GTF2H2D | - |
| hsa-mir-1289-2 | HGLibA_57624 | 182 | 0.511558919 | hsa-mir-1289-2 | - |
| hsa-mir-195 | HGLibA_58089 | 182 | 0.511558919 | hsa-mir-195 | - |
| hsa-mir-3936 | HGLibA_59603 | 182 | 0.511558919 | hsa-mir-3936 | - |
| hsa-mir-4294 | HGLibA_59888 | 182 | 0.511558919 | hsa-mir-4294 | - |
| hsa-mir-4488 | HGLibA_60370 | 182 | 0.511558919 | hsa-mir-4488 | - |
| hsa-mir-4735 | HGLibA_61036 | 182 | 0.511558919 | hsa-mir-4735 | - |
| hsa-mir-668 | HGLibA_63007 | 182 | 0.511558919 | hsa-mir-668 | - |
| OR9G9 | HGLibA_34574 | 182 | 0.511558919 | OR9G9 | - |
| C1orf111 | HGLibA_05746 | 181 | 0.502513576 | C1orf111 | - |
| ENSG00000011332 | HGLibA_13826 | 181 | 0.502513576 | DPF1 | double PHD fingers 1 [Source:HGNC Symbol;Acc:HGNC:20225] |
| ENSG00000011405 | HGLibA_36620 | 181 | 0.502513576 | PIK3C2A | phosphatidylinositol-4-phosphate 3-kinase catalytic subunit type 2 alpha [Source:HGNC Symbol;Acc:HGNC:8971] |
| ENSG00000049192 | HGLibA_00837 | 181 | 0.502513576 | ADAMTS6 | ADAM metallopeptidase with thrombospondin type 1 motif 6 [Source:HGNC Symbol;Acc:HGNC:222] |
| ENSG00000049768 | HGLibA_17936 | 181 | 0.502513576 | FOXP3 | forkhead box P3 [Source:HGNC Symbol;Acc:HGNC:6106] |
| ENSG00000059728 | HGLibA_30609 | 181 | 0.502513576 | MXD1 | MAX dimerization protein 1 [Source:HGNC Symbol;Acc:HGNC:6761] |
| ENSG00000060762 | HGLibA_29701 | 181 | 0.502513576 | MPC1 | mitochondrial pyruvate carrier 1 [Source:HGNC Symbol;Acc:HGNC:21606] |
| ENSG00000065268 | HGLibA_54041 | 181 | 0.502513576 | WDR18 | WD repeat domain 18 [Source:HGNC Symbol;Acc:HGNC:17956] |
| ENSG00000068001 | HGLibA_22439 | 181 | 0.502513576 | HYAL2 | hyaluronidase 2 [Source:HGNC Symbol;Acc:HGNC:5321] |
| ENSG00000083799 | HGLibA_12049 | 181 | 0.502513576 | CYLD | CYLD lysine 63 deubiquitinase [Source:HGNC Symbol;Acc:HGNC:2584] |
| ENSG00000084207 | HGLibA_20493 | 181 | 0.502513576 | GSTP1 | glutathione S-transferase pi 1 [Source:HGNC Symbol;Acc:HGNC:4638] |
| ENSG00000099814 | HGLibA_09119 | 181 | 0.502513576 | CEP170B | centrosomal protein 170B [Source:HGNC Symbol;Acc:HGNC:20362] |
| ENSG00000100206 | HGLibA_13399 | 181 | 0.502513576 | DMC1 | DNA meiotic recombinase 1 [Source:HGNC Symbol;Acc:HGNC:2927] |
| ENSG00000101343 | HGLibA_11203 | 181 | 0.502513576 | CRNKL1 | crooked neck pre-mRNA splicing factor 1 [Source:HGNC Symbol;Acc:HGNC:15762] |
| ENSG00000102230 | HGLibA_35703 | 181 | 0.502513576 | PCYT1B | beta [Source:HGNC Symbol;Acc:HGNC:8755]" |
| ENSG00000103089 | HGLibA_15899 | 181 | 0.502513576 | FA2H | fatty acid 2-hydroxylase [Source:HGNC Symbol;Acc:HGNC:21197] |
| ENSG00000103160 | HGLibA_22214 | 181 | 0.502513576 | HSDL1 | hydroxysteroid dehydrogenase like 1 [Source:HGNC Symbol;Acc:HGNC:16475] |
| ENSG00000106397 | HGLibA_37214 | 181 | 0.502513576 | PLOD3 | 2-oxoglutarate 5-dioxygenase 3 [Source:HGNC Symbol;Acc:HGNC:9083]" |
| ENSG00000107789 | HGLibA_29281 | 181 | 0.502513576 | MINPP1 | multiple inositol-polyphosphate phosphatase 1 [Source:HGNC Symbol;Acc:HGNC:7102] |
| ENSG00000107902 | HGLibA_26444 | 181 | 0.502513576 | LHPP | phospholysine phosphohistidine inorganic pyrophosphate phosphatase [Source:HGNC Symbol;Acc:HGNC:30042] |
| ENSG00000112799 | HGLibA_27687 | 181 | 0.502513576 | LY86 | lymphocyte antigen 86 [Source:HGNC Symbol;Acc:HGNC:16837] |
| ENSG00000114019 | HGLibA_01820 | 181 | 0.502513576 | AMOTL2 | angiomotin like 2 [Source:HGNC Symbol;Acc:HGNC:17812] |
| ENSG00000115486 | HGLibA_18994 | 181 | 0.502513576 | GGCX | gamma-glutamyl carboxylase [Source:HGNC Symbol;Acc:HGNC:4247] |
| ENSG00000116903 | HGLibA_15762 | 181 | 0.502513576 | EXOC8 | exocyst complex component 8 [Source:HGNC Symbol;Acc:HGNC:24659] |
| ENSG00000117650 | HGLibA_31646 | 181 | 0.502513576 | NEK2 | NIMA related kinase 2 [Source:HGNC Symbol;Acc:HGNC:7745] |
| ENSG00000119707 | HGLibA_40519 | 181 | 0.502513576 | RBM25 | RNA binding motif protein 25 [Source:HGNC Symbol;Acc:HGNC:23244] |
| ENSG00000120563 | HGLibA_27786 | 181 | 0.502513576 | LYZL1 | lysozyme like 1 [Source:HGNC Symbol;Acc:HGNC:30502] |
| ENSG00000122126 | HGLibA_33264 | 181 | 0.502513576 | OCRL | inositol polyphosphate-5-phosphatase [Source:HGNC Symbol;Acc:HGNC:8108]" |
| ENSG00000124222 | HGLibA_47625 | 181 | 0.502513576 | STX16 | syntaxin 16 [Source:HGNC Symbol;Acc:HGNC:11431] |
| ENSG00000124786 | HGLibA_44964 | 181 | 0.502513576 | SLC35B3 | solute carrier family 35 member B3 [Source:HGNC Symbol;Acc:HGNC:21601] |
| ENSG00000126562 | HGLibA_54340 | 181 | 0.502513576 | WNK4 | WNK lysine deficient protein kinase 4 [Source:HGNC Symbol;Acc:HGNC:14544] |
| ENSG00000128463 | HGLibA_15014 | 181 | 0.502513576 | EMC4 | ER membrane protein complex subunit 4 [Source:HGNC Symbol;Acc:HGNC:28032] |
| ENSG00000133124 | HGLibA_23625 | 181 | 0.502513576 | IRS4 | insulin receptor substrate 4 [Source:HGNC Symbol;Acc:HGNC:6128] |
| ENSG00000133265 | HGLibA_22327 | 181 | 0.502513576 | HSPBP1 | HSPA (Hsp70) binding protein 1 [Source:HGNC Symbol;Acc:HGNC:24989] |
| ENSG00000133627 | HGLibA_00663 | 181 | 0.502513576 | ACTR3B | ARP3 actin related protein 3 homolog B [Source:HGNC Symbol;Acc:HGNC:17256] |
| ENSG00000134333 | HGLibA_26221 | 181 | 0.502513576 | LDHA | lactate dehydrogenase A [Source:HGNC Symbol;Acc:HGNC:6535] |
| ENSG00000134365 | HGLibA_09271 | 181 | 0.502513576 | CFHR4 | complement factor H related 4 [Source:HGNC Symbol;Acc:HGNC:16979] |
| ENSG00000135063 | HGLibA_16354 | 181 | 0.502513576 | FAM189A2 | family with sequence similarity 189 member A2 [Source:HGNC Symbol;Acc:HGNC:24820] |

| | | | | | |
|---|---|---|---|---|---|
| ENSG00000135631 | HGLibA_39805 | 181 | 0.502513576 | RAB11FIP5 | RAB11 family interacting protein 5 [Source:HGNC Symbol;Acc:HGNC:24845] |
| ENSG00000136100 | HGLibA_53772 | 181 | 0.502513576 | VPS36 | vacuolar protein sorting 36 homolog [Source:HGNC Symbol;Acc:HGNC:20312] |
| ENSG00000137393 | HGLibA_41529 | 181 | 0.502513576 | RNF144B | ring finger protein 144B [Source:HGNC Symbol;Acc:HGNC:21578] |
| ENSG00000138439 | HGLibA_16046 | 181 | 0.502513576 | FAM117B | family with sequence similarity 117 member B [Source:HGNC Symbol;Acc:HGNC:14440] |
| ENSG00000141741 | HGLibA_29250 | 181 | 0.502513576 | MIEN1 | migration and invasion enhancer 1 [Source:HGNC Symbol;Acc:HGNC:28230] |
| ENSG00000142156 | HGLibA_10595 | 181 | 0.502513576 | COL6A1 | collagen type VI alpha 1 chain [Source:HGNC Symbol;Acc:HGNC:2211] |
| ENSG00000145309 | HGLibA_06864 | 181 | 0.502513576 | CABS1 | spermatid associated 1 [Source:HGNC Symbol;Acc:HGNC:30710]" |
| ENSG00000146192 | HGLibA_17346 | 181 | 0.502513576 | FGD2 | RhoGEF and PH domain containing 2 [Source:HGNC Symbol;Acc:HGNC:3664]" |
| ENSG00000147164 | HGLibA_46033 | 181 | 0.502513576 | SNX12 | sorting nexin 12 [Source:HGNC Symbol;Acc:HGNC:14976] |
| ENSG00000149948 | HGLibA_21686 | 181 | 0.502513576 | HMGA2 | high mobility group AT-hook 2 [Source:HGNC Symbol;Acc:HGNC:5009] |
| ENSG00000150750 | HGLibA_05155 | 181 | 0.502513576 | C11orf53 | chromosome 11 open reading frame 53 [Source:HGNC Symbol;Acc:HGNC:30527] |
| ENSG00000155304 | HGLibA_22264 | 181 | 0.502513576 | HSPA13 | heat shock protein family A (Hsp70) member 13 [Source:HGNC Symbol;Acc:HGNC:11375] |
| ENSG00000156873 | HGLibA_36415 | 181 | 0.502513576 | PHKG2 | phosphorylase kinase catalytic subunit gamma 2 [Source:HGNC Symbol;Acc:HGNC:8931] |
| ENSG00000157456 | HGLibA_08064 | 181 | 0.502513576 | CCNB2 | cyclin B2 [Source:HGNC Symbol;Acc:HGNC:1580] |
| ENSG00000158050 | HGLibA_14158 | 181 | 0.502513576 | DUSP2 | dual specificity phosphatase 2 [Source:HGNC Symbol;Acc:HGNC:3068] |
| ENSG00000158062 | HGLibA_52780 | 181 | 0.502513576 | UBXN11 | UBX domain protein 11 [Source:HGNC Symbol;Acc:HGNC:30600] |
| ENSG00000159348 | HGLibA_12001 | 181 | 0.502513576 | CYB5R1 | cytochrome b5 reductase 1 [Source:HGNC Symbol;Acc:HGNC:13397] |
| ENSG00000159723 | HGLibA_01253 | 181 | 0.502513576 | AGRP | agouti related neuropeptide [Source:HGNC Symbol;Acc:HGNC:330] |
| ENSG00000162383 | HGLibA_44525 | 181 | 0.502513576 | SLC1A7 | solute carrier family 1 member 7 [Source:HGNC Symbol;Acc:HGNC:10945] |
| ENSG00000165312 | HGLibA_34759 | 181 | 0.502513576 | OTUD1 | OTU deubiquitinase 1 [Source:HGNC Symbol;Acc:HGNC:27346] |
| ENSG00000165805 | HGLibA_05260 | 181 | 0.502513576 | C12orf50 | chromosome 12 open reading frame 50 [Source:HGNC Symbol;Acc:HGNC:26665] |
| ENSG00000166435 | HGLibA_54609 | 181 | 0.502513576 | XRRA1 | X-ray radiation resistance associated 1 [Source:HGNC Symbol;Acc:HGNC:18868] |
| ENSG00000166676 | HGLibA_52358 | 181 | 0.502513576 | TVP23A | trans-golgi network vesicle protein 23 homolog A [Source:HGNC Symbol;Acc:HGNC:20398] |
| ENSG00000166912 | HGLibA_30412 | 181 | 0.502513576 | MTMR10 | myotubularin related protein 10 [Source:HGNC Symbol;Acc:HGNC:25999] |
| ENSG00000167588 | HGLibA_19784 | 181 | 0.502513576 | GPD1 | glycerol-3-phosphate dehydrogenase 1 [Source:HGNC Symbol;Acc:HGNC:4455] |
| ENSG00000167995 | HGLibA_04420 | 181 | 0.502513576 | BEST1 | bestrophin 1 [Source:HGNC Symbol;Acc:HGNC:12703] |
| ENSG00000168026 | HGLibA_52069 | 181 | 0.502513576 | TTC21A | tetratricopeptide repeat domain 21A [Source:HGNC Symbol;Acc:HGNC:30761] |
| ENSG00000169016 | HGLibA_14322 | 181 | 0.502513576 | E2F6 | E2F transcription factor 6 [Source:HGNC Symbol;Acc:HGNC:3120] |
| ENSG00000169297 | HGLibA_32568 | 181 | 0.502513576 | NR0B1 | nuclear receptor subfamily 0 group B member 1 [Source:HGNC Symbol;Acc:HGNC:7960] |
| ENSG00000169925 | HGLibA_04777 | 181 | 0.502513576 | BRD3 | bromodomain containing 3 [Source:HGNC Symbol;Acc:HGNC:1104] |
| ENSG00000169946 | HGLibA_55256 | 181 | 0.502513576 | ZFPM2 | FOG family member 2 [Source:HGNC Symbol;Acc:HGNC:16700]" |
| ENSG00000170085 | HGLibA_44181 | 181 | 0.502513576 | SIMC1 | SUMO interacting motifs containing 1 [Source:HGNC Symbol;Acc:HGNC:24779] |
| ENSG00000170396 | HGLibA_56788 | 181 | 0.502513576 | ZNF804A | zinc finger protein 804A [Source:HGNC Symbol;Acc:HGNC:21711] |
| ENSG00000171634 | HGLibA_04741 | 181 | 0.502513576 | BPTF | bromodomain PHD finger transcription factor [Source:HGNC Symbol;Acc:HGNC:3581] |
| ENSG00000172199 | HGLibA_34557 | 181 | 0.502513576 | OR8U1 | olfactory receptor family 8 subfamily U member 1 [Source:HGNC Symbol;Acc:HGNC:19611] |
| ENSG00000172927 | HGLibA_30691 | 181 | 0.502513576 | MYEOV | myeloma overexpressed [Source:HGNC Symbol;Acc:HGNC:7563] |
| ENSG00000175274 | HGLibA_51061 | 181 | 0.502513576 | TP53I11 | tumor protein p53 inducible protein 11 [Source:HGNC Symbol;Acc:HGNC:16842] |
| ENSG00000175518 | HGLibA_52745 | 181 | 0.502513576 | UBQLNL | ubiquilin like [Source:HGNC Symbol;Acc:HGNC:28294] |
| ENSG00000179407 | HGLibA_13556 | 181 | 0.502513576 | DNAJB8 | DnaJ heat shock protein family (Hsp40) member B8 [Source:HGNC Symbol;Acc:HGNC:23699] |
| ENSG00000180953 | HGLibA_47231 | 181 | 0.502513576 | ST20 | suppressor of tumorigenicity 20 [Source:HGNC Symbol;Acc:HGNC:33520] |
| ENSG00000181396 | HGLibA_33315 | 181 | 0.502513576 | OGFOD3 | 2-oxoglutarate and iron dependent oxygenase domain containing 3 [Source:HGNC Symbol;Acc:HGNC:26174] |
| ENSG00000183150 | HGLibA_19992 | 181 | 0.502513576 | GPR19 | G protein-coupled receptor 19 [Source:HGNC Symbol;Acc:HGNC:4473] |
| ENSG00000183439 | HGLibA_51566 | 181 | 0.502513576 | TRIM61 | tripartite motif containing 61 [Source:HGNC Symbol;Acc:HGNC:24339] |
| ENSG00000184545 | HGLibA_14194 | 181 | 0.502513576 | DUSP8 | dual specificity phosphatase 8 [Source:HGNC Symbol;Acc:HGNC:3074] |
| ENSG00000187391 | HGLibA_27997 | 181 | 0.502513576 | MAGI2 | WW and PDZ domain containing 2 [Source:HGNC Symbol;Acc:HGNC:18957]" |
| ENSG00000196975 | HGLibA_02240 | 181 | 0.502513576 | ANXA4 | annexin A4 [Source:HGNC Symbol;Acc:HGNC:542] |
| ENSG00000197122 | HGLibA_46929 | 181 | 0.502513576 | SRC | non-receptor tyrosine kinase [Source:HGNC Symbol;Acc:HGNC:11283]" |
| ENSG00000198538 | HGLibA_55719 | 181 | 0.502513576 | ZNF28 | zinc finger protein 28 [Source:HGNC Symbol;Acc:HGNC:13073] |
| ENSG00000205085 | HGLibA_16704 | 181 | 0.502513576 | FAM71F2 | family with sequence similarity 71 member F2 [Source:HGNC Symbol;Acc:HGNC:27998] |
| ENSG00000213215 | HGLibA_33787 | 181 | 0.502513576 | OR2F1 | olfactory receptor family 2 subfamily F member 1 (gene/pseudogene) [Source:HGNC Symbol;Acc:HGNC:8246] |
| ENSG00000241106 | HGLibA_21612 | 181 | 0.502513576 | HLA-DOB | DO beta [Source:HGNC Symbol;Acc:HGNC:4937]" |
| ENSG00000273777 | HGLibA_08933 | 181 | 0.502513576 | CEACAM20 | carcinoembryonic antigen related cell adhesion molecule 20 [Source:HGNC Symbol;Acc:HGNC:24879] |
| ENSG00000274600 | HGLibA_41264 | 181 | 0.502513576 | RIMBP3B | RIMS binding protein 3B [Source:HGNC Symbol;Acc:HGNC:33891] |
| ENSG00000275183 | HGLibA_26304 | 181 | 0.502513576 | LENG9 | leukocyte receptor cluster member 9 [Source:HGNC Symbol;Acc:HGNC:16306] |
| ENSG00000275395 | HGLibA_17179 | 181 | 0.502513576 | FCGBP | Fc fragment of IgG binding protein [Source:HGNC Symbol;Acc:HGNC:13572] |
| ENSG00000275722 | HGLibA_27794 | 181 | 0.502513576 | LYZL6 | lysozyme like 6 [Source:HGNC Symbol;Acc:HGNC:29614] |
| FBXO18 | HGLibA_17038 | 181 | 0.502513576 | FBXO18 | - |
| HIST1H4E | HGLibA_21498 | 181 | 0.502513576 | HIST1H4E | - |
| hsa-mir-1226 | HGLibA_57302 | 181 | 0.502513576 | hsa-mir-1226 | - |
| hsa-mir-1251 | HGLibA_57402 | 181 | 0.502513576 | hsa-mir-1251 | - |
| hsa-mir-1282 | HGLibA_57583 | 181 | 0.502513576 | hsa-mir-1282 | - |
| hsa-mir-2114 | HGLibA_58229 | 181 | 0.502513576 | hsa-mir-2114 | - |
| hsa-mir-367 | HGLibA_59229 | 181 | 0.502513576 | hsa-mir-367 | - |
| hsa-mir-4418 | HGLibA_60055 | 181 | 0.502513576 | hsa-mir-4418 | - |
| hsa-mir-4801 | HGLibA_61308 | 181 | 0.502513576 | hsa-mir-4801 | - |
| hsa-mir-5011 | HGLibA_61460 | 181 | 0.502513576 | hsa-mir-5011 | - |
| hsa-mir-521-1 | HGLibA_61766 | 181 | 0.502513576 | hsa-mir-521-1 | - |
| hsa-mir-5687 | HGLibA_62265 | 181 | 0.502513576 | hsa-mir-5687 | - |
| hsa-mir-5699 | HGLibA_62328 | 181 | 0.502513576 | hsa-mir-5699 | - |
| hsa-mir-648 | HGLibA_62836 | 181 | 0.502513576 | hsa-mir-648 | - |
| hsa-mir-6870 | HGLibA_63665 | 181 | 0.502513576 | hsa-mir-6870 | - |
| hsa-mir-7843 | HGLibA_63961 | 181 | 0.502513576 | hsa-mir-7843 | - |
| hsa-mir-943 | HGLibA_64357 | 181 | 0.502513576 | hsa-mir-943 | - |
| LOC440335 | HGLibA_26944 | 181 | 0.502513576 | LOC440335 | - |
| PRSS42 | HGLibA_38940 | 181 | 0.502513576 | PRSS42 | - |
| USMG5 | HGLibA_53186 | 181 | 0.502513576 | USMG5 | - |
| ADPRHL2 | HGLibA_01031 | 180 | 0.493468233 | ADPRHL2 | - |
| C7orf73 | HGLibA_06589 | 180 | 0.493468233 | C7orf73 | - |
| CXorf36 | HGLibA_11918 | 180 | 0.493468233 | CXorf36 | - |
| ENSG00000013297 | HGLibA_09852 | 180 | 0.493468233 | CLDN11 | claudin 11 [Source:HGNC Symbol;Acc:HGNC:8514] |
| ENSG00000061656 | HGLibA_46358 | 180 | 0.493468233 | SPAG4 | sperm associated antigen 4 [Source:HGNC Symbol;Acc:HGNC:11214] |
| ENSG00000076242 | HGLibA_29381 | 180 | 0.493468233 | MLH1 | mutL homolog 1 [Source:HGNC Symbol;Acc:HGNC:7127] |
| ENSG00000077327 | HGLibA_46364 | 180 | 0.493468233 | SPAG6 | sperm associated antigen 6 [Source:HGNC Symbol;Acc:HGNC:11215] |
| ENSG00000100249 | HGLibA_06071 | 180 | 0.493468233 | C22orf31 | chromosome 22 open reading frame 31 [Source:HGNC Symbol;Acc:HGNC:26931] |
| ENSG00000100583 | HGLibA_42678 | 180 | 0.493468233 | SAMD15 | sterile alpha motif domain containing 15 [Source:HGNC Symbol;Acc:HGNC:18631] |
| ENSG00000101115 | HGLibA_42657 | 180 | 0.493468233 | SALL4 | spalt like transcription factor 4 [Source:HGNC Symbol;Acc:HGNC:15924] |
| ENSG00000101342 | HGLibA_49604 | 180 | 0.493468233 | TLDC2 | TBC/LysM-associated domain containing 2 [Source:HGNC Symbol;Acc:HGNC:16112] |
| ENSG00000102393 | HGLibA_19214 | 180 | 0.493468233 | GLA | galactosidase alpha [Source:HGNC Symbol;Acc:HGNC:4296] |
| ENSG00000103145 | HGLibA_20962 | 180 | 0.493468233 | HCFC1R1 | host cell factor C1 regulator 1 [Source:HGNC Symbol;Acc:HGNC:21198] |
| ENSG00000104231 | HGLibA_55144 | 180 | 0.493468233 | ZFAND1 | zinc finger AN1-type containing 1 [Source:HGNC Symbol;Acc:HGNC:25858] |
| ENSG00000104435 | HGLibA_47518 | 180 | 0.493468233 | STMN2 | stathmin 2 [Source:HGNC Symbol;Acc:HGNC:10577] |
| ENSG00000107821 | HGLibA_24087 | 180 | 0.493468233 | KAZALD1 | Kazal type serine peptidase inhibitor domain 1 [Source:HGNC Symbol;Acc:HGNC:25460] |
| ENSG00000109736 | HGLibA_29085 | 180 | 0.493468233 | MFSD10 | major facilitator superfamily domain containing 10 [Source:HGNC Symbol;Acc:HGNC:16894] |
| ENSG00000110756 | HGLibA_22056 | 180 | 0.493468233 | HPS5 | biogenesis of lysosomal organelles complex 2 subunit 2 [Source:HGNC Symbol;Acc:HGNC:17022]" |
| ENSG00000112530 | HGLibA_34949 | 180 | 0.493468233 | PACRG | parkin coregulated [Source:HGNC Symbol;Acc:HGNC:19152] |
| ENSG00000113088 | HGLibA_20728 | 180 | 0.493468233 | GZMK | granzyme K [Source:HGNC Symbol;Acc:HGNC:4711] |
| ENSG00000113302 | HGLibA_22961 | 180 | 0.493468233 | IL12B | interleukin 12B [Source:HGNC Symbol;Acc:HGNC:5970] |
| ENSG00000115216 | HGLibA_32654 | 180 | 0.493468233 | NRBP1 | nuclear receptor binding protein 1 [Source:HGNC Symbol;Acc:HGNC:7993] |
| ENSG00000115361 | HGLibA_00365 | 180 | 0.493468233 | ACADL | acyl-CoA dehydrogenase long chain [Source:HGNC Symbol;Acc:HGNC:88] |
| ENSG00000120594 | HGLibA_37254 | 180 | 0.493468233 | PLXDC2 | plexin domain containing 2 [Source:HGNC Symbol;Acc:HGNC:21013] |
| ENSG00000120669 | HGLibA_46165 | 180 | 0.493468233 | SOHLH2 | spermatogenesis and oogenesis specific basic helix-loop-helix 2 [Source:HGNC Symbol;Acc:HGNC:26026] |
| ENSG00000122033 | HGLibA_30400 | 180 | 0.493468233 | MTIF3 | mitochondrial translational initiation factor 3 [Source:HGNC Symbol;Acc:HGNC:29788] |
| ENSG00000122512 | HGLibA_37335 | 180 | 0.493468233 | PMS2 | mismatch repair system component [Source:HGNC Symbol;Acc:HGNC:9122]" |
| ENSG00000122728 | HGLibA_48224 | 180 | 0.493468233 | TAF1L | TATA-box binding protein associated factor 1 like [Source:HGNC Symbol;Acc:HGNC:18056] |
| ENSG00000127084 | HGLibA_17348 | 180 | 0.493468233 | FGD3 | RhoGEF and PH domain containing 3 [Source:HGNC Symbol;Acc:HGNC:16027]" |
| ENSG00000129988 | HGLibA_26080 | 180 | 0.493468233 | LBP | lipopolysaccharide binding protein [Source:HGNC Symbol;Acc:HGNC:6517] |
| ENSG00000131050 | HGLibA_04713 | 180 | 0.493468233 | BPIFA2 | BPI fold containing family A member 2 [Source:HGNC Symbol;Acc:HGNC:16203] |
| ENSG00000131055 | HGLibA_10833 | 180 | 0.493468233 | COX4I2 | cytochrome c oxidase subunit 4I2 [Source:HGNC Symbol;Acc:HGNC:16232] |
| ENSG00000131508 | HGLibA_52585 | 180 | 0.493468233 | UBE2D2 | ubiquitin conjugating enzyme E2 D2 [Source:HGNC Symbol;Acc:HGNC:12475] |
| ENSG00000134007 | HGLibA_00752 | 180 | 0.493468233 | ADAM20 | ADAM metallopeptidase domain 20 [Source:HGNC Symbol;Acc:HGNC:199] |
| ENSG00000134470 | HGLibA_22982 | 180 | 0.493468233 | IL15RA | interleukin 15 receptor subunit alpha [Source:HGNC Symbol;Acc:HGNC:5978] |
| ENSG00000135097 | HGLibA_30206 | 180 | 0.493468233 | MSI1 | musashi RNA binding protein 1 [Source:HGNC Symbol;Acc:HGNC:7330] |
| ENSG00000135437 | HGLibA_40749 | 180 | 0.493468233 | RDH5 | retinol dehydrogenase 5 [Source:HGNC Symbol;Acc:HGNC:9940] |
| ENSG00000135899 | HGLibA_46287 | 180 | 0.493468233 | SP110 | SP110 nuclear body protein [Source:HGNC Symbol;Acc:HGNC:5401] |
| ENSG00000138138 | HGLibA_03402 | 180 | 0.493468233 | ATAD1 | AAA domain containing 1 [Source:HGNC Symbol;Acc:HGNC:25903]" |
| ENSG00000139641 | HGLibA_15605 | 180 | 0.493468233 | ESYT1 | extended synaptotagmin 1 [Source:HGNC Symbol;Acc:HGNC:29534] |
| ENSG00000140093 | HGLibA_43509 | 180 | 0.493468233 | SERPINA10 | serpin family A member 10 [Source:HGNC Symbol;Acc:HGNC:15996] |
| ENSG00000140297 | HGLibA_18815 | 180 | 0.493468233 | GCNT3 | mucin type [Source:HGNC Symbol;Acc:HGNC:4205]" |
| ENSG00000142892 | HGLibA_36561 | 180 | 0.493468233 | PIGK | phosphatidylinositol glycan anchor biosynthesis class K [Source:HGNC Symbol;Acc:HGNC:8965] |
| ENSG00000143520 | HGLibA_17612 | 180 | 0.493468233 | FLG2 | filaggrin family member 2 [Source:HGNC Symbol;Acc:HGNC:33276] |
| ENSG00000146678 | HGLibA_22815 | 180 | 0.493468233 | IGFBP1 | insulin like growth factor binding protein 1 [Source:HGNC Symbol;Acc:HGNC:5469] |
| ENSG00000146830 | HGLibA_19064 | 180 | 0.493468233 | GIGYF1 | GRB10 interacting GYF protein 1 [Source:HGNC Symbol;Acc:HGNC:9126] |
| ENSG00000147180 | HGLibA_56615 | 180 | 0.493468233 | ZNF711 | zinc finger protein 711 [Source:HGNC Symbol;Acc:HGNC:13128] |
| ENSG00000151240 | HGLibA_13253 | 180 | 0.493468233 | DIP2C | disco interacting protein 2 homolog C [Source:HGNC Symbol;Acc:HGNC:29150] |
| ENSG00000152229 | HGLibA_39240 | 180 | 0.493468233 | PSTPIP2 | proline-serine-threonine phosphatase interacting protein 2 [Source:HGNC Symbol;Acc:HGNC:9581] |
| ENSG00000157045 | HGLibA_32836 | 180 | 0.493468233 | NTAN1 | N-terminal asparagine amidase [Source:HGNC Symbol;Acc:HGNC:29909] |
| ENSG00000158156 | HGLibA_54532 | 180 | 0.493468233 | XKR8 | XK related 8 [Source:HGNC Symbol;Acc:HGNC:25508] |
| ENSG00000158258 | HGLibA_10136 | 180 | 0.493468233 | CLSTN2 | calsyntenin 2 [Source:HGNC Symbol;Acc:HGNC:17448] |
| ENSG00000162814 | HGLibA_46435 | 180 | 0.493468233 | SPATA17 | spermatogenesis associated 17 [Source:HGNC Symbol;Acc:HGNC:25184] |
| ENSG00000163848 | HGLibA_55497 | 180 | 0.493468233 | ZNF148 | zinc finger protein 148 [Source:HGNC Symbol;Acc:HGNC:12933] |
| ENSG00000166884 | HGLibA_33979 | 180 | 0.493468233 | OR4D6 | olfactory receptor family 4 subfamily D member 6 [Source:HGNC Symbol;Acc:HGNC:15175] |
| ENSG00000166961 | HGLibA_30141 | 180 | 0.493468233 | MS4A15 | membrane spanning 4-domains A15 [Source:HGNC Symbol;Acc:HGNC:28573] |
| ENSG00000170054 | HGLibA_43532 | 180 | 0.493468233 | SERPINA9 | serpin family A member 9 [Source:HGNC Symbol;Acc:HGNC:15995] |
| ENSG00000170917 | HGLibA_32986 | 180 | 0.493468233 | NUDT6 | nudix hydrolase 6 [Source:HGNC Symbol;Acc:HGNC:8053] |
| ENSG00000171487 | HGLibA_32123 | 180 | 0.493468233 | NLRP5 | NLR family pyrin domain containing 5 [Source:HGNC Symbol;Acc:HGNC:21269] |

| | | | | | |
|---|---|---|---|---|---|
| ENSG00000171517 | HGLibA_27059 | 180 | 0.493468233 | LPAR3 | lysophosphatidic acid receptor 3 [Source:HGNC Symbol;Acc:HGNC:14298] |
| ENSG00000173621 | HGLibA_27160 | 180 | 0.493468233 | LRFN4 | leucine rich repeat and fibronectin type III domain containing 4 [Source:HGNC Symbol;Acc:HGNC:28456] |
| ENSG00000173699 | HGLibA_46465 | 180 | 0.493468233 | SPATA3 | spermatogenesis associated 3 [Source:HGNC Symbol;Acc:HGNC:17884] |
| ENSG00000173826 | HGLibA_24224 | 180 | 0.493468233 | KCNH6 | potassium voltage-gated channel subfamily H member 6 [Source:HGNC Symbol;Acc:HGNC:18862] |
| ENSG00000174326 | HGLibA_44424 | 180 | 0.493468233 | SLC16A11 | solute carrier family 16 member 11 [Source:HGNC Symbol;Acc:HGNC:23093] |
| ENSG00000174738 | HGLibA_32578 | 180 | 0.493468233 | NR1D2 | nuclear receptor subfamily 1 group D member 2 [Source:HGNC Symbol;Acc:HGNC:7963] |
| ENSG00000175548 | HGLibA_01607 | 180 | 0.493468233 | ALG10B | 2-glucosyltransferase [Source:HGNC Symbol;Acc:HGNC:31088]" |
| ENSG00000176101 | HGLibA_47145 | 180 | 0.493468233 | SSNA1 | SS nuclear autoantigen 1 [Source:HGNC Symbol;Acc:HGNC:11321] |
| ENSG00000180424 | HGLibA_12873 | 180 | 0.493468233 | DEFB123 | defensin beta 123 [Source:HGNC Symbol;Acc:HGNC:18103] |
| ENSG00000182393 | HGLibA_22719 | 180 | 0.493468233 | IFNL1 | interferon lambda 1 [Source:HGNC Symbol;Acc:HGNC:18363] |
| ENSG00000183318 | HGLibA_46594 | 180 | 0.493468233 | SPDYE4 | speedy/RINGO cell cycle regulator family member E4 [Source:HGNC Symbol;Acc:HGNC:35463] |
| ENSG00000184530 | HGLibA_06501 | 180 | 0.493468233 | C6orf58 | chromosome 6 open reading frame 58 [Source:HGNC Symbol;Acc:HGNC:20960] |
| ENSG00000185198 | HGLibA_38969 | 180 | 0.493468233 | PRSS57 | serine protease 57 [Source:HGNC Symbol;Acc:HGNC:31397] |
| ENSG00000186094 | HGLibA_01175 | 180 | 0.493468233 | AGBL4 | ATP/GTP binding protein like 4 [Source:HGNC Symbol;Acc:HGNC:25892] |
| ENSG00000187017 | HGLibA_15576 | 180 | 0.493468233 | ESPN | espin [Source:HGNC Symbol;Acc:HGNC:13281] |
| ENSG00000188483 | HGLibA_22583 | 180 | 0.493468233 | IER5L | immediate early response 5 like [Source:HGNC Symbol;Acc:HGNC:23679] |
| ENSG00000188707 | HGLibA_54804 | 180 | 0.493468233 | ZBED6CL | ZBED6 C-terminal like [Source:HGNC Symbol;Acc:HGNC:21720] |
| ENSG00000196712 | HGLibA_31757 | 180 | 0.493468233 | NF1 | neurofibromin 1 [Source:HGNC Symbol;Acc:HGNC:7765] |
| ENSG00000197766 | HGLibA_09255 | 180 | 0.493468233 | CFD | complement factor D [Source:HGNC Symbol;Acc:HGNC:2771] |
| ENSG00000197841 | HGLibA_55541 | 180 | 0.493468233 | ZNF181 | zinc finger protein 181 [Source:HGNC Symbol;Acc:HGNC:12971] |
| ENSG00000197905 | HGLibA_48934 | 180 | 0.493468233 | TEAD4 | TEA domain transcription factor 4 [Source:HGNC Symbol;Acc:HGNC:11717] |
| ENSG00000204583 | HGLibA_27150 | 180 | 0.493468233 | LRCOL1 | leucine rich colipase like 1 [Source:HGNC Symbol;Acc:HGNC:44160] |
| ENSG00000221963 | HGLibA_02543 | 180 | 0.493468233 | APOL6 | apolipoprotein L6 [Source:HGNC Symbol;Acc:HGNC:14870] |
| ENSG00000229183 | HGLibA_36188 | 180 | 0.493468233 | PGA4 | group I (pepsinogen A) [Source:HGNC Symbol;Acc:HGNC:8886]" |
| ENSG00000229474 | HGLibA_35285 | 180 | 0.493468233 | PATL2 | PAT1 homolog 2 [Source:HGNC Symbol;Acc:HGNC:33630] |
| ENSG00000259494 | HGLibA_29997 | 180 | 0.493468233 | MRPL46 | mitochondrial ribosomal protein L46 [Source:HGNC Symbol;Acc:HGNC:1192] |
| ENSG00000275410 | HGLibA_21776 | 180 | 0.493468233 | HNF1B | HNF1 homeobox B [Source:HGNC Symbol;Acc:HGNC:11630] |
| hsa-let-7f-1 | HGLibA_57125 | 180 | 0.493468233 | hsa-let-7f-1 | - |
| hsa-mir-1203 | HGLibA_57269 | 180 | 0.493468233 | hsa-mir-1203 | - |
| hsa-mir-3689c | HGLibA_59321 | 180 | 0.493468233 | hsa-mir-3689c | - |
| hsa-mir-4274 | HGLibA_59801 | 180 | 0.493468233 | hsa-mir-4274 | - |
| hsa-mir-4284 | HGLibA_59843 | 180 | 0.493468233 | hsa-mir-4284 | - |
| hsa-mir-6732 | HGLibA_63080 | 180 | 0.493468233 | hsa-mir-6732 | - |
| hsa-mir-6771 | HGLibA_63259 | 180 | 0.493468233 | hsa-mir-6771 | - |
| hsa-mir-8068 | HGLibA_64112 | 180 | 0.493468233 | hsa-mir-8068 | - |
| C17orf62 | HGLibA_05555 | 179 | 0.48442289 | C17orf62 | - |
| CCL3L1 | HGLibA_08020 | 179 | 0.48442289 | CCL3L1 | - |
| ENSG00000006625 | HGLibA_18993 | 179 | 0.48442289 | GGCT | gamma-glutamylcyclotransferase [Source:HGNC Symbol;Acc:HGNC:21705] |
| ENSG00000011243 | HGLibA_01448 | 179 | 0.48442289 | AKAP8L | A-kinase anchoring protein 8 like [Source:HGNC Symbol;Acc:HGNC:29857] |
| ENSG00000056487 | HGLibA_36371 | 179 | 0.48442289 | PHF21B | PHD finger protein 21B [Source:HGNC Symbol;Acc:HGNC:25161] |
| ENSG00000061676 | HGLibA_31312 | 179 | 0.48442289 | NCKAP1 | NCK associated protein 1 [Source:HGNC Symbol;Acc:HGNC:7666] |
| ENSG00000074211 | HGLibA_38153 | 179 | 0.48442289 | PPP2R2C | protein phosphatase 2 regulatory subunit Bgamma [Source:HGNC Symbol;Acc:HGNC:9306] |
| ENSG00000075539 | HGLibA_18055 | 179 | 0.48442289 | FRYL | FRY like transcription coactivator [Source:HGNC Symbol;Acc:HGNC:29127] |
| ENSG00000078403 | HGLibA_29412 | 179 | 0.48442289 | MLLT10 | histone lysine methyltransferase DOT1L cofactor [Source:HGNC Symbol;Acc:HGNC:16063]" |
| ENSG00000085721 | HGLibA_42259 | 179 | 0.48442289 | RRN3 | RNA polymerase I transcription factor [Source:HGNC Symbol;Acc:HGNC:30346]" |
| ENSG00000099860 | HGLibA_18418 | 179 | 0.48442289 | GADD45B | growth arrest and DNA damage inducible beta [Source:HGNC Symbol;Acc:HGNC:4096] |
| ENSG00000099899 | HGLibA_51684 | 179 | 0.48442289 | TRMT2A | tRNA methyltransferase 2 homolog A [Source:HGNC Symbol;Acc:HGNC:24974] |
| ENSG00000100314 | HGLibA_06859 | 179 | 0.48442289 | CABP7 | calcium binding protein 7 [Source:HGNC Symbol;Acc:HGNC:20834] |
| ENSG00000100362 | HGLibA_39613 | 179 | 0.48442289 | PVALB | parvalbumin [Source:HGNC Symbol;Acc:HGNC:9704] |
| ENSG00000100372 | HGLibA_44656 | 179 | 0.48442289 | SLC25A17 | solute carrier family 25 member 17 [Source:HGNC Symbol;Acc:HGNC:10987] |
| ENSG00000100897 | HGLibA_12416 | 179 | 0.48442289 | DCAF11 | DDB1 and CUL4 associated factor 11 [Source:HGNC Symbol;Acc:HGNC:20258] |
| ENSG00000104059 | HGLibA_16352 | 179 | 0.48442289 | FAM189A1 | family with sequence similarity 189 member A1 [Source:HGNC Symbol;Acc:HGNC:29075] |
| ENSG00000105879 | HGLibA_07402 | 179 | 0.48442289 | CBLL1 | Cbl proto-oncogene like 1 [Source:HGNC Symbol;Acc:HGNC:21225] |
| ENSG00000106367 | HGLibA_02297 | 179 | 0.48442289 | AP1S1 | adaptor related protein complex 1 subunit sigma 1 [Source:HGNC Symbol;Acc:HGNC:559] |
| ENSG00000110076 | HGLibA_32735 | 179 | 0.48442289 | NRXN2 | neurexin 2 [Source:HGNC Symbol;Acc:HGNC:8009] |
| ENSG00000110077 | HGLibA_30158 | 179 | 0.48442289 | MS4A6A | membrane spanning 4-domains A6A [Source:HGNC Symbol;Acc:HGNC:13375] |
| ENSG00000115966 | HGLibA_03435 | 179 | 0.48442289 | ATF2 | activating transcription factor 2 [Source:HGNC Symbol;Acc:HGNC:784] |
| ENSG00000117533 | HGLibA_53498 | 179 | 0.48442289 | VAMP4 | vesicle associated membrane protein 4 [Source:HGNC Symbol;Acc:HGNC:12645] |
| ENSG00000120440 | HGLibA_52199 | 179 | 0.48442289 | TTLL2 | tubulin tyrosine ligase like 2 [Source:HGNC Symbol;Acc:HGNC:21211] |
| ENSG00000120688 | HGLibA_53982 | 179 | 0.48442289 | WBP4 | WW domain binding protein 4 [Source:HGNC Symbol;Acc:HGNC:12739] |
| ENSG00000122711 | HGLibA_46695 | 179 | 0.48442289 | SPINK4 | Kazal type 4 [Source:HGNC Symbol;Acc:HGNC:16646]" |
| ENSG00000122873 | HGLibA_09727 | 179 | 0.48442289 | CISD1 | CDGSH iron sulfur domain 1 [Source:HGNC Symbol;Acc:HGNC:30880] |
| ENSG00000124493 | HGLibA_20349 | 179 | 0.48442289 | GRM4 | glutamate metabotropic receptor 4 [Source:HGNC Symbol;Acc:HGNC:4596] |
| ENSG00000125962 | HGLibA_03050 | 179 | 0.48442289 | ARMCX5 | armadillo repeat containing X-linked 5 [Source:HGNC Symbol;Acc:HGNC:25772] |
| ENSG00000127603 | HGLibA_27833 | 179 | 0.48442289 | MACF1 | microtubule-actin crosslinking factor 1 [Source:HGNC Symbol;Acc:HGNC:13664] |
| ENSG00000130584 | HGLibA_54897 | 179 | 0.48442289 | ZBTB46 | zinc finger and BTB domain containing 46 [Source:HGNC Symbol;Acc:HGNC:16094] |
| ENSG00000130764 | HGLibA_27370 | 179 | 0.48442289 | LRRC47 | leucine rich repeat containing 47 [Source:HGNC Symbol;Acc:HGNC:29207] |
| ENSG00000135473 | HGLibA_35092 | 179 | 0.48442289 | PAN2 | poly(A) specific ribonuclease subunit PAN2 [Source:HGNC Symbol;Acc:HGNC:20074] |
| ENSG00000135622 | HGLibA_43352 | 179 | 0.48442289 | SEMA4F | ssemaphorin 4F [Source:HGNC Symbol;Acc:HGNC:10734] |
| ENSG00000135845 | HGLibA_36548 | 179 | 0.48442289 | PIGC | phosphatidylinositol glycan anchor biosynthesis class C [Source:HGNC Symbol;Acc:HGNC:8960] |
| ENSG00000137218 | HGLibA_18049 | 179 | 0.48442289 | FRS3 | fibroblast growth factor receptor substrate 3 [Source:HGNC Symbol;Acc:HGNC:16970] |
| ENSG00000142937 | HGLibA_42191 | 179 | 0.48442289 | RPS8 | ribosomal protein S8 [Source:HGNC Symbol;Acc:HGNC:10441] |
| ENSG00000145217 | HGLibA_44780 | 179 | 0.48442289 | SLC26A1 | solute carrier family 26 member 1 [Source:HGNC Symbol;Acc:HGNC:10993] |
| ENSG00000147256 | HGLibA_02767 | 179 | 0.48442289 | ARHGAP36 | Rho GTPase activating protein 36 [Source:HGNC Symbol;Acc:HGNC:26388] |
| ENSG00000147485 | HGLibA_39652 | 179 | 0.48442289 | PXDNL | peroxidasin like [Source:HGNC Symbol;Acc:HGNC:26359] |
| ENSG00000149256 | HGLibA_49005 | 179 | 0.48442289 | TENM4 | teneurin transmembrane protein 4 [Source:HGNC Symbol;Acc:HGNC:29945] |
| ENSG00000149929 | HGLibA_21354 | 179 | 0.48442289 | HIRIP3 | HIRA interacting protein 3 [Source:HGNC Symbol;Acc:HGNC:4917] |
| ENSG00000150630 | HGLibA_53616 | 179 | 0.48442289 | VEGFC | vascular endothelial growth factor C [Source:HGNC Symbol;Acc:HGNC:12682] |
| ENSG00000150995 | HGLibA_23852 | 179 | 0.48442289 | ITPR1 | 5-trisphosphate receptor type 1 [Source:HGNC Symbol;Acc:HGNC:6180]" |
| ENSG00000154721 | HGLibA_23931 | 179 | 0.48442289 | JAM2 | junctional adhesion molecule 2 [Source:HGNC Symbol;Acc:HGNC:14686] |
| ENSG00000157077 | HGLibA_55294 | 179 | 0.48442289 | ZFYVE9 | zinc finger FYVE-type containing 9 [Source:HGNC Symbol;Acc:HGNC:6775] |
| ENSG00000157510 | HGLibA_01099 | 179 | 0.48442289 | AFAP1L1 | actin filament associated protein 1 like 1 [Source:HGNC Symbol;Acc:HGNC:26714] |
| ENSG00000158715 | HGLibA_45179 | 179 | 0.48442289 | SLC45A3 | solute carrier family 45 member 3 [Source:HGNC Symbol;Acc:HGNC:8642] |
| ENSG00000160781 | HGLibA_35166 | 179 | 0.48442289 | PAQR6 | progestin and adipoQ receptor family member 6 [Source:HGNC Symbol;Acc:HGNC:30132] |
| ENSG00000162409 | HGLibA_38493 | 179 | 0.48442289 | PRKAA2 | protein kinase AMP-activated catalytic subunit alpha 2 [Source:HGNC Symbol;Acc:HGNC:9377] |
| ENSG00000164253 | HGLibA_54089 | 179 | 0.48442289 | WDR41 | WD repeat domain 41 [Source:HGNC Symbol;Acc:HGNC:25601] |
| ENSG00000164713 | HGLibA_04804 | 179 | 0.48442289 | BRI3 | brain protein I3 [Source:HGNC Symbol;Acc:HGNC:1109] |
| ENSG00000164794 | HGLibA_24402 | 179 | 0.48442289 | KCNV1 | potassium voltage-gated channel modifier subfamily V member 1 [Source:HGNC Symbol;Acc:HGNC:18861] |
| ENSG00000164822 | HGLibA_12806 | 179 | 0.48442289 | DEFA6 | defensin alpha 6 [Source:HGNC Symbol;Acc:HGNC:2765] |
| ENSG00000165138 | HGLibA_02162 | 179 | 0.48442289 | ANKS6 | ankyrin repeat and sterile alpha motif domain containing 6 [Source:HGNC Symbol;Acc:HGNC:26724] |
| ENSG00000165512 | HGLibA_55605 | 179 | 0.48442289 | ZNF22 | zinc finger protein 22 [Source:HGNC Symbol;Acc:HGNC:13012] |
| ENSG00000165799 | HGLibA_41414 | 179 | 0.48442289 | RNASE7 | ribonuclease A family member 7 [Source:HGNC Symbol;Acc:HGNC:19278] |
| ENSG00000167202 | HGLibA_48526 | 179 | 0.48442289 | TBC1D2B | TBC1 domain family member 2B [Source:HGNC Symbol;Acc:HGNC:29183] |
| ENSG00000171060 | HGLibA_06643 | 179 | 0.48442289 | C8orf74 | chromosome 8 open reading frame 74 [Source:HGNC Symbol;Acc:HGNC:32296] |
| ENSG00000171587 | HGLibA_13997 | 179 | 0.48442289 | DSCAM | DS cell adhesion molecule [Source:HGNC Symbol;Acc:HGNC:3039] |
| ENSG00000172367 | HGLibA_35975 | 179 | 0.48442289 | PDZD3 | PDZ domain containing 3 [Source:HGNC Symbol;Acc:HGNC:19891] |
| ENSG00000172640 | HGLibA_33478 | 179 | 0.48442289 | OR10AD1 | olfactory receptor family 10 subfamily AD member 1 [Source:HGNC Symbol;Acc:HGNC:14819] |
| ENSG00000173762 | HGLibA_08434 | 179 | 0.48442289 | CD7 | CD7 molecule [Source:HGNC Symbol;Acc:HGNC:1695] |
| ENSG00000175220 | HGLibA_02698 | 179 | 0.48442289 | ARHGAP1 | Rho GTPase activating protein 1 [Source:HGNC Symbol;Acc:HGNC:673] |
| ENSG00000175727 | HGLibA_29442 | 179 | 0.48442289 | MLXIP | MLX interacting protein [Source:HGNC Symbol;Acc:HGNC:17055] |
| ENSG00000176293 | HGLibA_55472 | 179 | 0.48442289 | ZNF135 | zinc finger protein 135 [Source:HGNC Symbol;Acc:HGNC:12919] |
| ENSG00000176697 | HGLibA_04387 | 179 | 0.48442289 | BDNF | brain derived neurotrophic factor [Source:HGNC Symbol;Acc:HGNC:1033] |
| ENSG00000179776 | HGLibA_08682 | 179 | 0.48442289 | CDH5 | cadherin 5 [Source:HGNC Symbol;Acc:HGNC:1764] |
| ENSG00000181939 | HGLibA_33948 | 179 | 0.48442289 | OR4C15 | olfactory receptor family 4 subfamily C member 15 [Source:HGNC Symbol;Acc:HGNC:15171] |
| ENSG00000182261 | HGLibA_32101 | 179 | 0.48442289 | NLRP10 | NLR family pyrin domain containing 10 [Source:HGNC Symbol;Acc:HGNC:21464] |
| ENSG00000183423 | HGLibA_27192 | 179 | 0.48442289 | LRIT3 | Ig-like and transmembrane domains 3 [Source:HGNC Symbol;Acc:HGNC:24783]" |
| ENSG00000184677 | HGLibA_54880 | 179 | 0.48442289 | ZBTB40 | zinc finger and BTB domain containing 40 [Source:HGNC Symbol;Acc:HGNC:29045] |
| ENSG00000186017 | HGLibA_56260 | 179 | 0.48442289 | ZNF566 | zinc finger protein 566 [Source:HGNC Symbol;Acc:HGNC:25919] |
| ENSG00000187498 | HGLibA_10567 | 179 | 0.48442289 | COL4A1 | collagen type IV alpha 1 chain [Source:HGNC Symbol;Acc:HGNC:2202] |
| ENSG00000187821 | HGLibA_21120 | 179 | 0.48442289 | HELT | helt bHLH transcription factor [Source:HGNC Symbol;Acc:HGNC:33783] |
| ENSG00000188620 | HGLibA_21765 | 179 | 0.48442289 | HMX3 | H6 family homeobox 3 [Source:HGNC Symbol;Acc:HGNC:5019] |
| ENSG00000189090 | HGLibA_16559 | 179 | 0.48442289 | FAM25G | family with sequence similarity 25 member G [Source:HGNC Symbol;Acc:HGNC:23590] |
| ENSG00000197437 | HGLibA_33619 | 179 | 0.48442289 | OR13G1 | olfactory receptor family 13 subfamily G member 1 [Source:HGNC Symbol;Acc:HGNC:14999] |
| ENSG00000198354 | HGLibA_12425 | 179 | 0.48442289 | DCAF12L2 | DDB1 and CUL4 associated factor 12 like 2 [Source:HGNC Symbol;Acc:HGNC:32950] |
| ENSG00000204386 | HGLibA_31710 | 179 | 0.48442289 | NEU1 | neuraminidase 1 [Source:HGNC Symbol;Acc:HGNC:7758] |
| ENSG00000205758 | HGLibA_11298 | 179 | 0.48442289 | CRYZL1 | crystallin zeta like 1 [Source:HGNC Symbol;Acc:HGNC:2420] |
| ENSG00000213694 | HGLibA_42598 | 179 | 0.48442289 | S1PR3 | sphingosine-1-phosphate receptor 3 [Source:HGNC Symbol;Acc:HGNC:3167] |
| ENSG00000218336 | HGLibA_49002 | 179 | 0.48442289 | TENM3 | teneurin transmembrane protein 3 [Source:HGNC Symbol;Acc:HGNC:29944] |
| ENSG00000230178 | HGLibA_34003 | 179 | 0.48442289 | OR4F3 | olfactory receptor family 4 subfamily F member 3 [Source:HGNC Symbol;Acc:HGNC:8300] |
| ENSG00000244411 | HGLibA_25806 | 179 | 0.48442289 | KRTAP5-7 | keratin associated protein 5-7 [Source:HGNC Symbol;Acc:HGNC:23602] |
| ENSG00000253117 | HGLibA_33236 | 179 | 0.48442289 | OC90 | otoconin 90 [Source:HGNC Symbol;Acc:HGNC:8100] |
| ENSG00000255072 | HGLibA_36603 | 179 | 0.48442289 | PIGY | phosphatidylinositol glycan anchor biosynthesis class Y [Source:HGNC Symbol;Acc:HGNC:28213] |
| ENSG00000262628 | HGLibA_33656 | 179 | 0.48442289 | OR1D5 | olfactory receptor family 1 subfamily D member 5 [Source:HGNC Symbol;Acc:HGNC:8186] |
| ENSG00000272333 | HGLibA_25325 | 179 | 0.48442289 | KMT2B | lysine methyltransferase 2B [Source:HGNC Symbol;Acc:HGNC:15840] |
| FAM160A1 | HGLibA_16204 | 179 | 0.48442289 | FAM160A1 | - |
| FAM65B | HGLibA_16670 | 179 | 0.48442289 | FAM65B | - |
| FTH1P18 | HGLibA_18118 | 179 | 0.48442289 | FTH1P18 | - |
| hsa-mir-101-1 | HGLibA_57154 | 179 | 0.48442289 | hsa-mir-101-1 | - |
| hsa-mir-3680-1 | HGLibA_59271 | 179 | 0.48442289 | hsa-mir-3680-1 | - |
| hsa-mir-3689a | HGLibA_59314 | 179 | 0.48442289 | hsa-mir-3689a | - |
| hsa-mir-3907 | HGLibA_59491 | 179 | 0.48442289 | hsa-mir-3907 | - |
| hsa-mir-4297 | HGLibA_59901 | 179 | 0.48442289 | hsa-mir-4297 | - |
| hsa-mir-4330 | HGLibA_60048 | 179 | 0.48442289 | hsa-mir-4330 | - |
| hsa-mir-503 | HGLibA_61466 | 179 | 0.48442289 | hsa-mir-503 | - |
| hsa-mir-5196 | HGLibA_61702 | 179 | 0.48442289 | hsa-mir-5196 | - |

| | | | | | |
|---|---|---|---|---|---|
| hsa-mir-548ag-1 | HGLibA_61873 | 179 | 0.48442289 | hsa-mir-548ag-1 | - |
| hsa-mir-6515 | HGLibA_62933 | 179 | 0.48442289 | hsa-mir-6515 | - |
| ADC | HGLibA_00886 | 178 | 0.475377547 | ADC | - |
| ALPPL2 | HGLibA_01715 | 178 | 0.475377547 | ALPPL2 | - |
| APITD1 | HGLibA_02425 | 178 | 0.475377547 | APITD1 | - |
| ATP5G2 | HGLibA_03658 | 178 | 0.475377547 | ATP5G2 | - |
| ENSG00000007384 | HGLibA_41133 | 178 | 0.475377547 | RHBDF1 | rhomboid 5 homolog 1 [Source:HGNC Symbol;Acc:HGNC:20561] |
| ENSG00000007541 | HGLibA_36580 | 178 | 0.475377547 | PIGQ | phosphatidylinositol glycan anchor biosynthesis class Q [Source:HGNC Symbol;Acc:HGNC:14135] |
| ENSG00000042286 | HGLibA_01341 | 178 | 0.475377547 | AIFM2 | mitochondria associated 2 [Source:HGNC Symbol;Acc:HGNC:21411]" |
| ENSG00000051523 | HGLibA_12016 | 178 | 0.475377547 | CYBA | cytochrome b-245 alpha chain [Source:HGNC Symbol;Acc:HGNC:2577] |
| ENSG00000063244 | HGLibA_52497 | 178 | 0.475377547 | U2AF2 | U2 small nuclear RNA auxiliary factor 2 [Source:HGNC Symbol;Acc:HGNC:23156] |
| ENSG00000064961 | HGLibA_21682 | 178 | 0.475377547 | HMG20B | high mobility group 20B [Source:HGNC Symbol;Acc:HGNC:5002] |
| ENSG00000067066 | HGLibA_46284 | 178 | 0.475377547 | SP100 | SP100 nuclear antigen [Source:HGNC Symbol;Acc:HGNC:11206] |
| ENSG00000073792 | HGLibA_22803 | 178 | 0.475377547 | IGF2BP2 | insulin like growth factor 2 mRNA binding protein 2 [Source:HGNC Symbol;Acc:HGNC:28867] |
| ENSG00000080371 | HGLibA_39839 | 178 | 0.475377547 | RAB21 | member RAS oncogene family [Source:HGNC Symbol;Acc:HGNC:18263]" |
| ENSG00000095370 | HGLibA_43895 | 178 | 0.475377547 | SH2D3C | SH2 domain containing 3C [Source:HGNC Symbol;Acc:HGNC:16884] |
| ENSG00000100311 | HGLibA_35845 | 178 | 0.475377547 | PDGFB | platelet derived growth factor subunit B [Source:HGNC Symbol;Acc:HGNC:8800] |
| ENSG00000102312 | HGLibA_37709 | 178 | 0.475377547 | PORCN | porcupine O-acyltransferase [Source:HGNC Symbol;Acc:HGNC:17652] |
| ENSG00000103066 | HGLibA_36875 | 178 | 0.475377547 | PLA2G15 | phospholipase A2 group XV [Source:HGNC Symbol;Acc:HGNC:17163] |
| ENSG00000104825 | HGLibA_31825 | 178 | 0.475377547 | NFKBIB | NFKB inhibitor beta [Source:HGNC Symbol;Acc:HGNC:7798] |
| ENSG00000104946 | HGLibA_48488 | 178 | 0.475377547 | TBC1D17 | TBC1 domain family member 17 [Source:HGNC Symbol;Acc:HGNC:25699] |
| ENSG00000105409 | HGLibA_03588 | 178 | 0.475377547 | ATP1A3 | ATPase Na+/K+ transporting subunit alpha 3 [Source:HGNC Symbol;Acc:HGNC:801] |
| ENSG00000107140 | HGLibA_49027 | 178 | 0.475377547 | TESK1 | testis associated actin remodelling kinase 1 [Source:HGNC Symbol;Acc:HGNC:11731] |
| ENSG00000108433 | HGLibA_19687 | 178 | 0.475377547 | GOSR2 | golgi SNAP receptor complex member 2 [Source:HGNC Symbol;Acc:HGNC:4431] |
| ENSG00000108578 | HGLibA_04551 | 178 | 0.475377547 | BLMH | bleomycin hydrolase [Source:HGNC Symbol;Acc:HGNC:1059] |
| ENSG00000111361 | HGLibA_14734 | 178 | 0.475377547 | EIF2B1 | eukaryotic translation initiation factor 2B subunit alpha [Source:HGNC Symbol;Acc:HGNC:3257] |
| ENSG00000111554 | HGLibA_28694 | 178 | 0.475377547 | MDM1 | Mdm1 nuclear protein [Source:HGNC Symbol;Acc:HGNC:29917] |
| ENSG00000111907 | HGLibA_51114 | 178 | 0.475377547 | TPD52L1 | TPD52 like 1 [Source:HGNC Symbol;Acc:HGNC:12006] |
| ENSG00000112312 | HGLibA_19417 | 178 | 0.475377547 | GMNN | DNA replication inhibitor [Source:HGNC Symbol;Acc:HGNC:17493]" |
| ENSG00000113070 | HGLibA_20921 | 178 | 0.475377547 | HBEGF | heparin binding EGF like growth factor [Source:HGNC Symbol;Acc:HGNC:3059] |
| ENSG00000116014 | HGLibA_25011 | 178 | 0.475377547 | KISS1R | KISS1 receptor [Source:HGNC Symbol;Acc:HGNC:4510] |
| ENSG00000117228 | HGLibA_18728 | 178 | 0.475377547 | GBP1 | guanylate binding protein 1 [Source:HGNC Symbol;Acc:HGNC:4182] |
| ENSG00000117500 | HGLibA_49814 | 178 | 0.475377547 | TMED5 | transmembrane p24 trafficking protein 5 [Source:HGNC Symbol;Acc:HGNC:24251] |
| ENSG00000118017 | HGLibA_00020 | 178 | 0.475377547 | A4GNT | 4-N-acetylglucosaminyltransferase [Source:HGNC Symbol;Acc:HGNC:17968]" |
| ENSG00000118242 | HGLibA_29825 | 178 | 0.475377547 | MREG | melanoregulin [Source:HGNC Symbol;Acc:HGNC:25478] |
| ENSG00000119650 | HGLibA_22758 | 178 | 0.475377547 | IFT43 | intraflagellar transport 43 [Source:HGNC Symbol;Acc:HGNC:29669] |
| ENSG00000119714 | HGLibA_20076 | 178 | 0.475377547 | GPR68 | G protein-coupled receptor 68 [Source:HGNC Symbol;Acc:HGNC:4519] |
| ENSG00000120705 | HGLibA_15616 | 178 | 0.475377547 | ETF1 | eukaryotic translation termination factor 1 [Source:HGNC Symbol;Acc:HGNC:3477] |
| ENSG00000124102 | HGLibA_36490 | 178 | 0.475377547 | PI3 | peptidase inhibitor 3 [Source:HGNC Symbol;Acc:HGNC:8947] |
| ENSG00000125246 | HGLibA_10173 | 178 | 0.475377547 | CLYBL | citrate lyase beta like [Source:HGNC Symbol;Acc:HGNC:18355] |
| ENSG00000128829 | HGLibA_14731 | 178 | 0.475377547 | EIF2AK4 | eukaryotic translation initiation factor 2 alpha kinase 4 [Source:HGNC Symbol;Acc:HGNC:19687] |
| ENSG00000130313 | HGLibA_36247 | 178 | 0.475377547 | PGLS | 6-phosphogluconolactonase [Source:HGNC Symbol;Acc:HGNC:8903] |
| ENSG00000130487 | HGLibA_25107 | 178 | 0.475377547 | KLHDC7B | kelch domain containing 7B [Source:HGNC Symbol;Acc:HGNC:25145] |
| ENSG00000131389 | HGLibA_45334 | 178 | 0.475377547 | SLC6A6 | solute carrier family 6 member 6 [Source:HGNC Symbol;Acc:HGNC:11052] |
| ENSG00000133115 | HGLibA_47541 | 178 | 0.475377547 | STOML3 | stomatin like 3 [Source:HGNC Symbol;Acc:HGNC:19420] |
| ENSG00000136738 | HGLibA_47331 | 178 | 0.475377547 | STAM | signal transducing adaptor molecule [Source:HGNC Symbol;Acc:HGNC:11357] |
| ENSG00000136859 | HGLibA_01913 | 178 | 0.475377547 | ANGPTL2 | angiopoietin like 2 [Source:HGNC Symbol;Acc:HGNC:490] |
| ENSG00000137634 | HGLibA_33159 | 178 | 0.475377547 | NXPE4 | neurexophilin and PC-esterase domain family member 4 [Source:HGNC Symbol;Acc:HGNC:23117] |
| ENSG00000139197 | HGLibA_36117 | 178 | 0.475377547 | PEX5 | peroxisomal biogenesis factor 5 [Source:HGNC Symbol;Acc:HGNC:9719] |
| ENSG00000140488 | HGLibA_09019 | 178 | 0.475377547 | CELF6 | CUGBP Elav-like family member 6 [Source:HGNC Symbol;Acc:HGNC:14059] |
| ENSG00000140836 | HGLibA_55175 | 178 | 0.475377547 | ZFHX3 | zinc finger homeobox 3 [Source:HGNC Symbol;Acc:HGNC:777] |
| ENSG00000142347 | HGLibA_30835 | 178 | 0.475377547 | MYO1F | myosin IF [Source:HGNC Symbol;Acc:HGNC:7600] |
| ENSG00000142867 | HGLibA_04287 | 178 | 0.475377547 | BCL10 | B cell CLL/lymphoma 10 [Source:HGNC Symbol;Acc:HGNC:989] |
| ENSG00000143924 | HGLibA_15065 | 178 | 0.475377547 | EML4 | echinoderm microtubule associated protein like 4 [Source:HGNC Symbol;Acc:HGNC:1316] |
| ENSG00000144891 | HGLibA_01263 | 178 | 0.475377547 | AGTR1 | angiotensin II receptor type 1 [Source:HGNC Symbol;Acc:HGNC:336] |
| ENSG00000145244 | HGLibA_10769 | 178 | 0.475377547 | CORIN | serine peptidase [Source:HGNC Symbol;Acc:HGNC:19012]" |
| ENSG00000146540 | HGLibA_06550 | 178 | 0.475377547 | C7orf50 | chromosome 7 open reading frame 50 [Source:HGNC Symbol;Acc:HGNC:22421] |
| ENSG00000146674 | HGLibA_22822 | 178 | 0.475377547 | IGFBP3 | insulin like growth factor binding protein 3 [Source:HGNC Symbol;Acc:HGNC:5472] |
| ENSG00000148180 | HGLibA_20437 | 178 | 0.475377547 | GSN | gelsolin [Source:HGNC Symbol;Acc:HGNC:4620] |
| ENSG00000152467 | HGLibA_56984 | 178 | 0.475377547 | ZSCAN1 | zinc finger and SCAN domain containing 1 [Source:HGNC Symbol;Acc:HGNC:23712] |
| ENSG00000153207 | HGLibA_01282 | 178 | 0.475377547 | AHCTF1 | AT-hook containing transcription factor 1 [Source:HGNC Symbol;Acc:HGNC:24618] |
| ENSG00000154493 | HGLibA_05103 | 178 | 0.475377547 | C10orf90 | chromosome 10 open reading frame 90 [Source:HGNC Symbol;Acc:HGNC:26563] |
| ENSG00000154553 | HGLibA_35912 | 178 | 0.475377547 | PDLIM3 | PDZ and LIM domain 3 [Source:HGNC Symbol;Acc:HGNC:20767] |
| ENSG00000154783 | HGLibA_17354 | 178 | 0.475377547 | FGD5 | RhoGEF and PH domain containing 5 [Source:HGNC Symbol;Acc:HGNC:19117]" |
| ENSG00000156873 | HGLibA_36414 | 178 | 0.475377547 | PHKG2 | phosphorylase kinase catalytic subunit gamma 2 [Source:HGNC Symbol;Acc:HGNC:8931] |
| ENSG00000157152 | HGLibA_47957 | 178 | 0.475377547 | SYN2 | synapsin II [Source:HGNC Symbol;Acc:HGNC:11495] |
| ENSG00000157625 | HGLibA_48148 | 178 | 0.475377547 | TAB3 | TGF-beta activated kinase 1 (MAP3K7) binding protein 3 [Source:HGNC Symbol;Acc:HGNC:30681] |
| ENSG00000158402 | HGLibA_08534 | 178 | 0.475377547 | CDC25C | cell division cycle 25C [Source:HGNC Symbol;Acc:HGNC:1727] |
| ENSG00000159648 | HGLibA_49011 | 178 | 0.475377547 | TEPP | prostate and placenta expressed [Source:HGNC Symbol;Acc:HGNC:33745]" |
| ENSG00000161940 | HGLibA_04336 | 178 | 0.475377547 | BCL6B | B cell CLL/lymphoma 6B [Source:HGNC Symbol;Acc:HGNC:1002] |
| ENSG00000162571 | HGLibA_52190 | 178 | 0.475377547 | TTLL10 | tubulin tyrosine ligase like 10 [Source:HGNC Symbol;Acc:HGNC:26693] |
| ENSG00000163297 | HGLibA_02215 | 178 | 0.475377547 | ANTXR2 | ANTXR cell adhesion molecule 2 [Source:HGNC Symbol;Acc:HGNC:21732] |
| ENSG00000163682 | HGLibA_42006 | 178 | 0.475377547 | RPL9 | ribosomal protein L9 [Source:HGNC Symbol;Acc:HGNC:10369] |
| ENSG00000164190 | HGLibA_31969 | 178 | 0.475377547 | NIPBL | cohesin loading factor [Source:HGNC Symbol;Acc:HGNC:28862]" |
| ENSG00000164256 | HGLibA_38404 | 178 | 0.475377547 | PRDM9 | PR/SET domain 9 [Source:HGNC Symbol;Acc:HGNC:13994] |
| ENSG00000164342 | HGLibA_49648 | 178 | 0.475377547 | TLR3 | toll like receptor 3 [Source:HGNC Symbol;Acc:HGNC:11849] |
| ENSG00000166947 | HGLibA_15259 | 178 | 0.475377547 | EPB42 | erythrocyte membrane protein band 4.2 [Source:HGNC Symbol;Acc:HGNC:3381] |
| ENSG00000169618 | HGLibA_38670 | 178 | 0.475377547 | PROKR1 | prokineticin receptor 1 [Source:HGNC Symbol;Acc:HGNC:4524] |
| ENSG00000173253 | HGLibA_13421 | 178 | 0.475377547 | DMRT2 | doublesex and mab-3 related transcription factor 2 [Source:HGNC Symbol;Acc:HGNC:2935] |
| ENSG00000174516 | HGLibA_36040 | 178 | 0.475377547 | PELI3 | pellino E3 ubiquitin protein ligase family member 3 [Source:HGNC Symbol;Acc:HGNC:30010] |
| ENSG00000176472 | HGLibA_56291 | 178 | 0.475377547 | ZNF575 | zinc finger protein 575 [Source:HGNC Symbol;Acc:HGNC:27606] |
| ENSG00000178722 | HGLibA_06422 | 178 | 0.475377547 | C5orf64 | chromosome 5 open reading frame 64 [Source:HGNC Symbol;Acc:HGNC:26744] |
| ENSG00000181903 | HGLibA_33961 | 178 | 0.475377547 | OR4C6 | olfactory receptor family 4 subfamily C member 6 [Source:HGNC Symbol;Acc:HGNC:14743] |
| ENSG00000183621 | HGLibA_55994 | 178 | 0.475377547 | ZNF438 | zinc finger protein 438 [Source:HGNC Symbol;Acc:HGNC:21029] |
| ENSG00000184271 | HGLibA_37796 | 178 | 0.475377547 | POU6F1 | POU class 6 homeobox 1 [Source:HGNC Symbol;Acc:HGNC:9224] |
| ENSG00000184345 | HGLibA_23505 | 178 | 0.475377547 | IQCF2 | IQ motif containing F2 [Source:HGNC Symbol;Acc:HGNC:31815] |
| ENSG00000185758 | HGLibA_09888 | 178 | 0.475377547 | CLDN24 | claudin 24 [Source:HGNC Symbol;Acc:HGNC:37200] |
| ENSG00000185909 | HGLibA_25114 | 178 | 0.475377547 | KLHDC8B | kelch domain containing 8B [Source:HGNC Symbol;Acc:HGNC:28557] |
| ENSG00000186265 | HGLibA_04957 | 178 | 0.475377547 | BTLA | B and T lymphocyte associated [Source:HGNC Symbol;Acc:HGNC:21087] |
| ENSG00000186493 | HGLibA_06381 | 178 | 0.475377547 | C5orf38 | chromosome 5 open reading frame 38 [Source:HGNC Symbol;Acc:HGNC:24226] |
| ENSG00000186998 | HGLibA_15044 | 178 | 0.475377547 | EMID1 | EMI domain containing 1 [Source:HGNC Symbol;Acc:HGNC:18036] |
| ENSG00000187950 | HGLibA_34791 | 178 | 0.475377547 | OVCH1 | ovochymase 1 [Source:HGNC Symbol;Acc:HGNC:23080] |
| ENSG00000196526 | HGLibA_01096 | 178 | 0.475377547 | AFAP1 | actin filament associated protein 1 [Source:HGNC Symbol;Acc:HGNC:24017] |
| ENSG00000197561 | HGLibA_14876 | 178 | 0.475377547 | ELANE | neutrophil expressed [Source:HGNC Symbol;Acc:HGNC:3309]" |
| ENSG00000204248 | HGLibA_10503 | 178 | 0.475377547 | COL11A2 | collagen type XI alpha 2 chain [Source:HGNC Symbol;Acc:HGNC:2187] |
| ENSG00000204659 | HGLibA_07473 | 178 | 0.475377547 | CBY3 | chibby family member 3 [Source:HGNC Symbol;Acc:HGNC:33278] |
| ENSG00000215045 | HGLibA_20278 | 178 | 0.475377547 | GRID2IP | Grid2 interacting protein [Source:HGNC Symbol;Acc:HGNC:18464] |
| ENSG00000250486 | HGLibA_16494 | 178 | 0.475377547 | FAM218A | family with sequence similarity 218 member A [Source:HGNC Symbol;Acc:HGNC:26466] |
| ENSG00000256453 | HGLibA_13671 | 178 | 0.475377547 | DND1 | DND microRNA-mediated repression inhibitor 1 [Source:HGNC Symbol;Acc:HGNC:23799] |
| ENSG00000272414 | HGLibA_16620 | 178 | 0.475377547 | FAM47E-STBD1 | FAM47E-STBD1 readthrough [Source:HGNC Symbol;Acc:HGNC:44667] |
| ENSG00000276085 | HGLibA_08023 | 178 | 0.475377547 | CCL3L3 | C-C motif chemokine ligand 3 like 1 [Source:HGNC Symbol;Acc:HGNC:10628] |
| ENTHD2 | HGLibA_15195 | 178 | 0.475377547 | ENTHD2 | - |
| GSG2 | HGLibA_20424 | 178 | 0.475377547 | GSG2 | - |
| hsa-mir-1283-1 | HGLibA_57588 | 178 | 0.475377547 | hsa-mir-1283-1 | - |
| hsa-mir-3158-1 | HGLibA_58705 | 178 | 0.475377547 | hsa-mir-3158-1 | - |
| hsa-mir-4793 | HGLibA_61278 | 178 | 0.475377547 | hsa-mir-4793 | - |
| hsa-mir-5586 | HGLibA_62185 | 178 | 0.475377547 | hsa-mir-5586 | - |
| hsa-mir-608 | HGLibA_62578 | 178 | 0.475377547 | hsa-mir-608 | - |
| hsa-mir-634 | HGLibA_62775 | 178 | 0.475377547 | hsa-mir-634 | - |
| hsa-mir-7706 | HGLibA_63956 | 178 | 0.475377547 | hsa-mir-7706 | - |
| LHFP | HGLibA_26425 | 178 | 0.475377547 | LHFP | - |
| LOC100133267 | HGLibA_26809 | 178 | 0.475377547 | LOC100133267 | - |
| LPPR2 | HGLibA_27117 | 178 | 0.475377547 | LPPR2 | - |
| P2RY8_X | HGLibA_34893 | 178 | 0.475377547 | P2RY8_X | - |
| PROL1 | HGLibA_38676 | 178 | 0.475377547 | PROL1 | - |
| SMEK1 | HGLibA_45693 | 178 | 0.475377547 | SMEK1 | - |
| TMEM155 | HGLibA_50008 | 178 | 0.475377547 | TMEM155 | - |
| BAGE5 | HGLibA_04105 | 177 | 0.466332204 | BAGE5 | - |
| C11orf85 | HGLibA_05200 | 177 | 0.466332204 | C11orf85 | - |
| C16orf11 | HGLibA_05438 | 177 | 0.466332204 | C16orf11 | - |
| C16orf93 | HGLibA_05505 | 177 | 0.466332204 | C16orf93 | - |
| C18orf42 | HGLibA_05615 | 177 | 0.466332204 | C18orf42 | - |
| C7orf62 | HGLibA_06568 | 177 | 0.466332204 | C7orf62 | - |
| ENSG00000008441 | HGLibA_31811 | 177 | 0.466332204 | NFIX | nuclear factor I X [Source:HGNC Symbol;Acc:HGNC:7788] |
| ENSG00000026297 | HGLibA_41443 | 177 | 0.466332204 | RNASET2 | ribonuclease T2 [Source:HGNC Symbol;Acc:HGNC:21686] |
| ENSG00000051128 | HGLibA_21870 | 177 | 0.466332204 | HOMER3 | homer scaffold protein 3 [Source:HGNC Symbol;Acc:HGNC:17514] |
| ENSG00000057935 | HGLibA_30315 | 177 | 0.466332204 | MTA3 | metastasis associated 1 family member 3 [Source:HGNC Symbol;Acc:HGNC:23784] |
| ENSG00000064195 | HGLibA_13378 | 177 | 0.466332204 | DLX3 | distal-less homeobox 3 [Source:HGNC Symbol;Acc:HGNC:2916] |
| ENSG00000064989 | HGLibA_07007 | 177 | 0.466332204 | CALCRL | calcitonin receptor like receptor [Source:HGNC Symbol;Acc:HGNC:16709] |
| ENSG00000066923 | HGLibA_47327 | 177 | 0.466332204 | STAG3 | stromal antigen 3 [Source:HGNC Symbol;Acc:HGNC:11356] |
| ENSG00000080824 | HGLibA_22249 | 177 | 0.466332204 | HSP90AA1 | heat shock protein 90 alpha family class A member 1 [Source:HGNC Symbol;Acc:HGNC:5253] |
| ENSG00000084110 | HGLibA_20821 | 177 | 0.466332204 | HAL | histidine ammonia-lyase [Source:HGNC Symbol;Acc:HGNC:4806] |
| ENSG00000096060 | HGLibA_17578 | 177 | 0.466332204 | FKBP5 | FK506 binding protein 5 [Source:HGNC Symbol;Acc:HGNC:3721] |
| ENSG00000101342 | HGLibA_49603 | 177 | 0.466332204 | TLDC2 | TBC/LysM-associated domain containing 2 [Source:HGNC Symbol;Acc:HGNC:16112] |
| ENSG00000104549 | HGLibA_46913 | 177 | 0.466332204 | SQLE | squalene epoxidase [Source:HGNC Symbol;Acc:HGNC:11279] |

| | | | | | |
|---|---|---|---|---|---|
| ENSG00000105398 | HGLibA_47759 | 177 | 0.466332204 | SULT2A1 | sulfotransferase family 2A member 1 [Source:HGNC Symbol;Acc:HGNC:11458] |
| ENSG00000105852 | HGLibA_37684 | 177 | 0.466332204 | PON3 | paraoxonase 3 [Source:HGNC Symbol;Acc:HGNC:9206] |
| ENSG00000106772 | HGLibA_38988 | 177 | 0.466332204 | PRUNE2 | prune homolog 2 [Source:HGNC Symbol;Acc:HGNC:25209] |
| ENSG00000108001 | HGLibA_14354 | 177 | 0.466332204 | EBF3 | EBF transcription factor 3 [Source:HGNC Symbol;Acc:HGNC:19087] |
| ENSG00000108622 | HGLibA_22496 | 177 | 0.466332204 | ICAM2 | intercellular adhesion molecule 2 [Source:HGNC Symbol;Acc:HGNC:5345] |
| ENSG00000108799 | HGLibA_15831 | 177 | 0.466332204 | EZH1 | enhancer of zeste 1 polycomb repressive complex 2 subunit [Source:HGNC Symbol;Acc:HGNC:3526] |
| ENSG00000110025 | HGLibA_46042 | 177 | 0.466332204 | SNX15 | sorting nexin 15 [Source:HGNC Symbol;Acc:HGNC:14978] |
| ENSG00000111639 | HGLibA_30010 | 177 | 0.466332204 | MRPL51 | mitochondrial ribosomal protein L51 [Source:HGNC Symbol;Acc:HGNC:14044] |
| ENSG00000111728 | HGLibA_47287 | 177 | 0.466332204 | ST8SIA1 | 8-sialyltransferase 1 [Source:HGNC Symbol;Acc:HGNC:10869]" |
| ENSG00000111731 | HGLibA_06106 | 177 | 0.466332204 | C2CD5 | C2 calcium dependent domain containing 5 [Source:HGNC Symbol;Acc:HGNC:29062] |
| ENSG00000112343 | HGLibA_51479 | 177 | 0.466332204 | TRIM38 | tripartite motif containing 38 [Source:HGNC Symbol;Acc:HGNC:10059] |
| ENSG00000112539 | HGLibA_06436 | 177 | 0.466332204 | C6orf118 | chromosome 6 open reading frame 118 [Source:HGNC Symbol;Acc:HGNC:21233] |
| ENSG00000112874 | HGLibA_32939 | 177 | 0.466332204 | NUDT12 | nudix hydrolase 12 [Source:HGNC Symbol;Acc:HGNC:18826] |
| ENSG00000113196 | HGLibA_20825 | 177 | 0.466332204 | HAND1 | heart and neural crest derivatives expressed 1 [Source:HGNC Symbol;Acc:HGNC:4807] |
| ENSG00000114491 | HGLibA_52985 | 177 | 0.466332204 | UMPS | uridine monophosphate synthetase [Source:HGNC Symbol;Acc:HGNC:12563] |
| ENSG00000116218 | HGLibA_32457 | 177 | 0.466332204 | NPHS2 | podocin [Source:HGNC Symbol;Acc:HGNC:13394]" |
| ENSG00000117226 | HGLibA_18735 | 177 | 0.466332204 | GBP3 | guanylate binding protein 3 [Source:HGNC Symbol;Acc:HGNC:4184] |
| ENSG00000118946 | HGLibA_35403 | 177 | 0.466332204 | PCDH17 | protocadherin 17 [Source:HGNC Symbol;Acc:HGNC:14267] |
| ENSG00000124749 | HGLibA_10540 | 177 | 0.466332204 | COL21A1 | collagen type XXI alpha 1 chain [Source:HGNC Symbol;Acc:HGNC:17025] |
| ENSG00000125337 | HGLibA_24913 | 177 | 0.466332204 | KIF25 | kinesin family member 25 [Source:HGNC Symbol;Acc:HGNC:6390] |
| ENSG00000127364 | HGLibA_48398 | 177 | 0.466332204 | TAS2R4 | taste 2 receptor member 4 [Source:HGNC Symbol;Acc:HGNC:14911] |
| ENSG00000127418 | HGLibA_17452 | 177 | 0.466332204 | FGFRL1 | fibroblast growth factor receptor like 1 [Source:HGNC Symbol;Acc:HGNC:3693] |
| ENSG00000130856 | HGLibA_55652 | 177 | 0.466332204 | ZNF236 | zinc finger protein 236 [Source:HGNC Symbol;Acc:HGNC:13028] |
| ENSG00000130988 | HGLibA_41018 | 177 | 0.466332204 | RGN | regucalcin [Source:HGNC Symbol;Acc:HGNC:9989] |
| ENSG00000133115 | HGLibA_47539 | 177 | 0.466332204 | STOML3 | stomatin like 3 [Source:HGNC Symbol;Acc:HGNC:19420] |
| ENSG00000134909 | HGLibA_02758 | 177 | 0.466332204 | ARHGAP32 | Rho GTPase activating protein 32 [Source:HGNC Symbol;Acc:HGNC:17399] |
| ENSG00000136161 | HGLibA_40681 | 177 | 0.466332204 | RCBTB2 | RCC1 and BTB domain containing protein 2 [Source:HGNC Symbol;Acc:HGNC:1914] |
| ENSG00000136305 | HGLibA_09701 | 177 | 0.466332204 | CIDEB | cell death-inducing DFFA-like effector b [Source:HGNC Symbol;Acc:HGNC:1977] |
| ENSG00000136514 | HGLibA_42431 | 177 | 0.466332204 | RTP4 | receptor transporter protein 4 [Source:HGNC Symbol;Acc:HGNC:23992] |
| ENSG00000137500 | HGLibA_07921 | 177 | 0.466332204 | CCDC90B | coiled-coil domain containing 90B [Source:HGNC Symbol;Acc:HGNC:28108] |
| ENSG00000139832 | HGLibA_39834 | 177 | 0.466332204 | RAB20 | member RAS oncogene family [Source:HGNC Symbol;Acc:HGNC:18260]" |
| ENSG00000140545 | HGLibA_29060 | 177 | 0.466332204 | MFGE8 | milk fat globule-EGF factor 8 protein [Source:HGNC Symbol;Acc:HGNC:7036] |
| ENSG00000141576 | HGLibA_41552 | 177 | 0.466332204 | RNF157 | ring finger protein 157 [Source:HGNC Symbol;Acc:HGNC:29402] |
| ENSG00000142700 | HGLibA_13428 | 177 | 0.466332204 | DMRTA2 | DMRT like family A2 [Source:HGNC Symbol;Acc:HGNC:13908] |
| ENSG00000145979 | HGLibA_48562 | 177 | 0.466332204 | TBC1D7 | TBC1 domain family member 7 [Source:HGNC Symbol;Acc:HGNC:21066] |
| ENSG00000147059 | HGLibA_46671 | 177 | 0.466332204 | SPIN2A | spindlin family member 2A [Source:HGNC Symbol;Acc:HGNC:20694] |
| ENSG00000158869 | HGLibA_17172 | 177 | 0.466332204 | FCER1G | Fc fragment of IgE receptor Ig [Source:HGNC Symbol;Acc:HGNC:3611] |
| ENSG00000160801 | HGLibA_39368 | 177 | 0.466332204 | PTH1R | parathyroid hormone 1 receptor [Source:HGNC Symbol;Acc:HGNC:9608] |
| ENSG00000161542 | HGLibA_38764 | 177 | 0.466332204 | PRPSAP1 | phosphoribosyl pyrophosphate synthetase associated protein 1 [Source:HGNC Symbol;Acc:HGNC:9466] |
| ENSG00000161929 | HGLibA_42943 | 177 | 0.466332204 | SCIMP | SLP adaptor and CSK interacting membrane protein [Source:HGNC Symbol;Acc:HGNC:33504] |
| ENSG00000162670 | HGLibA_04818 | 177 | 0.466332204 | BRINP3 | BMP/retinoic acid inducible neural specific 3 [Source:HGNC Symbol;Acc:HGNC:22393] |
| ENSG00000162728 | HGLibA_24290 | 177 | 0.466332204 | KCNJ9 | potassium voltage-gated channel subfamily J member 9 [Source:HGNC Symbol;Acc:HGNC:6270] |
| ENSG00000163376 | HGLibA_24118 | 177 | 0.466332204 | KBTBD8 | kelch repeat and BTB domain containing 8 [Source:HGNC Symbol;Acc:HGNC:30691] |
| ENSG00000163501 | HGLibA_22907 | 177 | 0.466332204 | IHH | indian hedgehog [Source:HGNC Symbol;Acc:HGNC:5956] |
| ENSG00000165102 | HGLibA_21250 | 177 | 0.466332204 | HGSNAT | heparan-alpha-glucosaminide N-acetyltransferase [Source:HGNC Symbol;Acc:HGNC:26527] |
| ENSG00000165125 | HGLibA_51797 | 177 | 0.466332204 | TRPV6 | transient receptor potential cation channel subfamily V member 6 [Source:HGNC Symbol;Acc:HGNC:14006] |
| ENSG00000165861 | HGLibA_55271 | 177 | 0.466332204 | ZFYVE1 | zinc finger FYVE-type containing 1 [Source:HGNC Symbol;Acc:HGNC:13180] |
| ENSG00000166211 | HGLibA_46659 | 177 | 0.466332204 | SPIC | Spi-C transcription factor [Source:HGNC Symbol;Acc:HGNC:29549] |
| ENSG00000166263 | HGLibA_47673 | 177 | 0.466332204 | STXBP4 | syntaxin binding protein 4 [Source:HGNC Symbol;Acc:HGNC:19694] |
| ENSG00000166401 | HGLibA_43566 | 177 | 0.466332204 | SERPINB8 | serpin family B member 8 [Source:HGNC Symbol;Acc:HGNC:8952] |
| ENSG00000166676 | HGLibA_52356 | 177 | 0.466332204 | TVP23A | trans-golgi network vesicle protein 23 homolog A [Source:HGNC Symbol;Acc:HGNC:20398] |
| ENSG00000167114 | HGLibA_44822 | 177 | 0.466332204 | SLC27A4 | solute carrier family 27 member 4 [Source:HGNC Symbol;Acc:HGNC:10998] |
| ENSG00000169247 | HGLibA_43983 | 177 | 0.466332204 | SH3TC2 | SH3 domain and tetratricopeptide repeats 2 [Source:HGNC Symbol;Acc:HGNC:29427] |
| ENSG00000170365 | HGLibA_45570 | 177 | 0.466332204 | SMAD1 | SMAD family member 1 [Source:HGNC Symbol;Acc:HGNC:6767] |
| ENSG00000170909 | HGLibA_34671 | 177 | 0.466332204 | OSCAR | immunoglobulin-like receptor [Source:HGNC Symbol;Acc:HGNC:29960]" |
| ENSG00000173467 | HGLibA_01246 | 177 | 0.466332204 | AGR3 | protein disulphide isomerase family member [Source:HGNC Symbol;Acc:HGNC:24167]" |
| ENSG00000174791 | HGLibA_41288 | 177 | 0.466332204 | RIN1 | Ras and Rab interactor 1 [Source:HGNC Symbol;Acc:HGNC:18749] |
| ENSG00000174799 | HGLibA_09108 | 177 | 0.466332204 | CEP135 | centrosomal protein 135 [Source:HGNC Symbol;Acc:HGNC:29086] |
| ENSG00000174839 | HGLibA_12965 | 177 | 0.466332204 | DENND6A | DENN domain containing 6A [Source:HGNC Symbol;Acc:HGNC:26635] |
| ENSG00000176884 | HGLibA_20295 | 177 | 0.466332204 | GRIN1 | glutamate ionotropic receptor NMDA type subunit 1 [Source:HGNC Symbol;Acc:HGNC:4584] |
| ENSG00000177103 | HGLibA_14000 | 177 | 0.466332204 | DSCAML1 | DS cell adhesion molecule like 1 [Source:HGNC Symbol;Acc:HGNC:14656] |
| ENSG00000177990 | HGLibA_13907 | 177 | 0.466332204 | DPY19L2 | dpy-19 like 2 [Source:HGNC Symbol;Acc:HGNC:19414] |
| ENSG00000177994 | HGLibA_06180 | 177 | 0.466332204 | C2orf73 | chromosome 2 open reading frame 73 [Source:HGNC Symbol;Acc:HGNC:26861] |
| ENSG00000181982 | HGLibA_07638 | 177 | 0.466332204 | CCDC149 | coiled-coil domain containing 149 [Source:HGNC Symbol;Acc:HGNC:25405] |
| ENSG00000183691 | HGLibA_32252 | 177 | 0.466332204 | NOG | noggin [Source:HGNC Symbol;Acc:HGNC:7866] |
| ENSG00000183914 | HGLibA_13482 | 177 | 0.466332204 | DNAH2 | dynein axonemal heavy chain 2 [Source:HGNC Symbol;Acc:HGNC:2948] |
| ENSG00000185088 | HGLibA_42129 | 177 | 0.466332204 | RPS27L | ribosomal protein S27 like [Source:HGNC Symbol;Acc:HGNC:18476] |
| ENSG00000185176 | HGLibA_02588 | 177 | 0.466332204 | AQP12B | aquaporin 12B [Source:HGNC Symbol;Acc:HGNC:6096] |
| ENSG00000185482 | HGLibA_47319 | 177 | 0.466332204 | STAC3 | SH3 and cysteine rich domain 3 [Source:HGNC Symbol;Acc:HGNC:28423] |
| ENSG00000186479 | HGLibA_41105 | 177 | 0.466332204 | RGS7BP | regulator of G protein signaling 7 binding protein [Source:HGNC Symbol;Acc:HGNC:23271] |
| ENSG00000189090 | HGLibA_16557 | 177 | 0.466332204 | FAM25G | family with sequence similarity 25 member G [Source:HGNC Symbol;Acc:HGNC:23590] |
| ENSG00000196188 | HGLibA_11701 | 177 | 0.466332204 | CTSE | cathepsin E [Source:HGNC Symbol;Acc:HGNC:2530] |
| ENSG00000196715 | HGLibA_53680 | 177 | 0.466332204 | VKORC1L1 | vitamin K epoxide reductase complex subunit 1 like 1 [Source:HGNC Symbol;Acc:HGNC:21492] |
| ENSG00000204345 | HGLibA_08339 | 177 | 0.466332204 | CD300LD | CD300 molecule like family member d [Source:HGNC Symbol;Acc:HGNC:16848] |
| ENSG00000204424 | HGLibA_27670 | 177 | 0.466332204 | LY6G6F | lymphocyte antigen 6 family member G6F [Source:HGNC Symbol;Acc:HGNC:13933] |
| ENSG00000204501 | HGLibA_38294 | 177 | 0.466332204 | PRAMEF15 | PRAME family member 15 [Source:HGNC Symbol;Acc:HGNC:26764] |
| ENSG00000204542 | HGLibA_06452 | 177 | 0.466332204 | C6orf15 | chromosome 6 open reading frame 15 [Source:HGNC Symbol;Acc:HGNC:13927] |
| ENSG00000241123 | HGLibA_25611 | 177 | 0.466332204 | KRTAP10-5 | keratin associated protein 10-5 [Source:HGNC Symbol;Acc:HGNC:22969] |
| ENSG00000242441 | HGLibA_20524 | 177 | 0.466332204 | GTF2A1L | general transcription factor IIA subunit 1 like [Source:HGNC Symbol;Acc:HGNC:30727] |
| ENSG00000243414 | HGLibA_49449 | 177 | 0.466332204 | TICAM2 | toll like receptor adaptor molecule 2 [Source:HGNC Symbol;Acc:HGNC:21354] |
| ENSG00000244057 | HGLibA_26135 | 177 | 0.466332204 | LCE3C | late cornified envelope 3C [Source:HGNC Symbol;Acc:HGNC:16612] |
| ENSG00000250741 | HGLibA_32808 | 177 | 0.466332204 | NT5C1B-RDH14 | NT5C1B-RDH14 readthrough [Source:HGNC Symbol;Acc:HGNC:38831] |
| ENSG00000258453 | HGLibA_33576 | 177 | 0.466332204 | OR11H2 | olfactory receptor family 11 subfamily H member 2 [Source:HGNC Symbol;Acc:HGNC:14716] |
| ENSG00000282608 | HGLibA_01020 | 177 | 0.466332204 | ADORA3 | adenosine A3 receptor [Source:HGNC Symbol;Acc:HGNC:268] |
| ENSG00000284609 | HGLibA_34500 | 177 | 0.466332204 | OR8B3 | olfactory receptor family 8 subfamily B member 3 [Source:HGNC Symbol;Acc:HGNC:8472] |
| FLJ44313 | HGLibA_17629 | 177 | 0.466332204 | FLJ44313 | - |
| hsa-mir-105-1 | HGLibA_57178 | 177 | 0.466332204 | hsa-mir-105-1 | - |
| hsa-mir-217 | HGLibA_58266 | 177 | 0.466332204 | hsa-mir-217 | - |
| hsa-mir-30b | HGLibA_58475 | 177 | 0.466332204 | hsa-mir-30b | - |
| hsa-mir-32 | HGLibA_58904 | 177 | 0.466332204 | hsa-mir-32 | - |
| hsa-mir-362 | HGLibA_59109 | 177 | 0.466332204 | hsa-mir-362 | - |
| hsa-mir-4330 | HGLibA_60047 | 177 | 0.466332204 | hsa-mir-4330 | - |
| hsa-mir-4711 | HGLibA_60944 | 177 | 0.466332204 | hsa-mir-4711 | - |
| hsa-mir-483 | HGLibA_61324 | 177 | 0.466332204 | hsa-mir-483 | - |
| hsa-mir-487b | HGLibA_61349 | 177 | 0.466332204 | hsa-mir-487b | - |
| hsa-mir-506 | HGLibA_61484 | 177 | 0.466332204 | hsa-mir-506 | - |
| hsa-mir-514a-3 | HGLibA_61594 | 177 | 0.466332204 | hsa-mir-514a-3 | - |
| hsa-mir-548ba | HGLibA_61954 | 177 | 0.466332204 | hsa-mir-548ba | - |
| hsa-mir-6715b | HGLibA_63021 | 177 | 0.466332204 | hsa-mir-6715b | - |
| hsa-mir-7113 | HGLibA_63819 | 177 | 0.466332204 | hsa-mir-7113 | - |
| LOC401052 | HGLibA_26935 | 177 | 0.466332204 | LOC401052 | - |
| LPPR1 | HGLibA_27113 | 177 | 0.466332204 | LPPR1 | - |
| PCNXL3 | HGLibA_35649 | 177 | 0.466332204 | PCNXL3 | - |
| BAGE4 | HGLibA_04102 | 176 | 0.457286861 | BAGE4 | - |
| C1orf177 | HGLibA_05800 | 176 | 0.457286861 | C1orf177 | - |
| C9orf156 | HGLibA_06694 | 176 | 0.457286861 | C9orf156 | - |
| ENSG00000008394 | HGLibA_29174 | 176 | 0.457286861 | MGST1 | microsomal glutathione S-transferase 1 [Source:HGNC Symbol;Acc:HGNC:7061] |
| ENSG00000010072 | HGLibA_46833 | 176 | 0.457286861 | SPRTN | SprT-like N-terminal domain [Source:HGNC Symbol;Acc:HGNC:25356] |
| ENSG00000027644 | HGLibA_23402 | 176 | 0.457286861 | INSRR | insulin receptor related receptor [Source:HGNC Symbol;Acc:HGNC:6093] |
| ENSG00000034971 | HGLibA_30873 | 176 | 0.457286861 | MYOC | myocilin [Source:HGNC Symbol;Acc:HGNC:7610] |
| ENSG00000065320 | HGLibA_32852 | 176 | 0.457286861 | NTN1 | netrin 1 [Source:HGNC Symbol;Acc:HGNC:8029] |
| ENSG00000065911 | HGLibA_30381 | 176 | 0.457286861 | MTHFD2 | methenyltetrahydrofolate cyclohydrolase [Source:HGNC Symbol;Acc:HGNC:7434]" |
| ENSG00000066422 | HGLibA_54816 | 176 | 0.457286861 | ZBTB11 | zinc finger and BTB domain containing 11 [Source:HGNC Symbol;Acc:HGNC:16740] |
| ENSG00000068903 | HGLibA_44220 | 176 | 0.457286861 | SIRT2 | sirtuin 2 [Source:HGNC Symbol;Acc:HGNC:10886] |
| ENSG00000073417 | HGLibA_35829 | 176 | 0.457286861 | PDE8A | phosphodiesterase 8A [Source:HGNC Symbol;Acc:HGNC:8793] |
| ENSG00000100867 | HGLibA_13129 | 176 | 0.457286861 | DHRS2 | dehydrogenase/reductase 2 [Source:HGNC Symbol;Acc:HGNC:18349] |
| ENSG00000103490 | HGLibA_39669 | 176 | 0.457286861 | PYCARD | PYD and CARD domain containing [Source:HGNC Symbol;Acc:HGNC:16608] |
| ENSG00000105088 | HGLibA_33347 | 176 | 0.457286861 | OLFM2 | olfactomedin 2 [Source:HGNC Symbol;Acc:HGNC:17189] |
| ENSG00000105576 | HGLibA_50902 | 176 | 0.457286861 | TNPO2 | transportin 2 [Source:HGNC Symbol;Acc:HGNC:19998] |
| ENSG00000105583 | HGLibA_54195 | 176 | 0.457286861 | WDR83OS | WD repeat domain 83 opposite strand [Source:HGNC Symbol;Acc:HGNC:30203] |
| ENSG00000111801 | HGLibA_04975 | 176 | 0.457286861 | BTN3A3 | butyrophilin subfamily 3 member A3 [Source:HGNC Symbol;Acc:HGNC:1140] |
| ENSG00000111816 | HGLibA_18006 | 176 | 0.457286861 | FRK | fyn related Src family tyrosine kinase [Source:HGNC Symbol;Acc:HGNC:3955] |
| ENSG00000114771 | HGLibA_00028 | 176 | 0.457286861 | AADAC | arylacetamide deacetylase [Source:HGNC Symbol;Acc:HGNC:17] |
| ENSG00000115361 | HGLibA_00366 | 176 | 0.457286861 | ACADL | acyl-CoA dehydrogenase long chain [Source:HGNC Symbol;Acc:HGNC:88] |
| ENSG00000116329 | HGLibA_33442 | 176 | 0.457286861 | OPRD1 | opioid receptor delta 1 [Source:HGNC Symbol;Acc:HGNC:8153] |
| ENSG00000116729 | HGLibA_54331 | 176 | 0.457286861 | WLS | wntless Wnt ligand secretion mediator [Source:HGNC Symbol;Acc:HGNC:30238] |
| ENSG00000117479 | HGLibA_44500 | 176 | 0.457286861 | SLC19A2 | solute carrier family 19 member 2 [Source:HGNC Symbol;Acc:HGNC:10938] |
| ENSG00000118231 | HGLibA_11280 | 176 | 0.457286861 | CRYGD | crystallin gamma D [Source:HGNC Symbol;Acc:HGNC:2411] |
| ENSG00000118733 | HGLibA_33351 | 176 | 0.457286861 | OLFM3 | olfactomedin 3 [Source:HGNC Symbol;Acc:HGNC:17990] |
| ENSG00000119185 | HGLibA_23772 | 176 | 0.457286861 | ITGB1BP1 | integrin subunit beta 1 binding protein 1 [Source:HGNC Symbol;Acc:HGNC:23927] |
| ENSG00000119688 | HGLibA_00201 | 176 | 0.457286861 | ABCD4 | ATP binding cassette subfamily D member 4 [Source:HGNC Symbol;Acc:HGNC:68] |
| ENSG00000121644 | HGLibA_13009 | 176 | 0.457286861 | DESI2 | desumoylating isopeptidase 2 [Source:HGNC Symbol;Acc:HGNC:24264] |
| ENSG00000121940 | HGLibA_09815 | 176 | 0.457286861 | CLCC1 | chloride channel CLIC like 1 [Source:HGNC Symbol;Acc:HGNC:29675] |
| ENSG00000124193 | HGLibA_47085 | 176 | 0.457286861 | SRSF6 | serine and arginine rich splicing factor 6 [Source:HGNC Symbol;Acc:HGNC:10788] |
| ENSG00000124785 | HGLibA_32709 | 176 | 0.457286861 | NRN1 | neuritin 1 [Source:HGNC Symbol;Acc:HGNC:17972] |

| | | | | | |
|---|---|---|---|---|---|
| ENSG00000124939 | HGLibA_42923 | 176 | 0.457286861 | SCGB2A1 | secretoglobin family 2A member 1 [Source:HGNC Symbol;Acc:HGNC:7051] |
| ENSG00000125144 | HGLibA_30289 | 176 | 0.457286861 | MT1G | metallothionein 1G [Source:HGNC Symbol;Acc:HGNC:7399] |
| ENSG00000125363 | HGLibA_01765 | 176 | 0.457286861 | AMELX | amelogenin X-linked [Source:HGNC Symbol;Acc:HGNC:461] |
| ENSG00000126464 | HGLibA_38772 | 176 | 0.457286861 | PRR12 | proline rich 12 [Source:HGNC Symbol;Acc:HGNC:29217] |
| ENSG00000133488 | HGLibA_43199 | 176 | 0.457286861 | SEC14L4 | SEC14 like lipid binding 4 [Source:HGNC Symbol;Acc:HGNC:20627] |
| ENSG00000133742 | HGLibA_06785 | 176 | 0.457286861 | CA1 | carbonic anhydrase 1 [Source:HGNC Symbol;Acc:HGNC:1368] |
| ENSG00000134882 | HGLibA_52537 | 176 | 0.457286861 | UBAC2 | UBA domain containing 2 [Source:HGNC Symbol;Acc:HGNC:20486] |
| ENSG00000135426 | HGLibA_49035 | 176 | 0.457286861 | TESPA1 | positive selection associated 1 [Source:HGNC Symbol;Acc:HGNC:29109]" |
| ENSG00000136485 | HGLibA_12453 | 176 | 0.457286861 | DCAF7 | DDB1 and CUL4 associated factor 7 [Source:HGNC Symbol;Acc:HGNC:30915] |
| ENSG00000136870 | HGLibA_55552 | 176 | 0.457286861 | ZNF189 | zinc finger protein 189 [Source:HGNC Symbol;Acc:HGNC:12980] |
| ENSG00000137809 | HGLibA_23720 | 176 | 0.457286861 | ITGA11 | integrin subunit alpha 11 [Source:HGNC Symbol;Acc:HGNC:6136] |
| ENSG00000138030 | HGLibA_24575 | 176 | 0.457286861 | KHK | ketohexokinase [Source:HGNC Symbol;Acc:HGNC:6315] |
| ENSG00000138180 | HGLibA_09142 | 176 | 0.457286861 | CEP55 | centrosomal protein 55 [Source:HGNC Symbol;Acc:HGNC:1161] |
| ENSG00000138587 | HGLibA_29572 | 176 | 0.457286861 | MNS1 | meiosis specific nuclear structural 1 [Source:HGNC Symbol;Acc:HGNC:29636] |
| ENSG00000139624 | HGLibA_09211 | 176 | 0.457286861 | CERS5 | ceramide synthase 5 [Source:HGNC Symbol;Acc:HGNC:23749] |
| ENSG00000139737 | HGLibA_44306 | 176 | 0.457286861 | SLAIN1 | SLAIN motif family member 1 [Source:HGNC Symbol;Acc:HGNC:26387] |
| ENSG00000144354 | HGLibA_08614 | 176 | 0.457286861 | CDCA7 | cell division cycle associated 7 [Source:HGNC Symbol;Acc:HGNC:14628] |
| ENSG00000144868 | HGLibA_49865 | 176 | 0.457286861 | TMEM108 | transmembrane protein 108 [Source:HGNC Symbol;Acc:HGNC:28451] |
| ENSG00000147044 | HGLibA_07287 | 176 | 0.457286861 | CASK | calcium/calmodulin dependent serine protein kinase [Source:HGNC Symbol;Acc:HGNC:1497] |
| ENSG00000149435 | HGLibA_19021 | 176 | 0.457286861 | GGTLC1 | gamma-glutamyltransferase light chain 1 [Source:HGNC Symbol;Acc:HGNC:16437] |
| ENSG00000151233 | HGLibA_20681 | 176 | 0.457286861 | GXYLT1 | glucoside xylosyltransferase 1 [Source:HGNC Symbol;Acc:HGNC:27482] |
| ENSG00000156675 | HGLibA_39793 | 176 | 0.457286861 | RAB11FIP1 | RAB11 family interacting protein 1 [Source:HGNC Symbol;Acc:HGNC:30265] |
| ENSG00000158578 | HGLibA_01525 | 176 | 0.457286861 | ALAS2 | 5'-aminolevulinate synthase 2 [Source:HGNC Symbol;Acc:HGNC:397] |
| ENSG00000162927 | HGLibA_39598 | 176 | 0.457286861 | PUS10 | pseudouridine synthase 10 [Source:HGNC Symbol;Acc:HGNC:26505] |
| ENSG00000162944 | HGLibA_40952 | 176 | 0.457286861 | RFTN2 | raftlin family member 2 [Source:HGNC Symbol;Acc:HGNC:26402] |
| ENSG00000163479 | HGLibA_47157 | 176 | 0.457286861 | SSR2 | signal sequence receptor subunit 2 [Source:HGNC Symbol;Acc:HGNC:11324] |
| ENSG00000164512 | HGLibA_02126 | 176 | 0.457286861 | ANKRD55 | ankyrin repeat domain 55 [Source:HGNC Symbol;Acc:HGNC:25681] |
| ENSG00000165138 | HGLibA_02163 | 176 | 0.457286861 | ANKS6 | ankyrin repeat and sterile alpha motif domain containing 6 [Source:HGNC Symbol;Acc:HGNC:26724] |
| ENSG00000166225 | HGLibA_18047 | 176 | 0.457286861 | FRS2 | fibroblast growth factor receptor substrate 2 [Source:HGNC Symbol;Acc:HGNC:16971] |
| ENSG00000167881 | HGLibA_47009 | 176 | 0.457286861 | SRP68 | signal recognition particle 68 [Source:HGNC Symbol;Acc:HGNC:11302] |
| ENSG00000169871 | HGLibA_51550 | 176 | 0.457286861 | TRIM56 | tripartite motif containing 56 [Source:HGNC Symbol;Acc:HGNC:19028] |
| ENSG00000171365 | HGLibA_09831 | 176 | 0.457286861 | CLCN5 | chloride voltage-gated channel 5 [Source:HGNC Symbol;Acc:HGNC:2023] |
| ENSG00000171487 | HGLibA_32124 | 176 | 0.457286861 | NLRP5 | NLR family pyrin domain containing 5 [Source:HGNC Symbol;Acc:HGNC:21269] |
| ENSG00000173171 | HGLibA_30512 | 176 | 0.457286861 | MTX1 | metaxin 1 [Source:HGNC Symbol;Acc:HGNC:7504] |
| ENSG00000173950 | HGLibA_54612 | 176 | 0.457286861 | XXYLT1 | xyloside xylosyltransferase 1 [Source:HGNC Symbol;Acc:HGNC:26639] |
| ENSG00000174482 | HGLibA_26589 | 176 | 0.457286861 | LINGO2 | leucine rich repeat and Ig domain containing 2 [Source:HGNC Symbol;Acc:HGNC:21207] |
| ENSG00000175265 | HGLibA_19639 | 176 | 0.457286861 | GOLGA8A | golgin A8 family member A [Source:HGNC Symbol;Acc:HGNC:31972] |
| ENSG00000177000 | HGLibA_30389 | 176 | 0.457286861 | MTHFR | methylenetetrahydrofolate reductase [Source:HGNC Symbol;Acc:HGNC:7436] |
| ENSG00000177732 | HGLibA_46233 | 176 | 0.457286861 | SOX12 | SRY-box 12 [Source:HGNC Symbol;Acc:HGNC:11198] |
| ENSG00000181826 | HGLibA_40817 | 176 | 0.457286861 | RELL1 | RELT like 1 [Source:HGNC Symbol;Acc:HGNC:27379] |
| ENSG00000182170 | HGLibA_29842 | 176 | 0.457286861 | MRGPRG | MAS related GPR family member G [Source:HGNC Symbol;Acc:HGNC:24829] |
| ENSG00000183960 | HGLibA_24232 | 176 | 0.457286861 | KCNH8 | potassium voltage-gated channel subfamily H member 8 [Source:HGNC Symbol;Acc:HGNC:18864] |
| ENSG00000184313 | HGLibA_29876 | 176 | 0.457286861 | MROH7 | maestro heat like repeat family member 7 [Source:HGNC Symbol;Acc:HGNC:24802] |
| ENSG00000185800 | HGLibA_13450 | 176 | 0.457286861 | DMWD | WD repeat containing [Source:HGNC Symbol;Acc:HGNC:2936]" |
| ENSG00000186790 | HGLibA_17861 | 176 | 0.457286861 | FOXE3 | forkhead box E3 [Source:HGNC Symbol;Acc:HGNC:3808] |
| ENSG00000186943 | HGLibA_33607 | 176 | 0.457286861 | OR13C8 | olfactory receptor family 13 subfamily C member 8 [Source:HGNC Symbol;Acc:HGNC:15103] |
| ENSG00000187238 | HGLibA_26134 | 176 | 0.457286861 | LCE3B | late cornified envelope 3B [Source:HGNC Symbol;Acc:HGNC:29462] |
| ENSG00000187288 | HGLibA_09702 | 176 | 0.457286861 | CIDEC | cell death inducing DFFA like effector c [Source:HGNC Symbol;Acc:HGNC:24229] |
| ENSG00000196123 | HGLibA_24661 | 176 | 0.457286861 | KIAA0895L | KIAA0895 like [Source:HGNC Symbol;Acc:HGNC:34408] |
| ENSG00000197134 | HGLibA_55681 | 176 | 0.457286861 | ZNF257 | zinc finger protein 257 [Source:HGNC Symbol;Acc:HGNC:13498] |
| ENSG00000198464 | HGLibA_56071 | 176 | 0.457286861 | ZNF480 | zinc finger protein 480 [Source:HGNC Symbol;Acc:HGNC:23305] |
| ENSG00000204962 | HGLibA_35456 | 176 | 0.457286861 | PCDHA8 | protocadherin alpha 8 [Source:HGNC Symbol;Acc:HGNC:8674] |
| ENSG00000205667 | HGLibA_03146 | 176 | 0.457286861 | ARSH | arylsulfatase family member H [Source:HGNC Symbol;Acc:HGNC:32488] |
| ENSG00000249861 | HGLibA_26357 | 176 | 0.457286861 | LGALS16 | galectin 16 [Source:HGNC Symbol;Acc:HGNC:40039] |
| ENSG00000257218 | HGLibA_18692 | 176 | 0.457286861 | GATC | glutamyl-tRNA amidotransferase subunit C [Source:HGNC Symbol;Acc:HGNC:25068] |
| ENSG00000259803 | HGLibA_44586 | 176 | 0.457286861 | SLC22A31 | solute carrier family 22 member 31 [Source:HGNC Symbol;Acc:HGNC:27091] |
| ENSG00000268223 | HGLibA_02941 | 176 | 0.457286861 | ARL14EPL | ADP ribosylation factor like GTPase 14 effector protein like [Source:HGNC Symbol;Acc:HGNC:44201] |
| ENSG00000275152 | HGLibA_07976 | 176 | 0.457286861 | CCL16 | C-C motif chemokine ligand 16 [Source:HGNC Symbol;Acc:HGNC:10614] |
| FAM49A | HGLibA_16623 | 176 | 0.457286861 | FAM49A | - |
| HIST1H2BJ | HGLibA_21438 | 176 | 0.457286861 | HIST1H2BJ | - |
| hsa-mir-133a-2 | HGLibA_57777 | 176 | 0.457286861 | hsa-mir-133a-2 | - |
| hsa-mir-148a | HGLibA_57879 | 176 | 0.457286861 | hsa-mir-148a | - |
| hsa-mir-3680-1 | HGLibA_59270 | 176 | 0.457286861 | hsa-mir-3680-1 | - |
| hsa-mir-4320 | HGLibA_60005 | 176 | 0.457286861 | hsa-mir-4320 | - |
| hsa-mir-4473 | HGLibA_60301 | 176 | 0.457286861 | hsa-mir-4473 | - |
| hsa-mir-4682 | HGLibA_60838 | 176 | 0.457286861 | hsa-mir-4682 | - |
| hsa-mir-548d-2 | HGLibA_61965 | 176 | 0.457286861 | hsa-mir-548d-2 | - |
| hsa-mir-6767 | HGLibA_63229 | 176 | 0.457286861 | hsa-mir-6767 | - |
| hsa-mir-6829 | HGLibA_63495 | 176 | 0.457286861 | hsa-mir-6829 | - |
| hsa-mir-6851 | HGLibA_63578 | 176 | 0.457286861 | hsa-mir-6851 | - |
| hsa-mir-7153 | HGLibA_63840 | 176 | 0.457286861 | hsa-mir-7153 | - |
| hsa-mir-937 | HGLibA_64324 | 176 | 0.457286861 | hsa-mir-937 | - |
| KIAA2022 | HGLibA_24843 | 176 | 0.457286861 | KIAA2022 | - |
| LINS | HGLibA_26598 | 176 | 0.457286861 | LINS | - |
| LOC650293 | HGLibA_26983 | 176 | 0.457286861 | LOC650293 | - |
| PPAP2C | HGLibA_37822 | 176 | 0.457286861 | PPAP2C | - |
| TUSC5 | HGLibA_52351 | 176 | 0.457286861 | TUSC5 | - |
| 4-Mar | HGLibA_28336 | 175 | 0.448241518 | 4-Mar | - |
| ASMTL_X | HGLibA_03326 | 175 | 0.448241518 | ASMTL_X | - |
| C16orf11 | HGLibA_05437 | 175 | 0.448241518 | C16orf11 | - |
| C1orf106 | HGLibA_05737 | 175 | 0.448241518 | C1orf106 | - |
| ENSG00000007968 | HGLibA_14311 | 175 | 0.448241518 | E2F2 | E2F transcription factor 2 [Source:HGNC Symbol;Acc:HGNC:3114] |
| ENSG00000022355 | HGLibA_18357 | 175 | 0.448241518 | GABRA1 | gamma-aminobutyric acid type A receptor alpha1 subunit [Source:HGNC Symbol;Acc:HGNC:4075] |
| ENSG00000049246 | HGLibA_36061 | 175 | 0.448241518 | PER3 | period circadian regulator 3 [Source:HGNC Symbol;Acc:HGNC:8847] |
| ENSG00000063176 | HGLibA_46645 | 175 | 0.448241518 | SPHK2 | sphingosine kinase 2 [Source:HGNC Symbol;Acc:HGNC:18859] |
| ENSG00000064961 | HGLibA_21681 | 175 | 0.448241518 | HMG20B | high mobility group 20B [Source:HGNC Symbol;Acc:HGNC:5002] |
| ENSG00000065054 | HGLibA_45412 | 175 | 0.448241518 | SLC9A3R2 | SLC9A3 regulator 2 [Source:HGNC Symbol;Acc:HGNC:11076] |
| ENSG00000065615 | HGLibA_12011 | 175 | 0.448241518 | CYB5R4 | cytochrome b5 reductase 4 [Source:HGNC Symbol;Acc:HGNC:20147] |
| ENSG00000070814 | HGLibA_48827 | 175 | 0.448241518 | TCOF1 | treacle ribosome biogenesis factor 1 [Source:HGNC Symbol;Acc:HGNC:11654] |
| ENSG00000071967 | HGLibA_12022 | 175 | 0.448241518 | CYBRD1 | cytochrome b reductase 1 [Source:HGNC Symbol;Acc:HGNC:20797] |
| ENSG00000072694 | HGLibA_17191 | 175 | 0.448241518 | FCGR2B | Fc fragment of IgG receptor IIb [Source:HGNC Symbol;Acc:HGNC:3618] |
| ENSG00000082269 | HGLibA_16132 | 175 | 0.448241518 | FAM135A | family with sequence similarity 135 member A [Source:HGNC Symbol;Acc:HGNC:21084] |
| ENSG00000087245 | HGLibA_29506 | 175 | 0.448241518 | MMP2 | matrix metallopeptidase 2 [Source:HGNC Symbol;Acc:HGNC:7166] |
| ENSG00000089091 | HGLibA_14294 | 175 | 0.448241518 | DZANK1 | double zinc ribbon and ankyrin repeat domains 1 [Source:HGNC Symbol;Acc:HGNC:15858] |
| ENSG00000092020 | HGLibA_38164 | 175 | 0.448241518 | PPP2R3C | protein phosphatase 2 regulatory subunit B''gamma [Source:HGNC Symbol;Acc:HGNC:17485] |
| ENSG00000100241 | HGLibA_42793 | 175 | 0.448241518 | SBF1 | SET binding factor 1 [Source:HGNC Symbol;Acc:HGNC:10542] |
| ENSG00000100601 | HGLibA_01645 | 175 | 0.448241518 | ALKBH1 | histone H2A dioxygenase [Source:HGNC Symbol;Acc:HGNC:17911]" |
| ENSG00000101856 | HGLibA_36289 | 175 | 0.448241518 | PGRMC1 | progesterone receptor membrane component 1 [Source:HGNC Symbol;Acc:HGNC:16090] |
| ENSG00000102302 | HGLibA_17342 | 175 | 0.448241518 | FGD1 | RhoGEF and PH domain containing 1 [Source:HGNC Symbol;Acc:HGNC:3663]" |
| ENSG00000103043 | HGLibA_53485 | 175 | 0.448241518 | VAC14 | PIKFYVE complex component [Source:HGNC Symbol;Acc:HGNC:25507]" |
| ENSG00000103111 | HGLibA_29642 | 175 | 0.448241518 | MON1B | secretory trafficking associated [Source:HGNC Symbol;Acc:HGNC:25020]" |
| ENSG00000104915 | HGLibA_47616 | 175 | 0.448241518 | STX10 | syntaxin 10 [Source:HGNC Symbol;Acc:HGNC:11428] |
| ENSG00000105428 | HGLibA_56947 | 175 | 0.448241518 | ZNRF4 | zinc and ring finger 4 [Source:HGNC Symbol;Acc:HGNC:17726] |
| ENSG00000108924 | HGLibA_21655 | 175 | 0.448241518 | HLF | PAR bZIP transcription factor [Source:HGNC Symbol;Acc:HGNC:4977]" |
| ENSG00000109381 | HGLibA_14893 | 175 | 0.448241518 | ELF2 | E74 like ETS transcription factor 2 [Source:HGNC Symbol;Acc:HGNC:3317] |
| ENSG00000115446 | HGLibA_53012 | 175 | 0.448241518 | UNC50 | unc-50 inner nuclear membrane RNA binding protein [Source:HGNC Symbol;Acc:HGNC:16046] |
| ENSG00000115738 | HGLibA_22526 | 175 | 0.448241518 | ID2 | inhibitor of DNA binding 2 [Source:HGNC Symbol;Acc:HGNC:5361] |
| ENSG00000122012 | HGLibA_47880 | 175 | 0.448241518 | SV2C | synaptic vesicle glycoprotein 2C [Source:HGNC Symbol;Acc:HGNC:30670] |
| ENSG00000125868 | HGLibA_14046 | 175 | 0.448241518 | DSTN | actin depolymerizing factor [Source:HGNC Symbol;Acc:HGNC:15750]" |
| ENSG00000127220 | HGLibA_00287 | 175 | 0.448241518 | ABHD8 | abhydrolase domain containing 8 [Source:HGNC Symbol;Acc:HGNC:23759] |
| ENSG00000127663 | HGLibA_24508 | 175 | 0.448241518 | KDM4B | lysine demethylase 4B [Source:HGNC Symbol;Acc:HGNC:29136] |
| ENSG00000129197 | HGLibA_41812 | 175 | 0.448241518 | RPAIN | RPA interacting protein [Source:HGNC Symbol;Acc:HGNC:28641] |
| ENSG00000129749 | HGLibA_09566 | 175 | 0.448241518 | CHRNA10 | cholinergic receptor nicotinic alpha 10 subunit [Source:HGNC Symbol;Acc:HGNC:13800] |
| ENSG00000130158 | HGLibA_13751 | 175 | 0.448241518 | DOCK6 | dedicator of cytokinesis 6 [Source:HGNC Symbol;Acc:HGNC:19189] |
| ENSG00000131196 | HGLibA_31773 | 175 | 0.448241518 | NFATC1 | nuclear factor of activated T cells 1 [Source:HGNC Symbol;Acc:HGNC:7775] |
| ENSG00000134028 | HGLibA_00789 | 175 | 0.448241518 | ADAMDEC1 | ADAM like decysin 1 [Source:HGNC Symbol;Acc:HGNC:16299] |
| ENSG00000139292 | HGLibA_26410 | 175 | 0.448241518 | LGR5 | leucine rich repeat containing G protein-coupled receptor 5 [Source:HGNC Symbol;Acc:HGNC:4504] |
| ENSG00000140853 | HGLibA_32093 | 175 | 0.448241518 | NLRC5 | NLR family CARD domain containing 5 [Source:HGNC Symbol;Acc:HGNC:29933] |
| ENSG00000140983 | HGLibA_41204 | 175 | 0.448241518 | RHOT2 | ras homolog family member T2 [Source:HGNC Symbol;Acc:HGNC:21169] |
| ENSG00000143013 | HGLibA_26710 | 175 | 0.448241518 | LMO4 | LIM domain only 4 [Source:HGNC Symbol;Acc:HGNC:6644] |
| ENSG00000143858 | HGLibA_48073 | 175 | 0.448241518 | SYT2 | synaptotagmin 2 [Source:HGNC Symbol;Acc:HGNC:11510] |
| ENSG00000144136 | HGLibA_44527 | 175 | 0.448241518 | SLC20A1 | solute carrier family 20 member 1 [Source:HGNC Symbol;Acc:HGNC:10946] |
| ENSG00000145861 | HGLibA_05939 | 175 | 0.448241518 | C1QTNF2 | C1q and TNF related 2 [Source:HGNC Symbol;Acc:HGNC:14325] |
| ENSG00000146674 | HGLibA_22820 | 175 | 0.448241518 | IGFBP3 | insulin like growth factor binding protein 3 [Source:HGNC Symbol;Acc:HGNC:5472] |
| ENSG00000147885 | HGLibA_22669 | 175 | 0.448241518 | IFNA16 | interferon alpha 16 [Source:HGNC Symbol;Acc:HGNC:5421] |
| ENSG00000148426 | HGLibA_38697 | 175 | 0.448241518 | PROSER2 | proline and serine rich 2 [Source:HGNC Symbol;Acc:HGNC:23728] |
| ENSG00000156273 | HGLibA_04064 | 175 | 0.448241518 | BACH1 | BTB domain and CNC homolog 1 [Source:HGNC Symbol;Acc:HGNC:935] |
| ENSG00000156804 | HGLibA_17071 | 175 | 0.448241518 | FBXO32 | F-box protein 32 [Source:HGNC Symbol;Acc:HGNC:16731] |
| ENSG00000156858 | HGLibA_38778 | 175 | 0.448241518 | PRR14 | proline rich 14 [Source:HGNC Symbol;Acc:HGNC:28458] |
| ENSG00000158828 | HGLibA_36690 | 175 | 0.448241518 | PINK1 | PTEN induced kinase 1 [Source:HGNC Symbol;Acc:HGNC:14581] |
| ENSG00000159339 | HGLibA_34979 | 175 | 0.448241518 | PADI4 | peptidyl arginine deiminase 4 [Source:HGNC Symbol;Acc:HGNC:18368] |
| ENSG00000162804 | HGLibA_45923 | 175 | 0.448241518 | SNED1 | nidogen and EGF like domains 1 [Source:HGNC Symbol;Acc:HGNC:24696]" |
| ENSG00000163219 | HGLibA_02738 | 175 | 0.448241518 | ARHGAP25 | Rho GTPase activating protein 25 [Source:HGNC Symbol;Acc:HGNC:28951] |
| ENSG00000166200 | HGLibA_10714 | 175 | 0.448241518 | COPS2 | COP9 signalosome subunit 2 [Source:HGNC Symbol;Acc:HGNC:30747] |
| ENSG00000166478 | HGLibA_55493 | 175 | 0.448241518 | ZNF143 | zinc finger protein 143 [Source:HGNC Symbol;Acc:HGNC:12928] |

| | | | | | |
|---|---|---|---|---|---|
| ENSG00000166796 | HGLibA_26231 | 175 | 0.448241518 | LDHC | lactate dehydrogenase C [Source:HGNC Symbol;Acc:HGNC:6544] |
| ENSG00000169550 | HGLibA_30526 | 175 | 0.448241518 | MUC15 | cell surface associated [Source:HGNC Symbol;Acc:HGNC:14956]" |
| ENSG00000170322 | HGLibA_31840 | 175 | 0.448241518 | NFRKB | nuclear factor related to kappaB binding protein [Source:HGNC Symbol;Acc:HGNC:7802] |
| ENSG00000175691 | HGLibA_56702 | 175 | 0.448241518 | ZNF77 | zinc finger protein 77 [Source:HGNC Symbol;Acc:HGNC:13150] |
| ENSG00000179761 | HGLibA_36725 | 175 | 0.448241518 | PIPOX | pipecolic acid and sarcosine oxidase [Source:HGNC Symbol;Acc:HGNC:17804] |
| ENSG00000180772 | HGLibA_01265 | 175 | 0.448241518 | AGTR2 | angiotensin II receptor type 2 [Source:HGNC Symbol;Acc:HGNC:338] |
| ENSG00000181143 | HGLibA_30530 | 175 | 0.448241518 | MUC16 | cell surface associated [Source:HGNC Symbol;Acc:HGNC:15582]" |
| ENSG00000181374 | HGLibA_07967 | 175 | 0.448241518 | CCL13 | C-C motif chemokine ligand 13 [Source:HGNC Symbol;Acc:HGNC:10611] |
| ENSG00000185069 | HGLibA_25541 | 175 | 0.448241518 | KRT76 | keratin 76 [Source:HGNC Symbol;Acc:HGNC:24430] |
| ENSG00000185760 | HGLibA_24378 | 175 | 0.448241518 | KCNQ5 | potassium voltage-gated channel subfamily Q member 5 [Source:HGNC Symbol;Acc:HGNC:6299] |
| ENSG00000186575 | HGLibA_31760 | 175 | 0.448241518 | NF2 | neurofibromin 2 [Source:HGNC Symbol;Acc:HGNC:7773] |
| ENSG00000187513 | HGLibA_19144 | 175 | 0.448241518 | GJA4 | gap junction protein alpha 4 [Source:HGNC Symbol;Acc:HGNC:4278] |
| ENSG00000188878 | HGLibA_16934 | 175 | 0.448241518 | FBF1 | Fas binding factor 1 [Source:HGNC Symbol;Acc:HGNC:24674] |
| ENSG00000196353 | HGLibA_10963 | 175 | 0.448241518 | CPNE4 | copine 4 [Source:HGNC Symbol;Acc:HGNC:2317] |
| ENSG00000197363 | HGLibA_56145 | 175 | 0.448241518 | ZNF517 | zinc finger protein 517 [Source:HGNC Symbol;Acc:HGNC:27984] |
| ENSG00000197487 | HGLibA_18583 | 175 | 0.448241518 | GALP | galanin like peptide [Source:HGNC Symbol;Acc:HGNC:24840] |
| ENSG00000198521 | HGLibA_55975 | 175 | 0.448241518 | ZNF43 | zinc finger protein 43 [Source:HGNC Symbol;Acc:HGNC:13109] |
| ENSG00000198643 | HGLibA_16585 | 175 | 0.448241518 | FAM3D | family with sequence similarity 3 member D [Source:HGNC Symbol;Acc:HGNC:18665] |
| ENSG00000198732 | HGLibA_45795 | 175 | 0.448241518 | SMOC1 | SPARC related modular calcium binding 1 [Source:HGNC Symbol;Acc:HGNC:20318] |
| ENSG00000198788 | HGLibA_30536 | 175 | 0.448241518 | MUC2 | oligomeric mucus/gel-forming [Source:HGNC Symbol;Acc:HGNC:7512]" |
| ENSG00000198930 | HGLibA_11306 | 175 | 0.448241518 | CSAG1 | chondrosarcoma associated gene 1 [Source:HGNC Symbol;Acc:HGNC:24294] |
| ENSG00000198964 | HGLibA_43835 | 175 | 0.448241518 | SGMS1 | sphingomyelin synthase 1 [Source:HGNC Symbol;Acc:HGNC:29799] |
| ENSG00000203942 | HGLibA_05083 | 175 | 0.448241518 | C10orf62 | chromosome 10 open reading frame 62 [Source:HGNC Symbol;Acc:HGNC:23294] |
| ENSG00000204386 | HGLibA_31709 | 175 | 0.448241518 | NEU1 | neuraminidase 1 [Source:HGNC Symbol;Acc:HGNC:7758] |
| ENSG00000205978 | HGLibA_33186 | 175 | 0.448241518 | NYNRIN | NYN domain and retroviral integrase containing [Source:HGNC Symbol;Acc:HGNC:20165] |
| ENSG00000206102 | HGLibA_25685 | 175 | 0.448241518 | KRTAP19-8 | keratin associated protein 19-8 [Source:HGNC Symbol;Acc:HGNC:33898] |
| ENSG00000206104 | HGLibA_25706 | 175 | 0.448241518 | KRTAP20-3 | keratin associated protein 20-3 [Source:HGNC Symbol;Acc:HGNC:34001] |
| ENSG00000206140 | HGLibA_50148 | 175 | 0.448241518 | TMEM191C | transmembrane protein 191C [Source:HGNC Symbol;Acc:HGNC:33601] |
| ENSG00000213901 | HGLibA_44611 | 175 | 0.448241518 | SLC23A3 | solute carrier family 23 member 3 [Source:HGNC Symbol;Acc:HGNC:20601] |
| ENSG00000213996 | HGLibA_49713 | 175 | 0.448241518 | TM6SF2 | transmembrane 6 superfamily member 2 [Source:HGNC Symbol;Acc:HGNC:11861] |
| ENSG00000214413 | HGLibA_04200 | 175 | 0.448241518 | BBIP1 | BBSome interacting protein 1 [Source:HGNC Symbol;Acc:HGNC:28093] |
| ENSG00000215455 | HGLibA_25592 | 175 | 0.448241518 | KRTAP10-1 | keratin associated protein 10-1 [Source:HGNC Symbol;Acc:HGNC:22966] |
| ENSG00000215784 | HGLibA_16712 | 175 | 0.448241518 | FAM72D | family with sequence similarity 72 member D [Source:HGNC Symbol;Acc:HGNC:33593] |
| ENSG00000218823 | HGLibA_35139 | 175 | 0.448241518 | PAPOLB | poly(A) polymerase beta [Source:HGNC Symbol;Acc:HGNC:15970] |
| ENSG00000223731 | HGLibA_47807 | 175 | 0.448241518 | SUPT20HL1 | SUPT20H like 1 [Source:HGNC Symbol;Acc:HGNC:30773] |
| ENSG00000240583 | HGLibA_02577 | 175 | 0.448241518 | AQP1 | aquaporin 1 (Colton blood group) [Source:HGNC Symbol;Acc:HGNC:633] |
| ENSG00000253797 | HGLibA_53437 | 175 | 0.448241518 | UTP14C | small subunit processome component [Source:HGNC Symbol;Acc:HGNC:20321]" |
| ENSG00000255833 | HGLibA_49460 | 175 | 0.448241518 | TIFAB | TIFA inhibitor [Source:HGNC Symbol;Acc:HGNC:34024] |
| ENSG00000259431 | HGLibA_49411 | 175 | 0.448241518 | THTPA | thiamine triphosphatase [Source:HGNC Symbol;Acc:HGNC:18987] |
| ENSG00000279111 | HGLibA_33558 | 175 | 0.448241518 | OR10X1 | olfactory receptor family 10 subfamily X member 1 (gene/pseudogene) [Source:HGNC Symbol;Acc:HGNC:14995] |
| FAM206A | HGLibA_16426 | 175 | 0.448241518 | FAM206A | - |
| H1F0 | HGLibA_20732 | 175 | 0.448241518 | H1F0 | - |
| HIST3H2BB | HGLibA_21558 | 175 | 0.448241518 | HIST3H2BB | - |
| hsa-mir-194-2 | HGLibA_58083 | 175 | 0.448241518 | hsa-mir-194-2 | - |
| hsa-mir-208a | HGLibA_58196 | 175 | 0.448241518 | hsa-mir-208a | - |
| hsa-mir-2467 | HGLibA_58349 | 175 | 0.448241518 | hsa-mir-2467 | - |
| hsa-mir-3915 | HGLibA_59536 | 175 | 0.448241518 | hsa-mir-3915 | - |
| hsa-mir-4645 | HGLibA_60682 | 175 | 0.448241518 | hsa-mir-4645 | - |
| hsa-mir-552 | HGLibA_62127 | 175 | 0.448241518 | hsa-mir-552 | - |
| hsa-mir-577 | HGLibA_62402 | 175 | 0.448241518 | hsa-mir-577 | - |
| hsa-mir-648 | HGLibA_62834 | 175 | 0.448241518 | hsa-mir-648 | - |
| hsa-mir-6788 | HGLibA_63331 | 175 | 0.448241518 | hsa-mir-6788 | - |
| hsa-mir-6841 | HGLibA_63541 | 175 | 0.448241518 | hsa-mir-6841 | - |
| LOC100505841 | HGLibA_26854 | 175 | 0.448241518 | LOC100505841 | - |
| TUSC5 | HGLibA_52352 | 175 | 0.448241518 | TUSC5 | - |
| WAPAL | HGLibA_53941 | 175 | 0.448241518 | WAPAL | - |
| C1orf192 | HGLibA_05813 | 174 | 0.439196176 | C1orf192 | - |
| CXorf22 | HGLibA_11905 | 174 | 0.439196176 | CXorf22 | - |
| ENSG00000007306 | HGLibA_08951 | 174 | 0.439196176 | CEACAM7 | carcinoembryonic antigen related cell adhesion molecule 7 [Source:HGNC Symbol;Acc:HGNC:1819] |
| ENSG00000008300 | HGLibA_09028 | 174 | 0.439196176 | CELSR3 | cadherin EGF LAG seven-pass G-type receptor 3 [Source:HGNC Symbol;Acc:HGNC:3230] |
| ENSG00000022267 | HGLibA_17481 | 174 | 0.439196176 | FHL1 | four and a half LIM domains 1 [Source:HGNC Symbol;Acc:HGNC:3702] |
| ENSG00000036565 | HGLibA_44486 | 174 | 0.439196176 | SLC18A1 | solute carrier family 18 member A1 [Source:HGNC Symbol;Acc:HGNC:10934] |
| ENSG00000078142 | HGLibA_36631 | 174 | 0.439196176 | PIK3C3 | phosphatidylinositol 3-kinase catalytic subunit type 3 [Source:HGNC Symbol;Acc:HGNC:8974] |
| ENSG00000086061 | HGLibA_13510 | 174 | 0.439196176 | DNAJA1 | DnaJ heat shock protein family (Hsp40) member A1 [Source:HGNC Symbol;Acc:HGNC:5229] |
| ENSG00000087903 | HGLibA_40964 | 174 | 0.439196176 | RFX2 | regulatory factor X2 [Source:HGNC Symbol;Acc:HGNC:9983] |
| ENSG00000088876 | HGLibA_55843 | 174 | 0.439196176 | ZNF343 | zinc finger protein 343 [Source:HGNC Symbol;Acc:HGNC:16017] |
| ENSG00000089048 | HGLibA_15565 | 174 | 0.439196176 | ESF1 | ESF1 nucleolar pre-rRNA processing protein homolog [Source:HGNC Symbol;Acc:HGNC:15898] |
| ENSG00000101199 | HGLibA_02661 | 174 | 0.439196176 | ARFGAP1 | ADP ribosylation factor GTPase activating protein 1 [Source:HGNC Symbol;Acc:HGNC:15852] |
| ENSG00000101966 | HGLibA_54507 | 174 | 0.439196176 | XIAP | X-linked inhibitor of apoptosis [Source:HGNC Symbol;Acc:HGNC:592] |
| ENSG00000105352 | HGLibA_08942 | 174 | 0.439196176 | CEACAM4 | carcinoembryonic antigen related cell adhesion molecule 4 [Source:HGNC Symbol;Acc:HGNC:1816] |
| ENSG00000106609 | HGLibA_50308 | 174 | 0.439196176 | TMEM248 | transmembrane protein 248 [Source:HGNC Symbol;Acc:HGNC:25476] |
| ENSG00000107819 | HGLibA_43786 | 174 | 0.439196176 | SFXN3 | sideroflexin 3 [Source:HGNC Symbol;Acc:HGNC:16087] |
| ENSG00000109113 | HGLibA_39885 | 174 | 0.439196176 | RAB34 | member RAS oncogene family [Source:HGNC Symbol;Acc:HGNC:16519]" |
| ENSG00000110987 | HGLibA_04337 | 174 | 0.439196176 | BCL7A | BCL tumor suppressor 7A [Source:HGNC Symbol;Acc:HGNC:1004] |
| ENSG00000112337 | HGLibA_44463 | 174 | 0.439196176 | SLC17A2 | solute carrier family 17 member 2 [Source:HGNC Symbol;Acc:HGNC:10930] |
| ENSG00000113494 | HGLibA_38605 | 174 | 0.439196176 | PRLR | prolactin receptor [Source:HGNC Symbol;Acc:HGNC:9446] |
| ENSG00000115421 | HGLibA_35143 | 174 | 0.439196176 | PAPOLG | poly(A) polymerase gamma [Source:HGNC Symbol;Acc:HGNC:14982] |
| ENSG00000120699 | HGLibA_15793 | 174 | 0.439196176 | EXOSC8 | exosome component 8 [Source:HGNC Symbol;Acc:HGNC:17035] |
| ENSG00000123388 | HGLibA_21959 | 174 | 0.439196176 | HOXC11 | homeobox C11 [Source:HGNC Symbol;Acc:HGNC:5123] |
| ENSG00000124207 | HGLibA_11319 | 174 | 0.439196176 | CSE1L | chromosome segregation 1 like [Source:HGNC Symbol;Acc:HGNC:2431] |
| ENSG00000131174 | HGLibA_10867 | 174 | 0.439196176 | COX7B | cytochrome c oxidase subunit 7B [Source:HGNC Symbol;Acc:HGNC:2291] |
| ENSG00000132823 | HGLibA_34679 | 174 | 0.439196176 | OSER1 | oxidative stress responsive serine rich 1 [Source:HGNC Symbol;Acc:HGNC:16105] |
| ENSG00000133048 | HGLibA_09437 | 174 | 0.439196176 | CHI3L1 | chitinase 3 like 1 [Source:HGNC Symbol;Acc:HGNC:1932] |
| ENSG00000134884 | HGLibA_02695 | 174 | 0.439196176 | ARGLU1 | arginine and glutamate rich 1 [Source:HGNC Symbol;Acc:HGNC:25482] |
| ENSG00000135108 | HGLibA_17046 | 174 | 0.439196176 | FBXO21 | F-box protein 21 [Source:HGNC Symbol;Acc:HGNC:13592] |
| ENSG00000137074 | HGLibA_02575 | 174 | 0.439196176 | APTX | aprataxin [Source:HGNC Symbol;Acc:HGNC:15984] |
| ENSG00000139053 | HGLibA_35821 | 174 | 0.439196176 | PDE6H | phosphodiesterase 6H [Source:HGNC Symbol;Acc:HGNC:8790] |
| ENSG00000141252 | HGLibA_53810 | 174 | 0.439196176 | VPS53 | GARP complex subunit [Source:HGNC Symbol;Acc:HGNC:25608]" |
| ENSG00000141526 | HGLibA_44439 | 174 | 0.439196176 | SLC16A3 | solute carrier family 16 member 3 [Source:HGNC Symbol;Acc:HGNC:10924] |
| ENSG00000143006 | HGLibA_13433 | 174 | 0.439196176 | DMRTB1 | DMRT like family B with proline rich C-terminal 1 [Source:HGNC Symbol;Acc:HGNC:13913] |
| ENSG00000143569 | HGLibA_52556 | 174 | 0.439196176 | UBAP2L | ubiquitin associated protein 2 like [Source:HGNC Symbol;Acc:HGNC:29877] |
| ENSG00000145362 | HGLibA_01936 | 174 | 0.439196176 | ANK2 | ankyrin 2 [Source:HGNC Symbol;Acc:HGNC:493] |
| ENSG00000147614 | HGLibA_03722 | 174 | 0.439196176 | ATP6V0D2 | ATPase H+ transporting V0 subunit d2 [Source:HGNC Symbol;Acc:HGNC:18266] |
| ENSG00000148483 | HGLibA_50273 | 174 | 0.439196176 | TMEM236 | transmembrane protein 236 [Source:HGNC Symbol;Acc:HGNC:23473] |
| ENSG00000149262 | HGLibA_23421 | 174 | 0.439196176 | INTS4 | integrator complex subunit 4 [Source:HGNC Symbol;Acc:HGNC:25048] |
| ENSG00000151923 | HGLibA_49437 | 174 | 0.439196176 | TIAL1 | TIA1 cytotoxic granule associated RNA binding protein like 1 [Source:HGNC Symbol;Acc:HGNC:11804] |
| ENSG00000155719 | HGLibA_34722 | 174 | 0.439196176 | OTOA | otoancorin [Source:HGNC Symbol;Acc:HGNC:16378] |
| ENSG00000156398 | HGLibA_43783 | 174 | 0.439196176 | SFXN2 | sideroflexin 2 [Source:HGNC Symbol;Acc:HGNC:16086] |
| ENSG00000156427 | HGLibA_17383 | 174 | 0.439196176 | FGF18 | fibroblast growth factor 18 [Source:HGNC Symbol;Acc:HGNC:3674] |
| ENSG00000157060 | HGLibA_44014 | 174 | 0.439196176 | SHCBP1L | SHC binding and spindle associated 1 like [Source:HGNC Symbol;Acc:HGNC:16788] |
| ENSG00000159069 | HGLibA_17150 | 174 | 0.439196176 | FBXW5 | F-box and WD repeat domain containing 5 [Source:HGNC Symbol;Acc:HGNC:13613] |
| ENSG00000160716 | HGLibA_09593 | 174 | 0.439196176 | CHRNB2 | cholinergic receptor nicotinic beta 2 subunit [Source:HGNC Symbol;Acc:HGNC:1962] |
| ENSG00000161944 | HGLibA_03291 | 174 | 0.439196176 | ASGR2 | asialoglycoprotein receptor 2 [Source:HGNC Symbol;Acc:HGNC:743] |
| ENSG00000162994 | HGLibA_10017 | 174 | 0.439196176 | CLHC1 | clathrin heavy chain linker domain containing 1 [Source:HGNC Symbol;Acc:HGNC:26453] |
| ENSG00000163154 | HGLibA_50723 | 174 | 0.439196176 | TNFAIP8L2 | TNF alpha induced protein 8 like 2 [Source:HGNC Symbol;Acc:HGNC:26277] |
| ENSG00000164897 | HGLibA_50682 | 174 | 0.439196176 | TMUB1 | transmembrane and ubiquitin like domain containing 1 [Source:HGNC Symbol;Acc:HGNC:21709] |
| ENSG00000164967 | HGLibA_42033 | 174 | 0.439196176 | RPP25L | ribonuclease P/MRP subunit p25 like [Source:HGNC Symbol;Acc:HGNC:19909] |
| ENSG00000165495 | HGLibA_36850 | 174 | 0.439196176 | PKNOX2 | PBX/knotted 1 homeobox 2 [Source:HGNC Symbol;Acc:HGNC:16714] |
| ENSG00000165501 | HGLibA_27242 | 174 | 0.439196176 | LRR1 | leucine rich repeat protein 1 [Source:HGNC Symbol;Acc:HGNC:19742] |
| ENSG00000166206 | HGLibA_18379 | 174 | 0.439196176 | GABRB3 | gamma-aminobutyric acid type A receptor beta3 subunit [Source:HGNC Symbol;Acc:HGNC:4083] |
| ENSG00000166363 | HGLibA_33470 | 174 | 0.439196176 | OR10A5 | olfactory receptor family 10 subfamily A member 5 [Source:HGNC Symbol;Acc:HGNC:15131] |
| ENSG00000166428 | HGLibA_37043 | 174 | 0.439196176 | PLD4 | phospholipase D family member 4 [Source:HGNC Symbol;Acc:HGNC:23792] |
| ENSG00000166821 | HGLibA_36084 | 174 | 0.439196176 | PEX11A | peroxisomal biogenesis factor 11 alpha [Source:HGNC Symbol;Acc:HGNC:8852] |
| ENSG00000167419 | HGLibA_27106 | 174 | 0.439196176 | LPO | lactoperoxidase [Source:HGNC Symbol;Acc:HGNC:6678] |
| ENSG00000167851 | HGLibA_08334 | 174 | 0.439196176 | CD300A | CD300a molecule [Source:HGNC Symbol;Acc:HGNC:19319] |
| ENSG00000168216 | HGLibA_26675 | 174 | 0.439196176 | LMBRD1 | LMBR1 domain containing 1 [Source:HGNC Symbol;Acc:HGNC:23038] |
| ENSG00000168575 | HGLibA_44531 | 174 | 0.439196176 | SLC20A2 | solute carrier family 20 member 2 [Source:HGNC Symbol;Acc:HGNC:10947] |
| ENSG00000168710 | HGLibA_01289 | 174 | 0.439196176 | AHCYL1 | adenosylhomocysteinase like 1 [Source:HGNC Symbol;Acc:HGNC:344] |
| ENSG00000169777 | HGLibA_48362 | 174 | 0.439196176 | TAS2R1 | taste 2 receptor member 1 [Source:HGNC Symbol;Acc:HGNC:14909] |
| ENSG00000170260 | HGLibA_55589 | 174 | 0.439196176 | ZNF212 | zinc finger protein 212 [Source:HGNC Symbol;Acc:HGNC:13004] |
| ENSG00000170542 | HGLibA_43568 | 174 | 0.439196176 | SERPINB9 | serpin family B member 9 [Source:HGNC Symbol;Acc:HGNC:8955] |
| ENSG00000171310 | HGLibA_09617 | 174 | 0.439196176 | CHST11 | carbohydrate sulfotransferase 11 [Source:HGNC Symbol;Acc:HGNC:17422] |
| ENSG00000173950 | HGLibA_54613 | 174 | 0.439196176 | XXYLT1 | xyloside xylosyltransferase 1 [Source:HGNC Symbol;Acc:HGNC:26639] |
| ENSG00000175329 | HGLibA_23692 | 174 | 0.439196176 | ISX | intestine specific homeobox [Source:HGNC Symbol;Acc:HGNC:28084] |
| ENSG00000177119 | HGLibA_02187 | 174 | 0.439196176 | ANO6 | anoctamin 6 [Source:HGNC Symbol;Acc:HGNC:25240] |
| ENSG00000177692 | HGLibA_13612 | 174 | 0.439196176 | DNAJC28 | DnaJ heat shock protein family (Hsp40) member C28 [Source:HGNC Symbol;Acc:HGNC:1297] |
| ENSG00000178498 | HGLibA_14086 | 174 | 0.439196176 | DTX3 | deltex E3 ubiquitin ligase 3 [Source:HGNC Symbol;Acc:HGNC:24457] |
| ENSG00000178764 | HGLibA_55315 | 174 | 0.439196176 | ZHX2 | zinc fingers and homeoboxes 2 [Source:HGNC Symbol;Acc:HGNC:18513] |
| ENSG00000185894 | HGLibA_04750 | 174 | 0.439196176 | BPY2C | basic charge Y-linked 2C [Source:HGNC Symbol;Acc:HGNC:18225] |
| ENSG00000189007 | HGLibA_00881 | 174 | 0.439196176 | ADAT2 | tRNA specific 2 [Source:HGNC Symbol;Acc:HGNC:21172]" |
| ENSG00000204531 | HGLibA_37787 | 174 | 0.439196176 | POU5F1 | POU class 5 homeobox 1 [Source:HGNC Symbol;Acc:HGNC:9221] |
| ENSG00000204577 | HGLibA_26512 | 174 | 0.439196176 | LILRB3 | leukocyte immunoglobulin like receptor B3 [Source:HGNC Symbol;Acc:HGNC:6607] |
| ENSG00000205138 | HGLibA_43135 | 174 | 0.439196176 | SDHAF1 | succinate dehydrogenase complex assembly factor 1 [Source:HGNC Symbol;Acc:HGNC:33867] |
| ENSG00000237172 | HGLibA_04006 | 174 | 0.439196176 | B3GNT9 | 3-N-acetylglucosaminyltransferase 9 [Source:HGNC Symbol;Acc:HGNC:28714]" |
| ENSG00000243678 | HGLibA_32156 | 174 | 0.439196176 | NME2 | NME/NM23 nucleoside diphosphate kinase 2 [Source:HGNC Symbol;Acc:HGNC:7850] |

| | | | | | |
|---|---|---|---|---|---|
| ENSG00000248905 | HGLibA_17686 | 174 | 0.439196176 | FMN1 | formin 1 [Source:HGNC Symbol;Acc:HGNC:3768] |
| ENSG00000269743 | HGLibA_44771 | 174 | 0.439196176 | SLC25A53 | solute carrier family 25 member 53 [Source:HGNC Symbol;Acc:HGNC:31894] |
| ENSG00000270757 | HGLibA_22338 | 174 | 0.439196176 | HSPE1-MOB4 | HSPE1-MOB4 readthrough [Source:HGNC Symbol;Acc:HGNC:49184] |
| ENSG00000278259 | HGLibA_30817 | 174 | 0.439196176 | MYO19 | myosin XIX [Source:HGNC Symbol;Acc:HGNC:26234] |
| ENSG00000278619 | HGLibA_29861 | 174 | 0.439196176 | MRM1 | mitochondrial rRNA methyltransferase 1 [Source:HGNC Symbol;Acc:HGNC:26202] |
| H3F3A | HGLibA_20778 | 174 | 0.439196176 | H3F3A | - |
| hsa-mir-1267 | HGLibA_57492 | 174 | 0.439196176 | hsa-mir-1267 | - |
| hsa-mir-3155a | HGLibA_58678 | 174 | 0.439196176 | hsa-mir-3155a | - |
| hsa-mir-4423 | HGLibA_60081 | 174 | 0.439196176 | hsa-mir-4423 | - |
| hsa-mir-4644 | HGLibA_60679 | 174 | 0.439196176 | hsa-mir-4644 | - |
| hsa-mir-4675 | HGLibA_60818 | 174 | 0.439196176 | hsa-mir-4675 | - |
| hsa-mir-548aw | HGLibA_61937 | 174 | 0.439196176 | hsa-mir-548aw | - |
| hsa-mir-5587 | HGLibA_62190 | 174 | 0.439196176 | hsa-mir-5587 | - |
| hsa-mir-6715b | HGLibA_63020 | 174 | 0.439196176 | hsa-mir-6715b | - |
| KIAA1377 | HGLibA_24746 | 174 | 0.439196176 | KIAA1377 | - |
| LOC100506422 | HGLibA_26860 | 174 | 0.439196176 | LOC100506422 | - |
| NIM1 | HGLibA_31932 | 174 | 0.439196176 | NIM1 | - |
| C12orf10 | HGLibA_05224 | 173 | 0.430150833 | C12orf10 | - |
| ENSG00000006638 | HGLibA_48680 | 173 | 0.430150833 | TBXA2R | thromboxane A2 receptor [Source:HGNC Symbol;Acc:HGNC:11608] |
| ENSG00000007062 | HGLibA_38681 | 173 | 0.430150833 | PROM1 | prominin 1 [Source:HGNC Symbol;Acc:HGNC:9454] |
| ENSG00000008838 | HGLibA_28783 | 173 | 0.430150833 | MED24 | mediator complex subunit 24 [Source:HGNC Symbol;Acc:HGNC:22963] |
| ENSG00000027847 | HGLibA_04040 | 173 | 0.430150833 | B4GALT7 | 4-galactosyltransferase 7 [Source:HGNC Symbol;Acc:HGNC:930]" |
| ENSG00000054690 | HGLibA_37125 | 173 | 0.430150833 | PLEKHH1 | MyTH4 and FERM domain containing H1 [Source:HGNC Symbol;Acc:HGNC:17733]" |
| ENSG00000062485 | HGLibA_11300 | 173 | 0.430150833 | CS | citrate synthase [Source:HGNC Symbol;Acc:HGNC:2422] |
| ENSG00000069482 | HGLibA_18493 | 173 | 0.430150833 | GAL | galanin and GMAP prepropeptide [Source:HGNC Symbol;Acc:HGNC:4114] |
| ENSG00000070756 | HGLibA_34921 | 173 | 0.430150833 | PABPC1 | poly(A) binding protein cytoplasmic 1 [Source:HGNC Symbol;Acc:HGNC:8554] |
| ENSG00000072803 | HGLibA_17138 | 173 | 0.430150833 | FBXW11 | F-box and WD repeat domain containing 11 [Source:HGNC Symbol;Acc:HGNC:13607] |
| ENSG00000079841 | HGLibA_41277 | 173 | 0.430150833 | RIMS1 | regulating synaptic membrane exocytosis 1 [Source:HGNC Symbol;Acc:HGNC:17282] |
| ENSG00000099817 | HGLibA_37576 | 173 | 0.430150833 | POLR2E | RNA polymerase II subunit E [Source:HGNC Symbol;Acc:HGNC:9192] |
| ENSG00000100335 | HGLibA_29242 | 173 | 0.430150833 | MIEF1 | mitochondrial elongation factor 1 [Source:HGNC Symbol;Acc:HGNC:25979] |
| ENSG00000100528 | HGLibA_10284 | 173 | 0.430150833 | CNIH1 | cornichon family AMPA receptor auxiliary protein 1 [Source:HGNC Symbol;Acc:HGNC:19431] |
| ENSG00000101417 | HGLibA_39661 | 173 | 0.430150833 | PXMP4 | peroxisomal membrane protein 4 [Source:HGNC Symbol;Acc:HGNC:15920] |
| ENSG00000101417 | HGLibA_39660 | 173 | 0.430150833 | PXMP4 | peroxisomal membrane protein 4 [Source:HGNC Symbol;Acc:HGNC:15920] |
| ENSG00000102904 | HGLibA_51890 | 173 | 0.430150833 | TSNAXIP1 | translin associated factor X interacting protein 1 [Source:HGNC Symbol;Acc:HGNC:18586] |
| ENSG00000103037 | HGLibA_43654 | 173 | 0.430150833 | SETD6 | SET domain containing 6 [Source:HGNC Symbol;Acc:HGNC:26116] |
| ENSG00000104059 | HGLibA_16350 | 173 | 0.430150833 | FAM189A1 | family with sequence similarity 189 member A1 [Source:HGNC Symbol;Acc:HGNC:29075] |
| ENSG00000106336 | HGLibA_17052 | 173 | 0.430150833 | FBXO24 | F-box protein 24 [Source:HGNC Symbol;Acc:HGNC:13595] |
| ENSG00000113396 | HGLibA_44828 | 173 | 0.430150833 | SLC27A6 | solute carrier family 27 member 6 [Source:HGNC Symbol;Acc:HGNC:11000] |
| ENSG00000113851 | HGLibA_11078 | 173 | 0.430150833 | CRBN | cereblon [Source:HGNC Symbol;Acc:HGNC:30185] |
| ENSG00000115652 | HGLibA_53478 | 173 | 0.430150833 | UXS1 | UDP-glucuronate decarboxylase 1 [Source:HGNC Symbol;Acc:HGNC:17729] |
| ENSG00000115657 | HGLibA_00147 | 173 | 0.430150833 | ABCB6 | ATP binding cassette subfamily B member 6 (Langereis blood group) [Source:HGNC Symbol;Acc:HGNC:47] |
| ENSG00000117724 | HGLibA_09054 | 173 | 0.430150833 | CENPF | centromere protein F [Source:HGNC Symbol;Acc:HGNC:1857] |
| ENSG00000120063 | HGLibA_19443 | 173 | 0.430150833 | GNA13 | G protein subunit alpha 13 [Source:HGNC Symbol;Acc:HGNC:4381] |
| ENSG00000120162 | HGLibA_29597 | 173 | 0.430150833 | MOB3B | MOB kinase activator 3B [Source:HGNC Symbol;Acc:HGNC:23825] |
| ENSG00000121634 | HGLibA_19150 | 173 | 0.430150833 | GJA8 | gap junction protein alpha 8 [Source:HGNC Symbol;Acc:HGNC:4281] |
| ENSG00000122644 | HGLibA_02965 | 173 | 0.430150833 | ARL4A | ADP ribosylation factor like GTPase 4A [Source:HGNC Symbol;Acc:HGNC:695] |
| ENSG00000124116 | HGLibA_54266 | 173 | 0.430150833 | WFDC3 | WAP four-disulfide core domain 3 [Source:HGNC Symbol;Acc:HGNC:15957] |
| ENSG00000124203 | HGLibA_56835 | 173 | 0.430150833 | ZNF831 | zinc finger protein 831 [Source:HGNC Symbol;Acc:HGNC:16167] |
| ENSG00000128699 | HGLibA_34626 | 173 | 0.430150833 | ORMDL1 | ORMDL sphingolipid biosynthesis regulator 1 [Source:HGNC Symbol;Acc:HGNC:16036] |
| ENSG00000129317 | HGLibA_39606 | 173 | 0.430150833 | PUS7L | pseudouridine synthase 7 like [Source:HGNC Symbol;Acc:HGNC:25276] |
| ENSG00000129968 | HGLibA_00263 | 173 | 0.430150833 | ABHD17A | abhydrolase domain containing 17A [Source:HGNC Symbol;Acc:HGNC:28756] |
| ENSG00000133619 | HGLibA_25386 | 173 | 0.430150833 | KRBA1 | KRAB-A domain containing 1 [Source:HGNC Symbol;Acc:HGNC:22228] |
| ENSG00000135929 | HGLibA_12093 | 173 | 0.430150833 | CYP27A1 | cytochrome P450 family 27 subfamily A member 1 [Source:HGNC Symbol;Acc:HGNC:2605] |
| ENSG00000137947 | HGLibA_20529 | 173 | 0.430150833 | GTF2B | general transcription factor IIB [Source:HGNC Symbol;Acc:HGNC:4648] |
| ENSG00000138798 | HGLibA_14620 | 173 | 0.430150833 | EGF | epidermal growth factor [Source:HGNC Symbol;Acc:HGNC:3229] |
| ENSG00000139278 | HGLibA_19258 | 173 | 0.430150833 | GLIPR1 | GLI pathogenesis related 1 [Source:HGNC Symbol;Acc:HGNC:17001] |
| ENSG00000139572 | HGLibA_20094 | 173 | 0.430150833 | GPR84 | G protein-coupled receptor 84 [Source:HGNC Symbol;Acc:HGNC:4535] |
| ENSG00000140254 | HGLibA_14103 | 173 | 0.430150833 | DUOXA1 | dual oxidase maturation factor 1 [Source:HGNC Symbol;Acc:HGNC:26507] |
| ENSG00000143032 | HGLibA_04160 | 173 | 0.430150833 | BARHL2 | BarH like homeobox 2 [Source:HGNC Symbol;Acc:HGNC:954] |
| ENSG00000143499 | HGLibA_45853 | 173 | 0.430150833 | SMYD2 | SET and MYND domain containing 2 [Source:HGNC Symbol;Acc:HGNC:20982] |
| ENSG00000143543 | HGLibA_24000 | 173 | 0.430150833 | JTB | jumping translocation breakpoint [Source:HGNC Symbol;Acc:HGNC:6201] |
| ENSG00000145632 | HGLibA_37192 | 173 | 0.430150833 | PLK2 | polo like kinase 2 [Source:HGNC Symbol;Acc:HGNC:19699] |
| ENSG00000145794 | HGLibA_28847 | 173 | 0.430150833 | MEGF10 | multiple EGF like domains 10 [Source:HGNC Symbol;Acc:HGNC:29634] |
| ENSG00000147852 | HGLibA_53684 | 173 | 0.430150833 | VLDLR | very low density lipoprotein receptor [Source:HGNC Symbol;Acc:HGNC:12698] |
| ENSG00000152582 | HGLibA_46612 | 173 | 0.430150833 | SPEF2 | sperm flagellar 2 [Source:HGNC Symbol;Acc:HGNC:26293] |
| ENSG00000154719 | HGLibA_29971 | 173 | 0.430150833 | MRPL39 | mitochondrial ribosomal protein L39 [Source:HGNC Symbol;Acc:HGNC:14027] |
| ENSG00000156345 | HGLibA_08759 | 173 | 0.430150833 | CDK20 | cyclin dependent kinase 20 [Source:HGNC Symbol;Acc:HGNC:21420] |
| ENSG00000157653 | HGLibA_06733 | 173 | 0.430150833 | C9orf43 | chromosome 9 open reading frame 43 [Source:HGNC Symbol;Acc:HGNC:23570] |
| ENSG00000160182 | HGLibA_49169 | 173 | 0.430150833 | TFF1 | trefoil factor 1 [Source:HGNC Symbol;Acc:HGNC:11755] |
| ENSG00000162687 | HGLibA_24397 | 173 | 0.430150833 | KCNT2 | potassium sodium-activated channel subfamily T member 2 [Source:HGNC Symbol;Acc:HGNC:18866] |
| ENSG00000162999 | HGLibA_14154 | 173 | 0.430150833 | DUSP19 | dual specificity phosphatase 19 [Source:HGNC Symbol;Acc:HGNC:18894] |
| ENSG00000164106 | HGLibA_43042 | 173 | 0.430150833 | SCRG1 | stimulator of chondrogenesis 1 [Source:HGNC Symbol;Acc:HGNC:17036] |
| ENSG00000165621 | HGLibA_34823 | 173 | 0.430150833 | OXGR1 | oxoglutarate receptor 1 [Source:HGNC Symbol;Acc:HGNC:4531] |
| ENSG00000165985 | HGLibA_05931 | 173 | 0.430150833 | C1QL3 | complement C1q like 3 [Source:HGNC Symbol;Acc:HGNC:19359] |
| ENSG00000166501 | HGLibA_38535 | 173 | 0.430150833 | PRKCB | protein kinase C beta [Source:HGNC Symbol;Acc:HGNC:9395] |
| ENSG00000168329 | HGLibA_11835 | 173 | 0.430150833 | CX3CR1 | C-X3-C motif chemokine receptor 1 [Source:HGNC Symbol;Acc:HGNC:2558] |
| ENSG00000169224 | HGLibA_18831 | 173 | 0.430150833 | GCSAML | germinal center associated signaling and motility like [Source:HGNC Symbol;Acc:HGNC:29583] |
| ENSG00000169427 | HGLibA_24335 | 173 | 0.430150833 | KCNK9 | potassium two pore domain channel subfamily K member 9 [Source:HGNC Symbol;Acc:HGNC:6283] |
| ENSG00000171241 | HGLibA_44011 | 173 | 0.430150833 | SHCBP1 | SHC binding and spindle associated 1 [Source:HGNC Symbol;Acc:HGNC:29547] |
| ENSG00000172289 | HGLibA_33552 | 173 | 0.430150833 | OR10V1 | olfactory receptor family 10 subfamily V member 1 [Source:HGNC Symbol;Acc:HGNC:15136] |
| ENSG00000174776 | HGLibA_54111 | 173 | 0.430150833 | WDR49 | WD repeat domain 49 [Source:HGNC Symbol;Acc:HGNC:26587] |
| ENSG00000175619 | HGLibA_33936 | 173 | 0.430150833 | OR4B1 | olfactory receptor family 4 subfamily B member 1 [Source:HGNC Symbol;Acc:HGNC:8290] |
| ENSG00000176531 | HGLibA_36433 | 173 | 0.430150833 | PHLDB3 | pleckstrin homology like domain family B member 3 [Source:HGNC Symbol;Acc:HGNC:30499] |
| ENSG00000177613 | HGLibA_11490 | 173 | 0.430150833 | CSTF2T | cleavage stimulation factor subunit 2 tau variant [Source:HGNC Symbol;Acc:HGNC:17086] |
| ENSG00000178279 | HGLibA_50895 | 173 | 0.430150833 | TNP2 | transition protein 2 [Source:HGNC Symbol;Acc:HGNC:11952] |
| ENSG00000179002 | HGLibA_48356 | 173 | 0.430150833 | TAS1R2 | taste 1 receptor member 2 [Source:HGNC Symbol;Acc:HGNC:14905] |
| ENSG00000179673 | HGLibA_42055 | 173 | 0.430150833 | RPRML | reprimo like [Source:HGNC Symbol;Acc:HGNC:32422] |
| ENSG00000180660 | HGLibA_27822 | 173 | 0.430150833 | MAB21L1 | mab-21 like 1 [Source:HGNC Symbol;Acc:HGNC:6757] |
| ENSG00000182325 | HGLibA_17016 | 173 | 0.430150833 | FBXL6 | F-box and leucine rich repeat protein 6 [Source:HGNC Symbol;Acc:HGNC:13603] |
| ENSG00000184304 | HGLibA_38565 | 173 | 0.430150833 | PRKD1 | protein kinase D1 [Source:HGNC Symbol;Acc:HGNC:9407] |
| ENSG00000186431 | HGLibA_17164 | 173 | 0.430150833 | FCAR | Fc fragment of IgA receptor [Source:HGNC Symbol;Acc:HGNC:3608] |
| ENSG00000187624 | HGLibA_05597 | 173 | 0.430150833 | C17orf97 | chromosome 17 open reading frame 97 [Source:HGNC Symbol;Acc:HGNC:33800] |
| ENSG00000188643 | HGLibA_42548 | 173 | 0.430150833 | S100A16 | S100 calcium binding protein A16 [Source:HGNC Symbol;Acc:HGNC:20441] |
| ENSG00000189369 | HGLibA_20442 | 173 | 0.430150833 | GSPT2 | G1 to S phase transition 2 [Source:HGNC Symbol;Acc:HGNC:4622] |
| ENSG00000196218 | HGLibA_42520 | 173 | 0.430150833 | RYR1 | ryanodine receptor 1 [Source:HGNC Symbol;Acc:HGNC:10483] |
| ENSG00000196290 | HGLibA_31928 | 173 | 0.430150833 | NIF3L1 | NGG1 interacting factor 3 like 1 [Source:HGNC Symbol;Acc:HGNC:13390] |
| ENSG00000197375 | HGLibA_44592 | 173 | 0.430150833 | SLC22A5 | solute carrier family 22 member 5 [Source:HGNC Symbol;Acc:HGNC:10969] |
| ENSG00000198721 | HGLibA_14410 | 173 | 0.430150833 | ECI2 | enoyl-CoA delta isomerase 2 [Source:HGNC Symbol;Acc:HGNC:14601] |
| ENSG00000198876 | HGLibA_12417 | 173 | 0.430150833 | DCAF12 | DDB1 and CUL4 associated factor 12 [Source:HGNC Symbol;Acc:HGNC:19911] |
| ENSG00000206013 | HGLibA_22651 | 173 | 0.430150833 | IFITM5 | interferon induced transmembrane protein 5 [Source:HGNC Symbol;Acc:HGNC:16644] |
| ENSG00000215695 | HGLibA_42307 | 173 | 0.430150833 | RSC1A1 | regulator of solute carriers 1 [Source:HGNC Symbol;Acc:HGNC:10458] |
| ENSG00000224389 | HGLibA_06284 | 173 | 0.430150833 | C4B | complement C4B (Chido blood group) [Source:HGNC Symbol;Acc:HGNC:1324] |
| ENSG00000227471 | HGLibA_01477 | 173 | 0.430150833 | AKR1B15 | aldo-keto reductase family 1 member B15 [Source:HGNC Symbol;Acc:HGNC:37281] |
| ENSG00000233198 | HGLibA_41649 | 173 | 0.430150833 | RNF224 | ring finger protein 224 [Source:HGNC Symbol;Acc:HGNC:41912] |
| ENSG00000256235 | HGLibA_45754 | 173 | 0.430150833 | SMIM3 | small integral membrane protein 3 [Source:HGNC Symbol;Acc:HGNC:30248] |
| ENSG00000257365 | HGLibA_17773 | 173 | 0.430150833 | FNTB | beta [Source:HGNC Symbol;Acc:HGNC:3785]" |
| ENSG00000259803 | HGLibA_44584 | 173 | 0.430150833 | SLC22A31 | solute carrier family 22 member 31 [Source:HGNC Symbol;Acc:HGNC:27091] |
| ENSG00000265190 | HGLibA_02253 | 173 | 0.430150833 | ANXA8 | annexin A8 [Source:HGNC Symbol;Acc:HGNC:546] |
| FAM213B | HGLibA_16475 | 173 | 0.430150833 | FAM213B | - |
| hsa-mir-1228 | HGLibA_57311 | 173 | 0.430150833 | hsa-mir-1228 | - |
| hsa-mir-15a | HGLibA_57939 | 173 | 0.430150833 | hsa-mir-15a | - |
| hsa-mir-4763 | HGLibA_61147 | 173 | 0.430150833 | hsa-mir-4763 | - |
| hsa-mir-5190 | HGLibA_61679 | 173 | 0.430150833 | hsa-mir-5190 | - |
| hsa-mir-6084 | HGLibA_62595 | 173 | 0.430150833 | hsa-mir-6084 | - |
| hsa-mir-6739 | HGLibA_63109 | 173 | 0.430150833 | hsa-mir-6739 | - |
| hsa-mir-941-3 | HGLibA_64345 | 173 | 0.430150833 | hsa-mir-941-3 | - |
| KIAA1804 | HGLibA_24821 | 173 | 0.430150833 | KIAA1804 | - |
| PPP2R3B_X | HGLibA_38157 | 173 | 0.430150833 | PPP2R3B_X | - |
| C1orf111 | HGLibA_05747 | 172 | 0.42110549 | C1orf111 | - |
| C1orf64 | HGLibA_05885 | 172 | 0.42110549 | C1orf64 | - |
| ENSG00000008118 | HGLibA_07064 | 172 | 0.42110549 | CAMK1G | calcium/calmodulin dependent protein kinase IG [Source:HGNC Symbol;Acc:HGNC:14585] |
| ENSG00000054282 | HGLibA_43119 | 172 | 0.42110549 | SDCCAG8 | serologically defined colon cancer antigen 8 [Source:HGNC Symbol;Acc:HGNC:10671] |
| ENSG00000066855 | HGLibA_30360 | 172 | 0.42110549 | MTFR1 | mitochondrial fission regulator 1 [Source:HGNC Symbol;Acc:HGNC:29510] |
| ENSG00000072195 | HGLibA_46615 | 172 | 0.42110549 | SPEG | SPEG complex locus [Source:HGNC Symbol;Acc:HGNC:16901] |
| ENSG00000072364 | HGLibA_01116 | 172 | 0.42110549 | AFF4 | AF4/FMR2 family member 4 [Source:HGNC Symbol;Acc:HGNC:17869] |
| ENSG00000078403 | HGLibA_29410 | 172 | 0.42110549 | MLLT10 | histone lysine methyltransferase DOT1L cofactor [Source:HGNC Symbol;Acc:HGNC:16063]" |
| ENSG00000080007 | HGLibA_12718 | 172 | 0.42110549 | DDX43 | DEAD-box helicase 43 [Source:HGNC Symbol;Acc:HGNC:18677] |
| ENSG00000083838 | HGLibA_56019 | 172 | 0.42110549 | ZNF446 | zinc finger protein 446 [Source:HGNC Symbol;Acc:HGNC:21036] |
| ENSG00000085274 | HGLibA_30799 | 172 | 0.42110549 | MYNN | myoneurin [Source:HGNC Symbol;Acc:HGNC:14955] |
| ENSG00000089159 | HGLibA_39665 | 172 | 0.42110549 | PXN | paxillin [Source:HGNC Symbol;Acc:HGNC:9718] |
| ENSG00000100426 | HGLibA_54800 | 172 | 0.42110549 | ZBED4 | zinc finger BED-type containing 4 [Source:HGNC Symbol;Acc:HGNC:20721] |
| ENSG00000106004 | HGLibA_21914 | 172 | 0.42110549 | HOXA5 | homeobox A5 [Source:HGNC Symbol;Acc:HGNC:5106] |
| ENSG00000106246 | HGLibA_39259 | 172 | 0.42110549 | PTCD1 | pentatricopeptide repeat domain 1 [Source:HGNC Symbol;Acc:HGNC:22198] |
| ENSG00000106799 | HGLibA_49223 | 172 | 0.42110549 | TGFBR1 | transforming growth factor beta receptor 1 [Source:HGNC Symbol;Acc:HGNC:11772] |
| ENSG00000106991 | HGLibA_15124 | 172 | 0.42110549 | ENG | endoglin [Source:HGNC Symbol;Acc:HGNC:3349] |

| | | | | | |
|---|---|---|---|---|---|
| ENSG00000107854 | HGLibA_50855 | 172 | 0.42110549 | TNKS2 | tankyrase 2 [Source:HGNC Symbol;Acc:HGNC:15677] |
| ENSG00000110243 | HGLibA_02462 | 172 | 0.42110549 | APOA5 | apolipoprotein A5 [Source:HGNC Symbol;Acc:HGNC:17288] |
| ENSG00000110321 | HGLibA_14843 | 172 | 0.42110549 | EIF4G2 | eukaryotic translation initiation factor 4 gamma 2 [Source:HGNC Symbol;Acc:HGNC:3297] |
| ENSG00000111752 | HGLibA_36319 | 172 | 0.42110549 | PHC1 | polyhomeotic homolog 1 [Source:HGNC Symbol;Acc:HGNC:3182] |
| ENSG00000112149 | HGLibA_08461 | 172 | 0.42110549 | CD83 | CD83 molecule [Source:HGNC Symbol;Acc:HGNC:1703] |
| ENSG00000112238 | HGLibA_38373 | 172 | 0.42110549 | PRDM13 | PR/SET domain 13 [Source:HGNC Symbol;Acc:HGNC:13998] |
| ENSG00000114541 | HGLibA_18017 | 172 | 0.42110549 | FRMD4B | FERM domain containing 4B [Source:HGNC Symbol;Acc:HGNC:24886] |
| ENSG00000119138 | HGLibA_25088 | 172 | 0.42110549 | KLF9 | Kruppel like factor 9 [Source:HGNC Symbol;Acc:HGNC:1123] |
| ENSG00000121207 | HGLibA_27132 | 172 | 0.42110549 | LRAT | lecithin retinol acyltransferase [Source:HGNC Symbol;Acc:HGNC:6685] |
| ENSG00000122068 | HGLibA_18256 | 172 | 0.42110549 | FYTTD1 | forty-two-three domain containing 1 [Source:HGNC Symbol;Acc:HGNC:25407] |
| ENSG00000124275 | HGLibA_30494 | 172 | 0.42110549 | MTRR | 5-methyltetrahydrofolate-homocysteine methyltransferase reductase [Source:HGNC Symbol;Acc:HGNC:7473] |
| ENSG00000126264 | HGLibA_20994 | 172 | 0.42110549 | HCST | hematopoietic cell signal transducer [Source:HGNC Symbol;Acc:HGNC:16977] |
| ENSG00000128487 | HGLibA_46604 | 172 | 0.42110549 | SPECC1 | sperm antigen with calponin homology and coiled-coil domains 1 [Source:HGNC Symbol;Acc:HGNC:30615] |
| ENSG00000130540 | HGLibA_47763 | 172 | 0.42110549 | SULT4A1 | sulfotransferase family 4A member 1 [Source:HGNC Symbol;Acc:HGNC:14903] |
| ENSG00000132017 | HGLibA_12431 | 172 | 0.42110549 | DCAF15 | DDB1 and CUL4 associated factor 15 [Source:HGNC Symbol;Acc:HGNC:25095] |
| ENSG00000132406 | HGLibA_49920 | 172 | 0.42110549 | TMEM128 | transmembrane protein 128 [Source:HGNC Symbol;Acc:HGNC:28201] |
| ENSG00000134049 | HGLibA_22577 | 172 | 0.42110549 | IER3IP1 | immediate early response 3 interacting protein 1 [Source:HGNC Symbol;Acc:HGNC:18550] |
| ENSG00000138380 | HGLibA_07245 | 172 | 0.42110549 | CARF | calcium responsive transcription factor [Source:HGNC Symbol;Acc:HGNC:14435] |
| ENSG00000138622 | HGLibA_20983 | 172 | 0.42110549 | HCN4 | hyperpolarization activated cyclic nucleotide gated potassium channel 4 [Source:HGNC Symbol;Acc:HGNC:16882] |
| ENSG00000140374 | HGLibA_15620 | 172 | 0.42110549 | ETFA | electron transfer flavoprotein subunit alpha [Source:HGNC Symbol;Acc:HGNC:3481] |
| ENSG00000143469 | HGLibA_48059 | 172 | 0.42110549 | SYT14 | synaptotagmin 14 [Source:HGNC Symbol;Acc:HGNC:23143] |
| ENSG00000144119 | HGLibA_05928 | 172 | 0.42110549 | C1QL2 | complement C1q like 2 [Source:HGNC Symbol;Acc:HGNC:24181] |
| ENSG00000144214 | HGLibA_27698 | 172 | 0.42110549 | LYG1 | lysozyme g1 [Source:HGNC Symbol;Acc:HGNC:27014] |
| ENSG00000144642 | HGLibA_40592 | 172 | 0.42110549 | RBMS3 | RNA binding motif single stranded interacting protein 3 [Source:HGNC Symbol;Acc:HGNC:13427] |
| ENSG00000144895 | HGLibA_14720 | 172 | 0.42110549 | EIF2A | eukaryotic translation initiation factor 2A [Source:HGNC Symbol;Acc:HGNC:3254] |
| ENSG00000146842 | HGLibA_50205 | 172 | 0.42110549 | TMEM209 | transmembrane protein 209 [Source:HGNC Symbol;Acc:HGNC:21898] |
| ENSG00000148303 | HGLibA_41996 | 172 | 0.42110549 | RPL7A | ribosomal protein L7a [Source:HGNC Symbol;Acc:HGNC:10364] |
| ENSG00000148516 | HGLibA_55135 | 172 | 0.42110549 | ZEB1 | zinc finger E-box binding homeobox 1 [Source:HGNC Symbol;Acc:HGNC:11642] |
| ENSG00000148604 | HGLibA_41045 | 172 | 0.42110549 | RGR | retinal G protein coupled receptor [Source:HGNC Symbol;Acc:HGNC:9990] |
| ENSG00000148704 | HGLibA_53556 | 172 | 0.42110549 | VAX1 | ventral anterior homeobox 1 [Source:HGNC Symbol;Acc:HGNC:12660] |
| ENSG00000149054 | HGLibA_55596 | 172 | 0.42110549 | ZNF215 | zinc finger protein 215 [Source:HGNC Symbol;Acc:HGNC:13007] |
| ENSG00000153064 | HGLibA_04147 | 172 | 0.42110549 | BANK1 | B cell scaffold protein with ankyrin repeats 1 [Source:HGNC Symbol;Acc:HGNC:18233] |
| ENSG00000153531 | HGLibA_01028 | 172 | 0.42110549 | ADPRHL1 | ADP-ribosylhydrolase like 1 [Source:HGNC Symbol;Acc:HGNC:21303] |
| ENSG00000153989 | HGLibA_33092 | 172 | 0.42110549 | NUS1 | dehydrodolichyl diphosphate synthase subunit [Source:HGNC Symbol;Acc:HGNC:21042]" |
| ENSG00000157456 | HGLibA_08062 | 172 | 0.42110549 | CCNB2 | cyclin B2 [Source:HGNC Symbol;Acc:HGNC:1580] |
| ENSG00000159788 | HGLibA_41057 | 172 | 0.42110549 | RGS12 | regulator of G protein signaling 12 [Source:HGNC Symbol;Acc:HGNC:9994] |
| ENSG00000160505 | HGLibA_32120 | 172 | 0.42110549 | NLRP4 | NLR family pyrin domain containing 4 [Source:HGNC Symbol;Acc:HGNC:22943] |
| ENSG00000165055 | HGLibA_29002 | 172 | 0.42110549 | METTL2B | methyltransferase like 2B [Source:HGNC Symbol;Acc:HGNC:18272] |
| ENSG00000166025 | HGLibA_01818 | 172 | 0.42110549 | AMOTL1 | angiomotin like 1 [Source:HGNC Symbol;Acc:HGNC:17811] |
| ENSG00000167476 | HGLibA_23996 | 172 | 0.42110549 | JSRP1 | junctional sarcoplasmic reticulum protein 1 [Source:HGNC Symbol;Acc:HGNC:24963] |
| ENSG00000168140 | HGLibA_53536 | 172 | 0.42110549 | VASN | vasorin [Source:HGNC Symbol;Acc:HGNC:18517] |
| ENSG00000168243 | HGLibA_19527 | 172 | 0.42110549 | GNG4 | G protein subunit gamma 4 [Source:HGNC Symbol;Acc:HGNC:4407] |
| ENSG00000169418 | HGLibA_32509 | 172 | 0.42110549 | NPR1 | natriuretic peptide receptor 1 [Source:HGNC Symbol;Acc:HGNC:7943] |
| ENSG00000170989 | HGLibA_42592 | 172 | 0.42110549 | S1PR1 | sphingosine-1-phosphate receptor 1 [Source:HGNC Symbol;Acc:HGNC:3165] |
| ENSG00000172915 | HGLibA_31206 | 172 | 0.42110549 | NBEA | neurobeachin [Source:HGNC Symbol;Acc:HGNC:7648] |
| ENSG00000174327 | HGLibA_44430 | 172 | 0.42110549 | SLC16A13 | solute carrier family 16 member 13 [Source:HGNC Symbol;Acc:HGNC:31037] |
| ENSG00000174842 | HGLibA_19279 | 172 | 0.42110549 | GLMN | FKBP associated protein [Source:HGNC Symbol;Acc:HGNC:14373]" |
| ENSG00000178188 | HGLibA_43875 | 172 | 0.42110549 | SH2B1 | SH2B adaptor protein 1 [Source:HGNC Symbol;Acc:HGNC:30417] |
| ENSG00000179761 | HGLibA_36727 | 172 | 0.42110549 | PIPOX | pipecolic acid and sarcosine oxidase [Source:HGNC Symbol;Acc:HGNC:17804] |
| ENSG00000180509 | HGLibA_24183 | 172 | 0.42110549 | KCNE1 | potassium voltage-gated channel subfamily E regulatory subunit 1 [Source:HGNC Symbol;Acc:HGNC:6240] |
| ENSG00000180626 | HGLibA_56343 | 172 | 0.42110549 | ZNF594 | zinc finger protein 594 [Source:HGNC Symbol;Acc:HGNC:29392] |
| ENSG00000181035 | HGLibA_44741 | 172 | 0.42110549 | SLC25A42 | solute carrier family 25 member 42 [Source:HGNC Symbol;Acc:HGNC:28380] |
| ENSG00000183977 | HGLibA_37802 | 172 | 0.42110549 | PP2D1 | protein phosphatase 2C like domain containing 1 [Source:HGNC Symbol;Acc:HGNC:28406] |
| ENSG00000186350 | HGLibA_42506 | 172 | 0.42110549 | RXRA | retinoid X receptor alpha [Source:HGNC Symbol;Acc:HGNC:10477] |
| ENSG00000186860 | HGLibA_25661 | 172 | 0.42110549 | KRTAP17-1 | keratin associated protein 17-1 [Source:HGNC Symbol;Acc:HGNC:18917] |
| ENSG00000187105 | HGLibA_21076 | 172 | 0.42110549 | HEATR4 | HEAT repeat containing 4 [Source:HGNC Symbol;Acc:HGNC:16761] |
| ENSG00000188408 | HGLibA_27954 | 172 | 0.42110549 | MAGEB5 | MAGE family member B5 [Source:HGNC Symbol;Acc:HGNC:23795] |
| ENSG00000188505 | HGLibA_31287 | 172 | 0.42110549 | NCCRP1 | non-specific cytotoxic cell receptor protein 1 homolog (zebrafish) [Source:HGNC Symbol;Acc:HGNC:33739] |
| ENSG00000196119 | HGLibA_34491 | 172 | 0.42110549 | OR8A1 | olfactory receptor family 8 subfamily A member 1 [Source:HGNC Symbol;Acc:HGNC:8469] |
| ENSG00000196460 | HGLibA_40983 | 172 | 0.42110549 | RFX8 | lacking RFX DNA binding domain [Source:HGNC Symbol;Acc:HGNC:37253]" |
| ENSG00000196646 | HGLibA_55475 | 172 | 0.42110549 | ZNF136 | zinc finger protein 136 [Source:HGNC Symbol;Acc:HGNC:12920] |
| ENSG00000196917 | HGLibA_20946 | 172 | 0.42110549 | HCAR1 | hydroxycarboxylic acid receptor 1 [Source:HGNC Symbol;Acc:HGNC:4532] |
| ENSG00000197608 | HGLibA_56853 | 172 | 0.42110549 | ZNF841 | zinc finger protein 841 [Source:HGNC Symbol;Acc:HGNC:27611] |
| ENSG00000198732 | HGLibA_45793 | 172 | 0.42110549 | SMOC1 | SPARC related modular calcium binding 1 [Source:HGNC Symbol;Acc:HGNC:20318] |
| ENSG00000198856 | HGLibA_34708 | 172 | 0.42110549 | OSTC | oligosaccharyltransferase complex non-catalytic subunit [Source:HGNC Symbol;Acc:HGNC:24448] |
| ENSG00000205923 | HGLibA_09032 | 172 | 0.42110549 | CEMP1 | cementum protein 1 [Source:HGNC Symbol;Acc:HGNC:32553] |
| ENSG00000214642 | HGLibA_12853 | 172 | 0.42110549 | DEFB113 | defensin beta 113 [Source:HGNC Symbol;Acc:HGNC:18094] |
| ENSG00000221852 | HGLibA_25587 | 172 | 0.42110549 | KRTAP1-5 | keratin associated protein 1-5 [Source:HGNC Symbol;Acc:HGNC:16777] |
| ENSG00000221916 | HGLibA_05706 | 172 | 0.42110549 | C19orf73 | chromosome 19 open reading frame 73 [Source:HGNC Symbol;Acc:HGNC:25534] |
| ENSG00000221937 | HGLibA_48403 | 172 | 0.42110549 | TAS2R40 | taste 2 receptor member 40 [Source:HGNC Symbol;Acc:HGNC:18885] |
| ENSG00000231213 | HGLibA_37244 | 172 | 0.42110549 | PLSCR5 | phospholipid scramblase family member 5 [Source:HGNC Symbol;Acc:HGNC:19952] |
| ENSG00000242802 | HGLibA_02363 | 172 | 0.42110549 | AP5Z1 | adaptor related protein complex 5 subunit zeta 1 [Source:HGNC Symbol;Acc:HGNC:22197] |
| ENSG00000242866 | HGLibA_47585 | 172 | 0.42110549 | STRC | stereocilin [Source:HGNC Symbol;Acc:HGNC:16035] |
| ENSG00000244025 | HGLibA_25669 | 172 | 0.42110549 | KRTAP19-3 | keratin associated protein 19-3 [Source:HGNC Symbol;Acc:HGNC:18938] |
| ENSG00000255408 | HGLibA_35441 | 172 | 0.42110549 | PCDHA3 | protocadherin alpha 3 [Source:HGNC Symbol;Acc:HGNC:8669] |
| ENSG00000255587 | HGLibA_39953 | 172 | 0.42110549 | RAB44 | member RAS oncogene family [Source:HGNC Symbol;Acc:HGNC:21068]" |
| ENSG00000256294 | HGLibA_55622 | 172 | 0.42110549 | ZNF225 | zinc finger protein 225 [Source:HGNC Symbol;Acc:HGNC:13018] |
| ENSG00000274749 | HGLibA_25824 | 172 | 0.42110549 | KRTAP7-1 | keratin associated protein 7-1 (gene/pseudogene) [Source:HGNC Symbol;Acc:HGNC:18934] |
| ENSG00000275074 | HGLibA_32960 | 172 | 0.42110549 | NUDT18 | nudix hydrolase 18 [Source:HGNC Symbol;Acc:HGNC:26194] |
| GPR114 | HGLibA_19859 | 172 | 0.42110549 | GPR114 | - |
| hsa-mir-146b | HGLibA_57860 | 172 | 0.42110549 | hsa-mir-146b | - |
| hsa-mir-196a-1 | HGLibA_58093 | 172 | 0.42110549 | hsa-mir-196a-1 | - |
| hsa-mir-301a | HGLibA_58428 | 172 | 0.42110549 | hsa-mir-301a | - |
| hsa-mir-3166 | HGLibA_58742 | 172 | 0.42110549 | hsa-mir-3166 | - |
| hsa-mir-3911 | HGLibA_59511 | 172 | 0.42110549 | hsa-mir-3911 | - |
| hsa-mir-4530 | HGLibA_60579 | 172 | 0.42110549 | hsa-mir-4530 | - |
| hsa-mir-511 | HGLibA_61555 | 172 | 0.42110549 | hsa-mir-511 | - |
| hsa-mir-563 | HGLibA_62214 | 172 | 0.42110549 | hsa-mir-563 | - |
| hsa-mir-652 | HGLibA_62940 | 172 | 0.42110549 | hsa-mir-652 | - |
| hsa-mir-6715a | HGLibA_63017 | 172 | 0.42110549 | hsa-mir-6715a | - |
| hsa-mir-6785 | HGLibA_63318 | 172 | 0.42110549 | hsa-mir-6785 | - |
| hsa-mir-7973-2 | HGLibA_64022 | 172 | 0.42110549 | hsa-mir-7973-2 | - |
| hsa-mir-8080 | HGLibA_64161 | 172 | 0.42110549 | hsa-mir-8080 | - |
| AKAP17A_X | HGLibA_01421 | 171 | 0.412060147 | AKAP17A_X | - |
| C6orf48 | HGLibA_06492 | 171 | 0.412060147 | C6orf48 | - |
| C6orf70 | HGLibA_06508 | 171 | 0.412060147 | C6orf70 | - |
| C9orf171 | HGLibA_06710 | 171 | 0.412060147 | C9orf171 | - |
| ENSG00000047634 | HGLibA_42958 | 171 | 0.412060147 | SCML1 | Scm polycomb group protein like 1 [Source:HGNC Symbol;Acc:HGNC:10580] |
| ENSG00000060982 | HGLibA_04263 | 171 | 0.412060147 | BCAT1 | branched chain amino acid transaminase 1 [Source:HGNC Symbol;Acc:HGNC:976] |
| ENSG00000063761 | HGLibA_00891 | 171 | 0.412060147 | ADCK1 | aarF domain containing kinase 1 [Source:HGNC Symbol;Acc:HGNC:19038] |
| ENSG00000072110 | HGLibA_00639 | 171 | 0.412060147 | ACTN1 | actinin alpha 1 [Source:HGNC Symbol;Acc:HGNC:163] |
| ENSG00000080493 | HGLibA_45219 | 171 | 0.412060147 | SLC4A4 | solute carrier family 4 member 4 [Source:HGNC Symbol;Acc:HGNC:11030] |
| ENSG00000085978 | HGLibA_03472 | 171 | 0.412060147 | ATG16L1 | autophagy related 16 like 1 [Source:HGNC Symbol;Acc:HGNC:21498] |
| ENSG00000087303 | HGLibA_31925 | 171 | 0.412060147 | NID2 | nidogen 2 [Source:HGNC Symbol;Acc:HGNC:13389] |
| ENSG00000087365 | HGLibA_43705 | 171 | 0.412060147 | SF3B2 | splicing factor 3b subunit 2 [Source:HGNC Symbol;Acc:HGNC:10769] |
| ENSG00000088320 | HGLibA_40831 | 171 | 0.412060147 | REM1 | RRAD and GEM like GTPase 1 [Source:HGNC Symbol;Acc:HGNC:15922] |
| ENSG00000088832 | HGLibA_17564 | 171 | 0.412060147 | FKBP1A | FK506 binding protein 1A [Source:HGNC Symbol;Acc:HGNC:3711] |
| ENSG00000089505 | HGLibA_10208 | 171 | 0.412060147 | CMTM1 | CKLF like MARVEL transmembrane domain containing 1 [Source:HGNC Symbol;Acc:HGNC:19172] |
| ENSG00000095585 | HGLibA_04553 | 171 | 0.412060147 | BLNK | B cell linker [Source:HGNC Symbol;Acc:HGNC:14211] |
| ENSG00000107560 | HGLibA_39796 | 171 | 0.412060147 | RAB11FIP2 | RAB11 family interacting protein 2 [Source:HGNC Symbol;Acc:HGNC:29152] |
| ENSG00000107862 | HGLibA_18723 | 171 | 0.412060147 | GBF1 | golgi brefeldin A resistant guanine nucleotide exchange factor 1 [Source:HGNC Symbol;Acc:HGNC:4181] |
| ENSG00000108691 | HGLibA_07989 | 171 | 0.412060147 | CCL2 | C-C motif chemokine ligand 2 [Source:HGNC Symbol;Acc:HGNC:10618] |
| ENSG00000109270 | HGLibA_25994 | 171 | 0.412060147 | LAMTOR3 | MAPK and MTOR activator 3 [Source:HGNC Symbol;Acc:HGNC:15606]" |
| ENSG00000111879 | HGLibA_16326 | 171 | 0.412060147 | FAM184A | family with sequence similarity 184 member A [Source:HGNC Symbol;Acc:HGNC:20991] |
| ENSG00000114648 | HGLibA_25145 | 171 | 0.412060147 | KLHL18 | kelch like family member 18 [Source:HGNC Symbol;Acc:HGNC:29120] |
| ENSG00000115935 | HGLibA_54305 | 171 | 0.412060147 | WIPF1 | WAS/WASL interacting protein family member 1 [Source:HGNC Symbol;Acc:HGNC:12736] |
| ENSG00000116039 | HGLibA_03734 | 171 | 0.412060147 | ATP6V1B1 | ATPase H+ transporting V1 subunit B1 [Source:HGNC Symbol;Acc:HGNC:853] |
| ENSG00000118257 | HGLibA_32717 | 171 | 0.412060147 | NRP2 | neuropilin 2 [Source:HGNC Symbol;Acc:HGNC:8005] |
| ENSG00000119446 | HGLibA_40501 | 171 | 0.412060147 | RBM18 | RNA binding motif protein 18 [Source:HGNC Symbol;Acc:HGNC:28413] |
| ENSG00000121578 | HGLibA_04031 | 171 | 0.412060147 | B4GALT4 | 4-galactosyltransferase 4 [Source:HGNC Symbol;Acc:HGNC:927]" |
| ENSG00000121966 | HGLibA_11889 | 171 | 0.412060147 | CXCR4 | C-X-C motif chemokine receptor 4 [Source:HGNC Symbol;Acc:HGNC:2561] |
| ENSG00000125285 | HGLibA_46255 | 171 | 0.412060147 | SOX21 | SRY-box 21 [Source:HGNC Symbol;Acc:HGNC:11197] |
| ENSG00000127920 | HGLibA_19512 | 171 | 0.412060147 | GNG11 | G protein subunit gamma 11 [Source:HGNC Symbol;Acc:HGNC:4403] |
| ENSG00000129538 | HGLibA_41387 | 171 | 0.412060147 | RNASE1 | pancreatic [Source:HGNC Symbol;Acc:HGNC:10044]" |
| ENSG00000130383 | HGLibA_18198 | 171 | 0.412060147 | FUT5 | fucosyltransferase 5 [Source:HGNC Symbol;Acc:HGNC:4016] |
| ENSG00000130812 | HGLibA_01925 | 171 | 0.412060147 | ANGPTL6 | angiopoietin like 6 [Source:HGNC Symbol;Acc:HGNC:23140] |
| ENSG00000131778 | HGLibA_09385 | 171 | 0.412060147 | CHD1L | chromodomain helicase DNA binding protein 1 like [Source:HGNC Symbol;Acc:HGNC:1916] |
| ENSG00000132563 | HGLibA_40778 | 171 | 0.412060147 | REEP2 | receptor accessory protein 2 [Source:HGNC Symbol;Acc:HGNC:17975] |
| ENSG00000134265 | HGLibA_31135 | 171 | 0.412060147 | NAPG | NSF attachment protein gamma [Source:HGNC Symbol;Acc:HGNC:7642] |
| ENSG00000134285 | HGLibA_17554 | 171 | 0.412060147 | FKBP11 | FK506 binding protein 11 [Source:HGNC Symbol;Acc:HGNC:18624] |
| ENSG00000135114 | HGLibA_33206 | 171 | 0.412060147 | OASL | 2'-5'-oligoadenylate synthetase like [Source:HGNC Symbol;Acc:HGNC:8090] |
| ENSG00000135778 | HGLibA_32871 | 171 | 0.412060147 | NTPCR | cancer-related [Source:HGNC Symbol;Acc:HGNC:28204]" |
| ENSG00000138813 | HGLibA_06297 | 171 | 0.412060147 | C4orf17 | chromosome 4 open reading frame 17 [Source:HGNC Symbol;Acc:HGNC:25274] |
| ENSG00000141052 | HGLibA_30876 | 171 | 0.412060147 | MYOCD | myocardin [Source:HGNC Symbol;Acc:HGNC:16067] |
| ENSG00000141295 | HGLibA_43052 | 171 | 0.412060147 | SCRN2 | secernin 2 [Source:HGNC Symbol;Acc:HGNC:30381] |

| | | | | | |
|---|---|---|---|---|---|
| ENSG00000141522 | HGLibA_02795 | 171 | 0.412060147 | ARHGDIA | Rho GDP dissociation inhibitor alpha [Source:HGNC Symbol;Acc:HGNC:678] |
| ENSG00000143252 | HGLibA_43145 | 171 | 0.412060147 | SDHC | succinate dehydrogenase complex subunit C [Source:HGNC Symbol;Acc:HGNC:10682] |
| ENSG00000143322 | HGLibA_00306 | 171 | 0.412060147 | ABL2 | non-receptor tyrosine kinase [Source:HGNC Symbol;Acc:HGNC:77]" |
| ENSG00000143575 | HGLibA_20904 | 171 | 0.412060147 | HAX1 | HCLS1 associated protein X-1 [Source:HGNC Symbol;Acc:HGNC:16915] |
| ENSG00000143727 | HGLibA_00508 | 171 | 0.412060147 | ACP1 | acid phosphatase 1 [Source:HGNC Symbol;Acc:HGNC:122] |
| ENSG00000145293 | HGLibA_15151 | 171 | 0.412060147 | ENOPH1 | enolase-phosphatase 1 [Source:HGNC Symbol;Acc:HGNC:24599] |
| ENSG00000145850 | HGLibA_49487 | 171 | 0.412060147 | TIMD4 | T cell immunoglobulin and mucin domain containing 4 [Source:HGNC Symbol;Acc:HGNC:25132] |
| ENSG00000146221 | HGLibA_48856 | 171 | 0.412060147 | TCTE1 | t-complex-associated-testis-expressed 1 [Source:HGNC Symbol;Acc:HGNC:11693] |
| ENSG00000147256 | HGLibA_02769 | 171 | 0.412060147 | ARHGAP36 | Rho GTPase activating protein 36 [Source:HGNC Symbol;Acc:HGNC:26388] |
| ENSG00000147408 | HGLibA_11346 | 171 | 0.412060147 | CSGALNACT1 | chondroitin sulfate N-acetylgalactosaminyltransferase 1 [Source:HGNC Symbol;Acc:HGNC:24290] |
| ENSG00000151729 | HGLibA_44731 | 171 | 0.412060147 | SLC25A4 | solute carrier family 25 member 4 [Source:HGNC Symbol;Acc:HGNC:10990] |
| ENSG00000151962 | HGLibA_40564 | 171 | 0.412060147 | RBM46 | RNA binding motif protein 46 [Source:HGNC Symbol;Acc:HGNC:28401] |
| ENSG00000153495 | HGLibA_49090 | 171 | 0.412060147 | TEX29 | testis expressed 29 [Source:HGNC Symbol;Acc:HGNC:20370] |
| ENSG00000156671 | HGLibA_42696 | 171 | 0.412060147 | SAMD8 | sterile alpha motif domain containing 8 [Source:HGNC Symbol;Acc:HGNC:26320] |
| ENSG00000160570 | HGLibA_12782 | 171 | 0.412060147 | DEDD2 | death effector domain containing 2 [Source:HGNC Symbol;Acc:HGNC:24450] |
| ENSG00000162076 | HGLibA_17684 | 171 | 0.412060147 | FLYWCH2 | FLYWCH family member 2 [Source:HGNC Symbol;Acc:HGNC:25178] |
| ENSG00000162755 | HGLibA_25116 | 171 | 0.412060147 | KLHDC9 | kelch domain containing 9 [Source:HGNC Symbol;Acc:HGNC:28489] |
| ENSG00000164185 | HGLibA_56061 | 171 | 0.412060147 | ZNF474 | zinc finger protein 474 [Source:HGNC Symbol;Acc:HGNC:23245] |
| ENSG00000164236 | HGLibA_02071 | 171 | 0.412060147 | ANKRD33B | ankyrin repeat domain 33B [Source:HGNC Symbol;Acc:HGNC:35240] |
| ENSG00000165695 | HGLibA_01398 | 171 | 0.412060147 | AK8 | adenylate kinase 8 [Source:HGNC Symbol;Acc:HGNC:26526] |
| ENSG00000166455 | HGLibA_05451 | 171 | 0.412060147 | C16orf46 | chromosome 16 open reading frame 46 [Source:HGNC Symbol;Acc:HGNC:26525] |
| ENSG00000167332 | HGLibA_34099 | 171 | 0.412060147 | OR51E2 | olfactory receptor family 51 subfamily E member 2 [Source:HGNC Symbol;Acc:HGNC:15195] |
| ENSG00000170523 | HGLibA_25564 | 171 | 0.412060147 | KRT83 | keratin 83 [Source:HGNC Symbol;Acc:HGNC:6460] |
| ENSG00000170734 | HGLibA_37528 | 171 | 0.412060147 | POLH | DNA polymerase eta [Source:HGNC Symbol;Acc:HGNC:9181] |
| ENSG00000172403 | HGLibA_48022 | 171 | 0.412060147 | SYNPO2 | synaptopodin 2 [Source:HGNC Symbol;Acc:HGNC:17732] |
| ENSG00000174989 | HGLibA_17155 | 171 | 0.412060147 | FBXW8 | F-box and WD repeat domain containing 8 [Source:HGNC Symbol;Acc:HGNC:13597] |
| ENSG00000175664 | HGLibA_49080 | 171 | 0.412060147 | TEX26 | testis expressed 26 [Source:HGNC Symbol;Acc:HGNC:28622] |
| ENSG00000176225 | HGLibA_42435 | 171 | 0.412060147 | RTTN | rotatin [Source:HGNC Symbol;Acc:HGNC:18654] |
| ENSG00000177212 | HGLibA_33875 | 171 | 0.412060147 | OR2T33 | olfactory receptor family 2 subfamily T member 33 [Source:HGNC Symbol;Acc:HGNC:31255] |
| ENSG00000177324 | HGLibA_04400 | 171 | 0.412060147 | BEND2 | BEN domain containing 2 [Source:HGNC Symbol;Acc:HGNC:28509] |
| ENSG00000177989 | HGLibA_33287 | 171 | 0.412060147 | ODF3B | outer dense fiber of sperm tails 3B [Source:HGNC Symbol;Acc:HGNC:34388] |
| ENSG00000178764 | HGLibA_55316 | 171 | 0.412060147 | ZHX2 | zinc fingers and homeoboxes 2 [Source:HGNC Symbol;Acc:HGNC:18513] |
| ENSG00000179562 | HGLibA_18764 | 171 | 0.412060147 | GCC1 | GRIP and coiled-coil domain containing 1 [Source:HGNC Symbol;Acc:HGNC:19095] |
| ENSG00000180929 | HGLibA_20065 | 171 | 0.412060147 | GPR62 | G protein-coupled receptor 62 [Source:HGNC Symbol;Acc:HGNC:13301] |
| ENSG00000181852 | HGLibA_41681 | 171 | 0.412060147 | RNF41 | ring finger protein 41 [Source:HGNC Symbol;Acc:HGNC:18401] |
| ENSG00000182621 | HGLibA_36979 | 171 | 0.412060147 | PLCB1 | phospholipase C beta 1 [Source:HGNC Symbol;Acc:HGNC:15917] |
| ENSG00000182931 | HGLibA_54249 | 171 | 0.412060147 | WFDC10B | WAP four-disulfide core domain 10B [Source:HGNC Symbol;Acc:HGNC:20479] |
| ENSG00000183643 | HGLibA_05385 | 171 | 0.412060147 | C15orf32 | chromosome 15 open reading frame 32 [Source:HGNC Symbol;Acc:HGNC:26549] |
| ENSG00000185448 | HGLibA_16609 | 171 | 0.412060147 | FAM47A | family with sequence similarity 47 member A [Source:HGNC Symbol;Acc:HGNC:29962] |
| ENSG00000185940 | HGLibA_25800 | 171 | 0.412060147 | KRTAP5-5 | keratin associated protein 5-5 [Source:HGNC Symbol;Acc:HGNC:23601] |
| ENSG00000187144 | HGLibA_46450 | 171 | 0.412060147 | SPATA21 | spermatogenesis associated 21 [Source:HGNC Symbol;Acc:HGNC:28026] |
| ENSG00000189169 | HGLibA_25600 | 171 | 0.412060147 | KRTAP10-12 | keratin associated protein 10-12 [Source:HGNC Symbol;Acc:HGNC:20533] |
| ENSG00000196171 | HGLibA_34417 | 171 | 0.412060147 | OR6K2 | olfactory receptor family 6 subfamily K member 2 [Source:HGNC Symbol;Acc:HGNC:15029] |
| ENSG00000197948 | HGLibA_17211 | 171 | 0.412060147 | FCHSD1 | FCH and double SH3 domains 1 [Source:HGNC Symbol;Acc:HGNC:25463] |
| ENSG00000197959 | HGLibA_13691 | 171 | 0.412060147 | DNM3 | dynamin 3 [Source:HGNC Symbol;Acc:HGNC:29125] |
| ENSG00000197982 | HGLibA_05758 | 171 | 0.412060147 | C1orf122 | chromosome 1 open reading frame 122 [Source:HGNC Symbol;Acc:HGNC:24789] |
| ENSG00000198286 | HGLibA_07219 | 171 | 0.412060147 | CARD11 | caspase recruitment domain family member 11 [Source:HGNC Symbol;Acc:HGNC:16393] |
| ENSG00000206013 | HGLibA_22649 | 171 | 0.412060147 | IFITM5 | interferon induced transmembrane protein 5 [Source:HGNC Symbol;Acc:HGNC:16644] |
| ENSG00000213215 | HGLibA_33788 | 171 | 0.412060147 | OR2F1 | olfactory receptor family 2 subfamily F member 1 (gene/pseudogene) [Source:HGNC Symbol;Acc:HGNC:8246] |
| ENSG00000244476 | HGLibA_15540 | 171 | 0.412060147 | ERVFRD-1 | envelope [Source:HGNC Symbol;Acc:HGNC:33823]" |
| ENSG00000249861 | HGLibA_26358 | 171 | 0.412060147 | LGALS16 | galectin 16 [Source:HGNC Symbol;Acc:HGNC:40039] |
| ENSG00000254834 | HGLibA_34331 | 171 | 0.412060147 | OR5M10 | olfactory receptor family 5 subfamily M member 10 [Source:HGNC Symbol;Acc:HGNC:15290] |
| ENSG00000258817 | HGLibA_33943 | 171 | 0.412060147 | OR4C13 | olfactory receptor family 4 subfamily C member 13 [Source:HGNC Symbol;Acc:HGNC:15169] |
| ENSG00000264230 | HGLibA_02255 | 171 | 0.412060147 | ANXA8L1 | annexin A8 like 1 [Source:HGNC Symbol;Acc:HGNC:23334] |
| ENSG00000266560 | HGLibA_27897 | 171 | 0.412060147 | MAGEA10-MAGEA5 | MAGEA10-MAGEA5 readthrough [Source:NCBI gene;Acc:100533997] |
| ENSG00000278889 | HGLibA_33848 | 171 | 0.412060147 | OR2S2 | olfactory receptor family 2 subfamily S member 2 (gene/pseudogene) [Source:HGNC Symbol;Acc:HGNC:8276] |
| ENSG00000284662 | HGLibA_33993 | 171 | 0.412060147 | OR4F16 | olfactory receptor family 4 subfamily F member 16 [Source:HGNC Symbol;Acc:HGNC:15079] |
| FAM203A | HGLibA_16417 | 171 | 0.412060147 | FAM203A | - |
| GTDC2 | HGLibA_20516 | 171 | 0.412060147 | GTDC2 | - |
| hsa-mir-17 | HGLibA_57953 | 171 | 0.412060147 | hsa-mir-17 | - |
| hsa-mir-3688-1 | HGLibA_59305 | 171 | 0.412060147 | hsa-mir-3688-1 | - |
| hsa-mir-4708 | HGLibA_60931 | 171 | 0.412060147 | hsa-mir-4708 | - |
| hsa-mir-514a-2 | HGLibA_61590 | 171 | 0.412060147 | hsa-mir-514a-2 | - |
| hsa-mir-5579 | HGLibA_62155 | 171 | 0.412060147 | hsa-mir-5579 | - |
| hsa-mir-6845 | HGLibA_63552 | 171 | 0.412060147 | hsa-mir-6845 | - |
| hsa-mir-758 | HGLibA_63894 | 171 | 0.412060147 | hsa-mir-758 | - |
| ENSG00000007174 | HGLibA_13500 | 170 | 0.403014804 | DNAH9 | dynein axonemal heavy chain 9 [Source:HGNC Symbol;Acc:HGNC:2953] |
| ENSG00000057608 | HGLibA_18885 | 170 | 0.403014804 | GDI2 | GDP dissociation inhibitor 2 [Source:HGNC Symbol;Acc:HGNC:4227] |
| ENSG00000064835 | HGLibA_37751 | 170 | 0.403014804 | POU1F1 | POU class 1 homeobox 1 [Source:HGNC Symbol;Acc:HGNC:9210] |
| ENSG00000071575 | HGLibA_51394 | 170 | 0.403014804 | TRIB2 | tribbles pseudokinase 2 [Source:HGNC Symbol;Acc:HGNC:30809] |
| ENSG00000076513 | HGLibA_01999 | 170 | 0.403014804 | ANKRD13A | ankyrin repeat domain 13A [Source:HGNC Symbol;Acc:HGNC:21268] |
| ENSG00000079785 | HGLibA_12647 | 170 | 0.403014804 | DDX1 | DEAD-box helicase 1 [Source:HGNC Symbol;Acc:HGNC:2734] |
| ENSG00000084710 | HGLibA_14609 | 170 | 0.403014804 | EFR3B | EFR3 homolog B [Source:HGNC Symbol;Acc:HGNC:29155] |
| ENSG00000088002 | HGLibA_47761 | 170 | 0.403014804 | SULT2B1 | sulfotransferase family 2B member 1 [Source:HGNC Symbol;Acc:HGNC:11459] |
| ENSG00000088756 | HGLibA_02746 | 170 | 0.403014804 | ARHGAP28 | Rho GTPase activating protein 28 [Source:HGNC Symbol;Acc:HGNC:25509] |
| ENSG00000091622 | HGLibA_36756 | 170 | 0.403014804 | PITPNM3 | PITPNM family member 3 [Source:HGNC Symbol;Acc:HGNC:21043] |
| ENSG00000100908 | HGLibA_15026 | 170 | 0.403014804 | EMC9 | ER membrane protein complex subunit 9 [Source:HGNC Symbol;Acc:HGNC:20273] |
| ENSG00000101446 | HGLibA_46717 | 170 | 0.403014804 | SPINT3 | Kunitz type 3 [Source:HGNC Symbol;Acc:HGNC:11248]" |
| ENSG00000102239 | HGLibA_04844 | 170 | 0.403014804 | BRS3 | bombesin receptor subtype 3 [Source:HGNC Symbol;Acc:HGNC:1113] |
| ENSG00000102302 | HGLibA_17341 | 170 | 0.403014804 | FGD1 | RhoGEF and PH domain containing 1 [Source:HGNC Symbol;Acc:HGNC:3663]" |
| ENSG00000103248 | HGLibA_30395 | 170 | 0.403014804 | MTHFSD | methenyltetrahydrofolate synthetase domain containing [Source:HGNC Symbol;Acc:HGNC:25778] |
| ENSG00000103657 | HGLibA_21153 | 170 | 0.403014804 | HERC1 | HECT and RLD domain containing E3 ubiquitin protein ligase family member 1 [Source:HGNC Symbol;Acc:HGNC:4867] |
| ENSG00000104904 | HGLibA_33214 | 170 | 0.403014804 | OAZ1 | ornithine decarboxylase antizyme 1 [Source:HGNC Symbol;Acc:HGNC:8095] |
| ENSG00000105974 | HGLibA_07376 | 170 | 0.403014804 | CAV1 | caveolin 1 [Source:HGNC Symbol;Acc:HGNC:1527] |
| ENSG00000106993 | HGLibA_08549 | 170 | 0.403014804 | CDC37L1 | cell division cycle 37 like 1 [Source:HGNC Symbol;Acc:HGNC:17179] |
| ENSG00000107262 | HGLibA_04073 | 170 | 0.403014804 | BAG1 | BCL2 associated athanogene 1 [Source:HGNC Symbol;Acc:HGNC:937] |
| ENSG00000108846 | HGLibA_00173 | 170 | 0.403014804 | ABCC3 | ATP binding cassette subfamily C member 3 [Source:HGNC Symbol;Acc:HGNC:54] |
| ENSG00000111252 | HGLibA_43881 | 170 | 0.403014804 | SH2B3 | SH2B adaptor protein 3 [Source:HGNC Symbol;Acc:HGNC:29605] |
| ENSG00000113248 | HGLibA_35486 | 170 | 0.403014804 | PCDHB15 | protocadherin beta 15 [Source:HGNC Symbol;Acc:HGNC:8686] |
| ENSG00000113389 | HGLibA_32515 | 170 | 0.403014804 | NPR3 | natriuretic peptide receptor 3 [Source:HGNC Symbol;Acc:HGNC:7945] |
| ENSG00000114770 | HGLibA_00178 | 170 | 0.403014804 | ABCC5 | ATP binding cassette subfamily C member 5 [Source:HGNC Symbol;Acc:HGNC:56] |
| ENSG00000117620 | HGLibA_44953 | 170 | 0.403014804 | SLC35A3 | solute carrier family 35 member A3 [Source:HGNC Symbol;Acc:HGNC:11023] |
| ENSG00000119401 | HGLibA_51463 | 170 | 0.403014804 | TRIM32 | tripartite motif containing 32 [Source:HGNC Symbol;Acc:HGNC:16380] |
| ENSG00000121898 | HGLibA_11040 | 170 | 0.403014804 | CPXM2 | M14 family member 2 [Source:HGNC Symbol;Acc:HGNC:26977]" |
| ENSG00000123901 | HGLibA_20091 | 170 | 0.403014804 | GPR83 | G protein-coupled receptor 83 [Source:HGNC Symbol;Acc:HGNC:4523] |
| ENSG00000124091 | HGLibA_18822 | 170 | 0.403014804 | GCNT7 | glucosaminyl (N-acetyl) transferase family member 7 [Source:HGNC Symbol;Acc:HGNC:16099] |
| ENSG00000125434 | HGLibA_44718 | 170 | 0.403014804 | SLC25A35 | solute carrier family 25 member 35 [Source:HGNC Symbol;Acc:HGNC:31921] |
| ENSG00000125735 | HGLibA_50814 | 170 | 0.403014804 | TNFSF14 | TNF superfamily member 14 [Source:HGNC Symbol;Acc:HGNC:11930] |
| ENSG00000125877 | HGLibA_23837 | 170 | 0.403014804 | ITPA | inosine triphosphatase [Source:HGNC Symbol;Acc:HGNC:6176] |
| ENSG00000126266 | HGLibA_17323 | 170 | 0.403014804 | FFAR1 | free fatty acid receptor 1 [Source:HGNC Symbol;Acc:HGNC:4498] |
| ENSG00000128228 | HGLibA_43127 | 170 | 0.403014804 | SDF2L1 | stromal cell derived factor 2 like 1 [Source:HGNC Symbol;Acc:HGNC:10676] |
| ENSG00000130158 | HGLibA_13753 | 170 | 0.403014804 | DOCK6 | dedicator of cytokinesis 6 [Source:HGNC Symbol;Acc:HGNC:19189] |
| ENSG00000130402 | HGLibA_00644 | 170 | 0.403014804 | ACTN4 | actinin alpha 4 [Source:HGNC Symbol;Acc:HGNC:166] |
| ENSG00000130528 | HGLibA_22085 | 170 | 0.403014804 | HRC | histidine rich calcium binding protein [Source:HGNC Symbol;Acc:HGNC:5178] |
| ENSG00000131165 | HGLibA_09471 | 170 | 0.403014804 | CHMP1A | charged multivesicular body protein 1A [Source:HGNC Symbol;Acc:HGNC:8740] |
| ENSG00000133878 | HGLibA_14168 | 170 | 0.403014804 | DUSP26 | dual specificity phosphatase 26 [Source:HGNC Symbol;Acc:HGNC:28161] |
| ENSG00000137090 | HGLibA_13418 | 170 | 0.403014804 | DMRT1 | doublesex and mab-3 related transcription factor 1 [Source:HGNC Symbol;Acc:HGNC:2934] |
| ENSG00000137757 | HGLibA_07315 | 170 | 0.403014804 | CASP5 | caspase 5 [Source:HGNC Symbol;Acc:HGNC:1506] |
| ENSG00000138136 | HGLibA_26084 | 170 | 0.403014804 | LBX1 | ladybird homeobox 1 [Source:HGNC Symbol;Acc:HGNC:16960] |
| ENSG00000138271 | HGLibA_20100 | 170 | 0.403014804 | GPR87 | G protein-coupled receptor 87 [Source:HGNC Symbol;Acc:HGNC:4538] |
| ENSG00000138821 | HGLibA_45123 | 170 | 0.403014804 | SLC39A8 | solute carrier family 39 member 8 [Source:HGNC Symbol;Acc:HGNC:20862] |
| ENSG00000139193 | HGLibA_08315 | 170 | 0.403014804 | CD27 | CD27 molecule [Source:HGNC Symbol;Acc:HGNC:11922] |
| ENSG00000144395 | HGLibA_07644 | 170 | 0.403014804 | CCDC150 | coiled-coil domain containing 150 [Source:HGNC Symbol;Acc:HGNC:26834] |
| ENSG00000147571 | HGLibA_11136 | 170 | 0.403014804 | CRH | corticotropin releasing hormone [Source:HGNC Symbol;Acc:HGNC:2355] |
| ENSG00000152475 | HGLibA_56844 | 170 | 0.403014804 | ZNF837 | zinc finger protein 837 [Source:HGNC Symbol;Acc:HGNC:25164] |
| ENSG00000155966 | HGLibA_01108 | 170 | 0.403014804 | AFF2 | AF4/FMR2 family member 2 [Source:HGNC Symbol;Acc:HGNC:3776] |
| ENSG00000158352 | HGLibA_44097 | 170 | 0.403014804 | SHROOM4 | shroom family member 4 [Source:HGNC Symbol;Acc:HGNC:29215] |
| ENSG00000160007 | HGLibA_02766 | 170 | 0.403014804 | ARHGAP35 | Rho GTPase activating protein 35 [Source:HGNC Symbol;Acc:HGNC:4591] |
| ENSG00000160613 | HGLibA_35684 | 170 | 0.403014804 | PCSK7 | proprotein convertase subtilisin/kexin type 7 [Source:HGNC Symbol;Acc:HGNC:8748] |
| ENSG00000160882 | HGLibA_12054 | 170 | 0.403014804 | CYP11B1 | cytochrome P450 family 11 subfamily B member 1 [Source:HGNC Symbol;Acc:HGNC:2591] |
| ENSG00000161281 | HGLibA_10857 | 170 | 0.403014804 | COX7A1 | cytochrome c oxidase subunit 7A1 [Source:HGNC Symbol;Acc:HGNC:2287] |
| ENSG00000162437 | HGLibA_40405 | 170 | 0.403014804 | RAVER2 | PTB binding 2 [Source:HGNC Symbol;Acc:HGNC:25577]" |
| ENSG00000162817 | HGLibA_05753 | 170 | 0.403014804 | C1orf115 | chromosome 1 open reading frame 115 [Source:HGNC Symbol;Acc:HGNC:25873] |
| ENSG00000163412 | HGLibA_14824 | 170 | 0.403014804 | EIF4E3 | eukaryotic translation initiation factor 4E family member 3 [Source:HGNC Symbol;Acc:HGNC:31837] |
| ENSG00000164187 | HGLibA_26679 | 170 | 0.403014804 | LMBRD2 | LMBR1 domain containing 2 [Source:HGNC Symbol;Acc:HGNC:25287] |
| ENSG00000164610 | HGLibA_41796 | 170 | 0.403014804 | RP9 | pre-mRNA splicing factor [Source:HGNC Symbol;Acc:HGNC:10288]" |
| ENSG00000164754 | HGLibA_40064 | 170 | 0.403014804 | RAD21 | RAD21 cohesin complex component [Source:HGNC Symbol;Acc:HGNC:9811] |
| ENSG00000166432 | HGLibA_55366 | 170 | 0.403014804 | ZMAT1 | zinc finger matrin-type 1 [Source:HGNC Symbol;Acc:HGNC:29377] |
| ENSG00000166575 | HGLibA_49948 | 170 | 0.403014804 | TMEM135 | transmembrane protein 135 [Source:HGNC Symbol;Acc:HGNC:26167] |
| ENSG00000167535 | HGLibA_06924 | 170 | 0.403014804 | CACNB3 | calcium voltage-gated channel auxiliary subunit beta 3 [Source:HGNC Symbol;Acc:HGNC:1403] |
| ENSG00000168769 | HGLibA_49041 | 170 | 0.403014804 | TET2 | tet methylcytosine dioxygenase 2 [Source:HGNC Symbol;Acc:HGNC:25941] |
| ENSG00000169291 | HGLibA_44020 | 170 | 0.403014804 | SHE | Src homology 2 domain containing E [Source:HGNC Symbol;Acc:HGNC:27004] |
| ENSG00000169515 | HGLibA_07873 | 170 | 0.403014804 | CCDC8 | coiled-coil domain containing 8 [Source:HGNC Symbol;Acc:HGNC:25367] |
| ENSG00000172005 | HGLibA_28023 | 170 | 0.403014804 | MAL | T cell differentiation protein [Source:HGNC Symbol;Acc:HGNC:6817]" |
| ENSG00000174776 | HGLibA_54113 | 170 | 0.403014804 | WDR49 | WD repeat domain 49 [Source:HGNC Symbol;Acc:HGNC:26587] |

| | | | | | |
|---|---|---|---|---|---|
| ENSG00000174903 | HGLibA_39831 | 170 | 0.403014804 | RAB1B | member RAS oncogene family [Source:HGNC Symbol;Acc:HGNC:18370]" |
| ENSG00000174992 | HGLibA_55296 | 170 | 0.403014804 | ZG16 | zymogen granule protein 16 [Source:HGNC Symbol;Acc:HGNC:30961] |
| ENSG00000176105 | HGLibA_54662 | 170 | 0.403014804 | YES1 | Src family tyrosine kinase [Source:HGNC Symbol;Acc:HGNC:12841]" |
| ENSG00000176988 | HGLibA_17723 | 170 | 0.403014804 | FMR1NB | FMR1 neighbor [Source:HGNC Symbol;Acc:HGNC:26372] |
| ENSG00000177047 | HGLibA_22733 | 170 | 0.403014804 | IFNW1 | interferon omega 1 [Source:HGNC Symbol;Acc:HGNC:5448] |
| ENSG00000179242 | HGLibA_08680 | 170 | 0.403014804 | CDH4 | cadherin 4 [Source:HGNC Symbol;Acc:HGNC:1763] |
| ENSG00000180318 | HGLibA_01734 | 170 | 0.403014804 | ALX1 | ALX homeobox 1 [Source:HGNC Symbol;Acc:HGNC:1494] |
| ENSG00000180592 | HGLibA_44278 | 170 | 0.403014804 | SKIDA1 | SKI/DACH domain containing 1 [Source:HGNC Symbol;Acc:HGNC:32697] |
| ENSG00000180817 | HGLibA_37804 | 170 | 0.403014804 | PPA1 | pyrophosphatase (inorganic) 1 [Source:HGNC Symbol;Acc:HGNC:9226] |
| ENSG00000181885 | HGLibA_09906 | 170 | 0.403014804 | CLDN7 | claudin 7 [Source:HGNC Symbol;Acc:HGNC:2049] |
| ENSG00000182938 | HGLibA_34740 | 170 | 0.403014804 | OTOP3 | otopetrin 3 [Source:HGNC Symbol;Acc:HGNC:19658] |
| ENSG00000183310 | HGLibA_33878 | 170 | 0.403014804 | OR2T34 | olfactory receptor family 2 subfamily T member 34 [Source:HGNC Symbol;Acc:HGNC:31256] |
| ENSG00000183313 | HGLibA_34186 | 170 | 0.403014804 | OR52L1 | olfactory receptor family 52 subfamily L member 1 [Source:HGNC Symbol;Acc:HGNC:14785] |
| ENSG00000184647 | HGLibA_38961 | 170 | 0.403014804 | PRSS55 | serine protease 55 [Source:HGNC Symbol;Acc:HGNC:30824] |
| ENSG00000184831 | HGLibA_02550 | 170 | 0.403014804 | APOO | apolipoprotein O [Source:HGNC Symbol;Acc:HGNC:28727] |
| ENSG00000185420 | HGLibA_45858 | 170 | 0.403014804 | SMYD3 | SET and MYND domain containing 3 [Source:HGNC Symbol;Acc:HGNC:15513] |
| ENSG00000185624 | HGLibA_34908 | 170 | 0.403014804 | P4HB | prolyl 4-hydroxylase subunit beta [Source:HGNC Symbol;Acc:HGNC:8548] |
| ENSG00000186654 | HGLibA_38842 | 170 | 0.403014804 | PRR5 | proline rich 5 [Source:HGNC Symbol;Acc:HGNC:31682] |
| ENSG00000186862 | HGLibA_35981 | 170 | 0.403014804 | PDZD7 | PDZ domain containing 7 [Source:HGNC Symbol;Acc:HGNC:26257] |
| ENSG00000186897 | HGLibA_05932 | 170 | 0.403014804 | C1QL4 | complement C1q like 4 [Source:HGNC Symbol;Acc:HGNC:31416] |
| ENSG00000187238 | HGLibA_26133 | 170 | 0.403014804 | LCE3B | late cornified envelope 3B [Source:HGNC Symbol;Acc:HGNC:29462] |
| ENSG00000188338 | HGLibA_45067 | 170 | 0.403014804 | SLC38A3 | solute carrier family 38 member 3 [Source:HGNC Symbol;Acc:HGNC:18044] |
| ENSG00000188730 | HGLibA_53924 | 170 | 0.403014804 | VWC2 | von Willebrand factor C domain containing 2 [Source:HGNC Symbol;Acc:HGNC:30200] |
| ENSG00000188735 | HGLibA_49898 | 170 | 0.403014804 | TMEM120B | transmembrane protein 120B [Source:HGNC Symbol;Acc:HGNC:32008] |
| ENSG00000196132 | HGLibA_30925 | 170 | 0.403014804 | MYT1 | myelin transcription factor 1 [Source:HGNC Symbol;Acc:HGNC:7622] |
| ENSG00000197933 | HGLibA_56822 | 170 | 0.403014804 | ZNF823 | zinc finger protein 823 [Source:HGNC Symbol;Acc:HGNC:30936] |
| ENSG00000198445 | HGLibA_08219 | 170 | 0.403014804 | CCT8L2 | chaperonin containing TCP1 subunit 8 like 2 [Source:HGNC Symbol;Acc:HGNC:15553] |
| ENSG00000198612 | HGLibA_10734 | 170 | 0.403014804 | COPS8 | COP9 signalosome subunit 8 [Source:HGNC Symbol;Acc:HGNC:24335] |
| ENSG00000198765 | HGLibA_47928 | 170 | 0.403014804 | SYCP1 | synaptonemal complex protein 1 [Source:HGNC Symbol;Acc:HGNC:11487] |
| ENSG00000198797 | HGLibA_04815 | 170 | 0.403014804 | BRINP2 | BMP/retinoic acid inducible neural specific 2 [Source:HGNC Symbol;Acc:HGNC:13746] |
| ENSG00000198948 | HGLibA_29048 | 170 | 0.403014804 | MFAP3L | microfibril associated protein 3 like [Source:HGNC Symbol;Acc:HGNC:29083] |
| ENSG00000204071 | HGLibA_48719 | 170 | 0.403014804 | TCEAL6 | transcription elongation factor A like 6 [Source:HGNC Symbol;Acc:HGNC:24553] |
| ENSG00000205659 | HGLibA_26568 | 170 | 0.403014804 | LIN52 | lin-52 DREAM MuvB core complex component [Source:HGNC Symbol;Acc:HGNC:19856] |
| ENSG00000214414 | HGLibA_51611 | 170 | 0.403014804 | TRIM77 | tripartite motif containing 77 [Source:HGNC Symbol;Acc:HGNC:34228] |
| ENSG00000215712 | HGLibA_50291 | 170 | 0.403014804 | TMEM242 | transmembrane protein 242 [Source:HGNC Symbol;Acc:HGNC:17206] |
| ENSG00000221931 | HGLibA_34451 | 170 | 0.403014804 | OR6X1 | olfactory receptor family 6 subfamily X member 1 [Source:HGNC Symbol;Acc:HGNC:14737] |
| ENSG00000223601 | HGLibA_14365 | 170 | 0.403014804 | EBLN1 | endogenous Bornavirus like nucleoprotein 1 [Source:HGNC Symbol;Acc:HGNC:39430] |
| ENSG00000241322 | HGLibA_08873 | 170 | 0.403014804 | CDRT1 | CMT1A duplicated region transcript 1 [Source:HGNC Symbol;Acc:HGNC:14379] |
| ENSG00000258818 | HGLibA_41409 | 170 | 0.403014804 | RNASE4 | ribonuclease A family member 4 [Source:HGNC Symbol;Acc:HGNC:10047] |
| ENSG00000259803 | HGLibA_44585 | 170 | 0.403014804 | SLC22A31 | solute carrier family 22 member 31 [Source:HGNC Symbol;Acc:HGNC:27091] |
| ENSG00000261678 | HGLibA_43057 | 170 | 0.403014804 | SCRT1 | scratch family transcriptional repressor 1 [Source:HGNC Symbol;Acc:HGNC:15950] |
| FAM173B | HGLibA_16278 | 170 | 0.403014804 | FAM173B | - |
| H2AFX | HGLibA_20760 | 170 | 0.403014804 | H2AFX | - |
| hsa-mir-1288 | HGLibA_57616 | 170 | 0.403014804 | hsa-mir-1288 | - |
| hsa-mir-1297 | HGLibA_57669 | 170 | 0.403014804 | hsa-mir-1297 | - |
| hsa-mir-190b | HGLibA_58037 | 170 | 0.403014804 | hsa-mir-190b | - |
| hsa-mir-197 | HGLibA_58106 | 170 | 0.403014804 | hsa-mir-197 | - |
| hsa-mir-3198-2 | HGLibA_58894 | 170 | 0.403014804 | hsa-mir-3198-2 | - |
| hsa-mir-3918 | HGLibA_59547 | 170 | 0.403014804 | hsa-mir-3918 | - |
| hsa-mir-4306 | HGLibA_59935 | 170 | 0.403014804 | hsa-mir-4306 | - |
| hsa-mir-4642 | HGLibA_60671 | 170 | 0.403014804 | hsa-mir-4642 | - |
| hsa-mir-559 | HGLibA_62201 | 170 | 0.403014804 | hsa-mir-559 | - |
| hsa-mir-6165 | HGLibA_62703 | 170 | 0.403014804 | hsa-mir-6165 | - |
| hsa-mir-6784 | HGLibA_63312 | 170 | 0.403014804 | hsa-mir-6784 | - |
| hsa-mir-6834 | HGLibA_63512 | 170 | 0.403014804 | hsa-mir-6834 | - |
| hsa-mir-7161 | HGLibA_63873 | 170 | 0.403014804 | hsa-mir-7161 | - |
| hsa-mir-7706 | HGLibA_63959 | 170 | 0.403014804 | hsa-mir-7706 | - |
| PNMAL2 | HGLibA_37386 | 170 | 0.403014804 | PNMAL2 | - |
| PRR21 | HGLibA_38816 | 170 | 0.403014804 | PRR21 | - |
| PVRL4 | HGLibA_39632 | 170 | 0.403014804 | PVRL4 | - |
| WDR65 | HGLibA_54152 | 170 | 0.403014804 | WDR65 | - |
| ANXA8L2 | HGLibA_02258 | 169 | 0.393969461 | ANXA8L2 | - |
| C10orf10 | HGLibA_05020 | 169 | 0.393969461 | C10orf10 | - |
| C12orf61 | HGLibA_05276 | 169 | 0.393969461 | C12orf61 | - |
| C1orf192 | HGLibA_05814 | 169 | 0.393969461 | C1orf192 | - |
| C2orf61 | HGLibA_06156 | 169 | 0.393969461 | C2orf61 | - |
| ENSG00000006576 | HGLibA_36464 | 169 | 0.393969461 | PHTF2 | putative homeodomain transcription factor 2 [Source:HGNC Symbol;Acc:HGNC:13411] |
| ENSG00000006747 | HGLibA_42947 | 169 | 0.393969461 | SCIN | scinderin [Source:HGNC Symbol;Acc:HGNC:21695] |
| ENSG00000012171 | HGLibA_43322 | 169 | 0.393969461 | SEMA3B | semaphorin 3B [Source:HGNC Symbol;Acc:HGNC:10724] |
| ENSG00000021776 | HGLibA_02616 | 169 | 0.393969461 | AQR | aquarius intron-binding spliceosomal factor [Source:HGNC Symbol;Acc:HGNC:29513] |
| ENSG00000034533 | HGLibA_03373 | 169 | 0.393969461 | ASTE1 | asteroid homolog 1 [Source:HGNC Symbol;Acc:HGNC:25021] |
| ENSG00000049656 | HGLibA_10117 | 169 | 0.393969461 | CLPTM1L | CLPTM1 like [Source:HGNC Symbol;Acc:HGNC:24308] |
| ENSG00000072736 | HGLibA_31783 | 169 | 0.393969461 | NFATC3 | nuclear factor of activated T cells 3 [Source:HGNC Symbol;Acc:HGNC:7777] |
| ENSG00000085999 | HGLibA_40101 | 169 | 0.393969461 | RAD54L | RAD54 like [Source:HGNC Symbol;Acc:HGNC:9826] |
| ENSG00000100109 | HGLibA_49182 | 169 | 0.393969461 | TFIP11 | tuftelin interacting protein 11 [Source:HGNC Symbol;Acc:HGNC:17165] |
| ENSG00000100342 | HGLibA_02527 | 169 | 0.393969461 | APOL1 | apolipoprotein L1 [Source:HGNC Symbol;Acc:HGNC:618] |
| ENSG00000101811 | HGLibA_11486 | 169 | 0.393969461 | CSTF2 | cleavage stimulation factor subunit 2 [Source:HGNC Symbol;Acc:HGNC:2484] |
| ENSG00000102543 | HGLibA_08500 | 169 | 0.393969461 | CDADC1 | cytidine and dCMP deaminase domain containing 1 [Source:HGNC Symbol;Acc:HGNC:20299] |
| ENSG00000102878 | HGLibA_22228 | 169 | 0.393969461 | HSF4 | heat shock transcription factor 4 [Source:HGNC Symbol;Acc:HGNC:5227] |
| ENSG00000104368 | HGLibA_36961 | 169 | 0.393969461 | PLAT | tissue type [Source:HGNC Symbol;Acc:HGNC:9051]" |
| ENSG00000105186 | HGLibA_02052 | 169 | 0.393969461 | ANKRD27 | ankyrin repeat domain 27 [Source:HGNC Symbol;Acc:HGNC:25310] |
| ENSG00000105647 | HGLibA_36650 | 169 | 0.393969461 | PIK3R2 | phosphoinositide-3-kinase regulatory subunit 2 [Source:HGNC Symbol;Acc:HGNC:8980] |
| ENSG00000109991 | HGLibA_34853 | 169 | 0.393969461 | P2RX3 | purinergic receptor P2X 3 [Source:HGNC Symbol;Acc:HGNC:8534] |
| ENSG00000111058 | HGLibA_00594 | 169 | 0.393969461 | ACSS3 | acyl-CoA synthetase short chain family member 3 [Source:HGNC Symbol;Acc:HGNC:24723] |
| ENSG00000111224 | HGLibA_35218 | 169 | 0.393969461 | PARP11 | poly(ADP-ribose) polymerase family member 11 [Source:HGNC Symbol;Acc:HGNC:1186] |
| ENSG00000113555 | HGLibA_35396 | 169 | 0.393969461 | PCDH12 | protocadherin 12 [Source:HGNC Symbol;Acc:HGNC:8657] |
| ENSG00000114209 | HGLibA_35718 | 169 | 0.393969461 | PDCD10 | programmed cell death 10 [Source:HGNC Symbol;Acc:HGNC:8761] |
| ENSG00000115306 | HGLibA_46881 | 169 | 0.393969461 | SPTBN1 | non-erythrocytic 1 [Source:HGNC Symbol;Acc:HGNC:11275]" |
| ENSG00000116726 | HGLibA_38286 | 169 | 0.393969461 | PRAMEF12 | PRAME family member 12 [Source:HGNC Symbol;Acc:HGNC:22125] |
| ENSG00000116906 | HGLibA_19561 | 169 | 0.393969461 | GNPAT | glyceronephosphate O-acyltransferase [Source:HGNC Symbol;Acc:HGNC:4416] |
| ENSG00000117148 | HGLibA_00631 | 169 | 0.393969461 | ACTL8 | actin like 8 [Source:HGNC Symbol;Acc:HGNC:24018] |
| ENSG00000121410 | HGLibA_00002 | 169 | 0.393969461 | A1BG | alpha-1-B glycoprotein [Source:HGNC Symbol;Acc:HGNC:5] |
| ENSG00000123154 | HGLibA_54194 | 169 | 0.393969461 | WDR83 | WD repeat domain 83 [Source:HGNC Symbol;Acc:HGNC:32672] |
| ENSG00000123159 | HGLibA_19119 | 169 | 0.393969461 | GIPC1 | GIPC PDZ domain containing family member 1 [Source:HGNC Symbol;Acc:HGNC:1226] |
| ENSG00000123353 | HGLibA_34628 | 169 | 0.393969461 | ORMDL2 | ORMDL sphingolipid biosynthesis regulator 2 [Source:HGNC Symbol;Acc:HGNC:16037] |
| ENSG00000124194 | HGLibA_18841 | 169 | 0.393969461 | GDAP1L1 | ganglioside induced differentiation associated protein 1 like 1 [Source:HGNC Symbol;Acc:HGNC:4213] |
| ENSG00000124275 | HGLibA_30493 | 169 | 0.393969461 | MTRR | 5-methyltetrahydrofolate-homocysteine methyltransferase reductase [Source:HGNC Symbol;Acc:HGNC:7473] |
| ENSG00000124784 | HGLibA_41307 | 169 | 0.393969461 | RIOK1 | RIO kinase 1 [Source:HGNC Symbol;Acc:HGNC:18656] |
| ENSG00000124818 | HGLibA_33441 | 169 | 0.393969461 | OPN5 | opsin 5 [Source:HGNC Symbol;Acc:HGNC:19992] |
| ENSG00000125571 | HGLibA_23157 | 169 | 0.393969461 | IL37 | interleukin 37 [Source:HGNC Symbol;Acc:HGNC:15563] |
| ENSG00000125879 | HGLibA_34744 | 169 | 0.393969461 | OTOR | otoraplin [Source:HGNC Symbol;Acc:HGNC:8517] |
| ENSG00000127743 | HGLibA_22992 | 169 | 0.393969461 | IL17B | interleukin 17B [Source:HGNC Symbol;Acc:HGNC:5982] |
| ENSG00000130429 | HGLibA_03078 | 169 | 0.393969461 | ARPC1B | actin related protein 2/3 complex subunit 1B [Source:HGNC Symbol;Acc:HGNC:704] |
| ENSG00000130643 | HGLibA_07055 | 169 | 0.393969461 | CALY | calcyon neuron specific vesicular protein [Source:HGNC Symbol;Acc:HGNC:17938] |
| ENSG00000132164 | HGLibA_45291 | 169 | 0.393969461 | SLC6A11 | solute carrier family 6 member 11 [Source:HGNC Symbol;Acc:HGNC:11044] |
| ENSG00000135480 | HGLibA_25523 | 169 | 0.393969461 | KRT7 | keratin 7 [Source:HGNC Symbol;Acc:HGNC:6445] |
| ENSG00000136371 | HGLibA_30390 | 169 | 0.393969461 | MTHFS | methenyltetrahydrofolate synthetase [Source:HGNC Symbol;Acc:HGNC:7437] |
| ENSG00000138336 | HGLibA_49038 | 169 | 0.393969461 | TET1 | tet methylcytosine dioxygenase 1 [Source:HGNC Symbol;Acc:HGNC:29484] |
| ENSG00000139549 | HGLibA_13110 | 169 | 0.393969461 | DHH | desert hedgehog [Source:HGNC Symbol;Acc:HGNC:2865] |
| ENSG00000141965 | HGLibA_17272 | 169 | 0.393969461 | FEM1A | fem-1 homolog A [Source:HGNC Symbol;Acc:HGNC:16934] |
| ENSG00000142634 | HGLibA_14578 | 169 | 0.393969461 | EFHD2 | EF-hand domain family member D2 [Source:HGNC Symbol;Acc:HGNC:28670] |
| ENSG00000143815 | HGLibA_26081 | 169 | 0.393969461 | LBR | lamin B receptor [Source:HGNC Symbol;Acc:HGNC:6518] |
| ENSG00000146856 | HGLibA_01173 | 169 | 0.393969461 | AGBL3 | ATP/GTP binding protein like 3 [Source:HGNC Symbol;Acc:HGNC:27981] |
| ENSG00000147224 | HGLibA_38755 | 169 | 0.393969461 | PRPS1 | phosphoribosyl pyrophosphate synthetase 1 [Source:HGNC Symbol;Acc:HGNC:9462] |
| ENSG00000147789 | HGLibA_56568 | 169 | 0.393969461 | ZNF7 | zinc finger protein 7 [Source:HGNC Symbol;Acc:HGNC:13139] |
| ENSG00000148297 | HGLibA_28777 | 169 | 0.393969461 | MED22 | mediator complex subunit 22 [Source:HGNC Symbol;Acc:HGNC:11477] |
| ENSG00000149633 | HGLibA_24817 | 169 | 0.393969461 | KIAA1755 | KIAA1755 [Source:HGNC Symbol;Acc:HGNC:29372] |
| ENSG00000155265 | HGLibA_19638 | 169 | 0.393969461 | GOLGA7B | golgin A7 family member B [Source:HGNC Symbol;Acc:HGNC:31668] |
| ENSG00000156486 | HGLibA_24387 | 169 | 0.393969461 | KCNS2 | potassium voltage-gated channel modifier subfamily S member 2 [Source:HGNC Symbol;Acc:HGNC:6301] |
| ENSG00000158480 | HGLibA_46443 | 169 | 0.393969461 | SPATA2 | spermatogenesis associated 2 [Source:HGNC Symbol;Acc:HGNC:14681] |
| ENSG00000158901 | HGLibA_54275 | 169 | 0.393969461 | WFDC8 | WAP four-disulfide core domain 8 [Source:HGNC Symbol;Acc:HGNC:16163] |
| ENSG00000159176 | HGLibA_11433 | 169 | 0.393969461 | CSRP1 | cysteine and glycine rich protein 1 [Source:HGNC Symbol;Acc:HGNC:2469] |
| ENSG00000159516 | HGLibA_46824 | 169 | 0.393969461 | SPRR2G | small proline rich protein 2G [Source:HGNC Symbol;Acc:HGNC:11267] |
| ENSG00000160856 | HGLibA_17232 | 169 | 0.393969461 | FCRL3 | Fc receptor like 3 [Source:HGNC Symbol;Acc:HGNC:18506] |
| ENSG00000164440 | HGLibA_52392 | 169 | 0.393969461 | TXLNB | taxilin beta [Source:HGNC Symbol;Acc:HGNC:21617] |
| ENSG00000164609 | HGLibA_45550 | 169 | 0.393969461 | SLU7 | splicing factor [Source:HGNC Symbol;Acc:HGNC:16939]" |
| ENSG00000165572 | HGLibA_24112 | 169 | 0.393969461 | KBTBD6 | kelch repeat and BTB domain containing 6 [Source:HGNC Symbol;Acc:HGNC:25340] |
| ENSG00000165970 | HGLibA_45330 | 169 | 0.393969461 | SLC6A5 | solute carrier family 6 member 5 [Source:HGNC Symbol;Acc:HGNC:11051] |
| ENSG00000166025 | HGLibA_01816 | 169 | 0.393969461 | AMOTL1 | angiomotin like 1 [Source:HGNC Symbol;Acc:HGNC:17811] |
| ENSG00000167740 | HGLibA_11997 | 169 | 0.393969461 | CYB5D2 | cytochrome b5 domain containing 2 [Source:HGNC Symbol;Acc:HGNC:28471] |
| ENSG00000170525 | HGLibA_36160 | 169 | 0.393969461 | PFKFB3 | 6-biphosphatase 3 [Source:HGNC Symbol;Acc:HGNC:8874]" |
| ENSG00000170561 | HGLibA_23632 | 169 | 0.393969461 | IRX2 | iroquois homeobox 2 [Source:HGNC Symbol;Acc:HGNC:14359] |
| ENSG00000171462 | HGLibA_13358 | 169 | 0.393969461 | DLK2 | delta like non-canonical Notch ligand 2 [Source:HGNC Symbol;Acc:HGNC:21113] |
| ENSG00000172322 | HGLibA_09927 | 169 | 0.393969461 | CLEC12A | C-type lectin domain family 12 member A [Source:HGNC Symbol;Acc:HGNC:31713] |
| ENSG00000172345 | HGLibA_47365 | 169 | 0.393969461 | STARD5 | StAR related lipid transfer domain containing 5 [Source:HGNC Symbol;Acc:HGNC:18065] |

| | | | | | |
|---|---|---|---|---|---|
| ENSG00000172572 | HGLibA_35782 | 169 | 0.393969461 | PDE3A | phosphodiesterase 3A [Source:HGNC Symbol;Acc:HGNC:8778] |
| ENSG00000175387 | HGLibA_45571 | 169 | 0.393969461 | SMAD2 | SMAD family member 2 [Source:HGNC Symbol;Acc:HGNC:6768] |
| ENSG00000175646 | HGLibA_38609 | 169 | 0.393969461 | PRM1 | protamine 1 [Source:HGNC Symbol;Acc:HGNC:9447] |
| ENSG00000176136 | HGLibA_28552 | 169 | 0.393969461 | MC5R | melanocortin 5 receptor [Source:HGNC Symbol;Acc:HGNC:6933] |
| ENSG00000176495 | HGLibA_34242 | 169 | 0.393969461 | OR5AN1 | olfactory receptor family 5 subfamily AN member 1 [Source:HGNC Symbol;Acc:HGNC:15255] |
| ENSG00000177191 | HGLibA_04003 | 169 | 0.393969461 | B3GNT8 | 3-N-acetylglucosaminyltransferase 8 [Source:HGNC Symbol;Acc:HGNC:24139]" |
| ENSG00000177383 | HGLibA_27986 | 169 | 0.393969461 | MAGEF1 | MAGE family member F1 [Source:HGNC Symbol;Acc:HGNC:29639] |
| ENSG00000180340 | HGLibA_18267 | 169 | 0.393969461 | FZD2 | frizzled class receptor 2 [Source:HGNC Symbol;Acc:HGNC:4040] |
| ENSG00000181544 | HGLibA_16832 | 169 | 0.393969461 | FANCB | FA complementation group B [Source:HGNC Symbol;Acc:HGNC:3583] |
| ENSG00000182782 | HGLibA_20948 | 169 | 0.393969461 | HCAR2 | hydroxycarboxylic acid receptor 2 [Source:HGNC Symbol;Acc:HGNC:24827] |
| ENSG00000184005 | HGLibA_47271 | 169 | 0.393969461 | ST6GALNAC3 | 6-sialyltransferase 3 [Source:HGNC Symbol;Acc:HGNC:19343]" |
| ENSG00000184047 | HGLibA_13215 | 169 | 0.393969461 | DIABLO | diablo IAP-binding mitochondrial protein [Source:HGNC Symbol;Acc:HGNC:21528] |
| ENSG00000184933 | HGLibA_34369 | 169 | 0.393969461 | OR6A2 | olfactory receptor family 6 subfamily A member 2 [Source:HGNC Symbol;Acc:HGNC:15301] |
| ENSG00000186513 | HGLibA_34586 | 169 | 0.393969461 | OR9Q2 | olfactory receptor family 9 subfamily Q member 2 [Source:HGNC Symbol;Acc:HGNC:15328] |
| ENSG00000188334 | HGLibA_04876 | 169 | 0.393969461 | BSPH1 | binder of sperm protein homolog 1 [Source:HGNC Symbol;Acc:HGNC:33906] |
| ENSG00000196172 | HGLibA_56527 | 169 | 0.393969461 | ZNF681 | zinc finger protein 681 [Source:HGNC Symbol;Acc:HGNC:26457] |
| ENSG00000196639 | HGLibA_22095 | 169 | 0.393969461 | HRH1 | histamine receptor H1 [Source:HGNC Symbol;Acc:HGNC:5182] |
| ENSG00000197641 | HGLibA_43545 | 169 | 0.393969461 | SERPINB13 | serpin family B member 13 [Source:HGNC Symbol;Acc:HGNC:8944] |
| ENSG00000198860 | HGLibA_51829 | 169 | 0.393969461 | TSEN15 | tRNA splicing endonuclease subunit 15 [Source:HGNC Symbol;Acc:HGNC:16791] |
| ENSG00000213401 | HGLibA_27901 | 169 | 0.393969461 | MAGEA12 | MAGE family member A12 [Source:HGNC Symbol;Acc:HGNC:6799] |
| ENSG00000213578 | HGLibA_10937 | 169 | 0.393969461 | CPLX3 | complexin 3 [Source:HGNC Symbol;Acc:HGNC:27652] |
| ENSG00000213859 | HGLibA_24418 | 169 | 0.393969461 | KCTD11 | potassium channel tetramerization domain containing 11 [Source:HGNC Symbol;Acc:HGNC:21302] |
| ENSG00000227802 | HGLibA_13541 | 169 | 0.393969461 | DNAJB3 | DnaJ heat shock protein family (Hsp40) member B3 [Source:HGNC Symbol;Acc:HGNC:32397] |
| ENSG00000237693 | HGLibA_23612 | 169 | 0.393969461 | IRGM | immunity related GTPase M [Source:HGNC Symbol;Acc:HGNC:29597] |
| ENSG00000256394 | HGLibA_03312 | 169 | 0.393969461 | ASIC5 | acid sensing ion channel subunit family member 5 [Source:HGNC Symbol;Acc:HGNC:17537] |
| ENSG00000256797 | HGLibA_25307 | 169 | 0.393969461 | KLRF2 | killer cell lectin like receptor F2 [Source:HGNC Symbol;Acc:HGNC:37646] |
| ENSG00000278195 | HGLibA_47181 | 169 | 0.393969461 | SSTR3 | somatostatin receptor 3 [Source:HGNC Symbol;Acc:HGNC:11332] |
| GPR64 | HGLibA_20072 | 169 | 0.393969461 | GPR64 | - |
| HARS | HGLibA_20857 | 169 | 0.393969461 | HARS | - |
| HIST1H3A | HGLibA_21455 | 169 | 0.393969461 | HIST1H3A | - |
| hsa-let-7c | HGLibA_57114 | 169 | 0.393969461 | hsa-let-7c | - |
| hsa-mir-224 | HGLibA_58303 | 169 | 0.393969461 | hsa-mir-224 | - |
| hsa-mir-320e | HGLibA_58951 | 169 | 0.393969461 | hsa-mir-320e | - |
| hsa-mir-3920 | HGLibA_59557 | 169 | 0.393969461 | hsa-mir-3920 | - |
| hsa-mir-449c | HGLibA_60423 | 169 | 0.393969461 | hsa-mir-449c | - |
| hsa-mir-4700 | HGLibA_60910 | 169 | 0.393969461 | hsa-mir-4700 | - |
| hsa-mir-511 | HGLibA_61560 | 169 | 0.393969461 | hsa-mir-511 | - |
| hsa-mir-518b | HGLibA_61659 | 169 | 0.393969461 | hsa-mir-518b | - |
| hsa-mir-573 | HGLibA_62381 | 169 | 0.393969461 | hsa-mir-573 | - |
| hsa-mir-6814 | HGLibA_63433 | 169 | 0.393969461 | hsa-mir-6814 | - |
| hsa-mir-8054 | HGLibA_64053 | 169 | 0.393969461 | hsa-mir-8054 | - |
| KAL1 | HGLibA_24019 | 169 | 0.393969461 | KAL1 | - |
| PLAC1L | HGLibA_36936 | 169 | 0.393969461 | PLAC1L | - |
| QARS | HGLibA_39719 | 169 | 0.393969461 | QARS | - |
| QTRTD1 | HGLibA_39763 | 169 | 0.393969461 | QTRTD1 | - |
| ATP5L | HGLibA_03678 | 168 | 0.384924118 | ATP5L | - |
| C1orf186 | HGLibA_05806 | 168 | 0.384924118 | C1orf186 | - |
| ENSG00000002834 | HGLibA_26056 | 168 | 0.384924118 | LASP1 | LIM and SH3 protein 1 [Source:HGNC Symbol;Acc:HGNC:6513] |
| ENSG00000006071 | HGLibA_00185 | 168 | 0.384924118 | ABCC8 | ATP binding cassette subfamily C member 8 [Source:HGNC Symbol;Acc:HGNC:59] |
| ENSG00000007372 | HGLibA_35311 | 168 | 0.384924118 | PAX6 | paired box 6 [Source:HGNC Symbol;Acc:HGNC:8620] |
| ENSG00000013725 | HGLibA_08422 | 168 | 0.384924118 | CD6 | CD6 molecule [Source:HGNC Symbol;Acc:HGNC:1691] |
| ENSG00000056487 | HGLibA_36372 | 168 | 0.384924118 | PHF21B | PHD finger protein 21B [Source:HGNC Symbol;Acc:HGNC:25161] |
| ENSG00000067208 | HGLibA_15693 | 168 | 0.384924118 | EVI5 | ecotropic viral integration site 5 [Source:HGNC Symbol;Acc:HGNC:3501] |
| ENSG00000067829 | HGLibA_22550 | 168 | 0.384924118 | IDH3G | isocitrate dehydrogenase 3 (NAD(+)) gamma [Source:HGNC Symbol;Acc:HGNC:5386] |
| ENSG00000071246 | HGLibA_53531 | 168 | 0.384924118 | VASH1 | vasohibin 1 [Source:HGNC Symbol;Acc:HGNC:19964] |
| ENSG00000086289 | HGLibA_15276 | 168 | 0.384924118 | EPDR1 | ependymin related 1 [Source:HGNC Symbol;Acc:HGNC:17572] |
| ENSG00000088543 | HGLibA_06214 | 168 | 0.384924118 | C3orf18 | chromosome 3 open reading frame 18 [Source:HGNC Symbol;Acc:HGNC:24837] |
| ENSG00000090376 | HGLibA_23562 | 168 | 0.384924118 | IRAK3 | interleukin 1 receptor associated kinase 3 [Source:HGNC Symbol;Acc:HGNC:17020] |
| ENSG00000099822 | HGLibA_20977 | 168 | 0.384924118 | HCN2 | hyperpolarization activated cyclic nucleotide gated potassium and sodium channel 2 [Source:HGNC Symbol;Acc:HGNC:4846] |
| ENSG00000100979 | HGLibA_37247 | 168 | 0.384924118 | PLTP | phospholipid transfer protein [Source:HGNC Symbol;Acc:HGNC:9093] |
| ENSG00000101132 | HGLibA_36144 | 168 | 0.384924118 | PFDN4 | prefoldin subunit 4 [Source:HGNC Symbol;Acc:HGNC:8868] |
| ENSG00000101353 | HGLibA_29879 | 168 | 0.384924118 | MROH8 | maestro heat like repeat family member 8 [Source:HGNC Symbol;Acc:HGNC:16125] |
| ENSG00000101413 | HGLibA_42047 | 168 | 0.384924118 | RPRD1B | regulation of nuclear pre-mRNA domain containing 1B [Source:HGNC Symbol;Acc:HGNC:16209] |
| ENSG00000104267 | HGLibA_06804 | 168 | 0.384924118 | CA2 | carbonic anhydrase 2 [Source:HGNC Symbol;Acc:HGNC:1373] |
| ENSG00000104755 | HGLibA_00748 | 168 | 0.384924118 | ADAM2 | ADAM metallopeptidase domain 2 [Source:HGNC Symbol;Acc:HGNC:198] |
| ENSG00000104812 | HGLibA_20710 | 168 | 0.384924118 | GYS1 | glycogen synthase 1 [Source:HGNC Symbol;Acc:HGNC:4706] |
| ENSG00000105438 | HGLibA_24480 | 168 | 0.384924118 | KDELR1 | KDEL endoplasmic reticulum protein retention receptor 1 [Source:HGNC Symbol;Acc:HGNC:6304] |
| ENSG00000109689 | HGLibA_47445 | 168 | 0.384924118 | STIM2 | stromal interaction molecule 2 [Source:HGNC Symbol;Acc:HGNC:19205] |
| ENSG00000112837 | HGLibA_48649 | 168 | 0.384924118 | TBX18 | T-box 18 [Source:HGNC Symbol;Acc:HGNC:11595] |
| ENSG00000113249 | HGLibA_20898 | 168 | 0.384924118 | HAVCR1 | hepatitis A virus cellular receptor 1 [Source:HGNC Symbol;Acc:HGNC:17866] |
| ENSG00000113597 | HGLibA_51308 | 168 | 0.384924118 | TRAPPC13 | trafficking protein particle complex 13 [Source:HGNC Symbol;Acc:HGNC:25828] |
| ENSG00000116731 | HGLibA_38385 | 168 | 0.384924118 | PRDM2 | PR/SET domain 2 [Source:HGNC Symbol;Acc:HGNC:9347] |
| ENSG00000117091 | HGLibA_08399 | 168 | 0.384924118 | CD48 | CD48 molecule [Source:HGNC Symbol;Acc:HGNC:1683] |
| ENSG00000118503 | HGLibA_50713 | 168 | 0.384924118 | TNFAIP3 | TNF alpha induced protein 3 [Source:HGNC Symbol;Acc:HGNC:11896] |
| ENSG00000119402 | HGLibA_17144 | 168 | 0.384924118 | FBXW2 | F-box and WD repeat domain containing 2 [Source:HGNC Symbol;Acc:HGNC:13608] |
| ENSG00000122729 | HGLibA_00460 | 168 | 0.384924118 | ACO1 | aconitase 1 [Source:HGNC Symbol;Acc:HGNC:117] |
| ENSG00000122786 | HGLibA_07011 | 168 | 0.384924118 | CALD1 | caldesmon 1 [Source:HGNC Symbol;Acc:HGNC:1441] |
| ENSG00000123975 | HGLibA_09789 | 168 | 0.384924118 | CKS2 | CDC28 protein kinase regulatory subunit 2 [Source:HGNC Symbol;Acc:HGNC:2000] |
| ENSG00000125878 | HGLibA_48763 | 168 | 0.384924118 | TCF15 | transcription factor 15 [Source:HGNC Symbol;Acc:HGNC:11627] |
| ENSG00000126705 | HGLibA_01294 | 168 | 0.384924118 | AHDC1 | AT-hook DNA binding motif containing 1 [Source:HGNC Symbol;Acc:HGNC:25230] |
| ENSG00000129221 | HGLibA_01364 | 168 | 0.384924118 | AIPL1 | aryl hydrocarbon receptor interacting protein like 1 [Source:HGNC Symbol;Acc:HGNC:359] |
| ENSG00000131721 | HGLibA_41216 | 168 | 0.384924118 | RHOXF2 | Rhox homeobox family member 2 [Source:HGNC Symbol;Acc:HGNC:30011] |
| ENSG00000133661 | HGLibA_43777 | 168 | 0.384924118 | SFTPD | surfactant protein D [Source:HGNC Symbol;Acc:HGNC:10803] |
| ENSG00000134222 | HGLibA_39233 | 168 | 0.384924118 | PSRC1 | proline and serine rich coiled-coil 1 [Source:HGNC Symbol;Acc:HGNC:24472] |
| ENSG00000134438 | HGLibA_40408 | 168 | 0.384924118 | RAX | retina and anterior neural fold homeobox [Source:HGNC Symbol;Acc:HGNC:18662] |
| ENSG00000137878 | HGLibA_18825 | 168 | 0.384924118 | GCOM1 | GRINL1A complex locus 1 [Source:HGNC Symbol;Acc:HGNC:26424] |
| ENSG00000138092 | HGLibA_09078 | 168 | 0.384924118 | CENPO | centromere protein O [Source:HGNC Symbol;Acc:HGNC:28152] |
| ENSG00000139352 | HGLibA_03266 | 168 | 0.384924118 | ASCL1 | achaete-scute family bHLH transcription factor 1 [Source:HGNC Symbol;Acc:HGNC:738] |
| ENSG00000140743 | HGLibA_08865 | 168 | 0.384924118 | CDR2 | cerebellar degeneration related protein 2 [Source:HGNC Symbol;Acc:HGNC:1799] |
| ENSG00000141295 | HGLibA_43053 | 168 | 0.384924118 | SCRN2 | secernin 2 [Source:HGNC Symbol;Acc:HGNC:30381] |
| ENSG00000141542 | HGLibA_39937 | 168 | 0.384924118 | RAB40B | member RAS oncogene family [Source:HGNC Symbol;Acc:HGNC:18284]" |
| ENSG00000141736 | HGLibA_15412 | 168 | 0.384924118 | ERBB2 | erb-b2 receptor tyrosine kinase 2 [Source:HGNC Symbol;Acc:HGNC:3430] |
| ENSG00000149403 | HGLibA_20291 | 168 | 0.384924118 | GRIK4 | glutamate ionotropic receptor kainate type subunit 4 [Source:HGNC Symbol;Acc:HGNC:4582] |
| ENSG00000150269 | HGLibA_34343 | 168 | 0.384924118 | OR5M9 | olfactory receptor family 5 subfamily M member 9 [Source:HGNC Symbol;Acc:HGNC:15294] |
| ENSG00000150628 | HGLibA_46512 | 168 | 0.384924118 | SPATA4 | spermatogenesis associated 4 [Source:HGNC Symbol;Acc:HGNC:17333] |
| ENSG00000150628 | HGLibA_46513 | 168 | 0.384924118 | SPATA4 | spermatogenesis associated 4 [Source:HGNC Symbol;Acc:HGNC:17333] |
| ENSG00000154640 | HGLibA_04946 | 168 | 0.384924118 | BTG3 | BTG anti-proliferation factor 3 [Source:HGNC Symbol;Acc:HGNC:1132] |
| ENSG00000156127 | HGLibA_04173 | 168 | 0.384924118 | BATF | basic leucine zipper ATF-like transcription factor [Source:HGNC Symbol;Acc:HGNC:958] |
| ENSG00000156282 | HGLibA_09869 | 168 | 0.384924118 | CLDN17 | claudin 17 [Source:HGNC Symbol;Acc:HGNC:2038] |
| ENSG00000157800 | HGLibA_45049 | 168 | 0.384924118 | SLC37A3 | solute carrier family 37 member 3 [Source:HGNC Symbol;Acc:HGNC:20651] |
| ENSG00000158805 | HGLibA_55714 | 168 | 0.384924118 | ZNF276 | zinc finger protein 276 [Source:HGNC Symbol;Acc:HGNC:23330] |
| ENSG00000159495 | HGLibA_49272 | 168 | 0.384924118 | TGM7 | transglutaminase 7 [Source:HGNC Symbol;Acc:HGNC:30790] |
| ENSG00000161929 | HGLibA_42944 | 168 | 0.384924118 | SCIMP | SLP adaptor and CSK interacting membrane protein [Source:HGNC Symbol;Acc:HGNC:33504] |
| ENSG00000162004 | HGLibA_07870 | 168 | 0.384924118 | CCDC78 | coiled-coil domain containing 78 [Source:HGNC Symbol;Acc:HGNC:14153] |
| ENSG00000162836 | HGLibA_00519 | 168 | 0.384924118 | ACP6 | lysophosphatidic [Source:HGNC Symbol;Acc:HGNC:29609]" |
| ENSG00000164107 | HGLibA_20828 | 168 | 0.384924118 | HAND2 | heart and neural crest derivatives expressed 2 [Source:HGNC Symbol;Acc:HGNC:4808] |
| ENSG00000164303 | HGLibA_15181 | 168 | 0.384924118 | ENPP6 | ectonucleotide pyrophosphatase/phosphodiesterase 6 [Source:HGNC Symbol;Acc:HGNC:23409] |
| ENSG00000165246 | HGLibA_32079 | 168 | 0.384924118 | NLGN4Y | neuroligin 4 Y-linked [Source:HGNC Symbol;Acc:HGNC:15529] |
| ENSG00000165695 | HGLibA_01397 | 168 | 0.384924118 | AK8 | adenylate kinase 8 [Source:HGNC Symbol;Acc:HGNC:26526] |
| ENSG00000168646 | HGLibA_03924 | 168 | 0.384924118 | AXIN2 | axin 2 [Source:HGNC Symbol;Acc:HGNC:904] |
| ENSG00000169067 | HGLibA_00606 | 168 | 0.384924118 | ACTBL2 | beta like 2 [Source:HGNC Symbol;Acc:HGNC:17780]" |
| ENSG00000170381 | HGLibA_43331 | 168 | 0.384924118 | SEMA3E | semaphorin 3E [Source:HGNC Symbol;Acc:HGNC:10727] |
| ENSG00000170852 | HGLibA_24103 | 168 | 0.384924118 | KBTBD2 | kelch repeat and BTB domain containing 2 [Source:HGNC Symbol;Acc:HGNC:21751] |
| ENSG00000174358 | HGLibA_45315 | 168 | 0.384924118 | SLC6A19 | solute carrier family 6 member 19 [Source:HGNC Symbol;Acc:HGNC:27960] |
| ENSG00000174957 | HGLibA_34307 | 168 | 0.384924118 | OR5J2 | olfactory receptor family 5 subfamily J member 2 [Source:HGNC Symbol;Acc:HGNC:19612] |
| ENSG00000176567 | HGLibA_34066 | 168 | 0.384924118 | OR4X1 | olfactory receptor family 4 subfamily X member 1 (gene/pseudogene) [Source:HGNC Symbol;Acc:HGNC:14854] |
| ENSG00000177098 | HGLibA_42996 | 168 | 0.384924118 | SCN4B | sodium voltage-gated channel beta subunit 4 [Source:HGNC Symbol;Acc:HGNC:10592] |
| ENSG00000178814 | HGLibA_33418 | 168 | 0.384924118 | OPLAH | ATP-hydrolysing [Source:HGNC Symbol;Acc:HGNC:8149]" |
| ENSG00000180720 | HGLibA_09556 | 168 | 0.384924118 | CHRM4 | cholinergic receptor muscarinic 4 [Source:HGNC Symbol;Acc:HGNC:1953] |
| ENSG00000181019 | HGLibA_32563 | 168 | 0.384924118 | NQO1 | NAD(P)H quinone dehydrogenase 1 [Source:HGNC Symbol;Acc:HGNC:2874] |
| ENSG00000181323 | HGLibA_46617 | 168 | 0.384924118 | SPEM1 | spermatid maturation 1 [Source:HGNC Symbol;Acc:HGNC:32429] |
| ENSG00000182108 | HGLibA_13017 | 168 | 0.384924118 | DEXI | Dexi homolog [Source:HGNC Symbol;Acc:HGNC:13267] |
| ENSG00000182326 | HGLibA_05975 | 168 | 0.384924118 | C1S | complement C1s [Source:HGNC Symbol;Acc:HGNC:1247] |
| ENSG00000183908 | HGLibA_27390 | 168 | 0.384924118 | LRRC55 | leucine rich repeat containing 55 [Source:HGNC Symbol;Acc:HGNC:32324] |
| ENSG00000184113 | HGLibA_09901 | 168 | 0.384924118 | CLDN5 | claudin 5 [Source:HGNC Symbol;Acc:HGNC:2047] |
| ENSG00000185000 | HGLibA_13033 | 168 | 0.384924118 | DGAT1 | diacylglycerol O-acyltransferase 1 [Source:HGNC Symbol;Acc:HGNC:2843] |
| ENSG00000185652 | HGLibA_32838 | 168 | 0.384924118 | NTF3 | neurotrophin 3 [Source:HGNC Symbol;Acc:HGNC:8023] |
| ENSG00000186470 | HGLibA_04972 | 168 | 0.384924118 | BTN3A2 | butyrophilin subfamily 3 member A2 [Source:HGNC Symbol;Acc:HGNC:1139] |
| ENSG00000186566 | HGLibA_19750 | 168 | 0.384924118 | GPATCH8 | G-patch domain containing 8 [Source:HGNC Symbol;Acc:HGNC:29066] |
| ENSG00000187581 | HGLibA_10879 | 168 | 0.384924118 | COX8C | cytochrome c oxidase subunit 8C [Source:HGNC Symbol;Acc:HGNC:24382] |
| ENSG00000187713 | HGLibA_50185 | 168 | 0.384924118 | TMEM203 | transmembrane protein 203 [Source:HGNC Symbol;Acc:HGNC:28217] |
| ENSG00000188042 | HGLibA_02968 | 168 | 0.384924118 | ARL4C | ADP ribosylation factor like GTPase 4C [Source:HGNC Symbol;Acc:HGNC:698] |
| ENSG00000188133 | HGLibA_50219 | 168 | 0.384924118 | TMEM215 | transmembrane protein 215 [Source:HGNC Symbol;Acc:HGNC:33816] |
| ENSG00000196262 | HGLibA_37893 | 168 | 0.384924118 | PPIA | peptidylprolyl isomerase A [Source:HGNC Symbol;Acc:HGNC:9253] |
| ENSG00000198793 | HGLibA_30455 | 168 | 0.384924118 | MTOR | mechanistic target of rapamycin kinase [Source:HGNC Symbol;Acc:HGNC:3942] |

| | | | | | |
|---|---|---|---|---|---|
| ENSG00000204006 | HGLibA_05804 | 168 | 0.384924118 | C1orf185 | chromosome 1 open reading frame 185 [Source:HGNC Symbol;Acc:HGNC:28096] |
| ENSG00000204099 | HGLibA_31718 | 168 | 0.384924118 | NEU4 | neuraminidase 4 [Source:HGNC Symbol;Acc:HGNC:21328] |
| ENSG00000204449 | HGLibA_51528 | 168 | 0.384924118 | TRIM49C | tripartite motif containing 49C [Source:HGNC Symbol;Acc:HGNC:38877] |
| ENSG00000204849 | HGLibA_46468 | 168 | 0.384924118 | SPATA31A1 | SPATA31 subfamily A member 1 [Source:HGNC Symbol;Acc:HGNC:23394] |
| ENSG00000221963 | HGLibA_02542 | 168 | 0.384924118 | APOL6 | apolipoprotein L6 [Source:HGNC Symbol;Acc:HGNC:14870] |
| ENSG00000250510 | HGLibA_19961 | 168 | 0.384924118 | GPR162 | G protein-coupled receptor 162 [Source:HGNC Symbol;Acc:HGNC:16693] |
| ENSG00000254470 | HGLibA_02352 | 168 | 0.384924118 | AP5B1 | adaptor related protein complex 5 subunit beta 1 [Source:HGNC Symbol;Acc:HGNC:25104] |
| ENSG00000255307 | HGLibA_34146 | 168 | 0.384924118 | OR52B2 | olfactory receptor family 52 subfamily B member 2 [Source:HGNC Symbol;Acc:HGNC:15207] |
| ENSG00000255359 | HGLibA_07719 | 168 | 0.384924118 | CCDC179 | coiled-coil domain containing 179 [Source:HGNC Symbol;Acc:HGNC:44653] |
| ENSG00000258644 | HGLibA_48011 | 168 | 0.384924118 | SYNJ2BP-COX16 | SYNJ2BP-COX16 readthrough [Source:HGNC Symbol;Acc:HGNC:48350] |
| ENSG00000288642 | HGLibA_08862 | 168 | 0.384924118 | CDR1 | - |
| ENSG00000288784 | HGLibA_11574 | 168 | 0.384924118 | CTAGE4 | - |
| GATS | HGLibA_18697 | 168 | 0.384924118 | GATS | - |
| GPR75-ASB3 | HGLibA_20083 | 168 | 0.384924118 | GPR75-ASB3 | - |
| hsa-mir-217 | HGLibA_58264 | 168 | 0.384924118 | hsa-mir-217 | - |
| hsa-mir-448 | HGLibA_60335 | 168 | 0.384924118 | hsa-mir-448 | - |
| hsa-mir-4701 | HGLibA_60912 | 168 | 0.384924118 | hsa-mir-4701 | - |
| hsa-mir-526b | HGLibA_61798 | 168 | 0.384924118 | hsa-mir-526b | - |
| hsa-mir-6508 | HGLibA_62880 | 168 | 0.384924118 | hsa-mir-6508 | - |
| hsa-mir-6729 | HGLibA_63070 | 168 | 0.384924118 | hsa-mir-6729 | - |
| hsa-mir-6790 | HGLibA_63337 | 168 | 0.384924118 | hsa-mir-6790 | - |
| hsa-mir-8079 | HGLibA_64159 | 168 | 0.384924118 | hsa-mir-8079 | - |
| LOC729059 | HGLibA_26998 | 168 | 0.384924118 | LOC729059 | - |
| MLL2 | HGLibA_29395 | 168 | 0.384924118 | MLL2 | - |
| NADKD1 | HGLibA_31051 | 168 | 0.384924118 | NADKD1 | - |
| PRAMEF22 | HGLibA_38315 | 168 | 0.384924118 | PRAMEF22 | - |
| AGPAT6 | HGLibA_01231 | 167 | 0.375878775 | AGPAT6 | - |
| C15orf43 | HGLibA_05402 | 167 | 0.375878775 | C15orf43 | - |
| C5orf28 | HGLibA_06371 | 167 | 0.375878775 | C5orf28 | - |
| ENSG00000025156 | HGLibA_22223 | 167 | 0.375878775 | HSF2 | heat shock transcription factor 2 [Source:HGNC Symbol;Acc:HGNC:5225] |
| ENSG00000034152 | HGLibA_28146 | 167 | 0.375878775 | MAP2K3 | mitogen-activated protein kinase kinase 3 [Source:HGNC Symbol;Acc:HGNC:6843] |
| ENSG00000066322 | HGLibA_14956 | 167 | 0.375878775 | ELOVL1 | ELOVL fatty acid elongase 1 [Source:HGNC Symbol;Acc:HGNC:14418] |
| ENSG00000077454 | HGLibA_27146 | 167 | 0.375878775 | LRCH4 | leucine rich repeats and calponin homology domain containing 4 [Source:HGNC Symbol;Acc:HGNC:6691] |
| ENSG00000080839 | HGLibA_40468 | 167 | 0.375878775 | RBL1 | RB transcriptional corepressor like 1 [Source:HGNC Symbol;Acc:HGNC:9893] |
| ENSG00000089127 | HGLibA_33199 | 167 | 0.375878775 | OAS1 | 2'-5'-oligoadenylate synthetase 1 [Source:HGNC Symbol;Acc:HGNC:8086] |
| ENSG00000089472 | HGLibA_21143 | 167 | 0.375878775 | HEPH | hephaestin [Source:HGNC Symbol;Acc:HGNC:4866] |
| ENSG00000090060 | HGLibA_35136 | 167 | 0.375878775 | PAPOLA | poly(A) polymerase alpha [Source:HGNC Symbol;Acc:HGNC:14981] |
| ENSG00000091732 | HGLibA_54998 | 167 | 0.375878775 | ZC3HC1 | zinc finger C3HC-type containing 1 [Source:HGNC Symbol;Acc:HGNC:29913] |
| ENSG00000096006 | HGLibA_11171 | 167 | 0.375878775 | CRISP3 | cysteine rich secretory protein 3 [Source:HGNC Symbol;Acc:HGNC:16904] |
| ENSG00000100033 | HGLibA_38659 | 167 | 0.375878775 | PRODH | proline dehydrogenase 1 [Source:HGNC Symbol;Acc:HGNC:9453] |
| ENSG00000100106 | HGLibA_51629 | 167 | 0.375878775 | TRIOBP | TRIO and F-actin binding protein [Source:HGNC Symbol;Acc:HGNC:17009] |
| ENSG00000103064 | HGLibA_45372 | 167 | 0.375878775 | SLC7A6 | solute carrier family 7 member 6 [Source:HGNC Symbol;Acc:HGNC:11064] |
| ENSG00000103067 | HGLibA_15590 | 167 | 0.375878775 | ESRP2 | epithelial splicing regulatory protein 2 [Source:HGNC Symbol;Acc:HGNC:26152] |
| ENSG00000104267 | HGLibA_06803 | 167 | 0.375878775 | CA2 | carbonic anhydrase 2 [Source:HGNC Symbol;Acc:HGNC:1373] |
| ENSG00000104892 | HGLibA_25033 | 167 | 0.375878775 | KLC3 | kinesin light chain 3 [Source:HGNC Symbol;Acc:HGNC:20717] |
| ENSG00000105229 | HGLibA_36516 | 167 | 0.375878775 | PIAS4 | protein inhibitor of activated STAT 4 [Source:HGNC Symbol;Acc:HGNC:17002] |
| ENSG00000106605 | HGLibA_04576 | 167 | 0.375878775 | BLVRA | biliverdin reductase A [Source:HGNC Symbol;Acc:HGNC:1062] |
| ENSG00000108819 | HGLibA_38130 | 167 | 0.375878775 | PPP1R9B | protein phosphatase 1 regulatory subunit 9B [Source:HGNC Symbol;Acc:HGNC:9298] |
| ENSG00000112077 | HGLibA_41120 | 167 | 0.375878775 | RHAG | Rh associated glycoprotein [Source:HGNC Symbol;Acc:HGNC:10006] |
| ENSG00000114023 | HGLibA_16222 | 167 | 0.375878775 | FAM162A | family with sequence similarity 162 member A [Source:HGNC Symbol;Acc:HGNC:17865] |
| ENSG00000116031 | HGLibA_08290 | 167 | 0.375878775 | CD207 | CD207 molecule [Source:HGNC Symbol;Acc:HGNC:17935] |
| ENSG00000117318 | HGLibA_22529 | 167 | 0.375878775 | ID3 | HLH protein [Source:HGNC Symbol;Acc:HGNC:5362]" |
| ENSG00000119715 | HGLibA_15597 | 167 | 0.375878775 | ESRRB | estrogen related receptor beta [Source:HGNC Symbol;Acc:HGNC:3473] |
| ENSG00000120725 | HGLibA_44172 | 167 | 0.375878775 | SIL1 | SIL1 nucleotide exchange factor [Source:HGNC Symbol;Acc:HGNC:24624] |
| ENSG00000122203 | HGLibA_24707 | 167 | 0.375878775 | KIAA1191 | KIAA1191 [Source:HGNC Symbol;Acc:HGNC:29209] |
| ENSG00000125967 | HGLibA_31591 | 167 | 0.375878775 | NECAB3 | N-terminal EF-hand calcium binding protein 3 [Source:HGNC Symbol;Acc:HGNC:15851] |
| ENSG00000130755 | HGLibA_19406 | 167 | 0.375878775 | GMFG | glia maturation factor gamma [Source:HGNC Symbol;Acc:HGNC:4374] |
| ENSG00000131378 | HGLibA_40949 | 167 | 0.375878775 | RFTN1 | lipid raft linker 1 [Source:HGNC Symbol;Acc:HGNC:30278]" |
| ENSG00000133265 | HGLibA_22328 | 167 | 0.375878775 | HSPBP1 | HSPA (Hsp70) binding protein 1 [Source:HGNC Symbol;Acc:HGNC:24989] |
| ENSG00000134109 | HGLibA_14453 | 167 | 0.375878775 | EDEM1 | ER degradation enhancing alpha-mannosidase like protein 1 [Source:HGNC Symbol;Acc:HGNC:18967] |
| ENSG00000136874 | HGLibA_47627 | 167 | 0.375878775 | STX17 | syntaxin 17 [Source:HGNC Symbol;Acc:HGNC:11432] |
| ENSG00000137145 | HGLibA_12956 | 167 | 0.375878775 | DENND4C | DENN domain containing 4C [Source:HGNC Symbol;Acc:HGNC:26079] |
| ENSG00000137166 | HGLibA_17938 | 167 | 0.375878775 | FOXP4 | forkhead box P4 [Source:HGNC Symbol;Acc:HGNC:20842] |
| ENSG00000137801 | HGLibA_49322 | 167 | 0.375878775 | THBS1 | thrombospondin 1 [Source:HGNC Symbol;Acc:HGNC:11785] |
| ENSG00000137872 | HGLibA_43374 | 167 | 0.375878775 | SEMA6D | semaphorin 6D [Source:HGNC Symbol;Acc:HGNC:16770] |
| ENSG00000138175 | HGLibA_02962 | 167 | 0.375878775 | ARL3 | ADP ribosylation factor like GTPase 3 [Source:HGNC Symbol;Acc:HGNC:694] |
| ENSG00000138376 | HGLibA_04154 | 167 | 0.375878775 | BARD1 | BRCA1 associated RING domain 1 [Source:HGNC Symbol;Acc:HGNC:952] |
| ENSG00000138771 | HGLibA_44093 | 167 | 0.375878775 | SHROOM3 | shroom family member 3 [Source:HGNC Symbol;Acc:HGNC:30422] |
| ENSG00000138814 | HGLibA_38184 | 167 | 0.375878775 | PPP3CA | protein phosphatase 3 catalytic subunit alpha [Source:HGNC Symbol;Acc:HGNC:9314] |
| ENSG00000139194 | HGLibA_40640 | 167 | 0.375878775 | RBP5 | retinol binding protein 5 [Source:HGNC Symbol;Acc:HGNC:15847] |
| ENSG00000139874 | HGLibA_47174 | 167 | 0.375878775 | SSTR1 | somatostatin receptor 1 [Source:HGNC Symbol;Acc:HGNC:11330] |
| ENSG00000142235 | HGLibA_26729 | 167 | 0.375878775 | LMTK3 | lemur tyrosine kinase 3 [Source:HGNC Symbol;Acc:HGNC:19295] |
| ENSG00000142546 | HGLibA_32334 | 167 | 0.375878775 | NOSIP | nitric oxide synthase interacting protein [Source:HGNC Symbol;Acc:HGNC:17946] |
| ENSG00000142623 | HGLibA_34968 | 167 | 0.375878775 | PADI1 | peptidyl arginine deiminase 1 [Source:HGNC Symbol;Acc:HGNC:18367] |
| ENSG00000146350 | HGLibA_48540 | 167 | 0.375878775 | TBC1D32 | TBC1 domain family member 32 [Source:HGNC Symbol;Acc:HGNC:21485] |
| ENSG00000146411 | HGLibA_44860 | 167 | 0.375878775 | SLC2A12 | solute carrier family 2 member 12 [Source:HGNC Symbol;Acc:HGNC:18067] |
| ENSG00000147168 | HGLibA_23125 | 167 | 0.375878775 | IL2RG | interleukin 2 receptor subunit gamma [Source:HGNC Symbol;Acc:HGNC:6010] |
| ENSG00000151617 | HGLibA_14478 | 167 | 0.375878775 | EDNRA | endothelin receptor type A [Source:HGNC Symbol;Acc:HGNC:3179] |
| ENSG00000151882 | HGLibA_08014 | 167 | 0.375878775 | CCL28 | C-C motif chemokine ligand 28 [Source:HGNC Symbol;Acc:HGNC:17700] |
| ENSG00000155542 | HGLibA_43664 | 167 | 0.375878775 | SETD9 | SET domain containing 9 [Source:HGNC Symbol;Acc:HGNC:28508] |
| ENSG00000157827 | HGLibA_17697 | 167 | 0.375878775 | FMNL2 | formin like 2 [Source:HGNC Symbol;Acc:HGNC:18267] |
| ENSG00000158987 | HGLibA_40261 | 167 | 0.375878775 | RAPGEF6 | Rap guanine nucleotide exchange factor 6 [Source:HGNC Symbol;Acc:HGNC:20655] |
| ENSG00000160339 | HGLibA_17219 | 167 | 0.375878775 | FCN2 | ficolin 2 [Source:HGNC Symbol;Acc:HGNC:3624] |
| ENSG00000165120 | HGLibA_47140 | 167 | 0.375878775 | SSMEM1 | serine rich single-pass membrane protein 1 [Source:HGNC Symbol;Acc:HGNC:29580] |
| ENSG00000166439 | HGLibA_41569 | 167 | 0.375878775 | RNF169 | ring finger protein 169 [Source:HGNC Symbol;Acc:HGNC:26961] |
| ENSG00000167208 | HGLibA_46060 | 167 | 0.375878775 | SNX20 | sorting nexin 20 [Source:HGNC Symbol;Acc:HGNC:30390] |
| ENSG00000168743 | HGLibA_32496 | 167 | 0.375878775 | NPNT | nephronectin [Source:HGNC Symbol;Acc:HGNC:27405] |
| ENSG00000168884 | HGLibA_50837 | 167 | 0.375878775 | TNIP2 | TNFAIP3 interacting protein 2 [Source:HGNC Symbol;Acc:HGNC:19118] |
| ENSG00000170325 | HGLibA_38364 | 167 | 0.375878775 | PRDM10 | PR/SET domain 10 [Source:HGNC Symbol;Acc:HGNC:13995] |
| ENSG00000170624 | HGLibA_43802 | 167 | 0.375878775 | SGCD | sarcoglycan delta [Source:HGNC Symbol;Acc:HGNC:10807] |
| ENSG00000171055 | HGLibA_17314 | 167 | 0.375878775 | FEZ2 | fasciculation and elongation protein zeta 2 [Source:HGNC Symbol;Acc:HGNC:3660] |
| ENSG00000171431 | HGLibA_25446 | 167 | 0.375878775 | KRT20 | keratin 20 [Source:HGNC Symbol;Acc:HGNC:20412] |
| ENSG00000172000 | HGLibA_56227 | 167 | 0.375878775 | ZNF556 | zinc finger protein 556 [Source:HGNC Symbol;Acc:HGNC:25669] |
| ENSG00000172009 | HGLibA_49381 | 167 | 0.375878775 | THOP1 | thimet oligopeptidase 1 [Source:HGNC Symbol;Acc:HGNC:11793] |
| ENSG00000172113 | HGLibA_32170 | 167 | 0.375878775 | NME6 | NME/NM23 nucleoside diphosphate kinase 6 [Source:HGNC Symbol;Acc:HGNC:20567] |
| ENSG00000172350 | HGLibA_00220 | 167 | 0.375878775 | ABCG4 | ATP binding cassette subfamily G member 4 [Source:HGNC Symbol;Acc:HGNC:13884] |
| ENSG00000172458 | HGLibA_22998 | 167 | 0.375878775 | IL17D | interleukin 17D [Source:HGNC Symbol;Acc:HGNC:5984] |
| ENSG00000173638 | HGLibA_44497 | 167 | 0.375878775 | SLC19A1 | solute carrier family 19 member 1 [Source:HGNC Symbol;Acc:HGNC:10937] |
| ENSG00000174600 | HGLibA_10197 | 167 | 0.375878775 | CMKLR1 | chemerin chemokine-like receptor 1 [Source:HGNC Symbol;Acc:HGNC:2121] |
| ENSG00000174628 | HGLibA_23530 | 167 | 0.375878775 | IQCK | IQ motif containing K [Source:HGNC Symbol;Acc:HGNC:28556] |
| ENSG00000175065 | HGLibA_14031 | 167 | 0.375878775 | DSG4 | desmoglein 4 [Source:HGNC Symbol;Acc:HGNC:21307] |
| ENSG00000175182 | HGLibA_16104 | 167 | 0.375878775 | FAM131A | family with sequence similarity 131 member A [Source:HGNC Symbol;Acc:HGNC:28308] |
| ENSG00000177504 | HGLibA_53583 | 167 | 0.375878775 | VCX2 | variable charge X-linked 2 [Source:HGNC Symbol;Acc:HGNC:18158] |
| ENSG00000180929 | HGLibA_20066 | 167 | 0.375878775 | GPR62 | G protein-coupled receptor 62 [Source:HGNC Symbol;Acc:HGNC:13301] |
| ENSG00000181291 | HGLibA_49941 | 167 | 0.375878775 | TMEM132E | transmembrane protein 132E [Source:HGNC Symbol;Acc:HGNC:26991] |
| ENSG00000182973 | HGLibA_10330 | 167 | 0.375878775 | CNOT10 | CCR4-NOT transcription complex subunit 10 [Source:HGNC Symbol;Acc:HGNC:23817] |
| ENSG00000183914 | HGLibA_13484 | 167 | 0.375878775 | DNAH2 | dynein axonemal heavy chain 2 [Source:HGNC Symbol;Acc:HGNC:2948] |
| ENSG00000184486 | HGLibA_37769 | 167 | 0.375878775 | POU3F2 | POU class 3 homeobox 2 [Source:HGNC Symbol;Acc:HGNC:9215] |
| ENSG00000185085 | HGLibA_23425 | 167 | 0.375878775 | INTS5 | integrator complex subunit 5 [Source:HGNC Symbol;Acc:HGNC:29352] |
| ENSG00000186115 | HGLibA_12188 | 167 | 0.375878775 | CYP4F2 | cytochrome P450 family 4 subfamily F member 2 [Source:HGNC Symbol;Acc:HGNC:2645] |
| ENSG00000189127 | HGLibA_02079 | 167 | 0.375878775 | ANKRD34B | ankyrin repeat domain 34B [Source:HGNC Symbol;Acc:HGNC:33736] |
| ENSG00000189308 | HGLibA_26571 | 167 | 0.375878775 | LIN54 | lin-54 DREAM MuvB core complex component [Source:HGNC Symbol;Acc:HGNC:25397] |
| ENSG00000196422 | HGLibA_38079 | 167 | 0.375878775 | PPP1R26 | protein phosphatase 1 regulatory subunit 26 [Source:HGNC Symbol;Acc:HGNC:29089] |
| ENSG00000196422 | HGLibA_38080 | 167 | 0.375878775 | PPP1R26 | protein phosphatase 1 regulatory subunit 26 [Source:HGNC Symbol;Acc:HGNC:29089] |
| ENSG00000198625 | HGLibA_28701 | 167 | 0.375878775 | MDM4 | p53 regulator [Source:HGNC Symbol;Acc:HGNC:6974]" |
| ENSG00000203705 | HGLibA_48446 | 167 | 0.375878775 | TATDN3 | TatD DNase domain containing 3 [Source:HGNC Symbol;Acc:HGNC:27010] |
| ENSG00000204381 | HGLibA_26072 | 167 | 0.375878775 | LAYN | layilin [Source:HGNC Symbol;Acc:HGNC:29471] |
| ENSG00000213316 | HGLibA_27606 | 167 | 0.375878775 | LTC4S | leukotriene C4 synthase [Source:HGNC Symbol;Acc:HGNC:6719] |
| ENSG00000214595 | HGLibA_15070 | 167 | 0.375878775 | EML6 | echinoderm microtubule associated protein like 6 [Source:HGNC Symbol;Acc:HGNC:35412] |
| ENSG00000221859 | HGLibA_25593 | 167 | 0.375878775 | KRTAP10-10 | keratin associated protein 10-10 [Source:HGNC Symbol;Acc:HGNC:22972] |
| ENSG00000250709 | HGLibA_07685 | 167 | 0.375878775 | CCDC169-SOHLH2 | CCDC169-SOHLH2 readthrough [Source:HGNC Symbol;Acc:HGNC:38866] |
| ENSG00000260811 | HGLibA_33957 | 167 | 0.375878775 | OR4C45 | olfactory receptor family 4 subfamily C member 45 (gene/pseudogene) [Source:HGNC Symbol;Acc:HGNC:31270] |
| ENSG00000277494 | HGLibA_19810 | 167 | 0.375878775 | GPIHBP1 | glycosylphosphatidylinositol anchored high density lipoprotein binding protein 1 [Source:HGNC Symbol;Acc:HGNC:24945] |
| ENSG00000277556 | HGLibA_33605 | 167 | 0.375878775 | OR13C5 | olfactory receptor family 13 subfamily C member 5 [Source:HGNC Symbol;Acc:HGNC:15100] |
| ENSG00000288649 | HGLibA_00617 | 167 | 0.375878775 | ACTL10 | - |
| hsa-mir-1296 | HGLibA_57664 | 167 | 0.375878775 | hsa-mir-1296 | - |
| hsa-mir-3920 | HGLibA_59555 | 167 | 0.375878775 | hsa-mir-3920 | - |
| hsa-mir-3941 | HGLibA_59626 | 167 | 0.375878775 | hsa-mir-3941 | - |
| hsa-mir-3944 | HGLibA_59636 | 167 | 0.375878775 | hsa-mir-3944 | - |
| hsa-mir-4260 | HGLibA_59748 | 167 | 0.375878775 | hsa-mir-4260 | - |
| hsa-mir-4283-2 | HGLibA_59838 | 167 | 0.375878775 | hsa-mir-4283-2 | - |
| hsa-mir-4737 | HGLibA_61047 | 167 | 0.375878775 | hsa-mir-4737 | - |
| hsa-mir-637 | HGLibA_62787 | 167 | 0.375878775 | hsa-mir-637 | - |
| hsa-mir-647 | HGLibA_62832 | 167 | 0.375878775 | hsa-mir-647 | - |
| hsa-mir-6752 | HGLibA_63166 | 167 | 0.375878775 | hsa-mir-6752 | - |

| | | | | | |
|---|---|---|---|---|---|
| hsa-mir-8059 | HGLibA_64073 | 167 | 0.375878775 | hsa-mir-8059 | - |
| KIAA1211 | HGLibA_24716 | 167 | 0.375878775 | KIAA1211 | - |
| LEPREL2 | HGLibA_26322 | 167 | 0.375878775 | LEPREL2 | - |
| LOC100287177 | HGLibA_26818 | 167 | 0.375878775 | LOC100287177 | - |
| SMIM11 | HGLibA_45721 | 167 | 0.375878775 | SMIM11 | - |
| TARSL2 | HGLibA_48352 | 167 | 0.375878775 | TARSL2 | - |
| C21orf59 | HGLibA_06045 | 166 | 0.366833432 | C21orf59 | - |
| ENSG00000006530 | HGLibA_01193 | 166 | 0.366833432 | AGK | acylglycerol kinase [Source:HGNC Symbol;Acc:HGNC:21869] |
| ENSG00000007062 | HGLibA_38679 | 166 | 0.366833432 | PROM1 | prominin 1 [Source:HGNC Symbol;Acc:HGNC:9454] |
| ENSG00000059145 | HGLibA_53051 | 166 | 0.366833432 | UNKL | unkempt family like zinc finger [Source:HGNC Symbol;Acc:HGNC:14184] |
| ENSG00000064012 | HGLibA_07326 | 166 | 0.366833432 | CASP8 | caspase 8 [Source:HGNC Symbol;Acc:HGNC:1509] |
| ENSG00000064115 | HGLibA_49718 | 166 | 0.366833432 | TM7SF3 | transmembrane 7 superfamily member 3 [Source:HGNC Symbol;Acc:HGNC:23049] |
| ENSG00000088888 | HGLibA_28445 | 166 | 0.366833432 | MAVS | mitochondrial antiviral signaling protein [Source:HGNC Symbol;Acc:HGNC:29233] |
| ENSG00000095303 | HGLibA_39357 | 166 | 0.366833432 | PTGS1 | prostaglandin-endoperoxide synthase 1 [Source:HGNC Symbol;Acc:HGNC:9604] |
| ENSG00000099954 | HGLibA_08977 | 166 | 0.366833432 | CECR2 | histone acetyl-lysine reader [Source:HGNC Symbol;Acc:HGNC:1840]" |
| ENSG00000100721 | HGLibA_48815 | 166 | 0.366833432 | TCL1A | T cell leukemia/lymphoma 1A [Source:HGNC Symbol;Acc:HGNC:11648] |
| ENSG00000102468 | HGLibA_22370 | 166 | 0.366833432 | HTR2A | 5-hydroxytryptamine receptor 2A [Source:HGNC Symbol;Acc:HGNC:5293] |
| ENSG00000103145 | HGLibA_20961 | 166 | 0.366833432 | HCFC1R1 | host cell factor C1 regulator 1 [Source:HGNC Symbol;Acc:HGNC:21198] |
| ENSG00000104808 | HGLibA_13101 | 166 | 0.366833432 | DHDH | dihydrodiol dehydrogenase [Source:HGNC Symbol;Acc:HGNC:17887] |
| ENSG00000108576 | HGLibA_45327 | 166 | 0.366833432 | SLC6A4 | solute carrier family 6 member 4 [Source:HGNC Symbol;Acc:HGNC:11050] |
| ENSG00000108771 | HGLibA_13204 | 166 | 0.366833432 | DHX58 | DExH-box helicase 58 [Source:HGNC Symbol;Acc:HGNC:29517] |
| ENSG00000109084 | HGLibA_50566 | 166 | 0.366833432 | TMEM97 | transmembrane protein 97 [Source:HGNC Symbol;Acc:HGNC:28106] |
| ENSG00000110013 | HGLibA_44101 | 166 | 0.366833432 | SIAE | sialic acid acetylesterase [Source:HGNC Symbol;Acc:HGNC:18187] |
| ENSG00000111907 | HGLibA_51113 | 166 | 0.366833432 | TPD52L1 | TPD52 like 1 [Source:HGNC Symbol;Acc:HGNC:12006] |
| ENSG00000112208 | HGLibA_04078 | 166 | 0.366833432 | BAG2 | BCL2 associated athanogene 2 [Source:HGNC Symbol;Acc:HGNC:938] |
| ENSG00000112499 | HGLibA_44567 | 166 | 0.366833432 | SLC22A2 | solute carrier family 22 member 2 [Source:HGNC Symbol;Acc:HGNC:10966] |
| ENSG00000114757 | HGLibA_36120 | 166 | 0.366833432 | PEX5L | peroxisomal biogenesis factor 5 like [Source:HGNC Symbol;Acc:HGNC:30024] |
| ENSG00000115241 | HGLibA_37979 | 166 | 0.366833432 | PPM1G | Mg2+/Mn2+ dependent 1G [Source:HGNC Symbol;Acc:HGNC:9278]" |
| ENSG00000115419 | HGLibA_19326 | 166 | 0.366833432 | GLS | glutaminase [Source:HGNC Symbol;Acc:HGNC:4331] |
| ENSG00000115446 | HGLibA_53011 | 166 | 0.366833432 | UNC50 | unc-50 inner nuclear membrane RNA binding protein [Source:HGNC Symbol;Acc:HGNC:16046] |
| ENSG00000115844 | HGLibA_13374 | 166 | 0.366833432 | DLX2 | distal-less homeobox 2 [Source:HGNC Symbol;Acc:HGNC:2915] |
| ENSG00000115884 | HGLibA_43098 | 166 | 0.366833432 | SDC1 | syndecan 1 [Source:HGNC Symbol;Acc:HGNC:10658] |
| ENSG00000117643 | HGLibA_28068 | 166 | 0.366833432 | MAN1C1 | mannosidase alpha class 1C member 1 [Source:HGNC Symbol;Acc:HGNC:19080] |
| ENSG00000119632 | HGLibA_22601 | 166 | 0.366833432 | IFI27L2 | interferon alpha inducible protein 27 like 2 [Source:HGNC Symbol;Acc:HGNC:19753] |
| ENSG00000120440 | HGLibA_52198 | 166 | 0.366833432 | TTLL2 | tubulin tyrosine ligase like 2 [Source:HGNC Symbol;Acc:HGNC:21211] |
| ENSG00000123908 | HGLibA_01209 | 166 | 0.366833432 | AGO2 | RISC catalytic component [Source:HGNC Symbol;Acc:HGNC:3263]" |
| ENSG00000125798 | HGLibA_17809 | 166 | 0.366833432 | FOXA2 | forkhead box A2 [Source:HGNC Symbol;Acc:HGNC:5022] |
| ENSG00000128487 | HGLibA_46603 | 166 | 0.366833432 | SPECC1 | sperm antigen with calponin homology and coiled-coil domains 1 [Source:HGNC Symbol;Acc:HGNC:30615] |
| ENSG00000131233 | HGLibA_19154 | 166 | 0.366833432 | GJA9 | gap junction protein alpha 9 [Source:HGNC Symbol;Acc:HGNC:19155] |
| ENSG00000132405 | HGLibA_48478 | 166 | 0.366833432 | TBC1D14 | TBC1 domain family member 14 [Source:HGNC Symbol;Acc:HGNC:29246] |
| ENSG00000132912 | HGLibA_12554 | 166 | 0.366833432 | DCTN4 | dynactin subunit 4 [Source:HGNC Symbol;Acc:HGNC:15518] |
| ENSG00000133317 | HGLibA_26347 | 166 | 0.366833432 | LGALS12 | galectin 12 [Source:HGNC Symbol;Acc:HGNC:15788] |
| ENSG00000137225 | HGLibA_07149 | 166 | 0.366833432 | CAPN11 | calpain 11 [Source:HGNC Symbol;Acc:HGNC:1478] |
| ENSG00000137801 | HGLibA_49321 | 166 | 0.366833432 | THBS1 | thrombospondin 1 [Source:HGNC Symbol;Acc:HGNC:11785] |
| ENSG00000138185 | HGLibA_15196 | 166 | 0.366833432 | ENTPD1 | ectonucleoside triphosphate diphosphohydrolase 1 [Source:HGNC Symbol;Acc:HGNC:3363] |
| ENSG00000138760 | HGLibA_42871 | 166 | 0.366833432 | SCARB2 | scavenger receptor class B member 2 [Source:HGNC Symbol;Acc:HGNC:1665] |
| ENSG00000139053 | HGLibA_35819 | 166 | 0.366833432 | PDE6H | phosphodiesterase 6H [Source:HGNC Symbol;Acc:HGNC:8790] |
| ENSG00000139323 | HGLibA_37443 | 166 | 0.366833432 | POC1B | POC1 centriolar protein B [Source:HGNC Symbol;Acc:HGNC:30836] |
| ENSG00000140400 | HGLibA_28081 | 166 | 0.366833432 | MAN2C1 | mannosidase alpha class 2C member 1 [Source:HGNC Symbol;Acc:HGNC:6827] |
| ENSG00000140743 | HGLibA_08867 | 166 | 0.366833432 | CDR2 | cerebellar degeneration related protein 2 [Source:HGNC Symbol;Acc:HGNC:1799] |
| ENSG00000141200 | HGLibA_24927 | 166 | 0.366833432 | KIF2B | kinesin family member 2B [Source:HGNC Symbol;Acc:HGNC:29443] |
| ENSG00000141552 | HGLibA_01869 | 166 | 0.366833432 | ANAPC11 | anaphase promoting complex subunit 11 [Source:HGNC Symbol;Acc:HGNC:14452] |
| ENSG00000144035 | HGLibA_31181 | 166 | 0.366833432 | NAT8 | N-acetyltransferase 8 (putative) [Source:HGNC Symbol;Acc:HGNC:18069] |
| ENSG00000145864 | HGLibA_18378 | 166 | 0.366833432 | GABRB2 | gamma-aminobutyric acid type A receptor beta2 subunit [Source:HGNC Symbol;Acc:HGNC:4082] |
| ENSG00000148604 | HGLibA_41047 | 166 | 0.366833432 | RGR | retinal G protein coupled receptor [Source:HGNC Symbol;Acc:HGNC:9990] |
| ENSG00000149043 | HGLibA_48090 | 166 | 0.366833432 | SYT8 | synaptotagmin 8 [Source:HGNC Symbol;Acc:HGNC:19264] |
| ENSG00000149305 | HGLibA_22384 | 166 | 0.366833432 | HTR3B | 5-hydroxytryptamine receptor 3B [Source:HGNC Symbol;Acc:HGNC:5298] |
| ENSG00000151612 | HGLibA_56825 | 166 | 0.366833432 | ZNF827 | zinc finger protein 827 [Source:HGNC Symbol;Acc:HGNC:27193] |
| ENSG00000151789 | HGLibA_55904 | 166 | 0.366833432 | ZNF385D | zinc finger protein 385D [Source:HGNC Symbol;Acc:HGNC:26191] |
| ENSG00000153347 | HGLibA_16736 | 166 | 0.366833432 | FAM81B | family with sequence similarity 81 member B [Source:HGNC Symbol;Acc:HGNC:26335] |
| ENSG00000154380 | HGLibA_15104 | 166 | 0.366833432 | ENAH | actin regulator [Source:HGNC Symbol;Acc:HGNC:18271]" |
| ENSG00000154856 | HGLibA_02395 | 166 | 0.366833432 | APCDD1 | APC down-regulated 1 [Source:HGNC Symbol;Acc:HGNC:15718] |
| ENSG00000156049 | HGLibA_19445 | 166 | 0.366833432 | GNA14 | G protein subunit alpha 14 [Source:HGNC Symbol;Acc:HGNC:4382] |
| ENSG00000157005 | HGLibA_47172 | 166 | 0.366833432 | SST | somatostatin [Source:HGNC Symbol;Acc:HGNC:11329] |
| ENSG00000158467 | HGLibA_01293 | 166 | 0.366833432 | AHCYL2 | adenosylhomocysteinase like 2 [Source:HGNC Symbol;Acc:HGNC:22204] |
| ENSG00000158955 | HGLibA_54396 | 166 | 0.366833432 | WNT9B | Wnt family member 9B [Source:HGNC Symbol;Acc:HGNC:12779] |
| ENSG00000159128 | HGLibA_22713 | 166 | 0.366833432 | IFNGR2 | interferon gamma receptor 2 [Source:HGNC Symbol;Acc:HGNC:5440] |
| ENSG00000159182 | HGLibA_38263 | 166 | 0.366833432 | PRAC1 | PRAC1 small nuclear protein [Source:HGNC Symbol;Acc:HGNC:30591] |
| ENSG00000159873 | HGLibA_07559 | 166 | 0.366833432 | CCDC117 | coiled-coil domain containing 117 [Source:HGNC Symbol;Acc:HGNC:26599] |
| ENSG00000161807 | HGLibA_34481 | 166 | 0.366833432 | OR7G1 | olfactory receptor family 7 subfamily G member 1 [Source:HGNC Symbol;Acc:HGNC:8465] |
| ENSG00000162383 | HGLibA_44526 | 166 | 0.366833432 | SLC1A7 | solute carrier family 1 member 7 [Source:HGNC Symbol;Acc:HGNC:10945] |
| ENSG00000163719 | HGLibA_30421 | 166 | 0.366833432 | MTMR14 | myotubularin related protein 14 [Source:HGNC Symbol;Acc:HGNC:26190] |
| ENSG00000164946 | HGLibA_17984 | 166 | 0.366833432 | FREM1 | FRAS1 related extracellular matrix 1 [Source:HGNC Symbol;Acc:HGNC:23399] |
| ENSG00000166527 | HGLibA_09984 | 166 | 0.366833432 | CLEC4D | C-type lectin domain family 4 member D [Source:HGNC Symbol;Acc:HGNC:14554] |
| ENSG00000167548 | HGLibA_25329 | 166 | 0.366833432 | KMT2D | lysine methyltransferase 2D [Source:HGNC Symbol;Acc:HGNC:7133] |
| ENSG00000167555 | HGLibA_56171 | 166 | 0.366833432 | ZNF528 | zinc finger protein 528 [Source:HGNC Symbol;Acc:HGNC:29384] |
| ENSG00000167785 | HGLibA_56232 | 166 | 0.366833432 | ZNF558 | zinc finger protein 558 [Source:HGNC Symbol;Acc:HGNC:26422] |
| ENSG00000167815 | HGLibA_38409 | 166 | 0.366833432 | PRDX2 | peroxiredoxin 2 [Source:HGNC Symbol;Acc:HGNC:9353] |
| ENSG00000168175 | HGLibA_28268 | 166 | 0.366833432 | MAPK1IP1L | mitogen-activated protein kinase 1 interacting protein 1 like [Source:HGNC Symbol;Acc:HGNC:19840] |
| ENSG00000168273 | HGLibA_45758 | 166 | 0.366833432 | SMIM4 | small integral membrane protein 4 [Source:HGNC Symbol;Acc:HGNC:37257] |
| ENSG00000168993 | HGLibA_10931 | 166 | 0.366833432 | CPLX1 | complexin 1 [Source:HGNC Symbol;Acc:HGNC:2309] |
| ENSG00000169877 | HGLibA_01321 | 166 | 0.366833432 | AHSP | alpha hemoglobin stabilizing protein [Source:HGNC Symbol;Acc:HGNC:18075] |
| ENSG00000172530 | HGLibA_04148 | 166 | 0.366833432 | BANP | BTG3 associated nuclear protein [Source:HGNC Symbol;Acc:HGNC:13450] |
| ENSG00000172554 | HGLibA_46009 | 166 | 0.366833432 | SNTG2 | syntrophin gamma 2 [Source:HGNC Symbol;Acc:HGNC:13741] |
| ENSG00000173269 | HGLibA_29548 | 166 | 0.366833432 | MMRN2 | multimerin 2 [Source:HGNC Symbol;Acc:HGNC:19888] |
| ENSG00000174740 | HGLibA_34939 | 166 | 0.366833432 | PABPC5 | poly(A) binding protein cytoplasmic 5 [Source:HGNC Symbol;Acc:HGNC:13629] |
| ENSG00000174842 | HGLibA_19280 | 166 | 0.366833432 | GLMN | FKBP associated protein [Source:HGNC Symbol;Acc:HGNC:14373]" |
| ENSG00000175352 | HGLibA_32698 | 166 | 0.366833432 | NRIP3 | nuclear receptor interacting protein 3 [Source:HGNC Symbol;Acc:HGNC:1167] |
| ENSG00000176024 | HGLibA_56385 | 166 | 0.366833432 | ZNF613 | zinc finger protein 613 [Source:HGNC Symbol;Acc:HGNC:25827] |
| ENSG00000177675 | HGLibA_08246 | 166 | 0.366833432 | CD163L1 | CD163 molecule like 1 [Source:HGNC Symbol;Acc:HGNC:30375] |
| ENSG00000178971 | HGLibA_11592 | 166 | 0.366833432 | CTC1 | CST telomere replication complex component 1 [Source:HGNC Symbol;Acc:HGNC:26169] |
| ENSG00000180245 | HGLibA_42247 | 166 | 0.366833432 | RRH | retinal pigment epithelium-derived rhodopsin homolog [Source:HGNC Symbol;Acc:HGNC:10450] |
| ENSG00000181026 | HGLibA_01090 | 166 | 0.366833432 | AEN | apoptosis enhancing nuclease [Source:HGNC Symbol;Acc:HGNC:25722] |
| ENSG00000182247 | HGLibA_52598 | 166 | 0.366833432 | UBE2E2 | ubiquitin conjugating enzyme E2 E2 [Source:HGNC Symbol;Acc:HGNC:12478] |
| ENSG00000182463 | HGLibA_51865 | 166 | 0.366833432 | TSHZ2 | teashirt zinc finger homeobox 2 [Source:HGNC Symbol;Acc:HGNC:13010] |
| ENSG00000185104 | HGLibA_15947 | 166 | 0.366833432 | FAF1 | Fas associated factor 1 [Source:HGNC Symbol;Acc:HGNC:3578] |
| ENSG00000185372 | HGLibA_33897 | 166 | 0.366833432 | OR2V1 | olfactory receptor family 2 subfamily V member 1 [Source:HGNC Symbol;Acc:HGNC:8280] |
| ENSG00000186866 | HGLibA_37472 | 166 | 0.366833432 | POFUT2 | protein O-fucosyltransferase 2 [Source:HGNC Symbol;Acc:HGNC:14683] |
| ENSG00000198034 | HGLibA_42142 | 166 | 0.366833432 | RPS4X | ribosomal protein S4 X-linked [Source:HGNC Symbol;Acc:HGNC:10424] |
| ENSG00000198498 | HGLibA_49734 | 166 | 0.366833432 | TMA16 | translation machinery associated 16 homolog [Source:HGNC Symbol;Acc:HGNC:25638] |
| ENSG00000198624 | HGLibA_07843 | 166 | 0.366833432 | CCDC69 | coiled-coil domain containing 69 [Source:HGNC Symbol;Acc:HGNC:24487] |
| ENSG00000203772 | HGLibA_46799 | 166 | 0.366833432 | SPRN | shadow of prion protein [Source:HGNC Symbol;Acc:HGNC:16871] |
| ENSG00000204271 | HGLibA_46676 | 166 | 0.366833432 | SPIN3 | spindlin family member 3 [Source:HGNC Symbol;Acc:HGNC:27272] |
| ENSG00000205359 | HGLibA_45479 | 166 | 0.366833432 | SLCO6A1 | solute carrier organic anion transporter family member 6A1 [Source:HGNC Symbol;Acc:HGNC:23613] |
| ENSG00000206562 | HGLibA_29016 | 166 | 0.366833432 | METTL6 | methyltransferase like 6 [Source:HGNC Symbol;Acc:HGNC:28343] |
| ENSG00000214456 | HGLibA_37181 | 166 | 0.366833432 | PLIN5 | perilipin 5 [Source:HGNC Symbol;Acc:HGNC:33196] |
| ENSG00000215475 | HGLibA_44110 | 166 | 0.366833432 | SIAH3 | siah E3 ubiquitin protein ligase family member 3 [Source:HGNC Symbol;Acc:HGNC:30553] |
| ENSG00000237541 | HGLibA_21623 | 166 | 0.366833432 | HLA-DQA2 | DQ alpha 2 [Source:HGNC Symbol;Acc:HGNC:4943]" |
| ENSG00000242019 | HGLibA_24997 | 166 | 0.366833432 | KIR3DL3 | three Ig domains and long cytoplasmic tail 3 [Source:HGNC Symbol;Acc:HGNC:16312]" |
| ENSG00000250486 | HGLibA_16495 | 166 | 0.366833432 | FAM218A | family with sequence similarity 218 member A [Source:HGNC Symbol;Acc:HGNC:26466] |
| ENSG00000256436 | HGLibA_48390 | 166 | 0.366833432 | TAS2R31 | taste 2 receptor member 31 [Source:HGNC Symbol;Acc:HGNC:19113] |
| ENSG00000256713 | HGLibA_36191 | 166 | 0.366833432 | PGA5 | group I (pepsinogen A) [Source:HGNC Symbol;Acc:HGNC:8887]" |
| ENSG00000263639 | HGLibA_30224 | 166 | 0.366833432 | MSMB | microseminoprotein beta [Source:HGNC Symbol;Acc:HGNC:7372] |
| ENSG00000272636 | HGLibA_13729 | 166 | 0.366833432 | DOC2B | double C2 domain beta [Source:HGNC Symbol;Acc:HGNC:2986] |
| ENSG00000275074 | HGLibA_32961 | 166 | 0.366833432 | NUDT18 | nudix hydrolase 18 [Source:HGNC Symbol;Acc:HGNC:26194] |
| hsa-mir-1182 | HGLibA_57223 | 166 | 0.366833432 | hsa-mir-1182 | - |
| hsa-mir-3135a | HGLibA_58595 | 166 | 0.366833432 | hsa-mir-3135a | - |
| hsa-mir-320b-2 | HGLibA_58931 | 166 | 0.366833432 | hsa-mir-320b-2 | - |
| hsa-mir-378a | HGLibA_59426 | 166 | 0.366833432 | hsa-mir-378a | - |
| hsa-mir-4315-1 | HGLibA_59976 | 166 | 0.366833432 | hsa-mir-4315-1 | - |
| hsa-mir-4704 | HGLibA_60918 | 166 | 0.366833432 | hsa-mir-4704 | - |
| hsa-mir-5706 | HGLibA_62365 | 166 | 0.366833432 | hsa-mir-5706 | - |
| hsa-mir-589 | HGLibA_62453 | 166 | 0.366833432 | hsa-mir-589 | - |
| hsa-mir-593 | HGLibA_62470 | 166 | 0.366833432 | hsa-mir-593 | - |
| hsa-mir-621 | HGLibA_62725 | 166 | 0.366833432 | hsa-mir-621 | - |
| hsa-mir-6772 | HGLibA_63261 | 166 | 0.366833432 | hsa-mir-6772 | - |
| hsa-mir-8084 | HGLibA_64178 | 166 | 0.366833432 | hsa-mir-8084 | - |
| NEURL | HGLibA_31723 | 166 | 0.366833432 | NEURL | - |
| P2RY8_Y | HGLibA_34897 | 166 | 0.366833432 | P2RY8_Y | - |
| SRCRB4D | HGLibA_46937 | 166 | 0.366833432 | SRCRB4D | - |
| WDR67 | HGLibA_54158 | 166 | 0.366833432 | WDR67 | - |
| ATP5SL | HGLibA_03691 | 165 | 0.357788089 | ATP5SL | - |
| CCDC114 | HGLibA_07549 | 165 | 0.357788089 | CCDC114 | - |
| DEFB131 | HGLibA_12896 | 165 | 0.357788089 | DEFB131 | - |
| ENSG00000009950 | HGLibA_29443 | 165 | 0.357788089 | MLXIPL | MLX interacting protein like [Source:HGNC Symbol;Acc:HGNC:12744] |

| | | | | | |
|---|---|---|---|---|---|
| ENSG00000036448 | HGLibA_30893 | 165 | 0.357788089 | MYOM2 | myomesin 2 [Source:HGNC Symbol;Acc:HGNC:7614] |
| ENSG00000055163 | HGLibA_12035 | 165 | 0.357788089 | CYFIP2 | cytoplasmic FMR1 interacting protein 2 [Source:HGNC Symbol;Acc:HGNC:13760] |
| ENSG00000073008 | HGLibA_39617 | 165 | 0.357788089 | PVR | poliovirus receptor [Source:HGNC Symbol;Acc:HGNC:9705] |
| ENSG00000078898 | HGLibA_04721 | 165 | 0.357788089 | BPIFB2 | BPI fold containing family B member 2 [Source:HGNC Symbol;Acc:HGNC:16177] |
| ENSG00000081386 | HGLibA_56126 | 165 | 0.357788089 | ZNF510 | zinc finger protein 510 [Source:HGNC Symbol;Acc:HGNC:29161] |
| ENSG00000083312 | HGLibA_50899 | 165 | 0.357788089 | TNPO1 | transportin 1 [Source:HGNC Symbol;Acc:HGNC:6401] |
| ENSG00000091656 | HGLibA_55177 | 165 | 0.357788089 | ZFHX4 | zinc finger homeobox 4 [Source:HGNC Symbol;Acc:HGNC:30939] |
| ENSG00000099203 | HGLibA_49798 | 165 | 0.357788089 | TMED1 | transmembrane p24 trafficking protein 1 [Source:HGNC Symbol;Acc:HGNC:17291] |
| ENSG00000099956 | HGLibA_45621 | 165 | 0.357788089 | SMARCB1 | member 1 [Source:HGNC Symbol;Acc:HGNC:11103]" |
| ENSG00000100526 | HGLibA_08848 | 165 | 0.357788089 | CDKN3 | cyclin dependent kinase inhibitor 3 [Source:HGNC Symbol;Acc:HGNC:1791] |
| ENSG00000100605 | HGLibA_23841 | 165 | 0.357788089 | ITPK1 | inositol-tetrakisphosphate 1-kinase [Source:HGNC Symbol;Acc:HGNC:6177] |
| ENSG00000102010 | HGLibA_04641 | 165 | 0.357788089 | BMX | BMX non-receptor tyrosine kinase [Source:HGNC Symbol;Acc:HGNC:1079] |
| ENSG00000102524 | HGLibA_50812 | 165 | 0.357788089 | TNFSF13B | TNF superfamily member 13b [Source:HGNC Symbol;Acc:HGNC:11929] |
| ENSG00000104432 | HGLibA_23190 | 165 | 0.357788089 | IL7 | interleukin 7 [Source:HGNC Symbol;Acc:HGNC:6023] |
| ENSG00000105552 | HGLibA_04266 | 165 | 0.357788089 | BCAT2 | branched chain amino acid transaminase 2 [Source:HGNC Symbol;Acc:HGNC:977] |
| ENSG00000106546 | HGLibA_01307 | 165 | 0.357788089 | AHR | aryl hydrocarbon receptor [Source:HGNC Symbol;Acc:HGNC:348] |
| ENSG00000106692 | HGLibA_17601 | 165 | 0.357788089 | FKTN | fukutin [Source:HGNC Symbol;Acc:HGNC:3622] |
| ENSG00000110723 | HGLibA_15799 | 165 | 0.357788089 | EXPH5 | exophilin 5 [Source:HGNC Symbol;Acc:HGNC:30578] |
| ENSG00000110925 | HGLibA_11426 | 165 | 0.357788089 | CSRNP2 | cysteine and serine rich nuclear protein 2 [Source:HGNC Symbol;Acc:HGNC:16006] |
| ENSG00000112715 | HGLibA_53612 | 165 | 0.357788089 | VEGFA | vascular endothelial growth factor A [Source:HGNC Symbol;Acc:HGNC:12680] |
| ENSG00000113369 | HGLibA_03120 | 165 | 0.357788089 | ARRDC3 | arrestin domain containing 3 [Source:HGNC Symbol;Acc:HGNC:29263] |
| ENSG00000115840 | HGLibA_44641 | 165 | 0.357788089 | SLC25A12 | solute carrier family 25 member 12 [Source:HGNC Symbol;Acc:HGNC:10982] |
| ENSG00000119242 | HGLibA_07929 | 165 | 0.357788089 | CCDC92 | coiled-coil domain containing 92 [Source:HGNC Symbol;Acc:HGNC:29563] |
| ENSG00000120329 | HGLibA_44666 | 165 | 0.357788089 | SLC25A2 | solute carrier family 25 member 2 [Source:HGNC Symbol;Acc:HGNC:22921] |
| ENSG00000120756 | HGLibA_37225 | 165 | 0.357788089 | PLS1 | plastin 1 [Source:HGNC Symbol;Acc:HGNC:9090] |
| ENSG00000123159 | HGLibA_19118 | 165 | 0.357788089 | GIPC1 | GIPC PDZ domain containing family member 1 [Source:HGNC Symbol;Acc:HGNC:1226] |
| ENSG00000123407 | HGLibA_21961 | 165 | 0.357788089 | HOXC12 | homeobox C12 [Source:HGNC Symbol;Acc:HGNC:5124] |
| ENSG00000124249 | HGLibA_24305 | 165 | 0.357788089 | KCNK15 | potassium two pore domain channel subfamily K member 15 [Source:HGNC Symbol;Acc:HGNC:13814] |
| ENSG00000124469 | HGLibA_08954 | 165 | 0.357788089 | CEACAM8 | carcinoembryonic antigen related cell adhesion molecule 8 [Source:HGNC Symbol;Acc:HGNC:1820] |
| ENSG00000126107 | HGLibA_21098 | 165 | 0.357788089 | HECTD3 | HECT domain E3 ubiquitin protein ligase 3 [Source:HGNC Symbol;Acc:HGNC:26117] |
| ENSG00000126746 | HGLibA_55893 | 165 | 0.357788089 | ZNF384 | zinc finger protein 384 [Source:HGNC Symbol;Acc:HGNC:11955] |
| ENSG00000127152 | HGLibA_04294 | 165 | 0.357788089 | BCL11B | B cell CLL/lymphoma 11B [Source:HGNC Symbol;Acc:HGNC:13222] |
| ENSG00000127903 | HGLibA_56840 | 165 | 0.357788089 | ZNF835 | zinc finger protein 835 [Source:HGNC Symbol;Acc:HGNC:34332] |
| ENSG00000129292 | HGLibA_36365 | 165 | 0.357788089 | PHF20L1 | PHD finger protein 20 like 1 [Source:HGNC Symbol;Acc:HGNC:24280] |
| ENSG00000131016 | HGLibA_01411 | 165 | 0.357788089 | AKAP12 | A-kinase anchoring protein 12 [Source:HGNC Symbol;Acc:HGNC:370] |
| ENSG00000132109 | HGLibA_51429 | 165 | 0.357788089 | TRIM21 | tripartite motif containing 21 [Source:HGNC Symbol;Acc:HGNC:11312] |
| ENSG00000132801 | HGLibA_57043 | 165 | 0.357788089 | ZSWIM3 | zinc finger SWIM-type containing 3 [Source:HGNC Symbol;Acc:HGNC:16157] |
| ENSG00000133083 | HGLibA_12500 | 165 | 0.357788089 | DCLK1 | doublecortin like kinase 1 [Source:HGNC Symbol;Acc:HGNC:2700] |
| ENSG00000133246 | HGLibA_38272 | 165 | 0.357788089 | PRAM1 | PML-RARA regulated adaptor molecule 1 [Source:HGNC Symbol;Acc:HGNC:30091] |
| ENSG00000137474 | HGLibA_30863 | 165 | 0.357788089 | MYO7A | myosin VIIA [Source:HGNC Symbol;Acc:HGNC:7606] |
| ENSG00000140396 | HGLibA_31338 | 165 | 0.357788089 | NCOA2 | nuclear receptor coactivator 2 [Source:HGNC Symbol;Acc:HGNC:7669] |
| ENSG00000140678 | HGLibA_23767 | 165 | 0.357788089 | ITGAX | integrin subunit alpha X [Source:HGNC Symbol;Acc:HGNC:6152] |
| ENSG00000146278 | HGLibA_37434 | 165 | 0.357788089 | PNRC1 | proline rich nuclear receptor coactivator 1 [Source:HGNC Symbol;Acc:HGNC:17278] |
| ENSG00000146463 | HGLibA_55399 | 165 | 0.357788089 | ZMYM4 | zinc finger MYM-type containing 4 [Source:HGNC Symbol;Acc:HGNC:13055] |
| ENSG00000146859 | HGLibA_49960 | 165 | 0.357788089 | TMEM140 | transmembrane protein 140 [Source:HGNC Symbol;Acc:HGNC:21870] |
| ENSG00000146904 | HGLibA_15283 | 165 | 0.357788089 | EPHA1 | EPH receptor A1 [Source:HGNC Symbol;Acc:HGNC:3385] |
| ENSG00000148541 | HGLibA_16147 | 165 | 0.357788089 | FAM13C | family with sequence similarity 13 member C [Source:HGNC Symbol;Acc:HGNC:19371] |
| ENSG00000151348 | HGLibA_15805 | 165 | 0.357788089 | EXT2 | exostosin glycosyltransferase 2 [Source:HGNC Symbol;Acc:HGNC:3513] |
| ENSG00000151715 | HGLibA_50410 | 165 | 0.357788089 | TMEM45B | transmembrane protein 45B [Source:HGNC Symbol;Acc:HGNC:25194] |
| ENSG00000152082 | HGLibA_30946 | 165 | 0.357788089 | MZT2B | mitotic spindle organizing protein 2B [Source:HGNC Symbol;Acc:HGNC:25886] |
| ENSG00000155729 | HGLibA_24438 | 165 | 0.357788089 | KCTD18 | potassium channel tetramerization domain containing 18 [Source:HGNC Symbol;Acc:HGNC:26446] |
| ENSG00000158470 | HGLibA_04035 | 165 | 0.357788089 | B4GALT5 | 4-galactosyltransferase 5 [Source:HGNC Symbol;Acc:HGNC:928]" |
| ENSG00000159423 | HGLibA_01575 | 165 | 0.357788089 | ALDH4A1 | aldehyde dehydrogenase 4 family member A1 [Source:HGNC Symbol;Acc:HGNC:406] |
| ENSG00000162267 | HGLibA_23812 | 165 | 0.357788089 | ITIH3 | inter-alpha-trypsin inhibitor heavy chain 3 [Source:HGNC Symbol;Acc:HGNC:6168] |
| ENSG00000162438 | HGLibA_11682 | 165 | 0.357788089 | CTRC | chymotrypsin C [Source:HGNC Symbol;Acc:HGNC:2523] |
| ENSG00000162624 | HGLibA_26465 | 165 | 0.357788089 | LHX8 | LIM homeobox 8 [Source:HGNC Symbol;Acc:HGNC:28838] |
| ENSG00000163170 | HGLibA_04688 | 165 | 0.357788089 | BOLA3 | bolA family member 3 [Source:HGNC Symbol;Acc:HGNC:24415] |
| ENSG00000163704 | HGLibA_38886 | 165 | 0.357788089 | PRRT3 | proline rich transmembrane protein 3 [Source:HGNC Symbol;Acc:HGNC:26591] |
| ENSG00000164088 | HGLibA_37994 | 165 | 0.357788089 | PPM1M | Mg2+/Mn2+ dependent 1M [Source:HGNC Symbol;Acc:HGNC:26506]" |
| ENSG00000165115 | HGLibA_24923 | 165 | 0.357788089 | KIF27 | kinesin family member 27 [Source:HGNC Symbol;Acc:HGNC:18632] |
| ENSG00000165192 | HGLibA_03210 | 165 | 0.357788089 | ASB11 | ankyrin repeat and SOCS box containing 11 [Source:HGNC Symbol;Acc:HGNC:17186] |
| ENSG00000166959 | HGLibA_30167 | 165 | 0.357788089 | MS4A8 | membrane spanning 4-domains A8 [Source:HGNC Symbol;Acc:HGNC:13380] |
| ENSG00000167634 | HGLibA_32131 | 165 | 0.357788089 | NLRP7 | NLR family pyrin domain containing 7 [Source:HGNC Symbol;Acc:HGNC:22947] |
| ENSG00000171017 | HGLibA_27439 | 165 | 0.357788089 | LRRC8E | leucine rich repeat containing 8 VRAC subunit E [Source:HGNC Symbol;Acc:HGNC:26272] |
| ENSG00000171102 | HGLibA_33229 | 165 | 0.357788089 | OBP2B | odorant binding protein 2B [Source:HGNC Symbol;Acc:HGNC:23381] |
| ENSG00000171792 | HGLibA_41161 | 165 | 0.357788089 | RHNO1 | RAD9-HUS1-RAD1 interacting nuclear orphan 1 [Source:HGNC Symbol;Acc:HGNC:28206] |
| ENSG00000172377 | HGLibA_34577 | 165 | 0.357788089 | OR9I1 | olfactory receptor family 9 subfamily I member 1 [Source:HGNC Symbol;Acc:HGNC:14718] |
| ENSG00000173889 | HGLibA_36324 | 165 | 0.357788089 | PHC3 | polyhomeotic homolog 3 [Source:HGNC Symbol;Acc:HGNC:15682] |
| ENSG00000174482 | HGLibA_26587 | 165 | 0.357788089 | LINGO2 | leucine rich repeat and Ig domain containing 2 [Source:HGNC Symbol;Acc:HGNC:21207] |
| ENSG00000174564 | HGLibA_23078 | 165 | 0.357788089 | IL20RB | interleukin 20 receptor subunit beta [Source:HGNC Symbol;Acc:HGNC:6004] |
| ENSG00000175309 | HGLibA_36480 | 165 | 0.357788089 | PHYKPL | 5-phosphohydroxy-L-lysine phospho-lyase [Source:HGNC Symbol;Acc:HGNC:28249] |
| ENSG00000175315 | HGLibA_11461 | 165 | 0.357788089 | CST6 | cystatin E/M [Source:HGNC Symbol;Acc:HGNC:2478] |
| ENSG00000175697 | HGLibA_19945 | 165 | 0.357788089 | GPR156 | G protein-coupled receptor 156 [Source:HGNC Symbol;Acc:HGNC:20844] |
| ENSG00000176988 | HGLibA_17722 | 165 | 0.357788089 | FMR1NB | FMR1 neighbor [Source:HGNC Symbol;Acc:HGNC:26372] |
| ENSG00000178567 | HGLibA_15338 | 165 | 0.357788089 | EPM2AIP1 | EPM2A interacting protein 1 [Source:HGNC Symbol;Acc:HGNC:19735] |
| ENSG00000179841 | HGLibA_01437 | 165 | 0.357788089 | AKAP5 | A-kinase anchoring protein 5 [Source:HGNC Symbol;Acc:HGNC:375] |
| ENSG00000181619 | HGLibA_19895 | 165 | 0.357788089 | GPR135 | G protein-coupled receptor 135 [Source:HGNC Symbol;Acc:HGNC:19991] |
| ENSG00000181856 | HGLibA_44876 | 165 | 0.357788089 | SLC2A4 | solute carrier family 2 member 4 [Source:HGNC Symbol;Acc:HGNC:11009] |
| ENSG00000182986 | HGLibA_55794 | 165 | 0.357788089 | ZNF320 | zinc finger protein 320 [Source:HGNC Symbol;Acc:HGNC:13842] |
| ENSG00000183765 | HGLibA_09422 | 165 | 0.357788089 | CHEK2 | checkpoint kinase 2 [Source:HGNC Symbol;Acc:HGNC:16627] |
| ENSG00000183826 | HGLibA_04926 | 165 | 0.357788089 | BTBD9 | BTB domain containing 9 [Source:HGNC Symbol;Acc:HGNC:21228] |
| ENSG00000185149 | HGLibA_32552 | 165 | 0.357788089 | NPY2R | neuropeptide Y receptor Y2 [Source:HGNC Symbol;Acc:HGNC:7957] |
| ENSG00000186952 | HGLibA_50261 | 165 | 0.357788089 | TMEM232 | transmembrane protein 232 [Source:HGNC Symbol;Acc:HGNC:37270] |
| ENSG00000187862 | HGLibA_52085 | 165 | 0.357788089 | TTC24 | tetratricopeptide repeat domain 24 [Source:HGNC Symbol;Acc:HGNC:32348] |
| ENSG00000189233 | HGLibA_33007 | 165 | 0.357788089 | NUGGC | germinal center associated [Source:HGNC Symbol;Acc:HGNC:33550]" |
| ENSG00000196290 | HGLibA_31929 | 165 | 0.357788089 | NIF3L1 | NGG1 interacting factor 3 like 1 [Source:HGNC Symbol;Acc:HGNC:13390] |
| ENSG00000196504 | HGLibA_38733 | 165 | 0.357788089 | PRPF40A | pre-mRNA processing factor 40 homolog A [Source:HGNC Symbol;Acc:HGNC:16463] |
| ENSG00000196505 | HGLibA_18845 | 165 | 0.357788089 | GDAP2 | ganglioside induced differentiation associated protein 2 [Source:HGNC Symbol;Acc:HGNC:18010] |
| ENSG00000196659 | HGLibA_52109 | 165 | 0.357788089 | TTC30B | tetratricopeptide repeat domain 30B [Source:HGNC Symbol;Acc:HGNC:26425] |
| ENSG00000197860 | HGLibA_43871 | 165 | 0.357788089 | SGTB | small glutamine rich tetratricopeptide repeat containing beta [Source:HGNC Symbol;Acc:HGNC:23567] |
| ENSG00000198570 | HGLibA_40725 | 165 | 0.357788089 | RD3 | GUCY2D regulator [Source:HGNC Symbol;Acc:HGNC:19689]" |
| ENSG00000203724 | HGLibA_05870 | 165 | 0.357788089 | C1orf53 | chromosome 1 open reading frame 53 [Source:HGNC Symbol;Acc:HGNC:30003] |
| ENSG00000204165 | HGLibA_11960 | 165 | 0.357788089 | CXorf65 | chromosome X open reading frame 65 [Source:HGNC Symbol;Acc:HGNC:33713] |
| ENSG00000204542 | HGLibA_06453 | 165 | 0.357788089 | C6orf15 | chromosome 6 open reading frame 15 [Source:HGNC Symbol;Acc:HGNC:13927] |
| ENSG00000204618 | HGLibA_41674 | 165 | 0.357788089 | RNF39 | ring finger protein 39 [Source:HGNC Symbol;Acc:HGNC:18064] |
| ENSG00000206503 | HGLibA_21595 | 165 | 0.357788089 | HLA-A | A [Source:HGNC Symbol;Acc:HGNC:4931]" |
| ENSG00000213699 | HGLibA_45014 | 165 | 0.357788089 | SLC35F6 | solute carrier family 35 member F6 [Source:HGNC Symbol;Acc:HGNC:26055] |
| ENSG00000214655 | HGLibA_57058 | 165 | 0.357788089 | ZSWIM8 | zinc finger SWIM-type containing 8 [Source:HGNC Symbol;Acc:HGNC:23528] |
| ENSG00000229937 | HGLibA_38757 | 165 | 0.357788089 | PRPS1L1 | phosphoribosyl pyrophosphate synthetase 1-like 1 [Source:HGNC Symbol;Acc:HGNC:9463] |
| ENSG00000232423 | HGLibA_38323 | 165 | 0.357788089 | PRAMEF6 | PRAME family member 6 [Source:HGNC Symbol;Acc:HGNC:30583] |
| ENSG00000242866 | HGLibA_47586 | 165 | 0.357788089 | STRC | stereocilin [Source:HGNC Symbol;Acc:HGNC:16035] |
| ENSG00000249481 | HGLibA_46542 | 165 | 0.357788089 | SPATS1 | spermatogenesis associated serine rich 1 [Source:HGNC Symbol;Acc:HGNC:22957] |
| ENSG00000254737 | HGLibA_33493 | 165 | 0.357788089 | OR10G4 | olfactory receptor family 10 subfamily G member 4 [Source:HGNC Symbol;Acc:HGNC:14809] |
| ENSG00000261272 | HGLibA_30543 | 165 | 0.357788089 | MUC22 | mucin 22 [Source:HGNC Symbol;Acc:HGNC:39755] |
| FAM84B | HGLibA_16767 | 165 | 0.357788089 | FAM84B | - |
| hsa-mir-153-1 | HGLibA_57908 | 165 | 0.357788089 | hsa-mir-153-1 | - |
| hsa-mir-1587 | HGLibA_57936 | 165 | 0.357788089 | hsa-mir-1587 | - |
| hsa-mir-181b-2 | HGLibA_57971 | 165 | 0.357788089 | hsa-mir-181b-2 | - |
| hsa-mir-425 | HGLibA_59708 | 165 | 0.357788089 | hsa-mir-425 | - |
| hsa-mir-4496 | HGLibA_60402 | 165 | 0.357788089 | hsa-mir-4496 | - |
| hsa-mir-4522 | HGLibA_60546 | 165 | 0.357788089 | hsa-mir-4522 | - |
| hsa-mir-4694 | HGLibA_60884 | 165 | 0.357788089 | hsa-mir-4694 | - |
| hsa-mir-4730 | HGLibA_61017 | 165 | 0.357788089 | hsa-mir-4730 | - |
| hsa-mir-5006 | HGLibA_61428 | 165 | 0.357788089 | hsa-mir-5006 | - |
| hsa-mir-564 | HGLibA_62221 | 165 | 0.357788089 | hsa-mir-564 | - |
| hsa-mir-567 | HGLibA_62226 | 165 | 0.357788089 | hsa-mir-567 | - |
| hsa-mir-653 | HGLibA_62944 | 165 | 0.357788089 | hsa-mir-653 | - |
| hsa-mir-711 | HGLibA_63800 | 165 | 0.357788089 | hsa-mir-711 | - |
| hsa-mir-7973-1 | HGLibA_64016 | 165 | 0.357788089 | hsa-mir-7973-1 | - |
| LOC554223 | HGLibA_26954 | 165 | 0.357788089 | LOC554223 | - |
| LOC643802 | HGLibA_26973 | 165 | 0.357788089 | LOC643802 | - |
| SEPP1 | HGLibA_43420 | 165 | 0.357788089 | SEPP1 | - |
| TMEM55A | HGLibA_50443 | 165 | 0.357788089 | TMEM55A | - |
| C7orf72 | HGLibA_06588 | 164 | 0.348742746 | C7orf72 | - |
| CPSF3L | HGLibA_11004 | 164 | 0.348742746 | CPSF3L | - |
| ENSG00000001561 | HGLibA_15177 | 164 | 0.348742746 | ENPP4 | ectonucleotide pyrophosphatase/phosphodiesterase 4 [Source:HGNC Symbol;Acc:HGNC:3359] |
| ENSG00000006468 | HGLibA_15647 | 164 | 0.348742746 | ETV1 | ETS variant 1 [Source:HGNC Symbol;Acc:HGNC:3490] |
| ENSG00000021300 | HGLibA_37085 | 164 | 0.348742746 | PLEKHB1 | pleckstrin homology domain containing B1 [Source:HGNC Symbol;Acc:HGNC:19079] |
| ENSG00000022355 | HGLibA_18355 | 164 | 0.348742746 | GABRA1 | gamma-aminobutyric acid type A receptor alpha1 subunit [Source:HGNC Symbol;Acc:HGNC:4075] |
| ENSG00000026297 | HGLibA_41442 | 164 | 0.348742746 | RNASET2 | ribonuclease T2 [Source:HGNC Symbol;Acc:HGNC:21686] |
| ENSG00000046653 | HGLibA_19822 | 164 | 0.348742746 | GPM6B | glycoprotein M6B [Source:HGNC Symbol;Acc:HGNC:4461] |
| ENSG00000050130 | HGLibA_23949 | 164 | 0.348742746 | JKAMP | JNK1/MAPK8 associated membrane protein [Source:HGNC Symbol;Acc:HGNC:20184] |
| ENSG00000057704 | HGLibA_49779 | 164 | 0.348742746 | TMCC3 | transmembrane and coiled-coil domain family 3 [Source:HGNC Symbol;Acc:HGNC:29199] |
| ENSG00000066117 | HGLibA_45629 | 164 | 0.348742746 | SMARCD1 | member 1 [Source:HGNC Symbol;Acc:HGNC:11106]" |
| ENSG00000072786 | HGLibA_47451 | 164 | 0.348742746 | STK10 | serine/threonine kinase 10 [Source:HGNC Symbol;Acc:HGNC:11388] |

| | | | | | |
|---|---|---|---|---|---|
| ENSG00000079134 | HGLibA_49363 | 164 | 0.348742746 | THOC1 | THO complex 1 [Source:HGNC Symbol;Acc:HGNC:19070] |
| ENSG00000084093 | HGLibA_40868 | 164 | 0.348742746 | REST | RE1 silencing transcription factor [Source:HGNC Symbol;Acc:HGNC:9966] |
| ENSG00000091106 | HGLibA_32091 | 164 | 0.348742746 | NLRC4 | NLR family CARD domain containing 4 [Source:HGNC Symbol;Acc:HGNC:16412] |
| ENSG00000091129 | HGLibA_32661 | 164 | 0.348742746 | NRCAM | neuronal cell adhesion molecule [Source:HGNC Symbol;Acc:HGNC:7994] |
| ENSG00000094631 | HGLibA_21019 | 164 | 0.348742746 | HDAC6 | histone deacetylase 6 [Source:HGNC Symbol;Acc:HGNC:14064] |
| ENSG00000099866 | HGLibA_27855 | 164 | 0.348742746 | MADCAM1 | mucosal vascular addressin cell adhesion molecule 1 [Source:HGNC Symbol;Acc:HGNC:6765] |
| ENSG00000100564 | HGLibA_36557 | 164 | 0.348742746 | PIGH | phosphatidylinositol glycan anchor biosynthesis class H [Source:HGNC Symbol;Acc:HGNC:8964] |
| ENSG00000101493 | HGLibA_56143 | 164 | 0.348742746 | ZNF516 | zinc finger protein 516 [Source:HGNC Symbol;Acc:HGNC:28990] |
| ENSG00000102854 | HGLibA_30219 | 164 | 0.348742746 | MSLN | mesothelin [Source:HGNC Symbol;Acc:HGNC:7371] |
| ENSG00000103978 | HGLibA_50531 | 164 | 0.348742746 | TMEM87A | transmembrane protein 87A [Source:HGNC Symbol;Acc:HGNC:24522] |
| ENSG00000104375 | HGLibA_47476 | 164 | 0.348742746 | STK3 | serine/threonine kinase 3 [Source:HGNC Symbol;Acc:HGNC:11406] |
| ENSG00000106723 | HGLibA_46667 | 164 | 0.348742746 | SPIN1 | spindlin 1 [Source:HGNC Symbol;Acc:HGNC:11243] |
| ENSG00000112159 | HGLibA_28703 | 164 | 0.348742746 | MDN1 | midasin AAA ATPase 1 [Source:HGNC Symbol;Acc:HGNC:18302] |
| ENSG00000112559 | HGLibA_28670 | 164 | 0.348742746 | MDFI | MyoD family inhibitor [Source:HGNC Symbol;Acc:HGNC:6967] |
| ENSG00000119121 | HGLibA_51766 | 164 | 0.348742746 | TRPM6 | transient receptor potential cation channel subfamily M member 6 [Source:HGNC Symbol;Acc:HGNC:17995] |
| ENSG00000122591 | HGLibA_16082 | 164 | 0.348742746 | FAM126A | family with sequence similarity 126 member A [Source:HGNC Symbol;Acc:HGNC:24587] |
| ENSG00000124444 | HGLibA_56294 | 164 | 0.348742746 | ZNF576 | zinc finger protein 576 [Source:HGNC Symbol;Acc:HGNC:28357] |
| ENSG00000124818 | HGLibA_33439 | 164 | 0.348742746 | OPN5 | opsin 5 [Source:HGNC Symbol;Acc:HGNC:19992] |
| ENSG00000125454 | HGLibA_44664 | 164 | 0.348742746 | SLC25A19 | solute carrier family 25 member 19 [Source:HGNC Symbol;Acc:HGNC:14409] |
| ENSG00000132256 | HGLibA_51534 | 164 | 0.348742746 | TRIM5 | tripartite motif containing 5 [Source:HGNC Symbol;Acc:HGNC:16276] |
| ENSG00000133019 | HGLibA_09552 | 164 | 0.348742746 | CHRM3 | cholinergic receptor muscarinic 3 [Source:HGNC Symbol;Acc:HGNC:1952] |
| ENSG00000134851 | HGLibA_50037 | 164 | 0.348742746 | TMEM165 | transmembrane protein 165 [Source:HGNC Symbol;Acc:HGNC:30760] |
| ENSG00000135424 | HGLibA_23741 | 164 | 0.348742746 | ITGA7 | integrin subunit alpha 7 [Source:HGNC Symbol;Acc:HGNC:6143] |
| ENSG00000136286 | HGLibA_30838 | 164 | 0.348742746 | MYO1G | myosin IG [Source:HGNC Symbol;Acc:HGNC:13880] |
| ENSG00000136297 | HGLibA_29465 | 164 | 0.348742746 | MMD2 | monocyte to macrophage differentiation associated 2 [Source:HGNC Symbol;Acc:HGNC:30133] |
| ENSG00000139182 | HGLibA_10141 | 164 | 0.348742746 | CLSTN3 | calsyntenin 3 [Source:HGNC Symbol;Acc:HGNC:18371] |
| ENSG00000139354 | HGLibA_18651 | 164 | 0.348742746 | GAS2L3 | growth arrest specific 2 like 3 [Source:HGNC Symbol;Acc:HGNC:27475] |
| ENSG00000140718 | HGLibA_18131 | 164 | 0.348742746 | FTO | alpha-ketoglutarate dependent dioxygenase [Source:HGNC Symbol;Acc:HGNC:24678]" |
| ENSG00000142252 | HGLibA_18924 | 164 | 0.348742746 | GEMIN7 | gem nuclear organelle associated protein 7 [Source:HGNC Symbol;Acc:HGNC:20045] |
| ENSG00000143536 | HGLibA_11207 | 164 | 0.348742746 | CRNN | cornulin [Source:HGNC Symbol;Acc:HGNC:1230] |
| ENSG00000145246 | HGLibA_03552 | 164 | 0.348742746 | ATP10D | ATPase phospholipid transporting 10D (putative) [Source:HGNC Symbol;Acc:HGNC:13549] |
| ENSG00000146276 | HGLibA_18405 | 164 | 0.348742746 | GABRR1 | gamma-aminobutyric acid type A receptor rho1 subunit [Source:HGNC Symbol;Acc:HGNC:4090] |
| ENSG00000147223 | HGLibA_41327 | 164 | 0.348742746 | RIPPLY1 | ripply transcriptional repressor 1 [Source:HGNC Symbol;Acc:HGNC:25117] |
| ENSG00000150457 | HGLibA_26068 | 164 | 0.348742746 | LATS2 | large tumor suppressor kinase 2 [Source:HGNC Symbol;Acc:HGNC:6515] |
| ENSG00000153157 | HGLibA_47933 | 164 | 0.348742746 | SYCP2L | synaptonemal complex protein 2 like [Source:HGNC Symbol;Acc:HGNC:21537] |
| ENSG00000157578 | HGLibA_26093 | 164 | 0.348742746 | LCA5L | lebercilin like [Source:HGNC Symbol;Acc:HGNC:1255]" |
| ENSG00000158125 | HGLibA_54503 | 164 | 0.348742746 | XDH | xanthine dehydrogenase [Source:HGNC Symbol;Acc:HGNC:12805] |
| ENSG00000158955 | HGLibA_54398 | 164 | 0.348742746 | WNT9B | Wnt family member 9B [Source:HGNC Symbol;Acc:HGNC:12779] |
| ENSG00000161610 | HGLibA_20986 | 164 | 0.348742746 | HCRT | hypocretin neuropeptide precursor [Source:HGNC Symbol;Acc:HGNC:4847] |
| ENSG00000161649 | HGLibA_08345 | 164 | 0.348742746 | CD300LG | CD300 molecule like family member g [Source:HGNC Symbol;Acc:HGNC:30455] |
| ENSG00000162032 | HGLibA_46865 | 164 | 0.348742746 | SPSB3 | splA/ryanodine receptor domain and SOCS box containing 3 [Source:HGNC Symbol;Acc:HGNC:30629] |
| ENSG00000162552 | HGLibA_54370 | 164 | 0.348742746 | WNT4 | Wnt family member 4 [Source:HGNC Symbol;Acc:HGNC:12783] |
| ENSG00000164822 | HGLibA_12808 | 164 | 0.348742746 | DEFA6 | defensin alpha 6 [Source:HGNC Symbol;Acc:HGNC:2765] |
| ENSG00000165985 | HGLibA_05929 | 164 | 0.348742746 | C1QL3 | complement C1q like 3 [Source:HGNC Symbol;Acc:HGNC:19359] |
| ENSG00000166634 | HGLibA_43541 | 164 | 0.348742746 | SERPINB12 | serpin family B member 12 [Source:HGNC Symbol;Acc:HGNC:14220] |
| ENSG00000166987 | HGLibA_28493 | 164 | 0.348742746 | MBD6 | methyl-CpG binding domain protein 6 [Source:HGNC Symbol;Acc:HGNC:20445] |
| ENSG00000167995 | HGLibA_04418 | 164 | 0.348742746 | BEST1 | bestrophin 1 [Source:HGNC Symbol;Acc:HGNC:12703] |
| ENSG00000168461 | HGLibA_39874 | 164 | 0.348742746 | RAB31 | member RAS oncogene family [Source:HGNC Symbol;Acc:HGNC:9771]" |
| ENSG00000168658 | HGLibA_53905 | 164 | 0.348742746 | VWA3B | von Willebrand factor A domain containing 3B [Source:HGNC Symbol;Acc:HGNC:28385] |
| ENSG00000170310 | HGLibA_47661 | 164 | 0.348742746 | STX8 | syntaxin 8 [Source:HGNC Symbol;Acc:HGNC:11443] |
| ENSG00000170364 | HGLibA_43673 | 164 | 0.348742746 | SETMAR | SET domain and mariner transposase fusion gene [Source:HGNC Symbol;Acc:HGNC:10762] |
| ENSG00000170837 | HGLibA_20012 | 164 | 0.348742746 | GPR27 | G protein-coupled receptor 27 [Source:HGNC Symbol;Acc:HGNC:4482] |
| ENSG00000172059 | HGLibA_25046 | 164 | 0.348742746 | KLF11 | Kruppel like factor 11 [Source:HGNC Symbol;Acc:HGNC:11811] |
| ENSG00000172156 | HGLibA_07965 | 164 | 0.348742746 | CCL11 | C-C motif chemokine ligand 11 [Source:HGNC Symbol;Acc:HGNC:10610] |
| ENSG00000172466 | HGLibA_55657 | 164 | 0.348742746 | ZNF24 | zinc finger protein 24 [Source:HGNC Symbol;Acc:HGNC:13032] |
| ENSG00000174485 | HGLibA_12949 | 164 | 0.348742746 | DENND4A | DENN domain containing 4A [Source:HGNC Symbol;Acc:HGNC:24321] |
| ENSG00000176406 | HGLibA_41281 | 164 | 0.348742746 | RIMS2 | regulating synaptic membrane exocytosis 2 [Source:HGNC Symbol;Acc:HGNC:17283] |
| ENSG00000176678 | HGLibA_17900 | 164 | 0.348742746 | FOXL1 | forkhead box L1 [Source:HGNC Symbol;Acc:HGNC:3817] |
| ENSG00000178015 | HGLibA_19930 | 164 | 0.348742746 | GPR150 | G protein-coupled receptor 150 [Source:HGNC Symbol;Acc:HGNC:23628] |
| ENSG00000179873 | HGLibA_32103 | 164 | 0.348742746 | NLRP11 | NLR family pyrin domain containing 11 [Source:HGNC Symbol;Acc:HGNC:22945] |
| ENSG00000179988 | HGLibA_39235 | 164 | 0.348742746 | PSTK | phosphoseryl-tRNA kinase [Source:HGNC Symbol;Acc:HGNC:28578] |
| ENSG00000180089 | HGLibA_50528 | 164 | 0.348742746 | TMEM86B | transmembrane protein 86B [Source:HGNC Symbol;Acc:HGNC:28448] |
| ENSG00000182463 | HGLibA_51866 | 164 | 0.348742746 | TSHZ2 | teashirt zinc finger homeobox 2 [Source:HGNC Symbol;Acc:HGNC:13010] |
| ENSG00000183647 | HGLibA_56176 | 164 | 0.348742746 | ZNF530 | zinc finger protein 530 [Source:HGNC Symbol;Acc:HGNC:29297] |
| ENSG00000184140 | HGLibA_34014 | 164 | 0.348742746 | OR4F6 | olfactory receptor family 4 subfamily F member 6 [Source:HGNC Symbol;Acc:HGNC:15372] |
| ENSG00000184517 | HGLibA_55180 | 164 | 0.348742746 | ZFP1 | ZFP1 zinc finger protein [Source:HGNC Symbol;Acc:HGNC:23328] |
| ENSG00000186867 | HGLibA_39738 | 164 | 0.348742746 | QRFPR | pyroglutamylated RFamide peptide receptor [Source:HGNC Symbol;Acc:HGNC:15565] |
| ENSG00000187242 | HGLibA_25419 | 164 | 0.348742746 | KRT12 | keratin 12 [Source:HGNC Symbol;Acc:HGNC:6414] |
| ENSG00000196169 | HGLibA_24880 | 164 | 0.348742746 | KIF19 | kinesin family member 19 [Source:HGNC Symbol;Acc:HGNC:26735] |
| ENSG00000196757 | HGLibA_56574 | 164 | 0.348742746 | ZNF700 | zinc finger protein 700 [Source:HGNC Symbol;Acc:HGNC:25292] |
| ENSG00000197479 | HGLibA_35476 | 164 | 0.348742746 | PCDHB11 | protocadherin beta 11 [Source:HGNC Symbol;Acc:HGNC:8682] |
| ENSG00000197683 | HGLibA_25732 | 164 | 0.348742746 | KRTAP26-1 | keratin associated protein 26-1 [Source:HGNC Symbol;Acc:HGNC:33760] |
| ENSG00000198246 | HGLibA_44846 | 164 | 0.348742746 | SLC29A3 | solute carrier family 29 member 3 [Source:HGNC Symbol;Acc:HGNC:23096] |
| ENSG00000204764 | HGLibA_40197 | 164 | 0.348742746 | RANBP17 | RAN binding protein 17 [Source:HGNC Symbol;Acc:HGNC:14428] |
| ENSG00000205436 | HGLibA_15747 | 164 | 0.348742746 | EXOC3L4 | exocyst complex component 3 like 4 [Source:HGNC Symbol;Acc:HGNC:20120] |
| ENSG00000213160 | HGLibA_25160 | 164 | 0.348742746 | KLHL23 | kelch like family member 23 [Source:HGNC Symbol;Acc:HGNC:27506] |
| ENSG00000214814 | HGLibA_17287 | 164 | 0.348742746 | FER1L6 | fer-1 like family member 6 [Source:HGNC Symbol;Acc:HGNC:28065] |
| ENSG00000215356 | HGLibA_56591 | 164 | 0.348742746 | ZNF705B | zinc finger protein 705B [Source:HGNC Symbol;Acc:HGNC:32284] |
| ENSG00000232258 | HGLibA_49881 | 164 | 0.348742746 | TMEM114 | transmembrane protein 114 [Source:HGNC Symbol;Acc:HGNC:33227] |
| ENSG00000243725 | HGLibA_52142 | 164 | 0.348742746 | TTC4 | tetratricopeptide repeat domain 4 [Source:HGNC Symbol;Acc:HGNC:12394] |
| ENSG00000248672 | HGLibA_27682 | 164 | 0.348742746 | LY75-CD302 | LY75-CD302 readthrough [Source:HGNC Symbol;Acc:HGNC:38828] |
| ENSG00000250479 | HGLibA_09361 | 164 | 0.348742746 | CHCHD10 | coiled-coil-helix-coiled-coil-helix domain containing 10 [Source:HGNC Symbol;Acc:HGNC:15559] |
| ENSG00000257923 | HGLibA_11804 | 164 | 0.348742746 | CUX1 | cut like homeobox 1 [Source:HGNC Symbol;Acc:HGNC:2557] |
| ENSG00000260456 | HGLibA_05508 | 164 | 0.348742746 | C16orf95 | chromosome 16 open reading frame 95 [Source:HGNC Symbol;Acc:HGNC:40033] |
| ENSG00000270011 | HGLibA_56240 | 164 | 0.348742746 | ZNF559-ZNF177 | ZNF559-ZNF177 readthrough [Source:HGNC Symbol;Acc:HGNC:42964] |
| FAM160B2 | HGLibA_16214 | 164 | 0.348742746 | FAM160B2 | - |
| HIST1H3A | HGLibA_21457 | 164 | 0.348742746 | HIST1H3A | - |
| HIST1H4B | HGLibA_21490 | 164 | 0.348742746 | HIST1H4B | - |
| HIST1H4E | HGLibA_21497 | 164 | 0.348742746 | HIST1H4E | - |
| hsa-mir-1276 | HGLibA_57560 | 164 | 0.348742746 | hsa-mir-1276 | - |
| hsa-mir-222 | HGLibA_58297 | 164 | 0.348742746 | hsa-mir-222 | - |
| hsa-mir-30d | HGLibA_58489 | 164 | 0.348742746 | hsa-mir-30d | - |
| hsa-mir-3197 | HGLibA_58887 | 164 | 0.348742746 | hsa-mir-3197 | - |
| hsa-mir-378d-1 | HGLibA_59438 | 164 | 0.348742746 | hsa-mir-378d-1 | - |
| hsa-mir-3907 | HGLibA_59494 | 164 | 0.348742746 | hsa-mir-3907 | - |
| hsa-mir-4292 | HGLibA_59880 | 164 | 0.348742746 | hsa-mir-4292 | - |
| hsa-mir-4316 | HGLibA_59985 | 164 | 0.348742746 | hsa-mir-4316 | - |
| hsa-mir-4445 | HGLibA_60186 | 164 | 0.348742746 | hsa-mir-4445 | - |
| hsa-mir-4660 | HGLibA_60748 | 164 | 0.348742746 | hsa-mir-4660 | - |
| hsa-mir-5091 | HGLibA_61522 | 164 | 0.348742746 | hsa-mir-5091 | - |
| hsa-mir-521-1 | HGLibA_61765 | 164 | 0.348742746 | hsa-mir-521-1 | - |
| hsa-mir-7113 | HGLibA_63821 | 164 | 0.348742746 | hsa-mir-7113 | - |
| hsa-mir-7156 | HGLibA_63852 | 164 | 0.348742746 | hsa-mir-7156 | - |
| hsa-mir-8063 | HGLibA_64089 | 164 | 0.348742746 | hsa-mir-8063 | - |
| KIAA2022 | HGLibA_24844 | 164 | 0.348742746 | KIAA2022 | - |
| PLK1S1 | HGLibA_37187 | 164 | 0.348742746 | PLK1S1 | - |
| RAB7L1 | HGLibA_39983 | 164 | 0.348742746 | RAB7L1 | - |
| RNMTL1 | HGLibA_41722 | 164 | 0.348742746 | RNMTL1 | - |
| TCTE3 | HGLibA_48861 | 164 | 0.348742746 | TCTE3 | - |
| C10orf114 | HGLibA_05036 | 163 | 0.339697404 | C10orf114 | - |
| C9orf173 | HGLibA_06717 | 163 | 0.339697404 | C9orf173 | - |
| CCDC129 | HGLibA_07585 | 163 | 0.339697404 | CCDC129 | - |
| ENSG00000006451 | HGLibA_40147 | 163 | 0.339697404 | RALA | RAS like proto-oncogene A [Source:HGNC Symbol;Acc:HGNC:9839] |
| ENSG00000008438 | HGLibA_36249 | 163 | 0.339697404 | PGLYRP1 | peptidoglycan recognition protein 1 [Source:HGNC Symbol;Acc:HGNC:8904] |
| ENSG00000039068 | HGLibA_08630 | 163 | 0.339697404 | CDH1 | cadherin 1 [Source:HGNC Symbol;Acc:HGNC:1748] |
| ENSG00000052126 | HGLibA_37073 | 163 | 0.339697404 | PLEKHA5 | pleckstrin homology domain containing A5 [Source:HGNC Symbol;Acc:HGNC:30036] |
| ENSG00000070778 | HGLibA_39457 | 163 | 0.339697404 | PTPN21 | non-receptor type 21 [Source:HGNC Symbol;Acc:HGNC:9651]" |
| ENSG00000073464 | HGLibA_09828 | 163 | 0.339697404 | CLCN4 | chloride voltage-gated channel 4 [Source:HGNC Symbol;Acc:HGNC:2022] |
| ENSG00000074657 | HGLibA_56179 | 163 | 0.339697404 | ZNF532 | zinc finger protein 532 [Source:HGNC Symbol;Acc:HGNC:30940] |
| ENSG00000075340 | HGLibA_00944 | 163 | 0.339697404 | ADD2 | adducin 2 [Source:HGNC Symbol;Acc:HGNC:244] |
| ENSG00000076351 | HGLibA_45185 | 163 | 0.339697404 | SLC46A1 | solute carrier family 46 member 1 [Source:HGNC Symbol;Acc:HGNC:30521] |
| ENSG00000080007 | HGLibA_12717 | 163 | 0.339697404 | DDX43 | DEAD-box helicase 43 [Source:HGNC Symbol;Acc:HGNC:18677] |
| ENSG00000084453 | HGLibA_45445 | 163 | 0.339697404 | SLCO1A2 | solute carrier organic anion transporter family member 1A2 [Source:HGNC Symbol;Acc:HGNC:10956] |
| ENSG00000087258 | HGLibA_19464 | 163 | 0.339697404 | GNAO1 | G protein subunit alpha o1 [Source:HGNC Symbol;Acc:HGNC:4389] |
| ENSG00000092850 | HGLibA_48973 | 163 | 0.339697404 | TEKT2 | tektin 2 [Source:HGNC Symbol;Acc:HGNC:11725] |
| ENSG00000100294 | HGLibA_28558 | 163 | 0.339697404 | MCAT | malonyl-CoA-acyl carrier protein transacylase [Source:HGNC Symbol;Acc:HGNC:29622] |
| ENSG00000100532 | HGLibA_09330 | 163 | 0.339697404 | CGRRF1 | cell growth regulator with ring finger domain 1 [Source:HGNC Symbol;Acc:HGNC:15528] |
| ENSG00000102048 | HGLibA_03253 | 163 | 0.339697404 | ASB9 | ankyrin repeat and SOCS box containing 9 [Source:HGNC Symbol;Acc:HGNC:17184] |
| ENSG00000104313 | HGLibA_15817 | 163 | 0.339697404 | EYA1 | EYA transcriptional coactivator and phosphatase 1 [Source:HGNC Symbol;Acc:HGNC:3519] |
| ENSG00000105011 | HGLibA_03283 | 163 | 0.339697404 | ASF1B | anti-silencing function 1B histone chaperone [Source:HGNC Symbol;Acc:HGNC:20996] |
| ENSG00000105063 | HGLibA_38220 | 163 | 0.339697404 | PPP6R1 | protein phosphatase 6 regulatory subunit 1 [Source:HGNC Symbol;Acc:HGNC:29195] |
| ENSG00000105737 | HGLibA_20294 | 163 | 0.339697404 | GRIK5 | glutamate ionotropic receptor kainate type subunit 5 [Source:HGNC Symbol;Acc:HGNC:4583] |
| ENSG00000107018 | HGLibA_41351 | 163 | 0.339697404 | RLN1 | relaxin 1 [Source:HGNC Symbol;Acc:HGNC:10026] |
| ENSG00000108370 | HGLibA_41112 | 163 | 0.339697404 | RGS9 | regulator of G protein signaling 9 [Source:HGNC Symbol;Acc:HGNC:10004] |
| ENSG00000114315 | HGLibA_21176 | 163 | 0.339697404 | HES1 | hes family bHLH transcription factor 1 [Source:HGNC Symbol;Acc:HGNC:5192] |

| | | | | | |
|---|---|---|---|---|---|
| ENSG00000115271 | HGLibA_18757 | 163 | 0.339697404 | GCA | grancalcin [Source:HGNC Symbol;Acc:HGNC:15990] |
| ENSG00000115468 | HGLibA_14575 | 163 | 0.339697404 | EFHD1 | EF-hand domain family member D1 [Source:HGNC Symbol;Acc:HGNC:29556] |
| ENSG00000116918 | HGLibA_51887 | 163 | 0.339697404 | TSNAX | translin associated factor X [Source:HGNC Symbol;Acc:HGNC:12380] |
| ENSG00000117000 | HGLibA_41345 | 163 | 0.339697404 | RLF | rearranged L-myc fusion [Source:HGNC Symbol;Acc:HGNC:10025] |
| ENSG00000117758 | HGLibA_47622 | 163 | 0.339697404 | STX12 | syntaxin 12 [Source:HGNC Symbol;Acc:HGNC:11430] |
| ENSG00000118298 | HGLibA_06800 | 163 | 0.339697404 | CA14 | carbonic anhydrase 14 [Source:HGNC Symbol;Acc:HGNC:1372] |
| ENSG00000120251 | HGLibA_20264 | 163 | 0.339697404 | GRIA2 | glutamate ionotropic receptor AMPA type subunit 2 [Source:HGNC Symbol;Acc:HGNC:4572] |
| ENSG00000120645 | HGLibA_23547 | 163 | 0.339697404 | IQSEC3 | IQ motif and Sec7 domain 3 [Source:HGNC Symbol;Acc:HGNC:29193] |
| ENSG00000123500 | HGLibA_10498 | 163 | 0.339697404 | COL10A1 | collagen type X alpha 1 chain [Source:HGNC Symbol;Acc:HGNC:2185] |
| ENSG00000125485 | HGLibA_12692 | 163 | 0.339697404 | DDX31 | DEAD-box helicase 31 [Source:HGNC Symbol;Acc:HGNC:16715] |
| ENSG00000125731 | HGLibA_43892 | 163 | 0.339697404 | SH2D3A | SH2 domain containing 3A [Source:HGNC Symbol;Acc:HGNC:16885] |
| ENSG00000127743 | HGLibA_22991 | 163 | 0.339697404 | IL17B | interleukin 17B [Source:HGNC Symbol;Acc:HGNC:5982] |
| ENSG00000133619 | HGLibA_25387 | 163 | 0.339697404 | KRBA1 | KRAB-A domain containing 1 [Source:HGNC Symbol;Acc:HGNC:22228] |
| ENSG00000134020 | HGLibA_36014 | 163 | 0.339697404 | PEBP4 | phosphatidylethanolamine binding protein 4 [Source:HGNC Symbol;Acc:HGNC:28319] |
| ENSG00000135605 | HGLibA_48938 | 163 | 0.339697404 | TEC | tec protein tyrosine kinase [Source:HGNC Symbol;Acc:HGNC:11719] |
| ENSG00000136371 | HGLibA_30392 | 163 | 0.339697404 | MTHFS | methenyltetrahydrofolate synthetase [Source:HGNC Symbol;Acc:HGNC:7437] |
| ENSG00000136574 | HGLibA_18674 | 163 | 0.339697404 | GATA4 | GATA binding protein 4 [Source:HGNC Symbol;Acc:HGNC:4173] |
| ENSG00000137135 | HGLibA_02861 | 163 | 0.339697404 | ARHGEF39 | Rho guanine nucleotide exchange factor 39 [Source:HGNC Symbol;Acc:HGNC:25909] |
| ENSG00000137496 | HGLibA_23025 | 163 | 0.339697404 | IL18BP | interleukin 18 binding protein [Source:HGNC Symbol;Acc:HGNC:5987] |
| ENSG00000139537 | HGLibA_07833 | 163 | 0.339697404 | CCDC65 | coiled-coil domain containing 65 [Source:HGNC Symbol;Acc:HGNC:29937] |
| ENSG00000140092 | HGLibA_16949 | 163 | 0.339697404 | FBLN5 | fibulin 5 [Source:HGNC Symbol;Acc:HGNC:3602] |
| ENSG00000141560 | HGLibA_17733 | 163 | 0.339697404 | FN3KRP | fructosamine 3 kinase related protein [Source:HGNC Symbol;Acc:HGNC:25700] |
| ENSG00000143811 | HGLibA_39675 | 163 | 0.339697404 | PYCR2 | pyrroline-5-carboxylate reductase 2 [Source:HGNC Symbol;Acc:HGNC:30262] |
| ENSG00000147853 | HGLibA_01381 | 163 | 0.339697404 | AK3 | adenylate kinase 3 [Source:HGNC Symbol;Acc:HGNC:17376] |
| ENSG00000148700 | HGLibA_00948 | 163 | 0.339697404 | ADD3 | adducin 3 [Source:HGNC Symbol;Acc:HGNC:245] |
| ENSG00000148985 | HGLibA_36211 | 163 | 0.339697404 | PGAP2 | post-GPI attachment to proteins 2 [Source:HGNC Symbol;Acc:HGNC:17893] |
| ENSG00000149289 | HGLibA_54958 | 163 | 0.339697404 | ZC3H12C | zinc finger CCCH-type containing 12C [Source:HGNC Symbol;Acc:HGNC:29362] |
| ENSG00000151322 | HGLibA_32401 | 163 | 0.339697404 | NPAS3 | neuronal PAS domain protein 3 [Source:HGNC Symbol;Acc:HGNC:19311] |
| ENSG00000151498 | HGLibA_00359 | 163 | 0.339697404 | ACAD8 | acyl-CoA dehydrogenase family member 8 [Source:HGNC Symbol;Acc:HGNC:87] |
| ENSG00000152601 | HGLibA_28509 | 163 | 0.339697404 | MBNL1 | muscleblind like splicing regulator 1 [Source:HGNC Symbol;Acc:HGNC:6923] |
| ENSG00000153902 | HGLibA_26402 | 163 | 0.339697404 | LGI4 | leucine rich repeat LGI family member 4 [Source:HGNC Symbol;Acc:HGNC:18712] |
| ENSG00000153933 | HGLibA_13066 | 163 | 0.339697404 | DGKE | diacylglycerol kinase epsilon [Source:HGNC Symbol;Acc:HGNC:2852] |
| ENSG00000156076 | HGLibA_54302 | 163 | 0.339697404 | WIF1 | WNT inhibitory factor 1 [Source:HGNC Symbol;Acc:HGNC:18081] |
| ENSG00000160993 | HGLibA_01654 | 163 | 0.339697404 | ALKBH4 | lysine demethylase [Source:HGNC Symbol;Acc:HGNC:21900]" |
| ENSG00000162241 | HGLibA_44750 | 163 | 0.339697404 | SLC25A45 | solute carrier family 25 member 45 [Source:HGNC Symbol;Acc:HGNC:27442] |
| ENSG00000162402 | HGLibA_53308 | 163 | 0.339697404 | USP24 | ubiquitin specific peptidase 24 [Source:HGNC Symbol;Acc:HGNC:12623] |
| ENSG00000163683 | HGLibA_45731 | 163 | 0.339697404 | SMIM14 | small integral membrane protein 14 [Source:HGNC Symbol;Acc:HGNC:27321] |
| ENSG00000164175 | HGLibA_45176 | 163 | 0.339697404 | SLC45A2 | solute carrier family 45 member 2 [Source:HGNC Symbol;Acc:HGNC:16472] |
| ENSG00000165118 | HGLibA_06750 | 163 | 0.339697404 | C9orf64 | chromosome 9 open reading frame 64 [Source:HGNC Symbol;Acc:HGNC:28144] |
| ENSG00000165516 | HGLibA_25096 | 163 | 0.339697404 | KLHDC2 | kelch domain containing 2 [Source:HGNC Symbol;Acc:HGNC:20231] |
| ENSG00000165801 | HGLibA_02868 | 163 | 0.339697404 | ARHGEF40 | Rho guanine nucleotide exchange factor 40 [Source:HGNC Symbol;Acc:HGNC:25516] |
| ENSG00000166295 | HGLibA_01876 | 163 | 0.339697404 | ANAPC16 | anaphase promoting complex subunit 16 [Source:HGNC Symbol;Acc:HGNC:26976] |
| ENSG00000166984 | HGLibA_48840 | 163 | 0.339697404 | TCP10L2 | t-complex 10 like 2 [Source:HGNC Symbol;Acc:HGNC:21254] |
| ENSG00000167491 | HGLibA_18685 | 163 | 0.339697404 | GATAD2A | GATA zinc finger domain containing 2A [Source:HGNC Symbol;Acc:HGNC:29989] |
| ENSG00000168066 | HGLibA_43688 | 163 | 0.339697404 | SF1 | splicing factor 1 [Source:HGNC Symbol;Acc:HGNC:12950] |
| ENSG00000169344 | HGLibA_52978 | 163 | 0.339697404 | UMOD | uromodulin [Source:HGNC Symbol;Acc:HGNC:12559] |
| ENSG00000169981 | HGLibA_55855 | 163 | 0.339697404 | ZNF35 | zinc finger protein 35 [Source:HGNC Symbol;Acc:HGNC:13099] |
| ENSG00000170419 | HGLibA_53861 | 163 | 0.339697404 | VSTM2A | V-set and transmembrane domain containing 2A [Source:HGNC Symbol;Acc:HGNC:28499] |
| ENSG00000170608 | HGLibA_17812 | 163 | 0.339697404 | FOXA3 | forkhead box A3 [Source:HGNC Symbol;Acc:HGNC:5023] |
| ENSG00000171246 | HGLibA_32533 | 163 | 0.339697404 | NPTX1 | neuronal pentraxin 1 [Source:HGNC Symbol;Acc:HGNC:7952] |
| ENSG00000172380 | HGLibA_19514 | 163 | 0.339697404 | GNG12 | G protein subunit gamma 12 [Source:HGNC Symbol;Acc:HGNC:19663] |
| ENSG00000172459 | HGLibA_34246 | 163 | 0.339697404 | OR5AR1 | olfactory receptor family 5 subfamily AR member 1 (gene/pseudogene) [Source:HGNC Symbol;Acc:HGNC:15260] |
| ENSG00000177464 | HGLibA_20042 | 163 | 0.339697404 | GPR4 | G protein-coupled receptor 4 [Source:HGNC Symbol;Acc:HGNC:4497] |
| ENSG00000179314 | HGLibA_54422 | 163 | 0.339697404 | WSCD1 | WSC domain containing 1 [Source:HGNC Symbol;Acc:HGNC:29060] |
| ENSG00000180884 | HGLibA_56769 | 163 | 0.339697404 | ZNF792 | zinc finger protein 792 [Source:HGNC Symbol;Acc:HGNC:24751] |
| ENSG00000182253 | HGLibA_48015 | 163 | 0.339697404 | SYNM | synemin [Source:HGNC Symbol;Acc:HGNC:24466] |
| ENSG00000182890 | HGLibA_19365 | 163 | 0.339697404 | GLUD2 | glutamate dehydrogenase 2 [Source:HGNC Symbol;Acc:HGNC:4336] |
| ENSG00000183305 | HGLibA_27909 | 163 | 0.339697404 | MAGEA2B | MAGE family member A2B [Source:HGNC Symbol;Acc:HGNC:19340] |
| ENSG00000185090 | HGLibA_28093 | 163 | 0.339697404 | MANEAL | mannosidase endo-alpha like [Source:HGNC Symbol;Acc:HGNC:26452] |
| ENSG00000186049 | HGLibA_25531 | 163 | 0.339697404 | KRT73 | keratin 73 [Source:HGNC Symbol;Acc:HGNC:28928] |
| ENSG00000187266 | HGLibA_15353 | 163 | 0.339697404 | EPOR | erythropoietin receptor [Source:HGNC Symbol;Acc:HGNC:3416] |
| ENSG00000187583 | HGLibA_37147 | 163 | 0.339697404 | PLEKHN1 | pleckstrin homology domain containing N1 [Source:HGNC Symbol;Acc:HGNC:25284] |
| ENSG00000188050 | HGLibA_41509 | 163 | 0.339697404 | RNF133 | ring finger protein 133 [Source:HGNC Symbol;Acc:HGNC:21154] |
| ENSG00000198346 | HGLibA_56805 | 163 | 0.339697404 | ZNF813 | zinc finger protein 813 [Source:HGNC Symbol;Acc:HGNC:33257] |
| ENSG00000205693 | HGLibA_28102 | 163 | 0.339697404 | MANSC4 | MANSC domain containing 4 [Source:HGNC Symbol;Acc:HGNC:40023] |
| ENSG00000206561 | HGLibA_10648 | 163 | 0.339697404 | COLQ | collagen like tail subunit of asymmetric acetylcholinesterase [Source:HGNC Symbol;Acc:HGNC:2226] |
| ENSG00000221910 | HGLibA_33792 | 163 | 0.339697404 | OR2F2 | olfactory receptor family 2 subfamily F member 2 [Source:HGNC Symbol;Acc:HGNC:8247] |
| ENSG00000243232 | HGLibA_35465 | 163 | 0.339697404 | PCDHAC2 | 2 [Source:HGNC Symbol;Acc:HGNC:8677]" |
| ENSG00000248099 | HGLibA_23383 | 163 | 0.339697404 | INSL3 | insulin like 3 [Source:HGNC Symbol;Acc:HGNC:6086] |
| ENSG00000258484 | HGLibA_46627 | 163 | 0.339697404 | SPESP1 | sperm equatorial segment protein 1 [Source:HGNC Symbol;Acc:HGNC:15570] |
| ENSG00000259332 | HGLibA_47233 | 163 | 0.339697404 | ST20-MTHFS | ST20-MTHFS readthrough [Source:HGNC Symbol;Acc:HGNC:44655] |
| ENSG00000273111 | HGLibA_27722 | 163 | 0.339697404 | LYPD4 | LY6/PLAUR domain containing 4 [Source:HGNC Symbol;Acc:HGNC:28659] |
| ENSG00000277149 | HGLibA_52484 | 163 | 0.339697404 | TYW1B | tRNA-yW synthesizing protein 1 homolog B [Source:HGNC Symbol;Acc:HGNC:33908] |
| ENSG00000278023 | HGLibA_40755 | 163 | 0.339697404 | RDM1 | RAD52 motif containing 1 [Source:HGNC Symbol;Acc:HGNC:19950] |
| ENSG00000279395 | HGLibA_34322 | 163 | 0.339697404 | OR5L1 | olfactory receptor family 5 subfamily L member 1 (gene/pseudogene) [Source:HGNC Symbol;Acc:HGNC:8350] |
| HIST2H3D | HGLibA_21546 | 163 | 0.339697404 | HIST2H3D | - |
| hsa-mir-105-2 | HGLibA_57183 | 163 | 0.339697404 | hsa-mir-105-2 | - |
| hsa-mir-1469 | HGLibA_57854 | 163 | 0.339697404 | hsa-mir-1469 | - |
| hsa-mir-15a | HGLibA_57940 | 163 | 0.339697404 | hsa-mir-15a | - |
| hsa-mir-302a | HGLibA_58436 | 163 | 0.339697404 | hsa-mir-302a | - |
| hsa-mir-4449 | HGLibA_60201 | 163 | 0.339697404 | hsa-mir-4449 | - |
| hsa-mir-4687 | HGLibA_60858 | 163 | 0.339697404 | hsa-mir-4687 | - |
| hsa-mir-4718 | HGLibA_60974 | 163 | 0.339697404 | hsa-mir-4718 | - |
| hsa-mir-4804 | HGLibA_61320 | 163 | 0.339697404 | hsa-mir-4804 | - |
| hsa-mir-496 | HGLibA_61386 | 163 | 0.339697404 | hsa-mir-496 | - |
| hsa-mir-5186 | HGLibA_61636 | 163 | 0.339697404 | hsa-mir-5186 | - |
| hsa-mir-603 | HGLibA_62504 | 163 | 0.339697404 | hsa-mir-603 | - |
| hsa-mir-615 | HGLibA_62695 | 163 | 0.339697404 | hsa-mir-615 | - |
| hsa-mir-641 | HGLibA_62804 | 163 | 0.339697404 | hsa-mir-641 | - |
| hsa-mir-6854 | HGLibA_63587 | 163 | 0.339697404 | hsa-mir-6854 | - |
| hsa-mir-8079 | HGLibA_64157 | 163 | 0.339697404 | hsa-mir-8079 | - |
| LOC100289561 | HGLibA_26843 | 163 | 0.339697404 | LOC100289561 | - |
| MTERFD3 | HGLibA_30346 | 163 | 0.339697404 | MTERFD3 | - |
| PVRL4 | HGLibA_39630 | 163 | 0.339697404 | PVRL4 | - |
| AES | HGLibA_01094 | 162 | 0.330652061 | AES | - |
| ANKRD32 | HGLibA_02066 | 162 | 0.330652061 | ANKRD32 | - |
| C17orf102 | HGLibA_05520 | 162 | 0.330652061 | C17orf102 | - |
| C22orf43 | HGLibA_06081 | 162 | 0.330652061 | C22orf43 | - |
| CCDC151 | HGLibA_07646 | 162 | 0.330652061 | CCDC151 | - |
| EMR1 | HGLibA_15082 | 162 | 0.330652061 | EMR1 | - |
| ENSG00000005187 | HGLibA_00578 | 162 | 0.330652061 | ACSM3 | acyl-CoA synthetase medium chain family member 3 [Source:HGNC Symbol;Acc:HGNC:10522] |
| ENSG00000010256 | HGLibA_53113 | 162 | 0.330652061 | UQCRC1 | ubiquinol-cytochrome c reductase core protein 1 [Source:HGNC Symbol;Acc:HGNC:12585] |
| ENSG00000017797 | HGLibA_40154 | 162 | 0.330652061 | RALBP1 | ralA binding protein 1 [Source:HGNC Symbol;Acc:HGNC:9841] |
| ENSG00000021488 | HGLibA_45385 | 162 | 0.330652061 | SLC7A9 | solute carrier family 7 member 9 [Source:HGNC Symbol;Acc:HGNC:11067] |
| ENSG00000032444 | HGLibA_37419 | 162 | 0.330652061 | PNPLA6 | patatin like phospholipase domain containing 6 [Source:HGNC Symbol;Acc:HGNC:16268] |
| ENSG00000039319 | HGLibA_55272 | 162 | 0.330652061 | ZFYVE16 | zinc finger FYVE-type containing 16 [Source:HGNC Symbol;Acc:HGNC:20756] |
| ENSG00000049540 | HGLibA_14952 | 162 | 0.330652061 | ELN | elastin [Source:HGNC Symbol;Acc:HGNC:3327] |
| ENSG00000074047 | HGLibA_19251 | 162 | 0.330652061 | GLI2 | GLI family zinc finger 2 [Source:HGNC Symbol;Acc:HGNC:4318] |
| ENSG00000079102 | HGLibA_42458 | 162 | 0.330652061 | RUNX1T1 | RUNX1 translocation partner 1 [Source:HGNC Symbol;Acc:HGNC:1535] |
| ENSG00000079150 | HGLibA_17585 | 162 | 0.330652061 | FKBP7 | FK506 binding protein 7 [Source:HGNC Symbol;Acc:HGNC:3723] |
| ENSG00000082438 | HGLibA_10464 | 162 | 0.330652061 | COBLL1 | cordon-bleu WH2 repeat protein like 1 [Source:HGNC Symbol;Acc:HGNC:23571] |
| ENSG00000084652 | HGLibA_52389 | 162 | 0.330652061 | TXLNA | taxilin alpha [Source:HGNC Symbol;Acc:HGNC:30685] |
| ENSG00000086015 | HGLibA_28402 | 162 | 0.330652061 | MAST2 | microtubule associated serine/threonine kinase 2 [Source:HGNC Symbol;Acc:HGNC:19035] |
| ENSG00000087085 | HGLibA_00436 | 162 | 0.330652061 | ACHE | acetylcholinesterase (Cartwright blood group) [Source:HGNC Symbol;Acc:HGNC:108] |
| ENSG00000090686 | HGLibA_53383 | 162 | 0.330652061 | USP48 | ubiquitin specific peptidase 48 [Source:HGNC Symbol;Acc:HGNC:18533] |
| ENSG00000091482 | HGLibA_45822 | 162 | 0.330652061 | SMPX | small muscle protein X-linked [Source:HGNC Symbol;Acc:HGNC:11122] |
| ENSG00000092009 | HGLibA_10176 | 162 | 0.330652061 | CMA1 | chymase 1 [Source:HGNC Symbol;Acc:HGNC:2097] |
| ENSG00000099995 | HGLibA_43692 | 162 | 0.330652061 | SF3A1 | splicing factor 3a subunit 1 [Source:HGNC Symbol;Acc:HGNC:10765] |
| ENSG00000100341 | HGLibA_37417 | 162 | 0.330652061 | PNPLA5 | patatin like phospholipase domain containing 5 [Source:HGNC Symbol;Acc:HGNC:24888] |
| ENSG00000101464 | HGLibA_36592 | 162 | 0.330652061 | PIGU | phosphatidylinositol glycan anchor biosynthesis class U [Source:HGNC Symbol;Acc:HGNC:15791] |
| ENSG00000102931 | HGLibA_02960 | 162 | 0.330652061 | ARL2BP | ADP ribosylation factor like GTPase 2 binding protein [Source:HGNC Symbol;Acc:HGNC:17146] |
| ENSG00000105290 | HGLibA_02440 | 162 | 0.330652061 | APLP1 | amyloid beta precursor like protein 1 [Source:HGNC Symbol;Acc:HGNC:597] |
| ENSG00000105612 | HGLibA_13664 | 162 | 0.330652061 | DNASE2 | lysosomal [Source:HGNC Symbol;Acc:HGNC:2960]" |
| ENSG00000105851 | HGLibA_36642 | 162 | 0.330652061 | PIK3CG | 5-bisphosphate 3-kinase catalytic subunit gamma [Source:HGNC Symbol;Acc:HGNC:8978]" |
| ENSG00000106948 | HGLibA_01463 | 162 | 0.330652061 | AKNA | AT-hook transcription factor [Source:HGNC Symbol;Acc:HGNC:24108] |
| ENSG00000108349 | HGLibA_07273 | 162 | 0.330652061 | CASC3 | exon junction complex subunit [Source:HGNC Symbol;Acc:HGNC:17040]" |
| ENSG00000111142 | HGLibA_28935 | 162 | 0.330652061 | METAP2 | methionyl aminopeptidase 2 [Source:HGNC Symbol;Acc:HGNC:16672] |
| ENSG00000112578 | HGLibA_05006 | 162 | 0.330652061 | BYSL | bystin like [Source:HGNC Symbol;Acc:HGNC:1157] |
| ENSG00000112769 | HGLibA_25950 | 162 | 0.330652061 | LAMA4 | laminin subunit alpha 4 [Source:HGNC Symbol;Acc:HGNC:6484] |
| ENSG00000113327 | HGLibA_18391 | 162 | 0.330652061 | GABRG2 | gamma-aminobutyric acid type A receptor gamma2 subunit [Source:HGNC Symbol;Acc:HGNC:4087] |
| ENSG00000115042 | HGLibA_15957 | 162 | 0.330652061 | FAHD2A | fumarylacetoacetate hydrolase domain containing 2A [Source:HGNC Symbol;Acc:HGNC:24252] |
| ENSG00000115317 | HGLibA_22410 | 162 | 0.330652061 | HTRA2 | HtrA serine peptidase 2 [Source:HGNC Symbol;Acc:HGNC:14348] |
| ENSG00000115514 | HGLibA_52428 | 162 | 0.330652061 | TXNDC9 | thioredoxin domain containing 9 [Source:HGNC Symbol;Acc:HGNC:24110] |
| ENSG00000117090 | HGLibA_44312 | 162 | 0.330652061 | SLAMF1 | signaling lymphocytic activation molecule family member 1 [Source:HGNC Symbol;Acc:HGNC:10903] |
| ENSG00000119508 | HGLibA_32632 | 162 | 0.330652061 | NR4A3 | nuclear receptor subfamily 4 group A member 3 [Source:HGNC Symbol;Acc:HGNC:7982] |

| | | | | | |
|---|---|---|---|---|---|
| ENSG00000120896 | HGLibA_46181 | 162 | 0.330652061 | SORBS3 | sorbin and SH3 domain containing 3 [Source:HGNC Symbol;Acc:HGNC:30907] |
| ENSG00000122971 | HGLibA_00370 | 162 | 0.330652061 | ACADS | acyl-CoA dehydrogenase short chain [Source:HGNC Symbol;Acc:HGNC:90] |
| ENSG00000126368 | HGLibA_32573 | 162 | 0.330652061 | NR1D1 | nuclear receptor subfamily 1 group D member 1 [Source:HGNC Symbol;Acc:HGNC:7962] |
| ENSG00000126432 | HGLibA_38420 | 162 | 0.330652061 | PRDX5 | peroxiredoxin 5 [Source:HGNC Symbol;Acc:HGNC:9355] |
| ENSG00000126460 | HGLibA_38873 | 162 | 0.330652061 | PRRG2 | proline rich and Gla domain 2 [Source:HGNC Symbol;Acc:HGNC:9470] |
| ENSG00000126746 | HGLibA_55895 | 162 | 0.330652061 | ZNF384 | zinc finger protein 384 [Source:HGNC Symbol;Acc:HGNC:11955] |
| ENSG00000129151 | HGLibA_04204 | 162 | 0.330652061 | BBOX1 | gamma-butyrobetaine hydroxylase 1 [Source:HGNC Symbol;Acc:HGNC:964] |
| ENSG00000129538 | HGLibA_41388 | 162 | 0.330652061 | RNASE1 | pancreatic [Source:HGNC Symbol;Acc:HGNC:10044]" |
| ENSG00000131050 | HGLibA_04714 | 162 | 0.330652061 | BPIFA2 | BPI fold containing family A member 2 [Source:HGNC Symbol;Acc:HGNC:16203] |
| ENSG00000131080 | HGLibA_14432 | 162 | 0.330652061 | EDA2R | ectodysplasin A2 receptor [Source:HGNC Symbol;Acc:HGNC:17756] |
| ENSG00000131127 | HGLibA_55487 | 162 | 0.330652061 | ZNF141 | zinc finger protein 141 [Source:HGNC Symbol;Acc:HGNC:12926] |
| ENSG00000131148 | HGLibA_15022 | 162 | 0.330652061 | EMC8 | ER membrane protein complex subunit 8 [Source:HGNC Symbol;Acc:HGNC:7864] |
| ENSG00000132275 | HGLibA_42283 | 162 | 0.330652061 | RRP8 | ribosomal RNA processing 8 [Source:HGNC Symbol;Acc:HGNC:29030] |
| ENSG00000134463 | HGLibA_14400 | 162 | 0.330652061 | ECHDC3 | enoyl-CoA hydratase domain containing 3 [Source:HGNC Symbol;Acc:HGNC:23489] |
| ENSG00000135111 | HGLibA_48667 | 162 | 0.330652061 | TBX3 | T-box 3 [Source:HGNC Symbol;Acc:HGNC:11602] |
| ENSG00000135476 | HGLibA_15572 | 162 | 0.330652061 | ESPL1 | separase [Source:HGNC Symbol;Acc:HGNC:16856]" |
| ENSG00000136848 | HGLibA_12270 | 162 | 0.330652061 | DAB2IP | DAB2 interacting protein [Source:HGNC Symbol;Acc:HGNC:17294] |
| ENSG00000137726 | HGLibA_18240 | 162 | 0.330652061 | FXYD6 | FXYD domain containing ion transport regulator 6 [Source:HGNC Symbol;Acc:HGNC:4030] |
| ENSG00000137817 | HGLibA_35242 | 162 | 0.330652061 | PARP6 | poly(ADP-ribose) polymerase family member 6 [Source:HGNC Symbol;Acc:HGNC:26921] |
| ENSG00000139180 | HGLibA_31475 | 162 | 0.330652061 | NDUFA9 | NADH:ubiquinone oxidoreductase subunit A9 [Source:HGNC Symbol;Acc:HGNC:7693] |
| ENSG00000139211 | HGLibA_01795 | 162 | 0.330652061 | AMIGO2 | adhesion molecule with Ig like domain 2 [Source:HGNC Symbol;Acc:HGNC:24073] |
| ENSG00000142606 | HGLibA_29470 | 162 | 0.330652061 | MMEL1 | membrane metalloendopeptidase like 1 [Source:HGNC Symbol;Acc:HGNC:14668] |
| ENSG00000143226 | HGLibA_17188 | 162 | 0.330652061 | FCGR2A | Fc fragment of IgG receptor IIa [Source:HGNC Symbol;Acc:HGNC:3616] |
| ENSG00000144647 | HGLibA_37659 | 162 | 0.330652061 | POMGNT2 | 4-) [Source:HGNC Symbol;Acc:HGNC:25902]" |
| ENSG00000145331 | HGLibA_51661 | 162 | 0.330652061 | TRMT10A | tRNA methyltransferase 10A [Source:HGNC Symbol;Acc:HGNC:28403] |
| ENSG00000150457 | HGLibA_26067 | 162 | 0.330652061 | LATS2 | large tumor suppressor kinase 2 [Source:HGNC Symbol;Acc:HGNC:6515] |
| ENSG00000152102 | HGLibA_16249 | 162 | 0.330652061 | FAM168B | family with sequence similarity 168 member B [Source:HGNC Symbol;Acc:HGNC:27016] |
| ENSG00000153896 | HGLibA_56359 | 162 | 0.330652061 | ZNF599 | zinc finger protein 599 [Source:HGNC Symbol;Acc:HGNC:26408] |
| ENSG00000154305 | HGLibA_29188 | 162 | 0.330652061 | MIA3 | MIA SH3 domain ER export factor 3 [Source:HGNC Symbol;Acc:HGNC:24008] |
| ENSG00000155890 | HGLibA_51499 | 162 | 0.330652061 | TRIM42 | tripartite motif containing 42 [Source:HGNC Symbol;Acc:HGNC:19014] |
| ENSG00000156171 | HGLibA_13947 | 162 | 0.330652061 | DRAM2 | DNA damage regulated autophagy modulator 2 [Source:HGNC Symbol;Acc:HGNC:28769] |
| ENSG00000157653 | HGLibA_06734 | 162 | 0.330652061 | C9orf43 | chromosome 9 open reading frame 43 [Source:HGNC Symbol;Acc:HGNC:23570] |
| ENSG00000159692 | HGLibA_11584 | 162 | 0.330652061 | CTBP1 | C-terminal binding protein 1 [Source:HGNC Symbol;Acc:HGNC:2494] |
| ENSG00000160161 | HGLibA_09713 | 162 | 0.330652061 | CILP2 | cartilage intermediate layer protein 2 [Source:HGNC Symbol;Acc:HGNC:24213] |
| ENSG00000161677 | HGLibA_23977 | 162 | 0.330652061 | JOSD2 | Josephin domain containing 2 [Source:HGNC Symbol;Acc:HGNC:28853] |
| ENSG00000161973 | HGLibA_07780 | 162 | 0.330652061 | CCDC42 | coiled-coil domain containing 42 [Source:HGNC Symbol;Acc:HGNC:26528] |
| ENSG00000162222 | HGLibA_52165 | 162 | 0.330652061 | TTC9C | tetratricopeptide repeat domain 9C [Source:HGNC Symbol;Acc:HGNC:28432] |
| ENSG00000162402 | HGLibA_53307 | 162 | 0.330652061 | USP24 | ubiquitin specific peptidase 24 [Source:HGNC Symbol;Acc:HGNC:12623] |
| ENSG00000162896 | HGLibA_36581 | 162 | 0.330652061 | PIGR | polymeric immunoglobulin receptor [Source:HGNC Symbol;Acc:HGNC:8968] |
| ENSG00000163071 | HGLibA_46439 | 162 | 0.330652061 | SPATA18 | spermatogenesis associated 18 [Source:HGNC Symbol;Acc:HGNC:29579] |
| ENSG00000163283 | HGLibA_01713 | 162 | 0.330652061 | ALPP | placental [Source:HGNC Symbol;Acc:HGNC:439]" |
| ENSG00000163565 | HGLibA_22592 | 162 | 0.330652061 | IFI16 | interferon gamma inducible protein 16 [Source:HGNC Symbol;Acc:HGNC:5395] |
| ENSG00000164924 | HGLibA_54753 | 162 | 0.330652061 | YWHAZ | tyrosine 3-monooxygenase/tryptophan 5-monooxygenase activation protein zeta [Source:HGNC Symbol;Acc:HGNC:12855] |
| ENSG00000165496 | HGLibA_41858 | 162 | 0.330652061 | RPL10L | ribosomal protein L10 like [Source:HGNC Symbol;Acc:HGNC:17976] |
| ENSG00000166922 | HGLibA_42905 | 162 | 0.330652061 | SCG5 | secretogranin V [Source:HGNC Symbol;Acc:HGNC:10816] |
| ENSG00000167608 | HGLibA_49757 | 162 | 0.330652061 | TMC4 | transmembrane channel like 4 [Source:HGNC Symbol;Acc:HGNC:22998] |
| ENSG00000169397 | HGLibA_41407 | 162 | 0.330652061 | RNASE3 | ribonuclease A family member 3 [Source:HGNC Symbol;Acc:HGNC:10046] |
| ENSG00000169884 | HGLibA_54349 | 162 | 0.330652061 | WNT10B | Wnt family member 10B [Source:HGNC Symbol;Acc:HGNC:12775] |
| ENSG00000169981 | HGLibA_55854 | 162 | 0.330652061 | ZNF35 | zinc finger protein 35 [Source:HGNC Symbol;Acc:HGNC:13099] |
| ENSG00000170836 | HGLibA_37969 | 162 | 0.330652061 | PPM1D | Mg2+/Mn2+ dependent 1D [Source:HGNC Symbol;Acc:HGNC:9277]" |
| ENSG00000171772 | HGLibA_47912 | 162 | 0.330652061 | SYCE1 | synaptonemal complex central element protein 1 [Source:HGNC Symbol;Acc:HGNC:28852] |
| ENSG00000172410 | HGLibA_23389 | 162 | 0.330652061 | INSL5 | insulin like 5 [Source:HGNC Symbol;Acc:HGNC:6088] |
| ENSG00000175662 | HGLibA_50951 | 162 | 0.330652061 | TOM1L2 | target of myb1 like 2 membrane trafficking protein [Source:HGNC Symbol;Acc:HGNC:11984] |
| ENSG00000177025 | HGLibA_05633 | 162 | 0.330652061 | C19orf18 | chromosome 19 open reading frame 18 [Source:HGNC Symbol;Acc:HGNC:28642] |
| ENSG00000177830 | HGLibA_09451 | 162 | 0.330652061 | CHID1 | chitinase domain containing 1 [Source:HGNC Symbol;Acc:HGNC:28474] |
| ENSG00000179546 | HGLibA_22361 | 162 | 0.330652061 | HTR1D | 5-hydroxytryptamine receptor 1D [Source:HGNC Symbol;Acc:HGNC:5289] |
| ENSG00000181552 | HGLibA_14452 | 162 | 0.330652061 | EDDM3B | epididymal protein 3B [Source:HGNC Symbol;Acc:HGNC:19223] |
| ENSG00000183206 | HGLibA_37722 | 162 | 0.330652061 | POTEC | POTE ankyrin domain family member C [Source:HGNC Symbol;Acc:HGNC:33894] |
| ENSG00000183576 | HGLibA_43647 | 162 | 0.330652061 | SETD3 | SET domain containing 3 [Source:HGNC Symbol;Acc:HGNC:20493] |
| ENSG00000184613 | HGLibA_31687 | 162 | 0.330652061 | NELL2 | neural EGFL like 2 [Source:HGNC Symbol;Acc:HGNC:7751] |
| ENSG00000185668 | HGLibA_37764 | 162 | 0.330652061 | POU3F1 | POU class 3 homeobox 1 [Source:HGNC Symbol;Acc:HGNC:9214] |
| ENSG00000186642 | HGLibA_35779 | 162 | 0.330652061 | PDE2A | phosphodiesterase 2A [Source:HGNC Symbol;Acc:HGNC:8777] |
| ENSG00000186867 | HGLibA_39739 | 162 | 0.330652061 | QRFPR | pyroglutamylated RFamide peptide receptor [Source:HGNC Symbol;Acc:HGNC:15565] |
| ENSG00000187118 | HGLibA_10184 | 162 | 0.330652061 | CMC1 | C-X9-C motif containing 1 [Source:HGNC Symbol;Acc:HGNC:28783] |
| ENSG00000188352 | HGLibA_17777 | 162 | 0.330652061 | FOCAD | focadhesin [Source:HGNC Symbol;Acc:HGNC:23377] |
| ENSG00000196083 | HGLibA_23051 | 162 | 0.330652061 | IL1RAP | interleukin 1 receptor accessory protein [Source:HGNC Symbol;Acc:HGNC:5995] |
| ENSG00000196266 | HGLibA_33523 | 162 | 0.330652061 | OR10J3 | olfactory receptor family 10 subfamily J member 3 [Source:HGNC Symbol;Acc:HGNC:14992] |
| ENSG00000196666 | HGLibA_16314 | 162 | 0.330652061 | FAM180B | family with sequence similarity 180 member B [Source:HGNC Symbol;Acc:HGNC:34451] |
| ENSG00000197724 | HGLibA_36361 | 162 | 0.330652061 | PHF2 | PHD finger protein 2 [Source:HGNC Symbol;Acc:HGNC:8920] |
| ENSG00000204478 | HGLibA_38308 | 162 | 0.330652061 | PRAMEF20 | PRAME family member 20 [Source:HGNC Symbol;Acc:HGNC:25224] |
| ENSG00000204946 | HGLibA_56742 | 162 | 0.330652061 | ZNF783 | zinc finger family member 783 [Source:HGNC Symbol;Acc:HGNC:27222] |
| ENSG00000205108 | HGLibA_16424 | 162 | 0.330652061 | FAM205A | family with sequence similarity 205 member A [Source:HGNC Symbol;Acc:HGNC:41911] |
| ENSG00000205129 | HGLibA_06338 | 162 | 0.330652061 | C4orf47 | chromosome 4 open reading frame 47 [Source:HGNC Symbol;Acc:HGNC:34346] |
| ENSG00000205710 | HGLibA_05528 | 162 | 0.330652061 | C17orf107 | chromosome 17 open reading frame 107 [Source:HGNC Symbol;Acc:HGNC:37238] |
| ENSG00000205929 | HGLibA_06046 | 162 | 0.330652061 | C21orf62 | chromosome 21 open reading frame 62 [Source:HGNC Symbol;Acc:HGNC:1305] |
| ENSG00000213625 | HGLibA_26326 | 162 | 0.330652061 | LEPROT | leptin receptor overlapping transcript [Source:HGNC Symbol;Acc:HGNC:29477] |
| ENSG00000214530 | HGLibA_47351 | 162 | 0.330652061 | STARD10 | StAR related lipid transfer domain containing 10 [Source:HGNC Symbol;Acc:HGNC:10666] |
| ENSG00000214891 | HGLibA_51581 | 162 | 0.330652061 | TRIM64C | tripartite motif containing 64C [Source:HGNC Symbol;Acc:HGNC:37148] |
| ENSG00000222014 | HGLibA_39977 | 162 | 0.330652061 | RAB6C | member RAS oncogene family [Source:HGNC Symbol;Acc:HGNC:16525]" |
| ENSG00000230062 | HGLibA_02142 | 162 | 0.330652061 | ANKRD66 | ankyrin repeat domain 66 [Source:HGNC Symbol;Acc:HGNC:44669] |
| ENSG00000240583 | HGLibA_02578 | 162 | 0.330652061 | AQP1 | aquaporin 1 (Colton blood group) [Source:HGNC Symbol;Acc:HGNC:633] |
| ENSG00000241598 | HGLibA_25797 | 162 | 0.330652061 | KRTAP5-4 | keratin associated protein 5-4 [Source:HGNC Symbol;Acc:HGNC:23599] |
| ENSG00000243509 | HGLibA_50786 | 162 | 0.330652061 | TNFRSF6B | TNF receptor superfamily member 6b [Source:HGNC Symbol;Acc:HGNC:11921] |
| ENSG00000256453 | HGLibA_13672 | 162 | 0.330652061 | DND1 | DND microRNA-mediated repression inhibitor 1 [Source:HGNC Symbol;Acc:HGNC:23799] |
| ENSG00000259332 | HGLibA_47235 | 162 | 0.330652061 | ST20-MTHFS | ST20-MTHFS readthrough [Source:HGNC Symbol;Acc:HGNC:44655] |
| ENSG00000266412 | HGLibA_31344 | 162 | 0.330652061 | NCOA4 | nuclear receptor coactivator 4 [Source:HGNC Symbol;Acc:HGNC:7671] |
| ENSG00000278318 | HGLibA_55630 | 162 | 0.330652061 | ZNF229 | zinc finger protein 229 [Source:HGNC Symbol;Acc:HGNC:13022] |
| ENSG00000284662 | HGLibA_33992 | 162 | 0.330652061 | OR4F16 | olfactory receptor family 4 subfamily F member 16 [Source:HGNC Symbol;Acc:HGNC:15079] |
| hsa-mir-1296 | HGLibA_57663 | 162 | 0.330652061 | hsa-mir-1296 | - |
| hsa-mir-184 | HGLibA_57998 | 162 | 0.330652061 | hsa-mir-184 | - |
| hsa-mir-298 | HGLibA_58400 | 162 | 0.330652061 | hsa-mir-298 | - |
| hsa-mir-3126 | HGLibA_58557 | 162 | 0.330652061 | hsa-mir-3126 | - |
| hsa-mir-3181 | HGLibA_58821 | 162 | 0.330652061 | hsa-mir-3181 | - |
| hsa-mir-3189 | HGLibA_58854 | 162 | 0.330652061 | hsa-mir-3189 | - |
| hsa-mir-3939 | HGLibA_59616 | 162 | 0.330652061 | hsa-mir-3939 | - |
| hsa-mir-4253 | HGLibA_59718 | 162 | 0.330652061 | hsa-mir-4253 | - |
| hsa-mir-4522 | HGLibA_60545 | 162 | 0.330652061 | hsa-mir-4522 | - |
| hsa-mir-4538 | HGLibA_60612 | 162 | 0.330652061 | hsa-mir-4538 | - |
| hsa-mir-4770 | HGLibA_61177 | 162 | 0.330652061 | hsa-mir-4770 | - |
| hsa-mir-548h-3 | HGLibA_62001 | 162 | 0.330652061 | hsa-mir-548h-3 | - |
| hsa-mir-593 | HGLibA_62467 | 162 | 0.330652061 | hsa-mir-593 | - |
| hsa-mir-6761 | HGLibA_63205 | 162 | 0.330652061 | hsa-mir-6761 | - |
| ICK | HGLibA_22509 | 162 | 0.330652061 | ICK | - |
| KIAA1407 | HGLibA_24749 | 162 | 0.330652061 | KIAA1407 | - |
| XAGE1D | HGLibA_54475 | 162 | 0.330652061 | XAGE1D | - |
| ACPT | HGLibA_00528 | 161 | 0.321606718 | ACPT | - |
| AGPAT9 | HGLibA_01236 | 161 | 0.321606718 | AGPAT9 | - |
| C20orf197 | HGLibA_06007 | 161 | 0.321606718 | C20orf197 | - |
| C8orf22 | HGLibA_06603 | 161 | 0.321606718 | C8orf22 | - |
| ENSG00000005249 | HGLibA_38529 | 161 | 0.321606718 | PRKAR2B | protein kinase cAMP-dependent type II regulatory subunit beta [Source:HGNC Symbol;Acc:HGNC:9392] |
| ENSG00000065150 | HGLibA_23468 | 161 | 0.321606718 | IPO5 | importin 5 [Source:HGNC Symbol;Acc:HGNC:6402] |
| ENSG00000065457 | HGLibA_00879 | 161 | 0.321606718 | ADAT1 | tRNA specific 1 [Source:HGNC Symbol;Acc:HGNC:228]" |
| ENSG00000066427 | HGLibA_03844 | 161 | 0.321606718 | ATXN3 | ataxin 3 [Source:HGNC Symbol;Acc:HGNC:7106] |
| ENSG00000068438 | HGLibA_18133 | 161 | 0.321606718 | FTSJ1 | FtsJ RNA methyltransferase homolog 1 [Source:HGNC Symbol;Acc:HGNC:13254] |
| ENSG00000069329 | HGLibA_53769 | 161 | 0.321606718 | VPS35 | retromer complex component [Source:HGNC Symbol;Acc:HGNC:13487]" |
| ENSG00000071051 | HGLibA_31307 | 161 | 0.321606718 | NCK2 | NCK adaptor protein 2 [Source:HGNC Symbol;Acc:HGNC:7665] |
| ENSG00000075568 | HGLibA_49929 | 161 | 0.321606718 | TMEM131 | transmembrane protein 131 [Source:HGNC Symbol;Acc:HGNC:30366] |
| ENSG00000076706 | HGLibA_28557 | 161 | 0.321606718 | MCAM | melanoma cell adhesion molecule [Source:HGNC Symbol;Acc:HGNC:6934] |
| ENSG00000083454 | HGLibA_34858 | 161 | 0.321606718 | P2RX5 | purinergic receptor P2X 5 [Source:HGNC Symbol;Acc:HGNC:8536] |
| ENSG00000091129 | HGLibA_32660 | 161 | 0.321606718 | NRCAM | neuronal cell adhesion molecule [Source:HGNC Symbol;Acc:HGNC:7994] |
| ENSG00000103264 | HGLibA_17068 | 161 | 0.321606718 | FBXO31 | F-box protein 31 [Source:HGNC Symbol;Acc:HGNC:16510] |
| ENSG00000104755 | HGLibA_00750 | 161 | 0.321606718 | ADAM2 | ADAM metallopeptidase domain 2 [Source:HGNC Symbol;Acc:HGNC:198] |
| ENSG00000106443 | HGLibA_36345 | 161 | 0.321606718 | PHF14 | PHD finger protein 14 [Source:HGNC Symbol;Acc:HGNC:22203] |
| ENSG00000106692 | HGLibA_17600 | 161 | 0.321606718 | FKTN | fukutin [Source:HGNC Symbol;Acc:HGNC:3622] |
| ENSG00000107317 | HGLibA_39309 | 161 | 0.321606718 | PTGDS | prostaglandin D2 synthase [Source:HGNC Symbol;Acc:HGNC:9592] |
| ENSG00000111644 | HGLibA_00534 | 161 | 0.321606718 | ACRBP | acrosin binding protein [Source:HGNC Symbol;Acc:HGNC:17195] |
| ENSG00000115468 | HGLibA_14577 | 161 | 0.321606718 | EFHD1 | EF-hand domain family member D1 [Source:HGNC Symbol;Acc:HGNC:29556] |
| ENSG00000118432 | HGLibA_10373 | 161 | 0.321606718 | CNR1 | cannabinoid receptor 1 [Source:HGNC Symbol;Acc:HGNC:2159] |
| ENSG00000118729 | HGLibA_07337 | 161 | 0.321606718 | CASQ2 | calsequestrin 2 [Source:HGNC Symbol;Acc:HGNC:1513] |
| ENSG00000120440 | HGLibA_52197 | 161 | 0.321606718 | TTLL2 | tubulin tyrosine ligase like 2 [Source:HGNC Symbol;Acc:HGNC:21211] |
| ENSG00000123562 | HGLibA_29664 | 161 | 0.321606718 | MORF4L2 | mortality factor 4 like 2 [Source:HGNC Symbol;Acc:HGNC:16849] |
| ENSG00000124215 | HGLibA_08674 | 161 | 0.321606718 | CDH26 | cadherin 26 [Source:HGNC Symbol;Acc:HGNC:15902] |
| ENSG00000124496 | HGLibA_51368 | 161 | 0.321606718 | TRERF1 | transcriptional regulating factor 1 [Source:HGNC Symbol;Acc:HGNC:18273] |
| ENSG00000128519 | HGLibA_48376 | 161 | 0.321606718 | TAS2R16 | taste 2 receptor member 16 [Source:HGNC Symbol;Acc:HGNC:14921] |
| ENSG00000128607 | HGLibA_25092 | 161 | 0.321606718 | KLHDC10 | kelch domain containing 10 [Source:HGNC Symbol;Acc:HGNC:22194] |

| | | | | | |
|---|---|---|---|---|---|
| ENSG00000129151 | HGLibA_04202 | 161 | 0.321606718 | BBOX1 | gamma-butyrobetaine hydroxylase 1 [Source:HGNC Symbol;Acc:HGNC:964] |
| ENSG00000130702 | HGLibA_25953 | 161 | 0.321606718 | LAMA5 | laminin subunit alpha 5 [Source:HGNC Symbol;Acc:HGNC:6485] |
| ENSG00000130762 | HGLibA_02821 | 161 | 0.321606718 | ARHGEF16 | Rho guanine nucleotide exchange factor 16 [Source:HGNC Symbol;Acc:HGNC:15515] |
| ENSG00000131127 | HGLibA_55486 | 161 | 0.321606718 | ZNF141 | zinc finger protein 141 [Source:HGNC Symbol;Acc:HGNC:12926] |
| ENSG00000131711 | HGLibA_28119 | 161 | 0.321606718 | MAP1B | microtubule associated protein 1B [Source:HGNC Symbol;Acc:HGNC:6836] |
| ENSG00000136861 | HGLibA_08786 | 161 | 0.321606718 | CDK5RAP2 | CDK5 regulatory subunit associated protein 2 [Source:HGNC Symbol;Acc:HGNC:18672] |
| ENSG00000140488 | HGLibA_09020 | 161 | 0.321606718 | CELF6 | CUGBP Elav-like family member 6 [Source:HGNC Symbol;Acc:HGNC:14059] |
| ENSG00000141194 | HGLibA_33964 | 161 | 0.321606718 | OR4D1 | olfactory receptor family 4 subfamily D member 1 [Source:HGNC Symbol;Acc:HGNC:8293] |
| ENSG00000143318 | HGLibA_07333 | 161 | 0.321606718 | CASQ1 | calsequestrin 1 [Source:HGNC Symbol;Acc:HGNC:1512] |
| ENSG00000144331 | HGLibA_55900 | 161 | 0.321606718 | ZNF385B | zinc finger protein 385B [Source:HGNC Symbol;Acc:HGNC:26332] |
| ENSG00000146858 | HGLibA_54994 | 161 | 0.321606718 | ZC3HAV1L | antiviral 1 like [Source:HGNC Symbol;Acc:HGNC:22423]" |
| ENSG00000148296 | HGLibA_47840 | 161 | 0.321606718 | SURF6 | surfeit 6 [Source:HGNC Symbol;Acc:HGNC:11478] |
| ENSG00000148399 | HGLibA_13855 | 161 | 0.321606718 | DPH7 | diphthamide biosynthesis 7 [Source:HGNC Symbol;Acc:HGNC:25199] |
| ENSG00000148680 | HGLibA_22405 | 161 | 0.321606718 | HTR7 | 5-hydroxytryptamine receptor 7 [Source:HGNC Symbol;Acc:HGNC:5302] |
| ENSG00000151834 | HGLibA_18359 | 161 | 0.321606718 | GABRA2 | gamma-aminobutyric acid type A receptor alpha2 subunit [Source:HGNC Symbol;Acc:HGNC:4076] |
| ENSG00000151952 | HGLibA_49936 | 161 | 0.321606718 | TMEM132D | transmembrane protein 132D [Source:HGNC Symbol;Acc:HGNC:29411] |
| ENSG00000152795 | HGLibA_21816 | 161 | 0.321606718 | HNRNPDL | heterogeneous nuclear ribonucleoprotein D like [Source:HGNC Symbol;Acc:HGNC:5037] |
| ENSG00000155897 | HGLibA_00930 | 161 | 0.321606718 | ADCY8 | adenylate cyclase 8 [Source:HGNC Symbol;Acc:HGNC:239] |
| ENSG00000155918 | HGLibA_40127 | 161 | 0.321606718 | RAET1L | retinoic acid early transcript 1L [Source:HGNC Symbol;Acc:HGNC:16798] |
| ENSG00000156140 | HGLibA_00826 | 161 | 0.321606718 | ADAMTS3 | ADAM metallopeptidase with thrombospondin type 1 motif 3 [Source:HGNC Symbol;Acc:HGNC:219] |
| ENSG00000156395 | HGLibA_46188 | 161 | 0.321606718 | SORCS3 | sortilin related VPS10 domain containing receptor 3 [Source:HGNC Symbol;Acc:HGNC:16699] |
| ENSG00000157426 | HGLibA_00076 | 161 | 0.321606718 | AASDH | aminoadipate-semialdehyde dehydrogenase [Source:HGNC Symbol;Acc:HGNC:23993] |
| ENSG00000157827 | HGLibA_17696 | 161 | 0.321606718 | FMNL2 | formin like 2 [Source:HGNC Symbol;Acc:HGNC:18267] |
| ENSG00000162777 | HGLibA_12944 | 161 | 0.321606718 | DENND2D | DENN domain containing 2D [Source:HGNC Symbol;Acc:HGNC:26192] |
| ENSG00000163444 | HGLibA_50106 | 161 | 0.321606718 | TMEM183A | transmembrane protein 183A [Source:HGNC Symbol;Acc:HGNC:20173] |
| ENSG00000163874 | HGLibA_54952 | 161 | 0.321606718 | ZC3H12A | zinc finger CCCH-type containing 12A [Source:HGNC Symbol;Acc:HGNC:26259] |
| ENSG00000164418 | HGLibA_20284 | 161 | 0.321606718 | GRIK2 | glutamate ionotropic receptor kainate type subunit 2 [Source:HGNC Symbol;Acc:HGNC:4580] |
| ENSG00000164978 | HGLibA_32968 | 161 | 0.321606718 | NUDT2 | nudix hydrolase 2 [Source:HGNC Symbol;Acc:HGNC:8049] |
| ENSG00000165059 | HGLibA_38509 | 161 | 0.321606718 | PRKACG | protein kinase cAMP-activated catalytic subunit gamma [Source:HGNC Symbol;Acc:HGNC:9382] |
| ENSG00000166926 | HGLibA_30160 | 161 | 0.321606718 | MS4A6E | membrane spanning 4-domains A6E [Source:HGNC Symbol;Acc:HGNC:14285] |
| ENSG00000166960 | HGLibA_07716 | 161 | 0.321606718 | CCDC178 | coiled-coil domain containing 178 [Source:HGNC Symbol;Acc:HGNC:29588] |
| ENSG00000167173 | HGLibA_05393 | 161 | 0.321606718 | C15orf39 | chromosome 15 open reading frame 39 [Source:HGNC Symbol;Acc:HGNC:24497] |
| ENSG00000167614 | HGLibA_52230 | 161 | 0.321606718 | TTYH1 | tweety family member 1 [Source:HGNC Symbol;Acc:HGNC:13476] |
| ENSG00000167842 | HGLibA_29302 | 161 | 0.321606718 | MIS12 | kinetochore complex component [Source:HGNC Symbol;Acc:HGNC:24967]" |
| ENSG00000168930 | HGLibA_51521 | 161 | 0.321606718 | TRIM49 | tripartite motif containing 49 [Source:HGNC Symbol;Acc:HGNC:13431] |
| ENSG00000169314 | HGLibA_06052 | 161 | 0.321606718 | C22orf15 | chromosome 22 open reading frame 15 [Source:HGNC Symbol;Acc:HGNC:15558] |
| ENSG00000171148 | HGLibA_48191 | 161 | 0.321606718 | TADA3 | transcriptional adaptor 3 [Source:HGNC Symbol;Acc:HGNC:19422] |
| ENSG00000171475 | HGLibA_54308 | 161 | 0.321606718 | WIPF2 | WAS/WASL interacting protein family member 2 [Source:HGNC Symbol;Acc:HGNC:30923] |
| ENSG00000171561 | HGLibA_33760 | 161 | 0.321606718 | OR2AT4 | olfactory receptor family 2 subfamily AT member 4 [Source:HGNC Symbol;Acc:HGNC:19620] |
| ENSG00000171561 | HGLibA_33761 | 161 | 0.321606718 | OR2AT4 | olfactory receptor family 2 subfamily AT member 4 [Source:HGNC Symbol;Acc:HGNC:19620] |
| ENSG00000176204 | HGLibA_27495 | 161 | 0.321606718 | LRRTM4 | leucine rich repeat transmembrane neuronal 4 [Source:HGNC Symbol;Acc:HGNC:19411] |
| ENSG00000176381 | HGLibA_38794 | 161 | 0.321606718 | PRR18 | proline rich 18 [Source:HGNC Symbol;Acc:HGNC:28574] |
| ENSG00000179630 | HGLibA_25908 | 161 | 0.321606718 | LACC1 | laccase domain containing 1 [Source:HGNC Symbol;Acc:HGNC:26789] |
| ENSG00000180772 | HGLibA_01264 | 161 | 0.321606718 | AGTR2 | angiotensin II receptor type 2 [Source:HGNC Symbol;Acc:HGNC:338] |
| ENSG00000181577 | HGLibA_06478 | 161 | 0.321606718 | C6orf223 | chromosome 6 open reading frame 223 [Source:HGNC Symbol;Acc:HGNC:28692] |
| ENSG00000182197 | HGLibA_15803 | 161 | 0.321606718 | EXT1 | exostosin glycosyltransferase 1 [Source:HGNC Symbol;Acc:HGNC:3512] |
| ENSG00000182854 | HGLibA_33990 | 161 | 0.321606718 | OR4F15 | olfactory receptor family 4 subfamily F member 15 [Source:HGNC Symbol;Acc:HGNC:15078] |
| ENSG00000183718 | HGLibA_51541 | 161 | 0.321606718 | TRIM52 | tripartite motif containing 52 [Source:HGNC Symbol;Acc:HGNC:19024] |
| ENSG00000185344 | HGLibA_03706 | 161 | 0.321606718 | ATP6V0A2 | ATPase H+ transporting V0 subunit a2 [Source:HGNC Symbol;Acc:HGNC:18481] |
| ENSG00000185668 | HGLibA_37765 | 161 | 0.321606718 | POU3F1 | POU class 3 homeobox 1 [Source:HGNC Symbol;Acc:HGNC:9214] |
| ENSG00000185808 | HGLibA_36577 | 161 | 0.321606718 | PIGP | phosphatidylinositol glycan anchor biosynthesis class P [Source:HGNC Symbol;Acc:HGNC:3046] |
| ENSG00000185899 | HGLibA_48424 | 161 | 0.321606718 | TAS2R60 | taste 2 receptor member 60 [Source:HGNC Symbol;Acc:HGNC:20639] |
| ENSG00000186103 | HGLibA_02690 | 161 | 0.321606718 | ARGFX | arginine-fifty homeobox [Source:HGNC Symbol;Acc:HGNC:30146] |
| ENSG00000187054 | HGLibA_50612 | 161 | 0.321606718 | TMPRSS11A | transmembrane serine protease 11A [Source:HGNC Symbol;Acc:HGNC:27954] |
| ENSG00000187950 | HGLibA_34792 | 161 | 0.321606718 | OVCH1 | ovochymase 1 [Source:HGNC Symbol;Acc:HGNC:23080] |
| ENSG00000189283 | HGLibA_17478 | 161 | 0.321606718 | FHIT | fragile histidine triad [Source:HGNC Symbol;Acc:HGNC:3701] |
| ENSG00000196748 | HGLibA_10111 | 161 | 0.321606718 | CLPSL2 | colipase like 2 [Source:HGNC Symbol;Acc:HGNC:21250] |
| ENSG00000197321 | HGLibA_47886 | 161 | 0.321606718 | SVIL | supervillin [Source:HGNC Symbol;Acc:HGNC:11480] |
| ENSG00000198173 | HGLibA_16615 | 161 | 0.321606718 | FAM47C | family with sequence similarity 47 member C [Source:HGNC Symbol;Acc:HGNC:25301] |
| ENSG00000198271 | HGLibA_25767 | 161 | 0.321606718 | KRTAP4-5 | keratin associated protein 4-5 [Source:HGNC Symbol;Acc:HGNC:18899] |
| ENSG00000198856 | HGLibA_34707 | 161 | 0.321606718 | OSTC | oligosaccharyltransferase complex non-catalytic subunit [Source:HGNC Symbol;Acc:HGNC:24448] |
| ENSG00000204390 | HGLibA_22276 | 161 | 0.321606718 | HSPA1L | heat shock protein family A (Hsp70) member 1 like [Source:HGNC Symbol;Acc:HGNC:5234] |
| ENSG00000213145 | HGLibA_11150 | 161 | 0.321606718 | CRIP1 | cysteine rich protein 1 [Source:HGNC Symbol;Acc:HGNC:2360] |
| ENSG00000214022 | HGLibA_40844 | 161 | 0.321606718 | REPIN1 | replication initiator 1 [Source:HGNC Symbol;Acc:HGNC:17922] |
| ENSG00000214078 | HGLibA_10954 | 161 | 0.321606718 | CPNE1 | copine 1 [Source:HGNC Symbol;Acc:HGNC:2314] |
| ENSG00000221989 | HGLibA_33729 | 161 | 0.321606718 | OR2A2 | olfactory receptor family 2 subfamily A member 2 [Source:HGNC Symbol;Acc:HGNC:8230] |
| ENSG00000258839 | HGLibA_28541 | 161 | 0.321606718 | MC1R | melanocortin 1 receptor [Source:HGNC Symbol;Acc:HGNC:6929] |
| hsa-mir-128-2 | HGLibA_57578 | 161 | 0.321606718 | hsa-mir-128-2 | - |
| hsa-mir-1468 | HGLibA_57850 | 161 | 0.321606718 | hsa-mir-1468 | - |
| hsa-mir-181a-2 | HGLibA_57961 | 161 | 0.321606718 | hsa-mir-181a-2 | - |
| hsa-mir-222 | HGLibA_58295 | 161 | 0.321606718 | hsa-mir-222 | - |
| hsa-mir-3195 | HGLibA_58879 | 161 | 0.321606718 | hsa-mir-3195 | - |
| hsa-mir-3650 | HGLibA_59146 | 161 | 0.321606718 | hsa-mir-3650 | - |
| hsa-mir-4284 | HGLibA_59844 | 161 | 0.321606718 | hsa-mir-4284 | - |
| hsa-mir-4322 | HGLibA_60010 | 161 | 0.321606718 | hsa-mir-4322 | - |
| hsa-mir-4323 | HGLibA_60017 | 161 | 0.321606718 | hsa-mir-4323 | - |
| hsa-mir-4454 | HGLibA_60223 | 161 | 0.321606718 | hsa-mir-4454 | - |
| hsa-mir-4635 | HGLibA_60647 | 161 | 0.321606718 | hsa-mir-4635 | - |
| hsa-mir-514a-1 | HGLibA_61588 | 161 | 0.321606718 | hsa-mir-514a-1 | - |
| hsa-mir-514a-2 | HGLibA_61591 | 161 | 0.321606718 | hsa-mir-514a-2 | - |
| hsa-mir-551a | HGLibA_62120 | 161 | 0.321606718 | hsa-mir-551a | - |
| hsa-mir-584 | HGLibA_62435 | 161 | 0.321606718 | hsa-mir-584 | - |
| hsa-mir-7-1 | HGLibA_63769 | 161 | 0.321606718 | hsa-mir-7-1 | - |
| hsa-mir-7153 | HGLibA_63841 | 161 | 0.321606718 | hsa-mir-7153 | - |
| LOC649330 | HGLibA_26982 | 161 | 0.321606718 | LOC649330 | - |
| SCXA | HGLibA_43079 | 161 | 0.321606718 | SCXA | - |
| 7-Mar | HGLibA_28346 | 160 | 0.312561375 | 7-Mar | - |
| AGXT2L1 | HGLibA_01276 | 160 | 0.312561375 | AGXT2L1 | - |
| C12orf68 | HGLibA_05286 | 160 | 0.312561375 | C12orf68 | - |
| C14orf166B | HGLibA_05337 | 160 | 0.312561375 | C14orf166B | - |
| C1orf111 | HGLibA_05748 | 160 | 0.312561375 | C1orf111 | - |
| C1orf63 | HGLibA_05881 | 160 | 0.312561375 | C1orf63 | - |
| CENPC1 | HGLibA_09047 | 160 | 0.312561375 | CENPC1 | - |
| ENSG00000009765 | HGLibA_23889 | 160 | 0.312561375 | IYD | iodotyrosine deiodinase [Source:HGNC Symbol;Acc:HGNC:21071] |
| ENSG00000034510 | HGLibA_50658 | 160 | 0.312561375 | TMSB10 | thymosin beta 10 [Source:HGNC Symbol;Acc:HGNC:11879] |
| ENSG00000036672 | HGLibA_53295 | 160 | 0.312561375 | USP2 | ubiquitin specific peptidase 2 [Source:HGNC Symbol;Acc:HGNC:12618] |
| ENSG00000036828 | HGLibA_07340 | 160 | 0.312561375 | CASR | calcium sensing receptor [Source:HGNC Symbol;Acc:HGNC:1514] |
| ENSG00000055950 | HGLibA_29988 | 160 | 0.312561375 | MRPL43 | mitochondrial ribosomal protein L43 [Source:HGNC Symbol;Acc:HGNC:14517] |
| ENSG00000064012 | HGLibA_07324 | 160 | 0.312561375 | CASP8 | caspase 8 [Source:HGNC Symbol;Acc:HGNC:1509] |
| ENSG00000065491 | HGLibA_48505 | 160 | 0.312561375 | TBC1D22B | TBC1 domain family member 22B [Source:HGNC Symbol;Acc:HGNC:21602] |
| ENSG00000068912 | HGLibA_15492 | 160 | 0.312561375 | ERLEC1 | endoplasmic reticulum lectin 1 [Source:HGNC Symbol;Acc:HGNC:25222] |
| ENSG00000070610 | HGLibA_18712 | 160 | 0.312561375 | GBA2 | glucosylceramidase beta 2 [Source:HGNC Symbol;Acc:HGNC:18986] |
| ENSG00000076003 | HGLibA_28619 | 160 | 0.312561375 | MCM6 | minichromosome maintenance complex component 6 [Source:HGNC Symbol;Acc:HGNC:6949] |
| ENSG00000078177 | HGLibA_30952 | 160 | 0.312561375 | N4BP2 | NEDD4 binding protein 2 [Source:HGNC Symbol;Acc:HGNC:29851] |
| ENSG00000079974 | HGLibA_40029 | 160 | 0.312561375 | RABL2B | member of RAS oncogene family like 2B [Source:HGNC Symbol;Acc:HGNC:9800]" |
| ENSG00000090054 | HGLibA_46891 | 160 | 0.312561375 | SPTLC1 | serine palmitoyltransferase long chain base subunit 1 [Source:HGNC Symbol;Acc:HGNC:11277] |
| ENSG00000099864 | HGLibA_35066 | 160 | 0.312561375 | PALM | paralemmin [Source:HGNC Symbol;Acc:HGNC:8594] |
| ENSG00000101294 | HGLibA_21662 | 160 | 0.312561375 | HM13 | histocompatibility minor 13 [Source:HGNC Symbol;Acc:HGNC:16435] |
| ENSG00000101888 | HGLibA_33176 | 160 | 0.312561375 | NXT2 | nuclear transport factor 2 like export factor 2 [Source:HGNC Symbol;Acc:HGNC:18151] |
| ENSG00000103253 | HGLibA_20817 | 160 | 0.312561375 | HAGHL | hydroxyacylglutathione hydrolase like [Source:HGNC Symbol;Acc:HGNC:14177] |
| ENSG00000104960 | HGLibA_39401 | 160 | 0.312561375 | PTOV1 | prostate tumor overexpressed 1 [Source:HGNC Symbol;Acc:HGNC:9632] |
| ENSG00000105254 | HGLibA_48582 | 160 | 0.312561375 | TBCB | tubulin folding cofactor B [Source:HGNC Symbol;Acc:HGNC:1989] |
| ENSG00000105429 | HGLibA_28856 | 160 | 0.312561375 | MEGF8 | multiple EGF like domains 8 [Source:HGNC Symbol;Acc:HGNC:3233] |
| ENSG00000107551 | HGLibA_40382 | 160 | 0.312561375 | RASSF4 | Ras association domain family member 4 [Source:HGNC Symbol;Acc:HGNC:20793] |
| ENSG00000109771 | HGLibA_27216 | 160 | 0.312561375 | LRP2BP | LRP2 binding protein [Source:HGNC Symbol;Acc:HGNC:25434] |
| ENSG00000112116 | HGLibA_23000 | 160 | 0.312561375 | IL17F | interleukin 17F [Source:HGNC Symbol;Acc:HGNC:16404] |
| ENSG00000120451 | HGLibA_46055 | 160 | 0.312561375 | SNX19 | sorting nexin 19 [Source:HGNC Symbol;Acc:HGNC:21532] |
| ENSG00000120616 | HGLibA_15265 | 160 | 0.312561375 | EPC1 | enhancer of polycomb homolog 1 [Source:HGNC Symbol;Acc:HGNC:19876] |
| ENSG00000126768 | HGLibA_49506 | 160 | 0.312561375 | TIMM17B | translocase of inner mitochondrial membrane 17B [Source:HGNC Symbol;Acc:HGNC:17310] |
| ENSG00000127329 | HGLibA_39487 | 160 | 0.312561375 | PTPRB | receptor type B [Source:HGNC Symbol;Acc:HGNC:9665]" |
| ENSG00000127423 | HGLibA_03868 | 160 | 0.312561375 | AUNIP | aurora kinase A and ninein interacting protein [Source:HGNC Symbol;Acc:HGNC:28363] |
| ENSG00000127838 | HGLibA_37347 | 160 | 0.312561375 | PNKD | MBL domain containing [Source:HGNC Symbol;Acc:HGNC:9153]" |
| ENSG00000130234 | HGLibA_00425 | 160 | 0.312561375 | ACE2 | angiotensin I converting enzyme 2 [Source:HGNC Symbol;Acc:HGNC:13557] |
| ENSG00000140983 | HGLibA_41205 | 160 | 0.312561375 | RHOT2 | ras homolog family member T2 [Source:HGNC Symbol;Acc:HGNC:21169] |
| ENSG00000143862 | HGLibA_02997 | 160 | 0.312561375 | ARL8A | ADP ribosylation factor like GTPase 8A [Source:HGNC Symbol;Acc:HGNC:25192] |
| ENSG00000145348 | HGLibA_48599 | 160 | 0.312561375 | TBCK | TBC1 domain containing kinase [Source:HGNC Symbol;Acc:HGNC:28261] |
| ENSG00000146834 | HGLibA_28900 | 160 | 0.312561375 | MEPCE | methylphosphate capping enzyme [Source:HGNC Symbol;Acc:HGNC:20247] |
| ENSG00000147036 | HGLibA_26011 | 160 | 0.312561375 | LANCL3 | LanC like 3 [Source:HGNC Symbol;Acc:HGNC:24767] |
| ENSG00000148516 | HGLibA_55136 | 160 | 0.312561375 | ZEB1 | zinc finger E-box binding homeobox 1 [Source:HGNC Symbol;Acc:HGNC:11642] |
| ENSG00000149527 | HGLibA_37012 | 160 | 0.312561375 | PLCH2 | phospholipase C eta 2 [Source:HGNC Symbol;Acc:HGNC:29037] |
| ENSG00000151491 | HGLibA_15373 | 160 | 0.312561375 | EPS8 | epidermal growth factor receptor pathway substrate 8 [Source:HGNC Symbol;Acc:HGNC:3420] |
| ENSG00000152076 | HGLibA_07864 | 160 | 0.312561375 | CCDC74B | coiled-coil domain containing 74B [Source:HGNC Symbol;Acc:HGNC:25267] |
| ENSG00000154736 | HGLibA_00832 | 160 | 0.312561375 | ADAMTS5 | ADAM metallopeptidase with thrombospondin type 1 motif 5 [Source:HGNC Symbol;Acc:HGNC:221] |
| ENSG00000155755 | HGLibA_50275 | 160 | 0.312561375 | TMEM237 | transmembrane protein 237 [Source:HGNC Symbol;Acc:HGNC:14432] |
| ENSG00000156515 | HGLibA_21580 | 160 | 0.312561375 | HK1 | hexokinase 1 [Source:HGNC Symbol;Acc:HGNC:4922] |

| | | | | | |
|---|---|---|---|---|---|
| ENSG00000157060 | HGLibA_44015 | 160 | 0.312561375 | SHCBP1L | SHC binding and spindle associated 1 like [Source:HGNC Symbol;Acc:HGNC:16788] |
| ENSG00000158966 | HGLibA_06871 | 160 | 0.312561375 | CACHD1 | cache domain containing 1 [Source:HGNC Symbol;Acc:HGNC:29314] |
| ENSG00000162695 | HGLibA_44918 | 160 | 0.312561375 | SLC30A7 | solute carrier family 30 member 7 [Source:HGNC Symbol;Acc:HGNC:19306] |
| ENSG00000163599 | HGLibA_11635 | 160 | 0.312561375 | CTLA4 | cytotoxic T-lymphocyte associated protein 4 [Source:HGNC Symbol;Acc:HGNC:2505] |
| ENSG00000164007 | HGLibA_09874 | 160 | 0.312561375 | CLDN19 | claudin 19 [Source:HGNC Symbol;Acc:HGNC:2040] |
| ENSG00000164823 | HGLibA_34691 | 160 | 0.312561375 | OSGIN2 | oxidative stress induced growth inhibitor family member 2 [Source:HGNC Symbol;Acc:HGNC:1355] |
| ENSG00000166069 | HGLibA_49794 | 160 | 0.312561375 | TMCO5A | transmembrane and coiled-coil domains 5A [Source:HGNC Symbol;Acc:HGNC:28558] |
| ENSG00000166340 | HGLibA_51160 | 160 | 0.312561375 | TPP1 | tripeptidyl peptidase 1 [Source:HGNC Symbol;Acc:HGNC:2073] |
| ENSG00000166828 | HGLibA_43021 | 160 | 0.312561375 | SCNN1G | sodium channel epithelial 1 gamma subunit [Source:HGNC Symbol;Acc:HGNC:10602] |
| ENSG00000166974 | HGLibA_28317 | 160 | 0.312561375 | MAPRE2 | microtubule associated protein RP/EB family member 2 [Source:HGNC Symbol;Acc:HGNC:6891] |
| ENSG00000168539 | HGLibA_09547 | 160 | 0.312561375 | CHRM1 | cholinergic receptor muscarinic 1 [Source:HGNC Symbol;Acc:HGNC:1950] |
| ENSG00000171425 | HGLibA_56307 | 160 | 0.312561375 | ZNF581 | zinc finger protein 581 [Source:HGNC Symbol;Acc:HGNC:25017] |
| ENSG00000171428 | HGLibA_31163 | 160 | 0.312561375 | NAT1 | N-acetyltransferase 1 [Source:HGNC Symbol;Acc:HGNC:7645] |
| ENSG00000172073 | HGLibA_49106 | 160 | 0.312561375 | TEX37 | testis expressed 37 [Source:HGNC Symbol;Acc:HGNC:26341] |
| ENSG00000172208 | HGLibA_34071 | 160 | 0.312561375 | OR4X2 | olfactory receptor family 4 subfamily X member 2 (gene/pseudogene) [Source:HGNC Symbol;Acc:HGNC:15184] |
| ENSG00000172551 | HGLibA_30560 | 160 | 0.312561375 | MUCL1 | mucin like 1 [Source:HGNC Symbol;Acc:HGNC:30588] |
| ENSG00000174080 | HGLibA_11704 | 160 | 0.312561375 | CTSF | cathepsin F [Source:HGNC Symbol;Acc:HGNC:2531] |
| ENSG00000174607 | HGLibA_52938 | 160 | 0.312561375 | UGT8 | UDP glycosyltransferase 8 [Source:HGNC Symbol;Acc:HGNC:12555] |
| ENSG00000175718 | HGLibA_40609 | 160 | 0.312561375 | RBMXL3 | RBMX like 3 [Source:HGNC Symbol;Acc:HGNC:26859] |
| ENSG00000176153 | HGLibA_20172 | 160 | 0.312561375 | GPX2 | glutathione peroxidase 2 [Source:HGNC Symbol;Acc:HGNC:4554] |
| ENSG00000176945 | HGLibA_30538 | 160 | 0.312561375 | MUC20 | cell surface associated [Source:HGNC Symbol;Acc:HGNC:23282]" |
| ENSG00000177076 | HGLibA_00430 | 160 | 0.312561375 | ACER2 | alkaline ceramidase 2 [Source:HGNC Symbol;Acc:HGNC:23675] |
| ENSG00000177283 | HGLibA_18283 | 160 | 0.312561375 | FZD8 | frizzled class receptor 8 [Source:HGNC Symbol;Acc:HGNC:4046] |
| ENSG00000177468 | HGLibA_33375 | 160 | 0.312561375 | OLIG3 | oligodendrocyte transcription factor 3 [Source:HGNC Symbol;Acc:HGNC:18003] |
| ENSG00000177910 | HGLibA_46491 | 160 | 0.312561375 | SPATA31C2 | SPATA31 subfamily C member 2 [Source:HGNC Symbol;Acc:HGNC:24508] |
| ENSG00000180304 | HGLibA_33215 | 160 | 0.312561375 | OAZ2 | ornithine decarboxylase antizyme 2 [Source:HGNC Symbol;Acc:HGNC:8096] |
| ENSG00000180708 | HGLibA_33532 | 160 | 0.312561375 | OR10K2 | olfactory receptor family 10 subfamily K member 2 [Source:HGNC Symbol;Acc:HGNC:14826] |
| ENSG00000180988 | HGLibA_34195 | 160 | 0.312561375 | OR52N2 | olfactory receptor family 52 subfamily N member 2 [Source:HGNC Symbol;Acc:HGNC:15228] |
| ENSG00000181023 | HGLibA_34222 | 160 | 0.312561375 | OR56B1 | olfactory receptor family 56 subfamily B member 1 [Source:HGNC Symbol;Acc:HGNC:15245] |
| ENSG00000182158 | HGLibA_11097 | 160 | 0.312561375 | CREB3L2 | cAMP responsive element binding protein 3 like 2 [Source:HGNC Symbol;Acc:HGNC:23720] |
| ENSG00000182534 | HGLibA_30626 | 160 | 0.312561375 | MXRA7 | matrix remodeling associated 7 [Source:HGNC Symbol;Acc:HGNC:7541] |
| ENSG00000183172 | HGLibA_45689 | 160 | 0.312561375 | SMDT1 | single-pass membrane protein with aspartate rich tail 1 [Source:HGNC Symbol;Acc:HGNC:25055] |
| ENSG00000183579 | HGLibA_56945 | 160 | 0.312561375 | ZNRF3 | zinc and ring finger 3 [Source:HGNC Symbol;Acc:HGNC:18126] |
| ENSG00000184508 | HGLibA_21036 | 160 | 0.312561375 | HDDC3 | HD domain containing 3 [Source:HGNC Symbol;Acc:HGNC:30522] |
| ENSG00000185915 | HGLibA_25193 | 160 | 0.312561375 | KLHL34 | kelch like family member 34 [Source:HGNC Symbol;Acc:HGNC:26634] |
| ENSG00000186832 | HGLibA_25432 | 160 | 0.312561375 | KRT16 | keratin 16 [Source:HGNC Symbol;Acc:HGNC:6423] |
| ENSG00000186891 | HGLibA_50765 | 160 | 0.312561375 | TNFRSF18 | TNF receptor superfamily member 18 [Source:HGNC Symbol;Acc:HGNC:11914] |
| ENSG00000187714 | HGLibA_44491 | 160 | 0.312561375 | SLC18A3 | solute carrier family 18 member A3 [Source:HGNC Symbol;Acc:HGNC:10936] |
| ENSG00000196341 | HGLibA_34508 | 160 | 0.312561375 | OR8D1 | olfactory receptor family 8 subfamily D member 1 [Source:HGNC Symbol;Acc:HGNC:8481] |
| ENSG00000196975 | HGLibA_02241 | 160 | 0.312561375 | ANXA4 | annexin A4 [Source:HGNC Symbol;Acc:HGNC:542] |
| ENSG00000197265 | HGLibA_20535 | 160 | 0.312561375 | GTF2E2 | general transcription factor IIE subunit 2 [Source:HGNC Symbol;Acc:HGNC:4651] |
| ENSG00000198146 | HGLibA_56703 | 160 | 0.312561375 | ZNF770 | zinc finger protein 770 [Source:HGNC Symbol;Acc:HGNC:26061] |
| ENSG00000204382 | HGLibA_54468 | 160 | 0.312561375 | XAGE1B | X antigen family member 1B [Source:HGNC Symbol;Acc:HGNC:25400] |
| ENSG00000204687 | HGLibA_28392 | 160 | 0.312561375 | MAS1L | G protein-coupled receptor [Source:HGNC Symbol;Acc:HGNC:13961]" |
| ENSG00000213658 | HGLibA_26057 | 160 | 0.312561375 | LAT | linker for activation of T cells [Source:HGNC Symbol;Acc:HGNC:18874] |
| ENSG00000231500 | HGLibA_42093 | 160 | 0.312561375 | RPS18 | ribosomal protein S18 [Source:HGNC Symbol;Acc:HGNC:10401] |
| ENSG00000240247 | HGLibA_12794 | 160 | 0.312561375 | DEFA1B | defensin alpha 1B [Source:HGNC Symbol;Acc:HGNC:33596] |
| ENSG00000240764 | HGLibA_35577 | 160 | 0.312561375 | PCDHGC5 | 5 [Source:HGNC Symbol;Acc:HGNC:8718]" |
| ENSG00000269190 | HGLibA_17035 | 160 | 0.312561375 | FBXO17 | F-box protein 17 [Source:HGNC Symbol;Acc:HGNC:18754] |
| HIST1H2AJ | HGLibA_21398 | 160 | 0.312561375 | HIST1H2AJ | - |
| hsa-mir-1285-2 | HGLibA_57603 | 160 | 0.312561375 | hsa-mir-1285-2 | - |
| hsa-mir-146b | HGLibA_57862 | 160 | 0.312561375 | hsa-mir-146b | - |
| hsa-mir-153-1 | HGLibA_57907 | 160 | 0.312561375 | hsa-mir-153-1 | - |
| hsa-mir-186 | HGLibA_58005 | 160 | 0.312561375 | hsa-mir-186 | - |
| hsa-mir-195 | HGLibA_58088 | 160 | 0.312561375 | hsa-mir-195 | - |
| hsa-mir-205 | HGLibA_58178 | 160 | 0.312561375 | hsa-mir-205 | - |
| hsa-mir-3683 | HGLibA_59286 | 160 | 0.312561375 | hsa-mir-3683 | - |
| hsa-mir-548x-2 | HGLibA_62083 | 160 | 0.312561375 | hsa-mir-548x-2 | - |
| hsa-mir-6513 | HGLibA_62927 | 160 | 0.312561375 | hsa-mir-6513 | - |
| hsa-mir-6775 | HGLibA_63274 | 160 | 0.312561375 | hsa-mir-6775 | - |
| RSG1 | HGLibA_42314 | 160 | 0.312561375 | RSG1 | - |
| ACRC | HGLibA_00537 | 159 | 0.303516032 | ACRC | - |
| C14orf105 | HGLibA_05318 | 159 | 0.303516032 | C14orf105 | - |
| C2orf54 | HGLibA_06148 | 159 | 0.303516032 | C2orf54 | - |
| C6orf57 | HGLibA_06498 | 159 | 0.303516032 | C6orf57 | - |
| C9orf96 | HGLibA_06781 | 159 | 0.303516032 | C9orf96 | - |
| CYR61 | HGLibA_12219 | 159 | 0.303516032 | CYR61 | - |
| ENSG00000002587 | HGLibA_22122 | 159 | 0.303516032 | HS3ST1 | heparan sulfate-glucosamine 3-sulfotransferase 1 [Source:HGNC Symbol;Acc:HGNC:5194] |
| ENSG00000003393 | HGLibA_01718 | 159 | 0.303516032 | ALS2 | alsin Rho guanine nucleotide exchange factor [Source:HGNC Symbol;Acc:HGNC:443]" |
| ENSG00000003402 | HGLibA_09286 | 159 | 0.303516032 | CFLAR | CASP8 and FADD like apoptosis regulator [Source:HGNC Symbol;Acc:HGNC:1876] |
| ENSG00000004700 | HGLibA_40768 | 159 | 0.303516032 | RECQL | RecQ like helicase [Source:HGNC Symbol;Acc:HGNC:9948] |
| ENSG00000012660 | HGLibA_14969 | 159 | 0.303516032 | ELOVL5 | ELOVL fatty acid elongase 5 [Source:HGNC Symbol;Acc:HGNC:21308] |
| ENSG00000030582 | HGLibA_20366 | 159 | 0.303516032 | GRN | granulin precursor [Source:HGNC Symbol;Acc:HGNC:4601] |
| ENSG00000062716 | HGLibA_53695 | 159 | 0.303516032 | VMP1 | vacuole membrane protein 1 [Source:HGNC Symbol;Acc:HGNC:29559] |
| ENSG00000068308 | HGLibA_34767 | 159 | 0.303516032 | OTUD5 | OTU deubiquitinase 5 [Source:HGNC Symbol;Acc:HGNC:25402] |
| ENSG00000074603 | HGLibA_13882 | 159 | 0.303516032 | DPP8 | dipeptidyl peptidase 8 [Source:HGNC Symbol;Acc:HGNC:16490] |
| ENSG00000075884 | HGLibA_02710 | 159 | 0.303516032 | ARHGAP15 | Rho GTPase activating protein 15 [Source:HGNC Symbol;Acc:HGNC:21030] |
| ENSG00000077080 | HGLibA_00624 | 159 | 0.303516032 | ACTL6B | actin like 6B [Source:HGNC Symbol;Acc:HGNC:160] |
| ENSG00000078900 | HGLibA_51096 | 159 | 0.303516032 | TP73 | tumor protein p73 [Source:HGNC Symbol;Acc:HGNC:12003] |
| ENSG00000082805 | HGLibA_15424 | 159 | 0.303516032 | ERC1 | ELKS/RAB6-interacting/CAST family member 1 [Source:HGNC Symbol;Acc:HGNC:17072] |
| ENSG00000084093 | HGLibA_40870 | 159 | 0.303516032 | REST | RE1 silencing transcription factor [Source:HGNC Symbol;Acc:HGNC:9966] |
| ENSG00000100258 | HGLibA_26687 | 159 | 0.303516032 | LMF2 | lipase maturation factor 2 [Source:HGNC Symbol;Acc:HGNC:25096] |
| ENSG00000101850 | HGLibA_19916 | 159 | 0.303516032 | GPR143 | G protein-coupled receptor 143 [Source:HGNC Symbol;Acc:HGNC:20145] |
| ENSG00000102109 | HGLibA_35669 | 159 | 0.303516032 | PCSK1N | proprotein convertase subtilisin/kexin type 1 inhibitor [Source:HGNC Symbol;Acc:HGNC:17301] |
| ENSG00000102189 | HGLibA_14483 | 159 | 0.303516032 | EEA1 | early endosome antigen 1 [Source:HGNC Symbol;Acc:HGNC:3185] |
| ENSG00000103313 | HGLibA_28843 | 159 | 0.303516032 | MEFV | pyrin innate immunity regulator [Source:HGNC Symbol;Acc:HGNC:6998]" |
| ENSG00000105245 | HGLibA_33015 | 159 | 0.303516032 | NUMBL | endocytic adaptor protein [Source:HGNC Symbol;Acc:HGNC:8061]" |
| ENSG00000105483 | HGLibA_07239 | 159 | 0.303516032 | CARD8 | caspase recruitment domain family member 8 [Source:HGNC Symbol;Acc:HGNC:17057] |
| ENSG00000105519 | HGLibA_07196 | 159 | 0.303516032 | CAPS | calcyphosine [Source:HGNC Symbol;Acc:HGNC:1487] |
| ENSG00000105723 | HGLibA_20427 | 159 | 0.303516032 | GSK3A | glycogen synthase kinase 3 alpha [Source:HGNC Symbol;Acc:HGNC:4616] |
| ENSG00000105953 | HGLibA_33301 | 159 | 0.303516032 | OGDH | oxoglutarate dehydrogenase [Source:HGNC Symbol;Acc:HGNC:8124] |
| ENSG00000106780 | HGLibA_28860 | 159 | 0.303516032 | MEGF9 | multiple EGF like domains 9 [Source:HGNC Symbol;Acc:HGNC:3234] |
| ENSG00000108384 | HGLibA_40089 | 159 | 0.303516032 | RAD51C | RAD51 paralog C [Source:HGNC Symbol;Acc:HGNC:9820] |
| ENSG00000110011 | HGLibA_13622 | 159 | 0.303516032 | DNAJC4 | DnaJ heat shock protein family (Hsp40) member C4 [Source:HGNC Symbol;Acc:HGNC:5271] |
| ENSG00000112667 | HGLibA_13712 | 159 | 0.303516032 | DNPH1 | 2'-deoxynucleoside 5'-phosphate N-hydrolase 1 [Source:HGNC Symbol;Acc:HGNC:21218] |
| ENSG00000115548 | HGLibA_24500 | 159 | 0.303516032 | KDM3A | lysine demethylase 3A [Source:HGNC Symbol;Acc:HGNC:20815] |
| ENSG00000117971 | HGLibA_09598 | 159 | 0.303516032 | CHRNB4 | cholinergic receptor nicotinic beta 4 subunit [Source:HGNC Symbol;Acc:HGNC:1964] |
| ENSG00000118156 | HGLibA_56192 | 159 | 0.303516032 | ZNF541 | zinc finger protein 541 [Source:HGNC Symbol;Acc:HGNC:25294] |
| ENSG00000119396 | HGLibA_39814 | 159 | 0.303516032 | RAB14 | member RAS oncogene family [Source:HGNC Symbol;Acc:HGNC:16524]" |
| ENSG00000119396 | HGLibA_39815 | 159 | 0.303516032 | RAB14 | member RAS oncogene family [Source:HGNC Symbol;Acc:HGNC:16524]" |
| ENSG00000122375 | HGLibA_33437 | 159 | 0.303516032 | OPN4 | opsin 4 [Source:HGNC Symbol;Acc:HGNC:14449] |
| ENSG00000123843 | HGLibA_06292 | 159 | 0.303516032 | C4BPB | complement component 4 binding protein beta [Source:HGNC Symbol;Acc:HGNC:1328] |
| ENSG00000124302 | HGLibA_09649 | 159 | 0.303516032 | CHST8 | carbohydrate sulfotransferase 8 [Source:HGNC Symbol;Acc:HGNC:15993] |
| ENSG00000125827 | HGLibA_50696 | 159 | 0.303516032 | TMX4 | thioredoxin related transmembrane protein 4 [Source:HGNC Symbol;Acc:HGNC:25237] |
| ENSG00000126091 | HGLibA_47243 | 159 | 0.303516032 | ST3GAL3 | 3-sialyltransferase 3 [Source:HGNC Symbol;Acc:HGNC:10866]" |
| ENSG00000128052 | HGLibA_24542 | 159 | 0.303516032 | KDR | kinase insert domain receptor [Source:HGNC Symbol;Acc:HGNC:6307] |
| ENSG00000128268 | HGLibA_29139 | 159 | 0.303516032 | MGAT3 | 4-N-acetylglucosaminyltransferase [Source:HGNC Symbol;Acc:HGNC:7046]" |
| ENSG00000130764 | HGLibA_27371 | 159 | 0.303516032 | LRRC47 | leucine rich repeat containing 47 [Source:HGNC Symbol;Acc:HGNC:29207] |
| ENSG00000131591 | HGLibA_05783 | 159 | 0.303516032 | C1orf159 | chromosome 1 open reading frame 159 [Source:HGNC Symbol;Acc:HGNC:26062] |
| ENSG00000134463 | HGLibA_14401 | 159 | 0.303516032 | ECHDC3 | enoyl-CoA hydratase domain containing 3 [Source:HGNC Symbol;Acc:HGNC:23489] |
| ENSG00000134827 | HGLibA_48821 | 159 | 0.303516032 | TCN1 | transcobalamin 1 [Source:HGNC Symbol;Acc:HGNC:11652] |
| ENSG00000135903 | HGLibA_35302 | 159 | 0.303516032 | PAX3 | paired box 3 [Source:HGNC Symbol;Acc:HGNC:8617] |
| ENSG00000137154 | HGLibA_42156 | 159 | 0.303516032 | RPS6 | ribosomal protein S6 [Source:HGNC Symbol;Acc:HGNC:10429] |
| ENSG00000137513 | HGLibA_31157 | 159 | 0.303516032 | NARS2 | mitochondrial [Source:HGNC Symbol;Acc:HGNC:26274]" |
| ENSG00000137558 | HGLibA_36483 | 159 | 0.303516032 | PI15 | peptidase inhibitor 15 [Source:HGNC Symbol;Acc:HGNC:8946] |
| ENSG00000138080 | HGLibA_15048 | 159 | 0.303516032 | EMILIN1 | elastin microfibril interfacer 1 [Source:HGNC Symbol;Acc:HGNC:19880] |
| ENSG00000138180 | HGLibA_09143 | 159 | 0.303516032 | CEP55 | centrosomal protein 55 [Source:HGNC Symbol;Acc:HGNC:1161] |
| ENSG00000138468 | HGLibA_43401 | 159 | 0.303516032 | SENP7 | SUMO specific peptidase 7 [Source:HGNC Symbol;Acc:HGNC:30402] |
| ENSG00000139354 | HGLibA_18650 | 159 | 0.303516032 | GAS2L3 | growth arrest specific 2 like 3 [Source:HGNC Symbol;Acc:HGNC:27475] |
| ENSG00000140043 | HGLibA_39356 | 159 | 0.303516032 | PTGR2 | prostaglandin reductase 2 [Source:HGNC Symbol;Acc:HGNC:20149] |
| ENSG00000140332 | HGLibA_49614 | 159 | 0.303516032 | TLE3 | transducin like enhancer of split 3 [Source:HGNC Symbol;Acc:HGNC:11839] |
| ENSG00000140839 | HGLibA_09947 | 159 | 0.303516032 | CLEC18B | C-type lectin domain family 18 member B [Source:HGNC Symbol;Acc:HGNC:33849] |
| ENSG00000141002 | HGLibA_48782 | 159 | 0.303516032 | TCF25 | transcription factor 25 [Source:HGNC Symbol;Acc:HGNC:29181] |
| ENSG00000141013 | HGLibA_18660 | 159 | 0.303516032 | GAS8 | growth arrest specific 8 [Source:HGNC Symbol;Acc:HGNC:4166] |
| ENSG00000141570 | HGLibA_07465 | 159 | 0.303516032 | CBX8 | chromobox 8 [Source:HGNC Symbol;Acc:HGNC:15962] |
| ENSG00000141668 | HGLibA_07410 | 159 | 0.303516032 | CBLN2 | cerebellin 2 precursor [Source:HGNC Symbol;Acc:HGNC:1544] |
| ENSG00000142515 | HGLibA_25254 | 159 | 0.303516032 | KLK3 | kallikrein related peptidase 3 [Source:HGNC Symbol;Acc:HGNC:6364] |
| ENSG00000144355 | HGLibA_13373 | 159 | 0.303516032 | DLX1 | distal-less homeobox 1 [Source:HGNC Symbol;Acc:HGNC:2914] |
| ENSG00000144401 | HGLibA_28975 | 159 | 0.303516032 | METTL21A | methyltransferase like 21A [Source:HGNC Symbol;Acc:HGNC:30476] |
| ENSG00000146376 | HGLibA_02717 | 159 | 0.303516032 | ARHGAP18 | Rho GTPase activating protein 18 [Source:HGNC Symbol;Acc:HGNC:21035] |
| ENSG00000148290 | HGLibA_47833 | 159 | 0.303516032 | SURF1 | cytochrome c oxidase assembly factor [Source:HGNC Symbol;Acc:HGNC:11474]" |
| ENSG00000150636 | HGLibA_07511 | 159 | 0.303516032 | CCDC102B | coiled-coil domain containing 102B [Source:HGNC Symbol;Acc:HGNC:26295] |
| ENSG00000155100 | HGLibA_34774 | 159 | 0.303516032 | OTUD6B | OTU domain containing 6B [Source:HGNC Symbol;Acc:HGNC:24281] |
| ENSG00000155719 | HGLibA_34724 | 159 | 0.303516032 | OTOA | otoancorin [Source:HGNC Symbol;Acc:HGNC:16378] |
| ENSG00000156284 | HGLibA_09910 | 159 | 0.303516032 | CLDN8 | claudin 8 [Source:HGNC Symbol;Acc:HGNC:2050] |

| | | | | | |
|---|---|---|---|---|---|
| ENSG00000158164 | HGLibA_50661 | 159 | 0.303516032 | TMSB15A | thymosin beta 15a [Source:HGNC Symbol;Acc:HGNC:30744] |
| ENSG00000159167 | HGLibA_47413 | 159 | 0.303516032 | STC1 | stanniocalcin 1 [Source:HGNC Symbol;Acc:HGNC:11373] |
| ENSG00000160182 | HGLibA_49170 | 159 | 0.303516032 | TFF1 | trefoil factor 1 [Source:HGNC Symbol;Acc:HGNC:11755] |
| ENSG00000161653 | HGLibA_31076 | 159 | 0.303516032 | NAGS | N-acetylglutamate synthase [Source:HGNC Symbol;Acc:HGNC:17996] |
| ENSG00000162702 | HGLibA_55736 | 159 | 0.303516032 | ZNF281 | zinc finger protein 281 [Source:HGNC Symbol;Acc:HGNC:13075] |
| ENSG00000162739 | HGLibA_44315 | 159 | 0.303516032 | SLAMF6 | SLAM family member 6 [Source:HGNC Symbol;Acc:HGNC:21392] |
| ENSG00000165588 | HGLibA_34786 | 159 | 0.303516032 | OTX2 | orthodenticle homeobox 2 [Source:HGNC Symbol;Acc:HGNC:8522] |
| ENSG00000165650 | HGLibA_35984 | 159 | 0.303516032 | PDZD8 | PDZ domain containing 8 [Source:HGNC Symbol;Acc:HGNC:26974] |
| ENSG00000165879 | HGLibA_17977 | 159 | 0.303516032 | FRAT1 | WNT signaling pathway regulator [Source:HGNC Symbol;Acc:HGNC:3944]" |
| ENSG00000167522 | HGLibA_01994 | 159 | 0.303516032 | ANKRD11 | ankyrin repeat domain 11 [Source:HGNC Symbol;Acc:HGNC:21316] |
| ENSG00000167635 | HGLibA_55495 | 159 | 0.303516032 | ZNF146 | zinc finger protein 146 [Source:HGNC Symbol;Acc:HGNC:12931] |
| ENSG00000167910 | HGLibA_12212 | 159 | 0.303516032 | CYP7A1 | cytochrome P450 family 7 subfamily A member 1 [Source:HGNC Symbol;Acc:HGNC:2651] |
| ENSG00000168209 | HGLibA_12617 | 159 | 0.303516032 | DDIT4 | DNA damage inducible transcript 4 [Source:HGNC Symbol;Acc:HGNC:24944] |
| ENSG00000168259 | HGLibA_13635 | 159 | 0.303516032 | DNAJC7 | DnaJ heat shock protein family (Hsp40) member C7 [Source:HGNC Symbol;Acc:HGNC:12392] |
| ENSG00000168913 | HGLibA_15130 | 159 | 0.303516032 | ENHO | energy homeostasis associated [Source:HGNC Symbol;Acc:HGNC:24838] |
| ENSG00000170044 | HGLibA_56967 | 159 | 0.303516032 | ZPLD1 | zona pellucida like domain containing 1 [Source:HGNC Symbol;Acc:HGNC:27022] |
| ENSG00000171135 | HGLibA_23910 | 159 | 0.303516032 | JAGN1 | jagunal homolog 1 [Source:HGNC Symbol;Acc:HGNC:26926] |
| ENSG00000171219 | HGLibA_08562 | 159 | 0.303516032 | CDC42BPG | CDC42 binding protein kinase gamma [Source:HGNC Symbol;Acc:HGNC:29829] |
| ENSG00000171501 | HGLibA_33706 | 159 | 0.303516032 | OR1N2 | olfactory receptor family 1 subfamily N member 2 [Source:HGNC Symbol;Acc:HGNC:15111] |
| ENSG00000171847 | HGLibA_16790 | 159 | 0.303516032 | FAM90A1 | family with sequence similarity 90 member A1 [Source:HGNC Symbol;Acc:HGNC:25526] |
| ENSG00000173464 | HGLibA_41394 | 159 | 0.303516032 | RNASE11 | ribonuclease A family member 11 (inactive) [Source:HGNC Symbol;Acc:HGNC:19269] |
| ENSG00000173821 | HGLibA_41621 | 159 | 0.303516032 | RNF213 | ring finger protein 213 [Source:HGNC Symbol;Acc:HGNC:14539] |
| ENSG00000174599 | HGLibA_51285 | 159 | 0.303516032 | TRAM1L1 | translocation associated membrane protein 1 like 1 [Source:HGNC Symbol;Acc:HGNC:28371] |
| ENSG00000175564 | HGLibA_52838 | 159 | 0.303516032 | UCP3 | uncoupling protein 3 [Source:HGNC Symbol;Acc:HGNC:12519] |
| ENSG00000176383 | HGLibA_03991 | 159 | 0.303516032 | B3GNT4 | 3-N-acetylglucosaminyltransferase 4 [Source:HGNC Symbol;Acc:HGNC:15683]" |
| ENSG00000176435 | HGLibA_09933 | 159 | 0.303516032 | CLEC14A | C-type lectin domain containing 14A [Source:HGNC Symbol;Acc:HGNC:19832] |
| ENSG00000176909 | HGLibA_28056 | 159 | 0.303516032 | MAMSTR | MEF2 activating motif and SAP domain containing transcriptional regulator [Source:HGNC Symbol;Acc:HGNC:26689] |
| ENSG00000179172 | HGLibA_21811 | 159 | 0.303516032 | HNRNPCL1 | heterogeneous nuclear ribonucleoprotein C-like 1 [Source:HGNC Symbol;Acc:HGNC:29295] |
| ENSG00000182218 | HGLibA_21263 | 159 | 0.303516032 | HHIPL1 | HHIP like 1 [Source:HGNC Symbol;Acc:HGNC:19710] |
| ENSG00000182871 | HGLibA_10524 | 159 | 0.303516032 | COL18A1 | collagen type XVIII alpha 1 chain [Source:HGNC Symbol;Acc:HGNC:2195] |
| ENSG00000184588 | HGLibA_35790 | 159 | 0.303516032 | PDE4B | phosphodiesterase 4B [Source:HGNC Symbol;Acc:HGNC:8781] |
| ENSG00000185803 | HGLibA_45246 | 159 | 0.303516032 | SLC52A2 | solute carrier family 52 member 2 [Source:HGNC Symbol;Acc:HGNC:30224] |
| ENSG00000186652 | HGLibA_38452 | 159 | 0.303516032 | PRG2 | pro eosinophil major basic protein [Source:HGNC Symbol;Acc:HGNC:9362]" |
| ENSG00000187942 | HGLibA_26244 | 159 | 0.303516032 | LDLRAD2 | low density lipoprotein receptor class A domain containing 2 [Source:HGNC Symbol;Acc:HGNC:32071] |
| ENSG00000188404 | HGLibA_43295 | 159 | 0.303516032 | SELL | selectin L [Source:HGNC Symbol;Acc:HGNC:10720] |
| ENSG00000188779 | HGLibA_44290 | 159 | 0.303516032 | SKOR1 | SKI family transcriptional corepressor 1 [Source:HGNC Symbol;Acc:HGNC:21326] |
| ENSG00000198298 | HGLibA_56079 | 159 | 0.303516032 | ZNF485 | zinc finger protein 485 [Source:HGNC Symbol;Acc:HGNC:23440] |
| ENSG00000203883 | HGLibA_46248 | 159 | 0.303516032 | SOX18 | SRY-box 18 [Source:HGNC Symbol;Acc:HGNC:11194] |
| ENSG00000204001 | HGLibA_26183 | 159 | 0.303516032 | LCN8 | lipocalin 8 [Source:HGNC Symbol;Acc:HGNC:27038] |
| ENSG00000213551 | HGLibA_13642 | 159 | 0.303516032 | DNAJC9 | DnaJ heat shock protein family (Hsp40) member C9 [Source:HGNC Symbol;Acc:HGNC:19123] |
| ENSG00000213619 | HGLibA_31552 | 159 | 0.303516032 | NDUFS3 | NADH:ubiquinone oxidoreductase core subunit S3 [Source:HGNC Symbol;Acc:HGNC:7710] |
| ENSG00000213676 | HGLibA_03448 | 159 | 0.303516032 | ATF6B | activating transcription factor 6 beta [Source:HGNC Symbol;Acc:HGNC:2349] |
| ENSG00000225828 | HGLibA_16539 | 159 | 0.303516032 | FAM229A | family with sequence similarity 229 member A [Source:HGNC Symbol;Acc:HGNC:44652] |
| ENSG00000235699 | HGLibA_11941 | 159 | 0.303516032 | CXorf51B | chromosome X open reading frame 51B [Source:HGNC Symbol;Acc:HGNC:42787] |
| ENSG00000277399 | HGLibA_19977 | 159 | 0.303516032 | GPR179 | G protein-coupled receptor 179 [Source:HGNC Symbol;Acc:HGNC:31371] |
| HIST4H4 | HGLibA_21565 | 159 | 0.303516032 | HIST4H4 | - |
| hsa-mir-155 | HGLibA_57932 | 159 | 0.303516032 | hsa-mir-155 | - |
| hsa-mir-224 | HGLibA_58306 | 159 | 0.303516032 | hsa-mir-224 | - |
| hsa-mir-3150a | HGLibA_58654 | 159 | 0.303516032 | hsa-mir-3150a | - |
| hsa-mir-3186 | HGLibA_58843 | 159 | 0.303516032 | hsa-mir-3186 | - |
| hsa-mir-320b-1 | HGLibA_58928 | 159 | 0.303516032 | hsa-mir-320b-1 | - |
| hsa-mir-320d-2 | HGLibA_58946 | 159 | 0.303516032 | hsa-mir-320d-2 | - |
| hsa-mir-3620 | HGLibA_59113 | 159 | 0.303516032 | hsa-mir-3620 | - |
| hsa-mir-4474 | HGLibA_60305 | 159 | 0.303516032 | hsa-mir-4474 | - |
| hsa-mir-5192 | HGLibA_61685 | 159 | 0.303516032 | hsa-mir-5192 | - |
| hsa-mir-6782 | HGLibA_63307 | 159 | 0.303516032 | hsa-mir-6782 | - |
| hsa-mir-6847 | HGLibA_63559 | 159 | 0.303516032 | hsa-mir-6847 | - |
| hsa-mir-7106 | HGLibA_63783 | 159 | 0.303516032 | hsa-mir-7106 | - |
| KCNE1L | HGLibA_24187 | 159 | 0.303516032 | KCNE1L | - |
| LOC100287534 | HGLibA_26826 | 159 | 0.303516032 | LOC100287534 | - |
| LOC339862 | HGLibA_26922 | 159 | 0.303516032 | LOC339862 | - |
| SUV420H2 | HGLibA_47868 | 159 | 0.303516032 | SUV420H2 | - |
| TCEB3 | HGLibA_48741 | 159 | 0.303516032 | TCEB3 | - |
| TMEM133 | HGLibA_49942 | 159 | 0.303516032 | TMEM133 | - |
| TMEM8C | HGLibA_50553 | 159 | 0.303516032 | TMEM8C | - |
| 9-Mar | HGLibA_28353 | 158 | 0.294470689 | 9-Mar | - |
| AAED1 | HGLibA_00044 | 158 | 0.294470689 | AAED1 | - |
| C16orf13 | HGLibA_05442 | 158 | 0.294470689 | C16orf13 | - |
| C4orf40 | HGLibA_06330 | 158 | 0.294470689 | C4orf40 | - |
| ENSG00000005844 | HGLibA_23758 | 158 | 0.294470689 | ITGAL | integrin subunit alpha L [Source:HGNC Symbol;Acc:HGNC:6148] |
| ENSG00000008869 | HGLibA_21079 | 158 | 0.294470689 | HEATR5B | HEAT repeat containing 5B [Source:HGNC Symbol;Acc:HGNC:29273] |
| ENSG00000023287 | HGLibA_40417 | 158 | 0.294470689 | RB1CC1 | RB1 inducible coiled-coil 1 [Source:HGNC Symbol;Acc:HGNC:15574] |
| ENSG00000040531 | HGLibA_11665 | 158 | 0.294470689 | CTNS | lysosomal cystine transporter [Source:HGNC Symbol;Acc:HGNC:2518]" |
| ENSG00000056736 | HGLibA_23008 | 158 | 0.294470689 | IL17RB | interleukin 17 receptor B [Source:HGNC Symbol;Acc:HGNC:18015] |
| ENSG00000069011 | HGLibA_36761 | 158 | 0.294470689 | PITX1 | paired like homeodomain 1 [Source:HGNC Symbol;Acc:HGNC:9004] |
| ENSG00000072062 | HGLibA_38504 | 158 | 0.294470689 | PRKACA | protein kinase cAMP-activated catalytic subunit alpha [Source:HGNC Symbol;Acc:HGNC:9380] |
| ENSG00000072133 | HGLibA_42174 | 158 | 0.294470689 | RPS6KA6 | ribosomal protein S6 kinase A6 [Source:HGNC Symbol;Acc:HGNC:10435] |
| ENSG00000085265 | HGLibA_17215 | 158 | 0.294470689 | FCN1 | ficolin 1 [Source:HGNC Symbol;Acc:HGNC:3623] |
| ENSG00000090989 | HGLibA_15731 | 158 | 0.294470689 | EXOC1 | exocyst complex component 1 [Source:HGNC Symbol;Acc:HGNC:30380] |
| ENSG00000092969 | HGLibA_49215 | 158 | 0.294470689 | TGFB2 | transforming growth factor beta 2 [Source:HGNC Symbol;Acc:HGNC:11768] |
| ENSG00000095713 | HGLibA_11220 | 158 | 0.294470689 | CRTAC1 | cartilage acidic protein 1 [Source:HGNC Symbol;Acc:HGNC:14882] |
| ENSG00000097033 | HGLibA_43954 | 158 | 0.294470689 | SH3GLB1 | endophilin B1 [Source:HGNC Symbol;Acc:HGNC:10833]" |
| ENSG00000099282 | HGLibA_51908 | 158 | 0.294470689 | TSPAN15 | tetraspanin 15 [Source:HGNC Symbol;Acc:HGNC:23298] |
| ENSG00000100121 | HGLibA_19026 | 158 | 0.294470689 | GGTLC2 | gamma-glutamyltransferase light chain 2 [Source:HGNC Symbol;Acc:HGNC:18596] |
| ENSG00000102870 | HGLibA_56431 | 158 | 0.294470689 | ZNF629 | zinc finger protein 629 [Source:HGNC Symbol;Acc:HGNC:29008] |
| ENSG00000105143 | HGLibA_44522 | 158 | 0.294470689 | SLC1A6 | solute carrier family 1 member 6 [Source:HGNC Symbol;Acc:HGNC:10944] |
| ENSG00000105290 | HGLibA_02441 | 158 | 0.294470689 | APLP1 | amyloid beta precursor like protein 1 [Source:HGNC Symbol;Acc:HGNC:597] |
| ENSG00000106477 | HGLibA_09136 | 158 | 0.294470689 | CEP41 | centrosomal protein 41 [Source:HGNC Symbol;Acc:HGNC:12370] |
| ENSG00000108830 | HGLibA_41447 | 158 | 0.294470689 | RND2 | Rho family GTPase 2 [Source:HGNC Symbol;Acc:HGNC:18315] |
| ENSG00000111726 | HGLibA_10178 | 158 | 0.294470689 | CMAS | cytidine monophosphate N-acetylneuraminic acid synthetase [Source:HGNC Symbol;Acc:HGNC:18290] |
| ENSG00000113194 | HGLibA_15950 | 158 | 0.294470689 | FAF2 | Fas associated factor family member 2 [Source:HGNC Symbol;Acc:HGNC:24666] |
| ENSG00000113575 | HGLibA_38133 | 158 | 0.294470689 | PPP2CA | protein phosphatase 2 catalytic subunit alpha [Source:HGNC Symbol;Acc:HGNC:9299] |
| ENSG00000114554 | HGLibA_37257 | 158 | 0.294470689 | PLXNA1 | plexin A1 [Source:HGNC Symbol;Acc:HGNC:9099] |
| ENSG00000115392 | HGLibA_16858 | 158 | 0.294470689 | FANCL | FA complementation group L [Source:HGNC Symbol;Acc:HGNC:20748] |
| ENSG00000119408 | HGLibA_31660 | 158 | 0.294470689 | NEK6 | NIMA related kinase 6 [Source:HGNC Symbol;Acc:HGNC:7749] |
| ENSG00000119650 | HGLibA_22757 | 158 | 0.294470689 | IFT43 | intraflagellar transport 43 [Source:HGNC Symbol;Acc:HGNC:29669] |
| ENSG00000120075 | HGLibA_21940 | 158 | 0.294470689 | HOXB5 | homeobox B5 [Source:HGNC Symbol;Acc:HGNC:5116] |
| ENSG00000120458 | HGLibA_30173 | 158 | 0.294470689 | MSANTD2 | Myb/SANT DNA binding domain containing 2 [Source:HGNC Symbol;Acc:HGNC:26266] |
| ENSG00000120662 | HGLibA_30465 | 158 | 0.294470689 | MTRF1 | mitochondrial translation release factor 1 [Source:HGNC Symbol;Acc:HGNC:7469] |
| ENSG00000122543 | HGLibA_33257 | 158 | 0.294470689 | OCM | oncomodulin [Source:HGNC Symbol;Acc:HGNC:8105] |
| ENSG00000124222 | HGLibA_47623 | 158 | 0.294470689 | STX16 | syntaxin 16 [Source:HGNC Symbol;Acc:HGNC:11431] |
| ENSG00000124562 | HGLibA_45973 | 158 | 0.294470689 | SNRPC | small nuclear ribonucleoprotein polypeptide C [Source:HGNC Symbol;Acc:HGNC:11157] |
| ENSG00000128739 | HGLibA_45994 | 158 | 0.294470689 | SNRPN | small nuclear ribonucleoprotein polypeptide N [Source:HGNC Symbol;Acc:HGNC:11164] |
| ENSG00000130066 | HGLibA_42769 | 158 | 0.294470689 | SAT1 | spermidine/spermine N1-acetyltransferase 1 [Source:HGNC Symbol;Acc:HGNC:10540] |
| ENSG00000132781 | HGLibA_30587 | 158 | 0.294470689 | MUTYH | mutY DNA glycosylase [Source:HGNC Symbol;Acc:HGNC:7527] |
| ENSG00000132801 | HGLibA_57042 | 158 | 0.294470689 | ZSWIM3 | zinc finger SWIM-type containing 3 [Source:HGNC Symbol;Acc:HGNC:16157] |
| ENSG00000133805 | HGLibA_01829 | 158 | 0.294470689 | AMPD3 | adenosine monophosphate deaminase 3 [Source:HGNC Symbol;Acc:HGNC:470] |
| ENSG00000134247 | HGLibA_39342 | 158 | 0.294470689 | PTGFRN | prostaglandin F2 receptor inhibitor [Source:HGNC Symbol;Acc:HGNC:9601] |
| ENSG00000134533 | HGLibA_40860 | 158 | 0.294470689 | RERG | RAS like estrogen regulated growth inhibitor [Source:HGNC Symbol;Acc:HGNC:15980] |
| ENSG00000135452 | HGLibA_51929 | 158 | 0.294470689 | TSPAN31 | tetraspanin 31 [Source:HGNC Symbol;Acc:HGNC:10539] |
| ENSG00000135452 | HGLibA_51927 | 158 | 0.294470689 | TSPAN31 | tetraspanin 31 [Source:HGNC Symbol;Acc:HGNC:10539] |
| ENSG00000135974 | HGLibA_06140 | 158 | 0.294470689 | C2orf49 | chromosome 2 open reading frame 49 [Source:HGNC Symbol;Acc:HGNC:28772] |
| ENSG00000137161 | HGLibA_10367 | 158 | 0.294470689 | CNPY3 | canopy FGF signaling regulator 3 [Source:HGNC Symbol;Acc:HGNC:11968] |
| ENSG00000138207 | HGLibA_40639 | 158 | 0.294470689 | RBP4 | retinol binding protein 4 [Source:HGNC Symbol;Acc:HGNC:9922] |
| ENSG00000138411 | HGLibA_21105 | 158 | 0.294470689 | HECW2 | C2 and WW domain containing E3 ubiquitin protein ligase 2 [Source:HGNC Symbol;Acc:HGNC:29853]" |
| ENSG00000139719 | HGLibA_53763 | 158 | 0.294470689 | VPS33A | CORVET/HOPS core subunit [Source:HGNC Symbol;Acc:HGNC:18179]" |
| ENSG00000140451 | HGLibA_36536 | 158 | 0.294470689 | PIF1 | PIF1 5'-to-3' DNA helicase [Source:HGNC Symbol;Acc:HGNC:26220] |
| ENSG00000143155 | HGLibA_49563 | 158 | 0.294470689 | TIPRL | TOR signaling pathway regulator [Source:HGNC Symbol;Acc:HGNC:30231] |
| ENSG00000143341 | HGLibA_21676 | 158 | 0.294470689 | HMCN1 | hemicentin 1 [Source:HGNC Symbol;Acc:HGNC:19194] |
| ENSG00000143515 | HGLibA_03782 | 158 | 0.294470689 | ATP8B2 | ATPase phospholipid transporting 8B2 [Source:HGNC Symbol;Acc:HGNC:13534] |
| ENSG00000143751 | HGLibA_43121 | 158 | 0.294470689 | SDE2 | SDE2 telomere maintenance homolog [Source:HGNC Symbol;Acc:HGNC:26643] |
| ENSG00000144455 | HGLibA_47770 | 158 | 0.294470689 | SUMF1 | sulfatase modifying factor 1 [Source:HGNC Symbol;Acc:HGNC:20376] |
| ENSG00000145428 | HGLibA_41576 | 158 | 0.294470689 | RNF175 | ring finger protein 175 [Source:HGNC Symbol;Acc:HGNC:27735] |
| ENSG00000145916 | HGLibA_41384 | 158 | 0.294470689 | RMND5B | required for meiotic nuclear division 5 homolog B [Source:HGNC Symbol;Acc:HGNC:26181] |
| ENSG00000146955 | HGLibA_39827 | 158 | 0.294470689 | RAB19 | member RAS oncogene family [Source:HGNC Symbol;Acc:HGNC:19982]" |
| ENSG00000147027 | HGLibA_50415 | 158 | 0.294470689 | TMEM47 | transmembrane protein 47 [Source:HGNC Symbol;Acc:HGNC:18515] |
| ENSG00000152778 | HGLibA_22634 | 158 | 0.294470689 | IFIT5 | interferon induced protein with tetratricopeptide repeats 5 [Source:HGNC Symbol;Acc:HGNC:13328] |
| ENSG00000153162 | HGLibA_04614 | 158 | 0.294470689 | BMP6 | bone morphogenetic protein 6 [Source:HGNC Symbol;Acc:HGNC:1073] |
| ENSG00000154330 | HGLibA_36272 | 158 | 0.294470689 | PGM5 | phosphoglucomutase 5 [Source:HGNC Symbol;Acc:HGNC:8908] |
| ENSG00000154975 | HGLibA_06788 | 158 | 0.294470689 | CA10 | carbonic anhydrase 10 [Source:HGNC Symbol;Acc:HGNC:1369] |
| ENSG00000157734 | HGLibA_46067 | 158 | 0.294470689 | SNX22 | sorting nexin 22 [Source:HGNC Symbol;Acc:HGNC:16315] |
| ENSG00000161649 | HGLibA_08346 | 158 | 0.294470689 | CD300LG | CD300 molecule like family member g [Source:HGNC Symbol;Acc:HGNC:30455] |
| ENSG00000161914 | HGLibA_56464 | 158 | 0.294470689 | ZNF653 | zinc finger protein 653 [Source:HGNC Symbol;Acc:HGNC:25196] |
| ENSG00000162188 | HGLibA_19523 | 158 | 0.294470689 | GNG3 | G protein subunit gamma 3 [Source:HGNC Symbol;Acc:HGNC:4405] |
| ENSG00000163320 | HGLibA_09318 | 158 | 0.294470689 | CGGBP1 | CGG triplet repeat binding protein 1 [Source:HGNC Symbol;Acc:HGNC:1888] |

| | | | | | |
|---|---|---|---|---|---|
| ENSG00000163687 | HGLibA_13661 | 158 | 0.294470689 | DNASE1L3 | deoxyribonuclease 1 like 3 [Source:HGNC Symbol;Acc:HGNC:2959] |
| ENSG00000163719 | HGLibA_30420 | 158 | 0.294470689 | MTMR14 | myotubularin related protein 14 [Source:HGNC Symbol;Acc:HGNC:26190] |
| ENSG00000164047 | HGLibA_07103 | 158 | 0.294470689 | CAMP | cathelicidin antimicrobial peptide [Source:HGNC Symbol;Acc:HGNC:1472] |
| ENSG00000164330 | HGLibA_14350 | 158 | 0.294470689 | EBF1 | EBF transcription factor 1 [Source:HGNC Symbol;Acc:HGNC:3126] |
| ENSG00000164485 | HGLibA_23093 | 158 | 0.294470689 | IL22RA2 | interleukin 22 receptor subunit alpha 2 [Source:HGNC Symbol;Acc:HGNC:14901] |
| ENSG00000164893 | HGLibA_45356 | 158 | 0.294470689 | SLC7A13 | solute carrier family 7 member 13 [Source:HGNC Symbol;Acc:HGNC:23092] |
| ENSG00000165029 | HGLibA_00096 | 158 | 0.294470689 | ABCA1 | ATP binding cassette subfamily A member 1 [Source:HGNC Symbol;Acc:HGNC:29] |
| ENSG00000166527 | HGLibA_09986 | 158 | 0.294470689 | CLEC4D | C-type lectin domain family 4 member D [Source:HGNC Symbol;Acc:HGNC:14554] |
| ENSG00000167962 | HGLibA_56355 | 158 | 0.294470689 | ZNF598 | zinc finger protein 598 [Source:HGNC Symbol;Acc:HGNC:28079] |
| ENSG00000169230 | HGLibA_38428 | 158 | 0.294470689 | PRELID1 | PRELI domain containing 1 [Source:HGNC Symbol;Acc:HGNC:30255] |
| ENSG00000169933 | HGLibA_18039 | 158 | 0.294470689 | FRMPD4 | FERM and PDZ domain containing 4 [Source:HGNC Symbol;Acc:HGNC:29007] |
| ENSG00000170667 | HGLibA_40308 | 158 | 0.294470689 | RASA4B | RAS p21 protein activator 4B [Source:HGNC Symbol;Acc:HGNC:35202] |
| ENSG00000171262 | HGLibA_16812 | 158 | 0.294470689 | FAM98B | family with sequence similarity 98 member B [Source:HGNC Symbol;Acc:HGNC:26773] |
| ENSG00000171867 | HGLibA_38645 | 158 | 0.294470689 | PRNP | prion protein [Source:HGNC Symbol;Acc:HGNC:9449] |
| ENSG00000173153 | HGLibA_15593 | 158 | 0.294470689 | ESRRA | estrogen related receptor alpha [Source:HGNC Symbol;Acc:HGNC:3471] |
| ENSG00000173641 | HGLibA_22316 | 158 | 0.294470689 | HSPB7 | heat shock protein family B (small) member 7 [Source:HGNC Symbol;Acc:HGNC:5249] |
| ENSG00000174276 | HGLibA_56927 | 158 | 0.294470689 | ZNHIT2 | zinc finger HIT-type containing 2 [Source:HGNC Symbol;Acc:HGNC:1177] |
| ENSG00000174442 | HGLibA_57066 | 158 | 0.294470689 | ZWILCH | zwilch kinetochore protein [Source:HGNC Symbol;Acc:HGNC:25468] |
| ENSG00000175077 | HGLibA_42423 | 158 | 0.294470689 | RTP1 | receptor transporter protein 1 [Source:HGNC Symbol;Acc:HGNC:28580] |
| ENSG00000175899 | HGLibA_00008 | 158 | 0.294470689 | A2M | alpha-2-macroglobulin [Source:HGNC Symbol;Acc:HGNC:7] |
| ENSG00000176269 | HGLibA_33998 | 158 | 0.294470689 | OR4F21 | olfactory receptor family 4 subfamily F member 21 [Source:HGNC Symbol;Acc:HGNC:19583] |
| ENSG00000180776 | HGLibA_55098 | 158 | 0.294470689 | ZDHHC20 | zinc finger DHHC-type containing 20 [Source:HGNC Symbol;Acc:HGNC:20749] |
| ENSG00000181649 | HGLibA_36421 | 158 | 0.294470689 | PHLDA2 | pleckstrin homology like domain family A member 2 [Source:HGNC Symbol;Acc:HGNC:12385] |
| ENSG00000182318 | HGLibA_57003 | 158 | 0.294470689 | ZSCAN22 | zinc finger and SCAN domain containing 22 [Source:HGNC Symbol;Acc:HGNC:4929] |
| ENSG00000183597 | HGLibA_48297 | 158 | 0.294470689 | TANGO2 | transport and golgi organization 2 homolog [Source:HGNC Symbol;Acc:HGNC:25439] |
| ENSG00000183734 | HGLibA_03269 | 158 | 0.294470689 | ASCL2 | achaete-scute family bHLH transcription factor 2 [Source:HGNC Symbol;Acc:HGNC:739] |
| ENSG00000184845 | HGLibA_13960 | 158 | 0.294470689 | DRD1 | dopamine receptor D1 [Source:HGNC Symbol;Acc:HGNC:3020] |
| ENSG00000185272 | HGLibA_40475 | 158 | 0.294470689 | RBM11 | RNA binding motif protein 11 [Source:HGNC Symbol;Acc:HGNC:9897] |
| ENSG00000186314 | HGLibA_38430 | 158 | 0.294470689 | PRELID2 | PRELI domain containing 2 [Source:HGNC Symbol;Acc:HGNC:28306] |
| ENSG00000186452 | HGLibA_50628 | 158 | 0.294470689 | TMPRSS12 | transmembrane serine protease 12 [Source:HGNC Symbol;Acc:HGNC:28779] |
| ENSG00000187492 | HGLibA_08708 | 158 | 0.294470689 | CDHR4 | cadherin related family member 4 [Source:HGNC Symbol;Acc:HGNC:34527] |
| ENSG00000187790 | HGLibA_16861 | 158 | 0.294470689 | FANCM | FA complementation group M [Source:HGNC Symbol;Acc:HGNC:23168] |
| ENSG00000188559 | HGLibA_40159 | 158 | 0.294470689 | RALGAPA2 | Ral GTPase activating protein catalytic alpha subunit 2 [Source:HGNC Symbol;Acc:HGNC:16207] |
| ENSG00000188883 | HGLibA_25313 | 158 | 0.294470689 | KLRG2 | killer cell lectin like receptor G2 [Source:HGNC Symbol;Acc:HGNC:24778] |
| ENSG00000189180 | HGLibA_55832 | 158 | 0.294470689 | ZNF33A | zinc finger protein 33A [Source:HGNC Symbol;Acc:HGNC:13096] |
| ENSG00000196290 | HGLibA_31930 | 158 | 0.294470689 | NIF3L1 | NGG1 interacting factor 3 like 1 [Source:HGNC Symbol;Acc:HGNC:13390] |
| ENSG00000196660 | HGLibA_44900 | 158 | 0.294470689 | SLC30A10 | solute carrier family 30 member 10 [Source:HGNC Symbol;Acc:HGNC:25355] |
| ENSG00000196724 | HGLibA_55951 | 158 | 0.294470689 | ZNF418 | zinc finger protein 418 [Source:HGNC Symbol;Acc:HGNC:20647] |
| ENSG00000197123 | HGLibA_56520 | 158 | 0.294470689 | ZNF679 | zinc finger protein 679 [Source:HGNC Symbol;Acc:HGNC:28650] |
| ENSG00000197233 | HGLibA_33676 | 158 | 0.294470689 | OR1J2 | olfactory receptor family 1 subfamily J member 2 [Source:HGNC Symbol;Acc:HGNC:8209] |
| ENSG00000197428 | HGLibA_34094 | 158 | 0.294470689 | OR51D1 | olfactory receptor family 51 subfamily D member 1 [Source:HGNC Symbol;Acc:HGNC:15193] |
| ENSG00000198099 | HGLibA_00955 | 158 | 0.294470689 | ADH4 | pi polypeptide [Source:HGNC Symbol;Acc:HGNC:252]" |
| ENSG00000198128 | HGLibA_33824 | 158 | 0.294470689 | OR2L3 | olfactory receptor family 2 subfamily L member 3 [Source:HGNC Symbol;Acc:HGNC:15009] |
| ENSG00000198185 | HGLibA_55823 | 158 | 0.294470689 | ZNF334 | zinc finger protein 334 [Source:HGNC Symbol;Acc:HGNC:15806] |
| ENSG00000198471 | HGLibA_42424 | 158 | 0.294470689 | RTP2 | receptor transporter protein 2 [Source:HGNC Symbol;Acc:HGNC:32486] |
| ENSG00000204880 | HGLibA_25777 | 158 | 0.294470689 | KRTAP4-8 | keratin associated protein 4-8 [Source:HGNC Symbol;Acc:HGNC:17230] |
| ENSG00000213760 | HGLibA_03761 | 158 | 0.294470689 | ATP6V1G2 | ATPase H+ transporting V1 subunit G2 [Source:HGNC Symbol;Acc:HGNC:862] |
| ENSG00000213892 | HGLibA_08926 | 158 | 0.294470689 | CEACAM16 | carcinoembryonic antigen related cell adhesion molecule 16 [Source:HGNC Symbol;Acc:HGNC:31948] |
| ENSG00000219607 | HGLibA_38117 | 158 | 0.294470689 | PPP1R3G | protein phosphatase 1 regulatory subunit 3G [Source:HGNC Symbol;Acc:HGNC:14945] |
| ENSG00000238269 | HGLibA_35011 | 158 | 0.294470689 | PAGE2B | PAGE family member 2B [Source:HGNC Symbol;Acc:HGNC:31805] |
| ENSG00000249242 | HGLibA_49996 | 158 | 0.294470689 | TMEM150C | transmembrane protein 150C [Source:HGNC Symbol;Acc:HGNC:37263] |
| ENSG00000254221 | HGLibA_35551 | 158 | 0.294470689 | PCDHGB1 | 1 [Source:HGNC Symbol;Acc:HGNC:8708]" |
| ENSG00000254999 | HGLibA_04827 | 158 | 0.294470689 | BRK1 | SCAR/WAVE actin nucleating complex subunit [Source:HGNC Symbol;Acc:HGNC:23057]" |
| ENSG00000266074 | HGLibA_04108 | 158 | 0.294470689 | BAHCC1 | BAH domain and coiled-coil containing 1 [Source:HGNC Symbol;Acc:HGNC:29279] |
| ENSG00000268223 | HGLibA_02942 | 158 | 0.294470689 | ARL14EPL | ADP ribosylation factor like GTPase 14 effector protein like [Source:HGNC Symbol;Acc:HGNC:44201] |
| ENSG00000269743 | HGLibA_44770 | 158 | 0.294470689 | SLC25A53 | solute carrier family 25 member 53 [Source:HGNC Symbol;Acc:HGNC:31894] |
| ENSG00000274211 | HGLibA_46142 | 158 | 0.294470689 | SOCS7 | suppressor of cytokine signaling 7 [Source:HGNC Symbol;Acc:HGNC:29846] |
| ENSG00000276409 | HGLibA_07970 | 158 | 0.294470689 | CCL14 | C-C motif chemokine ligand 14 [Source:HGNC Symbol;Acc:HGNC:10612] |
| FAM134A | HGLibA_16122 | 158 | 0.294470689 | FAM134A | - |
| hsa-mir-1-2 | HGLibA_57147 | 158 | 0.294470689 | hsa-mir-1-2 | - |
| hsa-mir-147b | HGLibA_57875 | 158 | 0.294470689 | hsa-mir-147b | - |
| hsa-mir-200b | HGLibA_58153 | 158 | 0.294470689 | hsa-mir-200b | - |
| hsa-mir-210 | HGLibA_58213 | 158 | 0.294470689 | hsa-mir-210 | - |
| hsa-mir-2681 | HGLibA_58358 | 158 | 0.294470689 | hsa-mir-2681 | - |
| hsa-mir-339 | HGLibA_59008 | 158 | 0.294470689 | hsa-mir-339 | - |
| hsa-mir-3714 | HGLibA_59371 | 158 | 0.294470689 | hsa-mir-3714 | - |
| hsa-mir-4282 | HGLibA_59830 | 158 | 0.294470689 | hsa-mir-4282 | - |
| hsa-mir-4444-1 | HGLibA_60177 | 158 | 0.294470689 | hsa-mir-4444-1 | - |
| hsa-mir-4652 | HGLibA_60714 | 158 | 0.294470689 | hsa-mir-4652 | - |
| hsa-mir-4753 | HGLibA_61109 | 158 | 0.294470689 | hsa-mir-4753 | - |
| hsa-mir-583 | HGLibA_62432 | 158 | 0.294470689 | hsa-mir-583 | - |
| hsa-mir-584 | HGLibA_62433 | 158 | 0.294470689 | hsa-mir-584 | - |
| hsa-mir-602 | HGLibA_62501 | 158 | 0.294470689 | hsa-mir-602 | - |
| hsa-mir-627 | HGLibA_62748 | 158 | 0.294470689 | hsa-mir-627 | - |
| KIAA1244 | HGLibA_24726 | 158 | 0.294470689 | KIAA1244 | - |
| RARRES3 | HGLibA_40287 | 158 | 0.294470689 | RARRES3 | - |
| T | HGLibA_48120 | 158 | 0.294470689 | T | - |
| ALS2CR11 | HGLibA_01725 | 157 | 0.285425346 | ALS2CR11 | - |
| AQPEP | HGLibA_02615 | 157 | 0.285425346 | AQPEP | - |
| C4B_2 | HGLibA_06286 | 157 | 0.285425346 | C4B_2 | - |
| CRAMP1L | HGLibA_11064 | 157 | 0.285425346 | CRAMP1L | - |
| CXCR7 | HGLibA_11899 | 157 | 0.285425346 | CXCR7 | - |
| ENSG00000005961 | HGLibA_23727 | 157 | 0.285425346 | ITGA2B | integrin subunit alpha 2b [Source:HGNC Symbol;Acc:HGNC:6138] |
| ENSG00000017427 | HGLibA_22790 | 157 | 0.285425346 | IGF1 | insulin like growth factor 1 [Source:HGNC Symbol;Acc:HGNC:5464] |
| ENSG00000019186 | HGLibA_12082 | 157 | 0.285425346 | CYP24A1 | cytochrome P450 family 24 subfamily A member 1 [Source:HGNC Symbol;Acc:HGNC:2602] |
| ENSG00000047849 | HGLibA_28209 | 157 | 0.285425346 | MAP4 | microtubule associated protein 4 [Source:HGNC Symbol;Acc:HGNC:6862] |
| ENSG00000049449 | HGLibA_40701 | 157 | 0.285425346 | RCN1 | reticulocalbin 1 [Source:HGNC Symbol;Acc:HGNC:9934] |
| ENSG00000054148 | HGLibA_36455 | 157 | 0.285425346 | PHPT1 | phosphohistidine phosphatase 1 [Source:HGNC Symbol;Acc:HGNC:30033] |
| ENSG00000054219 | HGLibA_27679 | 157 | 0.285425346 | LY75 | lymphocyte antigen 75 [Source:HGNC Symbol;Acc:HGNC:6729] |
| ENSG00000059588 | HGLibA_48331 | 157 | 0.285425346 | TARBP1 | TAR (HIV-1) RNA binding protein 1 [Source:HGNC Symbol;Acc:HGNC:11568] |
| ENSG00000068079 | HGLibA_22607 | 157 | 0.285425346 | IFI35 | interferon induced protein 35 [Source:HGNC Symbol;Acc:HGNC:5399] |
| ENSG00000080644 | HGLibA_09572 | 157 | 0.285425346 | CHRNA3 | cholinergic receptor nicotinic alpha 3 subunit [Source:HGNC Symbol;Acc:HGNC:1957] |
| ENSG00000084731 | HGLibA_24940 | 157 | 0.285425346 | KIF3C | kinesin family member 3C [Source:HGNC Symbol;Acc:HGNC:6321] |
| ENSG00000086544 | HGLibA_23850 | 157 | 0.285425346 | ITPKC | inositol-trisphosphate 3-kinase C [Source:HGNC Symbol;Acc:HGNC:14897] |
| ENSG00000088826 | HGLibA_45801 | 157 | 0.285425346 | SMOX | spermine oxidase [Source:HGNC Symbol;Acc:HGNC:15862] |
| ENSG00000089199 | HGLibA_09433 | 157 | 0.285425346 | CHGB | chromogranin B [Source:HGNC Symbol;Acc:HGNC:1930] |
| ENSG00000091583 | HGLibA_02523 | 157 | 0.285425346 | APOH | apolipoprotein H [Source:HGNC Symbol;Acc:HGNC:616] |
| ENSG00000100207 | HGLibA_48770 | 157 | 0.285425346 | TCF20 | transcription factor 20 [Source:HGNC Symbol;Acc:HGNC:11631] |
| ENSG00000101000 | HGLibA_38657 | 157 | 0.285425346 | PROCR | protein C receptor [Source:HGNC Symbol;Acc:HGNC:9452] |
| ENSG00000101448 | HGLibA_15357 | 157 | 0.285425346 | EPPIN | epididymal peptidase inhibitor [Source:HGNC Symbol;Acc:HGNC:15932] |
| ENSG00000102100 | HGLibA_44951 | 157 | 0.285425346 | SLC35A2 | solute carrier family 35 member A2 [Source:HGNC Symbol;Acc:HGNC:11022] |
| ENSG00000102317 | HGLibA_40529 | 157 | 0.285425346 | RBM3 | RNA binding motif protein 3 [Source:HGNC Symbol;Acc:HGNC:9900] |
| ENSG00000102595 | HGLibA_52869 | 157 | 0.285425346 | UGGT2 | UDP-glucose glycoprotein glucosyltransferase 2 [Source:HGNC Symbol;Acc:HGNC:15664] |
| ENSG00000102879 | HGLibA_10772 | 157 | 0.285425346 | CORO1A | coronin 1A [Source:HGNC Symbol;Acc:HGNC:2252] |
| ENSG00000103024 | HGLibA_32161 | 157 | 0.285425346 | NME3 | NME/NM23 nucleoside diphosphate kinase 3 [Source:HGNC Symbol;Acc:HGNC:7851] |
| ENSG00000105402 | HGLibA_31127 | 157 | 0.285425346 | NAPA | NSF attachment protein alpha [Source:HGNC Symbol;Acc:HGNC:7641] |
| ENSG00000105825 | HGLibA_49186 | 157 | 0.285425346 | TFPI2 | tissue factor pathway inhibitor 2 [Source:HGNC Symbol;Acc:HGNC:11761] |
| ENSG00000107829 | HGLibA_17146 | 157 | 0.285425346 | FBXW4 | F-box and WD repeat domain containing 4 [Source:HGNC Symbol;Acc:HGNC:10847] |
| ENSG00000109625 | HGLibA_11042 | 157 | 0.285425346 | CPZ | carboxypeptidase Z [Source:HGNC Symbol;Acc:HGNC:2333] |
| ENSG00000109787 | HGLibA_25069 | 157 | 0.285425346 | KLF3 | Kruppel like factor 3 [Source:HGNC Symbol;Acc:HGNC:16516] |
| ENSG00000112078 | HGLibA_24447 | 157 | 0.285425346 | KCTD20 | potassium channel tetramerization domain containing 20 [Source:HGNC Symbol;Acc:HGNC:21052] |
| ENSG00000112335 | HGLibA_46080 | 157 | 0.285425346 | SNX3 | sorting nexin 3 [Source:HGNC Symbol;Acc:HGNC:11174] |
| ENSG00000113100 | HGLibA_08696 | 157 | 0.285425346 | CDH9 | cadherin 9 [Source:HGNC Symbol;Acc:HGNC:1768] |
| ENSG00000113249 | HGLibA_20899 | 157 | 0.285425346 | HAVCR1 | hepatitis A virus cellular receptor 1 [Source:HGNC Symbol;Acc:HGNC:17866] |
| ENSG00000114735 | HGLibA_21132 | 157 | 0.285425346 | HEMK1 | HemK methyltransferase family member 1 [Source:HGNC Symbol;Acc:HGNC:24923] |
| ENSG00000115234 | HGLibA_46049 | 157 | 0.285425346 | SNX17 | sorting nexin 17 [Source:HGNC Symbol;Acc:HGNC:14979] |
| ENSG00000115339 | HGLibA_18557 | 157 | 0.285425346 | GALNT3 | polypeptide N-acetylgalactosaminyltransferase 3 [Source:HGNC Symbol;Acc:HGNC:4125] |
| ENSG00000115998 | HGLibA_06126 | 157 | 0.285425346 | C2orf42 | chromosome 2 open reading frame 42 [Source:HGNC Symbol;Acc:HGNC:26056] |
| ENSG00000118193 | HGLibA_24861 | 157 | 0.285425346 | KIF14 | kinesin family member 14 [Source:HGNC Symbol;Acc:HGNC:19181] |
| ENSG00000121318 | HGLibA_48365 | 157 | 0.285425346 | TAS2R10 | taste 2 receptor member 10 [Source:HGNC Symbol;Acc:HGNC:14918] |
| ENSG00000125257 | HGLibA_00176 | 157 | 0.285425346 | ABCC4 | ATP binding cassette subfamily C member 4 [Source:HGNC Symbol;Acc:HGNC:55] |
| ENSG00000125285 | HGLibA_46254 | 157 | 0.285425346 | SOX21 | SRY-box 21 [Source:HGNC Symbol;Acc:HGNC:11197] |
| ENSG00000125944 | HGLibA_21843 | 157 | 0.285425346 | HNRNPR | heterogeneous nuclear ribonucleoprotein R [Source:HGNC Symbol;Acc:HGNC:5047] |
| ENSG00000127980 | HGLibA_36077 | 157 | 0.285425346 | PEX1 | peroxisomal biogenesis factor 1 [Source:HGNC Symbol;Acc:HGNC:8850] |
| ENSG00000129473 | HGLibA_04323 | 157 | 0.285425346 | BCL2L2 | BCL2 like 2 [Source:HGNC Symbol;Acc:HGNC:995] |
| ENSG00000130222 | HGLibA_18422 | 157 | 0.285425346 | GADD45G | growth arrest and DNA damage inducible gamma [Source:HGNC Symbol;Acc:HGNC:4097] |
| ENSG00000131115 | HGLibA_55627 | 157 | 0.285425346 | ZNF227 | zinc finger protein 227 [Source:HGNC Symbol;Acc:HGNC:13020] |
| ENSG00000131142 | HGLibA_08005 | 157 | 0.285425346 | CCL25 | C-C motif chemokine ligand 25 [Source:HGNC Symbol;Acc:HGNC:10624] |
| ENSG00000131730 | HGLibA_09783 | 157 | 0.285425346 | CKMT2 | mitochondrial 2 [Source:HGNC Symbol;Acc:HGNC:1996]" |
| ENSG00000132763 | HGLibA_29457 | 157 | 0.285425346 | MMACHC | metabolism of cobalamin associated C [Source:HGNC Symbol;Acc:HGNC:24525] |
| ENSG00000133101 | HGLibA_08051 | 157 | 0.285425346 | CCNA1 | cyclin A1 [Source:HGNC Symbol;Acc:HGNC:1577] |
| ENSG00000134987 | HGLibA_54076 | 157 | 0.285425346 | WDR36 | WD repeat domain 36 [Source:HGNC Symbol;Acc:HGNC:30696] |
| ENSG00000135740 | HGLibA_45419 | 157 | 0.285425346 | SLC9A5 | solute carrier family 9 member A5 [Source:HGNC Symbol;Acc:HGNC:11078] |
| ENSG00000135823 | HGLibA_47655 | 157 | 0.285425346 | STX6 | syntaxin 6 [Source:HGNC Symbol;Acc:HGNC:11441] |
| ENSG00000135945 | HGLibA_40883 | 157 | 0.285425346 | REV1 | DNA directed polymerase [Source:HGNC Symbol;Acc:HGNC:14060]" |

| | | | | | |
|---|---|---|---|---|---|
| ENSG00000136002 | HGLibA_02865 | 157 | 0.285425346 | ARHGEF4 | Rho guanine nucleotide exchange factor 4 [Source:HGNC Symbol;Acc:HGNC:684] |
| ENSG00000136717 | HGLibA_04507 | 157 | 0.285425346 | BIN1 | bridging integrator 1 [Source:HGNC Symbol;Acc:HGNC:1052] |
| ENSG00000136720 | HGLibA_22141 | 157 | 0.285425346 | HS6ST1 | heparan sulfate 6-O-sulfotransferase 1 [Source:HGNC Symbol;Acc:HGNC:5201] |
| ENSG00000139132 | HGLibA_17352 | 157 | 0.285425346 | FGD4 | RhoGEF and PH domain containing 4 [Source:HGNC Symbol;Acc:HGNC:19125]" |
| ENSG00000139278 | HGLibA_19260 | 157 | 0.285425346 | GLIPR1 | GLI pathogenesis related 1 [Source:HGNC Symbol;Acc:HGNC:17001] |
| ENSG00000139722 | HGLibA_53779 | 157 | 0.285425346 | VPS37B | ESCRT-I subunit [Source:HGNC Symbol;Acc:HGNC:25754]" |
| ENSG00000140450 | HGLibA_03122 | 157 | 0.285425346 | ARRDC4 | arrestin domain containing 4 [Source:HGNC Symbol;Acc:HGNC:28087] |
| ENSG00000140795 | HGLibA_30790 | 157 | 0.285425346 | MYLK3 | myosin light chain kinase 3 [Source:HGNC Symbol;Acc:HGNC:29826] |
| ENSG00000144668 | HGLibA_23747 | 157 | 0.285425346 | ITGA9 | integrin subunit alpha 9 [Source:HGNC Symbol;Acc:HGNC:6145] |
| ENSG00000145014 | HGLibA_50404 | 157 | 0.285425346 | TMEM44 | transmembrane protein 44 [Source:HGNC Symbol;Acc:HGNC:25120] |
| ENSG00000145147 | HGLibA_45508 | 157 | 0.285425346 | SLIT2 | slit guidance ligand 2 [Source:HGNC Symbol;Acc:HGNC:11086] |
| ENSG00000145390 | HGLibA_53399 | 157 | 0.285425346 | USP53 | ubiquitin specific peptidase 53 [Source:HGNC Symbol;Acc:HGNC:29255] |
| ENSG00000146416 | HGLibA_01346 | 157 | 0.285425346 | AIG1 | androgen induced 1 [Source:HGNC Symbol;Acc:HGNC:21607] |
| ENSG00000148429 | HGLibA_53407 | 157 | 0.285425346 | USP6NL | USP6 N-terminal like [Source:HGNC Symbol;Acc:HGNC:16858] |
| ENSG00000148541 | HGLibA_16146 | 157 | 0.285425346 | FAM13C | family with sequence similarity 13 member C [Source:HGNC Symbol;Acc:HGNC:19371] |
| ENSG00000148835 | HGLibA_48240 | 157 | 0.285425346 | TAF5 | TATA-box binding protein associated factor 5 [Source:HGNC Symbol;Acc:HGNC:11539] |
| ENSG00000148925 | HGLibA_04895 | 157 | 0.285425346 | BTBD10 | BTB domain containing 10 [Source:HGNC Symbol;Acc:HGNC:21445] |
| ENSG00000151835 | HGLibA_42628 | 157 | 0.285425346 | SACS | sacsin molecular chaperone [Source:HGNC Symbol;Acc:HGNC:10519] |
| ENSG00000152457 | HGLibA_12513 | 157 | 0.285425346 | DCLRE1C | DNA cross-link repair 1C [Source:HGNC Symbol;Acc:HGNC:17642] |
| ENSG00000159388 | HGLibA_04944 | 157 | 0.285425346 | BTG2 | BTG anti-proliferation factor 2 [Source:HGNC Symbol;Acc:HGNC:1131] |
| ENSG00000160190 | HGLibA_45043 | 157 | 0.285425346 | SLC37A1 | solute carrier family 37 member 1 [Source:HGNC Symbol;Acc:HGNC:11024] |
| ENSG00000161082 | HGLibA_09015 | 157 | 0.285425346 | CELF5 | CUGBP Elav-like family member 5 [Source:HGNC Symbol;Acc:HGNC:14058] |
| ENSG00000162975 | HGLibA_24196 | 157 | 0.285425346 | KCNF1 | potassium voltage-gated channel modifier subfamily F member 1 [Source:HGNC Symbol;Acc:HGNC:6246] |
| ENSG00000163157 | HGLibA_50604 | 157 | 0.285425346 | TMOD4 | tropomodulin 4 [Source:HGNC Symbol;Acc:HGNC:11874] |
| ENSG00000163833 | HGLibA_17092 | 157 | 0.285425346 | FBXO40 | F-box protein 40 [Source:HGNC Symbol;Acc:HGNC:29816] |
| ENSG00000164692 | HGLibA_10532 | 157 | 0.285425346 | COL1A2 | collagen type I alpha 2 chain [Source:HGNC Symbol;Acc:HGNC:2198] |
| ENSG00000164776 | HGLibA_36411 | 157 | 0.285425346 | PHKG1 | phosphorylase kinase catalytic subunit gamma 1 [Source:HGNC Symbol;Acc:HGNC:8930] |
| ENSG00000164889 | HGLibA_45214 | 157 | 0.285425346 | SLC4A2 | solute carrier family 4 member 2 [Source:HGNC Symbol;Acc:HGNC:11028] |
| ENSG00000165650 | HGLibA_35985 | 157 | 0.285425346 | PDZD8 | PDZ domain containing 8 [Source:HGNC Symbol;Acc:HGNC:26974] |
| ENSG00000166024 | HGLibA_39769 | 157 | 0.285425346 | R3HCC1L | R3H domain and coiled-coil containing 1 like [Source:HGNC Symbol;Acc:HGNC:23512] |
| ENSG00000166167 | HGLibA_04985 | 157 | 0.285425346 | BTRC | beta-transducin repeat containing E3 ubiquitin protein ligase [Source:HGNC Symbol;Acc:HGNC:1144] |
| ENSG00000166407 | HGLibA_26701 | 157 | 0.285425346 | LMO1 | LIM domain only 1 [Source:HGNC Symbol;Acc:HGNC:6641] |
| ENSG00000167565 | HGLibA_43610 | 157 | 0.285425346 | SERTAD3 | SERTA domain containing 3 [Source:HGNC Symbol;Acc:HGNC:17931] |
| ENSG00000167850 | HGLibA_08337 | 157 | 0.285425346 | CD300C | CD300c molecule [Source:HGNC Symbol;Acc:HGNC:19320] |
| ENSG00000168209 | HGLibA_12619 | 157 | 0.285425346 | DDIT4 | DNA damage inducible transcript 4 [Source:HGNC Symbol;Acc:HGNC:24944] |
| ENSG00000168398 | HGLibA_04383 | 157 | 0.285425346 | BDKRB2 | bradykinin receptor B2 [Source:HGNC Symbol;Acc:HGNC:1030] |
| ENSG00000169035 | HGLibA_25268 | 157 | 0.285425346 | KLK7 | kallikrein related peptidase 7 [Source:HGNC Symbol;Acc:HGNC:6368] |
| ENSG00000169180 | HGLibA_54571 | 157 | 0.285425346 | XPO6 | exportin 6 [Source:HGNC Symbol;Acc:HGNC:19733] |
| ENSG00000169684 | HGLibA_09578 | 157 | 0.285425346 | CHRNA5 | cholinergic receptor nicotinic alpha 5 subunit [Source:HGNC Symbol;Acc:HGNC:1959] |
| ENSG00000172116 | HGLibA_08474 | 157 | 0.285425346 | CD8B | CD8b molecule [Source:HGNC Symbol;Acc:HGNC:1707] |
| ENSG00000172867 | HGLibA_25445 | 157 | 0.285425346 | KRT2 | keratin 2 [Source:HGNC Symbol;Acc:HGNC:6439] |
| ENSG00000173145 | HGLibA_32237 | 157 | 0.285425346 | NOC3L | NOC3 like DNA replication regulator [Source:HGNC Symbol;Acc:HGNC:24034] |
| ENSG00000174132 | HGLibA_16283 | 157 | 0.285425346 | FAM174A | family with sequence similarity 174 member A [Source:HGNC Symbol;Acc:HGNC:24943] |
| ENSG00000174255 | HGLibA_56781 | 157 | 0.285425346 | ZNF80 | zinc finger protein 80 [Source:HGNC Symbol;Acc:HGNC:13155] |
| ENSG00000175467 | HGLibA_42754 | 157 | 0.285425346 | SART1 | recruiter of U4/U6.U5 tri-snRNP [Source:HGNC Symbol;Acc:HGNC:10538]" |
| ENSG00000175792 | HGLibA_42472 | 157 | 0.285425346 | RUVBL1 | RuvB like AAA ATPase 1 [Source:HGNC Symbol;Acc:HGNC:10474] |
| ENSG00000176407 | HGLibA_24119 | 157 | 0.285425346 | KCMF1 | potassium channel modulatory factor 1 [Source:HGNC Symbol;Acc:HGNC:20589] |
| ENSG00000178409 | HGLibA_04403 | 157 | 0.285425346 | BEND3 | BEN domain containing 3 [Source:HGNC Symbol;Acc:HGNC:23040] |
| ENSG00000178662 | HGLibA_11428 | 157 | 0.285425346 | CSRNP3 | cysteine and serine rich nuclear protein 3 [Source:HGNC Symbol;Acc:HGNC:30729] |
| ENSG00000179855 | HGLibA_19123 | 157 | 0.285425346 | GIPC3 | GIPC PDZ domain containing family member 3 [Source:HGNC Symbol;Acc:HGNC:18183] |
| ENSG00000182185 | HGLibA_40087 | 157 | 0.285425346 | RAD51B | RAD51 paralog B [Source:HGNC Symbol;Acc:HGNC:9822] |
| ENSG00000183134 | HGLibA_39306 | 157 | 0.285425346 | PTGDR2 | prostaglandin D2 receptor 2 [Source:HGNC Symbol;Acc:HGNC:4502] |
| ENSG00000183305 | HGLibA_27907 | 157 | 0.285425346 | MAGEA2B | MAGE family member A2B [Source:HGNC Symbol;Acc:HGNC:19340] |
| ENSG00000184925 | HGLibA_26171 | 157 | 0.285425346 | LCN12 | lipocalin 12 [Source:HGNC Symbol;Acc:HGNC:28733] |
| ENSG00000185669 | HGLibA_45872 | 157 | 0.285425346 | SNAI3 | snail family transcriptional repressor 3 [Source:HGNC Symbol;Acc:HGNC:18411] |
| ENSG00000186007 | HGLibA_26285 | 157 | 0.285425346 | LEMD1 | LEM domain containing 1 [Source:HGNC Symbol;Acc:HGNC:18725] |
| ENSG00000186660 | HGLibA_55243 | 157 | 0.285425346 | ZFP91 | ZFP91 zinc finger protein [Source:HGNC Symbol;Acc:HGNC:14983] |
| ENSG00000188171 | HGLibA_56423 | 157 | 0.285425346 | ZNF626 | zinc finger protein 626 [Source:HGNC Symbol;Acc:HGNC:30461] |
| ENSG00000196578 | HGLibA_34236 | 157 | 0.285425346 | OR5AC2 | olfactory receptor family 5 subfamily AC member 2 [Source:HGNC Symbol;Acc:HGNC:15431] |
| ENSG00000196998 | HGLibA_54097 | 157 | 0.285425346 | WDR45 | WD repeat domain 45 [Source:HGNC Symbol;Acc:HGNC:28912] |
| ENSG00000197651 | HGLibA_07944 | 157 | 0.285425346 | CCER1 | coiled-coil glutamate rich protein 1 [Source:HGNC Symbol;Acc:HGNC:28373] |
| ENSG00000197798 | HGLibA_16051 | 157 | 0.285425346 | FAM118B | family with sequence similarity 118 member B [Source:HGNC Symbol;Acc:HGNC:26110] |
| ENSG00000198889 | HGLibA_12420 | 157 | 0.285425346 | DCAF12L1 | DDB1 and CUL4 associated factor 12 like 1 [Source:HGNC Symbol;Acc:HGNC:29395] |
| ENSG00000203667 | HGLibA_10826 | 157 | 0.285425346 | COX20 | cytochrome c oxidase assembly factor COX20 [Source:HGNC Symbol;Acc:HGNC:26970] |
| ENSG00000213585 | HGLibA_53599 | 157 | 0.285425346 | VDAC1 | voltage dependent anion channel 1 [Source:HGNC Symbol;Acc:HGNC:12669] |
| ENSG00000214688 | HGLibA_05021 | 157 | 0.285425346 | C10orf105 | chromosome 10 open reading frame 105 [Source:HGNC Symbol;Acc:HGNC:20304] |
| ENSG00000234545 | HGLibA_16121 | 157 | 0.285425346 | FAM133B | family with sequence similarity 133 member B [Source:HGNC Symbol;Acc:HGNC:28629] |
| ENSG00000234906 | HGLibA_02507 | 157 | 0.285425346 | APOC2 | apolipoprotein C2 [Source:HGNC Symbol;Acc:HGNC:609] |
| ENSG00000238083 | HGLibA_27330 | 157 | 0.285425346 | LRRC37A2 | leucine rich repeat containing 37 member A2 [Source:HGNC Symbol;Acc:HGNC:32404] |
| ENSG00000240857 | HGLibA_40743 | 157 | 0.285425346 | RDH14 | retinol dehydrogenase 14 [Source:HGNC Symbol;Acc:HGNC:19979] |
| ENSG00000254737 | HGLibA_33494 | 157 | 0.285425346 | OR10G4 | olfactory receptor family 10 subfamily G member 4 [Source:HGNC Symbol;Acc:HGNC:14809] |
| ENSG00000256980 | HGLibA_24559 | 157 | 0.285425346 | KHDC1L | KH domain containing 1 like [Source:HGNC Symbol;Acc:HGNC:37274] |
| ENSG00000272398 | HGLibA_08302 | 157 | 0.285425346 | CD24 | CD24 molecule [Source:HGNC Symbol;Acc:HGNC:1645] |
| ENSG00000279000 | HGLibA_33472 | 157 | 0.285425346 | OR10A6 | olfactory receptor family 10 subfamily A member 6 (gene/pseudogene) [Source:HGNC Symbol;Acc:HGNC:15132] |
| HIST1H3A | HGLibA_21456 | 157 | 0.285425346 | HIST1H3A | - |
| hsa-mir-101-1 | HGLibA_57153 | 157 | 0.285425346 | hsa-mir-101-1 | - |
| hsa-mir-1287 | HGLibA_57612 | 157 | 0.285425346 | hsa-mir-1287 | - |
| hsa-mir-193b | HGLibA_58076 | 157 | 0.285425346 | hsa-mir-193b | - |
| hsa-mir-3128 | HGLibA_58566 | 157 | 0.285425346 | hsa-mir-3128 | - |
| hsa-mir-3680-1 | HGLibA_59268 | 157 | 0.285425346 | hsa-mir-3680-1 | - |
| hsa-mir-3689d-1 | HGLibA_59324 | 157 | 0.285425346 | hsa-mir-3689d-1 | - |
| hsa-mir-3714 | HGLibA_59368 | 157 | 0.285425346 | hsa-mir-3714 | - |
| hsa-mir-4286 | HGLibA_59852 | 157 | 0.285425346 | hsa-mir-4286 | - |
| hsa-mir-4470 | HGLibA_60286 | 157 | 0.285425346 | hsa-mir-4470 | - |
| hsa-mir-4516 | HGLibA_60506 | 157 | 0.285425346 | hsa-mir-4516 | - |
| hsa-mir-454 | HGLibA_60623 | 157 | 0.285425346 | hsa-mir-454 | - |
| hsa-mir-518a-2 | HGLibA_61657 | 157 | 0.285425346 | hsa-mir-518a-2 | - |
| hsa-mir-544a | HGLibA_61825 | 157 | 0.285425346 | hsa-mir-544a | - |
| hsa-mir-562 | HGLibA_62212 | 157 | 0.285425346 | hsa-mir-562 | - |
| hsa-mir-6134 | HGLibA_62687 | 157 | 0.285425346 | hsa-mir-6134 | - |
| hsa-mir-653 | HGLibA_62947 | 157 | 0.285425346 | hsa-mir-653 | - |
| hsa-mir-6868 | HGLibA_63655 | 157 | 0.285425346 | hsa-mir-6868 | - |
| hsa-mir-873 | HGLibA_64202 | 157 | 0.285425346 | hsa-mir-873 | - |
| hsa-mir-875 | HGLibA_64212 | 157 | 0.285425346 | hsa-mir-875 | - |
| KIAA0556 | HGLibA_24645 | 157 | 0.285425346 | KIAA0556 | - |
| LSMD1 | HGLibA_27554 | 157 | 0.285425346 | LSMD1 | - |
| PALM2-AKAP2 | HGLibA_35072 | 157 | 0.285425346 | PALM2-AKAP2 | - |
| PLCXD1_X | HGLibA_37021 | 157 | 0.285425346 | PLCXD1_X | - |
| C1orf177 | HGLibA_05801 | 156 | 0.276380003 | C1orf177 | - |
| C9orf91 | HGLibA_06777 | 156 | 0.276380003 | C9orf91 | - |
| ENSG00000005700 | HGLibA_22485 | 156 | 0.276380003 | IBTK | inhibitor of Bruton tyrosine kinase [Source:HGNC Symbol;Acc:HGNC:17853] |
| ENSG00000041357 | HGLibA_39079 | 156 | 0.276380003 | PSMA4 | proteasome subunit alpha 4 [Source:HGNC Symbol;Acc:HGNC:9533] |
| ENSG00000044012 | HGLibA_20646 | 156 | 0.276380003 | GUCA2B | guanylate cyclase activator 2B [Source:HGNC Symbol;Acc:HGNC:4683] |
| ENSG00000053254 | HGLibA_17916 | 156 | 0.276380003 | FOXN3 | forkhead box N3 [Source:HGNC Symbol;Acc:HGNC:1928] |
| ENSG00000067167 | HGLibA_51283 | 156 | 0.276380003 | TRAM1 | translocation associated membrane protein 1 [Source:HGNC Symbol;Acc:HGNC:20568] |
| ENSG00000067560 | HGLibA_41167 | 156 | 0.276380003 | RHOA | ras homolog family member A [Source:HGNC Symbol;Acc:HGNC:667] |
| ENSG00000070961 | HGLibA_03615 | 156 | 0.276380003 | ATP2B1 | ATPase plasma membrane Ca2+ transporting 1 [Source:HGNC Symbol;Acc:HGNC:814] |
| ENSG00000076554 | HGLibA_51110 | 156 | 0.276380003 | TPD52 | tumor protein D52 [Source:HGNC Symbol;Acc:HGNC:12005] |
| ENSG00000082014 | HGLibA_45635 | 156 | 0.276380003 | SMARCD3 | member 3 [Source:HGNC Symbol;Acc:HGNC:11108]" |
| ENSG00000092931 | HGLibA_29087 | 156 | 0.276380003 | MFSD11 | major facilitator superfamily domain containing 11 [Source:HGNC Symbol;Acc:HGNC:25458] |
| ENSG00000099783 | HGLibA_21839 | 156 | 0.276380003 | HNRNPM | heterogeneous nuclear ribonucleoprotein M [Source:HGNC Symbol;Acc:HGNC:5046] |
| ENSG00000101605 | HGLibA_30889 | 156 | 0.276380003 | MYOM1 | myomesin 1 [Source:HGNC Symbol;Acc:HGNC:7613] |
| ENSG00000101911 | HGLibA_38760 | 156 | 0.276380003 | PRPS2 | phosphoribosyl pyrophosphate synthetase 2 [Source:HGNC Symbol;Acc:HGNC:9465] |
| ENSG00000102119 | HGLibA_15031 | 156 | 0.276380003 | EMD | emerin [Source:HGNC Symbol;Acc:HGNC:3331] |
| ENSG00000104833 | HGLibA_52286 | 156 | 0.276380003 | TUBB4A | tubulin beta 4A class IVa [Source:HGNC Symbol;Acc:HGNC:20774] |
| ENSG00000105255 | HGLibA_18076 | 156 | 0.276380003 | FSD1 | fibronectin type III and SPRY domain containing 1 [Source:HGNC Symbol;Acc:HGNC:13745] |
| ENSG00000106328 | HGLibA_18074 | 156 | 0.276380003 | FSCN3 | fascin actin-bundling protein 3 [Source:HGNC Symbol;Acc:HGNC:3961] |
| ENSG00000106477 | HGLibA_09138 | 156 | 0.276380003 | CEP41 | centrosomal protein 41 [Source:HGNC Symbol;Acc:HGNC:12370] |
| ENSG00000112218 | HGLibA_20069 | 156 | 0.276380003 | GPR63 | G protein-coupled receptor 63 [Source:HGNC Symbol;Acc:HGNC:13302] |
| ENSG00000114054 | HGLibA_35383 | 156 | 0.276380003 | PCCB | propionyl-CoA carboxylase subunit beta [Source:HGNC Symbol;Acc:HGNC:8654] |
| ENSG00000114115 | HGLibA_40630 | 156 | 0.276380003 | RBP1 | retinol binding protein 1 [Source:HGNC Symbol;Acc:HGNC:9919] |
| ENSG00000114127 | HGLibA_54603 | 156 | 0.276380003 | XRN1 | 5'-3' exoribonuclease 1 [Source:HGNC Symbol;Acc:HGNC:30654] |
| ENSG00000115596 | HGLibA_54378 | 156 | 0.276380003 | WNT6 | Wnt family member 6 [Source:HGNC Symbol;Acc:HGNC:12785] |
| ENSG00000115687 | HGLibA_35268 | 156 | 0.276380003 | PASK | PAS domain containing serine/threonine kinase [Source:HGNC Symbol;Acc:HGNC:17270] |
| ENSG00000115760 | HGLibA_04525 | 156 | 0.276380003 | BIRC6 | baculoviral IAP repeat containing 6 [Source:HGNC Symbol;Acc:HGNC:13516] |
| ENSG00000116786 | HGLibA_37139 | 156 | 0.276380003 | PLEKHM2 | pleckstrin homology and RUN domain containing M2 [Source:HGNC Symbol;Acc:HGNC:29131] |
| ENSG00000116885 | HGLibA_34674 | 156 | 0.276380003 | OSCP1 | organic solute carrier partner 1 [Source:HGNC Symbol;Acc:HGNC:29971] |
| ENSG00000116962 | HGLibA_31922 | 156 | 0.276380003 | NID1 | nidogen 1 [Source:HGNC Symbol;Acc:HGNC:7821] |
| ENSG00000119121 | HGLibA_51768 | 156 | 0.276380003 | TRPM6 | transient receptor potential cation channel subfamily M member 6 [Source:HGNC Symbol;Acc:HGNC:17995] |
| ENSG00000119686 | HGLibA_17678 | 156 | 0.276380003 | FLVCR2 | feline leukemia virus subgroup C cellular receptor family member 2 [Source:HGNC Symbol;Acc:HGNC:20105] |
| ENSG00000120278 | HGLibA_37101 | 156 | 0.276380003 | PLEKHG1 | pleckstrin homology and RhoGEF domain containing G1 [Source:HGNC Symbol;Acc:HGNC:20884] |
| ENSG00000120328 | HGLibA_35478 | 156 | 0.276380003 | PCDHB12 | protocadherin beta 12 [Source:HGNC Symbol;Acc:HGNC:8683] |
| ENSG00000120498 | HGLibA_49051 | 156 | 0.276380003 | TEX11 | testis expressed 11 [Source:HGNC Symbol;Acc:HGNC:11733] |
| ENSG00000122679 | HGLibA_40186 | 156 | 0.276380003 | RAMP3 | receptor activity modifying protein 3 [Source:HGNC Symbol;Acc:HGNC:9845] |
| ENSG00000124467 | HGLibA_39054 | 156 | 0.276380003 | PSG8 | pregnancy specific beta-1-glycoprotein 8 [Source:HGNC Symbol;Acc:HGNC:9525] |

| | | | | | |
|---|---|---|---|---|---|
| ENSG00000126890 | HGLibA_11563 | 156 | 0.276380003 | CTAG2 | cancer/testis antigen 2 [Source:HGNC Symbol;Acc:HGNC:2492] |
| ENSG00000127074 | HGLibA_41060 | 156 | 0.276380003 | RGS13 | regulator of G protein signaling 13 [Source:HGNC Symbol;Acc:HGNC:9995] |
| ENSG00000127540 | HGLibA_53107 | 156 | 0.276380003 | UQCR11 | complex III subunit XI [Source:HGNC Symbol;Acc:HGNC:30862]" |
| ENSG00000127948 | HGLibA_37706 | 156 | 0.276380003 | POR | cytochrome p450 oxidoreductase [Source:HGNC Symbol;Acc:HGNC:9208] |
| ENSG00000128322 | HGLibA_22866 | 156 | 0.276380003 | IGLL1 | immunoglobulin lambda like polypeptide 1 [Source:HGNC Symbol;Acc:HGNC:5870] |
| ENSG00000131183 | HGLibA_44937 | 156 | 0.276380003 | SLC34A1 | solute carrier family 34 member 1 [Source:HGNC Symbol;Acc:HGNC:11019] |
| ENSG00000131788 | HGLibA_36512 | 156 | 0.276380003 | PIAS3 | protein inhibitor of activated STAT 3 [Source:HGNC Symbol;Acc:HGNC:16861] |
| ENSG00000133597 | HGLibA_00893 | 156 | 0.276380003 | ADCK2 | aarF domain containing kinase 2 [Source:HGNC Symbol;Acc:HGNC:19039] |
| ENSG00000135373 | HGLibA_14678 | 156 | 0.276380003 | EHF | ETS homologous factor [Source:HGNC Symbol;Acc:HGNC:3246] |
| ENSG00000135778 | HGLibA_32870 | 156 | 0.276380003 | NTPCR | cancer-related [Source:HGNC Symbol;Acc:HGNC:28204]" |
| ENSG00000136463 | HGLibA_48169 | 156 | 0.276380003 | TACO1 | translational activator of cytochrome c oxidase I [Source:HGNC Symbol;Acc:HGNC:24316] |
| ENSG00000136688 | HGLibA_23150 | 156 | 0.276380003 | IL36G | interleukin 36 gamma [Source:HGNC Symbol;Acc:HGNC:15741] |
| ENSG00000137656 | HGLibA_04998 | 156 | 0.276380003 | BUD13 | BUD13 homolog [Source:HGNC Symbol;Acc:HGNC:28199] |
| ENSG00000137845 | HGLibA_00728 | 156 | 0.276380003 | ADAM10 | ADAM metallopeptidase domain 10 [Source:HGNC Symbol;Acc:HGNC:188] |
| ENSG00000138193 | HGLibA_37000 | 156 | 0.276380003 | PLCE1 | phospholipase C epsilon 1 [Source:HGNC Symbol;Acc:HGNC:17175] |
| ENSG00000139725 | HGLibA_41188 | 156 | 0.276380003 | RHOF | filopodia associated [Source:HGNC Symbol;Acc:HGNC:15703]" |
| ENSG00000140577 | HGLibA_11233 | 156 | 0.276380003 | CRTC3 | CREB regulated transcription coactivator 3 [Source:HGNC Symbol;Acc:HGNC:26148] |
| ENSG00000143375 | HGLibA_09321 | 156 | 0.276380003 | CGN | cingulin [Source:HGNC Symbol;Acc:HGNC:17429] |
| ENSG00000146243 | HGLibA_23556 | 156 | 0.276380003 | IRAK1BP1 | interleukin 1 receptor associated kinase 1 binding protein 1 [Source:HGNC Symbol;Acc:HGNC:17368] |
| ENSG00000146731 | HGLibA_08208 | 156 | 0.276380003 | CCT6A | chaperonin containing TCP1 subunit 6A [Source:HGNC Symbol;Acc:HGNC:1620] |
| ENSG00000154016 | HGLibA_20212 | 156 | 0.276380003 | GRAP | GRB2 related adaptor protein [Source:HGNC Symbol;Acc:HGNC:4562] |
| ENSG00000154133 | HGLibA_41742 | 156 | 0.276380003 | ROBO4 | roundabout guidance receptor 4 [Source:HGNC Symbol;Acc:HGNC:17985] |
| ENSG00000154545 | HGLibA_27974 | 156 | 0.276380003 | MAGED4 | MAGE family member D4 [Source:HGNC Symbol;Acc:HGNC:23793] |
| ENSG00000154764 | HGLibA_54382 | 156 | 0.276380003 | WNT7A | Wnt family member 7A [Source:HGNC Symbol;Acc:HGNC:12786] |
| ENSG00000156970 | HGLibA_04992 | 156 | 0.276380003 | BUB1B | BUB1 mitotic checkpoint serine/threonine kinase B [Source:HGNC Symbol;Acc:HGNC:1149] |
| ENSG00000158301 | HGLibA_20122 | 156 | 0.276380003 | GPRASP2 | G protein-coupled receptor associated sorting protein 2 [Source:HGNC Symbol;Acc:HGNC:25169] |
| ENSG00000162623 | HGLibA_52485 | 156 | 0.276380003 | TYW3 | tRNA-yW synthesizing protein 3 homolog [Source:HGNC Symbol;Acc:HGNC:24757] |
| ENSG00000162664 | HGLibA_55806 | 156 | 0.276380003 | ZNF326 | zinc finger protein 326 [Source:HGNC Symbol;Acc:HGNC:14104] |
| ENSG00000163218 | HGLibA_36259 | 156 | 0.276380003 | PGLYRP4 | peptidoglycan recognition protein 4 [Source:HGNC Symbol;Acc:HGNC:30015] |
| ENSG00000163527 | HGLibA_47609 | 156 | 0.276380003 | STT3B | catalytic subunit of the oligosaccharyltransferase complex [Source:HGNC Symbol;Acc:HGNC:30611]" |
| ENSG00000163687 | HGLibA_13662 | 156 | 0.276380003 | DNASE1L3 | deoxyribonuclease 1 like 3 [Source:HGNC Symbol;Acc:HGNC:2959] |
| ENSG00000164294 | HGLibA_20191 | 156 | 0.276380003 | GPX8 | glutathione peroxidase 8 (putative) [Source:HGNC Symbol;Acc:HGNC:33100] |
| ENSG00000164691 | HGLibA_48268 | 156 | 0.276380003 | TAGAP | T cell activation RhoGTPase activating protein [Source:HGNC Symbol;Acc:HGNC:15669] |
| ENSG00000164893 | HGLibA_45354 | 156 | 0.276380003 | SLC7A13 | solute carrier family 7 member 13 [Source:HGNC Symbol;Acc:HGNC:23092] |
| ENSG00000165168 | HGLibA_12020 | 156 | 0.276380003 | CYBB | cytochrome b-245 beta chain [Source:HGNC Symbol;Acc:HGNC:2578] |
| ENSG00000167842 | HGLibA_29303 | 156 | 0.276380003 | MIS12 | kinetochore complex component [Source:HGNC Symbol;Acc:HGNC:24967]" |
| ENSG00000170345 | HGLibA_17794 | 156 | 0.276380003 | FOS | AP-1 transcription factor subunit [Source:HGNC Symbol;Acc:HGNC:3796]" |
| ENSG00000170743 | HGLibA_48094 | 156 | 0.276380003 | SYT9 | synaptotagmin 9 [Source:HGNC Symbol;Acc:HGNC:19265] |
| ENSG00000172324 | HGLibA_34231 | 156 | 0.276380003 | OR5A2 | olfactory receptor family 5 subfamily A member 2 [Source:HGNC Symbol;Acc:HGNC:15249] |
| ENSG00000173166 | HGLibA_40266 | 156 | 0.276380003 | RAPH1 | Ras association (RalGDS/AF-6) and pleckstrin homology domains 1 [Source:HGNC Symbol;Acc:HGNC:14436] |
| ENSG00000175197 | HGLibA_12615 | 156 | 0.276380003 | DDIT3 | DNA damage inducible transcript 3 [Source:HGNC Symbol;Acc:HGNC:2726] |
| ENSG00000176635 | HGLibA_21891 | 156 | 0.276380003 | HORMAD2 | HORMA domain containing 2 [Source:HGNC Symbol;Acc:HGNC:28383] |
| ENSG00000176922 | HGLibA_34131 | 156 | 0.276380003 | OR51S1 | olfactory receptor family 51 subfamily S member 1 [Source:HGNC Symbol;Acc:HGNC:15204] |
| ENSG00000178397 | HGLibA_16512 | 156 | 0.276380003 | FAM220A | family with sequence similarity 220 member A [Source:HGNC Symbol;Acc:HGNC:22422] |
| ENSG00000180828 | HGLibA_04475 | 156 | 0.276380003 | BHLHE22 | basic helix-loop-helix family member e22 [Source:HGNC Symbol;Acc:HGNC:11963] |
| ENSG00000181444 | HGLibA_56043 | 156 | 0.276380003 | ZNF467 | zinc finger protein 467 [Source:HGNC Symbol;Acc:HGNC:23154] |
| ENSG00000185792 | HGLibA_32137 | 156 | 0.276380003 | NLRP9 | NLR family pyrin domain containing 9 [Source:HGNC Symbol;Acc:HGNC:22941] |
| ENSG00000196700 | HGLibA_56133 | 156 | 0.276380003 | ZNF512B | zinc finger protein 512B [Source:HGNC Symbol;Acc:HGNC:29212] |
| ENSG00000197471 | HGLibA_46731 | 156 | 0.276380003 | SPN | sialophorin [Source:HGNC Symbol;Acc:HGNC:11249] |
| ENSG00000198908 | HGLibA_04472 | 156 | 0.276380003 | BHLHB9 | basic helix-loop-helix family member b9 [Source:HGNC Symbol;Acc:HGNC:29353] |
| ENSG00000204267 | HGLibA_48318 | 156 | 0.276380003 | TAP2 | ATP binding cassette subfamily B member [Source:HGNC Symbol;Acc:HGNC:44]" |
| ENSG00000204505 | HGLibA_38332 | 156 | 0.276380003 | PRAMEF9 | PRAME family member 9 [Source:HGNC Symbol;Acc:HGNC:27996] |
| ENSG00000204571 | HGLibA_25787 | 156 | 0.276380003 | KRTAP5-11 | keratin associated protein 5-11 [Source:HGNC Symbol;Acc:HGNC:23606] |
| ENSG00000204920 | HGLibA_55503 | 156 | 0.276380003 | ZNF155 | zinc finger protein 155 [Source:HGNC Symbol;Acc:HGNC:12940] |
| ENSG00000214415 | HGLibA_19477 | 156 | 0.276380003 | GNAT3 | G protein subunit alpha transducin 3 [Source:HGNC Symbol;Acc:HGNC:22800] |
| ENSG00000214941 | HGLibA_57054 | 156 | 0.276380003 | ZSWIM7 | zinc finger SWIM-type containing 7 [Source:HGNC Symbol;Acc:HGNC:26993] |
| ENSG00000221968 | HGLibA_15941 | 156 | 0.276380003 | FADS3 | fatty acid desaturase 3 [Source:HGNC Symbol;Acc:HGNC:3576] |
| ENSG00000231738 | HGLibA_51919 | 156 | 0.276380003 | TSPAN19 | tetraspanin 19 [Source:HGNC Symbol;Acc:HGNC:31886] |
| ENSG00000268738 | HGLibA_22237 | 156 | 0.276380003 | HSFX2 | X-linked 2 [Source:HGNC Symbol;Acc:HGNC:32701]" |
| ENSG00000273274 | HGLibA_54928 | 156 | 0.276380003 | ZBTB8B | zinc finger and BTB domain containing 8B [Source:HGNC Symbol;Acc:HGNC:37057] |
| ENSG00000279761 | HGLibA_34274 | 156 | 0.276380003 | OR5D13 | olfactory receptor family 5 subfamily D member 13 (gene/pseudogene) [Source:HGNC Symbol;Acc:HGNC:15280] |
| FAM195B | HGLibA_16378 | 156 | 0.276380003 | FAM195B | - |
| FAM46B | HGLibA_16599 | 156 | 0.276380003 | FAM46B | - |
| GATSL3 | HGLibA_18707 | 156 | 0.276380003 | GATSL3 | - |
| H3F3A | HGLibA_20777 | 156 | 0.276380003 | H3F3A | - |
| HIST1H3E | HGLibA_21467 | 156 | 0.276380003 | HIST1H3E | - |
| hsa-mir-1295b | HGLibA_57662 | 156 | 0.276380003 | hsa-mir-1295b | - |
| hsa-mir-150 | HGLibA_57890 | 156 | 0.276380003 | hsa-mir-150 | - |
| hsa-mir-200a | HGLibA_58152 | 156 | 0.276380003 | hsa-mir-200a | - |
| hsa-mir-211 | HGLibA_58216 | 156 | 0.276380003 | hsa-mir-211 | - |
| hsa-mir-2115 | HGLibA_58233 | 156 | 0.276380003 | hsa-mir-2115 | - |
| hsa-mir-4316 | HGLibA_59984 | 156 | 0.276380003 | hsa-mir-4316 | - |
| hsa-mir-4452 | HGLibA_60213 | 156 | 0.276380003 | hsa-mir-4452 | - |
| hsa-mir-449c | HGLibA_60426 | 156 | 0.276380003 | hsa-mir-449c | - |
| hsa-mir-450b | HGLibA_60479 | 156 | 0.276380003 | hsa-mir-450b | - |
| hsa-mir-4531 | HGLibA_60584 | 156 | 0.276380003 | hsa-mir-4531 | - |
| hsa-mir-4768 | HGLibA_61169 | 156 | 0.276380003 | hsa-mir-4768 | - |
| hsa-mir-4792 | HGLibA_61276 | 156 | 0.276380003 | hsa-mir-4792 | - |
| hsa-mir-541 | HGLibA_61815 | 156 | 0.276380003 | hsa-mir-541 | - |
| hsa-mir-6785 | HGLibA_63317 | 156 | 0.276380003 | hsa-mir-6785 | - |
| hsa-mir-7-2 | HGLibA_63772 | 156 | 0.276380003 | hsa-mir-7-2 | - |
| hsa-mir-941-4 | HGLibA_64349 | 156 | 0.276380003 | hsa-mir-941-4 | - |
| KIAA1715 | HGLibA_24807 | 156 | 0.276380003 | KIAA1715 | - |
| LOC100130357 | HGLibA_26780 | 156 | 0.276380003 | LOC100130357 | - |
| LPPR4 | HGLibA_27122 | 156 | 0.276380003 | LPPR4 | - |
| TMEM189-UBE2V1 | HGLibA_50135 | 156 | 0.276380003 | TMEM189-UBE2V1 | - |
| AKAP17A_Y | HGLibA_01424 | 155 | 0.26733466 | AKAP17A_Y | - |
| C1orf168 | HGLibA_05790 | 155 | 0.26733466 | C1orf168 | - |
| ELMSAN1 | HGLibA_14949 | 155 | 0.26733466 | ELMSAN1 | - |
| ENSG00000034152 | HGLibA_28145 | 155 | 0.26733466 | MAP2K3 | mitogen-activated protein kinase kinase 3 [Source:HGNC Symbol;Acc:HGNC:6843] |
| ENSG00000054803 | HGLibA_07416 | 155 | 0.26733466 | CBLN4 | cerebellin 4 precursor [Source:HGNC Symbol;Acc:HGNC:16231] |
| ENSG00000056558 | HGLibA_51240 | 155 | 0.26733466 | TRAF1 | TNF receptor associated factor 1 [Source:HGNC Symbol;Acc:HGNC:12031] |
| ENSG00000057019 | HGLibA_12473 | 155 | 0.26733466 | DCBLD2 | CUB and LCCL domain containing 2 [Source:HGNC Symbol;Acc:HGNC:24627]" |
| ENSG00000065328 | HGLibA_28602 | 155 | 0.26733466 | MCM10 | minichromosome maintenance 10 replication initiation factor [Source:HGNC Symbol;Acc:HGNC:18043] |
| ENSG00000070748 | HGLibA_09355 | 155 | 0.26733466 | CHAT | choline O-acetyltransferase [Source:HGNC Symbol;Acc:HGNC:1912] |
| ENSG00000073146 | HGLibA_29696 | 155 | 0.26733466 | MOV10L1 | Mov10 like RISC complex RNA helicase 1 [Source:HGNC Symbol;Acc:HGNC:7201] |
| ENSG00000079156 | HGLibA_34662 | 155 | 0.26733466 | OSBPL6 | oxysterol binding protein like 6 [Source:HGNC Symbol;Acc:HGNC:16388] |
| ENSG00000080371 | HGLibA_39838 | 155 | 0.26733466 | RAB21 | member RAS oncogene family [Source:HGNC Symbol;Acc:HGNC:18263]" |
| ENSG00000089116 | HGLibA_26460 | 155 | 0.26733466 | LHX5 | LIM homeobox 5 [Source:HGNC Symbol;Acc:HGNC:14216] |
| ENSG00000092067 | HGLibA_08966 | 155 | 0.26733466 | CEBPE | CCAAT enhancer binding protein epsilon [Source:HGNC Symbol;Acc:HGNC:1836] |
| ENSG00000100201 | HGLibA_12657 | 155 | 0.26733466 | DDX17 | DEAD-box helicase 17 [Source:HGNC Symbol;Acc:HGNC:2740] |
| ENSG00000100442 | HGLibA_17574 | 155 | 0.26733466 | FKBP3 | FK506 binding protein 3 [Source:HGNC Symbol;Acc:HGNC:3719] |
| ENSG00000101457 | HGLibA_13720 | 155 | 0.26733466 | DNTTIP1 | deoxynucleotidyltransferase terminal interacting protein 1 [Source:HGNC Symbol;Acc:HGNC:16160] |
| ENSG00000103507 | HGLibA_04284 | 155 | 0.26733466 | BCKDK | branched chain ketoacid dehydrogenase kinase [Source:HGNC Symbol;Acc:HGNC:16902] |
| ENSG00000105717 | HGLibA_35351 | 155 | 0.26733466 | PBX4 | PBX homeobox 4 [Source:HGNC Symbol;Acc:HGNC:13403] |
| ENSG00000107984 | HGLibA_13295 | 155 | 0.26733466 | DKK1 | dickkopf WNT signaling pathway inhibitor 1 [Source:HGNC Symbol;Acc:HGNC:2891] |
| ENSG00000111052 | HGLibA_26574 | 155 | 0.26733466 | LIN7A | crumbs cell polarity complex component [Source:HGNC Symbol;Acc:HGNC:17787]" |
| ENSG00000112276 | HGLibA_05003 | 155 | 0.26733466 | BVES | blood vessel epicardial substance [Source:HGNC Symbol;Acc:HGNC:1152] |
| ENSG00000113494 | HGLibA_38606 | 155 | 0.26733466 | PRLR | prolactin receptor [Source:HGNC Symbol;Acc:HGNC:9446] |
| ENSG00000113721 | HGLibA_35857 | 155 | 0.26733466 | PDGFRB | platelet derived growth factor receptor beta [Source:HGNC Symbol;Acc:HGNC:8804] |
| ENSG00000113790 | HGLibA_14682 | 155 | 0.26733466 | EHHADH | enoyl-CoA hydratase and 3-hydroxyacyl CoA dehydrogenase [Source:HGNC Symbol;Acc:HGNC:3247] |
| ENSG00000115919 | HGLibA_25881 | 155 | 0.26733466 | KYNU | kynureninase [Source:HGNC Symbol;Acc:HGNC:6469] |
| ENSG00000117281 | HGLibA_08240 | 155 | 0.26733466 | CD160 | CD160 molecule [Source:HGNC Symbol;Acc:HGNC:17013] |
| ENSG00000120645 | HGLibA_23548 | 155 | 0.26733466 | IQSEC3 | IQ motif and Sec7 domain 3 [Source:HGNC Symbol;Acc:HGNC:29193] |
| ENSG00000122642 | HGLibA_17591 | 155 | 0.26733466 | FKBP9 | FK506 binding protein 9 [Source:HGNC Symbol;Acc:HGNC:3725] |
| ENSG00000123453 | HGLibA_42742 | 155 | 0.26733466 | SARDH | sarcosine dehydrogenase [Source:HGNC Symbol;Acc:HGNC:10536] |
| ENSG00000123584 | HGLibA_27924 | 155 | 0.26733466 | MAGEA9 | MAGE family member A9 [Source:HGNC Symbol;Acc:HGNC:6807] |
| ENSG00000124827 | HGLibA_18802 | 155 | 0.26733466 | GCM2 | glial cells missing homolog 2 [Source:HGNC Symbol;Acc:HGNC:4198] |
| ENSG00000125820 | HGLibA_32035 | 155 | 0.26733466 | NKX2-2 | NK2 homeobox 2 [Source:HGNC Symbol;Acc:HGNC:7835] |
| ENSG00000127529 | HGLibA_34470 | 155 | 0.26733466 | OR7C2 | olfactory receptor family 7 subfamily C member 2 [Source:HGNC Symbol;Acc:HGNC:8374] |
| ENSG00000128510 | HGLibA_10892 | 155 | 0.26733466 | CPA4 | carboxypeptidase A4 [Source:HGNC Symbol;Acc:HGNC:15740] |
| ENSG00000132932 | HGLibA_03778 | 155 | 0.26733466 | ATP8A2 | ATPase phospholipid transporting 8A2 [Source:HGNC Symbol;Acc:HGNC:13533] |
| ENSG00000133636 | HGLibA_32882 | 155 | 0.26733466 | NTS | neurotensin [Source:HGNC Symbol;Acc:HGNC:8038] |
| ENSG00000135702 | HGLibA_09639 | 155 | 0.26733466 | CHST5 | carbohydrate sulfotransferase 5 [Source:HGNC Symbol;Acc:HGNC:1973] |
| ENSG00000136267 | HGLibA_13061 | 155 | 0.26733466 | DGKB | diacylglycerol kinase beta [Source:HGNC Symbol;Acc:HGNC:2850] |
| ENSG00000136827 | HGLibA_51015 | 155 | 0.26733466 | TOR1A | torsin family 1 member A [Source:HGNC Symbol;Acc:HGNC:3098] |
| ENSG00000137700 | HGLibA_45052 | 155 | 0.26733466 | SLC37A4 | solute carrier family 37 member 4 [Source:HGNC Symbol;Acc:HGNC:4061] |
| ENSG00000139269 | HGLibA_23309 | 155 | 0.26733466 | INHBE | inhibin subunit beta E [Source:HGNC Symbol;Acc:HGNC:24029] |
| ENSG00000140830 | HGLibA_52441 | 155 | 0.26733466 | TXNL4B | thioredoxin like 4B [Source:HGNC Symbol;Acc:HGNC:26041] |
| ENSG00000142530 | HGLibA_16698 | 155 | 0.26733466 | FAM71E1 | family with sequence similarity 71 member E1 [Source:HGNC Symbol;Acc:HGNC:25107] |
| ENSG00000144840 | HGLibA_40034 | 155 | 0.26733466 | RABL3 | member of RAS oncogene family like 3 [Source:HGNC Symbol;Acc:HGNC:18072]" |
| ENSG00000145708 | HGLibA_11139 | 155 | 0.26733466 | CRHBP | corticotropin releasing hormone binding protein [Source:HGNC Symbol;Acc:HGNC:2356] |
| ENSG00000149043 | HGLibA_48089 | 155 | 0.26733466 | SYT8 | synaptotagmin 8 [Source:HGNC Symbol;Acc:HGNC:19264] |
| ENSG00000149124 | HGLibA_19371 | 155 | 0.26733466 | GLYAT | glycine-N-acyltransferase [Source:HGNC Symbol;Acc:HGNC:13734] |
| ENSG00000150556 | HGLibA_27730 | 155 | 0.26733466 | LYPD6B | LY6/PLAUR domain containing 6B [Source:HGNC Symbol;Acc:HGNC:27018] |

| | | | | | |
|---|---|---|---|---|---|
| ENSG00000153179 | HGLibA_40379 | 155 | 0.26733466 | RASSF3 | Ras association domain family member 3 [Source:HGNC Symbol;Acc:HGNC:14271] |
| ENSG00000157036 | HGLibA_15767 | 155 | 0.26733466 | EXOG | exo/endonuclease G [Source:HGNC Symbol;Acc:HGNC:3347] |
| ENSG00000159714 | HGLibA_55066 | 155 | 0.26733466 | ZDHHC1 | zinc finger DHHC-type containing 1 [Source:HGNC Symbol;Acc:HGNC:17916] |
| ENSG00000162733 | HGLibA_12637 | 155 | 0.26733466 | DDR2 | discoidin domain receptor tyrosine kinase 2 [Source:HGNC Symbol;Acc:HGNC:2731] |
| ENSG00000163040 | HGLibA_07863 | 155 | 0.26733466 | CCDC74A | coiled-coil domain containing 74A [Source:HGNC Symbol;Acc:HGNC:25197] |
| ENSG00000163093 | HGLibA_04221 | 155 | 0.26733466 | BBS5 | Bardet-Biedl syndrome 5 [Source:HGNC Symbol;Acc:HGNC:970] |
| ENSG00000163602 | HGLibA_42516 | 155 | 0.26733466 | RYBP | RING1 and YY1 binding protein [Source:HGNC Symbol;Acc:HGNC:10480] |
| ENSG00000163630 | HGLibA_48028 | 155 | 0.26733466 | SYNPR | synaptoporin [Source:HGNC Symbol;Acc:HGNC:16507] |
| ENSG00000164070 | HGLibA_22286 | 155 | 0.26733466 | HSPA4L | heat shock protein family A (Hsp70) member 4 like [Source:HGNC Symbol;Acc:HGNC:17041] |
| ENSG00000164291 | HGLibA_03156 | 155 | 0.26733466 | ARSK | arylsulfatase family member K [Source:HGNC Symbol;Acc:HGNC:25239] |
| ENSG00000164896 | HGLibA_16898 | 155 | 0.26733466 | FASTK | Fas activated serine/threonine kinase [Source:HGNC Symbol;Acc:HGNC:24676] |
| ENSG00000164924 | HGLibA_54755 | 155 | 0.26733466 | YWHAZ | tyrosine 3-monooxygenase/tryptophan 5-monooxygenase activation protein zeta [Source:HGNC Symbol;Acc:HGNC:12855] |
| ENSG00000165071 | HGLibA_50504 | 155 | 0.26733466 | TMEM71 | transmembrane protein 71 [Source:HGNC Symbol;Acc:HGNC:26572] |
| ENSG00000167971 | HGLibA_07289 | 155 | 0.26733466 | CASKIN1 | CASK interacting protein 1 [Source:HGNC Symbol;Acc:HGNC:20879] |
| ENSG00000168118 | HGLibA_39955 | 155 | 0.26733466 | RAB4A | member RAS oncogene family [Source:HGNC Symbol;Acc:HGNC:9781]" |
| ENSG00000169062 | HGLibA_53061 | 155 | 0.26733466 | UPF3A | regulator of nonsense mediated mRNA decay [Source:HGNC Symbol;Acc:HGNC:20332]" |
| ENSG00000169488 | HGLibA_34024 | 155 | 0.26733466 | OR4K15 | olfactory receptor family 4 subfamily K member 15 [Source:HGNC Symbol;Acc:HGNC:15353] |
| ENSG00000169592 | HGLibA_23331 | 155 | 0.26733466 | INO80E | INO80 complex subunit E [Source:HGNC Symbol;Acc:HGNC:26905] |
| ENSG00000171497 | HGLibA_37925 | 155 | 0.26733466 | PPID | peptidylprolyl isomerase D [Source:HGNC Symbol;Acc:HGNC:9257] |
| ENSG00000172188 | HGLibA_33939 | 155 | 0.26733466 | OR4C11 | olfactory receptor family 4 subfamily C member 11 [Source:HGNC Symbol;Acc:HGNC:15167] |
| ENSG00000172717 | HGLibA_16696 | 155 | 0.26733466 | FAM71D | family with sequence similarity 71 member D [Source:HGNC Symbol;Acc:HGNC:20101] |
| ENSG00000173276 | HGLibA_54840 | 155 | 0.26733466 | ZBTB21 | zinc finger and BTB domain containing 21 [Source:HGNC Symbol;Acc:HGNC:13083] |
| ENSG00000174943 | HGLibA_24423 | 155 | 0.26733466 | KCTD13 | potassium channel tetramerization domain containing 13 [Source:HGNC Symbol;Acc:HGNC:22234] |
| ENSG00000175155 | HGLibA_54710 | 155 | 0.26733466 | YPEL2 | yippee like 2 [Source:HGNC Symbol;Acc:HGNC:18326] |
| ENSG00000176774 | HGLibA_27940 | 155 | 0.26733466 | MAGEB18 | MAGE family member B18 [Source:HGNC Symbol;Acc:HGNC:28515] |
| ENSG00000176945 | HGLibA_30537 | 155 | 0.26733466 | MUC20 | cell surface associated [Source:HGNC Symbol;Acc:HGNC:23282]" |
| ENSG00000177494 | HGLibA_54793 | 155 | 0.26733466 | ZBED2 | zinc finger BED-type containing 2 [Source:HGNC Symbol;Acc:HGNC:20710] |
| ENSG00000177830 | HGLibA_09450 | 155 | 0.26733466 | CHID1 | chitinase domain containing 1 [Source:HGNC Symbol;Acc:HGNC:28474] |
| ENSG00000178445 | HGLibA_19236 | 155 | 0.26733466 | GLDC | glycine decarboxylase [Source:HGNC Symbol;Acc:HGNC:4313] |
| ENSG00000178522 | HGLibA_01747 | 155 | 0.26733466 | AMBN | ameloblastin [Source:HGNC Symbol;Acc:HGNC:452] |
| ENSG00000179399 | HGLibA_19773 | 155 | 0.26733466 | GPC5 | glypican 5 [Source:HGNC Symbol;Acc:HGNC:4453] |
| ENSG00000180209 | HGLibA_30797 | 155 | 0.26733466 | MYLPF | fast skeletal muscle [Source:HGNC Symbol;Acc:HGNC:29824]" |
| ENSG00000181135 | HGLibA_56599 | 155 | 0.26733466 | ZNF707 | zinc finger protein 707 [Source:HGNC Symbol;Acc:HGNC:27815] |
| ENSG00000181444 | HGLibA_56044 | 155 | 0.26733466 | ZNF467 | zinc finger protein 467 [Source:HGNC Symbol;Acc:HGNC:23154] |
| ENSG00000181754 | HGLibA_01794 | 155 | 0.26733466 | AMIGO1 | adhesion molecule with Ig like domain 1 [Source:HGNC Symbol;Acc:HGNC:20824] |
| ENSG00000183690 | HGLibA_14574 | 155 | 0.26733466 | EFHC2 | EF-hand domain containing 2 [Source:HGNC Symbol;Acc:HGNC:26233] |
| ENSG00000183977 | HGLibA_37801 | 155 | 0.26733466 | PP2D1 | protein phosphatase 2C like domain containing 1 [Source:HGNC Symbol;Acc:HGNC:28406] |
| ENSG00000184155 | HGLibA_33526 | 155 | 0.26733466 | OR10J5 | olfactory receptor family 10 subfamily J member 5 [Source:HGNC Symbol;Acc:HGNC:14993] |
| ENSG00000184210 | HGLibA_13041 | 155 | 0.26733466 | DGAT2L6 | diacylglycerol O-acyltransferase 2 like 6 [Source:HGNC Symbol;Acc:HGNC:23250] |
| ENSG00000184344 | HGLibA_18867 | 155 | 0.26733466 | GDF3 | growth differentiation factor 3 [Source:HGNC Symbol;Acc:HGNC:4218] |
| ENSG00000184502 | HGLibA_18663 | 155 | 0.26733466 | GAST | gastrin [Source:HGNC Symbol;Acc:HGNC:4164] |
| ENSG00000184867 | HGLibA_03040 | 155 | 0.26733466 | ARMCX2 | armadillo repeat containing X-linked 2 [Source:HGNC Symbol;Acc:HGNC:16869] |
| ENSG00000185008 | HGLibA_41736 | 155 | 0.26733466 | ROBO2 | roundabout guidance receptor 2 [Source:HGNC Symbol;Acc:HGNC:10250] |
| ENSG00000185985 | HGLibA_45516 | 155 | 0.26733466 | SLITRK2 | SLIT and NTRK like family member 2 [Source:HGNC Symbol;Acc:HGNC:13449] |
| ENSG00000188997 | HGLibA_24449 | 155 | 0.26733466 | KCTD21 | potassium channel tetramerization domain containing 21 [Source:HGNC Symbol;Acc:HGNC:27452] |
| ENSG00000196187 | HGLibA_50474 | 155 | 0.26733466 | TMEM63A | transmembrane protein 63A [Source:HGNC Symbol;Acc:HGNC:29118] |
| ENSG00000196535 | HGLibA_30811 | 155 | 0.26733466 | MYO18A | myosin XVIIIA [Source:HGNC Symbol;Acc:HGNC:31104] |
| ENSG00000197147 | HGLibA_27431 | 155 | 0.26733466 | LRRC8B | leucine rich repeat containing 8 VRAC subunit B [Source:HGNC Symbol;Acc:HGNC:30692] |
| ENSG00000197622 | HGLibA_08580 | 155 | 0.26733466 | CDC42SE1 | CDC42 small effector 1 [Source:HGNC Symbol;Acc:HGNC:17719] |
| ENSG00000204020 | HGLibA_26631 | 155 | 0.26733466 | LIPN | lipase family member N [Source:HGNC Symbol;Acc:HGNC:23452] |
| ENSG00000204505 | HGLibA_38333 | 155 | 0.26733466 | PRAMEF9 | PRAME family member 9 [Source:HGNC Symbol;Acc:HGNC:27996] |
| ENSG00000206172 | HGLibA_20907 | 155 | 0.26733466 | HBA1 | hemoglobin subunit alpha 1 [Source:HGNC Symbol;Acc:HGNC:4823] |
| ENSG00000214022 | HGLibA_40845 | 155 | 0.26733466 | REPIN1 | replication initiator 1 [Source:HGNC Symbol;Acc:HGNC:17922] |
| ENSG00000214087 | HGLibA_02948 | 155 | 0.26733466 | ARL16 | ADP ribosylation factor like GTPase 16 [Source:HGNC Symbol;Acc:HGNC:27902] |
| ENSG00000214827 | HGLibA_30332 | 155 | 0.26733466 | MTCP1 | mature T cell proliferation 1 [Source:HGNC Symbol;Acc:HGNC:7423] |
| ENSG00000241233 | HGLibA_25810 | 155 | 0.26733466 | KRTAP5-8 | keratin associated protein 5-8 [Source:HGNC Symbol;Acc:HGNC:23603] |
| ENSG00000278224 | HGLibA_38478 | 155 | 0.26733466 | PRICKLE4 | prickle planar cell polarity protein 4 [Source:HGNC Symbol;Acc:HGNC:16805] |
| ENSG00000280236 | HGLibA_33586 | 155 | 0.26733466 | OR12D2 | olfactory receptor family 12 subfamily D member 2 (gene/pseudogene) [Source:HGNC Symbol;Acc:HGNC:8178] |
| ENSG00000284609 | HGLibA_34499 | 155 | 0.26733466 | OR8B3 | olfactory receptor family 8 subfamily B member 3 [Source:HGNC Symbol;Acc:HGNC:8472] |
| hsa-mir-4252 | HGLibA_59715 | 155 | 0.26733466 | hsa-mir-4252 | - |
| hsa-mir-4470 | HGLibA_60288 | 155 | 0.26733466 | hsa-mir-4470 | - |
| hsa-mir-486-2 | HGLibA_61339 | 155 | 0.26733466 | hsa-mir-486-2 | - |
| hsa-mir-5585 | HGLibA_62181 | 155 | 0.26733466 | hsa-mir-5585 | - |
| hsa-mir-5683 | HGLibA_62253 | 155 | 0.26733466 | hsa-mir-5683 | - |
| hsa-mir-941-2 | HGLibA_64341 | 155 | 0.26733466 | hsa-mir-941-2 | - |
| LOC100129083 | HGLibA_26753 | 155 | 0.26733466 | LOC100129083 | - |
| MGEA5 | HGLibA_29157 | 155 | 0.26733466 | MGEA5 | - |
| PAK7 | HGLibA_35055 | 155 | 0.26733466 | PAK7 | - |
| SLC24A6 | HGLibA_44629 | 155 | 0.26733466 | SLC24A6 | - |
| WISP1 | HGLibA_54318 | 155 | 0.26733466 | WISP1 | - |
| WRB | HGLibA_54406 | 155 | 0.26733466 | WRB | - |
| ACN9 | HGLibA_00458 | 154 | 0.258289317 | ACN9 | - |
| C15orf54 | HGLibA_05413 | 154 | 0.258289317 | C15orf54 | - |
| ENSG00000006062 | HGLibA_28176 | 154 | 0.258289317 | MAP3K14 | mitogen-activated protein kinase kinase kinase 14 [Source:HGNC Symbol;Acc:HGNC:6853] |
| ENSG00000010610 | HGLibA_08380 | 154 | 0.258289317 | CD4 | CD4 molecule [Source:HGNC Symbol;Acc:HGNC:1678] |
| ENSG00000012223 | HGLibA_27607 | 154 | 0.258289317 | LTF | lactotransferrin [Source:HGNC Symbol;Acc:HGNC:6720] |
| ENSG00000036828 | HGLibA_07339 | 154 | 0.258289317 | CASR | calcium sensing receptor [Source:HGNC Symbol;Acc:HGNC:1514] |
| ENSG00000050438 | HGLibA_45230 | 154 | 0.258289317 | SLC4A8 | solute carrier family 4 member 8 [Source:HGNC Symbol;Acc:HGNC:11034] |
| ENSG00000059145 | HGLibA_53049 | 154 | 0.258289317 | UNKL | unkempt family like zinc finger [Source:HGNC Symbol;Acc:HGNC:14184] |
| ENSG00000060749 | HGLibA_39752 | 154 | 0.258289317 | QSER1 | glutamine and serine rich 1 [Source:HGNC Symbol;Acc:HGNC:26154] |
| ENSG00000063244 | HGLibA_52499 | 154 | 0.258289317 | U2AF2 | U2 small nuclear RNA auxiliary factor 2 [Source:HGNC Symbol;Acc:HGNC:23156] |
| ENSG00000068697 | HGLibA_26015 | 154 | 0.258289317 | LAPTM4A | lysosomal protein transmembrane 4 alpha [Source:HGNC Symbol;Acc:HGNC:6924] |
| ENSG00000074966 | HGLibA_52386 | 154 | 0.258289317 | TXK | TXK tyrosine kinase [Source:HGNC Symbol;Acc:HGNC:12434] |
| ENSG00000079616 | HGLibA_24903 | 154 | 0.258289317 | KIF22 | kinesin family member 22 [Source:HGNC Symbol;Acc:HGNC:6391] |
| ENSG00000095970 | HGLibA_51358 | 154 | 0.258289317 | TREM2 | triggering receptor expressed on myeloid cells 2 [Source:HGNC Symbol;Acc:HGNC:17761] |
| ENSG00000100225 | HGLibA_17125 | 154 | 0.258289317 | FBXO7 | F-box protein 7 [Source:HGNC Symbol;Acc:HGNC:13586] |
| ENSG00000102313 | HGLibA_23818 | 154 | 0.258289317 | ITIH6 | inter-alpha-trypsin inhibitor heavy chain family member 6 [Source:HGNC Symbol;Acc:HGNC:28907] |
| ENSG00000105639 | HGLibA_23918 | 154 | 0.258289317 | JAK3 | Janus kinase 3 [Source:HGNC Symbol;Acc:HGNC:6193] |
| ENSG00000105698 | HGLibA_53170 | 154 | 0.258289317 | USF2 | c-fos interacting [Source:HGNC Symbol;Acc:HGNC:12594]" |
| ENSG00000106591 | HGLibA_29951 | 154 | 0.258289317 | MRPL32 | mitochondrial ribosomal protein L32 [Source:HGNC Symbol;Acc:HGNC:14035] |
| ENSG00000109819 | HGLibA_37845 | 154 | 0.258289317 | PPARGC1A | PPARG coactivator 1 alpha [Source:HGNC Symbol;Acc:HGNC:9237] |
| ENSG00000110002 | HGLibA_53907 | 154 | 0.258289317 | VWA5A | von Willebrand factor A domain containing 5A [Source:HGNC Symbol;Acc:HGNC:6658] |
| ENSG00000114850 | HGLibA_47160 | 154 | 0.258289317 | SSR3 | signal sequence receptor subunit 3 [Source:HGNC Symbol;Acc:HGNC:11325] |
| ENSG00000115758 | HGLibA_33273 | 154 | 0.258289317 | ODC1 | ornithine decarboxylase 1 [Source:HGNC Symbol;Acc:HGNC:8109] |
| ENSG00000117593 | HGLibA_12344 | 154 | 0.258289317 | DARS2 | mitochondrial [Source:HGNC Symbol;Acc:HGNC:25538]" |
| ENSG00000118418 | HGLibA_21722 | 154 | 0.258289317 | HMGN3 | high mobility group nucleosomal binding domain 3 [Source:HGNC Symbol;Acc:HGNC:12312] |
| ENSG00000118849 | HGLibA_40281 | 154 | 0.258289317 | RARRES1 | retinoic acid receptor responder 1 [Source:HGNC Symbol;Acc:HGNC:9867] |
| ENSG00000119514 | HGLibA_18536 | 154 | 0.258289317 | GALNT12 | polypeptide N-acetylgalactosaminyltransferase 12 [Source:HGNC Symbol;Acc:HGNC:19877] |
| ENSG00000120686 | HGLibA_52852 | 154 | 0.258289317 | UFM1 | ubiquitin fold modifier 1 [Source:HGNC Symbol;Acc:HGNC:20597] |
| ENSG00000121335 | HGLibA_38341 | 154 | 0.258289317 | PRB2 | proline rich protein BstNI subfamily 2 [Source:HGNC Symbol;Acc:HGNC:9338] |
| ENSG00000122304 | HGLibA_38612 | 154 | 0.258289317 | PRM2 | protamine 2 [Source:HGNC Symbol;Acc:HGNC:9448] |
| ENSG00000124233 | HGLibA_43379 | 154 | 0.258289317 | SEMG1 | semenogelin 1 [Source:HGNC Symbol;Acc:HGNC:10742] |
| ENSG00000126561 | HGLibA_47394 | 154 | 0.258289317 | STAT5A | signal transducer and activator of transcription 5A [Source:HGNC Symbol;Acc:HGNC:11366] |
| ENSG00000128052 | HGLibA_24543 | 154 | 0.258289317 | KDR | kinase insert domain receptor [Source:HGNC Symbol;Acc:HGNC:6307] |
| ENSG00000128342 | HGLibA_26474 | 154 | 0.258289317 | LIF | interleukin 6 family cytokine [Source:HGNC Symbol;Acc:HGNC:6596]" |
| ENSG00000128965 | HGLibA_09336 | 154 | 0.258289317 | CHAC1 | ChaC glutathione specific gamma-glutamylcyclotransferase 1 [Source:HGNC Symbol;Acc:HGNC:28680] |
| ENSG00000130066 | HGLibA_42770 | 154 | 0.258289317 | SAT1 | spermidine/spermine N1-acetyltransferase 1 [Source:HGNC Symbol;Acc:HGNC:10540] |
| ENSG00000131042 | HGLibA_26511 | 154 | 0.258289317 | LILRB2 | leukocyte immunoglobulin like receptor B2 [Source:HGNC Symbol;Acc:HGNC:6606] |
| ENSG00000136878 | HGLibA_53300 | 154 | 0.258289317 | USP20 | ubiquitin specific peptidase 20 [Source:HGNC Symbol;Acc:HGNC:12619] |
| ENSG00000137185 | HGLibA_57033 | 154 | 0.258289317 | ZSCAN9 | zinc finger and SCAN domain containing 9 [Source:HGNC Symbol;Acc:HGNC:12984] |
| ENSG00000137992 | HGLibA_12407 | 154 | 0.258289317 | DBT | dihydrolipoamide branched chain transacylase E2 [Source:HGNC Symbol;Acc:HGNC:2698] |
| ENSG00000144908 | HGLibA_01554 | 154 | 0.258289317 | ALDH1L1 | aldehyde dehydrogenase 1 family member L1 [Source:HGNC Symbol;Acc:HGNC:3978] |
| ENSG00000146950 | HGLibA_44090 | 154 | 0.258289317 | SHROOM2 | shroom family member 2 [Source:HGNC Symbol;Acc:HGNC:630] |
| ENSG00000147454 | HGLibA_44724 | 154 | 0.258289317 | SLC25A37 | solute carrier family 25 member 37 [Source:HGNC Symbol;Acc:HGNC:29786] |
| ENSG00000148300 | HGLibA_40899 | 154 | 0.258289317 | REXO4 | 3'-5' exonuclease [Source:HGNC Symbol;Acc:HGNC:12820]" |
| ENSG00000148600 | HGLibA_08698 | 154 | 0.258289317 | CDHR1 | cadherin related family member 1 [Source:HGNC Symbol;Acc:HGNC:14550] |
| ENSG00000149573 | HGLibA_29799 | 154 | 0.258289317 | MPZL2 | myelin protein zero like 2 [Source:HGNC Symbol;Acc:HGNC:3496] |
| ENSG00000151500 | HGLibA_49426 | 154 | 0.258289317 | THYN1 | thymocyte nuclear protein 1 [Source:HGNC Symbol;Acc:HGNC:29560] |
| ENSG00000152086 | HGLibA_52257 | 154 | 0.258289317 | TUBA3E | tubulin alpha 3e [Source:HGNC Symbol;Acc:HGNC:20765] |
| ENSG00000155744 | HGLibA_16084 | 154 | 0.258289317 | FAM126B | family with sequence similarity 126 member B [Source:HGNC Symbol;Acc:HGNC:28593] |
| ENSG00000155959 | HGLibA_53564 | 154 | 0.258289317 | VBP1 | VHL binding protein 1 [Source:HGNC Symbol;Acc:HGNC:12662] |
| ENSG00000159527 | HGLibA_36254 | 154 | 0.258289317 | PGLYRP3 | peptidoglycan recognition protein 3 [Source:HGNC Symbol;Acc:HGNC:30014] |
| ENSG00000159640 | HGLibA_00423 | 154 | 0.258289317 | ACE | angiotensin I converting enzyme [Source:HGNC Symbol;Acc:HGNC:2707] |
| ENSG00000160307 | HGLibA_42579 | 154 | 0.258289317 | S100B | S100 calcium binding protein B [Source:HGNC Symbol;Acc:HGNC:10500] |
| ENSG00000160392 | HGLibA_05669 | 154 | 0.258289317 | C19orf47 | chromosome 19 open reading frame 47 [Source:HGNC Symbol;Acc:HGNC:26723] |
| ENSG00000160551 | HGLibA_48307 | 154 | 0.258289317 | TAOK1 | TAO kinase 1 [Source:HGNC Symbol;Acc:HGNC:29259] |
| ENSG00000162723 | HGLibA_44324 | 154 | 0.258289317 | SLAMF9 | SLAM family member 9 [Source:HGNC Symbol;Acc:HGNC:18430] |
| ENSG00000164211 | HGLibA_47363 | 154 | 0.258289317 | STARD4 | StAR related lipid transfer domain containing 4 [Source:HGNC Symbol;Acc:HGNC:18058] |
| ENSG00000165496 | HGLibA_41860 | 154 | 0.258289317 | RPL10L | ribosomal protein L10 like [Source:HGNC Symbol;Acc:HGNC:17976] |
| ENSG00000166526 | HGLibA_55766 | 154 | 0.258289317 | ZNF3 | zinc finger protein 3 [Source:HGNC Symbol;Acc:HGNC:13089] |
| ENSG00000166863 | HGLibA_48153 | 154 | 0.258289317 | TAC3 | tachykinin 3 [Source:HGNC Symbol;Acc:HGNC:11521] |
| ENSG00000167104 | HGLibA_04730 | 154 | 0.258289317 | BPIFB6 | BPI fold containing family B member 6 [Source:HGNC Symbol;Acc:HGNC:16504] |
| ENSG00000168209 | HGLibA_12618 | 154 | 0.258289317 | DDIT4 | DNA damage inducible transcript 4 [Source:HGNC Symbol;Acc:HGNC:24944] |
| ENSG00000168546 | HGLibA_18968 | 154 | 0.258289317 | GFRA2 | GDNF family receptor alpha 2 [Source:HGNC Symbol;Acc:HGNC:4244] |
| ENSG00000168781 | HGLibA_37954 | 154 | 0.258289317 | PPIP5K1 | diphosphoinositol pentakisphosphate kinase 1 [Source:HGNC Symbol;Acc:HGNC:29023] |

| | | | | | |
|---|---|---|---|---|---|
| ENSG00000170498 | HGLibA_25008 | 154 | 0.258289317 | KISS1 | KiSS-1 metastasis suppressor [Source:HGNC Symbol;Acc:HGNC:6341] |
| ENSG00000172840 | HGLibA_35927 | 154 | 0.258289317 | PDP2 | pyruvate dehyrogenase phosphatase catalytic subunit 2 [Source:HGNC Symbol;Acc:HGNC:30263] |
| ENSG00000172878 | HGLibA_28931 | 154 | 0.258289317 | METAP1D | mitochondrial [Source:HGNC Symbol;Acc:HGNC:32583]" |
| ENSG00000173535 | HGLibA_50740 | 154 | 0.258289317 | TNFRSF10C | TNF receptor superfamily member 10c [Source:HGNC Symbol;Acc:HGNC:11906] |
| ENSG00000174600 | HGLibA_10198 | 154 | 0.258289317 | CMKLR1 | chemerin chemokine-like receptor 1 [Source:HGNC Symbol;Acc:HGNC:2121] |
| ENSG00000174721 | HGLibA_17432 | 154 | 0.258289317 | FGFBP3 | fibroblast growth factor binding protein 3 [Source:HGNC Symbol;Acc:HGNC:23428] |
| ENSG00000175573 | HGLibA_05172 | 154 | 0.258289317 | C11orf68 | chromosome 11 open reading frame 68 [Source:HGNC Symbol;Acc:HGNC:28801] |
| ENSG00000176299 | HGLibA_34041 | 154 | 0.258289317 | OR4M1 | olfactory receptor family 4 subfamily M member 1 [Source:HGNC Symbol;Acc:HGNC:14735] |
| ENSG00000178075 | HGLibA_20201 | 154 | 0.258289317 | GRAMD1C | GRAM domain containing 1C [Source:HGNC Symbol;Acc:HGNC:25252] |
| ENSG00000178761 | HGLibA_16501 | 154 | 0.258289317 | FAM219B | family with sequence similarity 219 member B [Source:HGNC Symbol;Acc:HGNC:24695] |
| ENSG00000179833 | HGLibA_43606 | 154 | 0.258289317 | SERTAD2 | SERTA domain containing 2 [Source:HGNC Symbol;Acc:HGNC:30784] |
| ENSG00000179950 | HGLibA_39578 | 154 | 0.258289317 | PUF60 | poly(U) binding splicing factor 60 [Source:HGNC Symbol;Acc:HGNC:17042] |
| ENSG00000182170 | HGLibA_29843 | 154 | 0.258289317 | MRGPRG | MAS related GPR family member G [Source:HGNC Symbol;Acc:HGNC:24829] |
| ENSG00000183655 | HGLibA_25166 | 154 | 0.258289317 | KLHL25 | kelch like family member 25 [Source:HGNC Symbol;Acc:HGNC:25732] |
| ENSG00000185385 | HGLibA_34459 | 154 | 0.258289317 | OR7A17 | olfactory receptor family 7 subfamily A member 17 [Source:HGNC Symbol;Acc:HGNC:8363] |
| ENSG00000186001 | HGLibA_27142 | 154 | 0.258289317 | LRCH3 | leucine rich repeats and calponin homology domain containing 3 [Source:HGNC Symbol;Acc:HGNC:28637] |
| ENSG00000186281 | HGLibA_19733 | 154 | 0.258289317 | GPAT2 | mitochondrial [Source:HGNC Symbol;Acc:HGNC:27168]" |
| ENSG00000186526 | HGLibA_12195 | 154 | 0.258289317 | CYP4F8 | cytochrome P450 family 4 subfamily F member 8 [Source:HGNC Symbol;Acc:HGNC:2648] |
| ENSG00000187630 | HGLibA_13142 | 154 | 0.258289317 | DHRS4L2 | dehydrogenase/reductase 4 like 2 [Source:HGNC Symbol;Acc:HGNC:19731] |
| ENSG00000188895 | HGLibA_30211 | 154 | 0.258289317 | MSL1 | male specific lethal 1 homolog [Source:HGNC Symbol;Acc:HGNC:27905] |
| ENSG00000189132 | HGLibA_16612 | 154 | 0.258289317 | FAM47B | family with sequence similarity 47 member B [Source:HGNC Symbol;Acc:HGNC:26659] |
| ENSG00000197150 | HGLibA_00151 | 154 | 0.258289317 | ABCB8 | ATP binding cassette subfamily B member 8 [Source:HGNC Symbol;Acc:HGNC:49] |
| ENSG00000197892 | HGLibA_24860 | 154 | 0.258289317 | KIF13B | kinesin family member 13B [Source:HGNC Symbol;Acc:HGNC:14405] |
| ENSG00000198932 | HGLibA_20118 | 154 | 0.258289317 | GPRASP1 | G protein-coupled receptor associated sorting protein 1 [Source:HGNC Symbol;Acc:HGNC:24834] |
| ENSG00000198961 | HGLibA_36787 | 154 | 0.258289317 | PJA2 | praja ring finger ubiquitin ligase 2 [Source:HGNC Symbol;Acc:HGNC:17481] |
| ENSG00000204969 | HGLibA_35440 | 154 | 0.258289317 | PCDHA2 | protocadherin alpha 2 [Source:HGNC Symbol;Acc:HGNC:8668] |
| ENSG00000205642 | HGLibA_53590 | 154 | 0.258289317 | VCX3B | variable charge X-linked 3B [Source:HGNC Symbol;Acc:HGNC:31838] |
| ENSG00000213064 | HGLibA_43755 | 154 | 0.258289317 | SFT2D2 | SFT2 domain containing 2 [Source:HGNC Symbol;Acc:HGNC:25140] |
| ENSG00000221813 | HGLibA_34373 | 154 | 0.258289317 | OR6B1 | olfactory receptor family 6 subfamily B member 1 [Source:HGNC Symbol;Acc:HGNC:8354] |
| ENSG00000241839 | HGLibA_37152 | 154 | 0.258289317 | PLEKHO2 | pleckstrin homology domain containing O2 [Source:HGNC Symbol;Acc:HGNC:30026] |
| ENSG00000244045 | HGLibA_50166 | 154 | 0.258289317 | TMEM199 | transmembrane protein 199 [Source:HGNC Symbol;Acc:HGNC:18085] |
| ENSG00000244474 | HGLibA_52885 | 154 | 0.258289317 | UGT1A4 | UDP glucuronosyltransferase family 1 member A4 [Source:HGNC Symbol;Acc:HGNC:12536] |
| ENSG00000267206 | HGLibA_26182 | 154 | 0.258289317 | LCN6 | lipocalin 6 [Source:HGNC Symbol;Acc:HGNC:17337] |
| ENSG00000276409 | HGLibA_07971 | 154 | 0.258289317 | CCL14 | C-C motif chemokine ligand 14 [Source:HGNC Symbol;Acc:HGNC:10612] |
| ENSG00000278057 | HGLibA_49089 | 154 | 0.258289317 | TEX28 | testis expressed 28 [Source:HGNC Symbol;Acc:HGNC:2563] |
| ENSG00000288705 | HGLibA_52888 | 154 | 0.258289317 | UGT1A5 | - |
| HIST2H3C | HGLibA_21542 | 154 | 0.258289317 | HIST2H3C | - |
| HIST3H3 | HGLibA_21560 | 154 | 0.258289317 | HIST3H3 | - |
| hsa-mir-199a-1 | HGLibA_58127 | 154 | 0.258289317 | hsa-mir-199a-1 | - |
| hsa-mir-219a-2 | HGLibA_58280 | 154 | 0.258289317 | hsa-mir-219a-2 | - |
| hsa-mir-3135a | HGLibA_58594 | 154 | 0.258289317 | hsa-mir-3135a | - |
| hsa-mir-3153 | HGLibA_58670 | 154 | 0.258289317 | hsa-mir-3153 | - |
| hsa-mir-3939 | HGLibA_59618 | 154 | 0.258289317 | hsa-mir-3939 | - |
| hsa-mir-4524b | HGLibA_60558 | 154 | 0.258289317 | hsa-mir-4524b | - |
| hsa-mir-4656 | HGLibA_60733 | 154 | 0.258289317 | hsa-mir-4656 | - |
| hsa-mir-5002 | HGLibA_61416 | 154 | 0.258289317 | hsa-mir-5002 | - |
| hsa-mir-514a-1 | HGLibA_61587 | 154 | 0.258289317 | hsa-mir-514a-1 | - |
| hsa-mir-5695 | HGLibA_62315 | 154 | 0.258289317 | hsa-mir-5695 | - |
| hsa-mir-6511b-2 | HGLibA_62919 | 154 | 0.258289317 | hsa-mir-6511b-2 | - |
| hsa-mir-6844 | HGLibA_63550 | 154 | 0.258289317 | hsa-mir-6844 | - |
| hsa-mir-6875 | HGLibA_63686 | 154 | 0.258289317 | hsa-mir-6875 | - |
| hsa-mir-6889 | HGLibA_63740 | 154 | 0.258289317 | hsa-mir-6889 | - |
| hsa-mir-7974 | HGLibA_64025 | 154 | 0.258289317 | hsa-mir-7974 | - |
| hsa-mir-890 | HGLibA_64240 | 154 | 0.258289317 | hsa-mir-890 | - |
| MPP6 | HGLibA_29761 | 154 | 0.258289317 | MPP6 | - |
| C10orf35 | HGLibA_05071 | 153 | 0.249243975 | C10orf35 | - |
| C19orf70 | HGLibA_05699 | 153 | 0.249243975 | C19orf70 | - |
| C22orf29 | HGLibA_06067 | 153 | 0.249243975 | C22orf29 | - |
| C9orf170 | HGLibA_06708 | 153 | 0.249243975 | C9orf170 | - |
| CXorf36 | HGLibA_11917 | 153 | 0.249243975 | CXorf36 | - |
| DEFB130 | HGLibA_12893 | 153 | 0.249243975 | DEFB130 | - |
| ENSG00000059573 | HGLibA_01537 | 153 | 0.249243975 | ALDH18A1 | aldehyde dehydrogenase 18 family member A1 [Source:HGNC Symbol;Acc:HGNC:9722] |
| ENSG00000064666 | HGLibA_10309 | 153 | 0.249243975 | CNN2 | calponin 2 [Source:HGNC Symbol;Acc:HGNC:2156] |
| ENSG00000066084 | HGLibA_13249 | 153 | 0.249243975 | DIP2B | disco interacting protein 2 homolog B [Source:HGNC Symbol;Acc:HGNC:29284] |
| ENSG00000066468 | HGLibA_17444 | 153 | 0.249243975 | FGFR2 | fibroblast growth factor receptor 2 [Source:HGNC Symbol;Acc:HGNC:3689] |
| ENSG00000067955 | HGLibA_07391 | 153 | 0.249243975 | CBFB | core-binding factor subunit beta [Source:HGNC Symbol;Acc:HGNC:1539] |
| ENSG00000072121 | HGLibA_55285 | 153 | 0.249243975 | ZFYVE26 | zinc finger FYVE-type containing 26 [Source:HGNC Symbol;Acc:HGNC:20761] |
| ENSG00000072364 | HGLibA_01115 | 153 | 0.249243975 | AFF4 | AF4/FMR2 family member 4 [Source:HGNC Symbol;Acc:HGNC:17869] |
| ENSG00000074603 | HGLibA_13881 | 153 | 0.249243975 | DPP8 | dipeptidyl peptidase 8 [Source:HGNC Symbol;Acc:HGNC:16490] |
| ENSG00000074771 | HGLibA_32372 | 153 | 0.249243975 | NOX3 | NADPH oxidase 3 [Source:HGNC Symbol;Acc:HGNC:7890] |
| ENSG00000077279 | HGLibA_12581 | 153 | 0.249243975 | DCX | doublecortin [Source:HGNC Symbol;Acc:HGNC:2714] |
| ENSG00000086506 | HGLibA_20938 | 153 | 0.249243975 | HBQ1 | hemoglobin subunit theta 1 [Source:HGNC Symbol;Acc:HGNC:4833] |
| ENSG00000090674 | HGLibA_28637 | 153 | 0.249243975 | MCOLN1 | mucolipin 1 [Source:HGNC Symbol;Acc:HGNC:13356] |
| ENSG00000100652 | HGLibA_44329 | 153 | 0.249243975 | SLC10A1 | solute carrier family 10 member 1 [Source:HGNC Symbol;Acc:HGNC:10905] |
| ENSG00000100714 | HGLibA_30376 | 153 | 0.249243975 | MTHFD1 | cyclohydrolase and formyltetrahydrofolate synthetase 1 [Source:HGNC Symbol;Acc:HGNC:7432]" |
| ENSG00000103876 | HGLibA_15951 | 153 | 0.249243975 | FAH | fumarylacetoacetate hydrolase [Source:HGNC Symbol;Acc:HGNC:3579] |
| ENSG00000105143 | HGLibA_44521 | 153 | 0.249243975 | SLC1A6 | solute carrier family 1 member 6 [Source:HGNC Symbol;Acc:HGNC:10944] |
| ENSG00000105281 | HGLibA_44519 | 153 | 0.249243975 | SLC1A5 | solute carrier family 1 member 5 [Source:HGNC Symbol;Acc:HGNC:10943] |
| ENSG00000105467 | HGLibA_48001 | 153 | 0.249243975 | SYNGR4 | synaptogyrin 4 [Source:HGNC Symbol;Acc:HGNC:11502] |
| ENSG00000106025 | HGLibA_51899 | 153 | 0.249243975 | TSPAN12 | tetraspanin 12 [Source:HGNC Symbol;Acc:HGNC:21641] |
| ENSG00000106617 | HGLibA_38515 | 153 | 0.249243975 | PRKAG2 | protein kinase AMP-activated non-catalytic subunit gamma 2 [Source:HGNC Symbol;Acc:HGNC:9386] |
| ENSG00000107897 | HGLibA_00405 | 153 | 0.249243975 | ACBD5 | acyl-CoA binding domain containing 5 [Source:HGNC Symbol;Acc:HGNC:23338] |
| ENSG00000111665 | HGLibA_08604 | 153 | 0.249243975 | CDCA3 | cell division cycle associated 3 [Source:HGNC Symbol;Acc:HGNC:14624] |
| ENSG00000111666 | HGLibA_09530 | 153 | 0.249243975 | CHPT1 | choline phosphotransferase 1 [Source:HGNC Symbol;Acc:HGNC:17852] |
| ENSG00000116663 | HGLibA_17123 | 153 | 0.249243975 | FBXO6 | F-box protein 6 [Source:HGNC Symbol;Acc:HGNC:13585] |
| ENSG00000117971 | HGLibA_09599 | 153 | 0.249243975 | CHRNB4 | cholinergic receptor nicotinic beta 4 subunit [Source:HGNC Symbol;Acc:HGNC:1964] |
| ENSG00000119522 | HGLibA_12929 | 153 | 0.249243975 | DENND1A | DENN domain containing 1A [Source:HGNC Symbol;Acc:HGNC:29324] |
| ENSG00000119865 | HGLibA_10379 | 153 | 0.249243975 | CNRIP1 | cannabinoid receptor interacting protein 1 [Source:HGNC Symbol;Acc:HGNC:24546] |
| ENSG00000121314 | HGLibA_48428 | 153 | 0.249243975 | TAS2R8 | taste 2 receptor member 8 [Source:HGNC Symbol;Acc:HGNC:14915] |
| ENSG00000123171 | HGLibA_07849 | 153 | 0.249243975 | CCDC70 | coiled-coil domain containing 70 [Source:HGNC Symbol;Acc:HGNC:25303] |
| ENSG00000126243 | HGLibA_27158 | 153 | 0.249243975 | LRFN3 | leucine rich repeat and fibronectin type III domain containing 3 [Source:HGNC Symbol;Acc:HGNC:28370] |
| ENSG00000131969 | HGLibA_00242 | 153 | 0.249243975 | ABHD12B | abhydrolase domain containing 12B [Source:HGNC Symbol;Acc:HGNC:19837] |
| ENSG00000133665 | HGLibA_14224 | 153 | 0.249243975 | DYDC2 | DPY30 domain containing 2 [Source:HGNC Symbol;Acc:HGNC:23468] |
| ENSG00000134107 | HGLibA_04480 | 153 | 0.249243975 | BHLHE40 | basic helix-loop-helix family member e40 [Source:HGNC Symbol;Acc:HGNC:1046] |
| ENSG00000135409 | HGLibA_01788 | 153 | 0.249243975 | AMHR2 | anti-Mullerian hormone receptor type 2 [Source:HGNC Symbol;Acc:HGNC:465] |
| ENSG00000137338 | HGLibA_36215 | 153 | 0.249243975 | PGBD1 | piggyBac transposable element derived 1 [Source:HGNC Symbol;Acc:HGNC:19398] |
| ENSG00000137558 | HGLibA_36484 | 153 | 0.249243975 | PI15 | peptidase inhibitor 15 [Source:HGNC Symbol;Acc:HGNC:8946] |
| ENSG00000137875 | HGLibA_04305 | 153 | 0.249243975 | BCL2L10 | BCL2 like 10 [Source:HGNC Symbol;Acc:HGNC:993] |
| ENSG00000138668 | HGLibA_21812 | 153 | 0.249243975 | HNRNPD | heterogeneous nuclear ribonucleoprotein D [Source:HGNC Symbol;Acc:HGNC:5036] |
| ENSG00000139197 | HGLibA_36118 | 153 | 0.249243975 | PEX5 | peroxisomal biogenesis factor 5 [Source:HGNC Symbol;Acc:HGNC:9719] |
| ENSG00000139890 | HGLibA_40832 | 153 | 0.249243975 | REM2 | RRAD and GEM like GTPase 2 [Source:HGNC Symbol;Acc:HGNC:20248] |
| ENSG00000140853 | HGLibA_32094 | 153 | 0.249243975 | NLRC5 | NLR family CARD domain containing 5 [Source:HGNC Symbol;Acc:HGNC:29933] |
| ENSG00000141068 | HGLibA_25862 | 153 | 0.249243975 | KSR1 | kinase suppressor of ras 1 [Source:HGNC Symbol;Acc:HGNC:6465] |
| ENSG00000142556 | HGLibA_56390 | 153 | 0.249243975 | ZNF614 | zinc finger protein 614 [Source:HGNC Symbol;Acc:HGNC:24722] |
| ENSG00000142675 | HGLibA_10297 | 153 | 0.249243975 | CNKSR1 | connector enhancer of kinase suppressor of Ras 1 [Source:HGNC Symbol;Acc:HGNC:19700] |
| ENSG00000143028 | HGLibA_48040 | 153 | 0.249243975 | SYPL2 | synaptophysin like 2 [Source:HGNC Symbol;Acc:HGNC:27638] |
| ENSG00000144504 | HGLibA_01980 | 153 | 0.249243975 | ANKMY1 | ankyrin repeat and MYND domain containing 1 [Source:HGNC Symbol;Acc:HGNC:20987] |
| ENSG00000145384 | HGLibA_15914 | 153 | 0.249243975 | FABP2 | fatty acid binding protein 2 [Source:HGNC Symbol;Acc:HGNC:3556] |
| ENSG00000151376 | HGLibA_28715 | 153 | 0.249243975 | ME3 | malic enzyme 3 [Source:HGNC Symbol;Acc:HGNC:6985] |
| ENSG00000153179 | HGLibA_40380 | 153 | 0.249243975 | RASSF3 | Ras association domain family member 3 [Source:HGNC Symbol;Acc:HGNC:14271] |
| ENSG00000154277 | HGLibA_52800 | 153 | 0.249243975 | UCHL1 | ubiquitin C-terminal hydrolase L1 [Source:HGNC Symbol;Acc:HGNC:12513] |
| ENSG00000156599 | HGLibA_55119 | 153 | 0.249243975 | ZDHHC5 | zinc finger DHHC-type containing 5 [Source:HGNC Symbol;Acc:HGNC:18472] |
| ENSG00000160145 | HGLibA_24021 | 153 | 0.249243975 | KALRN | kalirin RhoGEF kinase [Source:HGNC Symbol;Acc:HGNC:4814] |
| ENSG00000160999 | HGLibA_43877 | 153 | 0.249243975 | SH2B2 | SH2B adaptor protein 2 [Source:HGNC Symbol;Acc:HGNC:17381] |
| ENSG00000162415 | HGLibA_57049 | 153 | 0.249243975 | ZSWIM5 | zinc finger SWIM-type containing 5 [Source:HGNC Symbol;Acc:HGNC:29299] |
| ENSG00000162520 | HGLibA_47963 | 153 | 0.249243975 | SYNC | intermediate filament protein [Source:HGNC Symbol;Acc:HGNC:28897]" |
| ENSG00000162975 | HGLibA_24195 | 153 | 0.249243975 | KCNF1 | potassium voltage-gated channel modifier subfamily F member 1 [Source:HGNC Symbol;Acc:HGNC:6246] |
| ENSG00000164241 | HGLibA_06420 | 153 | 0.249243975 | C5orf63 | chromosome 5 open reading frame 63 [Source:HGNC Symbol;Acc:HGNC:40051] |
| ENSG00000164509 | HGLibA_23134 | 153 | 0.249243975 | IL31RA | interleukin 31 receptor A [Source:HGNC Symbol;Acc:HGNC:18969] |
| ENSG00000164919 | HGLibA_10854 | 153 | 0.249243975 | COX6C | cytochrome c oxidase subunit 6C [Source:HGNC Symbol;Acc:HGNC:2285] |
| ENSG00000165124 | HGLibA_47883 | 153 | 0.249243975 | SVEP1 | EGF and pentraxin domain containing 1 [Source:HGNC Symbol;Acc:HGNC:15985]" |
| ENSG00000166387 | HGLibA_37889 | 153 | 0.249243975 | PPFIBP2 | PPFIA binding protein 2 [Source:HGNC Symbol;Acc:HGNC:9250] |
| ENSG00000166949 | HGLibA_45574 | 153 | 0.249243975 | SMAD3 | SMAD family member 3 [Source:HGNC Symbol;Acc:HGNC:6769] |
| ENSG00000167617 | HGLibA_08577 | 153 | 0.249243975 | CDC42EP5 | CDC42 effector protein 5 [Source:HGNC Symbol;Acc:HGNC:17408] |
| ENSG00000167733 | HGLibA_22162 | 153 | 0.249243975 | HSD11B1L | hydroxysteroid 11-beta dehydrogenase 1 like [Source:HGNC Symbol;Acc:HGNC:30419] |
| ENSG00000168594 | HGLibA_00768 | 153 | 0.249243975 | ADAM29 | ADAM metallopeptidase domain 29 [Source:HGNC Symbol;Acc:HGNC:207] |
| ENSG00000171735 | HGLibA_07114 | 153 | 0.249243975 | CAMTA1 | calmodulin binding transcription activator 1 [Source:HGNC Symbol;Acc:HGNC:18806] |
| ENSG00000171843 | HGLibA_29417 | 153 | 0.249243975 | MLLT3 | super elongation complex subunit [Source:HGNC Symbol;Acc:HGNC:7136]" |
| ENSG00000171989 | HGLibA_26225 | 153 | 0.249243975 | LDHAL6B | lactate dehydrogenase A like 6B [Source:HGNC Symbol;Acc:HGNC:21481] |
| ENSG00000172058 | HGLibA_43469 | 153 | 0.249243975 | SERF1A | small EDRK-rich factor 1A [Source:HGNC Symbol;Acc:HGNC:10755] |
| ENSG00000172362 | HGLibA_34256 | 153 | 0.249243975 | OR5B12 | olfactory receptor family 5 subfamily B member 12 [Source:HGNC Symbol;Acc:HGNC:15432] |
| ENSG00000173269 | HGLibA_29549 | 153 | 0.249243975 | MMRN2 | multimerin 2 [Source:HGNC Symbol;Acc:HGNC:19888] |
| ENSG00000174032 | HGLibA_44701 | 153 | 0.249243975 | SLC25A30 | solute carrier family 25 member 30 [Source:HGNC Symbol;Acc:HGNC:27371] |
| ENSG00000174276 | HGLibA_56925 | 153 | 0.249243975 | ZNHIT2 | zinc finger HIT-type containing 2 [Source:HGNC Symbol;Acc:HGNC:1177] |
| ENSG00000174808 | HGLibA_04928 | 153 | 0.249243975 | BTC | betacellulin [Source:HGNC Symbol;Acc:HGNC:1121] |

| | | | | | |
|---|---|---|---|---|---|
| ENSG00000175697 | HGLibA_19944 | 153 | 0.249243975 | GPR156 | G protein-coupled receptor 156 [Source:HGNC Symbol;Acc:HGNC:20844] |
| ENSG00000176809 | HGLibA_27332 | 153 | 0.249243975 | LRRC37A3 | leucine rich repeat containing 37 member A3 [Source:HGNC Symbol;Acc:HGNC:32427] |
| ENSG00000177663 | HGLibA_23003 | 153 | 0.249243975 | IL17RA | interleukin 17 receptor A [Source:HGNC Symbol;Acc:HGNC:5985] |
| ENSG00000178597 | HGLibA_39005 | 153 | 0.249243975 | PSAPL1 | prosaposin like 1 (gene/pseudogene) [Source:HGNC Symbol;Acc:HGNC:33131] |
| ENSG00000179314 | HGLibA_54421 | 153 | 0.249243975 | WSCD1 | WSC domain containing 1 [Source:HGNC Symbol;Acc:HGNC:29060] |
| ENSG00000180383 | HGLibA_12877 | 153 | 0.249243975 | DEFB124 | defensin beta 124 [Source:HGNC Symbol;Acc:HGNC:18104] |
| ENSG00000181609 | HGLibA_34155 | 153 | 0.249243975 | OR52D1 | olfactory receptor family 52 subfamily D member 1 [Source:HGNC Symbol;Acc:HGNC:15212] |
| ENSG00000182022 | HGLibA_09627 | 153 | 0.249243975 | CHST15 | carbohydrate sulfotransferase 15 [Source:HGNC Symbol;Acc:HGNC:18137] |
| ENSG00000182050 | HGLibA_29148 | 153 | 0.249243975 | MGAT4C | MGAT4 family member C [Source:HGNC Symbol;Acc:HGNC:30871] |
| ENSG00000182195 | HGLibA_26255 | 153 | 0.249243975 | LDOC1 | regulator of NFKB signaling [Source:HGNC Symbol;Acc:HGNC:6548]" |
| ENSG00000182346 | HGLibA_12313 | 153 | 0.249243975 | DAOA | D-amino acid oxidase activator [Source:HGNC Symbol;Acc:HGNC:21191] |
| ENSG00000184210 | HGLibA_13039 | 153 | 0.249243975 | DGAT2L6 | diacylglycerol O-acyltransferase 2 like 6 [Source:HGNC Symbol;Acc:HGNC:23250] |
| ENSG00000184459 | HGLibA_04734 | 153 | 0.249243975 | BPIFC | BPI fold containing family C [Source:HGNC Symbol;Acc:HGNC:16503] |
| ENSG00000184698 | HGLibA_34124 | 153 | 0.249243975 | OR51M1 | olfactory receptor family 51 subfamily M member 1 [Source:HGNC Symbol;Acc:HGNC:14847] |
| ENSG00000187855 | HGLibA_03274 | 153 | 0.249243975 | ASCL4 | achaete-scute family bHLH transcription factor 4 [Source:HGNC Symbol;Acc:HGNC:24311] |
| ENSG00000188033 | HGLibA_56090 | 153 | 0.249243975 | ZNF490 | zinc finger protein 490 [Source:HGNC Symbol;Acc:HGNC:23705] |
| ENSG00000188582 | HGLibA_35176 | 153 | 0.249243975 | PAQR9 | progestin and adipoQ receptor family member 9 [Source:HGNC Symbol;Acc:HGNC:30131] |
| ENSG00000188859 | HGLibA_16730 | 153 | 0.249243975 | FAM78B | family with sequence similarity 78 member B [Source:HGNC Symbol;Acc:HGNC:13495] |
| ENSG00000196345 | HGLibA_55360 | 153 | 0.249243975 | ZKSCAN7 | zinc finger with KRAB and SCAN domains 7 [Source:HGNC Symbol;Acc:HGNC:12955] |
| ENSG00000196911 | HGLibA_25365 | 153 | 0.249243975 | KPNA5 | karyopherin subunit alpha 5 [Source:HGNC Symbol;Acc:HGNC:6398] |
| ENSG00000197857 | HGLibA_56000 | 153 | 0.249243975 | ZNF44 | zinc finger protein 44 [Source:HGNC Symbol;Acc:HGNC:13110] |
| ENSG00000198931 | HGLibA_02572 | 153 | 0.249243975 | APRT | adenine phosphoribosyltransferase [Source:HGNC Symbol;Acc:HGNC:626] |
| ENSG00000198944 | HGLibA_46214 | 153 | 0.249243975 | SOWAHA | sosondowah ankyrin repeat domain family member A [Source:HGNC Symbol;Acc:HGNC:27033] |
| ENSG00000205413 | HGLibA_42697 | 153 | 0.249243975 | SAMD9 | sterile alpha motif domain containing 9 [Source:HGNC Symbol;Acc:HGNC:1348] |
| ENSG00000205730 | HGLibA_23869 | 153 | 0.249243975 | ITPRIPL2 | ITPRIP like 2 [Source:HGNC Symbol;Acc:HGNC:27257] |
| ENSG00000205978 | HGLibA_33185 | 153 | 0.249243975 | NYNRIN | NYN domain and retroviral integrase containing [Source:HGNC Symbol;Acc:HGNC:20165] |
| ENSG00000213341 | HGLibA_09669 | 153 | 0.249243975 | CHUK | conserved helix-loop-helix ubiquitous kinase [Source:HGNC Symbol;Acc:HGNC:1974] |
| ENSG00000221923 | HGLibA_56893 | 153 | 0.249243975 | ZNF880 | zinc finger protein 880 [Source:HGNC Symbol;Acc:HGNC:37249] |
| ENSG00000248385 | HGLibA_48340 | 153 | 0.249243975 | TARM1 | activating receptor on myeloid cells 1 [Source:HGNC Symbol;Acc:HGNC:37250]" |
| ENSG00000261221 | HGLibA_56885 | 153 | 0.249243975 | ZNF865 | zinc finger protein 865 [Source:HGNC Symbol;Acc:HGNC:38705] |
| ENSG00000267795 | HGLibA_45752 | 153 | 0.249243975 | SMIM22 | small integral membrane protein 22 [Source:HGNC Symbol;Acc:HGNC:48329] |
| ENSG00000268089 | HGLibA_18402 | 153 | 0.249243975 | GABRQ | gamma-aminobutyric acid type A receptor theta subunit [Source:HGNC Symbol;Acc:HGNC:14454] |
| ENSG00000268738 | HGLibA_22239 | 153 | 0.249243975 | HSFX2 | X-linked 2 [Source:HGNC Symbol;Acc:HGNC:32701]" |
| ENSG00000274391 | HGLibA_51212 | 153 | 0.249243975 | TPTE | transmembrane phosphatase with tensin homology [Source:HGNC Symbol;Acc:HGNC:12023] |
| ENSG00000276581 | HGLibA_46479 | 153 | 0.249243975 | SPATA31A5 | SPATA31 subfamily A member 5 [Source:HGNC Symbol;Acc:HGNC:32005] |
| ENSG00000279761 | HGLibA_34275 | 153 | 0.249243975 | OR5D13 | olfactory receptor family 5 subfamily D member 13 (gene/pseudogene) [Source:HGNC Symbol;Acc:HGNC:15280] |
| FAM73B | HGLibA_16719 | 153 | 0.249243975 | FAM73B | - |
| H2AFX | HGLibA_20759 | 153 | 0.249243975 | H2AFX | - |
| HIST1H2AD | HGLibA_21383 | 153 | 0.249243975 | HIST1H2AD | - |
| hsa-mir-302d | HGLibA_58447 | 153 | 0.249243975 | hsa-mir-302d | - |
| hsa-mir-3180-3 | HGLibA_58810 | 153 | 0.249243975 | hsa-mir-3180-3 | - |
| hsa-mir-320e | HGLibA_58953 | 153 | 0.249243975 | hsa-mir-320e | - |
| hsa-mir-340 | HGLibA_59018 | 153 | 0.249243975 | hsa-mir-340 | - |
| hsa-mir-3666 | HGLibA_59214 | 153 | 0.249243975 | hsa-mir-3666 | - |
| hsa-mir-3680-2 | HGLibA_59275 | 153 | 0.249243975 | hsa-mir-3680-2 | - |
| hsa-mir-4325 | HGLibA_60024 | 153 | 0.249243975 | hsa-mir-4325 | - |
| hsa-mir-4329 | HGLibA_60041 | 153 | 0.249243975 | hsa-mir-4329 | - |
| hsa-mir-4718 | HGLibA_60971 | 153 | 0.249243975 | hsa-mir-4718 | - |
| hsa-mir-4776-1 | HGLibA_61209 | 153 | 0.249243975 | hsa-mir-4776-1 | - |
| hsa-mir-4789 | HGLibA_61263 | 153 | 0.249243975 | hsa-mir-4789 | - |
| hsa-mir-514a-3 | HGLibA_61595 | 153 | 0.249243975 | hsa-mir-514a-3 | - |
| hsa-mir-661 | HGLibA_62979 | 153 | 0.249243975 | hsa-mir-661 | - |
| hsa-mir-7112-2 | HGLibA_63816 | 153 | 0.249243975 | hsa-mir-7112-2 | - |
| hsa-mir-8056 | HGLibA_64063 | 153 | 0.249243975 | hsa-mir-8056 | - |
| hsa-mir-891a | HGLibA_64242 | 153 | 0.249243975 | hsa-mir-891a | - |
| LOC100129924 | HGLibA_26770 | 153 | 0.249243975 | LOC100129924 | - |
| PCDP1 | HGLibA_35580 | 153 | 0.249243975 | PCDP1 | - |
| PNMAL2 | HGLibA_37388 | 153 | 0.249243975 | PNMAL2 | - |
| TARP | HGLibA_48341 | 153 | 0.249243975 | TARP | - |
| C10orf32 | HGLibA_05068 | 152 | 0.240198632 | C10orf32 | - |
| C19orf43 | HGLibA_05660 | 152 | 0.240198632 | C19orf43 | - |
| C6orf99 | HGLibA_06514 | 152 | 0.240198632 | C6orf99 | - |
| ENSG00000007968 | HGLibA_14309 | 152 | 0.240198632 | E2F2 | E2F transcription factor 2 [Source:HGNC Symbol;Acc:HGNC:3114] |
| ENSG00000032219 | HGLibA_02901 | 152 | 0.240198632 | ARID4A | AT-rich interaction domain 4A [Source:HGNC Symbol;Acc:HGNC:9885] |
| ENSG00000044012 | HGLibA_20647 | 152 | 0.240198632 | GUCA2B | guanylate cyclase activator 2B [Source:HGNC Symbol;Acc:HGNC:4683] |
| ENSG00000054611 | HGLibA_48502 | 152 | 0.240198632 | TBC1D22A | TBC1 domain family member 22A [Source:HGNC Symbol;Acc:HGNC:1309] |
| ENSG00000055813 | HGLibA_07892 | 152 | 0.240198632 | CCDC85A | coiled-coil domain containing 85A [Source:HGNC Symbol;Acc:HGNC:29400] |
| ENSG00000064652 | HGLibA_46068 | 152 | 0.240198632 | SNX24 | sorting nexin 24 [Source:HGNC Symbol;Acc:HGNC:21533] |
| ENSG00000073756 | HGLibA_39361 | 152 | 0.240198632 | PTGS2 | prostaglandin-endoperoxide synthase 2 [Source:HGNC Symbol;Acc:HGNC:9605] |
| ENSG00000083544 | HGLibA_48905 | 152 | 0.240198632 | TDRD3 | tudor domain containing 3 [Source:HGNC Symbol;Acc:HGNC:20612] |
| ENSG00000089692 | HGLibA_25926 | 152 | 0.240198632 | LAG3 | lymphocyte activating 3 [Source:HGNC Symbol;Acc:HGNC:6476] |
| ENSG00000100934 | HGLibA_43219 | 152 | 0.240198632 | SEC23A | coat complex II component [Source:HGNC Symbol;Acc:HGNC:10701]" |
| ENSG00000101290 | HGLibA_08885 | 152 | 0.240198632 | CDS2 | CDP-diacylglycerol synthase 2 [Source:HGNC Symbol;Acc:HGNC:1801] |
| ENSG00000102048 | HGLibA_03255 | 152 | 0.240198632 | ASB9 | ankyrin repeat and SOCS box containing 9 [Source:HGNC Symbol;Acc:HGNC:17184] |
| ENSG00000102226 | HGLibA_53196 | 152 | 0.240198632 | USP11 | ubiquitin specific peptidase 11 [Source:HGNC Symbol;Acc:HGNC:12609] |
| ENSG00000103528 | HGLibA_48070 | 152 | 0.240198632 | SYT17 | synaptotagmin 17 [Source:HGNC Symbol;Acc:HGNC:24119] |
| ENSG00000104894 | HGLibA_08362 | 152 | 0.240198632 | CD37 | CD37 molecule [Source:HGNC Symbol;Acc:HGNC:1666] |
| ENSG00000104972 | HGLibA_26507 | 152 | 0.240198632 | LILRB1 | leukocyte immunoglobulin like receptor B1 [Source:HGNC Symbol;Acc:HGNC:6605] |
| ENSG00000105146 | HGLibA_03883 | 152 | 0.240198632 | AURKC | aurora kinase C [Source:HGNC Symbol;Acc:HGNC:11391] |
| ENSG00000105366 | HGLibA_44151 | 152 | 0.240198632 | SIGLEC8 | sialic acid binding Ig like lectin 8 [Source:HGNC Symbol;Acc:HGNC:10877] |
| ENSG00000106004 | HGLibA_21913 | 152 | 0.240198632 | HOXA5 | homeobox A5 [Source:HGNC Symbol;Acc:HGNC:5106] |
| ENSG00000109163 | HGLibA_19586 | 152 | 0.240198632 | GNRHR | gonadotropin releasing hormone receptor [Source:HGNC Symbol;Acc:HGNC:4421] |
| ENSG00000109208 | HGLibA_45824 | 152 | 0.240198632 | SMR3A | submaxillary gland androgen regulated protein 3A [Source:HGNC Symbol;Acc:HGNC:19216] |
| ENSG00000112539 | HGLibA_06438 | 152 | 0.240198632 | C6orf118 | chromosome 6 open reading frame 118 [Source:HGNC Symbol;Acc:HGNC:21233] |
| ENSG00000116885 | HGLibA_34676 | 152 | 0.240198632 | OSCP1 | organic solute carrier partner 1 [Source:HGNC Symbol;Acc:HGNC:29971] |
| ENSG00000122417 | HGLibA_33281 | 152 | 0.240198632 | ODF2L | outer dense fiber of sperm tails 2 like [Source:HGNC Symbol;Acc:HGNC:29225] |
| ENSG00000122824 | HGLibA_32935 | 152 | 0.240198632 | NUDT10 | nudix hydrolase 10 [Source:HGNC Symbol;Acc:HGNC:17621] |
| ENSG00000125903 | HGLibA_12890 | 152 | 0.240198632 | DEFB129 | defensin beta 129 [Source:HGNC Symbol;Acc:HGNC:16218] |
| ENSG00000126070 | HGLibA_01211 | 152 | 0.240198632 | AGO3 | RISC catalytic component [Source:HGNC Symbol;Acc:HGNC:18421]" |
| ENSG00000127528 | HGLibA_25066 | 152 | 0.240198632 | KLF2 | Kruppel like factor 2 [Source:HGNC Symbol;Acc:HGNC:6347] |
| ENSG00000127533 | HGLibA_15872 | 152 | 0.240198632 | F2RL3 | F2R like thrombin or trypsin receptor 3 [Source:HGNC Symbol;Acc:HGNC:3540] |
| ENSG00000129167 | HGLibA_51129 | 152 | 0.240198632 | TPH1 | tryptophan hydroxylase 1 [Source:HGNC Symbol;Acc:HGNC:12008] |
| ENSG00000130300 | HGLibA_37249 | 152 | 0.240198632 | PLVAP | plasmalemma vesicle associated protein [Source:HGNC Symbol;Acc:HGNC:13635] |
| ENSG00000131188 | HGLibA_38852 | 152 | 0.240198632 | PRR7 | synaptic [Source:HGNC Symbol;Acc:HGNC:28130]" |
| ENSG00000131196 | HGLibA_31772 | 152 | 0.240198632 | NFATC1 | nuclear factor of activated T cells 1 [Source:HGNC Symbol;Acc:HGNC:7775] |
| ENSG00000132205 | HGLibA_15051 | 152 | 0.240198632 | EMILIN2 | elastin microfibril interfacer 2 [Source:HGNC Symbol;Acc:HGNC:19881] |
| ENSG00000133574 | HGLibA_19078 | 152 | 0.240198632 | GIMAP4 | IMAP family member 4 [Source:HGNC Symbol;Acc:HGNC:21872]" |
| ENSG00000133636 | HGLibA_32884 | 152 | 0.240198632 | NTS | neurotensin [Source:HGNC Symbol;Acc:HGNC:8038] |
| ENSG00000134057 | HGLibA_08057 | 152 | 0.240198632 | CCNB1 | cyclin B1 [Source:HGNC Symbol;Acc:HGNC:1579] |
| ENSG00000134258 | HGLibA_53883 | 152 | 0.240198632 | VTCN1 | V-set domain containing T cell activation inhibitor 1 [Source:HGNC Symbol;Acc:HGNC:28873] |
| ENSG00000135622 | HGLibA_43351 | 152 | 0.240198632 | SEMA4F | ssemaphorin 4F [Source:HGNC Symbol;Acc:HGNC:10734] |
| ENSG00000135763 | HGLibA_53137 | 152 | 0.240198632 | URB2 | URB2 ribosome biogenesis homolog [Source:HGNC Symbol;Acc:HGNC:28967] |
| ENSG00000137709 | HGLibA_37763 | 152 | 0.240198632 | POU2F3 | POU class 2 homeobox 3 [Source:HGNC Symbol;Acc:HGNC:19864] |
| ENSG00000138316 | HGLibA_00803 | 152 | 0.240198632 | ADAMTS14 | ADAM metallopeptidase with thrombospondin type 1 motif 14 [Source:HGNC Symbol;Acc:HGNC:14899] |
| ENSG00000139832 | HGLibA_39836 | 152 | 0.240198632 | RAB20 | member RAS oncogene family [Source:HGNC Symbol;Acc:HGNC:18260]" |
| ENSG00000143036 | HGLibA_45163 | 152 | 0.240198632 | SLC44A3 | solute carrier family 44 member 3 [Source:HGNC Symbol;Acc:HGNC:28689] |
| ENSG00000143147 | HGLibA_19958 | 152 | 0.240198632 | GPR161 | G protein-coupled receptor 161 [Source:HGNC Symbol;Acc:HGNC:23694] |
| ENSG00000144468 | HGLibA_41124 | 152 | 0.240198632 | RHBDD1 | rhomboid domain containing 1 [Source:HGNC Symbol;Acc:HGNC:23081] |
| ENSG00000144589 | HGLibA_47456 | 152 | 0.240198632 | STK11IP | serine/threonine kinase 11 interacting protein [Source:HGNC Symbol;Acc:HGNC:19184] |
| ENSG00000144791 | HGLibA_26532 | 152 | 0.240198632 | LIMD1 | LIM domains containing 1 [Source:HGNC Symbol;Acc:HGNC:6612] |
| ENSG00000145293 | HGLibA_15152 | 152 | 0.240198632 | ENOPH1 | enolase-phosphatase 1 [Source:HGNC Symbol;Acc:HGNC:24599] |
| ENSG00000145692 | HGLibA_04484 | 152 | 0.240198632 | BHMT | betaine--homocysteine S-methyltransferase [Source:HGNC Symbol;Acc:HGNC:1047] |
| ENSG00000145708 | HGLibA_11137 | 152 | 0.240198632 | CRHBP | corticotropin releasing hormone binding protein [Source:HGNC Symbol;Acc:HGNC:2356] |
| ENSG00000147724 | HGLibA_16134 | 152 | 0.240198632 | FAM135B | family with sequence similarity 135 member B [Source:HGNC Symbol;Acc:HGNC:28029] |
| ENSG00000147799 | HGLibA_02770 | 152 | 0.240198632 | ARHGAP39 | Rho GTPase activating protein 39 [Source:HGNC Symbol;Acc:HGNC:29351] |
| ENSG00000149179 | HGLibA_05147 | 152 | 0.240198632 | C11orf49 | chromosome 11 open reading frame 49 [Source:HGNC Symbol;Acc:HGNC:28720] |
| ENSG00000150867 | HGLibA_36705 | 152 | 0.240198632 | PIP4K2A | phosphatidylinositol-5-phosphate 4-kinase type 2 alpha [Source:HGNC Symbol;Acc:HGNC:8997] |
| ENSG00000153982 | HGLibA_18889 | 152 | 0.240198632 | GDPD1 | glycerophosphodiester phosphodiesterase domain containing 1 [Source:HGNC Symbol;Acc:HGNC:20883] |
| ENSG00000160446 | HGLibA_55072 | 152 | 0.240198632 | ZDHHC12 | zinc finger DHHC-type containing 12 [Source:HGNC Symbol;Acc:HGNC:19159] |
| ENSG00000160838 | HGLibA_27419 | 152 | 0.240198632 | LRRC71 | leucine rich repeat containing 71 [Source:HGNC Symbol;Acc:HGNC:26556] |
| ENSG00000162068 | HGLibA_32856 | 152 | 0.240198632 | NTN3 | netrin 3 [Source:HGNC Symbol;Acc:HGNC:8030] |
| ENSG00000164078 | HGLibA_30258 | 152 | 0.240198632 | MST1R | macrophage stimulating 1 receptor [Source:HGNC Symbol;Acc:HGNC:7381] |
| ENSG00000165898 | HGLibA_23650 | 152 | 0.240198632 | ISCA2 | iron-sulfur cluster assembly 2 [Source:HGNC Symbol;Acc:HGNC:19857] |
| ENSG00000166049 | HGLibA_35266 | 152 | 0.240198632 | PASD1 | PAS domain containing repressor 1 [Source:HGNC Symbol;Acc:HGNC:20686] |
| ENSG00000166664 | HGLibA_09544 | 152 | 0.240198632 | CHRFAM7A | CHRNA7 (exons 5-10) and FAM7A (exons A-E) fusion [Source:HGNC Symbol;Acc:HGNC:15781] |
| ENSG00000167165 | HGLibA_52891 | 152 | 0.240198632 | UGT1A6 | UDP glucuronosyltransferase family 1 member A6 [Source:HGNC Symbol;Acc:HGNC:12538] |
| ENSG00000167799 | HGLibA_32990 | 152 | 0.240198632 | NUDT8 | nudix hydrolase 8 [Source:HGNC Symbol;Acc:HGNC:8055] |
| ENSG00000168826 | HGLibA_54907 | 152 | 0.240198632 | ZBTB49 | zinc finger and BTB domain containing 49 [Source:HGNC Symbol;Acc:HGNC:19883] |
| ENSG00000169789 | HGLibA_38995 | 152 | 0.240198632 | PRY | PTPN13 like Y-linked [Source:HGNC Symbol;Acc:HGNC:14024] |
| ENSG00000169906 | HGLibA_42580 | 152 | 0.240198632 | S100G | S100 calcium binding protein G [Source:HGNC Symbol;Acc:HGNC:1436] |
| ENSG00000170092 | HGLibA_46598 | 152 | 0.240198632 | SPDYE5 | speedy/RINGO cell cycle regulator family member E5 [Source:HGNC Symbol;Acc:HGNC:35464] |
| ENSG00000170917 | HGLibA_32985 | 152 | 0.240198632 | NUDT6 | nudix hydrolase 6 [Source:HGNC Symbol;Acc:HGNC:8053] |
| ENSG00000171291 | HGLibA_55997 | 152 | 0.240198632 | ZNF439 | zinc finger protein 439 [Source:HGNC Symbol;Acc:HGNC:20873] |
| ENSG00000171815 | HGLibA_35470 | 152 | 0.240198632 | PCDHB1 | protocadherin beta 1 [Source:HGNC Symbol;Acc:HGNC:8680] |
| ENSG00000172264 | HGLibA_27840 | 152 | 0.240198632 | MACROD2 | MACRO domain containing 2 [Source:HGNC Symbol;Acc:HGNC:16126] |

| | | | | | |
|---|---|---|---|---|---|
| ENSG00000172487 | HGLibA_34539 | 152 | 0.240198632 | OR8J1 | olfactory receptor family 8 subfamily J member 1 [Source:HGNC Symbol;Acc:HGNC:14855] |
| ENSG00000173200 | HGLibA_35225 | 152 | 0.240198632 | PARP15 | poly(ADP-ribose) polymerase family member 15 [Source:HGNC Symbol;Acc:HGNC:26876] |
| ENSG00000173391 | HGLibA_33378 | 152 | 0.240198632 | OLR1 | oxidized low density lipoprotein receptor 1 [Source:HGNC Symbol;Acc:HGNC:8133] |
| ENSG00000173933 | HGLibA_40545 | 152 | 0.240198632 | RBM4 | RNA binding motif protein 4 [Source:HGNC Symbol;Acc:HGNC:9901] |
| ENSG00000175175 | HGLibA_37971 | 152 | 0.240198632 | PPM1E | Mg2+/Mn2+ dependent 1E [Source:HGNC Symbol;Acc:HGNC:19322]" |
| ENSG00000175264 | HGLibA_09610 | 152 | 0.240198632 | CHST1 | carbohydrate sulfotransferase 1 [Source:HGNC Symbol;Acc:HGNC:1969] |
| ENSG00000175591 | HGLibA_34884 | 152 | 0.240198632 | P2RY2 | purinergic receptor P2Y2 [Source:HGNC Symbol;Acc:HGNC:8541] |
| ENSG00000176358 | HGLibA_48157 | 152 | 0.240198632 | TAC4 | tachykinin 4 [Source:HGNC Symbol;Acc:HGNC:16641] |
| ENSG00000176749 | HGLibA_08775 | 152 | 0.240198632 | CDK5R1 | cyclin dependent kinase 5 regulatory subunit 1 [Source:HGNC Symbol;Acc:HGNC:1775] |
| ENSG00000177674 | HGLibA_01269 | 152 | 0.240198632 | AGTRAP | angiotensin II receptor associated protein [Source:HGNC Symbol;Acc:HGNC:13539] |
| ENSG00000178764 | HGLibA_55314 | 152 | 0.240198632 | ZHX2 | zinc fingers and homeoboxes 2 [Source:HGNC Symbol;Acc:HGNC:18513] |
| ENSG00000180304 | HGLibA_33216 | 152 | 0.240198632 | OAZ2 | ornithine decarboxylase antizyme 2 [Source:HGNC Symbol;Acc:HGNC:8096] |
| ENSG00000182326 | HGLibA_05974 | 152 | 0.240198632 | C1S | complement C1s [Source:HGNC Symbol;Acc:HGNC:1247] |
| ENSG00000182601 | HGLibA_22133 | 152 | 0.240198632 | HS3ST4 | heparan sulfate-glucosamine 3-sulfotransferase 4 [Source:HGNC Symbol;Acc:HGNC:5200] |
| ENSG00000182645 | HGLibA_07697 | 152 | 0.240198632 | CCDC172 | coiled-coil domain containing 172 [Source:HGNC Symbol;Acc:HGNC:30524] |
| ENSG00000185900 | HGLibA_37663 | 152 | 0.240198632 | POMK | protein-O-mannose kinase [Source:HGNC Symbol;Acc:HGNC:26267] |
| ENSG00000188807 | HGLibA_50181 | 152 | 0.240198632 | TMEM201 | transmembrane protein 201 [Source:HGNC Symbol;Acc:HGNC:33719] |
| ENSG00000189051 | HGLibA_41642 | 152 | 0.240198632 | RNF222 | ring finger protein 222 [Source:HGNC Symbol;Acc:HGNC:34517] |
| ENSG00000196131 | HGLibA_53702 | 152 | 0.240198632 | VN1R2 | vomeronasal 1 receptor 2 [Source:HGNC Symbol;Acc:HGNC:19872] |
| ENSG00000196748 | HGLibA_10110 | 152 | 0.240198632 | CLPSL2 | colipase like 2 [Source:HGNC Symbol;Acc:HGNC:21250] |
| ENSG00000196811 | HGLibA_09606 | 152 | 0.240198632 | CHRNG | cholinergic receptor nicotinic gamma subunit [Source:HGNC Symbol;Acc:HGNC:1967] |
| ENSG00000196967 | HGLibA_56320 | 152 | 0.240198632 | ZNF585A | zinc finger protein 585A [Source:HGNC Symbol;Acc:HGNC:26305] |
| ENSG00000197933 | HGLibA_56820 | 152 | 0.240198632 | ZNF823 | zinc finger protein 823 [Source:HGNC Symbol;Acc:HGNC:30936] |
| ENSG00000198182 | HGLibA_56371 | 152 | 0.240198632 | ZNF607 | zinc finger protein 607 [Source:HGNC Symbol;Acc:HGNC:28192] |
| ENSG00000198443 | HGLibA_25750 | 152 | 0.240198632 | KRTAP4-1 | keratin associated protein 4-1 [Source:HGNC Symbol;Acc:HGNC:18907] |
| ENSG00000204165 | HGLibA_11959 | 152 | 0.240198632 | CXorf65 | chromosome X open reading frame 65 [Source:HGNC Symbol;Acc:HGNC:33713] |
| ENSG00000204351 | HGLibA_44286 | 152 | 0.240198632 | SKIV2L | Ski2 like RNA helicase [Source:HGNC Symbol;Acc:HGNC:10898] |
| ENSG00000204694 | HGLibA_33562 | 152 | 0.240198632 | OR11A1 | olfactory receptor family 11 subfamily A member 1 [Source:HGNC Symbol;Acc:HGNC:8176] |
| ENSG00000213658 | HGLibA_26059 | 152 | 0.240198632 | LAT | linker for activation of T cells [Source:HGNC Symbol;Acc:HGNC:18874] |
| ENSG00000213983 | HGLibA_02287 | 152 | 0.240198632 | AP1G2 | adaptor related protein complex 1 subunit gamma 2 [Source:HGNC Symbol;Acc:HGNC:556] |
| ENSG00000229859 | HGLibA_36185 | 152 | 0.240198632 | PGA3 | group I (pepsinogen A) [Source:HGNC Symbol;Acc:HGNC:8885]" |
| ENSG00000234560 | HGLibA_33499 | 152 | 0.240198632 | OR10G8 | olfactory receptor family 10 subfamily G member 8 [Source:HGNC Symbol;Acc:HGNC:14845] |
| ENSG00000236609 | HGLibA_56874 | 152 | 0.240198632 | ZNF853 | zinc finger protein 853 [Source:HGNC Symbol;Acc:HGNC:21767] |
| ENSG00000237515 | HGLibA_44055 | 152 | 0.240198632 | SHISA9 | shisa family member 9 [Source:HGNC Symbol;Acc:HGNC:37231] |
| ENSG00000239961 | HGLibA_26498 | 152 | 0.240198632 | LILRA4 | leukocyte immunoglobulin like receptor A4 [Source:HGNC Symbol;Acc:HGNC:15503] |
| FAM134B | HGLibA_16126 | 152 | 0.240198632 | FAM134B | - |
| FAM154B | HGLibA_16176 | 152 | 0.240198632 | FAM154B | - |
| HMP19 | HGLibA_21752 | 152 | 0.240198632 | HMP19 | - |
| hsa-mir-181d | HGLibA_57980 | 152 | 0.240198632 | hsa-mir-181d | - |
| hsa-mir-3164 | HGLibA_58736 | 152 | 0.240198632 | hsa-mir-3164 | - |
| hsa-mir-3201 | HGLibA_58912 | 152 | 0.240198632 | hsa-mir-3201 | - |
| hsa-mir-3622b | HGLibA_59127 | 152 | 0.240198632 | hsa-mir-3622b | - |
| hsa-mir-363 | HGLibA_59130 | 152 | 0.240198632 | hsa-mir-363 | - |
| hsa-mir-3928 | HGLibA_59589 | 152 | 0.240198632 | hsa-mir-3928 | - |
| hsa-mir-4505 | HGLibA_60448 | 152 | 0.240198632 | hsa-mir-4505 | - |
| hsa-mir-4639 | HGLibA_60662 | 152 | 0.240198632 | hsa-mir-4639 | - |
| hsa-mir-4692 | HGLibA_60879 | 152 | 0.240198632 | hsa-mir-4692 | - |
| hsa-mir-4715 | HGLibA_60962 | 152 | 0.240198632 | hsa-mir-4715 | - |
| hsa-mir-511 | HGLibA_61556 | 152 | 0.240198632 | hsa-mir-511 | - |
| hsa-mir-6715a | HGLibA_63016 | 152 | 0.240198632 | hsa-mir-6715a | - |
| hsa-mir-6747 | HGLibA_63142 | 152 | 0.240198632 | hsa-mir-6747 | - |
| hsa-mir-6804 | HGLibA_63395 | 152 | 0.240198632 | hsa-mir-6804 | - |
| KIAA0146 | HGLibA_24596 | 152 | 0.240198632 | KIAA0146 | - |
| PIDD | HGLibA_36531 | 152 | 0.240198632 | PIDD | - |
| PPAPDC1A | HGLibA_37825 | 152 | 0.240198632 | PPAPDC1A | - |
| TROVE2 | HGLibA_51723 | 152 | 0.240198632 | TROVE2 | - |
| C5orf45 | HGLibA_06385 | 151 | 0.231153289 | C5orf45 | - |
| ENSG00000008394 | HGLibA_29175 | 151 | 0.231153289 | MGST1 | microsomal glutathione S-transferase 1 [Source:HGNC Symbol;Acc:HGNC:7061] |
| ENSG00000010626 | HGLibA_27285 | 151 | 0.231153289 | LRRC23 | leucine rich repeat containing 23 [Source:HGNC Symbol;Acc:HGNC:19138] |
| ENSG00000050628 | HGLibA_39318 | 151 | 0.231153289 | PTGER3 | prostaglandin E receptor 3 [Source:HGNC Symbol;Acc:HGNC:9595] |
| ENSG00000053438 | HGLibA_32221 | 151 | 0.231153289 | NNAT | neuronatin [Source:HGNC Symbol;Acc:HGNC:7860] |
| ENSG00000057149 | HGLibA_43549 | 151 | 0.231153289 | SERPINB3 | serpin family B member 3 [Source:HGNC Symbol;Acc:HGNC:10569] |
| ENSG00000064995 | HGLibA_48202 | 151 | 0.231153289 | TAF11 | TATA-box binding protein associated factor 11 [Source:HGNC Symbol;Acc:HGNC:11544] |
| ENSG00000066185 | HGLibA_55416 | 151 | 0.231153289 | ZMYND12 | zinc finger MYND-type containing 12 [Source:HGNC Symbol;Acc:HGNC:21192] |
| ENSG00000069399 | HGLibA_04328 | 151 | 0.231153289 | BCL3 | B cell CLL/lymphoma 3 [Source:HGNC Symbol;Acc:HGNC:998] |
| ENSG00000069431 | HGLibA_00187 | 151 | 0.231153289 | ABCC9 | ATP binding cassette subfamily C member 9 [Source:HGNC Symbol;Acc:HGNC:60] |
| ENSG00000075413 | HGLibA_28370 | 151 | 0.231153289 | MARK3 | microtubule affinity regulating kinase 3 [Source:HGNC Symbol;Acc:HGNC:6897] |
| ENSG00000078043 | HGLibA_36511 | 151 | 0.231153289 | PIAS2 | protein inhibitor of activated STAT 2 [Source:HGNC Symbol;Acc:HGNC:17311] |
| ENSG00000078902 | HGLibA_50942 | 151 | 0.231153289 | TOLLIP | toll interacting protein [Source:HGNC Symbol;Acc:HGNC:16476] |
| ENSG00000079277 | HGLibA_29341 | 151 | 0.231153289 | MKNK1 | MAP kinase interacting serine/threonine kinase 1 [Source:HGNC Symbol;Acc:HGNC:7110] |
| ENSG00000084072 | HGLibA_37928 | 151 | 0.231153289 | PPIE | peptidylprolyl isomerase E [Source:HGNC Symbol;Acc:HGNC:9258] |
| ENSG00000095981 | HGLibA_24308 | 151 | 0.231153289 | KCNK16 | potassium two pore domain channel subfamily K member 16 [Source:HGNC Symbol;Acc:HGNC:14464] |
| ENSG00000100813 | HGLibA_00439 | 151 | 0.231153289 | ACIN1 | apoptotic chromatin condensation inducer 1 [Source:HGNC Symbol;Acc:HGNC:17066] |
| ENSG00000101361 | HGLibA_32314 | 151 | 0.231153289 | NOP56 | NOP56 ribonucleoprotein [Source:HGNC Symbol;Acc:HGNC:15911] |
| ENSG00000102539 | HGLibA_29428 | 151 | 0.231153289 | MLNR | motilin receptor [Source:HGNC Symbol;Acc:HGNC:4495] |
| ENSG00000104043 | HGLibA_03789 | 151 | 0.231153289 | ATP8B4 | ATPase phospholipid transporting 8B4 (putative) [Source:HGNC Symbol;Acc:HGNC:13536] |
| ENSG00000105737 | HGLibA_20292 | 151 | 0.231153289 | GRIK5 | glutamate ionotropic receptor kainate type subunit 5 [Source:HGNC Symbol;Acc:HGNC:4583] |
| ENSG00000106013 | HGLibA_02143 | 151 | 0.231153289 | ANKRD7 | ankyrin repeat domain 7 [Source:HGNC Symbol;Acc:HGNC:18588] |
| ENSG00000109762 | HGLibA_46071 | 151 | 0.231153289 | SNX25 | sorting nexin 25 [Source:HGNC Symbol;Acc:HGNC:21883] |
| ENSG00000109956 | HGLibA_03971 | 151 | 0.231153289 | B3GAT1 | 3-glucuronyltransferase 1 [Source:HGNC Symbol;Acc:HGNC:921]" |
| ENSG00000110435 | HGLibA_35870 | 151 | 0.231153289 | PDHX | pyruvate dehydrogenase complex component X [Source:HGNC Symbol;Acc:HGNC:21350] |
| ENSG00000111481 | HGLibA_10736 | 151 | 0.231153289 | COPZ1 | coatomer protein complex subunit zeta 1 [Source:HGNC Symbol;Acc:HGNC:2243] |
| ENSG00000112237 | HGLibA_08070 | 151 | 0.231153289 | CCNC | cyclin C [Source:HGNC Symbol;Acc:HGNC:1581] |
| ENSG00000112877 | HGLibA_09161 | 151 | 0.231153289 | CEP72 | centrosomal protein 72 [Source:HGNC Symbol;Acc:HGNC:25547] |
| ENSG00000113100 | HGLibA_08694 | 151 | 0.231153289 | CDH9 | cadherin 9 [Source:HGNC Symbol;Acc:HGNC:1768] |
| ENSG00000113600 | HGLibA_06658 | 151 | 0.231153289 | C9 | complement C9 [Source:HGNC Symbol;Acc:HGNC:1358] |
| ENSG00000114302 | HGLibA_38526 | 151 | 0.231153289 | PRKAR2A | protein kinase cAMP-dependent type II regulatory subunit alpha [Source:HGNC Symbol;Acc:HGNC:9391] |
| ENSG00000114698 | HGLibA_37241 | 151 | 0.231153289 | PLSCR4 | phospholipid scramblase 4 [Source:HGNC Symbol;Acc:HGNC:16497] |
| ENSG00000118217 | HGLibA_03447 | 151 | 0.231153289 | ATF6 | activating transcription factor 6 [Source:HGNC Symbol;Acc:HGNC:791] |
| ENSG00000120725 | HGLibA_44171 | 151 | 0.231153289 | SIL1 | SIL1 nucleotide exchange factor [Source:HGNC Symbol;Acc:HGNC:24624] |
| ENSG00000122870 | HGLibA_04492 | 151 | 0.231153289 | BICC1 | BicC family RNA binding protein 1 [Source:HGNC Symbol;Acc:HGNC:19351] |
| ENSG00000124380 | HGLibA_45947 | 151 | 0.231153289 | SNRNP27 | small nuclear ribonucleoprotein U4/U6.U5 subunit 27 [Source:HGNC Symbol;Acc:HGNC:30240] |
| ENSG00000124749 | HGLibA_10538 | 151 | 0.231153289 | COL21A1 | collagen type XXI alpha 1 chain [Source:HGNC Symbol;Acc:HGNC:17025] |
| ENSG00000125888 | HGLibA_04143 | 151 | 0.231153289 | BANF2 | barrier to autointegration factor 2 [Source:HGNC Symbol;Acc:HGNC:16172] |
| ENSG00000126785 | HGLibA_41197 | 151 | 0.231153289 | RHOJ | ras homolog family member J [Source:HGNC Symbol;Acc:HGNC:688] |
| ENSG00000126804 | HGLibA_54811 | 151 | 0.231153289 | ZBTB1 | zinc finger and BTB domain containing 1 [Source:HGNC Symbol;Acc:HGNC:20259] |
| ENSG00000128165 | HGLibA_00996 | 151 | 0.231153289 | ADM2 | adrenomedullin 2 [Source:HGNC Symbol;Acc:HGNC:28898] |
| ENSG00000131779 | HGLibA_36088 | 151 | 0.231153289 | PEX11B | peroxisomal biogenesis factor 11 beta [Source:HGNC Symbol;Acc:HGNC:8853] |
| ENSG00000132164 | HGLibA_45293 | 151 | 0.231153289 | SLC6A11 | solute carrier family 6 member 11 [Source:HGNC Symbol;Acc:HGNC:11044] |
| ENSG00000135924 | HGLibA_13536 | 151 | 0.231153289 | DNAJB2 | DnaJ heat shock protein family (Hsp40) member B2 [Source:HGNC Symbol;Acc:HGNC:5228] |
| ENSG00000136250 | HGLibA_02264 | 151 | 0.231153289 | AOAH | acyloxyacyl hydrolase [Source:HGNC Symbol;Acc:HGNC:548] |
| ENSG00000136560 | HGLibA_48304 | 151 | 0.231153289 | TANK | TRAF family member associated NFKB activator [Source:HGNC Symbol;Acc:HGNC:11562] |
| ENSG00000139178 | HGLibA_05972 | 151 | 0.231153289 | C1RL | complement C1r subcomponent like [Source:HGNC Symbol;Acc:HGNC:21265] |
| ENSG00000139651 | HGLibA_56663 | 151 | 0.231153289 | ZNF740 | zinc finger protein 740 [Source:HGNC Symbol;Acc:HGNC:27465] |
| ENSG00000141086 | HGLibA_11686 | 151 | 0.231153289 | CTRL | chymotrypsin like [Source:HGNC Symbol;Acc:HGNC:2524] |
| ENSG00000141194 | HGLibA_33966 | 151 | 0.231153289 | OR4D1 | olfactory receptor family 4 subfamily D member 1 [Source:HGNC Symbol;Acc:HGNC:8293] |
| ENSG00000141738 | HGLibA_20232 | 151 | 0.231153289 | GRB7 | growth factor receptor bound protein 7 [Source:HGNC Symbol;Acc:HGNC:4567] |
| ENSG00000142959 | HGLibA_04427 | 151 | 0.231153289 | BEST4 | bestrophin 4 [Source:HGNC Symbol;Acc:HGNC:17106] |
| ENSG00000143816 | HGLibA_54395 | 151 | 0.231153289 | WNT9A | Wnt family member 9A [Source:HGNC Symbol;Acc:HGNC:12778] |
| ENSG00000145244 | HGLibA_10770 | 151 | 0.231153289 | CORIN | serine peptidase [Source:HGNC Symbol;Acc:HGNC:19012]" |
| ENSG00000145864 | HGLibA_18376 | 151 | 0.231153289 | GABRB2 | gamma-aminobutyric acid type A receptor beta2 subunit [Source:HGNC Symbol;Acc:HGNC:4082] |
| ENSG00000146285 | HGLibA_42966 | 151 | 0.231153289 | SCML4 | Scm polycomb group protein like 4 [Source:HGNC Symbol;Acc:HGNC:21397] |
| ENSG00000147488 | HGLibA_47227 | 151 | 0.231153289 | ST18 | C2H2C-type zinc finger [Source:HGNC Symbol;Acc:HGNC:18695]" |
| ENSG00000148335 | HGLibA_32851 | 151 | 0.231153289 | NTMT1 | N-terminal Xaa-Pro-Lys N-methyltransferase 1 [Source:HGNC Symbol;Acc:HGNC:23373] |
| ENSG00000148700 | HGLibA_00947 | 151 | 0.231153289 | ADD3 | adducin 3 [Source:HGNC Symbol;Acc:HGNC:245] |
| ENSG00000152700 | HGLibA_42741 | 151 | 0.231153289 | SAR1B | secretion associated Ras related GTPase 1B [Source:HGNC Symbol;Acc:HGNC:10535] |
| ENSG00000153767 | HGLibA_20533 | 151 | 0.231153289 | GTF2E1 | general transcription factor IIE subunit 1 [Source:HGNC Symbol;Acc:HGNC:4650] |
| ENSG00000156076 | HGLibA_54301 | 151 | 0.231153289 | WIF1 | WNT inhibitory factor 1 [Source:HGNC Symbol;Acc:HGNC:18081] |
| ENSG00000156136 | HGLibA_12495 | 151 | 0.231153289 | DCK | deoxycytidine kinase [Source:HGNC Symbol;Acc:HGNC:2704] |
| ENSG00000157429 | HGLibA_55554 | 151 | 0.231153289 | ZNF19 | zinc finger protein 19 [Source:HGNC Symbol;Acc:HGNC:12981] |
| ENSG00000157445 | HGLibA_06912 | 151 | 0.231153289 | CACNA2D3 | calcium voltage-gated channel auxiliary subunit alpha2delta 3 [Source:HGNC Symbol;Acc:HGNC:15460] |
| ENSG00000157557 | HGLibA_15643 | 151 | 0.231153289 | ETS2 | transcription factor [Source:HGNC Symbol;Acc:HGNC:3489]" |
| ENSG00000159792 | HGLibA_39063 | 151 | 0.231153289 | PSKH1 | protein serine kinase H1 [Source:HGNC Symbol;Acc:HGNC:9529] |
| ENSG00000162236 | HGLibA_47651 | 151 | 0.231153289 | STX5 | syntaxin 5 [Source:HGNC Symbol;Acc:HGNC:11440] |
| ENSG00000163254 | HGLibA_11276 | 151 | 0.231153289 | CRYGC | crystallin gamma C [Source:HGNC Symbol;Acc:HGNC:2410] |
| ENSG00000164107 | HGLibA_20830 | 151 | 0.231153289 | HAND2 | heart and neural crest derivatives expressed 2 [Source:HGNC Symbol;Acc:HGNC:4808] |
| ENSG00000164318 | HGLibA_14632 | 151 | 0.231153289 | EGFLAM | fibronectin type III and laminin G domains [Source:HGNC Symbol;Acc:HGNC:26810]" |
| ENSG00000164808 | HGLibA_46665 | 151 | 0.231153289 | SPIDR | scaffold protein involved in DNA repair [Source:HGNC Symbol;Acc:HGNC:28971] |
| ENSG00000165643 | HGLibA_46162 | 151 | 0.231153289 | SOHLH1 | spermatogenesis and oogenesis specific basic helix-loop-helix 1 [Source:HGNC Symbol;Acc:HGNC:27845] |
| ENSG00000166024 | HGLibA_39770 | 151 | 0.231153289 | R3HCC1L | R3H domain and coiled-coil containing 1 like [Source:HGNC Symbol;Acc:HGNC:23512] |
| ENSG00000167543 | HGLibA_51062 | 151 | 0.231153289 | TP53I13 | tumor protein p53 inducible protein 13 [Source:HGNC Symbol;Acc:HGNC:25102] |
| ENSG00000167748 | HGLibA_25231 | 151 | 0.231153289 | KLK1 | kallikrein 1 [Source:HGNC Symbol;Acc:HGNC:6357] |
| ENSG00000168610 | HGLibA_47386 | 151 | 0.231153289 | STAT3 | signal transducer and activator of transcription 3 [Source:HGNC Symbol;Acc:HGNC:11364] |
| ENSG00000168779 | HGLibA_44076 | 151 | 0.231153289 | SHOX2 | short stature homeobox 2 [Source:HGNC Symbol;Acc:HGNC:10854] |
| ENSG00000169258 | HGLibA_20140 | 151 | 0.231153289 | GPRIN1 | G protein regulated inducer of neurite outgrowth 1 [Source:HGNC Symbol;Acc:HGNC:24835] |

| | | | | | |
|---|---|---|---|---|---|
| ENSG00000169432 | HGLibA_43006 | 151 | 0.231153289 | SCN9A | sodium voltage-gated channel alpha subunit 9 [Source:HGNC Symbol;Acc:HGNC:10597] |
| ENSG00000169583 | HGLibA_10028 | 151 | 0.231153289 | CLIC3 | chloride intracellular channel 3 [Source:HGNC Symbol;Acc:HGNC:2064] |
| ENSG00000170929 | HGLibA_33700 | 151 | 0.231153289 | OR1M1 | olfactory receptor family 1 subfamily M member 1 [Source:HGNC Symbol;Acc:HGNC:8220] |
| ENSG00000171649 | HGLibA_55336 | 151 | 0.231153289 | ZIK1 | zinc finger protein interacting with K protein 1 [Source:HGNC Symbol;Acc:HGNC:33104] |
| ENSG00000172367 | HGLibA_35976 | 151 | 0.231153289 | PDZD3 | PDZ domain containing 3 [Source:HGNC Symbol;Acc:HGNC:19891] |
| ENSG00000173480 | HGLibA_55947 | 151 | 0.231153289 | ZNF417 | zinc finger protein 417 [Source:HGNC Symbol;Acc:HGNC:20646] |
| ENSG00000175220 | HGLibA_02696 | 151 | 0.231153289 | ARHGAP1 | Rho GTPase activating protein 1 [Source:HGNC Symbol;Acc:HGNC:673] |
| ENSG00000176239 | HGLibA_34092 | 151 | 0.231153289 | OR51B6 | olfactory receptor family 51 subfamily B member 6 [Source:HGNC Symbol;Acc:HGNC:19600] |
| ENSG00000177106 | HGLibA_15379 | 151 | 0.231153289 | EPS8L2 | EPS8 like 2 [Source:HGNC Symbol;Acc:HGNC:21296] |
| ENSG00000177494 | HGLibA_54794 | 151 | 0.231153289 | ZBED2 | zinc finger BED-type containing 2 [Source:HGNC Symbol;Acc:HGNC:20710] |
| ENSG00000179008 | HGLibA_05367 | 151 | 0.231153289 | C14orf39 | chromosome 14 open reading frame 39 [Source:HGNC Symbol;Acc:HGNC:19849] |
| ENSG00000180934 | HGLibA_34210 | 151 | 0.231153289 | OR56A1 | olfactory receptor family 56 subfamily A member 1 [Source:HGNC Symbol;Acc:HGNC:14781] |
| ENSG00000181585 | HGLibA_50581 | 151 | 0.231153289 | TMIE | transmembrane inner ear [Source:HGNC Symbol;Acc:HGNC:30800] |
| ENSG00000181885 | HGLibA_09907 | 151 | 0.231153289 | CLDN7 | claudin 7 [Source:HGNC Symbol;Acc:HGNC:2049] |
| ENSG00000182308 | HGLibA_12442 | 151 | 0.231153289 | DCAF4L1 | DDB1 and CUL4 associated factor 4 like 1 [Source:HGNC Symbol;Acc:HGNC:27723] |
| ENSG00000185046 | HGLibA_02154 | 151 | 0.231153289 | ANKS1B | ankyrin repeat and sterile alpha motif domain containing 1B [Source:HGNC Symbol;Acc:HGNC:24600] |
| ENSG00000185608 | HGLibA_29979 | 151 | 0.231153289 | MRPL40 | mitochondrial ribosomal protein L40 [Source:HGNC Symbol;Acc:HGNC:14491] |
| ENSG00000186599 | HGLibA_12828 | 151 | 0.231153289 | DEFB105B | defensin beta 105B [Source:HGNC Symbol;Acc:HGNC:29930] |
| ENSG00000187082 | HGLibA_12834 | 151 | 0.231153289 | DEFB106B | defensin beta 106B [Source:HGNC Symbol;Acc:HGNC:28879] |
| ENSG00000187772 | HGLibA_26562 | 151 | 0.231153289 | LIN28B | lin-28 homolog B [Source:HGNC Symbol;Acc:HGNC:32207] |
| ENSG00000188613 | HGLibA_31095 | 151 | 0.231153289 | NANOS1 | nanos C2HC-type zinc finger 1 [Source:HGNC Symbol;Acc:HGNC:23044] |
| ENSG00000188785 | HGLibA_56205 | 151 | 0.231153289 | ZNF548 | zinc finger protein 548 [Source:HGNC Symbol;Acc:HGNC:26561] |
| ENSG00000196652 | HGLibA_55356 | 151 | 0.231153289 | ZKSCAN5 | zinc finger with KRAB and SCAN domains 5 [Source:HGNC Symbol;Acc:HGNC:12867] |
| ENSG00000197562 | HGLibA_39940 | 151 | 0.231153289 | RAB40C | member RAS oncogene family [Source:HGNC Symbol;Acc:HGNC:18285]" |
| ENSG00000197888 | HGLibA_52921 | 151 | 0.231153289 | UGT2B17 | UDP glucuronosyltransferase family 2 member B17 [Source:HGNC Symbol;Acc:HGNC:12547] |
| ENSG00000198818 | HGLibA_43750 | 151 | 0.231153289 | SFT2D1 | SFT2 domain containing 1 [Source:HGNC Symbol;Acc:HGNC:21102] |
| ENSG00000198836 | HGLibA_33404 | 151 | 0.231153289 | OPA1 | mitochondrial dynamin like GTPase [Source:HGNC Symbol;Acc:HGNC:8140]" |
| ENSG00000203896 | HGLibA_26537 | 151 | 0.231153289 | LIME1 | Lck interacting transmembrane adaptor 1 [Source:HGNC Symbol;Acc:HGNC:26016] |
| ENSG00000204071 | HGLibA_48720 | 151 | 0.231153289 | TCEAL6 | transcription elongation factor A like 6 [Source:HGNC Symbol;Acc:HGNC:24553] |
| ENSG00000211584 | HGLibA_45200 | 151 | 0.231153289 | SLC48A1 | solute carrier family 48 member 1 [Source:HGNC Symbol;Acc:HGNC:26035] |
| ENSG00000213096 | HGLibA_55674 | 151 | 0.231153289 | ZNF254 | zinc finger protein 254 [Source:HGNC Symbol;Acc:HGNC:13047] |
| ENSG00000213221 | HGLibA_13679 | 151 | 0.231153289 | DNLZ | DNL-type zinc finger [Source:HGNC Symbol;Acc:HGNC:33879] |
| ENSG00000214113 | HGLibA_27752 | 151 | 0.231153289 | LYRM4 | LYR motif containing 4 [Source:HGNC Symbol;Acc:HGNC:21365] |
| ENSG00000215203 | HGLibA_20390 | 151 | 0.231153289 | GRXCR1 | glutaredoxin and cysteine rich domain containing 1 [Source:HGNC Symbol;Acc:HGNC:31673] |
| ENSG00000226372 | HGLibA_12459 | 151 | 0.231153289 | DCAF8L1 | DDB1 and CUL4 associated factor 8 like 1 [Source:HGNC Symbol;Acc:HGNC:31810] |
| ENSG00000229292 | HGLibA_40941 | 151 | 0.231153289 | RFPL4AL1 | ret finger protein like 4A like 1 [Source:HGNC Symbol;Acc:HGNC:45147] |
| ENSG00000242221 | HGLibA_39041 | 151 | 0.231153289 | PSG2 | pregnancy specific beta-1-glycoprotein 2 [Source:HGNC Symbol;Acc:HGNC:9519] |
| ENSG00000251692 | HGLibA_39574 | 151 | 0.231153289 | PTX4 | pentraxin 4 [Source:HGNC Symbol;Acc:HGNC:14171] |
| ENSG00000256812 | HGLibA_07187 | 151 | 0.231153289 | CAPNS2 | calpain small subunit 2 [Source:HGNC Symbol;Acc:HGNC:16371] |
| ENSG00000269437 | HGLibA_33132 | 151 | 0.231153289 | NXF2B | nuclear RNA export factor 2B [Source:HGNC Symbol;Acc:HGNC:23984] |
| ENSG00000278023 | HGLibA_40754 | 151 | 0.231153289 | RDM1 | RAD52 motif containing 1 [Source:HGNC Symbol;Acc:HGNC:19950] |
| FAM19A2 | HGLibA_16399 | 151 | 0.231153289 | FAM19A2 | - |
| FAM86A | HGLibA_16769 | 151 | 0.231153289 | FAM86A | - |
| hsa-mir-17 | HGLibA_57956 | 151 | 0.231153289 | hsa-mir-17 | - |
| hsa-mir-218-2 | HGLibA_58274 | 151 | 0.231153289 | hsa-mir-218-2 | - |
| hsa-mir-363 | HGLibA_59129 | 151 | 0.231153289 | hsa-mir-363 | - |
| hsa-mir-3689f | HGLibA_59339 | 151 | 0.231153289 | hsa-mir-3689f | - |
| hsa-mir-454 | HGLibA_60621 | 151 | 0.231153289 | hsa-mir-454 | - |
| hsa-mir-4633 | HGLibA_60639 | 151 | 0.231153289 | hsa-mir-4633 | - |
| hsa-mir-4726 | HGLibA_61000 | 151 | 0.231153289 | hsa-mir-4726 | - |
| hsa-mir-611 | HGLibA_62637 | 151 | 0.231153289 | hsa-mir-611 | - |
| hsa-mir-6766 | HGLibA_63225 | 151 | 0.231153289 | hsa-mir-6766 | - |
| hsa-mir-6793 | HGLibA_63348 | 151 | 0.231153289 | hsa-mir-6793 | - |
| hsa-mir-6857 | HGLibA_63601 | 151 | 0.231153289 | hsa-mir-6857 | - |
| hsa-mir-8088 | HGLibA_64196 | 151 | 0.231153289 | hsa-mir-8088 | - |
| hsa-mir-921 | HGLibA_64278 | 151 | 0.231153289 | hsa-mir-921 | - |
| LEPREL4 | HGLibA_26323 | 151 | 0.231153289 | LEPREL4 | - |
| NADKD1 | HGLibA_31052 | 151 | 0.231153289 | NADKD1 | - |
| OR8G2 | HGLibA_34519 | 151 | 0.231153289 | OR8G2 | - |
| SELRC1 | HGLibA_43309 | 151 | 0.231153289 | SELRC1 | - |
| C12orf49 | HGLibA_05253 | 150 | 0.222107946 | C12orf49 | - |
| C9orf3 | HGLibA_06721 | 150 | 0.222107946 | C9orf3 | - |
| ENSG00000006059 | HGLibA_25481 | 150 | 0.222107946 | KRT33A | keratin 33A [Source:HGNC Symbol;Acc:HGNC:6450] |
| ENSG00000018189 | HGLibA_42444 | 150 | 0.222107946 | RUFY3 | RUN and FYVE domain containing 3 [Source:HGNC Symbol;Acc:HGNC:30285] |
| ENSG00000019186 | HGLibA_12081 | 150 | 0.222107946 | CYP24A1 | cytochrome P450 family 24 subfamily A member 1 [Source:HGNC Symbol;Acc:HGNC:2602] |
| ENSG00000026025 | HGLibA_53656 | 150 | 0.222107946 | VIM | vimentin [Source:HGNC Symbol;Acc:HGNC:12692] |
| ENSG00000058729 | HGLibA_41310 | 150 | 0.222107946 | RIOK2 | RIO kinase 2 [Source:HGNC Symbol;Acc:HGNC:18999] |
| ENSG00000060718 | HGLibA_10501 | 150 | 0.222107946 | COL11A1 | collagen type XI alpha 1 chain [Source:HGNC Symbol;Acc:HGNC:2186] |
| ENSG00000064989 | HGLibA_07006 | 150 | 0.222107946 | CALCRL | calcitonin receptor like receptor [Source:HGNC Symbol;Acc:HGNC:16709] |
| ENSG00000068394 | HGLibA_19814 | 150 | 0.222107946 | GPKOW | G-patch domain and KOW motifs [Source:HGNC Symbol;Acc:HGNC:30677] |
| ENSG00000073712 | HGLibA_17296 | 150 | 0.222107946 | FERMT2 | fermitin family member 2 [Source:HGNC Symbol;Acc:HGNC:15767] |
| ENSG00000074370 | HGLibA_03610 | 150 | 0.222107946 | ATP2A3 | ATPase sarcoplasmic/endoplasmic reticulum Ca2+ transporting 3 [Source:HGNC Symbol;Acc:HGNC:813] |
| ENSG00000077348 | HGLibA_15785 | 150 | 0.222107946 | EXOSC5 | exosome component 5 [Source:HGNC Symbol;Acc:HGNC:24662] |
| ENSG00000078401 | HGLibA_14469 | 150 | 0.222107946 | EDN1 | endothelin 1 [Source:HGNC Symbol;Acc:HGNC:3176] |
| ENSG00000079156 | HGLibA_34660 | 150 | 0.222107946 | OSBPL6 | oxysterol binding protein like 6 [Source:HGNC Symbol;Acc:HGNC:16388] |
| ENSG00000100221 | HGLibA_23974 | 150 | 0.222107946 | JOSD1 | Josephin domain containing 1 [Source:HGNC Symbol;Acc:HGNC:28953] |
| ENSG00000100342 | HGLibA_02526 | 150 | 0.222107946 | APOL1 | apolipoprotein L1 [Source:HGNC Symbol;Acc:HGNC:618] |
| ENSG00000101425 | HGLibA_04707 | 150 | 0.222107946 | BPI | bactericidal permeability increasing protein [Source:HGNC Symbol;Acc:HGNC:1095] |
| ENSG00000103196 | HGLibA_11178 | 150 | 0.222107946 | CRISPLD2 | cysteine rich secretory protein LCCL domain containing 2 [Source:HGNC Symbol;Acc:HGNC:25248] |
| ENSG00000107371 | HGLibA_15779 | 150 | 0.222107946 | EXOSC3 | exosome component 3 [Source:HGNC Symbol;Acc:HGNC:17944] |
| ENSG00000108679 | HGLibA_26366 | 150 | 0.222107946 | LGALS3BP | galectin 3 binding protein [Source:HGNC Symbol;Acc:HGNC:6564] |
| ENSG00000110887 | HGLibA_12310 | 150 | 0.222107946 | DAO | D-amino acid oxidase [Source:HGNC Symbol;Acc:HGNC:2671] |
| ENSG00000111752 | HGLibA_36318 | 150 | 0.222107946 | PHC1 | polyhomeotic homolog 1 [Source:HGNC Symbol;Acc:HGNC:3182] |
| ENSG00000113312 | HGLibA_52044 | 150 | 0.222107946 | TTC1 | tetratricopeptide repeat domain 1 [Source:HGNC Symbol;Acc:HGNC:12391] |
| ENSG00000114670 | HGLibA_31644 | 150 | 0.222107946 | NEK11 | NIMA related kinase 11 [Source:HGNC Symbol;Acc:HGNC:18593] |
| ENSG00000114956 | HGLibA_13088 | 150 | 0.222107946 | DGUOK | deoxyguanosine kinase [Source:HGNC Symbol;Acc:HGNC:2858] |
| ENSG00000115607 | HGLibA_23031 | 150 | 0.222107946 | IL18RAP | interleukin 18 receptor accessory protein [Source:HGNC Symbol;Acc:HGNC:5989] |
| ENSG00000117245 | HGLibA_24871 | 150 | 0.222107946 | KIF17 | kinesin family member 17 [Source:HGNC Symbol;Acc:HGNC:19167] |
| ENSG00000117569 | HGLibA_39253 | 150 | 0.222107946 | PTBP2 | polypyrimidine tract binding protein 2 [Source:HGNC Symbol;Acc:HGNC:17662] |
| ENSG00000117592 | HGLibA_38423 | 150 | 0.222107946 | PRDX6 | peroxiredoxin 6 [Source:HGNC Symbol;Acc:HGNC:16753] |
| ENSG00000119231 | HGLibA_43394 | 150 | 0.222107946 | SENP5 | SUMO specific peptidase 5 [Source:HGNC Symbol;Acc:HGNC:28407] |
| ENSG00000119899 | HGLibA_44471 | 150 | 0.222107946 | SLC17A5 | solute carrier family 17 member 5 [Source:HGNC Symbol;Acc:HGNC:10933] |
| ENSG00000124159 | HGLibA_28436 | 150 | 0.222107946 | MATN4 | matrilin 4 [Source:HGNC Symbol;Acc:HGNC:6910] |
| ENSG00000124203 | HGLibA_56836 | 150 | 0.222107946 | ZNF831 | zinc finger protein 831 [Source:HGNC Symbol;Acc:HGNC:16167] |
| ENSG00000124935 | HGLibA_42917 | 150 | 0.222107946 | SCGB1D2 | secretoglobin family 1D member 2 [Source:HGNC Symbol;Acc:HGNC:18396] |
| ENSG00000125998 | HGLibA_16747 | 150 | 0.222107946 | FAM83C | family with sequence similarity 83 member C [Source:HGNC Symbol;Acc:HGNC:16121] |
| ENSG00000127616 | HGLibA_45609 | 150 | 0.222107946 | SMARCA4 | member 4 [Source:HGNC Symbol;Acc:HGNC:11100]" |
| ENSG00000129173 | HGLibA_14327 | 150 | 0.222107946 | E2F8 | E2F transcription factor 8 [Source:HGNC Symbol;Acc:HGNC:24727] |
| ENSG00000131864 | HGLibA_53324 | 150 | 0.222107946 | USP29 | ubiquitin specific peptidase 29 [Source:HGNC Symbol;Acc:HGNC:18563] |
| ENSG00000134262 | HGLibA_02342 | 150 | 0.222107946 | AP4B1 | adaptor related protein complex 4 subunit beta 1 [Source:HGNC Symbol;Acc:HGNC:572] |
| ENSG00000134910 | HGLibA_47606 | 150 | 0.222107946 | STT3A | catalytic subunit of the oligosaccharyltransferase complex [Source:HGNC Symbol;Acc:HGNC:6172]" |
| ENSG00000135898 | HGLibA_20052 | 150 | 0.222107946 | GPR55 | G protein-coupled receptor 55 [Source:HGNC Symbol;Acc:HGNC:4511] |
| ENSG00000136205 | HGLibA_50924 | 150 | 0.222107946 | TNS3 | tensin 3 [Source:HGNC Symbol;Acc:HGNC:21616] |
| ENSG00000136944 | HGLibA_26736 | 150 | 0.222107946 | LMX1B | LIM homeobox transcription factor 1 beta [Source:HGNC Symbol;Acc:HGNC:6654] |
| ENSG00000137815 | HGLibA_42382 | 150 | 0.222107946 | RTF1 | Paf1/RNA polymerase II complex component [Source:HGNC Symbol;Acc:HGNC:28996]" |
| ENSG00000138615 | HGLibA_09710 | 150 | 0.222107946 | CILP | cartilage intermediate layer protein [Source:HGNC Symbol;Acc:HGNC:1980] |
| ENSG00000139330 | HGLibA_24554 | 150 | 0.222107946 | KERA | keratocan [Source:HGNC Symbol;Acc:HGNC:6309] |
| ENSG00000143156 | HGLibA_32173 | 150 | 0.222107946 | NME7 | NME/NM23 family member 7 [Source:HGNC Symbol;Acc:HGNC:20461] |
| ENSG00000143556 | HGLibA_42565 | 150 | 0.222107946 | S100A7 | S100 calcium binding protein A7 [Source:HGNC Symbol;Acc:HGNC:10497] |
| ENSG00000148488 | HGLibA_47302 | 150 | 0.222107946 | ST8SIA6 | 8-sialyltransferase 6 [Source:HGNC Symbol;Acc:HGNC:23317]" |
| ENSG00000149591 | HGLibA_48270 | 150 | 0.222107946 | TAGLN | transgelin [Source:HGNC Symbol;Acc:HGNC:11553] |
| ENSG00000149970 | HGLibA_10299 | 150 | 0.222107946 | CNKSR2 | connector enhancer of kinase suppressor of Ras 2 [Source:HGNC Symbol;Acc:HGNC:19701] |
| ENSG00000150433 | HGLibA_50227 | 150 | 0.222107946 | TMEM218 | transmembrane protein 218 [Source:HGNC Symbol;Acc:HGNC:27344] |
| ENSG00000151287 | HGLibA_49093 | 150 | 0.222107946 | TEX30 | testis expressed 30 [Source:HGNC Symbol;Acc:HGNC:25188] |
| ENSG00000152939 | HGLibA_28381 | 150 | 0.222107946 | MARVELD2 | MARVEL domain containing 2 [Source:HGNC Symbol;Acc:HGNC:26401] |
| ENSG00000157613 | HGLibA_11094 | 150 | 0.222107946 | CREB3L1 | cAMP responsive element binding protein 3 like 1 [Source:HGNC Symbol;Acc:HGNC:18856] |
| ENSG00000158553 | HGLibA_37652 | 150 | 0.222107946 | POM121L2 | POM121 transmembrane nucleoporin like 2 [Source:HGNC Symbol;Acc:HGNC:13973] |
| ENSG00000159720 | HGLibA_03718 | 150 | 0.222107946 | ATP6V0D1 | ATPase H+ transporting V0 subunit d1 [Source:HGNC Symbol;Acc:HGNC:13724] |
| ENSG00000160233 | HGLibA_27306 | 150 | 0.222107946 | LRRC3 | leucine rich repeat containing 3 [Source:HGNC Symbol;Acc:HGNC:14965] |
| ENSG00000162551 | HGLibA_01709 | 150 | 0.222107946 | ALPL | biomineralization associated [Source:HGNC Symbol;Acc:HGNC:438]" |
| ENSG00000162704 | HGLibA_03091 | 150 | 0.222107946 | ARPC5 | actin related protein 2/3 complex subunit 5 [Source:HGNC Symbol;Acc:HGNC:708] |
| ENSG00000163081 | HGLibA_07617 | 150 | 0.222107946 | CCDC140 | coiled-coil domain containing 140 [Source:HGNC Symbol;Acc:HGNC:26514] |
| ENSG00000164136 | HGLibA_22980 | 150 | 0.222107946 | IL15 | interleukin 15 [Source:HGNC Symbol;Acc:HGNC:5977] |
| ENSG00000165304 | HGLibA_28881 | 150 | 0.222107946 | MELK | maternal embryonic leucine zipper kinase [Source:HGNC Symbol;Acc:HGNC:16870] |
| ENSG00000165476 | HGLibA_40783 | 150 | 0.222107946 | REEP3 | receptor accessory protein 3 [Source:HGNC Symbol;Acc:HGNC:23711] |
| ENSG00000165516 | HGLibA_25095 | 150 | 0.222107946 | KLHDC2 | kelch domain containing 2 [Source:HGNC Symbol;Acc:HGNC:20231] |
| ENSG00000165682 | HGLibA_09958 | 150 | 0.222107946 | CLEC1B | C-type lectin domain family 1 member B [Source:HGNC Symbol;Acc:HGNC:24356] |
| ENSG00000166159 | HGLibA_27502 | 150 | 0.222107946 | LRTM2 | leucine rich repeats and transmembrane domains 2 [Source:HGNC Symbol;Acc:HGNC:32443] |
| ENSG00000166446 | HGLibA_08919 | 150 | 0.222107946 | CDYL2 | chromodomain Y like 2 [Source:HGNC Symbol;Acc:HGNC:23030] |
| ENSG00000167186 | HGLibA_10765 | 150 | 0.222107946 | COQ7 | hydroxylase [Source:HGNC Symbol;Acc:HGNC:2244]" |
| ENSG00000170291 | HGLibA_14986 | 150 | 0.222107946 | ELP5 | elongator acetyltransferase complex subunit 5 [Source:HGNC Symbol;Acc:HGNC:30617] |
| ENSG00000170523 | HGLibA_25565 | 150 | 0.222107946 | KRT83 | keratin 83 [Source:HGNC Symbol;Acc:HGNC:6460] |
| ENSG00000171405 | HGLibA_54486 | 150 | 0.222107946 | XAGE5 | X antigen family member 5 [Source:HGNC Symbol;Acc:HGNC:30930] |
| ENSG00000171714 | HGLibA_02182 | 150 | 0.222107946 | ANO5 | anoctamin 5 [Source:HGNC Symbol;Acc:HGNC:27337] |
| ENSG00000171867 | HGLibA_38643 | 150 | 0.222107946 | PRNP | prion protein [Source:HGNC Symbol;Acc:HGNC:9449] |

| | | | | | |
|---|---|---|---|---|---|
| ENSG00000172640 | HGLibA_33479 | 150 | 0.222107946 | OR10AD1 | olfactory receptor family 10 subfamily AD member 1 [Source:HGNC Symbol;Acc:HGNC:14819] |
| ENSG00000172680 | HGLibA_29680 | 150 | 0.222107946 | MOS | serine/threonine kinase [Source:HGNC Symbol;Acc:HGNC:7199]" |
| ENSG00000172893 | HGLibA_13095 | 150 | 0.222107946 | DHCR7 | 7-dehydrocholesterol reductase [Source:HGNC Symbol;Acc:HGNC:2860] |
| ENSG00000173905 | HGLibA_19652 | 150 | 0.222107946 | GOLIM4 | golgi integral membrane protein 4 [Source:HGNC Symbol;Acc:HGNC:15448] |
| ENSG00000174243 | HGLibA_12675 | 150 | 0.222107946 | DDX23 | DEAD-box helicase 23 [Source:HGNC Symbol;Acc:HGNC:17347] |
| ENSG00000174460 | HGLibA_55009 | 150 | 0.222107946 | ZCCHC12 | zinc finger CCHC-type containing 12 [Source:HGNC Symbol;Acc:HGNC:27273] |
| ENSG00000174500 | HGLibA_18827 | 150 | 0.222107946 | GCSAM | germinal center associated signaling and motility [Source:HGNC Symbol;Acc:HGNC:20253] |
| ENSG00000174898 | HGLibA_07370 | 150 | 0.222107946 | CATSPERD | cation channel sperm associated auxiliary subunit delta [Source:HGNC Symbol;Acc:HGNC:28598] |
| ENSG00000175216 | HGLibA_09762 | 150 | 0.222107946 | CKAP5 | cytoskeleton associated protein 5 [Source:HGNC Symbol;Acc:HGNC:28959] |
| ENSG00000175782 | HGLibA_44995 | 150 | 0.222107946 | SLC35E3 | solute carrier family 35 member E3 [Source:HGNC Symbol;Acc:HGNC:20864] |
| ENSG00000178821 | HGLibA_50433 | 150 | 0.222107946 | TMEM52 | transmembrane protein 52 [Source:HGNC Symbol;Acc:HGNC:27916] |
| ENSG00000178852 | HGLibA_14528 | 150 | 0.222107946 | EFCAB13 | EF-hand calcium binding domain 13 [Source:HGNC Symbol;Acc:HGNC:26864] |
| ENSG00000179526 | HGLibA_43991 | 150 | 0.222107946 | SHARPIN | SHANK associated RH domain interactor [Source:HGNC Symbol;Acc:HGNC:25321] |
| ENSG00000180611 | HGLibA_28461 | 150 | 0.222107946 | MB21D2 | Mab-21 domain containing 2 [Source:HGNC Symbol;Acc:HGNC:30438] |
| ENSG00000181036 | HGLibA_17240 | 150 | 0.222107946 | FCRL6 | Fc receptor like 6 [Source:HGNC Symbol;Acc:HGNC:31910] |
| ENSG00000182585 | HGLibA_15280 | 150 | 0.222107946 | EPGN | epithelial mitogen [Source:HGNC Symbol;Acc:HGNC:17470] |
| ENSG00000184601 | HGLibA_05347 | 150 | 0.222107946 | C14orf180 | chromosome 14 open reading frame 180 [Source:HGNC Symbol;Acc:HGNC:33795] |
| ENSG00000185220 | HGLibA_36218 | 150 | 0.222107946 | PGBD2 | piggyBac transposable element derived 2 [Source:HGNC Symbol;Acc:HGNC:19399] |
| ENSG00000185475 | HGLibA_50089 | 150 | 0.222107946 | TMEM179B | transmembrane protein 179B [Source:HGNC Symbol;Acc:HGNC:33744] |
| ENSG00000187556 | HGLibA_31103 | 150 | 0.222107946 | NANOS3 | nanos C2HC-type zinc finger 3 [Source:HGNC Symbol;Acc:HGNC:22048] |
| ENSG00000187942 | HGLibA_26243 | 150 | 0.222107946 | LDLRAD2 | low density lipoprotein receptor class A domain containing 2 [Source:HGNC Symbol;Acc:HGNC:32071] |
| ENSG00000196224 | HGLibA_25795 | 150 | 0.222107946 | KRTAP5-3 | keratin associated protein 5-3 [Source:HGNC Symbol;Acc:HGNC:23598] |
| ENSG00000196586 | HGLibA_30860 | 150 | 0.222107946 | MYO6 | myosin VI [Source:HGNC Symbol;Acc:HGNC:7605] |
| ENSG00000197416 | HGLibA_15909 | 150 | 0.222107946 | FABP12 | fatty acid binding protein 12 [Source:HGNC Symbol;Acc:HGNC:34524] |
| ENSG00000197580 | HGLibA_04360 | 150 | 0.222107946 | BCO2 | beta-carotene oxygenase 2 [Source:HGNC Symbol;Acc:HGNC:18503] |
| ENSG00000197915 | HGLibA_22108 | 150 | 0.222107946 | HRNR | hornerin [Source:HGNC Symbol;Acc:HGNC:20846] |
| ENSG00000198246 | HGLibA_44847 | 150 | 0.222107946 | SLC29A3 | solute carrier family 29 member 3 [Source:HGNC Symbol;Acc:HGNC:23096] |
| ENSG00000198755 | HGLibA_41855 | 150 | 0.222107946 | RPL10A | ribosomal protein L10a [Source:HGNC Symbol;Acc:HGNC:10299] |
| ENSG00000204130 | HGLibA_42441 | 150 | 0.222107946 | RUFY2 | RUN and FYVE domain containing 2 [Source:HGNC Symbol;Acc:HGNC:19761] |
| ENSG00000204612 | HGLibA_17818 | 150 | 0.222107946 | FOXB2 | forkhead box B2 [Source:HGNC Symbol;Acc:HGNC:23315] |
| ENSG00000213722 | HGLibA_12592 | 150 | 0.222107946 | DDAH2 | dimethylarginine dimethylaminohydrolase 2 [Source:HGNC Symbol;Acc:HGNC:2716] |
| ENSG00000228567 | HGLibA_53703 | 150 | 0.222107946 | VN1R4 | vomeronasal 1 receptor 4 [Source:HGNC Symbol;Acc:HGNC:19871] |
| ENSG00000241360 | HGLibA_35964 | 150 | 0.222107946 | PDXP | pyridoxal phosphatase [Source:HGNC Symbol;Acc:HGNC:30259] |
| ENSG00000241404 | HGLibA_14630 | 150 | 0.222107946 | EGFL8 | EGF like domain multiple 8 [Source:HGNC Symbol;Acc:HGNC:13944] |
| ENSG00000242366 | HGLibA_52897 | 150 | 0.222107946 | UGT1A8 | UDP glucuronosyltransferase family 1 member A8 [Source:HGNC Symbol;Acc:HGNC:12540] |
| ENSG00000254550 | HGLibA_33390 | 150 | 0.222107946 | OMP | olfactory marker protein [Source:HGNC Symbol;Acc:HGNC:8136] |
| ENSG00000257335 | HGLibA_29126 | 150 | 0.222107946 | MGAM | maltase-glucoamylase [Source:HGNC Symbol;Acc:HGNC:7043] |
| ENSG00000257591 | HGLibA_56418 | 150 | 0.222107946 | ZNF625 | zinc finger protein 625 [Source:HGNC Symbol;Acc:HGNC:30571] |
| ENSG00000261652 | HGLibA_05436 | 150 | 0.222107946 | C15orf65 | chromosome 15 open reading frame 65 [Source:HGNC Symbol;Acc:HGNC:44654] |
| ENSG00000275183 | HGLibA_26302 | 150 | 0.222107946 | LENG9 | leukocyte receptor cluster member 9 [Source:HGNC Symbol;Acc:HGNC:16306] |
| GPR98 | HGLibA_20115 | 150 | 0.222107946 | GPR98 | - |
| H1FX | HGLibA_20742 | 150 | 0.222107946 | H1FX | - |
| HIST1H2BM | HGLibA_21446 | 150 | 0.222107946 | HIST1H2BM | - |
| hsa-mir-1471 | HGLibA_57867 | 150 | 0.222107946 | hsa-mir-1471 | - |
| hsa-mir-3616 | HGLibA_59093 | 150 | 0.222107946 | hsa-mir-3616 | - |
| hsa-mir-429 | HGLibA_59869 | 150 | 0.222107946 | hsa-mir-429 | - |
| hsa-mir-4511 | HGLibA_60488 | 150 | 0.222107946 | hsa-mir-4511 | - |
| hsa-mir-4650-2 | HGLibA_60709 | 150 | 0.222107946 | hsa-mir-4650-2 | - |
| hsa-mir-4777 | HGLibA_61215 | 150 | 0.222107946 | hsa-mir-4777 | - |
| hsa-mir-657 | HGLibA_62963 | 150 | 0.222107946 | hsa-mir-657 | - |
| hsa-mir-6830 | HGLibA_63496 | 150 | 0.222107946 | hsa-mir-6830 | - |
| hsa-mir-6851 | HGLibA_63577 | 150 | 0.222107946 | hsa-mir-6851 | - |
| LYRM5 | HGLibA_27754 | 150 | 0.222107946 | LYRM5 | - |
| MESDC1 | HGLibA_28909 | 150 | 0.222107946 | MESDC1 | - |
| P2RY8_X | HGLibA_34894 | 150 | 0.222107946 | P2RY8_X | - |
| ACRC | HGLibA_00535 | 149 | 0.213062603 | ACRC | - |
| C3orf58 | HGLibA_06255 | 149 | 0.213062603 | C3orf58 | - |
| C8orf4 | HGLibA_06616 | 149 | 0.213062603 | C8orf4 | - |
| CASC10 | HGLibA_07270 | 149 | 0.213062603 | CASC10 | - |
| DUPD1 | HGLibA_14108 | 149 | 0.213062603 | DUPD1 | - |
| ENSG00000008438 | HGLibA_36250 | 149 | 0.213062603 | PGLYRP1 | peptidoglycan recognition protein 1 [Source:HGNC Symbol;Acc:HGNC:8904] |
| ENSG00000010319 | HGLibA_43336 | 149 | 0.213062603 | SEMA3G | semaphorin 3G [Source:HGNC Symbol;Acc:HGNC:30400] |
| ENSG00000010327 | HGLibA_47305 | 149 | 0.213062603 | STAB1 | stabilin 1 [Source:HGNC Symbol;Acc:HGNC:18628] |
| ENSG00000035664 | HGLibA_12326 | 149 | 0.213062603 | DAPK2 | death associated protein kinase 2 [Source:HGNC Symbol;Acc:HGNC:2675] |
| ENSG00000070526 | HGLibA_47263 | 149 | 0.213062603 | ST6GALNAC1 | 6-sialyltransferase 1 [Source:HGNC Symbol;Acc:HGNC:23614]" |
| ENSG00000076067 | HGLibA_40591 | 149 | 0.213062603 | RBMS2 | RNA binding motif single stranded interacting protein 2 [Source:HGNC Symbol;Acc:HGNC:9909] |
| ENSG00000085831 | HGLibA_52131 | 149 | 0.213062603 | TTC39A | tetratricopeptide repeat domain 39A [Source:HGNC Symbol;Acc:HGNC:18657] |
| ENSG00000089050 | HGLibA_40445 | 149 | 0.213062603 | RBBP9 | serine hydrolase [Source:HGNC Symbol;Acc:HGNC:9892]" |
| ENSG00000099203 | HGLibA_49799 | 149 | 0.213062603 | TMED1 | transmembrane p24 trafficking protein 1 [Source:HGNC Symbol;Acc:HGNC:17291] |
| ENSG00000099899 | HGLibA_51683 | 149 | 0.213062603 | TRMT2A | tRNA methyltransferase 2 homolog A [Source:HGNC Symbol;Acc:HGNC:24974] |
| ENSG00000100079 | HGLibA_26359 | 149 | 0.213062603 | LGALS2 | galectin 2 [Source:HGNC Symbol;Acc:HGNC:6562] |
| ENSG00000100522 | HGLibA_19571 | 149 | 0.213062603 | GNPNAT1 | glucosamine-phosphate N-acetyltransferase 1 [Source:HGNC Symbol;Acc:HGNC:19980] |
| ENSG00000100626 | HGLibA_18547 | 149 | 0.213062603 | GALNT16 | polypeptide N-acetylgalactosaminyltransferase 16 [Source:HGNC Symbol;Acc:HGNC:23233] |
| ENSG00000100852 | HGLibA_02783 | 149 | 0.213062603 | ARHGAP5 | Rho GTPase activating protein 5 [Source:HGNC Symbol;Acc:HGNC:675] |
| ENSG00000101665 | HGLibA_45587 | 149 | 0.213062603 | SMAD7 | SMAD family member 7 [Source:HGNC Symbol;Acc:HGNC:6773] |
| ENSG00000103227 | HGLibA_26683 | 149 | 0.213062603 | LMF1 | lipase maturation factor 1 [Source:HGNC Symbol;Acc:HGNC:14154] |
| ENSG00000104969 | HGLibA_43868 | 149 | 0.213062603 | SGTA | small glutamine rich tetratricopeptide repeat containing alpha [Source:HGNC Symbol;Acc:HGNC:10819] |
| ENSG00000106009 | HGLibA_04758 | 149 | 0.213062603 | BRAT1 | BRCA1 associated ATM activator 1 [Source:HGNC Symbol;Acc:HGNC:21701] |
| ENSG00000107175 | HGLibA_11089 | 149 | 0.213062603 | CREB3 | cAMP responsive element binding protein 3 [Source:HGNC Symbol;Acc:HGNC:2347] |
| ENSG00000107968 | HGLibA_28202 | 149 | 0.213062603 | MAP3K8 | mitogen-activated protein kinase kinase kinase 8 [Source:HGNC Symbol;Acc:HGNC:6860] |
| ENSG00000110344 | HGLibA_52690 | 149 | 0.213062603 | UBE4A | ubiquitination factor E4A [Source:HGNC Symbol;Acc:HGNC:12499] |
| ENSG00000113645 | HGLibA_54438 | 149 | 0.213062603 | WWC1 | WW and C2 domain containing 1 [Source:HGNC Symbol;Acc:HGNC:29435] |
| ENSG00000114026 | HGLibA_33323 | 149 | 0.213062603 | OGG1 | 8-oxoguanine DNA glycosylase [Source:HGNC Symbol;Acc:HGNC:8125] |
| ENSG00000115523 | HGLibA_19556 | 149 | 0.213062603 | GNLY | granulysin [Source:HGNC Symbol;Acc:HGNC:4414] |
| ENSG00000116044 | HGLibA_31793 | 149 | 0.213062603 | NFE2L2 | erythroid 2 like 2 [Source:HGNC Symbol;Acc:HGNC:7782]" |
| ENSG00000117143 | HGLibA_52508 | 149 | 0.213062603 | UAP1 | UDP-N-acetylglucosamine pyrophosphorylase 1 [Source:HGNC Symbol;Acc:HGNC:12457] |
| ENSG00000117400 | HGLibA_29735 | 149 | 0.213062603 | MPL | thrombopoietin receptor [Source:HGNC Symbol;Acc:HGNC:7217]" |
| ENSG00000117640 | HGLibA_30364 | 149 | 0.213062603 | MTFR1L | mitochondrial fission regulator 1 like [Source:HGNC Symbol;Acc:HGNC:28836] |
| ENSG00000119411 | HGLibA_04877 | 149 | 0.213062603 | BSPRY | B-box and SPRY domain containing [Source:HGNC Symbol;Acc:HGNC:18232] |
| ENSG00000121931 | HGLibA_27174 | 149 | 0.213062603 | LRIF1 | ligand dependent nuclear receptor interacting factor 1 [Source:HGNC Symbol;Acc:HGNC:30299] |
| ENSG00000123171 | HGLibA_07850 | 149 | 0.213062603 | CCDC70 | coiled-coil domain containing 70 [Source:HGNC Symbol;Acc:HGNC:25303] |
| ENSG00000123329 | HGLibA_02792 | 149 | 0.213062603 | ARHGAP9 | Rho GTPase activating protein 9 [Source:HGNC Symbol;Acc:HGNC:14130] |
| ENSG00000124104 | HGLibA_46062 | 149 | 0.213062603 | SNX21 | sorting nexin family member 21 [Source:HGNC Symbol;Acc:HGNC:16154] |
| ENSG00000124743 | HGLibA_25183 | 149 | 0.213062603 | KLHL31 | kelch like family member 31 [Source:HGNC Symbol;Acc:HGNC:21353] |
| ENSG00000125386 | HGLibA_16362 | 149 | 0.213062603 | FAM193A | family with sequence similarity 193 member A [Source:HGNC Symbol;Acc:HGNC:16822] |
| ENSG00000129595 | HGLibA_15252 | 149 | 0.213062603 | EPB41L4A | erythrocyte membrane protein band 4.1 like 4A [Source:HGNC Symbol;Acc:HGNC:13278] |
| ENSG00000130244 | HGLibA_16814 | 149 | 0.213062603 | FAM98C | family with sequence similarity 98 member C [Source:HGNC Symbol;Acc:HGNC:27119] |
| ENSG00000131910 | HGLibA_32571 | 149 | 0.213062603 | NR0B2 | nuclear receptor subfamily 0 group B member 2 [Source:HGNC Symbol;Acc:HGNC:7961] |
| ENSG00000132259 | HGLibA_10271 | 149 | 0.213062603 | CNGA4 | cyclic nucleotide gated channel alpha 4 [Source:HGNC Symbol;Acc:HGNC:2152] |
| ENSG00000132478 | HGLibA_53048 | 149 | 0.213062603 | UNK | unkempt family zinc finger [Source:HGNC Symbol;Acc:HGNC:29369] |
| ENSG00000135040 | HGLibA_30990 | 149 | 0.213062603 | NAA35 | NatC auxiliary subunit [Source:HGNC Symbol;Acc:HGNC:24340]" |
| ENSG00000136267 | HGLibA_13060 | 149 | 0.213062603 | DGKB | diacylglycerol kinase beta [Source:HGNC Symbol;Acc:HGNC:2850] |
| ENSG00000136488 | HGLibA_11350 | 149 | 0.213062603 | CSH1 | chorionic somatomammotropin hormone 1 [Source:HGNC Symbol;Acc:HGNC:2440] |
| ENSG00000138315 | HGLibA_33335 | 149 | 0.213062603 | OIT3 | oncoprotein induced transcript 3 [Source:HGNC Symbol;Acc:HGNC:29953] |
| ENSG00000139517 | HGLibA_26748 | 149 | 0.213062603 | LNX2 | ligand of numb-protein X 2 [Source:HGNC Symbol;Acc:HGNC:20421] |
| ENSG00000139597 | HGLibA_30954 | 149 | 0.213062603 | N4BP2L1 | NEDD4 binding protein 2 like 1 [Source:HGNC Symbol;Acc:HGNC:25037] |
| ENSG00000140575 | HGLibA_23533 | 149 | 0.213062603 | IQGAP1 | IQ motif containing GTPase activating protein 1 [Source:HGNC Symbol;Acc:HGNC:6110] |
| ENSG00000140948 | HGLibA_55014 | 149 | 0.213062603 | ZCCHC14 | zinc finger CCHC-type containing 14 [Source:HGNC Symbol;Acc:HGNC:24134] |
| ENSG00000144061 | HGLibA_32444 | 149 | 0.213062603 | NPHP1 | nephrocystin 1 [Source:HGNC Symbol;Acc:HGNC:7905] |
| ENSG00000144597 | HGLibA_14333 | 149 | 0.213062603 | EAF1 | ELL associated factor 1 [Source:HGNC Symbol;Acc:HGNC:20907] |
| ENSG00000145850 | HGLibA_49488 | 149 | 0.213062603 | TIMD4 | T cell immunoglobulin and mucin domain containing 4 [Source:HGNC Symbol;Acc:HGNC:25132] |
| ENSG00000146232 | HGLibA_31830 | 149 | 0.213062603 | NFKBIE | NFKB inhibitor epsilon [Source:HGNC Symbol;Acc:HGNC:7799] |
| ENSG00000146540 | HGLibA_06551 | 149 | 0.213062603 | C7orf50 | chromosome 7 open reading frame 50 [Source:HGNC Symbol;Acc:HGNC:22421] |
| ENSG00000148175 | HGLibA_47531 | 149 | 0.213062603 | STOM | stomatin [Source:HGNC Symbol;Acc:HGNC:3383] |
| ENSG00000148429 | HGLibA_53408 | 149 | 0.213062603 | USP6NL | USP6 N-terminal like [Source:HGNC Symbol;Acc:HGNC:16858] |
| ENSG00000150045 | HGLibA_25303 | 149 | 0.213062603 | KLRF1 | killer cell lectin like receptor F1 [Source:HGNC Symbol;Acc:HGNC:13342] |
| ENSG00000152443 | HGLibA_56722 | 149 | 0.213062603 | ZNF776 | zinc finger protein 776 [Source:HGNC Symbol;Acc:HGNC:26765] |
| ENSG00000152894 | HGLibA_39513 | 149 | 0.213062603 | PTPRK | receptor type K [Source:HGNC Symbol;Acc:HGNC:9674]" |
| ENSG00000153233 | HGLibA_39531 | 149 | 0.213062603 | PTPRR | receptor type R [Source:HGNC Symbol;Acc:HGNC:9680]" |
| ENSG00000154114 | HGLibA_48596 | 149 | 0.213062603 | TBCEL | tubulin folding cofactor E like [Source:HGNC Symbol;Acc:HGNC:28115] |
| ENSG00000157119 | HGLibA_25208 | 149 | 0.213062603 | KLHL40 | kelch like family member 40 [Source:HGNC Symbol;Acc:HGNC:30372] |
| ENSG00000158169 | HGLibA_16835 | 149 | 0.213062603 | FANCC | FA complementation group C [Source:HGNC Symbol;Acc:HGNC:3584] |
| ENSG00000158955 | HGLibA_54397 | 149 | 0.213062603 | WNT9B | Wnt family member 9B [Source:HGNC Symbol;Acc:HGNC:12779] |
| ENSG00000160870 | HGLibA_12164 | 149 | 0.213062603 | CYP3A7 | cytochrome P450 family 3 subfamily A member 7 [Source:HGNC Symbol;Acc:HGNC:2640] |
| ENSG00000162004 | HGLibA_07872 | 149 | 0.213062603 | CCDC78 | coiled-coil domain containing 78 [Source:HGNC Symbol;Acc:HGNC:14153] |
| ENSG00000162889 | HGLibA_28301 | 149 | 0.213062603 | MAPKAPK2 | mitogen-activated protein kinase-activated protein kinase 2 [Source:HGNC Symbol;Acc:HGNC:6887] |
| ENSG00000163393 | HGLibA_44552 | 149 | 0.213062603 | SLC22A15 | solute carrier family 22 member 15 [Source:HGNC Symbol;Acc:HGNC:20301] |
| ENSG00000163694 | HGLibA_40566 | 149 | 0.213062603 | RBM47 | RNA binding motif protein 47 [Source:HGNC Symbol;Acc:HGNC:30358] |
| ENSG00000163710 | HGLibA_35656 | 149 | 0.213062603 | PCOLCE2 | procollagen C-endopeptidase enhancer 2 [Source:HGNC Symbol;Acc:HGNC:8739] |
| ENSG00000164197 | HGLibA_41581 | 149 | 0.213062603 | RNF180 | ring finger protein 180 [Source:HGNC Symbol;Acc:HGNC:27752] |
| ENSG00000165832 | HGLibA_51802 | 149 | 0.213062603 | TRUB1 | TruB pseudouridine synthase family member 1 [Source:HGNC Symbol;Acc:HGNC:16060] |
| ENSG00000166922 | HGLibA_42904 | 149 | 0.213062603 | SCG5 | secretogranin V [Source:HGNC Symbol;Acc:HGNC:10816] |
| ENSG00000167612 | HGLibA_02069 | 149 | 0.213062603 | ANKRD33 | ankyrin repeat domain 33 [Source:HGNC Symbol;Acc:HGNC:13788] |
| ENSG00000167711 | HGLibA_43590 | 149 | 0.213062603 | SERPINF2 | serpin family F member 2 [Source:HGNC Symbol;Acc:HGNC:9075] |
| ENSG00000167797 | HGLibA_08765 | 149 | 0.213062603 | CDK2AP2 | cyclin dependent kinase 2 associated protein 2 [Source:HGNC Symbol;Acc:HGNC:30833] |

| | | | | | |
|---|---|---|---|---|---|
| ENSG00000168071 | HGLibA_07911 | 149 | 0.213062603 | CCDC88B | coiled-coil domain containing 88B [Source:HGNC Symbol;Acc:HGNC:26757] |
| ENSG00000170075 | HGLibA_20036 | 149 | 0.213062603 | GPR37L1 | G protein-coupled receptor 37 like 1 [Source:HGNC Symbol;Acc:HGNC:14923] |
| ENSG00000172320 | HGLibA_34228 | 149 | 0.213062603 | OR5A1 | olfactory receptor family 5 subfamily A member 1 [Source:HGNC Symbol;Acc:HGNC:8319] |
| ENSG00000172487 | HGLibA_34537 | 149 | 0.213062603 | OR8J1 | olfactory receptor family 8 subfamily J member 1 [Source:HGNC Symbol;Acc:HGNC:14855] |
| ENSG00000172888 | HGLibA_56406 | 149 | 0.213062603 | ZNF621 | zinc finger protein 621 [Source:HGNC Symbol;Acc:HGNC:24787] |
| ENSG00000172995 | HGLibA_03102 | 149 | 0.213062603 | ARPP21 | cAMP regulated phosphoprotein 21 [Source:HGNC Symbol;Acc:HGNC:16968] |
| ENSG00000174339 | HGLibA_33907 | 149 | 0.213062603 | OR2Y1 | olfactory receptor family 2 subfamily Y member 1 [Source:HGNC Symbol;Acc:HGNC:14837] |
| ENSG00000176894 | HGLibA_39659 | 149 | 0.213062603 | PXMP2 | peroxisomal membrane protein 2 [Source:HGNC Symbol;Acc:HGNC:9716] |
| ENSG00000177352 | HGLibA_07854 | 149 | 0.213062603 | CCDC71 | coiled-coil domain containing 71 [Source:HGNC Symbol;Acc:HGNC:25760] |
| ENSG00000177383 | HGLibA_27987 | 149 | 0.213062603 | MAGEF1 | MAGE family member F1 [Source:HGNC Symbol;Acc:HGNC:29639] |
| ENSG00000177791 | HGLibA_30902 | 149 | 0.213062603 | MYOZ1 | myozenin 1 [Source:HGNC Symbol;Acc:HGNC:13752] |
| ENSG00000177839 | HGLibA_35515 | 149 | 0.213062603 | PCDHB9 | protocadherin beta 9 [Source:HGNC Symbol;Acc:HGNC:8694] |
| ENSG00000178623 | HGLibA_20029 | 149 | 0.213062603 | GPR35 | G protein-coupled receptor 35 [Source:HGNC Symbol;Acc:HGNC:4492] |
| ENSG00000178913 | HGLibA_48253 | 149 | 0.213062603 | TAF7 | TATA-box binding protein associated factor 7 [Source:HGNC Symbol;Acc:HGNC:11541] |
| ENSG00000183762 | HGLibA_25401 | 149 | 0.213062603 | KREMEN1 | kringle containing transmembrane protein 1 [Source:HGNC Symbol;Acc:HGNC:17550] |
| ENSG00000183891 | HGLibA_52114 | 149 | 0.213062603 | TTC32 | tetratricopeptide repeat domain 32 [Source:HGNC Symbol;Acc:HGNC:32954] |
| ENSG00000184271 | HGLibA_37794 | 149 | 0.213062603 | POU6F1 | POU class 6 homeobox 1 [Source:HGNC Symbol;Acc:HGNC:9224] |
| ENSG00000184451 | HGLibA_08146 | 149 | 0.213062603 | CCR10 | C-C motif chemokine receptor 10 [Source:HGNC Symbol;Acc:HGNC:4474] |
| ENSG00000184984 | HGLibA_09560 | 149 | 0.213062603 | CHRM5 | cholinergic receptor muscarinic 5 [Source:HGNC Symbol;Acc:HGNC:1954] |
| ENSG00000186716 | HGLibA_04368 | 149 | 0.213062603 | BCR | RhoGEF and GTPase activating protein [Source:HGNC Symbol;Acc:HGNC:1014]" |
| ENSG00000187486 | HGLibA_24251 | 149 | 0.213062603 | KCNJ11 | potassium voltage-gated channel subfamily J member 11 [Source:HGNC Symbol;Acc:HGNC:6257] |
| ENSG00000187942 | HGLibA_26245 | 149 | 0.213062603 | LDLRAD2 | low density lipoprotein receptor class A domain containing 2 [Source:HGNC Symbol;Acc:HGNC:32071] |
| ENSG00000188322 | HGLibA_42801 | 149 | 0.213062603 | SBK1 | SH3 domain binding kinase 1 [Source:HGNC Symbol;Acc:HGNC:17699] |
| ENSG00000188655 | HGLibA_41422 | 149 | 0.213062603 | RNASE9 | ribonuclease A family member 9 (inactive) [Source:HGNC Symbol;Acc:HGNC:20673] |
| ENSG00000196611 | HGLibA_29476 | 149 | 0.213062603 | MMP1 | matrix metallopeptidase 1 [Source:HGNC Symbol;Acc:HGNC:7155] |
| ENSG00000197381 | HGLibA_00872 | 149 | 0.213062603 | ADARB1 | RNA specific B1 [Source:HGNC Symbol;Acc:HGNC:226]" |
| ENSG00000197724 | HGLibA_36360 | 149 | 0.213062603 | PHF2 | PHD finger protein 2 [Source:HGNC Symbol;Acc:HGNC:8920] |
| ENSG00000198429 | HGLibA_56548 | 149 | 0.213062603 | ZNF69 | zinc finger protein 69 [Source:HGNC Symbol;Acc:HGNC:13138] |
| ENSG00000198707 | HGLibA_09132 | 149 | 0.213062603 | CEP290 | centrosomal protein 290 [Source:HGNC Symbol;Acc:HGNC:29021] |
| ENSG00000198919 | HGLibA_14303 | 149 | 0.213062603 | DZIP3 | DAZ interacting zinc finger protein 3 [Source:HGNC Symbol;Acc:HGNC:30938] |
| ENSG00000203989 | HGLibA_41221 | 149 | 0.213062603 | RHOXF2B | Rhox homeobox family member 2B [Source:HGNC Symbol;Acc:HGNC:33519] |
| ENSG00000204065 | HGLibA_48715 | 149 | 0.213062603 | TCEAL5 | transcription elongation factor A like 5 [Source:HGNC Symbol;Acc:HGNC:22282] |
| ENSG00000204703 | HGLibA_33771 | 149 | 0.213062603 | OR2B3 | olfactory receptor family 2 subfamily B member 3 [Source:HGNC Symbol;Acc:HGNC:8238] |
| ENSG00000205445 | HGLibA_25603 | 149 | 0.213062603 | KRTAP10-2 | keratin associated protein 10-2 [Source:HGNC Symbol;Acc:HGNC:22967] |
| ENSG00000205726 | HGLibA_23871 | 149 | 0.213062603 | ITSN1 | intersectin 1 [Source:HGNC Symbol;Acc:HGNC:6183] |
| ENSG00000212659 | HGLibA_25841 | 149 | 0.213062603 | KRTAP9-6 | keratin associated protein 9-6 [Source:HGNC Symbol;Acc:HGNC:18914] |
| ENSG00000215454 | HGLibA_25609 | 149 | 0.213062603 | KRTAP10-4 | keratin associated protein 10-4 [Source:HGNC Symbol;Acc:HGNC:20521] |
| ENSG00000235568 | HGLibA_31763 | 149 | 0.213062603 | NFAM1 | NFAT activating protein with ITAM motif 1 [Source:HGNC Symbol;Acc:HGNC:29872] |
| ENSG00000240038 | HGLibA_01853 | 149 | 0.213062603 | AMY2B | alpha 2B (pancreatic) [Source:HGNC Symbol;Acc:HGNC:478]" |
| ENSG00000256045 | HGLibA_30474 | 149 | 0.213062603 | MTRNR2L10 | MT-RNR2 like 10 [Source:HGNC Symbol;Acc:HGNC:37167] |
| ENSG00000256463 | HGLibA_42652 | 149 | 0.213062603 | SALL3 | spalt like transcription factor 3 [Source:HGNC Symbol;Acc:HGNC:10527] |
| ENSG00000261934 | HGLibA_35547 | 149 | 0.213062603 | PCDHGA9 | 9 [Source:HGNC Symbol;Acc:HGNC:8707]" |
| ENSG00000266265 | HGLibA_25054 | 149 | 0.213062603 | KLF14 | Kruppel like factor 14 [Source:HGNC Symbol;Acc:HGNC:23025] |
| ENSG00000268500 | HGLibA_44142 | 149 | 0.213062603 | SIGLEC5 | sense intronic to AC018755.18" |
| ENSG00000275832 | HGLibA_02732 | 149 | 0.213062603 | ARHGAP23 | Rho GTPase activating protein 23 [Source:HGNC Symbol;Acc:HGNC:29293] |
| EPB49 | HGLibA_15262 | 149 | 0.213062603 | EPB49 | - |
| FAM192A | HGLibA_16361 | 149 | 0.213062603 | FAM192A | - |
| hsa-mir-2116 | HGLibA_58237 | 149 | 0.213062603 | hsa-mir-2116 | - |
| hsa-mir-2392 | HGLibA_58323 | 149 | 0.213062603 | hsa-mir-2392 | - |
| hsa-mir-3117 | HGLibA_58510 | 149 | 0.213062603 | hsa-mir-3117 | - |
| hsa-mir-3129 | HGLibA_58567 | 149 | 0.213062603 | hsa-mir-3129 | - |
| hsa-mir-3618 | HGLibA_59103 | 149 | 0.213062603 | hsa-mir-3618 | - |
| hsa-mir-3620 | HGLibA_59115 | 149 | 0.213062603 | hsa-mir-3620 | - |
| hsa-mir-4419a | HGLibA_60059 | 149 | 0.213062603 | hsa-mir-4419a | - |
| hsa-mir-4712 | HGLibA_60950 | 149 | 0.213062603 | hsa-mir-4712 | - |
| hsa-mir-548x-2 | HGLibA_62081 | 149 | 0.213062603 | hsa-mir-548x-2 | - |
| hsa-mir-598 | HGLibA_62483 | 149 | 0.213062603 | hsa-mir-598 | - |
| hsa-mir-6069 | HGLibA_62524 | 149 | 0.213062603 | hsa-mir-6069 | - |
| hsa-mir-6730 | HGLibA_63072 | 149 | 0.213062603 | hsa-mir-6730 | - |
| hsa-mir-6766 | HGLibA_63227 | 149 | 0.213062603 | hsa-mir-6766 | - |
| hsa-mir-6851 | HGLibA_63576 | 149 | 0.213062603 | hsa-mir-6851 | - |
| hsa-mir-6876 | HGLibA_63689 | 149 | 0.213062603 | hsa-mir-6876 | - |
| hsa-mir-8069 | HGLibA_64114 | 149 | 0.213062603 | hsa-mir-8069 | - |
| LOC152586 | HGLibA_26894 | 149 | 0.213062603 | LOC152586 | - |
| TTC25 | HGLibA_52088 | 149 | 0.213062603 | TTC25 | - |
| XAGE1E | HGLibA_54478 | 149 | 0.213062603 | XAGE1E | - |
| C19orf24 | HGLibA_05638 | 148 | 0.20401726 | C19orf24 | - |
| ENSG00000010030 | HGLibA_15667 | 148 | 0.20401726 | ETV7 | ETS variant 7 [Source:HGNC Symbol;Acc:HGNC:18160] |
| ENSG00000015285 | HGLibA_53951 | 148 | 0.20401726 | WAS | Wiskott-Aldrich syndrome [Source:HGNC Symbol;Acc:HGNC:12731] |
| ENSG00000041988 | HGLibA_49299 | 148 | 0.20401726 | THAP3 | THAP domain containing 3 [Source:HGNC Symbol;Acc:HGNC:20855] |
| ENSG00000046774 | HGLibA_27961 | 148 | 0.20401726 | MAGEC2 | MAGE family member C2 [Source:HGNC Symbol;Acc:HGNC:13574] |
| ENSG00000047579 | HGLibA_14071 | 148 | 0.20401726 | DTNBP1 | dystrobrevin binding protein 1 [Source:HGNC Symbol;Acc:HGNC:17328] |
| ENSG00000067646 | HGLibA_55268 | 148 | 0.20401726 | ZFY | zinc finger protein Y-linked [Source:HGNC Symbol;Acc:HGNC:12870] |
| ENSG00000071073 | HGLibA_29142 | 148 | 0.20401726 | MGAT4A | 3-mannosyl-glycoprotein 4-beta-N-acetylglucosaminyltransferase A [Source:HGNC Symbol;Acc:HGNC:7047]" |
| ENSG00000080910 | HGLibA_09266 | 148 | 0.20401726 | CFHR2 | complement factor H related 2 [Source:HGNC Symbol;Acc:HGNC:4890] |
| ENSG00000083807 | HGLibA_44824 | 148 | 0.20401726 | SLC27A5 | solute carrier family 27 member 5 [Source:HGNC Symbol;Acc:HGNC:10999] |
| ENSG00000085465 | HGLibA_34798 | 148 | 0.20401726 | OVGP1 | oviductal glycoprotein 1 [Source:HGNC Symbol;Acc:HGNC:8524] |
| ENSG00000085871 | HGLibA_29176 | 148 | 0.20401726 | MGST2 | microsomal glutathione S-transferase 2 [Source:HGNC Symbol;Acc:HGNC:7063] |
| ENSG00000089723 | HGLibA_34757 | 148 | 0.20401726 | OTUB2 | ubiquitin aldehyde binding 2 [Source:HGNC Symbol;Acc:HGNC:20351]" |
| ENSG00000092203 | HGLibA_51044 | 148 | 0.20401726 | TOX4 | TOX high mobility group box family member 4 [Source:HGNC Symbol;Acc:HGNC:20161] |
| ENSG00000095564 | HGLibA_04891 | 148 | 0.20401726 | BTAF1 | B-TFIID TATA-box binding protein associated factor 1 [Source:HGNC Symbol;Acc:HGNC:17307] |
| ENSG00000096092 | HGLibA_49978 | 148 | 0.20401726 | TMEM14A | transmembrane protein 14A [Source:HGNC Symbol;Acc:HGNC:21076] |
| ENSG00000101109 | HGLibA_47511 | 148 | 0.20401726 | STK4 | serine/threonine kinase 4 [Source:HGNC Symbol;Acc:HGNC:11408] |
| ENSG00000101216 | HGLibA_19399 | 148 | 0.20401726 | GMEB2 | glucocorticoid modulatory element binding protein 2 [Source:HGNC Symbol;Acc:HGNC:4371] |
| ENSG00000101438 | HGLibA_44932 | 148 | 0.20401726 | SLC32A1 | solute carrier family 32 member 1 [Source:HGNC Symbol;Acc:HGNC:11018] |
| ENSG00000102755 | HGLibA_17662 | 148 | 0.20401726 | FLT1 | fms related tyrosine kinase 1 [Source:HGNC Symbol;Acc:HGNC:3763] |
| ENSG00000105509 | HGLibA_20862 | 148 | 0.20401726 | HAS1 | hyaluronan synthase 1 [Source:HGNC Symbol;Acc:HGNC:4818] |
| ENSG00000106266 | HGLibA_46107 | 148 | 0.20401726 | SNX8 | sorting nexin 8 [Source:HGNC Symbol;Acc:HGNC:14972] |
| ENSG00000106538 | HGLibA_40284 | 148 | 0.20401726 | RARRES2 | retinoic acid receptor responder 2 [Source:HGNC Symbol;Acc:HGNC:9868] |
| ENSG00000109072 | HGLibA_53892 | 148 | 0.20401726 | VTN | vitronectin [Source:HGNC Symbol;Acc:HGNC:12724] |
| ENSG00000110315 | HGLibA_41522 | 148 | 0.20401726 | RNF141 | ring finger protein 141 [Source:HGNC Symbol;Acc:HGNC:21159] |
| ENSG00000111249 | HGLibA_11805 | 148 | 0.20401726 | CUX2 | cut like homeobox 2 [Source:HGNC Symbol;Acc:HGNC:19347] |
| ENSG00000112077 | HGLibA_41122 | 148 | 0.20401726 | RHAG | Rh associated glycoprotein [Source:HGNC Symbol;Acc:HGNC:10006] |
| ENSG00000112200 | HGLibA_56028 | 148 | 0.20401726 | ZNF451 | zinc finger protein 451 [Source:HGNC Symbol;Acc:HGNC:21091] |
| ENSG00000112303 | HGLibA_53714 | 148 | 0.20401726 | VNN2 | vanin 2 [Source:HGNC Symbol;Acc:HGNC:12706] |
| ENSG00000112658 | HGLibA_46961 | 148 | 0.20401726 | SRF | serum response factor [Source:HGNC Symbol;Acc:HGNC:11291] |
| ENSG00000113269 | HGLibA_41505 | 148 | 0.20401726 | RNF130 | ring finger protein 130 [Source:HGNC Symbol;Acc:HGNC:18280] |
| ENSG00000113448 | HGLibA_35796 | 148 | 0.20401726 | PDE4D | phosphodiesterase 4D [Source:HGNC Symbol;Acc:HGNC:8783] |
| ENSG00000115221 | HGLibA_23794 | 148 | 0.20401726 | ITGB6 | integrin subunit beta 6 [Source:HGNC Symbol;Acc:HGNC:6161] |
| ENSG00000118985 | HGLibA_14925 | 148 | 0.20401726 | ELL2 | elongation factor for RNA polymerase II 2 [Source:HGNC Symbol;Acc:HGNC:17064] |
| ENSG00000121005 | HGLibA_11175 | 148 | 0.20401726 | CRISPLD1 | cysteine rich secretory protein LCCL domain containing 1 [Source:HGNC Symbol;Acc:HGNC:18206] |
| ENSG00000122176 | HGLibA_17716 | 148 | 0.20401726 | FMOD | fibromodulin [Source:HGNC Symbol;Acc:HGNC:3774] |
| ENSG00000125746 | HGLibA_15059 | 148 | 0.20401726 | EML2 | echinoderm microtubule associated protein like 2 [Source:HGNC Symbol;Acc:HGNC:18035] |
| ENSG00000127526 | HGLibA_44986 | 148 | 0.20401726 | SLC35E1 | solute carrier family 35 member E1 [Source:HGNC Symbol;Acc:HGNC:20803] |
| ENSG00000128285 | HGLibA_28590 | 148 | 0.20401726 | MCHR1 | melanin concentrating hormone receptor 1 [Source:HGNC Symbol;Acc:HGNC:4479] |
| ENSG00000130182 | HGLibA_56985 | 148 | 0.20401726 | ZSCAN10 | zinc finger and SCAN domain containing 10 [Source:HGNC Symbol;Acc:HGNC:12997] |
| ENSG00000131781 | HGLibA_17714 | 148 | 0.20401726 | FMO5 | flavin containing monooxygenase 5 [Source:HGNC Symbol;Acc:HGNC:3773] |
| ENSG00000132535 | HGLibA_13333 | 148 | 0.20401726 | DLG4 | discs large MAGUK scaffold protein 4 [Source:HGNC Symbol;Acc:HGNC:2903] |
| ENSG00000132640 | HGLibA_04914 | 148 | 0.20401726 | BTBD3 | BTB domain containing 3 [Source:HGNC Symbol;Acc:HGNC:15854] |
| ENSG00000134057 | HGLibA_08058 | 148 | 0.20401726 | CCNB1 | cyclin B1 [Source:HGNC Symbol;Acc:HGNC:1579] |
| ENSG00000134775 | HGLibA_17494 | 148 | 0.20401726 | FHOD3 | formin homology 2 domain containing 3 [Source:HGNC Symbol;Acc:HGNC:26178] |
| ENSG00000135063 | HGLibA_16355 | 148 | 0.20401726 | FAM189A2 | family with sequence similarity 189 member A2 [Source:HGNC Symbol;Acc:HGNC:24820] |
| ENSG00000135837 | HGLibA_09133 | 148 | 0.20401726 | CEP350 | centrosomal protein 350 [Source:HGNC Symbol;Acc:HGNC:24238] |
| ENSG00000136160 | HGLibA_14481 | 148 | 0.20401726 | EDNRB | endothelin receptor type B [Source:HGNC Symbol;Acc:HGNC:3180] |
| ENSG00000136634 | HGLibA_22945 | 148 | 0.20401726 | IL10 | interleukin 10 [Source:HGNC Symbol;Acc:HGNC:5962] |
| ENSG00000139508 | HGLibA_45190 | 148 | 0.20401726 | SLC46A3 | solute carrier family 46 member 3 [Source:HGNC Symbol;Acc:HGNC:27501] |
| ENSG00000139973 | HGLibA_48065 | 148 | 0.20401726 | SYT16 | synaptotagmin 16 [Source:HGNC Symbol;Acc:HGNC:23142] |
| ENSG00000140575 | HGLibA_23532 | 148 | 0.20401726 | IQGAP1 | IQ motif containing GTPase activating protein 1 [Source:HGNC Symbol;Acc:HGNC:6110] |
| ENSG00000141101 | HGLibA_32233 | 148 | 0.20401726 | NOB1 | NIN1 (RPN12) binding protein 1 homolog [Source:HGNC Symbol;Acc:HGNC:29540] |
| ENSG00000143226 | HGLibA_17190 | 148 | 0.20401726 | FCGR2A | Fc fragment of IgG receptor IIa [Source:HGNC Symbol;Acc:HGNC:3616] |
| ENSG00000143971 | HGLibA_15613 | 148 | 0.20401726 | ETAA1 | ATR kinase activator [Source:HGNC Symbol;Acc:HGNC:24648]" |
| ENSG00000143995 | HGLibA_28871 | 148 | 0.20401726 | MEIS1 | Meis homeobox 1 [Source:HGNC Symbol;Acc:HGNC:7000] |
| ENSG00000145975 | HGLibA_16488 | 148 | 0.20401726 | FAM217A | family with sequence similarity 217 member A [Source:HGNC Symbol;Acc:HGNC:21362] |
| ENSG00000147894 | HGLibA_06758 | 148 | 0.20401726 | C9orf72 | chromosome 9 open reading frame 72 [Source:HGNC Symbol;Acc:HGNC:28337] |
| ENSG00000149218 | HGLibA_15112 | 148 | 0.20401726 | ENDOD1 | endonuclease domain containing 1 [Source:HGNC Symbol;Acc:HGNC:29129] |
| ENSG00000149231 | HGLibA_07882 | 148 | 0.20401726 | CCDC82 | coiled-coil domain containing 82 [Source:HGNC Symbol;Acc:HGNC:26282] |
| ENSG00000150347 | HGLibA_02909 | 148 | 0.20401726 | ARID5B | AT-rich interaction domain 5B [Source:HGNC Symbol;Acc:HGNC:17362] |
| ENSG00000151718 | HGLibA_54443 | 148 | 0.20401726 | WWC2 | WW and C2 domain containing 2 [Source:HGNC Symbol;Acc:HGNC:24148] |
| ENSG00000151962 | HGLibA_40563 | 148 | 0.20401726 | RBM46 | RNA binding motif protein 46 [Source:HGNC Symbol;Acc:HGNC:28401] |
| ENSG00000152104 | HGLibA_39441 | 148 | 0.20401726 | PTPN14 | non-receptor type 14 [Source:HGNC Symbol;Acc:HGNC:9647]" |
| ENSG00000152207 | HGLibA_12230 | 148 | 0.20401726 | CYSLTR2 | cysteinyl leukotriene receptor 2 [Source:HGNC Symbol;Acc:HGNC:18274] |
| ENSG00000152944 | HGLibA_28775 | 148 | 0.20401726 | MED21 | mediator complex subunit 21 [Source:HGNC Symbol;Acc:HGNC:11473] |
| ENSG00000152953 | HGLibA_47486 | 148 | 0.20401726 | STK32B | serine/threonine kinase 32B [Source:HGNC Symbol;Acc:HGNC:14217] |
| ENSG00000154122 | HGLibA_01962 | 148 | 0.20401726 | ANKH | ANKH inorganic pyrophosphate transport regulator [Source:HGNC Symbol;Acc:HGNC:15492] |
| ENSG00000156804 | HGLibA_17072 | 148 | 0.20401726 | FBXO32 | F-box protein 32 [Source:HGNC Symbol;Acc:HGNC:16731] |

| | | | | | |
|---|---|---|---|---|---|
| ENSG00000159176 | HGLibA_11431 | 148 | 0.20401726 | CSRP1 | cysteine and glycine rich protein 1 [Source:HGNC Symbol;Acc:HGNC:2469] |
| ENSG00000159692 | HGLibA_11585 | 148 | 0.20401726 | CTBP1 | C-terminal binding protein 1 [Source:HGNC Symbol;Acc:HGNC:2494] |
| ENSG00000160460 | HGLibA_46887 | 148 | 0.20401726 | SPTBN4 | non-erythrocytic 4 [Source:HGNC Symbol;Acc:HGNC:14896]" |
| ENSG00000163126 | HGLibA_02043 | 148 | 0.20401726 | ANKRD23 | ankyrin repeat domain 23 [Source:HGNC Symbol;Acc:HGNC:24470] |
| ENSG00000163378 | HGLibA_15225 | 148 | 0.20401726 | EOGT | EGF domain specific O-linked N-acetylglucosamine transferase [Source:HGNC Symbol;Acc:HGNC:28526] |
| ENSG00000164251 | HGLibA_15864 | 148 | 0.20401726 | F2RL1 | F2R like trypsin receptor 1 [Source:HGNC Symbol;Acc:HGNC:3538] |
| ENSG00000164366 | HGLibA_07582 | 148 | 0.20401726 | CCDC127 | coiled-coil domain containing 127 [Source:HGNC Symbol;Acc:HGNC:30520] |
| ENSG00000166257 | HGLibA_42990 | 148 | 0.20401726 | SCN3B | sodium voltage-gated channel beta subunit 3 [Source:HGNC Symbol;Acc:HGNC:20665] |
| ENSG00000167604 | HGLibA_31826 | 148 | 0.20401726 | NFKBID | NFKB inhibitor delta [Source:HGNC Symbol;Acc:HGNC:15671] |
| ENSG00000167632 | HGLibA_51340 | 148 | 0.20401726 | TRAPPC9 | trafficking protein particle complex 9 [Source:HGNC Symbol;Acc:HGNC:30832] |
| ENSG00000168477 | HGLibA_50930 | 148 | 0.20401726 | TNXB | tenascin XB [Source:HGNC Symbol;Acc:HGNC:11976] |
| ENSG00000169271 | HGLibA_22311 | 148 | 0.20401726 | HSPB3 | heat shock protein family B (small) member 3 [Source:HGNC Symbol;Acc:HGNC:5248] |
| ENSG00000169800 | HGLibA_40623 | 148 | 0.20401726 | RBMY1F | member F [Source:HGNC Symbol;Acc:HGNC:23974]" |
| ENSG00000170881 | HGLibA_41518 | 148 | 0.20401726 | RNF139 | ring finger protein 139 [Source:HGNC Symbol;Acc:HGNC:17023] |
| ENSG00000170927 | HGLibA_36814 | 148 | 0.20401726 | PKHD1 | fibrocystin/polyductin [Source:HGNC Symbol;Acc:HGNC:9016]" |
| ENSG00000171314 | HGLibA_36194 | 148 | 0.20401726 | PGAM1 | phosphoglycerate mutase 1 [Source:HGNC Symbol;Acc:HGNC:8888] |
| ENSG00000171505 | HGLibA_33705 | 148 | 0.20401726 | OR1N1 | olfactory receptor family 1 subfamily N member 1 [Source:HGNC Symbol;Acc:HGNC:8221] |
| ENSG00000174038 | HGLibA_06678 | 148 | 0.20401726 | C9orf131 | chromosome 9 open reading frame 131 [Source:HGNC Symbol;Acc:HGNC:31418] |
| ENSG00000174282 | HGLibA_54878 | 148 | 0.20401726 | ZBTB4 | zinc finger and BTB domain containing 4 [Source:HGNC Symbol;Acc:HGNC:23847] |
| ENSG00000174640 | HGLibA_45460 | 148 | 0.20401726 | SLCO2A1 | solute carrier organic anion transporter family member 2A1 [Source:HGNC Symbol;Acc:HGNC:10955] |
| ENSG00000177025 | HGLibA_05634 | 148 | 0.20401726 | C19orf18 | chromosome 19 open reading frame 18 [Source:HGNC Symbol;Acc:HGNC:28642] |
| ENSG00000178209 | HGLibA_37053 | 148 | 0.20401726 | PLEC | plectin [Source:HGNC Symbol;Acc:HGNC:9069] |
| ENSG00000178307 | HGLibA_49872 | 148 | 0.20401726 | TMEM11 | transmembrane protein 11 [Source:HGNC Symbol;Acc:HGNC:16823] |
| ENSG00000179922 | HGLibA_56747 | 148 | 0.20401726 | ZNF784 | zinc finger protein 784 [Source:HGNC Symbol;Acc:HGNC:33111] |
| ENSG00000183323 | HGLibA_07576 | 148 | 0.20401726 | CCDC125 | coiled-coil domain containing 125 [Source:HGNC Symbol;Acc:HGNC:28924] |
| ENSG00000184058 | HGLibA_48641 | 148 | 0.20401726 | TBX1 | T-box 1 [Source:HGNC Symbol;Acc:HGNC:11592] |
| ENSG00000184185 | HGLibA_24254 | 148 | 0.20401726 | KCNJ12 | potassium voltage-gated channel subfamily J member 12 [Source:HGNC Symbol;Acc:HGNC:6258] |
| ENSG00000185046 | HGLibA_02152 | 148 | 0.20401726 | ANKS1B | ankyrin repeat and sterile alpha motif domain containing 1B [Source:HGNC Symbol;Acc:HGNC:24600] |
| ENSG00000185722 | HGLibA_01957 | 148 | 0.20401726 | ANKFY1 | ankyrin repeat and FYVE domain containing 1 [Source:HGNC Symbol;Acc:HGNC:20763] |
| ENSG00000187170 | HGLibA_26146 | 148 | 0.20401726 | LCE4A | late cornified envelope 4A [Source:HGNC Symbol;Acc:HGNC:16613] |
| ENSG00000187239 | HGLibA_17734 | 148 | 0.20401726 | FNBP1 | formin binding protein 1 [Source:HGNC Symbol;Acc:HGNC:17069] |
| ENSG00000189079 | HGLibA_02887 | 148 | 0.20401726 | ARID2 | AT-rich interaction domain 2 [Source:HGNC Symbol;Acc:HGNC:18037] |
| ENSG00000196505 | HGLibA_18844 | 148 | 0.20401726 | GDAP2 | ganglioside induced differentiation associated protein 2 [Source:HGNC Symbol;Acc:HGNC:18010] |
| ENSG00000196839 | HGLibA_00717 | 148 | 0.20401726 | ADA | adenosine deaminase [Source:HGNC Symbol;Acc:HGNC:186] |
| ENSG00000197724 | HGLibA_36359 | 148 | 0.20401726 | PHF2 | PHD finger protein 2 [Source:HGNC Symbol;Acc:HGNC:8920] |
| ENSG00000197919 | HGLibA_22656 | 148 | 0.20401726 | IFNA1 | interferon alpha 1 [Source:HGNC Symbol;Acc:HGNC:5417] |
| ENSG00000198131 | HGLibA_56197 | 148 | 0.20401726 | ZNF544 | zinc finger protein 544 [Source:HGNC Symbol;Acc:HGNC:16759] |
| ENSG00000198914 | HGLibA_37771 | 148 | 0.20401726 | POU3F3 | POU class 3 homeobox 3 [Source:HGNC Symbol;Acc:HGNC:9216] |
| ENSG00000204300 | HGLibA_50246 | 148 | 0.20401726 | TMEM225 | transmembrane protein 225 [Source:HGNC Symbol;Acc:HGNC:32390] |
| ENSG00000204351 | HGLibA_44284 | 148 | 0.20401726 | SKIV2L | Ski2 like RNA helicase [Source:HGNC Symbol;Acc:HGNC:10898] |
| ENSG00000205710 | HGLibA_05527 | 148 | 0.20401726 | C17orf107 | chromosome 17 open reading frame 107 [Source:HGNC Symbol;Acc:HGNC:37238] |
| ENSG00000212721 | HGLibA_25753 | 148 | 0.20401726 | KRTAP4-11 | keratin associated protein 4-11 [Source:HGNC Symbol;Acc:HGNC:18911] |
| ENSG00000214336 | HGLibA_17881 | 148 | 0.20401726 | FOXI3 | forkhead box I3 [Source:HGNC Symbol;Acc:HGNC:35123] |
| ENSG00000241322 | HGLibA_08871 | 148 | 0.20401726 | CDRT1 | CMT1A duplicated region transcript 1 [Source:HGNC Symbol;Acc:HGNC:14379] |
| ENSG00000251349 | HGLibA_30178 | 148 | 0.20401726 | MSANTD3-TMEFF1 | MSANTD3-TMEFF1 readthrough [Source:HGNC Symbol;Acc:HGNC:38838] |
| ENSG00000261308 | HGLibA_17525 | 148 | 0.20401726 | FIGNL2 | fidgetin like 2 [Source:HGNC Symbol;Acc:HGNC:13287] |
| ENSG00000263528 | HGLibA_22923 | 148 | 0.20401726 | IKBKE | inhibitor of nuclear factor kappa B kinase subunit epsilon [Source:HGNC Symbol;Acc:HGNC:14552] |
| ENSG00000269858 | HGLibA_14643 | 148 | 0.20401726 | EGLN2 | egl-9 family hypoxia inducible factor 2 [Source:HGNC Symbol;Acc:HGNC:14660] |
| ENSG00000272395 | HGLibA_22728 | 148 | 0.20401726 | IFNL4 | interferon lambda 4 (gene/pseudogene) [Source:HGNC Symbol;Acc:HGNC:44480] |
| ENSG00000273841 | HGLibA_48261 | 148 | 0.20401726 | TAF9 | TATA-box binding protein associated factor 9 [Source:HGNC Symbol;Acc:HGNC:11542] |
| ENSG00000275835 | HGLibA_52319 | 148 | 0.20401726 | TUBGCP5 | tubulin gamma complex associated protein 5 [Source:HGNC Symbol;Acc:HGNC:18600] |
| ENSG00000277893 | HGLibA_46943 | 148 | 0.20401726 | SRD5A2 | steroid 5 alpha-reductase 2 [Source:HGNC Symbol;Acc:HGNC:11285] |
| hsa-mir-1260a | HGLibA_57460 | 148 | 0.20401726 | hsa-mir-1260a | - |
| hsa-mir-140 | HGLibA_57823 | 148 | 0.20401726 | hsa-mir-140 | - |
| hsa-mir-140 | HGLibA_57825 | 148 | 0.20401726 | hsa-mir-140 | - |
| hsa-mir-1976 | HGLibA_58120 | 148 | 0.20401726 | hsa-mir-1976 | - |
| hsa-mir-2467 | HGLibA_58348 | 148 | 0.20401726 | hsa-mir-2467 | - |
| hsa-mir-3679 | HGLibA_59267 | 148 | 0.20401726 | hsa-mir-3679 | - |
| hsa-mir-3690-2 | HGLibA_59350 | 148 | 0.20401726 | hsa-mir-3690-2 | - |
| hsa-mir-4299 | HGLibA_59908 | 148 | 0.20401726 | hsa-mir-4299 | - |
| hsa-mir-552 | HGLibA_62128 | 148 | 0.20401726 | hsa-mir-552 | - |
| hsa-mir-598 | HGLibA_62486 | 148 | 0.20401726 | hsa-mir-598 | - |
| hsa-mir-6770-3 | HGLibA_63253 | 148 | 0.20401726 | hsa-mir-6770-3 | - |
| hsa-mir-6806 | HGLibA_63402 | 148 | 0.20401726 | hsa-mir-6806 | - |
| hsa-mir-6817 | HGLibA_63445 | 148 | 0.20401726 | hsa-mir-6817 | - |
| hsa-mir-770 | HGLibA_63941 | 148 | 0.20401726 | hsa-mir-770 | - |
| hsa-mir-8072 | HGLibA_64129 | 148 | 0.20401726 | hsa-mir-8072 | - |
| KDELC2 | HGLibA_24476 | 148 | 0.20401726 | KDELC2 | - |
| PPAPDC1B | HGLibA_37829 | 148 | 0.20401726 | PPAPDC1B | - |
| RAB7L1 | HGLibA_39981 | 148 | 0.20401726 | RAB7L1 | - |
| SSPO | HGLibA_47149 | 148 | 0.20401726 | SSPO | - |
| C10orf129 | HGLibA_05053 | 147 | 0.194971917 | C10orf129 | - |
| C14orf169 | HGLibA_05340 | 147 | 0.194971917 | C14orf169 | - |
| C20orf112 | HGLibA_05983 | 147 | 0.194971917 | C20orf112 | - |
| CCDC104 | HGLibA_07518 | 147 | 0.194971917 | CCDC104 | - |
| CTGF | HGLibA_11622 | 147 | 0.194971917 | CTGF | - |
| ENSG00000011422 | HGLibA_36966 | 147 | 0.194971917 | PLAUR | urokinase receptor [Source:HGNC Symbol;Acc:HGNC:9053]" |
| ENSG00000013561 | HGLibA_41519 | 147 | 0.194971917 | RNF14 | ring finger protein 14 [Source:HGNC Symbol;Acc:HGNC:10058] |
| ENSG00000029534 | HGLibA_01933 | 147 | 0.194971917 | ANK1 | ankyrin 1 [Source:HGNC Symbol;Acc:HGNC:492] |
| ENSG00000029559 | HGLibA_22481 | 147 | 0.194971917 | IBSP | integrin binding sialoprotein [Source:HGNC Symbol;Acc:HGNC:5341] |
| ENSG00000047849 | HGLibA_28207 | 147 | 0.194971917 | MAP4 | microtubule associated protein 4 [Source:HGNC Symbol;Acc:HGNC:6862] |
| ENSG00000064835 | HGLibA_37750 | 147 | 0.194971917 | POU1F1 | POU class 1 homeobox 1 [Source:HGNC Symbol;Acc:HGNC:9210] |
| ENSG00000075413 | HGLibA_28371 | 147 | 0.194971917 | MARK3 | microtubule affinity regulating kinase 3 [Source:HGNC Symbol;Acc:HGNC:6897] |
| ENSG00000084754 | HGLibA_20808 | 147 | 0.194971917 | HADHA | hydroxyacyl-CoA dehydrogenase trifunctional multienzyme complex subunit alpha [Source:HGNC Symbol;Acc:HGNC:4801] |
| ENSG00000087448 | HGLibA_25214 | 147 | 0.194971917 | KLHL42 | kelch like family member 42 [Source:HGNC Symbol;Acc:HGNC:29252] |
| ENSG00000092439 | HGLibA_51770 | 147 | 0.194971917 | TRPM7 | transient receptor potential cation channel subfamily M member 7 [Source:HGNC Symbol;Acc:HGNC:17994] |
| ENSG00000100241 | HGLibA_42794 | 147 | 0.194971917 | SBF1 | SET binding factor 1 [Source:HGNC Symbol;Acc:HGNC:10542] |
| ENSG00000100852 | HGLibA_02784 | 147 | 0.194971917 | ARHGAP5 | Rho GTPase activating protein 5 [Source:HGNC Symbol;Acc:HGNC:675] |
| ENSG00000101000 | HGLibA_38656 | 147 | 0.194971917 | PROCR | protein C receptor [Source:HGNC Symbol;Acc:HGNC:9452] |
| ENSG00000102804 | HGLibA_51816 | 147 | 0.194971917 | TSC22D1 | TSC22 domain family member 1 [Source:HGNC Symbol;Acc:HGNC:16826] |
| ENSG00000103343 | HGLibA_55526 | 147 | 0.194971917 | ZNF174 | zinc finger protein 174 [Source:HGNC Symbol;Acc:HGNC:12963] |
| ENSG00000105963 | HGLibA_00864 | 147 | 0.194971917 | ADAP1 | ArfGAP with dual PH domains 1 [Source:HGNC Symbol;Acc:HGNC:16486] |
| ENSG00000107807 | HGLibA_49671 | 147 | 0.194971917 | TLX1 | T cell leukemia homeobox 1 [Source:HGNC Symbol;Acc:HGNC:5056] |
| ENSG00000107949 | HGLibA_04269 | 147 | 0.194971917 | BCCIP | BRCA2 and CDKN1A interacting protein [Source:HGNC Symbol;Acc:HGNC:978] |
| ENSG00000110218 | HGLibA_35110 | 147 | 0.194971917 | PANX1 | pannexin 1 [Source:HGNC Symbol;Acc:HGNC:8599] |
| ENSG00000112378 | HGLibA_36063 | 147 | 0.194971917 | PERP | TP53 apoptosis effector [Source:HGNC Symbol;Acc:HGNC:17637]" |
| ENSG00000112599 | HGLibA_20637 | 147 | 0.194971917 | GUCA1B | guanylate cyclase activator 1B [Source:HGNC Symbol;Acc:HGNC:4679] |
| ENSG00000114315 | HGLibA_21177 | 147 | 0.194971917 | HES1 | hes family bHLH transcription factor 1 [Source:HGNC Symbol;Acc:HGNC:5192] |
| ENSG00000114942 | HGLibA_14497 | 147 | 0.194971917 | EEF1B2 | eukaryotic translation elongation factor 1 beta 2 [Source:HGNC Symbol;Acc:HGNC:3208] |
| ENSG00000116703 | HGLibA_35713 | 147 | 0.194971917 | PDC | phosducin [Source:HGNC Symbol;Acc:HGNC:8759] |
| ENSG00000117691 | HGLibA_31692 | 147 | 0.194971917 | NENF | neudesin neurotrophic factor [Source:HGNC Symbol;Acc:HGNC:30384] |
| ENSG00000118407 | HGLibA_17529 | 147 | 0.194971917 | FILIP1 | filamin A interacting protein 1 [Source:HGNC Symbol;Acc:HGNC:21015] |
| ENSG00000119326 | HGLibA_11647 | 147 | 0.194971917 | CTNNAL1 | catenin alpha like 1 [Source:HGNC Symbol;Acc:HGNC:2512] |
| ENSG00000119782 | HGLibA_17567 | 147 | 0.194971917 | FKBP1B | FK506 binding protein 1B [Source:HGNC Symbol;Acc:HGNC:3712] |
| ENSG00000120833 | HGLibA_46127 | 147 | 0.194971917 | SOCS2 | suppressor of cytokine signaling 2 [Source:HGNC Symbol;Acc:HGNC:19382] |
| ENSG00000121314 | HGLibA_48430 | 147 | 0.194971917 | TAS2R8 | taste 2 receptor member 8 [Source:HGNC Symbol;Acc:HGNC:14915] |
| ENSG00000124104 | HGLibA_46064 | 147 | 0.194971917 | SNX21 | sorting nexin family member 21 [Source:HGNC Symbol;Acc:HGNC:16154] |
| ENSG00000125618 | HGLibA_35315 | 147 | 0.194971917 | PAX8 | paired box 8 [Source:HGNC Symbol;Acc:HGNC:8622] |
| ENSG00000125810 | HGLibA_08481 | 147 | 0.194971917 | CD93 | CD93 molecule [Source:HGNC Symbol;Acc:HGNC:15855] |
| ENSG00000125975 | HGLibA_05996 | 147 | 0.194971917 | C20orf173 | chromosome 20 open reading frame 173 [Source:HGNC Symbol;Acc:HGNC:16166] |
| ENSG00000126561 | HGLibA_47392 | 147 | 0.194971917 | STAT5A | signal transducer and activator of transcription 5A [Source:HGNC Symbol;Acc:HGNC:11366] |
| ENSG00000127780 | HGLibA_33661 | 147 | 0.194971917 | OR1E2 | olfactory receptor family 1 subfamily E member 2 [Source:HGNC Symbol;Acc:HGNC:8190] |
| ENSG00000132286 | HGLibA_49496 | 147 | 0.194971917 | TIMM10B | translocase of inner mitochondrial membrane 10B [Source:HGNC Symbol;Acc:HGNC:4022] |
| ENSG00000135094 | HGLibA_43171 | 147 | 0.194971917 | SDS | serine dehydratase [Source:HGNC Symbol;Acc:HGNC:10691] |
| ENSG00000135346 | HGLibA_09295 | 147 | 0.194971917 | CGA | alpha polypeptide [Source:HGNC Symbol;Acc:HGNC:1885]" |
| ENSG00000135587 | HGLibA_45807 | 147 | 0.194971917 | SMPD2 | sphingomyelin phosphodiesterase 2 [Source:HGNC Symbol;Acc:HGNC:11121] |
| ENSG00000136378 | HGLibA_00838 | 147 | 0.194971917 | ADAMTS7 | ADAM metallopeptidase with thrombospondin type 1 motif 7 [Source:HGNC Symbol;Acc:HGNC:223] |
| ENSG00000136811 | HGLibA_33279 | 147 | 0.194971917 | ODF2 | outer dense fiber of sperm tails 2 [Source:HGNC Symbol;Acc:HGNC:8114] |
| ENSG00000136986 | HGLibA_12996 | 147 | 0.194971917 | DERL1 | derlin 1 [Source:HGNC Symbol;Acc:HGNC:28454] |
| ENSG00000138435 | HGLibA_09562 | 147 | 0.194971917 | CHRNA1 | cholinergic receptor nicotinic alpha 1 subunit [Source:HGNC Symbol;Acc:HGNC:1955] |
| ENSG00000138606 | HGLibA_44025 | 147 | 0.194971917 | SHF | Src homology 2 domain containing F [Source:HGNC Symbol;Acc:HGNC:25116] |
| ENSG00000139687 | HGLibA_40412 | 147 | 0.194971917 | RB1 | RB transcriptional corepressor 1 [Source:HGNC Symbol;Acc:HGNC:9884] |
| ENSG00000139988 | HGLibA_40737 | 147 | 0.194971917 | RDH12 | retinol dehydrogenase 12 [Source:HGNC Symbol;Acc:HGNC:19977] |
| ENSG00000140948 | HGLibA_55015 | 147 | 0.194971917 | ZCCHC14 | zinc finger CCHC-type containing 14 [Source:HGNC Symbol;Acc:HGNC:24134] |
| ENSG00000140990 | HGLibA_31507 | 147 | 0.194971917 | NDUFB10 | NADH:ubiquinone oxidoreductase subunit B10 [Source:HGNC Symbol;Acc:HGNC:7696] |
| ENSG00000142449 | HGLibA_16960 | 147 | 0.194971917 | FBN3 | fibrillin 3 [Source:HGNC Symbol;Acc:HGNC:18794] |
| ENSG00000143315 | HGLibA_36568 | 147 | 0.194971917 | PIGM | phosphatidylinositol glycan anchor biosynthesis class M [Source:HGNC Symbol;Acc:HGNC:18858] |
| ENSG00000144191 | HGLibA_10268 | 147 | 0.194971917 | CNGA3 | cyclic nucleotide gated channel alpha 3 [Source:HGNC Symbol;Acc:HGNC:2150] |
| ENSG00000144218 | HGLibA_01113 | 147 | 0.194971917 | AFF3 | AF4/FMR2 family member 3 [Source:HGNC Symbol;Acc:HGNC:6473] |
| ENSG00000146757 | HGLibA_56907 | 147 | 0.194971917 | ZNF92 | zinc finger protein 92 [Source:HGNC Symbol;Acc:HGNC:13168] |
| ENSG00000147862 | HGLibA_31804 | 147 | 0.194971917 | NFIB | nuclear factor I B [Source:HGNC Symbol;Acc:HGNC:7785] |
| ENSG00000154134 | HGLibA_41738 | 147 | 0.194971917 | ROBO3 | roundabout guidance receptor 3 [Source:HGNC Symbol;Acc:HGNC:13433] |
| ENSG00000155269 | HGLibA_20086 | 147 | 0.194971917 | GPR78 | G protein-coupled receptor 78 [Source:HGNC Symbol;Acc:HGNC:4528] |
| ENSG00000155465 | HGLibA_45378 | 147 | 0.194971917 | SLC7A7 | solute carrier family 7 member 7 [Source:HGNC Symbol;Acc:HGNC:11065] |
| ENSG00000156219 | HGLibA_03162 | 147 | 0.194971917 | ART3 | ADP-ribosyltransferase 3 [Source:HGNC Symbol;Acc:HGNC:725] |

| | | | | | |
|---|---|---|---|---|---|
| ENSG00000157343 | HGLibA_03011 | 147 | 0.194971917 | ARMC12 | armadillo repeat containing 12 [Source:HGNC Symbol;Acc:HGNC:21099] |
| ENSG00000158445 | HGLibA_24157 | 147 | 0.194971917 | KCNB1 | potassium voltage-gated channel subfamily B member 1 [Source:HGNC Symbol;Acc:HGNC:6231] |
| ENSG00000158528 | HGLibA_38128 | 147 | 0.194971917 | PPP1R9A | protein phosphatase 1 regulatory subunit 9A [Source:HGNC Symbol;Acc:HGNC:14946] |
| ENSG00000160219 | HGLibA_18326 | 147 | 0.194971917 | GAB3 | GRB2 associated binding protein 3 [Source:HGNC Symbol;Acc:HGNC:17515] |
| ENSG00000163154 | HGLibA_50724 | 147 | 0.194971917 | TNFAIP8L2 | TNF alpha induced protein 8 like 2 [Source:HGNC Symbol;Acc:HGNC:26277] |
| ENSG00000163219 | HGLibA_02737 | 147 | 0.194971917 | ARHGAP25 | Rho GTPase activating protein 25 [Source:HGNC Symbol;Acc:HGNC:28951] |
| ENSG00000164089 | HGLibA_15637 | 147 | 0.194971917 | ETNPPL | ethanolamine-phosphate phospho-lyase [Source:HGNC Symbol;Acc:HGNC:14404] |
| ENSG00000164122 | HGLibA_03242 | 147 | 0.194971917 | ASB5 | ankyrin repeat and SOCS box containing 5 [Source:HGNC Symbol;Acc:HGNC:17180] |
| ENSG00000164687 | HGLibA_15922 | 147 | 0.194971917 | FABP5 | fatty acid binding protein 5 [Source:HGNC Symbol;Acc:HGNC:3560] |
| ENSG00000164736 | HGLibA_46245 | 147 | 0.194971917 | SOX17 | SRY-box 17 [Source:HGNC Symbol;Acc:HGNC:18122] |
| ENSG00000164841 | HGLibA_50511 | 147 | 0.194971917 | TMEM74 | transmembrane protein 74 [Source:HGNC Symbol;Acc:HGNC:26409] |
| ENSG00000164853 | HGLibA_53041 | 147 | 0.194971917 | UNCX | UNC homeobox [Source:HGNC Symbol;Acc:HGNC:33194] |
| ENSG00000165078 | HGLibA_10900 | 147 | 0.194971917 | CPA6 | carboxypeptidase A6 [Source:HGNC Symbol;Acc:HGNC:17245] |
| ENSG00000165175 | HGLibA_29234 | 147 | 0.194971917 | MID1IP1 | MID1 interacting protein 1 [Source:HGNC Symbol;Acc:HGNC:20715] |
| ENSG00000165392 | HGLibA_54409 | 147 | 0.194971917 | WRN | Werner syndrome RecQ like helicase [Source:HGNC Symbol;Acc:HGNC:12791] |
| ENSG00000169057 | HGLibA_28727 | 147 | 0.194971917 | MECP2 | methyl-CpG binding protein 2 [Source:HGNC Symbol;Acc:HGNC:6990] |
| ENSG00000169248 | HGLibA_11848 | 147 | 0.194971917 | CXCL11 | C-X-C motif chemokine ligand 11 [Source:HGNC Symbol;Acc:HGNC:10638] |
| ENSG00000169340 | HGLibA_35893 | 147 | 0.194971917 | PDILT | testis expressed [Source:HGNC Symbol;Acc:HGNC:27338]" |
| ENSG00000169393 | HGLibA_14994 | 147 | 0.194971917 | ELSPBP1 | epididymal sperm binding protein 1 [Source:HGNC Symbol;Acc:HGNC:14417] |
| ENSG00000170633 | HGLibA_41666 | 147 | 0.194971917 | RNF34 | ring finger protein 34 [Source:HGNC Symbol;Acc:HGNC:17297] |
| ENSG00000170802 | HGLibA_17913 | 147 | 0.194971917 | FOXN2 | forkhead box N2 [Source:HGNC Symbol;Acc:HGNC:5281] |
| ENSG00000171016 | HGLibA_39694 | 147 | 0.194971917 | PYGO1 | pygopus family PHD finger 1 [Source:HGNC Symbol;Acc:HGNC:30256] |
| ENSG00000171135 | HGLibA_23909 | 147 | 0.194971917 | JAGN1 | jagunal homolog 1 [Source:HGNC Symbol;Acc:HGNC:26926] |
| ENSG00000177106 | HGLibA_15381 | 147 | 0.194971917 | EPS8L2 | EPS8 like 2 [Source:HGNC Symbol;Acc:HGNC:21296] |
| ENSG00000177468 | HGLibA_33374 | 147 | 0.194971917 | OLIG3 | oligodendrocyte transcription factor 3 [Source:HGNC Symbol;Acc:HGNC:18003] |
| ENSG00000177565 | HGLibA_48612 | 147 | 0.194971917 | TBL1XR1 | transducin beta like 1 X-linked receptor 1 [Source:HGNC Symbol;Acc:HGNC:29529] |
| ENSG00000178127 | HGLibA_31571 | 147 | 0.194971917 | NDUFV2 | NADH:ubiquinone oxidoreductase core subunit V2 [Source:HGNC Symbol;Acc:HGNC:7717] |
| ENSG00000180257 | HGLibA_56813 | 147 | 0.194971917 | ZNF816 | zinc finger protein 816 [Source:HGNC Symbol;Acc:HGNC:26995] |
| ENSG00000180730 | HGLibA_44034 | 147 | 0.194971917 | SHISA2 | shisa family member 2 [Source:HGNC Symbol;Acc:HGNC:20366] |
| ENSG00000182534 | HGLibA_30625 | 147 | 0.194971917 | MXRA7 | matrix remodeling associated 7 [Source:HGNC Symbol;Acc:HGNC:7541] |
| ENSG00000183128 | HGLibA_07020 | 147 | 0.194971917 | CALHM3 | calcium homeostasis modulator 3 [Source:HGNC Symbol;Acc:HGNC:23458] |
| ENSG00000185013 | HGLibA_32804 | 147 | 0.194971917 | NT5C1B | cytosolic IB [Source:HGNC Symbol;Acc:HGNC:17818]" |
| ENSG00000185669 | HGLibA_45873 | 147 | 0.194971917 | SNAI3 | snail family transcriptional repressor 3 [Source:HGNC Symbol;Acc:HGNC:18411] |
| ENSG00000186566 | HGLibA_19751 | 147 | 0.194971917 | GPATCH8 | G-patch domain containing 8 [Source:HGNC Symbol;Acc:HGNC:29066] |
| ENSG00000187079 | HGLibA_48926 | 147 | 0.194971917 | TEAD1 | TEA domain transcription factor 1 [Source:HGNC Symbol;Acc:HGNC:11714] |
| ENSG00000187609 | HGLibA_15722 | 147 | 0.194971917 | EXD3 | exonuclease 3'-5' domain containing 3 [Source:HGNC Symbol;Acc:HGNC:26023] |
| ENSG00000188394 | HGLibA_20000 | 147 | 0.194971917 | GPR21 | G protein-coupled receptor 21 [Source:HGNC Symbol;Acc:HGNC:4476] |
| ENSG00000188611 | HGLibA_03189 | 147 | 0.194971917 | ASAH2 | N-acylsphingosine amidohydrolase 2 [Source:HGNC Symbol;Acc:HGNC:18860] |
| ENSG00000188992 | HGLibA_26617 | 147 | 0.194971917 | LIPI | lipase I [Source:HGNC Symbol;Acc:HGNC:18821] |
| ENSG00000196116 | HGLibA_48915 | 147 | 0.194971917 | TDRD7 | tudor domain containing 7 [Source:HGNC Symbol;Acc:HGNC:30831] |
| ENSG00000196465 | HGLibA_30773 | 147 | 0.194971917 | MYL6B | myosin light chain 6B [Source:HGNC Symbol;Acc:HGNC:29823] |
| ENSG00000196565 | HGLibA_20928 | 147 | 0.194971917 | HBG2 | hemoglobin subunit gamma 2 [Source:HGNC Symbol;Acc:HGNC:4832] |
| ENSG00000196693 | HGLibA_55834 | 147 | 0.194971917 | ZNF33B | zinc finger protein 33B [Source:HGNC Symbol;Acc:HGNC:13097] |
| ENSG00000196739 | HGLibA_10553 | 147 | 0.194971917 | COL27A1 | collagen type XXVII alpha 1 chain [Source:HGNC Symbol;Acc:HGNC:22986] |
| ENSG00000196844 | HGLibA_35275 | 147 | 0.194971917 | PATE2 | prostate and testis expressed 2 [Source:HGNC Symbol;Acc:HGNC:32249] |
| ENSG00000197858 | HGLibA_19721 | 147 | 0.194971917 | GPAA1 | glycosylphosphatidylinositol anchor attachment 1 [Source:HGNC Symbol;Acc:HGNC:4446] |
| ENSG00000197943 | HGLibA_37004 | 147 | 0.194971917 | PLCG2 | phospholipase C gamma 2 [Source:HGNC Symbol;Acc:HGNC:9066] |
| ENSG00000198160 | HGLibA_29252 | 147 | 0.194971917 | MIER1 | MIER1 transcriptional regulator [Source:HGNC Symbol;Acc:HGNC:29657] |
| ENSG00000198478 | HGLibA_43920 | 147 | 0.194971917 | SH3BGRL2 | SH3 domain binding glutamate rich protein like 2 [Source:HGNC Symbol;Acc:HGNC:15567] |
| ENSG00000198542 | HGLibA_23803 | 147 | 0.194971917 | ITGBL1 | integrin subunit beta like 1 [Source:HGNC Symbol;Acc:HGNC:6164] |
| ENSG00000204195 | HGLibA_03913 | 147 | 0.194971917 | AWAT1 | acyl-CoA wax alcohol acyltransferase 1 [Source:HGNC Symbol;Acc:HGNC:23252] |
| ENSG00000204252 | HGLibA_21608 | 147 | 0.194971917 | HLA-DOA | DO alpha [Source:HGNC Symbol;Acc:HGNC:4936]" |
| ENSG00000204478 | HGLibA_38307 | 147 | 0.194971917 | PRAMEF20 | PRAME family member 20 [Source:HGNC Symbol;Acc:HGNC:25224] |
| ENSG00000204913 | HGLibA_27348 | 147 | 0.194971917 | LRRC3C | leucine rich repeat containing 3C [Source:HGNC Symbol;Acc:HGNC:40034] |
| ENSG00000213265 | HGLibA_51854 | 147 | 0.194971917 | TSGA13 | testis specific 13 [Source:HGNC Symbol;Acc:HGNC:12369] |
| ENSG00000214046 | HGLibA_45766 | 147 | 0.194971917 | SMIM7 | small integral membrane protein 7 [Source:HGNC Symbol;Acc:HGNC:28419] |
| ENSG00000221829 | HGLibA_16852 | 147 | 0.194971917 | FANCG | FA complementation group G [Source:HGNC Symbol;Acc:HGNC:3588] |
| ENSG00000240386 | HGLibA_26114 | 147 | 0.194971917 | LCE1F | late cornified envelope 1F [Source:HGNC Symbol;Acc:HGNC:29467] |
| ENSG00000241119 | HGLibA_52901 | 147 | 0.194971917 | UGT1A9 | UDP glucuronosyltransferase family 1 member A9 [Source:HGNC Symbol;Acc:HGNC:12541] |
| ENSG00000256223 | HGLibA_55429 | 147 | 0.194971917 | ZNF10 | zinc finger protein 10 [Source:HGNC Symbol;Acc:HGNC:12879] |
| ENSG00000268964 | HGLibA_15549 | 147 | 0.194971917 | ERVV-2 | envelope [Source:HGNC Symbol;Acc:HGNC:39051]" |
| ENSG00000273899 | HGLibA_32262 | 147 | 0.194971917 | NOL12 | nucleolar protein 12 [Source:HGNC Symbol;Acc:HGNC:28585] |
| FAM84B | HGLibA_16766 | 147 | 0.194971917 | FAM84B | - |
| hsa-mir-106b | HGLibA_57190 | 147 | 0.194971917 | hsa-mir-106b | - |
| hsa-mir-1273f | HGLibA_57543 | 147 | 0.194971917 | hsa-mir-1273f | - |
| hsa-mir-2392 | HGLibA_58326 | 147 | 0.194971917 | hsa-mir-2392 | - |
| hsa-mir-3666 | HGLibA_59217 | 147 | 0.194971917 | hsa-mir-3666 | - |
| hsa-mir-4662b | HGLibA_60761 | 147 | 0.194971917 | hsa-mir-4662b | - |
| hsa-mir-506 | HGLibA_61482 | 147 | 0.194971917 | hsa-mir-506 | - |
| hsa-mir-6728 | HGLibA_63066 | 147 | 0.194971917 | hsa-mir-6728 | - |
| hsa-mir-6735 | HGLibA_63095 | 147 | 0.194971917 | hsa-mir-6735 | - |
| hsa-mir-6878 | HGLibA_63698 | 147 | 0.194971917 | hsa-mir-6878 | - |
| LOC100129361 | HGLibA_26759 | 147 | 0.194971917 | LOC100129361 | - |
| NUTMF | HGLibA_33114 | 147 | 0.194971917 | NUTMF | - |
| SMCR9 | HGLibA_45686 | 147 | 0.194971917 | SMCR9 | - |
| SPANXB2 | HGLibA_46387 | 147 | 0.194971917 | SPANXB2 | - |
| TCTE3 | HGLibA_48860 | 147 | 0.194971917 | TCTE3 | - |
| CCDC135 | HGLibA_07601 | 146 | 0.185926574 | CCDC135 | - |
| ENSG00000002919 | HGLibA_46030 | 146 | 0.185926574 | SNX11 | sorting nexin 11 [Source:HGNC Symbol;Acc:HGNC:14975] |
| ENSG00000006118 | HGLibA_49930 | 146 | 0.185926574 | TMEM132A | transmembrane protein 132A [Source:HGNC Symbol;Acc:HGNC:31092] |
| ENSG00000006576 | HGLibA_36466 | 146 | 0.185926574 | PHTF2 | putative homeodomain transcription factor 2 [Source:HGNC Symbol;Acc:HGNC:13411] |
| ENSG00000008083 | HGLibA_23937 | 146 | 0.185926574 | JARID2 | jumonji and AT-rich interaction domain containing 2 [Source:HGNC Symbol;Acc:HGNC:6196] |
| ENSG00000008513 | HGLibA_47236 | 146 | 0.185926574 | ST3GAL1 | 3-sialyltransferase 1 [Source:HGNC Symbol;Acc:HGNC:10862]" |
| ENSG00000011405 | HGLibA_36621 | 146 | 0.185926574 | PIK3C2A | phosphatidylinositol-4-phosphate 3-kinase catalytic subunit type 2 alpha [Source:HGNC Symbol;Acc:HGNC:8971] |
| ENSG00000026751 | HGLibA_44318 | 146 | 0.185926574 | SLAMF7 | SLAM family member 7 [Source:HGNC Symbol;Acc:HGNC:21394] |
| ENSG00000049249 | HGLibA_50794 | 146 | 0.185926574 | TNFRSF9 | TNF receptor superfamily member 9 [Source:HGNC Symbol;Acc:HGNC:11924] |
| ENSG00000063241 | HGLibA_23684 | 146 | 0.185926574 | ISOC2 | isochorismatase domain containing 2 [Source:HGNC Symbol;Acc:HGNC:26278] |
| ENSG00000063601 | HGLibA_30409 | 146 | 0.185926574 | MTMR1 | myotubularin related protein 1 [Source:HGNC Symbol;Acc:HGNC:7449] |
| ENSG00000065802 | HGLibA_03202 | 146 | 0.185926574 | ASB1 | ankyrin repeat and SOCS box containing 1 [Source:HGNC Symbol;Acc:HGNC:16011] |
| ENSG00000072121 | HGLibA_55286 | 146 | 0.185926574 | ZFYVE26 | zinc finger FYVE-type containing 26 [Source:HGNC Symbol;Acc:HGNC:20761] |
| ENSG00000074590 | HGLibA_32891 | 146 | 0.185926574 | NUAK1 | NUAK family kinase 1 [Source:HGNC Symbol;Acc:HGNC:14311] |
| ENSG00000075043 | HGLibA_24369 | 146 | 0.185926574 | KCNQ2 | potassium voltage-gated channel subfamily Q member 2 [Source:HGNC Symbol;Acc:HGNC:6296] |
| ENSG00000075945 | HGLibA_24966 | 146 | 0.185926574 | KIFAP3 | kinesin associated protein 3 [Source:HGNC Symbol;Acc:HGNC:17060] |
| ENSG00000088682 | HGLibA_10766 | 146 | 0.185926574 | COQ9 | coenzyme Q9 [Source:HGNC Symbol;Acc:HGNC:25302] |
| ENSG00000099139 | HGLibA_35680 | 146 | 0.185926574 | PCSK5 | proprotein convertase subtilisin/kexin type 5 [Source:HGNC Symbol;Acc:HGNC:8747] |
| ENSG00000099849 | HGLibA_40391 | 146 | 0.185926574 | RASSF7 | Ras association domain family member 7 [Source:HGNC Symbol;Acc:HGNC:1166] |
| ENSG00000101019 | HGLibA_53099 | 146 | 0.185926574 | UQCC1 | ubiquinol-cytochrome c reductase complex assembly factor 1 [Source:HGNC Symbol;Acc:HGNC:15891] |
| ENSG00000101997 | HGLibA_07731 | 146 | 0.185926574 | CCDC22 | coiled-coil domain containing 22 [Source:HGNC Symbol;Acc:HGNC:28909] |
| ENSG00000103510 | HGLibA_24070 | 146 | 0.185926574 | KAT8 | lysine acetyltransferase 8 [Source:HGNC Symbol;Acc:HGNC:17933] |
| ENSG00000105711 | HGLibA_42976 | 146 | 0.185926574 | SCN1B | sodium voltage-gated channel beta subunit 1 [Source:HGNC Symbol;Acc:HGNC:10586] |
| ENSG00000106733 | HGLibA_32196 | 146 | 0.185926574 | NMRK1 | nicotinamide riboside kinase 1 [Source:HGNC Symbol;Acc:HGNC:26057] |
| ENSG00000107581 | HGLibA_14763 | 146 | 0.185926574 | EIF3A | eukaryotic translation initiation factor 3 subunit A [Source:HGNC Symbol;Acc:HGNC:3271] |
| ENSG00000107731 | HGLibA_53017 | 146 | 0.185926574 | UNC5B | unc-5 netrin receptor B [Source:HGNC Symbol;Acc:HGNC:12568] |
| ENSG00000108587 | HGLibA_19684 | 146 | 0.185926574 | GOSR1 | golgi SNAP receptor complex member 1 [Source:HGNC Symbol;Acc:HGNC:4430] |
| ENSG00000108774 | HGLibA_39968 | 146 | 0.185926574 | RAB5C | member RAS oncogene family [Source:HGNC Symbol;Acc:HGNC:9785]" |
| ENSG00000108848 | HGLibA_27627 | 146 | 0.185926574 | LUC7L3 | LUC7 like 3 pre-mRNA splicing factor [Source:HGNC Symbol;Acc:HGNC:24309] |
| ENSG00000110080 | HGLibA_47245 | 146 | 0.185926574 | ST3GAL4 | 3-sialyltransferase 4 [Source:HGNC Symbol;Acc:HGNC:10864]" |
| ENSG00000110321 | HGLibA_14844 | 146 | 0.185926574 | EIF4G2 | eukaryotic translation initiation factor 4 gamma 2 [Source:HGNC Symbol;Acc:HGNC:3297] |
| ENSG00000110900 | HGLibA_51896 | 146 | 0.185926574 | TSPAN11 | tetraspanin 11 [Source:HGNC Symbol;Acc:HGNC:30795] |
| ENSG00000113211 | HGLibA_35505 | 146 | 0.185926574 | PCDHB6 | protocadherin beta 6 [Source:HGNC Symbol;Acc:HGNC:8691] |
| ENSG00000113739 | HGLibA_47417 | 146 | 0.185926574 | STC2 | stanniocalcin 2 [Source:HGNC Symbol;Acc:HGNC:11374] |
| ENSG00000115282 | HGLibA_52112 | 146 | 0.185926574 | TTC31 | tetratricopeptide repeat domain 31 [Source:HGNC Symbol;Acc:HGNC:25759] |
| ENSG00000117472 | HGLibA_51893 | 146 | 0.185926574 | TSPAN1 | tetraspanin 1 [Source:HGNC Symbol;Acc:HGNC:20657] |
| ENSG00000123352 | HGLibA_46547 | 146 | 0.185926574 | SPATS2 | spermatogenesis associated serine rich 2 [Source:HGNC Symbol;Acc:HGNC:18650] |
| ENSG00000124523 | HGLibA_44229 | 146 | 0.185926574 | SIRT5 | sirtuin 5 [Source:HGNC Symbol;Acc:HGNC:14933] |
| ENSG00000125398 | HGLibA_46279 | 146 | 0.185926574 | SOX9 | SRY-box 9 [Source:HGNC Symbol;Acc:HGNC:11204] |
| ENSG00000130640 | HGLibA_52309 | 146 | 0.185926574 | TUBGCP2 | tubulin gamma complex associated protein 2 [Source:HGNC Symbol;Acc:HGNC:18599] |
| ENSG00000131096 | HGLibA_39713 | 146 | 0.185926574 | PYY | peptide YY [Source:HGNC Symbol;Acc:HGNC:9748] |
| ENSG00000131373 | HGLibA_20803 | 146 | 0.185926574 | HACL1 | 2-hydroxyacyl-CoA lyase 1 [Source:HGNC Symbol;Acc:HGNC:17856] |
| ENSG00000133665 | HGLibA_14223 | 146 | 0.185926574 | DYDC2 | DPY30 domain containing 2 [Source:HGNC Symbol;Acc:HGNC:23468] |
| ENSG00000136404 | HGLibA_49710 | 146 | 0.185926574 | TM6SF1 | transmembrane 6 superfamily member 1 [Source:HGNC Symbol;Acc:HGNC:11860] |
| ENSG00000136982 | HGLibA_14004 | 146 | 0.185926574 | DSCC1 | DNA replication and sister chromatid cohesion 1 [Source:HGNC Symbol;Acc:HGNC:24453] |
| ENSG00000137841 | HGLibA_36980 | 146 | 0.185926574 | PLCB2 | phospholipase C beta 2 [Source:HGNC Symbol;Acc:HGNC:9055] |
| ENSG00000139364 | HGLibA_49933 | 146 | 0.185926574 | TMEM132B | transmembrane protein 132B [Source:HGNC Symbol;Acc:HGNC:29397] |
| ENSG00000140939 | HGLibA_32266 | 146 | 0.185926574 | NOL3 | nucleolar protein 3 [Source:HGNC Symbol;Acc:HGNC:7869] |
| ENSG00000141294 | HGLibA_27368 | 146 | 0.185926574 | LRRC46 | leucine rich repeat containing 46 [Source:HGNC Symbol;Acc:HGNC:25047] |
| ENSG00000143365 | HGLibA_41782 | 146 | 0.185926574 | RORC | RAR related orphan receptor C [Source:HGNC Symbol;Acc:HGNC:10260] |
| ENSG00000143514 | HGLibA_51058 | 146 | 0.185926574 | TP53BP2 | tumor protein p53 binding protein 2 [Source:HGNC Symbol;Acc:HGNC:12000] |
| ENSG00000143847 | HGLibA_37881 | 146 | 0.185926574 | PPFIA4 | PTPRF interacting protein alpha 4 [Source:HGNC Symbol;Acc:HGNC:9248] |
| ENSG00000144868 | HGLibA_49864 | 146 | 0.185926574 | TMEM108 | transmembrane protein 108 [Source:HGNC Symbol;Acc:HGNC:28451] |
| ENSG00000145649 | HGLibA_20719 | 146 | 0.185926574 | GZMA | granzyme A [Source:HGNC Symbol;Acc:HGNC:4708] |
| ENSG00000146243 | HGLibA_23557 | 146 | 0.185926574 | IRAK1BP1 | interleukin 1 receptor associated kinase 1 binding protein 1 [Source:HGNC Symbol;Acc:HGNC:17368] |
| ENSG00000147065 | HGLibA_30232 | 146 | 0.185926574 | MSN | moesin [Source:HGNC Symbol;Acc:HGNC:7373] |
| ENSG00000149452 | HGLibA_44599 | 146 | 0.185926574 | SLC22A8 | solute carrier family 22 member 8 [Source:HGNC Symbol;Acc:HGNC:10972] |
| ENSG00000149577 | HGLibA_44118 | 146 | 0.185926574 | SIDT2 | SID1 transmembrane family member 2 [Source:HGNC Symbol;Acc:HGNC:24272] |

| | | | | | |
|---|---|---|---|---|---|
| ENSG00000150459 | HGLibA_42715 | 146 | 0.185926574 | SAP18 | Sin3A associated protein 18 [Source:HGNC Symbol;Acc:HGNC:10530] |
| ENSG00000151470 | HGLibA_06323 | 146 | 0.185926574 | C4orf33 | chromosome 4 open reading frame 33 [Source:HGNC Symbol;Acc:HGNC:27025] |
| ENSG00000155366 | HGLibA_41182 | 146 | 0.185926574 | RHOC | ras homolog family member C [Source:HGNC Symbol;Acc:HGNC:669] |
| ENSG00000158481 | HGLibA_08270 | 146 | 0.185926574 | CD1C | CD1c molecule [Source:HGNC Symbol;Acc:HGNC:1636] |
| ENSG00000160233 | HGLibA_27305 | 146 | 0.185926574 | LRRC3 | leucine rich repeat containing 3 [Source:HGNC Symbol;Acc:HGNC:14965] |
| ENSG00000160408 | HGLibA_47278 | 146 | 0.185926574 | ST6GALNAC6 | 6-sialyltransferase 6 [Source:HGNC Symbol;Acc:HGNC:23364]" |
| ENSG00000160808 | HGLibA_30759 | 146 | 0.185926574 | MYL3 | myosin light chain 3 [Source:HGNC Symbol;Acc:HGNC:7584] |
| ENSG00000162408 | HGLibA_32279 | 146 | 0.185926574 | NOL9 | nucleolar protein 9 [Source:HGNC Symbol;Acc:HGNC:26265] |
| ENSG00000163479 | HGLibA_47155 | 146 | 0.185926574 | SSR2 | signal sequence receptor subunit 2 [Source:HGNC Symbol;Acc:HGNC:11324] |
| ENSG00000164168 | HGLibA_50117 | 146 | 0.185926574 | TMEM184C | transmembrane protein 184C [Source:HGNC Symbol;Acc:HGNC:25587] |
| ENSG00000165555 | HGLibA_32387 | 146 | 0.185926574 | NOXRED1 | NADP dependent oxidoreductase domain containing 1 [Source:HGNC Symbol;Acc:HGNC:20487] |
| ENSG00000165724 | HGLibA_55424 | 146 | 0.185926574 | ZMYND19 | zinc finger MYND-type containing 19 [Source:HGNC Symbol;Acc:HGNC:21146] |
| ENSG00000166454 | HGLibA_03528 | 146 | 0.185926574 | ATMIN | ATM interactor [Source:HGNC Symbol;Acc:HGNC:29034] |
| ENSG00000166959 | HGLibA_30166 | 146 | 0.185926574 | MS4A8 | membrane spanning 4-domains A8 [Source:HGNC Symbol;Acc:HGNC:13380] |
| ENSG00000168010 | HGLibA_03475 | 146 | 0.185926574 | ATG16L2 | autophagy related 16 like 2 [Source:HGNC Symbol;Acc:HGNC:25464] |
| ENSG00000168412 | HGLibA_30444 | 146 | 0.185926574 | MTNR1A | melatonin receptor 1A [Source:HGNC Symbol;Acc:HGNC:7463] |
| ENSG00000168676 | HGLibA_24442 | 146 | 0.185926574 | KCTD19 | potassium channel tetramerization domain containing 19 [Source:HGNC Symbol;Acc:HGNC:24753] |
| ENSG00000168916 | HGLibA_56374 | 146 | 0.185926574 | ZNF608 | zinc finger protein 608 [Source:HGNC Symbol;Acc:HGNC:29238] |
| ENSG00000168958 | HGLibA_29056 | 146 | 0.185926574 | MFF | mitochondrial fission factor [Source:HGNC Symbol;Acc:HGNC:24858] |
| ENSG00000170390 | HGLibA_12503 | 146 | 0.185926574 | DCLK2 | doublecortin like kinase 2 [Source:HGNC Symbol;Acc:HGNC:19002] |
| ENSG00000171148 | HGLibA_48192 | 146 | 0.185926574 | TADA3 | transcriptional adaptor 3 [Source:HGNC Symbol;Acc:HGNC:19422] |
| ENSG00000171540 | HGLibA_34750 | 146 | 0.185926574 | OTP | orthopedia homeobox [Source:HGNC Symbol;Acc:HGNC:8518] |
| ENSG00000171561 | HGLibA_33762 | 146 | 0.185926574 | OR2AT4 | olfactory receptor family 2 subfamily AT member 4 [Source:HGNC Symbol;Acc:HGNC:19620] |
| ENSG00000172748 | HGLibA_56349 | 146 | 0.185926574 | ZNF596 | zinc finger protein 596 [Source:HGNC Symbol;Acc:HGNC:27268] |
| ENSG00000173193 | HGLibA_35222 | 146 | 0.185926574 | PARP14 | poly(ADP-ribose) polymerase family member 14 [Source:HGNC Symbol;Acc:HGNC:29232] |
| ENSG00000173198 | HGLibA_12226 | 146 | 0.185926574 | CYSLTR1 | cysteinyl leukotriene receptor 1 [Source:HGNC Symbol;Acc:HGNC:17451] |
| ENSG00000173227 | HGLibA_48053 | 146 | 0.185926574 | SYT12 | synaptotagmin 12 [Source:HGNC Symbol;Acc:HGNC:18381] |
| ENSG00000174989 | HGLibA_17156 | 146 | 0.185926574 | FBXW8 | F-box and WD repeat domain containing 8 [Source:HGNC Symbol;Acc:HGNC:13597] |
| ENSG00000175556 | HGLibA_27026 | 146 | 0.185926574 | LONRF3 | LON peptidase N-terminal domain and ring finger 3 [Source:HGNC Symbol;Acc:HGNC:21152] |
| ENSG00000176230 | HGLibA_34028 | 146 | 0.185926574 | OR4K17 | olfactory receptor family 4 subfamily K member 17 [Source:HGNC Symbol;Acc:HGNC:15355] |
| ENSG00000176253 | HGLibA_34019 | 146 | 0.185926574 | OR4K13 | olfactory receptor family 4 subfamily K member 13 [Source:HGNC Symbol;Acc:HGNC:15351] |
| ENSG00000176679 | HGLibA_49251 | 146 | 0.185926574 | TGIF2LY | TGFB induced factor homeobox 2 like Y-linked [Source:HGNC Symbol;Acc:HGNC:18569] |
| ENSG00000179021 | HGLibA_06241 | 146 | 0.185926574 | C3orf38 | chromosome 3 open reading frame 38 [Source:HGNC Symbol;Acc:HGNC:28384] |
| ENSG00000179477 | HGLibA_01678 | 146 | 0.185926574 | ALOX12B | 12R type [Source:HGNC Symbol;Acc:HGNC:430]" |
| ENSG00000179869 | HGLibA_00105 | 146 | 0.185926574 | ABCA13 | ATP binding cassette subfamily A member 13 [Source:HGNC Symbol;Acc:HGNC:14638] |
| ENSG00000180071 | HGLibA_02019 | 146 | 0.185926574 | ANKRD18A | ankyrin repeat domain 18A [Source:HGNC Symbol;Acc:HGNC:23643] |
| ENSG00000182240 | HGLibA_04062 | 146 | 0.185926574 | BACE2 | beta-secretase 2 [Source:HGNC Symbol;Acc:HGNC:934] |
| ENSG00000183020 | HGLibA_02309 | 146 | 0.185926574 | AP2A2 | adaptor related protein complex 2 subunit alpha 2 [Source:HGNC Symbol;Acc:HGNC:562] |
| ENSG00000184261 | HGLibA_24300 | 146 | 0.185926574 | KCNK12 | potassium two pore domain channel subfamily K member 12 [Source:HGNC Symbol;Acc:HGNC:6274] |
| ENSG00000185352 | HGLibA_22147 | 146 | 0.185926574 | HS6ST3 | heparan sulfate 6-O-sulfotransferase 3 [Source:HGNC Symbol;Acc:HGNC:19134] |
| ENSG00000186132 | HGLibA_06182 | 146 | 0.185926574 | C2orf76 | chromosome 2 open reading frame 76 [Source:HGNC Symbol;Acc:HGNC:27017] |
| ENSG00000186288 | HGLibA_34928 | 146 | 0.185926574 | PABPC1L2A | poly(A) binding protein cytoplasmic 1 like 2A [Source:HGNC Symbol;Acc:HGNC:27989] |
| ENSG00000186501 | HGLibA_50241 | 146 | 0.185926574 | TMEM222 | transmembrane protein 222 [Source:HGNC Symbol;Acc:HGNC:25363] |
| ENSG00000186980 | HGLibA_25723 | 146 | 0.185926574 | KRTAP23-1 | keratin associated protein 23-1 [Source:HGNC Symbol;Acc:HGNC:18928] |
| ENSG00000188177 | HGLibA_54980 | 146 | 0.185926574 | ZC3H6 | zinc finger CCCH-type containing 6 [Source:HGNC Symbol;Acc:HGNC:24762] |
| ENSG00000188766 | HGLibA_46795 | 146 | 0.185926574 | SPRED3 | sprouty related EVH1 domain containing 3 [Source:HGNC Symbol;Acc:HGNC:31041] |
| ENSG00000197046 | HGLibA_44137 | 146 | 0.185926574 | SIGLEC15 | sialic acid binding Ig like lectin 15 [Source:HGNC Symbol;Acc:HGNC:27596] |
| ENSG00000197479 | HGLibA_35474 | 146 | 0.185926574 | PCDHB11 | protocadherin beta 11 [Source:HGNC Symbol;Acc:HGNC:8682] |
| ENSG00000197563 | HGLibA_36569 | 146 | 0.185926574 | PIGN | phosphatidylinositol glycan anchor biosynthesis class N [Source:HGNC Symbol;Acc:HGNC:8967] |
| ENSG00000198010 | HGLibA_13342 | 146 | 0.185926574 | DLGAP2 | DLG associated protein 2 [Source:HGNC Symbol;Acc:HGNC:2906] |
| ENSG00000198162 | HGLibA_28062 | 146 | 0.185926574 | MAN1A2 | mannosidase alpha class 1A member 2 [Source:HGNC Symbol;Acc:HGNC:6822] |
| ENSG00000198203 | HGLibA_47745 | 146 | 0.185926574 | SULT1C2 | sulfotransferase family 1C member 2 [Source:HGNC Symbol;Acc:HGNC:11456] |
| ENSG00000198734 | HGLibA_15876 | 146 | 0.185926574 | F5 | coagulation factor V [Source:HGNC Symbol;Acc:HGNC:3542] |
| ENSG00000203872 | HGLibA_06454 | 146 | 0.185926574 | C6orf163 | chromosome 6 open reading frame 163 [Source:HGNC Symbol;Acc:HGNC:21403] |
| ENSG00000205593 | HGLibA_12969 | 146 | 0.185926574 | DENND6B | DENN domain containing 6B [Source:HGNC Symbol;Acc:HGNC:32690] |
| ENSG00000205923 | HGLibA_09031 | 146 | 0.185926574 | CEMP1 | cementum protein 1 [Source:HGNC Symbol;Acc:HGNC:32553] |
| ENSG00000206047 | HGLibA_12791 | 146 | 0.185926574 | DEFA1 | defensin alpha 1 [Source:HGNC Symbol;Acc:HGNC:2761] |
| ENSG00000215915 | HGLibA_03415 | 146 | 0.185926574 | ATAD3C | AAA domain containing 3C [Source:HGNC Symbol;Acc:HGNC:32151]" |
| ENSG00000217236 | HGLibA_46318 | 146 | 0.185926574 | SP9 | Sp9 transcription factor [Source:HGNC Symbol;Acc:HGNC:30690] |
| ENSG00000221994 | HGLibA_56435 | 146 | 0.185926574 | ZNF630 | zinc finger protein 630 [Source:HGNC Symbol;Acc:HGNC:28855] |
| ENSG00000235194 | HGLibA_38110 | 146 | 0.185926574 | PPP1R3E | protein phosphatase 1 regulatory subunit 3E [Source:HGNC Symbol;Acc:HGNC:14943] |
| ENSG00000240542 | HGLibA_25829 | 146 | 0.185926574 | KRTAP9-1 | keratin associated protein 9-1 [Source:HGNC Symbol;Acc:HGNC:18912] |
| ENSG00000240871 | HGLibA_25773 | 146 | 0.185926574 | KRTAP4-7 | keratin associated protein 4-7 [Source:HGNC Symbol;Acc:HGNC:18898] |
| ENSG00000242852 | HGLibA_56605 | 146 | 0.185926574 | ZNF709 | zinc finger protein 709 [Source:HGNC Symbol;Acc:HGNC:20629] |
| ENSG00000243244 | HGLibA_47543 | 146 | 0.185926574 | STON1 | stonin 1 [Source:HGNC Symbol;Acc:HGNC:17003] |
| GPR113 | HGLibA_19856 | 146 | 0.185926574 | GPR113 | - |
| hsa-mir-136 | HGLibA_57806 | 146 | 0.185926574 | hsa-mir-136 | - |
| hsa-mir-1976 | HGLibA_58121 | 146 | 0.185926574 | hsa-mir-1976 | - |
| hsa-mir-3183 | HGLibA_58828 | 146 | 0.185926574 | hsa-mir-3183 | - |
| hsa-mir-3650 | HGLibA_59145 | 146 | 0.185926574 | hsa-mir-3650 | - |
| hsa-mir-4288 | HGLibA_59860 | 146 | 0.185926574 | hsa-mir-4288 | - |
| hsa-mir-4434 | HGLibA_60127 | 146 | 0.185926574 | hsa-mir-4434 | - |
| hsa-mir-4647 | HGLibA_60690 | 146 | 0.185926574 | hsa-mir-4647 | - |
| hsa-mir-4780 | HGLibA_61228 | 146 | 0.185926574 | hsa-mir-4780 | - |
| hsa-mir-5704 | HGLibA_62356 | 146 | 0.185926574 | hsa-mir-5704 | - |
| hsa-mir-572 | HGLibA_62380 | 146 | 0.185926574 | hsa-mir-572 | - |
| hsa-mir-584 | HGLibA_62436 | 146 | 0.185926574 | hsa-mir-584 | - |
| hsa-mir-6085 | HGLibA_62602 | 146 | 0.185926574 | hsa-mir-6085 | - |
| hsa-mir-6511a-1 | HGLibA_62898 | 146 | 0.185926574 | hsa-mir-6511a-1 | - |
| hsa-mir-664b | HGLibA_62997 | 146 | 0.185926574 | hsa-mir-664b | - |
| hsa-mir-6726 | HGLibA_63059 | 146 | 0.185926574 | hsa-mir-6726 | - |
| hsa-mir-6773 | HGLibA_63266 | 146 | 0.185926574 | hsa-mir-6773 | - |
| hsa-mir-6798 | HGLibA_63370 | 146 | 0.185926574 | hsa-mir-6798 | - |
| hsa-mir-6837 | HGLibA_63523 | 146 | 0.185926574 | hsa-mir-6837 | - |
| hsa-mir-6866 | HGLibA_63650 | 146 | 0.185926574 | hsa-mir-6866 | - |
| hsa-mir-6883 | HGLibA_63715 | 146 | 0.185926574 | hsa-mir-6883 | - |
| hsa-mir-7973-2 | HGLibA_64020 | 146 | 0.185926574 | hsa-mir-7973-2 | - |
| hsa-mir-873 | HGLibA_64204 | 146 | 0.185926574 | hsa-mir-873 | - |
| hsa-mir-92b | HGLibA_64300 | 146 | 0.185926574 | hsa-mir-92b | - |
| LOC100288814 | HGLibA_26838 | 146 | 0.185926574 | LOC100288814 | - |
| RRNAD1 | HGLibA_42264 | 146 | 0.185926574 | RRNAD1 | - |
| TMEM189 | HGLibA_50132 | 146 | 0.185926574 | TMEM189 | - |
| WDR85 | HGLibA_54198 | 146 | 0.185926574 | WDR85 | - |
| ENSG00000001167 | HGLibA_31853 | 145 | 0.176881231 | NFYA | nuclear transcription factor Y subunit alpha [Source:HGNC Symbol;Acc:HGNC:7804] |
| ENSG00000004059 | HGLibA_02654 | 145 | 0.176881231 | ARF5 | ADP ribosylation factor 5 [Source:HGNC Symbol;Acc:HGNC:658] |
| ENSG00000011198 | HGLibA_00282 | 145 | 0.176881231 | ABHD5 | abhydrolase domain containing 5 [Source:HGNC Symbol;Acc:HGNC:21396] |
| ENSG00000060971 | HGLibA_00341 | 145 | 0.176881231 | ACAA1 | acetyl-CoA acyltransferase 1 [Source:HGNC Symbol;Acc:HGNC:82] |
| ENSG00000064726 | HGLibA_04893 | 145 | 0.176881231 | BTBD1 | BTB domain containing 1 [Source:HGNC Symbol;Acc:HGNC:1120] |
| ENSG00000077498 | HGLibA_52465 | 145 | 0.176881231 | TYR | tyrosinase [Source:HGNC Symbol;Acc:HGNC:12442] |
| ENSG00000081913 | HGLibA_36436 | 145 | 0.176881231 | PHLPP1 | PH domain and leucine rich repeat protein phosphatase 1 [Source:HGNC Symbol;Acc:HGNC:20610] |
| ENSG00000086848 | HGLibA_01640 | 145 | 0.176881231 | ALG9 | 2-mannosyltransferase [Source:HGNC Symbol;Acc:HGNC:15672]" |
| ENSG00000091640 | HGLibA_46365 | 145 | 0.176881231 | SPAG7 | sperm associated antigen 7 [Source:HGNC Symbol;Acc:HGNC:11216] |
| ENSG00000096401 | HGLibA_08591 | 145 | 0.176881231 | CDC5L | cell division cycle 5 like [Source:HGNC Symbol;Acc:HGNC:1743] |
| ENSG00000100012 | HGLibA_43195 | 145 | 0.176881231 | SEC14L3 | SEC14 like lipid binding 3 [Source:HGNC Symbol;Acc:HGNC:18655] |
| ENSG00000100344 | HGLibA_37410 | 145 | 0.176881231 | PNPLA3 | patatin like phospholipase domain containing 3 [Source:HGNC Symbol;Acc:HGNC:18590] |
| ENSG00000100784 | HGLibA_42171 | 145 | 0.176881231 | RPS6KA5 | ribosomal protein S6 kinase A5 [Source:HGNC Symbol;Acc:HGNC:10434] |
| ENSG00000101306 | HGLibA_30787 | 145 | 0.176881231 | MYLK2 | myosin light chain kinase 2 [Source:HGNC Symbol;Acc:HGNC:16243] |
| ENSG00000102034 | HGLibA_14899 | 145 | 0.176881231 | ELF4 | E74 like ETS transcription factor 4 [Source:HGNC Symbol;Acc:HGNC:3319] |
| ENSG00000105085 | HGLibA_28790 | 145 | 0.176881231 | MED26 | mediator complex subunit 26 [Source:HGNC Symbol;Acc:HGNC:2376] |
| ENSG00000105388 | HGLibA_08945 | 145 | 0.176881231 | CEACAM5 | carcinoembryonic antigen related cell adhesion molecule 5 [Source:HGNC Symbol;Acc:HGNC:1817] |
| ENSG00000105717 | HGLibA_35353 | 145 | 0.176881231 | PBX4 | PBX homeobox 4 [Source:HGNC Symbol;Acc:HGNC:13403] |
| ENSG00000105997 | HGLibA_21908 | 145 | 0.176881231 | HOXA3 | homeobox A3 [Source:HGNC Symbol;Acc:HGNC:5104] |
| ENSG00000106785 | HGLibA_51410 | 145 | 0.176881231 | TRIM14 | tripartite motif containing 14 [Source:HGNC Symbol;Acc:HGNC:16283] |
| ENSG00000108474 | HGLibA_36565 | 145 | 0.176881231 | PIGL | phosphatidylinositol glycan anchor biosynthesis class L [Source:HGNC Symbol;Acc:HGNC:8966] |
| ENSG00000110203 | HGLibA_17788 | 145 | 0.176881231 | FOLR3 | folate receptor 3 [Source:HGNC Symbol;Acc:HGNC:3795] |
| ENSG00000111011 | HGLibA_42361 | 145 | 0.176881231 | RSRC2 | arginine and serine rich coiled-coil 2 [Source:HGNC Symbol;Acc:HGNC:30559] |
| ENSG00000111145 | HGLibA_14914 | 145 | 0.176881231 | ELK3 | ETS transcription factor [Source:HGNC Symbol;Acc:HGNC:3325]" |
| ENSG00000112212 | HGLibA_51960 | 145 | 0.176881231 | TSPO2 | translocator protein 2 [Source:HGNC Symbol;Acc:HGNC:21256] |
| ENSG00000112667 | HGLibA_13713 | 145 | 0.176881231 | DNPH1 | 2'-deoxynucleoside 5'-phosphate N-hydrolase 1 [Source:HGNC Symbol;Acc:HGNC:21218] |
| ENSG00000112992 | HGLibA_32227 | 145 | 0.176881231 | NNT | nicotinamide nucleotide transhydrogenase [Source:HGNC Symbol;Acc:HGNC:7863] |
| ENSG00000116824 | HGLibA_08279 | 145 | 0.176881231 | CD2 | CD2 molecule [Source:HGNC Symbol;Acc:HGNC:1639] |
| ENSG00000119599 | HGLibA_12438 | 145 | 0.176881231 | DCAF4 | DDB1 and CUL4 associated factor 4 [Source:HGNC Symbol;Acc:HGNC:20229] |
| ENSG00000119801 | HGLibA_54718 | 145 | 0.176881231 | YPEL5 | yippee like 5 [Source:HGNC Symbol;Acc:HGNC:18329] |
| ENSG00000123612 | HGLibA_00692 | 145 | 0.176881231 | ACVR1C | activin A receptor type 1C [Source:HGNC Symbol;Acc:HGNC:18123] |
| ENSG00000125434 | HGLibA_44716 | 145 | 0.176881231 | SLC25A35 | solute carrier family 25 member 35 [Source:HGNC Symbol;Acc:HGNC:31921] |
| ENSG00000125975 | HGLibA_05995 | 145 | 0.176881231 | C20orf173 | chromosome 20 open reading frame 173 [Source:HGNC Symbol;Acc:HGNC:16166] |
| ENSG00000126246 | HGLibA_22852 | 145 | 0.176881231 | IGFLR1 | IGF like family receptor 1 [Source:HGNC Symbol;Acc:HGNC:23620] |
| ENSG00000126581 | HGLibA_04394 | 145 | 0.176881231 | BECN1 | beclin 1 [Source:HGNC Symbol;Acc:HGNC:1034] |
| ENSG00000127399 | HGLibA_27406 | 145 | 0.176881231 | LRRC61 | leucine rich repeat containing 61 [Source:HGNC Symbol;Acc:HGNC:21704] |
| ENSG00000129990 | HGLibA_48082 | 145 | 0.176881231 | SYT5 | synaptotagmin 5 [Source:HGNC Symbol;Acc:HGNC:11513] |
| ENSG00000130522 | HGLibA_24009 | 145 | 0.176881231 | JUND | AP-1 transcription factor subunit [Source:HGNC Symbol;Acc:HGNC:6206]" |
| ENSG00000131187 | HGLibA_15851 | 145 | 0.176881231 | F12 | coagulation factor XII [Source:HGNC Symbol;Acc:HGNC:3530] |
| ENSG00000131669 | HGLibA_31939 | 145 | 0.176881231 | NINJ1 | ninjurin 1 [Source:HGNC Symbol;Acc:HGNC:7824] |
| ENSG00000132382 | HGLibA_30640 | 145 | 0.176881231 | MYBBP1A | MYB binding protein 1a [Source:HGNC Symbol;Acc:HGNC:7546] |
| ENSG00000132661 | HGLibA_33174 | 145 | 0.176881231 | NXT1 | nuclear transport factor 2 like export factor 1 [Source:HGNC Symbol;Acc:HGNC:15913] |

| | | | | | |
|---|---|---|---|---|---|
| ENSG00000132801 | HGLibA_57044 | 145 | 0.176881231 | ZSWIM3 | zinc finger SWIM-type containing 3 [Source:HGNC Symbol;Acc:HGNC:16157] |
| ENSG00000133124 | HGLibA_23624 | 145 | 0.176881231 | IRS4 | insulin receptor substrate 4 [Source:HGNC Symbol;Acc:HGNC:6128] |
| ENSG00000134443 | HGLibA_20368 | 145 | 0.176881231 | GRP | gastrin releasing peptide [Source:HGNC Symbol;Acc:HGNC:4605] |
| ENSG00000134551 | HGLibA_38465 | 145 | 0.176881231 | PRH2 | proline rich protein HaeIII subfamily 2 [Source:HGNC Symbol;Acc:HGNC:9367] |
| ENSG00000135406 | HGLibA_38748 | 145 | 0.176881231 | PRPH | peripherin [Source:HGNC Symbol;Acc:HGNC:9461] |
| ENSG00000135636 | HGLibA_14286 | 145 | 0.176881231 | DYSF | dysferlin [Source:HGNC Symbol;Acc:HGNC:3097] |
| ENSG00000136193 | HGLibA_43048 | 145 | 0.176881231 | SCRN1 | secernin 1 [Source:HGNC Symbol;Acc:HGNC:22192] |
| ENSG00000136750 | HGLibA_18412 | 145 | 0.176881231 | GAD2 | glutamate decarboxylase 2 [Source:HGNC Symbol;Acc:HGNC:4093] |
| ENSG00000136872 | HGLibA_01596 | 145 | 0.176881231 | ALDOB | fructose-bisphosphate B [Source:HGNC Symbol;Acc:HGNC:417]" |
| ENSG00000137203 | HGLibA_49120 | 145 | 0.176881231 | TFAP2A | transcription factor AP-2 alpha [Source:HGNC Symbol;Acc:HGNC:11742] |
| ENSG00000137496 | HGLibA_23026 | 145 | 0.176881231 | IL18BP | interleukin 18 binding protein [Source:HGNC Symbol;Acc:HGNC:5987] |
| ENSG00000138080 | HGLibA_15046 | 145 | 0.176881231 | EMILIN1 | elastin microfibril interfacer 1 [Source:HGNC Symbol;Acc:HGNC:19880] |
| ENSG00000139613 | HGLibA_45625 | 145 | 0.176881231 | SMARCC2 | actin dependent regulator of chromatin subfamily c member 2 [Source:HGNC Symbol;Acc:HGNC:11105]" |
| ENSG00000140274 | HGLibA_14107 | 145 | 0.176881231 | DUOXA2 | dual oxidase maturation factor 2 [Source:HGNC Symbol;Acc:HGNC:32698] |
| ENSG00000140368 | HGLibA_39237 | 145 | 0.176881231 | PSTPIP1 | proline-serine-threonine phosphatase interacting protein 1 [Source:HGNC Symbol;Acc:HGNC:9580] |
| ENSG00000142619 | HGLibA_34976 | 145 | 0.176881231 | PADI3 | peptidyl arginine deiminase 3 [Source:HGNC Symbol;Acc:HGNC:18337] |
| ENSG00000142973 | HGLibA_12179 | 145 | 0.176881231 | CYP4B1 | cytochrome P450 family 4 subfamily B member 1 [Source:HGNC Symbol;Acc:HGNC:2644] |
| ENSG00000146378 | HGLibA_48127 | 145 | 0.176881231 | TAAR2 | trace amine associated receptor 2 (gene/pseudogene) [Source:HGNC Symbol;Acc:HGNC:4514] |
| ENSG00000148019 | HGLibA_09166 | 145 | 0.176881231 | CEP78 | centrosomal protein 78 [Source:HGNC Symbol;Acc:HGNC:25740] |
| ENSG00000151789 | HGLibA_55902 | 145 | 0.176881231 | ZNF385D | zinc finger protein 385D [Source:HGNC Symbol;Acc:HGNC:26191] |
| ENSG00000152291 | HGLibA_49273 | 145 | 0.176881231 | TGOLN2 | trans-golgi network protein 2 [Source:HGNC Symbol;Acc:HGNC:15450] |
| ENSG00000154258 | HGLibA_00127 | 145 | 0.176881231 | ABCA9 | ATP binding cassette subfamily A member 9 [Source:HGNC Symbol;Acc:HGNC:39] |
| ENSG00000154358 | HGLibA_33232 | 145 | 0.176881231 | OBSCN | cytoskeletal calmodulin and titin-interacting RhoGEF [Source:HGNC Symbol;Acc:HGNC:15719]" |
| ENSG00000155097 | HGLibA_03740 | 145 | 0.176881231 | ATP6V1C1 | ATPase H+ transporting V1 subunit C1 [Source:HGNC Symbol;Acc:HGNC:856] |
| ENSG00000156194 | HGLibA_37869 | 145 | 0.176881231 | PPEF2 | protein phosphatase with EF-hand domain 2 [Source:HGNC Symbol;Acc:HGNC:9244] |
| ENSG00000156675 | HGLibA_39792 | 145 | 0.176881231 | RAB11FIP1 | RAB11 family interacting protein 1 [Source:HGNC Symbol;Acc:HGNC:30265] |
| ENSG00000157837 | HGLibA_46783 | 145 | 0.176881231 | SPPL3 | signal peptide peptidase like 3 [Source:HGNC Symbol;Acc:HGNC:30424] |
| ENSG00000158711 | HGLibA_14919 | 145 | 0.176881231 | ELK4 | ETS transcription factor [Source:HGNC Symbol;Acc:HGNC:3326]" |
| ENSG00000159398 | HGLibA_09229 | 145 | 0.176881231 | CES5A | carboxylesterase 5A [Source:HGNC Symbol;Acc:HGNC:26459] |
| ENSG00000160299 | HGLibA_35641 | 145 | 0.176881231 | PCNT | pericentrin [Source:HGNC Symbol;Acc:HGNC:16068] |
| ENSG00000163017 | HGLibA_00615 | 145 | 0.176881231 | ACTG2 | enteric [Source:HGNC Symbol;Acc:HGNC:145]" |
| ENSG00000163754 | HGLibA_20687 | 145 | 0.176881231 | GYG1 | glycogenin 1 [Source:HGNC Symbol;Acc:HGNC:4699] |
| ENSG00000163870 | HGLibA_51178 | 145 | 0.176881231 | TPRA1 | transmembrane protein adipocyte associated 1 [Source:HGNC Symbol;Acc:HGNC:30413] |
| ENSG00000164597 | HGLibA_10481 | 145 | 0.176881231 | COG5 | component of oligomeric golgi complex 5 [Source:HGNC Symbol;Acc:HGNC:14857] |
| ENSG00000165804 | HGLibA_55602 | 145 | 0.176881231 | ZNF219 | zinc finger protein 219 [Source:HGNC Symbol;Acc:HGNC:13011] |
| ENSG00000166002 | HGLibA_45670 | 145 | 0.176881231 | SMCO4 | single-pass membrane protein with coiled-coil domains 4 [Source:HGNC Symbol;Acc:HGNC:24810] |
| ENSG00000166908 | HGLibA_36712 | 145 | 0.176881231 | PIP4K2C | phosphatidylinositol-5-phosphate 4-kinase type 2 gamma [Source:HGNC Symbol;Acc:HGNC:23786] |
| ENSG00000167676 | HGLibA_37180 | 145 | 0.176881231 | PLIN4 | perilipin 4 [Source:HGNC Symbol;Acc:HGNC:29393] |
| ENSG00000168269 | HGLibA_17877 | 145 | 0.176881231 | FOXI1 | forkhead box I1 [Source:HGNC Symbol;Acc:HGNC:3815] |
| ENSG00000168938 | HGLibA_37921 | 145 | 0.176881231 | PPIC | peptidylprolyl isomerase C [Source:HGNC Symbol;Acc:HGNC:9256] |
| ENSG00000169641 | HGLibA_27639 | 145 | 0.176881231 | LUZP1 | leucine zipper protein 1 [Source:HGNC Symbol;Acc:HGNC:14985] |
| ENSG00000169807 | HGLibA_38998 | 145 | 0.176881231 | PRY2 | PTPN13 like Y-linked 2 [Source:HGNC Symbol;Acc:HGNC:21504] |
| ENSG00000170801 | HGLibA_22414 | 145 | 0.176881231 | HTRA3 | HtrA serine peptidase 3 [Source:HGNC Symbol;Acc:HGNC:30406] |
| ENSG00000171217 | HGLibA_09879 | 145 | 0.176881231 | CLDN20 | claudin 20 [Source:HGNC Symbol;Acc:HGNC:2042] |
| ENSG00000172594 | HGLibA_45815 | 145 | 0.176881231 | SMPDL3A | sphingomyelin phosphodiesterase acid like 3A [Source:HGNC Symbol;Acc:HGNC:17389] |
| ENSG00000173226 | HGLibA_23490 | 145 | 0.176881231 | IQCB1 | IQ motif containing B1 [Source:HGNC Symbol;Acc:HGNC:28949] |
| ENSG00000175315 | HGLibA_11462 | 145 | 0.176881231 | CST6 | cystatin E/M [Source:HGNC Symbol;Acc:HGNC:2478] |
| ENSG00000175550 | HGLibA_13951 | 145 | 0.176881231 | DRAP1 | DR1 associated protein 1 [Source:HGNC Symbol;Acc:HGNC:3019] |
| ENSG00000177542 | HGLibA_44675 | 145 | 0.176881231 | SLC25A22 | solute carrier family 25 member 22 [Source:HGNC Symbol;Acc:HGNC:19954] |
| ENSG00000177606 | HGLibA_24004 | 145 | 0.176881231 | JUN | AP-1 transcription factor subunit [Source:HGNC Symbol;Acc:HGNC:6204]" |
| ENSG00000178177 | HGLibA_26195 | 145 | 0.176881231 | LCORL | ligand dependent nuclear receptor corepressor like [Source:HGNC Symbol;Acc:HGNC:30776] |
| ENSG00000178951 | HGLibA_54917 | 145 | 0.176881231 | ZBTB7A | zinc finger and BTB domain containing 7A [Source:HGNC Symbol;Acc:HGNC:18078] |
| ENSG00000179218 | HGLibA_07047 | 145 | 0.176881231 | CALR | calreticulin [Source:HGNC Symbol;Acc:HGNC:1455] |
| ENSG00000184497 | HGLibA_50332 | 145 | 0.176881231 | TMEM255B | transmembrane protein 255B [Source:HGNC Symbol;Acc:HGNC:28297] |
| ENSG00000186038 | HGLibA_22391 | 145 | 0.176881231 | HTR3E | 5-hydroxytryptamine receptor 3E [Source:HGNC Symbol;Acc:HGNC:24005] |
| ENSG00000187474 | HGLibA_17968 | 145 | 0.176881231 | FPR3 | formyl peptide receptor 3 [Source:HGNC Symbol;Acc:HGNC:3828] |
| ENSG00000188725 | HGLibA_45734 | 145 | 0.176881231 | SMIM15 | small integral membrane protein 15 [Source:HGNC Symbol;Acc:HGNC:33861] |
| ENSG00000197106 | HGLibA_45309 | 145 | 0.176881231 | SLC6A17 | solute carrier family 6 member 17 [Source:HGNC Symbol;Acc:HGNC:31399] |
| ENSG00000198171 | HGLibA_12639 | 145 | 0.176881231 | DDRGK1 | DDRGK domain containing 1 [Source:HGNC Symbol;Acc:HGNC:16110] |
| ENSG00000198535 | HGLibA_06094 | 145 | 0.176881231 | C2CD4A | C2 calcium dependent domain containing 4A [Source:HGNC Symbol;Acc:HGNC:33627] |
| ENSG00000198932 | HGLibA_20120 | 145 | 0.176881231 | GPRASP1 | G protein-coupled receptor associated sorting protein 1 [Source:HGNC Symbol;Acc:HGNC:24834] |
| ENSG00000203909 | HGLibA_13895 | 145 | 0.176881231 | DPPA5 | developmental pluripotency associated 5 [Source:HGNC Symbol;Acc:HGNC:19201] |
| ENSG00000204300 | HGLibA_50247 | 145 | 0.176881231 | TMEM225 | transmembrane protein 225 [Source:HGNC Symbol;Acc:HGNC:32390] |
| ENSG00000205038 | HGLibA_36817 | 145 | 0.176881231 | PKHD1L1 | PKHD1 like 1 [Source:HGNC Symbol;Acc:HGNC:20313] |
| ENSG00000213390 | HGLibA_02719 | 145 | 0.176881231 | ARHGAP19 | Rho GTPase activating protein 19 [Source:HGNC Symbol;Acc:HGNC:23724] |
| ENSG00000213445 | HGLibA_44189 | 145 | 0.176881231 | SIPA1 | signal-induced proliferation-associated 1 [Source:HGNC Symbol;Acc:HGNC:10885] |
| ENSG00000213722 | HGLibA_12590 | 145 | 0.176881231 | DDAH2 | dimethylarginine dimethylaminohydrolase 2 [Source:HGNC Symbol;Acc:HGNC:2716] |
| ENSG00000214491 | HGLibA_43206 | 145 | 0.176881231 | SEC14L6 | SEC14 like lipid binding 6 [Source:HGNC Symbol;Acc:HGNC:40047] |
| ENSG00000222011 | HGLibA_16334 | 145 | 0.176881231 | FAM185A | family with sequence similarity 185 member A [Source:HGNC Symbol;Acc:HGNC:22412] |
| ENSG00000223443 | HGLibA_53243 | 145 | 0.176881231 | USP17L2 | ubiquitin specific peptidase 17-like family member 2 [Source:HGNC Symbol;Acc:HGNC:34434] |
| ENSG00000233838 | HGLibA_13845 | 145 | 0.176881231 | DPH3P1 | diphthamide biosynthesis 3 pseudogene 1 [Source:HGNC Symbol;Acc:HGNC:16136] |
| ENSG00000239886 | HGLibA_25832 | 145 | 0.176881231 | KRTAP9-2 | keratin associated protein 9-2 [Source:HGNC Symbol;Acc:HGNC:16926] |
| ENSG00000243073 | HGLibA_38316 | 145 | 0.176881231 | PRAMEF4 | PRAME family member 4 [Source:HGNC Symbol;Acc:HGNC:31971] |
| ENSG00000255529 | HGLibA_37605 | 145 | 0.176881231 | POLR2M | RNA polymerase II subunit M [Source:HGNC Symbol;Acc:HGNC:14862] |
| ENSG00000280789 | HGLibA_35020 | 145 | 0.176881231 | PAGR1 | PAXIP1 associated glutamate rich protein 1 [Source:HGNC Symbol;Acc:HGNC:28707] |
| H2AFV | HGLibA_20757 | 145 | 0.176881231 | H2AFV | - |
| HNRPDL | HGLibA_21855 | 145 | 0.176881231 | HNRPDL | - |
| hsa-mir-1469 | HGLibA_57853 | 145 | 0.176881231 | hsa-mir-1469 | - |
| hsa-mir-1470 | HGLibA_57866 | 145 | 0.176881231 | hsa-mir-1470 | - |
| hsa-mir-3529 | HGLibA_59046 | 145 | 0.176881231 | hsa-mir-3529 | - |
| hsa-mir-3938 | HGLibA_59611 | 145 | 0.176881231 | hsa-mir-3938 | - |
| hsa-mir-4299 | HGLibA_59907 | 145 | 0.176881231 | hsa-mir-4299 | - |
| hsa-mir-4491 | HGLibA_60381 | 145 | 0.176881231 | hsa-mir-4491 | - |
| hsa-mir-4494 | HGLibA_60395 | 145 | 0.176881231 | hsa-mir-4494 | - |
| hsa-mir-518e | HGLibA_61673 | 145 | 0.176881231 | hsa-mir-518e | - |
| hsa-mir-541 | HGLibA_61813 | 145 | 0.176881231 | hsa-mir-541 | - |
| hsa-mir-574 | HGLibA_62390 | 145 | 0.176881231 | hsa-mir-574 | - |
| hsa-mir-604 | HGLibA_62508 | 145 | 0.176881231 | hsa-mir-604 | - |
| hsa-mir-636 | HGLibA_62785 | 145 | 0.176881231 | hsa-mir-636 | - |
| hsa-mir-6810 | HGLibA_63419 | 145 | 0.176881231 | hsa-mir-6810 | - |
| hsa-mir-7107 | HGLibA_63788 | 145 | 0.176881231 | hsa-mir-7107 | - |
| LOC81691 | HGLibA_27009 | 145 | 0.176881231 | LOC81691 | - |
| NUPR1L | HGLibA_33091 | 145 | 0.176881231 | NUPR1L | - |
| SMCR9 | HGLibA_45685 | 145 | 0.176881231 | SMCR9 | - |
| ASMTL_X | HGLibA_03325 | 144 | 0.167835888 | ASMTL_X | - |
| C4orf40 | HGLibA_06328 | 144 | 0.167835888 | C4orf40 | - |
| ENSG00000011143 | HGLibA_29358 | 144 | 0.167835888 | MKS1 | type 1 [Source:HGNC Symbol;Acc:HGNC:7121]" |
| ENSG00000013523 | HGLibA_01894 | 144 | 0.167835888 | ANGEL1 | angel homolog 1 [Source:HGNC Symbol;Acc:HGNC:19961] |
| ENSG00000037749 | HGLibA_29045 | 144 | 0.167835888 | MFAP3 | microfibril associated protein 3 [Source:HGNC Symbol;Acc:HGNC:7034] |
| ENSG00000054796 | HGLibA_46743 | 144 | 0.167835888 | SPO11 | initiator of meiotic double stranded breaks [Source:HGNC Symbol;Acc:HGNC:11250]" |
| ENSG00000065923 | HGLibA_45423 | 144 | 0.167835888 | SLC9A7 | solute carrier family 9 member A7 [Source:HGNC Symbol;Acc:HGNC:17123] |
| ENSG00000066230 | HGLibA_45406 | 144 | 0.167835888 | SLC9A3 | solute carrier family 9 member A3 [Source:HGNC Symbol;Acc:HGNC:11073] |
| ENSG00000069424 | HGLibA_24151 | 144 | 0.167835888 | KCNAB2 | potassium voltage-gated channel subfamily A regulatory beta subunit 2 [Source:HGNC Symbol;Acc:HGNC:6229] |
| ENSG00000077238 | HGLibA_23171 | 144 | 0.167835888 | IL4R | interleukin 4 receptor [Source:HGNC Symbol;Acc:HGNC:6015] |
| ENSG00000077463 | HGLibA_44230 | 144 | 0.167835888 | SIRT6 | sirtuin 6 [Source:HGNC Symbol;Acc:HGNC:14934] |
| ENSG00000077984 | HGLibA_11465 | 144 | 0.167835888 | CST7 | cystatin F [Source:HGNC Symbol;Acc:HGNC:2479] |
| ENSG00000078061 | HGLibA_02621 | 144 | 0.167835888 | ARAF | serine/threonine kinase [Source:HGNC Symbol;Acc:HGNC:646]" |
| ENSG00000079263 | HGLibA_46290 | 144 | 0.167835888 | SP140 | SP140 nuclear body protein [Source:HGNC Symbol;Acc:HGNC:17133] |
| ENSG00000081041 | HGLibA_11865 | 144 | 0.167835888 | CXCL2 | C-X-C motif chemokine ligand 2 [Source:HGNC Symbol;Acc:HGNC:4603] |
| ENSG00000081320 | HGLibA_47465 | 144 | 0.167835888 | STK17B | serine/threonine kinase 17b [Source:HGNC Symbol;Acc:HGNC:11396] |
| ENSG00000088682 | HGLibA_10768 | 144 | 0.167835888 | COQ9 | coenzyme Q9 [Source:HGNC Symbol;Acc:HGNC:25302] |
| ENSG00000089356 | HGLibA_18229 | 144 | 0.167835888 | FXYD3 | FXYD domain containing ion transport regulator 3 [Source:HGNC Symbol;Acc:HGNC:4027] |
| ENSG00000091972 | HGLibA_08282 | 144 | 0.167835888 | CD200 | CD200 molecule [Source:HGNC Symbol;Acc:HGNC:7203] |
| ENSG00000100151 | HGLibA_36525 | 144 | 0.167835888 | PICK1 | protein interacting with PRKCA 1 [Source:HGNC Symbol;Acc:HGNC:9394] |
| ENSG00000100359 | HGLibA_43865 | 144 | 0.167835888 | SGSM3 | small G protein signaling modulator 3 [Source:HGNC Symbol;Acc:HGNC:25228] |
| ENSG00000100485 | HGLibA_46203 | 144 | 0.167835888 | SOS2 | SOS Ras/Rho guanine nucleotide exchange factor 2 [Source:HGNC Symbol;Acc:HGNC:11188] |
| ENSG00000100504 | HGLibA_39687 | 144 | 0.167835888 | PYGL | glycogen phosphorylase L [Source:HGNC Symbol;Acc:HGNC:9725] |
| ENSG00000100526 | HGLibA_08849 | 144 | 0.167835888 | CDKN3 | cyclin dependent kinase inhibitor 3 [Source:HGNC Symbol;Acc:HGNC:1791] |
| ENSG00000100665 | HGLibA_43520 | 144 | 0.167835888 | SERPINA4 | serpin family A member 4 [Source:HGNC Symbol;Acc:HGNC:8948] |
| ENSG00000100697 | HGLibA_13225 | 144 | 0.167835888 | DICER1 | ribonuclease III [Source:HGNC Symbol;Acc:HGNC:17098]" |
| ENSG00000101363 | HGLibA_28088 | 144 | 0.167835888 | MANBAL | mannosidase beta like [Source:HGNC Symbol;Acc:HGNC:15799] |
| ENSG00000101945 | HGLibA_47858 | 144 | 0.167835888 | SUV39H1 | suppressor of variegation 3-9 homolog 1 [Source:HGNC Symbol;Acc:HGNC:11479] |
| ENSG00000102010 | HGLibA_04642 | 144 | 0.167835888 | BMX | BMX non-receptor tyrosine kinase [Source:HGNC Symbol;Acc:HGNC:1079] |
| ENSG00000102144 | HGLibA_36241 | 144 | 0.167835888 | PGK1 | phosphoglycerate kinase 1 [Source:HGNC Symbol;Acc:HGNC:8896] |
| ENSG00000102384 | HGLibA_09058 | 144 | 0.167835888 | CENPI | centromere protein I [Source:HGNC Symbol;Acc:HGNC:3968] |
| ENSG00000103423 | HGLibA_13517 | 144 | 0.167835888 | DNAJA3 | DnaJ heat shock protein family (Hsp40) member A3 [Source:HGNC Symbol;Acc:HGNC:11808] |
| ENSG00000105854 | HGLibA_37682 | 144 | 0.167835888 | PON2 | paraoxonase 2 [Source:HGNC Symbol;Acc:HGNC:9205] |
| ENSG00000105866 | HGLibA_46304 | 144 | 0.167835888 | SP4 | Sp4 transcription factor [Source:HGNC Symbol;Acc:HGNC:11209] |
| ENSG00000110436 | HGLibA_44509 | 144 | 0.167835888 | SLC1A2 | solute carrier family 1 member 2 [Source:HGNC Symbol;Acc:HGNC:10940] |
| ENSG00000110484 | HGLibA_42926 | 144 | 0.167835888 | SCGB2A2 | secretoglobin family 2A member 2 [Source:HGNC Symbol;Acc:HGNC:7050] |
| ENSG00000110768 | HGLibA_20544 | 144 | 0.167835888 | GTF2H1 | general transcription factor IIH subunit 1 [Source:HGNC Symbol;Acc:HGNC:4655] |
| ENSG00000112977 | HGLibA_12316 | 144 | 0.167835888 | DAP | death associated protein [Source:HGNC Symbol;Acc:HGNC:2672] |
| ENSG00000114405 | HGLibA_06209 | 144 | 0.167835888 | C3orf14 | chromosome 3 open reading frame 14 [Source:HGNC Symbol;Acc:HGNC:25024] |
| ENSG00000114650 | HGLibA_42858 | 144 | 0.167835888 | SCAP | SREBF chaperone [Source:HGNC Symbol;Acc:HGNC:30634] |
| ENSG00000117215 | HGLibA_36888 | 144 | 0.167835888 | PLA2G2D | phospholipase A2 group IID [Source:HGNC Symbol;Acc:HGNC:9033] |

| | | | | | |
|---|---|---|---|---|---|
| ENSG00000119326 | HGLibA_11646 | 144 | 0.167835888 | CTNNAL1 | catenin alpha like 1 [Source:HGNC Symbol;Acc:HGNC:2512] |
| ENSG00000120436 | HGLibA_20017 | 144 | 0.167835888 | GPR31 | G protein-coupled receptor 31 [Source:HGNC Symbol;Acc:HGNC:4486] |
| ENSG00000123989 | HGLibA_09524 | 144 | 0.167835888 | CHPF | chondroitin polymerizing factor [Source:HGNC Symbol;Acc:HGNC:24291] |
| ENSG00000124196 | HGLibA_20631 | 144 | 0.167835888 | GTSF1L | gametocyte specific factor 1 like [Source:HGNC Symbol;Acc:HGNC:16198] |
| ENSG00000125944 | HGLibA_21844 | 144 | 0.167835888 | HNRNPR | heterogeneous nuclear ribonucleoprotein R [Source:HGNC Symbol;Acc:HGNC:5047] |
| ENSG00000127561 | HGLibA_47997 | 144 | 0.167835888 | SYNGR3 | synaptogyrin 3 [Source:HGNC Symbol;Acc:HGNC:11501] |
| ENSG00000128815 | HGLibA_54013 | 144 | 0.167835888 | WDFY4 | WDFY family member 4 [Source:HGNC Symbol;Acc:HGNC:29323] |
| ENSG00000133317 | HGLibA_26349 | 144 | 0.167835888 | LGALS12 | galectin 12 [Source:HGNC Symbol;Acc:HGNC:15788] |
| ENSG00000134363 | HGLibA_18099 | 144 | 0.167835888 | FST | follistatin [Source:HGNC Symbol;Acc:HGNC:3971] |
| ENSG00000135211 | HGLibA_50465 | 144 | 0.167835888 | TMEM60 | transmembrane protein 60 [Source:HGNC Symbol;Acc:HGNC:21754] |
| ENSG00000135722 | HGLibA_17021 | 144 | 0.167835888 | FBXL8 | F-box and leucine rich repeat protein 8 [Source:HGNC Symbol;Acc:HGNC:17875] |
| ENSG00000135740 | HGLibA_45417 | 144 | 0.167835888 | SLC9A5 | solute carrier family 9 member A5 [Source:HGNC Symbol;Acc:HGNC:11078] |
| ENSG00000137252 | HGLibA_20991 | 144 | 0.167835888 | HCRTR2 | hypocretin receptor 2 [Source:HGNC Symbol;Acc:HGNC:4849] |
| ENSG00000137265 | HGLibA_23590 | 144 | 0.167835888 | IRF4 | interferon regulatory factor 4 [Source:HGNC Symbol;Acc:HGNC:6119] |
| ENSG00000137675 | HGLibA_29528 | 144 | 0.167835888 | MMP27 | matrix metallopeptidase 27 [Source:HGNC Symbol;Acc:HGNC:14250] |
| ENSG00000141526 | HGLibA_44437 | 144 | 0.167835888 | SLC16A3 | solute carrier family 16 member 3 [Source:HGNC Symbol;Acc:HGNC:10924] |
| ENSG00000141655 | HGLibA_50744 | 144 | 0.167835888 | TNFRSF11A | TNF receptor superfamily member 11a [Source:HGNC Symbol;Acc:HGNC:11908] |
| ENSG00000142065 | HGLibA_55189 | 144 | 0.167835888 | ZFP14 | ZFP14 zinc finger protein [Source:HGNC Symbol;Acc:HGNC:29312] |
| ENSG00000143376 | HGLibA_46075 | 144 | 0.167835888 | SNX27 | sorting nexin family member 27 [Source:HGNC Symbol;Acc:HGNC:20073] |
| ENSG00000143753 | HGLibA_12919 | 144 | 0.167835888 | DEGS1 | sphingolipid 1 [Source:HGNC Symbol;Acc:HGNC:13709]" |
| ENSG00000145321 | HGLibA_18755 | 144 | 0.167835888 | GC | vitamin D binding protein [Source:HGNC Symbol;Acc:HGNC:4187]" |
| ENSG00000145908 | HGLibA_55770 | 144 | 0.167835888 | ZNF300 | zinc finger protein 300 [Source:HGNC Symbol;Acc:HGNC:13091] |
| ENSG00000147130 | HGLibA_55397 | 144 | 0.167835888 | ZMYM3 | zinc finger MYM-type containing 3 [Source:HGNC Symbol;Acc:HGNC:13054] |
| ENSG00000147576 | HGLibA_00968 | 144 | 0.167835888 | ADHFE1 | iron containing 1 [Source:HGNC Symbol;Acc:HGNC:16354]" |
| ENSG00000149547 | HGLibA_14689 | 144 | 0.167835888 | EI24 | autophagy associated transmembrane protein [Source:HGNC Symbol;Acc:HGNC:13276]" |
| ENSG00000149761 | HGLibA_32974 | 144 | 0.167835888 | NUDT22 | nudix hydrolase 22 [Source:HGNC Symbol;Acc:HGNC:28189] |
| ENSG00000150051 | HGLibA_29361 | 144 | 0.167835888 | MKX | mohawk homeobox [Source:HGNC Symbol;Acc:HGNC:23729] |
| ENSG00000151702 | HGLibA_17616 | 144 | 0.167835888 | FLI1 | ETS transcription factor [Source:HGNC Symbol;Acc:HGNC:3749]" |
| ENSG00000151729 | HGLibA_44732 | 144 | 0.167835888 | SLC25A4 | solute carrier family 25 member 4 [Source:HGNC Symbol;Acc:HGNC:10990] |
| ENSG00000152591 | HGLibA_14041 | 144 | 0.167835888 | DSPP | dentin sialophosphoprotein [Source:HGNC Symbol;Acc:HGNC:3054] |
| ENSG00000152910 | HGLibA_10432 | 144 | 0.167835888 | CNTNAP4 | contactin associated protein like 4 [Source:HGNC Symbol;Acc:HGNC:18747] |
| ENSG00000157551 | HGLibA_24265 | 144 | 0.167835888 | KCNJ15 | potassium voltage-gated channel subfamily J member 15 [Source:HGNC Symbol;Acc:HGNC:6261] |
| ENSG00000157800 | HGLibA_45048 | 144 | 0.167835888 | SLC37A3 | solute carrier family 37 member 3 [Source:HGNC Symbol;Acc:HGNC:20651] |
| ENSG00000157837 | HGLibA_46784 | 144 | 0.167835888 | SPPL3 | signal peptide peptidase like 3 [Source:HGNC Symbol;Acc:HGNC:30424] |
| ENSG00000158717 | HGLibA_41559 | 144 | 0.167835888 | RNF166 | ring finger protein 166 [Source:HGNC Symbol;Acc:HGNC:28856] |
| ENSG00000160131 | HGLibA_53687 | 144 | 0.167835888 | VMA21 | vacuolar ATPase assembly factor [Source:HGNC Symbol;Acc:HGNC:22082]" |
| ENSG00000163519 | HGLibA_51343 | 144 | 0.167835888 | TRAT1 | T cell receptor associated transmembrane adaptor 1 [Source:HGNC Symbol;Acc:HGNC:30698] |
| ENSG00000164056 | HGLibA_46836 | 144 | 0.167835888 | SPRY1 | sprouty RTK signaling antagonist 1 [Source:HGNC Symbol;Acc:HGNC:11269] |
| ENSG00000164161 | HGLibA_21261 | 144 | 0.167835888 | HHIP | hedgehog interacting protein [Source:HGNC Symbol;Acc:HGNC:14866] |
| ENSG00000164362 | HGLibA_49019 | 144 | 0.167835888 | TERT | telomerase reverse transcriptase [Source:HGNC Symbol;Acc:HGNC:11730] |
| ENSG00000164588 | HGLibA_20973 | 144 | 0.167835888 | HCN1 | hyperpolarization activated cyclic nucleotide gated potassium channel 1 [Source:HGNC Symbol;Acc:HGNC:4845] |
| ENSG00000164694 | HGLibA_17743 | 144 | 0.167835888 | FNDC1 | fibronectin type III domain containing 1 [Source:HGNC Symbol;Acc:HGNC:21184] |
| ENSG00000165182 | HGLibA_11950 | 144 | 0.167835888 | CXorf58 | chromosome X open reading frame 58 [Source:HGNC Symbol;Acc:HGNC:26356] |
| ENSG00000165462 | HGLibA_36451 | 144 | 0.167835888 | PHOX2A | paired like homeobox 2A [Source:HGNC Symbol;Acc:HGNC:691] |
| ENSG00000167136 | HGLibA_15115 | 144 | 0.167835888 | ENDOG | endonuclease G [Source:HGNC Symbol;Acc:HGNC:3346] |
| ENSG00000167759 | HGLibA_25242 | 144 | 0.167835888 | KLK13 | kallikrein related peptidase 13 [Source:HGNC Symbol;Acc:HGNC:6361] |
| ENSG00000168538 | HGLibA_51304 | 144 | 0.167835888 | TRAPPC11 | trafficking protein particle complex 11 [Source:HGNC Symbol;Acc:HGNC:25751] |
| ENSG00000169393 | HGLibA_14992 | 144 | 0.167835888 | ELSPBP1 | epididymal sperm binding protein 1 [Source:HGNC Symbol;Acc:HGNC:14417] |
| ENSG00000170166 | HGLibA_22002 | 144 | 0.167835888 | HOXD4 | homeobox D4 [Source:HGNC Symbol;Acc:HGNC:5138] |
| ENSG00000170890 | HGLibA_36881 | 144 | 0.167835888 | PLA2G1B | phospholipase A2 group IB [Source:HGNC Symbol;Acc:HGNC:9030] |
| ENSG00000170959 | HGLibA_12480 | 144 | 0.167835888 | DCDC1 | doublecortin domain containing 1 [Source:HGNC Symbol;Acc:HGNC:20625] |
| ENSG00000170965 | HGLibA_36933 | 144 | 0.167835888 | PLAC1 | placenta specific 1 [Source:HGNC Symbol;Acc:HGNC:9044] |
| ENSG00000171204 | HGLibA_49914 | 144 | 0.167835888 | TMEM126B | transmembrane protein 126B [Source:HGNC Symbol;Acc:HGNC:30883] |
| ENSG00000171551 | HGLibA_14389 | 144 | 0.167835888 | ECEL1 | endothelin converting enzyme like 1 [Source:HGNC Symbol;Acc:HGNC:3147] |
| ENSG00000172238 | HGLibA_03533 | 144 | 0.167835888 | ATOH1 | atonal bHLH transcription factor 1 [Source:HGNC Symbol;Acc:HGNC:797] |
| ENSG00000175414 | HGLibA_02924 | 144 | 0.167835888 | ARL10 | ADP ribosylation factor like GTPase 10 [Source:HGNC Symbol;Acc:HGNC:22042] |
| ENSG00000175745 | HGLibA_32609 | 144 | 0.167835888 | NR2F1 | nuclear receptor subfamily 2 group F member 1 [Source:HGNC Symbol;Acc:HGNC:7975] |
| ENSG00000177201 | HGLibA_33860 | 144 | 0.167835888 | OR2T12 | olfactory receptor family 2 subfamily T member 12 [Source:HGNC Symbol;Acc:HGNC:19592] |
| ENSG00000178187 | HGLibA_56032 | 144 | 0.167835888 | ZNF454 | zinc finger protein 454 [Source:HGNC Symbol;Acc:HGNC:21200] |
| ENSG00000178776 | HGLibA_06389 | 144 | 0.167835888 | C5orf46 | chromosome 5 open reading frame 46 [Source:HGNC Symbol;Acc:HGNC:33768] |
| ENSG00000180440 | HGLibA_43616 | 144 | 0.167835888 | SERTM1 | serine rich and transmembrane domain containing 1 [Source:HGNC Symbol;Acc:HGNC:33792] |
| ENSG00000180479 | HGLibA_56277 | 144 | 0.167835888 | ZNF571 | zinc finger protein 571 [Source:HGNC Symbol;Acc:HGNC:25000] |
| ENSG00000180891 | HGLibA_11768 | 144 | 0.167835888 | CUEDC1 | CUE domain containing 1 [Source:HGNC Symbol;Acc:HGNC:31350] |
| ENSG00000181773 | HGLibA_20013 | 144 | 0.167835888 | GPR3 | G protein-coupled receptor 3 [Source:HGNC Symbol;Acc:HGNC:4484] |
| ENSG00000182224 | HGLibA_11996 | 144 | 0.167835888 | CYB5D1 | cytochrome b5 domain containing 1 [Source:HGNC Symbol;Acc:HGNC:26516] |
| ENSG00000182271 | HGLibA_50584 | 144 | 0.167835888 | TMIGD1 | transmembrane and immunoglobulin domain containing 1 [Source:HGNC Symbol;Acc:HGNC:32431] |
| ENSG00000183389 | HGLibA_34218 | 144 | 0.167835888 | OR56A4 | olfactory receptor family 56 subfamily A member 4 [Source:HGNC Symbol;Acc:HGNC:14791] |
| ENSG00000184371 | HGLibA_11320 | 144 | 0.167835888 | CSF1 | colony stimulating factor 1 [Source:HGNC Symbol;Acc:HGNC:2432] |
| ENSG00000185567 | HGLibA_01304 | 144 | 0.167835888 | AHNAK2 | AHNAK nucleoprotein 2 [Source:HGNC Symbol;Acc:HGNC:20125] |
| ENSG00000185885 | HGLibA_22637 | 144 | 0.167835888 | IFITM1 | interferon induced transmembrane protein 1 [Source:HGNC Symbol;Acc:HGNC:5412] |
| ENSG00000185888 | HGLibA_38939 | 144 | 0.167835888 | PRSS38 | serine protease 38 [Source:HGNC Symbol;Acc:HGNC:29625] |
| ENSG00000186231 | HGLibA_25186 | 144 | 0.167835888 | KLHL32 | kelch like family member 32 [Source:HGNC Symbol;Acc:HGNC:21221] |
| ENSG00000188321 | HGLibA_56237 | 144 | 0.167835888 | ZNF559 | zinc finger protein 559 [Source:HGNC Symbol;Acc:HGNC:28197] |
| ENSG00000196449 | HGLibA_54721 | 144 | 0.167835888 | YRDC | yrdC N6-threonylcarbamoyltransferase domain containing [Source:HGNC Symbol;Acc:HGNC:28905] |
| ENSG00000197275 | HGLibA_40098 | 144 | 0.167835888 | RAD54B | RAD54 homolog B [Source:HGNC Symbol;Acc:HGNC:17228] |
| ENSG00000198791 | HGLibA_10351 | 144 | 0.167835888 | CNOT7 | CCR4-NOT transcription complex subunit 7 [Source:HGNC Symbol;Acc:HGNC:14101] |
| ENSG00000203772 | HGLibA_46800 | 144 | 0.167835888 | SPRN | shadow of prion protein [Source:HGNC Symbol;Acc:HGNC:16871] |
| ENSG00000203797 | HGLibA_12626 | 144 | 0.167835888 | DDO | D-aspartate oxidase [Source:HGNC Symbol;Acc:HGNC:2727] |
| ENSG00000204538 | HGLibA_39219 | 144 | 0.167835888 | PSORS1C2 | psoriasis susceptibility 1 candidate 2 [Source:HGNC Symbol;Acc:HGNC:17199] |
| ENSG00000205116 | HGLibA_50541 | 144 | 0.167835888 | TMEM88B | transmembrane protein 88B [Source:HGNC Symbol;Acc:HGNC:37099] |
| ENSG00000205328 | HGLibA_34397 | 144 | 0.167835888 | OR6C65 | olfactory receptor family 6 subfamily C member 65 [Source:HGNC Symbol;Acc:HGNC:31295] |
| ENSG00000205439 | HGLibA_25635 | 144 | 0.167835888 | KRTAP12-3 | keratin associated protein 12-3 [Source:HGNC Symbol;Acc:HGNC:20531] |
| ENSG00000213699 | HGLibA_45013 | 144 | 0.167835888 | SLC35F6 | solute carrier family 35 member F6 [Source:HGNC Symbol;Acc:HGNC:26055] |
| ENSG00000214046 | HGLibA_45767 | 144 | 0.167835888 | SMIM7 | small integral membrane protein 7 [Source:HGNC Symbol;Acc:HGNC:28419] |
| ENSG00000216937 | HGLibA_07846 | 144 | 0.167835888 | CCDC7 | coiled-coil domain containing 7 [Source:HGNC Symbol;Acc:HGNC:26533] |
| ENSG00000229474 | HGLibA_35287 | 144 | 0.167835888 | PATL2 | PAT1 homolog 2 [Source:HGNC Symbol;Acc:HGNC:33630] |
| ENSG00000230062 | HGLibA_02140 | 144 | 0.167835888 | ANKRD66 | ankyrin repeat domain 66 [Source:HGNC Symbol;Acc:HGNC:44669] |
| ENSG00000236398 | HGLibA_48396 | 144 | 0.167835888 | TAS2R39 | taste 2 receptor member 39 [Source:HGNC Symbol;Acc:HGNC:18886] |
| ENSG00000237649 | HGLibA_24971 | 144 | 0.167835888 | KIFC1 | kinesin family member C1 [Source:HGNC Symbol;Acc:HGNC:6389] |
| ENSG00000239839 | HGLibA_12797 | 144 | 0.167835888 | DEFA3 | defensin alpha 3 [Source:HGNC Symbol;Acc:HGNC:2762] |
| ENSG00000241878 | HGLibA_36736 | 144 | 0.167835888 | PISD | phosphatidylserine decarboxylase [Source:HGNC Symbol;Acc:HGNC:8999] |
| ENSG00000254997 | HGLibA_25813 | 144 | 0.167835888 | KRTAP5-9 | keratin associated protein 5-9 [Source:HGNC Symbol;Acc:HGNC:23604] |
| hsa-mir-124-1 | HGLibA_57345 | 144 | 0.167835888 | hsa-mir-124-1 | - |
| hsa-mir-1294 | HGLibA_57652 | 144 | 0.167835888 | hsa-mir-1294 | - |
| hsa-mir-147a | HGLibA_57874 | 144 | 0.167835888 | hsa-mir-147a | - |
| hsa-mir-296 | HGLibA_58396 | 144 | 0.167835888 | hsa-mir-296 | - |
| hsa-mir-3156-3 | HGLibA_58696 | 144 | 0.167835888 | hsa-mir-3156-3 | - |
| hsa-mir-3177 | HGLibA_58780 | 144 | 0.167835888 | hsa-mir-3177 | - |
| hsa-mir-3688-2 | HGLibA_59309 | 144 | 0.167835888 | hsa-mir-3688-2 | - |
| hsa-mir-3691 | HGLibA_59355 | 144 | 0.167835888 | hsa-mir-3691 | - |
| hsa-mir-4274 | HGLibA_59800 | 144 | 0.167835888 | hsa-mir-4274 | - |
| hsa-mir-4286 | HGLibA_59853 | 144 | 0.167835888 | hsa-mir-4286 | - |
| hsa-mir-4297 | HGLibA_59899 | 144 | 0.167835888 | hsa-mir-4297 | - |
| hsa-mir-4299 | HGLibA_59909 | 144 | 0.167835888 | hsa-mir-4299 | - |
| hsa-mir-4643 | HGLibA_60675 | 144 | 0.167835888 | hsa-mir-4643 | - |
| hsa-mir-4704 | HGLibA_60917 | 144 | 0.167835888 | hsa-mir-4704 | - |
| hsa-mir-502 | HGLibA_61464 | 144 | 0.167835888 | hsa-mir-502 | - |
| hsa-mir-548o-2 | HGLibA_62054 | 144 | 0.167835888 | hsa-mir-548o-2 | - |
| hsa-mir-5682 | HGLibA_62248 | 144 | 0.167835888 | hsa-mir-5682 | - |
| hsa-mir-637 | HGLibA_62789 | 144 | 0.167835888 | hsa-mir-637 | - |
| hsa-mir-656 | HGLibA_62957 | 144 | 0.167835888 | hsa-mir-656 | - |
| hsa-mir-6813 | HGLibA_63429 | 144 | 0.167835888 | hsa-mir-6813 | - |
| KIAA1024L | HGLibA_24681 | 144 | 0.167835888 | KIAA1024L | - |
| PCNX | HGLibA_35642 | 144 | 0.167835888 | PCNX | - |
| PTCHD2 | HGLibA_39276 | 144 | 0.167835888 | PTCHD2 | - |
| PTPLA | HGLibA_39415 | 144 | 0.167835888 | PTPLA | - |
| TCTEX1D4 | HGLibA_48870 | 144 | 0.167835888 | TCTEX1D4 | - |
| BAGE | HGLibA_04092 | 143 | 0.158790546 | BAGE | - |
| C12orf66 | HGLibA_05283 | 143 | 0.158790546 | C12orf66 | - |
| ENSG00000002834 | HGLibA_26054 | 143 | 0.158790546 | LASP1 | LIM and SH3 protein 1 [Source:HGNC Symbol;Acc:HGNC:6513] |
| ENSG00000049246 | HGLibA_36059 | 143 | 0.158790546 | PER3 | period circadian regulator 3 [Source:HGNC Symbol;Acc:HGNC:8847] |
| ENSG00000062096 | HGLibA_03140 | 143 | 0.158790546 | ARSF | arylsulfatase F [Source:HGNC Symbol;Acc:HGNC:721] |
| ENSG00000064300 | HGLibA_31874 | 143 | 0.158790546 | NGFR | nerve growth factor receptor [Source:HGNC Symbol;Acc:HGNC:7809] |
| ENSG00000065135 | HGLibA_19458 | 143 | 0.158790546 | GNAI3 | G protein subunit alpha i3 [Source:HGNC Symbol;Acc:HGNC:4387] |
| ENSG00000067606 | HGLibA_38563 | 143 | 0.158790546 | PRKCZ | protein kinase C zeta [Source:HGNC Symbol;Acc:HGNC:9412] |
| ENSG00000070814 | HGLibA_48826 | 143 | 0.158790546 | TCOF1 | treacle ribosome biogenesis factor 1 [Source:HGNC Symbol;Acc:HGNC:11654] |
| ENSG00000072756 | HGLibA_51713 | 143 | 0.158790546 | TRNT1 | tRNA nucleotidyl transferase 1 [Source:HGNC Symbol;Acc:HGNC:17341] |
| ENSG00000077238 | HGLibA_23172 | 143 | 0.158790546 | IL4R | interleukin 4 receptor [Source:HGNC Symbol;Acc:HGNC:6015] |
| ENSG00000084234 | HGLibA_02444 | 143 | 0.158790546 | APLP2 | amyloid beta precursor like protein 2 [Source:HGNC Symbol;Acc:HGNC:598] |
| ENSG00000089169 | HGLibA_41845 | 143 | 0.158790546 | RPH3A | rabphilin 3A [Source:HGNC Symbol;Acc:HGNC:17056] |
| ENSG00000092439 | HGLibA_51769 | 143 | 0.158790546 | TRPM7 | transient receptor potential cation channel subfamily M member 7 [Source:HGNC Symbol;Acc:HGNC:17994] |
| ENSG00000092607 | HGLibA_48648 | 143 | 0.158790546 | TBX15 | T-box 15 [Source:HGNC Symbol;Acc:HGNC:11594] |
| ENSG00000100084 | HGLibA_21351 | 143 | 0.158790546 | HIRA | histone cell cycle regulator [Source:HGNC Symbol;Acc:HGNC:4916] |
| ENSG00000100324 | HGLibA_48141 | 143 | 0.158790546 | TAB1 | TGF-beta activated kinase 1 (MAP3K7) binding protein 1 [Source:HGNC Symbol;Acc:HGNC:18157] |
| ENSG00000100603 | HGLibA_46021 | 143 | 0.158790546 | SNW1 | SNW domain containing 1 [Source:HGNC Symbol;Acc:HGNC:16696] |

| | | | | | |
|---|---|---|---|---|---|
| ENSG00000101825 | HGLibA_30621 | 143 | 0.158790546 | MXRA5 | matrix remodeling associated 5 [Source:HGNC Symbol;Acc:HGNC:7539] |
| ENSG00000102178 | HGLibA_52705 | 143 | 0.158790546 | UBL4A | ubiquitin like 4A [Source:HGNC Symbol;Acc:HGNC:12505] |
| ENSG00000103485 | HGLibA_39734 | 143 | 0.158790546 | QPRT | quinolinate phosphoribosyltransferase [Source:HGNC Symbol;Acc:HGNC:9755] |
| ENSG00000105131 | HGLibA_15328 | 143 | 0.158790546 | EPHX3 | epoxide hydrolase 3 [Source:HGNC Symbol;Acc:HGNC:23760] |
| ENSG00000106261 | HGLibA_55345 | 143 | 0.158790546 | ZKSCAN1 | zinc finger with KRAB and SCAN domains 1 [Source:HGNC Symbol;Acc:HGNC:13101] |
| ENSG00000111704 | HGLibA_31091 | 143 | 0.158790546 | NANOG | Nanog homeobox [Source:HGNC Symbol;Acc:HGNC:20857] |
| ENSG00000113269 | HGLibA_41504 | 143 | 0.158790546 | RNF130 | ring finger protein 130 [Source:HGNC Symbol;Acc:HGNC:18280] |
| ENSG00000113758 | HGLibA_12388 | 143 | 0.158790546 | DBN1 | drebrin 1 [Source:HGNC Symbol;Acc:HGNC:2695] |
| ENSG00000113758 | HGLibA_12387 | 143 | 0.158790546 | DBN1 | drebrin 1 [Source:HGNC Symbol;Acc:HGNC:2695] |
| ENSG00000114349 | HGLibA_19472 | 143 | 0.158790546 | GNAT1 | G protein subunit alpha transducin 1 [Source:HGNC Symbol;Acc:HGNC:4393] |
| ENSG00000116711 | HGLibA_36900 | 143 | 0.158790546 | PLA2G4A | phospholipase A2 group IVA [Source:HGNC Symbol;Acc:HGNC:9035] |
| ENSG00000118997 | HGLibA_13495 | 143 | 0.158790546 | DNAH7 | dynein axonemal heavy chain 7 [Source:HGNC Symbol;Acc:HGNC:18661] |
| ENSG00000119401 | HGLibA_51461 | 143 | 0.158790546 | TRIM32 | tripartite motif containing 32 [Source:HGNC Symbol;Acc:HGNC:16380] |
| ENSG00000119685 | HGLibA_52207 | 143 | 0.158790546 | TTLL5 | tubulin tyrosine ligase like 5 [Source:HGNC Symbol;Acc:HGNC:19963] |
| ENSG00000122585 | HGLibA_32546 | 143 | 0.158790546 | NPY | neuropeptide Y [Source:HGNC Symbol;Acc:HGNC:7955] |
| ENSG00000125788 | HGLibA_12883 | 143 | 0.158790546 | DEFB126 | defensin beta 126 [Source:HGNC Symbol;Acc:HGNC:15900] |
| ENSG00000126218 | HGLibA_15841 | 143 | 0.158790546 | F10 | coagulation factor X [Source:HGNC Symbol;Acc:HGNC:3528] |
| ENSG00000127152 | HGLibA_04293 | 143 | 0.158790546 | BCL11B | B cell CLL/lymphoma 11B [Source:HGNC Symbol;Acc:HGNC:13222] |
| ENSG00000127831 | HGLibA_53651 | 143 | 0.158790546 | VIL1 | villin 1 [Source:HGNC Symbol;Acc:HGNC:12690] |
| ENSG00000128011 | HGLibA_27153 | 143 | 0.158790546 | LRFN1 | leucine rich repeat and fibronectin type III domain containing 1 [Source:HGNC Symbol;Acc:HGNC:29290] |
| ENSG00000128881 | HGLibA_52042 | 143 | 0.158790546 | TTBK2 | tau tubulin kinase 2 [Source:HGNC Symbol;Acc:HGNC:19141] |
| ENSG00000129465 | HGLibA_41321 | 143 | 0.158790546 | RIPK3 | receptor interacting serine/threonine kinase 3 [Source:HGNC Symbol;Acc:HGNC:10021] |
| ENSG00000131389 | HGLibA_45333 | 143 | 0.158790546 | SLC6A6 | solute carrier family 6 member 6 [Source:HGNC Symbol;Acc:HGNC:11052] |
| ENSG00000131409 | HGLibA_27381 | 143 | 0.158790546 | LRRC4B | leucine rich repeat containing 4B [Source:HGNC Symbol;Acc:HGNC:25042] |
| ENSG00000131759 | HGLibA_40271 | 143 | 0.158790546 | RARA | retinoic acid receptor alpha [Source:HGNC Symbol;Acc:HGNC:9864] |
| ENSG00000133812 | HGLibA_42797 | 143 | 0.158790546 | SBF2 | SET binding factor 2 [Source:HGNC Symbol;Acc:HGNC:2135] |
| ENSG00000134760 | HGLibA_14022 | 143 | 0.158790546 | DSG1 | desmoglein 1 [Source:HGNC Symbol;Acc:HGNC:3048] |
| ENSG00000137857 | HGLibA_14096 | 143 | 0.158790546 | DUOX1 | dual oxidase 1 [Source:HGNC Symbol;Acc:HGNC:3062] |
| ENSG00000139726 | HGLibA_12971 | 143 | 0.158790546 | DENR | density regulated re-initiation and release factor [Source:HGNC Symbol;Acc:HGNC:2769] |
| ENSG00000140093 | HGLibA_43508 | 143 | 0.158790546 | SERPINA10 | serpin family A member 10 [Source:HGNC Symbol;Acc:HGNC:15996] |
| ENSG00000140459 | HGLibA_12052 | 143 | 0.158790546 | CYP11A1 | cytochrome P450 family 11 subfamily A member 1 [Source:HGNC Symbol;Acc:HGNC:2590] |
| ENSG00000141096 | HGLibA_13823 | 143 | 0.158790546 | DPEP3 | dipeptidase 3 [Source:HGNC Symbol;Acc:HGNC:23029] |
| ENSG00000141540 | HGLibA_52233 | 143 | 0.158790546 | TTYH2 | tweety family member 2 [Source:HGNC Symbol;Acc:HGNC:13877] |
| ENSG00000143416 | HGLibA_43290 | 143 | 0.158790546 | SELENBP1 | selenium binding protein 1 [Source:HGNC Symbol;Acc:HGNC:10719] |
| ENSG00000143595 | HGLibA_02580 | 143 | 0.158790546 | AQP10 | aquaporin 10 [Source:HGNC Symbol;Acc:HGNC:16029] |
| ENSG00000143921 | HGLibA_00228 | 143 | 0.158790546 | ABCG8 | ATP binding cassette subfamily G member 8 [Source:HGNC Symbol;Acc:HGNC:13887] |
| ENSG00000144815 | HGLibA_33155 | 143 | 0.158790546 | NXPE3 | neurexophilin and PC-esterase domain family member 3 [Source:HGNC Symbol;Acc:HGNC:28238] |
| ENSG00000145216 | HGLibA_17533 | 143 | 0.158790546 | FIP1L1 | factor interacting with PAPOLA and CPSF1 [Source:HGNC Symbol;Acc:HGNC:19124] |
| ENSG00000146414 | HGLibA_44081 | 143 | 0.158790546 | SHPRH | SNF2 histone linker PHD RING helicase [Source:HGNC Symbol;Acc:HGNC:19336] |
| ENSG00000147383 | HGLibA_32747 | 143 | 0.158790546 | NSDHL | NAD(P) dependent steroid dehydrogenase-like [Source:HGNC Symbol;Acc:HGNC:13398] |
| ENSG00000148399 | HGLibA_13854 | 143 | 0.158790546 | DPH7 | diphthamide biosynthesis 7 [Source:HGNC Symbol;Acc:HGNC:25199] |
| ENSG00000149016 | HGLibA_52353 | 143 | 0.158790546 | TUT1 | U6 snRNA-specific [Source:HGNC Symbol;Acc:HGNC:26184]" |
| ENSG00000152254 | HGLibA_18309 | 143 | 0.158790546 | G6PC2 | glucose-6-phosphatase catalytic subunit 2 [Source:HGNC Symbol;Acc:HGNC:28906] |
| ENSG00000152689 | HGLibA_40349 | 143 | 0.158790546 | RASGRP3 | RAS guanyl releasing protein 3 [Source:HGNC Symbol;Acc:HGNC:14545] |
| ENSG00000153790 | HGLibA_06534 | 143 | 0.158790546 | C7orf31 | chromosome 7 open reading frame 31 [Source:HGNC Symbol;Acc:HGNC:21722] |
| ENSG00000154655 | HGLibA_25906 | 143 | 0.158790546 | L3MBTL4 | histone methyl-lysine binding protein [Source:HGNC Symbol;Acc:HGNC:26677]" |
| ENSG00000157093 | HGLibA_27792 | 143 | 0.158790546 | LYZL4 | lysozyme like 4 [Source:HGNC Symbol;Acc:HGNC:28387] |
| ENSG00000157216 | HGLibA_47121 | 143 | 0.158790546 | SSBP3 | single stranded DNA binding protein 3 [Source:HGNC Symbol;Acc:HGNC:15674] |
| ENSG00000157823 | HGLibA_02338 | 143 | 0.158790546 | AP3S2 | adaptor related protein complex 3 subunit sigma 2 [Source:HGNC Symbol;Acc:HGNC:571] |
| ENSG00000158258 | HGLibA_10138 | 143 | 0.158790546 | CLSTN2 | calsyntenin 2 [Source:HGNC Symbol;Acc:HGNC:17448] |
| ENSG00000160339 | HGLibA_17218 | 143 | 0.158790546 | FCN2 | ficolin 2 [Source:HGNC Symbol;Acc:HGNC:3624] |
| ENSG00000160716 | HGLibA_09591 | 143 | 0.158790546 | CHRNB2 | cholinergic receptor nicotinic beta 2 subunit [Source:HGNC Symbol;Acc:HGNC:1962] |
| ENSG00000161021 | HGLibA_28044 | 143 | 0.158790546 | MAML1 | mastermind like transcriptional coactivator 1 [Source:HGNC Symbol;Acc:HGNC:13632] |
| ENSG00000162599 | HGLibA_31800 | 143 | 0.158790546 | NFIA | nuclear factor I A [Source:HGNC Symbol;Acc:HGNC:7784] |
| ENSG00000162747 | HGLibA_17197 | 143 | 0.158790546 | FCGR3B | Fc fragment of IgG receptor IIIb [Source:HGNC Symbol;Acc:HGNC:3620] |
| ENSG00000166508 | HGLibA_28623 | 143 | 0.158790546 | MCM7 | minichromosome maintenance complex component 7 [Source:HGNC Symbol;Acc:HGNC:6950] |
| ENSG00000167580 | HGLibA_02590 | 143 | 0.158790546 | AQP2 | aquaporin 2 [Source:HGNC Symbol;Acc:HGNC:634] |
| ENSG00000168152 | HGLibA_49316 | 143 | 0.158790546 | THAP9 | THAP domain containing 9 [Source:HGNC Symbol;Acc:HGNC:23192] |
| ENSG00000168306 | HGLibA_00500 | 143 | 0.158790546 | ACOX2 | acyl-CoA oxidase 2 [Source:HGNC Symbol;Acc:HGNC:120] |
| ENSG00000169021 | HGLibA_53119 | 143 | 0.158790546 | UQCRFS1 | Rieske iron-sulfur polypeptide 1 [Source:HGNC Symbol;Acc:HGNC:12587]" |
| ENSG00000169208 | HGLibA_33492 | 143 | 0.158790546 | OR10G3 | olfactory receptor family 10 subfamily G member 3 [Source:HGNC Symbol;Acc:HGNC:8171] |
| ENSG00000169410 | HGLibA_39481 | 143 | 0.158790546 | PTPN9 | non-receptor type 9 [Source:HGNC Symbol;Acc:HGNC:9661]" |
| ENSG00000169783 | HGLibA_26586 | 143 | 0.158790546 | LINGO1 | leucine rich repeat and Ig domain containing 1 [Source:HGNC Symbol;Acc:HGNC:21205] |
| ENSG00000170484 | HGLibA_25533 | 143 | 0.158790546 | KRT74 | keratin 74 [Source:HGNC Symbol;Acc:HGNC:28929] |
| ENSG00000171459 | HGLibA_33695 | 143 | 0.158790546 | OR1L6 | olfactory receptor family 1 subfamily L member 6 [Source:HGNC Symbol;Acc:HGNC:8218] |
| ENSG00000171649 | HGLibA_55335 | 143 | 0.158790546 | ZIK1 | zinc finger protein interacting with K protein 1 [Source:HGNC Symbol;Acc:HGNC:33104] |
| ENSG00000171777 | HGLibA_40353 | 143 | 0.158790546 | RASGRP4 | RAS guanyl releasing protein 4 [Source:HGNC Symbol;Acc:HGNC:18958] |
| ENSG00000172243 | HGLibA_10007 | 143 | 0.158790546 | CLEC7A | C-type lectin domain containing 7A [Source:HGNC Symbol;Acc:HGNC:14558] |
| ENSG00000172889 | HGLibA_14628 | 143 | 0.158790546 | EGFL7 | EGF like domain multiple 7 [Source:HGNC Symbol;Acc:HGNC:20594] |
| ENSG00000173137 | HGLibA_00902 | 143 | 0.158790546 | ADCK5 | aarF domain containing kinase 5 [Source:HGNC Symbol;Acc:HGNC:21738] |
| ENSG00000173611 | HGLibA_42832 | 143 | 0.158790546 | SCAI | suppressor of cancer cell invasion [Source:HGNC Symbol;Acc:HGNC:26709] |
| ENSG00000173638 | HGLibA_44499 | 143 | 0.158790546 | SLC19A1 | solute carrier family 19 member 1 [Source:HGNC Symbol;Acc:HGNC:10937] |
| ENSG00000173917 | HGLibA_21931 | 143 | 0.158790546 | HOXB2 | homeobox B2 [Source:HGNC Symbol;Acc:HGNC:5113] |
| ENSG00000175514 | HGLibA_19936 | 143 | 0.158790546 | GPR152 | G protein-coupled receptor 152 [Source:HGNC Symbol;Acc:HGNC:23622] |
| ENSG00000176595 | HGLibA_24092 | 143 | 0.158790546 | KBTBD11 | kelch repeat and BTB domain containing 11 [Source:HGNC Symbol;Acc:HGNC:29104] |
| ENSG00000181072 | HGLibA_09549 | 143 | 0.158790546 | CHRM2 | cholinergic receptor muscarinic 2 [Source:HGNC Symbol;Acc:HGNC:1951] |
| ENSG00000182816 | HGLibA_25645 | 143 | 0.158790546 | KRTAP13-2 | keratin associated protein 13-2 [Source:HGNC Symbol;Acc:HGNC:18923] |
| ENSG00000184108 | HGLibA_51620 | 143 | 0.158790546 | TRIML1 | tripartite motif family like 1 [Source:HGNC Symbol;Acc:HGNC:26698] |
| ENSG00000185561 | HGLibA_49598 | 143 | 0.158790546 | TLCD2 | TLC domain containing 2 [Source:HGNC Symbol;Acc:HGNC:33522] |
| ENSG00000186442 | HGLibA_25470 | 143 | 0.158790546 | KRT3 | keratin 3 [Source:HGNC Symbol;Acc:HGNC:6440] |
| ENSG00000196189 | HGLibA_43340 | 143 | 0.158790546 | SEMA4A | semaphorin 4A [Source:HGNC Symbol;Acc:HGNC:10729] |
| ENSG00000196576 | HGLibA_37274 | 143 | 0.158790546 | PLXNB2 | plexin B2 [Source:HGNC Symbol;Acc:HGNC:9104] |
| ENSG00000196843 | HGLibA_02907 | 143 | 0.158790546 | ARID5A | AT-rich interaction domain 5A [Source:HGNC Symbol;Acc:HGNC:17361] |
| ENSG00000197915 | HGLibA_22109 | 143 | 0.158790546 | HRNR | hornerin [Source:HGNC Symbol;Acc:HGNC:20846] |
| ENSG00000197921 | HGLibA_21188 | 143 | 0.158790546 | HES5 | hes family bHLH transcription factor 5 [Source:HGNC Symbol;Acc:HGNC:19764] |
| ENSG00000198908 | HGLibA_04473 | 143 | 0.158790546 | BHLHB9 | basic helix-loop-helix family member b9 [Source:HGNC Symbol;Acc:HGNC:29353] |
| ENSG00000198963 | HGLibA_41779 | 143 | 0.158790546 | RORB | RAR related orphan receptor B [Source:HGNC Symbol;Acc:HGNC:10259] |
| ENSG00000204482 | HGLibA_27571 | 143 | 0.158790546 | LST1 | leukocyte specific transcript 1 [Source:HGNC Symbol;Acc:HGNC:14189] |
| ENSG00000204501 | HGLibA_38292 | 143 | 0.158790546 | PRAMEF15 | PRAME family member 15 [Source:HGNC Symbol;Acc:HGNC:26764] |
| ENSG00000204640 | HGLibA_32201 | 143 | 0.158790546 | NMS | neuromedin S [Source:HGNC Symbol;Acc:HGNC:32203] |
| ENSG00000205328 | HGLibA_34396 | 143 | 0.158790546 | OR6C65 | olfactory receptor family 6 subfamily C member 65 [Source:HGNC Symbol;Acc:HGNC:31295] |
| ENSG00000205456 | HGLibA_51088 | 143 | 0.158790546 | TP53TG3D | TP53 target 3D [Source:HGNC Symbol;Acc:HGNC:44657] |
| ENSG00000215568 | HGLibA_18329 | 143 | 0.158790546 | GAB4 | GRB2 associated binding protein family member 4 [Source:HGNC Symbol;Acc:HGNC:18325] |
| ENSG00000225614 | HGLibA_56050 | 143 | 0.158790546 | ZNF469 | zinc finger protein 469 [Source:HGNC Symbol;Acc:HGNC:23216] |
| ENSG00000230301 | HGLibA_34302 | 143 | 0.158790546 | OR5H6 | olfactory receptor family 5 subfamily H member 6 (gene/pseudogene) [Source:HGNC Symbol;Acc:HGNC:14767] |
| ENSG00000234745 | HGLibA_21598 | 143 | 0.158790546 | HLA-B | B [Source:HGNC Symbol;Acc:HGNC:4932]" |
| ENSG00000243244 | HGLibA_47544 | 143 | 0.158790546 | STON1 | stonin 1 [Source:HGNC Symbol;Acc:HGNC:17003] |
| ENSG00000249751 | HGLibA_14418 | 143 | 0.158790546 | ECSCR | endothelial cell surface expressed chemotaxis and apoptosis regulator [Source:HGNC Symbol;Acc:HGNC:35454] |
| ENSG00000284733 | HGLibA_34000 | 143 | 0.158790546 | OR4F29 | olfactory receptor family 4 subfamily F member 29 [Source:HGNC Symbol;Acc:HGNC:31275] |
| HIST1H2BA | HGLibA_21412 | 143 | 0.158790546 | HIST1H2BA | - |
| hsa-mir-1273f | HGLibA_57545 | 143 | 0.158790546 | hsa-mir-1273f | - |
| hsa-mir-221 | HGLibA_58292 | 143 | 0.158790546 | hsa-mir-221 | - |
| hsa-mir-3144 | HGLibA_58634 | 143 | 0.158790546 | hsa-mir-3144 | - |
| hsa-mir-3158-2 | HGLibA_58708 | 143 | 0.158790546 | hsa-mir-3158-2 | - |
| hsa-mir-320c-1 | HGLibA_58935 | 143 | 0.158790546 | hsa-mir-320c-1 | - |
| hsa-mir-3650 | HGLibA_59147 | 143 | 0.158790546 | hsa-mir-3650 | - |
| hsa-mir-4747 | HGLibA_61084 | 143 | 0.158790546 | hsa-mir-4747 | - |
| hsa-mir-6804 | HGLibA_63392 | 143 | 0.158790546 | hsa-mir-6804 | - |
| hsa-mir-6857 | HGLibA_63599 | 143 | 0.158790546 | hsa-mir-6857 | - |
| hsa-mir-6865 | HGLibA_63643 | 143 | 0.158790546 | hsa-mir-6865 | - |
| MPP6 | HGLibA_29762 | 143 | 0.158790546 | MPP6 | - |
| NARS | HGLibA_31154 | 143 | 0.158790546 | NARS | - |
| PLA2G16 | HGLibA_36879 | 143 | 0.158790546 | PLA2G16 | - |
| SELV | HGLibA_43317 | 143 | 0.158790546 | SELV | - |
| CCDC155 | HGLibA_07658 | 142 | 0.149745203 | CCDC155 | - |
| ENSG00000004660 | HGLibA_07087 | 142 | 0.149745203 | CAMKK1 | calcium/calmodulin dependent protein kinase kinase 1 [Source:HGNC Symbol;Acc:HGNC:1469] |
| ENSG00000005844 | HGLibA_23756 | 142 | 0.149745203 | ITGAL | integrin subunit alpha L [Source:HGNC Symbol;Acc:HGNC:6148] |
| ENSG00000007264 | HGLibA_28425 | 142 | 0.149745203 | MATK | megakaryocyte-associated tyrosine kinase [Source:HGNC Symbol;Acc:HGNC:6906] |
| ENSG00000018189 | HGLibA_42443 | 142 | 0.149745203 | RUFY3 | RUN and FYVE domain containing 3 [Source:HGNC Symbol;Acc:HGNC:30285] |
| ENSG00000023909 | HGLibA_18798 | 142 | 0.149745203 | GCLM | glutamate-cysteine ligase modifier subunit [Source:HGNC Symbol;Acc:HGNC:4312] |
| ENSG00000053770 | HGLibA_02356 | 142 | 0.149745203 | AP5M1 | adaptor related protein complex 5 subunit mu 1 [Source:HGNC Symbol;Acc:HGNC:20192] |
| ENSG00000058085 | HGLibA_25971 | 142 | 0.149745203 | LAMC2 | laminin subunit gamma 2 [Source:HGNC Symbol;Acc:HGNC:6493] |
| ENSG00000072182 | HGLibA_03308 | 142 | 0.149745203 | ASIC4 | acid sensing ion channel subunit family member 4 [Source:HGNC Symbol;Acc:HGNC:21263] |
| ENSG00000077454 | HGLibA_27145 | 142 | 0.149745203 | LRCH4 | leucine rich repeats and calponin homology domain containing 4 [Source:HGNC Symbol;Acc:HGNC:6691] |
| ENSG00000079482 | HGLibA_33417 | 142 | 0.149745203 | OPHN1 | oligophrenin 1 [Source:HGNC Symbol;Acc:HGNC:8148] |
| ENSG00000087086 | HGLibA_18124 | 142 | 0.149745203 | FTL | ferritin light chain [Source:HGNC Symbol;Acc:HGNC:3999] |
| ENSG00000089091 | HGLibA_14296 | 142 | 0.149745203 | DZANK1 | double zinc ribbon and ankyrin repeat domains 1 [Source:HGNC Symbol;Acc:HGNC:15858] |
| ENSG00000092036 | HGLibA_20882 | 142 | 0.149745203 | HAUS4 | HAUS augmin like complex subunit 4 [Source:HGNC Symbol;Acc:HGNC:20163] |
| ENSG00000099282 | HGLibA_51906 | 142 | 0.149745203 | TSPAN15 | tetraspanin 15 [Source:HGNC Symbol;Acc:HGNC:23298] |
| ENSG00000100311 | HGLibA_35844 | 142 | 0.149745203 | PDGFB | platelet derived growth factor subunit B [Source:HGNC Symbol;Acc:HGNC:8800] |
| ENSG00000101220 | HGLibA_06022 | 142 | 0.149745203 | C20orf27 | chromosome 20 open reading frame 27 [Source:HGNC Symbol;Acc:HGNC:15873] |
| ENSG00000101608 | HGLibA_30751 | 142 | 0.149745203 | MYL12A | myosin light chain 12A [Source:HGNC Symbol;Acc:HGNC:16701] |
| ENSG00000102119 | HGLibA_15033 | 142 | 0.149745203 | EMD | emerin [Source:HGNC Symbol;Acc:HGNC:3331] |
| ENSG00000104883 | HGLibA_36091 | 142 | 0.149745203 | PEX11G | peroxisomal biogenesis factor 11 gamma [Source:HGNC Symbol;Acc:HGNC:20208] |
| ENSG00000105261 | HGLibA_34808 | 142 | 0.149745203 | OVOL3 | ovo like zinc finger 3 [Source:HGNC Symbol;Acc:HGNC:14186] |

| | | | | | |
|---|---|---|---|---|---|
| ENSG00000106733 | HGLibA_32195 | 142 | 0.149745203 | NMRK1 | nicotinamide riboside kinase 1 [Source:HGNC Symbol;Acc:HGNC:26057] |
| ENSG00000112877 | HGLibA_09162 | 142 | 0.149745203 | CEP72 | centrosomal protein 72 [Source:HGNC Symbol;Acc:HGNC:25547] |
| ENSG00000113073 | HGLibA_45232 | 142 | 0.149745203 | SLC4A9 | solute carrier family 4 member 9 [Source:HGNC Symbol;Acc:HGNC:11035] |
| ENSG00000113302 | HGLibA_22962 | 142 | 0.149745203 | IL12B | interleukin 12B [Source:HGNC Symbol;Acc:HGNC:5970] |
| ENSG00000113719 | HGLibA_15466 | 142 | 0.149745203 | ERGIC1 | endoplasmic reticulum-golgi intermediate compartment 1 [Source:HGNC Symbol;Acc:HGNC:29205] |
| ENSG00000114098 | HGLibA_03031 | 142 | 0.149745203 | ARMC8 | armadillo repeat containing 8 [Source:HGNC Symbol;Acc:HGNC:24999] |
| ENSG00000115596 | HGLibA_54380 | 142 | 0.149745203 | WNT6 | Wnt family member 6 [Source:HGNC Symbol;Acc:HGNC:12785] |
| ENSG00000116157 | HGLibA_20189 | 142 | 0.149745203 | GPX7 | glutathione peroxidase 7 [Source:HGNC Symbol;Acc:HGNC:4559] |
| ENSG00000116667 | HGLibA_05824 | 142 | 0.149745203 | C1orf21 | chromosome 1 open reading frame 21 [Source:HGNC Symbol;Acc:HGNC:15494] |
| ENSG00000116882 | HGLibA_20835 | 142 | 0.149745203 | HAO2 | hydroxyacid oxidase 2 [Source:HGNC Symbol;Acc:HGNC:4810] |
| ENSG00000117139 | HGLibA_24525 | 142 | 0.149745203 | KDM5B | lysine demethylase 5B [Source:HGNC Symbol;Acc:HGNC:18039] |
| ENSG00000120742 | HGLibA_43499 | 142 | 0.149745203 | SERP1 | stress associated endoplasmic reticulum protein 1 [Source:HGNC Symbol;Acc:HGNC:10759] |
| ENSG00000124074 | HGLibA_15133 | 142 | 0.149745203 | ENKD1 | enkurin domain containing 1 [Source:HGNC Symbol;Acc:HGNC:25246] |
| ENSG00000125266 | HGLibA_14601 | 142 | 0.149745203 | EFNB2 | ephrin B2 [Source:HGNC Symbol;Acc:HGNC:3227] |
| ENSG00000127074 | HGLibA_41062 | 142 | 0.149745203 | RGS13 | regulator of G protein signaling 13 [Source:HGNC Symbol;Acc:HGNC:9995] |
| ENSG00000128245 | HGLibA_54747 | 142 | 0.149745203 | YWHAH | tyrosine 3-monooxygenase/tryptophan 5-monooxygenase activation protein eta [Source:HGNC Symbol;Acc:HGNC:12853] |
| ENSG00000131089 | HGLibA_02878 | 142 | 0.149745203 | ARHGEF9 | Cdc42 guanine nucleotide exchange factor 9 [Source:HGNC Symbol;Acc:HGNC:14561] |
| ENSG00000131165 | HGLibA_09473 | 142 | 0.149745203 | CHMP1A | charged multivesicular body protein 1A [Source:HGNC Symbol;Acc:HGNC:8740] |
| ENSG00000132702 | HGLibA_20845 | 142 | 0.149745203 | HAPLN2 | hyaluronan and proteoglycan link protein 2 [Source:HGNC Symbol;Acc:HGNC:17410] |
| ENSG00000132879 | HGLibA_17104 | 142 | 0.149745203 | FBXO44 | F-box protein 44 [Source:HGNC Symbol;Acc:HGNC:24847] |
| ENSG00000134545 | HGLibA_25286 | 142 | 0.149745203 | KLRC1 | killer cell lectin like receptor C1 [Source:HGNC Symbol;Acc:HGNC:6374] |
| ENSG00000135472 | HGLibA_15968 | 142 | 0.149745203 | FAIM2 | Fas apoptotic inhibitory molecule 2 [Source:HGNC Symbol;Acc:HGNC:17067] |
| ENSG00000135835 | HGLibA_24792 | 142 | 0.149745203 | KIAA1614 | KIAA1614 [Source:HGNC Symbol;Acc:HGNC:29327] |
| ENSG00000135924 | HGLibA_13538 | 142 | 0.149745203 | DNAJB2 | DnaJ heat shock protein family (Hsp40) member B2 [Source:HGNC Symbol;Acc:HGNC:5228] |
| ENSG00000136045 | HGLibA_39634 | 142 | 0.149745203 | PWP1 | endonuclein [Source:HGNC Symbol;Acc:HGNC:17015]" |
| ENSG00000137814 | HGLibA_20877 | 142 | 0.149745203 | HAUS2 | HAUS augmin like complex subunit 2 [Source:HGNC Symbol;Acc:HGNC:25530] |
| ENSG00000138399 | HGLibA_16901 | 142 | 0.149745203 | FASTKD1 | FAST kinase domains 1 [Source:HGNC Symbol;Acc:HGNC:26150] |
| ENSG00000138794 | HGLibA_07319 | 142 | 0.149745203 | CASP6 | caspase 6 [Source:HGNC Symbol;Acc:HGNC:1507] |
| ENSG00000139835 | HGLibA_20382 | 142 | 0.149745203 | GRTP1 | growth hormone regulated TBC protein 1 [Source:HGNC Symbol;Acc:HGNC:20310] |
| ENSG00000141384 | HGLibA_48237 | 142 | 0.149745203 | TAF4B | TATA-box binding protein associated factor 4b [Source:HGNC Symbol;Acc:HGNC:11538] |
| ENSG00000142973 | HGLibA_12178 | 142 | 0.149745203 | CYP4B1 | cytochrome P450 family 4 subfamily B member 1 [Source:HGNC Symbol;Acc:HGNC:2644] |
| ENSG00000144120 | HGLibA_50078 | 142 | 0.149745203 | TMEM177 | transmembrane protein 177 [Source:HGNC Symbol;Acc:HGNC:28143] |
| ENSG00000144749 | HGLibA_27175 | 142 | 0.149745203 | LRIG1 | leucine rich repeats and immunoglobulin like domains 1 [Source:HGNC Symbol;Acc:HGNC:17360] |
| ENSG00000145861 | HGLibA_05938 | 142 | 0.149745203 | C1QTNF2 | C1q and TNF related 2 [Source:HGNC Symbol;Acc:HGNC:14325] |
| ENSG00000147257 | HGLibA_19768 | 142 | 0.149745203 | GPC3 | glypican 3 [Source:HGNC Symbol;Acc:HGNC:4451] |
| ENSG00000153130 | HGLibA_43031 | 142 | 0.149745203 | SCOC | short coiled-coil protein [Source:HGNC Symbol;Acc:HGNC:20335] |
| ENSG00000153879 | HGLibA_08969 | 142 | 0.149745203 | CEBPG | CCAAT enhancer binding protein gamma [Source:HGNC Symbol;Acc:HGNC:1837] |
| ENSG00000156831 | HGLibA_32768 | 142 | 0.149745203 | NSMCE2 | SMC5-SMC6 complex SUMO ligase [Source:HGNC Symbol;Acc:HGNC:26513]" |
| ENSG00000157087 | HGLibA_03617 | 142 | 0.149745203 | ATP2B2 | ATPase plasma membrane Ca2+ transporting 2 [Source:HGNC Symbol;Acc:HGNC:815] |
| ENSG00000160284 | HGLibA_46539 | 142 | 0.149745203 | SPATC1L | spermatogenesis and centriole associated 1 like [Source:HGNC Symbol;Acc:HGNC:1298] |
| ENSG00000162814 | HGLibA_46434 | 142 | 0.149745203 | SPATA17 | spermatogenesis associated 17 [Source:HGNC Symbol;Acc:HGNC:25184] |
| ENSG00000162927 | HGLibA_39599 | 142 | 0.149745203 | PUS10 | pseudouridine synthase 10 [Source:HGNC Symbol;Acc:HGNC:26505] |
| ENSG00000163823 | HGLibA_08143 | 142 | 0.149745203 | CCR1 | C-C motif chemokine receptor 1 [Source:HGNC Symbol;Acc:HGNC:1602] |
| ENSG00000164022 | HGLibA_01355 | 142 | 0.149745203 | AIMP1 | aminoacyl tRNA synthetase complex interacting multifunctional protein 1 [Source:HGNC Symbol;Acc:HGNC:10648] |
| ENSG00000164117 | HGLibA_17128 | 142 | 0.149745203 | FBXO8 | F-box protein 8 [Source:HGNC Symbol;Acc:HGNC:13587] |
| ENSG00000164270 | HGLibA_22396 | 142 | 0.149745203 | HTR4 | 5-hydroxytryptamine receptor 4 [Source:HGNC Symbol;Acc:HGNC:5299] |
| ENSG00000165097 | HGLibA_24492 | 142 | 0.149745203 | KDM1B | lysine demethylase 1B [Source:HGNC Symbol;Acc:HGNC:21577] |
| ENSG00000165392 | HGLibA_54408 | 142 | 0.149745203 | WRN | Werner syndrome RecQ like helicase [Source:HGNC Symbol;Acc:HGNC:12791] |
| ENSG00000166135 | HGLibA_21301 | 142 | 0.149745203 | HIF1AN | hypoxia inducible factor 1 subunit alpha inhibitor [Source:HGNC Symbol;Acc:HGNC:17113] |
| ENSG00000166351 | HGLibA_37725 | 142 | 0.149745203 | POTED | POTE ankyrin domain family member D [Source:HGNC Symbol;Acc:HGNC:23822] |
| ENSG00000167355 | HGLibA_34088 | 142 | 0.149745203 | OR51B5 | olfactory receptor family 51 subfamily B member 5 [Source:HGNC Symbol;Acc:HGNC:19599] |
| ENSG00000168137 | HGLibA_43652 | 142 | 0.149745203 | SETD5 | SET domain containing 5 [Source:HGNC Symbol;Acc:HGNC:25566] |
| ENSG00000169807 | HGLibA_38997 | 142 | 0.149745203 | PRY2 | PTPN13 like Y-linked 2 [Source:HGNC Symbol;Acc:HGNC:21504] |
| ENSG00000170074 | HGLibA_16169 | 142 | 0.149745203 | FAM153A | family with sequence similarity 153 member A [Source:HGNC Symbol;Acc:HGNC:29940] |
| ENSG00000170142 | HGLibA_52594 | 142 | 0.149745203 | UBE2E1 | ubiquitin conjugating enzyme E2 E1 [Source:HGNC Symbol;Acc:HGNC:12477] |
| ENSG00000170482 | HGLibA_44606 | 142 | 0.149745203 | SLC23A1 | solute carrier family 23 member 1 [Source:HGNC Symbol;Acc:HGNC:10974] |
| ENSG00000171180 | HGLibA_33840 | 142 | 0.149745203 | OR2M4 | olfactory receptor family 2 subfamily M member 4 [Source:HGNC Symbol;Acc:HGNC:8270] |
| ENSG00000172969 | HGLibA_18001 | 142 | 0.149745203 | FRG2C | FSHD region gene 2 family member C [Source:HGNC Symbol;Acc:HGNC:33626] |
| ENSG00000174010 | HGLibA_25137 | 142 | 0.149745203 | KLHL15 | kelch like family member 15 [Source:HGNC Symbol;Acc:HGNC:29347] |
| ENSG00000176769 | HGLibA_48759 | 142 | 0.149745203 | TCERG1L | transcription elongation regulator 1 like [Source:HGNC Symbol;Acc:HGNC:23533] |
| ENSG00000179241 | HGLibA_26248 | 142 | 0.149745203 | LDLRAD3 | low density lipoprotein receptor class A domain containing 3 [Source:HGNC Symbol;Acc:HGNC:27046] |
| ENSG00000182552 | HGLibA_42490 | 142 | 0.149745203 | RWDD4 | RWD domain containing 4 [Source:HGNC Symbol;Acc:HGNC:23750] |
| ENSG00000182985 | HGLibA_06965 | 142 | 0.149745203 | CADM1 | cell adhesion molecule 1 [Source:HGNC Symbol;Acc:HGNC:5951] |
| ENSG00000183077 | HGLibA_01125 | 142 | 0.149745203 | AFMID | arylformamidase [Source:HGNC Symbol;Acc:HGNC:20910] |
| ENSG00000183632 | HGLibA_51077 | 142 | 0.149745203 | TP53TG3 | TP53 target 3 [Source:HGNC Symbol;Acc:HGNC:30759] |
| ENSG00000185722 | HGLibA_01959 | 142 | 0.149745203 | ANKFY1 | ankyrin repeat and FYVE domain containing 1 [Source:HGNC Symbol;Acc:HGNC:20763] |
| ENSG00000185730 | HGLibA_56559 | 142 | 0.149745203 | ZNF696 | zinc finger protein 696 [Source:HGNC Symbol;Acc:HGNC:25872] |
| ENSG00000185761 | HGLibA_00860 | 142 | 0.149745203 | ADAMTSL5 | ADAMTS like 5 [Source:HGNC Symbol;Acc:HGNC:27912] |
| ENSG00000186687 | HGLibA_27759 | 142 | 0.149745203 | LYRM7 | LYR motif containing 7 [Source:HGNC Symbol;Acc:HGNC:28072] |
| ENSG00000187193 | HGLibA_30299 | 142 | 0.149745203 | MT1X | metallothionein 1X [Source:HGNC Symbol;Acc:HGNC:7405] |
| ENSG00000188312 | HGLibA_09081 | 142 | 0.149745203 | CENPP | centromere protein P [Source:HGNC Symbol;Acc:HGNC:32933] |
| ENSG00000188352 | HGLibA_17776 | 142 | 0.149745203 | FOCAD | focadhesin [Source:HGNC Symbol;Acc:HGNC:23377] |
| ENSG00000188559 | HGLibA_40158 | 142 | 0.149745203 | RALGAPA2 | Ral GTPase activating protein catalytic alpha subunit 2 [Source:HGNC Symbol;Acc:HGNC:16207] |
| ENSG00000189127 | HGLibA_02078 | 142 | 0.149745203 | ANKRD34B | ankyrin repeat domain 34B [Source:HGNC Symbol;Acc:HGNC:33736] |
| ENSG00000189195 | HGLibA_04924 | 142 | 0.149745203 | BTBD8 | BTB domain containing 8 [Source:HGNC Symbol;Acc:HGNC:21019] |
| ENSG00000189233 | HGLibA_33005 | 142 | 0.149745203 | NUGGC | germinal center associated [Source:HGNC Symbol;Acc:HGNC:33550]" |
| ENSG00000197125 | HGLibA_34504 | 142 | 0.149745203 | OR8B8 | olfactory receptor family 8 subfamily B member 8 [Source:HGNC Symbol;Acc:HGNC:8477] |
| ENSG00000197487 | HGLibA_18584 | 142 | 0.149745203 | GALP | galanin like peptide [Source:HGNC Symbol;Acc:HGNC:24840] |
| ENSG00000197555 | HGLibA_44192 | 142 | 0.149745203 | SIPA1L1 | signal induced proliferation associated 1 like 1 [Source:HGNC Symbol;Acc:HGNC:20284] |
| ENSG00000198390 | HGLibA_25641 | 142 | 0.149745203 | KRTAP13-1 | keratin associated protein 13-1 [Source:HGNC Symbol;Acc:HGNC:18924] |
| ENSG00000198826 | HGLibA_02702 | 142 | 0.149745203 | ARHGAP11A | Rho GTPase activating protein 11A [Source:HGNC Symbol;Acc:HGNC:15783] |
| ENSG00000203883 | HGLibA_46250 | 142 | 0.149745203 | SOX18 | SRY-box 18 [Source:HGNC Symbol;Acc:HGNC:11194] |
| ENSG00000204314 | HGLibA_38882 | 142 | 0.149745203 | PRRT1 | proline rich transmembrane protein 1 [Source:HGNC Symbol;Acc:HGNC:13943] |
| ENSG00000204510 | HGLibA_38325 | 142 | 0.149745203 | PRAMEF7 | PRAME family member 7 [Source:HGNC Symbol;Acc:HGNC:28415] |
| ENSG00000204688 | HGLibA_33803 | 142 | 0.149745203 | OR2H1 | olfactory receptor family 2 subfamily H member 1 [Source:HGNC Symbol;Acc:HGNC:8252] |
| ENSG00000204839 | HGLibA_29874 | 142 | 0.149745203 | MROH6 | maestro heat like repeat family member 6 [Source:HGNC Symbol;Acc:HGNC:27814] |
| ENSG00000204843 | HGLibA_12545 | 142 | 0.149745203 | DCTN1 | dynactin subunit 1 [Source:HGNC Symbol;Acc:HGNC:2711] |
| ENSG00000213203 | HGLibA_19069 | 142 | 0.149745203 | GIMAP1 | IMAP family member 1 [Source:HGNC Symbol;Acc:HGNC:23237]" |
| ENSG00000213397 | HGLibA_20893 | 142 | 0.149745203 | HAUS7 | HAUS augmin like complex subunit 7 [Source:HGNC Symbol;Acc:HGNC:32979] |
| ENSG00000213462 | HGLibA_15535 | 142 | 0.149745203 | ERV3-1 | envelope [Source:HGNC Symbol;Acc:HGNC:3454]" |
| ENSG00000213809 | HGLibA_25315 | 142 | 0.149745203 | KLRK1 | killer cell lectin like receptor K1 [Source:HGNC Symbol;Acc:HGNC:18788] |
| ENSG00000214652 | HGLibA_56634 | 142 | 0.149745203 | ZNF727 | zinc finger protein 727 [Source:HGNC Symbol;Acc:HGNC:22785] |
| ENSG00000214706 | HGLibA_22740 | 142 | 0.149745203 | IFRD2 | interferon related developmental regulator 2 [Source:HGNC Symbol;Acc:HGNC:5457] |
| ENSG00000215029 | HGLibA_48852 | 142 | 0.149745203 | TCP11X2 | X-linked 2 [Source:HGNC Symbol;Acc:HGNC:48335]" |
| ENSG00000230797 | HGLibA_54762 | 142 | 0.149745203 | YY2 | YY2 transcription factor [Source:HGNC Symbol;Acc:HGNC:31684] |
| ENSG00000231068 | HGLibA_25713 | 142 | 0.149745203 | KRTAP21-3 | keratin associated protein 21-3 [Source:HGNC Symbol;Acc:HGNC:34216] |
| ENSG00000232258 | HGLibA_49880 | 142 | 0.149745203 | TMEM114 | transmembrane protein 114 [Source:HGNC Symbol;Acc:HGNC:33227] |
| ENSG00000233803 | HGLibA_51973 | 142 | 0.149745203 | TSPY4 | testis specific protein Y-linked 4 [Source:HGNC Symbol;Acc:HGNC:37287] |
| ENSG00000253309 | HGLibA_43582 | 142 | 0.149745203 | SERPINE3 | serpin family E member 3 [Source:HGNC Symbol;Acc:HGNC:24774] |
| ENSG00000262664 | HGLibA_34789 | 142 | 0.149745203 | OVCA2 | serine hydrolase domain containing [Source:HGNC Symbol;Acc:HGNC:24203]" |
| ENSG00000272414 | HGLibA_16619 | 142 | 0.149745203 | FAM47E-STBD1 | FAM47E-STBD1 readthrough [Source:HGNC Symbol;Acc:HGNC:44667] |
| ENSG00000279051 | HGLibA_34440 | 142 | 0.149745203 | OR6Q1 | olfactory receptor family 6 subfamily Q member 1 (gene/pseudogene) [Source:HGNC Symbol;Acc:HGNC:15302] |
| HDGFRP3 | HGLibA_21049 | 142 | 0.149745203 | HDGFRP3 | - |
| hsa-mir-10a | HGLibA_57199 | 142 | 0.149745203 | hsa-mir-10a | - |
| hsa-mir-153-1 | HGLibA_57906 | 142 | 0.149745203 | hsa-mir-153-1 | - |
| hsa-mir-3136 | HGLibA_58601 | 142 | 0.149745203 | hsa-mir-3136 | - |
| hsa-mir-325 | HGLibA_58967 | 142 | 0.149745203 | hsa-mir-325 | - |
| hsa-mir-4309 | HGLibA_59947 | 142 | 0.149745203 | hsa-mir-4309 | - |
| hsa-mir-4501 | HGLibA_60431 | 142 | 0.149745203 | hsa-mir-4501 | - |
| hsa-mir-486 | HGLibA_61335 | 142 | 0.149745203 | hsa-mir-486 | - |
| hsa-mir-5680 | HGLibA_62235 | 142 | 0.149745203 | hsa-mir-5680 | - |
| hsa-mir-6741 | HGLibA_63119 | 142 | 0.149745203 | hsa-mir-6741 | - |
| hsa-mir-6773 | HGLibA_63265 | 142 | 0.149745203 | hsa-mir-6773 | - |
| hsa-mir-764 | HGLibA_63913 | 142 | 0.149745203 | hsa-mir-764 | - |
| LOC100130301 | HGLibA_26775 | 142 | 0.149745203 | LOC100130301 | - |
| C9orf172 | HGLibA_06714 | 141 | 0.14069986 | C9orf172 | - |
| ENSG00000008311 | HGLibA_00084 | 141 | 0.14069986 | AASS | aminoadipate-semialdehyde synthase [Source:HGNC Symbol;Acc:HGNC:17366] |
| ENSG00000013375 | HGLibA_36271 | 141 | 0.14069986 | PGM3 | phosphoglucomutase 3 [Source:HGNC Symbol;Acc:HGNC:8907] |
| ENSG00000029153 | HGLibA_03070 | 141 | 0.14069986 | ARNTL2 | aryl hydrocarbon receptor nuclear translocator like 2 [Source:HGNC Symbol;Acc:HGNC:18984] |
| ENSG00000044090 | HGLibA_11791 | 141 | 0.14069986 | CUL7 | cullin 7 [Source:HGNC Symbol;Acc:HGNC:21024] |
| ENSG00000052795 | HGLibA_17767 | 141 | 0.14069986 | FNIP2 | folliculin interacting protein 2 [Source:HGNC Symbol;Acc:HGNC:29280] |
| ENSG00000065413 | HGLibA_02103 | 141 | 0.14069986 | ANKRD44 | ankyrin repeat domain 44 [Source:HGNC Symbol;Acc:HGNC:25259] |
| ENSG00000066230 | HGLibA_45405 | 141 | 0.14069986 | SLC9A3 | solute carrier family 9 member A3 [Source:HGNC Symbol;Acc:HGNC:11073] |
| ENSG00000070019 | HGLibA_20660 | 141 | 0.14069986 | GUCY2C | guanylate cyclase 2C [Source:HGNC Symbol;Acc:HGNC:4688] |
| ENSG00000076258 | HGLibA_17710 | 141 | 0.14069986 | FMO4 | flavin containing monooxygenase 4 [Source:HGNC Symbol;Acc:HGNC:3772] |
| ENSG00000084676 | HGLibA_31335 | 141 | 0.14069986 | NCOA1 | nuclear receptor coactivator 1 [Source:HGNC Symbol;Acc:HGNC:7668] |
| ENSG00000100078 | HGLibA_36897 | 141 | 0.14069986 | PLA2G3 | phospholipase A2 group III [Source:HGNC Symbol;Acc:HGNC:17934] |
| ENSG00000101977 | HGLibA_28578 | 141 | 0.14069986 | MCF2 | MCF.2 cell line derived transforming sequence [Source:HGNC Symbol;Acc:HGNC:6940] |
| ENSG00000102924 | HGLibA_07406 | 141 | 0.14069986 | CBLN1 | cerebellin 1 precursor [Source:HGNC Symbol;Acc:HGNC:1543] |
| ENSG00000104915 | HGLibA_47614 | 141 | 0.14069986 | STX10 | syntaxin 10 [Source:HGNC Symbol;Acc:HGNC:11428] |
| ENSG00000108255 | HGLibA_11251 | 141 | 0.14069986 | CRYBA1 | crystallin beta A1 [Source:HGNC Symbol;Acc:HGNC:2394] |
| ENSG00000108691 | HGLibA_07988 | 141 | 0.14069986 | CCL2 | C-C motif chemokine ligand 2 [Source:HGNC Symbol;Acc:HGNC:10618] |
| ENSG00000108950 | HGLibA_16444 | 141 | 0.14069986 | FAM20A | golgi associated secretory pathway pseudokinase [Source:HGNC Symbol;Acc:HGNC:23015]" |
| ENSG00000109536 | HGLibA_17993 | 141 | 0.14069986 | FRG1 | FSHD region gene 1 [Source:HGNC Symbol;Acc:HGNC:3954] |
| ENSG00000112183 | HGLibA_40514 | 141 | 0.14069986 | RBM24 | RNA binding motif protein 24 [Source:HGNC Symbol;Acc:HGNC:21539] |

| | | | | | |
|---|---|---|---|---|---|
| ENSG00000112695 | HGLibA_10859 | 141 | 0.14069986 | COX7A2 | cytochrome c oxidase subunit 7A2 [Source:HGNC Symbol;Acc:HGNC:2288] |
| ENSG00000113583 | HGLibA_06360 | 141 | 0.14069986 | C5orf15 | chromosome 5 open reading frame 15 [Source:HGNC Symbol;Acc:HGNC:20656] |
| ENSG00000115155 | HGLibA_34726 | 141 | 0.14069986 | OTOF | otoferlin [Source:HGNC Symbol;Acc:HGNC:8515] |
| ENSG00000115252 | HGLibA_35770 | 141 | 0.14069986 | PDE1A | phosphodiesterase 1A [Source:HGNC Symbol;Acc:HGNC:8774] |
| ENSG00000119720 | HGLibA_32668 | 141 | 0.14069986 | NRDE2 | domain containing [Source:HGNC Symbol;Acc:HGNC:20186]" |
| ENSG00000120215 | HGLibA_29363 | 141 | 0.14069986 | MLANA | melan-A [Source:HGNC Symbol;Acc:HGNC:7124] |
| ENSG00000123933 | HGLibA_30616 | 141 | 0.14069986 | MXD4 | MAX dimerization protein 4 [Source:HGNC Symbol;Acc:HGNC:13906] |
| ENSG00000125651 | HGLibA_20539 | 141 | 0.14069986 | GTF2F1 | general transcription factor IIF subunit 1 [Source:HGNC Symbol;Acc:HGNC:4652] |
| ENSG00000125798 | HGLibA_17810 | 141 | 0.14069986 | FOXA2 | forkhead box A2 [Source:HGNC Symbol;Acc:HGNC:5022] |
| ENSG00000128272 | HGLibA_03441 | 141 | 0.14069986 | ATF4 | activating transcription factor 4 [Source:HGNC Symbol;Acc:HGNC:786] |
| ENSG00000129988 | HGLibA_26079 | 141 | 0.14069986 | LBP | lipopolysaccharide binding protein [Source:HGNC Symbol;Acc:HGNC:6517] |
| ENSG00000130479 | HGLibA_28132 | 141 | 0.14069986 | MAP1S | microtubule associated protein 1S [Source:HGNC Symbol;Acc:HGNC:15715] |
| ENSG00000130957 | HGLibA_16966 | 141 | 0.14069986 | FBP2 | fructose-bisphosphatase 2 [Source:HGNC Symbol;Acc:HGNC:3607] |
| ENSG00000131015 | HGLibA_52963 | 141 | 0.14069986 | ULBP2 | UL16 binding protein 2 [Source:HGNC Symbol;Acc:HGNC:14894] |
| ENSG00000131462 | HGLibA_52302 | 141 | 0.14069986 | TUBG1 | tubulin gamma 1 [Source:HGNC Symbol;Acc:HGNC:12417] |
| ENSG00000131969 | HGLibA_00241 | 141 | 0.14069986 | ABHD12B | abhydrolase domain containing 12B [Source:HGNC Symbol;Acc:HGNC:19837] |
| ENSG00000132698 | HGLibA_39851 | 141 | 0.14069986 | RAB25 | member RAS oncogene family [Source:HGNC Symbol;Acc:HGNC:18238]" |
| ENSG00000133574 | HGLibA_19079 | 141 | 0.14069986 | GIMAP4 | IMAP family member 4 [Source:HGNC Symbol;Acc:HGNC:21872]" |
| ENSG00000133704 | HGLibA_23474 | 141 | 0.14069986 | IPO8 | importin 8 [Source:HGNC Symbol;Acc:HGNC:9853] |
| ENSG00000134007 | HGLibA_00753 | 141 | 0.14069986 | ADAM20 | ADAM metallopeptidase domain 20 [Source:HGNC Symbol;Acc:HGNC:199] |
| ENSG00000134294 | HGLibA_45064 | 141 | 0.14069986 | SLC38A2 | solute carrier family 38 member 2 [Source:HGNC Symbol;Acc:HGNC:13448] |
| ENSG00000134970 | HGLibA_49821 | 141 | 0.14069986 | TMED7 | transmembrane p24 trafficking protein 7 [Source:HGNC Symbol;Acc:HGNC:24253] |
| ENSG00000134996 | HGLibA_34712 | 141 | 0.14069986 | OSTF1 | osteoclast stimulating factor 1 [Source:HGNC Symbol;Acc:HGNC:8510] |
| ENSG00000135333 | HGLibA_15304 | 141 | 0.14069986 | EPHA7 | EPH receptor A7 [Source:HGNC Symbol;Acc:HGNC:3390] |
| ENSG00000135341 | HGLibA_28197 | 141 | 0.14069986 | MAP3K7 | mitogen-activated protein kinase kinase kinase 7 [Source:HGNC Symbol;Acc:HGNC:6859] |
| ENSG00000135686 | HGLibA_25199 | 141 | 0.14069986 | KLHL36 | kelch like family member 36 [Source:HGNC Symbol;Acc:HGNC:17844] |
| ENSG00000138246 | HGLibA_13573 | 141 | 0.14069986 | DNAJC13 | DnaJ heat shock protein family (Hsp40) member C13 [Source:HGNC Symbol;Acc:HGNC:30343] |
| ENSG00000140937 | HGLibA_08636 | 141 | 0.14069986 | CDH11 | cadherin 11 [Source:HGNC Symbol;Acc:HGNC:1750] |
| ENSG00000144642 | HGLibA_40593 | 141 | 0.14069986 | RBMS3 | RNA binding motif single stranded interacting protein 3 [Source:HGNC Symbol;Acc:HGNC:13427] |
| ENSG00000151651 | HGLibA_00782 | 141 | 0.14069986 | ADAM8 | ADAM metallopeptidase domain 8 [Source:HGNC Symbol;Acc:HGNC:215] |
| ENSG00000154556 | HGLibA_46176 | 141 | 0.14069986 | SORBS2 | sorbin and SH3 domain containing 2 [Source:HGNC Symbol;Acc:HGNC:24098] |
| ENSG00000156509 | HGLibA_17102 | 141 | 0.14069986 | FBXO43 | F-box protein 43 [Source:HGNC Symbol;Acc:HGNC:28521] |
| ENSG00000157326 | HGLibA_13136 | 141 | 0.14069986 | DHRS4 | dehydrogenase/reductase 4 [Source:HGNC Symbol;Acc:HGNC:16985] |
| ENSG00000157778 | HGLibA_39212 | 141 | 0.14069986 | PSMG3 | proteasome assembly chaperone 3 [Source:HGNC Symbol;Acc:HGNC:22420] |
| ENSG00000157985 | HGLibA_01136 | 141 | 0.14069986 | AGAP1 | ankyrin repeat and PH domain 1 [Source:HGNC Symbol;Acc:HGNC:16922]" |
| ENSG00000158815 | HGLibA_17381 | 141 | 0.14069986 | FGF17 | fibroblast growth factor 17 [Source:HGNC Symbol;Acc:HGNC:3673] |
| ENSG00000159905 | HGLibA_55610 | 141 | 0.14069986 | ZNF221 | zinc finger protein 221 [Source:HGNC Symbol;Acc:HGNC:13014] |
| ENSG00000160862 | HGLibA_03930 | 141 | 0.14069986 | AZGP1 | zinc-binding [Source:HGNC Symbol;Acc:HGNC:910]" |
| ENSG00000163060 | HGLibA_48979 | 141 | 0.14069986 | TEKT4 | tektin 4 [Source:HGNC Symbol;Acc:HGNC:31012] |
| ENSG00000163251 | HGLibA_18275 | 141 | 0.14069986 | FZD5 | frizzled class receptor 5 [Source:HGNC Symbol;Acc:HGNC:4043] |
| ENSG00000163421 | HGLibA_38668 | 141 | 0.14069986 | PROK2 | prokineticin 2 [Source:HGNC Symbol;Acc:HGNC:18455] |
| ENSG00000163646 | HGLibA_10122 | 141 | 0.14069986 | CLRN1 | clarin 1 [Source:HGNC Symbol;Acc:HGNC:12605] |
| ENSG00000163737 | HGLibA_36130 | 141 | 0.14069986 | PF4 | platelet factor 4 [Source:HGNC Symbol;Acc:HGNC:8861] |
| ENSG00000164841 | HGLibA_50510 | 141 | 0.14069986 | TMEM74 | transmembrane protein 74 [Source:HGNC Symbol;Acc:HGNC:26409] |
| ENSG00000165246 | HGLibA_32080 | 141 | 0.14069986 | NLGN4Y | neuroligin 4 Y-linked [Source:HGNC Symbol;Acc:HGNC:15529] |
| ENSG00000166133 | HGLibA_42210 | 141 | 0.14069986 | RPUSD2 | RNA pseudouridine synthase domain containing 2 [Source:HGNC Symbol;Acc:HGNC:24180] |
| ENSG00000166405 | HGLibA_41231 | 141 | 0.14069986 | RIC3 | RIC3 acetylcholine receptor chaperone [Source:HGNC Symbol;Acc:HGNC:30338] |
| ENSG00000166823 | HGLibA_28916 | 141 | 0.14069986 | MESP1 | mesoderm posterior bHLH transcription factor 1 [Source:HGNC Symbol;Acc:HGNC:29658] |
| ENSG00000167654 | HGLibA_03425 | 141 | 0.14069986 | ATCAY | caytaxin [Source:HGNC Symbol;Acc:HGNC:779]" |
| ENSG00000168528 | HGLibA_43488 | 141 | 0.14069986 | SERINC2 | serine incorporator 2 [Source:HGNC Symbol;Acc:HGNC:23231] |
| ENSG00000168843 | HGLibA_18110 | 141 | 0.14069986 | FSTL5 | follistatin like 5 [Source:HGNC Symbol;Acc:HGNC:21386] |
| ENSG00000168918 | HGLibA_23350 | 141 | 0.14069986 | INPP5D | inositol polyphosphate-5-phosphatase D [Source:HGNC Symbol;Acc:HGNC:6079] |
| ENSG00000169105 | HGLibA_09625 | 141 | 0.14069986 | CHST14 | carbohydrate sulfotransferase 14 [Source:HGNC Symbol;Acc:HGNC:24464] |
| ENSG00000169154 | HGLibA_19692 | 141 | 0.14069986 | GOT1L1 | glutamic-oxaloacetic transaminase 1 like 1 [Source:HGNC Symbol;Acc:HGNC:28487] |
| ENSG00000169251 | HGLibA_32147 | 141 | 0.14069986 | NMD3 | NMD3 ribosome export adaptor [Source:HGNC Symbol;Acc:HGNC:24250] |
| ENSG00000170373 | HGLibA_11445 | 141 | 0.14069986 | CST1 | cystatin SN [Source:HGNC Symbol;Acc:HGNC:2473] |
| ENSG00000170638 | HGLibA_51228 | 141 | 0.14069986 | TRABD | TraB domain containing [Source:HGNC Symbol;Acc:HGNC:28805] |
| ENSG00000170779 | HGLibA_08608 | 141 | 0.14069986 | CDCA4 | cell division cycle associated 4 [Source:HGNC Symbol;Acc:HGNC:14625] |
| ENSG00000170965 | HGLibA_36934 | 141 | 0.14069986 | PLAC1 | placenta specific 1 [Source:HGNC Symbol;Acc:HGNC:9044] |
| ENSG00000171862 | HGLibA_39296 | 141 | 0.14069986 | PTEN | phosphatase and tensin homolog [Source:HGNC Symbol;Acc:HGNC:9588] |
| ENSG00000172967 | HGLibA_54521 | 141 | 0.14069986 | XKR3 | XK related 3 [Source:HGNC Symbol;Acc:HGNC:28778] |
| ENSG00000173786 | HGLibA_10355 | 141 | 0.14069986 | CNP | 3'-cyclic nucleotide 3' phosphodiesterase [Source:HGNC Symbol;Acc:HGNC:2158]" |
| ENSG00000176125 | HGLibA_52854 | 141 | 0.14069986 | UFSP1 | UFM1 specific peptidase 1 (inactive) [Source:HGNC Symbol;Acc:HGNC:33821] |
| ENSG00000176302 | HGLibA_17944 | 141 | 0.14069986 | FOXR1 | forkhead box R1 [Source:HGNC Symbol;Acc:HGNC:29980] |
| ENSG00000177888 | HGLibA_54884 | 141 | 0.14069986 | ZBTB41 | zinc finger and BTB domain containing 41 [Source:HGNC Symbol;Acc:HGNC:24819] |
| ENSG00000178338 | HGLibA_55863 | 141 | 0.14069986 | ZNF354B | zinc finger protein 354B [Source:HGNC Symbol;Acc:HGNC:17197] |
| ENSG00000178982 | HGLibA_14795 | 141 | 0.14069986 | EIF3K | eukaryotic translation initiation factor 3 subunit K [Source:HGNC Symbol;Acc:HGNC:24656] |
| ENSG00000180549 | HGLibA_18204 | 141 | 0.14069986 | FUT7 | fucosyltransferase 7 [Source:HGNC Symbol;Acc:HGNC:4018] |
| ENSG00000180772 | HGLibA_01266 | 141 | 0.14069986 | AGTR2 | angiotensin II receptor type 2 [Source:HGNC Symbol;Acc:HGNC:338] |
| ENSG00000181284 | HGLibA_49843 | 141 | 0.14069986 | TMEM102 | transmembrane protein 102 [Source:HGNC Symbol;Acc:HGNC:26722] |
| ENSG00000182362 | HGLibA_54640 | 141 | 0.14069986 | YBEY | ybeY metalloendoribonuclease [Source:HGNC Symbol;Acc:HGNC:1299] |
| ENSG00000183625 | HGLibA_08152 | 141 | 0.14069986 | CCR3 | C-C motif chemokine receptor 3 [Source:HGNC Symbol;Acc:HGNC:1604] |
| ENSG00000183862 | HGLibA_10265 | 141 | 0.14069986 | CNGA2 | cyclic nucleotide gated channel alpha 2 [Source:HGNC Symbol;Acc:HGNC:2149] |
| ENSG00000185156 | HGLibA_29110 | 141 | 0.14069986 | MFSD6L | major facilitator superfamily domain containing 6 like [Source:HGNC Symbol;Acc:HGNC:26656] |
| ENSG00000185739 | HGLibA_46990 | 141 | 0.14069986 | SRL | sarcalumenin [Source:HGNC Symbol;Acc:HGNC:11295] |
| ENSG00000186625 | HGLibA_24071 | 141 | 0.14069986 | KATNA1 | katanin catalytic subunit A1 [Source:HGNC Symbol;Acc:HGNC:6216] |
| ENSG00000186803 | HGLibA_22658 | 141 | 0.14069986 | IFNA10 | interferon alpha 10 [Source:HGNC Symbol;Acc:HGNC:5418] |
| ENSG00000187026 | HGLibA_25712 | 141 | 0.14069986 | KRTAP21-2 | keratin associated protein 21-2 [Source:HGNC Symbol;Acc:HGNC:18946] |
| ENSG00000187325 | HGLibA_48263 | 141 | 0.14069986 | TAF9B | TATA-box binding protein associated factor 9b [Source:HGNC Symbol;Acc:HGNC:17306] |
| ENSG00000188613 | HGLibA_31097 | 141 | 0.14069986 | NANOS1 | nanos C2HC-type zinc finger 1 [Source:HGNC Symbol;Acc:HGNC:23044] |
| ENSG00000189190 | HGLibA_56362 | 141 | 0.14069986 | ZNF600 | zinc finger protein 600 [Source:HGNC Symbol;Acc:HGNC:30951] |
| ENSG00000196628 | HGLibA_48787 | 141 | 0.14069986 | TCF4 | transcription factor 4 [Source:HGNC Symbol;Acc:HGNC:11634] |
| ENSG00000198062 | HGLibA_37737 | 141 | 0.14069986 | POTEH | POTE ankyrin domain family member H [Source:HGNC Symbol;Acc:HGNC:133] |
| ENSG00000198270 | HGLibA_49886 | 141 | 0.14069986 | TMEM116 | transmembrane protein 116 [Source:HGNC Symbol;Acc:HGNC:25084] |
| ENSG00000198670 | HGLibA_27049 | 141 | 0.14069986 | LPA | lipoprotein(a) [Source:HGNC Symbol;Acc:HGNC:6667] |
| ENSG00000198829 | HGLibA_47705 | 141 | 0.14069986 | SUCNR1 | succinate receptor 1 [Source:HGNC Symbol;Acc:HGNC:4542] |
| ENSG00000198885 | HGLibA_23866 | 141 | 0.14069986 | ITPRIPL1 | ITPRIP like 1 [Source:HGNC Symbol;Acc:HGNC:29371] |
| ENSG00000203727 | HGLibA_42688 | 141 | 0.14069986 | SAMD5 | sterile alpha motif domain containing 5 [Source:HGNC Symbol;Acc:HGNC:21180] |
| ENSG00000204379 | HGLibA_54466 | 141 | 0.14069986 | XAGE1A | X antigen family member 1A [Source:HGNC Symbol;Acc:HGNC:4111] |
| ENSG00000204479 | HGLibA_38300 | 141 | 0.14069986 | PRAMEF17 | PRAME family member 17 [Source:HGNC Symbol;Acc:HGNC:29485] |
| ENSG00000204711 | HGLibA_06679 | 141 | 0.14069986 | C9orf135 | chromosome 9 open reading frame 135 [Source:HGNC Symbol;Acc:HGNC:31422] |
| ENSG00000221823 | HGLibA_38194 | 141 | 0.14069986 | PPP3R1 | alpha [Source:HGNC Symbol;Acc:HGNC:9317]" |
| ENSG00000221996 | HGLibA_34149 | 141 | 0.14069986 | OR52B4 | olfactory receptor family 52 subfamily B member 4 (gene/pseudogene) [Source:HGNC Symbol;Acc:HGNC:15209] |
| ENSG00000229972 | HGLibA_23507 | 141 | 0.14069986 | IQCF3 | IQ motif containing F3 [Source:HGNC Symbol;Acc:HGNC:31816] |
| ENSG00000235169 | HGLibA_45717 | 141 | 0.14069986 | SMIM1 | small integral membrane protein 1 (Vel blood group) [Source:HGNC Symbol;Acc:HGNC:44204] |
| ENSG00000241635 | HGLibA_52876 | 141 | 0.14069986 | UGT1A1 | UDP glucuronosyltransferase family 1 member A1 [Source:HGNC Symbol;Acc:HGNC:12530] |
| ENSG00000242515 | HGLibA_52879 | 141 | 0.14069986 | UGT1A10 | UDP glucuronosyltransferase family 1 member A10 [Source:HGNC Symbol;Acc:HGNC:12531] |
| ENSG00000253313 | HGLibA_05829 | 141 | 0.14069986 | C1orf210 | chromosome 1 open reading frame 210 [Source:HGNC Symbol;Acc:HGNC:28755] |
| ENSG00000253831 | HGLibA_15656 | 141 | 0.14069986 | ETV3L | ETS variant 3 like [Source:HGNC Symbol;Acc:HGNC:33834] |
| ENSG00000260903 | HGLibA_54530 | 141 | 0.14069986 | XKR7 | XK related 7 [Source:HGNC Symbol;Acc:HGNC:23062] |
| ENSG00000270757 | HGLibA_22336 | 141 | 0.14069986 | HSPE1-MOB4 | HSPE1-MOB4 readthrough [Source:HGNC Symbol;Acc:HGNC:49184] |
| ENSG00000272325 | HGLibA_32977 | 141 | 0.14069986 | NUDT3 | nudix hydrolase 3 [Source:HGNC Symbol;Acc:HGNC:8050] |
| ENSG00000279514 | HGLibA_33951 | 141 | 0.14069986 | OR4C16 | olfactory receptor family 4 subfamily C member 16 (gene/pseudogene) [Source:HGNC Symbol;Acc:HGNC:15172] |
| GPR112 | HGLibA_19851 | 141 | 0.14069986 | GPR112 | - |
| hsa-mir-1282 | HGLibA_57585 | 141 | 0.14069986 | hsa-mir-1282 | - |
| hsa-mir-1302-7 | HGLibA_57714 | 141 | 0.14069986 | hsa-mir-1302-7 | - |
| hsa-mir-1322 | HGLibA_57762 | 141 | 0.14069986 | hsa-mir-1322 | - |
| hsa-mir-138-2 | HGLibA_57817 | 141 | 0.14069986 | hsa-mir-138-2 | - |
| hsa-mir-222 | HGLibA_58296 | 141 | 0.14069986 | hsa-mir-222 | - |
| hsa-mir-3158-1 | HGLibA_58704 | 141 | 0.14069986 | hsa-mir-3158-1 | - |
| hsa-mir-3174 | HGLibA_58769 | 141 | 0.14069986 | hsa-mir-3174 | - |
| hsa-mir-449a | HGLibA_60417 | 141 | 0.14069986 | hsa-mir-449a | - |
| hsa-mir-4769 | HGLibA_61172 | 141 | 0.14069986 | hsa-mir-4769 | - |
| hsa-mir-5090 | HGLibA_61518 | 141 | 0.14069986 | hsa-mir-5090 | - |
| hsa-mir-5197 | HGLibA_61705 | 141 | 0.14069986 | hsa-mir-5197 | - |
| hsa-mir-544b | HGLibA_61827 | 141 | 0.14069986 | hsa-mir-544b | - |
| hsa-mir-7851 | HGLibA_63992 | 141 | 0.14069986 | hsa-mir-7851 | - |
| MGEA5 | HGLibA_29155 | 141 | 0.14069986 | MGEA5 | - |
| PALM2 | HGLibA_35069 | 141 | 0.14069986 | PALM2 | - |
| SPANXF1 | HGLibA_46398 | 141 | 0.14069986 | SPANXF1 | - |
| TMEM8A | HGLibA_50545 | 141 | 0.14069986 | TMEM8A | - |
| C1orf63 | HGLibA_05883 | 140 | 0.131654517 | C1orf63 | - |
| C3orf43 | HGLibA_06246 | 140 | 0.131654517 | C3orf43 | - |
| C8orf47 | HGLibA_06631 | 140 | 0.131654517 | C8orf47 | - |
| ENSG00000007341 | HGLibA_47285 | 140 | 0.131654517 | ST7L | suppression of tumorigenicity 7 like [Source:HGNC Symbol;Acc:HGNC:18441] |
| ENSG00000012171 | HGLibA_43321 | 140 | 0.131654517 | SEMA3B | semaphorin 3B [Source:HGNC Symbol;Acc:HGNC:10724] |
| ENSG00000021826 | HGLibA_10992 | 140 | 0.131654517 | CPS1 | carbamoyl-phosphate synthase 1 [Source:HGNC Symbol;Acc:HGNC:2323] |
| ENSG00000035141 | HGLibA_16139 | 140 | 0.131654517 | FAM136A | family with sequence similarity 136 member A [Source:HGNC Symbol;Acc:HGNC:25911] |
| ENSG00000067369 | HGLibA_51053 | 140 | 0.131654517 | TP53BP1 | tumor protein p53 binding protein 1 [Source:HGNC Symbol;Acc:HGNC:11999] |
| ENSG00000072315 | HGLibA_51741 | 140 | 0.131654517 | TRPC5 | transient receptor potential cation channel subfamily C member 5 [Source:HGNC Symbol;Acc:HGNC:12337] |
| ENSG00000074054 | HGLibA_09793 | 140 | 0.131654517 | CLASP1 | cytoplasmic linker associated protein 1 [Source:HGNC Symbol;Acc:HGNC:17088] |
| ENSG00000081479 | HGLibA_27211 | 140 | 0.131654517 | LRP2 | LDL receptor related protein 2 [Source:HGNC Symbol;Acc:HGNC:6694] |
| ENSG00000082258 | HGLibA_08129 | 140 | 0.131654517 | CCNT2 | cyclin T2 [Source:HGNC Symbol;Acc:HGNC:1600] |
| ENSG00000086289 | HGLibA_15274 | 140 | 0.131654517 | EPDR1 | ependymin related 1 [Source:HGNC Symbol;Acc:HGNC:17572] |
| ENSG00000089157 | HGLibA_42007 | 140 | 0.131654517 | RPLP0 | ribosomal protein lateral stalk subunit P0 [Source:HGNC Symbol;Acc:HGNC:10371] |

| | | | | | |
|---|---|---|---|---|---|
| ENSG00000091844 | HGLibA_41069 | 140 | 0.131654517 | RGS17 | regulator of G protein signaling 17 [Source:HGNC Symbol;Acc:HGNC:14088] |
| ENSG00000092978 | HGLibA_19737 | 140 | 0.131654517 | GPATCH2 | G-patch domain containing 2 [Source:HGNC Symbol;Acc:HGNC:25499] |
| ENSG00000094975 | HGLibA_47709 | 140 | 0.131654517 | SUCO | SUN domain containing ossification factor [Source:HGNC Symbol;Acc:HGNC:1240] |
| ENSG00000099937 | HGLibA_43574 | 140 | 0.131654517 | SERPIND1 | serpin family D member 1 [Source:HGNC Symbol;Acc:HGNC:4838] |
| ENSG00000100219 | HGLibA_54490 | 140 | 0.131654517 | XBP1 | X-box binding protein 1 [Source:HGNC Symbol;Acc:HGNC:12801] |
| ENSG00000100600 | HGLibA_26405 | 140 | 0.131654517 | LGMN | legumain [Source:HGNC Symbol;Acc:HGNC:9472] |
| ENSG00000101974 | HGLibA_03560 | 140 | 0.131654517 | ATP11C | ATPase phospholipid transporting 11C [Source:HGNC Symbol;Acc:HGNC:13554] |
| ENSG00000105767 | HGLibA_06974 | 140 | 0.131654517 | CADM4 | cell adhesion molecule 4 [Source:HGNC Symbol;Acc:HGNC:30825] |
| ENSG00000108262 | HGLibA_19129 | 140 | 0.131654517 | GIT1 | GIT ArfGAP 1 [Source:HGNC Symbol;Acc:HGNC:4272] |
| ENSG00000112232 | HGLibA_24569 | 140 | 0.131654517 | KHDRBS2 | signal transduction associated 2 [Source:HGNC Symbol;Acc:HGNC:18114]" |
| ENSG00000117595 | HGLibA_23594 | 140 | 0.131654517 | IRF6 | interferon regulatory factor 6 [Source:HGNC Symbol;Acc:HGNC:6121] |
| ENSG00000124789 | HGLibA_33023 | 140 | 0.131654517 | NUP153 | nucleoporin 153 [Source:HGNC Symbol;Acc:HGNC:8062] |
| ENSG00000125148 | HGLibA_30302 | 140 | 0.131654517 | MT2A | metallothionein 2A [Source:HGNC Symbol;Acc:HGNC:7406] |
| ENSG00000126787 | HGLibA_13351 | 140 | 0.131654517 | DLGAP5 | DLG associated protein 5 [Source:HGNC Symbol;Acc:HGNC:16864] |
| ENSG00000127334 | HGLibA_14278 | 140 | 0.131654517 | DYRK2 | dual specificity tyrosine phosphorylation regulated kinase 2 [Source:HGNC Symbol;Acc:HGNC:3093] |
| ENSG00000128185 | HGLibA_13053 | 140 | 0.131654517 | DGCR6L | DiGeorge syndrome critical region gene 6 like [Source:HGNC Symbol;Acc:HGNC:18551] |
| ENSG00000129170 | HGLibA_11441 | 140 | 0.131654517 | CSRP3 | cysteine and glycine rich protein 3 [Source:HGNC Symbol;Acc:HGNC:2472] |
| ENSG00000131791 | HGLibA_38499 | 140 | 0.131654517 | PRKAB2 | protein kinase AMP-activated non-catalytic subunit beta 2 [Source:HGNC Symbol;Acc:HGNC:9379] |
| ENSG00000135049 | HGLibA_01260 | 140 | 0.131654517 | AGTPBP1 | ATP/GTP binding protein 1 [Source:HGNC Symbol;Acc:HGNC:17258] |
| ENSG00000135773 | HGLibA_07182 | 140 | 0.131654517 | CAPN9 | calpain 9 [Source:HGNC Symbol;Acc:HGNC:1486] |
| ENSG00000136040 | HGLibA_37278 | 140 | 0.131654517 | PLXNC1 | plexin C1 [Source:HGNC Symbol;Acc:HGNC:9106] |
| ENSG00000137070 | HGLibA_22956 | 140 | 0.131654517 | IL11RA | interleukin 11 receptor subunit alpha [Source:HGNC Symbol;Acc:HGNC:5967] |
| ENSG00000137975 | HGLibA_09808 | 140 | 0.131654517 | CLCA2 | chloride channel accessory 2 [Source:HGNC Symbol;Acc:HGNC:2016] |
| ENSG00000140939 | HGLibA_32265 | 140 | 0.131654517 | NOL3 | nucleolar protein 3 [Source:HGNC Symbol;Acc:HGNC:7869] |
| ENSG00000141040 | HGLibA_55757 | 140 | 0.131654517 | ZNF287 | zinc finger protein 287 [Source:HGNC Symbol;Acc:HGNC:13502] |
| ENSG00000141458 | HGLibA_32418 | 140 | 0.131654517 | NPC1 | NPC intracellular cholesterol transporter 1 [Source:HGNC Symbol;Acc:HGNC:7897] |
| ENSG00000142627 | HGLibA_15291 | 140 | 0.131654517 | EPHA2 | EPH receptor A2 [Source:HGNC Symbol;Acc:HGNC:3386] |
| ENSG00000144724 | HGLibA_39506 | 140 | 0.131654517 | PTPRG | receptor type G [Source:HGNC Symbol;Acc:HGNC:9671]" |
| ENSG00000147889 | HGLibA_08830 | 140 | 0.131654517 | CDKN2A | cyclin dependent kinase inhibitor 2A [Source:HGNC Symbol;Acc:HGNC:1787] |
| ENSG00000149503 | HGLibA_23278 | 140 | 0.131654517 | INCENP | inner centromere protein [Source:HGNC Symbol;Acc:HGNC:6058] |
| ENSG00000150201 | HGLibA_18232 | 140 | 0.131654517 | FXYD4 | FXYD domain containing ion transport regulator 4 [Source:HGNC Symbol;Acc:HGNC:4028] |
| ENSG00000151967 | HGLibA_42942 | 140 | 0.131654517 | SCHIP1 | schwannomin interacting protein 1 [Source:HGNC Symbol;Acc:HGNC:15678] |
| ENSG00000152082 | HGLibA_30945 | 140 | 0.131654517 | MZT2B | mitotic spindle organizing protein 2B [Source:HGNC Symbol;Acc:HGNC:25886] |
| ENSG00000154162 | HGLibA_08638 | 140 | 0.131654517 | CDH12 | cadherin 12 [Source:HGNC Symbol;Acc:HGNC:1751] |
| ENSG00000154479 | HGLibA_07699 | 140 | 0.131654517 | CCDC173 | coiled-coil domain containing 173 [Source:HGNC Symbol;Acc:HGNC:25064] |
| ENSG00000157107 | HGLibA_17208 | 140 | 0.131654517 | FCHO2 | FCH domain only 2 [Source:HGNC Symbol;Acc:HGNC:25180] |
| ENSG00000157514 | HGLibA_51823 | 140 | 0.131654517 | TSC22D3 | TSC22 domain family member 3 [Source:HGNC Symbol;Acc:HGNC:3051] |
| ENSG00000157890 | HGLibA_28850 | 140 | 0.131654517 | MEGF11 | multiple EGF like domains 11 [Source:HGNC Symbol;Acc:HGNC:29635] |
| ENSG00000158402 | HGLibA_08533 | 140 | 0.131654517 | CDC25C | cell division cycle 25C [Source:HGNC Symbol;Acc:HGNC:1727] |
| ENSG00000160294 | HGLibA_28609 | 140 | 0.131654517 | MCM3AP | minichromosome maintenance complex component 3 associated protein [Source:HGNC Symbol;Acc:HGNC:6946] |
| ENSG00000162039 | HGLibA_28868 | 140 | 0.131654517 | MEIOB | meiosis specific with OB domains [Source:HGNC Symbol;Acc:HGNC:28569] |
| ENSG00000163536 | HGLibA_43597 | 140 | 0.131654517 | SERPINI1 | serpin family I member 1 [Source:HGNC Symbol;Acc:HGNC:8943] |
| ENSG00000164237 | HGLibA_10181 | 140 | 0.131654517 | CMBL | carboxymethylenebutenolidase homolog [Source:HGNC Symbol;Acc:HGNC:25090] |
| ENSG00000166111 | HGLibA_47893 | 140 | 0.131654517 | SVOP | SV2 related protein [Source:HGNC Symbol;Acc:HGNC:25417] |
| ENSG00000166342 | HGLibA_31703 | 140 | 0.131654517 | NETO1 | neuropilin and tolloid like 1 [Source:HGNC Symbol;Acc:HGNC:13823] |
| ENSG00000166501 | HGLibA_38537 | 140 | 0.131654517 | PRKCB | protein kinase C beta [Source:HGNC Symbol;Acc:HGNC:9395] |
| ENSG00000167461 | HGLibA_39984 | 140 | 0.131654517 | RAB8A | member RAS oncogene family [Source:HGNC Symbol;Acc:HGNC:7007]" |
| ENSG00000167549 | HGLibA_10787 | 140 | 0.131654517 | CORO6 | coronin 6 [Source:HGNC Symbol;Acc:HGNC:21356] |
| ENSG00000168256 | HGLibA_32019 | 140 | 0.131654517 | NKIRAS2 | NFKB inhibitor interacting Ras like 2 [Source:HGNC Symbol;Acc:HGNC:17898] |
| ENSG00000169800 | HGLibA_40624 | 140 | 0.131654517 | RBMY1F | member F [Source:HGNC Symbol;Acc:HGNC:23974]" |
| ENSG00000170153 | HGLibA_41543 | 140 | 0.131654517 | RNF150 | ring finger protein 150 [Source:HGNC Symbol;Acc:HGNC:23138] |
| ENSG00000170191 | HGLibA_31106 | 140 | 0.131654517 | NANP | N-acetylneuraminic acid phosphatase [Source:HGNC Symbol;Acc:HGNC:16140] |
| ENSG00000170262 | HGLibA_29806 | 140 | 0.131654517 | MRAP | melanocortin 2 receptor accessory protein [Source:HGNC Symbol;Acc:HGNC:1304] |
| ENSG00000170458 | HGLibA_08234 | 140 | 0.131654517 | CD14 | CD14 molecule [Source:HGNC Symbol;Acc:HGNC:1628] |
| ENSG00000170571 | HGLibA_15000 | 140 | 0.131654517 | EMB | embigin [Source:HGNC Symbol;Acc:HGNC:30465] |
| ENSG00000170748 | HGLibA_40605 | 140 | 0.131654517 | RBMXL2 | RBMX like 2 [Source:HGNC Symbol;Acc:HGNC:17886] |
| ENSG00000170961 | HGLibA_20864 | 140 | 0.131654517 | HAS2 | hyaluronan synthase 2 [Source:HGNC Symbol;Acc:HGNC:4819] |
| ENSG00000171885 | HGLibA_02597 | 140 | 0.131654517 | AQP4 | aquaporin 4 [Source:HGNC Symbol;Acc:HGNC:637] |
| ENSG00000172188 | HGLibA_33938 | 140 | 0.131654517 | OR4C11 | olfactory receptor family 4 subfamily C member 11 [Source:HGNC Symbol;Acc:HGNC:15167] |
| ENSG00000174370 | HGLibA_05143 | 140 | 0.131654517 | C11orf45 | chromosome 11 open reading frame 45 [Source:HGNC Symbol;Acc:HGNC:28584] |
| ENSG00000174607 | HGLibA_52939 | 140 | 0.131654517 | UGT8 | UDP glycosyltransferase 8 [Source:HGNC Symbol;Acc:HGNC:12555] |
| ENSG00000175220 | HGLibA_02697 | 140 | 0.131654517 | ARHGAP1 | Rho GTPase activating protein 1 [Source:HGNC Symbol;Acc:HGNC:673] |
| ENSG00000175445 | HGLibA_27105 | 140 | 0.131654517 | LPL | lipoprotein lipase [Source:HGNC Symbol;Acc:HGNC:6677] |
| ENSG00000176182 | HGLibA_30913 | 140 | 0.131654517 | MYPOP | partner of profilin [Source:HGNC Symbol;Acc:HGNC:20178]" |
| ENSG00000176715 | HGLibA_00551 | 140 | 0.131654517 | ACSF3 | acyl-CoA synthetase family member 3 [Source:HGNC Symbol;Acc:HGNC:27288] |
| ENSG00000177689 | HGLibA_27933 | 140 | 0.131654517 | MAGEB10 | MAGE family member B10 [Source:HGNC Symbol;Acc:HGNC:25377] |
| ENSG00000179331 | HGLibA_39902 | 140 | 0.131654517 | RAB39A | member RAS oncogene family [Source:HGNC Symbol;Acc:HGNC:16521]" |
| ENSG00000180974 | HGLibA_34161 | 140 | 0.131654517 | OR52E4 | olfactory receptor family 52 subfamily E member 4 [Source:HGNC Symbol;Acc:HGNC:15213] |
| ENSG00000185985 | HGLibA_45517 | 140 | 0.131654517 | SLITRK2 | SLIT and NTRK like family member 2 [Source:HGNC Symbol;Acc:HGNC:13449] |
| ENSG00000186897 | HGLibA_05933 | 140 | 0.131654517 | C1QL4 | complement C1q like 4 [Source:HGNC Symbol;Acc:HGNC:31416] |
| ENSG00000188869 | HGLibA_49753 | 140 | 0.131654517 | TMC3 | transmembrane channel like 3 [Source:HGNC Symbol;Acc:HGNC:22995] |
| ENSG00000189367 | HGLibA_24636 | 140 | 0.131654517 | KIAA0408 | KIAA0408 [Source:HGNC Symbol;Acc:HGNC:21636] |
| ENSG00000197496 | HGLibA_44854 | 140 | 0.131654517 | SLC2A10 | solute carrier family 2 member 10 [Source:HGNC Symbol;Acc:HGNC:13444] |
| ENSG00000198081 | HGLibA_54823 | 140 | 0.131654517 | ZBTB14 | zinc finger and BTB domain containing 14 [Source:HGNC Symbol;Acc:HGNC:12860] |
| ENSG00000205835 | HGLibA_19416 | 140 | 0.131654517 | GMNC | geminin coiled-coil domain containing [Source:HGNC Symbol;Acc:HGNC:40049] |
| ENSG00000213973 | HGLibA_56917 | 140 | 0.131654517 | ZNF99 | zinc finger protein 99 [Source:HGNC Symbol;Acc:HGNC:13175] |
| ENSG00000214530 | HGLibA_47352 | 140 | 0.131654517 | STARD10 | StAR related lipid transfer domain containing 10 [Source:HGNC Symbol;Acc:HGNC:10666] |
| ENSG00000214575 | HGLibA_10917 | 140 | 0.131654517 | CPEB1 | cytoplasmic polyadenylation element binding protein 1 [Source:HGNC Symbol;Acc:HGNC:21744] |
| ENSG00000244122 | HGLibA_52894 | 140 | 0.131654517 | UGT1A7 | UDP glucuronosyltransferase family 1 member A7 [Source:HGNC Symbol;Acc:HGNC:12539] |
| ENTHD2 | HGLibA_15194 | 140 | 0.131654517 | ENTHD2 | - |
| FAM203A | HGLibA_16418 | 140 | 0.131654517 | FAM203A | - |
| HIST1H2AB | HGLibA_21377 | 140 | 0.131654517 | HIST1H2AB | - |
| HIST1H4G | HGLibA_21504 | 140 | 0.131654517 | HIST1H4G | - |
| HIST2H2AC | HGLibA_21530 | 140 | 0.131654517 | HIST2H2AC | - |
| hsa-mir-103a-2 | HGLibA_57165 | 140 | 0.131654517 | hsa-mir-103a-2 | - |
| hsa-mir-1244-1 | HGLibA_57361 | 140 | 0.131654517 | hsa-mir-1244-1 | - |
| hsa-mir-125b-2 | HGLibA_57452 | 140 | 0.131654517 | hsa-mir-125b-2 | - |
| hsa-mir-16-1 | HGLibA_57948 | 140 | 0.131654517 | hsa-mir-16-1 | - |
| hsa-mir-4436a | HGLibA_60140 | 140 | 0.131654517 | hsa-mir-4436a | - |
| hsa-mir-499a | HGLibA_61401 | 140 | 0.131654517 | hsa-mir-499a | - |
| hsa-mir-5702 | HGLibA_62349 | 140 | 0.131654517 | hsa-mir-5702 | - |
| hsa-mir-600 | HGLibA_62491 | 140 | 0.131654517 | hsa-mir-600 | - |
| hsa-mir-6504 | HGLibA_62866 | 140 | 0.131654517 | hsa-mir-6504 | - |
| hsa-mir-6776 | HGLibA_63278 | 140 | 0.131654517 | hsa-mir-6776 | - |
| hsa-mir-6797 | HGLibA_63365 | 140 | 0.131654517 | hsa-mir-6797 | - |
| hsa-mir-6842 | HGLibA_63545 | 140 | 0.131654517 | hsa-mir-6842 | - |
| hsa-mir-7155 | HGLibA_63849 | 140 | 0.131654517 | hsa-mir-7155 | - |
| WISP3 | HGLibA_54325 | 140 | 0.131654517 | WISP3 | - |
| 3-Mar | HGLibA_28333 | 139 | 0.122609174 | 3-Mar | - |
| AGPAT6 | HGLibA_01233 | 139 | 0.122609174 | AGPAT6 | - |
| C22orf24 | HGLibA_06059 | 139 | 0.122609174 | C22orf24 | - |
| CCDC11 | HGLibA_07535 | 139 | 0.122609174 | CCDC11 | - |
| ENSG00000006282 | HGLibA_46447 | 139 | 0.122609174 | SPATA20 | spermatogenesis associated 20 [Source:HGNC Symbol;Acc:HGNC:26125] |
| ENSG00000012504 | HGLibA_32586 | 139 | 0.122609174 | NR1H4 | nuclear receptor subfamily 1 group H member 4 [Source:HGNC Symbol;Acc:HGNC:7967] |
| ENSG00000015285 | HGLibA_53950 | 139 | 0.122609174 | WAS | Wiskott-Aldrich syndrome [Source:HGNC Symbol;Acc:HGNC:12731] |
| ENSG00000033122 | HGLibA_27413 | 139 | 0.122609174 | LRRC7 | leucine rich repeat containing 7 [Source:HGNC Symbol;Acc:HGNC:18531] |
| ENSG00000054523 | HGLibA_24887 | 139 | 0.122609174 | KIF1B | kinesin family member 1B [Source:HGNC Symbol;Acc:HGNC:16636] |
| ENSG00000064651 | HGLibA_44360 | 139 | 0.122609174 | SLC12A2 | solute carrier family 12 member 2 [Source:HGNC Symbol;Acc:HGNC:10911] |
| ENSG00000064835 | HGLibA_37749 | 139 | 0.122609174 | POU1F1 | POU class 1 homeobox 1 [Source:HGNC Symbol;Acc:HGNC:9210] |
| ENSG00000067445 | HGLibA_51716 | 139 | 0.122609174 | TRO | trophinin [Source:HGNC Symbol;Acc:HGNC:12326] |
| ENSG00000067829 | HGLibA_22552 | 139 | 0.122609174 | IDH3G | isocitrate dehydrogenase 3 (NAD(+)) gamma [Source:HGNC Symbol;Acc:HGNC:5386] |
| ENSG00000070404 | HGLibA_18104 | 139 | 0.122609174 | FSTL3 | follistatin like 3 [Source:HGNC Symbol;Acc:HGNC:3973] |
| ENSG00000085491 | HGLibA_44680 | 139 | 0.122609174 | SLC25A24 | solute carrier family 25 member 24 [Source:HGNC Symbol;Acc:HGNC:20662] |
| ENSG00000090539 | HGLibA_09534 | 139 | 0.122609174 | CHRD | chordin [Source:HGNC Symbol;Acc:HGNC:1949] |
| ENSG00000090932 | HGLibA_13362 | 139 | 0.122609174 | DLL3 | delta like canonical Notch ligand 3 [Source:HGNC Symbol;Acc:HGNC:2909] |
| ENSG00000095585 | HGLibA_04555 | 139 | 0.122609174 | BLNK | B cell linker [Source:HGNC Symbol;Acc:HGNC:14211] |
| ENSG00000099866 | HGLibA_27853 | 139 | 0.122609174 | MADCAM1 | mucosal vascular addressin cell adhesion molecule 1 [Source:HGNC Symbol;Acc:HGNC:6765] |
| ENSG00000100122 | HGLibA_11261 | 139 | 0.122609174 | CRYBB1 | crystallin beta B1 [Source:HGNC Symbol;Acc:HGNC:2397] |
| ENSG00000100304 | HGLibA_52196 | 139 | 0.122609174 | TTLL12 | tubulin tyrosine ligase like 12 [Source:HGNC Symbol;Acc:HGNC:28974] |
| ENSG00000100312 | HGLibA_00529 | 139 | 0.122609174 | ACR | acrosin [Source:HGNC Symbol;Acc:HGNC:126] |
| ENSG00000100764 | HGLibA_39128 | 139 | 0.122609174 | PSMC1 | ATPase 1 [Source:HGNC Symbol;Acc:HGNC:9547]" |
| ENSG00000100804 | HGLibA_39112 | 139 | 0.122609174 | PSMB5 | proteasome subunit beta 5 [Source:HGNC Symbol;Acc:HGNC:9542] |
| ENSG00000101440 | HGLibA_03314 | 139 | 0.122609174 | ASIP | agouti signaling protein [Source:HGNC Symbol;Acc:HGNC:745] |
| ENSG00000101544 | HGLibA_01005 | 139 | 0.122609174 | ADNP2 | ADNP homeobox 2 [Source:HGNC Symbol;Acc:HGNC:23803] |
| ENSG00000103174 | HGLibA_31073 | 139 | 0.122609174 | NAGPA | N-acetylglucosamine-1-phosphodiester alpha-N-acetylglucosaminidase [Source:HGNC Symbol;Acc:HGNC:17378] |
| ENSG00000105939 | HGLibA_54990 | 139 | 0.122609174 | ZC3HAV1 | antiviral 1 [Source:HGNC Symbol;Acc:HGNC:23721]" |
| ENSG00000106080 | HGLibA_17558 | 139 | 0.122609174 | FKBP14 | FK506 binding protein 14 [Source:HGNC Symbol;Acc:HGNC:18625] |
| ENSG00000107959 | HGLibA_36759 | 139 | 0.122609174 | PITRM1 | pitrilysin metallopeptidase 1 [Source:HGNC Symbol;Acc:HGNC:17663] |
| ENSG00000108821 | HGLibA_10530 | 139 | 0.122609174 | COL1A1 | collagen type I alpha 1 chain [Source:HGNC Symbol;Acc:HGNC:2197] |
| ENSG00000109255 | HGLibA_32210 | 139 | 0.122609174 | NMU | neuromedin U [Source:HGNC Symbol;Acc:HGNC:7859] |
| ENSG00000110245 | HGLibA_02510 | 139 | 0.122609174 | APOC3 | apolipoprotein C3 [Source:HGNC Symbol;Acc:HGNC:610] |
| ENSG00000111785 | HGLibA_41238 | 139 | 0.122609174 | RIC8B | RIC8 guanine nucleotide exchange factor B [Source:HGNC Symbol;Acc:HGNC:25555] |
| ENSG00000112697 | HGLibA_50359 | 139 | 0.122609174 | TMEM30A | transmembrane protein 30A [Source:HGNC Symbol;Acc:HGNC:16667] |
| ENSG00000113391 | HGLibA_16274 | 139 | 0.122609174 | FAM172A | family with sequence similarity 172 member A [Source:HGNC Symbol;Acc:HGNC:25365] |
| ENSG00000115207 | HGLibA_20583 | 139 | 0.122609174 | GTF3C2 | general transcription factor IIIC subunit 2 [Source:HGNC Symbol;Acc:HGNC:4665] |

| | | | | | |
|---|---|---|---|---|---|
| ENSG00000115318 | HGLibA_27045 | 139 | 0.122609174 | LOXL3 | lysyl oxidase like 3 [Source:HGNC Symbol;Acc:HGNC:13869] |
| ENSG00000117153 | HGLibA_25130 | 139 | 0.122609174 | KLHL12 | kelch like family member 12 [Source:HGNC Symbol;Acc:HGNC:19360] |
| ENSG00000117632 | HGLibA_47517 | 139 | 0.122609174 | STMN1 | stathmin 1 [Source:HGNC Symbol;Acc:HGNC:6510] |
| ENSG00000120937 | HGLibA_32501 | 139 | 0.122609174 | NPPB | natriuretic peptide B [Source:HGNC Symbol;Acc:HGNC:7940] |
| ENSG00000125657 | HGLibA_50830 | 139 | 0.122609174 | TNFSF9 | TNF superfamily member 9 [Source:HGNC Symbol;Acc:HGNC:11939] |
| ENSG00000125734 | HGLibA_19847 | 139 | 0.122609174 | GPR108 | G protein-coupled receptor 108 [Source:HGNC Symbol;Acc:HGNC:17829] |
| ENSG00000125843 | HGLibA_02360 | 139 | 0.122609174 | AP5S1 | adaptor related protein complex 5 subunit sigma 1 [Source:HGNC Symbol;Acc:HGNC:15875] |
| ENSG00000126803 | HGLibA_22281 | 139 | 0.122609174 | HSPA2 | heat shock protein family A (Hsp70) member 2 [Source:HGNC Symbol;Acc:HGNC:5235] |
| ENSG00000127585 | HGLibA_16985 | 139 | 0.122609174 | FBXL16 | F-box and leucine rich repeat protein 16 [Source:HGNC Symbol;Acc:HGNC:14150] |
| ENSG00000130176 | HGLibA_10305 | 139 | 0.122609174 | CNN1 | calponin 1 [Source:HGNC Symbol;Acc:HGNC:2155] |
| ENSG00000131409 | HGLibA_27379 | 139 | 0.122609174 | LRRC4B | leucine rich repeat containing 4B [Source:HGNC Symbol;Acc:HGNC:25042] |
| ENSG00000131462 | HGLibA_52304 | 139 | 0.122609174 | TUBG1 | tubulin gamma 1 [Source:HGNC Symbol;Acc:HGNC:12417] |
| ENSG00000132010 | HGLibA_55568 | 139 | 0.122609174 | ZNF20 | zinc finger protein 20 [Source:HGNC Symbol;Acc:HGNC:12992] |
| ENSG00000132622 | HGLibA_22263 | 139 | 0.122609174 | HSPA12B | heat shock protein family A (Hsp70) member 12B [Source:HGNC Symbol;Acc:HGNC:16193] |
| ENSG00000133937 | HGLibA_20399 | 139 | 0.122609174 | GSC | goosecoid homeobox [Source:HGNC Symbol;Acc:HGNC:4612] |
| ENSG00000136143 | HGLibA_47697 | 139 | 0.122609174 | SUCLA2 | succinate-CoA ligase ADP-forming beta subunit [Source:HGNC Symbol;Acc:HGNC:11448] |
| ENSG00000136450 | HGLibA_47060 | 139 | 0.122609174 | SRSF1 | serine and arginine rich splicing factor 1 [Source:HGNC Symbol;Acc:HGNC:10780] |
| ENSG00000136573 | HGLibA_04545 | 139 | 0.122609174 | BLK | Src family tyrosine kinase [Source:HGNC Symbol;Acc:HGNC:1057]" |
| ENSG00000138381 | HGLibA_03337 | 139 | 0.122609174 | ASNSD1 | asparagine synthetase domain containing 1 [Source:HGNC Symbol;Acc:HGNC:24910] |
| ENSG00000141449 | HGLibA_20238 | 139 | 0.122609174 | GREB1L | GREB1 like retinoic acid receptor coactivator [Source:HGNC Symbol;Acc:HGNC:31042] |
| ENSG00000142166 | HGLibA_22696 | 139 | 0.122609174 | IFNAR1 | interferon alpha and beta receptor subunit 1 [Source:HGNC Symbol;Acc:HGNC:5432] |
| ENSG00000143549 | HGLibA_51148 | 139 | 0.122609174 | TPM3 | tropomyosin 3 [Source:HGNC Symbol;Acc:HGNC:12012] |
| ENSG00000143554 | HGLibA_44818 | 139 | 0.122609174 | SLC27A3 | solute carrier family 27 member 3 [Source:HGNC Symbol;Acc:HGNC:10997] |
| ENSG00000144040 | HGLibA_43793 | 139 | 0.122609174 | SFXN5 | sideroflexin 5 [Source:HGNC Symbol;Acc:HGNC:16073] |
| ENSG00000145214 | HGLibA_13082 | 139 | 0.122609174 | DGKQ | diacylglycerol kinase theta [Source:HGNC Symbol;Acc:HGNC:2856] |
| ENSG00000148935 | HGLibA_18645 | 139 | 0.122609174 | GAS2 | growth arrest specific 2 [Source:HGNC Symbol;Acc:HGNC:4167] |
| ENSG00000153071 | HGLibA_12267 | 139 | 0.122609174 | DAB2 | clathrin adaptor protein [Source:HGNC Symbol;Acc:HGNC:2662]" |
| ENSG00000158445 | HGLibA_24155 | 139 | 0.122609174 | KCNB1 | potassium voltage-gated channel subfamily B member 1 [Source:HGNC Symbol;Acc:HGNC:6231] |
| ENSG00000163125 | HGLibA_42051 | 139 | 0.122609174 | RPRD2 | regulation of nuclear pre-mRNA domain containing 2 [Source:HGNC Symbol;Acc:HGNC:29039] |
| ENSG00000163625 | HGLibA_54009 | 139 | 0.122609174 | WDFY3 | WD repeat and FYVE domain containing 3 [Source:HGNC Symbol;Acc:HGNC:20751] |
| ENSG00000163683 | HGLibA_45730 | 139 | 0.122609174 | SMIM14 | small integral membrane protein 14 [Source:HGNC Symbol;Acc:HGNC:27321] |
| ENSG00000164066 | HGLibA_23440 | 139 | 0.122609174 | INTU | inturned planar cell polarity protein [Source:HGNC Symbol;Acc:HGNC:29239] |
| ENSG00000164756 | HGLibA_44919 | 139 | 0.122609174 | SLC30A8 | solute carrier family 30 member 8 [Source:HGNC Symbol;Acc:HGNC:20303] |
| ENSG00000165304 | HGLibA_28880 | 139 | 0.122609174 | MELK | maternal embryonic leucine zipper kinase [Source:HGNC Symbol;Acc:HGNC:16870] |
| ENSG00000166579 | HGLibA_31388 | 139 | 0.122609174 | NDEL1 | nudE neurodevelopment protein 1 like 1 [Source:HGNC Symbol;Acc:HGNC:17620] |
| ENSG00000168621 | HGLibA_18888 | 139 | 0.122609174 | GDNF | glial cell derived neurotrophic factor [Source:HGNC Symbol;Acc:HGNC:4232] |
| ENSG00000169032 | HGLibA_28139 | 139 | 0.122609174 | MAP2K1 | mitogen-activated protein kinase kinase 1 [Source:HGNC Symbol;Acc:HGNC:6840] |
| ENSG00000171044 | HGLibA_54526 | 139 | 0.122609174 | XKR6 | XK related 6 [Source:HGNC Symbol;Acc:HGNC:27806] |
| ENSG00000171462 | HGLibA_13357 | 139 | 0.122609174 | DLK2 | delta like non-canonical Notch ligand 2 [Source:HGNC Symbol;Acc:HGNC:21113] |
| ENSG00000171827 | HGLibA_56276 | 139 | 0.122609174 | ZNF570 | zinc finger protein 570 [Source:HGNC Symbol;Acc:HGNC:26416] |
| ENSG00000172508 | HGLibA_07255 | 139 | 0.122609174 | CARNS1 | carnosine synthase 1 [Source:HGNC Symbol;Acc:HGNC:29268] |
| ENSG00000173281 | HGLibA_38102 | 139 | 0.122609174 | PPP1R3B | protein phosphatase 1 regulatory subunit 3B [Source:HGNC Symbol;Acc:HGNC:14942] |
| ENSG00000173546 | HGLibA_11413 | 139 | 0.122609174 | CSPG4 | chondroitin sulfate proteoglycan 4 [Source:HGNC Symbol;Acc:HGNC:2466] |
| ENSG00000175931 | HGLibA_52641 | 139 | 0.122609174 | UBE2O | ubiquitin conjugating enzyme E2 O [Source:HGNC Symbol;Acc:HGNC:29554] |
| ENSG00000177311 | HGLibA_54872 | 139 | 0.122609174 | ZBTB38 | zinc finger and BTB domain containing 38 [Source:HGNC Symbol;Acc:HGNC:26636] |
| ENSG00000178802 | HGLibA_29732 | 139 | 0.122609174 | MPI | mannose phosphate isomerase [Source:HGNC Symbol;Acc:HGNC:7216] |
| ENSG00000179023 | HGLibA_25104 | 139 | 0.122609174 | KLHDC7A | kelch domain containing 7A [Source:HGNC Symbol;Acc:HGNC:26791] |
| ENSG00000183785 | HGLibA_52264 | 139 | 0.122609174 | TUBA8 | tubulin alpha 8 [Source:HGNC Symbol;Acc:HGNC:12410] |
| ENSG00000184232 | HGLibA_33192 | 139 | 0.122609174 | OAF | out at first homolog [Source:HGNC Symbol;Acc:HGNC:28752] |
| ENSG00000184922 | HGLibA_17694 | 139 | 0.122609174 | FMNL1 | formin like 1 [Source:HGNC Symbol;Acc:HGNC:1212] |
| ENSG00000185053 | HGLibA_43810 | 139 | 0.122609174 | SGCZ | sarcoglycan zeta [Source:HGNC Symbol;Acc:HGNC:14075] |
| ENSG00000186047 | HGLibA_13322 | 139 | 0.122609174 | DLEU7 | deleted in lymphocytic leukemia 7 [Source:HGNC Symbol;Acc:HGNC:17567] |
| ENSG00000196367 | HGLibA_51799 | 139 | 0.122609174 | TRRAP | transformation/transcription domain associated protein [Source:HGNC Symbol;Acc:HGNC:12347] |
| ENSG00000196772 | HGLibA_33630 | 139 | 0.122609174 | OR14A16 | olfactory receptor family 14 subfamily A member 16 [Source:HGNC Symbol;Acc:HGNC:15022] |
| ENSG00000198092 | HGLibA_50623 | 139 | 0.122609174 | TMPRSS11F | transmembrane serine protease 11F [Source:HGNC Symbol;Acc:HGNC:29994] |
| ENSG00000198812 | HGLibA_27249 | 139 | 0.122609174 | LRRC10 | leucine rich repeat containing 10 [Source:HGNC Symbol;Acc:HGNC:20264] |
| ENSG00000198846 | HGLibA_51037 | 139 | 0.122609174 | TOX | thymocyte selection associated high mobility group box [Source:HGNC Symbol;Acc:HGNC:18988] |
| ENSG00000204444 | HGLibA_02548 | 139 | 0.122609174 | APOM | apolipoprotein M [Source:HGNC Symbol;Acc:HGNC:13916] |
| ENSG00000205089 | HGLibA_08106 | 139 | 0.122609174 | CCNI2 | cyclin I family member 2 [Source:HGNC Symbol;Acc:HGNC:33869] |
| ENSG00000206190 | HGLibA_03546 | 139 | 0.122609174 | ATP10A | ATPase phospholipid transporting 10A (putative) [Source:HGNC Symbol;Acc:HGNC:13542] |
| ENSG00000212900 | HGLibA_25744 | 139 | 0.122609174 | KRTAP3-2 | keratin associated protein 3-2 [Source:HGNC Symbol;Acc:HGNC:16779] |
| ENSG00000214456 | HGLibA_37183 | 139 | 0.122609174 | PLIN5 | perilipin 5 [Source:HGNC Symbol;Acc:HGNC:33196] |
| ENSG00000215595 | HGLibA_06015 | 139 | 0.122609174 | C20orf202 | chromosome 20 open reading frame 202 [Source:HGNC Symbol;Acc:HGNC:37254] |
| ENSG00000229549 | HGLibA_51976 | 139 | 0.122609174 | TSPY8 | testis specific protein Y-linked 8 [Source:HGNC Symbol;Acc:HGNC:37471] |
| ENSG00000236737 | HGLibA_18436 | 139 | 0.122609174 | GAGE12B | G antigen 12B [Source:HGNC Symbol;Acc:HGNC:26779] |
| ENSG00000260314 | HGLibA_29815 | 139 | 0.122609174 | MRC1 | mannose receptor C-type 1 [Source:HGNC Symbol;Acc:HGNC:7228] |
| ENSG00000270647 | HGLibA_48210 | 139 | 0.122609174 | TAF15 | TATA-box binding protein associated factor 15 [Source:HGNC Symbol;Acc:HGNC:11547] |
| ENSG00000279301 | HGLibA_33857 | 139 | 0.122609174 | OR2T11 | olfactory receptor family 2 subfamily T member 11 (gene/pseudogene) [Source:HGNC Symbol;Acc:HGNC:19574] |
| GPR112 | HGLibA_19853 | 139 | 0.122609174 | GPR112 | - |
| GPR97 | HGLibA_20112 | 139 | 0.122609174 | GPR97 | - |
| HIST1H2AA | HGLibA_21375 | 139 | 0.122609174 | HIST1H2AA | - |
| hsa-mir-107 | HGLibA_57195 | 139 | 0.122609174 | hsa-mir-107 | - |
| hsa-mir-3150b | HGLibA_58658 | 139 | 0.122609174 | hsa-mir-3150b | - |
| hsa-mir-3155b | HGLibA_58684 | 139 | 0.122609174 | hsa-mir-3155b | - |
| hsa-mir-3194 | HGLibA_58875 | 139 | 0.122609174 | hsa-mir-3194 | - |
| hsa-mir-323a | HGLibA_58956 | 139 | 0.122609174 | hsa-mir-323a | - |
| hsa-mir-337 | HGLibA_58998 | 139 | 0.122609174 | hsa-mir-337 | - |
| hsa-mir-337 | HGLibA_59001 | 139 | 0.122609174 | hsa-mir-337 | - |
| hsa-mir-3655 | HGLibA_59167 | 139 | 0.122609174 | hsa-mir-3655 | - |
| hsa-mir-3659 | HGLibA_59180 | 139 | 0.122609174 | hsa-mir-3659 | - |
| hsa-mir-374a | HGLibA_59388 | 139 | 0.122609174 | hsa-mir-374a | - |
| hsa-mir-4306 | HGLibA_59936 | 139 | 0.122609174 | hsa-mir-4306 | - |
| hsa-mir-4518 | HGLibA_60515 | 139 | 0.122609174 | hsa-mir-4518 | - |
| hsa-mir-4749 | HGLibA_61092 | 139 | 0.122609174 | hsa-mir-4749 | - |
| hsa-mir-7973-1 | HGLibA_64018 | 139 | 0.122609174 | hsa-mir-7973-1 | - |
| LOC100288255 | HGLibA_26827 | 139 | 0.122609174 | LOC100288255 | - |
| LOC149373 | HGLibA_26892 | 139 | 0.122609174 | LOC149373 | - |
| PRR24 | HGLibA_38830 | 139 | 0.122609174 | PRR24 | - |
| REXO1L1 | HGLibA_40894 | 139 | 0.122609174 | REXO1L1 | - |
| SLC22A20 | HGLibA_44571 | 139 | 0.122609174 | SLC22A20 | - |
| TMEM56 | HGLibA_50450 | 139 | 0.122609174 | TMEM56 | - |
| WDR66 | HGLibA_54153 | 139 | 0.122609174 | WDR66 | - |
| C17orf82 | HGLibA_05584 | 138 | 0.113563831 | C17orf82 | - |
| ENSG00000005020 | HGLibA_44273 | 138 | 0.113563831 | SKAP2 | src kinase associated phosphoprotein 2 [Source:HGNC Symbol;Acc:HGNC:15687] |
| ENSG00000008197 | HGLibA_49130 | 138 | 0.113563831 | TFAP2D | transcription factor AP-2 delta [Source:HGNC Symbol;Acc:HGNC:15581] |
| ENSG00000011083 | HGLibA_45336 | 138 | 0.113563831 | SLC6A7 | solute carrier family 6 member 7 [Source:HGNC Symbol;Acc:HGNC:11054] |
| ENSG00000034677 | HGLibA_41600 | 138 | 0.113563831 | RNF19A | RBR E3 ubiquitin protein ligase [Source:HGNC Symbol;Acc:HGNC:13432]" |
| ENSG00000055813 | HGLibA_07891 | 138 | 0.113563831 | CCDC85A | coiled-coil domain containing 85A [Source:HGNC Symbol;Acc:HGNC:29400] |
| ENSG00000065559 | HGLibA_28149 | 138 | 0.113563831 | MAP2K4 | mitogen-activated protein kinase kinase 4 [Source:HGNC Symbol;Acc:HGNC:6844] |
| ENSG00000066855 | HGLibA_30362 | 138 | 0.113563831 | MTFR1 | mitochondrial fission regulator 1 [Source:HGNC Symbol;Acc:HGNC:29510] |
| ENSG00000067840 | HGLibA_35979 | 138 | 0.113563831 | PDZD4 | PDZ domain containing 4 [Source:HGNC Symbol;Acc:HGNC:21167] |
| ENSG00000073350 | HGLibA_26650 | 138 | 0.113563831 | LLGL2 | scribble cell polarity complex component [Source:HGNC Symbol;Acc:HGNC:6629]" |
| ENSG00000075618 | HGLibA_18068 | 138 | 0.113563831 | FSCN1 | fascin actin-bundling protein 1 [Source:HGNC Symbol;Acc:HGNC:11148] |
| ENSG00000082269 | HGLibA_16131 | 138 | 0.113563831 | FAM135A | family with sequence similarity 135 member A [Source:HGNC Symbol;Acc:HGNC:21084] |
| ENSG00000099904 | HGLibA_55128 | 138 | 0.113563831 | ZDHHC8 | zinc finger DHHC-type containing 8 [Source:HGNC Symbol;Acc:HGNC:18474] |
| ENSG00000100592 | HGLibA_12258 | 138 | 0.113563831 | DAAM1 | dishevelled associated activator of morphogenesis 1 [Source:HGNC Symbol;Acc:HGNC:18142] |
| ENSG00000100604 | HGLibA_09430 | 138 | 0.113563831 | CHGA | chromogranin A [Source:HGNC Symbol;Acc:HGNC:1929] |
| ENSG00000101558 | HGLibA_53518 | 138 | 0.113563831 | VAPA | VAMP associated protein A [Source:HGNC Symbol;Acc:HGNC:12648] |
| ENSG00000101574 | HGLibA_29009 | 138 | 0.113563831 | METTL4 | methyltransferase like 4 [Source:HGNC Symbol;Acc:HGNC:24726] |
| ENSG00000101986 | HGLibA_00191 | 138 | 0.113563831 | ABCD1 | ATP binding cassette subfamily D member 1 [Source:HGNC Symbol;Acc:HGNC:61] |
| ENSG00000102104 | HGLibA_42294 | 138 | 0.113563831 | RS1 | retinoschisin 1 [Source:HGNC Symbol;Acc:HGNC:10457] |
| ENSG00000102195 | HGLibA_20046 | 138 | 0.113563831 | GPR50 | G protein-coupled receptor 50 [Source:HGNC Symbol;Acc:HGNC:4506] |
| ENSG00000106484 | HGLibA_28921 | 138 | 0.113563831 | MEST | mesoderm specific transcript [Source:HGNC Symbol;Acc:HGNC:7028] |
| ENSG00000109654 | HGLibA_51425 | 138 | 0.113563831 | TRIM2 | tripartite motif containing 2 [Source:HGNC Symbol;Acc:HGNC:15974] |
| ENSG00000109771 | HGLibA_27214 | 138 | 0.113563831 | LRP2BP | LRP2 binding protein [Source:HGNC Symbol;Acc:HGNC:25434] |
| ENSG00000111291 | HGLibA_20133 | 138 | 0.113563831 | GPRC5D | G protein-coupled receptor class C group 5 member D [Source:HGNC Symbol;Acc:HGNC:13310] |
| ENSG00000112210 | HGLibA_39845 | 138 | 0.113563831 | RAB23 | member RAS oncogene family [Source:HGNC Symbol;Acc:HGNC:14263]" |
| ENSG00000112531 | HGLibA_39724 | 138 | 0.113563831 | QKI | KH domain containing RNA binding [Source:HGNC Symbol;Acc:HGNC:21100]" |
| ENSG00000112640 | HGLibA_38178 | 138 | 0.113563831 | PPP2R5D | protein phosphatase 2 regulatory subunit B'delta [Source:HGNC Symbol;Acc:HGNC:9312] |
| ENSG00000113430 | HGLibA_23638 | 138 | 0.113563831 | IRX4 | iroquois homeobox 4 [Source:HGNC Symbol;Acc:HGNC:6129] |
| ENSG00000114573 | HGLibA_03729 | 138 | 0.113563831 | ATP6V1A | ATPase H+ transporting V1 subunit A [Source:HGNC Symbol;Acc:HGNC:851] |
| ENSG00000115295 | HGLibA_10050 | 138 | 0.113563831 | CLIP4 | CAP-Gly domain containing linker protein family member 4 [Source:HGNC Symbol;Acc:HGNC:26108] |
| ENSG00000115944 | HGLibA_10862 | 138 | 0.113563831 | COX7A2L | cytochrome c oxidase subunit 7A2 like [Source:HGNC Symbol;Acc:HGNC:2289] |
| ENSG00000116819 | HGLibA_49132 | 138 | 0.113563831 | TFAP2E | transcription factor AP-2 epsilon [Source:HGNC Symbol;Acc:HGNC:30774] |
| ENSG00000116833 | HGLibA_32638 | 138 | 0.113563831 | NR5A2 | nuclear receptor subfamily 5 group A member 2 [Source:HGNC Symbol;Acc:HGNC:7984] |
| ENSG00000119004 | HGLibA_12075 | 138 | 0.113563831 | CYP20A1 | cytochrome P450 family 20 subfamily A member 1 [Source:HGNC Symbol;Acc:HGNC:20576] |
| ENSG00000121022 | HGLibA_10722 | 138 | 0.113563831 | COPS5 | COP9 signalosome subunit 5 [Source:HGNC Symbol;Acc:HGNC:2240] |
| ENSG00000123388 | HGLibA_21958 | 138 | 0.113563831 | HOXC11 | homeobox C11 [Source:HGNC Symbol;Acc:HGNC:5123] |
| ENSG00000126003 | HGLibA_36956 | 138 | 0.113563831 | PLAGL2 | PLAG1 like zinc finger 2 [Source:HGNC Symbol;Acc:HGNC:9047] |
| ENSG00000126752 | HGLibA_47194 | 138 | 0.113563831 | SSX1 | SSX family member 1 [Source:HGNC Symbol;Acc:HGNC:11335] |
| ENSG00000127586 | HGLibA_09661 | 138 | 0.113563831 | CHTF18 | chromosome transmission fidelity factor 18 [Source:HGNC Symbol;Acc:HGNC:18435] |
| ENSG00000130713 | HGLibA_15776 | 138 | 0.113563831 | EXOSC2 | exosome component 2 [Source:HGNC Symbol;Acc:HGNC:17097] |
| ENSG00000131116 | HGLibA_55968 | 138 | 0.113563831 | ZNF428 | zinc finger protein 428 [Source:HGNC Symbol;Acc:HGNC:20804] |
| ENSG00000131558 | HGLibA_15750 | 138 | 0.113563831 | EXOC4 | exocyst complex component 4 [Source:HGNC Symbol;Acc:HGNC:30389] |
| ENSG00000132819 | HGLibA_40539 | 138 | 0.113563831 | RBM38 | RNA binding motif protein 38 [Source:HGNC Symbol;Acc:HGNC:15818] |

| | | | | | |
|---|---|---|---|---|---|
| ENSG00000136696 | HGLibA_23147 | 138 | 0.113563831 | IL36B | interleukin 36 beta [Source:HGNC Symbol;Acc:HGNC:15564] |
| ENSG00000137628 | HGLibA_12762 | 138 | 0.113563831 | DDX60 | DExD/H-box helicase 60 [Source:HGNC Symbol;Acc:HGNC:25942] |
| ENSG00000138136 | HGLibA_26086 | 138 | 0.113563831 | LBX1 | ladybird homeobox 1 [Source:HGNC Symbol;Acc:HGNC:16960] |
| ENSG00000138433 | HGLibA_09719 | 138 | 0.113563831 | CIR1 | 1 [Source:HGNC Symbol;Acc:HGNC:24217]" |
| ENSG00000141337 | HGLibA_03143 | 138 | 0.113563831 | ARSG | arylsulfatase G [Source:HGNC Symbol;Acc:HGNC:24102] |
| ENSG00000143520 | HGLibA_17611 | 138 | 0.113563831 | FLG2 | filaggrin family member 2 [Source:HGNC Symbol;Acc:HGNC:33276] |
| ENSG00000143774 | HGLibA_20673 | 138 | 0.113563831 | GUK1 | guanylate kinase 1 [Source:HGNC Symbol;Acc:HGNC:4693] |
| ENSG00000146963 | HGLibA_27624 | 138 | 0.113563831 | LUC7L2 | pre-mRNA splicing factor [Source:HGNC Symbol;Acc:HGNC:21608]" |
| ENSG00000149609 | HGLibA_05990 | 138 | 0.113563831 | C20orf144 | chromosome 20 open reading frame 144 [Source:HGNC Symbol;Acc:HGNC:16137] |
| ENSG00000151239 | HGLibA_52370 | 138 | 0.113563831 | TWF1 | twinfilin actin binding protein 1 [Source:HGNC Symbol;Acc:HGNC:9620] |
| ENSG00000153214 | HGLibA_50535 | 138 | 0.113563831 | TMEM87B | transmembrane protein 87B [Source:HGNC Symbol;Acc:HGNC:25913] |
| ENSG00000155966 | HGLibA_01109 | 138 | 0.113563831 | AFF2 | AF4/FMR2 family member 2 [Source:HGNC Symbol;Acc:HGNC:3776] |
| ENSG00000157827 | HGLibA_17695 | 138 | 0.113563831 | FMNL2 | formin like 2 [Source:HGNC Symbol;Acc:HGNC:18267] |
| ENSG00000158458 | HGLibA_32679 | 138 | 0.113563831 | NRG2 | neuregulin 2 [Source:HGNC Symbol;Acc:HGNC:7998] |
| ENSG00000159339 | HGLibA_34977 | 138 | 0.113563831 | PADI4 | peptidyl arginine deiminase 4 [Source:HGNC Symbol;Acc:HGNC:18368] |
| ENSG00000160293 | HGLibA_53552 | 138 | 0.113563831 | VAV2 | vav guanine nucleotide exchange factor 2 [Source:HGNC Symbol;Acc:HGNC:12658] |
| ENSG00000161013 | HGLibA_29145 | 138 | 0.113563831 | MGAT4B | 3-mannosyl-glycoprotein 4-beta-N-acetylglucosaminyltransferase B [Source:HGNC Symbol;Acc:HGNC:7048]" |
| ENSG00000161270 | HGLibA_32454 | 138 | 0.113563831 | NPHS1 | nephrin [Source:HGNC Symbol;Acc:HGNC:7908]" |
| ENSG00000161618 | HGLibA_01535 | 138 | 0.113563831 | ALDH16A1 | aldehyde dehydrogenase 16 family member A1 [Source:HGNC Symbol;Acc:HGNC:28114] |
| ENSG00000162490 | HGLibA_13953 | 138 | 0.113563831 | DRAXIN | dorsal inhibitory axon guidance protein [Source:HGNC Symbol;Acc:HGNC:25054] |
| ENSG00000162727 | HGLibA_33841 | 138 | 0.113563831 | OR2M5 | olfactory receptor family 2 subfamily M member 5 [Source:HGNC Symbol;Acc:HGNC:19576] |
| ENSG00000163914 | HGLibA_41163 | 138 | 0.113563831 | RHO | rhodopsin [Source:HGNC Symbol;Acc:HGNC:10012] |
| ENSG00000164078 | HGLibA_30260 | 138 | 0.113563831 | MST1R | macrophage stimulating 1 receptor [Source:HGNC Symbol;Acc:HGNC:7381] |
| ENSG00000164221 | HGLibA_07544 | 138 | 0.113563831 | CCDC112 | coiled-coil domain containing 112 [Source:HGNC Symbol;Acc:HGNC:28599] |
| ENSG00000164543 | HGLibA_47463 | 138 | 0.113563831 | STK17A | serine/threonine kinase 17a [Source:HGNC Symbol;Acc:HGNC:11395] |
| ENSG00000165752 | HGLibA_47488 | 138 | 0.113563831 | STK32C | serine/threonine kinase 32C [Source:HGNC Symbol;Acc:HGNC:21332] |
| ENSG00000167065 | HGLibA_14152 | 138 | 0.113563831 | DUSP18 | dual specificity phosphatase 18 [Source:HGNC Symbol;Acc:HGNC:18484] |
| ENSG00000167701 | HGLibA_20163 | 138 | 0.113563831 | GPT | glutamic--pyruvic transaminase [Source:HGNC Symbol;Acc:HGNC:4552] |
| ENSG00000169032 | HGLibA_28140 | 138 | 0.113563831 | MAP2K1 | mitogen-activated protein kinase kinase 1 [Source:HGNC Symbol;Acc:HGNC:6840] |
| ENSG00000169562 | HGLibA_19158 | 138 | 0.113563831 | GJB1 | gap junction protein beta 1 [Source:HGNC Symbol;Acc:HGNC:4283] |
| ENSG00000169813 | HGLibA_21819 | 138 | 0.113563831 | HNRNPF | heterogeneous nuclear ribonucleoprotein F [Source:HGNC Symbol;Acc:HGNC:5039] |
| ENSG00000170035 | HGLibA_52599 | 138 | 0.113563831 | UBE2E3 | ubiquitin conjugating enzyme E2 E3 [Source:HGNC Symbol;Acc:HGNC:12479] |
| ENSG00000171853 | HGLibA_51307 | 138 | 0.113563831 | TRAPPC12 | trafficking protein particle complex 12 [Source:HGNC Symbol;Acc:HGNC:24284] |
| ENSG00000172154 | HGLibA_34535 | 138 | 0.113563831 | OR8I2 | olfactory receptor family 8 subfamily I member 2 [Source:HGNC Symbol;Acc:HGNC:15310] |
| ENSG00000172208 | HGLibA_34069 | 138 | 0.113563831 | OR4X2 | olfactory receptor family 4 subfamily X member 2 (gene/pseudogene) [Source:HGNC Symbol;Acc:HGNC:15184] |
| ENSG00000173673 | HGLibA_21183 | 138 | 0.113563831 | HES3 | hes family bHLH transcription factor 3 [Source:HGNC Symbol;Acc:HGNC:26226] |
| ENSG00000173679 | HGLibA_33685 | 138 | 0.113563831 | OR1L1 | olfactory receptor family 1 subfamily L member 1 [Source:HGNC Symbol;Acc:HGNC:8213] |
| ENSG00000173692 | HGLibA_39147 | 138 | 0.113563831 | PSMD1 | non-ATPase 1 [Source:HGNC Symbol;Acc:HGNC:9554]" |
| ENSG00000175336 | HGLibA_02522 | 138 | 0.113563831 | APOF | apolipoprotein F [Source:HGNC Symbol;Acc:HGNC:615] |
| ENSG00000176547 | HGLibA_33952 | 138 | 0.113563831 | OR4C3 | olfactory receptor family 4 subfamily C member 3 [Source:HGNC Symbol;Acc:HGNC:14697] |
| ENSG00000177324 | HGLibA_04402 | 138 | 0.113563831 | BEND2 | BEN domain containing 2 [Source:HGNC Symbol;Acc:HGNC:28509] |
| ENSG00000177425 | HGLibA_35293 | 138 | 0.113563831 | PAWR | pro-apoptotic WT1 regulator [Source:HGNC Symbol;Acc:HGNC:8614] |
| ENSG00000177646 | HGLibA_00363 | 138 | 0.113563831 | ACAD9 | acyl-CoA dehydrogenase family member 9 [Source:HGNC Symbol;Acc:HGNC:21497] |
| ENSG00000177839 | HGLibA_35514 | 138 | 0.113563831 | PCDHB9 | protocadherin beta 9 [Source:HGNC Symbol;Acc:HGNC:8694] |
| ENSG00000178591 | HGLibA_12880 | 138 | 0.113563831 | DEFB125 | defensin beta 125 [Source:HGNC Symbol;Acc:HGNC:18105] |
| ENSG00000178796 | HGLibA_41248 | 138 | 0.113563831 | RIIAD1 | regulatory subunit of type II PKA R-subunit (RIIa) domain containing 1 [Source:HGNC Symbol;Acc:HGNC:26686] |
| ENSG00000180964 | HGLibA_48725 | 138 | 0.113563831 | TCEAL8 | transcription elongation factor A like 8 [Source:HGNC Symbol;Acc:HGNC:28683] |
| ENSG00000180988 | HGLibA_34197 | 138 | 0.113563831 | OR52N2 | olfactory receptor family 52 subfamily N member 2 [Source:HGNC Symbol;Acc:HGNC:15228] |
| ENSG00000182759 | HGLibA_27871 | 138 | 0.113563831 | MAFA | MAF bZIP transcription factor A [Source:HGNC Symbol;Acc:HGNC:23145] |
| ENSG00000183060 | HGLibA_27773 | 138 | 0.113563831 | LYSMD4 | LysM domain containing 4 [Source:HGNC Symbol;Acc:HGNC:26571] |
| ENSG00000183303 | HGLibA_34347 | 138 | 0.113563831 | OR5P2 | olfactory receptor family 5 subfamily P member 2 [Source:HGNC Symbol;Acc:HGNC:14783] |
| ENSG00000183979 | HGLibA_32410 | 138 | 0.113563831 | NPB | neuropeptide B [Source:HGNC Symbol;Acc:HGNC:30099] |
| ENSG00000184032 | HGLibA_25701 | 138 | 0.113563831 | KRTAP20-2 | keratin associated protein 20-2 [Source:HGNC Symbol;Acc:HGNC:18944] |
| ENSG00000184999 | HGLibA_44537 | 138 | 0.113563831 | SLC22A10 | solute carrier family 22 member 10 [Source:HGNC Symbol;Acc:HGNC:18057] |
| ENSG00000185798 | HGLibA_54122 | 138 | 0.113563831 | WDR53 | WD repeat domain 53 [Source:HGNC Symbol;Acc:HGNC:28786] |
| ENSG00000186912 | HGLibA_34888 | 138 | 0.113563831 | P2RY4 | pyrimidinergic receptor P2Y4 [Source:HGNC Symbol;Acc:HGNC:8542] |
| ENSG00000188176 | HGLibA_45835 | 138 | 0.113563831 | SMTNL2 | smoothelin like 2 [Source:HGNC Symbol;Acc:HGNC:24764] |
| ENSG00000188211 | HGLibA_31372 | 138 | 0.113563831 | NCR3LG1 | natural killer cell cytotoxicity receptor 3 ligand 1 [Source:HGNC Symbol;Acc:HGNC:42400] |
| ENSG00000189410 | HGLibA_43904 | 138 | 0.113563831 | SH2D5 | SH2 domain containing 5 [Source:HGNC Symbol;Acc:HGNC:28819] |
| ENSG00000197859 | HGLibA_00851 | 138 | 0.113563831 | ADAMTSL2 | ADAMTS like 2 [Source:HGNC Symbol;Acc:HGNC:14631] |
| ENSG00000198088 | HGLibA_33067 | 138 | 0.113563831 | NUP62CL | nucleoporin 62 C-terminal like [Source:HGNC Symbol;Acc:HGNC:25960] |
| ENSG00000198483 | HGLibA_02085 | 138 | 0.113563831 | ANKRD35 | ankyrin repeat domain 35 [Source:HGNC Symbol;Acc:HGNC:26323] |
| ENSG00000198758 | HGLibA_15382 | 138 | 0.113563831 | EPS8L3 | EPS8 like 3 [Source:HGNC Symbol;Acc:HGNC:21297] |
| ENSG00000198892 | HGLibA_44038 | 138 | 0.113563831 | SHISA4 | shisa family member 4 [Source:HGNC Symbol;Acc:HGNC:27139] |
| ENSG00000204396 | HGLibA_53915 | 138 | 0.113563831 | VWA7 | von Willebrand factor A domain containing 7 [Source:HGNC Symbol;Acc:HGNC:13939] |
| ENSG00000204822 | HGLibA_30017 | 138 | 0.113563831 | MRPL53 | mitochondrial ribosomal protein L53 [Source:HGNC Symbol;Acc:HGNC:16684] |
| ENSG00000204852 | HGLibA_48872 | 138 | 0.113563831 | TCTN1 | tectonic family member 1 [Source:HGNC Symbol;Acc:HGNC:26113] |
| ENSG00000205045 | HGLibA_45488 | 138 | 0.113563831 | SLFN12L | schlafen family member 12 like [Source:HGNC Symbol;Acc:HGNC:33920] |
| ENSG00000205138 | HGLibA_43137 | 138 | 0.113563831 | SDHAF1 | succinate dehydrogenase complex assembly factor 1 [Source:HGNC Symbol;Acc:HGNC:33867] |
| ENSG00000206503 | HGLibA_21594 | 138 | 0.113563831 | HLA-A | A [Source:HGNC Symbol;Acc:HGNC:4931]" |
| ENSG00000213988 | HGLibA_56901 | 138 | 0.113563831 | ZNF90 | zinc finger protein 90 [Source:HGNC Symbol;Acc:HGNC:13165] |
| ENSG00000225190 | HGLibA_37138 | 138 | 0.113563831 | PLEKHM1 | pleckstrin homology and RUN domain containing M1 [Source:HGNC Symbol;Acc:HGNC:29017] |
| ENSG00000226650 | HGLibA_24945 | 138 | 0.113563831 | KIF4B | kinesin family member 4B [Source:HGNC Symbol;Acc:HGNC:6322] |
| ENSG00000240563 | HGLibA_25886 | 138 | 0.113563831 | L1TD1 | LINE1 type transposase domain containing 1 [Source:HGNC Symbol;Acc:HGNC:25595] |
| ENSG00000242515 | HGLibA_52880 | 138 | 0.113563831 | UGT1A10 | UDP glucuronosyltransferase family 1 member A10 [Source:HGNC Symbol;Acc:HGNC:12531] |
| ENSG00000243772 | HGLibA_24986 | 138 | 0.113563831 | KIR2DL3 | two Ig domains and long cytoplasmic tail 3 [Source:HGNC Symbol;Acc:HGNC:6331]" |
| ENSG00000254997 | HGLibA_25811 | 138 | 0.113563831 | KRTAP5-9 | keratin associated protein 5-9 [Source:HGNC Symbol;Acc:HGNC:23604] |
| ENSG00000261456 | HGLibA_52293 | 138 | 0.113563831 | TUBB8 | tubulin beta 8 class VIII [Source:HGNC Symbol;Acc:HGNC:20773] |
| ENSG00000261456 | HGLibA_52294 | 138 | 0.113563831 | TUBB8 | tubulin beta 8 class VIII [Source:HGNC Symbol;Acc:HGNC:20773] |
| ENSG00000276409 | HGLibA_07969 | 138 | 0.113563831 | CCL14 | C-C motif chemokine ligand 14 [Source:HGNC Symbol;Acc:HGNC:10612] |
| FAM109B | HGLibA_16009 | 138 | 0.113563831 | FAM109B | - |
| FAM127B | HGLibA_16091 | 138 | 0.113563831 | FAM127B | - |
| hsa-mir-146a | HGLibA_57858 | 138 | 0.113563831 | hsa-mir-146a | - |
| hsa-mir-4328 | HGLibA_60037 | 138 | 0.113563831 | hsa-mir-4328 | - |
| hsa-mir-4765 | HGLibA_61156 | 138 | 0.113563831 | hsa-mir-4765 | - |
| hsa-mir-4790 | HGLibA_61268 | 138 | 0.113563831 | hsa-mir-4790 | - |
| hsa-mir-484 | HGLibA_61329 | 138 | 0.113563831 | hsa-mir-484 | - |
| hsa-mir-485 | HGLibA_61331 | 138 | 0.113563831 | hsa-mir-485 | - |
| hsa-mir-591 | HGLibA_62461 | 138 | 0.113563831 | hsa-mir-591 | - |
| hsa-mir-621 | HGLibA_62722 | 138 | 0.113563831 | hsa-mir-621 | - |
| hsa-mir-8056 | HGLibA_64061 | 138 | 0.113563831 | hsa-mir-8056 | - |
| KIAA0196 | HGLibA_24601 | 138 | 0.113563831 | KIAA0196 | - |
| METTL12 | HGLibA_28953 | 138 | 0.113563831 | METTL12 | - |
| PPAP2B | HGLibA_37819 | 138 | 0.113563831 | PPAP2B | - |
| REXO1L1 | HGLibA_40892 | 138 | 0.113563831 | REXO1L1 | - |
| RTFDC1 | HGLibA_42386 | 138 | 0.113563831 | RTFDC1 | - |
| SEPW1 | HGLibA_43461 | 138 | 0.113563831 | SEPW1 | - |
| USMG5 | HGLibA_53185 | 138 | 0.113563831 | USMG5 | - |
| WBSCR17 | HGLibA_53992 | 138 | 0.113563831 | WBSCR17 | - |
| C12orf39 | HGLibA_05233 | 137 | 0.104518488 | C12orf39 | - |
| C7orf10 | HGLibA_06520 | 137 | 0.104518488 | C7orf10 | - |
| C9orf9 | HGLibA_06773 | 137 | 0.104518488 | C9orf9 | - |
| DBC1 | HGLibA_12372 | 137 | 0.104518488 | DBC1 | - |
| ENSG00000009335 | HGLibA_52684 | 137 | 0.104518488 | UBE3C | ubiquitin protein ligase E3C [Source:HGNC Symbol;Acc:HGNC:16803] |
| ENSG00000013392 | HGLibA_42483 | 137 | 0.104518488 | RWDD2A | RWD domain containing 2A [Source:HGNC Symbol;Acc:HGNC:21385] |
| ENSG00000028277 | HGLibA_37760 | 137 | 0.104518488 | POU2F2 | POU class 2 homeobox 2 [Source:HGNC Symbol;Acc:HGNC:9213] |
| ENSG00000031691 | HGLibA_09084 | 137 | 0.104518488 | CENPQ | centromere protein Q [Source:HGNC Symbol;Acc:HGNC:21347] |
| ENSG00000047579 | HGLibA_14070 | 137 | 0.104518488 | DTNBP1 | dystrobrevin binding protein 1 [Source:HGNC Symbol;Acc:HGNC:17328] |
| ENSG00000071889 | HGLibA_16577 | 137 | 0.104518488 | FAM3A | family with sequence similarity 3 member A [Source:HGNC Symbol;Acc:HGNC:13749] |
| ENSG00000073111 | HGLibA_28605 | 137 | 0.104518488 | MCM2 | minichromosome maintenance complex component 2 [Source:HGNC Symbol;Acc:HGNC:6944] |
| ENSG00000100242 | HGLibA_47791 | 137 | 0.104518488 | SUN2 | Sad1 and UNC84 domain containing 2 [Source:HGNC Symbol;Acc:HGNC:14210] |
| ENSG00000100478 | HGLibA_02351 | 137 | 0.104518488 | AP4S1 | adaptor related protein complex 4 subunit sigma 1 [Source:HGNC Symbol;Acc:HGNC:575] |
| ENSG00000100632 | HGLibA_15475 | 137 | 0.104518488 | ERH | mRNA splicing and mitosis factor [Source:HGNC Symbol;Acc:HGNC:3447]" |
| ENSG00000102554 | HGLibA_25074 | 137 | 0.104518488 | KLF5 | Kruppel like factor 5 [Source:HGNC Symbol;Acc:HGNC:6349] |
| ENSG00000104818 | HGLibA_09304 | 137 | 0.104518488 | CGB2 | chorionic gonadotropin subunit beta 2 [Source:HGNC Symbol;Acc:HGNC:16722] |
| ENSG00000105722 | HGLibA_15462 | 137 | 0.104518488 | ERF | ETS2 repressor factor [Source:HGNC Symbol;Acc:HGNC:3444] |
| ENSG00000107281 | HGLibA_32426 | 137 | 0.104518488 | NPDC1 | differentiation and control 1 [Source:HGNC Symbol;Acc:HGNC:7899]" |
| ENSG00000107282 | HGLibA_02367 | 137 | 0.104518488 | APBA1 | amyloid beta precursor protein binding family A member 1 [Source:HGNC Symbol;Acc:HGNC:578] |
| ENSG00000107736 | HGLibA_08669 | 137 | 0.104518488 | CDH23 | cadherin related 23 [Source:HGNC Symbol;Acc:HGNC:13733] |
| ENSG00000108439 | HGLibA_37430 | 137 | 0.104518488 | PNPO | pyridoxamine 5'-phosphate oxidase [Source:HGNC Symbol;Acc:HGNC:30260] |
| ENSG00000108561 | HGLibA_05917 | 137 | 0.104518488 | C1QBP | complement C1q binding protein [Source:HGNC Symbol;Acc:HGNC:1243] |
| ENSG00000108819 | HGLibA_38132 | 137 | 0.104518488 | PPP1R9B | protein phosphatase 1 regulatory subunit 9B [Source:HGNC Symbol;Acc:HGNC:9298] |
| ENSG00000108828 | HGLibA_53542 | 137 | 0.104518488 | VAT1 | vesicle amine transport 1 [Source:HGNC Symbol;Acc:HGNC:16919] |
| ENSG00000109171 | HGLibA_44308 | 137 | 0.104518488 | SLAIN2 | SLAIN motif family member 2 [Source:HGNC Symbol;Acc:HGNC:29282] |
| ENSG00000109208 | HGLibA_45823 | 137 | 0.104518488 | SMR3A | submaxillary gland androgen regulated protein 3A [Source:HGNC Symbol;Acc:HGNC:19216] |
| ENSG00000111266 | HGLibA_14148 | 137 | 0.104518488 | DUSP16 | dual specificity phosphatase 16 [Source:HGNC Symbol;Acc:HGNC:17909] |
| ENSG00000111536 | HGLibA_23110 | 137 | 0.104518488 | IL26 | interleukin 26 [Source:HGNC Symbol;Acc:HGNC:17119] |
| ENSG00000111732 | HGLibA_01324 | 137 | 0.104518488 | AICDA | activation induced cytidine deaminase [Source:HGNC Symbol;Acc:HGNC:13203] |
| ENSG00000117245 | HGLibA_24870 | 137 | 0.104518488 | KIF17 | kinesin family member 17 [Source:HGNC Symbol;Acc:HGNC:19167] |
| ENSG00000120254 | HGLibA_30379 | 137 | 0.104518488 | MTHFD1L | methylenetetrahydrofolate dehydrogenase (NADP+ dependent) 1 like [Source:HGNC Symbol;Acc:HGNC:21055] |
| ENSG00000125775 | HGLibA_43111 | 137 | 0.104518488 | SDCBP2 | syndecan binding protein 2 [Source:HGNC Symbol;Acc:HGNC:15756] |
| ENSG00000127914 | HGLibA_01451 | 137 | 0.104518488 | AKAP9 | A-kinase anchoring protein 9 [Source:HGNC Symbol;Acc:HGNC:379] |
| ENSG00000128512 | HGLibA_13746 | 137 | 0.104518488 | DOCK4 | dedicator of cytokinesis 4 [Source:HGNC Symbol;Acc:HGNC:19192] |
| ENSG00000132323 | HGLibA_23223 | 137 | 0.104518488 | ILKAP | ILK associated serine/threonine phosphatase [Source:HGNC Symbol;Acc:HGNC:15566] |
| ENSG00000132514 | HGLibA_09921 | 137 | 0.104518488 | CLEC10A | C-type lectin domain containing 10A [Source:HGNC Symbol;Acc:HGNC:16916] |

| | | | | | |
|---|---|---|---|---|---|
| ENSG00000132688 | HGLibA_31698 | 137 | 0.104518488 | NES | nestin [Source:HGNC Symbol;Acc:HGNC:7756] |
| ENSG00000132911 | HGLibA_32217 | 137 | 0.104518488 | NMUR2 | neuromedin U receptor 2 [Source:HGNC Symbol;Acc:HGNC:16454] |
| ENSG00000135116 | HGLibA_22107 | 137 | 0.104518488 | HRK | BCL2 interacting protein [Source:HGNC Symbol;Acc:HGNC:5185]" |
| ENSG00000135549 | HGLibA_36823 | 137 | 0.104518488 | PKIB | cAMP-dependent protein kinase inhibitor beta [Source:HGNC Symbol;Acc:HGNC:9018] |
| ENSG00000135766 | HGLibA_14638 | 137 | 0.104518488 | EGLN1 | egl-9 family hypoxia inducible factor 1 [Source:HGNC Symbol;Acc:HGNC:1232] |
| ENSG00000135821 | HGLibA_19368 | 137 | 0.104518488 | GLUL | glutamate-ammonia ligase [Source:HGNC Symbol;Acc:HGNC:4341] |
| ENSG00000136098 | HGLibA_31651 | 137 | 0.104518488 | NEK3 | NIMA related kinase 3 [Source:HGNC Symbol;Acc:HGNC:7746] |
| ENSG00000136352 | HGLibA_32030 | 137 | 0.104518488 | NKX2-1 | NK2 homeobox 1 [Source:HGNC Symbol;Acc:HGNC:11825] |
| ENSG00000136720 | HGLibA_22143 | 137 | 0.104518488 | HS6ST1 | heparan sulfate 6-O-sulfotransferase 1 [Source:HGNC Symbol;Acc:HGNC:5201] |
| ENSG00000138068 | HGLibA_47768 | 137 | 0.104518488 | SULT6B1 | sulfotransferase family 6B member 1 [Source:HGNC Symbol;Acc:HGNC:33433] |
| ENSG00000139438 | HGLibA_16523 | 137 | 0.104518488 | FAM222A | family with sequence similarity 222 member A [Source:HGNC Symbol;Acc:HGNC:25915] |
| ENSG00000142856 | HGLibA_23785 | 137 | 0.104518488 | ITGB3BP | integrin subunit beta 3 binding protein [Source:HGNC Symbol;Acc:HGNC:6157] |
| ENSG00000143006 | HGLibA_13431 | 137 | 0.104518488 | DMRTB1 | DMRT like family B with proline rich C-terminal 1 [Source:HGNC Symbol;Acc:HGNC:13913] |
| ENSG00000143494 | HGLibA_53533 | 137 | 0.104518488 | VASH2 | vasohibin 2 [Source:HGNC Symbol;Acc:HGNC:25723] |
| ENSG00000145362 | HGLibA_01938 | 137 | 0.104518488 | ANK2 | ankyrin 2 [Source:HGNC Symbol;Acc:HGNC:493] |
| ENSG00000146574 | HGLibA_08226 | 137 | 0.104518488 | CCZ1B | vacuolar protein trafficking and biogenesis associated [Source:HGNC Symbol;Acc:HGNC:21717]" |
| ENSG00000148331 | HGLibA_03246 | 137 | 0.104518488 | ASB6 | ankyrin repeat and SOCS box containing 6 [Source:HGNC Symbol;Acc:HGNC:17181] |
| ENSG00000150477 | HGLibA_24743 | 137 | 0.104518488 | KIAA1328 | KIAA1328 [Source:HGNC Symbol;Acc:HGNC:29248] |
| ENSG00000153029 | HGLibA_29803 | 137 | 0.104518488 | MR1 | class I-related [Source:HGNC Symbol;Acc:HGNC:4975]" |
| ENSG00000153140 | HGLibA_09239 | 137 | 0.104518488 | CETN3 | centrin 3 [Source:HGNC Symbol;Acc:HGNC:1868] |
| ENSG00000153157 | HGLibA_47935 | 137 | 0.104518488 | SYCP2L | synaptonemal complex protein 2 like [Source:HGNC Symbol;Acc:HGNC:21537] |
| ENSG00000153201 | HGLibA_40200 | 137 | 0.104518488 | RANBP2 | RAN binding protein 2 [Source:HGNC Symbol;Acc:HGNC:9848] |
| ENSG00000153253 | HGLibA_42987 | 137 | 0.104518488 | SCN3A | sodium voltage-gated channel alpha subunit 3 [Source:HGNC Symbol;Acc:HGNC:10590] |
| ENSG00000153707 | HGLibA_39496 | 137 | 0.104518488 | PTPRD | receptor type D [Source:HGNC Symbol;Acc:HGNC:9668]" |
| ENSG00000158805 | HGLibA_55715 | 137 | 0.104518488 | ZNF276 | zinc finger protein 276 [Source:HGNC Symbol;Acc:HGNC:23330] |
| ENSG00000159197 | HGLibA_24190 | 137 | 0.104518488 | KCNE2 | potassium voltage-gated channel subfamily E regulatory subunit 2 [Source:HGNC Symbol;Acc:HGNC:6242] |
| ENSG00000160285 | HGLibA_27568 | 137 | 0.104518488 | LSS | lanosterol synthase [Source:HGNC Symbol;Acc:HGNC:6708] |
| ENSG00000160856 | HGLibA_17230 | 137 | 0.104518488 | FCRL3 | Fc receptor like 3 [Source:HGNC Symbol;Acc:HGNC:18506] |
| ENSG00000161850 | HGLibA_25562 | 137 | 0.104518488 | KRT82 | keratin 82 [Source:HGNC Symbol;Acc:HGNC:6459] |
| ENSG00000161960 | HGLibA_14803 | 137 | 0.104518488 | EIF4A1 | eukaryotic translation initiation factor 4A1 [Source:HGNC Symbol;Acc:HGNC:3282] |
| ENSG00000162627 | HGLibA_46106 | 137 | 0.104518488 | SNX7 | sorting nexin 7 [Source:HGNC Symbol;Acc:HGNC:14971] |
| ENSG00000163012 | HGLibA_57039 | 137 | 0.104518488 | ZSWIM2 | zinc finger SWIM-type containing 2 [Source:HGNC Symbol;Acc:HGNC:30990] |
| ENSG00000163884 | HGLibA_25057 | 137 | 0.104518488 | KLF15 | Kruppel like factor 15 [Source:HGNC Symbol;Acc:HGNC:14536] |
| ENSG00000165661 | HGLibA_39757 | 137 | 0.104518488 | QSOX2 | quiescin sulfhydryl oxidase 2 [Source:HGNC Symbol;Acc:HGNC:30249] |
| ENSG00000166192 | HGLibA_43402 | 137 | 0.104518488 | SENP8 | NEDD8 specific [Source:HGNC Symbol;Acc:HGNC:22992]" |
| ENSG00000166925 | HGLibA_51826 | 137 | 0.104518488 | TSC22D4 | TSC22 domain family member 4 [Source:HGNC Symbol;Acc:HGNC:21696] |
| ENSG00000167257 | HGLibA_41625 | 137 | 0.104518488 | RNF214 | ring finger protein 214 [Source:HGNC Symbol;Acc:HGNC:25335] |
| ENSG00000167291 | HGLibA_48485 | 137 | 0.104518488 | TBC1D16 | TBC1 domain family member 16 [Source:HGNC Symbol;Acc:HGNC:28356] |
| ENSG00000168081 | HGLibA_37399 | 137 | 0.104518488 | PNOC | prepronociceptin [Source:HGNC Symbol;Acc:HGNC:9163] |
| ENSG00000168228 | HGLibA_55035 | 137 | 0.104518488 | ZCCHC4 | zinc finger CCHC-type containing 4 [Source:HGNC Symbol;Acc:HGNC:22917] |
| ENSG00000168824 | HGLibA_32758 | 137 | 0.104518488 | NSG1 | neuronal vesicle trafficking associated 1 [Source:HGNC Symbol;Acc:HGNC:18790] |
| ENSG00000171109 | HGLibA_29069 | 137 | 0.104518488 | MFN1 | mitofusin 1 [Source:HGNC Symbol;Acc:HGNC:18262] |
| ENSG00000171956 | HGLibA_17816 | 137 | 0.104518488 | FOXB1 | forkhead box B1 [Source:HGNC Symbol;Acc:HGNC:3799] |
| ENSG00000172663 | HGLibA_49945 | 137 | 0.104518488 | TMEM134 | transmembrane protein 134 [Source:HGNC Symbol;Acc:HGNC:26142] |
| ENSG00000173581 | HGLibA_07524 | 137 | 0.104518488 | CCDC106 | coiled-coil domain containing 106 [Source:HGNC Symbol;Acc:HGNC:30181] |
| ENSG00000175161 | HGLibA_06968 | 137 | 0.104518488 | CADM2 | cell adhesion molecule 2 [Source:HGNC Symbol;Acc:HGNC:29849] |
| ENSG00000175575 | HGLibA_34919 | 137 | 0.104518488 | PAAF1 | proteasomal ATPase associated factor 1 [Source:HGNC Symbol;Acc:HGNC:25687] |
| ENSG00000175984 | HGLibA_12941 | 137 | 0.104518488 | DENND2C | DENN domain containing 2C [Source:HGNC Symbol;Acc:HGNC:24748] |
| ENSG00000176246 | HGLibA_34038 | 137 | 0.104518488 | OR4L1 | olfactory receptor family 4 subfamily L member 1 [Source:HGNC Symbol;Acc:HGNC:15356] |
| ENSG00000182264 | HGLibA_23893 | 137 | 0.104518488 | IZUMO1 | izumo sperm-egg fusion 1 [Source:HGNC Symbol;Acc:HGNC:28539] |
| ENSG00000182687 | HGLibA_18591 | 137 | 0.104518488 | GALR2 | galanin receptor 2 [Source:HGNC Symbol;Acc:HGNC:4133] |
| ENSG00000183304 | HGLibA_16817 | 137 | 0.104518488 | FAM9A | family with sequence similarity 9 member A [Source:HGNC Symbol;Acc:HGNC:18403] |
| ENSG00000183597 | HGLibA_48296 | 137 | 0.104518488 | TANGO2 | transport and golgi organization 2 homolog [Source:HGNC Symbol;Acc:HGNC:25439] |
| ENSG00000184602 | HGLibA_45931 | 137 | 0.104518488 | SNN | stannin [Source:HGNC Symbol;Acc:HGNC:11149] |
| ENSG00000185019 | HGLibA_52725 | 137 | 0.104518488 | UBOX5 | U-box domain containing 5 [Source:HGNC Symbol;Acc:HGNC:17777] |
| ENSG00000185753 | HGLibA_11919 | 137 | 0.104518488 | CXorf38 | chromosome X open reading frame 38 [Source:HGNC Symbol;Acc:HGNC:28589] |
| ENSG00000185917 | HGLibA_43648 | 137 | 0.104518488 | SETD4 | SET domain containing 4 [Source:HGNC Symbol;Acc:HGNC:1258] |
| ENSG00000186272 | HGLibA_55521 | 137 | 0.104518488 | ZNF17 | zinc finger protein 17 [Source:HGNC Symbol;Acc:HGNC:12958] |
| ENSG00000188321 | HGLibA_56235 | 137 | 0.104518488 | ZNF559 | zinc finger protein 559 [Source:HGNC Symbol;Acc:HGNC:28197] |
| ENSG00000196182 | HGLibA_47512 | 137 | 0.104518488 | STK40 | serine/threonine kinase 40 [Source:HGNC Symbol;Acc:HGNC:21373] |
| ENSG00000196233 | HGLibA_26194 | 137 | 0.104518488 | LCOR | ligand dependent nuclear receptor corepressor [Source:HGNC Symbol;Acc:HGNC:29503] |
| ENSG00000196363 | HGLibA_54115 | 137 | 0.104518488 | WDR5 | WD repeat domain 5 [Source:HGNC Symbol;Acc:HGNC:12757] |
| ENSG00000196458 | HGLibA_56366 | 137 | 0.104518488 | ZNF605 | zinc finger protein 605 [Source:HGNC Symbol;Acc:HGNC:28068] |
| ENSG00000196754 | HGLibA_42551 | 137 | 0.104518488 | S100A2 | S100 calcium binding protein A2 [Source:HGNC Symbol;Acc:HGNC:10492] |
| ENSG00000197233 | HGLibA_33678 | 137 | 0.104518488 | OR1J2 | olfactory receptor family 1 subfamily J member 2 [Source:HGNC Symbol;Acc:HGNC:8209] |
| ENSG00000197323 | HGLibA_51466 | 137 | 0.104518488 | TRIM33 | tripartite motif containing 33 [Source:HGNC Symbol;Acc:HGNC:16290] |
| ENSG00000198183 | HGLibA_04711 | 137 | 0.104518488 | BPIFA1 | BPI fold containing family A member 1 [Source:HGNC Symbol;Acc:HGNC:15749] |
| ENSG00000198756 | HGLibA_10644 | 137 | 0.104518488 | COLGALT2 | collagen beta(1-O)galactosyltransferase 2 [Source:HGNC Symbol;Acc:HGNC:16790] |
| ENSG00000205858 | HGLibA_27421 | 137 | 0.104518488 | LRRC72 | leucine rich repeat containing 72 [Source:HGNC Symbol;Acc:HGNC:42972] |
| ENSG00000206104 | HGLibA_25705 | 137 | 0.104518488 | KRTAP20-3 | keratin associated protein 20-3 [Source:HGNC Symbol;Acc:HGNC:34001] |
| ENSG00000211455 | HGLibA_47503 | 137 | 0.104518488 | STK38L | serine/threonine kinase 38 like [Source:HGNC Symbol;Acc:HGNC:17848] |
| ENSG00000213185 | HGLibA_16548 | 137 | 0.104518488 | FAM24B | family with sequence similarity 24 member B [Source:HGNC Symbol;Acc:HGNC:23475] |
| ENSG00000214891 | HGLibA_51582 | 137 | 0.104518488 | TRIM64C | tripartite motif containing 64C [Source:HGNC Symbol;Acc:HGNC:37148] |
| ENSG00000215788 | HGLibA_50780 | 137 | 0.104518488 | TNFRSF25 | TNF receptor superfamily member 25 [Source:HGNC Symbol;Acc:HGNC:11910] |
| ENSG00000239389 | HGLibA_35437 | 137 | 0.104518488 | PCDHA13 | protocadherin alpha 13 [Source:HGNC Symbol;Acc:HGNC:8667] |
| ENSG00000243725 | HGLibA_52141 | 137 | 0.104518488 | TTC4 | tetratricopeptide repeat domain 4 [Source:HGNC Symbol;Acc:HGNC:12394] |
| ENSG00000260458 | HGLibA_24269 | 137 | 0.104518488 | KCNJ18 | potassium voltage-gated channel subfamily J member 18 [Source:HGNC Symbol;Acc:HGNC:39080] |
| ENSG00000269791 | HGLibA_47213 | 137 | 0.104518488 | SSX4B | SSX family member 4B [Source:HGNC Symbol;Acc:HGNC:16880] |
| ENSG00000270601 | HGLibA_38319 | 137 | 0.104518488 | PRAMEF5 | PRAME family member 5 [Source:HGNC Symbol;Acc:HGNC:27995] |
| ENSG00000277556 | HGLibA_33604 | 137 | 0.104518488 | OR13C5 | olfactory receptor family 13 subfamily C member 5 [Source:HGNC Symbol;Acc:HGNC:15100] |
| ENSG00000278259 | HGLibA_30816 | 137 | 0.104518488 | MYO19 | myosin XIX [Source:HGNC Symbol;Acc:HGNC:26234] |
| FAM122A | HGLibA_16066 | 137 | 0.104518488 | FAM122A | - |
| FAM127C | HGLibA_16092 | 137 | 0.104518488 | FAM127C | - |
| HIST1H4C | HGLibA_21492 | 137 | 0.104518488 | HIST1H4C | - |
| hsa-mir-153-2 | HGLibA_57910 | 137 | 0.104518488 | hsa-mir-153-2 | - |
| hsa-mir-198 | HGLibA_58126 | 137 | 0.104518488 | hsa-mir-198 | - |
| hsa-mir-199b | HGLibA_58138 | 137 | 0.104518488 | hsa-mir-199b | - |
| hsa-mir-28 | HGLibA_58386 | 137 | 0.104518488 | hsa-mir-28 | - |
| hsa-mir-3150b | HGLibA_58661 | 137 | 0.104518488 | hsa-mir-3150b | - |
| hsa-mir-3152 | HGLibA_58668 | 137 | 0.104518488 | hsa-mir-3152 | - |
| hsa-mir-375 | HGLibA_59402 | 137 | 0.104518488 | hsa-mir-375 | - |
| hsa-mir-4296 | HGLibA_59894 | 137 | 0.104518488 | hsa-mir-4296 | - |
| hsa-mir-4711 | HGLibA_60946 | 137 | 0.104518488 | hsa-mir-4711 | - |
| hsa-mir-4743 | HGLibA_61068 | 137 | 0.104518488 | hsa-mir-4743 | - |
| hsa-mir-5000 | HGLibA_61407 | 137 | 0.104518488 | hsa-mir-5000 | - |
| hsa-mir-520d | HGLibA_61745 | 137 | 0.104518488 | hsa-mir-520d | - |
| hsa-mir-601 | HGLibA_62498 | 137 | 0.104518488 | hsa-mir-601 | - |
| hsa-mir-668 | HGLibA_63004 | 137 | 0.104518488 | hsa-mir-668 | - |
| hsa-mir-6717 | HGLibA_63031 | 137 | 0.104518488 | hsa-mir-6717 | - |
| hsa-mir-6733 | HGLibA_63085 | 137 | 0.104518488 | hsa-mir-6733 | - |
| hsa-mir-6794 | HGLibA_63354 | 137 | 0.104518488 | hsa-mir-6794 | - |
| hsa-mir-8067 | HGLibA_64108 | 137 | 0.104518488 | hsa-mir-8067 | - |
| LEPREL1 | HGLibA_26317 | 137 | 0.104518488 | LEPREL1 | - |
| LOC100129083 | HGLibA_26754 | 137 | 0.104518488 | LOC100129083 | - |
| APOPT1 | HGLibA_02557 | 136 | 0.095473145 | APOPT1 | - |
| C1orf233 | HGLibA_05846 | 136 | 0.095473145 | C1orf233 | - |
| C20orf166 | HGLibA_05992 | 136 | 0.095473145 | C20orf166 | - |
| ENSG00000001461 | HGLibA_31963 | 136 | 0.095473145 | NIPAL3 | NIPA like domain containing 3 [Source:HGNC Symbol;Acc:HGNC:25233] |
| ENSG00000004468 | HGLibA_08365 | 136 | 0.095473145 | CD38 | CD38 molecule [Source:HGNC Symbol;Acc:HGNC:1667] |
| ENSG00000006453 | HGLibA_04124 | 136 | 0.095473145 | BAIAP2L1 | BAI1 associated protein 2 like 1 [Source:HGNC Symbol;Acc:HGNC:21649] |
| ENSG00000011454 | HGLibA_40010 | 136 | 0.095473145 | RABGAP1 | RAB GTPase activating protein 1 [Source:HGNC Symbol;Acc:HGNC:17155] |
| ENSG00000012211 | HGLibA_38475 | 136 | 0.095473145 | PRICKLE3 | prickle planar cell polarity protein 3 [Source:HGNC Symbol;Acc:HGNC:6645] |
| ENSG00000018869 | HGLibA_56310 | 136 | 0.095473145 | ZNF582 | zinc finger protein 582 [Source:HGNC Symbol;Acc:HGNC:26421] |
| ENSG00000042317 | HGLibA_46527 | 136 | 0.095473145 | SPATA7 | spermatogenesis associated 7 [Source:HGNC Symbol;Acc:HGNC:20423] |
| ENSG00000042317 | HGLibA_46529 | 136 | 0.095473145 | SPATA7 | spermatogenesis associated 7 [Source:HGNC Symbol;Acc:HGNC:20423] |
| ENSG00000048545 | HGLibA_20635 | 136 | 0.095473145 | GUCA1A | guanylate cyclase activator 1A [Source:HGNC Symbol;Acc:HGNC:4678] |
| ENSG00000060656 | HGLibA_39540 | 136 | 0.095473145 | PTPRU | receptor type U [Source:HGNC Symbol;Acc:HGNC:9683]" |
| ENSG00000066027 | HGLibA_38170 | 136 | 0.095473145 | PPP2R5A | protein phosphatase 2 regulatory subunit B'alpha [Source:HGNC Symbol;Acc:HGNC:9309] |
| ENSG00000067057 | HGLibA_36171 | 136 | 0.095473145 | PFKP | platelet [Source:HGNC Symbol;Acc:HGNC:8878]" |
| ENSG00000068985 | HGLibA_35004 | 136 | 0.095473145 | PAGE1 | PAGE family member 1 [Source:HGNC Symbol;Acc:HGNC:4107] |
| ENSG00000069943 | HGLibA_36546 | 136 | 0.095473145 | PIGB | phosphatidylinositol glycan anchor biosynthesis class B [Source:HGNC Symbol;Acc:HGNC:8959] |
| ENSG00000081248 | HGLibA_06901 | 136 | 0.095473145 | CACNA1S | calcium voltage-gated channel subunit alpha1 S [Source:HGNC Symbol;Acc:HGNC:1397] |
| ENSG00000082293 | HGLibA_10527 | 136 | 0.095473145 | COL19A1 | collagen type XIX alpha 1 chain [Source:HGNC Symbol;Acc:HGNC:2196] |
| ENSG00000090020 | HGLibA_45400 | 136 | 0.095473145 | SLC9A1 | solute carrier family 9 member A1 [Source:HGNC Symbol;Acc:HGNC:11071] |
| ENSG00000090097 | HGLibA_35377 | 136 | 0.095473145 | PCBP4 | poly(rC) binding protein 4 [Source:HGNC Symbol;Acc:HGNC:8652] |
| ENSG00000095002 | HGLibA_30191 | 136 | 0.095473145 | MSH2 | mutS homolog 2 [Source:HGNC Symbol;Acc:HGNC:7325] |
| ENSG00000095739 | HGLibA_04138 | 136 | 0.095473145 | BAMBI | BMP and activin membrane bound inhibitor [Source:HGNC Symbol;Acc:HGNC:30251] |
| ENSG00000096264 | HGLibA_31366 | 136 | 0.095473145 | NCR2 | natural cytotoxicity triggering receptor 2 [Source:HGNC Symbol;Acc:HGNC:6732] |
| ENSG00000099256 | HGLibA_38977 | 136 | 0.095473145 | PRTFDC1 | phosphoribosyl transferase domain containing 1 [Source:HGNC Symbol;Acc:HGNC:23333] |
| ENSG00000100207 | HGLibA_48769 | 136 | 0.095473145 | TCF20 | transcription factor 20 [Source:HGNC Symbol;Acc:HGNC:11631] |
| ENSG00000100276 | HGLibA_40358 | 136 | 0.095473145 | RASL10A | RAS like family 10 member A [Source:HGNC Symbol;Acc:HGNC:16954] |
| ENSG00000100453 | HGLibA_20722 | 136 | 0.095473145 | GZMB | granzyme B [Source:HGNC Symbol;Acc:HGNC:4709] |
| ENSG00000101152 | HGLibA_13623 | 136 | 0.095473145 | DNAJC5 | DnaJ heat shock protein family (Hsp40) member C5 [Source:HGNC Symbol;Acc:HGNC:16235] |
| ENSG00000101311 | HGLibA_17294 | 136 | 0.095473145 | FERMT1 | fermitin family member 1 [Source:HGNC Symbol;Acc:HGNC:15889] |
| ENSG00000101665 | HGLibA_45588 | 136 | 0.095473145 | SMAD7 | SMAD family member 7 [Source:HGNC Symbol;Acc:HGNC:6773] |

| | | | | | |
|---|---|---|---|---|---|
| ENSG00000102241 | HGLibA_22350 | 136 | 0.095473145 | HTATSF1 | HIV-1 Tat specific factor 1 [Source:HGNC Symbol;Acc:HGNC:5276] |
| ENSG00000102678 | HGLibA_17424 | 136 | 0.095473145 | FGF9 | fibroblast growth factor 9 [Source:HGNC Symbol;Acc:HGNC:3687] |
| ENSG00000104499 | HGLibA_19411 | 136 | 0.095473145 | GML | glycosylphosphatidylinositol anchored molecule like [Source:HGNC Symbol;Acc:HGNC:4375] |
| ENSG00000104848 | HGLibA_24144 | 136 | 0.095473145 | KCNA7 | potassium voltage-gated channel subfamily A member 7 [Source:HGNC Symbol;Acc:HGNC:6226] |
| ENSG00000104883 | HGLibA_36089 | 136 | 0.095473145 | PEX11G | peroxisomal biogenesis factor 11 gamma [Source:HGNC Symbol;Acc:HGNC:20208] |
| ENSG00000105641 | HGLibA_45274 | 136 | 0.095473145 | SLC5A5 | solute carrier family 5 member 5 [Source:HGNC Symbol;Acc:HGNC:11040] |
| ENSG00000106546 | HGLibA_01308 | 136 | 0.095473145 | AHR | aryl hydrocarbon receptor [Source:HGNC Symbol;Acc:HGNC:348] |
| ENSG00000106631 | HGLibA_30774 | 136 | 0.095473145 | MYL7 | myosin light chain 7 [Source:HGNC Symbol;Acc:HGNC:21719] |
| ENSG00000107796 | HGLibA_00599 | 136 | 0.095473145 | ACTA2 | aorta [Source:HGNC Symbol;Acc:HGNC:130]" |
| ENSG00000108465 | HGLibA_08787 | 136 | 0.095473145 | CDK5RAP3 | CDK5 regulatory subunit associated protein 3 [Source:HGNC Symbol;Acc:HGNC:18673] |
| ENSG00000108576 | HGLibA_45329 | 136 | 0.095473145 | SLC6A4 | solute carrier family 6 member 4 [Source:HGNC Symbol;Acc:HGNC:11050] |
| ENSG00000108651 | HGLibA_53456 | 136 | 0.095473145 | UTP6 | small subunit processome component [Source:HGNC Symbol;Acc:HGNC:18279]" |
| ENSG00000110880 | HGLibA_10779 | 136 | 0.095473145 | CORO1C | coronin 1C [Source:HGNC Symbol;Acc:HGNC:2254] |
| ENSG00000113851 | HGLibA_11079 | 136 | 0.095473145 | CRBN | cereblon [Source:HGNC Symbol;Acc:HGNC:30185] |
| ENSG00000115137 | HGLibA_13610 | 136 | 0.095473145 | DNAJC27 | DnaJ heat shock protein family (Hsp40) member C27 [Source:HGNC Symbol;Acc:HGNC:30290] |
| ENSG00000116285 | HGLibA_15532 | 136 | 0.095473145 | ERRFI1 | ERBB receptor feedback inhibitor 1 [Source:HGNC Symbol;Acc:HGNC:18185] |
| ENSG00000117118 | HGLibA_43142 | 136 | 0.095473145 | SDHB | succinate dehydrogenase complex iron sulfur subunit B [Source:HGNC Symbol;Acc:HGNC:10681] |
| ENSG00000120539 | HGLibA_28411 | 136 | 0.095473145 | MASTL | microtubule associated serine/threonine kinase like [Source:HGNC Symbol;Acc:HGNC:19042] |
| ENSG00000121446 | HGLibA_41117 | 136 | 0.095473145 | RGSL1 | regulator of G protein signaling like 1 [Source:HGNC Symbol;Acc:HGNC:18636] |
| ENSG00000123268 | HGLibA_03431 | 136 | 0.095473145 | ATF1 | activating transcription factor 1 [Source:HGNC Symbol;Acc:HGNC:783] |
| ENSG00000123607 | HGLibA_52073 | 136 | 0.095473145 | TTC21B | tetratricopeptide repeat domain 21B [Source:HGNC Symbol;Acc:HGNC:25660] |
| ENSG00000123977 | HGLibA_12346 | 136 | 0.095473145 | DAW1 | dynein assembly factor with WD repeats 1 [Source:HGNC Symbol;Acc:HGNC:26383] |
| ENSG00000125944 | HGLibA_21842 | 136 | 0.095473145 | HNRNPR | heterogeneous nuclear ribonucleoprotein R [Source:HGNC Symbol;Acc:HGNC:5047] |
| ENSG00000126106 | HGLibA_50438 | 136 | 0.095473145 | TMEM53 | transmembrane protein 53 [Source:HGNC Symbol;Acc:HGNC:26186] |
| ENSG00000127022 | HGLibA_07129 | 136 | 0.095473145 | CANX | calnexin [Source:HGNC Symbol;Acc:HGNC:1473] |
| ENSG00000128016 | HGLibA_55203 | 136 | 0.095473145 | ZFP36 | ZFP36 ring finger protein [Source:HGNC Symbol;Acc:HGNC:12862] |
| ENSG00000130775 | HGLibA_49353 | 136 | 0.095473145 | THEMIS2 | thymocyte selection associated family member 2 [Source:HGNC Symbol;Acc:HGNC:16839] |
| ENSG00000132199 | HGLibA_15155 | 136 | 0.095473145 | ENOSF1 | enolase superfamily member 1 [Source:HGNC Symbol;Acc:HGNC:30365] |
| ENSG00000132429 | HGLibA_37702 | 136 | 0.095473145 | POPDC3 | popeye domain containing 3 [Source:HGNC Symbol;Acc:HGNC:17649] |
| ENSG00000134532 | HGLibA_46267 | 136 | 0.095473145 | SOX5 | SRY-box 5 [Source:HGNC Symbol;Acc:HGNC:11201] |
| ENSG00000134940 | HGLibA_00539 | 136 | 0.095473145 | ACRV1 | acrosomal vesicle protein 1 [Source:HGNC Symbol;Acc:HGNC:127] |
| ENSG00000135114 | HGLibA_33208 | 136 | 0.095473145 | OASL | 2'-5'-oligoadenylate synthetase like [Source:HGNC Symbol;Acc:HGNC:8090] |
| ENSG00000135916 | HGLibA_23835 | 136 | 0.095473145 | ITM2C | integral membrane protein 2C [Source:HGNC Symbol;Acc:HGNC:6175] |
| ENSG00000136045 | HGLibA_39633 | 136 | 0.095473145 | PWP1 | endonuclein [Source:HGNC Symbol;Acc:HGNC:17015]" |
| ENSG00000137642 | HGLibA_46195 | 136 | 0.095473145 | SORL1 | sortilin related receptor 1 [Source:HGNC Symbol;Acc:HGNC:11185] |
| ENSG00000137962 | HGLibA_02750 | 136 | 0.095473145 | ARHGAP29 | Rho GTPase activating protein 29 [Source:HGNC Symbol;Acc:HGNC:30207] |
| ENSG00000139926 | HGLibA_18023 | 136 | 0.095473145 | FRMD6 | FERM domain containing 6 [Source:HGNC Symbol;Acc:HGNC:19839] |
| ENSG00000140455 | HGLibA_53327 | 136 | 0.095473145 | USP3 | ubiquitin specific peptidase 3 [Source:HGNC Symbol;Acc:HGNC:12626] |
| ENSG00000141447 | HGLibA_34650 | 136 | 0.095473145 | OSBPL1A | oxysterol binding protein like 1A [Source:HGNC Symbol;Acc:HGNC:16398] |
| ENSG00000142528 | HGLibA_56059 | 136 | 0.095473145 | ZNF473 | zinc finger protein 473 [Source:HGNC Symbol;Acc:HGNC:23239] |
| ENSG00000142583 | HGLibA_44882 | 136 | 0.095473145 | SLC2A5 | solute carrier family 2 member 5 [Source:HGNC Symbol;Acc:HGNC:11010] |
| ENSG00000143554 | HGLibA_44819 | 136 | 0.095473145 | SLC27A3 | solute carrier family 27 member 3 [Source:HGNC Symbol;Acc:HGNC:10997] |
| ENSG00000143702 | HGLibA_09117 | 136 | 0.095473145 | CEP170 | centrosomal protein 170 [Source:HGNC Symbol;Acc:HGNC:28920] |
| ENSG00000143994 | HGLibA_00229 | 136 | 0.095473145 | ABHD1 | abhydrolase domain containing 1 [Source:HGNC Symbol;Acc:HGNC:17553] |
| ENSG00000144045 | HGLibA_13938 | 136 | 0.095473145 | DQX1 | DEAQ-box RNA dependent ATPase 1 [Source:HGNC Symbol;Acc:HGNC:20410] |
| ENSG00000144566 | HGLibA_39962 | 136 | 0.095473145 | RAB5A | member RAS oncogene family [Source:HGNC Symbol;Acc:HGNC:9783]" |
| ENSG00000144847 | HGLibA_22882 | 136 | 0.095473145 | IGSF11 | immunoglobulin superfamily member 11 [Source:HGNC Symbol;Acc:HGNC:16669] |
| ENSG00000145826 | HGLibA_26267 | 136 | 0.095473145 | LECT2 | leukocyte cell derived chemotaxin 2 [Source:HGNC Symbol;Acc:HGNC:6550] |
| ENSG00000148019 | HGLibA_09168 | 136 | 0.095473145 | CEP78 | centrosomal protein 78 [Source:HGNC Symbol;Acc:HGNC:25740] |
| ENSG00000151577 | HGLibA_13966 | 136 | 0.095473145 | DRD3 | dopamine receptor D3 [Source:HGNC Symbol;Acc:HGNC:3024] |
| ENSG00000151892 | HGLibA_18964 | 136 | 0.095473145 | GFRA1 | GDNF family receptor alpha 1 [Source:HGNC Symbol;Acc:HGNC:4243] |
| ENSG00000152795 | HGLibA_21817 | 136 | 0.095473145 | HNRNPDL | heterogeneous nuclear ribonucleoprotein D like [Source:HGNC Symbol;Acc:HGNC:5037] |
| ENSG00000153071 | HGLibA_12268 | 136 | 0.095473145 | DAB2 | clathrin adaptor protein [Source:HGNC Symbol;Acc:HGNC:2662]" |
| ENSG00000155329 | HGLibA_55003 | 136 | 0.095473145 | ZCCHC10 | zinc finger CCHC-type containing 10 [Source:HGNC Symbol;Acc:HGNC:25954] |
| ENSG00000156006 | HGLibA_31175 | 136 | 0.095473145 | NAT2 | N-acetyltransferase 2 [Source:HGNC Symbol;Acc:HGNC:7646] |
| ENSG00000157191 | HGLibA_31597 | 136 | 0.095473145 | NECAP2 | NECAP endocytosis associated 2 [Source:HGNC Symbol;Acc:HGNC:25528] |
| ENSG00000157426 | HGLibA_00078 | 136 | 0.095473145 | AASDH | aminoadipate-semialdehyde dehydrogenase [Source:HGNC Symbol;Acc:HGNC:23993] |
| ENSG00000162482 | HGLibA_01502 | 136 | 0.095473145 | AKR7A3 | aldo-keto reductase family 7 member A3 [Source:HGNC Symbol;Acc:HGNC:390] |
| ENSG00000162521 | HGLibA_40429 | 136 | 0.095473145 | RBBP4 | chromatin remodeling factor [Source:HGNC Symbol;Acc:HGNC:9887]" |
| ENSG00000163209 | HGLibA_46827 | 136 | 0.095473145 | SPRR3 | small proline rich protein 3 [Source:HGNC Symbol;Acc:HGNC:11268] |
| ENSG00000163399 | HGLibA_03581 | 136 | 0.095473145 | ATP1A1 | ATPase Na+/K+ transporting subunit alpha 1 [Source:HGNC Symbol;Acc:HGNC:799] |
| ENSG00000163519 | HGLibA_51342 | 136 | 0.095473145 | TRAT1 | T cell receptor associated transmembrane adaptor 1 [Source:HGNC Symbol;Acc:HGNC:30698] |
| ENSG00000164400 | HGLibA_11327 | 136 | 0.095473145 | CSF2 | colony stimulating factor 2 [Source:HGNC Symbol;Acc:HGNC:2434] |
| ENSG00000165675 | HGLibA_15161 | 136 | 0.095473145 | ENOX2 | ecto-NOX disulfide-thiol exchanger 2 [Source:HGNC Symbol;Acc:HGNC:2259] |
| ENSG00000166068 | HGLibA_46789 | 136 | 0.095473145 | SPRED1 | sprouty related EVH1 domain containing 1 [Source:HGNC Symbol;Acc:HGNC:20249] |
| ENSG00000166912 | HGLibA_30411 | 136 | 0.095473145 | MTMR10 | myotubularin related protein 10 [Source:HGNC Symbol;Acc:HGNC:25999] |
| ENSG00000167098 | HGLibA_47796 | 136 | 0.095473145 | SUN5 | Sad1 and UNC84 domain containing 5 [Source:HGNC Symbol;Acc:HGNC:16252] |
| ENSG00000168062 | HGLibA_04176 | 136 | 0.095473145 | BATF2 | basic leucine zipper ATF-like transcription factor 2 [Source:HGNC Symbol;Acc:HGNC:25163] |
| ENSG00000171467 | HGLibA_55786 | 136 | 0.095473145 | ZNF318 | zinc finger protein 318 [Source:HGNC Symbol;Acc:HGNC:13578] |
| ENSG00000171611 | HGLibA_39285 | 136 | 0.095473145 | PTCRA | pre T cell antigen receptor alpha [Source:HGNC Symbol;Acc:HGNC:21290] |
| ENSG00000172818 | HGLibA_34801 | 136 | 0.095473145 | OVOL1 | ovo like transcriptional repressor 1 [Source:HGNC Symbol;Acc:HGNC:8525] |
| ENSG00000177181 | HGLibA_41272 | 136 | 0.095473145 | RIMKLA | ribosomal modification protein rimK like family member A [Source:HGNC Symbol;Acc:HGNC:28725] |
| ENSG00000177303 | HGLibA_07293 | 136 | 0.095473145 | CASKIN2 | CASK interacting protein 2 [Source:HGNC Symbol;Acc:HGNC:18200] |
| ENSG00000177693 | HGLibA_34008 | 136 | 0.095473145 | OR4F4 | olfactory receptor family 4 subfamily F member 4 [Source:HGNC Symbol;Acc:HGNC:8301] |
| ENSG00000178538 | HGLibA_06823 | 136 | 0.095473145 | CA8 | carbonic anhydrase 8 [Source:HGNC Symbol;Acc:HGNC:1382] |
| ENSG00000179142 | HGLibA_12057 | 136 | 0.095473145 | CYP11B2 | cytochrome P450 family 11 subfamily B member 2 [Source:HGNC Symbol;Acc:HGNC:2592] |
| ENSG00000182208 | HGLibA_29592 | 136 | 0.095473145 | MOB2 | MOB kinase activator 2 [Source:HGNC Symbol;Acc:HGNC:24904] |
| ENSG00000182224 | HGLibA_11994 | 136 | 0.095473145 | CYB5D1 | cytochrome b5 domain containing 1 [Source:HGNC Symbol;Acc:HGNC:26516] |
| ENSG00000183978 | HGLibA_10447 | 136 | 0.095473145 | COA3 | cytochrome c oxidase assembly factor 3 [Source:HGNC Symbol;Acc:HGNC:24990] |
| ENSG00000184033 | HGLibA_11562 | 136 | 0.095473145 | CTAG1B | cancer/testis antigen 1B [Source:HGNC Symbol;Acc:HGNC:2491] |
| ENSG00000184492 | HGLibA_17841 | 136 | 0.095473145 | FOXD4L1 | forkhead box D4 like 1 [Source:HGNC Symbol;Acc:HGNC:18521] |
| ENSG00000184611 | HGLibA_24229 | 136 | 0.095473145 | KCNH7 | potassium voltage-gated channel subfamily H member 7 [Source:HGNC Symbol;Acc:HGNC:18863] |
| ENSG00000185565 | HGLibA_27512 | 136 | 0.095473145 | LSAMP | limbic system associated membrane protein [Source:HGNC Symbol;Acc:HGNC:6705] |
| ENSG00000185862 | HGLibA_15690 | 136 | 0.095473145 | EVI2B | ecotropic viral integration site 2B [Source:HGNC Symbol;Acc:HGNC:3500] |
| ENSG00000186111 | HGLibA_36719 | 136 | 0.095473145 | PIP5K1C | phosphatidylinositol-4-phosphate 5-kinase type 1 gamma [Source:HGNC Symbol;Acc:HGNC:8996] |
| ENSG00000187268 | HGLibA_16825 | 136 | 0.095473145 | FAM9C | family with sequence similarity 9 member C [Source:HGNC Symbol;Acc:HGNC:18405] |
| ENSG00000187612 | HGLibA_34368 | 136 | 0.095473145 | OR5W2 | olfactory receptor family 5 subfamily W member 2 [Source:HGNC Symbol;Acc:HGNC:15299] |
| ENSG00000188603 | HGLibA_10075 | 136 | 0.095473145 | CLN3 | battenin [Source:HGNC Symbol;Acc:HGNC:2074]" |
| ENSG00000196329 | HGLibA_19082 | 136 | 0.095473145 | GIMAP5 | IMAP family member 5 [Source:HGNC Symbol;Acc:HGNC:18005]" |
| ENSG00000196689 | HGLibA_51783 | 136 | 0.095473145 | TRPV1 | transient receptor potential cation channel subfamily V member 1 [Source:HGNC Symbol;Acc:HGNC:12716] |
| ENSG00000197870 | HGLibA_38343 | 136 | 0.095473145 | PRB3 | proline rich protein BstNI subfamily 3 [Source:HGNC Symbol;Acc:HGNC:9339] |
| ENSG00000198964 | HGLibA_43834 | 136 | 0.095473145 | SGMS1 | sphingomyelin synthase 1 [Source:HGNC Symbol;Acc:HGNC:29799] |
| ENSG00000203722 | HGLibA_40123 | 136 | 0.095473145 | RAET1G | retinoic acid early transcript 1G [Source:HGNC Symbol;Acc:HGNC:16795] |
| ENSG00000204347 | HGLibA_04905 | 136 | 0.095473145 | BTBD17 | BTB domain containing 17 [Source:HGNC Symbol;Acc:HGNC:33758] |
| ENSG00000206538 | HGLibA_53642 | 136 | 0.095473145 | VGLL3 | vestigial like family member 3 [Source:HGNC Symbol;Acc:HGNC:24327] |
| ENSG00000263639 | HGLibA_30223 | 136 | 0.095473145 | MSMB | microseminoprotein beta [Source:HGNC Symbol;Acc:HGNC:7372] |
| ENSG00000270765 | HGLibA_18648 | 136 | 0.095473145 | GAS2L2 | growth arrest specific 2 like 2 [Source:HGNC Symbol;Acc:HGNC:24846] |
| ENSG00000288611 | HGLibA_32413 | 136 | 0.095473145 | NPBWR1 | - |
| hsa-mir-20b | HGLibA_58203 | 136 | 0.095473145 | hsa-mir-20b | - |
| hsa-mir-3619 | HGLibA_59105 | 136 | 0.095473145 | hsa-mir-3619 | - |
| hsa-mir-4261 | HGLibA_59752 | 136 | 0.095473145 | hsa-mir-4261 | - |
| hsa-mir-454 | HGLibA_60620 | 136 | 0.095473145 | hsa-mir-454 | - |
| hsa-mir-4635 | HGLibA_60646 | 136 | 0.095473145 | hsa-mir-4635 | - |
| hsa-mir-4690 | HGLibA_60871 | 136 | 0.095473145 | hsa-mir-4690 | - |
| hsa-mir-4723 | HGLibA_60990 | 136 | 0.095473145 | hsa-mir-4723 | - |
| hsa-mir-5187 | HGLibA_61640 | 136 | 0.095473145 | hsa-mir-5187 | - |
| hsa-mir-599 | HGLibA_62490 | 136 | 0.095473145 | hsa-mir-599 | - |
| hsa-mir-601 | HGLibA_62497 | 136 | 0.095473145 | hsa-mir-601 | - |
| hsa-mir-6511a-2 | HGLibA_62902 | 136 | 0.095473145 | hsa-mir-6511a-2 | - |
| hsa-mir-6515 | HGLibA_62932 | 136 | 0.095473145 | hsa-mir-6515 | - |
| hsa-mir-6767 | HGLibA_63228 | 136 | 0.095473145 | hsa-mir-6767 | - |
| hsa-mir-6772 | HGLibA_63262 | 136 | 0.095473145 | hsa-mir-6772 | - |
| hsa-mir-6879 | HGLibA_63701 | 136 | 0.095473145 | hsa-mir-6879 | - |
| C14orf79 | HGLibA_05370 | 135 | 0.086427802 | C14orf79 | - |
| CNIH | HGLibA_10280 | 135 | 0.086427802 | CNIH | - |
| CRLF2_Y | HGLibA_11192 | 135 | 0.086427802 | CRLF2_Y | - |
| ENSG00000005421 | HGLibA_37678 | 135 | 0.086427802 | PON1 | paraoxonase 1 [Source:HGNC Symbol;Acc:HGNC:9204] |
| ENSG00000008869 | HGLibA_21077 | 135 | 0.086427802 | HEATR5B | HEAT repeat containing 5B [Source:HGNC Symbol;Acc:HGNC:29273] |
| ENSG00000009790 | HGLibA_51254 | 135 | 0.086427802 | TRAF3IP3 | TRAF3 interacting protein 3 [Source:HGNC Symbol;Acc:HGNC:30766] |
| ENSG00000011426 | HGLibA_02169 | 135 | 0.086427802 | ANLN | anillin actin binding protein [Source:HGNC Symbol;Acc:HGNC:14082] |
| ENSG00000023171 | HGLibA_20197 | 135 | 0.086427802 | GRAMD1B | GRAM domain containing 1B [Source:HGNC Symbol;Acc:HGNC:29214] |
| ENSG00000055070 | HGLibA_48113 | 135 | 0.086427802 | SZRD1 | SUZ RNA binding domain containing 1 [Source:HGNC Symbol;Acc:HGNC:30232] |
| ENSG00000056558 | HGLibA_51241 | 135 | 0.086427802 | TRAF1 | TNF receptor associated factor 1 [Source:HGNC Symbol;Acc:HGNC:12031] |
| ENSG00000065485 | HGLibA_35883 | 135 | 0.086427802 | PDIA5 | protein disulfide isomerase family A member 5 [Source:HGNC Symbol;Acc:HGNC:24811] |
| ENSG00000065675 | HGLibA_38556 | 135 | 0.086427802 | PRKCQ | protein kinase C theta [Source:HGNC Symbol;Acc:HGNC:9410] |
| ENSG00000065833 | HGLibA_28708 | 135 | 0.086427802 | ME1 | malic enzyme 1 [Source:HGNC Symbol;Acc:HGNC:6983] |
| ENSG00000077943 | HGLibA_23744 | 135 | 0.086427802 | ITGA8 | integrin subunit alpha 8 [Source:HGNC Symbol;Acc:HGNC:6144] |
| ENSG00000078596 | HGLibA_23828 | 135 | 0.086427802 | ITM2A | integral membrane protein 2A [Source:HGNC Symbol;Acc:HGNC:6173] |
| ENSG00000083642 | HGLibA_35947 | 135 | 0.086427802 | PDS5B | PDS5 cohesin associated factor B [Source:HGNC Symbol;Acc:HGNC:20418] |
| ENSG00000087128 | HGLibA_50621 | 135 | 0.086427802 | TMPRSS11E | transmembrane serine protease 11E [Source:HGNC Symbol;Acc:HGNC:24465] |
| ENSG00000100288 | HGLibA_09460 | 135 | 0.086427802 | CHKB | choline kinase beta [Source:HGNC Symbol;Acc:HGNC:1938] |
| ENSG00000101150 | HGLibA_51117 | 135 | 0.086427802 | TPD52L2 | TPD52 like 2 [Source:HGNC Symbol;Acc:HGNC:12007] |
| ENSG00000102078 | HGLibA_44647 | 135 | 0.086427802 | SLC25A14 | solute carrier family 25 member 14 [Source:HGNC Symbol;Acc:HGNC:10984] |
| ENSG00000103187 | HGLibA_10801 | 135 | 0.086427802 | COTL1 | coactosin like F-actin binding protein 1 [Source:HGNC Symbol;Acc:HGNC:18304] |
| ENSG00000103942 | HGLibA_21868 | 135 | 0.086427802 | HOMER2 | homer scaffold protein 2 [Source:HGNC Symbol;Acc:HGNC:17513] |

| | | | | | |
|---|---|---|---|---|---|
| ENSG00000104368 | HGLibA_36960 | 135 | 0.086427802 | PLAT | tissue type [Source:HGNC Symbol;Acc:HGNC:9051]" |
| ENSG00000104635 | HGLibA_45104 | 135 | 0.086427802 | SLC39A14 | solute carrier family 39 member 14 [Source:HGNC Symbol;Acc:HGNC:20858] |
| ENSG00000106346 | HGLibA_53365 | 135 | 0.086427802 | USP42 | ubiquitin specific peptidase 42 [Source:HGNC Symbol;Acc:HGNC:20068] |
| ENSG00000106701 | HGLibA_18080 | 135 | 0.086427802 | FSD1L | fibronectin type III and SPRY domain containing 1 like [Source:HGNC Symbol;Acc:HGNC:13753] |
| ENSG00000108176 | HGLibA_13569 | 135 | 0.086427802 | DNAJC12 | DnaJ heat shock protein family (Hsp40) member C12 [Source:HGNC Symbol;Acc:HGNC:28908] |
| ENSG00000108352 | HGLibA_40263 | 135 | 0.086427802 | RAPGEFL1 | Rap guanine nucleotide exchange factor like 1 [Source:HGNC Symbol;Acc:HGNC:17428] |
| ENSG00000109189 | HGLibA_53378 | 135 | 0.086427802 | USP46 | ubiquitin specific peptidase 46 [Source:HGNC Symbol;Acc:HGNC:20075] |
| ENSG00000110934 | HGLibA_04509 | 135 | 0.086427802 | BIN2 | bridging integrator 2 [Source:HGNC Symbol;Acc:HGNC:1053] |
| ENSG00000115415 | HGLibA_47382 | 135 | 0.086427802 | STAT1 | signal transducer and activator of transcription 1 [Source:HGNC Symbol;Acc:HGNC:11362] |
| ENSG00000115993 | HGLibA_51279 | 135 | 0.086427802 | TRAK2 | trafficking kinesin protein 2 [Source:HGNC Symbol;Acc:HGNC:13206] |
| ENSG00000118412 | HGLibA_07329 | 135 | 0.086427802 | CASP8AP2 | caspase 8 associated protein 2 [Source:HGNC Symbol;Acc:HGNC:1510] |
| ENSG00000119688 | HGLibA_00200 | 135 | 0.086427802 | ABCD4 | ATP binding cassette subfamily D member 4 [Source:HGNC Symbol;Acc:HGNC:68] |
| ENSG00000119737 | HGLibA_20081 | 135 | 0.086427802 | GPR75 | G protein-coupled receptor 75 [Source:HGNC Symbol;Acc:HGNC:4526] |
| ENSG00000120889 | HGLibA_50735 | 135 | 0.086427802 | TNFRSF10B | TNF receptor superfamily member 10b [Source:HGNC Symbol;Acc:HGNC:11905] |
| ENSG00000121005 | HGLibA_11173 | 135 | 0.086427802 | CRISPLD1 | cysteine rich secretory protein LCCL domain containing 1 [Source:HGNC Symbol;Acc:HGNC:18206] |
| ENSG00000121594 | HGLibA_08453 | 135 | 0.086427802 | CD80 | CD80 molecule [Source:HGNC Symbol;Acc:HGNC:1700] |
| ENSG00000125703 | HGLibA_03493 | 135 | 0.086427802 | ATG4C | autophagy related 4C cysteine peptidase [Source:HGNC Symbol;Acc:HGNC:16040] |
| ENSG00000126453 | HGLibA_04310 | 135 | 0.086427802 | BCL2L12 | BCL2 like 12 [Source:HGNC Symbol;Acc:HGNC:13787] |
| ENSG00000126549 | HGLibA_47401 | 135 | 0.086427802 | STATH | statherin [Source:HGNC Symbol;Acc:HGNC:11369] |
| ENSG00000128609 | HGLibA_31463 | 135 | 0.086427802 | NDUFA5 | NADH:ubiquinone oxidoreductase subunit A5 [Source:HGNC Symbol;Acc:HGNC:7688] |
| ENSG00000130312 | HGLibA_29957 | 135 | 0.086427802 | MRPL34 | mitochondrial ribosomal protein L34 [Source:HGNC Symbol;Acc:HGNC:14488] |
| ENSG00000130513 | HGLibA_18860 | 135 | 0.086427802 | GDF15 | growth differentiation factor 15 [Source:HGNC Symbol;Acc:HGNC:30142] |
| ENSG00000132424 | HGLibA_37344 | 135 | 0.086427802 | PNISR | PNN interacting serine and arginine rich protein [Source:HGNC Symbol;Acc:HGNC:21222] |
| ENSG00000132681 | HGLibA_03589 | 135 | 0.086427802 | ATP1A4 | ATPase Na+/K+ transporting subunit alpha 4 [Source:HGNC Symbol;Acc:HGNC:14073] |
| ENSG00000132938 | HGLibA_30508 | 135 | 0.086427802 | MTUS2 | microtubule associated scaffold protein 2 [Source:HGNC Symbol;Acc:HGNC:20595] |
| ENSG00000134324 | HGLibA_27096 | 135 | 0.086427802 | LPIN1 | lipin 1 [Source:HGNC Symbol;Acc:HGNC:13345] |
| ENSG00000134575 | HGLibA_00512 | 135 | 0.086427802 | ACP2 | lysosomal [Source:HGNC Symbol;Acc:HGNC:123]" |
| ENSG00000134917 | HGLibA_00841 | 135 | 0.086427802 | ADAMTS8 | ADAM metallopeptidase with thrombospondin type 1 motif 8 [Source:HGNC Symbol;Acc:HGNC:224] |
| ENSG00000135269 | HGLibA_49021 | 135 | 0.086427802 | TES | testin LIM domain protein [Source:HGNC Symbol;Acc:HGNC:14620] |
| ENSG00000135316 | HGLibA_47967 | 135 | 0.086427802 | SYNCRIP | synaptotagmin binding cytoplasmic RNA interacting protein [Source:HGNC Symbol;Acc:HGNC:16918] |
| ENSG00000135931 | HGLibA_03035 | 135 | 0.086427802 | ARMC9 | armadillo repeat containing 9 [Source:HGNC Symbol;Acc:HGNC:20730] |
| ENSG00000136929 | HGLibA_21129 | 135 | 0.086427802 | HEMGN | hemogen [Source:HGNC Symbol;Acc:HGNC:17509] |
| ENSG00000138182 | HGLibA_24895 | 135 | 0.086427802 | KIF20B | kinesin family member 20B [Source:HGNC Symbol;Acc:HGNC:7212] |
| ENSG00000139914 | HGLibA_17540 | 135 | 0.086427802 | FITM1 | fat storage inducing transmembrane protein 1 [Source:HGNC Symbol;Acc:HGNC:33714] |
| ENSG00000140750 | HGLibA_02715 | 135 | 0.086427802 | ARHGAP17 | Rho GTPase activating protein 17 [Source:HGNC Symbol;Acc:HGNC:18239] |
| ENSG00000143977 | HGLibA_45992 | 135 | 0.086427802 | SNRPG | small nuclear ribonucleoprotein polypeptide G [Source:HGNC Symbol;Acc:HGNC:11163] |
| ENSG00000144026 | HGLibA_56139 | 135 | 0.086427802 | ZNF514 | zinc finger protein 514 [Source:HGNC Symbol;Acc:HGNC:25894] |
| ENSG00000144040 | HGLibA_43792 | 135 | 0.086427802 | SFXN5 | sideroflexin 5 [Source:HGNC Symbol;Acc:HGNC:16073] |
| ENSG00000146267 | HGLibA_16927 | 135 | 0.086427802 | FAXC | failed axon connections homolog [Source:HGNC Symbol;Acc:HGNC:20742] |
| ENSG00000149177 | HGLibA_39510 | 135 | 0.086427802 | PTPRJ | receptor type J [Source:HGNC Symbol;Acc:HGNC:9673]" |
| ENSG00000150656 | HGLibA_10251 | 135 | 0.086427802 | CNDP1 | carnosine dipeptidase 1 [Source:HGNC Symbol;Acc:HGNC:20675] |
| ENSG00000151364 | HGLibA_24426 | 135 | 0.086427802 | KCTD14 | potassium channel tetramerization domain containing 14 [Source:HGNC Symbol;Acc:HGNC:23295] |
| ENSG00000151418 | HGLibA_03764 | 135 | 0.086427802 | ATP6V1G3 | ATPase H+ transporting V1 subunit G3 [Source:HGNC Symbol;Acc:HGNC:18265] |
| ENSG00000153234 | HGLibA_32629 | 135 | 0.086427802 | NR4A2 | nuclear receptor subfamily 4 group A member 2 [Source:HGNC Symbol;Acc:HGNC:7981] |
| ENSG00000155428 | HGLibA_51609 | 135 | 0.086427802 | TRIM74 | tripartite motif containing 74 [Source:HGNC Symbol;Acc:HGNC:17453] |
| ENSG00000156735 | HGLibA_04084 | 135 | 0.086427802 | BAG4 | BCL2 associated athanogene 4 [Source:HGNC Symbol;Acc:HGNC:940] |
| ENSG00000157368 | HGLibA_23141 | 135 | 0.086427802 | IL34 | interleukin 34 [Source:HGNC Symbol;Acc:HGNC:28529] |
| ENSG00000158296 | HGLibA_44389 | 135 | 0.086427802 | SLC13A3 | solute carrier family 13 member 3 [Source:HGNC Symbol;Acc:HGNC:14430] |
| ENSG00000160224 | HGLibA_01367 | 135 | 0.086427802 | AIRE | autoimmune regulator [Source:HGNC Symbol;Acc:HGNC:360] |
| ENSG00000160271 | HGLibA_40166 | 135 | 0.086427802 | RALGDS | ral guanine nucleotide dissociation stimulator [Source:HGNC Symbol;Acc:HGNC:9842] |
| ENSG00000160948 | HGLibA_53758 | 135 | 0.086427802 | VPS28 | ESCRT-I subunit [Source:HGNC Symbol;Acc:HGNC:18178]" |
| ENSG00000161634 | HGLibA_12479 | 135 | 0.086427802 | DCD | dermcidin [Source:HGNC Symbol;Acc:HGNC:14669] |
| ENSG00000161654 | HGLibA_27527 | 135 | 0.086427802 | LSM12 | LSM12 homolog [Source:HGNC Symbol;Acc:HGNC:26407] |
| ENSG00000161860 | HGLibA_47919 | 135 | 0.086427802 | SYCE2 | synaptonemal complex central element protein 2 [Source:HGNC Symbol;Acc:HGNC:27411] |
| ENSG00000161904 | HGLibA_26290 | 135 | 0.086427802 | LEMD2 | LEM domain containing 2 [Source:HGNC Symbol;Acc:HGNC:21244] |
| ENSG00000162722 | HGLibA_51553 | 135 | 0.086427802 | TRIM58 | tripartite motif containing 58 [Source:HGNC Symbol;Acc:HGNC:24150] |
| ENSG00000165322 | HGLibA_02708 | 135 | 0.086427802 | ARHGAP12 | Rho GTPase activating protein 12 [Source:HGNC Symbol;Acc:HGNC:16348] |
| ENSG00000165502 | HGLibA_41960 | 135 | 0.086427802 | RPL36AL | ribosomal protein L36a like [Source:HGNC Symbol;Acc:HGNC:10346] |
| ENSG00000166147 | HGLibA_16955 | 135 | 0.086427802 | FBN1 | fibrillin 1 [Source:HGNC Symbol;Acc:HGNC:3603] |
| ENSG00000166888 | HGLibA_47398 | 135 | 0.086427802 | STAT6 | signal transducer and activator of transcription 6 [Source:HGNC Symbol;Acc:HGNC:11368] |
| ENSG00000167608 | HGLibA_49758 | 135 | 0.086427802 | TMC4 | transmembrane channel like 4 [Source:HGNC Symbol;Acc:HGNC:22998] |
| ENSG00000168101 | HGLibA_32956 | 135 | 0.086427802 | NUDT16L1 | nudix hydrolase 16 like 1 [Source:HGNC Symbol;Acc:HGNC:28154] |
| ENSG00000168917 | HGLibA_45019 | 135 | 0.086427802 | SLC35G2 | solute carrier family 35 member G2 [Source:HGNC Symbol;Acc:HGNC:28480] |
| ENSG00000171133 | HGLibA_33814 | 135 | 0.086427802 | OR2K2 | olfactory receptor family 2 subfamily K member 2 [Source:HGNC Symbol;Acc:HGNC:8264] |
| ENSG00000172061 | HGLibA_27260 | 135 | 0.086427802 | LRRC15 | leucine rich repeat containing 15 [Source:HGNC Symbol;Acc:HGNC:20818] |
| ENSG00000172500 | HGLibA_17505 | 135 | 0.086427802 | FIBP | FGF1 intracellular binding protein [Source:HGNC Symbol;Acc:HGNC:3705] |
| ENSG00000173535 | HGLibA_50738 | 135 | 0.086427802 | TNFRSF10C | TNF receptor superfamily member 10c [Source:HGNC Symbol;Acc:HGNC:11906] |
| ENSG00000174453 | HGLibA_53925 | 135 | 0.086427802 | VWC2L | von Willebrand factor C domain containing 2 like [Source:HGNC Symbol;Acc:HGNC:37203] |
| ENSG00000174695 | HGLibA_50040 | 135 | 0.086427802 | TMEM167A | transmembrane protein 167A [Source:HGNC Symbol;Acc:HGNC:28330] |
| ENSG00000175387 | HGLibA_45572 | 135 | 0.086427802 | SMAD2 | SMAD family member 2 [Source:HGNC Symbol;Acc:HGNC:6768] |
| ENSG00000175691 | HGLibA_56700 | 135 | 0.086427802 | ZNF77 | zinc finger protein 77 [Source:HGNC Symbol;Acc:HGNC:13150] |
| ENSG00000175928 | HGLibA_27470 | 135 | 0.086427802 | LRRN1 | leucine rich repeat neuronal 1 [Source:HGNC Symbol;Acc:HGNC:20980] |
| ENSG00000176230 | HGLibA_34029 | 135 | 0.086427802 | OR4K17 | olfactory receptor family 4 subfamily K member 17 [Source:HGNC Symbol;Acc:HGNC:15355] |
| ENSG00000182489 | HGLibA_54538 | 135 | 0.086427802 | XKRX | XK related X-linked [Source:HGNC Symbol;Acc:HGNC:29845] |
| ENSG00000183114 | HGLibA_16589 | 135 | 0.086427802 | FAM43B | family with sequence similarity 43 member B [Source:HGNC Symbol;Acc:HGNC:31791] |
| ENSG00000183386 | HGLibA_17485 | 135 | 0.086427802 | FHL3 | four and a half LIM domains 3 [Source:HGNC Symbol;Acc:HGNC:3704] |
| ENSG00000184574 | HGLibA_27065 | 135 | 0.086427802 | LPAR5 | lysophosphatidic acid receptor 5 [Source:HGNC Symbol;Acc:HGNC:13307] |
| ENSG00000185278 | HGLibA_54869 | 135 | 0.086427802 | ZBTB37 | zinc finger and BTB domain containing 37 [Source:HGNC Symbol;Acc:HGNC:28365] |
| ENSG00000185477 | HGLibA_20146 | 135 | 0.086427802 | GPRIN3 | GPRIN family member 3 [Source:HGNC Symbol;Acc:HGNC:27733] |
| ENSG00000186212 | HGLibA_46215 | 135 | 0.086427802 | SOWAHB | sosondowah ankyrin repeat domain family member B [Source:HGNC Symbol;Acc:HGNC:32958] |
| ENSG00000186377 | HGLibA_12202 | 135 | 0.086427802 | CYP4X1 | cytochrome P450 family 4 subfamily X member 1 [Source:HGNC Symbol;Acc:HGNC:20244] |
| ENSG00000186562 | HGLibA_12824 | 135 | 0.086427802 | DEFB105A | defensin beta 105A [Source:HGNC Symbol;Acc:HGNC:18087] |
| ENSG00000187049 | HGLibA_50221 | 135 | 0.086427802 | TMEM216 | transmembrane protein 216 [Source:HGNC Symbol;Acc:HGNC:25018] |
| ENSG00000187173 | HGLibA_26119 | 135 | 0.086427802 | LCE2A | late cornified envelope 2A [Source:HGNC Symbol;Acc:HGNC:29469] |
| ENSG00000187210 | HGLibA_18808 | 135 | 0.086427802 | GCNT1 | core 2 [Source:HGNC Symbol;Acc:HGNC:4203]" |
| ENSG00000187753 | HGLibA_06692 | 135 | 0.086427802 | C9orf153 | chromosome 9 open reading frame 153 [Source:HGNC Symbol;Acc:HGNC:31456] |
| ENSG00000196090 | HGLibA_39539 | 135 | 0.086427802 | PTPRT | receptor type T [Source:HGNC Symbol;Acc:HGNC:9682]" |
| ENSG00000198162 | HGLibA_28060 | 135 | 0.086427802 | MAN1A2 | mannosidase alpha class 1A member 2 [Source:HGNC Symbol;Acc:HGNC:6822] |
| ENSG00000205302 | HGLibA_46058 | 135 | 0.086427802 | SNX2 | sorting nexin 2 [Source:HGNC Symbol;Acc:HGNC:11173] |
| ENSG00000214102 | HGLibA_54240 | 135 | 0.086427802 | WEE2 | WEE1 homolog 2 [Source:HGNC Symbol;Acc:HGNC:19684] |
| ENSG00000221988 | HGLibA_38237 | 135 | 0.086427802 | PPT2 | palmitoyl-protein thioesterase 2 [Source:HGNC Symbol;Acc:HGNC:9326] |
| ENSG00000223496 | HGLibA_15786 | 135 | 0.086427802 | EXOSC6 | exosome component 6 [Source:HGNC Symbol;Acc:HGNC:19055] |
| ENSG00000223569 | HGLibA_53227 | 135 | 0.086427802 | USP17L15 | ubiquitin specific peptidase 17-like family member 15 [Source:HGNC Symbol;Acc:HGNC:44443] |
| ENSG00000235711 | HGLibA_02081 | 135 | 0.086427802 | ANKRD34C | ankyrin repeat domain 34C [Source:HGNC Symbol;Acc:HGNC:33888] |
| ENSG00000241119 | HGLibA_52900 | 135 | 0.086427802 | UGT1A9 | UDP glucuronosyltransferase family 1 member A9 [Source:HGNC Symbol;Acc:HGNC:12541] |
| ENSG00000241852 | HGLibA_06638 | 135 | 0.086427802 | C8orf58 | chromosome 8 open reading frame 58 [Source:HGNC Symbol;Acc:HGNC:32233] |
| ENSG00000253293 | HGLibA_21897 | 135 | 0.086427802 | HOXA10 | homeobox A10 [Source:HGNC Symbol;Acc:HGNC:5100] |
| ENSG00000267060 | HGLibA_39333 | 135 | 0.086427802 | PTGES3L | prostaglandin E synthase 3 like [Source:HGNC Symbol;Acc:HGNC:43943] |
| ENSG00000273749 | HGLibA_12031 | 135 | 0.086427802 | CYFIP1 | cytoplasmic FMR1 interacting protein 1 [Source:HGNC Symbol;Acc:HGNC:13759] |
| ENSG00000283706 | HGLibA_38953 | 135 | 0.086427802 | PRSS50 | serine protease 50 [Source:NCBI gene;Acc:29122] |
| ENSG00000288702 | HGLibA_52882 | 135 | 0.086427802 | UGT1A3 | - |
| ENSG00000288705 | HGLibA_52889 | 135 | 0.086427802 | UGT1A5 | - |
| FAM69B | HGLibA_16681 | 135 | 0.086427802 | FAM69B | - |
| FBXO18 | HGLibA_17039 | 135 | 0.086427802 | FBXO18 | - |
| hsa-mir-132 | HGLibA_57755 | 135 | 0.086427802 | hsa-mir-132 | - |
| hsa-mir-148b | HGLibA_57882 | 135 | 0.086427802 | hsa-mir-148b | - |
| hsa-mir-185 | HGLibA_58003 | 135 | 0.086427802 | hsa-mir-185 | - |
| hsa-mir-1913 | HGLibA_58056 | 135 | 0.086427802 | hsa-mir-1913 | - |
| hsa-mir-320c-1 | HGLibA_58936 | 135 | 0.086427802 | hsa-mir-320c-1 | - |
| hsa-mir-376a-1 | HGLibA_59404 | 135 | 0.086427802 | hsa-mir-376a-1 | - |
| hsa-mir-3909 | HGLibA_59499 | 135 | 0.086427802 | hsa-mir-3909 | - |
| hsa-mir-3976 | HGLibA_59665 | 135 | 0.086427802 | hsa-mir-3976 | - |
| hsa-mir-4281 | HGLibA_59827 | 135 | 0.086427802 | hsa-mir-4281 | - |
| hsa-mir-4509-3 | HGLibA_60471 | 135 | 0.086427802 | hsa-mir-4509-3 | - |
| hsa-mir-4758 | HGLibA_61131 | 135 | 0.086427802 | hsa-mir-4758 | - |
| hsa-mir-514a-2 | HGLibA_61592 | 135 | 0.086427802 | hsa-mir-514a-2 | - |
| hsa-mir-548a-1 | HGLibA_61837 | 135 | 0.086427802 | hsa-mir-548a-1 | - |
| hsa-mir-548ay | HGLibA_61945 | 135 | 0.086427802 | hsa-mir-548ay | - |
| hsa-mir-5692b | HGLibA_62294 | 135 | 0.086427802 | hsa-mir-5692b | - |
| hsa-mir-6068 | HGLibA_62520 | 135 | 0.086427802 | hsa-mir-6068 | - |
| hsa-mir-6128 | HGLibA_62660 | 135 | 0.086427802 | hsa-mir-6128 | - |
| hsa-mir-6741 | HGLibA_63116 | 135 | 0.086427802 | hsa-mir-6741 | - |
| SDPR | HGLibA_43158 | 135 | 0.086427802 | SDPR | - |
| SMCR7L | HGLibA_45681 | 135 | 0.086427802 | SMCR7L | - |
| TMEM2 | HGLibA_50167 | 135 | 0.086427802 | TMEM2 | - |
| USP17L1P | HGLibA_53238 | 135 | 0.086427802 | USP17L1P | - |
| ADSSL1 | HGLibA_01078 | 134 | 0.077382459 | ADSSL1 | - |
| AKAP17A_Y | HGLibA_01423 | 134 | 0.077382459 | AKAP17A_Y | - |
| C15orf52 | HGLibA_05409 | 134 | 0.077382459 | C15orf52 | - |
| C21orf33 | HGLibA_06038 | 134 | 0.077382459 | C21orf33 | - |
| C6orf7 | HGLibA_06505 | 134 | 0.077382459 | C6orf7 | - |
| C7orf55 | HGLibA_06555 | 134 | 0.077382459 | C7orf55 | - |
| DSCR3 | HGLibA_14008 | 134 | 0.077382459 | DSCR3 | - |
| DSCR6 | HGLibA_14014 | 134 | 0.077382459 | DSCR6 | - |
| ENSG00000007168 | HGLibA_34989 | 134 | 0.077382459 | PAFAH1B1 | platelet activating factor acetylhydrolase 1b regulatory subunit 1 [Source:HGNC Symbol;Acc:HGNC:8574] |

| | | | | | |
|---|---|---|---|---|---|
| ENSG00000007264 | HGLibA_28423 | 134 | 0.077382459 | MATK | megakaryocyte-associated tyrosine kinase [Source:HGNC Symbol;Acc:HGNC:6906] |
| ENSG00000008277 | HGLibA_00759 | 134 | 0.077382459 | ADAM22 | ADAM metallopeptidase domain 22 [Source:HGNC Symbol;Acc:HGNC:201] |
| ENSG00000033170 | HGLibA_18206 | 134 | 0.077382459 | FUT8 | fucosyltransferase 8 [Source:HGNC Symbol;Acc:HGNC:4019] |
| ENSG00000068781 | HGLibA_47546 | 134 | 0.077382459 | STON1-GTF2A1L | STON1-GTF2A1L readthrough [Source:HGNC Symbol;Acc:HGNC:30651] |
| ENSG00000076242 | HGLibA_29382 | 134 | 0.077382459 | MLH1 | mutL homolog 1 [Source:HGNC Symbol;Acc:HGNC:7127] |
| ENSG00000079257 | HGLibA_27650 | 134 | 0.077382459 | LXN | latexin [Source:HGNC Symbol;Acc:HGNC:13347] |
| ENSG00000080845 | HGLibA_13348 | 134 | 0.077382459 | DLGAP4 | DLG associated protein 4 [Source:HGNC Symbol;Acc:HGNC:24476] |
| ENSG00000081842 | HGLibA_35452 | 134 | 0.077382459 | PCDHA6 | protocadherin alpha 6 [Source:HGNC Symbol;Acc:HGNC:8672] |
| ENSG00000084636 | HGLibA_10519 | 134 | 0.077382459 | COL16A1 | collagen type XVI alpha 1 chain [Source:HGNC Symbol;Acc:HGNC:2193] |
| ENSG00000089225 | HGLibA_48673 | 134 | 0.077382459 | TBX5 | T-box 5 [Source:HGNC Symbol;Acc:HGNC:11604] |
| ENSG00000089820 | HGLibA_02774 | 134 | 0.077382459 | ARHGAP4 | Rho GTPase activating protein 4 [Source:HGNC Symbol;Acc:HGNC:674] |
| ENSG00000095627 | HGLibA_48896 | 134 | 0.077382459 | TDRD1 | tudor domain containing 1 [Source:HGNC Symbol;Acc:HGNC:11712] |
| ENSG00000102575 | HGLibA_00516 | 134 | 0.077382459 | ACP5 | tartrate resistant [Source:HGNC Symbol;Acc:HGNC:124]" |
| ENSG00000102699 | HGLibA_35237 | 134 | 0.077382459 | PARP4 | poly(ADP-ribose) polymerase family member 4 [Source:HGNC Symbol;Acc:HGNC:271] |
| ENSG00000102710 | HGLibA_47805 | 134 | 0.077382459 | SUPT20H | SAGA complex component [Source:HGNC Symbol;Acc:HGNC:20596]" |
| ENSG00000103056 | HGLibA_45810 | 134 | 0.077382459 | SMPD3 | sphingomyelin phosphodiesterase 3 [Source:HGNC Symbol;Acc:HGNC:14240] |
| ENSG00000103876 | HGLibA_15952 | 134 | 0.077382459 | FAH | fumarylacetoacetate hydrolase [Source:HGNC Symbol;Acc:HGNC:3579] |
| ENSG00000104497 | HGLibA_46044 | 134 | 0.077382459 | SNX16 | sorting nexin 16 [Source:HGNC Symbol;Acc:HGNC:14980] |
| ENSG00000106066 | HGLibA_11031 | 134 | 0.077382459 | CPVL | vitellogenic like [Source:HGNC Symbol;Acc:HGNC:14399]" |
| ENSG00000106605 | HGLibA_04575 | 134 | 0.077382459 | BLVRA | biliverdin reductase A [Source:HGNC Symbol;Acc:HGNC:1062] |
| ENSG00000112812 | HGLibA_38904 | 134 | 0.077382459 | PRSS16 | serine protease 16 [Source:HGNC Symbol;Acc:HGNC:9480] |
| ENSG00000114745 | HGLibA_19678 | 134 | 0.077382459 | GORASP1 | golgi reassembly stacking protein 1 [Source:HGNC Symbol;Acc:HGNC:16769] |
| ENSG00000115145 | HGLibA_47334 | 134 | 0.077382459 | STAM2 | signal transducing adaptor molecule 2 [Source:HGNC Symbol;Acc:HGNC:11358] |
| ENSG00000117862 | HGLibA_52407 | 134 | 0.077382459 | TXNDC12 | thioredoxin domain containing 12 [Source:HGNC Symbol;Acc:HGNC:24626] |
| ENSG00000120738 | HGLibA_14648 | 134 | 0.077382459 | EGR1 | early growth response 1 [Source:HGNC Symbol;Acc:HGNC:3238] |
| ENSG00000120784 | HGLibA_55200 | 134 | 0.077382459 | ZFP30 | ZFP30 zinc finger protein [Source:HGNC Symbol;Acc:HGNC:29555] |
| ENSG00000123352 | HGLibA_46546 | 134 | 0.077382459 | SPATS2 | spermatogenesis associated serine rich 2 [Source:HGNC Symbol;Acc:HGNC:18650] |
| ENSG00000125166 | HGLibA_19697 | 134 | 0.077382459 | GOT2 | glutamic-oxaloacetic transaminase 2 [Source:HGNC Symbol;Acc:HGNC:4433] |
| ENSG00000125772 | HGLibA_19779 | 134 | 0.077382459 | GPCPD1 | glycerophosphocholine phosphodiesterase 1 [Source:HGNC Symbol;Acc:HGNC:26957] |
| ENSG00000128346 | HGLibA_06057 | 134 | 0.077382459 | C22orf23 | chromosome 22 open reading frame 23 [Source:HGNC Symbol;Acc:HGNC:18589] |
| ENSG00000128510 | HGLibA_10894 | 134 | 0.077382459 | CPA4 | carboxypeptidase A4 [Source:HGNC Symbol;Acc:HGNC:15740] |
| ENSG00000128917 | HGLibA_13366 | 134 | 0.077382459 | DLL4 | delta like canonical Notch ligand 4 [Source:HGNC Symbol;Acc:HGNC:2910] |
| ENSG00000129675 | HGLibA_02874 | 134 | 0.077382459 | ARHGEF6 | Rac/Cdc42 guanine nucleotide exchange factor 6 [Source:HGNC Symbol;Acc:HGNC:685] |
| ENSG00000130032 | HGLibA_38876 | 134 | 0.077382459 | PRRG3 | proline rich and Gla domain 3 [Source:HGNC Symbol;Acc:HGNC:30798] |
| ENSG00000130956 | HGLibA_20797 | 134 | 0.077382459 | HABP4 | hyaluronan binding protein 4 [Source:HGNC Symbol;Acc:HGNC:17062] |
| ENSG00000131097 | HGLibA_21308 | 134 | 0.077382459 | HIGD1B | HIG1 hypoxia inducible domain family member 1B [Source:HGNC Symbol;Acc:HGNC:24318] |
| ENSG00000131748 | HGLibA_47358 | 134 | 0.077382459 | STARD3 | StAR related lipid transfer domain containing 3 [Source:HGNC Symbol;Acc:HGNC:17579] |
| ENSG00000133027 | HGLibA_36045 | 134 | 0.077382459 | PEMT | phosphatidylethanolamine N-methyltransferase [Source:HGNC Symbol;Acc:HGNC:8830] |
| ENSG00000134049 | HGLibA_22579 | 134 | 0.077382459 | IER3IP1 | immediate early response 3 interacting protein 1 [Source:HGNC Symbol;Acc:HGNC:18550] |
| ENSG00000135446 | HGLibA_08769 | 134 | 0.077382459 | CDK4 | cyclin dependent kinase 4 [Source:HGNC Symbol;Acc:HGNC:1773] |
| ENSG00000136487 | HGLibA_19030 | 134 | 0.077382459 | GH2 | growth hormone 2 [Source:HGNC Symbol;Acc:HGNC:4262] |
| ENSG00000136839 | HGLibA_33612 | 134 | 0.077382459 | OR13C9 | olfactory receptor family 13 subfamily C member 9 [Source:HGNC Symbol;Acc:HGNC:15104] |
| ENSG00000138286 | HGLibA_16153 | 134 | 0.077382459 | FAM149B1 | family with sequence similarity 149 member B1 [Source:HGNC Symbol;Acc:HGNC:29162] |
| ENSG00000138964 | HGLibA_35261 | 134 | 0.077382459 | PARVG | parvin gamma [Source:HGNC Symbol;Acc:HGNC:14654] |
| ENSG00000140157 | HGLibA_31954 | 134 | 0.077382459 | NIPA2 | NIPA magnesium transporter 2 [Source:HGNC Symbol;Acc:HGNC:17044] |
| ENSG00000140526 | HGLibA_00272 | 134 | 0.077382459 | ABHD2 | abhydrolase domain containing 2 [Source:HGNC Symbol;Acc:HGNC:18717] |
| ENSG00000141574 | HGLibA_43271 | 134 | 0.077382459 | SECTM1 | secreted and transmembrane 1 [Source:HGNC Symbol;Acc:HGNC:10707] |
| ENSG00000141744 | HGLibA_37391 | 134 | 0.077382459 | PNMT | phenylethanolamine N-methyltransferase [Source:HGNC Symbol;Acc:HGNC:9160] |
| ENSG00000141977 | HGLibA_09687 | 134 | 0.077382459 | CIB3 | calcium and integrin binding family member 3 [Source:HGNC Symbol;Acc:HGNC:24580] |
| ENSG00000143543 | HGLibA_24001 | 134 | 0.077382459 | JTB | jumping translocation breakpoint [Source:HGNC Symbol;Acc:HGNC:6201] |
| ENSG00000143569 | HGLibA_52555 | 134 | 0.077382459 | UBAP2L | ubiquitin associated protein 2 like [Source:HGNC Symbol;Acc:HGNC:29877] |
| ENSG00000144355 | HGLibA_13372 | 134 | 0.077382459 | DLX1 | distal-less homeobox 1 [Source:HGNC Symbol;Acc:HGNC:2914] |
| ENSG00000145975 | HGLibA_16490 | 134 | 0.077382459 | FAM217A | family with sequence similarity 217 member A [Source:HGNC Symbol;Acc:HGNC:21362] |
| ENSG00000146166 | HGLibA_26417 | 134 | 0.077382459 | LGSN | lens protein with glutamine synthetase domain [Source:HGNC Symbol;Acc:HGNC:21016]" |
| ENSG00000148842 | HGLibA_10316 | 134 | 0.077382459 | CNNM2 | cyclin and CBS domain divalent metal cation transport mediator 2 [Source:HGNC Symbol;Acc:HGNC:103] |
| ENSG00000154451 | HGLibA_18741 | 134 | 0.077382459 | GBP5 | guanylate binding protein 5 [Source:HGNC Symbol;Acc:HGNC:19895] |
| ENSG00000158578 | HGLibA_01526 | 134 | 0.077382459 | ALAS2 | 5'-aminolevulinate synthase 2 [Source:HGNC Symbol;Acc:HGNC:397] |
| ENSG00000160181 | HGLibA_49171 | 134 | 0.077382459 | TFF2 | trefoil factor 2 [Source:HGNC Symbol;Acc:HGNC:11756] |
| ENSG00000162490 | HGLibA_13952 | 134 | 0.077382459 | DRAXIN | dorsal inhibitory axon guidance protein [Source:HGNC Symbol;Acc:HGNC:25054] |
| ENSG00000163002 | HGLibA_33049 | 134 | 0.077382459 | NUP35 | nucleoporin 35 [Source:HGNC Symbol;Acc:HGNC:29797] |
| ENSG00000163249 | HGLibA_08136 | 134 | 0.077382459 | CCNYL1 | cyclin Y like 1 [Source:HGNC Symbol;Acc:HGNC:26868] |
| ENSG00000163898 | HGLibA_26615 | 134 | 0.077382459 | LIPH | lipase H [Source:HGNC Symbol;Acc:HGNC:18483] |
| ENSG00000165138 | HGLibA_02161 | 134 | 0.077382459 | ANKS6 | ankyrin repeat and sterile alpha motif domain containing 6 [Source:HGNC Symbol;Acc:HGNC:26724] |
| ENSG00000166689 | HGLibA_37080 | 134 | 0.077382459 | PLEKHA7 | pleckstrin homology domain containing A7 [Source:HGNC Symbol;Acc:HGNC:27049] |
| ENSG00000167074 | HGLibA_48962 | 134 | 0.077382459 | TEF | PAR bZIP transcription factor [Source:HGNC Symbol;Acc:HGNC:11722]" |
| ENSG00000167286 | HGLibA_08370 | 134 | 0.077382459 | CD3D | CD3d molecule [Source:HGNC Symbol;Acc:HGNC:1673] |
| ENSG00000167703 | HGLibA_45152 | 134 | 0.077382459 | SLC43A2 | solute carrier family 43 member 2 [Source:HGNC Symbol;Acc:HGNC:23087] |
| ENSG00000167880 | HGLibA_15701 | 134 | 0.077382459 | EVPL | envoplakin [Source:HGNC Symbol;Acc:HGNC:3503] |
| ENSG00000167904 | HGLibA_50496 | 134 | 0.077382459 | TMEM68 | transmembrane protein 68 [Source:HGNC Symbol;Acc:HGNC:26510] |
| ENSG00000168454 | HGLibA_52419 | 134 | 0.077382459 | TXNDC2 | thioredoxin domain containing 2 [Source:HGNC Symbol;Acc:HGNC:16470] |
| ENSG00000168757 | HGLibA_51968 | 134 | 0.077382459 | TSPY2 | testis specific protein Y-linked 2 [Source:HGNC Symbol;Acc:HGNC:23924] |
| ENSG00000169604 | HGLibA_02213 | 134 | 0.077382459 | ANTXR1 | ANTXR cell adhesion molecule 1 [Source:HGNC Symbol;Acc:HGNC:21014] |
| ENSG00000170581 | HGLibA_47383 | 134 | 0.077382459 | STAT2 | signal transducer and activator of transcription 2 [Source:HGNC Symbol;Acc:HGNC:11363] |
| ENSG00000170876 | HGLibA_50403 | 134 | 0.077382459 | TMEM43 | transmembrane protein 43 [Source:HGNC Symbol;Acc:HGNC:28472] |
| ENSG00000172380 | HGLibA_19515 | 134 | 0.077382459 | GNG12 | G protein subunit gamma 12 [Source:HGNC Symbol;Acc:HGNC:19663] |
| ENSG00000173210 | HGLibA_00315 | 134 | 0.077382459 | ABLIM3 | actin binding LIM protein family member 3 [Source:HGNC Symbol;Acc:HGNC:29132] |
| ENSG00000173627 | HGLibA_02497 | 134 | 0.077382459 | APOBEC4 | apolipoprotein B mRNA editing enzyme catalytic polypeptide like 4 [Source:HGNC Symbol;Acc:HGNC:32152] |
| ENSG00000176771 | HGLibA_31318 | 134 | 0.077382459 | NCKAP5 | NCK associated protein 5 [Source:HGNC Symbol;Acc:HGNC:29847] |
| ENSG00000177174 | HGLibA_33632 | 134 | 0.077382459 | OR14C36 | olfactory receptor family 14 subfamily C member 36 [Source:HGNC Symbol;Acc:HGNC:15026] |
| ENSG00000177683 | HGLibA_49303 | 134 | 0.077382459 | THAP5 | THAP domain containing 5 [Source:HGNC Symbol;Acc:HGNC:23188] |
| ENSG00000177938 | HGLibA_07211 | 134 | 0.077382459 | CAPZA3 | capping actin protein of muscle Z-line subunit alpha 3 [Source:HGNC Symbol;Acc:HGNC:24205] |
| ENSG00000180767 | HGLibA_09621 | 134 | 0.077382459 | CHST13 | carbohydrate sulfotransferase 13 [Source:HGNC Symbol;Acc:HGNC:21755] |
| ENSG00000182040 | HGLibA_53177 | 134 | 0.077382459 | USH1G | USH1 protein network component sans [Source:HGNC Symbol;Acc:HGNC:16356] |
| ENSG00000182447 | HGLibA_34732 | 134 | 0.077382459 | OTOL1 | otolin 1 [Source:HGNC Symbol;Acc:HGNC:34071] |
| ENSG00000183048 | HGLibA_44636 | 134 | 0.077382459 | SLC25A10 | solute carrier family 25 member 10 [Source:HGNC Symbol;Acc:HGNC:10980] |
| ENSG00000183397 | HGLibA_05702 | 134 | 0.077382459 | C19orf71 | chromosome 19 open reading frame 71 [Source:HGNC Symbol;Acc:HGNC:34496] |
| ENSG00000183628 | HGLibA_13048 | 134 | 0.077382459 | DGCR6 | DiGeorge syndrome critical region gene 6 [Source:HGNC Symbol;Acc:HGNC:2846] |
| ENSG00000185070 | HGLibA_17657 | 134 | 0.077382459 | FLRT2 | fibronectin leucine rich transmembrane protein 2 [Source:HGNC Symbol;Acc:HGNC:3761] |
| ENSG00000185513 | HGLibA_25897 | 134 | 0.077382459 | L3MBTL1 | histone methyl-lysine binding protein [Source:HGNC Symbol;Acc:HGNC:15905]" |
| ENSG00000185728 | HGLibA_54736 | 134 | 0.077382459 | YTHDF3 | YTH N6-methyladenosine RNA binding protein 3 [Source:HGNC Symbol;Acc:HGNC:26465] |
| ENSG00000186297 | HGLibA_18367 | 134 | 0.077382459 | GABRA5 | gamma-aminobutyric acid type A receptor alpha5 subunit [Source:HGNC Symbol;Acc:HGNC:4079] |
| ENSG00000186377 | HGLibA_12203 | 134 | 0.077382459 | CYP4X1 | cytochrome P450 family 4 subfamily X member 1 [Source:HGNC Symbol;Acc:HGNC:20244] |
| ENSG00000186448 | HGLibA_55561 | 134 | 0.077382459 | ZNF197 | zinc finger protein 197 [Source:HGNC Symbol;Acc:HGNC:12988] |
| ENSG00000186943 | HGLibA_33609 | 134 | 0.077382459 | OR13C8 | olfactory receptor family 13 subfamily C member 8 [Source:HGNC Symbol;Acc:HGNC:15103] |
| ENSG00000188001 | HGLibA_51179 | 134 | 0.077382459 | TPRG1 | tumor protein p63 regulated 1 [Source:HGNC Symbol;Acc:HGNC:24759] |
| ENSG00000188155 | HGLibA_25614 | 134 | 0.077382459 | KRTAP10-6 | keratin associated protein 10-6 [Source:HGNC Symbol;Acc:HGNC:20523] |
| ENSG00000189283 | HGLibA_17476 | 134 | 0.077382459 | FHIT | fragile histidine triad [Source:HGNC Symbol;Acc:HGNC:3701] |
| ENSG00000196218 | HGLibA_42521 | 134 | 0.077382459 | RYR1 | ryanodine receptor 1 [Source:HGNC Symbol;Acc:HGNC:10483] |
| ENSG00000196639 | HGLibA_22094 | 134 | 0.077382459 | HRH1 | histamine receptor H1 [Source:HGNC Symbol;Acc:HGNC:5182] |
| ENSG00000197045 | HGLibA_19403 | 134 | 0.077382459 | GMFB | glia maturation factor beta [Source:HGNC Symbol;Acc:HGNC:4373] |
| ENSG00000197165 | HGLibA_47734 | 134 | 0.077382459 | SULT1A2 | sulfotransferase family 1A member 2 [Source:HGNC Symbol;Acc:HGNC:11454] |
| ENSG00000197965 | HGLibA_29795 | 134 | 0.077382459 | MPZL1 | myelin protein zero like 1 [Source:HGNC Symbol;Acc:HGNC:7226] |
| ENSG00000198077 | HGLibA_12109 | 134 | 0.077382459 | CYP2A7 | cytochrome P450 family 2 subfamily A member 7 [Source:HGNC Symbol;Acc:HGNC:2611] |
| ENSG00000203909 | HGLibA_13897 | 134 | 0.077382459 | DPPA5 | developmental pluripotency associated 5 [Source:HGNC Symbol;Acc:HGNC:19201] |
| ENSG00000221883 | HGLibA_02919 | 134 | 0.077382459 | ARIH2OS | ariadne RBR E3 ubiquitin protein ligase 2 opposite strand [Source:HGNC Symbol;Acc:HGNC:34425] |
| ENSG00000228727 | HGLibA_42731 | 134 | 0.077382459 | SAPCD1 | suppressor APC domain containing 1 [Source:HGNC Symbol;Acc:HGNC:13938] |
| ENSG00000234414 | HGLibA_40612 | 134 | 0.077382459 | RBMY1A1 | member A1 [Source:HGNC Symbol;Acc:HGNC:9912]" |
| ENSG00000237515 | HGLibA_44054 | 134 | 0.077382459 | SHISA9 | shisa family member 9 [Source:HGNC Symbol;Acc:HGNC:37231] |
| ENSG00000244624 | HGLibA_25698 | 134 | 0.077382459 | KRTAP20-1 | keratin associated protein 20-1 [Source:HGNC Symbol;Acc:HGNC:18943] |
| ENSG00000248098 | HGLibA_04277 | 134 | 0.077382459 | BCKDHA | alpha polypeptide [Source:HGNC Symbol;Acc:HGNC:986]" |
| ENSG00000257594 | HGLibA_18560 | 134 | 0.077382459 | GALNT4 | polypeptide N-acetylgalactosaminyltransferase 4 [Source:HGNC Symbol;Acc:HGNC:4126] |
| ENSG00000258223 | HGLibA_38971 | 134 | 0.077382459 | PRSS58 | serine protease 58 [Source:HGNC Symbol;Acc:HGNC:39125] |
| hsa-mir-151a | HGLibA_57895 | 134 | 0.077382459 | hsa-mir-151a | - |
| hsa-mir-1539 | HGLibA_57921 | 134 | 0.077382459 | hsa-mir-1539 | - |
| hsa-mir-3118-3 | HGLibA_58516 | 134 | 0.077382459 | hsa-mir-3118-3 | - |
| hsa-mir-3151 | HGLibA_58665 | 134 | 0.077382459 | hsa-mir-3151 | - |
| hsa-mir-34b | HGLibA_59041 | 134 | 0.077382459 | hsa-mir-34b | - |
| hsa-mir-4496 | HGLibA_60401 | 134 | 0.077382459 | hsa-mir-4496 | - |
| hsa-mir-512-1 | HGLibA_61564 | 134 | 0.077382459 | hsa-mir-512-1 | - |
| hsa-mir-513a-1 | HGLibA_61570 | 134 | 0.077382459 | hsa-mir-513a-1 | - |
| hsa-mir-519e | HGLibA_61732 | 134 | 0.077382459 | hsa-mir-519e | - |
| hsa-mir-584 | HGLibA_62434 | 134 | 0.077382459 | hsa-mir-584 | - |
| hsa-mir-6089-2 | HGLibA_62618 | 134 | 0.077382459 | hsa-mir-6089-2 | - |
| hsa-mir-6134 | HGLibA_62686 | 134 | 0.077382459 | hsa-mir-6134 | - |
| hsa-mir-622 | HGLibA_62726 | 134 | 0.077382459 | hsa-mir-622 | - |
| hsa-mir-6753 | HGLibA_63169 | 134 | 0.077382459 | hsa-mir-6753 | - |
| hsa-mir-6864 | HGLibA_63639 | 134 | 0.077382459 | hsa-mir-6864 | - |
| hsa-mir-7158 | HGLibA_63860 | 134 | 0.077382459 | hsa-mir-7158 | - |
| C6orf48 | HGLibA_06490 | 133 | 0.068337116 | C6orf48 | - |
| DOPEY2 | HGLibA_13803 | 133 | 0.068337116 | DOPEY2 | - |
| ENSG00000004939 | HGLibA_45202 | 133 | 0.068337116 | SLC4A1 | solute carrier family 4 member 1 (Diego blood group) [Source:HGNC Symbol;Acc:HGNC:11027] |
| ENSG00000007392 | HGLibA_27619 | 133 | 0.068337116 | LUC7L | LUC7 like [Source:HGNC Symbol;Acc:HGNC:6723] |
| ENSG00000036565 | HGLibA_44487 | 133 | 0.068337116 | SLC18A1 | solute carrier family 18 member A1 [Source:HGNC Symbol;Acc:HGNC:10934] |
| ENSG00000065320 | HGLibA_32854 | 133 | 0.068337116 | NTN1 | netrin 1 [Source:HGNC Symbol;Acc:HGNC:8029] |
| ENSG00000065548 | HGLibA_54966 | 133 | 0.068337116 | ZC3H15 | zinc finger CCCH-type containing 15 [Source:HGNC Symbol;Acc:HGNC:29528] |

| | | | | | |
|---|---|---|---|---|---|
| ENSG00000066855 | HGLibA_30361 | 133 | 0.068337116 | MTFR1 | mitochondrial fission regulator 1 [Source:HGNC Symbol;Acc:HGNC:29510] |
| ENSG00000068615 | HGLibA_40776 | 133 | 0.068337116 | REEP1 | receptor accessory protein 1 [Source:HGNC Symbol;Acc:HGNC:25786] |
| ENSG00000069956 | HGLibA_28277 | 133 | 0.068337116 | MAPK6 | mitogen-activated protein kinase 6 [Source:HGNC Symbol;Acc:HGNC:6879] |
| ENSG00000069956 | HGLibA_28278 | 133 | 0.068337116 | MAPK6 | mitogen-activated protein kinase 6 [Source:HGNC Symbol;Acc:HGNC:6879] |
| ENSG00000070182 | HGLibA_46876 | 133 | 0.068337116 | SPTB | erythrocytic [Source:HGNC Symbol;Acc:HGNC:11274]" |
| ENSG00000077009 | HGLibA_32198 | 133 | 0.068337116 | NMRK2 | nicotinamide riboside kinase 2 [Source:HGNC Symbol;Acc:HGNC:17871] |
| ENSG00000077458 | HGLibA_16726 | 133 | 0.068337116 | FAM76B | family with sequence similarity 76 member B [Source:HGNC Symbol;Acc:HGNC:28492] |
| ENSG00000081923 | HGLibA_03781 | 133 | 0.068337116 | ATP8B1 | ATPase phospholipid transporting 8B1 [Source:HGNC Symbol;Acc:HGNC:3706] |
| ENSG00000083099 | HGLibA_27750 | 133 | 0.068337116 | LYRM2 | LYR motif containing 2 [Source:HGNC Symbol;Acc:HGNC:25229] |
| ENSG00000084453 | HGLibA_45446 | 133 | 0.068337116 | SLCO1A2 | solute carrier organic anion transporter family member 1A2 [Source:HGNC Symbol;Acc:HGNC:10956] |
| ENSG00000085276 | HGLibA_28725 | 133 | 0.068337116 | MECOM | MDS1 and EVI1 complex locus [Source:HGNC Symbol;Acc:HGNC:3498] |
| ENSG00000100219 | HGLibA_54491 | 133 | 0.068337116 | XBP1 | X-box binding protein 1 [Source:HGNC Symbol;Acc:HGNC:12801] |
| ENSG00000100284 | HGLibA_50946 | 133 | 0.068337116 | TOM1 | target of myb1 membrane trafficking protein [Source:HGNC Symbol;Acc:HGNC:11982] |
| ENSG00000100350 | HGLibA_17953 | 133 | 0.068337116 | FOXRED2 | FAD dependent oxidoreductase domain containing 2 [Source:HGNC Symbol;Acc:HGNC:26264] |
| ENSG00000100744 | HGLibA_20434 | 133 | 0.068337116 | GSKIP | GSK3B interacting protein [Source:HGNC Symbol;Acc:HGNC:20343] |
| ENSG00000101198 | HGLibA_31997 | 133 | 0.068337116 | NKAIN4 | sodium/potassium transporting ATPase interacting 4 [Source:HGNC Symbol;Acc:HGNC:16191] |
| ENSG00000101638 | HGLibA_47299 | 133 | 0.068337116 | ST8SIA5 | 8-sialyltransferase 5 [Source:HGNC Symbol;Acc:HGNC:17827]" |
| ENSG00000102038 | HGLibA_45601 | 133 | 0.068337116 | SMARCA1 | member 1 [Source:HGNC Symbol;Acc:HGNC:11097]" |
| ENSG00000102984 | HGLibA_56819 | 133 | 0.068337116 | ZNF821 | zinc finger protein 821 [Source:HGNC Symbol;Acc:HGNC:28043] |
| ENSG00000104714 | HGLibA_15487 | 133 | 0.068337116 | ERICH1 | glutamate rich 1 [Source:HGNC Symbol;Acc:HGNC:27234] |
| ENSG00000105281 | HGLibA_44520 | 133 | 0.068337116 | SLC1A5 | solute carrier family 1 member 5 [Source:HGNC Symbol;Acc:HGNC:10943] |
| ENSG00000106804 | HGLibA_06349 | 133 | 0.068337116 | C5 | complement C5 [Source:HGNC Symbol;Acc:HGNC:1331] |
| ENSG00000107863 | HGLibA_02727 | 133 | 0.068337116 | ARHGAP21 | Rho GTPase activating protein 21 [Source:HGNC Symbol;Acc:HGNC:23725] |
| ENSG00000111245 | HGLibA_30757 | 133 | 0.068337116 | MYL2 | myosin light chain 2 [Source:HGNC Symbol;Acc:HGNC:7583] |
| ENSG00000111540 | HGLibA_39963 | 133 | 0.068337116 | RAB5B | member RAS oncogene family [Source:HGNC Symbol;Acc:HGNC:9784]" |
| ENSG00000112031 | HGLibA_30469 | 133 | 0.068337116 | MTRF1L | mitochondrial translational release factor 1 like [Source:HGNC Symbol;Acc:HGNC:21051] |
| ENSG00000114378 | HGLibA_22438 | 133 | 0.068337116 | HYAL1 | hyaluronidase 1 [Source:HGNC Symbol;Acc:HGNC:5320] |
| ENSG00000114988 | HGLibA_26665 | 133 | 0.068337116 | LMAN2L | mannose binding 2 like [Source:HGNC Symbol;Acc:HGNC:19263]" |
| ENSG00000115073 | HGLibA_00653 | 133 | 0.068337116 | ACTR1B | ARP1 actin related protein 1 homolog B [Source:HGNC Symbol;Acc:HGNC:168] |
| ENSG00000116035 | HGLibA_53559 | 133 | 0.068337116 | VAX2 | ventral anterior homeobox 2 [Source:HGNC Symbol;Acc:HGNC:12661] |
| ENSG00000116213 | HGLibA_54403 | 133 | 0.068337116 | WRAP73 | antisense to TP73 [Source:HGNC Symbol;Acc:HGNC:12759]" |
| ENSG00000119004 | HGLibA_12076 | 133 | 0.068337116 | CYP20A1 | cytochrome P450 family 20 subfamily A member 1 [Source:HGNC Symbol;Acc:HGNC:20576] |
| ENSG00000119280 | HGLibA_05818 | 133 | 0.068337116 | C1orf198 | chromosome 1 open reading frame 198 [Source:HGNC Symbol;Acc:HGNC:25900] |
| ENSG00000119608 | HGLibA_38705 | 133 | 0.068337116 | PROX2 | prospero homeobox 2 [Source:HGNC Symbol;Acc:HGNC:26715] |
| ENSG00000123989 | HGLibA_09523 | 133 | 0.068337116 | CHPF | chondroitin polymerizing factor [Source:HGNC Symbol;Acc:HGNC:24291] |
| ENSG00000124496 | HGLibA_51370 | 133 | 0.068337116 | TRERF1 | transcriptional regulating factor 1 [Source:HGNC Symbol;Acc:HGNC:18273] |
| ENSG00000125492 | HGLibA_04157 | 133 | 0.068337116 | BARHL1 | BarH like homeobox 1 [Source:HGNC Symbol;Acc:HGNC:953] |
| ENSG00000126067 | HGLibA_39103 | 133 | 0.068337116 | PSMB2 | proteasome subunit beta 2 [Source:HGNC Symbol;Acc:HGNC:9539] |
| ENSG00000126217 | HGLibA_28580 | 133 | 0.068337116 | MCF2L | MCF.2 cell line derived transforming sequence like [Source:HGNC Symbol;Acc:HGNC:14576] |
| ENSG00000126778 | HGLibA_44244 | 133 | 0.068337116 | SIX1 | SIX homeobox 1 [Source:HGNC Symbol;Acc:HGNC:10887] |
| ENSG00000127083 | HGLibA_33383 | 133 | 0.068337116 | OMD | osteomodulin [Source:HGNC Symbol;Acc:HGNC:8134] |
| ENSG00000127526 | HGLibA_44985 | 133 | 0.068337116 | SLC35E1 | solute carrier family 35 member E1 [Source:HGNC Symbol;Acc:HGNC:20803] |
| ENSG00000128039 | HGLibA_46945 | 133 | 0.068337116 | SRD5A3 | steroid 5 alpha-reductase 3 [Source:HGNC Symbol;Acc:HGNC:25812] |
| ENSG00000130167 | HGLibA_51909 | 133 | 0.068337116 | TSPAN16 | tetraspanin 16 [Source:HGNC Symbol;Acc:HGNC:30725] |
| ENSG00000132275 | HGLibA_42285 | 133 | 0.068337116 | RRP8 | ribosomal RNA processing 8 [Source:HGNC Symbol;Acc:HGNC:29030] |
| ENSG00000132549 | HGLibA_53733 | 133 | 0.068337116 | VPS13B | vacuolar protein sorting 13 homolog B [Source:HGNC Symbol;Acc:HGNC:2183] |
| ENSG00000132612 | HGLibA_53796 | 133 | 0.068337116 | VPS4A | vacuolar protein sorting 4 homolog A [Source:HGNC Symbol;Acc:HGNC:13488] |
| ENSG00000132821 | HGLibA_53866 | 133 | 0.068337116 | VSTM2L | V-set and transmembrane domain containing 2 like [Source:HGNC Symbol;Acc:HGNC:16096] |
| ENSG00000133111 | HGLibA_40988 | 133 | 0.068337116 | RFXAP | regulatory factor X associated protein [Source:HGNC Symbol;Acc:HGNC:9988] |
| ENSG00000135968 | HGLibA_18767 | 133 | 0.068337116 | GCC2 | GRIP and coiled-coil domain containing 2 [Source:HGNC Symbol;Acc:HGNC:23218] |
| ENSG00000136240 | HGLibA_24481 | 133 | 0.068337116 | KDELR2 | KDEL endoplasmic reticulum protein retention receptor 2 [Source:HGNC Symbol;Acc:HGNC:6305] |
| ENSG00000136783 | HGLibA_31973 | 133 | 0.068337116 | NIPSNAP3A | nipsnap homolog 3A [Source:HGNC Symbol;Acc:HGNC:23619] |
| ENSG00000137414 | HGLibA_16787 | 133 | 0.068337116 | FAM8A1 | family with sequence similarity 8 member A1 [Source:HGNC Symbol;Acc:HGNC:16372] |
| ENSG00000137942 | HGLibA_17739 | 133 | 0.068337116 | FNBP1L | formin binding protein 1 like [Source:HGNC Symbol;Acc:HGNC:20851] |
| ENSG00000139629 | HGLibA_18567 | 133 | 0.068337116 | GALNT6 | polypeptide N-acetylgalactosaminyltransferase 6 [Source:HGNC Symbol;Acc:HGNC:4128] |
| ENSG00000141127 | HGLibA_38767 | 133 | 0.068337116 | PRPSAP2 | phosphoribosyl pyrophosphate synthetase associated protein 2 [Source:HGNC Symbol;Acc:HGNC:9467] |
| ENSG00000144290 | HGLibA_45204 | 133 | 0.068337116 | SLC4A10 | solute carrier family 4 member 10 [Source:HGNC Symbol;Acc:HGNC:13811] |
| ENSG00000144476 | HGLibA_00445 | 133 | 0.068337116 | ACKR3 | atypical chemokine receptor 3 [Source:HGNC Symbol;Acc:HGNC:23692] |
| ENSG00000144504 | HGLibA_01979 | 133 | 0.068337116 | ANKMY1 | ankyrin repeat and MYND domain containing 1 [Source:HGNC Symbol;Acc:HGNC:20987] |
| ENSG00000145337 | HGLibA_39710 | 133 | 0.068337116 | PYURF | PIGY upstream reading frame [Source:HGNC Symbol;Acc:HGNC:44317] |
| ENSG00000146707 | HGLibA_37675 | 133 | 0.068337116 | POMZP3 | POM121 and ZP3 fusion [Source:HGNC Symbol;Acc:HGNC:9203] |
| ENSG00000146755 | HGLibA_51536 | 133 | 0.068337116 | TRIM50 | tripartite motif containing 50 [Source:HGNC Symbol;Acc:HGNC:19017] |
| ENSG00000148950 | HGLibA_23229 | 133 | 0.068337116 | IMMP1L | inner mitochondrial membrane peptidase subunit 1 [Source:HGNC Symbol;Acc:HGNC:26317] |
| ENSG00000150760 | HGLibA_13730 | 133 | 0.068337116 | DOCK1 | dedicator of cytokinesis 1 [Source:HGNC Symbol;Acc:HGNC:2987] |
| ENSG00000150977 | HGLibA_41256 | 133 | 0.068337116 | RILPL2 | Rab interacting lysosomal protein like 2 [Source:HGNC Symbol;Acc:HGNC:28787] |
| ENSG00000155093 | HGLibA_39522 | 133 | 0.068337116 | PTPRN2 | receptor type N2 [Source:HGNC Symbol;Acc:HGNC:9677]" |
| ENSG00000155657 | HGLibA_52219 | 133 | 0.068337116 | TTN | titin [Source:HGNC Symbol;Acc:HGNC:12403] |
| ENSG00000155849 | HGLibA_14931 | 133 | 0.068337116 | ELMO1 | engulfment and cell motility 1 [Source:HGNC Symbol;Acc:HGNC:16286] |
| ENSG00000157423 | HGLibA_22448 | 133 | 0.068337116 | HYDIN | axonemal central pair apparatus protein [Source:HGNC Symbol;Acc:HGNC:19368]" |
| ENSG00000158517 | HGLibA_31295 | 133 | 0.068337116 | NCF1 | neutrophil cytosolic factor 1 [Source:HGNC Symbol;Acc:HGNC:7660] |
| ENSG00000159650 | HGLibA_53153 | 133 | 0.068337116 | UROC1 | urocanate hydratase 1 [Source:HGNC Symbol;Acc:HGNC:26444] |
| ENSG00000160392 | HGLibA_05668 | 133 | 0.068337116 | C19orf47 | chromosome 19 open reading frame 47 [Source:HGNC Symbol;Acc:HGNC:26723] |
| ENSG00000161036 | HGLibA_27508 | 133 | 0.068337116 | LRWD1 | leucine rich repeats and WD repeat domain containing 1 [Source:HGNC Symbol;Acc:HGNC:21769] |
| ENSG00000162267 | HGLibA_23811 | 133 | 0.068337116 | ITIH3 | inter-alpha-trypsin inhibitor heavy chain 3 [Source:HGNC Symbol;Acc:HGNC:6168] |
| ENSG00000162711 | HGLibA_32117 | 133 | 0.068337116 | NLRP3 | NLR family pyrin domain containing 3 [Source:HGNC Symbol;Acc:HGNC:16400] |
| ENSG00000163499 | HGLibA_11254 | 133 | 0.068337116 | CRYBA2 | crystallin beta A2 [Source:HGNC Symbol;Acc:HGNC:2395] |
| ENSG00000163545 | HGLibA_32896 | 133 | 0.068337116 | NUAK2 | NUAK family kinase 2 [Source:HGNC Symbol;Acc:HGNC:29558] |
| ENSG00000164743 | HGLibA_06636 | 133 | 0.068337116 | C8orf48 | chromosome 8 open reading frame 48 [Source:HGNC Symbol;Acc:HGNC:26345] |
| ENSG00000165084 | HGLibA_06612 | 133 | 0.068337116 | C8orf34 | chromosome 8 open reading frame 34 [Source:HGNC Symbol;Acc:HGNC:30905] |
| ENSG00000165195 | HGLibA_36542 | 133 | 0.068337116 | PIGA | phosphatidylinositol glycan anchor biosynthesis class A [Source:HGNC Symbol;Acc:HGNC:8957] |
| ENSG00000165887 | HGLibA_02024 | 133 | 0.068337116 | ANKRD2 | ankyrin repeat domain 2 [Source:HGNC Symbol;Acc:HGNC:495] |
| ENSG00000166341 | HGLibA_12491 | 133 | 0.068337116 | DCHS1 | dachsous cadherin-related 1 [Source:HGNC Symbol;Acc:HGNC:13681] |
| ENSG00000166401 | HGLibA_43565 | 133 | 0.068337116 | SERPINB8 | serpin family B member 8 [Source:HGNC Symbol;Acc:HGNC:8952] |
| ENSG00000166979 | HGLibA_15677 | 133 | 0.068337116 | EVA1C | eva-1 homolog C [Source:HGNC Symbol;Acc:HGNC:13239] |
| ENSG00000167740 | HGLibA_11999 | 133 | 0.068337116 | CYB5D2 | cytochrome b5 domain containing 2 [Source:HGNC Symbol;Acc:HGNC:28471] |
| ENSG00000169248 | HGLibA_11849 | 133 | 0.068337116 | CXCL11 | C-X-C motif chemokine ligand 11 [Source:HGNC Symbol;Acc:HGNC:10638] |
| ENSG00000169756 | HGLibA_26547 | 133 | 0.068337116 | LIMS1 | LIM zinc finger domain containing 1 [Source:HGNC Symbol;Acc:HGNC:6616] |
| ENSG00000170044 | HGLibA_56968 | 133 | 0.068337116 | ZPLD1 | zona pellucida like domain containing 1 [Source:HGNC Symbol;Acc:HGNC:27022] |
| ENSG00000170315 | HGLibA_52564 | 133 | 0.068337116 | UBB | ubiquitin B [Source:HGNC Symbol;Acc:HGNC:12463] |
| ENSG00000171044 | HGLibA_54527 | 133 | 0.068337116 | XKR6 | XK related 6 [Source:HGNC Symbol;Acc:HGNC:27806] |
| ENSG00000171467 | HGLibA_55787 | 133 | 0.068337116 | ZNF318 | zinc finger protein 318 [Source:HGNC Symbol;Acc:HGNC:13578] |
| ENSG00000172487 | HGLibA_34538 | 133 | 0.068337116 | OR8J1 | olfactory receptor family 8 subfamily J member 1 [Source:HGNC Symbol;Acc:HGNC:14855] |
| ENSG00000172638 | HGLibA_14565 | 133 | 0.068337116 | EFEMP2 | EGF containing fibulin extracellular matrix protein 2 [Source:HGNC Symbol;Acc:HGNC:3219] |
| ENSG00000173212 | HGLibA_27826 | 133 | 0.068337116 | MAB21L3 | mab-21 like 3 [Source:HGNC Symbol;Acc:HGNC:26787] |
| ENSG00000173673 | HGLibA_21182 | 133 | 0.068337116 | HES3 | hes family bHLH transcription factor 3 [Source:HGNC Symbol;Acc:HGNC:26226] |
| ENSG00000174125 | HGLibA_49641 | 133 | 0.068337116 | TLR1 | toll like receptor 1 [Source:HGNC Symbol;Acc:HGNC:11847] |
| ENSG00000175970 | HGLibA_52990 | 133 | 0.068337116 | UNC119B | unc-119 lipid binding chaperone B [Source:HGNC Symbol;Acc:HGNC:16488] |
| ENSG00000176281 | HGLibA_34033 | 133 | 0.068337116 | OR4K5 | olfactory receptor family 4 subfamily K member 5 [Source:HGNC Symbol;Acc:HGNC:14745] |
| ENSG00000176401 | HGLibA_14699 | 133 | 0.068337116 | EID2B | EP300 interacting inhibitor of differentiation 2B [Source:HGNC Symbol;Acc:HGNC:26796] |
| ENSG00000177301 | HGLibA_24129 | 133 | 0.068337116 | KCNA2 | potassium voltage-gated channel subfamily A member 2 [Source:HGNC Symbol;Acc:HGNC:6220] |
| ENSG00000178295 | HGLibA_18929 | 133 | 0.068337116 | GEN1 | Holliday junction 5' flap endonuclease [Source:HGNC Symbol;Acc:HGNC:26881]" |
| ENSG00000182141 | HGLibA_56602 | 133 | 0.068337116 | ZNF708 | zinc finger protein 708 [Source:HGNC Symbol;Acc:HGNC:12945] |
| ENSG00000182158 | HGLibA_11095 | 133 | 0.068337116 | CREB3L2 | cAMP responsive element binding protein 3 like 2 [Source:HGNC Symbol;Acc:HGNC:23720] |
| ENSG00000182177 | HGLibA_03230 | 133 | 0.068337116 | ASB18 | ankyrin repeat and SOCS box containing 18 [Source:HGNC Symbol;Acc:HGNC:19770] |
| ENSG00000183035 | HGLibA_12043 | 133 | 0.068337116 | CYLC1 | cylicin 1 [Source:HGNC Symbol;Acc:HGNC:2582] |
| ENSG00000183386 | HGLibA_17486 | 133 | 0.068337116 | FHL3 | four and a half LIM domains 3 [Source:HGNC Symbol;Acc:HGNC:3704] |
| ENSG00000183530 | HGLibA_38781 | 133 | 0.068337116 | PRR14L | proline rich 14 like [Source:HGNC Symbol;Acc:HGNC:28738] |
| ENSG00000185352 | HGLibA_22148 | 133 | 0.068337116 | HS6ST3 | heparan sulfate 6-O-sulfotransferase 3 [Source:HGNC Symbol;Acc:HGNC:19134] |
| ENSG00000185634 | HGLibA_44010 | 133 | 0.068337116 | SHC4 | SHC adaptor protein 4 [Source:HGNC Symbol;Acc:HGNC:16743] |
| ENSG00000185888 | HGLibA_38937 | 133 | 0.068337116 | PRSS38 | serine protease 38 [Source:HGNC Symbol;Acc:HGNC:29625] |
| ENSG00000187017 | HGLibA_15575 | 133 | 0.068337116 | ESPN | espin [Source:HGNC Symbol;Acc:HGNC:13281] |
| ENSG00000187151 | HGLibA_01922 | 133 | 0.068337116 | ANGPTL5 | angiopoietin like 5 [Source:HGNC Symbol;Acc:HGNC:19705] |
| ENSG00000187260 | HGLibA_54202 | 133 | 0.068337116 | WDR86 | WD repeat domain 86 [Source:HGNC Symbol;Acc:HGNC:28020] |
| ENSG00000187957 | HGLibA_13674 | 133 | 0.068337116 | DNER | delta/notch like EGF repeat containing [Source:HGNC Symbol;Acc:HGNC:24456] |
| ENSG00000188153 | HGLibA_10581 | 133 | 0.068337116 | COL4A5 | collagen type IV alpha 5 chain [Source:HGNC Symbol;Acc:HGNC:2207] |
| ENSG00000188536 | HGLibA_20910 | 133 | 0.068337116 | HBA2 | hemoglobin subunit alpha 2 [Source:HGNC Symbol;Acc:HGNC:4824] |
| ENSG00000189366 | HGLibA_01621 | 133 | 0.068337116 | ALG1L | chitobiosyldiphosphodolichol beta-mannosyltransferase like [Source:HGNC Symbol;Acc:HGNC:33721]" |
| ENSG00000196123 | HGLibA_24662 | 133 | 0.068337116 | KIAA0895L | KIAA0895 like [Source:HGNC Symbol;Acc:HGNC:34408] |
| ENSG00000196263 | HGLibA_56055 | 133 | 0.068337116 | ZNF471 | zinc finger protein 471 [Source:HGNC Symbol;Acc:HGNC:23226] |
| ENSG00000197079 | HGLibA_25488 | 133 | 0.068337116 | KRT35 | keratin 35 [Source:HGNC Symbol;Acc:HGNC:6453] |
| ENSG00000197386 | HGLibA_22418 | 133 | 0.068337116 | HTT | huntingtin [Source:HGNC Symbol;Acc:HGNC:4851] |
| ENSG00000197430 | HGLibA_33410 | 133 | 0.068337116 | OPALIN | oligodendrocytic myelin paranodal and inner loop protein [Source:HGNC Symbol;Acc:HGNC:20707] |
| ENSG00000197483 | HGLibA_56429 | 133 | 0.068337116 | ZNF628 | zinc finger protein 628 [Source:HGNC Symbol;Acc:HGNC:28054] |
| ENSG00000198721 | HGLibA_14409 | 133 | 0.068337116 | ECI2 | enoyl-CoA delta isomerase 2 [Source:HGNC Symbol;Acc:HGNC:14601] |
| ENSG00000213221 | HGLibA_13680 | 133 | 0.068337116 | DNLZ | DNL-type zinc finger [Source:HGNC Symbol;Acc:HGNC:33879] |
| ENSG00000227051 | HGLibA_05324 | 133 | 0.068337116 | C14orf132 | chromosome 14 open reading frame 132 [Source:HGNC Symbol;Acc:HGNC:20346] |
| ENSG00000233013 | HGLibA_16195 | 133 | 0.068337116 | FAM157B | family with sequence similarity 157 member B [Source:HGNC Symbol;Acc:HGNC:34080] |
| ENSG00000241106 | HGLibA_21611 | 133 | 0.068337116 | HLA-DOB | DO beta [Source:HGNC Symbol;Acc:HGNC:4937]" |
| ENSG00000260238 | HGLibA_37307 | 133 | 0.068337116 | PMF1-BGLAP | PMF1-BGLAP readthrough [Source:HGNC Symbol;Acc:HGNC:42953] |
| ENSG00000263761 | HGLibA_18863 | 133 | 0.068337116 | GDF2 | growth differentiation factor 2 [Source:HGNC Symbol;Acc:HGNC:4217] |
| ENSG00000264364 | HGLibA_14255 | 133 | 0.068337116 | DYNLL2 | dynein light chain LC8-type 2 [Source:HGNC Symbol;Acc:HGNC:24596] |
| ENSG00000275113 | HGLibA_18478 | 133 | 0.068337116 | GAGE2E | G antigen 2E [Source:HGNC Symbol;Acc:HGNC:31960] |
| FAM129C | HGLibA_16102 | 133 | 0.068337116 | FAM129C | - |
| FTSJD2 | HGLibA_18145 | 133 | 0.068337116 | FTSJD2 | - |
| GPR56 | HGLibA_20057 | 133 | 0.068337116 | GPR56 | - |

| | | | | | |
|---|---|---|---|---|---|
| HIST1H2AE | HGLibA_21388 | 133 | 0.068337116 | HIST1H2AE | - |
| hsa-mir-1288 | HGLibA_57615 | 133 | 0.068337116 | hsa-mir-1288 | - |
| hsa-mir-138-2 | HGLibA_57818 | 133 | 0.068337116 | hsa-mir-138-2 | - |
| hsa-mir-3119-1 | HGLibA_58524 | 133 | 0.068337116 | hsa-mir-3119-1 | - |
| hsa-mir-3192 | HGLibA_58867 | 133 | 0.068337116 | hsa-mir-3192 | - |
| hsa-mir-3201 | HGLibA_58910 | 133 | 0.068337116 | hsa-mir-3201 | - |
| hsa-mir-3622a | HGLibA_59122 | 133 | 0.068337116 | hsa-mir-3622a | - |
| hsa-mir-382 | HGLibA_59482 | 133 | 0.068337116 | hsa-mir-382 | - |
| hsa-mir-3918 | HGLibA_59548 | 133 | 0.068337116 | hsa-mir-3918 | - |
| hsa-mir-4515 | HGLibA_60502 | 133 | 0.068337116 | hsa-mir-4515 | - |
| hsa-mir-4641 | HGLibA_60670 | 133 | 0.068337116 | hsa-mir-4641 | - |
| hsa-mir-4671 | HGLibA_60800 | 133 | 0.068337116 | hsa-mir-4671 | - |
| hsa-mir-580 | HGLibA_62419 | 133 | 0.068337116 | hsa-mir-580 | - |
| hsa-mir-606 | HGLibA_62517 | 133 | 0.068337116 | hsa-mir-606 | - |
| hsa-mir-6079 | HGLibA_62572 | 133 | 0.068337116 | hsa-mir-6079 | - |
| hsa-mir-6754 | HGLibA_63172 | 133 | 0.068337116 | hsa-mir-6754 | - |
| hsa-mir-6809 | HGLibA_63413 | 133 | 0.068337116 | hsa-mir-6809 | - |
| hsa-mir-6865 | HGLibA_63644 | 133 | 0.068337116 | hsa-mir-6865 | - |
| hsa-mir-7158 | HGLibA_63861 | 133 | 0.068337116 | hsa-mir-7158 | - |
| LOC100862671 | HGLibA_26880 | 133 | 0.068337116 | LOC100862671 | - |
| TCEB3B | HGLibA_48742 | 133 | 0.068337116 | TCEB3B | - |
| TMEM56-RWDD3 | HGLibA_50452 | 133 | 0.068337116 | TMEM56-RWDD3 | - |
| WDYHV1 | HGLibA_54235 | 133 | 0.068337116 | WDYHV1 | - |
| WDYHV1 | HGLibA_54234 | 133 | 0.068337116 | WDYHV1 | - |
| C16orf55 | HGLibA_05460 | 132 | 0.059291774 | C16orf55 | - |
| CASC10 | HGLibA_07271 | 132 | 0.059291774 | CASC10 | - |
| ENSG00000007520 | HGLibA_52001 | 132 | 0.059291774 | TSR3 | acp transferase ribosome maturation factor [Source:HGNC Symbol;Acc:HGNC:14175]" |
| ENSG00000044115 | HGLibA_11639 | 132 | 0.059291774 | CTNNA1 | catenin alpha 1 [Source:HGNC Symbol;Acc:HGNC:2509] |
| ENSG00000047621 | HGLibA_05236 | 132 | 0.059291774 | C12orf4 | chromosome 12 open reading frame 4 [Source:HGNC Symbol;Acc:HGNC:1184] |
| ENSG00000087338 | HGLibA_19391 | 132 | 0.059291774 | GMCL1 | spermatogenesis associated 1 [Source:HGNC Symbol;Acc:HGNC:23843]" |
| ENSG00000088882 | HGLibA_11035 | 132 | 0.059291774 | CPXM1 | M14 family member 1 [Source:HGNC Symbol;Acc:HGNC:15771]" |
| ENSG00000088882 | HGLibA_11037 | 132 | 0.059291774 | CPXM1 | M14 family member 1 [Source:HGNC Symbol;Acc:HGNC:15771]" |
| ENSG00000091136 | HGLibA_25955 | 132 | 0.059291774 | LAMB1 | laminin subunit beta 1 [Source:HGNC Symbol;Acc:HGNC:6486] |
| ENSG00000099875 | HGLibA_29344 | 132 | 0.059291774 | MKNK2 | MAP kinase interacting serine/threonine kinase 2 [Source:HGNC Symbol;Acc:HGNC:7111] |
| ENSG00000100100 | HGLibA_36646 | 132 | 0.059291774 | PIK3IP1 | phosphoinositide-3-kinase interacting protein 1 [Source:HGNC Symbol;Acc:HGNC:24942] |
| ENSG00000100427 | HGLibA_29367 | 132 | 0.059291774 | MLC1 | megalencephalic leukoencephalopathy with subcortical cysts 1 [Source:HGNC Symbol;Acc:HGNC:17082] |
| ENSG00000100991 | HGLibA_51738 | 132 | 0.059291774 | TRPC4AP | transient receptor potential cation channel subfamily C member 4 associated protein [Source:HGNC Symbol;Acc:HGNC:16181] |
| ENSG00000101188 | HGLibA_32885 | 132 | 0.059291774 | NTSR1 | neurotensin receptor 1 [Source:HGNC Symbol;Acc:HGNC:8039] |
| ENSG00000101199 | HGLibA_02662 | 132 | 0.059291774 | ARFGAP1 | ADP ribosylation factor GTPase activating protein 1 [Source:HGNC Symbol;Acc:HGNC:15852] |
| ENSG00000102524 | HGLibA_50811 | 132 | 0.059291774 | TNFSF13B | TNF superfamily member 13b [Source:HGNC Symbol;Acc:HGNC:11929] |
| ENSG00000104112 | HGLibA_42901 | 132 | 0.059291774 | SCG3 | secretogranin III [Source:HGNC Symbol;Acc:HGNC:13707] |
| ENSG00000104973 | HGLibA_28786 | 132 | 0.059291774 | MED25 | mediator complex subunit 25 [Source:HGNC Symbol;Acc:HGNC:28845] |
| ENSG00000107679 | HGLibA_37062 | 132 | 0.059291774 | PLEKHA1 | pleckstrin homology domain containing A1 [Source:HGNC Symbol;Acc:HGNC:14335] |
| ENSG00000109084 | HGLibA_50568 | 132 | 0.059291774 | TMEM97 | transmembrane protein 97 [Source:HGNC Symbol;Acc:HGNC:28106] |
| ENSG00000110090 | HGLibA_11017 | 132 | 0.059291774 | CPT1A | carnitine palmitoyltransferase 1A [Source:HGNC Symbol;Acc:HGNC:2328] |
| ENSG00000111203 | HGLibA_23708 | 132 | 0.059291774 | ITFG2 | integrin alpha FG-GAP repeat containing 2 [Source:HGNC Symbol;Acc:HGNC:30879] |
| ENSG00000111215 | HGLibA_38839 | 132 | 0.059291774 | PRR4 | proline rich 4 [Source:HGNC Symbol;Acc:HGNC:18020] |
| ENSG00000116016 | HGLibA_15237 | 132 | 0.059291774 | EPAS1 | endothelial PAS domain protein 1 [Source:HGNC Symbol;Acc:HGNC:3374] |
| ENSG00000119013 | HGLibA_31516 | 132 | 0.059291774 | NDUFB3 | NADH:ubiquinone oxidoreductase subunit B3 [Source:HGNC Symbol;Acc:HGNC:7698] |
| ENSG00000120697 | HGLibA_01630 | 132 | 0.059291774 | ALG5 | dolichyl-phosphate beta-glucosyltransferase [Source:HGNC Symbol;Acc:HGNC:20266]" |
| ENSG00000120903 | HGLibA_09568 | 132 | 0.059291774 | CHRNA2 | cholinergic receptor nicotinic alpha 2 subunit [Source:HGNC Symbol;Acc:HGNC:1956] |
| ENSG00000126062 | HGLibA_49884 | 132 | 0.059291774 | TMEM115 | transmembrane protein 115 [Source:HGNC Symbol;Acc:HGNC:30055] |
| ENSG00000127329 | HGLibA_39488 | 132 | 0.059291774 | PTPRB | receptor type B [Source:HGNC Symbol;Acc:HGNC:9665]" |
| ENSG00000127578 | HGLibA_54281 | 132 | 0.059291774 | WFIKKN1 | kunitz and netrin domain containing 1 [Source:HGNC Symbol;Acc:HGNC:30912]" |
| ENSG00000127580 | HGLibA_54048 | 132 | 0.059291774 | WDR24 | WD repeat domain 24 [Source:HGNC Symbol;Acc:HGNC:20852] |
| ENSG00000128645 | HGLibA_21983 | 132 | 0.059291774 | HOXD1 | homeobox D1 [Source:HGNC Symbol;Acc:HGNC:5132] |
| ENSG00000130377 | HGLibA_00545 | 132 | 0.059291774 | ACSBG2 | acyl-CoA synthetase bubblegum family member 2 [Source:HGNC Symbol;Acc:HGNC:24174] |
| ENSG00000131480 | HGLibA_02269 | 132 | 0.059291774 | AOC2 | copper containing 2 [Source:HGNC Symbol;Acc:HGNC:549]" |
| ENSG00000132446 | HGLibA_18122 | 132 | 0.059291774 | FTHL17 | ferritin heavy chain like 17 [Source:HGNC Symbol;Acc:HGNC:3987] |
| ENSG00000132746 | HGLibA_01571 | 132 | 0.059291774 | ALDH3B2 | aldehyde dehydrogenase 3 family member B2 [Source:HGNC Symbol;Acc:HGNC:411] |
| ENSG00000132855 | HGLibA_01917 | 132 | 0.059291774 | ANGPTL3 | angiopoietin like 3 [Source:HGNC Symbol;Acc:HGNC:491] |
| ENSG00000133641 | HGLibA_05230 | 132 | 0.059291774 | C12orf29 | chromosome 12 open reading frame 29 [Source:HGNC Symbol;Acc:HGNC:25322] |
| ENSG00000134202 | HGLibA_20478 | 132 | 0.059291774 | GSTM3 | glutathione S-transferase mu 3 [Source:HGNC Symbol;Acc:HGNC:4635] |
| ENSG00000134851 | HGLibA_50036 | 132 | 0.059291774 | TMEM165 | transmembrane protein 165 [Source:HGNC Symbol;Acc:HGNC:30760] |
| ENSG00000136305 | HGLibA_09700 | 132 | 0.059291774 | CIDEB | cell death-inducing DFFA-like effector b [Source:HGNC Symbol;Acc:HGNC:1977] |
| ENSG00000136731 | HGLibA_52866 | 132 | 0.059291774 | UGGT1 | UDP-glucose glycoprotein glucosyltransferase 1 [Source:HGNC Symbol;Acc:HGNC:15663] |
| ENSG00000137177 | HGLibA_24857 | 132 | 0.059291774 | KIF13A | kinesin family member 13A [Source:HGNC Symbol;Acc:HGNC:14566] |
| ENSG00000137709 | HGLibA_37762 | 132 | 0.059291774 | POU2F3 | POU class 2 homeobox 3 [Source:HGNC Symbol;Acc:HGNC:19864] |
| ENSG00000138101 | HGLibA_14068 | 132 | 0.059291774 | DTNB | dystrobrevin beta [Source:HGNC Symbol;Acc:HGNC:3058] |
| ENSG00000138109 | HGLibA_12125 | 132 | 0.059291774 | CYP2C9 | cytochrome P450 family 2 subfamily C member 9 [Source:HGNC Symbol;Acc:HGNC:2623] |
| ENSG00000140367 | HGLibA_52649 | 132 | 0.059291774 | UBE2Q2 | ubiquitin conjugating enzyme E2 Q2 [Source:HGNC Symbol;Acc:HGNC:19248] |
| ENSG00000143158 | HGLibA_29708 | 132 | 0.059291774 | MPC2 | mitochondrial pyruvate carrier 2 [Source:HGNC Symbol;Acc:HGNC:24515] |
| ENSG00000146205 | HGLibA_02190 | 132 | 0.059291774 | ANO7 | anoctamin 7 [Source:HGNC Symbol;Acc:HGNC:31677] |
| ENSG00000146540 | HGLibA_06552 | 132 | 0.059291774 | C7orf50 | chromosome 7 open reading frame 50 [Source:HGNC Symbol;Acc:HGNC:22421] |
| ENSG00000151322 | HGLibA_32399 | 132 | 0.059291774 | NPAS3 | neuronal PAS domain protein 3 [Source:HGNC Symbol;Acc:HGNC:19311] |
| ENSG00000151498 | HGLibA_00358 | 132 | 0.059291774 | ACAD8 | acyl-CoA dehydrogenase family member 8 [Source:HGNC Symbol;Acc:HGNC:87] |
| ENSG00000154146 | HGLibA_32688 | 132 | 0.059291774 | NRGN | neurogranin [Source:HGNC Symbol;Acc:HGNC:8000] |
| ENSG00000154217 | HGLibA_36747 | 132 | 0.059291774 | PITPNC1 | phosphatidylinositol transfer protein cytoplasmic 1 [Source:HGNC Symbol;Acc:HGNC:21045] |
| ENSG00000157014 | HGLibA_48444 | 132 | 0.059291774 | TATDN2 | TatD DNase domain containing 2 [Source:HGNC Symbol;Acc:HGNC:28988] |
| ENSG00000157796 | HGLibA_54043 | 132 | 0.059291774 | WDR19 | WD repeat domain 19 [Source:HGNC Symbol;Acc:HGNC:18340] |
| ENSG00000158874 | HGLibA_02456 | 132 | 0.059291774 | APOA2 | apolipoprotein A2 [Source:HGNC Symbol;Acc:HGNC:601] |
| ENSG00000161692 | HGLibA_12380 | 132 | 0.059291774 | DBF4B | DBF4 zinc finger B [Source:HGNC Symbol;Acc:HGNC:17883] |
| ENSG00000162551 | HGLibA_01708 | 132 | 0.059291774 | ALPL | biomineralization associated [Source:HGNC Symbol;Acc:HGNC:438]" |
| ENSG00000163319 | HGLibA_30055 | 132 | 0.059291774 | MRPS18C | mitochondrial ribosomal protein S18C [Source:HGNC Symbol;Acc:HGNC:16633] |
| ENSG00000163659 | HGLibA_49555 | 132 | 0.059291774 | TIPARP | TCDD inducible poly(ADP-ribose) polymerase [Source:HGNC Symbol;Acc:HGNC:23696] |
| ENSG00000163739 | HGLibA_11842 | 132 | 0.059291774 | CXCL1 | C-X-C motif chemokine ligand 1 [Source:HGNC Symbol;Acc:HGNC:4602] |
| ENSG00000166152 | HGLibA_05484 | 132 | 0.059291774 | C16orf78 | chromosome 16 open reading frame 78 [Source:HGNC Symbol;Acc:HGNC:28479] |
| ENSG00000166582 | HGLibA_09088 | 132 | 0.059291774 | CENPV | centromere protein V [Source:HGNC Symbol;Acc:HGNC:29920] |
| ENSG00000167034 | HGLibA_32048 | 132 | 0.059291774 | NKX3-1 | NK3 homeobox 1 [Source:HGNC Symbol;Acc:HGNC:7838] |
| ENSG00000167747 | HGLibA_05671 | 132 | 0.059291774 | C19orf48 | chromosome 19 open reading frame 48 [Source:HGNC Symbol;Acc:HGNC:29667] |
| ENSG00000168904 | HGLibA_27300 | 132 | 0.059291774 | LRRC28 | leucine rich repeat containing 28 [Source:HGNC Symbol;Acc:HGNC:28355] |
| ENSG00000169255 | HGLibA_03949 | 132 | 0.059291774 | B3GALNT1 | 3-N-acetylgalactosaminyltransferase 1 (globoside blood group) [Source:HGNC Symbol;Acc:HGNC:918]" |
| ENSG00000169618 | HGLibA_38671 | 132 | 0.059291774 | PROKR1 | prokineticin receptor 1 [Source:HGNC Symbol;Acc:HGNC:4524] |
| ENSG00000170322 | HGLibA_31839 | 132 | 0.059291774 | NFRKB | nuclear factor related to kappaB binding protein [Source:HGNC Symbol;Acc:HGNC:7802] |
| ENSG00000171121 | HGLibA_24347 | 132 | 0.059291774 | KCNMB3 | potassium calcium-activated channel subfamily M regulatory beta subunit 3 [Source:HGNC Symbol;Acc:HGNC:6287] |
| ENSG00000171621 | HGLibA_46859 | 132 | 0.059291774 | SPSB1 | splA/ryanodine receptor domain and SOCS box containing 1 [Source:HGNC Symbol;Acc:HGNC:30628] |
| ENSG00000172766 | HGLibA_30980 | 132 | 0.059291774 | NAA16 | NatA auxiliary subunit [Source:HGNC Symbol;Acc:HGNC:26164]" |
| ENSG00000174938 | HGLibA_43684 | 132 | 0.059291774 | SEZ6L2 | seizure related 6 homolog like 2 [Source:HGNC Symbol;Acc:HGNC:30844] |
| ENSG00000175224 | HGLibA_03467 | 132 | 0.059291774 | ATG13 | autophagy related 13 [Source:HGNC Symbol;Acc:HGNC:29091] |
| ENSG00000175376 | HGLibA_14708 | 132 | 0.059291774 | EIF1AD | eukaryotic translation initiation factor 1A domain containing [Source:HGNC Symbol;Acc:HGNC:28147] |
| ENSG00000176024 | HGLibA_56386 | 132 | 0.059291774 | ZNF613 | zinc finger protein 613 [Source:HGNC Symbol;Acc:HGNC:25827] |
| ENSG00000177119 | HGLibA_02186 | 132 | 0.059291774 | ANO6 | anoctamin 6 [Source:HGNC Symbol;Acc:HGNC:25240] |
| ENSG00000178425 | HGLibA_32820 | 132 | 0.059291774 | NT5DC1 | 5'-nucleotidase domain containing 1 [Source:HGNC Symbol;Acc:HGNC:21556] |
| ENSG00000179168 | HGLibA_19002 | 132 | 0.059291774 | GGN | gametogenetin [Source:HGNC Symbol;Acc:HGNC:18869] |
| ENSG00000179630 | HGLibA_25907 | 132 | 0.059291774 | LACC1 | laccase domain containing 1 [Source:HGNC Symbol;Acc:HGNC:26789] |
| ENSG00000180011 | HGLibA_54769 | 132 | 0.059291774 | ZADH2 | zinc binding alcohol dehydrogenase domain containing 2 [Source:HGNC Symbol;Acc:HGNC:28697] |
| ENSG00000181191 | HGLibA_36783 | 132 | 0.059291774 | PJA1 | praja ring finger ubiquitin ligase 1 [Source:HGNC Symbol;Acc:HGNC:16648] |
| ENSG00000181315 | HGLibA_55799 | 132 | 0.059291774 | ZNF322 | zinc finger protein 322 [Source:HGNC Symbol;Acc:HGNC:23640] |
| ENSG00000182218 | HGLibA_21264 | 132 | 0.059291774 | HHIPL1 | HHIP like 1 [Source:HGNC Symbol;Acc:HGNC:19710] |
| ENSG00000184903 | HGLibA_23233 | 132 | 0.059291774 | IMMP2L | inner mitochondrial membrane peptidase subunit 2 [Source:HGNC Symbol;Acc:HGNC:14598] |
| ENSG00000185324 | HGLibA_08723 | 132 | 0.059291774 | CDK10 | cyclin dependent kinase 10 [Source:HGNC Symbol;Acc:HGNC:1770] |
| ENSG00000186118 | HGLibA_49110 | 132 | 0.059291774 | TEX38 | testis expressed 38 [Source:HGNC Symbol;Acc:HGNC:29589] |
| ENSG00000197696 | HGLibA_32142 | 132 | 0.059291774 | NMB | neuromedin B [Source:HGNC Symbol;Acc:HGNC:7842] |
| ENSG00000198189 | HGLibA_22176 | 132 | 0.059291774 | HSD17B11 | hydroxysteroid 17-beta dehydrogenase 11 [Source:HGNC Symbol;Acc:HGNC:22960] |
| ENSG00000198218 | HGLibA_39743 | 132 | 0.059291774 | QRICH1 | glutamine rich 1 [Source:HGNC Symbol;Acc:HGNC:24713] |
| ENSG00000198959 | HGLibA_49255 | 132 | 0.059291774 | TGM2 | transglutaminase 2 [Source:HGNC Symbol;Acc:HGNC:11778] |
| ENSG00000205089 | HGLibA_08105 | 132 | 0.059291774 | CCNI2 | cyclin I family member 2 [Source:HGNC Symbol;Acc:HGNC:33869] |
| ENSG00000205457 | HGLibA_51084 | 132 | 0.059291774 | TP53TG3C | TP53 target 3C [Source:HGNC Symbol;Acc:HGNC:42962] |
| ENSG00000205496 | HGLibA_34072 | 132 | 0.059291774 | OR51A2 | olfactory receptor family 51 subfamily A member 2 [Source:HGNC Symbol;Acc:HGNC:14764] |
| ENSG00000214367 | HGLibA_20880 | 132 | 0.059291774 | HAUS3 | HAUS augmin like complex subunit 3 [Source:HGNC Symbol;Acc:HGNC:28719] |
| ENSG00000214941 | HGLibA_57055 | 132 | 0.059291774 | ZSWIM7 | zinc finger SWIM-type containing 7 [Source:HGNC Symbol;Acc:HGNC:26993] |
| ENSG00000221916 | HGLibA_05704 | 132 | 0.059291774 | C19orf73 | chromosome 19 open reading frame 73 [Source:HGNC Symbol;Acc:HGNC:25534] |
| ENSG00000221988 | HGLibA_38235 | 132 | 0.059291774 | PPT2 | palmitoyl-protein thioesterase 2 [Source:HGNC Symbol;Acc:HGNC:9326] |
| ENSG00000224982 | HGLibA_50263 | 132 | 0.059291774 | TMEM233 | transmembrane protein 233 [Source:HGNC Symbol;Acc:HGNC:37219] |
| ENSG00000226941 | HGLibA_40627 | 132 | 0.059291774 | RBMY1J | member J [Source:HGNC Symbol;Acc:HGNC:23917]" |
| ENSG00000237440 | HGLibA_56655 | 132 | 0.059291774 | ZNF737 | zinc finger protein 737 [Source:HGNC Symbol;Acc:HGNC:32468] |
| ENSG00000243489 | HGLibA_25597 | 132 | 0.059291774 | KRTAP10-11 | keratin associated protein 10-11 [Source:HGNC Symbol;Acc:HGNC:20528] |
| ENSG00000248405 | HGLibA_38846 | 132 | 0.059291774 | PRR5-ARHGAP8 | PRR5-ARHGAP8 readthrough [Source:HGNC Symbol;Acc:HGNC:34512] |
| ENSG00000256087 | HGLibA_55984 | 132 | 0.059291774 | ZNF432 | zinc finger protein 432 [Source:HGNC Symbol;Acc:HGNC:20810] |
| ENSG00000256162 | HGLibA_45779 | 132 | 0.059291774 | SMLR1 | small leucine rich protein 1 [Source:HGNC Symbol;Acc:HGNC:44670] |
| ENSG00000261371 | HGLibA_36019 | 132 | 0.059291774 | PECAM1 | platelet and endothelial cell adhesion molecule 1 [Source:HGNC Symbol;Acc:HGNC:8823] |
| ENSG00000267041 | HGLibA_56873 | 132 | 0.059291774 | ZNF850 | zinc finger protein 850 [Source:HGNC Symbol;Acc:HGNC:27994] |
| ENSG00000267855 | HGLibA_31469 | 132 | 0.059291774 | NDUFA7 | NADH:ubiquinone oxidoreductase subunit A7 [Source:HGNC Symbol;Acc:HGNC:7691] |
| ENSG00000269556 | HGLibA_50120 | 132 | 0.059291774 | TMEM185A | transmembrane protein 185A [Source:HGNC Symbol;Acc:HGNC:17125] |
| ENSG00000273540 | HGLibA_01167 | 132 | 0.059291774 | AGBL1 | ATP/GTP binding protein like 1 [Source:HGNC Symbol;Acc:HGNC:26504] |

| | | | | | |
|---|---|---|---|---|---|
| ENSG00000281887 | HGLibA_19073 | 132 | 0.059291774 | GIMAP1-GIMAP5 | GIMAP1-GIMAP5 readthrough [Source:HGNC Symbol;Acc:HGNC:51257] |
| ENSG00000288702 | HGLibA_52883 | 132 | 0.059291774 | UGT1A3 | - |
| FIGF | HGLibA_17513 | 132 | 0.059291774 | FIGF | - |
| GPR75-ASB3 | HGLibA_20084 | 132 | 0.059291774 | GPR75-ASB3 | - |
| H2BFM | HGLibA_20772 | 132 | 0.059291774 | H2BFM | - |
| HGC6.3 | HGLibA_21233 | 132 | 0.059291774 | HGC6.3 | - |
| hsa-mir-144 | HGLibA_57839 | 132 | 0.059291774 | hsa-mir-144 | - |
| hsa-mir-299 | HGLibA_58404 | 132 | 0.059291774 | hsa-mir-299 | - |
| hsa-mir-3181 | HGLibA_58822 | 132 | 0.059291774 | hsa-mir-3181 | - |
| hsa-mir-3184 | HGLibA_58835 | 132 | 0.059291774 | hsa-mir-3184 | - |
| hsa-mir-3665 | HGLibA_59213 | 132 | 0.059291774 | hsa-mir-3665 | - |
| hsa-mir-383 | HGLibA_59483 | 132 | 0.059291774 | hsa-mir-383 | - |
| hsa-mir-425 | HGLibA_59706 | 132 | 0.059291774 | hsa-mir-425 | - |
| hsa-mir-4302 | HGLibA_59919 | 132 | 0.059291774 | hsa-mir-4302 | - |
| hsa-mir-4734 | HGLibA_61035 | 132 | 0.059291774 | hsa-mir-4734 | - |
| hsa-mir-4773-1 | HGLibA_61191 | 132 | 0.059291774 | hsa-mir-4773-1 | - |
| hsa-mir-499b | HGLibA_61405 | 132 | 0.059291774 | hsa-mir-499b | - |
| hsa-mir-520a | HGLibA_61733 | 132 | 0.059291774 | hsa-mir-520a | - |
| hsa-mir-526a-2 | HGLibA_61795 | 132 | 0.059291774 | hsa-mir-526a-2 | - |
| hsa-mir-545 | HGLibA_61834 | 132 | 0.059291774 | hsa-mir-545 | - |
| hsa-mir-592 | HGLibA_62465 | 132 | 0.059291774 | hsa-mir-592 | - |
| hsa-mir-6733 | HGLibA_63086 | 132 | 0.059291774 | hsa-mir-6733 | - |
| hsa-mir-7107 | HGLibA_63790 | 132 | 0.059291774 | hsa-mir-7107 | - |
| hsa-mir-7703 | HGLibA_63949 | 132 | 0.059291774 | hsa-mir-7703 | - |
| RTDR1 | HGLibA_42378 | 132 | 0.059291774 | RTDR1 | - |
| SETD8 | HGLibA_43662 | 132 | 0.059291774 | SETD8 | - |
| TMEM159 | HGLibA_50017 | 132 | 0.059291774 | TMEM159 | - |
| XAGE1C | HGLibA_54471 | 132 | 0.059291774 | XAGE1C | - |
| C17orf96 | HGLibA_05595 | 131 | 0.050246431 | C17orf96 | - |
| C20orf26 | HGLibA_06021 | 131 | 0.050246431 | C20orf26 | - |
| ENSG00000006704 | HGLibA_20567 | 131 | 0.050246431 | GTF2IRD1 | GTF2I repeat domain containing 1 [Source:HGNC Symbol;Acc:HGNC:4661] |
| ENSG00000008300 | HGLibA_09027 | 131 | 0.050246431 | CELSR3 | cadherin EGF LAG seven-pass G-type receptor 3 [Source:HGNC Symbol;Acc:HGNC:3230] |
| ENSG00000010818 | HGLibA_21569 | 131 | 0.050246431 | HIVEP2 | human immunodeficiency virus type I enhancer binding protein 2 [Source:HGNC Symbol;Acc:HGNC:4921] |
| ENSG00000048545 | HGLibA_20634 | 131 | 0.050246431 | GUCA1A | guanylate cyclase activator 1A [Source:HGNC Symbol;Acc:HGNC:4678] |
| ENSG00000052841 | HGLibA_52061 | 131 | 0.050246431 | TTC17 | tetratricopeptide repeat domain 17 [Source:HGNC Symbol;Acc:HGNC:25596] |
| ENSG00000058453 | HGLibA_11210 | 131 | 0.050246431 | CROCC | rootletin [Source:HGNC Symbol;Acc:HGNC:21299]" |
| ENSG00000065615 | HGLibA_12010 | 131 | 0.050246431 | CYB5R4 | cytochrome b5 reductase 4 [Source:HGNC Symbol;Acc:HGNC:20147] |
| ENSG00000066294 | HGLibA_08464 | 131 | 0.050246431 | CD84 | CD84 molecule [Source:HGNC Symbol;Acc:HGNC:1704] |
| ENSG00000069020 | HGLibA_28409 | 131 | 0.050246431 | MAST4 | microtubule associated serine/threonine kinase family member 4 [Source:HGNC Symbol;Acc:HGNC:19037] |
| ENSG00000070081 | HGLibA_32913 | 131 | 0.050246431 | NUCB2 | nucleobindin 2 [Source:HGNC Symbol;Acc:HGNC:8044] |
| ENSG00000075643 | HGLibA_29608 | 131 | 0.050246431 | MOCOS | molybdenum cofactor sulfurase [Source:HGNC Symbol;Acc:HGNC:18234] |
| ENSG00000077150 | HGLibA_31818 | 131 | 0.050246431 | NFKB2 | nuclear factor kappa B subunit 2 [Source:HGNC Symbol;Acc:HGNC:7795] |
| ENSG00000077279 | HGLibA_12580 | 131 | 0.050246431 | DCX | doublecortin [Source:HGNC Symbol;Acc:HGNC:2714] |
| ENSG00000080371 | HGLibA_39837 | 131 | 0.050246431 | RAB21 | member RAS oncogene family [Source:HGNC Symbol;Acc:HGNC:18263]" |
| ENSG00000080823 | HGLibA_29635 | 131 | 0.050246431 | MOK | MOK protein kinase [Source:HGNC Symbol;Acc:HGNC:9833] |
| ENSG00000092758 | HGLibA_10627 | 131 | 0.050246431 | COL9A3 | collagen type IX alpha 3 chain [Source:HGNC Symbol;Acc:HGNC:2219] |
| ENSG00000092841 | HGLibA_30769 | 131 | 0.050246431 | MYL6 | myosin light chain 6 [Source:HGNC Symbol;Acc:HGNC:7587] |
| ENSG00000100038 | HGLibA_51004 | 131 | 0.050246431 | TOP3B | DNA topoisomerase III beta [Source:HGNC Symbol;Acc:HGNC:11993] |
| ENSG00000100558 | HGLibA_37059 | 131 | 0.050246431 | PLEK2 | pleckstrin 2 [Source:HGNC Symbol;Acc:HGNC:19238] |
| ENSG00000101276 | HGLibA_45251 | 131 | 0.050246431 | SLC52A3 | solute carrier family 52 member 3 [Source:HGNC Symbol;Acc:HGNC:16187] |
| ENSG00000101871 | HGLibA_29231 | 131 | 0.050246431 | MID1 | midline 1 [Source:HGNC Symbol;Acc:HGNC:7095] |
| ENSG00000102349 | HGLibA_25083 | 131 | 0.050246431 | KLF8 | Kruppel like factor 8 [Source:HGNC Symbol;Acc:HGNC:6351] |
| ENSG00000102362 | HGLibA_48104 | 131 | 0.050246431 | SYTL4 | synaptotagmin like 4 [Source:HGNC Symbol;Acc:HGNC:15588] |
| ENSG00000106526 | HGLibA_00666 | 131 | 0.050246431 | ACTR3C | ARP3 actin related protein 3 homolog C [Source:HGNC Symbol;Acc:HGNC:37282] |
| ENSG00000107438 | HGLibA_35907 | 131 | 0.050246431 | PDLIM1 | PDZ and LIM domain 1 [Source:HGNC Symbol;Acc:HGNC:2067] |
| ENSG00000108784 | HGLibA_31068 | 131 | 0.050246431 | NAGLU | N-acetyl-alpha-glucosaminidase [Source:HGNC Symbol;Acc:HGNC:7632] |
| ENSG00000109065 | HGLibA_31192 | 131 | 0.050246431 | NAT9 | N-acetyltransferase 9 (putative) [Source:HGNC Symbol;Acc:HGNC:23133] |
| ENSG00000110011 | HGLibA_13621 | 131 | 0.050246431 | DNAJC4 | DnaJ heat shock protein family (Hsp40) member C4 [Source:HGNC Symbol;Acc:HGNC:5271] |
| ENSG00000111701 | HGLibA_02468 | 131 | 0.050246431 | APOBEC1 | apolipoprotein B mRNA editing enzyme catalytic subunit 1 [Source:HGNC Symbol;Acc:HGNC:604] |
| ENSG00000112053 | HGLibA_44806 | 131 | 0.050246431 | SLC26A8 | solute carrier family 26 member 8 [Source:HGNC Symbol;Acc:HGNC:14468] |
| ENSG00000115380 | HGLibA_14561 | 131 | 0.050246431 | EFEMP1 | EGF containing fibulin extracellular matrix protein 1 [Source:HGNC Symbol;Acc:HGNC:3218] |
| ENSG00000115592 | HGLibA_38518 | 131 | 0.050246431 | PRKAG3 | protein kinase AMP-activated non-catalytic subunit gamma 3 [Source:HGNC Symbol;Acc:HGNC:9387] |
| ENSG00000116874 | HGLibA_53948 | 131 | 0.050246431 | WARS2 | mitochondrial [Source:HGNC Symbol;Acc:HGNC:12730]" |
| ENSG00000120500 | HGLibA_03104 | 131 | 0.050246431 | ARR3 | arrestin 3 [Source:HGNC Symbol;Acc:HGNC:710] |
| ENSG00000124159 | HGLibA_28435 | 131 | 0.050246431 | MATN4 | matrilin 4 [Source:HGNC Symbol;Acc:HGNC:6910] |
| ENSG00000124782 | HGLibA_42244 | 131 | 0.050246431 | RREB1 | ras responsive element binding protein 1 [Source:HGNC Symbol;Acc:HGNC:10449] |
| ENSG00000125995 | HGLibA_41756 | 131 | 0.050246431 | ROMO1 | reactive oxygen species modulator 1 [Source:HGNC Symbol;Acc:HGNC:16185] |
| ENSG00000126860 | HGLibA_15686 | 131 | 0.050246431 | EVI2A | ecotropic viral integration site 2A [Source:HGNC Symbol;Acc:HGNC:3499] |
| ENSG00000127311 | HGLibA_21110 | 131 | 0.050246431 | HELB | DNA helicase B [Source:HGNC Symbol;Acc:HGNC:17196] |
| ENSG00000128000 | HGLibA_56734 | 131 | 0.050246431 | ZNF780B | zinc finger protein 780B [Source:HGNC Symbol;Acc:HGNC:33109] |
| ENSG00000130150 | HGLibA_29688 | 131 | 0.050246431 | MOSPD2 | motile sperm domain containing 2 [Source:HGNC Symbol;Acc:HGNC:28381] |
| ENSG00000130812 | HGLibA_01924 | 131 | 0.050246431 | ANGPTL6 | angiopoietin like 6 [Source:HGNC Symbol;Acc:HGNC:23140] |
| ENSG00000132950 | HGLibA_55402 | 131 | 0.050246431 | ZMYM5 | zinc finger MYM-type containing 5 [Source:HGNC Symbol;Acc:HGNC:13029] |
| ENSG00000133858 | HGLibA_55168 | 131 | 0.050246431 | ZFC3H1 | zinc finger C3H1-type containing [Source:HGNC Symbol;Acc:HGNC:28328] |
| ENSG00000134291 | HGLibA_49859 | 131 | 0.050246431 | TMEM106C | transmembrane protein 106C [Source:HGNC Symbol;Acc:HGNC:28775] |
| ENSG00000135046 | HGLibA_02219 | 131 | 0.050246431 | ANXA1 | annexin A1 [Source:HGNC Symbol;Acc:HGNC:533] |
| ENSG00000135454 | HGLibA_04012 | 131 | 0.050246431 | B4GALNT1 | 4-N-acetyl-galactosaminyltransferase 1 [Source:HGNC Symbol;Acc:HGNC:4117]" |
| ENSG00000136059 | HGLibA_53654 | 131 | 0.050246431 | VILL | villin like [Source:HGNC Symbol;Acc:HGNC:30906] |
| ENSG00000136155 | HGLibA_42891 | 131 | 0.050246431 | SCEL | sciellin [Source:HGNC Symbol;Acc:HGNC:10573] |
| ENSG00000136279 | HGLibA_12398 | 131 | 0.050246431 | DBNL | drebrin like [Source:HGNC Symbol;Acc:HGNC:2696] |
| ENSG00000136840 | HGLibA_47272 | 131 | 0.050246431 | ST6GALNAC4 | 6-sialyltransferase 4 [Source:HGNC Symbol;Acc:HGNC:17846]" |
| ENSG00000137154 | HGLibA_42155 | 131 | 0.050246431 | RPS6 | ribosomal protein S6 [Source:HGNC Symbol;Acc:HGNC:10429] |
| ENSG00000137273 | HGLibA_17867 | 131 | 0.050246431 | FOXF2 | forkhead box F2 [Source:HGNC Symbol;Acc:HGNC:3810] |
| ENSG00000137760 | HGLibA_01667 | 131 | 0.050246431 | ALKBH8 | tRNA methyltransferase [Source:HGNC Symbol;Acc:HGNC:25189]" |
| ENSG00000138750 | HGLibA_33061 | 131 | 0.050246431 | NUP54 | nucleoporin 54 [Source:HGNC Symbol;Acc:HGNC:17359] |
| ENSG00000142789 | HGLibA_08999 | 131 | 0.050246431 | CELA3A | chymotrypsin like elastase family member 3A [Source:HGNC Symbol;Acc:HGNC:15944] |
| ENSG00000143110 | HGLibA_05787 | 131 | 0.050246431 | C1orf162 | chromosome 1 open reading frame 162 [Source:HGNC Symbol;Acc:HGNC:28344] |
| ENSG00000143942 | HGLibA_09338 | 131 | 0.050246431 | CHAC2 | ChaC cation transport regulator homolog 2 [Source:HGNC Symbol;Acc:HGNC:32363] |
| ENSG00000144460 | HGLibA_33182 | 131 | 0.050246431 | NYAP2 | neuronal tyrosine-phosphorylated phosphoinositide-3-kinase adaptor 2 [Source:HGNC Symbol;Acc:HGNC:29291] |
| ENSG00000145423 | HGLibA_43738 | 131 | 0.050246431 | SFRP2 | secreted frizzled related protein 2 [Source:HGNC Symbol;Acc:HGNC:10777] |
| ENSG00000145623 | HGLibA_34697 | 131 | 0.050246431 | OSMR | oncostatin M receptor [Source:HGNC Symbol;Acc:HGNC:8507] |
| ENSG00000146094 | HGLibA_13774 | 131 | 0.050246431 | DOK3 | docking protein 3 [Source:HGNC Symbol;Acc:HGNC:24583] |
| ENSG00000147381 | HGLibA_27915 | 131 | 0.050246431 | MAGEA4 | MAGE family member A4 [Source:HGNC Symbol;Acc:HGNC:6802] |
| ENSG00000147614 | HGLibA_03721 | 131 | 0.050246431 | ATP6V0D2 | ATPase H+ transporting V0 subunit d2 [Source:HGNC Symbol;Acc:HGNC:18266] |
| ENSG00000147894 | HGLibA_06759 | 131 | 0.050246431 | C9orf72 | chromosome 9 open reading frame 72 [Source:HGNC Symbol;Acc:HGNC:28337] |
| ENSG00000148057 | HGLibA_22559 | 131 | 0.050246431 | IDNK | gluconokinase [Source:HGNC Symbol;Acc:HGNC:31367]" |
| ENSG00000148335 | HGLibA_32850 | 131 | 0.050246431 | NTMT1 | N-terminal Xaa-Pro-Lys N-methyltransferase 1 [Source:HGNC Symbol;Acc:HGNC:23373] |
| ENSG00000148704 | HGLibA_53557 | 131 | 0.050246431 | VAX1 | ventral anterior homeobox 1 [Source:HGNC Symbol;Acc:HGNC:12660] |
| ENSG00000148773 | HGLibA_29324 | 131 | 0.050246431 | MKI67 | marker of proliferation Ki-67 [Source:HGNC Symbol;Acc:HGNC:7107] |
| ENSG00000149133 | HGLibA_34286 | 131 | 0.050246431 | OR5F1 | olfactory receptor family 5 subfamily F member 1 [Source:HGNC Symbol;Acc:HGNC:8343] |
| ENSG00000152782 | HGLibA_35096 | 131 | 0.050246431 | PANK1 | pantothenate kinase 1 [Source:HGNC Symbol;Acc:HGNC:8598] |
| ENSG00000154007 | HGLibA_03227 | 131 | 0.050246431 | ASB17 | ankyrin repeat and SOCS box containing 17 [Source:HGNC Symbol;Acc:HGNC:19769] |
| ENSG00000156958 | HGLibA_18519 | 131 | 0.050246431 | GALK2 | galactokinase 2 [Source:HGNC Symbol;Acc:HGNC:4119] |
| ENSG00000157429 | HGLibA_55555 | 131 | 0.050246431 | ZNF19 | zinc finger protein 19 [Source:HGNC Symbol;Acc:HGNC:12981] |
| ENSG00000157617 | HGLibA_06085 | 131 | 0.050246431 | C2CD2 | C2 calcium dependent domain containing 2 [Source:HGNC Symbol;Acc:HGNC:1266] |
| ENSG00000160007 | HGLibA_02765 | 131 | 0.050246431 | ARHGAP35 | Rho GTPase activating protein 35 [Source:HGNC Symbol;Acc:HGNC:4591] |
| ENSG00000160803 | HGLibA_52742 | 131 | 0.050246431 | UBQLN4 | ubiquilin 4 [Source:HGNC Symbol;Acc:HGNC:1237] |
| ENSG00000160856 | HGLibA_17231 | 131 | 0.050246431 | FCRL3 | Fc receptor like 3 [Source:HGNC Symbol;Acc:HGNC:18506] |
| ENSG00000161031 | HGLibA_36252 | 131 | 0.050246431 | PGLYRP2 | peptidoglycan recognition protein 2 [Source:HGNC Symbol;Acc:HGNC:30013] |
| ENSG00000161642 | HGLibA_55897 | 131 | 0.050246431 | ZNF385A | zinc finger protein 385A [Source:HGNC Symbol;Acc:HGNC:17521] |
| ENSG00000163530 | HGLibA_13888 | 131 | 0.050246431 | DPPA2 | developmental pluripotency associated 2 [Source:HGNC Symbol;Acc:HGNC:19197] |
| ENSG00000164733 | HGLibA_11691 | 131 | 0.050246431 | CTSB | cathepsin B [Source:HGNC Symbol;Acc:HGNC:2527] |
| ENSG00000165376 | HGLibA_09876 | 131 | 0.050246431 | CLDN2 | claudin 2 [Source:HGNC Symbol;Acc:HGNC:2041] |
| ENSG00000165626 | HGLibA_04417 | 131 | 0.050246431 | BEND7 | BEN domain containing 7 [Source:HGNC Symbol;Acc:HGNC:23514] |
| ENSG00000166359 | HGLibA_54208 | 131 | 0.050246431 | WDR88 | WD repeat domain 88 [Source:HGNC Symbol;Acc:HGNC:26999] |
| ENSG00000166800 | HGLibA_26223 | 131 | 0.050246431 | LDHAL6A | lactate dehydrogenase A like 6A [Source:HGNC Symbol;Acc:HGNC:28335] |
| ENSG00000166869 | HGLibA_09520 | 131 | 0.050246431 | CHP2 | calcineurin like EF-hand protein 2 [Source:HGNC Symbol;Acc:HGNC:24927] |
| ENSG00000166913 | HGLibA_54738 | 131 | 0.050246431 | YWHAB | tyrosine 3-monooxygenase/tryptophan 5-monooxygenase activation protein beta [Source:HGNC Symbol;Acc:HGNC:12849] |
| ENSG00000167553 | HGLibA_52248 | 131 | 0.050246431 | TUBA1C | tubulin alpha 1c [Source:HGNC Symbol;Acc:HGNC:20768] |
| ENSG00000167977 | HGLibA_24457 | 131 | 0.050246431 | KCTD5 | potassium channel tetramerization domain containing 5 [Source:HGNC Symbol;Acc:HGNC:21423] |
| ENSG00000168096 | HGLibA_02155 | 131 | 0.050246431 | ANKS3 | ankyrin repeat and sterile alpha motif domain containing 3 [Source:HGNC Symbol;Acc:HGNC:29422] |
| ENSG00000168461 | HGLibA_39875 | 131 | 0.050246431 | RAB31 | member RAS oncogene family [Source:HGNC Symbol;Acc:HGNC:9771]" |
| ENSG00000168818 | HGLibA_47631 | 131 | 0.050246431 | STX18 | syntaxin 18 [Source:HGNC Symbol;Acc:HGNC:15942] |
| ENSG00000170465 | HGLibA_25519 | 131 | 0.050246431 | KRT6C | keratin 6C [Source:HGNC Symbol;Acc:HGNC:20406] |
| ENSG00000170522 | HGLibA_14971 | 131 | 0.050246431 | ELOVL6 | ELOVL fatty acid elongase 6 [Source:HGNC Symbol;Acc:HGNC:15829] |
| ENSG00000170782 | HGLibA_33468 | 131 | 0.050246431 | OR10A4 | olfactory receptor family 10 subfamily A member 4 [Source:HGNC Symbol;Acc:HGNC:15130] |
| ENSG00000171476 | HGLibA_21884 | 131 | 0.050246431 | HOPX | HOP homeobox [Source:HGNC Symbol;Acc:HGNC:24961] |
| ENSG00000172238 | HGLibA_03534 | 131 | 0.050246431 | ATOH1 | atonal bHLH transcription factor 1 [Source:HGNC Symbol;Acc:HGNC:797] |
| ENSG00000173908 | HGLibA_25469 | 131 | 0.050246431 | KRT28 | keratin 28 [Source:HGNC Symbol;Acc:HGNC:30842] |
| ENSG00000175183 | HGLibA_11436 | 131 | 0.050246431 | CSRP2 | cysteine and glycine rich protein 2 [Source:HGNC Symbol;Acc:HGNC:2470] |
| ENSG00000175538 | HGLibA_24191 | 131 | 0.050246431 | KCNE3 | potassium voltage-gated channel subfamily E regulatory subunit 3 [Source:HGNC Symbol;Acc:HGNC:6243] |
| ENSG00000175711 | HGLibA_04007 | 131 | 0.050246431 | B3GNTL1 | 3-N-acetylglucosaminyltransferase like 1 [Source:HGNC Symbol;Acc:HGNC:21727]" |
| ENSG00000176920 | HGLibA_18187 | 131 | 0.050246431 | FUT2 | fucosyltransferase 2 [Source:HGNC Symbol;Acc:HGNC:4013] |
| ENSG00000178188 | HGLibA_43874 | 131 | 0.050246431 | SH2B1 | SH2B adaptor protein 1 [Source:HGNC Symbol;Acc:HGNC:30417] |
| ENSG00000178372 | HGLibA_07037 | 131 | 0.050246431 | CALML5 | calmodulin like 5 [Source:HGNC Symbol;Acc:HGNC:18180] |

| | | | | | |
|---|---|---|---|---|---|
| ENSG00000178445 | HGLibA_19234 | 131 | 0.050246431 | GLDC | glycine decarboxylase [Source:HGNC Symbol;Acc:HGNC:4313] |
| ENSG00000179772 | HGLibA_17956 | 131 | 0.050246431 | FOXS1 | forkhead box S1 [Source:HGNC Symbol;Acc:HGNC:3735] |
| ENSG00000182070 | HGLibA_34138 | 131 | 0.050246431 | OR52A1 | olfactory receptor family 52 subfamily A member 1 [Source:HGNC Symbol;Acc:HGNC:8318] |
| ENSG00000182103 | HGLibA_16322 | 131 | 0.050246431 | FAM181B | family with sequence similarity 181 member B [Source:HGNC Symbol;Acc:HGNC:28512] |
| ENSG00000182621 | HGLibA_36977 | 131 | 0.050246431 | PLCB1 | phospholipase C beta 1 [Source:HGNC Symbol;Acc:HGNC:15917] |
| ENSG00000182898 | HGLibA_48805 | 131 | 0.050246431 | TCHHL1 | trichohyalin like 1 [Source:HGNC Symbol;Acc:HGNC:31796] |
| ENSG00000183172 | HGLibA_45688 | 131 | 0.050246431 | SMDT1 | single-pass membrane protein with aspartate rich tail 1 [Source:HGNC Symbol;Acc:HGNC:25055] |
| ENSG00000183580 | HGLibA_17017 | 131 | 0.050246431 | FBXL7 | F-box and leucine rich repeat protein 7 [Source:HGNC Symbol;Acc:HGNC:13604] |
| ENSG00000184022 | HGLibA_33855 | 131 | 0.050246431 | OR2T10 | olfactory receptor family 2 subfamily T member 10 [Source:HGNC Symbol;Acc:HGNC:19573] |
| ENSG00000185085 | HGLibA_23423 | 131 | 0.050246431 | INTS5 | integrator complex subunit 5 [Source:HGNC Symbol;Acc:HGNC:29352] |
| ENSG00000185339 | HGLibA_48824 | 131 | 0.050246431 | TCN2 | transcobalamin 2 [Source:HGNC Symbol;Acc:HGNC:11653] |
| ENSG00000185896 | HGLibA_25978 | 131 | 0.050246431 | LAMP1 | lysosomal associated membrane protein 1 [Source:HGNC Symbol;Acc:HGNC:6499] |
| ENSG00000185942 | HGLibA_31995 | 131 | 0.050246431 | NKAIN3 | sodium/potassium transporting ATPase interacting 3 [Source:HGNC Symbol;Acc:HGNC:26829] |
| ENSG00000186038 | HGLibA_22392 | 131 | 0.050246431 | HTR3E | 5-hydroxytryptamine receptor 3E [Source:HGNC Symbol;Acc:HGNC:24005] |
| ENSG00000187555 | HGLibA_53410 | 131 | 0.050246431 | USP7 | ubiquitin specific peptidase 7 [Source:HGNC Symbol;Acc:HGNC:12630] |
| ENSG00000187678 | HGLibA_46846 | 131 | 0.050246431 | SPRY4 | sprouty RTK signaling antagonist 4 [Source:HGNC Symbol;Acc:HGNC:15533] |
| ENSG00000188725 | HGLibA_45733 | 131 | 0.050246431 | SMIM15 | small integral membrane protein 15 [Source:HGNC Symbol;Acc:HGNC:33861] |
| ENSG00000188760 | HGLibA_50163 | 131 | 0.050246431 | TMEM198 | transmembrane protein 198 [Source:HGNC Symbol;Acc:HGNC:33704] |
| ENSG00000189253 | HGLibA_51579 | 131 | 0.050246431 | TRIM64B | tripartite motif containing 64B [Source:HGNC Symbol;Acc:HGNC:37147] |
| ENSG00000196683 | HGLibA_50980 | 131 | 0.050246431 | TOMM7 | translocase of outer mitochondrial membrane 7 [Source:HGNC Symbol;Acc:HGNC:21648] |
| ENSG00000197343 | HGLibA_56469 | 131 | 0.050246431 | ZNF655 | zinc finger protein 655 [Source:HGNC Symbol;Acc:HGNC:30899] |
| ENSG00000197978 | HGLibA_19632 | 131 | 0.050246431 | GOLGA6L9 | golgin A6 family-like 9 [Source:HGNC Symbol;Acc:HGNC:37229] |
| ENSG00000198133 | HGLibA_50253 | 131 | 0.050246431 | TMEM229B | transmembrane protein 229B [Source:HGNC Symbol;Acc:HGNC:20130] |
| ENSG00000198283 | HGLibA_34266 | 131 | 0.050246431 | OR5B21 | olfactory receptor family 5 subfamily B member 21 [Source:HGNC Symbol;Acc:HGNC:19616] |
| ENSG00000198417 | HGLibA_30286 | 131 | 0.050246431 | MT1F | metallothionein 1F [Source:HGNC Symbol;Acc:HGNC:7398] |
| ENSG00000198521 | HGLibA_55974 | 131 | 0.050246431 | ZNF43 | zinc finger protein 43 [Source:HGNC Symbol;Acc:HGNC:13109] |
| ENSG00000204604 | HGLibA_56047 | 131 | 0.050246431 | ZNF468 | zinc finger protein 468 [Source:HGNC Symbol;Acc:HGNC:33105] |
| ENSG00000204713 | HGLibA_51447 | 131 | 0.050246431 | TRIM27 | tripartite motif containing 27 [Source:HGNC Symbol;Acc:HGNC:9975] |
| ENSG00000204873 | HGLibA_25835 | 131 | 0.050246431 | KRTAP9-3 | keratin associated protein 9-3 [Source:HGNC Symbol;Acc:HGNC:16927] |
| ENSG00000205423 | HGLibA_10257 | 131 | 0.050246431 | CNEP1R1 | CTD nuclear envelope phosphatase 1 regulatory subunit 1 [Source:HGNC Symbol;Acc:HGNC:26759] |
| ENSG00000213030 | HGLibA_09315 | 131 | 0.050246431 | CGB8 | chorionic gonadotropin subunit beta 8 [Source:HGNC Symbol;Acc:HGNC:16453] |
| ENSG00000213585 | HGLibA_53598 | 131 | 0.050246431 | VDAC1 | voltage dependent anion channel 1 [Source:HGNC Symbol;Acc:HGNC:12669] |
| ENSG00000213973 | HGLibA_56916 | 131 | 0.050246431 | ZNF99 | zinc finger protein 99 [Source:HGNC Symbol;Acc:HGNC:13175] |
| ENSG00000214415 | HGLibA_19479 | 131 | 0.050246431 | GNAT3 | G protein subunit alpha transducin 3 [Source:HGNC Symbol;Acc:HGNC:22800] |
| ENSG00000228198 | HGLibA_33836 | 131 | 0.050246431 | OR2M3 | olfactory receptor family 2 subfamily M member 3 [Source:HGNC Symbol;Acc:HGNC:8269] |
| ENSG00000249471 | HGLibA_55805 | 131 | 0.050246431 | ZNF324B | zinc finger protein 324B [Source:HGNC Symbol;Acc:HGNC:33107] |
| H1FX | HGLibA_20741 | 131 | 0.050246431 | H1FX | - |
| hsa-mir-1208 | HGLibA_57287 | 131 | 0.050246431 | hsa-mir-1208 | - |
| hsa-mir-138-1 | HGLibA_57814 | 131 | 0.050246431 | hsa-mir-138-1 | - |
| hsa-mir-218-2 | HGLibA_58272 | 131 | 0.050246431 | hsa-mir-218-2 | - |
| hsa-mir-2682 | HGLibA_58360 | 131 | 0.050246431 | hsa-mir-2682 | - |
| hsa-mir-300 | HGLibA_58425 | 131 | 0.050246431 | hsa-mir-300 | - |
| hsa-mir-4509-1 | HGLibA_60463 | 131 | 0.050246431 | hsa-mir-4509-1 | - |
| hsa-mir-4681 | HGLibA_60835 | 131 | 0.050246431 | hsa-mir-4681 | - |
| hsa-mir-4682 | HGLibA_60839 | 131 | 0.050246431 | hsa-mir-4682 | - |
| hsa-mir-4689 | HGLibA_60868 | 131 | 0.050246431 | hsa-mir-4689 | - |
| hsa-mir-513b | HGLibA_61580 | 131 | 0.050246431 | hsa-mir-513b | - |
| hsa-mir-6767 | HGLibA_63231 | 131 | 0.050246431 | hsa-mir-6767 | - |
| hsa-mir-7112-2 | HGLibA_63815 | 131 | 0.050246431 | hsa-mir-7112-2 | - |
| KIAA0355 | HGLibA_24625 | 131 | 0.050246431 | KIAA0355 | - |
| KIAA1324L | HGLibA_24740 | 131 | 0.050246431 | KIAA1324L | - |
| LOC100129083 | HGLibA_26752 | 131 | 0.050246431 | LOC100129083 | - |
| LOC100131094 | HGLibA_26802 | 131 | 0.050246431 | LOC100131094 | - |
| LPPR1 | HGLibA_27114 | 131 | 0.050246431 | LPPR1 | - |
| PPIAL4B | HGLibA_37901 | 131 | 0.050246431 | PPIAL4B | - |
| PRUNE | HGLibA_38987 | 131 | 0.050246431 | PRUNE | - |
| RTDR1 | HGLibA_42376 | 131 | 0.050246431 | RTDR1 | - |
| ADCK3 | HGLibA_00895 | 130 | 0.041201088 | ADCK3 | - |
| C4B_2 | HGLibA_06288 | 130 | 0.041201088 | C4B_2 | - |
| C4orf32 | HGLibA_06320 | 130 | 0.041201088 | C4orf32 | - |
| C8orf42 | HGLibA_06620 | 130 | 0.041201088 | C8orf42 | - |
| CCDC111 | HGLibA_07541 | 130 | 0.041201088 | CCDC111 | - |
| CCDC64 | HGLibA_07825 | 130 | 0.041201088 | CCDC64 | - |
| CXorf61 | HGLibA_11954 | 130 | 0.041201088 | CXorf61 | - |
| ENSG00000004455 | HGLibA_01379 | 130 | 0.041201088 | AK2 | adenylate kinase 2 [Source:HGNC Symbol;Acc:HGNC:362] |
| ENSG00000011132 | HGLibA_02374 | 130 | 0.041201088 | APBA3 | amyloid beta precursor protein binding family A member 3 [Source:HGNC Symbol;Acc:HGNC:580] |
| ENSG00000035681 | HGLibA_32763 | 130 | 0.041201088 | NSMAF | neutral sphingomyelinase activation associated factor [Source:HGNC Symbol;Acc:HGNC:8017] |
| ENSG00000049323 | HGLibA_27590 | 130 | 0.041201088 | LTBP1 | latent transforming growth factor beta binding protein 1 [Source:HGNC Symbol;Acc:HGNC:6714] |
| ENSG00000058063 | HGLibA_03556 | 130 | 0.041201088 | ATP11B | ATPase phospholipid transporting 11B (putative) [Source:HGNC Symbol;Acc:HGNC:13553] |
| ENSG00000065325 | HGLibA_19295 | 130 | 0.041201088 | GLP2R | glucagon like peptide 2 receptor [Source:HGNC Symbol;Acc:HGNC:4325] |
| ENSG00000066294 | HGLibA_08466 | 130 | 0.041201088 | CD84 | CD84 molecule [Source:HGNC Symbol;Acc:HGNC:1704] |
| ENSG00000072201 | HGLibA_26743 | 130 | 0.041201088 | LNX1 | ligand of numb-protein X 1 [Source:HGNC Symbol;Acc:HGNC:6657] |
| ENSG00000074370 | HGLibA_03611 | 130 | 0.041201088 | ATP2A3 | ATPase sarcoplasmic/endoplasmic reticulum Ca2+ transporting 3 [Source:HGNC Symbol;Acc:HGNC:813] |
| ENSG00000078589 | HGLibA_34871 | 130 | 0.041201088 | P2RY10 | P2Y receptor family member 10 [Source:HGNC Symbol;Acc:HGNC:19906] |
| ENSG00000086189 | HGLibA_13236 | 130 | 0.041201088 | DIMT1 | DIM1 dimethyladenosine transferase 1 homolog [Source:HGNC Symbol;Acc:HGNC:30217] |
| ENSG00000087077 | HGLibA_51647 | 130 | 0.041201088 | TRIP6 | thyroid hormone receptor interactor 6 [Source:HGNC Symbol;Acc:HGNC:12311] |
| ENSG00000089486 | HGLibA_08714 | 130 | 0.041201088 | CDIP1 | cell death inducing p53 target 1 [Source:HGNC Symbol;Acc:HGNC:13234] |
| ENSG00000089775 | HGLibA_54849 | 130 | 0.041201088 | ZBTB25 | zinc finger and BTB domain containing 25 [Source:HGNC Symbol;Acc:HGNC:13112] |
| ENSG00000091536 | HGLibA_30805 | 130 | 0.041201088 | MYO15A | myosin XVA [Source:HGNC Symbol;Acc:HGNC:7594] |
| ENSG00000091542 | HGLibA_01657 | 130 | 0.041201088 | ALKBH5 | RNA demethylase [Source:HGNC Symbol;Acc:HGNC:25996]" |
| ENSG00000101435 | HGLibA_11473 | 130 | 0.041201088 | CST9L | cystatin 9 like [Source:HGNC Symbol;Acc:HGNC:16233] |
| ENSG00000101868 | HGLibA_37484 | 130 | 0.041201088 | POLA1 | catalytic subunit [Source:HGNC Symbol;Acc:HGNC:9173]" |
| ENSG00000103187 | HGLibA_10800 | 130 | 0.041201088 | COTL1 | coactosin like F-actin binding protein 1 [Source:HGNC Symbol;Acc:HGNC:18304] |
| ENSG00000104332 | HGLibA_43736 | 130 | 0.041201088 | SFRP1 | secreted frizzled related protein 1 [Source:HGNC Symbol;Acc:HGNC:10776] |
| ENSG00000105671 | HGLibA_12727 | 130 | 0.041201088 | DDX49 | DEAD-box helicase 49 [Source:HGNC Symbol;Acc:HGNC:18684] |
| ENSG00000105997 | HGLibA_21909 | 130 | 0.041201088 | HOXA3 | homeobox A3 [Source:HGNC Symbol;Acc:HGNC:5104] |
| ENSG00000107201 | HGLibA_12752 | 130 | 0.041201088 | DDX58 | DExD/H-box helicase 58 [Source:HGNC Symbol;Acc:HGNC:19102] |
| ENSG00000108849 | HGLibA_38246 | 130 | 0.041201088 | PPY | pancreatic polypeptide [Source:HGNC Symbol;Acc:HGNC:9327] |
| ENSG00000110244 | HGLibA_02458 | 130 | 0.041201088 | APOA4 | apolipoprotein A4 [Source:HGNC Symbol;Acc:HGNC:602] |
| ENSG00000110665 | HGLibA_05122 | 130 | 0.041201088 | C11orf21 | chromosome 11 open reading frame 21 [Source:HGNC Symbol;Acc:HGNC:13231] |
| ENSG00000111875 | HGLibA_03280 | 130 | 0.041201088 | ASF1A | anti-silencing function 1A histone chaperone [Source:HGNC Symbol;Acc:HGNC:20995] |
| ENSG00000112116 | HGLibA_23002 | 130 | 0.041201088 | IL17F | interleukin 17F [Source:HGNC Symbol;Acc:HGNC:16404] |
| ENSG00000112855 | HGLibA_20860 | 130 | 0.041201088 | HARS2 | mitochondrial [Source:HGNC Symbol;Acc:HGNC:4817]" |
| ENSG00000113812 | HGLibA_00675 | 130 | 0.041201088 | ACTR8 | ARP8 actin related protein 8 homolog [Source:HGNC Symbol;Acc:HGNC:14672] |
| ENSG00000114631 | HGLibA_37462 | 130 | 0.041201088 | PODXL2 | podocalyxin like 2 [Source:HGNC Symbol;Acc:HGNC:17936] |
| ENSG00000115590 | HGLibA_23049 | 130 | 0.041201088 | IL1R2 | interleukin 1 receptor type 2 [Source:HGNC Symbol;Acc:HGNC:5994] |
| ENSG00000117281 | HGLibA_08241 | 130 | 0.041201088 | CD160 | CD160 molecule [Source:HGNC Symbol;Acc:HGNC:17013] |
| ENSG00000117481 | HGLibA_32786 | 130 | 0.041201088 | NSUN4 | NOP2/Sun RNA methyltransferase family member 4 [Source:HGNC Symbol;Acc:HGNC:31802] |
| ENSG00000122705 | HGLibA_10142 | 130 | 0.041201088 | CLTA | clathrin light chain A [Source:HGNC Symbol;Acc:HGNC:2090] |
| ENSG00000124313 | HGLibA_23543 | 130 | 0.041201088 | IQSEC2 | IQ motif and Sec7 domain 2 [Source:HGNC Symbol;Acc:HGNC:29059] |
| ENSG00000124490 | HGLibA_11168 | 130 | 0.041201088 | CRISP2 | cysteine rich secretory protein 2 [Source:HGNC Symbol;Acc:HGNC:12024] |
| ENSG00000126337 | HGLibA_25492 | 130 | 0.041201088 | KRT36 | keratin 36 [Source:HGNC Symbol;Acc:HGNC:6454] |
| ENSG00000127022 | HGLibA_07130 | 130 | 0.041201088 | CANX | calnexin [Source:HGNC Symbol;Acc:HGNC:1473] |
| ENSG00000127528 | HGLibA_25067 | 130 | 0.041201088 | KLF2 | Kruppel like factor 2 [Source:HGNC Symbol;Acc:HGNC:6347] |
| ENSG00000127870 | HGLibA_41695 | 130 | 0.041201088 | RNF6 | ring finger protein 6 [Source:HGNC Symbol;Acc:HGNC:10069] |
| ENSG00000128714 | HGLibA_21996 | 130 | 0.041201088 | HOXD13 | homeobox D13 [Source:HGNC Symbol;Acc:HGNC:5136] |
| ENSG00000129515 | HGLibA_46103 | 130 | 0.041201088 | SNX6 | sorting nexin 6 [Source:HGNC Symbol;Acc:HGNC:14970] |
| ENSG00000130234 | HGLibA_00426 | 130 | 0.041201088 | ACE2 | angiotensin I converting enzyme 2 [Source:HGNC Symbol;Acc:HGNC:13557] |
| ENSG00000130427 | HGLibA_15350 | 130 | 0.041201088 | EPO | erythropoietin [Source:HGNC Symbol;Acc:HGNC:3415] |
| ENSG00000131943 | HGLibA_05631 | 130 | 0.041201088 | C19orf12 | chromosome 19 open reading frame 12 [Source:HGNC Symbol;Acc:HGNC:25443] |
| ENSG00000132031 | HGLibA_28432 | 130 | 0.041201088 | MATN3 | matrilin 3 [Source:HGNC Symbol;Acc:HGNC:6909] |
| ENSG00000134291 | HGLibA_49860 | 130 | 0.041201088 | TMEM106C | transmembrane protein 106C [Source:HGNC Symbol;Acc:HGNC:28775] |
| ENSG00000134686 | HGLibA_36321 | 130 | 0.041201088 | PHC2 | polyhomeotic homolog 2 [Source:HGNC Symbol;Acc:HGNC:3183] |
| ENSG00000135018 | HGLibA_52732 | 130 | 0.041201088 | UBQLN1 | ubiquilin 1 [Source:HGNC Symbol;Acc:HGNC:12508] |
| ENSG00000135338 | HGLibA_26091 | 130 | 0.041201088 | LCA5 | lebercilin [Source:HGNC Symbol;Acc:HGNC:31923]" |
| ENSG00000135625 | HGLibA_14657 | 130 | 0.041201088 | EGR4 | early growth response 4 [Source:HGNC Symbol;Acc:HGNC:3241] |
| ENSG00000135776 | HGLibA_00134 | 130 | 0.041201088 | ABCB10 | ATP binding cassette subfamily B member 10 [Source:HGNC Symbol;Acc:HGNC:41] |
| ENSG00000135914 | HGLibA_22374 | 130 | 0.041201088 | HTR2B | 5-hydroxytryptamine receptor 2B [Source:HGNC Symbol;Acc:HGNC:5294] |
| ENSG00000137441 | HGLibA_17428 | 130 | 0.041201088 | FGFBP2 | fibroblast growth factor binding protein 2 [Source:HGNC Symbol;Acc:HGNC:29451] |
| ENSG00000137574 | HGLibA_49278 | 130 | 0.041201088 | TGS1 | trimethylguanosine synthase 1 [Source:HGNC Symbol;Acc:HGNC:17843] |
| ENSG00000138028 | HGLibA_09327 | 130 | 0.041201088 | CGREF1 | cell growth regulator with EF-hand domain 1 [Source:HGNC Symbol;Acc:HGNC:16962] |
| ENSG00000138100 | HGLibA_51544 | 130 | 0.041201088 | TRIM54 | tripartite motif containing 54 [Source:HGNC Symbol;Acc:HGNC:16008] |
| ENSG00000138193 | HGLibA_36998 | 130 | 0.041201088 | PLCE1 | phospholipase C epsilon 1 [Source:HGNC Symbol;Acc:HGNC:17175] |
| ENSG00000138399 | HGLibA_16900 | 130 | 0.041201088 | FASTKD1 | FAST kinase domains 1 [Source:HGNC Symbol;Acc:HGNC:26150] |
| ENSG00000140525 | HGLibA_16855 | 130 | 0.041201088 | FANCI | FA complementation group I [Source:HGNC Symbol;Acc:HGNC:25568] |
| ENSG00000140600 | HGLibA_43952 | 130 | 0.041201088 | SH3GL3 | endophilin A3 [Source:HGNC Symbol;Acc:HGNC:10832]" |
| ENSG00000140986 | HGLibA_41979 | 130 | 0.041201088 | RPL3L | ribosomal protein L3 like [Source:HGNC Symbol;Acc:HGNC:10351] |
| ENSG00000141219 | HGLibA_05581 | 130 | 0.041201088 | C17orf80 | chromosome 17 open reading frame 80 [Source:HGNC Symbol;Acc:HGNC:29601] |
| ENSG00000141582 | HGLibA_07454 | 130 | 0.041201088 | CBX4 | chromobox 4 [Source:HGNC Symbol;Acc:HGNC:1554] |
| ENSG00000142192 | HGLibA_02561 | 130 | 0.041201088 | APP | amyloid beta precursor protein [Source:HGNC Symbol;Acc:HGNC:620] |
| ENSG00000143222 | HGLibA_52842 | 130 | 0.041201088 | UFC1 | ubiquitin-fold modifier conjugating enzyme 1 [Source:HGNC Symbol;Acc:HGNC:26941] |
| ENSG00000144451 | HGLibA_46351 | 130 | 0.041201088 | SPAG16 | sperm associated antigen 16 [Source:HGNC Symbol;Acc:HGNC:23225] |
| ENSG00000145002 | HGLibA_16775 | 130 | 0.041201088 | FAM86B2 | family with sequence similarity 86 member B2 [Source:HGNC Symbol;Acc:HGNC:32222] |
| ENSG00000145649 | HGLibA_20718 | 130 | 0.041201088 | GZMA | granzyme A [Source:HGNC Symbol;Acc:HGNC:4708] |
| ENSG00000145649 | HGLibA_20717 | 130 | 0.041201088 | GZMA | granzyme A [Source:HGNC Symbol;Acc:HGNC:4708] |

| | | | | | |
|---|---|---|---|---|---|
| ENSG00000146352 | HGLibA_10170 | 130 | 0.041201088 | CLVS2 | clavesin 2 [Source:HGNC Symbol;Acc:HGNC:23046] |
| ENSG00000149346 | HGLibA_45565 | 130 | 0.041201088 | SLX4IP | SLX4 interacting protein [Source:HGNC Symbol;Acc:HGNC:16225] |
| ENSG00000149923 | HGLibA_38199 | 130 | 0.041201088 | PPP4C | protein phosphatase 4 catalytic subunit [Source:HGNC Symbol;Acc:HGNC:9319] |
| ENSG00000155111 | HGLibA_08751 | 130 | 0.041201088 | CDK19 | cyclin dependent kinase 19 [Source:HGNC Symbol;Acc:HGNC:19338] |
| ENSG00000155792 | HGLibA_12990 | 130 | 0.041201088 | DEPTOR | DEP domain containing MTOR interacting protein [Source:HGNC Symbol;Acc:HGNC:22953] |
| ENSG00000156535 | HGLibA_08230 | 130 | 0.041201088 | CD109 | CD109 molecule [Source:HGNC Symbol;Acc:HGNC:21685] |
| ENSG00000157554 | HGLibA_15463 | 130 | 0.041201088 | ERG | ETS transcription factor ERG [Source:HGNC Symbol;Acc:HGNC:3446] |
| ENSG00000158008 | HGLibA_15809 | 130 | 0.041201088 | EXTL1 | exostosin like glycosyltransferase 1 [Source:HGNC Symbol;Acc:HGNC:3515] |
| ENSG00000158887 | HGLibA_29792 | 130 | 0.041201088 | MPZ | myelin protein zero [Source:HGNC Symbol;Acc:HGNC:7225] |
| ENSG00000159023 | HGLibA_15240 | 130 | 0.041201088 | EPB41 | erythrocyte membrane protein band 4.1 [Source:HGNC Symbol;Acc:HGNC:3377] |
| ENSG00000161202 | HGLibA_14215 | 130 | 0.041201088 | DVL3 | dishevelled segment polarity protein 3 [Source:HGNC Symbol;Acc:HGNC:3087] |
| ENSG00000161265 | HGLibA_52495 | 130 | 0.041201088 | U2AF1L4 | U2 small nuclear RNA auxiliary factor 1 like 4 [Source:HGNC Symbol;Acc:HGNC:23020] |
| ENSG00000162222 | HGLibA_52164 | 130 | 0.041201088 | TTC9C | tetratricopeptide repeat domain 9C [Source:HGNC Symbol;Acc:HGNC:28432] |
| ENSG00000162889 | HGLibA_28300 | 130 | 0.041201088 | MAPKAPK2 | mitogen-activated protein kinase-activated protein kinase 2 [Source:HGNC Symbol;Acc:HGNC:6887] |
| ENSG00000162931 | HGLibA_51423 | 130 | 0.041201088 | TRIM17 | tripartite motif containing 17 [Source:HGNC Symbol;Acc:HGNC:13430] |
| ENSG00000163126 | HGLibA_02041 | 130 | 0.041201088 | ANKRD23 | ankyrin repeat domain 23 [Source:HGNC Symbol;Acc:HGNC:24470] |
| ENSG00000163352 | HGLibA_26293 | 130 | 0.041201088 | LENEP | lens epithelial protein [Source:HGNC Symbol;Acc:HGNC:14429] |
| ENSG00000163430 | HGLibA_18100 | 130 | 0.041201088 | FSTL1 | follistatin like 1 [Source:HGNC Symbol;Acc:HGNC:3972] |
| ENSG00000164304 | HGLibA_06982 | 130 | 0.041201088 | CAGE1 | cancer antigen 1 [Source:HGNC Symbol;Acc:HGNC:21622] |
| ENSG00000164574 | HGLibA_18529 | 130 | 0.041201088 | GALNT10 | polypeptide N-acetylgalactosaminyltransferase 10 [Source:HGNC Symbol;Acc:HGNC:19873] |
| ENSG00000165092 | HGLibA_01542 | 130 | 0.041201088 | ALDH1A1 | aldehyde dehydrogenase 1 family member A1 [Source:HGNC Symbol;Acc:HGNC:402] |
| ENSG00000165204 | HGLibA_33684 | 130 | 0.041201088 | OR1K1 | olfactory receptor family 1 subfamily K member 1 [Source:HGNC Symbol;Acc:HGNC:8212] |
| ENSG00000168924 | HGLibA_26333 | 130 | 0.041201088 | LETM1 | leucine zipper and EF-hand containing transmembrane protein 1 [Source:HGNC Symbol;Acc:HGNC:6556] |
| ENSG00000168995 | HGLibA_44146 | 130 | 0.041201088 | SIGLEC7 | sialic acid binding Ig like lectin 7 [Source:HGNC Symbol;Acc:HGNC:10876] |
| ENSG00000171864 | HGLibA_38640 | 130 | 0.041201088 | PRND | prion like protein doppel [Source:HGNC Symbol;Acc:HGNC:15748] |
| ENSG00000171903 | HGLibA_12180 | 130 | 0.041201088 | CYP4F11 | cytochrome P450 family 4 subfamily F member 11 [Source:HGNC Symbol;Acc:HGNC:13265] |
| ENSG00000173542 | HGLibA_29588 | 130 | 0.041201088 | MOB1B | MOB kinase activator 1B [Source:HGNC Symbol;Acc:HGNC:29801] |
| ENSG00000174914 | HGLibA_34568 | 130 | 0.041201088 | OR9G1 | olfactory receptor family 9 subfamily G member 1 [Source:HGNC Symbol;Acc:HGNC:15319] |
| ENSG00000174944 | HGLibA_34881 | 130 | 0.041201088 | P2RY14 | purinergic receptor P2Y14 [Source:HGNC Symbol;Acc:HGNC:16442] |
| ENSG00000176597 | HGLibA_03992 | 130 | 0.041201088 | B3GNT5 | 3-N-acetylglucosaminyltransferase 5 [Source:HGNC Symbol;Acc:HGNC:15684]" |
| ENSG00000178252 | HGLibA_54135 | 130 | 0.041201088 | WDR6 | WD repeat domain 6 [Source:HGNC Symbol;Acc:HGNC:12758] |
| ENSG00000178257 | HGLibA_38615 | 130 | 0.041201088 | PRM3 | protamine 3 [Source:HGNC Symbol;Acc:HGNC:13732] |
| ENSG00000179284 | HGLibA_12307 | 130 | 0.041201088 | DAND5 | DAN domain BMP antagonist family member 5 [Source:HGNC Symbol;Acc:HGNC:26780] |
| ENSG00000179954 | HGLibA_47125 | 130 | 0.041201088 | SSC5D | scavenger receptor cysteine rich family member with 5 domains [Source:HGNC Symbol;Acc:HGNC:26641] |
| ENSG00000182117 | HGLibA_32301 | 130 | 0.041201088 | NOP10 | NOP10 ribonucleoprotein [Source:HGNC Symbol;Acc:HGNC:14378] |
| ENSG00000182473 | HGLibA_15761 | 130 | 0.041201088 | EXOC7 | exocyst complex component 7 [Source:HGNC Symbol;Acc:HGNC:23214] |
| ENSG00000183309 | HGLibA_56412 | 130 | 0.041201088 | ZNF623 | zinc finger protein 623 [Source:HGNC Symbol;Acc:HGNC:29084] |
| ENSG00000184058 | HGLibA_48642 | 130 | 0.041201088 | TBX1 | T-box 1 [Source:HGNC Symbol;Acc:HGNC:11592] |
| ENSG00000184828 | HGLibA_54923 | 130 | 0.041201088 | ZBTB7C | zinc finger and BTB domain containing 7C [Source:HGNC Symbol;Acc:HGNC:31700] |
| ENSG00000184905 | HGLibA_48707 | 130 | 0.041201088 | TCEAL2 | transcription elongation factor A like 2 [Source:HGNC Symbol;Acc:HGNC:29818] |
| ENSG00000185262 | HGLibA_52542 | 130 | 0.041201088 | UBALD2 | UBA like domain containing 2 [Source:HGNC Symbol;Acc:HGNC:28438] |
| ENSG00000185518 | HGLibA_47877 | 130 | 0.041201088 | SV2B | synaptic vesicle glycoprotein 2B [Source:HGNC Symbol;Acc:HGNC:16874] |
| ENSG00000186283 | HGLibA_51030 | 130 | 0.041201088 | TOR3A | torsin family 3 member A [Source:HGNC Symbol;Acc:HGNC:11997] |
| ENSG00000186329 | HGLibA_50209 | 130 | 0.041201088 | TMEM212 | transmembrane protein 212 [Source:HGNC Symbol;Acc:HGNC:34295] |
| ENSG00000187097 | HGLibA_15208 | 130 | 0.041201088 | ENTPD5 | ectonucleoside triphosphate diphosphohydrolase 5 [Source:HGNC Symbol;Acc:HGNC:3367] |
| ENSG00000196242 | HGLibA_33780 | 130 | 0.041201088 | OR2C3 | olfactory receptor family 2 subfamily C member 3 [Source:HGNC Symbol;Acc:HGNC:15005] |
| ENSG00000196376 | HGLibA_45000 | 130 | 0.041201088 | SLC35F1 | solute carrier family 35 member F1 [Source:HGNC Symbol;Acc:HGNC:21483] |
| ENSG00000197472 | HGLibA_56556 | 130 | 0.041201088 | ZNF695 | zinc finger protein 695 [Source:HGNC Symbol;Acc:HGNC:30954] |
| ENSG00000197579 | HGLibA_51012 | 130 | 0.041201088 | TOPORS | TOP1 binding arginine/serine rich protein [Source:HGNC Symbol;Acc:HGNC:21653] |
| ENSG00000197980 | HGLibA_26279 | 130 | 0.041201088 | LEKR1 | glutamate and lysine rich 1 [Source:HGNC Symbol;Acc:HGNC:33765]" |
| ENSG00000198169 | HGLibA_55670 | 130 | 0.041201088 | ZNF251 | zinc finger protein 251 [Source:HGNC Symbol;Acc:HGNC:13045] |
| ENSG00000198198 | HGLibA_48118 | 130 | 0.041201088 | SZT2 | KICSTOR complex subunit [Source:HGNC Symbol;Acc:HGNC:29040]" |
| ENSG00000198551 | HGLibA_56424 | 130 | 0.041201088 | ZNF627 | zinc finger protein 627 [Source:HGNC Symbol;Acc:HGNC:30570] |
| ENSG00000204323 | HGLibA_45762 | 130 | 0.041201088 | SMIM5 | small integral membrane protein 5 [Source:HGNC Symbol;Acc:HGNC:40030] |
| ENSG00000205221 | HGLibA_53675 | 130 | 0.041201088 | VIT | vitrin [Source:HGNC Symbol;Acc:HGNC:12697] |
| ENSG00000205502 | HGLibA_06098 | 130 | 0.041201088 | C2CD4B | C2 calcium dependent domain containing 4B [Source:HGNC Symbol;Acc:HGNC:33628] |
| ENSG00000212127 | HGLibA_48373 | 130 | 0.041201088 | TAS2R14 | taste 2 receptor member 14 [Source:HGNC Symbol;Acc:HGNC:14920] |
| ENSG00000212901 | HGLibA_25741 | 130 | 0.041201088 | KRTAP3-1 | keratin associated protein 3-1 [Source:HGNC Symbol;Acc:HGNC:16778] |
| ENSG00000213741 | HGLibA_42134 | 130 | 0.041201088 | RPS29 | ribosomal protein S29 [Source:HGNC Symbol;Acc:HGNC:10419] |
| ENSG00000242220 | HGLibA_48836 | 130 | 0.041201088 | TCP10L | t-complex 10 like [Source:HGNC Symbol;Acc:HGNC:11657] |
| ENSG00000244617 | HGLibA_03363 | 130 | 0.041201088 | ASPRV1 | aspartic peptidase retroviral like 1 [Source:HGNC Symbol;Acc:HGNC:26321] |
| ENSG00000253797 | HGLibA_53438 | 130 | 0.041201088 | UTP14C | small subunit processome component [Source:HGNC Symbol;Acc:HGNC:20321]" |
| ENSG00000255221 | HGLibA_07229 | 130 | 0.041201088 | CARD17 | caspase recruitment domain family member 17 [Source:HGNC Symbol;Acc:HGNC:33827] |
| ENSG00000255346 | HGLibA_32378 | 130 | 0.041201088 | NOX5 | NADPH oxidase 5 [Source:HGNC Symbol;Acc:HGNC:14874] |
| ENSG00000258992 | HGLibA_51964 | 130 | 0.041201088 | TSPY1 | testis specific protein Y-linked 1 [Source:HGNC Symbol;Acc:HGNC:12381] |
| ENSG00000260001 | HGLibA_49231 | 130 | 0.041201088 | TGFBR3L | transforming growth factor beta receptor 3 like [Source:HGNC Symbol;Acc:HGNC:44152] |
| ENSG00000268221 | HGLibA_33425 | 130 | 0.041201088 | OPN1MW | medium wave sensitive [Source:HGNC Symbol;Acc:HGNC:4206]" |
| FAM208B | HGLibA_16436 | 130 | 0.041201088 | FAM208B | - |
| HDGFRP2 | HGLibA_21046 | 130 | 0.041201088 | HDGFRP2 | - |
| hsa-let-7a-2 | HGLibA_57101 | 130 | 0.041201088 | hsa-let-7a-2 | - |
| hsa-mir-1184-1 | HGLibA_57228 | 130 | 0.041201088 | hsa-mir-1184-1 | - |
| hsa-mir-1237 | HGLibA_57339 | 130 | 0.041201088 | hsa-mir-1237 | - |
| hsa-mir-301a | HGLibA_58430 | 130 | 0.041201088 | hsa-mir-301a | - |
| hsa-mir-340 | HGLibA_59021 | 130 | 0.041201088 | hsa-mir-340 | - |
| hsa-mir-3677 | HGLibA_59257 | 130 | 0.041201088 | hsa-mir-3677 | - |
| hsa-mir-3908 | HGLibA_59498 | 130 | 0.041201088 | hsa-mir-3908 | - |
| hsa-mir-4436b-2 | HGLibA_60146 | 130 | 0.041201088 | hsa-mir-4436b-2 | - |
| hsa-mir-4456 | HGLibA_60232 | 130 | 0.041201088 | hsa-mir-4456 | - |
| hsa-mir-4488 | HGLibA_60372 | 130 | 0.041201088 | hsa-mir-4488 | - |
| hsa-mir-4648 | HGLibA_60696 | 130 | 0.041201088 | hsa-mir-4648 | - |
| hsa-mir-4700 | HGLibA_60909 | 130 | 0.041201088 | hsa-mir-4700 | - |
| hsa-mir-4736 | HGLibA_61041 | 130 | 0.041201088 | hsa-mir-4736 | - |
| hsa-mir-488 | HGLibA_61354 | 130 | 0.041201088 | hsa-mir-488 | - |
| hsa-mir-514a-1 | HGLibA_61585 | 130 | 0.041201088 | hsa-mir-514a-1 | - |
| hsa-mir-649 | HGLibA_62839 | 130 | 0.041201088 | hsa-mir-649 | - |
| hsa-mir-6748 | HGLibA_63146 | 130 | 0.041201088 | hsa-mir-6748 | - |
| hsa-mir-6834 | HGLibA_63513 | 130 | 0.041201088 | hsa-mir-6834 | - |
| hsa-mir-7106 | HGLibA_63786 | 130 | 0.041201088 | hsa-mir-7106 | - |
| hsa-mir-7-3 | HGLibA_63776 | 130 | 0.041201088 | hsa-mir-7-3 | - |
| hsa-mir-943 | HGLibA_64358 | 130 | 0.041201088 | hsa-mir-943 | - |
| PNMA6C | HGLibA_37382 | 130 | 0.041201088 | PNMA6C | - |
| TCEB3CL2 | HGLibA_48751 | 130 | 0.041201088 | TCEB3CL2 | - |
| ZNF259 | HGLibA_55685 | 130 | 0.041201088 | ZNF259 | - |
| 10-Mar | HGLibA_28328 | 129 | 0.032155745 | 10-Mar | - |
| ATP5L | HGLibA_03680 | 129 | 0.032155745 | ATP5L | - |
| C20orf197 | HGLibA_06009 | 129 | 0.032155745 | C20orf197 | - |
| ENSG00000008086 | HGLibA_08819 | 129 | 0.032155745 | CDKL5 | cyclin dependent kinase like 5 [Source:HGNC Symbol;Acc:HGNC:11411] |
| ENSG00000008323 | HGLibA_37120 | 129 | 0.032155745 | PLEKHG6 | pleckstrin homology and RhoGEF domain containing G6 [Source:HGNC Symbol;Acc:HGNC:25562] |
| ENSG00000071909 | HGLibA_30846 | 129 | 0.032155745 | MYO3B | myosin IIIB [Source:HGNC Symbol;Acc:HGNC:15576] |
| ENSG00000071967 | HGLibA_12021 | 129 | 0.032155745 | CYBRD1 | cytochrome b reductase 1 [Source:HGNC Symbol;Acc:HGNC:20797] |
| ENSG00000072840 | HGLibA_15679 | 129 | 0.032155745 | EVC | EvC ciliary complex subunit 1 [Source:HGNC Symbol;Acc:HGNC:3497] |
| ENSG00000084207 | HGLibA_20492 | 129 | 0.032155745 | GSTP1 | glutathione S-transferase pi 1 [Source:HGNC Symbol;Acc:HGNC:4638] |
| ENSG00000088812 | HGLibA_03819 | 129 | 0.032155745 | ATRN | attractin [Source:HGNC Symbol;Acc:HGNC:885] |
| ENSG00000088836 | HGLibA_45207 | 129 | 0.032155745 | SLC4A11 | solute carrier family 4 member 11 [Source:HGNC Symbol;Acc:HGNC:16438] |
| ENSG00000088930 | HGLibA_54607 | 129 | 0.032155745 | XRN2 | 5'-3' exoribonuclease 2 [Source:HGNC Symbol;Acc:HGNC:12836] |
| ENSG00000089639 | HGLibA_19410 | 129 | 0.032155745 | GMIP | GEM interacting protein [Source:HGNC Symbol;Acc:HGNC:24852] |
| ENSG00000094631 | HGLibA_21017 | 129 | 0.032155745 | HDAC6 | histone deacetylase 6 [Source:HGNC Symbol;Acc:HGNC:14064] |
| ENSG00000095596 | HGLibA_12085 | 129 | 0.032155745 | CYP26A1 | cytochrome P450 family 26 subfamily A member 1 [Source:HGNC Symbol;Acc:HGNC:2603] |
| ENSG00000099364 | HGLibA_16993 | 129 | 0.032155745 | FBXL19 | F-box and leucine rich repeat protein 19 [Source:HGNC Symbol;Acc:HGNC:25300] |
| ENSG00000099365 | HGLibA_47639 | 129 | 0.032155745 | STX1B | syntaxin 1B [Source:HGNC Symbol;Acc:HGNC:18539] |
| ENSG00000099889 | HGLibA_03175 | 129 | 0.032155745 | ARVCF | delta catenin family member [Source:HGNC Symbol;Acc:HGNC:728]" |
| ENSG00000100354 | HGLibA_50915 | 129 | 0.032155745 | TNRC6B | trinucleotide repeat containing 6B [Source:HGNC Symbol;Acc:HGNC:29190] |
| ENSG00000101220 | HGLibA_06023 | 129 | 0.032155745 | C20orf27 | chromosome 20 open reading frame 27 [Source:HGNC Symbol;Acc:HGNC:15873] |
| ENSG00000101265 | HGLibA_40378 | 129 | 0.032155745 | RASSF2 | Ras association domain family member 2 [Source:HGNC Symbol;Acc:HGNC:9883] |
| ENSG00000103064 | HGLibA_45374 | 129 | 0.032155745 | SLC7A6 | solute carrier family 7 member 6 [Source:HGNC Symbol;Acc:HGNC:11064] |
| ENSG00000104866 | HGLibA_38096 | 129 | 0.032155745 | PPP1R37 | protein phosphatase 1 regulatory subunit 37 [Source:HGNC Symbol;Acc:HGNC:27607] |
| ENSG00000105641 | HGLibA_45273 | 129 | 0.032155745 | SLC5A5 | solute carrier family 5 member 5 [Source:HGNC Symbol;Acc:HGNC:11040] |
| ENSG00000105699 | HGLibA_27565 | 129 | 0.032155745 | LSR | lipolysis stimulated lipoprotein receptor [Source:HGNC Symbol;Acc:HGNC:29572] |
| ENSG00000106080 | HGLibA_17557 | 129 | 0.032155745 | FKBP14 | FK506 binding protein 14 [Source:HGNC Symbol;Acc:HGNC:18625] |
| ENSG00000106178 | HGLibA_08004 | 129 | 0.032155745 | CCL24 | C-C motif chemokine ligand 24 [Source:HGNC Symbol;Acc:HGNC:10623] |
| ENSG00000106648 | HGLibA_18577 | 129 | 0.032155745 | GALNTL5 | polypeptide N-acetylgalactosaminyltransferase like 5 [Source:HGNC Symbol;Acc:HGNC:21725] |
| ENSG00000108961 | HGLibA_40218 | 129 | 0.032155745 | RANGRF | RAN guanine nucleotide release factor [Source:HGNC Symbol;Acc:HGNC:17679] |
| ENSG00000110031 | HGLibA_27129 | 129 | 0.032155745 | LPXN | leupaxin [Source:HGNC Symbol;Acc:HGNC:14061] |
| ENSG00000111012 | HGLibA_12098 | 129 | 0.032155745 | CYP27B1 | cytochrome P450 family 27 subfamily B member 1 [Source:HGNC Symbol;Acc:HGNC:2606] |
| ENSG00000111371 | HGLibA_45054 | 129 | 0.032155745 | SLC38A1 | solute carrier family 38 member 1 [Source:HGNC Symbol;Acc:HGNC:13447] |
| ENSG00000111371 | HGLibA_45055 | 129 | 0.032155745 | SLC38A1 | solute carrier family 38 member 1 [Source:HGNC Symbol;Acc:HGNC:13447] |
| ENSG00000113140 | HGLibA_46418 | 129 | 0.032155745 | SPARC | secreted protein acidic and cysteine rich [Source:HGNC Symbol;Acc:HGNC:11219] |
| ENSG00000113460 | HGLibA_04824 | 129 | 0.032155745 | BRIX1 | biogenesis of ribosomes [Source:HGNC Symbol;Acc:HGNC:24170]" |
| ENSG00000114019 | HGLibA_01821 | 129 | 0.032155745 | AMOTL2 | angiomotin like 2 [Source:HGNC Symbol;Acc:HGNC:17812] |
| ENSG00000114450 | HGLibA_19499 | 129 | 0.032155745 | GNB4 | G protein subunit beta 4 [Source:HGNC Symbol;Acc:HGNC:20731] |
| ENSG00000115353 | HGLibA_48172 | 129 | 0.032155745 | TACR1 | tachykinin receptor 1 [Source:HGNC Symbol;Acc:HGNC:11526] |
| ENSG00000115602 | HGLibA_23061 | 129 | 0.032155745 | IL1RL1 | interleukin 1 receptor like 1 [Source:HGNC Symbol;Acc:HGNC:5998] |
| ENSG00000117151 | HGLibA_11589 | 129 | 0.032155745 | CTBS | chitobiase [Source:HGNC Symbol;Acc:HGNC:2496] |
| ENSG00000121316 | HGLibA_36971 | 129 | 0.032155745 | PLBD1 | phospholipase B domain containing 1 [Source:HGNC Symbol;Acc:HGNC:26215] |
| ENSG00000121542 | HGLibA_43214 | 129 | 0.032155745 | SEC22A | vesicle trafficking protein [Source:HGNC Symbol;Acc:HGNC:20260]" |

| | | | | | |
|---|---|---|---|---|---|
| ENSG00000121900 | HGLibA_50440 | 129 | 0.032155745 | TMEM54 | transmembrane protein 54 [Source:HGNC Symbol;Acc:HGNC:24143] |
| ENSG00000122126 | HGLibA_33263 | 129 | 0.032155745 | OCRL | inositol polyphosphate-5-phosphatase [Source:HGNC Symbol;Acc:HGNC:8108]" |
| ENSG00000123453 | HGLibA_42744 | 129 | 0.032155745 | SARDH | sarcosine dehydrogenase [Source:HGNC Symbol;Acc:HGNC:10536] |
| ENSG00000125787 | HGLibA_19583 | 129 | 0.032155745 | GNRH2 | gonadotropin releasing hormone 2 [Source:HGNC Symbol;Acc:HGNC:4420] |
| ENSG00000125812 | HGLibA_20715 | 129 | 0.032155745 | GZF1 | GDNF inducible zinc finger protein 1 [Source:HGNC Symbol;Acc:HGNC:15808] |
| ENSG00000126233 | HGLibA_45553 | 129 | 0.032155745 | SLURP1 | secreted LY6/PLAUR domain containing 1 [Source:HGNC Symbol;Acc:HGNC:18746] |
| ENSG00000127362 | HGLibA_48385 | 129 | 0.032155745 | TAS2R3 | taste 2 receptor member 3 [Source:HGNC Symbol;Acc:HGNC:14910] |
| ENSG00000129083 | HGLibA_10694 | 129 | 0.032155745 | COPB1 | coatomer protein complex subunit beta 1 [Source:HGNC Symbol;Acc:HGNC:2231] |
| ENSG00000130589 | HGLibA_21125 | 129 | 0.032155745 | HELZ2 | helicase with zinc finger 2 [Source:HGNC Symbol;Acc:HGNC:30021] |
| ENSG00000131013 | HGLibA_37949 | 129 | 0.032155745 | PPIL4 | peptidylprolyl isomerase like 4 [Source:HGNC Symbol;Acc:HGNC:15702] |
| ENSG00000131069 | HGLibA_00589 | 129 | 0.032155745 | ACSS2 | acyl-CoA synthetase short chain family member 2 [Source:HGNC Symbol;Acc:HGNC:15814] |
| ENSG00000131686 | HGLibA_06817 | 129 | 0.032155745 | CA6 | carbonic anhydrase 6 [Source:HGNC Symbol;Acc:HGNC:1380] |
| ENSG00000132975 | HGLibA_19871 | 129 | 0.032155745 | GPR12 | G protein-coupled receptor 12 [Source:HGNC Symbol;Acc:HGNC:4466] |
| ENSG00000133110 | HGLibA_37712 | 129 | 0.032155745 | POSTN | periostin [Source:HGNC Symbol;Acc:HGNC:16953] |
| ENSG00000133597 | HGLibA_00894 | 129 | 0.032155745 | ADCK2 | aarF domain containing kinase 2 [Source:HGNC Symbol;Acc:HGNC:19039] |
| ENSG00000137872 | HGLibA_43373 | 129 | 0.032155745 | SEMA6D | semaphorin 6D [Source:HGNC Symbol;Acc:HGNC:16770] |
| ENSG00000139344 | HGLibA_01761 | 129 | 0.032155745 | AMDHD1 | amidohydrolase domain containing 1 [Source:HGNC Symbol;Acc:HGNC:28577] |
| ENSG00000140332 | HGLibA_49613 | 129 | 0.032155745 | TLE3 | transducin like enhancer of split 3 [Source:HGNC Symbol;Acc:HGNC:11839] |
| ENSG00000142784 | HGLibA_54233 | 129 | 0.032155745 | WDTC1 | WD and tetratricopeptide repeats 1 [Source:HGNC Symbol;Acc:HGNC:29175] |
| ENSG00000143889 | HGLibA_21836 | 129 | 0.032155745 | HNRNPLL | heterogeneous nuclear ribonucleoprotein L like [Source:HGNC Symbol;Acc:HGNC:25127] |
| ENSG00000143951 | HGLibA_54018 | 129 | 0.032155745 | WDPCP | WD repeat containing planar cell polarity effector [Source:HGNC Symbol;Acc:HGNC:28027] |
| ENSG00000144010 | HGLibA_51505 | 129 | 0.032155745 | TRIM43B | tripartite motif containing 43B [Source:HGNC Symbol;Acc:HGNC:37146] |
| ENSG00000144040 | HGLibA_43794 | 129 | 0.032155745 | SFXN5 | sideroflexin 5 [Source:HGNC Symbol;Acc:HGNC:16073] |
| ENSG00000144579 | HGLibA_11608 | 129 | 0.032155745 | CTDSP1 | CTD small phosphatase 1 [Source:HGNC Symbol;Acc:HGNC:21614] |
| ENSG00000148450 | HGLibA_30243 | 129 | 0.032155745 | MSRB2 | methionine sulfoxide reductase B2 [Source:HGNC Symbol;Acc:HGNC:17061] |
| ENSG00000149571 | HGLibA_25006 | 129 | 0.032155745 | KIRREL3 | kirre like nephrin family adhesion molecule 3 [Source:HGNC Symbol;Acc:HGNC:23204] |
| ENSG00000150907 | HGLibA_17921 | 129 | 0.032155745 | FOXO1 | forkhead box O1 [Source:HGNC Symbol;Acc:HGNC:3819] |
| ENSG00000151287 | HGLibA_49094 | 129 | 0.032155745 | TEX30 | testis expressed 30 [Source:HGNC Symbol;Acc:HGNC:25188] |
| ENSG00000154920 | HGLibA_15035 | 129 | 0.032155745 | EME1 | essential meiotic structure-specific endonuclease 1 [Source:HGNC Symbol;Acc:HGNC:24965] |
| ENSG00000159189 | HGLibA_05921 | 129 | 0.032155745 | C1QC | complement C1q C chain [Source:HGNC Symbol;Acc:HGNC:1245] |
| ENSG00000161981 | HGLibA_45944 | 129 | 0.032155745 | SNRNP25 | small nuclear ribonucleoprotein U11/U12 subunit 25 [Source:HGNC Symbol;Acc:HGNC:14161] |
| ENSG00000162444 | HGLibA_40644 | 129 | 0.032155745 | RBP7 | retinol binding protein 7 [Source:HGNC Symbol;Acc:HGNC:30316] |
| ENSG00000162599 | HGLibA_31801 | 129 | 0.032155745 | NFIA | nuclear factor I A [Source:HGNC Symbol;Acc:HGNC:7784] |
| ENSG00000162704 | HGLibA_03093 | 129 | 0.032155745 | ARPC5 | actin related protein 2/3 complex subunit 5 [Source:HGNC Symbol;Acc:HGNC:708] |
| ENSG00000163312 | HGLibA_21117 | 129 | 0.032155745 | HELQ | POLQ like [Source:HGNC Symbol;Acc:HGNC:18536]" |
| ENSG00000163661 | HGLibA_39571 | 129 | 0.032155745 | PTX3 | pentraxin 3 [Source:HGNC Symbol;Acc:HGNC:9692] |
| ENSG00000164209 | HGLibA_44752 | 129 | 0.032155745 | SLC25A46 | solute carrier family 25 member 46 [Source:HGNC Symbol;Acc:HGNC:25198] |
| ENSG00000164520 | HGLibA_40121 | 129 | 0.032155745 | RAET1E | retinoic acid early transcript 1E [Source:HGNC Symbol;Acc:HGNC:16793] |
| ENSG00000165572 | HGLibA_24111 | 129 | 0.032155745 | KBTBD6 | kelch repeat and BTB domain containing 6 [Source:HGNC Symbol;Acc:HGNC:25340] |
| ENSG00000166049 | HGLibA_35264 | 129 | 0.032155745 | PASD1 | PAS domain containing repressor 1 [Source:HGNC Symbol;Acc:HGNC:20686] |
| ENSG00000166317 | HGLibA_48024 | 129 | 0.032155745 | SYNPO2L | synaptopodin 2 like [Source:HGNC Symbol;Acc:HGNC:23532] |
| ENSG00000166923 | HGLibA_20243 | 129 | 0.032155745 | GREM1 | DAN family BMP antagonist [Source:HGNC Symbol;Acc:HGNC:2001]" |
| ENSG00000167625 | HGLibA_56164 | 129 | 0.032155745 | ZNF526 | zinc finger protein 526 [Source:HGNC Symbol;Acc:HGNC:29415] |
| ENSG00000167840 | HGLibA_55638 | 129 | 0.032155745 | ZNF232 | zinc finger protein 232 [Source:HGNC Symbol;Acc:HGNC:13026] |
| ENSG00000168612 | HGLibA_57037 | 129 | 0.032155745 | ZSWIM1 | zinc finger SWIM-type containing 1 [Source:HGNC Symbol;Acc:HGNC:16155] |
| ENSG00000168634 | HGLibA_54258 | 129 | 0.032155745 | WFDC13 | WAP four-disulfide core domain 13 [Source:HGNC Symbol;Acc:HGNC:16131] |
| ENSG00000168671 | HGLibA_52935 | 129 | 0.032155745 | UGT3A2 | UDP glycosyltransferase family 3 member A2 [Source:HGNC Symbol;Acc:HGNC:27266] |
| ENSG00000171873 | HGLibA_01042 | 129 | 0.032155745 | ADRA1D | adrenoceptor alpha 1D [Source:HGNC Symbol;Acc:HGNC:280] |
| ENSG00000172432 | HGLibA_20603 | 129 | 0.032155745 | GTPBP2 | GTP binding protein 2 [Source:HGNC Symbol;Acc:HGNC:4670] |
| ENSG00000173093 | HGLibA_07824 | 129 | 0.032155745 | CCDC63 | coiled-coil domain containing 63 [Source:HGNC Symbol;Acc:HGNC:26669] |
| ENSG00000174775 | HGLibA_22073 | 129 | 0.032155745 | HRAS | GTPase [Source:HGNC Symbol;Acc:HGNC:5173]" |
| ENSG00000174970 | HGLibA_33481 | 129 | 0.032155745 | OR10AG1 | olfactory receptor family 10 subfamily AG member 1 [Source:HGNC Symbol;Acc:HGNC:19607] |
| ENSG00000175564 | HGLibA_52836 | 129 | 0.032155745 | UCP3 | uncoupling protein 3 [Source:HGNC Symbol;Acc:HGNC:12519] |
| ENSG00000176040 | HGLibA_50652 | 129 | 0.032155745 | TMPRSS7 | transmembrane serine protease 7 [Source:HGNC Symbol;Acc:HGNC:30846] |
| ENSG00000178403 | HGLibA_31750 | 129 | 0.032155745 | NEUROG2 | neurogenin 2 [Source:HGNC Symbol;Acc:HGNC:13805] |
| ENSG00000178460 | HGLibA_28635 | 129 | 0.032155745 | MCMDC2 | minichromosome maintenance domain containing 2 [Source:HGNC Symbol;Acc:HGNC:26368] |
| ENSG00000179468 | HGLibA_34563 | 129 | 0.032155745 | OR9A2 | olfactory receptor family 9 subfamily A member 2 [Source:HGNC Symbol;Acc:HGNC:15093] |
| ENSG00000182866 | HGLibA_26153 | 129 | 0.032155745 | LCK | Src family tyrosine kinase [Source:HGNC Symbol;Acc:HGNC:6524]" |
| ENSG00000182973 | HGLibA_10328 | 129 | 0.032155745 | CNOT10 | CCR4-NOT transcription complex subunit 10 [Source:HGNC Symbol;Acc:HGNC:23817] |
| ENSG00000184207 | HGLibA_36277 | 129 | 0.032155745 | PGP | phosphoglycolate phosphatase [Source:HGNC Symbol;Acc:HGNC:8909] |
| ENSG00000184895 | HGLibA_47099 | 129 | 0.032155745 | SRY | sex determining region Y [Source:HGNC Symbol;Acc:HGNC:11311] |
| ENSG00000185069 | HGLibA_25540 | 129 | 0.032155745 | KRT76 | keratin 76 [Source:HGNC Symbol;Acc:HGNC:24430] |
| ENSG00000185129 | HGLibA_39586 | 129 | 0.032155745 | PURA | purine rich element binding protein A [Source:HGNC Symbol;Acc:HGNC:9701] |
| ENSG00000185250 | HGLibA_37951 | 129 | 0.032155745 | PPIL6 | peptidylprolyl isomerase like 6 [Source:HGNC Symbol;Acc:HGNC:21557] |
| ENSG00000185640 | HGLibA_25548 | 129 | 0.032155745 | KRT79 | keratin 79 [Source:HGNC Symbol;Acc:HGNC:28930] |
| ENSG00000186583 | HGLibA_46538 | 129 | 0.032155745 | SPATC1 | spermatogenesis and centriole associated 1 [Source:HGNC Symbol;Acc:HGNC:30510] |
| ENSG00000186834 | HGLibA_21211 | 129 | 0.032155745 | HEXIM1 | hexamethylene bisacetamide inducible 1 [Source:HGNC Symbol;Acc:HGNC:24953] |
| ENSG00000188428 | HGLibA_04568 | 129 | 0.032155745 | BLOC1S5 | biogenesis of lysosomal organelles complex 1 subunit 5 [Source:HGNC Symbol;Acc:HGNC:18561] |
| ENSG00000188906 | HGLibA_27468 | 129 | 0.032155745 | LRRK2 | leucine rich repeat kinase 2 [Source:HGNC Symbol;Acc:HGNC:18618] |
| ENSG00000189064 | HGLibA_18466 | 129 | 0.032155745 | GAGE2A | G antigen 2A [Source:HGNC Symbol;Acc:HGNC:4099] |
| ENSG00000196230 | HGLibA_52271 | 129 | 0.032155745 | TUBB | tubulin beta class I [Source:HGNC Symbol;Acc:HGNC:20778] |
| ENSG00000196531 | HGLibA_31029 | 129 | 0.032155745 | NACA | nascent polypeptide associated complex subunit alpha [Source:HGNC Symbol;Acc:HGNC:7629] |
| ENSG00000196663 | HGLibA_48945 | 129 | 0.032155745 | TECPR2 | tectonin beta-propeller repeat containing 2 [Source:HGNC Symbol;Acc:HGNC:19957] |
| ENSG00000197013 | HGLibA_55972 | 129 | 0.032155745 | ZNF429 | zinc finger protein 429 [Source:HGNC Symbol;Acc:HGNC:20817] |
| ENSG00000197375 | HGLibA_44590 | 129 | 0.032155745 | SLC22A5 | solute carrier family 22 member 5 [Source:HGNC Symbol;Acc:HGNC:10969] |
| ENSG00000198130 | HGLibA_21286 | 129 | 0.032155745 | HIBCH | 3-hydroxyisobutyryl-CoA hydrolase [Source:HGNC Symbol;Acc:HGNC:4908] |
| ENSG00000203985 | HGLibA_26240 | 129 | 0.032155745 | LDLRAD1 | low density lipoprotein receptor class A domain containing 1 [Source:HGNC Symbol;Acc:HGNC:32069] |
| ENSG00000212901 | HGLibA_25739 | 129 | 0.032155745 | KRTAP3-1 | keratin associated protein 3-1 [Source:HGNC Symbol;Acc:HGNC:16778] |
| ENSG00000239305 | HGLibA_41457 | 129 | 0.032155745 | RNF103 | ring finger protein 103 [Source:HGNC Symbol;Acc:HGNC:12859] |
| ENSG00000240230 | HGLibA_10823 | 129 | 0.032155745 | COX19 | cytochrome c oxidase assembly factor COX19 [Source:HGNC Symbol;Acc:HGNC:28074] |
| ENSG00000242441 | HGLibA_20523 | 129 | 0.032155745 | GTF2A1L | general transcription factor IIA subunit 1 like [Source:HGNC Symbol;Acc:HGNC:30727] |
| ENSG00000244694 | HGLibA_39282 | 129 | 0.032155745 | PTCHD4 | patched domain containing 4 [Source:HGNC Symbol;Acc:HGNC:21345] |
| ENSG00000255974 | HGLibA_12105 | 129 | 0.032155745 | CYP2A6 | cytochrome P450 family 2 subfamily A member 6 [Source:HGNC Symbol;Acc:HGNC:2610] |
| ENSG00000258890 | HGLibA_09179 | 129 | 0.032155745 | CEP95 | centrosomal protein 95 [Source:HGNC Symbol;Acc:HGNC:25141] |
| ENSG00000274588 | HGLibA_13080 | 129 | 0.032155745 | DGKK | diacylglycerol kinase kappa [Source:HGNC Symbol;Acc:HGNC:32395] |
| ENSG00000280314 | HGLibA_34546 | 129 | 0.032155745 | OR8K3 | olfactory receptor family 8 subfamily K member 3 (gene/pseudogene) [Source:HGNC Symbol;Acc:HGNC:15313] |
| FAM21C | HGLibA_16510 | 129 | 0.032155745 | FAM21C | - |
| GAGE7 | HGLibA_18487 | 129 | 0.032155745 | GAGE7 | - |
| HIST1H3D | HGLibA_21465 | 129 | 0.032155745 | HIST1H3D | - |
| HIST1H3F | HGLibA_21472 | 129 | 0.032155745 | HIST1H3F | - |
| hsa-mir-1587 | HGLibA_57933 | 129 | 0.032155745 | hsa-mir-1587 | - |
| hsa-mir-3155a | HGLibA_58680 | 129 | 0.032155745 | hsa-mir-3155a | - |
| hsa-mir-3689e | HGLibA_59334 | 129 | 0.032155745 | hsa-mir-3689e | - |
| hsa-mir-4445 | HGLibA_60188 | 129 | 0.032155745 | hsa-mir-4445 | - |
| hsa-mir-4524a | HGLibA_60553 | 129 | 0.032155745 | hsa-mir-4524a | - |
| hsa-mir-4730 | HGLibA_61018 | 129 | 0.032155745 | hsa-mir-4730 | - |
| hsa-mir-526b | HGLibA_61800 | 129 | 0.032155745 | hsa-mir-526b | - |
| hsa-mir-6796 | HGLibA_63360 | 129 | 0.032155745 | hsa-mir-6796 | - |
| hsa-mir-6843 | HGLibA_63546 | 129 | 0.032155745 | hsa-mir-6843 | - |
| hsa-mir-7156 | HGLibA_63851 | 129 | 0.032155745 | hsa-mir-7156 | - |
| KIAA1257 | HGLibA_24730 | 129 | 0.032155745 | KIAA1257 | - |
| LOC147646 | HGLibA_26888 | 129 | 0.032155745 | LOC147646 | - |
| LPPR1 | HGLibA_27112 | 129 | 0.032155745 | LPPR1 | - |
| PHOSPHO2-KLHL23 | HGLibA_36448 | 129 | 0.032155745 | PHOSPHO2-KLHL23 | - |
| TXNRD3NB | HGLibA_52452 | 129 | 0.032155745 | TXNRD3NB | - |
| WDR34 | HGLibA_54071 | 129 | 0.032155745 | WDR34 | - |
| C14orf169 | HGLibA_05338 | 128 | 0.023110402 | C14orf169 | - |
| C8orf46 | HGLibA_06630 | 128 | 0.023110402 | C8orf46 | - |
| CNIH | HGLibA_10282 | 128 | 0.023110402 | CNIH | - |
| ENSG00000005513 | HGLibA_46277 | 128 | 0.023110402 | SOX8 | SRY-box 8 [Source:HGNC Symbol;Acc:HGNC:11203] |
| ENSG00000006025 | HGLibA_34663 | 128 | 0.023110402 | OSBPL7 | oxysterol binding protein like 7 [Source:HGNC Symbol;Acc:HGNC:16387] |
| ENSG00000019991 | HGLibA_21239 | 128 | 0.023110402 | HGF | hepatocyte growth factor [Source:HGNC Symbol;Acc:HGNC:4893] |
| ENSG00000023902 | HGLibA_37149 | 128 | 0.023110402 | PLEKHO1 | pleckstrin homology domain containing O1 [Source:HGNC Symbol;Acc:HGNC:24310] |
| ENSG00000025772 | HGLibA_50963 | 128 | 0.023110402 | TOMM34 | translocase of outer mitochondrial membrane 34 [Source:HGNC Symbol;Acc:HGNC:15746] |
| ENSG00000034693 | HGLibA_36113 | 128 | 0.023110402 | PEX3 | peroxisomal biogenesis factor 3 [Source:HGNC Symbol;Acc:HGNC:8858] |
| ENSG00000037280 | HGLibA_17672 | 128 | 0.023110402 | FLT4 | fms related tyrosine kinase 4 [Source:HGNC Symbol;Acc:HGNC:3767] |
| ENSG00000058668 | HGLibA_03623 | 128 | 0.023110402 | ATP2B4 | ATPase plasma membrane Ca2+ transporting 4 [Source:HGNC Symbol;Acc:HGNC:817] |
| ENSG00000059378 | HGLibA_35220 | 128 | 0.023110402 | PARP12 | poly(ADP-ribose) polymerase family member 12 [Source:HGNC Symbol;Acc:HGNC:21919] |
| ENSG00000065150 | HGLibA_23470 | 128 | 0.023110402 | IPO5 | importin 5 [Source:HGNC Symbol;Acc:HGNC:6402] |
| ENSG00000071189 | HGLibA_46035 | 128 | 0.023110402 | SNX13 | sorting nexin 13 [Source:HGNC Symbol;Acc:HGNC:21335] |
| ENSG00000071859 | HGLibA_16630 | 128 | 0.023110402 | FAM50A | family with sequence similarity 50 member A [Source:HGNC Symbol;Acc:HGNC:18786] |
| ENSG00000075711 | HGLibA_13323 | 128 | 0.023110402 | DLG1 | discs large MAGUK scaffold protein 1 [Source:HGNC Symbol;Acc:HGNC:2900] |
| ENSG00000080839 | HGLibA_40467 | 128 | 0.023110402 | RBL1 | RB transcriptional corepressor like 1 [Source:HGNC Symbol;Acc:HGNC:9893] |
| ENSG00000088053 | HGLibA_19710 | 128 | 0.023110402 | GP6 | glycoprotein VI platelet [Source:HGNC Symbol;Acc:HGNC:14388] |
| ENSG00000100490 | HGLibA_08807 | 128 | 0.023110402 | CDKL1 | cyclin dependent kinase like 1 [Source:HGNC Symbol;Acc:HGNC:1781] |
| ENSG00000101266 | HGLibA_11401 | 128 | 0.023110402 | CSNK2A1 | casein kinase 2 alpha 1 [Source:HGNC Symbol;Acc:HGNC:2457] |
| ENSG00000101307 | HGLibA_44203 | 128 | 0.023110402 | SIRPB1 | signal regulatory protein beta 1 [Source:HGNC Symbol;Acc:HGNC:15928] |
| ENSG00000101928 | HGLibA_29684 | 128 | 0.023110402 | MOSPD1 | motile sperm domain containing 1 [Source:HGNC Symbol;Acc:HGNC:25235] |
| ENSG00000102003 | HGLibA_48033 | 128 | 0.023110402 | SYP | synaptophysin [Source:HGNC Symbol;Acc:HGNC:11506] |
| ENSG00000103479 | HGLibA_40470 | 128 | 0.023110402 | RBL2 | RB transcriptional corepressor like 2 [Source:HGNC Symbol;Acc:HGNC:9894] |
| ENSG00000103496 | HGLibA_47647 | 128 | 0.023110402 | STX4 | syntaxin 4 [Source:HGNC Symbol;Acc:HGNC:11439] |
| ENSG00000104325 | HGLibA_12775 | 128 | 0.023110402 | DECR1 | 4-dienoyl-CoA reductase 1 [Source:HGNC Symbol;Acc:HGNC:2753]" |
| ENSG00000104848 | HGLibA_24145 | 128 | 0.023110402 | KCNA7 | potassium voltage-gated channel subfamily A member 7 [Source:HGNC Symbol;Acc:HGNC:6226] |
| ENSG00000105171 | HGLibA_37690 | 128 | 0.023110402 | POP4 | ribonuclease P/MRP subunit [Source:HGNC Symbol;Acc:HGNC:30081]" |
| ENSG00000108786 | HGLibA_22168 | 128 | 0.023110402 | HSD17B1 | hydroxysteroid 17-beta dehydrogenase 1 [Source:HGNC Symbol;Acc:HGNC:5210] |

| | | | | | |
|---|---|---|---|---|---|
| ENSG00000109466 | HGLibA_25148 | 128 | 0.023110402 | KLHL2 | kelch like family member 2 [Source:HGNC Symbol;Acc:HGNC:6353] |
| ENSG00000110660 | HGLibA_45003 | 128 | 0.023110402 | SLC35F2 | solute carrier family 35 member F2 [Source:HGNC Symbol;Acc:HGNC:23615] |
| ENSG00000111537 | HGLibA_22707 | 128 | 0.023110402 | IFNG | interferon gamma [Source:HGNC Symbol;Acc:HGNC:5438] |
| ENSG00000112214 | HGLibA_17488 | 128 | 0.023110402 | FHL5 | four and a half LIM domains 5 [Source:HGNC Symbol;Acc:HGNC:17371] |
| ENSG00000113600 | HGLibA_06660 | 128 | 0.023110402 | C9 | complement C9 [Source:HGNC Symbol;Acc:HGNC:1358] |
| ENSG00000114115 | HGLibA_40629 | 128 | 0.023110402 | RBP1 | retinol binding protein 1 [Source:HGNC Symbol;Acc:HGNC:9919] |
| ENSG00000114251 | HGLibA_54373 | 128 | 0.023110402 | WNT5A | Wnt family member 5A [Source:HGNC Symbol;Acc:HGNC:12784] |
| ENSG00000114779 | HGLibA_00251 | 128 | 0.023110402 | ABHD14B | abhydrolase domain containing 14B [Source:HGNC Symbol;Acc:HGNC:28235] |
| ENSG00000116254 | HGLibA_09398 | 128 | 0.023110402 | CHD5 | chromodomain helicase DNA binding protein 5 [Source:HGNC Symbol;Acc:HGNC:16816] |
| ENSG00000116783 | HGLibA_50880 | 128 | 0.023110402 | TNNI3K | TNNI3 interacting kinase [Source:HGNC Symbol;Acc:HGNC:19661] |
| ENSG00000117143 | HGLibA_52507 | 128 | 0.023110402 | UAP1 | UDP-N-acetylglucosamine pyrophosphorylase 1 [Source:HGNC Symbol;Acc:HGNC:12457] |
| ENSG00000117148 | HGLibA_00633 | 128 | 0.023110402 | ACTL8 | actin like 8 [Source:HGNC Symbol;Acc:HGNC:24018] |
| ENSG00000125870 | HGLibA_45970 | 128 | 0.023110402 | SNRPB2 | small nuclear ribonucleoprotein polypeptide B2 [Source:HGNC Symbol;Acc:HGNC:11155] |
| ENSG00000127314 | HGLibA_40225 | 128 | 0.023110402 | RAP1B | member of RAS oncogene family [Source:HGNC Symbol;Acc:HGNC:9857]" |
| ENSG00000132671 | HGLibA_47183 | 128 | 0.023110402 | SSTR4 | somatostatin receptor 4 [Source:HGNC Symbol;Acc:HGNC:11333] |
| ENSG00000133805 | HGLibA_01830 | 128 | 0.023110402 | AMPD3 | adenosine monophosphate deaminase 3 [Source:HGNC Symbol;Acc:HGNC:470] |
| ENSG00000137075 | HGLibA_41671 | 128 | 0.023110402 | RNF38 | ring finger protein 38 [Source:HGNC Symbol;Acc:HGNC:18052] |
| ENSG00000137693 | HGLibA_54631 | 128 | 0.023110402 | YAP1 | Yes associated protein 1 [Source:HGNC Symbol;Acc:HGNC:16262] |
| ENSG00000139200 | HGLibA_36505 | 128 | 0.023110402 | PIANP | PILR alpha associated neural protein [Source:HGNC Symbol;Acc:HGNC:25338] |
| ENSG00000140263 | HGLibA_46193 | 128 | 0.023110402 | SORD | sorbitol dehydrogenase [Source:HGNC Symbol;Acc:HGNC:11184] |
| ENSG00000141449 | HGLibA_20239 | 128 | 0.023110402 | GREB1L | GREB1 like retinoic acid receptor coactivator [Source:HGNC Symbol;Acc:HGNC:31042] |
| ENSG00000141524 | HGLibA_49764 | 128 | 0.023110402 | TMC6 | transmembrane channel like 6 [Source:HGNC Symbol;Acc:HGNC:18021] |
| ENSG00000142252 | HGLibA_18922 | 128 | 0.023110402 | GEMIN7 | gem nuclear organelle associated protein 7 [Source:HGNC Symbol;Acc:HGNC:20045] |
| ENSG00000142459 | HGLibA_15696 | 128 | 0.023110402 | EVI5L | ecotropic viral integration site 5 like [Source:HGNC Symbol;Acc:HGNC:30464] |
| ENSG00000144031 | HGLibA_02119 | 128 | 0.023110402 | ANKRD53 | ankyrin repeat domain 53 [Source:HGNC Symbol;Acc:HGNC:25691] |
| ENSG00000146197 | HGLibA_43077 | 128 | 0.023110402 | SCUBE3 | CUB domain and EGF like domain containing 3 [Source:HGNC Symbol;Acc:HGNC:13655]" |
| ENSG00000148175 | HGLibA_47532 | 128 | 0.023110402 | STOM | stomatin [Source:HGNC Symbol;Acc:HGNC:3383] |
| ENSG00000151929 | HGLibA_04079 | 128 | 0.023110402 | BAG3 | BCL2 associated athanogene 3 [Source:HGNC Symbol;Acc:HGNC:939] |
| ENSG00000152086 | HGLibA_52258 | 128 | 0.023110402 | TUBA3E | tubulin alpha 3e [Source:HGNC Symbol;Acc:HGNC:20765] |
| ENSG00000152377 | HGLibA_46748 | 128 | 0.023110402 | SPOCK1 | cwcv and kazal like domains proteoglycan 1 [Source:HGNC Symbol;Acc:HGNC:11251]" |
| ENSG00000153029 | HGLibA_29804 | 128 | 0.023110402 | MR1 | class I-related [Source:HGNC Symbol;Acc:HGNC:4975]" |
| ENSG00000153187 | HGLibA_21845 | 128 | 0.023110402 | HNRNPU | heterogeneous nuclear ribonucleoprotein U [Source:HGNC Symbol;Acc:HGNC:5048] |
| ENSG00000153944 | HGLibA_30207 | 128 | 0.023110402 | MSI2 | musashi RNA binding protein 2 [Source:HGNC Symbol;Acc:HGNC:18585] |
| ENSG00000154370 | HGLibA_51404 | 128 | 0.023110402 | TRIM11 | tripartite motif containing 11 [Source:HGNC Symbol;Acc:HGNC:16281] |
| ENSG00000157954 | HGLibA_54316 | 128 | 0.023110402 | WIPI2 | phosphoinositide interacting 2 [Source:HGNC Symbol;Acc:HGNC:32225]" |
| ENSG00000162542 | HGLibA_49789 | 128 | 0.023110402 | TMCO4 | transmembrane and coiled-coil domains 4 [Source:HGNC Symbol;Acc:HGNC:27393] |
| ENSG00000163319 | HGLibA_30056 | 128 | 0.023110402 | MRPS18C | mitochondrial ribosomal protein S18C [Source:HGNC Symbol;Acc:HGNC:16633] |
| ENSG00000163825 | HGLibA_42428 | 128 | 0.023110402 | RTP3 | receptor transporter protein 3 [Source:HGNC Symbol;Acc:HGNC:15572] |
| ENSG00000164123 | HGLibA_06333 | 128 | 0.023110402 | C4orf45 | chromosome 4 open reading frame 45 [Source:HGNC Symbol;Acc:HGNC:26342] |
| ENSG00000165699 | HGLibA_51812 | 128 | 0.023110402 | TSC1 | TSC complex subunit 1 [Source:HGNC Symbol;Acc:HGNC:12362] |
| ENSG00000166450 | HGLibA_38981 | 128 | 0.023110402 | PRTG | protogenin [Source:HGNC Symbol;Acc:HGNC:26373] |
| ENSG00000166866 | HGLibA_30821 | 128 | 0.023110402 | MYO1A | myosin IA [Source:HGNC Symbol;Acc:HGNC:7595] |
| ENSG00000167165 | HGLibA_52892 | 128 | 0.023110402 | UGT1A6 | UDP glucuronosyltransferase family 1 member A6 [Source:HGNC Symbol;Acc:HGNC:12538] |
| ENSG00000167641 | HGLibA_38034 | 128 | 0.023110402 | PPP1R14A | protein phosphatase 1 regulatory inhibitor subunit 14A [Source:HGNC Symbol;Acc:HGNC:14871] |
| ENSG00000168530 | HGLibA_30744 | 128 | 0.023110402 | MYL1 | myosin light chain 1 [Source:HGNC Symbol;Acc:HGNC:7582] |
| ENSG00000169122 | HGLibA_16015 | 128 | 0.023110402 | FAM110B | family with sequence similarity 110 member B [Source:HGNC Symbol;Acc:HGNC:28587] |
| ENSG00000169220 | HGLibA_41063 | 128 | 0.023110402 | RGS14 | regulator of G protein signaling 14 [Source:HGNC Symbol;Acc:HGNC:9996] |
| ENSG00000169789 | HGLibA_38994 | 128 | 0.023110402 | PRY | PTPN13 like Y-linked [Source:HGNC Symbol;Acc:HGNC:14024] |
| ENSG00000169908 | HGLibA_49691 | 128 | 0.023110402 | TM4SF1 | transmembrane 4 L six family member 1 [Source:HGNC Symbol;Acc:HGNC:11853] |
| ENSG00000170419 | HGLibA_53859 | 128 | 0.023110402 | VSTM2A | V-set and transmembrane domain containing 2A [Source:HGNC Symbol;Acc:HGNC:28499] |
| ENSG00000170832 | HGLibA_53334 | 128 | 0.023110402 | USP32 | ubiquitin specific peptidase 32 [Source:HGNC Symbol;Acc:HGNC:19143] |
| ENSG00000171503 | HGLibA_15625 | 128 | 0.023110402 | ETFDH | electron transfer flavoprotein dehydrogenase [Source:HGNC Symbol;Acc:HGNC:3483] |
| ENSG00000172183 | HGLibA_23658 | 128 | 0.023110402 | ISG20 | interferon stimulated exonuclease gene 20 [Source:HGNC Symbol;Acc:HGNC:6130] |
| ENSG00000172538 | HGLibA_16261 | 128 | 0.023110402 | FAM170B | family with sequence similarity 170 member B [Source:HGNC Symbol;Acc:HGNC:19736] |
| ENSG00000173889 | HGLibA_36325 | 128 | 0.023110402 | PHC3 | polyhomeotic homolog 3 [Source:HGNC Symbol;Acc:HGNC:15682] |
| ENSG00000174231 | HGLibA_38745 | 128 | 0.023110402 | PRPF8 | pre-mRNA processing factor 8 [Source:HGNC Symbol;Acc:HGNC:17340] |
| ENSG00000175895 | HGLibA_37097 | 128 | 0.023110402 | PLEKHF2 | pleckstrin homology and FYVE domain containing 2 [Source:HGNC Symbol;Acc:HGNC:20757] |
| ENSG00000176194 | HGLibA_09697 | 128 | 0.023110402 | CIDEA | cell death-inducing DFFA-like effector a [Source:HGNC Symbol;Acc:HGNC:1976] |
| ENSG00000176269 | HGLibA_33999 | 128 | 0.023110402 | OR4F21 | olfactory receptor family 4 subfamily F member 21 [Source:HGNC Symbol;Acc:HGNC:19583] |
| ENSG00000177873 | HGLibA_56401 | 128 | 0.023110402 | ZNF619 | zinc finger protein 619 [Source:HGNC Symbol;Acc:HGNC:26910] |
| ENSG00000177951 | HGLibA_04434 | 128 | 0.023110402 | BET1L | Bet1 golgi vesicular membrane trafficking protein like [Source:HGNC Symbol;Acc:HGNC:19348] |
| ENSG00000179059 | HGLibA_55219 | 128 | 0.023110402 | ZFP42 | ZFP42 zinc finger protein [Source:HGNC Symbol;Acc:HGNC:30949] |
| ENSG00000179604 | HGLibA_08575 | 128 | 0.023110402 | CDC42EP4 | CDC42 effector protein 4 [Source:HGNC Symbol;Acc:HGNC:17147] |
| ENSG00000181609 | HGLibA_34154 | 128 | 0.023110402 | OR52D1 | olfactory receptor family 52 subfamily D member 1 [Source:HGNC Symbol;Acc:HGNC:15212] |
| ENSG00000182450 | HGLibA_24324 | 128 | 0.023110402 | KCNK4 | potassium two pore domain channel subfamily K member 4 [Source:HGNC Symbol;Acc:HGNC:6279] |
| ENSG00000183389 | HGLibA_34217 | 128 | 0.023110402 | OR56A4 | olfactory receptor family 56 subfamily A member 4 [Source:HGNC Symbol;Acc:HGNC:14791] |
| ENSG00000183615 | HGLibA_16243 | 128 | 0.023110402 | FAM167B | family with sequence similarity 167 member B [Source:HGNC Symbol;Acc:HGNC:28133] |
| ENSG00000183828 | HGLibA_32946 | 128 | 0.023110402 | NUDT14 | nudix hydrolase 14 [Source:HGNC Symbol;Acc:HGNC:20141] |
| ENSG00000183840 | HGLibA_20037 | 128 | 0.023110402 | GPR39 | G protein-coupled receptor 39 [Source:HGNC Symbol;Acc:HGNC:4496] |
| ENSG00000185585 | HGLibA_33360 | 128 | 0.023110402 | OLFML2A | olfactomedin like 2A [Source:HGNC Symbol;Acc:HGNC:27270] |
| ENSG00000185630 | HGLibA_35342 | 128 | 0.023110402 | PBX1 | PBX homeobox 1 [Source:HGNC Symbol;Acc:HGNC:8632] |
| ENSG00000187391 | HGLibA_27998 | 128 | 0.023110402 | MAGI2 | WW and PDZ domain containing 2 [Source:HGNC Symbol;Acc:HGNC:18957]" |
| ENSG00000188467 | HGLibA_44628 | 128 | 0.023110402 | SLC24A5 | solute carrier family 24 member 5 [Source:HGNC Symbol;Acc:HGNC:20611] |
| ENSG00000188554 | HGLibA_31247 | 128 | 0.023110402 | NBR1 | autophagy cargo receptor [Source:HGNC Symbol;Acc:HGNC:6746]" |
| ENSG00000196431 | HGLibA_11259 | 128 | 0.023110402 | CRYBA4 | crystallin beta A4 [Source:HGNC Symbol;Acc:HGNC:2396] |
| ENSG00000196655 | HGLibA_51325 | 128 | 0.023110402 | TRAPPC4 | trafficking protein particle complex 4 [Source:HGNC Symbol;Acc:HGNC:19943] |
| ENSG00000196961 | HGLibA_02304 | 128 | 0.023110402 | AP2A1 | adaptor related protein complex 2 subunit alpha 1 [Source:HGNC Symbol;Acc:HGNC:561] |
| ENSG00000197223 | HGLibA_05719 | 128 | 0.023110402 | C1D | C1D nuclear receptor corepressor [Source:HGNC Symbol;Acc:HGNC:29911] |
| ENSG00000198542 | HGLibA_23801 | 128 | 0.023110402 | ITGBL1 | integrin subunit beta like 1 [Source:HGNC Symbol;Acc:HGNC:6164] |
| ENSG00000198551 | HGLibA_56425 | 128 | 0.023110402 | ZNF627 | zinc finger protein 627 [Source:HGNC Symbol;Acc:HGNC:30570] |
| ENSG00000198844 | HGLibA_02820 | 128 | 0.023110402 | ARHGEF15 | Rho guanine nucleotide exchange factor 15 [Source:HGNC Symbol;Acc:HGNC:15590] |
| ENSG00000198855 | HGLibA_17507 | 128 | 0.023110402 | FICD | FIC domain containing [Source:HGNC Symbol;Acc:HGNC:18416] |
| ENSG00000198889 | HGLibA_12421 | 128 | 0.023110402 | DCAF12L1 | DDB1 and CUL4 associated factor 12 like 1 [Source:HGNC Symbol;Acc:HGNC:29395] |
| ENSG00000203722 | HGLibA_40122 | 128 | 0.023110402 | RAET1G | retinoic acid early transcript 1G [Source:HGNC Symbol;Acc:HGNC:16795] |
| ENSG00000204632 | HGLibA_21649 | 128 | 0.023110402 | HLA-G | G [Source:HGNC Symbol;Acc:HGNC:4964]" |
| ENSG00000204695 | HGLibA_33638 | 128 | 0.023110402 | OR14J1 | olfactory receptor family 14 subfamily J member 1 [Source:HGNC Symbol;Acc:HGNC:13971] |
| ENSG00000214711 | HGLibA_07158 | 128 | 0.023110402 | CAPN14 | calpain 14 [Source:HGNC Symbol;Acc:HGNC:16664] |
| ENSG00000214946 | HGLibA_48517 | 128 | 0.023110402 | TBC1D26 | TBC1 domain family member 26 [Source:HGNC Symbol;Acc:HGNC:28745] |
| ENSG00000215045 | HGLibA_20277 | 128 | 0.023110402 | GRID2IP | Grid2 interacting protein [Source:HGNC Symbol;Acc:HGNC:18464] |
| ENSG00000215271 | HGLibA_21873 | 128 | 0.023110402 | HOMEZ | homeobox and leucine zipper encoding [Source:HGNC Symbol;Acc:HGNC:20164] |
| ENSG00000225556 | HGLibA_06103 | 128 | 0.023110402 | C2CD4D | C2 calcium dependent domain containing 4D [Source:HGNC Symbol;Acc:HGNC:37210] |
| ENSG00000259112 | HGLibA_31541 | 128 | 0.023110402 | NDUFC2-KCTD14 | NDUFC2-KCTD14 readthrough [Source:HGNC Symbol;Acc:HGNC:42956] |
| FAM207A | HGLibA_16430 | 128 | 0.023110402 | FAM207A | - |
| FAM213B | HGLibA_16474 | 128 | 0.023110402 | FAM213B | - |
| hsa-mir-1199 | HGLibA_57257 | 128 | 0.023110402 | hsa-mir-1199 | - |
| hsa-mir-1243 | HGLibA_57359 | 128 | 0.023110402 | hsa-mir-1243 | - |
| hsa-mir-181a-2 | HGLibA_57964 | 128 | 0.023110402 | hsa-mir-181a-2 | - |
| hsa-mir-192 | HGLibA_58069 | 128 | 0.023110402 | hsa-mir-192 | - |
| hsa-mir-218-1 | HGLibA_58267 | 128 | 0.023110402 | hsa-mir-218-1 | - |
| hsa-mir-2682 | HGLibA_58359 | 128 | 0.023110402 | hsa-mir-2682 | - |
| hsa-mir-27b | HGLibA_58379 | 128 | 0.023110402 | hsa-mir-27b | - |
| hsa-mir-3156-1 | HGLibA_58688 | 128 | 0.023110402 | hsa-mir-3156-1 | - |
| hsa-mir-328 | HGLibA_58976 | 128 | 0.023110402 | hsa-mir-328 | - |
| hsa-mir-34c | HGLibA_59045 | 128 | 0.023110402 | hsa-mir-34c | - |
| hsa-mir-3677 | HGLibA_59258 | 128 | 0.023110402 | hsa-mir-3677 | - |
| hsa-mir-4785 | HGLibA_61249 | 128 | 0.023110402 | hsa-mir-4785 | - |
| hsa-mir-501 | HGLibA_61453 | 128 | 0.023110402 | hsa-mir-501 | - |
| hsa-mir-5590 | HGLibA_62202 | 128 | 0.023110402 | hsa-mir-5590 | - |
| hsa-mir-569 | HGLibA_62275 | 128 | 0.023110402 | hsa-mir-569 | - |
| hsa-mir-571 | HGLibA_62374 | 128 | 0.023110402 | hsa-mir-571 | - |
| hsa-mir-578 | HGLibA_62406 | 128 | 0.023110402 | hsa-mir-578 | - |
| hsa-mir-6782 | HGLibA_63305 | 128 | 0.023110402 | hsa-mir-6782 | - |
| hsa-mir-6894 | HGLibA_63760 | 128 | 0.023110402 | hsa-mir-6894 | - |
| MB21D1 | HGLibA_28457 | 128 | 0.023110402 | MB21D1 | - |
| MUM1L1 | HGLibA_30567 | 128 | 0.023110402 | MUM1L1 | - |
| SMEK2 | HGLibA_45695 | 128 | 0.023110402 | SMEK2 | - |
| UTP11L | HGLibA_53430 | 128 | 0.023110402 | UTP11L | - |
| WARS | HGLibA_53945 | 128 | 0.023110402 | WARS | - |
| WDR52 | HGLibA_54118 | 128 | 0.023110402 | WDR52 | - |
| ZFYVE20 | HGLibA_55278 | 128 | 0.023110402 | ZFYVE20 | - |
| 1-Dec | HGLibA_12770 | 127 | 0.014065059 | 1-Dec | - |
| C17orf105 | HGLibA_05525 | 127 | 0.014065059 | C17orf105 | - |
| C1orf123 | HGLibA_05761 | 127 | 0.014065059 | C1orf123 | - |
| CSF2RA_Y | HGLibA_11334 | 127 | 0.014065059 | CSF2RA_Y | - |
| EFCAB4B | HGLibA_14545 | 127 | 0.014065059 | EFCAB4B | - |
| ENSG00000011478 | HGLibA_39731 | 127 | 0.014065059 | QPCTL | glutaminyl-peptide cyclotransferase like [Source:HGNC Symbol;Acc:HGNC:25952] |
| ENSG00000015532 | HGLibA_54621 | 127 | 0.014065059 | XYLT2 | xylosyltransferase 2 [Source:HGNC Symbol;Acc:HGNC:15517] |
| ENSG00000036054 | HGLibA_48508 | 127 | 0.014065059 | TBC1D23 | TBC1 domain family member 23 [Source:HGNC Symbol;Acc:HGNC:25622] |
| ENSG00000043462 | HGLibA_26201 | 127 | 0.014065059 | LCP2 | lymphocyte cytosolic protein 2 [Source:HGNC Symbol;Acc:HGNC:6529] |
| ENSG00000044090 | HGLibA_11792 | 127 | 0.014065059 | CUL7 | cullin 7 [Source:HGNC Symbol;Acc:HGNC:21024] |
| ENSG00000044524 | HGLibA_15293 | 127 | 0.014065059 | EPHA3 | EPH receptor A3 [Source:HGNC Symbol;Acc:HGNC:3387] |
| ENSG00000049239 | HGLibA_20786 | 127 | 0.014065059 | H6PD | hexose-6-phosphate dehydrogenase/glucose 1-dehydrogenase [Source:HGNC Symbol;Acc:HGNC:4795] |
| ENSG00000063322 | HGLibA_28799 | 127 | 0.014065059 | MED29 | mediator complex subunit 29 [Source:HGNC Symbol;Acc:HGNC:23074] |
| ENSG00000065371 | HGLibA_41759 | 127 | 0.014065059 | ROPN1 | rhophilin associated tail protein 1 [Source:HGNC Symbol;Acc:HGNC:17692] |
| ENSG00000066923 | HGLibA_47328 | 127 | 0.014065059 | STAG3 | stromal antigen 3 [Source:HGNC Symbol;Acc:HGNC:11356] |

| | | | | | |
|---|---|---|---|---|---|
| ENSG00000073754 | HGLibA_08420 | 127 | 0.014065059 | CD5L | CD5 molecule like [Source:HGNC Symbol;Acc:HGNC:1690] |
| ENSG00000075429 | HGLibA_06940 | 127 | 0.014065059 | CACNG5 | calcium voltage-gated channel auxiliary subunit gamma 5 [Source:HGNC Symbol;Acc:HGNC:1409] |
| ENSG00000076321 | HGLibA_25150 | 127 | 0.014065059 | KLHL20 | kelch like family member 20 [Source:HGNC Symbol;Acc:HGNC:25056] |
| ENSG00000083093 | HGLibA_35057 | 127 | 0.014065059 | PALB2 | partner and localizer of BRCA2 [Source:HGNC Symbol;Acc:HGNC:26144] |
| ENSG00000083099 | HGLibA_27749 | 127 | 0.014065059 | LYRM2 | LYR motif containing 2 [Source:HGNC Symbol;Acc:HGNC:25229] |
| ENSG00000099715 | HGLibA_35393 | 127 | 0.014065059 | PCDH11Y | protocadherin 11 Y-linked [Source:HGNC Symbol;Acc:HGNC:15813] |
| ENSG00000100629 | HGLibA_09104 | 127 | 0.014065059 | CEP128 | centrosomal protein 128 [Source:HGNC Symbol;Acc:HGNC:20359] |
| ENSG00000100938 | HGLibA_19429 | 127 | 0.014065059 | GMPR2 | guanosine monophosphate reductase 2 [Source:HGNC Symbol;Acc:HGNC:4377] |
| ENSG00000100997 | HGLibA_00238 | 127 | 0.014065059 | ABHD12 | abhydrolase domain containing 12 [Source:HGNC Symbol;Acc:HGNC:15868] |
| ENSG00000101843 | HGLibA_39150 | 127 | 0.014065059 | PSMD10 | non-ATPase 10 [Source:HGNC Symbol;Acc:HGNC:9555]" |
| ENSG00000103035 | HGLibA_39180 | 127 | 0.014065059 | PSMD7 | non-ATPase 7 [Source:HGNC Symbol;Acc:HGNC:9565]" |
| ENSG00000103353 | HGLibA_52696 | 127 | 0.014065059 | UBFD1 | ubiquitin family domain containing 1 [Source:HGNC Symbol;Acc:HGNC:30565] |
| ENSG00000103404 | HGLibA_53332 | 127 | 0.014065059 | USP31 | ubiquitin specific peptidase 31 [Source:HGNC Symbol;Acc:HGNC:20060] |
| ENSG00000103546 | HGLibA_45318 | 127 | 0.014065059 | SLC6A2 | solute carrier family 6 member 2 [Source:HGNC Symbol;Acc:HGNC:11048] |
| ENSG00000104880 | HGLibA_02829 | 127 | 0.014065059 | ARHGEF18 | Rho/Rac guanine nucleotide exchange factor 18 [Source:HGNC Symbol;Acc:HGNC:17090] |
| ENSG00000105287 | HGLibA_38569 | 127 | 0.014065059 | PRKD2 | protein kinase D2 [Source:HGNC Symbol;Acc:HGNC:17293] |
| ENSG00000105376 | HGLibA_22507 | 127 | 0.014065059 | ICAM5 | intercellular adhesion molecule 5 [Source:HGNC Symbol;Acc:HGNC:5348] |
| ENSG00000105393 | HGLibA_04056 | 127 | 0.014065059 | BABAM1 | BRISC and BRCA1 A complex member 1 [Source:HGNC Symbol;Acc:HGNC:25008] |
| ENSG00000106034 | HGLibA_10928 | 127 | 0.014065059 | CPED1 | cadherin like and PC-esterase domain containing 1 [Source:HGNC Symbol;Acc:HGNC:26159] |
| ENSG00000106404 | HGLibA_09862 | 127 | 0.014065059 | CLDN15 | claudin 15 [Source:HGNC Symbol;Acc:HGNC:2036] |
| ENSG00000108590 | HGLibA_28804 | 127 | 0.014065059 | MED31 | mediator complex subunit 31 [Source:HGNC Symbol;Acc:HGNC:24260] |
| ENSG00000108666 | HGLibA_05574 | 127 | 0.014065059 | C17orf75 | chromosome 17 open reading frame 75 [Source:HGNC Symbol;Acc:HGNC:30173] |
| ENSG00000109758 | HGLibA_21244 | 127 | 0.014065059 | HGFAC | HGF activator [Source:HGNC Symbol;Acc:HGNC:4894] |
| ENSG00000110046 | HGLibA_03480 | 127 | 0.014065059 | ATG2A | autophagy related 2A [Source:HGNC Symbol;Acc:HGNC:29028] |
| ENSG00000110330 | HGLibA_04515 | 127 | 0.014065059 | BIRC2 | baculoviral IAP repeat containing 2 [Source:HGNC Symbol;Acc:HGNC:590] |
| ENSG00000110911 | HGLibA_44354 | 127 | 0.014065059 | SLC11A2 | solute carrier family 11 member 2 [Source:HGNC Symbol;Acc:HGNC:10908] |
| ENSG00000112115 | HGLibA_22989 | 127 | 0.014065059 | IL17A | interleukin 17A [Source:HGNC Symbol;Acc:HGNC:5981] |
| ENSG00000113504 | HGLibA_44375 | 127 | 0.014065059 | SLC12A7 | solute carrier family 12 member 7 [Source:HGNC Symbol;Acc:HGNC:10915] |
| ENSG00000113734 | HGLibA_04650 | 127 | 0.014065059 | BNIP1 | BCL2 interacting protein 1 [Source:HGNC Symbol;Acc:HGNC:1082] |
| ENSG00000114473 | HGLibA_23516 | 127 | 0.014065059 | IQCG | IQ motif containing G [Source:HGNC Symbol;Acc:HGNC:25251] |
| ENSG00000114853 | HGLibA_54900 | 127 | 0.014065059 | ZBTB47 | zinc finger and BTB domain containing 47 [Source:HGNC Symbol;Acc:HGNC:26955] |
| ENSG00000116745 | HGLibA_41827 | 127 | 0.014065059 | RPE65 | retinoid isomerohydrolase [Source:HGNC Symbol;Acc:HGNC:10294]" |
| ENSG00000116791 | HGLibA_11293 | 127 | 0.014065059 | CRYZ | crystallin zeta [Source:HGNC Symbol;Acc:HGNC:2419] |
| ENSG00000117519 | HGLibA_10312 | 127 | 0.014065059 | CNN3 | calponin 3 [Source:HGNC Symbol;Acc:HGNC:2157] |
| ENSG00000117650 | HGLibA_31648 | 127 | 0.014065059 | NEK2 | NIMA related kinase 2 [Source:HGNC Symbol;Acc:HGNC:7745] |
| ENSG00000121236 | HGLibA_51558 | 127 | 0.014065059 | TRIM6 | tripartite motif containing 6 [Source:HGNC Symbol;Acc:HGNC:16277] |
| ENSG00000123200 | HGLibA_54960 | 127 | 0.014065059 | ZC3H13 | zinc finger CCCH-type containing 13 [Source:HGNC Symbol;Acc:HGNC:20368] |
| ENSG00000123240 | HGLibA_33459 | 127 | 0.014065059 | OPTN | optineurin [Source:HGNC Symbol;Acc:HGNC:17142] |
| ENSG00000124089 | HGLibA_28547 | 127 | 0.014065059 | MC3R | melanocortin 3 receptor [Source:HGNC Symbol;Acc:HGNC:6931] |
| ENSG00000125845 | HGLibA_04600 | 127 | 0.014065059 | BMP2 | bone morphogenetic protein 2 [Source:HGNC Symbol;Acc:HGNC:1069] |
| ENSG00000127527 | HGLibA_15370 | 127 | 0.014065059 | EPS15L1 | epidermal growth factor receptor pathway substrate 15 like 1 [Source:HGNC Symbol;Acc:HGNC:24634] |
| ENSG00000128242 | HGLibA_18497 | 127 | 0.014065059 | GAL3ST1 | galactose-3-O-sulfotransferase 1 [Source:HGNC Symbol;Acc:HGNC:24240] |
| ENSG00000128604 | HGLibA_23592 | 127 | 0.014065059 | IRF5 | interferon regulatory factor 5 [Source:HGNC Symbol;Acc:HGNC:6120] |
| ENSG00000129235 | HGLibA_52417 | 127 | 0.014065059 | TXNDC17 | thioredoxin domain containing 17 [Source:HGNC Symbol;Acc:HGNC:28218] |
| ENSG00000130159 | HGLibA_14420 | 127 | 0.014065059 | ECSIT | ECSIT signalling integrator [Source:HGNC Symbol;Acc:HGNC:29548] |
| ENSG00000130725 | HGLibA_52632 | 127 | 0.014065059 | UBE2M | ubiquitin conjugating enzyme E2 M [Source:HGNC Symbol;Acc:HGNC:12491] |
| ENSG00000131653 | HGLibA_51267 | 127 | 0.014065059 | TRAF7 | TNF receptor associated factor 7 [Source:HGNC Symbol;Acc:HGNC:20456] |
| ENSG00000132837 | HGLibA_13404 | 127 | 0.014065059 | DMGDH | dimethylglycine dehydrogenase [Source:HGNC Symbol;Acc:HGNC:24475] |
| ENSG00000132842 | HGLibA_02320 | 127 | 0.014065059 | AP3B1 | adaptor related protein complex 3 subunit beta 1 [Source:HGNC Symbol;Acc:HGNC:566] |
| ENSG00000133067 | HGLibA_26415 | 127 | 0.014065059 | LGR6 | leucine rich repeat containing G protein-coupled receptor 6 [Source:HGNC Symbol;Acc:HGNC:19719] |
| ENSG00000134138 | HGLibA_28873 | 127 | 0.014065059 | MEIS2 | Meis homeobox 2 [Source:HGNC Symbol;Acc:HGNC:7001] |
| ENSG00000134287 | HGLibA_02648 | 127 | 0.014065059 | ARF3 | ADP ribosylation factor 3 [Source:HGNC Symbol;Acc:HGNC:654] |
| ENSG00000135218 | HGLibA_08360 | 127 | 0.014065059 | CD36 | CD36 molecule [Source:HGNC Symbol;Acc:HGNC:1663] |
| ENSG00000136634 | HGLibA_22943 | 127 | 0.014065059 | IL10 | interleukin 10 [Source:HGNC Symbol;Acc:HGNC:5962] |
| ENSG00000136698 | HGLibA_09248 | 127 | 0.014065059 | CFC1 | cryptic family 1 [Source:HGNC Symbol;Acc:HGNC:18292]" |
| ENSG00000137198 | HGLibA_19428 | 127 | 0.014065059 | GMPR | guanosine monophosphate reductase [Source:HGNC Symbol;Acc:HGNC:4376] |
| ENSG00000137392 | HGLibA_10104 | 127 | 0.014065059 | CLPS | colipase [Source:HGNC Symbol;Acc:HGNC:2085] |
| ENSG00000137869 | HGLibA_12063 | 127 | 0.014065059 | CYP19A1 | cytochrome P450 family 19 subfamily A member 1 [Source:HGNC Symbol;Acc:HGNC:2594] |
| ENSG00000138185 | HGLibA_15197 | 127 | 0.014065059 | ENTPD1 | ectonucleoside triphosphate diphosphohydrolase 1 [Source:HGNC Symbol;Acc:HGNC:3363] |
| ENSG00000138780 | HGLibA_20466 | 127 | 0.014065059 | GSTCD | glutathione S-transferase C-terminal domain containing [Source:HGNC Symbol;Acc:HGNC:25806] |
| ENSG00000138795 | HGLibA_26271 | 127 | 0.014065059 | LEF1 | lymphoid enhancer binding factor 1 [Source:HGNC Symbol;Acc:HGNC:6551] |
| ENSG00000140488 | HGLibA_09018 | 127 | 0.014065059 | CELF6 | CUGBP Elav-like family member 6 [Source:HGNC Symbol;Acc:HGNC:14059] |
| ENSG00000142784 | HGLibA_54232 | 127 | 0.014065059 | WDTC1 | WD and tetratricopeptide repeats 1 [Source:HGNC Symbol;Acc:HGNC:29175] |
| ENSG00000144659 | HGLibA_44725 | 127 | 0.014065059 | SLC25A38 | solute carrier family 25 member 38 [Source:HGNC Symbol;Acc:HGNC:26054] |
| ENSG00000145022 | HGLibA_48855 | 127 | 0.014065059 | TCTA | T cell leukemia translocation altered [Source:HGNC Symbol;Acc:HGNC:11692] |
| ENSG00000147419 | HGLibA_07738 | 127 | 0.014065059 | CCDC25 | coiled-coil domain containing 25 [Source:HGNC Symbol;Acc:HGNC:25591] |
| ENSG00000148136 | HGLibA_33601 | 127 | 0.014065059 | OR13C4 | olfactory receptor family 13 subfamily C member 4 [Source:HGNC Symbol;Acc:HGNC:14722] |
| ENSG00000148346 | HGLibA_26179 | 127 | 0.014065059 | LCN2 | lipocalin 2 [Source:HGNC Symbol;Acc:HGNC:6526] |
| ENSG00000148942 | HGLibA_45261 | 127 | 0.014065059 | SLC5A12 | solute carrier family 5 member 12 [Source:HGNC Symbol;Acc:HGNC:28750] |
| ENSG00000149260 | HGLibA_07168 | 127 | 0.014065059 | CAPN5 | calpain 5 [Source:HGNC Symbol;Acc:HGNC:1482] |
| ENSG00000149452 | HGLibA_44600 | 127 | 0.014065059 | SLC22A8 | solute carrier family 22 member 8 [Source:HGNC Symbol;Acc:HGNC:10972] |
| ENSG00000149742 | HGLibA_44604 | 127 | 0.014065059 | SLC22A9 | solute carrier family 22 member 9 [Source:HGNC Symbol;Acc:HGNC:16261] |
| ENSG00000150627 | HGLibA_54038 | 127 | 0.014065059 | WDR17 | WD repeat domain 17 [Source:HGNC Symbol;Acc:HGNC:16661] |
| ENSG00000151655 | HGLibA_23809 | 127 | 0.014065059 | ITIH2 | inter-alpha-trypsin inhibitor heavy chain 2 [Source:HGNC Symbol;Acc:HGNC:6167] |
| ENSG00000151883 | HGLibA_35245 | 127 | 0.014065059 | PARP8 | poly(ADP-ribose) polymerase family member 8 [Source:HGNC Symbol;Acc:HGNC:26124] |
| ENSG00000152128 | HGLibA_50030 | 127 | 0.014065059 | TMEM163 | transmembrane protein 163 [Source:HGNC Symbol;Acc:HGNC:25380] |
| ENSG00000152556 | HGLibA_36167 | 127 | 0.014065059 | PFKM | muscle [Source:HGNC Symbol;Acc:HGNC:8877]" |
| ENSG00000154889 | HGLibA_29767 | 127 | 0.014065059 | MPPE1 | metallophosphoesterase 1 [Source:HGNC Symbol;Acc:HGNC:15988] |
| ENSG00000161981 | HGLibA_45945 | 127 | 0.014065059 | SNRNP25 | small nuclear ribonucleoprotein U11/U12 subunit 25 [Source:HGNC Symbol;Acc:HGNC:14161] |
| ENSG00000163584 | HGLibA_41901 | 127 | 0.014065059 | RPL22L1 | ribosomal protein L22 like 1 [Source:HGNC Symbol;Acc:HGNC:27610] |
| ENSG00000163710 | HGLibA_35654 | 127 | 0.014065059 | PCOLCE2 | procollagen C-endopeptidase enhancer 2 [Source:HGNC Symbol;Acc:HGNC:8739] |
| ENSG00000164104 | HGLibA_21692 | 127 | 0.014065059 | HMGB2 | high mobility group box 2 [Source:HGNC Symbol;Acc:HGNC:5000] |
| ENSG00000164400 | HGLibA_11328 | 127 | 0.014065059 | CSF2 | colony stimulating factor 2 [Source:HGNC Symbol;Acc:HGNC:2434] |
| ENSG00000164742 | HGLibA_00905 | 127 | 0.014065059 | ADCY1 | adenylate cyclase 1 [Source:HGNC Symbol;Acc:HGNC:232] |
| ENSG00000165272 | HGLibA_02593 | 127 | 0.014065059 | AQP3 | aquaporin 3 (Gill blood group) [Source:HGNC Symbol;Acc:HGNC:636] |
| ENSG00000165678 | HGLibA_19038 | 127 | 0.014065059 | GHITM | growth hormone inducible transmembrane protein [Source:HGNC Symbol;Acc:HGNC:17281] |
| ENSG00000166573 | HGLibA_18587 | 127 | 0.014065059 | GALR1 | galanin receptor 1 [Source:HGNC Symbol;Acc:HGNC:4132] |
| ENSG00000167613 | HGLibA_25933 | 127 | 0.014065059 | LAIR1 | leukocyte associated immunoglobulin like receptor 1 [Source:HGNC Symbol;Acc:HGNC:6477] |
| ENSG00000167992 | HGLibA_53928 | 127 | 0.014065059 | VWCE | von Willebrand factor C and EGF domains [Source:HGNC Symbol;Acc:HGNC:26487] |
| ENSG00000169583 | HGLibA_10026 | 127 | 0.014065059 | CLIC3 | chloride intracellular channel 3 [Source:HGNC Symbol;Acc:HGNC:2064] |
| ENSG00000170234 | HGLibA_39639 | 127 | 0.014065059 | PWWP2A | PWWP domain containing 2A [Source:HGNC Symbol;Acc:HGNC:29406] |
| ENSG00000170423 | HGLibA_25546 | 127 | 0.014065059 | KRT78 | keratin 78 [Source:HGNC Symbol;Acc:HGNC:28926] |
| ENSG00000172943 | HGLibA_36389 | 127 | 0.014065059 | PHF8 | PHD finger protein 8 [Source:HGNC Symbol;Acc:HGNC:20672] |
| ENSG00000173581 | HGLibA_07523 | 127 | 0.014065059 | CCDC106 | coiled-coil domain containing 106 [Source:HGNC Symbol;Acc:HGNC:30181] |
| ENSG00000173905 | HGLibA_19651 | 127 | 0.014065059 | GOLIM4 | golgi integral membrane protein 4 [Source:HGNC Symbol;Acc:HGNC:15448] |
| ENSG00000173918 | HGLibA_05937 | 127 | 0.014065059 | C1QTNF1 | C1q and TNF related 1 [Source:HGNC Symbol;Acc:HGNC:14324] |
| ENSG00000174123 | HGLibA_49644 | 127 | 0.014065059 | TLR10 | toll like receptor 10 [Source:HGNC Symbol;Acc:HGNC:15634] |
| ENSG00000174292 | HGLibA_50845 | 127 | 0.014065059 | TNK1 | tyrosine kinase non receptor 1 [Source:HGNC Symbol;Acc:HGNC:11940] |
| ENSG00000174996 | HGLibA_25030 | 127 | 0.014065059 | KLC2 | kinesin light chain 2 [Source:HGNC Symbol;Acc:HGNC:20716] |
| ENSG00000175536 | HGLibA_26637 | 127 | 0.014065059 | LIPT2 | lipoyl(octanoyl) transferase 2 [Source:HGNC Symbol;Acc:HGNC:37216] |
| ENSG00000175820 | HGLibA_07679 | 127 | 0.014065059 | CCDC168 | coiled-coil domain containing 168 [Source:HGNC Symbol;Acc:HGNC:26851] |
| ENSG00000176160 | HGLibA_22232 | 127 | 0.014065059 | HSF5 | heat shock transcription factor 5 [Source:HGNC Symbol;Acc:HGNC:26862] |
| ENSG00000176692 | HGLibA_17825 | 127 | 0.014065059 | FOXC2 | forkhead box C2 [Source:HGNC Symbol;Acc:HGNC:3801] |
| ENSG00000177243 | HGLibA_12816 | 127 | 0.014065059 | DEFB103B | defensin beta 103B [Source:HGNC Symbol;Acc:HGNC:31702] |
| ENSG00000177731 | HGLibA_17617 | 127 | 0.014065059 | FLII | actin remodeling protein [Source:HGNC Symbol;Acc:HGNC:3750]" |
| ENSG00000178904 | HGLibA_13911 | 127 | 0.014065059 | DPY19L3 | dpy-19 like C-mannosyltransferase 3 [Source:HGNC Symbol;Acc:HGNC:27120] |
| ENSG00000179751 | HGLibA_47926 | 127 | 0.014065059 | SYCN | syncollin [Source:HGNC Symbol;Acc:HGNC:18442] |
| ENSG00000180730 | HGLibA_44032 | 127 | 0.014065059 | SHISA2 | shisa family member 2 [Source:HGNC Symbol;Acc:HGNC:20366] |
| ENSG00000181617 | HGLibA_17248 | 127 | 0.014065059 | FDCSP | follicular dendritic cell secreted protein [Source:HGNC Symbol;Acc:HGNC:19215] |
| ENSG00000181939 | HGLibA_33946 | 127 | 0.014065059 | OR4C15 | olfactory receptor family 4 subfamily C member 15 [Source:HGNC Symbol;Acc:HGNC:15171] |
| ENSG00000182054 | HGLibA_22542 | 127 | 0.014065059 | IDH2 | mitochondrial [Source:HGNC Symbol;Acc:HGNC:5383]" |
| ENSG00000186174 | HGLibA_04349 | 127 | 0.014065059 | BCL9L | B cell CLL/lymphoma 9 like [Source:HGNC Symbol;Acc:HGNC:23688] |
| ENSG00000186335 | HGLibA_45034 | 127 | 0.014065059 | SLC36A2 | solute carrier family 36 member 2 [Source:HGNC Symbol;Acc:HGNC:18762] |
| ENSG00000186453 | HGLibA_16535 | 127 | 0.014065059 | FAM228A | family with sequence similarity 228 member A [Source:HGNC Symbol;Acc:HGNC:34418] |
| ENSG00000187510 | HGLibA_37121 | 127 | 0.014065059 | PLEKHG7 | pleckstrin homology and RhoGEF domain containing G7 [Source:HGNC Symbol;Acc:HGNC:33829] |
| ENSG00000188243 | HGLibA_10672 | 127 | 0.014065059 | COMMD6 | COMM domain containing 6 [Source:HGNC Symbol;Acc:HGNC:24015] |
| ENSG00000189181 | HGLibA_33634 | 127 | 0.014065059 | OR14I1 | olfactory receptor family 14 subfamily I member 1 [Source:HGNC Symbol;Acc:HGNC:19575] |
| ENSG00000189252 | HGLibA_46407 | 127 | 0.014065059 | SPANXN3 | SPANX family member N3 [Source:HGNC Symbol;Acc:HGNC:33176] |
| ENSG00000196159 | HGLibA_16918 | 127 | 0.014065059 | FAT4 | FAT atypical cadherin 4 [Source:HGNC Symbol;Acc:HGNC:23109] |
| ENSG00000196329 | HGLibA_19081 | 127 | 0.014065059 | GIMAP5 | IMAP family member 5 [Source:HGNC Symbol;Acc:HGNC:18005]" |
| ENSG00000196793 | HGLibA_55655 | 127 | 0.014065059 | ZNF239 | zinc finger protein 239 [Source:HGNC Symbol;Acc:HGNC:13031] |
| ENSG00000197121 | HGLibA_36207 | 127 | 0.014065059 | PGAP1 | post-GPI attachment to proteins 1 [Source:HGNC Symbol;Acc:HGNC:25712] |
| ENSG00000197849 | HGLibA_34518 | 127 | 0.014065059 | OR8G1 | olfactory receptor family 8 subfamily G member 1 (gene/pseudogene) [Source:HGNC Symbol;Acc:HGNC:8484] |
| ENSG00000198242 | HGLibA_41907 | 127 | 0.014065059 | RPL23A | ribosomal protein L23a [Source:HGNC Symbol;Acc:HGNC:10317] |
| ENSG00000203795 | HGLibA_16547 | 127 | 0.014065059 | FAM24A | family with sequence similarity 24 member A [Source:HGNC Symbol;Acc:HGNC:23470] |
| ENSG00000203870 | HGLibA_45774 | 127 | 0.014065059 | SMIM9 | small integral membrane protein 9 [Source:HGNC Symbol;Acc:HGNC:41915] |
| ENSG00000204217 | HGLibA_04636 | 127 | 0.014065059 | BMPR2 | bone morphogenetic protein receptor type 2 [Source:HGNC Symbol;Acc:HGNC:1078] |
| ENSG00000204323 | HGLibA_45760 | 127 | 0.014065059 | SMIM5 | small integral membrane protein 5 [Source:HGNC Symbol;Acc:HGNC:40030] |
| ENSG00000204344 | HGLibA_47467 | 127 | 0.014065059 | STK19 | serine/threonine kinase 19 [Source:HGNC Symbol;Acc:HGNC:11398] |
| ENSG00000204352 | HGLibA_06675 | 127 | 0.014065059 | C9orf129 | chromosome 9 open reading frame 129 [Source:HGNC Symbol;Acc:HGNC:31116] |
| ENSG00000204866 | HGLibA_22841 | 127 | 0.014065059 | IGFL2 | IGF like family member 2 [Source:HGNC Symbol;Acc:HGNC:32929] |
| ENSG00000212993 | HGLibA_37789 | 127 | 0.014065059 | POU5F1B | POU class 5 homeobox 1B [Source:HGNC Symbol;Acc:HGNC:9223] |
| ENSG00000213231 | HGLibA_48817 | 127 | 0.014065059 | TCL1B | T cell leukemia/lymphoma 1B [Source:HGNC Symbol;Acc:HGNC:11649] |
| ENSG00000213465 | HGLibA_02956 | 127 | 0.014065059 | ARL2 | ADP ribosylation factor like GTPase 2 [Source:HGNC Symbol;Acc:HGNC:693] |
| ENSG00000214686 | HGLibA_23514 | 127 | 0.014065059 | IQCF6 | IQ motif containing F6 [Source:HGNC Symbol;Acc:HGNC:35158] |

| | | | | | |
|---|---|---|---|---|---|
| ENSG00000219200 | HGLibA_41437 | 127 | 0.014065059 | RNASEK | ribonuclease K [Source:HGNC Symbol;Acc:HGNC:33911] |
| ENSG00000221859 | HGLibA_25595 | 127 | 0.014065059 | KRTAP10-10 | keratin associated protein 10-10 [Source:HGNC Symbol;Acc:HGNC:22972] |
| ENSG00000221874 | HGLibA_56816 | 127 | 0.014065059 | ZNF816-ZNF321P | ZNF816-ZNF321P readthrough [Source:HGNC Symbol;Acc:HGNC:38879] |
| ENSG00000228927 | HGLibA_51971 | 127 | 0.014065059 | TSPY3 | testis specific protein Y-linked 3 [Source:HGNC Symbol;Acc:HGNC:33876] |
| ENSG00000231068 | HGLibA_25714 | 127 | 0.014065059 | KRTAP21-3 | keratin associated protein 21-3 [Source:HGNC Symbol;Acc:HGNC:34216] |
| ENSG00000237412 | HGLibA_38965 | 127 | 0.014065059 | PRSS56 | serine protease 56 [Source:HGNC Symbol;Acc:HGNC:39433] |
| ENSG00000240038 | HGLibA_01852 | 127 | 0.014065059 | AMY2B | alpha 2B (pancreatic) [Source:HGNC Symbol;Acc:HGNC:478]" |
| ENSG00000244476 | HGLibA_15539 | 127 | 0.014065059 | ERVFRD-1 | envelope [Source:HGNC Symbol;Acc:HGNC:33823]" |
| ENSG00000244754 | HGLibA_30958 | 127 | 0.014065059 | N4BP2L2 | NEDD4 binding protein 2 like 2 [Source:HGNC Symbol;Acc:HGNC:26916] |
| ENSG00000258405 | HGLibA_56300 | 127 | 0.014065059 | ZNF578 | zinc finger protein 578 [Source:HGNC Symbol;Acc:HGNC:26449] |
| ENSG00000258436 | HGLibA_41397 | 127 | 0.014065059 | RNASE12 | ribonuclease A family member 12 (inactive) [Source:HGNC Symbol;Acc:HGNC:24211] |
| ENSG00000261509 | HGLibA_51081 | 127 | 0.014065059 | TP53TG3B | TP53 target 3B [Source:HGNC Symbol;Acc:HGNC:37202] |
| ENSG00000267680 | HGLibA_55617 | 127 | 0.014065059 | ZNF224 | zinc finger protein 224 [Source:HGNC Symbol;Acc:HGNC:13017] |
| ENSG00000270231 | HGLibA_31243 | 127 | 0.014065059 | NBPF8 | NBPF member 8 [Source:HGNC Symbol;Acc:HGNC:31990] |
| ENSG00000277586 | HGLibA_31620 | 127 | 0.014065059 | NEFL | neurofilament light [Source:HGNC Symbol;Acc:HGNC:7739] |
| FAM86C1 | HGLibA_16779 | 127 | 0.014065059 | FAM86C1 | - |
| GUCY1A3 | HGLibA_20654 | 127 | 0.014065059 | GUCY1A3 | - |
| HIST1H3D | HGLibA_21464 | 127 | 0.014065059 | HIST1H3D | - |
| hsa-mir-3127 | HGLibA_58561 | 127 | 0.014065059 | hsa-mir-3127 | - |
| hsa-mir-4312 | HGLibA_59962 | 127 | 0.014065059 | hsa-mir-4312 | - |
| hsa-mir-4329 | HGLibA_60039 | 127 | 0.014065059 | hsa-mir-4329 | - |
| hsa-mir-4506 | HGLibA_60451 | 127 | 0.014065059 | hsa-mir-4506 | - |
| hsa-mir-4520a | HGLibA_60535 | 127 | 0.014065059 | hsa-mir-4520a | - |
| hsa-mir-4653 | HGLibA_60720 | 127 | 0.014065059 | hsa-mir-4653 | - |
| hsa-mir-4784 | HGLibA_61245 | 127 | 0.014065059 | hsa-mir-4784 | - |
| hsa-mir-4802 | HGLibA_61313 | 127 | 0.014065059 | hsa-mir-4802 | - |
| hsa-mir-512-2 | HGLibA_61568 | 127 | 0.014065059 | hsa-mir-512-2 | - |
| hsa-mir-548f-1 | HGLibA_61971 | 127 | 0.014065059 | hsa-mir-548f-1 | - |
| hsa-mir-5689 | HGLibA_62272 | 127 | 0.014065059 | hsa-mir-5689 | - |
| hsa-mir-6501 | HGLibA_62854 | 127 | 0.014065059 | hsa-mir-6501 | - |
| hsa-mir-7843 | HGLibA_63963 | 127 | 0.014065059 | hsa-mir-7843 | - |
| hsa-mir-8055 | HGLibA_64060 | 127 | 0.014065059 | hsa-mir-8055 | - |
| hsa-mir-875 | HGLibA_64211 | 127 | 0.014065059 | hsa-mir-875 | - |
| hsa-mir-93 | HGLibA_64303 | 127 | 0.014065059 | hsa-mir-93 | - |
| MURC | HGLibA_30570 | 127 | 0.014065059 | MURC | - |
| TMEM183B | HGLibA_50109 | 127 | 0.014065059 | TMEM183B | - |
| TMEM56 | HGLibA_50449 | 127 | 0.014065059 | TMEM56 | - |
| AMICA1 | HGLibA_01789 | 126 | 0.005019716 | AMICA1 | - |
| C10orf107 | HGLibA_05024 | 126 | 0.005019716 | C10orf107 | - |
| C11orf82 | HGLibA_05191 | 126 | 0.005019716 | C11orf82 | - |
| C15orf53 | HGLibA_05411 | 126 | 0.005019716 | C15orf53 | - |
| C7orf41 | HGLibA_06542 | 126 | 0.005019716 | C7orf41 | - |
| C8orf31 | HGLibA_06604 | 126 | 0.005019716 | C8orf31 | - |
| CCDC19 | HGLibA_07726 | 126 | 0.005019716 | CCDC19 | - |
| DHRSX_X | HGLibA_13158 | 126 | 0.005019716 | DHRSX_X | - |
| ENSG00000005194 | HGLibA_09678 | 126 | 0.005019716 | CIAPIN1 | cytokine induced apoptosis inhibitor 1 [Source:HGNC Symbol;Acc:HGNC:28050] |
| ENSG00000006377 | HGLibA_13387 | 126 | 0.005019716 | DLX6 | distal-less homeobox 6 [Source:HGNC Symbol;Acc:HGNC:2919] |
| ENSG00000009765 | HGLibA_23890 | 126 | 0.005019716 | IYD | iodotyrosine deiodinase [Source:HGNC Symbol;Acc:HGNC:21071] |
| ENSG00000011600 | HGLibA_52471 | 126 | 0.005019716 | TYROBP | TYRO protein tyrosine kinase binding protein [Source:HGNC Symbol;Acc:HGNC:12449] |
| ENSG00000012660 | HGLibA_14970 | 126 | 0.005019716 | ELOVL5 | ELOVL fatty acid elongase 5 [Source:HGNC Symbol;Acc:HGNC:21308] |
| ENSG00000054796 | HGLibA_46742 | 126 | 0.005019716 | SPO11 | initiator of meiotic double stranded breaks [Source:HGNC Symbol;Acc:HGNC:11250]" |
| ENSG00000056998 | HGLibA_20691 | 126 | 0.005019716 | GYG2 | glycogenin 2 [Source:HGNC Symbol;Acc:HGNC:4700] |
| ENSG00000065060 | HGLibA_52948 | 126 | 0.005019716 | UHRF1BP1 | UHRF1 binding protein 1 [Source:HGNC Symbol;Acc:HGNC:21216] |
| ENSG00000081237 | HGLibA_39491 | 126 | 0.005019716 | PTPRC | receptor type C [Source:HGNC Symbol;Acc:HGNC:9666]" |
| ENSG00000081985 | HGLibA_22967 | 126 | 0.005019716 | IL12RB2 | interleukin 12 receptor subunit beta 2 [Source:HGNC Symbol;Acc:HGNC:5972] |
| ENSG00000085741 | HGLibA_54352 | 126 | 0.005019716 | WNT11 | Wnt family member 11 [Source:HGNC Symbol;Acc:HGNC:12776] |
| ENSG00000095209 | HGLibA_50381 | 126 | 0.005019716 | TMEM38B | transmembrane protein 38B [Source:HGNC Symbol;Acc:HGNC:25535] |
| ENSG00000099282 | HGLibA_51907 | 126 | 0.005019716 | TSPAN15 | tetraspanin 15 [Source:HGNC Symbol;Acc:HGNC:23298] |
| ENSG00000100593 | HGLibA_23679 | 126 | 0.005019716 | ISM2 | isthmin 2 [Source:HGNC Symbol;Acc:HGNC:23176] |
| ENSG00000101222 | HGLibA_46609 | 126 | 0.005019716 | SPEF1 | sperm flagellar 1 [Source:HGNC Symbol;Acc:HGNC:15874] |
| ENSG00000101639 | HGLibA_09126 | 126 | 0.005019716 | CEP192 | centrosomal protein 192 [Source:HGNC Symbol;Acc:HGNC:25515] |
| ENSG00000101751 | HGLibA_37532 | 126 | 0.005019716 | POLI | DNA polymerase iota [Source:HGNC Symbol;Acc:HGNC:9182] |
| ENSG00000102034 | HGLibA_14901 | 126 | 0.005019716 | ELF4 | E74 like ETS transcription factor 4 [Source:HGNC Symbol;Acc:HGNC:3319] |
| ENSG00000103035 | HGLibA_39181 | 126 | 0.005019716 | PSMD7 | non-ATPase 7 [Source:HGNC Symbol;Acc:HGNC:9565]" |
| ENSG00000104140 | HGLibA_41210 | 126 | 0.005019716 | RHOV | ras homolog family member V [Source:HGNC Symbol;Acc:HGNC:18313] |
| ENSG00000105443 | HGLibA_12238 | 126 | 0.005019716 | CYTH2 | cytohesin 2 [Source:HGNC Symbol;Acc:HGNC:9502] |
| ENSG00000108515 | HGLibA_15147 | 126 | 0.005019716 | ENO3 | enolase 3 [Source:HGNC Symbol;Acc:HGNC:3354] |
| ENSG00000108557 | HGLibA_40137 | 126 | 0.005019716 | RAI1 | retinoic acid induced 1 [Source:HGNC Symbol;Acc:HGNC:9834] |
| ENSG00000108947 | HGLibA_14604 | 126 | 0.005019716 | EFNB3 | ephrin B3 [Source:HGNC Symbol;Acc:HGNC:3228] |
| ENSG00000111058 | HGLibA_00593 | 126 | 0.005019716 | ACSS3 | acyl-CoA synthetase short chain family member 3 [Source:HGNC Symbol;Acc:HGNC:24723] |
| ENSG00000111684 | HGLibA_27076 | 126 | 0.005019716 | LPCAT3 | lysophosphatidylcholine acyltransferase 3 [Source:HGNC Symbol;Acc:HGNC:30244] |
| ENSG00000113108 | HGLibA_02387 | 126 | 0.005019716 | APBB3 | amyloid beta precursor protein binding family B member 3 [Source:HGNC Symbol;Acc:HGNC:20708] |
| ENSG00000113391 | HGLibA_16273 | 126 | 0.005019716 | FAM172A | family with sequence similarity 172 member A [Source:HGNC Symbol;Acc:HGNC:25365] |
| ENSG00000113712 | HGLibA_11382 | 126 | 0.005019716 | CSNK1A1 | casein kinase 1 alpha 1 [Source:HGNC Symbol;Acc:HGNC:2451] |
| ENSG00000115977 | HGLibA_00051 | 126 | 0.005019716 | AAK1 | AP2 associated kinase 1 [Source:HGNC Symbol;Acc:HGNC:19679] |
| ENSG00000116721 | HGLibA_38279 | 126 | 0.005019716 | PRAMEF1 | PRAME family member 1 [Source:HGNC Symbol;Acc:HGNC:28840] |
| ENSG00000120942 | HGLibA_52699 | 126 | 0.005019716 | UBIAD1 | UbiA prenyltransferase domain containing 1 [Source:HGNC Symbol;Acc:HGNC:30791] |
| ENSG00000125037 | HGLibA_15010 | 126 | 0.005019716 | EMC3 | ER membrane protein complex subunit 3 [Source:HGNC Symbol;Acc:HGNC:23999] |
| ENSG00000125089 | HGLibA_43980 | 126 | 0.005019716 | SH3TC1 | SH3 domain and tetratricopeptide repeats 1 [Source:HGNC Symbol;Acc:HGNC:26009] |
| ENSG00000130544 | HGLibA_56230 | 126 | 0.005019716 | ZNF557 | zinc finger protein 557 [Source:HGNC Symbol;Acc:HGNC:28632] |
| ENSG00000130726 | HGLibA_51451 | 126 | 0.005019716 | TRIM28 | tripartite motif containing 28 [Source:HGNC Symbol;Acc:HGNC:16384] |
| ENSG00000131055 | HGLibA_10832 | 126 | 0.005019716 | COX4I2 | cytochrome c oxidase subunit 4I2 [Source:HGNC Symbol;Acc:HGNC:16232] |
| ENSG00000131171 | HGLibA_43916 | 126 | 0.005019716 | SH3BGRL | SH3 domain binding glutamate rich protein like [Source:HGNC Symbol;Acc:HGNC:10823] |
| ENSG00000131626 | HGLibA_37872 | 126 | 0.005019716 | PPFIA1 | PTPRF interacting protein alpha 1 [Source:HGNC Symbol;Acc:HGNC:9245] |
| ENSG00000132182 | HGLibA_33040 | 126 | 0.005019716 | NUP210 | nucleoporin 210 [Source:HGNC Symbol;Acc:HGNC:30052] |
| ENSG00000134575 | HGLibA_00511 | 126 | 0.005019716 | ACP2 | lysosomal [Source:HGNC Symbol;Acc:HGNC:123]" |
| ENSG00000134986 | HGLibA_32671 | 126 | 0.005019716 | NREP | neuronal regeneration related protein [Source:HGNC Symbol;Acc:HGNC:16834] |
| ENSG00000135211 | HGLibA_50466 | 126 | 0.005019716 | TMEM60 | transmembrane protein 60 [Source:HGNC Symbol;Acc:HGNC:21754] |
| ENSG00000135299 | HGLibA_02130 | 126 | 0.005019716 | ANKRD6 | ankyrin repeat domain 6 [Source:HGNC Symbol;Acc:HGNC:17280] |
| ENSG00000135441 | HGLibA_04557 | 126 | 0.005019716 | BLOC1S1 | biogenesis of lysosomal organelles complex 1 subunit 1 [Source:HGNC Symbol;Acc:HGNC:4200] |
| ENSG00000135472 | HGLibA_15966 | 126 | 0.005019716 | FAIM2 | Fas apoptotic inhibitory molecule 2 [Source:HGNC Symbol;Acc:HGNC:17067] |
| ENSG00000136147 | HGLibA_36335 | 126 | 0.005019716 | PHF11 | PHD finger protein 11 [Source:HGNC Symbol;Acc:HGNC:17024] |
| ENSG00000138311 | HGLibA_55877 | 126 | 0.005019716 | ZNF365 | zinc finger protein 365 [Source:HGNC Symbol;Acc:HGNC:18194] |
| ENSG00000138653 | HGLibA_31433 | 126 | 0.005019716 | NDST4 | N-deacetylase and N-sulfotransferase 4 [Source:HGNC Symbol;Acc:HGNC:20779] |
| ENSG00000139505 | HGLibA_30432 | 126 | 0.005019716 | MTMR6 | myotubularin related protein 6 [Source:HGNC Symbol;Acc:HGNC:7453] |
| ENSG00000139714 | HGLibA_29671 | 126 | 0.005019716 | MORN3 | MORN repeat containing 3 [Source:HGNC Symbol;Acc:HGNC:29807] |
| ENSG00000141956 | HGLibA_38379 | 126 | 0.005019716 | PRDM15 | PR/SET domain 15 [Source:HGNC Symbol;Acc:HGNC:13999] |
| ENSG00000142632 | HGLibA_02830 | 126 | 0.005019716 | ARHGEF19 | Rho guanine nucleotide exchange factor 19 [Source:HGNC Symbol;Acc:HGNC:26604] |
| ENSG00000143341 | HGLibA_21674 | 126 | 0.005019716 | HMCN1 | hemicentin 1 [Source:HGNC Symbol;Acc:HGNC:19194] |
| ENSG00000144233 | HGLibA_01805 | 126 | 0.005019716 | AMMECR1L | AMMECR1 like [Source:HGNC Symbol;Acc:HGNC:28658] |
| ENSG00000145050 | HGLibA_28098 | 126 | 0.005019716 | MANF | mesencephalic astrocyte derived neurotrophic factor [Source:HGNC Symbol;Acc:HGNC:15461] |
| ENSG00000147133 | HGLibA_48196 | 126 | 0.005019716 | TAF1 | TATA-box binding protein associated factor 1 [Source:HGNC Symbol;Acc:HGNC:11535] |
| ENSG00000149635 | HGLibA_33267 | 126 | 0.005019716 | OCSTAMP | osteoclast stimulatory transmembrane protein [Source:HGNC Symbol;Acc:HGNC:16116] |
| ENSG00000150045 | HGLibA_25302 | 126 | 0.005019716 | KLRF1 | killer cell lectin like receptor F1 [Source:HGNC Symbol;Acc:HGNC:13342] |
| ENSG00000150867 | HGLibA_36706 | 126 | 0.005019716 | PIP4K2A | phosphatidylinositol-5-phosphate 4-kinase type 2 alpha [Source:HGNC Symbol;Acc:HGNC:8997] |
| ENSG00000152804 | HGLibA_21258 | 126 | 0.005019716 | HHEX | hematopoietically expressed homeobox [Source:HGNC Symbol;Acc:HGNC:4901] |
| ENSG00000158411 | HGLibA_29315 | 126 | 0.005019716 | MITD1 | microtubule interacting and trafficking domain containing 1 [Source:HGNC Symbol;Acc:HGNC:25207] |
| ENSG00000159640 | HGLibA_00421 | 126 | 0.005019716 | ACE | angiotensin I converting enzyme [Source:HGNC Symbol;Acc:HGNC:2707] |
| ENSG00000162236 | HGLibA_47652 | 126 | 0.005019716 | STX5 | syntaxin 5 [Source:HGNC Symbol;Acc:HGNC:11440] |
| ENSG00000162456 | HGLibA_25335 | 126 | 0.005019716 | KNCN | kinocilin [Source:HGNC Symbol;Acc:HGNC:26488] |
| ENSG00000164438 | HGLibA_49680 | 126 | 0.005019716 | TLX3 | T cell leukemia homeobox 3 [Source:HGNC Symbol;Acc:HGNC:13532] |
| ENSG00000165471 | HGLibA_28499 | 126 | 0.005019716 | MBL2 | mannose binding lectin 2 [Source:HGNC Symbol;Acc:HGNC:6922] |
| ENSG00000166167 | HGLibA_04987 | 126 | 0.005019716 | BTRC | beta-transducin repeat containing E3 ubiquitin protein ligase [Source:HGNC Symbol;Acc:HGNC:1144] |
| ENSG00000166323 | HGLibA_05168 | 126 | 0.005019716 | C11orf65 | chromosome 11 open reading frame 65 [Source:HGNC Symbol;Acc:HGNC:28519] |
| ENSG00000166526 | HGLibA_55764 | 126 | 0.005019716 | ZNF3 | zinc finger protein 3 [Source:HGNC Symbol;Acc:HGNC:13089] |
| ENSG00000166816 | HGLibA_26234 | 126 | 0.005019716 | LDHD | lactate dehydrogenase D [Source:HGNC Symbol;Acc:HGNC:19708] |
| ENSG00000167992 | HGLibA_53930 | 126 | 0.005019716 | VWCE | von Willebrand factor C and EGF domains [Source:HGNC Symbol;Acc:HGNC:26487] |
| ENSG00000168036 | HGLibA_11650 | 126 | 0.005019716 | CTNNB1 | catenin beta 1 [Source:HGNC Symbol;Acc:HGNC:2514] |
| ENSG00000168060 | HGLibA_31011 | 126 | 0.005019716 | NAALADL1 | N-acetylated alpha-linked acidic dipeptidase like 1 [Source:HGNC Symbol;Acc:HGNC:23536] |
| ENSG00000168348 | HGLibA_23398 | 126 | 0.005019716 | INSM2 | INSM transcriptional repressor 2 [Source:HGNC Symbol;Acc:HGNC:17539] |
| ENSG00000169231 | HGLibA_49327 | 126 | 0.005019716 | THBS3 | thrombospondin 3 [Source:HGNC Symbol;Acc:HGNC:11787] |
| ENSG00000169902 | HGLibA_51205 | 126 | 0.005019716 | TPST1 | tyrosylprotein sulfotransferase 1 [Source:HGNC Symbol;Acc:HGNC:12020] |
| ENSG00000170775 | HGLibA_20033 | 126 | 0.005019716 | GPR37 | G protein-coupled receptor 37 [Source:HGNC Symbol;Acc:HGNC:4494] |
| ENSG00000170881 | HGLibA_41517 | 126 | 0.005019716 | RNF139 | ring finger protein 139 [Source:HGNC Symbol;Acc:HGNC:17023] |
| ENSG00000171408 | HGLibA_35826 | 126 | 0.005019716 | PDE7B | phosphodiesterase 7B [Source:HGNC Symbol;Acc:HGNC:8792] |
| ENSG00000171595 | HGLibA_13508 | 126 | 0.005019716 | DNAI2 | dynein axonemal intermediate chain 2 [Source:HGNC Symbol;Acc:HGNC:18744] |
| ENSG00000171928 | HGLibA_52361 | 126 | 0.005019716 | TVP23B | trans-golgi network vesicle protein 23 homolog B [Source:HGNC Symbol;Acc:HGNC:20399] |
| ENSG00000172215 | HGLibA_11897 | 126 | 0.005019716 | CXCR6 | C-X-C motif chemokine receptor 6 [Source:HGNC Symbol;Acc:HGNC:16647] |
| ENSG00000173402 | HGLibA_12292 | 126 | 0.005019716 | DAG1 | dystroglycan 1 [Source:HGNC Symbol;Acc:HGNC:2666] |
| ENSG00000173846 | HGLibA_37195 | 126 | 0.005019716 | PLK3 | polo like kinase 3 [Source:HGNC Symbol;Acc:HGNC:2154] |
| ENSG00000174527 | HGLibA_30841 | 126 | 0.005019716 | MYO1H | myosin IH [Source:HGNC Symbol;Acc:HGNC:13879] |
| ENSG00000174780 | HGLibA_47011 | 126 | 0.005019716 | SRP72 | signal recognition particle 72 [Source:HGNC Symbol;Acc:HGNC:11303] |
| ENSG00000175283 | HGLibA_13789 | 126 | 0.005019716 | DOLK | dolichol kinase [Source:HGNC Symbol;Acc:HGNC:23406] |
| ENSG00000175322 | HGLibA_56154 | 126 | 0.005019716 | ZNF519 | zinc finger protein 519 [Source:HGNC Symbol;Acc:HGNC:30574] |
| ENSG00000175463 | HGLibA_48470 | 126 | 0.005019716 | TBC1D10C | TBC1 domain family member 10C [Source:HGNC Symbol;Acc:HGNC:24702] |

| | | | | | |
|---|---|---|---|---|---|
| ENSG00000175497 | HGLibA_13867 | 126 | 0.005019716 | DPP10 | dipeptidyl peptidase like 10 [Source:HGNC Symbol;Acc:HGNC:20823] |
| ENSG00000176454 | HGLibA_27080 | 126 | 0.005019716 | LPCAT4 | lysophosphatidylcholine acyltransferase 4 [Source:HGNC Symbol;Acc:HGNC:30059] |
| ENSG00000176769 | HGLibA_48757 | 126 | 0.005019716 | TCERG1L | transcription elongation regulator 1 like [Source:HGNC Symbol;Acc:HGNC:23533] |
| ENSG00000181481 | HGLibA_41512 | 126 | 0.005019716 | RNF135 | ring finger protein 135 [Source:HGNC Symbol;Acc:HGNC:21158] |
| ENSG00000181852 | HGLibA_41682 | 126 | 0.005019716 | RNF41 | ring finger protein 41 [Source:HGNC Symbol;Acc:HGNC:18401] |
| ENSG00000183337 | HGLibA_04362 | 126 | 0.005019716 | BCOR | BCL6 corepressor [Source:HGNC Symbol;Acc:HGNC:20893] |
| ENSG00000184898 | HGLibA_40553 | 126 | 0.005019716 | RBM43 | RNA binding motif protein 43 [Source:HGNC Symbol;Acc:HGNC:24790] |
| ENSG00000185559 | HGLibA_13354 | 126 | 0.005019716 | DLK1 | delta like non-canonical Notch ligand 1 [Source:HGNC Symbol;Acc:HGNC:2907] |
| ENSG00000185792 | HGLibA_32136 | 126 | 0.005019716 | NLRP9 | NLR family pyrin domain containing 9 [Source:HGNC Symbol;Acc:HGNC:22941] |
| ENSG00000186115 | HGLibA_12186 | 126 | 0.005019716 | CYP4F2 | cytochrome P450 family 4 subfamily F member 2 [Source:HGNC Symbol;Acc:HGNC:2645] |
| ENSG00000186222 | HGLibA_04567 | 126 | 0.005019716 | BLOC1S4 | biogenesis of lysosomal organelles complex 1 subunit 4 [Source:HGNC Symbol;Acc:HGNC:24206] |
| ENSG00000186487 | HGLibA_30929 | 126 | 0.005019716 | MYT1L | myelin transcription factor 1 like [Source:HGNC Symbol;Acc:HGNC:7623] |
| ENSG00000187048 | HGLibA_12173 | 126 | 0.005019716 | CYP4A11 | cytochrome P450 family 4 subfamily A member 11 [Source:HGNC Symbol;Acc:HGNC:2642] |
| ENSG00000187821 | HGLibA_21121 | 126 | 0.005019716 | HELT | helt bHLH transcription factor [Source:HGNC Symbol;Acc:HGNC:33783] |
| ENSG00000187912 | HGLibA_09941 | 126 | 0.005019716 | CLEC17A | C-type lectin domain containing 17A [Source:HGNC Symbol;Acc:HGNC:34520] |
| ENSG00000188691 | HGLibA_34220 | 126 | 0.005019716 | OR56A5 | olfactory receptor family 56 subfamily A member 5 [Source:HGNC Symbol;Acc:HGNC:14792] |
| ENSG00000189167 | HGLibA_54782 | 126 | 0.005019716 | ZAR1L | zygote arrest 1 like [Source:HGNC Symbol;Acc:HGNC:37116] |
| ENSG00000197261 | HGLibA_06448 | 126 | 0.005019716 | C6orf141 | chromosome 6 open reading frame 141 [Source:HGNC Symbol;Acc:HGNC:21351] |
| ENSG00000197312 | HGLibA_12611 | 126 | 0.005019716 | DDI2 | DNA damage inducible 1 homolog 2 [Source:HGNC Symbol;Acc:HGNC:24578] |
| ENSG00000197471 | HGLibA_46729 | 126 | 0.005019716 | SPN | sialophorin [Source:HGNC Symbol;Acc:HGNC:11249] |
| ENSG00000203747 | HGLibA_17194 | 126 | 0.005019716 | FCGR3A | Fc fragment of IgG receptor IIIa [Source:HGNC Symbol;Acc:HGNC:3619] |
| ENSG00000203780 | HGLibA_16862 | 126 | 0.005019716 | FANK1 | fibronectin type III and ankyrin repeat domains 1 [Source:HGNC Symbol;Acc:HGNC:23527] |
| ENSG00000204228 | HGLibA_22203 | 126 | 0.005019716 | HSD17B8 | hydroxysteroid 17-beta dehydrogenase 8 [Source:HGNC Symbol;Acc:HGNC:3554] |
| ENSG00000204344 | HGLibA_47468 | 126 | 0.005019716 | STK19 | serine/threonine kinase 19 [Source:HGNC Symbol;Acc:HGNC:11398] |
| ENSG00000205084 | HGLibA_50257 | 126 | 0.005019716 | TMEM231 | transmembrane protein 231 [Source:HGNC Symbol;Acc:HGNC:37234] |
| ENSG00000205413 | HGLibA_42698 | 126 | 0.005019716 | SAMD9 | sterile alpha motif domain containing 9 [Source:HGNC Symbol;Acc:HGNC:1348] |
| ENSG00000205439 | HGLibA_25636 | 126 | 0.005019716 | KRTAP12-3 | keratin associated protein 12-3 [Source:HGNC Symbol;Acc:HGNC:20531] |
| ENSG00000205777 | HGLibA_18430 | 126 | 0.005019716 | GAGE1 | G antigen 1 [Source:HGNC Symbol;Acc:HGNC:4098] |
| ENSG00000214753 | HGLibA_21851 | 126 | 0.005019716 | HNRNPUL2 | heterogeneous nuclear ribonucleoprotein U like 2 [Source:HGNC Symbol;Acc:HGNC:25451] |
| ENSG00000215187 | HGLibA_16238 | 126 | 0.005019716 | FAM166B | family with sequence similarity 166 member B [Source:HGNC Symbol;Acc:HGNC:34242] |
| ENSG00000226979 | HGLibA_27574 | 126 | 0.005019716 | LTA | lymphotoxin alpha [Source:HGNC Symbol;Acc:HGNC:6709] |
| ENSG00000228696 | HGLibA_02954 | 126 | 0.005019716 | ARL17B | ADP ribosylation factor like GTPase 17B [Source:HGNC Symbol;Acc:HGNC:32387] |
| ENSG00000236699 | HGLibA_02858 | 126 | 0.005019716 | ARHGEF38 | Rho guanine nucleotide exchange factor 38 [Source:HGNC Symbol;Acc:HGNC:25968] |
| ENSG00000241852 | HGLibA_06639 | 126 | 0.005019716 | C8orf58 | chromosome 8 open reading frame 58 [Source:HGNC Symbol;Acc:HGNC:32233] |
| ENSG00000243729 | HGLibA_34363 | 126 | 0.005019716 | OR5V1 | olfactory receptor family 5 subfamily V member 1 [Source:HGNC Symbol;Acc:HGNC:13972] |
| ENSG00000244537 | HGLibA_25759 | 126 | 0.005019716 | KRTAP4-2 | keratin associated protein 4-2 [Source:HGNC Symbol;Acc:HGNC:18900] |
| ENSG00000254087 | HGLibA_27707 | 126 | 0.005019716 | LYN | Src family tyrosine kinase [Source:HGNC Symbol;Acc:HGNC:6735]" |
| ENSG00000254788 | HGLibA_09773 | 126 | 0.005019716 | CKLF-CMTM1 | CKLF-CMTM1 readthrough [Source:HGNC Symbol;Acc:HGNC:39977] |
| ENSG00000255819 | HGLibA_25297 | 126 | 0.005019716 | KLRC4-KLRK1 | KLRC4-KLRK1 readthrough [Source:HGNC Symbol;Acc:HGNC:48357] |
| ENSG00000256043 | HGLibA_11726 | 126 | 0.005019716 | CTSO | cathepsin O [Source:HGNC Symbol;Acc:HGNC:2542] |
| ENSG00000258366 | HGLibA_42380 | 126 | 0.005019716 | RTEL1 | regulator of telomere elongation helicase 1 [Source:HGNC Symbol;Acc:HGNC:15888] |
| ENSG00000265491 | HGLibA_41482 | 126 | 0.005019716 | RNF115 | ring finger protein 115 [Source:HGNC Symbol;Acc:HGNC:18154] |
| ENSG00000268104 | HGLibA_45300 | 126 | 0.005019716 | SLC6A14 | solute carrier family 6 member 14 [Source:HGNC Symbol;Acc:HGNC:11047] |
| ENSG00000270765 | HGLibA_18646 | 126 | 0.005019716 | GAS2L2 | growth arrest specific 2 like 2 [Source:HGNC Symbol;Acc:HGNC:24846] |
| ENSG00000272031 | HGLibA_02074 | 126 | 0.005019716 | ANKRD34A | ankyrin repeat domain 34A [Source:HGNC Symbol;Acc:HGNC:27639] |
| FAM175B | HGLibA_16290 | 126 | 0.005019716 | FAM175B | - |
| GPER | HGLibA_19793 | 126 | 0.005019716 | GPER | - |
| HKR1 | HGLibA_21592 | 126 | 0.005019716 | HKR1 | - |
| hsa-mir-1256 | HGLibA_57433 | 126 | 0.005019716 | hsa-mir-1256 | - |
| hsa-mir-133a-2 | HGLibA_57778 | 126 | 0.005019716 | hsa-mir-133a-2 | - |
| hsa-mir-196b | HGLibA_58099 | 126 | 0.005019716 | hsa-mir-196b | - |
| hsa-mir-204 | HGLibA_58174 | 126 | 0.005019716 | hsa-mir-204 | - |
| hsa-mir-3920 | HGLibA_59556 | 126 | 0.005019716 | hsa-mir-3920 | - |
| hsa-mir-3925 | HGLibA_59574 | 126 | 0.005019716 | hsa-mir-3925 | - |
| hsa-mir-4274 | HGLibA_59798 | 126 | 0.005019716 | hsa-mir-4274 | - |
| hsa-mir-4483 | HGLibA_60350 | 126 | 0.005019716 | hsa-mir-4483 | - |
| hsa-mir-4640 | HGLibA_60663 | 126 | 0.005019716 | hsa-mir-4640 | - |
| hsa-mir-4739 | HGLibA_61052 | 126 | 0.005019716 | hsa-mir-4739 | - |
| hsa-mir-5002 | HGLibA_61417 | 126 | 0.005019716 | hsa-mir-5002 | - |
| hsa-mir-514a-2 | HGLibA_61589 | 126 | 0.005019716 | hsa-mir-514a-2 | - |
| hsa-mir-514a-3 | HGLibA_61596 | 126 | 0.005019716 | hsa-mir-514a-3 | - |
| hsa-mir-520e | HGLibA_61751 | 126 | 0.005019716 | hsa-mir-520e | - |
| hsa-mir-5707 | HGLibA_62368 | 126 | 0.005019716 | hsa-mir-5707 | - |
| hsa-mir-579 | HGLibA_62416 | 126 | 0.005019716 | hsa-mir-579 | - |
| hsa-mir-6075 | HGLibA_62554 | 126 | 0.005019716 | hsa-mir-6075 | - |
| hsa-mir-654 | HGLibA_62951 | 126 | 0.005019716 | hsa-mir-654 | - |
| hsa-mir-6797 | HGLibA_63366 | 126 | 0.005019716 | hsa-mir-6797 | - |
| hsa-mir-6872 | HGLibA_63674 | 126 | 0.005019716 | hsa-mir-6872 | - |
| hsa-mir-7849 | HGLibA_63986 | 126 | 0.005019716 | hsa-mir-7849 | - |
| hsa-mir-887 | HGLibA_64225 | 126 | 0.005019716 | hsa-mir-887 | - |
| hsa-mir-889 | HGLibA_64233 | 126 | 0.005019716 | hsa-mir-889 | - |
| KIAA1456 | HGLibA_24759 | 126 | 0.005019716 | KIAA1456 | - |
| UTP11L | HGLibA_53432 | 126 | 0.005019716 | UTP11L | - |
| C10orf11 | HGLibA_05028 | 125 | -0.004025627 | C10orf11 | - |
| C3orf79 | HGLibA_06270 | 125 | -0.004025627 | C3orf79 | - |
| ENSG00000005700 | HGLibA_22484 | 125 | -0.004025627 | IBTK | inhibitor of Bruton tyrosine kinase [Source:HGNC Symbol;Acc:HGNC:17853] |
| ENSG00000008952 | HGLibA_43258 | 125 | -0.004025627 | SEC62 | preprotein translocation factor [Source:HGNC Symbol;Acc:HGNC:11846]" |
| ENSG00000027697 | HGLibA_22709 | 125 | -0.004025627 | IFNGR1 | interferon gamma receptor 1 [Source:HGNC Symbol;Acc:HGNC:5439] |
| ENSG00000048342 | HGLibA_07480 | 125 | -0.004025627 | CC2D2A | coiled-coil and C2 domain containing 2A [Source:HGNC Symbol;Acc:HGNC:29253] |
| ENSG00000077585 | HGLibA_19901 | 125 | -0.004025627 | GPR137B | G protein-coupled receptor 137B [Source:HGNC Symbol;Acc:HGNC:11862] |
| ENSG00000077800 | HGLibA_17581 | 125 | -0.004025627 | FKBP6 | FK506 binding protein 6 [Source:HGNC Symbol;Acc:HGNC:3722] |
| ENSG00000079277 | HGLibA_29343 | 125 | -0.004025627 | MKNK1 | MAP kinase interacting serine/threonine kinase 1 [Source:HGNC Symbol;Acc:HGNC:7110] |
| ENSG00000081148 | HGLibA_23266 | 125 | -0.004025627 | IMPG2 | interphotoreceptor matrix proteoglycan 2 [Source:HGNC Symbol;Acc:HGNC:18362] |
| ENSG00000084754 | HGLibA_20809 | 125 | -0.004025627 | HADHA | hydroxyacyl-CoA dehydrogenase trifunctional multienzyme complex subunit alpha [Source:HGNC Symbol;Acc:HGNC:4801] |
| ENSG00000086200 | HGLibA_23461 | 125 | -0.004025627 | IPO11 | importin 11 [Source:HGNC Symbol;Acc:HGNC:20628] |
| ENSG00000088256 | HGLibA_19436 | 125 | -0.004025627 | GNA11 | G protein subunit alpha 11 [Source:HGNC Symbol;Acc:HGNC:4379] |
| ENSG00000089169 | HGLibA_41843 | 125 | -0.004025627 | RPH3A | rabphilin 3A [Source:HGNC Symbol;Acc:HGNC:17056] |
| ENSG00000095015 | HGLibA_28159 | 125 | -0.004025627 | MAP3K1 | mitogen-activated protein kinase kinase kinase 1 [Source:HGNC Symbol;Acc:HGNC:6848] |
| ENSG00000099957 | HGLibA_34861 | 125 | -0.004025627 | P2RX6 | purinergic receptor P2X 6 [Source:HGNC Symbol;Acc:HGNC:8538] |
| ENSG00000100243 | HGLibA_12007 | 125 | -0.004025627 | CYB5R3 | cytochrome b5 reductase 3 [Source:HGNC Symbol;Acc:HGNC:2873] |
| ENSG00000100246 | HGLibA_13647 | 125 | -0.004025627 | DNAL4 | dynein axonemal light chain 4 [Source:HGNC Symbol;Acc:HGNC:2955] |
| ENSG00000101224 | HGLibA_08529 | 125 | -0.004025627 | CDC25B | cell division cycle 25B [Source:HGNC Symbol;Acc:HGNC:1726] |
| ENSG00000101310 | HGLibA_43224 | 125 | -0.004025627 | SEC23B | coat complex II component [Source:HGNC Symbol;Acc:HGNC:10702]" |
| ENSG00000101542 | HGLibA_08661 | 125 | -0.004025627 | CDH20 | cadherin 20 [Source:HGNC Symbol;Acc:HGNC:1760] |
| ENSG00000102390 | HGLibA_35328 | 125 | -0.004025627 | PBDC1 | polysaccharide biosynthesis domain containing 1 [Source:HGNC Symbol;Acc:HGNC:28790] |
| ENSG00000105967 | HGLibA_49165 | 125 | -0.004025627 | TFEC | transcription factor EC [Source:HGNC Symbol;Acc:HGNC:11754] |
| ENSG00000106258 | HGLibA_12160 | 125 | -0.004025627 | CYP3A5 | cytochrome P450 family 3 subfamily A member 5 [Source:HGNC Symbol;Acc:HGNC:2638] |
| ENSG00000109832 | HGLibA_12682 | 125 | -0.004025627 | DDX25 | DEAD-box helicase 25 [Source:HGNC Symbol;Acc:HGNC:18698] |
| ENSG00000110786 | HGLibA_39473 | 125 | -0.004025627 | PTPN5 | non-receptor type 5 [Source:HGNC Symbol;Acc:HGNC:9657]" |
| ENSG00000112276 | HGLibA_05004 | 125 | -0.004025627 | BVES | blood vessel epicardial substance [Source:HGNC Symbol;Acc:HGNC:1152] |
| ENSG00000113621 | HGLibA_52410 | 125 | -0.004025627 | TXNDC15 | thioredoxin domain containing 15 [Source:HGNC Symbol;Acc:HGNC:20652] |
| ENSG00000115756 | HGLibA_22019 | 125 | -0.004025627 | HPCAL1 | hippocalcin like 1 [Source:HGNC Symbol;Acc:HGNC:5145] |
| ENSG00000115844 | HGLibA_13376 | 125 | -0.004025627 | DLX2 | distal-less homeobox 2 [Source:HGNC Symbol;Acc:HGNC:2915] |
| ENSG00000116525 | HGLibA_51569 | 125 | -0.004025627 | TRIM62 | tripartite motif containing 62 [Source:HGNC Symbol;Acc:HGNC:25574] |
| ENSG00000117983 | HGLibA_30550 | 125 | -0.004025627 | MUC5B | oligomeric mucus/gel-forming [Source:HGNC Symbol;Acc:HGNC:7516]" |
| ENSG00000119446 | HGLibA_40499 | 125 | -0.004025627 | RBM18 | RNA binding motif protein 18 [Source:HGNC Symbol;Acc:HGNC:28413] |
| ENSG00000119522 | HGLibA_12928 | 125 | -0.004025627 | DENND1A | DENN domain containing 1A [Source:HGNC Symbol;Acc:HGNC:29324] |
| ENSG00000119917 | HGLibA_22633 | 125 | -0.004025627 | IFIT3 | interferon induced protein with tetratricopeptide repeats 3 [Source:HGNC Symbol;Acc:HGNC:5411] |
| ENSG00000120318 | HGLibA_02630 | 125 | -0.004025627 | ARAP3 | ankyrin repeat and PH domain 3 [Source:HGNC Symbol;Acc:HGNC:24097]" |
| ENSG00000120820 | HGLibA_19341 | 125 | -0.004025627 | GLT8D2 | glycosyltransferase 8 domain containing 2 [Source:HGNC Symbol;Acc:HGNC:24890] |
| ENSG00000121390 | HGLibA_39223 | 125 | -0.004025627 | PSPC1 | paraspeckle component 1 [Source:HGNC Symbol;Acc:HGNC:20320] |
| ENSG00000121769 | HGLibA_15917 | 125 | -0.004025627 | FABP3 | fatty acid binding protein 3 [Source:HGNC Symbol;Acc:HGNC:3557] |
| ENSG00000122691 | HGLibA_52376 | 125 | -0.004025627 | TWIST1 | twist family bHLH transcription factor 1 [Source:HGNC Symbol;Acc:HGNC:12428] |
| ENSG00000124098 | HGLibA_16457 | 125 | -0.004025627 | FAM210B | family with sequence similarity 210 member B [Source:HGNC Symbol;Acc:HGNC:16102] |
| ENSG00000125746 | HGLibA_15058 | 125 | -0.004025627 | EML2 | echinoderm microtubule associated protein like 2 [Source:HGNC Symbol;Acc:HGNC:18035] |
| ENSG00000125821 | HGLibA_14053 | 125 | -0.004025627 | DTD1 | D-tyrosyl-tRNA deacylase 1 [Source:HGNC Symbol;Acc:HGNC:16219] |
| ENSG00000128294 | HGLibA_51207 | 125 | -0.004025627 | TPST2 | tyrosylprotein sulfotransferase 2 [Source:HGNC Symbol;Acc:HGNC:12021] |
| ENSG00000128908 | HGLibA_23320 | 125 | -0.004025627 | INO80 | INO80 complex subunit [Source:HGNC Symbol;Acc:HGNC:26956] |
| ENSG00000129932 | HGLibA_13763 | 125 | -0.004025627 | DOHH | deoxyhypusine hydroxylase [Source:HGNC Symbol;Acc:HGNC:28662] |
| ENSG00000130830 | HGLibA_29748 | 125 | -0.004025627 | MPP1 | membrane palmitoylated protein 1 [Source:HGNC Symbol;Acc:HGNC:7219] |
| ENSG00000133020 | HGLibA_30739 | 125 | -0.004025627 | MYH8 | myosin heavy chain 8 [Source:HGNC Symbol;Acc:HGNC:7578] |
| ENSG00000133884 | HGLibA_13831 | 125 | -0.004025627 | DPF2 | double PHD fingers 2 [Source:HGNC Symbol;Acc:HGNC:9964] |
| ENSG00000134249 | HGLibA_00771 | 125 | -0.004025627 | ADAM30 | ADAM metallopeptidase domain 30 [Source:HGNC Symbol;Acc:HGNC:208] |
| ENSG00000134326 | HGLibA_10202 | 125 | -0.004025627 | CMPK2 | cytidine/uridine monophosphate kinase 2 [Source:HGNC Symbol;Acc:HGNC:27015] |
| ENSG00000135083 | HGLibA_08111 | 125 | -0.004025627 | CCNJL | cyclin J like [Source:HGNC Symbol;Acc:HGNC:25876] |
| ENSG00000135723 | HGLibA_17491 | 125 | -0.004025627 | FHOD1 | formin homology 2 domain containing 1 [Source:HGNC Symbol;Acc:HGNC:17905] |
| ENSG00000137478 | HGLibA_17212 | 125 | -0.004025627 | FCHSD2 | FCH and double SH3 domains 2 [Source:HGNC Symbol;Acc:HGNC:29114] |
| ENSG00000142528 | HGLibA_56060 | 125 | -0.004025627 | ZNF473 | zinc finger protein 473 [Source:HGNC Symbol;Acc:HGNC:23239] |
| ENSG00000143624 | HGLibA_23417 | 125 | -0.004025627 | INTS3 | integrator complex subunit 3 [Source:HGNC Symbol;Acc:HGNC:26153] |
| ENSG00000144644 | HGLibA_18427 | 125 | -0.004025627 | GADL1 | glutamate decarboxylase like 1 [Source:HGNC Symbol;Acc:HGNC:27949] |
| ENSG00000145741 | HGLibA_04936 | 125 | -0.004025627 | BTF3 | basic transcription factor 3 [Source:HGNC Symbol;Acc:HGNC:1125] |
| ENSG00000148400 | HGLibA_32341 | 125 | -0.004025627 | NOTCH1 | notch 1 [Source:HGNC Symbol;Acc:HGNC:7881] |
| ENSG00000151632 | HGLibA_01483 | 125 | -0.004025627 | AKR1C2 | aldo-keto reductase family 1 member C2 [Source:HGNC Symbol;Acc:HGNC:385] |
| ENSG00000152266 | HGLibA_39363 | 125 | -0.004025627 | PTH | parathyroid hormone [Source:HGNC Symbol;Acc:HGNC:9606] |
| ENSG00000152672 | HGLibA_09990 | 125 | -0.004025627 | CLEC4F | C-type lectin domain family 4 member F [Source:HGNC Symbol;Acc:HGNC:25357] |

| | | | | | |
|---|---|---|---|---|---|
| ENSG00000154237 | HGLibA_27465 | 125 | -0.004025627 | LRRK1 | leucine rich repeat kinase 1 [Source:HGNC Symbol;Acc:HGNC:18608] |
| ENSG00000154263 | HGLibA_00097 | 125 | -0.004025627 | ABCA10 | ATP binding cassette subfamily A member 10 [Source:HGNC Symbol;Acc:HGNC:30] |
| ENSG00000155087 | HGLibA_33276 | 125 | -0.004025627 | ODF1 | outer dense fiber of sperm tails 1 [Source:HGNC Symbol;Acc:HGNC:8113] |
| ENSG00000160999 | HGLibA_43876 | 125 | -0.004025627 | SH2B2 | SH2B adaptor protein 2 [Source:HGNC Symbol;Acc:HGNC:17381] |
| ENSG00000161813 | HGLibA_26034 | 125 | -0.004025627 | LARP4 | La ribonucleoprotein domain family member 4 [Source:HGNC Symbol;Acc:HGNC:24320] |
| ENSG00000162104 | HGLibA_00931 | 125 | -0.004025627 | ADCY9 | adenylate cyclase 9 [Source:HGNC Symbol;Acc:HGNC:240] |
| ENSG00000162545 | HGLibA_07080 | 125 | -0.004025627 | CAMK2N1 | calcium/calmodulin dependent protein kinase II inhibitor 1 [Source:HGNC Symbol;Acc:HGNC:24190] |
| ENSG00000162783 | HGLibA_22580 | 125 | -0.004025627 | IER5 | immediate early response 5 [Source:HGNC Symbol;Acc:HGNC:5393] |
| ENSG00000163013 | HGLibA_17096 | 125 | -0.004025627 | FBXO41 | F-box protein 41 [Source:HGNC Symbol;Acc:HGNC:29409] |
| ENSG00000163815 | HGLibA_09976 | 125 | -0.004025627 | CLEC3B | C-type lectin domain family 3 member B [Source:HGNC Symbol;Acc:HGNC:11891] |
| ENSG00000163864 | HGLibA_32190 | 125 | -0.004025627 | NMNAT3 | nicotinamide nucleotide adenylyltransferase 3 [Source:HGNC Symbol;Acc:HGNC:20989] |
| ENSG00000164542 | HGLibA_24657 | 125 | -0.004025627 | KIAA0895 | KIAA0895 [Source:HGNC Symbol;Acc:HGNC:22206] |
| ENSG00000165355 | HGLibA_17074 | 125 | -0.004025627 | FBXO33 | F-box protein 33 [Source:HGNC Symbol;Acc:HGNC:19833] |
| ENSG00000165802 | HGLibA_32775 | 125 | -0.004025627 | NSMF | NMDA receptor synaptonuclear signaling and neuronal migration factor [Source:HGNC Symbol;Acc:HGNC:29843] |
| ENSG00000166278 | HGLibA_05977 | 125 | -0.004025627 | C2 | complement C2 [Source:HGNC Symbol;Acc:HGNC:1248] |
| ENSG00000166321 | HGLibA_32944 | 125 | -0.004025627 | NUDT13 | nudix hydrolase 13 [Source:HGNC Symbol;Acc:HGNC:18827] |
| ENSG00000166557 | HGLibA_49808 | 125 | -0.004025627 | TMED3 | transmembrane p24 trafficking protein 3 [Source:HGNC Symbol;Acc:HGNC:28889] |
| ENSG00000167914 | HGLibA_20405 | 125 | -0.004025627 | GSDMA | gasdermin A [Source:HGNC Symbol;Acc:HGNC:13311] |
| ENSG00000169696 | HGLibA_03364 | 125 | -0.004025627 | ASPSCR1 | UBX domain containing tether for SLC2A4 [Source:HGNC Symbol;Acc:HGNC:13825]" |
| ENSG00000169918 | HGLibA_34777 | 125 | -0.004025627 | OTUD7A | OTU deubiquitinase 7A [Source:HGNC Symbol;Acc:HGNC:20718] |
| ENSG00000170324 | HGLibA_18034 | 125 | -0.004025627 | FRMPD2 | FERM and PDZ domain containing 2 [Source:HGNC Symbol;Acc:HGNC:28572] |
| ENSG00000170745 | HGLibA_24391 | 125 | -0.004025627 | KCNS3 | potassium voltage-gated channel modifier subfamily S member 3 [Source:HGNC Symbol;Acc:HGNC:6302] |
| ENSG00000170893 | HGLibA_51377 | 125 | -0.004025627 | TRH | thyrotropin releasing hormone [Source:HGNC Symbol;Acc:HGNC:12298] |
| ENSG00000171451 | HGLibA_14020 | 125 | -0.004025627 | DSEL | dermatan sulfate epimerase like [Source:HGNC Symbol;Acc:HGNC:18144] |
| ENSG00000172006 | HGLibA_56220 | 125 | -0.004025627 | ZNF554 | zinc finger protein 554 [Source:HGNC Symbol;Acc:HGNC:26629] |
| ENSG00000173457 | HGLibA_38037 | 125 | -0.004025627 | PPP1R14B | protein phosphatase 1 regulatory inhibitor subunit 14B [Source:HGNC Symbol;Acc:HGNC:9057] |
| ENSG00000176177 | HGLibA_15191 | 125 | -0.004025627 | ENTHD1 | ENTH domain containing 1 [Source:HGNC Symbol;Acc:HGNC:26352] |
| ENSG00000177106 | HGLibA_15380 | 125 | -0.004025627 | EPS8L2 | EPS8 like 2 [Source:HGNC Symbol;Acc:HGNC:21296] |
| ENSG00000177150 | HGLibA_16452 | 125 | -0.004025627 | FAM210A | family with sequence similarity 210 member A [Source:HGNC Symbol;Acc:HGNC:28346] |
| ENSG00000177291 | HGLibA_19193 | 125 | -0.004025627 | GJD4 | gap junction protein delta 4 [Source:HGNC Symbol;Acc:HGNC:23296] |
| ENSG00000178177 | HGLibA_26196 | 125 | -0.004025627 | LCORL | ligand dependent nuclear receptor corepressor like [Source:HGNC Symbol;Acc:HGNC:30776] |
| ENSG00000178921 | HGLibA_36135 | 125 | -0.004025627 | PFAS | phosphoribosylformylglycinamidine synthase [Source:HGNC Symbol;Acc:HGNC:8863] |
| ENSG00000179889 | HGLibA_35958 | 125 | -0.004025627 | PDXDC1 | pyridoxal dependent decarboxylase domain containing 1 [Source:HGNC Symbol;Acc:HGNC:28995] |
| ENSG00000181016 | HGLibA_27558 | 125 | -0.004025627 | LSMEM1 | leucine rich single-pass membrane protein 1 [Source:HGNC Symbol;Acc:HGNC:22036] |
| ENSG00000183765 | HGLibA_09423 | 125 | -0.004025627 | CHEK2 | checkpoint kinase 2 [Source:HGNC Symbol;Acc:HGNC:16627] |
| ENSG00000184305 | HGLibA_08188 | 125 | -0.004025627 | CCSER1 | coiled-coil serine rich protein 1 [Source:HGNC Symbol;Acc:HGNC:29349] |
| ENSG00000184345 | HGLibA_23504 | 125 | -0.004025627 | IQCF2 | IQ motif containing F2 [Source:HGNC Symbol;Acc:HGNC:31815] |
| ENSG00000184650 | HGLibA_33297 | 125 | -0.004025627 | ODF4 | outer dense fiber of sperm tails 4 [Source:HGNC Symbol;Acc:HGNC:19056] |
| ENSG00000184900 | HGLibA_47782 | 125 | -0.004025627 | SUMO3 | small ubiquitin-like modifier 3 [Source:HGNC Symbol;Acc:HGNC:11124] |
| ENSG00000186094 | HGLibA_01174 | 125 | -0.004025627 | AGBL4 | ATP/GTP binding protein like 4 [Source:HGNC Symbol;Acc:HGNC:25892] |
| ENSG00000186652 | HGLibA_38453 | 125 | -0.004025627 | PRG2 | pro eosinophil major basic protein [Source:HGNC Symbol;Acc:HGNC:9362]" |
| ENSG00000186832 | HGLibA_25433 | 125 | -0.004025627 | KRT16 | keratin 16 [Source:HGNC Symbol;Acc:HGNC:6423] |
| ENSG00000187416 | HGLibA_26436 | 125 | -0.004025627 | LHFPL3 | LHFPL tetraspan subfamily member 3 [Source:HGNC Symbol;Acc:HGNC:6589] |
| ENSG00000187758 | HGLibA_00951 | 125 | -0.004025627 | ADH1A | alpha polypeptide [Source:HGNC Symbol;Acc:HGNC:249]" |
| ENSG00000187954 | HGLibA_12040 | 125 | -0.004025627 | CYHR1 | cysteine and histidine rich 1 [Source:HGNC Symbol;Acc:HGNC:17806] |
| ENSG00000187959 | HGLibA_11010 | 125 | -0.004025627 | CPSF4L | cleavage and polyadenylation specific factor 4 like [Source:HGNC Symbol;Acc:HGNC:33632] |
| ENSG00000188124 | HGLibA_33751 | 125 | -0.004025627 | OR2AG2 | olfactory receptor family 2 subfamily AG member 2 [Source:HGNC Symbol;Acc:HGNC:15143] |
| ENSG00000188610 | HGLibA_16709 | 125 | -0.004025627 | FAM72B | family with sequence similarity 72 member B [Source:HGNC Symbol;Acc:HGNC:24805] |
| ENSG00000188629 | HGLibA_55531 | 125 | -0.004025627 | ZNF177 | zinc finger protein 177 [Source:HGNC Symbol;Acc:HGNC:12966] |
| ENSG00000196136 | HGLibA_43517 | 125 | -0.004025627 | SERPINA3 | serpin family A member 3 [Source:HGNC Symbol;Acc:HGNC:16] |
| ENSG00000196267 | HGLibA_56841 | 125 | -0.004025627 | ZNF836 | zinc finger protein 836 [Source:HGNC Symbol;Acc:HGNC:34333] |
| ENSG00000196923 | HGLibA_35921 | 125 | -0.004025627 | PDLIM7 | PDZ and LIM domain 7 [Source:HGNC Symbol;Acc:HGNC:22958] |
| ENSG00000197417 | HGLibA_44078 | 125 | -0.004025627 | SHPK | sedoheptulokinase [Source:HGNC Symbol;Acc:HGNC:1492] |
| ENSG00000197497 | HGLibA_56488 | 125 | -0.004025627 | ZNF665 | zinc finger protein 665 [Source:HGNC Symbol;Acc:HGNC:25885] |
| ENSG00000198173 | HGLibA_16613 | 125 | -0.004025627 | FAM47C | family with sequence similarity 47 member C [Source:HGNC Symbol;Acc:HGNC:25301] |
| ENSG00000198390 | HGLibA_25643 | 125 | -0.004025627 | KRTAP13-1 | keratin associated protein 13-1 [Source:HGNC Symbol;Acc:HGNC:18924] |
| ENSG00000198625 | HGLibA_28700 | 125 | -0.004025627 | MDM4 | p53 regulator [Source:HGNC Symbol;Acc:HGNC:6974]" |
| ENSG00000213918 | HGLibA_13652 | 125 | -0.004025627 | DNASE1 | deoxyribonuclease 1 [Source:HGNC Symbol;Acc:HGNC:2956] |
| ENSG00000214819 | HGLibA_08879 | 125 | -0.004025627 | CDRT15L2 | CMT1A duplicated region transcript 15 like 2 [Source:HGNC Symbol;Acc:HGNC:34075] |
| ENSG00000215305 | HGLibA_53742 | 125 | -0.004025627 | VPS16 | CORVET/HOPS core subunit [Source:HGNC Symbol;Acc:HGNC:14584]" |
| ENSG00000221821 | HGLibA_06481 | 125 | -0.004025627 | C6orf226 | chromosome 6 open reading frame 226 [Source:HGNC Symbol;Acc:HGNC:34431] |
| ENSG00000228927 | HGLibA_51970 | 125 | -0.004025627 | TSPY3 | testis specific protein Y-linked 3 [Source:HGNC Symbol;Acc:HGNC:33876] |
| ENSG00000233816 | HGLibA_22661 | 125 | -0.004025627 | IFNA13 | interferon alpha 13 [Source:HGNC Symbol;Acc:HGNC:5419] |
| ENSG00000243364 | HGLibA_14590 | 125 | -0.004025627 | EFNA4 | ephrin A4 [Source:HGNC Symbol;Acc:HGNC:3224] |
| ENSG00000244486 | HGLibA_42877 | 125 | -0.004025627 | SCARF2 | scavenger receptor class F member 2 [Source:HGNC Symbol;Acc:HGNC:19869] |
| ENSG00000253148 | HGLibA_41084 | 125 | -0.004025627 | RGS21 | regulator of G protein signaling 21 [Source:HGNC Symbol;Acc:HGNC:26839] |
| ENSG00000253304 | HGLibA_50173 | 125 | -0.004025627 | TMEM200B | transmembrane protein 200B [Source:HGNC Symbol;Acc:HGNC:33785] |
| ENSG00000255071 | HGLibA_42615 | 125 | -0.004025627 | SAA2-SAA4 | SAA2-SAA4 readthrough [Source:HGNC Symbol;Acc:HGNC:39550] |
| ENSG00000270601 | HGLibA_38320 | 125 | -0.004025627 | PRAMEF5 | PRAME family member 5 [Source:HGNC Symbol;Acc:HGNC:27995] |
| FAM129A | HGLibA_16096 | 125 | -0.004025627 | FAM129A | - |
| HIST1H2BC | HGLibA_21418 | 125 | -0.004025627 | HIST1H2BC | - |
| hsa-mir-1231 | HGLibA_57319 | 125 | -0.004025627 | hsa-mir-1231 | - |
| hsa-mir-1236 | HGLibA_57335 | 125 | -0.004025627 | hsa-mir-1236 | - |
| hsa-mir-135a-1 | HGLibA_57792 | 125 | -0.004025627 | hsa-mir-135a-1 | - |
| hsa-mir-181a-1 | HGLibA_57958 | 125 | -0.004025627 | hsa-mir-181a-1 | - |
| hsa-mir-3183 | HGLibA_58829 | 125 | -0.004025627 | hsa-mir-3183 | - |
| hsa-mir-3605 | HGLibA_59057 | 125 | -0.004025627 | hsa-mir-3605 | - |
| hsa-mir-378c | HGLibA_59435 | 125 | -0.004025627 | hsa-mir-378c | - |
| hsa-mir-4774 | HGLibA_61201 | 125 | -0.004025627 | hsa-mir-4774 | - |
| hsa-mir-548ag-1 | HGLibA_61876 | 125 | -0.004025627 | hsa-mir-548ag-1 | - |
| hsa-mir-5685 | HGLibA_62261 | 125 | -0.004025627 | hsa-mir-5685 | - |
| hsa-mir-581 | HGLibA_62421 | 125 | -0.004025627 | hsa-mir-581 | - |
| hsa-mir-6511a-3 | HGLibA_62906 | 125 | -0.004025627 | hsa-mir-6511a-3 | - |
| hsa-mir-654 | HGLibA_62949 | 125 | -0.004025627 | hsa-mir-654 | - |
| hsa-mir-6798 | HGLibA_63369 | 125 | -0.004025627 | hsa-mir-6798 | - |
| hsa-mir-6803 | HGLibA_63390 | 125 | -0.004025627 | hsa-mir-6803 | - |
| hsa-mir-6807 | HGLibA_63404 | 125 | -0.004025627 | hsa-mir-6807 | - |
| hsa-mir-7853 | HGLibA_64003 | 125 | -0.004025627 | hsa-mir-7853 | - |
| hsa-mir-938 | HGLibA_64327 | 125 | -0.004025627 | hsa-mir-938 | - |
| LOC100505841 | HGLibA_26856 | 125 | -0.004025627 | LOC100505841 | - |
| LRRC33 | HGLibA_27317 | 125 | -0.004025627 | LRRC33 | - |
| MYEOV2 | HGLibA_30693 | 125 | -0.004025627 | MYEOV2 | - |
| C10orf35 | HGLibA_05069 | 124 | -0.01307097 | C10orf35 | - |
| C14orf159 | HGLibA_05331 | 124 | -0.01307097 | C14orf159 | - |
| C1orf64 | HGLibA_05884 | 124 | -0.01307097 | C1orf64 | - |
| C9orf139 | HGLibA_06684 | 124 | -0.01307097 | C9orf139 | - |
| C9orf37 | HGLibA_06725 | 124 | -0.01307097 | C9orf37 | - |
| C9orf66 | HGLibA_06751 | 124 | -0.01307097 | C9orf66 | - |
| C9orf89 | HGLibA_06769 | 124 | -0.01307097 | C9orf89 | - |
| CASC1 | HGLibA_07267 | 124 | -0.01307097 | CASC1 | - |
| CRLF2_X | HGLibA_11189 | 124 | -0.01307097 | CRLF2_X | - |
| DFNA5 | HGLibA_13025 | 124 | -0.01307097 | DFNA5 | - |
| DSCR3 | HGLibA_14007 | 124 | -0.01307097 | DSCR3 | - |
| ENSG00000000457 | HGLibA_43090 | 124 | -0.01307097 | SCYL3 | SCY1 like pseudokinase 3 [Source:HGNC Symbol;Acc:HGNC:19285] |
| ENSG00000003147 | HGLibA_22487 | 124 | -0.01307097 | ICA1 | islet cell autoantigen 1 [Source:HGNC Symbol;Acc:HGNC:5343] |
| ENSG00000004848 | HGLibA_03179 | 124 | -0.01307097 | ARX | aristaless related homeobox [Source:HGNC Symbol;Acc:HGNC:18060] |
| ENSG00000005339 | HGLibA_11108 | 124 | -0.01307097 | CREBBP | CREB binding protein [Source:HGNC Symbol;Acc:HGNC:2348] |
| ENSG00000006747 | HGLibA_42948 | 124 | -0.01307097 | SCIN | scinderin [Source:HGNC Symbol;Acc:HGNC:21695] |
| ENSG00000013374 | HGLibA_32899 | 124 | -0.01307097 | NUB1 | negative regulator of ubiquitin like proteins 1 [Source:HGNC Symbol;Acc:HGNC:17623] |
| ENSG00000042445 | HGLibA_40882 | 124 | -0.01307097 | RETSAT | retinol saturase [Source:HGNC Symbol;Acc:HGNC:25991] |
| ENSG00000042980 | HGLibA_00765 | 124 | -0.01307097 | ADAM28 | ADAM metallopeptidase domain 28 [Source:HGNC Symbol;Acc:HGNC:206] |
| ENSG00000056972 | HGLibA_51251 | 124 | -0.01307097 | TRAF3IP2 | TRAF3 interacting protein 2 [Source:HGNC Symbol;Acc:HGNC:1343] |
| ENSG00000066248 | HGLibA_31869 | 124 | -0.01307097 | NGEF | neuronal guanine nucleotide exchange factor [Source:HGNC Symbol;Acc:HGNC:7807] |
| ENSG00000068796 | HGLibA_24926 | 124 | -0.01307097 | KIF2A | kinesin family member 2A [Source:HGNC Symbol;Acc:HGNC:6318] |
| ENSG00000069966 | HGLibA_19503 | 124 | -0.01307097 | GNB5 | G protein subunit beta 5 [Source:HGNC Symbol;Acc:HGNC:4401] |
| ENSG00000077274 | HGLibA_07172 | 124 | -0.01307097 | CAPN6 | calpain 6 [Source:HGNC Symbol;Acc:HGNC:1483] |
| ENSG00000081041 | HGLibA_11867 | 124 | -0.01307097 | CXCL2 | C-X-C motif chemokine ligand 2 [Source:HGNC Symbol;Acc:HGNC:4603] |
| ENSG00000081760 | HGLibA_00026 | 124 | -0.01307097 | AACS | acetoacetyl-CoA synthetase [Source:HGNC Symbol;Acc:HGNC:21298] |
| ENSG00000085185 | HGLibA_04364 | 124 | -0.01307097 | BCORL1 | BCL6 corepressor like 1 [Source:HGNC Symbol;Acc:HGNC:25657] |
| ENSG00000085449 | HGLibA_54004 | 124 | -0.01307097 | WDFY1 | WD repeat and FYVE domain containing 1 [Source:HGNC Symbol;Acc:HGNC:20451] |
| ENSG00000086062 | HGLibA_04022 | 124 | -0.01307097 | B4GALT1 | 4-galactosyltransferase 1 [Source:HGNC Symbol;Acc:HGNC:924]" |
| ENSG00000087088 | HGLibA_04182 | 124 | -0.01307097 | BAX | apoptosis regulator [Source:HGNC Symbol;Acc:HGNC:959]" |
| ENSG00000089486 | HGLibA_08713 | 124 | -0.01307097 | CDIP1 | cell death inducing p53 target 1 [Source:HGNC Symbol;Acc:HGNC:13234] |
| ENSG00000089847 | HGLibA_02044 | 124 | -0.01307097 | ANKRD24 | ankyrin repeat domain 24 [Source:HGNC Symbol;Acc:HGNC:29424] |
| ENSG00000091137 | HGLibA_44795 | 124 | -0.01307097 | SLC26A4 | solute carrier family 26 member 4 [Source:HGNC Symbol;Acc:HGNC:8818] |
| ENSG00000093217 | HGLibA_54615 | 124 | -0.01307097 | XYLB | xylulokinase [Source:HGNC Symbol;Acc:HGNC:12839] |
| ENSG00000101180 | HGLibA_22100 | 124 | -0.01307097 | HRH3 | histamine receptor H3 [Source:HGNC Symbol;Acc:HGNC:5184] |
| ENSG00000101443 | HGLibA_54262 | 124 | -0.01307097 | WFDC2 | WAP four-disulfide core domain 2 [Source:HGNC Symbol;Acc:HGNC:15939] |
| ENSG00000102054 | HGLibA_40437 | 124 | -0.01307097 | RBBP7 | chromatin remodeling factor [Source:HGNC Symbol;Acc:HGNC:9890]" |
| ENSG00000102096 | HGLibA_36677 | 124 | -0.01307097 | PIM2 | serine/threonine kinase [Source:HGNC Symbol;Acc:HGNC:8987]" |
| ENSG00000104695 | HGLibA_38136 | 124 | -0.01307097 | PPP2CB | protein phosphatase 2 catalytic subunit beta [Source:HGNC Symbol;Acc:HGNC:9300] |
| ENSG00000104756 | HGLibA_24471 | 124 | -0.01307097 | KCTD9 | potassium channel tetramerization domain containing 9 [Source:HGNC Symbol;Acc:HGNC:22401] |
| ENSG00000105518 | HGLibA_50191 | 124 | -0.01307097 | TMEM205 | transmembrane protein 205 [Source:HGNC Symbol;Acc:HGNC:29631] |
| ENSG00000105550 | HGLibA_17396 | 124 | -0.01307097 | FGF21 | fibroblast growth factor 21 [Source:HGNC Symbol;Acc:HGNC:3678] |
| ENSG00000106484 | HGLibA_28923 | 124 | -0.01307097 | MEST | mesoderm specific transcript [Source:HGNC Symbol;Acc:HGNC:7028] |

| | | | | | |
|---|---|---|---|---|---|
| ENSG00000106868 | HGLibA_47845 | 124 | -0.01307097 | SUSD1 | sushi domain containing 1 [Source:HGNC Symbol;Acc:HGNC:25413] |
| ENSG00000107929 | HGLibA_26036 | 124 | -0.01307097 | LARP4B | La ribonucleoprotein domain family member 4B [Source:HGNC Symbol;Acc:HGNC:28987] |
| ENSG00000108349 | HGLibA_07274 | 124 | -0.01307097 | CASC3 | exon junction complex subunit [Source:HGNC Symbol;Acc:HGNC:17040]" |
| ENSG00000110218 | HGLibA_35109 | 124 | -0.01307097 | PANX1 | pannexin 1 [Source:HGNC Symbol;Acc:HGNC:8599] |
| ENSG00000111049 | HGLibA_30696 | 124 | -0.01307097 | MYF5 | myogenic factor 5 [Source:HGNC Symbol;Acc:HGNC:7565] |
| ENSG00000119707 | HGLibA_40518 | 124 | -0.01307097 | RBM25 | RNA binding motif protein 25 [Source:HGNC Symbol;Acc:HGNC:23244] |
| ENSG00000120053 | HGLibA_19691 | 124 | -0.01307097 | GOT1 | glutamic-oxaloacetic transaminase 1 [Source:HGNC Symbol;Acc:HGNC:4432] |
| ENSG00000120159 | HGLibA_06830 | 124 | -0.01307097 | CAAP1 | caspase activity and apoptosis inhibitor 1 [Source:HGNC Symbol;Acc:HGNC:25834] |
| ENSG00000120693 | HGLibA_45589 | 124 | -0.01307097 | SMAD9 | SMAD family member 9 [Source:HGNC Symbol;Acc:HGNC:6774] |
| ENSG00000122591 | HGLibA_16080 | 124 | -0.01307097 | FAM126A | family with sequence similarity 126 member A [Source:HGNC Symbol;Acc:HGNC:24587] |
| ENSG00000124374 | HGLibA_35035 | 124 | -0.01307097 | PAIP2B | poly(A) binding protein interacting protein 2B [Source:HGNC Symbol;Acc:HGNC:29200] |
| ENSG00000124702 | HGLibA_25100 | 124 | -0.01307097 | KLHDC3 | kelch domain containing 3 [Source:HGNC Symbol;Acc:HGNC:20704] |
| ENSG00000125484 | HGLibA_20588 | 124 | -0.01307097 | GTF3C4 | general transcription factor IIIC subunit 4 [Source:HGNC Symbol;Acc:HGNC:4667] |
| ENSG00000128422 | HGLibA_25434 | 124 | -0.01307097 | KRT17 | keratin 17 [Source:HGNC Symbol;Acc:HGNC:6427] |
| ENSG00000129744 | HGLibA_03158 | 124 | -0.01307097 | ART1 | ADP-ribosyltransferase 1 [Source:HGNC Symbol;Acc:HGNC:723] |
| ENSG00000130203 | HGLibA_02517 | 124 | -0.01307097 | APOE | apolipoprotein E [Source:HGNC Symbol;Acc:HGNC:613] |
| ENSG00000131126 | HGLibA_49048 | 124 | -0.01307097 | TEX101 | testis expressed 101 [Source:HGNC Symbol;Acc:HGNC:30722] |
| ENSG00000133401 | HGLibA_35973 | 124 | -0.01307097 | PDZD2 | PDZ domain containing 2 [Source:HGNC Symbol;Acc:HGNC:18486] |
| ENSG00000133460 | HGLibA_44857 | 124 | -0.01307097 | SLC2A11 | solute carrier family 2 member 11 [Source:HGNC Symbol;Acc:HGNC:14239] |
| ENSG00000134780 | HGLibA_12295 | 124 | -0.01307097 | DAGLA | diacylglycerol lipase alpha [Source:HGNC Symbol;Acc:HGNC:1165] |
| ENSG00000134852 | HGLibA_10092 | 124 | -0.01307097 | CLOCK | clock circadian regulator [Source:HGNC Symbol;Acc:HGNC:2082] |
| ENSG00000136560 | HGLibA_48303 | 124 | -0.01307097 | TANK | TRAF family member associated NFKB activator [Source:HGNC Symbol;Acc:HGNC:11562] |
| ENSG00000137161 | HGLibA_10369 | 124 | -0.01307097 | CNPY3 | canopy FGF signaling regulator 3 [Source:HGNC Symbol;Acc:HGNC:11968] |
| ENSG00000139998 | HGLibA_39816 | 124 | -0.01307097 | RAB15 | member RAS oncogene family [Source:HGNC Symbol;Acc:HGNC:20150]" |
| ENSG00000140199 | HGLibA_44371 | 124 | -0.01307097 | SLC12A6 | solute carrier family 12 member 6 [Source:HGNC Symbol;Acc:HGNC:10914] |
| ENSG00000140749 | HGLibA_22900 | 124 | -0.01307097 | IGSF6 | immunoglobulin superfamily member 6 [Source:HGNC Symbol;Acc:HGNC:5953] |
| ENSG00000142252 | HGLibA_18923 | 124 | -0.01307097 | GEMIN7 | gem nuclear organelle associated protein 7 [Source:HGNC Symbol;Acc:HGNC:20045] |
| ENSG00000144230 | HGLibA_19962 | 124 | -0.01307097 | GPR17 | G protein-coupled receptor 17 [Source:HGNC Symbol;Acc:HGNC:4471] |
| ENSG00000145103 | HGLibA_23209 | 124 | -0.01307097 | ILDR1 | immunoglobulin like domain containing receptor 1 [Source:HGNC Symbol;Acc:HGNC:28741] |
| ENSG00000147160 | HGLibA_03914 | 124 | -0.01307097 | AWAT2 | acyl-CoA wax alcohol acyltransferase 2 [Source:HGNC Symbol;Acc:HGNC:23251] |
| ENSG00000150527 | HGLibA_29187 | 124 | -0.01307097 | MIA2 | MIA SH3 domain ER export factor 2 [Source:HGNC Symbol;Acc:HGNC:18432] |
| ENSG00000151445 | HGLibA_53665 | 124 | -0.01307097 | VIPAS39 | spe-39 homolog [Source:HGNC Symbol;Acc:HGNC:20347]" |
| ENSG00000151882 | HGLibA_08016 | 124 | -0.01307097 | CCL28 | C-C motif chemokine ligand 28 [Source:HGNC Symbol;Acc:HGNC:17700] |
| ENSG00000152208 | HGLibA_20276 | 124 | -0.01307097 | GRID2 | glutamate ionotropic receptor delta type subunit 2 [Source:HGNC Symbol;Acc:HGNC:4576] |
| ENSG00000152766 | HGLibA_02040 | 124 | -0.01307097 | ANKRD22 | ankyrin repeat domain 22 [Source:HGNC Symbol;Acc:HGNC:28321] |
| ENSG00000153002 | HGLibA_10906 | 124 | -0.01307097 | CPB1 | carboxypeptidase B1 [Source:HGNC Symbol;Acc:HGNC:2299] |
| ENSG00000153113 | HGLibA_07347 | 124 | -0.01307097 | CAST | calpastatin [Source:HGNC Symbol;Acc:HGNC:1515] |
| ENSG00000153283 | HGLibA_08482 | 124 | -0.01307097 | CD96 | CD96 molecule [Source:HGNC Symbol;Acc:HGNC:16892] |
| ENSG00000153721 | HGLibA_10301 | 124 | -0.01307097 | CNKSR3 | CNKSR family member 3 [Source:HGNC Symbol;Acc:HGNC:23034] |
| ENSG00000154678 | HGLibA_35775 | 124 | -0.01307097 | PDE1C | phosphodiesterase 1C [Source:HGNC Symbol;Acc:HGNC:8776] |
| ENSG00000156265 | HGLibA_28198 | 124 | -0.01307097 | MAP3K7CL | MAP3K7 C-terminal like [Source:HGNC Symbol;Acc:HGNC:16457] |
| ENSG00000158156 | HGLibA_54533 | 124 | -0.01307097 | XKR8 | XK related 8 [Source:HGNC Symbol;Acc:HGNC:25508] |
| ENSG00000159374 | HGLibA_27813 | 124 | -0.01307097 | M1AP | meiosis 1 associated protein [Source:HGNC Symbol;Acc:HGNC:25183] |
| ENSG00000160813 | HGLibA_38089 | 124 | -0.01307097 | PPP1R35 | protein phosphatase 1 regulatory subunit 35 [Source:HGNC Symbol;Acc:HGNC:28320] |
| ENSG00000161798 | HGLibA_02600 | 124 | -0.01307097 | AQP5 | aquaporin 5 [Source:HGNC Symbol;Acc:HGNC:638] |
| ENSG00000162456 | HGLibA_25336 | 124 | -0.01307097 | KNCN | kinocilin [Source:HGNC Symbol;Acc:HGNC:26488] |
| ENSG00000162783 | HGLibA_22582 | 124 | -0.01307097 | IER5 | immediate early response 5 [Source:HGNC Symbol;Acc:HGNC:5393] |
| ENSG00000162971 | HGLibA_52489 | 124 | -0.01307097 | TYW5 | tRNA-yW synthesizing protein 5 [Source:HGNC Symbol;Acc:HGNC:26754] |
| ENSG00000163092 | HGLibA_54515 | 124 | -0.01307097 | XIRP2 | xin actin binding repeat containing 2 [Source:HGNC Symbol;Acc:HGNC:14303] |
| ENSG00000164181 | HGLibA_14974 | 124 | -0.01307097 | ELOVL7 | ELOVL fatty acid elongase 7 [Source:HGNC Symbol;Acc:HGNC:26292] |
| ENSG00000165192 | HGLibA_03209 | 124 | -0.01307097 | ASB11 | ankyrin repeat and SOCS box containing 11 [Source:HGNC Symbol;Acc:HGNC:17186] |
| ENSG00000165244 | HGLibA_55881 | 124 | -0.01307097 | ZNF367 | zinc finger protein 367 [Source:HGNC Symbol;Acc:HGNC:18320] |
| ENSG00000165672 | HGLibA_38414 | 124 | -0.01307097 | PRDX3 | peroxiredoxin 3 [Source:HGNC Symbol;Acc:HGNC:9354] |
| ENSG00000165694 | HGLibA_18025 | 124 | -0.01307097 | FRMD7 | FERM domain containing 7 [Source:HGNC Symbol;Acc:HGNC:8079] |
| ENSG00000166822 | HGLibA_50055 | 124 | -0.01307097 | TMEM170A | transmembrane protein 170A [Source:HGNC Symbol;Acc:HGNC:29577] |
| ENSG00000166984 | HGLibA_48838 | 124 | -0.01307097 | TCP10L2 | t-complex 10 like 2 [Source:HGNC Symbol;Acc:HGNC:21254] |
| ENSG00000167094 | HGLibA_52057 | 124 | -0.01307097 | TTC16 | tetratricopeptide repeat domain 16 [Source:HGNC Symbol;Acc:HGNC:26536] |
| ENSG00000167394 | HGLibA_56494 | 124 | -0.01307097 | ZNF668 | zinc finger protein 668 [Source:HGNC Symbol;Acc:HGNC:25821] |
| ENSG00000167526 | HGLibA_41868 | 124 | -0.01307097 | RPL13 | ribosomal protein L13 [Source:HGNC Symbol;Acc:HGNC:10303] |
| ENSG00000168491 | HGLibA_07538 | 124 | -0.01307097 | CCDC110 | coiled-coil domain containing 110 [Source:HGNC Symbol;Acc:HGNC:28504] |
| ENSG00000169918 | HGLibA_34776 | 124 | -0.01307097 | OTUD7A | OTU deubiquitinase 7A [Source:HGNC Symbol;Acc:HGNC:20718] |
| ENSG00000170370 | HGLibA_15094 | 124 | -0.01307097 | EMX2 | empty spiracles homeobox 2 [Source:HGNC Symbol;Acc:HGNC:3341] |
| ENSG00000170604 | HGLibA_23578 | 124 | -0.01307097 | IRF2BP1 | interferon regulatory factor 2 binding protein 1 [Source:HGNC Symbol;Acc:HGNC:21728] |
| ENSG00000170953 | HGLibA_34493 | 124 | -0.01307097 | OR8B12 | olfactory receptor family 8 subfamily B member 12 [Source:HGNC Symbol;Acc:HGNC:15307] |
| ENSG00000171115 | HGLibA_19091 | 124 | -0.01307097 | GIMAP8 | IMAP family member 8 [Source:HGNC Symbol;Acc:HGNC:21792]" |
| ENSG00000171295 | HGLibA_56002 | 124 | -0.01307097 | ZNF440 | zinc finger protein 440 [Source:HGNC Symbol;Acc:HGNC:20874] |
| ENSG00000171596 | HGLibA_32214 | 124 | -0.01307097 | NMUR1 | neuromedin U receptor 1 [Source:HGNC Symbol;Acc:HGNC:4518] |
| ENSG00000172538 | HGLibA_16262 | 124 | -0.01307097 | FAM170B | family with sequence similarity 170 member B [Source:HGNC Symbol;Acc:HGNC:19736] |
| ENSG00000172572 | HGLibA_35780 | 124 | -0.01307097 | PDE3A | phosphodiesterase 3A [Source:HGNC Symbol;Acc:HGNC:8778] |
| ENSG00000174939 | HGLibA_03352 | 124 | -0.01307097 | ASPHD1 | aspartate beta-hydroxylase domain containing 1 [Source:HGNC Symbol;Acc:HGNC:27380] |
| ENSG00000177455 | HGLibA_08261 | 124 | -0.01307097 | CD19 | CD19 molecule [Source:HGNC Symbol;Acc:HGNC:1633] |
| ENSG00000177489 | HGLibA_33795 | 124 | -0.01307097 | OR2G2 | olfactory receptor family 2 subfamily G member 2 [Source:HGNC Symbol;Acc:HGNC:15007] |
| ENSG00000177710 | HGLibA_45026 | 124 | -0.01307097 | SLC35G5 | solute carrier family 35 member G5 [Source:HGNC Symbol;Acc:HGNC:15546] |
| ENSG00000177938 | HGLibA_07212 | 124 | -0.01307097 | CAPZA3 | capping actin protein of muscle Z-line subunit alpha 3 [Source:HGNC Symbol;Acc:HGNC:24205] |
| ENSG00000177994 | HGLibA_06179 | 124 | -0.01307097 | C2orf73 | chromosome 2 open reading frame 73 [Source:HGNC Symbol;Acc:HGNC:26861] |
| ENSG00000178473 | HGLibA_52829 | 124 | -0.01307097 | UCN3 | urocortin 3 [Source:HGNC Symbol;Acc:HGNC:17781] |
| ENSG00000179615 | HGLibA_33759 | 124 | -0.01307097 | OR2AP1 | olfactory receptor family 2 subfamily AP member 1 [Source:HGNC Symbol;Acc:HGNC:15335] |
| ENSG00000180773 | HGLibA_45041 | 124 | -0.01307097 | SLC36A4 | solute carrier family 36 member 4 [Source:HGNC Symbol;Acc:HGNC:19660] |
| ENSG00000182220 | HGLibA_03700 | 124 | -0.01307097 | ATP6AP2 | ATPase H+ transporting accessory protein 2 [Source:HGNC Symbol;Acc:HGNC:18305] |
| ENSG00000182389 | HGLibA_06927 | 124 | -0.01307097 | CACNB4 | calcium voltage-gated channel auxiliary subunit beta 4 [Source:HGNC Symbol;Acc:HGNC:1404] |
| ENSG00000182511 | HGLibA_17304 | 124 | -0.01307097 | FES | tyrosine kinase [Source:HGNC Symbol;Acc:HGNC:3657]" |
| ENSG00000182718 | HGLibA_02231 | 124 | -0.01307097 | ANXA2 | annexin A2 [Source:HGNC Symbol;Acc:HGNC:537] |
| ENSG00000183032 | HGLibA_44672 | 124 | -0.01307097 | SLC25A21 | solute carrier family 25 member 21 [Source:HGNC Symbol;Acc:HGNC:14411] |
| ENSG00000183807 | HGLibA_16224 | 124 | -0.01307097 | FAM162B | family with sequence similarity 162 member B [Source:HGNC Symbol;Acc:HGNC:21549] |
| ENSG00000183977 | HGLibA_37800 | 124 | -0.01307097 | PP2D1 | protein phosphatase 2C like domain containing 1 [Source:HGNC Symbol;Acc:HGNC:28406] |
| ENSG00000184845 | HGLibA_13959 | 124 | -0.01307097 | DRD1 | dopamine receptor D1 [Source:HGNC Symbol;Acc:HGNC:3020] |
| ENSG00000187848 | HGLibA_34849 | 124 | -0.01307097 | P2RX2 | purinergic receptor P2X 2 [Source:HGNC Symbol;Acc:HGNC:15459] |
| ENSG00000196431 | HGLibA_11257 | 124 | -0.01307097 | CRYBA4 | crystallin beta A4 [Source:HGNC Symbol;Acc:HGNC:2396] |
| ENSG00000196605 | HGLibA_56867 | 124 | -0.01307097 | ZNF846 | zinc finger protein 846 [Source:HGNC Symbol;Acc:HGNC:27260] |
| ENSG00000197956 | HGLibA_42563 | 124 | -0.01307097 | S100A6 | S100 calcium binding protein A6 [Source:HGNC Symbol;Acc:HGNC:10496] |
| ENSG00000198125 | HGLibA_28455 | 124 | -0.01307097 | MB | myoglobin [Source:HGNC Symbol;Acc:HGNC:6915] |
| ENSG00000198276 | HGLibA_52816 | 124 | -0.01307097 | UCKL1 | uridine-cytidine kinase 1 like 1 [Source:HGNC Symbol;Acc:HGNC:15938] |
| ENSG00000198573 | HGLibA_46389 | 124 | -0.01307097 | SPANXC | SPANX family member C [Source:HGNC Symbol;Acc:HGNC:14331] |
| ENSG00000198746 | HGLibA_19743 | 124 | -0.01307097 | GPATCH3 | G-patch domain containing 3 [Source:HGNC Symbol;Acc:HGNC:25720] |
| ENSG00000204406 | HGLibA_28490 | 124 | -0.01307097 | MBD5 | methyl-CpG binding domain protein 5 [Source:HGNC Symbol;Acc:HGNC:20444] |
| ENSG00000205076 | HGLibA_26373 | 124 | -0.01307097 | LGALS7 | galectin 7 [Source:HGNC Symbol;Acc:HGNC:6568] |
| ENSG00000205710 | HGLibA_05529 | 124 | -0.01307097 | C17orf107 | chromosome 17 open reading frame 107 [Source:HGNC Symbol;Acc:HGNC:37238] |
| ENSG00000213699 | HGLibA_45012 | 124 | -0.01307097 | SLC35F6 | solute carrier family 35 member F6 [Source:HGNC Symbol;Acc:HGNC:26055] |
| ENSG00000213886 | HGLibA_52569 | 124 | -0.01307097 | UBD | ubiquitin D [Source:HGNC Symbol;Acc:HGNC:18795] |
| ENSG00000236699 | HGLibA_02857 | 124 | -0.01307097 | ARHGEF38 | Rho guanine nucleotide exchange factor 38 [Source:HGNC Symbol;Acc:HGNC:25968] |
| ENSG00000237441 | HGLibA_41005 | 124 | -0.01307097 | RGL2 | ral guanine nucleotide dissociation stimulator like 2 [Source:HGNC Symbol;Acc:HGNC:9769] |
| ENSG00000239779 | HGLibA_53967 | 124 | -0.01307097 | WBP1 | WW domain binding protein 1 [Source:HGNC Symbol;Acc:HGNC:12737] |
| ENSG00000244694 | HGLibA_39284 | 124 | -0.01307097 | PTCHD4 | patched domain containing 4 [Source:HGNC Symbol;Acc:HGNC:21345] |
| ENSG00000251692 | HGLibA_39573 | 124 | -0.01307097 | PTX4 | pentraxin 4 [Source:HGNC Symbol;Acc:HGNC:14171] |
| ENSG00000254470 | HGLibA_02353 | 124 | -0.01307097 | AP5B1 | adaptor related protein complex 5 subunit beta 1 [Source:HGNC Symbol;Acc:HGNC:25104] |
| ENSG00000255690 | HGLibA_51400 | 124 | -0.01307097 | TRIL | TLR4 interactor with leucine rich repeats [Source:HGNC Symbol;Acc:HGNC:22200] |
| ENSG00000263761 | HGLibA_18862 | 124 | -0.01307097 | GDF2 | growth differentiation factor 2 [Source:HGNC Symbol;Acc:HGNC:4217] |
| ENSG00000280987 | HGLibA_28440 | 124 | -0.01307097 | MATR3 | matrin 3 [Source:NCBI gene;Acc:9782] |
| ENSG00000284680 | HGLibA_34495 | 124 | -0.01307097 | OR8B2 | olfactory receptor family 8 subfamily B member 2 [Source:HGNC Symbol;Acc:HGNC:8471] |
| FAM212B | HGLibA_16467 | 124 | -0.01307097 | FAM212B | - |
| GAGE6 | HGLibA_18484 | 124 | -0.01307097 | GAGE6 | - |
| HIST1H2AL | HGLibA_21405 | 124 | -0.01307097 | HIST1H2AL | - |
| HNRPDL | HGLibA_21856 | 124 | -0.01307097 | HNRPDL | - |
| hsa-mir-3131 | HGLibA_58581 | 124 | -0.01307097 | hsa-mir-3131 | - |
| hsa-mir-3141 | HGLibA_58623 | 124 | -0.01307097 | hsa-mir-3141 | - |
| hsa-mir-34a | HGLibA_59036 | 124 | -0.01307097 | hsa-mir-34a | - |
| hsa-mir-376a-2 | HGLibA_59409 | 124 | -0.01307097 | hsa-mir-376a-2 | - |
| hsa-mir-432 | HGLibA_60001 | 124 | -0.01307097 | hsa-mir-432 | - |
| hsa-mir-4327 | HGLibA_60031 | 124 | -0.01307097 | hsa-mir-4327 | - |
| hsa-mir-4436b-2 | HGLibA_60145 | 124 | -0.01307097 | hsa-mir-4436b-2 | - |
| hsa-mir-4538 | HGLibA_60613 | 124 | -0.01307097 | hsa-mir-4538 | - |
| hsa-mir-4656 | HGLibA_60730 | 124 | -0.01307097 | hsa-mir-4656 | - |
| hsa-mir-4707 | HGLibA_60930 | 124 | -0.01307097 | hsa-mir-4707 | - |
| hsa-mir-5586 | HGLibA_62187 | 124 | -0.01307097 | hsa-mir-5586 | - |
| hsa-mir-600 | HGLibA_62492 | 124 | -0.01307097 | hsa-mir-600 | - |
| hsa-mir-6794 | HGLibA_63352 | 124 | -0.01307097 | hsa-mir-6794 | - |
| hsa-mir-6838 | HGLibA_63529 | 124 | -0.01307097 | hsa-mir-6838 | - |
| hsa-mir-7108 | HGLibA_63792 | 124 | -0.01307097 | hsa-mir-7108 | - |
| hsa-mir-8076 | HGLibA_64145 | 124 | -0.01307097 | hsa-mir-8076 | - |
| KIAA1257 | HGLibA_24731 | 124 | -0.01307097 | KIAA1257 | - |
| MKL1 | HGLibA_29333 | 124 | -0.01307097 | MKL1 | - |
| 2-Sep | HGLibA_43437 | 124 | -0.01307097 | 2-Sep | - |
| TCEB3B | HGLibA_48743 | 124 | -0.01307097 | TCEB3B | - |
| TCTEX1D1 | HGLibA_48863 | 124 | -0.01307097 | TCTEX1D1 | - |
| TMEM246 | HGLibA_50303 | 124 | -0.01307097 | TMEM246 | - |

| | | | | | |
|---|---|---|---|---|---|
| TROVE2 | HGLibA_51722 | 124 | -0.01307097 | TROVE2 | - |
| ACN9 | HGLibA_00457 | 123 | -0.022116313 | ACN9 | - |
| C11orf88 | HGLibA_05209 | 123 | -0.022116313 | C11orf88 | - |
| C1orf204 | HGLibA_05822 | 123 | -0.022116313 | C1orf204 | - |
| CCRL1 | HGLibA_08173 | 123 | -0.022116313 | CCRL1 | - |
| ENSG00000006788 | HGLibA_30713 | 123 | -0.022116313 | MYH13 | myosin heavy chain 13 [Source:HGNC Symbol;Acc:HGNC:7571] |
| ENSG00000019549 | HGLibA_45869 | 123 | -0.022116313 | SNAI2 | snail family transcriptional repressor 2 [Source:HGNC Symbol;Acc:HGNC:11094] |
| ENSG00000064218 | HGLibA_13424 | 123 | -0.022116313 | DMRT3 | doublesex and mab-3 related transcription factor 3 [Source:HGNC Symbol;Acc:HGNC:13909] |
| ENSG00000064300 | HGLibA_31875 | 123 | -0.022116313 | NGFR | nerve growth factor receptor [Source:HGNC Symbol;Acc:HGNC:7809] |
| ENSG00000091129 | HGLibA_32662 | 123 | -0.022116313 | NRCAM | neuronal cell adhesion molecule [Source:HGNC Symbol;Acc:HGNC:7994] |
| ENSG00000095319 | HGLibA_33032 | 123 | -0.022116313 | NUP188 | nucleoporin 188 [Source:HGNC Symbol;Acc:HGNC:17859] |
| ENSG00000100226 | HGLibA_20597 | 123 | -0.022116313 | GTPBP1 | GTP binding protein 1 [Source:HGNC Symbol;Acc:HGNC:4669] |
| ENSG00000100523 | HGLibA_12604 | 123 | -0.022116313 | DDHD1 | DDHD domain containing 1 [Source:HGNC Symbol;Acc:HGNC:19714] |
| ENSG00000100941 | HGLibA_37393 | 123 | -0.022116313 | PNN | desmosome associated protein [Source:HGNC Symbol;Acc:HGNC:9162]" |
| ENSG00000100985 | HGLibA_29542 | 123 | -0.022116313 | MMP9 | matrix metallopeptidase 9 [Source:HGNC Symbol;Acc:HGNC:7176] |
| ENSG00000100994 | HGLibA_39686 | 123 | -0.022116313 | PYGB | glycogen phosphorylase B [Source:HGNC Symbol;Acc:HGNC:9723] |
| ENSG00000101306 | HGLibA_30786 | 123 | -0.022116313 | MYLK2 | myosin light chain kinase 2 [Source:HGNC Symbol;Acc:HGNC:16243] |
| ENSG00000101986 | HGLibA_00190 | 123 | -0.022116313 | ABCD1 | ATP binding cassette subfamily D member 1 [Source:HGNC Symbol;Acc:HGNC:61] |
| ENSG00000102466 | HGLibA_17374 | 123 | -0.022116313 | FGF14 | fibroblast growth factor 14 [Source:HGNC Symbol;Acc:HGNC:3671] |
| ENSG00000103355 | HGLibA_38926 | 123 | -0.022116313 | PRSS33 | serine protease 33 [Source:HGNC Symbol;Acc:HGNC:30405] |
| ENSG00000104154 | HGLibA_44910 | 123 | -0.022116313 | SLC30A4 | solute carrier family 30 member 4 [Source:HGNC Symbol;Acc:HGNC:11015] |
| ENSG00000104529 | HGLibA_14500 | 123 | -0.022116313 | EEF1D | eukaryotic translation elongation factor 1 delta [Source:HGNC Symbol;Acc:HGNC:3211] |
| ENSG00000105447 | HGLibA_20386 | 123 | -0.022116313 | GRWD1 | glutamate rich WD repeat containing 1 [Source:HGNC Symbol;Acc:HGNC:21270] |
| ENSG00000105880 | HGLibA_13384 | 123 | -0.022116313 | DLX5 | distal-less homeobox 5 [Source:HGNC Symbol;Acc:HGNC:2918] |
| ENSG00000105889 | HGLibA_47424 | 123 | -0.022116313 | STEAP1B | STEAP family member 1B [Source:HGNC Symbol;Acc:HGNC:41907] |
| ENSG00000106290 | HGLibA_48247 | 123 | -0.022116313 | TAF6 | TATA-box binding protein associated factor 6 [Source:HGNC Symbol;Acc:HGNC:11540] |
| ENSG00000106327 | HGLibA_49193 | 123 | -0.022116313 | TFR2 | transferrin receptor 2 [Source:HGNC Symbol;Acc:HGNC:11762] |
| ENSG00000106541 | HGLibA_01243 | 123 | -0.022116313 | AGR2 | protein disulphide isomerase family member [Source:HGNC Symbol;Acc:HGNC:328]" |
| ENSG00000108395 | HGLibA_51478 | 123 | -0.022116313 | TRIM37 | tripartite motif containing 37 [Source:HGNC Symbol;Acc:HGNC:7523] |
| ENSG00000108406 | HGLibA_13200 | 123 | -0.022116313 | DHX40 | DEAH-box helicase 40 [Source:HGNC Symbol;Acc:HGNC:18018] |
| ENSG00000111266 | HGLibA_14147 | 123 | -0.022116313 | DUSP16 | dual specificity phosphatase 16 [Source:HGNC Symbol;Acc:HGNC:17909] |
| ENSG00000111276 | HGLibA_08824 | 123 | -0.022116313 | CDKN1B | cyclin dependent kinase inhibitor 1B [Source:HGNC Symbol;Acc:HGNC:1785] |
| ENSG00000111537 | HGLibA_22706 | 123 | -0.022116313 | IFNG | interferon gamma [Source:HGNC Symbol;Acc:HGNC:5438] |
| ENSG00000112902 | HGLibA_43359 | 123 | -0.022116313 | SEMA5A | semaphorin 5A [Source:HGNC Symbol;Acc:HGNC:10736] |
| ENSG00000114248 | HGLibA_27311 | 123 | -0.022116313 | LRRC31 | leucine rich repeat containing 31 [Source:HGNC Symbol;Acc:HGNC:26261] |
| ENSG00000115718 | HGLibA_38649 | 123 | -0.022116313 | PROC | inactivator of coagulation factors Va and VIIIa [Source:HGNC Symbol;Acc:HGNC:9451]" |
| ENSG00000116221 | HGLibA_29966 | 123 | -0.022116313 | MRPL37 | mitochondrial ribosomal protein L37 [Source:HGNC Symbol;Acc:HGNC:14034] |
| ENSG00000117054 | HGLibA_00369 | 123 | -0.022116313 | ACADM | acyl-CoA dehydrogenase medium chain [Source:HGNC Symbol;Acc:HGNC:89] |
| ENSG00000118491 | HGLibA_54940 | 123 | -0.022116313 | ZC2HC1B | zinc finger C2HC-type containing 1B [Source:HGNC Symbol;Acc:HGNC:21174] |
| ENSG00000119403 | HGLibA_36357 | 123 | -0.022116313 | PHF19 | PHD finger protein 19 [Source:HGNC Symbol;Acc:HGNC:24566] |
| ENSG00000119698 | HGLibA_38210 | 123 | -0.022116313 | PPP4R4 | protein phosphatase 4 regulatory subunit 4 [Source:HGNC Symbol;Acc:HGNC:23788] |
| ENSG00000120093 | HGLibA_21936 | 123 | -0.022116313 | HOXB3 | homeobox B3 [Source:HGNC Symbol;Acc:HGNC:5114] |
| ENSG00000120337 | HGLibA_50819 | 123 | -0.022116313 | TNFSF18 | TNF superfamily member 18 [Source:HGNC Symbol;Acc:HGNC:11932] |
| ENSG00000120693 | HGLibA_45591 | 123 | -0.022116313 | SMAD9 | SMAD family member 9 [Source:HGNC Symbol;Acc:HGNC:6774] |
| ENSG00000121417 | HGLibA_55586 | 123 | -0.022116313 | ZNF211 | zinc finger protein 211 [Source:HGNC Symbol;Acc:HGNC:13003] |
| ENSG00000121653 | HGLibA_28286 | 123 | -0.022116313 | MAPK8IP1 | mitogen-activated protein kinase 8 interacting protein 1 [Source:HGNC Symbol;Acc:HGNC:6882] |
| ENSG00000124074 | HGLibA_15134 | 123 | -0.022116313 | ENKD1 | enkurin domain containing 1 [Source:HGNC Symbol;Acc:HGNC:25246] |
| ENSG00000124143 | HGLibA_02778 | 123 | -0.022116313 | ARHGAP40 | Rho GTPase activating protein 40 [Source:HGNC Symbol;Acc:HGNC:16226] |
| ENSG00000126247 | HGLibA_07183 | 123 | -0.022116313 | CAPNS1 | calpain small subunit 1 [Source:HGNC Symbol;Acc:HGNC:1481] |
| ENSG00000127720 | HGLibA_28998 | 123 | -0.022116313 | METTL25 | methyltransferase like 25 [Source:HGNC Symbol;Acc:HGNC:26228] |
| ENSG00000128652 | HGLibA_21998 | 123 | -0.022116313 | HOXD3 | homeobox D3 [Source:HGNC Symbol;Acc:HGNC:5137] |
| ENSG00000130822 | HGLibA_37343 | 123 | -0.022116313 | PNCK | pregnancy up-regulated nonubiquitous CaM kinase [Source:HGNC Symbol;Acc:HGNC:13415] |
| ENSG00000131650 | HGLibA_25406 | 123 | -0.022116313 | KREMEN2 | kringle containing transmembrane protein 2 [Source:HGNC Symbol;Acc:HGNC:18797] |
| ENSG00000132026 | HGLibA_42369 | 123 | -0.022116313 | RTBDN | retbindin [Source:HGNC Symbol;Acc:HGNC:30310] |
| ENSG00000132196 | HGLibA_22198 | 123 | -0.022116313 | HSD17B7 | hydroxysteroid 17-beta dehydrogenase 7 [Source:HGNC Symbol;Acc:HGNC:5215] |
| ENSG00000133433 | HGLibA_20502 | 123 | -0.022116313 | GSTT2B | glutathione S-transferase theta 2B (gene/pseudogene) [Source:HGNC Symbol;Acc:HGNC:33437] |
| ENSG00000133816 | HGLibA_29204 | 123 | -0.022116313 | MICAL2 | calponin and LIM domain containing 2 [Source:HGNC Symbol;Acc:HGNC:24693]" |
| ENSG00000136052 | HGLibA_45143 | 123 | -0.022116313 | SLC41A2 | solute carrier family 41 member 2 [Source:HGNC Symbol;Acc:HGNC:31045] |
| ENSG00000136144 | HGLibA_40678 | 123 | -0.022116313 | RCBTB1 | RCC1 and BTB domain containing protein 1 [Source:HGNC Symbol;Acc:HGNC:18243] |
| ENSG00000136193 | HGLibA_43049 | 123 | -0.022116313 | SCRN1 | secernin 1 [Source:HGNC Symbol;Acc:HGNC:22192] |
| ENSG00000136235 | HGLibA_19834 | 123 | -0.022116313 | GPNMB | glycoprotein nmb [Source:HGNC Symbol;Acc:HGNC:4462] |
| ENSG00000136492 | HGLibA_04821 | 123 | -0.022116313 | BRIP1 | BRCA1 interacting protein C-terminal helicase 1 [Source:HGNC Symbol;Acc:HGNC:20473] |
| ENSG00000137078 | HGLibA_44237 | 123 | -0.022116313 | SIT1 | signaling threshold regulating transmembrane adaptor 1 [Source:HGNC Symbol;Acc:HGNC:17710] |
| ENSG00000138002 | HGLibA_22748 | 123 | -0.022116313 | IFT172 | intraflagellar transport 172 [Source:HGNC Symbol;Acc:HGNC:30391] |
| ENSG00000138593 | HGLibA_43267 | 123 | -0.022116313 | SECISBP2L | SECIS binding protein 2 like [Source:HGNC Symbol;Acc:HGNC:28997] |
| ENSG00000140043 | HGLibA_39354 | 123 | -0.022116313 | PTGR2 | prostaglandin reductase 2 [Source:HGNC Symbol;Acc:HGNC:20149] |
| ENSG00000141503 | HGLibA_29277 | 123 | -0.022116313 | MINK1 | misshapen like kinase 1 [Source:HGNC Symbol;Acc:HGNC:17565] |
| ENSG00000144410 | HGLibA_10980 | 123 | -0.022116313 | CPO | carboxypeptidase O [Source:HGNC Symbol;Acc:HGNC:21011] |
| ENSG00000145246 | HGLibA_03550 | 123 | -0.022116313 | ATP10D | ATPase phospholipid transporting 10D (putative) [Source:HGNC Symbol;Acc:HGNC:13549] |
| ENSG00000146233 | HGLibA_12152 | 123 | -0.022116313 | CYP39A1 | cytochrome P450 family 39 subfamily A member 1 [Source:HGNC Symbol;Acc:HGNC:17449] |
| ENSG00000147119 | HGLibA_09646 | 123 | -0.022116313 | CHST7 | carbohydrate sulfotransferase 7 [Source:HGNC Symbol;Acc:HGNC:13817] |
| ENSG00000147324 | HGLibA_29064 | 123 | -0.022116313 | MFHAS1 | malignant fibrous histiocytoma amplified sequence 1 [Source:HGNC Symbol;Acc:HGNC:16982] |
| ENSG00000147481 | HGLibA_46006 | 123 | -0.022116313 | SNTG1 | syntrophin gamma 1 [Source:HGNC Symbol;Acc:HGNC:13740] |
| ENSG00000147592 | HGLibA_25919 | 123 | -0.022116313 | LACTB2 | lactamase beta 2 [Source:HGNC Symbol;Acc:HGNC:18512] |
| ENSG00000147854 | HGLibA_52953 | 123 | -0.022116313 | UHRF2 | ubiquitin like with PHD and ring finger domains 2 [Source:HGNC Symbol;Acc:HGNC:12557] |
| ENSG00000148297 | HGLibA_28778 | 123 | -0.022116313 | MED22 | mediator complex subunit 22 [Source:HGNC Symbol;Acc:HGNC:11477] |
| ENSG00000156689 | HGLibA_19375 | 123 | -0.022116313 | GLYATL2 | glycine-N-acyltransferase like 2 [Source:HGNC Symbol;Acc:HGNC:24178] |
| ENSG00000157350 | HGLibA_47239 | 123 | -0.022116313 | ST3GAL2 | 3-sialyltransferase 2 [Source:HGNC Symbol;Acc:HGNC:10863]" |
| ENSG00000158006 | HGLibA_34998 | 123 | -0.022116313 | PAFAH2 | platelet activating factor acetylhydrolase 2 [Source:HGNC Symbol;Acc:HGNC:8579] |
| ENSG00000160445 | HGLibA_55140 | 123 | -0.022116313 | ZER1 | zyg-11 related cell cycle regulator [Source:HGNC Symbol;Acc:HGNC:30960] |
| ENSG00000160753 | HGLibA_42466 | 123 | -0.022116313 | RUSC1 | RUN and SH3 domain containing 1 [Source:HGNC Symbol;Acc:HGNC:17153] |
| ENSG00000161031 | HGLibA_36251 | 123 | -0.022116313 | PGLYRP2 | peptidoglycan recognition protein 2 [Source:HGNC Symbol;Acc:HGNC:30013] |
| ENSG00000162458 | HGLibA_16939 | 123 | -0.022116313 | FBLIM1 | filamin binding LIM protein 1 [Source:HGNC Symbol;Acc:HGNC:24686] |
| ENSG00000162753 | HGLibA_45443 | 123 | -0.022116313 | SLC9C2 | solute carrier family 9 member C2 (putative) [Source:HGNC Symbol;Acc:HGNC:28664] |
| ENSG00000163814 | HGLibA_08622 | 123 | -0.022116313 | CDCP1 | CUB domain containing protein 1 [Source:HGNC Symbol;Acc:HGNC:24357] |
| ENSG00000164176 | HGLibA_14467 | 123 | -0.022116313 | EDIL3 | EGF like repeats and discoidin domains 3 [Source:HGNC Symbol;Acc:HGNC:3173] |
| ENSG00000164871 | HGLibA_46349 | 123 | -0.022116313 | SPAG11B | sperm associated antigen 11B [Source:HGNC Symbol;Acc:HGNC:14534] |
| ENSG00000164889 | HGLibA_45215 | 123 | -0.022116313 | SLC4A2 | solute carrier family 4 member 2 [Source:HGNC Symbol;Acc:HGNC:11028] |
| ENSG00000164902 | HGLibA_36309 | 123 | -0.022116313 | PHAX | phosphorylated adaptor for RNA export [Source:HGNC Symbol;Acc:HGNC:10241] |
| ENSG00000164983 | HGLibA_50486 | 123 | -0.022116313 | TMEM65 | transmembrane protein 65 [Source:HGNC Symbol;Acc:HGNC:25203] |
| ENSG00000166160 | HGLibA_33429 | 123 | -0.022116313 | OPN1MW2 | medium wave sensitive 2 [Source:HGNC Symbol;Acc:HGNC:26952]" |
| ENSG00000167077 | HGLibA_28862 | 123 | -0.022116313 | MEI1 | meiotic double-stranded break formation protein 1 [Source:HGNC Symbol;Acc:HGNC:28613] |
| ENSG00000167202 | HGLibA_48528 | 123 | -0.022116313 | TBC1D2B | TBC1 domain family member 2B [Source:HGNC Symbol;Acc:HGNC:29183] |
| ENSG00000167996 | HGLibA_18115 | 123 | -0.022116313 | FTH1 | ferritin heavy chain 1 [Source:HGNC Symbol;Acc:HGNC:3976] |
| ENSG00000168067 | HGLibA_28215 | 123 | -0.022116313 | MAP4K2 | mitogen-activated protein kinase kinase kinase kinase 2 [Source:HGNC Symbol;Acc:HGNC:6864] |
| ENSG00000168621 | HGLibA_18887 | 123 | -0.022116313 | GDNF | glial cell derived neurotrophic factor [Source:HGNC Symbol;Acc:HGNC:4232] |
| ENSG00000168671 | HGLibA_52936 | 123 | -0.022116313 | UGT3A2 | UDP glycosyltransferase family 3 member A2 [Source:HGNC Symbol;Acc:HGNC:27266] |
| ENSG00000169397 | HGLibA_41405 | 123 | -0.022116313 | RNASE3 | ribonuclease A family member 3 [Source:HGNC Symbol;Acc:HGNC:10046] |
| ENSG00000169903 | HGLibA_49703 | 123 | -0.022116313 | TM4SF4 | transmembrane 4 L six family member 4 [Source:HGNC Symbol;Acc:HGNC:11856] |
| ENSG00000170734 | HGLibA_37529 | 123 | -0.022116313 | POLH | DNA polymerase eta [Source:HGNC Symbol;Acc:HGNC:9181] |
| ENSG00000172269 | HGLibA_13810 | 123 | -0.022116313 | DPAGT1 | dolichyl-phosphate N-acetylglucosaminephosphotransferase 1 [Source:HGNC Symbol;Acc:HGNC:2995] |
| ENSG00000172757 | HGLibA_09282 | 123 | -0.022116313 | CFL1 | cofilin 1 [Source:HGNC Symbol;Acc:HGNC:1874] |
| ENSG00000174886 | HGLibA_31443 | 123 | -0.022116313 | NDUFA11 | NADH:ubiquinone oxidoreductase subunit A11 [Source:HGNC Symbol;Acc:HGNC:20371] |
| ENSG00000176842 | HGLibA_23641 | 123 | -0.022116313 | IRX5 | iroquois homeobox 5 [Source:HGNC Symbol;Acc:HGNC:14361] |
| ENSG00000176903 | HGLibA_37367 | 123 | -0.022116313 | PNMA1 | PNMA family member 1 [Source:HGNC Symbol;Acc:HGNC:9158] |
| ENSG00000177294 | HGLibA_17086 | 123 | -0.022116313 | FBXO39 | F-box protein 39 [Source:HGNC Symbol;Acc:HGNC:28565] |
| ENSG00000177669 | HGLibA_28523 | 123 | -0.022116313 | MBOAT4 | membrane bound O-acyltransferase domain containing 4 [Source:HGNC Symbol;Acc:HGNC:32311] |
| ENSG00000178776 | HGLibA_06388 | 123 | -0.022116313 | C5orf46 | chromosome 5 open reading frame 46 [Source:HGNC Symbol;Acc:HGNC:33768] |
| ENSG00000179165 | HGLibA_39666 | 123 | -0.022116313 | PXT1 | peroxisomal testis enriched protein 1 [Source:HGNC Symbol;Acc:HGNC:18312] |
| ENSG00000181773 | HGLibA_20014 | 123 | -0.022116313 | GPR3 | G protein-coupled receptor 3 [Source:HGNC Symbol;Acc:HGNC:4484] |
| ENSG00000183682 | HGLibA_04619 | 123 | -0.022116313 | BMP8A | bone morphogenetic protein 8a [Source:HGNC Symbol;Acc:HGNC:21650] |
| ENSG00000184347 | HGLibA_45510 | 123 | -0.022116313 | SLIT3 | slit guidance ligand 3 [Source:HGNC Symbol;Acc:HGNC:11087] |
| ENSG00000185437 | HGLibA_43914 | 123 | -0.022116313 | SH3BGR | SH3 domain binding glutamate rich protein [Source:HGNC Symbol;Acc:HGNC:10822] |
| ENSG00000185823 | HGLibA_32392 | 123 | -0.022116313 | NPAP1 | nuclear pore associated protein 1 [Source:HGNC Symbol;Acc:HGNC:1190] |
| ENSG00000186280 | HGLibA_24514 | 123 | -0.022116313 | KDM4D | lysine demethylase 4D [Source:HGNC Symbol;Acc:HGNC:25498] |
| ENSG00000186474 | HGLibA_25240 | 123 | -0.022116313 | KLK12 | kallikrein related peptidase 12 [Source:HGNC Symbol;Acc:HGNC:6360] |
| ENSG00000187391 | HGLibA_27999 | 123 | -0.022116313 | MAGI2 | WW and PDZ domain containing 2 [Source:HGNC Symbol;Acc:HGNC:18957]" |
| ENSG00000196341 | HGLibA_34507 | 123 | -0.022116313 | OR8D1 | olfactory receptor family 8 subfamily D member 1 [Source:HGNC Symbol;Acc:HGNC:8481] |
| ENSG00000197044 | HGLibA_56005 | 123 | -0.022116313 | ZNF441 | zinc finger protein 441 [Source:HGNC Symbol;Acc:HGNC:20875] |
| ENSG00000198794 | HGLibA_42847 | 123 | -0.022116313 | SCAMP5 | secretory carrier membrane protein 5 [Source:HGNC Symbol;Acc:HGNC:30386] |
| ENSG00000203970 | HGLibA_12847 | 123 | -0.022116313 | DEFB110 | defensin beta 110 [Source:HGNC Symbol;Acc:HGNC:18091] |
| ENSG00000204103 | HGLibA_27875 | 123 | -0.022116313 | MAFB | MAF bZIP transcription factor B [Source:HGNC Symbol;Acc:HGNC:6408] |
| ENSG00000204193 | HGLibA_52426 | 123 | -0.022116313 | TXNDC8 | thioredoxin domain containing 8 [Source:HGNC Symbol;Acc:HGNC:31454] |
| ENSG00000205629 | HGLibA_26161 | 123 | -0.022116313 | LCMT1 | leucine carboxyl methyltransferase 1 [Source:HGNC Symbol;Acc:HGNC:17557] |
| ENSG00000213512 | HGLibA_18746 | 123 | -0.022116313 | GBP7 | guanylate binding protein 7 [Source:HGNC Symbol;Acc:HGNC:29606] |
| ENSG00000213886 | HGLibA_52570 | 123 | -0.022116313 | UBD | ubiquitin D [Source:HGNC Symbol;Acc:HGNC:18795] |
| ENSG00000213977 | HGLibA_48453 | 123 | -0.022116313 | TAX1BP3 | Tax1 binding protein 3 [Source:HGNC Symbol;Acc:HGNC:30684] |
| ENSG00000214309 | HGLibA_28501 | 123 | -0.022116313 | MBLAC1 | metallo-beta-lactamase domain containing 1 [Source:HGNC Symbol;Acc:HGNC:22180] |
| ENSG00000254585 | HGLibA_27991 | 123 | -0.022116313 | MAGEL2 | MAGE family member L2 [Source:HGNC Symbol;Acc:HGNC:6814] |
| ENSG00000257103 | HGLibA_27530 | 123 | -0.022116313 | LSM14A | mRNA processing body assembly factor [Source:HGNC Symbol;Acc:HGNC:24489]" |
| ENSG00000258289 | HGLibA_09672 | 123 | -0.022116313 | CHURC1 | churchill domain containing 1 [Source:HGNC Symbol;Acc:HGNC:20099] |
| ENSG00000266919 | HGLibA_59700 | 123 | -0.022116313 | hsa-mir-423 | microRNA 3184 |
| ENSG00000267534 | HGLibA_42597 | 123 | -0.022116313 | S1PR2 | sphingosine-1-phosphate receptor 2 [Source:HGNC Symbol;Acc:HGNC:3169] |
| ENSG00000272804 | HGLibA_25617 | 123 | -0.022116313 | KRTAP10-7 | keratin associated protein 10-7 [Source:HGNC Symbol;Acc:HGNC:22970] |
| ENSG00000277791 | HGLibA_39105 | 123 | -0.022116313 | PSMB3 | proteasome subunit beta 3 [Source:HGNC Symbol;Acc:HGNC:9540] |

| | | | | | |
|---|---|---|---|---|---|
| GPR64 | HGLibA_20071 | 123 | -0.022116313 | GPR64 | - |
| GUCY1A3 | HGLibA_20656 | 123 | -0.022116313 | GUCY1A3 | - |
| H2AFY2 | HGLibA_20766 | 123 | -0.022116313 | H2AFY2 | - |
| HDGFRP2 | HGLibA_21045 | 123 | -0.022116313 | HDGFRP2 | - |
| hsa-mir-1204 | HGLibA_57275 | 123 | -0.022116313 | hsa-mir-1204 | - |
| hsa-mir-1915 | HGLibA_58064 | 123 | -0.022116313 | hsa-mir-1915 | - |
| hsa-mir-2355 | HGLibA_58322 | 123 | -0.022116313 | hsa-mir-2355 | - |
| hsa-mir-3190 | HGLibA_58859 | 123 | -0.022116313 | hsa-mir-3190 | - |
| hsa-mir-3191 | HGLibA_58861 | 123 | -0.022116313 | hsa-mir-3191 | - |
| hsa-mir-3605 | HGLibA_59054 | 123 | -0.022116313 | hsa-mir-3605 | - |
| hsa-mir-4318 | HGLibA_59991 | 123 | -0.022116313 | hsa-mir-4318 | - |
| hsa-mir-4430 | HGLibA_60106 | 123 | -0.022116313 | hsa-mir-4430 | - |
| hsa-mir-4663 | HGLibA_60765 | 123 | -0.022116313 | hsa-mir-4663 | - |
| hsa-mir-4674 | HGLibA_60814 | 123 | -0.022116313 | hsa-mir-4674 | - |
| hsa-mir-4727 | HGLibA_61005 | 123 | -0.022116313 | hsa-mir-4727 | - |
| hsa-mir-5089 | HGLibA_61503 | 123 | -0.022116313 | hsa-mir-5089 | - |
| hsa-mir-6771 | HGLibA_63256 | 123 | -0.022116313 | hsa-mir-6771 | - |
| hsa-mir-6790 | HGLibA_63336 | 123 | -0.022116313 | hsa-mir-6790 | - |
| LOC402160 | HGLibA_26940 | 123 | -0.022116313 | LOC402160 | - |
| NAPRT1 | HGLibA_31136 | 123 | -0.022116313 | NAPRT1 | - |
| PARK2 | HGLibA_35196 | 123 | -0.022116313 | PARK2 | - |
| TARP | HGLibA_48343 | 123 | -0.022116313 | TARP | - |
| XAGE1C | HGLibA_54473 | 123 | -0.022116313 | XAGE1C | - |
| C16orf13 | HGLibA_05440 | 122 | -0.031161655 | C16orf13 | - |
| C19orf59 | HGLibA_05686 | 122 | -0.031161655 | C19orf59 | - |
| C1orf204 | HGLibA_05821 | 122 | -0.031161655 | C1orf204 | - |
| C9orf117 | HGLibA_06668 | 122 | -0.031161655 | C9orf117 | - |
| CXorf40B | HGLibA_11925 | 122 | -0.031161655 | CXorf40B | - |
| ENSG00000000938 | HGLibA_17469 | 122 | -0.031161655 | FGR | Src family tyrosine kinase [Source:HGNC Symbol;Acc:HGNC:3697]" |
| ENSG00000007129 | HGLibA_08936 | 122 | -0.031161655 | CEACAM21 | carcinoembryonic antigen related cell adhesion molecule 21 [Source:HGNC Symbol;Acc:HGNC:28834] |
| ENSG00000012779 | HGLibA_01689 | 122 | -0.031161655 | ALOX5 | arachidonate 5-lipoxygenase [Source:HGNC Symbol;Acc:HGNC:435] |
| ENSG00000013364 | HGLibA_30602 | 122 | -0.031161655 | MVP | major vault protein [Source:HGNC Symbol;Acc:HGNC:7531] |
| ENSG00000036530 | HGLibA_12169 | 122 | -0.031161655 | CYP46A1 | cytochrome P450 family 46 subfamily A member 1 [Source:HGNC Symbol;Acc:HGNC:2641] |
| ENSG00000036672 | HGLibA_53297 | 122 | -0.031161655 | USP2 | ubiquitin specific peptidase 2 [Source:HGNC Symbol;Acc:HGNC:12618] |
| ENSG00000056487 | HGLibA_36373 | 122 | -0.031161655 | PHF21B | PHD finger protein 21B [Source:HGNC Symbol;Acc:HGNC:25161] |
| ENSG00000068354 | HGLibA_48516 | 122 | -0.031161655 | TBC1D25 | TBC1 domain family member 25 [Source:HGNC Symbol;Acc:HGNC:8092] |
| ENSG00000071054 | HGLibA_28221 | 122 | -0.031161655 | MAP4K4 | mitogen-activated protein kinase kinase kinase kinase 4 [Source:HGNC Symbol;Acc:HGNC:6866] |
| ENSG00000072609 | HGLibA_09429 | 122 | -0.031161655 | CHFR | checkpoint with forkhead and ring finger domains [Source:HGNC Symbol;Acc:HGNC:20455] |
| ENSG00000072778 | HGLibA_00376 | 122 | -0.031161655 | ACADVL | acyl-CoA dehydrogenase very long chain [Source:HGNC Symbol;Acc:HGNC:92] |
| ENSG00000075415 | HGLibA_44700 | 122 | -0.031161655 | SLC25A3 | solute carrier family 25 member 3 [Source:HGNC Symbol;Acc:HGNC:10989] |
| ENSG00000081051 | HGLibA_01127 | 122 | -0.031161655 | AFP | alpha fetoprotein [Source:HGNC Symbol;Acc:HGNC:317] |
| ENSG00000087842 | HGLibA_36729 | 122 | -0.031161655 | PIR | pirin [Source:HGNC Symbol;Acc:HGNC:30048] |
| ENSG00000091490 | HGLibA_43282 | 122 | -0.031161655 | SEL1L3 | SEL1L family member 3 [Source:HGNC Symbol;Acc:HGNC:29108] |
| ENSG00000092445 | HGLibA_52468 | 122 | -0.031161655 | TYRO3 | TYRO3 protein tyrosine kinase [Source:HGNC Symbol;Acc:HGNC:12446] |
| ENSG00000095777 | HGLibA_30843 | 122 | -0.031161655 | MYO3A | myosin IIIA [Source:HGNC Symbol;Acc:HGNC:7601] |
| ENSG00000100097 | HGLibA_26346 | 122 | -0.031161655 | LGALS1 | galectin 1 [Source:HGNC Symbol;Acc:HGNC:6561] |
| ENSG00000101294 | HGLibA_21664 | 122 | -0.031161655 | HM13 | histocompatibility minor 13 [Source:HGNC Symbol;Acc:HGNC:16435] |
| ENSG00000101435 | HGLibA_11474 | 122 | -0.031161655 | CST9L | cystatin 9 like [Source:HGNC Symbol;Acc:HGNC:16233] |
| ENSG00000102743 | HGLibA_44651 | 122 | -0.031161655 | SLC25A15 | solute carrier family 25 member 15 [Source:HGNC Symbol;Acc:HGNC:10985] |
| ENSG00000102921 | HGLibA_30948 | 122 | -0.031161655 | N4BP1 | NEDD4 binding protein 1 [Source:HGNC Symbol;Acc:HGNC:29850] |
| ENSG00000103175 | HGLibA_54244 | 122 | -0.031161655 | WFDC1 | WAP four-disulfide core domain 1 [Source:HGNC Symbol;Acc:HGNC:15466] |
| ENSG00000103351 | HGLibA_10159 | 122 | -0.031161655 | CLUAP1 | clusterin associated protein 1 [Source:HGNC Symbol;Acc:HGNC:19009] |
| ENSG00000104728 | HGLibA_02806 | 122 | -0.031161655 | ARHGEF10 | Rho guanine nucleotide exchange factor 10 [Source:HGNC Symbol;Acc:HGNC:14103] |
| ENSG00000105401 | HGLibA_08544 | 122 | -0.031161655 | CDC37 | cell division cycle 37 [Source:HGNC Symbol;Acc:HGNC:1735] |
| ENSG00000105639 | HGLibA_23919 | 122 | -0.031161655 | JAK3 | Janus kinase 3 [Source:HGNC Symbol;Acc:HGNC:6193] |
| ENSG00000106302 | HGLibA_22445 | 122 | -0.031161655 | HYAL4 | hyaluronidase 4 [Source:HGNC Symbol;Acc:HGNC:5323] |
| ENSG00000106344 | HGLibA_40528 | 122 | -0.031161655 | RBM28 | RNA binding motif protein 28 [Source:HGNC Symbol;Acc:HGNC:21863] |
| ENSG00000106415 | HGLibA_19230 | 122 | -0.031161655 | GLCCI1 | glucocorticoid induced 1 [Source:HGNC Symbol;Acc:HGNC:18713] |
| ENSG00000107758 | HGLibA_38188 | 122 | -0.031161655 | PPP3CB | protein phosphatase 3 catalytic subunit beta [Source:HGNC Symbol;Acc:HGNC:9315] |
| ENSG00000108671 | HGLibA_39154 | 122 | -0.031161655 | PSMD11 | non-ATPase 11 [Source:HGNC Symbol;Acc:HGNC:9556]" |
| ENSG00000111642 | HGLibA_09395 | 122 | -0.031161655 | CHD4 | chromodomain helicase DNA binding protein 4 [Source:HGNC Symbol;Acc:HGNC:1919] |
| ENSG00000113742 | HGLibA_10925 | 122 | -0.031161655 | CPEB4 | cytoplasmic polyadenylation element binding protein 4 [Source:HGNC Symbol;Acc:HGNC:21747] |
| ENSG00000113763 | HGLibA_53015 | 122 | -0.031161655 | UNC5A | unc-5 netrin receptor A [Source:HGNC Symbol;Acc:HGNC:12567] |
| ENSG00000113966 | HGLibA_02984 | 122 | -0.031161655 | ARL6 | ADP ribosylation factor like GTPase 6 [Source:HGNC Symbol;Acc:HGNC:13210] |
| ENSG00000115232 | HGLibA_23732 | 122 | -0.031161655 | ITGA4 | integrin subunit alpha 4 [Source:HGNC Symbol;Acc:HGNC:6140] |
| ENSG00000115523 | HGLibA_19555 | 122 | -0.031161655 | GNLY | granulysin [Source:HGNC Symbol;Acc:HGNC:4414] |
| ENSG00000115677 | HGLibA_21061 | 122 | -0.031161655 | HDLBP | high density lipoprotein binding protein [Source:HGNC Symbol;Acc:HGNC:4857] |
| ENSG00000116521 | HGLibA_42842 | 122 | -0.031161655 | SCAMP3 | secretory carrier membrane protein 3 [Source:HGNC Symbol;Acc:HGNC:10565] |
| ENSG00000116981 | HGLibA_32802 | 122 | -0.031161655 | NT5C1A | cytosolic IA [Source:HGNC Symbol;Acc:HGNC:17819]" |
| ENSG00000117228 | HGLibA_18729 | 122 | -0.031161655 | GBP1 | guanylate binding protein 1 [Source:HGNC Symbol;Acc:HGNC:4182] |
| ENSG00000120784 | HGLibA_55201 | 122 | -0.031161655 | ZFP30 | ZFP30 zinc finger protein [Source:HGNC Symbol;Acc:HGNC:29555] |
| ENSG00000124181 | HGLibA_37002 | 122 | -0.031161655 | PLCG1 | phospholipase C gamma 1 [Source:HGNC Symbol;Acc:HGNC:9065] |
| ENSG00000124596 | HGLibA_33195 | 122 | -0.031161655 | OARD1 | O-acyl-ADP-ribose deacylase 1 [Source:HGNC Symbol;Acc:HGNC:21257] |
| ENSG00000124767 | HGLibA_19284 | 122 | -0.031161655 | GLO1 | glyoxalase I [Source:HGNC Symbol;Acc:HGNC:4323] |
| ENSG00000124839 | HGLibA_39819 | 122 | -0.031161655 | RAB17 | member RAS oncogene family [Source:HGNC Symbol;Acc:HGNC:16523]" |
| ENSG00000128739 | HGLibA_45993 | 122 | -0.031161655 | SNRPN | small nuclear ribonucleoprotein polypeptide N [Source:HGNC Symbol;Acc:HGNC:11164] |
| ENSG00000130475 | HGLibA_17204 | 122 | -0.031161655 | FCHO1 | FCH domain only 1 [Source:HGNC Symbol;Acc:HGNC:29002] |
| ENSG00000130558 | HGLibA_33344 | 122 | -0.031161655 | OLFM1 | olfactomedin 1 [Source:HGNC Symbol;Acc:HGNC:17187] |
| ENSG00000130649 | HGLibA_12131 | 122 | -0.031161655 | CYP2E1 | cytochrome P450 family 2 subfamily E member 1 [Source:HGNC Symbol;Acc:HGNC:2631] |
| ENSG00000130950 | HGLibA_33108 | 122 | -0.031161655 | NUTM2F | NUT family member 2F [Source:HGNC Symbol;Acc:HGNC:23450] |
| ENSG00000131668 | HGLibA_04163 | 122 | -0.031161655 | BARX1 | BARX homeobox 1 [Source:HGNC Symbol;Acc:HGNC:955] |
| ENSG00000132563 | HGLibA_40780 | 122 | -0.031161655 | REEP2 | receptor accessory protein 2 [Source:HGNC Symbol;Acc:HGNC:17975] |
| ENSG00000134259 | HGLibA_31873 | 122 | -0.031161655 | NGF | nerve growth factor [Source:HGNC Symbol;Acc:HGNC:7808] |
| ENSG00000134287 | HGLibA_02649 | 122 | -0.031161655 | ARF3 | ADP ribosylation factor 3 [Source:HGNC Symbol;Acc:HGNC:654] |
| ENSG00000135124 | HGLibA_34856 | 122 | -0.031161655 | P2RX4 | purinergic receptor P2X 4 [Source:HGNC Symbol;Acc:HGNC:8535] |
| ENSG00000135773 | HGLibA_07180 | 122 | -0.031161655 | CAPN9 | calpain 9 [Source:HGNC Symbol;Acc:HGNC:1486] |
| ENSG00000135973 | HGLibA_20043 | 122 | -0.031161655 | GPR45 | G protein-coupled receptor 45 [Source:HGNC Symbol;Acc:HGNC:4503] |
| ENSG00000136367 | HGLibA_55171 | 122 | -0.031161655 | ZFHX2 | zinc finger homeobox 2 [Source:HGNC Symbol;Acc:HGNC:20152] |
| ENSG00000136535 | HGLibA_48631 | 122 | -0.031161655 | TBR1 | brain 1 [Source:HGNC Symbol;Acc:HGNC:11590]" |
| ENSG00000136931 | HGLibA_32633 | 122 | -0.031161655 | NR5A1 | nuclear receptor subfamily 5 group A member 1 [Source:HGNC Symbol;Acc:HGNC:7983] |
| ENSG00000137221 | HGLibA_49568 | 122 | -0.031161655 | TJAP1 | tight junction associated protein 1 [Source:HGNC Symbol;Acc:HGNC:17949] |
| ENSG00000138801 | HGLibA_35151 | 122 | -0.031161655 | PAPSS1 | 3'-phosphoadenosine 5'-phosphosulfate synthase 1 [Source:HGNC Symbol;Acc:HGNC:8603] |
| ENSG00000139631 | HGLibA_11304 | 122 | -0.031161655 | CSAD | cysteine sulfinic acid decarboxylase [Source:HGNC Symbol;Acc:HGNC:18966] |
| ENSG00000140264 | HGLibA_43476 | 122 | -0.031161655 | SERF2 | small EDRK-rich factor 2 [Source:HGNC Symbol;Acc:HGNC:10757] |
| ENSG00000141026 | HGLibA_28819 | 122 | -0.031161655 | MED9 | mediator complex subunit 9 [Source:HGNC Symbol;Acc:HGNC:25487] |
| ENSG00000142511 | HGLibA_20021 | 122 | -0.031161655 | GPR32 | G protein-coupled receptor 32 [Source:HGNC Symbol;Acc:HGNC:4487] |
| ENSG00000143756 | HGLibA_17059 | 122 | -0.031161655 | FBXO28 | F-box protein 28 [Source:HGNC Symbol;Acc:HGNC:29046] |
| ENSG00000144647 | HGLibA_37660 | 122 | -0.031161655 | POMGNT2 | 4-) [Source:HGNC Symbol;Acc:HGNC:25902]" |
| ENSG00000145780 | HGLibA_17280 | 122 | -0.031161655 | FEM1C | fem-1 homolog C [Source:HGNC Symbol;Acc:HGNC:16933] |
| ENSG00000148384 | HGLibA_23352 | 122 | -0.031161655 | INPP5E | inositol polyphosphate-5-phosphatase E [Source:HGNC Symbol;Acc:HGNC:21474] |
| ENSG00000149599 | HGLibA_14144 | 122 | -0.031161655 | DUSP15 | dual specificity phosphatase 15 [Source:HGNC Symbol;Acc:HGNC:16236] |
| ENSG00000150687 | HGLibA_38916 | 122 | -0.031161655 | PRSS23 | serine protease 23 [Source:HGNC Symbol;Acc:HGNC:14370] |
| ENSG00000150750 | HGLibA_05153 | 122 | -0.031161655 | C11orf53 | chromosome 11 open reading frame 53 [Source:HGNC Symbol;Acc:HGNC:30527] |
| ENSG00000151413 | HGLibA_32906 | 122 | -0.031161655 | NUBPL | nucleotide binding protein like [Source:HGNC Symbol;Acc:HGNC:20278] |
| ENSG00000152291 | HGLibA_49274 | 122 | -0.031161655 | TGOLN2 | trans-golgi network protein 2 [Source:HGNC Symbol;Acc:HGNC:15450] |
| ENSG00000153303 | HGLibA_18009 | 122 | -0.031161655 | FRMD1 | FERM domain containing 1 [Source:HGNC Symbol;Acc:HGNC:21240] |
| ENSG00000153574 | HGLibA_41851 | 122 | -0.031161655 | RPIA | ribose 5-phosphate isomerase A [Source:HGNC Symbol;Acc:HGNC:10297] |
| ENSG00000153896 | HGLibA_56360 | 122 | -0.031161655 | ZNF599 | zinc finger protein 599 [Source:HGNC Symbol;Acc:HGNC:26408] |
| ENSG00000157191 | HGLibA_31596 | 122 | -0.031161655 | NECAP2 | NECAP endocytosis associated 2 [Source:HGNC Symbol;Acc:HGNC:25528] |
| ENSG00000157404 | HGLibA_25015 | 122 | -0.031161655 | KIT | KIT proto-oncogene receptor tyrosine kinase [Source:HGNC Symbol;Acc:HGNC:6342] |
| ENSG00000159592 | HGLibA_19759 | 122 | -0.031161655 | GPBP1L1 | GC-rich promoter binding protein 1 like 1 [Source:HGNC Symbol;Acc:HGNC:28843] |
| ENSG00000159592 | HGLibA_19760 | 122 | -0.031161655 | GPBP1L1 | GC-rich promoter binding protein 1 like 1 [Source:HGNC Symbol;Acc:HGNC:28843] |
| ENSG00000159714 | HGLibA_55065 | 122 | -0.031161655 | ZDHHC1 | zinc finger DHHC-type containing 1 [Source:HGNC Symbol;Acc:HGNC:17916] |
| ENSG00000160255 | HGLibA_23778 | 122 | -0.031161655 | ITGB2 | integrin subunit beta 2 [Source:HGNC Symbol;Acc:HGNC:6155] |
| ENSG00000160716 | HGLibA_09592 | 122 | -0.031161655 | CHRNB2 | cholinergic receptor nicotinic beta 2 subunit [Source:HGNC Symbol;Acc:HGNC:1962] |
| ENSG00000160783 | HGLibA_37302 | 122 | -0.031161655 | PMF1 | polyamine modulated factor 1 [Source:HGNC Symbol;Acc:HGNC:9112] |
| ENSG00000160801 | HGLibA_39367 | 122 | -0.031161655 | PTH1R | parathyroid hormone 1 receptor [Source:HGNC Symbol;Acc:HGNC:9608] |
| ENSG00000161888 | HGLibA_46553 | 122 | -0.031161655 | SPC24 | NDC80 kinetochore complex component [Source:HGNC Symbol;Acc:HGNC:26913]" |
| ENSG00000162735 | HGLibA_36104 | 122 | -0.031161655 | PEX19 | peroxisomal biogenesis factor 19 [Source:HGNC Symbol;Acc:HGNC:9713] |
| ENSG00000162753 | HGLibA_45442 | 122 | -0.031161655 | SLC9C2 | solute carrier family 9 member C2 (putative) [Source:HGNC Symbol;Acc:HGNC:28664] |
| ENSG00000163069 | HGLibA_43799 | 122 | -0.031161655 | SGCB | sarcoglycan beta [Source:HGNC Symbol;Acc:HGNC:10806] |
| ENSG00000163206 | HGLibA_45674 | 122 | -0.031161655 | SMCP | sperm mitochondria associated cysteine rich protein [Source:HGNC Symbol;Acc:HGNC:6962] |
| ENSG00000163703 | HGLibA_11127 | 122 | -0.031161655 | CRELD1 | cysteine rich with EGF like domains 1 [Source:HGNC Symbol;Acc:HGNC:14630] |
| ENSG00000164400 | HGLibA_11326 | 122 | -0.031161655 | CSF2 | colony stimulating factor 2 [Source:HGNC Symbol;Acc:HGNC:2434] |
| ENSG00000165810 | HGLibA_04982 | 122 | -0.031161655 | BTNL9 | butyrophilin like 9 [Source:HGNC Symbol;Acc:HGNC:24176] |
| ENSG00000166123 | HGLibA_20168 | 122 | -0.031161655 | GPT2 | glutamic--pyruvic transaminase 2 [Source:HGNC Symbol;Acc:HGNC:18062] |
| ENSG00000166664 | HGLibA_09543 | 122 | -0.031161655 | CHRFAM7A | CHRNA7 (exons 5-10) and FAM7A (exons A-E) fusion [Source:HGNC Symbol;Acc:HGNC:15781] |
| ENSG00000166747 | HGLibA_02283 | 122 | -0.031161655 | AP1G1 | adaptor related protein complex 1 subunit gamma 1 [Source:HGNC Symbol;Acc:HGNC:555] |
| ENSG00000166847 | HGLibA_12557 | 122 | -0.031161655 | DCTN5 | dynactin subunit 5 [Source:HGNC Symbol;Acc:HGNC:24594] |
| ENSG00000167619 | HGLibA_49972 | 122 | -0.031161655 | TMEM145 | transmembrane protein 145 [Source:HGNC Symbol;Acc:HGNC:26912] |
| ENSG00000168016 | HGLibA_51290 | 122 | -0.031161655 | TRANK1 | tetratricopeptide repeat and ankyrin repeat containing 1 [Source:HGNC Symbol;Acc:HGNC:29011] |
| ENSG00000169402 | HGLibA_42330 | 122 | -0.031161655 | RSPH10B2 | radial spoke head 10 homolog B2 [Source:HGNC Symbol;Acc:HGNC:34385] |
| ENSG00000169418 | HGLibA_32508 | 122 | -0.031161655 | NPR1 | natriuretic peptide receptor 1 [Source:HGNC Symbol;Acc:HGNC:7943] |
| ENSG00000169604 | HGLibA_02214 | 122 | -0.031161655 | ANTXR1 | ANTXR cell adhesion molecule 1 [Source:HGNC Symbol;Acc:HGNC:21014] |
| ENSG00000172421 | HGLibA_14537 | 122 | -0.031161655 | EFCAB3 | EF-hand calcium binding domain 3 [Source:HGNC Symbol;Acc:HGNC:26379] |

| | | | | | |
|---|---|---|---|---|---|
| ENSG00000173572 | HGLibA_32108 | 122 | -0.031161655 | NLRP13 | NLR family pyrin domain containing 13 [Source:HGNC Symbol;Acc:HGNC:22937] |
| ENSG00000174123 | HGLibA_49642 | 122 | -0.031161655 | TLR10 | toll like receptor 10 [Source:HGNC Symbol;Acc:HGNC:15634] |
| ENSG00000174498 | HGLibA_22784 | 122 | -0.031161655 | IGDCC3 | immunoglobulin superfamily DCC subclass member 3 [Source:HGNC Symbol;Acc:HGNC:9700] |
| ENSG00000176383 | HGLibA_03989 | 122 | -0.031161655 | B3GNT4 | 3-N-acetylglucosaminyltransferase 4 [Source:HGNC Symbol;Acc:HGNC:15683]" |
| ENSG00000176946 | HGLibA_49300 | 122 | -0.031161655 | THAP4 | THAP domain containing 4 [Source:HGNC Symbol;Acc:HGNC:23187] |
| ENSG00000177599 | HGLibA_56093 | 122 | -0.031161655 | ZNF491 | zinc finger protein 491 [Source:HGNC Symbol;Acc:HGNC:23706] |
| ENSG00000178718 | HGLibA_42028 | 122 | -0.031161655 | RPP25 | ribonuclease P and MRP subunit p25 [Source:HGNC Symbol;Acc:HGNC:30361] |
| ENSG00000179583 | HGLibA_09707 | 122 | -0.031161655 | CIITA | class II major histocompatibility complex transactivator [Source:HGNC Symbol;Acc:HGNC:7067] |
| ENSG00000179832 | HGLibA_29866 | 122 | -0.031161655 | MROH1 | maestro heat like repeat family member 1 [Source:HGNC Symbol;Acc:HGNC:26958] |
| ENSG00000180263 | HGLibA_17357 | 122 | -0.031161655 | FGD6 | RhoGEF and PH domain containing 6 [Source:HGNC Symbol;Acc:HGNC:21740]" |
| ENSG00000182393 | HGLibA_22720 | 122 | -0.031161655 | IFNL1 | interferon lambda 1 [Source:HGNC Symbol;Acc:HGNC:18363] |
| ENSG00000183971 | HGLibA_32543 | 122 | -0.031161655 | NPW | neuropeptide W [Source:HGNC Symbol;Acc:HGNC:30509] |
| ENSG00000184144 | HGLibA_10405 | 122 | -0.031161655 | CNTN2 | contactin 2 [Source:HGNC Symbol;Acc:HGNC:2172] |
| ENSG00000185559 | HGLibA_13355 | 122 | -0.031161655 | DLK1 | delta like non-canonical Notch ligand 1 [Source:HGNC Symbol;Acc:HGNC:2907] |
| ENSG00000186074 | HGLibA_08341 | 122 | -0.031161655 | CD300LF | CD300 molecule like family member f [Source:HGNC Symbol;Acc:HGNC:29883] |
| ENSG00000187005 | HGLibA_25707 | 122 | -0.031161655 | KRTAP21-1 | keratin associated protein 21-1 [Source:HGNC Symbol;Acc:HGNC:18945] |
| ENSG00000187720 | HGLibA_49403 | 122 | -0.031161655 | THSD4 | thrombospondin type 1 domain containing 4 [Source:HGNC Symbol;Acc:HGNC:25835] |
| ENSG00000187997 | HGLibA_05603 | 122 | -0.031161655 | C17orf99 | chromosome 17 open reading frame 99 [Source:HGNC Symbol;Acc:HGNC:34490] |
| ENSG00000189052 | HGLibA_09308 | 122 | -0.031161655 | CGB5 | chorionic gonadotropin subunit beta 5 [Source:HGNC Symbol;Acc:HGNC:16452] |
| ENSG00000196431 | HGLibA_11258 | 122 | -0.031161655 | CRYBA4 | crystallin beta A4 [Source:HGNC Symbol;Acc:HGNC:2396] |
| ENSG00000196653 | HGLibA_56112 | 122 | -0.031161655 | ZNF502 | zinc finger protein 502 [Source:HGNC Symbol;Acc:HGNC:23718] |
| ENSG00000197233 | HGLibA_33677 | 122 | -0.031161655 | OR1J2 | olfactory receptor family 1 subfamily J member 2 [Source:HGNC Symbol;Acc:HGNC:8209] |
| ENSG00000197980 | HGLibA_26281 | 122 | -0.031161655 | LEKR1 | glutamate and lysine rich 1 [Source:HGNC Symbol;Acc:HGNC:33765]" |
| ENSG00000204228 | HGLibA_22201 | 122 | -0.031161655 | HSD17B8 | hydroxysteroid 17-beta dehydrogenase 8 [Source:HGNC Symbol;Acc:HGNC:3554] |
| ENSG00000204379 | HGLibA_54467 | 122 | -0.031161655 | XAGE1A | X antigen family member 1A [Source:HGNC Symbol;Acc:HGNC:4111] |
| ENSG00000204427 | HGLibA_00257 | 122 | -0.031161655 | ABHD16A | abhydrolase domain containing 16A [Source:HGNC Symbol;Acc:HGNC:13921] |
| ENSG00000205420 | HGLibA_25513 | 122 | -0.031161655 | KRT6A | keratin 6A [Source:HGNC Symbol;Acc:HGNC:6443] |
| ENSG00000214688 | HGLibA_05023 | 122 | -0.031161655 | C10orf105 | chromosome 10 open reading frame 105 [Source:HGNC Symbol;Acc:HGNC:20304] |
| ENSG00000223601 | HGLibA_14363 | 122 | -0.031161655 | EBLN1 | endogenous Bornavirus like nucleoprotein 1 [Source:HGNC Symbol;Acc:HGNC:39430] |
| ENSG00000227488 | HGLibA_18442 | 122 | -0.031161655 | GAGE12D | G antigen 12D [Source:HGNC Symbol;Acc:HGNC:31904] |
| ENSG00000233412 | HGLibA_34294 | 122 | -0.031161655 | OR5H15 | olfactory receptor family 5 subfamily H member 15 [Source:HGNC Symbol;Acc:HGNC:31287] |
| ENSG00000233803 | HGLibA_51972 | 122 | -0.031161655 | TSPY4 | testis specific protein Y-linked 4 [Source:HGNC Symbol;Acc:HGNC:37287] |
| ENSG00000234444 | HGLibA_56653 | 122 | -0.031161655 | ZNF736 | zinc finger protein 736 [Source:HGNC Symbol;Acc:HGNC:32467] |
| ENSG00000253313 | HGLibA_05827 | 122 | -0.031161655 | C1orf210 | chromosome 1 open reading frame 210 [Source:HGNC Symbol;Acc:HGNC:28755] |
| ENSG00000256683 | HGLibA_55858 | 122 | -0.031161655 | ZNF350 | zinc finger protein 350 [Source:HGNC Symbol;Acc:HGNC:16656] |
| ENSG00000266173 | HGLibA_47574 | 122 | -0.031161655 | STRADA | STE20-related kinase adaptor alpha [Source:HGNC Symbol;Acc:HGNC:30172] |
| ENSG00000279116 | HGLibA_34512 | 122 | -0.031161655 | OR8D2 | olfactory receptor family 8 subfamily D member 2 (gene/pseudogene) [Source:HGNC Symbol;Acc:HGNC:8482] |
| FAM19A5 | HGLibA_16407 | 122 | -0.031161655 | FAM19A5 | - |
| FAM65B | HGLibA_16672 | 122 | -0.031161655 | FAM65B | - |
| FAM65C | HGLibA_16675 | 122 | -0.031161655 | FAM65C | - |
| hsa-mir-122 | HGLibA_57290 | 122 | -0.031161655 | hsa-mir-122 | - |
| hsa-mir-138-1 | HGLibA_57813 | 122 | -0.031161655 | hsa-mir-138-1 | - |
| hsa-mir-145 | HGLibA_57843 | 122 | -0.031161655 | hsa-mir-145 | - |
| hsa-mir-3163 | HGLibA_58732 | 122 | -0.031161655 | hsa-mir-3163 | - |
| hsa-mir-3671 | HGLibA_59240 | 122 | -0.031161655 | hsa-mir-3671 | - |
| hsa-mir-3927 | HGLibA_59583 | 122 | -0.031161655 | hsa-mir-3927 | - |
| hsa-mir-4292 | HGLibA_59881 | 122 | -0.031161655 | hsa-mir-4292 | - |
| hsa-mir-4436b-1 | HGLibA_60143 | 122 | -0.031161655 | hsa-mir-4436b-1 | - |
| hsa-mir-4526 | HGLibA_60565 | 122 | -0.031161655 | hsa-mir-4526 | - |
| hsa-mir-4632 | HGLibA_60633 | 122 | -0.031161655 | hsa-mir-4632 | - |
| hsa-mir-4724 | HGLibA_60994 | 122 | -0.031161655 | hsa-mir-4724 | - |
| hsa-mir-548a-1 | HGLibA_61835 | 122 | -0.031161655 | hsa-mir-548a-1 | - |
| hsa-mir-556 | HGLibA_62139 | 122 | -0.031161655 | hsa-mir-556 | - |
| hsa-mir-608 | HGLibA_62576 | 122 | -0.031161655 | hsa-mir-608 | - |
| hsa-mir-635 | HGLibA_62779 | 122 | -0.031161655 | hsa-mir-635 | - |
| hsa-mir-876 | HGLibA_64213 | 122 | -0.031161655 | hsa-mir-876 | - |
| KIAA1524 | HGLibA_24775 | 122 | -0.031161655 | KIAA1524 | - |
| PTCHD2 | HGLibA_39278 | 122 | -0.031161655 | PTCHD2 | - |
| 9-Sep | HGLibA_43458 | 122 | -0.031161655 | 9-Sep | - |
| TMEM173 | HGLibA_50064 | 122 | -0.031161655 | TMEM173 | - |
| TMEM56 | HGLibA_50451 | 122 | -0.031161655 | TMEM56 | - |
| C17orf59 | HGLibA_05552 | 121 | -0.040206998 | C17orf59 | - |
| C1orf51 | HGLibA_05864 | 121 | -0.040206998 | C1orf51 | - |
| C6orf203 | HGLibA_06470 | 121 | -0.040206998 | C6orf203 | - |
| C7orf29 | HGLibA_06529 | 121 | -0.040206998 | C7orf29 | - |
| EMR2 | HGLibA_15087 | 121 | -0.040206998 | EMR2 | - |
| ENSG00000000005 | HGLibA_50858 | 121 | -0.040206998 | TNMD | tenomodulin [Source:HGNC Symbol;Acc:HGNC:17757] |
| ENSG00000006432 | HGLibA_28204 | 121 | -0.040206998 | MAP3K9 | mitogen-activated protein kinase kinase kinase 9 [Source:HGNC Symbol;Acc:HGNC:6861] |
| ENSG00000006530 | HGLibA_01194 | 121 | -0.040206998 | AGK | acylglycerol kinase [Source:HGNC Symbol;Acc:HGNC:21869] |
| ENSG00000011590 | HGLibA_54860 | 121 | -0.040206998 | ZBTB32 | zinc finger and BTB domain containing 32 [Source:HGNC Symbol;Acc:HGNC:16763] |
| ENSG00000012061 | HGLibA_15432 | 121 | -0.040206998 | ERCC1 | endonuclease non-catalytic subunit [Source:HGNC Symbol;Acc:HGNC:3433]" |
| ENSG00000013441 | HGLibA_10055 | 121 | -0.040206998 | CLK1 | CDC like kinase 1 [Source:HGNC Symbol;Acc:HGNC:2068] |
| ENSG00000027001 | HGLibA_29294 | 121 | -0.040206998 | MIPEP | mitochondrial intermediate peptidase [Source:HGNC Symbol;Acc:HGNC:7104] |
| ENSG00000046653 | HGLibA_19823 | 121 | -0.040206998 | GPM6B | glycoprotein M6B [Source:HGNC Symbol;Acc:HGNC:4461] |
| ENSG00000047932 | HGLibA_19673 | 121 | -0.040206998 | GOPC | golgi associated PDZ and coiled-coil motif containing [Source:HGNC Symbol;Acc:HGNC:17643] |
| ENSG00000051108 | HGLibA_21172 | 121 | -0.040206998 | HERPUD1 | homocysteine inducible ER protein with ubiquitin like domain 1 [Source:HGNC Symbol;Acc:HGNC:13744] |
| ENSG00000065057 | HGLibA_32845 | 121 | -0.040206998 | NTHL1 | nth like DNA glycosylase 1 [Source:HGNC Symbol;Acc:HGNC:8028] |
| ENSG00000067182 | HGLibA_50772 | 121 | -0.040206998 | TNFRSF1A | TNF receptor superfamily member 1A [Source:HGNC Symbol;Acc:HGNC:11916] |
| ENSG00000070366 | HGLibA_45705 | 121 | -0.040206998 | SMG6 | nonsense mediated mRNA decay factor [Source:HGNC Symbol;Acc:HGNC:17809]" |
| ENSG00000070808 | HGLibA_07066 | 121 | -0.040206998 | CAMK2A | calcium/calmodulin dependent protein kinase II alpha [Source:HGNC Symbol;Acc:HGNC:1460] |
| ENSG00000074071 | HGLibA_30096 | 121 | -0.040206998 | MRPS34 | mitochondrial ribosomal protein S34 [Source:HGNC Symbol;Acc:HGNC:16618] |
| ENSG00000074695 | HGLibA_26658 | 121 | -0.040206998 | LMAN1 | mannose binding 1 [Source:HGNC Symbol;Acc:HGNC:6631]" |
| ENSG00000075073 | HGLibA_48175 | 121 | -0.040206998 | TACR2 | tachykinin receptor 2 [Source:HGNC Symbol;Acc:HGNC:11527] |
| ENSG00000075884 | HGLibA_02711 | 121 | -0.040206998 | ARHGAP15 | Rho GTPase activating protein 15 [Source:HGNC Symbol;Acc:HGNC:21030] |
| ENSG00000077522 | HGLibA_00642 | 121 | -0.040206998 | ACTN2 | actinin alpha 2 [Source:HGNC Symbol;Acc:HGNC:164] |
| ENSG00000078668 | HGLibA_53605 | 121 | -0.040206998 | VDAC3 | voltage dependent anion channel 3 [Source:HGNC Symbol;Acc:HGNC:12674] |
| ENSG00000078808 | HGLibA_43129 | 121 | -0.040206998 | SDF4 | stromal cell derived factor 4 [Source:HGNC Symbol;Acc:HGNC:24188] |
| ENSG00000082458 | HGLibA_13329 | 121 | -0.040206998 | DLG3 | discs large MAGUK scaffold protein 3 [Source:HGNC Symbol;Acc:HGNC:2902] |
| ENSG00000085063 | HGLibA_08418 | 121 | -0.040206998 | CD59 | CD59 molecule (CD59 blood group) [Source:HGNC Symbol;Acc:HGNC:1689] |
| ENSG00000085831 | HGLibA_52133 | 121 | -0.040206998 | TTC39A | tetratricopeptide repeat domain 39A [Source:HGNC Symbol;Acc:HGNC:18657] |
| ENSG00000086991 | HGLibA_32375 | 121 | -0.040206998 | NOX4 | NADPH oxidase 4 [Source:HGNC Symbol;Acc:HGNC:7891] |
| ENSG00000090612 | HGLibA_55704 | 121 | -0.040206998 | ZNF268 | zinc finger protein 268 [Source:HGNC Symbol;Acc:HGNC:13061] |
| ENSG00000092421 | HGLibA_43363 | 121 | -0.040206998 | SEMA6A | semaphorin 6A [Source:HGNC Symbol;Acc:HGNC:10738] |
| ENSG00000100036 | HGLibA_44998 | 121 | -0.040206998 | SLC35E4 | solute carrier family 35 member E4 [Source:HGNC Symbol;Acc:HGNC:17058] |
| ENSG00000100116 | HGLibA_18762 | 121 | -0.040206998 | GCAT | glycine C-acetyltransferase [Source:HGNC Symbol;Acc:HGNC:4188] |
| ENSG00000100156 | HGLibA_44454 | 121 | -0.040206998 | SLC16A8 | solute carrier family 16 member 8 [Source:HGNC Symbol;Acc:HGNC:16270] |
| ENSG00000100429 | HGLibA_21000 | 121 | -0.040206998 | HDAC10 | histone deacetylase 10 [Source:HGNC Symbol;Acc:HGNC:18128] |
| ENSG00000101230 | HGLibA_23676 | 121 | -0.040206998 | ISM1 | isthmin 1 [Source:HGNC Symbol;Acc:HGNC:16213] |
| ENSG00000103257 | HGLibA_45370 | 121 | -0.040206998 | SLC7A5 | solute carrier family 7 member 5 [Source:HGNC Symbol;Acc:HGNC:11063] |
| ENSG00000105568 | HGLibA_38141 | 121 | -0.040206998 | PPP2R1A | protein phosphatase 2 scaffold subunit Aalpha [Source:HGNC Symbol;Acc:HGNC:9302] |
| ENSG00000107485 | HGLibA_18670 | 121 | -0.040206998 | GATA3 | GATA binding protein 3 [Source:HGNC Symbol;Acc:HGNC:4172] |
| ENSG00000107651 | HGLibA_43226 | 121 | -0.040206998 | SEC23IP | SEC23 interacting protein [Source:HGNC Symbol;Acc:HGNC:17018] |
| ENSG00000108684 | HGLibA_03301 | 121 | -0.040206998 | ASIC2 | acid sensing ion channel subunit 2 [Source:HGNC Symbol;Acc:HGNC:99] |
| ENSG00000108830 | HGLibA_41449 | 121 | -0.040206998 | RND2 | Rho family GTPase 2 [Source:HGNC Symbol;Acc:HGNC:18315] |
| ENSG00000111879 | HGLibA_16327 | 121 | -0.040206998 | FAM184A | family with sequence similarity 184 member A [Source:HGNC Symbol;Acc:HGNC:20991] |
| ENSG00000112208 | HGLibA_04077 | 121 | -0.040206998 | BAG2 | BCL2 associated athanogene 2 [Source:HGNC Symbol;Acc:HGNC:938] |
| ENSG00000113361 | HGLibA_08686 | 121 | -0.040206998 | CDH6 | cadherin 6 [Source:HGNC Symbol;Acc:HGNC:1765] |
| ENSG00000115524 | HGLibA_43701 | 121 | -0.040206998 | SF3B1 | splicing factor 3b subunit 1 [Source:HGNC Symbol;Acc:HGNC:10768] |
| ENSG00000115598 | HGLibA_23065 | 121 | -0.040206998 | IL1RL2 | interleukin 1 receptor like 2 [Source:HGNC Symbol;Acc:HGNC:5999] |
| ENSG00000115616 | HGLibA_45403 | 121 | -0.040206998 | SLC9A2 | solute carrier family 9 member A2 [Source:HGNC Symbol;Acc:HGNC:11072] |
| ENSG00000116981 | HGLibA_32801 | 121 | -0.040206998 | NT5C1A | cytosolic IA [Source:HGNC Symbol;Acc:HGNC:17819]" |
| ENSG00000117362 | HGLibA_02413 | 121 | -0.040206998 | APH1A | gamma-secretase subunit [Source:HGNC Symbol;Acc:HGNC:29509]" |
| ENSG00000119820 | HGLibA_54678 | 121 | -0.040206998 | YIPF4 | Yip1 domain family member 4 [Source:HGNC Symbol;Acc:HGNC:28145] |
| ENSG00000120333 | HGLibA_30038 | 121 | -0.040206998 | MRPS14 | mitochondrial ribosomal protein S14 [Source:HGNC Symbol;Acc:HGNC:14049] |
| ENSG00000121742 | HGLibA_19173 | 121 | -0.040206998 | GJB6 | gap junction protein beta 6 [Source:HGNC Symbol;Acc:HGNC:4288] |
| ENSG00000121766 | HGLibA_55021 | 121 | -0.040206998 | ZCCHC17 | zinc finger CCHC-type containing 17 [Source:HGNC Symbol;Acc:HGNC:30246] |
| ENSG00000124019 | HGLibA_16077 | 121 | -0.040206998 | FAM124B | family with sequence similarity 124 member B [Source:HGNC Symbol;Acc:HGNC:26224] |
| ENSG00000124260 | HGLibA_27892 | 121 | -0.040206998 | MAGEA10 | MAGE family member A10 [Source:HGNC Symbol;Acc:HGNC:6797] |
| ENSG00000125952 | HGLibA_28448 | 121 | -0.040206998 | MAX | MYC associated factor X [Source:HGNC Symbol;Acc:HGNC:6913] |
| ENSG00000126749 | HGLibA_15042 | 121 | -0.040206998 | EMG1 | N1-specific pseudouridine methyltransferase [Source:HGNC Symbol;Acc:HGNC:16912]" |
| ENSG00000127124 | HGLibA_21574 | 121 | -0.040206998 | HIVEP3 | human immunodeficiency virus type I enhancer binding protein 3 [Source:HGNC Symbol;Acc:HGNC:13561] |
| ENSG00000127366 | HGLibA_48417 | 121 | -0.040206998 | TAS2R5 | taste 2 receptor member 5 [Source:HGNC Symbol;Acc:HGNC:14912] |
| ENSG00000129646 | HGLibA_39746 | 121 | -0.040206998 | QRICH2 | glutamine rich 2 [Source:HGNC Symbol;Acc:HGNC:25326] |
| ENSG00000129932 | HGLibA_13765 | 121 | -0.040206998 | DOHH | deoxyhypusine hydroxylase [Source:HGNC Symbol;Acc:HGNC:28662] |
| ENSG00000131737 | HGLibA_25486 | 121 | -0.040206998 | KRT34 | keratin 34 [Source:HGNC Symbol;Acc:HGNC:6452] |
| ENSG00000132517 | HGLibA_45245 | 121 | -0.040206998 | SLC52A1 | solute carrier family 52 member 1 [Source:HGNC Symbol;Acc:HGNC:30225] |
| ENSG00000134873 | HGLibA_09850 | 121 | -0.040206998 | CLDN10 | claudin 10 [Source:HGNC Symbol;Acc:HGNC:2033] |
| ENSG00000134917 | HGLibA_00843 | 121 | -0.040206998 | ADAMTS8 | ADAM metallopeptidase with thrombospondin type 1 motif 8 [Source:HGNC Symbol;Acc:HGNC:224] |
| ENSG00000135720 | HGLibA_14244 | 121 | -0.040206998 | DYNC1LI2 | dynein cytoplasmic 1 light intermediate chain 2 [Source:HGNC Symbol;Acc:HGNC:2966] |
| ENSG00000135956 | HGLibA_49916 | 121 | -0.040206998 | TMEM127 | transmembrane protein 127 [Source:HGNC Symbol;Acc:HGNC:26038] |
| ENSG00000136856 | HGLibA_44891 | 121 | -0.040206998 | SLC2A8 | solute carrier family 2 member 8 [Source:HGNC Symbol;Acc:HGNC:13812] |
| ENSG00000136870 | HGLibA_55553 | 121 | -0.040206998 | ZNF189 | zinc finger protein 189 [Source:HGNC Symbol;Acc:HGNC:12980] |
| ENSG00000138604 | HGLibA_19233 | 121 | -0.040206998 | GLCE | glucuronic acid epimerase [Source:HGNC Symbol;Acc:HGNC:17855] |
| ENSG00000139173 | HGLibA_49890 | 121 | -0.040206998 | TMEM117 | transmembrane protein 117 [Source:HGNC Symbol;Acc:HGNC:25308] |
| ENSG00000139725 | HGLibA_41187 | 121 | -0.040206998 | RHOF | filopodia associated [Source:HGNC Symbol;Acc:HGNC:15703]" |

| | | | | | |
|---|---|---|---|---|---|
| ENSG00000140057 | HGLibA_01394 | 121 | -0.040206998 | AK7 | adenylate kinase 7 [Source:HGNC Symbol;Acc:HGNC:20091] |
| ENSG00000140090 | HGLibA_44624 | 121 | -0.040206998 | SLC24A4 | solute carrier family 24 member 4 [Source:HGNC Symbol;Acc:HGNC:10978] |
| ENSG00000141505 | HGLibA_03287 | 121 | -0.040206998 | ASGR1 | asialoglycoprotein receptor 1 [Source:HGNC Symbol;Acc:HGNC:742] |
| ENSG00000142611 | HGLibA_38382 | 121 | -0.040206998 | PRDM16 | PR/SET domain 16 [Source:HGNC Symbol;Acc:HGNC:14000] |
| ENSG00000142669 | HGLibA_43922 | 121 | -0.040206998 | SH3BGRL3 | SH3 domain binding glutamate rich protein like 3 [Source:HGNC Symbol;Acc:HGNC:15568] |
| ENSG00000143374 | HGLibA_48347 | 121 | -0.040206998 | TARS2 | mitochondrial [Source:HGNC Symbol;Acc:HGNC:30740]" |
| ENSG00000143669 | HGLibA_27776 | 121 | -0.040206998 | LYST | lysosomal trafficking regulator [Source:HGNC Symbol;Acc:HGNC:1968] |
| ENSG00000146276 | HGLibA_18404 | 121 | -0.040206998 | GABRR1 | gamma-aminobutyric acid type A receptor rho1 subunit [Source:HGNC Symbol;Acc:HGNC:4090] |
| ENSG00000146425 | HGLibA_14266 | 121 | -0.040206998 | DYNLT1 | dynein light chain Tctex-type 1 [Source:HGNC Symbol;Acc:HGNC:11697] |
| ENSG00000146587 | HGLibA_40420 | 121 | -0.040206998 | RBAK | RB associated KRAB zinc finger [Source:HGNC Symbol;Acc:HGNC:17680] |
| ENSG00000147127 | HGLibA_39944 | 121 | -0.040206998 | RAB41 | member RAS oncogene family [Source:HGNC Symbol;Acc:HGNC:18293]" |
| ENSG00000151320 | HGLibA_01440 | 121 | -0.040206998 | AKAP6 | A-kinase anchoring protein 6 [Source:HGNC Symbol;Acc:HGNC:376] |
| ENSG00000151466 | HGLibA_42949 | 121 | -0.040206998 | SCLT1 | sodium channel and clathrin linker 1 [Source:HGNC Symbol;Acc:HGNC:26406] |
| ENSG00000152484 | HGLibA_53201 | 121 | -0.040206998 | USP12 | ubiquitin specific peptidase 12 [Source:HGNC Symbol;Acc:HGNC:20485] |
| ENSG00000152689 | HGLibA_40350 | 121 | -0.040206998 | RASGRP3 | RAS guanyl releasing protein 3 [Source:HGNC Symbol;Acc:HGNC:14545] |
| ENSG00000153774 | HGLibA_09256 | 121 | -0.040206998 | CFDP1 | craniofacial development protein 1 [Source:HGNC Symbol;Acc:HGNC:1873] |
| ENSG00000156170 | HGLibA_31496 | 121 | -0.040206998 | NDUFAF6 | NADH:ubiquinone oxidoreductase complex assembly factor 6 [Source:HGNC Symbol;Acc:HGNC:28625] |
| ENSG00000158292 | HGLibA_19939 | 121 | -0.040206998 | GPR153 | G protein-coupled receptor 153 [Source:HGNC Symbol;Acc:HGNC:23618] |
| ENSG00000159784 | HGLibA_16108 | 121 | -0.040206998 | FAM131B | family with sequence similarity 131 member B [Source:HGNC Symbol;Acc:HGNC:22202] |
| ENSG00000160551 | HGLibA_48306 | 121 | -0.040206998 | TAOK1 | TAO kinase 1 [Source:HGNC Symbol;Acc:HGNC:29259] |
| ENSG00000162139 | HGLibA_31717 | 121 | -0.040206998 | NEU3 | neuraminidase 3 [Source:HGNC Symbol;Acc:HGNC:7760] |
| ENSG00000162490 | HGLibA_13954 | 121 | -0.040206998 | DRAXIN | dorsal inhibitory axon guidance protein [Source:HGNC Symbol;Acc:HGNC:25054] |
| ENSG00000162757 | HGLibA_05895 | 121 | -0.040206998 | C1orf74 | chromosome 1 open reading frame 74 [Source:HGNC Symbol;Acc:HGNC:26319] |
| ENSG00000162849 | HGLibA_24919 | 121 | -0.040206998 | KIF26B | kinesin family member 26B [Source:HGNC Symbol;Acc:HGNC:25484] |
| ENSG00000162931 | HGLibA_51424 | 121 | -0.040206998 | TRIM17 | tripartite motif containing 17 [Source:HGNC Symbol;Acc:HGNC:13430] |
| ENSG00000163331 | HGLibA_12330 | 121 | -0.040206998 | DAPL1 | death associated protein like 1 [Source:HGNC Symbol;Acc:HGNC:21490] |
| ENSG00000163577 | HGLibA_14859 | 121 | -0.040206998 | EIF5A2 | eukaryotic translation initiation factor 5A2 [Source:HGNC Symbol;Acc:HGNC:3301] |
| ENSG00000163737 | HGLibA_36129 | 121 | -0.040206998 | PF4 | platelet factor 4 [Source:HGNC Symbol;Acc:HGNC:8861] |
| ENSG00000163818 | HGLibA_27800 | 121 | -0.040206998 | LZTFL1 | leucine zipper transcription factor like 1 [Source:HGNC Symbol;Acc:HGNC:6741] |
| ENSG00000163823 | HGLibA_08144 | 121 | -0.040206998 | CCR1 | C-C motif chemokine receptor 1 [Source:HGNC Symbol;Acc:HGNC:1602] |
| ENSG00000164244 | HGLibA_38856 | 121 | -0.040206998 | PRRC1 | proline rich coiled-coil 1 [Source:HGNC Symbol;Acc:HGNC:28164] |
| ENSG00000165066 | HGLibA_32060 | 121 | -0.040206998 | NKX6-3 | NK6 homeobox 3 [Source:HGNC Symbol;Acc:HGNC:26328] |
| ENSG00000165684 | HGLibA_45898 | 121 | -0.040206998 | SNAPC4 | small nuclear RNA activating complex polypeptide 4 [Source:HGNC Symbol;Acc:HGNC:11137] |
| ENSG00000165810 | HGLibA_04984 | 121 | -0.040206998 | BTNL9 | butyrophilin like 9 [Source:HGNC Symbol;Acc:HGNC:24176] |
| ENSG00000166220 | HGLibA_48457 | 121 | -0.040206998 | TBATA | brain and testes associated [Source:HGNC Symbol;Acc:HGNC:23511]" |
| ENSG00000167528 | HGLibA_56444 | 121 | -0.040206998 | ZNF641 | zinc finger protein 641 [Source:HGNC Symbol;Acc:HGNC:31834] |
| ENSG00000167654 | HGLibA_03426 | 121 | -0.040206998 | ATCAY | caytaxin [Source:HGNC Symbol;Acc:HGNC:779]" |
| ENSG00000167749 | HGLibA_25257 | 121 | -0.040206998 | KLK4 | kallikrein related peptidase 4 [Source:HGNC Symbol;Acc:HGNC:6365] |
| ENSG00000167910 | HGLibA_12210 | 121 | -0.040206998 | CYP7A1 | cytochrome P450 family 7 subfamily A member 1 [Source:HGNC Symbol;Acc:HGNC:2651] |
| ENSG00000168026 | HGLibA_52070 | 121 | -0.040206998 | TTC21A | tetratricopeptide repeat domain 21A [Source:HGNC Symbol;Acc:HGNC:30761] |
| ENSG00000169439 | HGLibA_43100 | 121 | -0.040206998 | SDC2 | syndecan 2 [Source:HGNC Symbol;Acc:HGNC:10659] |
| ENSG00000169710 | HGLibA_16896 | 121 | -0.040206998 | FASN | fatty acid synthase [Source:HGNC Symbol;Acc:HGNC:3594] |
| ENSG00000170142 | HGLibA_52593 | 121 | -0.040206998 | UBE2E1 | ubiquitin conjugating enzyme E2 E1 [Source:HGNC Symbol;Acc:HGNC:12477] |
| ENSG00000171014 | HGLibA_33977 | 121 | -0.040206998 | OR4D5 | olfactory receptor family 4 subfamily D member 5 [Source:HGNC Symbol;Acc:HGNC:14852] |
| ENSG00000171206 | HGLibA_51615 | 121 | -0.040206998 | TRIM8 | tripartite motif containing 8 [Source:HGNC Symbol;Acc:HGNC:15579] |
| ENSG00000171488 | HGLibA_27434 | 121 | -0.040206998 | LRRC8C | leucine rich repeat containing 8 VRAC subunit C [Source:HGNC Symbol;Acc:HGNC:25075] |
| ENSG00000171503 | HGLibA_15627 | 121 | -0.040206998 | ETFDH | electron transfer flavoprotein dehydrogenase [Source:HGNC Symbol;Acc:HGNC:3483] |
| ENSG00000171853 | HGLibA_51305 | 121 | -0.040206998 | TRAPPC12 | trafficking protein particle complex 12 [Source:HGNC Symbol;Acc:HGNC:24284] |
| ENSG00000172594 | HGLibA_45814 | 121 | -0.040206998 | SMPDL3A | sphingomyelin phosphodiesterase acid like 3A [Source:HGNC Symbol;Acc:HGNC:17389] |
| ENSG00000173212 | HGLibA_27827 | 121 | -0.040206998 | MAB21L3 | mab-21 like 3 [Source:HGNC Symbol;Acc:HGNC:26787] |
| ENSG00000173269 | HGLibA_29550 | 121 | -0.040206998 | MMRN2 | multimerin 2 [Source:HGNC Symbol;Acc:HGNC:19888] |
| ENSG00000173275 | HGLibA_56023 | 121 | -0.040206998 | ZNF449 | zinc finger protein 449 [Source:HGNC Symbol;Acc:HGNC:21039] |
| ENSG00000174038 | HGLibA_06677 | 121 | -0.040206998 | C9orf131 | chromosome 9 open reading frame 131 [Source:HGNC Symbol;Acc:HGNC:31418] |
| ENSG00000175294 | HGLibA_07356 | 121 | -0.040206998 | CATSPER1 | cation channel sperm associated 1 [Source:HGNC Symbol;Acc:HGNC:17116] |
| ENSG00000176393 | HGLibA_41727 | 121 | -0.040206998 | RNPEP | arginyl aminopeptidase [Source:HGNC Symbol;Acc:HGNC:10078] |
| ENSG00000176422 | HGLibA_46853 | 121 | -0.040206998 | SPRYD4 | SPRY domain containing 4 [Source:HGNC Symbol;Acc:HGNC:27468] |
| ENSG00000176853 | HGLibA_16795 | 121 | -0.040206998 | FAM91A1 | family with sequence similarity 91 member A1 [Source:HGNC Symbol;Acc:HGNC:26306] |
| ENSG00000177272 | HGLibA_24131 | 121 | -0.040206998 | KCNA3 | potassium voltage-gated channel subfamily A member 3 [Source:HGNC Symbol;Acc:HGNC:6221] |
| ENSG00000178401 | HGLibA_13599 | 121 | -0.040206998 | DNAJC22 | DnaJ heat shock protein family (Hsp40) member C22 [Source:HGNC Symbol;Acc:HGNC:25802] |
| ENSG00000182272 | HGLibA_04020 | 121 | -0.040206998 | B4GALNT4 | 4-N-acetyl-galactosaminyltransferase 4 [Source:HGNC Symbol;Acc:HGNC:26315]" |
| ENSG00000183155 | HGLibA_40024 | 121 | -0.040206998 | RABIF | RAB interacting factor [Source:HGNC Symbol;Acc:HGNC:9797] |
| ENSG00000183287 | HGLibA_07494 | 121 | -0.040206998 | CCBE1 | collagen and calcium binding EGF domains 1 [Source:HGNC Symbol;Acc:HGNC:29426] |
| ENSG00000183726 | HGLibA_50422 | 121 | -0.040206998 | TMEM50A | transmembrane protein 50A [Source:HGNC Symbol;Acc:HGNC:30590] |
| ENSG00000183741 | HGLibA_07460 | 121 | -0.040206998 | CBX6 | chromobox 6 [Source:HGNC Symbol;Acc:HGNC:1556] |
| ENSG00000185261 | HGLibA_24656 | 121 | -0.040206998 | KIAA0825 | KIAA0825 [Source:HGNC Symbol;Acc:HGNC:28532] |
| ENSG00000185829 | HGLibA_02952 | 121 | -0.040206998 | ARL17A | ADP ribosylation factor like GTPase 17A [Source:HGNC Symbol;Acc:HGNC:24096] |
| ENSG00000186298 | HGLibA_38010 | 121 | -0.040206998 | PPP1CC | protein phosphatase 1 catalytic subunit gamma [Source:HGNC Symbol;Acc:HGNC:9283] |
| ENSG00000186350 | HGLibA_42507 | 121 | -0.040206998 | RXRA | retinoid X receptor alpha [Source:HGNC Symbol;Acc:HGNC:10477] |
| ENSG00000187553 | HGLibA_12091 | 121 | -0.040206998 | CYP26C1 | cytochrome P450 family 26 subfamily C member 1 [Source:HGNC Symbol;Acc:HGNC:20577] |
| ENSG00000189007 | HGLibA_00880 | 121 | -0.040206998 | ADAT2 | tRNA specific 2 [Source:HGNC Symbol;Acc:HGNC:21172]" |
| ENSG00000189376 | HGLibA_06648 | 121 | -0.040206998 | C8orf76 | chromosome 8 open reading frame 76 [Source:HGNC Symbol;Acc:HGNC:25924] |
| ENSG00000196156 | HGLibA_25762 | 121 | -0.040206998 | KRTAP4-3 | keratin associated protein 4-3 [Source:HGNC Symbol;Acc:HGNC:18908] |
| ENSG00000197506 | HGLibA_44837 | 121 | -0.040206998 | SLC28A3 | solute carrier family 28 member 3 [Source:HGNC Symbol;Acc:HGNC:16484] |
| ENSG00000197601 | HGLibA_16869 | 121 | -0.040206998 | FAR1 | fatty acyl-CoA reductase 1 [Source:HGNC Symbol;Acc:HGNC:26222] |
| ENSG00000197614 | HGLibA_29055 | 121 | -0.040206998 | MFAP5 | microfibril associated protein 5 [Source:HGNC Symbol;Acc:HGNC:29673] |
| ENSG00000198049 | HGLibA_03906 | 121 | -0.040206998 | AVPR1B | arginine vasopressin receptor 1B [Source:HGNC Symbol;Acc:HGNC:896] |
| ENSG00000198088 | HGLibA_33066 | 121 | -0.040206998 | NUP62CL | nucleoporin 62 C-terminal like [Source:HGNC Symbol;Acc:HGNC:25960] |
| ENSG00000198626 | HGLibA_42524 | 121 | -0.040206998 | RYR2 | ryanodine receptor 2 [Source:HGNC Symbol;Acc:HGNC:10484] |
| ENSG00000198729 | HGLibA_38041 | 121 | -0.040206998 | PPP1R14C | protein phosphatase 1 regulatory inhibitor subunit 14C [Source:HGNC Symbol;Acc:HGNC:14952] |
| ENSG00000203877 | HGLibA_41332 | 121 | -0.040206998 | RIPPLY2 | ripply transcriptional repressor 2 [Source:HGNC Symbol;Acc:HGNC:21390] |
| ENSG00000204531 | HGLibA_37785 | 121 | -0.040206998 | POU5F1 | POU class 5 homeobox 1 [Source:HGNC Symbol;Acc:HGNC:9221] |
| ENSG00000204673 | HGLibA_01509 | 121 | -0.040206998 | AKT1S1 | AKT1 substrate 1 [Source:HGNC Symbol;Acc:HGNC:28426] |
| ENSG00000206106 | HGLibA_25719 | 121 | -0.040206998 | KRTAP22-2 | keratin associated protein 22-2 [Source:HGNC Symbol;Acc:HGNC:37091] |
| ENSG00000206536 | HGLibA_34315 | 121 | -0.040206998 | OR5K3 | olfactory receptor family 5 subfamily K member 3 [Source:HGNC Symbol;Acc:HGNC:31290] |
| ENSG00000213341 | HGLibA_09671 | 121 | -0.040206998 | CHUK | conserved helix-loop-helix ubiquitous kinase [Source:HGNC Symbol;Acc:HGNC:1974] |
| ENSG00000231852 | HGLibA_12080 | 121 | -0.040206998 | CYP21A2 | cytochrome P450 family 21 subfamily A member 2 [Source:HGNC Symbol;Acc:HGNC:2600] |
| ENSG00000236637 | HGLibA_22681 | 121 | -0.040206998 | IFNA4 | interferon alpha 4 [Source:HGNC Symbol;Acc:HGNC:5425] |
| ENSG00000243989 | HGLibA_00704 | 121 | -0.040206998 | ACY1 | aminoacylase 1 [Source:HGNC Symbol;Acc:HGNC:177] |
| ENSG00000244462 | HGLibA_40479 | 121 | -0.040206998 | RBM12 | RNA binding motif protein 12 [Source:HGNC Symbol;Acc:HGNC:9898] |
| ENSG00000249693 | HGLibA_49336 | 121 | -0.040206998 | THEGL | theg spermatid protein like [Source:HGNC Symbol;Acc:HGNC:43771] |
| ENSG00000251623 | HGLibA_16171 | 121 | -0.040206998 | FAM153B | - |
| ENSG00000259120 | HGLibA_45765 | 121 | -0.040206998 | SMIM6 | small integral membrane protein 6 [Source:HGNC Symbol;Acc:HGNC:40032] |
| ENSG00000259224 | HGLibA_45029 | 121 | -0.040206998 | SLC35G6 | solute carrier family 35 member G6 [Source:HGNC Symbol;Acc:HGNC:31351] |
| ENSG00000269526 | HGLibA_15546 | 121 | -0.040206998 | ERVV-1 | envelope [Source:HGNC Symbol;Acc:HGNC:26501]" |
| ENSG00000278505 | HGLibA_05578 | 121 | -0.040206998 | C17orf78 | chromosome 17 open reading frame 78 [Source:HGNC Symbol;Acc:HGNC:26831] |
| hsa-mir-103b-2 | HGLibA_57173 | 121 | -0.040206998 | hsa-mir-103b-2 | - |
| hsa-mir-1324 | HGLibA_57770 | 121 | -0.040206998 | hsa-mir-1324 | - |
| hsa-mir-152 | HGLibA_57904 | 121 | -0.040206998 | hsa-mir-152 | - |
| hsa-mir-181b-2 | HGLibA_57970 | 121 | -0.040206998 | hsa-mir-181b-2 | - |
| hsa-mir-187 | HGLibA_58012 | 121 | -0.040206998 | hsa-mir-187 | - |
| hsa-mir-204 | HGLibA_58173 | 121 | -0.040206998 | hsa-mir-204 | - |
| hsa-mir-29a | HGLibA_58411 | 121 | -0.040206998 | hsa-mir-29a | - |
| hsa-mir-3197 | HGLibA_58884 | 121 | -0.040206998 | hsa-mir-3197 | - |
| hsa-mir-362 | HGLibA_59110 | 121 | -0.040206998 | hsa-mir-362 | - |
| hsa-mir-4429 | HGLibA_60102 | 121 | -0.040206998 | hsa-mir-4429 | - |
| hsa-mir-4689 | HGLibA_60866 | 121 | -0.040206998 | hsa-mir-4689 | - |
| hsa-mir-4762 | HGLibA_61143 | 121 | -0.040206998 | hsa-mir-4762 | - |
| hsa-mir-5000 | HGLibA_61406 | 121 | -0.040206998 | hsa-mir-5000 | - |
| hsa-mir-6082 | HGLibA_62589 | 121 | -0.040206998 | hsa-mir-6082 | - |
| hsa-mir-647 | HGLibA_62830 | 121 | -0.040206998 | hsa-mir-647 | - |
| hsa-mir-6717 | HGLibA_63029 | 121 | -0.040206998 | hsa-mir-6717 | - |
| hsa-mir-6769b | HGLibA_63242 | 121 | -0.040206998 | hsa-mir-6769b | - |
| hsa-mir-6853 | HGLibA_63586 | 121 | -0.040206998 | hsa-mir-6853 | - |
| hsa-mir-921 | HGLibA_64279 | 121 | -0.040206998 | hsa-mir-921 | - |
| KIAA1211L | HGLibA_24719 | 121 | -0.040206998 | KIAA1211L | - |
| KIAA1407 | HGLibA_24748 | 121 | -0.040206998 | KIAA1407 | - |
| LOC100287399 | HGLibA_26821 | 121 | -0.040206998 | LOC100287399 | - |
| LOC100507003 | HGLibA_26866 | 121 | -0.040206998 | LOC100507003 | - |
| SUV420H1 | HGLibA_47866 | 121 | -0.040206998 | SUV420H1 | - |
| SUV420H2 | HGLibA_47869 | 121 | -0.040206998 | SUV420H2 | - |
| WDR34 | HGLibA_54070 | 121 | -0.040206998 | WDR34 | - |
| YAE1D1 | HGLibA_54626 | 121 | -0.040206998 | YAE1D1 | - |
| ZHX1-C8ORF76 | HGLibA_55313 | 121 | -0.040206998 | ZHX1-C8ORF76 | - |
| 4-Mar | HGLibA_28337 | 120 | -0.049252341 | 4-Mar | - |
| ASMTL_Y | HGLibA_03329 | 120 | -0.049252341 | ASMTL_Y | - |
| C17orf47 | HGLibA_05535 | 120 | -0.049252341 | C17orf47 | - |
| C2orf57 | HGLibA_06151 | 120 | -0.049252341 | C2orf57 | - |
| C6orf106 | HGLibA_06433 | 120 | -0.049252341 | C6orf106 | - |
| ENSG00000005156 | HGLibA_26482 | 120 | -0.049252341 | LIG3 | DNA ligase 3 [Source:HGNC Symbol;Acc:HGNC:6600] |
| ENSG00000040633 | HGLibA_36374 | 120 | -0.049252341 | PHF23 | PHD finger protein 23 [Source:HGNC Symbol;Acc:HGNC:28428] |
| ENSG00000048828 | HGLibA_16055 | 120 | -0.049252341 | FAM120A | family with sequence similarity 120A [Source:HGNC Symbol;Acc:HGNC:13247] |
| ENSG00000060749 | HGLibA_39751 | 120 | -0.049252341 | QSER1 | glutamine and serine rich 1 [Source:HGNC Symbol;Acc:HGNC:26154] |
| ENSG00000069482 | HGLibA_18494 | 120 | -0.049252341 | GAL | galanin and GMAP prepropeptide [Source:HGNC Symbol;Acc:HGNC:4114] |
| ENSG00000078053 | HGLibA_01832 | 120 | -0.049252341 | AMPH | amphiphysin [Source:HGNC Symbol;Acc:HGNC:471] |
| ENSG00000078967 | HGLibA_52591 | 120 | -0.049252341 | UBE2D4 | ubiquitin conjugating enzyme E2 D4 (putative) [Source:HGNC Symbol;Acc:HGNC:21647] |

| | | | | | |
|---|---|---|---|---|---|
| ENSG00000082641 | HGLibA_31791 | 120 | -0.049252341 | NFE2L1 | erythroid 2 like 1 [Source:HGNC Symbol;Acc:HGNC:7781]" |
| ENSG00000087111 | HGLibA_36584 | 120 | -0.049252341 | PIGS | phosphatidylinositol glycan anchor biosynthesis class S [Source:HGNC Symbol;Acc:HGNC:14937] |
| ENSG00000090539 | HGLibA_09535 | 120 | -0.049252341 | CHRD | chordin [Source:HGNC Symbol;Acc:HGNC:1949] |
| ENSG00000092096 | HGLibA_44558 | 120 | -0.049252341 | SLC22A17 | solute carrier family 22 member 17 [Source:HGNC Symbol;Acc:HGNC:23095] |
| ENSG00000099721 | HGLibA_01768 | 120 | -0.049252341 | AMELY | amelogenin Y-linked [Source:HGNC Symbol;Acc:HGNC:462] |
| ENSG00000099901 | HGLibA_40192 | 120 | -0.049252341 | RANBP1 | RAN binding protein 1 [Source:HGNC Symbol;Acc:HGNC:9847] |
| ENSG00000100060 | HGLibA_29074 | 120 | -0.049252341 | MFNG | MFNG O-fucosylpeptide 3-beta-N-acetylglucosaminyltransferase [Source:HGNC Symbol;Acc:HGNC:7038] |
| ENSG00000100319 | HGLibA_55378 | 120 | -0.049252341 | ZMAT5 | zinc finger matrin-type 5 [Source:HGNC Symbol;Acc:HGNC:28046] |
| ENSG00000100320 | HGLibA_40459 | 120 | -0.049252341 | RBFOX2 | RNA binding fox-1 homolog 2 [Source:HGNC Symbol;Acc:HGNC:9906] |
| ENSG00000101294 | HGLibA_21663 | 120 | -0.049252341 | HM13 | histocompatibility minor 13 [Source:HGNC Symbol;Acc:HGNC:16435] |
| ENSG00000104611 | HGLibA_43897 | 120 | -0.049252341 | SH2D4A | SH2 domain containing 4A [Source:HGNC Symbol;Acc:HGNC:26102] |
| ENSG00000105664 | HGLibA_10684 | 120 | -0.049252341 | COMP | cartilage oligomeric matrix protein [Source:HGNC Symbol;Acc:HGNC:2227] |
| ENSG00000106049 | HGLibA_21281 | 120 | -0.049252341 | HIBADH | 3-hydroxyisobutyrate dehydrogenase [Source:HGNC Symbol;Acc:HGNC:4907] |
| ENSG00000106341 | HGLibA_38058 | 120 | -0.049252341 | PPP1R17 | protein phosphatase 1 regulatory subunit 17 [Source:HGNC Symbol;Acc:HGNC:16973] |
| ENSG00000106948 | HGLibA_01462 | 120 | -0.049252341 | AKNA | AT-hook transcription factor [Source:HGNC Symbol;Acc:HGNC:24108] |
| ENSG00000107796 | HGLibA_00600 | 120 | -0.049252341 | ACTA2 | aorta [Source:HGNC Symbol;Acc:HGNC:130]" |
| ENSG00000109654 | HGLibA_51427 | 120 | -0.049252341 | TRIM2 | tripartite motif containing 2 [Source:HGNC Symbol;Acc:HGNC:15974] |
| ENSG00000110107 | HGLibA_38714 | 120 | -0.049252341 | PRPF19 | pre-mRNA processing factor 19 [Source:HGNC Symbol;Acc:HGNC:17896] |
| ENSG00000110446 | HGLibA_44410 | 120 | -0.049252341 | SLC15A3 | solute carrier family 15 member 3 [Source:HGNC Symbol;Acc:HGNC:18068] |
| ENSG00000111254 | HGLibA_01431 | 120 | -0.049252341 | AKAP3 | A-kinase anchoring protein 3 [Source:HGNC Symbol;Acc:HGNC:373] |
| ENSG00000111711 | HGLibA_19666 | 120 | -0.049252341 | GOLT1B | golgi transport 1B [Source:HGNC Symbol;Acc:HGNC:20175] |
| ENSG00000114374 | HGLibA_53418 | 120 | -0.049252341 | USP9Y | ubiquitin specific peptidase 9 Y-linked [Source:HGNC Symbol;Acc:HGNC:12633] |
| ENSG00000115461 | HGLibA_22826 | 120 | -0.049252341 | IGFBP5 | insulin like growth factor binding protein 5 [Source:HGNC Symbol;Acc:HGNC:5474] |
| ENSG00000116954 | HGLibA_42230 | 120 | -0.049252341 | RRAGC | Ras related GTP binding C [Source:HGNC Symbol;Acc:HGNC:19902] |
| ENSG00000118162 | HGLibA_25381 | 120 | -0.049252341 | KPTN | actin binding protein [Source:HGNC Symbol;Acc:HGNC:6404]" |
| ENSG00000119682 | HGLibA_02643 | 120 | -0.049252341 | AREL1 | apoptosis resistant E3 ubiquitin protein ligase 1 [Source:HGNC Symbol;Acc:HGNC:20363] |
| ENSG00000120451 | HGLibA_46053 | 120 | -0.049252341 | SNX19 | sorting nexin 19 [Source:HGNC Symbol;Acc:HGNC:21532] |
| ENSG00000121552 | HGLibA_11477 | 120 | -0.049252341 | CSTA | cystatin A [Source:HGNC Symbol;Acc:HGNC:2481] |
| ENSG00000121807 | HGLibA_08150 | 120 | -0.049252341 | CCR2 | C-C motif chemokine receptor 2 [Source:HGNC Symbol;Acc:HGNC:1603] |
| ENSG00000121903 | HGLibA_56997 | 120 | -0.049252341 | ZSCAN20 | zinc finger and SCAN domain containing 20 [Source:HGNC Symbol;Acc:HGNC:13093] |
| ENSG00000122121 | HGLibA_54556 | 120 | -0.049252341 | XPNPEP2 | X-prolyl aminopeptidase 2 [Source:HGNC Symbol;Acc:HGNC:12823] |
| ENSG00000122641 | HGLibA_23302 | 120 | -0.049252341 | INHBA | inhibin subunit beta A [Source:HGNC Symbol;Acc:HGNC:6066] |
| ENSG00000123179 | HGLibA_14375 | 120 | -0.049252341 | EBPL | EBP like [Source:HGNC Symbol;Acc:HGNC:18061] |
| ENSG00000124091 | HGLibA_18820 | 120 | -0.049252341 | GCNT7 | glucosaminyl (N-acetyl) transferase family member 7 [Source:HGNC Symbol;Acc:HGNC:16099] |
| ENSG00000124107 | HGLibA_45545 | 120 | -0.049252341 | SLPI | secretory leukocyte peptidase inhibitor [Source:HGNC Symbol;Acc:HGNC:11092] |
| ENSG00000124226 | HGLibA_41478 | 120 | -0.049252341 | RNF114 | ring finger protein 114 [Source:HGNC Symbol;Acc:HGNC:13094] |
| ENSG00000125386 | HGLibA_16364 | 120 | -0.049252341 | FAM193A | family with sequence similarity 193 member A [Source:HGNC Symbol;Acc:HGNC:16822] |
| ENSG00000126581 | HGLibA_04396 | 120 | -0.049252341 | BECN1 | beclin 1 [Source:HGNC Symbol;Acc:HGNC:1034] |
| ENSG00000127129 | HGLibA_14471 | 120 | -0.049252341 | EDN2 | endothelin 2 [Source:HGNC Symbol;Acc:HGNC:3177] |
| ENSG00000128242 | HGLibA_18498 | 120 | -0.049252341 | GAL3ST1 | galactose-3-O-sulfotransferase 1 [Source:HGNC Symbol;Acc:HGNC:24240] |
| ENSG00000128595 | HGLibA_07051 | 120 | -0.049252341 | CALU | calumenin [Source:HGNC Symbol;Acc:HGNC:1458] |
| ENSG00000128815 | HGLibA_54014 | 120 | -0.049252341 | WDFY4 | WDFY family member 4 [Source:HGNC Symbol;Acc:HGNC:29323] |
| ENSG00000130254 | HGLibA_42638 | 120 | -0.049252341 | SAFB2 | scaffold attachment factor B2 [Source:HGNC Symbol;Acc:HGNC:21605] |
| ENSG00000133401 | HGLibA_35972 | 120 | -0.049252341 | PDZD2 | PDZ domain containing 2 [Source:HGNC Symbol;Acc:HGNC:18486] |
| ENSG00000134222 | HGLibA_39232 | 120 | -0.049252341 | PSRC1 | proline and serine rich coiled-coil 1 [Source:HGNC Symbol;Acc:HGNC:24472] |
| ENSG00000135378 | HGLibA_38878 | 120 | -0.049252341 | PRRG4 | proline rich and Gla domain 4 [Source:HGNC Symbol;Acc:HGNC:30799] |
| ENSG00000135632 | HGLibA_45863 | 120 | -0.049252341 | SMYD5 | SMYD family member 5 [Source:HGNC Symbol;Acc:HGNC:16258] |
| ENSG00000135919 | HGLibA_43581 | 120 | -0.049252341 | SERPINE2 | serpin family E member 2 [Source:HGNC Symbol;Acc:HGNC:8951] |
| ENSG00000136542 | HGLibA_18563 | 120 | -0.049252341 | GALNT5 | polypeptide N-acetylgalactosaminyltransferase 5 [Source:HGNC Symbol;Acc:HGNC:4127] |
| ENSG00000137628 | HGLibA_12761 | 120 | -0.049252341 | DDX60 | DExD/H-box helicase 60 [Source:HGNC Symbol;Acc:HGNC:25942] |
| ENSG00000137965 | HGLibA_22610 | 120 | -0.049252341 | IFI44 | interferon induced protein 44 [Source:HGNC Symbol;Acc:HGNC:16938] |
| ENSG00000139209 | HGLibA_45069 | 120 | -0.049252341 | SLC38A4 | solute carrier family 38 member 4 [Source:HGNC Symbol;Acc:HGNC:14679] |
| ENSG00000139613 | HGLibA_45627 | 120 | -0.049252341 | SMARCC2 | actin dependent regulator of chromatin subfamily c member 2 [Source:HGNC Symbol;Acc:HGNC:11105]" |
| ENSG00000139624 | HGLibA_09213 | 120 | -0.049252341 | CERS5 | ceramide synthase 5 [Source:HGNC Symbol;Acc:HGNC:23749] |
| ENSG00000140521 | HGLibA_37522 | 120 | -0.049252341 | POLG | catalytic subunit [Source:HGNC Symbol;Acc:HGNC:9179]" |
| ENSG00000141744 | HGLibA_37389 | 120 | -0.049252341 | PNMT | phenylethanolamine N-methyltransferase [Source:HGNC Symbol;Acc:HGNC:9160] |
| ENSG00000142677 | HGLibA_23092 | 120 | -0.049252341 | IL22RA1 | interleukin 22 receptor subunit alpha 1 [Source:HGNC Symbol;Acc:HGNC:13700] |
| ENSG00000143001 | HGLibA_50469 | 120 | -0.049252341 | TMEM61 | transmembrane protein 61 [Source:HGNC Symbol;Acc:HGNC:27296] |
| ENSG00000143158 | HGLibA_29707 | 120 | -0.049252341 | MPC2 | mitochondrial pyruvate carrier 2 [Source:HGNC Symbol;Acc:HGNC:24515] |
| ENSG00000143184 | HGLibA_54493 | 120 | -0.049252341 | XCL1 | X-C motif chemokine ligand 1 [Source:HGNC Symbol;Acc:HGNC:10645] |
| ENSG00000143816 | HGLibA_54394 | 120 | -0.049252341 | WNT9A | Wnt family member 9A [Source:HGNC Symbol;Acc:HGNC:12778] |
| ENSG00000144026 | HGLibA_56140 | 120 | -0.049252341 | ZNF514 | zinc finger protein 514 [Source:HGNC Symbol;Acc:HGNC:25894] |
| ENSG00000144802 | HGLibA_31835 | 120 | -0.049252341 | NFKBIZ | NFKB inhibitor zeta [Source:HGNC Symbol;Acc:HGNC:29805] |
| ENSG00000148660 | HGLibA_07075 | 120 | -0.049252341 | CAMK2G | calcium/calmodulin dependent protein kinase II gamma [Source:HGNC Symbol;Acc:HGNC:1463] |
| ENSG00000150593 | HGLibA_35732 | 120 | -0.049252341 | PDCD4 | programmed cell death 4 [Source:HGNC Symbol;Acc:HGNC:8763] |
| ENSG00000151164 | HGLibA_40111 | 120 | -0.049252341 | RAD9B | RAD9 checkpoint clamp component B [Source:HGNC Symbol;Acc:HGNC:21700] |
| ENSG00000151623 | HGLibA_32622 | 120 | -0.049252341 | NR3C2 | nuclear receptor subfamily 3 group C member 2 [Source:HGNC Symbol;Acc:HGNC:7979] |
| ENSG00000156299 | HGLibA_49440 | 120 | -0.049252341 | TIAM1 | T cell lymphoma invasion and metastasis 1 [Source:HGNC Symbol;Acc:HGNC:11805] |
| ENSG00000156886 | HGLibA_23750 | 120 | -0.049252341 | ITGAD | integrin subunit alpha D [Source:HGNC Symbol;Acc:HGNC:6146] |
| ENSG00000158234 | HGLibA_15963 | 120 | -0.049252341 | FAIM | Fas apoptotic inhibitory molecule [Source:HGNC Symbol;Acc:HGNC:18703] |
| ENSG00000160972 | HGLibA_38053 | 120 | -0.049252341 | PPP1R16A | protein phosphatase 1 regulatory subunit 16A [Source:HGNC Symbol;Acc:HGNC:14941] |
| ENSG00000162300 | HGLibA_55250 | 120 | -0.049252341 | ZFPL1 | zinc finger protein like 1 [Source:HGNC Symbol;Acc:HGNC:12868] |
| ENSG00000162391 | HGLibA_16161 | 120 | -0.049252341 | FAM151A | family with sequence similarity 151 member A [Source:HGNC Symbol;Acc:HGNC:25032] |
| ENSG00000162763 | HGLibA_27387 | 120 | -0.049252341 | LRRC52 | leucine rich repeat containing 52 [Source:HGNC Symbol;Acc:HGNC:32156] |
| ENSG00000163157 | HGLibA_50603 | 120 | -0.049252341 | TMOD4 | tropomodulin 4 [Source:HGNC Symbol;Acc:HGNC:11874] |
| ENSG00000163644 | HGLibA_37988 | 120 | -0.049252341 | PPM1K | Mg2+/Mn2+ dependent 1K [Source:HGNC Symbol;Acc:HGNC:25415]" |
| ENSG00000163736 | HGLibA_37856 | 120 | -0.049252341 | PPBP | pro-platelet basic protein [Source:HGNC Symbol;Acc:HGNC:9240] |
| ENSG00000164035 | HGLibA_15029 | 120 | -0.049252341 | EMCN | endomucin [Source:HGNC Symbol;Acc:HGNC:16041] |
| ENSG00000164743 | HGLibA_06634 | 120 | -0.049252341 | C8orf48 | chromosome 8 open reading frame 48 [Source:HGNC Symbol;Acc:HGNC:26345] |
| ENSG00000166135 | HGLibA_21299 | 120 | -0.049252341 | HIF1AN | hypoxia inducible factor 1 subunit alpha inhibitor [Source:HGNC Symbol;Acc:HGNC:17113] |
| ENSG00000166562 | HGLibA_43183 | 120 | -0.049252341 | SEC11C | signal peptidase complex subunit [Source:HGNC Symbol;Acc:HGNC:23400]" |
| ENSG00000167523 | HGLibA_46510 | 120 | -0.049252341 | SPATA33 | spermatogenesis associated 33 [Source:HGNC Symbol;Acc:HGNC:26463] |
| ENSG00000168517 | HGLibA_21213 | 120 | -0.049252341 | HEXIM2 | hexamethylene bisacetamide inducible 2 [Source:HGNC Symbol;Acc:HGNC:28591] |
| ENSG00000168528 | HGLibA_43486 | 120 | -0.049252341 | SERINC2 | serine incorporator 2 [Source:HGNC Symbol;Acc:HGNC:23231] |
| ENSG00000169953 | HGLibA_22243 | 120 | -0.049252341 | HSFY2 | heat shock transcription factor Y-linked 2 [Source:HGNC Symbol;Acc:HGNC:23950] |
| ENSG00000170144 | HGLibA_21802 | 120 | -0.049252341 | HNRNPA3 | heterogeneous nuclear ribonucleoprotein A3 [Source:HGNC Symbol;Acc:HGNC:24941] |
| ENSG00000170289 | HGLibA_10279 | 120 | -0.049252341 | CNGB3 | cyclic nucleotide gated channel beta 3 [Source:HGNC Symbol;Acc:HGNC:2153] |
| ENSG00000170370 | HGLibA_15096 | 120 | -0.049252341 | EMX2 | empty spiracles homeobox 2 [Source:HGNC Symbol;Acc:HGNC:3341] |
| ENSG00000171295 | HGLibA_56001 | 120 | -0.049252341 | ZNF440 | zinc finger protein 440 [Source:HGNC Symbol;Acc:HGNC:20874] |
| ENSG00000172936 | HGLibA_30686 | 120 | -0.049252341 | MYD88 | innate immune signal transduction adaptor [Source:HGNC Symbol;Acc:HGNC:7562]" |
| ENSG00000174226 | HGLibA_46088 | 120 | -0.049252341 | SNX31 | sorting nexin 31 [Source:HGNC Symbol;Acc:HGNC:28605] |
| ENSG00000174370 | HGLibA_05142 | 120 | -0.049252341 | C11orf45 | chromosome 11 open reading frame 45 [Source:HGNC Symbol;Acc:HGNC:28584] |
| ENSG00000177556 | HGLibA_03543 | 120 | -0.049252341 | ATOX1 | antioxidant 1 copper chaperone [Source:HGNC Symbol;Acc:HGNC:798] |
| ENSG00000178104 | HGLibA_35800 | 120 | -0.049252341 | PDE4DIP | phosphodiesterase 4D interacting protein [Source:HGNC Symbol;Acc:HGNC:15580] |
| ENSG00000178719 | HGLibA_20318 | 120 | -0.049252341 | GRINA | glutamate ionotropic receptor NMDA type subunit associated protein 1 [Source:HGNC Symbol;Acc:HGNC:4589] |
| ENSG00000180011 | HGLibA_54768 | 120 | -0.049252341 | ZADH2 | zinc binding alcohol dehydrogenase domain containing 2 [Source:HGNC Symbol;Acc:HGNC:28697] |
| ENSG00000181192 | HGLibA_13163 | 120 | -0.049252341 | DHTKD1 | dehydrogenase E1 and transketolase domain containing 1 [Source:HGNC Symbol;Acc:HGNC:23537] |
| ENSG00000183773 | HGLibA_01344 | 120 | -0.049252341 | AIFM3 | mitochondria associated 3 [Source:HGNC Symbol;Acc:HGNC:26398]" |
| ENSG00000186787 | HGLibA_46674 | 120 | -0.049252341 | SPIN2B | spindlin family member 2B [Source:HGNC Symbol;Acc:HGNC:33147] |
| ENSG00000196313 | HGLibA_37643 | 120 | -0.049252341 | POM121 | POM121 transmembrane nucleoporin [Source:HGNC Symbol;Acc:HGNC:19702] |
| ENSG00000197016 | HGLibA_56054 | 120 | -0.049252341 | ZNF470 | zinc finger protein 470 [Source:HGNC Symbol;Acc:HGNC:22220] |
| ENSG00000197057 | HGLibA_14057 | 120 | -0.049252341 | DTHD1 | death domain containing 1 [Source:HGNC Symbol;Acc:HGNC:37261] |
| ENSG00000198171 | HGLibA_12638 | 120 | -0.049252341 | DDRGK1 | DDRGK domain containing 1 [Source:HGNC Symbol;Acc:HGNC:16110] |
| ENSG00000198522 | HGLibA_19825 | 120 | -0.049252341 | GPN1 | GPN-loop GTPase 1 [Source:HGNC Symbol;Acc:HGNC:17030] |
| ENSG00000198952 | HGLibA_45702 | 120 | -0.049252341 | SMG5 | nonsense mediated mRNA decay factor [Source:HGNC Symbol;Acc:HGNC:24644]" |
| ENSG00000204472 | HGLibA_01330 | 120 | -0.049252341 | AIF1 | allograft inflammatory factor 1 [Source:HGNC Symbol;Acc:HGNC:352] |
| ENSG00000204548 | HGLibA_12869 | 120 | -0.049252341 | DEFB121 | defensin beta 121 [Source:HGNC Symbol;Acc:HGNC:18101] |
| ENSG00000205730 | HGLibA_23867 | 120 | -0.049252341 | ITPRIPL2 | ITPRIP like 2 [Source:HGNC Symbol;Acc:HGNC:27257] |
| ENSG00000215595 | HGLibA_06014 | 120 | -0.049252341 | C20orf202 | chromosome 20 open reading frame 202 [Source:HGNC Symbol;Acc:HGNC:37254] |
| ENSG00000222038 | HGLibA_37744 | 120 | -0.049252341 | POTEJ | POTE ankyrin domain family member J [Source:HGNC Symbol;Acc:HGNC:37094] |
| ENSG00000224440 | HGLibA_11937 | 120 | -0.049252341 | CXorf51A | chromosome X open reading frame 51A [Source:HGNC Symbol;Acc:HGNC:30533] |
| ENSG00000235863 | HGLibA_03961 | 120 | -0.049252341 | B3GALT4 | 3-galactosyltransferase 4 [Source:HGNC Symbol;Acc:HGNC:919]" |
| ENSG00000236609 | HGLibA_56876 | 120 | -0.049252341 | ZNF853 | zinc finger protein 853 [Source:HGNC Symbol;Acc:HGNC:21767] |
| ENSG00000242366 | HGLibA_52898 | 120 | -0.049252341 | UGT1A8 | UDP glucuronosyltransferase family 1 member A8 [Source:HGNC Symbol;Acc:HGNC:12540] |
| ENSG00000243943 | HGLibA_56132 | 120 | -0.049252341 | ZNF512 | zinc finger protein 512 [Source:HGNC Symbol;Acc:HGNC:29380] |
| ENSG00000267467 | HGLibA_02511 | 120 | -0.049252341 | APOC4 | apolipoprotein C4 [Source:HGNC Symbol;Acc:HGNC:611] |
| ENSG00000272968 | HGLibA_40426 | 120 | -0.049252341 | RBAK-RBAKDN | RBAK-RBAKDN readthrough [Source:HGNC Symbol;Acc:HGNC:42971] |
| HIST1H2BD | HGLibA_21420 | 120 | -0.049252341 | HIST1H2BD | - |
| HIST4H4 | HGLibA_21564 | 120 | -0.049252341 | HIST4H4 | - |
| hsa-mir-1204 | HGLibA_57272 | 120 | -0.049252341 | hsa-mir-1204 | - |
| hsa-mir-1244-2 | HGLibA_57366 | 120 | -0.049252341 | hsa-mir-1244-2 | - |
| hsa-mir-1295a | HGLibA_57655 | 120 | -0.049252341 | hsa-mir-1295a | - |
| hsa-mir-181d | HGLibA_57978 | 120 | -0.049252341 | hsa-mir-181d | - |
| hsa-mir-2116 | HGLibA_58238 | 120 | -0.049252341 | hsa-mir-2116 | - |
| hsa-mir-29a | HGLibA_58408 | 120 | -0.049252341 | hsa-mir-29a | - |
| hsa-mir-3138 | HGLibA_58612 | 120 | -0.049252341 | hsa-mir-3138 | - |
| hsa-mir-4635 | HGLibA_60644 | 120 | -0.049252341 | hsa-mir-4635 | - |
| hsa-mir-4715 | HGLibA_60960 | 120 | -0.049252341 | hsa-mir-4715 | - |
| hsa-mir-4735 | HGLibA_61038 | 120 | -0.049252341 | hsa-mir-4735 | - |
| hsa-mir-519a-2 | HGLibA_61713 | 120 | -0.049252341 | hsa-mir-519a-2 | - |
| hsa-mir-548h-5 | HGLibA_62009 | 120 | -0.049252341 | hsa-mir-548h-5 | - |
| hsa-mir-5693 | HGLibA_62306 | 120 | -0.049252341 | hsa-mir-5693 | - |
| hsa-mir-624 | HGLibA_62737 | 120 | -0.049252341 | hsa-mir-624 | - |
| hsa-mir-7154 | HGLibA_63846 | 120 | -0.049252341 | hsa-mir-7154 | - |
| hsa-mir-7975 | HGLibA_64029 | 120 | -0.049252341 | hsa-mir-7975 | - |

| | | | | | |
|---|---|---|---|---|---|
| LOC730183 | HGLibA_27006 | 120 | -0.049252341 | LOC730183 | - |
| MTSS1L | HGLibA_30500 | 120 | -0.049252341 | MTSS1L | - |
| OR8G2 | HGLibA_34521 | 120 | -0.049252341 | OR8G2 | - |
| OR8G2 | HGLibA_34520 | 120 | -0.049252341 | OR8G2 | - |
| PVRL1 | HGLibA_39622 | 120 | -0.049252341 | PVRL1 | - |
| RRNAD1 | HGLibA_42262 | 120 | -0.049252341 | RRNAD1 | - |
| TMEM257 | HGLibA_50340 | 120 | -0.049252341 | TMEM257 | - |
| ZMYM6NB | HGLibA_55408 | 120 | -0.049252341 | ZMYM6NB | - |
| C17orf104 | HGLibA_05523 | 119 | -0.058297684 | C17orf104 | - |
| C17orf112 | HGLibA_05532 | 119 | -0.058297684 | C17orf112 | - |
| C19orf45 | HGLibA_05667 | 119 | -0.058297684 | C19orf45 | - |
| C6orf1 | HGLibA_06427 | 119 | -0.058297684 | C6orf1 | - |
| ENSG00000002549 | HGLibA_26012 | 119 | -0.058297684 | LAP3 | leucine aminopeptidase 3 [Source:HGNC Symbol;Acc:HGNC:18449] |
| ENSG00000006659 | HGLibA_26355 | 119 | -0.058297684 | LGALS14 | galectin 14 [Source:HGNC Symbol;Acc:HGNC:30054] |
| ENSG00000007516 | HGLibA_04132 | 119 | -0.058297684 | BAIAP3 | BAI1 associated protein 3 [Source:HGNC Symbol;Acc:HGNC:948] |
| ENSG00000011454 | HGLibA_40008 | 119 | -0.058297684 | RABGAP1 | RAB GTPase activating protein 1 [Source:HGNC Symbol;Acc:HGNC:17155] |
| ENSG00000013441 | HGLibA_10053 | 119 | -0.058297684 | CLK1 | CDC like kinase 1 [Source:HGNC Symbol;Acc:HGNC:2068] |
| ENSG00000016402 | HGLibA_23077 | 119 | -0.058297684 | IL20RA | interleukin 20 receptor subunit alpha [Source:HGNC Symbol;Acc:HGNC:6003] |
| ENSG00000032742 | HGLibA_22778 | 119 | -0.058297684 | IFT88 | intraflagellar transport 88 [Source:HGNC Symbol;Acc:HGNC:20606] |
| ENSG00000047315 | HGLibA_37566 | 119 | -0.058297684 | POLR2B | RNA polymerase II subunit B [Source:HGNC Symbol;Acc:HGNC:9188] |
| ENSG00000054282 | HGLibA_43118 | 119 | -0.058297684 | SDCCAG8 | serologically defined colon cancer antigen 8 [Source:HGNC Symbol;Acc:HGNC:10671] |
| ENSG00000059122 | HGLibA_17680 | 119 | -0.058297684 | FLYWCH1 | FLYWCH-type zinc finger 1 [Source:HGNC Symbol;Acc:HGNC:25404] |
| ENSG00000065621 | HGLibA_20489 | 119 | -0.058297684 | GSTO2 | glutathione S-transferase omega 2 [Source:HGNC Symbol;Acc:HGNC:23064] |
| ENSG00000067365 | HGLibA_28989 | 119 | -0.058297684 | METTL22 | methyltransferase like 22 [Source:HGNC Symbol;Acc:HGNC:28368] |
| ENSG00000068781 | HGLibA_47545 | 119 | -0.058297684 | STON1-GTF2A1L | STON1-GTF2A1L readthrough [Source:HGNC Symbol;Acc:HGNC:30651] |
| ENSG00000071994 | HGLibA_35728 | 119 | -0.058297684 | PDCD2 | programmed cell death 2 [Source:HGNC Symbol;Acc:HGNC:8762] |
| ENSG00000072110 | HGLibA_00637 | 119 | -0.058297684 | ACTN1 | actinin alpha 1 [Source:HGNC Symbol;Acc:HGNC:163] |
| ENSG00000076706 | HGLibA_28556 | 119 | -0.058297684 | MCAM | melanoma cell adhesion molecule [Source:HGNC Symbol;Acc:HGNC:6934] |
| ENSG00000078725 | HGLibA_04812 | 119 | -0.058297684 | BRINP1 | BMP/retinoic acid inducible neural specific 1 [Source:HGNC Symbol;Acc:HGNC:2687] |
| ENSG00000079841 | HGLibA_41276 | 119 | -0.058297684 | RIMS1 | regulating synaptic membrane exocytosis 1 [Source:HGNC Symbol;Acc:HGNC:17282] |
| ENSG00000081800 | HGLibA_44384 | 119 | -0.058297684 | SLC13A1 | solute carrier family 13 member 1 [Source:HGNC Symbol;Acc:HGNC:10916] |
| ENSG00000087250 | HGLibA_30305 | 119 | -0.058297684 | MT3 | metallothionein 3 [Source:HGNC Symbol;Acc:HGNC:7408] |
| ENSG00000099812 | HGLibA_29311 | 119 | -0.058297684 | MISP | mitotic spindle positioning [Source:HGNC Symbol;Acc:HGNC:27000] |
| ENSG00000100982 | HGLibA_35610 | 119 | -0.058297684 | PCIF1 | PDX1 C-terminal inhibiting factor 1 [Source:HGNC Symbol;Acc:HGNC:16200] |
| ENSG00000101004 | HGLibA_31945 | 119 | -0.058297684 | NINL | ninein like [Source:HGNC Symbol;Acc:HGNC:29163] |
| ENSG00000101282 | HGLibA_42352 | 119 | -0.058297684 | RSPO4 | R-spondin 4 [Source:HGNC Symbol;Acc:HGNC:16175] |
| ENSG00000103174 | HGLibA_31072 | 119 | -0.058297684 | NAGPA | N-acetylglucosamine-1-phosphodiester alpha-N-acetylglucosaminidase [Source:HGNC Symbol;Acc:HGNC:17378] |
| ENSG00000103199 | HGLibA_56106 | 119 | -0.058297684 | ZNF500 | zinc finger protein 500 [Source:HGNC Symbol;Acc:HGNC:23716] |
| ENSG00000103356 | HGLibA_14343 | 119 | -0.058297684 | EARS2 | mitochondrial [Source:HGNC Symbol;Acc:HGNC:29419]" |
| ENSG00000104133 | HGLibA_46630 | 119 | -0.058297684 | SPG11 | spatacsin vesicle trafficking associated [Source:HGNC Symbol;Acc:HGNC:11226]" |
| ENSG00000105650 | HGLibA_35793 | 119 | -0.058297684 | PDE4C | phosphodiesterase 4C [Source:HGNC Symbol;Acc:HGNC:8782] |
| ENSG00000108854 | HGLibA_45849 | 119 | -0.058297684 | SMURF2 | SMAD specific E3 ubiquitin protein ligase 2 [Source:HGNC Symbol;Acc:HGNC:16809] |
| ENSG00000109674 | HGLibA_31636 | 119 | -0.058297684 | NEIL3 | nei like DNA glycosylase 3 [Source:HGNC Symbol;Acc:HGNC:24573] |
| ENSG00000109686 | HGLibA_43940 | 119 | -0.058297684 | SH3D19 | SH3 domain containing 19 [Source:HGNC Symbol;Acc:HGNC:30418] |
| ENSG00000110665 | HGLibA_05121 | 119 | -0.058297684 | C11orf21 | chromosome 11 open reading frame 21 [Source:HGNC Symbol;Acc:HGNC:13231] |
| ENSG00000111261 | HGLibA_28099 | 119 | -0.058297684 | MANSC1 | MANSC domain containing 1 [Source:HGNC Symbol;Acc:HGNC:25505] |
| ENSG00000111348 | HGLibA_02799 | 119 | -0.058297684 | ARHGDIB | Rho GDP dissociation inhibitor beta [Source:HGNC Symbol;Acc:HGNC:679] |
| ENSG00000111834 | HGLibA_42335 | 119 | -0.058297684 | RSPH4A | radial spoke head component 4A [Source:HGNC Symbol;Acc:HGNC:21558] |
| ENSG00000112208 | HGLibA_04076 | 119 | -0.058297684 | BAG2 | BCL2 associated athanogene 2 [Source:HGNC Symbol;Acc:HGNC:938] |
| ENSG00000113100 | HGLibA_08695 | 119 | -0.058297684 | CDH9 | cadherin 9 [Source:HGNC Symbol;Acc:HGNC:1768] |
| ENSG00000115414 | HGLibA_17727 | 119 | -0.058297684 | FN1 | fibronectin 1 [Source:HGNC Symbol;Acc:HGNC:3778] |
| ENSG00000115488 | HGLibA_31713 | 119 | -0.058297684 | NEU2 | neuraminidase 2 [Source:HGNC Symbol;Acc:HGNC:7759] |
| ENSG00000116062 | HGLibA_30203 | 119 | -0.058297684 | MSH6 | mutS homolog 6 [Source:HGNC Symbol;Acc:HGNC:7329] |
| ENSG00000116132 | HGLibA_38893 | 119 | -0.058297684 | PRRX1 | paired related homeobox 1 [Source:HGNC Symbol;Acc:HGNC:9142] |
| ENSG00000124103 | HGLibA_16439 | 119 | -0.058297684 | FAM209A | family with sequence similarity 209 member A [Source:HGNC Symbol;Acc:HGNC:16100] |
| ENSG00000124785 | HGLibA_32708 | 119 | -0.058297684 | NRN1 | neuritin 1 [Source:HGNC Symbol;Acc:HGNC:17972] |
| ENSG00000125148 | HGLibA_30301 | 119 | -0.058297684 | MT2A | metallothionein 2A [Source:HGNC Symbol;Acc:HGNC:7406] |
| ENSG00000125900 | HGLibA_44209 | 119 | -0.058297684 | SIRPD | signal regulatory protein delta [Source:HGNC Symbol;Acc:HGNC:16248] |
| ENSG00000129226 | HGLibA_08428 | 119 | -0.058297684 | CD68 | CD68 molecule [Source:HGNC Symbol;Acc:HGNC:1693] |
| ENSG00000130023 | HGLibA_15504 | 119 | -0.058297684 | ERMARD | ER membrane associated RNA degradation [Source:HGNC Symbol;Acc:HGNC:21056] |
| ENSG00000130590 | HGLibA_42661 | 119 | -0.058297684 | SAMD10 | sterile alpha motif domain containing 10 [Source:HGNC Symbol;Acc:HGNC:16129] |
| ENSG00000132313 | HGLibA_29960 | 119 | -0.058297684 | MRPL35 | mitochondrial ribosomal protein L35 [Source:HGNC Symbol;Acc:HGNC:14489] |
| ENSG00000132639 | HGLibA_45878 | 119 | -0.058297684 | SNAP25 | synaptosome associated protein 25 [Source:HGNC Symbol;Acc:HGNC:11132] |
| ENSG00000133103 | HGLibA_10484 | 119 | -0.058297684 | COG6 | component of oligomeric golgi complex 6 [Source:HGNC Symbol;Acc:HGNC:18621] |
| ENSG00000133800 | HGLibA_27779 | 119 | -0.058297684 | LYVE1 | lymphatic vessel endothelial hyaluronan receptor 1 [Source:HGNC Symbol;Acc:HGNC:14687] |
| ENSG00000134769 | HGLibA_14064 | 119 | -0.058297684 | DTNA | dystrobrevin alpha [Source:HGNC Symbol;Acc:HGNC:3057] |
| ENSG00000136111 | HGLibA_48557 | 119 | -0.058297684 | TBC1D4 | TBC1 domain family member 4 [Source:HGNC Symbol;Acc:HGNC:19165] |
| ENSG00000137269 | HGLibA_27245 | 119 | -0.058297684 | LRRC1 | leucine rich repeat containing 1 [Source:HGNC Symbol;Acc:HGNC:14307] |
| ENSG00000138622 | HGLibA_20982 | 119 | -0.058297684 | HCN4 | hyperpolarization activated cyclic nucleotide gated potassium channel 4 [Source:HGNC Symbol;Acc:HGNC:16882] |
| ENSG00000140443 | HGLibA_22793 | 119 | -0.058297684 | IGF1R | insulin like growth factor 1 receptor [Source:HGNC Symbol;Acc:HGNC:5465] |
| ENSG00000142677 | HGLibA_23091 | 119 | -0.058297684 | IL22RA1 | interleukin 22 receptor subunit alpha 1 [Source:HGNC Symbol;Acc:HGNC:13700] |
| ENSG00000143442 | HGLibA_37479 | 119 | -0.058297684 | POGZ | pogo transposable element derived with ZNF domain [Source:HGNC Symbol;Acc:HGNC:18801] |
| ENSG00000144120 | HGLibA_50079 | 119 | -0.058297684 | TMEM177 | transmembrane protein 177 [Source:HGNC Symbol;Acc:HGNC:28143] |
| ENSG00000144674 | HGLibA_19602 | 119 | -0.058297684 | GOLGA4 | golgin A4 [Source:HGNC Symbol;Acc:HGNC:4427] |
| ENSG00000145014 | HGLibA_50405 | 119 | -0.058297684 | TMEM44 | transmembrane protein 44 [Source:HGNC Symbol;Acc:HGNC:25120] |
| ENSG00000147894 | HGLibA_06757 | 119 | -0.058297684 | C9orf72 | chromosome 9 open reading frame 72 [Source:HGNC Symbol;Acc:HGNC:28337] |
| ENSG00000149273 | HGLibA_42137 | 119 | -0.058297684 | RPS3 | ribosomal protein S3 [Source:HGNC Symbol;Acc:HGNC:10420] |
| ENSG00000151665 | HGLibA_36551 | 119 | -0.058297684 | PIGF | phosphatidylinositol glycan anchor biosynthesis class F [Source:HGNC Symbol;Acc:HGNC:8962] |
| ENSG00000156959 | HGLibA_26437 | 119 | -0.058297684 | LHFPL4 | LHFPL tetraspan subfamily member 4 [Source:HGNC Symbol;Acc:HGNC:29568] |
| ENSG00000158089 | HGLibA_18543 | 119 | -0.058297684 | GALNT14 | polypeptide N-acetylgalactosaminyltransferase 14 [Source:HGNC Symbol;Acc:HGNC:22946] |
| ENSG00000158815 | HGLibA_17380 | 119 | -0.058297684 | FGF17 | fibroblast growth factor 17 [Source:HGNC Symbol;Acc:HGNC:3673] |
| ENSG00000160213 | HGLibA_11480 | 119 | -0.058297684 | CSTB | cystatin B [Source:HGNC Symbol;Acc:HGNC:2482] |
| ENSG00000160753 | HGLibA_42468 | 119 | -0.058297684 | RUSC1 | RUN and SH3 domain containing 1 [Source:HGNC Symbol;Acc:HGNC:17153] |
| ENSG00000161265 | HGLibA_52494 | 119 | -0.058297684 | U2AF1L4 | U2 small nuclear RNA auxiliary factor 1 like 4 [Source:HGNC Symbol;Acc:HGNC:23020] |
| ENSG00000163357 | HGLibA_12528 | 119 | -0.058297684 | DCST1 | DC-STAMP domain containing 1 [Source:HGNC Symbol;Acc:HGNC:26539] |
| ENSG00000163623 | HGLibA_32055 | 119 | -0.058297684 | NKX6-1 | NK6 homeobox 1 [Source:HGNC Symbol;Acc:HGNC:7839] |
| ENSG00000163635 | HGLibA_03849 | 119 | -0.058297684 | ATXN7 | ataxin 7 [Source:HGNC Symbol;Acc:HGNC:10560] |
| ENSG00000163683 | HGLibA_45732 | 119 | -0.058297684 | SMIM14 | small integral membrane protein 14 [Source:HGNC Symbol;Acc:HGNC:27321] |
| ENSG00000163746 | HGLibA_37233 | 119 | -0.058297684 | PLSCR2 | phospholipid scramblase 2 [Source:HGNC Symbol;Acc:HGNC:16494] |
| ENSG00000165078 | HGLibA_10899 | 119 | -0.058297684 | CPA6 | carboxypeptidase A6 [Source:HGNC Symbol;Acc:HGNC:17245] |
| ENSG00000166278 | HGLibA_05978 | 119 | -0.058297684 | C2 | complement C2 [Source:HGNC Symbol;Acc:HGNC:1248] |
| ENSG00000166619 | HGLibA_04540 | 119 | -0.058297684 | BLCAP | apoptosis inducing factor [Source:HGNC Symbol;Acc:HGNC:1055]" |
| ENSG00000167131 | HGLibA_07514 | 119 | -0.058297684 | CCDC103 | coiled-coil domain containing 103 [Source:HGNC Symbol;Acc:HGNC:32700] |
| ENSG00000167216 | HGLibA_24078 | 119 | -0.058297684 | KATNAL2 | katanin catalytic subunit A1 like 2 [Source:HGNC Symbol;Acc:HGNC:25387] |
| ENSG00000167363 | HGLibA_17730 | 119 | -0.058297684 | FN3K | fructosamine 3 kinase [Source:HGNC Symbol;Acc:HGNC:24822] |
| ENSG00000167941 | HGLibA_46207 | 119 | -0.058297684 | SOST | sclerostin [Source:HGNC Symbol;Acc:HGNC:13771] |
| ENSG00000168135 | HGLibA_24278 | 119 | -0.058297684 | KCNJ4 | potassium voltage-gated channel subfamily J member 4 [Source:HGNC Symbol;Acc:HGNC:6265] |
| ENSG00000169181 | HGLibA_20421 | 119 | -0.058297684 | GSG1L | GSG1 like [Source:HGNC Symbol;Acc:HGNC:28283] |
| ENSG00000169375 | HGLibA_44182 | 119 | -0.058297684 | SIN3A | SIN3 transcription regulator family member A [Source:HGNC Symbol;Acc:HGNC:19353] |
| ENSG00000169692 | HGLibA_01220 | 119 | -0.058297684 | AGPAT2 | 1-acylglycerol-3-phosphate O-acyltransferase 2 [Source:HGNC Symbol;Acc:HGNC:325] |
| ENSG00000170426 | HGLibA_43169 | 119 | -0.058297684 | SDR9C7 | short chain dehydrogenase/reductase family 9C member 7 [Source:HGNC Symbol;Acc:HGNC:29958] |
| ENSG00000170777 | HGLibA_51121 | 119 | -0.058297684 | TPD52L3 | TPD52 like 3 [Source:HGNC Symbol;Acc:HGNC:23382] |
| ENSG00000171126 | HGLibA_24203 | 119 | -0.058297684 | KCNG3 | potassium voltage-gated channel modifier subfamily G member 3 [Source:HGNC Symbol;Acc:HGNC:18306] |
| ENSG00000172340 | HGLibA_47703 | 119 | -0.058297684 | SUCLG2 | succinate-CoA ligase GDP-forming beta subunit [Source:HGNC Symbol;Acc:HGNC:11450] |
| ENSG00000173852 | HGLibA_13904 | 119 | -0.058297684 | DPY19L1 | dpy-19 like C-mannosyltransferase 1 [Source:HGNC Symbol;Acc:HGNC:22205] |
| ENSG00000175449 | HGLibA_40918 | 119 | -0.058297684 | RFESD | Rieske Fe-S domain containing [Source:HGNC Symbol;Acc:HGNC:29587] |
| ENSG00000176273 | HGLibA_45017 | 119 | -0.058297684 | SLC35G1 | solute carrier family 35 member G1 [Source:HGNC Symbol;Acc:HGNC:26607] |
| ENSG00000176401 | HGLibA_14700 | 119 | -0.058297684 | EID2B | EP300 interacting inhibitor of differentiation 2B [Source:HGNC Symbol;Acc:HGNC:26796] |
| ENSG00000176532 | HGLibA_38784 | 119 | -0.058297684 | PRR15 | proline rich 15 [Source:HGNC Symbol;Acc:HGNC:22310] |
| ENSG00000176619 | HGLibA_26699 | 119 | -0.058297684 | LMNB2 | lamin B2 [Source:HGNC Symbol;Acc:HGNC:6638] |
| ENSG00000177156 | HGLibA_48285 | 119 | -0.058297684 | TALDO1 | transaldolase 1 [Source:HGNC Symbol;Acc:HGNC:11559] |
| ENSG00000177548 | HGLibA_40003 | 119 | -0.058297684 | RABEP2 | RAB GTPase binding effector protein 2 [Source:HGNC Symbol;Acc:HGNC:24817]" |
| ENSG00000178175 | HGLibA_55878 | 119 | -0.058297684 | ZNF366 | zinc finger protein 366 [Source:HGNC Symbol;Acc:HGNC:18316] |
| ENSG00000178860 | HGLibA_30185 | 119 | -0.058297684 | MSC | musculin [Source:HGNC Symbol;Acc:HGNC:7321] |
| ENSG00000179094 | HGLibA_36055 | 119 | -0.058297684 | PER1 | period circadian regulator 1 [Source:HGNC Symbol;Acc:HGNC:8845] |
| ENSG00000181588 | HGLibA_29035 | 119 | -0.058297684 | MEX3D | mex-3 RNA binding family member D [Source:HGNC Symbol;Acc:HGNC:16734] |
| ENSG00000182010 | HGLibA_42391 | 119 | -0.058297684 | RTKN2 | rhotekin 2 [Source:HGNC Symbol;Acc:HGNC:19364] |
| ENSG00000182718 | HGLibA_02232 | 119 | -0.058297684 | ANXA2 | annexin A2 [Source:HGNC Symbol;Acc:HGNC:537] |
| ENSG00000182810 | HGLibA_12689 | 119 | -0.058297684 | DDX28 | DEAD-box helicase 28 [Source:HGNC Symbol;Acc:HGNC:17330] |
| ENSG00000182916 | HGLibA_48721 | 119 | -0.058297684 | TCEAL7 | transcription elongation factor A like 7 [Source:HGNC Symbol;Acc:HGNC:28336] |
| ENSG00000183401 | HGLibA_07666 | 119 | -0.058297684 | CCDC159 | coiled-coil domain containing 159 [Source:HGNC Symbol;Acc:HGNC:26996] |
| ENSG00000183559 | HGLibA_05045 | 119 | -0.058297684 | C10orf120 | chromosome 10 open reading frame 120 [Source:HGNC Symbol;Acc:HGNC:25707] |
| ENSG00000184194 | HGLibA_19968 | 119 | -0.058297684 | GPR173 | G protein-coupled receptor 173 [Source:HGNC Symbol;Acc:HGNC:18186] |
| ENSG00000185303 | HGLibA_43769 | 119 | -0.058297684 | SFTPA2 | surfactant protein A2 [Source:HGNC Symbol;Acc:HGNC:10799] |
| ENSG00000186795 | HGLibA_24316 | 119 | -0.058297684 | KCNK18 | potassium two pore domain channel subfamily K member 18 [Source:HGNC Symbol;Acc:HGNC:19439] |
| ENSG00000186910 | HGLibA_43512 | 119 | -0.058297684 | SERPINA11 | serpin family A member 11 [Source:HGNC Symbol;Acc:HGNC:19193] |
| ENSG00000188076 | HGLibA_42912 | 119 | -0.058297684 | SCGB1C1 | secretoglobin family 1C member 1 [Source:HGNC Symbol;Acc:HGNC:18394] |
| ENSG00000196155 | HGLibA_37109 | 119 | -0.058297684 | PLEKHG4 | pleckstrin homology and RhoGEF domain containing G4 [Source:HGNC Symbol;Acc:HGNC:24501] |
| ENSG00000197372 | HGLibA_56513 | 119 | -0.058297684 | ZNF675 | zinc finger protein 675 [Source:HGNC Symbol;Acc:HGNC:30768] |
| ENSG00000197576 | HGLibA_21912 | 119 | -0.058297684 | HOXA4 | homeobox A4 [Source:HGNC Symbol;Acc:HGNC:5105] |
| ENSG00000197629 | HGLibA_29718 | 119 | -0.058297684 | MPEG1 | macrophage expressed 1 [Source:HGNC Symbol;Acc:HGNC:29619] |
| ENSG00000204498 | HGLibA_31833 | 119 | -0.058297684 | NFKBIL1 | NFKB inhibitor like 1 [Source:HGNC Symbol;Acc:HGNC:7800] |
| ENSG00000205045 | HGLibA_45486 | 119 | -0.058297684 | SLFN12L | schlafen family member 12 like [Source:HGNC Symbol;Acc:HGNC:33920] |
| ENSG00000205495 | HGLibA_34178 | 119 | -0.058297684 | OR52J3 | olfactory receptor family 52 subfamily J member 3 [Source:HGNC Symbol;Acc:HGNC:14799] |
| ENSG00000213199 | HGLibA_03304 | 119 | -0.058297684 | ASIC3 | acid sensing ion channel subunit 3 [Source:HGNC Symbol;Acc:HGNC:101] |

| | | | | | |
|---|---|---|---|---|---|
| ENSG00000221923 | HGLibA_56892 | 119 | -0.058297684 | ZNF880 | zinc finger protein 880 [Source:HGNC Symbol;Acc:HGNC:37249] |
| ENSG00000244474 | HGLibA_52886 | 119 | -0.058297684 | UGT1A4 | UDP glucuronosyltransferase family 1 member A4 [Source:HGNC Symbol;Acc:HGNC:12536] |
| ENSG00000255150 | HGLibA_14703 | 119 | -0.058297684 | EID3 | EP300 interacting inhibitor of differentiation 3 [Source:HGNC Symbol;Acc:HGNC:32961] |
| ENSG00000256394 | HGLibA_03311 | 119 | -0.058297684 | ASIC5 | acid sensing ion channel subunit family member 5 [Source:HGNC Symbol;Acc:HGNC:17537] |
| ENSG00000260456 | HGLibA_05507 | 119 | -0.058297684 | C16orf95 | chromosome 16 open reading frame 95 [Source:HGNC Symbol;Acc:HGNC:40033] |
| ENSG00000271303 | HGLibA_47095 | 119 | -0.058297684 | SRXN1 | sulfiredoxin 1 [Source:HGNC Symbol;Acc:HGNC:16132] |
| ENSG00000273540 | HGLibA_01166 | 119 | -0.058297684 | AGBL1 | ATP/GTP binding protein like 1 [Source:HGNC Symbol;Acc:HGNC:26504] |
| ENSG00000277893 | HGLibA_46942 | 119 | -0.058297684 | SRD5A2 | steroid 5 alpha-reductase 2 [Source:HGNC Symbol;Acc:HGNC:11285] |
| FAM196B | HGLibA_16384 | 119 | -0.058297684 | FAM196B | - |
| HIST1H4D | HGLibA_21496 | 119 | -0.058297684 | HIST1H4D | - |
| HIST2H3A | HGLibA_21540 | 119 | -0.058297684 | HIST2H3A | - |
| hsa-mir-1204 | HGLibA_57273 | 119 | -0.058297684 | hsa-mir-1204 | - |
| hsa-mir-1228 | HGLibA_57309 | 119 | -0.058297684 | hsa-mir-1228 | - |
| hsa-mir-1244-3 | HGLibA_57372 | 119 | -0.058297684 | hsa-mir-1244-3 | - |
| hsa-mir-130a | HGLibA_57748 | 119 | -0.058297684 | hsa-mir-130a | - |
| hsa-mir-26b | HGLibA_58373 | 119 | -0.058297684 | hsa-mir-26b | - |
| hsa-mir-3148 | HGLibA_58650 | 119 | -0.058297684 | hsa-mir-3148 | - |
| hsa-mir-3201 | HGLibA_58913 | 119 | -0.058297684 | hsa-mir-3201 | - |
| hsa-mir-3915 | HGLibA_59537 | 119 | -0.058297684 | hsa-mir-3915 | - |
| hsa-mir-4288 | HGLibA_59858 | 119 | -0.058297684 | hsa-mir-4288 | - |
| hsa-mir-4424 | HGLibA_60082 | 119 | -0.058297684 | hsa-mir-4424 | - |
| hsa-mir-4438 | HGLibA_60155 | 119 | -0.058297684 | hsa-mir-4438 | - |
| hsa-mir-4462 | HGLibA_60254 | 119 | -0.058297684 | hsa-mir-4462 | - |
| hsa-mir-4480 | HGLibA_60339 | 119 | -0.058297684 | hsa-mir-4480 | - |
| hsa-mir-4521 | HGLibA_60543 | 119 | -0.058297684 | hsa-mir-4521 | - |
| hsa-mir-4650-1 | HGLibA_60702 | 119 | -0.058297684 | hsa-mir-4650-1 | - |
| hsa-mir-591 | HGLibA_62463 | 119 | -0.058297684 | hsa-mir-591 | - |
| hsa-mir-650 | HGLibA_62848 | 119 | -0.058297684 | hsa-mir-650 | - |
| hsa-mir-6862-1 | HGLibA_63628 | 119 | -0.058297684 | hsa-mir-6862-1 | - |
| hsa-mir-6869 | HGLibA_63661 | 119 | -0.058297684 | hsa-mir-6869 | - |
| hsa-mir-708 | HGLibA_63780 | 119 | -0.058297684 | hsa-mir-708 | - |
| hsa-mir-7847 | HGLibA_63977 | 119 | -0.058297684 | hsa-mir-7847 | - |
| PRKCDBP | HGLibA_38541 | 119 | -0.058297684 | PRKCDBP | - |
| C11orf85 | HGLibA_05198 | 118 | -0.067343027 | C11orf85 | - |
| C6orf195 | HGLibA_06464 | 118 | -0.067343027 | C6orf195 | - |
| C8orf22 | HGLibA_06601 | 118 | -0.067343027 | C8orf22 | - |
| DOPEY1 | HGLibA_13801 | 118 | -0.067343027 | DOPEY1 | - |
| ENSG00000001497 | HGLibA_26053 | 118 | -0.067343027 | LAS1L | ribosome biogenesis factor [Source:HGNC Symbol;Acc:HGNC:25726]" |
| ENSG00000005801 | HGLibA_55557 | 118 | -0.067343027 | ZNF195 | zinc finger protein 195 [Source:HGNC Symbol;Acc:HGNC:12986] |
| ENSG00000006453 | HGLibA_04125 | 118 | -0.067343027 | BAIAP2L1 | BAI1 associated protein 2 like 1 [Source:HGNC Symbol;Acc:HGNC:21649] |
| ENSG00000009724 | HGLibA_28396 | 118 | -0.067343027 | MASP2 | mannan binding lectin serine peptidase 2 [Source:HGNC Symbol;Acc:HGNC:6902] |
| ENSG00000019144 | HGLibA_36426 | 118 | -0.067343027 | PHLDB1 | pleckstrin homology like domain family B member 1 [Source:HGNC Symbol;Acc:HGNC:23697] |
| ENSG00000029153 | HGLibA_03072 | 118 | -0.067343027 | ARNTL2 | aryl hydrocarbon receptor nuclear translocator like 2 [Source:HGNC Symbol;Acc:HGNC:18984] |
| ENSG00000049245 | HGLibA_53493 | 118 | -0.067343027 | VAMP3 | vesicle associated membrane protein 3 [Source:HGNC Symbol;Acc:HGNC:12644] |
| ENSG00000049249 | HGLibA_50793 | 118 | -0.067343027 | TNFRSF9 | TNF receptor superfamily member 9 [Source:HGNC Symbol;Acc:HGNC:11924] |
| ENSG00000049323 | HGLibA_27589 | 118 | -0.067343027 | LTBP1 | latent transforming growth factor beta binding protein 1 [Source:HGNC Symbol;Acc:HGNC:6714] |
| ENSG00000059804 | HGLibA_44872 | 118 | -0.067343027 | SLC2A3 | solute carrier family 2 member 3 [Source:HGNC Symbol;Acc:HGNC:11007] |
| ENSG00000062716 | HGLibA_53696 | 118 | -0.067343027 | VMP1 | vacuole membrane protein 1 [Source:HGNC Symbol;Acc:HGNC:29559] |
| ENSG00000064199 | HGLibA_46320 | 118 | -0.067343027 | SPA17 | sperm autoantigenic protein 17 [Source:HGNC Symbol;Acc:HGNC:11210] |
| ENSG00000069696 | HGLibA_13969 | 118 | -0.067343027 | DRD4 | dopamine receptor D4 [Source:HGNC Symbol;Acc:HGNC:3025] |
| ENSG00000075886 | HGLibA_52254 | 118 | -0.067343027 | TUBA3D | tubulin alpha 3d [Source:HGNC Symbol;Acc:HGNC:24071] |
| ENSG00000075975 | HGLibA_29352 | 118 | -0.067343027 | MKRN2 | makorin ring finger protein 2 [Source:HGNC Symbol;Acc:HGNC:7113] |
| ENSG00000077080 | HGLibA_00623 | 118 | -0.067343027 | ACTL6B | actin like 6B [Source:HGNC Symbol;Acc:HGNC:160] |
| ENSG00000078687 | HGLibA_50919 | 118 | -0.067343027 | TNRC6C | trinucleotide repeat containing 6C [Source:HGNC Symbol;Acc:HGNC:29318] |
| ENSG00000089335 | HGLibA_55774 | 118 | -0.067343027 | ZNF302 | zinc finger protein 302 [Source:HGNC Symbol;Acc:HGNC:13848] |
| ENSG00000090534 | HGLibA_49386 | 118 | -0.067343027 | THPO | thrombopoietin [Source:HGNC Symbol;Acc:HGNC:11795] |
| ENSG00000090659 | HGLibA_08295 | 118 | -0.067343027 | CD209 | CD209 molecule [Source:HGNC Symbol;Acc:HGNC:1641] |
| ENSG00000090857 | HGLibA_35936 | 118 | -0.067343027 | PDPR | pyruvate dehydrogenase phosphatase regulatory subunit [Source:HGNC Symbol;Acc:HGNC:30264] |
| ENSG00000091732 | HGLibA_54997 | 118 | -0.067343027 | ZC3HC1 | zinc finger C3HC-type containing 1 [Source:HGNC Symbol;Acc:HGNC:29913] |
| ENSG00000099250 | HGLibA_32716 | 118 | -0.067343027 | NRP1 | neuropilin 1 [Source:HGNC Symbol;Acc:HGNC:8004] |
| ENSG00000100346 | HGLibA_06900 | 118 | -0.067343027 | CACNA1I | calcium voltage-gated channel subunit alpha1 I [Source:HGNC Symbol;Acc:HGNC:1396] |
| ENSG00000101082 | HGLibA_44304 | 118 | -0.067343027 | SLA2 | Src like adaptor 2 [Source:HGNC Symbol;Acc:HGNC:17329] |
| ENSG00000101391 | HGLibA_08783 | 118 | -0.067343027 | CDK5RAP1 | CDK5 regulatory subunit associated protein 1 [Source:HGNC Symbol;Acc:HGNC:15880] |
| ENSG00000102241 | HGLibA_22348 | 118 | -0.067343027 | HTATSF1 | HIV-1 Tat specific factor 1 [Source:HGNC Symbol;Acc:HGNC:5276] |
| ENSG00000102755 | HGLibA_17664 | 118 | -0.067343027 | FLT1 | fms related tyrosine kinase 1 [Source:HGNC Symbol;Acc:HGNC:3763] |
| ENSG00000102921 | HGLibA_30949 | 118 | -0.067343027 | N4BP1 | NEDD4 binding protein 1 [Source:HGNC Symbol;Acc:HGNC:29850] |
| ENSG00000103966 | HGLibA_14674 | 118 | -0.067343027 | EHD4 | EH domain containing 4 [Source:HGNC Symbol;Acc:HGNC:3245] |
| ENSG00000104826 | HGLibA_26421 | 118 | -0.067343027 | LHB | luteinizing hormone beta polypeptide [Source:HGNC Symbol;Acc:HGNC:6584] |
| ENSG00000105204 | HGLibA_14274 | 118 | -0.067343027 | DYRK1B | dual specificity tyrosine phosphorylation regulated kinase 1B [Source:HGNC Symbol;Acc:HGNC:3092] |
| ENSG00000105352 | HGLibA_08941 | 118 | -0.067343027 | CEACAM4 | carcinoembryonic antigen related cell adhesion molecule 4 [Source:HGNC Symbol;Acc:HGNC:1816] |
| ENSG00000106460 | HGLibA_49855 | 118 | -0.067343027 | TMEM106B | transmembrane protein 106B [Source:HGNC Symbol;Acc:HGNC:22407] |
| ENSG00000106538 | HGLibA_40283 | 118 | -0.067343027 | RARRES2 | retinoic acid receptor responder 2 [Source:HGNC Symbol;Acc:HGNC:9868] |
| ENSG00000107719 | HGLibA_35060 | 118 | -0.067343027 | PALD1 | phosphatase domain containing paladin 1 [Source:HGNC Symbol;Acc:HGNC:23530] |
| ENSG00000107771 | HGLibA_08191 | 118 | -0.067343027 | CCSER2 | coiled-coil serine rich protein 2 [Source:HGNC Symbol;Acc:HGNC:29197] |
| ENSG00000107833 | HGLibA_32494 | 118 | -0.067343027 | NPM3 | nucleophosmin/nucleoplasmin 3 [Source:HGNC Symbol;Acc:HGNC:7931] |
| ENSG00000108312 | HGLibA_52771 | 118 | -0.067343027 | UBTF | upstream binding transcription factor [Source:HGNC Symbol;Acc:HGNC:12511] |
| ENSG00000108523 | HGLibA_41561 | 118 | -0.067343027 | RNF167 | ring finger protein 167 [Source:HGNC Symbol;Acc:HGNC:24544] |
| ENSG00000108602 | HGLibA_01561 | 118 | -0.067343027 | ALDH3A1 | aldehyde dehydrogenase 3 family member A1 [Source:HGNC Symbol;Acc:HGNC:405] |
| ENSG00000109501 | HGLibA_54285 | 118 | -0.067343027 | WFS1 | wolframin ER transmembrane glycoprotein [Source:HGNC Symbol;Acc:HGNC:12762] |
| ENSG00000109906 | HGLibA_54826 | 118 | -0.067343027 | ZBTB16 | zinc finger and BTB domain containing 16 [Source:HGNC Symbol;Acc:HGNC:12930] |
| ENSG00000110888 | HGLibA_07193 | 118 | -0.067343027 | CAPRIN2 | caprin family member 2 [Source:HGNC Symbol;Acc:HGNC:21259] |
| ENSG00000111305 | HGLibA_20418 | 118 | -0.067343027 | GSG1 | germ cell associated 1 [Source:HGNC Symbol;Acc:HGNC:19716] |
| ENSG00000112320 | HGLibA_46119 | 118 | -0.067343027 | SOBP | sine oculis binding protein homolog [Source:HGNC Symbol;Acc:HGNC:29256] |
| ENSG00000112679 | HGLibA_14164 | 118 | -0.067343027 | DUSP22 | dual specificity phosphatase 22 [Source:HGNC Symbol;Acc:HGNC:16077] |
| ENSG00000113282 | HGLibA_10038 | 118 | -0.067343027 | CLINT1 | clathrin interactor 1 [Source:HGNC Symbol;Acc:HGNC:23186] |
| ENSG00000113525 | HGLibA_23176 | 118 | -0.067343027 | IL5 | interleukin 5 [Source:HGNC Symbol;Acc:HGNC:6016] |
| ENSG00000113649 | HGLibA_48754 | 118 | -0.067343027 | TCERG1 | transcription elongation regulator 1 [Source:HGNC Symbol;Acc:HGNC:15630] |
| ENSG00000113761 | HGLibA_55848 | 118 | -0.067343027 | ZNF346 | zinc finger protein 346 [Source:HGNC Symbol;Acc:HGNC:16403] |
| ENSG00000115425 | HGLibA_36022 | 118 | -0.067343027 | PECR | peroxisomal trans-2-enoyl-CoA reductase [Source:HGNC Symbol;Acc:HGNC:18281] |
| ENSG00000116771 | HGLibA_01198 | 118 | -0.067343027 | AGMAT | agmatinase [Source:HGNC Symbol;Acc:HGNC:18407] |
| ENSG00000117122 | HGLibA_29043 | 118 | -0.067343027 | MFAP2 | microfibril associated protein 2 [Source:HGNC Symbol;Acc:HGNC:7033] |
| ENSG00000120669 | HGLibA_46166 | 118 | -0.067343027 | SOHLH2 | spermatogenesis and oogenesis specific basic helix-loop-helix 2 [Source:HGNC Symbol;Acc:HGNC:26026] |
| ENSG00000121989 | HGLibA_00696 | 118 | -0.067343027 | ACVR2A | activin A receptor type 2A [Source:HGNC Symbol;Acc:HGNC:173] |
| ENSG00000123064 | HGLibA_12744 | 118 | -0.067343027 | DDX54 | DEAD-box helicase 54 [Source:HGNC Symbol;Acc:HGNC:20084] |
| ENSG00000124159 | HGLibA_28437 | 118 | -0.067343027 | MATN4 | matrilin 4 [Source:HGNC Symbol;Acc:HGNC:6910] |
| ENSG00000125384 | HGLibA_39316 | 118 | -0.067343027 | PTGER2 | prostaglandin E receptor 2 [Source:HGNC Symbol;Acc:HGNC:9594] |
| ENSG00000125900 | HGLibA_44211 | 118 | -0.067343027 | SIRPD | signal regulatory protein delta [Source:HGNC Symbol;Acc:HGNC:16248] |
| ENSG00000127311 | HGLibA_21112 | 118 | -0.067343027 | HELB | DNA helicase B [Source:HGNC Symbol;Acc:HGNC:17196] |
| ENSG00000128928 | HGLibA_23878 | 118 | -0.067343027 | IVD | isovaleryl-CoA dehydrogenase [Source:HGNC Symbol;Acc:HGNC:6186] |
| ENSG00000129071 | HGLibA_28486 | 118 | -0.067343027 | MBD4 | DNA glycosylase [Source:HGNC Symbol;Acc:HGNC:6919]" |
| ENSG00000130669 | HGLibA_35049 | 118 | -0.067343027 | PAK4 | p21 (RAC1) activated kinase 4 [Source:HGNC Symbol;Acc:HGNC:16059] |
| ENSG00000134308 | HGLibA_54750 | 118 | -0.067343027 | YWHAQ | tyrosine 3-monooxygenase/tryptophan 5-monooxygenase activation protein theta [Source:HGNC Symbol;Acc:HGNC:12854] |
| ENSG00000134533 | HGLibA_40859 | 118 | -0.067343027 | RERG | RAS like estrogen regulated growth inhibitor [Source:HGNC Symbol;Acc:HGNC:15980] |
| ENSG00000135773 | HGLibA_07181 | 118 | -0.067343027 | CAPN9 | calpain 9 [Source:HGNC Symbol;Acc:HGNC:1486] |
| ENSG00000137073 | HGLibA_52552 | 118 | -0.067343027 | UBAP2 | ubiquitin associated protein 2 [Source:HGNC Symbol;Acc:HGNC:14185] |
| ENSG00000137860 | HGLibA_44835 | 118 | -0.067343027 | SLC28A2 | solute carrier family 28 member 2 [Source:HGNC Symbol;Acc:HGNC:11002] |
| ENSG00000138483 | HGLibA_07801 | 118 | -0.067343027 | CCDC54 | coiled-coil domain containing 54 [Source:HGNC Symbol;Acc:HGNC:30703] |
| ENSG00000139800 | HGLibA_55334 | 118 | -0.067343027 | ZIC5 | Zic family member 5 [Source:HGNC Symbol;Acc:HGNC:20322] |
| ENSG00000141759 | HGLibA_52438 | 118 | -0.067343027 | TXNL4A | thioredoxin like 4A [Source:HGNC Symbol;Acc:HGNC:30551] |
| ENSG00000142230 | HGLibA_42633 | 118 | -0.067343027 | SAE1 | SUMO1 activating enzyme subunit 1 [Source:HGNC Symbol;Acc:HGNC:30660] |
| ENSG00000142330 | HGLibA_07145 | 118 | -0.067343027 | CAPN10 | calpain 10 [Source:HGNC Symbol;Acc:HGNC:1477] |
| ENSG00000144366 | HGLibA_20675 | 118 | -0.067343027 | GULP1 | GULP PTB domain containing engulfment adaptor 1 [Source:HGNC Symbol;Acc:HGNC:18649] |
| ENSG00000145808 | HGLibA_00819 | 118 | -0.067343027 | ADAMTS19 | ADAM metallopeptidase with thrombospondin type 1 motif 19 [Source:HGNC Symbol;Acc:HGNC:17111] |
| ENSG00000147127 | HGLibA_39942 | 118 | -0.067343027 | RAB41 | member RAS oncogene family [Source:HGNC Symbol;Acc:HGNC:18293]" |
| ENSG00000147883 | HGLibA_08839 | 118 | -0.067343027 | CDKN2B | cyclin dependent kinase inhibitor 2B [Source:HGNC Symbol;Acc:HGNC:1788] |
| ENSG00000151093 | HGLibA_34835 | 118 | -0.067343027 | OXSM | mitochondrial [Source:HGNC Symbol;Acc:HGNC:26063]" |
| ENSG00000151790 | HGLibA_48887 | 118 | -0.067343027 | TDO2 | 3-dioxygenase [Source:HGNC Symbol;Acc:HGNC:11708]" |
| ENSG00000152484 | HGLibA_53200 | 118 | -0.067343027 | USP12 | ubiquitin specific peptidase 12 [Source:HGNC Symbol;Acc:HGNC:20485] |
| ENSG00000152953 | HGLibA_47485 | 118 | -0.067343027 | STK32B | serine/threonine kinase 32B [Source:HGNC Symbol;Acc:HGNC:14217] |
| ENSG00000154493 | HGLibA_05104 | 118 | -0.067343027 | C10orf90 | chromosome 10 open reading frame 90 [Source:HGNC Symbol;Acc:HGNC:26563] |
| ENSG00000156282 | HGLibA_09868 | 118 | -0.067343027 | CLDN17 | claudin 17 [Source:HGNC Symbol;Acc:HGNC:2038] |
| ENSG00000156510 | HGLibA_21589 | 118 | -0.067343027 | HKDC1 | hexokinase domain containing 1 [Source:HGNC Symbol;Acc:HGNC:23302] |
| ENSG00000156976 | HGLibA_14807 | 118 | -0.067343027 | EIF4A2 | eukaryotic translation initiation factor 4A2 [Source:HGNC Symbol;Acc:HGNC:3284] |
| ENSG00000158901 | HGLibA_54274 | 118 | -0.067343027 | WFDC8 | WAP four-disulfide core domain 8 [Source:HGNC Symbol;Acc:HGNC:16163] |
| ENSG00000160224 | HGLibA_01366 | 118 | -0.067343027 | AIRE | autoimmune regulator [Source:HGNC Symbol;Acc:HGNC:360] |
| ENSG00000160293 | HGLibA_53551 | 118 | -0.067343027 | VAV2 | vav guanine nucleotide exchange factor 2 [Source:HGNC Symbol;Acc:HGNC:12658] |
| ENSG00000160570 | HGLibA_12784 | 118 | -0.067343027 | DEDD2 | death effector domain containing 2 [Source:HGNC Symbol;Acc:HGNC:24450] |
| ENSG00000160886 | HGLibA_27678 | 118 | -0.067343027 | LY6K | lymphocyte antigen 6 family member K [Source:HGNC Symbol;Acc:HGNC:24225] |
| ENSG00000162627 | HGLibA_46104 | 118 | -0.067343027 | SNX7 | sorting nexin 7 [Source:HGNC Symbol;Acc:HGNC:14971] |
| ENSG00000163288 | HGLibA_18373 | 118 | -0.067343027 | GABRB1 | gamma-aminobutyric acid type A receptor beta1 subunit [Source:HGNC Symbol;Acc:HGNC:4081] |
| ENSG00000163964 | HGLibA_36600 | 118 | -0.067343027 | PIGX | phosphatidylinositol glycan anchor biosynthesis class X [Source:HGNC Symbol;Acc:HGNC:26046] |
| ENSG00000164542 | HGLibA_24658 | 118 | -0.067343027 | KIAA0895 | KIAA0895 [Source:HGNC Symbol;Acc:HGNC:22206] |
| ENSG00000164764 | HGLibA_42814 | 118 | -0.067343027 | SBSPON | somatomedin B and thrombospondin type 1 domain containing [Source:HGNC Symbol;Acc:HGNC:30362] |
| ENSG00000165124 | HGLibA_47884 | 118 | -0.067343027 | SVEP1 | EGF and pentraxin domain containing 1 [Source:HGNC Symbol;Acc:HGNC:15985]" |
| ENSG00000165525 | HGLibA_31688 | 118 | -0.067343027 | NEMF | nuclear export mediator factor [Source:HGNC Symbol;Acc:HGNC:10663] |

| | | | | | |
|---|---|---|---|---|---|
| ENSG00000166532 | HGLibA_41274 | 118 | -0.067343027 | RIMKLB | ribosomal modification protein rimK like family member B [Source:HGNC Symbol;Acc:HGNC:29228] |
| ENSG00000168071 | HGLibA_07909 | 118 | -0.067343027 | CCDC88B | coiled-coil domain containing 88B [Source:HGNC Symbol;Acc:HGNC:26757] |
| ENSG00000168476 | HGLibA_40786 | 118 | -0.067343027 | REEP4 | receptor accessory protein 4 [Source:HGNC Symbol;Acc:HGNC:26176] |
| ENSG00000168734 | HGLibA_36826 | 118 | -0.067343027 | PKIG | cAMP-dependent protein kinase inhibitor gamma [Source:HGNC Symbol;Acc:HGNC:9019] |
| ENSG00000169169 | HGLibA_11023 | 118 | -0.067343027 | CPT1C | carnitine palmitoyltransferase 1C [Source:HGNC Symbol;Acc:HGNC:18540] |
| ENSG00000171124 | HGLibA_18192 | 118 | -0.067343027 | FUT3 | fucosyltransferase 3 (Lewis blood group) [Source:HGNC Symbol;Acc:HGNC:4014] |
| ENSG00000171368 | HGLibA_51166 | 118 | -0.067343027 | TPPP | tubulin polymerization promoting protein [Source:HGNC Symbol;Acc:HGNC:24164] |
| ENSG00000172020 | HGLibA_18611 | 118 | -0.067343027 | GAP43 | growth associated protein 43 [Source:HGNC Symbol;Acc:HGNC:4140] |
| ENSG00000174951 | HGLibA_18180 | 118 | -0.067343027 | FUT1 | fucosyltransferase 1 (H blood group) [Source:HGNC Symbol;Acc:HGNC:4012] |
| ENSG00000176148 | HGLibA_48846 | 118 | -0.067343027 | TCP11L1 | t-complex 11 like 1 [Source:HGNC Symbol;Acc:HGNC:25655] |
| ENSG00000176555 | HGLibA_34062 | 118 | -0.067343027 | OR4S1 | olfactory receptor family 4 subfamily S member 1 [Source:HGNC Symbol;Acc:HGNC:14705] |
| ENSG00000176635 | HGLibA_21890 | 118 | -0.067343027 | HORMAD2 | HORMA domain containing 2 [Source:HGNC Symbol;Acc:HGNC:28383] |
| ENSG00000177212 | HGLibA_33874 | 118 | -0.067343027 | OR2T33 | olfactory receptor family 2 subfamily T member 33 [Source:HGNC Symbol;Acc:HGNC:31255] |
| ENSG00000178522 | HGLibA_01748 | 118 | -0.067343027 | AMBN | ameloblastin [Source:HGNC Symbol;Acc:HGNC:452] |
| ENSG00000179841 | HGLibA_01435 | 118 | -0.067343027 | AKAP5 | A-kinase anchoring protein 5 [Source:HGNC Symbol;Acc:HGNC:375] |
| ENSG00000182870 | HGLibA_18574 | 118 | -0.067343027 | GALNT9 | polypeptide N-acetylgalactosaminyltransferase 9 [Source:HGNC Symbol;Acc:HGNC:4131] |
| ENSG00000183246 | HGLibA_41269 | 118 | -0.067343027 | RIMBP3C | RIMS binding protein 3C [Source:HGNC Symbol;Acc:HGNC:33892] |
| ENSG00000184182 | HGLibA_52602 | 118 | -0.067343027 | UBE2F | ubiquitin conjugating enzyme E2 F (putative) [Source:HGNC Symbol;Acc:HGNC:12480] |
| ENSG00000184203 | HGLibA_38075 | 118 | -0.067343027 | PPP1R2 | protein phosphatase 1 regulatory inhibitor subunit 2 [Source:HGNC Symbol;Acc:HGNC:9288] |
| ENSG00000184956 | HGLibA_30553 | 118 | -0.067343027 | MUC6 | oligomeric mucus/gel-forming [Source:HGNC Symbol;Acc:HGNC:7517]" |
| ENSG00000185156 | HGLibA_29112 | 118 | -0.067343027 | MFSD6L | major facilitator superfamily domain containing 6 like [Source:HGNC Symbol;Acc:HGNC:26656] |
| ENSG00000186818 | HGLibA_26515 | 118 | -0.067343027 | LILRB4 | leukocyte immunoglobulin like receptor B4 [Source:HGNC Symbol;Acc:HGNC:6608] |
| ENSG00000187257 | HGLibA_42306 | 118 | -0.067343027 | RSBN1L | round spermatid basic protein 1 like [Source:HGNC Symbol;Acc:HGNC:24765] |
| ENSG00000188517 | HGLibA_10552 | 118 | -0.067343027 | COL25A1 | collagen type XXV alpha 1 chain [Source:HGNC Symbol;Acc:HGNC:18603] |
| ENSG00000189339 | HGLibA_44991 | 118 | -0.067343027 | SLC35E2B | solute carrier family 35 member E2B [Source:HGNC Symbol;Acc:HGNC:33941] |
| ENSG00000189409 | HGLibA_29516 | 118 | -0.067343027 | MMP23B | matrix metallopeptidase 23B [Source:HGNC Symbol;Acc:HGNC:7171] |
| ENSG00000196704 | HGLibA_01858 | 118 | -0.067343027 | AMZ2 | archaelysin family metallopeptidase 2 [Source:HGNC Symbol;Acc:HGNC:28041] |
| ENSG00000197343 | HGLibA_56471 | 118 | -0.067343027 | ZNF655 | zinc finger protein 655 [Source:HGNC Symbol;Acc:HGNC:30899] |
| ENSG00000197437 | HGLibA_33620 | 118 | -0.067343027 | OR13G1 | olfactory receptor family 13 subfamily G member 1 [Source:HGNC Symbol;Acc:HGNC:14999] |
| ENSG00000197915 | HGLibA_22110 | 118 | -0.067343027 | HRNR | hornerin [Source:HGNC Symbol;Acc:HGNC:20846] |
| ENSG00000198168 | HGLibA_47890 | 118 | -0.067343027 | SVIP | small VCP interacting protein [Source:HGNC Symbol;Acc:HGNC:25238] |
| ENSG00000198373 | HGLibA_54454 | 118 | -0.067343027 | WWP2 | WW domain containing E3 ubiquitin protein ligase 2 [Source:HGNC Symbol;Acc:HGNC:16804] |
| ENSG00000198722 | HGLibA_52997 | 118 | -0.067343027 | UNC13B | unc-13 homolog B [Source:HGNC Symbol;Acc:HGNC:12566] |
| ENSG00000198765 | HGLibA_47927 | 118 | -0.067343027 | SYCP1 | synaptonemal complex protein 1 [Source:HGNC Symbol;Acc:HGNC:11487] |
| ENSG00000198854 | HGLibA_05892 | 118 | -0.067343027 | C1orf68 | chromosome 1 open reading frame 68 [Source:HGNC Symbol;Acc:HGNC:29468] |
| ENSG00000204525 | HGLibA_21601 | 118 | -0.067343027 | HLA-C | C [Source:HGNC Symbol;Acc:HGNC:4933]" |
| ENSG00000205155 | HGLibA_39031 | 118 | -0.067343027 | PSENEN | gamma-secretase subunit [Source:HGNC Symbol;Acc:HGNC:30100]" |
| ENSG00000205856 | HGLibA_06076 | 118 | -0.067343027 | C22orf42 | chromosome 22 open reading frame 42 [Source:HGNC Symbol;Acc:HGNC:27160] |
| ENSG00000213463 | HGLibA_48009 | 118 | -0.067343027 | SYNJ2BP | synaptojanin 2 binding protein [Source:HGNC Symbol;Acc:HGNC:18955] |
| ENSG00000213638 | HGLibA_00884 | 118 | -0.067343027 | ADAT3 | tRNA specific 3 [Source:HGNC Symbol;Acc:HGNC:25151]" |
| ENSG00000221864 | HGLibA_25633 | 118 | -0.067343027 | KRTAP12-2 | keratin associated protein 12-2 [Source:HGNC Symbol;Acc:HGNC:20530] |
| ENSG00000228567 | HGLibA_53705 | 118 | -0.067343027 | VN1R4 | vomeronasal 1 receptor 4 [Source:HGNC Symbol;Acc:HGNC:19871] |
| ENSG00000240505 | HGLibA_50753 | 118 | -0.067343027 | TNFRSF13B | TNF receptor superfamily member 13B [Source:HGNC Symbol;Acc:HGNC:18153] |
| ENSG00000249709 | HGLibA_56253 | 118 | -0.067343027 | ZNF564 | zinc finger protein 564 [Source:HGNC Symbol;Acc:HGNC:31106] |
| ENSG00000251369 | HGLibA_56211 | 118 | -0.067343027 | ZNF550 | zinc finger protein 550 [Source:HGNC Symbol;Acc:HGNC:28643] |
| ENSG00000255423 | HGLibA_14366 | 118 | -0.067343027 | EBLN2 | endogenous Bornavirus like nucleoprotein 2 [Source:HGNC Symbol;Acc:HGNC:25493] |
| ENSG00000257138 | HGLibA_48394 | 118 | -0.067343027 | TAS2R38 | taste 2 receptor member 38 [Source:HGNC Symbol;Acc:HGNC:9584] |
| ENSG00000269897 | HGLibA_10661 | 118 | -0.067343027 | COMMD3-BMI1 | COMMD3-BMI1 readthrough [Source:HGNC Symbol;Acc:HGNC:48326] |
| ENSG00000288867 | HGLibA_37903 | 118 | -0.067343027 | PPIAL4C | - |
| HIST1H4E | HGLibA_21499 | 118 | -0.067343027 | HIST1H4E | - |
| HIST3H2A | HGLibA_21556 | 118 | -0.067343027 | HIST3H2A | - |
| hsa-mir-30b | HGLibA_58477 | 118 | -0.067343027 | hsa-mir-30b | - |
| hsa-mir-4253 | HGLibA_59719 | 118 | -0.067343027 | hsa-mir-4253 | - |
| hsa-mir-431 | HGLibA_59950 | 118 | -0.067343027 | hsa-mir-431 | - |
| hsa-mir-4417 | HGLibA_60051 | 118 | -0.067343027 | hsa-mir-4417 | - |
| hsa-mir-4441 | HGLibA_60168 | 118 | -0.067343027 | hsa-mir-4441 | - |
| hsa-mir-4691 | HGLibA_60873 | 118 | -0.067343027 | hsa-mir-4691 | - |
| hsa-mir-4710 | HGLibA_60940 | 118 | -0.067343027 | hsa-mir-4710 | - |
| hsa-mir-4773-2 | HGLibA_61195 | 118 | -0.067343027 | hsa-mir-4773-2 | - |
| hsa-mir-4790 | HGLibA_61266 | 118 | -0.067343027 | hsa-mir-4790 | - |
| hsa-mir-5100 | HGLibA_61551 | 118 | -0.067343027 | hsa-mir-5100 | - |
| hsa-mir-514a-3 | HGLibA_61593 | 118 | -0.067343027 | hsa-mir-514a-3 | - |
| hsa-mir-610 | HGLibA_62632 | 118 | -0.067343027 | hsa-mir-610 | - |
| hsa-mir-612 | HGLibA_62639 | 118 | -0.067343027 | hsa-mir-612 | - |
| hsa-mir-6124 | HGLibA_62645 | 118 | -0.067343027 | hsa-mir-6124 | - |
| hsa-mir-657 | HGLibA_62960 | 118 | -0.067343027 | hsa-mir-657 | - |
| hsa-mir-6748 | HGLibA_63145 | 118 | -0.067343027 | hsa-mir-6748 | - |
| hsa-mir-6786 | HGLibA_63323 | 118 | -0.067343027 | hsa-mir-6786 | - |
| hsa-mir-7158 | HGLibA_63859 | 118 | -0.067343027 | hsa-mir-7158 | - |
| LOC100144595 | HGLibA_26814 | 118 | -0.067343027 | LOC100144595 | - |
| PALM2 | HGLibA_35071 | 118 | -0.067343027 | PALM2 | - |
| SOGA2 | HGLibA_46157 | 118 | -0.067343027 | SOGA2 | - |
| TXNRD3NB | HGLibA_52453 | 118 | -0.067343027 | TXNRD3NB | - |
| C16orf45 | HGLibA_05448 | 117 | -0.07638837 | C16orf45 | - |
| C2orf48 | HGLibA_06137 | 117 | -0.07638837 | C2orf48 | - |
| C2orf53 | HGLibA_06147 | 117 | -0.07638837 | C2orf53 | - |
| CCDC101 | HGLibA_07505 | 117 | -0.07638837 | CCDC101 | - |
| ENSG00000008226 | HGLibA_13317 | 117 | -0.07638837 | DLEC1 | cilia and flagella associated protein [Source:HGNC Symbol;Acc:HGNC:2899]" |
| ENSG00000022840 | HGLibA_41455 | 117 | -0.07638837 | RNF10 | ring finger protein 10 [Source:HGNC Symbol;Acc:HGNC:10055] |
| ENSG00000028310 | HGLibA_04788 | 117 | -0.07638837 | BRD9 | bromodomain containing 9 [Source:HGNC Symbol;Acc:HGNC:25818] |
| ENSG00000036054 | HGLibA_48509 | 117 | -0.07638837 | TBC1D23 | TBC1 domain family member 23 [Source:HGNC Symbol;Acc:HGNC:25622] |
| ENSG00000054219 | HGLibA_27680 | 117 | -0.07638837 | LY75 | lymphocyte antigen 75 [Source:HGNC Symbol;Acc:HGNC:6729] |
| ENSG00000054796 | HGLibA_46741 | 117 | -0.07638837 | SPO11 | initiator of meiotic double stranded breaks [Source:HGNC Symbol;Acc:HGNC:11250]" |
| ENSG00000060069 | HGLibA_11606 | 117 | -0.07638837 | CTDP1 | CTD phosphatase subunit 1 [Source:HGNC Symbol;Acc:HGNC:2498] |
| ENSG00000065665 | HGLibA_43251 | 117 | -0.07638837 | SEC61A2 | Sec61 translocon alpha 2 subunit [Source:HGNC Symbol;Acc:HGNC:17702] |
| ENSG00000070718 | HGLibA_02333 | 117 | -0.07638837 | AP3M2 | adaptor related protein complex 3 subunit mu 2 [Source:HGNC Symbol;Acc:HGNC:570] |
| ENSG00000071203 | HGLibA_30132 | 117 | -0.07638837 | MS4A12 | membrane spanning 4-domains A12 [Source:HGNC Symbol;Acc:HGNC:13370] |
| ENSG00000073060 | HGLibA_42870 | 117 | -0.07638837 | SCARB1 | scavenger receptor class B member 1 [Source:HGNC Symbol;Acc:HGNC:1664] |
| ENSG00000081800 | HGLibA_44383 | 117 | -0.07638837 | SLC13A1 | solute carrier family 13 member 1 [Source:HGNC Symbol;Acc:HGNC:10916] |
| ENSG00000083720 | HGLibA_34812 | 117 | -0.07638837 | OXCT1 | 3-oxoacid CoA-transferase 1 [Source:HGNC Symbol;Acc:HGNC:8527] |
| ENSG00000086102 | HGLibA_31849 | 117 | -0.07638837 | NFX1 | X-box binding 1 [Source:HGNC Symbol;Acc:HGNC:7803]" |
| ENSG00000086548 | HGLibA_08948 | 117 | -0.07638837 | CEACAM6 | carcinoembryonic antigen related cell adhesion molecule 6 [Source:HGNC Symbol;Acc:HGNC:1818] |
| ENSG00000091128 | HGLibA_25966 | 117 | -0.07638837 | LAMB4 | laminin subunit beta 4 [Source:HGNC Symbol;Acc:HGNC:6491] |
| ENSG00000096384 | HGLibA_22252 | 117 | -0.07638837 | HSP90AB1 | heat shock protein 90 alpha family class B member 1 [Source:HGNC Symbol;Acc:HGNC:5258] |
| ENSG00000099377 | HGLibA_22212 | 117 | -0.07638837 | HSD3B7 | 3 beta- and steroid delta-isomerase 7 [Source:HGNC Symbol;Acc:HGNC:18324]" |
| ENSG00000100012 | HGLibA_43197 | 117 | -0.07638837 | SEC14L3 | SEC14 like lipid binding 3 [Source:HGNC Symbol;Acc:HGNC:18655] |
| ENSG00000100095 | HGLibA_43681 | 117 | -0.07638837 | SEZ6L | seizure related 6 homolog like [Source:HGNC Symbol;Acc:HGNC:10763] |
| ENSG00000101782 | HGLibA_41313 | 117 | -0.07638837 | RIOK3 | RIO kinase 3 [Source:HGNC Symbol;Acc:HGNC:11451] |
| ENSG00000101842 | HGLibA_53836 | 117 | -0.07638837 | VSIG1 | V-set and immunoglobulin domain containing 1 [Source:HGNC Symbol;Acc:HGNC:28675] |
| ENSG00000102471 | HGLibA_31395 | 117 | -0.07638837 | NDFIP2 | Nedd4 family interacting protein 2 [Source:HGNC Symbol;Acc:HGNC:18537] |
| ENSG00000102683 | HGLibA_43809 | 117 | -0.07638837 | SGCG | sarcoglycan gamma [Source:HGNC Symbol;Acc:HGNC:10809] |
| ENSG00000104356 | HGLibA_37687 | 117 | -0.07638837 | POP1 | ribonuclease P/MRP subunit [Source:HGNC Symbol;Acc:HGNC:30129]" |
| ENSG00000104853 | HGLibA_10114 | 117 | -0.07638837 | CLPTM1 | transmembrane protein [Source:HGNC Symbol;Acc:HGNC:2087]" |
| ENSG00000105185 | HGLibA_35735 | 117 | -0.07638837 | PDCD5 | programmed cell death 5 [Source:HGNC Symbol;Acc:HGNC:8764] |
| ENSG00000105605 | HGLibA_06947 | 117 | -0.07638837 | CACNG7 | calcium voltage-gated channel auxiliary subunit gamma 7 [Source:HGNC Symbol;Acc:HGNC:13626] |
| ENSG00000105877 | HGLibA_13475 | 117 | -0.07638837 | DNAH11 | dynein axonemal heavy chain 11 [Source:HGNC Symbol;Acc:HGNC:2942] |
| ENSG00000106351 | HGLibA_01186 | 117 | -0.07638837 | AGFG2 | ArfGAP with FG repeats 2 [Source:HGNC Symbol;Acc:HGNC:5177] |
| ENSG00000108219 | HGLibA_51904 | 117 | -0.07638837 | TSPAN14 | tetraspanin 14 [Source:HGNC Symbol;Acc:HGNC:23303] |
| ENSG00000108588 | HGLibA_07791 | 117 | -0.07638837 | CCDC47 | coiled-coil domain containing 47 [Source:HGNC Symbol;Acc:HGNC:24856] |
| ENSG00000110057 | HGLibA_53039 | 117 | -0.07638837 | UNC93B1 | TLR signaling regulator [Source:HGNC Symbol;Acc:HGNC:13481]" |
| ENSG00000111785 | HGLibA_41239 | 117 | -0.07638837 | RIC8B | RIC8 guanine nucleotide exchange factor B [Source:HGNC Symbol;Acc:HGNC:25555] |
| ENSG00000111877 | HGLibA_28627 | 117 | -0.07638837 | MCM9 | minichromosome maintenance 9 homologous recombination repair factor [Source:HGNC Symbol;Acc:HGNC:21484] |
| ENSG00000112539 | HGLibA_06437 | 117 | -0.07638837 | C6orf118 | chromosome 6 open reading frame 118 [Source:HGNC Symbol;Acc:HGNC:21233] |
| ENSG00000113716 | HGLibA_21731 | 117 | -0.07638837 | HMGXB3 | HMG-box containing 3 [Source:HGNC Symbol;Acc:HGNC:28982] |
| ENSG00000114626 | HGLibA_00336 | 117 | -0.07638837 | ABTB1 | ankyrin repeat and BTB domain containing 1 [Source:HGNC Symbol;Acc:HGNC:18275] |
| ENSG00000115594 | HGLibA_23047 | 117 | -0.07638837 | IL1R1 | interleukin 1 receptor type 1 [Source:HGNC Symbol;Acc:HGNC:5993] |
| ENSG00000115649 | HGLibA_10360 | 117 | -0.07638837 | CNPPD1 | cyclin Pas1/PHO80 domain containing 1 [Source:HGNC Symbol;Acc:HGNC:25220] |
| ENSG00000115944 | HGLibA_10863 | 117 | -0.07638837 | COX7A2L | cytochrome c oxidase subunit 7A2 like [Source:HGNC Symbol;Acc:HGNC:2289] |
| ENSG00000117602 | HGLibA_40673 | 117 | -0.07638837 | RCAN3 | RCAN family member 3 [Source:HGNC Symbol;Acc:HGNC:3042] |
| ENSG00000120008 | HGLibA_54026 | 117 | -0.07638837 | WDR11 | WD repeat domain 11 [Source:HGNC Symbol;Acc:HGNC:13831] |
| ENSG00000122359 | HGLibA_02225 | 117 | -0.07638837 | ANXA11 | annexin A11 [Source:HGNC Symbol;Acc:HGNC:535] |
| ENSG00000122507 | HGLibA_04228 | 117 | -0.07638837 | BBS9 | Bardet-Biedl syndrome 9 [Source:HGNC Symbol;Acc:HGNC:30000] |
| ENSG00000123171 | HGLibA_07851 | 117 | -0.07638837 | CCDC70 | coiled-coil domain containing 70 [Source:HGNC Symbol;Acc:HGNC:25303] |
| ENSG00000124466 | HGLibA_27720 | 117 | -0.07638837 | LYPD3 | LY6/PLAUR domain containing 3 [Source:HGNC Symbol;Acc:HGNC:24880] |
| ENSG00000127540 | HGLibA_53108 | 117 | -0.07638837 | UQCR11 | complex III subunit XI [Source:HGNC Symbol;Acc:HGNC:30862]" |
| ENSG00000127804 | HGLibA_28965 | 117 | -0.07638837 | METTL16 | methyltransferase like 16 [Source:HGNC Symbol;Acc:HGNC:28484] |
| ENSG00000129009 | HGLibA_23670 | 117 | -0.07638837 | ISLR | immunoglobulin superfamily containing leucine rich repeat [Source:HGNC Symbol;Acc:HGNC:6133] |
| ENSG00000130775 | HGLibA_49352 | 117 | -0.07638837 | THEMIS2 | thymocyte selection associated family member 2 [Source:HGNC Symbol;Acc:HGNC:16839] |
| ENSG00000131849 | HGLibA_55462 | 117 | -0.07638837 | ZNF132 | zinc finger protein 132 [Source:HGNC Symbol;Acc:HGNC:12916] |
| ENSG00000135521 | HGLibA_27618 | 117 | -0.07638837 | LTV1 | LTV1 ribosome biogenesis factor [Source:HGNC Symbol;Acc:HGNC:21173] |
| ENSG00000135966 | HGLibA_49236 | 117 | -0.07638837 | TGFBRAP1 | transforming growth factor beta receptor associated protein 1 [Source:HGNC Symbol;Acc:HGNC:16836] |
| ENSG00000136010 | HGLibA_01557 | 117 | -0.07638837 | ALDH1L2 | aldehyde dehydrogenase 1 family member L2 [Source:HGNC Symbol;Acc:HGNC:26777] |
| ENSG00000137441 | HGLibA_17429 | 117 | -0.07638837 | FGFBP2 | fibroblast growth factor binding protein 2 [Source:HGNC Symbol;Acc:HGNC:29451] |
| ENSG00000138166 | HGLibA_14183 | 117 | -0.07638837 | DUSP5 | dual specificity phosphatase 5 [Source:HGNC Symbol;Acc:HGNC:3071] |
| ENSG00000139318 | HGLibA_14187 | 117 | -0.07638837 | DUSP6 | dual specificity phosphatase 6 [Source:HGNC Symbol;Acc:HGNC:3072] |

| | | | | | |
|---|---|---|---|---|---|
| ENSG00000139597 | HGLibA_30956 | 117 | -0.07638837 | N4BP2L1 | NEDD4 binding protein 2 like 1 [Source:HGNC Symbol;Acc:HGNC:25037] |
| ENSG00000141294 | HGLibA_27367 | 117 | -0.07638837 | LRRC46 | leucine rich repeat containing 46 [Source:HGNC Symbol;Acc:HGNC:25047] |
| ENSG00000143149 | HGLibA_01588 | 117 | -0.07638837 | ALDH9A1 | aldehyde dehydrogenase 9 family member A1 [Source:HGNC Symbol;Acc:HGNC:412] |
| ENSG00000143294 | HGLibA_38353 | 117 | -0.07638837 | PRCC | proline rich mitotic checkpoint control factor [Source:HGNC Symbol;Acc:HGNC:9343] |
| ENSG00000144711 | HGLibA_23540 | 117 | -0.07638837 | IQSEC1 | IQ motif and Sec7 domain 1 [Source:HGNC Symbol;Acc:HGNC:29112] |
| ENSG00000146910 | HGLibA_10363 | 117 | -0.07638837 | CNPY1 | canopy FGF signaling regulator 1 [Source:HGNC Symbol;Acc:HGNC:27786] |
| ENSG00000147439 | HGLibA_04511 | 117 | -0.07638837 | BIN3 | bridging integrator 3 [Source:HGNC Symbol;Acc:HGNC:1054] |
| ENSG00000147573 | HGLibA_51546 | 117 | -0.07638837 | TRIM55 | tripartite motif containing 55 [Source:HGNC Symbol;Acc:HGNC:14215] |
| ENSG00000148541 | HGLibA_16148 | 117 | -0.07638837 | FAM13C | family with sequence similarity 13 member C [Source:HGNC Symbol;Acc:HGNC:19371] |
| ENSG00000151414 | HGLibA_31662 | 117 | -0.07638837 | NEK7 | NIMA related kinase 7 [Source:HGNC Symbol;Acc:HGNC:13386] |
| ENSG00000152104 | HGLibA_39443 | 117 | -0.07638837 | PTPN14 | non-receptor type 14 [Source:HGNC Symbol;Acc:HGNC:9647]" |
| ENSG00000152214 | HGLibA_41341 | 117 | -0.07638837 | RIT2 | Ras like without CAAX 2 [Source:HGNC Symbol;Acc:HGNC:10017] |
| ENSG00000152315 | HGLibA_24302 | 117 | -0.07638837 | KCNK13 | potassium two pore domain channel subfamily K member 13 [Source:HGNC Symbol;Acc:HGNC:6275] |
| ENSG00000152767 | HGLibA_16875 | 117 | -0.07638837 | FARP1 | ARH/RhoGEF and pleckstrin domain protein 1 [Source:HGNC Symbol;Acc:HGNC:3591]" |
| ENSG00000154928 | HGLibA_15310 | 117 | -0.07638837 | EPHB1 | EPH receptor B1 [Source:HGNC Symbol;Acc:HGNC:3392] |
| ENSG00000156482 | HGLibA_41935 | 117 | -0.07638837 | RPL30 | ribosomal protein L30 [Source:HGNC Symbol;Acc:HGNC:10333] |
| ENSG00000157350 | HGLibA_47241 | 117 | -0.07638837 | ST3GAL2 | 3-sialyltransferase 2 [Source:HGNC Symbol;Acc:HGNC:10863]" |
| ENSG00000157765 | HGLibA_44941 | 117 | -0.07638837 | SLC34A2 | solute carrier family 34 member 2 [Source:HGNC Symbol;Acc:HGNC:11020] |
| ENSG00000158158 | HGLibA_10323 | 117 | -0.07638837 | CNNM4 | cyclin and CBS domain divalent metal cation transport mediator 4 [Source:HGNC Symbol;Acc:HGNC:105] |
| ENSG00000158796 | HGLibA_12781 | 117 | -0.07638837 | DEDD | death effector domain containing [Source:HGNC Symbol;Acc:HGNC:2755] |
| ENSG00000160007 | HGLibA_02764 | 117 | -0.07638837 | ARHGAP35 | Rho GTPase activating protein 35 [Source:HGNC Symbol;Acc:HGNC:4591] |
| ENSG00000160298 | HGLibA_06042 | 117 | -0.07638837 | C21orf58 | chromosome 21 open reading frame 58 [Source:HGNC Symbol;Acc:HGNC:1300] |
| ENSG00000160877 | HGLibA_31039 | 117 | -0.07638837 | NACC1 | nucleus accumbens associated 1 [Source:HGNC Symbol;Acc:HGNC:20967] |
| ENSG00000160917 | HGLibA_11006 | 117 | -0.07638837 | CPSF4 | cleavage and polyadenylation specific factor 4 [Source:HGNC Symbol;Acc:HGNC:2327] |
| ENSG00000161544 | HGLibA_12037 | 117 | -0.07638837 | CYGB | cytoglobin [Source:HGNC Symbol;Acc:HGNC:16505] |
| ENSG00000163166 | HGLibA_23885 | 117 | -0.07638837 | IWS1 | SUPT6H interacting protein [Source:HGNC Symbol;Acc:HGNC:25467]" |
| ENSG00000163611 | HGLibA_46661 | 117 | -0.07638837 | SPICE1 | spindle and centriole associated protein 1 [Source:HGNC Symbol;Acc:HGNC:25083] |
| ENSG00000163811 | HGLibA_54090 | 117 | -0.07638837 | WDR43 | WD repeat domain 43 [Source:HGNC Symbol;Acc:HGNC:28945] |
| ENSG00000164304 | HGLibA_06983 | 117 | -0.07638837 | CAGE1 | cancer antigen 1 [Source:HGNC Symbol;Acc:HGNC:21622] |
| ENSG00000164850 | HGLibA_19795 | 117 | -0.07638837 | GPER1 | G protein-coupled estrogen receptor 1 [Source:HGNC Symbol;Acc:HGNC:4485] |
| ENSG00000164932 | HGLibA_11628 | 117 | -0.07638837 | CTHRC1 | collagen triple helix repeat containing 1 [Source:HGNC Symbol;Acc:HGNC:18831] |
| ENSG00000165527 | HGLibA_02658 | 117 | -0.07638837 | ARF6 | ADP ribosylation factor 6 [Source:HGNC Symbol;Acc:HGNC:659] |
| ENSG00000166160 | HGLibA_33428 | 117 | -0.07638837 | OPN1MW2 | medium wave sensitive 2 [Source:HGNC Symbol;Acc:HGNC:26952]" |
| ENSG00000166529 | HGLibA_57002 | 117 | -0.07638837 | ZSCAN21 | zinc finger and SCAN domain containing 21 [Source:HGNC Symbol;Acc:HGNC:13104] |
| ENSG00000167535 | HGLibA_06922 | 117 | -0.07638837 | CACNB3 | calcium voltage-gated channel auxiliary subunit beta 3 [Source:HGNC Symbol;Acc:HGNC:1403] |
| ENSG00000168291 | HGLibA_35867 | 117 | -0.07638837 | PDHB | pyruvate dehydrogenase E1 beta subunit [Source:HGNC Symbol;Acc:HGNC:8808] |
| ENSG00000168481 | HGLibA_26399 | 117 | -0.07638837 | LGI3 | leucine rich repeat LGI family member 3 [Source:HGNC Symbol;Acc:HGNC:18711] |
| ENSG00000168481 | HGLibA_26400 | 117 | -0.07638837 | LGI3 | leucine rich repeat LGI family member 3 [Source:HGNC Symbol;Acc:HGNC:18711] |
| ENSG00000168679 | HGLibA_44442 | 117 | -0.07638837 | SLC16A4 | solute carrier family 16 member 4 [Source:HGNC Symbol;Acc:HGNC:10925] |
| ENSG00000168806 | HGLibA_26163 | 117 | -0.07638837 | LCMT2 | leucine carboxyl methyltransferase 2 [Source:HGNC Symbol;Acc:HGNC:17558] |
| ENSG00000169826 | HGLibA_11347 | 117 | -0.07638837 | CSGALNACT2 | chondroitin sulfate N-acetylgalactosaminyltransferase 2 [Source:HGNC Symbol;Acc:HGNC:24292] |
| ENSG00000169860 | HGLibA_34867 | 117 | -0.07638837 | P2RY1 | purinergic receptor P2Y1 [Source:HGNC Symbol;Acc:HGNC:8539] |
| ENSG00000170417 | HGLibA_50102 | 117 | -0.07638837 | TMEM182 | transmembrane protein 182 [Source:HGNC Symbol;Acc:HGNC:26391] |
| ENSG00000170525 | HGLibA_36158 | 117 | -0.07638837 | PFKFB3 | 6-biphosphatase 3 [Source:HGNC Symbol;Acc:HGNC:8874]" |
| ENSG00000171954 | HGLibA_12191 | 117 | -0.07638837 | CYP4F22 | cytochrome P450 family 4 subfamily F member 22 [Source:HGNC Symbol;Acc:HGNC:26820] |
| ENSG00000171954 | HGLibA_12190 | 117 | -0.07638837 | CYP4F22 | cytochrome P450 family 4 subfamily F member 22 [Source:HGNC Symbol;Acc:HGNC:26820] |
| ENSG00000172155 | HGLibA_26109 | 117 | -0.07638837 | LCE1D | late cornified envelope 1D [Source:HGNC Symbol;Acc:HGNC:29465] |
| ENSG00000172260 | HGLibA_31626 | 117 | -0.07638837 | NEGR1 | neuronal growth regulator 1 [Source:HGNC Symbol;Acc:HGNC:17302] |
| ENSG00000173706 | HGLibA_21107 | 117 | -0.07638837 | HEG1 | heart development protein with EGF like domains 1 [Source:HGNC Symbol;Acc:HGNC:29227] |
| ENSG00000174844 | HGLibA_13478 | 117 | -0.07638837 | DNAH12 | dynein axonemal heavy chain 12 [Source:HGNC Symbol;Acc:HGNC:2943] |
| ENSG00000175785 | HGLibA_38488 | 117 | -0.07638837 | PRIMA1 | proline rich membrane anchor 1 [Source:HGNC Symbol;Acc:HGNC:18319] |
| ENSG00000176014 | HGLibA_52290 | 117 | -0.07638837 | TUBB6 | tubulin beta 6 class V [Source:HGNC Symbol;Acc:HGNC:20776] |
| ENSG00000176567 | HGLibA_34068 | 117 | -0.07638837 | OR4X1 | olfactory receptor family 4 subfamily X member 1 (gene/pseudogene) [Source:HGNC Symbol;Acc:HGNC:14854] |
| ENSG00000176915 | HGLibA_01975 | 117 | -0.07638837 | ANKLE2 | ankyrin repeat and LEM domain containing 2 [Source:HGNC Symbol;Acc:HGNC:29101] |
| ENSG00000176979 | HGLibA_51564 | 117 | -0.07638837 | TRIM60 | tripartite motif containing 60 [Source:HGNC Symbol;Acc:HGNC:21162] |
| ENSG00000179091 | HGLibA_12024 | 117 | -0.07638837 | CYC1 | cytochrome c1 [Source:HGNC Symbol;Acc:HGNC:2579] |
| ENSG00000179409 | HGLibA_18914 | 117 | -0.07638837 | GEMIN4 | gem nuclear organelle associated protein 4 [Source:HGNC Symbol;Acc:HGNC:15717] |
| ENSG00000181472 | HGLibA_54835 | 117 | -0.07638837 | ZBTB2 | zinc finger and BTB domain containing 2 [Source:HGNC Symbol;Acc:HGNC:20868] |
| ENSG00000181626 | HGLibA_02135 | 117 | -0.07638837 | ANKRD62 | ankyrin repeat domain 62 [Source:HGNC Symbol;Acc:HGNC:35241] |
| ENSG00000183091 | HGLibA_31577 | 117 | -0.07638837 | NEB | nebulin [Source:HGNC Symbol;Acc:HGNC:7720] |
| ENSG00000183310 | HGLibA_33877 | 117 | -0.07638837 | OR2T34 | olfactory receptor family 2 subfamily T member 34 [Source:HGNC Symbol;Acc:HGNC:31256] |
| ENSG00000183317 | HGLibA_15287 | 117 | -0.07638837 | EPHA10 | EPH receptor A10 [Source:HGNC Symbol;Acc:HGNC:19987] |
| ENSG00000183747 | HGLibA_00572 | 117 | -0.07638837 | ACSM2A | acyl-CoA synthetase medium chain family member 2A [Source:HGNC Symbol;Acc:HGNC:32017] |
| ENSG00000184144 | HGLibA_10404 | 117 | -0.07638837 | CNTN2 | contactin 2 [Source:HGNC Symbol;Acc:HGNC:2172] |
| ENSG00000184465 | HGLibA_54057 | 117 | -0.07638837 | WDR27 | WD repeat domain 27 [Source:HGNC Symbol;Acc:HGNC:21248] |
| ENSG00000184575 | HGLibA_54578 | 117 | -0.07638837 | XPOT | exportin for tRNA [Source:HGNC Symbol;Acc:HGNC:12826] |
| ENSG00000186106 | HGLibA_02108 | 117 | -0.07638837 | ANKRD46 | ankyrin repeat domain 46 [Source:HGNC Symbol;Acc:HGNC:27229] |
| ENSG00000186188 | HGLibA_17333 | 117 | -0.07638837 | FFAR4 | free fatty acid receptor 4 [Source:HGNC Symbol;Acc:HGNC:19061] |
| ENSG00000186191 | HGLibA_04729 | 117 | -0.07638837 | BPIFB4 | BPI fold containing family B member 4 [Source:HGNC Symbol;Acc:HGNC:16179] |
| ENSG00000186198 | HGLibA_45241 | 117 | -0.07638837 | SLC51B | solute carrier family 51 beta subunit [Source:HGNC Symbol;Acc:HGNC:29956] |
| ENSG00000186230 | HGLibA_56672 | 117 | -0.07638837 | ZNF749 | zinc finger protein 749 [Source:HGNC Symbol;Acc:HGNC:32783] |
| ENSG00000186472 | HGLibA_35620 | 117 | -0.07638837 | PCLO | piccolo presynaptic cytomatrix protein [Source:HGNC Symbol;Acc:HGNC:13406] |
| ENSG00000188419 | HGLibA_09465 | 117 | -0.07638837 | CHM | Rab escort protein 1 [Source:HGNC Symbol;Acc:HGNC:1940]" |
| ENSG00000188629 | HGLibA_55532 | 117 | -0.07638837 | ZNF177 | zinc finger protein 177 [Source:HGNC Symbol;Acc:HGNC:12966] |
| ENSG00000188785 | HGLibA_56207 | 117 | -0.07638837 | ZNF548 | zinc finger protein 548 [Source:HGNC Symbol;Acc:HGNC:26561] |
| ENSG00000189167 | HGLibA_54780 | 117 | -0.07638837 | ZAR1L | zygote arrest 1 like [Source:HGNC Symbol;Acc:HGNC:37116] |
| ENSG00000196990 | HGLibA_16232 | 117 | -0.07638837 | FAM163B | family with sequence similarity 163 member B [Source:HGNC Symbol;Acc:HGNC:33277] |
| ENSG00000197157 | HGLibA_45921 | 117 | -0.07638837 | SND1 | staphylococcal nuclease and tudor domain containing 1 [Source:HGNC Symbol;Acc:HGNC:30646] |
| ENSG00000198252 | HGLibA_47686 | 117 | -0.07638837 | STYX | serine/threonine/tyrosine interacting protein [Source:HGNC Symbol;Acc:HGNC:11447] |
| ENSG00000204176 | HGLibA_48063 | 117 | -0.07638837 | SYT15 | synaptotagmin 15 [Source:HGNC Symbol;Acc:HGNC:17167] |
| ENSG00000204444 | HGLibA_02547 | 117 | -0.07638837 | APOM | apolipoprotein M [Source:HGNC Symbol;Acc:HGNC:13916] |
| ENSG00000214414 | HGLibA_51612 | 117 | -0.07638837 | TRIM77 | tripartite motif containing 77 [Source:HGNC Symbol;Acc:HGNC:34228] |
| ENSG00000221938 | HGLibA_33725 | 117 | -0.07638837 | OR2A14 | olfactory receptor family 2 subfamily A member 14 [Source:HGNC Symbol;Acc:HGNC:15084] |
| ENSG00000221989 | HGLibA_33728 | 117 | -0.07638837 | OR2A2 | olfactory receptor family 2 subfamily A member 2 [Source:HGNC Symbol;Acc:HGNC:8230] |
| ENSG00000225327 | HGLibA_53272 | 117 | -0.07638837 | USP17L3 | ubiquitin specific peptidase 17-like family member 3 [Source:HGNC Symbol;Acc:HGNC:37175] |
| ENSG00000229453 | HGLibA_46705 | 117 | -0.07638837 | SPINK8 | Kazal type 8 (putative) [Source:HGNC Symbol;Acc:HGNC:33160]" |
| ENSG00000231861 | HGLibA_34312 | 117 | -0.07638837 | OR5K2 | olfactory receptor family 5 subfamily K member 2 [Source:HGNC Symbol;Acc:HGNC:14774] |
| ENSG00000239887 | HGLibA_05835 | 117 | -0.07638837 | C1orf226 | chromosome 1 open reading frame 226 [Source:HGNC Symbol;Acc:HGNC:34351] |
| ENSG00000243137 | HGLibA_39046 | 117 | -0.07638837 | PSG4 | pregnancy specific beta-1-glycoprotein 4 [Source:HGNC Symbol;Acc:HGNC:9521] |
| ENSG00000256683 | HGLibA_55857 | 117 | -0.07638837 | ZNF350 | zinc finger protein 350 [Source:HGNC Symbol;Acc:HGNC:16656] |
| ENSG00000263001 | HGLibA_20564 | 117 | -0.07638837 | GTF2I | general transcription factor IIi [Source:HGNC Symbol;Acc:HGNC:4659] |
| ENSG00000267978 | HGLibA_27927 | 117 | -0.07638837 | MAGEA9B | MAGE family member A9B [Source:HGNC Symbol;Acc:HGNC:31909] |
| hsa-mir-125a | HGLibA_57445 | 117 | -0.07638837 | hsa-mir-125a | - |
| hsa-mir-155 | HGLibA_57930 | 117 | -0.07638837 | hsa-mir-155 | - |
| hsa-mir-182 | HGLibA_57984 | 117 | -0.07638837 | hsa-mir-182 | - |
| hsa-mir-1913 | HGLibA_58055 | 117 | -0.07638837 | hsa-mir-1913 | - |
| hsa-mir-1914 | HGLibA_58062 | 117 | -0.07638837 | hsa-mir-1914 | - |
| hsa-mir-25 | HGLibA_58353 | 117 | -0.07638837 | hsa-mir-25 | - |
| hsa-mir-3121 | HGLibA_58538 | 117 | -0.07638837 | hsa-mir-3121 | - |
| hsa-mir-363 | HGLibA_59131 | 117 | -0.07638837 | hsa-mir-363 | - |
| hsa-mir-378i | HGLibA_59460 | 117 | -0.07638837 | hsa-mir-378i | - |
| hsa-mir-409 | HGLibA_59676 | 117 | -0.07638837 | hsa-mir-409 | - |
| hsa-mir-4261 | HGLibA_59751 | 117 | -0.07638837 | hsa-mir-4261 | - |
| hsa-mir-4269 | HGLibA_59784 | 117 | -0.07638837 | hsa-mir-4269 | - |
| hsa-mir-4661 | HGLibA_60755 | 117 | -0.07638837 | hsa-mir-4661 | - |
| hsa-mir-4738 | HGLibA_61049 | 117 | -0.07638837 | hsa-mir-4738 | - |
| hsa-mir-514a-1 | HGLibA_61586 | 117 | -0.07638837 | hsa-mir-514a-1 | - |
| hsa-mir-5700 | HGLibA_62335 | 117 | -0.07638837 | hsa-mir-5700 | - |
| hsa-mir-6810 | HGLibA_63417 | 117 | -0.07638837 | hsa-mir-6810 | - |
| hsa-mir-6832 | HGLibA_63506 | 117 | -0.07638837 | hsa-mir-6832 | - |
| PPAP2B | HGLibA_37818 | 117 | -0.07638837 | PPAP2B | - |
| SMEK1 | HGLibA_45691 | 117 | -0.07638837 | SMEK1 | - |
| C10orf12 | HGLibA_05043 | 116 | -0.085433713 | C10orf12 | - |
| C12orf77 | HGLibA_05304 | 116 | -0.085433713 | C12orf77 | - |
| C16orf97 | HGLibA_05513 | 116 | -0.085433713 | C16orf97 | - |
| C7orf63 | HGLibA_06572 | 116 | -0.085433713 | C7orf63 | - |
| DARC | HGLibA_12336 | 116 | -0.085433713 | DARC | - |
| ENSG00000003056 | HGLibA_27814 | 116 | -0.085433713 | M6PR | cation dependent [Source:HGNC Symbol;Acc:HGNC:6752]" |
| ENSG00000008256 | HGLibA_12240 | 116 | -0.085433713 | CYTH3 | cytohesin 3 [Source:HGNC Symbol;Acc:HGNC:9504] |
| ENSG00000009844 | HGLibA_53881 | 116 | -0.085433713 | VTA1 | vesicle trafficking 1 [Source:HGNC Symbol;Acc:HGNC:20954] |
| ENSG00000011295 | HGLibA_52065 | 116 | -0.085433713 | TTC19 | tetratricopeptide repeat domain 19 [Source:HGNC Symbol;Acc:HGNC:26006] |
| ENSG00000016391 | HGLibA_09415 | 116 | -0.085433713 | CHDH | choline dehydrogenase [Source:HGNC Symbol;Acc:HGNC:24288] |
| ENSG00000026559 | HGLibA_24199 | 116 | -0.085433713 | KCNG1 | potassium voltage-gated channel modifier subfamily G member 1 [Source:HGNC Symbol;Acc:HGNC:6248] |
| ENSG00000031823 | HGLibA_40203 | 116 | -0.085433713 | RANBP3 | RAN binding protein 3 [Source:HGNC Symbol;Acc:HGNC:9850] |
| ENSG00000036565 | HGLibA_44485 | 116 | -0.085433713 | SLC18A1 | solute carrier family 18 member A1 [Source:HGNC Symbol;Acc:HGNC:10934] |
| ENSG00000038295 | HGLibA_49629 | 116 | -0.085433713 | TLL1 | tolloid like 1 [Source:HGNC Symbol;Acc:HGNC:11843] |
| ENSG00000042781 | HGLibA_53178 | 116 | -0.085433713 | USH2A | usherin [Source:HGNC Symbol;Acc:HGNC:12601] |
| ENSG00000052126 | HGLibA_37075 | 116 | -0.085433713 | PLEKHA5 | pleckstrin homology domain containing A5 [Source:HGNC Symbol;Acc:HGNC:30036] |
| ENSG00000065833 | HGLibA_28709 | 116 | -0.085433713 | ME1 | malic enzyme 1 [Source:HGNC Symbol;Acc:HGNC:6983] |
| ENSG00000067840 | HGLibA_35980 | 116 | -0.085433713 | PDZD4 | PDZ domain containing 4 [Source:HGNC Symbol;Acc:HGNC:21167] |
| ENSG00000070193 | HGLibA_17364 | 116 | -0.085433713 | FGF10 | fibroblast growth factor 10 [Source:HGNC Symbol;Acc:HGNC:3666] |
| ENSG00000076685 | HGLibA_32811 | 116 | -0.085433713 | NT5C2 | cytosolic II [Source:HGNC Symbol;Acc:HGNC:8022]" |
| ENSG00000082556 | HGLibA_33447 | 116 | -0.085433713 | OPRK1 | opioid receptor kappa 1 [Source:HGNC Symbol;Acc:HGNC:8154] |
| ENSG00000091622 | HGLibA_36757 | 116 | -0.085433713 | PITPNM3 | PITPNM family member 3 [Source:HGNC Symbol;Acc:HGNC:21043] |
| ENSG00000092068 | HGLibA_45382 | 116 | -0.085433713 | SLC7A8 | solute carrier family 7 member 8 [Source:HGNC Symbol;Acc:HGNC:11066] |

| | | | | | |
|---|---|---|---|---|---|
| ENSG00000093167 | HGLibA_27452 | 116 | -0.085433713 | LRRFIP2 | LRR binding FLII interacting protein 2 [Source:HGNC Symbol;Acc:HGNC:6703] |
| ENSG00000099260 | HGLibA_35080 | 116 | -0.085433713 | PALMD | palmdelphin [Source:HGNC Symbol;Acc:HGNC:15846] |
| ENSG00000100350 | HGLibA_17955 | 116 | -0.085433713 | FOXRED2 | FAD dependent oxidoreductase domain containing 2 [Source:HGNC Symbol;Acc:HGNC:26264] |
| ENSG00000100528 | HGLibA_10283 | 116 | -0.085433713 | CNIH1 | cornichon family AMPA receptor auxiliary protein 1 [Source:HGNC Symbol;Acc:HGNC:19431] |
| ENSG00000100749 | HGLibA_53823 | 116 | -0.085433713 | VRK1 | VRK serine/threonine kinase 1 [Source:HGNC Symbol;Acc:HGNC:12718] |
| ENSG00000104936 | HGLibA_13413 | 116 | -0.085433713 | DMPK | DM1 protein kinase [Source:HGNC Symbol;Acc:HGNC:2933] |
| ENSG00000105438 | HGLibA_24478 | 116 | -0.085433713 | KDELR1 | KDEL endoplasmic reticulum protein retention receptor 1 [Source:HGNC Symbol;Acc:HGNC:6304] |
| ENSG00000105664 | HGLibA_10682 | 116 | -0.085433713 | COMP | cartilage oligomeric matrix protein [Source:HGNC Symbol;Acc:HGNC:2227] |
| ENSG00000105708 | HGLibA_55479 | 116 | -0.085433713 | ZNF14 | zinc finger protein 14 [Source:HGNC Symbol;Acc:HGNC:12924] |
| ENSG00000109684 | HGLibA_10086 | 116 | -0.085433713 | CLNK | cytokine dependent hematopoietic cell linker [Source:HGNC Symbol;Acc:HGNC:17438] |
| ENSG00000110057 | HGLibA_53037 | 116 | -0.085433713 | UNC93B1 | TLR signaling regulator [Source:HGNC Symbol;Acc:HGNC:13481]" |
| ENSG00000110330 | HGLibA_04514 | 116 | -0.085433713 | BIRC2 | baculoviral IAP repeat containing 2 [Source:HGNC Symbol;Acc:HGNC:590] |
| ENSG00000111729 | HGLibA_09978 | 116 | -0.085433713 | CLEC4A | C-type lectin domain family 4 member A [Source:HGNC Symbol;Acc:HGNC:13257] |
| ENSG00000112139 | HGLibA_28676 | 116 | -0.085433713 | MDGA1 | MAM domain containing glycosylphosphatidylinositol anchor 1 [Source:HGNC Symbol;Acc:HGNC:19267] |
| ENSG00000115194 | HGLibA_44907 | 116 | -0.085433713 | SLC30A3 | solute carrier family 30 member 3 [Source:HGNC Symbol;Acc:HGNC:11014] |
| ENSG00000115423 | HGLibA_13493 | 116 | -0.085433713 | DNAH6 | dynein axonemal heavy chain 6 [Source:HGNC Symbol;Acc:HGNC:2951] |
| ENSG00000116754 | HGLibA_47066 | 116 | -0.085433713 | SRSF11 | serine and arginine rich splicing factor 11 [Source:HGNC Symbol;Acc:HGNC:10782] |
| ENSG00000119938 | HGLibA_38105 | 116 | -0.085433713 | PPP1R3C | protein phosphatase 1 regulatory subunit 3C [Source:HGNC Symbol;Acc:HGNC:9293] |
| ENSG00000120318 | HGLibA_02632 | 116 | -0.085433713 | ARAP3 | ankyrin repeat and PH domain 3 [Source:HGNC Symbol;Acc:HGNC:24097]" |
| ENSG00000121335 | HGLibA_38342 | 116 | -0.085433713 | PRB2 | proline rich protein BstNI subfamily 2 [Source:HGNC Symbol;Acc:HGNC:9338] |
| ENSG00000123064 | HGLibA_12745 | 116 | -0.085433713 | DDX54 | DEAD-box helicase 54 [Source:HGNC Symbol;Acc:HGNC:20084] |
| ENSG00000124212 | HGLibA_39350 | 116 | -0.085433713 | PTGIS | prostaglandin I2 synthase [Source:HGNC Symbol;Acc:HGNC:9603] |
| ENSG00000125869 | HGLibA_25985 | 116 | -0.085433713 | LAMP5 | lysosomal associated membrane protein family member 5 [Source:HGNC Symbol;Acc:HGNC:16097] |
| ENSG00000125962 | HGLibA_03051 | 116 | -0.085433713 | ARMCX5 | armadillo repeat containing X-linked 5 [Source:HGNC Symbol;Acc:HGNC:25772] |
| ENSG00000126262 | HGLibA_17327 | 116 | -0.085433713 | FFAR2 | free fatty acid receptor 2 [Source:HGNC Symbol;Acc:HGNC:4501] |
| ENSG00000128709 | HGLibA_22007 | 116 | -0.085433713 | HOXD9 | homeobox D9 [Source:HGNC Symbol;Acc:HGNC:5140] |
| ENSG00000129514 | HGLibA_17807 | 116 | -0.085433713 | FOXA1 | forkhead box A1 [Source:HGNC Symbol;Acc:HGNC:5021] |
| ENSG00000130167 | HGLibA_51910 | 116 | -0.085433713 | TSPAN16 | tetraspanin 16 [Source:HGNC Symbol;Acc:HGNC:30725] |
| ENSG00000130347 | HGLibA_42409 | 116 | -0.085433713 | RTN4IP1 | reticulon 4 interacting protein 1 [Source:HGNC Symbol;Acc:HGNC:18647] |
| ENSG00000130816 | HGLibA_13698 | 116 | -0.085433713 | DNMT1 | DNA methyltransferase 1 [Source:HGNC Symbol;Acc:HGNC:2976] |
| ENSG00000131236 | HGLibA_07133 | 116 | -0.085433713 | CAP1 | cyclase associated actin cytoskeleton regulatory protein 1 [Source:HGNC Symbol;Acc:HGNC:20040] |
| ENSG00000131238 | HGLibA_38233 | 116 | -0.085433713 | PPT1 | palmitoyl-protein thioesterase 1 [Source:HGNC Symbol;Acc:HGNC:9325] |
| ENSG00000131732 | HGLibA_55051 | 116 | -0.085433713 | ZCCHC9 | zinc finger CCHC-type containing 9 [Source:HGNC Symbol;Acc:HGNC:25424] |
| ENSG00000131910 | HGLibA_32570 | 116 | -0.085433713 | NR0B2 | nuclear receptor subfamily 0 group B member 2 [Source:HGNC Symbol;Acc:HGNC:7961] |
| ENSG00000136928 | HGLibA_18343 | 116 | -0.085433713 | GABBR2 | gamma-aminobutyric acid type B receptor subunit 2 [Source:HGNC Symbol;Acc:HGNC:4507] |
| ENSG00000137563 | HGLibA_18998 | 116 | -0.085433713 | GGH | gamma-glutamyl hydrolase [Source:HGNC Symbol;Acc:HGNC:4248] |
| ENSG00000138115 | HGLibA_12120 | 116 | -0.085433713 | CYP2C8 | cytochrome P450 family 2 subfamily C member 8 [Source:HGNC Symbol;Acc:HGNC:2622] |
| ENSG00000140632 | HGLibA_19385 | 116 | -0.085433713 | GLYR1 | glyoxylate reductase 1 homolog [Source:HGNC Symbol;Acc:HGNC:24434] |
| ENSG00000141338 | HGLibA_00125 | 116 | -0.085433713 | ABCA8 | ATP binding cassette subfamily A member 8 [Source:HGNC Symbol;Acc:HGNC:38] |
| ENSG00000141499 | HGLibA_54401 | 116 | -0.085433713 | WRAP53 | WD repeat containing antisense to TP53 [Source:HGNC Symbol;Acc:HGNC:25522] |
| ENSG00000142530 | HGLibA_16697 | 116 | -0.085433713 | FAM71E1 | family with sequence similarity 71 member E1 [Source:HGNC Symbol;Acc:HGNC:25107] |
| ENSG00000143393 | HGLibA_36501 | 116 | -0.085433713 | PI4KB | phosphatidylinositol 4-kinase beta [Source:HGNC Symbol;Acc:HGNC:8984] |
| ENSG00000145934 | HGLibA_48998 | 116 | -0.085433713 | TENM2 | teneurin transmembrane protein 2 [Source:HGNC Symbol;Acc:HGNC:29943] |
| ENSG00000146757 | HGLibA_56909 | 116 | -0.085433713 | ZNF92 | zinc finger protein 92 [Source:HGNC Symbol;Acc:HGNC:13168] |
| ENSG00000147459 | HGLibA_13750 | 116 | -0.085433713 | DOCK5 | dedicator of cytokinesis 5 [Source:HGNC Symbol;Acc:HGNC:23476] |
| ENSG00000148459 | HGLibA_35948 | 116 | -0.085433713 | PDSS1 | decaprenyl diphosphate synthase subunit 1 [Source:HGNC Symbol;Acc:HGNC:17759] |
| ENSG00000152086 | HGLibA_52259 | 116 | -0.085433713 | TUBA3E | tubulin alpha 3e [Source:HGNC Symbol;Acc:HGNC:20765] |
| ENSG00000153064 | HGLibA_04145 | 116 | -0.085433713 | BANK1 | B cell scaffold protein with ankyrin repeats 1 [Source:HGNC Symbol;Acc:HGNC:18233] |
| ENSG00000153406 | HGLibA_32192 | 116 | -0.085433713 | NMRAL1 | NmrA like redox sensor 1 [Source:HGNC Symbol;Acc:HGNC:24987] |
| ENSG00000154262 | HGLibA_00120 | 116 | -0.085433713 | ABCA6 | ATP binding cassette subfamily A member 6 [Source:HGNC Symbol;Acc:HGNC:36] |
| ENSG00000155495 | HGLibA_27960 | 116 | -0.085433713 | MAGEC1 | MAGE family member C1 [Source:HGNC Symbol;Acc:HGNC:6812] |
| ENSG00000155975 | HGLibA_53777 | 116 | -0.085433713 | VPS37A | ESCRT-I subunit [Source:HGNC Symbol;Acc:HGNC:24928]" |
| ENSG00000156150 | HGLibA_01736 | 116 | -0.085433713 | ALX3 | ALX homeobox 3 [Source:HGNC Symbol;Acc:HGNC:449] |
| ENSG00000159314 | HGLibA_02743 | 116 | -0.085433713 | ARHGAP27 | Rho GTPase activating protein 27 [Source:HGNC Symbol;Acc:HGNC:31813] |
| ENSG00000159388 | HGLibA_04943 | 116 | -0.085433713 | BTG2 | BTG anti-proliferation factor 2 [Source:HGNC Symbol;Acc:HGNC:1131] |
| ENSG00000160781 | HGLibA_35165 | 116 | -0.085433713 | PAQR6 | progestin and adipoQ receptor family member 6 [Source:HGNC Symbol;Acc:HGNC:30132] |
| ENSG00000160973 | HGLibA_17872 | 116 | -0.085433713 | FOXH1 | forkhead box H1 [Source:HGNC Symbol;Acc:HGNC:3814] |
| ENSG00000163352 | HGLibA_26295 | 116 | -0.085433713 | LENEP | lens epithelial protein [Source:HGNC Symbol;Acc:HGNC:14429] |
| ENSG00000163357 | HGLibA_12529 | 116 | -0.085433713 | DCST1 | DC-STAMP domain containing 1 [Source:HGNC Symbol;Acc:HGNC:26539] |
| ENSG00000163867 | HGLibA_55405 | 116 | -0.085433713 | ZMYM6 | zinc finger MYM-type containing 6 [Source:HGNC Symbol;Acc:HGNC:13050] |
| ENSG00000163913 | HGLibA_22742 | 116 | -0.085433713 | IFT122 | intraflagellar transport 122 [Source:HGNC Symbol;Acc:HGNC:13556] |
| ENSG00000165970 | HGLibA_45331 | 116 | -0.085433713 | SLC6A5 | solute carrier family 6 member 5 [Source:HGNC Symbol;Acc:HGNC:11051] |
| ENSG00000166736 | HGLibA_22380 | 116 | -0.085433713 | HTR3A | 5-hydroxytryptamine receptor 3A [Source:HGNC Symbol;Acc:HGNC:5297] |
| ENSG00000167005 | HGLibA_32969 | 116 | -0.085433713 | NUDT21 | nudix hydrolase 21 [Source:HGNC Symbol;Acc:HGNC:13870] |
| ENSG00000167699 | HGLibA_19286 | 116 | -0.085433713 | GLOD4 | glyoxalase domain containing 4 [Source:HGNC Symbol;Acc:HGNC:14111] |
| ENSG00000168283 | HGLibA_04588 | 116 | -0.085433713 | BMI1 | polycomb ring finger [Source:HGNC Symbol;Acc:HGNC:1066]" |
| ENSG00000169047 | HGLibA_23620 | 116 | -0.085433713 | IRS1 | insulin receptor substrate 1 [Source:HGNC Symbol;Acc:HGNC:6125] |
| ENSG00000171033 | HGLibA_36820 | 116 | -0.085433713 | PKIA | cAMP-dependent protein kinase inhibitor alpha [Source:HGNC Symbol;Acc:HGNC:9017] |
| ENSG00000171054 | HGLibA_33622 | 116 | -0.085433713 | OR13H1 | olfactory receptor family 13 subfamily H member 1 [Source:HGNC Symbol;Acc:HGNC:14755] |
| ENSG00000171346 | HGLibA_25428 | 116 | -0.085433713 | KRT15 | keratin 15 [Source:HGNC Symbol;Acc:HGNC:6421] |
| ENSG00000171815 | HGLibA_35468 | 116 | -0.085433713 | PCDHB1 | protocadherin beta 1 [Source:HGNC Symbol;Acc:HGNC:8680] |
| ENSG00000172244 | HGLibA_06377 | 116 | -0.085433713 | C5orf34 | chromosome 5 open reading frame 34 [Source:HGNC Symbol;Acc:HGNC:24738] |
| ENSG00000172260 | HGLibA_31625 | 116 | -0.085433713 | NEGR1 | neuronal growth regulator 1 [Source:HGNC Symbol;Acc:HGNC:17302] |
| ENSG00000174738 | HGLibA_32576 | 116 | -0.085433713 | NR1D2 | nuclear receptor subfamily 1 group D member 2 [Source:HGNC Symbol;Acc:HGNC:7963] |
| ENSG00000176463 | HGLibA_45465 | 116 | -0.085433713 | SLCO3A1 | solute carrier organic anion transporter family member 3A1 [Source:HGNC Symbol;Acc:HGNC:10952] |
| ENSG00000176956 | HGLibA_27673 | 116 | -0.085433713 | LY6H | lymphocyte antigen 6 family member H [Source:HGNC Symbol;Acc:HGNC:6728] |
| ENSG00000176978 | HGLibA_13878 | 116 | -0.085433713 | DPP7 | dipeptidyl peptidase 7 [Source:HGNC Symbol;Acc:HGNC:14892] |
| ENSG00000177575 | HGLibA_08243 | 116 | -0.085433713 | CD163 | CD163 molecule [Source:HGNC Symbol;Acc:HGNC:1631] |
| ENSG00000177697 | HGLibA_08237 | 116 | -0.085433713 | CD151 | CD151 molecule (Raph blood group) [Source:HGNC Symbol;Acc:HGNC:1630] |
| ENSG00000177710 | HGLibA_45025 | 116 | -0.085433713 | SLC35G5 | solute carrier family 35 member G5 [Source:HGNC Symbol;Acc:HGNC:15546] |
| ENSG00000177854 | HGLibA_50129 | 116 | -0.085433713 | TMEM187 | transmembrane protein 187 [Source:HGNC Symbol;Acc:HGNC:13705] |
| ENSG00000178096 | HGLibA_04679 | 116 | -0.085433713 | BOLA1 | bolA family member 1 [Source:HGNC Symbol;Acc:HGNC:24263] |
| ENSG00000178568 | HGLibA_15423 | 116 | -0.085433713 | ERBB4 | erb-b2 receptor tyrosine kinase 4 [Source:HGNC Symbol;Acc:HGNC:3432] |
| ENSG00000181690 | HGLibA_36952 | 116 | -0.085433713 | PLAG1 | PLAG1 zinc finger [Source:HGNC Symbol;Acc:HGNC:9045] |
| ENSG00000182150 | HGLibA_15452 | 116 | -0.085433713 | ERCC6L2 | ERCC excision repair 6 like 2 [Source:HGNC Symbol;Acc:HGNC:26922] |
| ENSG00000182240 | HGLibA_04061 | 116 | -0.085433713 | BACE2 | beta-secretase 2 [Source:HGNC Symbol;Acc:HGNC:934] |
| ENSG00000182667 | HGLibA_32847 | 116 | -0.085433713 | NTM | neurotrimin [Source:HGNC Symbol;Acc:HGNC:17941] |
| ENSG00000184507 | HGLibA_33103 | 116 | -0.085433713 | NUTM1 | NUT midline carcinoma family member 1 [Source:HGNC Symbol;Acc:HGNC:29919] |
| ENSG00000184557 | HGLibA_46128 | 116 | -0.085433713 | SOCS3 | suppressor of cytokine signaling 3 [Source:HGNC Symbol;Acc:HGNC:19391] |
| ENSG00000185610 | HGLibA_12408 | 116 | -0.085433713 | DBX2 | developing brain homeobox 2 [Source:HGNC Symbol;Acc:HGNC:33186] |
| ENSG00000185800 | HGLibA_13451 | 116 | -0.085433713 | DMWD | WD repeat containing [Source:HGNC Symbol;Acc:HGNC:2936]" |
| ENSG00000186566 | HGLibA_19749 | 116 | -0.085433713 | GPATCH8 | G-patch domain containing 8 [Source:HGNC Symbol;Acc:HGNC:29066] |
| ENSG00000187513 | HGLibA_19146 | 116 | -0.085433713 | GJA4 | gap junction protein alpha 4 [Source:HGNC Symbol;Acc:HGNC:4278] |
| ENSG00000187714 | HGLibA_44492 | 116 | -0.085433713 | SLC18A3 | solute carrier family 18 member A3 [Source:HGNC Symbol;Acc:HGNC:10936] |
| ENSG00000187922 | HGLibA_26170 | 116 | -0.085433713 | LCN10 | lipocalin 10 [Source:HGNC Symbol;Acc:HGNC:20892] |
| ENSG00000189067 | HGLibA_26640 | 116 | -0.085433713 | LITAF | lipopolysaccharide induced TNF factor [Source:HGNC Symbol;Acc:HGNC:16841] |
| ENSG00000189186 | HGLibA_12462 | 116 | -0.085433713 | DCAF8L2 | DDB1 and CUL4 associated factor 8 like 2 [Source:HGNC Symbol;Acc:HGNC:31811] |
| ENSG00000196584 | HGLibA_54585 | 116 | -0.085433713 | XRCC2 | X-ray repair cross complementing 2 [Source:HGNC Symbol;Acc:HGNC:12829] |
| ENSG00000196814 | HGLibA_30591 | 116 | -0.085433713 | MVB12B | multivesicular body subunit 12B [Source:HGNC Symbol;Acc:HGNC:23368] |
| ENSG00000196832 | HGLibA_33566 | 116 | -0.085433713 | OR11G2 | olfactory receptor family 11 subfamily G member 2 [Source:HGNC Symbol;Acc:HGNC:15346] |
| ENSG00000196914 | HGLibA_02816 | 116 | -0.085433713 | ARHGEF12 | Rho guanine nucleotide exchange factor 12 [Source:HGNC Symbol;Acc:HGNC:14193] |
| ENSG00000198113 | HGLibA_51033 | 116 | -0.085433713 | TOR4A | torsin family 4 member A [Source:HGNC Symbol;Acc:HGNC:25981] |
| ENSG00000198208 | HGLibA_42185 | 116 | -0.085433713 | RPS6KL1 | ribosomal protein S6 kinase like 1 [Source:HGNC Symbol;Acc:HGNC:20222] |
| ENSG00000198573 | HGLibA_46391 | 116 | -0.085433713 | SPANXC | SPANX family member C [Source:HGNC Symbol;Acc:HGNC:14331] |
| ENSG00000198912 | HGLibA_05798 | 116 | -0.085433713 | C1orf174 | chromosome 1 open reading frame 174 [Source:HGNC Symbol;Acc:HGNC:27915] |
| ENSG00000205832 | HGLibA_05511 | 116 | -0.085433713 | C16orf96 | chromosome 16 open reading frame 96 [Source:HGNC Symbol;Acc:HGNC:40031] |
| ENSG00000221887 | HGLibA_21755 | 116 | -0.085433713 | HMSD | histocompatibility minor serpin domain containing [Source:HGNC Symbol;Acc:HGNC:23037] |
| ENSG00000222046 | HGLibA_12486 | 116 | -0.085433713 | DCDC2B | doublecortin domain containing 2B [Source:HGNC Symbol;Acc:HGNC:32576] |
| ENSG00000226650 | HGLibA_24947 | 116 | -0.085433713 | KIF4B | kinesin family member 4B [Source:HGNC Symbol;Acc:HGNC:6322] |
| ENSG00000229549 | HGLibA_51977 | 116 | -0.085433713 | TSPY8 | testis specific protein Y-linked 8 [Source:HGNC Symbol;Acc:HGNC:37471] |
| ENSG00000244362 | HGLibA_25680 | 116 | -0.085433713 | KRTAP19-7 | keratin associated protein 19-7 [Source:HGNC Symbol;Acc:HGNC:18942] |
| ENSG00000255833 | HGLibA_49459 | 116 | -0.085433713 | TIFAB | TIFA inhibitor [Source:HGNC Symbol;Acc:HGNC:34024] |
| ENSG00000257727 | HGLibA_10364 | 116 | -0.085433713 | CNPY2 | canopy FGF signaling regulator 2 [Source:HGNC Symbol;Acc:HGNC:13529] |
| ENSG00000258992 | HGLibA_51963 | 116 | -0.085433713 | TSPY1 | testis specific protein Y-linked 1 [Source:HGNC Symbol;Acc:HGNC:12381] |
| ENSG00000263639 | HGLibA_30222 | 116 | -0.085433713 | MSMB | microseminoprotein beta [Source:HGNC Symbol;Acc:HGNC:7372] |
| FAM154A | HGLibA_16175 | 116 | -0.085433713 | FAM154A | - |
| FAM57A | HGLibA_16644 | 116 | -0.085433713 | FAM57A | - |
| HIST1H3G | HGLibA_21474 | 116 | -0.085433713 | HIST1H3G | - |
| hsa-let-7b | HGLibA_57108 | 116 | -0.085433713 | hsa-let-7b | - |
| hsa-mir-1250 | HGLibA_57400 | 116 | -0.085433713 | hsa-mir-1250 | - |
| hsa-mir-1289-1 | HGLibA_57620 | 116 | -0.085433713 | hsa-mir-1289-1 | - |
| hsa-mir-3150a | HGLibA_58657 | 116 | -0.085433713 | hsa-mir-3150a | - |
| hsa-mir-3180-2 | HGLibA_58805 | 116 | -0.085433713 | hsa-mir-3180-2 | - |
| hsa-mir-3674 | HGLibA_59250 | 116 | -0.085433713 | hsa-mir-3674 | - |
| hsa-mir-3913-2 | HGLibA_59524 | 116 | -0.085433713 | hsa-mir-3913-2 | - |
| hsa-mir-4290 | HGLibA_59872 | 116 | -0.085433713 | hsa-mir-4290 | - |
| hsa-mir-4295 | HGLibA_59892 | 116 | -0.085433713 | hsa-mir-4295 | - |
| hsa-mir-4493 | HGLibA_60392 | 116 | -0.085433713 | hsa-mir-4493 | - |
| hsa-mir-4674 | HGLibA_60811 | 116 | -0.085433713 | hsa-mir-4674 | - |
| hsa-mir-5088 | HGLibA_61499 | 116 | -0.085433713 | hsa-mir-5088 | - |
| hsa-mir-6070 | HGLibA_62533 | 116 | -0.085433713 | hsa-mir-6070 | - |
| hsa-mir-6074 | HGLibA_62549 | 116 | -0.085433713 | hsa-mir-6074 | - |
| hsa-mir-6503 | HGLibA_62864 | 116 | -0.085433713 | hsa-mir-6503 | - |
| hsa-mir-655 | HGLibA_62954 | 116 | -0.085433713 | hsa-mir-655 | - |
| hsa-mir-7976 | HGLibA_64034 | 116 | -0.085433713 | hsa-mir-7976 | - |

| | | | | | |
|---|---|---|---|---|---|
| hsa-mir-7976 | HGLibA_64033 | 116 | -0.085433713 | hsa-mir-7976 | - |
| hsa-mir-92a-2 | HGLibA_64296 | 116 | -0.085433713 | hsa-mir-92a-2 | - |
| LOC401052 | HGLibA_26937 | 116 | -0.085433713 | LOC401052 | - |
| PTCHD2 | HGLibA_39277 | 116 | -0.085433713 | PTCHD2 | - |
| SCXB | HGLibA_43082 | 116 | -0.085433713 | SCXB | - |
| TOMM70A | HGLibA_50982 | 116 | -0.085433713 | TOMM70A | - |
| C15orf27 | HGLibA_05380 | 115 | -0.094479056 | C15orf27 | - |
| C20orf196 | HGLibA_06005 | 115 | -0.094479056 | C20orf196 | - |
| C7orf49 | HGLibA_06547 | 115 | -0.094479056 | C7orf49 | - |
| CSF2RA_X | HGLibA_11331 | 115 | -0.094479056 | CSF2RA_X | - |
| DSCR6 | HGLibA_14012 | 115 | -0.094479056 | DSCR6 | - |
| ENSG00000001461 | HGLibA_31961 | 115 | -0.094479056 | NIPAL3 | NIPA like domain containing 3 [Source:HGNC Symbol;Acc:HGNC:25233] |
| ENSG00000005513 | HGLibA_46275 | 115 | -0.094479056 | SOX8 | SRY-box 8 [Source:HGNC Symbol;Acc:HGNC:11203] |
| ENSG00000007129 | HGLibA_08934 | 115 | -0.094479056 | CEACAM21 | carcinoembryonic antigen related cell adhesion molecule 21 [Source:HGNC Symbol;Acc:HGNC:28834] |
| ENSG00000012171 | HGLibA_43323 | 115 | -0.094479056 | SEMA3B | semaphorin 3B [Source:HGNC Symbol;Acc:HGNC:10724] |
| ENSG00000021300 | HGLibA_37087 | 115 | -0.094479056 | PLEKHB1 | pleckstrin homology domain containing B1 [Source:HGNC Symbol;Acc:HGNC:19079] |
| ENSG00000043355 | HGLibA_55323 | 115 | -0.094479056 | ZIC2 | Zic family member 2 [Source:HGNC Symbol;Acc:HGNC:12873] |
| ENSG00000049192 | HGLibA_00835 | 115 | -0.094479056 | ADAMTS6 | ADAM metallopeptidase with thrombospondin type 1 motif 6 [Source:HGNC Symbol;Acc:HGNC:222] |
| ENSG00000058272 | HGLibA_38021 | 115 | -0.094479056 | PPP1R12A | protein phosphatase 1 regulatory subunit 12A [Source:HGNC Symbol;Acc:HGNC:7618] |
| ENSG00000064393 | HGLibA_21341 | 115 | -0.094479056 | HIPK2 | homeodomain interacting protein kinase 2 [Source:HGNC Symbol;Acc:HGNC:14402] |
| ENSG00000068383 | HGLibA_23343 | 115 | -0.094479056 | INPP5A | inositol polyphosphate-5-phosphatase A [Source:HGNC Symbol;Acc:HGNC:6076] |
| ENSG00000068903 | HGLibA_44218 | 115 | -0.094479056 | SIRT2 | sirtuin 2 [Source:HGNC Symbol;Acc:HGNC:10886] |
| ENSG00000069248 | HGLibA_33020 | 115 | -0.094479056 | NUP133 | nucleoporin 133 [Source:HGNC Symbol;Acc:HGNC:18016] |
| ENSG00000072315 | HGLibA_51740 | 115 | -0.094479056 | TRPC5 | transient receptor potential cation channel subfamily C member 5 [Source:HGNC Symbol;Acc:HGNC:12337] |
| ENSG00000079462 | HGLibA_34994 | 115 | -0.094479056 | PAFAH1B3 | platelet activating factor acetylhydrolase 1b catalytic subunit 3 [Source:HGNC Symbol;Acc:HGNC:8576] |
| ENSG00000088247 | HGLibA_24581 | 115 | -0.094479056 | KHSRP | KH-type splicing regulatory protein [Source:HGNC Symbol;Acc:HGNC:6316] |
| ENSG00000090263 | HGLibA_30092 | 115 | -0.094479056 | MRPS33 | mitochondrial ribosomal protein S33 [Source:HGNC Symbol;Acc:HGNC:16634] |
| ENSG00000092871 | HGLibA_40920 | 115 | -0.094479056 | RFFL | ring finger and FYVE like domain containing E3 ubiquitin protein ligase [Source:HGNC Symbol;Acc:HGNC:24821] |
| ENSG00000100228 | HGLibA_39893 | 115 | -0.094479056 | RAB36 | member RAS oncogene family [Source:HGNC Symbol;Acc:HGNC:9775]" |
| ENSG00000101052 | HGLibA_22765 | 115 | -0.094479056 | IFT52 | intraflagellar transport 52 [Source:HGNC Symbol;Acc:HGNC:15901] |
| ENSG00000103089 | HGLibA_15898 | 115 | -0.094479056 | FA2H | fatty acid 2-hydroxylase [Source:HGNC Symbol;Acc:HGNC:21197] |
| ENSG00000103507 | HGLibA_04283 | 115 | -0.094479056 | BCKDK | branched chain ketoacid dehydrogenase kinase [Source:HGNC Symbol;Acc:HGNC:16902] |
| ENSG00000105617 | HGLibA_26298 | 115 | -0.094479056 | LENG1 | leukocyte receptor cluster member 1 [Source:HGNC Symbol;Acc:HGNC:15502] |
| ENSG00000106628 | HGLibA_37494 | 115 | -0.094479056 | POLD2 | accessory subunit [Source:HGNC Symbol;Acc:HGNC:9176]" |
| ENSG00000106785 | HGLibA_51411 | 115 | -0.094479056 | TRIM14 | tripartite motif containing 14 [Source:HGNC Symbol;Acc:HGNC:16283] |
| ENSG00000107105 | HGLibA_14881 | 115 | -0.094479056 | ELAVL2 | ELAV like RNA binding protein 2 [Source:HGNC Symbol;Acc:HGNC:3313] |
| ENSG00000107819 | HGLibA_43788 | 115 | -0.094479056 | SFXN3 | sideroflexin 3 [Source:HGNC Symbol;Acc:HGNC:16087] |
| ENSG00000109181 | HGLibA_52912 | 115 | -0.094479056 | UGT2B10 | UDP glucuronosyltransferase family 2 member B10 [Source:HGNC Symbol;Acc:HGNC:12544] |
| ENSG00000109193 | HGLibA_47754 | 115 | -0.094479056 | SULT1E1 | sulfotransferase family 1E member 1 [Source:HGNC Symbol;Acc:HGNC:11377] |
| ENSG00000112273 | HGLibA_21043 | 115 | -0.094479056 | HDGFL1 | HDGF like 1 [Source:HGNC Symbol;Acc:HGNC:21095] |
| ENSG00000113810 | HGLibA_45653 | 115 | -0.094479056 | SMC4 | structural maintenance of chromosomes 4 [Source:HGNC Symbol;Acc:HGNC:14013] |
| ENSG00000114125 | HGLibA_41698 | 115 | -0.094479056 | RNF7 | ring finger protein 7 [Source:HGNC Symbol;Acc:HGNC:10070] |
| ENSG00000114270 | HGLibA_10610 | 115 | -0.094479056 | COL7A1 | collagen type VII alpha 1 chain [Source:HGNC Symbol;Acc:HGNC:2214] |
| ENSG00000114904 | HGLibA_31654 | 115 | -0.094479056 | NEK4 | NIMA related kinase 4 [Source:HGNC Symbol;Acc:HGNC:11399] |
| ENSG00000115827 | HGLibA_12436 | 115 | -0.094479056 | DCAF17 | DDB1 and CUL4 associated factor 17 [Source:HGNC Symbol;Acc:HGNC:25784] |
| ENSG00000116981 | HGLibA_32803 | 115 | -0.094479056 | NT5C1A | cytosolic IA [Source:HGNC Symbol;Acc:HGNC:17819]" |
| ENSG00000117408 | HGLibA_23462 | 115 | -0.094479056 | IPO13 | importin 13 [Source:HGNC Symbol;Acc:HGNC:16853] |
| ENSG00000117523 | HGLibA_38866 | 115 | -0.094479056 | PRRC2C | proline rich coiled-coil 2C [Source:HGNC Symbol;Acc:HGNC:24903] |
| ENSG00000118217 | HGLibA_03446 | 115 | -0.094479056 | ATF6 | activating transcription factor 6 [Source:HGNC Symbol;Acc:HGNC:791] |
| ENSG00000118507 | HGLibA_01441 | 115 | -0.094479056 | AKAP7 | A-kinase anchoring protein 7 [Source:HGNC Symbol;Acc:HGNC:377] |
| ENSG00000118557 | HGLibA_37310 | 115 | -0.094479056 | PMFBP1 | polyamine modulated factor 1 binding protein 1 [Source:HGNC Symbol;Acc:HGNC:17728] |
| ENSG00000119335 | HGLibA_43632 | 115 | -0.094479056 | SET | SET nuclear proto-oncogene [Source:HGNC Symbol;Acc:HGNC:10760] |
| ENSG00000119640 | HGLibA_00711 | 115 | -0.094479056 | ACYP1 | acylphosphatase 1 [Source:HGNC Symbol;Acc:HGNC:179] |
| ENSG00000119977 | HGLibA_48877 | 115 | -0.094479056 | TCTN3 | tectonic family member 3 [Source:HGNC Symbol;Acc:HGNC:24519] |
| ENSG00000120805 | HGLibA_02921 | 115 | -0.094479056 | ARL1 | ADP ribosylation factor like GTPase 1 [Source:HGNC Symbol;Acc:HGNC:692] |
| ENSG00000123500 | HGLibA_10496 | 115 | -0.094479056 | COL10A1 | collagen type X alpha 1 chain [Source:HGNC Symbol;Acc:HGNC:2185] |
| ENSG00000124249 | HGLibA_24307 | 115 | -0.094479056 | KCNK15 | potassium two pore domain channel subfamily K member 15 [Source:HGNC Symbol;Acc:HGNC:13814] |
| ENSG00000125703 | HGLibA_03495 | 115 | -0.094479056 | ATG4C | autophagy related 4C cysteine peptidase [Source:HGNC Symbol;Acc:HGNC:16040] |
| ENSG00000128165 | HGLibA_00995 | 115 | -0.094479056 | ADM2 | adrenomedullin 2 [Source:HGNC Symbol;Acc:HGNC:28898] |
| ENSG00000128965 | HGLibA_09335 | 115 | -0.094479056 | CHAC1 | ChaC glutathione specific gamma-glutamylcyclotransferase 1 [Source:HGNC Symbol;Acc:HGNC:28680] |
| ENSG00000129353 | HGLibA_45160 | 115 | -0.094479056 | SLC44A2 | solute carrier family 44 member 2 [Source:HGNC Symbol;Acc:HGNC:17292] |
| ENSG00000129437 | HGLibA_25245 | 115 | -0.094479056 | KLK14 | kallikrein related peptidase 14 [Source:HGNC Symbol;Acc:HGNC:6362] |
| ENSG00000129450 | HGLibA_44154 | 115 | -0.094479056 | SIGLEC9 | sialic acid binding Ig like lectin 9 [Source:HGNC Symbol;Acc:HGNC:10878] |
| ENSG00000130385 | HGLibA_04595 | 115 | -0.094479056 | BMP15 | bone morphogenetic protein 15 [Source:HGNC Symbol;Acc:HGNC:1068] |
| ENSG00000131470 | HGLibA_39135 | 115 | -0.094479056 | PSMC3IP | PSMC3 interacting protein [Source:HGNC Symbol;Acc:HGNC:17928] |
| ENSG00000133678 | HGLibA_50328 | 115 | -0.094479056 | TMEM254 | transmembrane protein 254 [Source:HGNC Symbol;Acc:HGNC:25804] |
| ENSG00000134030 | HGLibA_11632 | 115 | -0.094479056 | CTIF | cap binding complex dependent translation initiation factor [Source:HGNC Symbol;Acc:HGNC:23925] |
| ENSG00000134247 | HGLibA_39343 | 115 | -0.094479056 | PTGFRN | prostaglandin F2 receptor inhibitor [Source:HGNC Symbol;Acc:HGNC:9601] |
| ENSG00000134698 | HGLibA_01213 | 115 | -0.094479056 | AGO4 | RISC catalytic component [Source:HGNC Symbol;Acc:HGNC:18424]" |
| ENSG00000134757 | HGLibA_14029 | 115 | -0.094479056 | DSG3 | desmoglein 3 [Source:HGNC Symbol;Acc:HGNC:3050] |
| ENSG00000134871 | HGLibA_10568 | 115 | -0.094479056 | COL4A2 | collagen type IV alpha 2 chain [Source:HGNC Symbol;Acc:HGNC:2203] |
| ENSG00000135249 | HGLibA_41303 | 115 | -0.094479056 | RINT1 | RAD50 interactor 1 [Source:HGNC Symbol;Acc:HGNC:21876] |
| ENSG00000135312 | HGLibA_22360 | 115 | -0.094479056 | HTR1B | 5-hydroxytryptamine receptor 1B [Source:HGNC Symbol;Acc:HGNC:5287] |
| ENSG00000136720 | HGLibA_22142 | 115 | -0.094479056 | HS6ST1 | heparan sulfate 6-O-sulfotransferase 1 [Source:HGNC Symbol;Acc:HGNC:5201] |
| ENSG00000137154 | HGLibA_42154 | 115 | -0.094479056 | RPS6 | ribosomal protein S6 [Source:HGNC Symbol;Acc:HGNC:10429] |
| ENSG00000137501 | HGLibA_48100 | 115 | -0.094479056 | SYTL2 | synaptotagmin like 2 [Source:HGNC Symbol;Acc:HGNC:15585] |
| ENSG00000138375 | HGLibA_45616 | 115 | -0.094479056 | SMARCAL1 | subfamily a like 1 [Source:HGNC Symbol;Acc:HGNC:11102]" |
| ENSG00000138375 | HGLibA_45617 | 115 | -0.094479056 | SMARCAL1 | subfamily a like 1 [Source:HGNC Symbol;Acc:HGNC:11102]" |
| ENSG00000138640 | HGLibA_16141 | 115 | -0.094479056 | FAM13A | family with sequence similarity 13 member A [Source:HGNC Symbol;Acc:HGNC:19367] |
| ENSG00000139324 | HGLibA_50675 | 115 | -0.094479056 | TMTC3 | transmembrane and tetratricopeptide repeat containing 3 [Source:HGNC Symbol;Acc:HGNC:26899] |
| ENSG00000139826 | HGLibA_00246 | 115 | -0.094479056 | ABHD13 | abhydrolase domain containing 13 [Source:HGNC Symbol;Acc:HGNC:20293] |
| ENSG00000140548 | HGLibA_56611 | 115 | -0.094479056 | ZNF710 | zinc finger protein 710 [Source:HGNC Symbol;Acc:HGNC:25352] |
| ENSG00000142512 | HGLibA_44126 | 115 | -0.094479056 | SIGLEC10 | sialic acid binding Ig like lectin 10 [Source:HGNC Symbol;Acc:HGNC:15620] |
| ENSG00000142556 | HGLibA_56388 | 115 | -0.094479056 | ZNF614 | zinc finger protein 614 [Source:HGNC Symbol;Acc:HGNC:24722] |
| ENSG00000143793 | HGLibA_05856 | 115 | -0.094479056 | C1orf35 | chromosome 1 open reading frame 35 [Source:HGNC Symbol;Acc:HGNC:19032] |
| ENSG00000144834 | HGLibA_48275 | 115 | -0.094479056 | TAGLN3 | transgelin 3 [Source:HGNC Symbol;Acc:HGNC:29868] |
| ENSG00000145192 | HGLibA_01318 | 115 | -0.094479056 | AHSG | alpha 2-HS glycoprotein [Source:HGNC Symbol;Acc:HGNC:349] |
| ENSG00000146433 | HGLibA_50099 | 115 | -0.094479056 | TMEM181 | transmembrane protein 181 [Source:HGNC Symbol;Acc:HGNC:20958] |
| ENSG00000148444 | HGLibA_10658 | 115 | -0.094479056 | COMMD3 | COMM domain containing 3 [Source:HGNC Symbol;Acc:HGNC:23332] |
| ENSG00000149084 | HGLibA_22179 | 115 | -0.094479056 | HSD17B12 | hydroxysteroid 17-beta dehydrogenase 12 [Source:HGNC Symbol;Acc:HGNC:18646] |
| ENSG00000149573 | HGLibA_29798 | 115 | -0.094479056 | MPZL2 | myelin protein zero like 2 [Source:HGNC Symbol;Acc:HGNC:3496] |
| ENSG00000152256 | HGLibA_35895 | 115 | -0.094479056 | PDK1 | pyruvate dehydrogenase kinase 1 [Source:HGNC Symbol;Acc:HGNC:8809] |
| ENSG00000152430 | HGLibA_04693 | 115 | -0.094479056 | BOLL | RNA binding protein [Source:HGNC Symbol;Acc:HGNC:14273]" |
| ENSG00000154839 | HGLibA_44262 | 115 | -0.094479056 | SKA1 | spindle and kinetochore associated complex subunit 1 [Source:HGNC Symbol;Acc:HGNC:28109] |
| ENSG00000158220 | HGLibA_15612 | 115 | -0.094479056 | ESYT3 | extended synaptotagmin 3 [Source:HGNC Symbol;Acc:HGNC:24295] |
| ENSG00000160298 | HGLibA_06040 | 115 | -0.094479056 | C21orf58 | chromosome 21 open reading frame 58 [Source:HGNC Symbol;Acc:HGNC:1300] |
| ENSG00000161204 | HGLibA_00212 | 115 | -0.094479056 | ABCF3 | ATP binding cassette subfamily F member 3 [Source:HGNC Symbol;Acc:HGNC:72] |
| ENSG00000162188 | HGLibA_19522 | 115 | -0.094479056 | GNG3 | G protein subunit gamma 3 [Source:HGNC Symbol;Acc:HGNC:4405] |
| ENSG00000162367 | HGLibA_48278 | 115 | -0.094479056 | TAL1 | erythroid differentiation factor [Source:HGNC Symbol;Acc:HGNC:11556]" |
| ENSG00000162378 | HGLibA_57085 | 115 | -0.094479056 | ZYG11B | cell cycle regulator [Source:HGNC Symbol;Acc:HGNC:25820]" |
| ENSG00000162601 | HGLibA_30923 | 115 | -0.094479056 | MYSM1 | SWIRM and MPN domains 1 [Source:HGNC Symbol;Acc:HGNC:29401]" |
| ENSG00000162729 | HGLibA_22901 | 115 | -0.094479056 | IGSF8 | immunoglobulin superfamily member 8 [Source:HGNC Symbol;Acc:HGNC:17813] |
| ENSG00000163104 | HGLibA_45613 | 115 | -0.094479056 | SMARCAD1 | containing DEAD/H box 1 [Source:HGNC Symbol;Acc:HGNC:18398]" |
| ENSG00000163633 | HGLibA_06325 | 115 | -0.094479056 | C4orf36 | chromosome 4 open reading frame 36 [Source:HGNC Symbol;Acc:HGNC:28386] |
| ENSG00000164932 | HGLibA_11630 | 115 | -0.094479056 | CTHRC1 | collagen triple helix repeat containing 1 [Source:HGNC Symbol;Acc:HGNC:18831] |
| ENSG00000164938 | HGLibA_51069 | 115 | -0.094479056 | TP53INP1 | tumor protein p53 inducible nuclear protein 1 [Source:HGNC Symbol;Acc:HGNC:18022] |
| ENSG00000165105 | HGLibA_40326 | 115 | -0.094479056 | RASEF | RAS and EF-hand domain containing [Source:HGNC Symbol;Acc:HGNC:26464] |
| ENSG00000166446 | HGLibA_08921 | 115 | -0.094479056 | CDYL2 | chromodomain Y like 2 [Source:HGNC Symbol;Acc:HGNC:23030] |
| ENSG00000166523 | HGLibA_09988 | 115 | -0.094479056 | CLEC4E | C-type lectin domain family 4 member E [Source:HGNC Symbol;Acc:HGNC:14555] |
| ENSG00000167104 | HGLibA_04731 | 115 | -0.094479056 | BPIFB6 | BPI fold containing family B member 6 [Source:HGNC Symbol;Acc:HGNC:16504] |
| ENSG00000168158 | HGLibA_33775 | 115 | -0.094479056 | OR2C1 | olfactory receptor family 2 subfamily C member 1 [Source:HGNC Symbol;Acc:HGNC:8242] |
| ENSG00000169750 | HGLibA_40047 | 115 | -0.094479056 | RAC3 | Rac family small GTPase 3 [Source:HGNC Symbol;Acc:HGNC:9803] |
| ENSG00000170734 | HGLibA_37527 | 115 | -0.094479056 | POLH | DNA polymerase eta [Source:HGNC Symbol;Acc:HGNC:9181] |
| ENSG00000171388 | HGLibA_02434 | 115 | -0.094479056 | APLN | apelin [Source:HGNC Symbol;Acc:HGNC:16665] |
| ENSG00000171603 | HGLibA_10133 | 115 | -0.094479056 | CLSTN1 | calsyntenin 1 [Source:HGNC Symbol;Acc:HGNC:17447] |
| ENSG00000172534 | HGLibA_20959 | 115 | -0.094479056 | HCFC1 | host cell factor C1 [Source:HGNC Symbol;Acc:HGNC:4839] |
| ENSG00000173714 | HGLibA_54284 | 115 | -0.094479056 | WFIKKN2 | kunitz and netrin domain containing 2 [Source:HGNC Symbol;Acc:HGNC:30916]" |
| ENSG00000174903 | HGLibA_39833 | 115 | -0.094479056 | RAB1B | member RAS oncogene family [Source:HGNC Symbol;Acc:HGNC:18370]" |
| ENSG00000175727 | HGLibA_29440 | 115 | -0.094479056 | MLXIP | MLX interacting protein [Source:HGNC Symbol;Acc:HGNC:17055] |
| ENSG00000175820 | HGLibA_07680 | 115 | -0.094479056 | CCDC168 | coiled-coil domain containing 168 [Source:HGNC Symbol;Acc:HGNC:26851] |
| ENSG00000176198 | HGLibA_33578 | 115 | -0.094479056 | OR11H4 | olfactory receptor family 11 subfamily H member 4 [Source:HGNC Symbol;Acc:HGNC:15347] |
| ENSG00000176797 | HGLibA_12813 | 115 | -0.094479056 | DEFB103A | defensin beta 103A [Source:HGNC Symbol;Acc:HGNC:15967] |
| ENSG00000177105 | HGLibA_41191 | 115 | -0.094479056 | RHOG | ras homolog family member G [Source:HGNC Symbol;Acc:HGNC:672] |
| ENSG00000177669 | HGLibA_28524 | 115 | -0.094479056 | MBOAT4 | membrane bound O-acyltransferase domain containing 4 [Source:HGNC Symbol;Acc:HGNC:32311] |
| ENSG00000177947 | HGLibA_33283 | 115 | -0.094479056 | ODF3 | outer dense fiber of sperm tails 3 [Source:HGNC Symbol;Acc:HGNC:19905] |
| ENSG00000178252 | HGLibA_54136 | 115 | -0.094479056 | WDR6 | WD repeat domain 6 [Source:HGNC Symbol;Acc:HGNC:12758] |
| ENSG00000178381 | HGLibA_55147 | 115 | -0.094479056 | ZFAND2A | zinc finger AN1-type containing 2A [Source:HGNC Symbol;Acc:HGNC:28073] |
| ENSG00000179151 | HGLibA_14443 | 115 | -0.094479056 | EDC3 | enhancer of mRNA decapping 3 [Source:HGNC Symbol;Acc:HGNC:26114] |
| ENSG00000179262 | HGLibA_40070 | 115 | -0.094479056 | RAD23A | nucleotide excision repair protein [Source:HGNC Symbol;Acc:HGNC:9812]" |
| ENSG00000180483 | HGLibA_12866 | 115 | -0.094479056 | DEFB119 | defensin beta 119 [Source:HGNC Symbol;Acc:HGNC:18099] |
| ENSG00000181396 | HGLibA_33313 | 115 | -0.094479056 | OGFOD3 | 2-oxoglutarate and iron dependent oxygenase domain containing 3 [Source:HGNC Symbol;Acc:HGNC:26174] |
| ENSG00000181827 | HGLibA_40981 | 115 | -0.094479056 | RFX7 | regulatory factor X7 [Source:HGNC Symbol;Acc:HGNC:25777] |
| ENSG00000182634 | HGLibA_33498 | 115 | -0.094479056 | OR10G7 | olfactory receptor family 10 subfamily G member 7 [Source:HGNC Symbol;Acc:HGNC:14842] |
| ENSG00000182963 | HGLibA_19177 | 115 | -0.094479056 | GJC1 | gap junction protein gamma 1 [Source:HGNC Symbol;Acc:HGNC:4280] |
| ENSG00000186509 | HGLibA_34584 | 115 | -0.094479056 | OR9Q1 | olfactory receptor family 9 subfamily Q member 1 [Source:HGNC Symbol;Acc:HGNC:14724] |

| | | | | | |
|---|---|---|---|---|---|
| ENSG00000186714 | HGLibA_07860 | 115 | -0.094479056 | CCDC73 | coiled-coil domain containing 73 [Source:HGNC Symbol;Acc:HGNC:23261] |
| ENSG00000187243 | HGLibA_27978 | 115 | -0.094479056 | MAGED4B | MAGE family member D4B [Source:HGNC Symbol;Acc:HGNC:22880] |
| ENSG00000188269 | HGLibA_34464 | 115 | -0.094479056 | OR7A5 | olfactory receptor family 7 subfamily A member 5 [Source:HGNC Symbol;Acc:HGNC:8368] |
| ENSG00000188878 | HGLibA_16935 | 115 | -0.094479056 | FBF1 | Fas binding factor 1 [Source:HGNC Symbol;Acc:HGNC:24674] |
| ENSG00000189064 | HGLibA_18468 | 115 | -0.094479056 | GAGE2A | G antigen 2A [Source:HGNC Symbol;Acc:HGNC:4099] |
| ENSG00000189253 | HGLibA_51578 | 115 | -0.094479056 | TRIM64B | tripartite motif containing 64B [Source:HGNC Symbol;Acc:HGNC:37147] |
| ENSG00000189334 | HGLibA_42545 | 115 | -0.094479056 | S100A14 | S100 calcium binding protein A14 [Source:HGNC Symbol;Acc:HGNC:18901] |
| ENSG00000196531 | HGLibA_31031 | 115 | -0.094479056 | NACA | nascent polypeptide associated complex subunit alpha [Source:HGNC Symbol;Acc:HGNC:7629] |
| ENSG00000196539 | HGLibA_33871 | 115 | -0.094479056 | OR2T3 | olfactory receptor family 2 subfamily T member 3 [Source:HGNC Symbol;Acc:HGNC:14727] |
| ENSG00000196646 | HGLibA_55474 | 115 | -0.094479056 | ZNF136 | zinc finger protein 136 [Source:HGNC Symbol;Acc:HGNC:12920] |
| ENSG00000197273 | HGLibA_20642 | 115 | -0.094479056 | GUCA2A | guanylate cyclase activator 2A [Source:HGNC Symbol;Acc:HGNC:4682] |
| ENSG00000197568 | HGLibA_21272 | 115 | -0.094479056 | HHLA3 | HERV-H LTR-associating 3 [Source:HGNC Symbol;Acc:HGNC:4906] |
| ENSG00000197785 | HGLibA_03410 | 115 | -0.094479056 | ATAD3A | AAA domain containing 3A [Source:HGNC Symbol;Acc:HGNC:25567]" |
| ENSG00000198668 | HGLibA_07022 | 115 | -0.094479056 | CALM1 | calmodulin 1 [Source:HGNC Symbol;Acc:HGNC:1442] |
| ENSG00000198805 | HGLibA_37402 | 115 | -0.094479056 | PNP | purine nucleoside phosphorylase [Source:HGNC Symbol;Acc:HGNC:7892] |
| ENSG00000198844 | HGLibA_02818 | 115 | -0.094479056 | ARHGEF15 | Rho guanine nucleotide exchange factor 15 [Source:HGNC Symbol;Acc:HGNC:15590] |
| ENSG00000204257 | HGLibA_21603 | 115 | -0.094479056 | HLA-DMA | DM alpha [Source:HGNC Symbol;Acc:HGNC:4934]" |
| ENSG00000204428 | HGLibA_27663 | 115 | -0.094479056 | LY6G5C | lymphocyte antigen 6 family member G5C [Source:HGNC Symbol;Acc:HGNC:13932] |
| ENSG00000204983 | HGLibA_38899 | 115 | -0.094479056 | PRSS1 | serine protease 1 [Source:HGNC Symbol;Acc:HGNC:9475] |
| ENSG00000205981 | HGLibA_13591 | 115 | -0.094479056 | DNAJC19 | DnaJ heat shock protein family (Hsp40) member C19 [Source:HGNC Symbol;Acc:HGNC:30528] |
| ENSG00000206069 | HGLibA_50208 | 115 | -0.094479056 | TMEM211 | transmembrane protein 211 [Source:HGNC Symbol;Acc:HGNC:33725] |
| ENSG00000211448 | HGLibA_13242 | 115 | -0.094479056 | DIO2 | iodothyronine deiodinase 2 [Source:HGNC Symbol;Acc:HGNC:2884] |
| ENSG00000212935 | HGLibA_25607 | 115 | -0.094479056 | KRTAP10-3 | keratin associated protein 10-3 [Source:HGNC Symbol;Acc:HGNC:22968] |
| ENSG00000213639 | HGLibA_38009 | 115 | -0.094479056 | PPP1CB | protein phosphatase 1 catalytic subunit beta [Source:HGNC Symbol;Acc:HGNC:9282] |
| ENSG00000213931 | HGLibA_20920 | 115 | -0.094479056 | HBE1 | hemoglobin subunit epsilon 1 [Source:HGNC Symbol;Acc:HGNC:4830] |
| ENSG00000221994 | HGLibA_56433 | 115 | -0.094479056 | ZNF630 | zinc finger protein 630 [Source:HGNC Symbol;Acc:HGNC:28855] |
| ENSG00000226430 | HGLibA_53285 | 115 | -0.094479056 | USP17L7 | ubiquitin specific peptidase 17-like family member 7 [Source:HGNC Symbol;Acc:HGNC:37180] |
| ENSG00000247315 | HGLibA_55032 | 115 | -0.094479056 | ZCCHC3 | zinc finger CCHC-type containing 3 [Source:HGNC Symbol;Acc:HGNC:16230] |
| ENSG00000251258 | HGLibA_40944 | 115 | -0.094479056 | RFPL4B | ret finger protein like 4B [Source:HGNC Symbol;Acc:HGNC:33264] |
| ENSG00000269437 | HGLibA_33131 | 115 | -0.094479056 | NXF2B | nuclear RNA export factor 2B [Source:HGNC Symbol;Acc:HGNC:23984] |
| ENSG00000275302 | HGLibA_08028 | 115 | -0.094479056 | CCL4 | C-C motif chemokine ligand 4 [Source:HGNC Symbol;Acc:HGNC:10630] |
| ENSG00000281887 | HGLibA_19072 | 115 | -0.094479056 | GIMAP1-GIMAP5 | GIMAP1-GIMAP5 readthrough [Source:HGNC Symbol;Acc:HGNC:51257] |
| HIST1H1T | HGLibA_21373 | 115 | -0.094479056 | HIST1H1T | - |
| HIST1H2BO | HGLibA_21454 | 115 | -0.094479056 | HIST1H2BO | - |
| hsa-mir-124-1 | HGLibA_57348 | 115 | -0.094479056 | hsa-mir-124-1 | - |
| hsa-mir-16-1 | HGLibA_57945 | 115 | -0.094479056 | hsa-mir-16-1 | - |
| hsa-mir-1914 | HGLibA_58061 | 115 | -0.094479056 | hsa-mir-1914 | - |
| hsa-mir-27a | HGLibA_58375 | 115 | -0.094479056 | hsa-mir-27a | - |
| hsa-mir-3617 | HGLibA_59097 | 115 | -0.094479056 | hsa-mir-3617 | - |
| hsa-mir-4256 | HGLibA_59730 | 115 | -0.094479056 | hsa-mir-4256 | - |
| hsa-mir-4435-2 | HGLibA_60134 | 115 | -0.094479056 | hsa-mir-4435-2 | - |
| hsa-mir-4458 | HGLibA_60237 | 115 | -0.094479056 | hsa-mir-4458 | - |
| hsa-mir-4645 | HGLibA_60683 | 115 | -0.094479056 | hsa-mir-4645 | - |
| hsa-mir-4714 | HGLibA_60955 | 115 | -0.094479056 | hsa-mir-4714 | - |
| hsa-mir-5011 | HGLibA_61458 | 115 | -0.094479056 | hsa-mir-5011 | - |
| hsa-mir-645 | HGLibA_62823 | 115 | -0.094479056 | hsa-mir-645 | - |
| hsa-mir-6837 | HGLibA_63525 | 115 | -0.094479056 | hsa-mir-6837 | - |
| hsa-mir-6867 | HGLibA_63653 | 115 | -0.094479056 | hsa-mir-6867 | - |
| hsa-mir-7847 | HGLibA_63978 | 115 | -0.094479056 | hsa-mir-7847 | - |
| hsa-mir-98 | HGLibA_64372 | 115 | -0.094479056 | hsa-mir-98 | - |
| LOC100129520 | HGLibA_26766 | 115 | -0.094479056 | LOC100129520 | - |
| NOV | HGLibA_32360 | 115 | -0.094479056 | NOV | - |
| PAPD7 | HGLibA_35128 | 115 | -0.094479056 | PAPD7 | - |
| PLK1S1 | HGLibA_37189 | 115 | -0.094479056 | PLK1S1 | - |
| PRSS45 | HGLibA_38945 | 115 | -0.094479056 | PRSS45 | - |
| SPANXB2 | HGLibA_46388 | 115 | -0.094479056 | SPANXB2 | - |
| C13orf35 | HGLibA_05309 | 114 | -0.103524399 | C13orf35 | - |
| C1orf186 | HGLibA_05808 | 114 | -0.103524399 | C1orf186 | - |
| CXorf61 | HGLibA_11953 | 114 | -0.103524399 | CXorf61 | - |
| DBC1 | HGLibA_12374 | 114 | -0.103524399 | DBC1 | - |
| ENSG00000004478 | HGLibA_17577 | 114 | -0.103524399 | FKBP4 | FK506 binding protein 4 [Source:HGNC Symbol;Acc:HGNC:3720] |
| ENSG00000009413 | HGLibA_40888 | 114 | -0.103524399 | REV3L | DNA directed polymerase zeta catalytic subunit [Source:HGNC Symbol;Acc:HGNC:9968]" |
| ENSG00000011451 | HGLibA_54327 | 114 | -0.103524399 | WIZ | widely interspaced zinc finger motifs [Source:HGNC Symbol;Acc:HGNC:30917] |
| ENSG00000016602 | HGLibA_09810 | 114 | -0.103524399 | CLCA4 | chloride channel accessory 4 [Source:HGNC Symbol;Acc:HGNC:2018] |
| ENSG00000017483 | HGLibA_45073 | 114 | -0.103524399 | SLC38A5 | solute carrier family 38 member 5 [Source:HGNC Symbol;Acc:HGNC:18070] |
| ENSG00000026508 | HGLibA_08389 | 114 | -0.103524399 | CD44 | CD44 molecule (Indian blood group) [Source:HGNC Symbol;Acc:HGNC:1681] |
| ENSG00000041988 | HGLibA_49297 | 114 | -0.103524399 | THAP3 | THAP domain containing 3 [Source:HGNC Symbol;Acc:HGNC:20855] |
| ENSG00000042286 | HGLibA_01340 | 114 | -0.103524399 | AIFM2 | mitochondria associated 2 [Source:HGNC Symbol;Acc:HGNC:21411]" |
| ENSG00000065675 | HGLibA_38557 | 114 | -0.103524399 | PRKCQ | protein kinase C theta [Source:HGNC Symbol;Acc:HGNC:9410] |
| ENSG00000066827 | HGLibA_55164 | 114 | -0.103524399 | ZFAT | zinc finger and AT-hook domain containing [Source:HGNC Symbol;Acc:HGNC:19899] |
| ENSG00000067533 | HGLibA_42273 | 114 | -0.103524399 | RRP15 | ribosomal RNA processing 15 homolog [Source:HGNC Symbol;Acc:HGNC:24255] |
| ENSG00000073969 | HGLibA_32750 | 114 | -0.103524399 | NSF | vesicle fusing ATPase [Source:HGNC Symbol;Acc:HGNC:8016]" |
| ENSG00000075399 | HGLibA_53821 | 114 | -0.103524399 | VPS9D1 | VPS9 domain containing 1 [Source:HGNC Symbol;Acc:HGNC:13526] |
| ENSG00000076321 | HGLibA_25149 | 114 | -0.103524399 | KLHL20 | kelch like family member 20 [Source:HGNC Symbol;Acc:HGNC:25056] |
| ENSG00000077097 | HGLibA_50997 | 114 | -0.103524399 | TOP2B | DNA topoisomerase II beta [Source:HGNC Symbol;Acc:HGNC:11990] |
| ENSG00000077721 | HGLibA_52574 | 114 | -0.103524399 | UBE2A | ubiquitin conjugating enzyme E2 A [Source:HGNC Symbol;Acc:HGNC:12472] |
| ENSG00000081052 | HGLibA_10579 | 114 | -0.103524399 | COL4A4 | collagen type IV alpha 4 chain [Source:HGNC Symbol;Acc:HGNC:2206] |
| ENSG00000084764 | HGLibA_28320 | 114 | -0.103524399 | MAPRE3 | microtubule associated protein RP/EB family member 3 [Source:HGNC Symbol;Acc:HGNC:6892] |
| ENSG00000089053 | HGLibA_01886 | 114 | -0.103524399 | ANAPC5 | anaphase promoting complex subunit 5 [Source:HGNC Symbol;Acc:HGNC:15713] |
| ENSG00000091592 | HGLibA_32098 | 114 | -0.103524399 | NLRP1 | NLR family pyrin domain containing 1 [Source:HGNC Symbol;Acc:HGNC:14374] |
| ENSG00000092295 | HGLibA_49252 | 114 | -0.103524399 | TGM1 | transglutaminase 1 [Source:HGNC Symbol;Acc:HGNC:11777] |
| ENSG00000099308 | HGLibA_28405 | 114 | -0.103524399 | MAST3 | microtubule associated serine/threonine kinase 3 [Source:HGNC Symbol;Acc:HGNC:19036] |
| ENSG00000099960 | HGLibA_45366 | 114 | -0.103524399 | SLC7A4 | solute carrier family 7 member 4 [Source:HGNC Symbol;Acc:HGNC:11062] |
| ENSG00000099974 | HGLibA_12646 | 114 | -0.103524399 | DDTL | D-dopachrome tautomerase like [Source:HGNC Symbol;Acc:HGNC:33446] |
| ENSG00000100558 | HGLibA_37060 | 114 | -0.103524399 | PLEK2 | pleckstrin 2 [Source:HGNC Symbol;Acc:HGNC:19238] |
| ENSG00000103353 | HGLibA_52697 | 114 | -0.103524399 | UBFD1 | ubiquitin family domain containing 1 [Source:HGNC Symbol;Acc:HGNC:30565] |
| ENSG00000105655 | HGLibA_23699 | 114 | -0.103524399 | ISYNA1 | inositol-3-phosphate synthase 1 [Source:HGNC Symbol;Acc:HGNC:29821] |
| ENSG00000106003 | HGLibA_26342 | 114 | -0.103524399 | LFNG | LFNG O-fucosylpeptide 3-beta-N-acetylglucosaminyltransferase [Source:HGNC Symbol;Acc:HGNC:6560] |
| ENSG00000109270 | HGLibA_25995 | 114 | -0.103524399 | LAMTOR3 | MAPK and MTOR activator 3 [Source:HGNC Symbol;Acc:HGNC:15606]" |
| ENSG00000110092 | HGLibA_08072 | 114 | -0.103524399 | CCND1 | cyclin D1 [Source:HGNC Symbol;Acc:HGNC:1582] |
| ENSG00000111961 | HGLibA_42760 | 114 | -0.103524399 | SASH1 | SAM and SH3 domain containing 1 [Source:HGNC Symbol;Acc:HGNC:19182] |
| ENSG00000112276 | HGLibA_05005 | 114 | -0.103524399 | BVES | blood vessel epicardial substance [Source:HGNC Symbol;Acc:HGNC:1152] |
| ENSG00000112739 | HGLibA_38739 | 114 | -0.103524399 | PRPF4B | pre-mRNA processing factor 4B [Source:HGNC Symbol;Acc:HGNC:17346] |
| ENSG00000113272 | HGLibA_49355 | 114 | -0.103524399 | THG1L | tRNA-histidine guanylyltransferase 1 like [Source:HGNC Symbol;Acc:HGNC:26053] |
| ENSG00000113456 | HGLibA_40054 | 114 | -0.103524399 | RAD1 | RAD1 checkpoint DNA exonuclease [Source:HGNC Symbol;Acc:HGNC:9806] |
| ENSG00000114388 | HGLibA_32516 | 114 | -0.103524399 | NPRL2 | GATOR1 complex subunit [Source:HGNC Symbol;Acc:HGNC:24969]" |
| ENSG00000114784 | HGLibA_14717 | 114 | -0.103524399 | EIF1B | eukaryotic translation initiation factor 1B [Source:HGNC Symbol;Acc:HGNC:30792] |
| ENSG00000115271 | HGLibA_18758 | 114 | -0.103524399 | GCA | grancalcin [Source:HGNC Symbol;Acc:HGNC:15990] |
| ENSG00000115556 | HGLibA_36997 | 114 | -0.103524399 | PLCD4 | phospholipase C delta 4 [Source:HGNC Symbol;Acc:HGNC:9062] |
| ENSG00000115839 | HGLibA_39918 | 114 | -0.103524399 | RAB3GAP1 | RAB3 GTPase activating protein catalytic subunit 1 [Source:HGNC Symbol;Acc:HGNC:17063] |
| ENSG00000116157 | HGLibA_20188 | 114 | -0.103524399 | GPX7 | glutathione peroxidase 7 [Source:HGNC Symbol;Acc:HGNC:4559] |
| ENSG00000118971 | HGLibA_08076 | 114 | -0.103524399 | CCND2 | cyclin D2 [Source:HGNC Symbol;Acc:HGNC:1583] |
| ENSG00000120242 | HGLibA_22692 | 114 | -0.103524399 | IFNA8 | interferon alpha 8 [Source:HGNC Symbol;Acc:HGNC:5429] |
| ENSG00000122359 | HGLibA_02224 | 114 | -0.103524399 | ANXA11 | annexin A11 [Source:HGNC Symbol;Acc:HGNC:535] |
| ENSG00000122707 | HGLibA_40764 | 114 | -0.103524399 | RECK | reversion inducing cysteine rich protein with kazal motifs [Source:HGNC Symbol;Acc:HGNC:11345] |
| ENSG00000125266 | HGLibA_14600 | 114 | -0.103524399 | EFNB2 | ephrin B2 [Source:HGNC Symbol;Acc:HGNC:3227] |
| ENSG00000125652 | HGLibA_01665 | 114 | -0.103524399 | ALKBH7 | alkB homolog 7 [Source:HGNC Symbol;Acc:HGNC:21306] |
| ENSG00000125834 | HGLibA_47496 | 114 | -0.103524399 | STK35 | serine/threonine kinase 35 [Source:HGNC Symbol;Acc:HGNC:16254] |
| ENSG00000126088 | HGLibA_53155 | 114 | -0.103524399 | UROD | uroporphyrinogen decarboxylase [Source:HGNC Symbol;Acc:HGNC:12591] |
| ENSG00000126259 | HGLibA_25004 | 114 | -0.103524399 | KIRREL2 | kirre like nephrin family adhesion molecule 2 [Source:HGNC Symbol;Acc:HGNC:18816] |
| ENSG00000128313 | HGLibA_02538 | 114 | -0.103524399 | APOL5 | apolipoprotein L5 [Source:HGNC Symbol;Acc:HGNC:14869] |
| ENSG00000130856 | HGLibA_55651 | 114 | -0.103524399 | ZNF236 | zinc finger protein 236 [Source:HGNC Symbol;Acc:HGNC:13028] |
| ENSG00000131686 | HGLibA_06819 | 114 | -0.103524399 | CA6 | carbonic anhydrase 6 [Source:HGNC Symbol;Acc:HGNC:1380] |
| ENSG00000133731 | HGLibA_23243 | 114 | -0.103524399 | IMPA1 | inositol monophosphatase 1 [Source:HGNC Symbol;Acc:HGNC:6050] |
| ENSG00000135090 | HGLibA_48313 | 114 | -0.103524399 | TAOK3 | TAO kinase 3 [Source:HGNC Symbol;Acc:HGNC:18133] |
| ENSG00000135175 | HGLibA_33260 | 114 | -0.103524399 | OCM2 | oncomodulin 2 [Source:HGNC Symbol;Acc:HGNC:34396] |
| ENSG00000136161 | HGLibA_40680 | 114 | -0.103524399 | RCBTB2 | RCC1 and BTB domain containing protein 2 [Source:HGNC Symbol;Acc:HGNC:1914] |
| ENSG00000136237 | HGLibA_40258 | 114 | -0.103524399 | RAPGEF5 | Rap guanine nucleotide exchange factor 5 [Source:HGNC Symbol;Acc:HGNC:16862] |
| ENSG00000137309 | HGLibA_21683 | 114 | -0.103524399 | HMGA1 | high mobility group AT-hook 1 [Source:HGNC Symbol;Acc:HGNC:5010] |
| ENSG00000137486 | HGLibA_03106 | 114 | -0.103524399 | ARRB1 | arrestin beta 1 [Source:HGNC Symbol;Acc:HGNC:711] |
| ENSG00000137806 | HGLibA_31482 | 114 | -0.103524399 | NDUFAF1 | NADH:ubiquinone oxidoreductase complex assembly factor 1 [Source:HGNC Symbol;Acc:HGNC:18828] |
| ENSG00000139192 | HGLibA_48323 | 114 | -0.103524399 | TAPBPL | TAP binding protein like [Source:HGNC Symbol;Acc:HGNC:30683] |
| ENSG00000139641 | HGLibA_15606 | 114 | -0.103524399 | ESYT1 | extended synaptotagmin 1 [Source:HGNC Symbol;Acc:HGNC:29534] |
| ENSG00000140548 | HGLibA_56610 | 114 | -0.103524399 | ZNF710 | zinc finger protein 710 [Source:HGNC Symbol;Acc:HGNC:25352] |
| ENSG00000140557 | HGLibA_47291 | 114 | -0.103524399 | ST8SIA2 | 8-sialyltransferase 2 [Source:HGNC Symbol;Acc:HGNC:10870]" |
| ENSG00000140937 | HGLibA_08635 | 114 | -0.103524399 | CDH11 | cadherin 11 [Source:HGNC Symbol;Acc:HGNC:1750] |
| ENSG00000140937 | HGLibA_08634 | 114 | -0.103524399 | CDH11 | cadherin 11 [Source:HGNC Symbol;Acc:HGNC:1750] |
| ENSG00000141519 | HGLibA_07776 | 114 | -0.103524399 | CCDC40 | coiled-coil domain containing 40 [Source:HGNC Symbol;Acc:HGNC:26090] |
| ENSG00000141965 | HGLibA_17273 | 114 | -0.103524399 | FEM1A | fem-1 homolog A [Source:HGNC Symbol;Acc:HGNC:16934] |
| ENSG00000143153 | HGLibA_03594 | 114 | -0.103524399 | ATP1B1 | ATPase Na+/K+ transporting subunit beta 1 [Source:HGNC Symbol;Acc:HGNC:804] |
| ENSG00000143452 | HGLibA_21888 | 114 | -0.103524399 | HORMAD1 | HORMA domain containing 1 [Source:HGNC Symbol;Acc:HGNC:25245] |
| ENSG00000148334 | HGLibA_39329 | 114 | -0.103524399 | PTGES2 | prostaglandin E synthase 2 [Source:HGNC Symbol;Acc:HGNC:17822] |
| ENSG00000149591 | HGLibA_48271 | 114 | -0.103524399 | TAGLN | transgelin [Source:HGNC Symbol;Acc:HGNC:11553] |
| ENSG00000149634 | HGLibA_46459 | 114 | -0.103524399 | SPATA25 | spermatogenesis associated 25 [Source:HGNC Symbol;Acc:HGNC:16158] |
| ENSG00000150433 | HGLibA_50228 | 114 | -0.103524399 | TMEM218 | transmembrane protein 218 [Source:HGNC Symbol;Acc:HGNC:27344] |

| | | | | | |
|---|---|---|---|---|---|
| ENSG00000151715 | HGLibA_50412 | 114 | -0.103524399 | TMEM45B | transmembrane protein 45B [Source:HGNC Symbol;Acc:HGNC:25194] |
| ENSG00000153208 | HGLibA_28908 | 114 | -0.103524399 | MERTK | tyrosine kinase [Source:HGNC Symbol;Acc:HGNC:7027]" |
| ENSG00000153879 | HGLibA_08967 | 114 | -0.103524399 | CEBPG | CCAAT enhancer binding protein gamma [Source:HGNC Symbol;Acc:HGNC:1837] |
| ENSG00000153933 | HGLibA_13068 | 114 | -0.103524399 | DGKE | diacylglycerol kinase epsilon [Source:HGNC Symbol;Acc:HGNC:2852] |
| ENSG00000154188 | HGLibA_01902 | 114 | -0.103524399 | ANGPT1 | angiopoietin 1 [Source:HGNC Symbol;Acc:HGNC:484] |
| ENSG00000156886 | HGLibA_23751 | 114 | -0.103524399 | ITGAD | integrin subunit alpha D [Source:HGNC Symbol;Acc:HGNC:6146] |
| ENSG00000159224 | HGLibA_19114 | 114 | -0.103524399 | GIP | gastric inhibitory polypeptide [Source:HGNC Symbol;Acc:HGNC:4270] |
| ENSG00000160214 | HGLibA_42267 | 114 | -0.103524399 | RRP1 | ribosomal RNA processing 1 [Source:HGNC Symbol;Acc:HGNC:18785] |
| ENSG00000161798 | HGLibA_02598 | 114 | -0.103524399 | AQP5 | aquaporin 5 [Source:HGNC Symbol;Acc:HGNC:638] |
| ENSG00000162614 | HGLibA_31755 | 114 | -0.103524399 | NEXN | nexilin F-actin binding protein [Source:HGNC Symbol;Acc:HGNC:29557] |
| ENSG00000163348 | HGLibA_39698 | 114 | -0.103524399 | PYGO2 | pygopus family PHD finger 2 [Source:HGNC Symbol;Acc:HGNC:30257] |
| ENSG00000163815 | HGLibA_09977 | 114 | -0.103524399 | CLEC3B | C-type lectin domain family 3 member B [Source:HGNC Symbol;Acc:HGNC:11891] |
| ENSG00000164506 | HGLibA_47675 | 114 | -0.103524399 | STXBP5 | syntaxin binding protein 5 [Source:HGNC Symbol;Acc:HGNC:19665] |
| ENSG00000164620 | HGLibA_40821 | 114 | -0.103524399 | RELL2 | RELT like 2 [Source:HGNC Symbol;Acc:HGNC:26902] |
| ENSG00000165828 | HGLibA_38334 | 114 | -0.103524399 | PRAP1 | proline rich acidic protein 1 [Source:HGNC Symbol;Acc:HGNC:23304] |
| ENSG00000166153 | HGLibA_12979 | 114 | -0.103524399 | DEPDC4 | DEP domain containing 4 [Source:HGNC Symbol;Acc:HGNC:22952] |
| ENSG00000166326 | HGLibA_51508 | 114 | -0.103524399 | TRIM44 | tripartite motif containing 44 [Source:HGNC Symbol;Acc:HGNC:19016] |
| ENSG00000167378 | HGLibA_23616 | 114 | -0.103524399 | IRGQ | immunity related GTPase Q [Source:HGNC Symbol;Acc:HGNC:24868] |
| ENSG00000167757 | HGLibA_25236 | 114 | -0.103524399 | KLK11 | kallikrein related peptidase 11 [Source:HGNC Symbol;Acc:HGNC:6359] |
| ENSG00000168135 | HGLibA_24280 | 114 | -0.103524399 | KCNJ4 | potassium voltage-gated channel subfamily J member 4 [Source:HGNC Symbol;Acc:HGNC:6265] |
| ENSG00000168140 | HGLibA_53535 | 114 | -0.103524399 | VASN | vasorin [Source:HGNC Symbol;Acc:HGNC:18517] |
| ENSG00000169495 | HGLibA_22417 | 114 | -0.103524399 | HTRA4 | HtrA serine peptidase 4 [Source:HGNC Symbol;Acc:HGNC:26909] |
| ENSG00000169762 | HGLibA_48327 | 114 | -0.103524399 | TAPT1 | transmembrane anterior posterior transformation 1 [Source:HGNC Symbol;Acc:HGNC:26887] |
| ENSG00000170537 | HGLibA_49767 | 114 | -0.103524399 | TMC7 | transmembrane channel like 7 [Source:HGNC Symbol;Acc:HGNC:23000] |
| ENSG00000170633 | HGLibA_41668 | 114 | -0.103524399 | RNF34 | ring finger protein 34 [Source:HGNC Symbol;Acc:HGNC:17297] |
| ENSG00000171133 | HGLibA_33815 | 114 | -0.103524399 | OR2K2 | olfactory receptor family 2 subfamily K member 2 [Source:HGNC Symbol;Acc:HGNC:8264] |
| ENSG00000171169 | HGLibA_31079 | 114 | -0.103524399 | NAIF1 | nuclear apoptosis inducing factor 1 [Source:HGNC Symbol;Acc:HGNC:25446] |
| ENSG00000172458 | HGLibA_22999 | 114 | -0.103524399 | IL17D | interleukin 17D [Source:HGNC Symbol;Acc:HGNC:5984] |
| ENSG00000172771 | HGLibA_14525 | 114 | -0.103524399 | EFCAB12 | EF-hand calcium binding domain 12 [Source:HGNC Symbol;Acc:HGNC:28061] |
| ENSG00000173262 | HGLibA_44867 | 114 | -0.103524399 | SLC2A14 | solute carrier family 2 member 14 [Source:HGNC Symbol;Acc:HGNC:18301] |
| ENSG00000174442 | HGLibA_57067 | 114 | -0.103524399 | ZWILCH | zwilch kinetochore protein [Source:HGNC Symbol;Acc:HGNC:25468] |
| ENSG00000174928 | HGLibA_06229 | 114 | -0.103524399 | C3orf33 | chromosome 3 open reading frame 33 [Source:HGNC Symbol;Acc:HGNC:26434] |
| ENSG00000175344 | HGLibA_09582 | 114 | -0.103524399 | CHRNA7 | cholinergic receptor nicotinic alpha 7 subunit [Source:HGNC Symbol;Acc:HGNC:1960] |
| ENSG00000175485 | HGLibA_34209 | 114 | -0.103524399 | OR52W1 | olfactory receptor family 52 subfamily W member 1 [Source:HGNC Symbol;Acc:HGNC:15239] |
| ENSG00000179083 | HGLibA_16116 | 114 | -0.103524399 | FAM133A | family with sequence similarity 133 member A [Source:HGNC Symbol;Acc:HGNC:26748] |
| ENSG00000179218 | HGLibA_07045 | 114 | -0.103524399 | CALR | calreticulin [Source:HGNC Symbol;Acc:HGNC:1455] |
| ENSG00000179403 | HGLibA_53896 | 114 | -0.103524399 | VWA1 | von Willebrand factor A domain containing 1 [Source:HGNC Symbol;Acc:HGNC:30910] |
| ENSG00000180257 | HGLibA_56812 | 114 | -0.103524399 | ZNF816 | zinc finger protein 816 [Source:HGNC Symbol;Acc:HGNC:26995] |
| ENSG00000181718 | HGLibA_34359 | 114 | -0.103524399 | OR5T2 | olfactory receptor family 5 subfamily T member 2 [Source:HGNC Symbol;Acc:HGNC:15296] |
| ENSG00000181963 | HGLibA_34185 | 114 | -0.103524399 | OR52K2 | olfactory receptor family 52 subfamily K member 2 [Source:HGNC Symbol;Acc:HGNC:15223] |
| ENSG00000182613 | HGLibA_33899 | 114 | -0.103524399 | OR2V2 | olfactory receptor family 2 subfamily V member 2 [Source:HGNC Symbol;Acc:HGNC:15341] |
| ENSG00000182752 | HGLibA_35144 | 114 | -0.103524399 | PAPPA | pappalysin 1 [Source:HGNC Symbol;Acc:HGNC:8602] |
| ENSG00000183753 | HGLibA_04743 | 114 | -0.103524399 | BPY2 | basic charge Y-linked 2 [Source:HGNC Symbol;Acc:HGNC:13508] |
| ENSG00000184292 | HGLibA_48179 | 114 | -0.103524399 | TACSTD2 | tumor associated calcium signal transducer 2 [Source:HGNC Symbol;Acc:HGNC:11530] |
| ENSG00000184465 | HGLibA_54058 | 114 | -0.103524399 | WDR27 | WD repeat domain 27 [Source:HGNC Symbol;Acc:HGNC:21248] |
| ENSG00000185245 | HGLibA_19700 | 114 | -0.103524399 | GP1BA | glycoprotein Ib platelet subunit alpha [Source:HGNC Symbol;Acc:HGNC:4439] |
| ENSG00000186020 | HGLibA_56172 | 114 | -0.103524399 | ZNF529 | zinc finger protein 529 [Source:HGNC Symbol;Acc:HGNC:29328] |
| ENSG00000186790 | HGLibA_17860 | 114 | -0.103524399 | FOXE3 | forkhead box E3 [Source:HGNC Symbol;Acc:HGNC:3808] |
| ENSG00000187537 | HGLibA_37734 | 114 | -0.103524399 | POTEG | POTE ankyrin domain family member G [Source:HGNC Symbol;Acc:HGNC:33896] |
| ENSG00000187912 | HGLibA_09939 | 114 | -0.103524399 | CLEC17A | C-type lectin domain containing 17A [Source:HGNC Symbol;Acc:HGNC:34520] |
| ENSG00000196557 | HGLibA_06897 | 114 | -0.103524399 | CACNA1H | calcium voltage-gated channel subunit alpha1 H [Source:HGNC Symbol;Acc:HGNC:1395] |
| ENSG00000197461 | HGLibA_35840 | 114 | -0.103524399 | PDGFA | platelet derived growth factor subunit A [Source:HGNC Symbol;Acc:HGNC:8799] |
| ENSG00000197816 | HGLibA_07721 | 114 | -0.103524399 | CCDC180 | coiled-coil domain containing 180 [Source:HGNC Symbol;Acc:HGNC:29303] |
| ENSG00000197951 | HGLibA_56609 | 114 | -0.103524399 | ZNF71 | zinc finger protein 71 [Source:HGNC Symbol;Acc:HGNC:13141] |
| ENSG00000198042 | HGLibA_28019 | 114 | -0.103524399 | MAK16 | MAK16 homolog [Source:HGNC Symbol;Acc:HGNC:13703] |
| ENSG00000198798 | HGLibA_27947 | 114 | -0.103524399 | MAGEB3 | MAGE family member B3 [Source:HGNC Symbol;Acc:HGNC:6810] |
| ENSG00000198856 | HGLibA_34709 | 114 | -0.103524399 | OSTC | oligosaccharyltransferase complex non-catalytic subunit [Source:HGNC Symbol;Acc:HGNC:24448] |
| ENSG00000198862 | HGLibA_27615 | 114 | -0.103524399 | LTN1 | listerin E3 ubiquitin protein ligase 1 [Source:HGNC Symbol;Acc:HGNC:13082] |
| ENSG00000198879 | HGLibA_43725 | 114 | -0.103524399 | SFMBT2 | Scm like with four mbt domains 2 [Source:HGNC Symbol;Acc:HGNC:20256] |
| ENSG00000203995 | HGLibA_57081 | 114 | -0.103524399 | ZYG11A | cell cycle regulator [Source:HGNC Symbol;Acc:HGNC:32058]" |
| ENSG00000205030 | HGLibA_34326 | 114 | -0.103524399 | OR5L2 | olfactory receptor family 5 subfamily L member 2 [Source:HGNC Symbol;Acc:HGNC:8351] |
| ENSG00000205857 | HGLibA_31092 | 114 | -0.103524399 | NANOGNB | NANOG neighbor homeobox [Source:HGNC Symbol;Acc:HGNC:24958] |
| ENSG00000206073 | HGLibA_43553 | 114 | -0.103524399 | SERPINB4 | serpin family B member 4 [Source:HGNC Symbol;Acc:HGNC:10570] |
| ENSG00000213809 | HGLibA_25316 | 114 | -0.103524399 | KLRK1 | killer cell lectin like receptor K1 [Source:HGNC Symbol;Acc:HGNC:18788] |
| ENSG00000215454 | HGLibA_25608 | 114 | -0.103524399 | KRTAP10-4 | keratin associated protein 10-4 [Source:HGNC Symbol;Acc:HGNC:20521] |
| ENSG00000221996 | HGLibA_34147 | 114 | -0.103524399 | OR52B4 | olfactory receptor family 52 subfamily B member 4 (gene/pseudogene) [Source:HGNC Symbol;Acc:HGNC:15209] |
| ENSG00000226479 | HGLibA_50124 | 114 | -0.103524399 | TMEM185B | transmembrane protein 185B [Source:HGNC Symbol;Acc:HGNC:18896] |
| ENSG00000231637 | HGLibA_53269 | 114 | -0.103524399 | USP17L29 | ubiquitin specific peptidase 17-like family member 29 [Source:HGNC Symbol;Acc:HGNC:44457] |
| ENSG00000239713 | HGLibA_02491 | 114 | -0.103524399 | APOBEC3G | apolipoprotein B mRNA editing enzyme catalytic subunit 3G [Source:HGNC Symbol;Acc:HGNC:17357] |
| ENSG00000239732 | HGLibA_49668 | 114 | -0.103524399 | TLR9 | toll like receptor 9 [Source:HGNC Symbol;Acc:HGNC:15633] |
| ENSG00000243566 | HGLibA_53080 | 114 | -0.103524399 | UPK3B | uroplakin 3B [Source:HGNC Symbol;Acc:HGNC:21444] |
| ENSG00000248643 | HGLibA_40489 | 114 | -0.103524399 | RBM14-RBM4 | RBM14-RBM4 readthrough [Source:HGNC Symbol;Acc:HGNC:38840] |
| ENSG00000249709 | HGLibA_56254 | 114 | -0.103524399 | ZNF564 | zinc finger protein 564 [Source:HGNC Symbol;Acc:HGNC:31106] |
| ENSG00000262209 | HGLibA_35556 | 114 | -0.103524399 | PCDHGB3 | 3 [Source:HGNC Symbol;Acc:HGNC:8710]" |
| ENSG00000268221 | HGLibA_33424 | 114 | -0.103524399 | OPN1MW | medium wave sensitive [Source:HGNC Symbol;Acc:HGNC:4206]" |
| ENSG00000269343 | HGLibA_56331 | 114 | -0.103524399 | ZNF587B | zinc finger protein 587B [Source:HGNC Symbol;Acc:HGNC:37142] |
| ENSG00000279270 | HGLibA_34205 | 114 | -0.103524399 | OR52R1 | olfactory receptor family 52 subfamily R member 1 (gene/pseudogene) [Source:HGNC Symbol;Acc:HGNC:15235] |
| FAM129B | HGLibA_16098 | 114 | -0.103524399 | FAM129B | - |
| hsa-mir-1244-1 | HGLibA_57364 | 114 | -0.103524399 | hsa-mir-1244-1 | - |
| hsa-mir-196a-2 | HGLibA_58095 | 114 | -0.103524399 | hsa-mir-196a-2 | - |
| hsa-mir-202 | HGLibA_58162 | 114 | -0.103524399 | hsa-mir-202 | - |
| hsa-mir-3156-2 | HGLibA_58693 | 114 | -0.103524399 | hsa-mir-3156-2 | - |
| hsa-mir-3919 | HGLibA_59551 | 114 | -0.103524399 | hsa-mir-3919 | - |
| hsa-mir-4431 | HGLibA_60111 | 114 | -0.103524399 | hsa-mir-4431 | - |
| hsa-mir-4650-2 | HGLibA_60708 | 114 | -0.103524399 | hsa-mir-4650-2 | - |
| hsa-mir-4665 | HGLibA_60772 | 114 | -0.103524399 | hsa-mir-4665 | - |
| hsa-mir-4686 | HGLibA_60854 | 114 | -0.103524399 | hsa-mir-4686 | - |
| hsa-mir-4731 | HGLibA_61023 | 114 | -0.103524399 | hsa-mir-4731 | - |
| hsa-mir-510 | HGLibA_61545 | 114 | -0.103524399 | hsa-mir-510 | - |
| hsa-mir-548ab | HGLibA_61858 | 114 | -0.103524399 | hsa-mir-548ab | - |
| hsa-mir-548i-4 | HGLibA_62026 | 114 | -0.103524399 | hsa-mir-548i-4 | - |
| hsa-mir-551a | HGLibA_62118 | 114 | -0.103524399 | hsa-mir-551a | - |
| hsa-mir-577 | HGLibA_62403 | 114 | -0.103524399 | hsa-mir-577 | - |
| hsa-mir-653 | HGLibA_62945 | 114 | -0.103524399 | hsa-mir-653 | - |
| hsa-mir-653 | HGLibA_62946 | 114 | -0.103524399 | hsa-mir-653 | - |
| hsa-mir-6780b | HGLibA_63298 | 114 | -0.103524399 | hsa-mir-6780b | - |
| hsa-mir-6840 | HGLibA_63535 | 114 | -0.103524399 | hsa-mir-6840 | - |
| hsa-mir-6863 | HGLibA_63636 | 114 | -0.103524399 | hsa-mir-6863 | - |
| hsa-mir-9-3 | HGLibA_64269 | 114 | -0.103524399 | hsa-mir-9-3 | - |
| NUPL1 | HGLibA_33082 | 114 | -0.103524399 | NUPL1 | - |
| SMCR7L | HGLibA_45679 | 114 | -0.103524399 | SMCR7L | - |
| C10orf25 | HGLibA_05065 | 113 | -0.112569742 | C10orf25 | - |
| C14orf159 | HGLibA_05329 | 113 | -0.112569742 | C14orf159 | - |
| C17orf85 | HGLibA_05589 | 113 | -0.112569742 | C17orf85 | - |
| C6orf10 | HGLibA_06430 | 113 | -0.112569742 | C6orf10 | - |
| C8orf37 | HGLibA_06613 | 113 | -0.112569742 | C8orf37 | - |
| CCDC114 | HGLibA_07550 | 113 | -0.112569742 | CCDC114 | - |
| CGB | HGLibA_09300 | 113 | -0.112569742 | CGB | - |
| CLLU1OS | HGLibA_10066 | 113 | -0.112569742 | CLLU1OS | - |
| ENSG00000001617 | HGLibA_43334 | 113 | -0.112569742 | SEMA3F | semaphorin 3F [Source:HGNC Symbol;Acc:HGNC:10728] |
| ENSG00000006210 | HGLibA_11834 | 113 | -0.112569742 | CX3CL1 | C-X3-C motif chemokine ligand 1 [Source:HGNC Symbol;Acc:HGNC:10647] |
| ENSG00000006327 | HGLibA_50750 | 113 | -0.112569742 | TNFRSF12A | TNF receptor superfamily member 12A [Source:HGNC Symbol;Acc:HGNC:18152] |
| ENSG00000008394 | HGLibA_29173 | 113 | -0.112569742 | MGST1 | microsomal glutathione S-transferase 1 [Source:HGNC Symbol;Acc:HGNC:7061] |
| ENSG00000010322 | HGLibA_31981 | 113 | -0.112569742 | NISCH | nischarin [Source:HGNC Symbol;Acc:HGNC:18006] |
| ENSG00000027869 | HGLibA_43888 | 113 | -0.112569742 | SH2D2A | SH2 domain containing 2A [Source:HGNC Symbol;Acc:HGNC:10821] |
| ENSG00000038295 | HGLibA_49627 | 113 | -0.112569742 | TLL1 | tolloid like 1 [Source:HGNC Symbol;Acc:HGNC:11843] |
| ENSG00000042304 | HGLibA_06195 | 113 | -0.112569742 | C2orf83 | chromosome 2 open reading frame 83 [Source:HGNC Symbol;Acc:HGNC:25344] |
| ENSG00000049192 | HGLibA_00836 | 113 | -0.112569742 | ADAMTS6 | ADAM metallopeptidase with thrombospondin type 1 motif 6 [Source:HGNC Symbol;Acc:HGNC:222] |
| ENSG00000055483 | HGLibA_53347 | 113 | -0.112569742 | USP36 | ubiquitin specific peptidase 36 [Source:HGNC Symbol;Acc:HGNC:20062] |
| ENSG00000065883 | HGLibA_08734 | 113 | -0.112569742 | CDK13 | cyclin dependent kinase 13 [Source:HGNC Symbol;Acc:HGNC:1733] |
| ENSG00000068615 | HGLibA_40775 | 113 | -0.112569742 | REEP1 | receptor accessory protein 1 [Source:HGNC Symbol;Acc:HGNC:25786] |
| ENSG00000068724 | HGLibA_52151 | 113 | -0.112569742 | TTC7A | tetratricopeptide repeat domain 7A [Source:HGNC Symbol;Acc:HGNC:19750] |
| ENSG00000070495 | HGLibA_23958 | 113 | -0.112569742 | JMJD6 | arginine demethylase and lysine hydroxylase [Source:HGNC Symbol;Acc:HGNC:19355]" |
| ENSG00000072133 | HGLibA_42172 | 113 | -0.112569742 | RPS6KA6 | ribosomal protein S6 kinase A6 [Source:HGNC Symbol;Acc:HGNC:10435] |
| ENSG00000075643 | HGLibA_29610 | 113 | -0.112569742 | MOCOS | molybdenum cofactor sulfurase [Source:HGNC Symbol;Acc:HGNC:18234] |
| ENSG00000081692 | HGLibA_23954 | 113 | -0.112569742 | JMJD4 | jumonji domain containing 4 [Source:HGNC Symbol;Acc:HGNC:25724] |
| ENSG00000087191 | HGLibA_39142 | 113 | -0.112569742 | PSMC5 | ATPase 5 [Source:HGNC Symbol;Acc:HGNC:9552]" |
| ENSG00000088881 | HGLibA_14359 | 113 | -0.112569742 | EBF4 | EBF family member 4 [Source:HGNC Symbol;Acc:HGNC:29278] |
| ENSG00000092295 | HGLibA_49253 | 113 | -0.112569742 | TGM1 | transglutaminase 1 [Source:HGNC Symbol;Acc:HGNC:11777] |
| ENSG00000099810 | HGLibA_30319 | 113 | -0.112569742 | MTAP | methylthioadenosine phosphorylase [Source:HGNC Symbol;Acc:HGNC:7413] |
| ENSG00000099860 | HGLibA_18419 | 113 | -0.112569742 | GADD45B | growth arrest and DNA damage inducible beta [Source:HGNC Symbol;Acc:HGNC:4096] |
| ENSG00000100433 | HGLibA_24296 | 113 | -0.112569742 | KCNK10 | potassium two pore domain channel subfamily K member 10 [Source:HGNC Symbol;Acc:HGNC:6273] |
| ENSG00000101160 | HGLibA_11738 | 113 | -0.112569742 | CTSZ | cathepsin Z [Source:HGNC Symbol;Acc:HGNC:2547] |
| ENSG00000102595 | HGLibA_52870 | 113 | -0.112569742 | UGGT2 | UDP-glucose glycoprotein glucosyltransferase 2 [Source:HGNC Symbol;Acc:HGNC:15664] |
| ENSG00000102931 | HGLibA_02959 | 113 | -0.112569742 | ARL2BP | ADP ribosylation factor like GTPase 2 binding protein [Source:HGNC Symbol;Acc:HGNC:17146] |

| | | | | | |
|---|---|---|---|---|---|
| ENSG00000103152 | HGLibA_29719 | 113 | -0.112569742 | MPG | N-methylpurine DNA glycosylase [Source:HGNC Symbol;Acc:HGNC:7211] |
| ENSG00000104972 | HGLibA_26506 | 113 | -0.112569742 | LILRB1 | leukocyte immunoglobulin like receptor B1 [Source:HGNC Symbol;Acc:HGNC:6605] |
| ENSG00000105610 | HGLibA_25039 | 113 | -0.112569742 | KLF1 | Kruppel like factor 1 [Source:HGNC Symbol;Acc:HGNC:6345] |
| ENSG00000106511 | HGLibA_28892 | 113 | -0.112569742 | MEOX2 | mesenchyme homeobox 2 [Source:HGNC Symbol;Acc:HGNC:7014] |
| ENSG00000106560 | HGLibA_19076 | 113 | -0.112569742 | GIMAP2 | IMAP family member 2 [Source:HGNC Symbol;Acc:HGNC:21789]" |
| ENSG00000106686 | HGLibA_46526 | 113 | -0.112569742 | SPATA6L | spermatogenesis associated 6 like [Source:HGNC Symbol;Acc:HGNC:25472] |
| ENSG00000107625 | HGLibA_12731 | 113 | -0.112569742 | DDX50 | DExD-box helicase 50 [Source:HGNC Symbol;Acc:HGNC:17906] |
| ENSG00000108406 | HGLibA_13199 | 113 | -0.112569742 | DHX40 | DEAH-box helicase 40 [Source:HGNC Symbol;Acc:HGNC:18018] |
| ENSG00000108823 | HGLibA_43795 | 113 | -0.112569742 | SGCA | sarcoglycan alpha [Source:HGNC Symbol;Acc:HGNC:10805] |
| ENSG00000112062 | HGLibA_28262 | 113 | -0.112569742 | MAPK14 | mitogen-activated protein kinase 14 [Source:HGNC Symbol;Acc:HGNC:6876] |
| ENSG00000118520 | HGLibA_02686 | 113 | -0.112569742 | ARG1 | arginase 1 [Source:HGNC Symbol;Acc:HGNC:663] |
| ENSG00000119403 | HGLibA_36356 | 113 | -0.112569742 | PHF19 | PHD finger protein 19 [Source:HGNC Symbol;Acc:HGNC:24566] |
| ENSG00000123213 | HGLibA_32085 | 113 | -0.112569742 | NLN | neurolysin [Source:HGNC Symbol;Acc:HGNC:16058] |
| ENSG00000123595 | HGLibA_39992 | 113 | -0.112569742 | RAB9A | member RAS oncogene family [Source:HGNC Symbol;Acc:HGNC:9792]" |
| ENSG00000124564 | HGLibA_44464 | 113 | -0.112569742 | SLC17A3 | solute carrier family 17 member 3 [Source:HGNC Symbol;Acc:HGNC:10931] |
| ENSG00000125485 | HGLibA_12694 | 113 | -0.112569742 | DDX31 | DEAD-box helicase 31 [Source:HGNC Symbol;Acc:HGNC:16715] |
| ENSG00000125869 | HGLibA_25987 | 113 | -0.112569742 | LAMP5 | lysosomal associated membrane protein family member 5 [Source:HGNC Symbol;Acc:HGNC:16097] |
| ENSG00000125962 | HGLibA_03049 | 113 | -0.112569742 | ARMCX5 | armadillo repeat containing X-linked 5 [Source:HGNC Symbol;Acc:HGNC:25772] |
| ENSG00000128309 | HGLibA_29781 | 113 | -0.112569742 | MPST | mercaptopyruvate sulfurtransferase [Source:HGNC Symbol;Acc:HGNC:7223] |
| ENSG00000128311 | HGLibA_52025 | 113 | -0.112569742 | TST | thiosulfate sulfurtransferase [Source:HGNC Symbol;Acc:HGNC:12388] |
| ENSG00000131914 | HGLibA_26558 | 113 | -0.112569742 | LIN28A | lin-28 homolog A [Source:HGNC Symbol;Acc:HGNC:15986] |
| ENSG00000131931 | HGLibA_49285 | 113 | -0.112569742 | THAP1 | THAP domain containing 1 [Source:HGNC Symbol;Acc:HGNC:20856] |
| ENSG00000131979 | HGLibA_18782 | 113 | -0.112569742 | GCH1 | GTP cyclohydrolase 1 [Source:HGNC Symbol;Acc:HGNC:4193] |
| ENSG00000132581 | HGLibA_43123 | 113 | -0.112569742 | SDF2 | stromal cell derived factor 2 [Source:HGNC Symbol;Acc:HGNC:10675] |
| ENSG00000132953 | HGLibA_54565 | 113 | -0.112569742 | XPO4 | exportin 4 [Source:HGNC Symbol;Acc:HGNC:17796] |
| ENSG00000134013 | HGLibA_27042 | 113 | -0.112569742 | LOXL2 | lysyl oxidase like 2 [Source:HGNC Symbol;Acc:HGNC:6666] |
| ENSG00000134242 | HGLibA_39461 | 113 | -0.112569742 | PTPN22 | non-receptor type 22 [Source:HGNC Symbol;Acc:HGNC:9652]" |
| ENSG00000134363 | HGLibA_18097 | 113 | -0.112569742 | FST | follistatin [Source:HGNC Symbol;Acc:HGNC:3971] |
| ENSG00000134986 | HGLibA_32669 | 113 | -0.112569742 | NREP | neuronal regeneration related protein [Source:HGNC Symbol;Acc:HGNC:16834] |
| ENSG00000136114 | HGLibA_49401 | 113 | -0.112569742 | THSD1 | thrombospondin type 1 domain containing 1 [Source:HGNC Symbol;Acc:HGNC:17754] |
| ENSG00000136231 | HGLibA_22806 | 113 | -0.112569742 | IGF2BP3 | insulin like growth factor 2 mRNA binding protein 3 [Source:HGNC Symbol;Acc:HGNC:28868] |
| ENSG00000137310 | HGLibA_48766 | 113 | -0.112569742 | TCF19 | transcription factor 19 [Source:HGNC Symbol;Acc:HGNC:11629] |
| ENSG00000137720 | HGLibA_05115 | 113 | -0.112569742 | C11orf1 | chromosome 11 open reading frame 1 [Source:HGNC Symbol;Acc:HGNC:1163] |
| ENSG00000137960 | HGLibA_19121 | 113 | -0.112569742 | GIPC2 | GIPC PDZ domain containing family member 2 [Source:HGNC Symbol;Acc:HGNC:18177] |
| ENSG00000138083 | HGLibA_44249 | 113 | -0.112569742 | SIX3 | SIX homeobox 3 [Source:HGNC Symbol;Acc:HGNC:10889] |
| ENSG00000138722 | HGLibA_29547 | 113 | -0.112569742 | MMRN1 | multimerin 1 [Source:HGNC Symbol;Acc:HGNC:7178] |
| ENSG00000139726 | HGLibA_12970 | 113 | -0.112569742 | DENR | density regulated re-initiation and release factor [Source:HGNC Symbol;Acc:HGNC:2769] |
| ENSG00000140543 | HGLibA_13014 | 113 | -0.112569742 | DET1 | COP1 ubiquitin ligase partner [Source:HGNC Symbol;Acc:HGNC:25477]" |
| ENSG00000142233 | HGLibA_32862 | 113 | -0.112569742 | NTN5 | netrin 5 [Source:HGNC Symbol;Acc:HGNC:25208] |
| ENSG00000142515 | HGLibA_25256 | 113 | -0.112569742 | KLK3 | kallikrein related peptidase 3 [Source:HGNC Symbol;Acc:HGNC:6364] |
| ENSG00000143919 | HGLibA_07093 | 113 | -0.112569742 | CAMKMT | calmodulin-lysine N-methyltransferase [Source:HGNC Symbol;Acc:HGNC:26276] |
| ENSG00000143924 | HGLibA_15064 | 113 | -0.112569742 | EML4 | echinoderm microtubule associated protein like 4 [Source:HGNC Symbol;Acc:HGNC:1316] |
| ENSG00000144451 | HGLibA_46350 | 113 | -0.112569742 | SPAG16 | sperm associated antigen 16 [Source:HGNC Symbol;Acc:HGNC:23225] |
| ENSG00000145242 | HGLibA_15300 | 113 | -0.112569742 | EPHA5 | EPH receptor A5 [Source:HGNC Symbol;Acc:HGNC:3389] |
| ENSG00000147650 | HGLibA_27206 | 113 | -0.112569742 | LRP12 | LDL receptor related protein 12 [Source:HGNC Symbol;Acc:HGNC:31708] |
| ENSG00000148377 | HGLibA_22558 | 113 | -0.112569742 | IDI2 | isopentenyl-diphosphate delta isomerase 2 [Source:HGNC Symbol;Acc:HGNC:23487] |
| ENSG00000149090 | HGLibA_35087 | 113 | -0.112569742 | PAMR1 | peptidase domain containing associated with muscle regeneration 1 [Source:HGNC Symbol;Acc:HGNC:24554] |
| ENSG00000149557 | HGLibA_17312 | 113 | -0.112569742 | FEZ1 | fasciculation and elongation protein zeta 1 [Source:HGNC Symbol;Acc:HGNC:3659] |
| ENSG00000150051 | HGLibA_29359 | 113 | -0.112569742 | MKX | mohawk homeobox [Source:HGNC Symbol;Acc:HGNC:23729] |
| ENSG00000153130 | HGLibA_43032 | 113 | -0.112569742 | SCOC | short coiled-coil protein [Source:HGNC Symbol;Acc:HGNC:20335] |
| ENSG00000155008 | HGLibA_02555 | 113 | -0.112569742 | APOOL | apolipoprotein O like [Source:HGNC Symbol;Acc:HGNC:24009] |
| ENSG00000155304 | HGLibA_22265 | 113 | -0.112569742 | HSPA13 | heat shock protein family A (Hsp70) member 13 [Source:HGNC Symbol;Acc:HGNC:11375] |
| ENSG00000156009 | HGLibA_27921 | 113 | -0.112569742 | MAGEA8 | MAGE family member A8 [Source:HGNC Symbol;Acc:HGNC:6806] |
| ENSG00000157212 | HGLibA_35324 | 113 | -0.112569742 | PAXIP1 | PAX interacting protein 1 [Source:HGNC Symbol;Acc:HGNC:8624] |
| ENSG00000159251 | HGLibA_00607 | 113 | -0.112569742 | ACTC1 | cardiac muscle 1 [Source:HGNC Symbol;Acc:HGNC:143]" |
| ENSG00000159842 | HGLibA_00323 | 113 | -0.112569742 | ABR | RhoGEF and GTPase activating protein [Source:HGNC Symbol;Acc:HGNC:81]" |
| ENSG00000160961 | HGLibA_55818 | 113 | -0.112569742 | ZNF333 | zinc finger protein 333 [Source:HGNC Symbol;Acc:HGNC:15624] |
| ENSG00000161298 | HGLibA_55887 | 113 | -0.112569742 | ZNF382 | zinc finger protein 382 [Source:HGNC Symbol;Acc:HGNC:17409] |
| ENSG00000161328 | HGLibA_27391 | 113 | -0.112569742 | LRRC56 | leucine rich repeat containing 56 [Source:HGNC Symbol;Acc:HGNC:25430] |
| ENSG00000162129 | HGLibA_10097 | 113 | -0.112569742 | CLPB | mitochondrial AAA ATPase chaperonin [Source:HGNC Symbol;Acc:HGNC:30664]" |
| ENSG00000162493 | HGLibA_35935 | 113 | -0.112569742 | PDPN | podoplanin [Source:HGNC Symbol;Acc:HGNC:29602] |
| ENSG00000162694 | HGLibA_15811 | 113 | -0.112569742 | EXTL2 | exostosin like glycosyltransferase 2 [Source:HGNC Symbol;Acc:HGNC:3516] |
| ENSG00000162928 | HGLibA_36095 | 113 | -0.112569742 | PEX13 | peroxisomal biogenesis factor 13 [Source:HGNC Symbol;Acc:HGNC:8855] |
| ENSG00000163389 | HGLibA_37477 | 113 | -0.112569742 | POGLUT1 | protein O-glucosyltransferase 1 [Source:HGNC Symbol;Acc:HGNC:22954] |
| ENSG00000163596 | HGLibA_22491 | 113 | -0.112569742 | ICA1L | islet cell autoantigen 1 like [Source:HGNC Symbol;Acc:HGNC:14442] |
| ENSG00000163687 | HGLibA_13663 | 113 | -0.112569742 | DNASE1L3 | deoxyribonuclease 1 like 3 [Source:HGNC Symbol;Acc:HGNC:2959] |
| ENSG00000163888 | HGLibA_07081 | 113 | -0.112569742 | CAMK2N2 | calcium/calmodulin dependent protein kinase II inhibitor 2 [Source:HGNC Symbol;Acc:HGNC:24197] |
| ENSG00000164124 | HGLibA_49971 | 113 | -0.112569742 | TMEM144 | transmembrane protein 144 [Source:HGNC Symbol;Acc:HGNC:25633] |
| ENSG00000165671 | HGLibA_32744 | 113 | -0.112569742 | NSD1 | nuclear receptor binding SET domain protein 1 [Source:HGNC Symbol;Acc:HGNC:14234] |
| ENSG00000165985 | HGLibA_05930 | 113 | -0.112569742 | C1QL3 | complement C1q like 3 [Source:HGNC Symbol;Acc:HGNC:19359] |
| ENSG00000166685 | HGLibA_10471 | 113 | -0.112569742 | COG1 | component of oligomeric golgi complex 1 [Source:HGNC Symbol;Acc:HGNC:6545] |
| ENSG00000166833 | HGLibA_31196 | 113 | -0.112569742 | NAV2 | neuron navigator 2 [Source:HGNC Symbol;Acc:HGNC:15997] |
| ENSG00000166863 | HGLibA_48154 | 113 | -0.112569742 | TAC3 | tachykinin 3 [Source:HGNC Symbol;Acc:HGNC:11521] |
| ENSG00000166938 | HGLibA_13275 | 113 | -0.112569742 | DIS3L | DIS3 like exosome 3'-5' exoribonuclease [Source:HGNC Symbol;Acc:HGNC:28698] |
| ENSG00000167232 | HGLibA_56904 | 113 | -0.112569742 | ZNF91 | zinc finger protein 91 [Source:HGNC Symbol;Acc:HGNC:13166] |
| ENSG00000168079 | HGLibA_42867 | 113 | -0.112569742 | SCARA5 | scavenger receptor class A member 5 [Source:HGNC Symbol;Acc:HGNC:28701] |
| ENSG00000168589 | HGLibA_14263 | 113 | -0.112569742 | DYNLRB2 | dynein light chain roadblock-type 2 [Source:HGNC Symbol;Acc:HGNC:15467] |
| ENSG00000168619 | HGLibA_00743 | 113 | -0.112569742 | ADAM18 | ADAM metallopeptidase domain 18 [Source:HGNC Symbol;Acc:HGNC:196] |
| ENSG00000171307 | HGLibA_55083 | 113 | -0.112569742 | ZDHHC16 | zinc finger DHHC-type containing 16 [Source:HGNC Symbol;Acc:HGNC:20714] |
| ENSG00000172037 | HGLibA_25960 | 113 | -0.112569742 | LAMB2 | laminin subunit beta 2 [Source:HGNC Symbol;Acc:HGNC:6487] |
| ENSG00000172508 | HGLibA_07256 | 113 | -0.112569742 | CARNS1 | carnosine synthase 1 [Source:HGNC Symbol;Acc:HGNC:29268] |
| ENSG00000172548 | HGLibA_31964 | 113 | -0.112569742 | NIPAL4 | NIPA like domain containing 4 [Source:HGNC Symbol;Acc:HGNC:28018] |
| ENSG00000172986 | HGLibA_20684 | 113 | -0.112569742 | GXYLT2 | glucoside xylosyltransferase 2 [Source:HGNC Symbol;Acc:HGNC:33383] |
| ENSG00000173338 | HGLibA_24332 | 113 | -0.112569742 | KCNK7 | potassium two pore domain channel subfamily K member 7 [Source:HGNC Symbol;Acc:HGNC:6282] |
| ENSG00000174951 | HGLibA_18178 | 113 | -0.112569742 | FUT1 | fucosyltransferase 1 (H blood group) [Source:HGNC Symbol;Acc:HGNC:4012] |
| ENSG00000175938 | HGLibA_34596 | 113 | -0.112569742 | ORAI3 | ORAI calcium release-activated calcium modulator 3 [Source:HGNC Symbol;Acc:HGNC:28185] |
| ENSG00000177463 | HGLibA_32598 | 113 | -0.112569742 | NR2C2 | nuclear receptor subfamily 2 group C member 2 [Source:HGNC Symbol;Acc:HGNC:7972] |
| ENSG00000178217 | HGLibA_43902 | 113 | -0.112569742 | SH2D4B | SH2 domain containing 4B [Source:HGNC Symbol;Acc:HGNC:31440] |
| ENSG00000178537 | HGLibA_44668 | 113 | -0.112569742 | SLC25A20 | solute carrier family 25 member 20 [Source:HGNC Symbol;Acc:HGNC:1421] |
| ENSG00000178904 | HGLibA_13912 | 113 | -0.112569742 | DPY19L3 | dpy-19 like C-mannosyltransferase 3 [Source:HGNC Symbol;Acc:HGNC:27120] |
| ENSG00000179813 | HGLibA_16486 | 113 | -0.112569742 | FAM216B | family with sequence similarity 216 member B [Source:HGNC Symbol;Acc:HGNC:26883] |
| ENSG00000179855 | HGLibA_19124 | 113 | -0.112569742 | GIPC3 | GIPC PDZ domain containing family member 3 [Source:HGNC Symbol;Acc:HGNC:18183] |
| ENSG00000180425 | HGLibA_05179 | 113 | -0.112569742 | C11orf71 | chromosome 11 open reading frame 71 [Source:HGNC Symbol;Acc:HGNC:25937] |
| ENSG00000182308 | HGLibA_12443 | 113 | -0.112569742 | DCAF4L1 | DDB1 and CUL4 associated factor 4 like 1 [Source:HGNC Symbol;Acc:HGNC:27723] |
| ENSG00000182979 | HGLibA_30310 | 113 | -0.112569742 | MTA1 | metastasis associated 1 [Source:HGNC Symbol;Acc:HGNC:7410] |
| ENSG00000183715 | HGLibA_33414 | 113 | -0.112569742 | OPCML | opioid binding protein/cell adhesion molecule like [Source:HGNC Symbol;Acc:HGNC:8143] |
| ENSG00000183780 | HGLibA_45007 | 113 | -0.112569742 | SLC35F3 | solute carrier family 35 member F3 [Source:HGNC Symbol;Acc:HGNC:23616] |
| ENSG00000184182 | HGLibA_52603 | 113 | -0.112569742 | UBE2F | ubiquitin conjugating enzyme E2 F (putative) [Source:HGNC Symbol;Acc:HGNC:12480] |
| ENSG00000185088 | HGLibA_42127 | 113 | -0.112569742 | RPS27L | ribosomal protein S27 like [Source:HGNC Symbol;Acc:HGNC:18476] |
| ENSG00000185149 | HGLibA_32553 | 113 | -0.112569742 | NPY2R | neuropeptide Y receptor Y2 [Source:HGNC Symbol;Acc:HGNC:7957] |
| ENSG00000185324 | HGLibA_08722 | 113 | -0.112569742 | CDK10 | cyclin dependent kinase 10 [Source:HGNC Symbol;Acc:HGNC:1770] |
| ENSG00000185905 | HGLibA_05457 | 113 | -0.112569742 | C16orf54 | chromosome 16 open reading frame 54 [Source:HGNC Symbol;Acc:HGNC:26649] |
| ENSG00000186579 | HGLibA_12832 | 113 | -0.112569742 | DEFB106A | defensin beta 106A [Source:HGNC Symbol;Acc:HGNC:18088] |
| ENSG00000186638 | HGLibA_24909 | 113 | -0.112569742 | KIF24 | kinesin family member 24 [Source:HGNC Symbol;Acc:HGNC:19916] |
| ENSG00000187094 | HGLibA_07953 | 113 | -0.112569742 | CCK | cholecystokinin [Source:HGNC Symbol;Acc:HGNC:1569] |
| ENSG00000188324 | HGLibA_34395 | 113 | -0.112569742 | OR6C6 | olfactory receptor family 6 subfamily C member 6 [Source:HGNC Symbol;Acc:HGNC:31293] |
| ENSG00000188613 | HGLibA_31096 | 113 | -0.112569742 | NANOS1 | nanos C2HC-type zinc finger 1 [Source:HGNC Symbol;Acc:HGNC:23044] |
| ENSG00000188647 | HGLibA_39248 | 113 | -0.112569742 | PTAR1 | protein prenyltransferase alpha subunit repeat containing 1 [Source:HGNC Symbol;Acc:HGNC:30449] |
| ENSG00000189420 | HGLibA_55247 | 113 | -0.112569742 | ZFP92 | ZFP92 zinc finger protein [Source:HGNC Symbol;Acc:HGNC:12865] |
| ENSG00000196344 | HGLibA_00965 | 113 | -0.112569742 | ADH7 | mu or sigma polypeptide [Source:HGNC Symbol;Acc:HGNC:256]" |
| ENSG00000196604 | HGLibA_37732 | 113 | -0.112569742 | POTEF | POTE ankyrin domain family member F [Source:HGNC Symbol;Acc:HGNC:33905] |
| ENSG00000197272 | HGLibA_23112 | 113 | -0.112569742 | IL27 | interleukin 27 [Source:HGNC Symbol;Acc:HGNC:19157] |
| ENSG00000197360 | HGLibA_56915 | 113 | -0.112569742 | ZNF98 | zinc finger protein 98 [Source:HGNC Symbol;Acc:HGNC:13174] |
| ENSG00000197712 | HGLibA_16031 | 113 | -0.112569742 | FAM114A1 | family with sequence similarity 114 member A1 [Source:HGNC Symbol;Acc:HGNC:25087] |
| ENSG00000197822 | HGLibA_33255 | 113 | -0.112569742 | OCLN | occludin [Source:HGNC Symbol;Acc:HGNC:8104] |
| ENSG00000197958 | HGLibA_41866 | 113 | -0.112569742 | RPL12 | ribosomal protein L12 [Source:HGNC Symbol;Acc:HGNC:10302] |
| ENSG00000198561 | HGLibA_11660 | 113 | -0.112569742 | CTNND1 | catenin delta 1 [Source:HGNC Symbol;Acc:HGNC:2515] |
| ENSG00000198830 | HGLibA_21719 | 113 | -0.112569742 | HMGN2 | high mobility group nucleosomal binding domain 2 [Source:HGNC Symbol;Acc:HGNC:4986] |
| ENSG00000204397 | HGLibA_07225 | 113 | -0.112569742 | CARD16 | caspase recruitment domain family member 16 [Source:HGNC Symbol;Acc:HGNC:33701] |
| ENSG00000204560 | HGLibA_13168 | 113 | -0.112569742 | DHX16 | DEAH-box helicase 16 [Source:HGNC Symbol;Acc:HGNC:2739] |
| ENSG00000204595 | HGLibA_13899 | 113 | -0.112569742 | DPRX | divergent-paired related homeobox [Source:HGNC Symbol;Acc:HGNC:32166] |
| ENSG00000205442 | HGLibA_23899 | 113 | -0.112569742 | IZUMO3 | IZUMO family member 3 [Source:HGNC Symbol;Acc:HGNC:31421] |
| ENSG00000211445 | HGLibA_20175 | 113 | -0.112569742 | GPX3 | glutathione peroxidase 3 [Source:HGNC Symbol;Acc:HGNC:4555] |
| ENSG00000213927 | HGLibA_08012 | 113 | -0.112569742 | CCL27 | C-C motif chemokine ligand 27 [Source:HGNC Symbol;Acc:HGNC:10626] |
| ENSG00000213967 | HGLibA_56633 | 113 | -0.112569742 | ZNF726 | zinc finger protein 726 [Source:HGNC Symbol;Acc:HGNC:32462] |
| ENSG00000213983 | HGLibA_02288 | 113 | -0.112569742 | AP1G2 | adaptor related protein complex 1 subunit gamma 2 [Source:HGNC Symbol;Acc:HGNC:556] |
| ENSG00000214415 | HGLibA_19478 | 113 | -0.112569742 | GNAT3 | G protein subunit alpha transducin 3 [Source:HGNC Symbol;Acc:HGNC:22800] |
| ENSG00000216649 | HGLibA_18445 | 113 | -0.112569742 | GAGE12E | G antigen 12E [Source:HGNC Symbol;Acc:HGNC:31905] |
| ENSG00000221880 | HGLibA_25583 | 113 | -0.112569742 | KRTAP1-3 | keratin associated protein 1-3 [Source:HGNC Symbol;Acc:HGNC:16771] |
| ENSG00000224982 | HGLibA_50264 | 113 | -0.112569742 | TMEM233 | transmembrane protein 233 [Source:HGNC Symbol;Acc:HGNC:37219] |
| ENSG00000227234 | HGLibA_46384 | 113 | -0.112569742 | SPANXB1 | SPANX family member B1 [Source:HGNC Symbol;Acc:HGNC:14329] |
| ENSG00000227234 | HGLibA_46385 | 113 | -0.112569742 | SPANXB1 | SPANX family member B1 [Source:HGNC Symbol;Acc:HGNC:14329] |
| ENSG00000232264 | HGLibA_53254 | 113 | -0.112569742 | USP17L24 | ubiquitin specific peptidase 17-like family member 24 [Source:HGNC Symbol;Acc:HGNC:44453] |
| ENSG00000242389 | HGLibA_40620 | 113 | -0.112569742 | RBMY1E | member E [Source:HGNC Symbol;Acc:HGNC:23916]" |
| ENSG00000243543 | HGLibA_54272 | 113 | -0.112569742 | WFDC6 | WAP four-disulfide core domain 6 [Source:HGNC Symbol;Acc:HGNC:16164] |

| | | | | | |
|---|---|---|---|---|---|
| ENSG00000259207 | HGLibA_23782 | 113 | -0.112569742 | ITGB3 | integrin subunit beta 3 [Source:HGNC Symbol;Acc:HGNC:6156] |
| ENSG00000261236 | HGLibA_04695 | 113 | -0.112569742 | BOP1 | block of proliferation 1 [Source:HGNC Symbol;Acc:HGNC:15519] |
| ENSG00000273820 | HGLibA_53316 | 113 | -0.112569742 | USP27X | ubiquitin specific peptidase 27 X-linked [Source:HGNC Symbol;Acc:HGNC:13486] |
| ENSG00000274749 | HGLibA_25825 | 113 | -0.112569742 | KRTAP7-1 | keratin associated protein 7-1 (gene/pseudogene) [Source:HGNC Symbol;Acc:HGNC:18934] |
| ENSG00000276644 | HGLibA_12273 | 113 | -0.112569742 | DACH1 | dachshund family transcription factor 1 [Source:HGNC Symbol;Acc:HGNC:2663] |
| GPR115 | HGLibA_19862 | 113 | -0.112569742 | GPR115 | - |
| H2BFWT | HGLibA_20774 | 113 | -0.112569742 | H2BFWT | - |
| H3F3C | HGLibA_20784 | 113 | -0.112569742 | H3F3C | - |
| HIST1H4I | HGLibA_21511 | 113 | -0.112569742 | HIST1H4I | - |
| hsa-let-7a-1 | HGLibA_57098 | 113 | -0.112569742 | hsa-let-7a-1 | - |
| hsa-mir-1178 | HGLibA_57204 | 113 | -0.112569742 | hsa-mir-1178 | - |
| hsa-mir-1289-2 | HGLibA_57623 | 113 | -0.112569742 | hsa-mir-1289-2 | - |
| hsa-mir-19b-2 | HGLibA_58147 | 113 | -0.112569742 | hsa-mir-19b-2 | - |
| hsa-mir-202 | HGLibA_58164 | 113 | -0.112569742 | hsa-mir-202 | - |
| hsa-mir-302e | HGLibA_58454 | 113 | -0.112569742 | hsa-mir-302e | - |
| hsa-mir-3130-2 | HGLibA_58574 | 113 | -0.112569742 | hsa-mir-3130-2 | - |
| hsa-mir-3193 | HGLibA_58870 | 113 | -0.112569742 | hsa-mir-3193 | - |
| hsa-mir-3614 | HGLibA_59084 | 113 | -0.112569742 | hsa-mir-3614 | - |
| hsa-mir-3651 | HGLibA_59151 | 113 | -0.112569742 | hsa-mir-3651 | - |
| hsa-mir-3692 | HGLibA_59358 | 113 | -0.112569742 | hsa-mir-3692 | - |
| hsa-mir-381 | HGLibA_59478 | 113 | -0.112569742 | hsa-mir-381 | - |
| hsa-mir-3909 | HGLibA_59500 | 113 | -0.112569742 | hsa-mir-3909 | - |
| hsa-mir-4440 | HGLibA_60163 | 113 | -0.112569742 | hsa-mir-4440 | - |
| hsa-mir-4684 | HGLibA_60848 | 113 | -0.112569742 | hsa-mir-4684 | - |
| hsa-mir-4722 | HGLibA_60986 | 113 | -0.112569742 | hsa-mir-4722 | - |
| hsa-mir-509-3 | HGLibA_61516 | 113 | -0.112569742 | hsa-mir-509-3 | - |
| hsa-mir-511 | HGLibA_61553 | 113 | -0.112569742 | hsa-mir-511 | - |
| hsa-mir-550a-3 | HGLibA_62106 | 113 | -0.112569742 | hsa-mir-550a-3 | - |
| hsa-mir-6812 | HGLibA_63427 | 113 | -0.112569742 | hsa-mir-6812 | - |
| hsa-mir-7112-1 | HGLibA_63811 | 113 | -0.112569742 | hsa-mir-7112-1 | - |
| hsa-mir-7848 | HGLibA_63981 | 113 | -0.112569742 | hsa-mir-7848 | - |
| KIAA1919 | HGLibA_24826 | 113 | -0.112569742 | KIAA1919 | - |
| LOC100129216 | HGLibA_26757 | 113 | -0.112569742 | LOC100129216 | - |
| LOC100287036 | HGLibA_26816 | 113 | -0.112569742 | LOC100287036 | - |
| OCLM | HGLibA_33252 | 113 | -0.112569742 | OCLM | - |
| WTH3DI | HGLibA_54434 | 113 | -0.112569742 | WTH3DI | - |
| C10orf114 | HGLibA_05037 | 112 | -0.121615085 | C10orf114 | - |
| C12orf5 | HGLibA_05255 | 112 | -0.121615085 | C12orf5 | - |
| C16orf52 | HGLibA_05454 | 112 | -0.121615085 | C16orf52 | - |
| C20orf201 | HGLibA_06011 | 112 | -0.121615085 | C20orf201 | - |
| C22orf26 | HGLibA_06062 | 112 | -0.121615085 | C22orf26 | - |
| ENSG00000013288 | HGLibA_28078 | 112 | -0.121615085 | MAN2B2 | mannosidase alpha class 2B member 2 [Source:HGNC Symbol;Acc:HGNC:29623] |
| ENSG00000018236 | HGLibA_10401 | 112 | -0.121615085 | CNTN1 | contactin 1 [Source:HGNC Symbol;Acc:HGNC:2171] |
| ENSG00000023318 | HGLibA_15529 | 112 | -0.121615085 | ERP44 | endoplasmic reticulum protein 44 [Source:HGNC Symbol;Acc:HGNC:18311] |
| ENSG00000034053 | HGLibA_02370 | 112 | -0.121615085 | APBA2 | amyloid beta precursor protein binding family A member 2 [Source:HGNC Symbol;Acc:HGNC:579] |
| ENSG00000038358 | HGLibA_14444 | 112 | -0.121615085 | EDC4 | enhancer of mRNA decapping 4 [Source:HGNC Symbol;Acc:HGNC:17157] |
| ENSG00000039560 | HGLibA_40140 | 112 | -0.121615085 | RAI14 | retinoic acid induced 14 [Source:HGNC Symbol;Acc:HGNC:14873] |
| ENSG00000050730 | HGLibA_50841 | 112 | -0.121615085 | TNIP3 | TNFAIP3 interacting protein 3 [Source:HGNC Symbol;Acc:HGNC:19315] |
| ENSG00000050767 | HGLibA_10546 | 112 | -0.121615085 | COL23A1 | collagen type XXIII alpha 1 chain [Source:HGNC Symbol;Acc:HGNC:22990] |
| ENSG00000060558 | HGLibA_19448 | 112 | -0.121615085 | GNA15 | G protein subunit alpha 15 [Source:HGNC Symbol;Acc:HGNC:4383] |
| ENSG00000061492 | HGLibA_54388 | 112 | -0.121615085 | WNT8A | Wnt family member 8A [Source:HGNC Symbol;Acc:HGNC:12788] |
| ENSG00000062038 | HGLibA_08678 | 112 | -0.121615085 | CDH3 | cadherin 3 [Source:HGNC Symbol;Acc:HGNC:1762] |
| ENSG00000066056 | HGLibA_49455 | 112 | -0.121615085 | TIE1 | tyrosine kinase with immunoglobulin like and EGF like domains 1 [Source:HGNC Symbol;Acc:HGNC:11809] |
| ENSG00000066827 | HGLibA_55166 | 112 | -0.121615085 | ZFAT | zinc finger and AT-hook domain containing [Source:HGNC Symbol;Acc:HGNC:19899] |
| ENSG00000083812 | HGLibA_55800 | 112 | -0.121615085 | ZNF324 | zinc finger protein 324 [Source:HGNC Symbol;Acc:HGNC:14096] |
| ENSG00000088305 | HGLibA_13703 | 112 | -0.121615085 | DNMT3B | DNA methyltransferase 3 beta [Source:HGNC Symbol;Acc:HGNC:2979] |
| ENSG00000091128 | HGLibA_25965 | 112 | -0.121615085 | LAMB4 | laminin subunit beta 4 [Source:HGNC Symbol;Acc:HGNC:6491] |
| ENSG00000099899 | HGLibA_51682 | 112 | -0.121615085 | TRMT2A | tRNA methyltransferase 2 homolog A [Source:HGNC Symbol;Acc:HGNC:24974] |
| ENSG00000102890 | HGLibA_14935 | 112 | -0.121615085 | ELMO3 | engulfment and cell motility 3 [Source:HGNC Symbol;Acc:HGNC:17289] |
| ENSG00000103264 | HGLibA_17070 | 112 | -0.121615085 | FBXO31 | F-box protein 31 [Source:HGNC Symbol;Acc:HGNC:16510] |
| ENSG00000103710 | HGLibA_40370 | 112 | -0.121615085 | RASL12 | RAS like family 12 [Source:HGNC Symbol;Acc:HGNC:30289] |
| ENSG00000105559 | HGLibA_37072 | 112 | -0.121615085 | PLEKHA4 | pleckstrin homology domain containing A4 [Source:HGNC Symbol;Acc:HGNC:14339] |
| ENSG00000105696 | HGLibA_50461 | 112 | -0.121615085 | TMEM59L | transmembrane protein 59 like [Source:HGNC Symbol;Acc:HGNC:13237] |
| ENSG00000106588 | HGLibA_39074 | 112 | -0.121615085 | PSMA2 | proteasome subunit alpha 2 [Source:HGNC Symbol;Acc:HGNC:9531] |
| ENSG00000110958 | HGLibA_39332 | 112 | -0.121615085 | PTGES3 | prostaglandin E synthase 3 [Source:HGNC Symbol;Acc:HGNC:16049] |
| ENSG00000111087 | HGLibA_19246 | 112 | -0.121615085 | GLI1 | GLI family zinc finger 1 [Source:HGNC Symbol;Acc:HGNC:4317] |
| ENSG00000111261 | HGLibA_28100 | 112 | -0.121615085 | MANSC1 | MANSC domain containing 1 [Source:HGNC Symbol;Acc:HGNC:25505] |
| ENSG00000112303 | HGLibA_53712 | 112 | -0.121615085 | VNN2 | vanin 2 [Source:HGNC Symbol;Acc:HGNC:12706] |
| ENSG00000117475 | HGLibA_04582 | 112 | -0.121615085 | BLZF1 | basic leucine zipper nuclear factor 1 [Source:HGNC Symbol;Acc:HGNC:1065] |
| ENSG00000118496 | HGLibA_17067 | 112 | -0.121615085 | FBXO30 | F-box protein 30 [Source:HGNC Symbol;Acc:HGNC:15600] |
| ENSG00000120156 | HGLibA_48967 | 112 | -0.121615085 | TEK | TEK receptor tyrosine kinase [Source:HGNC Symbol;Acc:HGNC:11724] |
| ENSG00000121653 | HGLibA_28287 | 112 | -0.121615085 | MAPK8IP1 | mitogen-activated protein kinase 8 interacting protein 1 [Source:HGNC Symbol;Acc:HGNC:6882] |
| ENSG00000122543 | HGLibA_33256 | 112 | -0.121615085 | OCM | oncomodulin [Source:HGNC Symbol;Acc:HGNC:8105] |
| ENSG00000125510 | HGLibA_33448 | 112 | -0.121615085 | OPRL1 | opioid related nociceptin receptor 1 [Source:HGNC Symbol;Acc:HGNC:8155] |
| ENSG00000126653 | HGLibA_32777 | 112 | -0.121615085 | NSRP1 | nuclear speckle splicing regulatory protein 1 [Source:HGNC Symbol;Acc:HGNC:25305] |
| ENSG00000128268 | HGLibA_29138 | 112 | -0.121615085 | MGAT3 | 4-N-acetylglucosaminyltransferase [Source:HGNC Symbol;Acc:HGNC:7046]" |
| ENSG00000129007 | HGLibA_07034 | 112 | -0.121615085 | CALML4 | calmodulin like 4 [Source:HGNC Symbol;Acc:HGNC:18445] |
| ENSG00000129028 | HGLibA_49289 | 112 | -0.121615085 | THAP10 | THAP domain containing 10 [Source:HGNC Symbol;Acc:HGNC:23193] |
| ENSG00000129667 | HGLibA_41136 | 112 | -0.121615085 | RHBDF2 | rhomboid 5 homolog 2 [Source:HGNC Symbol;Acc:HGNC:20788] |
| ENSG00000130449 | HGLibA_57053 | 112 | -0.121615085 | ZSWIM6 | zinc finger SWIM-type containing 6 [Source:HGNC Symbol;Acc:HGNC:29316] |
| ENSG00000130528 | HGLibA_22086 | 112 | -0.121615085 | HRC | histidine rich calcium binding protein [Source:HGNC Symbol;Acc:HGNC:5178] |
| ENSG00000130772 | HGLibA_28767 | 112 | -0.121615085 | MED18 | mediator complex subunit 18 [Source:HGNC Symbol;Acc:HGNC:25944] |
| ENSG00000133627 | HGLibA_00661 | 112 | -0.121615085 | ACTR3B | ARP3 actin related protein 3 homolog B [Source:HGNC Symbol;Acc:HGNC:17256] |
| ENSG00000134686 | HGLibA_36320 | 112 | -0.121615085 | PHC2 | polyhomeotic homolog 2 [Source:HGNC Symbol;Acc:HGNC:3183] |
| ENSG00000135913 | HGLibA_53350 | 112 | -0.121615085 | USP37 | ubiquitin specific peptidase 37 [Source:HGNC Symbol;Acc:HGNC:20063] |
| ENSG00000136695 | HGLibA_23153 | 112 | -0.121615085 | IL36RN | interleukin 36 receptor antagonist [Source:HGNC Symbol;Acc:HGNC:15561] |
| ENSG00000136834 | HGLibA_33673 | 112 | -0.121615085 | OR1J1 | olfactory receptor family 1 subfamily J member 1 [Source:HGNC Symbol;Acc:HGNC:8208] |
| ENSG00000136872 | HGLibA_01595 | 112 | -0.121615085 | ALDOB | fructose-bisphosphate B [Source:HGNC Symbol;Acc:HGNC:417]" |
| ENSG00000137656 | HGLibA_04999 | 112 | -0.121615085 | BUD13 | BUD13 homolog [Source:HGNC Symbol;Acc:HGNC:28199] |
| ENSG00000137824 | HGLibA_41371 | 112 | -0.121615085 | RMDN3 | regulator of microtubule dynamics 3 [Source:HGNC Symbol;Acc:HGNC:25550] |
| ENSG00000137869 | HGLibA_12065 | 112 | -0.121615085 | CYP19A1 | cytochrome P450 family 19 subfamily A member 1 [Source:HGNC Symbol;Acc:HGNC:2594] |
| ENSG00000138162 | HGLibA_48161 | 112 | -0.121615085 | TACC2 | transforming acidic coiled-coil containing protein 2 [Source:HGNC Symbol;Acc:HGNC:11523] |
| ENSG00000138640 | HGLibA_16140 | 112 | -0.121615085 | FAM13A | family with sequence similarity 13 member A [Source:HGNC Symbol;Acc:HGNC:19367] |
| ENSG00000139131 | HGLibA_54637 | 112 | -0.121615085 | YARS2 | tyrosyl-tRNA synthetase 2 [Source:HGNC Symbol;Acc:HGNC:24249] |
| ENSG00000139684 | HGLibA_15564 | 112 | -0.121615085 | ESD | esterase D [Source:HGNC Symbol;Acc:HGNC:3465] |
| ENSG00000141034 | HGLibA_19056 | 112 | -0.121615085 | GID4 | GID complex subunit 4 homolog [Source:HGNC Symbol;Acc:HGNC:28453] |
| ENSG00000141480 | HGLibA_03110 | 112 | -0.121615085 | ARRB2 | arrestin beta 2 [Source:HGNC Symbol;Acc:HGNC:712] |
| ENSG00000143333 | HGLibA_41068 | 112 | -0.121615085 | RGS16 | regulator of G protein signaling 16 [Source:HGNC Symbol;Acc:HGNC:9997] |
| ENSG00000143727 | HGLibA_00509 | 112 | -0.121615085 | ACP1 | acid phosphatase 1 [Source:HGNC Symbol;Acc:HGNC:122] |
| ENSG00000145087 | HGLibA_47679 | 112 | -0.121615085 | STXBP5L | syntaxin binding protein 5 like [Source:HGNC Symbol;Acc:HGNC:30757] |
| ENSG00000145781 | HGLibA_10652 | 112 | -0.121615085 | COMMD10 | COMM domain containing 10 [Source:HGNC Symbol;Acc:HGNC:30201] |
| ENSG00000146776 | HGLibA_03852 | 112 | -0.121615085 | ATXN7L1 | ataxin 7 like 1 [Source:HGNC Symbol;Acc:HGNC:22210] |
| ENSG00000148672 | HGLibA_19361 | 112 | -0.121615085 | GLUD1 | glutamate dehydrogenase 1 [Source:HGNC Symbol;Acc:HGNC:4335] |
| ENSG00000150764 | HGLibA_13287 | 112 | -0.121615085 | DIXDC1 | DIX domain containing 1 [Source:HGNC Symbol;Acc:HGNC:23695] |
| ENSG00000150967 | HGLibA_00154 | 112 | -0.121615085 | ABCB9 | ATP binding cassette subfamily B member 9 [Source:HGNC Symbol;Acc:HGNC:50] |
| ENSG00000151150 | HGLibA_01941 | 112 | -0.121615085 | ANK3 | ankyrin 3 [Source:HGNC Symbol;Acc:HGNC:494] |
| ENSG00000154330 | HGLibA_36274 | 112 | -0.121615085 | PGM5 | phosphoglucomutase 5 [Source:HGNC Symbol;Acc:HGNC:8908] |
| ENSG00000155011 | HGLibA_13297 | 112 | -0.121615085 | DKK2 | dickkopf WNT signaling pathway inhibitor 2 [Source:HGNC Symbol;Acc:HGNC:2892] |
| ENSG00000155542 | HGLibA_43663 | 112 | -0.121615085 | SETD9 | SET domain containing 9 [Source:HGNC Symbol;Acc:HGNC:28508] |
| ENSG00000156256 | HGLibA_53212 | 112 | -0.121615085 | USP16 | ubiquitin specific peptidase 16 [Source:HGNC Symbol;Acc:HGNC:12614] |
| ENSG00000157823 | HGLibA_02337 | 112 | -0.121615085 | AP3S2 | adaptor related protein complex 3 subunit sigma 2 [Source:HGNC Symbol;Acc:HGNC:571] |
| ENSG00000158006 | HGLibA_34997 | 112 | -0.121615085 | PAFAH2 | platelet activating factor acetylhydrolase 2 [Source:HGNC Symbol;Acc:HGNC:8579] |
| ENSG00000160789 | HGLibA_26693 | 112 | -0.121615085 | LMNA | lamin A/C [Source:HGNC Symbol;Acc:HGNC:6636] |
| ENSG00000161328 | HGLibA_27393 | 112 | -0.121615085 | LRRC56 | leucine rich repeat containing 56 [Source:HGNC Symbol;Acc:HGNC:25430] |
| ENSG00000163217 | HGLibA_04594 | 112 | -0.121615085 | BMP10 | bone morphogenetic protein 10 [Source:HGNC Symbol;Acc:HGNC:20869] |
| ENSG00000163283 | HGLibA_01712 | 112 | -0.121615085 | ALPP | placental [Source:HGNC Symbol;Acc:HGNC:439]" |
| ENSG00000163293 | HGLibA_31955 | 112 | -0.121615085 | NIPAL1 | NIPA like domain containing 1 [Source:HGNC Symbol;Acc:HGNC:27194] |
| ENSG00000163349 | HGLibA_21338 | 112 | -0.121615085 | HIPK1 | homeodomain interacting protein kinase 1 [Source:HGNC Symbol;Acc:HGNC:19006] |
| ENSG00000163463 | HGLibA_25855 | 112 | -0.121615085 | KRTCAP2 | keratinocyte associated protein 2 [Source:HGNC Symbol;Acc:HGNC:28942] |
| ENSG00000163735 | HGLibA_11871 | 112 | -0.121615085 | CXCL5 | C-X-C motif chemokine ligand 5 [Source:HGNC Symbol;Acc:HGNC:10642] |
| ENSG00000164056 | HGLibA_46834 | 112 | -0.121615085 | SPRY1 | sprouty RTK signaling antagonist 1 [Source:HGNC Symbol;Acc:HGNC:11269] |
| ENSG00000164120 | HGLibA_22031 | 112 | -0.121615085 | HPGD | 15-hydroxyprostaglandin dehydrogenase [Source:HGNC Symbol;Acc:HGNC:5154] |
| ENSG00000165186 | HGLibA_39275 | 112 | -0.121615085 | PTCHD1 | patched domain containing 1 [Source:HGNC Symbol;Acc:HGNC:26392] |
| ENSG00000165802 | HGLibA_32774 | 112 | -0.121615085 | NSMF | NMDA receptor synaptonuclear signaling and neuronal migration factor [Source:HGNC Symbol;Acc:HGNC:29843] |
| ENSG00000166278 | HGLibA_05979 | 112 | -0.121615085 | C2 | complement C2 [Source:HGNC Symbol;Acc:HGNC:1248] |
| ENSG00000166946 | HGLibA_08081 | 112 | -0.121615085 | CCNDBP1 | cyclin D1 binding protein 1 [Source:HGNC Symbol;Acc:HGNC:1587] |
| ENSG00000169814 | HGLibA_04931 | 112 | -0.121615085 | BTD | biotinidase [Source:HGNC Symbol;Acc:HGNC:1122] |
| ENSG00000170558 | HGLibA_08658 | 112 | -0.121615085 | CDH2 | cadherin 2 [Source:HGNC Symbol;Acc:HGNC:1759] |
| ENSG00000170632 | HGLibA_03007 | 112 | -0.121615085 | ARMC10 | armadillo repeat containing 10 [Source:HGNC Symbol;Acc:HGNC:21706] |
| ENSG00000170820 | HGLibA_18089 | 112 | -0.121615085 | FSHR | follicle stimulating hormone receptor [Source:HGNC Symbol;Acc:HGNC:3969] |
| ENSG00000171405 | HGLibA_54488 | 112 | -0.121615085 | XAGE5 | X antigen family member 5 [Source:HGNC Symbol;Acc:HGNC:30930] |
| ENSG00000171916 | HGLibA_26386 | 112 | -0.121615085 | LGALS9C | galectin 9C [Source:HGNC Symbol;Acc:HGNC:33874] |
| ENSG00000172346 | HGLibA_11312 | 112 | -0.121615085 | CSDC2 | cold shock domain containing C2 [Source:HGNC Symbol;Acc:HGNC:30359] |
| ENSG00000174405 | HGLibA_26486 | 112 | -0.121615085 | LIG4 | DNA ligase 4 [Source:HGNC Symbol;Acc:HGNC:6601] |
| ENSG00000174697 | HGLibA_26308 | 112 | -0.121615085 | LEP | leptin [Source:HGNC Symbol;Acc:HGNC:6553] |

| | | | | | |
|---|---|---|---|---|---|
| ENSG00000174939 | HGLibA_03353 | 112 | -0.121615085 | ASPHD1 | aspartate beta-hydroxylase domain containing 1 [Source:HGNC Symbol;Acc:HGNC:27380] |
| ENSG00000175206 | HGLibA_32499 | 112 | -0.121615085 | NPPA | natriuretic peptide A [Source:HGNC Symbol;Acc:HGNC:7939] |
| ENSG00000175874 | HGLibA_11123 | 112 | -0.121615085 | CREG2 | cellular repressor of E1A stimulated genes 2 [Source:HGNC Symbol;Acc:HGNC:14272] |
| ENSG00000176105 | HGLibA_54660 | 112 | -0.121615085 | YES1 | Src family tyrosine kinase [Source:HGNC Symbol;Acc:HGNC:12841]" |
| ENSG00000176244 | HGLibA_00411 | 112 | -0.121615085 | ACBD7 | acyl-CoA binding domain containing 7 [Source:HGNC Symbol;Acc:HGNC:17715] |
| ENSG00000178235 | HGLibA_45514 | 112 | -0.121615085 | SLITRK1 | SLIT and NTRK like family member 1 [Source:HGNC Symbol;Acc:HGNC:20297] |
| ENSG00000179213 | HGLibA_44155 | 112 | -0.121615085 | SIGLECL1 | SIGLEC family like 1 [Source:HGNC Symbol;Acc:HGNC:26856] |
| ENSG00000180481 | HGLibA_19264 | 112 | -0.121615085 | GLIPR1L2 | GLIPR1 like 2 [Source:HGNC Symbol;Acc:HGNC:28592] |
| ENSG00000181690 | HGLibA_36950 | 112 | -0.121615085 | PLAG1 | PLAG1 zinc finger [Source:HGNC Symbol;Acc:HGNC:9045] |
| ENSG00000181778 | HGLibA_50320 | 112 | -0.121615085 | TMEM252 | transmembrane protein 252 [Source:HGNC Symbol;Acc:HGNC:28537] |
| ENSG00000181786 | HGLibA_00634 | 112 | -0.121615085 | ACTL9 | actin like 9 [Source:HGNC Symbol;Acc:HGNC:28494] |
| ENSG00000182083 | HGLibA_34377 | 112 | -0.121615085 | OR6B2 | olfactory receptor family 6 subfamily B member 2 [Source:HGNC Symbol;Acc:HGNC:15041] |
| ENSG00000182400 | HGLibA_51334 | 112 | -0.121615085 | TRAPPC6B | trafficking protein particle complex 6B [Source:HGNC Symbol;Acc:HGNC:23066] |
| ENSG00000182518 | HGLibA_15991 | 112 | -0.121615085 | FAM104B | family with sequence similarity 104 member B [Source:HGNC Symbol;Acc:HGNC:25085] |
| ENSG00000182890 | HGLibA_19363 | 112 | -0.121615085 | GLUD2 | glutamate dehydrogenase 2 [Source:HGNC Symbol;Acc:HGNC:4336] |
| ENSG00000184478 | HGLibA_34213 | 112 | -0.121615085 | OR56A3 | olfactory receptor family 56 subfamily A member 3 [Source:HGNC Symbol;Acc:HGNC:14786] |
| ENSG00000186572 | HGLibA_12838 | 112 | -0.121615085 | DEFB107A | defensin beta 107A [Source:HGNC Symbol;Acc:HGNC:18086] |
| ENSG00000186792 | HGLibA_22444 | 112 | -0.121615085 | HYAL3 | hyaluronidase 3 [Source:HGNC Symbol;Acc:HGNC:5322] |
| ENSG00000187808 | HGLibA_46223 | 112 | -0.121615085 | SOWAHD | sosondowah ankyrin repeat domain family member D [Source:HGNC Symbol;Acc:HGNC:32960] |
| ENSG00000188342 | HGLibA_20540 | 112 | -0.121615085 | GTF2F2 | general transcription factor IIF subunit 2 [Source:HGNC Symbol;Acc:HGNC:4653] |
| ENSG00000188822 | HGLibA_10378 | 112 | -0.121615085 | CNR2 | cannabinoid receptor 2 [Source:HGNC Symbol;Acc:HGNC:2160] |
| ENSG00000197769 | HGLibA_28130 | 112 | -0.121615085 | MAP1LC3C | microtubule associated protein 1 light chain 3 gamma [Source:HGNC Symbol;Acc:HGNC:13353] |
| ENSG00000198087 | HGLibA_08326 | 112 | -0.121615085 | CD2AP | CD2 associated protein [Source:HGNC Symbol;Acc:HGNC:14258] |
| ENSG00000198570 | HGLibA_40724 | 112 | -0.121615085 | RD3 | GUCY2D regulator [Source:HGNC Symbol;Acc:HGNC:19689]" |
| ENSG00000198858 | HGLibA_39777 | 112 | -0.121615085 | R3HDM4 | R3H domain containing 4 [Source:HGNC Symbol;Acc:HGNC:28270] |
| ENSG00000228716 | HGLibA_13102 | 112 | -0.121615085 | DHFR | dihydrofolate reductase [Source:HGNC Symbol;Acc:HGNC:2861] |
| ENSG00000228741 | HGLibA_46430 | 112 | -0.121615085 | SPATA13 | spermatogenesis associated 13 [Source:NCBI gene;Acc:221178] |
| ENSG00000233701 | HGLibA_38827 | 112 | -0.121615085 | PRR23C | proline rich 23C [Source:HGNC Symbol;Acc:HGNC:37173] |
| ENSG00000244588 | HGLibA_40067 | 112 | -0.121615085 | RAD21L1 | RAD21 cohesin complex component like 1 [Source:HGNC Symbol;Acc:HGNC:16271] |
| ENSG00000251369 | HGLibA_56213 | 112 | -0.121615085 | ZNF550 | zinc finger protein 550 [Source:HGNC Symbol;Acc:HGNC:28643] |
| ENSG00000256771 | HGLibA_55671 | 112 | -0.121615085 | ZNF253 | zinc finger protein 253 [Source:HGNC Symbol;Acc:HGNC:13497] |
| ENSG00000269113 | HGLibA_51235 | 112 | -0.121615085 | TRABD2B | TraB domain containing 2B [Source:HGNC Symbol;Acc:HGNC:44200] |
| ENSG00000274600 | HGLibA_41265 | 112 | -0.121615085 | RIMBP3B | RIMS binding protein 3B [Source:HGNC Symbol;Acc:HGNC:33891] |
| ENSG00000283706 | HGLibA_38954 | 112 | -0.121615085 | PRSS50 | serine protease 50 [Source:NCBI gene;Acc:29122] |
| ENSG00000288547 | HGLibA_06237 | 112 | -0.121615085 | C3orf36 | - |
| FAM129A | HGLibA_16095 | 112 | -0.121615085 | FAM129A | - |
| HIST1H3D | HGLibA_21466 | 112 | -0.121615085 | HIST1H3D | - |
| hsa-let-7a-1 | HGLibA_57096 | 112 | -0.121615085 | hsa-let-7a-1 | - |
| hsa-mir-1182 | HGLibA_57222 | 112 | -0.121615085 | hsa-mir-1182 | - |
| hsa-mir-1184-1 | HGLibA_57230 | 112 | -0.121615085 | hsa-mir-1184-1 | - |
| hsa-mir-1295a | HGLibA_57658 | 112 | -0.121615085 | hsa-mir-1295a | - |
| hsa-mir-139 | HGLibA_57819 | 112 | -0.121615085 | hsa-mir-139 | - |
| hsa-mir-1587 | HGLibA_57935 | 112 | -0.121615085 | hsa-mir-1587 | - |
| hsa-mir-23c | HGLibA_58336 | 112 | -0.121615085 | hsa-mir-23c | - |
| hsa-mir-3158-2 | HGLibA_58709 | 112 | -0.121615085 | hsa-mir-3158-2 | - |
| hsa-mir-3194 | HGLibA_58872 | 112 | -0.121615085 | hsa-mir-3194 | - |
| hsa-mir-3940 | HGLibA_59620 | 112 | -0.121615085 | hsa-mir-3940 | - |
| hsa-mir-4262 | HGLibA_59757 | 112 | -0.121615085 | hsa-mir-4262 | - |
| hsa-mir-4640 | HGLibA_60666 | 112 | -0.121615085 | hsa-mir-4640 | - |
| hsa-mir-4770 | HGLibA_61178 | 112 | -0.121615085 | hsa-mir-4770 | - |
| hsa-mir-5194 | HGLibA_61695 | 112 | -0.121615085 | hsa-mir-5194 | - |
| hsa-mir-525 | HGLibA_61786 | 112 | -0.121615085 | hsa-mir-525 | - |
| hsa-mir-5588 | HGLibA_62192 | 112 | -0.121615085 | hsa-mir-5588 | - |
| hsa-mir-6125 | HGLibA_62648 | 112 | -0.121615085 | hsa-mir-6125 | - |
| hsa-mir-6824 | HGLibA_63472 | 112 | -0.121615085 | hsa-mir-6824 | - |
| hsa-mir-6863 | HGLibA_63638 | 112 | -0.121615085 | hsa-mir-6863 | - |
| hsa-mir-940 | HGLibA_64333 | 112 | -0.121615085 | hsa-mir-940 | - |
| KIAA0020 | HGLibA_24584 | 112 | -0.121615085 | KIAA0020 | - |
| KIAA1033 | HGLibA_24685 | 112 | -0.121615085 | KIAA1033 | - |
| MLL2 | HGLibA_29397 | 112 | -0.121615085 | MLL2 | - |
| NAT6 | HGLibA_31180 | 112 | -0.121615085 | NAT6 | - |
| PDDC1 | HGLibA_35756 | 112 | -0.121615085 | PDDC1 | - |
| RBAK-LOC389458 | HGLibA_40423 | 112 | -0.121615085 | RBAK-LOC389458 | - |
| SELM | HGLibA_43298 | 112 | -0.121615085 | SELM | - |
| TCEB3 | HGLibA_48739 | 112 | -0.121615085 | TCEB3 | - |
| C17orf51 | HGLibA_05544 | 111 | -0.130660427 | C17orf51 | - |
| C3orf67 | HGLibA_06260 | 111 | -0.130660427 | C3orf67 | - |
| DHRSX_Y | HGLibA_13161 | 111 | -0.130660427 | DHRSX_Y | - |
| ENSG00000000971 | HGLibA_09261 | 111 | -0.130660427 | CFH | complement factor H [Source:HGNC Symbol;Acc:HGNC:4883] |
| ENSG00000007402 | HGLibA_06908 | 111 | -0.130660427 | CACNA2D2 | calcium voltage-gated channel auxiliary subunit alpha2delta 2 [Source:HGNC Symbol;Acc:HGNC:1400] |
| ENSG00000016490 | HGLibA_09806 | 111 | -0.130660427 | CLCA1 | chloride channel accessory 1 [Source:HGNC Symbol;Acc:HGNC:2015] |
| ENSG00000025770 | HGLibA_31278 | 111 | -0.130660427 | NCAPH2 | non-SMC condensin II complex subunit H2 [Source:HGNC Symbol;Acc:HGNC:25071] |
| ENSG00000029559 | HGLibA_22483 | 111 | -0.130660427 | IBSP | integrin binding sialoprotein [Source:HGNC Symbol;Acc:HGNC:5341] |
| ENSG00000035499 | HGLibA_12977 | 111 | -0.130660427 | DEPDC1B | DEP domain containing 1B [Source:HGNC Symbol;Acc:HGNC:24902] |
| ENSG00000036473 | HGLibA_34720 | 111 | -0.130660427 | OTC | ornithine carbamoyltransferase [Source:HGNC Symbol;Acc:HGNC:8512] |
| ENSG00000037757 | HGLibA_29859 | 111 | -0.130660427 | MRI1 | methylthioribose-1-phosphate isomerase 1 [Source:HGNC Symbol;Acc:HGNC:28469] |
| ENSG00000038427 | HGLibA_53570 | 111 | -0.130660427 | VCAN | versican [Source:HGNC Symbol;Acc:HGNC:2464] |
| ENSG00000046774 | HGLibA_27962 | 111 | -0.130660427 | MAGEC2 | MAGE family member C2 [Source:HGNC Symbol;Acc:HGNC:13574] |
| ENSG00000056277 | HGLibA_55729 | 111 | -0.130660427 | ZNF280C | zinc finger protein 280C [Source:HGNC Symbol;Acc:HGNC:25955] |
| ENSG00000061936 | HGLibA_43747 | 111 | -0.130660427 | SFSWAP | splicing factor SWAP [Source:HGNC Symbol;Acc:HGNC:10790] |
| ENSG00000064313 | HGLibA_48227 | 111 | -0.130660427 | TAF2 | TATA-box binding protein associated factor 2 [Source:HGNC Symbol;Acc:HGNC:11536] |
| ENSG00000064393 | HGLibA_21342 | 111 | -0.130660427 | HIPK2 | homeodomain interacting protein kinase 2 [Source:HGNC Symbol;Acc:HGNC:14402] |
| ENSG00000074071 | HGLibA_30095 | 111 | -0.130660427 | MRPS34 | mitochondrial ribosomal protein S34 [Source:HGNC Symbol;Acc:HGNC:16618] |
| ENSG00000075292 | HGLibA_56438 | 111 | -0.130660427 | ZNF638 | zinc finger protein 638 [Source:HGNC Symbol;Acc:HGNC:17894] |
| ENSG00000085644 | HGLibA_55590 | 111 | -0.130660427 | ZNF213 | zinc finger protein 213 [Source:HGNC Symbol;Acc:HGNC:13005] |
| ENSG00000087076 | HGLibA_22184 | 111 | -0.130660427 | HSD17B14 | hydroxysteroid 17-beta dehydrogenase 14 [Source:HGNC Symbol;Acc:HGNC:23238] |
| ENSG00000088926 | HGLibA_15845 | 111 | -0.130660427 | F11 | coagulation factor XI [Source:HGNC Symbol;Acc:HGNC:3529] |
| ENSG00000091317 | HGLibA_10224 | 111 | -0.130660427 | CMTM6 | CKLF like MARVEL transmembrane domain containing 6 [Source:HGNC Symbol;Acc:HGNC:19177] |
| ENSG00000094755 | HGLibA_18397 | 111 | -0.130660427 | GABRP | gamma-aminobutyric acid type A receptor pi subunit [Source:HGNC Symbol;Acc:HGNC:4089] |
| ENSG00000095203 | HGLibA_15253 | 111 | -0.130660427 | EPB41L4B | erythrocyte membrane protein band 4.1 like 4B [Source:HGNC Symbol;Acc:HGNC:19818] |
| ENSG00000099331 | HGLibA_30872 | 111 | -0.130660427 | MYO9B | myosin IXB [Source:HGNC Symbol;Acc:HGNC:7609] |
| ENSG00000100591 | HGLibA_01313 | 111 | -0.130660427 | AHSA1 | activator of HSP90 ATPase activity 1 [Source:HGNC Symbol;Acc:HGNC:1189] |
| ENSG00000100804 | HGLibA_39111 | 111 | -0.130660427 | PSMB5 | proteasome subunit beta 5 [Source:HGNC Symbol;Acc:HGNC:9542] |
| ENSG00000100983 | HGLibA_20449 | 111 | -0.130660427 | GSS | glutathione synthetase [Source:HGNC Symbol;Acc:HGNC:4624] |
| ENSG00000101074 | HGLibA_39782 | 111 | -0.130660427 | R3HDML | R3H domain containing like [Source:HGNC Symbol;Acc:HGNC:16249] |
| ENSG00000101966 | HGLibA_54508 | 111 | -0.130660427 | XIAP | X-linked inhibitor of apoptosis [Source:HGNC Symbol;Acc:HGNC:592] |
| ENSG00000102287 | HGLibA_18387 | 111 | -0.130660427 | GABRE | gamma-aminobutyric acid type A receptor epsilon subunit [Source:HGNC Symbol;Acc:HGNC:4085] |
| ENSG00000103546 | HGLibA_45319 | 111 | -0.130660427 | SLC6A2 | solute carrier family 6 member 2 [Source:HGNC Symbol;Acc:HGNC:11048] |
| ENSG00000103550 | HGLibA_25346 | 111 | -0.130660427 | KNOP1 | lysine rich nucleolar protein 1 [Source:HGNC Symbol;Acc:HGNC:34404] |
| ENSG00000104312 | HGLibA_41319 | 111 | -0.130660427 | RIPK2 | receptor interacting serine/threonine kinase 2 [Source:HGNC Symbol;Acc:HGNC:10020] |
| ENSG00000104907 | HGLibA_51658 | 111 | -0.130660427 | TRMT1 | tRNA methyltransferase 1 [Source:HGNC Symbol;Acc:HGNC:25980] |
| ENSG00000105397 | HGLibA_52457 | 111 | -0.130660427 | TYK2 | tyrosine kinase 2 [Source:HGNC Symbol;Acc:HGNC:12440] |
| ENSG00000105419 | HGLibA_28877 | 111 | -0.130660427 | MEIS3 | Meis homeobox 3 [Source:HGNC Symbol;Acc:HGNC:29537] |
| ENSG00000105679 | HGLibA_18617 | 111 | -0.130660427 | GAPDHS | spermatogenic [Source:HGNC Symbol;Acc:HGNC:24864]" |
| ENSG00000109667 | HGLibA_44893 | 111 | -0.130660427 | SLC2A9 | solute carrier family 2 member 9 [Source:HGNC Symbol;Acc:HGNC:13446] |
| ENSG00000109736 | HGLibA_29083 | 111 | -0.130660427 | MFSD10 | major facilitator superfamily domain containing 10 [Source:HGNC Symbol;Acc:HGNC:16894] |
| ENSG00000110315 | HGLibA_41524 | 111 | -0.130660427 | RNF141 | ring finger protein 141 [Source:HGNC Symbol;Acc:HGNC:21159] |
| ENSG00000111249 | HGLibA_11807 | 111 | -0.130660427 | CUX2 | cut like homeobox 2 [Source:HGNC Symbol;Acc:HGNC:19347] |
| ENSG00000114784 | HGLibA_14718 | 111 | -0.130660427 | EIF1B | eukaryotic translation initiation factor 1B [Source:HGNC Symbol;Acc:HGNC:30792] |
| ENSG00000115760 | HGLibA_04523 | 111 | -0.130660427 | BIRC6 | baculoviral IAP repeat containing 6 [Source:HGNC Symbol;Acc:HGNC:13516] |
| ENSG00000115884 | HGLibA_43097 | 111 | -0.130660427 | SDC1 | syndecan 1 [Source:HGNC Symbol;Acc:HGNC:10658] |
| ENSG00000116147 | HGLibA_50907 | 111 | -0.130660427 | TNR | tenascin R [Source:HGNC Symbol;Acc:HGNC:11953] |
| ENSG00000116489 | HGLibA_07206 | 111 | -0.130660427 | CAPZA1 | capping actin protein of muscle Z-line subunit alpha 1 [Source:HGNC Symbol;Acc:HGNC:1488] |
| ENSG00000116678 | HGLibA_26313 | 111 | -0.130660427 | LEPR | leptin receptor [Source:HGNC Symbol;Acc:HGNC:6554] |
| ENSG00000117010 | HGLibA_56537 | 111 | -0.130660427 | ZNF684 | zinc finger protein 684 [Source:HGNC Symbol;Acc:HGNC:28418] |
| ENSG00000117155 | HGLibA_47205 | 111 | -0.130660427 | SSX2IP | SSX family member 2 interacting protein [Source:HGNC Symbol;Acc:HGNC:16509] |
| ENSG00000117362 | HGLibA_02412 | 111 | -0.130660427 | APH1A | gamma-secretase subunit [Source:HGNC Symbol;Acc:HGNC:29509]" |
| ENSG00000118046 | HGLibA_47454 | 111 | -0.130660427 | STK11 | serine/threonine kinase 11 [Source:HGNC Symbol;Acc:HGNC:11389] |
| ENSG00000118689 | HGLibA_17925 | 111 | -0.130660427 | FOXO3 | forkhead box O3 [Source:HGNC Symbol;Acc:HGNC:3821] |
| ENSG00000119280 | HGLibA_05819 | 111 | -0.130660427 | C1orf198 | chromosome 1 open reading frame 198 [Source:HGNC Symbol;Acc:HGNC:25900] |
| ENSG00000121413 | HGLibA_56991 | 111 | -0.130660427 | ZSCAN18 | zinc finger and SCAN domain containing 18 [Source:HGNC Symbol;Acc:HGNC:21037] |
| ENSG00000122861 | HGLibA_36962 | 111 | -0.130660427 | PLAU | urokinase [Source:HGNC Symbol;Acc:HGNC:9052]" |
| ENSG00000124134 | HGLibA_24384 | 111 | -0.130660427 | KCNS1 | potassium voltage-gated channel modifier subfamily S member 1 [Source:HGNC Symbol;Acc:HGNC:6300] |
| ENSG00000125522 | HGLibA_32415 | 111 | -0.130660427 | NPBWR2 | neuropeptides B and W receptor 2 [Source:HGNC Symbol;Acc:HGNC:4530] |
| ENSG00000125703 | HGLibA_03494 | 111 | -0.130660427 | ATG4C | autophagy related 4C cysteine peptidase [Source:HGNC Symbol;Acc:HGNC:16040] |
| ENSG00000125826 | HGLibA_40450 | 111 | -0.130660427 | RBCK1 | RANBP2-type and C3HC4-type zinc finger containing 1 [Source:HGNC Symbol;Acc:HGNC:15864] |
| ENSG00000126337 | HGLibA_25491 | 111 | -0.130660427 | KRT36 | keratin 36 [Source:HGNC Symbol;Acc:HGNC:6454] |
| ENSG00000126903 | HGLibA_44337 | 111 | -0.130660427 | SLC10A3 | solute carrier family 10 member 3 [Source:HGNC Symbol;Acc:HGNC:22979] |
| ENSG00000127586 | HGLibA_09662 | 111 | -0.130660427 | CHTF18 | chromosome transmission fidelity factor 18 [Source:HGNC Symbol;Acc:HGNC:18435] |
| ENSG00000128250 | HGLibA_40928 | 111 | -0.130660427 | RFPL1 | ret finger protein like 1 [Source:HGNC Symbol;Acc:HGNC:9977] |
| ENSG00000129158 | HGLibA_43478 | 111 | -0.130660427 | SERGEF | secretion regulating guanine nucleotide exchange factor [Source:HGNC Symbol;Acc:HGNC:17499] |
| ENSG00000129680 | HGLibA_28240 | 111 | -0.130660427 | MAP7D3 | MAP7 domain containing 3 [Source:HGNC Symbol;Acc:HGNC:25742] |
| ENSG00000130244 | HGLibA_16816 | 111 | -0.130660427 | FAM98C | family with sequence similarity 98 member C [Source:HGNC Symbol;Acc:HGNC:27119] |
| ENSG00000130749 | HGLibA_54976 | 111 | -0.130660427 | ZC3H4 | zinc finger CCCH-type containing 4 [Source:HGNC Symbol;Acc:HGNC:17808] |
| ENSG00000132155 | HGLibA_40130 | 111 | -0.130660427 | RAF1 | serine/threonine kinase [Source:HGNC Symbol;Acc:HGNC:9829]" |

| | | | | | |
|---|---|---|---|---|---|
| ENSG00000132664 | HGLibA_37625 | 111 | -0.130660427 | POLR3F | RNA polymerase III subunit F [Source:HGNC Symbol;Acc:HGNC:15763] |
| ENSG00000132964 | HGLibA_08796 | 111 | -0.130660427 | CDK8 | cyclin dependent kinase 8 [Source:HGNC Symbol;Acc:HGNC:1779] |
| ENSG00000134086 | HGLibA_53648 | 111 | -0.130660427 | VHL | von Hippel-Lindau tumor suppressor [Source:HGNC Symbol;Acc:HGNC:12687] |
| ENSG00000134245 | HGLibA_54360 | 111 | -0.130660427 | WNT2B | Wnt family member 2B [Source:HGNC Symbol;Acc:HGNC:12781] |
| ENSG00000135249 | HGLibA_41305 | 111 | -0.130660427 | RINT1 | RAD50 interactor 1 [Source:HGNC Symbol;Acc:HGNC:21876] |
| ENSG00000135299 | HGLibA_02129 | 111 | -0.130660427 | ANKRD6 | ankyrin repeat domain 6 [Source:HGNC Symbol;Acc:HGNC:17280] |
| ENSG00000135698 | HGLibA_29726 | 111 | -0.130660427 | MPHOSPH6 | M-phase phosphoprotein 6 [Source:HGNC Symbol;Acc:HGNC:7214] |
| ENSG00000135736 | HGLibA_07507 | 111 | -0.130660427 | CCDC102A | coiled-coil domain containing 102A [Source:HGNC Symbol;Acc:HGNC:28097] |
| ENSG00000136267 | HGLibA_13062 | 111 | -0.130660427 | DGKB | diacylglycerol kinase beta [Source:HGNC Symbol;Acc:HGNC:2850] |
| ENSG00000136731 | HGLibA_52868 | 111 | -0.130660427 | UGGT1 | UDP-glucose glycoprotein glucosyltransferase 1 [Source:HGNC Symbol;Acc:HGNC:15663] |
| ENSG00000137392 | HGLibA_10105 | 111 | -0.130660427 | CLPS | colipase [Source:HGNC Symbol;Acc:HGNC:2085] |
| ENSG00000138604 | HGLibA_19231 | 111 | -0.130660427 | GLCE | glucuronic acid epimerase [Source:HGNC Symbol;Acc:HGNC:17855] |
| ENSG00000140553 | HGLibA_53005 | 111 | -0.130660427 | UNC45A | unc-45 myosin chaperone A [Source:HGNC Symbol;Acc:HGNC:30594] |
| ENSG00000140987 | HGLibA_57021 | 111 | -0.130660427 | ZSCAN32 | zinc finger and SCAN domain containing 32 [Source:HGNC Symbol;Acc:HGNC:20812] |
| ENSG00000141371 | HGLibA_05559 | 111 | -0.130660427 | C17orf64 | chromosome 17 open reading frame 64 [Source:HGNC Symbol;Acc:HGNC:26990] |
| ENSG00000141429 | HGLibA_18527 | 111 | -0.130660427 | GALNT1 | polypeptide N-acetylgalactosaminyltransferase 1 [Source:HGNC Symbol;Acc:HGNC:4123] |
| ENSG00000142230 | HGLibA_42632 | 111 | -0.130660427 | SAE1 | SUMO1 activating enzyme subunit 1 [Source:HGNC Symbol;Acc:HGNC:30660] |
| ENSG00000142330 | HGLibA_07144 | 111 | -0.130660427 | CAPN10 | calpain 10 [Source:HGNC Symbol;Acc:HGNC:1477] |
| ENSG00000142549 | HGLibA_22872 | 111 | -0.130660427 | IGLON5 | IgLON family member 5 [Source:HGNC Symbol;Acc:HGNC:34550] |
| ENSG00000143869 | HGLibA_18874 | 111 | -0.130660427 | GDF7 | growth differentiation factor 7 [Source:HGNC Symbol;Acc:HGNC:4222] |
| ENSG00000144406 | HGLibA_53032 | 111 | -0.130660427 | UNC80 | NALCN channel complex subunit [Source:HGNC Symbol;Acc:HGNC:26582]" |
| ENSG00000148248 | HGLibA_47838 | 111 | -0.130660427 | SURF4 | surfeit 4 [Source:HGNC Symbol;Acc:HGNC:11476] |
| ENSG00000149489 | HGLibA_41754 | 111 | -0.130660427 | ROM1 | retinal outer segment membrane protein 1 [Source:HGNC Symbol;Acc:HGNC:10254] |
| ENSG00000149654 | HGLibA_08664 | 111 | -0.130660427 | CDH22 | cadherin 22 [Source:HGNC Symbol;Acc:HGNC:13251] |
| ENSG00000150667 | HGLibA_18093 | 111 | -0.130660427 | FSIP1 | fibrous sheath interacting protein 1 [Source:HGNC Symbol;Acc:HGNC:21674] |
| ENSG00000151468 | HGLibA_07750 | 111 | -0.130660427 | CCDC3 | coiled-coil domain containing 3 [Source:HGNC Symbol;Acc:HGNC:23813] |
| ENSG00000152804 | HGLibA_21257 | 111 | -0.130660427 | HHEX | hematopoietically expressed homeobox [Source:HGNC Symbol;Acc:HGNC:4901] |
| ENSG00000153827 | HGLibA_51640 | 111 | -0.130660427 | TRIP12 | thyroid hormone receptor interactor 12 [Source:HGNC Symbol;Acc:HGNC:12306] |
| ENSG00000156463 | HGLibA_43974 | 111 | -0.130660427 | SH3RF2 | SH3 domain containing ring finger 2 [Source:HGNC Symbol;Acc:HGNC:26299] |
| ENSG00000158055 | HGLibA_20255 | 111 | -0.130660427 | GRHL3 | grainyhead like transcription factor 3 [Source:HGNC Symbol;Acc:HGNC:25839] |
| ENSG00000158473 | HGLibA_08273 | 111 | -0.130660427 | CD1D | CD1d molecule [Source:HGNC Symbol;Acc:HGNC:1637] |
| ENSG00000159588 | HGLibA_07687 | 111 | -0.130660427 | CCDC17 | coiled-coil domain containing 17 [Source:HGNC Symbol;Acc:HGNC:26574] |
| ENSG00000160695 | HGLibA_53729 | 111 | -0.130660427 | VPS11 | CORVET/HOPS core subunit [Source:HGNC Symbol;Acc:HGNC:14583]" |
| ENSG00000161265 | HGLibA_52496 | 111 | -0.130660427 | U2AF1L4 | U2 small nuclear RNA auxiliary factor 1 like 4 [Source:HGNC Symbol;Acc:HGNC:23020] |
| ENSG00000161638 | HGLibA_23737 | 111 | -0.130660427 | ITGA5 | integrin subunit alpha 5 [Source:HGNC Symbol;Acc:HGNC:6141] |
| ENSG00000161940 | HGLibA_04334 | 111 | -0.130660427 | BCL6B | B cell CLL/lymphoma 6B [Source:HGNC Symbol;Acc:HGNC:1002] |
| ENSG00000163875 | HGLibA_28720 | 111 | -0.130660427 | MEAF6 | MYST/Esa1 associated factor 6 [Source:HGNC Symbol;Acc:HGNC:25674] |
| ENSG00000164294 | HGLibA_20192 | 111 | -0.130660427 | GPX8 | glutathione peroxidase 8 (putative) [Source:HGNC Symbol;Acc:HGNC:33100] |
| ENSG00000164406 | HGLibA_26262 | 111 | -0.130660427 | LEAP2 | liver enriched antimicrobial peptide 2 [Source:HGNC Symbol;Acc:HGNC:29571] |
| ENSG00000164821 | HGLibA_12801 | 111 | -0.130660427 | DEFA4 | defensin alpha 4 [Source:HGNC Symbol;Acc:HGNC:2763] |
| ENSG00000167081 | HGLibA_35348 | 111 | -0.130660427 | PBX3 | PBX homeobox 3 [Source:HGNC Symbol;Acc:HGNC:8634] |
| ENSG00000167280 | HGLibA_15127 | 111 | -0.130660427 | ENGASE | endo-beta-N-acetylglucosaminidase [Source:HGNC Symbol;Acc:HGNC:24622] |
| ENSG00000168003 | HGLibA_45134 | 111 | -0.130660427 | SLC3A2 | solute carrier family 3 member 2 [Source:HGNC Symbol;Acc:HGNC:11026] |
| ENSG00000168040 | HGLibA_15930 | 111 | -0.130660427 | FADD | Fas associated via death domain [Source:HGNC Symbol;Acc:HGNC:3573] |
| ENSG00000168958 | HGLibA_29057 | 111 | -0.130660427 | MFF | mitochondrial fission factor [Source:HGNC Symbol;Acc:HGNC:24858] |
| ENSG00000170043 | HGLibA_51296 | 111 | -0.130660427 | TRAPPC1 | trafficking protein particle complex 1 [Source:HGNC Symbol;Acc:HGNC:19894] |
| ENSG00000170439 | HGLibA_29021 | 111 | -0.130660427 | METTL7B | methyltransferase like 7B [Source:HGNC Symbol;Acc:HGNC:28276] |
| ENSG00000170782 | HGLibA_33466 | 111 | -0.130660427 | OR10A4 | olfactory receptor family 10 subfamily A member 4 [Source:HGNC Symbol;Acc:HGNC:15130] |
| ENSG00000171174 | HGLibA_40464 | 111 | -0.130660427 | RBKS | ribokinase [Source:HGNC Symbol;Acc:HGNC:30325] |
| ENSG00000171823 | HGLibA_16979 | 111 | -0.130660427 | FBXL14 | F-box and leucine rich repeat protein 14 [Source:HGNC Symbol;Acc:HGNC:28624] |
| ENSG00000172671 | HGLibA_55155 | 111 | -0.130660427 | ZFAND4 | zinc finger AN1-type containing 4 [Source:HGNC Symbol;Acc:HGNC:23504] |
| ENSG00000173157 | HGLibA_00825 | 111 | -0.130660427 | ADAMTS20 | ADAM metallopeptidase with thrombospondin type 1 motif 20 [Source:HGNC Symbol;Acc:HGNC:17178] |
| ENSG00000173473 | HGLibA_45622 | 111 | -0.130660427 | SMARCC1 | actin dependent regulator of chromatin subfamily c member 1 [Source:HGNC Symbol;Acc:HGNC:11104]" |
| ENSG00000173473 | HGLibA_45624 | 111 | -0.130660427 | SMARCC1 | actin dependent regulator of chromatin subfamily c member 1 [Source:HGNC Symbol;Acc:HGNC:11104]" |
| ENSG00000173914 | HGLibA_40572 | 111 | -0.130660427 | RBM4B | RNA binding motif protein 4B [Source:HGNC Symbol;Acc:HGNC:28842] |
| ENSG00000174123 | HGLibA_49643 | 111 | -0.130660427 | TLR10 | toll like receptor 10 [Source:HGNC Symbol;Acc:HGNC:15634] |
| ENSG00000175390 | HGLibA_14780 | 111 | -0.130660427 | EIF3F | eukaryotic translation initiation factor 3 subunit F [Source:HGNC Symbol;Acc:HGNC:3275] |
| ENSG00000177548 | HGLibA_40004 | 111 | -0.130660427 | RABEP2 | RAB GTPase binding effector protein 2 [Source:HGNC Symbol;Acc:HGNC:24817]" |
| ENSG00000177992 | HGLibA_46504 | 111 | -0.130660427 | SPATA31E1 | SPATA31 subfamily E member 1 [Source:HGNC Symbol;Acc:HGNC:26672] |
| ENSG00000178467 | HGLibA_34913 | 111 | -0.130660427 | P4HTM | transmembrane [Source:HGNC Symbol;Acc:HGNC:28858]" |
| ENSG00000179165 | HGLibA_39668 | 111 | -0.130660427 | PXT1 | peroxisomal testis enriched protein 1 [Source:HGNC Symbol;Acc:HGNC:18312] |
| ENSG00000179934 | HGLibA_08169 | 111 | -0.130660427 | CCR8 | C-C motif chemokine receptor 8 [Source:HGNC Symbol;Acc:HGNC:1609] |
| ENSG00000181045 | HGLibA_44786 | 111 | -0.130660427 | SLC26A11 | solute carrier family 26 member 11 [Source:HGNC Symbol;Acc:HGNC:14471] |
| ENSG00000181634 | HGLibA_50816 | 111 | -0.130660427 | TNFSF15 | TNF superfamily member 15 [Source:HGNC Symbol;Acc:HGNC:11931] |
| ENSG00000183395 | HGLibA_37294 | 111 | -0.130660427 | PMCH | pro-melanin concentrating hormone [Source:HGNC Symbol;Acc:HGNC:9109] |
| ENSG00000183423 | HGLibA_27190 | 111 | -0.130660427 | LRIT3 | Ig-like and transmembrane domains 3 [Source:HGNC Symbol;Acc:HGNC:24783]" |
| ENSG00000183795 | HGLibA_04745 | 111 | -0.130660427 | BPY2B | basic charge Y-linked 2B [Source:HGNC Symbol;Acc:HGNC:25449] |
| ENSG00000184428 | HGLibA_50992 | 111 | -0.130660427 | TOP1MT | DNA topoisomerase I mitochondrial [Source:HGNC Symbol;Acc:HGNC:29787] |
| ENSG00000184545 | HGLibA_14193 | 111 | -0.130660427 | DUSP8 | dual specificity phosphatase 8 [Source:HGNC Symbol;Acc:HGNC:3074] |
| ENSG00000185231 | HGLibA_28545 | 111 | -0.130660427 | MC2R | melanocortin 2 receptor [Source:HGNC Symbol;Acc:HGNC:6930] |
| ENSG00000185634 | HGLibA_44009 | 111 | -0.130660427 | SHC4 | SHC adaptor protein 4 [Source:HGNC Symbol;Acc:HGNC:16743] |
| ENSG00000185745 | HGLibA_22623 | 111 | -0.130660427 | IFIT1 | interferon induced protein with tetratricopeptide repeats 1 [Source:HGNC Symbol;Acc:HGNC:5407] |
| ENSG00000185947 | HGLibA_55701 | 111 | -0.130660427 | ZNF267 | zinc finger protein 267 [Source:HGNC Symbol;Acc:HGNC:13060] |
| ENSG00000186665 | HGLibA_05549 | 111 | -0.130660427 | C17orf58 | chromosome 17 open reading frame 58 [Source:HGNC Symbol;Acc:HGNC:27568] |
| ENSG00000187135 | HGLibA_53864 | 111 | -0.130660427 | VSTM2B | V-set and transmembrane domain containing 2B [Source:HGNC Symbol;Acc:HGNC:33595] |
| ENSG00000187735 | HGLibA_48696 | 111 | -0.130660427 | TCEA1 | transcription elongation factor A1 [Source:HGNC Symbol;Acc:HGNC:11612] |
| ENSG00000187736 | HGLibA_31886 | 111 | -0.130660427 | NHEJ1 | non-homologous end joining factor 1 [Source:HGNC Symbol;Acc:HGNC:25737] |
| ENSG00000187742 | HGLibA_43265 | 111 | -0.130660427 | SECISBP2 | SECIS binding protein 2 [Source:HGNC Symbol;Acc:HGNC:30972] |
| ENSG00000188833 | HGLibA_15218 | 111 | -0.130660427 | ENTPD8 | ectonucleoside triphosphate diphosphohydrolase 8 [Source:HGNC Symbol;Acc:HGNC:24860] |
| ENSG00000196391 | HGLibA_56715 | 111 | -0.130660427 | ZNF774 | zinc finger protein 774 [Source:HGNC Symbol;Acc:HGNC:33108] |
| ENSG00000196781 | HGLibA_49606 | 111 | -0.130660427 | TLE1 | transducin like enhancer of split 1 [Source:HGNC Symbol;Acc:HGNC:11837] |
| ENSG00000196859 | HGLibA_25501 | 111 | -0.130660427 | KRT39 | keratin 39 [Source:HGNC Symbol;Acc:HGNC:32971] |
| ENSG00000197037 | HGLibA_57010 | 111 | -0.130660427 | ZSCAN25 | zinc finger and SCAN domain containing 25 [Source:HGNC Symbol;Acc:HGNC:21961] |
| ENSG00000197122 | HGLibA_46928 | 111 | -0.130660427 | SRC | non-receptor tyrosine kinase [Source:HGNC Symbol;Acc:HGNC:11283]" |
| ENSG00000197562 | HGLibA_39941 | 111 | -0.130660427 | RAB40C | member RAS oncogene family [Source:HGNC Symbol;Acc:HGNC:18285]" |
| ENSG00000198453 | HGLibA_56266 | 111 | -0.130660427 | ZNF568 | zinc finger protein 568 [Source:HGNC Symbol;Acc:HGNC:25392] |
| ENSG00000203907 | HGLibA_33401 | 111 | -0.130660427 | OOEP | oocyte expressed protein [Source:HGNC Symbol;Acc:HGNC:21382] |
| ENSG00000206432 | HGLibA_50178 | 111 | -0.130660427 | TMEM200C | transmembrane protein 200C [Source:HGNC Symbol;Acc:HGNC:37208] |
| ENSG00000213190 | HGLibA_29413 | 111 | -0.130660427 | MLLT11 | transcription factor 7 cofactor [Source:HGNC Symbol;Acc:HGNC:16997]" |
| ENSG00000231924 | HGLibA_39035 | 111 | -0.130660427 | PSG1 | pregnancy specific beta-1-glycoprotein 1 [Source:HGNC Symbol;Acc:HGNC:9514] |
| ENSG00000236104 | HGLibA_54845 | 111 | -0.130660427 | ZBTB22 | zinc finger and BTB domain containing 22 [Source:HGNC Symbol;Acc:HGNC:13085] |
| ENSG00000244122 | HGLibA_52895 | 111 | -0.130660427 | UGT1A7 | UDP glucuronosyltransferase family 1 member A7 [Source:HGNC Symbol;Acc:HGNC:12539] |
| ENSG00000257520 | HGLibA_43764 | 111 | -0.130660427 | SFTA3 | surfactant associated 3 [Source:NCBI gene;Acc:253970] |
| ENSG00000258644 | HGLibA_48012 | 111 | -0.130660427 | SYNJ2BP-COX16 | SYNJ2BP-COX16 readthrough [Source:HGNC Symbol;Acc:HGNC:48350] |
| ENSG00000269437 | HGLibA_33133 | 111 | -0.130660427 | NXF2B | nuclear RNA export factor 2B [Source:HGNC Symbol;Acc:HGNC:23984] |
| ENSG00000272602 | HGLibA_56347 | 111 | -0.130660427 | ZNF595 | zinc finger protein 595 [Source:HGNC Symbol;Acc:HGNC:27196] |
| ENSG00000279514 | HGLibA_33950 | 111 | -0.130660427 | OR4C16 | olfactory receptor family 4 subfamily C member 16 (gene/pseudogene) [Source:HGNC Symbol;Acc:HGNC:15172] |
| ENSG00000280109 | HGLibA_36940 | 111 | -0.130660427 | PLAC4 | placenta specific 4 [Source:HGNC Symbol;Acc:HGNC:14616] |
| H1F0 | HGLibA_20734 | 111 | -0.130660427 | H1F0 | - |
| hsa-mir-103a-1 | HGLibA_57162 | 111 | -0.130660427 | hsa-mir-103a-1 | - |
| hsa-mir-1302-8 | HGLibA_57719 | 111 | -0.130660427 | hsa-mir-1302-8 | - |
| hsa-mir-130b | HGLibA_57750 | 111 | -0.130660427 | hsa-mir-130b | - |
| hsa-mir-181b-1 | HGLibA_57966 | 111 | -0.130660427 | hsa-mir-181b-1 | - |
| hsa-mir-1827 | HGLibA_57992 | 111 | -0.130660427 | hsa-mir-1827 | - |
| hsa-mir-2115 | HGLibA_58232 | 111 | -0.130660427 | hsa-mir-2115 | - |
| hsa-mir-214 | HGLibA_58250 | 111 | -0.130660427 | hsa-mir-214 | - |
| hsa-mir-3175 | HGLibA_58773 | 111 | -0.130660427 | hsa-mir-3175 | - |
| hsa-mir-3191 | HGLibA_58863 | 111 | -0.130660427 | hsa-mir-3191 | - |
| hsa-mir-328 | HGLibA_58975 | 111 | -0.130660427 | hsa-mir-328 | - |
| hsa-mir-3660 | HGLibA_59192 | 111 | -0.130660427 | hsa-mir-3660 | - |
| hsa-mir-3661 | HGLibA_59198 | 111 | -0.130660427 | hsa-mir-3661 | - |
| hsa-mir-378i | HGLibA_59461 | 111 | -0.130660427 | hsa-mir-378i | - |
| hsa-mir-4273 | HGLibA_59796 | 111 | -0.130660427 | hsa-mir-4273 | - |
| hsa-mir-4455 | HGLibA_60226 | 111 | -0.130660427 | hsa-mir-4455 | - |
| hsa-mir-5008 | HGLibA_61435 | 111 | -0.130660427 | hsa-mir-5008 | - |
| hsa-mir-513c | HGLibA_61583 | 111 | -0.130660427 | hsa-mir-513c | - |
| hsa-mir-548w | HGLibA_62080 | 111 | -0.130660427 | hsa-mir-548w | - |
| hsa-mir-6829 | HGLibA_63493 | 111 | -0.130660427 | hsa-mir-6829 | - |
| hsa-mir-6865 | HGLibA_63645 | 111 | -0.130660427 | hsa-mir-6865 | - |
| hsa-mir-7160 | HGLibA_63869 | 111 | -0.130660427 | hsa-mir-7160 | - |
| JHDM1D | HGLibA_23945 | 111 | -0.130660427 | JHDM1D | - |
| KIAA1239 | HGLibA_24724 | 111 | -0.130660427 | KIAA1239 | - |
| LRRC48 | HGLibA_27375 | 111 | -0.130660427 | LRRC48 | - |
| PLCXD1_X | HGLibA_37019 | 111 | -0.130660427 | PLCXD1_X | - |
| PLCXD1_Y | HGLibA_37022 | 111 | -0.130660427 | PLCXD1_Y | - |
| PQLC3 | HGLibA_38257 | 111 | -0.130660427 | PQLC3 | - |
| C10orf113 | HGLibA_05033 | 110 | -0.13970577 | C10orf113 | - |
| C17orf66 | HGLibA_05562 | 110 | -0.13970577 | C17orf66 | - |
| CCDC135 | HGLibA_07602 | 110 | -0.13970577 | CCDC135 | - |
| ENSG00000000419 | HGLibA_13858 | 110 | -0.13970577 | DPM1 | catalytic [Source:HGNC Symbol;Acc:HGNC:3005]" |
| ENSG00000004142 | HGLibA_37504 | 110 | -0.13970577 | POLDIP2 | DNA polymerase delta interacting protein 2 [Source:HGNC Symbol;Acc:HGNC:23781] |
| ENSG00000004478 | HGLibA_17576 | 110 | -0.13970577 | FKBP4 | FK506 binding protein 4 [Source:HGNC Symbol;Acc:HGNC:3720] |
| ENSG00000005486 | HGLibA_41127 | 110 | -0.13970577 | RHBDD2 | rhomboid domain containing 2 [Source:HGNC Symbol;Acc:HGNC:23082] |
| ENSG00000007923 | HGLibA_13567 | 110 | -0.13970577 | DNAJC11 | DnaJ heat shock protein family (Hsp40) member C11 [Source:HGNC Symbol;Acc:HGNC:25570] |
| ENSG00000010219 | HGLibA_14284 | 110 | -0.13970577 | DYRK4 | dual specificity tyrosine phosphorylation regulated kinase 4 [Source:HGNC Symbol;Acc:HGNC:3095] |

| | | | | | |
|---|---|---|---|---|---|
| ENSG00000026652 | HGLibA_01226 | 110 | -0.13970577 | AGPAT4 | 1-acylglycerol-3-phosphate O-acyltransferase 4 [Source:HGNC Symbol;Acc:HGNC:20885] |
| ENSG00000036257 | HGLibA_11780 | 110 | -0.13970577 | CUL3 | cullin 3 [Source:HGNC Symbol;Acc:HGNC:2553] |
| ENSG00000040608 | HGLibA_42414 | 110 | -0.13970577 | RTN4R | reticulon 4 receptor [Source:HGNC Symbol;Acc:HGNC:18601] |
| ENSG00000041880 | HGLibA_35236 | 110 | -0.13970577 | PARP3 | poly(ADP-ribose) polymerase family member 3 [Source:HGNC Symbol;Acc:HGNC:273] |
| ENSG00000067334 | HGLibA_13722 | 110 | -0.13970577 | DNTTIP2 | deoxynucleotidyltransferase terminal interacting protein 2 [Source:HGNC Symbol;Acc:HGNC:24013] |
| ENSG00000068078 | HGLibA_17447 | 110 | -0.13970577 | FGFR3 | fibroblast growth factor receptor 3 [Source:HGNC Symbol;Acc:HGNC:3690] |
| ENSG00000070831 | HGLibA_08553 | 110 | -0.13970577 | CDC42 | cell division cycle 42 [Source:HGNC Symbol;Acc:HGNC:1736] |
| ENSG00000071054 | HGLibA_28219 | 110 | -0.13970577 | MAP4K4 | mitogen-activated protein kinase kinase kinase kinase 4 [Source:HGNC Symbol;Acc:HGNC:6866] |
| ENSG00000071655 | HGLibA_28468 | 110 | -0.13970577 | MBD3 | methyl-CpG binding domain protein 3 [Source:HGNC Symbol;Acc:HGNC:6918] |
| ENSG00000076555 | HGLibA_00350 | 110 | -0.13970577 | ACACB | acetyl-CoA carboxylase beta [Source:HGNC Symbol;Acc:HGNC:85] |
| ENSG00000079102 | HGLibA_42457 | 110 | -0.13970577 | RUNX1T1 | RUNX1 translocation partner 1 [Source:HGNC Symbol;Acc:HGNC:1535] |
| ENSG00000079257 | HGLibA_27649 | 110 | -0.13970577 | LXN | latexin [Source:HGNC Symbol;Acc:HGNC:13347] |
| ENSG00000084628 | HGLibA_31988 | 110 | -0.13970577 | NKAIN1 | sodium/potassium transporting ATPase interacting 1 [Source:HGNC Symbol;Acc:HGNC:25743] |
| ENSG00000092531 | HGLibA_45874 | 110 | -0.13970577 | SNAP23 | synaptosome associated protein 23 [Source:HGNC Symbol;Acc:HGNC:11131] |
| ENSG00000100266 | HGLibA_34963 | 110 | -0.13970577 | PACSIN2 | protein kinase C and casein kinase substrate in neurons 2 [Source:HGNC Symbol;Acc:HGNC:8571] |
| ENSG00000100804 | HGLibA_39113 | 110 | -0.13970577 | PSMB5 | proteasome subunit beta 5 [Source:HGNC Symbol;Acc:HGNC:9542] |
| ENSG00000100897 | HGLibA_12415 | 110 | -0.13970577 | DCAF11 | DDB1 and CUL4 associated factor 11 [Source:HGNC Symbol;Acc:HGNC:20258] |
| ENSG00000100918 | HGLibA_40761 | 110 | -0.13970577 | REC8 | REC8 meiotic recombination protein [Source:HGNC Symbol;Acc:HGNC:16879] |
| ENSG00000102024 | HGLibA_37227 | 110 | -0.13970577 | PLS3 | plastin 3 [Source:HGNC Symbol;Acc:HGNC:9091] |
| ENSG00000102226 | HGLibA_53197 | 110 | -0.13970577 | USP11 | ubiquitin specific peptidase 11 [Source:HGNC Symbol;Acc:HGNC:12609] |
| ENSG00000104435 | HGLibA_47520 | 110 | -0.13970577 | STMN2 | stathmin 2 [Source:HGNC Symbol;Acc:HGNC:10577] |
| ENSG00000104856 | HGLibA_40816 | 110 | -0.13970577 | RELB | NF-kB subunit [Source:HGNC Symbol;Acc:HGNC:9956]" |
| ENSG00000104979 | HGLibA_05679 | 110 | -0.13970577 | C19orf53 | chromosome 19 open reading frame 53 [Source:HGNC Symbol;Acc:HGNC:24991] |
| ENSG00000107831 | HGLibA_17421 | 110 | -0.13970577 | FGF8 | fibroblast growth factor 8 [Source:HGNC Symbol;Acc:HGNC:3686] |
| ENSG00000108846 | HGLibA_00172 | 110 | -0.13970577 | ABCC3 | ATP binding cassette subfamily C member 3 [Source:HGNC Symbol;Acc:HGNC:54] |
| ENSG00000109920 | HGLibA_17742 | 110 | -0.13970577 | FNBP4 | formin binding protein 4 [Source:HGNC Symbol;Acc:HGNC:19752] |
| ENSG00000111596 | HGLibA_10336 | 110 | -0.13970577 | CNOT2 | CCR4-NOT transcription complex subunit 2 [Source:HGNC Symbol;Acc:HGNC:7878] |
| ENSG00000111642 | HGLibA_09396 | 110 | -0.13970577 | CHD4 | chromodomain helicase DNA binding protein 4 [Source:HGNC Symbol;Acc:HGNC:1919] |
| ENSG00000111711 | HGLibA_19665 | 110 | -0.13970577 | GOLT1B | golgi transport 1B [Source:HGNC Symbol;Acc:HGNC:20175] |
| ENSG00000111817 | HGLibA_14016 | 110 | -0.13970577 | DSE | dermatan sulfate epimerase [Source:HGNC Symbol;Acc:HGNC:21144] |
| ENSG00000112273 | HGLibA_21042 | 110 | -0.13970577 | HDGFL1 | HDGF like 1 [Source:HGNC Symbol;Acc:HGNC:21095] |
| ENSG00000112796 | HGLibA_15179 | 110 | -0.13970577 | ENPP5 | ectonucleotide pyrophosphatase/phosphodiesterase 5 (putative) [Source:HGNC Symbol;Acc:HGNC:13717] |
| ENSG00000114455 | HGLibA_21271 | 110 | -0.13970577 | HHLA2 | HERV-H LTR-associating 2 [Source:HGNC Symbol;Acc:HGNC:4905] |
| ENSG00000114812 | HGLibA_53667 | 110 | -0.13970577 | VIPR1 | vasoactive intestinal peptide receptor 1 [Source:HGNC Symbol;Acc:HGNC:12694] |
| ENSG00000115355 | HGLibA_07906 | 110 | -0.13970577 | CCDC88A | coiled-coil domain containing 88A [Source:HGNC Symbol;Acc:HGNC:25523] |
| ENSG00000115419 | HGLibA_19324 | 110 | -0.13970577 | GLS | glutaminase [Source:HGNC Symbol;Acc:HGNC:4331] |
| ENSG00000115946 | HGLibA_37397 | 110 | -0.13970577 | PNO1 | partner of NOB1 homolog [Source:HGNC Symbol;Acc:HGNC:32790] |
| ENSG00000115966 | HGLibA_03433 | 110 | -0.13970577 | ATF2 | activating transcription factor 2 [Source:HGNC Symbol;Acc:HGNC:784] |
| ENSG00000116176 | HGLibA_51201 | 110 | -0.13970577 | TPSG1 | tryptase gamma 1 [Source:HGNC Symbol;Acc:HGNC:14134] |
| ENSG00000116205 | HGLibA_48731 | 110 | -0.13970577 | TCEANC2 | transcription elongation factor A N-terminal and central domain containing 2 [Source:HGNC Symbol;Acc:HGNC:26494] |
| ENSG00000118515 | HGLibA_43818 | 110 | -0.13970577 | SGK1 | serum/glucocorticoid regulated kinase 1 [Source:HGNC Symbol;Acc:HGNC:10810] |
| ENSG00000119685 | HGLibA_52208 | 110 | -0.13970577 | TTLL5 | tubulin tyrosine ligase like 5 [Source:HGNC Symbol;Acc:HGNC:19963] |
| ENSG00000122188 | HGLibA_26071 | 110 | -0.13970577 | LAX1 | lymphocyte transmembrane adaptor 1 [Source:HGNC Symbol;Acc:HGNC:26005] |
| ENSG00000122507 | HGLibA_04226 | 110 | -0.13970577 | BBS9 | Bardet-Biedl syndrome 9 [Source:HGNC Symbol;Acc:HGNC:30000] |
| ENSG00000123364 | HGLibA_21966 | 110 | -0.13970577 | HOXC13 | homeobox C13 [Source:HGNC Symbol;Acc:HGNC:5125] |
| ENSG00000124574 | HGLibA_00160 | 110 | -0.13970577 | ABCC10 | ATP binding cassette subfamily C member 10 [Source:HGNC Symbol;Acc:HGNC:52] |
| ENSG00000128253 | HGLibA_40932 | 110 | -0.13970577 | RFPL2 | ret finger protein like 2 [Source:HGNC Symbol;Acc:HGNC:9979] |
| ENSG00000130338 | HGLibA_52339 | 110 | -0.13970577 | TULP4 | tubby like protein 4 [Source:HGNC Symbol;Acc:HGNC:15530] |
| ENSG00000130414 | HGLibA_31440 | 110 | -0.13970577 | NDUFA10 | NADH:ubiquinone oxidoreductase subunit A10 [Source:HGNC Symbol;Acc:HGNC:7684] |
| ENSG00000130997 | HGLibA_37542 | 110 | -0.13970577 | POLN | DNA polymerase nu [Source:HGNC Symbol;Acc:HGNC:18870] |
| ENSG00000131100 | HGLibA_03748 | 110 | -0.13970577 | ATP6V1E1 | ATPase H+ transporting V1 subunit E1 [Source:HGNC Symbol;Acc:HGNC:857] |
| ENSG00000132005 | HGLibA_40963 | 110 | -0.13970577 | RFX1 | regulatory factor X1 [Source:HGNC Symbol;Acc:HGNC:9982] |
| ENSG00000133997 | HGLibA_28810 | 110 | -0.13970577 | MED6 | mediator complex subunit 6 [Source:HGNC Symbol;Acc:HGNC:19970] |
| ENSG00000134250 | HGLibA_32342 | 110 | -0.13970577 | NOTCH2 | notch 2 [Source:HGNC Symbol;Acc:HGNC:7882] |
| ENSG00000135100 | HGLibA_21773 | 110 | -0.13970577 | HNF1A | HNF1 homeobox A [Source:HGNC Symbol;Acc:HGNC:11621] |
| ENSG00000135316 | HGLibA_47968 | 110 | -0.13970577 | SYNCRIP | synaptotagmin binding cytoplasmic RNA interacting protein [Source:HGNC Symbol;Acc:HGNC:16918] |
| ENSG00000136048 | HGLibA_13943 | 110 | -0.13970577 | DRAM1 | DNA damage regulated autophagy modulator 1 [Source:HGNC Symbol;Acc:HGNC:25645] |
| ENSG00000136114 | HGLibA_49400 | 110 | -0.13970577 | THSD1 | thrombospondin type 1 domain containing 1 [Source:HGNC Symbol;Acc:HGNC:17754] |
| ENSG00000136689 | HGLibA_23067 | 110 | -0.13970577 | IL1RN | interleukin 1 receptor antagonist [Source:HGNC Symbol;Acc:HGNC:6000] |
| ENSG00000136758 | HGLibA_54699 | 110 | -0.13970577 | YME1L1 | YME1 like 1 ATPase [Source:HGNC Symbol;Acc:HGNC:12843] |
| ENSG00000136826 | HGLibA_25073 | 110 | -0.13970577 | KLF4 | Kruppel like factor 4 [Source:HGNC Symbol;Acc:HGNC:6348] |
| ENSG00000137266 | HGLibA_44572 | 110 | -0.13970577 | SLC22A23 | solute carrier family 22 member 23 [Source:HGNC Symbol;Acc:HGNC:21106] |
| ENSG00000137936 | HGLibA_04249 | 110 | -0.13970577 | BCAR3 | NSP family adaptor protein [Source:HGNC Symbol;Acc:HGNC:973]" |
| ENSG00000138100 | HGLibA_51542 | 110 | -0.13970577 | TRIM54 | tripartite motif containing 54 [Source:HGNC Symbol;Acc:HGNC:16008] |
| ENSG00000139517 | HGLibA_26747 | 110 | -0.13970577 | LNX2 | ligand of numb-protein X 2 [Source:HGNC Symbol;Acc:HGNC:20421] |
| ENSG00000140030 | HGLibA_20075 | 110 | -0.13970577 | GPR65 | G protein-coupled receptor 65 [Source:HGNC Symbol;Acc:HGNC:4517] |
| ENSG00000141505 | HGLibA_03286 | 110 | -0.13970577 | ASGR1 | asialoglycoprotein receptor 1 [Source:HGNC Symbol;Acc:HGNC:742] |
| ENSG00000142700 | HGLibA_13429 | 110 | -0.13970577 | DMRTA2 | DMRT like family A2 [Source:HGNC Symbol;Acc:HGNC:13908] |
| ENSG00000144036 | HGLibA_15757 | 110 | -0.13970577 | EXOC6B | exocyst complex component 6B [Source:HGNC Symbol;Acc:HGNC:17085] |
| ENSG00000144115 | HGLibA_49360 | 110 | -0.13970577 | THNSL2 | threonine synthase like 2 [Source:HGNC Symbol;Acc:HGNC:25602] |
| ENSG00000144285 | HGLibA_42973 | 110 | -0.13970577 | SCN1A | sodium voltage-gated channel alpha subunit 1 [Source:HGNC Symbol;Acc:HGNC:10585] |
| ENSG00000144821 | HGLibA_30719 | 110 | -0.13970577 | MYH15 | myosin heavy chain 15 [Source:HGNC Symbol;Acc:HGNC:31073] |
| ENSG00000144848 | HGLibA_03485 | 110 | -0.13970577 | ATG3 | autophagy related 3 [Source:HGNC Symbol;Acc:HGNC:20962] |
| ENSG00000145103 | HGLibA_23207 | 110 | -0.13970577 | ILDR1 | immunoglobulin like domain containing receptor 1 [Source:HGNC Symbol;Acc:HGNC:28741] |
| ENSG00000145715 | HGLibA_40295 | 110 | -0.13970577 | RASA1 | RAS p21 protein activator 1 [Source:HGNC Symbol;Acc:HGNC:9871] |
| ENSG00000147262 | HGLibA_19866 | 110 | -0.13970577 | GPR119 | G protein-coupled receptor 119 [Source:HGNC Symbol;Acc:HGNC:19060] |
| ENSG00000147481 | HGLibA_46005 | 110 | -0.13970577 | SNTG1 | syntrophin gamma 1 [Source:HGNC Symbol;Acc:HGNC:13740] |
| ENSG00000148483 | HGLibA_50272 | 110 | -0.13970577 | TMEM236 | transmembrane protein 236 [Source:HGNC Symbol;Acc:HGNC:23473] |
| ENSG00000148803 | HGLibA_18169 | 110 | -0.13970577 | FUOM | fucose mutarotase [Source:HGNC Symbol;Acc:HGNC:24733] |
| ENSG00000153012 | HGLibA_26396 | 110 | -0.13970577 | LGI2 | leucine rich repeat LGI family member 2 [Source:HGNC Symbol;Acc:HGNC:18710] |
| ENSG00000155755 | HGLibA_50276 | 110 | -0.13970577 | TMEM237 | transmembrane protein 237 [Source:HGNC Symbol;Acc:HGNC:14432] |
| ENSG00000157766 | HGLibA_00381 | 110 | -0.13970577 | ACAN | aggrecan [Source:HGNC Symbol;Acc:HGNC:319] |
| ENSG00000158714 | HGLibA_44322 | 110 | -0.13970577 | SLAMF8 | SLAM family member 8 [Source:HGNC Symbol;Acc:HGNC:21391] |
| ENSG00000161328 | HGLibA_27392 | 110 | -0.13970577 | LRRC56 | leucine rich repeat containing 56 [Source:HGNC Symbol;Acc:HGNC:25430] |
| ENSG00000161610 | HGLibA_20985 | 110 | -0.13970577 | HCRT | hypocretin neuropeptide precursor [Source:HGNC Symbol;Acc:HGNC:4847] |
| ENSG00000163584 | HGLibA_41900 | 110 | -0.13970577 | RPL22L1 | ribosomal protein L22 like 1 [Source:HGNC Symbol;Acc:HGNC:27610] |
| ENSG00000163605 | HGLibA_38207 | 110 | -0.13970577 | PPP4R2 | protein phosphatase 4 regulatory subunit 2 [Source:HGNC Symbol;Acc:HGNC:18296] |
| ENSG00000164532 | HGLibA_48659 | 110 | -0.13970577 | TBX20 | T-box 20 [Source:HGNC Symbol;Acc:HGNC:11598] |
| ENSG00000164743 | HGLibA_06635 | 110 | -0.13970577 | C8orf48 | chromosome 8 open reading frame 48 [Source:HGNC Symbol;Acc:HGNC:26345] |
| ENSG00000164972 | HGLibA_06720 | 110 | -0.13970577 | C9orf24 | chromosome 9 open reading frame 24 [Source:HGNC Symbol;Acc:HGNC:19919] |
| ENSG00000165553 | HGLibA_31863 | 110 | -0.13970577 | NGB | neuroglobin [Source:HGNC Symbol;Acc:HGNC:14077] |
| ENSG00000166598 | HGLibA_22255 | 110 | -0.13970577 | HSP90B1 | heat shock protein 90 beta family member 1 [Source:HGNC Symbol;Acc:HGNC:12028] |
| ENSG00000167566 | HGLibA_31320 | 110 | -0.13970577 | NCKAP5L | NCK associated protein 5 like [Source:HGNC Symbol;Acc:HGNC:29321] |
| ENSG00000168395 | HGLibA_23294 | 110 | -0.13970577 | ING5 | inhibitor of growth family member 5 [Source:HGNC Symbol;Acc:HGNC:19421] |
| ENSG00000168653 | HGLibA_31558 | 110 | -0.13970577 | NDUFS5 | NADH:ubiquinone oxidoreductase subunit S5 [Source:HGNC Symbol;Acc:HGNC:7712] |
| ENSG00000168876 | HGLibA_02111 | 110 | -0.13970577 | ANKRD49 | ankyrin repeat domain 49 [Source:HGNC Symbol;Acc:HGNC:25970] |
| ENSG00000169087 | HGLibA_22324 | 110 | -0.13970577 | HSPBAP1 | HSPB1 associated protein 1 [Source:HGNC Symbol;Acc:HGNC:16389] |
| ENSG00000169764 | HGLibA_52874 | 110 | -0.13970577 | UGP2 | UDP-glucose pyrophosphorylase 2 [Source:HGNC Symbol;Acc:HGNC:12527] |
| ENSG00000169992 | HGLibA_32071 | 110 | -0.13970577 | NLGN2 | neuroligin 2 [Source:HGNC Symbol;Acc:HGNC:14290] |
| ENSG00000170786 | HGLibA_43159 | 110 | -0.13970577 | SDR16C5 | short chain dehydrogenase/reductase family 16C member 5 [Source:HGNC Symbol;Acc:HGNC:30311] |
| ENSG00000172023 | HGLibA_40798 | 110 | -0.13970577 | REG1B | regenerating family member 1 beta [Source:HGNC Symbol;Acc:HGNC:9952] |
| ENSG00000172150 | HGLibA_33644 | 110 | -0.13970577 | OR1A2 | olfactory receptor family 1 subfamily A member 2 [Source:HGNC Symbol;Acc:HGNC:8180] |
| ENSG00000172716 | HGLibA_45481 | 110 | -0.13970577 | SLFN11 | schlafen family member 11 [Source:HGNC Symbol;Acc:HGNC:26633] |
| ENSG00000174175 | HGLibA_43304 | 110 | -0.13970577 | SELP | selectin P [Source:HGNC Symbol;Acc:HGNC:10721] |
| ENSG00000174903 | HGLibA_39832 | 110 | -0.13970577 | RAB1B | member RAS oncogene family [Source:HGNC Symbol;Acc:HGNC:18370]" |
| ENSG00000175471 | HGLibA_28653 | 110 | -0.13970577 | MCTP1 | multiple C2 and transmembrane domain containing 1 [Source:HGNC Symbol;Acc:HGNC:26183] |
| ENSG00000176095 | HGLibA_23444 | 110 | -0.13970577 | IP6K1 | inositol hexakisphosphate kinase 1 [Source:HGNC Symbol;Acc:HGNC:18360] |
| ENSG00000178177 | HGLibA_26197 | 110 | -0.13970577 | LCORL | ligand dependent nuclear receptor corepressor like [Source:HGNC Symbol;Acc:HGNC:30776] |
| ENSG00000179520 | HGLibA_44480 | 110 | -0.13970577 | SLC17A8 | solute carrier family 17 member 8 [Source:HGNC Symbol;Acc:HGNC:20151] |
| ENSG00000180210 | HGLibA_15860 | 110 | -0.13970577 | F2 | thrombin [Source:HGNC Symbol;Acc:HGNC:3535]" |
| ENSG00000180383 | HGLibA_12875 | 110 | -0.13970577 | DEFB124 | defensin beta 124 [Source:HGNC Symbol;Acc:HGNC:18104] |
| ENSG00000181523 | HGLibA_43857 | 110 | -0.13970577 | SGSH | N-sulfoglucosamine sulfohydrolase [Source:HGNC Symbol;Acc:HGNC:10818] |
| ENSG00000182255 | HGLibA_24134 | 110 | -0.13970577 | KCNA4 | potassium voltage-gated channel subfamily A member 4 [Source:HGNC Symbol;Acc:HGNC:6222] |
| ENSG00000183145 | HGLibA_41333 | 110 | -0.13970577 | RIPPLY3 | ripply transcriptional repressor 3 [Source:HGNC Symbol;Acc:HGNC:3047] |
| ENSG00000183340 | HGLibA_23995 | 110 | -0.13970577 | JRKL | JRK like [Source:HGNC Symbol;Acc:HGNC:6200] |
| ENSG00000183476 | HGLibA_43909 | 110 | -0.13970577 | SH2D7 | SH2 domain containing 7 [Source:HGNC Symbol;Acc:HGNC:34549] |
| ENSG00000184009 | HGLibA_00610 | 110 | -0.13970577 | ACTG1 | actin gamma 1 [Source:HGNC Symbol;Acc:HGNC:144] |
| ENSG00000184148 | HGLibA_46830 | 110 | -0.13970577 | SPRR4 | small proline rich protein 4 [Source:HGNC Symbol;Acc:HGNC:23173] |
| ENSG00000184368 | HGLibA_28239 | 110 | -0.13970577 | MAP7D2 | MAP7 domain containing 2 [Source:HGNC Symbol;Acc:HGNC:25899] |
| ENSG00000185070 | HGLibA_17656 | 110 | -0.13970577 | FLRT2 | fibronectin leucine rich transmembrane protein 2 [Source:HGNC Symbol;Acc:HGNC:3761] |
| ENSG00000185681 | HGLibA_29679 | 110 | -0.13970577 | MORN5 | MORN repeat containing 5 [Source:HGNC Symbol;Acc:HGNC:17841] |
| ENSG00000185697 | HGLibA_30644 | 110 | -0.13970577 | MYBL1 | MYB proto-oncogene like 1 [Source:HGNC Symbol;Acc:HGNC:7547] |
| ENSG00000186222 | HGLibA_04566 | 110 | -0.13970577 | BLOC1S4 | biogenesis of lysosomal organelles complex 1 subunit 4 [Source:HGNC Symbol;Acc:HGNC:24206] |
| ENSG00000187260 | HGLibA_54203 | 110 | -0.13970577 | WDR86 | WD repeat domain 86 [Source:HGNC Symbol;Acc:HGNC:28020] |
| ENSG00000187268 | HGLibA_16824 | 110 | -0.13970577 | FAM9C | family with sequence similarity 9 member C [Source:HGNC Symbol;Acc:HGNC:18405] |
| ENSG00000187678 | HGLibA_46847 | 110 | -0.13970577 | SPRY4 | sprouty RTK signaling antagonist 4 [Source:HGNC Symbol;Acc:HGNC:15533] |
| ENSG00000187764 | HGLibA_43349 | 110 | -0.13970577 | SEMA4D | semaphorin 4D [Source:HGNC Symbol;Acc:HGNC:10732] |
| ENSG00000187959 | HGLibA_11009 | 110 | -0.13970577 | CPSF4L | cleavage and polyadenylation specific factor 4 like [Source:HGNC Symbol;Acc:HGNC:33632] |
| ENSG00000196338 | HGLibA_32073 | 110 | -0.13970577 | NLGN3 | neuroligin 3 [Source:HGNC Symbol;Acc:HGNC:14289] |
| ENSG00000196361 | HGLibA_14886 | 110 | -0.13970577 | ELAVL3 | ELAV like RNA binding protein 3 [Source:HGNC Symbol;Acc:HGNC:3314] |
| ENSG00000196361 | HGLibA_14885 | 110 | -0.13970577 | ELAVL3 | ELAV like RNA binding protein 3 [Source:HGNC Symbol;Acc:HGNC:3314] |
| ENSG00000197162 | HGLibA_56748 | 110 | -0.13970577 | ZNF785 | zinc finger protein 785 [Source:HGNC Symbol;Acc:HGNC:26496] |
| ENSG00000198015 | HGLibA_29985 | 110 | -0.13970577 | MRPL42 | mitochondrial ribosomal protein L42 [Source:HGNC Symbol;Acc:HGNC:14493] |
| ENSG00000198464 | HGLibA_56070 | 110 | -0.13970577 | ZNF480 | zinc finger protein 480 [Source:HGNC Symbol;Acc:HGNC:23305] |
| ENSG00000204195 | HGLibA_03911 | 110 | -0.13970577 | AWAT1 | acyl-CoA wax alcohol acyltransferase 1 [Source:HGNC Symbol;Acc:HGNC:23252] |

| | | | | | |
|---|---|---|---|---|---|
| ENSG00000204438 | HGLibA_19730 | 110 | -0.13970577 | GPANK1 | G-patch domain and ankyrin repeats 1 [Source:HGNC Symbol;Acc:HGNC:13920] |
| ENSG00000204611 | HGLibA_56394 | 110 | -0.13970577 | ZNF616 | zinc finger protein 616 [Source:HGNC Symbol;Acc:HGNC:28062] |
| ENSG00000204961 | HGLibA_35461 | 110 | -0.13970577 | PCDHA9 | protocadherin alpha 9 [Source:HGNC Symbol;Acc:HGNC:8675] |
| ENSG00000214376 | HGLibA_53872 | 110 | -0.13970577 | VSTM5 | V-set and transmembrane domain containing 5 [Source:HGNC Symbol;Acc:HGNC:34443] |
| ENSG00000215217 | HGLibA_06397 | 110 | -0.13970577 | C5orf49 | chromosome 5 open reading frame 49 [Source:HGNC Symbol;Acc:HGNC:27028] |
| ENSG00000228856 | HGLibA_53275 | 110 | -0.13970577 | USP17L30 | ubiquitin specific peptidase 17-like family member 30 [Source:HGNC Symbol;Acc:HGNC:44458] |
| ENSG00000231396 | HGLibA_53215 | 110 | -0.13970577 | USP17L10 | ubiquitin specific peptidase 17-like family member 10 [Source:HGNC Symbol;Acc:HGNC:44438] |
| ENSG00000239810 | HGLibA_38284 | 110 | -0.13970577 | PRAMEF11 | PRAME family member 11 [Source:HGNC Symbol;Acc:HGNC:14086] |
| ENSG00000243244 | HGLibA_47542 | 110 | -0.13970577 | STON1 | stonin 1 [Source:HGNC Symbol;Acc:HGNC:17003] |
| ENSG00000253368 | HGLibA_51710 | 110 | -0.13970577 | TRNP1 | TMF1-regulated nuclear protein 1 [Source:HGNC Symbol;Acc:HGNC:34348] |
| ENSG00000269897 | HGLibA_10663 | 110 | -0.13970577 | COMMD3-BMI1 | COMMD3-BMI1 readthrough [Source:HGNC Symbol;Acc:HGNC:48326] |
| FAM19A4 | HGLibA_16406 | 110 | -0.13970577 | FAM19A4 | - |
| FAM212B | HGLibA_16468 | 110 | -0.13970577 | FAM212B | - |
| hsa-mir-1234 | HGLibA_57332 | 110 | -0.13970577 | hsa-mir-1234 | - |
| hsa-mir-142 | HGLibA_57834 | 110 | -0.13970577 | hsa-mir-142 | - |
| hsa-mir-1909 | HGLibA_58031 | 110 | -0.13970577 | hsa-mir-1909 | - |
| hsa-mir-320d-1 | HGLibA_58943 | 110 | -0.13970577 | hsa-mir-320d-1 | - |
| hsa-mir-3658 | HGLibA_59176 | 110 | -0.13970577 | hsa-mir-3658 | - |
| hsa-mir-365b | HGLibA_59191 | 110 | -0.13970577 | hsa-mir-365b | - |
| hsa-mir-3660 | HGLibA_59194 | 110 | -0.13970577 | hsa-mir-3660 | - |
| hsa-mir-4256 | HGLibA_59731 | 110 | -0.13970577 | hsa-mir-4256 | - |
| hsa-mir-4329 | HGLibA_60038 | 110 | -0.13970577 | hsa-mir-4329 | - |
| hsa-mir-4482 | HGLibA_60347 | 110 | -0.13970577 | hsa-mir-4482 | - |
| hsa-mir-4540 | HGLibA_60625 | 110 | -0.13970577 | hsa-mir-4540 | - |
| hsa-mir-4786 | HGLibA_61250 | 110 | -0.13970577 | hsa-mir-4786 | - |
| hsa-mir-4789 | HGLibA_61264 | 110 | -0.13970577 | hsa-mir-4789 | - |
| hsa-mir-4804 | HGLibA_61321 | 110 | -0.13970577 | hsa-mir-4804 | - |
| hsa-mir-511 | HGLibA_61558 | 110 | -0.13970577 | hsa-mir-511 | - |
| hsa-mir-6069 | HGLibA_62525 | 110 | -0.13970577 | hsa-mir-6069 | - |
| hsa-mir-623 | HGLibA_62731 | 110 | -0.13970577 | hsa-mir-623 | - |
| hsa-mir-675 | HGLibA_63154 | 110 | -0.13970577 | hsa-mir-675 | - |
| hsa-mir-6773 | HGLibA_63267 | 110 | -0.13970577 | hsa-mir-6773 | - |
| hsa-mir-758 | HGLibA_63891 | 110 | -0.13970577 | hsa-mir-758 | - |
| hsa-mir-7706 | HGLibA_63958 | 110 | -0.13970577 | hsa-mir-7706 | - |
| PLCXD1_X | HGLibA_37020 | 110 | -0.13970577 | PLCXD1_X | - |
| PTPLA | HGLibA_39414 | 110 | -0.13970577 | PTPLA | - |
| C20orf196 | HGLibA_06006 | 109 | -0.148751113 | C20orf196 | - |
| C9orf169 | HGLibA_06704 | 109 | -0.148751113 | C9orf169 | - |
| CCDC94 | HGLibA_07934 | 109 | -0.148751113 | CCDC94 | - |
| DOPEY2 | HGLibA_13802 | 109 | -0.148751113 | DOPEY2 | - |
| ENSG00000004809 | HGLibA_44554 | 109 | -0.148751113 | SLC22A16 | solute carrier family 22 member 16 [Source:HGNC Symbol;Acc:HGNC:20302] |
| ENSG00000004975 | HGLibA_14210 | 109 | -0.148751113 | DVL2 | dishevelled segment polarity protein 2 [Source:HGNC Symbol;Acc:HGNC:3086] |
| ENSG00000006740 | HGLibA_02779 | 109 | -0.148751113 | ARHGAP44 | Rho GTPase activating protein 44 [Source:HGNC Symbol;Acc:HGNC:29096] |
| ENSG00000007541 | HGLibA_36579 | 109 | -0.148751113 | PIGQ | phosphatidylinositol glycan anchor biosynthesis class Q [Source:HGNC Symbol;Acc:HGNC:14135] |
| ENSG00000010165 | HGLibA_28956 | 109 | -0.148751113 | METTL13 | eEF1A lysine and N-terminal methyltransferase [Source:HGNC Symbol;Acc:HGNC:24248] |
| ENSG00000010219 | HGLibA_14283 | 109 | -0.148751113 | DYRK4 | dual specificity tyrosine phosphorylation regulated kinase 4 [Source:HGNC Symbol;Acc:HGNC:3095] |
| ENSG00000013573 | HGLibA_12654 | 109 | -0.148751113 | DDX11 | DEAD/H-box helicase 11 [Source:HGNC Symbol;Acc:HGNC:2736] |
| ENSG00000037241 | HGLibA_41915 | 109 | -0.148751113 | RPL26L1 | ribosomal protein L26 like 1 [Source:HGNC Symbol;Acc:HGNC:17050] |
| ENSG00000037757 | HGLibA_29858 | 109 | -0.148751113 | MRI1 | methylthioribose-1-phosphate isomerase 1 [Source:HGNC Symbol;Acc:HGNC:28469] |
| ENSG00000038210 | HGLibA_36494 | 109 | -0.148751113 | PI4K2B | phosphatidylinositol 4-kinase type 2 beta [Source:HGNC Symbol;Acc:HGNC:18215] |
| ENSG00000054654 | HGLibA_47979 | 109 | -0.148751113 | SYNE2 | spectrin repeat containing nuclear envelope protein 2 [Source:HGNC Symbol;Acc:HGNC:17084] |
| ENSG00000062194 | HGLibA_19757 | 109 | -0.148751113 | GPBP1 | GC-rich promoter binding protein 1 [Source:HGNC Symbol;Acc:HGNC:29520] |
| ENSG00000065970 | HGLibA_17888 | 109 | -0.148751113 | FOXJ2 | forkhead box J2 [Source:HGNC Symbol;Acc:HGNC:24818] |
| ENSG00000066455 | HGLibA_19604 | 109 | -0.148751113 | GOLGA5 | golgin A5 [Source:HGNC Symbol;Acc:HGNC:4428] |
| ENSG00000070190 | HGLibA_12333 | 109 | -0.148751113 | DAPP1 | dual adaptor of phosphotyrosine and 3-phosphoinositides 1 [Source:HGNC Symbol;Acc:HGNC:16500] |
| ENSG00000074621 | HGLibA_44616 | 109 | -0.148751113 | SLC24A1 | solute carrier family 24 member 1 [Source:HGNC Symbol;Acc:HGNC:10975] |
| ENSG00000078098 | HGLibA_16865 | 109 | -0.148751113 | FAP | fibroblast activation protein alpha [Source:HGNC Symbol;Acc:HGNC:3590] |
| ENSG00000083844 | HGLibA_55697 | 109 | -0.148751113 | ZNF264 | zinc finger protein 264 [Source:HGNC Symbol;Acc:HGNC:13057] |
| ENSG00000088833 | HGLibA_32754 | 109 | -0.148751113 | NSFL1C | NSFL1 cofactor [Source:HGNC Symbol;Acc:HGNC:15912] |
| ENSG00000090487 | HGLibA_46635 | 109 | -0.148751113 | SPG21 | maspardin [Source:HGNC Symbol;Acc:HGNC:20373]" |
| ENSG00000099219 | HGLibA_15508 | 109 | -0.148751113 | ERMP1 | endoplasmic reticulum metallopeptidase 1 [Source:HGNC Symbol;Acc:HGNC:23703] |
| ENSG00000099910 | HGLibA_25157 | 109 | -0.148751113 | KLHL22 | kelch like family member 22 [Source:HGNC Symbol;Acc:HGNC:25888] |
| ENSG00000100228 | HGLibA_39892 | 109 | -0.148751113 | RAB36 | member RAS oncogene family [Source:HGNC Symbol;Acc:HGNC:9775]" |
| ENSG00000101144 | HGLibA_04618 | 109 | -0.148751113 | BMP7 | bone morphogenetic protein 7 [Source:HGNC Symbol;Acc:HGNC:1074] |
| ENSG00000101441 | HGLibA_11455 | 109 | -0.148751113 | CST4 | cystatin S [Source:HGNC Symbol;Acc:HGNC:2476] |
| ENSG00000102539 | HGLibA_29430 | 109 | -0.148751113 | MLNR | motilin receptor [Source:HGNC Symbol;Acc:HGNC:4495] |
| ENSG00000103253 | HGLibA_20818 | 109 | -0.148751113 | HAGHL | hydroxyacylglutathione hydrolase like [Source:HGNC Symbol;Acc:HGNC:14177] |
| ENSG00000105393 | HGLibA_04055 | 109 | -0.148751113 | BABAM1 | BRISC and BRCA1 A complex member 1 [Source:HGNC Symbol;Acc:HGNC:25008] |
| ENSG00000105514 | HGLibA_39917 | 109 | -0.148751113 | RAB3D | member RAS oncogene family [Source:HGNC Symbol;Acc:HGNC:9779]" |
| ENSG00000106633 | HGLibA_18788 | 109 | -0.148751113 | GCK | glucokinase [Source:HGNC Symbol;Acc:HGNC:4195] |
| ENSG00000107187 | HGLibA_26452 | 109 | -0.148751113 | LHX3 | LIM homeobox 3 [Source:HGNC Symbol;Acc:HGNC:6595] |
| ENSG00000107957 | HGLibA_43964 | 109 | -0.148751113 | SH3PXD2A | SH3 and PX domains 2A [Source:HGNC Symbol;Acc:HGNC:23664] |
| ENSG00000108405 | HGLibA_34846 | 109 | -0.148751113 | P2RX1 | purinergic receptor P2X 1 [Source:HGNC Symbol;Acc:HGNC:8533] |
| ENSG00000108825 | HGLibA_39337 | 109 | -0.148751113 | PTGES3L-AARSD1 | PTGES3L-AARSD1 readthrough [Source:HGNC Symbol;Acc:HGNC:43946] |
| ENSG00000111252 | HGLibA_43879 | 109 | -0.148751113 | SH2B3 | SH2B adaptor protein 3 [Source:HGNC Symbol;Acc:HGNC:29605] |
| ENSG00000111837 | HGLibA_28017 | 109 | -0.148751113 | MAK | male germ cell associated kinase [Source:HGNC Symbol;Acc:HGNC:6816] |
| ENSG00000112699 | HGLibA_19393 | 109 | -0.148751113 | GMDS | 6-dehydratase [Source:HGNC Symbol;Acc:HGNC:4369]" |
| ENSG00000113460 | HGLibA_04825 | 109 | -0.148751113 | BRIX1 | biogenesis of ribosomes [Source:HGNC Symbol;Acc:HGNC:24170]" |
| ENSG00000114019 | HGLibA_01819 | 109 | -0.148751113 | AMOTL2 | angiomotin like 2 [Source:HGNC Symbol;Acc:HGNC:17812] |
| ENSG00000114933 | HGLibA_23327 | 109 | -0.148751113 | INO80D | INO80 complex subunit D [Source:HGNC Symbol;Acc:HGNC:25997] |
| ENSG00000117569 | HGLibA_39252 | 109 | -0.148751113 | PTBP2 | polypyrimidine tract binding protein 2 [Source:HGNC Symbol;Acc:HGNC:17662] |
| ENSG00000119431 | HGLibA_21057 | 109 | -0.148751113 | HDHD3 | haloacid dehalogenase like hydrolase domain containing 3 [Source:HGNC Symbol;Acc:HGNC:28171] |
| ENSG00000119900 | HGLibA_33319 | 109 | -0.148751113 | OGFRL1 | opioid growth factor receptor like 1 [Source:HGNC Symbol;Acc:HGNC:21378] |
| ENSG00000119986 | HGLibA_03900 | 109 | -0.148751113 | AVPI1 | arginine vasopressin induced 1 [Source:HGNC Symbol;Acc:HGNC:30898] |
| ENSG00000120937 | HGLibA_32503 | 109 | -0.148751113 | NPPB | natriuretic peptide B [Source:HGNC Symbol;Acc:HGNC:7940] |
| ENSG00000121690 | HGLibA_12985 | 109 | -0.148751113 | DEPDC7 | DEP domain containing 7 [Source:HGNC Symbol;Acc:HGNC:29899] |
| ENSG00000122741 | HGLibA_12413 | 109 | -0.148751113 | DCAF10 | DDB1 and CUL4 associated factor 10 [Source:HGNC Symbol;Acc:HGNC:23686] |
| ENSG00000126756 | HGLibA_53483 | 109 | -0.148751113 | UXT | ubiquitously expressed prefoldin like chaperone [Source:HGNC Symbol;Acc:HGNC:12641] |
| ENSG00000126861 | HGLibA_33387 | 109 | -0.148751113 | OMG | oligodendrocyte myelin glycoprotein [Source:HGNC Symbol;Acc:HGNC:8135] |
| ENSG00000127418 | HGLibA_17454 | 109 | -0.148751113 | FGFRL1 | fibroblast growth factor receptor like 1 [Source:HGNC Symbol;Acc:HGNC:3693] |
| ENSG00000128617 | HGLibA_33432 | 109 | -0.148751113 | OPN1SW | short wave sensitive [Source:HGNC Symbol;Acc:HGNC:1012]" |
| ENSG00000132635 | HGLibA_35583 | 109 | -0.148751113 | PCED1A | PC-esterase domain containing 1A [Source:HGNC Symbol;Acc:HGNC:16212] |
| ENSG00000132669 | HGLibA_41292 | 109 | -0.148751113 | RIN2 | Ras and Rab interactor 2 [Source:HGNC Symbol;Acc:HGNC:18750] |
| ENSG00000132906 | HGLibA_07332 | 109 | -0.148751113 | CASP9 | caspase 9 [Source:HGNC Symbol;Acc:HGNC:1511] |
| ENSG00000133315 | HGLibA_27836 | 109 | -0.148751113 | MACROD1 | MACRO domain containing 1 [Source:HGNC Symbol;Acc:HGNC:29598] |
| ENSG00000135248 | HGLibA_16702 | 109 | -0.148751113 | FAM71F1 | family with sequence similarity 71 member F1 [Source:HGNC Symbol;Acc:HGNC:30704] |
| ENSG00000135482 | HGLibA_54947 | 109 | -0.148751113 | ZC3H10 | zinc finger CCCH-type containing 10 [Source:HGNC Symbol;Acc:HGNC:25893] |
| ENSG00000135519 | HGLibA_24217 | 109 | -0.148751113 | KCNH3 | potassium voltage-gated channel subfamily H member 3 [Source:HGNC Symbol;Acc:HGNC:6252] |
| ENSG00000135597 | HGLibA_40848 | 109 | -0.148751113 | REPS1 | RALBP1 associated Eps domain containing 1 [Source:HGNC Symbol;Acc:HGNC:15578] |
| ENSG00000136111 | HGLibA_48556 | 109 | -0.148751113 | TBC1D4 | TBC1 domain family member 4 [Source:HGNC Symbol;Acc:HGNC:19165] |
| ENSG00000136603 | HGLibA_44283 | 109 | -0.148751113 | SKIL | SKI like proto-oncogene [Source:HGNC Symbol;Acc:HGNC:10897] |
| ENSG00000136942 | HGLibA_41945 | 109 | -0.148751113 | RPL35 | ribosomal protein L35 [Source:HGNC Symbol;Acc:HGNC:10344] |
| ENSG00000138813 | HGLibA_06296 | 109 | -0.148751113 | C4orf17 | chromosome 4 open reading frame 17 [Source:HGNC Symbol;Acc:HGNC:25274] |
| ENSG00000140262 | HGLibA_48761 | 109 | -0.148751113 | TCF12 | transcription factor 12 [Source:HGNC Symbol;Acc:HGNC:11623] |
| ENSG00000140740 | HGLibA_53117 | 109 | -0.148751113 | UQCRC2 | ubiquinol-cytochrome c reductase core protein 2 [Source:HGNC Symbol;Acc:HGNC:12586] |
| ENSG00000140987 | HGLibA_57023 | 109 | -0.148751113 | ZSCAN32 | zinc finger and SCAN domain containing 32 [Source:HGNC Symbol;Acc:HGNC:20812] |
| ENSG00000141294 | HGLibA_27369 | 109 | -0.148751113 | LRRC46 | leucine rich repeat containing 46 [Source:HGNC Symbol;Acc:HGNC:25047] |
| ENSG00000141526 | HGLibA_44438 | 109 | -0.148751113 | SLC16A3 | solute carrier family 16 member 3 [Source:HGNC Symbol;Acc:HGNC:10924] |
| ENSG00000143162 | HGLibA_11120 | 109 | -0.148751113 | CREG1 | cellular repressor of E1A stimulated genes 1 [Source:HGNC Symbol;Acc:HGNC:2351] |
| ENSG00000143537 | HGLibA_00737 | 109 | -0.148751113 | ADAM15 | ADAM metallopeptidase domain 15 [Source:HGNC Symbol;Acc:HGNC:193] |
| ENSG00000143590 | HGLibA_14588 | 109 | -0.148751113 | EFNA3 | ephrin A3 [Source:HGNC Symbol;Acc:HGNC:3223] |
| ENSG00000144278 | HGLibA_18539 | 109 | -0.148751113 | GALNT13 | polypeptide N-acetylgalactosaminyltransferase 13 [Source:HGNC Symbol;Acc:HGNC:23242] |
| ENSG00000144645 | HGLibA_34642 | 109 | -0.148751113 | OSBPL10 | oxysterol binding protein like 10 [Source:HGNC Symbol;Acc:HGNC:16395] |
| ENSG00000147869 | HGLibA_09188 | 109 | -0.148751113 | CER1 | DAN family BMP antagonist [Source:HGNC Symbol;Acc:HGNC:1862]" |
| ENSG00000147872 | HGLibA_37174 | 109 | -0.148751113 | PLIN2 | perilipin 2 [Source:HGNC Symbol;Acc:HGNC:248] |
| ENSG00000148200 | HGLibA_32641 | 109 | -0.148751113 | NR6A1 | nuclear receptor subfamily 6 group A member 1 [Source:HGNC Symbol;Acc:HGNC:7985] |
| ENSG00000148671 | HGLibA_00987 | 109 | -0.148751113 | ADIRF | adipogenesis regulatory factor [Source:HGNC Symbol;Acc:HGNC:24043] |
| ENSG00000149182 | HGLibA_02663 | 109 | -0.148751113 | ARFGAP2 | ADP ribosylation factor GTPase activating protein 2 [Source:HGNC Symbol;Acc:HGNC:13504] |
| ENSG00000149541 | HGLibA_03977 | 109 | -0.148751113 | B3GAT3 | 3-glucuronyltransferase 3 [Source:HGNC Symbol;Acc:HGNC:923]" |
| ENSG00000150403 | HGLibA_49787 | 109 | -0.148751113 | TMCO3 | transmembrane and coiled-coil domains 3 [Source:HGNC Symbol;Acc:HGNC:20329] |
| ENSG00000150760 | HGLibA_13731 | 109 | -0.148751113 | DOCK1 | dedicator of cytokinesis 1 [Source:HGNC Symbol;Acc:HGNC:2987] |
| ENSG00000151065 | HGLibA_12520 | 109 | -0.148751113 | DCP1B | decapping mRNA 1B [Source:HGNC Symbol;Acc:HGNC:24451] |
| ENSG00000152475 | HGLibA_56846 | 109 | -0.148751113 | ZNF837 | zinc finger protein 837 [Source:HGNC Symbol;Acc:HGNC:25164] |
| ENSG00000152689 | HGLibA_40351 | 109 | -0.148751113 | RASGRP3 | RAS guanyl releasing protein 3 [Source:HGNC Symbol;Acc:HGNC:14545] |
| ENSG00000155287 | HGLibA_44692 | 109 | -0.148751113 | SLC25A28 | solute carrier family 25 member 28 [Source:HGNC Symbol;Acc:HGNC:23472] |
| ENSG00000156853 | HGLibA_56546 | 109 | -0.148751113 | ZNF689 | zinc finger protein 689 [Source:HGNC Symbol;Acc:HGNC:25173] |
| ENSG00000157542 | HGLibA_24285 | 109 | -0.148751113 | KCNJ6 | potassium voltage-gated channel subfamily J member 6 [Source:HGNC Symbol;Acc:HGNC:6267] |
| ENSG00000157554 | HGLibA_15464 | 109 | -0.148751113 | ERG | ETS transcription factor ERG [Source:HGNC Symbol;Acc:HGNC:3446] |
| ENSG00000157617 | HGLibA_06086 | 109 | -0.148751113 | C2CD2 | C2 calcium dependent domain containing 2 [Source:HGNC Symbol;Acc:HGNC:1266] |
| ENSG00000157657 | HGLibA_56398 | 109 | -0.148751113 | ZNF618 | zinc finger protein 618 [Source:HGNC Symbol;Acc:HGNC:29416] |
| ENSG00000157978 | HGLibA_26254 | 109 | -0.148751113 | LDLRAP1 | low density lipoprotein receptor adaptor protein 1 [Source:HGNC Symbol;Acc:HGNC:18640] |
| ENSG00000158423 | HGLibA_41229 | 109 | -0.148751113 | RIBC1 | RIB43A domain with coiled-coils 1 [Source:HGNC Symbol;Acc:HGNC:26537] |
| ENSG00000159166 | HGLibA_25923 | 109 | -0.148751113 | LAD1 | ladinin 1 [Source:HGNC Symbol;Acc:HGNC:6472] |
| ENSG00000162727 | HGLibA_33843 | 109 | -0.148751113 | OR2M5 | olfactory receptor family 2 subfamily M member 5 [Source:HGNC Symbol;Acc:HGNC:19576] |
| ENSG00000163104 | HGLibA_45614 | 109 | -0.148751113 | SMARCAD1 | containing DEAD/H box 1 [Source:HGNC Symbol;Acc:HGNC:18398]" |

| | | | | | |
|---|---|---|---|---|---|
| ENSG00000163513 | HGLibA_49226 | 109 | -0.148751113 | TGFBR2 | transforming growth factor beta receptor 2 [Source:HGNC Symbol;Acc:HGNC:11773] |
| ENSG00000163636 | HGLibA_39179 | 109 | -0.148751113 | PSMD6 | non-ATPase 6 [Source:HGNC Symbol;Acc:HGNC:9564]" |
| ENSG00000164023 | HGLibA_43839 | 109 | -0.148751113 | SGMS2 | sphingomyelin synthase 2 [Source:HGNC Symbol;Acc:HGNC:28395] |
| ENSG00000164684 | HGLibA_56585 | 109 | -0.148751113 | ZNF704 | zinc finger protein 704 [Source:HGNC Symbol;Acc:HGNC:32291] |
| ENSG00000165029 | HGLibA_00094 | 109 | -0.148751113 | ABCA1 | ATP binding cassette subfamily A member 1 [Source:HGNC Symbol;Acc:HGNC:29] |
| ENSG00000165140 | HGLibA_16963 | 109 | -0.148751113 | FBP1 | fructose-bisphosphatase 1 [Source:HGNC Symbol;Acc:HGNC:3606] |
| ENSG00000167378 | HGLibA_23617 | 109 | -0.148751113 | IRGQ | immunity related GTPase Q [Source:HGNC Symbol;Acc:HGNC:24868] |
| ENSG00000167552 | HGLibA_52242 | 109 | -0.148751113 | TUBA1A | tubulin alpha 1a [Source:HGNC Symbol;Acc:HGNC:20766] |
| ENSG00000168010 | HGLibA_03476 | 109 | -0.148751113 | ATG16L2 | autophagy related 16 like 2 [Source:HGNC Symbol;Acc:HGNC:25464] |
| ENSG00000169359 | HGLibA_44934 | 109 | -0.148751113 | SLC33A1 | solute carrier family 33 member 1 [Source:HGNC Symbol;Acc:HGNC:95] |
| ENSG00000170396 | HGLibA_56789 | 109 | -0.148751113 | ZNF804A | zinc finger protein 804A [Source:HGNC Symbol;Acc:HGNC:21711] |
| ENSG00000170743 | HGLibA_48093 | 109 | -0.148751113 | SYT9 | synaptotagmin 9 [Source:HGNC Symbol;Acc:HGNC:19265] |
| ENSG00000170782 | HGLibA_33467 | 109 | -0.148751113 | OR10A4 | olfactory receptor family 10 subfamily A member 4 [Source:HGNC Symbol;Acc:HGNC:15130] |
| ENSG00000170802 | HGLibA_17912 | 109 | -0.148751113 | FOXN2 | forkhead box N2 [Source:HGNC Symbol;Acc:HGNC:5281] |
| ENSG00000171067 | HGLibA_05123 | 109 | -0.148751113 | C11orf24 | chromosome 11 open reading frame 24 [Source:HGNC Symbol;Acc:HGNC:1174] |
| ENSG00000172016 | HGLibA_40800 | 109 | -0.148751113 | REG3A | regenerating family member 3 alpha [Source:HGNC Symbol;Acc:HGNC:8601] |
| ENSG00000173253 | HGLibA_13420 | 109 | -0.148751113 | DMRT2 | doublesex and mab-3 related transcription factor 2 [Source:HGNC Symbol;Acc:HGNC:2935] |
| ENSG00000175216 | HGLibA_09764 | 109 | -0.148751113 | CKAP5 | cytoskeleton associated protein 5 [Source:HGNC Symbol;Acc:HGNC:28959] |
| ENSG00000176956 | HGLibA_27675 | 109 | -0.148751113 | LY6H | lymphocyte antigen 6 family member H [Source:HGNC Symbol;Acc:HGNC:6728] |
| ENSG00000177489 | HGLibA_33793 | 109 | -0.148751113 | OR2G2 | olfactory receptor family 2 subfamily G member 2 [Source:HGNC Symbol;Acc:HGNC:15007] |
| ENSG00000177706 | HGLibA_16451 | 109 | -0.148751113 | FAM20C | golgi associated secretory pathway kinase [Source:HGNC Symbol;Acc:HGNC:22140]" |
| ENSG00000178084 | HGLibA_22387 | 109 | -0.148751113 | HTR3C | 5-hydroxytryptamine receptor 3C [Source:HGNC Symbol;Acc:HGNC:24003] |
| ENSG00000179091 | HGLibA_12026 | 109 | -0.148751113 | CYC1 | cytochrome c1 [Source:HGNC Symbol;Acc:HGNC:2579] |
| ENSG00000180044 | HGLibA_06271 | 109 | -0.148751113 | C3orf80 | chromosome 3 open reading frame 80 [Source:HGNC Symbol;Acc:HGNC:40048] |
| ENSG00000183044 | HGLibA_00093 | 109 | -0.148751113 | ABAT | 4-aminobutyrate aminotransferase [Source:HGNC Symbol;Acc:HGNC:23] |
| ENSG00000183763 | HGLibA_51274 | 109 | -0.148751113 | TRAIP | TRAF interacting protein [Source:HGNC Symbol;Acc:HGNC:30764] |
| ENSG00000183795 | HGLibA_04747 | 109 | -0.148751113 | BPY2B | basic charge Y-linked 2B [Source:HGNC Symbol;Acc:HGNC:25449] |
| ENSG00000183850 | HGLibA_56645 | 109 | -0.148751113 | ZNF730 | zinc finger protein 730 [Source:HGNC Symbol;Acc:HGNC:32470] |
| ENSG00000185009 | HGLibA_02328 | 109 | -0.148751113 | AP3M1 | adaptor related protein complex 3 subunit mu 1 [Source:HGNC Symbol;Acc:HGNC:569] |
| ENSG00000185122 | HGLibA_22221 | 109 | -0.148751113 | HSF1 | heat shock transcription factor 1 [Source:HGNC Symbol;Acc:HGNC:5224] |
| ENSG00000185219 | HGLibA_56017 | 109 | -0.148751113 | ZNF445 | zinc finger protein 445 [Source:HGNC Symbol;Acc:HGNC:21018] |
| ENSG00000185686 | HGLibA_38275 | 109 | -0.148751113 | PRAME | preferentially expressed antigen in melanoma [Source:HGNC Symbol;Acc:HGNC:9336] |
| ENSG00000185737 | HGLibA_32682 | 109 | -0.148751113 | NRG3 | neuregulin 3 [Source:HGNC Symbol;Acc:HGNC:7999] |
| ENSG00000186564 | HGLibA_17830 | 109 | -0.148751113 | FOXD2 | forkhead box D2 [Source:HGNC Symbol;Acc:HGNC:3803] |
| ENSG00000186666 | HGLibA_04271 | 109 | -0.148751113 | BCDIN3D | BCDIN3 domain containing RNA methyltransferase [Source:HGNC Symbol;Acc:HGNC:27050] |
| ENSG00000187522 | HGLibA_22268 | 109 | -0.148751113 | HSPA14 | heat shock protein family A (Hsp70) member 14 [Source:HGNC Symbol;Acc:HGNC:29526] |
| ENSG00000187554 | HGLibA_49656 | 109 | -0.148751113 | TLR5 | toll like receptor 5 [Source:HGNC Symbol;Acc:HGNC:11851] |
| ENSG00000187808 | HGLibA_46221 | 109 | -0.148751113 | SOWAHD | sosondowah ankyrin repeat domain family member D [Source:HGNC Symbol;Acc:HGNC:32960] |
| ENSG00000187848 | HGLibA_34850 | 109 | -0.148751113 | P2RX2 | purinergic receptor P2X 2 [Source:HGNC Symbol;Acc:HGNC:15459] |
| ENSG00000188611 | HGLibA_03188 | 109 | -0.148751113 | ASAH2 | N-acylsphingosine amidohydrolase 2 [Source:HGNC Symbol;Acc:HGNC:18860] |
| ENSG00000188647 | HGLibA_39246 | 109 | -0.148751113 | PTAR1 | protein prenyltransferase alpha subunit repeat containing 1 [Source:HGNC Symbol;Acc:HGNC:30449] |
| ENSG00000188784 | HGLibA_36891 | 109 | -0.148751113 | PLA2G2E | phospholipase A2 group IIE [Source:HGNC Symbol;Acc:HGNC:13414] |
| ENSG00000188817 | HGLibA_46013 | 109 | -0.148751113 | SNTN | cilia apical structure protein [Source:HGNC Symbol;Acc:HGNC:33706]" |
| ENSG00000188996 | HGLibA_22428 | 109 | -0.148751113 | HUS1B | HUS1 checkpoint clamp component B [Source:HGNC Symbol;Acc:HGNC:16485] |
| ENSG00000196456 | HGLibA_56718 | 109 | -0.148751113 | ZNF775 | zinc finger protein 775 [Source:HGNC Symbol;Acc:HGNC:28501] |
| ENSG00000196700 | HGLibA_56135 | 109 | -0.148751113 | ZNF512B | zinc finger protein 512B [Source:HGNC Symbol;Acc:HGNC:29212] |
| ENSG00000197353 | HGLibA_27717 | 109 | -0.148751113 | LYPD2 | LY6/PLAUR domain containing 2 [Source:HGNC Symbol;Acc:HGNC:25215] |
| ENSG00000197361 | HGLibA_17003 | 109 | -0.148751113 | FBXL22 | F-box and leucine rich repeat protein 22 [Source:HGNC Symbol;Acc:HGNC:27537] |
| ENSG00000197763 | HGLibA_52450 | 109 | -0.148751113 | TXNRD3 | thioredoxin reductase 3 [Source:HGNC Symbol;Acc:HGNC:20667] |
| ENSG00000197887 | HGLibA_33716 | 109 | -0.148751113 | OR1S2 | olfactory receptor family 1 subfamily S member 2 [Source:HGNC Symbol;Acc:HGNC:15141] |
| ENSG00000203907 | HGLibA_33402 | 109 | -0.148751113 | OOEP | oocyte expressed protein [Source:HGNC Symbol;Acc:HGNC:21382] |
| ENSG00000204103 | HGLibA_27876 | 109 | -0.148751113 | MAFB | MAF bZIP transcription factor B [Source:HGNC Symbol;Acc:HGNC:6408] |
| ENSG00000204518 | HGLibA_00037 | 109 | -0.148751113 | AADACL4 | arylacetamide deacetylase like 4 [Source:HGNC Symbol;Acc:HGNC:32038] |
| ENSG00000205212 | HGLibA_07626 | 109 | -0.148751113 | CCDC144NL | N-terminal like [Source:HGNC Symbol;Acc:HGNC:33735]" |
| ENSG00000205330 | HGLibA_34383 | 109 | -0.148751113 | OR6C1 | olfactory receptor family 6 subfamily C member 1 [Source:HGNC Symbol;Acc:HGNC:8355] |
| ENSG00000213614 | HGLibA_21201 | 109 | -0.148751113 | HEXA | hexosaminidase subunit alpha [Source:HGNC Symbol;Acc:HGNC:4878] |
| ENSG00000213923 | HGLibA_11390 | 109 | -0.148751113 | CSNK1E | casein kinase 1 epsilon [Source:HGNC Symbol;Acc:HGNC:2453] |
| ENSG00000214946 | HGLibA_48519 | 109 | -0.148751113 | TBC1D26 | TBC1 domain family member 26 [Source:HGNC Symbol;Acc:HGNC:28745] |
| ENSG00000215269 | HGLibA_18451 | 109 | -0.148751113 | GAGE12G | G antigen 12G [Source:HGNC Symbol;Acc:HGNC:31907] |
| ENSG00000220008 | HGLibA_26591 | 109 | -0.148751113 | LINGO3 | leucine rich repeat and Ig domain containing 3 [Source:HGNC Symbol;Acc:HGNC:21206] |
| ENSG00000221968 | HGLibA_15939 | 109 | -0.148751113 | FADS3 | fatty acid desaturase 3 [Source:HGNC Symbol;Acc:HGNC:3576] |
| ENSG00000224982 | HGLibA_50265 | 109 | -0.148751113 | TMEM233 | transmembrane protein 233 [Source:HGNC Symbol;Acc:HGNC:37219] |
| ENSG00000232399 | HGLibA_53225 | 109 | -0.148751113 | USP17L13 | ubiquitin specific peptidase 17-like family member 13 [Source:HGNC Symbol;Acc:HGNC:44441] |
| ENSG00000233608 | HGLibA_52379 | 109 | -0.148751113 | TWIST2 | twist family bHLH transcription factor 2 [Source:HGNC Symbol;Acc:HGNC:20670] |
| ENSG00000233670 | HGLibA_36733 | 109 | -0.148751113 | PIRT | phosphoinositide interacting regulator of transient receptor potential channels [Source:HGNC Symbol;Acc:HGNC:37239] |
| ENSG00000236362 | HGLibA_18448 | 109 | -0.148751113 | GAGE12F | G antigen 12F [Source:HGNC Symbol;Acc:HGNC:31906] |
| ENSG00000242485 | HGLibA_29924 | 109 | -0.148751113 | MRPL20 | mitochondrial ribosomal protein L20 [Source:HGNC Symbol;Acc:HGNC:14478] |
| ENSG00000255374 | HGLibA_48411 | 109 | -0.148751113 | TAS2R43 | taste 2 receptor member 43 [Source:HGNC Symbol;Acc:HGNC:18875] |
| ENSG00000263465 | HGLibA_47090 | 109 | -0.148751113 | SRSF8 | serine and arginine rich splicing factor 8 [Source:HGNC Symbol;Acc:HGNC:16988] |
| ENSG00000269226 | HGLibA_50663 | 109 | -0.148751113 | TMSB15B | thymosin beta 15B [Source:NCBI gene;Acc:286527] |
| ENSG00000271601 | HGLibA_26644 | 109 | -0.148751113 | LIX1L | limb and CNS expressed 1 like [Source:HGNC Symbol;Acc:HGNC:28715] |
| ENSG00000279514 | HGLibA_33949 | 109 | -0.148751113 | OR4C16 | olfactory receptor family 4 subfamily C member 16 (gene/pseudogene) [Source:HGNC Symbol;Acc:HGNC:15172] |
| ENSG00000289549 | HGLibA_37907 | 109 | -0.148751113 | PPIAL4D | - |
| FAM109A | HGLibA_16007 | 109 | -0.148751113 | FAM109A | - |
| H2AFY2 | HGLibA_20765 | 109 | -0.148751113 | H2AFY2 | - |
| hsa-mir-1247 | HGLibA_57386 | 109 | -0.148751113 | hsa-mir-1247 | - |
| hsa-mir-1248 | HGLibA_57391 | 109 | -0.148751113 | hsa-mir-1248 | - |
| hsa-mir-217 | HGLibA_58263 | 109 | -0.148751113 | hsa-mir-217 | - |
| hsa-mir-3122 | HGLibA_58539 | 109 | -0.148751113 | hsa-mir-3122 | - |
| hsa-mir-3160-2 | HGLibA_58719 | 109 | -0.148751113 | hsa-mir-3160-2 | - |
| hsa-mir-3174 | HGLibA_58768 | 109 | -0.148751113 | hsa-mir-3174 | - |
| hsa-mir-3180-1 | HGLibA_58802 | 109 | -0.148751113 | hsa-mir-3180-1 | - |
| hsa-mir-3662 | HGLibA_59199 | 109 | -0.148751113 | hsa-mir-3662 | - |
| hsa-mir-3690-1 | HGLibA_59346 | 109 | -0.148751113 | hsa-mir-3690-1 | - |
| hsa-mir-378h | HGLibA_59458 | 109 | -0.148751113 | hsa-mir-378h | - |
| hsa-mir-382 | HGLibA_59481 | 109 | -0.148751113 | hsa-mir-382 | - |
| hsa-mir-4513 | HGLibA_60494 | 109 | -0.148751113 | hsa-mir-4513 | - |
| hsa-mir-4767 | HGLibA_61165 | 109 | -0.148751113 | hsa-mir-4767 | - |
| hsa-mir-4772 | HGLibA_61190 | 109 | -0.148751113 | hsa-mir-4772 | - |
| hsa-mir-548ad | HGLibA_61863 | 109 | -0.148751113 | hsa-mir-548ad | - |
| hsa-mir-576 | HGLibA_62399 | 109 | -0.148751113 | hsa-mir-576 | - |
| hsa-mir-6074 | HGLibA_62547 | 109 | -0.148751113 | hsa-mir-6074 | - |
| hsa-mir-6739 | HGLibA_63110 | 109 | -0.148751113 | hsa-mir-6739 | - |
| hsa-mir-6828 | HGLibA_63491 | 109 | -0.148751113 | hsa-mir-6828 | - |
| hsa-mir-6848 | HGLibA_63565 | 109 | -0.148751113 | hsa-mir-6848 | - |
| hsa-mir-8060 | HGLibA_64080 | 109 | -0.148751113 | hsa-mir-8060 | - |
| KIAA1644 | HGLibA_24795 | 109 | -0.148751113 | KIAA1644 | - |
| OBFC1 | HGLibA_33222 | 109 | -0.148751113 | OBFC1 | - |
| SMCR7L | HGLibA_45680 | 109 | -0.148751113 | SMCR7L | - |
| TMEM56-RWDD3 | HGLibA_50453 | 109 | -0.148751113 | TMEM56-RWDD3 | - |
| WDR78 | HGLibA_54185 | 109 | -0.148751113 | WDR78 | - |
| ZNF664-FAM101A | HGLibA_56485 | 109 | -0.148751113 | ZNF664-FAM101A | - |
| C7orf72 | HGLibA_06586 | 108 | -0.157796456 | C7orf72 | - |
| C8orf59 | HGLibA_06642 | 108 | -0.157796456 | C8orf59 | - |
| ENSG00000006042 | HGLibA_50571 | 108 | -0.157796456 | TMEM98 | transmembrane protein 98 [Source:HGNC Symbol;Acc:HGNC:24529] |
| ENSG00000007047 | HGLibA_28372 | 108 | -0.157796456 | MARK4 | microtubule affinity regulating kinase 4 [Source:HGNC Symbol;Acc:HGNC:13538] |
| ENSG00000011347 | HGLibA_48087 | 108 | -0.157796456 | SYT7 | synaptotagmin 7 [Source:HGNC Symbol;Acc:HGNC:11514] |
| ENSG00000025039 | HGLibA_42233 | 108 | -0.157796456 | RRAGD | Ras related GTP binding D [Source:HGNC Symbol;Acc:HGNC:19903] |
| ENSG00000025708 | HGLibA_52458 | 108 | -0.157796456 | TYMP | thymidine phosphorylase [Source:HGNC Symbol;Acc:HGNC:3148] |
| ENSG00000046604 | HGLibA_14026 | 108 | -0.157796456 | DSG2 | desmoglein 2 [Source:HGNC Symbol;Acc:HGNC:3049] |
| ENSG00000053108 | HGLibA_18107 | 108 | -0.157796456 | FSTL4 | follistatin like 4 [Source:HGNC Symbol;Acc:HGNC:21389] |
| ENSG00000068831 | HGLibA_40347 | 108 | -0.157796456 | RASGRP2 | RAS guanyl releasing protein 2 [Source:HGNC Symbol;Acc:HGNC:9879] |
| ENSG00000070718 | HGLibA_02332 | 108 | -0.157796456 | AP3M2 | adaptor related protein complex 3 subunit mu 2 [Source:HGNC Symbol;Acc:HGNC:570] |
| ENSG00000077420 | HGLibA_02381 | 108 | -0.157796456 | APBB1IP | amyloid beta precursor protein binding family B member 1 interacting protein [Source:HGNC Symbol;Acc:HGNC:17379] |
| ENSG00000077522 | HGLibA_00640 | 108 | -0.157796456 | ACTN2 | actinin alpha 2 [Source:HGNC Symbol;Acc:HGNC:164] |
| ENSG00000091947 | HGLibA_49840 | 108 | -0.157796456 | TMEM101 | transmembrane protein 101 [Source:HGNC Symbol;Acc:HGNC:28653] |
| ENSG00000100234 | HGLibA_49541 | 108 | -0.157796456 | TIMP3 | TIMP metallopeptidase inhibitor 3 [Source:HGNC Symbol;Acc:HGNC:11822] |
| ENSG00000100341 | HGLibA_37418 | 108 | -0.157796456 | PNPLA5 | patatin like phospholipase domain containing 5 [Source:HGNC Symbol;Acc:HGNC:24888] |
| ENSG00000100445 | HGLibA_43162 | 108 | -0.157796456 | SDR39U1 | short chain dehydrogenase/reductase family 39U member 1 [Source:HGNC Symbol;Acc:HGNC:20275] |
| ENSG00000100985 | HGLibA_29544 | 108 | -0.157796456 | MMP9 | matrix metallopeptidase 9 [Source:HGNC Symbol;Acc:HGNC:7176] |
| ENSG00000101331 | HGLibA_08049 | 108 | -0.157796456 | CCM2L | CCM2 like scaffold protein [Source:HGNC Symbol;Acc:HGNC:16153] |
| ENSG00000101445 | HGLibA_38057 | 108 | -0.157796456 | PPP1R16B | protein phosphatase 1 regulatory subunit 16B [Source:HGNC Symbol;Acc:HGNC:15850] |
| ENSG00000102081 | HGLibA_17721 | 108 | -0.157796456 | FMR1 | fragile X mental retardation 1 [Source:HGNC Symbol;Acc:HGNC:3775] |
| ENSG00000102547 | HGLibA_06837 | 108 | -0.157796456 | CAB39L | calcium binding protein 39 like [Source:HGNC Symbol;Acc:HGNC:20290] |
| ENSG00000102908 | HGLibA_31769 | 108 | -0.157796456 | NFAT5 | nuclear factor of activated T cells 5 [Source:HGNC Symbol;Acc:HGNC:7774] |
| ENSG00000103671 | HGLibA_51645 | 108 | -0.157796456 | TRIP4 | thyroid hormone receptor interactor 4 [Source:HGNC Symbol;Acc:HGNC:12310] |
| ENSG00000104131 | HGLibA_14791 | 108 | -0.157796456 | EIF3J | eukaryotic translation initiation factor 3 subunit J [Source:HGNC Symbol;Acc:HGNC:3270] |
| ENSG00000104381 | HGLibA_18839 | 108 | -0.157796456 | GDAP1 | ganglioside induced differentiation associated protein 1 [Source:HGNC Symbol;Acc:HGNC:15968] |
| ENSG00000104899 | HGLibA_01783 | 108 | -0.157796456 | AMH | anti-Mullerian hormone [Source:HGNC Symbol;Acc:HGNC:464] |
| ENSG00000105649 | HGLibA_39908 | 108 | -0.157796456 | RAB3A | member RAS oncogene family [Source:HGNC Symbol;Acc:HGNC:9777]" |
| ENSG00000105982 | HGLibA_41663 | 108 | -0.157796456 | RNF32 | ring finger protein 32 [Source:HGNC Symbol;Acc:HGNC:17118] |
| ENSG00000107295 | HGLibA_43950 | 108 | -0.157796456 | SH3GL2 | endophilin A1 [Source:HGNC Symbol;Acc:HGNC:10831]" |
| ENSG00000109787 | HGLibA_25070 | 108 | -0.157796456 | KLF3 | Kruppel like factor 3 [Source:HGNC Symbol;Acc:HGNC:16516] |
| ENSG00000111142 | HGLibA_28933 | 108 | -0.157796456 | METAP2 | methionyl aminopeptidase 2 [Source:HGNC Symbol;Acc:HGNC:16672] |
| ENSG00000113140 | HGLibA_46416 | 108 | -0.157796456 | SPARC | secreted protein acidic and cysteine rich [Source:HGNC Symbol;Acc:HGNC:11219] |

| | | | | | |
|---|---|---|---|---|---|
| ENSG00000115514 | HGLibA_52430 | 108 | -0.157796456 | TXNDC9 | thioredoxin domain containing 9 [Source:HGNC Symbol;Acc:HGNC:24110] |
| ENSG00000115593 | HGLibA_45851 | 108 | -0.157796456 | SMYD1 | SET and MYND domain containing 1 [Source:HGNC Symbol;Acc:HGNC:20986] |
| ENSG00000116729 | HGLibA_54332 | 108 | -0.157796456 | WLS | wntless Wnt ligand secretion mediator [Source:HGNC Symbol;Acc:HGNC:30238] |
| ENSG00000117305 | HGLibA_21703 | 108 | -0.157796456 | HMGCL | 3-hydroxy-3-methylglutaryl-CoA lyase [Source:HGNC Symbol;Acc:HGNC:5005] |
| ENSG00000119414 | HGLibA_38217 | 108 | -0.157796456 | PPP6C | protein phosphatase 6 catalytic subunit [Source:HGNC Symbol;Acc:HGNC:9323] |
| ENSG00000119699 | HGLibA_49218 | 108 | -0.157796456 | TGFB3 | transforming growth factor beta 3 [Source:HGNC Symbol;Acc:HGNC:11769] |
| ENSG00000119703 | HGLibA_54943 | 108 | -0.157796456 | ZC2HC1C | zinc finger C2HC-type containing 1C [Source:HGNC Symbol;Acc:HGNC:20354] |
| ENSG00000121797 | HGLibA_08177 | 108 | -0.157796456 | CCRL2 | C-C motif chemokine receptor like 2 [Source:HGNC Symbol;Acc:HGNC:1612] |
| ENSG00000125430 | HGLibA_22129 | 108 | -0.157796456 | HS3ST3B1 | heparan sulfate-glucosamine 3-sulfotransferase 3B1 [Source:HGNC Symbol;Acc:HGNC:5198] |
| ENSG00000126733 | HGLibA_12278 | 108 | -0.157796456 | DACH2 | dachshund family transcription factor 2 [Source:HGNC Symbol;Acc:HGNC:16814] |
| ENSG00000127903 | HGLibA_56838 | 108 | -0.157796456 | ZNF835 | zinc finger protein 835 [Source:HGNC Symbol;Acc:HGNC:34332] |
| ENSG00000127928 | HGLibA_19537 | 108 | -0.157796456 | GNGT1 | G protein subunit gamma transducin 1 [Source:HGNC Symbol;Acc:HGNC:4411] |
| ENSG00000130035 | HGLibA_18571 | 108 | -0.157796456 | GALNT8 | polypeptide N-acetylgalactosaminyltransferase 8 [Source:HGNC Symbol;Acc:HGNC:4130] |
| ENSG00000131148 | HGLibA_15024 | 108 | -0.157796456 | EMC8 | ER membrane protein complex subunit 8 [Source:HGNC Symbol;Acc:HGNC:7864] |
| ENSG00000131153 | HGLibA_19105 | 108 | -0.157796456 | GINS2 | GINS complex subunit 2 [Source:HGNC Symbol;Acc:HGNC:24575] |
| ENSG00000132530 | HGLibA_54463 | 108 | -0.157796456 | XAF1 | XIAP associated factor 1 [Source:HGNC Symbol;Acc:HGNC:30932] |
| ENSG00000134470 | HGLibA_22984 | 108 | -0.157796456 | IL15RA | interleukin 15 receptor subunit alpha [Source:HGNC Symbol;Acc:HGNC:5978] |
| ENSG00000134802 | HGLibA_45154 | 108 | -0.157796456 | SLC43A3 | solute carrier family 43 member 3 [Source:HGNC Symbol;Acc:HGNC:17466] |
| ENSG00000134815 | HGLibA_13183 | 108 | -0.157796456 | DHX34 | DExH-box helicase 34 [Source:HGNC Symbol;Acc:HGNC:16719] |
| ENSG00000136026 | HGLibA_09759 | 108 | -0.157796456 | CKAP4 | cytoskeleton associated protein 4 [Source:HGNC Symbol;Acc:HGNC:16991] |
| ENSG00000136943 | HGLibA_11732 | 108 | -0.157796456 | CTSV | cathepsin V [Source:HGNC Symbol;Acc:HGNC:2538] |
| ENSG00000138134 | HGLibA_47338 | 108 | -0.157796456 | STAMBPL1 | STAM binding protein like 1 [Source:HGNC Symbol;Acc:HGNC:24105] |
| ENSG00000140905 | HGLibA_18834 | 108 | -0.157796456 | GCSH | glycine cleavage system protein H [Source:HGNC Symbol;Acc:HGNC:4208] |
| ENSG00000141437 | HGLibA_44767 | 108 | -0.157796456 | SLC25A52 | solute carrier family 25 member 52 [Source:HGNC Symbol;Acc:HGNC:23324] |
| ENSG00000141485 | HGLibA_44397 | 108 | -0.157796456 | SLC13A5 | solute carrier family 13 member 5 [Source:HGNC Symbol;Acc:HGNC:23089] |
| ENSG00000142408 | HGLibA_06950 | 108 | -0.157796456 | CACNG8 | calcium voltage-gated channel auxiliary subunit gamma 8 [Source:HGNC Symbol;Acc:HGNC:13628] |
| ENSG00000143387 | HGLibA_11712 | 108 | -0.157796456 | CTSK | cathepsin K [Source:HGNC Symbol;Acc:HGNC:2536] |
| ENSG00000143801 | HGLibA_39027 | 108 | -0.157796456 | PSEN2 | presenilin 2 [Source:HGNC Symbol;Acc:HGNC:9509] |
| ENSG00000145536 | HGLibA_00810 | 108 | -0.157796456 | ADAMTS16 | ADAM metallopeptidase with thrombospondin type 1 motif 16 [Source:HGNC Symbol;Acc:HGNC:17108] |
| ENSG00000145832 | HGLibA_44758 | 108 | -0.157796456 | SLC25A48 | solute carrier family 25 member 48 [Source:HGNC Symbol;Acc:HGNC:30451] |
| ENSG00000146013 | HGLibA_18972 | 108 | -0.157796456 | GFRA3 | GDNF family receptor alpha 3 [Source:HGNC Symbol;Acc:HGNC:4245] |
| ENSG00000147121 | HGLibA_25396 | 108 | -0.157796456 | KRBOX4 | KRAB box domain containing 4 [Source:HGNC Symbol;Acc:HGNC:26007] |
| ENSG00000148935 | HGLibA_18644 | 108 | -0.157796456 | GAS2 | growth arrest specific 2 [Source:HGNC Symbol;Acc:HGNC:4167] |
| ENSG00000150712 | HGLibA_30418 | 108 | -0.157796456 | MTMR12 | myotubularin related protein 12 [Source:HGNC Symbol;Acc:HGNC:18191] |
| ENSG00000151693 | HGLibA_03197 | 108 | -0.157796456 | ASAP2 | ankyrin repeat and PH domain 2 [Source:HGNC Symbol;Acc:HGNC:2721]" |
| ENSG00000152556 | HGLibA_36169 | 108 | -0.157796456 | PFKM | muscle [Source:HGNC Symbol;Acc:HGNC:8877]" |
| ENSG00000152785 | HGLibA_04605 | 108 | -0.157796456 | BMP3 | bone morphogenetic protein 3 [Source:HGNC Symbol;Acc:HGNC:1070] |
| ENSG00000153558 | HGLibA_16997 | 108 | -0.157796456 | FBXL2 | F-box and leucine rich repeat protein 2 [Source:HGNC Symbol;Acc:HGNC:13598] |
| ENSG00000153560 | HGLibA_52729 | 108 | -0.157796456 | UBP1 | upstream binding protein 1 [Source:HGNC Symbol;Acc:HGNC:12507] |
| ENSG00000154438 | HGLibA_03398 | 108 | -0.157796456 | ASZ1 | SAM and basic leucine zipper domain containing 1 [Source:HGNC Symbol;Acc:HGNC:1350]" |
| ENSG00000154485 | HGLibA_29513 | 108 | -0.157796456 | MMP21 | matrix metallopeptidase 21 [Source:HGNC Symbol;Acc:HGNC:14357] |
| ENSG00000154646 | HGLibA_50633 | 108 | -0.157796456 | TMPRSS15 | transmembrane serine protease 15 [Source:HGNC Symbol;Acc:HGNC:9490] |
| ENSG00000156509 | HGLibA_17101 | 108 | -0.157796456 | FBXO43 | F-box protein 43 [Source:HGNC Symbol;Acc:HGNC:28521] |
| ENSG00000156973 | HGLibA_35815 | 108 | -0.157796456 | PDE6D | phosphodiesterase 6D [Source:HGNC Symbol;Acc:HGNC:8788] |
| ENSG00000156976 | HGLibA_14808 | 108 | -0.157796456 | EIF4A2 | eukaryotic translation initiation factor 4A2 [Source:HGNC Symbol;Acc:HGNC:3284] |
| ENSG00000158008 | HGLibA_15807 | 108 | -0.157796456 | EXTL1 | exostosin like glycosyltransferase 1 [Source:HGNC Symbol;Acc:HGNC:3515] |
| ENSG00000160201 | HGLibA_52492 | 108 | -0.157796456 | U2AF1 | U2 small nuclear RNA auxiliary factor 1 [Source:HGNC Symbol;Acc:HGNC:12453] |
| ENSG00000160323 | HGLibA_00801 | 108 | -0.157796456 | ADAMTS13 | ADAM metallopeptidase with thrombospondin type 1 motif 13 [Source:HGNC Symbol;Acc:HGNC:1366] |
| ENSG00000162231 | HGLibA_33125 | 108 | -0.157796456 | NXF1 | nuclear RNA export factor 1 [Source:HGNC Symbol;Acc:HGNC:8071] |
| ENSG00000162231 | HGLibA_33126 | 108 | -0.157796456 | NXF1 | nuclear RNA export factor 1 [Source:HGNC Symbol;Acc:HGNC:8071] |
| ENSG00000162545 | HGLibA_07078 | 108 | -0.157796456 | CAMK2N1 | calcium/calmodulin dependent protein kinase II inhibitor 1 [Source:HGNC Symbol;Acc:HGNC:24190] |
| ENSG00000162601 | HGLibA_30922 | 108 | -0.157796456 | MYSM1 | SWIRM and MPN domains 1 [Source:HGNC Symbol;Acc:HGNC:29401]" |
| ENSG00000162998 | HGLibA_18059 | 108 | -0.157796456 | FRZB | frizzled related protein [Source:HGNC Symbol;Acc:HGNC:3959] |
| ENSG00000163064 | HGLibA_15098 | 108 | -0.157796456 | EN1 | engrailed homeobox 1 [Source:HGNC Symbol;Acc:HGNC:3342] |
| ENSG00000163138 | HGLibA_34950 | 108 | -0.157796456 | PACRGL | parkin coregulated like [Source:HGNC Symbol;Acc:HGNC:28442] |
| ENSG00000163599 | HGLibA_11634 | 108 | -0.157796456 | CTLA4 | cytotoxic T-lymphocyte associated protein 4 [Source:HGNC Symbol;Acc:HGNC:2505] |
| ENSG00000163611 | HGLibA_46662 | 108 | -0.157796456 | SPICE1 | spindle and centriole associated protein 1 [Source:HGNC Symbol;Acc:HGNC:25083] |
| ENSG00000163751 | HGLibA_10889 | 108 | -0.157796456 | CPA3 | carboxypeptidase A3 [Source:HGNC Symbol;Acc:HGNC:2298] |
| ENSG00000163898 | HGLibA_26614 | 108 | -0.157796456 | LIPH | lipase H [Source:HGNC Symbol;Acc:HGNC:18483] |
| ENSG00000164175 | HGLibA_45175 | 108 | -0.157796456 | SLC45A2 | solute carrier family 45 member 2 [Source:HGNC Symbol;Acc:HGNC:16472] |
| ENSG00000164305 | HGLibA_07310 | 108 | -0.157796456 | CASP3 | caspase 3 [Source:HGNC Symbol;Acc:HGNC:1504] |
| ENSG00000165195 | HGLibA_36544 | 108 | -0.157796456 | PIGA | phosphatidylinositol glycan anchor biosynthesis class A [Source:HGNC Symbol;Acc:HGNC:8957] |
| ENSG00000165923 | HGLibA_01169 | 108 | -0.157796456 | AGBL2 | ATP/GTP binding protein like 2 [Source:HGNC Symbol;Acc:HGNC:26296] |
| ENSG00000165997 | HGLibA_02978 | 108 | -0.157796456 | ARL5B | ADP ribosylation factor like GTPase 5B [Source:HGNC Symbol;Acc:HGNC:23052] |
| ENSG00000166439 | HGLibA_41567 | 108 | -0.157796456 | RNF169 | ring finger protein 169 [Source:HGNC Symbol;Acc:HGNC:26961] |
| ENSG00000167769 | HGLibA_00428 | 108 | -0.157796456 | ACER1 | alkaline ceramidase 1 [Source:HGNC Symbol;Acc:HGNC:18356] |
| ENSG00000168356 | HGLibA_42970 | 108 | -0.157796456 | SCN11A | sodium voltage-gated channel alpha subunit 11 [Source:HGNC Symbol;Acc:HGNC:10583] |
| ENSG00000169306 | HGLibA_23055 | 108 | -0.157796456 | IL1RAPL1 | interleukin 1 receptor accessory protein like 1 [Source:HGNC Symbol;Acc:HGNC:5996] |
| ENSG00000169372 | HGLibA_11060 | 108 | -0.157796456 | CRADD | CASP2 and RIPK1 domain containing adaptor with death domain [Source:HGNC Symbol;Acc:HGNC:2340] |
| ENSG00000169605 | HGLibA_19209 | 108 | -0.157796456 | GKN1 | gastrokine 1 [Source:HGNC Symbol;Acc:HGNC:23217] |
| ENSG00000169714 | HGLibA_10249 | 108 | -0.157796456 | CNBP | CCHC-type zinc finger nucleic acid binding protein [Source:HGNC Symbol;Acc:HGNC:13164] |
| ENSG00000169744 | HGLibA_26215 | 108 | -0.157796456 | LDB2 | LIM domain binding 2 [Source:HGNC Symbol;Acc:HGNC:6533] |
| ENSG00000170100 | HGLibA_56727 | 108 | -0.157796456 | ZNF778 | zinc finger protein 778 [Source:HGNC Symbol;Acc:HGNC:26479] |
| ENSG00000170949 | HGLibA_55513 | 108 | -0.157796456 | ZNF160 | zinc finger protein 160 [Source:HGNC Symbol;Acc:HGNC:12948] |
| ENSG00000170954 | HGLibA_55943 | 108 | -0.157796456 | ZNF415 | zinc finger protein 415 [Source:HGNC Symbol;Acc:HGNC:20636] |
| ENSG00000170962 | HGLibA_35849 | 108 | -0.157796456 | PDGFD | platelet derived growth factor D [Source:HGNC Symbol;Acc:HGNC:30620] |
| ENSG00000171163 | HGLibA_56555 | 108 | -0.157796456 | ZNF692 | zinc finger protein 692 [Source:HGNC Symbol;Acc:HGNC:26049] |
| ENSG00000172037 | HGLibA_25959 | 108 | -0.157796456 | LAMB2 | laminin subunit beta 2 [Source:HGNC Symbol;Acc:HGNC:6487] |
| ENSG00000172794 | HGLibA_39895 | 108 | -0.157796456 | RAB37 | member RAS oncogene family [Source:HGNC Symbol;Acc:HGNC:30268]" |
| ENSG00000174243 | HGLibA_12676 | 108 | -0.157796456 | DDX23 | DEAD-box helicase 23 [Source:HGNC Symbol;Acc:HGNC:17347] |
| ENSG00000174343 | HGLibA_09587 | 108 | -0.157796456 | CHRNA9 | cholinergic receptor nicotinic alpha 9 subunit [Source:HGNC Symbol;Acc:HGNC:14079] |
| ENSG00000174498 | HGLibA_22785 | 108 | -0.157796456 | IGDCC3 | immunoglobulin superfamily DCC subclass member 3 [Source:HGNC Symbol;Acc:HGNC:9700] |
| ENSG00000175097 | HGLibA_40136 | 108 | -0.157796456 | RAG2 | recombination activating 2 [Source:HGNC Symbol;Acc:HGNC:9832] |
| ENSG00000176040 | HGLibA_50651 | 108 | -0.157796456 | TMPRSS7 | transmembrane serine protease 7 [Source:HGNC Symbol;Acc:HGNC:30846] |
| ENSG00000176049 | HGLibA_23925 | 108 | -0.157796456 | JAKMIP2 | janus kinase and microtubule interacting protein 2 [Source:HGNC Symbol;Acc:HGNC:29067] |
| ENSG00000177932 | HGLibA_55868 | 108 | -0.157796456 | ZNF354C | zinc finger protein 354C [Source:HGNC Symbol;Acc:HGNC:16736] |
| ENSG00000178015 | HGLibA_19929 | 108 | -0.157796456 | GPR150 | G protein-coupled receptor 150 [Source:HGNC Symbol;Acc:HGNC:23628] |
| ENSG00000178358 | HGLibA_33785 | 108 | -0.157796456 | OR2D3 | olfactory receptor family 2 subfamily D member 3 [Source:HGNC Symbol;Acc:HGNC:15146] |
| ENSG00000178776 | HGLibA_06390 | 108 | -0.157796456 | C5orf46 | chromosome 5 open reading frame 46 [Source:HGNC Symbol;Acc:HGNC:33768] |
| ENSG00000179564 | HGLibA_27559 | 108 | -0.157796456 | LSMEM2 | leucine rich single-pass membrane protein 2 [Source:HGNC Symbol;Acc:HGNC:26781] |
| ENSG00000179709 | HGLibA_32133 | 108 | -0.157796456 | NLRP8 | NLR family pyrin domain containing 8 [Source:HGNC Symbol;Acc:HGNC:22940] |
| ENSG00000179751 | HGLibA_47924 | 108 | -0.157796456 | SYCN | syncollin [Source:HGNC Symbol;Acc:HGNC:18442] |
| ENSG00000181847 | HGLibA_49485 | 108 | -0.157796456 | TIGIT | T cell immunoreceptor with Ig and ITIM domains [Source:HGNC Symbol;Acc:HGNC:26838] |
| ENSG00000182070 | HGLibA_34139 | 108 | -0.157796456 | OR52A1 | olfactory receptor family 52 subfamily A member 1 [Source:HGNC Symbol;Acc:HGNC:8318] |
| ENSG00000182511 | HGLibA_17302 | 108 | -0.157796456 | FES | tyrosine kinase [Source:HGNC Symbol;Acc:HGNC:3657]" |
| ENSG00000182902 | HGLibA_44660 | 108 | -0.157796456 | SLC25A18 | solute carrier family 25 member 18 [Source:HGNC Symbol;Acc:HGNC:10988] |
| ENSG00000183323 | HGLibA_07577 | 108 | -0.157796456 | CCDC125 | coiled-coil domain containing 125 [Source:HGNC Symbol;Acc:HGNC:28924] |
| ENSG00000183850 | HGLibA_56644 | 108 | -0.157796456 | ZNF730 | zinc finger protein 730 [Source:HGNC Symbol;Acc:HGNC:32470] |
| ENSG00000184292 | HGLibA_48181 | 108 | -0.157796456 | TACSTD2 | tumor associated calcium signal transducer 2 [Source:HGNC Symbol;Acc:HGNC:11530] |
| ENSG00000185272 | HGLibA_40476 | 108 | -0.157796456 | RBM11 | RNA binding motif protein 11 [Source:HGNC Symbol;Acc:HGNC:9897] |
| ENSG00000186105 | HGLibA_27417 | 108 | -0.157796456 | LRRC70 | leucine rich repeat containing 70 [Source:HGNC Symbol;Acc:HGNC:35155] |
| ENSG00000186471 | HGLibA_01419 | 108 | -0.157796456 | AKAP14 | A-kinase anchoring protein 14 [Source:HGNC Symbol;Acc:HGNC:24061] |
| ENSG00000187720 | HGLibA_49402 | 108 | -0.157796456 | THSD4 | thrombospondin type 1 domain containing 4 [Source:HGNC Symbol;Acc:HGNC:25835] |
| ENSG00000187806 | HGLibA_50183 | 108 | -0.157796456 | TMEM202 | transmembrane protein 202 [Source:HGNC Symbol;Acc:HGNC:33733] |
| ENSG00000188163 | HGLibA_16233 | 108 | -0.157796456 | FAM166A | family with sequence similarity 166 member A [Source:HGNC Symbol;Acc:HGNC:33818] |
| ENSG00000188293 | HGLibA_22840 | 108 | -0.157796456 | IGFL1 | IGF like family member 1 [Source:HGNC Symbol;Acc:HGNC:24093] |
| ENSG00000188818 | HGLibA_55069 | 108 | -0.157796456 | ZDHHC11 | zinc finger DHHC-type containing 11 [Source:HGNC Symbol;Acc:HGNC:19158] |
| ENSG00000189221 | HGLibA_28106 | 108 | -0.157796456 | MAOA | monoamine oxidase A [Source:HGNC Symbol;Acc:HGNC:6833] |
| ENSG00000197045 | HGLibA_19402 | 108 | -0.157796456 | GMFB | glia maturation factor beta [Source:HGNC Symbol;Acc:HGNC:4373] |
| ENSG00000198967 | HGLibA_33559 | 108 | -0.157796456 | OR10Z1 | olfactory receptor family 10 subfamily Z member 1 [Source:HGNC Symbol;Acc:HGNC:14996] |
| ENSG00000203727 | HGLibA_42690 | 108 | -0.157796456 | SAMD5 | sterile alpha motif domain containing 5 [Source:HGNC Symbol;Acc:HGNC:21180] |
| ENSG00000203837 | HGLibA_37363 | 108 | -0.157796456 | PNLIPRP3 | pancreatic lipase related protein 3 [Source:HGNC Symbol;Acc:HGNC:23492] |
| ENSG00000204020 | HGLibA_26630 | 108 | -0.157796456 | LIPN | lipase family member N [Source:HGNC Symbol;Acc:HGNC:23452] |
| ENSG00000204227 | HGLibA_41298 | 108 | -0.157796456 | RING1 | ring finger protein 1 [Source:HGNC Symbol;Acc:HGNC:10018] |
| ENSG00000214107 | HGLibA_27930 | 108 | -0.157796456 | MAGEB1 | MAGE family member B1 [Source:HGNC Symbol;Acc:HGNC:6808] |
| ENSG00000214944 | HGLibA_02843 | 108 | -0.157796456 | ARHGEF28 | Rho guanine nucleotide exchange factor 28 [Source:HGNC Symbol;Acc:HGNC:30322] |
| ENSG00000230453 | HGLibA_02021 | 108 | -0.157796456 | ANKRD18B | ankyrin repeat domain 18B [Source:HGNC Symbol;Acc:HGNC:23644] |
| ENSG00000237440 | HGLibA_56656 | 108 | -0.157796456 | ZNF737 | zinc finger protein 737 [Source:HGNC Symbol;Acc:HGNC:32468] |
| ENSG00000243709 | HGLibA_26275 | 108 | -0.157796456 | LEFTY1 | left-right determination factor 1 [Source:HGNC Symbol;Acc:HGNC:6552] |
| ENSG00000243927 | HGLibA_30108 | 108 | -0.157796456 | MRPS6 | mitochondrial ribosomal protein S6 [Source:HGNC Symbol;Acc:HGNC:14051] |
| ENSG00000255690 | HGLibA_51399 | 108 | -0.157796456 | TRIL | TLR4 interactor with leucine rich repeats [Source:HGNC Symbol;Acc:HGNC:22200] |
| ENSG00000255823 | HGLibA_30491 | 108 | -0.157796456 | MTRNR2L8 | MT-RNR2 like 8 [Source:HGNC Symbol;Acc:HGNC:37165] |
| ENSG00000256762 | HGLibA_47434 | 108 | -0.157796456 | STH | saitohin [Source:HGNC Symbol;Acc:HGNC:18839] |
| ENSG00000258102 | HGLibA_28126 | 108 | -0.157796456 | MAP1LC3B2 | microtubule associated protein 1 light chain 3 beta 2 [Source:HGNC Symbol;Acc:HGNC:34390] |
| ENSG00000258315 | HGLibA_05538 | 108 | -0.157796456 | C17orf49 | chromosome 17 open reading frame 49 [Source:HGNC Symbol;Acc:HGNC:28737] |
| FAM101A | HGLibA_15974 | 108 | -0.157796456 | FAM101A | - |
| FAM35A | HGLibA_16572 | 108 | -0.157796456 | FAM35A | - |
| FUK | HGLibA_18162 | 108 | -0.157796456 | FUK | - |
| GAGE2C | HGLibA_18472 | 108 | -0.157796456 | GAGE2C | - |
| H2BFWT | HGLibA_20776 | 108 | -0.157796456 | H2BFWT | - |
| HIST2H4A | HGLibA_21548 | 108 | -0.157796456 | HIST2H4A | - |
| hsa-mir-1184-3 | HGLibA_57238 | 108 | -0.157796456 | hsa-mir-1184-3 | - |
| hsa-mir-1292 | HGLibA_57643 | 108 | -0.157796456 | hsa-mir-1292 | - |
| hsa-mir-1343 | HGLibA_57788 | 108 | -0.157796456 | hsa-mir-1343 | - |
| hsa-mir-2276 | HGLibA_58309 | 108 | -0.157796456 | hsa-mir-2276 | - |

| | | | | | |
|---|---|---|---|---|---|
| hsa-mir-2276 | HGLibA_58310 | 108 | -0.157796456 | hsa-mir-2276 | - |
| hsa-mir-4490 | HGLibA_60380 | 108 | -0.157796456 | hsa-mir-4490 | - |
| hsa-mir-4756 | HGLibA_61123 | 108 | -0.157796456 | hsa-mir-4756 | - |
| hsa-mir-4763 | HGLibA_61148 | 108 | -0.157796456 | hsa-mir-4763 | - |
| hsa-mir-4787 | HGLibA_61254 | 108 | -0.157796456 | hsa-mir-4787 | - |
| hsa-mir-513a-2 | HGLibA_61575 | 108 | -0.157796456 | hsa-mir-513a-2 | - |
| hsa-mir-518a-1 | HGLibA_61652 | 108 | -0.157796456 | hsa-mir-518a-1 | - |
| hsa-mir-548y | HGLibA_62085 | 108 | -0.157796456 | hsa-mir-548y | - |
| hsa-mir-577 | HGLibA_62404 | 108 | -0.157796456 | hsa-mir-577 | - |
| hsa-mir-605 | HGLibA_62513 | 108 | -0.157796456 | hsa-mir-605 | - |
| hsa-mir-6078 | HGLibA_62570 | 108 | -0.157796456 | hsa-mir-6078 | - |
| hsa-mir-6502 | HGLibA_62858 | 108 | -0.157796456 | hsa-mir-6502 | - |
| hsa-mir-6774 | HGLibA_63268 | 108 | -0.157796456 | hsa-mir-6774 | - |
| hsa-mir-6854 | HGLibA_63589 | 108 | -0.157796456 | hsa-mir-6854 | - |
| hsa-mir-6872 | HGLibA_63672 | 108 | -0.157796456 | hsa-mir-6872 | - |
| hsa-mir-8066 | HGLibA_64104 | 108 | -0.157796456 | hsa-mir-8066 | - |
| hsa-mir-922 | HGLibA_64283 | 108 | -0.157796456 | hsa-mir-922 | - |
| LOC100133267 | HGLibA_26810 | 108 | -0.157796456 | LOC100133267 | - |
| LOC440335 | HGLibA_26945 | 108 | -0.157796456 | LOC440335 | - |
| PQLC2 | HGLibA_38255 | 108 | -0.157796456 | PQLC2 | - |
| PRAC | HGLibA_38260 | 108 | -0.157796456 | PRAC | - |
| PTPN20A | HGLibA_39452 | 108 | -0.157796456 | PTPN20A | - |
| SELT | HGLibA_43314 | 108 | -0.157796456 | SELT | - |
| 10-Sep | HGLibA_43426 | 108 | -0.157796456 | 10-Sep | - |
| TROVE2 | HGLibA_51721 | 108 | -0.157796456 | TROVE2 | - |
| C15orf38 | HGLibA_05387 | 107 | -0.166841799 | C15orf38 | - |
| ENSG00000008128 | HGLibA_08725 | 107 | -0.166841799 | CDK11A | cyclin dependent kinase 11A [Source:HGNC Symbol;Acc:HGNC:1730] |
| ENSG00000011451 | HGLibA_54329 | 107 | -0.166841799 | WIZ | widely interspaced zinc finger motifs [Source:HGNC Symbol;Acc:HGNC:30917] |
| ENSG00000012983 | HGLibA_28222 | 107 | -0.166841799 | MAP4K5 | mitogen-activated protein kinase kinase kinase kinase 5 [Source:HGNC Symbol;Acc:HGNC:6867] |
| ENSG00000015592 | HGLibA_47524 | 107 | -0.166841799 | STMN4 | stathmin 4 [Source:HGNC Symbol;Acc:HGNC:16078] |
| ENSG00000016402 | HGLibA_23075 | 107 | -0.166841799 | IL20RA | interleukin 20 receptor subunit alpha [Source:HGNC Symbol;Acc:HGNC:6003] |
| ENSG00000018236 | HGLibA_10400 | 107 | -0.166841799 | CNTN1 | contactin 1 [Source:HGNC Symbol;Acc:HGNC:2171] |
| ENSG00000022567 | HGLibA_45182 | 107 | -0.166841799 | SLC45A4 | solute carrier family 45 member 4 [Source:HGNC Symbol;Acc:HGNC:29196] |
| ENSG00000027847 | HGLibA_04041 | 107 | -0.166841799 | B4GALT7 | 4-galactosyltransferase 7 [Source:HGNC Symbol;Acc:HGNC:930]" |
| ENSG00000047648 | HGLibA_02787 | 107 | -0.166841799 | ARHGAP6 | Rho GTPase activating protein 6 [Source:HGNC Symbol;Acc:HGNC:676] |
| ENSG00000050130 | HGLibA_23950 | 107 | -0.166841799 | JKAMP | JNK1/MAPK8 associated membrane protein [Source:HGNC Symbol;Acc:HGNC:20184] |
| ENSG00000055950 | HGLibA_29986 | 107 | -0.166841799 | MRPL43 | mitochondrial ribosomal protein L43 [Source:HGNC Symbol;Acc:HGNC:14517] |
| ENSG00000057252 | HGLibA_46114 | 107 | -0.166841799 | SOAT1 | sterol O-acyltransferase 1 [Source:HGNC Symbol;Acc:HGNC:11177] |
| ENSG00000063660 | HGLibA_19761 | 107 | -0.166841799 | GPC1 | glypican 1 [Source:HGNC Symbol;Acc:HGNC:4449] |
| ENSG00000073754 | HGLibA_08419 | 107 | -0.166841799 | CD5L | CD5 molecule like [Source:HGNC Symbol;Acc:HGNC:1690] |
| ENSG00000075131 | HGLibA_49559 | 107 | -0.166841799 | TIPIN | TIMELESS interacting protein [Source:HGNC Symbol;Acc:HGNC:30750] |
| ENSG00000079785 | HGLibA_12649 | 107 | -0.166841799 | DDX1 | DEAD-box helicase 1 [Source:HGNC Symbol;Acc:HGNC:2734] |
| ENSG00000081237 | HGLibA_39489 | 107 | -0.166841799 | PTPRC | receptor type C [Source:HGNC Symbol;Acc:HGNC:9666]" |
| ENSG00000083457 | HGLibA_23753 | 107 | -0.166841799 | ITGAE | integrin subunit alpha E [Source:HGNC Symbol;Acc:HGNC:6147] |
| ENSG00000100325 | HGLibA_03259 | 107 | -0.166841799 | ASCC2 | activating signal cointegrator 1 complex subunit 2 [Source:HGNC Symbol;Acc:HGNC:24103] |
| ENSG00000100526 | HGLibA_08847 | 107 | -0.166841799 | CDKN3 | cyclin dependent kinase inhibitor 3 [Source:HGNC Symbol;Acc:HGNC:1791] |
| ENSG00000100564 | HGLibA_36558 | 107 | -0.166841799 | PIGH | phosphatidylinositol glycan anchor biosynthesis class H [Source:HGNC Symbol;Acc:HGNC:8964] |
| ENSG00000101266 | HGLibA_11403 | 107 | -0.166841799 | CSNK2A1 | casein kinase 2 alpha 1 [Source:HGNC Symbol;Acc:HGNC:2457] |
| ENSG00000101438 | HGLibA_44933 | 107 | -0.166841799 | SLC32A1 | solute carrier family 32 member 1 [Source:HGNC Symbol;Acc:HGNC:11018] |
| ENSG00000101457 | HGLibA_13719 | 107 | -0.166841799 | DNTTIP1 | deoxynucleotidyltransferase terminal interacting protein 1 [Source:HGNC Symbol;Acc:HGNC:16160] |
| ENSG00000102245 | HGLibA_08386 | 107 | -0.166841799 | CD40LG | CD40 ligand [Source:HGNC Symbol;Acc:HGNC:11935] |
| ENSG00000102595 | HGLibA_52871 | 107 | -0.166841799 | UGGT2 | UDP-glucose glycoprotein glucosyltransferase 2 [Source:HGNC Symbol;Acc:HGNC:15664] |
| ENSG00000103018 | HGLibA_11991 | 107 | -0.166841799 | CYB5B | cytochrome b5 type B [Source:HGNC Symbol;Acc:HGNC:24374] |
| ENSG00000105339 | HGLibA_12946 | 107 | -0.166841799 | DENND3 | DENN domain containing 3 [Source:HGNC Symbol;Acc:HGNC:29134] |
| ENSG00000107566 | HGLibA_15493 | 107 | -0.166841799 | ERLIN1 | ER lipid raft associated 1 [Source:HGNC Symbol;Acc:HGNC:16947] |
| ENSG00000107816 | HGLibA_27810 | 107 | -0.166841799 | LZTS2 | leucine zipper tumor suppressor 2 [Source:HGNC Symbol;Acc:HGNC:29381] |
| ENSG00000108947 | HGLibA_14602 | 107 | -0.166841799 | EFNB3 | ephrin B3 [Source:HGNC Symbol;Acc:HGNC:3228] |
| ENSG00000108947 | HGLibA_14603 | 107 | -0.166841799 | EFNB3 | ephrin B3 [Source:HGNC Symbol;Acc:HGNC:3228] |
| ENSG00000110148 | HGLibA_07958 | 107 | -0.166841799 | CCKBR | cholecystokinin B receptor [Source:HGNC Symbol;Acc:HGNC:1571] |
| ENSG00000111046 | HGLibA_30700 | 107 | -0.166841799 | MYF6 | myogenic factor 6 [Source:HGNC Symbol;Acc:HGNC:7566] |
| ENSG00000112584 | HGLibA_16061 | 107 | -0.166841799 | FAM120B | family with sequence similarity 120B [Source:HGNC Symbol;Acc:HGNC:21109] |
| ENSG00000114473 | HGLibA_23518 | 107 | -0.166841799 | IQCG | IQ motif containing G [Source:HGNC Symbol;Acc:HGNC:25251] |
| ENSG00000114742 | HGLibA_54109 | 107 | -0.166841799 | WDR48 | WD repeat domain 48 [Source:HGNC Symbol;Acc:HGNC:30914] |
| ENSG00000115540 | HGLibA_29603 | 107 | -0.166841799 | MOB4 | phocein [Source:HGNC Symbol;Acc:HGNC:17261]" |
| ENSG00000116254 | HGLibA_09397 | 107 | -0.166841799 | CHD5 | chromodomain helicase DNA binding protein 5 [Source:HGNC Symbol;Acc:HGNC:16816] |
| ENSG00000119682 | HGLibA_02642 | 107 | -0.166841799 | AREL1 | apoptosis resistant E3 ubiquitin protein ligase 1 [Source:HGNC Symbol;Acc:HGNC:20363] |
| ENSG00000119912 | HGLibA_22537 | 107 | -0.166841799 | IDE | insulin degrading enzyme [Source:HGNC Symbol;Acc:HGNC:5381] |
| ENSG00000120913 | HGLibA_35910 | 107 | -0.166841799 | PDLIM2 | PDZ and LIM domain 2 [Source:HGNC Symbol;Acc:HGNC:13992] |
| ENSG00000121316 | HGLibA_36972 | 107 | -0.166841799 | PLBD1 | phospholipase B domain containing 1 [Source:HGNC Symbol;Acc:HGNC:26215] |
| ENSG00000122786 | HGLibA_07009 | 107 | -0.166841799 | CALD1 | caldesmon 1 [Source:HGNC Symbol;Acc:HGNC:1441] |
| ENSG00000123496 | HGLibA_22978 | 107 | -0.166841799 | IL13RA2 | interleukin 13 receptor subunit alpha 2 [Source:HGNC Symbol;Acc:HGNC:5975] |
| ENSG00000124207 | HGLibA_11318 | 107 | -0.166841799 | CSE1L | chromosome segregation 1 like [Source:HGNC Symbol;Acc:HGNC:2431] |
| ENSG00000124225 | HGLibA_37301 | 107 | -0.166841799 | PMEPA1 | androgen induced 1 [Source:HGNC Symbol;Acc:HGNC:14107]" |
| ENSG00000124302 | HGLibA_09648 | 107 | -0.166841799 | CHST8 | carbohydrate sulfotransferase 8 [Source:HGNC Symbol;Acc:HGNC:15993] |
| ENSG00000125503 | HGLibA_38025 | 107 | -0.166841799 | PPP1R12C | protein phosphatase 1 regulatory subunit 12C [Source:HGNC Symbol;Acc:HGNC:14947] |
| ENSG00000125966 | HGLibA_29520 | 107 | -0.166841799 | MMP24 | matrix metallopeptidase 24 [Source:HGNC Symbol;Acc:HGNC:7172] |
| ENSG00000127366 | HGLibA_48416 | 107 | -0.166841799 | TAS2R5 | taste 2 receptor member 5 [Source:HGNC Symbol;Acc:HGNC:14912] |
| ENSG00000129654 | HGLibA_17884 | 107 | -0.166841799 | FOXJ1 | forkhead box J1 [Source:HGNC Symbol;Acc:HGNC:3816] |
| ENSG00000129911 | HGLibA_25059 | 107 | -0.166841799 | KLF16 | Kruppel like factor 16 [Source:HGNC Symbol;Acc:HGNC:16857] |
| ENSG00000131096 | HGLibA_39711 | 107 | -0.166841799 | PYY | peptide YY [Source:HGNC Symbol;Acc:HGNC:9748] |
| ENSG00000132824 | HGLibA_43491 | 107 | -0.166841799 | SERINC3 | serine incorporator 3 [Source:HGNC Symbol;Acc:HGNC:11699] |
| ENSG00000134242 | HGLibA_39459 | 107 | -0.166841799 | PTPN22 | non-receptor type 22 [Source:HGNC Symbol;Acc:HGNC:9652]" |
| ENSG00000137806 | HGLibA_31483 | 107 | -0.166841799 | NDUFAF1 | NADH:ubiquinone oxidoreductase complex assembly factor 1 [Source:HGNC Symbol;Acc:HGNC:18828] |
| ENSG00000137825 | HGLibA_23844 | 107 | -0.166841799 | ITPKA | inositol-trisphosphate 3-kinase A [Source:HGNC Symbol;Acc:HGNC:6178] |
| ENSG00000138778 | HGLibA_09052 | 107 | -0.166841799 | CENPE | centromere protein E [Source:HGNC Symbol;Acc:HGNC:1856] |
| ENSG00000143320 | HGLibA_11056 | 107 | -0.166841799 | CRABP2 | cellular retinoic acid binding protein 2 [Source:HGNC Symbol;Acc:HGNC:2339] |
| ENSG00000143450 | HGLibA_33220 | 107 | -0.166841799 | OAZ3 | ornithine decarboxylase antizyme 3 [Source:HGNC Symbol;Acc:HGNC:8097] |
| ENSG00000143842 | HGLibA_46236 | 107 | -0.166841799 | SOX13 | SRY-box 13 [Source:HGNC Symbol;Acc:HGNC:11192] |
| ENSG00000144214 | HGLibA_27697 | 107 | -0.166841799 | LYG1 | lysozyme g1 [Source:HGNC Symbol;Acc:HGNC:27014] |
| ENSG00000144357 | HGLibA_52754 | 107 | -0.166841799 | UBR3 | ubiquitin protein ligase E3 component n-recognin 3 [Source:HGNC Symbol;Acc:HGNC:30467] |
| ENSG00000144550 | HGLibA_10976 | 107 | -0.166841799 | CPNE9 | copine family member 9 [Source:HGNC Symbol;Acc:HGNC:24336] |
| ENSG00000144711 | HGLibA_23541 | 107 | -0.166841799 | IQSEC1 | IQ motif and Sec7 domain 1 [Source:HGNC Symbol;Acc:HGNC:29112] |
| ENSG00000145777 | HGLibA_51876 | 107 | -0.166841799 | TSLP | thymic stromal lymphopoietin [Source:HGNC Symbol;Acc:HGNC:30743] |
| ENSG00000146063 | HGLibA_51496 | 107 | -0.166841799 | TRIM41 | tripartite motif containing 41 [Source:HGNC Symbol;Acc:HGNC:19013] |
| ENSG00000147121 | HGLibA_25395 | 107 | -0.166841799 | KRBOX4 | KRAB box domain containing 4 [Source:HGNC Symbol;Acc:HGNC:26007] |
| ENSG00000147223 | HGLibA_41329 | 107 | -0.166841799 | RIPPLY1 | ripply transcriptional repressor 1 [Source:HGNC Symbol;Acc:HGNC:25117] |
| ENSG00000152270 | HGLibA_35785 | 107 | -0.166841799 | PDE3B | phosphodiesterase 3B [Source:HGNC Symbol;Acc:HGNC:8779] |
| ENSG00000153779 | HGLibA_49248 | 107 | -0.166841799 | TGIF2LX | TGFB induced factor homeobox 2 like X-linked [Source:HGNC Symbol;Acc:HGNC:18570] |
| ENSG00000154277 | HGLibA_52802 | 107 | -0.166841799 | UCHL1 | ubiquitin C-terminal hydrolase L1 [Source:HGNC Symbol;Acc:HGNC:12513] |
| ENSG00000154451 | HGLibA_18739 | 107 | -0.166841799 | GBP5 | guanylate binding protein 5 [Source:HGNC Symbol;Acc:HGNC:19895] |
| ENSG00000154734 | HGLibA_00791 | 107 | -0.166841799 | ADAMTS1 | ADAM metallopeptidase with thrombospondin type 1 motif 1 [Source:HGNC Symbol;Acc:HGNC:217] |
| ENSG00000157601 | HGLibA_30604 | 107 | -0.166841799 | MX1 | MX dynamin like GTPase 1 [Source:HGNC Symbol;Acc:HGNC:7532] |
| ENSG00000158164 | HGLibA_50660 | 107 | -0.166841799 | TMSB15A | thymosin beta 15a [Source:HGNC Symbol;Acc:HGNC:30744] |
| ENSG00000159433 | HGLibA_47377 | 107 | -0.166841799 | STARD9 | StAR related lipid transfer domain containing 9 [Source:HGNC Symbol;Acc:HGNC:19162] |
| ENSG00000162511 | HGLibA_26023 | 107 | -0.166841799 | LAPTM5 | lysosomal protein transmembrane 5 [Source:HGNC Symbol;Acc:HGNC:29612] |
| ENSG00000163114 | HGLibA_35865 | 107 | -0.166841799 | PDHA2 | pyruvate dehydrogenase E1 alpha 2 subunit [Source:HGNC Symbol;Acc:HGNC:8807] |
| ENSG00000163659 | HGLibA_49557 | 107 | -0.166841799 | TIPARP | TCDD inducible poly(ADP-ribose) polymerase [Source:HGNC Symbol;Acc:HGNC:23696] |
| ENSG00000163697 | HGLibA_02383 | 107 | -0.166841799 | APBB2 | amyloid beta precursor protein binding family B member 2 [Source:HGNC Symbol;Acc:HGNC:582] |
| ENSG00000164167 | HGLibA_27549 | 107 | -0.166841799 | LSM6 | U6 small nuclear RNA and mRNA degradation associated [Source:HGNC Symbol;Acc:HGNC:17017]" |
| ENSG00000164172 | HGLibA_29616 | 107 | -0.166841799 | MOCS2 | molybdenum cofactor synthesis 2 [Source:HGNC Symbol;Acc:HGNC:7193] |
| ENSG00000164318 | HGLibA_14634 | 107 | -0.166841799 | EGFLAM | fibronectin type III and laminin G domains [Source:HGNC Symbol;Acc:HGNC:26810]" |
| ENSG00000164715 | HGLibA_26725 | 107 | -0.166841799 | LMTK2 | lemur tyrosine kinase 2 [Source:HGNC Symbol;Acc:HGNC:17880] |
| ENSG00000164916 | HGLibA_17894 | 107 | -0.166841799 | FOXK1 | forkhead box K1 [Source:HGNC Symbol;Acc:HGNC:23480] |
| ENSG00000166311 | HGLibA_45804 | 107 | -0.166841799 | SMPD1 | sphingomyelin phosphodiesterase 1 [Source:HGNC Symbol;Acc:HGNC:11120] |
| ENSG00000166813 | HGLibA_24961 | 107 | -0.166841799 | KIF7 | kinesin family member 7 [Source:HGNC Symbol;Acc:HGNC:30497] |
| ENSG00000166926 | HGLibA_30159 | 107 | -0.166841799 | MS4A6E | membrane spanning 4-domains A6E [Source:HGNC Symbol;Acc:HGNC:14285] |
| ENSG00000166961 | HGLibA_30143 | 107 | -0.166841799 | MS4A15 | membrane spanning 4-domains A15 [Source:HGNC Symbol;Acc:HGNC:28573] |
| ENSG00000167889 | HGLibA_29154 | 107 | -0.166841799 | MGAT5B | 6-mannosylglycoprotein 6-beta-N-acetylglucosaminyltransferase B [Source:HGNC Symbol;Acc:HGNC:24140]" |
| ENSG00000168461 | HGLibA_39873 | 107 | -0.166841799 | RAB31 | member RAS oncogene family [Source:HGNC Symbol;Acc:HGNC:9771]" |
| ENSG00000169169 | HGLibA_11024 | 107 | -0.166841799 | CPT1C | carnitine palmitoyltransferase 1C [Source:HGNC Symbol;Acc:HGNC:18540] |
| ENSG00000169926 | HGLibA_25051 | 107 | -0.166841799 | KLF13 | Kruppel like factor 13 [Source:HGNC Symbol;Acc:HGNC:13672] |
| ENSG00000170122 | HGLibA_17838 | 107 | -0.166841799 | FOXD4 | forkhead box D4 [Source:HGNC Symbol;Acc:HGNC:3805] |
| ENSG00000170231 | HGLibA_15926 | 107 | -0.166841799 | FABP6 | fatty acid binding protein 6 [Source:HGNC Symbol;Acc:HGNC:3561] |
| ENSG00000170293 | HGLibA_10229 | 107 | -0.166841799 | CMTM8 | CKLF like MARVEL transmembrane domain containing 8 [Source:HGNC Symbol;Acc:HGNC:19179] |
| ENSG00000170791 | HGLibA_09380 | 107 | -0.166841799 | CHCHD7 | coiled-coil-helix-coiled-coil-helix domain containing 7 [Source:HGNC Symbol;Acc:HGNC:28314] |
| ENSG00000171130 | HGLibA_03728 | 107 | -0.166841799 | ATP6V0E2 | ATPase H+ transporting V0 subunit e2 [Source:HGNC Symbol;Acc:HGNC:21723] |
| ENSG00000172830 | HGLibA_47137 | 107 | -0.166841799 | SSH3 | slingshot protein phosphatase 3 [Source:HGNC Symbol;Acc:HGNC:30581] |
| ENSG00000173175 | HGLibA_00921 | 107 | -0.166841799 | ADCY5 | adenylate cyclase 5 [Source:HGNC Symbol;Acc:HGNC:236] |
| ENSG00000173212 | HGLibA_27828 | 107 | -0.166841799 | MAB21L3 | mab-21 like 3 [Source:HGNC Symbol;Acc:HGNC:26787] |
| ENSG00000173451 | HGLibA_49295 | 107 | -0.166841799 | THAP2 | THAP domain containing 2 [Source:HGNC Symbol;Acc:HGNC:20854] |
| ENSG00000176842 | HGLibA_23639 | 107 | -0.166841799 | IRX5 | iroquois homeobox 5 [Source:HGNC Symbol;Acc:HGNC:14361] |
| ENSG00000180155 | HGLibA_27711 | 107 | -0.166841799 | LYNX1 | Ly6/neurotoxin 1 [Source:HGNC Symbol;Acc:HGNC:29604] |
| ENSG00000180353 | HGLibA_20969 | 107 | -0.166841799 | HCLS1 | hematopoietic cell-specific Lyn substrate 1 [Source:HGNC Symbol;Acc:HGNC:4844] |
| ENSG00000182010 | HGLibA_42392 | 107 | -0.166841799 | RTKN2 | rhotekin 2 [Source:HGNC Symbol;Acc:HGNC:19364] |
| ENSG00000182168 | HGLibA_53019 | 107 | -0.166841799 | UNC5C | unc-5 netrin receptor C [Source:HGNC Symbol;Acc:HGNC:12569] |

| | | | | | |
|---|---|---|---|---|---|
| ENSG00000182566 | HGLibA_09993 | 107 | -0.166841799 | CLEC4G | C-type lectin domain family 4 member G [Source:HGNC Symbol;Acc:HGNC:24591] |
| ENSG00000182583 | HGLibA_53580 | 107 | -0.166841799 | VCX | variable charge X-linked [Source:HGNC Symbol;Acc:HGNC:12667] |
| ENSG00000182809 | HGLibA_11154 | 107 | -0.166841799 | CRIP2 | cysteine rich protein 2 [Source:HGNC Symbol;Acc:HGNC:2361] |
| ENSG00000183208 | HGLibA_18904 | 107 | -0.166841799 | GDPGP1 | GDP-D-glucose phosphorylase 1 [Source:HGNC Symbol;Acc:HGNC:34360] |
| ENSG00000183379 | HGLibA_47973 | 107 | -0.166841799 | SYNDIG1L | synapse differentiation inducing 1 like [Source:HGNC Symbol;Acc:HGNC:32388] |
| ENSG00000183779 | HGLibA_56582 | 107 | -0.166841799 | ZNF703 | zinc finger protein 703 [Source:HGNC Symbol;Acc:HGNC:25883] |
| ENSG00000184530 | HGLibA_06500 | 107 | -0.166841799 | C6orf58 | chromosome 6 open reading frame 58 [Source:HGNC Symbol;Acc:HGNC:20960] |
| ENSG00000186150 | HGLibA_52709 | 107 | -0.166841799 | UBL4B | ubiquitin like 4B [Source:HGNC Symbol;Acc:HGNC:32309] |
| ENSG00000186451 | HGLibA_46427 | 107 | -0.166841799 | SPATA12 | spermatogenesis associated 12 [Source:HGNC Symbol;Acc:HGNC:23221] |
| ENSG00000186827 | HGLibA_50783 | 107 | -0.166841799 | TNFRSF4 | TNF receptor superfamily member 4 [Source:HGNC Symbol;Acc:HGNC:11918] |
| ENSG00000187091 | HGLibA_36990 | 107 | -0.166841799 | PLCD1 | phospholipase C delta 1 [Source:HGNC Symbol;Acc:HGNC:9060] |
| ENSG00000187672 | HGLibA_15429 | 107 | -0.166841799 | ERC2 | ELKS/RAB6-interacting/CAST family member 2 [Source:HGNC Symbol;Acc:HGNC:31922] |
| ENSG00000188322 | HGLibA_42799 | 107 | -0.166841799 | SBK1 | SH3 domain binding kinase 1 [Source:HGNC Symbol;Acc:HGNC:17699] |
| ENSG00000189377 | HGLibA_11862 | 107 | -0.166841799 | CXCL17 | C-X-C motif chemokine ligand 17 [Source:HGNC Symbol;Acc:HGNC:19232] |
| ENSG00000196083 | HGLibA_23053 | 107 | -0.166841799 | IL1RAP | interleukin 1 receptor accessory protein [Source:HGNC Symbol;Acc:HGNC:5995] |
| ENSG00000196214 | HGLibA_56694 | 107 | -0.166841799 | ZNF766 | zinc finger protein 766 [Source:HGNC Symbol;Acc:HGNC:28063] |
| ENSG00000196850 | HGLibA_38240 | 107 | -0.166841799 | PPTC7 | PTC7 protein phosphatase homolog [Source:HGNC Symbol;Acc:HGNC:30695] |
| ENSG00000196860 | HGLibA_50957 | 107 | -0.166841799 | TOMM20L | translocase of outer mitochondrial membrane 20 like [Source:HGNC Symbol;Acc:HGNC:33752] |
| ENSG00000197008 | HGLibA_55476 | 107 | -0.166841799 | ZNF138 | zinc finger protein 138 [Source:HGNC Symbol;Acc:HGNC:12922] |
| ENSG00000197016 | HGLibA_56052 | 107 | -0.166841799 | ZNF470 | zinc finger protein 470 [Source:HGNC Symbol;Acc:HGNC:22220] |
| ENSG00000197647 | HGLibA_55986 | 107 | -0.166841799 | ZNF433 | zinc finger protein 433 [Source:HGNC Symbol;Acc:HGNC:20811] |
| ENSG00000197993 | HGLibA_24551 | 107 | -0.166841799 | KEL | Kell metallo-endopeptidase (Kell blood group) [Source:HGNC Symbol;Acc:HGNC:6308] |
| ENSG00000198062 | HGLibA_37738 | 107 | -0.166841799 | POTEH | POTE ankyrin domain family member H [Source:HGNC Symbol;Acc:HGNC:133] |
| ENSG00000198515 | HGLibA_10262 | 107 | -0.166841799 | CNGA1 | cyclic nucleotide gated channel alpha 1 [Source:HGNC Symbol;Acc:HGNC:2148] |
| ENSG00000198700 | HGLibA_23477 | 107 | -0.166841799 | IPO9 | importin 9 [Source:HGNC Symbol;Acc:HGNC:19425] |
| ENSG00000198919 | HGLibA_14305 | 107 | -0.166841799 | DZIP3 | DAZ interacting zinc finger protein 3 [Source:HGNC Symbol;Acc:HGNC:30938] |
| ENSG00000204130 | HGLibA_42439 | 107 | -0.166841799 | RUFY2 | RUN and FYVE domain containing 2 [Source:HGNC Symbol;Acc:HGNC:19761] |
| ENSG00000204382 | HGLibA_54470 | 107 | -0.166841799 | XAGE1B | X antigen family member 1B [Source:HGNC Symbol;Acc:HGNC:25400] |
| ENSG00000204970 | HGLibA_35424 | 107 | -0.166841799 | PCDHA1 | protocadherin alpha 1 [Source:HGNC Symbol;Acc:HGNC:8663] |
| ENSG00000205076 | HGLibA_26371 | 107 | -0.166841799 | LGALS7 | galectin 7 [Source:HGNC Symbol;Acc:HGNC:6568] |
| ENSG00000205177 | HGLibA_05211 | 107 | -0.166841799 | C11orf91 | chromosome 11 open reading frame 91 [Source:HGNC Symbol;Acc:HGNC:34444] |
| ENSG00000205329 | HGLibA_34387 | 107 | -0.166841799 | OR6C3 | olfactory receptor family 6 subfamily C member 3 [Source:HGNC Symbol;Acc:HGNC:15437] |
| ENSG00000213185 | HGLibA_16549 | 107 | -0.166841799 | FAM24B | family with sequence similarity 24 member B [Source:HGNC Symbol;Acc:HGNC:23475] |
| ENSG00000215218 | HGLibA_52650 | 107 | -0.166841799 | UBE2QL1 | ubiquitin conjugating enzyme E2 Q family like 1 [Source:HGNC Symbol;Acc:HGNC:37269] |
| ENSG00000219607 | HGLibA_38115 | 107 | -0.166841799 | PPP1R3G | protein phosphatase 1 regulatory subunit 3G [Source:HGNC Symbol;Acc:HGNC:14945] |
| ENSG00000224440 | HGLibA_11939 | 107 | -0.166841799 | CXorf51A | chromosome X open reading frame 51A [Source:HGNC Symbol;Acc:HGNC:30533] |
| ENSG00000228075 | HGLibA_04674 | 107 | -0.166841799 | BOD1L2 | biorientation of chromosomes in cell division 1 like 2 [Source:HGNC Symbol;Acc:HGNC:28505] |
| ENSG00000230031 | HGLibA_37719 | 107 | -0.166841799 | POTEB2 | POTE ankyrin domain family member B2 [Source:HGNC Symbol;Acc:HGNC:48327] |
| ENSG00000233838 | HGLibA_13846 | 107 | -0.166841799 | DPH3P1 | diphthamide biosynthesis 3 pseudogene 1 [Source:HGNC Symbol;Acc:HGNC:16136] |
| ENSG00000235863 | HGLibA_03959 | 107 | -0.166841799 | B3GALT4 | 3-galactosyltransferase 4 [Source:HGNC Symbol;Acc:HGNC:919]" |
| ENSG00000236980 | HGLibA_06278 | 107 | -0.166841799 | C3orf84 | chromosome 3 open reading frame 84 [Source:HGNC Symbol;Acc:HGNC:44666] |
| ENSG00000240065 | HGLibA_39123 | 107 | -0.166841799 | PSMB9 | proteasome subunit beta 9 [Source:HGNC Symbol;Acc:HGNC:9546] |
| ENSG00000241186 | HGLibA_48884 | 107 | -0.166841799 | TDGF1 | teratocarcinoma-derived growth factor 1 [Source:HGNC Symbol;Acc:HGNC:11701] |
| ENSG00000243649 | HGLibA_09245 | 107 | -0.166841799 | CFB | complement factor B [Source:HGNC Symbol;Acc:HGNC:1037] |
| ENSG00000262628 | HGLibA_33655 | 107 | -0.166841799 | OR1D5 | olfactory receptor family 1 subfamily D member 5 [Source:HGNC Symbol;Acc:HGNC:8186] |
| ENSG00000264522 | HGLibA_34781 | 107 | -0.166841799 | OTUD7B | OTU deubiquitinase 7B [Source:HGNC Symbol;Acc:HGNC:16683] |
| ENSG00000267534 | HGLibA_42596 | 107 | -0.166841799 | S1PR2 | sphingosine-1-phosphate receptor 2 [Source:HGNC Symbol;Acc:HGNC:3169] |
| GPR1 | HGLibA_19836 | 107 | -0.166841799 | GPR1 | - |
| GPR133 | HGLibA_19890 | 107 | -0.166841799 | GPR133 | - |
| HIST1H2BK | HGLibA_21441 | 107 | -0.166841799 | HIST1H2BK | - |
| hsa-mir-1269a | HGLibA_57502 | 107 | -0.166841799 | hsa-mir-1269a | - |
| hsa-mir-146b | HGLibA_57861 | 107 | -0.166841799 | hsa-mir-146b | - |
| hsa-mir-151b | HGLibA_57899 | 107 | -0.166841799 | hsa-mir-151b | - |
| hsa-mir-3119-2 | HGLibA_58528 | 107 | -0.166841799 | hsa-mir-3119-2 | - |
| hsa-mir-345 | HGLibA_59027 | 107 | -0.166841799 | hsa-mir-345 | - |
| hsa-mir-3691 | HGLibA_59353 | 107 | -0.166841799 | hsa-mir-3691 | - |
| hsa-mir-378a | HGLibA_59427 | 107 | -0.166841799 | hsa-mir-378a | - |
| hsa-mir-3972 | HGLibA_59646 | 107 | -0.166841799 | hsa-mir-3972 | - |
| hsa-mir-4271 | HGLibA_59792 | 107 | -0.166841799 | hsa-mir-4271 | - |
| hsa-mir-4448 | HGLibA_60198 | 107 | -0.166841799 | hsa-mir-4448 | - |
| hsa-mir-449c | HGLibA_60425 | 107 | -0.166841799 | hsa-mir-449c | - |
| hsa-mir-4776-2 | HGLibA_61213 | 107 | -0.166841799 | hsa-mir-4776-2 | - |
| hsa-mir-4796 | HGLibA_61291 | 107 | -0.166841799 | hsa-mir-4796 | - |
| hsa-mir-548ax | HGLibA_61938 | 107 | -0.166841799 | hsa-mir-548ax | - |
| hsa-mir-5680 | HGLibA_62234 | 107 | -0.166841799 | hsa-mir-5680 | - |
| hsa-mir-610 | HGLibA_62631 | 107 | -0.166841799 | hsa-mir-610 | - |
| hsa-mir-889 | HGLibA_64236 | 107 | -0.166841799 | hsa-mir-889 | - |
| hsa-mir-95 | HGLibA_64364 | 107 | -0.166841799 | hsa-mir-95 | - |
| PQLC2 | HGLibA_38254 | 107 | -0.166841799 | PQLC2 | - |
| SDPR | HGLibA_43157 | 107 | -0.166841799 | SDPR | - |
| 6-Sep | HGLibA_43447 | 107 | -0.166841799 | 6-Sep | - |
| SGK110 | HGLibA_43819 | 107 | -0.166841799 | SGK110 | - |
| TMEM56-RWDD3 | HGLibA_50454 | 107 | -0.166841799 | TMEM56-RWDD3 | - |
| WBSCR22 | HGLibA_53996 | 107 | -0.166841799 | WBSCR22 | - |
| XAGE1E | HGLibA_54479 | 107 | -0.166841799 | XAGE1E | - |
| ADPRHL2 | HGLibA_01030 | 106 | -0.175887142 | ADPRHL2 | - |
| C11orf70 | HGLibA_05176 | 106 | -0.175887142 | C11orf70 | - |
| C16orf55 | HGLibA_05459 | 106 | -0.175887142 | C16orf55 | - |
| C1orf101 | HGLibA_05733 | 106 | -0.175887142 | C1orf101 | - |
| CCL4L1 | HGLibA_08030 | 106 | -0.175887142 | CCL4L1 | - |
| ENSG00000026652 | HGLibA_01227 | 106 | -0.175887142 | AGPAT4 | 1-acylglycerol-3-phosphate O-acyltransferase 4 [Source:HGNC Symbol;Acc:HGNC:20885] |
| ENSG00000042832 | HGLibA_49198 | 106 | -0.175887142 | TG | thyroglobulin [Source:HGNC Symbol;Acc:HGNC:11764] |
| ENSG00000060339 | HGLibA_07488 | 106 | -0.175887142 | CCAR1 | cell division cycle and apoptosis regulator 1 [Source:HGNC Symbol;Acc:HGNC:24236] |
| ENSG00000062282 | HGLibA_13037 | 106 | -0.175887142 | DGAT2 | diacylglycerol O-acyltransferase 2 [Source:HGNC Symbol;Acc:HGNC:16940] |
| ENSG00000065135 | HGLibA_19456 | 106 | -0.175887142 | GNAI3 | G protein subunit alpha i3 [Source:HGNC Symbol;Acc:HGNC:4387] |
| ENSG00000070759 | HGLibA_49031 | 106 | -0.175887142 | TESK2 | testis associated actin remodelling kinase 2 [Source:HGNC Symbol;Acc:HGNC:11732] |
| ENSG00000078596 | HGLibA_23830 | 106 | -0.175887142 | ITM2A | integral membrane protein 2A [Source:HGNC Symbol;Acc:HGNC:6173] |
| ENSG00000085719 | HGLibA_10958 | 106 | -0.175887142 | CPNE3 | copine 3 [Source:HGNC Symbol;Acc:HGNC:2316] |
| ENSG00000087884 | HGLibA_00054 | 106 | -0.175887142 | AAMDC | adipogenesis associated Mth938 domain containing [Source:HGNC Symbol;Acc:HGNC:30205] |
| ENSG00000095794 | HGLibA_11131 | 106 | -0.175887142 | CREM | cAMP responsive element modulator [Source:HGNC Symbol;Acc:HGNC:2352] |
| ENSG00000100461 | HGLibA_40511 | 106 | -0.175887142 | RBM23 | RNA binding motif protein 23 [Source:HGNC Symbol;Acc:HGNC:20155] |
| ENSG00000102531 | HGLibA_17747 | 106 | -0.175887142 | FNDC3A | fibronectin type III domain containing 3A [Source:HGNC Symbol;Acc:HGNC:20296] |
| ENSG00000103034 | HGLibA_31422 | 106 | -0.175887142 | NDRG4 | NDRG family member 4 [Source:HGNC Symbol;Acc:HGNC:14466] |
| ENSG00000104055 | HGLibA_49266 | 106 | -0.175887142 | TGM5 | transglutaminase 5 [Source:HGNC Symbol;Acc:HGNC:11781] |
| ENSG00000104490 | HGLibA_31251 | 106 | -0.175887142 | NCALD | neurocalcin delta [Source:HGNC Symbol;Acc:HGNC:7655] |
| ENSG00000105229 | HGLibA_36517 | 106 | -0.175887142 | PIAS4 | protein inhibitor of activated STAT 4 [Source:HGNC Symbol;Acc:HGNC:17002] |
| ENSG00000105991 | HGLibA_21894 | 106 | -0.175887142 | HOXA1 | homeobox A1 [Source:HGNC Symbol;Acc:HGNC:5099] |
| ENSG00000106638 | HGLibA_48616 | 106 | -0.175887142 | TBL2 | transducin beta like 2 [Source:HGNC Symbol;Acc:HGNC:11586] |
| ENSG00000106853 | HGLibA_39352 | 106 | -0.175887142 | PTGR1 | prostaglandin reductase 1 [Source:HGNC Symbol;Acc:HGNC:18429] |
| ENSG00000107537 | HGLibA_36467 | 106 | -0.175887142 | PHYH | phytanoyl-CoA 2-hydroxylase [Source:HGNC Symbol;Acc:HGNC:8940] |
| ENSG00000108528 | HGLibA_44640 | 106 | -0.175887142 | SLC25A11 | solute carrier family 25 member 11 [Source:HGNC Symbol;Acc:HGNC:10981] |
| ENSG00000109063 | HGLibA_30725 | 106 | -0.175887142 | MYH3 | myosin heavy chain 3 [Source:HGNC Symbol;Acc:HGNC:7573] |
| ENSG00000109436 | HGLibA_48572 | 106 | -0.175887142 | TBC1D9 | TBC1 domain family member 9 [Source:HGNC Symbol;Acc:HGNC:21710] |
| ENSG00000110063 | HGLibA_12527 | 106 | -0.175887142 | DCPS | scavenger [Source:HGNC Symbol;Acc:HGNC:29812]" |
| ENSG00000110092 | HGLibA_08073 | 106 | -0.175887142 | CCND1 | cyclin D1 [Source:HGNC Symbol;Acc:HGNC:1582] |
| ENSG00000111664 | HGLibA_19495 | 106 | -0.175887142 | GNB3 | G protein subunit beta 3 [Source:HGNC Symbol;Acc:HGNC:4400] |
| ENSG00000112305 | HGLibA_45596 | 106 | -0.175887142 | SMAP1 | small ArfGAP 1 [Source:HGNC Symbol;Acc:HGNC:19651] |
| ENSG00000113583 | HGLibA_06359 | 106 | -0.175887142 | C5orf15 | chromosome 5 open reading frame 15 [Source:HGNC Symbol;Acc:HGNC:20656] |
| ENSG00000114738 | HGLibA_28303 | 106 | -0.175887142 | MAPKAPK3 | mitogen-activated protein kinase-activated protein kinase 3 [Source:HGNC Symbol;Acc:HGNC:6888] |
| ENSG00000115415 | HGLibA_47381 | 106 | -0.175887142 | STAT1 | signal transducer and activator of transcription 1 [Source:HGNC Symbol;Acc:HGNC:11362] |
| ENSG00000116212 | HGLibA_27358 | 106 | -0.175887142 | LRRC42 | leucine rich repeat containing 42 [Source:HGNC Symbol;Acc:HGNC:28792] |
| ENSG00000117013 | HGLibA_24375 | 106 | -0.175887142 | KCNQ4 | potassium voltage-gated channel subfamily Q member 4 [Source:HGNC Symbol;Acc:HGNC:6298] |
| ENSG00000117266 | HGLibA_08750 | 106 | -0.175887142 | CDK18 | cyclin dependent kinase 18 [Source:HGNC Symbol;Acc:HGNC:8751] |
| ENSG00000122958 | HGLibA_53751 | 106 | -0.175887142 | VPS26A | retromer complex component A [Source:HGNC Symbol;Acc:HGNC:12711]" |
| ENSG00000123213 | HGLibA_32086 | 106 | -0.175887142 | NLN | neurolysin [Source:HGNC Symbol;Acc:HGNC:16058] |
| ENSG00000124356 | HGLibA_47336 | 106 | -0.175887142 | STAMBP | STAM binding protein [Source:HGNC Symbol;Acc:HGNC:16950] |
| ENSG00000124374 | HGLibA_35033 | 106 | -0.175887142 | PAIP2B | poly(A) binding protein interacting protein 2B [Source:HGNC Symbol;Acc:HGNC:29200] |
| ENSG00000125675 | HGLibA_20265 | 106 | -0.175887142 | GRIA3 | glutamate ionotropic receptor AMPA type subunit 3 [Source:HGNC Symbol;Acc:HGNC:4573] |
| ENSG00000126821 | HGLibA_43851 | 106 | -0.175887142 | SGPP1 | sphingosine-1-phosphate phosphatase 1 [Source:HGNC Symbol;Acc:HGNC:17720] |
| ENSG00000130520 | HGLibA_27542 | 106 | -0.175887142 | LSM4 | U6 small nuclear RNA and mRNA degradation associated [Source:HGNC Symbol;Acc:HGNC:17259]" |
| ENSG00000132507 | HGLibA_14854 | 106 | -0.175887142 | EIF5A | eukaryotic translation initiation factor 5A [Source:HGNC Symbol;Acc:HGNC:3300] |
| ENSG00000134765 | HGLibA_13990 | 106 | -0.175887142 | DSC1 | desmocollin 1 [Source:HGNC Symbol;Acc:HGNC:3035] |
| ENSG00000134970 | HGLibA_49819 | 106 | -0.175887142 | TMED7 | transmembrane p24 trafficking protein 7 [Source:HGNC Symbol;Acc:HGNC:24253] |
| ENSG00000135175 | HGLibA_33259 | 106 | -0.175887142 | OCM2 | oncomodulin 2 [Source:HGNC Symbol;Acc:HGNC:34396] |
| ENSG00000135248 | HGLibA_16701 | 106 | -0.175887142 | FAM71F1 | family with sequence similarity 71 member F1 [Source:HGNC Symbol;Acc:HGNC:30704] |
| ENSG00000136040 | HGLibA_37280 | 106 | -0.175887142 | PLXNC1 | plexin C1 [Source:HGNC Symbol;Acc:HGNC:9106] |
| ENSG00000137288 | HGLibA_53102 | 106 | -0.175887142 | UQCC2 | ubiquinol-cytochrome c reductase complex assembly factor 2 [Source:HGNC Symbol;Acc:HGNC:21237] |
| ENSG00000138152 | HGLibA_04902 | 106 | -0.175887142 | BTBD16 | BTB domain containing 16 [Source:HGNC Symbol;Acc:HGNC:26340] |
| ENSG00000138686 | HGLibA_04223 | 106 | -0.175887142 | BBS7 | Bardet-Biedl syndrome 7 [Source:HGNC Symbol;Acc:HGNC:18758] |
| ENSG00000138757 | HGLibA_18302 | 106 | -0.175887142 | G3BP2 | G3BP stress granule assembly factor 2 [Source:HGNC Symbol;Acc:HGNC:30291] |
| ENSG00000139044 | HGLibA_04018 | 106 | -0.175887142 | B4GALNT3 | 4-N-acetyl-galactosaminyltransferase 3 [Source:HGNC Symbol;Acc:HGNC:24137]" |
| ENSG00000139223 | HGLibA_02205 | 106 | -0.175887142 | ANP32D | acidic nuclear phosphoprotein 32 family member D [Source:HGNC Symbol;Acc:HGNC:16676] |
| ENSG00000140557 | HGLibA_47290 | 106 | -0.175887142 | ST8SIA2 | 8-sialyltransferase 2 [Source:HGNC Symbol;Acc:HGNC:10870]" |
| ENSG00000140945 | HGLibA_08640 | 106 | -0.175887142 | CDH13 | cadherin 13 [Source:HGNC Symbol;Acc:HGNC:1753] |
| ENSG00000141551 | HGLibA_11387 | 106 | -0.175887142 | CSNK1D | casein kinase 1 delta [Source:HGNC Symbol;Acc:HGNC:2452] |

| | | | | | |
|---|---|---|---|---|---|
| ENSG00000141664 | HGLibA_55028 | 106 | -0.175887142 | ZCCHC2 | zinc finger CCHC-type containing 2 [Source:HGNC Symbol;Acc:HGNC:22916] |
| ENSG00000143921 | HGLibA_00226 | 106 | -0.175887142 | ABCG8 | ATP binding cassette subfamily G member 8 [Source:HGNC Symbol;Acc:HGNC:13887] |
| ENSG00000145321 | HGLibA_18756 | 106 | -0.175887142 | GC | vitamin D binding protein [Source:HGNC Symbol;Acc:HGNC:4187]" |
| ENSG00000146070 | HGLibA_36924 | 106 | -0.175887142 | PLA2G7 | phospholipase A2 group VII [Source:HGNC Symbol;Acc:HGNC:9040] |
| ENSG00000147394 | HGLibA_55549 | 106 | -0.175887142 | ZNF185 | zinc finger protein 185 with LIM domain [Source:HGNC Symbol;Acc:HGNC:12976] |
| ENSG00000147400 | HGLibA_09235 | 106 | -0.175887142 | CETN2 | centrin 2 [Source:HGNC Symbol;Acc:HGNC:1867] |
| ENSG00000150275 | HGLibA_35401 | 106 | -0.175887142 | PCDH15 | protocadherin related 15 [Source:HGNC Symbol;Acc:HGNC:14674] |
| ENSG00000151650 | HGLibA_53620 | 106 | -0.175887142 | VENTX | VENT homeobox [Source:HGNC Symbol;Acc:HGNC:13639] |
| ENSG00000151657 | HGLibA_24979 | 106 | -0.175887142 | KIN | Kin17 DNA and RNA binding protein [Source:HGNC Symbol;Acc:HGNC:6327] |
| ENSG00000153498 | HGLibA_46339 | 106 | -0.175887142 | SPACA7 | sperm acrosome associated 7 [Source:HGNC Symbol;Acc:HGNC:29575] |
| ENSG00000154099 | HGLibA_13460 | 106 | -0.175887142 | DNAAF1 | dynein axonemal assembly factor 1 [Source:HGNC Symbol;Acc:HGNC:30539] |
| ENSG00000156006 | HGLibA_31177 | 106 | -0.175887142 | NAT2 | N-acetyltransferase 2 [Source:HGNC Symbol;Acc:HGNC:7646] |
| ENSG00000160396 | HGLibA_21349 | 106 | -0.175887142 | HIPK4 | homeodomain interacting protein kinase 4 [Source:HGNC Symbol;Acc:HGNC:19007] |
| ENSG00000160949 | HGLibA_50985 | 106 | -0.175887142 | TONSL | DNA repair protein [Source:HGNC Symbol;Acc:HGNC:7801]" |
| ENSG00000162753 | HGLibA_45441 | 106 | -0.175887142 | SLC9C2 | solute carrier family 9 member C2 (putative) [Source:HGNC Symbol;Acc:HGNC:28664] |
| ENSG00000162804 | HGLibA_45924 | 106 | -0.175887142 | SNED1 | nidogen and EGF like domains 1 [Source:HGNC Symbol;Acc:HGNC:24696]" |
| ENSG00000162989 | HGLibA_24276 | 106 | -0.175887142 | KCNJ3 | potassium voltage-gated channel subfamily J member 3 [Source:HGNC Symbol;Acc:HGNC:6264] |
| ENSG00000163145 | HGLibA_05954 | 106 | -0.175887142 | C1QTNF7 | C1q and TNF related 7 [Source:HGNC Symbol;Acc:HGNC:14342] |
| ENSG00000163349 | HGLibA_21339 | 106 | -0.175887142 | HIPK1 | homeodomain interacting protein kinase 1 [Source:HGNC Symbol;Acc:HGNC:19006] |
| ENSG00000164070 | HGLibA_22285 | 106 | -0.175887142 | HSPA4L | heat shock protein family A (Hsp70) member 4 like [Source:HGNC Symbol;Acc:HGNC:17041] |
| ENSG00000164411 | HGLibA_19174 | 106 | -0.175887142 | GJB7 | gap junction protein beta 7 [Source:HGNC Symbol;Acc:HGNC:16690] |
| ENSG00000164576 | HGLibA_42727 | 106 | -0.175887142 | SAP30L | SAP30 like [Source:HGNC Symbol;Acc:HGNC:25663] |
| ENSG00000166046 | HGLibA_48848 | 106 | -0.175887142 | TCP11L2 | t-complex 11 like 2 [Source:HGNC Symbol;Acc:HGNC:28627] |
| ENSG00000166333 | HGLibA_23219 | 106 | -0.175887142 | ILK | integrin linked kinase [Source:HGNC Symbol;Acc:HGNC:6040] |
| ENSG00000166527 | HGLibA_09985 | 106 | -0.175887142 | CLEC4D | C-type lectin domain family 4 member D [Source:HGNC Symbol;Acc:HGNC:14554] |
| ENSG00000167165 | HGLibA_52890 | 106 | -0.175887142 | UGT1A6 | UDP glucuronosyltransferase family 1 member A6 [Source:HGNC Symbol;Acc:HGNC:12538] |
| ENSG00000167608 | HGLibA_49756 | 106 | -0.175887142 | TMC4 | transmembrane channel like 4 [Source:HGNC Symbol;Acc:HGNC:22998] |
| ENSG00000167985 | HGLibA_43138 | 106 | -0.175887142 | SDHAF2 | succinate dehydrogenase complex assembly factor 2 [Source:HGNC Symbol;Acc:HGNC:26034] |
| ENSG00000168131 | HGLibA_33767 | 106 | -0.175887142 | OR2B2 | olfactory receptor family 2 subfamily B member 2 [Source:HGNC Symbol;Acc:HGNC:13966] |
| ENSG00000168246 | HGLibA_52769 | 106 | -0.175887142 | UBTD2 | ubiquitin domain containing 2 [Source:HGNC Symbol;Acc:HGNC:24463] |
| ENSG00000169789 | HGLibA_38996 | 106 | -0.175887142 | PRY | PTPN13 like Y-linked [Source:HGNC Symbol;Acc:HGNC:14024] |
| ENSG00000169895 | HGLibA_47907 | 106 | -0.175887142 | SYAP1 | synapse associated protein 1 [Source:HGNC Symbol;Acc:HGNC:16273] |
| ENSG00000170099 | HGLibA_43525 | 106 | -0.175887142 | SERPINA6 | serpin family A member 6 [Source:HGNC Symbol;Acc:HGNC:1540] |
| ENSG00000170779 | HGLibA_08607 | 106 | -0.175887142 | CDCA4 | cell division cycle associated 4 [Source:HGNC Symbol;Acc:HGNC:14625] |
| ENSG00000170891 | HGLibA_12249 | 106 | -0.175887142 | CYTL1 | cytokine like 1 [Source:HGNC Symbol;Acc:HGNC:24435] |
| ENSG00000171476 | HGLibA_21885 | 106 | -0.175887142 | HOPX | HOP homeobox [Source:HGNC Symbol;Acc:HGNC:24961] |
| ENSG00000171724 | HGLibA_53545 | 106 | -0.175887142 | VAT1L | vesicle amine transport 1 like [Source:HGNC Symbol;Acc:HGNC:29315] |
| ENSG00000172270 | HGLibA_04867 | 106 | -0.175887142 | BSG | basigin (Ok blood group) [Source:HGNC Symbol;Acc:HGNC:1116] |
| ENSG00000172352 | HGLibA_08907 | 106 | -0.175887142 | CDY1B | chromodomain Y-linked 1B [Source:HGNC Symbol;Acc:HGNC:23920] |
| ENSG00000172578 | HGLibA_25219 | 106 | -0.175887142 | KLHL6 | kelch like family member 6 [Source:HGNC Symbol;Acc:HGNC:18653] |
| ENSG00000173110 | HGLibA_22293 | 106 | -0.175887142 | HSPA6 | heat shock protein family A (Hsp70) member 6 [Source:HGNC Symbol;Acc:HGNC:5239] |
| ENSG00000175106 | HGLibA_52364 | 106 | -0.175887142 | TVP23C | trans-golgi network vesicle protein 23 homolog C [Source:HGNC Symbol;Acc:HGNC:30453] |
| ENSG00000176219 | HGLibA_33580 | 106 | -0.175887142 | OR11H6 | olfactory receptor family 11 subfamily H member 6 [Source:HGNC Symbol;Acc:HGNC:15349] |
| ENSG00000177283 | HGLibA_18284 | 106 | -0.175887142 | FZD8 | frizzled class receptor 8 [Source:HGNC Symbol;Acc:HGNC:4046] |
| ENSG00000180205 | HGLibA_54277 | 106 | -0.175887142 | WFDC9 | WAP four-disulfide core domain 9 [Source:HGNC Symbol;Acc:HGNC:20380] |
| ENSG00000180626 | HGLibA_56344 | 106 | -0.175887142 | ZNF594 | zinc finger protein 594 [Source:HGNC Symbol;Acc:HGNC:29392] |
| ENSG00000181467 | HGLibA_40238 | 106 | -0.175887142 | RAP2B | member of RAS oncogene family [Source:HGNC Symbol;Acc:HGNC:9862]" |
| ENSG00000182224 | HGLibA_11995 | 106 | -0.175887142 | CYB5D1 | cytochrome b5 domain containing 1 [Source:HGNC Symbol;Acc:HGNC:26516] |
| ENSG00000182308 | HGLibA_12441 | 106 | -0.175887142 | DCAF4L1 | DDB1 and CUL4 associated factor 4 like 1 [Source:HGNC Symbol;Acc:HGNC:27723] |
| ENSG00000182704 | HGLibA_51874 | 106 | -0.175887142 | TSKU | small leucine rich proteoglycan [Source:HGNC Symbol;Acc:HGNC:28850]" |
| ENSG00000183475 | HGLibA_03248 | 106 | -0.175887142 | ASB7 | ankyrin repeat and SOCS box containing 7 [Source:HGNC Symbol;Acc:HGNC:17182] |
| ENSG00000183753 | HGLibA_04744 | 106 | -0.175887142 | BPY2 | basic charge Y-linked 2 [Source:HGNC Symbol;Acc:HGNC:13508] |
| ENSG00000184194 | HGLibA_19970 | 106 | -0.175887142 | GPR173 | G protein-coupled receptor 173 [Source:HGNC Symbol;Acc:HGNC:18186] |
| ENSG00000184216 | HGLibA_23552 | 106 | -0.175887142 | IRAK1 | interleukin 1 receptor associated kinase 1 [Source:HGNC Symbol;Acc:HGNC:6112] |
| ENSG00000184470 | HGLibA_52448 | 106 | -0.175887142 | TXNRD2 | thioredoxin reductase 2 [Source:HGNC Symbol;Acc:HGNC:18155] |
| ENSG00000184672 | HGLibA_40176 | 106 | -0.175887142 | RALYL | RALY RNA binding protein like [Source:HGNC Symbol;Acc:HGNC:27036] |
| ENSG00000185049 | HGLibA_31672 | 106 | -0.175887142 | NELFA | negative elongation factor complex member A [Source:HGNC Symbol;Acc:HGNC:12768] |
| ENSG00000186222 | HGLibA_04565 | 106 | -0.175887142 | BLOC1S4 | biogenesis of lysosomal organelles complex 1 subunit 4 [Source:HGNC Symbol;Acc:HGNC:24206] |
| ENSG00000186329 | HGLibA_50211 | 106 | -0.175887142 | TMEM212 | transmembrane protein 212 [Source:HGNC Symbol;Acc:HGNC:34295] |
| ENSG00000186334 | HGLibA_45037 | 106 | -0.175887142 | SLC36A3 | solute carrier family 36 member 3 [Source:HGNC Symbol;Acc:HGNC:19659] |
| ENSG00000186994 | HGLibA_24030 | 106 | -0.175887142 | KANK3 | KN motif and ankyrin repeat domains 3 [Source:HGNC Symbol;Acc:HGNC:24796] |
| ENSG00000187272 | HGLibA_25849 | 106 | -0.175887142 | KRTAP9-8 | keratin associated protein 9-8 [Source:HGNC Symbol;Acc:HGNC:17231] |
| ENSG00000187715 | HGLibA_24095 | 106 | -0.175887142 | KBTBD12 | kelch repeat and BTB domain containing 12 [Source:HGNC Symbol;Acc:HGNC:25731] |
| ENSG00000188373 | HGLibA_05112 | 106 | -0.175887142 | C10orf99 | chromosome 10 open reading frame 99 [Source:HGNC Symbol;Acc:HGNC:31428] |
| ENSG00000188542 | HGLibA_14175 | 106 | -0.175887142 | DUSP28 | dual specificity phosphatase 28 [Source:HGNC Symbol;Acc:HGNC:33237] |
| ENSG00000196227 | HGLibA_16492 | 106 | -0.175887142 | FAM217B | family with sequence similarity 217 member B [Source:HGNC Symbol;Acc:HGNC:16170] |
| ENSG00000197110 | HGLibA_22726 | 106 | -0.175887142 | IFNL3 | interferon lambda 3 [Source:HGNC Symbol;Acc:HGNC:18365] |
| ENSG00000197446 | HGLibA_12134 | 106 | -0.175887142 | CYP2F1 | cytochrome P450 family 2 subfamily F member 1 [Source:HGNC Symbol;Acc:HGNC:2632] |
| ENSG00000197782 | HGLibA_56732 | 106 | -0.175887142 | ZNF780A | zinc finger protein 780A [Source:HGNC Symbol;Acc:HGNC:27603] |
| ENSG00000197838 | HGLibA_12104 | 106 | -0.175887142 | CYP2A13 | cytochrome P450 family 2 subfamily A member 13 [Source:HGNC Symbol;Acc:HGNC:2608] |
| ENSG00000198585 | HGLibA_32953 | 106 | -0.175887142 | NUDT16 | nudix hydrolase 16 [Source:HGNC Symbol;Acc:HGNC:26442] |
| ENSG00000198791 | HGLibA_10350 | 106 | -0.175887142 | CNOT7 | CCR4-NOT transcription complex subunit 7 [Source:HGNC Symbol;Acc:HGNC:14101] |
| ENSG00000198911 | HGLibA_46953 | 106 | -0.175887142 | SREBF2 | sterol regulatory element binding transcription factor 2 [Source:HGNC Symbol;Acc:HGNC:11290] |
| ENSG00000203783 | HGLibA_38853 | 106 | -0.175887142 | PRR9 | proline rich 9 [Source:HGNC Symbol;Acc:HGNC:32057] |
| ENSG00000204052 | HGLibA_27424 | 106 | -0.175887142 | LRRC73 | leucine rich repeat containing 73 [Source:HGNC Symbol;Acc:HGNC:21375] |
| ENSG00000206106 | HGLibA_25720 | 106 | -0.175887142 | KRTAP22-2 | keratin associated protein 22-2 [Source:HGNC Symbol;Acc:HGNC:37091] |
| ENSG00000214706 | HGLibA_22739 | 106 | -0.175887142 | IFRD2 | interferon related developmental regulator 2 [Source:HGNC Symbol;Acc:HGNC:5457] |
| ENSG00000221836 | HGLibA_33740 | 106 | -0.175887142 | OR2A5 | olfactory receptor family 2 subfamily A member 5 [Source:HGNC Symbol;Acc:HGNC:8232] |
| ENSG00000227140 | HGLibA_53282 | 106 | -0.175887142 | USP17L5 | ubiquitin specific peptidase 17-like family member 5 [Source:HGNC Symbol;Acc:HGNC:37177] |
| ENSG00000227234 | HGLibA_46383 | 106 | -0.175887142 | SPANXB1 | SPANX family member B1 [Source:HGNC Symbol;Acc:HGNC:14329] |
| ENSG00000230430 | HGLibA_53257 | 106 | -0.175887142 | USP17L25 | ubiquitin specific peptidase 17-like family member 25 [Source:HGNC Symbol;Acc:HGNC:44452] |
| ENSG00000233932 | HGLibA_11758 | 106 | -0.175887142 | CTXN2 | cortexin 2 [Source:HGNC Symbol;Acc:HGNC:31109] |
| ENSG00000239382 | HGLibA_01660 | 106 | -0.175887142 | ALKBH6 | alkB homolog 6 [Source:HGNC Symbol;Acc:HGNC:28243] |
| ENSG00000239590 | HGLibA_33680 | 106 | -0.175887142 | OR1J4 | olfactory receptor family 1 subfamily J member 4 [Source:HGNC Symbol;Acc:HGNC:8211] |
| ENSG00000242366 | HGLibA_52896 | 106 | -0.175887142 | UGT1A8 | UDP glucuronosyltransferase family 1 member A8 [Source:HGNC Symbol;Acc:HGNC:12540] |
| ENSG00000243279 | HGLibA_38270 | 106 | -0.175887142 | PRAF2 | PRA1 domain family member 2 [Source:HGNC Symbol;Acc:HGNC:28911] |
| ENSG00000244734 | HGLibA_20914 | 106 | -0.175887142 | HBB | hemoglobin subunit beta [Source:HGNC Symbol;Acc:HGNC:4827] |
| ENSG00000251623 | HGLibA_16172 | 106 | -0.175887142 | FAM153B | - |
| ENSG00000253276 | HGLibA_07855 | 106 | -0.175887142 | CCDC71L | coiled-coil domain containing 71 like [Source:HGNC Symbol;Acc:HGNC:26685] |
| ENSG00000253305 | HGLibA_35564 | 106 | -0.175887142 | PCDHGB6 | 6 [Source:HGNC Symbol;Acc:HGNC:8713]" |
| ENSG00000257115 | HGLibA_33572 | 106 | -0.175887142 | OR11H12 | olfactory receptor family 11 subfamily H member 12 [Source:HGNC Symbol;Acc:HGNC:30738] |
| ENSG00000258289 | HGLibA_09673 | 106 | -0.175887142 | CHURC1 | churchill domain containing 1 [Source:HGNC Symbol;Acc:HGNC:20099] |
| ENSG00000258436 | HGLibA_41398 | 106 | -0.175887142 | RNASE12 | ribonuclease A family member 12 (inactive) [Source:HGNC Symbol;Acc:HGNC:24211] |
| ENSG00000259224 | HGLibA_45028 | 106 | -0.175887142 | SLC35G6 | solute carrier family 35 member G6 [Source:HGNC Symbol;Acc:HGNC:31351] |
| ENSG00000259571 | HGLibA_04542 | 106 | -0.175887142 | BLID | cell death inducer [Source:HGNC Symbol;Acc:HGNC:33495]" |
| ENSG00000263353 | HGLibA_37898 | 106 | -0.175887142 | PPIAL4A | peptidylprolyl isomerase A like 4A [Source:HGNC Symbol;Acc:HGNC:24369] |
| ENSG00000269335 | HGLibA_22926 | 106 | -0.175887142 | IKBKG | inhibitor of nuclear factor kappa B kinase subunit gamma [Source:HGNC Symbol;Acc:HGNC:5961] |
| ENSG00000271079 | HGLibA_11570 | 106 | -0.175887142 | CTAGE15 | CTAGE family member 15 [Source:HGNC Symbol;Acc:HGNC:37295] |
| ENSG00000272602 | HGLibA_56346 | 106 | -0.175887142 | ZNF595 | zinc finger protein 595 [Source:HGNC Symbol;Acc:HGNC:27196] |
| ENSG00000286237 | HGLibA_03052 | 106 | -0.175887142 | ARMCX5-GPRASP2 | - |
| FAM173B | HGLibA_16280 | 106 | -0.175887142 | FAM173B | - |
| H2AFB1 | HGLibA_20746 | 106 | -0.175887142 | H2AFB1 | - |
| H2AFB3 | HGLibA_20750 | 106 | -0.175887142 | H2AFB3 | - |
| hsa-let-7a-1 | HGLibA_57099 | 106 | -0.175887142 | hsa-let-7a-1 | - |
| hsa-mir-1302-1 | HGLibA_57684 | 106 | -0.175887142 | hsa-mir-1302-1 | - |
| hsa-mir-130b | HGLibA_57752 | 106 | -0.175887142 | hsa-mir-130b | - |
| hsa-mir-188 | HGLibA_58014 | 106 | -0.175887142 | hsa-mir-188 | - |
| hsa-mir-2278 | HGLibA_58316 | 106 | -0.175887142 | hsa-mir-2278 | - |
| hsa-mir-23c | HGLibA_58337 | 106 | -0.175887142 | hsa-mir-23c | - |
| hsa-mir-3130-1 | HGLibA_58571 | 106 | -0.175887142 | hsa-mir-3130-1 | - |
| hsa-mir-3160-1 | HGLibA_58715 | 106 | -0.175887142 | hsa-mir-3160-1 | - |
| hsa-mir-326 | HGLibA_58973 | 106 | -0.175887142 | hsa-mir-326 | - |
| hsa-mir-3713 | HGLibA_59366 | 106 | -0.175887142 | hsa-mir-3713 | - |
| hsa-mir-4278 | HGLibA_59814 | 106 | -0.175887142 | hsa-mir-4278 | - |
| hsa-mir-4425 | HGLibA_60089 | 106 | -0.175887142 | hsa-mir-4425 | - |
| hsa-mir-4441 | HGLibA_60167 | 106 | -0.175887142 | hsa-mir-4441 | - |
| hsa-mir-4778 | HGLibA_61220 | 106 | -0.175887142 | hsa-mir-4778 | - |
| hsa-mir-5091 | HGLibA_61524 | 106 | -0.175887142 | hsa-mir-5091 | - |
| hsa-mir-5187 | HGLibA_61638 | 106 | -0.175887142 | hsa-mir-5187 | - |
| hsa-mir-526b | HGLibA_61797 | 106 | -0.175887142 | hsa-mir-526b | - |
| hsa-mir-6820 | HGLibA_63456 | 106 | -0.175887142 | hsa-mir-6820 | - |
| hsa-mir-7111 | HGLibA_63810 | 106 | -0.175887142 | hsa-mir-7111 | - |
| hsa-mir-7846 | HGLibA_63975 | 106 | -0.175887142 | hsa-mir-7846 | - |
| hsa-mir-98 | HGLibA_64373 | 106 | -0.175887142 | hsa-mir-98 | - |
| KIAA0101 | HGLibA_24589 | 106 | -0.175887142 | KIAA0101 | - |
| LARGE | HGLibA_26026 | 106 | -0.175887142 | LARGE | - |
| LOC389895 | HGLibA_26931 | 106 | -0.175887142 | LOC389895 | - |
| MRE11A | HGLibA_29822 | 106 | -0.175887142 | MRE11A | - |
| RLTPR | HGLibA_41362 | 106 | -0.175887142 | RLTPR | - |
| C12orf74 | HGLibA_05293 | 105 | -0.184932485 | C12orf74 | - |
| C14orf2 | HGLibA_05358 | 105 | -0.184932485 | C14orf2 | - |
| C2orf54 | HGLibA_06150 | 105 | -0.184932485 | C2orf54 | - |
| C9orf62 | HGLibA_06747 | 105 | -0.184932485 | C9orf62 | - |
| ENSG00000005469 | HGLibA_11212 | 105 | -0.184932485 | CROT | carnitine O-octanoyltransferase [Source:HGNC Symbol;Acc:HGNC:2366] |
| ENSG00000008513 | HGLibA_47237 | 105 | -0.184932485 | ST3GAL1 | 3-sialyltransferase 1 [Source:HGNC Symbol;Acc:HGNC:10862]" |

| | | | | | |
|---|---|---|---|---|---|
| ENSG00000047230 | HGLibA_11670 | 105 | -0.184932485 | CTPS2 | CTP synthase 2 [Source:HGNC Symbol;Acc:HGNC:2520] |
| ENSG00000050327 | HGLibA_02870 | 105 | -0.184932485 | ARHGEF5 | Rho guanine nucleotide exchange factor 5 [Source:HGNC Symbol;Acc:HGNC:13209] |
| ENSG00000055955 | HGLibA_23813 | 105 | -0.184932485 | ITIH4 | inter-alpha-trypsin inhibitor heavy chain family member 4 [Source:HGNC Symbol;Acc:HGNC:6169] |
| ENSG00000059804 | HGLibA_44873 | 105 | -0.184932485 | SLC2A3 | solute carrier family 2 member 3 [Source:HGNC Symbol;Acc:HGNC:11007] |
| ENSG00000060566 | HGLibA_11099 | 105 | -0.184932485 | CREB3L3 | cAMP responsive element binding protein 3 like 3 [Source:HGNC Symbol;Acc:HGNC:18855] |
| ENSG00000063978 | HGLibA_41676 | 105 | -0.184932485 | RNF4 | ring finger protein 4 [Source:HGNC Symbol;Acc:HGNC:10067] |
| ENSG00000068650 | HGLibA_03554 | 105 | -0.184932485 | ATP11A | ATPase phospholipid transporting 11A [Source:HGNC Symbol;Acc:HGNC:13552] |
| ENSG00000073008 | HGLibA_39615 | 105 | -0.184932485 | PVR | poliovirus receptor [Source:HGNC Symbol;Acc:HGNC:9705] |
| ENSG00000074855 | HGLibA_02192 | 105 | -0.184932485 | ANO8 | anoctamin 8 [Source:HGNC Symbol;Acc:HGNC:29329] |
| ENSG00000075886 | HGLibA_52255 | 105 | -0.184932485 | TUBA3D | tubulin alpha 3d [Source:HGNC Symbol;Acc:HGNC:24071] |
| ENSG00000076662 | HGLibA_22500 | 105 | -0.184932485 | ICAM3 | intercellular adhesion molecule 3 [Source:HGNC Symbol;Acc:HGNC:5346] |
| ENSG00000089177 | HGLibA_24867 | 105 | -0.184932485 | KIF16B | kinesin family member 16B [Source:HGNC Symbol;Acc:HGNC:15869] |
| ENSG00000090382 | HGLibA_27783 | 105 | -0.184932485 | LYZ | lysozyme [Source:HGNC Symbol;Acc:HGNC:6740] |
| ENSG00000099797 | HGLibA_48947 | 105 | -0.184932485 | TECR | 3-enoyl-CoA reductase [Source:HGNC Symbol;Acc:HGNC:4551]" |
| ENSG00000100330 | HGLibA_30427 | 105 | -0.184932485 | MTMR3 | myotubularin related protein 3 [Source:HGNC Symbol;Acc:HGNC:7451] |
| ENSG00000101361 | HGLibA_32312 | 105 | -0.184932485 | NOP56 | NOP56 ribonucleoprotein [Source:HGNC Symbol;Acc:HGNC:15911] |
| ENSG00000102387 | HGLibA_48256 | 105 | -0.184932485 | TAF7L | TATA-box binding protein associated factor 7 like [Source:HGNC Symbol;Acc:HGNC:11548] |
| ENSG00000104290 | HGLibA_18269 | 105 | -0.184932485 | FZD3 | frizzled class receptor 3 [Source:HGNC Symbol;Acc:HGNC:4041] |
| ENSG00000104613 | HGLibA_23408 | 105 | -0.184932485 | INTS10 | integrator complex subunit 10 [Source:HGNC Symbol;Acc:HGNC:25548] |
| ENSG00000104946 | HGLibA_48487 | 105 | -0.184932485 | TBC1D17 | TBC1 domain family member 17 [Source:HGNC Symbol;Acc:HGNC:25699] |
| ENSG00000105516 | HGLibA_12399 | 105 | -0.184932485 | DBP | D-box binding PAR bZIP transcription factor [Source:HGNC Symbol;Acc:HGNC:2697] |
| ENSG00000105662 | HGLibA_11229 | 105 | -0.184932485 | CRTC1 | CREB regulated transcription coactivator 1 [Source:HGNC Symbol;Acc:HGNC:16062] |
| ENSG00000105697 | HGLibA_20822 | 105 | -0.184932485 | HAMP | hepcidin antimicrobial peptide [Source:HGNC Symbol;Acc:HGNC:15598] |
| ENSG00000105808 | HGLibA_40304 | 105 | -0.184932485 | RASA4 | RAS p21 protein activator 4 [Source:HGNC Symbol;Acc:HGNC:23181] |
| ENSG00000110200 | HGLibA_01873 | 105 | -0.184932485 | ANAPC15 | anaphase promoting complex subunit 15 [Source:HGNC Symbol;Acc:HGNC:24531] |
| ENSG00000110237 | HGLibA_02825 | 105 | -0.184932485 | ARHGEF17 | Rho guanine nucleotide exchange factor 17 [Source:HGNC Symbol;Acc:HGNC:21726] |
| ENSG00000111218 | HGLibA_38637 | 105 | -0.184932485 | PRMT8 | protein arginine methyltransferase 8 [Source:HGNC Symbol;Acc:HGNC:5188] |
| ENSG00000111726 | HGLibA_10179 | 105 | -0.184932485 | CMAS | cytidine monophosphate N-acetylneuraminic acid synthetase [Source:HGNC Symbol;Acc:HGNC:18290] |
| ENSG00000112280 | HGLibA_10620 | 105 | -0.184932485 | COL9A1 | collagen type IX alpha 1 chain [Source:HGNC Symbol;Acc:HGNC:2217] |
| ENSG00000112494 | HGLibA_53034 | 105 | -0.184932485 | UNC93A | unc-93 homolog A [Source:HGNC Symbol;Acc:HGNC:12570] |
| ENSG00000115520 | HGLibA_10746 | 105 | -0.184932485 | COQ10B | coenzyme Q10B [Source:HGNC Symbol;Acc:HGNC:25819] |
| ENSG00000116514 | HGLibA_41605 | 105 | -0.184932485 | RNF19B | ring finger protein 19B [Source:HGNC Symbol;Acc:HGNC:26886] |
| ENSG00000116809 | HGLibA_54829 | 105 | -0.184932485 | ZBTB17 | zinc finger and BTB domain containing 17 [Source:HGNC Symbol;Acc:HGNC:12936] |
| ENSG00000120235 | HGLibA_22687 | 105 | -0.184932485 | IFNA6 | interferon alpha 6 [Source:HGNC Symbol;Acc:HGNC:5427] |
| ENSG00000120686 | HGLibA_52853 | 105 | -0.184932485 | UFM1 | ubiquitin fold modifier 1 [Source:HGNC Symbol;Acc:HGNC:20597] |
| ENSG00000120805 | HGLibA_02920 | 105 | -0.184932485 | ARL1 | ADP ribosylation factor like GTPase 1 [Source:HGNC Symbol;Acc:HGNC:692] |
| ENSG00000121764 | HGLibA_20988 | 105 | -0.184932485 | HCRTR1 | hypocretin receptor 1 [Source:HGNC Symbol;Acc:HGNC:4848] |
| ENSG00000121964 | HGLibA_20513 | 105 | -0.184932485 | GTDC1 | glycosyltransferase like domain containing 1 [Source:HGNC Symbol;Acc:HGNC:20887] |
| ENSG00000122574 | HGLibA_54311 | 105 | -0.184932485 | WIPF3 | WAS/WASL interacting protein family member 3 [Source:HGNC Symbol;Acc:HGNC:22004] |
| ENSG00000123561 | HGLibA_43529 | 105 | -0.184932485 | SERPINA7 | serpin family A member 7 [Source:HGNC Symbol;Acc:HGNC:11583] |
| ENSG00000124257 | HGLibA_31728 | 105 | -0.184932485 | NEURL2 | neuralized E3 ubiquitin protein ligase 2 [Source:HGNC Symbol;Acc:HGNC:16156] |
| ENSG00000124574 | HGLibA_00161 | 105 | -0.184932485 | ABCC10 | ATP binding cassette subfamily C member 10 [Source:HGNC Symbol;Acc:HGNC:52] |
| ENSG00000127241 | HGLibA_28394 | 105 | -0.184932485 | MASP1 | mannan binding lectin serine peptidase 1 [Source:HGNC Symbol;Acc:HGNC:6901] |
| ENSG00000130309 | HGLibA_10642 | 105 | -0.184932485 | COLGALT1 | collagen beta(1-O)galactosyltransferase 1 [Source:HGNC Symbol;Acc:HGNC:26182] |
| ENSG00000131375 | HGLibA_07174 | 105 | -0.184932485 | CAPN7 | calpain 7 [Source:HGNC Symbol;Acc:HGNC:1484] |
| ENSG00000132661 | HGLibA_33175 | 105 | -0.184932485 | NXT1 | nuclear transport factor 2 like export factor 1 [Source:HGNC Symbol;Acc:HGNC:15913] |
| ENSG00000133107 | HGLibA_51735 | 105 | -0.184932485 | TRPC4 | transient receptor potential cation channel subfamily C member 4 [Source:HGNC Symbol;Acc:HGNC:12336] |
| ENSG00000133121 | HGLibA_47355 | 105 | -0.184932485 | STARD13 | StAR related lipid transfer domain containing 13 [Source:HGNC Symbol;Acc:HGNC:19164] |
| ENSG00000133858 | HGLibA_55167 | 105 | -0.184932485 | ZFC3H1 | zinc finger C3H1-type containing [Source:HGNC Symbol;Acc:HGNC:28328] |
| ENSG00000135063 | HGLibA_16353 | 105 | -0.184932485 | FAM189A2 | family with sequence similarity 189 member A2 [Source:HGNC Symbol;Acc:HGNC:24820] |
| ENSG00000135541 | HGLibA_01299 | 105 | -0.184932485 | AHI1 | Abelson helper integration site 1 [Source:HGNC Symbol;Acc:HGNC:21575] |
| ENSG00000135698 | HGLibA_29725 | 105 | -0.184932485 | MPHOSPH6 | M-phase phosphoprotein 6 [Source:HGNC Symbol;Acc:HGNC:7214] |
| ENSG00000137673 | HGLibA_29537 | 105 | -0.184932485 | MMP7 | matrix metallopeptidase 7 [Source:HGNC Symbol;Acc:HGNC:7174] |
| ENSG00000138050 | HGLibA_49417 | 105 | -0.184932485 | THUMPD2 | THUMP domain containing 2 [Source:HGNC Symbol;Acc:HGNC:14890] |
| ENSG00000139437 | HGLibA_48809 | 105 | -0.184932485 | TCHP | trichoplein keratin filament binding [Source:HGNC Symbol;Acc:HGNC:28135] |
| ENSG00000139644 | HGLibA_49744 | 105 | -0.184932485 | TMBIM6 | transmembrane BAX inhibitor motif containing 6 [Source:HGNC Symbol;Acc:HGNC:11723] |
| ENSG00000140945 | HGLibA_08641 | 105 | -0.184932485 | CDH13 | cadherin 13 [Source:HGNC Symbol;Acc:HGNC:1753] |
| ENSG00000141279 | HGLibA_32432 | 105 | -0.184932485 | NPEPPS | aminopeptidase puromycin sensitive [Source:HGNC Symbol;Acc:HGNC:7900] |
| ENSG00000142512 | HGLibA_44127 | 105 | -0.184932485 | SIGLEC10 | sialic acid binding Ig like lectin 10 [Source:HGNC Symbol;Acc:HGNC:15620] |
| ENSG00000143061 | HGLibA_22894 | 105 | -0.184932485 | IGSF3 | immunoglobulin superfamily member 3 [Source:HGNC Symbol;Acc:HGNC:5950] |
| ENSG00000143452 | HGLibA_21887 | 105 | -0.184932485 | HORMAD1 | HORMA domain containing 1 [Source:HGNC Symbol;Acc:HGNC:25245] |
| ENSG00000145555 | HGLibA_30801 | 105 | -0.184932485 | MYO10 | myosin X [Source:HGNC Symbol;Acc:HGNC:7593] |
| ENSG00000155868 | HGLibA_28815 | 105 | -0.184932485 | MED7 | mediator complex subunit 7 [Source:HGNC Symbol;Acc:HGNC:2378] |
| ENSG00000156097 | HGLibA_20063 | 105 | -0.184932485 | GPR61 | G protein-coupled receptor 61 [Source:HGNC Symbol;Acc:HGNC:13300] |
| ENSG00000160014 | HGLibA_07028 | 105 | -0.184932485 | CALM3 | calmodulin 3 [Source:HGNC Symbol;Acc:HGNC:1449] |
| ENSG00000162458 | HGLibA_16941 | 105 | -0.184932485 | FBLIM1 | filamin binding LIM protein 1 [Source:HGNC Symbol;Acc:HGNC:24686] |
| ENSG00000162664 | HGLibA_55807 | 105 | -0.184932485 | ZNF326 | zinc finger protein 326 [Source:HGNC Symbol;Acc:HGNC:14104] |
| ENSG00000162910 | HGLibA_30023 | 105 | -0.184932485 | MRPL55 | mitochondrial ribosomal protein L55 [Source:HGNC Symbol;Acc:HGNC:16686] |
| ENSG00000163207 | HGLibA_23879 | 105 | -0.184932485 | IVL | involucrin [Source:HGNC Symbol;Acc:HGNC:6187] |
| ENSG00000163359 | HGLibA_10601 | 105 | -0.184932485 | COL6A3 | collagen type VI alpha 3 chain [Source:HGNC Symbol;Acc:HGNC:2213] |
| ENSG00000163536 | HGLibA_43598 | 105 | -0.184932485 | SERPINI1 | serpin family I member 1 [Source:HGNC Symbol;Acc:HGNC:8943] |
| ENSG00000163818 | HGLibA_27799 | 105 | -0.184932485 | LZTFL1 | leucine zipper transcription factor like 1 [Source:HGNC Symbol;Acc:HGNC:6741] |
| ENSG00000164849 | HGLibA_19917 | 105 | -0.184932485 | GPR146 | G protein-coupled receptor 146 [Source:HGNC Symbol;Acc:HGNC:21718] |
| ENSG00000164877 | HGLibA_29216 | 105 | -0.184932485 | MICALL2 | MICAL like 2 [Source:HGNC Symbol;Acc:HGNC:29672] |
| ENSG00000165409 | HGLibA_51859 | 105 | -0.184932485 | TSHR | thyroid stimulating hormone receptor [Source:HGNC Symbol;Acc:HGNC:12373] |
| ENSG00000167359 | HGLibA_34114 | 105 | -0.184932485 | OR51I1 | olfactory receptor family 51 subfamily I member 1 [Source:HGNC Symbol;Acc:HGNC:15200] |
| ENSG00000168546 | HGLibA_18969 | 105 | -0.184932485 | GFRA2 | GDNF family receptor alpha 2 [Source:HGNC Symbol;Acc:HGNC:4244] |
| ENSG00000169777 | HGLibA_48363 | 105 | -0.184932485 | TAS2R1 | taste 2 receptor member 1 [Source:HGNC Symbol;Acc:HGNC:14909] |
| ENSG00000170190 | HGLibA_44443 | 105 | -0.184932485 | SLC16A5 | solute carrier family 16 member 5 [Source:HGNC Symbol;Acc:HGNC:10926] |
| ENSG00000171056 | HGLibA_46272 | 105 | -0.184932485 | SOX7 | SRY-box 7 [Source:HGNC Symbol;Acc:HGNC:18196] |
| ENSG00000171262 | HGLibA_16813 | 105 | -0.184932485 | FAM98B | family with sequence similarity 98 member B [Source:HGNC Symbol;Acc:HGNC:26773] |
| ENSG00000171478 | HGLibA_46335 | 105 | -0.184932485 | SPACA5B | sperm acrosome associated 5B [Source:HGNC Symbol;Acc:HGNC:19142] |
| ENSG00000171551 | HGLibA_14388 | 105 | -0.184932485 | ECEL1 | endothelin converting enzyme like 1 [Source:HGNC Symbol;Acc:HGNC:3147] |
| ENSG00000171914 | HGLibA_49638 | 105 | -0.184932485 | TLN2 | talin 2 [Source:HGNC Symbol;Acc:HGNC:15447] |
| ENSG00000172005 | HGLibA_28021 | 105 | -0.184932485 | MAL | T cell differentiation protein [Source:HGNC Symbol;Acc:HGNC:6817]" |
| ENSG00000173080 | HGLibA_42503 | 105 | -0.184932485 | RXFP4 | relaxin family peptide/INSL5 receptor 4 [Source:HGNC Symbol;Acc:HGNC:14666] |
| ENSG00000174130 | HGLibA_49659 | 105 | -0.184932485 | TLR6 | toll like receptor 6 [Source:HGNC Symbol;Acc:HGNC:16711] |
| ENSG00000174606 | HGLibA_01898 | 105 | -0.184932485 | ANGEL2 | angel homolog 2 [Source:HGNC Symbol;Acc:HGNC:30534] |
| ENSG00000175482 | HGLibA_37501 | 105 | -0.184932485 | POLD4 | accessory subunit [Source:HGNC Symbol;Acc:HGNC:14106]" |
| ENSG00000176083 | HGLibA_56532 | 105 | -0.184932485 | ZNF683 | zinc finger protein 683 [Source:HGNC Symbol;Acc:HGNC:28495] |
| ENSG00000178607 | HGLibA_15513 | 105 | -0.184932485 | ERN1 | endoplasmic reticulum to nucleus signaling 1 [Source:HGNC Symbol;Acc:HGNC:3449] |
| ENSG00000180938 | HGLibA_56281 | 105 | -0.184932485 | ZNF572 | zinc finger protein 572 [Source:HGNC Symbol;Acc:HGNC:26758] |
| ENSG00000181408 | HGLibA_53466 | 105 | -0.184932485 | UTS2R | urotensin 2 receptor [Source:HGNC Symbol;Acc:HGNC:4468] |
| ENSG00000182645 | HGLibA_07698 | 105 | -0.184932485 | CCDC172 | coiled-coil domain containing 172 [Source:HGNC Symbol;Acc:HGNC:30524] |
| ENSG00000183303 | HGLibA_34346 | 105 | -0.184932485 | OR5P2 | olfactory receptor family 5 subfamily P member 2 [Source:HGNC Symbol;Acc:HGNC:14783] |
| ENSG00000183795 | HGLibA_04746 | 105 | -0.184932485 | BPY2B | basic charge Y-linked 2B [Source:HGNC Symbol;Acc:HGNC:25449] |
| ENSG00000184022 | HGLibA_33854 | 105 | -0.184932485 | OR2T10 | olfactory receptor family 2 subfamily T member 10 [Source:HGNC Symbol;Acc:HGNC:19573] |
| ENSG00000184492 | HGLibA_17839 | 105 | -0.184932485 | FOXD4L1 | forkhead box D4 like 1 [Source:HGNC Symbol;Acc:HGNC:18521] |
| ENSG00000185775 | HGLibA_46482 | 105 | -0.184932485 | SPATA31A6 | SPATA31 subfamily A member 6 [Source:HGNC Symbol;Acc:HGNC:32006] |
| ENSG00000185798 | HGLibA_54120 | 105 | -0.184932485 | WDR53 | WD repeat domain 53 [Source:HGNC Symbol;Acc:HGNC:28786] |
| ENSG00000185813 | HGLibA_35707 | 105 | -0.184932485 | PCYT2 | ethanolamine [Source:HGNC Symbol;Acc:HGNC:8756]" |
| ENSG00000186075 | HGLibA_56964 | 105 | -0.184932485 | ZPBP2 | zona pellucida binding protein 2 [Source:HGNC Symbol;Acc:HGNC:20678] |
| ENSG00000186907 | HGLibA_42418 | 105 | -0.184932485 | RTN4RL2 | reticulon 4 receptor like 2 [Source:HGNC Symbol;Acc:HGNC:23053] |
| ENSG00000187546 | HGLibA_01201 | 105 | -0.184932485 | AGMO | alkylglycerol monooxygenase [Source:HGNC Symbol;Acc:HGNC:33784] |
| ENSG00000196187 | HGLibA_50475 | 105 | -0.184932485 | TMEM63A | transmembrane protein 63A [Source:HGNC Symbol;Acc:HGNC:29118] |
| ENSG00000196498 | HGLibA_31359 | 105 | -0.184932485 | NCOR2 | nuclear receptor corepressor 2 [Source:HGNC Symbol;Acc:HGNC:7673] |
| ENSG00000196923 | HGLibA_35922 | 105 | -0.184932485 | PDLIM7 | PDZ and LIM domain 7 [Source:HGNC Symbol;Acc:HGNC:22958] |
| ENSG00000197891 | HGLibA_44543 | 105 | -0.184932485 | SLC22A12 | solute carrier family 22 member 12 [Source:HGNC Symbol;Acc:HGNC:17989] |
| ENSG00000197893 | HGLibA_32643 | 105 | -0.184932485 | NRAP | nebulin related anchoring protein [Source:HGNC Symbol;Acc:HGNC:7988] |
| ENSG00000198033 | HGLibA_52253 | 105 | -0.184932485 | TUBA3C | tubulin alpha 3c [Source:HGNC Symbol;Acc:HGNC:12408] |
| ENSG00000198300 | HGLibA_36029 | 105 | -0.184932485 | PEG3 | paternally expressed 3 [Source:HGNC Symbol;Acc:HGNC:8826] |
| ENSG00000198736 | HGLibA_30241 | 105 | -0.184932485 | MSRB1 | methionine sulfoxide reductase B1 [Source:HGNC Symbol;Acc:HGNC:14133] |
| ENSG00000198829 | HGLibA_47704 | 105 | -0.184932485 | SUCNR1 | succinate receptor 1 [Source:HGNC Symbol;Acc:HGNC:4542] |
| ENSG00000203797 | HGLibA_12627 | 105 | -0.184932485 | DDO | D-aspartate oxidase [Source:HGNC Symbol;Acc:HGNC:2727] |
| ENSG00000204120 | HGLibA_19066 | 105 | -0.184932485 | GIGYF2 | GRB10 interacting GYF protein 2 [Source:HGNC Symbol;Acc:HGNC:11960] |
| ENSG00000204217 | HGLibA_04634 | 105 | -0.184932485 | BMPR2 | bone morphogenetic protein receptor type 2 [Source:HGNC Symbol;Acc:HGNC:1078] |
| ENSG00000205364 | HGLibA_30294 | 105 | -0.184932485 | MT1M | metallothionein 1M [Source:HGNC Symbol;Acc:HGNC:14296] |
| ENSG00000205549 | HGLibA_06780 | 105 | -0.184932485 | C9orf92 | chromosome 9 open reading frame 92 [Source:HGNC Symbol;Acc:HGNC:19054] |
| ENSG00000221909 | HGLibA_16410 | 105 | -0.184932485 | FAM200A | family with sequence similarity 200 member A [Source:HGNC Symbol;Acc:HGNC:25401] |
| ENSG00000242259 | HGLibA_06075 | 105 | -0.184932485 | C22orf39 | chromosome 22 open reading frame 39 [Source:HGNC Symbol;Acc:HGNC:27012] |
| ENSG00000243543 | HGLibA_54271 | 105 | -0.184932485 | WFDC6 | WAP four-disulfide core domain 6 [Source:HGNC Symbol;Acc:HGNC:16164] |
| ENSG00000243646 | HGLibA_22950 | 105 | -0.184932485 | IL10RB | interleukin 10 receptor subunit beta [Source:HGNC Symbol;Acc:HGNC:5965] |
| ENSG00000261236 | HGLibA_04696 | 105 | -0.184932485 | BOP1 | block of proliferation 1 [Source:HGNC Symbol;Acc:HGNC:15519] |
| ENSG00000261934 | HGLibA_35546 | 105 | -0.184932485 | PCDHGA9 | 9 [Source:HGNC Symbol;Acc:HGNC:8707]" |
| ENSG00000268221 | HGLibA_33426 | 105 | -0.184932485 | OPN1MW | medium wave sensitive [Source:HGNC Symbol;Acc:HGNC:4206]" |
| ENSG00000269405 | HGLibA_33128 | 105 | -0.184932485 | NXF2 | nuclear RNA export factor 2 [Source:HGNC Symbol;Acc:HGNC:8072] |
| ENSG00000276045 | HGLibA_34588 | 105 | -0.184932485 | ORAI1 | ORAI calcium release-activated calcium modulator 1 [Source:HGNC Symbol;Acc:HGNC:25896] |
| ENSG00000286237 | HGLibA_03053 | 105 | -0.184932485 | ARMCX5-GPRASP2 | - |
| FAM69A | HGLibA_16678 | 105 | -0.184932485 | FAM69A | - |
| HIST1H4H | HGLibA_21508 | 105 | -0.184932485 | HIST1H4H | - |
| hsa-let-7f-2 | HGLibA_57130 | 105 | -0.184932485 | hsa-let-7f-2 | - |
| hsa-mir-1208 | HGLibA_57285 | 105 | -0.184932485 | hsa-mir-1208 | - |
| hsa-mir-1290 | HGLibA_57636 | 105 | -0.184932485 | hsa-mir-1290 | - |
| hsa-mir-1295b | HGLibA_57659 | 105 | -0.184932485 | hsa-mir-1295b | - |
| hsa-mir-1471 | HGLibA_57869 | 105 | -0.184932485 | hsa-mir-1471 | - |

| | | | | | |
|---|---|---|---|---|---|
| hsa-mir-185 | HGLibA_58001 | 105 | -0.184932485 | hsa-mir-185 | - |
| hsa-mir-302c | HGLibA_58444 | 105 | -0.184932485 | hsa-mir-302c | - |
| hsa-mir-3940 | HGLibA_59622 | 105 | -0.184932485 | hsa-mir-3940 | - |
| hsa-mir-4318 | HGLibA_59992 | 105 | -0.184932485 | hsa-mir-4318 | - |
| hsa-mir-4420 | HGLibA_60069 | 105 | -0.184932485 | hsa-mir-4420 | - |
| hsa-mir-4667 | HGLibA_60784 | 105 | -0.184932485 | hsa-mir-4667 | - |
| hsa-mir-4762 | HGLibA_61144 | 105 | -0.184932485 | hsa-mir-4762 | - |
| hsa-mir-5701-1 | HGLibA_62341 | 105 | -0.184932485 | hsa-mir-5701-1 | - |
| hsa-mir-6129 | HGLibA_62662 | 105 | -0.184932485 | hsa-mir-6129 | - |
| hsa-mir-620 | HGLibA_62720 | 105 | -0.184932485 | hsa-mir-620 | - |
| hsa-mir-664a | HGLibA_62995 | 105 | -0.184932485 | hsa-mir-664a | - |
| hsa-mir-6716 | HGLibA_63025 | 105 | -0.184932485 | hsa-mir-6716 | - |
| hsa-mir-6858 | HGLibA_63604 | 105 | -0.184932485 | hsa-mir-6858 | - |
| hsa-mir-92b | HGLibA_64297 | 105 | -0.184932485 | hsa-mir-92b | - |
| LOC388849 | HGLibA_26926 | 105 | -0.184932485 | LOC388849 | - |
| NOTCH2NL | HGLibA_32347 | 105 | -0.184932485 | NOTCH2NL | - |
| PAPD5 | HGLibA_35125 | 105 | -0.184932485 | PAPD5 | - |
| SELT | HGLibA_43312 | 105 | -0.184932485 | SELT | - |
| SPATA31A4 | HGLibA_46476 | 105 | -0.184932485 | SPATA31A4 | - |
| ADCK4 | HGLibA_00900 | 104 | -0.193977828 | ADCK4 | - |
| C10orf32 | HGLibA_05067 | 104 | -0.193977828 | C10orf32 | - |
| C18orf42 | HGLibA_05616 | 104 | -0.193977828 | C18orf42 | - |
| CNTD2 | HGLibA_10390 | 104 | -0.193977828 | CNTD2 | - |
| CXorf40A | HGLibA_11924 | 104 | -0.193977828 | CXorf40A | - |
| DFNA5 | HGLibA_13026 | 104 | -0.193977828 | DFNA5 | - |
| ENSG00000051596 | HGLibA_49369 | 104 | -0.193977828 | THOC3 | THO complex 3 [Source:HGNC Symbol;Acc:HGNC:19072] |
| ENSG00000054118 | HGLibA_49391 | 104 | -0.193977828 | THRAP3 | thyroid hormone receptor associated protein 3 [Source:HGNC Symbol;Acc:HGNC:22964] |
| ENSG00000063127 | HGLibA_45306 | 104 | -0.193977828 | SLC6A16 | solute carrier family 6 member 16 [Source:HGNC Symbol;Acc:HGNC:13622] |
| ENSG00000065609 | HGLibA_45887 | 104 | -0.193977828 | SNAP91 | synaptosome associated protein 91 [Source:HGNC Symbol;Acc:HGNC:14986] |
| ENSG00000072682 | HGLibA_34903 | 104 | -0.193977828 | P4HA2 | prolyl 4-hydroxylase subunit alpha 2 [Source:HGNC Symbol;Acc:HGNC:8547] |
| ENSG00000073282 | HGLibA_51092 | 104 | -0.193977828 | TP63 | tumor protein p63 [Source:HGNC Symbol;Acc:HGNC:15979] |
| ENSG00000075223 | HGLibA_43326 | 104 | -0.193977828 | SEMA3C | semaphorin 3C [Source:HGNC Symbol;Acc:HGNC:10725] |
| ENSG00000082512 | HGLibA_51261 | 104 | -0.193977828 | TRAF5 | TNF receptor associated factor 5 [Source:HGNC Symbol;Acc:HGNC:12035] |
| ENSG00000090615 | HGLibA_19598 | 104 | -0.193977828 | GOLGA3 | golgin A3 [Source:HGNC Symbol;Acc:HGNC:4426] |
| ENSG00000097021 | HGLibA_00488 | 104 | -0.193977828 | ACOT7 | acyl-CoA thioesterase 7 [Source:HGNC Symbol;Acc:HGNC:24157] |
| ENSG00000100142 | HGLibA_37578 | 104 | -0.193977828 | POLR2F | RNA polymerase II subunit F [Source:HGNC Symbol;Acc:HGNC:9193] |
| ENSG00000100290 | HGLibA_04503 | 104 | -0.193977828 | BIK | BCL2 interacting killer [Source:HGNC Symbol;Acc:HGNC:1051] |
| ENSG00000100842 | HGLibA_14613 | 104 | -0.193977828 | EFS | embryonal Fyn-associated substrate [Source:HGNC Symbol;Acc:HGNC:16898] |
| ENSG00000100979 | HGLibA_37246 | 104 | -0.193977828 | PLTP | phospholipid transfer protein [Source:HGNC Symbol;Acc:HGNC:9093] |
| ENSG00000102893 | HGLibA_36409 | 104 | -0.193977828 | PHKB | phosphorylase kinase regulatory subunit beta [Source:HGNC Symbol;Acc:HGNC:8927] |
| ENSG00000103202 | HGLibA_32162 | 104 | -0.193977828 | NME4 | NME/NM23 nucleoside diphosphate kinase 4 [Source:HGNC Symbol;Acc:HGNC:7852] |
| ENSG00000103512 | HGLibA_32290 | 104 | -0.193977828 | NOMO1 | NODAL modulator 1 [Source:HGNC Symbol;Acc:HGNC:30060] |
| ENSG00000105327 | HGLibA_04198 | 104 | -0.193977828 | BBC3 | BCL2 binding component 3 [Source:HGNC Symbol;Acc:HGNC:17868] |
| ENSG00000105559 | HGLibA_37070 | 104 | -0.193977828 | PLEKHA4 | pleckstrin homology domain containing A4 [Source:HGNC Symbol;Acc:HGNC:14339] |
| ENSG00000106608 | HGLibA_53139 | 104 | -0.193977828 | URGCP | upregulator of cell proliferation [Source:HGNC Symbol;Acc:HGNC:30890] |
| ENSG00000107290 | HGLibA_43675 | 104 | -0.193977828 | SETX | senataxin [Source:HGNC Symbol;Acc:HGNC:445] |
| ENSG00000108759 | HGLibA_25477 | 104 | -0.193977828 | KRT32 | keratin 32 [Source:HGNC Symbol;Acc:HGNC:6449] |
| ENSG00000109016 | HGLibA_13147 | 104 | -0.193977828 | DHRS7B | dehydrogenase/reductase 7B [Source:HGNC Symbol;Acc:HGNC:24547] |
| ENSG00000109046 | HGLibA_54416 | 104 | -0.193977828 | WSB1 | WD repeat and SOCS box containing 1 [Source:HGNC Symbol;Acc:HGNC:19221] |
| ENSG00000111087 | HGLibA_19247 | 104 | -0.193977828 | GLI1 | GLI family zinc finger 1 [Source:HGNC Symbol;Acc:HGNC:4317] |
| ENSG00000111674 | HGLibA_15142 | 104 | -0.193977828 | ENO2 | enolase 2 [Source:HGNC Symbol;Acc:HGNC:3353] |
| ENSG00000111707 | HGLibA_47710 | 104 | -0.193977828 | SUDS3 | SIN3A corepressor complex component [Source:HGNC Symbol;Acc:HGNC:29545]" |
| ENSG00000113303 | HGLibA_04981 | 104 | -0.193977828 | BTNL8 | butyrophilin like 8 [Source:HGNC Symbol;Acc:HGNC:26131] |
| ENSG00000115363 | HGLibA_15671 | 104 | -0.193977828 | EVA1A | regulator of programmed cell death [Source:HGNC Symbol;Acc:HGNC:25816]" |
| ENSG00000115365 | HGLibA_26004 | 104 | -0.193977828 | LANCL1 | LanC like 1 [Source:HGNC Symbol;Acc:HGNC:6508] |
| ENSG00000116741 | HGLibA_41080 | 104 | -0.193977828 | RGS2 | regulator of G protein signaling 2 [Source:HGNC Symbol;Acc:HGNC:9998] |
| ENSG00000117868 | HGLibA_15608 | 104 | -0.193977828 | ESYT2 | extended synaptotagmin 2 [Source:HGNC Symbol;Acc:HGNC:22211] |
| ENSG00000119787 | HGLibA_03518 | 104 | -0.193977828 | ATL2 | atlastin GTPase 2 [Source:HGNC Symbol;Acc:HGNC:24047] |
| ENSG00000121486 | HGLibA_51680 | 104 | -0.193977828 | TRMT1L | tRNA methyltransferase 1 like [Source:HGNC Symbol;Acc:HGNC:16782] |
| ENSG00000121577 | HGLibA_37698 | 104 | -0.193977828 | POPDC2 | popeye domain containing 2 [Source:HGNC Symbol;Acc:HGNC:17648] |
| ENSG00000122477 | HGLibA_27340 | 104 | -0.193977828 | LRRC39 | leucine rich repeat containing 39 [Source:HGNC Symbol;Acc:HGNC:28228] |
| ENSG00000122642 | HGLibA_17592 | 104 | -0.193977828 | FKBP9 | FK506 binding protein 9 [Source:HGNC Symbol;Acc:HGNC:3725] |
| ENSG00000125304 | HGLibA_49724 | 104 | -0.193977828 | TM9SF2 | transmembrane 9 superfamily member 2 [Source:HGNC Symbol;Acc:HGNC:11865] |
| ENSG00000125637 | HGLibA_39021 | 104 | -0.193977828 | PSD4 | pleckstrin and Sec7 domain containing 4 [Source:HGNC Symbol;Acc:HGNC:19096] |
| ENSG00000126467 | HGLibA_51871 | 104 | -0.193977828 | TSKS | testis specific serine kinase substrate [Source:HGNC Symbol;Acc:HGNC:30719] |
| ENSG00000126856 | HGLibA_38397 | 104 | -0.193977828 | PRDM7 | PR/SET domain 7 [Source:HGNC Symbol;Acc:HGNC:9351] |
| ENSG00000127580 | HGLibA_54049 | 104 | -0.193977828 | WDR24 | WD repeat domain 24 [Source:HGNC Symbol;Acc:HGNC:20852] |
| ENSG00000129255 | HGLibA_29712 | 104 | -0.193977828 | MPDU1 | mannose-P-dolichol utilization defect 1 [Source:HGNC Symbol;Acc:HGNC:7207] |
| ENSG00000130037 | HGLibA_24138 | 104 | -0.193977828 | KCNA5 | potassium voltage-gated channel subfamily A member 5 [Source:HGNC Symbol;Acc:HGNC:6224] |
| ENSG00000131470 | HGLibA_39136 | 104 | -0.193977828 | PSMC3IP | PSMC3 interacting protein [Source:HGNC Symbol;Acc:HGNC:17928] |
| ENSG00000131503 | HGLibA_01963 | 104 | -0.193977828 | ANKHD1 | ankyrin repeat and KH domain containing 1 [Source:HGNC Symbol;Acc:HGNC:24714] |
| ENSG00000132300 | HGLibA_39266 | 104 | -0.193977828 | PTCD3 | pentatricopeptide repeat domain 3 [Source:HGNC Symbol;Acc:HGNC:24717] |
| ENSG00000133138 | HGLibA_48570 | 104 | -0.193977828 | TBC1D8B | TBC1 domain family member 8B [Source:HGNC Symbol;Acc:HGNC:24715] |
| ENSG00000134830 | HGLibA_06356 | 104 | -0.193977828 | C5AR2 | complement component 5a receptor 2 [Source:HGNC Symbol;Acc:HGNC:4527] |
| ENSG00000136487 | HGLibA_19032 | 104 | -0.193977828 | GH2 | growth hormone 2 [Source:HGNC Symbol;Acc:HGNC:4262] |
| ENSG00000136643 | HGLibA_42181 | 104 | -0.193977828 | RPS6KC1 | ribosomal protein S6 kinase C1 [Source:HGNC Symbol;Acc:HGNC:10439] |
| ENSG00000137100 | HGLibA_12549 | 104 | -0.193977828 | DCTN3 | dynactin subunit 3 [Source:HGNC Symbol;Acc:HGNC:2713] |
| ENSG00000137210 | HGLibA_49982 | 104 | -0.193977828 | TMEM14B | transmembrane protein 14B [Source:HGNC Symbol;Acc:HGNC:21384] |
| ENSG00000137210 | HGLibA_49981 | 104 | -0.193977828 | TMEM14B | transmembrane protein 14B [Source:HGNC Symbol;Acc:HGNC:21384] |
| ENSG00000137675 | HGLibA_29529 | 104 | -0.193977828 | MMP27 | matrix metallopeptidase 27 [Source:HGNC Symbol;Acc:HGNC:14250] |
| ENSG00000137766 | HGLibA_52999 | 104 | -0.193977828 | UNC13C | unc-13 homolog C [Source:HGNC Symbol;Acc:HGNC:23149] |
| ENSG00000137843 | HGLibA_35051 | 104 | -0.193977828 | PAK6 | p21 (RAC1) activated kinase 6 [Source:HGNC Symbol;Acc:HGNC:16061] |
| ENSG00000138735 | HGLibA_35803 | 104 | -0.193977828 | PDE5A | phosphodiesterase 5A [Source:HGNC Symbol;Acc:HGNC:8784] |
| ENSG00000140279 | HGLibA_14099 | 104 | -0.193977828 | DUOX2 | dual oxidase 2 [Source:HGNC Symbol;Acc:HGNC:13273] |
| ENSG00000140993 | HGLibA_49480 | 104 | -0.193977828 | TIGD7 | tigger transposable element derived 7 [Source:HGNC Symbol;Acc:HGNC:18331] |
| ENSG00000141002 | HGLibA_48781 | 104 | -0.193977828 | TCF25 | transcription factor 25 [Source:HGNC Symbol;Acc:HGNC:29181] |
| ENSG00000142178 | HGLibA_44161 | 104 | -0.193977828 | SIK1 | salt inducible kinase 1 [Source:HGNC Symbol;Acc:HGNC:11142] |
| ENSG00000143028 | HGLibA_48038 | 104 | -0.193977828 | SYPL2 | synaptophysin like 2 [Source:HGNC Symbol;Acc:HGNC:27638] |
| ENSG00000143196 | HGLibA_13903 | 104 | -0.193977828 | DPT | dermatopontin [Source:HGNC Symbol;Acc:HGNC:3011] |
| ENSG00000143256 | HGLibA_36142 | 104 | -0.193977828 | PFDN2 | prefoldin subunit 2 [Source:HGNC Symbol;Acc:HGNC:8867] |
| ENSG00000144229 | HGLibA_49410 | 104 | -0.193977828 | THSD7B | thrombospondin type 1 domain containing 7B [Source:HGNC Symbol;Acc:HGNC:29348] |
| ENSG00000144959 | HGLibA_31294 | 104 | -0.193977828 | NCEH1 | neutral cholesterol ester hydrolase 1 [Source:HGNC Symbol;Acc:HGNC:29260] |
| ENSG00000145248 | HGLibA_44339 | 104 | -0.193977828 | SLC10A4 | solute carrier family 10 member 4 [Source:HGNC Symbol;Acc:HGNC:22980] |
| ENSG00000145715 | HGLibA_40296 | 104 | -0.193977828 | RASA1 | RAS p21 protein activator 1 [Source:HGNC Symbol;Acc:HGNC:9871] |
| ENSG00000146013 | HGLibA_18970 | 104 | -0.193977828 | GFRA3 | GDNF family receptor alpha 3 [Source:HGNC Symbol;Acc:HGNC:4245] |
| ENSG00000146909 | HGLibA_32285 | 104 | -0.193977828 | NOM1 | nucleolar protein with MIF4G domain 1 [Source:HGNC Symbol;Acc:HGNC:13244] |
| ENSG00000147140 | HGLibA_32297 | 104 | -0.193977828 | NONO | non-POU domain containing octamer binding [Source:HGNC Symbol;Acc:HGNC:7871] |
| ENSG00000149948 | HGLibA_21687 | 104 | -0.193977828 | HMGA2 | high mobility group AT-hook 2 [Source:HGNC Symbol;Acc:HGNC:5009] |
| ENSG00000150768 | HGLibA_13310 | 104 | -0.193977828 | DLAT | dihydrolipoamide S-acetyltransferase [Source:HGNC Symbol;Acc:HGNC:2896] |
| ENSG00000150873 | HGLibA_06143 | 104 | -0.193977828 | C2orf50 | chromosome 2 open reading frame 50 [Source:HGNC Symbol;Acc:HGNC:26324] |
| ENSG00000150991 | HGLibA_52567 | 104 | -0.193977828 | UBC | ubiquitin C [Source:HGNC Symbol;Acc:HGNC:12468] |
| ENSG00000151414 | HGLibA_31663 | 104 | -0.193977828 | NEK7 | NIMA related kinase 7 [Source:HGNC Symbol;Acc:HGNC:13386] |
| ENSG00000152213 | HGLibA_02928 | 104 | -0.193977828 | ARL11 | ADP ribosylation factor like GTPase 11 [Source:HGNC Symbol;Acc:HGNC:24046] |
| ENSG00000153786 | HGLibA_55127 | 104 | -0.193977828 | ZDHHC7 | zinc finger DHHC-type containing 7 [Source:HGNC Symbol;Acc:HGNC:18459] |
| ENSG00000154099 | HGLibA_13459 | 104 | -0.193977828 | DNAAF1 | dynein axonemal assembly factor 1 [Source:HGNC Symbol;Acc:HGNC:30539] |
| ENSG00000154429 | HGLibA_08186 | 104 | -0.193977828 | CCSAP | cilia and spindle associated protein [Source:HGNC Symbol;Acc:HGNC:29578]" |
| ENSG00000154548 | HGLibA_47068 | 104 | -0.193977828 | SRSF12 | serine and arginine rich splicing factor 12 [Source:HGNC Symbol;Acc:HGNC:21220] |
| ENSG00000156234 | HGLibA_11854 | 104 | -0.193977828 | CXCL13 | C-X-C motif chemokine ligand 13 [Source:HGNC Symbol;Acc:HGNC:10639] |
| ENSG00000156508 | HGLibA_14491 | 104 | -0.193977828 | EEF1A1 | eukaryotic translation elongation factor 1 alpha 1 [Source:HGNC Symbol;Acc:HGNC:3189] |
| ENSG00000156564 | HGLibA_27156 | 104 | -0.193977828 | LRFN2 | leucine rich repeat and fibronectin type III domain containing 2 [Source:HGNC Symbol;Acc:HGNC:21226] |
| ENSG00000158106 | HGLibA_41223 | 104 | -0.193977828 | RHPN1 | rhophilin Rho GTPase binding protein 1 [Source:HGNC Symbol;Acc:HGNC:19973] |
| ENSG00000158457 | HGLibA_51933 | 104 | -0.193977828 | TSPAN33 | tetraspanin 33 [Source:HGNC Symbol;Acc:HGNC:28743] |
| ENSG00000158764 | HGLibA_23826 | 104 | -0.193977828 | ITLN2 | intelectin 2 [Source:HGNC Symbol;Acc:HGNC:20599] |
| ENSG00000160321 | HGLibA_55582 | 104 | -0.193977828 | ZNF208 | zinc finger protein 208 [Source:HGNC Symbol;Acc:HGNC:12999] |
| ENSG00000161956 | HGLibA_43392 | 104 | -0.193977828 | SENP3 | SUMO specific peptidase 3 [Source:HGNC Symbol;Acc:HGNC:17862] |
| ENSG00000162063 | HGLibA_08089 | 104 | -0.193977828 | CCNF | cyclin F [Source:HGNC Symbol;Acc:HGNC:1591] |
| ENSG00000162191 | HGLibA_52773 | 104 | -0.193977828 | UBXN1 | UBX domain protein 1 [Source:HGNC Symbol;Acc:HGNC:18402] |
| ENSG00000162819 | HGLibA_04835 | 104 | -0.193977828 | BROX | BRO1 domain and CAAX motif containing [Source:HGNC Symbol;Acc:HGNC:26512] |
| ENSG00000162852 | HGLibA_10384 | 104 | -0.193977828 | CNST | connexin sorting protein [Source:HGNC Symbol;Acc:HGNC:26486]" |
| ENSG00000163219 | HGLibA_02739 | 104 | -0.193977828 | ARHGAP25 | Rho GTPase activating protein 25 [Source:HGNC Symbol;Acc:HGNC:28951] |
| ENSG00000163263 | HGLibA_05810 | 104 | -0.193977828 | C1orf189 | chromosome 1 open reading frame 189 [Source:HGNC Symbol;Acc:HGNC:32305] |
| ENSG00000163453 | HGLibA_22832 | 104 | -0.193977828 | IGFBP7 | insulin like growth factor binding protein 7 [Source:HGNC Symbol;Acc:HGNC:5476] |
| ENSG00000163554 | HGLibA_46872 | 104 | -0.193977828 | SPTA1 | erythrocytic 1 [Source:HGNC Symbol;Acc:HGNC:11272]" |
| ENSG00000163618 | HGLibA_06977 | 104 | -0.193977828 | CADPS | calcium dependent secretion activator [Source:HGNC Symbol;Acc:HGNC:1426] |
| ENSG00000163762 | HGLibA_49695 | 104 | -0.193977828 | TM4SF18 | transmembrane 4 L six family member 18 [Source:HGNC Symbol;Acc:HGNC:25181] |
| ENSG00000166793 | HGLibA_54716 | 104 | -0.193977828 | YPEL4 | yippee like 4 [Source:HGNC Symbol;Acc:HGNC:18328] |
| ENSG00000166889 | HGLibA_35284 | 104 | -0.193977828 | PATL1 | processing body mRNA decay factor [Source:HGNC Symbol;Acc:HGNC:26721]" |
| ENSG00000167085 | HGLibA_36312 | 104 | -0.193977828 | PHB | prohibitin [Source:HGNC Symbol;Acc:HGNC:8912] |
| ENSG00000167785 | HGLibA_56233 | 104 | -0.193977828 | ZNF558 | zinc finger protein 558 [Source:HGNC Symbol;Acc:HGNC:26422] |
| ENSG00000168439 | HGLibA_47446 | 104 | -0.193977828 | STIP1 | stress induced phosphoprotein 1 [Source:HGNC Symbol;Acc:HGNC:11387] |
| ENSG00000168806 | HGLibA_26164 | 104 | -0.193977828 | LCMT2 | leucine carboxyl methyltransferase 2 [Source:HGNC Symbol;Acc:HGNC:17558] |
| ENSG00000168994 | HGLibA_39647 | 104 | -0.193977828 | PXDC1 | PX domain containing 1 [Source:HGNC Symbol;Acc:HGNC:21361] |
| ENSG00000169379 | HGLibA_02934 | 104 | -0.193977828 | ARL13B | ADP ribosylation factor like GTPase 13B [Source:HGNC Symbol;Acc:HGNC:25419] |
| ENSG00000170925 | HGLibA_49062 | 104 | -0.193977828 | TEX13B | testis expressed 13B [Source:HGNC Symbol;Acc:HGNC:11736] |
| ENSG00000171051 | HGLibA_17964 | 104 | -0.193977828 | FPR1 | formyl peptide receptor 1 [Source:HGNC Symbol;Acc:HGNC:3826] |

| | | | | | |
|---|---|---|---|---|---|
| ENSG00000174672 | HGLibA_04851 | 104 | -0.193977828 | BRSK2 | BR serine/threonine kinase 2 [Source:HGNC Symbol;Acc:HGNC:11405] |
| ENSG00000175203 | HGLibA_12547 | 104 | -0.193977828 | DCTN2 | dynactin subunit 2 [Source:HGNC Symbol;Acc:HGNC:2712] |
| ENSG00000177409 | HGLibA_42701 | 104 | -0.193977828 | SAMD9L | sterile alpha motif domain containing 9 like [Source:HGNC Symbol;Acc:HGNC:1349] |
| ENSG00000179304 | HGLibA_16190 | 104 | -0.193977828 | FAM156B | family with sequence similarity 156 member B [Source:HGNC Symbol;Acc:HGNC:31962] |
| ENSG00000179674 | HGLibA_02935 | 104 | -0.193977828 | ARL14 | ADP ribosylation factor like GTPase 14 [Source:HGNC Symbol;Acc:HGNC:22974] |
| ENSG00000180537 | HGLibA_41585 | 104 | -0.193977828 | RNF182 | ring finger protein 182 [Source:HGNC Symbol;Acc:HGNC:28522] |
| ENSG00000182652 | HGLibA_34057 | 104 | -0.193977828 | OR4Q3 | olfactory receptor family 4 subfamily Q member 3 [Source:HGNC Symbol;Acc:HGNC:15426] |
| ENSG00000183753 | HGLibA_04742 | 104 | -0.193977828 | BPY2 | basic charge Y-linked 2 [Source:HGNC Symbol;Acc:HGNC:13508] |
| ENSG00000183793 | HGLibA_32471 | 104 | -0.193977828 | NPIPA5 | nuclear pore complex interacting protein family member A5 [Source:HGNC Symbol;Acc:HGNC:41980] |
| ENSG00000184330 | HGLibA_42570 | 104 | -0.193977828 | S100A7A | S100 calcium binding protein A7A [Source:HGNC Symbol;Acc:HGNC:21657] |
| ENSG00000184470 | HGLibA_52446 | 104 | -0.193977828 | TXNRD2 | thioredoxin reductase 2 [Source:HGNC Symbol;Acc:HGNC:18155] |
| ENSG00000185379 | HGLibA_40094 | 104 | -0.193977828 | RAD51D | RAD51 paralog D [Source:HGNC Symbol;Acc:HGNC:9823] |
| ENSG00000185962 | HGLibA_26129 | 104 | -0.193977828 | LCE3A | late cornified envelope 3A [Source:HGNC Symbol;Acc:HGNC:29461] |
| ENSG00000186448 | HGLibA_55560 | 104 | -0.193977828 | ZNF197 | zinc finger protein 197 [Source:HGNC Symbol;Acc:HGNC:12988] |
| ENSG00000186675 | HGLibA_27982 | 104 | -0.193977828 | MAGEE2 | MAGE family member E2 [Source:HGNC Symbol;Acc:HGNC:24935] |
| ENSG00000187239 | HGLibA_17736 | 104 | -0.193977828 | FNBP1 | formin binding protein 1 [Source:HGNC Symbol;Acc:HGNC:17069] |
| ENSG00000188306 | HGLibA_27462 | 104 | -0.193977828 | LRRIQ4 | leucine rich repeats and IQ motif containing 4 [Source:HGNC Symbol;Acc:HGNC:34298] |
| ENSG00000188389 | HGLibA_35714 | 104 | -0.193977828 | PDCD1 | programmed cell death 1 [Source:HGNC Symbol;Acc:HGNC:8760] |
| ENSG00000189058 | HGLibA_02515 | 104 | -0.193977828 | APOD | apolipoprotein D [Source:HGNC Symbol;Acc:HGNC:612] |
| ENSG00000196171 | HGLibA_34419 | 104 | -0.193977828 | OR6K2 | olfactory receptor family 6 subfamily K member 2 [Source:HGNC Symbol;Acc:HGNC:15029] |
| ENSG00000196405 | HGLibA_15699 | 104 | -0.193977828 | EVL | Enah/Vasp-like [Source:HGNC Symbol;Acc:HGNC:20234] |
| ENSG00000197050 | HGLibA_55956 | 104 | -0.193977828 | ZNF420 | zinc finger protein 420 [Source:HGNC Symbol;Acc:HGNC:20649] |
| ENSG00000198586 | HGLibA_49623 | 104 | -0.193977828 | TLK1 | tousled like kinase 1 [Source:HGNC Symbol;Acc:HGNC:11841] |
| ENSG00000203985 | HGLibA_26241 | 104 | -0.193977828 | LDLRAD1 | low density lipoprotein receptor class A domain containing 1 [Source:HGNC Symbol;Acc:HGNC:32069] |
| ENSG00000204979 | HGLibA_30137 | 104 | -0.193977828 | MS4A13 | membrane spanning 4-domains A13 [Source:HGNC Symbol;Acc:HGNC:16674] |
| ENSG00000205078 | HGLibA_47916 | 104 | -0.193977828 | SYCE1L | synaptonemal complex central element protein 1 like [Source:HGNC Symbol;Acc:HGNC:37236] |
| ENSG00000205649 | HGLibA_22354 | 104 | -0.193977828 | HTN3 | histatin 3 [Source:HGNC Symbol;Acc:HGNC:5284] |
| ENSG00000212722 | HGLibA_25780 | 104 | -0.193977828 | KRTAP4-9 | keratin associated protein 4-9 [Source:HGNC Symbol;Acc:HGNC:18910] |
| ENSG00000213218 | HGLibA_11353 | 104 | -0.193977828 | CSH2 | chorionic somatomammotropin hormone 2 [Source:HGNC Symbol;Acc:HGNC:2441] |
| ENSG00000213760 | HGLibA_03760 | 104 | -0.193977828 | ATP6V1G2 | ATPase H+ transporting V1 subunit G2 [Source:HGNC Symbol;Acc:HGNC:862] |
| ENSG00000215912 | HGLibA_52121 | 104 | -0.193977828 | TTC34 | tetratricopeptide repeat domain 34 [Source:HGNC Symbol;Acc:HGNC:34297] |
| ENSG00000221843 | HGLibA_06112 | 104 | -0.193977828 | C2orf16 | chromosome 2 open reading frame 16 [Source:HGNC Symbol;Acc:HGNC:25275] |
| ENSG00000221855 | HGLibA_48406 | 104 | -0.193977828 | TAS2R41 | taste 2 receptor member 41 [Source:HGNC Symbol;Acc:HGNC:18883] |
| ENSG00000221910 | HGLibA_33791 | 104 | -0.193977828 | OR2F2 | olfactory receptor family 2 subfamily F member 2 [Source:HGNC Symbol;Acc:HGNC:8247] |
| ENSG00000224586 | HGLibA_20181 | 104 | -0.193977828 | GPX5 | glutathione peroxidase 5 [Source:HGNC Symbol;Acc:HGNC:4557] |
| ENSG00000224659 | HGLibA_18461 | 104 | -0.193977828 | GAGE12J | G antigen 12J [Source:HGNC Symbol;Acc:HGNC:17778] |
| ENSG00000225614 | HGLibA_56049 | 104 | -0.193977828 | ZNF469 | zinc finger protein 469 [Source:HGNC Symbol;Acc:HGNC:23216] |
| ENSG00000233608 | HGLibA_52377 | 104 | -0.193977828 | TWIST2 | twist family bHLH transcription factor 2 [Source:HGNC Symbol;Acc:HGNC:20670] |
| ENSG00000235711 | HGLibA_02080 | 104 | -0.193977828 | ANKRD34C | ankyrin repeat domain 34C [Source:HGNC Symbol;Acc:HGNC:33888] |
| ENSG00000239697 | HGLibA_50802 | 104 | -0.193977828 | TNFSF12 | TNF superfamily member 12 [Source:HGNC Symbol;Acc:HGNC:11927] |
| ENSG00000244476 | HGLibA_15538 | 104 | -0.193977828 | ERVFRD-1 | envelope [Source:HGNC Symbol;Acc:HGNC:33823]" |
| ENSG00000248167 | HGLibA_51486 | 104 | -0.193977828 | TRIM39-RPP21 | TRIM39-RPP21 readthrough [Source:HGNC Symbol;Acc:HGNC:38845] |
| ENSG00000255587 | HGLibA_39952 | 104 | -0.193977828 | RAB44 | member RAS oncogene family [Source:HGNC Symbol;Acc:HGNC:21068]" |
| ENSG00000256574 | HGLibA_33594 | 104 | -0.193977828 | OR13A1 | olfactory receptor family 13 subfamily A member 1 [Source:HGNC Symbol;Acc:HGNC:14772] |
| ENSG00000259075 | HGLibA_37448 | 104 | -0.193977828 | POC1B-GALNT4 | POC1B-GALNT4 readthrough [Source:HGNC Symbol;Acc:HGNC:42957] |
| ENSG00000275385 | HGLibA_07981 | 104 | -0.193977828 | CCL18 | C-C motif chemokine ligand 18 [Source:HGNC Symbol;Acc:HGNC:10616] |
| ENSG00000277363 | HGLibA_46933 | 104 | -0.193977828 | SRCIN1 | SRC kinase signaling inhibitor 1 [Source:HGNC Symbol;Acc:HGNC:29506] |
| FAM19A1 | HGLibA_16395 | 104 | -0.193977828 | FAM19A1 | - |
| FAM207A | HGLibA_16428 | 104 | -0.193977828 | FAM207A | - |
| FAM69A | HGLibA_16676 | 104 | -0.193977828 | FAM69A | - |
| hsa-mir-1281 | HGLibA_57580 | 104 | -0.193977828 | hsa-mir-1281 | - |
| hsa-mir-3184 | HGLibA_58833 | 104 | -0.193977828 | hsa-mir-3184 | - |
| hsa-mir-4277 | HGLibA_59812 | 104 | -0.193977828 | hsa-mir-4277 | - |
| hsa-mir-4430 | HGLibA_60105 | 104 | -0.193977828 | hsa-mir-4430 | - |
| hsa-mir-4433 | HGLibA_60120 | 104 | -0.193977828 | hsa-mir-4433 | - |
| hsa-mir-4523 | HGLibA_60549 | 104 | -0.193977828 | hsa-mir-4523 | - |
| hsa-mir-4666a | HGLibA_60776 | 104 | -0.193977828 | hsa-mir-4666a | - |
| hsa-mir-4676 | HGLibA_60821 | 104 | -0.193977828 | hsa-mir-4676 | - |
| hsa-mir-4758 | HGLibA_61128 | 104 | -0.193977828 | hsa-mir-4758 | - |
| hsa-mir-548p | HGLibA_62055 | 104 | -0.193977828 | hsa-mir-548p | - |
| hsa-mir-5708 | HGLibA_62371 | 104 | -0.193977828 | hsa-mir-5708 | - |
| hsa-mir-6081 | HGLibA_62583 | 104 | -0.193977828 | hsa-mir-6081 | - |
| hsa-mir-7-1 | HGLibA_63767 | 104 | -0.193977828 | hsa-mir-7-1 | - |
| INADL | HGLibA_23272 | 104 | -0.193977828 | INADL | - |
| JHDM1D | HGLibA_23947 | 104 | -0.193977828 | JHDM1D | - |
| KIAA1984 | HGLibA_24836 | 104 | -0.193977828 | KIAA1984 | - |
| LOC441155 | HGLibA_26952 | 104 | -0.193977828 | LOC441155 | - |
| PTPN20B | HGLibA_39455 | 104 | -0.193977828 | PTPN20B | - |
| SSPO | HGLibA_47151 | 104 | -0.193977828 | SSPO | - |
| TMEM57 | HGLibA_50456 | 104 | -0.193977828 | TMEM57 | - |
| XAGE1D | HGLibA_54476 | 104 | -0.193977828 | XAGE1D | - |
| ZCCHC11 | HGLibA_55006 | 104 | -0.193977828 | ZCCHC11 | - |
| ATP5B | HGLibA_03642 | 103 | -0.203023171 | ATP5B | - |
| ATP5F1 | HGLibA_03651 | 103 | -0.203023171 | ATP5F1 | - |
| C11orf57 | HGLibA_05160 | 103 | -0.203023171 | C11orf57 | - |
| C11orf88 | HGLibA_05207 | 103 | -0.203023171 | C11orf88 | - |
| C15orf27 | HGLibA_05381 | 103 | -0.203023171 | C15orf27 | - |
| CCDC111 | HGLibA_07540 | 103 | -0.203023171 | CCDC111 | - |
| DDX26B | HGLibA_12685 | 103 | -0.203023171 | DDX26B | - |
| ENSG00000004779 | HGLibA_31480 | 103 | -0.203023171 | NDUFAB1 | NADH:ubiquinone oxidoreductase subunit AB1 [Source:HGNC Symbol;Acc:HGNC:7694] |
| ENSG00000004948 | HGLibA_07003 | 103 | -0.203023171 | CALCR | calcitonin receptor [Source:HGNC Symbol;Acc:HGNC:1440] |
| ENSG00000005448 | HGLibA_54125 | 103 | -0.203023171 | WDR54 | WD repeat domain 54 [Source:HGNC Symbol;Acc:HGNC:25770] |
| ENSG00000008283 | HGLibA_11977 | 103 | -0.203023171 | CYB561 | cytochrome b561 [Source:HGNC Symbol;Acc:HGNC:2571] |
| ENSG00000010310 | HGLibA_19126 | 103 | -0.203023171 | GIPR | gastric inhibitory polypeptide receptor [Source:HGNC Symbol;Acc:HGNC:4271] |
| ENSG00000013297 | HGLibA_09854 | 103 | -0.203023171 | CLDN11 | claudin 11 [Source:HGNC Symbol;Acc:HGNC:8514] |
| ENSG00000023516 | HGLibA_01408 | 103 | -0.203023171 | AKAP11 | A-kinase anchoring protein 11 [Source:HGNC Symbol;Acc:HGNC:369] |
| ENSG00000028116 | HGLibA_53828 | 103 | -0.203023171 | VRK2 | VRK serine/threonine kinase 2 [Source:HGNC Symbol;Acc:HGNC:12719] |
| ENSG00000029639 | HGLibA_49138 | 103 | -0.203023171 | TFB1M | mitochondrial [Source:HGNC Symbol;Acc:HGNC:17037]" |
| ENSG00000051523 | HGLibA_12017 | 103 | -0.203023171 | CYBA | cytochrome b-245 alpha chain [Source:HGNC Symbol;Acc:HGNC:2577] |
| ENSG00000054611 | HGLibA_48504 | 103 | -0.203023171 | TBC1D22A | TBC1 domain family member 22A [Source:HGNC Symbol;Acc:HGNC:1309] |
| ENSG00000055955 | HGLibA_23815 | 103 | -0.203023171 | ITIH4 | inter-alpha-trypsin inhibitor heavy chain family member 4 [Source:HGNC Symbol;Acc:HGNC:6169] |
| ENSG00000058866 | HGLibA_13070 | 103 | -0.203023171 | DGKG | diacylglycerol kinase gamma [Source:HGNC Symbol;Acc:HGNC:2853] |
| ENSG00000070366 | HGLibA_45704 | 103 | -0.203023171 | SMG6 | nonsense mediated mRNA decay factor [Source:HGNC Symbol;Acc:HGNC:17809]" |
| ENSG00000075388 | HGLibA_17409 | 103 | -0.203023171 | FGF4 | fibroblast growth factor 4 [Source:HGNC Symbol;Acc:HGNC:3682] |
| ENSG00000079335 | HGLibA_08510 | 103 | -0.203023171 | CDC14A | cell division cycle 14A [Source:HGNC Symbol;Acc:HGNC:1718] |
| ENSG00000084636 | HGLibA_10517 | 103 | -0.203023171 | COL16A1 | collagen type XVI alpha 1 chain [Source:HGNC Symbol;Acc:HGNC:2193] |
| ENSG00000085719 | HGLibA_10959 | 103 | -0.203023171 | CPNE3 | copine 3 [Source:HGNC Symbol;Acc:HGNC:2316] |
| ENSG00000089060 | HGLibA_45396 | 103 | -0.203023171 | SLC8B1 | solute carrier family 8 member B1 [Source:HGNC Symbol;Acc:HGNC:26175] |
| ENSG00000092529 | HGLibA_07167 | 103 | -0.203023171 | CAPN3 | calpain 3 [Source:HGNC Symbol;Acc:HGNC:1480] |
| ENSG00000100099 | HGLibA_22053 | 103 | -0.203023171 | HPS4 | biogenesis of lysosomal organelles complex 3 subunit 2 [Source:HGNC Symbol;Acc:HGNC:15844]" |
| ENSG00000100626 | HGLibA_18549 | 103 | -0.203023171 | GALNT16 | polypeptide N-acetylgalactosaminyltransferase 16 [Source:HGNC Symbol;Acc:HGNC:23233] |
| ENSG00000101152 | HGLibA_13624 | 103 | -0.203023171 | DNAJC5 | DnaJ heat shock protein family (Hsp40) member C5 [Source:HGNC Symbol;Acc:HGNC:16235] |
| ENSG00000101307 | HGLibA_44204 | 103 | -0.203023171 | SIRPB1 | signal regulatory protein beta 1 [Source:HGNC Symbol;Acc:HGNC:15928] |
| ENSG00000101916 | HGLibA_49664 | 103 | -0.203023171 | TLR8 | toll like receptor 8 [Source:HGNC Symbol;Acc:HGNC:15632] |
| ENSG00000104812 | HGLibA_20708 | 103 | -0.203023171 | GYS1 | glycogen synthase 1 [Source:HGNC Symbol;Acc:HGNC:4706] |
| ENSG00000104918 | HGLibA_40874 | 103 | -0.203023171 | RETN | resistin [Source:HGNC Symbol;Acc:HGNC:20389] |
| ENSG00000105370 | HGLibA_26522 | 103 | -0.203023171 | LIM2 | lens intrinsic membrane protein 2 [Source:HGNC Symbol;Acc:HGNC:6610] |
| ENSG00000105374 | HGLibA_32014 | 103 | -0.203023171 | NKG7 | natural killer cell granule protein 7 [Source:HGNC Symbol;Acc:HGNC:7830] |
| ENSG00000105607 | HGLibA_18771 | 103 | -0.203023171 | GCDH | glutaryl-CoA dehydrogenase [Source:HGNC Symbol;Acc:HGNC:4189] |
| ENSG00000105755 | HGLibA_15630 | 103 | -0.203023171 | ETHE1 | persulfide dioxygenase [Source:HGNC Symbol;Acc:HGNC:23287]" |
| ENSG00000107625 | HGLibA_12733 | 103 | -0.203023171 | DDX50 | DExD-box helicase 50 [Source:HGNC Symbol;Acc:HGNC:17906] |
| ENSG00000107854 | HGLibA_50857 | 103 | -0.203023171 | TNKS2 | tankyrase 2 [Source:HGNC Symbol;Acc:HGNC:15677] |
| ENSG00000110077 | HGLibA_30156 | 103 | -0.203023171 | MS4A6A | membrane spanning 4-domains A6A [Source:HGNC Symbol;Acc:HGNC:13375] |
| ENSG00000112031 | HGLibA_30468 | 103 | -0.203023171 | MTRF1L | mitochondrial translational release factor 1 like [Source:HGNC Symbol;Acc:HGNC:21051] |
| ENSG00000112304 | HGLibA_00476 | 103 | -0.203023171 | ACOT13 | acyl-CoA thioesterase 13 [Source:HGNC Symbol;Acc:HGNC:20999] |
| ENSG00000112333 | HGLibA_32604 | 103 | -0.203023171 | NR2E1 | nuclear receptor subfamily 2 group E member 1 [Source:HGNC Symbol;Acc:HGNC:7973] |
| ENSG00000112877 | HGLibA_09160 | 103 | -0.203023171 | CEP72 | centrosomal protein 72 [Source:HGNC Symbol;Acc:HGNC:25547] |
| ENSG00000113356 | HGLibA_37627 | 103 | -0.203023171 | POLR3G | RNA polymerase III subunit G [Source:HGNC Symbol;Acc:HGNC:30075] |
| ENSG00000114771 | HGLibA_00030 | 103 | -0.203023171 | AADAC | arylacetamide deacetylase [Source:HGNC Symbol;Acc:HGNC:17] |
| ENSG00000114779 | HGLibA_00252 | 103 | -0.203023171 | ABHD14B | abhydrolase domain containing 14B [Source:HGNC Symbol;Acc:HGNC:28235] |
| ENSG00000115738 | HGLibA_22528 | 103 | -0.203023171 | ID2 | inhibitor of DNA binding 2 [Source:HGNC Symbol;Acc:HGNC:5361] |
| ENSG00000116194 | HGLibA_01911 | 103 | -0.203023171 | ANGPTL1 | angiopoietin like 1 [Source:HGNC Symbol;Acc:HGNC:489] |
| ENSG00000116260 | HGLibA_39754 | 103 | -0.203023171 | QSOX1 | quiescin sulfhydryl oxidase 1 [Source:HGNC Symbol;Acc:HGNC:9756] |
| ENSG00000117450 | HGLibA_38407 | 103 | -0.203023171 | PRDX1 | peroxiredoxin 1 [Source:HGNC Symbol;Acc:HGNC:9352] |
| ENSG00000118160 | HGLibA_45391 | 103 | -0.203023171 | SLC8A2 | solute carrier family 8 member A2 [Source:HGNC Symbol;Acc:HGNC:11069] |
| ENSG00000118246 | HGLibA_16903 | 103 | -0.203023171 | FASTKD2 | FAST kinase domains 2 [Source:HGNC Symbol;Acc:HGNC:29160] |
| ENSG00000118292 | HGLibA_05872 | 103 | -0.203023171 | C1orf54 | chromosome 1 open reading frame 54 [Source:HGNC Symbol;Acc:HGNC:26258] |
| ENSG00000118689 | HGLibA_17923 | 103 | -0.203023171 | FOXO3 | forkhead box O3 [Source:HGNC Symbol;Acc:HGNC:3821] |
| ENSG00000118873 | HGLibA_39921 | 103 | -0.203023171 | RAB3GAP2 | RAB3 GTPase activating non-catalytic protein subunit 2 [Source:HGNC Symbol;Acc:HGNC:17168] |
| ENSG00000119912 | HGLibA_22536 | 103 | -0.203023171 | IDE | insulin degrading enzyme [Source:HGNC Symbol;Acc:HGNC:5381] |
| ENSG00000120662 | HGLibA_30467 | 103 | -0.203023171 | MTRF1 | mitochondrial translation release factor 1 [Source:HGNC Symbol;Acc:HGNC:7469] |
| ENSG00000124126 | HGLibA_38444 | 103 | -0.203023171 | PREX1 | 5-trisphosphate dependent Rac exchange factor 1 [Source:HGNC Symbol;Acc:HGNC:32594]" |
| ENSG00000125144 | HGLibA_30288 | 103 | -0.203023171 | MT1G | metallothionein 1G [Source:HGNC Symbol;Acc:HGNC:7399] |
| ENSG00000126756 | HGLibA_53481 | 103 | -0.203023171 | UXT | ubiquitously expressed prefoldin like chaperone [Source:HGNC Symbol;Acc:HGNC:12641] |
| ENSG00000127377 | HGLibA_11281 | 103 | -0.203023171 | CRYGN | crystallin gamma N [Source:HGNC Symbol;Acc:HGNC:20458] |

| | | | | | |
|---|---|---|---|---|---|
| ENSG00000128253 | HGLibA_40931 | 103 | -0.203023171 | RFPL2 | ret finger protein like 2 [Source:HGNC Symbol;Acc:HGNC:9979] |
| ENSG00000128645 | HGLibA_21984 | 103 | -0.203023171 | HOXD1 | homeobox D1 [Source:HGNC Symbol;Acc:HGNC:5132] |
| ENSG00000128833 | HGLibA_30855 | 103 | -0.203023171 | MYO5C | myosin VC [Source:HGNC Symbol;Acc:HGNC:7604] |
| ENSG00000129028 | HGLibA_49288 | 103 | -0.203023171 | THAP10 | THAP domain containing 10 [Source:HGNC Symbol;Acc:HGNC:23193] |
| ENSG00000129315 | HGLibA_08127 | 103 | -0.203023171 | CCNT1 | cyclin T1 [Source:HGNC Symbol;Acc:HGNC:1599] |
| ENSG00000131115 | HGLibA_55626 | 103 | -0.203023171 | ZNF227 | zinc finger protein 227 [Source:HGNC Symbol;Acc:HGNC:13020] |
| ENSG00000131620 | HGLibA_02172 | 103 | -0.203023171 | ANO1 | anoctamin 1 [Source:HGNC Symbol;Acc:HGNC:21625] |
| ENSG00000132382 | HGLibA_30641 | 103 | -0.203023171 | MYBBP1A | MYB binding protein 1a [Source:HGNC Symbol;Acc:HGNC:7546] |
| ENSG00000134569 | HGLibA_27222 | 103 | -0.203023171 | LRP4 | LDL receptor related protein 4 [Source:HGNC Symbol;Acc:HGNC:6696] |
| ENSG00000135372 | HGLibA_31168 | 103 | -0.203023171 | NAT10 | N-acetyltransferase 10 [Source:HGNC Symbol;Acc:HGNC:29830] |
| ENSG00000135655 | HGLibA_53208 | 103 | -0.203023171 | USP15 | ubiquitin specific peptidase 15 [Source:HGNC Symbol;Acc:HGNC:12613] |
| ENSG00000138316 | HGLibA_00804 | 103 | -0.203023171 | ADAMTS14 | ADAM metallopeptidase with thrombospondin type 1 motif 14 [Source:HGNC Symbol;Acc:HGNC:14899] |
| ENSG00000138709 | HGLibA_26032 | 103 | -0.203023171 | LARP1B | La ribonucleoprotein domain family member 1B [Source:HGNC Symbol;Acc:HGNC:24704] |
| ENSG00000140403 | HGLibA_13518 | 103 | -0.203023171 | DNAJA4 | DnaJ heat shock protein family (Hsp40) member A4 [Source:HGNC Symbol;Acc:HGNC:14885] |
| ENSG00000140848 | HGLibA_10956 | 103 | -0.203023171 | CPNE2 | copine 2 [Source:HGNC Symbol;Acc:HGNC:2315] |
| ENSG00000141646 | HGLibA_45579 | 103 | -0.203023171 | SMAD4 | SMAD family member 4 [Source:HGNC Symbol;Acc:HGNC:6770] |
| ENSG00000142484 | HGLibA_49706 | 103 | -0.203023171 | TM4SF5 | transmembrane 4 L six family member 5 [Source:HGNC Symbol;Acc:HGNC:11857] |
| ENSG00000143006 | HGLibA_13432 | 103 | -0.203023171 | DMRTB1 | DMRT like family B with proline rich C-terminal 1 [Source:HGNC Symbol;Acc:HGNC:13913] |
| ENSG00000143321 | HGLibA_21040 | 103 | -0.203023171 | HDGF | heparin binding growth factor [Source:HGNC Symbol;Acc:HGNC:4856] |
| ENSG00000144152 | HGLibA_16952 | 103 | -0.203023171 | FBLN7 | fibulin 7 [Source:HGNC Symbol;Acc:HGNC:26740] |
| ENSG00000145451 | HGLibA_19305 | 103 | -0.203023171 | GLRA3 | glycine receptor alpha 3 [Source:HGNC Symbol;Acc:HGNC:4328] |
| ENSG00000145779 | HGLibA_50719 | 103 | -0.203023171 | TNFAIP8 | TNF alpha induced protein 8 [Source:HGNC Symbol;Acc:HGNC:17260] |
| ENSG00000147874 | HGLibA_20890 | 103 | -0.203023171 | HAUS6 | HAUS augmin like complex subunit 6 [Source:HGNC Symbol;Acc:HGNC:25948] |
| ENSG00000148985 | HGLibA_36210 | 103 | -0.203023171 | PGAP2 | post-GPI attachment to proteins 2 [Source:HGNC Symbol;Acc:HGNC:17893] |
| ENSG00000149503 | HGLibA_23276 | 103 | -0.203023171 | INCENP | inner centromere protein [Source:HGNC Symbol;Acc:HGNC:6058] |
| ENSG00000151360 | HGLibA_01671 | 103 | -0.203023171 | ALLC | allantoicase [Source:HGNC Symbol;Acc:HGNC:17377] |
| ENSG00000151743 | HGLibA_01810 | 103 | -0.203023171 | AMN1 | antagonist of mitotic exit network 1 homolog [Source:HGNC Symbol;Acc:HGNC:27281] |
| ENSG00000155592 | HGLibA_55349 | 103 | -0.203023171 | ZKSCAN2 | zinc finger with KRAB and SCAN domains 2 [Source:HGNC Symbol;Acc:HGNC:25677] |
| ENSG00000155744 | HGLibA_16083 | 103 | -0.203023171 | FAM126B | family with sequence similarity 126 member B [Source:HGNC Symbol;Acc:HGNC:28593] |
| ENSG00000156049 | HGLibA_19444 | 103 | -0.203023171 | GNA14 | G protein subunit alpha 14 [Source:HGNC Symbol;Acc:HGNC:4382] |
| ENSG00000157259 | HGLibA_18684 | 103 | -0.203023171 | GATAD1 | GATA zinc finger domain containing 1 [Source:HGNC Symbol;Acc:HGNC:29941] |
| ENSG00000158113 | HGLibA_27363 | 103 | -0.203023171 | LRRC43 | leucine rich repeat containing 43 [Source:HGNC Symbol;Acc:HGNC:28562] |
| ENSG00000158163 | HGLibA_14301 | 103 | -0.203023171 | DZIP1L | DAZ interacting zinc finger protein 1 like [Source:HGNC Symbol;Acc:HGNC:26551] |
| ENSG00000158470 | HGLibA_04036 | 103 | -0.203023171 | B4GALT5 | 4-galactosyltransferase 5 [Source:HGNC Symbol;Acc:HGNC:928]" |
| ENSG00000159228 | HGLibA_07417 | 103 | -0.203023171 | CBR1 | carbonyl reductase 1 [Source:HGNC Symbol;Acc:HGNC:1548] |
| ENSG00000159346 | HGLibA_00980 | 103 | -0.203023171 | ADIPOR1 | adiponectin receptor 1 [Source:HGNC Symbol;Acc:HGNC:24040] |
| ENSG00000159346 | HGLibA_00979 | 103 | -0.203023171 | ADIPOR1 | adiponectin receptor 1 [Source:HGNC Symbol;Acc:HGNC:24040] |
| ENSG00000160404 | HGLibA_51027 | 103 | -0.203023171 | TOR2A | torsin family 2 member A [Source:HGNC Symbol;Acc:HGNC:11996] |
| ENSG00000162511 | HGLibA_26022 | 103 | -0.203023171 | LAPTM5 | lysosomal protein transmembrane 5 [Source:HGNC Symbol;Acc:HGNC:29612] |
| ENSG00000162543 | HGLibA_52778 | 103 | -0.203023171 | UBXN10 | UBX domain protein 10 [Source:HGNC Symbol;Acc:HGNC:26354] |
| ENSG00000162739 | HGLibA_44316 | 103 | -0.203023171 | SLAMF6 | SLAM family member 6 [Source:HGNC Symbol;Acc:HGNC:21392] |
| ENSG00000163069 | HGLibA_43800 | 103 | -0.203023171 | SGCB | sarcoglycan beta [Source:HGNC Symbol;Acc:HGNC:10806] |
| ENSG00000163131 | HGLibA_11729 | 103 | -0.203023171 | CTSS | cathepsin S [Source:HGNC Symbol;Acc:HGNC:2545] |
| ENSG00000164604 | HGLibA_20098 | 103 | -0.203023171 | GPR85 | G protein-coupled receptor 85 [Source:HGNC Symbol;Acc:HGNC:4536] |
| ENSG00000165023 | HGLibA_13260 | 103 | -0.203023171 | DIRAS2 | DIRAS family GTPase 2 [Source:HGNC Symbol;Acc:HGNC:19323] |
| ENSG00000165209 | HGLibA_47581 | 103 | -0.203023171 | STRBP | spermatid perinuclear RNA binding protein [Source:HGNC Symbol;Acc:HGNC:16462] |
| ENSG00000165383 | HGLibA_27272 | 103 | -0.203023171 | LRRC18 | leucine rich repeat containing 18 [Source:HGNC Symbol;Acc:HGNC:23199] |
| ENSG00000165724 | HGLibA_55423 | 103 | -0.203023171 | ZMYND19 | zinc finger MYND-type containing 19 [Source:HGNC Symbol;Acc:HGNC:21146] |
| ENSG00000166105 | HGLibA_19225 | 103 | -0.203023171 | GLB1L3 | galactosidase beta 1 like 3 [Source:HGNC Symbol;Acc:HGNC:25147] |
| ENSG00000166126 | HGLibA_01807 | 103 | -0.203023171 | AMN | amnion associated transmembrane protein [Source:HGNC Symbol;Acc:HGNC:14604] |
| ENSG00000166441 | HGLibA_41922 | 103 | -0.203023171 | RPL27A | ribosomal protein L27a [Source:HGNC Symbol;Acc:HGNC:10329] |
| ENSG00000167359 | HGLibA_34115 | 103 | -0.203023171 | OR51I1 | olfactory receptor family 51 subfamily I member 1 [Source:HGNC Symbol;Acc:HGNC:15200] |
| ENSG00000168830 | HGLibA_22366 | 103 | -0.203023171 | HTR1E | 5-hydroxytryptamine receptor 1E [Source:HGNC Symbol;Acc:HGNC:5291] |
| ENSG00000168916 | HGLibA_56373 | 103 | -0.203023171 | ZNF608 | zinc finger protein 608 [Source:HGNC Symbol;Acc:HGNC:29238] |
| ENSG00000169926 | HGLibA_25050 | 103 | -0.203023171 | KLF13 | Kruppel like factor 13 [Source:HGNC Symbol;Acc:HGNC:13672] |
| ENSG00000170222 | HGLibA_01035 | 103 | -0.203023171 | ADPRM | manganese dependent [Source:HGNC Symbol;Acc:HGNC:30925]" |
| ENSG00000171466 | HGLibA_56247 | 103 | -0.203023171 | ZNF562 | zinc finger protein 562 [Source:HGNC Symbol;Acc:HGNC:25950] |
| ENSG00000171517 | HGLibA_27058 | 103 | -0.203023171 | LPAR3 | lysophosphatidic acid receptor 3 [Source:HGNC Symbol;Acc:HGNC:14298] |
| ENSG00000171603 | HGLibA_10135 | 103 | -0.203023171 | CLSTN1 | calsyntenin 1 [Source:HGNC Symbol;Acc:HGNC:17447] |
| ENSG00000172171 | HGLibA_48964 | 103 | -0.203023171 | TEFM | mitochondrial [Source:HGNC Symbol;Acc:HGNC:26223]" |
| ENSG00000172288 | HGLibA_08905 | 103 | -0.203023171 | CDY1 | chromodomain Y-linked 1 [Source:HGNC Symbol;Acc:HGNC:1809] |
| ENSG00000172346 | HGLibA_11311 | 103 | -0.203023171 | CSDC2 | cold shock domain containing C2 [Source:HGNC Symbol;Acc:HGNC:30359] |
| ENSG00000172489 | HGLibA_34360 | 103 | -0.203023171 | OR5T3 | olfactory receptor family 5 subfamily T member 3 [Source:HGNC Symbol;Acc:HGNC:15297] |
| ENSG00000172967 | HGLibA_54519 | 103 | -0.203023171 | XKR3 | XK related 3 [Source:HGNC Symbol;Acc:HGNC:28778] |
| ENSG00000173442 | HGLibA_14662 | 103 | -0.203023171 | EHBP1L1 | EH domain binding protein 1 like 1 [Source:HGNC Symbol;Acc:HGNC:30682] |
| ENSG00000173610 | HGLibA_52902 | 103 | -0.203023171 | UGT2A1 | UDP glucuronosyltransferase family 2 member A1 complex locus [Source:HGNC Symbol;Acc:HGNC:12542] |
| ENSG00000174004 | HGLibA_32720 | 103 | -0.203023171 | NRROS | negative regulator of reactive oxygen species [Source:HGNC Symbol;Acc:HGNC:24613] |
| ENSG00000174343 | HGLibA_09586 | 103 | -0.203023171 | CHRNA9 | cholinergic receptor nicotinic alpha 9 subunit [Source:HGNC Symbol;Acc:HGNC:14079] |
| ENSG00000175445 | HGLibA_27104 | 103 | -0.203023171 | LPL | lipoprotein lipase [Source:HGNC Symbol;Acc:HGNC:6677] |
| ENSG00000175573 | HGLibA_05173 | 103 | -0.203023171 | C11orf68 | chromosome 11 open reading frame 68 [Source:HGNC Symbol;Acc:HGNC:28801] |
| ENSG00000176381 | HGLibA_38793 | 103 | -0.203023171 | PRR18 | proline rich 18 [Source:HGNC Symbol;Acc:HGNC:28574] |
| ENSG00000176533 | HGLibA_19531 | 103 | -0.203023171 | GNG7 | G protein subunit gamma 7 [Source:HGNC Symbol;Acc:HGNC:4410] |
| ENSG00000178462 | HGLibA_52268 | 103 | -0.203023171 | TUBAL3 | tubulin alpha like 3 [Source:HGNC Symbol;Acc:HGNC:23534] |
| ENSG00000179943 | HGLibA_17545 | 103 | -0.203023171 | FIZ1 | FLT3 interacting zinc finger 1 [Source:HGNC Symbol;Acc:HGNC:25917] |
| ENSG00000180875 | HGLibA_20246 | 103 | -0.203023171 | GREM2 | DAN family BMP antagonist [Source:HGNC Symbol;Acc:HGNC:17655]" |
| ENSG00000183034 | HGLibA_34738 | 103 | -0.203023171 | OTOP2 | otopetrin 2 [Source:HGNC Symbol;Acc:HGNC:19657] |
| ENSG00000183273 | HGLibA_07814 | 103 | -0.203023171 | CCDC60 | coiled-coil domain containing 60 [Source:HGNC Symbol;Acc:HGNC:28610] |
| ENSG00000183513 | HGLibA_10451 | 103 | -0.203023171 | COA5 | cytochrome c oxidase assembly factor 5 [Source:HGNC Symbol;Acc:HGNC:33848] |
| ENSG00000183576 | HGLibA_43645 | 103 | -0.203023171 | SETD3 | SET domain containing 3 [Source:HGNC Symbol;Acc:HGNC:20493] |
| ENSG00000185926 | HGLibA_33958 | 103 | -0.203023171 | OR4C46 | olfactory receptor family 4 subfamily C member 46 [Source:HGNC Symbol;Acc:HGNC:31271] |
| ENSG00000185989 | HGLibA_40301 | 103 | -0.203023171 | RASA3 | RAS p21 protein activator 3 [Source:HGNC Symbol;Acc:HGNC:20331] |
| ENSG00000186439 | HGLibA_51349 | 103 | -0.203023171 | TRDN | triadin [Source:HGNC Symbol;Acc:HGNC:12261] |
| ENSG00000186451 | HGLibA_46425 | 103 | -0.203023171 | SPATA12 | spermatogenesis associated 12 [Source:HGNC Symbol;Acc:HGNC:23221] |
| ENSG00000186591 | HGLibA_52611 | 103 | -0.203023171 | UBE2H | ubiquitin conjugating enzyme E2 H [Source:HGNC Symbol;Acc:HGNC:12484] |
| ENSG00000186854 | HGLibA_51232 | 103 | -0.203023171 | TRABD2A | TraB domain containing 2A [Source:HGNC Symbol;Acc:HGNC:27013] |
| ENSG00000187838 | HGLibA_37236 | 103 | -0.203023171 | PLSCR3 | phospholipid scramblase 3 [Source:HGNC Symbol;Acc:HGNC:16495] |
| ENSG00000196154 | HGLibA_42557 | 103 | -0.203023171 | S100A4 | S100 calcium binding protein A4 [Source:HGNC Symbol;Acc:HGNC:10494] |
| ENSG00000197057 | HGLibA_14058 | 103 | -0.203023171 | DTHD1 | death domain containing 1 [Source:HGNC Symbol;Acc:HGNC:37261] |
| ENSG00000198794 | HGLibA_42849 | 103 | -0.203023171 | SCAMP5 | secretory carrier membrane protein 5 [Source:HGNC Symbol;Acc:HGNC:30386] |
| ENSG00000204952 | HGLibA_17113 | 103 | -0.203023171 | FBXO47 | F-box protein 47 [Source:HGNC Symbol;Acc:HGNC:31969] |
| ENSG00000205358 | HGLibA_30292 | 103 | -0.203023171 | MT1H | metallothionein 1H [Source:HGNC Symbol;Acc:HGNC:7400] |
| ENSG00000213799 | HGLibA_56863 | 103 | -0.203023171 | ZNF845 | zinc finger protein 845 [Source:HGNC Symbol;Acc:HGNC:25112] |
| ENSG00000214160 | HGLibA_01629 | 103 | -0.203023171 | ALG3 | 3- mannosyltransferase [Source:HGNC Symbol;Acc:HGNC:23056]" |
| ENSG00000225781 | HGLibA_34448 | 103 | -0.203023171 | OR6V1 | olfactory receptor family 6 subfamily V member 1 [Source:HGNC Symbol;Acc:HGNC:15090] |
| ENSG00000233917 | HGLibA_37716 | 103 | -0.203023171 | POTEB | POTE ankyrin domain family member B [Source:HGNC Symbol;Acc:HGNC:33734] |
| ENSG00000237671 | HGLibA_18439 | 103 | -0.203023171 | GAGE12C | G antigen 12C [Source:HGNC Symbol;Acc:HGNC:28402] |
| ENSG00000242515 | HGLibA_52878 | 103 | -0.203023171 | UGT1A10 | UDP glucuronosyltransferase family 1 member A10 [Source:HGNC Symbol;Acc:HGNC:12531] |
| ENSG00000244482 | HGLibA_26503 | 103 | -0.203023171 | LILRA6 | leukocyte immunoglobulin like receptor A6 [Source:HGNC Symbol;Acc:HGNC:15495] |
| ENSG00000244509 | HGLibA_02482 | 103 | -0.203023171 | APOBEC3C | apolipoprotein B mRNA editing enzyme catalytic subunit 3C [Source:HGNC Symbol;Acc:HGNC:17353] |
| ENSG00000244646 | HGLibA_54544 | 103 | -0.203023171 | XKRY2 | Y-linked 2 [Source:HGNC Symbol;Acc:HGNC:23922]" |
| ENSG00000261587 | HGLibA_50311 | 103 | -0.203023171 | TMEM249 | transmembrane protein 249 [Source:HGNC Symbol;Acc:HGNC:44155] |
| ENSG00000271605 | HGLibA_29269 | 103 | -0.203023171 | MILR1 | mast cell immunoglobulin like receptor 1 [Source:HGNC Symbol;Acc:HGNC:27570] |
| ENSG00000274102 | HGLibA_34042 | 103 | -0.203023171 | OR4M2 | olfactory receptor family 4 subfamily M member 2 [Source:HGNC Symbol;Acc:HGNC:15373] |
| ENSG00000275152 | HGLibA_07975 | 103 | -0.203023171 | CCL16 | C-C motif chemokine ligand 16 [Source:HGNC Symbol;Acc:HGNC:10614] |
| ENSG00000277161 | HGLibA_36598 | 103 | -0.203023171 | PIGW | phosphatidylinositol glycan anchor biosynthesis class W [Source:HGNC Symbol;Acc:HGNC:23213] |
| ENSG00000277322 | HGLibA_19627 | 103 | -0.203023171 | GOLGA6L6 | golgin A6 family-like 6 [Source:HGNC Symbol;Acc:HGNC:37225] |
| ENSG00000278224 | HGLibA_38479 | 103 | -0.203023171 | PRICKLE4 | prickle planar cell polarity protein 4 [Source:HGNC Symbol;Acc:HGNC:16805] |
| FAM69B | HGLibA_16680 | 103 | -0.203023171 | FAM69B | - |
| GAGE12I | HGLibA_18457 | 103 | -0.203023171 | GAGE12I | - |
| GAGE2D | HGLibA_18475 | 103 | -0.203023171 | GAGE2D | - |
| GIF | HGLibA_19061 | 103 | -0.203023171 | GIF | - |
| hsa-mir-1184-2 | HGLibA_57234 | 103 | -0.203023171 | hsa-mir-1184-2 | - |
| hsa-mir-1185-2 | HGLibA_57246 | 103 | -0.203023171 | hsa-mir-1185-2 | - |
| hsa-mir-18a | HGLibA_58019 | 103 | -0.203023171 | hsa-mir-18a | - |
| hsa-mir-192 | HGLibA_58067 | 103 | -0.203023171 | hsa-mir-192 | - |
| hsa-mir-3130-2 | HGLibA_58575 | 103 | -0.203023171 | hsa-mir-3130-2 | - |
| hsa-mir-329-2 | HGLibA_58982 | 103 | -0.203023171 | hsa-mir-329-2 | - |
| hsa-mir-3664 | HGLibA_59208 | 103 | -0.203023171 | hsa-mir-3664 | - |
| hsa-mir-3692 | HGLibA_59359 | 103 | -0.203023171 | hsa-mir-3692 | - |
| hsa-mir-3925 | HGLibA_59571 | 103 | -0.203023171 | hsa-mir-3925 | - |
| hsa-mir-3976 | HGLibA_59662 | 103 | -0.203023171 | hsa-mir-3976 | - |
| hsa-mir-4307 | HGLibA_59941 | 103 | -0.203023171 | hsa-mir-4307 | - |
| hsa-mir-4473 | HGLibA_60302 | 103 | -0.203023171 | hsa-mir-4473 | - |
| hsa-mir-4525 | HGLibA_60563 | 103 | -0.203023171 | hsa-mir-4525 | - |
| hsa-mir-4775 | HGLibA_61204 | 103 | -0.203023171 | hsa-mir-4775 | - |
| hsa-mir-522 | HGLibA_61776 | 103 | -0.203023171 | hsa-mir-522 | - |
| hsa-mir-576 | HGLibA_62397 | 103 | -0.203023171 | hsa-mir-576 | - |
| hsa-mir-6717 | HGLibA_63030 | 103 | -0.203023171 | hsa-mir-6717 | - |
| hsa-mir-876 | HGLibA_64216 | 103 | -0.203023171 | hsa-mir-876 | - |
| LINS | HGLibA_26596 | 103 | -0.203023171 | LINS | - |
| LOC100288332 | HGLibA_26832 | 103 | -0.203023171 | LOC100288332 | - |
| MFSD4 | HGLibA_29103 | 103 | -0.203023171 | MFSD4 | - |
| NEURL | HGLibA_31721 | 103 | -0.203023171 | NEURL | - |
| PTPLB | HGLibA_39423 | 103 | -0.203023171 | PTPLB | - |
| RFWD2 | HGLibA_40957 | 103 | -0.203023171 | RFWD2 | - |

| | | | | | |
|---|---|---|---|---|---|
| SDCCAG3 | HGLibA_43116 | 103 | -0.203023171 | SDCCAG3 | - |
| SHOX_X | HGLibA_44070 | 103 | -0.203023171 | SHOX_X | - |
| TMEM27 | HGLibA_50358 | 103 | -0.203023171 | TMEM27 | - |
| ALS2CR8 | HGLibA_01730 | 102 | -0.212068514 | ALS2CR8 | - |
| C12orf44 | HGLibA_05248 | 102 | -0.212068514 | C12orf44 | - |
| C20orf112 | HGLibA_05984 | 102 | -0.212068514 | C20orf112 | - |
| CXorf64 | HGLibA_11957 | 102 | -0.212068514 | CXorf64 | - |
| ENSG00000002834 | HGLibA_26055 | 102 | -0.212068514 | LASP1 | LIM and SH3 protein 1 [Source:HGNC Symbol;Acc:HGNC:6513] |
| ENSG00000003096 | HGLibA_25131 | 102 | -0.212068514 | KLHL13 | kelch like family member 13 [Source:HGNC Symbol;Acc:HGNC:22931] |
| ENSG00000005844 | HGLibA_23757 | 102 | -0.212068514 | ITGAL | integrin subunit alpha L [Source:HGNC Symbol;Acc:HGNC:6148] |
| ENSG00000008516 | HGLibA_29523 | 102 | -0.212068514 | MMP25 | matrix metallopeptidase 25 [Source:HGNC Symbol;Acc:HGNC:14246] |
| ENSG00000035499 | HGLibA_12976 | 102 | -0.212068514 | DEPDC1B | DEP domain containing 1B [Source:HGNC Symbol;Acc:HGNC:24902] |
| ENSG00000055332 | HGLibA_14726 | 102 | -0.212068514 | EIF2AK2 | eukaryotic translation initiation factor 2 alpha kinase 2 [Source:HGNC Symbol;Acc:HGNC:9437] |
| ENSG00000057294 | HGLibA_36855 | 102 | -0.212068514 | PKP2 | plakophilin 2 [Source:HGNC Symbol;Acc:HGNC:9024] |
| ENSG00000072506 | HGLibA_22173 | 102 | -0.212068514 | HSD17B10 | hydroxysteroid 17-beta dehydrogenase 10 [Source:HGNC Symbol;Acc:HGNC:4800] |
| ENSG00000084731 | HGLibA_24939 | 102 | -0.212068514 | KIF3C | kinesin family member 3C [Source:HGNC Symbol;Acc:HGNC:6321] |
| ENSG00000084734 | HGLibA_18791 | 102 | -0.212068514 | GCKR | glucokinase regulator [Source:HGNC Symbol;Acc:HGNC:4196] |
| ENSG00000087303 | HGLibA_31927 | 102 | -0.212068514 | NID2 | nidogen 2 [Source:HGNC Symbol;Acc:HGNC:13389] |
| ENSG00000088881 | HGLibA_14357 | 102 | -0.212068514 | EBF4 | EBF family member 4 [Source:HGNC Symbol;Acc:HGNC:29278] |
| ENSG00000089351 | HGLibA_20194 | 102 | -0.212068514 | GRAMD1A | GRAM domain containing 1A [Source:HGNC Symbol;Acc:HGNC:29305] |
| ENSG00000099998 | HGLibA_19012 | 102 | -0.212068514 | GGT5 | gamma-glutamyltransferase 5 [Source:HGNC Symbol;Acc:HGNC:4260] |
| ENSG00000100604 | HGLibA_09431 | 102 | -0.212068514 | CHGA | chromogranin A [Source:HGNC Symbol;Acc:HGNC:1929] |
| ENSG00000100823 | HGLibA_02406 | 102 | -0.212068514 | APEX1 | apurinic/apyrimidinic endodeoxyribonuclease 1 [Source:HGNC Symbol;Acc:HGNC:587] |
| ENSG00000101323 | HGLibA_20831 | 102 | -0.212068514 | HAO1 | hydroxyacid oxidase 1 [Source:HGNC Symbol;Acc:HGNC:4809] |
| ENSG00000101951 | HGLibA_35013 | 102 | -0.212068514 | PAGE4 | PAGE family member 4 [Source:HGNC Symbol;Acc:HGNC:4108] |
| ENSG00000103023 | HGLibA_38960 | 102 | -0.212068514 | PRSS54 | serine protease 54 [Source:HGNC Symbol;Acc:HGNC:26336] |
| ENSG00000103550 | HGLibA_25345 | 102 | -0.212068514 | KNOP1 | lysine rich nucleolar protein 1 [Source:HGNC Symbol;Acc:HGNC:34404] |
| ENSG00000103769 | HGLibA_39786 | 102 | -0.212068514 | RAB11A | member RAS oncogene family [Source:HGNC Symbol;Acc:HGNC:9760]" |
| ENSG00000105374 | HGLibA_32012 | 102 | -0.212068514 | NKG7 | natural killer cell granule protein 7 [Source:HGNC Symbol;Acc:HGNC:7830] |
| ENSG00000105726 | HGLibA_03566 | 102 | -0.212068514 | ATP13A1 | ATPase 13A1 [Source:HGNC Symbol;Acc:HGNC:24215] |
| ENSG00000106009 | HGLibA_04757 | 102 | -0.212068514 | BRAT1 | BRCA1 associated ATM activator 1 [Source:HGNC Symbol;Acc:HGNC:21701] |
| ENSG00000108384 | HGLibA_40090 | 102 | -0.212068514 | RAD51C | RAD51 paralog C [Source:HGNC Symbol;Acc:HGNC:9820] |
| ENSG00000108518 | HGLibA_36174 | 102 | -0.212068514 | PFN1 | profilin 1 [Source:HGNC Symbol;Acc:HGNC:8881] |
| ENSG00000108551 | HGLibA_40320 | 102 | -0.212068514 | RASD1 | ras related dexamethasone induced 1 [Source:HGNC Symbol;Acc:HGNC:15828] |
| ENSG00000108819 | HGLibA_38131 | 102 | -0.212068514 | PPP1R9B | protein phosphatase 1 regulatory subunit 9B [Source:HGNC Symbol;Acc:HGNC:9298] |
| ENSG00000108924 | HGLibA_21654 | 102 | -0.212068514 | HLF | PAR bZIP transcription factor [Source:HGNC Symbol;Acc:HGNC:4977]" |
| ENSG00000109381 | HGLibA_14894 | 102 | -0.212068514 | ELF2 | E74 like ETS transcription factor 2 [Source:HGNC Symbol;Acc:HGNC:3317] |
| ENSG00000110696 | HGLibA_05164 | 102 | -0.212068514 | C11orf58 | chromosome 11 open reading frame 58 [Source:HGNC Symbol;Acc:HGNC:16990] |
| ENSG00000111271 | HGLibA_00354 | 102 | -0.212068514 | ACAD10 | acyl-CoA dehydrogenase family member 10 [Source:HGNC Symbol;Acc:HGNC:21597] |
| ENSG00000111670 | HGLibA_19575 | 102 | -0.212068514 | GNPTAB | N-acetylglucosamine-1-phosphate transferase subunits alpha and beta [Source:HGNC Symbol;Acc:HGNC:29670] |
| ENSG00000113721 | HGLibA_35856 | 102 | -0.212068514 | PDGFRB | platelet derived growth factor receptor beta [Source:HGNC Symbol;Acc:HGNC:8804] |
| ENSG00000113749 | HGLibA_22097 | 102 | -0.212068514 | HRH2 | histamine receptor H2 [Source:HGNC Symbol;Acc:HGNC:5183] |
| ENSG00000114251 | HGLibA_54374 | 102 | -0.212068514 | WNT5A | Wnt family member 5A [Source:HGNC Symbol;Acc:HGNC:12784] |
| ENSG00000115592 | HGLibA_38519 | 102 | -0.212068514 | PRKAG3 | protein kinase AMP-activated non-catalytic subunit gamma 3 [Source:HGNC Symbol;Acc:HGNC:9387] |
| ENSG00000115616 | HGLibA_45402 | 102 | -0.212068514 | SLC9A2 | solute carrier family 9 member A2 [Source:HGNC Symbol;Acc:HGNC:11072] |
| ENSG00000117586 | HGLibA_50824 | 102 | -0.212068514 | TNFSF4 | TNF superfamily member 4 [Source:HGNC Symbol;Acc:HGNC:11934] |
| ENSG00000120334 | HGLibA_09068 | 102 | -0.212068514 | CENPL | centromere protein L [Source:HGNC Symbol;Acc:HGNC:17879] |
| ENSG00000120875 | HGLibA_14182 | 102 | -0.212068514 | DUSP4 | dual specificity phosphatase 4 [Source:HGNC Symbol;Acc:HGNC:3070] |
| ENSG00000121005 | HGLibA_11174 | 102 | -0.212068514 | CRISPLD1 | cysteine rich secretory protein LCCL domain containing 1 [Source:HGNC Symbol;Acc:HGNC:18206] |
| ENSG00000122482 | HGLibA_56446 | 102 | -0.212068514 | ZNF644 | zinc finger protein 644 [Source:HGNC Symbol;Acc:HGNC:29222] |
| ENSG00000122678 | HGLibA_37541 | 102 | -0.212068514 | POLM | DNA polymerase mu [Source:HGNC Symbol;Acc:HGNC:9185] |
| ENSG00000122971 | HGLibA_00371 | 102 | -0.212068514 | ACADS | acyl-CoA dehydrogenase short chain [Source:HGNC Symbol;Acc:HGNC:90] |
| ENSG00000126231 | HGLibA_38707 | 102 | -0.212068514 | PROZ | vitamin K dependent plasma glycoprotein [Source:HGNC Symbol;Acc:HGNC:9460]" |
| ENSG00000126351 | HGLibA_49389 | 102 | -0.212068514 | THRA | thyroid hormone receptor alpha [Source:HGNC Symbol;Acc:HGNC:11796] |
| ENSG00000128383 | HGLibA_02474 | 102 | -0.212068514 | APOBEC3A | apolipoprotein B mRNA editing enzyme catalytic subunit 3A [Source:HGNC Symbol;Acc:HGNC:17343] |
| ENSG00000129038 | HGLibA_27039 | 102 | -0.212068514 | LOXL1 | lysyl oxidase like 1 [Source:HGNC Symbol;Acc:HGNC:6665] |
| ENSG00000129116 | HGLibA_35065 | 102 | -0.212068514 | PALLD | cytoskeletal associated protein [Source:HGNC Symbol;Acc:HGNC:17068]" |
| ENSG00000130283 | HGLibA_18852 | 102 | -0.212068514 | GDF1 | growth differentiation factor 1 [Source:HGNC Symbol;Acc:HGNC:4214] |
| ENSG00000130717 | HGLibA_52809 | 102 | -0.212068514 | UCK1 | uridine-cytidine kinase 1 [Source:HGNC Symbol;Acc:HGNC:14859] |
| ENSG00000130881 | HGLibA_27218 | 102 | -0.212068514 | LRP3 | LDL receptor related protein 3 [Source:HGNC Symbol;Acc:HGNC:6695] |
| ENSG00000131374 | HGLibA_48561 | 102 | -0.212068514 | TBC1D5 | TBC1 domain family member 5 [Source:HGNC Symbol;Acc:HGNC:19166] |
| ENSG00000132563 | HGLibA_40779 | 102 | -0.212068514 | REEP2 | receptor accessory protein 2 [Source:HGNC Symbol;Acc:HGNC:17975] |
| ENSG00000132906 | HGLibA_07330 | 102 | -0.212068514 | CASP9 | caspase 9 [Source:HGNC Symbol;Acc:HGNC:1511] |
| ENSG00000135093 | HGLibA_53329 | 102 | -0.212068514 | USP30 | ubiquitin specific peptidase 30 [Source:HGNC Symbol;Acc:HGNC:20065] |
| ENSG00000135902 | HGLibA_09600 | 102 | -0.212068514 | CHRND | cholinergic receptor nicotinic delta subunit [Source:HGNC Symbol;Acc:HGNC:1965] |
| ENSG00000136425 | HGLibA_09684 | 102 | -0.212068514 | CIB2 | calcium and integrin binding family member 2 [Source:HGNC Symbol;Acc:HGNC:24579] |
| ENSG00000137133 | HGLibA_21326 | 102 | -0.212068514 | HINT2 | histidine triad nucleotide binding protein 2 [Source:HGNC Symbol;Acc:HGNC:18344] |
| ENSG00000138623 | HGLibA_43376 | 102 | -0.212068514 | SEMA7A | semaphorin 7A (John Milton Hagen blood group) [Source:HGNC Symbol;Acc:HGNC:10741] |
| ENSG00000139200 | HGLibA_36503 | 102 | -0.212068514 | PIANP | PILR alpha associated neural protein [Source:HGNC Symbol;Acc:HGNC:25338] |
| ENSG00000139910 | HGLibA_32364 | 102 | -0.212068514 | NOVA1 | NOVA alternative splicing regulator 1 [Source:HGNC Symbol;Acc:HGNC:7886] |
| ENSG00000139926 | HGLibA_18022 | 102 | -0.212068514 | FRMD6 | FERM domain containing 6 [Source:HGNC Symbol;Acc:HGNC:19839] |
| ENSG00000143178 | HGLibA_48652 | 102 | -0.212068514 | TBX19 | T-box 19 [Source:HGNC Symbol;Acc:HGNC:11596] |
| ENSG00000144681 | HGLibA_47313 | 102 | -0.212068514 | STAC | SH3 and cysteine rich domain [Source:HGNC Symbol;Acc:HGNC:11353] |
| ENSG00000145832 | HGLibA_44759 | 102 | -0.212068514 | SLC25A48 | solute carrier family 25 member 48 [Source:HGNC Symbol;Acc:HGNC:30451] |
| ENSG00000146776 | HGLibA_03851 | 102 | -0.212068514 | ATXN7L1 | ataxin 7 like 1 [Source:HGNC Symbol;Acc:HGNC:22210] |
| ENSG00000148384 | HGLibA_23351 | 102 | -0.212068514 | INPP5E | inositol polyphosphate-5-phosphatase E [Source:HGNC Symbol;Acc:HGNC:21474] |
| ENSG00000149922 | HGLibA_48676 | 102 | -0.212068514 | TBX6 | T-box 6 [Source:HGNC Symbol;Acc:HGNC:11605] |
| ENSG00000150625 | HGLibA_19818 | 102 | -0.212068514 | GPM6A | glycoprotein M6A [Source:HGNC Symbol;Acc:HGNC:4460] |
| ENSG00000150628 | HGLibA_46514 | 102 | -0.212068514 | SPATA4 | spermatogenesis associated 4 [Source:HGNC Symbol;Acc:HGNC:17333] |
| ENSG00000151474 | HGLibA_18015 | 102 | -0.212068514 | FRMD4A | FERM domain containing 4A [Source:HGNC Symbol;Acc:HGNC:25491] |
| ENSG00000153790 | HGLibA_06532 | 102 | -0.212068514 | C7orf31 | chromosome 7 open reading frame 31 [Source:HGNC Symbol;Acc:HGNC:21722] |
| ENSG00000154127 | HGLibA_52561 | 102 | -0.212068514 | UBASH3B | ubiquitin associated and SH3 domain containing B [Source:HGNC Symbol;Acc:HGNC:29884] |
| ENSG00000155792 | HGLibA_12988 | 102 | -0.212068514 | DEPTOR | DEP domain containing MTOR interacting protein [Source:HGNC Symbol;Acc:HGNC:22953] |
| ENSG00000155876 | HGLibA_42224 | 102 | -0.212068514 | RRAGA | Ras related GTP binding A [Source:HGNC Symbol;Acc:HGNC:16963] |
| ENSG00000156521 | HGLibA_52476 | 102 | -0.212068514 | TYSND1 | trypsin domain containing 1 [Source:HGNC Symbol;Acc:HGNC:28531] |
| ENSG00000158104 | HGLibA_22024 | 102 | -0.212068514 | HPD | 4-hydroxyphenylpyruvate dioxygenase [Source:HGNC Symbol;Acc:HGNC:5147] |
| ENSG00000158578 | HGLibA_01527 | 102 | -0.212068514 | ALAS2 | 5'-aminolevulinate synthase 2 [Source:HGNC Symbol;Acc:HGNC:397] |
| ENSG00000159176 | HGLibA_11432 | 102 | -0.212068514 | CSRP1 | cysteine and glycine rich protein 1 [Source:HGNC Symbol;Acc:HGNC:2469] |
| ENSG00000159184 | HGLibA_21930 | 102 | -0.212068514 | HOXB13 | homeobox B13 [Source:HGNC Symbol;Acc:HGNC:5112] |
| ENSG00000160886 | HGLibA_27677 | 102 | -0.212068514 | LY6K | lymphocyte antigen 6 family member K [Source:HGNC Symbol;Acc:HGNC:24225] |
| ENSG00000161791 | HGLibA_17699 | 102 | -0.212068514 | FMNL3 | formin like 3 [Source:HGNC Symbol;Acc:HGNC:23698] |
| ENSG00000164252 | HGLibA_01190 | 102 | -0.212068514 | AGGF1 | angiogenic factor with G-patch and FHA domains 1 [Source:HGNC Symbol;Acc:HGNC:24684] |
| ENSG00000166069 | HGLibA_49793 | 102 | -0.212068514 | TMCO5A | transmembrane and coiled-coil domains 5A [Source:HGNC Symbol;Acc:HGNC:28558] |
| ENSG00000166250 | HGLibA_10071 | 102 | -0.212068514 | CLMP | CXADR like membrane protein [Source:HGNC Symbol;Acc:HGNC:24039] |
| ENSG00000167280 | HGLibA_15129 | 102 | -0.212068514 | ENGASE | endo-beta-N-acetylglucosaminidase [Source:HGNC Symbol;Acc:HGNC:24622] |
| ENSG00000169860 | HGLibA_34868 | 102 | -0.212068514 | P2RY1 | purinergic receptor P2Y1 [Source:HGNC Symbol;Acc:HGNC:8539] |
| ENSG00000170323 | HGLibA_15920 | 102 | -0.212068514 | FABP4 | fatty acid binding protein 4 [Source:HGNC Symbol;Acc:HGNC:3559] |
| ENSG00000171201 | HGLibA_45827 | 102 | -0.212068514 | SMR3B | submaxillary gland androgen regulated protein 3B [Source:HGNC Symbol;Acc:HGNC:17326] |
| ENSG00000171759 | HGLibA_35022 | 102 | -0.212068514 | PAH | phenylalanine hydroxylase [Source:HGNC Symbol;Acc:HGNC:8582] |
| ENSG00000171798 | HGLibA_25339 | 102 | -0.212068514 | KNDC1 | kinase non-catalytic C-lobe domain containing 1 [Source:HGNC Symbol;Acc:HGNC:29374] |
| ENSG00000172209 | HGLibA_20003 | 102 | -0.212068514 | GPR22 | G protein-coupled receptor 22 [Source:HGNC Symbol;Acc:HGNC:4477] |
| ENSG00000172717 | HGLibA_16695 | 102 | -0.212068514 | FAM71D | family with sequence similarity 71 member D [Source:HGNC Symbol;Acc:HGNC:20101] |
| ENSG00000172955 | HGLibA_00963 | 102 | -0.212068514 | ADH6 | alcohol dehydrogenase 6 (class V) [Source:HGNC Symbol;Acc:HGNC:255] |
| ENSG00000173327 | HGLibA_28165 | 102 | -0.212068514 | MAP3K11 | mitogen-activated protein kinase kinase kinase 11 [Source:HGNC Symbol;Acc:HGNC:6850] |
| ENSG00000173597 | HGLibA_47744 | 102 | -0.212068514 | SULT1B1 | sulfotransferase family 1B member 1 [Source:HGNC Symbol;Acc:HGNC:17845] |
| ENSG00000174799 | HGLibA_09107 | 102 | -0.212068514 | CEP135 | centrosomal protein 135 [Source:HGNC Symbol;Acc:HGNC:29086] |
| ENSG00000174953 | HGLibA_13189 | 102 | -0.212068514 | DHX36 | DEAH-box helicase 36 [Source:HGNC Symbol;Acc:HGNC:14410] |
| ENSG00000175213 | HGLibA_55931 | 102 | -0.212068514 | ZNF408 | zinc finger protein 408 [Source:HGNC Symbol;Acc:HGNC:20041] |
| ENSG00000175868 | HGLibA_06994 | 102 | -0.212068514 | CALCB | calcitonin related polypeptide beta [Source:HGNC Symbol;Acc:HGNC:1438] |
| ENSG00000177432 | HGLibA_31123 | 102 | -0.212068514 | NAP1L5 | nucleosome assembly protein 1 like 5 [Source:HGNC Symbol;Acc:HGNC:19968] |
| ENSG00000178171 | HGLibA_01778 | 102 | -0.212068514 | AMER3 | APC membrane recruitment protein 3 [Source:HGNC Symbol;Acc:HGNC:26771] |
| ENSG00000178607 | HGLibA_15512 | 102 | -0.212068514 | ERN1 | endoplasmic reticulum to nucleus signaling 1 [Source:HGNC Symbol;Acc:HGNC:3449] |
| ENSG00000178997 | HGLibA_15717 | 102 | -0.212068514 | EXD1 | exonuclease 3'-5' domain containing 1 [Source:HGNC Symbol;Acc:HGNC:28507] |
| ENSG00000179178 | HGLibA_49907 | 102 | -0.212068514 | TMEM125 | transmembrane protein 125 [Source:HGNC Symbol;Acc:HGNC:28275] |
| ENSG00000180044 | HGLibA_06272 | 102 | -0.212068514 | C3orf80 | chromosome 3 open reading frame 80 [Source:HGNC Symbol;Acc:HGNC:40048] |
| ENSG00000182541 | HGLibA_26544 | 102 | -0.212068514 | LIMK2 | LIM domain kinase 2 [Source:HGNC Symbol;Acc:HGNC:6614] |
| ENSG00000182747 | HGLibA_44984 | 102 | -0.212068514 | SLC35D3 | solute carrier family 35 member D3 [Source:HGNC Symbol;Acc:HGNC:15621] |
| ENSG00000183024 | HGLibA_33667 | 102 | -0.212068514 | OR1G1 | olfactory receptor family 1 subfamily G member 1 [Source:HGNC Symbol;Acc:HGNC:8204] |
| ENSG00000185278 | HGLibA_54868 | 102 | -0.212068514 | ZBTB37 | zinc finger and BTB domain containing 37 [Source:HGNC Symbol;Acc:HGNC:28365] |
| ENSG00000185689 | HGLibA_06466 | 102 | -0.212068514 | C6orf201 | chromosome 6 open reading frame 201 [Source:HGNC Symbol;Acc:HGNC:21620] |
| ENSG00000185838 | HGLibA_19488 | 102 | -0.212068514 | GNB1L | G protein subunit beta 1 like [Source:HGNC Symbol;Acc:HGNC:4397] |
| ENSG00000186187 | HGLibA_56937 | 102 | -0.212068514 | ZNRF1 | zinc and ring finger 1 [Source:HGNC Symbol;Acc:HGNC:18452] |
| ENSG00000186803 | HGLibA_22660 | 102 | -0.212068514 | IFNA10 | interferon alpha 10 [Source:HGNC Symbol;Acc:HGNC:5418] |
| ENSG00000186967 | HGLibA_25673 | 102 | -0.212068514 | KRTAP19-4 | keratin associated protein 19-4 [Source:HGNC Symbol;Acc:HGNC:18939] |
| ENSG00000187021 | HGLibA_37358 | 102 | -0.212068514 | PNLIPRP1 | pancreatic lipase related protein 1 [Source:HGNC Symbol;Acc:HGNC:9156] |
| ENSG00000187514 | HGLibA_39390 | 102 | -0.212068514 | PTMA | prothymosin alpha [Source:HGNC Symbol;Acc:HGNC:9623] |
| ENSG00000196150 | HGLibA_55666 | 102 | -0.212068514 | ZNF250 | zinc finger protein 250 [Source:HGNC Symbol;Acc:HGNC:13044] |
| ENSG00000196734 | HGLibA_26102 | 102 | -0.212068514 | LCE1B | late cornified envelope 1B [Source:HGNC Symbol;Acc:HGNC:16611] |
| ENSG00000197296 | HGLibA_17543 | 102 | -0.212068514 | FITM2 | fat storage inducing transmembrane protein 2 [Source:HGNC Symbol;Acc:HGNC:16135] |
| ENSG00000197429 | HGLibA_23480 | 102 | -0.212068514 | IPP | intracisternal A particle-promoted polypeptide [Source:HGNC Symbol;Acc:HGNC:6108] |
| ENSG00000198021 | HGLibA_46379 | 102 | -0.212068514 | SPANXA1 | family member A1 [Source:HGNC Symbol;Acc:HGNC:11218]" |
| ENSG00000198104 | HGLibA_33890 | 102 | -0.212068514 | OR2T6 | olfactory receptor family 2 subfamily T member 6 [Source:HGNC Symbol;Acc:HGNC:15018] |
| ENSG00000198353 | HGLibA_21967 | 102 | -0.212068514 | HOXC4 | homeobox C4 [Source:HGNC Symbol;Acc:HGNC:5126] |
| ENSG00000198909 | HGLibA_28183 | 102 | -0.212068514 | MAP3K3 | mitogen-activated protein kinase kinase kinase 3 [Source:HGNC Symbol;Acc:HGNC:6855] |
| ENSG00000204371 | HGLibA_14686 | 102 | -0.212068514 | EHMT2 | euchromatic histone lysine methyltransferase 2 [Source:HGNC Symbol;Acc:HGNC:14129] |

| | | | | | |
|---|---|---|---|---|---|
| ENSG00000205097 | HGLibA_17995 | 102 | -0.212068514 | FRG2 | FSHD region gene 2 [Source:HGNC Symbol;Acc:HGNC:19136] |
| ENSG00000205221 | HGLibA_53674 | 102 | -0.212068514 | VIT | vitrin [Source:HGNC Symbol;Acc:HGNC:12697] |
| ENSG00000205403 | HGLibA_09279 | 102 | -0.212068514 | CFI | complement factor I [Source:HGNC Symbol;Acc:HGNC:5394] |
| ENSG00000205899 | HGLibA_04470 | 102 | -0.212068514 | BHLHA9 | basic helix-loop-helix family member a9 [Source:HGNC Symbol;Acc:HGNC:35126] |
| ENSG00000221914 | HGLibA_38147 | 102 | -0.212068514 | PPP2R2A | protein phosphatase 2 regulatory subunit Balpha [Source:HGNC Symbol;Acc:HGNC:9304] |
| ENSG00000221955 | HGLibA_44377 | 102 | -0.212068514 | SLC12A8 | solute carrier family 12 member 8 [Source:HGNC Symbol;Acc:HGNC:15595] |
| ENSG00000234511 | HGLibA_06414 | 102 | -0.212068514 | C5orf58 | chromosome 5 open reading frame 58 [Source:HGNC Symbol;Acc:HGNC:37272] |
| ENSG00000236032 | HGLibA_34293 | 102 | -0.212068514 | OR5H14 | olfactory receptor family 5 subfamily H member 14 [Source:HGNC Symbol;Acc:HGNC:31286] |
| ENSG00000255307 | HGLibA_34145 | 102 | -0.212068514 | OR52B2 | olfactory receptor family 52 subfamily B member 2 [Source:HGNC Symbol;Acc:HGNC:15207] |
| ENSG00000258366 | HGLibA_42381 | 102 | -0.212068514 | RTEL1 | regulator of telomere elongation helicase 1 [Source:HGNC Symbol;Acc:HGNC:15888] |
| ENSG00000259431 | HGLibA_49413 | 102 | -0.212068514 | THTPA | thiamine triphosphatase [Source:HGNC Symbol;Acc:HGNC:18987] |
| ENSG00000268651 | HGLibA_11559 | 102 | -0.212068514 | CTAG1A | cancer/testis antigen 1A [Source:HGNC Symbol;Acc:HGNC:24198] |
| ENSG00000280969 | HGLibA_42149 | 102 | -0.212068514 | RPS4Y2 | ribosomal protein S4 Y-linked 2 [Source:HGNC Symbol;Acc:HGNC:18501] |
| ENSG00000288722 | HGLibA_15886 | 102 | -0.212068514 | F8A1 | - |
| HNRPLL | HGLibA_21858 | 102 | -0.212068514 | HNRPLL | - |
| hsa-mir-1302-4 | HGLibA_57703 | 102 | -0.212068514 | hsa-mir-1302-4 | - |
| hsa-mir-139 | HGLibA_57822 | 102 | -0.212068514 | hsa-mir-139 | - |
| hsa-mir-197 | HGLibA_58104 | 102 | -0.212068514 | hsa-mir-197 | - |
| hsa-mir-2277 | HGLibA_58313 | 102 | -0.212068514 | hsa-mir-2277 | - |
| hsa-mir-3123 | HGLibA_58545 | 102 | -0.212068514 | hsa-mir-3123 | - |
| hsa-mir-3140 | HGLibA_58620 | 102 | -0.212068514 | hsa-mir-3140 | - |
| hsa-mir-3170 | HGLibA_58759 | 102 | -0.212068514 | hsa-mir-3170 | - |
| hsa-mir-3179-3 | HGLibA_58799 | 102 | -0.212068514 | hsa-mir-3179-3 | - |
| hsa-mir-3186 | HGLibA_58841 | 102 | -0.212068514 | hsa-mir-3186 | - |
| hsa-mir-3613 | HGLibA_59080 | 102 | -0.212068514 | hsa-mir-3613 | - |
| hsa-mir-3913-1 | HGLibA_59520 | 102 | -0.212068514 | hsa-mir-3913-1 | - |
| hsa-mir-3921 | HGLibA_59561 | 102 | -0.212068514 | hsa-mir-3921 | - |
| hsa-mir-4326 | HGLibA_60028 | 102 | -0.212068514 | hsa-mir-4326 | - |
| hsa-mir-4636 | HGLibA_60651 | 102 | -0.212068514 | hsa-mir-4636 | - |
| hsa-mir-4772 | HGLibA_61189 | 102 | -0.212068514 | hsa-mir-4772 | - |
| hsa-mir-499a | HGLibA_61400 | 102 | -0.212068514 | hsa-mir-499a | - |
| hsa-mir-5195 | HGLibA_61698 | 102 | -0.212068514 | hsa-mir-5195 | - |
| hsa-mir-581 | HGLibA_62424 | 102 | -0.212068514 | hsa-mir-581 | - |
| hsa-mir-6727 | HGLibA_63062 | 102 | -0.212068514 | hsa-mir-6727 | - |
| hsa-mir-6749 | HGLibA_63148 | 102 | -0.212068514 | hsa-mir-6749 | - |
| hsa-mir-6854 | HGLibA_63590 | 102 | -0.212068514 | hsa-mir-6854 | - |
| hsa-mir-6862-1 | HGLibA_63630 | 102 | -0.212068514 | hsa-mir-6862-1 | - |
| hsa-mir-744 | HGLibA_63885 | 102 | -0.212068514 | hsa-mir-744 | - |
| hsa-mir-8085 | HGLibA_64184 | 102 | -0.212068514 | hsa-mir-8085 | - |
| hsa-mir-891a | HGLibA_64243 | 102 | -0.212068514 | hsa-mir-891a | - |
| MINA | HGLibA_29273 | 102 | -0.212068514 | MINA | - |
| MTL5 | HGLibA_30402 | 102 | -0.212068514 | MTL5 | - |
| 12-Sep | HGLibA_43432 | 102 | -0.212068514 | 12-Sep | - |
| SGK223 | HGLibA_43830 | 102 | -0.212068514 | SGK223 | - |
| SLC35E2 | HGLibA_44988 | 102 | -0.212068514 | SLC35E2 | - |
| TMEM189-UBE2V1 | HGLibA_50134 | 102 | -0.212068514 | TMEM189-UBE2V1 | - |
| C17orf66 | HGLibA_05560 | 101 | -0.221113856 | C17orf66 | - |
| C2orf70 | HGLibA_06169 | 101 | -0.221113856 | C2orf70 | - |
| C7orf49 | HGLibA_06549 | 101 | -0.221113856 | C7orf49 | - |
| DHFRL1 | HGLibA_13105 | 101 | -0.221113856 | DHFRL1 | - |
| ENSG00000006659 | HGLibA_26354 | 101 | -0.221113856 | LGALS14 | galectin 14 [Source:HGNC Symbol;Acc:HGNC:30054] |
| ENSG00000023191 | HGLibA_41711 | 101 | -0.221113856 | RNH1 | ribonuclease/angiogenin inhibitor 1 [Source:HGNC Symbol;Acc:HGNC:10074] |
| ENSG00000041988 | HGLibA_49298 | 101 | -0.221113856 | THAP3 | THAP domain containing 3 [Source:HGNC Symbol;Acc:HGNC:20855] |
| ENSG00000050393 | HGLibA_28664 | 101 | -0.221113856 | MCUR1 | mitochondrial calcium uniporter regulator 1 [Source:HGNC Symbol;Acc:HGNC:21097] |
| ENSG00000059377 | HGLibA_48683 | 101 | -0.221113856 | TBXAS1 | thromboxane A synthase 1 [Source:HGNC Symbol;Acc:HGNC:11609] |
| ENSG00000062485 | HGLibA_11299 | 101 | -0.221113856 | CS | citrate synthase [Source:HGNC Symbol;Acc:HGNC:2422] |
| ENSG00000064601 | HGLibA_11690 | 101 | -0.221113856 | CTSA | cathepsin A [Source:HGNC Symbol;Acc:HGNC:9251] |
| ENSG00000066185 | HGLibA_55417 | 101 | -0.221113856 | ZMYND12 | zinc finger MYND-type containing 12 [Source:HGNC Symbol;Acc:HGNC:21192] |
| ENSG00000066654 | HGLibA_49415 | 101 | -0.221113856 | THUMPD1 | THUMP domain containing 1 [Source:HGNC Symbol;Acc:HGNC:23807] |
| ENSG00000070614 | HGLibA_31426 | 101 | -0.221113856 | NDST1 | N-deacetylase and N-sulfotransferase 1 [Source:HGNC Symbol;Acc:HGNC:7680] |
| ENSG00000072518 | HGLibA_28366 | 101 | -0.221113856 | MARK2 | microtubule affinity regulating kinase 2 [Source:HGNC Symbol;Acc:HGNC:3332] |
| ENSG00000075142 | HGLibA_46988 | 101 | -0.221113856 | SRI | sorcin [Source:HGNC Symbol;Acc:HGNC:11292] |
| ENSG00000076984 | HGLibA_28157 | 101 | -0.221113856 | MAP2K7 | mitogen-activated protein kinase kinase 7 [Source:HGNC Symbol;Acc:HGNC:6847] |
| ENSG00000077380 | HGLibA_14237 | 101 | -0.221113856 | DYNC1I2 | dynein cytoplasmic 1 intermediate chain 2 [Source:HGNC Symbol;Acc:HGNC:2964] |
| ENSG00000077721 | HGLibA_52573 | 101 | -0.221113856 | UBE2A | ubiquitin conjugating enzyme E2 A [Source:HGNC Symbol;Acc:HGNC:12472] |
| ENSG00000082212 | HGLibA_28713 | 101 | -0.221113856 | ME2 | malic enzyme 2 [Source:HGNC Symbol;Acc:HGNC:6984] |
| ENSG00000083123 | HGLibA_04280 | 101 | -0.221113856 | BCKDHB | branched chain keto acid dehydrogenase E1 subunit beta [Source:HGNC Symbol;Acc:HGNC:987] |
| ENSG00000086598 | HGLibA_49804 | 101 | -0.221113856 | TMED2 | transmembrane p24 trafficking protein 2 [Source:HGNC Symbol;Acc:HGNC:16996] |
| ENSG00000088386 | HGLibA_44406 | 101 | -0.221113856 | SLC15A1 | solute carrier family 15 member 1 [Source:HGNC Symbol;Acc:HGNC:10920] |
| ENSG00000089682 | HGLibA_40547 | 101 | -0.221113856 | RBM41 | RNA binding motif protein 41 [Source:HGNC Symbol;Acc:HGNC:25617] |
| ENSG00000089876 | HGLibA_13179 | 101 | -0.221113856 | DHX32 | DEAH-box helicase 32 (putative) [Source:HGNC Symbol;Acc:HGNC:16717] |
| ENSG00000099330 | HGLibA_33244 | 101 | -0.221113856 | OCEL1 | occludin/ELL domain containing 1 [Source:HGNC Symbol;Acc:HGNC:26221] |
| ENSG00000099960 | HGLibA_45368 | 101 | -0.221113856 | SLC7A4 | solute carrier family 7 member 4 [Source:HGNC Symbol;Acc:HGNC:11062] |
| ENSG00000100104 | HGLibA_47041 | 101 | -0.221113856 | SRRD | SRR1 domain containing [Source:HGNC Symbol;Acc:HGNC:33910] |
| ENSG00000101346 | HGLibA_37467 | 101 | -0.221113856 | POFUT1 | protein O-fucosyltransferase 1 [Source:HGNC Symbol;Acc:HGNC:14988] |
| ENSG00000101347 | HGLibA_42705 | 101 | -0.221113856 | SAMHD1 | SAM and HD domain containing deoxynucleoside triphosphate triphosphohydrolase 1 [Source:HGNC Symbol;Acc:HGNC:15925] |
| ENSG00000102271 | HGLibA_25204 | 101 | -0.221113856 | KLHL4 | kelch like family member 4 [Source:HGNC Symbol;Acc:HGNC:6355] |
| ENSG00000103249 | HGLibA_09837 | 101 | -0.221113856 | CLCN7 | chloride voltage-gated channel 7 [Source:HGNC Symbol;Acc:HGNC:2025] |
| ENSG00000104312 | HGLibA_41318 | 101 | -0.221113856 | RIPK2 | receptor interacting serine/threonine kinase 2 [Source:HGNC Symbol;Acc:HGNC:10020] |
| ENSG00000104863 | HGLibA_26577 | 101 | -0.221113856 | LIN7B | crumbs cell polarity complex component [Source:HGNC Symbol;Acc:HGNC:17788]" |
| ENSG00000104879 | HGLibA_09774 | 101 | -0.221113856 | CKM | M-type [Source:HGNC Symbol;Acc:HGNC:1994]" |
| ENSG00000105136 | HGLibA_55955 | 101 | -0.221113856 | ZNF419 | zinc finger protein 419 [Source:HGNC Symbol;Acc:HGNC:20648] |
| ENSG00000105618 | HGLibA_38718 | 101 | -0.221113856 | PRPF31 | pre-mRNA processing factor 31 [Source:HGNC Symbol;Acc:HGNC:15446] |
| ENSG00000106100 | HGLibA_32244 | 101 | -0.221113856 | NOD1 | nucleotide binding oligomerization domain containing 1 [Source:HGNC Symbol;Acc:HGNC:16390] |
| ENSG00000106355 | HGLibA_27544 | 101 | -0.221113856 | LSM5 | U6 small nuclear RNA and mRNA degradation associated [Source:HGNC Symbol;Acc:HGNC:17162]" |
| ENSG00000107521 | HGLibA_22047 | 101 | -0.221113856 | HPS1 | biogenesis of lysosomal organelles complex 3 subunit 1 [Source:HGNC Symbol;Acc:HGNC:5163]" |
| ENSG00000107562 | HGLibA_11851 | 101 | -0.221113856 | CXCL12 | C-X-C motif chemokine ligand 12 [Source:HGNC Symbol;Acc:HGNC:10672] |
| ENSG00000108684 | HGLibA_03302 | 101 | -0.221113856 | ASIC2 | acid sensing ion channel subunit 2 [Source:HGNC Symbol;Acc:HGNC:99] |
| ENSG00000109062 | HGLibA_45409 | 101 | -0.221113856 | SLC9A3R1 | SLC9A3 regulator 1 [Source:HGNC Symbol;Acc:HGNC:11075] |
| ENSG00000109063 | HGLibA_30723 | 101 | -0.221113856 | MYH3 | myosin heavy chain 3 [Source:HGNC Symbol;Acc:HGNC:7573] |
| ENSG00000109667 | HGLibA_44895 | 101 | -0.221113856 | SLC2A9 | solute carrier family 2 member 9 [Source:HGNC Symbol;Acc:HGNC:13446] |
| ENSG00000109911 | HGLibA_14985 | 101 | -0.221113856 | ELP4 | elongator acetyltransferase complex subunit 4 [Source:HGNC Symbol;Acc:HGNC:1171] |
| ENSG00000110060 | HGLibA_39600 | 101 | -0.221113856 | PUS3 | pseudouridine synthase 3 [Source:HGNC Symbol;Acc:HGNC:25461] |
| ENSG00000112425 | HGLibA_15335 | 101 | -0.221113856 | EPM2A | laforin glucan phosphatase [Source:HGNC Symbol;Acc:HGNC:3413]" |
| ENSG00000113805 | HGLibA_10407 | 101 | -0.221113856 | CNTN3 | contactin 3 [Source:HGNC Symbol;Acc:HGNC:2173] |
| ENSG00000114491 | HGLibA_52984 | 101 | -0.221113856 | UMPS | uridine monophosphate synthetase [Source:HGNC Symbol;Acc:HGNC:12563] |
| ENSG00000115423 | HGLibA_13492 | 101 | -0.221113856 | DNAH6 | dynein axonemal heavy chain 6 [Source:HGNC Symbol;Acc:HGNC:2951] |
| ENSG00000115568 | HGLibA_55489 | 101 | -0.221113856 | ZNF142 | zinc finger protein 142 [Source:HGNC Symbol;Acc:HGNC:12927] |
| ENSG00000116005 | HGLibA_35694 | 101 | -0.221113856 | PCYOX1 | prenylcysteine oxidase 1 [Source:HGNC Symbol;Acc:HGNC:20588] |
| ENSG00000116396 | HGLibA_24172 | 101 | -0.221113856 | KCNC4 | potassium voltage-gated channel subfamily C member 4 [Source:HGNC Symbol;Acc:HGNC:6236] |
| ENSG00000119508 | HGLibA_32630 | 101 | -0.221113856 | NR4A3 | nuclear receptor subfamily 4 group A member 3 [Source:HGNC Symbol;Acc:HGNC:7982] |
| ENSG00000120756 | HGLibA_37226 | 101 | -0.221113856 | PLS1 | plastin 1 [Source:HGNC Symbol;Acc:HGNC:9090] |
| ENSG00000122420 | HGLibA_39340 | 101 | -0.221113856 | PTGFR | prostaglandin F receptor [Source:HGNC Symbol;Acc:HGNC:9600] |
| ENSG00000122557 | HGLibA_21175 | 101 | -0.221113856 | HERPUD2 | HERPUD family member 2 [Source:HGNC Symbol;Acc:HGNC:21915] |
| ENSG00000123124 | HGLibA_54451 | 101 | -0.221113856 | WWP1 | WW domain containing E3 ubiquitin protein ligase 1 [Source:HGNC Symbol;Acc:HGNC:17004] |
| ENSG00000124198 | HGLibA_02673 | 101 | -0.221113856 | ARFGEF2 | ADP ribosylation factor guanine nucleotide exchange factor 2 [Source:HGNC Symbol;Acc:HGNC:15853] |
| ENSG00000124253 | HGLibA_35612 | 101 | -0.221113856 | PCK1 | phosphoenolpyruvate carboxykinase 1 [Source:HGNC Symbol;Acc:HGNC:8724] |
| ENSG00000124942 | HGLibA_01302 | 101 | -0.221113856 | AHNAK | AHNAK nucleoprotein [Source:HGNC Symbol;Acc:HGNC:347] |
| ENSG00000127561 | HGLibA_47996 | 101 | -0.221113856 | SYNGR3 | synaptogyrin 3 [Source:HGNC Symbol;Acc:HGNC:11501] |
| ENSG00000130347 | HGLibA_42410 | 101 | -0.221113856 | RTN4IP1 | reticulon 4 interacting protein 1 [Source:HGNC Symbol;Acc:HGNC:18647] |
| ENSG00000132781 | HGLibA_30585 | 101 | -0.221113856 | MUTYH | mutY DNA glycosylase [Source:HGNC Symbol;Acc:HGNC:7527] |
| ENSG00000134824 | HGLibA_15938 | 101 | -0.221113856 | FADS2 | fatty acid desaturase 2 [Source:HGNC Symbol;Acc:HGNC:3575] |
| ENSG00000135502 | HGLibA_44784 | 101 | -0.221113856 | SLC26A10 | solute carrier family 26 member 10 [Source:HGNC Symbol;Acc:HGNC:14470] |
| ENSG00000136261 | HGLibA_05015 | 101 | -0.221113856 | BZW2 | basic leucine zipper and W2 domains 2 [Source:HGNC Symbol;Acc:HGNC:18808] |
| ENSG00000136297 | HGLibA_29464 | 101 | -0.221113856 | MMD2 | monocyte to macrophage differentiation associated 2 [Source:HGNC Symbol;Acc:HGNC:30133] |
| ENSG00000137673 | HGLibA_29538 | 101 | -0.221113856 | MMP7 | matrix metallopeptidase 7 [Source:HGNC Symbol;Acc:HGNC:7174] |
| ENSG00000137766 | HGLibA_53000 | 101 | -0.221113856 | UNC13C | unc-13 homolog C [Source:HGNC Symbol;Acc:HGNC:23149] |
| ENSG00000138744 | HGLibA_31006 | 101 | -0.221113856 | NAAA | N-acylethanolamine acid amidase [Source:HGNC Symbol;Acc:HGNC:736] |
| ENSG00000139220 | HGLibA_37876 | 101 | -0.221113856 | PPFIA2 | PTPRF interacting protein alpha 2 [Source:HGNC Symbol;Acc:HGNC:9246] |
| ENSG00000139946 | HGLibA_36035 | 101 | -0.221113856 | PELI2 | pellino E3 ubiquitin protein ligase family member 2 [Source:HGNC Symbol;Acc:HGNC:8828] |
| ENSG00000140391 | HGLibA_51924 | 101 | -0.221113856 | TSPAN3 | tetraspanin 3 [Source:HGNC Symbol;Acc:HGNC:17752] |
| ENSG00000140463 | HGLibA_04219 | 101 | -0.221113856 | BBS4 | Bardet-Biedl syndrome 4 [Source:HGNC Symbol;Acc:HGNC:969] |
| ENSG00000140836 | HGLibA_55173 | 101 | -0.221113856 | ZFHX3 | zinc finger homeobox 3 [Source:HGNC Symbol;Acc:HGNC:777] |
| ENSG00000141682 | HGLibA_37290 | 101 | -0.221113856 | PMAIP1 | phorbol-12-myristate-13-acetate-induced protein 1 [Source:HGNC Symbol;Acc:HGNC:9108] |
| ENSG00000143373 | HGLibA_56538 | 101 | -0.221113856 | ZNF687 | zinc finger protein 687 [Source:HGNC Symbol;Acc:HGNC:29277] |
| ENSG00000145020 | HGLibA_01834 | 101 | -0.221113856 | AMT | aminomethyltransferase [Source:HGNC Symbol;Acc:HGNC:473] |
| ENSG00000145476 | HGLibA_12199 | 101 | -0.221113856 | CYP4V2 | cytochrome P450 family 4 subfamily V member 2 [Source:HGNC Symbol;Acc:HGNC:23198] |
| ENSG00000147383 | HGLibA_32748 | 101 | -0.221113856 | NSDHL | NAD(P) dependent steroid dehydrogenase-like [Source:HGNC Symbol;Acc:HGNC:13398] |
| ENSG00000147905 | HGLibA_55045 | 101 | -0.221113856 | ZCCHC7 | zinc finger CCHC-type containing 7 [Source:HGNC Symbol;Acc:HGNC:26209] |
| ENSG00000148834 | HGLibA_20487 | 101 | -0.221113856 | GSTO1 | glutathione S-transferase omega 1 [Source:HGNC Symbol;Acc:HGNC:13312] |
| ENSG00000149485 | HGLibA_15934 | 101 | -0.221113856 | FADS1 | fatty acid desaturase 1 [Source:HGNC Symbol;Acc:HGNC:3574] |
| ENSG00000149488 | HGLibA_49752 | 101 | -0.221113856 | TMC2 | transmembrane channel like 2 [Source:HGNC Symbol;Acc:HGNC:16527] |
| ENSG00000151743 | HGLibA_01812 | 101 | -0.221113856 | AMN1 | antagonist of mitotic exit network 1 homolog [Source:HGNC Symbol;Acc:HGNC:27281] |
| ENSG00000152977 | HGLibA_55320 | 101 | -0.221113856 | ZIC1 | Zic family member 1 [Source:HGNC Symbol;Acc:HGNC:12872] |
| ENSG00000153029 | HGLibA_29805 | 101 | -0.221113856 | MR1 | class I-related [Source:HGNC Symbol;Acc:HGNC:4975]" |

| | | | | | |
|---|---|---|---|---|---|
| ENSG00000156873 | HGLibA_36413 | 101 | -0.221113856 | PHKG2 | phosphorylase kinase catalytic subunit gamma 2 [Source:HGNC Symbol;Acc:HGNC:8931] |
| ENSG00000158158 | HGLibA_10324 | 101 | -0.221113856 | CNNM4 | cyclin and CBS domain divalent metal cation transport mediator 4 [Source:HGNC Symbol;Acc:HGNC:105] |
| ENSG00000160072 | HGLibA_03414 | 101 | -0.221113856 | ATAD3B | AAA domain containing 3B [Source:HGNC Symbol;Acc:HGNC:24007]" |
| ENSG00000160349 | HGLibA_26165 | 101 | -0.221113856 | LCN1 | lipocalin 1 [Source:HGNC Symbol;Acc:HGNC:6525] |
| ENSG00000161542 | HGLibA_38765 | 101 | -0.221113856 | PRPSAP1 | phosphoribosyl pyrophosphate synthetase associated protein 1 [Source:HGNC Symbol;Acc:HGNC:9466] |
| ENSG00000162399 | HGLibA_04872 | 101 | -0.221113856 | BSND | barttin CLCNK type accessory beta subunit [Source:HGNC Symbol;Acc:HGNC:16512] |
| ENSG00000162604 | HGLibA_49683 | 101 | -0.221113856 | TM2D1 | TM2 domain containing 1 [Source:HGNC Symbol;Acc:HGNC:24142] |
| ENSG00000162931 | HGLibA_51422 | 101 | -0.221113856 | TRIM17 | tripartite motif containing 17 [Source:HGNC Symbol;Acc:HGNC:13430] |
| ENSG00000163666 | HGLibA_21197 | 101 | -0.221113856 | HESX1 | HESX homeobox 1 [Source:HGNC Symbol;Acc:HGNC:4877] |
| ENSG00000163904 | HGLibA_43387 | 101 | -0.221113856 | SENP2 | SUMO specific peptidase 2 [Source:HGNC Symbol;Acc:HGNC:23116] |
| ENSG00000163918 | HGLibA_40912 | 101 | -0.221113856 | RFC4 | replication factor C subunit 4 [Source:HGNC Symbol;Acc:HGNC:9972] |
| ENSG00000164039 | HGLibA_04378 | 101 | -0.221113856 | BDH2 | 3-hydroxybutyrate dehydrogenase 2 [Source:HGNC Symbol;Acc:HGNC:32389] |
| ENSG00000164619 | HGLibA_04626 | 101 | -0.221113856 | BMPER | BMP binding endothelial regulator [Source:HGNC Symbol;Acc:HGNC:24154] |
| ENSG00000164808 | HGLibA_46664 | 101 | -0.221113856 | SPIDR | scaffold protein involved in DNA repair [Source:HGNC Symbol;Acc:HGNC:28971] |
| ENSG00000165568 | HGLibA_01497 | 101 | -0.221113856 | AKR1E2 | aldo-keto reductase family 1 member E2 [Source:HGNC Symbol;Acc:HGNC:23437] |
| ENSG00000165959 | HGLibA_10068 | 101 | -0.221113856 | CLMN | calmin [Source:HGNC Symbol;Acc:HGNC:19972] |
| ENSG00000166634 | HGLibA_43542 | 101 | -0.221113856 | SERPINB12 | serpin family B member 12 [Source:HGNC Symbol;Acc:HGNC:14220] |
| ENSG00000167468 | HGLibA_20178 | 101 | -0.221113856 | GPX4 | glutathione peroxidase 4 [Source:HGNC Symbol;Acc:HGNC:4556] |
| ENSG00000167633 | HGLibA_24991 | 101 | -0.221113856 | KIR3DL1 | three Ig domains and long cytoplasmic tail 1 [Source:HGNC Symbol;Acc:HGNC:6338]" |
| ENSG00000167741 | HGLibA_19017 | 101 | -0.221113856 | GGT6 | gamma-glutamyltransferase 6 [Source:HGNC Symbol;Acc:HGNC:26891] |
| ENSG00000167775 | HGLibA_08352 | 101 | -0.221113856 | CD320 | CD320 molecule [Source:HGNC Symbol;Acc:HGNC:16692] |
| ENSG00000167792 | HGLibA_31570 | 101 | -0.221113856 | NDUFV1 | NADH:ubiquinone oxidoreductase core subunit V1 [Source:HGNC Symbol;Acc:HGNC:7716] |
| ENSG00000168267 | HGLibA_39302 | 101 | -0.221113856 | PTF1A | pancreas associated transcription factor 1a [Source:HGNC Symbol;Acc:HGNC:23734] |
| ENSG00000168282 | HGLibA_29134 | 101 | -0.221113856 | MGAT2 | 2-N-acetylglucosaminyltransferase [Source:HGNC Symbol;Acc:HGNC:7045]" |
| ENSG00000168310 | HGLibA_23575 | 101 | -0.221113856 | IRF2 | interferon regulatory factor 2 [Source:HGNC Symbol;Acc:HGNC:6117] |
| ENSG00000168564 | HGLibA_08833 | 101 | -0.221113856 | CDKN2AIP | CDKN2A interacting protein [Source:HGNC Symbol;Acc:HGNC:24325] |
| ENSG00000169826 | HGLibA_11349 | 101 | -0.221113856 | CSGALNACT2 | chondroitin sulfate N-acetylgalactosaminyltransferase 2 [Source:HGNC Symbol;Acc:HGNC:24292] |
| ENSG00000170166 | HGLibA_22001 | 101 | -0.221113856 | HOXD4 | homeobox D4 [Source:HGNC Symbol;Acc:HGNC:5138] |
| ENSG00000170627 | HGLibA_20627 | 101 | -0.221113856 | GTSF1 | gametocyte specific factor 1 [Source:HGNC Symbol;Acc:HGNC:26565] |
| ENSG00000170743 | HGLibA_48092 | 101 | -0.221113856 | SYT9 | synaptotagmin 9 [Source:HGNC Symbol;Acc:HGNC:19265] |
| ENSG00000171109 | HGLibA_29070 | 101 | -0.221113856 | MFN1 | mitofusin 1 [Source:HGNC Symbol;Acc:HGNC:18262] |
| ENSG00000171435 | HGLibA_25866 | 101 | -0.221113856 | KSR2 | kinase suppressor of ras 2 [Source:HGNC Symbol;Acc:HGNC:18610] |
| ENSG00000173083 | HGLibA_22061 | 101 | -0.221113856 | HPSE | heparanase [Source:HGNC Symbol;Acc:HGNC:5164] |
| ENSG00000173585 | HGLibA_08170 | 101 | -0.221113856 | CCR9 | C-C motif chemokine receptor 9 [Source:HGNC Symbol;Acc:HGNC:1610] |
| ENSG00000173852 | HGLibA_13906 | 101 | -0.221113856 | DPY19L1 | dpy-19 like C-mannosyltransferase 1 [Source:HGNC Symbol;Acc:HGNC:22205] |
| ENSG00000174417 | HGLibA_51383 | 101 | -0.221113856 | TRHR | thyrotropin releasing hormone receptor [Source:HGNC Symbol;Acc:HGNC:12299] |
| ENSG00000174485 | HGLibA_12950 | 101 | -0.221113856 | DENND4A | DENN domain containing 4A [Source:HGNC Symbol;Acc:HGNC:24321] |
| ENSG00000174547 | HGLibA_29895 | 101 | -0.221113856 | MRPL11 | mitochondrial ribosomal protein L11 [Source:HGNC Symbol;Acc:HGNC:14042] |
| ENSG00000174721 | HGLibA_17433 | 101 | -0.221113856 | FGFBP3 | fibroblast growth factor binding protein 3 [Source:HGNC Symbol;Acc:HGNC:23428] |
| ENSG00000177352 | HGLibA_07853 | 101 | -0.221113856 | CCDC71 | coiled-coil domain containing 71 [Source:HGNC Symbol;Acc:HGNC:25760] |
| ENSG00000177504 | HGLibA_53584 | 101 | -0.221113856 | VCX2 | variable charge X-linked 2 [Source:HGNC Symbol;Acc:HGNC:18158] |
| ENSG00000177888 | HGLibA_54883 | 101 | -0.221113856 | ZBTB41 | zinc finger and BTB domain containing 41 [Source:HGNC Symbol;Acc:HGNC:24819] |
| ENSG00000178502 | HGLibA_25126 | 101 | -0.221113856 | KLHL11 | kelch like family member 11 [Source:HGNC Symbol;Acc:HGNC:19008] |
| ENSG00000180745 | HGLibA_10127 | 101 | -0.221113856 | CLRN3 | clarin 3 [Source:HGNC Symbol;Acc:HGNC:20795] |
| ENSG00000181392 | HGLibA_47985 | 101 | -0.221113856 | SYNE4 | spectrin repeat containing nuclear envelope family member 4 [Source:HGNC Symbol;Acc:HGNC:26703] |
| ENSG00000183844 | HGLibA_16578 | 101 | -0.221113856 | FAM3B | family with sequence similarity 3 member B [Source:HGNC Symbol;Acc:HGNC:1253] |
| ENSG00000184564 | HGLibA_45527 | 101 | -0.221113856 | SLITRK6 | SLIT and NTRK like family member 6 [Source:HGNC Symbol;Acc:HGNC:23503] |
| ENSG00000184619 | HGLibA_25390 | 101 | -0.221113856 | KRBA2 | KRAB-A domain containing 2 [Source:HGNC Symbol;Acc:HGNC:26989] |
| ENSG00000185015 | HGLibA_06797 | 101 | -0.221113856 | CA13 | carbonic anhydrase 13 [Source:HGNC Symbol;Acc:HGNC:14914] |
| ENSG00000186188 | HGLibA_17332 | 101 | -0.221113856 | FFAR4 | free fatty acid receptor 4 [Source:HGNC Symbol;Acc:HGNC:19061] |
| ENSG00000188379 | HGLibA_22675 | 101 | -0.221113856 | IFNA2 | interferon alpha 2 [Source:HGNC Symbol;Acc:HGNC:5423] |
| ENSG00000188566 | HGLibA_31408 | 101 | -0.221113856 | NDOR1 | NADPH dependent diflavin oxidoreductase 1 [Source:HGNC Symbol;Acc:HGNC:29838] |
| ENSG00000189369 | HGLibA_20443 | 101 | -0.221113856 | GSPT2 | G1 to S phase transition 2 [Source:HGNC Symbol;Acc:HGNC:4622] |
| ENSG00000196072 | HGLibA_04559 | 101 | -0.221113856 | BLOC1S2 | biogenesis of lysosomal organelles complex 1 subunit 2 [Source:HGNC Symbol;Acc:HGNC:20984] |
| ENSG00000196865 | HGLibA_31900 | 101 | -0.221113856 | NHLRC2 | NHL repeat containing 2 [Source:HGNC Symbol;Acc:HGNC:24731] |
| ENSG00000197892 | HGLibA_24858 | 101 | -0.221113856 | KIF13B | kinesin family member 13B [Source:HGNC Symbol;Acc:HGNC:14405] |
| ENSG00000198601 | HGLibA_33833 | 101 | -0.221113856 | OR2M2 | olfactory receptor family 2 subfamily M member 2 [Source:HGNC Symbol;Acc:HGNC:8268] |
| ENSG00000198730 | HGLibA_11673 | 101 | -0.221113856 | CTR9 | Paf1/RNA polymerase II complex component [Source:HGNC Symbol;Acc:HGNC:16850]" |
| ENSG00000204371 | HGLibA_14688 | 101 | -0.221113856 | EHMT2 | euchromatic histone lysine methyltransferase 2 [Source:HGNC Symbol;Acc:HGNC:14129] |
| ENSG00000206075 | HGLibA_43557 | 101 | -0.221113856 | SERPINB5 | serpin family B member 5 [Source:HGNC Symbol;Acc:HGNC:8949] |
| ENSG00000206102 | HGLibA_25684 | 101 | -0.221113856 | KRTAP19-8 | keratin associated protein 19-8 [Source:HGNC Symbol;Acc:HGNC:33898] |
| ENSG00000206535 | HGLibA_26739 | 101 | -0.221113856 | LNP1 | leukemia NUP98 fusion partner 1 [Source:HGNC Symbol;Acc:HGNC:28014] |
| ENSG00000212657 | HGLibA_25656 | 101 | -0.221113856 | KRTAP16-1 | keratin associated protein 16-1 [Source:HGNC Symbol;Acc:HGNC:18916] |
| ENSG00000224902 | HGLibA_18456 | 101 | -0.221113856 | GAGE12H | G antigen 12H [Source:HGNC Symbol;Acc:HGNC:31908] |
| ENSG00000244646 | HGLibA_54545 | 101 | -0.221113856 | XKRY2 | Y-linked 2 [Source:HGNC Symbol;Acc:HGNC:23922]" |
| ENSG00000248672 | HGLibA_27684 | 101 | -0.221113856 | LY75-CD302 | LY75-CD302 readthrough [Source:HGNC Symbol;Acc:HGNC:38828] |
| ENSG00000265681 | HGLibA_41881 | 101 | -0.221113856 | RPL17 | ribosomal protein L17 [Source:HGNC Symbol;Acc:HGNC:10307] |
| ENSG00000272325 | HGLibA_32976 | 101 | -0.221113856 | NUDT3 | nudix hydrolase 3 [Source:HGNC Symbol;Acc:HGNC:8050] |
| ENSG00000278224 | HGLibA_38480 | 101 | -0.221113856 | PRICKLE4 | prickle planar cell polarity protein 4 [Source:HGNC Symbol;Acc:HGNC:16805] |
| ENSG00000279301 | HGLibA_33858 | 101 | -0.221113856 | OR2T11 | olfactory receptor family 2 subfamily T member 11 (gene/pseudogene) [Source:HGNC Symbol;Acc:HGNC:19574] |
| GAGE5 | HGLibA_18481 | 101 | -0.221113856 | GAGE5 | - |
| HIST1H3I | HGLibA_21479 | 101 | -0.221113856 | HIST1H3I | - |
| hsa-mir-29a | HGLibA_58410 | 101 | -0.221113856 | hsa-mir-29a | - |
| hsa-mir-3180-1 | HGLibA_58800 | 101 | -0.221113856 | hsa-mir-3180-1 | - |
| hsa-mir-3663 | HGLibA_59203 | 101 | -0.221113856 | hsa-mir-3663 | - |
| hsa-mir-381 | HGLibA_59479 | 101 | -0.221113856 | hsa-mir-381 | - |
| hsa-mir-3910-1 | HGLibA_59504 | 101 | -0.221113856 | hsa-mir-3910-1 | - |
| hsa-mir-4298 | HGLibA_59903 | 101 | -0.221113856 | hsa-mir-4298 | - |
| hsa-mir-4533 | HGLibA_60591 | 101 | -0.221113856 | hsa-mir-4533 | - |
| hsa-mir-4686 | HGLibA_60856 | 101 | -0.221113856 | hsa-mir-4686 | - |
| hsa-mir-4742 | HGLibA_61067 | 101 | -0.221113856 | hsa-mir-4742 | - |
| hsa-mir-4778 | HGLibA_61218 | 101 | -0.221113856 | hsa-mir-4778 | - |
| hsa-mir-4791 | HGLibA_61270 | 101 | -0.221113856 | hsa-mir-4791 | - |
| hsa-mir-516b-1 | HGLibA_61618 | 101 | -0.221113856 | hsa-mir-516b-1 | - |
| hsa-mir-6082 | HGLibA_62590 | 101 | -0.221113856 | hsa-mir-6082 | - |
| hsa-mir-6720 | HGLibA_63040 | 101 | -0.221113856 | hsa-mir-6720 | - |
| hsa-mir-6728 | HGLibA_63064 | 101 | -0.221113856 | hsa-mir-6728 | - |
| hsa-mir-7849 | HGLibA_63984 | 101 | -0.221113856 | hsa-mir-7849 | - |
| hsa-mir-8081 | HGLibA_64168 | 101 | -0.221113856 | hsa-mir-8081 | - |
| MTERFD1 | HGLibA_30341 | 101 | -0.221113856 | MTERFD1 | - |
| RARS | HGLibA_40290 | 101 | -0.221113856 | RARS | - |
| TLDC1 | HGLibA_49601 | 101 | -0.221113856 | TLDC1 | - |
| UQCC | HGLibA_53096 | 101 | -0.221113856 | UQCC | - |
| VAMP7_Y | HGLibA_53507 | 101 | -0.221113856 | VAMP7_Y | - |
| C17orf104 | HGLibA_05522 | 100 | -0.230159199 | C17orf104 | - |
| C20orf194 | HGLibA_05999 | 100 | -0.230159199 | C20orf194 | - |
| C20orf195 | HGLibA_06001 | 100 | -0.230159199 | C20orf195 | - |
| EFCAB4A | HGLibA_14540 | 100 | -0.230159199 | EFCAB4A | - |
| ENSG00000021355 | HGLibA_43536 | 100 | -0.230159199 | SERPINB1 | serpin family B member 1 [Source:HGNC Symbol;Acc:HGNC:3311] |
| ENSG00000047648 | HGLibA_02786 | 100 | -0.230159199 | ARHGAP6 | Rho GTPase activating protein 6 [Source:HGNC Symbol;Acc:HGNC:676] |
| ENSG00000050748 | HGLibA_28296 | 100 | -0.230159199 | MAPK9 | mitogen-activated protein kinase 9 [Source:HGNC Symbol;Acc:HGNC:6886] |
| ENSG00000058335 | HGLibA_40337 | 100 | -0.230159199 | RASGRF1 | Ras protein specific guanine nucleotide releasing factor 1 [Source:HGNC Symbol;Acc:HGNC:9875] |
| ENSG00000064313 | HGLibA_48228 | 100 | -0.230159199 | TAF2 | TATA-box binding protein associated factor 2 [Source:HGNC Symbol;Acc:HGNC:11536] |
| ENSG00000070190 | HGLibA_12334 | 100 | -0.230159199 | DAPP1 | dual adaptor of phosphotyrosine and 3-phosphoinositides 1 [Source:HGNC Symbol;Acc:HGNC:16500] |
| ENSG00000071991 | HGLibA_08657 | 100 | -0.230159199 | CDH19 | cadherin 19 [Source:HGNC Symbol;Acc:HGNC:1758] |
| ENSG00000072163 | HGLibA_26548 | 100 | -0.230159199 | LIMS2 | LIM zinc finger domain containing 2 [Source:HGNC Symbol;Acc:HGNC:16084] |
| ENSG00000073146 | HGLibA_29697 | 100 | -0.230159199 | MOV10L1 | Mov10 like RISC complex RNA helicase 1 [Source:HGNC Symbol;Acc:HGNC:7201] |
| ENSG00000087460 | HGLibA_19468 | 100 | -0.230159199 | GNAS | GNAS complex locus [Source:HGNC Symbol;Acc:HGNC:4392] |
| ENSG00000089327 | HGLibA_18236 | 100 | -0.230159199 | FXYD5 | FXYD domain containing ion transport regulator 5 [Source:HGNC Symbol;Acc:HGNC:4029] |
| ENSG00000099968 | HGLibA_04313 | 100 | -0.230159199 | BCL2L13 | BCL2 like 13 [Source:HGNC Symbol;Acc:HGNC:17164] |
| ENSG00000101236 | HGLibA_41652 | 100 | -0.230159199 | RNF24 | ring finger protein 24 [Source:HGNC Symbol;Acc:HGNC:13779] |
| ENSG00000101846 | HGLibA_47602 | 100 | -0.230159199 | STS | steroid sulfatase [Source:HGNC Symbol;Acc:HGNC:11425] |
| ENSG00000102699 | HGLibA_35238 | 100 | -0.230159199 | PARP4 | poly(ADP-ribose) polymerase family member 4 [Source:HGNC Symbol;Acc:HGNC:271] |
| ENSG00000102996 | HGLibA_29494 | 100 | -0.230159199 | MMP15 | matrix metallopeptidase 15 [Source:HGNC Symbol;Acc:HGNC:7161] |
| ENSG00000103067 | HGLibA_15591 | 100 | -0.230159199 | ESRP2 | epithelial splicing regulatory protein 2 [Source:HGNC Symbol;Acc:HGNC:26152] |
| ENSG00000103067 | HGLibA_15589 | 100 | -0.230159199 | ESRP2 | epithelial splicing regulatory protein 2 [Source:HGNC Symbol;Acc:HGNC:26152] |
| ENSG00000103591 | HGLibA_00047 | 100 | -0.230159199 | AAGAB | alpha and gamma adaptin binding protein [Source:HGNC Symbol;Acc:HGNC:25662] |
| ENSG00000104901 | HGLibA_13306 | 100 | -0.230159199 | DKKL1 | dickkopf like acrosomal protein 1 [Source:HGNC Symbol;Acc:HGNC:16528] |
| ENSG00000105402 | HGLibA_31126 | 100 | -0.230159199 | NAPA | NSF attachment protein alpha [Source:HGNC Symbol;Acc:HGNC:7641] |
| ENSG00000105889 | HGLibA_47423 | 100 | -0.230159199 | STEAP1B | STEAP family member 1B [Source:HGNC Symbol;Acc:HGNC:41907] |
| ENSG00000106025 | HGLibA_51897 | 100 | -0.230159199 | TSPAN12 | tetraspanin 12 [Source:HGNC Symbol;Acc:HGNC:21641] |
| ENSG00000106153 | HGLibA_09365 | 100 | -0.230159199 | CHCHD2 | coiled-coil-helix-coiled-coil-helix domain containing 2 [Source:HGNC Symbol;Acc:HGNC:21645] |
| ENSG00000107362 | HGLibA_00267 | 100 | -0.230159199 | ABHD17B | abhydrolase domain containing 17B [Source:HGNC Symbol;Acc:HGNC:24278] |
| ENSG00000108578 | HGLibA_04550 | 100 | -0.230159199 | BLMH | bleomycin hydrolase [Source:HGNC Symbol;Acc:HGNC:1059] |
| ENSG00000110536 | HGLibA_39428 | 100 | -0.230159199 | PTPMT1 | mitochondrial 1 [Source:HGNC Symbol;Acc:HGNC:26965]" |
| ENSG00000111490 | HGLibA_48532 | 100 | -0.230159199 | TBC1D30 | TBC1 domain family member 30 [Source:HGNC Symbol;Acc:HGNC:29164] |
| ENSG00000112078 | HGLibA_24448 | 100 | -0.230159199 | KCTD20 | potassium channel tetramerization domain containing 20 [Source:HGNC Symbol;Acc:HGNC:21052] |
| ENSG00000112462 | HGLibA_33589 | 100 | -0.230159199 | OR12D3 | olfactory receptor family 12 subfamily D member 3 [Source:HGNC Symbol;Acc:HGNC:13963] |
| ENSG00000113248 | HGLibA_35487 | 100 | -0.230159199 | PCDHB15 | protocadherin beta 15 [Source:HGNC Symbol;Acc:HGNC:8686] |
| ENSG00000114948 | HGLibA_00762 | 100 | -0.230159199 | ADAM23 | ADAM metallopeptidase domain 23 [Source:HGNC Symbol;Acc:HGNC:202] |
| ENSG00000115008 | HGLibA_23036 | 100 | -0.230159199 | IL1A | interleukin 1 alpha [Source:HGNC Symbol;Acc:HGNC:5991] |
| ENSG00000115041 | HGLibA_24239 | 100 | -0.230159199 | KCNIP3 | potassium voltage-gated channel interacting protein 3 [Source:HGNC Symbol;Acc:HGNC:15523] |
| ENSG00000116750 | HGLibA_52806 | 100 | -0.230159199 | UCHL5 | ubiquitin C-terminal hydrolase L5 [Source:HGNC Symbol;Acc:HGNC:19678] |
| ENSG00000116771 | HGLibA_01200 | 100 | -0.230159199 | AGMAT | agmatinase [Source:HGNC Symbol;Acc:HGNC:18407] |
| ENSG00000116906 | HGLibA_19563 | 100 | -0.230159199 | GNPAT | glyceronephosphate O-acyltransferase [Source:HGNC Symbol;Acc:HGNC:4416] |

| | | | | | |
|---|---|---|---|---|---|
| ENSG00000117425 | HGLibA_39270 | 100 | -0.230159199 | PTCH2 | patched 2 [Source:HGNC Symbol;Acc:HGNC:9586] |
| ENSG00000118245 | HGLibA_50893 | 100 | -0.230159199 | TNP1 | transition protein 1 [Source:HGNC Symbol;Acc:HGNC:11951] |
| ENSG00000119185 | HGLibA_23771 | 100 | -0.230159199 | ITGB1BP1 | integrin subunit beta 1 binding protein 1 [Source:HGNC Symbol;Acc:HGNC:23927] |
| ENSG00000119862 | HGLibA_26391 | 100 | -0.230159199 | LGALSL | galectin like [Source:HGNC Symbol;Acc:HGNC:25012] |
| ENSG00000120322 | HGLibA_35512 | 100 | -0.230159199 | PCDHB8 | protocadherin beta 8 [Source:HGNC Symbol;Acc:HGNC:8693] |
| ENSG00000123119 | HGLibA_31583 | 100 | -0.230159199 | NECAB1 | N-terminal EF-hand calcium binding protein 1 [Source:HGNC Symbol;Acc:HGNC:20983] |
| ENSG00000123131 | HGLibA_38415 | 100 | -0.230159199 | PRDX4 | peroxiredoxin 4 [Source:HGNC Symbol;Acc:HGNC:17169] |
| ENSG00000124469 | HGLibA_08953 | 100 | -0.230159199 | CEACAM8 | carcinoembryonic antigen related cell adhesion molecule 8 [Source:HGNC Symbol;Acc:HGNC:1820] |
| ENSG00000125520 | HGLibA_44879 | 100 | -0.230159199 | SLC2A4RG | SLC2A4 regulator [Source:HGNC Symbol;Acc:HGNC:15930] |
| ENSG00000125676 | HGLibA_49366 | 100 | -0.230159199 | THOC2 | THO complex 2 [Source:HGNC Symbol;Acc:HGNC:19073] |
| ENSG00000128011 | HGLibA_27151 | 100 | -0.230159199 | LRFN1 | leucine rich repeat and fibronectin type III domain containing 1 [Source:HGNC Symbol;Acc:HGNC:29290] |
| ENSG00000128918 | HGLibA_01544 | 100 | -0.230159199 | ALDH1A2 | aldehyde dehydrogenase 1 family member A2 [Source:HGNC Symbol;Acc:HGNC:15472] |
| ENSG00000128973 | HGLibA_10080 | 100 | -0.230159199 | CLN6 | transmembrane ER protein [Source:HGNC Symbol;Acc:HGNC:2077]" |
| ENSG00000130695 | HGLibA_09171 | 100 | -0.230159199 | CEP85 | centrosomal protein 85 [Source:HGNC Symbol;Acc:HGNC:25309] |
| ENSG00000131626 | HGLibA_37873 | 100 | -0.230159199 | PPFIA1 | PTPRF interacting protein alpha 1 [Source:HGNC Symbol;Acc:HGNC:9245] |
| ENSG00000132915 | HGLibA_35804 | 100 | -0.230159199 | PDE6A | phosphodiesterase 6A [Source:HGNC Symbol;Acc:HGNC:8785] |
| ENSG00000135074 | HGLibA_00746 | 100 | -0.230159199 | ADAM19 | ADAM metallopeptidase domain 19 [Source:HGNC Symbol;Acc:HGNC:197] |
| ENSG00000135220 | HGLibA_52908 | 100 | -0.230159199 | UGT2A3 | UDP glucuronosyltransferase family 2 member A3 [Source:HGNC Symbol;Acc:HGNC:28528] |
| ENSG00000135678 | HGLibA_10943 | 100 | -0.230159199 | CPM | carboxypeptidase M [Source:HGNC Symbol;Acc:HGNC:2311] |
| ENSG00000137252 | HGLibA_20992 | 100 | -0.230159199 | HCRTR2 | hypocretin receptor 2 [Source:HGNC Symbol;Acc:HGNC:4849] |
| ENSG00000139372 | HGLibA_48880 | 100 | -0.230159199 | TDG | thymine DNA glycosylase [Source:HGNC Symbol;Acc:HGNC:11700] |
| ENSG00000140297 | HGLibA_18814 | 100 | -0.230159199 | GCNT3 | mucin type [Source:HGNC Symbol;Acc:HGNC:4205]" |
| ENSG00000141026 | HGLibA_28820 | 100 | -0.230159199 | MED9 | mediator complex subunit 9 [Source:HGNC Symbol;Acc:HGNC:25487] |
| ENSG00000141293 | HGLibA_44269 | 100 | -0.230159199 | SKAP1 | src kinase associated phosphoprotein 1 [Source:HGNC Symbol;Acc:HGNC:15605] |
| ENSG00000143398 | HGLibA_36713 | 100 | -0.230159199 | PIP5K1A | phosphatidylinositol-4-phosphate 5-kinase type 1 alpha [Source:HGNC Symbol;Acc:HGNC:8994] |
| ENSG00000145050 | HGLibA_28097 | 100 | -0.230159199 | MANF | mesencephalic astrocyte derived neurotrophic factor [Source:HGNC Symbol;Acc:HGNC:15461] |
| ENSG00000145626 | HGLibA_52934 | 100 | -0.230159199 | UGT3A1 | UDP glycosyltransferase family 3 member A1 [Source:HGNC Symbol;Acc:HGNC:26625] |
| ENSG00000146066 | HGLibA_21315 | 100 | -0.230159199 | HIGD2A | HIG1 hypoxia inducible domain family member 2A [Source:HGNC Symbol;Acc:HGNC:28311] |
| ENSG00000148396 | HGLibA_43209 | 100 | -0.230159199 | SEC16A | endoplasmic reticulum export factor [Source:HGNC Symbol;Acc:HGNC:29006]" |
| ENSG00000148803 | HGLibA_18171 | 100 | -0.230159199 | FUOM | fucose mutarotase [Source:HGNC Symbol;Acc:HGNC:24733] |
| ENSG00000151136 | HGLibA_04900 | 100 | -0.230159199 | BTBD11 | BTB domain containing 11 [Source:HGNC Symbol;Acc:HGNC:23844] |
| ENSG00000151500 | HGLibA_49428 | 100 | -0.230159199 | THYN1 | thymocyte nuclear protein 1 [Source:HGNC Symbol;Acc:HGNC:29560] |
| ENSG00000152214 | HGLibA_41340 | 100 | -0.230159199 | RIT2 | Ras like without CAAX 2 [Source:HGNC Symbol;Acc:HGNC:10017] |
| ENSG00000154165 | HGLibA_19928 | 100 | -0.230159199 | GPR15 | G protein-coupled receptor 15 [Source:HGNC Symbol;Acc:HGNC:4469] |
| ENSG00000156096 | HGLibA_52927 | 100 | -0.230159199 | UGT2B4 | UDP glucuronosyltransferase family 2 member B4 [Source:HGNC Symbol;Acc:HGNC:12553] |
| ENSG00000158525 | HGLibA_10895 | 100 | -0.230159199 | CPA5 | carboxypeptidase A5 [Source:HGNC Symbol;Acc:HGNC:15722] |
| ENSG00000160588 | HGLibA_29800 | 100 | -0.230159199 | MPZL3 | myelin protein zero like 3 [Source:HGNC Symbol;Acc:HGNC:27279] |
| ENSG00000160783 | HGLibA_37303 | 100 | -0.230159199 | PMF1 | polyamine modulated factor 1 [Source:HGNC Symbol;Acc:HGNC:9112] |
| ENSG00000160932 | HGLibA_27656 | 100 | -0.230159199 | LY6E | lymphocyte antigen 6 family member E [Source:HGNC Symbol;Acc:HGNC:6727] |
| ENSG00000161714 | HGLibA_36993 | 100 | -0.230159199 | PLCD3 | phospholipase C delta 3 [Source:HGNC Symbol;Acc:HGNC:9061] |
| ENSG00000164038 | HGLibA_45437 | 100 | -0.230159199 | SLC9B2 | solute carrier family 9 member B2 [Source:HGNC Symbol;Acc:HGNC:25143] |
| ENSG00000164574 | HGLibA_18530 | 100 | -0.230159199 | GALNT10 | polypeptide N-acetylgalactosaminyltransferase 10 [Source:HGNC Symbol;Acc:HGNC:19873] |
| ENSG00000164647 | HGLibA_47421 | 100 | -0.230159199 | STEAP1 | STEAP family member 1 [Source:HGNC Symbol;Acc:HGNC:11378] |
| ENSG00000165685 | HGLibA_50435 | 100 | -0.230159199 | TMEM52B | transmembrane protein 52B [Source:HGNC Symbol;Acc:HGNC:26438] |
| ENSG00000166118 | HGLibA_46442 | 100 | -0.230159199 | SPATA19 | spermatogenesis associated 19 [Source:HGNC Symbol;Acc:HGNC:30614] |
| ENSG00000166948 | HGLibA_49269 | 100 | -0.230159199 | TGM6 | transglutaminase 6 [Source:HGNC Symbol;Acc:HGNC:16255] |
| ENSG00000167657 | HGLibA_12329 | 100 | -0.230159199 | DAPK3 | death associated protein kinase 3 [Source:HGNC Symbol;Acc:HGNC:2676] |
| ENSG00000167711 | HGLibA_43589 | 100 | -0.230159199 | SERPINF2 | serpin family F member 2 [Source:HGNC Symbol;Acc:HGNC:9075] |
| ENSG00000167766 | HGLibA_56831 | 100 | -0.230159199 | ZNF83 | zinc finger protein 83 [Source:HGNC Symbol;Acc:HGNC:13158] |
| ENSG00000168676 | HGLibA_24441 | 100 | -0.230159199 | KCTD19 | potassium channel tetramerization domain containing 19 [Source:HGNC Symbol;Acc:HGNC:24753] |
| ENSG00000168803 | HGLibA_00724 | 100 | -0.230159199 | ADAL | adenosine deaminase like [Source:HGNC Symbol;Acc:HGNC:31853] |
| ENSG00000168884 | HGLibA_50839 | 100 | -0.230159199 | TNIP2 | TNFAIP3 interacting protein 2 [Source:HGNC Symbol;Acc:HGNC:19118] |
| ENSG00000169019 | HGLibA_10678 | 100 | -0.230159199 | COMMD8 | COMM domain containing 8 [Source:HGNC Symbol;Acc:HGNC:26036] |
| ENSG00000169136 | HGLibA_03442 | 100 | -0.230159199 | ATF5 | activating transcription factor 5 [Source:HGNC Symbol;Acc:HGNC:790] |
| ENSG00000169302 | HGLibA_47483 | 100 | -0.230159199 | STK32A | serine/threonine kinase 32A [Source:HGNC Symbol;Acc:HGNC:28317] |
| ENSG00000169439 | HGLibA_43101 | 100 | -0.230159199 | SDC2 | syndecan 2 [Source:HGNC Symbol;Acc:HGNC:10659] |
| ENSG00000169933 | HGLibA_18037 | 100 | -0.230159199 | FRMPD4 | FERM and PDZ domain containing 4 [Source:HGNC Symbol;Acc:HGNC:29007] |
| ENSG00000170439 | HGLibA_29020 | 100 | -0.230159199 | METTL7B | methyltransferase like 7B [Source:HGNC Symbol;Acc:HGNC:28276] |
| ENSG00000171401 | HGLibA_25423 | 100 | -0.230159199 | KRT13 | keratin 13 [Source:HGNC Symbol;Acc:HGNC:6415] |
| ENSG00000171943 | HGLibA_46975 | 100 | -0.230159199 | SRGAP2C | SLIT-ROBO Rho GTPase activating protein 2C [Source:HGNC Symbol;Acc:HGNC:30584] |
| ENSG00000173597 | HGLibA_47743 | 100 | -0.230159199 | SULT1B1 | sulfotransferase family 1B member 1 [Source:HGNC Symbol;Acc:HGNC:17845] |
| ENSG00000173757 | HGLibA_47395 | 100 | -0.230159199 | STAT5B | signal transducer and activator of transcription 5B [Source:HGNC Symbol;Acc:HGNC:11367] |
| ENSG00000174780 | HGLibA_47012 | 100 | -0.230159199 | SRP72 | signal recognition particle 72 [Source:HGNC Symbol;Acc:HGNC:11303] |
| ENSG00000174839 | HGLibA_12966 | 100 | -0.230159199 | DENND6A | DENN domain containing 6A [Source:HGNC Symbol;Acc:HGNC:26635] |
| ENSG00000175567 | HGLibA_52833 | 100 | -0.230159199 | UCP2 | uncoupling protein 2 [Source:HGNC Symbol;Acc:HGNC:12518] |
| ENSG00000175606 | HGLibA_50501 | 100 | -0.230159199 | TMEM70 | transmembrane protein 70 [Source:HGNC Symbol;Acc:HGNC:26050] |
| ENSG00000176623 | HGLibA_41365 | 100 | -0.230159199 | RMDN1 | regulator of microtubule dynamics 1 [Source:HGNC Symbol;Acc:HGNC:24285] |
| ENSG00000178988 | HGLibA_29831 | 100 | -0.230159199 | MRFAP1L1 | Morf4 family associated protein 1 like 1 [Source:HGNC Symbol;Acc:HGNC:28796] |
| ENSG00000180383 | HGLibA_12876 | 100 | -0.230159199 | DEFB124 | defensin beta 124 [Source:HGNC Symbol;Acc:HGNC:18104] |
| ENSG00000180611 | HGLibA_28459 | 100 | -0.230159199 | MB21D2 | Mab-21 domain containing 2 [Source:HGNC Symbol;Acc:HGNC:30438] |
| ENSG00000180628 | HGLibA_35601 | 100 | -0.230159199 | PCGF5 | polycomb group ring finger 5 [Source:HGNC Symbol;Acc:HGNC:28264] |
| ENSG00000180872 | HGLibA_12850 | 100 | -0.230159199 | DEFB112 | defensin beta 112 [Source:HGNC Symbol;Acc:HGNC:18093] |
| ENSG00000181371 | HGLibA_34339 | 100 | -0.230159199 | OR5M8 | olfactory receptor family 5 subfamily M member 8 [Source:HGNC Symbol;Acc:HGNC:14846] |
| ENSG00000181418 | HGLibA_12623 | 100 | -0.230159199 | DDN | dendrin [Source:HGNC Symbol;Acc:HGNC:24458] |
| ENSG00000182521 | HGLibA_48630 | 100 | -0.230159199 | TBPL2 | TATA-box binding protein like 2 [Source:HGNC Symbol;Acc:HGNC:19841] |
| ENSG00000182871 | HGLibA_10523 | 100 | -0.230159199 | COL18A1 | collagen type XVIII alpha 1 chain [Source:HGNC Symbol;Acc:HGNC:2195] |
| ENSG00000183072 | HGLibA_32044 | 100 | -0.230159199 | NKX2-5 | NK2 homeobox 5 [Source:HGNC Symbol;Acc:HGNC:2488] |
| ENSG00000185245 | HGLibA_19698 | 100 | -0.230159199 | GP1BA | glycoprotein Ib platelet subunit alpha [Source:HGNC Symbol;Acc:HGNC:4439] |
| ENSG00000185527 | HGLibA_35817 | 100 | -0.230159199 | PDE6G | phosphodiesterase 6G [Source:HGNC Symbol;Acc:HGNC:8789] |
| ENSG00000185962 | HGLibA_26130 | 100 | -0.230159199 | LCE3A | late cornified envelope 3A [Source:HGNC Symbol;Acc:HGNC:29461] |
| ENSG00000186723 | HGLibA_33505 | 100 | -0.230159199 | OR10H1 | olfactory receptor family 10 subfamily H member 1 [Source:HGNC Symbol;Acc:HGNC:8172] |
| ENSG00000186815 | HGLibA_51106 | 100 | -0.230159199 | TPCN1 | two pore segment channel 1 [Source:HGNC Symbol;Acc:HGNC:18182] |
| ENSG00000186930 | HGLibA_25819 | 100 | -0.230159199 | KRTAP6-2 | keratin associated protein 6-2 [Source:HGNC Symbol;Acc:HGNC:18932] |
| ENSG00000187151 | HGLibA_01921 | 100 | -0.230159199 | ANGPTL5 | angiopoietin like 5 [Source:HGNC Symbol;Acc:HGNC:19705] |
| ENSG00000187173 | HGLibA_26118 | 100 | -0.230159199 | LCE2A | late cornified envelope 2A [Source:HGNC Symbol;Acc:HGNC:29469] |
| ENSG00000187527 | HGLibA_03579 | 100 | -0.230159199 | ATP13A5 | ATPase 13A5 [Source:HGNC Symbol;Acc:HGNC:31789] |
| ENSG00000187699 | HGLibA_06198 | 100 | -0.230159199 | C2orf88 | chromosome 2 open reading frame 88 [Source:HGNC Symbol;Acc:HGNC:28191] |
| ENSG00000187742 | HGLibA_43264 | 100 | -0.230159199 | SECISBP2 | SECIS binding protein 2 [Source:HGNC Symbol;Acc:HGNC:30972] |
| ENSG00000187772 | HGLibA_26560 | 100 | -0.230159199 | LIN28B | lin-28 homolog B [Source:HGNC Symbol;Acc:HGNC:32207] |
| ENSG00000188191 | HGLibA_38525 | 100 | -0.230159199 | PRKAR1B | protein kinase cAMP-dependent type I regulatory subunit beta [Source:HGNC Symbol;Acc:HGNC:9390] |
| ENSG00000188219 | HGLibA_37729 | 100 | -0.230159199 | POTEE | POTE ankyrin domain family member E [Source:HGNC Symbol;Acc:HGNC:33895] |
| ENSG00000189181 | HGLibA_33635 | 100 | -0.230159199 | OR14I1 | olfactory receptor family 14 subfamily I member 1 [Source:HGNC Symbol;Acc:HGNC:19575] |
| ENSG00000189366 | HGLibA_01622 | 100 | -0.230159199 | ALG1L | chitobiosyldiphosphodolichol beta-mannosyltransferase like [Source:HGNC Symbol;Acc:HGNC:33721]" |
| ENSG00000196366 | HGLibA_06702 | 100 | -0.230159199 | C9orf163 | chromosome 9 open reading frame 163 [Source:HGNC Symbol;Acc:HGNC:26718] |
| ENSG00000196437 | HGLibA_56269 | 100 | -0.230159199 | ZNF569 | zinc finger protein 569 [Source:HGNC Symbol;Acc:HGNC:24737] |
| ENSG00000196586 | HGLibA_30859 | 100 | -0.230159199 | MYO6 | myosin VI [Source:HGNC Symbol;Acc:HGNC:7605] |
| ENSG00000197162 | HGLibA_56749 | 100 | -0.230159199 | ZNF785 | zinc finger protein 785 [Source:HGNC Symbol;Acc:HGNC:26496] |
| ENSG00000197747 | HGLibA_42533 | 100 | -0.230159199 | S100A10 | S100 calcium binding protein A10 [Source:HGNC Symbol;Acc:HGNC:10487] |
| ENSG00000197965 | HGLibA_29796 | 100 | -0.230159199 | MPZL1 | myelin protein zero like 1 [Source:HGNC Symbol;Acc:HGNC:7226] |
| ENSG00000198326 | HGLibA_50282 | 100 | -0.230159199 | TMEM239 | transmembrane protein 239 [Source:HGNC Symbol;Acc:HGNC:40044] |
| ENSG00000198331 | HGLibA_22458 | 100 | -0.230159199 | HYLS1 | centriolar and ciliogenesis associated [Source:HGNC Symbol;Acc:HGNC:26558]" |
| ENSG00000198881 | HGLibA_03213 | 100 | -0.230159199 | ASB12 | ankyrin repeat and SOCS box containing 12 [Source:HGNC Symbol;Acc:HGNC:19763] |
| ENSG00000198965 | HGLibA_33543 | 100 | -0.230159199 | OR10R2 | olfactory receptor family 10 subfamily R member 2 [Source:HGNC Symbol;Acc:HGNC:14820] |
| ENSG00000203952 | HGLibA_07671 | 100 | -0.230159199 | CCDC160 | coiled-coil domain containing 160 [Source:HGNC Symbol;Acc:HGNC:37286] |
| ENSG00000204120 | HGLibA_19067 | 100 | -0.230159199 | GIGYF2 | GRB10 interacting GYF protein 2 [Source:HGNC Symbol;Acc:HGNC:11960] |
| ENSG00000204174 | HGLibA_32556 | 100 | -0.230159199 | NPY4R | neuropeptide Y receptor Y4 [Source:HGNC Symbol;Acc:HGNC:9329] |
| ENSG00000204351 | HGLibA_44285 | 100 | -0.230159199 | SKIV2L | Ski2 like RNA helicase [Source:HGNC Symbol;Acc:HGNC:10898] |
| ENSG00000204642 | HGLibA_21644 | 100 | -0.230159199 | HLA-F | F [Source:HGNC Symbol;Acc:HGNC:4963]" |
| ENSG00000204889 | HGLibA_25508 | 100 | -0.230159199 | KRT40 | keratin 40 [Source:HGNC Symbol;Acc:HGNC:26707] |
| ENSG00000206127 | HGLibA_19647 | 100 | -0.230159199 | GOLGA8O | golgin A8 family member O [Source:HGNC Symbol;Acc:HGNC:44406] |
| ENSG00000214113 | HGLibA_27753 | 100 | -0.230159199 | LYRM4 | LYR motif containing 4 [Source:HGNC Symbol;Acc:HGNC:21365] |
| ENSG00000214338 | HGLibA_46159 | 100 | -0.230159199 | SOGA3 | SOGA family member 3 [Source:HGNC Symbol;Acc:HGNC:21494] |
| ENSG00000214814 | HGLibA_17289 | 100 | -0.230159199 | FER1L6 | fer-1 like family member 6 [Source:HGNC Symbol;Acc:HGNC:28065] |
| ENSG00000230657 | HGLibA_38347 | 100 | -0.230159199 | PRB4 | proline rich protein BstNI subfamily 4 [Source:HGNC Symbol;Acc:HGNC:9340] |
| ENSG00000234465 | HGLibA_36693 | 100 | -0.230159199 | PINLYP | phospholipase A2 inhibitor and LY6/PLAUR domain containing [Source:HGNC Symbol;Acc:HGNC:44206] |
| ENSG00000242110 | HGLibA_01746 | 100 | -0.230159199 | AMACR | alpha-methylacyl-CoA racemase [Source:HGNC Symbol;Acc:HGNC:451] |
| ENSG00000243232 | HGLibA_35467 | 100 | -0.230159199 | PCDHAC2 | 2 [Source:HGNC Symbol;Acc:HGNC:8677]" |
| ENSG00000244057 | HGLibA_26137 | 100 | -0.230159199 | LCE3C | late cornified envelope 3C [Source:HGNC Symbol;Acc:HGNC:16612] |
| ENSG00000258102 | HGLibA_28128 | 100 | -0.230159199 | MAP1LC3B2 | microtubule associated protein 1 light chain 3 beta 2 [Source:HGNC Symbol;Acc:HGNC:34390] |
| ENSG00000260027 | HGLibA_21947 | 100 | -0.230159199 | HOXB7 | homeobox B7 [Source:HGNC Symbol;Acc:HGNC:5118] |
| ENSG00000270806 | HGLibA_05540 | 100 | -0.230159199 | C17orf50 | chromosome 17 open reading frame 50 [Source:HGNC Symbol;Acc:HGNC:29581] |
| ENSG00000273749 | HGLibA_12030 | 100 | -0.230159199 | CYFIP1 | cytoplasmic FMR1 interacting protein 1 [Source:HGNC Symbol;Acc:HGNC:13759] |
| ENSG00000278662 | HGLibA_19621 | 100 | -0.230159199 | GOLGA6L10 | golgin A6 family-like 10 [Source:HGNC Symbol;Acc:HGNC:37228] |
| FAM196B | HGLibA_16385 | 100 | -0.230159199 | FAM196B | - |
| HIST2H2AB | HGLibA_21528 | 100 | -0.230159199 | HIST2H2AB | - |
| hsa-mir-1224 | HGLibA_57296 | 100 | -0.230159199 | hsa-mir-1224 | - |
| hsa-mir-1260b | HGLibA_57462 | 100 | -0.230159199 | hsa-mir-1260b | - |
| hsa-mir-1275 | HGLibA_57556 | 100 | -0.230159199 | hsa-mir-1275 | - |
| hsa-mir-1289-1 | HGLibA_57622 | 100 | -0.230159199 | hsa-mir-1289-1 | - |
| hsa-mir-130a | HGLibA_57747 | 100 | -0.230159199 | hsa-mir-130a | - |
| hsa-mir-19b-1 | HGLibA_58143 | 100 | -0.230159199 | hsa-mir-19b-1 | - |
| hsa-mir-3116-1 | HGLibA_58504 | 100 | -0.230159199 | hsa-mir-3116-1 | - |
| hsa-mir-3132 | HGLibA_58584 | 100 | -0.230159199 | hsa-mir-3132 | - |
| hsa-mir-3202-2 | HGLibA_58921 | 100 | -0.230159199 | hsa-mir-3202-2 | - |
| hsa-mir-330 | HGLibA_58986 | 100 | -0.230159199 | hsa-mir-330 | - |

| | | | | | |
|---|---|---|---|---|---|
| hsa-mir-3657 | HGLibA_59172 | 100 | -0.230159199 | hsa-mir-3657 | - |
| hsa-mir-378h | HGLibA_59456 | 100 | -0.230159199 | hsa-mir-378h | - |
| hsa-mir-3910-2 | HGLibA_59508 | 100 | -0.230159199 | hsa-mir-3910-2 | - |
| hsa-mir-3938 | HGLibA_59613 | 100 | -0.230159199 | hsa-mir-3938 | - |
| hsa-mir-4324 | HGLibA_60020 | 100 | -0.230159199 | hsa-mir-4324 | - |
| hsa-mir-4525 | HGLibA_60560 | 100 | -0.230159199 | hsa-mir-4525 | - |
| hsa-mir-4686 | HGLibA_60855 | 100 | -0.230159199 | hsa-mir-4686 | - |
| hsa-mir-4688 | HGLibA_60861 | 100 | -0.230159199 | hsa-mir-4688 | - |
| hsa-mir-4799 | HGLibA_61303 | 100 | -0.230159199 | hsa-mir-4799 | - |
| hsa-mir-516a-2 | HGLibA_61611 | 100 | -0.230159199 | hsa-mir-516a-2 | - |
| hsa-mir-5697 | HGLibA_62321 | 100 | -0.230159199 | hsa-mir-5697 | - |
| hsa-mir-643 | HGLibA_62814 | 100 | -0.230159199 | hsa-mir-643 | - |
| hsa-mir-6798 | HGLibA_63368 | 100 | -0.230159199 | hsa-mir-6798 | - |
| hsa-mir-6879 | HGLibA_63702 | 100 | -0.230159199 | hsa-mir-6879 | - |
| ICT1 | HGLibA_22520 | 100 | -0.230159199 | ICT1 | - |
| LOC100129216 | HGLibA_26756 | 100 | -0.230159199 | LOC100129216 | - |
| PTPLAD2 | HGLibA_39420 | 100 | -0.230159199 | PTPLAD2 | - |
| TCEB3B | HGLibA_48744 | 100 | -0.230159199 | TCEB3B | - |
| ZCCHC11 | HGLibA_55007 | 100 | -0.230159199 | ZCCHC11 | - |
| C14orf1 | HGLibA_05315 | 99 | -0.239204542 | C14orf1 | - |
| C3orf37 | HGLibA_06238 | 99 | -0.239204542 | C3orf37 | - |
| ENSG00000004799 | HGLibA_35905 | 99 | -0.239204542 | PDK4 | pyruvate dehydrogenase kinase 4 [Source:HGNC Symbol;Acc:HGNC:8812] |
| ENSG00000011009 | HGLibA_27741 | 99 | -0.239204542 | LYPLA2 | lysophospholipase II [Source:HGNC Symbol;Acc:HGNC:6738] |
| ENSG00000049283 | HGLibA_15346 | 99 | -0.239204542 | EPN3 | epsin 3 [Source:HGNC Symbol;Acc:HGNC:18235] |
| ENSG00000051108 | HGLibA_21171 | 99 | -0.239204542 | HERPUD1 | homocysteine inducible ER protein with ubiquitin like domain 1 [Source:HGNC Symbol;Acc:HGNC:13744] |
| ENSG00000058799 | HGLibA_54671 | 99 | -0.239204542 | YIPF1 | Yip1 domain family member 1 [Source:HGNC Symbol;Acc:HGNC:25231] |
| ENSG00000059769 | HGLibA_13607 | 99 | -0.239204542 | DNAJC25 | DnaJ heat shock protein family (Hsp40) member C25 [Source:HGNC Symbol;Acc:HGNC:34187] |
| ENSG00000065000 | HGLibA_02326 | 99 | -0.239204542 | AP3D1 | adaptor related protein complex 3 subunit delta 1 [Source:HGNC Symbol;Acc:HGNC:568] |
| ENSG00000065060 | HGLibA_52947 | 99 | -0.239204542 | UHRF1BP1 | UHRF1 binding protein 1 [Source:HGNC Symbol;Acc:HGNC:21216] |
| ENSG00000068438 | HGLibA_18134 | 99 | -0.239204542 | FTSJ1 | FtsJ RNA methyltransferase homolog 1 [Source:HGNC Symbol;Acc:HGNC:13254] |
| ENSG00000069018 | HGLibA_51749 | 99 | -0.239204542 | TRPC7 | transient receptor potential cation channel subfamily C member 7 [Source:HGNC Symbol;Acc:HGNC:20754] |
| ENSG00000070413 | HGLibA_13045 | 99 | -0.239204542 | DGCR2 | DiGeorge syndrome critical region gene 2 [Source:HGNC Symbol;Acc:HGNC:2845] |
| ENSG00000075239 | HGLibA_00392 | 99 | -0.239204542 | ACAT1 | acetyl-CoA acetyltransferase 1 [Source:HGNC Symbol;Acc:HGNC:93] |
| ENSG00000076716 | HGLibA_19770 | 99 | -0.239204542 | GPC4 | glypican 4 [Source:HGNC Symbol;Acc:HGNC:4452] |
| ENSG00000081760 | HGLibA_00025 | 99 | -0.239204542 | AACS | acetoacetyl-CoA synthetase [Source:HGNC Symbol;Acc:HGNC:21298] |
| ENSG00000089693 | HGLibA_29377 | 99 | -0.239204542 | MLF2 | myeloid leukemia factor 2 [Source:HGNC Symbol;Acc:HGNC:7126] |
| ENSG00000101938 | HGLibA_09539 | 99 | -0.239204542 | CHRDL1 | chordin like 1 [Source:HGNC Symbol;Acc:HGNC:29861] |
| ENSG00000102802 | HGLibA_28822 | 99 | -0.239204542 | MEDAG | mesenteric estrogen dependent adipogenesis [Source:HGNC Symbol;Acc:HGNC:25926] |
| ENSG00000102981 | HGLibA_35185 | 99 | -0.239204542 | PARD6A | par-6 family cell polarity regulator alpha [Source:HGNC Symbol;Acc:HGNC:15943] |
| ENSG00000103148 | HGLibA_32519 | 99 | -0.239204542 | NPRL3 | GATOR1 complex subunit [Source:HGNC Symbol;Acc:HGNC:14124]" |
| ENSG00000104112 | HGLibA_42902 | 99 | -0.239204542 | SCG3 | secretogranin III [Source:HGNC Symbol;Acc:HGNC:13707] |
| ENSG00000104388 | HGLibA_39866 | 99 | -0.239204542 | RAB2A | member RAS oncogene family [Source:HGNC Symbol;Acc:HGNC:9763]" |
| ENSG00000104687 | HGLibA_20445 | 99 | -0.239204542 | GSR | glutathione-disulfide reductase [Source:HGNC Symbol;Acc:HGNC:4623] |
| ENSG00000105369 | HGLibA_08446 | 99 | -0.239204542 | CD79A | CD79a molecule [Source:HGNC Symbol;Acc:HGNC:1698] |
| ENSG00000106018 | HGLibA_53671 | 99 | -0.239204542 | VIPR2 | vasoactive intestinal peptide receptor 2 [Source:HGNC Symbol;Acc:HGNC:12695] |
| ENSG00000106852 | HGLibA_26463 | 99 | -0.239204542 | LHX6 | LIM homeobox 6 [Source:HGNC Symbol;Acc:HGNC:21735] |
| ENSG00000108176 | HGLibA_13570 | 99 | -0.239204542 | DNAJC12 | DnaJ heat shock protein family (Hsp40) member C12 [Source:HGNC Symbol;Acc:HGNC:28908] |
| ENSG00000108523 | HGLibA_41563 | 99 | -0.239204542 | RNF167 | ring finger protein 167 [Source:HGNC Symbol;Acc:HGNC:24544] |
| ENSG00000111321 | HGLibA_27602 | 99 | -0.239204542 | LTBR | lymphotoxin beta receptor [Source:HGNC Symbol;Acc:HGNC:6718] |
| ENSG00000112115 | HGLibA_22988 | 99 | -0.239204542 | IL17A | interleukin 17A [Source:HGNC Symbol;Acc:HGNC:5981] |
| ENSG00000112559 | HGLibA_28669 | 99 | -0.239204542 | MDFI | MyoD family inhibitor [Source:HGNC Symbol;Acc:HGNC:6967] |
| ENSG00000112739 | HGLibA_38740 | 99 | -0.239204542 | PRPF4B | pre-mRNA processing factor 4B [Source:HGNC Symbol;Acc:HGNC:17346] |
| ENSG00000114316 | HGLibA_53360 | 99 | -0.239204542 | USP4 | ubiquitin specific peptidase 4 [Source:HGNC Symbol;Acc:HGNC:12627] |
| ENSG00000116032 | HGLibA_20314 | 99 | -0.239204542 | GRIN3B | glutamate ionotropic receptor NMDA type subunit 3B [Source:HGNC Symbol;Acc:HGNC:16768] |
| ENSG00000116752 | HGLibA_04253 | 99 | -0.239204542 | BCAS2 | pre-mRNA processing factor [Source:HGNC Symbol;Acc:HGNC:975]" |
| ENSG00000116774 | HGLibA_33366 | 99 | -0.239204542 | OLFML3 | olfactomedin like 3 [Source:HGNC Symbol;Acc:HGNC:24956] |
| ENSG00000117560 | HGLibA_16891 | 99 | -0.239204542 | FASLG | Fas ligand [Source:HGNC Symbol;Acc:HGNC:11936] |
| ENSG00000117625 | HGLibA_40717 | 99 | -0.239204542 | RCOR3 | REST corepressor 3 [Source:HGNC Symbol;Acc:HGNC:25594] |
| ENSG00000121797 | HGLibA_08176 | 99 | -0.239204542 | CCRL2 | C-C motif chemokine receptor like 2 [Source:HGNC Symbol;Acc:HGNC:1612] |
| ENSG00000123091 | HGLibA_41463 | 99 | -0.239204542 | RNF11 | ring finger protein 11 [Source:HGNC Symbol;Acc:HGNC:10056] |
| ENSG00000123999 | HGLibA_23298 | 99 | -0.239204542 | INHA | inhibin subunit alpha [Source:HGNC Symbol;Acc:HGNC:6065] |
| ENSG00000124641 | HGLibA_28773 | 99 | -0.239204542 | MED20 | mediator complex subunit 20 [Source:HGNC Symbol;Acc:HGNC:16840] |
| ENSG00000124664 | HGLibA_46567 | 99 | -0.239204542 | SPDEF | SAM pointed domain containing ETS transcription factor [Source:HGNC Symbol;Acc:HGNC:17257] |
| ENSG00000125864 | HGLibA_04454 | 99 | -0.239204542 | BFSP1 | beaded filament structural protein 1 [Source:HGNC Symbol;Acc:HGNC:1040] |
| ENSG00000126391 | HGLibA_18030 | 99 | -0.239204542 | FRMD8 | FERM domain containing 8 [Source:HGNC Symbol;Acc:HGNC:25462] |
| ENSG00000126759 | HGLibA_09291 | 99 | -0.239204542 | CFP | complement factor properdin [Source:HGNC Symbol;Acc:HGNC:8864] |
| ENSG00000126814 | HGLibA_51692 | 99 | -0.239204542 | TRMT5 | tRNA methyltransferase 5 [Source:HGNC Symbol;Acc:HGNC:23141] |
| ENSG00000127418 | HGLibA_17453 | 99 | -0.239204542 | FGFRL1 | fibroblast growth factor receptor like 1 [Source:HGNC Symbol;Acc:HGNC:3693] |
| ENSG00000129480 | HGLibA_14054 | 99 | -0.239204542 | DTD2 | D-tyrosyl-tRNA deacylase 2 (putative) [Source:HGNC Symbol;Acc:HGNC:20277] |
| ENSG00000129636 | HGLibA_23705 | 99 | -0.239204542 | ITFG1 | integrin alpha FG-GAP repeat containing 1 [Source:HGNC Symbol;Acc:HGNC:30697] |
| ENSG00000129993 | HGLibA_07389 | 99 | -0.239204542 | CBFA2T3 | CBFA2/RUNX1 translocation partner 3 [Source:HGNC Symbol;Acc:HGNC:1537] |
| ENSG00000130649 | HGLibA_12130 | 99 | -0.239204542 | CYP2E1 | cytochrome P450 family 2 subfamily E member 1 [Source:HGNC Symbol;Acc:HGNC:2631] |
| ENSG00000130720 | HGLibA_17498 | 99 | -0.239204542 | FIBCD1 | fibrinogen C domain containing 1 [Source:HGNC Symbol;Acc:HGNC:25922] |
| ENSG00000130779 | HGLibA_10041 | 99 | -0.239204542 | CLIP1 | CAP-Gly domain containing linker protein 1 [Source:HGNC Symbol;Acc:HGNC:10461] |
| ENSG00000131504 | HGLibA_13218 | 99 | -0.239204542 | DIAPH1 | diaphanous related formin 1 [Source:HGNC Symbol;Acc:HGNC:2876] |
| ENSG00000131620 | HGLibA_02171 | 99 | -0.239204542 | ANO1 | anoctamin 1 [Source:HGNC Symbol;Acc:HGNC:21625] |
| ENSG00000132510 | HGLibA_24535 | 99 | -0.239204542 | KDM6B | lysine demethylase 6B [Source:HGNC Symbol;Acc:HGNC:29012] |
| ENSG00000134278 | HGLibA_46724 | 99 | -0.239204542 | SPIRE1 | spire type actin nucleation factor 1 [Source:HGNC Symbol;Acc:HGNC:30622] |
| ENSG00000135596 | HGLibA_29200 | 99 | -0.239204542 | MICAL1 | calponin and LIM domain containing 1 [Source:HGNC Symbol;Acc:HGNC:20619]" |
| ENSG00000136868 | HGLibA_44927 | 99 | -0.239204542 | SLC31A1 | solute carrier family 31 member 1 [Source:HGNC Symbol;Acc:HGNC:11016] |
| ENSG00000138161 | HGLibA_11808 | 99 | -0.239204542 | CUZD1 | CUB and zona pellucida like domains 1 [Source:HGNC Symbol;Acc:HGNC:17937] |
| ENSG00000139725 | HGLibA_41186 | 99 | -0.239204542 | RHOF | filopodia associated [Source:HGNC Symbol;Acc:HGNC:15703]" |
| ENSG00000140395 | HGLibA_54143 | 99 | -0.239204542 | WDR61 | WD repeat domain 61 [Source:HGNC Symbol;Acc:HGNC:30300] |
| ENSG00000142494 | HGLibA_45192 | 99 | -0.239204542 | SLC47A1 | solute carrier family 47 member 1 [Source:HGNC Symbol;Acc:HGNC:25588] |
| ENSG00000143153 | HGLibA_03593 | 99 | -0.239204542 | ATP1B1 | ATPase Na+/K+ transporting subunit beta 1 [Source:HGNC Symbol;Acc:HGNC:804] |
| ENSG00000144366 | HGLibA_20677 | 99 | -0.239204542 | GULP1 | GULP PTB domain containing engulfment adaptor 1 [Source:HGNC Symbol;Acc:HGNC:18649] |
| ENSG00000144671 | HGLibA_44549 | 99 | -0.239204542 | SLC22A14 | solute carrier family 22 member 14 [Source:HGNC Symbol;Acc:HGNC:8495] |
| ENSG00000145912 | HGLibA_31907 | 99 | -0.239204542 | NHP2 | NHP2 ribonucleoprotein [Source:HGNC Symbol;Acc:HGNC:14377] |
| ENSG00000145934 | HGLibA_48999 | 99 | -0.239204542 | TENM2 | teneurin transmembrane protein 2 [Source:HGNC Symbol;Acc:HGNC:29943] |
| ENSG00000145936 | HGLibA_24341 | 99 | -0.239204542 | KCNMB1 | potassium calcium-activated channel subfamily M regulatory beta subunit 1 [Source:HGNC Symbol;Acc:HGNC:6285] |
| ENSG00000146350 | HGLibA_48538 | 99 | -0.239204542 | TBC1D32 | TBC1 domain family member 32 [Source:HGNC Symbol;Acc:HGNC:21485] |
| ENSG00000146833 | HGLibA_51488 | 99 | -0.239204542 | TRIM4 | tripartite motif containing 4 [Source:HGNC Symbol;Acc:HGNC:16275] |
| ENSG00000147041 | HGLibA_48107 | 99 | -0.239204542 | SYTL5 | synaptotagmin like 5 [Source:HGNC Symbol;Acc:HGNC:15589] |
| ENSG00000147852 | HGLibA_53682 | 99 | -0.239204542 | VLDLR | very low density lipoprotein receptor [Source:HGNC Symbol;Acc:HGNC:12698] |
| ENSG00000148154 | HGLibA_52861 | 99 | -0.239204542 | UGCG | UDP-glucose ceramide glucosyltransferase [Source:HGNC Symbol;Acc:HGNC:12524] |
| ENSG00000148248 | HGLibA_47837 | 99 | -0.239204542 | SURF4 | surfeit 4 [Source:HGNC Symbol;Acc:HGNC:11476] |
| ENSG00000149380 | HGLibA_34905 | 99 | -0.239204542 | P4HA3 | prolyl 4-hydroxylase subunit alpha 3 [Source:HGNC Symbol;Acc:HGNC:30135] |
| ENSG00000150361 | HGLibA_25120 | 99 | -0.239204542 | KLHL1 | kelch like family member 1 [Source:HGNC Symbol;Acc:HGNC:6352] |
| ENSG00000151632 | HGLibA_01484 | 99 | -0.239204542 | AKR1C2 | aldo-keto reductase family 1 member C2 [Source:HGNC Symbol;Acc:HGNC:385] |
| ENSG00000153234 | HGLibA_32627 | 99 | -0.239204542 | NR4A2 | nuclear receptor subfamily 4 group A member 2 [Source:HGNC Symbol;Acc:HGNC:7981] |
| ENSG00000153930 | HGLibA_01954 | 99 | -0.239204542 | ANKFN1 | ankyrin repeat and fibronectin type III domain containing 1 [Source:HGNC Symbol;Acc:HGNC:26766] |
| ENSG00000156510 | HGLibA_21587 | 99 | -0.239204542 | HKDC1 | hexokinase domain containing 1 [Source:HGNC Symbol;Acc:HGNC:23302] |
| ENSG00000159961 | HGLibA_33919 | 99 | -0.239204542 | OR3A3 | olfactory receptor family 3 subfamily A member 3 [Source:HGNC Symbol;Acc:HGNC:8284] |
| ENSG00000160472 | HGLibA_50141 | 99 | -0.239204542 | TMEM190 | transmembrane protein 190 [Source:HGNC Symbol;Acc:HGNC:29632] |
| ENSG00000161920 | HGLibA_28739 | 99 | -0.239204542 | MED11 | mediator complex subunit 11 [Source:HGNC Symbol;Acc:HGNC:32687] |
| ENSG00000162670 | HGLibA_04817 | 99 | -0.239204542 | BRINP3 | BMP/retinoic acid inducible neural specific 3 [Source:HGNC Symbol;Acc:HGNC:22393] |
| ENSG00000163249 | HGLibA_08134 | 99 | -0.239204542 | CCNYL1 | cyclin Y like 1 [Source:HGNC Symbol;Acc:HGNC:26868] |
| ENSG00000163320 | HGLibA_09316 | 99 | -0.239204542 | CGGBP1 | CGG triplet repeat binding protein 1 [Source:HGNC Symbol;Acc:HGNC:1888] |
| ENSG00000164077 | HGLibA_29639 | 99 | -0.239204542 | MON1A | secretory trafficking associated [Source:HGNC Symbol;Acc:HGNC:28207]" |
| ENSG00000164932 | HGLibA_11629 | 99 | -0.239204542 | CTHRC1 | collagen triple helix repeat containing 1 [Source:HGNC Symbol;Acc:HGNC:18831] |
| ENSG00000165682 | HGLibA_09957 | 99 | -0.239204542 | CLEC1B | C-type lectin domain family 1 member B [Source:HGNC Symbol;Acc:HGNC:24356] |
| ENSG00000167772 | HGLibA_01919 | 99 | -0.239204542 | ANGPTL4 | angiopoietin like 4 [Source:HGNC Symbol;Acc:HGNC:16039] |
| ENSG00000168447 | HGLibA_43017 | 99 | -0.239204542 | SCNN1B | sodium channel epithelial 1 beta subunit [Source:HGNC Symbol;Acc:HGNC:10600] |
| ENSG00000168830 | HGLibA_22364 | 99 | -0.239204542 | HTR1E | 5-hydroxytryptamine receptor 1E [Source:HGNC Symbol;Acc:HGNC:5291] |
| ENSG00000168928 | HGLibA_11680 | 99 | -0.239204542 | CTRB2 | chymotrypsinogen B2 [Source:HGNC Symbol;Acc:HGNC:2522] |
| ENSG00000168930 | HGLibA_51522 | 99 | -0.239204542 | TRIM49 | tripartite motif containing 49 [Source:HGNC Symbol;Acc:HGNC:13431] |
| ENSG00000169299 | HGLibA_36263 | 99 | -0.239204542 | PGM2 | phosphoglucomutase 2 [Source:HGNC Symbol;Acc:HGNC:8906] |
| ENSG00000170419 | HGLibA_53860 | 99 | -0.239204542 | VSTM2A | V-set and transmembrane domain containing 2A [Source:HGNC Symbol;Acc:HGNC:28499] |
| ENSG00000170581 | HGLibA_47385 | 99 | -0.239204542 | STAT2 | signal transducer and activator of transcription 2 [Source:HGNC Symbol;Acc:HGNC:11363] |
| ENSG00000170909 | HGLibA_34673 | 99 | -0.239204542 | OSCAR | immunoglobulin-like receptor [Source:HGNC Symbol;Acc:HGNC:29960]" |
| ENSG00000171566 | HGLibA_37221 | 99 | -0.239204542 | PLRG1 | pleiotropic regulator 1 [Source:HGNC Symbol;Acc:HGNC:9089] |
| ENSG00000171812 | HGLibA_10616 | 99 | -0.239204542 | COL8A2 | collagen type VIII alpha 2 chain [Source:HGNC Symbol;Acc:HGNC:2216] |
| ENSG00000172073 | HGLibA_49105 | 99 | -0.239204542 | TEX37 | testis expressed 37 [Source:HGNC Symbol;Acc:HGNC:26341] |
| ENSG00000172977 | HGLibA_24057 | 99 | -0.239204542 | KAT5 | lysine acetyltransferase 5 [Source:HGNC Symbol;Acc:HGNC:5275] |
| ENSG00000173679 | HGLibA_33687 | 99 | -0.239204542 | OR1L1 | olfactory receptor family 1 subfamily L member 1 [Source:HGNC Symbol;Acc:HGNC:8213] |
| ENSG00000174292 | HGLibA_50844 | 99 | -0.239204542 | TNK1 | tyrosine kinase non receptor 1 [Source:HGNC Symbol;Acc:HGNC:11940] |
| ENSG00000177051 | HGLibA_17110 | 99 | -0.239204542 | FBXO46 | F-box protein 46 [Source:HGNC Symbol;Acc:HGNC:25069] |
| ENSG00000178690 | HGLibA_14229 | 99 | -0.239204542 | DYNAP | dynactin associated protein [Source:HGNC Symbol;Acc:HGNC:26808] |
| ENSG00000178741 | HGLibA_10835 | 99 | -0.239204542 | COX5A | cytochrome c oxidase subunit 5A [Source:HGNC Symbol;Acc:HGNC:2267] |
| ENSG00000180329 | HGLibA_07787 | 99 | -0.239204542 | CCDC43 | coiled-coil domain containing 43 [Source:HGNC Symbol;Acc:HGNC:26472] |
| ENSG00000181004 | HGLibA_04211 | 99 | -0.239204542 | BBS12 | Bardet-Biedl syndrome 12 [Source:HGNC Symbol;Acc:HGNC:26648] |
| ENSG00000181885 | HGLibA_09908 | 99 | -0.239204542 | CLDN7 | claudin 7 [Source:HGNC Symbol;Acc:HGNC:2049] |
| ENSG00000182334 | HGLibA_34350 | 99 | -0.239204542 | OR5P3 | olfactory receptor family 5 subfamily P member 3 [Source:HGNC Symbol;Acc:HGNC:14784] |
| ENSG00000184164 | HGLibA_11129 | 99 | -0.239204542 | CRELD2 | cysteine rich with EGF like domains 2 [Source:HGNC Symbol;Acc:HGNC:28150] |
| ENSG00000184924 | HGLibA_39556 | 99 | -0.239204542 | PTRHD1 | peptidyl-tRNA hydrolase domain containing 1 [Source:HGNC Symbol;Acc:HGNC:33782] |

| | | | | | |
|---|---|---|---|---|---|
| ENSG00000186416 | HGLibA_32023 | 99 | -0.239204542 | NKRF | NFKB repressing factor [Source:HGNC Symbol;Acc:HGNC:19374] |
| ENSG00000186792 | HGLibA_22443 | 99 | -0.239204542 | HYAL3 | hyaluronidase 3 [Source:HGNC Symbol;Acc:HGNC:5322] |
| ENSG00000188293 | HGLibA_22839 | 99 | -0.239204542 | IGFL1 | IGF like family member 1 [Source:HGNC Symbol;Acc:HGNC:24093] |
| ENSG00000188827 | HGLibA_45562 | 99 | -0.239204542 | SLX4 | SLX4 structure-specific endonuclease subunit [Source:HGNC Symbol;Acc:HGNC:23845] |
| ENSG00000196408 | HGLibA_32386 | 99 | -0.239204542 | NOXO1 | NADPH oxidase organizer 1 [Source:HGNC Symbol;Acc:HGNC:19404] |
| ENSG00000196781 | HGLibA_49607 | 99 | -0.239204542 | TLE1 | transducin like enhancer of split 1 [Source:HGNC Symbol;Acc:HGNC:11837] |
| ENSG00000197530 | HGLibA_29196 | 99 | -0.239204542 | MIB2 | mindbomb E3 ubiquitin protein ligase 2 [Source:HGNC Symbol;Acc:HGNC:30577] |
| ENSG00000197816 | HGLibA_07720 | 99 | -0.239204542 | CCDC180 | coiled-coil domain containing 180 [Source:HGNC Symbol;Acc:HGNC:29303] |
| ENSG00000198680 | HGLibA_52343 | 99 | -0.239204542 | TUSC1 | tumor suppressor candidate 1 [Source:HGNC Symbol;Acc:HGNC:31010] |
| ENSG00000198752 | HGLibA_08559 | 99 | -0.239204542 | CDC42BPB | CDC42 binding protein kinase beta [Source:HGNC Symbol;Acc:HGNC:1738] |
| ENSG00000204681 | HGLibA_18340 | 99 | -0.239204542 | GABBR1 | gamma-aminobutyric acid type B receptor subunit 1 [Source:HGNC Symbol;Acc:HGNC:4070] |
| ENSG00000204947 | HGLibA_55962 | 99 | -0.239204542 | ZNF425 | zinc finger protein 425 [Source:HGNC Symbol;Acc:HGNC:20690] |
| ENSG00000204962 | HGLibA_35458 | 99 | -0.239204542 | PCDHA8 | protocadherin alpha 8 [Source:HGNC Symbol;Acc:HGNC:8674] |
| ENSG00000205212 | HGLibA_07625 | 99 | -0.239204542 | CCDC144NL | N-terminal like [Source:HGNC Symbol;Acc:HGNC:33735]" |
| ENSG00000205420 | HGLibA_25512 | 99 | -0.239204542 | KRT6A | keratin 6A [Source:HGNC Symbol;Acc:HGNC:6443] |
| ENSG00000213203 | HGLibA_19070 | 99 | -0.239204542 | GIMAP1 | IMAP family member 1 [Source:HGNC Symbol;Acc:HGNC:23237]" |
| ENSG00000215114 | HGLibA_52785 | 99 | -0.239204542 | UBXN2B | UBX domain protein 2B [Source:HGNC Symbol;Acc:HGNC:27035] |
| ENSG00000215883 | HGLibA_12014 | 99 | -0.239204542 | CYB5RL | cytochrome b5 reductase like [Source:HGNC Symbol;Acc:HGNC:32220] |
| ENSG00000231213 | HGLibA_37243 | 99 | -0.239204542 | PLSCR5 | phospholipid scramblase family member 5 [Source:HGNC Symbol;Acc:HGNC:19952] |
| ENSG00000238205 | HGLibA_29705 | 99 | -0.239204542 | MPC1L | mitochondrial pyruvate carrier 1 like [Source:HGNC Symbol;Acc:HGNC:44205] |
| ENSG00000240021 | HGLibA_49101 | 99 | -0.239204542 | TEX35 | testis expressed 35 [Source:HGNC Symbol;Acc:HGNC:25366] |
| ENSG00000240432 | HGLibA_25648 | 99 | -0.239204542 | KRTAP13-3 | keratin associated protein 13-3 [Source:HGNC Symbol;Acc:HGNC:18925] |
| ENSG00000241595 | HGLibA_25840 | 99 | -0.239204542 | KRTAP9-4 | keratin associated protein 9-4 [Source:HGNC Symbol;Acc:HGNC:18902] |
| ENSG00000241644 | HGLibA_23315 | 99 | -0.239204542 | INMT | indolethylamine N-methyltransferase [Source:HGNC Symbol;Acc:HGNC:6069] |
| ENSG00000241685 | HGLibA_03075 | 99 | -0.239204542 | ARPC1A | actin related protein 2/3 complex subunit 1A [Source:HGNC Symbol;Acc:HGNC:703] |
| ENSG00000244122 | HGLibA_52893 | 99 | -0.239204542 | UGT1A7 | UDP glucuronosyltransferase family 1 member A7 [Source:HGNC Symbol;Acc:HGNC:12539] |
| ENSG00000249860 | HGLibA_30483 | 99 | -0.239204542 | MTRNR2L5 | MT-RNR2 like 5 [Source:HGNC Symbol;Acc:HGNC:37162] |
| ENSG00000251258 | HGLibA_40943 | 99 | -0.239204542 | RFPL4B | ret finger protein like 4B [Source:HGNC Symbol;Acc:HGNC:33264] |
| ENSG00000254550 | HGLibA_33388 | 99 | -0.239204542 | OMP | olfactory marker protein [Source:HGNC Symbol;Acc:HGNC:8136] |
| ENSG00000256229 | HGLibA_56084 | 99 | -0.239204542 | ZNF486 | zinc finger protein 486 [Source:HGNC Symbol;Acc:HGNC:20807] |
| ENSG00000257017 | HGLibA_22010 | 99 | -0.239204542 | HP | haptoglobin [Source:HGNC Symbol;Acc:HGNC:5141] |
| ENSG00000258405 | HGLibA_56299 | 99 | -0.239204542 | ZNF578 | zinc finger protein 578 [Source:HGNC Symbol;Acc:HGNC:26449] |
| ENSG00000269405 | HGLibA_33129 | 99 | -0.239204542 | NXF2 | nuclear RNA export factor 2 [Source:HGNC Symbol;Acc:HGNC:8072] |
| GUCY1B3 | HGLibA_20657 | 99 | -0.239204542 | GUCY1B3 | - |
| hsa-mir-1178 | HGLibA_57206 | 99 | -0.239204542 | hsa-mir-1178 | - |
| hsa-mir-127 | HGLibA_57513 | 99 | -0.239204542 | hsa-mir-127 | - |
| hsa-mir-1307 | HGLibA_57743 | 99 | -0.239204542 | hsa-mir-1307 | - |
| hsa-mir-20a | HGLibA_58202 | 99 | -0.239204542 | hsa-mir-20a | - |
| hsa-mir-3143 | HGLibA_58632 | 99 | -0.239204542 | hsa-mir-3143 | - |
| hsa-mir-3146 | HGLibA_58640 | 99 | -0.239204542 | hsa-mir-3146 | - |
| hsa-mir-3156-2 | HGLibA_58692 | 99 | -0.239204542 | hsa-mir-3156-2 | - |
| hsa-mir-3177 | HGLibA_58783 | 99 | -0.239204542 | hsa-mir-3177 | - |
| hsa-mir-3197 | HGLibA_58885 | 99 | -0.239204542 | hsa-mir-3197 | - |
| hsa-mir-4320 | HGLibA_60004 | 99 | -0.239204542 | hsa-mir-4320 | - |
| hsa-mir-4321 | HGLibA_60007 | 99 | -0.239204542 | hsa-mir-4321 | - |
| hsa-mir-4420 | HGLibA_60068 | 99 | -0.239204542 | hsa-mir-4420 | - |
| hsa-mir-4426 | HGLibA_60092 | 99 | -0.239204542 | hsa-mir-4426 | - |
| hsa-mir-4740 | HGLibA_61057 | 99 | -0.239204542 | hsa-mir-4740 | - |
| hsa-mir-494 | HGLibA_61376 | 99 | -0.239204542 | hsa-mir-494 | - |
| hsa-mir-5089 | HGLibA_61501 | 99 | -0.239204542 | hsa-mir-5089 | - |
| hsa-mir-516b-2 | HGLibA_61622 | 99 | -0.239204542 | hsa-mir-516b-2 | - |
| hsa-mir-518a-1 | HGLibA_61650 | 99 | -0.239204542 | hsa-mir-518a-1 | - |
| hsa-mir-520f | HGLibA_61756 | 99 | -0.239204542 | hsa-mir-520f | - |
| hsa-mir-525 | HGLibA_61788 | 99 | -0.239204542 | hsa-mir-525 | - |
| hsa-mir-5587 | HGLibA_62189 | 99 | -0.239204542 | hsa-mir-5587 | - |
| hsa-mir-599 | HGLibA_62489 | 99 | -0.239204542 | hsa-mir-599 | - |
| hsa-mir-6077-2 | HGLibA_62566 | 99 | -0.239204542 | hsa-mir-6077-2 | - |
| hsa-mir-626 | HGLibA_62743 | 99 | -0.239204542 | hsa-mir-626 | - |
| hsa-mir-627 | HGLibA_62747 | 99 | -0.239204542 | hsa-mir-627 | - |
| hsa-mir-6514 | HGLibA_62930 | 99 | -0.239204542 | hsa-mir-6514 | - |
| hsa-mir-708 | HGLibA_63779 | 99 | -0.239204542 | hsa-mir-708 | - |
| hsa-mir-7155 | HGLibA_63850 | 99 | -0.239204542 | hsa-mir-7155 | - |
| hsa-mir-8075 | HGLibA_64141 | 99 | -0.239204542 | hsa-mir-8075 | - |
| hsa-mir-8085 | HGLibA_64181 | 99 | -0.239204542 | hsa-mir-8085 | - |
| KIAA1598 | HGLibA_24790 | 99 | -0.239204542 | KIAA1598 | - |
| PDDC1 | HGLibA_35757 | 99 | -0.239204542 | PDDC1 | - |
| XRCC6BP1 | HGLibA_54600 | 99 | -0.239204542 | XRCC6BP1 | - |
| ENSG00000010017 | HGLibA_40213 | 98 | -0.248249885 | RANBP9 | RAN binding protein 9 [Source:HGNC Symbol;Acc:HGNC:13727] |
| ENSG00000011295 | HGLibA_52066 | 98 | -0.248249885 | TTC19 | tetratricopeptide repeat domain 19 [Source:HGNC Symbol;Acc:HGNC:26006] |
| ENSG00000013619 | HGLibA_28053 | 98 | -0.248249885 | MAMLD1 | mastermind like domain containing 1 [Source:HGNC Symbol;Acc:HGNC:2568] |
| ENSG00000025423 | HGLibA_22197 | 98 | -0.248249885 | HSD17B6 | hydroxysteroid 17-beta dehydrogenase 6 [Source:HGNC Symbol;Acc:HGNC:23316] |
| ENSG00000030110 | HGLibA_04134 | 98 | -0.248249885 | BAK1 | BCL2 antagonist/killer 1 [Source:HGNC Symbol;Acc:HGNC:949] |
| ENSG00000058091 | HGLibA_08738 | 98 | -0.248249885 | CDK14 | cyclin dependent kinase 14 [Source:HGNC Symbol;Acc:HGNC:8883] |
| ENSG00000065361 | HGLibA_15419 | 98 | -0.248249885 | ERBB3 | erb-b2 receptor tyrosine kinase 3 [Source:HGNC Symbol;Acc:HGNC:3431] |
| ENSG00000067064 | HGLibA_22553 | 98 | -0.248249885 | IDI1 | isopentenyl-diphosphate delta isomerase 1 [Source:HGNC Symbol;Acc:HGNC:5387] |
| ENSG00000070031 | HGLibA_43064 | 98 | -0.248249885 | SCT | secretin [Source:HGNC Symbol;Acc:HGNC:10607] |
| ENSG00000072163 | HGLibA_26550 | 98 | -0.248249885 | LIMS2 | LIM zinc finger domain containing 2 [Source:HGNC Symbol;Acc:HGNC:16084] |
| ENSG00000084112 | HGLibA_47133 | 98 | -0.248249885 | SSH1 | slingshot protein phosphatase 1 [Source:HGNC Symbol;Acc:HGNC:30579] |
| ENSG00000086065 | HGLibA_09495 | 98 | -0.248249885 | CHMP5 | charged multivesicular body protein 5 [Source:HGNC Symbol;Acc:HGNC:26942] |
| ENSG00000086504 | HGLibA_29941 | 98 | -0.248249885 | MRPL28 | mitochondrial ribosomal protein L28 [Source:HGNC Symbol;Acc:HGNC:14484] |
| ENSG00000088836 | HGLibA_45209 | 98 | -0.248249885 | SLC4A11 | solute carrier family 4 member 11 [Source:HGNC Symbol;Acc:HGNC:16438] |
| ENSG00000090520 | HGLibA_13525 | 98 | -0.248249885 | DNAJB11 | DnaJ heat shock protein family (Hsp40) member B11 [Source:HGNC Symbol;Acc:HGNC:14889] |
| ENSG00000096717 | HGLibA_44215 | 98 | -0.248249885 | SIRT1 | sirtuin 1 [Source:HGNC Symbol;Acc:HGNC:14929] |
| ENSG00000100095 | HGLibA_43682 | 98 | -0.248249885 | SEZ6L | seizure related 6 homolog like [Source:HGNC Symbol;Acc:HGNC:10763] |
| ENSG00000100209 | HGLibA_22158 | 98 | -0.248249885 | HSCB | HscB mitochondrial iron-sulfur cluster cochaperone [Source:HGNC Symbol;Acc:HGNC:28913] |
| ENSG00000101003 | HGLibA_19102 | 98 | -0.248249885 | GINS1 | GINS complex subunit 1 [Source:HGNC Symbol;Acc:HGNC:28980] |
| ENSG00000103023 | HGLibA_38959 | 98 | -0.248249885 | PRSS54 | serine protease 54 [Source:HGNC Symbol;Acc:HGNC:26336] |
| ENSG00000103047 | HGLibA_48301 | 98 | -0.248249885 | TANGO6 | transport and golgi organization 6 homolog [Source:HGNC Symbol;Acc:HGNC:25749] |
| ENSG00000104432 | HGLibA_23189 | 98 | -0.248249885 | IL7 | interleukin 7 [Source:HGNC Symbol;Acc:HGNC:6023] |
| ENSG00000105289 | HGLibA_49578 | 98 | -0.248249885 | TJP3 | tight junction protein 3 [Source:HGNC Symbol;Acc:HGNC:11829] |
| ENSG00000105483 | HGLibA_07238 | 98 | -0.248249885 | CARD8 | caspase recruitment domain family member 8 [Source:HGNC Symbol;Acc:HGNC:17057] |
| ENSG00000106006 | HGLibA_21918 | 98 | -0.248249885 | HOXA6 | homeobox A6 [Source:HGNC Symbol;Acc:HGNC:5107] |
| ENSG00000107187 | HGLibA_26454 | 98 | -0.248249885 | LHX3 | LIM homeobox 3 [Source:HGNC Symbol;Acc:HGNC:6595] |
| ENSG00000107614 | HGLibA_51344 | 98 | -0.248249885 | TRDMT1 | tRNA aspartic acid methyltransferase 1 [Source:HGNC Symbol;Acc:HGNC:2977] |
| ENSG00000107937 | HGLibA_20609 | 98 | -0.248249885 | GTPBP4 | GTP binding protein 4 [Source:HGNC Symbol;Acc:HGNC:21535] |
| ENSG00000108389 | HGLibA_30430 | 98 | -0.248249885 | MTMR4 | myotubularin related protein 4 [Source:HGNC Symbol;Acc:HGNC:7452] |
| ENSG00000108443 | HGLibA_42176 | 98 | -0.248249885 | RPS6KB1 | ribosomal protein S6 kinase B1 [Source:HGNC Symbol;Acc:HGNC:10436] |
| ENSG00000110514 | HGLibA_27856 | 98 | -0.248249885 | MADD | MAP kinase activating death domain [Source:HGNC Symbol;Acc:HGNC:6766] |
| ENSG00000110844 | HGLibA_38738 | 98 | -0.248249885 | PRPF40B | pre-mRNA processing factor 40 homolog B [Source:HGNC Symbol;Acc:HGNC:25031] |
| ENSG00000111224 | HGLibA_35216 | 98 | -0.248249885 | PARP11 | poly(ADP-ribose) polymerase family member 11 [Source:HGNC Symbol;Acc:HGNC:1186] |
| ENSG00000112701 | HGLibA_43397 | 98 | -0.248249885 | SENP6 | SUMO specific peptidase 6 [Source:HGNC Symbol;Acc:HGNC:20944] |
| ENSG00000113048 | HGLibA_30081 | 98 | -0.248249885 | MRPS27 | mitochondrial ribosomal protein S27 [Source:HGNC Symbol;Acc:HGNC:14512] |
| ENSG00000115944 | HGLibA_10864 | 98 | -0.248249885 | COX7A2L | cytochrome c oxidase subunit 7A2 like [Source:HGNC Symbol;Acc:HGNC:2289] |
| ENSG00000118655 | HGLibA_12510 | 98 | -0.248249885 | DCLRE1B | DNA cross-link repair 1B [Source:HGNC Symbol;Acc:HGNC:17641] |
| ENSG00000119614 | HGLibA_53878 | 98 | -0.248249885 | VSX2 | visual system homeobox 2 [Source:HGNC Symbol;Acc:HGNC:1975] |
| ENSG00000120210 | HGLibA_23391 | 98 | -0.248249885 | INSL6 | insulin like 6 [Source:HGNC Symbol;Acc:HGNC:6089] |
| ENSG00000120334 | HGLibA_09069 | 98 | -0.248249885 | CENPL | centromere protein L [Source:HGNC Symbol;Acc:HGNC:17879] |
| ENSG00000120458 | HGLibA_30171 | 98 | -0.248249885 | MSANTD2 | Myb/SANT DNA binding domain containing 2 [Source:HGNC Symbol;Acc:HGNC:26266] |
| ENSG00000123178 | HGLibA_46857 | 98 | -0.248249885 | SPRYD7 | SPRY domain containing 7 [Source:HGNC Symbol;Acc:HGNC:14297] |
| ENSG00000124391 | HGLibA_22994 | 98 | -0.248249885 | IL17C | interleukin 17C [Source:HGNC Symbol;Acc:HGNC:5983] |
| ENSG00000125851 | HGLibA_35674 | 98 | -0.248249885 | PCSK2 | proprotein convertase subtilisin/kexin type 2 [Source:HGNC Symbol;Acc:HGNC:8744] |
| ENSG00000128595 | HGLibA_07052 | 98 | -0.248249885 | CALU | calumenin [Source:HGNC Symbol;Acc:HGNC:1458] |
| ENSG00000128602 | HGLibA_45790 | 98 | -0.248249885 | SMO | frizzled class receptor [Source:HGNC Symbol;Acc:HGNC:11119]" |
| ENSG00000129757 | HGLibA_08826 | 98 | -0.248249885 | CDKN1C | cyclin dependent kinase inhibitor 1C [Source:HGNC Symbol;Acc:HGNC:1786] |
| ENSG00000130711 | HGLibA_38372 | 98 | -0.248249885 | PRDM12 | PR/SET domain 12 [Source:HGNC Symbol;Acc:HGNC:13997] |
| ENSG00000131634 | HGLibA_50190 | 98 | -0.248249885 | TMEM204 | transmembrane protein 204 [Source:HGNC Symbol;Acc:HGNC:14158] |
| ENSG00000132405 | HGLibA_48480 | 98 | -0.248249885 | TBC1D14 | TBC1 domain family member 14 [Source:HGNC Symbol;Acc:HGNC:29246] |
| ENSG00000132485 | HGLibA_56975 | 98 | -0.248249885 | ZRANB2 | zinc finger RANBP2-type containing 2 [Source:HGNC Symbol;Acc:HGNC:13058] |
| ENSG00000134352 | HGLibA_23187 | 98 | -0.248249885 | IL6ST | interleukin 6 signal transducer [Source:HGNC Symbol;Acc:HGNC:6021] |
| ENSG00000134538 | HGLibA_45447 | 98 | -0.248249885 | SLCO1B1 | solute carrier organic anion transporter family member 1B1 [Source:HGNC Symbol;Acc:HGNC:10959] |
| ENSG00000134809 | HGLibA_49494 | 98 | -0.248249885 | TIMM10 | translocase of inner mitochondrial membrane 10 [Source:HGNC Symbol;Acc:HGNC:11814] |
| ENSG00000135631 | HGLibA_39806 | 98 | -0.248249885 | RAB11FIP5 | RAB11 family interacting protein 5 [Source:HGNC Symbol;Acc:HGNC:24845] |
| ENSG00000137965 | HGLibA_22611 | 98 | -0.248249885 | IFI44 | interferon induced protein 44 [Source:HGNC Symbol;Acc:HGNC:16938] |
| ENSG00000138101 | HGLibA_14067 | 98 | -0.248249885 | DTNB | dystrobrevin beta [Source:HGNC Symbol;Acc:HGNC:3058] |
| ENSG00000138131 | HGLibA_27046 | 98 | -0.248249885 | LOXL4 | lysyl oxidase like 4 [Source:HGNC Symbol;Acc:HGNC:17171] |
| ENSG00000140836 | HGLibA_55174 | 98 | -0.248249885 | ZFHX3 | zinc finger homeobox 3 [Source:HGNC Symbol;Acc:HGNC:777] |
| ENSG00000140992 | HGLibA_35930 | 98 | -0.248249885 | PDPK1 | 3-phosphoinositide dependent protein kinase 1 [Source:HGNC Symbol;Acc:HGNC:8816] |
| ENSG00000141030 | HGLibA_10715 | 98 | -0.248249885 | COPS3 | COP9 signalosome subunit 3 [Source:HGNC Symbol;Acc:HGNC:2239] |
| ENSG00000143858 | HGLibA_48071 | 98 | -0.248249885 | SYT2 | synaptotagmin 2 [Source:HGNC Symbol;Acc:HGNC:11510] |
| ENSG00000144741 | HGLibA_44686 | 98 | -0.248249885 | SLC25A26 | solute carrier family 25 member 26 [Source:HGNC Symbol;Acc:HGNC:20661] |
| ENSG00000144909 | HGLibA_34647 | 98 | -0.248249885 | OSBPL11 | oxysterol binding protein like 11 [Source:HGNC Symbol;Acc:HGNC:16397] |
| ENSG00000146383 | HGLibA_48132 | 98 | -0.248249885 | TAAR6 | trace amine associated receptor 6 [Source:HGNC Symbol;Acc:HGNC:20978] |
| ENSG00000146385 | HGLibA_48135 | 98 | -0.248249885 | TAAR8 | trace amine associated receptor 8 [Source:HGNC Symbol;Acc:HGNC:14964] |
| ENSG00000146834 | HGLibA_28901 | 98 | -0.248249885 | MEPCE | methylphosphate capping enzyme [Source:HGNC Symbol;Acc:HGNC:20247] |
| ENSG00000148680 | HGLibA_22403 | 98 | -0.248249885 | HTR7 | 5-hydroxytryptamine receptor 7 [Source:HGNC Symbol;Acc:HGNC:5302] |

| | | | | | |
|---|---|---|---|---|---|
| ENSG00000150316 | HGLibA_11812 | 98 | -0.248249885 | CWC15 | CWC15 spliceosome associated protein homolog [Source:HGNC Symbol;Acc:HGNC:26939] |
| ENSG00000153391 | HGLibA_23324 | 98 | -0.248249885 | INO80C | INO80 complex subunit C [Source:HGNC Symbol;Acc:HGNC:26994] |
| ENSG00000153531 | HGLibA_01029 | 98 | -0.248249885 | ADPRHL1 | ADP-ribosylhydrolase like 1 [Source:HGNC Symbol;Acc:HGNC:21303] |
| ENSG00000154007 | HGLibA_03228 | 98 | -0.248249885 | ASB17 | ankyrin repeat and SOCS box containing 17 [Source:HGNC Symbol;Acc:HGNC:19769] |
| ENSG00000156194 | HGLibA_37871 | 98 | -0.248249885 | PPEF2 | protein phosphatase with EF-hand domain 2 [Source:HGNC Symbol;Acc:HGNC:9244] |
| ENSG00000159082 | HGLibA_48004 | 98 | -0.248249885 | SYNJ1 | synaptojanin 1 [Source:HGNC Symbol;Acc:HGNC:11503] |
| ENSG00000159479 | HGLibA_28817 | 98 | -0.248249885 | MED8 | mediator complex subunit 8 [Source:HGNC Symbol;Acc:HGNC:19971] |
| ENSG00000159915 | HGLibA_55641 | 98 | -0.248249885 | ZNF233 | zinc finger protein 233 [Source:HGNC Symbol;Acc:HGNC:30946] |
| ENSG00000160951 | HGLibA_39314 | 98 | -0.248249885 | PTGER1 | prostaglandin E receptor 1 [Source:HGNC Symbol;Acc:HGNC:9593] |
| ENSG00000162852 | HGLibA_10383 | 98 | -0.248249885 | CNST | connexin sorting protein [Source:HGNC Symbol;Acc:HGNC:26486]" |
| ENSG00000164031 | HGLibA_13535 | 98 | -0.248249885 | DNAJB14 | DnaJ heat shock protein family (Hsp40) member B14 [Source:HGNC Symbol;Acc:HGNC:25881] |
| ENSG00000164111 | HGLibA_02243 | 98 | -0.248249885 | ANXA5 | annexin A5 [Source:HGNC Symbol;Acc:HGNC:543] |
| ENSG00000164946 | HGLibA_17985 | 98 | -0.248249885 | FREM1 | FRAS1 related extracellular matrix 1 [Source:HGNC Symbol;Acc:HGNC:23399] |
| ENSG00000165072 | HGLibA_28036 | 98 | -0.248249885 | MAMDC2 | MAM domain containing 2 [Source:HGNC Symbol;Acc:HGNC:23673] |
| ENSG00000166171 | HGLibA_13811 | 98 | -0.248249885 | DPCD | deleted in primary ciliary dyskinesia homolog (mouse) [Source:HGNC Symbol;Acc:HGNC:24542] |
| ENSG00000166685 | HGLibA_10470 | 98 | -0.248249885 | COG1 | component of oligomeric golgi complex 1 [Source:HGNC Symbol;Acc:HGNC:6545] |
| ENSG00000167196 | HGLibA_17047 | 98 | -0.248249885 | FBXO22 | F-box protein 22 [Source:HGNC Symbol;Acc:HGNC:13593] |
| ENSG00000167291 | HGLibA_48486 | 98 | -0.248249885 | TBC1D16 | TBC1 domain family member 16 [Source:HGNC Symbol;Acc:HGNC:28356] |
| ENSG00000168040 | HGLibA_15932 | 98 | -0.248249885 | FADD | Fas associated via death domain [Source:HGNC Symbol;Acc:HGNC:3573] |
| ENSG00000168237 | HGLibA_19383 | 98 | -0.248249885 | GLYCTK | glycerate kinase [Source:HGNC Symbol;Acc:HGNC:24247] |
| ENSG00000168591 | HGLibA_50686 | 98 | -0.248249885 | TMUB2 | transmembrane and ubiquitin like domain containing 2 [Source:HGNC Symbol;Acc:HGNC:28459] |
| ENSG00000168925 | HGLibA_11676 | 98 | -0.248249885 | CTRB1 | chymotrypsinogen B1 [Source:HGNC Symbol;Acc:HGNC:2521] |
| ENSG00000168930 | HGLibA_51523 | 98 | -0.248249885 | TRIM49 | tripartite motif containing 49 [Source:HGNC Symbol;Acc:HGNC:13431] |
| ENSG00000168995 | HGLibA_44148 | 98 | -0.248249885 | SIGLEC7 | sialic acid binding Ig like lectin 7 [Source:HGNC Symbol;Acc:HGNC:10876] |
| ENSG00000169347 | HGLibA_19704 | 98 | -0.248249885 | GP2 | glycoprotein 2 [Source:HGNC Symbol;Acc:HGNC:4441] |
| ENSG00000170004 | HGLibA_09391 | 98 | -0.248249885 | CHD3 | chromodomain helicase DNA binding protein 3 [Source:HGNC Symbol;Acc:HGNC:1918] |
| ENSG00000170044 | HGLibA_56969 | 98 | -0.248249885 | ZPLD1 | zona pellucida like domain containing 1 [Source:HGNC Symbol;Acc:HGNC:27022] |
| ENSG00000170425 | HGLibA_01017 | 98 | -0.248249885 | ADORA2B | adenosine A2b receptor [Source:HGNC Symbol;Acc:HGNC:264] |
| ENSG00000170683 | HGLibA_33464 | 98 | -0.248249885 | OR10A3 | olfactory receptor family 10 subfamily A member 3 [Source:HGNC Symbol;Acc:HGNC:8162] |
| ENSG00000171595 | HGLibA_13506 | 98 | -0.248249885 | DNAI2 | dynein axonemal intermediate chain 2 [Source:HGNC Symbol;Acc:HGNC:18744] |
| ENSG00000172831 | HGLibA_09221 | 98 | -0.248249885 | CES2 | carboxylesterase 2 [Source:HGNC Symbol;Acc:HGNC:1864] |
| ENSG00000173065 | HGLibA_16525 | 98 | -0.248249885 | FAM222B | family with sequence similarity 222 member B [Source:HGNC Symbol;Acc:HGNC:25563] |
| ENSG00000173239 | HGLibA_26626 | 98 | -0.248249885 | LIPM | lipase family member M [Source:HGNC Symbol;Acc:HGNC:23455] |
| ENSG00000173467 | HGLibA_01247 | 98 | -0.248249885 | AGR3 | protein disulphide isomerase family member [Source:HGNC Symbol;Acc:HGNC:24167]" |
| ENSG00000174358 | HGLibA_45317 | 98 | -0.248249885 | SLC6A19 | solute carrier family 6 member 19 [Source:HGNC Symbol;Acc:HGNC:27960] |
| ENSG00000174791 | HGLibA_41290 | 98 | -0.248249885 | RIN1 | Ras and Rab interactor 1 [Source:HGNC Symbol;Acc:HGNC:18749] |
| ENSG00000177300 | HGLibA_09883 | 98 | -0.248249885 | CLDN22 | claudin 22 [Source:HGNC Symbol;Acc:HGNC:2044] |
| ENSG00000178974 | HGLibA_17077 | 98 | -0.248249885 | FBXO34 | F-box protein 34 [Source:HGNC Symbol;Acc:HGNC:20201] |
| ENSG00000179958 | HGLibA_12562 | 98 | -0.248249885 | DCTPP1 | dCTP pyrophosphatase 1 [Source:HGNC Symbol;Acc:HGNC:28777] |
| ENSG00000180992 | HGLibA_29904 | 98 | -0.248249885 | MRPL14 | mitochondrial ribosomal protein L14 [Source:HGNC Symbol;Acc:HGNC:14279] |
| ENSG00000182103 | HGLibA_16320 | 98 | -0.248249885 | FAM181B | family with sequence similarity 181 member B [Source:HGNC Symbol;Acc:HGNC:28512] |
| ENSG00000182168 | HGLibA_53021 | 98 | -0.248249885 | UNC5C | unc-5 netrin receptor C [Source:HGNC Symbol;Acc:HGNC:12569] |
| ENSG00000182393 | HGLibA_22718 | 98 | -0.248249885 | IFNL1 | interferon lambda 1 [Source:HGNC Symbol;Acc:HGNC:18363] |
| ENSG00000182613 | HGLibA_33900 | 98 | -0.248249885 | OR2V2 | olfactory receptor family 2 subfamily V member 2 [Source:HGNC Symbol;Acc:HGNC:15341] |
| ENSG00000183161 | HGLibA_16849 | 98 | -0.248249885 | FANCF | FA complementation group F [Source:HGNC Symbol;Acc:HGNC:3587] |
| ENSG00000183696 | HGLibA_53086 | 98 | -0.248249885 | UPP1 | uridine phosphorylase 1 [Source:HGNC Symbol;Acc:HGNC:12576] |
| ENSG00000184203 | HGLibA_38074 | 98 | -0.248249885 | PPP1R2 | protein phosphatase 1 regulatory inhibitor subunit 2 [Source:HGNC Symbol;Acc:HGNC:9288] |
| ENSG00000185177 | HGLibA_56064 | 98 | -0.248249885 | ZNF479 | zinc finger protein 479 [Source:HGNC Symbol;Acc:HGNC:23258] |
| ENSG00000186908 | HGLibA_55088 | 98 | -0.248249885 | ZDHHC17 | zinc finger DHHC-type containing 17 [Source:HGNC Symbol;Acc:HGNC:18412] |
| ENSG00000187833 | HGLibA_06186 | 98 | -0.248249885 | C2orf78 | chromosome 2 open reading frame 78 [Source:HGNC Symbol;Acc:HGNC:34349] |
| ENSG00000187922 | HGLibA_26169 | 98 | -0.248249885 | LCN10 | lipocalin 10 [Source:HGNC Symbol;Acc:HGNC:20892] |
| ENSG00000188176 | HGLibA_45836 | 98 | -0.248249885 | SMTNL2 | smoothelin like 2 [Source:HGNC Symbol;Acc:HGNC:24764] |
| ENSG00000196189 | HGLibA_43341 | 98 | -0.248249885 | SEMA4A | semaphorin 4A [Source:HGNC Symbol;Acc:HGNC:10729] |
| ENSG00000196387 | HGLibA_55483 | 98 | -0.248249885 | ZNF140 | zinc finger protein 140 [Source:HGNC Symbol;Acc:HGNC:12925] |
| ENSG00000196924 | HGLibA_17638 | 98 | -0.248249885 | FLNA | filamin A [Source:HGNC Symbol;Acc:HGNC:3754] |
| ENSG00000196954 | HGLibA_07314 | 98 | -0.248249885 | CASP4 | caspase 4 [Source:HGNC Symbol;Acc:HGNC:1505] |
| ENSG00000197213 | HGLibA_57032 | 98 | -0.248249885 | ZSCAN5B | zinc finger and SCAN domain containing 5B [Source:HGNC Symbol;Acc:HGNC:34246] |
| ENSG00000197226 | HGLibA_48575 | 98 | -0.248249885 | TBC1D9B | TBC1 domain family member 9B [Source:HGNC Symbol;Acc:HGNC:29097] |
| ENSG00000197321 | HGLibA_47887 | 98 | -0.248249885 | SVIL | supervillin [Source:HGNC Symbol;Acc:HGNC:11480] |
| ENSG00000197617 | HGLibA_53707 | 98 | -0.248249885 | VN1R5 | vomeronasal 1 receptor 5 (gene/pseudogene) [Source:HGNC Symbol;Acc:HGNC:19870] |
| ENSG00000197705 | HGLibA_25135 | 98 | -0.248249885 | KLHL14 | kelch like family member 14 [Source:HGNC Symbol;Acc:HGNC:29266] |
| ENSG00000197857 | HGLibA_55998 | 98 | -0.248249885 | ZNF44 | zinc finger protein 44 [Source:HGNC Symbol;Acc:HGNC:13110] |
| ENSG00000197863 | HGLibA_56764 | 98 | -0.248249885 | ZNF790 | zinc finger protein 790 [Source:HGNC Symbol;Acc:HGNC:33114] |
| ENSG00000198216 | HGLibA_06887 | 98 | -0.248249885 | CACNA1E | calcium voltage-gated channel subunit alpha1 E [Source:HGNC Symbol;Acc:HGNC:1392] |
| ENSG00000198399 | HGLibA_23874 | 98 | -0.248249885 | ITSN2 | intersectin 2 [Source:HGNC Symbol;Acc:HGNC:6184] |
| ENSG00000198720 | HGLibA_02004 | 98 | -0.248249885 | ANKRD13B | ankyrin repeat domain 13B [Source:HGNC Symbol;Acc:HGNC:26363] |
| ENSG00000198837 | HGLibA_12954 | 98 | -0.248249885 | DENND4B | DENN domain containing 4B [Source:HGNC Symbol;Acc:HGNC:29044] |
| ENSG00000198914 | HGLibA_37772 | 98 | -0.248249885 | POU3F3 | POU class 3 homeobox 3 [Source:HGNC Symbol;Acc:HGNC:9216] |
| ENSG00000203872 | HGLibA_06455 | 98 | -0.248249885 | C6orf163 | chromosome 6 open reading frame 163 [Source:HGNC Symbol;Acc:HGNC:21403] |
| ENSG00000204505 | HGLibA_38331 | 98 | -0.248249885 | PRAMEF9 | PRAME family member 9 [Source:HGNC Symbol;Acc:HGNC:27996] |
| ENSG00000204599 | HGLibA_51482 | 98 | -0.248249885 | TRIM39 | tripartite motif containing 39 [Source:HGNC Symbol;Acc:HGNC:10065] |
| ENSG00000204619 | HGLibA_38017 | 98 | -0.248249885 | PPP1R11 | protein phosphatase 1 regulatory inhibitor subunit 11 [Source:HGNC Symbol;Acc:HGNC:9285] |
| ENSG00000205060 | HGLibA_44967 | 98 | -0.248249885 | SLC35B4 | solute carrier family 35 member B4 [Source:HGNC Symbol;Acc:HGNC:20584] |
| ENSG00000205629 | HGLibA_26159 | 98 | -0.248249885 | LCMT1 | leucine carboxyl methyltransferase 1 [Source:HGNC Symbol;Acc:HGNC:17557] |
| ENSG00000212127 | HGLibA_48372 | 98 | -0.248249885 | TAS2R14 | taste 2 receptor member 14 [Source:HGNC Symbol;Acc:HGNC:14920] |
| ENSG00000214212 | HGLibA_05655 | 98 | -0.248249885 | C19orf38 | chromosome 19 open reading frame 38 [Source:HGNC Symbol;Acc:HGNC:34073] |
| ENSG00000218891 | HGLibA_56301 | 98 | -0.248249885 | ZNF579 | zinc finger protein 579 [Source:HGNC Symbol;Acc:HGNC:26646] |
| ENSG00000221938 | HGLibA_33726 | 98 | -0.248249885 | OR2A14 | olfactory receptor family 2 subfamily A member 14 [Source:HGNC Symbol;Acc:HGNC:15084] |
| ENSG00000233041 | HGLibA_36396 | 98 | -0.248249885 | PHGR1 | histidine and glycine rich 1 [Source:HGNC Symbol;Acc:HGNC:37226]" |
| ENSG00000244537 | HGLibA_25757 | 98 | -0.248249885 | KRTAP4-2 | keratin associated protein 4-2 [Source:HGNC Symbol;Acc:HGNC:18900] |
| ENSG00000244754 | HGLibA_30959 | 98 | -0.248249885 | N4BP2L2 | NEDD4 binding protein 2 like 2 [Source:HGNC Symbol;Acc:HGNC:26916] |
| ENSG00000248643 | HGLibA_40487 | 98 | -0.248249885 | RBM14-RBM4 | RBM14-RBM4 readthrough [Source:HGNC Symbol;Acc:HGNC:38840] |
| ENSG00000251493 | HGLibA_17827 | 98 | -0.248249885 | FOXD1 | forkhead box D1 [Source:HGNC Symbol;Acc:HGNC:3802] |
| ENSG00000257446 | HGLibA_56888 | 98 | -0.248249885 | ZNF878 | zinc finger protein 878 [Source:HGNC Symbol;Acc:HGNC:37246] |
| ENSG00000272398 | HGLibA_08304 | 98 | -0.248249885 | CD24 | CD24 molecule [Source:HGNC Symbol;Acc:HGNC:1645] |
| ENSG00000272636 | HGLibA_13727 | 98 | -0.248249885 | DOC2B | double C2 domain beta [Source:HGNC Symbol;Acc:HGNC:2986] |
| ENSG00000274274 | HGLibA_18463 | 98 | -0.248249885 | GAGE13 | G antigen 13 [Source:HGNC Symbol;Acc:HGNC:29081] |
| ENSG00000278129 | HGLibA_56778 | 98 | -0.248249885 | ZNF8 | zinc finger protein 8 [Source:HGNC Symbol;Acc:HGNC:13154] |
| ENSG00000284723 | HGLibA_34554 | 98 | -0.248249885 | OR8S1 | olfactory receptor family 8 subfamily S member 1 [Source:HGNC Symbol;Acc:HGNC:19628] |
| FAM134C | HGLibA_16128 | 98 | -0.248249885 | FAM134C | - |
| GAREM | HGLibA_18627 | 98 | -0.248249885 | GAREM | - |
| HIST1H2BJ | HGLibA_21437 | 98 | -0.248249885 | HIST1H2BJ | - |
| hsa-mir-1286 | HGLibA_57607 | 98 | -0.248249885 | hsa-mir-1286 | - |
| hsa-mir-1295a | HGLibA_57657 | 98 | -0.248249885 | hsa-mir-1295a | - |
| hsa-mir-215 | HGLibA_58254 | 98 | -0.248249885 | hsa-mir-215 | - |
| hsa-mir-3130-1 | HGLibA_58570 | 98 | -0.248249885 | hsa-mir-3130-1 | - |
| hsa-mir-3190 | HGLibA_58857 | 98 | -0.248249885 | hsa-mir-3190 | - |
| hsa-mir-422a | HGLibA_59697 | 98 | -0.248249885 | hsa-mir-422a | - |
| hsa-mir-4261 | HGLibA_59753 | 98 | -0.248249885 | hsa-mir-4261 | - |
| hsa-mir-4293 | HGLibA_59883 | 98 | -0.248249885 | hsa-mir-4293 | - |
| hsa-mir-4635 | HGLibA_60645 | 98 | -0.248249885 | hsa-mir-4635 | - |
| hsa-mir-4672 | HGLibA_60805 | 98 | -0.248249885 | hsa-mir-4672 | - |
| hsa-mir-4748 | HGLibA_61089 | 98 | -0.248249885 | hsa-mir-4748 | - |
| hsa-mir-4793 | HGLibA_61277 | 98 | -0.248249885 | hsa-mir-4793 | - |
| hsa-mir-500a | HGLibA_61445 | 98 | -0.248249885 | hsa-mir-500a | - |
| hsa-mir-513a-2 | HGLibA_61574 | 98 | -0.248249885 | hsa-mir-513a-2 | - |
| hsa-mir-521-2 | HGLibA_61771 | 98 | -0.248249885 | hsa-mir-521-2 | - |
| hsa-mir-572 | HGLibA_62377 | 98 | -0.248249885 | hsa-mir-572 | - |
| hsa-mir-6778 | HGLibA_63285 | 98 | -0.248249885 | hsa-mir-6778 | - |
| hsa-mir-6890 | HGLibA_63744 | 98 | -0.248249885 | hsa-mir-6890 | - |
| hsa-mir-874 | HGLibA_64207 | 98 | -0.248249885 | hsa-mir-874 | - |
| hsa-mir-892b | HGLibA_64256 | 98 | -0.248249885 | hsa-mir-892b | - |
| KIAA1279 | HGLibA_24734 | 98 | -0.248249885 | KIAA1279 | - |
| LOC643355 | HGLibA_26966 | 98 | -0.248249885 | LOC643355 | - |
| LOC643355 | HGLibA_26967 | 98 | -0.248249885 | LOC643355 | - |
| MURC | HGLibA_30572 | 98 | -0.248249885 | MURC | - |
| 11-Mar | HGLibA_28332 | 97 | -0.257295228 | 11-Mar | - |
| C17orf74 | HGLibA_05571 | 97 | -0.257295228 | C17orf74 | - |
| CCBP2 | HGLibA_07502 | 97 | -0.257295228 | CCBP2 | - |
| DEFB130 | HGLibA_12895 | 97 | -0.257295228 | DEFB130 | - |
| ENSG00000006634 | HGLibA_12376 | 97 | -0.257295228 | DBF4 | DBF4 zinc finger [Source:HGNC Symbol;Acc:HGNC:17364] |
| ENSG00000007255 | HGLibA_51329 | 97 | -0.257295228 | TRAPPC6A | trafficking protein particle complex 6A [Source:HGNC Symbol;Acc:HGNC:23069] |
| ENSG00000017483 | HGLibA_45074 | 97 | -0.257295228 | SLC38A5 | solute carrier family 38 member 5 [Source:HGNC Symbol;Acc:HGNC:18070] |
| ENSG00000033030 | HGLibA_55049 | 97 | -0.257295228 | ZCCHC8 | zinc finger CCHC-type containing 8 [Source:HGNC Symbol;Acc:HGNC:25265] |
| ENSG00000035862 | HGLibA_49537 | 97 | -0.257295228 | TIMP2 | TIMP metallopeptidase inhibitor 2 [Source:HGNC Symbol;Acc:HGNC:11821] |
| ENSG00000043462 | HGLibA_26202 | 97 | -0.257295228 | LCP2 | lymphocyte cytosolic protein 2 [Source:HGNC Symbol;Acc:HGNC:6529] |
| ENSG00000047932 | HGLibA_19671 | 97 | -0.257295228 | GOPC | golgi associated PDZ and coiled-coil motif containing [Source:HGNC Symbol;Acc:HGNC:17643] |
| ENSG00000058056 | HGLibA_53202 | 97 | -0.257295228 | USP13 | ubiquitin specific peptidase 13 [Source:HGNC Symbol;Acc:HGNC:12611] |
| ENSG00000058063 | HGLibA_03557 | 97 | -0.257295228 | ATP11B | ATPase phospholipid transporting 11B (putative) [Source:HGNC Symbol;Acc:HGNC:13553] |
| ENSG00000058404 | HGLibA_07071 | 97 | -0.257295228 | CAMK2B | calcium/calmodulin dependent protein kinase II beta [Source:HGNC Symbol;Acc:HGNC:1461] |
| ENSG00000061455 | HGLibA_38395 | 97 | -0.257295228 | PRDM6 | PR/SET domain 6 [Source:HGNC Symbol;Acc:HGNC:9350] |
| ENSG00000063854 | HGLibA_20813 | 97 | -0.257295228 | HAGH | hydroxyacylglutathione hydrolase [Source:HGNC Symbol;Acc:HGNC:4805] |
| ENSG00000064042 | HGLibA_26529 | 97 | -0.257295228 | LIMCH1 | LIM and calponin homology domains 1 [Source:HGNC Symbol;Acc:HGNC:29191] |
| ENSG00000065802 | HGLibA_03203 | 97 | -0.257295228 | ASB1 | ankyrin repeat and SOCS box containing 1 [Source:HGNC Symbol;Acc:HGNC:16011] |
| ENSG00000066027 | HGLibA_38171 | 97 | -0.257295228 | PPP2R5A | protein phosphatase 2 regulatory subunit B'alpha [Source:HGNC Symbol;Acc:HGNC:9309] |

| | | | | | |
|---|---|---|---|---|---|
| ENSG00000066279 | HGLibA_03359 | 97 | -0.257295228 | ASPM | abnormal spindle microtubule assembly [Source:HGNC Symbol;Acc:HGNC:19048] |
| ENSG00000067842 | HGLibA_03621 | 97 | -0.257295228 | ATP2B3 | ATPase plasma membrane Ca2+ transporting 3 [Source:HGNC Symbol;Acc:HGNC:816] |
| ENSG00000068079 | HGLibA_22608 | 97 | -0.257295228 | IFI35 | interferon induced protein 35 [Source:HGNC Symbol;Acc:HGNC:5399] |
| ENSG00000071794 | HGLibA_21656 | 97 | -0.257295228 | HLTF | helicase like transcription factor [Source:HGNC Symbol;Acc:HGNC:11099] |
| ENSG00000074276 | HGLibA_08701 | 97 | -0.257295228 | CDHR2 | cadherin related family member 2 [Source:HGNC Symbol;Acc:HGNC:18231] |
| ENSG00000074803 | HGLibA_44357 | 97 | -0.257295228 | SLC12A1 | solute carrier family 12 member 1 [Source:HGNC Symbol;Acc:HGNC:10910] |
| ENSG00000075336 | HGLibA_49508 | 97 | -0.257295228 | TIMM21 | translocase of inner mitochondrial membrane 21 [Source:HGNC Symbol;Acc:HGNC:25010] |
| ENSG00000077147 | HGLibA_49728 | 97 | -0.257295228 | TM9SF3 | transmembrane 9 superfamily member 3 [Source:HGNC Symbol;Acc:HGNC:21529] |
| ENSG00000088808 | HGLibA_38030 | 97 | -0.257295228 | PPP1R13B | protein phosphatase 1 regulatory subunit 13B [Source:HGNC Symbol;Acc:HGNC:14950] |
| ENSG00000090863 | HGLibA_19244 | 97 | -0.257295228 | GLG1 | golgi glycoprotein 1 [Source:HGNC Symbol;Acc:HGNC:4316] |
| ENSG00000100347 | HGLibA_42706 | 97 | -0.257295228 | SAMM50 | SAMM50 sorting and assembly machinery component [Source:HGNC Symbol;Acc:HGNC:24276] |
| ENSG00000100379 | HGLibA_24435 | 97 | -0.257295228 | KCTD17 | potassium channel tetramerization domain containing 17 [Source:HGNC Symbol;Acc:HGNC:25705] |
| ENSG00000100644 | HGLibA_21298 | 97 | -0.257295228 | HIF1A | hypoxia inducible factor 1 subunit alpha [Source:HGNC Symbol;Acc:HGNC:4910] |
| ENSG00000100678 | HGLibA_45394 | 97 | -0.257295228 | SLC8A3 | solute carrier family 8 member A3 [Source:HGNC Symbol;Acc:HGNC:11070] |
| ENSG00000101363 | HGLibA_28089 | 97 | -0.257295228 | MANBAL | mannosidase beta like [Source:HGNC Symbol;Acc:HGNC:15799] |
| ENSG00000103184 | HGLibA_43201 | 97 | -0.257295228 | SEC14L5 | SEC14 like lipid binding 5 [Source:HGNC Symbol;Acc:HGNC:29032] |
| ENSG00000103260 | HGLibA_28937 | 97 | -0.257295228 | METRN | glial cell differentiation regulator [Source:HGNC Symbol;Acc:HGNC:14151]" |
| ENSG00000105246 | HGLibA_14362 | 97 | -0.257295228 | EBI3 | Epstein-Barr virus induced 3 [Source:HGNC Symbol;Acc:HGNC:3129] |
| ENSG00000105409 | HGLibA_03587 | 97 | -0.257295228 | ATP1A3 | ATPase Na+/K+ transporting subunit alpha 3 [Source:HGNC Symbol;Acc:HGNC:801] |
| ENSG00000105427 | HGLibA_10259 | 97 | -0.257295228 | CNFN | cornifelin [Source:HGNC Symbol;Acc:HGNC:30183] |
| ENSG00000105549 | HGLibA_49333 | 97 | -0.257295228 | THEG | theg spermatid protein [Source:HGNC Symbol;Acc:HGNC:13706] |
| ENSG00000107807 | HGLibA_49670 | 97 | -0.257295228 | TLX1 | T cell leukemia homeobox 1 [Source:HGNC Symbol;Acc:HGNC:5056] |
| ENSG00000109072 | HGLibA_53893 | 97 | -0.257295228 | VTN | vitronectin [Source:HGNC Symbol;Acc:HGNC:12724] |
| ENSG00000111196 | HGLibA_28011 | 97 | -0.257295228 | MAGOHB | exon junction complex subunit [Source:HGNC Symbol;Acc:HGNC:25504]" |
| ENSG00000112312 | HGLibA_19418 | 97 | -0.257295228 | GMNN | DNA replication inhibitor [Source:HGNC Symbol;Acc:HGNC:17493]" |
| ENSG00000113272 | HGLibA_49356 | 97 | -0.257295228 | THG1L | tRNA-histidine guanylyltransferase 1 like [Source:HGNC Symbol;Acc:HGNC:26053] |
| ENSG00000114200 | HGLibA_04274 | 97 | -0.257295228 | BCHE | butyrylcholinesterase [Source:HGNC Symbol;Acc:HGNC:983] |
| ENSG00000116001 | HGLibA_49431 | 97 | -0.257295228 | TIA1 | TIA1 cytotoxic granule associated RNA binding protein [Source:HGNC Symbol;Acc:HGNC:11802] |
| ENSG00000117481 | HGLibA_32787 | 97 | -0.257295228 | NSUN4 | NOP2/Sun RNA methyltransferase family member 4 [Source:HGNC Symbol;Acc:HGNC:31802] |
| ENSG00000119121 | HGLibA_51767 | 97 | -0.257295228 | TRPM6 | transient receptor potential cation channel subfamily M member 6 [Source:HGNC Symbol;Acc:HGNC:17995] |
| ENSG00000122512 | HGLibA_37337 | 97 | -0.257295228 | PMS2 | mismatch repair system component [Source:HGNC Symbol;Acc:HGNC:9122]" |
| ENSG00000122547 | HGLibA_14518 | 97 | -0.257295228 | EEPD1 | endonuclease/exonuclease/phosphatase family domain containing 1 [Source:HGNC Symbol;Acc:HGNC:22223] |
| ENSG00000123106 | HGLibA_07926 | 97 | -0.257295228 | CCDC91 | coiled-coil domain containing 91 [Source:HGNC Symbol;Acc:HGNC:24855] |
| ENSG00000124207 | HGLibA_11317 | 97 | -0.257295228 | CSE1L | chromosome segregation 1 like [Source:HGNC Symbol;Acc:HGNC:2431] |
| ENSG00000124743 | HGLibA_25182 | 97 | -0.257295228 | KLHL31 | kelch like family member 31 [Source:HGNC Symbol;Acc:HGNC:21353] |
| ENSG00000126822 | HGLibA_37108 | 97 | -0.257295228 | PLEKHG3 | pleckstrin homology and RhoGEF domain containing G3 [Source:HGNC Symbol;Acc:HGNC:20364] |
| ENSG00000126934 | HGLibA_28143 | 97 | -0.257295228 | MAP2K2 | mitogen-activated protein kinase kinase 2 [Source:HGNC Symbol;Acc:HGNC:6842] |
| ENSG00000128266 | HGLibA_19481 | 97 | -0.257295228 | GNAZ | G protein subunit alpha z [Source:HGNC Symbol;Acc:HGNC:4395] |
| ENSG00000128590 | HGLibA_13558 | 97 | -0.257295228 | DNAJB9 | DnaJ heat shock protein family (Hsp40) member B9 [Source:HGNC Symbol;Acc:HGNC:6968] |
| ENSG00000128656 | HGLibA_09504 | 97 | -0.257295228 | CHN1 | chimerin 1 [Source:HGNC Symbol;Acc:HGNC:1943] |
| ENSG00000131473 | HGLibA_00453 | 97 | -0.257295228 | ACLY | ATP citrate lyase [Source:HGNC Symbol;Acc:HGNC:115] |
| ENSG00000132436 | HGLibA_17523 | 97 | -0.257295228 | FIGNL1 | fidgetin like 1 [Source:HGNC Symbol;Acc:HGNC:13286] |
| ENSG00000133612 | HGLibA_01145 | 97 | -0.257295228 | AGAP3 | ankyrin repeat and PH domain 3 [Source:HGNC Symbol;Acc:HGNC:16923]" |
| ENSG00000133878 | HGLibA_14170 | 97 | -0.257295228 | DUSP26 | dual specificity phosphatase 26 [Source:HGNC Symbol;Acc:HGNC:28161] |
| ENSG00000134640 | HGLibA_30448 | 97 | -0.257295228 | MTNR1B | melatonin receptor 1B [Source:HGNC Symbol;Acc:HGNC:7464] |
| ENSG00000135318 | HGLibA_32828 | 97 | -0.257295228 | NT5E | 5'-nucleotidase ecto [Source:HGNC Symbol;Acc:HGNC:8021] |
| ENSG00000135919 | HGLibA_43579 | 97 | -0.257295228 | SERPINE2 | serpin family E member 2 [Source:HGNC Symbol;Acc:HGNC:8951] |
| ENSG00000136451 | HGLibA_53626 | 97 | -0.257295228 | VEZF1 | vascular endothelial zinc finger 1 [Source:HGNC Symbol;Acc:HGNC:12949] |
| ENSG00000136859 | HGLibA_01914 | 97 | -0.257295228 | ANGPTL2 | angiopoietin like 2 [Source:HGNC Symbol;Acc:HGNC:490] |
| ENSG00000137055 | HGLibA_36931 | 97 | -0.257295228 | PLAA | phospholipase A2 activating protein [Source:HGNC Symbol;Acc:HGNC:9043] |
| ENSG00000137210 | HGLibA_49983 | 97 | -0.257295228 | TMEM14B | transmembrane protein 14B [Source:HGNC Symbol;Acc:HGNC:21384] |
| ENSG00000138772 | HGLibA_02238 | 97 | -0.257295228 | ANXA3 | annexin A3 [Source:HGNC Symbol;Acc:HGNC:541] |
| ENSG00000138801 | HGLibA_35152 | 97 | -0.257295228 | PAPSS1 | 3'-phosphoadenosine 5'-phosphosulfate synthase 1 [Source:HGNC Symbol;Acc:HGNC:8603] |
| ENSG00000139508 | HGLibA_45191 | 97 | -0.257295228 | SLC46A3 | solute carrier family 46 member 3 [Source:HGNC Symbol;Acc:HGNC:27501] |
| ENSG00000141437 | HGLibA_44768 | 97 | -0.257295228 | SLC25A52 | solute carrier family 25 member 52 [Source:HGNC Symbol;Acc:HGNC:23324] |
| ENSG00000143315 | HGLibA_36566 | 97 | -0.257295228 | PIGM | phosphatidylinositol glycan anchor biosynthesis class M [Source:HGNC Symbol;Acc:HGNC:18858] |
| ENSG00000143751 | HGLibA_43122 | 97 | -0.257295228 | SDE2 | SDE2 telomere maintenance homolog [Source:HGNC Symbol;Acc:HGNC:26643] |
| ENSG00000143862 | HGLibA_02995 | 97 | -0.257295228 | ARL8A | ADP ribosylation factor like GTPase 8A [Source:HGNC Symbol;Acc:HGNC:25192] |
| ENSG00000145335 | HGLibA_45909 | 97 | -0.257295228 | SNCA | synuclein alpha [Source:HGNC Symbol;Acc:HGNC:11138] |
| ENSG00000145945 | HGLibA_16633 | 97 | -0.257295228 | FAM50B | family with sequence similarity 50 member B [Source:HGNC Symbol;Acc:HGNC:18789] |
| ENSG00000146013 | HGLibA_18971 | 97 | -0.257295228 | GFRA3 | GDNF family receptor alpha 3 [Source:HGNC Symbol;Acc:HGNC:4245] |
| ENSG00000148484 | HGLibA_42366 | 97 | -0.257295228 | RSU1 | Ras suppressor protein 1 [Source:HGNC Symbol;Acc:HGNC:10464] |
| ENSG00000148498 | HGLibA_35177 | 97 | -0.257295228 | PARD3 | par-3 family cell polarity regulator [Source:HGNC Symbol;Acc:HGNC:16051] |
| ENSG00000150275 | HGLibA_35399 | 97 | -0.257295228 | PCDH15 | protocadherin related 15 [Source:HGNC Symbol;Acc:HGNC:14674] |
| ENSG00000150281 | HGLibA_11619 | 97 | -0.257295228 | CTF1 | cardiotrophin 1 [Source:HGNC Symbol;Acc:HGNC:2499] |
| ENSG00000152669 | HGLibA_08123 | 97 | -0.257295228 | CCNO | cyclin O [Source:HGNC Symbol;Acc:HGNC:18576] |
| ENSG00000153246 | HGLibA_36927 | 97 | -0.257295228 | PLA2R1 | phospholipase A2 receptor 1 [Source:HGNC Symbol;Acc:HGNC:9042] |
| ENSG00000153815 | HGLibA_10195 | 97 | -0.257295228 | CMIP | c-Maf inducing protein [Source:HGNC Symbol;Acc:HGNC:24319] |
| ENSG00000153827 | HGLibA_51639 | 97 | -0.257295228 | TRIP12 | thyroid hormone receptor interactor 12 [Source:HGNC Symbol;Acc:HGNC:12306] |
| ENSG00000155096 | HGLibA_03940 | 97 | -0.257295228 | AZIN1 | antizyme inhibitor 1 [Source:HGNC Symbol;Acc:HGNC:16432] |
| ENSG00000155097 | HGLibA_03738 | 97 | -0.257295228 | ATP6V1C1 | ATPase H+ transporting V1 subunit C1 [Source:HGNC Symbol;Acc:HGNC:856] |
| ENSG00000155111 | HGLibA_08753 | 97 | -0.257295228 | CDK19 | cyclin dependent kinase 19 [Source:HGNC Symbol;Acc:HGNC:19338] |
| ENSG00000155252 | HGLibA_36493 | 97 | -0.257295228 | PI4K2A | phosphatidylinositol 4-kinase type 2 alpha [Source:HGNC Symbol;Acc:HGNC:30031] |
| ENSG00000156968 | HGLibA_29785 | 97 | -0.257295228 | MPV17L | MPV17 mitochondrial inner membrane protein like [Source:HGNC Symbol;Acc:HGNC:26827] |
| ENSG00000157087 | HGLibA_03616 | 97 | -0.257295228 | ATP2B2 | ATPase plasma membrane Ca2+ transporting 2 [Source:HGNC Symbol;Acc:HGNC:815] |
| ENSG00000157911 | HGLibA_36081 | 97 | -0.257295228 | PEX10 | peroxisomal biogenesis factor 10 [Source:HGNC Symbol;Acc:HGNC:8851] |
| ENSG00000160255 | HGLibA_23777 | 97 | -0.257295228 | ITGB2 | integrin subunit beta 2 [Source:HGNC Symbol;Acc:HGNC:6155] |
| ENSG00000162604 | HGLibA_49681 | 97 | -0.257295228 | TM2D1 | TM2 domain containing 1 [Source:HGNC Symbol;Acc:HGNC:24142] |
| ENSG00000163281 | HGLibA_19569 | 97 | -0.257295228 | GNPDA2 | glucosamine-6-phosphate deaminase 2 [Source:HGNC Symbol;Acc:HGNC:21526] |
| ENSG00000163913 | HGLibA_22744 | 97 | -0.257295228 | IFT122 | intraflagellar transport 122 [Source:HGNC Symbol;Acc:HGNC:13556] |
| ENSG00000164062 | HGLibA_02405 | 97 | -0.257295228 | APEH | acylaminoacyl-peptide hydrolase [Source:HGNC Symbol;Acc:HGNC:586] |
| ENSG00000164334 | HGLibA_16259 | 97 | -0.257295228 | FAM170A | family with sequence similarity 170 member A [Source:HGNC Symbol;Acc:HGNC:27963] |
| ENSG00000164900 | HGLibA_18749 | 97 | -0.257295228 | GBX1 | gastrulation brain homeobox 1 [Source:HGNC Symbol;Acc:HGNC:4185] |
| ENSG00000166153 | HGLibA_12981 | 97 | -0.257295228 | DEPDC4 | DEP domain containing 4 [Source:HGNC Symbol;Acc:HGNC:22952] |
| ENSG00000167447 | HGLibA_45709 | 97 | -0.257295228 | SMG8 | nonsense mediated mRNA decay factor [Source:HGNC Symbol;Acc:HGNC:25551]" |
| ENSG00000167965 | HGLibA_29434 | 97 | -0.257295228 | MLST8 | LST8 homolog [Source:HGNC Symbol;Acc:HGNC:24825]" |
| ENSG00000168427 | HGLibA_25181 | 97 | -0.257295228 | KLHL30 | kelch like family member 30 [Source:HGNC Symbol;Acc:HGNC:24770] |
| ENSG00000168505 | HGLibA_18751 | 97 | -0.257295228 | GBX2 | gastrulation brain homeobox 2 [Source:HGNC Symbol;Acc:HGNC:4186] |
| ENSG00000168685 | HGLibA_23193 | 97 | -0.257295228 | IL7R | interleukin 7 receptor [Source:HGNC Symbol;Acc:HGNC:6024] |
| ENSG00000168811 | HGLibA_22958 | 97 | -0.257295228 | IL12A | interleukin 12A [Source:HGNC Symbol;Acc:HGNC:5969] |
| ENSG00000169228 | HGLibA_39846 | 97 | -0.257295228 | RAB24 | member RAS oncogene family [Source:HGNC Symbol;Acc:HGNC:9765]" |
| ENSG00000169575 | HGLibA_53723 | 97 | -0.257295228 | VPREB1 | V-set pre-B cell surrogate light chain 1 [Source:HGNC Symbol;Acc:HGNC:12709] |
| ENSG00000169752 | HGLibA_32684 | 97 | -0.257295228 | NRG4 | neuregulin 4 [Source:HGNC Symbol;Acc:HGNC:29862] |
| ENSG00000170293 | HGLibA_10230 | 97 | -0.257295228 | CMTM8 | CKLF like MARVEL transmembrane domain containing 8 [Source:HGNC Symbol;Acc:HGNC:19179] |
| ENSG00000170516 | HGLibA_10869 | 97 | -0.257295228 | COX7B2 | cytochrome c oxidase subunit 7B2 [Source:HGNC Symbol;Acc:HGNC:24381] |
| ENSG00000171222 | HGLibA_42850 | 97 | -0.257295228 | SCAND1 | SCAN domain containing 1 [Source:HGNC Symbol;Acc:HGNC:10566] |
| ENSG00000171385 | HGLibA_24181 | 97 | -0.257295228 | KCND3 | potassium voltage-gated channel subfamily D member 3 [Source:HGNC Symbol;Acc:HGNC:6239] |
| ENSG00000171388 | HGLibA_02433 | 97 | -0.257295228 | APLN | apelin [Source:HGNC Symbol;Acc:HGNC:16665] |
| ENSG00000171604 | HGLibA_11973 | 97 | -0.257295228 | CXXC5 | CXXC finger protein 5 [Source:HGNC Symbol;Acc:HGNC:26943] |
| ENSG00000171703 | HGLibA_48698 | 97 | -0.257295228 | TCEA2 | transcription elongation factor A2 [Source:HGNC Symbol;Acc:HGNC:11614] |
| ENSG00000172172 | HGLibA_29899 | 97 | -0.257295228 | MRPL13 | mitochondrial ribosomal protein L13 [Source:HGNC Symbol;Acc:HGNC:14278] |
| ENSG00000172243 | HGLibA_10005 | 97 | -0.257295228 | CLEC7A | C-type lectin domain containing 7A [Source:HGNC Symbol;Acc:HGNC:14558] |
| ENSG00000172667 | HGLibA_55371 | 97 | -0.257295228 | ZMAT3 | zinc finger matrin-type 3 [Source:HGNC Symbol;Acc:HGNC:29983] |
| ENSG00000173517 | HGLibA_36006 | 97 | -0.257295228 | PEAK1 | pseudopodium enriched atypical kinase 1 [Source:HGNC Symbol;Acc:HGNC:29431] |
| ENSG00000173991 | HGLibA_48693 | 97 | -0.257295228 | TCAP | titin-cap [Source:HGNC Symbol;Acc:HGNC:11610] |
| ENSG00000174946 | HGLibA_19967 | 97 | -0.257295228 | GPR171 | G protein-coupled receptor 171 [Source:HGNC Symbol;Acc:HGNC:30057] |
| ENSG00000175115 | HGLibA_34954 | 97 | -0.257295228 | PACS1 | phosphofurin acidic cluster sorting protein 1 [Source:HGNC Symbol;Acc:HGNC:30032] |
| ENSG00000175143 | HGLibA_33851 | 97 | -0.257295228 | OR2T1 | olfactory receptor family 2 subfamily T member 1 [Source:HGNC Symbol;Acc:HGNC:8277] |
| ENSG00000176531 | HGLibA_36431 | 97 | -0.257295228 | PHLDB3 | pleckstrin homology like domain family B member 3 [Source:HGNC Symbol;Acc:HGNC:30499] |
| ENSG00000176919 | HGLibA_06599 | 97 | -0.257295228 | C8G | complement C8 gamma chain [Source:HGNC Symbol;Acc:HGNC:1354] |
| ENSG00000177732 | HGLibA_46234 | 97 | -0.257295228 | SOX12 | SRY-box 12 [Source:HGNC Symbol;Acc:HGNC:11198] |
| ENSG00000178031 | HGLibA_00848 | 97 | -0.257295228 | ADAMTSL1 | ADAMTS like 1 [Source:HGNC Symbol;Acc:HGNC:14632] |
| ENSG00000178826 | HGLibA_49959 | 97 | -0.257295228 | TMEM139 | transmembrane protein 139 [Source:HGNC Symbol;Acc:HGNC:22058] |
| ENSG00000179119 | HGLibA_46907 | 97 | -0.257295228 | SPTY2D1 | SPT2 chromatin protein domain containing 1 [Source:HGNC Symbol;Acc:HGNC:26818] |
| ENSG00000179242 | HGLibA_08681 | 97 | -0.257295228 | CDH4 | cadherin 4 [Source:HGNC Symbol;Acc:HGNC:1763] |
| ENSG00000179958 | HGLibA_12561 | 97 | -0.257295228 | DCTPP1 | dCTP pyrophosphatase 1 [Source:HGNC Symbol;Acc:HGNC:28777] |
| ENSG00000180287 | HGLibA_37048 | 97 | -0.257295228 | PLD5 | phospholipase D family member 5 [Source:HGNC Symbol;Acc:HGNC:26879] |
| ENSG00000180758 | HGLibA_19949 | 97 | -0.257295228 | GPR157 | G protein-coupled receptor 157 [Source:HGNC Symbol;Acc:HGNC:23687] |
| ENSG00000181323 | HGLibA_46618 | 97 | -0.257295228 | SPEM1 | spermatid maturation 1 [Source:HGNC Symbol;Acc:HGNC:32429] |
| ENSG00000181733 | HGLibA_33912 | 97 | -0.257295228 | OR2Z1 | olfactory receptor family 2 subfamily Z member 1 [Source:HGNC Symbol;Acc:HGNC:15391] |
| ENSG00000182177 | HGLibA_03229 | 97 | -0.257295228 | ASB18 | ankyrin repeat and SOCS box containing 18 [Source:HGNC Symbol;Acc:HGNC:19770] |
| ENSG00000182256 | HGLibA_18396 | 97 | -0.257295228 | GABRG3 | gamma-aminobutyric acid type A receptor gamma3 subunit [Source:HGNC Symbol;Acc:HGNC:4088] |
| ENSG00000183723 | HGLibA_10217 | 97 | -0.257295228 | CMTM4 | CKLF like MARVEL transmembrane domain containing 4 [Source:HGNC Symbol;Acc:HGNC:19175] |
| ENSG00000185344 | HGLibA_03707 | 97 | -0.257295228 | ATP6V0A2 | ATPase H+ transporting V0 subunit a2 [Source:HGNC Symbol;Acc:HGNC:18481] |
| ENSG00000185787 | HGLibA_29660 | 97 | -0.257295228 | MORF4L1 | mortality factor 4 like 1 [Source:HGNC Symbol;Acc:HGNC:16989] |
| ENSG00000186204 | HGLibA_12185 | 97 | -0.257295228 | CYP4F12 | cytochrome P450 family 4 subfamily F member 12 [Source:HGNC Symbol;Acc:HGNC:18857] |
| ENSG00000186714 | HGLibA_07859 | 97 | -0.257295228 | CCDC73 | coiled-coil domain containing 73 [Source:HGNC Symbol;Acc:HGNC:23261] |
| ENSG00000187189 | HGLibA_51985 | 97 | -0.257295228 | TSPYL4 | TSPY like 4 [Source:HGNC Symbol;Acc:HGNC:21559] |
| ENSG00000188706 | HGLibA_55133 | 97 | -0.257295228 | ZDHHC9 | zinc finger DHHC-type containing 9 [Source:HGNC Symbol;Acc:HGNC:18475] |
| ENSG00000189001 | HGLibA_42812 | 97 | -0.257295228 | SBSN | suprabasin [Source:HGNC Symbol;Acc:HGNC:24950] |
| ENSG00000189057 | HGLibA_16027 | 97 | -0.257295228 | FAM111B | family with sequence similarity 111 member B [Source:HGNC Symbol;Acc:HGNC:24200] |
| ENSG00000196659 | HGLibA_52108 | 97 | -0.257295228 | TTC30B | tetratricopeptide repeat domain 30B [Source:HGNC Symbol;Acc:HGNC:26425] |
| ENSG00000197279 | HGLibA_55517 | 97 | -0.257295228 | ZNF165 | zinc finger protein 165 [Source:HGNC Symbol;Acc:HGNC:12953] |
| ENSG00000197283 | HGLibA_47987 | 97 | -0.257295228 | SYNGAP1 | synaptic Ras GTPase activating protein 1 [Source:HGNC Symbol;Acc:HGNC:11497] |
| ENSG00000197454 | HGLibA_33826 | 97 | -0.257295228 | OR2L5 | olfactory receptor family 2 subfamily L member 5 [Source:HGNC Symbol;Acc:HGNC:15011] |

| | | | | | |
|---|---|---|---|---|---|
| ENSG00000198354 | HGLibA_12423 | 97 | -0.257295228 | DCAF12L2 | DDB1 and CUL4 associated factor 12 like 2 [Source:HGNC Symbol;Acc:HGNC:32950] |
| ENSG00000203784 | HGLibA_26283 | 97 | -0.257295228 | LELP1 | late cornified envelope like proline rich 1 [Source:HGNC Symbol;Acc:HGNC:32046] |
| ENSG00000204231 | HGLibA_42509 | 97 | -0.257295228 | RXRB | retinoid X receptor beta [Source:HGNC Symbol;Acc:HGNC:10478] |
| ENSG00000204287 | HGLibA_21633 | 97 | -0.257295228 | HLA-DRA | DR alpha [Source:HGNC Symbol;Acc:HGNC:4947]" |
| ENSG00000211460 | HGLibA_51880 | 97 | -0.257295228 | TSN | translin [Source:HGNC Symbol;Acc:HGNC:12379] |
| ENSG00000212710 | HGLibA_11567 | 97 | -0.257295228 | CTAGE1 | cutaneous T cell lymphoma-associated antigen 1 [Source:HGNC Symbol;Acc:HGNC:24346] |
| ENSG00000213889 | HGLibA_37996 | 97 | -0.257295228 | PPM1N | Mg2+/Mn2+ dependent 1N (putative) [Source:HGNC Symbol;Acc:HGNC:26845]" |
| ENSG00000213999 | HGLibA_28828 | 97 | -0.257295228 | MEF2B | myocyte enhancer factor 2B [Source:HGNC Symbol;Acc:HGNC:6995] |
| ENSG00000214160 | HGLibA_01628 | 97 | -0.257295228 | ALG3 | 3- mannosyltransferase [Source:HGNC Symbol;Acc:HGNC:23056]" |
| ENSG00000214860 | HGLibA_15703 | 97 | -0.257295228 | EVPLL | envoplakin like [Source:HGNC Symbol;Acc:HGNC:35236] |
| ENSG00000233276 | HGLibA_20170 | 97 | -0.257295228 | GPX1 | glutathione peroxidase 1 [Source:HGNC Symbol;Acc:HGNC:4553] |
| ENSG00000244395 | HGLibA_40617 | 97 | -0.257295228 | RBMY1D | member D [Source:HGNC Symbol;Acc:HGNC:23915]" |
| ENSG00000244607 | HGLibA_07589 | 97 | -0.257295228 | CCDC13 | coiled-coil domain containing 13 [Source:HGNC Symbol;Acc:HGNC:26358] |
| ENSG00000258839 | HGLibA_28540 | 97 | -0.257295228 | MC1R | melanocortin 1 receptor [Source:HGNC Symbol;Acc:HGNC:6929] |
| ENSG00000265763 | HGLibA_56086 | 97 | -0.257295228 | ZNF488 | zinc finger protein 488 [Source:HGNC Symbol;Acc:HGNC:23535] |
| ENSG00000278195 | HGLibA_47179 | 97 | -0.257295228 | SSTR3 | somatostatin receptor 3 [Source:HGNC Symbol;Acc:HGNC:11332] |
| FAM69C | HGLibA_16683 | 97 | -0.257295228 | FAM69C | - |
| FUK | HGLibA_18161 | 97 | -0.257295228 | FUK | - |
| GAGE2B | HGLibA_18469 | 97 | -0.257295228 | GAGE2B | - |
| H2AFJ | HGLibA_20753 | 97 | -0.257295228 | H2AFJ | - |
| hsa-mir-1184-2 | HGLibA_57232 | 97 | -0.257295228 | hsa-mir-1184-2 | - |
| hsa-mir-181a-1 | HGLibA_57960 | 97 | -0.257295228 | hsa-mir-181a-1 | - |
| hsa-mir-208b | HGLibA_58198 | 97 | -0.257295228 | hsa-mir-208b | - |
| hsa-mir-3157 | HGLibA_58701 | 97 | -0.257295228 | hsa-mir-3157 | - |
| hsa-mir-3615 | HGLibA_59089 | 97 | -0.257295228 | hsa-mir-3615 | - |
| hsa-mir-3657 | HGLibA_59175 | 97 | -0.257295228 | hsa-mir-3657 | - |
| hsa-mir-3689d-1 | HGLibA_59327 | 97 | -0.257295228 | hsa-mir-3689d-1 | - |
| hsa-mir-4283-1 | HGLibA_59837 | 97 | -0.257295228 | hsa-mir-4283-1 | - |
| hsa-mir-4505 | HGLibA_60447 | 97 | -0.257295228 | hsa-mir-4505 | - |
| hsa-mir-4632 | HGLibA_60634 | 97 | -0.257295228 | hsa-mir-4632 | - |
| hsa-mir-4762 | HGLibA_61145 | 97 | -0.257295228 | hsa-mir-4762 | - |
| hsa-mir-514b | HGLibA_61597 | 97 | -0.257295228 | hsa-mir-514b | - |
| hsa-mir-524 | HGLibA_61782 | 97 | -0.257295228 | hsa-mir-524 | - |
| hsa-mir-548ar | HGLibA_61917 | 97 | -0.257295228 | hsa-mir-548ar | - |
| hsa-mir-548i-2 | HGLibA_62015 | 97 | -0.257295228 | hsa-mir-548i-2 | - |
| hsa-mir-548t | HGLibA_62068 | 97 | -0.257295228 | hsa-mir-548t | - |
| hsa-mir-570 | HGLibA_62332 | 97 | -0.257295228 | hsa-mir-570 | - |
| hsa-mir-6762 | HGLibA_63211 | 97 | -0.257295228 | hsa-mir-6762 | - |
| hsa-mir-6764 | HGLibA_63216 | 97 | -0.257295228 | hsa-mir-6764 | - |
| hsa-mir-7515 | HGLibA_63888 | 97 | -0.257295228 | hsa-mir-7515 | - |
| hsa-mir-7976 | HGLibA_64032 | 97 | -0.257295228 | hsa-mir-7976 | - |
| hsa-mir-935 | HGLibA_64315 | 97 | -0.257295228 | hsa-mir-935 | - |
| LOC100996485 | HGLibA_26884 | 97 | -0.257295228 | LOC100996485 | - |
| LOC653486 | HGLibA_26986 | 97 | -0.257295228 | LOC653486 | - |
| LPHN3 | HGLibA_27091 | 97 | -0.257295228 | LPHN3 | - |
| NAT6 | HGLibA_31179 | 97 | -0.257295228 | NAT6 | - |
| RGAG1 | HGLibA_40991 | 97 | -0.257295228 | RGAG1 | - |
| SELK | HGLibA_43292 | 97 | -0.257295228 | SELK | - |
| SHOX_Y | HGLibA_44073 | 97 | -0.257295228 | SHOX_Y | - |
| SPDYE2L | HGLibA_46587 | 97 | -0.257295228 | SPDYE2L | - |
| USP17L1P | HGLibA_53240 | 97 | -0.257295228 | USP17L1P | - |
| VAMP7_Y | HGLibA_53506 | 97 | -0.257295228 | VAMP7_Y | - |
| C14orf183 | HGLibA_05353 | 96 | -0.266340571 | C14orf183 | - |
| C9orf66 | HGLibA_06752 | 96 | -0.266340571 | C9orf66 | - |
| CCDC36 | HGLibA_07764 | 96 | -0.266340571 | CCDC36 | - |
| CYR61 | HGLibA_12220 | 96 | -0.266340571 | CYR61 | - |
| DEFB131 | HGLibA_12898 | 96 | -0.266340571 | DEFB131 | - |
| ENSG00000007952 | HGLibA_32369 | 96 | -0.266340571 | NOX1 | NADPH oxidase 1 [Source:HGNC Symbol;Acc:HGNC:7889] |
| ENSG00000010256 | HGLibA_53112 | 96 | -0.266340571 | UQCRC1 | ubiquinol-cytochrome c reductase core protein 1 [Source:HGNC Symbol;Acc:HGNC:12585] |
| ENSG00000010310 | HGLibA_19127 | 96 | -0.266340571 | GIPR | gastric inhibitory polypeptide receptor [Source:HGNC Symbol;Acc:HGNC:4271] |
| ENSG00000013016 | HGLibA_14672 | 96 | -0.266340571 | EHD3 | EH domain containing 3 [Source:HGNC Symbol;Acc:HGNC:3244] |
| ENSG00000013288 | HGLibA_28079 | 96 | -0.266340571 | MAN2B2 | mannosidase alpha class 2B member 2 [Source:HGNC Symbol;Acc:HGNC:29623] |
| ENSG00000021762 | HGLibA_34659 | 96 | -0.266340571 | OSBPL5 | oxysterol binding protein like 5 [Source:HGNC Symbol;Acc:HGNC:16392] |
| ENSG00000033030 | HGLibA_55047 | 96 | -0.266340571 | ZCCHC8 | zinc finger CCHC-type containing 8 [Source:HGNC Symbol;Acc:HGNC:25265] |
| ENSG00000052802 | HGLibA_30226 | 96 | -0.266340571 | MSMO1 | methylsterol monooxygenase 1 [Source:HGNC Symbol;Acc:HGNC:10545] |
| ENSG00000055332 | HGLibA_14727 | 96 | -0.266340571 | EIF2AK2 | eukaryotic translation initiation factor 2 alpha kinase 2 [Source:HGNC Symbol;Acc:HGNC:9437] |
| ENSG00000055609 | HGLibA_25328 | 96 | -0.266340571 | KMT2C | lysine methyltransferase 2C [Source:HGNC Symbol;Acc:HGNC:13726] |
| ENSG00000066279 | HGLibA_03358 | 96 | -0.266340571 | ASPM | abnormal spindle microtubule assembly [Source:HGNC Symbol;Acc:HGNC:19048] |
| ENSG00000066629 | HGLibA_15057 | 96 | -0.266340571 | EML1 | echinoderm microtubule associated protein like 1 [Source:HGNC Symbol;Acc:HGNC:3330] |
| ENSG00000067225 | HGLibA_36830 | 96 | -0.266340571 | PKM | pyruvate kinase M1/2 [Source:HGNC Symbol;Acc:HGNC:9021] |
| ENSG00000067445 | HGLibA_51715 | 96 | -0.266340571 | TRO | trophinin [Source:HGNC Symbol;Acc:HGNC:12326] |
| ENSG00000071889 | HGLibA_16576 | 96 | -0.266340571 | FAM3A | family with sequence similarity 3 member A [Source:HGNC Symbol;Acc:HGNC:13749] |
| ENSG00000078902 | HGLibA_50944 | 96 | -0.266340571 | TOLLIP | toll interacting protein [Source:HGNC Symbol;Acc:HGNC:16476] |
| ENSG00000079974 | HGLibA_40031 | 96 | -0.266340571 | RABL2B | member of RAS oncogene family like 2B [Source:HGNC Symbol;Acc:HGNC:9800]" |
| ENSG00000086666 | HGLibA_55163 | 96 | -0.266340571 | ZFAND6 | zinc finger AN1-type containing 6 [Source:HGNC Symbol;Acc:HGNC:30164] |
| ENSG00000090447 | HGLibA_49137 | 96 | -0.266340571 | TFAP4 | transcription factor AP-4 [Source:HGNC Symbol;Acc:HGNC:11745] |
| ENSG00000095464 | HGLibA_35811 | 96 | -0.266340571 | PDE6C | phosphodiesterase 6C [Source:HGNC Symbol;Acc:HGNC:8787] |
| ENSG00000100462 | HGLibA_38628 | 96 | -0.266340571 | PRMT5 | protein arginine methyltransferase 5 [Source:HGNC Symbol;Acc:HGNC:10894] |
| ENSG00000103343 | HGLibA_55525 | 96 | -0.266340571 | ZNF174 | zinc finger protein 174 [Source:HGNC Symbol;Acc:HGNC:12963] |
| ENSG00000106665 | HGLibA_10044 | 96 | -0.266340571 | CLIP2 | CAP-Gly domain containing linker protein 2 [Source:HGNC Symbol;Acc:HGNC:2586] |
| ENSG00000107807 | HGLibA_49669 | 96 | -0.266340571 | TLX1 | T cell leukemia homeobox 1 [Source:HGNC Symbol;Acc:HGNC:5056] |
| ENSG00000107902 | HGLibA_26445 | 96 | -0.266340571 | LHPP | phospholysine phosphohistidine inorganic pyrophosphate phosphatase [Source:HGNC Symbol;Acc:HGNC:30042] |
| ENSG00000108946 | HGLibA_38521 | 96 | -0.266340571 | PRKAR1A | protein kinase cAMP-dependent type I regulatory subunit alpha [Source:HGNC Symbol;Acc:HGNC:9388] |
| ENSG00000109113 | HGLibA_39886 | 96 | -0.266340571 | RAB34 | member RAS oncogene family [Source:HGNC Symbol;Acc:HGNC:16519]" |
| ENSG00000110077 | HGLibA_30157 | 96 | -0.266340571 | MS4A6A | membrane spanning 4-domains A6A [Source:HGNC Symbol;Acc:HGNC:13375] |
| ENSG00000111339 | HGLibA_03165 | 96 | -0.266340571 | ART4 | ADP-ribosyltransferase 4 (Dombrock blood group) [Source:HGNC Symbol;Acc:HGNC:726] |
| ENSG00000111802 | HGLibA_48893 | 96 | -0.266340571 | TDP2 | tyrosyl-DNA phosphodiesterase 2 [Source:HGNC Symbol;Acc:HGNC:17768] |
| ENSG00000113387 | HGLibA_47694 | 96 | -0.266340571 | SUB1 | transcriptional regulator [Source:HGNC Symbol;Acc:HGNC:19985]" |
| ENSG00000114354 | HGLibA_49178 | 96 | -0.266340571 | TFG | TRK-fused gene [Source:HGNC Symbol;Acc:HGNC:11758] |
| ENSG00000115282 | HGLibA_52110 | 96 | -0.266340571 | TTC31 | tetratricopeptide repeat domain 31 [Source:HGNC Symbol;Acc:HGNC:25759] |
| ENSG00000115548 | HGLibA_24501 | 96 | -0.266340571 | KDM3A | lysine demethylase 3A [Source:HGNC Symbol;Acc:HGNC:20815] |
| ENSG00000116871 | HGLibA_28235 | 96 | -0.266340571 | MAP7D1 | MAP7 domain containing 1 [Source:HGNC Symbol;Acc:HGNC:25514] |
| ENSG00000119684 | HGLibA_29383 | 96 | -0.266340571 | MLH3 | mutL homolog 3 [Source:HGNC Symbol;Acc:HGNC:7128] |
| ENSG00000119969 | HGLibA_21113 | 96 | -0.266340571 | HELLS | lymphoid specific [Source:HGNC Symbol;Acc:HGNC:4861]" |
| ENSG00000120696 | HGLibA_24115 | 96 | -0.266340571 | KBTBD7 | kelch repeat and BTB domain containing 7 [Source:HGNC Symbol;Acc:HGNC:25266] |
| ENSG00000124151 | HGLibA_31340 | 96 | -0.266340571 | NCOA3 | nuclear receptor coactivator 3 [Source:HGNC Symbol;Acc:HGNC:7670] |
| ENSG00000124429 | HGLibA_37466 | 96 | -0.266340571 | POF1B | actin binding protein [Source:HGNC Symbol;Acc:HGNC:13711]" |
| ENSG00000125823 | HGLibA_11494 | 96 | -0.266340571 | CSTL1 | cystatin like 1 [Source:HGNC Symbol;Acc:HGNC:15958] |
| ENSG00000127561 | HGLibA_47998 | 96 | -0.266340571 | SYNGR3 | synaptogyrin 3 [Source:HGNC Symbol;Acc:HGNC:11501] |
| ENSG00000129521 | HGLibA_14644 | 96 | -0.266340571 | EGLN3 | egl-9 family hypoxia inducible factor 3 [Source:HGNC Symbol;Acc:HGNC:14661] |
| ENSG00000130052 | HGLibA_47374 | 96 | -0.266340571 | STARD8 | StAR related lipid transfer domain containing 8 [Source:HGNC Symbol;Acc:HGNC:19161] |
| ENSG00000130962 | HGLibA_38868 | 96 | -0.266340571 | PRRG1 | proline rich and Gla domain 1 [Source:HGNC Symbol;Acc:HGNC:9469] |
| ENSG00000132464 | HGLibA_15106 | 96 | -0.266340571 | ENAM | enamelin [Source:HGNC Symbol;Acc:HGNC:3344] |
| ENSG00000132466 | HGLibA_02016 | 96 | -0.266340571 | ANKRD17 | ankyrin repeat domain 17 [Source:HGNC Symbol;Acc:HGNC:23575] |
| ENSG00000132622 | HGLibA_22261 | 96 | -0.266340571 | HSPA12B | heat shock protein family A (Hsp70) member 12B [Source:HGNC Symbol;Acc:HGNC:16193] |
| ENSG00000132692 | HGLibA_04237 | 96 | -0.266340571 | BCAN | brevican [Source:HGNC Symbol;Acc:HGNC:23059] |
| ENSG00000132915 | HGLibA_35805 | 96 | -0.266340571 | PDE6A | phosphodiesterase 6A [Source:HGNC Symbol;Acc:HGNC:8785] |
| ENSG00000133138 | HGLibA_48569 | 96 | -0.266340571 | TBC1D8B | TBC1 domain family member 8B [Source:HGNC Symbol;Acc:HGNC:24715] |
| ENSG00000136261 | HGLibA_05017 | 96 | -0.266340571 | BZW2 | basic leucine zipper and W2 domains 2 [Source:HGNC Symbol;Acc:HGNC:18808] |
| ENSG00000136271 | HGLibA_12749 | 96 | -0.266340571 | DDX56 | DEAD-box helicase 56 [Source:HGNC Symbol;Acc:HGNC:18193] |
| ENSG00000136280 | HGLibA_08045 | 96 | -0.266340571 | CCM2 | CCM2 scaffold protein [Source:HGNC Symbol;Acc:HGNC:21708] |
| ENSG00000136943 | HGLibA_11730 | 96 | -0.266340571 | CTSV | cathepsin V [Source:HGNC Symbol;Acc:HGNC:2538] |
| ENSG00000137504 | HGLibA_11118 | 96 | -0.266340571 | CREBZF | CREB/ATF bZIP transcription factor [Source:HGNC Symbol;Acc:HGNC:24905] |
| ENSG00000138792 | HGLibA_15163 | 96 | -0.266340571 | ENPEP | glutamyl aminopeptidase [Source:HGNC Symbol;Acc:HGNC:3355] |
| ENSG00000139800 | HGLibA_55332 | 96 | -0.266340571 | ZIC5 | Zic family member 5 [Source:HGNC Symbol;Acc:HGNC:20322] |
| ENSG00000141446 | HGLibA_15557 | 96 | -0.266340571 | ESCO1 | establishment of sister chromatid cohesion N-acetyltransferase 1 [Source:HGNC Symbol;Acc:HGNC:24645] |
| ENSG00000141569 | HGLibA_51585 | 96 | -0.266340571 | TRIM65 | tripartite motif containing 65 [Source:HGNC Symbol;Acc:HGNC:27316] |
| ENSG00000141655 | HGLibA_50745 | 96 | -0.266340571 | TNFRSF11A | TNF receptor superfamily member 11a [Source:HGNC Symbol;Acc:HGNC:11908] |
| ENSG00000143278 | HGLibA_15856 | 96 | -0.266340571 | F13B | coagulation factor XIII B chain [Source:HGNC Symbol;Acc:HGNC:3534] |
| ENSG00000144029 | HGLibA_30104 | 96 | -0.266340571 | MRPS5 | mitochondrial ribosomal protein S5 [Source:HGNC Symbol;Acc:HGNC:14498] |
| ENSG00000144827 | HGLibA_00232 | 96 | -0.266340571 | ABHD10 | abhydrolase domain containing 10 [Source:HGNC Symbol;Acc:HGNC:25656] |
| ENSG00000146859 | HGLibA_49961 | 96 | -0.266340571 | TMEM140 | transmembrane protein 140 [Source:HGNC Symbol;Acc:HGNC:21870] |
| ENSG00000147434 | HGLibA_09580 | 96 | -0.266340571 | CHRNA6 | cholinergic receptor nicotinic alpha 6 subunit [Source:HGNC Symbol;Acc:HGNC:15963] |
| ENSG00000148356 | HGLibA_27498 | 96 | -0.266340571 | LRSAM1 | leucine rich repeat and sterile alpha motif containing 1 [Source:HGNC Symbol;Acc:HGNC:25135] |
| ENSG00000152137 | HGLibA_22319 | 96 | -0.266340571 | HSPB8 | heat shock protein family B (small) member 8 [Source:HGNC Symbol;Acc:HGNC:30171] |
| ENSG00000153823 | HGLibA_36527 | 96 | -0.266340571 | PID1 | phosphotyrosine interaction domain containing 1 [Source:HGNC Symbol;Acc:HGNC:26084] |
| ENSG00000154642 | HGLibA_06050 | 96 | -0.266340571 | C21orf91 | chromosome 21 open reading frame 91 [Source:HGNC Symbol;Acc:HGNC:16459] |
| ENSG00000154760 | HGLibA_45489 | 96 | -0.266340571 | SLFN13 | schlafen family member 13 [Source:HGNC Symbol;Acc:HGNC:26481] |
| ENSG00000154845 | HGLibA_38202 | 96 | -0.266340571 | PPP4R1 | protein phosphatase 4 regulatory subunit 1 [Source:HGNC Symbol;Acc:HGNC:9320] |
| ENSG00000154930 | HGLibA_00587 | 96 | -0.266340571 | ACSS1 | acyl-CoA synthetase short chain family member 1 [Source:HGNC Symbol;Acc:HGNC:16091] |
| ENSG00000155249 | HGLibA_34016 | 96 | -0.266340571 | OR4K1 | olfactory receptor family 4 subfamily K member 1 [Source:HGNC Symbol;Acc:HGNC:14726] |
| ENSG00000155657 | HGLibA_52218 | 96 | -0.266340571 | TTN | titin [Source:HGNC Symbol;Acc:HGNC:12403] |
| ENSG00000155755 | HGLibA_50277 | 96 | -0.266340571 | TMEM237 | transmembrane protein 237 [Source:HGNC Symbol;Acc:HGNC:14432] |
| ENSG00000156096 | HGLibA_52928 | 96 | -0.266340571 | UGT2B4 | UDP glucuronosyltransferase family 2 member B4 [Source:HGNC Symbol;Acc:HGNC:12553] |
| ENSG00000158710 | HGLibA_48274 | 96 | -0.266340571 | TAGLN2 | transgelin 2 [Source:HGNC Symbol;Acc:HGNC:11554] |

| | | | | | |
|---|---|---|---|---|---|
| ENSG00000158711 | HGLibA_14917 | 96 | -0.266340571 | ELK4 | ETS transcription factor [Source:HGNC Symbol;Acc:HGNC:3326]" |
| ENSG00000158792 | HGLibA_46461 | 96 | -0.266340571 | SPATA2L | spermatogenesis associated 2 like [Source:HGNC Symbol;Acc:HGNC:28393] |
| ENSG00000159423 | HGLibA_01573 | 96 | -0.266340571 | ALDH4A1 | aldehyde dehydrogenase 4 family member A1 [Source:HGNC Symbol;Acc:HGNC:406] |
| ENSG00000160202 | HGLibA_11245 | 96 | -0.266340571 | CRYAA | crystallin alpha A [Source:HGNC Symbol;Acc:HGNC:2388] |
| ENSG00000160908 | HGLibA_55909 | 96 | -0.266340571 | ZNF394 | zinc finger protein 394 [Source:HGNC Symbol;Acc:HGNC:18832] |
| ENSG00000162341 | HGLibA_51108 | 96 | -0.266340571 | TPCN2 | two pore segment channel 2 [Source:HGNC Symbol;Acc:HGNC:20820] |
| ENSG00000162517 | HGLibA_36025 | 96 | -0.266340571 | PEF1 | penta-EF-hand domain containing 1 [Source:HGNC Symbol;Acc:HGNC:30009] |
| ENSG00000162522 | HGLibA_24772 | 96 | -0.266340571 | KIAA1522 | KIAA1522 [Source:HGNC Symbol;Acc:HGNC:29301] |
| ENSG00000162595 | HGLibA_13261 | 96 | -0.266340571 | DIRAS3 | DIRAS family GTPase 3 [Source:HGNC Symbol;Acc:HGNC:687] |
| ENSG00000162745 | HGLibA_33361 | 96 | -0.266340571 | OLFML2B | olfactomedin like 2B [Source:HGNC Symbol;Acc:HGNC:24558] |
| ENSG00000163517 | HGLibA_21004 | 96 | -0.266340571 | HDAC11 | histone deacetylase 11 [Source:HGNC Symbol;Acc:HGNC:19086] |
| ENSG00000163638 | HGLibA_00845 | 96 | -0.266340571 | ADAMTS9 | ADAM metallopeptidase with thrombospondin type 1 motif 9 [Source:HGNC Symbol;Acc:HGNC:13202] |
| ENSG00000163737 | HGLibA_36128 | 96 | -0.266340571 | PF4 | platelet factor 4 [Source:HGNC Symbol;Acc:HGNC:8861] |
| ENSG00000164647 | HGLibA_47420 | 96 | -0.266340571 | STEAP1 | STEAP family member 1 [Source:HGNC Symbol;Acc:HGNC:11378] |
| ENSG00000164920 | HGLibA_34703 | 96 | -0.266340571 | OSR2 | odd-skipped related transciption factor 2 [Source:HGNC Symbol;Acc:HGNC:15830] |
| ENSG00000165644 | HGLibA_10689 | 96 | -0.266340571 | COMTD1 | catechol-O-methyltransferase domain containing 1 [Source:HGNC Symbol;Acc:HGNC:26309] |
| ENSG00000165959 | HGLibA_10070 | 96 | -0.266340571 | CLMN | calmin [Source:HGNC Symbol;Acc:HGNC:19972] |
| ENSG00000166471 | HGLibA_50397 | 96 | -0.266340571 | TMEM41B | transmembrane protein 41B [Source:HGNC Symbol;Acc:HGNC:28948] |
| ENSG00000166984 | HGLibA_48839 | 96 | -0.266340571 | TCP10L2 | t-complex 10 like 2 [Source:HGNC Symbol;Acc:HGNC:21254] |
| ENSG00000167525 | HGLibA_38654 | 96 | -0.266340571 | PROCA1 | protein interacting with cyclin A1 [Source:HGNC Symbol;Acc:HGNC:28600] |
| ENSG00000168002 | HGLibA_37581 | 96 | -0.266340571 | POLR2G | RNA polymerase II subunit G [Source:HGNC Symbol;Acc:HGNC:9194] |
| ENSG00000168421 | HGLibA_41193 | 96 | -0.266340571 | RHOH | ras homolog family member H [Source:HGNC Symbol;Acc:HGNC:686] |
| ENSG00000169327 | HGLibA_34253 | 96 | -0.266340571 | OR5AU1 | olfactory receptor family 5 subfamily AU member 1 [Source:HGNC Symbol;Acc:HGNC:15362] |
| ENSG00000169688 | HGLibA_30279 | 96 | -0.266340571 | MT1B | metallothionein 1B [Source:HGNC Symbol;Acc:HGNC:7394] |
| ENSG00000169876 | HGLibA_30532 | 96 | -0.266340571 | MUC17 | cell surface associated [Source:HGNC Symbol;Acc:HGNC:16800]" |
| ENSG00000170476 | HGLibA_30935 | 96 | -0.266340571 | MZB1 | marginal zone B and B1 cell specific protein [Source:HGNC Symbol;Acc:HGNC:30125] |
| ENSG00000173706 | HGLibA_21109 | 96 | -0.266340571 | HEG1 | heart development protein with EGF like domains 1 [Source:HGNC Symbol;Acc:HGNC:29227] |
| ENSG00000173992 | HGLibA_08182 | 96 | -0.266340571 | CCS | copper chaperone for superoxide dismutase [Source:HGNC Symbol;Acc:HGNC:1613] |
| ENSG00000174173 | HGLibA_51665 | 96 | -0.266340571 | TRMT10C | mitochondrial RNase P subunit [Source:HGNC Symbol;Acc:HGNC:26022]" |
| ENSG00000174175 | HGLibA_43305 | 96 | -0.266340571 | SELP | selectin P [Source:HGNC Symbol;Acc:HGNC:10721] |
| ENSG00000176797 | HGLibA_12812 | 96 | -0.266340571 | DEFB103A | defensin beta 103A [Source:HGNC Symbol;Acc:HGNC:15967] |
| ENSG00000179195 | HGLibA_56483 | 96 | -0.266340571 | ZNF664 | zinc finger protein 664 [Source:HGNC Symbol;Acc:HGNC:25406] |
| ENSG00000180957 | HGLibA_36745 | 96 | -0.266340571 | PITPNB | phosphatidylinositol transfer protein beta [Source:HGNC Symbol;Acc:HGNC:9002] |
| ENSG00000182134 | HGLibA_48920 | 96 | -0.266340571 | TDRKH | tudor and KH domain containing [Source:HGNC Symbol;Acc:HGNC:11713] |
| ENSG00000182175 | HGLibA_41014 | 96 | -0.266340571 | RGMA | repulsive guidance molecule BMP co-receptor a [Source:HGNC Symbol;Acc:HGNC:30308] |
| ENSG00000184162 | HGLibA_32600 | 96 | -0.266340571 | NR2C2AP | nuclear receptor 2C2 associated protein [Source:HGNC Symbol;Acc:HGNC:30763] |
| ENSG00000184302 | HGLibA_44258 | 96 | -0.266340571 | SIX6 | SIX homeobox 6 [Source:HGNC Symbol;Acc:HGNC:10892] |
| ENSG00000184724 | HGLibA_25814 | 96 | -0.266340571 | KRTAP6-1 | keratin associated protein 6-1 [Source:HGNC Symbol;Acc:HGNC:18931] |
| ENSG00000184988 | HGLibA_49853 | 96 | -0.266340571 | TMEM106A | transmembrane protein 106A [Source:HGNC Symbol;Acc:HGNC:28288] |
| ENSG00000185479 | HGLibA_25517 | 96 | -0.266340571 | KRT6B | keratin 6B [Source:HGNC Symbol;Acc:HGNC:6444] |
| ENSG00000187800 | HGLibA_36009 | 96 | -0.266340571 | PEAR1 | platelet endothelial aggregation receptor 1 [Source:HGNC Symbol;Acc:HGNC:33631] |
| ENSG00000187855 | HGLibA_03275 | 96 | -0.266340571 | ASCL4 | achaete-scute family bHLH transcription factor 4 [Source:HGNC Symbol;Acc:HGNC:24311] |
| ENSG00000188389 | HGLibA_35716 | 96 | -0.266340571 | PDCD1 | programmed cell death 1 [Source:HGNC Symbol;Acc:HGNC:8760] |
| ENSG00000189298 | HGLibA_55352 | 96 | -0.266340571 | ZKSCAN3 | zinc finger with KRAB and SCAN domains 3 [Source:HGNC Symbol;Acc:HGNC:13853] |
| ENSG00000196171 | HGLibA_34418 | 96 | -0.266340571 | OR6K2 | olfactory receptor family 6 subfamily K member 2 [Source:HGNC Symbol;Acc:HGNC:15029] |
| ENSG00000196712 | HGLibA_31759 | 96 | -0.266340571 | NF1 | neurofibromin 1 [Source:HGNC Symbol;Acc:HGNC:7765] |
| ENSG00000196917 | HGLibA_20945 | 96 | -0.266340571 | HCAR1 | hydroxycarboxylic acid receptor 1 [Source:HGNC Symbol;Acc:HGNC:4532] |
| ENSG00000196954 | HGLibA_07312 | 96 | -0.266340571 | CASP4 | caspase 4 [Source:HGNC Symbol;Acc:HGNC:1505] |
| ENSG00000197888 | HGLibA_52922 | 96 | -0.266340571 | UGT2B17 | UDP glucuronosyltransferase family 2 member B17 [Source:HGNC Symbol;Acc:HGNC:12547] |
| ENSG00000198182 | HGLibA_56370 | 96 | -0.266340571 | ZNF607 | zinc finger protein 607 [Source:HGNC Symbol;Acc:HGNC:28192] |
| ENSG00000198300 | HGLibA_36031 | 96 | -0.266340571 | PEG3 | paternally expressed 3 [Source:HGNC Symbol;Acc:HGNC:8826] |
| ENSG00000198342 | HGLibA_56009 | 96 | -0.266340571 | ZNF442 | zinc finger protein 442 [Source:HGNC Symbol;Acc:HGNC:20877] |
| ENSG00000198373 | HGLibA_54453 | 96 | -0.266340571 | WWP2 | WW domain containing E3 ubiquitin protein ligase 2 [Source:HGNC Symbol;Acc:HGNC:16804] |
| ENSG00000203786 | HGLibA_25378 | 96 | -0.266340571 | KPRP | keratinocyte proline rich protein [Source:HGNC Symbol;Acc:HGNC:31823] |
| ENSG00000204482 | HGLibA_27573 | 96 | -0.266340571 | LST1 | leukocyte specific transcript 1 [Source:HGNC Symbol;Acc:HGNC:14189] |
| ENSG00000204511 | HGLibA_28572 | 96 | -0.266340571 | MCCD1 | mitochondrial coiled-coil domain 1 [Source:HGNC Symbol;Acc:HGNC:20668] |
| ENSG00000204842 | HGLibA_03837 | 96 | -0.266340571 | ATXN2 | ataxin 2 [Source:HGNC Symbol;Acc:HGNC:10555] |
| ENSG00000204889 | HGLibA_25506 | 96 | -0.266340571 | KRT40 | keratin 40 [Source:HGNC Symbol;Acc:HGNC:26707] |
| ENSG00000204919 | HGLibA_38800 | 96 | -0.266340571 | PRR20A | proline rich 20A [Source:HGNC Symbol;Acc:HGNC:24754] |
| ENSG00000204963 | HGLibA_35454 | 96 | -0.266340571 | PCDHA7 | protocadherin alpha 7 [Source:HGNC Symbol;Acc:HGNC:8673] |
| ENSG00000205133 | HGLibA_51651 | 96 | -0.266340571 | TRIQK | triple QxxK/R motif containing [Source:HGNC Symbol;Acc:HGNC:27828] |
| ENSG00000212127 | HGLibA_48371 | 96 | -0.266340571 | TAS2R14 | taste 2 receptor member 14 [Source:HGNC Symbol;Acc:HGNC:14920] |
| ENSG00000213020 | HGLibA_56383 | 96 | -0.266340571 | ZNF611 | zinc finger protein 611 [Source:HGNC Symbol;Acc:HGNC:28766] |
| ENSG00000215115 | HGLibA_11932 | 96 | -0.266340571 | CXorf49 | chromosome X open reading frame 49 [Source:HGNC Symbol;Acc:HGNC:30891] |
| ENSG00000215301 | HGLibA_12703 | 96 | -0.266340571 | DDX3X | DEAD-box helicase 3 X-linked [Source:HGNC Symbol;Acc:HGNC:2745] |
| ENSG00000223731 | HGLibA_47808 | 96 | -0.266340571 | SUPT20HL1 | SUPT20H like 1 [Source:HGNC Symbol;Acc:HGNC:30773] |
| ENSG00000231051 | HGLibA_53266 | 96 | -0.266340571 | USP17L28 | ubiquitin specific peptidase 17-like family member 28 [Source:HGNC Symbol;Acc:HGNC:44456] |
| ENSG00000233436 | HGLibA_04908 | 96 | -0.266340571 | BTBD18 | BTB domain containing 18 [Source:HGNC Symbol;Acc:HGNC:37214] |
| ENSG00000243709 | HGLibA_26273 | 96 | -0.266340571 | LEFTY1 | left-right determination factor 1 [Source:HGNC Symbol;Acc:HGNC:6552] |
| ENSG00000244474 | HGLibA_52884 | 96 | -0.266340571 | UGT1A4 | UDP glucuronosyltransferase family 1 member A4 [Source:HGNC Symbol;Acc:HGNC:12536] |
| ENSG00000250565 | HGLibA_03750 | 96 | -0.266340571 | ATP6V1E2 | ATPase H+ transporting V1 subunit E2 [Source:HGNC Symbol;Acc:HGNC:18125] |
| ENSG00000256223 | HGLibA_55430 | 96 | -0.266340571 | ZNF10 | zinc finger protein 10 [Source:HGNC Symbol;Acc:HGNC:12879] |
| ENSG00000256235 | HGLibA_45756 | 96 | -0.266340571 | SMIM3 | small integral membrane protein 3 [Source:HGNC Symbol;Acc:HGNC:30248] |
| ENSG00000266173 | HGLibA_47572 | 96 | -0.266340571 | STRADA | STE20-related kinase adaptor alpha [Source:HGNC Symbol;Acc:HGNC:30172] |
| ENSG00000276747 | HGLibA_34982 | 96 | -0.266340571 | PADI6 | peptidyl arginine deiminase 6 [Source:HGNC Symbol;Acc:HGNC:20449] |
| ENSG00000288602 | HGLibA_06626 | 96 | -0.266340571 | C8orf44-SGK3 | - |
| FAM208A | HGLibA_16433 | 96 | -0.266340571 | FAM208A | - |
| FAM26E | HGLibA_16565 | 96 | -0.266340571 | FAM26E | - |
| FAM46A | HGLibA_16597 | 96 | -0.266340571 | FAM46A | - |
| hsa-mir-1229 | HGLibA_57314 | 96 | -0.266340571 | hsa-mir-1229 | - |
| hsa-mir-1302-6 | HGLibA_57713 | 96 | -0.266340571 | hsa-mir-1302-6 | - |
| hsa-mir-219a-1 | HGLibA_58275 | 96 | -0.266340571 | hsa-mir-219a-1 | - |
| hsa-mir-3120 | HGLibA_58532 | 96 | -0.266340571 | hsa-mir-3120 | - |
| hsa-mir-342 | HGLibA_59023 | 96 | -0.266340571 | hsa-mir-342 | - |
| hsa-mir-3649 | HGLibA_59143 | 96 | -0.266340571 | hsa-mir-3649 | - |
| hsa-mir-375 | HGLibA_59401 | 96 | -0.266340571 | hsa-mir-375 | - |
| hsa-mir-3945 | HGLibA_59639 | 96 | -0.266340571 | hsa-mir-3945 | - |
| hsa-mir-4426 | HGLibA_60091 | 96 | -0.266340571 | hsa-mir-4426 | - |
| hsa-mir-4500 | HGLibA_60427 | 96 | -0.266340571 | hsa-mir-4500 | - |
| hsa-mir-4650-2 | HGLibA_60707 | 96 | -0.266340571 | hsa-mir-4650-2 | - |
| hsa-mir-4654 | HGLibA_60725 | 96 | -0.266340571 | hsa-mir-4654 | - |
| hsa-mir-4700 | HGLibA_60908 | 96 | -0.266340571 | hsa-mir-4700 | - |
| hsa-mir-4707 | HGLibA_60927 | 96 | -0.266340571 | hsa-mir-4707 | - |
| hsa-mir-4774 | HGLibA_61202 | 96 | -0.266340571 | hsa-mir-4774 | - |
| hsa-mir-4800 | HGLibA_61305 | 96 | -0.266340571 | hsa-mir-4800 | - |
| hsa-mir-5191 | HGLibA_61684 | 96 | -0.266340571 | hsa-mir-5191 | - |
| hsa-mir-526a-1 | HGLibA_61789 | 96 | -0.266340571 | hsa-mir-526a-1 | - |
| hsa-mir-628 | HGLibA_62750 | 96 | -0.266340571 | hsa-mir-628 | - |
| hsa-mir-6855 | HGLibA_63593 | 96 | -0.266340571 | hsa-mir-6855 | - |
| hsa-mir-761 | HGLibA_63903 | 96 | -0.266340571 | hsa-mir-761 | - |
| hsa-mir-920 | HGLibA_64273 | 96 | -0.266340571 | hsa-mir-920 | - |
| hsa-mir-933 | HGLibA_64307 | 96 | -0.266340571 | hsa-mir-933 | - |
| MTERFD2 | HGLibA_30344 | 96 | -0.266340571 | MTERFD2 | - |
| PALM2-AKAP2 | HGLibA_35074 | 96 | -0.266340571 | PALM2-AKAP2 | - |
| RTDR1 | HGLibA_42377 | 96 | -0.266340571 | RTDR1 | - |
| ZCCHC5 | HGLibA_55038 | 96 | -0.266340571 | ZCCHC5 | - |
| APOA1BP | HGLibA_02451 | 95 | -0.275385914 | APOA1BP | - |
| C11orf82 | HGLibA_05189 | 95 | -0.275385914 | C11orf82 | - |
| C16orf3 | HGLibA_05444 | 95 | -0.275385914 | C16orf3 | - |
| C16orf55 | HGLibA_05458 | 95 | -0.275385914 | C16orf55 | - |
| C19orf80 | HGLibA_05710 | 95 | -0.275385914 | C19orf80 | - |
| C1orf61 | HGLibA_05878 | 95 | -0.275385914 | C1orf61 | - |
| C20orf78 | HGLibA_06026 | 95 | -0.275385914 | C20orf78 | - |
| DEFB130 | HGLibA_12894 | 95 | -0.275385914 | DEFB130 | - |
| ENSG00000005486 | HGLibA_41128 | 95 | -0.275385914 | RHBDD2 | rhomboid domain containing 2 [Source:HGNC Symbol;Acc:HGNC:23082] |
| ENSG00000006747 | HGLibA_42946 | 95 | -0.275385914 | SCIN | scinderin [Source:HGNC Symbol;Acc:HGNC:21695] |
| ENSG00000021826 | HGLibA_10991 | 95 | -0.275385914 | CPS1 | carbamoyl-phosphate synthase 1 [Source:HGNC Symbol;Acc:HGNC:2323] |
| ENSG00000023697 | HGLibA_12991 | 95 | -0.275385914 | DERA | deoxyribose-phosphate aldolase [Source:HGNC Symbol;Acc:HGNC:24269] |
| ENSG00000024526 | HGLibA_12975 | 95 | -0.275385914 | DEPDC1 | DEP domain containing 1 [Source:HGNC Symbol;Acc:HGNC:22949] |
| ENSG00000053900 | HGLibA_01884 | 95 | -0.275385914 | ANAPC4 | anaphase promoting complex subunit 4 [Source:HGNC Symbol;Acc:HGNC:19990] |
| ENSG00000054965 | HGLibA_16246 | 95 | -0.275385914 | FAM168A | family with sequence similarity 168 member A [Source:HGNC Symbol;Acc:HGNC:28999] |
| ENSG00000054967 | HGLibA_40826 | 95 | -0.275385914 | RELT | TNF receptor [Source:HGNC Symbol;Acc:HGNC:13764]" |
| ENSG00000065268 | HGLibA_54039 | 95 | -0.275385914 | WDR18 | WD repeat domain 18 [Source:HGNC Symbol;Acc:HGNC:17956] |
| ENSG00000070018 | HGLibA_27231 | 95 | -0.275385914 | LRP6 | LDL receptor related protein 6 [Source:HGNC Symbol;Acc:HGNC:6698] |
| ENSG00000071553 | HGLibA_03695 | 95 | -0.275385914 | ATP6AP1 | ATPase H+ transporting accessory protein 1 [Source:HGNC Symbol;Acc:HGNC:868] |
| ENSG00000072864 | HGLibA_31387 | 95 | -0.275385914 | NDE1 | nudE neurodevelopment protein 1 [Source:HGNC Symbol;Acc:HGNC:17619] |
| ENSG00000077522 | HGLibA_00641 | 95 | -0.275385914 | ACTN2 | actinin alpha 2 [Source:HGNC Symbol;Acc:HGNC:164] |
| ENSG00000078795 | HGLibA_36805 | 95 | -0.275385914 | PKD2L2 | transient receptor potential cation channel [Source:HGNC Symbol;Acc:HGNC:9012]" |
| ENSG00000086570 | HGLibA_16915 | 95 | -0.275385914 | FAT2 | FAT atypical cadherin 2 [Source:HGNC Symbol;Acc:HGNC:3596] |
| ENSG00000087250 | HGLibA_30304 | 95 | -0.275385914 | MT3 | metallothionein 3 [Source:HGNC Symbol;Acc:HGNC:7408] |
| ENSG00000089195 | HGLibA_51696 | 95 | -0.275385914 | TRMT6 | tRNA methyltransferase 6 [Source:HGNC Symbol;Acc:HGNC:20900] |
| ENSG00000089199 | HGLibA_09434 | 95 | -0.275385914 | CHGB | chromogranin B [Source:HGNC Symbol;Acc:HGNC:1930] |
| ENSG00000089737 | HGLibA_12678 | 95 | -0.275385914 | DDX24 | DEAD-box helicase 24 [Source:HGNC Symbol;Acc:HGNC:13266] |
| ENSG00000099834 | HGLibA_08710 | 95 | -0.275385914 | CDHR5 | cadherin related family member 5 [Source:HGNC Symbol;Acc:HGNC:7521] |
| ENSG00000100418 | HGLibA_13007 | 95 | -0.275385914 | DESI1 | desumoylating isopeptidase 1 [Source:HGNC Symbol;Acc:HGNC:24577] |

| | | | | | |
|---|---|---|---|---|---|
| ENSG00000100568 | HGLibA_53889 | 95 | -0.275385914 | VTI1B | vesicle transport through interaction with t-SNAREs 1B [Source:HGNC Symbol;Acc:HGNC:17793] |
| ENSG00000100991 | HGLibA_51737 | 95 | -0.275385914 | TRPC4AP | transient receptor potential cation channel subfamily C member 4 associated protein [Source:HGNC Symbol;Acc:HGNC:16181] |
| ENSG00000101842 | HGLibA_53835 | 95 | -0.275385914 | VSIG1 | V-set and immunoglobulin domain containing 1 [Source:HGNC Symbol;Acc:HGNC:28675] |
| ENSG00000101928 | HGLibA_29685 | 95 | -0.275385914 | MOSPD1 | motile sperm domain containing 1 [Source:HGNC Symbol;Acc:HGNC:25235] |
| ENSG00000102383 | HGLibA_55080 | 95 | -0.275385914 | ZDHHC15 | zinc finger DHHC-type containing 15 [Source:HGNC Symbol;Acc:HGNC:20342] |
| ENSG00000102897 | HGLibA_27745 | 95 | -0.275385914 | LYRM1 | LYR motif containing 1 [Source:HGNC Symbol;Acc:HGNC:25074] |
| ENSG00000103152 | HGLibA_29720 | 95 | -0.275385914 | MPG | N-methylpurine DNA glycosylase [Source:HGNC Symbol;Acc:HGNC:7211] |
| ENSG00000104221 | HGLibA_04800 | 95 | -0.275385914 | BRF2 | RNA polymerase III transcription initiation factor subunit [Source:HGNC Symbol;Acc:HGNC:17298]" |
| ENSG00000104613 | HGLibA_23410 | 95 | -0.275385914 | INTS10 | integrator complex subunit 10 [Source:HGNC Symbol;Acc:HGNC:25548] |
| ENSG00000105143 | HGLibA_44523 | 95 | -0.275385914 | SLC1A6 | solute carrier family 1 member 6 [Source:HGNC Symbol;Acc:HGNC:10944] |
| ENSG00000105146 | HGLibA_03881 | 95 | -0.275385914 | AURKC | aurora kinase C [Source:HGNC Symbol;Acc:HGNC:11391] |
| ENSG00000105357 | HGLibA_30715 | 95 | -0.275385914 | MYH14 | myosin heavy chain 14 [Source:HGNC Symbol;Acc:HGNC:23212] |
| ENSG00000105677 | HGLibA_49975 | 95 | -0.275385914 | TMEM147 | transmembrane protein 147 [Source:HGNC Symbol;Acc:HGNC:30414] |
| ENSG00000105983 | HGLibA_26670 | 95 | -0.275385914 | LMBR1 | limb development membrane protein 1 [Source:HGNC Symbol;Acc:HGNC:13243] |
| ENSG00000106392 | HGLibA_05724 | 95 | -0.275385914 | C1GALT1 | glycoprotein-N-acetylgalactosamine 3-beta-galactosyltransferase 1 [Source:HGNC Symbol;Acc:HGNC:24337]" |
| ENSG00000106823 | HGLibA_14415 | 95 | -0.275385914 | ECM2 | extracellular matrix protein 2 [Source:HGNC Symbol;Acc:HGNC:3154] |
| ENSG00000107404 | HGLibA_14209 | 95 | -0.275385914 | DVL1 | dishevelled segment polarity protein 1 [Source:HGNC Symbol;Acc:HGNC:3084] |
| ENSG00000108848 | HGLibA_27625 | 95 | -0.275385914 | LUC7L3 | LUC7 like 3 pre-mRNA splicing factor [Source:HGNC Symbol;Acc:HGNC:24309] |
| ENSG00000109072 | HGLibA_53894 | 95 | -0.275385914 | VTN | vitronectin [Source:HGNC Symbol;Acc:HGNC:12724] |
| ENSG00000109929 | HGLibA_42819 | 95 | -0.275385914 | SC5D | sterol-C5-desaturase [Source:HGNC Symbol;Acc:HGNC:10547] |
| ENSG00000109943 | HGLibA_11223 | 95 | -0.275385914 | CRTAM | cytotoxic and regulatory T cell molecule [Source:HGNC Symbol;Acc:HGNC:24313] |
| ENSG00000111181 | HGLibA_45296 | 95 | -0.275385914 | SLC6A12 | solute carrier family 6 member 12 [Source:HGNC Symbol;Acc:HGNC:11045] |
| ENSG00000111816 | HGLibA_18004 | 95 | -0.275385914 | FRK | fyn related Src family tyrosine kinase [Source:HGNC Symbol;Acc:HGNC:3955] |
| ENSG00000112246 | HGLibA_44173 | 95 | -0.275385914 | SIM1 | SIM bHLH transcription factor 1 [Source:HGNC Symbol;Acc:HGNC:10882] |
| ENSG00000112981 | HGLibA_32166 | 95 | -0.275385914 | NME5 | NME/NM23 family member 5 [Source:HGNC Symbol;Acc:HGNC:7853] |
| ENSG00000115266 | HGLibA_02392 | 95 | -0.275385914 | APC2 | WNT signaling pathway regulator [Source:HGNC Symbol;Acc:HGNC:24036]" |
| ENSG00000115286 | HGLibA_31562 | 95 | -0.275385914 | NDUFS7 | NADH:ubiquinone oxidoreductase core subunit S7 [Source:HGNC Symbol;Acc:HGNC:7714] |
| ENSG00000116704 | HGLibA_44976 | 95 | -0.275385914 | SLC35D1 | solute carrier family 35 member D1 [Source:HGNC Symbol;Acc:HGNC:20800] |
| ENSG00000117215 | HGLibA_36887 | 95 | -0.275385914 | PLA2G2D | phospholipase A2 group IID [Source:HGNC Symbol;Acc:HGNC:9033] |
| ENSG00000118939 | HGLibA_52805 | 95 | -0.275385914 | UCHL3 | ubiquitin C-terminal hydrolase L3 [Source:HGNC Symbol;Acc:HGNC:12515] |
| ENSG00000119684 | HGLibA_29385 | 95 | -0.275385914 | MLH3 | mutL homolog 3 [Source:HGNC Symbol;Acc:HGNC:7128] |
| ENSG00000119812 | HGLibA_16810 | 95 | -0.275385914 | FAM98A | family with sequence similarity 98 member A [Source:HGNC Symbol;Acc:HGNC:24520] |
| ENSG00000120738 | HGLibA_14649 | 95 | -0.275385914 | EGR1 | early growth response 1 [Source:HGNC Symbol;Acc:HGNC:3238] |
| ENSG00000121691 | HGLibA_07351 | 95 | -0.275385914 | CAT | catalase [Source:HGNC Symbol;Acc:HGNC:1516] |
| ENSG00000121769 | HGLibA_15915 | 95 | -0.275385914 | FABP3 | fatty acid binding protein 3 [Source:HGNC Symbol;Acc:HGNC:3557] |
| ENSG00000121853 | HGLibA_19052 | 95 | -0.275385914 | GHSR | growth hormone secretagogue receptor [Source:HGNC Symbol;Acc:HGNC:4267] |
| ENSG00000122705 | HGLibA_10143 | 95 | -0.275385914 | CLTA | clathrin light chain A [Source:HGNC Symbol;Acc:HGNC:2090] |
| ENSG00000123570 | HGLibA_39994 | 95 | -0.275385914 | RAB9B | member RAS oncogene family [Source:HGNC Symbol;Acc:HGNC:14090]" |
| ENSG00000123685 | HGLibA_04178 | 95 | -0.275385914 | BATF3 | basic leucine zipper ATF-like transcription factor 3 [Source:HGNC Symbol;Acc:HGNC:28915] |
| ENSG00000125823 | HGLibA_11496 | 95 | -0.275385914 | CSTL1 | cystatin like 1 [Source:HGNC Symbol;Acc:HGNC:15958] |
| ENSG00000126767 | HGLibA_14911 | 95 | -0.275385914 | ELK1 | ETS transcription factor [Source:HGNC Symbol;Acc:HGNC:3321]" |
| ENSG00000128298 | HGLibA_04127 | 95 | -0.275385914 | BAIAP2L2 | BAI1 associated protein 2 like 2 [Source:HGNC Symbol;Acc:HGNC:26203] |
| ENSG00000128645 | HGLibA_21982 | 95 | -0.275385914 | HOXD1 | homeobox D1 [Source:HGNC Symbol;Acc:HGNC:5132] |
| ENSG00000130958 | HGLibA_44980 | 95 | -0.275385914 | SLC35D2 | solute carrier family 35 member D2 [Source:HGNC Symbol;Acc:HGNC:20799] |
| ENSG00000132825 | HGLibA_38108 | 95 | -0.275385914 | PPP1R3D | protein phosphatase 1 regulatory subunit 3D [Source:HGNC Symbol;Acc:HGNC:9294] |
| ENSG00000133116 | HGLibA_25020 | 95 | -0.275385914 | KL | klotho [Source:HGNC Symbol;Acc:HGNC:6344] |
| ENSG00000133317 | HGLibA_26348 | 95 | -0.275385914 | LGALS12 | galectin 12 [Source:HGNC Symbol;Acc:HGNC:15788] |
| ENSG00000134717 | HGLibA_04937 | 95 | -0.275385914 | BTF3L4 | basic transcription factor 3 like 4 [Source:HGNC Symbol;Acc:HGNC:30547] |
| ENSG00000135318 | HGLibA_32830 | 95 | -0.275385914 | NT5E | 5'-nucleotidase ecto [Source:HGNC Symbol;Acc:HGNC:8021] |
| ENSG00000135469 | HGLibA_10743 | 95 | -0.275385914 | COQ10A | coenzyme Q10A [Source:HGNC Symbol;Acc:HGNC:26515] |
| ENSG00000136908 | HGLibA_13859 | 95 | -0.275385914 | DPM2 | regulatory [Source:HGNC Symbol;Acc:HGNC:3006]" |
| ENSG00000137674 | HGLibA_29510 | 95 | -0.275385914 | MMP20 | matrix metallopeptidase 20 [Source:HGNC Symbol;Acc:HGNC:7167] |
| ENSG00000138119 | HGLibA_30884 | 95 | -0.275385914 | MYOF | myoferlin [Source:HGNC Symbol;Acc:HGNC:3656] |
| ENSG00000138413 | HGLibA_22538 | 95 | -0.275385914 | IDH1 | cytosolic [Source:HGNC Symbol;Acc:HGNC:5382]" |
| ENSG00000138688 | HGLibA_24694 | 95 | -0.275385914 | KIAA1109 | KIAA1109 [Source:HGNC Symbol;Acc:HGNC:26953] |
| ENSG00000139131 | HGLibA_54636 | 95 | -0.275385914 | YARS2 | tyrosyl-tRNA synthetase 2 [Source:HGNC Symbol;Acc:HGNC:24249] |
| ENSG00000139155 | HGLibA_45458 | 95 | -0.275385914 | SLCO1C1 | solute carrier organic anion transporter family member 1C1 [Source:HGNC Symbol;Acc:HGNC:13819] |
| ENSG00000139173 | HGLibA_49889 | 95 | -0.275385914 | TMEM117 | transmembrane protein 117 [Source:HGNC Symbol;Acc:HGNC:25308] |
| ENSG00000140265 | HGLibA_57012 | 95 | -0.275385914 | ZSCAN29 | zinc finger and SCAN domain containing 29 [Source:HGNC Symbol;Acc:HGNC:26673] |
| ENSG00000140682 | HGLibA_49210 | 95 | -0.275385914 | TGFB1I1 | transforming growth factor beta 1 induced transcript 1 [Source:HGNC Symbol;Acc:HGNC:11767] |
| ENSG00000143862 | HGLibA_02996 | 95 | -0.275385914 | ARL8A | ADP ribosylation factor like GTPase 8A [Source:HGNC Symbol;Acc:HGNC:25192] |
| ENSG00000146938 | HGLibA_32076 | 95 | -0.275385914 | NLGN4X | neuroligin 4 X-linked [Source:HGNC Symbol;Acc:HGNC:14287] |
| ENSG00000148053 | HGLibA_32878 | 95 | -0.275385914 | NTRK2 | neurotrophic receptor tyrosine kinase 2 [Source:HGNC Symbol;Acc:HGNC:8032] |
| ENSG00000148090 | HGLibA_03864 | 95 | -0.275385914 | AUH | AU RNA binding methylglutaconyl-CoA hydratase [Source:HGNC Symbol;Acc:HGNC:890] |
| ENSG00000149582 | HGLibA_50315 | 95 | -0.275385914 | TMEM25 | transmembrane protein 25 [Source:HGNC Symbol;Acc:HGNC:25890] |
| ENSG00000149582 | HGLibA_50314 | 95 | -0.275385914 | TMEM25 | transmembrane protein 25 [Source:HGNC Symbol;Acc:HGNC:25890] |
| ENSG00000150593 | HGLibA_35734 | 95 | -0.275385914 | PDCD4 | programmed cell death 4 [Source:HGNC Symbol;Acc:HGNC:8763] |
| ENSG00000151012 | HGLibA_45351 | 95 | -0.275385914 | SLC7A11 | solute carrier family 7 member 11 [Source:HGNC Symbol;Acc:HGNC:11059] |
| ENSG00000151023 | HGLibA_15137 | 95 | -0.275385914 | ENKUR | TRPC channel interacting protein [Source:HGNC Symbol;Acc:HGNC:28388]" |
| ENSG00000151490 | HGLibA_39525 | 95 | -0.275385914 | PTPRO | receptor type O [Source:HGNC Symbol;Acc:HGNC:9678]" |
| ENSG00000152467 | HGLibA_56982 | 95 | -0.275385914 | ZSCAN1 | zinc finger and SCAN domain containing 1 [Source:HGNC Symbol;Acc:HGNC:23712] |
| ENSG00000153002 | HGLibA_10904 | 95 | -0.275385914 | CPB1 | carboxypeptidase B1 [Source:HGNC Symbol;Acc:HGNC:2299] |
| ENSG00000154319 | HGLibA_16240 | 95 | -0.275385914 | FAM167A | family with sequence similarity 167 member A [Source:HGNC Symbol;Acc:HGNC:15549] |
| ENSG00000154438 | HGLibA_03397 | 95 | -0.275385914 | ASZ1 | SAM and basic leucine zipper domain containing 1 [Source:HGNC Symbol;Acc:HGNC:1350]" |
| ENSG00000156170 | HGLibA_31497 | 95 | -0.275385914 | NDUFAF6 | NADH:ubiquinone oxidoreductase complex assembly factor 6 [Source:HGNC Symbol;Acc:HGNC:28625] |
| ENSG00000157570 | HGLibA_51915 | 95 | -0.275385914 | TSPAN18 | tetraspanin 18 [Source:HGNC Symbol;Acc:HGNC:20660] |
| ENSG00000160216 | HGLibA_01222 | 95 | -0.275385914 | AGPAT3 | 1-acylglycerol-3-phosphate O-acyltransferase 3 [Source:HGNC Symbol;Acc:HGNC:326] |
| ENSG00000162426 | HGLibA_45173 | 95 | -0.275385914 | SLC45A1 | solute carrier family 45 member 1 [Source:HGNC Symbol;Acc:HGNC:17939] |
| ENSG00000163626 | HGLibA_10822 | 95 | -0.275385914 | COX18 | cytochrome c oxidase assembly factor COX18 [Source:HGNC Symbol;Acc:HGNC:26801] |
| ENSG00000164182 | HGLibA_31486 | 95 | -0.275385914 | NDUFAF2 | NADH:ubiquinone oxidoreductase complex assembly factor 2 [Source:HGNC Symbol;Acc:HGNC:28086] |
| ENSG00000164404 | HGLibA_18878 | 95 | -0.275385914 | GDF9 | growth differentiation factor 9 [Source:HGNC Symbol;Acc:HGNC:4224] |
| ENSG00000165775 | HGLibA_18168 | 95 | -0.275385914 | FUNDC2 | FUN14 domain containing 2 [Source:HGNC Symbol;Acc:HGNC:24925] |
| ENSG00000166224 | HGLibA_43846 | 95 | -0.275385914 | SGPL1 | sphingosine-1-phosphate lyase 1 [Source:HGNC Symbol;Acc:HGNC:10817] |
| ENSG00000167548 | HGLibA_25330 | 95 | -0.275385914 | KMT2D | lysine methyltransferase 2D [Source:HGNC Symbol;Acc:HGNC:7133] |
| ENSG00000168275 | HGLibA_10454 | 95 | -0.275385914 | COA6 | cytochrome c oxidase assembly factor 6 [Source:HGNC Symbol;Acc:HGNC:18025] |
| ENSG00000170627 | HGLibA_20629 | 95 | -0.275385914 | GTSF1 | gametocyte specific factor 1 [Source:HGNC Symbol;Acc:HGNC:26565] |
| ENSG00000171053 | HGLibA_35271 | 95 | -0.275385914 | PATE1 | prostate and testis expressed 1 [Source:HGNC Symbol;Acc:HGNC:24664] |
| ENSG00000171067 | HGLibA_05124 | 95 | -0.275385914 | C11orf24 | chromosome 11 open reading frame 24 [Source:HGNC Symbol;Acc:HGNC:1174] |
| ENSG00000171729 | HGLibA_50428 | 95 | -0.275385914 | TMEM51 | transmembrane protein 51 [Source:HGNC Symbol;Acc:HGNC:25488] |
| ENSG00000171773 | HGLibA_33143 | 95 | -0.275385914 | NXNL1 | nucleoredoxin like 1 [Source:HGNC Symbol;Acc:HGNC:25179] |
| ENSG00000171815 | HGLibA_35469 | 95 | -0.275385914 | PCDHB1 | protocadherin beta 1 [Source:HGNC Symbol;Acc:HGNC:8680] |
| ENSG00000171873 | HGLibA_01044 | 95 | -0.275385914 | ADRA1D | adrenoceptor alpha 1D [Source:HGNC Symbol;Acc:HGNC:280] |
| ENSG00000172724 | HGLibA_07984 | 95 | -0.275385914 | CCL19 | C-C motif chemokine ligand 19 [Source:HGNC Symbol;Acc:HGNC:10617] |
| ENSG00000172817 | HGLibA_12215 | 95 | -0.275385914 | CYP7B1 | cytochrome P450 family 7 subfamily B member 1 [Source:HGNC Symbol;Acc:HGNC:2652] |
| ENSG00000172955 | HGLibA_00961 | 95 | -0.275385914 | ADH6 | alcohol dehydrogenase 6 (class V) [Source:HGNC Symbol;Acc:HGNC:255] |
| ENSG00000172985 | HGLibA_43976 | 95 | -0.275385914 | SH3RF3 | SH3 domain containing ring finger 3 [Source:HGNC Symbol;Acc:HGNC:24699] |
| ENSG00000174992 | HGLibA_55298 | 95 | -0.275385914 | ZG16 | zymogen granule protein 16 [Source:HGNC Symbol;Acc:HGNC:30961] |
| ENSG00000176454 | HGLibA_27081 | 95 | -0.275385914 | LPCAT4 | lysophosphatidylcholine acyltransferase 4 [Source:HGNC Symbol;Acc:HGNC:30059] |
| ENSG00000176531 | HGLibA_36432 | 95 | -0.275385914 | PHLDB3 | pleckstrin homology like domain family B member 3 [Source:HGNC Symbol;Acc:HGNC:30499] |
| ENSG00000176746 | HGLibA_27956 | 95 | -0.275385914 | MAGEB6 | MAGE family member B6 [Source:HGNC Symbol;Acc:HGNC:23796] |
| ENSG00000177666 | HGLibA_37407 | 95 | -0.275385914 | PNPLA2 | patatin like phospholipase domain containing 2 [Source:HGNC Symbol;Acc:HGNC:30802] |
| ENSG00000177692 | HGLibA_13613 | 95 | -0.275385914 | DNAJC28 | DnaJ heat shock protein family (Hsp40) member C28 [Source:HGNC Symbol;Acc:HGNC:1297] |
| ENSG00000178966 | HGLibA_41374 | 95 | -0.275385914 | RMI1 | RecQ mediated genome instability 1 [Source:HGNC Symbol;Acc:HGNC:25764] |
| ENSG00000180113 | HGLibA_48912 | 95 | -0.275385914 | TDRD6 | tudor domain containing 6 [Source:HGNC Symbol;Acc:HGNC:21339] |
| ENSG00000180190 | HGLibA_48923 | 95 | -0.275385914 | TDRP | testis development related protein [Source:HGNC Symbol;Acc:HGNC:26951] |
| ENSG00000180198 | HGLibA_40682 | 95 | -0.275385914 | RCC1 | regulator of chromosome condensation 1 [Source:HGNC Symbol;Acc:HGNC:1913] |
| ENSG00000180549 | HGLibA_18203 | 95 | -0.275385914 | FUT7 | fucosyltransferase 7 [Source:HGNC Symbol;Acc:HGNC:4018] |
| ENSG00000181778 | HGLibA_50321 | 95 | -0.275385914 | TMEM252 | transmembrane protein 252 [Source:HGNC Symbol;Acc:HGNC:28537] |
| ENSG00000182415 | HGLibA_08910 | 95 | -0.275385914 | CDY2A | chromodomain Y-linked 2A [Source:HGNC Symbol;Acc:HGNC:1810] |
| ENSG00000183145 | HGLibA_41335 | 95 | -0.275385914 | RIPPLY3 | ripply transcriptional repressor 3 [Source:HGNC Symbol;Acc:HGNC:3047] |
| ENSG00000183310 | HGLibA_33879 | 95 | -0.275385914 | OR2T34 | olfactory receptor family 2 subfamily T member 34 [Source:HGNC Symbol;Acc:HGNC:31256] |
| ENSG00000183542 | HGLibA_25295 | 95 | -0.275385914 | KLRC4 | killer cell lectin like receptor C4 [Source:HGNC Symbol;Acc:HGNC:6377] |
| ENSG00000184012 | HGLibA_50637 | 95 | -0.275385914 | TMPRSS2 | transmembrane serine protease 2 [Source:HGNC Symbol;Acc:HGNC:11876] |
| ENSG00000185507 | HGLibA_23597 | 95 | -0.275385914 | IRF7 | interferon regulatory factor 7 [Source:HGNC Symbol;Acc:HGNC:6122] |
| ENSG00000186432 | HGLibA_25363 | 95 | -0.275385914 | KPNA4 | karyopherin subunit alpha 4 [Source:HGNC Symbol;Acc:HGNC:6397] |
| ENSG00000186439 | HGLibA_51348 | 95 | -0.275385914 | TRDN | triadin [Source:HGNC Symbol;Acc:HGNC:12261] |
| ENSG00000186452 | HGLibA_50627 | 95 | -0.275385914 | TMPRSS12 | transmembrane serine protease 12 [Source:HGNC Symbol;Acc:HGNC:28779] |
| ENSG00000186871 | HGLibA_15450 | 95 | -0.275385914 | ERCC6L | spindle assembly checkpoint helicase [Source:HGNC Symbol;Acc:HGNC:20794]" |
| ENSG00000187080 | HGLibA_33754 | 95 | -0.275385914 | OR2AK2 | olfactory receptor family 2 subfamily AK member 2 [Source:HGNC Symbol;Acc:HGNC:19569] |
| ENSG00000187601 | HGLibA_27988 | 95 | -0.275385914 | MAGEH1 | MAGE family member H1 [Source:HGNC Symbol;Acc:HGNC:24092] |
| ENSG00000188032 | HGLibA_05697 | 95 | -0.275385914 | C19orf67 | chromosome 19 open reading frame 67 [Source:HGNC Symbol;Acc:HGNC:34354] |
| ENSG00000188243 | HGLibA_10671 | 95 | -0.275385914 | COMMD6 | COMM domain containing 6 [Source:HGNC Symbol;Acc:HGNC:24015] |
| ENSG00000188580 | HGLibA_31991 | 95 | -0.275385914 | NKAIN2 | sodium/potassium transporting ATPase interacting 2 [Source:HGNC Symbol;Acc:HGNC:16443] |
| ENSG00000188582 | HGLibA_35175 | 95 | -0.275385914 | PAQR9 | progestin and adipoQ receptor family member 9 [Source:HGNC Symbol;Acc:HGNC:30131] |
| ENSG00000188938 | HGLibA_16058 | 95 | -0.275385914 | FAM120AOS | family with sequence similarity 120A opposite strand [Source:HGNC Symbol;Acc:HGNC:23389] |
| ENSG00000189233 | HGLibA_33006 | 95 | -0.275385914 | NUGGC | germinal center associated [Source:HGNC Symbol;Acc:HGNC:33550]" |
| ENSG00000189266 | HGLibA_37438 | 95 | -0.275385914 | PNRC2 | proline rich nuclear receptor coactivator 2 [Source:HGNC Symbol;Acc:HGNC:23158] |
| ENSG00000196511 | HGLibA_51138 | 95 | -0.275385914 | TPK1 | thiamin pyrophosphokinase 1 [Source:HGNC Symbol;Acc:HGNC:17358] |
| ENSG00000197050 | HGLibA_55957 | 95 | -0.275385914 | ZNF420 | zinc finger protein 420 [Source:HGNC Symbol;Acc:HGNC:20649] |
| ENSG00000197256 | HGLibA_24026 | 95 | -0.275385914 | KANK2 | KN motif and ankyrin repeat domains 2 [Source:HGNC Symbol;Acc:HGNC:29300] |
| ENSG00000197405 | HGLibA_06353 | 95 | -0.275385914 | C5AR1 | complement C5a receptor 1 [Source:HGNC Symbol;Acc:HGNC:1338] |
| ENSG00000197451 | HGLibA_21804 | 95 | -0.275385914 | HNRNPAB | heterogeneous nuclear ribonucleoprotein A/B [Source:HGNC Symbol;Acc:HGNC:5034] |
| ENSG00000197580 | HGLibA_04358 | 95 | -0.275385914 | BCO2 | beta-carotene oxygenase 2 [Source:HGNC Symbol;Acc:HGNC:18503] |
| ENSG00000197608 | HGLibA_56855 | 95 | -0.275385914 | ZNF841 | zinc finger protein 841 [Source:HGNC Symbol;Acc:HGNC:27611] |
| ENSG00000197619 | HGLibA_56393 | 95 | -0.275385914 | ZNF615 | zinc finger protein 615 [Source:HGNC Symbol;Acc:HGNC:24740] |

| | | | | | |
|---|---|---|---|---|---|
| ENSG00000204516 | HGLibA_29220 | 95 | -0.275385914 | MICB | MHC class I polypeptide-related sequence B [Source:HGNC Symbol;Acc:HGNC:7091] |
| ENSG00000204694 | HGLibA_33563 | 95 | -0.275385914 | OR11A1 | olfactory receptor family 11 subfamily A member 1 [Source:HGNC Symbol;Acc:HGNC:8176] |
| ENSG00000205038 | HGLibA_36815 | 95 | -0.275385914 | PKHD1L1 | PKHD1 like 1 [Source:HGNC Symbol;Acc:HGNC:20313] |
| ENSG00000205060 | HGLibA_44969 | 95 | -0.275385914 | SLC35B4 | solute carrier family 35 member B4 [Source:HGNC Symbol;Acc:HGNC:20584] |
| ENSG00000205810 | HGLibA_25291 | 95 | -0.275385914 | KLRC3 | killer cell lectin like receptor C3 [Source:HGNC Symbol;Acc:HGNC:6376] |
| ENSG00000214842 | HGLibA_40085 | 95 | -0.275385914 | RAD51AP2 | RAD51 associated protein 2 [Source:HGNC Symbol;Acc:HGNC:34417] |
| ENSG00000215356 | HGLibA_56590 | 95 | -0.275385914 | ZNF705B | zinc finger protein 705B [Source:HGNC Symbol;Acc:HGNC:32284] |
| ENSG00000215784 | HGLibA_16714 | 95 | -0.275385914 | FAM72D | family with sequence similarity 72 member D [Source:HGNC Symbol;Acc:HGNC:33593] |
| ENSG00000221829 | HGLibA_16851 | 95 | -0.275385914 | FANCG | FA complementation group G [Source:HGNC Symbol;Acc:HGNC:3588] |
| ENSG00000221838 | HGLibA_02347 | 95 | -0.275385914 | AP4M1 | adaptor related protein complex 4 subunit mu 1 [Source:HGNC Symbol;Acc:HGNC:574] |
| ENSG00000225781 | HGLibA_34449 | 95 | -0.275385914 | OR6V1 | olfactory receptor family 6 subfamily V member 1 [Source:HGNC Symbol;Acc:HGNC:15090] |
| ENSG00000232995 | HGLibA_41097 | 95 | -0.275385914 | RGS5 | regulator of G protein signaling 5 [Source:NCBI gene;Acc:8490] |
| ENSG00000237136 | HGLibA_06343 | 95 | -0.275385914 | C4orf51 | chromosome 4 open reading frame 51 [Source:HGNC Symbol;Acc:HGNC:37264] |
| ENSG00000239474 | HGLibA_25210 | 95 | -0.275385914 | KLHL41 | kelch like family member 41 [Source:HGNC Symbol;Acc:HGNC:16905] |
| ENSG00000242110 | HGLibA_01745 | 95 | -0.275385914 | AMACR | alpha-methylacyl-CoA racemase [Source:HGNC Symbol;Acc:HGNC:451] |
| ENSG00000243364 | HGLibA_14591 | 95 | -0.275385914 | EFNA4 | ephrin A4 [Source:HGNC Symbol;Acc:HGNC:3224] |
| ENSG00000244646 | HGLibA_54543 | 95 | -0.275385914 | XKRY2 | Y-linked 2 [Source:HGNC Symbol;Acc:HGNC:23922]" |
| ENSG00000248672 | HGLibA_27683 | 95 | -0.275385914 | LY75-CD302 | LY75-CD302 readthrough [Source:HGNC Symbol;Acc:HGNC:38828] |
| ENSG00000248905 | HGLibA_17687 | 95 | -0.275385914 | FMN1 | formin 1 [Source:HGNC Symbol;Acc:HGNC:3768] |
| ENSG00000250510 | HGLibA_19960 | 95 | -0.275385914 | GPR162 | G protein-coupled receptor 162 [Source:HGNC Symbol;Acc:HGNC:16693] |
| ENSG00000253953 | HGLibA_35558 | 95 | -0.275385914 | PCDHGB4 | 4 [Source:HGNC Symbol;Acc:HGNC:8711]" |
| ENSG00000253953 | HGLibA_35560 | 95 | -0.275385914 | PCDHGB4 | 4 [Source:HGNC Symbol;Acc:HGNC:8711]" |
| ENSG00000254726 | HGLibA_29027 | 95 | -0.275385914 | MEX3A | mex-3 RNA binding family member A [Source:HGNC Symbol;Acc:HGNC:33482] |
| ENSG00000257923 | HGLibA_11802 | 95 | -0.275385914 | CUX1 | cut like homeobox 1 [Source:HGNC Symbol;Acc:HGNC:2557] |
| ENSG00000266094 | HGLibA_40387 | 95 | -0.275385914 | RASSF5 | Ras association domain family member 5 [Source:HGNC Symbol;Acc:HGNC:17609] |
| ENSG00000267508 | HGLibA_55747 | 95 | -0.275385914 | ZNF285 | zinc finger protein 285 [Source:HGNC Symbol;Acc:HGNC:13079] |
| ENSG00000269058 | HGLibA_07049 | 95 | -0.275385914 | CALR3 | calreticulin 3 [Source:HGNC Symbol;Acc:HGNC:20407] |
| ENSG00000272920 | HGLibA_57409 | 95 | -0.275385914 | hsa-mir-1253 | hsa-mir-1253 [Source:miRBase;Acc:MI0006387] |
| GUCY1B3 | HGLibA_20659 | 95 | -0.275385914 | GUCY1B3 | - |
| H2AFB2 | HGLibA_20748 | 95 | -0.275385914 | H2AFB2 | - |
| HRSP12 | HGLibA_22112 | 95 | -0.275385914 | HRSP12 | - |
| hsa-let-7e | HGLibA_57121 | 95 | -0.275385914 | hsa-let-7e | - |
| hsa-mir-105-2 | HGLibA_57181 | 95 | -0.275385914 | hsa-mir-105-2 | - |
| hsa-mir-1264 | HGLibA_57480 | 95 | -0.275385914 | hsa-mir-1264 | - |
| hsa-mir-1276 | HGLibA_57559 | 95 | -0.275385914 | hsa-mir-1276 | - |
| hsa-mir-1302-4 | HGLibA_57702 | 95 | -0.275385914 | hsa-mir-1302-4 | - |
| hsa-mir-150 | HGLibA_57891 | 95 | -0.275385914 | hsa-mir-150 | - |
| hsa-mir-205 | HGLibA_58179 | 95 | -0.275385914 | hsa-mir-205 | - |
| hsa-mir-30a | HGLibA_58472 | 95 | -0.275385914 | hsa-mir-30a | - |
| hsa-mir-3186 | HGLibA_58842 | 95 | -0.275385914 | hsa-mir-3186 | - |
| hsa-mir-33a | HGLibA_59013 | 95 | -0.275385914 | hsa-mir-33a | - |
| hsa-mir-3591 | HGLibA_59051 | 95 | -0.275385914 | hsa-mir-3591 | - |
| hsa-mir-3653 | HGLibA_59156 | 95 | -0.275385914 | hsa-mir-3653 | - |
| hsa-mir-4476 | HGLibA_60315 | 95 | -0.275385914 | hsa-mir-4476 | - |
| hsa-mir-4794 | HGLibA_61281 | 95 | -0.275385914 | hsa-mir-4794 | - |
| hsa-mir-489 | HGLibA_61358 | 95 | -0.275385914 | hsa-mir-489 | - |
| hsa-mir-548f-4 | HGLibA_61985 | 95 | -0.275385914 | hsa-mir-548f-4 | - |
| hsa-mir-5739 | HGLibA_62387 | 95 | -0.275385914 | hsa-mir-5739 | - |
| hsa-mir-624 | HGLibA_62736 | 95 | -0.275385914 | hsa-mir-624 | - |
| hsa-mir-631 | HGLibA_62762 | 95 | -0.275385914 | hsa-mir-631 | - |
| hsa-mir-6750 | HGLibA_63157 | 95 | -0.275385914 | hsa-mir-6750 | - |
| hsa-mir-6774 | HGLibA_63270 | 95 | -0.275385914 | hsa-mir-6774 | - |
| hsa-mir-6796 | HGLibA_63361 | 95 | -0.275385914 | hsa-mir-6796 | - |
| hsa-mir-8077 | HGLibA_64151 | 95 | -0.275385914 | hsa-mir-8077 | - |
| KIAA1324L | HGLibA_24739 | 95 | -0.275385914 | KIAA1324L | - |
| MRVI1 | HGLibA_30123 | 95 | -0.275385914 | MRVI1 | - |
| MTERF | HGLibA_30337 | 95 | -0.275385914 | MTERF | - |
| PVRL3 | HGLibA_39629 | 95 | -0.275385914 | PVRL3 | - |
| TMEM257 | HGLibA_50339 | 95 | -0.275385914 | TMEM257 | - |
| VAMP7_X | HGLibA_53504 | 95 | -0.275385914 | VAMP7_X | - |
| C2orf40 | HGLibA_06121 | 94 | -0.284431257 | C2orf40 | - |
| C2orf53 | HGLibA_06145 | 94 | -0.284431257 | C2orf53 | - |
| DPCR1 | HGLibA_13814 | 94 | -0.284431257 | DPCR1 | - |
| ENSG00000003393 | HGLibA_01719 | 94 | -0.284431257 | ALS2 | alsin Rho guanine nucleotide exchange factor [Source:HGNC Symbol;Acc:HGNC:443]" |
| ENSG00000005448 | HGLibA_54123 | 94 | -0.284431257 | WDR54 | WD repeat domain 54 [Source:HGNC Symbol;Acc:HGNC:25770] |
| ENSG00000011422 | HGLibA_36967 | 94 | -0.284431257 | PLAUR | urokinase receptor [Source:HGNC Symbol;Acc:HGNC:9053]" |
| ENSG00000020129 | HGLibA_31291 | 94 | -0.284431257 | NCDN | neurochondrin [Source:HGNC Symbol;Acc:HGNC:17597] |
| ENSG00000039987 | HGLibA_04423 | 94 | -0.284431257 | BEST2 | bestrophin 2 [Source:HGNC Symbol;Acc:HGNC:17107] |
| ENSG00000060069 | HGLibA_11604 | 94 | -0.284431257 | CTDP1 | CTD phosphatase subunit 1 [Source:HGNC Symbol;Acc:HGNC:2498] |
| ENSG00000060656 | HGLibA_39542 | 94 | -0.284431257 | PTPRU | receptor type U [Source:HGNC Symbol;Acc:HGNC:9683]" |
| ENSG00000063015 | HGLibA_43678 | 94 | -0.284431257 | SEZ6 | seizure related 6 homolog [Source:HGNC Symbol;Acc:HGNC:15955] |
| ENSG00000065054 | HGLibA_45411 | 94 | -0.284431257 | SLC9A3R2 | SLC9A3 regulator 2 [Source:HGNC Symbol;Acc:HGNC:11076] |
| ENSG00000065526 | HGLibA_46622 | 94 | -0.284431257 | SPEN | spen family transcriptional repressor [Source:HGNC Symbol;Acc:HGNC:17575] |
| ENSG00000068912 | HGLibA_15491 | 94 | -0.284431257 | ERLEC1 | endoplasmic reticulum lectin 1 [Source:HGNC Symbol;Acc:HGNC:25222] |
| ENSG00000072803 | HGLibA_17137 | 94 | -0.284431257 | FBXW11 | F-box and WD repeat domain containing 11 [Source:HGNC Symbol;Acc:HGNC:13607] |
| ENSG00000076053 | HGLibA_40580 | 94 | -0.284431257 | RBM7 | RNA binding motif protein 7 [Source:HGNC Symbol;Acc:HGNC:9904] |
| ENSG00000081237 | HGLibA_39490 | 94 | -0.284431257 | PTPRC | receptor type C [Source:HGNC Symbol;Acc:HGNC:9666]" |
| ENSG00000085377 | HGLibA_38438 | 94 | -0.284431257 | PREP | prolyl endopeptidase [Source:HGNC Symbol;Acc:HGNC:9358] |
| ENSG00000088756 | HGLibA_02748 | 94 | -0.284431257 | ARHGAP28 | Rho GTPase activating protein 28 [Source:HGNC Symbol;Acc:HGNC:25509] |
| ENSG00000089094 | HGLibA_24496 | 94 | -0.284431257 | KDM2B | lysine demethylase 2B [Source:HGNC Symbol;Acc:HGNC:13610] |
| ENSG00000092036 | HGLibA_20883 | 94 | -0.284431257 | HAUS4 | HAUS augmin like complex subunit 4 [Source:HGNC Symbol;Acc:HGNC:20163] |
| ENSG00000095110 | HGLibA_33150 | 94 | -0.284431257 | NXPE1 | neurexophilin and PC-esterase domain family member 1 [Source:HGNC Symbol;Acc:HGNC:28527] |
| ENSG00000099139 | HGLibA_35679 | 94 | -0.284431257 | PCSK5 | proprotein convertase subtilisin/kexin type 5 [Source:HGNC Symbol;Acc:HGNC:8747] |
| ENSG00000100121 | HGLibA_19024 | 94 | -0.284431257 | GGTLC2 | gamma-glutamyltransferase light chain 2 [Source:HGNC Symbol;Acc:HGNC:18596] |
| ENSG00000100376 | HGLibA_16048 | 94 | -0.284431257 | FAM118A | family with sequence similarity 118 member A [Source:HGNC Symbol;Acc:HGNC:1313] |
| ENSG00000101935 | HGLibA_01802 | 94 | -0.284431257 | AMMECR1 | midface hypoplasia and elliptocytosis chromosomal region gene 1 [Source:HGNC Symbol;Acc:HGNC:467]" |
| ENSG00000102978 | HGLibA_37571 | 94 | -0.284431257 | POLR2C | RNA polymerase II subunit C [Source:HGNC Symbol;Acc:HGNC:9189] |
| ENSG00000104290 | HGLibA_18268 | 94 | -0.284431257 | FZD3 | frizzled class receptor 3 [Source:HGNC Symbol;Acc:HGNC:4041] |
| ENSG00000104881 | HGLibA_38031 | 94 | -0.284431257 | PPP1R13L | protein phosphatase 1 regulatory subunit 13 like [Source:HGNC Symbol;Acc:HGNC:18838] |
| ENSG00000104946 | HGLibA_48489 | 94 | -0.284431257 | TBC1D17 | TBC1 domain family member 17 [Source:HGNC Symbol;Acc:HGNC:25699] |
| ENSG00000104974 | HGLibA_26490 | 94 | -0.284431257 | LILRA1 | leukocyte immunoglobulin like receptor A1 [Source:HGNC Symbol;Acc:HGNC:6602] |
| ENSG00000105643 | HGLibA_03116 | 94 | -0.284431257 | ARRDC2 | arrestin domain containing 2 [Source:HGNC Symbol;Acc:HGNC:25225] |
| ENSG00000106333 | HGLibA_35653 | 94 | -0.284431257 | PCOLCE | procollagen C-endopeptidase enhancer [Source:HGNC Symbol;Acc:HGNC:8738] |
| ENSG00000109424 | HGLibA_52830 | 94 | -0.284431257 | UCP1 | uncoupling protein 1 [Source:HGNC Symbol;Acc:HGNC:12517] |
| ENSG00000109606 | HGLibA_13166 | 94 | -0.284431257 | DHX15 | DEAH-box helicase 15 [Source:HGNC Symbol;Acc:HGNC:2738] |
| ENSG00000110931 | HGLibA_07091 | 94 | -0.284431257 | CAMKK2 | calcium/calmodulin dependent protein kinase kinase 2 [Source:HGNC Symbol;Acc:HGNC:1470] |
| ENSG00000111245 | HGLibA_30758 | 94 | -0.284431257 | MYL2 | myosin light chain 2 [Source:HGNC Symbol;Acc:HGNC:7583] |
| ENSG00000111729 | HGLibA_09980 | 94 | -0.284431257 | CLEC4A | C-type lectin domain family 4 member A [Source:HGNC Symbol;Acc:HGNC:13257] |
| ENSG00000113812 | HGLibA_00673 | 94 | -0.284431257 | ACTR8 | ARP8 actin related protein 8 homolog [Source:HGNC Symbol;Acc:HGNC:14672] |
| ENSG00000114126 | HGLibA_49155 | 94 | -0.284431257 | TFDP2 | transcription factor Dp-2 [Source:HGNC Symbol;Acc:HGNC:11751] |
| ENSG00000115993 | HGLibA_51280 | 94 | -0.284431257 | TRAK2 | trafficking kinesin protein 2 [Source:HGNC Symbol;Acc:HGNC:13206] |
| ENSG00000116017 | HGLibA_02890 | 94 | -0.284431257 | ARID3A | AT-rich interaction domain 3A [Source:HGNC Symbol;Acc:HGNC:3031] |
| ENSG00000116260 | HGLibA_39755 | 94 | -0.284431257 | QSOX1 | quiescin sulfhydryl oxidase 1 [Source:HGNC Symbol;Acc:HGNC:9756] |
| ENSG00000118514 | HGLibA_01585 | 94 | -0.284431257 | ALDH8A1 | aldehyde dehydrogenase 8 family member A1 [Source:HGNC Symbol;Acc:HGNC:15471] |
| ENSG00000119723 | HGLibA_10760 | 94 | -0.284431257 | COQ6 | monooxygenase [Source:HGNC Symbol;Acc:HGNC:20233]" |
| ENSG00000120063 | HGLibA_19441 | 94 | -0.284431257 | GNA13 | G protein subunit alpha 13 [Source:HGNC Symbol;Acc:HGNC:4381] |
| ENSG00000120215 | HGLibA_29364 | 94 | -0.284431257 | MLANA | melan-A [Source:HGNC Symbol;Acc:HGNC:7124] |
| ENSG00000121988 | HGLibA_56976 | 94 | -0.284431257 | ZRANB3 | zinc finger RANBP2-type containing 3 [Source:HGNC Symbol;Acc:HGNC:25249] |
| ENSG00000122547 | HGLibA_14517 | 94 | -0.284431257 | EEPD1 | endonuclease/exonuclease/phosphatase family domain containing 1 [Source:HGNC Symbol;Acc:HGNC:22223] |
| ENSG00000123411 | HGLibA_22938 | 94 | -0.284431257 | IKZF4 | IKAROS family zinc finger 4 [Source:HGNC Symbol;Acc:HGNC:13179] |
| ENSG00000123612 | HGLibA_00693 | 94 | -0.284431257 | ACVR1C | activin A receptor type 1C [Source:HGNC Symbol;Acc:HGNC:18123] |
| ENSG00000124772 | HGLibA_10964 | 94 | -0.284431257 | CPNE5 | copine 5 [Source:HGNC Symbol;Acc:HGNC:2318] |
| ENSG00000124788 | HGLibA_03828 | 94 | -0.284431257 | ATXN1 | ataxin 1 [Source:HGNC Symbol;Acc:HGNC:10548] |
| ENSG00000125815 | HGLibA_11467 | 94 | -0.284431257 | CST8 | cystatin 8 [Source:HGNC Symbol;Acc:HGNC:2480] |
| ENSG00000127720 | HGLibA_28997 | 94 | -0.284431257 | METTL25 | methyltransferase like 25 [Source:HGNC Symbol;Acc:HGNC:26228] |
| ENSG00000128709 | HGLibA_22008 | 94 | -0.284431257 | HOXD9 | homeobox D9 [Source:HGNC Symbol;Acc:HGNC:5140] |
| ENSG00000128805 | HGLibA_02729 | 94 | -0.284431257 | ARHGAP22 | Rho GTPase activating protein 22 [Source:HGNC Symbol;Acc:HGNC:30320] |
| ENSG00000129460 | HGLibA_31865 | 94 | -0.284431257 | NGDN | neuroguidin [Source:HGNC Symbol;Acc:HGNC:20271] |
| ENSG00000131738 | HGLibA_25482 | 94 | -0.284431257 | KRT33B | keratin 33B [Source:HGNC Symbol;Acc:HGNC:6451] |
| ENSG00000131941 | HGLibA_41226 | 94 | -0.284431257 | RHPN2 | rhophilin Rho GTPase binding protein 2 [Source:HGNC Symbol;Acc:HGNC:19974] |
| ENSG00000132554 | HGLibA_41087 | 94 | -0.284431257 | RGS22 | regulator of G protein signaling 22 [Source:HGNC Symbol;Acc:HGNC:24499] |
| ENSG00000133110 | HGLibA_37711 | 94 | -0.284431257 | POSTN | periostin [Source:HGNC Symbol;Acc:HGNC:16953] |
| ENSG00000133597 | HGLibA_00892 | 94 | -0.284431257 | ADCK2 | aarF domain containing kinase 2 [Source:HGNC Symbol;Acc:HGNC:19039] |
| ENSG00000134755 | HGLibA_13991 | 94 | -0.284431257 | DSC2 | desmocollin 2 [Source:HGNC Symbol;Acc:HGNC:3036] |
| ENSG00000136240 | HGLibA_24482 | 94 | -0.284431257 | KDELR2 | KDEL endoplasmic reticulum protein retention receptor 2 [Source:HGNC Symbol;Acc:HGNC:6305] |
| ENSG00000136463 | HGLibA_48168 | 94 | -0.284431257 | TACO1 | translational activator of cytochrome c oxidase I [Source:HGNC Symbol;Acc:HGNC:24316] |
| ENSG00000137507 | HGLibA_27313 | 94 | -0.284431257 | LRRC32 | leucine rich repeat containing 32 [Source:HGNC Symbol;Acc:HGNC:4161] |
| ENSG00000138172 | HGLibA_07015 | 94 | -0.284431257 | CALHM2 | calcium homeostasis modulator family member 2 [Source:HGNC Symbol;Acc:HGNC:23493] |
| ENSG00000138382 | HGLibA_29011 | 94 | -0.284431257 | METTL5 | methyltransferase like 5 [Source:HGNC Symbol;Acc:HGNC:25006] |
| ENSG00000139613 | HGLibA_45626 | 94 | -0.284431257 | SMARCC2 | actin dependent regulator of chromatin subfamily c member 2 [Source:HGNC Symbol;Acc:HGNC:11105]" |
| ENSG00000140274 | HGLibA_14105 | 94 | -0.284431257 | DUOXA2 | dual oxidase maturation factor 2 [Source:HGNC Symbol;Acc:HGNC:32698] |
| ENSG00000140859 | HGLibA_24975 | 94 | -0.284431257 | KIFC3 | kinesin family member C3 [Source:HGNC Symbol;Acc:HGNC:6326] |
| ENSG00000140993 | HGLibA_49481 | 94 | -0.284431257 | TIGD7 | tigger transposable element derived 7 [Source:HGNC Symbol;Acc:HGNC:18331] |
| ENSG00000141522 | HGLibA_02796 | 94 | -0.284431257 | ARHGDIA | Rho GDP dissociation inhibitor alpha [Source:HGNC Symbol;Acc:HGNC:678] |
| ENSG00000142599 | HGLibA_40858 | 94 | -0.284431257 | RERE | arginine-glutamic acid dipeptide repeats [Source:HGNC Symbol;Acc:HGNC:9965] |

| | | | | | |
|---|---|---|---|---|---|
| ENSG00000143353 | HGLibA_27743 | 94 | -0.284431257 | LYPLAL1 | lysophospholipase like 1 [Source:HGNC Symbol;Acc:HGNC:20440] |
| ENSG00000143545 | HGLibA_39812 | 94 | -0.284431257 | RAB13 | member RAS oncogene family [Source:HGNC Symbol;Acc:HGNC:9762]" |
| ENSG00000143867 | HGLibA_34700 | 94 | -0.284431257 | OSR1 | odd-skipped related transciption factor 1 [Source:HGNC Symbol;Acc:HGNC:8111] |
| ENSG00000143878 | HGLibA_41168 | 94 | -0.284431257 | RHOB | ras homolog family member B [Source:HGNC Symbol;Acc:HGNC:668] |
| ENSG00000145107 | HGLibA_49697 | 94 | -0.284431257 | TM4SF19 | transmembrane 4 L six family member 19 [Source:HGNC Symbol;Acc:HGNC:25167] |
| ENSG00000145708 | HGLibA_11138 | 94 | -0.284431257 | CRHBP | corticotropin releasing hormone binding protein [Source:HGNC Symbol;Acc:HGNC:2356] |
| ENSG00000147121 | HGLibA_25397 | 94 | -0.284431257 | KRBOX4 | KRAB box domain containing 4 [Source:HGNC Symbol;Acc:HGNC:26007] |
| ENSG00000148396 | HGLibA_43207 | 94 | -0.284431257 | SEC16A | endoplasmic reticulum export factor [Source:HGNC Symbol;Acc:HGNC:29006]" |
| ENSG00000151065 | HGLibA_12519 | 94 | -0.284431257 | DCP1B | decapping mRNA 1B [Source:HGNC Symbol;Acc:HGNC:24451] |
| ENSG00000151687 | HGLibA_01942 | 94 | -0.284431257 | ANKAR | ankyrin and armadillo repeat containing [Source:HGNC Symbol;Acc:HGNC:26350] |
| ENSG00000151718 | HGLibA_54442 | 94 | -0.284431257 | WWC2 | WW and C2 domain containing 2 [Source:HGNC Symbol;Acc:HGNC:24148] |
| ENSG00000151849 | HGLibA_09063 | 94 | -0.284431257 | CENPJ | centromere protein J [Source:HGNC Symbol;Acc:HGNC:17272] |
| ENSG00000152348 | HGLibA_03461 | 94 | -0.284431257 | ATG10 | autophagy related 10 [Source:HGNC Symbol;Acc:HGNC:20315] |
| ENSG00000152495 | HGLibA_07086 | 94 | -0.284431257 | CAMK4 | calcium/calmodulin dependent protein kinase IV [Source:HGNC Symbol;Acc:HGNC:1464] |
| ENSG00000152556 | HGLibA_36168 | 94 | -0.284431257 | PFKM | muscle [Source:HGNC Symbol;Acc:HGNC:8877]" |
| ENSG00000153976 | HGLibA_22128 | 94 | -0.284431257 | HS3ST3A1 | heparan sulfate-glucosamine 3-sulfotransferase 3A1 [Source:HGNC Symbol;Acc:HGNC:5196] |
| ENSG00000154330 | HGLibA_36273 | 94 | -0.284431257 | PGM5 | phosphoglucomutase 5 [Source:HGNC Symbol;Acc:HGNC:8908] |
| ENSG00000154678 | HGLibA_35776 | 94 | -0.284431257 | PDE1C | phosphodiesterase 1C [Source:HGNC Symbol;Acc:HGNC:8776] |
| ENSG00000157343 | HGLibA_03010 | 94 | -0.284431257 | ARMC12 | armadillo repeat containing 12 [Source:HGNC Symbol;Acc:HGNC:21099] |
| ENSG00000158077 | HGLibA_32111 | 94 | -0.284431257 | NLRP14 | NLR family pyrin domain containing 14 [Source:HGNC Symbol;Acc:HGNC:22939] |
| ENSG00000160058 | HGLibA_04862 | 94 | -0.284431257 | BSDC1 | BSD domain containing 1 [Source:HGNC Symbol;Acc:HGNC:25501] |
| ENSG00000160188 | HGLibA_42323 | 94 | -0.284431257 | RSPH1 | radial spoke head component 1 [Source:HGNC Symbol;Acc:HGNC:12371] |
| ENSG00000161249 | HGLibA_13407 | 94 | -0.284431257 | DMKN | dermokine [Source:HGNC Symbol;Acc:HGNC:25063] |
| ENSG00000162783 | HGLibA_22581 | 94 | -0.284431257 | IER5 | immediate early response 5 [Source:HGNC Symbol;Acc:HGNC:5393] |
| ENSG00000163206 | HGLibA_45673 | 94 | -0.284431257 | SMCP | sperm mitochondria associated cysteine rich protein [Source:HGNC Symbol;Acc:HGNC:6962] |
| ENSG00000163626 | HGLibA_10821 | 94 | -0.284431257 | COX18 | cytochrome c oxidase assembly factor COX18 [Source:HGNC Symbol;Acc:HGNC:26801] |
| ENSG00000163993 | HGLibA_42585 | 94 | -0.284431257 | S100P | S100 calcium binding protein P [Source:HGNC Symbol;Acc:HGNC:10504] |
| ENSG00000164211 | HGLibA_47362 | 94 | -0.284431257 | STARD4 | StAR related lipid transfer domain containing 4 [Source:HGNC Symbol;Acc:HGNC:18058] |
| ENSG00000164626 | HGLibA_24328 | 94 | -0.284431257 | KCNK5 | potassium two pore domain channel subfamily K member 5 [Source:HGNC Symbol;Acc:HGNC:6280] |
| ENSG00000164828 | HGLibA_47787 | 94 | -0.284431257 | SUN1 | Sad1 and UNC84 domain containing 1 [Source:HGNC Symbol;Acc:HGNC:18587] |
| ENSG00000165202 | HGLibA_33711 | 94 | -0.284431257 | OR1Q1 | olfactory receptor family 1 subfamily Q member 1 [Source:HGNC Symbol;Acc:HGNC:8223] |
| ENSG00000165983 | HGLibA_39299 | 94 | -0.284431257 | PTER | phosphotriesterase related [Source:HGNC Symbol;Acc:HGNC:9590] |
| ENSG00000166166 | HGLibA_51698 | 94 | -0.284431257 | TRMT61A | tRNA methyltransferase 61A [Source:HGNC Symbol;Acc:HGNC:23790] |
| ENSG00000166220 | HGLibA_48458 | 94 | -0.284431257 | TBATA | brain and testes associated [Source:HGNC Symbol;Acc:HGNC:23511]" |
| ENSG00000166716 | HGLibA_56338 | 94 | -0.284431257 | ZNF592 | zinc finger protein 592 [Source:HGNC Symbol;Acc:HGNC:28986] |
| ENSG00000169306 | HGLibA_23054 | 94 | -0.284431257 | IL1RAPL1 | interleukin 1 receptor accessory protein like 1 [Source:HGNC Symbol;Acc:HGNC:5996] |
| ENSG00000169621 | HGLibA_02432 | 94 | -0.284431257 | APLF | aprataxin and PNKP like factor [Source:HGNC Symbol;Acc:HGNC:28724] |
| ENSG00000170634 | HGLibA_00712 | 94 | -0.284431257 | ACYP2 | acylphosphatase 2 [Source:HGNC Symbol;Acc:HGNC:180] |
| ENSG00000171680 | HGLibA_37115 | 94 | -0.284431257 | PLEKHG5 | pleckstrin homology and RhoGEF domain containing G5 [Source:HGNC Symbol;Acc:HGNC:29105] |
| ENSG00000171812 | HGLibA_10618 | 94 | -0.284431257 | COL8A2 | collagen type VIII alpha 2 chain [Source:HGNC Symbol;Acc:HGNC:2216] |
| ENSG00000171819 | HGLibA_01929 | 94 | -0.284431257 | ANGPTL7 | angiopoietin like 7 [Source:HGNC Symbol;Acc:HGNC:24078] |
| ENSG00000175137 | HGLibA_43938 | 94 | -0.284431257 | SH3BP5L | SH3 binding domain protein 5 like [Source:HGNC Symbol;Acc:HGNC:29360] |
| ENSG00000176125 | HGLibA_52856 | 94 | -0.284431257 | UFSP1 | UFM1 specific peptidase 1 (inactive) [Source:HGNC Symbol;Acc:HGNC:33821] |
| ENSG00000177494 | HGLibA_54792 | 94 | -0.284431257 | ZBED2 | zinc finger BED-type containing 2 [Source:HGNC Symbol;Acc:HGNC:20710] |
| ENSG00000178562 | HGLibA_08324 | 94 | -0.284431257 | CD28 | CD28 molecule [Source:HGNC Symbol;Acc:HGNC:1653] |
| ENSG00000179151 | HGLibA_14442 | 94 | -0.284431257 | EDC3 | enhancer of mRNA decapping 3 [Source:HGNC Symbol;Acc:HGNC:26114] |
| ENSG00000180616 | HGLibA_47178 | 94 | -0.284431257 | SSTR2 | somatostatin receptor 2 [Source:HGNC Symbol;Acc:HGNC:11331] |
| ENSG00000182591 | HGLibA_25626 | 94 | -0.284431257 | KRTAP11-1 | keratin associated protein 11-1 [Source:HGNC Symbol;Acc:HGNC:18922] |
| ENSG00000182827 | HGLibA_00398 | 94 | -0.284431257 | ACBD3 | acyl-CoA binding domain containing 3 [Source:HGNC Symbol;Acc:HGNC:15453] |
| ENSG00000183034 | HGLibA_34739 | 94 | -0.284431257 | OTOP2 | otopetrin 2 [Source:HGNC Symbol;Acc:HGNC:19657] |
| ENSG00000183479 | HGLibA_51374 | 94 | -0.284431257 | TREX2 | three prime repair exonuclease 2 [Source:HGNC Symbol;Acc:HGNC:12270] |
| ENSG00000184925 | HGLibA_26172 | 94 | -0.284431257 | LCN12 | lipocalin 12 [Source:HGNC Symbol;Acc:HGNC:28733] |
| ENSG00000184954 | HGLibA_34404 | 94 | -0.284431257 | OR6C70 | olfactory receptor family 6 subfamily C member 70 [Source:HGNC Symbol;Acc:HGNC:31299] |
| ENSG00000185269 | HGLibA_32357 | 94 | -0.284431257 | NOTUM | palmitoleoyl-protein carboxylesterase [Source:HGNC Symbol;Acc:HGNC:27106]" |
| ENSG00000185821 | HGLibA_34412 | 94 | -0.284431257 | OR6C76 | olfactory receptor family 6 subfamily C member 76 [Source:HGNC Symbol;Acc:HGNC:31305] |
| ENSG00000185894 | HGLibA_04749 | 94 | -0.284431257 | BPY2C | basic charge Y-linked 2C [Source:HGNC Symbol;Acc:HGNC:18225] |
| ENSG00000185896 | HGLibA_25976 | 94 | -0.284431257 | LAMP1 | lysosomal associated membrane protein 1 [Source:HGNC Symbol;Acc:HGNC:6499] |
| ENSG00000186300 | HGLibA_56224 | 94 | -0.284431257 | ZNF555 | zinc finger protein 555 [Source:HGNC Symbol;Acc:HGNC:28382] |
| ENSG00000186310 | HGLibA_31117 | 94 | -0.284431257 | NAP1L3 | nucleosome assembly protein 1 like 3 [Source:HGNC Symbol;Acc:HGNC:7639] |
| ENSG00000186732 | HGLibA_29771 | 94 | -0.284431257 | MPPED1 | metallophosphoesterase domain containing 1 [Source:HGNC Symbol;Acc:HGNC:1306] |
| ENSG00000187550 | HGLibA_42804 | 94 | -0.284431257 | SBK2 | SH3 domain binding kinase family member 2 [Source:HGNC Symbol;Acc:HGNC:34416] |
| ENSG00000188603 | HGLibA_10076 | 94 | -0.284431257 | CLN3 | battenin [Source:HGNC Symbol;Acc:HGNC:2074]" |
| ENSG00000188761 | HGLibA_04319 | 94 | -0.284431257 | BCL2L15 | BCL2 like 15 [Source:HGNC Symbol;Acc:HGNC:33624] |
| ENSG00000197299 | HGLibA_04547 | 94 | -0.284431257 | BLM | BLM RecQ like helicase [Source:HGNC Symbol;Acc:HGNC:1058] |
| ENSG00000197763 | HGLibA_52451 | 94 | -0.284431257 | TXNRD3 | thioredoxin reductase 3 [Source:HGNC Symbol;Acc:HGNC:20667] |
| ENSG00000198131 | HGLibA_56196 | 94 | -0.284431257 | ZNF544 | zinc finger protein 544 [Source:HGNC Symbol;Acc:HGNC:16759] |
| ENSG00000198400 | HGLibA_32875 | 94 | -0.284431257 | NTRK1 | neurotrophic receptor tyrosine kinase 1 [Source:HGNC Symbol;Acc:HGNC:8031] |
| ENSG00000204540 | HGLibA_39216 | 94 | -0.284431257 | PSORS1C1 | psoriasis susceptibility 1 candidate 1 [Source:HGNC Symbol;Acc:HGNC:17202] |
| ENSG00000204610 | HGLibA_51413 | 94 | -0.284431257 | TRIM15 | tripartite motif containing 15 [Source:HGNC Symbol;Acc:HGNC:16284] |
| ENSG00000204920 | HGLibA_55505 | 94 | -0.284431257 | ZNF155 | zinc finger protein 155 [Source:HGNC Symbol;Acc:HGNC:12940] |
| ENSG00000205923 | HGLibA_09030 | 94 | -0.284431257 | CEMP1 | cementum protein 1 [Source:HGNC Symbol;Acc:HGNC:32553] |
| ENSG00000223443 | HGLibA_53241 | 94 | -0.284431257 | USP17L2 | ubiquitin specific peptidase 17-like family member 2 [Source:HGNC Symbol;Acc:HGNC:34434] |
| ENSG00000235780 | HGLibA_53263 | 94 | -0.284431257 | USP17L27 | ubiquitin specific peptidase 17-like family member 27 [Source:HGNC Symbol;Acc:HGNC:44455] |
| ENSG00000235961 | HGLibA_37379 | 94 | -0.284431257 | PNMA6A | PNMA family member 6A [Source:HGNC Symbol;Acc:HGNC:28248] |
| ENSG00000241119 | HGLibA_52899 | 94 | -0.284431257 | UGT1A9 | UDP glucuronosyltransferase family 1 member A9 [Source:HGNC Symbol;Acc:HGNC:12541] |
| ENSG00000244355 | HGLibA_27668 | 94 | -0.284431257 | LY6G6D | lymphocyte antigen 6 family member G6D [Source:HGNC Symbol;Acc:HGNC:13935] |
| ENSG00000249139 | HGLibA_15358 | 94 | -0.284431257 | EPPIN-WFDC6 | EPPIN-WFDC6 readthrough [Source:HGNC Symbol;Acc:HGNC:38825] |
| ENSG00000249581 | HGLibA_10125 | 94 | -0.284431257 | CLRN2 | clarin 2 [Source:HGNC Symbol;Acc:HGNC:33939] |
| ENSG00000250506 | HGLibA_08767 | 94 | -0.284431257 | CDK3 | cyclin dependent kinase 3 [Source:HGNC Symbol;Acc:HGNC:1772] |
| ENSG00000253846 | HGLibA_35519 | 94 | -0.284431257 | PCDHGA10 | 10 [Source:HGNC Symbol;Acc:HGNC:8697]" |
| ENSG00000253910 | HGLibA_35553 | 94 | -0.284431257 | PCDHGB2 | 2 [Source:HGNC Symbol;Acc:HGNC:8709]" |
| ENSG00000255112 | HGLibA_09476 | 94 | -0.284431257 | CHMP1B | charged multivesicular body protein 1B [Source:HGNC Symbol;Acc:HGNC:24287] |
| FAM19A3 | HGLibA_16402 | 94 | -0.284431257 | FAM19A3 | - |
| FAM211B | HGLibA_16462 | 94 | -0.284431257 | FAM211B | - |
| FLJ45513 | HGLibA_17636 | 94 | -0.284431257 | FLJ45513 | - |
| GYLTL1B | HGLibA_20695 | 94 | -0.284431257 | GYLTL1B | - |
| HIST1H2BG | HGLibA_21428 | 94 | -0.284431257 | HIST1H2BG | - |
| hsa-mir-1178 | HGLibA_57207 | 94 | -0.284431257 | hsa-mir-1178 | - |
| hsa-mir-1263 | HGLibA_57477 | 94 | -0.284431257 | hsa-mir-1263 | - |
| hsa-mir-1302-9 | HGLibA_57724 | 94 | -0.284431257 | hsa-mir-1302-9 | - |
| hsa-mir-133a-1 | HGLibA_57773 | 94 | -0.284431257 | hsa-mir-133a-1 | - |
| hsa-mir-211 | HGLibA_58218 | 94 | -0.284431257 | hsa-mir-211 | - |
| hsa-mir-30b | HGLibA_58474 | 94 | -0.284431257 | hsa-mir-30b | - |
| hsa-mir-3122 | HGLibA_58540 | 94 | -0.284431257 | hsa-mir-3122 | - |
| hsa-mir-3196 | HGLibA_58880 | 94 | -0.284431257 | hsa-mir-3196 | - |
| hsa-mir-320b-1 | HGLibA_58926 | 94 | -0.284431257 | hsa-mir-320b-1 | - |
| hsa-mir-3910-1 | HGLibA_59505 | 94 | -0.284431257 | hsa-mir-3910-1 | - |
| hsa-mir-3918 | HGLibA_59550 | 94 | -0.284431257 | hsa-mir-3918 | - |
| hsa-mir-4309 | HGLibA_59946 | 94 | -0.284431257 | hsa-mir-4309 | - |
| hsa-mir-4431 | HGLibA_60112 | 94 | -0.284431257 | hsa-mir-4431 | - |
| hsa-mir-450b | HGLibA_60480 | 94 | -0.284431257 | hsa-mir-450b | - |
| hsa-mir-450b | HGLibA_60482 | 94 | -0.284431257 | hsa-mir-450b | - |
| hsa-mir-4699 | HGLibA_60905 | 94 | -0.284431257 | hsa-mir-4699 | - |
| hsa-mir-4767 | HGLibA_61163 | 94 | -0.284431257 | hsa-mir-4767 | - |
| hsa-mir-5006 | HGLibA_61427 | 94 | -0.284431257 | hsa-mir-5006 | - |
| hsa-mir-589 | HGLibA_62454 | 94 | -0.284431257 | hsa-mir-589 | - |
| hsa-mir-6131 | HGLibA_62676 | 94 | -0.284431257 | hsa-mir-6131 | - |
| hsa-mir-6776 | HGLibA_63279 | 94 | -0.284431257 | hsa-mir-6776 | - |
| hsa-mir-8068 | HGLibA_64110 | 94 | -0.284431257 | hsa-mir-8068 | - |
| LOC149373 | HGLibA_26890 | 94 | -0.284431257 | LOC149373 | - |
| TMEM189 | HGLibA_50131 | 94 | -0.284431257 | TMEM189 | - |
| WIBG | HGLibA_54298 | 94 | -0.284431257 | WIBG | - |
| ATP5B | HGLibA_03640 | 93 | -0.2934766 | ATP5B | - |
| C12orf68 | HGLibA_05284 | 93 | -0.2934766 | C12orf68 | - |
| C16orf45 | HGLibA_05447 | 93 | -0.2934766 | C16orf45 | - |
| C19orf52 | HGLibA_05674 | 93 | -0.2934766 | C19orf52 | - |
| C22orf24 | HGLibA_06060 | 93 | -0.2934766 | C22orf24 | - |
| CCDC41 | HGLibA_07779 | 93 | -0.2934766 | CCDC41 | - |
| ENSG00000009307 | HGLibA_11316 | 93 | -0.2934766 | CSDE1 | cold shock domain containing E1 [Source:HGNC Symbol;Acc:HGNC:29905] |
| ENSG00000013392 | HGLibA_42481 | 93 | -0.2934766 | RWDD2A | RWD domain containing 2A [Source:HGNC Symbol;Acc:HGNC:21385] |
| ENSG00000030066 | HGLibA_33031 | 93 | -0.2934766 | NUP160 | nucleoporin 160 [Source:HGNC Symbol;Acc:HGNC:18017] |
| ENSG00000031081 | HGLibA_02756 | 93 | -0.2934766 | ARHGAP31 | Rho GTPase activating protein 31 [Source:HGNC Symbol;Acc:HGNC:29216] |
| ENSG00000038427 | HGLibA_53569 | 93 | -0.2934766 | VCAN | versican [Source:HGNC Symbol;Acc:HGNC:2464] |
| ENSG00000061273 | HGLibA_21021 | 93 | -0.2934766 | HDAC7 | histone deacetylase 7 [Source:HGNC Symbol;Acc:HGNC:14067] |
| ENSG00000061794 | HGLibA_30099 | 93 | -0.2934766 | MRPS35 | mitochondrial ribosomal protein S35 [Source:HGNC Symbol;Acc:HGNC:16635] |
| ENSG00000064225 | HGLibA_47251 | 93 | -0.2934766 | ST3GAL6 | 3-sialyltransferase 6 [Source:HGNC Symbol;Acc:HGNC:18080]" |
| ENSG00000069966 | HGLibA_19501 | 93 | -0.2934766 | GNB5 | G protein subunit beta 5 [Source:HGNC Symbol;Acc:HGNC:4401] |
| ENSG00000070087 | HGLibA_36178 | 93 | -0.2934766 | PFN2 | profilin 2 [Source:HGNC Symbol;Acc:HGNC:8882] |
| ENSG00000071677 | HGLibA_38600 | 93 | -0.2934766 | PRLH | prolactin releasing hormone [Source:HGNC Symbol;Acc:HGNC:17945] |
| ENSG00000078725 | HGLibA_04813 | 93 | -0.2934766 | BRINP1 | BMP/retinoic acid inducible neural specific 1 [Source:HGNC Symbol;Acc:HGNC:2687] |
| ENSG00000082126 | HGLibA_29755 | 93 | -0.2934766 | MPP4 | membrane palmitoylated protein 4 [Source:HGNC Symbol;Acc:HGNC:13680] |
| ENSG00000083635 | HGLibA_32999 | 93 | -0.2934766 | NUFIP1 | nuclear FMR1 interacting protein 1 [Source:HGNC Symbol;Acc:HGNC:8057] |
| ENSG00000085449 | HGLibA_54003 | 93 | -0.2934766 | WDFY1 | WD repeat and FYVE domain containing 1 [Source:HGNC Symbol;Acc:HGNC:20451] |
| ENSG00000086015 | HGLibA_28403 | 93 | -0.2934766 | MAST2 | microtubule associated serine/threonine kinase 2 [Source:HGNC Symbol;Acc:HGNC:19035] |
| ENSG00000086848 | HGLibA_01639 | 93 | -0.2934766 | ALG9 | 2-mannosyltransferase [Source:HGNC Symbol;Acc:HGNC:15672]" |

| | | | | | |
|---|---|---|---|---|---|
| ENSG00000100078 | HGLibA_36896 | 93 | -0.2934766 | PLA2G3 | phospholipase A2 group III [Source:HGNC Symbol;Acc:HGNC:17934] |
| ENSG00000100678 | HGLibA_45395 | 93 | -0.2934766 | SLC8A3 | solute carrier family 8 member A3 [Source:HGNC Symbol;Acc:HGNC:11070] |
| ENSG00000100911 | HGLibA_39194 | 93 | -0.2934766 | PSME2 | proteasome activator subunit 2 [Source:HGNC Symbol;Acc:HGNC:9569] |
| ENSG00000102048 | HGLibA_03254 | 93 | -0.2934766 | ASB9 | ankyrin repeat and SOCS box containing 9 [Source:HGNC Symbol;Acc:HGNC:17184] |
| ENSG00000102312 | HGLibA_37707 | 93 | -0.2934766 | PORCN | porcupine O-acyltransferase [Source:HGNC Symbol;Acc:HGNC:17652] |
| ENSG00000102981 | HGLibA_35183 | 93 | -0.2934766 | PARD6A | par-6 family cell polarity regulator alpha [Source:HGNC Symbol;Acc:HGNC:15943] |
| ENSG00000104327 | HGLibA_06986 | 93 | -0.2934766 | CALB1 | calbindin 1 [Source:HGNC Symbol;Acc:HGNC:1434] |
| ENSG00000104369 | HGLibA_23980 | 93 | -0.2934766 | JPH1 | junctophilin 1 [Source:HGNC Symbol;Acc:HGNC:14201] |
| ENSG00000105355 | HGLibA_37177 | 93 | -0.2934766 | PLIN3 | perilipin 3 [Source:HGNC Symbol;Acc:HGNC:16893] |
| ENSG00000105929 | HGLibA_03709 | 93 | -0.2934766 | ATP6V0A4 | ATPase H+ transporting V0 subunit a4 [Source:HGNC Symbol;Acc:HGNC:866] |
| ENSG00000107651 | HGLibA_43227 | 93 | -0.2934766 | SEC23IP | SEC23 interacting protein [Source:HGNC Symbol;Acc:HGNC:17018] |
| ENSG00000107745 | HGLibA_29223 | 93 | -0.2934766 | MICU1 | mitochondrial calcium uptake 1 [Source:HGNC Symbol;Acc:HGNC:1530] |
| ENSG00000108523 | HGLibA_41562 | 93 | -0.2934766 | RNF167 | ring finger protein 167 [Source:HGNC Symbol;Acc:HGNC:24544] |
| ENSG00000109787 | HGLibA_25068 | 93 | -0.2934766 | KLF3 | Kruppel like factor 3 [Source:HGNC Symbol;Acc:HGNC:16516] |
| ENSG00000110076 | HGLibA_32736 | 93 | -0.2934766 | NRXN2 | neurexin 2 [Source:HGNC Symbol;Acc:HGNC:8009] |
| ENSG00000110321 | HGLibA_14842 | 93 | -0.2934766 | EIF4G2 | eukaryotic translation initiation factor 4 gamma 2 [Source:HGNC Symbol;Acc:HGNC:3297] |
| ENSG00000111716 | HGLibA_26229 | 93 | -0.2934766 | LDHB | lactate dehydrogenase B [Source:HGNC Symbol;Acc:HGNC:6541] |
| ENSG00000112592 | HGLibA_48623 | 93 | -0.2934766 | TBP | TATA-box binding protein [Source:HGNC Symbol;Acc:HGNC:11588] |
| ENSG00000112818 | HGLibA_28894 | 93 | -0.2934766 | MEP1A | meprin A subunit alpha [Source:HGNC Symbol;Acc:HGNC:7015] |
| ENSG00000115414 | HGLibA_17726 | 93 | -0.2934766 | FN1 | fibronectin 1 [Source:HGNC Symbol;Acc:HGNC:3778] |
| ENSG00000116478 | HGLibA_20998 | 93 | -0.2934766 | HDAC1 | histone deacetylase 1 [Source:HGNC Symbol;Acc:HGNC:4852] |
| ENSG00000116667 | HGLibA_05825 | 93 | -0.2934766 | C1orf21 | chromosome 1 open reading frame 21 [Source:HGNC Symbol;Acc:HGNC:15494] |
| ENSG00000117586 | HGLibA_50823 | 93 | -0.2934766 | TNFSF4 | TNF superfamily member 4 [Source:HGNC Symbol;Acc:HGNC:11934] |
| ENSG00000117859 | HGLibA_34669 | 93 | -0.2934766 | OSBPL9 | oxysterol binding protein like 9 [Source:HGNC Symbol;Acc:HGNC:16386] |
| ENSG00000117971 | HGLibA_09597 | 93 | -0.2934766 | CHRNB4 | cholinergic receptor nicotinic beta 4 subunit [Source:HGNC Symbol;Acc:HGNC:1964] |
| ENSG00000118707 | HGLibA_49242 | 93 | -0.2934766 | TGIF2 | TGFB induced factor homeobox 2 [Source:HGNC Symbol;Acc:HGNC:15764] |
| ENSG00000119041 | HGLibA_20585 | 93 | -0.2934766 | GTF3C3 | general transcription factor IIIC subunit 3 [Source:HGNC Symbol;Acc:HGNC:4666] |
| ENSG00000119242 | HGLibA_07927 | 93 | -0.2934766 | CCDC92 | coiled-coil domain containing 92 [Source:HGNC Symbol;Acc:HGNC:29563] |
| ENSG00000121297 | HGLibA_51868 | 93 | -0.2934766 | TSHZ3 | teashirt zinc finger homeobox 3 [Source:HGNC Symbol;Acc:HGNC:30700] |
| ENSG00000121577 | HGLibA_37699 | 93 | -0.2934766 | POPDC2 | popeye domain containing 2 [Source:HGNC Symbol;Acc:HGNC:17648] |
| ENSG00000121680 | HGLibA_36102 | 93 | -0.2934766 | PEX16 | peroxisomal biogenesis factor 16 [Source:HGNC Symbol;Acc:HGNC:8857] |
| ENSG00000122550 | HGLibA_25222 | 93 | -0.2934766 | KLHL7 | kelch like family member 7 [Source:HGNC Symbol;Acc:HGNC:15646] |
| ENSG00000123191 | HGLibA_03771 | 93 | -0.2934766 | ATP7B | ATPase copper transporting beta [Source:HGNC Symbol;Acc:HGNC:870] |
| ENSG00000125388 | HGLibA_20329 | 93 | -0.2934766 | GRK4 | G protein-coupled receptor kinase 4 [Source:HGNC Symbol;Acc:HGNC:4543] |
| ENSG00000125676 | HGLibA_49368 | 93 | -0.2934766 | THOC2 | THO complex 2 [Source:HGNC Symbol;Acc:HGNC:19073] |
| ENSG00000125878 | HGLibA_48765 | 93 | -0.2934766 | TCF15 | transcription factor 15 [Source:HGNC Symbol;Acc:HGNC:11627] |
| ENSG00000126970 | HGLibA_55000 | 93 | -0.2934766 | ZC4H2 | zinc finger C4H2-type containing [Source:HGNC Symbol;Acc:HGNC:24931] |
| ENSG00000127528 | HGLibA_25065 | 93 | -0.2934766 | KLF2 | Kruppel like factor 2 [Source:HGNC Symbol;Acc:HGNC:6347] |
| ENSG00000128591 | HGLibA_17644 | 93 | -0.2934766 | FLNC | filamin C [Source:HGNC Symbol;Acc:HGNC:3756] |
| ENSG00000130635 | HGLibA_10586 | 93 | -0.2934766 | COL5A1 | collagen type V alpha 1 chain [Source:HGNC Symbol;Acc:HGNC:2209] |
| ENSG00000132323 | HGLibA_23224 | 93 | -0.2934766 | ILKAP | ILK associated serine/threonine phosphatase [Source:HGNC Symbol;Acc:HGNC:15566] |
| ENSG00000133019 | HGLibA_09554 | 93 | -0.2934766 | CHRM3 | cholinergic receptor muscarinic 3 [Source:HGNC Symbol;Acc:HGNC:1952] |
| ENSG00000134490 | HGLibA_50287 | 93 | -0.2934766 | TMEM241 | transmembrane protein 241 [Source:HGNC Symbol;Acc:HGNC:31723] |
| ENSG00000135362 | HGLibA_38847 | 93 | -0.2934766 | PRR5L | proline rich 5 like [Source:HGNC Symbol;Acc:HGNC:25878] |
| ENSG00000136695 | HGLibA_23155 | 93 | -0.2934766 | IL36RN | interleukin 36 receptor antagonist [Source:HGNC Symbol;Acc:HGNC:15561] |
| ENSG00000136828 | HGLibA_40168 | 93 | -0.2934766 | RALGPS1 | Ral GEF with PH domain and SH3 binding motif 1 [Source:HGNC Symbol;Acc:HGNC:16851] |
| ENSG00000137261 | HGLibA_24620 | 93 | -0.2934766 | KIAA0319 | KIAA0319 [Source:HGNC Symbol;Acc:HGNC:21580] |
| ENSG00000137474 | HGLibA_30862 | 93 | -0.2934766 | MYO7A | myosin VIIA [Source:HGNC Symbol;Acc:HGNC:7606] |
| ENSG00000137634 | HGLibA_33158 | 93 | -0.2934766 | NXPE4 | neurexophilin and PC-esterase domain family member 4 [Source:HGNC Symbol;Acc:HGNC:23117] |
| ENSG00000138039 | HGLibA_26424 | 93 | -0.2934766 | LHCGR | luteinizing hormone/choriogonadotropin receptor [Source:HGNC Symbol;Acc:HGNC:6585] |
| ENSG00000139329 | HGLibA_27629 | 93 | -0.2934766 | LUM | lumican [Source:HGNC Symbol;Acc:HGNC:6724] |
| ENSG00000140350 | HGLibA_02199 | 93 | -0.2934766 | ANP32A | acidic nuclear phosphoprotein 32 family member A [Source:HGNC Symbol;Acc:HGNC:13233] |
| ENSG00000143493 | HGLibA_23430 | 93 | -0.2934766 | INTS7 | integrator complex subunit 7 [Source:HGNC Symbol;Acc:HGNC:24484] |
| ENSG00000143553 | HGLibA_45904 | 93 | -0.2934766 | SNAPIN | SNAP associated protein [Source:HGNC Symbol;Acc:HGNC:17145] |
| ENSG00000145337 | HGLibA_39709 | 93 | -0.2934766 | PYURF | PIGY upstream reading frame [Source:HGNC Symbol;Acc:HGNC:44317] |
| ENSG00000145826 | HGLibA_26269 | 93 | -0.2934766 | LECT2 | leukocyte cell derived chemotaxin 2 [Source:HGNC Symbol;Acc:HGNC:6550] |
| ENSG00000146670 | HGLibA_08610 | 93 | -0.2934766 | CDCA5 | cell division cycle associated 5 [Source:HGNC Symbol;Acc:HGNC:14626] |
| ENSG00000147206 | HGLibA_33135 | 93 | -0.2934766 | NXF3 | nuclear RNA export factor 3 [Source:HGNC Symbol;Acc:HGNC:8073] |
| ENSG00000148700 | HGLibA_00946 | 93 | -0.2934766 | ADD3 | adducin 3 [Source:HGNC Symbol;Acc:HGNC:245] |
| ENSG00000151062 | HGLibA_06913 | 93 | -0.2934766 | CACNA2D4 | calcium voltage-gated channel auxiliary subunit alpha2delta 4 [Source:HGNC Symbol;Acc:HGNC:20202] |
| ENSG00000152582 | HGLibA_46611 | 93 | -0.2934766 | SPEF2 | sperm flagellar 2 [Source:HGNC Symbol;Acc:HGNC:26293] |
| ENSG00000153714 | HGLibA_27635 | 93 | -0.2934766 | LURAP1L | leucine rich adaptor protein 1 like [Source:HGNC Symbol;Acc:HGNC:31452] |
| ENSG00000154478 | HGLibA_20009 | 93 | -0.2934766 | GPR26 | G protein-coupled receptor 26 [Source:HGNC Symbol;Acc:HGNC:4481] |
| ENSG00000155980 | HGLibA_24950 | 93 | -0.2934766 | KIF5A | kinesin family member 5A [Source:HGNC Symbol;Acc:HGNC:6323] |
| ENSG00000158435 | HGLibA_10331 | 93 | -0.2934766 | CNOT11 | CCR4-NOT transcription complex subunit 11 [Source:HGNC Symbol;Acc:HGNC:25217] |
| ENSG00000159335 | HGLibA_39393 | 93 | -0.2934766 | PTMS | parathymosin [Source:HGNC Symbol;Acc:HGNC:9629] |
| ENSG00000160396 | HGLibA_21347 | 93 | -0.2934766 | HIPK4 | homeodomain interacting protein kinase 4 [Source:HGNC Symbol;Acc:HGNC:19007] |
| ENSG00000161956 | HGLibA_43391 | 93 | -0.2934766 | SENP3 | SUMO specific peptidase 3 [Source:HGNC Symbol;Acc:HGNC:17862] |
| ENSG00000163513 | HGLibA_49227 | 93 | -0.2934766 | TGFBR2 | transforming growth factor beta receptor 2 [Source:HGNC Symbol;Acc:HGNC:11773] |
| ENSG00000163785 | HGLibA_42519 | 93 | -0.2934766 | RYK | receptor-like tyrosine kinase [Source:HGNC Symbol;Acc:HGNC:10481] |
| ENSG00000163964 | HGLibA_36599 | 93 | -0.2934766 | PIGX | phosphatidylinositol glycan anchor biosynthesis class X [Source:HGNC Symbol;Acc:HGNC:26046] |
| ENSG00000164022 | HGLibA_01356 | 93 | -0.2934766 | AIMP1 | aminoacyl tRNA synthetase complex interacting multifunctional protein 1 [Source:HGNC Symbol;Acc:HGNC:10648] |
| ENSG00000164180 | HGLibA_50027 | 93 | -0.2934766 | TMEM161B | transmembrane protein 161B [Source:HGNC Symbol;Acc:HGNC:28483] |
| ENSG00000164591 | HGLibA_30908 | 93 | -0.2934766 | MYOZ3 | myozenin 3 [Source:HGNC Symbol;Acc:HGNC:18565] |
| ENSG00000164733 | HGLibA_11692 | 93 | -0.2934766 | CTSB | cathepsin B [Source:HGNC Symbol;Acc:HGNC:2527] |
| ENSG00000164879 | HGLibA_06807 | 93 | -0.2934766 | CA3 | carbonic anhydrase 3 [Source:HGNC Symbol;Acc:HGNC:1374] |
| ENSG00000165169 | HGLibA_14269 | 93 | -0.2934766 | DYNLT3 | dynein light chain Tctex-type 3 [Source:HGNC Symbol;Acc:HGNC:11694] |
| ENSG00000165188 | HGLibA_41588 | 93 | -0.2934766 | RNF183 | ring finger protein 183 [Source:HGNC Symbol;Acc:HGNC:28721] |
| ENSG00000166183 | HGLibA_03347 | 93 | -0.2934766 | ASPG | asparaginase [Source:HGNC Symbol;Acc:HGNC:20123] |
| ENSG00000166266 | HGLibA_11788 | 93 | -0.2934766 | CUL5 | cullin 5 [Source:HGNC Symbol;Acc:HGNC:2556] |
| ENSG00000166682 | HGLibA_50645 | 93 | -0.2934766 | TMPRSS5 | transmembrane serine protease 5 [Source:HGNC Symbol;Acc:HGNC:14908] |
| ENSG00000167633 | HGLibA_24990 | 93 | -0.2934766 | KIR3DL1 | three Ig domains and long cytoplasmic tail 1 [Source:HGNC Symbol;Acc:HGNC:6338]" |
| ENSG00000168566 | HGLibA_45956 | 93 | -0.2934766 | SNRNP48 | small nuclear ribonucleoprotein U11/U12 subunit 48 [Source:HGNC Symbol;Acc:HGNC:21368] |
| ENSG00000168612 | HGLibA_57038 | 93 | -0.2934766 | ZSWIM1 | zinc finger SWIM-type containing 1 [Source:HGNC Symbol;Acc:HGNC:16155] |
| ENSG00000169239 | HGLibA_06816 | 93 | -0.2934766 | CA5B | carbonic anhydrase 5B [Source:HGNC Symbol;Acc:HGNC:1378] |
| ENSG00000169594 | HGLibA_04645 | 93 | -0.2934766 | BNC1 | basonuclin 1 [Source:HGNC Symbol;Acc:HGNC:1081] |
| ENSG00000169851 | HGLibA_35414 | 93 | -0.2934766 | PCDH7 | protocadherin 7 [Source:HGNC Symbol;Acc:HGNC:8659] |
| ENSG00000171014 | HGLibA_33978 | 93 | -0.2934766 | OR4D5 | olfactory receptor family 4 subfamily D member 5 [Source:HGNC Symbol;Acc:HGNC:14852] |
| ENSG00000171060 | HGLibA_06645 | 93 | -0.2934766 | C8orf74 | chromosome 8 open reading frame 74 [Source:HGNC Symbol;Acc:HGNC:32296] |
| ENSG00000171135 | HGLibA_23911 | 93 | -0.2934766 | JAGN1 | jagunal homolog 1 [Source:HGNC Symbol;Acc:HGNC:26926] |
| ENSG00000171492 | HGLibA_27438 | 93 | -0.2934766 | LRRC8D | leucine rich repeat containing 8 VRAC subunit D [Source:HGNC Symbol;Acc:HGNC:16992] |
| ENSG00000171823 | HGLibA_16980 | 93 | -0.2934766 | FBXL14 | F-box and leucine rich repeat protein 14 [Source:HGNC Symbol;Acc:HGNC:28624] |
| ENSG00000173273 | HGLibA_50851 | 93 | -0.2934766 | TNKS | tankyrase [Source:HGNC Symbol;Acc:HGNC:11941] |
| ENSG00000174106 | HGLibA_26292 | 93 | -0.2934766 | LEMD3 | LEM domain containing 3 [Source:HGNC Symbol;Acc:HGNC:28887] |
| ENSG00000175826 | HGLibA_11601 | 93 | -0.2934766 | CTDNEP1 | CTD nuclear envelope phosphatase 1 [Source:HGNC Symbol;Acc:HGNC:19085] |
| ENSG00000175879 | HGLibA_22003 | 93 | -0.2934766 | HOXD8 | homeobox D8 [Source:HGNC Symbol;Acc:HGNC:5139] |
| ENSG00000175895 | HGLibA_37099 | 93 | -0.2934766 | PLEKHF2 | pleckstrin homology and FYVE domain containing 2 [Source:HGNC Symbol;Acc:HGNC:20757] |
| ENSG00000178338 | HGLibA_55865 | 93 | -0.2934766 | ZNF354B | zinc finger protein 354B [Source:HGNC Symbol;Acc:HGNC:17197] |
| ENSG00000178950 | HGLibA_18490 | 93 | -0.2934766 | GAK | cyclin G associated kinase [Source:HGNC Symbol;Acc:HGNC:4113] |
| ENSG00000179085 | HGLibA_13864 | 93 | -0.2934766 | DPM3 | dolichyl-phosphate mannosyltransferase subunit 3 [Source:HGNC Symbol;Acc:HGNC:3007] |
| ENSG00000179468 | HGLibA_34562 | 93 | -0.2934766 | OR9A2 | olfactory receptor family 9 subfamily A member 2 [Source:HGNC Symbol;Acc:HGNC:15093] |
| ENSG00000179930 | HGLibA_56454 | 93 | -0.2934766 | ZNF648 | zinc finger protein 648 [Source:HGNC Symbol;Acc:HGNC:18190] |
| ENSG00000180979 | HGLibA_27395 | 93 | -0.2934766 | LRRC57 | leucine rich repeat containing 57 [Source:HGNC Symbol;Acc:HGNC:26719] |
| ENSG00000181045 | HGLibA_44787 | 93 | -0.2934766 | SLC26A11 | solute carrier family 26 member 11 [Source:HGNC Symbol;Acc:HGNC:14471] |
| ENSG00000182973 | HGLibA_10329 | 93 | -0.2934766 | CNOT10 | CCR4-NOT transcription complex subunit 10 [Source:HGNC Symbol;Acc:HGNC:23817] |
| ENSG00000183023 | HGLibA_45387 | 93 | -0.2934766 | SLC8A1 | solute carrier family 8 member A1 [Source:HGNC Symbol;Acc:HGNC:11068] |
| ENSG00000183747 | HGLibA_00571 | 93 | -0.2934766 | ACSM2A | acyl-CoA synthetase medium chain family member 2A [Source:HGNC Symbol;Acc:HGNC:32017] |
| ENSG00000184602 | HGLibA_45932 | 93 | -0.2934766 | SNN | stannin [Source:HGNC Symbol;Acc:HGNC:11149] |
| ENSG00000184828 | HGLibA_54922 | 93 | -0.2934766 | ZBTB7C | zinc finger and BTB domain containing 7C [Source:HGNC Symbol;Acc:HGNC:31700] |
| ENSG00000186832 | HGLibA_25431 | 93 | -0.2934766 | KRT16 | keratin 16 [Source:HGNC Symbol;Acc:HGNC:6423] |
| ENSG00000187134 | HGLibA_01481 | 93 | -0.2934766 | AKR1C1 | aldo-keto reductase family 1 member C1 [Source:HGNC Symbol;Acc:HGNC:384] |
| ENSG00000187244 | HGLibA_04233 | 93 | -0.2934766 | BCAM | basal cell adhesion molecule (Lutheran blood group) [Source:HGNC Symbol;Acc:HGNC:6722] |
| ENSG00000187498 | HGLibA_10566 | 93 | -0.2934766 | COL4A1 | collagen type IV alpha 1 chain [Source:HGNC Symbol;Acc:HGNC:2202] |
| ENSG00000187758 | HGLibA_00950 | 93 | -0.2934766 | ADH1A | alpha polypeptide [Source:HGNC Symbol;Acc:HGNC:249]" |
| ENSG00000188010 | HGLibA_29670 | 93 | -0.2934766 | MORN2 | MORN repeat containing 2 [Source:HGNC Symbol;Acc:HGNC:30166] |
| ENSG00000188095 | HGLibA_28919 | 93 | -0.2934766 | MESP2 | mesoderm posterior bHLH transcription factor 2 [Source:HGNC Symbol;Acc:HGNC:29659] |
| ENSG00000188906 | HGLibA_27467 | 93 | -0.2934766 | LRRK2 | leucine rich repeat kinase 2 [Source:HGNC Symbol;Acc:HGNC:18618] |
| ENSG00000188993 | HGLibA_27409 | 93 | -0.2934766 | LRRC66 | leucine rich repeat containing 66 [Source:HGNC Symbol;Acc:HGNC:34299] |
| ENSG00000189334 | HGLibA_42546 | 93 | -0.2934766 | S100A14 | S100 calcium binding protein A14 [Source:HGNC Symbol;Acc:HGNC:18901] |
| ENSG00000196511 | HGLibA_51139 | 93 | -0.2934766 | TPK1 | thiamin pyrophosphokinase 1 [Source:HGNC Symbol;Acc:HGNC:17358] |
| ENSG00000196705 | HGLibA_55982 | 93 | -0.2934766 | ZNF431 | zinc finger protein 431 [Source:HGNC Symbol;Acc:HGNC:20809] |
| ENSG00000197535 | HGLibA_30851 | 93 | -0.2934766 | MYO5A | myosin VA [Source:HGNC Symbol;Acc:HGNC:7602] |
| ENSG00000197779 | HGLibA_56800 | 93 | -0.2934766 | ZNF81 | zinc finger protein 81 [Source:HGNC Symbol;Acc:HGNC:13156] |
| ENSG00000197978 | HGLibA_19631 | 93 | -0.2934766 | GOLGA6L9 | golgin A6 family-like 9 [Source:HGNC Symbol;Acc:HGNC:37229] |
| ENSG00000198554 | HGLibA_54015 | 93 | -0.2934766 | WDHD1 | WD repeat and HMG-box DNA binding protein 1 [Source:HGNC Symbol;Acc:HGNC:23170] |
| ENSG00000198689 | HGLibA_45421 | 93 | -0.2934766 | SLC9A6 | solute carrier family 9 member A6 [Source:HGNC Symbol;Acc:HGNC:11079] |
| ENSG00000198729 | HGLibA_38042 | 93 | -0.2934766 | PPP1R14C | protein phosphatase 1 regulatory inhibitor subunit 14C [Source:HGNC Symbol;Acc:HGNC:14952] |
| ENSG00000198930 | HGLibA_11305 | 93 | -0.2934766 | CSAG1 | chondrosarcoma associated gene 1 [Source:HGNC Symbol;Acc:HGNC:24294] |
| ENSG00000204086 | HGLibA_41809 | 93 | -0.2934766 | RPA4 | replication protein A4 [Source:HGNC Symbol;Acc:HGNC:30305] |
| ENSG00000204228 | HGLibA_22202 | 93 | -0.2934766 | HSD17B8 | hydroxysteroid 17-beta dehydrogenase 8 [Source:HGNC Symbol;Acc:HGNC:3554] |
| ENSG00000205765 | HGLibA_06400 | 93 | -0.2934766 | C5orf51 | chromosome 5 open reading frame 51 [Source:HGNC Symbol;Acc:HGNC:27750] |
| ENSG00000206536 | HGLibA_34317 | 93 | -0.2934766 | OR5K3 | olfactory receptor family 5 subfamily K member 3 [Source:HGNC Symbol;Acc:HGNC:31290] |
| ENSG00000215114 | HGLibA_52787 | 93 | -0.2934766 | UBXN2B | UBX domain protein 2B [Source:HGNC Symbol;Acc:HGNC:27035] |
| ENSG00000221890 | HGLibA_32537 | 93 | -0.2934766 | NPTXR | neuronal pentraxin receptor [Source:HGNC Symbol;Acc:HGNC:7954] |
| ENSG00000222014 | HGLibA_39975 | 93 | -0.2934766 | RAB6C | member RAS oncogene family [Source:HGNC Symbol;Acc:HGNC:16525]" |

| | | | | | |
|---|---|---|---|---|---|
| ENSG00000228517 | HGLibA_11542 | 93 | -0.2934766 | CT47A7 | member A7 [Source:HGNC Symbol;Acc:HGNC:33288]" |
| ENSG00000230347 | HGLibA_11545 | 93 | -0.2934766 | CT47A8 | member A8 [Source:HGNC Symbol;Acc:HGNC:33289]" |
| ENSG00000235169 | HGLibA_45715 | 93 | -0.2934766 | SMIM1 | small integral membrane protein 1 (Vel blood group) [Source:HGNC Symbol;Acc:HGNC:44204] |
| ENSG00000242173 | HGLibA_02801 | 93 | -0.2934766 | ARHGDIG | Rho GDP dissociation inhibitor gamma [Source:HGNC Symbol;Acc:HGNC:680] |
| ENSG00000248167 | HGLibA_51485 | 93 | -0.2934766 | TRIM39-RPP21 | TRIM39-RPP21 readthrough [Source:HGNC Symbol;Acc:HGNC:38845] |
| ENSG00000254221 | HGLibA_35550 | 93 | -0.2934766 | PCDHGB1 | 1 [Source:HGNC Symbol;Acc:HGNC:8708]" |
| ENSG00000254636 | HGLibA_03060 | 93 | -0.2934766 | ARMS2 | age-related maculopathy susceptibility 2 [Source:HGNC Symbol;Acc:HGNC:32685] |
| ENSG00000255823 | HGLibA_30490 | 93 | -0.2934766 | MTRNR2L8 | MT-RNR2 like 8 [Source:HGNC Symbol;Acc:HGNC:37165] |
| ENSG00000258484 | HGLibA_46628 | 93 | -0.2934766 | SPESP1 | sperm equatorial segment protein 1 [Source:HGNC Symbol;Acc:HGNC:15570] |
| ENSG00000259024 | HGLibA_52367 | 93 | -0.2934766 | TVP23C-CDRT4 | TVP23C-CDRT4 readthrough [Source:HGNC Symbol;Acc:HGNC:42961] |
| ENSG00000266265 | HGLibA_25055 | 93 | -0.2934766 | KLF14 | Kruppel like factor 14 [Source:HGNC Symbol;Acc:HGNC:23025] |
| ENSG00000266967 | HGLibA_00075 | 93 | -0.2934766 | AARSD1 | alanyl-tRNA synthetase domain containing 1 [Source:HGNC Symbol;Acc:HGNC:28417] |
| ENSG00000270647 | HGLibA_48209 | 93 | -0.2934766 | TAF15 | TATA-box binding protein associated factor 15 [Source:HGNC Symbol;Acc:HGNC:11547] |
| ENSG00000274749 | HGLibA_25823 | 93 | -0.2934766 | KRTAP7-1 | keratin associated protein 7-1 (gene/pseudogene) [Source:HGNC Symbol;Acc:HGNC:18934] |
| FAM46D | HGLibA_16604 | 93 | -0.2934766 | FAM46D | - |
| FAM5C | HGLibA_16652 | 93 | -0.2934766 | FAM5C | - |
| GTPBP5 | HGLibA_20613 | 93 | -0.2934766 | GTPBP5 | - |
| hsa-mir-3116-2 | HGLibA_58508 | 93 | -0.2934766 | hsa-mir-3116-2 | - |
| hsa-mir-320b-2 | HGLibA_58930 | 93 | -0.2934766 | hsa-mir-320b-2 | - |
| hsa-mir-345 | HGLibA_59026 | 93 | -0.2934766 | hsa-mir-345 | - |
| hsa-mir-34c | HGLibA_59043 | 93 | -0.2934766 | hsa-mir-34c | - |
| hsa-mir-3616 | HGLibA_59095 | 93 | -0.2934766 | hsa-mir-3616 | - |
| hsa-mir-3662 | HGLibA_59200 | 93 | -0.2934766 | hsa-mir-3662 | - |
| hsa-mir-4268 | HGLibA_59780 | 93 | -0.2934766 | hsa-mir-4268 | - |
| hsa-mir-4321 | HGLibA_60006 | 93 | -0.2934766 | hsa-mir-4321 | - |
| hsa-mir-4750 | HGLibA_61098 | 93 | -0.2934766 | hsa-mir-4750 | - |
| hsa-mir-4751 | HGLibA_61100 | 93 | -0.2934766 | hsa-mir-4751 | - |
| hsa-mir-4752 | HGLibA_61104 | 93 | -0.2934766 | hsa-mir-4752 | - |
| hsa-mir-518c | HGLibA_61665 | 93 | -0.2934766 | hsa-mir-518c | - |
| hsa-mir-518e | HGLibA_61670 | 93 | -0.2934766 | hsa-mir-518e | - |
| hsa-mir-548av | HGLibA_61933 | 93 | -0.2934766 | hsa-mir-548av | - |
| hsa-mir-548av | HGLibA_61932 | 93 | -0.2934766 | hsa-mir-548av | - |
| hsa-mir-548h-2 | HGLibA_61997 | 93 | -0.2934766 | hsa-mir-548h-2 | - |
| hsa-mir-552 | HGLibA_62126 | 93 | -0.2934766 | hsa-mir-552 | - |
| hsa-mir-5701-2 | HGLibA_62345 | 93 | -0.2934766 | hsa-mir-5701-2 | - |
| hsa-mir-5704 | HGLibA_62358 | 93 | -0.2934766 | hsa-mir-5704 | - |
| hsa-mir-6076 | HGLibA_62556 | 93 | -0.2934766 | hsa-mir-6076 | - |
| hsa-mir-6511a-1 | HGLibA_62899 | 93 | -0.2934766 | hsa-mir-6511a-1 | - |
| hsa-mir-6772 | HGLibA_63263 | 93 | -0.2934766 | hsa-mir-6772 | - |
| hsa-mir-6874 | HGLibA_63679 | 93 | -0.2934766 | hsa-mir-6874 | - |
| hsa-mir-7162 | HGLibA_63876 | 93 | -0.2934766 | hsa-mir-7162 | - |
| hsa-mir-7702 | HGLibA_63944 | 93 | -0.2934766 | hsa-mir-7702 | - |
| hsa-mir-8054 | HGLibA_64055 | 93 | -0.2934766 | hsa-mir-8054 | - |
| hsa-mir-95 | HGLibA_64366 | 93 | -0.2934766 | hsa-mir-95 | - |
| PLCXD1_Y | HGLibA_37023 | 93 | -0.2934766 | PLCXD1_Y | - |
| PNMA6C | HGLibA_37380 | 93 | -0.2934766 | PNMA6C | - |
| PTRF | HGLibA_39548 | 93 | -0.2934766 | PTRF | - |
| TOMM70A | HGLibA_50981 | 93 | -0.2934766 | TOMM70A | - |
| ARSE | HGLibA_03136 | 92 | -0.302521943 | ARSE | - |
| C15orf38-AP3S2 | HGLibA_05391 | 92 | -0.302521943 | C15orf38-AP3S2 | - |
| CCDC84 | HGLibA_07888 | 92 | -0.302521943 | CCDC84 | - |
| ENSG00000011114 | HGLibA_04919 | 92 | -0.302521943 | BTBD7 | BTB domain containing 7 [Source:HGNC Symbol;Acc:HGNC:18269] |
| ENSG00000012232 | HGLibA_15815 | 92 | -0.302521943 | EXTL3 | exostosin like glycosyltransferase 3 [Source:HGNC Symbol;Acc:HGNC:3518] |
| ENSG00000036530 | HGLibA_12168 | 92 | -0.302521943 | CYP46A1 | cytochrome P450 family 46 subfamily A member 1 [Source:HGNC Symbol;Acc:HGNC:2641] |
| ENSG00000038210 | HGLibA_36496 | 92 | -0.302521943 | PI4K2B | phosphatidylinositol 4-kinase type 2 beta [Source:HGNC Symbol;Acc:HGNC:18215] |
| ENSG00000040531 | HGLibA_11664 | 92 | -0.302521943 | CTNS | lysosomal cystine transporter [Source:HGNC Symbol;Acc:HGNC:2518]" |
| ENSG00000059145 | HGLibA_53050 | 92 | -0.302521943 | UNKL | unkempt family like zinc finger [Source:HGNC Symbol;Acc:HGNC:14184] |
| ENSG00000065135 | HGLibA_19457 | 92 | -0.302521943 | GNAI3 | G protein subunit alpha i3 [Source:HGNC Symbol;Acc:HGNC:4387] |
| ENSG00000070729 | HGLibA_10275 | 92 | -0.302521943 | CNGB1 | cyclic nucleotide gated channel beta 1 [Source:HGNC Symbol;Acc:HGNC:2151] |
| ENSG00000074201 | HGLibA_10090 | 92 | -0.302521943 | CLNS1A | chloride nucleotide-sensitive channel 1A [Source:HGNC Symbol;Acc:HGNC:2080] |
| ENSG00000077009 | HGLibA_32199 | 92 | -0.302521943 | NMRK2 | nicotinamide riboside kinase 2 [Source:HGNC Symbol;Acc:HGNC:17871] |
| ENSG00000078140 | HGLibA_52624 | 92 | -0.302521943 | UBE2K | ubiquitin conjugating enzyme E2 K [Source:HGNC Symbol;Acc:HGNC:4914] |
| ENSG00000079432 | HGLibA_09694 | 92 | -0.302521943 | CIC | capicua transcriptional repressor [Source:HGNC Symbol;Acc:HGNC:14214] |
| ENSG00000083720 | HGLibA_34813 | 92 | -0.302521943 | OXCT1 | 3-oxoacid CoA-transferase 1 [Source:HGNC Symbol;Acc:HGNC:8527] |
| ENSG00000090554 | HGLibA_17668 | 92 | -0.302521943 | FLT3LG | fms related tyrosine kinase 3 ligand [Source:HGNC Symbol;Acc:HGNC:3766] |
| ENSG00000091009 | HGLibA_40525 | 92 | -0.302521943 | RBM27 | RNA binding motif protein 27 [Source:HGNC Symbol;Acc:HGNC:29243] |
| ENSG00000099822 | HGLibA_20976 | 92 | -0.302521943 | HCN2 | hyperpolarization activated cyclic nucleotide gated potassium and sodium channel 2 [Source:HGNC Symbol;Acc:HGNC:4846] |
| ENSG00000101883 | HGLibA_41215 | 92 | -0.302521943 | RHOXF1 | Rhox homeobox family member 1 [Source:HGNC Symbol;Acc:HGNC:29993] |
| ENSG00000102890 | HGLibA_14936 | 92 | -0.302521943 | ELMO3 | engulfment and cell motility 3 [Source:HGNC Symbol;Acc:HGNC:17289] |
| ENSG00000104388 | HGLibA_39865 | 92 | -0.302521943 | RAB2A | member RAS oncogene family [Source:HGNC Symbol;Acc:HGNC:9763]" |
| ENSG00000104756 | HGLibA_24470 | 92 | -0.302521943 | KCTD9 | potassium channel tetramerization domain containing 9 [Source:HGNC Symbol;Acc:HGNC:22401] |
| ENSG00000104998 | HGLibA_23114 | 92 | -0.302521943 | IL27RA | interleukin 27 receptor subunit alpha [Source:HGNC Symbol;Acc:HGNC:17290] |
| ENSG00000106012 | HGLibA_23499 | 92 | -0.302521943 | IQCE | IQ motif containing E [Source:HGNC Symbol;Acc:HGNC:29171] |
| ENSG00000107165 | HGLibA_52474 | 92 | -0.302521943 | TYRP1 | tyrosinase related protein 1 [Source:HGNC Symbol;Acc:HGNC:12450] |
| ENSG00000108100 | HGLibA_08131 | 92 | -0.302521943 | CCNY | cyclin Y [Source:HGNC Symbol;Acc:HGNC:23354] |
| ENSG00000108187 | HGLibA_35334 | 92 | -0.302521943 | PBLD | phenazine biosynthesis like protein domain containing [Source:HGNC Symbol;Acc:HGNC:23301] |
| ENSG00000108469 | HGLibA_40773 | 92 | -0.302521943 | RECQL5 | RecQ like helicase 5 [Source:HGNC Symbol;Acc:HGNC:9950] |
| ENSG00000109794 | HGLibA_16149 | 92 | -0.302521943 | FAM149A | family with sequence similarity 149 member A [Source:HGNC Symbol;Acc:HGNC:24527] |
| ENSG00000109919 | HGLibA_30327 | 92 | -0.302521943 | MTCH2 | mitochondrial carrier 2 [Source:HGNC Symbol;Acc:HGNC:17587] |
| ENSG00000109943 | HGLibA_11221 | 92 | -0.302521943 | CRTAM | cytotoxic and regulatory T cell molecule [Source:HGNC Symbol;Acc:HGNC:24313] |
| ENSG00000110931 | HGLibA_07090 | 92 | -0.302521943 | CAMKK2 | calcium/calmodulin dependent protein kinase kinase 2 [Source:HGNC Symbol;Acc:HGNC:1470] |
| ENSG00000111537 | HGLibA_22708 | 92 | -0.302521943 | IFNG | interferon gamma [Source:HGNC Symbol;Acc:HGNC:5438] |
| ENSG00000112799 | HGLibA_27686 | 92 | -0.302521943 | LY86 | lymphocyte antigen 86 [Source:HGNC Symbol;Acc:HGNC:16837] |
| ENSG00000113263 | HGLibA_23820 | 92 | -0.302521943 | ITK | IL2 inducible T cell kinase [Source:HGNC Symbol;Acc:HGNC:6171] |
| ENSG00000113300 | HGLibA_10345 | 92 | -0.302521943 | CNOT6 | CCR4-NOT transcription complex subunit 6 [Source:HGNC Symbol;Acc:HGNC:14099] |
| ENSG00000113638 | HGLibA_52117 | 92 | -0.302521943 | TTC33 | tetratricopeptide repeat domain 33 [Source:HGNC Symbol;Acc:HGNC:29959] |
| ENSG00000114166 | HGLibA_24053 | 92 | -0.302521943 | KAT2B | lysine acetyltransferase 2B [Source:HGNC Symbol;Acc:HGNC:8638] |
| ENSG00000114354 | HGLibA_49179 | 92 | -0.302521943 | TFG | TRK-fused gene [Source:HGNC Symbol;Acc:HGNC:11758] |
| ENSG00000115459 | HGLibA_14944 | 92 | -0.302521943 | ELMOD3 | ELMO domain containing 3 [Source:HGNC Symbol;Acc:HGNC:26158] |
| ENSG00000116209 | HGLibA_50458 | 92 | -0.302521943 | TMEM59 | transmembrane protein 59 [Source:HGNC Symbol;Acc:HGNC:1239] |
| ENSG00000116678 | HGLibA_26312 | 92 | -0.302521943 | LEPR | leptin receptor [Source:HGNC Symbol;Acc:HGNC:6554] |
| ENSG00000116783 | HGLibA_50879 | 92 | -0.302521943 | TNNI3K | TNNI3 interacting kinase [Source:HGNC Symbol;Acc:HGNC:19661] |
| ENSG00000117643 | HGLibA_28066 | 92 | -0.302521943 | MAN1C1 | mannosidase alpha class 1C member 1 [Source:HGNC Symbol;Acc:HGNC:19080] |
| ENSG00000118420 | HGLibA_52686 | 92 | -0.302521943 | UBE3D | ubiquitin protein ligase E3D [Source:HGNC Symbol;Acc:HGNC:21381] |
| ENSG00000118939 | HGLibA_52803 | 92 | -0.302521943 | UCHL3 | ubiquitin C-terminal hydrolase L3 [Source:HGNC Symbol;Acc:HGNC:12515] |
| ENSG00000119041 | HGLibA_20587 | 92 | -0.302521943 | GTF3C3 | general transcription factor IIIC subunit 3 [Source:HGNC Symbol;Acc:HGNC:4666] |
| ENSG00000119431 | HGLibA_21058 | 92 | -0.302521943 | HDHD3 | haloacid dehalogenase like hydrolase domain containing 3 [Source:HGNC Symbol;Acc:HGNC:28171] |
| ENSG00000119705 | HGLibA_45503 | 92 | -0.302521943 | SLIRP | SRA stem-loop interacting RNA binding protein [Source:HGNC Symbol;Acc:HGNC:20495] |
| ENSG00000120727 | HGLibA_35032 | 92 | -0.302521943 | PAIP2 | poly(A) binding protein interacting protein 2 [Source:HGNC Symbol;Acc:HGNC:17970] |
| ENSG00000121207 | HGLibA_27130 | 92 | -0.302521943 | LRAT | lecithin retinol acyltransferase [Source:HGNC Symbol;Acc:HGNC:6685] |
| ENSG00000121440 | HGLibA_35997 | 92 | -0.302521943 | PDZRN3 | PDZ domain containing ring finger 3 [Source:HGNC Symbol;Acc:HGNC:17704] |
| ENSG00000122299 | HGLibA_54982 | 92 | -0.302521943 | ZC3H7A | zinc finger CCCH-type containing 7A [Source:HGNC Symbol;Acc:HGNC:30959] |
| ENSG00000122756 | HGLibA_10394 | 92 | -0.302521943 | CNTFR | ciliary neurotrophic factor receptor [Source:HGNC Symbol;Acc:HGNC:2170] |
| ENSG00000124171 | HGLibA_35187 | 92 | -0.302521943 | PARD6B | par-6 family cell polarity regulator beta [Source:HGNC Symbol;Acc:HGNC:16245] |
| ENSG00000124678 | HGLibA_48843 | 92 | -0.302521943 | TCP11 | t-complex 11 [Source:HGNC Symbol;Acc:HGNC:11658] |
| ENSG00000124942 | HGLibA_01301 | 92 | -0.302521943 | AHNAK | AHNAK nucleoprotein [Source:HGNC Symbol;Acc:HGNC:347] |
| ENSG00000125675 | HGLibA_20266 | 92 | -0.302521943 | GRIA3 | glutamate ionotropic receptor AMPA type subunit 3 [Source:HGNC Symbol;Acc:HGNC:4573] |
| ENSG00000125733 | HGLibA_51632 | 92 | -0.302521943 | TRIP10 | thyroid hormone receptor interactor 10 [Source:HGNC Symbol;Acc:HGNC:12304] |
| ENSG00000126215 | HGLibA_54589 | 92 | -0.302521943 | XRCC3 | X-ray repair cross complementing 3 [Source:HGNC Symbol;Acc:HGNC:12830] |
| ENSG00000126226 | HGLibA_35608 | 92 | -0.302521943 | PCID2 | PCI domain containing 2 [Source:HGNC Symbol;Acc:HGNC:25653] |
| ENSG00000128463 | HGLibA_15015 | 92 | -0.302521943 | EMC4 | ER membrane protein complex subunit 4 [Source:HGNC Symbol;Acc:HGNC:28032] |
| ENSG00000129116 | HGLibA_35063 | 92 | -0.302521943 | PALLD | cytoskeletal associated protein [Source:HGNC Symbol;Acc:HGNC:17068]" |
| ENSG00000129422 | HGLibA_30505 | 92 | -0.302521943 | MTUS1 | microtubule associated scaffold protein 1 [Source:HGNC Symbol;Acc:HGNC:29789] |
| ENSG00000130713 | HGLibA_15774 | 92 | -0.302521943 | EXOSC2 | exosome component 2 [Source:HGNC Symbol;Acc:HGNC:17097] |
| ENSG00000131096 | HGLibA_39712 | 92 | -0.302521943 | PYY | peptide YY [Source:HGNC Symbol;Acc:HGNC:9748] |
| ENSG00000131400 | HGLibA_31140 | 92 | -0.302521943 | NAPSA | napsin A aspartic peptidase [Source:HGNC Symbol;Acc:HGNC:13395] |
| ENSG00000131828 | HGLibA_35862 | 92 | -0.302521943 | PDHA1 | pyruvate dehydrogenase E1 alpha 1 subunit [Source:HGNC Symbol;Acc:HGNC:8806] |
| ENSG00000132481 | HGLibA_51515 | 92 | -0.302521943 | TRIM47 | tripartite motif containing 47 [Source:HGNC Symbol;Acc:HGNC:19020] |
| ENSG00000134115 | HGLibA_10416 | 92 | -0.302521943 | CNTN6 | contactin 6 [Source:HGNC Symbol;Acc:HGNC:2176] |
| ENSG00000135245 | HGLibA_21318 | 92 | -0.302521943 | HILPDA | hypoxia inducible lipid droplet associated [Source:HGNC Symbol;Acc:HGNC:28859] |
| ENSG00000136044 | HGLibA_02568 | 92 | -0.302521943 | APPL2 | phosphotyrosine interacting with PH domain and leucine zipper 2 [Source:HGNC Symbol;Acc:HGNC:18242]" |
| ENSG00000137171 | HGLibA_25036 | 92 | -0.302521943 | KLC4 | kinesin light chain 4 [Source:HGNC Symbol;Acc:HGNC:21624] |
| ENSG00000137574 | HGLibA_49277 | 92 | -0.302521943 | TGS1 | trimethylguanosine synthase 1 [Source:HGNC Symbol;Acc:HGNC:17843] |
| ENSG00000138031 | HGLibA_00915 | 92 | -0.302521943 | ADCY3 | adenylate cyclase 3 [Source:HGNC Symbol;Acc:HGNC:234] |
| ENSG00000138308 | HGLibA_36873 | 92 | -0.302521943 | PLA2G12B | phospholipase A2 group XIIB [Source:HGNC Symbol;Acc:HGNC:18555] |
| ENSG00000139112 | HGLibA_18336 | 92 | -0.302521943 | GABARAPL1 | GABA type A receptor associated protein like 1 [Source:HGNC Symbol;Acc:HGNC:4068] |
| ENSG00000139625 | HGLibA_28169 | 92 | -0.302521943 | MAP3K12 | mitogen-activated protein kinase kinase kinase 12 [Source:HGNC Symbol;Acc:HGNC:6851] |
| ENSG00000139899 | HGLibA_07413 | 92 | -0.302521943 | CBLN3 | cerebellin 3 precursor [Source:HGNC Symbol;Acc:HGNC:20146] |
| ENSG00000141084 | HGLibA_40194 | 92 | -0.302521943 | RANBP10 | RAN binding protein 10 [Source:HGNC Symbol;Acc:HGNC:29285] |
| ENSG00000141232 | HGLibA_50934 | 92 | -0.302521943 | TOB1 | 1 [Source:HGNC Symbol;Acc:HGNC:11979]" |
| ENSG00000141506 | HGLibA_36659 | 92 | -0.302521943 | PIK3R5 | phosphoinositide-3-kinase regulatory subunit 5 [Source:HGNC Symbol;Acc:HGNC:30035] |
| ENSG00000141985 | HGLibA_43947 | 92 | -0.302521943 | SH3GL1 | endophilin A2 [Source:HGNC Symbol;Acc:HGNC:10830]" |
| ENSG00000143553 | HGLibA_45905 | 92 | -0.302521943 | SNAPIN | SNAP associated protein [Source:HGNC Symbol;Acc:HGNC:17145] |
| ENSG00000144031 | HGLibA_02120 | 92 | -0.302521943 | ANKRD53 | ankyrin repeat domain 53 [Source:HGNC Symbol;Acc:HGNC:25691] |
| ENSG00000144134 | HGLibA_40028 | 92 | -0.302521943 | RABL2A | member of RAS oncogene family like 2A [Source:HGNC Symbol;Acc:HGNC:9799]" |

| | | | | | |
|---|---|---|---|---|---|
| ENSG00000144868 | HGLibA_49866 | 92 | -0.302521943 | TMEM108 | transmembrane protein 108 [Source:HGNC Symbol;Acc:HGNC:28451] |
| ENSG00000145040 | HGLibA_52825 | 92 | -0.302521943 | UCN2 | urocortin 2 [Source:HGNC Symbol;Acc:HGNC:18414] |
| ENSG00000147099 | HGLibA_21023 | 92 | -0.302521943 | HDAC8 | histone deacetylase 8 [Source:HGNC Symbol;Acc:HGNC:13315] |
| ENSG00000147789 | HGLibA_56570 | 92 | -0.302521943 | ZNF7 | zinc finger protein 7 [Source:HGNC Symbol;Acc:HGNC:13139] |
| ENSG00000147804 | HGLibA_45113 | 92 | -0.302521943 | SLC39A4 | solute carrier family 39 member 4 [Source:HGNC Symbol;Acc:HGNC:17129] |
| ENSG00000148814 | HGLibA_27296 | 92 | -0.302521943 | LRRC27 | leucine rich repeat containing 27 [Source:HGNC Symbol;Acc:HGNC:29346] |
| ENSG00000149090 | HGLibA_35089 | 92 | -0.302521943 | PAMR1 | peptidase domain containing associated with muscle regeneration 1 [Source:HGNC Symbol;Acc:HGNC:24554] |
| ENSG00000149131 | HGLibA_43591 | 92 | -0.302521943 | SERPING1 | serpin family G member 1 [Source:HGNC Symbol;Acc:HGNC:1228] |
| ENSG00000149179 | HGLibA_05148 | 92 | -0.302521943 | C11orf49 | chromosome 11 open reading frame 49 [Source:HGNC Symbol;Acc:HGNC:28720] |
| ENSG00000150048 | HGLibA_09955 | 92 | -0.302521943 | CLEC1A | C-type lectin domain family 1 member A [Source:HGNC Symbol;Acc:HGNC:24355] |
| ENSG00000150201 | HGLibA_18233 | 92 | -0.302521943 | FXYD4 | FXYD domain containing ion transport regulator 4 [Source:HGNC Symbol;Acc:HGNC:4028] |
| ENSG00000152409 | HGLibA_23969 | 92 | -0.302521943 | JMY | p53 cofactor [Source:HGNC Symbol;Acc:HGNC:28916]" |
| ENSG00000152611 | HGLibA_07201 | 92 | -0.302521943 | CAPSL | calcyphosine like [Source:HGNC Symbol;Acc:HGNC:28375] |
| ENSG00000152749 | HGLibA_19985 | 92 | -0.302521943 | GPR180 | G protein-coupled receptor 180 [Source:HGNC Symbol;Acc:HGNC:28899] |
| ENSG00000153930 | HGLibA_01955 | 92 | -0.302521943 | ANKFN1 | ankyrin repeat and fibronectin type III domain containing 1 [Source:HGNC Symbol;Acc:HGNC:26766] |
| ENSG00000154134 | HGLibA_41739 | 92 | -0.302521943 | ROBO3 | roundabout guidance receptor 3 [Source:HGNC Symbol;Acc:HGNC:13433] |
| ENSG00000157837 | HGLibA_46785 | 92 | -0.302521943 | SPPL3 | signal peptide peptidase like 3 [Source:HGNC Symbol;Acc:HGNC:30424] |
| ENSG00000160285 | HGLibA_27569 | 92 | -0.302521943 | LSS | lanosterol synthase [Source:HGNC Symbol;Acc:HGNC:6708] |
| ENSG00000160469 | HGLibA_04847 | 92 | -0.302521943 | BRSK1 | BR serine/threonine kinase 1 [Source:HGNC Symbol;Acc:HGNC:18994] |
| ENSG00000161217 | HGLibA_35700 | 92 | -0.302521943 | PCYT1A | alpha [Source:HGNC Symbol;Acc:HGNC:8754]" |
| ENSG00000162385 | HGLibA_28007 | 92 | -0.302521943 | MAGOH | exon junction complex subunit [Source:HGNC Symbol;Acc:HGNC:6815]" |
| ENSG00000163221 | HGLibA_42540 | 92 | -0.302521943 | S100A12 | S100 calcium binding protein A12 [Source:HGNC Symbol;Acc:HGNC:10489] |
| ENSG00000163539 | HGLibA_09797 | 92 | -0.302521943 | CLASP2 | cytoplasmic linker associated protein 2 [Source:HGNC Symbol;Acc:HGNC:17078] |
| ENSG00000163581 | HGLibA_44871 | 92 | -0.302521943 | SLC2A2 | solute carrier family 2 member 2 [Source:HGNC Symbol;Acc:HGNC:11006] |
| ENSG00000164124 | HGLibA_49970 | 92 | -0.302521943 | TMEM144 | transmembrane protein 144 [Source:HGNC Symbol;Acc:HGNC:25633] |
| ENSG00000164265 | HGLibA_42934 | 92 | -0.302521943 | SCGB3A2 | secretoglobin family 3A member 2 [Source:HGNC Symbol;Acc:HGNC:18391] |
| ENSG00000164434 | HGLibA_15929 | 92 | -0.302521943 | FABP7 | fatty acid binding protein 7 [Source:HGNC Symbol;Acc:HGNC:3562] |
| ENSG00000164849 | HGLibA_19919 | 92 | -0.302521943 | GPR146 | G protein-coupled receptor 146 [Source:HGNC Symbol;Acc:HGNC:21718] |
| ENSG00000165029 | HGLibA_00095 | 92 | -0.302521943 | ABCA1 | ATP binding cassette subfamily A member 1 [Source:HGNC Symbol;Acc:HGNC:29] |
| ENSG00000165168 | HGLibA_12018 | 92 | -0.302521943 | CYBB | cytochrome b-245 beta chain [Source:HGNC Symbol;Acc:HGNC:2578] |
| ENSG00000165312 | HGLibA_34760 | 92 | -0.302521943 | OTUD1 | OTU deubiquitinase 1 [Source:HGNC Symbol;Acc:HGNC:27346] |
| ENSG00000165621 | HGLibA_34821 | 92 | -0.302521943 | OXGR1 | oxoglutarate receptor 1 [Source:HGNC Symbol;Acc:HGNC:4531] |
| ENSG00000166173 | HGLibA_26039 | 92 | -0.302521943 | LARP6 | La ribonucleoprotein domain family member 6 [Source:HGNC Symbol;Acc:HGNC:24012] |
| ENSG00000166451 | HGLibA_09074 | 92 | -0.302521943 | CENPN | centromere protein N [Source:HGNC Symbol;Acc:HGNC:30873] |
| ENSG00000166831 | HGLibA_40656 | 92 | -0.302521943 | RBPMS2 | mRNA processing factor 2 [Source:HGNC Symbol;Acc:HGNC:19098]" |
| ENSG00000167325 | HGLibA_42250 | 92 | -0.302521943 | RRM1 | ribonucleotide reductase catalytic subunit M1 [Source:HGNC Symbol;Acc:HGNC:10451] |
| ENSG00000167815 | HGLibA_38411 | 92 | -0.302521943 | PRDX2 | peroxiredoxin 2 [Source:HGNC Symbol;Acc:HGNC:9353] |
| ENSG00000168158 | HGLibA_33777 | 92 | -0.302521943 | OR2C1 | olfactory receptor family 2 subfamily C member 1 [Source:HGNC Symbol;Acc:HGNC:8242] |
| ENSG00000168907 | HGLibA_36914 | 92 | -0.302521943 | PLA2G4F | phospholipase A2 group IVF [Source:HGNC Symbol;Acc:HGNC:27396] |
| ENSG00000169214 | HGLibA_34416 | 92 | -0.302521943 | OR6F1 | olfactory receptor family 6 subfamily F member 1 [Source:HGNC Symbol;Acc:HGNC:15027] |
| ENSG00000169857 | HGLibA_03888 | 92 | -0.302521943 | AVEN | apoptosis and caspase activation inhibitor [Source:HGNC Symbol;Acc:HGNC:13509] |
| ENSG00000170035 | HGLibA_52601 | 92 | -0.302521943 | UBE2E3 | ubiquitin conjugating enzyme E2 E3 [Source:HGNC Symbol;Acc:HGNC:12479] |
| ENSG00000170925 | HGLibA_49061 | 92 | -0.302521943 | TEX13B | testis expressed 13B [Source:HGNC Symbol;Acc:HGNC:11736] |
| ENSG00000171403 | HGLibA_25576 | 92 | -0.302521943 | KRT9 | keratin 9 [Source:HGNC Symbol;Acc:HGNC:6447] |
| ENSG00000172468 | HGLibA_22241 | 92 | -0.302521943 | HSFY1 | heat shock transcription factor Y-linked 1 [Source:HGNC Symbol;Acc:HGNC:18568] |
| ENSG00000173230 | HGLibA_19649 | 92 | -0.302521943 | GOLGB1 | golgin B1 [Source:HGNC Symbol;Acc:HGNC:4429] |
| ENSG00000173930 | HGLibA_45473 | 92 | -0.302521943 | SLCO4C1 | solute carrier organic anion transporter family member 4C1 [Source:HGNC Symbol;Acc:HGNC:23612] |
| ENSG00000174156 | HGLibA_20457 | 92 | -0.302521943 | GSTA3 | glutathione S-transferase alpha 3 [Source:HGNC Symbol;Acc:HGNC:4628] |
| ENSG00000175354 | HGLibA_39448 | 92 | -0.302521943 | PTPN2 | non-receptor type 2 [Source:HGNC Symbol;Acc:HGNC:9650]" |
| ENSG00000177752 | HGLibA_54688 | 92 | -0.302521943 | YIPF7 | Yip1 domain family member 7 [Source:HGNC Symbol;Acc:HGNC:26825] |
| ENSG00000178184 | HGLibA_35191 | 92 | -0.302521943 | PARD6G | par-6 family cell polarity regulator gamma [Source:HGNC Symbol;Acc:HGNC:16076] |
| ENSG00000178386 | HGLibA_55616 | 92 | -0.302521943 | ZNF223 | zinc finger protein 223 [Source:HGNC Symbol;Acc:HGNC:13016] |
| ENSG00000179213 | HGLibA_44156 | 92 | -0.302521943 | SIGLECL1 | SIGLEC family like 1 [Source:HGNC Symbol;Acc:HGNC:26856] |
| ENSG00000179331 | HGLibA_39900 | 92 | -0.302521943 | RAB39A | member RAS oncogene family [Source:HGNC Symbol;Acc:HGNC:16521]" |
| ENSG00000179593 | HGLibA_01684 | 92 | -0.302521943 | ALOX15B | type B [Source:HGNC Symbol;Acc:HGNC:434]" |
| ENSG00000179776 | HGLibA_08684 | 92 | -0.302521943 | CDH5 | cadherin 5 [Source:HGNC Symbol;Acc:HGNC:1764] |
| ENSG00000180353 | HGLibA_20971 | 92 | -0.302521943 | HCLS1 | hematopoietic cell-specific Lyn substrate 1 [Source:HGNC Symbol;Acc:HGNC:4844] |
| ENSG00000180660 | HGLibA_27821 | 92 | -0.302521943 | MAB21L1 | mab-21 like 1 [Source:HGNC Symbol;Acc:HGNC:6757] |
| ENSG00000181322 | HGLibA_32178 | 92 | -0.302521943 | NME9 | NME/NM23 family member 9 [Source:HGNC Symbol;Acc:HGNC:21343] |
| ENSG00000181371 | HGLibA_34340 | 92 | -0.302521943 | OR5M8 | olfactory receptor family 5 subfamily M member 8 [Source:HGNC Symbol;Acc:HGNC:14846] |
| ENSG00000182324 | HGLibA_24262 | 92 | -0.302521943 | KCNJ14 | potassium voltage-gated channel subfamily J member 14 [Source:HGNC Symbol;Acc:HGNC:6260] |
| ENSG00000183134 | HGLibA_39308 | 92 | -0.302521943 | PTGDR2 | prostaglandin D2 receptor 2 [Source:HGNC Symbol;Acc:HGNC:4502] |
| ENSG00000183628 | HGLibA_13050 | 92 | -0.302521943 | DGCR6 | DiGeorge syndrome critical region gene 6 [Source:HGNC Symbol;Acc:HGNC:2846] |
| ENSG00000184471 | HGLibA_05958 | 92 | -0.302521943 | C1QTNF8 | C1q and TNF related 8 [Source:HGNC Symbol;Acc:HGNC:31374] |
| ENSG00000184719 | HGLibA_41715 | 92 | -0.302521943 | RNLS | FAD dependent amine oxidase [Source:HGNC Symbol;Acc:HGNC:25641]" |
| ENSG00000184988 | HGLibA_49854 | 92 | -0.302521943 | TMEM106A | transmembrane protein 106A [Source:HGNC Symbol;Acc:HGNC:28288] |
| ENSG00000185056 | HGLibA_06392 | 92 | -0.302521943 | C5orf47 | chromosome 5 open reading frame 47 [Source:HGNC Symbol;Acc:HGNC:27026] |
| ENSG00000187268 | HGLibA_16823 | 92 | -0.302521943 | FAM9C | family with sequence similarity 9 member C [Source:HGNC Symbol;Acc:HGNC:18405] |
| ENSG00000187766 | HGLibA_25621 | 92 | -0.302521943 | KRTAP10-8 | keratin associated protein 10-8 [Source:HGNC Symbol;Acc:HGNC:20525] |
| ENSG00000187955 | HGLibA_10512 | 92 | -0.302521943 | COL14A1 | collagen type XIV alpha 1 chain [Source:HGNC Symbol;Acc:HGNC:2191] |
| ENSG00000187987 | HGLibA_57007 | 92 | -0.302521943 | ZSCAN23 | zinc finger and SCAN domain containing 23 [Source:HGNC Symbol;Acc:HGNC:21193] |
| ENSG00000188037 | HGLibA_09819 | 92 | -0.302521943 | CLCN1 | chloride voltage-gated channel 1 [Source:HGNC Symbol;Acc:HGNC:2019] |
| ENSG00000188295 | HGLibA_56498 | 92 | -0.302521943 | ZNF669 | zinc finger protein 669 [Source:HGNC Symbol;Acc:HGNC:25736] |
| ENSG00000188488 | HGLibA_43523 | 92 | -0.302521943 | SERPINA5 | serpin family A member 5 [Source:HGNC Symbol;Acc:HGNC:8723] |
| ENSG00000188732 | HGLibA_16515 | 92 | -0.302521943 | FAM221A | family with sequence similarity 221 member A [Source:HGNC Symbol;Acc:HGNC:27977] |
| ENSG00000189099 | HGLibA_38949 | 92 | -0.302521943 | PRSS48 | serine protease 48 [Source:HGNC Symbol;Acc:HGNC:24635] |
| ENSG00000196116 | HGLibA_48913 | 92 | -0.302521943 | TDRD7 | tudor domain containing 7 [Source:HGNC Symbol;Acc:HGNC:30831] |
| ENSG00000196214 | HGLibA_56695 | 92 | -0.302521943 | ZNF766 | zinc finger protein 766 [Source:HGNC Symbol;Acc:HGNC:28063] |
| ENSG00000196313 | HGLibA_37642 | 92 | -0.302521943 | POM121 | POM121 transmembrane nucleoporin [Source:HGNC Symbol;Acc:HGNC:19702] |
| ENSG00000196476 | HGLibA_06033 | 92 | -0.302521943 | C20orf96 | chromosome 20 open reading frame 96 [Source:HGNC Symbol;Acc:HGNC:16227] |
| ENSG00000196503 | HGLibA_03001 | 92 | -0.302521943 | ARL9 | ADP ribosylation factor like GTPase 9 [Source:HGNC Symbol;Acc:HGNC:23592] |
| ENSG00000196578 | HGLibA_34234 | 92 | -0.302521943 | OR5AC2 | olfactory receptor family 5 subfamily AC member 2 [Source:HGNC Symbol;Acc:HGNC:15431] |
| ENSG00000197894 | HGLibA_00958 | 92 | -0.302521943 | ADH5 | chi polypeptide [Source:HGNC Symbol;Acc:HGNC:253]" |
| ENSG00000198399 | HGLibA_23873 | 92 | -0.302521943 | ITSN2 | intersectin 2 [Source:HGNC Symbol;Acc:HGNC:6184] |
| ENSG00000198515 | HGLibA_10263 | 92 | -0.302521943 | CNGA1 | cyclic nucleotide gated channel alpha 1 [Source:HGNC Symbol;Acc:HGNC:2148] |
| ENSG00000198546 | HGLibA_56128 | 92 | -0.302521943 | ZNF511 | zinc finger protein 511 [Source:HGNC Symbol;Acc:HGNC:28445] |
| ENSG00000198753 | HGLibA_37275 | 92 | -0.302521943 | PLXNB3 | plexin B3 [Source:HGNC Symbol;Acc:HGNC:9105] |
| ENSG00000198915 | HGLibA_40330 | 92 | -0.302521943 | RASGEF1A | RasGEF domain family member 1A [Source:HGNC Symbol;Acc:HGNC:24246] |
| ENSG00000203667 | HGLibA_10828 | 92 | -0.302521943 | COX20 | cytochrome c oxidase assembly factor COX20 [Source:HGNC Symbol;Acc:HGNC:26970] |
| ENSG00000203780 | HGLibA_16864 | 92 | -0.302521943 | FANK1 | fibronectin type III and ankyrin repeat domains 1 [Source:HGNC Symbol;Acc:HGNC:23527] |
| ENSG00000204544 | HGLibA_30541 | 92 | -0.302521943 | MUC21 | cell surface associated [Source:HGNC Symbol;Acc:HGNC:21661]" |
| ENSG00000204576 | HGLibA_38836 | 92 | -0.302521943 | PRR3 | proline rich 3 [Source:HGNC Symbol;Acc:HGNC:21149] |
| ENSG00000204969 | HGLibA_35439 | 92 | -0.302521943 | PCDHA2 | protocadherin alpha 2 [Source:HGNC Symbol;Acc:HGNC:8668] |
| ENSG00000205899 | HGLibA_04471 | 92 | -0.302521943 | BHLHA9 | basic helix-loop-helix family member a9 [Source:HGNC Symbol;Acc:HGNC:35126] |
| ENSG00000213231 | HGLibA_48818 | 92 | -0.302521943 | TCL1B | T cell leukemia/lymphoma 1B [Source:HGNC Symbol;Acc:HGNC:11649] |
| ENSG00000213903 | HGLibA_27583 | 92 | -0.302521943 | LTB4R | leukotriene B4 receptor [Source:HGNC Symbol;Acc:HGNC:6713] |
| ENSG00000223802 | HGLibA_09200 | 92 | -0.302521943 | CERS1 | ceramide synthase 1 [Source:HGNC Symbol;Acc:HGNC:14253] |
| ENSG00000229474 | HGLibA_35286 | 92 | -0.302521943 | PATL2 | PAT1 homolog 2 [Source:HGNC Symbol;Acc:HGNC:33630] |
| ENSG00000242419 | HGLibA_35573 | 92 | -0.302521943 | PCDHGC4 | 4 [Source:HGNC Symbol;Acc:HGNC:8717]" |
| ENSG00000248712 | HGLibA_07653 | 92 | -0.302521943 | CCDC153 | coiled-coil domain containing 153 [Source:HGNC Symbol;Acc:HGNC:27446] |
| ENSG00000249115 | HGLibA_20886 | 92 | -0.302521943 | HAUS5 | HAUS augmin like complex subunit 5 [Source:HGNC Symbol;Acc:HGNC:29130] |
| ENSG00000250868 | HGLibA_54542 | 92 | -0.302521943 | XKRY | Y-linked [Source:HGNC Symbol;Acc:HGNC:18571]" |
| ENSG00000269699 | HGLibA_55338 | 92 | -0.302521943 | ZIM2 | zinc finger imprinted 2 [Source:HGNC Symbol;Acc:HGNC:12875] |
| ENSG00000276547 | HGLibA_35562 | 92 | -0.302521943 | PCDHGB5 | 5 [Source:HGNC Symbol;Acc:HGNC:8712]" |
| ENSG00000284723 | HGLibA_34552 | 92 | -0.302521943 | OR8S1 | olfactory receptor family 8 subfamily S member 1 [Source:HGNC Symbol;Acc:HGNC:19628] |
| ENSG00000288702 | HGLibA_52881 | 92 | -0.302521943 | UGT1A3 | - |
| ENSG00000288709 | HGLibA_15889 | 92 | -0.302521943 | F8A2 | - |
| FAM49A | HGLibA_16624 | 92 | -0.302521943 | FAM49A | - |
| FAM96B | HGLibA_16806 | 92 | -0.302521943 | FAM96B | - |
| GPR64 | HGLibA_20070 | 92 | -0.302521943 | GPR64 | - |
| hsa-mir-103b-1 | HGLibA_57171 | 92 | -0.302521943 | hsa-mir-103b-1 | - |
| hsa-mir-105-1 | HGLibA_57179 | 92 | -0.302521943 | hsa-mir-105-1 | - |
| hsa-mir-1-1 | HGLibA_57142 | 92 | -0.302521943 | hsa-mir-1-1 | - |
| hsa-mir-1471 | HGLibA_57868 | 92 | -0.302521943 | hsa-mir-1471 | - |
| hsa-mir-2355 | HGLibA_58319 | 92 | -0.302521943 | hsa-mir-2355 | - |
| hsa-mir-3160-2 | HGLibA_58718 | 92 | -0.302521943 | hsa-mir-3160-2 | - |
| hsa-mir-3618 | HGLibA_59101 | 92 | -0.302521943 | hsa-mir-3618 | - |
| hsa-mir-367 | HGLibA_59230 | 92 | -0.302521943 | hsa-mir-367 | - |
| hsa-mir-3682 | HGLibA_59280 | 92 | -0.302521943 | hsa-mir-3682 | - |
| hsa-mir-3907 | HGLibA_59492 | 92 | -0.302521943 | hsa-mir-3907 | - |
| hsa-mir-3914-2 | HGLibA_59532 | 92 | -0.302521943 | hsa-mir-3914-2 | - |
| hsa-mir-3973 | HGLibA_59651 | 92 | -0.302521943 | hsa-mir-3973 | - |
| hsa-mir-4258 | HGLibA_59738 | 92 | -0.302521943 | hsa-mir-4258 | - |
| hsa-mir-4315-1 | HGLibA_59974 | 92 | -0.302521943 | hsa-mir-4315-1 | - |
| hsa-mir-4479 | HGLibA_60331 | 92 | -0.302521943 | hsa-mir-4479 | - |
| hsa-mir-4492 | HGLibA_60386 | 92 | -0.302521943 | hsa-mir-4492 | - |
| hsa-mir-4688 | HGLibA_60863 | 92 | -0.302521943 | hsa-mir-4688 | - |
| hsa-mir-4708 | HGLibA_60933 | 92 | -0.302521943 | hsa-mir-4708 | - |
| hsa-mir-4727 | HGLibA_61006 | 92 | -0.302521943 | hsa-mir-4727 | - |
| hsa-mir-4733 | HGLibA_61030 | 92 | -0.302521943 | hsa-mir-4733 | - |
| hsa-mir-4757 | HGLibA_61126 | 92 | -0.302521943 | hsa-mir-4757 | - |
| hsa-mir-500a | HGLibA_61442 | 92 | -0.302521943 | hsa-mir-500a | - |
| hsa-mir-520a | HGLibA_61734 | 92 | -0.302521943 | hsa-mir-520a | - |
| hsa-mir-545 | HGLibA_61832 | 92 | -0.302521943 | hsa-mir-545 | - |
| hsa-mir-548v | HGLibA_62076 | 92 | -0.302521943 | hsa-mir-548v | - |

| | | | | | |
|---|---|---|---|---|---|
| hsa-mir-5571 | HGLibA_62149 | 92 | -0.302521943 | hsa-mir-5571 | - |
| hsa-mir-619 | HGLibA_62714 | 92 | -0.302521943 | hsa-mir-619 | - |
| hsa-mir-6770-2 | HGLibA_63250 | 92 | -0.302521943 | hsa-mir-6770-2 | - |
| hsa-mir-6779 | HGLibA_63288 | 92 | -0.302521943 | hsa-mir-6779 | - |
| hsa-mir-6852 | HGLibA_63582 | 92 | -0.302521943 | hsa-mir-6852 | - |
| hsa-mir-7843 | HGLibA_63960 | 92 | -0.302521943 | hsa-mir-7843 | - |
| hsa-mir-8066 | HGLibA_64103 | 92 | -0.302521943 | hsa-mir-8066 | - |
| hsa-mir-8076 | HGLibA_64147 | 92 | -0.302521943 | hsa-mir-8076 | - |
| LOC100129520 | HGLibA_26765 | 92 | -0.302521943 | LOC100129520 | - |
| LOC256021 | HGLibA_26906 | 92 | -0.302521943 | LOC256021 | - |
| PNMAL1 | HGLibA_37383 | 92 | -0.302521943 | PNMAL1 | - |
| PROSC | HGLibA_38691 | 92 | -0.302521943 | PROSC | - |
| PVRL2 | HGLibA_39625 | 92 | -0.302521943 | PVRL2 | - |
| 6-Sep | HGLibA_43448 | 92 | -0.302521943 | 6-Sep | - |
| SHOX_Y | HGLibA_44072 | 92 | -0.302521943 | SHOX_Y | - |
| SLC22A20 | HGLibA_44570 | 92 | -0.302521943 | SLC22A20 | - |
| TMEM180 | HGLibA_50097 | 92 | -0.302521943 | TMEM180 | - |
| TMEM55A | HGLibA_50445 | 92 | -0.302521943 | TMEM55A | - |
| VAMP7_X | HGLibA_53502 | 92 | -0.302521943 | VAMP7_X | - |
| AKAP17A_X | HGLibA_01420 | 91 | -0.311567285 | AKAP17A_X | - |
| ASNA1 | HGLibA_03333 | 91 | -0.311567285 | ASNA1 | - |
| C11orf85 | HGLibA_05199 | 91 | -0.311567285 | C11orf85 | - |
| C9orf170 | HGLibA_06707 | 91 | -0.311567285 | C9orf170 | - |
| C9orf96 | HGLibA_06782 | 91 | -0.311567285 | C9orf96 | - |
| CECR1 | HGLibA_08974 | 91 | -0.311567285 | CECR1 | - |
| ELMSAN1 | HGLibA_14947 | 91 | -0.311567285 | ELMSAN1 | - |
| ENSG00000002549 | HGLibA_26013 | 91 | -0.311567285 | LAP3 | leucine aminopeptidase 3 [Source:HGNC Symbol;Acc:HGNC:18449] |
| ENSG00000007080 | HGLibA_07574 | 91 | -0.311567285 | CCDC124 | coiled-coil domain containing 124 [Source:HGNC Symbol;Acc:HGNC:25171] |
| ENSG00000007952 | HGLibA_32371 | 91 | -0.311567285 | NOX1 | NADPH oxidase 1 [Source:HGNC Symbol;Acc:HGNC:7889] |
| ENSG00000039537 | HGLibA_06424 | 91 | -0.311567285 | C6 | complement C6 [Source:HGNC Symbol;Acc:HGNC:1339] |
| ENSG00000043093 | HGLibA_12565 | 91 | -0.311567285 | DCUN1D1 | defective in cullin neddylation 1 domain containing 1 [Source:HGNC Symbol;Acc:HGNC:18184] |
| ENSG00000052749 | HGLibA_42270 | 91 | -0.311567285 | RRP12 | ribosomal RNA processing 12 homolog [Source:HGNC Symbol;Acc:HGNC:29100] |
| ENSG00000061794 | HGLibA_30098 | 91 | -0.311567285 | MRPS35 | mitochondrial ribosomal protein S35 [Source:HGNC Symbol;Acc:HGNC:16635] |
| ENSG00000068001 | HGLibA_22440 | 91 | -0.311567285 | HYAL2 | hyaluronidase 2 [Source:HGNC Symbol;Acc:HGNC:5321] |
| ENSG00000074527 | HGLibA_32859 | 91 | -0.311567285 | NTN4 | netrin 4 [Source:HGNC Symbol;Acc:HGNC:13658] |
| ENSG00000074695 | HGLibA_26657 | 91 | -0.311567285 | LMAN1 | mannose binding 1 [Source:HGNC Symbol;Acc:HGNC:6631]" |
| ENSG00000075275 | HGLibA_09023 | 91 | -0.311567285 | CELSR1 | cadherin EGF LAG seven-pass G-type receptor 1 [Source:HGNC Symbol;Acc:HGNC:1850] |
| ENSG00000077150 | HGLibA_31817 | 91 | -0.311567285 | NFKB2 | nuclear factor kappa B subunit 2 [Source:HGNC Symbol;Acc:HGNC:7795] |
| ENSG00000081189 | HGLibA_28837 | 91 | -0.311567285 | MEF2C | myocyte enhancer factor 2C [Source:HGNC Symbol;Acc:HGNC:6996] |
| ENSG00000082258 | HGLibA_08130 | 91 | -0.311567285 | CCNT2 | cyclin T2 [Source:HGNC Symbol;Acc:HGNC:1600] |
| ENSG00000085365 | HGLibA_42835 | 91 | -0.311567285 | SCAMP1 | secretory carrier membrane protein 1 [Source:HGNC Symbol;Acc:HGNC:10563] |
| ENSG00000085514 | HGLibA_36670 | 91 | -0.311567285 | PILRA | paired immunoglobin like type 2 receptor alpha [Source:HGNC Symbol;Acc:HGNC:20396] |
| ENSG00000085733 | HGLibA_11741 | 91 | -0.311567285 | CTTN | cortactin [Source:HGNC Symbol;Acc:HGNC:3338] |
| ENSG00000090470 | HGLibA_35745 | 91 | -0.311567285 | PDCD7 | programmed cell death 7 [Source:HGNC Symbol;Acc:HGNC:8767] |
| ENSG00000091010 | HGLibA_37782 | 91 | -0.311567285 | POU4F3 | POU class 4 homeobox 3 [Source:HGNC Symbol;Acc:HGNC:9220] |
| ENSG00000100053 | HGLibA_11266 | 91 | -0.311567285 | CRYBB3 | crystallin beta B3 [Source:HGNC Symbol;Acc:HGNC:2400] |
| ENSG00000100348 | HGLibA_52403 | 91 | -0.311567285 | TXN2 | thioredoxin 2 [Source:HGNC Symbol;Acc:HGNC:17772] |
| ENSG00000101439 | HGLibA_11454 | 91 | -0.311567285 | CST3 | cystatin C [Source:HGNC Symbol;Acc:HGNC:2475] |
| ENSG00000101447 | HGLibA_16748 | 91 | -0.311567285 | FAM83D | family with sequence similarity 83 member D [Source:HGNC Symbol;Acc:HGNC:16122] |
| ENSG00000102007 | HGLibA_37219 | 91 | -0.311567285 | PLP2 | proteolipid protein 2 [Source:HGNC Symbol;Acc:HGNC:9087] |
| ENSG00000102100 | HGLibA_44950 | 91 | -0.311567285 | SLC35A2 | solute carrier family 35 member A2 [Source:HGNC Symbol;Acc:HGNC:11022] |
| ENSG00000102225 | HGLibA_08742 | 91 | -0.311567285 | CDK16 | cyclin dependent kinase 16 [Source:HGNC Symbol;Acc:HGNC:8749] |
| ENSG00000105298 | HGLibA_06952 | 91 | -0.311567285 | CACTIN | spliceosome C complex subunit [Source:HGNC Symbol;Acc:HGNC:29938]" |
| ENSG00000105374 | HGLibA_32013 | 91 | -0.311567285 | NKG7 | natural killer cell granule protein 7 [Source:HGNC Symbol;Acc:HGNC:7830] |
| ENSG00000106384 | HGLibA_29630 | 91 | -0.311567285 | MOGAT3 | monoacylglycerol O-acyltransferase 3 [Source:HGNC Symbol;Acc:HGNC:23249] |
| ENSG00000107890 | HGLibA_02049 | 91 | -0.311567285 | ANKRD26 | ankyrin repeat domain 26 [Source:HGNC Symbol;Acc:HGNC:29186] |
| ENSG00000108469 | HGLibA_40772 | 91 | -0.311567285 | RECQL5 | RecQ like helicase 5 [Source:HGNC Symbol;Acc:HGNC:9950] |
| ENSG00000108839 | HGLibA_01676 | 91 | -0.311567285 | ALOX12 | 12S type [Source:HGNC Symbol;Acc:HGNC:429]" |
| ENSG00000109458 | HGLibA_18321 | 91 | -0.311567285 | GAB1 | GRB2 associated binding protein 1 [Source:HGNC Symbol;Acc:HGNC:4066] |
| ENSG00000110848 | HGLibA_08432 | 91 | -0.311567285 | CD69 | CD69 molecule [Source:HGNC Symbol;Acc:HGNC:1694] |
| ENSG00000111300 | HGLibA_30985 | 91 | -0.311567285 | NAA25 | NatB auxiliary subunit [Source:HGNC Symbol;Acc:HGNC:25783]" |
| ENSG00000112238 | HGLibA_38374 | 91 | -0.311567285 | PRDM13 | PR/SET domain 13 [Source:HGNC Symbol;Acc:HGNC:13998] |
| ENSG00000115207 | HGLibA_20582 | 91 | -0.311567285 | GTF3C2 | general transcription factor IIIC subunit 2 [Source:HGNC Symbol;Acc:HGNC:4665] |
| ENSG00000116604 | HGLibA_28842 | 91 | -0.311567285 | MEF2D | myocyte enhancer factor 2D [Source:HGNC Symbol;Acc:HGNC:6997] |
| ENSG00000117016 | HGLibA_41283 | 91 | -0.311567285 | RIMS3 | regulating synaptic membrane exocytosis 3 [Source:HGNC Symbol;Acc:HGNC:21292] |
| ENSG00000117419 | HGLibA_15485 | 91 | -0.311567285 | ERI3 | ERI1 exoribonuclease family member 3 [Source:HGNC Symbol;Acc:HGNC:17276] |
| ENSG00000117640 | HGLibA_30365 | 91 | -0.311567285 | MTFR1L | mitochondrial fission regulator 1 like [Source:HGNC Symbol;Acc:HGNC:28836] |
| ENSG00000118777 | HGLibA_00218 | 91 | -0.311567285 | ABCG2 | ATP binding cassette subfamily G member 2 (Junior blood group) [Source:HGNC Symbol;Acc:HGNC:74] |
| ENSG00000118960 | HGLibA_22114 | 91 | -0.311567285 | HS1BP3 | HCLS1 binding protein 3 [Source:HGNC Symbol;Acc:HGNC:24979] |
| ENSG00000119547 | HGLibA_33395 | 91 | -0.311567285 | ONECUT2 | one cut homeobox 2 [Source:HGNC Symbol;Acc:HGNC:8139] |
| ENSG00000120256 | HGLibA_27202 | 91 | -0.311567285 | LRP11 | LDL receptor related protein 11 [Source:HGNC Symbol;Acc:HGNC:16936] |
| ENSG00000121578 | HGLibA_04032 | 91 | -0.311567285 | B4GALT4 | 4-galactosyltransferase 4 [Source:HGNC Symbol;Acc:HGNC:927]" |
| ENSG00000123838 | HGLibA_06291 | 91 | -0.311567285 | C4BPA | complement component 4 binding protein alpha [Source:HGNC Symbol;Acc:HGNC:1325] |
| ENSG00000124157 | HGLibA_43381 | 91 | -0.311567285 | SEMG2 | semenogelin 2 [Source:HGNC Symbol;Acc:HGNC:10743] |
| ENSG00000124570 | HGLibA_43558 | 91 | -0.311567285 | SERPINB6 | serpin family B member 6 [Source:HGNC Symbol;Acc:HGNC:8950] |
| ENSG00000125648 | HGLibA_44677 | 91 | -0.311567285 | SLC25A23 | solute carrier family 25 member 23 [Source:HGNC Symbol;Acc:HGNC:19375] |
| ENSG00000125726 | HGLibA_08437 | 91 | -0.311567285 | CD70 | CD70 molecule [Source:HGNC Symbol;Acc:HGNC:11937] |
| ENSG00000126016 | HGLibA_01815 | 91 | -0.311567285 | AMOT | angiomotin [Source:HGNC Symbol;Acc:HGNC:17810] |
| ENSG00000127951 | HGLibA_17465 | 91 | -0.311567285 | FGL2 | fibrinogen like 2 [Source:HGNC Symbol;Acc:HGNC:3696] |
| ENSG00000128383 | HGLibA_02472 | 91 | -0.311567285 | APOBEC3A | apolipoprotein B mRNA editing enzyme catalytic subunit 3A [Source:HGNC Symbol;Acc:HGNC:17343] |
| ENSG00000128791 | HGLibA_52384 | 91 | -0.311567285 | TWSG1 | twisted gastrulation BMP signaling modulator 1 [Source:HGNC Symbol;Acc:HGNC:12429] |
| ENSG00000129437 | HGLibA_25247 | 91 | -0.311567285 | KLK14 | kallikrein related peptidase 14 [Source:HGNC Symbol;Acc:HGNC:6362] |
| ENSG00000130032 | HGLibA_38875 | 91 | -0.311567285 | PRRG3 | proline rich and Gla domain 3 [Source:HGNC Symbol;Acc:HGNC:30798] |
| ENSG00000130303 | HGLibA_04885 | 91 | -0.311567285 | BST2 | bone marrow stromal cell antigen 2 [Source:HGNC Symbol;Acc:HGNC:1119] |
| ENSG00000130332 | HGLibA_27550 | 91 | -0.311567285 | LSM7 | U6 small nuclear RNA and mRNA degradation associated [Source:HGNC Symbol;Acc:HGNC:20470]" |
| ENSG00000130402 | HGLibA_00645 | 91 | -0.311567285 | ACTN4 | actinin alpha 4 [Source:HGNC Symbol;Acc:HGNC:166] |
| ENSG00000131095 | HGLibA_18935 | 91 | -0.311567285 | GFAP | glial fibrillary acidic protein [Source:HGNC Symbol;Acc:HGNC:4235] |
| ENSG00000132429 | HGLibA_37703 | 91 | -0.311567285 | POPDC3 | popeye domain containing 3 [Source:HGNC Symbol;Acc:HGNC:17649] |
| ENSG00000134061 | HGLibA_08259 | 91 | -0.311567285 | CD180 | CD180 molecule [Source:HGNC Symbol;Acc:HGNC:6726] |
| ENSG00000135617 | HGLibA_38266 | 91 | -0.311567285 | PRADC1 | protease associated domain containing 1 [Source:HGNC Symbol;Acc:HGNC:16047] |
| ENSG00000136986 | HGLibA_12994 | 91 | -0.311567285 | DERL1 | derlin 1 [Source:HGNC Symbol;Acc:HGNC:28454] |
| ENSG00000137200 | HGLibA_10234 | 91 | -0.311567285 | CMTR1 | cap methyltransferase 1 [Source:HGNC Symbol;Acc:HGNC:21077] |
| ENSG00000138079 | HGLibA_45131 | 91 | -0.311567285 | SLC3A1 | solute carrier family 3 member 1 [Source:HGNC Symbol;Acc:HGNC:11025] |
| ENSG00000138768 | HGLibA_53189 | 91 | -0.311567285 | USO1 | USO1 vesicle transport factor [Source:HGNC Symbol;Acc:HGNC:30904] |
| ENSG00000138769 | HGLibA_08809 | 91 | -0.311567285 | CDKL2 | cyclin dependent kinase like 2 [Source:HGNC Symbol;Acc:HGNC:1782] |
| ENSG00000140675 | HGLibA_45265 | 91 | -0.311567285 | SLC5A2 | solute carrier family 5 member 2 [Source:HGNC Symbol;Acc:HGNC:11037] |
| ENSG00000141316 | HGLibA_46326 | 91 | -0.311567285 | SPACA3 | sperm acrosome associated 3 [Source:HGNC Symbol;Acc:HGNC:16260] |
| ENSG00000142082 | HGLibA_44221 | 91 | -0.311567285 | SIRT3 | sirtuin 3 [Source:HGNC Symbol;Acc:HGNC:14931] |
| ENSG00000143458 | HGLibA_18354 | 91 | -0.311567285 | GABPB2 | GA binding protein transcription factor subunit beta 2 [Source:HGNC Symbol;Acc:HGNC:28441] |
| ENSG00000143552 | HGLibA_33041 | 91 | -0.311567285 | NUP210L | nucleoporin 210 like [Source:HGNC Symbol;Acc:HGNC:29915] |
| ENSG00000144655 | HGLibA_11423 | 91 | -0.311567285 | CSRNP1 | cysteine and serine rich nuclear protein 1 [Source:HGNC Symbol;Acc:HGNC:14300] |
| ENSG00000146707 | HGLibA_37676 | 91 | -0.311567285 | POMZP3 | POM121 and ZP3 fusion [Source:HGNC Symbol;Acc:HGNC:9203] |
| ENSG00000147155 | HGLibA_14373 | 91 | -0.311567285 | EBP | cholestenol delta-isomerase [Source:HGNC Symbol;Acc:HGNC:3133]" |
| ENSG00000148444 | HGLibA_10660 | 91 | -0.311567285 | COMMD3 | COMM domain containing 3 [Source:HGNC Symbol;Acc:HGNC:23332] |
| ENSG00000149480 | HGLibA_30313 | 91 | -0.311567285 | MTA2 | metastasis associated 1 family member 2 [Source:HGNC Symbol;Acc:HGNC:7411] |
| ENSG00000149646 | HGLibA_10245 | 91 | -0.311567285 | CNBD2 | cyclic nucleotide binding domain containing 2 [Source:HGNC Symbol;Acc:HGNC:16145] |
| ENSG00000151778 | HGLibA_43503 | 91 | -0.311567285 | SERP2 | stress associated endoplasmic reticulum protein family member 2 [Source:HGNC Symbol;Acc:HGNC:20607] |
| ENSG00000152595 | HGLibA_28905 | 91 | -0.311567285 | MEPE | matrix extracellular phosphoglycoprotein [Source:HGNC Symbol;Acc:HGNC:13361] |
| ENSG00000152766 | HGLibA_02039 | 91 | -0.311567285 | ANKRD22 | ankyrin repeat domain 22 [Source:HGNC Symbol;Acc:HGNC:28321] |
| ENSG00000153303 | HGLibA_18007 | 91 | -0.311567285 | FRMD1 | FERM domain containing 1 [Source:HGNC Symbol;Acc:HGNC:21240] |
| ENSG00000154856 | HGLibA_02396 | 91 | -0.311567285 | APCDD1 | APC down-regulated 1 [Source:HGNC Symbol;Acc:HGNC:15718] |
| ENSG00000155729 | HGLibA_24437 | 91 | -0.311567285 | KCTD18 | potassium channel tetramerization domain containing 18 [Source:HGNC Symbol;Acc:HGNC:26446] |
| ENSG00000155962 | HGLibA_10024 | 91 | -0.311567285 | CLIC2 | chloride intracellular channel 2 [Source:HGNC Symbol;Acc:HGNC:2063] |
| ENSG00000160218 | HGLibA_51301 | 91 | -0.311567285 | TRAPPC10 | trafficking protein particle complex 10 [Source:HGNC Symbol;Acc:HGNC:11868] |
| ENSG00000162572 | HGLibA_43019 | 91 | -0.311567285 | SCNN1D | sodium channel epithelial 1 delta subunit [Source:HGNC Symbol;Acc:HGNC:10601] |
| ENSG00000162692 | HGLibA_53565 | 91 | -0.311567285 | VCAM1 | vascular cell adhesion molecule 1 [Source:HGNC Symbol;Acc:HGNC:12663] |
| ENSG00000162736 | HGLibA_31376 | 91 | -0.311567285 | NCSTN | nicastrin [Source:HGNC Symbol;Acc:HGNC:17091] |
| ENSG00000162852 | HGLibA_10382 | 91 | -0.311567285 | CNST | connexin sorting protein [Source:HGNC Symbol;Acc:HGNC:26486]" |
| ENSG00000163623 | HGLibA_32054 | 91 | -0.311567285 | NKX6-1 | NK6 homeobox 1 [Source:HGNC Symbol;Acc:HGNC:7839] |
| ENSG00000163870 | HGLibA_51176 | 91 | -0.311567285 | TPRA1 | transmembrane protein adipocyte associated 1 [Source:HGNC Symbol;Acc:HGNC:30413] |
| ENSG00000165392 | HGLibA_54410 | 91 | -0.311567285 | WRN | Werner syndrome RecQ like helicase [Source:HGNC Symbol;Acc:HGNC:12791] |
| ENSG00000166123 | HGLibA_20167 | 91 | -0.311567285 | GPT2 | glutamic--pyruvic transaminase 2 [Source:HGNC Symbol;Acc:HGNC:18062] |
| ENSG00000166228 | HGLibA_35362 | 91 | -0.311567285 | PCBD1 | pterin-4 alpha-carbinolamine dehydratase 1 [Source:HGNC Symbol;Acc:HGNC:8646] |
| ENSG00000166869 | HGLibA_09521 | 91 | -0.311567285 | CHP2 | calcineurin like EF-hand protein 2 [Source:HGNC Symbol;Acc:HGNC:24927] |
| ENSG00000167618 | HGLibA_25934 | 91 | -0.311567285 | LAIR2 | leukocyte associated immunoglobulin like receptor 2 [Source:HGNC Symbol;Acc:HGNC:6478] |
| ENSG00000167664 | HGLibA_50589 | 91 | -0.311567285 | TMIGD2 | transmembrane and immunoglobulin domain containing 2 [Source:HGNC Symbol;Acc:HGNC:28324] |
| ENSG00000168032 | HGLibA_15204 | 91 | -0.311567285 | ENTPD3 | ectonucleoside triphosphate diphosphohydrolase 3 [Source:HGNC Symbol;Acc:HGNC:3365] |
| ENSG00000168621 | HGLibA_18886 | 91 | -0.311567285 | GDNF | glial cell derived neurotrophic factor [Source:HGNC Symbol;Acc:HGNC:4232] |
| ENSG00000169474 | HGLibA_46801 | 91 | -0.311567285 | SPRR1A | small proline rich protein 1A [Source:HGNC Symbol;Acc:HGNC:11259] |
| ENSG00000171772 | HGLibA_47913 | 91 | -0.311567285 | SYCE1 | synaptonemal complex central element protein 1 [Source:HGNC Symbol;Acc:HGNC:28852] |
| ENSG00000171786 | HGLibA_31890 | 91 | -0.311567285 | NHLH1 | nescient helix-loop-helix 1 [Source:HGNC Symbol;Acc:HGNC:7817] |
| ENSG00000171786 | HGLibA_31891 | 91 | -0.311567285 | NHLH1 | nescient helix-loop-helix 1 [Source:HGNC Symbol;Acc:HGNC:7817] |
| ENSG00000171858 | HGLibA_42107 | 91 | -0.311567285 | RPS21 | ribosomal protein S21 [Source:HGNC Symbol;Acc:HGNC:10409] |
| ENSG00000172150 | HGLibA_33645 | 91 | -0.311567285 | OR1A2 | olfactory receptor family 1 subfamily A member 2 [Source:HGNC Symbol;Acc:HGNC:8180] |
| ENSG00000172457 | HGLibA_34570 | 91 | -0.311567285 | OR9G4 | olfactory receptor family 9 subfamily G member 4 [Source:HGNC Symbol;Acc:HGNC:15322] |

| | | | | | |
|---|---|---|---|---|---|
| ENSG00000173660 | HGLibA_53122 | 91 | -0.311567285 | UQCRH | ubiquinol-cytochrome c reductase hinge protein [Source:HGNC Symbol;Acc:HGNC:12590] |
| ENSG00000174227 | HGLibA_36556 | 91 | -0.311567285 | PIGG | phosphatidylinositol glycan anchor biosynthesis class G [Source:HGNC Symbol;Acc:HGNC:25985] |
| ENSG00000174579 | HGLibA_30215 | 91 | -0.311567285 | MSL2 | MSL complex subunit 2 [Source:HGNC Symbol;Acc:HGNC:25544] |
| ENSG00000174928 | HGLibA_06231 | 91 | -0.311567285 | C3orf33 | chromosome 3 open reading frame 33 [Source:HGNC Symbol;Acc:HGNC:26434] |
| ENSG00000175264 | HGLibA_09611 | 91 | -0.311567285 | CHST1 | carbohydrate sulfotransferase 1 [Source:HGNC Symbol;Acc:HGNC:1969] |
| ENSG00000177951 | HGLibA_04435 | 91 | -0.311567285 | BET1L | Bet1 golgi vesicular membrane trafficking protein like [Source:HGNC Symbol;Acc:HGNC:19348] |
| ENSG00000179600 | HGLibA_19802 | 91 | -0.311567285 | GPHB5 | glycoprotein hormone beta 5 [Source:HGNC Symbol;Acc:HGNC:18055] |
| ENSG00000179615 | HGLibA_33757 | 91 | -0.311567285 | OR2AP1 | olfactory receptor family 2 subfamily AP member 1 [Source:HGNC Symbol;Acc:HGNC:15335] |
| ENSG00000182004 | HGLibA_45984 | 91 | -0.311567285 | SNRPE | small nuclear ribonucleoprotein polypeptide E [Source:HGNC Symbol;Acc:HGNC:11161] |
| ENSG00000182170 | HGLibA_29844 | 91 | -0.311567285 | MRGPRG | MAS related GPR family member G [Source:HGNC Symbol;Acc:HGNC:24829] |
| ENSG00000183682 | HGLibA_04621 | 91 | -0.311567285 | BMP8A | bone morphogenetic protein 8a [Source:HGNC Symbol;Acc:HGNC:21650] |
| ENSG00000184752 | HGLibA_31447 | 91 | -0.311567285 | NDUFA12 | NADH:ubiquinone oxidoreductase subunit A12 [Source:HGNC Symbol;Acc:HGNC:23987] |
| ENSG00000185019 | HGLibA_52726 | 91 | -0.311567285 | UBOX5 | U-box domain containing 5 [Source:HGNC Symbol;Acc:HGNC:17777] |
| ENSG00000185608 | HGLibA_29978 | 91 | -0.311567285 | MRPL40 | mitochondrial ribosomal protein L40 [Source:HGNC Symbol;Acc:HGNC:14491] |
| ENSG00000187957 | HGLibA_13675 | 91 | -0.311567285 | DNER | delta/notch like EGF repeat containing [Source:HGNC Symbol;Acc:HGNC:24456] |
| ENSG00000196156 | HGLibA_25761 | 91 | -0.311567285 | KRTAP4-3 | keratin associated protein 4-3 [Source:HGNC Symbol;Acc:HGNC:18908] |
| ENSG00000196456 | HGLibA_56719 | 91 | -0.311567285 | ZNF775 | zinc finger protein 775 [Source:HGNC Symbol;Acc:HGNC:28501] |
| ENSG00000197355 | HGLibA_52511 | 91 | -0.311567285 | UAP1L1 | UDP-N-acetylglucosamine pyrophosphorylase 1 like 1 [Source:HGNC Symbol;Acc:HGNC:28082] |
| ENSG00000198604 | HGLibA_04185 | 91 | -0.311567285 | BAZ1A | bromodomain adjacent to zinc finger domain 1A [Source:HGNC Symbol;Acc:HGNC:960] |
| ENSG00000198624 | HGLibA_07844 | 91 | -0.311567285 | CCDC69 | coiled-coil domain containing 69 [Source:HGNC Symbol;Acc:HGNC:24487] |
| ENSG00000198707 | HGLibA_09130 | 91 | -0.311567285 | CEP290 | centrosomal protein 290 [Source:HGNC Symbol;Acc:HGNC:29021] |
| ENSG00000198765 | HGLibA_47929 | 91 | -0.311567285 | SYCP1 | synaptonemal complex protein 1 [Source:HGNC Symbol;Acc:HGNC:11487] |
| ENSG00000198909 | HGLibA_28184 | 91 | -0.311567285 | MAP3K3 | mitogen-activated protein kinase kinase kinase 3 [Source:HGNC Symbol;Acc:HGNC:6855] |
| ENSG00000204262 | HGLibA_10590 | 91 | -0.311567285 | COL5A2 | collagen type V alpha 2 chain [Source:HGNC Symbol;Acc:HGNC:2210] |
| ENSG00000205832 | HGLibA_05510 | 91 | -0.311567285 | C16orf96 | chromosome 16 open reading frame 96 [Source:HGNC Symbol;Acc:HGNC:40031] |
| ENSG00000205863 | HGLibA_05962 | 91 | -0.311567285 | C1QTNF9B | C1q and TNF related 9B [Source:HGNC Symbol;Acc:HGNC:34072] |
| ENSG00000206559 | HGLibA_55059 | 91 | -0.311567285 | ZCWPW2 | zinc finger CW-type and PWWP domain containing 2 [Source:HGNC Symbol;Acc:HGNC:23574] |
| ENSG00000213186 | HGLibA_51556 | 91 | -0.311567285 | TRIM59 | tripartite motif containing 59 [Source:HGNC Symbol;Acc:HGNC:30834] |
| ENSG00000213588 | HGLibA_54933 | 91 | -0.311567285 | ZBTB9 | zinc finger and BTB domain containing 9 [Source:HGNC Symbol;Acc:HGNC:28323] |
| ENSG00000214274 | HGLibA_01893 | 91 | -0.311567285 | ANG | angiogenin [Source:HGNC Symbol;Acc:HGNC:483] |
| ENSG00000223614 | HGLibA_56650 | 91 | -0.311567285 | ZNF735 | zinc finger protein 735 [Source:HGNC Symbol;Acc:HGNC:32466] |
| ENSG00000229579 | HGLibA_53260 | 91 | -0.311567285 | USP17L26 | ubiquitin specific peptidase 17-like family member 26 [Source:HGNC Symbol;Acc:HGNC:44454] |
| ENSG00000232629 | HGLibA_21631 | 91 | -0.311567285 | HLA-DQB2 | DQ beta 2 [Source:HGNC Symbol;Acc:HGNC:4945]" |
| ENSG00000239389 | HGLibA_35436 | 91 | -0.311567285 | PCDHA13 | protocadherin alpha 13 [Source:HGNC Symbol;Acc:HGNC:8667] |
| ENSG00000240344 | HGLibA_37946 | 91 | -0.311567285 | PPIL3 | peptidylprolyl isomerase like 3 [Source:HGNC Symbol;Acc:HGNC:9262] |
| ENSG00000249115 | HGLibA_20885 | 91 | -0.311567285 | HAUS5 | HAUS augmin like complex subunit 5 [Source:HGNC Symbol;Acc:HGNC:29130] |
| ENSG00000269502 | HGLibA_13434 | 91 | -0.311567285 | DMRTC1 | DMRT like family C1 [Source:HGNC Symbol;Acc:HGNC:13910] |
| ENSG00000275718 | HGLibA_07973 | 91 | -0.311567285 | CCL15 | C-C motif chemokine ligand 15 [Source:HGNC Symbol;Acc:HGNC:10613] |
| ENSG00000286237 | HGLibA_03054 | 91 | -0.311567285 | ARMCX5-GPRASP2 | - |
| FAM160A2 | HGLibA_16207 | 91 | -0.311567285 | FAM160A2 | - |
| FAM188B | HGLibA_16348 | 91 | -0.311567285 | FAM188B | - |
| FAM69C | HGLibA_16682 | 91 | -0.311567285 | FAM69C | - |
| FAM96A | HGLibA_16804 | 91 | -0.311567285 | FAM96A | - |
| GPR123 | HGLibA_19873 | 91 | -0.311567285 | GPR123 | - |
| HIST2H2BE | HGLibA_21534 | 91 | -0.311567285 | HIST2H2BE | - |
| hsa-mir-22 | HGLibA_58290 | 91 | -0.311567285 | hsa-mir-22 | - |
| hsa-mir-23b | HGLibA_58334 | 91 | -0.311567285 | hsa-mir-23b | - |
| hsa-mir-3192 | HGLibA_58865 | 91 | -0.311567285 | hsa-mir-3192 | - |
| hsa-mir-3690-1 | HGLibA_59347 | 91 | -0.311567285 | hsa-mir-3690-1 | - |
| hsa-mir-378g | HGLibA_59455 | 91 | -0.311567285 | hsa-mir-378g | - |
| hsa-mir-3912 | HGLibA_59517 | 91 | -0.311567285 | hsa-mir-3912 | - |
| hsa-mir-4269 | HGLibA_59783 | 91 | -0.311567285 | hsa-mir-4269 | - |
| hsa-mir-449c | HGLibA_60424 | 91 | -0.311567285 | hsa-mir-449c | - |
| hsa-mir-4646 | HGLibA_60689 | 91 | -0.311567285 | hsa-mir-4646 | - |
| hsa-mir-5186 | HGLibA_61635 | 91 | -0.311567285 | hsa-mir-5186 | - |
| hsa-mir-548e | HGLibA_61970 | 91 | -0.311567285 | hsa-mir-548e | - |
| hsa-mir-5687 | HGLibA_62264 | 91 | -0.311567285 | hsa-mir-5687 | - |
| hsa-mir-592 | HGLibA_62466 | 91 | -0.311567285 | hsa-mir-592 | - |
| hsa-mir-632 | HGLibA_62769 | 91 | -0.311567285 | hsa-mir-632 | - |
| hsa-mir-6511a-3 | HGLibA_62907 | 91 | -0.311567285 | hsa-mir-6511a-3 | - |
| hsa-mir-668 | HGLibA_63005 | 91 | -0.311567285 | hsa-mir-668 | - |
| hsa-mir-6846 | HGLibA_63558 | 91 | -0.311567285 | hsa-mir-6846 | - |
| hsa-mir-6862-2 | HGLibA_63632 | 91 | -0.311567285 | hsa-mir-6862-2 | - |
| hsa-mir-764 | HGLibA_63911 | 91 | -0.311567285 | hsa-mir-764 | - |
| hsa-mir-95 | HGLibA_64365 | 91 | -0.311567285 | hsa-mir-95 | - |
| KIAA0317 | HGLibA_24617 | 91 | -0.311567285 | KIAA0317 | - |
| PHF16 | HGLibA_36351 | 91 | -0.311567285 | PHF16 | - |
| TCEB2 | HGLibA_48736 | 91 | -0.311567285 | TCEB2 | - |
| TCTEX1D4 | HGLibA_48869 | 91 | -0.311567285 | TCTEX1D4 | - |
| AGAP7 | HGLibA_01158 | 90 | -0.320612628 | AGAP7 | - |
| ASUN | HGLibA_03387 | 90 | -0.320612628 | ASUN | - |
| C1orf173 | HGLibA_05796 | 90 | -0.320612628 | C1orf173 | - |
| CCDC114 | HGLibA_07551 | 90 | -0.320612628 | CCDC114 | - |
| CPSF3L | HGLibA_11003 | 90 | -0.320612628 | CPSF3L | - |
| EMR3 | HGLibA_15090 | 90 | -0.320612628 | EMR3 | - |
| ENSG00000008197 | HGLibA_49131 | 90 | -0.320612628 | TFAP2D | transcription factor AP-2 delta [Source:HGNC Symbol;Acc:HGNC:15581] |
| ENSG00000011275 | HGLibA_41630 | 90 | -0.320612628 | RNF216 | ring finger protein 216 [Source:HGNC Symbol;Acc:HGNC:21698] |
| ENSG00000011347 | HGLibA_48088 | 90 | -0.320612628 | SYT7 | synaptotagmin 7 [Source:HGNC Symbol;Acc:HGNC:11514] |
| ENSG00000013306 | HGLibA_44730 | 90 | -0.320612628 | SLC25A39 | solute carrier family 25 member 39 [Source:HGNC Symbol;Acc:HGNC:24279] |
| ENSG00000028137 | HGLibA_50776 | 90 | -0.320612628 | TNFRSF1B | TNF receptor superfamily member 1B [Source:HGNC Symbol;Acc:HGNC:11917] |
| ENSG00000040731 | HGLibA_08632 | 90 | -0.320612628 | CDH10 | cadherin 10 [Source:HGNC Symbol;Acc:HGNC:1749] |
| ENSG00000044115 | HGLibA_11638 | 90 | -0.320612628 | CTNNA1 | catenin alpha 1 [Source:HGNC Symbol;Acc:HGNC:2509] |
| ENSG00000049769 | HGLibA_38113 | 90 | -0.320612628 | PPP1R3F | protein phosphatase 1 regulatory subunit 3F [Source:HGNC Symbol;Acc:HGNC:14944] |
| ENSG00000050767 | HGLibA_10545 | 90 | -0.320612628 | COL23A1 | collagen type XXIII alpha 1 chain [Source:HGNC Symbol;Acc:HGNC:22990] |
| ENSG00000051620 | HGLibA_21087 | 90 | -0.320612628 | HEBP2 | heme binding protein 2 [Source:HGNC Symbol;Acc:HGNC:15716] |
| ENSG00000058335 | HGLibA_40338 | 90 | -0.320612628 | RASGRF1 | Ras protein specific guanine nucleotide releasing factor 1 [Source:HGNC Symbol;Acc:HGNC:9875] |
| ENSG00000064309 | HGLibA_08856 | 90 | -0.320612628 | CDON | oncogene regulated [Source:HGNC Symbol;Acc:HGNC:17104]" |
| ENSG00000064490 | HGLibA_40987 | 90 | -0.320612628 | RFXANK | regulatory factor X associated ankyrin containing protein [Source:HGNC Symbol;Acc:HGNC:9987] |
| ENSG00000064687 | HGLibA_00121 | 90 | -0.320612628 | ABCA7 | ATP binding cassette subfamily A member 7 [Source:HGNC Symbol;Acc:HGNC:37] |
| ENSG00000064933 | HGLibA_37333 | 90 | -0.320612628 | PMS1 | mismatch repair system component [Source:HGNC Symbol;Acc:HGNC:9121]" |
| ENSG00000068305 | HGLibA_28825 | 90 | -0.320612628 | MEF2A | myocyte enhancer factor 2A [Source:HGNC Symbol;Acc:HGNC:6993] |
| ENSG00000071991 | HGLibA_08655 | 90 | -0.320612628 | CDH19 | cadherin 19 [Source:HGNC Symbol;Acc:HGNC:1758] |
| ENSG00000075826 | HGLibA_43244 | 90 | -0.320612628 | SEC31B | COPII coat complex component [Source:HGNC Symbol;Acc:HGNC:23197]" |
| ENSG00000076356 | HGLibA_37260 | 90 | -0.320612628 | PLXNA2 | plexin A2 [Source:HGNC Symbol;Acc:HGNC:9100] |
| ENSG00000077264 | HGLibA_35046 | 90 | -0.320612628 | PAK3 | p21 (RAC1) activated kinase 3 [Source:HGNC Symbol;Acc:HGNC:8592] |
| ENSG00000077463 | HGLibA_44232 | 90 | -0.320612628 | SIRT6 | sirtuin 6 [Source:HGNC Symbol;Acc:HGNC:14934] |
| ENSG00000079387 | HGLibA_43384 | 90 | -0.320612628 | SENP1 | SUMO specific peptidase 1 [Source:HGNC Symbol;Acc:HGNC:17927] |
| ENSG00000083168 | HGLibA_24060 | 90 | -0.320612628 | KAT6A | lysine acetyltransferase 6A [Source:HGNC Symbol;Acc:HGNC:13013] |
| ENSG00000087301 | HGLibA_52414 | 90 | -0.320612628 | TXNDC16 | thioredoxin domain containing 16 [Source:HGNC Symbol;Acc:HGNC:19965] |
| ENSG00000089902 | HGLibA_40710 | 90 | -0.320612628 | RCOR1 | REST corepressor 1 [Source:HGNC Symbol;Acc:HGNC:17441] |
| ENSG00000094914 | HGLibA_00022 | 90 | -0.320612628 | AAAS | aladin WD repeat nucleoporin [Source:HGNC Symbol;Acc:HGNC:13666] |
| ENSG00000099308 | HGLibA_28407 | 90 | -0.320612628 | MAST3 | microtubule associated serine/threonine kinase 3 [Source:HGNC Symbol;Acc:HGNC:19036] |
| ENSG00000100221 | HGLibA_23973 | 90 | -0.320612628 | JOSD1 | Josephin domain containing 1 [Source:HGNC Symbol;Acc:HGNC:28953] |
| ENSG00000100281 | HGLibA_21736 | 90 | -0.320612628 | HMGXB4 | HMG-box containing 4 [Source:HGNC Symbol;Acc:HGNC:5003] |
| ENSG00000101161 | HGLibA_38742 | 90 | -0.320612628 | PRPF6 | pre-mRNA processing factor 6 [Source:HGNC Symbol;Acc:HGNC:15860] |
| ENSG00000102218 | HGLibA_41793 | 90 | -0.320612628 | RP2 | ARL3 GTPase activating protein [Source:HGNC Symbol;Acc:HGNC:10274]" |
| ENSG00000102362 | HGLibA_48106 | 90 | -0.320612628 | SYTL4 | synaptotagmin like 4 [Source:HGNC Symbol;Acc:HGNC:15588] |
| ENSG00000102786 | HGLibA_23428 | 90 | -0.320612628 | INTS6 | integrator complex subunit 6 [Source:HGNC Symbol;Acc:HGNC:14879] |
| ENSG00000102882 | HGLibA_28270 | 90 | -0.320612628 | MAPK3 | mitogen-activated protein kinase 3 [Source:HGNC Symbol;Acc:HGNC:6877] |
| ENSG00000103175 | HGLibA_54243 | 90 | -0.320612628 | WFDC1 | WAP four-disulfide core domain 1 [Source:HGNC Symbol;Acc:HGNC:15466] |
| ENSG00000103227 | HGLibA_26684 | 90 | -0.320612628 | LMF1 | lipase maturation factor 1 [Source:HGNC Symbol;Acc:HGNC:14154] |
| ENSG00000104679 | HGLibA_39767 | 90 | -0.320612628 | R3HCC1 | R3H domain and coiled-coil containing 1 [Source:HGNC Symbol;Acc:HGNC:27329] |
| ENSG00000104823 | HGLibA_14392 | 90 | -0.320612628 | ECH1 | enoyl-CoA hydratase 1 [Source:HGNC Symbol;Acc:HGNC:3149] |
| ENSG00000106304 | HGLibA_46375 | 90 | -0.320612628 | SPAM1 | sperm adhesion molecule 1 [Source:HGNC Symbol;Acc:HGNC:11217] |
| ENSG00000107957 | HGLibA_43965 | 90 | -0.320612628 | SH3PXD2A | SH3 and PX domains 2A [Source:HGNC Symbol;Acc:HGNC:23664] |
| ENSG00000109061 | HGLibA_30704 | 90 | -0.320612628 | MYH1 | myosin heavy chain 1 [Source:HGNC Symbol;Acc:HGNC:7567] |
| ENSG00000113522 | HGLibA_40075 | 90 | -0.320612628 | RAD50 | RAD50 double strand break repair protein [Source:HGNC Symbol;Acc:HGNC:9816] |
| ENSG00000114491 | HGLibA_52983 | 90 | -0.320612628 | UMPS | uridine monophosphate synthetase [Source:HGNC Symbol;Acc:HGNC:12563] |
| ENSG00000116473 | HGLibA_40221 | 90 | -0.320612628 | RAP1A | member of RAS oncogene family [Source:HGNC Symbol;Acc:HGNC:9855]" |
| ENSG00000119487 | HGLibA_28298 | 90 | -0.320612628 | MAPKAP1 | mitogen-activated protein kinase associated protein 1 [Source:HGNC Symbol;Acc:HGNC:18752] |
| ENSG00000120471 | HGLibA_51052 | 90 | -0.320612628 | TP53AIP1 | tumor protein p53 regulated apoptosis inducing protein 1 [Source:HGNC Symbol;Acc:HGNC:29984] |
| ENSG00000120729 | HGLibA_30897 | 90 | -0.320612628 | MYOT | myotilin [Source:HGNC Symbol;Acc:HGNC:12399] |
| ENSG00000121690 | HGLibA_12986 | 90 | -0.320612628 | DEPDC7 | DEP domain containing 7 [Source:HGNC Symbol;Acc:HGNC:29899] |
| ENSG00000123453 | HGLibA_42743 | 90 | -0.320612628 | SARDH | sarcosine dehydrogenase [Source:HGNC Symbol;Acc:HGNC:10536] |
| ENSG00000124486 | HGLibA_53417 | 90 | -0.320612628 | USP9X | ubiquitin specific peptidase 9 X-linked [Source:HGNC Symbol;Acc:HGNC:12632] |
| ENSG00000124507 | HGLibA_34959 | 90 | -0.320612628 | PACSIN1 | protein kinase C and casein kinase substrate in neurons 1 [Source:HGNC Symbol;Acc:HGNC:8570] |
| ENSG00000124882 | HGLibA_15457 | 90 | -0.320612628 | EREG | epiregulin [Source:HGNC Symbol;Acc:HGNC:3443] |
| ENSG00000125826 | HGLibA_40449 | 90 | -0.320612628 | RBCK1 | RANBP2-type and C3HC4-type zinc finger containing 1 [Source:HGNC Symbol;Acc:HGNC:15864] |
| ENSG00000126562 | HGLibA_54341 | 90 | -0.320612628 | WNK4 | WNK lysine deficient protein kinase 4 [Source:HGNC Symbol;Acc:HGNC:14544] |
| ENSG00000126777 | HGLibA_25871 | 90 | -0.320612628 | KTN1 | kinectin 1 [Source:HGNC Symbol;Acc:HGNC:6467] |
| ENSG00000127220 | HGLibA_00286 | 90 | -0.320612628 | ABHD8 | abhydrolase domain containing 8 [Source:HGNC Symbol;Acc:HGNC:23759] |
| ENSG00000128513 | HGLibA_37715 | 90 | -0.320612628 | POT1 | protection of telomeres 1 [Source:HGNC Symbol;Acc:HGNC:17284] |
| ENSG00000130164 | HGLibA_26239 | 90 | -0.320612628 | LDLR | low density lipoprotein receptor [Source:HGNC Symbol;Acc:HGNC:6547] |
| ENSG00000130303 | HGLibA_04883 | 90 | -0.320612628 | BST2 | bone marrow stromal cell antigen 2 [Source:HGNC Symbol;Acc:HGNC:1119] |
| ENSG00000130714 | HGLibA_37668 | 90 | -0.320612628 | POMT1 | protein O-mannosyltransferase 1 [Source:HGNC Symbol;Acc:HGNC:9202] |

| | | | | | |
|---|---|---|---|---|---|
| ENSG00000131668 | HGLibA_04164 | 90 | -0.320612628 | BARX1 | BARX homeobox 1 [Source:HGNC Symbol;Acc:HGNC:955] |
| ENSG00000132274 | HGLibA_51432 | 90 | -0.320612628 | TRIM22 | tripartite motif containing 22 [Source:HGNC Symbol;Acc:HGNC:16379] |
| ENSG00000133026 | HGLibA_30706 | 90 | -0.320612628 | MYH10 | myosin heavy chain 10 [Source:HGNC Symbol;Acc:HGNC:7568] |
| ENSG00000134249 | HGLibA_00769 | 90 | -0.320612628 | ADAM30 | ADAM metallopeptidase domain 30 [Source:HGNC Symbol;Acc:HGNC:208] |
| ENSG00000134371 | HGLibA_08598 | 90 | -0.320612628 | CDC73 | cell division cycle 73 [Source:HGNC Symbol;Acc:HGNC:16783] |
| ENSG00000134686 | HGLibA_36322 | 90 | -0.320612628 | PHC2 | polyhomeotic homolog 2 [Source:HGNC Symbol;Acc:HGNC:3183] |
| ENSG00000134996 | HGLibA_34710 | 90 | -0.320612628 | OSTF1 | osteoclast stimulating factor 1 [Source:HGNC Symbol;Acc:HGNC:8510] |
| ENSG00000135686 | HGLibA_25198 | 90 | -0.320612628 | KLHL36 | kelch like family member 36 [Source:HGNC Symbol;Acc:HGNC:17844] |
| ENSG00000135776 | HGLibA_00135 | 90 | -0.320612628 | ABCB10 | ATP binding cassette subfamily B member 10 [Source:HGNC Symbol;Acc:HGNC:41] |
| ENSG00000135828 | HGLibA_41439 | 90 | -0.320612628 | RNASEL | ribonuclease L [Source:HGNC Symbol;Acc:HGNC:10050] |
| ENSG00000137731 | HGLibA_18228 | 90 | -0.320612628 | FXYD2 | FXYD domain containing ion transport regulator 2 [Source:HGNC Symbol;Acc:HGNC:4026] |
| ENSG00000138381 | HGLibA_03339 | 90 | -0.320612628 | ASNSD1 | asparagine synthetase domain containing 1 [Source:HGNC Symbol;Acc:HGNC:24910] |
| ENSG00000139433 | HGLibA_19343 | 90 | -0.320612628 | GLTP | glycolipid transfer protein [Source:HGNC Symbol;Acc:HGNC:24867] |
| ENSG00000139687 | HGLibA_40414 | 90 | -0.320612628 | RB1 | RB transcriptional corepressor 1 [Source:HGNC Symbol;Acc:HGNC:9884] |
| ENSG00000139832 | HGLibA_39835 | 90 | -0.320612628 | RAB20 | member RAS oncogene family [Source:HGNC Symbol;Acc:HGNC:18260]" |
| ENSG00000141576 | HGLibA_41553 | 90 | -0.320612628 | RNF157 | ring finger protein 157 [Source:HGNC Symbol;Acc:HGNC:29402] |
| ENSG00000142494 | HGLibA_45194 | 90 | -0.320612628 | SLC47A1 | solute carrier family 47 member 1 [Source:HGNC Symbol;Acc:HGNC:25588] |
| ENSG00000142669 | HGLibA_43923 | 90 | -0.320612628 | SH3BGRL3 | SH3 domain binding glutamate rich protein like 3 [Source:HGNC Symbol;Acc:HGNC:15568] |
| ENSG00000142694 | HGLibA_15675 | 90 | -0.320612628 | EVA1B | eva-1 homolog B [Source:HGNC Symbol;Acc:HGNC:25558] |
| ENSG00000143319 | HGLibA_23661 | 90 | -0.320612628 | ISG20L2 | interferon stimulated exonuclease gene 20 like 2 [Source:HGNC Symbol;Acc:HGNC:25745] |
| ENSG00000143995 | HGLibA_28872 | 90 | -0.320612628 | MEIS1 | Meis homeobox 1 [Source:HGNC Symbol;Acc:HGNC:7000] |
| ENSG00000145198 | HGLibA_53910 | 90 | -0.320612628 | VWA5B2 | von Willebrand factor A domain containing 5B2 [Source:HGNC Symbol;Acc:HGNC:25144] |
| ENSG00000145681 | HGLibA_20840 | 90 | -0.320612628 | HAPLN1 | hyaluronan and proteoglycan link protein 1 [Source:HGNC Symbol;Acc:HGNC:2380] |
| ENSG00000148671 | HGLibA_00986 | 90 | -0.320612628 | ADIRF | adipogenesis regulatory factor [Source:HGNC Symbol;Acc:HGNC:24043] |
| ENSG00000149651 | HGLibA_46721 | 90 | -0.320612628 | SPINT4 | Kunitz type 4 [Source:HGNC Symbol;Acc:HGNC:16130]" |
| ENSG00000152782 | HGLibA_35097 | 90 | -0.320612628 | PANK1 | pantothenate kinase 1 [Source:HGNC Symbol;Acc:HGNC:8598] |
| ENSG00000154914 | HGLibA_53368 | 90 | -0.320612628 | USP43 | ubiquitin specific peptidase 43 [Source:HGNC Symbol;Acc:HGNC:20072] |
| ENSG00000154930 | HGLibA_00586 | 90 | -0.320612628 | ACSS1 | acyl-CoA synthetase short chain family member 1 [Source:HGNC Symbol;Acc:HGNC:16091] |
| ENSG00000156928 | HGLibA_28030 | 90 | -0.320612628 | MALSU1 | mitochondrial assembly of ribosomal large subunit 1 [Source:HGNC Symbol;Acc:HGNC:21721] |
| ENSG00000158882 | HGLibA_50971 | 90 | -0.320612628 | TOMM40L | translocase of outer mitochondrial membrane 40 like [Source:HGNC Symbol;Acc:HGNC:25756] |
| ENSG00000159596 | HGLibA_50497 | 90 | -0.320612628 | TMEM69 | transmembrane protein 69 [Source:HGNC Symbol;Acc:HGNC:28035] |
| ENSG00000159961 | HGLibA_33920 | 90 | -0.320612628 | OR3A3 | olfactory receptor family 3 subfamily A member 3 [Source:HGNC Symbol;Acc:HGNC:8284] |
| ENSG00000160094 | HGLibA_55872 | 90 | -0.320612628 | ZNF362 | zinc finger protein 362 [Source:HGNC Symbol;Acc:HGNC:18079] |
| ENSG00000162438 | HGLibA_11683 | 90 | -0.320612628 | CTRC | chymotrypsin C [Source:HGNC Symbol;Acc:HGNC:2523] |
| ENSG00000163430 | HGLibA_18101 | 90 | -0.320612628 | FSTL1 | follistatin like 1 [Source:HGNC Symbol;Acc:HGNC:3972] |
| ENSG00000163463 | HGLibA_25854 | 90 | -0.320612628 | KRTCAP2 | keratinocyte associated protein 2 [Source:HGNC Symbol;Acc:HGNC:28942] |
| ENSG00000163734 | HGLibA_11868 | 90 | -0.320612628 | CXCL3 | C-X-C motif chemokine ligand 3 [Source:HGNC Symbol;Acc:HGNC:4604] |
| ENSG00000163803 | HGLibA_36968 | 90 | -0.320612628 | PLB1 | phospholipase B1 [Source:HGNC Symbol;Acc:HGNC:30041] |
| ENSG00000163882 | HGLibA_37586 | 90 | -0.320612628 | POLR2H | RNA polymerase II subunit H [Source:HGNC Symbol;Acc:HGNC:9195] |
| ENSG00000164022 | HGLibA_01354 | 90 | -0.320612628 | AIMP1 | aminoacyl tRNA synthetase complex interacting multifunctional protein 1 [Source:HGNC Symbol;Acc:HGNC:10648] |
| ENSG00000164073 | HGLibA_29118 | 90 | -0.320612628 | MFSD8 | major facilitator superfamily domain containing 8 [Source:HGNC Symbol;Acc:HGNC:28486] |
| ENSG00000164073 | HGLibA_29116 | 90 | -0.320612628 | MFSD8 | major facilitator superfamily domain containing 8 [Source:HGNC Symbol;Acc:HGNC:28486] |
| ENSG00000164122 | HGLibA_03241 | 90 | -0.320612628 | ASB5 | ankyrin repeat and SOCS box containing 5 [Source:HGNC Symbol;Acc:HGNC:17180] |
| ENSG00000164308 | HGLibA_15408 | 90 | -0.320612628 | ERAP2 | endoplasmic reticulum aminopeptidase 2 [Source:HGNC Symbol;Acc:HGNC:29499] |
| ENSG00000164597 | HGLibA_10483 | 90 | -0.320612628 | COG5 | component of oligomeric golgi complex 5 [Source:HGNC Symbol;Acc:HGNC:14857] |
| ENSG00000164920 | HGLibA_34701 | 90 | -0.320612628 | OSR2 | odd-skipped related transciption factor 2 [Source:HGNC Symbol;Acc:HGNC:15830] |
| ENSG00000164920 | HGLibA_34702 | 90 | -0.320612628 | OSR2 | odd-skipped related transciption factor 2 [Source:HGNC Symbol;Acc:HGNC:15830] |
| ENSG00000165609 | HGLibA_32982 | 90 | -0.320612628 | NUDT5 | nudix hydrolase 5 [Source:HGNC Symbol;Acc:HGNC:8052] |
| ENSG00000165966 | HGLibA_36000 | 90 | -0.320612628 | PDZRN4 | PDZ domain containing ring finger 4 [Source:HGNC Symbol;Acc:HGNC:30552] |
| ENSG00000166289 | HGLibA_37095 | 90 | -0.320612628 | PLEKHF1 | pleckstrin homology and FYVE domain containing 1 [Source:HGNC Symbol;Acc:HGNC:20764] |
| ENSG00000167193 | HGLibA_11179 | 90 | -0.320612628 | CRK | adaptor protein [Source:HGNC Symbol;Acc:HGNC:2362]" |
| ENSG00000167964 | HGLibA_39854 | 90 | -0.320612628 | RAB26 | member RAS oncogene family [Source:HGNC Symbol;Acc:HGNC:14259]" |
| ENSG00000168952 | HGLibA_47680 | 90 | -0.320612628 | STXBP6 | syntaxin binding protein 6 [Source:HGNC Symbol;Acc:HGNC:19666] |
| ENSG00000169006 | HGLibA_32888 | 90 | -0.320612628 | NTSR2 | neurotensin receptor 2 [Source:HGNC Symbol;Acc:HGNC:8040] |
| ENSG00000169504 | HGLibA_10030 | 90 | -0.320612628 | CLIC4 | chloride intracellular channel 4 [Source:HGNC Symbol;Acc:HGNC:13518] |
| ENSG00000170961 | HGLibA_20866 | 90 | -0.320612628 | HAS2 | hyaluronan synthase 2 [Source:HGNC Symbol;Acc:HGNC:4819] |
| ENSG00000171310 | HGLibA_09615 | 90 | -0.320612628 | CHST11 | carbohydrate sulfotransferase 11 [Source:HGNC Symbol;Acc:HGNC:17422] |
| ENSG00000171617 | HGLibA_15110 | 90 | -0.320612628 | ENC1 | ectodermal-neural cortex 1 [Source:HGNC Symbol;Acc:HGNC:3345] |
| ENSG00000172508 | HGLibA_07257 | 90 | -0.320612628 | CARNS1 | carnosine synthase 1 [Source:HGNC Symbol;Acc:HGNC:29268] |
| ENSG00000173221 | HGLibA_19312 | 90 | -0.320612628 | GLRX | glutaredoxin [Source:HGNC Symbol;Acc:HGNC:4330] |
| ENSG00000173517 | HGLibA_36007 | 90 | -0.320612628 | PEAK1 | pseudopodium enriched atypical kinase 1 [Source:HGNC Symbol;Acc:HGNC:29431] |
| ENSG00000173612 | HGLibA_20136 | 90 | -0.320612628 | GPRC6A | G protein-coupled receptor class C group 6 member A [Source:HGNC Symbol;Acc:HGNC:18510] |
| ENSG00000174586 | HGLibA_56105 | 90 | -0.320612628 | ZNF497 | zinc finger protein 497 [Source:HGNC Symbol;Acc:HGNC:23714] |
| ENSG00000174898 | HGLibA_07369 | 90 | -0.320612628 | CATSPERD | cation channel sperm associated auxiliary subunit delta [Source:HGNC Symbol;Acc:HGNC:28598] |
| ENSG00000175322 | HGLibA_56156 | 90 | -0.320612628 | ZNF519 | zinc finger protein 519 [Source:HGNC Symbol;Acc:HGNC:30574] |
| ENSG00000175329 | HGLibA_23691 | 90 | -0.320612628 | ISX | intestine specific homeobox [Source:HGNC Symbol;Acc:HGNC:28084] |
| ENSG00000177303 | HGLibA_07292 | 90 | -0.320612628 | CASKIN2 | CASK interacting protein 2 [Source:HGNC Symbol;Acc:HGNC:18200] |
| ENSG00000178279 | HGLibA_50894 | 90 | -0.320612628 | TNP2 | transition protein 2 [Source:HGNC Symbol;Acc:HGNC:11952] |
| ENSG00000180537 | HGLibA_41586 | 90 | -0.320612628 | RNF182 | ring finger protein 182 [Source:HGNC Symbol;Acc:HGNC:28522] |
| ENSG00000180875 | HGLibA_20245 | 90 | -0.320612628 | GREM2 | DAN family BMP antagonist [Source:HGNC Symbol;Acc:HGNC:17655]" |
| ENSG00000182179 | HGLibA_52530 | 90 | -0.320612628 | UBA7 | ubiquitin like modifier activating enzyme 7 [Source:HGNC Symbol;Acc:HGNC:12471] |
| ENSG00000183624 | HGLibA_21671 | 90 | -0.320612628 | HMCES | ES cell specific [Source:HGNC Symbol;Acc:HGNC:24446]" |
| ENSG00000184178 | HGLibA_42896 | 90 | -0.320612628 | SCFD2 | sec1 family domain containing 2 [Source:HGNC Symbol;Acc:HGNC:30676] |
| ENSG00000184908 | HGLibA_09845 | 90 | -0.320612628 | CLCNKB | chloride voltage-gated channel Kb [Source:HGNC Symbol;Acc:HGNC:2027] |
| ENSG00000184990 | HGLibA_44240 | 90 | -0.320612628 | SIVA1 | SIVA1 apoptosis inducing factor [Source:HGNC Symbol;Acc:HGNC:17712] |
| ENSG00000185000 | HGLibA_13035 | 90 | -0.320612628 | DGAT1 | diacylglycerol O-acyltransferase 1 [Source:HGNC Symbol;Acc:HGNC:2843] |
| ENSG00000185053 | HGLibA_43812 | 90 | -0.320612628 | SGCZ | sarcoglycan zeta [Source:HGNC Symbol;Acc:HGNC:14075] |
| ENSG00000185236 | HGLibA_39789 | 90 | -0.320612628 | RAB11B | member RAS oncogene family [Source:HGNC Symbol;Acc:HGNC:9761]" |
| ENSG00000185658 | HGLibA_04854 | 90 | -0.320612628 | BRWD1 | bromodomain and WD repeat domain containing 1 [Source:HGNC Symbol;Acc:HGNC:12760] |
| ENSG00000185739 | HGLibA_46992 | 90 | -0.320612628 | SRL | sarcalumenin [Source:HGNC Symbol;Acc:HGNC:11295] |
| ENSG00000185818 | HGLibA_31188 | 90 | -0.320612628 | NAT8L | N-acetyltransferase 8 like [Source:HGNC Symbol;Acc:HGNC:26742] |
| ENSG00000186288 | HGLibA_34926 | 90 | -0.320612628 | PABPC1L2A | poly(A) binding protein cytoplasmic 1 like 2A [Source:HGNC Symbol;Acc:HGNC:27989] |
| ENSG00000186451 | HGLibA_46426 | 90 | -0.320612628 | SPATA12 | spermatogenesis associated 12 [Source:HGNC Symbol;Acc:HGNC:23221] |
| ENSG00000188153 | HGLibA_10582 | 90 | -0.320612628 | COL4A5 | collagen type IV alpha 5 chain [Source:HGNC Symbol;Acc:HGNC:2207] |
| ENSG00000188372 | HGLibA_56956 | 90 | -0.320612628 | ZP3 | zona pellucida glycoprotein 3 [Source:HGNC Symbol;Acc:HGNC:13189] |
| ENSG00000196782 | HGLibA_28049 | 90 | -0.320612628 | MAML3 | mastermind like transcriptional coactivator 3 [Source:HGNC Symbol;Acc:HGNC:16272] |
| ENSG00000197885 | HGLibA_32015 | 90 | -0.320612628 | NKIRAS1 | NFKB inhibitor interacting Ras like 1 [Source:HGNC Symbol;Acc:HGNC:17899] |
| ENSG00000198075 | HGLibA_47751 | 90 | -0.320612628 | SULT1C4 | sulfotransferase family 1C member 4 [Source:HGNC Symbol;Acc:HGNC:11457] |
| ENSG00000198093 | HGLibA_56458 | 90 | -0.320612628 | ZNF649 | zinc finger protein 649 [Source:HGNC Symbol;Acc:HGNC:25741] |
| ENSG00000198169 | HGLibA_55668 | 90 | -0.320612628 | ZNF251 | zinc finger protein 251 [Source:HGNC Symbol;Acc:HGNC:13045] |
| ENSG00000204084 | HGLibA_23347 | 90 | -0.320612628 | INPP5B | inositol polyphosphate-5-phosphatase B [Source:HGNC Symbol;Acc:HGNC:6077] |
| ENSG00000204531 | HGLibA_37786 | 90 | -0.320612628 | POU5F1 | POU class 5 homeobox 1 [Source:HGNC Symbol;Acc:HGNC:9221] |
| ENSG00000204923 | HGLibA_17118 | 90 | -0.320612628 | FBXO48 | F-box protein 48 [Source:HGNC Symbol;Acc:HGNC:33857] |
| ENSG00000204961 | HGLibA_35460 | 90 | -0.320612628 | PCDHA9 | protocadherin alpha 9 [Source:HGNC Symbol;Acc:HGNC:8675] |
| ENSG00000224089 | HGLibA_11518 | 90 | -0.320612628 | CT47A10 | member A10 [Source:HGNC Symbol;Acc:HGNC:33291]" |
| ENSG00000225697 | HGLibA_44802 | 90 | -0.320612628 | SLC26A6 | solute carrier family 26 member 6 [Source:HGNC Symbol;Acc:HGNC:14472] |
| ENSG00000225830 | HGLibA_15447 | 90 | -0.320612628 | ERCC6 | chromatin remodeling factor [Source:HGNC Symbol;Acc:HGNC:3438]" |
| ENSG00000236126 | HGLibA_11530 | 90 | -0.320612628 | CT47A3 | member A3 [Source:HGNC Symbol;Acc:HGNC:33284]" |
| ENSG00000237693 | HGLibA_23614 | 90 | -0.320612628 | IRGM | immunity related GTPase M [Source:HGNC Symbol;Acc:HGNC:29597] |
| ENSG00000244414 | HGLibA_09262 | 90 | -0.320612628 | CFHR1 | complement factor H related 1 [Source:HGNC Symbol;Acc:HGNC:4888] |
| ENSG00000255561 | HGLibA_17264 | 90 | -0.320612628 | FDXACB1 | ferredoxin-fold anticodon binding domain containing 1 [Source:HGNC Symbol;Acc:HGNC:25110] |
| ENSG00000258102 | HGLibA_28127 | 90 | -0.320612628 | MAP1LC3B2 | microtubule associated protein 1 light chain 3 beta 2 [Source:HGNC Symbol;Acc:HGNC:34390] |
| ENSG00000258453 | HGLibA_33574 | 90 | -0.320612628 | OR11H2 | olfactory receptor family 11 subfamily H member 2 [Source:HGNC Symbol;Acc:HGNC:14716] |
| ENSG00000258659 | HGLibA_51467 | 90 | -0.320612628 | TRIM34 | tripartite motif containing 34 [Source:HGNC Symbol;Acc:HGNC:10063] |
| ENSG00000269096 | HGLibA_11504 | 90 | -0.320612628 | CT45A3 | cancer/testis antigen family 45 member A3 [Source:HGNC Symbol;Acc:HGNC:33268] |
| ENSG00000275954 | HGLibA_48549 | 90 | -0.320612628 | TBC1D3F | TBC1 domain family member 3F [Source:HGNC Symbol;Acc:HGNC:18257] |
| ENSG00000280204 | HGLibA_33714 | 90 | -0.320612628 | OR1S1 | olfactory receptor family 1 subfamily S member 1 (gene/pseudogene) [Source:HGNC Symbol;Acc:HGNC:8227] |
| FAM129C | HGLibA_16101 | 90 | -0.320612628 | FAM129C | - |
| HIST1H1B | HGLibA_21361 | 90 | -0.320612628 | HIST1H1B | - |
| hsa-mir-107 | HGLibA_57193 | 90 | -0.320612628 | hsa-mir-107 | - |
| hsa-mir-1200 | HGLibA_57262 | 90 | -0.320612628 | hsa-mir-1200 | - |
| hsa-mir-1238 | HGLibA_57344 | 90 | -0.320612628 | hsa-mir-1238 | - |
| hsa-mir-1298 | HGLibA_57673 | 90 | -0.320612628 | hsa-mir-1298 | - |
| hsa-mir-26a-1 | HGLibA_58363 | 90 | -0.320612628 | hsa-mir-26a-1 | - |
| hsa-mir-30c-2 | HGLibA_58483 | 90 | -0.320612628 | hsa-mir-30c-2 | - |
| hsa-mir-3669 | HGLibA_59225 | 90 | -0.320612628 | hsa-mir-3669 | - |
| hsa-mir-3926-1 | HGLibA_59577 | 90 | -0.320612628 | hsa-mir-3926-1 | - |
| hsa-mir-4286 | HGLibA_59851 | 90 | -0.320612628 | hsa-mir-4286 | - |
| hsa-mir-485 | HGLibA_61332 | 90 | -0.320612628 | hsa-mir-485 | - |
| hsa-mir-520d | HGLibA_61746 | 90 | -0.320612628 | hsa-mir-520d | - |
| hsa-mir-559 | HGLibA_62198 | 90 | -0.320612628 | hsa-mir-559 | - |
| hsa-mir-568 | HGLibA_62231 | 90 | -0.320612628 | hsa-mir-568 | - |
| hsa-mir-585 | HGLibA_62440 | 90 | -0.320612628 | hsa-mir-585 | - |
| hsa-mir-596 | HGLibA_62476 | 90 | -0.320612628 | hsa-mir-596 | - |
| hsa-mir-621 | HGLibA_62723 | 90 | -0.320612628 | hsa-mir-621 | - |
| hsa-mir-6738 | HGLibA_63105 | 90 | -0.320612628 | hsa-mir-6738 | - |
| hsa-mir-6750 | HGLibA_63158 | 90 | -0.320612628 | hsa-mir-6750 | - |
| hsa-mir-8064 | HGLibA_64096 | 90 | -0.320612628 | hsa-mir-8064 | - |
| MLLT4 | HGLibA_29421 | 90 | -0.320612628 | MLLT4 | - |
| MPP5 | HGLibA_29760 | 90 | -0.320612628 | MPP5 | - |
| SPANXF1 | HGLibA_46400 | 90 | -0.320612628 | SPANXF1 | - |
| TMEM206 | HGLibA_50196 | 90 | -0.320612628 | TMEM206 | - |
| WDR85 | HGLibA_54199 | 90 | -0.320612628 | WDR85 | - |
| ATP5O | HGLibA_03684 | 89 | -0.329657971 | ATP5O | - |
| C3orf43 | HGLibA_06245 | 89 | -0.329657971 | C3orf43 | - |

| | | | | | |
|---|---|---|---|---|---|
| C6orf25 | HGLibA_06486 | 89 | -0.329657971 | C6orf25 | - |
| CCDC104 | HGLibA_07517 | 89 | -0.329657971 | CCDC104 | - |
| CXorf22 | HGLibA_11904 | 89 | -0.329657971 | CXorf22 | - |
| ENSG00000007944 | HGLibA_30780 | 89 | -0.329657971 | MYLIP | myosin regulatory light chain interacting protein [Source:HGNC Symbol;Acc:HGNC:21155] |
| ENSG00000011021 | HGLibA_09836 | 89 | -0.329657971 | CLCN6 | chloride voltage-gated channel 6 [Source:HGNC Symbol;Acc:HGNC:2024] |
| ENSG00000014216 | HGLibA_07141 | 89 | -0.329657971 | CAPN1 | calpain 1 [Source:HGNC Symbol;Acc:HGNC:1476] |
| ENSG00000023041 | HGLibA_55124 | 89 | -0.329657971 | ZDHHC6 | zinc finger DHHC-type containing 6 [Source:HGNC Symbol;Acc:HGNC:19160] |
| ENSG00000039987 | HGLibA_04422 | 89 | -0.329657971 | BEST2 | bestrophin 2 [Source:HGNC Symbol;Acc:HGNC:17107] |
| ENSG00000046651 | HGLibA_33300 | 89 | -0.329657971 | OFD1 | centriole and centriolar satellite protein [Source:HGNC Symbol;Acc:HGNC:2567]" |
| ENSG00000048028 | HGLibA_53319 | 89 | -0.329657971 | USP28 | ubiquitin specific peptidase 28 [Source:HGNC Symbol;Acc:HGNC:12625] |
| ENSG00000048471 | HGLibA_46077 | 89 | -0.329657971 | SNX29 | sorting nexin 29 [Source:HGNC Symbol;Acc:HGNC:30542] |
| ENSG00000051382 | HGLibA_36636 | 89 | -0.329657971 | PIK3CB | 5-bisphosphate 3-kinase catalytic subunit beta [Source:HGNC Symbol;Acc:HGNC:8976]" |
| ENSG00000057252 | HGLibA_46113 | 89 | -0.329657971 | SOAT1 | sterol O-acyltransferase 1 [Source:HGNC Symbol;Acc:HGNC:11177] |
| ENSG00000066654 | HGLibA_49414 | 89 | -0.329657971 | THUMPD1 | THUMP domain containing 1 [Source:HGNC Symbol;Acc:HGNC:23807] |
| ENSG00000068489 | HGLibA_38770 | 89 | -0.329657971 | PRR11 | proline rich 11 [Source:HGNC Symbol;Acc:HGNC:25619] |
| ENSG00000068784 | HGLibA_46925 | 89 | -0.329657971 | SRBD1 | S1 RNA binding domain 1 [Source:HGNC Symbol;Acc:HGNC:25521] |
| ENSG00000071655 | HGLibA_28470 | 89 | -0.329657971 | MBD3 | methyl-CpG binding domain protein 3 [Source:HGNC Symbol;Acc:HGNC:6918] |
| ENSG00000072657 | HGLibA_51382 | 89 | -0.329657971 | TRHDE | thyrotropin releasing hormone degrading enzyme [Source:HGNC Symbol;Acc:HGNC:30748] |
| ENSG00000072778 | HGLibA_00377 | 89 | -0.329657971 | ACADVL | acyl-CoA dehydrogenase very long chain [Source:HGNC Symbol;Acc:HGNC:92] |
| ENSG00000079393 | HGLibA_14138 | 89 | -0.329657971 | DUSP13 | dual specificity phosphatase 13 [Source:HGNC Symbol;Acc:HGNC:19681] |
| ENSG00000079950 | HGLibA_47656 | 89 | -0.329657971 | STX7 | syntaxin 7 [Source:HGNC Symbol;Acc:HGNC:11442] |
| ENSG00000082781 | HGLibA_23789 | 89 | -0.329657971 | ITGB5 | integrin subunit beta 5 [Source:HGNC Symbol;Acc:HGNC:6160] |
| ENSG00000086189 | HGLibA_13234 | 89 | -0.329657971 | DIMT1 | DIM1 dimethyladenosine transferase 1 homolog [Source:HGNC Symbol;Acc:HGNC:30217] |
| ENSG00000087299 | HGLibA_25889 | 89 | -0.329657971 | L2HGDH | L-2-hydroxyglutarate dehydrogenase [Source:HGNC Symbol;Acc:HGNC:20499] |
| ENSG00000088035 | HGLibA_01635 | 89 | -0.329657971 | ALG6 | 3-glucosyltransferase [Source:HGNC Symbol;Acc:HGNC:23157]" |
| ENSG00000092964 | HGLibA_13926 | 89 | -0.329657971 | DPYSL2 | dihydropyrimidinase like 2 [Source:HGNC Symbol;Acc:HGNC:3014] |
| ENSG00000094631 | HGLibA_21018 | 89 | -0.329657971 | HDAC6 | histone deacetylase 6 [Source:HGNC Symbol;Acc:HGNC:14064] |
| ENSG00000097021 | HGLibA_00489 | 89 | -0.329657971 | ACOT7 | acyl-CoA thioesterase 7 [Source:HGNC Symbol;Acc:HGNC:24157] |
| ENSG00000100142 | HGLibA_37580 | 89 | -0.329657971 | POLR2F | RNA polymerase II subunit F [Source:HGNC Symbol;Acc:HGNC:9193] |
| ENSG00000100147 | HGLibA_07598 | 89 | -0.329657971 | CCDC134 | coiled-coil domain containing 134 [Source:HGNC Symbol;Acc:HGNC:26185] |
| ENSG00000100243 | HGLibA_12006 | 89 | -0.329657971 | CYB5R3 | cytochrome b5 reductase 3 [Source:HGNC Symbol;Acc:HGNC:2873] |
| ENSG00000100726 | HGLibA_48985 | 89 | -0.329657971 | TELO2 | telomere maintenance 2 [Source:HGNC Symbol;Acc:HGNC:29099] |
| ENSG00000100749 | HGLibA_53824 | 89 | -0.329657971 | VRK1 | VRK serine/threonine kinase 1 [Source:HGNC Symbol;Acc:HGNC:12718] |
| ENSG00000101782 | HGLibA_41314 | 89 | -0.329657971 | RIOK3 | RIO kinase 3 [Source:HGNC Symbol;Acc:HGNC:11451] |
| ENSG00000101849 | HGLibA_48608 | 89 | -0.329657971 | TBL1X | transducin beta like 1 X-linked [Source:HGNC Symbol;Acc:HGNC:11585] |
| ENSG00000104320 | HGLibA_31218 | 89 | -0.329657971 | NBN | nibrin [Source:HGNC Symbol;Acc:HGNC:7652] |
| ENSG00000106100 | HGLibA_32245 | 89 | -0.329657971 | NOD1 | nucleotide binding oligomerization domain containing 1 [Source:HGNC Symbol;Acc:HGNC:16390] |
| ENSG00000108443 | HGLibA_42177 | 89 | -0.329657971 | RPS6KB1 | ribosomal protein S6 kinase B1 [Source:HGNC Symbol;Acc:HGNC:10436] |
| ENSG00000109184 | HGLibA_12574 | 89 | -0.329657971 | DCUN1D4 | defective in cullin neddylation 1 domain containing 4 [Source:HGNC Symbol;Acc:HGNC:28998] |
| ENSG00000110245 | HGLibA_02508 | 89 | -0.329657971 | APOC3 | apolipoprotein C3 [Source:HGNC Symbol;Acc:HGNC:610] |
| ENSG00000110852 | HGLibA_09964 | 89 | -0.329657971 | CLEC2B | C-type lectin domain family 2 member B [Source:HGNC Symbol;Acc:HGNC:2053] |
| ENSG00000110900 | HGLibA_51894 | 89 | -0.329657971 | TSPAN11 | tetraspanin 11 [Source:HGNC Symbol;Acc:HGNC:30795] |
| ENSG00000111057 | HGLibA_25439 | 89 | -0.329657971 | KRT18 | keratin 18 [Source:HGNC Symbol;Acc:HGNC:6430] |
| ENSG00000111727 | HGLibA_20965 | 89 | -0.329657971 | HCFC2 | host cell factor C2 [Source:HGNC Symbol;Acc:HGNC:24972] |
| ENSG00000111799 | HGLibA_10505 | 89 | -0.329657971 | COL12A1 | collagen type XII alpha 1 chain [Source:HGNC Symbol;Acc:HGNC:2188] |
| ENSG00000111832 | HGLibA_42480 | 89 | -0.329657971 | RWDD1 | RWD domain containing 1 [Source:HGNC Symbol;Acc:HGNC:20993] |
| ENSG00000112964 | HGLibA_19041 | 89 | -0.329657971 | GHR | growth hormone receptor [Source:HGNC Symbol;Acc:HGNC:4263] |
| ENSG00000113209 | HGLibA_35502 | 89 | -0.329657971 | PCDHB5 | protocadherin beta 5 [Source:HGNC Symbol;Acc:HGNC:8690] |
| ENSG00000114779 | HGLibA_00250 | 89 | -0.329657971 | ABHD14B | abhydrolase domain containing 14B [Source:HGNC Symbol;Acc:HGNC:28235] |
| ENSG00000114796 | HGLibA_25162 | 89 | -0.329657971 | KLHL24 | kelch like family member 24 [Source:HGNC Symbol;Acc:HGNC:25947] |
| ENSG00000114857 | HGLibA_32025 | 89 | -0.329657971 | NKTR | natural killer cell triggering receptor [Source:HGNC Symbol;Acc:HGNC:7833] |
| ENSG00000115194 | HGLibA_44905 | 89 | -0.329657971 | SLC30A3 | solute carrier family 30 member 3 [Source:HGNC Symbol;Acc:HGNC:11014] |
| ENSG00000115239 | HGLibA_03236 | 89 | -0.329657971 | ASB3 | ankyrin repeat and SOCS box containing 3 [Source:HGNC Symbol;Acc:HGNC:16013] |
| ENSG00000115946 | HGLibA_37396 | 89 | -0.329657971 | PNO1 | partner of NOB1 homolog [Source:HGNC Symbol;Acc:HGNC:32790] |
| ENSG00000117394 | HGLibA_44853 | 89 | -0.329657971 | SLC2A1 | solute carrier family 2 member 1 [Source:HGNC Symbol;Acc:HGNC:11005] |
| ENSG00000118194 | HGLibA_50887 | 89 | -0.329657971 | TNNT2 | cardiac type [Source:HGNC Symbol;Acc:HGNC:11949]" |
| ENSG00000119509 | HGLibA_23441 | 89 | -0.329657971 | INVS | inversin [Source:HGNC Symbol;Acc:HGNC:17870] |
| ENSG00000119900 | HGLibA_33320 | 89 | -0.329657971 | OGFRL1 | opioid growth factor receptor like 1 [Source:HGNC Symbol;Acc:HGNC:21378] |
| ENSG00000120093 | HGLibA_21934 | 89 | -0.329657971 | HOXB3 | homeobox B3 [Source:HGNC Symbol;Acc:HGNC:5114] |
| ENSG00000121377 | HGLibA_48427 | 89 | -0.329657971 | TAS2R7 | taste 2 receptor member 7 [Source:HGNC Symbol;Acc:HGNC:14913] |
| ENSG00000121381 | HGLibA_48433 | 89 | -0.329657971 | TAS2R9 | taste 2 receptor member 9 [Source:HGNC Symbol;Acc:HGNC:14917] |
| ENSG00000121653 | HGLibA_28285 | 89 | -0.329657971 | MAPK8IP1 | mitogen-activated protein kinase 8 interacting protein 1 [Source:HGNC Symbol;Acc:HGNC:6882] |
| ENSG00000121671 | HGLibA_11244 | 89 | -0.329657971 | CRY2 | cryptochrome circadian regulator 2 [Source:HGNC Symbol;Acc:HGNC:2385] |
| ENSG00000121964 | HGLibA_20515 | 89 | -0.329657971 | GTDC1 | glycosyltransferase like domain containing 1 [Source:HGNC Symbol;Acc:HGNC:20887] |
| ENSG00000121966 | HGLibA_11891 | 89 | -0.329657971 | CXCR4 | C-X-C motif chemokine receptor 4 [Source:HGNC Symbol;Acc:HGNC:2561] |
| ENSG00000123268 | HGLibA_03432 | 89 | -0.329657971 | ATF1 | activating transcription factor 1 [Source:HGNC Symbol;Acc:HGNC:783] |
| ENSG00000123689 | HGLibA_18292 | 89 | -0.329657971 | G0S2 | G0/G1 switch 2 [Source:HGNC Symbol;Acc:HGNC:30229] |
| ENSG00000128713 | HGLibA_21988 | 89 | -0.329657971 | HOXD11 | homeobox D11 [Source:HGNC Symbol;Acc:HGNC:5134] |
| ENSG00000128713 | HGLibA_21990 | 89 | -0.329657971 | HOXD11 | homeobox D11 [Source:HGNC Symbol;Acc:HGNC:5134] |
| ENSG00000129873 | HGLibA_08913 | 89 | -0.329657971 | CDY2B | chromodomain Y-linked 2B [Source:HGNC Symbol;Acc:HGNC:23921] |
| ENSG00000131171 | HGLibA_43917 | 89 | -0.329657971 | SH3BGRL | SH3 domain binding glutamate rich protein like [Source:HGNC Symbol;Acc:HGNC:10823] |
| ENSG00000131437 | HGLibA_24935 | 89 | -0.329657971 | KIF3A | kinesin family member 3A [Source:HGNC Symbol;Acc:HGNC:6319] |
| ENSG00000133114 | HGLibA_19724 | 89 | -0.329657971 | GPALPP1 | GPALPP motifs containing 1 [Source:HGNC Symbol;Acc:HGNC:20298] |
| ENSG00000133275 | HGLibA_11397 | 89 | -0.329657971 | CSNK1G2 | casein kinase 1 gamma 2 [Source:HGNC Symbol;Acc:HGNC:2455] |
| ENSG00000134285 | HGLibA_17555 | 89 | -0.329657971 | FKBP11 | FK506 binding protein 11 [Source:HGNC Symbol;Acc:HGNC:18624] |
| ENSG00000134597 | HGLibA_40600 | 89 | -0.329657971 | RBMX2 | RNA binding motif protein X-linked 2 [Source:HGNC Symbol;Acc:HGNC:24282] |
| ENSG00000135124 | HGLibA_34855 | 89 | -0.329657971 | P2RX4 | purinergic receptor P2X 4 [Source:HGNC Symbol;Acc:HGNC:8535] |
| ENSG00000135569 | HGLibA_48130 | 89 | -0.329657971 | TAAR5 | trace amine associated receptor 5 [Source:HGNC Symbol;Acc:HGNC:30236] |
| ENSG00000135966 | HGLibA_49235 | 89 | -0.329657971 | TGFBRAP1 | transforming growth factor beta receptor associated protein 1 [Source:HGNC Symbol;Acc:HGNC:16836] |
| ENSG00000138594 | HGLibA_50601 | 89 | -0.329657971 | TMOD3 | tropomodulin 3 [Source:HGNC Symbol;Acc:HGNC:11873] |
| ENSG00000138670 | HGLibA_40332 | 89 | -0.329657971 | RASGEF1B | RasGEF domain family member 1B [Source:HGNC Symbol;Acc:HGNC:24881] |
| ENSG00000139915 | HGLibA_28680 | 89 | -0.329657971 | MDGA2 | MAM domain containing glycosylphosphatidylinositol anchor 2 [Source:HGNC Symbol;Acc:HGNC:19835] |
| ENSG00000141579 | HGLibA_56675 | 89 | -0.329657971 | ZNF750 | zinc finger protein 750 [Source:HGNC Symbol;Acc:HGNC:25843] |
| ENSG00000141642 | HGLibA_14869 | 89 | -0.329657971 | ELAC1 | elaC ribonuclease Z 1 [Source:HGNC Symbol;Acc:HGNC:14197] |
| ENSG00000142065 | HGLibA_55190 | 89 | -0.329657971 | ZFP14 | ZFP14 zinc finger protein [Source:HGNC Symbol;Acc:HGNC:29312] |
| ENSG00000142856 | HGLibA_23783 | 89 | -0.329657971 | ITGB3BP | integrin subunit beta 3 binding protein [Source:HGNC Symbol;Acc:HGNC:6157] |
| ENSG00000143167 | HGLibA_19717 | 89 | -0.329657971 | GPA33 | glycoprotein A33 [Source:HGNC Symbol;Acc:HGNC:4445] |
| ENSG00000143867 | HGLibA_34698 | 89 | -0.329657971 | OSR1 | odd-skipped related transciption factor 1 [Source:HGNC Symbol;Acc:HGNC:8111] |
| ENSG00000145781 | HGLibA_10654 | 89 | -0.329657971 | COMMD10 | COMM domain containing 10 [Source:HGNC Symbol;Acc:HGNC:30201] |
| ENSG00000149516 | HGLibA_30147 | 89 | -0.329657971 | MS4A3 | membrane spanning 4-domains A3 [Source:HGNC Symbol;Acc:HGNC:7317] |
| ENSG00000150401 | HGLibA_12567 | 89 | -0.329657971 | DCUN1D2 | defective in cullin neddylation 1 domain containing 2 [Source:HGNC Symbol;Acc:HGNC:20328] |
| ENSG00000150893 | HGLibA_17986 | 89 | -0.329657971 | FREM2 | FRAS1 related extracellular matrix protein 2 [Source:HGNC Symbol;Acc:HGNC:25396] |
| ENSG00000150938 | HGLibA_11147 | 89 | -0.329657971 | CRIM1 | cysteine rich transmembrane BMP regulator 1 [Source:HGNC Symbol;Acc:HGNC:2359] |
| ENSG00000151023 | HGLibA_15136 | 89 | -0.329657971 | ENKUR | TRPC channel interacting protein [Source:HGNC Symbol;Acc:HGNC:28388]" |
| ENSG00000151151 | HGLibA_23456 | 89 | -0.329657971 | IPMK | inositol polyphosphate multikinase [Source:HGNC Symbol;Acc:HGNC:20739] |
| ENSG00000157103 | HGLibA_45288 | 89 | -0.329657971 | SLC6A1 | solute carrier family 6 member 1 [Source:HGNC Symbol;Acc:HGNC:11042] |
| ENSG00000158555 | HGLibA_18903 | 89 | -0.329657971 | GDPD5 | glycerophosphodiester phosphodiesterase domain containing 5 [Source:HGNC Symbol;Acc:HGNC:28804] |
| ENSG00000159763 | HGLibA_36702 | 89 | -0.329657971 | PIP | prolactin induced protein [Source:HGNC Symbol;Acc:HGNC:8993] |
| ENSG00000161011 | HGLibA_46919 | 89 | -0.329657971 | SQSTM1 | sequestosome 1 [Source:HGNC Symbol;Acc:HGNC:11280] |
| ENSG00000161048 | HGLibA_31131 | 89 | -0.329657971 | NAPEPLD | N-acyl phosphatidylethanolamine phospholipase D [Source:HGNC Symbol;Acc:HGNC:21683] |
| ENSG00000161057 | HGLibA_39130 | 89 | -0.329657971 | PSMC2 | ATPase 2 [Source:HGNC Symbol;Acc:HGNC:9548]" |
| ENSG00000162542 | HGLibA_49790 | 89 | -0.329657971 | TMCO4 | transmembrane and coiled-coil domains 4 [Source:HGNC Symbol;Acc:HGNC:27393] |
| ENSG00000162771 | HGLibA_16687 | 89 | -0.329657971 | FAM71A | family with sequence similarity 71 member A [Source:HGNC Symbol;Acc:HGNC:26541] |
| ENSG00000163406 | HGLibA_44408 | 89 | -0.329657971 | SLC15A2 | solute carrier family 15 member 2 [Source:HGNC Symbol;Acc:HGNC:10921] |
| ENSG00000164076 | HGLibA_07096 | 89 | -0.329657971 | CAMKV | CaM kinase like vesicle associated [Source:HGNC Symbol;Acc:HGNC:28788] |
| ENSG00000164299 | HGLibA_46909 | 89 | -0.329657971 | SPZ1 | spermatogenic leucine zipper 1 [Source:HGNC Symbol;Acc:HGNC:30721] |
| ENSG00000165349 | HGLibA_45364 | 89 | -0.329657971 | SLC7A3 | solute carrier family 7 member 3 [Source:HGNC Symbol;Acc:HGNC:11061] |
| ENSG00000165804 | HGLibA_55604 | 89 | -0.329657971 | ZNF219 | zinc finger protein 219 [Source:HGNC Symbol;Acc:HGNC:13011] |
| ENSG00000166166 | HGLibA_51697 | 89 | -0.329657971 | TRMT61A | tRNA methyltransferase 61A [Source:HGNC Symbol;Acc:HGNC:23790] |
| ENSG00000166183 | HGLibA_03346 | 89 | -0.329657971 | ASPG | asparaginase [Source:HGNC Symbol;Acc:HGNC:20123] |
| ENSG00000166887 | HGLibA_53789 | 89 | -0.329657971 | VPS39 | HOPS complex subunit [Source:HGNC Symbol;Acc:HGNC:20593]" |
| ENSG00000167615 | HGLibA_26301 | 89 | -0.329657971 | LENG8 | leukocyte receptor cluster member 8 [Source:HGNC Symbol;Acc:HGNC:15500] |
| ENSG00000168065 | HGLibA_44539 | 89 | -0.329657971 | SLC22A11 | solute carrier family 22 member 11 [Source:HGNC Symbol;Acc:HGNC:18120] |
| ENSG00000168306 | HGLibA_00499 | 89 | -0.329657971 | ACOX2 | acyl-CoA oxidase 2 [Source:HGNC Symbol;Acc:HGNC:120] |
| ENSG00000168397 | HGLibA_03490 | 89 | -0.329657971 | ATG4B | autophagy related 4B cysteine peptidase [Source:HGNC Symbol;Acc:HGNC:20790] |
| ENSG00000168734 | HGLibA_36825 | 89 | -0.329657971 | PKIG | cAMP-dependent protein kinase inhibitor gamma [Source:HGNC Symbol;Acc:HGNC:9019] |
| ENSG00000168785 | HGLibA_51940 | 89 | -0.329657971 | TSPAN5 | tetraspanin 5 [Source:HGNC Symbol;Acc:HGNC:17753] |
| ENSG00000169733 | HGLibA_40925 | 89 | -0.329657971 | RFNG | RFNG O-fucosylpeptide 3-beta-N-acetylglucosaminyltransferase [Source:HGNC Symbol;Acc:HGNC:9974] |
| ENSG00000169891 | HGLibA_40851 | 89 | -0.329657971 | REPS2 | RALBP1 associated Eps domain containing 2 [Source:HGNC Symbol;Acc:HGNC:9963] |
| ENSG00000170412 | HGLibA_20130 | 89 | -0.329657971 | GPRC5C | G protein-coupled receptor class C group 5 member C [Source:HGNC Symbol;Acc:HGNC:13309] |
| ENSG00000170786 | HGLibA_43161 | 89 | -0.329657971 | SDR16C5 | short chain dehydrogenase/reductase family 16C member 5 [Source:HGNC Symbol;Acc:HGNC:30311] |
| ENSG00000170959 | HGLibA_12482 | 89 | -0.329657971 | DCDC1 | doublecortin domain containing 1 [Source:HGNC Symbol;Acc:HGNC:20625] |
| ENSG00000170965 | HGLibA_36932 | 89 | -0.329657971 | PLAC1 | placenta specific 1 [Source:HGNC Symbol;Acc:HGNC:9044] |
| ENSG00000171303 | HGLibA_24321 | 89 | -0.329657971 | KCNK3 | potassium two pore domain channel subfamily K member 3 [Source:HGNC Symbol;Acc:HGNC:6278] |
| ENSG00000171456 | HGLibA_03390 | 89 | -0.329657971 | ASXL1 | ASXL transcriptional regulator 1 [Source:HGNC Symbol;Acc:HGNC:18318] |
| ENSG00000172348 | HGLibA_40672 | 89 | -0.329657971 | RCAN2 | regulator of calcineurin 2 [Source:HGNC Symbol;Acc:HGNC:3041] |
| ENSG00000172590 | HGLibA_30015 | 89 | -0.329657971 | MRPL52 | mitochondrial ribosomal protein L52 [Source:HGNC Symbol;Acc:HGNC:16655] |
| ENSG00000174547 | HGLibA_29893 | 89 | -0.329657971 | MRPL11 | mitochondrial ribosomal protein L11 [Source:HGNC Symbol;Acc:HGNC:14042] |
| ENSG00000175294 | HGLibA_07355 | 89 | -0.329657971 | CATSPER1 | cation channel sperm associated 1 [Source:HGNC Symbol;Acc:HGNC:17116] |
| ENSG00000175518 | HGLibA_52744 | 89 | -0.329657971 | UBQLNL | ubiquilin like [Source:HGNC Symbol;Acc:HGNC:28294] |
| ENSG00000175906 | HGLibA_02972 | 89 | -0.329657971 | ARL4D | ADP ribosylation factor like GTPase 4D [Source:HGNC Symbol;Acc:HGNC:656] |
| ENSG00000176095 | HGLibA_23446 | 89 | -0.329657971 | IP6K1 | inositol hexakisphosphate kinase 1 [Source:HGNC Symbol;Acc:HGNC:18360] |
| ENSG00000178172 | HGLibA_46701 | 89 | -0.329657971 | SPINK6 | Kazal type 6 [Source:HGNC Symbol;Acc:HGNC:29486]" |
| ENSG00000178342 | HGLibA_24202 | 89 | -0.329657971 | KCNG2 | potassium voltage-gated channel modifier subfamily G member 2 [Source:HGNC Symbol;Acc:HGNC:6249] |
| ENSG00000179954 | HGLibA_47127 | 89 | -0.329657971 | SSC5D | scavenger receptor cysteine rich family member with 5 domains [Source:HGNC Symbol;Acc:HGNC:26641] |

| | | | | | |
|---|---|---|---|---|---|
| ENSG00000180919 | HGLibA_34227 | 89 | -0.329657971 | OR56B4 | olfactory receptor family 56 subfamily B member 4 [Source:HGNC Symbol;Acc:HGNC:15248] |
| ENSG00000181499 | HGLibA_34444 | 89 | -0.329657971 | OR6T1 | olfactory receptor family 6 subfamily T member 1 [Source:HGNC Symbol;Acc:HGNC:14848] |
| ENSG00000182791 | HGLibA_07903 | 89 | -0.329657971 | CCDC87 | coiled-coil domain containing 87 [Source:HGNC Symbol;Acc:HGNC:25579] |
| ENSG00000183549 | HGLibA_00583 | 89 | -0.329657971 | ACSM5 | acyl-CoA synthetase medium chain family member 5 [Source:HGNC Symbol;Acc:HGNC:26060] |
| ENSG00000183856 | HGLibA_23537 | 89 | -0.329657971 | IQGAP3 | IQ motif containing GTPase activating protein 3 [Source:HGNC Symbol;Acc:HGNC:20669] |
| ENSG00000183876 | HGLibA_03148 | 89 | -0.329657971 | ARSI | arylsulfatase family member I [Source:HGNC Symbol;Acc:HGNC:32521] |
| ENSG00000185518 | HGLibA_47878 | 89 | -0.329657971 | SV2B | synaptic vesicle glycoprotein 2B [Source:HGNC Symbol;Acc:HGNC:16874] |
| ENSG00000186038 | HGLibA_22393 | 89 | -0.329657971 | HTR3E | 5-hydroxytryptamine receptor 3E [Source:HGNC Symbol;Acc:HGNC:24005] |
| ENSG00000186642 | HGLibA_35778 | 89 | -0.329657971 | PDE2A | phosphodiesterase 2A [Source:HGNC Symbol;Acc:HGNC:8777] |
| ENSG00000187605 | HGLibA_49043 | 89 | -0.329657971 | TET3 | tet methylcytosine dioxygenase 3 [Source:HGNC Symbol;Acc:HGNC:28313] |
| ENSG00000187624 | HGLibA_05596 | 89 | -0.329657971 | C17orf97 | chromosome 17 open reading frame 97 [Source:HGNC Symbol;Acc:HGNC:33800] |
| ENSG00000188869 | HGLibA_49755 | 89 | -0.329657971 | TMC3 | transmembrane channel like 3 [Source:HGNC Symbol;Acc:HGNC:22995] |
| ENSG00000189132 | HGLibA_16610 | 89 | -0.329657971 | FAM47B | family with sequence similarity 47 member B [Source:HGNC Symbol;Acc:HGNC:26659] |
| ENSG00000197172 | HGLibA_27917 | 89 | -0.329657971 | MAGEA6 | MAGE family member A6 [Source:HGNC Symbol;Acc:HGNC:6804] |
| ENSG00000197885 | HGLibA_32016 | 89 | -0.329657971 | NKIRAS1 | NFKB inhibitor interacting Ras like 1 [Source:HGNC Symbol;Acc:HGNC:17899] |
| ENSG00000197992 | HGLibA_10010 | 89 | -0.329657971 | CLEC9A | C-type lectin domain containing 9A [Source:HGNC Symbol;Acc:HGNC:26705] |
| ENSG00000198865 | HGLibA_07650 | 89 | -0.329657971 | CCDC152 | coiled-coil domain containing 152 [Source:HGNC Symbol;Acc:HGNC:34438] |
| ENSG00000204449 | HGLibA_51527 | 89 | -0.329657971 | TRIM49C | tripartite motif containing 49C [Source:HGNC Symbol;Acc:HGNC:38877] |
| ENSG00000204577 | HGLibA_26513 | 89 | -0.329657971 | LILRB3 | leukocyte immunoglobulin like receptor B3 [Source:HGNC Symbol;Acc:HGNC:6607] |
| ENSG00000205784 | HGLibA_03124 | 89 | -0.329657971 | ARRDC5 | arrestin domain containing 5 [Source:HGNC Symbol;Acc:HGNC:31407] |
| ENSG00000214128 | HGLibA_50213 | 89 | -0.329657971 | TMEM213 | transmembrane protein 213 [Source:HGNC Symbol;Acc:HGNC:27220] |
| ENSG00000225190 | HGLibA_37137 | 89 | -0.329657971 | PLEKHM1 | pleckstrin homology and RUN domain containing M1 [Source:HGNC Symbol;Acc:HGNC:29017] |
| ENSG00000232263 | HGLibA_25728 | 89 | -0.329657971 | KRTAP25-1 | keratin associated protein 25-1 [Source:HGNC Symbol;Acc:HGNC:34003] |
| ENSG00000234602 | HGLibA_28595 | 89 | -0.329657971 | MCIDAS | multiciliate differentiation and DNA synthesis associated cell cycle protein [Source:HGNC Symbol;Acc:HGNC:40050] |
| ENSG00000235034 | HGLibA_05715 | 89 | -0.329657971 | C19orf81 | chromosome 19 open reading frame 81 [Source:HGNC Symbol;Acc:HGNC:40041] |
| ENSG00000240184 | HGLibA_35572 | 89 | -0.329657971 | PCDHGC3 | 3 [Source:HGNC Symbol;Acc:HGNC:8716]" |
| ENSG00000240654 | HGLibA_05960 | 89 | -0.329657971 | C1QTNF9 | C1q and TNF related 9 [Source:HGNC Symbol;Acc:HGNC:28732] |
| ENSG00000240764 | HGLibA_35576 | 89 | -0.329657971 | PCDHGC5 | 5 [Source:HGNC Symbol;Acc:HGNC:8718]" |
| ENSG00000241697 | HGLibA_49831 | 89 | -0.329657971 | TMEFF1 | transmembrane protein with EGF like and two follistatin like domains 1 [Source:HGNC Symbol;Acc:HGNC:11866] |
| ENSG00000253626 | HGLibA_14861 | 89 | -0.329657971 | EIF5AL1 | eukaryotic translation initiation factor 5A-like 1 [Source:HGNC Symbol;Acc:HGNC:17419] |
| ENSG00000257446 | HGLibA_56887 | 89 | -0.329657971 | ZNF878 | zinc finger protein 878 [Source:HGNC Symbol;Acc:HGNC:37246] |
| ENSG00000258724 | HGLibA_36695 | 89 | -0.329657971 | PINX1 | PIN2 (TERF1) interacting telomerase inhibitor 1 [Source:HGNC Symbol;Acc:HGNC:30046] |
| ENSG00000265972 | HGLibA_52433 | 89 | -0.329657971 | TXNIP | thioredoxin interacting protein [Source:HGNC Symbol;Acc:HGNC:16952] |
| ENSG00000277150 | HGLibA_15891 | 89 | -0.329657971 | F8A3 | coagulation factor VIII associated 3 [Source:HGNC Symbol;Acc:HGNC:31850] |
| ENSG00000288705 | HGLibA_52887 | 89 | -0.329657971 | UGT1A5 | - |
| FAM159A | HGLibA_16198 | 89 | -0.329657971 | FAM159A | - |
| FTSJD1 | HGLibA_18143 | 89 | -0.329657971 | FTSJD1 | - |
| GATSL1 | HGLibA_18701 | 89 | -0.329657971 | GATSL1 | - |
| hsa-mir-188 | HGLibA_58013 | 89 | -0.329657971 | hsa-mir-188 | - |
| hsa-mir-190b | HGLibA_58039 | 89 | -0.329657971 | hsa-mir-190b | - |
| hsa-mir-3937 | HGLibA_59608 | 89 | -0.329657971 | hsa-mir-3937 | - |
| hsa-mir-4325 | HGLibA_60025 | 89 | -0.329657971 | hsa-mir-4325 | - |
| hsa-mir-4433b | HGLibA_60124 | 89 | -0.329657971 | hsa-mir-4433b | - |
| hsa-mir-4486 | HGLibA_60362 | 89 | -0.329657971 | hsa-mir-4486 | - |
| hsa-mir-4539 | HGLibA_60618 | 89 | -0.329657971 | hsa-mir-4539 | - |
| hsa-mir-4667 | HGLibA_60786 | 89 | -0.329657971 | hsa-mir-4667 | - |
| hsa-mir-4678 | HGLibA_60828 | 89 | -0.329657971 | hsa-mir-4678 | - |
| hsa-mir-4684 | HGLibA_60845 | 89 | -0.329657971 | hsa-mir-4684 | - |
| hsa-mir-4696 | HGLibA_60893 | 89 | -0.329657971 | hsa-mir-4696 | - |
| hsa-mir-496 | HGLibA_61385 | 89 | -0.329657971 | hsa-mir-496 | - |
| hsa-mir-4999 | HGLibA_61397 | 89 | -0.329657971 | hsa-mir-4999 | - |
| hsa-mir-502 | HGLibA_61463 | 89 | -0.329657971 | hsa-mir-502 | - |
| hsa-mir-548h-1 | HGLibA_61994 | 89 | -0.329657971 | hsa-mir-548h-1 | - |
| hsa-mir-5581 | HGLibA_62163 | 89 | -0.329657971 | hsa-mir-5581 | - |
| hsa-mir-597 | HGLibA_62481 | 89 | -0.329657971 | hsa-mir-597 | - |
| hsa-mir-6128 | HGLibA_62661 | 89 | -0.329657971 | hsa-mir-6128 | - |
| hsa-mir-6511a-2 | HGLibA_62903 | 89 | -0.329657971 | hsa-mir-6511a-2 | - |
| hsa-mir-6737 | HGLibA_63101 | 89 | -0.329657971 | hsa-mir-6737 | - |
| hsa-mir-6744 | HGLibA_63130 | 89 | -0.329657971 | hsa-mir-6744 | - |
| hsa-mir-6770-3 | HGLibA_63255 | 89 | -0.329657971 | hsa-mir-6770-3 | - |
| hsa-mir-6787 | HGLibA_63324 | 89 | -0.329657971 | hsa-mir-6787 | - |
| hsa-mir-6798 | HGLibA_63371 | 89 | -0.329657971 | hsa-mir-6798 | - |
| hsa-mir-6866 | HGLibA_63647 | 89 | -0.329657971 | hsa-mir-6866 | - |
| hsa-mir-8089 | HGLibA_64199 | 89 | -0.329657971 | hsa-mir-8089 | - |
| hsa-mir-9-3 | HGLibA_64270 | 89 | -0.329657971 | hsa-mir-9-3 | - |
| hsa-mir-936 | HGLibA_64318 | 89 | -0.329657971 | hsa-mir-936 | - |
| SPANXB2 | HGLibA_46386 | 89 | -0.329657971 | SPANXB2 | - |
| WASH1 | HGLibA_53962 | 89 | -0.329657971 | WASH1 | - |
| WBSCR27 | HGLibA_53998 | 89 | -0.329657971 | WBSCR27 | - |
| ATP5SL | HGLibA_03692 | 88 | -0.338703314 | ATP5SL | - |
| C12orf23 | HGLibA_05227 | 88 | -0.338703314 | C12orf23 | - |
| C1orf61 | HGLibA_05879 | 88 | -0.338703314 | C1orf61 | - |
| C4orf21 | HGLibA_06303 | 88 | -0.338703314 | C4orf21 | - |
| C5orf30 | HGLibA_06373 | 88 | -0.338703314 | C5orf30 | - |
| C6orf211 | HGLibA_06474 | 88 | -0.338703314 | C6orf211 | - |
| CASC1 | HGLibA_07268 | 88 | -0.338703314 | CASC1 | - |
| DFNB59 | HGLibA_13030 | 88 | -0.338703314 | DFNB59 | - |
| EMR3 | HGLibA_15089 | 88 | -0.338703314 | EMR3 | - |
| ENSG00000005073 | HGLibA_21898 | 88 | -0.338703314 | HOXA11 | homeobox A11 [Source:HGNC Symbol;Acc:HGNC:5101] |
| ENSG00000005981 | HGLibA_03238 | 88 | -0.338703314 | ASB4 | ankyrin repeat and SOCS box containing 4 [Source:HGNC Symbol;Acc:HGNC:16009] |
| ENSG00000007237 | HGLibA_18655 | 88 | -0.338703314 | GAS7 | growth arrest specific 7 [Source:HGNC Symbol;Acc:HGNC:4169] |
| ENSG00000026950 | HGLibA_04967 | 88 | -0.338703314 | BTN3A1 | butyrophilin subfamily 3 member A1 [Source:HGNC Symbol;Acc:HGNC:1138] |
| ENSG00000042317 | HGLibA_46528 | 88 | -0.338703314 | SPATA7 | spermatogenesis associated 7 [Source:HGNC Symbol;Acc:HGNC:20423] |
| ENSG00000065308 | HGLibA_51287 | 88 | -0.338703314 | TRAM2 | translocation associated membrane protein 2 [Source:HGNC Symbol;Acc:HGNC:16855] |
| ENSG00000069345 | HGLibA_13514 | 88 | -0.338703314 | DNAJA2 | DnaJ heat shock protein family (Hsp40) member A2 [Source:HGNC Symbol;Acc:HGNC:14884] |
| ENSG00000075292 | HGLibA_56437 | 88 | -0.338703314 | ZNF638 | zinc finger protein 638 [Source:HGNC Symbol;Acc:HGNC:17894] |
| ENSG00000078401 | HGLibA_14468 | 88 | -0.338703314 | EDN1 | endothelin 1 [Source:HGNC Symbol;Acc:HGNC:3176] |
| ENSG00000090512 | HGLibA_17306 | 88 | -0.338703314 | FETUB | fetuin B [Source:HGNC Symbol;Acc:HGNC:3658] |
| ENSG00000091542 | HGLibA_01659 | 88 | -0.338703314 | ALKBH5 | RNA demethylase [Source:HGNC Symbol;Acc:HGNC:25996]" |
| ENSG00000092847 | HGLibA_01206 | 88 | -0.338703314 | AGO1 | RISC catalytic component [Source:HGNC Symbol;Acc:HGNC:3262]" |
| ENSG00000097033 | HGLibA_43956 | 88 | -0.338703314 | SH3GLB1 | endophilin B1 [Source:HGNC Symbol;Acc:HGNC:10833]" |
| ENSG00000099956 | HGLibA_45620 | 88 | -0.338703314 | SMARCB1 | member 1 [Source:HGNC Symbol;Acc:HGNC:11103]" |
| ENSG00000100003 | HGLibA_43192 | 88 | -0.338703314 | SEC14L2 | SEC14 like lipid binding 2 [Source:HGNC Symbol;Acc:HGNC:10699] |
| ENSG00000100234 | HGLibA_49540 | 88 | -0.338703314 | TIMP3 | TIMP metallopeptidase inhibitor 3 [Source:HGNC Symbol;Acc:HGNC:11822] |
| ENSG00000100297 | HGLibA_28615 | 88 | -0.338703314 | MCM5 | minichromosome maintenance complex component 5 [Source:HGNC Symbol;Acc:HGNC:6948] |
| ENSG00000100417 | HGLibA_37314 | 88 | -0.338703314 | PMM1 | phosphomannomutase 1 [Source:HGNC Symbol;Acc:HGNC:9114] |
| ENSG00000100461 | HGLibA_40513 | 88 | -0.338703314 | RBM23 | RNA binding motif protein 23 [Source:HGNC Symbol;Acc:HGNC:20155] |
| ENSG00000101057 | HGLibA_30647 | 88 | -0.338703314 | MYBL2 | MYB proto-oncogene like 2 [Source:HGNC Symbol;Acc:HGNC:7548] |
| ENSG00000102181 | HGLibA_08494 | 88 | -0.338703314 | CD99L2 | CD99 molecule like 2 [Source:HGNC Symbol;Acc:HGNC:18237] |
| ENSG00000102349 | HGLibA_25084 | 88 | -0.338703314 | KLF8 | Kruppel like factor 8 [Source:HGNC Symbol;Acc:HGNC:6351] |
| ENSG00000103018 | HGLibA_11993 | 88 | -0.338703314 | CYB5B | cytochrome b5 type B [Source:HGNC Symbol;Acc:HGNC:24374] |
| ENSG00000103932 | HGLibA_41813 | 88 | -0.338703314 | RPAP1 | RNA polymerase II associated protein 1 [Source:HGNC Symbol;Acc:HGNC:24567] |
| ENSG00000105649 | HGLibA_39907 | 88 | -0.338703314 | RAB3A | member RAS oncogene family [Source:HGNC Symbol;Acc:HGNC:9777]" |
| ENSG00000105722 | HGLibA_15461 | 88 | -0.338703314 | ERF | ETS2 repressor factor [Source:HGNC Symbol;Acc:HGNC:3444] |
| ENSG00000107821 | HGLibA_24086 | 88 | -0.338703314 | KAZALD1 | Kazal type serine peptidase inhibitor domain 1 [Source:HGNC Symbol;Acc:HGNC:25460] |
| ENSG00000108100 | HGLibA_08133 | 88 | -0.338703314 | CCNY | cyclin Y [Source:HGNC Symbol;Acc:HGNC:23354] |
| ENSG00000109323 | HGLibA_28086 | 88 | -0.338703314 | MANBA | mannosidase beta [Source:HGNC Symbol;Acc:HGNC:6831] |
| ENSG00000109743 | HGLibA_04882 | 88 | -0.338703314 | BST1 | bone marrow stromal cell antigen 1 [Source:HGNC Symbol;Acc:HGNC:1118] |
| ENSG00000109819 | HGLibA_37847 | 88 | -0.338703314 | PPARGC1A | PPARG coactivator 1 alpha [Source:HGNC Symbol;Acc:HGNC:9237] |
| ENSG00000109927 | HGLibA_48952 | 88 | -0.338703314 | TECTA | tectorin alpha [Source:HGNC Symbol;Acc:HGNC:11720] |
| ENSG00000111669 | HGLibA_51135 | 88 | -0.338703314 | TPI1 | triosephosphate isomerase 1 [Source:HGNC Symbol;Acc:HGNC:12009] |
| ENSG00000111877 | HGLibA_28629 | 88 | -0.338703314 | MCM9 | minichromosome maintenance 9 homologous recombination repair factor [Source:HGNC Symbol;Acc:HGNC:21484] |
| ENSG00000112242 | HGLibA_14312 | 88 | -0.338703314 | E2F3 | E2F transcription factor 3 [Source:HGNC Symbol;Acc:HGNC:3115] |
| ENSG00000112320 | HGLibA_46121 | 88 | -0.338703314 | SOBP | sine oculis binding protein homolog [Source:HGNC Symbol;Acc:HGNC:29256] |
| ENSG00000113712 | HGLibA_11381 | 88 | -0.338703314 | CSNK1A1 | casein kinase 1 alpha 1 [Source:HGNC Symbol;Acc:HGNC:2451] |
| ENSG00000113719 | HGLibA_15467 | 88 | -0.338703314 | ERGIC1 | endoplasmic reticulum-golgi intermediate compartment 1 [Source:HGNC Symbol;Acc:HGNC:29205] |
| ENSG00000114854 | HGLibA_50865 | 88 | -0.338703314 | TNNC1 | slow skeletal and cardiac type [Source:HGNC Symbol;Acc:HGNC:11943]" |
| ENSG00000115942 | HGLibA_34603 | 88 | -0.338703314 | ORC2 | origin recognition complex subunit 2 [Source:HGNC Symbol;Acc:HGNC:8488] |
| ENSG00000117122 | HGLibA_29041 | 88 | -0.338703314 | MFAP2 | microfibril associated protein 2 [Source:HGNC Symbol;Acc:HGNC:7033] |
| ENSG00000117228 | HGLibA_18727 | 88 | -0.338703314 | GBP1 | guanylate binding protein 1 [Source:HGNC Symbol;Acc:HGNC:4182] |
| ENSG00000117262 | HGLibA_20107 | 88 | -0.338703314 | GPR89A | G protein-coupled receptor 89A [Source:HGNC Symbol;Acc:HGNC:31984] |
| ENSG00000118518 | HGLibA_41534 | 88 | -0.338703314 | RNF146 | ring finger protein 146 [Source:HGNC Symbol;Acc:HGNC:21336] |
| ENSG00000118855 | HGLibA_29080 | 88 | -0.338703314 | MFSD1 | major facilitator superfamily domain containing 1 [Source:HGNC Symbol;Acc:HGNC:25874] |
| ENSG00000119655 | HGLibA_32424 | 88 | -0.338703314 | NPC2 | NPC intracellular cholesterol transporter 2 [Source:HGNC Symbol;Acc:HGNC:14537] |
| ENSG00000120647 | HGLibA_07867 | 88 | -0.338703314 | CCDC77 | coiled-coil domain containing 77 [Source:HGNC Symbol;Acc:HGNC:28203] |
| ENSG00000121152 | HGLibA_31276 | 88 | -0.338703314 | NCAPH | non-SMC condensin I complex subunit H [Source:HGNC Symbol;Acc:HGNC:1112] |
| ENSG00000122042 | HGLibA_52701 | 88 | -0.338703314 | UBL3 | ubiquitin like 3 [Source:HGNC Symbol;Acc:HGNC:12504] |
| ENSG00000123104 | HGLibA_23855 | 88 | -0.338703314 | ITPR2 | 5-trisphosphate receptor type 2 [Source:HGNC Symbol;Acc:HGNC:6181]" |
| ENSG00000124459 | HGLibA_56026 | 88 | -0.338703314 | ZNF45 | zinc finger protein 45 [Source:HGNC Symbol;Acc:HGNC:13111] |
| ENSG00000127314 | HGLibA_40224 | 88 | -0.338703314 | RAP1B | member of RAS oncogene family [Source:HGNC Symbol;Acc:HGNC:9857]" |
| ENSG00000129055 | HGLibA_01872 | 88 | -0.338703314 | ANAPC13 | anaphase promoting complex subunit 13 [Source:HGNC Symbol;Acc:HGNC:24540] |
| ENSG00000130164 | HGLibA_26238 | 88 | -0.338703314 | LDLR | low density lipoprotein receptor [Source:HGNC Symbol;Acc:HGNC:6547] |
| ENSG00000130545 | HGLibA_11075 | 88 | -0.338703314 | CRB3 | cell polarity complex component [Source:HGNC Symbol;Acc:HGNC:20237]" |
| ENSG00000132518 | HGLibA_20663 | 88 | -0.338703314 | GUCY2D | retinal [Source:HGNC Symbol;Acc:HGNC:4689]" |
| ENSG00000132670 | HGLibA_39483 | 88 | -0.338703314 | PTPRA | receptor type A [Source:HGNC Symbol;Acc:HGNC:9664]" |

| | | | | | |
|---|---|---|---|---|---|
| ENSG00000134160 | HGLibA_51753 | 88 | -0.338703314 | TRPM1 | transient receptor potential cation channel subfamily M member 1 [Source:HGNC Symbol;Acc:HGNC:7146] |
| ENSG00000135040 | HGLibA_30991 | 88 | -0.338703314 | NAA35 | NatC auxiliary subunit [Source:HGNC Symbol;Acc:HGNC:24340]" |
| ENSG00000136603 | HGLibA_44282 | 88 | -0.338703314 | SKIL | SKI like proto-oncogene [Source:HGNC Symbol;Acc:HGNC:10897] |
| ENSG00000136874 | HGLibA_47628 | 88 | -0.338703314 | STX17 | syntaxin 17 [Source:HGNC Symbol;Acc:HGNC:11432] |
| ENSG00000137509 | HGLibA_38358 | 88 | -0.338703314 | PRCP | prolylcarboxypeptidase [Source:HGNC Symbol;Acc:HGNC:9344] |
| ENSG00000137814 | HGLibA_20878 | 88 | -0.338703314 | HAUS2 | HAUS augmin like complex subunit 2 [Source:HGNC Symbol;Acc:HGNC:25530] |
| ENSG00000138684 | HGLibA_23081 | 88 | -0.338703314 | IL21 | interleukin 21 [Source:HGNC Symbol;Acc:HGNC:6005] |
| ENSG00000139144 | HGLibA_36626 | 88 | -0.338703314 | PIK3C2G | phosphatidylinositol-4-phosphate 3-kinase catalytic subunit type 2 gamma [Source:HGNC Symbol;Acc:HGNC:8973] |
| ENSG00000139410 | HGLibA_43175 | 88 | -0.338703314 | SDSL | serine dehydratase like [Source:HGNC Symbol;Acc:HGNC:30404] |
| ENSG00000139974 | HGLibA_45076 | 88 | -0.338703314 | SLC38A6 | solute carrier family 38 member 6 [Source:HGNC Symbol;Acc:HGNC:19863] |
| ENSG00000140274 | HGLibA_14106 | 88 | -0.338703314 | DUOXA2 | dual oxidase maturation factor 2 [Source:HGNC Symbol;Acc:HGNC:32698] |
| ENSG00000140525 | HGLibA_16854 | 88 | -0.338703314 | FANCI | FA complementation group I [Source:HGNC Symbol;Acc:HGNC:25568] |
| ENSG00000141200 | HGLibA_24929 | 88 | -0.338703314 | KIF2B | kinesin family member 2B [Source:HGNC Symbol;Acc:HGNC:29443] |
| ENSG00000141404 | HGLibA_19460 | 88 | -0.338703314 | GNAL | G protein subunit alpha L [Source:HGNC Symbol;Acc:HGNC:4388] |
| ENSG00000141497 | HGLibA_55420 | 88 | -0.338703314 | ZMYND15 | zinc finger MYND-type containing 15 [Source:HGNC Symbol;Acc:HGNC:20997] |
| ENSG00000142089 | HGLibA_22646 | 88 | -0.338703314 | IFITM3 | interferon induced transmembrane protein 3 [Source:HGNC Symbol;Acc:HGNC:5414] |
| ENSG00000142208 | HGLibA_01506 | 88 | -0.338703314 | AKT1 | AKT serine/threonine kinase 1 [Source:HGNC Symbol;Acc:HGNC:391] |
| ENSG00000143171 | HGLibA_42513 | 88 | -0.338703314 | RXRG | retinoid X receptor gamma [Source:HGNC Symbol;Acc:HGNC:10479] |
| ENSG00000143384 | HGLibA_28599 | 88 | -0.338703314 | MCL1 | BCL2 family apoptosis regulator [Source:HGNC Symbol;Acc:HGNC:6943]" |
| ENSG00000143387 | HGLibA_11713 | 88 | -0.338703314 | CTSK | cathepsin K [Source:HGNC Symbol;Acc:HGNC:2536] |
| ENSG00000144026 | HGLibA_56141 | 88 | -0.338703314 | ZNF514 | zinc finger protein 514 [Source:HGNC Symbol;Acc:HGNC:25894] |
| ENSG00000144566 | HGLibA_39960 | 88 | -0.338703314 | RAB5A | member RAS oncogene family [Source:HGNC Symbol;Acc:HGNC:9783]" |
| ENSG00000144648 | HGLibA_00443 | 88 | -0.338703314 | ACKR2 | atypical chemokine receptor 2 [Source:HGNC Symbol;Acc:HGNC:1565] |
| ENSG00000144747 | HGLibA_50578 | 88 | -0.338703314 | TMF1 | TATA element modulatory factor 1 [Source:HGNC Symbol;Acc:HGNC:11870] |
| ENSG00000145491 | HGLibA_41767 | 88 | -0.338703314 | ROPN1L | rhophilin associated tail protein 1 like [Source:HGNC Symbol;Acc:HGNC:24060] |
| ENSG00000145879 | HGLibA_46704 | 88 | -0.338703314 | SPINK7 | Kazal type 7 (putative) [Source:HGNC Symbol;Acc:HGNC:24643]" |
| ENSG00000146216 | HGLibA_52040 | 88 | -0.338703314 | TTBK1 | tau tubulin kinase 1 [Source:HGNC Symbol;Acc:HGNC:19140] |
| ENSG00000146592 | HGLibA_11106 | 88 | -0.338703314 | CREB5 | cAMP responsive element binding protein 5 [Source:HGNC Symbol;Acc:HGNC:16844] |
| ENSG00000147669 | HGLibA_37600 | 88 | -0.338703314 | POLR2K | RNA polymerase II subunit K [Source:HGNC Symbol;Acc:HGNC:9198] |
| ENSG00000148090 | HGLibA_03865 | 88 | -0.338703314 | AUH | AU RNA binding methylglutaconyl-CoA hydratase [Source:HGNC Symbol;Acc:HGNC:890] |
| ENSG00000149577 | HGLibA_44116 | 88 | -0.338703314 | SIDT2 | SID1 transmembrane family member 2 [Source:HGNC Symbol;Acc:HGNC:24272] |
| ENSG00000150681 | HGLibA_41072 | 88 | -0.338703314 | RGS18 | regulator of G protein signaling 18 [Source:HGNC Symbol;Acc:HGNC:14261] |
| ENSG00000151117 | HGLibA_50525 | 88 | -0.338703314 | TMEM86A | transmembrane protein 86A [Source:HGNC Symbol;Acc:HGNC:26890] |
| ENSG00000151240 | HGLibA_13254 | 88 | -0.338703314 | DIP2C | disco interacting protein 2 homolog C [Source:HGNC Symbol;Acc:HGNC:29150] |
| ENSG00000152527 | HGLibA_37127 | 88 | -0.338703314 | PLEKHH2 | MyTH4 and FERM domain containing H2 [Source:HGNC Symbol;Acc:HGNC:30506]" |
| ENSG00000154914 | HGLibA_53369 | 88 | -0.338703314 | USP43 | ubiquitin specific peptidase 43 [Source:HGNC Symbol;Acc:HGNC:20072] |
| ENSG00000155760 | HGLibA_18280 | 88 | -0.338703314 | FZD7 | frizzled class receptor 7 [Source:HGNC Symbol;Acc:HGNC:4045] |
| ENSG00000156885 | HGLibA_10846 | 88 | -0.338703314 | COX6A2 | cytochrome c oxidase subunit 6A2 [Source:HGNC Symbol;Acc:HGNC:2279] |
| ENSG00000157216 | HGLibA_47120 | 88 | -0.338703314 | SSBP3 | single stranded DNA binding protein 3 [Source:HGNC Symbol;Acc:HGNC:15674] |
| ENSG00000157368 | HGLibA_23143 | 88 | -0.338703314 | IL34 | interleukin 34 [Source:HGNC Symbol;Acc:HGNC:28529] |
| ENSG00000158710 | HGLibA_48272 | 88 | -0.338703314 | TAGLN2 | transgelin 2 [Source:HGNC Symbol;Acc:HGNC:11554] |
| ENSG00000159674 | HGLibA_46759 | 88 | -0.338703314 | SPON2 | spondin 2 [Source:HGNC Symbol;Acc:HGNC:11253] |
| ENSG00000160062 | HGLibA_54925 | 88 | -0.338703314 | ZBTB8A | zinc finger and BTB domain containing 8A [Source:HGNC Symbol;Acc:HGNC:24172] |
| ENSG00000160602 | HGLibA_31666 | 88 | -0.338703314 | NEK8 | NIMA related kinase 8 [Source:HGNC Symbol;Acc:HGNC:13387] |
| ENSG00000160746 | HGLibA_02174 | 88 | -0.338703314 | ANO10 | anoctamin 10 [Source:HGNC Symbol;Acc:HGNC:25519] |
| ENSG00000160801 | HGLibA_39366 | 88 | -0.338703314 | PTH1R | parathyroid hormone 1 receptor [Source:HGNC Symbol;Acc:HGNC:9608] |
| ENSG00000161800 | HGLibA_40050 | 88 | -0.338703314 | RACGAP1 | Rac GTPase activating protein 1 [Source:HGNC Symbol;Acc:HGNC:9804] |
| ENSG00000162078 | HGLibA_55301 | 88 | -0.338703314 | ZG16B | zymogen granule protein 16B [Source:HGNC Symbol;Acc:HGNC:30456] |
| ENSG00000162631 | HGLibA_32864 | 88 | -0.338703314 | NTNG1 | netrin G1 [Source:HGNC Symbol;Acc:HGNC:23319] |
| ENSG00000163481 | HGLibA_41654 | 88 | -0.338703314 | RNF25 | ring finger protein 25 [Source:HGNC Symbol;Acc:HGNC:14662] |
| ENSG00000164871 | HGLibA_46347 | 88 | -0.338703314 | SPAG11B | sperm associated antigen 11B [Source:HGNC Symbol;Acc:HGNC:14534] |
| ENSG00000164949 | HGLibA_18909 | 88 | -0.338703314 | GEM | GTP binding protein overexpressed in skeletal muscle [Source:HGNC Symbol;Acc:HGNC:4234] |
| ENSG00000165802 | HGLibA_32776 | 88 | -0.338703314 | NSMF | NMDA receptor synaptonuclear signaling and neuronal migration factor [Source:HGNC Symbol;Acc:HGNC:29843] |
| ENSG00000166558 | HGLibA_45081 | 88 | -0.338703314 | SLC38A8 | solute carrier family 38 member 8 [Source:HGNC Symbol;Acc:HGNC:32434] |
| ENSG00000167106 | HGLibA_15979 | 88 | -0.338703314 | FAM102A | family with sequence similarity 102 member A [Source:HGNC Symbol;Acc:HGNC:31419] |
| ENSG00000167721 | HGLibA_51995 | 88 | -0.338703314 | TSR1 | ribosome maturation factor [Source:HGNC Symbol;Acc:HGNC:25542]" |
| ENSG00000167770 | HGLibA_34754 | 88 | -0.338703314 | OTUB1 | ubiquitin aldehyde binding 1 [Source:HGNC Symbol;Acc:HGNC:23077]" |
| ENSG00000168000 | HGLibA_04861 | 88 | -0.338703314 | BSCL2 | seipin lipid droplet biogenesis associated [Source:HGNC Symbol;Acc:HGNC:15832]" |
| ENSG00000168928 | HGLibA_11679 | 88 | -0.338703314 | CTRB2 | chymotrypsinogen B2 [Source:HGNC Symbol;Acc:HGNC:2522] |
| ENSG00000169016 | HGLibA_14321 | 88 | -0.338703314 | E2F6 | E2F transcription factor 6 [Source:HGNC Symbol;Acc:HGNC:3120] |
| ENSG00000169375 | HGLibA_44183 | 88 | -0.338703314 | SIN3A | SIN3 transcription regulator family member A [Source:HGNC Symbol;Acc:HGNC:19353] |
| ENSG00000169499 | HGLibA_37065 | 88 | -0.338703314 | PLEKHA2 | pleckstrin homology domain containing A2 [Source:HGNC Symbol;Acc:HGNC:14336] |
| ENSG00000171163 | HGLibA_56553 | 88 | -0.338703314 | ZNF692 | zinc finger protein 692 [Source:HGNC Symbol;Acc:HGNC:26049] |
| ENSG00000171489 | HGLibA_46332 | 88 | -0.338703314 | SPACA5 | sperm acrosome associated 5 [Source:HGNC Symbol;Acc:HGNC:31353] |
| ENSG00000171777 | HGLibA_40352 | 88 | -0.338703314 | RASGRP4 | RAS guanyl releasing protein 4 [Source:HGNC Symbol;Acc:HGNC:18958] |
| ENSG00000172425 | HGLibA_52123 | 88 | -0.338703314 | TTC36 | tetratricopeptide repeat domain 36 [Source:HGNC Symbol;Acc:HGNC:33708] |
| ENSG00000172575 | HGLibA_40343 | 88 | -0.338703314 | RASGRP1 | RAS guanyl releasing protein 1 [Source:HGNC Symbol;Acc:HGNC:9878] |
| ENSG00000173530 | HGLibA_50743 | 88 | -0.338703314 | TNFRSF10D | TNF receptor superfamily member 10d [Source:HGNC Symbol;Acc:HGNC:11907] |
| ENSG00000173598 | HGLibA_32980 | 88 | -0.338703314 | NUDT4 | nudix hydrolase 4 [Source:HGNC Symbol;Acc:HGNC:8051] |
| ENSG00000174173 | HGLibA_51666 | 88 | -0.338703314 | TRMT10C | mitochondrial RNase P subunit [Source:HGNC Symbol;Acc:HGNC:26022]" |
| ENSG00000174640 | HGLibA_45461 | 88 | -0.338703314 | SLCO2A1 | solute carrier organic anion transporter family member 2A1 [Source:HGNC Symbol;Acc:HGNC:10955] |
| ENSG00000176555 | HGLibA_34060 | 88 | -0.338703314 | OR4S1 | olfactory receptor family 4 subfamily S member 1 [Source:HGNC Symbol;Acc:HGNC:14705] |
| ENSG00000176890 | HGLibA_52462 | 88 | -0.338703314 | TYMS | thymidylate synthetase [Source:HGNC Symbol;Acc:HGNC:12441] |
| ENSG00000176946 | HGLibA_49301 | 88 | -0.338703314 | THAP4 | THAP domain containing 4 [Source:HGNC Symbol;Acc:HGNC:23187] |
| ENSG00000177575 | HGLibA_08244 | 88 | -0.338703314 | CD163 | CD163 molecule [Source:HGNC Symbol;Acc:HGNC:1631] |
| ENSG00000178021 | HGLibA_51991 | 88 | -0.338703314 | TSPYL6 | TSPY like 6 [Source:HGNC Symbol;Acc:HGNC:14521] |
| ENSG00000180035 | HGLibA_56067 | 88 | -0.338703314 | ZNF48 | zinc finger protein 48 [Source:HGNC Symbol;Acc:HGNC:13114] |
| ENSG00000180974 | HGLibA_34159 | 88 | -0.338703314 | OR52E4 | olfactory receptor family 52 subfamily E member 4 [Source:HGNC Symbol;Acc:HGNC:15213] |
| ENSG00000181090 | HGLibA_14684 | 88 | -0.338703314 | EHMT1 | euchromatic histone lysine methyltransferase 1 [Source:HGNC Symbol;Acc:HGNC:24650] |
| ENSG00000181752 | HGLibA_34551 | 88 | -0.338703314 | OR8K5 | olfactory receptor family 8 subfamily K member 5 [Source:HGNC Symbol;Acc:HGNC:15315] |
| ENSG00000182054 | HGLibA_22543 | 88 | -0.338703314 | IDH2 | mitochondrial [Source:HGNC Symbol;Acc:HGNC:5383]" |
| ENSG00000183421 | HGLibA_41326 | 88 | -0.338703314 | RIPK4 | receptor interacting serine/threonine kinase 4 [Source:HGNC Symbol;Acc:HGNC:496] |
| ENSG00000183770 | HGLibA_17902 | 88 | -0.338703314 | FOXL2 | forkhead box L2 [Source:HGNC Symbol;Acc:HGNC:1092] |
| ENSG00000184330 | HGLibA_42569 | 88 | -0.338703314 | S100A7A | S100 calcium binding protein A7A [Source:HGNC Symbol;Acc:HGNC:21657] |
| ENSG00000184792 | HGLibA_34641 | 88 | -0.338703314 | OSBP2 | oxysterol binding protein 2 [Source:HGNC Symbol;Acc:HGNC:8504] |
| ENSG00000184916 | HGLibA_23907 | 88 | -0.338703314 | JAG2 | jagged 2 [Source:HGNC Symbol;Acc:HGNC:6189] |
| ENSG00000185305 | HGLibA_02944 | 88 | -0.338703314 | ARL15 | ADP ribosylation factor like GTPase 15 [Source:HGNC Symbol;Acc:HGNC:25945] |
| ENSG00000185313 | HGLibA_42967 | 88 | -0.338703314 | SCN10A | sodium voltage-gated channel alpha subunit 10 [Source:HGNC Symbol;Acc:HGNC:10582] |
| ENSG00000186226 | HGLibA_26113 | 88 | -0.338703314 | LCE1E | late cornified envelope 1E [Source:HGNC Symbol;Acc:HGNC:29466] |
| ENSG00000186439 | HGLibA_51347 | 88 | -0.338703314 | TRDN | triadin [Source:HGNC Symbol;Acc:HGNC:12261] |
| ENSG00000186788 | HGLibA_46497 | 88 | -0.338703314 | SPATA31D3 | SPATA31 subfamily D member 3 [Source:HGNC Symbol;Acc:HGNC:38603] |
| ENSG00000186881 | HGLibA_33616 | 88 | -0.338703314 | OR13F1 | olfactory receptor family 13 subfamily F member 1 [Source:HGNC Symbol;Acc:HGNC:14723] |
| ENSG00000186980 | HGLibA_25722 | 88 | -0.338703314 | KRTAP23-1 | keratin associated protein 23-1 [Source:HGNC Symbol;Acc:HGNC:18928] |
| ENSG00000187048 | HGLibA_12172 | 88 | -0.338703314 | CYP4A11 | cytochrome P450 family 4 subfamily A member 11 [Source:HGNC Symbol;Acc:HGNC:2642] |
| ENSG00000187569 | HGLibA_13891 | 88 | -0.338703314 | DPPA3 | developmental pluripotency associated 3 [Source:HGNC Symbol;Acc:HGNC:19199] |
| ENSG00000188056 | HGLibA_51367 | 88 | -0.338703314 | TREML4 | triggering receptor expressed on myeloid cells like 4 [Source:HGNC Symbol;Acc:HGNC:30807] |
| ENSG00000188100 | HGLibA_16551 | 88 | -0.338703314 | FAM25A | family with sequence similarity 25 member A [Source:HGNC Symbol;Acc:HGNC:23436] |
| ENSG00000188124 | HGLibA_33753 | 88 | -0.338703314 | OR2AG2 | olfactory receptor family 2 subfamily AG member 2 [Source:HGNC Symbol;Acc:HGNC:15143] |
| ENSG00000188379 | HGLibA_22674 | 88 | -0.338703314 | IFNA2 | interferon alpha 2 [Source:HGNC Symbol;Acc:HGNC:5423] |
| ENSG00000189134 | HGLibA_32003 | 88 | -0.338703314 | NKAPL | NFKB activating protein like [Source:HGNC Symbol;Acc:HGNC:21584] |
| ENSG00000196811 | HGLibA_09607 | 88 | -0.338703314 | CHRNG | cholinergic receptor nicotinic gamma subunit [Source:HGNC Symbol;Acc:HGNC:1967] |
| ENSG00000196850 | HGLibA_38238 | 88 | -0.338703314 | PPTC7 | PTC7 protein phosphatase homolog [Source:HGNC Symbol;Acc:HGNC:30695] |
| ENSG00000197586 | HGLibA_15213 | 88 | -0.338703314 | ENTPD6 | ectonucleoside triphosphate diphosphohydrolase 6 (putative) [Source:HGNC Symbol;Acc:HGNC:3368] |
| ENSG00000203784 | HGLibA_26282 | 88 | -0.338703314 | LELP1 | late cornified envelope like proline rich 1 [Source:HGNC Symbol;Acc:HGNC:32046] |
| ENSG00000204580 | HGLibA_12632 | 88 | -0.338703314 | DDR1 | discoidin domain receptor tyrosine kinase 1 [Source:HGNC Symbol;Acc:HGNC:2730] |
| ENSG00000204967 | HGLibA_35445 | 88 | -0.338703314 | PCDHA4 | protocadherin alpha 4 [Source:HGNC Symbol;Acc:HGNC:8670] |
| ENSG00000211445 | HGLibA_20177 | 88 | -0.338703314 | GPX3 | glutathione peroxidase 3 [Source:HGNC Symbol;Acc:HGNC:4555] |
| ENSG00000215183 | HGLibA_30229 | 88 | -0.338703314 | MSMP | prostate associated [Source:HGNC Symbol;Acc:HGNC:29663]" |
| ENSG00000215301 | HGLibA_12702 | 88 | -0.338703314 | DDX3X | DEAD-box helicase 3 X-linked [Source:HGNC Symbol;Acc:HGNC:2745] |
| ENSG00000227507 | HGLibA_27581 | 88 | -0.338703314 | LTB | lymphotoxin beta [Source:HGNC Symbol;Acc:HGNC:6711] |
| ENSG00000232810 | HGLibA_50703 | 88 | -0.338703314 | TNF | tumor necrosis factor [Source:HGNC Symbol;Acc:HGNC:11892] |
| ENSG00000235718 | HGLibA_29078 | 88 | -0.338703314 | MFRP | membrane frizzled-related protein [Source:HGNC Symbol;Acc:HGNC:18121] |
| ENSG00000237289 | HGLibA_09781 | 88 | -0.338703314 | CKMT1B | mitochondrial 1B [Source:HGNC Symbol;Acc:HGNC:1995]" |
| ENSG00000237649 | HGLibA_24970 | 88 | -0.338703314 | KIFC1 | kinesin family member C1 [Source:HGNC Symbol;Acc:HGNC:6389] |
| ENSG00000239886 | HGLibA_25834 | 88 | -0.338703314 | KRTAP9-2 | keratin associated protein 9-2 [Source:HGNC Symbol;Acc:HGNC:16926] |
| ENSG00000240053 | HGLibA_27658 | 88 | -0.338703314 | LY6G5B | lymphocyte antigen 6 family member G5B [Source:HGNC Symbol;Acc:HGNC:13931] |
| ENSG00000241484 | HGLibA_02788 | 88 | -0.338703314 | ARHGAP8 | Rho GTPase activating protein 8 [Source:HGNC Symbol;Acc:HGNC:677] |
| ENSG00000241553 | HGLibA_03087 | 88 | -0.338703314 | ARPC4 | actin related protein 2/3 complex subunit 4 [Source:HGNC Symbol;Acc:HGNC:707] |
| ENSG00000243709 | HGLibA_26274 | 88 | -0.338703314 | LEFTY1 | left-right determination factor 1 [Source:HGNC Symbol;Acc:HGNC:6552] |
| ENSG00000249581 | HGLibA_10124 | 88 | -0.338703314 | CLRN2 | clarin 2 [Source:HGNC Symbol;Acc:HGNC:33939] |
| ENSG00000253485 | HGLibA_35535 | 88 | -0.338703314 | PCDHGA5 | 5 [Source:HGNC Symbol;Acc:HGNC:8703]" |
| ENSG00000254521 | HGLibA_44131 | 88 | -0.338703314 | SIGLEC12 | sialic acid binding Ig like lectin 12 (gene/pseudogene) [Source:HGNC Symbol;Acc:HGNC:15482] |
| ENSG00000276234 | HGLibA_48187 | 88 | -0.338703314 | TADA2A | transcriptional adaptor 2A [Source:HGNC Symbol;Acc:HGNC:11531] |
| ENSG00000276644 | HGLibA_12274 | 88 | -0.338703314 | DACH1 | dachshund family transcription factor 1 [Source:HGNC Symbol;Acc:HGNC:2663] |
| ENSG00000284680 | HGLibA_34496 | 88 | -0.338703314 | OR8B2 | olfactory receptor family 8 subfamily B member 2 [Source:HGNC Symbol;Acc:HGNC:8471] |
| GLTSCR2 | HGLibA_19359 | 88 | -0.338703314 | GLTSCR2 | - |
| HIST1H1T | HGLibA_21372 | 88 | -0.338703314 | HIST1H1T | - |
| HIST1H2BJ | HGLibA_21439 | 88 | -0.338703314 | HIST1H2BJ | - |
| hsa-let-7c | HGLibA_57113 | 88 | -0.338703314 | hsa-let-7c | - |
| hsa-mir-132 | HGLibA_57756 | 88 | -0.338703314 | hsa-mir-132 | - |
| hsa-mir-146b | HGLibA_57859 | 88 | -0.338703314 | hsa-mir-146b | - |
| hsa-mir-151a | HGLibA_57894 | 88 | -0.338703314 | hsa-mir-151a | - |
| hsa-mir-153-2 | HGLibA_57913 | 88 | -0.338703314 | hsa-mir-153-2 | - |
| hsa-mir-2861 | HGLibA_58389 | 88 | -0.338703314 | hsa-mir-2861 | - |
| hsa-mir-34c | HGLibA_59044 | 88 | -0.338703314 | hsa-mir-34c | - |

| | | | | | |
|---|---|---|---|---|---|
| hsa-mir-3713 | HGLibA_59367 | 88 | -0.338703314 | hsa-mir-3713 | - |
| hsa-mir-4443 | HGLibA_60173 | 88 | -0.338703314 | hsa-mir-4443 | - |
| hsa-mir-4472-2 | HGLibA_60299 | 88 | -0.338703314 | hsa-mir-4472-2 | - |
| hsa-mir-4524a | HGLibA_60555 | 88 | -0.338703314 | hsa-mir-4524a | - |
| hsa-mir-4536-2 | HGLibA_60605 | 88 | -0.338703314 | hsa-mir-4536-2 | - |
| hsa-mir-4647 | HGLibA_60692 | 88 | -0.338703314 | hsa-mir-4647 | - |
| hsa-mir-500b | HGLibA_61448 | 88 | -0.338703314 | hsa-mir-500b | - |
| hsa-mir-518b | HGLibA_61661 | 88 | -0.338703314 | hsa-mir-518b | - |
| hsa-mir-578 | HGLibA_62408 | 88 | -0.338703314 | hsa-mir-578 | - |
| hsa-mir-6738 | HGLibA_63104 | 88 | -0.338703314 | hsa-mir-6738 | - |
| hsa-mir-6864 | HGLibA_63640 | 88 | -0.338703314 | hsa-mir-6864 | - |
| hsa-mir-8083 | HGLibA_64173 | 88 | -0.338703314 | hsa-mir-8083 | - |
| hsa-mir-8084 | HGLibA_64177 | 88 | -0.338703314 | hsa-mir-8084 | - |
| LOC100133267 | HGLibA_26811 | 88 | -0.338703314 | LOC100133267 | - |
| PHF16 | HGLibA_36352 | 88 | -0.338703314 | PHF16 | - |
| PHF17 | HGLibA_36353 | 88 | -0.338703314 | PHF17 | - |
| 1-Dec | HGLibA_12771 | 87 | -0.347748657 | 1-Dec | - |
| ATP5J2 | HGLibA_03673 | 87 | -0.347748657 | ATP5J2 | - |
| C15orf52 | HGLibA_05408 | 87 | -0.347748657 | C15orf52 | - |
| C2orf91 | HGLibA_06199 | 87 | -0.347748657 | C2orf91 | - |
| C3orf67 | HGLibA_06259 | 87 | -0.347748657 | C3orf67 | - |
| C6orf165 | HGLibA_06459 | 87 | -0.347748657 | C6orf165 | - |
| C7orf60 | HGLibA_06562 | 87 | -0.347748657 | C7orf60 | - |
| CCDC147 | HGLibA_07630 | 87 | -0.347748657 | CCDC147 | - |
| ENSG00000007350 | HGLibA_49588 | 87 | -0.347748657 | TKTL1 | transketolase like 1 [Source:HGNC Symbol;Acc:HGNC:11835] |
| ENSG00000011028 | HGLibA_29819 | 87 | -0.347748657 | MRC2 | mannose receptor C type 2 [Source:HGNC Symbol;Acc:HGNC:16875] |
| ENSG00000013561 | HGLibA_41521 | 87 | -0.347748657 | RNF14 | ring finger protein 14 [Source:HGNC Symbol;Acc:HGNC:10058] |
| ENSG00000021645 | HGLibA_32739 | 87 | -0.347748657 | NRXN3 | neurexin 3 [Source:HGNC Symbol;Acc:HGNC:8010] |
| ENSG00000034239 | HGLibA_14520 | 87 | -0.347748657 | EFCAB1 | EF-hand calcium binding domain 1 [Source:HGNC Symbol;Acc:HGNC:25678] |
| ENSG00000057757 | HGLibA_36737 | 87 | -0.347748657 | PITHD1 | PITH domain containing 1 [Source:HGNC Symbol;Acc:HGNC:25022] |
| ENSG00000060140 | HGLibA_47684 | 87 | -0.347748657 | STYK1 | serine/threonine/tyrosine kinase 1 [Source:HGNC Symbol;Acc:HGNC:18889] |
| ENSG00000064199 | HGLibA_46322 | 87 | -0.347748657 | SPA17 | sperm autoantigenic protein 17 [Source:HGNC Symbol;Acc:HGNC:11210] |
| ENSG00000072849 | HGLibA_12997 | 87 | -0.347748657 | DERL2 | derlin 2 [Source:HGNC Symbol;Acc:HGNC:17943] |
| ENSG00000075785 | HGLibA_39980 | 87 | -0.347748657 | RAB7A | member RAS oncogene family [Source:HGNC Symbol;Acc:HGNC:9788]" |
| ENSG00000084453 | HGLibA_45444 | 87 | -0.347748657 | SLCO1A2 | solute carrier organic anion transporter family member 1A2 [Source:HGNC Symbol;Acc:HGNC:10956] |
| ENSG00000085871 | HGLibA_29177 | 87 | -0.347748657 | MGST2 | microsomal glutathione S-transferase 2 [Source:HGNC Symbol;Acc:HGNC:7063] |
| ENSG00000086232 | HGLibA_14722 | 87 | -0.347748657 | EIF2AK1 | eukaryotic translation initiation factor 2 alpha kinase 1 [Source:HGNC Symbol;Acc:HGNC:24921] |
| ENSG00000091640 | HGLibA_46367 | 87 | -0.347748657 | SPAG7 | sperm associated antigen 7 [Source:HGNC Symbol;Acc:HGNC:11216] |
| ENSG00000092421 | HGLibA_43365 | 87 | -0.347748657 | SEMA6A | semaphorin 6A [Source:HGNC Symbol;Acc:HGNC:10738] |
| ENSG00000094804 | HGLibA_08594 | 87 | -0.347748657 | CDC6 | cell division cycle 6 [Source:HGNC Symbol;Acc:HGNC:1744] |
| ENSG00000096654 | HGLibA_55547 | 87 | -0.347748657 | ZNF184 | zinc finger protein 184 [Source:HGNC Symbol;Acc:HGNC:12975] |
| ENSG00000099840 | HGLibA_23901 | 87 | -0.347748657 | IZUMO4 | IZUMO family member 4 [Source:HGNC Symbol;Acc:HGNC:26950] |
| ENSG00000099958 | HGLibA_13001 | 87 | -0.347748657 | DERL3 | derlin 3 [Source:HGNC Symbol;Acc:HGNC:14236] |
| ENSG00000101197 | HGLibA_04528 | 87 | -0.347748657 | BIRC7 | baculoviral IAP repeat containing 7 [Source:HGNC Symbol;Acc:HGNC:13702] |
| ENSG00000101997 | HGLibA_07730 | 87 | -0.347748657 | CCDC22 | coiled-coil domain containing 22 [Source:HGNC Symbol;Acc:HGNC:28909] |
| ENSG00000103264 | HGLibA_17069 | 87 | -0.347748657 | FBXO31 | F-box protein 31 [Source:HGNC Symbol;Acc:HGNC:16510] |
| ENSG00000103375 | HGLibA_02608 | 87 | -0.347748657 | AQP8 | aquaporin 8 [Source:HGNC Symbol;Acc:HGNC:642] |
| ENSG00000104324 | HGLibA_10989 | 87 | -0.347748657 | CPQ | carboxypeptidase Q [Source:HGNC Symbol;Acc:HGNC:16910] |
| ENSG00000104763 | HGLibA_03185 | 87 | -0.347748657 | ASAH1 | N-acylsphingosine amidohydrolase 1 [Source:HGNC Symbol;Acc:HGNC:735] |
| ENSG00000104969 | HGLibA_43867 | 87 | -0.347748657 | SGTA | small glutamine rich tetratricopeptide repeat containing alpha [Source:HGNC Symbol;Acc:HGNC:10819] |
| ENSG00000105854 | HGLibA_37680 | 87 | -0.347748657 | PON2 | paraoxonase 2 [Source:HGNC Symbol;Acc:HGNC:9205] |
| ENSG00000106299 | HGLibA_53966 | 87 | -0.347748657 | WASL | Wiskott-Aldrich syndrome like [Source:HGNC Symbol;Acc:HGNC:12735] |
| ENSG00000108509 | HGLibA_07117 | 87 | -0.347748657 | CAMTA2 | calmodulin binding transcription activator 2 [Source:HGNC Symbol;Acc:HGNC:18807] |
| ENSG00000108651 | HGLibA_53454 | 87 | -0.347748657 | UTP6 | small subunit processome component [Source:HGNC Symbol;Acc:HGNC:18279]" |
| ENSG00000109089 | HGLibA_08869 | 87 | -0.347748657 | CDR2L | cerebellar degeneration related protein 2 like [Source:HGNC Symbol;Acc:HGNC:29999] |
| ENSG00000110906 | HGLibA_24414 | 87 | -0.347748657 | KCTD10 | potassium channel tetramerization domain containing 10 [Source:HGNC Symbol;Acc:HGNC:23236] |
| ENSG00000111275 | HGLibA_01559 | 87 | -0.347748657 | ALDH2 | aldehyde dehydrogenase 2 family member [Source:HGNC Symbol;Acc:HGNC:404] |
| ENSG00000111300 | HGLibA_30986 | 87 | -0.347748657 | NAA25 | NatB auxiliary subunit [Source:HGNC Symbol;Acc:HGNC:25783]" |
| ENSG00000112062 | HGLibA_28261 | 87 | -0.347748657 | MAPK14 | mitogen-activated protein kinase 14 [Source:HGNC Symbol;Acc:HGNC:6876] |
| ENSG00000112394 | HGLibA_44421 | 87 | -0.347748657 | SLC16A10 | solute carrier family 16 member 10 [Source:HGNC Symbol;Acc:HGNC:17027] |
| ENSG00000113073 | HGLibA_45233 | 87 | -0.347748657 | SLC4A9 | solute carrier family 4 member 9 [Source:HGNC Symbol;Acc:HGNC:11035] |
| ENSG00000115084 | HGLibA_45010 | 87 | -0.347748657 | SLC35F5 | solute carrier family 35 member F5 [Source:HGNC Symbol;Acc:HGNC:23617] |
| ENSG00000115138 | HGLibA_37653 | 87 | -0.347748657 | POMC | proopiomelanocortin [Source:HGNC Symbol;Acc:HGNC:9201] |
| ENSG00000115956 | HGLibA_37057 | 87 | -0.347748657 | PLEK | pleckstrin [Source:HGNC Symbol;Acc:HGNC:9070] |
| ENSG00000116266 | HGLibA_47670 | 87 | -0.347748657 | STXBP3 | syntaxin binding protein 3 [Source:HGNC Symbol;Acc:HGNC:11446] |
| ENSG00000116954 | HGLibA_42231 | 87 | -0.347748657 | RRAGC | Ras related GTP binding C [Source:HGNC Symbol;Acc:HGNC:19902] |
| ENSG00000117411 | HGLibA_04025 | 87 | -0.347748657 | B4GALT2 | 4-galactosyltransferase 2 [Source:HGNC Symbol;Acc:HGNC:925]" |
| ENSG00000117500 | HGLibA_49813 | 87 | -0.347748657 | TMED5 | transmembrane p24 trafficking protein 5 [Source:HGNC Symbol;Acc:HGNC:24251] |
| ENSG00000118322 | HGLibA_03548 | 87 | -0.347748657 | ATP10B | ATPase phospholipid transporting 10B (putative) [Source:HGNC Symbol;Acc:HGNC:13543] |
| ENSG00000118564 | HGLibA_17013 | 87 | -0.347748657 | FBXL5 | F-box and leucine rich repeat protein 5 [Source:HGNC Symbol;Acc:HGNC:13602] |
| ENSG00000118733 | HGLibA_33349 | 87 | -0.347748657 | OLFM3 | olfactomedin 3 [Source:HGNC Symbol;Acc:HGNC:17990] |
| ENSG00000119608 | HGLibA_38704 | 87 | -0.347748657 | PROX2 | prospero homeobox 2 [Source:HGNC Symbol;Acc:HGNC:26715] |
| ENSG00000119729 | HGLibA_41198 | 87 | -0.347748657 | RHOQ | ras homolog family member Q [Source:HGNC Symbol;Acc:HGNC:17736] |
| ENSG00000120896 | HGLibA_46180 | 87 | -0.347748657 | SORBS3 | sorbin and SH3 domain containing 3 [Source:HGNC Symbol;Acc:HGNC:30907] |
| ENSG00000121289 | HGLibA_09175 | 87 | -0.347748657 | CEP89 | centrosomal protein 89 [Source:HGNC Symbol;Acc:HGNC:25907] |
| ENSG00000122477 | HGLibA_27341 | 87 | -0.347748657 | LRRC39 | leucine rich repeat containing 39 [Source:HGNC Symbol;Acc:HGNC:28228] |
| ENSG00000123094 | HGLibA_40394 | 87 | -0.347748657 | RASSF8 | Ras association domain family member 8 [Source:HGNC Symbol;Acc:HGNC:13232] |
| ENSG00000124664 | HGLibA_46566 | 87 | -0.347748657 | SPDEF | SAM pointed domain containing ETS transcription factor [Source:HGNC Symbol;Acc:HGNC:17257] |
| ENSG00000126010 | HGLibA_20378 | 87 | -0.347748657 | GRPR | gastrin releasing peptide receptor [Source:HGNC Symbol;Acc:HGNC:4609] |
| ENSG00000126709 | HGLibA_22618 | 87 | -0.347748657 | IFI6 | interferon alpha inducible protein 6 [Source:HGNC Symbol;Acc:HGNC:4054] |
| ENSG00000127616 | HGLibA_45608 | 87 | -0.347748657 | SMARCA4 | member 4 [Source:HGNC Symbol;Acc:HGNC:11100]" |
| ENSG00000128394 | HGLibA_02488 | 87 | -0.347748657 | APOBEC3F | apolipoprotein B mRNA editing enzyme catalytic subunit 3F [Source:HGNC Symbol;Acc:HGNC:17356] |
| ENSG00000130413 | HGLibA_47492 | 87 | -0.347748657 | STK33 | serine/threonine kinase 33 [Source:HGNC Symbol;Acc:HGNC:14568] |
| ENSG00000130810 | HGLibA_37809 | 87 | -0.347748657 | PPAN | peter pan homolog [Source:HGNC Symbol;Acc:HGNC:9227] |
| ENSG00000132854 | HGLibA_24032 | 87 | -0.347748657 | KANK4 | KN motif and ankyrin repeat domains 4 [Source:HGNC Symbol;Acc:HGNC:27263] |
| ENSG00000133131 | HGLibA_29658 | 87 | -0.347748657 | MORC4 | MORC family CW-type zinc finger 4 [Source:HGNC Symbol;Acc:HGNC:23485] |
| ENSG00000133392 | HGLibA_30710 | 87 | -0.347748657 | MYH11 | myosin heavy chain 11 [Source:HGNC Symbol;Acc:HGNC:7569] |
| ENSG00000134042 | HGLibA_29864 | 87 | -0.347748657 | MRO | maestro [Source:HGNC Symbol;Acc:HGNC:24121] |
| ENSG00000134376 | HGLibA_11069 | 87 | -0.347748657 | CRB1 | cell polarity complex component [Source:HGNC Symbol;Acc:HGNC:2343]" |
| ENSG00000134954 | HGLibA_15642 | 87 | -0.347748657 | ETS1 | transcription factor [Source:HGNC Symbol;Acc:HGNC:3488]" |
| ENSG00000135241 | HGLibA_37426 | 87 | -0.347748657 | PNPLA8 | patatin like phospholipase domain containing 8 [Source:HGNC Symbol;Acc:HGNC:28900] |
| ENSG00000135355 | HGLibA_19138 | 87 | -0.347748657 | GJA10 | gap junction protein alpha 10 [Source:HGNC Symbol;Acc:HGNC:16995] |
| ENSG00000135374 | HGLibA_14902 | 87 | -0.347748657 | ELF5 | E74 like ETS transcription factor 5 [Source:HGNC Symbol;Acc:HGNC:3320] |
| ENSG00000135638 | HGLibA_15092 | 87 | -0.347748657 | EMX1 | empty spiracles homeobox 1 [Source:HGNC Symbol;Acc:HGNC:3340] |
| ENSG00000135837 | HGLibA_09134 | 87 | -0.347748657 | CEP350 | centrosomal protein 350 [Source:HGNC Symbol;Acc:HGNC:24238] |
| ENSG00000137075 | HGLibA_41669 | 87 | -0.347748657 | RNF38 | ring finger protein 38 [Source:HGNC Symbol;Acc:HGNC:18052] |
| ENSG00000137171 | HGLibA_25037 | 87 | -0.347748657 | KLC4 | kinesin light chain 4 [Source:HGNC Symbol;Acc:HGNC:21624] |
| ENSG00000138698 | HGLibA_40232 | 87 | -0.347748657 | RAP1GDS1 | Rap1 GTPase-GDP dissociation stimulator 1 [Source:HGNC Symbol;Acc:HGNC:9859] |
| ENSG00000138735 | HGLibA_35801 | 87 | -0.347748657 | PDE5A | phosphodiesterase 5A [Source:HGNC Symbol;Acc:HGNC:8784] |
| ENSG00000138834 | HGLibA_28293 | 87 | -0.347748657 | MAPK8IP3 | mitogen-activated protein kinase 8 interacting protein 3 [Source:HGNC Symbol;Acc:HGNC:6884] |
| ENSG00000139116 | HGLibA_24898 | 87 | -0.347748657 | KIF21A | kinesin family member 21A [Source:HGNC Symbol;Acc:HGNC:19349] |
| ENSG00000139908 | HGLibA_52017 | 87 | -0.347748657 | TSSK4 | testis specific serine kinase 4 [Source:HGNC Symbol;Acc:HGNC:19825] |
| ENSG00000141503 | HGLibA_29276 | 87 | -0.347748657 | MINK1 | misshapen like kinase 1 [Source:HGNC Symbol;Acc:HGNC:17565] |
| ENSG00000141510 | HGLibA_51048 | 87 | -0.347748657 | TP53 | tumor protein p53 [Source:HGNC Symbol;Acc:HGNC:11998] |
| ENSG00000141837 | HGLibA_06874 | 87 | -0.347748657 | CACNA1A | calcium voltage-gated channel subunit alpha1 A [Source:HGNC Symbol;Acc:HGNC:1388] |
| ENSG00000141971 | HGLibA_30589 | 87 | -0.347748657 | MVB12A | multivesicular body subunit 12A [Source:HGNC Symbol;Acc:HGNC:25153] |
| ENSG00000142945 | HGLibA_24930 | 87 | -0.347748657 | KIF2C | kinesin family member 2C [Source:HGNC Symbol;Acc:HGNC:6393] |
| ENSG00000143157 | HGLibA_37473 | 87 | -0.347748657 | POGK | pogo transposable element derived with KRAB domain [Source:HGNC Symbol;Acc:HGNC:18800] |
| ENSG00000143179 | HGLibA_52812 | 87 | -0.347748657 | UCK2 | uridine-cytidine kinase 2 [Source:HGNC Symbol;Acc:HGNC:12562] |
| ENSG00000143753 | HGLibA_12920 | 87 | -0.347748657 | DEGS1 | sphingolipid 1 [Source:HGNC Symbol;Acc:HGNC:13709]" |
| ENSG00000144118 | HGLibA_40150 | 87 | -0.347748657 | RALB | RAS like proto-oncogene B [Source:HGNC Symbol;Acc:HGNC:9840] |
| ENSG00000144455 | HGLibA_47769 | 87 | -0.347748657 | SUMF1 | sulfatase modifying factor 1 [Source:HGNC Symbol;Acc:HGNC:20376] |
| ENSG00000144681 | HGLibA_47311 | 87 | -0.347748657 | STAC | SH3 and cysteine rich domain [Source:HGNC Symbol;Acc:HGNC:11353] |
| ENSG00000145868 | HGLibA_17083 | 87 | -0.347748657 | FBXO38 | F-box protein 38 [Source:HGNC Symbol;Acc:HGNC:28844] |
| ENSG00000146411 | HGLibA_44861 | 87 | -0.347748657 | SLC2A12 | solute carrier family 2 member 12 [Source:HGNC Symbol;Acc:HGNC:18067] |
| ENSG00000147883 | HGLibA_08840 | 87 | -0.347748657 | CDKN2B | cyclin dependent kinase inhibitor 2B [Source:HGNC Symbol;Acc:HGNC:1788] |
| ENSG00000148399 | HGLibA_13853 | 87 | -0.347748657 | DPH7 | diphthamide biosynthesis 7 [Source:HGNC Symbol;Acc:HGNC:25199] |
| ENSG00000148572 | HGLibA_32653 | 87 | -0.347748657 | NRBF2 | nuclear receptor binding factor 2 [Source:HGNC Symbol;Acc:HGNC:19692] |
| ENSG00000149150 | HGLibA_45149 | 87 | -0.347748657 | SLC43A1 | solute carrier family 43 member 1 [Source:HGNC Symbol;Acc:HGNC:9225] |
| ENSG00000149257 | HGLibA_43596 | 87 | -0.347748657 | SERPINH1 | serpin family H member 1 [Source:HGNC Symbol;Acc:HGNC:1546] |
| ENSG00000149575 | HGLibA_42983 | 87 | -0.347748657 | SCN2B | sodium voltage-gated channel beta subunit 2 [Source:HGNC Symbol;Acc:HGNC:10589] |
| ENSG00000149972 | HGLibA_10413 | 87 | -0.347748657 | CNTN5 | contactin 5 [Source:HGNC Symbol;Acc:HGNC:2175] |
| ENSG00000151033 | HGLibA_27788 | 87 | -0.347748657 | LYZL2 | lysozyme like 2 [Source:HGNC Symbol;Acc:HGNC:29613] |
| ENSG00000151490 | HGLibA_39527 | 87 | -0.347748657 | PTPRO | receptor type O [Source:HGNC Symbol;Acc:HGNC:9678]" |
| ENSG00000151655 | HGLibA_23808 | 87 | -0.347748657 | ITIH2 | inter-alpha-trypsin inhibitor heavy chain 2 [Source:HGNC Symbol;Acc:HGNC:6167] |
| ENSG00000154813 | HGLibA_13842 | 87 | -0.347748657 | DPH3 | diphthamide biosynthesis 3 [Source:HGNC Symbol;Acc:HGNC:27717] |
| ENSG00000155011 | HGLibA_13296 | 87 | -0.347748657 | DKK2 | dickkopf WNT signaling pathway inhibitor 2 [Source:HGNC Symbol;Acc:HGNC:2892] |
| ENSG00000155087 | HGLibA_33275 | 87 | -0.347748657 | ODF1 | outer dense fiber of sperm tails 1 [Source:HGNC Symbol;Acc:HGNC:8113] |
| ENSG00000156575 | HGLibA_38456 | 87 | -0.347748657 | PRG3 | pro eosinophil major basic protein 2 [Source:HGNC Symbol;Acc:HGNC:9363]" |
| ENSG00000156973 | HGLibA_35813 | 87 | -0.347748657 | PDE6D | phosphodiesterase 6D [Source:HGNC Symbol;Acc:HGNC:8788] |
| ENSG00000157036 | HGLibA_15765 | 87 | -0.347748657 | EXOG | exo/endonuclease G [Source:HGNC Symbol;Acc:HGNC:3347] |
| ENSG00000158109 | HGLibA_51182 | 87 | -0.347748657 | TPRG1L | tumor protein p63 regulated 1 like [Source:HGNC Symbol;Acc:HGNC:27007] |
| ENSG00000158865 | HGLibA_45258 | 87 | -0.347748657 | SLC5A11 | solute carrier family 5 member 11 [Source:HGNC Symbol;Acc:HGNC:23091] |
| ENSG00000159289 | HGLibA_19607 | 87 | -0.347748657 | GOLGA6A | golgin A6 family member A [Source:HGNC Symbol;Acc:HGNC:13567] |
| ENSG00000160271 | HGLibA_40164 | 87 | -0.347748657 | RALGDS | ral guanine nucleotide dissociation stimulator [Source:HGNC Symbol;Acc:HGNC:9842] |

| | | | | | |
|---|---|---|---|---|---|
| ENSG00000162396 | HGLibA_35252 | 87 | -0.347748657 | PARS2 | mitochondrial [Source:HGNC Symbol;Acc:HGNC:30563]" |
| ENSG00000163428 | HGLibA_27397 | 87 | -0.347748657 | LRRC58 | leucine rich repeat containing 58 [Source:HGNC Symbol;Acc:HGNC:26968] |
| ENSG00000163501 | HGLibA_22908 | 87 | -0.347748657 | IHH | indian hedgehog [Source:HGNC Symbol;Acc:HGNC:5956] |
| ENSG00000163995 | HGLibA_00310 | 87 | -0.347748657 | ABLIM2 | actin binding LIM protein family member 2 [Source:HGNC Symbol;Acc:HGNC:19195] |
| ENSG00000164438 | HGLibA_49678 | 87 | -0.347748657 | TLX3 | T cell leukemia homeobox 3 [Source:HGNC Symbol;Acc:HGNC:13532] |
| ENSG00000165072 | HGLibA_28038 | 87 | -0.347748657 | MAMDC2 | MAM domain containing 2 [Source:HGNC Symbol;Acc:HGNC:23673] |
| ENSG00000165410 | HGLibA_09284 | 87 | -0.347748657 | CFL2 | cofilin 2 [Source:HGNC Symbol;Acc:HGNC:1875] |
| ENSG00000165841 | HGLibA_12119 | 87 | -0.347748657 | CYP2C19 | cytochrome P450 family 2 subfamily C member 19 [Source:HGNC Symbol;Acc:HGNC:2621] |
| ENSG00000165915 | HGLibA_45100 | 87 | -0.347748657 | SLC39A13 | solute carrier family 39 member 13 [Source:HGNC Symbol;Acc:HGNC:20859] |
| ENSG00000168792 | HGLibA_00255 | 87 | -0.347748657 | ABHD15 | abhydrolase domain containing 15 [Source:HGNC Symbol;Acc:HGNC:26971] |
| ENSG00000168959 | HGLibA_20353 | 87 | -0.347748657 | GRM5 | glutamate metabotropic receptor 5 [Source:HGNC Symbol;Acc:HGNC:4597] |
| ENSG00000169347 | HGLibA_19706 | 87 | -0.347748657 | GP2 | glycoprotein 2 [Source:HGNC Symbol;Acc:HGNC:4441] |
| ENSG00000170421 | HGLibA_25552 | 87 | -0.347748657 | KRT8 | keratin 8 [Source:HGNC Symbol;Acc:HGNC:6446] |
| ENSG00000170962 | HGLibA_35850 | 87 | -0.347748657 | PDGFD | platelet derived growth factor D [Source:HGNC Symbol;Acc:HGNC:30620] |
| ENSG00000171150 | HGLibA_46136 | 87 | -0.347748657 | SOCS5 | suppressor of cytokine signaling 5 [Source:HGNC Symbol;Acc:HGNC:16852] |
| ENSG00000171490 | HGLibA_42318 | 87 | -0.347748657 | RSL1D1 | ribosomal L1 domain containing 1 [Source:HGNC Symbol;Acc:HGNC:24534] |
| ENSG00000172352 | HGLibA_08909 | 87 | -0.347748657 | CDY1B | chromodomain Y-linked 1B [Source:HGNC Symbol;Acc:HGNC:23920] |
| ENSG00000172869 | HGLibA_13452 | 87 | -0.347748657 | DMXL1 | Dmx like 1 [Source:HGNC Symbol;Acc:HGNC:2937] |
| ENSG00000175756 | HGLibA_03876 | 87 | -0.347748657 | AURKAIP1 | aurora kinase A interacting protein 1 [Source:HGNC Symbol;Acc:HGNC:24114] |
| ENSG00000176014 | HGLibA_52292 | 87 | -0.347748657 | TUBB6 | tubulin beta 6 class V [Source:HGNC Symbol;Acc:HGNC:20776] |
| ENSG00000177045 | HGLibA_44254 | 87 | -0.347748657 | SIX5 | SIX homeobox 5 [Source:HGNC Symbol;Acc:HGNC:10891] |
| ENSG00000177045 | HGLibA_44256 | 87 | -0.347748657 | SIX5 | SIX homeobox 5 [Source:HGNC Symbol;Acc:HGNC:10891] |
| ENSG00000177409 | HGLibA_42700 | 87 | -0.347748657 | SAMD9L | sterile alpha motif domain containing 9 like [Source:HGNC Symbol;Acc:HGNC:1349] |
| ENSG00000177427 | HGLibA_29246 | 87 | -0.347748657 | MIEF2 | mitochondrial elongation factor 2 [Source:HGNC Symbol;Acc:HGNC:17920] |
| ENSG00000177854 | HGLibA_50128 | 87 | -0.347748657 | TMEM187 | transmembrane protein 187 [Source:HGNC Symbol;Acc:HGNC:13705] |
| ENSG00000178175 | HGLibA_55880 | 87 | -0.347748657 | ZNF366 | zinc finger protein 366 [Source:HGNC Symbol;Acc:HGNC:18316] |
| ENSG00000178860 | HGLibA_30184 | 87 | -0.347748657 | MSC | musculin [Source:HGNC Symbol;Acc:HGNC:7321] |
| ENSG00000179071 | HGLibA_07916 | 87 | -0.347748657 | CCDC89 | coiled-coil domain containing 89 [Source:HGNC Symbol;Acc:HGNC:26762] |
| ENSG00000181418 | HGLibA_12625 | 87 | -0.347748657 | DDN | dendrin [Source:HGNC Symbol;Acc:HGNC:24458] |
| ENSG00000182223 | HGLibA_54777 | 87 | -0.347748657 | ZAR1 | zygote arrest 1 [Source:HGNC Symbol;Acc:HGNC:20436] |
| ENSG00000182827 | HGLibA_00397 | 87 | -0.347748657 | ACBD3 | acyl-CoA binding domain containing 3 [Source:HGNC Symbol;Acc:HGNC:15453] |
| ENSG00000183741 | HGLibA_07461 | 87 | -0.347748657 | CBX6 | chromobox 6 [Source:HGNC Symbol;Acc:HGNC:1556] |
| ENSG00000184226 | HGLibA_35420 | 87 | -0.347748657 | PCDH9 | protocadherin 9 [Source:HGNC Symbol;Acc:HGNC:8661] |
| ENSG00000184515 | HGLibA_04449 | 87 | -0.347748657 | BEX5 | brain expressed X-linked 5 [Source:HGNC Symbol;Acc:HGNC:27990] |
| ENSG00000184908 | HGLibA_09843 | 87 | -0.347748657 | CLCNKB | chloride voltage-gated channel Kb [Source:HGNC Symbol;Acc:HGNC:2027] |
| ENSG00000185515 | HGLibA_04767 | 87 | -0.347748657 | BRCC3 | BRCA1/BRCA2-containing complex subunit 3 [Source:HGNC Symbol;Acc:HGNC:24185] |
| ENSG00000186185 | HGLibA_24876 | 87 | -0.347748657 | KIF18B | kinesin family member 18B [Source:HGNC Symbol;Acc:HGNC:27102] |
| ENSG00000186440 | HGLibA_34437 | 87 | -0.347748657 | OR6P1 | olfactory receptor family 6 subfamily P member 1 [Source:HGNC Symbol;Acc:HGNC:15036] |
| ENSG00000186795 | HGLibA_24315 | 87 | -0.347748657 | KCNK18 | potassium two pore domain channel subfamily K member 18 [Source:HGNC Symbol;Acc:HGNC:19439] |
| ENSG00000186891 | HGLibA_50766 | 87 | -0.347748657 | TNFRSF18 | TNF receptor superfamily member 18 [Source:HGNC Symbol;Acc:HGNC:11914] |
| ENSG00000186897 | HGLibA_05934 | 87 | -0.347748657 | C1QL4 | complement C1q like 4 [Source:HGNC Symbol;Acc:HGNC:31416] |
| ENSG00000186971 | HGLibA_25650 | 87 | -0.347748657 | KRTAP13-4 | keratin associated protein 13-4 [Source:HGNC Symbol;Acc:HGNC:18926] |
| ENSG00000187555 | HGLibA_53411 | 87 | -0.347748657 | USP7 | ubiquitin specific peptidase 7 [Source:HGNC Symbol;Acc:HGNC:12630] |
| ENSG00000188152 | HGLibA_33110 | 87 | -0.347748657 | NUTM2G | NUT family member 2G [Source:HGNC Symbol;Acc:HGNC:23449] |
| ENSG00000188786 | HGLibA_30348 | 87 | -0.347748657 | MTF1 | metal regulatory transcription factor 1 [Source:HGNC Symbol;Acc:HGNC:7428] |
| ENSG00000196132 | HGLibA_30926 | 87 | -0.347748657 | MYT1 | myelin transcription factor 1 [Source:HGNC Symbol;Acc:HGNC:7622] |
| ENSG00000196772 | HGLibA_33628 | 87 | -0.347748657 | OR14A16 | olfactory receptor family 14 subfamily A member 16 [Source:HGNC Symbol;Acc:HGNC:15022] |
| ENSG00000196914 | HGLibA_02815 | 87 | -0.347748657 | ARHGEF12 | Rho guanine nucleotide exchange factor 12 [Source:HGNC Symbol;Acc:HGNC:14193] |
| ENSG00000196924 | HGLibA_17639 | 87 | -0.347748657 | FLNA | filamin A [Source:HGNC Symbol;Acc:HGNC:3754] |
| ENSG00000197532 | HGLibA_34454 | 87 | -0.347748657 | OR6Y1 | olfactory receptor family 6 subfamily Y member 1 [Source:HGNC Symbol;Acc:HGNC:14823] |
| ENSG00000197928 | HGLibA_56517 | 87 | -0.347748657 | ZNF677 | zinc finger protein 677 [Source:HGNC Symbol;Acc:HGNC:28730] |
| ENSG00000198146 | HGLibA_56704 | 87 | -0.347748657 | ZNF770 | zinc finger protein 770 [Source:HGNC Symbol;Acc:HGNC:26061] |
| ENSG00000198585 | HGLibA_32952 | 87 | -0.347748657 | NUDT16 | nudix hydrolase 16 [Source:HGNC Symbol;Acc:HGNC:26442] |
| ENSG00000198860 | HGLibA_51830 | 87 | -0.347748657 | TSEN15 | tRNA splicing endonuclease subunit 15 [Source:HGNC Symbol;Acc:HGNC:16791] |
| ENSG00000204956 | HGLibA_35516 | 87 | -0.347748657 | PCDHGA1 | 1 [Source:HGNC Symbol;Acc:HGNC:8696]" |
| ENSG00000204965 | HGLibA_35449 | 87 | -0.347748657 | PCDHA5 | protocadherin alpha 5 [Source:HGNC Symbol;Acc:HGNC:8671] |
| ENSG00000205309 | HGLibA_32832 | 87 | -0.347748657 | NT5M | mitochondrial [Source:HGNC Symbol;Acc:HGNC:15769]" |
| ENSG00000213015 | HGLibA_56305 | 87 | -0.347748657 | ZNF580 | zinc finger protein 580 [Source:HGNC Symbol;Acc:HGNC:29473] |
| ENSG00000221869 | HGLibA_08962 | 87 | -0.347748657 | CEBPD | CCAAT enhancer binding protein delta [Source:HGNC Symbol;Acc:HGNC:1835] |
| ENSG00000234278 | HGLibA_38812 | 87 | -0.347748657 | PRR20E | proline rich 20E [Source:HGNC Symbol;Acc:HGNC:37223] |
| ENSG00000242574 | HGLibA_21606 | 87 | -0.347748657 | HLA-DMB | DM beta [Source:HGNC Symbol;Acc:HGNC:4935]" |
| ENSG00000243678 | HGLibA_32157 | 87 | -0.347748657 | NME2 | NME/NM23 nucleoside diphosphate kinase 2 [Source:HGNC Symbol;Acc:HGNC:7850] |
| ENSG00000250254 | HGLibA_39569 | 87 | -0.347748657 | PTTG2 | pituitary tumor-transforming 2 [Source:HGNC Symbol;Acc:HGNC:9691] |
| ENSG00000251192 | HGLibA_56508 | 87 | -0.347748657 | ZNF674 | zinc finger protein 674 [Source:HGNC Symbol;Acc:HGNC:17625] |
| ENSG00000267909 | HGLibA_07711 | 87 | -0.347748657 | CCDC177 | coiled-coil domain containing 177 [Source:HGNC Symbol;Acc:HGNC:23243] |
| ENSG00000272398 | HGLibA_08303 | 87 | -0.347748657 | CD24 | CD24 molecule [Source:HGNC Symbol;Acc:HGNC:1645] |
| GPR116 | HGLibA_19863 | 87 | -0.347748657 | GPR116 | - |
| HIST2H2BE | HGLibA_21533 | 87 | -0.347748657 | HIST2H2BE | - |
| HIST2H4B | HGLibA_21552 | 87 | -0.347748657 | HIST2H4B | - |
| hsa-mir-103b-1 | HGLibA_57169 | 87 | -0.347748657 | hsa-mir-103b-1 | - |
| hsa-mir-1302-3 | HGLibA_57700 | 87 | -0.347748657 | hsa-mir-1302-3 | - |
| hsa-mir-217 | HGLibA_58265 | 87 | -0.347748657 | hsa-mir-217 | - |
| hsa-mir-3064 | HGLibA_58458 | 87 | -0.347748657 | hsa-mir-3064 | - |
| hsa-mir-3116-1 | HGLibA_58502 | 87 | -0.347748657 | hsa-mir-3116-1 | - |
| hsa-mir-3153 | HGLibA_58672 | 87 | -0.347748657 | hsa-mir-3153 | - |
| hsa-mir-3922 | HGLibA_59566 | 87 | -0.347748657 | hsa-mir-3922 | - |
| hsa-mir-4315-2 | HGLibA_59978 | 87 | -0.347748657 | hsa-mir-4315-2 | - |
| hsa-mir-4326 | HGLibA_60027 | 87 | -0.347748657 | hsa-mir-4326 | - |
| hsa-mir-4446 | HGLibA_60191 | 87 | -0.347748657 | hsa-mir-4446 | - |
| hsa-mir-4711 | HGLibA_60945 | 87 | -0.347748657 | hsa-mir-4711 | - |
| hsa-mir-4779 | HGLibA_61224 | 87 | -0.347748657 | hsa-mir-4779 | - |
| hsa-mir-4803 | HGLibA_61318 | 87 | -0.347748657 | hsa-mir-4803 | - |
| hsa-mir-5092 | HGLibA_61526 | 87 | -0.347748657 | hsa-mir-5092 | - |
| hsa-mir-509-3 | HGLibA_61513 | 87 | -0.347748657 | hsa-mir-509-3 | - |
| hsa-mir-519b | HGLibA_61718 | 87 | -0.347748657 | hsa-mir-519b | - |
| hsa-mir-527 | HGLibA_61804 | 87 | -0.347748657 | hsa-mir-527 | - |
| hsa-mir-6089-1 | HGLibA_62617 | 87 | -0.347748657 | hsa-mir-6089-1 | - |
| hsa-mir-6826 | HGLibA_63481 | 87 | -0.347748657 | hsa-mir-6826 | - |
| hsa-mir-744 | HGLibA_63884 | 87 | -0.347748657 | hsa-mir-744 | - |
| hsa-mir-9-1 | HGLibA_64261 | 87 | -0.347748657 | hsa-mir-9-1 | - |
| KIAA0226L | HGLibA_24608 | 87 | -0.347748657 | KIAA0226L | - |
| KIAA1737 | HGLibA_24814 | 87 | -0.347748657 | KIAA1737 | - |
| LARGE | HGLibA_26024 | 87 | -0.347748657 | LARGE | - |
| LOC100130480 | HGLibA_26788 | 87 | -0.347748657 | LOC100130480 | - |
| LOC100132146 | HGLibA_26803 | 87 | -0.347748657 | LOC100132146 | - |
| LOC100288524 | HGLibA_26834 | 87 | -0.347748657 | LOC100288524 | - |
| LOC152586 | HGLibA_26895 | 87 | -0.347748657 | LOC152586 | - |
| LPPR3 | HGLibA_27118 | 87 | -0.347748657 | LPPR3 | - |
| MESDC1 | HGLibA_28910 | 87 | -0.347748657 | MESDC1 | - |
| PIH1D3 | HGLibA_36615 | 87 | -0.347748657 | PIH1D3 | - |
| TMEM180 | HGLibA_50095 | 87 | -0.347748657 | TMEM180 | - |
| WDR66 | HGLibA_54155 | 87 | -0.347748657 | WDR66 | - |
| ANKRD20A4 | HGLibA_02037 | 86 | -0.356794 | ANKRD20A4 | - |
| C17orf82 | HGLibA_05585 | 86 | -0.356794 | C17orf82 | - |
| C2orf61 | HGLibA_06155 | 86 | -0.356794 | C2orf61 | - |
| C5orf20 | HGLibA_06361 | 86 | -0.356794 | C5orf20 | - |
| CCDC111 | HGLibA_07542 | 86 | -0.356794 | CCDC111 | - |
| ENSG00000005001 | HGLibA_38915 | 86 | -0.356794 | PRSS22 | serine protease 22 [Source:HGNC Symbol;Acc:HGNC:14368] |
| ENSG00000007372 | HGLibA_35309 | 86 | -0.356794 | PAX6 | paired box 6 [Source:HGNC Symbol;Acc:HGNC:8620] |
| ENSG00000012817 | HGLibA_24531 | 86 | -0.356794 | KDM5D | lysine demethylase 5D [Source:HGNC Symbol;Acc:HGNC:11115] |
| ENSG00000015153 | HGLibA_54627 | 86 | -0.356794 | YAF2 | YY1 associated factor 2 [Source:HGNC Symbol;Acc:HGNC:17363] |
| ENSG00000051382 | HGLibA_36637 | 86 | -0.356794 | PIK3CB | 5-bisphosphate 3-kinase catalytic subunit beta [Source:HGNC Symbol;Acc:HGNC:8976]" |
| ENSG00000055732 | HGLibA_28643 | 86 | -0.356794 | MCOLN3 | mucolipin 3 [Source:HGNC Symbol;Acc:HGNC:13358] |
| ENSG00000072832 | HGLibA_11201 | 86 | -0.356794 | CRMP1 | collapsin response mediator protein 1 [Source:HGNC Symbol;Acc:HGNC:2365] |
| ENSG00000074211 | HGLibA_38151 | 86 | -0.356794 | PPP2R2C | protein phosphatase 2 regulatory subunit Bgamma [Source:HGNC Symbol;Acc:HGNC:9306] |
| ENSG00000075151 | HGLibA_14847 | 86 | -0.356794 | EIF4G3 | eukaryotic translation initiation factor 4 gamma 3 [Source:HGNC Symbol;Acc:HGNC:3298] |
| ENSG00000079435 | HGLibA_26605 | 86 | -0.356794 | LIPE | hormone sensitive type [Source:HGNC Symbol;Acc:HGNC:6621]" |
| ENSG00000079974 | HGLibA_40030 | 86 | -0.356794 | RABL2B | member of RAS oncogene family like 2B [Source:HGNC Symbol;Acc:HGNC:9800]" |
| ENSG00000082213 | HGLibA_06365 | 86 | -0.356794 | C5orf22 | chromosome 5 open reading frame 22 [Source:HGNC Symbol;Acc:HGNC:25639] |
| ENSG00000082515 | HGLibA_29930 | 86 | -0.356794 | MRPL22 | mitochondrial ribosomal protein L22 [Source:HGNC Symbol;Acc:HGNC:14480] |
| ENSG00000083896 | HGLibA_54724 | 86 | -0.356794 | YTHDC1 | YTH domain containing 1 [Source:HGNC Symbol;Acc:HGNC:30626] |
| ENSG00000085733 | HGLibA_11739 | 86 | -0.356794 | CTTN | cortactin [Source:HGNC Symbol;Acc:HGNC:3338] |
| ENSG00000087299 | HGLibA_25891 | 86 | -0.356794 | L2HGDH | L-2-hydroxyglutarate dehydrogenase [Source:HGNC Symbol;Acc:HGNC:20499] |
| ENSG00000088053 | HGLibA_19711 | 86 | -0.356794 | GP6 | glycoprotein VI platelet [Source:HGNC Symbol;Acc:HGNC:14388] |
| ENSG00000088298 | HGLibA_14457 | 86 | -0.356794 | EDEM2 | ER degradation enhancing alpha-mannosidase like protein 2 [Source:HGNC Symbol;Acc:HGNC:15877] |
| ENSG00000088782 | HGLibA_12886 | 86 | -0.356794 | DEFB127 | defensin beta 127 [Source:HGNC Symbol;Acc:HGNC:16206] |
| ENSG00000089157 | HGLibA_42008 | 86 | -0.356794 | RPLP0 | ribosomal protein lateral stalk subunit P0 [Source:HGNC Symbol;Acc:HGNC:10371] |
| ENSG00000090615 | HGLibA_19599 | 86 | -0.356794 | GOLGA3 | golgin A3 [Source:HGNC Symbol;Acc:HGNC:4426] |
| ENSG00000095321 | HGLibA_11067 | 86 | -0.356794 | CRAT | carnitine O-acetyltransferase [Source:HGNC Symbol;Acc:HGNC:2342] |
| ENSG00000100422 | HGLibA_09193 | 86 | -0.356794 | CERK | ceramide kinase [Source:HGNC Symbol;Acc:HGNC:19256] |
| ENSG00000100448 | HGLibA_11708 | 86 | -0.356794 | CTSG | cathepsin G [Source:HGNC Symbol;Acc:HGNC:2532] |
| ENSG00000101082 | HGLibA_44302 | 86 | -0.356794 | SLA2 | Src like adaptor 2 [Source:HGNC Symbol;Acc:HGNC:17329] |
| ENSG00000101276 | HGLibA_45250 | 86 | -0.356794 | SLC52A3 | solute carrier family 52 member 3 [Source:HGNC Symbol;Acc:HGNC:16187] |
| ENSG00000101901 | HGLibA_01617 | 86 | -0.356794 | ALG13 | UDP-N-acetylglucosaminyltransferase subunit [Source:HGNC Symbol;Acc:HGNC:30881]" |
| ENSG00000101981 | HGLibA_15896 | 86 | -0.356794 | F9 | coagulation factor IX [Source:HGNC Symbol;Acc:HGNC:3551] |

| | | | | | |
|---|---|---|---|---|---|
| ENSG00000102891 | HGLibA_30308 | 86 | -0.356794 | MT4 | metallothionein 4 [Source:HGNC Symbol;Acc:HGNC:18705] |
| ENSG00000103152 | HGLibA_29721 | 86 | -0.356794 | MPG | N-methylpurine DNA glycosylase [Source:HGNC Symbol;Acc:HGNC:7211] |
| ENSG00000105669 | HGLibA_10702 | 86 | -0.356794 | COPE | coatomer protein complex subunit epsilon [Source:HGNC Symbol;Acc:HGNC:2234] |
| ENSG00000105701 | HGLibA_17587 | 86 | -0.356794 | FKBP8 | FK506 binding protein 8 [Source:HGNC Symbol;Acc:HGNC:3724] |
| ENSG00000105865 | HGLibA_14123 | 86 | -0.356794 | DUS4L | dihydrouridine synthase 4 like [Source:HGNC Symbol;Acc:HGNC:21517] |
| ENSG00000105953 | HGLibA_33302 | 86 | -0.356794 | OGDH | oxoglutarate dehydrogenase [Source:HGNC Symbol;Acc:HGNC:8124] |
| ENSG00000106868 | HGLibA_47844 | 86 | -0.356794 | SUSD1 | sushi domain containing 1 [Source:HGNC Symbol;Acc:HGNC:25413] |
| ENSG00000106952 | HGLibA_50827 | 86 | -0.356794 | TNFSF8 | TNF superfamily member 8 [Source:HGNC Symbol;Acc:HGNC:11938] |
| ENSG00000107317 | HGLibA_39311 | 86 | -0.356794 | PTGDS | prostaglandin D2 synthase [Source:HGNC Symbol;Acc:HGNC:9592] |
| ENSG00000107859 | HGLibA_36767 | 86 | -0.356794 | PITX3 | paired like homeodomain 3 [Source:HGNC Symbol;Acc:HGNC:9006] |
| ENSG00000108064 | HGLibA_49119 | 86 | -0.356794 | TFAM | mitochondrial [Source:HGNC Symbol;Acc:HGNC:11741]" |
| ENSG00000108389 | HGLibA_30431 | 86 | -0.356794 | MTMR4 | myotubularin related protein 4 [Source:HGNC Symbol;Acc:HGNC:7452] |
| ENSG00000109046 | HGLibA_54414 | 86 | -0.356794 | WSB1 | WD repeat and SOCS box containing 1 [Source:HGNC Symbol;Acc:HGNC:19221] |
| ENSG00000110074 | HGLibA_17950 | 86 | -0.356794 | FOXRED1 | FAD dependent oxidoreductase domain containing 1 [Source:HGNC Symbol;Acc:HGNC:26927] |
| ENSG00000111335 | HGLibA_33200 | 86 | -0.356794 | OAS2 | 2'-5'-oligoadenylate synthetase 2 [Source:HGNC Symbol;Acc:HGNC:8087] |
| ENSG00000112319 | HGLibA_15826 | 86 | -0.356794 | EYA4 | EYA transcriptional coactivator and phosphatase 4 [Source:HGNC Symbol;Acc:HGNC:3522] |
| ENSG00000112462 | HGLibA_33590 | 86 | -0.356794 | OR12D3 | olfactory receptor family 12 subfamily D member 3 [Source:HGNC Symbol;Acc:HGNC:13963] |
| ENSG00000112992 | HGLibA_32226 | 86 | -0.356794 | NNT | nicotinamide nucleotide transhydrogenase [Source:HGNC Symbol;Acc:HGNC:7863] |
| ENSG00000113211 | HGLibA_35504 | 86 | -0.356794 | PCDHB6 | protocadherin beta 6 [Source:HGNC Symbol;Acc:HGNC:8691] |
| ENSG00000114737 | HGLibA_09736 | 86 | -0.356794 | CISH | cytokine inducible SH2 containing protein [Source:HGNC Symbol;Acc:HGNC:1984] |
| ENSG00000114796 | HGLibA_25163 | 86 | -0.356794 | KLHL24 | kelch like family member 24 [Source:HGNC Symbol;Acc:HGNC:25947] |
| ENSG00000115041 | HGLibA_24241 | 86 | -0.356794 | KCNIP3 | potassium voltage-gated channel interacting protein 3 [Source:HGNC Symbol;Acc:HGNC:15523] |
| ENSG00000117115 | HGLibA_34971 | 86 | -0.356794 | PADI2 | peptidyl arginine deiminase 2 [Source:HGNC Symbol;Acc:HGNC:18341] |
| ENSG00000117298 | HGLibA_14383 | 86 | -0.356794 | ECE1 | endothelin converting enzyme 1 [Source:HGNC Symbol;Acc:HGNC:3146] |
| ENSG00000117308 | HGLibA_18512 | 86 | -0.356794 | GALE | UDP-galactose-4-epimerase [Source:HGNC Symbol;Acc:HGNC:4116] |
| ENSG00000119787 | HGLibA_03519 | 86 | -0.356794 | ATL2 | atlastin GTPase 2 [Source:HGNC Symbol;Acc:HGNC:24047] |
| ENSG00000119866 | HGLibA_04289 | 86 | -0.356794 | BCL11A | B cell CLL/lymphoma 11A [Source:HGNC Symbol;Acc:HGNC:13221] |
| ENSG00000119969 | HGLibA_21115 | 86 | -0.356794 | HELLS | lymphoid specific [Source:HGNC Symbol;Acc:HGNC:4861]" |
| ENSG00000122741 | HGLibA_12412 | 86 | -0.356794 | DCAF10 | DDB1 and CUL4 associated factor 10 [Source:HGNC Symbol;Acc:HGNC:23686] |
| ENSG00000122779 | HGLibA_51439 | 86 | -0.356794 | TRIM24 | tripartite motif containing 24 [Source:HGNC Symbol;Acc:HGNC:11812] |
| ENSG00000123684 | HGLibA_27083 | 86 | -0.356794 | LPGAT1 | lysophosphatidylglycerol acyltransferase 1 [Source:HGNC Symbol;Acc:HGNC:28985] |
| ENSG00000124194 | HGLibA_18843 | 86 | -0.356794 | GDAP1L1 | ganglioside induced differentiation associated protein 1 like 1 [Source:HGNC Symbol;Acc:HGNC:4213] |
| ENSG00000124570 | HGLibA_43559 | 86 | -0.356794 | SERPINB6 | serpin family B member 6 [Source:HGNC Symbol;Acc:HGNC:8950] |
| ENSG00000124721 | HGLibA_13499 | 86 | -0.356794 | DNAH8 | dynein axonemal heavy chain 8 [Source:HGNC Symbol;Acc:HGNC:2952] |
| ENSG00000127511 | HGLibA_44187 | 86 | -0.356794 | SIN3B | SIN3 transcription regulator family member B [Source:HGNC Symbol;Acc:HGNC:19354] |
| ENSG00000127533 | HGLibA_15871 | 86 | -0.356794 | F2RL3 | F2R like thrombin or trypsin receptor 3 [Source:HGNC Symbol;Acc:HGNC:3540] |
| ENSG00000128607 | HGLibA_25093 | 86 | -0.356794 | KLHDC10 | kelch domain containing 10 [Source:HGNC Symbol;Acc:HGNC:22194] |
| ENSG00000129993 | HGLibA_07387 | 86 | -0.356794 | CBFA2T3 | CBFA2/RUNX1 translocation partner 3 [Source:HGNC Symbol;Acc:HGNC:1537] |
| ENSG00000130294 | HGLibA_24883 | 86 | -0.356794 | KIF1A | kinesin family member 1A [Source:HGNC Symbol;Acc:HGNC:888] |
| ENSG00000130772 | HGLibA_28765 | 86 | -0.356794 | MED18 | mediator complex subunit 18 [Source:HGNC Symbol;Acc:HGNC:25944] |
| ENSG00000132561 | HGLibA_28431 | 86 | -0.356794 | MATN2 | matrilin 2 [Source:HGNC Symbol;Acc:HGNC:6908] |
| ENSG00000132703 | HGLibA_02400 | 86 | -0.356794 | APCS | serum [Source:HGNC Symbol;Acc:HGNC:584]" |
| ENSG00000133069 | HGLibA_49775 | 86 | -0.356794 | TMCC2 | transmembrane and coiled-coil domain family 2 [Source:HGNC Symbol;Acc:HGNC:24239] |
| ENSG00000134398 | HGLibA_15514 | 86 | -0.356794 | ERN2 | endoplasmic reticulum to nucleus signaling 2 [Source:HGNC Symbol;Acc:HGNC:16942] |
| ENSG00000134716 | HGLibA_12137 | 86 | -0.356794 | CYP2J2 | cytochrome P450 family 2 subfamily J member 2 [Source:HGNC Symbol;Acc:HGNC:2634] |
| ENSG00000134769 | HGLibA_14065 | 86 | -0.356794 | DTNA | dystrobrevin alpha [Source:HGNC Symbol;Acc:HGNC:3057] |
| ENSG00000135387 | HGLibA_07189 | 86 | -0.356794 | CAPRIN1 | cell cycle associated protein 1 [Source:HGNC Symbol;Acc:HGNC:6743] |
| ENSG00000135525 | HGLibA_28232 | 86 | -0.356794 | MAP7 | microtubule associated protein 7 [Source:HGNC Symbol;Acc:HGNC:6869] |
| ENSG00000135540 | HGLibA_31918 | 86 | -0.356794 | NHSL1 | NHS like 1 [Source:HGNC Symbol;Acc:HGNC:21021] |
| ENSG00000135596 | HGLibA_29202 | 86 | -0.356794 | MICAL1 | calponin and LIM domain containing 1 [Source:HGNC Symbol;Acc:HGNC:20619]" |
| ENSG00000135824 | HGLibA_41109 | 86 | -0.356794 | RGS8 | regulator of G protein signaling 8 [Source:HGNC Symbol;Acc:HGNC:16810] |
| ENSG00000136158 | HGLibA_46839 | 86 | -0.356794 | SPRY2 | sprouty RTK signaling antagonist 2 [Source:HGNC Symbol;Acc:HGNC:11270] |
| ENSG00000136699 | HGLibA_45812 | 86 | -0.356794 | SMPD4 | sphingomyelin phosphodiesterase 4 [Source:HGNC Symbol;Acc:HGNC:32949] |
| ENSG00000136715 | HGLibA_42714 | 86 | -0.356794 | SAP130 | Sin3A associated protein 130 [Source:HGNC Symbol;Acc:HGNC:29813] |
| ENSG00000136738 | HGLibA_47330 | 86 | -0.356794 | STAM | signal transducing adaptor molecule [Source:HGNC Symbol;Acc:HGNC:11357] |
| ENSG00000136897 | HGLibA_30008 | 86 | -0.356794 | MRPL50 | mitochondrial ribosomal protein L50 [Source:HGNC Symbol;Acc:HGNC:16654] |
| ENSG00000140009 | HGLibA_15585 | 86 | -0.356794 | ESR2 | estrogen receptor 2 [Source:HGNC Symbol;Acc:HGNC:3468] |
| ENSG00000140299 | HGLibA_04652 | 86 | -0.356794 | BNIP2 | BCL2 interacting protein 2 [Source:HGNC Symbol;Acc:HGNC:1083] |
| ENSG00000140505 | HGLibA_12069 | 86 | -0.356794 | CYP1A2 | cytochrome P450 family 1 subfamily A member 2 [Source:HGNC Symbol;Acc:HGNC:2596] |
| ENSG00000140527 | HGLibA_54224 | 86 | -0.356794 | WDR93 | WD repeat domain 93 [Source:HGNC Symbol;Acc:HGNC:26924] |
| ENSG00000141316 | HGLibA_46328 | 86 | -0.356794 | SPACA3 | sperm acrosome associated 3 [Source:HGNC Symbol;Acc:HGNC:16260] |
| ENSG00000143105 | HGLibA_24127 | 86 | -0.356794 | KCNA10 | potassium voltage-gated channel subfamily A member 10 [Source:HGNC Symbol;Acc:HGNC:6219] |
| ENSG00000143162 | HGLibA_11121 | 86 | -0.356794 | CREG1 | cellular repressor of E1A stimulated genes 1 [Source:HGNC Symbol;Acc:HGNC:2351] |
| ENSG00000144749 | HGLibA_27176 | 86 | -0.356794 | LRIG1 | leucine rich repeats and immunoglobulin like domains 1 [Source:HGNC Symbol;Acc:HGNC:17360] |
| ENSG00000145214 | HGLibA_13081 | 86 | -0.356794 | DGKQ | diacylglycerol kinase theta [Source:HGNC Symbol;Acc:HGNC:2856] |
| ENSG00000146701 | HGLibA_28688 | 86 | -0.356794 | MDH2 | malate dehydrogenase 2 [Source:HGNC Symbol;Acc:HGNC:6971] |
| ENSG00000147869 | HGLibA_09187 | 86 | -0.356794 | CER1 | DAN family BMP antagonist [Source:HGNC Symbol;Acc:HGNC:1862]" |
| ENSG00000148344 | HGLibA_39324 | 86 | -0.356794 | PTGES | prostaglandin E synthase [Source:HGNC Symbol;Acc:HGNC:9599] |
| ENSG00000148408 | HGLibA_06878 | 86 | -0.356794 | CACNA1B | calcium voltage-gated channel subunit alpha1 B [Source:HGNC Symbol;Acc:HGNC:1389] |
| ENSG00000148908 | HGLibA_41051 | 86 | -0.356794 | RGS10 | regulator of G protein signaling 10 [Source:HGNC Symbol;Acc:HGNC:9992] |
| ENSG00000149115 | HGLibA_50854 | 86 | -0.356794 | TNKS1BP1 | tankyrase 1 binding protein 1 [Source:HGNC Symbol;Acc:HGNC:19081] |
| ENSG00000149262 | HGLibA_23422 | 86 | -0.356794 | INTS4 | integrator complex subunit 4 [Source:HGNC Symbol;Acc:HGNC:25048] |
| ENSG00000149564 | HGLibA_15555 | 86 | -0.356794 | ESAM | endothelial cell adhesion molecule [Source:HGNC Symbol;Acc:HGNC:17474] |
| ENSG00000150977 | HGLibA_41255 | 86 | -0.356794 | RILPL2 | Rab interacting lysosomal protein like 2 [Source:HGNC Symbol;Acc:HGNC:28787] |
| ENSG00000151690 | HGLibA_29108 | 86 | -0.356794 | MFSD6 | major facilitator superfamily domain containing 6 [Source:HGNC Symbol;Acc:HGNC:24711] |
| ENSG00000152404 | HGLibA_11827 | 86 | -0.356794 | CWF19L2 | CWF19 like cell cycle control factor 2 [Source:HGNC Symbol;Acc:HGNC:26508] |
| ENSG00000153956 | HGLibA_06904 | 86 | -0.356794 | CACNA2D1 | calcium voltage-gated channel auxiliary subunit alpha2delta 1 [Source:HGNC Symbol;Acc:HGNC:1399] |
| ENSG00000155760 | HGLibA_18282 | 86 | -0.356794 | FZD7 | frizzled class receptor 7 [Source:HGNC Symbol;Acc:HGNC:4045] |
| ENSG00000156735 | HGLibA_04082 | 86 | -0.356794 | BAG4 | BCL2 associated athanogene 4 [Source:HGNC Symbol;Acc:HGNC:940] |
| ENSG00000158006 | HGLibA_34999 | 86 | -0.356794 | PAFAH2 | platelet activating factor acetylhydrolase 2 [Source:HGNC Symbol;Acc:HGNC:8579] |
| ENSG00000159398 | HGLibA_09230 | 86 | -0.356794 | CES5A | carboxylesterase 5A [Source:HGNC Symbol;Acc:HGNC:26459] |
| ENSG00000160325 | HGLibA_06868 | 86 | -0.356794 | CACFD1 | calcium channel flower domain containing 1 [Source:HGNC Symbol;Acc:HGNC:1365] |
| ENSG00000160972 | HGLibA_38054 | 86 | -0.356794 | PPP1R16A | protein phosphatase 1 regulatory subunit 16A [Source:HGNC Symbol;Acc:HGNC:14941] |
| ENSG00000161973 | HGLibA_07781 | 86 | -0.356794 | CCDC42 | coiled-coil domain containing 42 [Source:HGNC Symbol;Acc:HGNC:26528] |
| ENSG00000162040 | HGLibA_22138 | 86 | -0.356794 | HS3ST6 | heparan sulfate-glucosamine 3-sulfotransferase 6 [Source:HGNC Symbol;Acc:HGNC:14178] |
| ENSG00000162073 | HGLibA_35159 | 86 | -0.356794 | PAQR4 | progestin and adipoQ receptor family member 4 [Source:HGNC Symbol;Acc:HGNC:26386] |
| ENSG00000163235 | HGLibA_49204 | 86 | -0.356794 | TGFA | transforming growth factor alpha [Source:HGNC Symbol;Acc:HGNC:11765] |
| ENSG00000163515 | HGLibA_40879 | 86 | -0.356794 | RETNLB | resistin like beta [Source:HGNC Symbol;Acc:HGNC:20388] |
| ENSG00000163607 | HGLibA_20622 | 86 | -0.356794 | GTPBP8 | GTP binding protein 8 (putative) [Source:HGNC Symbol;Acc:HGNC:25007] |
| ENSG00000163909 | HGLibA_21222 | 86 | -0.356794 | HEYL | hes related family bHLH transcription factor with YRPW motif-like [Source:HGNC Symbol;Acc:HGNC:4882] |
| ENSG00000164113 | HGLibA_00718 | 86 | -0.356794 | ADAD1 | adenosine deaminase domain containing 1 [Source:HGNC Symbol;Acc:HGNC:30713] |
| ENSG00000164114 | HGLibA_28243 | 86 | -0.356794 | MAP9 | microtubule associated protein 9 [Source:HGNC Symbol;Acc:HGNC:26118] |
| ENSG00000164512 | HGLibA_02125 | 86 | -0.356794 | ANKRD55 | ankyrin repeat domain 55 [Source:HGNC Symbol;Acc:HGNC:25681] |
| ENSG00000164985 | HGLibA_39062 | 86 | -0.356794 | PSIP1 | PC4 and SFRS1 interacting protein 1 [Source:HGNC Symbol;Acc:HGNC:9527] |
| ENSG00000166220 | HGLibA_48459 | 86 | -0.356794 | TBATA | brain and testes associated [Source:HGNC Symbol;Acc:HGNC:23511]" |
| ENSG00000166855 | HGLibA_10120 | 86 | -0.356794 | CLPX | caseinolytic mitochondrial matrix peptidase chaperone subunit [Source:HGNC Symbol;Acc:HGNC:2088] |
| ENSG00000166913 | HGLibA_54740 | 86 | -0.356794 | YWHAB | tyrosine 3-monooxygenase/tryptophan 5-monooxygenase activation protein beta [Source:HGNC Symbol;Acc:HGNC:12849] |
| ENSG00000166924 | HGLibA_33179 | 86 | -0.356794 | NYAP1 | neuronal tyrosine phosphorylated phosphoinositide-3-kinase adaptor 1 [Source:HGNC Symbol;Acc:HGNC:22009] |
| ENSG00000167525 | HGLibA_38653 | 86 | -0.356794 | PROCA1 | protein interacting with cyclin A1 [Source:HGNC Symbol;Acc:HGNC:28600] |
| ENSG00000167578 | HGLibA_39957 | 86 | -0.356794 | RAB4B | member RAS oncogene family [Source:HGNC Symbol;Acc:HGNC:9782]" |
| ENSG00000167635 | HGLibA_55496 | 86 | -0.356794 | ZNF146 | zinc finger protein 146 [Source:HGNC Symbol;Acc:HGNC:12931] |
| ENSG00000168515 | HGLibA_42913 | 86 | -0.356794 | SCGB1D1 | secretoglobin family 1D member 1 [Source:HGNC Symbol;Acc:HGNC:18395] |
| ENSG00000169136 | HGLibA_03444 | 86 | -0.356794 | ATF5 | activating transcription factor 5 [Source:HGNC Symbol;Acc:HGNC:790] |
| ENSG00000169245 | HGLibA_11846 | 86 | -0.356794 | CXCL10 | C-X-C motif chemokine ligand 10 [Source:HGNC Symbol;Acc:HGNC:10637] |
| ENSG00000169807 | HGLibA_38999 | 86 | -0.356794 | PRY2 | PTPN13 like Y-linked 2 [Source:HGNC Symbol;Acc:HGNC:21504] |
| ENSG00000170276 | HGLibA_22308 | 86 | -0.356794 | HSPB2 | heat shock protein family B (small) member 2 [Source:HGNC Symbol;Acc:HGNC:5247] |
| ENSG00000170579 | HGLibA_13339 | 86 | -0.356794 | DLGAP1 | DLG associated protein 1 [Source:HGNC Symbol;Acc:HGNC:2905] |
| ENSG00000170873 | HGLibA_30497 | 86 | -0.356794 | MTSS1 | I-BAR domain containing [Source:HGNC Symbol;Acc:HGNC:20443]" |
| ENSG00000171532 | HGLibA_31736 | 86 | -0.356794 | NEUROD2 | neuronal differentiation 2 [Source:HGNC Symbol;Acc:HGNC:7763] |
| ENSG00000171992 | HGLibA_48018 | 86 | -0.356794 | SYNPO | synaptopodin [Source:HGNC Symbol;Acc:HGNC:30672] |
| ENSG00000172179 | HGLibA_38597 | 86 | -0.356794 | PRL | prolactin [Source:HGNC Symbol;Acc:HGNC:9445] |
| ENSG00000172215 | HGLibA_11896 | 86 | -0.356794 | CXCR6 | C-X-C motif chemokine receptor 6 [Source:HGNC Symbol;Acc:HGNC:16647] |
| ENSG00000172244 | HGLibA_06378 | 86 | -0.356794 | C5orf34 | chromosome 5 open reading frame 34 [Source:HGNC Symbol;Acc:HGNC:24738] |
| ENSG00000172426 | HGLibA_42342 | 86 | -0.356794 | RSPH9 | radial spoke head 9 homolog [Source:HGNC Symbol;Acc:HGNC:21057] |
| ENSG00000173039 | HGLibA_40813 | 86 | -0.356794 | RELA | NF-kB subunit [Source:HGNC Symbol;Acc:HGNC:9955]" |
| ENSG00000173040 | HGLibA_15682 | 86 | -0.356794 | EVC2 | EvC ciliary complex subunit 2 [Source:HGNC Symbol;Acc:HGNC:19747] |
| ENSG00000175931 | HGLibA_52643 | 86 | -0.356794 | UBE2O | ubiquitin conjugating enzyme E2 O [Source:HGNC Symbol;Acc:HGNC:29554] |
| ENSG00000176978 | HGLibA_13877 | 86 | -0.356794 | DPP7 | dipeptidyl peptidase 7 [Source:HGNC Symbol;Acc:HGNC:14892] |
| ENSG00000177192 | HGLibA_39596 | 86 | -0.356794 | PUS1 | pseudouridine synthase 1 [Source:HGNC Symbol;Acc:HGNC:15508] |
| ENSG00000178188 | HGLibA_43873 | 86 | -0.356794 | SH2B1 | SH2B adaptor protein 1 [Source:HGNC Symbol;Acc:HGNC:30417] |
| ENSG00000178235 | HGLibA_45512 | 86 | -0.356794 | SLITRK1 | SLIT and NTRK like family member 1 [Source:HGNC Symbol;Acc:HGNC:20297] |
| ENSG00000179454 | HGLibA_25170 | 86 | -0.356794 | KLHL28 | kelch like family member 28 [Source:HGNC Symbol;Acc:HGNC:19741] |
| ENSG00000179542 | HGLibA_45522 | 86 | -0.356794 | SLITRK4 | SLIT and NTRK like family member 4 [Source:HGNC Symbol;Acc:HGNC:23502] |
| ENSG00000179934 | HGLibA_08168 | 86 | -0.356794 | CCR8 | C-C motif chemokine receptor 8 [Source:HGNC Symbol;Acc:HGNC:1609] |
| ENSG00000180988 | HGLibA_34196 | 86 | -0.356794 | OR52N2 | olfactory receptor family 52 subfamily N member 2 [Source:HGNC Symbol;Acc:HGNC:15228] |
| ENSG00000182986 | HGLibA_55796 | 86 | -0.356794 | ZNF320 | zinc finger protein 320 [Source:HGNC Symbol;Acc:HGNC:13842] |
| ENSG00000183665 | HGLibA_51675 | 86 | -0.356794 | TRMT12 | tRNA methyltransferase 12 homolog [Source:HGNC Symbol;Acc:HGNC:26091] |
| ENSG00000183668 | HGLibA_39059 | 86 | -0.356794 | PSG9 | pregnancy specific beta-1-glycoprotein 9 [Source:HGNC Symbol;Acc:HGNC:9526] |
| ENSG00000184564 | HGLibA_45528 | 86 | -0.356794 | SLITRK6 | SLIT and NTRK like family member 6 [Source:HGNC Symbol;Acc:HGNC:23503] |
| ENSG00000184924 | HGLibA_39555 | 86 | -0.356794 | PTRHD1 | peptidyl-tRNA hydrolase domain containing 1 [Source:HGNC Symbol;Acc:HGNC:33782] |
| ENSG00000184995 | HGLibA_22704 | 86 | -0.356794 | IFNE | interferon epsilon [Source:HGNC Symbol;Acc:HGNC:18163] |
| ENSG00000185842 | HGLibA_13480 | 86 | -0.356794 | DNAH14 | dynein axonemal heavy chain 14 [Source:HGNC Symbol;Acc:HGNC:2945] |
| ENSG00000186868 | HGLibA_28321 | 86 | -0.356794 | MAPT | microtubule associated protein tau [Source:HGNC Symbol;Acc:HGNC:6893] |
| ENSG00000188215 | HGLibA_12570 | 86 | -0.356794 | DCUN1D3 | defective in cullin neddylation 1 domain containing 3 [Source:HGNC Symbol;Acc:HGNC:28734] |
| ENSG00000188234 | HGLibA_01149 | 86 | -0.356794 | AGAP4 | ankyrin repeat and PH domain 4 [Source:HGNC Symbol;Acc:HGNC:23459]" |

| | | | | | |
|---|---|---|---|---|---|
| ENSG00000188386 | HGLibA_38198 | 86 | -0.356794 | PPP3R2 | beta [Source:HGNC Symbol;Acc:HGNC:9318]" |
| ENSG00000196220 | HGLibA_46983 | 86 | -0.356794 | SRGAP3 | SLIT-ROBO Rho GTPase activating protein 3 [Source:HGNC Symbol;Acc:HGNC:19744] |
| ENSG00000196542 | HGLibA_46905 | 86 | -0.356794 | SPTSSB | serine palmitoyltransferase small subunit B [Source:HGNC Symbol;Acc:HGNC:24045] |
| ENSG00000196943 | HGLibA_32320 | 86 | -0.356794 | NOP9 | NOP9 nucleolar protein [Source:HGNC Symbol;Acc:HGNC:19826] |
| ENSG00000197084 | HGLibA_26107 | 86 | -0.356794 | LCE1C | late cornified envelope 1C [Source:HGNC Symbol;Acc:HGNC:29464] |
| ENSG00000197961 | HGLibA_55454 | 86 | -0.356794 | ZNF121 | zinc finger protein 121 [Source:HGNC Symbol;Acc:HGNC:12904] |
| ENSG00000198128 | HGLibA_33825 | 86 | -0.356794 | OR2L3 | olfactory receptor family 2 subfamily L member 3 [Source:HGNC Symbol;Acc:HGNC:15009] |
| ENSG00000204147 | HGLibA_03190 | 86 | -0.356794 | ASAH2B | N-acylsphingosine amidohydrolase 2B [Source:HGNC Symbol;Acc:HGNC:23456] |
| ENSG00000204246 | HGLibA_33598 | 86 | -0.356794 | OR13C3 | olfactory receptor family 13 subfamily C member 3 [Source:HGNC Symbol;Acc:HGNC:14704] |
| ENSG00000205403 | HGLibA_09277 | 86 | -0.356794 | CFI | complement factor I [Source:HGNC Symbol;Acc:HGNC:5394] |
| ENSG00000213401 | HGLibA_27903 | 86 | -0.356794 | MAGEA12 | MAGE family member A12 [Source:HGNC Symbol;Acc:HGNC:6799] |
| ENSG00000213416 | HGLibA_25755 | 86 | -0.356794 | KRTAP4-12 | keratin associated protein 4-12 [Source:HGNC Symbol;Acc:HGNC:16776] |
| ENSG00000225968 | HGLibA_14906 | 86 | -0.356794 | ELFN1 | extracellular leucine rich repeat and fibronectin type III domain containing 1 [Source:HGNC Symbol;Acc:HGNC:33154] |
| ENSG00000230062 | HGLibA_02141 | 86 | -0.356794 | ANKRD66 | ankyrin repeat domain 66 [Source:HGNC Symbol;Acc:HGNC:44669] |
| ENSG00000232995 | HGLibA_41096 | 86 | -0.356794 | RGS5 | regulator of G protein signaling 5 [Source:NCBI gene;Acc:8490] |
| ENSG00000243137 | HGLibA_39047 | 86 | -0.356794 | PSG4 | pregnancy specific beta-1-glycoprotein 4 [Source:HGNC Symbol;Acc:HGNC:9521] |
| ENSG00000248871 | HGLibA_50804 | 86 | -0.356794 | TNFSF12-TNFSF13 | TNFSF12-TNFSF13 readthrough [Source:HGNC Symbol;Acc:HGNC:33537] |
| ENSG00000258873 | HGLibA_14204 | 86 | -0.356794 | DUXA | double homeobox A [Source:HGNC Symbol;Acc:HGNC:32179] |
| ENSG00000267041 | HGLibA_56872 | 86 | -0.356794 | ZNF850 | zinc finger protein 850 [Source:HGNC Symbol;Acc:HGNC:27994] |
| ENSG00000268988 | HGLibA_46405 | 86 | -0.356794 | SPANXN2 | SPANX family member N2 [Source:HGNC Symbol;Acc:HGNC:33175] |
| ENSG00000276070 | HGLibA_08032 | 86 | -0.356794 | CCL4L2 | C-C motif chemokine ligand 4 like 2 [Source:HGNC Symbol;Acc:HGNC:24066] |
| GPR125 | HGLibA_19878 | 86 | -0.356794 | GPR125 | - |
| HIST1H1C | HGLibA_21362 | 86 | -0.356794 | HIST1H1C | - |
| HIST1H1E | HGLibA_21369 | 86 | -0.356794 | HIST1H1E | - |
| HIST1H2AL | HGLibA_21406 | 86 | -0.356794 | HIST1H2AL | - |
| HIST1H2BO | HGLibA_21453 | 86 | -0.356794 | HIST1H2BO | - |
| HIST3H2BB | HGLibA_21557 | 86 | -0.356794 | HIST3H2BB | - |
| hsa-mir-124-2 | HGLibA_57350 | 86 | -0.356794 | hsa-mir-124-2 | - |
| hsa-mir-126 | HGLibA_57455 | 86 | -0.356794 | hsa-mir-126 | - |
| hsa-mir-129-1 | HGLibA_57630 | 86 | -0.356794 | hsa-mir-129-1 | - |
| hsa-mir-362 | HGLibA_59108 | 86 | -0.356794 | hsa-mir-362 | - |
| hsa-mir-3620 | HGLibA_59114 | 86 | -0.356794 | hsa-mir-3620 | - |
| hsa-mir-379 | HGLibA_59470 | 86 | -0.356794 | hsa-mir-379 | - |
| hsa-mir-3910-2 | HGLibA_59509 | 86 | -0.356794 | hsa-mir-3910-2 | - |
| hsa-mir-4288 | HGLibA_59861 | 86 | -0.356794 | hsa-mir-4288 | - |
| hsa-mir-4435-1 | HGLibA_60132 | 86 | -0.356794 | hsa-mir-4435-1 | - |
| hsa-mir-4517 | HGLibA_60512 | 86 | -0.356794 | hsa-mir-4517 | - |
| hsa-mir-4716 | HGLibA_60964 | 86 | -0.356794 | hsa-mir-4716 | - |
| hsa-mir-499a | HGLibA_61399 | 86 | -0.356794 | hsa-mir-499a | - |
| hsa-mir-519a-1 | HGLibA_61710 | 86 | -0.356794 | hsa-mir-519a-1 | - |
| hsa-mir-527 | HGLibA_61801 | 86 | -0.356794 | hsa-mir-527 | - |
| hsa-mir-602 | HGLibA_62499 | 86 | -0.356794 | hsa-mir-602 | - |
| hsa-mir-608 | HGLibA_62575 | 86 | -0.356794 | hsa-mir-608 | - |
| hsa-mir-642b | HGLibA_62813 | 86 | -0.356794 | hsa-mir-642b | - |
| hsa-mir-6741 | HGLibA_63117 | 86 | -0.356794 | hsa-mir-6741 | - |
| hsa-mir-936 | HGLibA_64319 | 86 | -0.356794 | hsa-mir-936 | - |
| LOC100996331 | HGLibA_26881 | 86 | -0.356794 | LOC100996331 | - |
| LPHN1 | HGLibA_27085 | 86 | -0.356794 | LPHN1 | - |
| MICALCL | HGLibA_29210 | 86 | -0.356794 | MICALCL | - |
| MLL | HGLibA_29392 | 86 | -0.356794 | MLL | - |
| MLL3 | HGLibA_29398 | 86 | -0.356794 | MLL3 | - |
| MPP5 | HGLibA_29759 | 86 | -0.356794 | MPP5 | - |
| SLC24A6 | HGLibA_44631 | 86 | -0.356794 | SLC24A6 | - |
| SPATA31A4 | HGLibA_46478 | 86 | -0.356794 | SPATA31A4 | - |
| APOBEC3A_B | HGLibA_02475 | 85 | -0.365839343 | APOBEC3A_B | - |
| C15orf27 | HGLibA_05382 | 85 | -0.365839343 | C15orf27 | - |
| C6orf48 | HGLibA_06491 | 85 | -0.365839343 | C6orf48 | - |
| CD97 | HGLibA_08485 | 85 | -0.365839343 | CD97 | - |
| CTSL1 | HGLibA_11719 | 85 | -0.365839343 | CTSL1 | - |
| DYX1C1 | HGLibA_14291 | 85 | -0.365839343 | DYX1C1 | - |
| ENSG00000002822 | HGLibA_27843 | 85 | -0.365839343 | MAD1L1 | mitotic arrest deficient 1 like 1 [Source:HGNC Symbol;Acc:HGNC:6762] |
| ENSG00000005243 | HGLibA_10739 | 85 | -0.365839343 | COPZ2 | coatomer protein complex subunit zeta 2 [Source:HGNC Symbol;Acc:HGNC:19356] |
| ENSG00000008988 | HGLibA_42105 | 85 | -0.365839343 | RPS20 | ribosomal protein S20 [Source:HGNC Symbol;Acc:HGNC:10405] |
| ENSG00000011677 | HGLibA_18362 | 85 | -0.365839343 | GABRA3 | gamma-aminobutyric acid type A receptor alpha3 subunit [Source:HGNC Symbol;Acc:HGNC:4077] |
| ENSG00000012174 | HGLibA_28539 | 85 | -0.365839343 | MBTPS2 | site 2 [Source:HGNC Symbol;Acc:HGNC:15455]" |
| ENSG00000018236 | HGLibA_10402 | 85 | -0.365839343 | CNTN1 | contactin 1 [Source:HGNC Symbol;Acc:HGNC:2171] |
| ENSG00000050344 | HGLibA_31797 | 85 | -0.365839343 | NFE2L3 | erythroid 2 like 3 [Source:HGNC Symbol;Acc:HGNC:7783]" |
| ENSG00000062582 | HGLibA_30072 | 85 | -0.365839343 | MRPS24 | mitochondrial ribosomal protein S24 [Source:HGNC Symbol;Acc:HGNC:14510] |
| ENSG00000071575 | HGLibA_51393 | 85 | -0.365839343 | TRIB2 | tribbles pseudokinase 2 [Source:HGNC Symbol;Acc:HGNC:30809] |
| ENSG00000075151 | HGLibA_14845 | 85 | -0.365839343 | EIF4G3 | eukaryotic translation initiation factor 4 gamma 3 [Source:HGNC Symbol;Acc:HGNC:3298] |
| ENSG00000075785 | HGLibA_39978 | 85 | -0.365839343 | RAB7A | member RAS oncogene family [Source:HGNC Symbol;Acc:HGNC:9788]" |
| ENSG00000079385 | HGLibA_08922 | 85 | -0.365839343 | CEACAM1 | carcinoembryonic antigen related cell adhesion molecule 1 [Source:HGNC Symbol;Acc:HGNC:1814] |
| ENSG00000080224 | HGLibA_15303 | 85 | -0.365839343 | EPHA6 | EPH receptor A6 [Source:HGNC Symbol;Acc:HGNC:19296] |
| ENSG00000085872 | HGLibA_09426 | 85 | -0.365839343 | CHERP | calcium homeostasis endoplasmic reticulum protein [Source:HGNC Symbol;Acc:HGNC:16930] |
| ENSG00000089163 | HGLibA_44226 | 85 | -0.365839343 | SIRT4 | sirtuin 4 [Source:HGNC Symbol;Acc:HGNC:14932] |
| ENSG00000090447 | HGLibA_49135 | 85 | -0.365839343 | TFAP4 | transcription factor AP-4 [Source:HGNC Symbol;Acc:HGNC:11745] |
| ENSG00000091527 | HGLibA_08894 | 85 | -0.365839343 | CDV3 | CDV3 homolog [Source:HGNC Symbol;Acc:HGNC:26928] |
| ENSG00000092068 | HGLibA_45381 | 85 | -0.365839343 | SLC7A8 | solute carrier family 7 member 8 [Source:HGNC Symbol;Acc:HGNC:11066] |
| ENSG00000092098 | HGLibA_41661 | 85 | -0.365839343 | RNF31 | ring finger protein 31 [Source:HGNC Symbol;Acc:HGNC:16031] |
| ENSG00000094755 | HGLibA_18399 | 85 | -0.365839343 | GABRP | gamma-aminobutyric acid type A receptor pi subunit [Source:HGNC Symbol;Acc:HGNC:4089] |
| ENSG00000095321 | HGLibA_11065 | 85 | -0.365839343 | CRAT | carnitine O-acetyltransferase [Source:HGNC Symbol;Acc:HGNC:2342] |
| ENSG00000095587 | HGLibA_49631 | 85 | -0.365839343 | TLL2 | tolloid like 2 [Source:HGNC Symbol;Acc:HGNC:11844] |
| ENSG00000096433 | HGLibA_23860 | 85 | -0.365839343 | ITPR3 | 5-trisphosphate receptor type 3 [Source:HGNC Symbol;Acc:HGNC:6182]" |
| ENSG00000099338 | HGLibA_07373 | 85 | -0.365839343 | CATSPERG | cation channel sperm associated auxiliary subunit gamma [Source:HGNC Symbol;Acc:HGNC:25243] |
| ENSG00000099904 | HGLibA_55130 | 85 | -0.365839343 | ZDHHC8 | zinc finger DHHC-type containing 8 [Source:HGNC Symbol;Acc:HGNC:18474] |
| ENSG00000100280 | HGLibA_02281 | 85 | -0.365839343 | AP1B1 | adaptor related protein complex 1 subunit beta 1 [Source:HGNC Symbol;Acc:HGNC:554] |
| ENSG00000100523 | HGLibA_12603 | 85 | -0.365839343 | DDHD1 | DDHD domain containing 1 [Source:HGNC Symbol;Acc:HGNC:19714] |
| ENSG00000100600 | HGLibA_26404 | 85 | -0.365839343 | LGMN | legumain [Source:HGNC Symbol;Acc:HGNC:9472] |
| ENSG00000100902 | HGLibA_39085 | 85 | -0.365839343 | PSMA6 | proteasome subunit alpha 6 [Source:HGNC Symbol;Acc:HGNC:9535] |
| ENSG00000101076 | HGLibA_21780 | 85 | -0.365839343 | HNF4A | hepatocyte nuclear factor 4 alpha [Source:HGNC Symbol;Acc:HGNC:5024] |
| ENSG00000101104 | HGLibA_34925 | 85 | -0.365839343 | PABPC1L | poly(A) binding protein cytoplasmic 1 like [Source:HGNC Symbol;Acc:HGNC:15797] |
| ENSG00000101247 | HGLibA_31495 | 85 | -0.365839343 | NDUFAF5 | NADH:ubiquinone oxidoreductase complex assembly factor 5 [Source:HGNC Symbol;Acc:HGNC:15899] |
| ENSG00000101407 | HGLibA_52175 | 85 | -0.365839343 | TTI1 | TELO2 interacting protein 1 [Source:HGNC Symbol;Acc:HGNC:29029] |
| ENSG00000102057 | HGLibA_24174 | 85 | -0.365839343 | KCND1 | potassium voltage-gated channel subfamily D member 1 [Source:HGNC Symbol;Acc:HGNC:6237] |
| ENSG00000104205 | HGLibA_43833 | 85 | -0.365839343 | SGK3 | serum/glucocorticoid regulated kinase family member 3 [Source:HGNC Symbol;Acc:HGNC:10812] |
| ENSG00000104218 | HGLibA_11421 | 85 | -0.365839343 | CSPP1 | centrosome and spindle pole associated protein 1 [Source:HGNC Symbol;Acc:HGNC:26193] |
| ENSG00000104312 | HGLibA_41320 | 85 | -0.365839343 | RIPK2 | receptor interacting serine/threonine kinase 2 [Source:HGNC Symbol;Acc:HGNC:10020] |
| ENSG00000106683 | HGLibA_26541 | 85 | -0.365839343 | LIMK1 | LIM domain kinase 1 [Source:HGNC Symbol;Acc:HGNC:6613] |
| ENSG00000107672 | HGLibA_32771 | 85 | -0.365839343 | NSMCE4A | SMC5-SMC6 complex component [Source:HGNC Symbol;Acc:HGNC:25935]" |
| ENSG00000109163 | HGLibA_19587 | 85 | -0.365839343 | GNRHR | gonadotropin releasing hormone receptor [Source:HGNC Symbol;Acc:HGNC:4421] |
| ENSG00000109680 | HGLibA_48490 | 85 | -0.365839343 | TBC1D19 | TBC1 domain family member 19 [Source:HGNC Symbol;Acc:HGNC:25624] |
| ENSG00000110203 | HGLibA_17789 | 85 | -0.365839343 | FOLR3 | folate receptor 3 [Source:HGNC Symbol;Acc:HGNC:3795] |
| ENSG00000110700 | HGLibA_42072 | 85 | -0.365839343 | RPS13 | ribosomal protein S13 [Source:HGNC Symbol;Acc:HGNC:10386] |
| ENSG00000112039 | HGLibA_16845 | 85 | -0.365839343 | FANCE | FA complementation group E [Source:HGNC Symbol;Acc:HGNC:3586] |
| ENSG00000112242 | HGLibA_14313 | 85 | -0.365839343 | E2F3 | E2F transcription factor 3 [Source:HGNC Symbol;Acc:HGNC:3115] |
| ENSG00000113073 | HGLibA_45231 | 85 | -0.365839343 | SLC4A9 | solute carrier family 4 member 9 [Source:HGNC Symbol;Acc:HGNC:11035] |
| ENSG00000113262 | HGLibA_20356 | 85 | -0.365839343 | GRM6 | glutamate metabotropic receptor 6 [Source:HGNC Symbol;Acc:HGNC:4598] |
| ENSG00000113520 | HGLibA_23165 | 85 | -0.365839343 | IL4 | interleukin 4 [Source:HGNC Symbol;Acc:HGNC:6014] |
| ENSG00000114026 | HGLibA_33322 | 85 | -0.365839343 | OGG1 | 8-oxoguanine DNA glycosylase [Source:HGNC Symbol;Acc:HGNC:8125] |
| ENSG00000115602 | HGLibA_23062 | 85 | -0.365839343 | IL1RL1 | interleukin 1 receptor like 1 [Source:HGNC Symbol;Acc:HGNC:5998] |
| ENSG00000116922 | HGLibA_05740 | 85 | -0.365839343 | C1orf109 | chromosome 1 open reading frame 109 [Source:HGNC Symbol;Acc:HGNC:26039] |
| ENSG00000117501 | HGLibA_29882 | 85 | -0.365839343 | MROH9 | maestro heat like repeat family member 9 [Source:HGNC Symbol;Acc:HGNC:26287] |
| ENSG00000119703 | HGLibA_54944 | 85 | -0.365839343 | ZC2HC1C | zinc finger C2HC-type containing 1C [Source:HGNC Symbol;Acc:HGNC:20354] |
| ENSG00000119878 | HGLibA_11163 | 85 | -0.365839343 | CRIPT | CXXC repeat containing interactor of PDZ3 domain [Source:HGNC Symbol;Acc:HGNC:14312] |
| ENSG00000121440 | HGLibA_35996 | 85 | -0.365839343 | PDZRN3 | PDZ domain containing ring finger 3 [Source:HGNC Symbol;Acc:HGNC:17704] |
| ENSG00000122033 | HGLibA_30401 | 85 | -0.365839343 | MTIF3 | mitochondrial translational initiation factor 3 [Source:HGNC Symbol;Acc:HGNC:29788] |
| ENSG00000123178 | HGLibA_46856 | 85 | -0.365839343 | SPRYD7 | SPRY domain containing 7 [Source:HGNC Symbol;Acc:HGNC:14297] |
| ENSG00000123353 | HGLibA_34629 | 85 | -0.365839343 | ORMDL2 | ORMDL sphingolipid biosynthesis regulator 2 [Source:HGNC Symbol;Acc:HGNC:16037] |
| ENSG00000124602 | HGLibA_53024 | 85 | -0.365839343 | UNC5CL | unc-5 family C-terminal like [Source:HGNC Symbol;Acc:HGNC:21203] |
| ENSG00000125686 | HGLibA_28734 | 85 | -0.365839343 | MED1 | mediator complex subunit 1 [Source:HGNC Symbol;Acc:HGNC:9234] |
| ENSG00000129862 | HGLibA_53597 | 85 | -0.365839343 | VCY1B | variable charge Y-linked 1B [Source:HGNC Symbol;Acc:HGNC:31751] |
| ENSG00000130558 | HGLibA_33345 | 85 | -0.365839343 | OLFM1 | olfactomedin 1 [Source:HGNC Symbol;Acc:HGNC:17187] |
| ENSG00000131203 | HGLibA_22563 | 85 | -0.365839343 | IDO1 | 3-dioxygenase 1 [Source:HGNC Symbol;Acc:HGNC:6059]" |
| ENSG00000132155 | HGLibA_40128 | 85 | -0.365839343 | RAF1 | serine/threonine kinase [Source:HGNC Symbol;Acc:HGNC:9829]" |
| ENSG00000132591 | HGLibA_15400 | 85 | -0.365839343 | ERAL1 | Era like 12S mitochondrial rRNA chaperone 1 [Source:HGNC Symbol;Acc:HGNC:3424] |
| ENSG00000133193 | HGLibA_15988 | 85 | -0.365839343 | FAM104A | family with sequence similarity 104 member A [Source:HGNC Symbol;Acc:HGNC:25918] |
| ENSG00000134013 | HGLibA_27041 | 85 | -0.365839343 | LOXL2 | lysyl oxidase like 2 [Source:HGNC Symbol;Acc:HGNC:6666] |
| ENSG00000136717 | HGLibA_04505 | 85 | -0.365839343 | BIN1 | bridging integrator 1 [Source:HGNC Symbol;Acc:HGNC:1052] |
| ENSG00000136866 | HGLibA_55212 | 85 | -0.365839343 | ZFP37 | ZFP37 zinc finger protein [Source:HGNC Symbol;Acc:HGNC:12863] |
| ENSG00000137200 | HGLibA_10233 | 85 | -0.365839343 | CMTR1 | cap methyltransferase 1 [Source:HGNC Symbol;Acc:HGNC:21077] |
| ENSG00000141905 | HGLibA_31806 | 85 | -0.365839343 | NFIC | nuclear factor I C [Source:HGNC Symbol;Acc:HGNC:7786] |
| ENSG00000141959 | HGLibA_36165 | 85 | -0.365839343 | PFKL | liver type [Source:HGNC Symbol;Acc:HGNC:8876]" |
| ENSG00000144791 | HGLibA_26531 | 85 | -0.365839343 | LIMD1 | LIM domains containing 1 [Source:HGNC Symbol;Acc:HGNC:6612] |
| ENSG00000145730 | HGLibA_35082 | 85 | -0.365839343 | PAM | peptidylglycine alpha-amidating monooxygenase [Source:HGNC Symbol;Acc:HGNC:8596] |
| ENSG00000146731 | HGLibA_08207 | 85 | -0.365839343 | CCT6A | chaperonin containing TCP1 subunit 6A [Source:HGNC Symbol;Acc:HGNC:1620] |

| | | | | | |
|---|---|---|---|---|---|
| ENSG00000146733 | HGLibA_39226 | 85 | -0.365839343 | PSPH | phosphoserine phosphatase [Source:HGNC Symbol;Acc:HGNC:9577] |
| ENSG00000147117 | HGLibA_55506 | 85 | -0.365839343 | ZNF157 | zinc finger protein 157 [Source:HGNC Symbol;Acc:HGNC:12942] |
| ENSG00000148660 | HGLibA_07076 | 85 | -0.365839343 | CAMK2G | calcium/calmodulin dependent protein kinase II gamma [Source:HGNC Symbol;Acc:HGNC:1463] |
| ENSG00000149823 | HGLibA_53803 | 85 | -0.365839343 | VPS51 | GARP complex subunit [Source:HGNC Symbol;Acc:HGNC:1172]" |
| ENSG00000150627 | HGLibA_54036 | 85 | -0.365839343 | WDR17 | WD repeat domain 17 [Source:HGNC Symbol;Acc:HGNC:16661] |
| ENSG00000150938 | HGLibA_11148 | 85 | -0.365839343 | CRIM1 | cysteine rich transmembrane BMP regulator 1 [Source:HGNC Symbol;Acc:HGNC:2359] |
| ENSG00000152207 | HGLibA_12228 | 85 | -0.365839343 | CYSLTR2 | cysteinyl leukotriene receptor 2 [Source:HGNC Symbol;Acc:HGNC:18274] |
| ENSG00000152558 | HGLibA_49903 | 85 | -0.365839343 | TMEM123 | transmembrane protein 123 [Source:HGNC Symbol;Acc:HGNC:30138] |
| ENSG00000153060 | HGLibA_48983 | 85 | -0.365839343 | TEKT5 | tektin 5 [Source:HGNC Symbol;Acc:HGNC:26554] |
| ENSG00000153266 | HGLibA_17322 | 85 | -0.365839343 | FEZF2 | FEZ family zinc finger 2 [Source:HGNC Symbol;Acc:HGNC:13506] |
| ENSG00000156304 | HGLibA_42827 | 85 | -0.365839343 | SCAF4 | SR-related CTD associated factor 4 [Source:HGNC Symbol;Acc:HGNC:19304] |
| ENSG00000157881 | HGLibA_35107 | 85 | -0.365839343 | PANK4 | pantothenate kinase 4 [Source:HGNC Symbol;Acc:HGNC:19366] |
| ENSG00000158109 | HGLibA_51183 | 85 | -0.365839343 | TPRG1L | tumor protein p63 regulated 1 like [Source:HGNC Symbol;Acc:HGNC:27007] |
| ENSG00000158716 | HGLibA_14166 | 85 | -0.365839343 | DUSP23 | dual specificity phosphatase 23 [Source:HGNC Symbol;Acc:HGNC:21480] |
| ENSG00000160326 | HGLibA_44884 | 85 | -0.365839343 | SLC2A6 | solute carrier family 2 member 6 [Source:HGNC Symbol;Acc:HGNC:11011] |
| ENSG00000160654 | HGLibA_08379 | 85 | -0.365839343 | CD3G | CD3g molecule [Source:HGNC Symbol;Acc:HGNC:1675] |
| ENSG00000160767 | HGLibA_16356 | 85 | -0.365839343 | FAM189B | family with sequence similarity 189 member B [Source:HGNC Symbol;Acc:HGNC:1233] |
| ENSG00000160883 | HGLibA_21585 | 85 | -0.365839343 | HK3 | hexokinase 3 [Source:HGNC Symbol;Acc:HGNC:4925] |
| ENSG00000161011 | HGLibA_46918 | 85 | -0.365839343 | SQSTM1 | sequestosome 1 [Source:HGNC Symbol;Acc:HGNC:11280] |
| ENSG00000161526 | HGLibA_42724 | 85 | -0.365839343 | SAP30BP | SAP30 binding protein [Source:HGNC Symbol;Acc:HGNC:30785] |
| ENSG00000161634 | HGLibA_12478 | 85 | -0.365839343 | DCD | dermcidin [Source:HGNC Symbol;Acc:HGNC:14669] |
| ENSG00000163098 | HGLibA_04531 | 85 | -0.365839343 | BIRC8 | baculoviral IAP repeat containing 8 [Source:HGNC Symbol;Acc:HGNC:14878] |
| ENSG00000163354 | HGLibA_12533 | 85 | -0.365839343 | DCST2 | DC-STAMP domain containing 2 [Source:HGNC Symbol;Acc:HGNC:26562] |
| ENSG00000163935 | HGLibA_43720 | 85 | -0.365839343 | SFMBT1 | Scm like with four mbt domains 1 [Source:HGNC Symbol;Acc:HGNC:20255] |
| ENSG00000164086 | HGLibA_14191 | 85 | -0.365839343 | DUSP7 | dual specificity phosphatase 7 [Source:HGNC Symbol;Acc:HGNC:3073] |
| ENSG00000164967 | HGLibA_42032 | 85 | -0.365839343 | RPP25L | ribonuclease P/MRP subunit p25 like [Source:HGNC Symbol;Acc:HGNC:19909] |
| ENSG00000165185 | HGLibA_24828 | 85 | -0.365839343 | KIAA1958 | KIAA1958 [Source:HGNC Symbol;Acc:HGNC:23427] |
| ENSG00000165678 | HGLibA_19037 | 85 | -0.365839343 | GHITM | growth hormone inducible transmembrane protein [Source:HGNC Symbol;Acc:HGNC:17281] |
| ENSG00000166411 | HGLibA_22545 | 85 | -0.365839343 | IDH3A | isocitrate dehydrogenase 3 (NAD(+)) alpha [Source:HGNC Symbol;Acc:HGNC:5384] |
| ENSG00000166860 | HGLibA_54874 | 85 | -0.365839343 | ZBTB39 | zinc finger and BTB domain containing 39 [Source:HGNC Symbol;Acc:HGNC:29014] |
| ENSG00000167394 | HGLibA_56495 | 85 | -0.365839343 | ZNF668 | zinc finger protein 668 [Source:HGNC Symbol;Acc:HGNC:25821] |
| ENSG00000168078 | HGLibA_35332 | 85 | -0.365839343 | PBK | PDZ binding kinase [Source:HGNC Symbol;Acc:HGNC:18282] |
| ENSG00000168703 | HGLibA_54257 | 85 | -0.365839343 | WFDC12 | WAP four-disulfide core domain 12 [Source:HGNC Symbol;Acc:HGNC:16115] |
| ENSG00000168807 | HGLibA_46002 | 85 | -0.365839343 | SNTB2 | syntrophin beta 2 [Source:HGNC Symbol;Acc:HGNC:11169] |
| ENSG00000169213 | HGLibA_39911 | 85 | -0.365839343 | RAB3B | member RAS oncogene family [Source:HGNC Symbol;Acc:HGNC:9778]" |
| ENSG00000170276 | HGLibA_22307 | 85 | -0.365839343 | HSPB2 | heat shock protein family B (small) member 2 [Source:HGNC Symbol;Acc:HGNC:5247] |
| ENSG00000171102 | HGLibA_33227 | 85 | -0.365839343 | OBP2B | odorant binding protein 2B [Source:HGNC Symbol;Acc:HGNC:23381] |
| ENSG00000172007 | HGLibA_39884 | 85 | -0.365839343 | RAB33B | member RAS oncogene family [Source:HGNC Symbol;Acc:HGNC:16075]" |
| ENSG00000172315 | HGLibA_51074 | 85 | -0.365839343 | TP53RK | TP53 regulating kinase [Source:HGNC Symbol;Acc:HGNC:16197] |
| ENSG00000174038 | HGLibA_06676 | 85 | -0.365839343 | C9orf131 | chromosome 9 open reading frame 131 [Source:HGNC Symbol;Acc:HGNC:31418] |
| ENSG00000174226 | HGLibA_46086 | 85 | -0.365839343 | SNX31 | sorting nexin 31 [Source:HGNC Symbol;Acc:HGNC:28605] |
| ENSG00000174502 | HGLibA_44811 | 85 | -0.365839343 | SLC26A9 | solute carrier family 26 member 9 [Source:HGNC Symbol;Acc:HGNC:14469] |
| ENSG00000175287 | HGLibA_36470 | 85 | -0.365839343 | PHYHD1 | phytanoyl-CoA dioxygenase domain containing 1 [Source:HGNC Symbol;Acc:HGNC:23396] |
| ENSG00000175866 | HGLibA_04122 | 85 | -0.365839343 | BAIAP2 | BAI1 associated protein 2 [Source:HGNC Symbol;Acc:HGNC:947] |
| ENSG00000176105 | HGLibA_54661 | 85 | -0.365839343 | YES1 | Src family tyrosine kinase [Source:HGNC Symbol;Acc:HGNC:12841]" |
| ENSG00000176393 | HGLibA_41726 | 85 | -0.365839343 | RNPEP | arginyl aminopeptidase [Source:HGNC Symbol;Acc:HGNC:10078] |
| ENSG00000176871 | HGLibA_54419 | 85 | -0.365839343 | WSB2 | WD repeat and SOCS box containing 2 [Source:HGNC Symbol;Acc:HGNC:19222] |
| ENSG00000177082 | HGLibA_54169 | 85 | -0.365839343 | WDR73 | WD repeat domain 73 [Source:HGNC Symbol;Acc:HGNC:25928] |
| ENSG00000177455 | HGLibA_08262 | 85 | -0.365839343 | CD19 | CD19 molecule [Source:HGNC Symbol;Acc:HGNC:1633] |
| ENSG00000178201 | HGLibA_53697 | 85 | -0.365839343 | VN1R1 | vomeronasal 1 receptor 1 [Source:HGNC Symbol;Acc:HGNC:13548] |
| ENSG00000178904 | HGLibA_13910 | 85 | -0.365839343 | DPY19L3 | dpy-19 like C-mannosyltransferase 3 [Source:HGNC Symbol;Acc:HGNC:27120] |
| ENSG00000181001 | HGLibA_34194 | 85 | -0.365839343 | OR52N1 | olfactory receptor family 52 subfamily N member 1 [Source:HGNC Symbol;Acc:HGNC:14853] |
| ENSG00000181322 | HGLibA_32177 | 85 | -0.365839343 | NME9 | NME/NM23 family member 9 [Source:HGNC Symbol;Acc:HGNC:21343] |
| ENSG00000182575 | HGLibA_33169 | 85 | -0.365839343 | NXPH3 | neurexophilin 3 [Source:HGNC Symbol;Acc:HGNC:8077] |
| ENSG00000183486 | HGLibA_30608 | 85 | -0.365839343 | MX2 | MX dynamin like GTPase 2 [Source:HGNC Symbol;Acc:HGNC:7533] |
| ENSG00000184056 | HGLibA_53767 | 85 | -0.365839343 | VPS33B | late endosome and lysosome associated [Source:HGNC Symbol;Acc:HGNC:12712]" |
| ENSG00000184344 | HGLibA_18866 | 85 | -0.365839343 | GDF3 | growth differentiation factor 3 [Source:HGNC Symbol;Acc:HGNC:4218] |
| ENSG00000184507 | HGLibA_33101 | 85 | -0.365839343 | NUTM1 | NUT midline carcinoma family member 1 [Source:HGNC Symbol;Acc:HGNC:29919] |
| ENSG00000185974 | HGLibA_20327 | 85 | -0.365839343 | GRK1 | G protein-coupled receptor kinase 1 [Source:HGNC Symbol;Acc:HGNC:10013] |
| ENSG00000186150 | HGLibA_52708 | 85 | -0.365839343 | UBL4B | ubiquitin like 4B [Source:HGNC Symbol;Acc:HGNC:32309] |
| ENSG00000186635 | HGLibA_02625 | 85 | -0.365839343 | ARAP1 | ankyrin repeat and PH domain 1 [Source:HGNC Symbol;Acc:HGNC:16925]" |
| ENSG00000186767 | HGLibA_46678 | 85 | -0.365839343 | SPIN4 | spindlin family member 4 [Source:HGNC Symbol;Acc:HGNC:27040] |
| ENSG00000188674 | HGLibA_06188 | 85 | -0.365839343 | C2orf80 | chromosome 2 open reading frame 80 [Source:HGNC Symbol;Acc:HGNC:34352] |
| ENSG00000196266 | HGLibA_33524 | 85 | -0.365839343 | OR10J3 | olfactory receptor family 10 subfamily J member 3 [Source:HGNC Symbol;Acc:HGNC:14992] |
| ENSG00000196767 | HGLibA_37774 | 85 | -0.365839343 | POU3F4 | POU class 3 homeobox 4 [Source:HGNC Symbol;Acc:HGNC:9217] |
| ENSG00000197506 | HGLibA_44836 | 85 | -0.365839343 | SLC28A3 | solute carrier family 28 member 3 [Source:HGNC Symbol;Acc:HGNC:16484] |
| ENSG00000197816 | HGLibA_07722 | 85 | -0.365839343 | CCDC180 | coiled-coil domain containing 180 [Source:HGNC Symbol;Acc:HGNC:29303] |
| ENSG00000198039 | HGLibA_55709 | 85 | -0.365839343 | ZNF273 | zinc finger protein 273 [Source:HGNC Symbol;Acc:HGNC:13067] |
| ENSG00000198176 | HGLibA_49151 | 85 | -0.365839343 | TFDP1 | transcription factor Dp-1 [Source:HGNC Symbol;Acc:HGNC:11749] |
| ENSG00000203710 | HGLibA_11045 | 85 | -0.365839343 | CR1 | complement C3b/C4b receptor 1 (Knops blood group) [Source:HGNC Symbol;Acc:HGNC:2334] |
| ENSG00000204256 | HGLibA_04772 | 85 | -0.365839343 | BRD2 | bromodomain containing 2 [Source:HGNC Symbol;Acc:HGNC:1103] |
| ENSG00000204450 | HGLibA_51576 | 85 | -0.365839343 | TRIM64 | tripartite motif containing 64 [Source:HGNC Symbol;Acc:HGNC:14663] |
| ENSG00000213316 | HGLibA_27604 | 85 | -0.365839343 | LTC4S | leukotriene C4 synthase [Source:HGNC Symbol;Acc:HGNC:6719] |
| ENSG00000214107 | HGLibA_27929 | 85 | -0.365839343 | MAGEB1 | MAGE family member B1 [Source:HGNC Symbol;Acc:HGNC:6808] |
| ENSG00000214819 | HGLibA_08878 | 85 | -0.365839343 | CDRT15L2 | CMT1A duplicated region transcript 15 like 2 [Source:HGNC Symbol;Acc:HGNC:34075] |
| ENSG00000215343 | HGLibA_56594 | 85 | -0.365839343 | ZNF705D | zinc finger protein 705D [Source:HGNC Symbol;Acc:HGNC:33202] |
| ENSG00000225921 | HGLibA_32273 | 85 | -0.365839343 | NOL7 | nucleolar protein 7 [Source:HGNC Symbol;Acc:HGNC:21040] |
| ENSG00000229809 | HGLibA_56543 | 85 | -0.365839343 | ZNF688 | zinc finger protein 688 [Source:HGNC Symbol;Acc:HGNC:30489] |
| ENSG00000230453 | HGLibA_02022 | 85 | -0.365839343 | ANKRD18B | ankyrin repeat domain 18B [Source:HGNC Symbol;Acc:HGNC:23644] |
| ENSG00000242362 | HGLibA_11527 | 85 | -0.365839343 | CT47A2 | member A2 [Source:HGNC Symbol;Acc:HGNC:33283]" |
| ENSG00000243927 | HGLibA_30107 | 85 | -0.365839343 | MRPS6 | mitochondrial ribosomal protein S6 [Source:HGNC Symbol;Acc:HGNC:14051] |
| ENSG00000249158 | HGLibA_35430 | 85 | -0.365839343 | PCDHA11 | protocadherin alpha 11 [Source:HGNC Symbol;Acc:HGNC:8665] |
| ENSG00000255298 | HGLibA_34522 | 85 | -0.365839343 | OR8G5 | olfactory receptor family 8 subfamily G member 5 [Source:HGNC Symbol;Acc:HGNC:19622] |
| ENSG00000260691 | HGLibA_02028 | 85 | -0.365839343 | ANKRD20A1 | ankyrin repeat domain 20 family member A1 [Source:HGNC Symbol;Acc:HGNC:23665] |
| ENSG00000265203 | HGLibA_40636 | 85 | -0.365839343 | RBP3 | retinol binding protein 3 [Source:HGNC Symbol;Acc:HGNC:9921] |
| ENSG00000266412 | HGLibA_31345 | 85 | -0.365839343 | NCOA4 | nuclear receptor coactivator 4 [Source:HGNC Symbol;Acc:HGNC:7671] |
| ENSG00000270011 | HGLibA_56238 | 85 | -0.365839343 | ZNF559-ZNF177 | ZNF559-ZNF177 readthrough [Source:HGNC Symbol;Acc:HGNC:42964] |
| ENSG00000288596 | HGLibA_06622 | 85 | -0.365839343 | C8orf44 | - |
| FLJ44635 | HGLibA_17632 | 85 | -0.365839343 | FLJ44635 | - |
| GPR110 | HGLibA_19850 | 85 | -0.365839343 | GPR110 | - |
| hsa-mir-1183 | HGLibA_57225 | 85 | -0.365839343 | hsa-mir-1183 | - |
| hsa-mir-1278 | HGLibA_57567 | 85 | -0.365839343 | hsa-mir-1278 | - |
| hsa-mir-1298 | HGLibA_57671 | 85 | -0.365839343 | hsa-mir-1298 | - |
| hsa-mir-3174 | HGLibA_58770 | 85 | -0.365839343 | hsa-mir-3174 | - |
| hsa-mir-3180-1 | HGLibA_58803 | 85 | -0.365839343 | hsa-mir-3180-1 | - |
| hsa-mir-3188 | HGLibA_58849 | 85 | -0.365839343 | hsa-mir-3188 | - |
| hsa-mir-4480 | HGLibA_60340 | 85 | -0.365839343 | hsa-mir-4480 | - |
| hsa-mir-4495 | HGLibA_60398 | 85 | -0.365839343 | hsa-mir-4495 | - |
| hsa-mir-4536-1 | HGLibA_60602 | 85 | -0.365839343 | hsa-mir-4536-1 | - |
| hsa-mir-4637 | HGLibA_60652 | 85 | -0.365839343 | hsa-mir-4637 | - |
| hsa-mir-4668 | HGLibA_60791 | 85 | -0.365839343 | hsa-mir-4668 | - |
| hsa-mir-4717 | HGLibA_60968 | 85 | -0.365839343 | hsa-mir-4717 | - |
| hsa-mir-488 | HGLibA_61351 | 85 | -0.365839343 | hsa-mir-488 | - |
| hsa-mir-5009 | HGLibA_61441 | 85 | -0.365839343 | hsa-mir-5009 | - |
| hsa-mir-5010 | HGLibA_61455 | 85 | -0.365839343 | hsa-mir-5010 | - |
| hsa-mir-5095 | HGLibA_61539 | 85 | -0.365839343 | hsa-mir-5095 | - |
| hsa-mir-516a-1 | HGLibA_61609 | 85 | -0.365839343 | hsa-mir-516a-1 | - |
| hsa-mir-524 | HGLibA_61784 | 85 | -0.365839343 | hsa-mir-524 | - |
| hsa-mir-548al | HGLibA_61896 | 85 | -0.365839343 | hsa-mir-548al | - |
| hsa-mir-554 | HGLibA_62131 | 85 | -0.365839343 | hsa-mir-554 | - |
| hsa-mir-607 | HGLibA_62528 | 85 | -0.365839343 | hsa-mir-607 | - |
| hsa-mir-6076 | HGLibA_62555 | 85 | -0.365839343 | hsa-mir-6076 | - |
| hsa-mir-6738 | HGLibA_63106 | 85 | -0.365839343 | hsa-mir-6738 | - |
| hsa-mir-6801 | HGLibA_63382 | 85 | -0.365839343 | hsa-mir-6801 | - |
| hsa-mir-6803 | HGLibA_63389 | 85 | -0.365839343 | hsa-mir-6803 | - |
| hsa-mir-6822 | HGLibA_63467 | 85 | -0.365839343 | hsa-mir-6822 | - |
| hsa-mir-6861 | HGLibA_63624 | 85 | -0.365839343 | hsa-mir-6861 | - |
| hsa-mir-6874 | HGLibA_63680 | 85 | -0.365839343 | hsa-mir-6874 | - |
| hsa-mir-8069 | HGLibA_64113 | 85 | -0.365839343 | hsa-mir-8069 | - |
| hsa-mir-8070 | HGLibA_64117 | 85 | -0.365839343 | hsa-mir-8070 | - |
| hsa-mir-8075 | HGLibA_64143 | 85 | -0.365839343 | hsa-mir-8075 | - |
| IL3RA_Y | HGLibA_23163 | 85 | -0.365839343 | IL3RA_Y | - |
| KIAA2018 | HGLibA_24841 | 85 | -0.365839343 | KIAA2018 | - |
| PPP2R4 | HGLibA_38167 | 85 | -0.365839343 | PPP2R4 | - |
| SPATA31A2 | HGLibA_46472 | 85 | -0.365839343 | SPATA31A2 | - |
| SRPR | HGLibA_47027 | 85 | -0.365839343 | SRPR | - |
| TMEM189 | HGLibA_50133 | 85 | -0.365839343 | TMEM189 | - |
| TSTA3 | HGLibA_52026 | 85 | -0.365839343 | TSTA3 | - |
| VARS | HGLibA_53523 | 85 | -0.365839343 | VARS | - |
| WDR60 | HGLibA_54138 | 85 | -0.365839343 | WDR60 | - |
| ABP1 | HGLibA_00319 | 84 | -0.374884686 | ABP1 | - |
| ATP5H | HGLibA_03665 | 84 | -0.374884686 | ATP5H | - |
| C17orf51 | HGLibA_05542 | 84 | -0.374884686 | C17orf51 | - |
| C1orf65 | HGLibA_05887 | 84 | -0.374884686 | C1orf65 | - |
| C21orf2 | HGLibA_06034 | 84 | -0.374884686 | C21orf2 | - |

| | | | | | |
|---|---|---|---|---|---|
| CXorf40B | HGLibA_11926 | 84 | -0.374884686 | CXorf40B | - |
| ENSG00000013374 | HGLibA_32897 | 84 | -0.374884686 | NUB1 | negative regulator of ubiquitin like proteins 1 [Source:HGNC Symbol;Acc:HGNC:17623] |
| ENSG00000014914 | HGLibA_30415 | 84 | -0.374884686 | MTMR11 | myotubularin related protein 11 [Source:HGNC Symbol;Acc:HGNC:24307] |
| ENSG00000031003 | HGLibA_16144 | 84 | -0.374884686 | FAM13B | family with sequence similarity 13 member B [Source:HGNC Symbol;Acc:HGNC:1335] |
| ENSG00000035499 | HGLibA_12978 | 84 | -0.374884686 | DEPDC1B | DEP domain containing 1B [Source:HGNC Symbol;Acc:HGNC:24902] |
| ENSG00000048462 | HGLibA_50762 | 84 | -0.374884686 | TNFRSF17 | TNF receptor superfamily member 17 [Source:HGNC Symbol;Acc:HGNC:11913] |
| ENSG00000049283 | HGLibA_15348 | 84 | -0.374884686 | EPN3 | epsin 3 [Source:HGNC Symbol;Acc:HGNC:18235] |
| ENSG00000053372 | HGLibA_30122 | 84 | -0.374884686 | MRTO4 | ribosome maturation factor [Source:HGNC Symbol;Acc:HGNC:18477]" |
| ENSG00000054116 | HGLibA_51319 | 84 | -0.374884686 | TRAPPC3 | trafficking protein particle complex 3 [Source:HGNC Symbol;Acc:HGNC:19942] |
| ENSG00000054116 | HGLibA_51318 | 84 | -0.374884686 | TRAPPC3 | trafficking protein particle complex 3 [Source:HGNC Symbol;Acc:HGNC:19942] |
| ENSG00000063660 | HGLibA_19762 | 84 | -0.374884686 | GPC1 | glypican 1 [Source:HGNC Symbol;Acc:HGNC:4449] |
| ENSG00000073670 | HGLibA_00730 | 84 | -0.374884686 | ADAM11 | ADAM metallopeptidase domain 11 [Source:HGNC Symbol;Acc:HGNC:189] |
| ENSG00000074317 | HGLibA_45913 | 84 | -0.374884686 | SNCB | synuclein beta [Source:HGNC Symbol;Acc:HGNC:11140] |
| ENSG00000076984 | HGLibA_28158 | 84 | -0.374884686 | MAP2K7 | mitogen-activated protein kinase kinase 7 [Source:HGNC Symbol;Acc:HGNC:6847] |
| ENSG00000077274 | HGLibA_07171 | 84 | -0.374884686 | CAPN6 | calpain 6 [Source:HGNC Symbol;Acc:HGNC:1483] |
| ENSG00000077800 | HGLibA_17583 | 84 | -0.374884686 | FKBP6 | FK506 binding protein 6 [Source:HGNC Symbol;Acc:HGNC:3722] |
| ENSG00000077984 | HGLibA_11466 | 84 | -0.374884686 | CST7 | cystatin F [Source:HGNC Symbol;Acc:HGNC:2479] |
| ENSG00000086102 | HGLibA_31848 | 84 | -0.374884686 | NFX1 | X-box binding 1 [Source:HGNC Symbol;Acc:HGNC:7803]" |
| ENSG00000089012 | HGLibA_44214 | 84 | -0.374884686 | SIRPG | signal regulatory protein gamma [Source:HGNC Symbol;Acc:HGNC:15757] |
| ENSG00000090013 | HGLibA_04579 | 84 | -0.374884686 | BLVRB | biliverdin reductase B [Source:HGNC Symbol;Acc:HGNC:1063] |
| ENSG00000096654 | HGLibA_55545 | 84 | -0.374884686 | ZNF184 | zinc finger protein 184 [Source:HGNC Symbol;Acc:HGNC:12975] |
| ENSG00000100139 | HGLibA_29213 | 84 | -0.374884686 | MICALL1 | MICAL like 1 [Source:HGNC Symbol;Acc:HGNC:29804] |
| ENSG00000100211 | HGLibA_07469 | 84 | -0.374884686 | CBY1 | beta catenin antagonist [Source:HGNC Symbol;Acc:HGNC:1307]" |
| ENSG00000104177 | HGLibA_30689 | 84 | -0.374884686 | MYEF2 | myelin expression factor 2 [Source:HGNC Symbol;Acc:HGNC:17940] |
| ENSG00000104825 | HGLibA_31824 | 84 | -0.374884686 | NFKBIB | NFKB inhibitor beta [Source:HGNC Symbol;Acc:HGNC:7798] |
| ENSG00000106648 | HGLibA_18578 | 84 | -0.374884686 | GALNTL5 | polypeptide N-acetylgalactosaminyltransferase like 5 [Source:HGNC Symbol;Acc:HGNC:21725] |
| ENSG00000106868 | HGLibA_47843 | 84 | -0.374884686 | SUSD1 | sushi domain containing 1 [Source:HGNC Symbol;Acc:HGNC:25413] |
| ENSG00000108375 | HGLibA_41684 | 84 | -0.374884686 | RNF43 | ring finger protein 43 [Source:HGNC Symbol;Acc:HGNC:18505] |
| ENSG00000108375 | HGLibA_41685 | 84 | -0.374884686 | RNF43 | ring finger protein 43 [Source:HGNC Symbol;Acc:HGNC:18505] |
| ENSG00000108395 | HGLibA_51477 | 84 | -0.374884686 | TRIM37 | tripartite motif containing 37 [Source:HGNC Symbol;Acc:HGNC:7523] |
| ENSG00000108515 | HGLibA_15145 | 84 | -0.374884686 | ENO3 | enolase 3 [Source:HGNC Symbol;Acc:HGNC:3354] |
| ENSG00000109475 | HGLibA_41942 | 84 | -0.374884686 | RPL34 | ribosomal protein L34 [Source:HGNC Symbol;Acc:HGNC:10340] |
| ENSG00000109674 | HGLibA_31634 | 84 | -0.374884686 | NEIL3 | nei like DNA glycosylase 3 [Source:HGNC Symbol;Acc:HGNC:24573] |
| ENSG00000110169 | HGLibA_22068 | 84 | -0.374884686 | HPX | hemopexin [Source:HGNC Symbol;Acc:HGNC:5171] |
| ENSG00000111087 | HGLibA_19248 | 84 | -0.374884686 | GLI1 | GLI family zinc finger 1 [Source:HGNC Symbol;Acc:HGNC:4317] |
| ENSG00000111364 | HGLibA_12746 | 84 | -0.374884686 | DDX55 | DEAD-box helicase 55 [Source:HGNC Symbol;Acc:HGNC:20085] |
| ENSG00000111850 | HGLibA_45769 | 84 | -0.374884686 | SMIM8 | small integral membrane protein 8 [Source:HGNC Symbol;Acc:HGNC:21401] |
| ENSG00000112175 | HGLibA_04612 | 84 | -0.374884686 | BMP5 | bone morphogenetic protein 5 [Source:HGNC Symbol;Acc:HGNC:1072] |
| ENSG00000114573 | HGLibA_03731 | 84 | -0.374884686 | ATP6V1A | ATPase H+ transporting V1 subunit A [Source:HGNC Symbol;Acc:HGNC:851] |
| ENSG00000115255 | HGLibA_40791 | 84 | -0.374884686 | REEP6 | receptor accessory protein 6 [Source:HGNC Symbol;Acc:HGNC:30078] |
| ENSG00000115380 | HGLibA_14562 | 84 | -0.374884686 | EFEMP1 | EGF containing fibulin extracellular matrix protein 1 [Source:HGNC Symbol;Acc:HGNC:3218] |
| ENSG00000115896 | HGLibA_37014 | 84 | -0.374884686 | PLCL1 | phospholipase C like 1 (inactive) [Source:HGNC Symbol;Acc:HGNC:9063] |
| ENSG00000116005 | HGLibA_35695 | 84 | -0.374884686 | PCYOX1 | prenylcysteine oxidase 1 [Source:HGNC Symbol;Acc:HGNC:20588] |
| ENSG00000116726 | HGLibA_38288 | 84 | -0.374884686 | PRAMEF12 | PRAME family member 12 [Source:HGNC Symbol;Acc:HGNC:22125] |
| ENSG00000116985 | HGLibA_04624 | 84 | -0.374884686 | BMP8B | bone morphogenetic protein 8b [Source:HGNC Symbol;Acc:HGNC:1075] |
| ENSG00000117298 | HGLibA_14382 | 84 | -0.374884686 | ECE1 | endothelin converting enzyme 1 [Source:HGNC Symbol;Acc:HGNC:3146] |
| ENSG00000118004 | HGLibA_10635 | 84 | -0.374884686 | COLEC11 | collectin subfamily member 11 [Source:HGNC Symbol;Acc:HGNC:17213] |
| ENSG00000118058 | HGLibA_25321 | 84 | -0.374884686 | KMT2A | lysine methyltransferase 2A [Source:HGNC Symbol;Acc:HGNC:7132] |
| ENSG00000118181 | HGLibA_42115 | 84 | -0.374884686 | RPS25 | ribosomal protein S25 [Source:HGNC Symbol;Acc:HGNC:10413] |
| ENSG00000118898 | HGLibA_37960 | 84 | -0.374884686 | PPL | periplakin [Source:HGNC Symbol;Acc:HGNC:9273] |
| ENSG00000120159 | HGLibA_06831 | 84 | -0.374884686 | CAAP1 | caspase activity and apoptosis inhibitor 1 [Source:HGNC Symbol;Acc:HGNC:25834] |
| ENSG00000120318 | HGLibA_02631 | 84 | -0.374884686 | ARAP3 | ankyrin repeat and PH domain 3 [Source:HGNC Symbol;Acc:HGNC:24097]" |
| ENSG00000120742 | HGLibA_43498 | 84 | -0.374884686 | SERP1 | stress associated endoplasmic reticulum protein 1 [Source:HGNC Symbol;Acc:HGNC:10759] |
| ENSG00000121766 | HGLibA_55022 | 84 | -0.374884686 | ZCCHC17 | zinc finger CCHC-type containing 17 [Source:HGNC Symbol;Acc:HGNC:30246] |
| ENSG00000122729 | HGLibA_00462 | 84 | -0.374884686 | ACO1 | aconitase 1 [Source:HGNC Symbol;Acc:HGNC:117] |
| ENSG00000123297 | HGLibA_51842 | 84 | -0.374884686 | TSFM | mitochondrial [Source:HGNC Symbol;Acc:HGNC:12367]" |
| ENSG00000123329 | HGLibA_02791 | 84 | -0.374884686 | ARHGAP9 | Rho GTPase activating protein 9 [Source:HGNC Symbol;Acc:HGNC:14130] |
| ENSG00000123444 | HGLibA_24107 | 84 | -0.374884686 | KBTBD4 | kelch repeat and BTB domain containing 4 [Source:HGNC Symbol;Acc:HGNC:23761] |
| ENSG00000123473 | HGLibA_47438 | 84 | -0.374884686 | STIL | centriolar assembly protein [Source:HGNC Symbol;Acc:HGNC:10879]" |
| ENSG00000124731 | HGLibA_51355 | 84 | -0.374884686 | TREM1 | triggering receptor expressed on myeloid cells 1 [Source:HGNC Symbol;Acc:HGNC:17760] |
| ENSG00000125337 | HGLibA_24912 | 84 | -0.374884686 | KIF25 | kinesin family member 25 [Source:HGNC Symbol;Acc:HGNC:6390] |
| ENSG00000125454 | HGLibA_44663 | 84 | -0.374884686 | SLC25A19 | solute carrier family 25 member 19 [Source:HGNC Symbol;Acc:HGNC:14409] |
| ENSG00000125780 | HGLibA_49258 | 84 | -0.374884686 | TGM3 | transglutaminase 3 [Source:HGNC Symbol;Acc:HGNC:11779] |
| ENSG00000125846 | HGLibA_55466 | 84 | -0.374884686 | ZNF133 | zinc finger protein 133 [Source:HGNC Symbol;Acc:HGNC:12917] |
| ENSG00000125999 | HGLibA_04719 | 84 | -0.374884686 | BPIFB1 | BPI fold containing family B member 1 [Source:HGNC Symbol;Acc:HGNC:16108] |
| ENSG00000126858 | HGLibA_41201 | 84 | -0.374884686 | RHOT1 | ras homolog family member T1 [Source:HGNC Symbol;Acc:HGNC:21168] |
| ENSG00000128310 | HGLibA_18593 | 84 | -0.374884686 | GALR3 | galanin receptor 3 [Source:HGNC Symbol;Acc:HGNC:4134] |
| ENSG00000128335 | HGLibA_02529 | 84 | -0.374884686 | APOL2 | apolipoprotein L2 [Source:HGNC Symbol;Acc:HGNC:619] |
| ENSG00000129480 | HGLibA_14056 | 84 | -0.374884686 | DTD2 | D-tyrosyl-tRNA deacylase 2 (putative) [Source:HGNC Symbol;Acc:HGNC:20277] |
| ENSG00000132963 | HGLibA_37665 | 84 | -0.374884686 | POMP | proteasome maturation protein [Source:HGNC Symbol;Acc:HGNC:20330] |
| ENSG00000135100 | HGLibA_21775 | 84 | -0.374884686 | HNF1A | HNF1 homeobox A [Source:HGNC Symbol;Acc:HGNC:11621] |
| ENSG00000135437 | HGLibA_40750 | 84 | -0.374884686 | RDH5 | retinol dehydrogenase 5 [Source:HGNC Symbol;Acc:HGNC:9940] |
| ENSG00000135473 | HGLibA_35091 | 84 | -0.374884686 | PAN2 | poly(A) specific ribonuclease subunit PAN2 [Source:HGNC Symbol;Acc:HGNC:20074] |
| ENSG00000135902 | HGLibA_09602 | 84 | -0.374884686 | CHRND | cholinergic receptor nicotinic delta subunit [Source:HGNC Symbol;Acc:HGNC:1965] |
| ENSG00000136449 | HGLibA_30671 | 84 | -0.374884686 | MYCBPAP | MYCBP associated protein [Source:HGNC Symbol;Acc:HGNC:19677] |
| ENSG00000137077 | HGLibA_07993 | 84 | -0.374884686 | CCL21 | C-C motif chemokine ligand 21 [Source:HGNC Symbol;Acc:HGNC:10620] |
| ENSG00000137462 | HGLibA_49645 | 84 | -0.374884686 | TLR2 | toll like receptor 2 [Source:HGNC Symbol;Acc:HGNC:11848] |
| ENSG00000137700 | HGLibA_45053 | 84 | -0.374884686 | SLC37A4 | solute carrier family 37 member 4 [Source:HGNC Symbol;Acc:HGNC:4061] |
| ENSG00000137818 | HGLibA_42012 | 84 | -0.374884686 | RPLP1 | ribosomal protein lateral stalk subunit P1 [Source:HGNC Symbol;Acc:HGNC:10372] |
| ENSG00000137942 | HGLibA_17737 | 84 | -0.374884686 | FNBP1L | formin binding protein 1 like [Source:HGNC Symbol;Acc:HGNC:20851] |
| ENSG00000138135 | HGLibA_09332 | 84 | -0.374884686 | CH25H | cholesterol 25-hydroxylase [Source:HGNC Symbol;Acc:HGNC:1907] |
| ENSG00000138175 | HGLibA_02964 | 84 | -0.374884686 | ARL3 | ADP ribosylation factor like GTPase 3 [Source:HGNC Symbol;Acc:HGNC:694] |
| ENSG00000138606 | HGLibA_44024 | 84 | -0.374884686 | SHF | Src homology 2 domain containing F [Source:HGNC Symbol;Acc:HGNC:25116] |
| ENSG00000138777 | HGLibA_37807 | 84 | -0.374884686 | PPA2 | pyrophosphatase (inorganic) 2 [Source:HGNC Symbol;Acc:HGNC:28883] |
| ENSG00000139636 | HGLibA_26672 | 84 | -0.374884686 | LMBR1L | limb development membrane protein 1 like [Source:HGNC Symbol;Acc:HGNC:18268] |
| ENSG00000140284 | HGLibA_44816 | 84 | -0.374884686 | SLC27A2 | solute carrier family 27 member 2 [Source:HGNC Symbol;Acc:HGNC:10996] |
| ENSG00000140450 | HGLibA_03121 | 84 | -0.374884686 | ARRDC4 | arrestin domain containing 4 [Source:HGNC Symbol;Acc:HGNC:28087] |
| ENSG00000140743 | HGLibA_08866 | 84 | -0.374884686 | CDR2 | cerebellar degeneration related protein 2 [Source:HGNC Symbol;Acc:HGNC:1799] |
| ENSG00000143119 | HGLibA_08409 | 84 | -0.374884686 | CD53 | CD53 molecule [Source:HGNC Symbol;Acc:HGNC:1686] |
| ENSG00000143340 | HGLibA_16227 | 84 | -0.374884686 | FAM163A | family with sequence similarity 163 member A [Source:HGNC Symbol;Acc:HGNC:28274] |
| ENSG00000143393 | HGLibA_36502 | 84 | -0.374884686 | PI4KB | phosphatidylinositol 4-kinase beta [Source:HGNC Symbol;Acc:HGNC:8984] |
| ENSG00000145979 | HGLibA_48563 | 84 | -0.374884686 | TBC1D7 | TBC1 domain family member 7 [Source:HGNC Symbol;Acc:HGNC:21066] |
| ENSG00000147316 | HGLibA_28645 | 84 | -0.374884686 | MCPH1 | microcephalin 1 [Source:HGNC Symbol;Acc:HGNC:6954] |
| ENSG00000148153 | HGLibA_23314 | 84 | -0.374884686 | INIP | INTS3 and NABP interacting protein [Source:HGNC Symbol;Acc:HGNC:24994] |
| ENSG00000148218 | HGLibA_01520 | 84 | -0.374884686 | ALAD | aminolevulinate dehydratase [Source:HGNC Symbol;Acc:HGNC:395] |
| ENSG00000148426 | HGLibA_38698 | 84 | -0.374884686 | PROSER2 | proline and serine rich 2 [Source:HGNC Symbol;Acc:HGNC:23728] |
| ENSG00000150045 | HGLibA_25304 | 84 | -0.374884686 | KLRF1 | killer cell lectin like receptor F1 [Source:HGNC Symbol;Acc:HGNC:13342] |
| ENSG00000152315 | HGLibA_24304 | 84 | -0.374884686 | KCNK13 | potassium two pore domain channel subfamily K member 13 [Source:HGNC Symbol;Acc:HGNC:6275] |
| ENSG00000153446 | HGLibA_05496 | 84 | -0.374884686 | C16orf89 | chromosome 16 open reading frame 89 [Source:HGNC Symbol;Acc:HGNC:28687] |
| ENSG00000153531 | HGLibA_01027 | 84 | -0.374884686 | ADPRHL1 | ADP-ribosylhydrolase like 1 [Source:HGNC Symbol;Acc:HGNC:21303] |
| ENSG00000153558 | HGLibA_16998 | 84 | -0.374884686 | FBXL2 | F-box and leucine rich repeat protein 2 [Source:HGNC Symbol;Acc:HGNC:13598] |
| ENSG00000153774 | HGLibA_09258 | 84 | -0.374884686 | CFDP1 | craniofacial development protein 1 [Source:HGNC Symbol;Acc:HGNC:1873] |
| ENSG00000154146 | HGLibA_32689 | 84 | -0.374884686 | NRGN | neurogranin [Source:HGNC Symbol;Acc:HGNC:8000] |
| ENSG00000157214 | HGLibA_47425 | 84 | -0.374884686 | STEAP2 | STEAP2 metalloreductase [Source:HGNC Symbol;Acc:HGNC:17885] |
| ENSG00000158258 | HGLibA_10137 | 84 | -0.374884686 | CLSTN2 | calsyntenin 2 [Source:HGNC Symbol;Acc:HGNC:17448] |
| ENSG00000159387 | HGLibA_23642 | 84 | -0.374884686 | IRX6 | iroquois homeobox 6 [Source:HGNC Symbol;Acc:HGNC:14675] |
| ENSG00000160679 | HGLibA_09666 | 84 | -0.374884686 | CHTOP | chromatin target of PRMT1 [Source:HGNC Symbol;Acc:HGNC:24511] |
| ENSG00000162438 | HGLibA_11684 | 84 | -0.374884686 | CTRC | chymotrypsin C [Source:HGNC Symbol;Acc:HGNC:2523] |
| ENSG00000162522 | HGLibA_24771 | 84 | -0.374884686 | KIAA1522 | KIAA1522 [Source:HGNC Symbol;Acc:HGNC:29301] |
| ENSG00000163935 | HGLibA_43722 | 84 | -0.374884686 | SFMBT1 | Scm like with four mbt domains 1 [Source:HGNC Symbol;Acc:HGNC:20255] |
| ENSG00000164134 | HGLibA_30975 | 84 | -0.374884686 | NAA15 | NatA auxiliary subunit [Source:HGNC Symbol;Acc:HGNC:30782]" |
| ENSG00000164535 | HGLibA_12299 | 84 | -0.374884686 | DAGLB | diacylglycerol lipase beta [Source:HGNC Symbol;Acc:HGNC:28923] |
| ENSG00000164816 | HGLibA_12805 | 84 | -0.374884686 | DEFA5 | defensin alpha 5 [Source:HGNC Symbol;Acc:HGNC:2764] |
| ENSG00000165935 | HGLibA_45665 | 84 | -0.374884686 | SMCO2 | single-pass membrane protein with coiled-coil domains 2 [Source:HGNC Symbol;Acc:HGNC:34448] |
| ENSG00000166529 | HGLibA_57001 | 84 | -0.374884686 | ZSCAN21 | zinc finger and SCAN domain containing 21 [Source:HGNC Symbol;Acc:HGNC:13104] |
| ENSG00000166535 | HGLibA_00010 | 84 | -0.374884686 | A2ML1 | alpha-2-macroglobulin like 1 [Source:HGNC Symbol;Acc:HGNC:23336] |
| ENSG00000166634 | HGLibA_43540 | 84 | -0.374884686 | SERPINB12 | serpin family B member 12 [Source:HGNC Symbol;Acc:HGNC:14220] |
| ENSG00000166670 | HGLibA_29479 | 84 | -0.374884686 | MMP10 | matrix metallopeptidase 10 [Source:HGNC Symbol;Acc:HGNC:7156] |
| ENSG00000167778 | HGLibA_46849 | 84 | -0.374884686 | SPRYD3 | SPRY domain containing 3 [Source:HGNC Symbol;Acc:HGNC:25920] |
| ENSG00000167978 | HGLibA_47049 | 84 | -0.374884686 | SRRM2 | serine/arginine repetitive matrix 2 [Source:HGNC Symbol;Acc:HGNC:16639] |
| ENSG00000168216 | HGLibA_26676 | 84 | -0.374884686 | LMBRD1 | LMBR1 domain containing 1 [Source:HGNC Symbol;Acc:HGNC:23038] |
| ENSG00000168282 | HGLibA_29136 | 84 | -0.374884686 | MGAT2 | 2-N-acetylglucosaminyltransferase [Source:HGNC Symbol;Acc:HGNC:7045]" |
| ENSG00000169733 | HGLibA_40926 | 84 | -0.374884686 | RFNG | RFNG O-fucosylpeptide 3-beta-N-acetylglucosaminyltransferase [Source:HGNC Symbol;Acc:HGNC:9974] |
| ENSG00000169905 | HGLibA_51020 | 84 | -0.374884686 | TOR1AIP2 | torsin 1A interacting protein 2 [Source:HGNC Symbol;Acc:HGNC:24055] |
| ENSG00000169918 | HGLibA_34778 | 84 | -0.374884686 | OTUD7A | OTU deubiquitinase 7A [Source:HGNC Symbol;Acc:HGNC:20718] |
| ENSG00000169994 | HGLibA_30866 | 84 | -0.374884686 | MYO7B | myosin VIIB [Source:HGNC Symbol;Acc:HGNC:7607] |
| ENSG00000170006 | HGLibA_50005 | 84 | -0.374884686 | TMEM154 | transmembrane protein 154 [Source:HGNC Symbol;Acc:HGNC:26489] |
| ENSG00000170255 | HGLibA_29847 | 84 | -0.374884686 | MRGPRX1 | MAS related GPR family member X1 [Source:HGNC Symbol;Acc:HGNC:17962] |
| ENSG00000170265 | HGLibA_55737 | 84 | -0.374884686 | ZNF282 | zinc finger protein 282 [Source:HGNC Symbol;Acc:HGNC:13076] |
| ENSG00000170324 | HGLibA_18035 | 84 | -0.374884686 | FRMPD2 | FERM and PDZ domain containing 2 [Source:HGNC Symbol;Acc:HGNC:28572] |
| ENSG00000170925 | HGLibA_49060 | 84 | -0.374884686 | TEX13B | testis expressed 13B [Source:HGNC Symbol;Acc:HGNC:11736] |
| ENSG00000171055 | HGLibA_17316 | 84 | -0.374884686 | FEZ2 | fasciculation and elongation protein zeta 2 [Source:HGNC Symbol;Acc:HGNC:3660] |
| ENSG00000171488 | HGLibA_27433 | 84 | -0.374884686 | LRRC8C | leucine rich repeat containing 8 VRAC subunit C [Source:HGNC Symbol;Acc:HGNC:25075] |
| ENSG00000172640 | HGLibA_33480 | 84 | -0.374884686 | OR10AD1 | olfactory receptor family 10 subfamily AD member 1 [Source:HGNC Symbol;Acc:HGNC:14819] |
| ENSG00000173769 | HGLibA_51005 | 84 | -0.374884686 | TOPAZ1 | testis and ovary specific PAZ domain containing 1 [Source:HGNC Symbol;Acc:HGNC:24746] |

| | | | | | |
|---|---|---|---|---|---|
| ENSG00000176454 | HGLibA_27079 | 84 | -0.374884686 | LPCAT4 | lysophosphatidylcholine acyltransferase 4 [Source:HGNC Symbol;Acc:HGNC:30059] |
| ENSG00000176547 | HGLibA_33953 | 84 | -0.374884686 | OR4C3 | olfactory receptor family 4 subfamily C member 3 [Source:HGNC Symbol;Acc:HGNC:14697] |
| ENSG00000177535 | HGLibA_33764 | 84 | -0.374884686 | OR2B11 | olfactory receptor family 2 subfamily B member 11 [Source:HGNC Symbol;Acc:HGNC:31249] |
| ENSG00000177684 | HGLibA_12855 | 84 | -0.374884686 | DEFB114 | defensin beta 114 [Source:HGNC Symbol;Acc:HGNC:18095] |
| ENSG00000179603 | HGLibA_20361 | 84 | -0.374884686 | GRM8 | glutamate metabotropic receptor 8 [Source:HGNC Symbol;Acc:HGNC:4600] |
| ENSG00000179636 | HGLibA_51167 | 84 | -0.374884686 | TPPP2 | tubulin polymerization promoting protein family member 2 [Source:HGNC Symbol;Acc:HGNC:19293] |
| ENSG00000179914 | HGLibA_23822 | 84 | -0.374884686 | ITLN1 | intelectin 1 [Source:HGNC Symbol;Acc:HGNC:18259] |
| ENSG00000181518 | HGLibA_34513 | 84 | -0.374884686 | OR8D4 | olfactory receptor family 8 subfamily D member 4 [Source:HGNC Symbol;Acc:HGNC:14840] |
| ENSG00000182810 | HGLibA_12690 | 84 | -0.374884686 | DDX28 | DEAD-box helicase 28 [Source:HGNC Symbol;Acc:HGNC:17330] |
| ENSG00000183559 | HGLibA_05047 | 84 | -0.374884686 | C10orf120 | chromosome 10 open reading frame 120 [Source:HGNC Symbol;Acc:HGNC:25707] |
| ENSG00000183690 | HGLibA_14573 | 84 | -0.374884686 | EFHC2 | EF-hand domain containing 2 [Source:HGNC Symbol;Acc:HGNC:26233] |
| ENSG00000184014 | HGLibA_12958 | 84 | -0.374884686 | DENND5A | DENN domain containing 5A [Source:HGNC Symbol;Acc:HGNC:19344] |
| ENSG00000184388 | HGLibA_34930 | 84 | -0.374884686 | PABPC1L2B | poly(A) binding protein cytoplasmic 1 like 2B [Source:HGNC Symbol;Acc:HGNC:31852] |
| ENSG00000184675 | HGLibA_01773 | 84 | -0.374884686 | AMER1 | APC membrane recruitment protein 1 [Source:HGNC Symbol;Acc:HGNC:26837] |
| ENSG00000186603 | HGLibA_22027 | 84 | -0.374884686 | HPDL | 4-hydroxyphenylpyruvate dioxygenase like [Source:HGNC Symbol;Acc:HGNC:28242] |
| ENSG00000186976 | HGLibA_14550 | 84 | -0.374884686 | EFCAB6 | EF-hand calcium binding domain 6 [Source:HGNC Symbol;Acc:HGNC:24204] |
| ENSG00000187325 | HGLibA_48265 | 84 | -0.374884686 | TAF9B | TATA-box binding protein associated factor 9b [Source:HGNC Symbol;Acc:HGNC:17306] |
| ENSG00000188958 | HGLibA_53465 | 84 | -0.374884686 | UTS2B | urotensin 2B [Source:HGNC Symbol;Acc:HGNC:30894] |
| ENSG00000189433 | HGLibA_19165 | 84 | -0.374884686 | GJB4 | gap junction protein beta 4 [Source:HGNC Symbol;Acc:HGNC:4286] |
| ENSG00000196266 | HGLibA_33525 | 84 | -0.374884686 | OR10J3 | olfactory receptor family 10 subfamily J member 3 [Source:HGNC Symbol;Acc:HGNC:14992] |
| ENSG00000196427 | HGLibA_31236 | 84 | -0.374884686 | NBPF4 | NBPF member 4 [Source:HGNC Symbol;Acc:HGNC:26550] |
| ENSG00000196502 | HGLibA_47731 | 84 | -0.374884686 | SULT1A1 | sulfotransferase family 1A member 1 [Source:HGNC Symbol;Acc:HGNC:11453] |
| ENSG00000196597 | HGLibA_56740 | 84 | -0.374884686 | ZNF782 | zinc finger protein 782 [Source:HGNC Symbol;Acc:HGNC:33110] |
| ENSG00000197110 | HGLibA_22724 | 84 | -0.374884686 | IFNL3 | interferon lambda 3 [Source:HGNC Symbol;Acc:HGNC:18365] |
| ENSG00000197119 | HGLibA_44696 | 84 | -0.374884686 | SLC25A29 | solute carrier family 25 member 29 [Source:HGNC Symbol;Acc:HGNC:20116] |
| ENSG00000197208 | HGLibA_44589 | 84 | -0.374884686 | SLC22A4 | solute carrier family 22 member 4 [Source:HGNC Symbol;Acc:HGNC:10968] |
| ENSG00000197561 | HGLibA_14875 | 84 | -0.374884686 | ELANE | neutrophil expressed [Source:HGNC Symbol;Acc:HGNC:3309]" |
| ENSG00000197584 | HGLibA_24345 | 84 | -0.374884686 | KCNMB2 | potassium calcium-activated channel subfamily M regulatory beta subunit 2 [Source:HGNC Symbol;Acc:HGNC:6286] |
| ENSG00000198756 | HGLibA_10645 | 84 | -0.374884686 | COLGALT2 | collagen beta(1-O)galactosyltransferase 2 [Source:HGNC Symbol;Acc:HGNC:16790] |
| ENSG00000198925 | HGLibA_03507 | 84 | -0.374884686 | ATG9A | autophagy related 9A [Source:HGNC Symbol;Acc:HGNC:22408] |
| ENSG00000203797 | HGLibA_12628 | 84 | -0.374884686 | DDO | D-aspartate oxidase [Source:HGNC Symbol;Acc:HGNC:2727] |
| ENSG00000204613 | HGLibA_51401 | 84 | -0.374884686 | TRIM10 | tripartite motif containing 10 [Source:HGNC Symbol;Acc:HGNC:10072] |
| ENSG00000204947 | HGLibA_55963 | 84 | -0.374884686 | ZNF425 | zinc finger protein 425 [Source:HGNC Symbol;Acc:HGNC:20690] |
| ENSG00000205327 | HGLibA_34401 | 84 | -0.374884686 | OR6C68 | olfactory receptor family 6 subfamily C member 68 [Source:HGNC Symbol;Acc:HGNC:31297] |
| ENSG00000213024 | HGLibA_33062 | 84 | -0.374884686 | NUP62 | nucleoporin 62 [Source:HGNC Symbol;Acc:HGNC:8066] |
| ENSG00000213064 | HGLibA_43753 | 84 | -0.374884686 | SFT2D2 | SFT2 domain containing 2 [Source:HGNC Symbol;Acc:HGNC:25140] |
| ENSG00000214216 | HGLibA_23522 | 84 | -0.374884686 | IQCJ | IQ motif containing J [Source:HGNC Symbol;Acc:HGNC:32406] |
| ENSG00000215853 | HGLibA_42200 | 84 | -0.374884686 | RPTN | repetin [Source:HGNC Symbol;Acc:HGNC:26809] |
| ENSG00000221838 | HGLibA_02348 | 84 | -0.374884686 | AP4M1 | adaptor related protein complex 4 subunit mu 1 [Source:HGNC Symbol;Acc:HGNC:574] |
| ENSG00000221880 | HGLibA_25582 | 84 | -0.374884686 | KRTAP1-3 | keratin associated protein 1-3 [Source:HGNC Symbol;Acc:HGNC:16771] |
| ENSG00000221978 | HGLibA_08119 | 84 | -0.374884686 | CCNL2 | cyclin L2 [Source:HGNC Symbol;Acc:HGNC:20570] |
| ENSG00000230124 | HGLibA_00408 | 84 | -0.374884686 | ACBD6 | acyl-CoA binding domain containing 6 [Source:HGNC Symbol;Acc:HGNC:23339] |
| ENSG00000249992 | HGLibA_50014 | 84 | -0.374884686 | TMEM158 | transmembrane protein 158 (gene/pseudogene) [Source:HGNC Symbol;Acc:HGNC:30293] |
| ENSG00000250120 | HGLibA_35428 | 84 | -0.374884686 | PCDHA10 | protocadherin alpha 10 [Source:HGNC Symbol;Acc:HGNC:8664] |
| ENSG00000253537 | HGLibA_35540 | 84 | -0.374884686 | PCDHGA7 | 7 [Source:HGNC Symbol;Acc:HGNC:8705]" |
| ENSG00000253731 | HGLibA_35538 | 84 | -0.374884686 | PCDHGA6 | 6 [Source:HGNC Symbol;Acc:HGNC:8704]" |
| ENSG00000264230 | HGLibA_02256 | 84 | -0.374884686 | ANXA8L1 | annexin A8 like 1 [Source:HGNC Symbol;Acc:HGNC:23334] |
| ENSG00000270617 | HGLibA_53142 | 84 | -0.374884686 | URGCP-MRPS24 | URGCP-MRPS24 readthrough [Source:HGNC Symbol;Acc:HGNC:49188] |
| FAM154A | HGLibA_16173 | 84 | -0.374884686 | FAM154A | - |
| GPR128 | HGLibA_19884 | 84 | -0.374884686 | GPR128 | - |
| GPR56 | HGLibA_20055 | 84 | -0.374884686 | GPR56 | - |
| HIST1H2AB | HGLibA_21379 | 84 | -0.374884686 | HIST1H2AB | - |
| hsa-mir-1200 | HGLibA_57261 | 84 | -0.374884686 | hsa-mir-1200 | - |
| hsa-mir-1226 | HGLibA_57301 | 84 | -0.374884686 | hsa-mir-1226 | - |
| hsa-mir-1283-1 | HGLibA_57587 | 84 | -0.374884686 | hsa-mir-1283-1 | - |
| hsa-mir-1302-10 | HGLibA_57686 | 84 | -0.374884686 | hsa-mir-1302-10 | - |
| hsa-mir-2277 | HGLibA_58311 | 84 | -0.374884686 | hsa-mir-2277 | - |
| hsa-mir-3121 | HGLibA_58537 | 84 | -0.374884686 | hsa-mir-3121 | - |
| hsa-mir-380 | HGLibA_59472 | 84 | -0.374884686 | hsa-mir-380 | - |
| hsa-mir-4286 | HGLibA_59850 | 84 | -0.374884686 | hsa-mir-4286 | - |
| hsa-mir-4453 | HGLibA_60218 | 84 | -0.374884686 | hsa-mir-4453 | - |
| hsa-mir-4466 | HGLibA_60270 | 84 | -0.374884686 | hsa-mir-4466 | - |
| hsa-mir-4524b | HGLibA_60557 | 84 | -0.374884686 | hsa-mir-4524b | - |
| hsa-mir-4658 | HGLibA_60741 | 84 | -0.374884686 | hsa-mir-4658 | - |
| hsa-mir-548ae-1 | HGLibA_61869 | 84 | -0.374884686 | hsa-mir-548ae-1 | - |
| hsa-mir-5697 | HGLibA_62320 | 84 | -0.374884686 | hsa-mir-5697 | - |
| hsa-mir-5703 | HGLibA_62353 | 84 | -0.374884686 | hsa-mir-5703 | - |
| hsa-mir-578 | HGLibA_62407 | 84 | -0.374884686 | hsa-mir-578 | - |
| hsa-mir-582 | HGLibA_62426 | 84 | -0.374884686 | hsa-mir-582 | - |
| hsa-mir-6799 | HGLibA_63374 | 84 | -0.374884686 | hsa-mir-6799 | - |
| hsa-mir-6855 | HGLibA_63594 | 84 | -0.374884686 | hsa-mir-6855 | - |
| hsa-mir-6880 | HGLibA_63706 | 84 | -0.374884686 | hsa-mir-6880 | - |
| hsa-mir-6887 | HGLibA_63731 | 84 | -0.374884686 | hsa-mir-6887 | - |
| hsa-mir-8057 | HGLibA_64066 | 84 | -0.374884686 | hsa-mir-8057 | - |
| hsa-mir-939 | HGLibA_64330 | 84 | -0.374884686 | hsa-mir-939 | - |
| KIAA0355 | HGLibA_24626 | 84 | -0.374884686 | KIAA0355 | - |
| LOC100131094 | HGLibA_26800 | 84 | -0.374884686 | LOC100131094 | - |
| LOC158434 | HGLibA_26901 | 84 | -0.374884686 | LOC158434 | - |
| RBAK-LOC389458 | HGLibA_40421 | 84 | -0.374884686 | RBAK-LOC389458 | - |
| SGK196 | HGLibA_43822 | 84 | -0.374884686 | SGK196 | - |
| SQRDL | HGLibA_46915 | 84 | -0.374884686 | SQRDL | - |
| TMEM66 | HGLibA_50490 | 84 | -0.374884686 | TMEM66 | - |
| 7-Mar | HGLibA_28345 | 83 | -0.383930029 | 7-Mar | - |
| ATP5G2 | HGLibA_03659 | 83 | -0.383930029 | ATP5G2 | - |
| C17orf70 | HGLibA_05563 | 83 | -0.383930029 | C17orf70 | - |
| C7orf34 | HGLibA_06538 | 83 | -0.383930029 | C7orf34 | - |
| C8orf87 | HGLibA_06657 | 83 | -0.383930029 | C8orf87 | - |
| CTSL2 | HGLibA_11723 | 83 | -0.383930029 | CTSL2 | - |
| ENSG00000001561 | HGLibA_15176 | 83 | -0.383930029 | ENPP4 | ectonucleotide pyrophosphatase/phosphodiesterase 4 [Source:HGNC Symbol;Acc:HGNC:3359] |
| ENSG00000004455 | HGLibA_01380 | 83 | -0.383930029 | AK2 | adenylate kinase 2 [Source:HGNC Symbol;Acc:HGNC:362] |
| ENSG00000008323 | HGLibA_37118 | 83 | -0.383930029 | PLEKHG6 | pleckstrin homology and RhoGEF domain containing G6 [Source:HGNC Symbol;Acc:HGNC:25562] |
| ENSG00000014123 | HGLibA_52850 | 83 | -0.383930029 | UFL1 | UFM1 specific ligase 1 [Source:HGNC Symbol;Acc:HGNC:23039] |
| ENSG00000033178 | HGLibA_52528 | 83 | -0.383930029 | UBA6 | ubiquitin like modifier activating enzyme 6 [Source:HGNC Symbol;Acc:HGNC:25581] |
| ENSG00000040275 | HGLibA_46571 | 83 | -0.383930029 | SPDL1 | spindle apparatus coiled-coil protein 1 [Source:HGNC Symbol;Acc:HGNC:26010] |
| ENSG00000042286 | HGLibA_01339 | 83 | -0.383930029 | AIFM2 | mitochondria associated 2 [Source:HGNC Symbol;Acc:HGNC:21411]" |
| ENSG00000043039 | HGLibA_04168 | 83 | -0.383930029 | BARX2 | BARX homeobox 2 [Source:HGNC Symbol;Acc:HGNC:956] |
| ENSG00000048828 | HGLibA_16053 | 83 | -0.383930029 | FAM120A | family with sequence similarity 120A [Source:HGNC Symbol;Acc:HGNC:13247] |
| ENSG00000049130 | HGLibA_25017 | 83 | -0.383930029 | KITLG | KIT ligand [Source:HGNC Symbol;Acc:HGNC:6343] |
| ENSG00000051341 | HGLibA_37545 | 83 | -0.383930029 | POLQ | DNA polymerase theta [Source:HGNC Symbol;Acc:HGNC:9186] |
| ENSG00000054356 | HGLibA_39520 | 83 | -0.383930029 | PTPRN | receptor type N [Source:HGNC Symbol;Acc:HGNC:9676]" |
| ENSG00000064300 | HGLibA_31876 | 83 | -0.383930029 | NGFR | nerve growth factor receptor [Source:HGNC Symbol;Acc:HGNC:7809] |
| ENSG00000066813 | HGLibA_00575 | 83 | -0.383930029 | ACSM2B | acyl-CoA synthetase medium chain family member 2B [Source:HGNC Symbol;Acc:HGNC:30931] |
| ENSG00000070159 | HGLibA_39467 | 83 | -0.383930029 | PTPN3 | non-receptor type 3 [Source:HGNC Symbol;Acc:HGNC:9655]" |
| ENSG00000070770 | HGLibA_11405 | 83 | -0.383930029 | CSNK2A2 | casein kinase 2 alpha 2 [Source:HGNC Symbol;Acc:HGNC:2459] |
| ENSG00000071242 | HGLibA_42162 | 83 | -0.383930029 | RPS6KA2 | ribosomal protein S6 kinase A2 [Source:HGNC Symbol;Acc:HGNC:10431] |
| ENSG00000076003 | HGLibA_28618 | 83 | -0.383930029 | MCM6 | minichromosome maintenance complex component 6 [Source:HGNC Symbol;Acc:HGNC:6949] |
| ENSG00000076864 | HGLibA_40227 | 83 | -0.383930029 | RAP1GAP | RAP1 GTPase activating protein [Source:HGNC Symbol;Acc:HGNC:9858] |
| ENSG00000080511 | HGLibA_40751 | 83 | -0.383930029 | RDH8 | retinol dehydrogenase 8 [Source:HGNC Symbol;Acc:HGNC:14423] |
| ENSG00000081026 | HGLibA_28000 | 83 | -0.383930029 | MAGI3 | WW and PDZ domain containing 3 [Source:HGNC Symbol;Acc:HGNC:29647]" |
| ENSG00000083814 | HGLibA_56504 | 83 | -0.383930029 | ZNF671 | zinc finger protein 671 [Source:HGNC Symbol;Acc:HGNC:26279] |
| ENSG00000087274 | HGLibA_00940 | 83 | -0.383930029 | ADD1 | adducin 1 [Source:HGNC Symbol;Acc:HGNC:243] |
| ENSG00000100504 | HGLibA_39689 | 83 | -0.383930029 | PYGL | glycogen phosphorylase L [Source:HGNC Symbol;Acc:HGNC:9725] |
| ENSG00000100593 | HGLibA_23680 | 83 | -0.383930029 | ISM2 | isthmin 2 [Source:HGNC Symbol;Acc:HGNC:23176] |
| ENSG00000100941 | HGLibA_37394 | 83 | -0.383930029 | PNN | desmosome associated protein [Source:HGNC Symbol;Acc:HGNC:9162]" |
| ENSG00000101680 | HGLibA_25940 | 83 | -0.383930029 | LAMA1 | laminin subunit alpha 1 [Source:HGNC Symbol;Acc:HGNC:6481] |
| ENSG00000101844 | HGLibA_03488 | 83 | -0.383930029 | ATG4A | autophagy related 4A cysteine peptidase [Source:HGNC Symbol;Acc:HGNC:16489] |
| ENSG00000102245 | HGLibA_08388 | 83 | -0.383930029 | CD40LG | CD40 ligand [Source:HGNC Symbol;Acc:HGNC:11935] |
| ENSG00000102753 | HGLibA_25360 | 83 | -0.383930029 | KPNA3 | karyopherin subunit alpha 3 [Source:HGNC Symbol;Acc:HGNC:6396] |
| ENSG00000104237 | HGLibA_41788 | 83 | -0.383930029 | RP1 | axonemal microtubule associated [Source:HGNC Symbol;Acc:HGNC:10263]" |
| ENSG00000104343 | HGLibA_52673 | 83 | -0.383930029 | UBE2W | ubiquitin conjugating enzyme E2 W [Source:HGNC Symbol;Acc:HGNC:25616] |
| ENSG00000104447 | HGLibA_51776 | 83 | -0.383930029 | TRPS1 | transcriptional repressor GATA binding 1 [Source:HGNC Symbol;Acc:HGNC:12340] |
| ENSG00000104853 | HGLibA_10112 | 83 | -0.383930029 | CLPTM1 | transmembrane protein [Source:HGNC Symbol;Acc:HGNC:2087]" |
| ENSG00000105085 | HGLibA_28789 | 83 | -0.383930029 | MED26 | mediator complex subunit 26 [Source:HGNC Symbol;Acc:HGNC:2376] |
| ENSG00000105221 | HGLibA_01512 | 83 | -0.383930029 | AKT2 | AKT serine/threonine kinase 2 [Source:HGNC Symbol;Acc:HGNC:392] |
| ENSG00000105499 | HGLibA_36905 | 83 | -0.383930029 | PLA2G4C | phospholipase A2 group IVC [Source:HGNC Symbol;Acc:HGNC:9037] |
| ENSG00000105516 | HGLibA_12400 | 83 | -0.383930029 | DBP | D-box binding PAR bZIP transcription factor [Source:HGNC Symbol;Acc:HGNC:2697] |
| ENSG00000105971 | HGLibA_07380 | 83 | -0.383930029 | CAV2 | caveolin 2 [Source:HGNC Symbol;Acc:HGNC:1528] |
| ENSG00000106144 | HGLibA_07307 | 83 | -0.383930029 | CASP2 | caspase 2 [Source:HGNC Symbol;Acc:HGNC:1503] |
| ENSG00000106178 | HGLibA_08003 | 83 | -0.383930029 | CCL24 | C-C motif chemokine ligand 24 [Source:HGNC Symbol;Acc:HGNC:10623] |
| ENSG00000108306 | HGLibA_17001 | 83 | -0.383930029 | FBXL20 | F-box and leucine rich repeat protein 20 [Source:HGNC Symbol;Acc:HGNC:24679] |
| ENSG00000108306 | HGLibA_17000 | 83 | -0.383930029 | FBXL20 | F-box and leucine rich repeat protein 20 [Source:HGNC Symbol;Acc:HGNC:24679] |
| ENSG00000108370 | HGLibA_41113 | 83 | -0.383930029 | RGS9 | regulator of G protein signaling 9 [Source:HGNC Symbol;Acc:HGNC:10004] |
| ENSG00000108641 | HGLibA_04044 | 83 | -0.383930029 | B9D1 | B9 domain containing 1 [Source:HGNC Symbol;Acc:HGNC:24123] |
| ENSG00000110243 | HGLibA_02461 | 83 | -0.383930029 | APOA5 | apolipoprotein A5 [Source:HGNC Symbol;Acc:HGNC:17288] |
| ENSG00000111404 | HGLibA_40864 | 83 | -0.383930029 | RERGL | RERG like [Source:HGNC Symbol;Acc:HGNC:26213] |

| | | | | | |
|---|---|---|---|---|---|
| ENSG00000111678 | HGLibA_05270 | 83 | -0.383930029 | C12orf57 | chromosome 12 open reading frame 57 [Source:HGNC Symbol;Acc:HGNC:29521] |
| ENSG00000114062 | HGLibA_52679 | 83 | -0.383930029 | UBE3A | ubiquitin protein ligase E3A [Source:HGNC Symbol;Acc:HGNC:12496] |
| ENSG00000114796 | HGLibA_25161 | 83 | -0.383930029 | KLHL24 | kelch like family member 24 [Source:HGNC Symbol;Acc:HGNC:25947] |
| ENSG00000114948 | HGLibA_00761 | 83 | -0.383930029 | ADAM23 | ADAM metallopeptidase domain 23 [Source:HGNC Symbol;Acc:HGNC:202] |
| ENSG00000117133 | HGLibA_41829 | 83 | -0.383930029 | RPF1 | ribosome production factor 1 homolog [Source:HGNC Symbol;Acc:HGNC:30350] |
| ENSG00000117222 | HGLibA_40430 | 83 | -0.383930029 | RBBP5 | histone lysine methyltransferase complex subunit [Source:HGNC Symbol;Acc:HGNC:9888]" |
| ENSG00000117676 | HGLibA_42159 | 83 | -0.383930029 | RPS6KA1 | ribosomal protein S6 kinase A1 [Source:HGNC Symbol;Acc:HGNC:10430] |
| ENSG00000120733 | HGLibA_24503 | 83 | -0.383930029 | KDM3B | lysine demethylase 3B [Source:HGNC Symbol;Acc:HGNC:1337] |
| ENSG00000120925 | HGLibA_41575 | 83 | -0.383930029 | RNF170 | ring finger protein 170 [Source:HGNC Symbol;Acc:HGNC:25358] |
| ENSG00000121281 | HGLibA_00925 | 83 | -0.383930029 | ADCY7 | adenylate cyclase 7 [Source:HGNC Symbol;Acc:HGNC:238] |
| ENSG00000121361 | HGLibA_24287 | 83 | -0.383930029 | KCNJ8 | potassium voltage-gated channel subfamily J member 8 [Source:HGNC Symbol;Acc:HGNC:6269] |
| ENSG00000121691 | HGLibA_07352 | 83 | -0.383930029 | CAT | catalase [Source:HGNC Symbol;Acc:HGNC:1516] |
| ENSG00000122194 | HGLibA_37159 | 83 | -0.383930029 | PLG | plasminogen [Source:HGNC Symbol;Acc:HGNC:9071] |
| ENSG00000123411 | HGLibA_22939 | 83 | -0.383930029 | IKZF4 | IKAROS family zinc finger 4 [Source:HGNC Symbol;Acc:HGNC:13179] |
| ENSG00000124126 | HGLibA_38442 | 83 | -0.383930029 | PREX1 | 5-trisphosphate dependent Rac exchange factor 1 [Source:HGNC Symbol;Acc:HGNC:32594]" |
| ENSG00000124657 | HGLibA_33772 | 83 | -0.383930029 | OR2B6 | olfactory receptor family 2 subfamily B member 6 [Source:HGNC Symbol;Acc:HGNC:8241] |
| ENSG00000125304 | HGLibA_49723 | 83 | -0.383930029 | TM9SF2 | transmembrane 9 superfamily member 2 [Source:HGNC Symbol;Acc:HGNC:11865] |
| ENSG00000126970 | HGLibA_54999 | 83 | -0.383930029 | ZC4H2 | zinc finger C4H2-type containing [Source:HGNC Symbol;Acc:HGNC:24931] |
| ENSG00000129864 | HGLibA_53594 | 83 | -0.383930029 | VCY | variable charge Y-linked [Source:HGNC Symbol;Acc:HGNC:12668] |
| ENSG00000130363 | HGLibA_42331 | 83 | -0.383930029 | RSPH3 | radial spoke head 3 homolog [Source:HGNC Symbol;Acc:HGNC:21054] |
| ENSG00000130520 | HGLibA_27543 | 83 | -0.383930029 | LSM4 | U6 small nuclear RNA and mRNA degradation associated [Source:HGNC Symbol;Acc:HGNC:17259]" |
| ENSG00000131808 | HGLibA_18087 | 83 | -0.383930029 | FSHB | follicle stimulating hormone subunit beta [Source:HGNC Symbol;Acc:HGNC:3964] |
| ENSG00000132424 | HGLibA_37345 | 83 | -0.383930029 | PNISR | PNN interacting serine and arginine rich protein [Source:HGNC Symbol;Acc:HGNC:21222] |
| ENSG00000134330 | HGLibA_22467 | 83 | -0.383930029 | IAH1 | isoamyl acetate hydrolyzing esterase 1 (putative) [Source:HGNC Symbol;Acc:HGNC:27696] |
| ENSG00000134882 | HGLibA_52536 | 83 | -0.383930029 | UBAC2 | UBA domain containing 2 [Source:HGNC Symbol;Acc:HGNC:20486] |
| ENSG00000134987 | HGLibA_54075 | 83 | -0.383930029 | WDR36 | WD repeat domain 36 [Source:HGNC Symbol;Acc:HGNC:30696] |
| ENSG00000136274 | HGLibA_31036 | 83 | -0.383930029 | NACAD | NAC alpha domain containing [Source:HGNC Symbol;Acc:HGNC:22196] |
| ENSG00000136457 | HGLibA_09340 | 83 | -0.383930029 | CHAD | chondroadherin [Source:HGNC Symbol;Acc:HGNC:1909] |
| ENSG00000136869 | HGLibA_49653 | 83 | -0.383930029 | TLR4 | toll like receptor 4 [Source:HGNC Symbol;Acc:HGNC:11850] |
| ENSG00000137266 | HGLibA_44574 | 83 | -0.383930029 | SLC22A23 | solute carrier family 22 member 23 [Source:HGNC Symbol;Acc:HGNC:21106] |
| ENSG00000137648 | HGLibA_50642 | 83 | -0.383930029 | TMPRSS4 | transmembrane serine protease 4 [Source:HGNC Symbol;Acc:HGNC:11878] |
| ENSG00000138759 | HGLibA_17975 | 83 | -0.383930029 | FRAS1 | Fraser extracellular matrix complex subunit 1 [Source:HGNC Symbol;Acc:HGNC:19185] |
| ENSG00000138823 | HGLibA_30501 | 83 | -0.383930029 | MTTP | microsomal triglyceride transfer protein [Source:HGNC Symbol;Acc:HGNC:7467] |
| ENSG00000139190 | HGLibA_53487 | 83 | -0.383930029 | VAMP1 | vesicle associated membrane protein 1 [Source:HGNC Symbol;Acc:HGNC:12642] |
| ENSG00000139211 | HGLibA_01796 | 83 | -0.383930029 | AMIGO2 | adhesion molecule with Ig like domain 2 [Source:HGNC Symbol;Acc:HGNC:24073] |
| ENSG00000139546 | HGLibA_48334 | 83 | -0.383930029 | TARBP2 | RISC loading complex RNA binding subunit [Source:HGNC Symbol;Acc:HGNC:11569]" |
| ENSG00000140285 | HGLibA_17416 | 83 | -0.383930029 | FGF7 | fibroblast growth factor 7 [Source:HGNC Symbol;Acc:HGNC:3685] |
| ENSG00000143621 | HGLibA_23214 | 83 | -0.383930029 | ILF2 | interleukin enhancer binding factor 2 [Source:HGNC Symbol;Acc:HGNC:6037] |
| ENSG00000143643 | HGLibA_52051 | 83 | -0.383930029 | TTC13 | tetratricopeptide repeat domain 13 [Source:HGNC Symbol;Acc:HGNC:26204] |
| ENSG00000144029 | HGLibA_30103 | 83 | -0.383930029 | MRPS5 | mitochondrial ribosomal protein S5 [Source:HGNC Symbol;Acc:HGNC:14498] |
| ENSG00000144357 | HGLibA_52752 | 83 | -0.383930029 | UBR3 | ubiquitin protein ligase E3 component n-recognin 3 [Source:HGNC Symbol;Acc:HGNC:30467] |
| ENSG00000144369 | HGLibA_16270 | 83 | -0.383930029 | FAM171B | family with sequence similarity 171 member B [Source:HGNC Symbol;Acc:HGNC:29412] |
| ENSG00000144554 | HGLibA_16838 | 83 | -0.383930029 | FANCD2 | FA complementation group D2 [Source:HGNC Symbol;Acc:HGNC:3585] |
| ENSG00000146007 | HGLibA_55370 | 83 | -0.383930029 | ZMAT2 | zinc finger matrin-type 2 [Source:HGNC Symbol;Acc:HGNC:26433] |
| ENSG00000147408 | HGLibA_11344 | 83 | -0.383930029 | CSGALNACT1 | chondroitin sulfate N-acetylgalactosaminyltransferase 1 [Source:HGNC Symbol;Acc:HGNC:24290] |
| ENSG00000147676 | HGLibA_28025 | 83 | -0.383930029 | MAL2 | T cell differentiation protein 2 (gene/pseudogene) [Source:HGNC Symbol;Acc:HGNC:13634]" |
| ENSG00000148677 | HGLibA_01989 | 83 | -0.383930029 | ANKRD1 | ankyrin repeat domain 1 [Source:HGNC Symbol;Acc:HGNC:15819] |
| ENSG00000149428 | HGLibA_22462 | 83 | -0.383930029 | HYOU1 | hypoxia up-regulated 1 [Source:HGNC Symbol;Acc:HGNC:16931] |
| ENSG00000149548 | HGLibA_07640 | 83 | -0.383930029 | CCDC15 | coiled-coil domain containing 15 [Source:HGNC Symbol;Acc:HGNC:25798] |
| ENSG00000150753 | HGLibA_08203 | 83 | -0.383930029 | CCT5 | chaperonin containing TCP1 subunit 5 [Source:HGNC Symbol;Acc:HGNC:1618] |
| ENSG00000150773 | HGLibA_36611 | 83 | -0.383930029 | PIH1D2 | PIH1 domain containing 2 [Source:HGNC Symbol;Acc:HGNC:25210] |
| ENSG00000151176 | HGLibA_36975 | 83 | -0.383930029 | PLBD2 | phospholipase B domain containing 2 [Source:HGNC Symbol;Acc:HGNC:27283] |
| ENSG00000151475 | HGLibA_44705 | 83 | -0.383930029 | SLC25A31 | solute carrier family 25 member 31 [Source:HGNC Symbol;Acc:HGNC:25319] |
| ENSG00000151532 | HGLibA_53888 | 83 | -0.383930029 | VTI1A | vesicle transport through interaction with t-SNAREs 1A [Source:HGNC Symbol;Acc:HGNC:17792] |
| ENSG00000151846 | HGLibA_34933 | 83 | -0.383930029 | PABPC3 | poly(A) binding protein cytoplasmic 3 [Source:HGNC Symbol;Acc:HGNC:8556] |
| ENSG00000153993 | HGLibA_43327 | 83 | -0.383930029 | SEMA3D | semaphorin 3D [Source:HGNC Symbol;Acc:HGNC:10726] |
| ENSG00000154252 | HGLibA_18499 | 83 | -0.383930029 | GAL3ST2 | galactose-3-O-sulfotransferase 2 [Source:HGNC Symbol;Acc:HGNC:24869] |
| ENSG00000154767 | HGLibA_54549 | 83 | -0.383930029 | XPC | DNA damage recognition and repair factor [Source:HGNC Symbol;Acc:HGNC:12816]" |
| ENSG00000157211 | HGLibA_08625 | 83 | -0.383930029 | CDCP2 | CUB domain containing protein 2 [Source:HGNC Symbol;Acc:HGNC:27297] |
| ENSG00000157869 | HGLibA_39862 | 83 | -0.383930029 | RAB28 | member RAS oncogene family [Source:HGNC Symbol;Acc:HGNC:9768]" |
| ENSG00000159403 | HGLibA_05970 | 83 | -0.383930029 | C1R | complement C1r [Source:HGNC Symbol;Acc:HGNC:1246] |
| ENSG00000160199 | HGLibA_36846 | 83 | -0.383930029 | PKNOX1 | PBX/knotted 1 homeobox 1 [Source:HGNC Symbol;Acc:HGNC:9022] |
| ENSG00000162063 | HGLibA_08090 | 83 | -0.383930029 | CCNF | cyclin F [Source:HGNC Symbol;Acc:HGNC:1591] |
| ENSG00000162552 | HGLibA_54369 | 83 | -0.383930029 | WNT4 | Wnt family member 4 [Source:HGNC Symbol;Acc:HGNC:12783] |
| ENSG00000162738 | HGLibA_53514 | 83 | -0.383930029 | VANGL2 | VANGL planar cell polarity protein 2 [Source:HGNC Symbol;Acc:HGNC:15511] |
| ENSG00000163347 | HGLibA_09846 | 83 | -0.383930029 | CLDN1 | claudin 1 [Source:HGNC Symbol;Acc:HGNC:2032] |
| ENSG00000163701 | HGLibA_23015 | 83 | -0.383930029 | IL17RE | interleukin 17 receptor E [Source:HGNC Symbol;Acc:HGNC:18439] |
| ENSG00000163738 | HGLibA_30386 | 83 | -0.383930029 | MTHFD2L | methylenetetrahydrofolate dehydrogenase (NADP+ dependent) 2 like [Source:HGNC Symbol;Acc:HGNC:31865] |
| ENSG00000163900 | HGLibA_50392 | 83 | -0.383930029 | TMEM41A | transmembrane protein 41A [Source:HGNC Symbol;Acc:HGNC:30544] |
| ENSG00000164683 | HGLibA_21217 | 83 | -0.383930029 | HEY1 | hes related family bHLH transcription factor with YRPW motif 1 [Source:HGNC Symbol;Acc:HGNC:4880] |
| ENSG00000165309 | HGLibA_03017 | 83 | -0.383930029 | ARMC3 | armadillo repeat containing 3 [Source:HGNC Symbol;Acc:HGNC:30964] |
| ENSG00000165434 | HGLibA_36268 | 83 | -0.383930029 | PGM2L1 | phosphoglucomutase 2 like 1 [Source:HGNC Symbol;Acc:HGNC:20898] |
| ENSG00000165458 | HGLibA_23365 | 83 | -0.383930029 | INPPL1 | inositol polyphosphate phosphatase like 1 [Source:HGNC Symbol;Acc:HGNC:6080] |
| ENSG00000166035 | HGLibA_26604 | 83 | -0.383930029 | LIPC | hepatic type [Source:HGNC Symbol;Acc:HGNC:6619]" |
| ENSG00000166160 | HGLibA_33427 | 83 | -0.383930029 | OPN1MW2 | medium wave sensitive 2 [Source:HGNC Symbol;Acc:HGNC:26952]" |
| ENSG00000166509 | HGLibA_09973 | 83 | -0.383930029 | CLEC3A | C-type lectin domain family 3 member A [Source:HGNC Symbol;Acc:HGNC:2052] |
| ENSG00000166856 | HGLibA_19988 | 83 | -0.383930029 | GPR182 | G protein-coupled receptor 182 [Source:HGNC Symbol;Acc:HGNC:13708] |
| ENSG00000166922 | HGLibA_42906 | 83 | -0.383930029 | SCG5 | secretogranin V [Source:HGNC Symbol;Acc:HGNC:10816] |
| ENSG00000167105 | HGLibA_50561 | 83 | -0.383930029 | TMEM92 | transmembrane protein 92 [Source:HGNC Symbol;Acc:HGNC:26579] |
| ENSG00000167658 | HGLibA_14509 | 83 | -0.383930029 | EEF2 | eukaryotic translation elongation factor 2 [Source:HGNC Symbol;Acc:HGNC:3214] |
| ENSG00000167723 | HGLibA_51787 | 83 | -0.383930029 | TRPV3 | transient receptor potential cation channel subfamily V member 3 [Source:HGNC Symbol;Acc:HGNC:18084] |
| ENSG00000167850 | HGLibA_08335 | 83 | -0.383930029 | CD300C | CD300c molecule [Source:HGNC Symbol;Acc:HGNC:19320] |
| ENSG00000169435 | HGLibA_40388 | 83 | -0.383930029 | RASSF6 | Ras association domain family member 6 [Source:HGNC Symbol;Acc:HGNC:20796] |
| ENSG00000169504 | HGLibA_10029 | 83 | -0.383930029 | CLIC4 | chloride intracellular channel 4 [Source:HGNC Symbol;Acc:HGNC:13518] |
| ENSG00000169599 | HGLibA_31846 | 83 | -0.383930029 | NFU1 | NFU1 iron-sulfur cluster scaffold [Source:HGNC Symbol;Acc:HGNC:16287] |
| ENSG00000169609 | HGLibA_05397 | 83 | -0.383930029 | C15orf40 | chromosome 15 open reading frame 40 [Source:HGNC Symbol;Acc:HGNC:28443] |
| ENSG00000169727 | HGLibA_20150 | 83 | -0.383930029 | GPS1 | G protein pathway suppressor 1 [Source:HGNC Symbol;Acc:HGNC:4549] |
| ENSG00000169738 | HGLibA_12582 | 83 | -0.383930029 | DCXR | dicarbonyl and L-xylulose reductase [Source:HGNC Symbol;Acc:HGNC:18985] |
| ENSG00000170214 | HGLibA_01039 | 83 | -0.383930029 | ADRA1B | adrenoceptor alpha 1B [Source:HGNC Symbol;Acc:HGNC:278] |
| ENSG00000170819 | HGLibA_04459 | 83 | -0.383930029 | BFSP2 | beaded filament structural protein 2 [Source:HGNC Symbol;Acc:HGNC:1041] |
| ENSG00000170903 | HGLibA_30182 | 83 | -0.383930029 | MSANTD4 | Myb/SANT DNA binding domain containing 4 with coiled-coils [Source:HGNC Symbol;Acc:HGNC:29383] |
| ENSG00000171100 | HGLibA_30405 | 83 | -0.383930029 | MTM1 | myotubularin 1 [Source:HGNC Symbol;Acc:HGNC:7448] |
| ENSG00000171365 | HGLibA_09833 | 83 | -0.383930029 | CLCN5 | chloride voltage-gated channel 5 [Source:HGNC Symbol;Acc:HGNC:2023] |
| ENSG00000171659 | HGLibA_20026 | 83 | -0.383930029 | GPR34 | G protein-coupled receptor 34 [Source:HGNC Symbol;Acc:HGNC:4490] |
| ENSG00000171819 | HGLibA_01927 | 83 | -0.383930029 | ANGPTL7 | angiopoietin like 7 [Source:HGNC Symbol;Acc:HGNC:24078] |
| ENSG00000171823 | HGLibA_16978 | 83 | -0.383930029 | FBXL14 | F-box and leucine rich repeat protein 14 [Source:HGNC Symbol;Acc:HGNC:28624] |
| ENSG00000172318 | HGLibA_03953 | 83 | -0.383930029 | B3GALT1 | 3-galactosyltransferase 1 [Source:HGNC Symbol;Acc:HGNC:916]" |
| ENSG00000172780 | HGLibA_39948 | 83 | -0.383930029 | RAB43 | member RAS oncogene family [Source:HGNC Symbol;Acc:HGNC:19983]" |
| ENSG00000174611 | HGLibA_25878 | 83 | -0.383930029 | KY | kyphoscoliosis peptidase [Source:HGNC Symbol;Acc:HGNC:26576] |
| ENSG00000175224 | HGLibA_03468 | 83 | -0.383930029 | ATG13 | autophagy related 13 [Source:HGNC Symbol;Acc:HGNC:29091] |
| ENSG00000175768 | HGLibA_50974 | 83 | -0.383930029 | TOMM5 | translocase of outer mitochondrial membrane 5 [Source:HGNC Symbol;Acc:HGNC:31369] |
| ENSG00000177202 | HGLibA_46329 | 83 | -0.383930029 | SPACA4 | sperm acrosome associated 4 [Source:HGNC Symbol;Acc:HGNC:16441] |
| ENSG00000177917 | HGLibA_02992 | 83 | -0.383930029 | ARL6IP6 | ADP ribosylation factor like GTPase 6 interacting protein 6 [Source:HGNC Symbol;Acc:HGNC:24048] |
| ENSG00000178773 | HGLibA_10970 | 83 | -0.383930029 | CPNE7 | copine 7 [Source:HGNC Symbol;Acc:HGNC:2320] |
| ENSG00000179919 | HGLibA_33477 | 83 | -0.383930029 | OR10A7 | olfactory receptor family 10 subfamily A member 7 [Source:HGNC Symbol;Acc:HGNC:15329] |
| ENSG00000180061 | HGLibA_49993 | 83 | -0.383930029 | TMEM150B | transmembrane protein 150B [Source:HGNC Symbol;Acc:HGNC:34415] |
| ENSG00000180138 | HGLibA_11384 | 83 | -0.383930029 | CSNK1A1L | casein kinase 1 alpha 1 like [Source:HGNC Symbol;Acc:HGNC:20289] |
| ENSG00000182551 | HGLibA_00970 | 83 | -0.383930029 | ADI1 | acireductone dioxygenase 1 [Source:HGNC Symbol;Acc:HGNC:30576] |
| ENSG00000183161 | HGLibA_16848 | 83 | -0.383930029 | FANCF | FA complementation group F [Source:HGNC Symbol;Acc:HGNC:3587] |
| ENSG00000183260 | HGLibA_00259 | 83 | -0.383930029 | ABHD16B | abhydrolase domain containing 16B [Source:HGNC Symbol;Acc:HGNC:16128] |
| ENSG00000183309 | HGLibA_56413 | 83 | -0.383930029 | ZNF623 | zinc finger protein 623 [Source:HGNC Symbol;Acc:HGNC:29084] |
| ENSG00000183963 | HGLibA_45834 | 83 | -0.383930029 | SMTN | smoothelin [Source:HGNC Symbol;Acc:HGNC:11126] |
| ENSG00000184860 | HGLibA_43165 | 83 | -0.383930029 | SDR42E1 | member 1 [Source:HGNC Symbol;Acc:HGNC:29834]" |
| ENSG00000185090 | HGLibA_28094 | 83 | -0.383930029 | MANEAL | mannosidase endo-alpha like [Source:HGNC Symbol;Acc:HGNC:26452] |
| ENSG00000185252 | HGLibA_56658 | 83 | -0.383930029 | ZNF74 | zinc finger protein 74 [Source:HGNC Symbol;Acc:HGNC:13144] |
| ENSG00000186376 | HGLibA_56681 | 83 | -0.383930029 | ZNF75D | zinc finger protein 75D [Source:HGNC Symbol;Acc:HGNC:13145] |
| ENSG00000187581 | HGLibA_10877 | 83 | -0.383930029 | COX8C | cytochrome c oxidase subunit 8C [Source:HGNC Symbol;Acc:HGNC:24382] |
| ENSG00000187957 | HGLibA_13673 | 83 | -0.383930029 | DNER | delta/notch like EGF repeat containing [Source:HGNC Symbol;Acc:HGNC:24456] |
| ENSG00000188467 | HGLibA_44626 | 83 | -0.383930029 | SLC24A5 | solute carrier family 24 member 5 [Source:HGNC Symbol;Acc:HGNC:20611] |
| ENSG00000189339 | HGLibA_44992 | 83 | -0.383930029 | SLC35E2B | solute carrier family 35 member E2B [Source:HGNC Symbol;Acc:HGNC:33941] |
| ENSG00000196119 | HGLibA_34489 | 83 | -0.383930029 | OR8A1 | olfactory receptor family 8 subfamily A member 1 [Source:HGNC Symbol;Acc:HGNC:8469] |
| ENSG00000196466 | HGLibA_56777 | 83 | -0.383930029 | ZNF799 | zinc finger protein 799 [Source:HGNC Symbol;Acc:HGNC:28071] |
| ENSG00000196547 | HGLibA_28072 | 83 | -0.383930029 | MAN2A2 | mannosidase alpha class 2A member 2 [Source:HGNC Symbol;Acc:HGNC:6825] |
| ENSG00000196562 | HGLibA_47729 | 83 | -0.383930029 | SULF2 | sulfatase 2 [Source:HGNC Symbol;Acc:HGNC:20392] |
| ENSG00000197385 | HGLibA_56879 | 83 | -0.383930029 | ZNF860 | zinc finger protein 860 [Source:HGNC Symbol;Acc:HGNC:34513] |
| ENSG00000198911 | HGLibA_46952 | 83 | -0.383930029 | SREBF2 | sterol regulatory element binding transcription factor 2 [Source:HGNC Symbol;Acc:HGNC:11290] |
| ENSG00000204669 | HGLibA_06743 | 83 | -0.383930029 | C9orf57 | chromosome 9 open reading frame 57 [Source:HGNC Symbol;Acc:HGNC:27037] |
| ENSG00000205643 | HGLibA_08861 | 83 | -0.383930029 | CDPF1 | cysteine rich DPF motif domain containing 1 [Source:HGNC Symbol;Acc:HGNC:33710] |
| ENSG00000213015 | HGLibA_56306 | 83 | -0.383930029 | ZNF580 | zinc finger protein 580 [Source:HGNC Symbol;Acc:HGNC:29473] |
| ENSG00000213918 | HGLibA_13653 | 83 | -0.383930029 | DNASE1 | deoxyribonuclease 1 [Source:HGNC Symbol;Acc:HGNC:2956] |
| ENSG00000227151 | HGLibA_38809 | 83 | -0.383930029 | PRR20D | proline rich 20D [Source:HGNC Symbol;Acc:HGNC:37222] |
| ENSG00000239306 | HGLibA_40484 | 83 | -0.383930029 | RBM14 | RNA binding motif protein 14 [Source:HGNC Symbol;Acc:HGNC:14219] |
| ENSG00000239998 | HGLibA_26493 | 83 | -0.383930029 | LILRA2 | leukocyte immunoglobulin like receptor A2 [Source:HGNC Symbol;Acc:HGNC:6603] |
| ENSG00000240247 | HGLibA_12796 | 83 | -0.383930029 | DEFA1B | defensin alpha 1B [Source:HGNC Symbol;Acc:HGNC:33596] |
| ENSG00000242265 | HGLibA_36028 | 83 | -0.383930029 | PEG10 | paternally expressed 10 [Source:HGNC Symbol;Acc:HGNC:14005] |

| | | | | | |
|---|---|---|---|---|---|
| ENSG00000242419 | HGLibA_35574 | 83 | -0.383930029 | PCDHGC4 | 4 [Source:HGNC Symbol;Acc:HGNC:8717]" |
| ENSG00000243489 | HGLibA_25598 | 83 | -0.383930029 | KRTAP10-11 | keratin associated protein 10-11 [Source:HGNC Symbol;Acc:HGNC:20528] |
| ENSG00000243896 | HGLibA_33742 | 83 | -0.383930029 | OR2A7 | olfactory receptor family 2 subfamily A member 7 [Source:HGNC Symbol;Acc:HGNC:8234] |
| ENSG00000244462 | HGLibA_40478 | 83 | -0.383930029 | RBM12 | RNA binding motif protein 12 [Source:HGNC Symbol;Acc:HGNC:9898] |
| ENSG00000246922 | HGLibA_52549 | 83 | -0.383930029 | UBAP1L | ubiquitin associated protein 1 like [Source:HGNC Symbol;Acc:HGNC:40028] |
| ENSG00000253767 | HGLibA_35544 | 83 | -0.383930029 | PCDHGA8 | 8 [Source:HGNC Symbol;Acc:HGNC:8706]" |
| ENSG00000254245 | HGLibA_35532 | 83 | -0.383930029 | PCDHGA3 | 3 [Source:HGNC Symbol;Acc:HGNC:8701]" |
| ENSG00000254772 | HGLibA_14506 | 83 | -0.383930029 | EEF1G | eukaryotic translation elongation factor 1 gamma [Source:HGNC Symbol;Acc:HGNC:3213] |
| ENSG00000266560 | HGLibA_27895 | 83 | -0.383930029 | MAGEA10-MAGEA5 | MAGEA10-MAGEA5 readthrough [Source:NCBI gene;Acc:100533997] |
| ENSG00000271271 | HGLibA_52905 | 83 | -0.383930029 | UGT2A2 | UDP glucuronosyltransferase family 2 member A2 [Source:HGNC Symbol;Acc:HGNC:28183] |
| ENSG00000285723 | HGLibA_29331 | 83 | -0.383930029 | MKKS | novel protein |
| H1FNT | HGLibA_20736 | 83 | -0.383930029 | H1FNT | - |
| hsa-mir-206 | HGLibA_58190 | 83 | -0.383930029 | hsa-mir-206 | - |
| hsa-mir-22 | HGLibA_58289 | 83 | -0.383930029 | hsa-mir-22 | - |
| hsa-mir-3914-2 | HGLibA_59534 | 83 | -0.383930029 | hsa-mir-3914-2 | - |
| hsa-mir-4436b-2 | HGLibA_60148 | 83 | -0.383930029 | hsa-mir-4436b-2 | - |
| hsa-mir-4659b | HGLibA_60745 | 83 | -0.383930029 | hsa-mir-4659b | - |
| hsa-mir-4773-1 | HGLibA_61193 | 83 | -0.383930029 | hsa-mir-4773-1 | - |
| hsa-mir-486 | HGLibA_61337 | 83 | -0.383930029 | hsa-mir-486 | - |
| hsa-mir-486-2 | HGLibA_61341 | 83 | -0.383930029 | hsa-mir-486-2 | - |
| hsa-mir-548ao | HGLibA_61907 | 83 | -0.383930029 | hsa-mir-548ao | - |
| hsa-mir-6721 | HGLibA_63044 | 83 | -0.383930029 | hsa-mir-6721 | - |
| hsa-mir-6815 | HGLibA_63438 | 83 | -0.383930029 | hsa-mir-6815 | - |
| hsa-mir-6832 | HGLibA_63507 | 83 | -0.383930029 | hsa-mir-6832 | - |
| hsa-mir-8073 | HGLibA_64133 | 83 | -0.383930029 | hsa-mir-8073 | - |
| hsa-mir-874 | HGLibA_64208 | 83 | -0.383930029 | hsa-mir-874 | - |
| KIAA0907 | HGLibA_24663 | 83 | -0.383930029 | KIAA0907 | - |
| LILRA3 | HGLibA_26494 | 83 | -0.383930029 | LILRA3 | - |
| LRRC16B | HGLibA_27265 | 83 | -0.383930029 | LRRC16B | - |
| PIDD | HGLibA_36532 | 83 | -0.383930029 | PIDD | - |
| SEPN1 | HGLibA_43417 | 83 | -0.383930029 | SEPN1 | - |
| ZUFSP | HGLibA_57061 | 83 | -0.383930029 | ZUFSP | - |
| ATP5A1 | HGLibA_03639 | 82 | -0.392975372 | ATP5A1 | - |
| C12orf65 | HGLibA_05278 | 82 | -0.392975372 | C12orf65 | - |
| C17orf59 | HGLibA_05553 | 82 | -0.392975372 | C17orf59 | - |
| CCDC53 | HGLibA_07799 | 82 | -0.392975372 | CCDC53 | - |
| CXXC11 | HGLibA_11967 | 82 | -0.392975372 | CXXC11 | - |
| ENSG00000010810 | HGLibA_18253 | 82 | -0.392975372 | FYN | Src family tyrosine kinase [Source:HGNC Symbol;Acc:HGNC:4037]" |
| ENSG00000012983 | HGLibA_28224 | 82 | -0.392975372 | MAP4K5 | mitogen-activated protein kinase kinase kinase kinase 5 [Source:HGNC Symbol;Acc:HGNC:6867] |
| ENSG00000024048 | HGLibA_52751 | 82 | -0.392975372 | UBR2 | ubiquitin protein ligase E3 component n-recognin 2 [Source:HGNC Symbol;Acc:HGNC:21289] |
| ENSG00000024422 | HGLibA_14669 | 82 | -0.392975372 | EHD2 | EH domain containing 2 [Source:HGNC Symbol;Acc:HGNC:3243] |
| ENSG00000038219 | HGLibA_04670 | 82 | -0.392975372 | BOD1L1 | biorientation of chromosomes in cell division 1 like 1 [Source:HGNC Symbol;Acc:HGNC:31792] |
| ENSG00000071553 | HGLibA_03694 | 82 | -0.392975372 | ATP6AP1 | ATPase H+ transporting accessory protein 1 [Source:HGNC Symbol;Acc:HGNC:868] |
| ENSG00000071991 | HGLibA_08656 | 82 | -0.392975372 | CDH19 | cadherin 19 [Source:HGNC Symbol;Acc:HGNC:1758] |
| ENSG00000072818 | HGLibA_00382 | 82 | -0.392975372 | ACAP1 | ankyrin repeat and PH domains 1 [Source:HGNC Symbol;Acc:HGNC:16467]" |
| ENSG00000073331 | HGLibA_01701 | 82 | -0.392975372 | ALPK1 | alpha kinase 1 [Source:HGNC Symbol;Acc:HGNC:20917] |
| ENSG00000075407 | HGLibA_55884 | 82 | -0.392975372 | ZNF37A | zinc finger protein 37A [Source:HGNC Symbol;Acc:HGNC:13102] |
| ENSG00000077463 | HGLibA_44231 | 82 | -0.392975372 | SIRT6 | sirtuin 6 [Source:HGNC Symbol;Acc:HGNC:14934] |
| ENSG00000080293 | HGLibA_43066 | 82 | -0.392975372 | SCTR | secretin receptor [Source:HGNC Symbol;Acc:HGNC:10608] |
| ENSG00000088726 | HGLibA_50390 | 82 | -0.392975372 | TMEM40 | transmembrane protein 40 [Source:HGNC Symbol;Acc:HGNC:25620] |
| ENSG00000090447 | HGLibA_49136 | 82 | -0.392975372 | TFAP4 | transcription factor AP-4 [Source:HGNC Symbol;Acc:HGNC:11745] |
| ENSG00000100395 | HGLibA_25900 | 82 | -0.392975372 | L3MBTL2 | polycomb repressive complex 1 subunit [Source:HGNC Symbol;Acc:HGNC:18594]" |
| ENSG00000100625 | HGLibA_44252 | 82 | -0.392975372 | SIX4 | SIX homeobox 4 [Source:HGNC Symbol;Acc:HGNC:10890] |
| ENSG00000101470 | HGLibA_50868 | 82 | -0.392975372 | TNNC2 | fast skeletal type [Source:HGNC Symbol;Acc:HGNC:11944]" |
| ENSG00000102452 | HGLibA_31083 | 82 | -0.392975372 | NALCN | non-selective [Source:HGNC Symbol;Acc:HGNC:19082]" |
| ENSG00000105894 | HGLibA_39397 | 82 | -0.392975372 | PTN | pleiotrophin [Source:HGNC Symbol;Acc:HGNC:9630] |
| ENSG00000106034 | HGLibA_10929 | 82 | -0.392975372 | CPED1 | cadherin like and PC-esterase domain containing 1 [Source:HGNC Symbol;Acc:HGNC:26159] |
| ENSG00000106268 | HGLibA_32930 | 82 | -0.392975372 | NUDT1 | nudix hydrolase 1 [Source:HGNC Symbol;Acc:HGNC:8048] |
| ENSG00000106541 | HGLibA_01244 | 82 | -0.392975372 | AGR2 | protein disulphide isomerase family member [Source:HGNC Symbol;Acc:HGNC:328]" |
| ENSG00000106565 | HGLibA_50075 | 82 | -0.392975372 | TMEM176B | transmembrane protein 176B [Source:HGNC Symbol;Acc:HGNC:29596] |
| ENSG00000106624 | HGLibA_01085 | 82 | -0.392975372 | AEBP1 | AE binding protein 1 [Source:HGNC Symbol;Acc:HGNC:303] |
| ENSG00000107371 | HGLibA_15777 | 82 | -0.392975372 | EXOSC3 | exosome component 3 [Source:HGNC Symbol;Acc:HGNC:17944] |
| ENSG00000107593 | HGLibA_36800 | 82 | -0.392975372 | PKD2L1 | transient receptor potential cation channel [Source:HGNC Symbol;Acc:HGNC:9011]" |
| ENSG00000109101 | HGLibA_17910 | 82 | -0.392975372 | FOXN1 | forkhead box N1 [Source:HGNC Symbol;Acc:HGNC:12765] |
| ENSG00000112038 | HGLibA_33453 | 82 | -0.392975372 | OPRM1 | opioid receptor mu 1 [Source:HGNC Symbol;Acc:HGNC:8156] |
| ENSG00000112367 | HGLibA_17511 | 82 | -0.392975372 | FIG4 | FIG4 phosphoinositide 5-phosphatase [Source:HGNC Symbol;Acc:HGNC:16873] |
| ENSG00000113578 | HGLibA_17360 | 82 | -0.392975372 | FGF1 | fibroblast growth factor 1 [Source:HGNC Symbol;Acc:HGNC:3665] |
| ENSG00000114812 | HGLibA_53668 | 82 | -0.392975372 | VIPR1 | vasoactive intestinal peptide receptor 1 [Source:HGNC Symbol;Acc:HGNC:12694] |
| ENSG00000117174 | HGLibA_56932 | 82 | -0.392975372 | ZNHIT6 | zinc finger HIT-type containing 6 [Source:HGNC Symbol;Acc:HGNC:26089] |
| ENSG00000117480 | HGLibA_15901 | 82 | -0.392975372 | FAAH | fatty acid amide hydrolase [Source:HGNC Symbol;Acc:HGNC:3553] |
| ENSG00000118113 | HGLibA_29541 | 82 | -0.392975372 | MMP8 | matrix metallopeptidase 8 [Source:HGNC Symbol;Acc:HGNC:7175] |
| ENSG00000118690 | HGLibA_03013 | 82 | -0.392975372 | ARMC2 | armadillo repeat containing 2 [Source:HGNC Symbol;Acc:HGNC:23045] |
| ENSG00000120055 | HGLibA_05108 | 82 | -0.392975372 | C10orf95 | chromosome 10 open reading frame 95 [Source:HGNC Symbol;Acc:HGNC:25880] |
| ENSG00000120458 | HGLibA_30172 | 82 | -0.392975372 | MSANTD2 | Myb/SANT DNA binding domain containing 2 [Source:HGNC Symbol;Acc:HGNC:26266] |
| ENSG00000121351 | HGLibA_22470 | 82 | -0.392975372 | IAPP | islet amyloid polypeptide [Source:HGNC Symbol;Acc:HGNC:5329] |
| ENSG00000122188 | HGLibA_26070 | 82 | -0.392975372 | LAX1 | lymphocyte transmembrane adaptor 1 [Source:HGNC Symbol;Acc:HGNC:26005] |
| ENSG00000122729 | HGLibA_00461 | 82 | -0.392975372 | ACO1 | aconitase 1 [Source:HGNC Symbol;Acc:HGNC:117] |
| ENSG00000124157 | HGLibA_43383 | 82 | -0.392975372 | SEMG2 | semenogelin 2 [Source:HGNC Symbol;Acc:HGNC:10743] |
| ENSG00000124212 | HGLibA_39349 | 82 | -0.392975372 | PTGIS | prostaglandin I2 synthase [Source:HGNC Symbol;Acc:HGNC:9603] |
| ENSG00000124444 | HGLibA_56293 | 82 | -0.392975372 | ZNF576 | zinc finger protein 576 [Source:HGNC Symbol;Acc:HGNC:28357] |
| ENSG00000124783 | HGLibA_47154 | 82 | -0.392975372 | SSR1 | signal sequence receptor subunit 1 [Source:HGNC Symbol;Acc:HGNC:11323] |
| ENSG00000126777 | HGLibA_25873 | 82 | -0.392975372 | KTN1 | kinectin 1 [Source:HGNC Symbol;Acc:HGNC:6467] |
| ENSG00000127481 | HGLibA_52757 | 82 | -0.392975372 | UBR4 | ubiquitin protein ligase E3 component n-recognin 4 [Source:HGNC Symbol;Acc:HGNC:30313] |
| ENSG00000128654 | HGLibA_30513 | 82 | -0.392975372 | MTX2 | metaxin 2 [Source:HGNC Symbol;Acc:HGNC:7506] |
| ENSG00000129596 | HGLibA_08855 | 82 | -0.392975372 | CDO1 | cysteine dioxygenase type 1 [Source:HGNC Symbol;Acc:HGNC:1795] |
| ENSG00000129657 | HGLibA_43190 | 82 | -0.392975372 | SEC14L1 | SEC14 like lipid binding 1 [Source:HGNC Symbol;Acc:HGNC:10698] |
| ENSG00000130640 | HGLibA_52310 | 82 | -0.392975372 | TUBGCP2 | tubulin gamma complex associated protein 2 [Source:HGNC Symbol;Acc:HGNC:18599] |
| ENSG00000130940 | HGLibA_07348 | 82 | -0.392975372 | CASZ1 | castor zinc finger 1 [Source:HGNC Symbol;Acc:HGNC:26002] |
| ENSG00000131899 | HGLibA_26648 | 82 | -0.392975372 | LLGL1 | scribble cell polarity complex component [Source:HGNC Symbol;Acc:HGNC:6628]" |
| ENSG00000133142 | HGLibA_48712 | 82 | -0.392975372 | TCEAL4 | transcription elongation factor A like 4 [Source:HGNC Symbol;Acc:HGNC:26121] |
| ENSG00000133703 | HGLibA_25385 | 82 | -0.392975372 | KRAS | GTPase [Source:HGNC Symbol;Acc:HGNC:6407]" |
| ENSG00000133961 | HGLibA_33013 | 82 | -0.392975372 | NUMB | endocytic adaptor protein [Source:HGNC Symbol;Acc:HGNC:8060]" |
| ENSG00000134193 | HGLibA_40807 | 82 | -0.392975372 | REG4 | regenerating family member 4 [Source:HGNC Symbol;Acc:HGNC:22977] |
| ENSG00000134207 | HGLibA_48085 | 82 | -0.392975372 | SYT6 | synaptotagmin 6 [Source:HGNC Symbol;Acc:HGNC:18638] |
| ENSG00000134897 | HGLibA_04533 | 82 | -0.392975372 | BIVM | immunoglobulin-like variable motif containing [Source:HGNC Symbol;Acc:HGNC:16034]" |
| ENSG00000135245 | HGLibA_21319 | 82 | -0.392975372 | HILPDA | hypoxia inducible lipid droplet associated [Source:HGNC Symbol;Acc:HGNC:28859] |
| ENSG00000135624 | HGLibA_08214 | 82 | -0.392975372 | CCT7 | chaperonin containing TCP1 subunit 7 [Source:HGNC Symbol;Acc:HGNC:1622] |
| ENSG00000137251 | HGLibA_49547 | 82 | -0.392975372 | TINAG | tubulointerstitial nephritis antigen [Source:HGNC Symbol;Acc:HGNC:14599] |
| ENSG00000137707 | HGLibA_04950 | 82 | -0.392975372 | BTG4 | BTG anti-proliferation factor 4 [Source:HGNC Symbol;Acc:HGNC:13862] |
| ENSG00000137731 | HGLibA_18227 | 82 | -0.392975372 | FXYD2 | FXYD domain containing ion transport regulator 2 [Source:HGNC Symbol;Acc:HGNC:4026] |
| ENSG00000137804 | HGLibA_33095 | 82 | -0.392975372 | NUSAP1 | nucleolar and spindle associated protein 1 [Source:HGNC Symbol;Acc:HGNC:18538] |
| ENSG00000138032 | HGLibA_37966 | 82 | -0.392975372 | PPM1B | Mg2+/Mn2+ dependent 1B [Source:HGNC Symbol;Acc:HGNC:9276]" |
| ENSG00000138400 | HGLibA_28685 | 82 | -0.392975372 | MDH1B | malate dehydrogenase 1B [Source:HGNC Symbol;Acc:HGNC:17836] |
| ENSG00000139547 | HGLibA_40747 | 82 | -0.392975372 | RDH16 | retinol dehydrogenase 16 [Source:HGNC Symbol;Acc:HGNC:29674] |
| ENSG00000140279 | HGLibA_14101 | 82 | -0.392975372 | DUOX2 | dual oxidase 2 [Source:HGNC Symbol;Acc:HGNC:13273] |
| ENSG00000141380 | HGLibA_47102 | 82 | -0.392975372 | SS18 | nBAF chromatin remodeling complex subunit [Source:HGNC Symbol;Acc:HGNC:11340]" |
| ENSG00000142731 | HGLibA_37196 | 82 | -0.392975372 | PLK4 | polo like kinase 4 [Source:HGNC Symbol;Acc:HGNC:11397] |
| ENSG00000143079 | HGLibA_11746 | 82 | -0.392975372 | CTTNBP2NL | CTTNBP2 N-terminal like [Source:HGNC Symbol;Acc:HGNC:25330] |
| ENSG00000143294 | HGLibA_38352 | 82 | -0.392975372 | PRCC | proline rich mitotic checkpoint control factor [Source:HGNC Symbol;Acc:HGNC:9343] |
| ENSG00000143382 | HGLibA_00858 | 82 | -0.392975372 | ADAMTSL4 | ADAMTS like 4 [Source:HGNC Symbol;Acc:HGNC:19706] |
| ENSG00000143776 | HGLibA_08558 | 82 | -0.392975372 | CDC42BPA | CDC42 binding protein kinase alpha [Source:HGNC Symbol;Acc:HGNC:1737] |
| ENSG00000145808 | HGLibA_00817 | 82 | -0.392975372 | ADAMTS19 | ADAM metallopeptidase with thrombospondin type 1 motif 19 [Source:HGNC Symbol;Acc:HGNC:17111] |
| ENSG00000145861 | HGLibA_05940 | 82 | -0.392975372 | C1QTNF2 | C1q and TNF related 2 [Source:HGNC Symbol;Acc:HGNC:14325] |
| ENSG00000146426 | HGLibA_49443 | 82 | -0.392975372 | TIAM2 | T cell lymphoma invasion and metastasis 2 [Source:HGNC Symbol;Acc:HGNC:11806] |
| ENSG00000147100 | HGLibA_44434 | 82 | -0.392975372 | SLC16A2 | solute carrier family 16 member 2 [Source:HGNC Symbol;Acc:HGNC:10923] |
| ENSG00000147118 | HGLibA_55543 | 82 | -0.392975372 | ZNF182 | zinc finger protein 182 [Source:HGNC Symbol;Acc:HGNC:13001] |
| ENSG00000147457 | HGLibA_09503 | 82 | -0.392975372 | CHMP7 | charged multivesicular body protein 7 [Source:HGNC Symbol;Acc:HGNC:28439] |
| ENSG00000147570 | HGLibA_13626 | 82 | -0.392975372 | DNAJC5B | DnaJ heat shock protein family (Hsp40) member C5 beta [Source:HGNC Symbol;Acc:HGNC:24138] |
| ENSG00000147905 | HGLibA_55046 | 82 | -0.392975372 | ZCCHC7 | zinc finger CCHC-type containing 7 [Source:HGNC Symbol;Acc:HGNC:26209] |
| ENSG00000149054 | HGLibA_55597 | 82 | -0.392975372 | ZNF215 | zinc finger protein 215 [Source:HGNC Symbol;Acc:HGNC:13007] |
| ENSG00000152208 | HGLibA_20274 | 82 | -0.392975372 | GRID2 | glutamate ionotropic receptor delta type subunit 2 [Source:HGNC Symbol;Acc:HGNC:4576] |
| ENSG00000154914 | HGLibA_53367 | 82 | -0.392975372 | USP43 | ubiquitin specific peptidase 43 [Source:HGNC Symbol;Acc:HGNC:20072] |
| ENSG00000155229 | HGLibA_29551 | 82 | -0.392975372 | MMS19 | cytosolic iron-sulfur assembly component [Source:HGNC Symbol;Acc:HGNC:13824]" |
| ENSG00000155561 | HGLibA_33036 | 82 | -0.392975372 | NUP205 | nucleoporin 205 [Source:HGNC Symbol;Acc:HGNC:18658] |
| ENSG00000156234 | HGLibA_11853 | 82 | -0.392975372 | CXCL13 | C-X-C motif chemokine ligand 13 [Source:HGNC Symbol;Acc:HGNC:10639] |
| ENSG00000157106 | HGLibA_45697 | 82 | -0.392975372 | SMG1 | nonsense mediated mRNA decay associated PI3K related kinase [Source:HGNC Symbol;Acc:HGNC:30045]" |
| ENSG00000158158 | HGLibA_10322 | 82 | -0.392975372 | CNNM4 | cyclin and CBS domain divalent metal cation transport mediator 4 [Source:HGNC Symbol;Acc:HGNC:105] |
| ENSG00000158874 | HGLibA_02455 | 82 | -0.392975372 | APOA2 | apolipoprotein A2 [Source:HGNC Symbol;Acc:HGNC:601] |
| ENSG00000160305 | HGLibA_13247 | 82 | -0.392975372 | DIP2A | disco interacting protein 2 homolog A [Source:HGNC Symbol;Acc:HGNC:17217] |
| ENSG00000160606 | HGLibA_49595 | 82 | -0.392975372 | TLCD1 | TLC domain containing 1 [Source:HGNC Symbol;Acc:HGNC:25177] |
| ENSG00000160613 | HGLibA_35686 | 82 | -0.392975372 | PCSK7 | proprotein convertase subtilisin/kexin type 7 [Source:HGNC Symbol;Acc:HGNC:8748] |
| ENSG00000160908 | HGLibA_55908 | 82 | -0.392975372 | ZNF394 | zinc finger protein 394 [Source:HGNC Symbol;Acc:HGNC:18832] |
| ENSG00000161860 | HGLibA_47920 | 82 | -0.392975372 | SYCE2 | synaptonemal complex central element protein 2 [Source:HGNC Symbol;Acc:HGNC:27411] |
| ENSG00000162366 | HGLibA_35994 | 82 | -0.392975372 | PDZK1IP1 | PDZK1 interacting protein 1 [Source:HGNC Symbol;Acc:HGNC:16887] |
| ENSG00000162722 | HGLibA_51552 | 82 | -0.392975372 | TRIM58 | tripartite motif containing 58 [Source:HGNC Symbol;Acc:HGNC:24150] |

| | | | | | |
|---|---|---|---|---|---|
| ENSG00000163155 | HGLibA_27763 | 82 | -0.392975372 | LYSMD1 | LysM domain containing 1 [Source:HGNC Symbol;Acc:HGNC:32070] |
| ENSG00000163629 | HGLibA_39440 | 82 | -0.392975372 | PTPN13 | non-receptor type 13 [Source:HGNC Symbol;Acc:HGNC:9646]" |
| ENSG00000163827 | HGLibA_27279 | 82 | -0.392975372 | LRRC2 | leucine rich repeat containing 2 [Source:HGNC Symbol;Acc:HGNC:14676] |
| ENSG00000164332 | HGLibA_52718 | 82 | -0.392975372 | UBLCP1 | ubiquitin like domain containing CTD phosphatase 1 [Source:HGNC Symbol;Acc:HGNC:28110] |
| ENSG00000164406 | HGLibA_26261 | 82 | -0.392975372 | LEAP2 | liver enriched antimicrobial peptide 2 [Source:HGNC Symbol;Acc:HGNC:29571] |
| ENSG00000164879 | HGLibA_06806 | 82 | -0.392975372 | CA3 | carbonic anhydrase 3 [Source:HGNC Symbol;Acc:HGNC:1374] |
| ENSG00000165240 | HGLibA_03770 | 82 | -0.392975372 | ATP7A | ATPase copper transporting alpha [Source:HGNC Symbol;Acc:HGNC:869] |
| ENSG00000166796 | HGLibA_26233 | 82 | -0.392975372 | LDHC | lactate dehydrogenase C [Source:HGNC Symbol;Acc:HGNC:6544] |
| ENSG00000167904 | HGLibA_50494 | 82 | -0.392975372 | TMEM68 | transmembrane protein 68 [Source:HGNC Symbol;Acc:HGNC:26510] |
| ENSG00000168310 | HGLibA_23573 | 82 | -0.392975372 | IRF2 | interferon regulatory factor 2 [Source:HGNC Symbol;Acc:HGNC:6117] |
| ENSG00000168314 | HGLibA_29607 | 82 | -0.392975372 | MOBP | myelin-associated oligodendrocyte basic protein [Source:HGNC Symbol;Acc:HGNC:7189] |
| ENSG00000168447 | HGLibA_43016 | 82 | -0.392975372 | SCNN1B | sodium channel epithelial 1 beta subunit [Source:HGNC Symbol;Acc:HGNC:10600] |
| ENSG00000169155 | HGLibA_54888 | 82 | -0.392975372 | ZBTB43 | zinc finger and BTB domain containing 43 [Source:HGNC Symbol;Acc:HGNC:17908] |
| ENSG00000169717 | HGLibA_00679 | 82 | -0.392975372 | ACTRT2 | actin related protein T2 [Source:HGNC Symbol;Acc:HGNC:24026] |
| ENSG00000169900 | HGLibA_39681 | 82 | -0.392975372 | PYDC1 | pyrin domain containing 1 [Source:HGNC Symbol;Acc:HGNC:30261] |
| ENSG00000169962 | HGLibA_48359 | 82 | -0.392975372 | TAS1R3 | taste 1 receptor member 3 [Source:HGNC Symbol;Acc:HGNC:15661] |
| ENSG00000170128 | HGLibA_20005 | 82 | -0.392975372 | GPR25 | G protein-coupled receptor 25 [Source:HGNC Symbol;Acc:HGNC:4480] |
| ENSG00000170248 | HGLibA_35741 | 82 | -0.392975372 | PDCD6IP | programmed cell death 6 interacting protein [Source:HGNC Symbol;Acc:HGNC:8766] |
| ENSG00000171433 | HGLibA_19288 | 82 | -0.392975372 | GLOD5 | glyoxalase domain containing 5 [Source:HGNC Symbol;Acc:HGNC:33358] |
| ENSG00000171505 | HGLibA_33704 | 82 | -0.392975372 | OR1N1 | olfactory receptor family 1 subfamily N member 1 [Source:HGNC Symbol;Acc:HGNC:8221] |
| ENSG00000171530 | HGLibA_48579 | 82 | -0.392975372 | TBCA | tubulin folding cofactor A [Source:HGNC Symbol;Acc:HGNC:11579] |
| ENSG00000171763 | HGLibA_46518 | 82 | -0.392975372 | SPATA5L1 | spermatogenesis associated 5 like 1 [Source:HGNC Symbol;Acc:HGNC:28762] |
| ENSG00000172239 | HGLibA_35028 | 82 | -0.392975372 | PAIP1 | poly(A) binding protein interacting protein 1 [Source:HGNC Symbol;Acc:HGNC:16945] |
| ENSG00000172673 | HGLibA_49348 | 82 | -0.392975372 | THEMIS | thymocyte selection associated [Source:HGNC Symbol;Acc:HGNC:21569] |
| ENSG00000172725 | HGLibA_10775 | 82 | -0.392975372 | CORO1B | coronin 1B [Source:HGNC Symbol;Acc:HGNC:2253] |
| ENSG00000172819 | HGLibA_40279 | 82 | -0.392975372 | RARG | retinoic acid receptor gamma [Source:HGNC Symbol;Acc:HGNC:9866] |
| ENSG00000173210 | HGLibA_00314 | 82 | -0.392975372 | ABLIM3 | actin binding LIM protein family member 3 [Source:HGNC Symbol;Acc:HGNC:29132] |
| ENSG00000173264 | HGLibA_19898 | 82 | -0.392975372 | GPR137 | G protein-coupled receptor 137 [Source:HGNC Symbol;Acc:HGNC:24300] |
| ENSG00000173267 | HGLibA_45916 | 82 | -0.392975372 | SNCG | synuclein gamma [Source:HGNC Symbol;Acc:HGNC:11141] |
| ENSG00000173692 | HGLibA_39148 | 82 | -0.392975372 | PSMD1 | non-ATPase 1 [Source:HGNC Symbol;Acc:HGNC:9554]" |
| ENSG00000173769 | HGLibA_51006 | 82 | -0.392975372 | TOPAZ1 | testis and ovary specific PAZ domain containing 1 [Source:HGNC Symbol;Acc:HGNC:24746] |
| ENSG00000174448 | HGLibA_47368 | 82 | -0.392975372 | STARD6 | StAR related lipid transfer domain containing 6 [Source:HGNC Symbol;Acc:HGNC:18066] |
| ENSG00000174844 | HGLibA_13476 | 82 | -0.392975372 | DNAH12 | dynein axonemal heavy chain 12 [Source:HGNC Symbol;Acc:HGNC:2943] |
| ENSG00000174982 | HGLibA_34065 | 82 | -0.392975372 | OR4S2 | olfactory receptor family 4 subfamily S member 2 [Source:HGNC Symbol;Acc:HGNC:15183] |
| ENSG00000175084 | HGLibA_13004 | 82 | -0.392975372 | DES | desmin [Source:HGNC Symbol;Acc:HGNC:2770] |
| ENSG00000176009 | HGLibA_03272 | 82 | -0.392975372 | ASCL3 | achaete-scute family bHLH transcription factor 3 [Source:HGNC Symbol;Acc:HGNC:740] |
| ENSG00000176058 | HGLibA_51188 | 82 | -0.392975372 | TPRN | taperin [Source:HGNC Symbol;Acc:HGNC:26894] |
| ENSG00000176171 | HGLibA_04655 | 82 | -0.392975372 | BNIP3 | BCL2 interacting protein 3 [Source:HGNC Symbol;Acc:HGNC:1084] |
| ENSG00000177606 | HGLibA_24003 | 82 | -0.392975372 | JUN | AP-1 transcription factor subunit [Source:HGNC Symbol;Acc:HGNC:6204]" |
| ENSG00000177684 | HGLibA_12856 | 82 | -0.392975372 | DEFB114 | defensin beta 114 [Source:HGNC Symbol;Acc:HGNC:18095] |
| ENSG00000178171 | HGLibA_01779 | 82 | -0.392975372 | AMER3 | APC membrane recruitment protein 3 [Source:HGNC Symbol;Acc:HGNC:26771] |
| ENSG00000178568 | HGLibA_15422 | 82 | -0.392975372 | ERBB4 | erb-b2 receptor tyrosine kinase 4 [Source:HGNC Symbol;Acc:HGNC:3432] |
| ENSG00000179115 | HGLibA_16884 | 82 | -0.392975372 | FARSA | phenylalanyl-tRNA synthetase subunit alpha [Source:HGNC Symbol;Acc:HGNC:3592] |
| ENSG00000179673 | HGLibA_42057 | 82 | -0.392975372 | RPRML | reprimo like [Source:HGNC Symbol;Acc:HGNC:32422] |
| ENSG00000180329 | HGLibA_07788 | 82 | -0.392975372 | CCDC43 | coiled-coil domain containing 43 [Source:HGNC Symbol;Acc:HGNC:26472] |
| ENSG00000180398 | HGLibA_28585 | 82 | -0.392975372 | MCFD2 | multiple coagulation factor deficiency 2 [Source:HGNC Symbol;Acc:HGNC:18451] |
| ENSG00000181817 | HGLibA_27521 | 82 | -0.392975372 | LSM10 | U7 small nuclear RNA associated [Source:HGNC Symbol;Acc:HGNC:17562]" |
| ENSG00000182333 | HGLibA_26608 | 82 | -0.392975372 | LIPF | gastric type [Source:HGNC Symbol;Acc:HGNC:6622]" |
| ENSG00000184347 | HGLibA_45511 | 82 | -0.392975372 | SLIT3 | slit guidance ligand 3 [Source:HGNC Symbol;Acc:HGNC:11087] |
| ENSG00000185112 | HGLibA_16588 | 82 | -0.392975372 | FAM43A | family with sequence similarity 43 member A [Source:HGNC Symbol;Acc:HGNC:26888] |
| ENSG00000185787 | HGLibA_29661 | 82 | -0.392975372 | MORF4L1 | mortality factor 4 like 1 [Source:HGNC Symbol;Acc:HGNC:16989] |
| ENSG00000185885 | HGLibA_22638 | 82 | -0.392975372 | IFITM1 | interferon induced transmembrane protein 1 [Source:HGNC Symbol;Acc:HGNC:5412] |
| ENSG00000186049 | HGLibA_25532 | 82 | -0.392975372 | KRT73 | keratin 73 [Source:HGNC Symbol;Acc:HGNC:28928] |
| ENSG00000186472 | HGLibA_35618 | 82 | -0.392975372 | PCLO | piccolo presynaptic cytomatrix protein [Source:HGNC Symbol;Acc:HGNC:13406] |
| ENSG00000186715 | HGLibA_30256 | 82 | -0.392975372 | MST1L | macrophage stimulating 1 like [Source:HGNC Symbol;Acc:HGNC:7390] |
| ENSG00000186815 | HGLibA_51104 | 82 | -0.392975372 | TPCN1 | two pore segment channel 1 [Source:HGNC Symbol;Acc:HGNC:18182] |
| ENSG00000188394 | HGLibA_19999 | 82 | -0.392975372 | GPR21 | G protein-coupled receptor 21 [Source:HGNC Symbol;Acc:HGNC:4476] |
| ENSG00000189046 | HGLibA_01649 | 82 | -0.392975372 | ALKBH2 | alpha-ketoglutarate dependent dioxygenase [Source:HGNC Symbol;Acc:HGNC:32487]" |
| ENSG00000196476 | HGLibA_06031 | 82 | -0.392975372 | C20orf96 | chromosome 20 open reading frame 96 [Source:HGNC Symbol;Acc:HGNC:16227] |
| ENSG00000196664 | HGLibA_49662 | 82 | -0.392975372 | TLR7 | toll like receptor 7 [Source:HGNC Symbol;Acc:HGNC:15631] |
| ENSG00000196782 | HGLibA_28050 | 82 | -0.392975372 | MAML3 | mastermind like transcriptional coactivator 3 [Source:HGNC Symbol;Acc:HGNC:16272] |
| ENSG00000197275 | HGLibA_40100 | 82 | -0.392975372 | RAD54B | RAD54 homolog B [Source:HGNC Symbol;Acc:HGNC:17228] |
| ENSG00000197776 | HGLibA_25091 | 82 | -0.392975372 | KLHDC1 | kelch domain containing 1 [Source:HGNC Symbol;Acc:HGNC:19836] |
| ENSG00000198818 | HGLibA_43752 | 82 | -0.392975372 | SFT2D1 | SFT2 domain containing 1 [Source:HGNC Symbol;Acc:HGNC:21102] |
| ENSG00000198961 | HGLibA_36785 | 82 | -0.392975372 | PJA2 | praja ring finger ubiquitin ligase 2 [Source:HGNC Symbol;Acc:HGNC:17481] |
| ENSG00000204539 | HGLibA_08887 | 82 | -0.392975372 | CDSN | corneodesmosin [Source:HGNC Symbol;Acc:HGNC:1802] |
| ENSG00000204941 | HGLibA_39050 | 82 | -0.392975372 | PSG5 | pregnancy specific beta-1-glycoprotein 5 [Source:HGNC Symbol;Acc:HGNC:9522] |
| ENSG00000204977 | HGLibA_51409 | 82 | -0.392975372 | TRIM13 | tripartite motif containing 13 [Source:HGNC Symbol;Acc:HGNC:9976] |
| ENSG00000205609 | HGLibA_14772 | 82 | -0.392975372 | EIF3CL | eukaryotic translation initiation factor 3 subunit C like [Source:HGNC Symbol;Acc:HGNC:26347] |
| ENSG00000205922 | HGLibA_33398 | 82 | -0.392975372 | ONECUT3 | one cut homeobox 3 [Source:HGNC Symbol;Acc:HGNC:13399] |
| ENSG00000206384 | HGLibA_10609 | 82 | -0.392975372 | COL6A6 | collagen type VI alpha 6 chain [Source:HGNC Symbol;Acc:HGNC:27023] |
| ENSG00000215472 | HGLibA_41884 | 82 | -0.392975372 | RPL17-C18orf32 | RPL17-C18orf32 readthrough [Source:HGNC Symbol;Acc:HGNC:44661] |
| ENSG00000226479 | HGLibA_50122 | 82 | -0.392975372 | TMEM185B | transmembrane protein 185B [Source:HGNC Symbol;Acc:HGNC:18896] |
| ENSG00000227471 | HGLibA_01478 | 82 | -0.392975372 | AKR1B15 | aldo-keto reductase family 1 member B15 [Source:HGNC Symbol;Acc:HGNC:37281] |
| ENSG00000241106 | HGLibA_21613 | 82 | -0.392975372 | HLA-DOB | DO beta [Source:HGNC Symbol;Acc:HGNC:4937]" |
| ENSG00000244362 | HGLibA_25681 | 82 | -0.392975372 | KRTAP19-7 | keratin associated protein 19-7 [Source:HGNC Symbol;Acc:HGNC:18942] |
| ENSG00000248905 | HGLibA_17688 | 82 | -0.392975372 | FMN1 | formin 1 [Source:HGNC Symbol;Acc:HGNC:3768] |
| ENSG00000250361 | HGLibA_20701 | 82 | -0.392975372 | GYPB | glycophorin B (MNS blood group) [Source:HGNC Symbol;Acc:HGNC:4703] |
| ENSG00000250510 | HGLibA_19959 | 82 | -0.392975372 | GPR162 | G protein-coupled receptor 162 [Source:HGNC Symbol;Acc:HGNC:16693] |
| ENSG00000250868 | HGLibA_54541 | 82 | -0.392975372 | XKRY | Y-linked [Source:HGNC Symbol;Acc:HGNC:18571]" |
| ENSG00000253958 | HGLibA_09886 | 82 | -0.392975372 | CLDN23 | claudin 23 [Source:HGNC Symbol;Acc:HGNC:17591] |
| ENSG00000254440 | HGLibA_35338 | 82 | -0.392975372 | PBOV1 | prostate and breast cancer overexpressed 1 [Source:HGNC Symbol;Acc:HGNC:21079] |
| ENSG00000254827 | HGLibA_44565 | 82 | -0.392975372 | SLC22A18AS | solute carrier family 22 member 18 antisense [Source:HGNC Symbol;Acc:HGNC:10965] |
| ENSG00000255582 | HGLibA_33489 | 82 | -0.392975372 | OR10G2 | olfactory receptor family 10 subfamily G member 2 [Source:HGNC Symbol;Acc:HGNC:8170] |
| ENSG00000270231 | HGLibA_31241 | 82 | -0.392975372 | NBPF8 | NBPF member 8 [Source:HGNC Symbol;Acc:HGNC:31990] |
| ENSG00000273611 | HGLibA_56930 | 82 | -0.392975372 | ZNHIT3 | zinc finger HIT-type containing 3 [Source:HGNC Symbol;Acc:HGNC:12309] |
| FAM101A | HGLibA_15972 | 82 | -0.392975372 | FAM101A | - |
| GPR128 | HGLibA_19885 | 82 | -0.392975372 | GPR128 | - |
| HGC6.3 | HGLibA_21234 | 82 | -0.392975372 | HGC6.3 | - |
| HKR1 | HGLibA_21590 | 82 | -0.392975372 | HKR1 | - |
| hsa-mir-106a | HGLibA_57187 | 82 | -0.392975372 | hsa-mir-106a | - |
| hsa-mir-1200 | HGLibA_57260 | 82 | -0.392975372 | hsa-mir-1200 | - |
| hsa-mir-124-3 | HGLibA_57356 | 82 | -0.392975372 | hsa-mir-124-3 | - |
| hsa-mir-1293 | HGLibA_57647 | 82 | -0.392975372 | hsa-mir-1293 | - |
| hsa-mir-3171 | HGLibA_58762 | 82 | -0.392975372 | hsa-mir-3171 | - |
| hsa-mir-3621 | HGLibA_59118 | 82 | -0.392975372 | hsa-mir-3621 | - |
| hsa-mir-3914-1 | HGLibA_59528 | 82 | -0.392975372 | hsa-mir-3914-1 | - |
| hsa-mir-4535 | HGLibA_60598 | 82 | -0.392975372 | hsa-mir-4535 | - |
| hsa-mir-4725 | HGLibA_60999 | 82 | -0.392975372 | hsa-mir-4725 | - |
| hsa-mir-4733 | HGLibA_61029 | 82 | -0.392975372 | hsa-mir-4733 | - |
| hsa-mir-4795 | HGLibA_61286 | 82 | -0.392975372 | hsa-mir-4795 | - |
| hsa-mir-514b | HGLibA_61598 | 82 | -0.392975372 | hsa-mir-514b | - |
| hsa-mir-555 | HGLibA_62135 | 82 | -0.392975372 | hsa-mir-555 | - |
| hsa-mir-569 | HGLibA_62274 | 82 | -0.392975372 | hsa-mir-569 | - |
| hsa-mir-6086 | HGLibA_62603 | 82 | -0.392975372 | hsa-mir-6086 | - |
| hsa-mir-640 | HGLibA_62798 | 82 | -0.392975372 | hsa-mir-640 | - |
| hsa-mir-659 | HGLibA_62968 | 82 | -0.392975372 | hsa-mir-659 | - |
| hsa-mir-7109 | HGLibA_63797 | 82 | -0.392975372 | hsa-mir-7109 | - |
| hsa-mir-8063 | HGLibA_64092 | 82 | -0.392975372 | hsa-mir-8063 | - |
| hsa-mir-8064 | HGLibA_64094 | 82 | -0.392975372 | hsa-mir-8064 | - |
| hsa-mir-8070 | HGLibA_64120 | 82 | -0.392975372 | hsa-mir-8070 | - |
| hsa-mir-92a-2 | HGLibA_64293 | 82 | -0.392975372 | hsa-mir-92a-2 | - |
| LOC730183 | HGLibA_27005 | 82 | -0.392975372 | LOC730183 | - |
| NARR | HGLibA_31153 | 82 | -0.392975372 | NARR | - |
| NUPR1L | HGLibA_33090 | 82 | -0.392975372 | NUPR1L | - |
| TMEM99 | HGLibA_50572 | 82 | -0.392975372 | TMEM99 | - |
| AIM1 | HGLibA_01349 | 81 | -0.402020715 | AIM1 | - |
| C10orf113 | HGLibA_05035 | 81 | -0.402020715 | C10orf113 | - |
| C12orf79 | HGLibA_05306 | 81 | -0.402020715 | C12orf79 | - |
| C1orf229 | HGLibA_05842 | 81 | -0.402020715 | C1orf229 | - |
| CCBL1 | HGLibA_07497 | 81 | -0.402020715 | CCBL1 | - |
| CRAMP1L | HGLibA_11062 | 81 | -0.402020715 | CRAMP1L | - |
| ENSG00000000457 | HGLibA_43091 | 81 | -0.402020715 | SCYL3 | SCY1 like pseudokinase 3 [Source:HGNC Symbol;Acc:HGNC:19285] |
| ENSG00000001617 | HGLibA_43335 | 81 | -0.402020715 | SEMA3F | semaphorin 3F [Source:HGNC Symbol;Acc:HGNC:10728] |
| ENSG00000006125 | HGLibA_02310 | 81 | -0.402020715 | AP2B1 | adaptor related protein complex 2 subunit beta 1 [Source:HGNC Symbol;Acc:HGNC:563] |
| ENSG00000006283 | HGLibA_06892 | 81 | -0.402020715 | CACNA1G | calcium voltage-gated channel subunit alpha1 G [Source:HGNC Symbol;Acc:HGNC:1394] |
| ENSG00000007202 | HGLibA_24588 | 81 | -0.402020715 | KIAA0100 | KIAA0100 [Source:HGNC Symbol;Acc:HGNC:28960] |
| ENSG00000010072 | HGLibA_46832 | 81 | -0.402020715 | SPRTN | SprT-like N-terminal domain [Source:HGNC Symbol;Acc:HGNC:25356] |
| ENSG00000010282 | HGLibA_21255 | 81 | -0.402020715 | HHATL | hedgehog acyltransferase like [Source:HGNC Symbol;Acc:HGNC:13242] |
| ENSG00000013016 | HGLibA_14671 | 81 | -0.402020715 | EHD3 | EH domain containing 3 [Source:HGNC Symbol;Acc:HGNC:3244] |
| ENSG00000027644 | HGLibA_23403 | 81 | -0.402020715 | INSRR | insulin receptor related receptor [Source:HGNC Symbol;Acc:HGNC:6093] |
| ENSG00000028528 | HGLibA_46023 | 81 | -0.402020715 | SNX1 | sorting nexin 1 [Source:HGNC Symbol;Acc:HGNC:11172] |
| ENSG00000053702 | HGLibA_32694 | 81 | -0.402020715 | NRIP2 | nuclear receptor interacting protein 2 [Source:HGNC Symbol;Acc:HGNC:23078] |
| ENSG00000058668 | HGLibA_03622 | 81 | -0.402020715 | ATP2B4 | ATPase plasma membrane Ca2+ transporting 4 [Source:HGNC Symbol;Acc:HGNC:817] |
| ENSG00000070778 | HGLibA_39456 | 81 | -0.402020715 | PTPN21 | non-receptor type 21 [Source:HGNC Symbol;Acc:HGNC:9651]" |

| | | | | | |
|---|---|---|---|---|---|
| ENSG00000072803 | HGLibA_17139 | 81 | -0.402020715 | FBXW11 | F-box and WD repeat domain containing 11 [Source:HGNC Symbol;Acc:HGNC:13607] |
| ENSG00000073756 | HGLibA_39360 | 81 | -0.402020715 | PTGS2 | prostaglandin-endoperoxide synthase 2 [Source:HGNC Symbol;Acc:HGNC:9605] |
| ENSG00000076356 | HGLibA_37262 | 81 | -0.402020715 | PLXNA2 | plexin A2 [Source:HGNC Symbol;Acc:HGNC:9100] |
| ENSG00000081189 | HGLibA_28839 | 81 | -0.402020715 | MEF2C | myocyte enhancer factor 2C [Source:HGNC Symbol;Acc:HGNC:6996] |
| ENSG00000082497 | HGLibA_43614 | 81 | -0.402020715 | SERTAD4 | SERTA domain containing 4 [Source:HGNC Symbol;Acc:HGNC:25236] |
| ENSG00000083307 | HGLibA_20252 | 81 | -0.402020715 | GRHL2 | grainyhead like transcription factor 2 [Source:HGNC Symbol;Acc:HGNC:2799] |
| ENSG00000083828 | HGLibA_56326 | 81 | -0.402020715 | ZNF586 | zinc finger protein 586 [Source:HGNC Symbol;Acc:HGNC:25949] |
| ENSG00000085741 | HGLibA_54353 | 81 | -0.402020715 | WNT11 | Wnt family member 11 [Source:HGNC Symbol;Acc:HGNC:12776] |
| ENSG00000088325 | HGLibA_51218 | 81 | -0.402020715 | TPX2 | microtubule nucleation factor [Source:HGNC Symbol;Acc:HGNC:1249]" |
| ENSG00000088726 | HGLibA_50389 | 81 | -0.402020715 | TMEM40 | transmembrane protein 40 [Source:HGNC Symbol;Acc:HGNC:25620] |
| ENSG00000091138 | HGLibA_44792 | 81 | -0.402020715 | SLC26A3 | solute carrier family 26 member 3 [Source:HGNC Symbol;Acc:HGNC:3018] |
| ENSG00000092108 | HGLibA_42893 | 81 | -0.402020715 | SCFD1 | sec1 family domain containing 1 [Source:HGNC Symbol;Acc:HGNC:20726] |
| ENSG00000092445 | HGLibA_52469 | 81 | -0.402020715 | TYRO3 | TYRO3 protein tyrosine kinase [Source:HGNC Symbol;Acc:HGNC:12446] |
| ENSG00000095917 | HGLibA_51197 | 81 | -0.402020715 | TPSD1 | tryptase delta 1 [Source:HGNC Symbol;Acc:HGNC:14118] |
| ENSG00000100147 | HGLibA_07599 | 81 | -0.402020715 | CCDC134 | coiled-coil domain containing 134 [Source:HGNC Symbol;Acc:HGNC:26185] |
| ENSG00000100321 | HGLibA_47990 | 81 | -0.402020715 | SYNGR1 | synaptogyrin 1 [Source:HGNC Symbol;Acc:HGNC:11498] |
| ENSG00000100652 | HGLibA_44330 | 81 | -0.402020715 | SLC10A1 | solute carrier family 10 member 1 [Source:HGNC Symbol;Acc:HGNC:10905] |
| ENSG00000100911 | HGLibA_39193 | 81 | -0.402020715 | PSME2 | proteasome activator subunit 2 [Source:HGNC Symbol;Acc:HGNC:9569] |
| ENSG00000101670 | HGLibA_26613 | 81 | -0.402020715 | LIPG | endothelial type [Source:HGNC Symbol;Acc:HGNC:6623]" |
| ENSG00000102057 | HGLibA_24175 | 81 | -0.402020715 | KCND1 | potassium voltage-gated channel subfamily D member 1 [Source:HGNC Symbol;Acc:HGNC:6237] |
| ENSG00000102967 | HGLibA_13113 | 81 | -0.402020715 | DHODH | dihydroorotate dehydrogenase (quinone) [Source:HGNC Symbol;Acc:HGNC:2867] |
| ENSG00000103355 | HGLibA_38927 | 81 | -0.402020715 | PRSS33 | serine protease 33 [Source:HGNC Symbol;Acc:HGNC:30405] |
| ENSG00000103485 | HGLibA_39733 | 81 | -0.402020715 | QPRT | quinolinate phosphoribosyltransferase [Source:HGNC Symbol;Acc:HGNC:9755] |
| ENSG00000104154 | HGLibA_44908 | 81 | -0.402020715 | SLC30A4 | solute carrier family 30 member 4 [Source:HGNC Symbol;Acc:HGNC:11015] |
| ENSG00000104213 | HGLibA_35858 | 81 | -0.402020715 | PDGFRL | platelet derived growth factor receptor like [Source:HGNC Symbol;Acc:HGNC:8805] |
| ENSG00000104343 | HGLibA_52672 | 81 | -0.402020715 | UBE2W | ubiquitin conjugating enzyme E2 W [Source:HGNC Symbol;Acc:HGNC:25616] |
| ENSG00000104356 | HGLibA_37688 | 81 | -0.402020715 | POP1 | ribonuclease P/MRP subunit [Source:HGNC Symbol;Acc:HGNC:30129]" |
| ENSG00000104824 | HGLibA_21834 | 81 | -0.402020715 | HNRNPL | heterogeneous nuclear ribonucleoprotein L [Source:HGNC Symbol;Acc:HGNC:5045] |
| ENSG00000105559 | HGLibA_37071 | 81 | -0.402020715 | PLEKHA4 | pleckstrin homology domain containing A4 [Source:HGNC Symbol;Acc:HGNC:14339] |
| ENSG00000105650 | HGLibA_35792 | 81 | -0.402020715 | PDE4C | phosphodiesterase 4C [Source:HGNC Symbol;Acc:HGNC:8782] |
| ENSG00000108242 | HGLibA_12116 | 81 | -0.402020715 | CYP2C18 | cytochrome P450 family 2 subfamily C member 18 [Source:HGNC Symbol;Acc:HGNC:2620] |
| ENSG00000108417 | HGLibA_25496 | 81 | -0.402020715 | KRT37 | keratin 37 [Source:HGNC Symbol;Acc:HGNC:6455] |
| ENSG00000108556 | HGLibA_09605 | 81 | -0.402020715 | CHRNE | cholinergic receptor nicotinic epsilon subunit [Source:HGNC Symbol;Acc:HGNC:1966] |
| ENSG00000108813 | HGLibA_13381 | 81 | -0.402020715 | DLX4 | distal-less homeobox 4 [Source:HGNC Symbol;Acc:HGNC:2917] |
| ENSG00000108950 | HGLibA_16445 | 81 | -0.402020715 | FAM20A | golgi associated secretory pathway pseudokinase [Source:HGNC Symbol;Acc:HGNC:23015]" |
| ENSG00000110900 | HGLibA_51895 | 81 | -0.402020715 | TSPAN11 | tetraspanin 11 [Source:HGNC Symbol;Acc:HGNC:30795] |
| ENSG00000112249 | HGLibA_03263 | 81 | -0.402020715 | ASCC3 | activating signal cointegrator 1 complex subunit 3 [Source:HGNC Symbol;Acc:HGNC:18697] |
| ENSG00000112305 | HGLibA_45595 | 81 | -0.402020715 | SMAP1 | small ArfGAP 1 [Source:HGNC Symbol;Acc:HGNC:19651] |
| ENSG00000112562 | HGLibA_45796 | 81 | -0.402020715 | SMOC2 | SPARC related modular calcium binding 2 [Source:HGNC Symbol;Acc:HGNC:20323] |
| ENSG00000113296 | HGLibA_49332 | 81 | -0.402020715 | THBS4 | thrombospondin 4 [Source:HGNC Symbol;Acc:HGNC:11788] |
| ENSG00000114626 | HGLibA_00334 | 81 | -0.402020715 | ABTB1 | ankyrin repeat and BTB domain containing 1 [Source:HGNC Symbol;Acc:HGNC:18275] |
| ENSG00000115107 | HGLibA_47428 | 81 | -0.402020715 | STEAP3 | STEAP3 metalloreductase [Source:HGNC Symbol;Acc:HGNC:24592] |
| ENSG00000115556 | HGLibA_36996 | 81 | -0.402020715 | PLCD4 | phospholipase C delta 4 [Source:HGNC Symbol;Acc:HGNC:9062] |
| ENSG00000116785 | HGLibA_09268 | 81 | -0.402020715 | CFHR3 | complement factor H related 3 [Source:HGNC Symbol;Acc:HGNC:16980] |
| ENSG00000116996 | HGLibA_56958 | 81 | -0.402020715 | ZP4 | zona pellucida glycoprotein 4 [Source:HGNC Symbol;Acc:HGNC:15770] |
| ENSG00000117152 | HGLibA_41093 | 81 | -0.402020715 | RGS4 | regulator of G protein signaling 4 [Source:HGNC Symbol;Acc:HGNC:10000] |
| ENSG00000119285 | HGLibA_21066 | 81 | -0.402020715 | HEATR1 | HEAT repeat containing 1 [Source:HGNC Symbol;Acc:HGNC:25517] |
| ENSG00000119899 | HGLibA_44470 | 81 | -0.402020715 | SLC17A5 | solute carrier family 17 member 5 [Source:HGNC Symbol;Acc:HGNC:10933] |
| ENSG00000120802 | HGLibA_50606 | 81 | -0.402020715 | TMPO | thymopoietin [Source:HGNC Symbol;Acc:HGNC:11875] |
| ENSG00000121879 | HGLibA_36633 | 81 | -0.402020715 | PIK3CA | 5-bisphosphate 3-kinase catalytic subunit alpha [Source:HGNC Symbol;Acc:HGNC:8975]" |
| ENSG00000122779 | HGLibA_51437 | 81 | -0.402020715 | TRIM24 | tripartite motif containing 24 [Source:HGNC Symbol;Acc:HGNC:11812] |
| ENSG00000123106 | HGLibA_07925 | 81 | -0.402020715 | CCDC91 | coiled-coil domain containing 91 [Source:HGNC Symbol;Acc:HGNC:24855] |
| ENSG00000124191 | HGLibA_51040 | 81 | -0.402020715 | TOX2 | TOX high mobility group box family member 2 [Source:HGNC Symbol;Acc:HGNC:16095] |
| ENSG00000125991 | HGLibA_15473 | 81 | -0.402020715 | ERGIC3 | ERGIC and golgi 3 [Source:HGNC Symbol;Acc:HGNC:15927] |
| ENSG00000126524 | HGLibA_42792 | 81 | -0.402020715 | SBDS | ribosome maturation factor [Source:HGNC Symbol;Acc:HGNC:19440]" |
| ENSG00000128285 | HGLibA_28589 | 81 | -0.402020715 | MCHR1 | melanin concentrating hormone receptor 1 [Source:HGNC Symbol;Acc:HGNC:4479] |
| ENSG00000129071 | HGLibA_28487 | 81 | -0.402020715 | MBD4 | DNA glycosylase [Source:HGNC Symbol;Acc:HGNC:6919]" |
| ENSG00000129654 | HGLibA_17886 | 81 | -0.402020715 | FOXJ1 | forkhead box J1 [Source:HGNC Symbol;Acc:HGNC:3816] |
| ENSG00000129673 | HGLibA_00058 | 81 | -0.402020715 | AANAT | aralkylamine N-acetyltransferase [Source:HGNC Symbol;Acc:HGNC:19] |
| ENSG00000130544 | HGLibA_56229 | 81 | -0.402020715 | ZNF557 | zinc finger protein 557 [Source:HGNC Symbol;Acc:HGNC:28632] |
| ENSG00000130751 | HGLibA_32394 | 81 | -0.402020715 | NPAS1 | neuronal PAS domain protein 1 [Source:HGNC Symbol;Acc:HGNC:7894] |
| ENSG00000131080 | HGLibA_14433 | 81 | -0.402020715 | EDA2R | ectodysplasin A2 receptor [Source:HGNC Symbol;Acc:HGNC:17756] |
| ENSG00000131943 | HGLibA_05630 | 81 | -0.402020715 | C19orf12 | chromosome 19 open reading frame 12 [Source:HGNC Symbol;Acc:HGNC:25443] |
| ENSG00000132330 | HGLibA_42952 | 81 | -0.402020715 | SCLY | selenocysteine lyase [Source:HGNC Symbol;Acc:HGNC:18161] |
| ENSG00000134240 | HGLibA_21713 | 81 | -0.402020715 | HMGCS2 | 3-hydroxy-3-methylglutaryl-CoA synthase 2 [Source:HGNC Symbol;Acc:HGNC:5008] |
| ENSG00000134627 | HGLibA_36780 | 81 | -0.402020715 | PIWIL4 | piwi like RNA-mediated gene silencing 4 [Source:HGNC Symbol;Acc:HGNC:18444] |
| ENSG00000134852 | HGLibA_10093 | 81 | -0.402020715 | CLOCK | clock circadian regulator [Source:HGNC Symbol;Acc:HGNC:2082] |
| ENSG00000135269 | HGLibA_49023 | 81 | -0.402020715 | TES | testin LIM domain protein [Source:HGNC Symbol;Acc:HGNC:14620] |
| ENSG00000135823 | HGLibA_47653 | 81 | -0.402020715 | STX6 | syntaxin 6 [Source:HGNC Symbol;Acc:HGNC:11441] |
| ENSG00000136573 | HGLibA_04544 | 81 | -0.402020715 | BLK | Src family tyrosine kinase [Source:HGNC Symbol;Acc:HGNC:1057]" |
| ENSG00000136709 | HGLibA_54067 | 81 | -0.402020715 | WDR33 | WD repeat domain 33 [Source:HGNC Symbol;Acc:HGNC:25651] |
| ENSG00000136839 | HGLibA_33611 | 81 | -0.402020715 | OR13C9 | olfactory receptor family 13 subfamily C member 9 [Source:HGNC Symbol;Acc:HGNC:15104] |
| ENSG00000137070 | HGLibA_22955 | 81 | -0.402020715 | IL11RA | interleukin 11 receptor subunit alpha [Source:HGNC Symbol;Acc:HGNC:5967] |
| ENSG00000137817 | HGLibA_35240 | 81 | -0.402020715 | PARP6 | poly(ADP-ribose) polymerase family member 6 [Source:HGNC Symbol;Acc:HGNC:26921] |
| ENSG00000138639 | HGLibA_02734 | 81 | -0.402020715 | ARHGAP24 | Rho GTPase activating protein 24 [Source:HGNC Symbol;Acc:HGNC:25361] |
| ENSG00000138964 | HGLibA_35262 | 81 | -0.402020715 | PARVG | parvin gamma [Source:HGNC Symbol;Acc:HGNC:14654] |
| ENSG00000139233 | HGLibA_26653 | 81 | -0.402020715 | LLPH | long-term synaptic facilitation factor [Source:HGNC Symbol;Acc:HGNC:28229]" |
| ENSG00000139372 | HGLibA_48882 | 81 | -0.402020715 | TDG | thymine DNA glycosylase [Source:HGNC Symbol;Acc:HGNC:11700] |
| ENSG00000139921 | HGLibA_50689 | 81 | -0.402020715 | TMX1 | thioredoxin related transmembrane protein 1 [Source:HGNC Symbol;Acc:HGNC:15487] |
| ENSG00000140287 | HGLibA_21030 | 81 | -0.402020715 | HDC | histidine decarboxylase [Source:HGNC Symbol;Acc:HGNC:4855] |
| ENSG00000141198 | HGLibA_50950 | 81 | -0.402020715 | TOM1L1 | target of myb1 like 1 membrane trafficking protein [Source:HGNC Symbol;Acc:HGNC:11983] |
| ENSG00000141448 | HGLibA_18681 | 81 | -0.402020715 | GATA6 | GATA binding protein 6 [Source:HGNC Symbol;Acc:HGNC:4174] |
| ENSG00000141644 | HGLibA_28464 | 81 | -0.402020715 | MBD1 | methyl-CpG binding domain protein 1 [Source:HGNC Symbol;Acc:HGNC:6916] |
| ENSG00000142546 | HGLibA_32333 | 81 | -0.402020715 | NOSIP | nitric oxide synthase interacting protein [Source:HGNC Symbol;Acc:HGNC:17946] |
| ENSG00000142686 | HGLibA_05832 | 81 | -0.402020715 | C1orf216 | chromosome 1 open reading frame 216 [Source:HGNC Symbol;Acc:HGNC:26800] |
| ENSG00000142687 | HGLibA_24624 | 81 | -0.402020715 | KIAA0319L | KIAA0319 like [Source:HGNC Symbol;Acc:HGNC:30071] |
| ENSG00000144895 | HGLibA_14719 | 81 | -0.402020715 | EIF2A | eukaryotic translation initiation factor 2A [Source:HGNC Symbol;Acc:HGNC:3254] |
| ENSG00000147381 | HGLibA_27914 | 81 | -0.402020715 | MAGEA4 | MAGE family member A4 [Source:HGNC Symbol;Acc:HGNC:6802] |
| ENSG00000147439 | HGLibA_04512 | 81 | -0.402020715 | BIN3 | bridging integrator 3 [Source:HGNC Symbol;Acc:HGNC:1054] |
| ENSG00000148730 | HGLibA_14832 | 81 | -0.402020715 | EIF4EBP2 | eukaryotic translation initiation factor 4E binding protein 2 [Source:HGNC Symbol;Acc:HGNC:3289] |
| ENSG00000148773 | HGLibA_29325 | 81 | -0.402020715 | MKI67 | marker of proliferation Ki-67 [Source:HGNC Symbol;Acc:HGNC:7107] |
| ENSG00000148848 | HGLibA_00735 | 81 | -0.402020715 | ADAM12 | ADAM metallopeptidase domain 12 [Source:HGNC Symbol;Acc:HGNC:190] |
| ENSG00000150093 | HGLibA_23768 | 81 | -0.402020715 | ITGB1 | integrin subunit beta 1 [Source:HGNC Symbol;Acc:HGNC:6153] |
| ENSG00000150261 | HGLibA_34544 | 81 | -0.402020715 | OR8K1 | olfactory receptor family 8 subfamily K member 1 [Source:HGNC Symbol;Acc:HGNC:14831] |
| ENSG00000150316 | HGLibA_11813 | 81 | -0.402020715 | CWC15 | CWC15 spliceosome associated protein homolog [Source:HGNC Symbol;Acc:HGNC:26939] |
| ENSG00000152315 | HGLibA_24303 | 81 | -0.402020715 | KCNK13 | potassium two pore domain channel subfamily K member 13 [Source:HGNC Symbol;Acc:HGNC:6275] |
| ENSG00000152683 | HGLibA_44915 | 81 | -0.402020715 | SLC30A6 | solute carrier family 30 member 6 [Source:HGNC Symbol;Acc:HGNC:19305] |
| ENSG00000152939 | HGLibA_28383 | 81 | -0.402020715 | MARVELD2 | MARVEL domain containing 2 [Source:HGNC Symbol;Acc:HGNC:26401] |
| ENSG00000153404 | HGLibA_37112 | 81 | -0.402020715 | PLEKHG4B | pleckstrin homology and RhoGEF domain containing G4B [Source:HGNC Symbol;Acc:HGNC:29399] |
| ENSG00000154162 | HGLibA_08637 | 81 | -0.402020715 | CDH12 | cadherin 12 [Source:HGNC Symbol;Acc:HGNC:1751] |
| ENSG00000156395 | HGLibA_46189 | 81 | -0.402020715 | SORCS3 | sortilin related VPS10 domain containing receptor 3 [Source:HGNC Symbol;Acc:HGNC:16699] |
| ENSG00000157557 | HGLibA_15645 | 81 | -0.402020715 | ETS2 | transcription factor [Source:HGNC Symbol;Acc:HGNC:3489]" |
| ENSG00000159063 | HGLibA_01637 | 81 | -0.402020715 | ALG8 | 3-glucosyltransferase [Source:HGNC Symbol;Acc:HGNC:23161]" |
| ENSG00000160789 | HGLibA_26694 | 81 | -0.402020715 | LMNA | lamin A/C [Source:HGNC Symbol;Acc:HGNC:6636] |
| ENSG00000160949 | HGLibA_50986 | 81 | -0.402020715 | TONSL | DNA repair protein [Source:HGNC Symbol;Acc:HGNC:7801]" |
| ENSG00000161036 | HGLibA_27510 | 81 | -0.402020715 | LRWD1 | leucine rich repeats and WD repeat domain containing 1 [Source:HGNC Symbol;Acc:HGNC:21769] |
| ENSG00000161381 | HGLibA_37253 | 81 | -0.402020715 | PLXDC1 | plexin domain containing 1 [Source:HGNC Symbol;Acc:HGNC:20945] |
| ENSG00000162188 | HGLibA_19524 | 81 | -0.402020715 | GNG3 | G protein subunit gamma 3 [Source:HGNC Symbol;Acc:HGNC:4405] |
| ENSG00000162592 | HGLibA_07743 | 81 | -0.402020715 | CCDC27 | coiled-coil domain containing 27 [Source:HGNC Symbol;Acc:HGNC:26546] |
| ENSG00000162694 | HGLibA_15810 | 81 | -0.402020715 | EXTL2 | exostosin like glycosyltransferase 2 [Source:HGNC Symbol;Acc:HGNC:3516] |
| ENSG00000162727 | HGLibA_33842 | 81 | -0.402020715 | OR2M5 | olfactory receptor family 2 subfamily M member 5 [Source:HGNC Symbol;Acc:HGNC:19576] |
| ENSG00000163032 | HGLibA_53853 | 81 | -0.402020715 | VSNL1 | visinin like 1 [Source:HGNC Symbol;Acc:HGNC:12722] |
| ENSG00000163141 | HGLibA_04661 | 81 | -0.402020715 | BNIPL | BCL2 interacting protein like [Source:HGNC Symbol;Acc:HGNC:16976] |
| ENSG00000163346 | HGLibA_35355 | 81 | -0.402020715 | PBXIP1 | PBX homeobox interacting protein 1 [Source:HGNC Symbol;Acc:HGNC:21199] |
| ENSG00000164066 | HGLibA_23439 | 81 | -0.402020715 | INTU | inturned planar cell polarity protein [Source:HGNC Symbol;Acc:HGNC:29239] |
| ENSG00000164776 | HGLibA_36412 | 81 | -0.402020715 | PHKG1 | phosphorylase kinase catalytic subunit gamma 1 [Source:HGNC Symbol;Acc:HGNC:8930] |
| ENSG00000165304 | HGLibA_28879 | 81 | -0.402020715 | MELK | maternal embryonic leucine zipper kinase [Source:HGNC Symbol;Acc:HGNC:16870] |
| ENSG00000166049 | HGLibA_35265 | 81 | -0.402020715 | PASD1 | PAS domain containing repressor 1 [Source:HGNC Symbol;Acc:HGNC:20686] |
| ENSG00000166927 | HGLibA_30164 | 81 | -0.402020715 | MS4A7 | membrane spanning 4-domains A7 [Source:HGNC Symbol;Acc:HGNC:13378] |
| ENSG00000166987 | HGLibA_28494 | 81 | -0.402020715 | MBD6 | methyl-CpG binding domain protein 6 [Source:HGNC Symbol;Acc:HGNC:20445] |
| ENSG00000167377 | HGLibA_55632 | 81 | -0.402020715 | ZNF23 | zinc finger protein 23 [Source:HGNC Symbol;Acc:HGNC:13023] |
| ENSG00000167755 | HGLibA_25264 | 81 | -0.402020715 | KLK6 | kallikrein related peptidase 6 [Source:HGNC Symbol;Acc:HGNC:6367] |
| ENSG00000168418 | HGLibA_24207 | 81 | -0.402020715 | KCNG4 | potassium voltage-gated channel modifier subfamily G member 4 [Source:HGNC Symbol;Acc:HGNC:19697] |
| ENSG00000168496 | HGLibA_17282 | 81 | -0.402020715 | FEN1 | flap structure-specific endonuclease 1 [Source:HGNC Symbol;Acc:HGNC:3650] |
| ENSG00000168661 | HGLibA_55767 | 81 | -0.402020715 | ZNF30 | zinc finger protein 30 [Source:HGNC Symbol;Acc:HGNC:13090] |
| ENSG00000168765 | HGLibA_20482 | 81 | -0.402020715 | GSTM4 | glutathione S-transferase mu 4 [Source:HGNC Symbol;Acc:HGNC:4636] |
| ENSG00000168925 | HGLibA_11677 | 81 | -0.402020715 | CTRB1 | chymotrypsinogen B1 [Source:HGNC Symbol;Acc:HGNC:2521] |
| ENSG00000169045 | HGLibA_21823 | 81 | -0.402020715 | HNRNPH1 | heterogeneous nuclear ribonucleoprotein H1 [Source:HGNC Symbol;Acc:HGNC:5041] |
| ENSG00000169499 | HGLibA_37064 | 81 | -0.402020715 | PLEKHA2 | pleckstrin homology domain containing A2 [Source:HGNC Symbol;Acc:HGNC:14336] |
| ENSG00000172380 | HGLibA_19513 | 81 | -0.402020715 | GNG12 | G protein subunit gamma 12 [Source:HGNC Symbol;Acc:HGNC:19663] |
| ENSG00000172586 | HGLibA_09360 | 81 | -0.402020715 | CHCHD1 | coiled-coil-helix-coiled-coil-helix domain containing 1 [Source:HGNC Symbol;Acc:HGNC:23518] |
| ENSG00000172803 | HGLibA_46091 | 81 | -0.402020715 | SNX32 | sorting nexin 32 [Source:HGNC Symbol;Acc:HGNC:26423] |
| ENSG00000173578 | HGLibA_54500 | 81 | -0.402020715 | XCR1 | X-C motif chemokine receptor 1 [Source:HGNC Symbol;Acc:HGNC:1625] |
| ENSG00000173890 | HGLibA_19953 | 81 | -0.402020715 | GPR160 | G protein-coupled receptor 160 [Source:HGNC Symbol;Acc:HGNC:23693] |
| ENSG00000176244 | HGLibA_00410 | 81 | -0.402020715 | ACBD7 | acyl-CoA binding domain containing 7 [Source:HGNC Symbol;Acc:HGNC:17715] |

| | | | | | |
|---|---|---|---|---|---|
| ENSG00000176269 | HGLibA_33997 | 81 | -0.402020715 | OR4F21 | olfactory receptor family 4 subfamily F member 21 [Source:HGNC Symbol;Acc:HGNC:19583] |
| ENSG00000176909 | HGLibA_28054 | 81 | -0.402020715 | MAMSTR | MEF2 activating motif and SAP domain containing transcriptional regulator [Source:HGNC Symbol;Acc:HGNC:26689] |
| ENSG00000177058 | HGLibA_45084 | 81 | -0.402020715 | SLC38A9 | solute carrier family 38 member 9 [Source:HGNC Symbol;Acc:HGNC:26907] |
| ENSG00000178974 | HGLibA_17079 | 81 | -0.402020715 | FBXO34 | F-box protein 34 [Source:HGNC Symbol;Acc:HGNC:20201] |
| ENSG00000179008 | HGLibA_05365 | 81 | -0.402020715 | C14orf39 | chromosome 14 open reading frame 39 [Source:HGNC Symbol;Acc:HGNC:19849] |
| ENSG00000179165 | HGLibA_39667 | 81 | -0.402020715 | PXT1 | peroxisomal testis enriched protein 1 [Source:HGNC Symbol;Acc:HGNC:18312] |
| ENSG00000180884 | HGLibA_56770 | 81 | -0.402020715 | ZNF792 | zinc finger protein 792 [Source:HGNC Symbol;Acc:HGNC:24751] |
| ENSG00000181867 | HGLibA_18128 | 81 | -0.402020715 | FTMT | ferritin mitochondrial [Source:HGNC Symbol;Acc:HGNC:17345] |
| ENSG00000182156 | HGLibA_15185 | 81 | -0.402020715 | ENPP7 | ectonucleotide pyrophosphatase/phosphodiesterase 7 [Source:HGNC Symbol;Acc:HGNC:23764] |
| ENSG00000182324 | HGLibA_24260 | 81 | -0.402020715 | KCNJ14 | potassium voltage-gated channel subfamily J member 14 [Source:HGNC Symbol;Acc:HGNC:6260] |
| ENSG00000182481 | HGLibA_25358 | 81 | -0.402020715 | KPNA2 | karyopherin subunit alpha 2 [Source:HGNC Symbol;Acc:HGNC:6395] |
| ENSG00000183318 | HGLibA_46595 | 81 | -0.402020715 | SPDYE4 | speedy/RINGO cell cycle regulator family member E4 [Source:HGNC Symbol;Acc:HGNC:35463] |
| ENSG00000184481 | HGLibA_17928 | 81 | -0.402020715 | FOXO4 | forkhead box O4 [Source:HGNC Symbol;Acc:HGNC:7139] |
| ENSG00000184515 | HGLibA_04448 | 81 | -0.402020715 | BEX5 | brain expressed X-linked 5 [Source:HGNC Symbol;Acc:HGNC:27990] |
| ENSG00000185010 | HGLibA_15883 | 81 | -0.402020715 | F8 | coagulation factor VIII [Source:HGNC Symbol;Acc:HGNC:3546] |
| ENSG00000185527 | HGLibA_35816 | 81 | -0.402020715 | PDE6G | phosphodiesterase 6G [Source:HGNC Symbol;Acc:HGNC:8789] |
| ENSG00000185894 | HGLibA_04748 | 81 | -0.402020715 | BPY2C | basic charge Y-linked 2C [Source:HGNC Symbol;Acc:HGNC:18225] |
| ENSG00000188760 | HGLibA_50162 | 81 | -0.402020715 | TMEM198 | transmembrane protein 198 [Source:HGNC Symbol;Acc:HGNC:33704] |
| ENSG00000188976 | HGLibA_32235 | 81 | -0.402020715 | NOC2L | NOC2 like nucleolar associated transcriptional repressor [Source:HGNC Symbol;Acc:HGNC:24517] |
| ENSG00000196098 | HGLibA_34318 | 81 | -0.402020715 | OR5K4 | olfactory receptor family 5 subfamily K member 4 [Source:HGNC Symbol;Acc:HGNC:31291] |
| ENSG00000196388 | HGLibA_23273 | 81 | -0.402020715 | INCA1 | cyclin A1 interacting protein 1 [Source:HGNC Symbol;Acc:HGNC:32224]" |
| ENSG00000197635 | HGLibA_13873 | 81 | -0.402020715 | DPP4 | dipeptidyl peptidase 4 [Source:HGNC Symbol;Acc:HGNC:3009] |
| ENSG00000197714 | HGLibA_56035 | 81 | -0.402020715 | ZNF460 | zinc finger protein 460 [Source:HGNC Symbol;Acc:HGNC:21628] |
| ENSG00000197860 | HGLibA_43872 | 81 | -0.402020715 | SGTB | small glutamine rich tetratricopeptide repeat containing beta [Source:HGNC Symbol;Acc:HGNC:23567] |
| ENSG00000198157 | HGLibA_21728 | 81 | -0.402020715 | HMGN5 | high mobility group nucleosome binding domain 5 [Source:HGNC Symbol;Acc:HGNC:8013] |
| ENSG00000198482 | HGLibA_56797 | 81 | -0.402020715 | ZNF808 | zinc finger protein 808 [Source:HGNC Symbol;Acc:HGNC:33230] |
| ENSG00000198650 | HGLibA_48438 | 81 | -0.402020715 | TAT | tyrosine aminotransferase [Source:HGNC Symbol;Acc:HGNC:11573] |
| ENSG00000204305 | HGLibA_01180 | 81 | -0.402020715 | AGER | advanced glycosylation end-product specific receptor [Source:HGNC Symbol;Acc:HGNC:320] |
| ENSG00000204681 | HGLibA_18342 | 81 | -0.402020715 | GABBR1 | gamma-aminobutyric acid type B receptor subunit 1 [Source:HGNC Symbol;Acc:HGNC:4070] |
| ENSG00000204923 | HGLibA_17116 | 81 | -0.402020715 | FBXO48 | F-box protein 48 [Source:HGNC Symbol;Acc:HGNC:33857] |
| ENSG00000204954 | HGLibA_05290 | 81 | -0.402020715 | C12orf73 | chromosome 12 open reading frame 73 [Source:HGNC Symbol;Acc:HGNC:34450] |
| ENSG00000205571 | HGLibA_45784 | 81 | -0.402020715 | SMN2 | centromeric [Source:HGNC Symbol;Acc:HGNC:11118]" |
| ENSG00000215440 | HGLibA_32431 | 81 | -0.402020715 | NPEPL1 | aminopeptidase like 1 [Source:HGNC Symbol;Acc:HGNC:16244] |
| ENSG00000221883 | HGLibA_02917 | 81 | -0.402020715 | ARIH2OS | ariadne RBR E3 ubiquitin protein ligase 2 opposite strand [Source:HGNC Symbol;Acc:HGNC:34425] |
| ENSG00000223547 | HGLibA_56860 | 81 | -0.402020715 | ZNF844 | zinc finger protein 844 [Source:HGNC Symbol;Acc:HGNC:25932] |
| ENSG00000229292 | HGLibA_40940 | 81 | -0.402020715 | RFPL4AL1 | ret finger protein like 4A like 1 [Source:HGNC Symbol;Acc:HGNC:45147] |
| ENSG00000229314 | HGLibA_34618 | 81 | -0.402020715 | ORM1 | orosomucoid 1 [Source:HGNC Symbol;Acc:HGNC:8498] |
| ENSG00000230873 | HGLibA_47527 | 81 | -0.402020715 | STMND1 | stathmin domain containing 1 [Source:HGNC Symbol;Acc:HGNC:44668] |
| ENSG00000230989 | HGLibA_22152 | 81 | -0.402020715 | HSBP1 | heat shock factor binding protein 1 [Source:HGNC Symbol;Acc:HGNC:5203] |
| ENSG00000233136 | HGLibA_53219 | 81 | -0.402020715 | USP17L11 | ubiquitin specific peptidase 17-like family member 11 [Source:HGNC Symbol;Acc:HGNC:44439] |
| ENSG00000239779 | HGLibA_53969 | 81 | -0.402020715 | WBP1 | WW domain binding protein 1 [Source:HGNC Symbol;Acc:HGNC:12737] |
| ENSG00000241186 | HGLibA_48885 | 81 | -0.402020715 | TDGF1 | teratocarcinoma-derived growth factor 1 [Source:HGNC Symbol;Acc:HGNC:11701] |
| ENSG00000241258 | HGLibA_11081 | 81 | -0.402020715 | CRCP | CGRP receptor component [Source:HGNC Symbol;Acc:HGNC:17888] |
| ENSG00000242612 | HGLibA_12776 | 81 | -0.402020715 | DECR2 | 4-dienoyl-CoA reductase 2 [Source:HGNC Symbol;Acc:HGNC:2754]" |
| ENSG00000248383 | HGLibA_35464 | 81 | -0.402020715 | PCDHAC1 | 1 [Source:HGNC Symbol;Acc:HGNC:8676]" |
| ENSG00000254466 | HGLibA_33968 | 81 | -0.402020715 | OR4D10 | olfactory receptor family 4 subfamily D member 10 [Source:HGNC Symbol;Acc:HGNC:15173] |
| ENSG00000269113 | HGLibA_51234 | 81 | -0.402020715 | TRABD2B | TraB domain containing 2B [Source:HGNC Symbol;Acc:HGNC:44200] |
| ENSG00000269699 | HGLibA_55340 | 81 | -0.402020715 | ZIM2 | zinc finger imprinted 2 [Source:HGNC Symbol;Acc:HGNC:12875] |
| ENSG00000273045 | HGLibA_06109 | 81 | -0.402020715 | C2orf15 | chromosome 2 open reading frame 15 [Source:HGNC Symbol;Acc:HGNC:28436] |
| ENSG00000276581 | HGLibA_46480 | 81 | -0.402020715 | SPATA31A5 | SPATA31 subfamily A member 5 [Source:HGNC Symbol;Acc:HGNC:32005] |
| ENSG00000277149 | HGLibA_52483 | 81 | -0.402020715 | TYW1B | tRNA-yW synthesizing protein 1 homolog B [Source:HGNC Symbol;Acc:HGNC:33908] |
| ENSG00000278570 | HGLibA_32608 | 81 | -0.402020715 | NR2E3 | nuclear receptor subfamily 2 group E member 3 [Source:HGNC Symbol;Acc:HGNC:7974] |
| ENSG00000280314 | HGLibA_34548 | 81 | -0.402020715 | OR8K3 | olfactory receptor family 8 subfamily K member 3 (gene/pseudogene) [Source:HGNC Symbol;Acc:HGNC:15313] |
| FTH1P18 | HGLibA_18119 | 81 | -0.402020715 | FTH1P18 | - |
| GPR110 | HGLibA_19849 | 81 | -0.402020715 | GPR110 | - |
| H1F0 | HGLibA_20733 | 81 | -0.402020715 | H1F0 | - |
| hsa-mir-1207 | HGLibA_57281 | 81 | -0.402020715 | hsa-mir-1207 | - |
| hsa-mir-1244-1 | HGLibA_57362 | 81 | -0.402020715 | hsa-mir-1244-1 | - |
| hsa-mir-1303 | HGLibA_57728 | 81 | -0.402020715 | hsa-mir-1303 | - |
| hsa-mir-133a-1 | HGLibA_57772 | 81 | -0.402020715 | hsa-mir-133a-1 | - |
| hsa-mir-197 | HGLibA_58105 | 81 | -0.402020715 | hsa-mir-197 | - |
| hsa-mir-302d | HGLibA_58449 | 81 | -0.402020715 | hsa-mir-302d | - |
| hsa-mir-3132 | HGLibA_58583 | 81 | -0.402020715 | hsa-mir-3132 | - |
| hsa-mir-324 | HGLibA_58962 | 81 | -0.402020715 | hsa-mir-324 | - |
| hsa-mir-3651 | HGLibA_59148 | 81 | -0.402020715 | hsa-mir-3651 | - |
| hsa-mir-3674 | HGLibA_59248 | 81 | -0.402020715 | hsa-mir-3674 | - |
| hsa-mir-3690-2 | HGLibA_59351 | 81 | -0.402020715 | hsa-mir-3690-2 | - |
| hsa-mir-4492 | HGLibA_60388 | 81 | -0.402020715 | hsa-mir-4492 | - |
| hsa-mir-4636 | HGLibA_60649 | 81 | -0.402020715 | hsa-mir-4636 | - |
| hsa-mir-4659a | HGLibA_60742 | 81 | -0.402020715 | hsa-mir-4659a | - |
| hsa-mir-4755 | HGLibA_61117 | 81 | -0.402020715 | hsa-mir-4755 | - |
| hsa-mir-4792 | HGLibA_61273 | 81 | -0.402020715 | hsa-mir-4792 | - |
| hsa-mir-487a | HGLibA_61346 | 81 | -0.402020715 | hsa-mir-487a | - |
| hsa-mir-493 | HGLibA_61371 | 81 | -0.402020715 | hsa-mir-493 | - |
| hsa-mir-504 | HGLibA_61471 | 81 | -0.402020715 | hsa-mir-504 | - |
| hsa-mir-548c | HGLibA_61955 | 81 | -0.402020715 | hsa-mir-548c | - |
| hsa-mir-586 | HGLibA_62443 | 81 | -0.402020715 | hsa-mir-586 | - |
| hsa-mir-646 | HGLibA_62828 | 81 | -0.402020715 | hsa-mir-646 | - |
| hsa-mir-6500 | HGLibA_62851 | 81 | -0.402020715 | hsa-mir-6500 | - |
| hsa-mir-711 | HGLibA_63801 | 81 | -0.402020715 | hsa-mir-711 | - |
| hsa-mir-7152 | HGLibA_63835 | 81 | -0.402020715 | hsa-mir-7152 | - |
| hsa-mir-7159 | HGLibA_63866 | 81 | -0.402020715 | hsa-mir-7159 | - |
| hsa-mir-942 | HGLibA_64356 | 81 | -0.402020715 | hsa-mir-942 | - |
| 8-Sep | HGLibA_43453 | 81 | -0.402020715 | 8-Sep | - |
| ALPPL2 | HGLibA_01714 | 80 | -0.411066057 | ALPPL2 | - |
| C10orf131 | HGLibA_05056 | 80 | -0.411066057 | C10orf131 | - |
| C19orf80 | HGLibA_05711 | 80 | -0.411066057 | C19orf80 | - |
| C3orf37 | HGLibA_06239 | 80 | -0.411066057 | C3orf37 | - |
| C4orf21 | HGLibA_06302 | 80 | -0.411066057 | C4orf21 | - |
| CRLF2_X | HGLibA_11190 | 80 | -0.411066057 | CRLF2_X | - |
| ENSG00000007908 | HGLibA_43286 | 80 | -0.411066057 | SELE | selectin E [Source:HGNC Symbol;Acc:HGNC:10718] |
| ENSG00000008196 | HGLibA_49123 | 80 | -0.411066057 | TFAP2B | transcription factor AP-2 beta [Source:HGNC Symbol;Acc:HGNC:11743] |
| ENSG00000008441 | HGLibA_31813 | 80 | -0.411066057 | NFIX | nuclear factor I X [Source:HGNC Symbol;Acc:HGNC:7788] |
| ENSG00000013288 | HGLibA_28080 | 80 | -0.411066057 | MAN2B2 | mannosidase alpha class 2B member 2 [Source:HGNC Symbol;Acc:HGNC:29623] |
| ENSG00000017797 | HGLibA_40152 | 80 | -0.411066057 | RALBP1 | ralA binding protein 1 [Source:HGNC Symbol;Acc:HGNC:9841] |
| ENSG00000048162 | HGLibA_32306 | 80 | -0.411066057 | NOP16 | NOP16 nucleolar protein [Source:HGNC Symbol;Acc:HGNC:26934] |
| ENSG00000049541 | HGLibA_40905 | 80 | -0.411066057 | RFC2 | replication factor C subunit 2 [Source:HGNC Symbol;Acc:HGNC:9970] |
| ENSG00000060688 | HGLibA_45954 | 80 | -0.411066057 | SNRNP40 | small nuclear ribonucleoprotein U5 subunit 40 [Source:HGNC Symbol;Acc:HGNC:30857] |
| ENSG00000069275 | HGLibA_32915 | 80 | -0.411066057 | NUCKS1 | nuclear casein kinase and cyclin dependent kinase substrate 1 [Source:HGNC Symbol;Acc:HGNC:29923] |
| ENSG00000072182 | HGLibA_03307 | 80 | -0.411066057 | ASIC4 | acid sensing ion channel subunit family member 4 [Source:HGNC Symbol;Acc:HGNC:21263] |
| ENSG00000074621 | HGLibA_44614 | 80 | -0.411066057 | SLC24A1 | solute carrier family 24 member 1 [Source:HGNC Symbol;Acc:HGNC:10975] |
| ENSG00000075290 | HGLibA_54392 | 80 | -0.411066057 | WNT8B | Wnt family member 8B [Source:HGNC Symbol;Acc:HGNC:12789] |
| ENSG00000079337 | HGLibA_40251 | 80 | -0.411066057 | RAPGEF3 | Rap guanine nucleotide exchange factor 3 [Source:HGNC Symbol;Acc:HGNC:16629] |
| ENSG00000081277 | HGLibA_36852 | 80 | -0.411066057 | PKP1 | plakophilin 1 [Source:HGNC Symbol;Acc:HGNC:9023] |
| ENSG00000082258 | HGLibA_08128 | 80 | -0.411066057 | CCNT2 | cyclin T2 [Source:HGNC Symbol;Acc:HGNC:1600] |
| ENSG00000085224 | HGLibA_03824 | 80 | -0.411066057 | ATRX | chromatin remodeler [Source:HGNC Symbol;Acc:HGNC:886]" |
| ENSG00000091164 | HGLibA_52436 | 80 | -0.411066057 | TXNL1 | thioredoxin like 1 [Source:HGNC Symbol;Acc:HGNC:12436] |
| ENSG00000091527 | HGLibA_08893 | 80 | -0.411066057 | CDV3 | CDV3 homolog [Source:HGNC Symbol;Acc:HGNC:26928] |
| ENSG00000092871 | HGLibA_40921 | 80 | -0.411066057 | RFFL | ring finger and FYVE like domain containing E3 ubiquitin protein ligase [Source:HGNC Symbol;Acc:HGNC:24821] |
| ENSG00000099338 | HGLibA_07372 | 80 | -0.411066057 | CATSPERG | cation channel sperm associated auxiliary subunit gamma [Source:HGNC Symbol;Acc:HGNC:25243] |
| ENSG00000100280 | HGLibA_02280 | 80 | -0.411066057 | AP1B1 | adaptor related protein complex 1 subunit beta 1 [Source:HGNC Symbol;Acc:HGNC:554] |
| ENSG00000101161 | HGLibA_38743 | 80 | -0.411066057 | PRPF6 | pre-mRNA processing factor 6 [Source:HGNC Symbol;Acc:HGNC:15860] |
| ENSG00000102096 | HGLibA_36678 | 80 | -0.411066057 | PIM2 | serine/threonine kinase [Source:HGNC Symbol;Acc:HGNC:8987]" |
| ENSG00000103351 | HGLibA_10157 | 80 | -0.411066057 | CLUAP1 | clusterin associated protein 1 [Source:HGNC Symbol;Acc:HGNC:19009] |
| ENSG00000105205 | HGLibA_09803 | 80 | -0.411066057 | CLC | Charcot-Leyden crystal galectin [Source:HGNC Symbol;Acc:HGNC:2014] |
| ENSG00000106299 | HGLibA_53964 | 80 | -0.411066057 | WASL | Wiskott-Aldrich syndrome like [Source:HGNC Symbol;Acc:HGNC:12735] |
| ENSG00000106688 | HGLibA_44507 | 80 | -0.411066057 | SLC1A1 | solute carrier family 1 member 1 [Source:HGNC Symbol;Acc:HGNC:10939] |
| ENSG00000108231 | HGLibA_26393 | 80 | -0.411066057 | LGI1 | leucine rich glioma inactivated 1 [Source:HGNC Symbol;Acc:HGNC:6572] |
| ENSG00000109158 | HGLibA_18366 | 80 | -0.411066057 | GABRA4 | gamma-aminobutyric acid type A receptor alpha4 subunit [Source:HGNC Symbol;Acc:HGNC:4078] |
| ENSG00000110330 | HGLibA_04516 | 80 | -0.411066057 | BIRC2 | baculoviral IAP repeat containing 2 [Source:HGNC Symbol;Acc:HGNC:590] |
| ENSG00000110711 | HGLibA_01361 | 80 | -0.411066057 | AIP | aryl hydrocarbon receptor interacting protein [Source:HGNC Symbol;Acc:HGNC:358] |
| ENSG00000111110 | HGLibA_37981 | 80 | -0.411066057 | PPM1H | Mg2+/Mn2+ dependent 1H [Source:HGNC Symbol;Acc:HGNC:18583]" |
| ENSG00000111731 | HGLibA_06107 | 80 | -0.411066057 | C2CD5 | C2 calcium dependent domain containing 5 [Source:HGNC Symbol;Acc:HGNC:29062] |
| ENSG00000111783 | HGLibA_40972 | 80 | -0.411066057 | RFX4 | regulatory factor X4 [Source:HGNC Symbol;Acc:HGNC:9985] |
| ENSG00000112195 | HGLibA_51363 | 80 | -0.411066057 | TREML2 | triggering receptor expressed on myeloid cells like 2 [Source:HGNC Symbol;Acc:HGNC:21092] |
| ENSG00000113722 | HGLibA_08896 | 80 | -0.411066057 | CDX1 | caudal type homeobox 1 [Source:HGNC Symbol;Acc:HGNC:1805] |
| ENSG00000114124 | HGLibA_20339 | 80 | -0.411066057 | GRK7 | G protein-coupled receptor kinase 7 [Source:HGNC Symbol;Acc:HGNC:17031] |
| ENSG00000116833 | HGLibA_32636 | 80 | -0.411066057 | NR5A2 | nuclear receptor subfamily 5 group A member 2 [Source:HGNC Symbol;Acc:HGNC:7984] |
| ENSG00000118564 | HGLibA_17012 | 80 | -0.411066057 | FBXL5 | F-box and leucine rich repeat protein 5 [Source:HGNC Symbol;Acc:HGNC:13602] |
| ENSG00000120457 | HGLibA_24281 | 80 | -0.411066057 | KCNJ5 | potassium voltage-gated channel subfamily J member 5 [Source:HGNC Symbol;Acc:HGNC:6266] |
| ENSG00000120798 | HGLibA_32595 | 80 | -0.411066057 | NR2C1 | nuclear receptor subfamily 2 group C member 1 [Source:HGNC Symbol;Acc:HGNC:7971] |
| ENSG00000121064 | HGLibA_43040 | 80 | -0.411066057 | SCPEP1 | serine carboxypeptidase 1 [Source:HGNC Symbol;Acc:HGNC:29507] |
| ENSG00000121289 | HGLibA_09177 | 80 | -0.411066057 | CEP89 | centrosomal protein 89 [Source:HGNC Symbol;Acc:HGNC:25907] |
| ENSG00000122133 | HGLibA_34984 | 80 | -0.411066057 | PAEP | progestagen associated endometrial protein [Source:HGNC Symbol;Acc:HGNC:8573] |
| ENSG00000122218 | HGLibA_10693 | 80 | -0.411066057 | COPA | coatomer protein complex subunit alpha [Source:HGNC Symbol;Acc:HGNC:2230] |

| | | | | | |
|---|---|---|---|---|---|
| ENSG00000122641 | HGLibA_23301 | 80 | -0.411066057 | INHBA | inhibin subunit beta A [Source:HGNC Symbol;Acc:HGNC:6066] |
| ENSG00000122787 | HGLibA_01493 | 80 | -0.411066057 | AKR1D1 | aldo-keto reductase family 1 member D1 [Source:HGNC Symbol;Acc:HGNC:388] |
| ENSG00000123594 | HGLibA_03846 | 80 | -0.411066057 | ATXN3L | ataxin 3 like [Source:HGNC Symbol;Acc:HGNC:24173] |
| ENSG00000123908 | HGLibA_01208 | 80 | -0.411066057 | AGO2 | RISC catalytic component [Source:HGNC Symbol;Acc:HGNC:3263]" |
| ENSG00000125363 | HGLibA_01767 | 80 | -0.411066057 | AMELX | amelogenin X-linked [Source:HGNC Symbol;Acc:HGNC:461] |
| ENSG00000125378 | HGLibA_04607 | 80 | -0.411066057 | BMP4 | bone morphogenetic protein 4 [Source:HGNC Symbol;Acc:HGNC:1071] |
| ENSG00000125848 | HGLibA_17659 | 80 | -0.411066057 | FLRT3 | fibronectin leucine rich transmembrane protein 3 [Source:HGNC Symbol;Acc:HGNC:3762] |
| ENSG00000125998 | HGLibA_16746 | 80 | -0.411066057 | FAM83C | family with sequence similarity 83 member C [Source:HGNC Symbol;Acc:HGNC:16121] |
| ENSG00000126214 | HGLibA_25026 | 80 | -0.411066057 | KLC1 | kinesin light chain 1 [Source:HGNC Symbol;Acc:HGNC:6387] |
| ENSG00000126934 | HGLibA_28142 | 80 | -0.411066057 | MAP2K2 | mitogen-activated protein kinase kinase 2 [Source:HGNC Symbol;Acc:HGNC:6842] |
| ENSG00000127870 | HGLibA_41694 | 80 | -0.411066057 | RNF6 | ring finger protein 6 [Source:HGNC Symbol;Acc:HGNC:10069] |
| ENSG00000130226 | HGLibA_13875 | 80 | -0.411066057 | DPP6 | dipeptidyl peptidase like 6 [Source:HGNC Symbol;Acc:HGNC:3010] |
| ENSG00000130717 | HGLibA_52810 | 80 | -0.411066057 | UCK1 | uridine-cytidine kinase 1 [Source:HGNC Symbol;Acc:HGNC:14859] |
| ENSG00000132518 | HGLibA_20665 | 80 | -0.411066057 | GUCY2D | retinal [Source:HGNC Symbol;Acc:HGNC:4689]" |
| ENSG00000132530 | HGLibA_54464 | 80 | -0.411066057 | XAF1 | XIAP associated factor 1 [Source:HGNC Symbol;Acc:HGNC:30932] |
| ENSG00000132702 | HGLibA_20843 | 80 | -0.411066057 | HAPLN2 | hyaluronan and proteoglycan link protein 2 [Source:HGNC Symbol;Acc:HGNC:17410] |
| ENSG00000134265 | HGLibA_31134 | 80 | -0.411066057 | NAPG | NSF attachment protein gamma [Source:HGNC Symbol;Acc:HGNC:7642] |
| ENSG00000135486 | HGLibA_21792 | 80 | -0.411066057 | HNRNPA1 | heterogeneous nuclear ribonucleoprotein A1 [Source:HGNC Symbol;Acc:HGNC:5031] |
| ENSG00000135898 | HGLibA_20053 | 80 | -0.411066057 | GPR55 | G protein-coupled receptor 55 [Source:HGNC Symbol;Acc:HGNC:4511] |
| ENSG00000137710 | HGLibA_40759 | 80 | -0.411066057 | RDX | radixin [Source:HGNC Symbol;Acc:HGNC:9944] |
| ENSG00000137968 | HGLibA_45168 | 80 | -0.411066057 | SLC44A5 | solute carrier family 44 member 5 [Source:HGNC Symbol;Acc:HGNC:28524] |
| ENSG00000138135 | HGLibA_09331 | 80 | -0.411066057 | CH25H | cholesterol 25-hydroxylase [Source:HGNC Symbol;Acc:HGNC:1907] |
| ENSG00000138759 | HGLibA_17976 | 80 | -0.411066057 | FRAS1 | Fraser extracellular matrix complex subunit 1 [Source:HGNC Symbol;Acc:HGNC:19185] |
| ENSG00000139055 | HGLibA_15524 | 80 | -0.411066057 | ERP27 | endoplasmic reticulum protein 27 [Source:HGNC Symbol;Acc:HGNC:26495] |
| ENSG00000139438 | HGLibA_16522 | 80 | -0.411066057 | FAM222A | family with sequence similarity 222 member A [Source:HGNC Symbol;Acc:HGNC:25915] |
| ENSG00000139567 | HGLibA_00701 | 80 | -0.411066057 | ACVRL1 | activin A receptor like type 1 [Source:HGNC Symbol;Acc:HGNC:175] |
| ENSG00000139734 | HGLibA_13222 | 80 | -0.411066057 | DIAPH3 | diaphanous related formin 3 [Source:HGNC Symbol;Acc:HGNC:15480] |
| ENSG00000140534 | HGLibA_49451 | 80 | -0.411066057 | TICRR | TOPBP1 interacting checkpoint and replication regulator [Source:HGNC Symbol;Acc:HGNC:28704] |
| ENSG00000140563 | HGLibA_28655 | 80 | -0.411066057 | MCTP2 | multiple C2 and transmembrane domain containing 2 [Source:HGNC Symbol;Acc:HGNC:25636] |
| ENSG00000140873 | HGLibA_00814 | 80 | -0.411066057 | ADAMTS18 | ADAM metallopeptidase with thrombospondin type 1 motif 18 [Source:HGNC Symbol;Acc:HGNC:17110] |
| ENSG00000141376 | HGLibA_04256 | 80 | -0.411066057 | BCAS3 | microtubule associated cell migration factor [Source:HGNC Symbol;Acc:HGNC:14347]" |
| ENSG00000141385 | HGLibA_01119 | 80 | -0.411066057 | AFG3L2 | AFG3 like matrix AAA peptidase subunit 2 [Source:HGNC Symbol;Acc:HGNC:315] |
| ENSG00000141551 | HGLibA_11388 | 80 | -0.411066057 | CSNK1D | casein kinase 1 delta [Source:HGNC Symbol;Acc:HGNC:2452] |
| ENSG00000141756 | HGLibA_17551 | 80 | -0.411066057 | FKBP10 | FK506 binding protein 10 [Source:HGNC Symbol;Acc:HGNC:18169] |
| ENSG00000143278 | HGLibA_15857 | 80 | -0.411066057 | F13B | coagulation factor XIII B chain [Source:HGNC Symbol;Acc:HGNC:3534] |
| ENSG00000143476 | HGLibA_14061 | 80 | -0.411066057 | DTL | denticleless E3 ubiquitin protein ligase homolog [Source:HGNC Symbol;Acc:HGNC:30288] |
| ENSG00000143727 | HGLibA_00510 | 80 | -0.411066057 | ACP1 | acid phosphatase 1 [Source:HGNC Symbol;Acc:HGNC:122] |
| ENSG00000145901 | HGLibA_50834 | 80 | -0.411066057 | TNIP1 | TNFAIP3 interacting protein 1 [Source:HGNC Symbol;Acc:HGNC:16903] |
| ENSG00000145901 | HGLibA_50836 | 80 | -0.411066057 | TNIP1 | TNFAIP3 interacting protein 1 [Source:HGNC Symbol;Acc:HGNC:16903] |
| ENSG00000146221 | HGLibA_48857 | 80 | -0.411066057 | TCTE1 | t-complex-associated-testis-expressed 1 [Source:HGNC Symbol;Acc:HGNC:11693] |
| ENSG00000147117 | HGLibA_55508 | 80 | -0.411066057 | ZNF157 | zinc finger protein 157 [Source:HGNC Symbol;Acc:HGNC:12942] |
| ENSG00000147862 | HGLibA_31802 | 80 | -0.411066057 | NFIB | nuclear factor I B [Source:HGNC Symbol;Acc:HGNC:7785] |
| ENSG00000148444 | HGLibA_10659 | 80 | -0.411066057 | COMMD3 | COMM domain containing 3 [Source:HGNC Symbol;Acc:HGNC:23332] |
| ENSG00000149635 | HGLibA_33266 | 80 | -0.411066057 | OCSTAMP | osteoclast stimulatory transmembrane protein [Source:HGNC Symbol;Acc:HGNC:16116] |
| ENSG00000151012 | HGLibA_45352 | 80 | -0.411066057 | SLC7A11 | solute carrier family 7 member 11 [Source:HGNC Symbol;Acc:HGNC:11059] |
| ENSG00000152518 | HGLibA_55211 | 80 | -0.411066057 | ZFP36L2 | ZFP36 ring finger protein like 2 [Source:HGNC Symbol;Acc:HGNC:1108] |
| ENSG00000152661 | HGLibA_19136 | 80 | -0.411066057 | GJA1 | gap junction protein alpha 1 [Source:HGNC Symbol;Acc:HGNC:4274] |
| ENSG00000153551 | HGLibA_10226 | 80 | -0.411066057 | CMTM7 | CKLF like MARVEL transmembrane domain containing 7 [Source:HGNC Symbol;Acc:HGNC:19178] |
| ENSG00000154165 | HGLibA_19927 | 80 | -0.411066057 | GPR15 | G protein-coupled receptor 15 [Source:HGNC Symbol;Acc:HGNC:4469] |
| ENSG00000154358 | HGLibA_33230 | 80 | -0.411066057 | OBSCN | cytoskeletal calmodulin and titin-interacting RhoGEF [Source:HGNC Symbol;Acc:HGNC:15719]" |
| ENSG00000157933 | HGLibA_44276 | 80 | -0.411066057 | SKI | SKI proto-oncogene [Source:HGNC Symbol;Acc:HGNC:10896] |
| ENSG00000158985 | HGLibA_08584 | 80 | -0.411066057 | CDC42SE2 | CDC42 small effector 2 [Source:HGNC Symbol;Acc:HGNC:18547] |
| ENSG00000160469 | HGLibA_04848 | 80 | -0.411066057 | BRSK1 | BR serine/threonine kinase 1 [Source:HGNC Symbol;Acc:HGNC:18994] |
| ENSG00000161180 | HGLibA_07556 | 80 | -0.411066057 | CCDC116 | coiled-coil domain containing 116 [Source:HGNC Symbol;Acc:HGNC:26688] |
| ENSG00000162365 | HGLibA_12176 | 80 | -0.411066057 | CYP4A22 | cytochrome P450 family 4 subfamily A member 22 [Source:HGNC Symbol;Acc:HGNC:20575] |
| ENSG00000164087 | HGLibA_37442 | 80 | -0.411066057 | POC1A | POC1 centriolar protein A [Source:HGNC Symbol;Acc:HGNC:24488] |
| ENSG00000164494 | HGLibA_35951 | 80 | -0.411066057 | PDSS2 | decaprenyl diphosphate synthase subunit 2 [Source:HGNC Symbol;Acc:HGNC:23041] |
| ENSG00000164626 | HGLibA_24327 | 80 | -0.411066057 | KCNK5 | potassium two pore domain channel subfamily K member 5 [Source:HGNC Symbol;Acc:HGNC:6280] |
| ENSG00000164627 | HGLibA_24959 | 80 | -0.411066057 | KIF6 | kinesin family member 6 [Source:HGNC Symbol;Acc:HGNC:21202] |
| ENSG00000165495 | HGLibA_36849 | 80 | -0.411066057 | PKNOX2 | PBX/knotted 1 homeobox 2 [Source:HGNC Symbol;Acc:HGNC:16714] |
| ENSG00000165655 | HGLibA_56115 | 80 | -0.411066057 | ZNF503 | zinc finger protein 503 [Source:HGNC Symbol;Acc:HGNC:23589] |
| ENSG00000166159 | HGLibA_27504 | 80 | -0.411066057 | LRTM2 | leucine rich repeats and transmembrane domains 2 [Source:HGNC Symbol;Acc:HGNC:32443] |
| ENSG00000166391 | HGLibA_29628 | 80 | -0.411066057 | MOGAT2 | monoacylglycerol O-acyltransferase 2 [Source:HGNC Symbol;Acc:HGNC:23248] |
| ENSG00000167286 | HGLibA_08369 | 80 | -0.411066057 | CD3D | CD3d molecule [Source:HGNC Symbol;Acc:HGNC:1673] |
| ENSG00000167653 | HGLibA_39011 | 80 | -0.411066057 | PSCA | prostate stem cell antigen [Source:HGNC Symbol;Acc:HGNC:9500] |
| ENSG00000167769 | HGLibA_00429 | 80 | -0.411066057 | ACER1 | alkaline ceramidase 1 [Source:HGNC Symbol;Acc:HGNC:18356] |
| ENSG00000168434 | HGLibA_10489 | 80 | -0.411066057 | COG7 | component of oligomeric golgi complex 7 [Source:HGNC Symbol;Acc:HGNC:18622] |
| ENSG00000168487 | HGLibA_04590 | 80 | -0.411066057 | BMP1 | bone morphogenetic protein 1 [Source:HGNC Symbol;Acc:HGNC:1067] |
| ENSG00000168522 | HGLibA_17772 | 80 | -0.411066057 | FNTA | alpha [Source:HGNC Symbol;Acc:HGNC:3782]" |
| ENSG00000168619 | HGLibA_00744 | 80 | -0.411066057 | ADAM18 | ADAM metallopeptidase domain 18 [Source:HGNC Symbol;Acc:HGNC:196] |
| ENSG00000168703 | HGLibA_54255 | 80 | -0.411066057 | WFDC12 | WAP four-disulfide core domain 12 [Source:HGNC Symbol;Acc:HGNC:16115] |
| ENSG00000168779 | HGLibA_44074 | 80 | -0.411066057 | SHOX2 | short stature homeobox 2 [Source:HGNC Symbol;Acc:HGNC:10854] |
| ENSG00000169288 | HGLibA_29887 | 80 | -0.411066057 | MRPL1 | mitochondrial ribosomal protein L1 [Source:HGNC Symbol;Acc:HGNC:14275] |
| ENSG00000169976 | HGLibA_43715 | 80 | -0.411066057 | SF3B5 | splicing factor 3b subunit 5 [Source:HGNC Symbol;Acc:HGNC:21083] |
| ENSG00000170558 | HGLibA_08659 | 80 | -0.411066057 | CDH2 | cadherin 2 [Source:HGNC Symbol;Acc:HGNC:1759] |
| ENSG00000171017 | HGLibA_27441 | 80 | -0.411066057 | LRRC8E | leucine rich repeat containing 8 VRAC subunit E [Source:HGNC Symbol;Acc:HGNC:26272] |
| ENSG00000171204 | HGLibA_49912 | 80 | -0.411066057 | TMEM126B | transmembrane protein 126B [Source:HGNC Symbol;Acc:HGNC:30883] |
| ENSG00000171408 | HGLibA_35827 | 80 | -0.411066057 | PDE7B | phosphodiesterase 7B [Source:HGNC Symbol;Acc:HGNC:8792] |
| ENSG00000171453 | HGLibA_37556 | 80 | -0.411066057 | POLR1C | RNA polymerase I and III subunit C [Source:HGNC Symbol;Acc:HGNC:20194] |
| ENSG00000171560 | HGLibA_17335 | 80 | -0.411066057 | FGA | fibrinogen alpha chain [Source:HGNC Symbol;Acc:HGNC:3661] |
| ENSG00000171766 | HGLibA_18695 | 80 | -0.411066057 | GATM | glycine amidinotransferase [Source:HGNC Symbol;Acc:HGNC:4175] |
| ENSG00000172456 | HGLibA_17460 | 80 | -0.411066057 | FGGY | FGGY carbohydrate kinase domain containing [Source:HGNC Symbol;Acc:HGNC:25610] |
| ENSG00000173237 | HGLibA_05202 | 80 | -0.411066057 | C11orf86 | chromosome 11 open reading frame 86 [Source:HGNC Symbol;Acc:HGNC:34442] |
| ENSG00000173653 | HGLibA_40693 | 80 | -0.411066057 | RCE1 | Ras converting CAAX endopeptidase 1 [Source:HGNC Symbol;Acc:HGNC:13721] |
| ENSG00000173660 | HGLibA_53121 | 80 | -0.411066057 | UQCRH | ubiquinol-cytochrome c reductase hinge protein [Source:HGNC Symbol;Acc:HGNC:12590] |
| ENSG00000173898 | HGLibA_46883 | 80 | -0.411066057 | SPTBN2 | non-erythrocytic 2 [Source:HGNC Symbol;Acc:HGNC:11276]" |
| ENSG00000174339 | HGLibA_33908 | 80 | -0.411066057 | OR2Y1 | olfactory receptor family 2 subfamily Y member 1 [Source:HGNC Symbol;Acc:HGNC:14837] |
| ENSG00000174898 | HGLibA_07371 | 80 | -0.411066057 | CATSPERD | cation channel sperm associated auxiliary subunit delta [Source:HGNC Symbol;Acc:HGNC:28598] |
| ENSG00000176022 | HGLibA_03965 | 80 | -0.411066057 | B3GALT6 | 3-galactosyltransferase 6 [Source:HGNC Symbol;Acc:HGNC:17978]" |
| ENSG00000176371 | HGLibA_56995 | 80 | -0.411066057 | ZSCAN2 | zinc finger and SCAN domain containing 2 [Source:HGNC Symbol;Acc:HGNC:20994] |
| ENSG00000176601 | HGLibA_28178 | 80 | -0.411066057 | MAP3K19 | mitogen-activated protein kinase kinase kinase 19 [Source:HGNC Symbol;Acc:HGNC:26249] |
| ENSG00000176715 | HGLibA_00552 | 80 | -0.411066057 | ACSF3 | acyl-CoA synthetase family member 3 [Source:HGNC Symbol;Acc:HGNC:27288] |
| ENSG00000177182 | HGLibA_10167 | 80 | -0.411066057 | CLVS1 | clavesin 1 [Source:HGNC Symbol;Acc:HGNC:23139] |
| ENSG00000177192 | HGLibA_39595 | 80 | -0.411066057 | PUS1 | pseudouridine synthase 1 [Source:HGNC Symbol;Acc:HGNC:15508] |
| ENSG00000181009 | HGLibA_34201 | 80 | -0.411066057 | OR52N5 | olfactory receptor family 52 subfamily N member 5 [Source:HGNC Symbol;Acc:HGNC:15231] |
| ENSG00000181585 | HGLibA_50582 | 80 | -0.411066057 | TMIE | transmembrane inner ear [Source:HGNC Symbol;Acc:HGNC:30800] |
| ENSG00000181873 | HGLibA_22478 | 80 | -0.411066057 | IBA57 | iron-sulfur cluster assembly [Source:HGNC Symbol;Acc:HGNC:27302]" |
| ENSG00000182489 | HGLibA_54537 | 80 | -0.411066057 | XKRX | XK related X-linked [Source:HGNC Symbol;Acc:HGNC:29845] |
| ENSG00000183010 | HGLibA_39673 | 80 | -0.411066057 | PYCR1 | pyrroline-5-carboxylate reductase 1 [Source:HGNC Symbol;Acc:HGNC:9721] |
| ENSG00000183067 | HGLibA_22896 | 80 | -0.411066057 | IGSF5 | immunoglobulin superfamily member 5 [Source:HGNC Symbol;Acc:HGNC:5952] |
| ENSG00000184117 | HGLibA_31971 | 80 | -0.411066057 | NIPSNAP1 | nipsnap homolog 1 [Source:HGNC Symbol;Acc:HGNC:7827] |
| ENSG00000184185 | HGLibA_24255 | 80 | -0.411066057 | KCNJ12 | potassium voltage-gated channel subfamily J member 12 [Source:HGNC Symbol;Acc:HGNC:6258] |
| ENSG00000185000 | HGLibA_13034 | 80 | -0.411066057 | DGAT1 | diacylglycerol O-acyltransferase 1 [Source:HGNC Symbol;Acc:HGNC:2843] |
| ENSG00000187987 | HGLibA_57006 | 80 | -0.411066057 | ZSCAN23 | zinc finger and SCAN domain containing 23 [Source:HGNC Symbol;Acc:HGNC:21193] |
| ENSG00000188152 | HGLibA_33112 | 80 | -0.411066057 | NUTM2G | NUT family member 2G [Source:HGNC Symbol;Acc:HGNC:23449] |
| ENSG00000188373 | HGLibA_05111 | 80 | -0.411066057 | C10orf99 | chromosome 10 open reading frame 99 [Source:HGNC Symbol;Acc:HGNC:31428] |
| ENSG00000188428 | HGLibA_04570 | 80 | -0.411066057 | BLOC1S5 | biogenesis of lysosomal organelles complex 1 subunit 5 [Source:HGNC Symbol;Acc:HGNC:18561] |
| ENSG00000188811 | HGLibA_31902 | 80 | -0.411066057 | NHLRC3 | NHL repeat containing 3 [Source:HGNC Symbol;Acc:HGNC:33751] |
| ENSG00000188917 | HGLibA_51685 | 80 | -0.411066057 | TRMT2B | tRNA methyltransferase 2 homolog B [Source:HGNC Symbol;Acc:HGNC:25748] |
| ENSG00000196209 | HGLibA_44206 | 80 | -0.411066057 | SIRPB2 | signal regulatory protein beta 2 [Source:HGNC Symbol;Acc:HGNC:16247] |
| ENSG00000197428 | HGLibA_34093 | 80 | -0.411066057 | OR51D1 | olfactory receptor family 51 subfamily D member 1 [Source:HGNC Symbol;Acc:HGNC:15193] |
| ENSG00000197498 | HGLibA_41831 | 80 | -0.411066057 | RPF2 | ribosome production factor 2 homolog [Source:HGNC Symbol;Acc:HGNC:20870] |
| ENSG00000197548 | HGLibA_03503 | 80 | -0.411066057 | ATG7 | autophagy related 7 [Source:HGNC Symbol;Acc:HGNC:16935] |
| ENSG00000198478 | HGLibA_43918 | 80 | -0.411066057 | SH3BGRL2 | SH3 domain binding glutamate rich protein like 2 [Source:HGNC Symbol;Acc:HGNC:15567] |
| ENSG00000198597 | HGLibA_56186 | 80 | -0.411066057 | ZNF536 | zinc finger protein 536 [Source:HGNC Symbol;Acc:HGNC:29025] |
| ENSG00000198601 | HGLibA_33832 | 80 | -0.411066057 | OR2M2 | olfactory receptor family 2 subfamily M member 2 [Source:HGNC Symbol;Acc:HGNC:8268] |
| ENSG00000198721 | HGLibA_14408 | 80 | -0.411066057 | ECI2 | enoyl-CoA delta isomerase 2 [Source:HGNC Symbol;Acc:HGNC:14601] |
| ENSG00000204930 | HGLibA_16519 | 80 | -0.411066057 | FAM221B | family with sequence similarity 221 member B [Source:HGNC Symbol;Acc:HGNC:30762] |
| ENSG00000205744 | HGLibA_12934 | 80 | -0.411066057 | DENND1C | DENN domain containing 1C [Source:HGNC Symbol;Acc:HGNC:26225] |
| ENSG00000205883 | HGLibA_12909 | 80 | -0.411066057 | DEFB135 | defensin beta 135 [Source:HGNC Symbol;Acc:HGNC:32400] |
| ENSG00000212659 | HGLibA_25842 | 80 | -0.411066057 | KRTAP9-6 | keratin associated protein 9-6 [Source:HGNC Symbol;Acc:HGNC:18914] |
| ENSG00000213079 | HGLibA_42831 | 80 | -0.411066057 | SCAF8 | SR-related CTD associated factor 8 [Source:HGNC Symbol;Acc:HGNC:20959] |
| ENSG00000213927 | HGLibA_08013 | 80 | -0.411066057 | CCL27 | C-C motif chemokine ligand 27 [Source:HGNC Symbol;Acc:HGNC:10626] |
| ENSG00000215788 | HGLibA_50781 | 80 | -0.411066057 | TNFRSF25 | TNF receptor superfamily member 25 [Source:HGNC Symbol;Acc:HGNC:11910] |
| ENSG00000221866 | HGLibA_37266 | 80 | -0.411066057 | PLXNA4 | plexin A4 [Source:HGNC Symbol;Acc:HGNC:9102] |
| ENSG00000225899 | HGLibA_17999 | 80 | -0.411066057 | FRG2B | FSHD region gene 2 family member B [Source:HGNC Symbol;Acc:HGNC:33518] |
| ENSG00000231389 | HGLibA_21614 | 80 | -0.411066057 | HLA-DPA1 | DP alpha 1 [Source:HGNC Symbol;Acc:HGNC:4938]" |
| ENSG00000236311 | HGLibA_49673 | 80 | -0.411066057 | TLX1NB | TLX1 neighbor [Source:HGNC Symbol;Acc:HGNC:37183] |
| ENSG00000250868 | HGLibA_54540 | 80 | -0.411066057 | XKRY | Y-linked [Source:HGNC Symbol;Acc:HGNC:18571]" |
| ENSG00000253293 | HGLibA_21896 | 80 | -0.411066057 | HOXA10 | homeobox A10 [Source:HGNC Symbol;Acc:HGNC:5100] |
| ENSG00000253304 | HGLibA_50175 | 80 | -0.411066057 | TMEM200B | transmembrane protein 200B [Source:HGNC Symbol;Acc:HGNC:33785] |
| ENSG00000253309 | HGLibA_43584 | 80 | -0.411066057 | SERPINE3 | serpin family E member 3 [Source:HGNC Symbol;Acc:HGNC:24774] |
| ENSG00000253485 | HGLibA_35536 | 80 | -0.411066057 | PCDHGA5 | 5 [Source:HGNC Symbol;Acc:HGNC:8703]" |
| ENSG00000254505 | HGLibA_09487 | 80 | -0.411066057 | CHMP4A | charged multivesicular body protein 4A [Source:HGNC Symbol;Acc:HGNC:20274] |
| ENSG00000260811 | HGLibA_33956 | 80 | -0.411066057 | OR4C45 | olfactory receptor family 4 subfamily C member 45 (gene/pseudogene) [Source:HGNC Symbol;Acc:HGNC:31270] |

| | | | | | |
|---|---|---|---|---|---|
| ENSG00000267060 | HGLibA_39334 | 80 | -0.411066057 | PTGES3L | prostaglandin E synthase 3 like [Source:HGNC Symbol;Acc:HGNC:43943] |
| ENSG00000276040 | HGLibA_46487 | 80 | -0.411066057 | SPATA31A7 | SPATA31 subfamily A member 7 [Source:HGNC Symbol;Acc:HGNC:32007] |
| ERBB2IP | HGLibA_15417 | 80 | -0.411066057 | ERBB2IP | - |
| H3F3A | HGLibA_20779 | 80 | -0.411066057 | H3F3A | - |
| HIST1H2BM | HGLibA_21447 | 80 | -0.411066057 | HIST1H2BM | - |
| hsa-mir-1266 | HGLibA_57486 | 80 | -0.411066057 | hsa-mir-1266 | - |
| hsa-mir-1295b | HGLibA_57660 | 80 | -0.411066057 | hsa-mir-1295b | - |
| hsa-mir-135a-2 | HGLibA_57795 | 80 | -0.411066057 | hsa-mir-135a-2 | - |
| hsa-mir-302b | HGLibA_58441 | 80 | -0.411066057 | hsa-mir-302b | - |
| hsa-mir-330 | HGLibA_58989 | 80 | -0.411066057 | hsa-mir-330 | - |
| hsa-mir-361 | HGLibA_59069 | 80 | -0.411066057 | hsa-mir-361 | - |
| hsa-mir-383 | HGLibA_59485 | 80 | -0.411066057 | hsa-mir-383 | - |
| hsa-mir-384 | HGLibA_59488 | 80 | -0.411066057 | hsa-mir-384 | - |
| hsa-mir-4327 | HGLibA_60033 | 80 | -0.411066057 | hsa-mir-4327 | - |
| hsa-mir-4475 | HGLibA_60311 | 80 | -0.411066057 | hsa-mir-4475 | - |
| hsa-mir-4636 | HGLibA_60648 | 80 | -0.411066057 | hsa-mir-4636 | - |
| hsa-mir-4706 | HGLibA_60925 | 80 | -0.411066057 | hsa-mir-4706 | - |
| hsa-mir-4766 | HGLibA_61160 | 80 | -0.411066057 | hsa-mir-4766 | - |
| hsa-mir-4773-2 | HGLibA_61197 | 80 | -0.411066057 | hsa-mir-4773-2 | - |
| hsa-mir-511 | HGLibA_61557 | 80 | -0.411066057 | hsa-mir-511 | - |
| hsa-mir-5696 | HGLibA_62316 | 80 | -0.411066057 | hsa-mir-5696 | - |
| hsa-mir-660 | HGLibA_62973 | 80 | -0.411066057 | hsa-mir-660 | - |
| hsa-mir-665 | HGLibA_63003 | 80 | -0.411066057 | hsa-mir-665 | - |
| hsa-mir-6849 | HGLibA_63569 | 80 | -0.411066057 | hsa-mir-6849 | - |
| hsa-mir-6880 | HGLibA_63703 | 80 | -0.411066057 | hsa-mir-6880 | - |
| hsa-mir-7856 | HGLibA_64012 | 80 | -0.411066057 | hsa-mir-7856 | - |
| hsa-mir-7974 | HGLibA_64026 | 80 | -0.411066057 | hsa-mir-7974 | - |
| hsa-mir-8071-1 | HGLibA_64122 | 80 | -0.411066057 | hsa-mir-8071-1 | - |
| LOH12CR1 | HGLibA_27010 | 80 | -0.411066057 | LOH12CR1 | - |
| LRRC33 | HGLibA_27316 | 80 | -0.411066057 | LRRC33 | - |
| MLL | HGLibA_29394 | 80 | -0.411066057 | MLL | - |
| OR5R1 | HGLibA_34353 | 80 | -0.411066057 | OR5R1 | - |
| SDPR | HGLibA_43156 | 80 | -0.411066057 | SDPR | - |
| TMEM66 | HGLibA_50488 | 80 | -0.411066057 | TMEM66 | - |
| VIMP | HGLibA_53660 | 80 | -0.411066057 | VIMP | - |
| C10orf76 | HGLibA_05093 | 79 | -0.4201114 | C10orf76 | - |
| C6orf170 | HGLibA_06460 | 79 | -0.4201114 | C6orf170 | - |
| CHDC2 | HGLibA_09412 | 79 | -0.4201114 | CHDC2 | - |
| CHDC2 | HGLibA_09414 | 79 | -0.4201114 | CHDC2 | - |
| CT45A4 | HGLibA_11508 | 79 | -0.4201114 | CT45A4 | - |
| CYP3A7-CYP3AP1 | HGLibA_12165 | 79 | -0.4201114 | CYP3A7-CYP3AP1 | - |
| ENSG00000004779 | HGLibA_31479 | 79 | -0.4201114 | NDUFAB1 | NADH:ubiquinone oxidoreductase subunit AB1 [Source:HGNC Symbol;Acc:HGNC:7694] |
| ENSG00000005238 | HGLibA_16480 | 79 | -0.4201114 | FAM214B | family with sequence similarity 214 member B [Source:HGNC Symbol;Acc:HGNC:25666] |
| ENSG00000006128 | HGLibA_48150 | 79 | -0.4201114 | TAC1 | tachykinin precursor 1 [Source:HGNC Symbol;Acc:HGNC:11517] |
| ENSG00000006756 | HGLibA_03134 | 79 | -0.4201114 | ARSD | arylsulfatase D [Source:HGNC Symbol;Acc:HGNC:717] |
| ENSG00000034510 | HGLibA_50657 | 79 | -0.4201114 | TMSB10 | thymosin beta 10 [Source:HGNC Symbol;Acc:HGNC:11879] |
| ENSG00000041982 | HGLibA_50699 | 79 | -0.4201114 | TNC | tenascin C [Source:HGNC Symbol;Acc:HGNC:5318] |
| ENSG00000047457 | HGLibA_10880 | 79 | -0.4201114 | CP | ceruloplasmin [Source:HGNC Symbol;Acc:HGNC:2295] |
| ENSG00000049167 | HGLibA_15455 | 79 | -0.4201114 | ERCC8 | CSA ubiquitin ligase complex subunit [Source:HGNC Symbol;Acc:HGNC:3439]" |
| ENSG00000050405 | HGLibA_26525 | 79 | -0.4201114 | LIMA1 | LIM domain and actin binding 1 [Source:HGNC Symbol;Acc:HGNC:24636] |
| ENSG00000061337 | HGLibA_27807 | 79 | -0.4201114 | LZTS1 | leucine zipper tumor suppressor 1 [Source:HGNC Symbol;Acc:HGNC:13861] |
| ENSG00000062194 | HGLibA_19756 | 79 | -0.4201114 | GPBP1 | GC-rich promoter binding protein 1 [Source:HGNC Symbol;Acc:HGNC:29520] |
| ENSG00000068137 | HGLibA_37130 | 79 | -0.4201114 | PLEKHH3 | MyTH4 and FERM domain containing H3 [Source:HGNC Symbol;Acc:HGNC:26105]" |
| ENSG00000074657 | HGLibA_56178 | 79 | -0.4201114 | ZNF532 | zinc finger protein 532 [Source:HGNC Symbol;Acc:HGNC:30940] |
| ENSG00000074706 | HGLibA_23454 | 79 | -0.4201114 | IPCEF1 | interaction protein for cytohesin exchange factors 1 [Source:HGNC Symbol;Acc:HGNC:21204] |
| ENSG00000075429 | HGLibA_06941 | 79 | -0.4201114 | CACNG5 | calcium voltage-gated channel auxiliary subunit gamma 5 [Source:HGNC Symbol;Acc:HGNC:1409] |
| ENSG00000077152 | HGLibA_52659 | 79 | -0.4201114 | UBE2T | ubiquitin conjugating enzyme E2 T [Source:HGNC Symbol;Acc:HGNC:25009] |
| ENSG00000077312 | HGLibA_45963 | 79 | -0.4201114 | SNRPA | small nuclear ribonucleoprotein polypeptide A [Source:HGNC Symbol;Acc:HGNC:11151] |
| ENSG00000077348 | HGLibA_15784 | 79 | -0.4201114 | EXOSC5 | exosome component 5 [Source:HGNC Symbol;Acc:HGNC:24662] |
| ENSG00000083782 | HGLibA_15395 | 79 | -0.4201114 | EPYC | epiphycan [Source:HGNC Symbol;Acc:HGNC:3053] |
| ENSG00000088808 | HGLibA_38029 | 79 | -0.4201114 | PPP1R13B | protein phosphatase 1 regulatory subunit 13B [Source:HGNC Symbol;Acc:HGNC:14950] |
| ENSG00000091879 | HGLibA_01903 | 79 | -0.4201114 | ANGPT2 | angiopoietin 2 [Source:HGNC Symbol;Acc:HGNC:485] |
| ENSG00000100368 | HGLibA_11335 | 79 | -0.4201114 | CSF2RB | colony stimulating factor 2 receptor beta common subunit [Source:HGNC Symbol;Acc:HGNC:2436] |
| ENSG00000100578 | HGLibA_24646 | 79 | -0.4201114 | KIAA0586 | KIAA0586 [Source:HGNC Symbol;Acc:HGNC:19960] |
| ENSG00000100629 | HGLibA_09105 | 79 | -0.4201114 | CEP128 | centrosomal protein 128 [Source:HGNC Symbol;Acc:HGNC:20359] |
| ENSG00000100714 | HGLibA_30377 | 79 | -0.4201114 | MTHFD1 | cyclohydrolase and formyltetrahydrofolate synthetase 1 [Source:HGNC Symbol;Acc:HGNC:7432]" |
| ENSG00000101265 | HGLibA_40377 | 79 | -0.4201114 | RASSF2 | Ras association domain family member 2 [Source:HGNC Symbol;Acc:HGNC:9883] |
| ENSG00000103222 | HGLibA_00159 | 79 | -0.4201114 | ABCC1 | ATP binding cassette subfamily C member 1 [Source:HGNC Symbol;Acc:HGNC:51] |
| ENSG00000103381 | HGLibA_10984 | 79 | -0.4201114 | CPPED1 | calcineurin like phosphoesterase domain containing 1 [Source:HGNC Symbol;Acc:HGNC:25632] |
| ENSG00000103404 | HGLibA_53333 | 79 | -0.4201114 | USP31 | ubiquitin specific peptidase 31 [Source:HGNC Symbol;Acc:HGNC:20060] |
| ENSG00000105464 | HGLibA_20309 | 79 | -0.4201114 | GRIN2D | glutamate ionotropic receptor NMDA type subunit 2D [Source:HGNC Symbol;Acc:HGNC:4588] |
| ENSG00000106078 | HGLibA_10462 | 79 | -0.4201114 | COBL | cordon-bleu WH2 repeat protein [Source:HGNC Symbol;Acc:HGNC:22199] |
| ENSG00000106328 | HGLibA_18075 | 79 | -0.4201114 | FSCN3 | fascin actin-bundling protein 3 [Source:HGNC Symbol;Acc:HGNC:3961] |
| ENSG00000106635 | HGLibA_04342 | 79 | -0.4201114 | BCL7B | BCL tumor suppressor 7B [Source:HGNC Symbol;Acc:HGNC:1005] |
| ENSG00000107562 | HGLibA_11850 | 79 | -0.4201114 | CXCL12 | C-X-C motif chemokine ligand 12 [Source:HGNC Symbol;Acc:HGNC:10672] |
| ENSG00000109762 | HGLibA_46072 | 79 | -0.4201114 | SNX25 | sorting nexin 25 [Source:HGNC Symbol;Acc:HGNC:21883] |
| ENSG00000110274 | HGLibA_09114 | 79 | -0.4201114 | CEP164 | centrosomal protein 164 [Source:HGNC Symbol;Acc:HGNC:29182] |
| ENSG00000110665 | HGLibA_05120 | 79 | -0.4201114 | C11orf21 | chromosome 11 open reading frame 21 [Source:HGNC Symbol;Acc:HGNC:13231] |
| ENSG00000112658 | HGLibA_46960 | 79 | -0.4201114 | SRF | serum response factor [Source:HGNC Symbol;Acc:HGNC:11291] |
| ENSG00000112742 | HGLibA_52181 | 79 | -0.4201114 | TTK | TTK protein kinase [Source:HGNC Symbol;Acc:HGNC:12401] |
| ENSG00000112787 | HGLibA_16969 | 79 | -0.4201114 | FBRSL1 | fibrosin like 1 [Source:HGNC Symbol;Acc:HGNC:29308] |
| ENSG00000113360 | HGLibA_13982 | 79 | -0.4201114 | DROSHA | drosha ribonuclease III [Source:HGNC Symbol;Acc:HGNC:17904] |
| ENSG00000114026 | HGLibA_33324 | 79 | -0.4201114 | OGG1 | 8-oxoguanine DNA glycosylase [Source:HGNC Symbol;Acc:HGNC:8125] |
| ENSG00000114062 | HGLibA_52678 | 79 | -0.4201114 | UBE3A | ubiquitin protein ligase E3A [Source:HGNC Symbol;Acc:HGNC:12496] |
| ENSG00000114784 | HGLibA_14716 | 79 | -0.4201114 | EIF1B | eukaryotic translation initiation factor 1B [Source:HGNC Symbol;Acc:HGNC:30792] |
| ENSG00000114904 | HGLibA_31653 | 79 | -0.4201114 | NEK4 | NIMA related kinase 4 [Source:HGNC Symbol;Acc:HGNC:11399] |
| ENSG00000115009 | HGLibA_07991 | 79 | -0.4201114 | CCL20 | C-C motif chemokine ligand 20 [Source:HGNC Symbol;Acc:HGNC:10619] |
| ENSG00000115526 | HGLibA_09612 | 79 | -0.4201114 | CHST10 | carbohydrate sulfotransferase 10 [Source:HGNC Symbol;Acc:HGNC:19650] |
| ENSG00000116213 | HGLibA_54404 | 79 | -0.4201114 | WRAP73 | antisense to TP73 [Source:HGNC Symbol;Acc:HGNC:12759]" |
| ENSG00000117419 | HGLibA_15484 | 79 | -0.4201114 | ERI3 | ERI1 exoribonuclease family member 3 [Source:HGNC Symbol;Acc:HGNC:17276] |
| ENSG00000117519 | HGLibA_10310 | 79 | -0.4201114 | CNN3 | calponin 3 [Source:HGNC Symbol;Acc:HGNC:2157] |
| ENSG00000118514 | HGLibA_01586 | 79 | -0.4201114 | ALDH8A1 | aldehyde dehydrogenase 8 family member A1 [Source:HGNC Symbol;Acc:HGNC:15471] |
| ENSG00000119866 | HGLibA_04291 | 79 | -0.4201114 | BCL11A | B cell CLL/lymphoma 11A [Source:HGNC Symbol;Acc:HGNC:13221] |
| ENSG00000122180 | HGLibA_30886 | 79 | -0.4201114 | MYOG | myogenin [Source:HGNC Symbol;Acc:HGNC:7612] |
| ENSG00000122965 | HGLibA_40503 | 79 | -0.4201114 | RBM19 | RNA binding motif protein 19 [Source:HGNC Symbol;Acc:HGNC:29098] |
| ENSG00000123977 | HGLibA_12347 | 79 | -0.4201114 | DAW1 | dynein assembly factor with WD repeats 1 [Source:HGNC Symbol;Acc:HGNC:26383] |
| ENSG00000124145 | HGLibA_43106 | 79 | -0.4201114 | SDC4 | syndecan 4 [Source:HGNC Symbol;Acc:HGNC:10661] |
| ENSG00000124237 | HGLibA_06030 | 79 | -0.4201114 | C20orf85 | chromosome 20 open reading frame 85 [Source:HGNC Symbol;Acc:HGNC:16216] |
| ENSG00000124493 | HGLibA_20351 | 79 | -0.4201114 | GRM4 | glutamate metabotropic receptor 4 [Source:HGNC Symbol;Acc:HGNC:4596] |
| ENSG00000125675 | HGLibA_20267 | 79 | -0.4201114 | GRIA3 | glutamate ionotropic receptor AMPA type subunit 3 [Source:HGNC Symbol;Acc:HGNC:4573] |
| ENSG00000126266 | HGLibA_17324 | 79 | -0.4201114 | FFAR1 | free fatty acid receptor 1 [Source:HGNC Symbol;Acc:HGNC:4498] |
| ENSG00000127952 | HGLibA_47690 | 79 | -0.4201114 | STYXL1 | serine/threonine/tyrosine interacting like 1 [Source:HGNC Symbol;Acc:HGNC:18165] |
| ENSG00000128594 | HGLibA_27351 | 79 | -0.4201114 | LRRC4 | leucine rich repeat containing 4 [Source:HGNC Symbol;Acc:HGNC:15586] |
| ENSG00000132329 | HGLibA_40180 | 79 | -0.4201114 | RAMP1 | receptor activity modifying protein 1 [Source:HGNC Symbol;Acc:HGNC:9843] |
| ENSG00000132356 | HGLibA_38492 | 79 | -0.4201114 | PRKAA1 | protein kinase AMP-activated catalytic subunit alpha 1 [Source:HGNC Symbol;Acc:HGNC:9376] |
| ENSG00000132406 | HGLibA_49919 | 79 | -0.4201114 | TMEM128 | transmembrane protein 128 [Source:HGNC Symbol;Acc:HGNC:28201] |
| ENSG00000132464 | HGLibA_15108 | 79 | -0.4201114 | ENAM | enamelin [Source:HGNC Symbol;Acc:HGNC:3344] |
| ENSG00000133105 | HGLibA_42497 | 79 | -0.4201114 | RXFP2 | relaxin family peptide receptor 2 [Source:HGNC Symbol;Acc:HGNC:17318] |
| ENSG00000135108 | HGLibA_17045 | 79 | -0.4201114 | FBXO21 | F-box protein 21 [Source:HGNC Symbol;Acc:HGNC:13592] |
| ENSG00000135297 | HGLibA_30452 | 79 | -0.4201114 | MTO1 | mitochondrial tRNA translation optimization 1 [Source:HGNC Symbol;Acc:HGNC:19261] |
| ENSG00000135824 | HGLibA_41110 | 79 | -0.4201114 | RGS8 | regulator of G protein signaling 8 [Source:HGNC Symbol;Acc:HGNC:16810] |
| ENSG00000135917 | HGLibA_44505 | 79 | -0.4201114 | SLC19A3 | solute carrier family 19 member 3 [Source:HGNC Symbol;Acc:HGNC:16266] |
| ENSG00000135951 | HGLibA_51846 | 79 | -0.4201114 | TSGA10 | testis specific 10 [Source:HGNC Symbol;Acc:HGNC:14927] |
| ENSG00000136141 | HGLibA_27138 | 79 | -0.4201114 | LRCH1 | leucine rich repeats and calponin homology domain containing 1 [Source:HGNC Symbol;Acc:HGNC:20309] |
| ENSG00000136159 | HGLibA_32948 | 79 | -0.4201114 | NUDT15 | nudix hydrolase 15 [Source:HGNC Symbol;Acc:HGNC:23063] |
| ENSG00000136244 | HGLibA_23182 | 79 | -0.4201114 | IL6 | interleukin 6 [Source:HGNC Symbol;Acc:HGNC:6018] |
| ENSG00000136631 | HGLibA_53793 | 79 | -0.4201114 | VPS45 | vacuolar protein sorting 45 homolog [Source:HGNC Symbol;Acc:HGNC:14579] |
| ENSG00000137221 | HGLibA_49569 | 79 | -0.4201114 | TJAP1 | tight junction associated protein 1 [Source:HGNC Symbol;Acc:HGNC:17949] |
| ENSG00000137310 | HGLibA_48768 | 79 | -0.4201114 | TCF19 | transcription factor 19 [Source:HGNC Symbol;Acc:HGNC:11629] |
| ENSG00000138623 | HGLibA_43375 | 79 | -0.4201114 | SEMA7A | semaphorin 7A (John Milton Hagen blood group) [Source:HGNC Symbol;Acc:HGNC:10741] |
| ENSG00000138794 | HGLibA_07320 | 79 | -0.4201114 | CASP6 | caspase 6 [Source:HGNC Symbol;Acc:HGNC:1507] |
| ENSG00000139793 | HGLibA_28512 | 79 | -0.4201114 | MBNL2 | muscleblind like splicing regulator 2 [Source:HGNC Symbol;Acc:HGNC:16746] |
| ENSG00000141469 | HGLibA_44399 | 79 | -0.4201114 | SLC14A1 | solute carrier family 14 member 1 (Kidd blood group) [Source:HGNC Symbol;Acc:HGNC:10918] |
| ENSG00000141867 | HGLibA_04778 | 79 | -0.4201114 | BRD4 | bromodomain containing 4 [Source:HGNC Symbol;Acc:HGNC:13575] |
| ENSG00000143125 | HGLibA_38664 | 79 | -0.4201114 | PROK1 | prokineticin 1 [Source:HGNC Symbol;Acc:HGNC:18454] |
| ENSG00000143578 | HGLibA_11103 | 79 | -0.4201114 | CREB3L4 | cAMP responsive element binding protein 3 like 4 [Source:HGNC Symbol;Acc:HGNC:18854] |
| ENSG00000143603 | HGLibA_24361 | 79 | -0.4201114 | KCNN3 | potassium calcium-activated channel subfamily N member 3 [Source:HGNC Symbol;Acc:HGNC:6292] |
| ENSG00000143641 | HGLibA_18555 | 79 | -0.4201114 | GALNT2 | polypeptide N-acetylgalactosaminyltransferase 2 [Source:HGNC Symbol;Acc:HGNC:4124] |
| ENSG00000143971 | HGLibA_15615 | 79 | -0.4201114 | ETAA1 | ATR kinase activator [Source:HGNC Symbol;Acc:HGNC:24648]" |
| ENSG00000143977 | HGLibA_45990 | 79 | -0.4201114 | SNRPG | small nuclear ribonucleoprotein polypeptide G [Source:HGNC Symbol;Acc:HGNC:11163] |
| ENSG00000144229 | HGLibA_49409 | 79 | -0.4201114 | THSD7B | thrombospondin type 1 domain containing 7B [Source:HGNC Symbol;Acc:HGNC:29348] |
| ENSG00000144535 | HGLibA_13277 | 79 | -0.4201114 | DIS3L2 | DIS3 like 3'-5' exoribonuclease 2 [Source:HGNC Symbol;Acc:HGNC:28648] |
| ENSG00000145723 | HGLibA_19098 | 79 | -0.4201114 | GIN1 | gypsy retrotransposon integrase 1 [Source:HGNC Symbol;Acc:HGNC:25959] |
| ENSG00000146802 | HGLibA_50044 | 79 | -0.4201114 | TMEM168 | transmembrane protein 168 [Source:HGNC Symbol;Acc:HGNC:25826] |
| ENSG00000148218 | HGLibA_01521 | 79 | -0.4201114 | ALAD | aminolevulinate dehydratase [Source:HGNC Symbol;Acc:HGNC:395] |

| | | | | | |
|---|---|---|---|---|---|
| ENSG00000151502 | HGLibA_53756 | 79 | -0.4201114 | VPS26B | retromer complex component B [Source:HGNC Symbol;Acc:HGNC:28119]" |
| ENSG00000152270 | HGLibA_35784 | 79 | -0.4201114 | PDE3B | phosphodiesterase 3B [Source:HGNC Symbol;Acc:HGNC:8779] |
| ENSG00000154814 | HGLibA_34828 | 79 | -0.4201114 | OXNAD1 | oxidoreductase NAD binding domain containing 1 [Source:HGNC Symbol;Acc:HGNC:25128] |
| ENSG00000158813 | HGLibA_14429 | 79 | -0.4201114 | EDA | ectodysplasin A [Source:HGNC Symbol;Acc:HGNC:3157] |
| ENSG00000160191 | HGLibA_35834 | 79 | -0.4201114 | PDE9A | phosphodiesterase 9A [Source:HGNC Symbol;Acc:HGNC:8795] |
| ENSG00000161551 | HGLibA_56295 | 79 | -0.4201114 | ZNF577 | zinc finger protein 577 [Source:HGNC Symbol;Acc:HGNC:28673] |
| ENSG00000162298 | HGLibA_48111 | 79 | -0.4201114 | SYVN1 | synoviolin 1 [Source:HGNC Symbol;Acc:HGNC:20738] |
| ENSG00000162341 | HGLibA_51109 | 79 | -0.4201114 | TPCN2 | two pore segment channel 2 [Source:HGNC Symbol;Acc:HGNC:20820] |
| ENSG00000162365 | HGLibA_12175 | 79 | -0.4201114 | CYP4A22 | cytochrome P450 family 4 subfamily A member 22 [Source:HGNC Symbol;Acc:HGNC:20575] |
| ENSG00000162706 | HGLibA_06970 | 79 | -0.4201114 | CADM3 | cell adhesion molecule 3 [Source:HGNC Symbol;Acc:HGNC:17601] |
| ENSG00000162836 | HGLibA_00518 | 79 | -0.4201114 | ACP6 | lysophosphatidic [Source:HGNC Symbol;Acc:HGNC:29609]" |
| ENSG00000163467 | HGLibA_51808 | 79 | -0.4201114 | TSACC | TSSK6 activating cochaperone [Source:HGNC Symbol;Acc:HGNC:30636] |
| ENSG00000163823 | HGLibA_08145 | 79 | -0.4201114 | CCR1 | C-C motif chemokine receptor 1 [Source:HGNC Symbol;Acc:HGNC:1602] |
| ENSG00000164045 | HGLibA_08526 | 79 | -0.4201114 | CDC25A | cell division cycle 25A [Source:HGNC Symbol;Acc:HGNC:1725] |
| ENSG00000164209 | HGLibA_44754 | 79 | -0.4201114 | SLC25A46 | solute carrier family 25 member 46 [Source:HGNC Symbol;Acc:HGNC:25198] |
| ENSG00000164506 | HGLibA_47676 | 79 | -0.4201114 | STXBP5 | syntaxin binding protein 5 [Source:HGNC Symbol;Acc:HGNC:19665] |
| ENSG00000164649 | HGLibA_08618 | 79 | -0.4201114 | CDCA7L | cell division cycle associated 7 like [Source:HGNC Symbol;Acc:HGNC:30777] |
| ENSG00000164707 | HGLibA_44394 | 79 | -0.4201114 | SLC13A4 | solute carrier family 13 member 4 [Source:HGNC Symbol;Acc:HGNC:15827] |
| ENSG00000164823 | HGLibA_34690 | 79 | -0.4201114 | OSGIN2 | oxidative stress induced growth inhibitor family member 2 [Source:HGNC Symbol;Acc:HGNC:1355] |
| ENSG00000165512 | HGLibA_55606 | 79 | -0.4201114 | ZNF22 | zinc finger protein 22 [Source:HGNC Symbol;Acc:HGNC:13012] |
| ENSG00000165732 | HGLibA_12673 | 79 | -0.4201114 | DDX21 | DExD-box helicase 21 [Source:HGNC Symbol;Acc:HGNC:2744] |
| ENSG00000166317 | HGLibA_48023 | 79 | -0.4201114 | SYNPO2L | synaptopodin 2 like [Source:HGNC Symbol;Acc:HGNC:23532] |
| ENSG00000166484 | HGLibA_28279 | 79 | -0.4201114 | MAPK7 | mitogen-activated protein kinase 7 [Source:HGNC Symbol;Acc:HGNC:6880] |
| ENSG00000166579 | HGLibA_31390 | 79 | -0.4201114 | NDEL1 | nudE neurodevelopment protein 1 like 1 [Source:HGNC Symbol;Acc:HGNC:17620] |
| ENSG00000167107 | HGLibA_00547 | 79 | -0.4201114 | ACSF2 | acyl-CoA synthetase family member 2 [Source:HGNC Symbol;Acc:HGNC:26101] |
| ENSG00000168062 | HGLibA_04175 | 79 | -0.4201114 | BATF2 | basic leucine zipper ATF-like transcription factor 2 [Source:HGNC Symbol;Acc:HGNC:25163] |
| ENSG00000169018 | HGLibA_17277 | 79 | -0.4201114 | FEM1B | fem-1 homolog B [Source:HGNC Symbol;Acc:HGNC:3649] |
| ENSG00000169564 | HGLibA_35366 | 79 | -0.4201114 | PCBP1 | poly(rC) binding protein 1 [Source:HGNC Symbol;Acc:HGNC:8647] |
| ENSG00000169926 | HGLibA_25052 | 79 | -0.4201114 | KLF13 | Kruppel like factor 13 [Source:HGNC Symbol;Acc:HGNC:13672] |
| ENSG00000170290 | HGLibA_45542 | 79 | -0.4201114 | SLN | sarcolipin [Source:HGNC Symbol;Acc:HGNC:11089] |
| ENSG00000170374 | HGLibA_46313 | 79 | -0.4201114 | SP7 | Sp7 transcription factor [Source:HGNC Symbol;Acc:HGNC:17321] |
| ENSG00000171189 | HGLibA_20282 | 79 | -0.4201114 | GRIK1 | glutamate ionotropic receptor kainate type subunit 1 [Source:HGNC Symbol;Acc:HGNC:4579] |
| ENSG00000171564 | HGLibA_17340 | 79 | -0.4201114 | FGB | fibrinogen beta chain [Source:HGNC Symbol;Acc:HGNC:3662] |
| ENSG00000171747 | HGLibA_26370 | 79 | -0.4201114 | LGALS4 | galectin 4 [Source:HGNC Symbol;Acc:HGNC:6565] |
| ENSG00000171790 | HGLibA_45498 | 79 | -0.4201114 | SLFNL1 | schlafen like 1 [Source:HGNC Symbol;Acc:HGNC:26313] |
| ENSG00000171847 | HGLibA_16791 | 79 | -0.4201114 | FAM90A1 | family with sequence similarity 90 member A1 [Source:HGNC Symbol;Acc:HGNC:25526] |
| ENSG00000171865 | HGLibA_41425 | 79 | -0.4201114 | RNASEH1 | ribonuclease H1 [Source:HGNC Symbol;Acc:HGNC:18466] |
| ENSG00000172458 | HGLibA_22997 | 79 | -0.4201114 | IL17D | interleukin 17D [Source:HGNC Symbol;Acc:HGNC:5984] |
| ENSG00000172733 | HGLibA_39591 | 79 | -0.4201114 | PURG | purine rich element binding protein G [Source:HGNC Symbol;Acc:HGNC:17930] |
| ENSG00000173093 | HGLibA_07822 | 79 | -0.4201114 | CCDC63 | coiled-coil domain containing 63 [Source:HGNC Symbol;Acc:HGNC:26669] |
| ENSG00000173432 | HGLibA_42607 | 79 | -0.4201114 | SAA1 | serum amyloid A1 [Source:HGNC Symbol;Acc:HGNC:10513] |
| ENSG00000173517 | HGLibA_36005 | 79 | -0.4201114 | PEAK1 | pseudopodium enriched atypical kinase 1 [Source:HGNC Symbol;Acc:HGNC:29431] |
| ENSG00000173757 | HGLibA_47397 | 79 | -0.4201114 | STAT5B | signal transducer and activator of transcription 5B [Source:HGNC Symbol;Acc:HGNC:11367] |
| ENSG00000174059 | HGLibA_08357 | 79 | -0.4201114 | CD34 | CD34 molecule [Source:HGNC Symbol;Acc:HGNC:1662] |
| ENSG00000174151 | HGLibA_11983 | 79 | -0.4201114 | CYB561D1 | cytochrome b561 family member D1 [Source:HGNC Symbol;Acc:HGNC:26804] |
| ENSG00000174945 | HGLibA_01857 | 79 | -0.4201114 | AMZ1 | archaelysin family metallopeptidase 1 [Source:HGNC Symbol;Acc:HGNC:22231] |
| ENSG00000177992 | HGLibA_46505 | 79 | -0.4201114 | SPATA31E1 | SPATA31 subfamily E member 1 [Source:HGNC Symbol;Acc:HGNC:26672] |
| ENSG00000178460 | HGLibA_28634 | 79 | -0.4201114 | MCMDC2 | minichromosome maintenance domain containing 2 [Source:HGNC Symbol;Acc:HGNC:26368] |
| ENSG00000180777 | HGLibA_02064 | 79 | -0.4201114 | ANKRD30B | ankyrin repeat domain 30B [Source:HGNC Symbol;Acc:HGNC:24165] |
| ENSG00000181143 | HGLibA_30529 | 79 | -0.4201114 | MUC16 | cell surface associated [Source:HGNC Symbol;Acc:HGNC:15582]" |
| ENSG00000181381 | HGLibA_12765 | 79 | -0.4201114 | DDX60L | DExD/H-box 60 like [Source:HGNC Symbol;Acc:HGNC:26429] |
| ENSG00000181767 | HGLibA_34529 | 79 | -0.4201114 | OR8H2 | olfactory receptor family 8 subfamily H member 2 [Source:HGNC Symbol;Acc:HGNC:15308] |
| ENSG00000182698 | HGLibA_40865 | 79 | -0.4201114 | RESP18 | regulated endocrine specific protein 18 [Source:HGNC Symbol;Acc:HGNC:33762] |
| ENSG00000182700 | HGLibA_22859 | 79 | -0.4201114 | IGIP | IgA inducing protein [Source:HGNC Symbol;Acc:HGNC:33847] |
| ENSG00000183378 | HGLibA_34794 | 79 | -0.4201114 | OVCH2 | ovochymase 2 (gene/pseudogene) [Source:HGNC Symbol;Acc:HGNC:29970] |
| ENSG00000183549 | HGLibA_00584 | 79 | -0.4201114 | ACSM5 | acyl-CoA synthetase medium chain family member 5 [Source:HGNC Symbol;Acc:HGNC:26060] |
| ENSG00000183570 | HGLibA_35372 | 79 | -0.4201114 | PCBP3 | poly(rC) binding protein 3 [Source:HGNC Symbol;Acc:HGNC:8651] |
| ENSG00000184384 | HGLibA_28047 | 79 | -0.4201114 | MAML2 | mastermind like transcriptional coactivator 2 [Source:HGNC Symbol;Acc:HGNC:16259] |
| ENSG00000185670 | HGLibA_54855 | 79 | -0.4201114 | ZBTB3 | zinc finger and BTB domain containing 3 [Source:HGNC Symbol;Acc:HGNC:22918] |
| ENSG00000186810 | HGLibA_11886 | 79 | -0.4201114 | CXCR3 | C-X-C motif chemokine receptor 3 [Source:HGNC Symbol;Acc:HGNC:4540] |
| ENSG00000187747 | HGLibA_34150 | 79 | -0.4201114 | OR52B6 | olfactory receptor family 52 subfamily B member 6 [Source:HGNC Symbol;Acc:HGNC:15211] |
| ENSG00000188112 | HGLibA_06444 | 79 | -0.4201114 | C6orf132 | chromosome 6 open reading frame 132 [Source:HGNC Symbol;Acc:HGNC:21288] |
| ENSG00000189114 | HGLibA_04563 | 79 | -0.4201114 | BLOC1S3 | biogenesis of lysosomal organelles complex 1 subunit 3 [Source:HGNC Symbol;Acc:HGNC:20914] |
| ENSG00000196458 | HGLibA_56365 | 79 | -0.4201114 | ZNF605 | zinc finger protein 605 [Source:HGNC Symbol;Acc:HGNC:28068] |
| ENSG00000196502 | HGLibA_47732 | 79 | -0.4201114 | SULT1A1 | sulfotransferase family 1A member 1 [Source:HGNC Symbol;Acc:HGNC:11453] |
| ENSG00000196581 | HGLibA_01371 | 79 | -0.4201114 | AJAP1 | adherens junctions associated protein 1 [Source:HGNC Symbol;Acc:HGNC:30801] |
| ENSG00000196950 | HGLibA_45091 | 79 | -0.4201114 | SLC39A10 | solute carrier family 39 member 10 [Source:HGNC Symbol;Acc:HGNC:20861] |
| ENSG00000196968 | HGLibA_18186 | 79 | -0.4201114 | FUT11 | fucosyltransferase 11 [Source:HGNC Symbol;Acc:HGNC:19233] |
| ENSG00000197124 | HGLibA_56529 | 79 | -0.4201114 | ZNF682 | zinc finger protein 682 [Source:HGNC Symbol;Acc:HGNC:28857] |
| ENSG00000197734 | HGLibA_05346 | 79 | -0.4201114 | C14orf178 | chromosome 14 open reading frame 178 [Source:HGNC Symbol;Acc:HGNC:26385] |
| ENSG00000197808 | HGLibA_56037 | 79 | -0.4201114 | ZNF461 | zinc finger protein 461 [Source:HGNC Symbol;Acc:HGNC:21629] |
| ENSG00000198398 | HGLibA_50197 | 79 | -0.4201114 | TMEM207 | transmembrane protein 207 [Source:HGNC Symbol;Acc:HGNC:33705] |
| ENSG00000198925 | HGLibA_03506 | 79 | -0.4201114 | ATG9A | autophagy related 9A [Source:HGNC Symbol;Acc:HGNC:22408] |
| ENSG00000204305 | HGLibA_01182 | 79 | -0.4201114 | AGER | advanced glycosylation end-product specific receptor [Source:HGNC Symbol;Acc:HGNC:320] |
| ENSG00000204540 | HGLibA_39218 | 79 | -0.4201114 | PSORS1C1 | psoriasis susceptibility 1 candidate 1 [Source:HGNC Symbol;Acc:HGNC:17202] |
| ENSG00000204979 | HGLibA_30136 | 79 | -0.4201114 | MS4A13 | membrane spanning 4-domains A13 [Source:HGNC Symbol;Acc:HGNC:16674] |
| ENSG00000205108 | HGLibA_16422 | 79 | -0.4201114 | FAM205A | family with sequence similarity 205 member A [Source:HGNC Symbol;Acc:HGNC:41911] |
| ENSG00000205307 | HGLibA_42720 | 79 | -0.4201114 | SAP25 | Sin3A associated protein 25 [Source:HGNC Symbol;Acc:HGNC:41908] |
| ENSG00000205502 | HGLibA_06099 | 79 | -0.4201114 | C2CD4B | C2 calcium dependent domain containing 4B [Source:HGNC Symbol;Acc:HGNC:33628] |
| ENSG00000213759 | HGLibA_52914 | 79 | -0.4201114 | UGT2B11 | UDP glucuronosyltransferase family 2 member B11 [Source:HGNC Symbol;Acc:HGNC:12545] |
| ENSG00000214013 | HGLibA_18608 | 79 | -0.4201114 | GANC | neutral C [Source:HGNC Symbol;Acc:HGNC:4139]" |
| ENSG00000214309 | HGLibA_28502 | 79 | -0.4201114 | MBLAC1 | metallo-beta-lactamase domain containing 1 [Source:HGNC Symbol;Acc:HGNC:22180] |
| ENSG00000226174 | HGLibA_49077 | 79 | -0.4201114 | TEX22 | testis expressed 22 [Source:HGNC Symbol;Acc:HGNC:40026] |
| ENSG00000226761 | HGLibA_48415 | 79 | -0.4201114 | TAS2R46 | taste 2 receptor member 46 [Source:HGNC Symbol;Acc:HGNC:18877] |
| ENSG00000230594 | HGLibA_11533 | 79 | -0.4201114 | CT47A4 | member A4 [Source:HGNC Symbol;Acc:HGNC:33285]" |
| ENSG00000234560 | HGLibA_33501 | 79 | -0.4201114 | OR10G8 | olfactory receptor family 10 subfamily G member 8 [Source:HGNC Symbol;Acc:HGNC:14845] |
| ENSG00000237957 | HGLibA_11536 | 79 | -0.4201114 | CT47A5 | member A5 [Source:HGNC Symbol;Acc:HGNC:33286]" |
| ENSG00000241635 | HGLibA_52875 | 79 | -0.4201114 | UGT1A1 | UDP glucuronosyltransferase family 1 member A1 [Source:HGNC Symbol;Acc:HGNC:12530] |
| ENSG00000244038 | HGLibA_12630 | 79 | -0.4201114 | DDOST | dolichyl-diphosphooligosaccharide--protein glycosyltransferase non-catalytic subunit [Source:HGNC Symbol;Acc:HGNC:2728] |
| ENSG00000254004 | HGLibA_55691 | 79 | -0.4201114 | ZNF260 | zinc finger protein 260 [Source:HGNC Symbol;Acc:HGNC:13499] |
| ENSG00000255529 | HGLibA_37606 | 79 | -0.4201114 | POLR2M | RNA polymerase II subunit M [Source:HGNC Symbol;Acc:HGNC:14862] |
| ENSG00000259024 | HGLibA_52366 | 79 | -0.4201114 | TVP23C-CDRT4 | TVP23C-CDRT4 readthrough [Source:HGNC Symbol;Acc:HGNC:42961] |
| ENSG00000259571 | HGLibA_04543 | 79 | -0.4201114 | BLID | cell death inducer [Source:HGNC Symbol;Acc:HGNC:33495]" |
| FAM195B | HGLibA_16379 | 79 | -0.4201114 | FAM195B | - |
| FLJ22184 | HGLibA_17622 | 79 | -0.4201114 | FLJ22184 | - |
| HIST1H3B | HGLibA_21458 | 79 | -0.4201114 | HIST1H3B | - |
| HIST1H4C | HGLibA_21493 | 79 | -0.4201114 | HIST1H4C | - |
| HIST1H4K | HGLibA_21515 | 79 | -0.4201114 | HIST1H4K | - |
| hsa-mir-101-1 | HGLibA_57155 | 79 | -0.4201114 | hsa-mir-101-1 | - |
| hsa-mir-1295b | HGLibA_57661 | 79 | -0.4201114 | hsa-mir-1295b | - |
| hsa-mir-2116 | HGLibA_58236 | 79 | -0.4201114 | hsa-mir-2116 | - |
| hsa-mir-27b | HGLibA_58382 | 79 | -0.4201114 | hsa-mir-27b | - |
| hsa-mir-3120 | HGLibA_58533 | 79 | -0.4201114 | hsa-mir-3120 | - |
| hsa-mir-3131 | HGLibA_58580 | 79 | -0.4201114 | hsa-mir-3131 | - |
| hsa-mir-3139 | HGLibA_58614 | 79 | -0.4201114 | hsa-mir-3139 | - |
| hsa-mir-33b | HGLibA_59016 | 79 | -0.4201114 | hsa-mir-33b | - |
| hsa-mir-3690-2 | HGLibA_59349 | 79 | -0.4201114 | hsa-mir-3690-2 | - |
| hsa-mir-4287 | HGLibA_59854 | 79 | -0.4201114 | hsa-mir-4287 | - |
| hsa-mir-4294 | HGLibA_59887 | 79 | -0.4201114 | hsa-mir-4294 | - |
| hsa-mir-4296 | HGLibA_59895 | 79 | -0.4201114 | hsa-mir-4296 | - |
| hsa-mir-4301 | HGLibA_59916 | 79 | -0.4201114 | hsa-mir-4301 | - |
| hsa-mir-4326 | HGLibA_60029 | 79 | -0.4201114 | hsa-mir-4326 | - |
| hsa-mir-4641 | HGLibA_60667 | 79 | -0.4201114 | hsa-mir-4641 | - |
| hsa-mir-4782 | HGLibA_61236 | 79 | -0.4201114 | hsa-mir-4782 | - |
| hsa-mir-4799 | HGLibA_61302 | 79 | -0.4201114 | hsa-mir-4799 | - |
| hsa-mir-4804 | HGLibA_61319 | 79 | -0.4201114 | hsa-mir-4804 | - |
| hsa-mir-548z | HGLibA_62089 | 79 | -0.4201114 | hsa-mir-548z | - |
| hsa-mir-6089-1 | HGLibA_62614 | 79 | -0.4201114 | hsa-mir-6089-1 | - |
| hsa-mir-6757 | HGLibA_63184 | 79 | -0.4201114 | hsa-mir-6757 | - |
| hsa-mir-6832 | HGLibA_63505 | 79 | -0.4201114 | hsa-mir-6832 | - |
| hsa-mir-873 | HGLibA_64201 | 79 | -0.4201114 | hsa-mir-873 | - |
| KIAA0317 | HGLibA_24616 | 79 | -0.4201114 | KIAA0317 | - |
| KIAA0391 | HGLibA_24632 | 79 | -0.4201114 | KIAA0391 | - |
| LOC100653515 | HGLibA_26876 | 79 | -0.4201114 | LOC100653515 | - |
| MKL1 | HGLibA_29334 | 79 | -0.4201114 | MKL1 | - |
| NARR | HGLibA_31151 | 79 | -0.4201114 | NARR | - |
| TCP10 | HGLibA_48834 | 79 | -0.4201114 | TCP10 | - |
| TMEM189-UBE2V1 | HGLibA_50136 | 79 | -0.4201114 | TMEM189-UBE2V1 | - |
| XRCC6BP1 | HGLibA_54601 | 79 | -0.4201114 | XRCC6BP1 | - |
| 4-Mar | HGLibA_28338 | 78 | -0.429156743 | 4-Mar | - |
| ABP1 | HGLibA_00321 | 78 | -0.429156743 | ABP1 | - |
| ALS2CR8 | HGLibA_01731 | 78 | -0.429156743 | ALS2CR8 | - |
| ATP5I | HGLibA_03668 | 78 | -0.429156743 | ATP5I | - |
| C20orf112 | HGLibA_05985 | 78 | -0.429156743 | C20orf112 | - |
| C6orf57 | HGLibA_06497 | 78 | -0.429156743 | C6orf57 | - |

| | | | | | |
|---|---|---|---|---|---|
| C7orf71 | HGLibA_06585 | 78 | -0.429156743 | C7orf71 | - |
| C9orf69 | HGLibA_06756 | 78 | -0.429156743 | C9orf69 | - |
| ENSG00000008300 | HGLibA_09029 | 78 | -0.429156743 | CELSR3 | cadherin EGF LAG seven-pass G-type receptor 3 [Source:HGNC Symbol;Acc:HGNC:3230] |
| ENSG00000008323 | HGLibA_37119 | 78 | -0.429156743 | PLEKHG6 | pleckstrin homology and RhoGEF domain containing G6 [Source:HGNC Symbol;Acc:HGNC:25562] |
| ENSG00000010932 | HGLibA_17702 | 78 | -0.429156743 | FMO1 | flavin containing monooxygenase 1 [Source:HGNC Symbol;Acc:HGNC:3769] |
| ENSG00000013275 | HGLibA_39138 | 78 | -0.429156743 | PSMC4 | ATPase 4 [Source:HGNC Symbol;Acc:HGNC:9551]" |
| ENSG00000018510 | HGLibA_01241 | 78 | -0.429156743 | AGPS | alkylglycerone phosphate synthase [Source:HGNC Symbol;Acc:HGNC:327] |
| ENSG00000020577 | HGLibA_42682 | 78 | -0.429156743 | SAMD4A | sterile alpha motif domain containing 4A [Source:HGNC Symbol;Acc:HGNC:23023] |
| ENSG00000023041 | HGLibA_55123 | 78 | -0.429156743 | ZDHHC6 | zinc finger DHHC-type containing 6 [Source:HGNC Symbol;Acc:HGNC:19160] |
| ENSG00000027697 | HGLibA_22711 | 78 | -0.429156743 | IFNGR1 | interferon gamma receptor 1 [Source:HGNC Symbol;Acc:HGNC:5439] |
| ENSG00000049247 | HGLibA_53461 | 78 | -0.429156743 | UTS2 | urotensin 2 [Source:HGNC Symbol;Acc:HGNC:12636] |
| ENSG00000052723 | HGLibA_44169 | 78 | -0.429156743 | SIKE1 | suppressor of IKBKE 1 [Source:HGNC Symbol;Acc:HGNC:26119] |
| ENSG00000058091 | HGLibA_08737 | 78 | -0.429156743 | CDK14 | cyclin dependent kinase 14 [Source:HGNC Symbol;Acc:HGNC:8883] |
| ENSG00000060709 | HGLibA_41258 | 78 | -0.429156743 | RIMBP2 | RIMS binding protein 2 [Source:HGNC Symbol;Acc:HGNC:30339] |
| ENSG00000064726 | HGLibA_04892 | 78 | -0.429156743 | BTBD1 | BTB domain containing 1 [Source:HGNC Symbol;Acc:HGNC:1120] |
| ENSG00000066557 | HGLibA_27354 | 78 | -0.429156743 | LRRC40 | leucine rich repeat containing 40 [Source:HGNC Symbol;Acc:HGNC:26004] |
| ENSG00000067836 | HGLibA_41751 | 78 | -0.429156743 | ROGDI | rogdi homolog [Source:HGNC Symbol;Acc:HGNC:29478] |
| ENSG00000070729 | HGLibA_10274 | 78 | -0.429156743 | CNGB1 | cyclic nucleotide gated channel beta 1 [Source:HGNC Symbol;Acc:HGNC:2151] |
| ENSG00000076650 | HGLibA_19735 | 78 | -0.429156743 | GPATCH1 | G-patch domain containing 1 [Source:HGNC Symbol;Acc:HGNC:24658] |
| ENSG00000078399 | HGLibA_21924 | 78 | -0.429156743 | HOXA9 | homeobox A9 [Source:HGNC Symbol;Acc:HGNC:5109] |
| ENSG00000081842 | HGLibA_35451 | 78 | -0.429156743 | PCDHA6 | protocadherin alpha 6 [Source:HGNC Symbol;Acc:HGNC:8672] |
| ENSG00000082196 | HGLibA_05942 | 78 | -0.429156743 | C1QTNF3 | C1q and TNF related 3 [Source:HGNC Symbol;Acc:HGNC:14326] |
| ENSG00000085491 | HGLibA_44682 | 78 | -0.429156743 | SLC25A24 | solute carrier family 25 member 24 [Source:HGNC Symbol;Acc:HGNC:20662] |
| ENSG00000085872 | HGLibA_09424 | 78 | -0.429156743 | CHERP | calcium homeostasis endoplasmic reticulum protein [Source:HGNC Symbol;Acc:HGNC:16930] |
| ENSG00000086205 | HGLibA_17781 | 78 | -0.429156743 | FOLH1 | folate hydrolase 1 [Source:HGNC Symbol;Acc:HGNC:3788] |
| ENSG00000087076 | HGLibA_22185 | 78 | -0.429156743 | HSD17B14 | hydroxysteroid 17-beta dehydrogenase 14 [Source:HGNC Symbol;Acc:HGNC:23238] |
| ENSG00000087502 | HGLibA_15470 | 78 | -0.429156743 | ERGIC2 | ERGIC and golgi 2 [Source:HGNC Symbol;Acc:HGNC:30208] |
| ENSG00000089639 | HGLibA_19409 | 78 | -0.429156743 | GMIP | GEM interacting protein [Source:HGNC Symbol;Acc:HGNC:24852] |
| ENSG00000092208 | HGLibA_18912 | 78 | -0.429156743 | GEMIN2 | gem nuclear organelle associated protein 2 [Source:HGNC Symbol;Acc:HGNC:10884] |
| ENSG00000099901 | HGLibA_40193 | 78 | -0.429156743 | RANBP1 | RAN binding protein 1 [Source:HGNC Symbol;Acc:HGNC:9847] |
| ENSG00000100985 | HGLibA_29543 | 78 | -0.429156743 | MMP9 | matrix metallopeptidase 9 [Source:HGNC Symbol;Acc:HGNC:7176] |
| ENSG00000100987 | HGLibA_53876 | 78 | -0.429156743 | VSX1 | visual system homeobox 1 [Source:HGNC Symbol;Acc:HGNC:12723] |
| ENSG00000101958 | HGLibA_19301 | 78 | -0.429156743 | GLRA2 | glycine receptor alpha 2 [Source:HGNC Symbol;Acc:HGNC:4327] |
| ENSG00000105738 | HGLibA_44199 | 78 | -0.429156743 | SIPA1L3 | signal induced proliferation associated 1 like 3 [Source:HGNC Symbol;Acc:HGNC:23801] |
| ENSG00000105835 | HGLibA_31086 | 78 | -0.429156743 | NAMPT | nicotinamide phosphoribosyltransferase [Source:HGNC Symbol;Acc:HGNC:30092] |
| ENSG00000106809 | HGLibA_33325 | 78 | -0.429156743 | OGN | osteoglycin [Source:HGNC Symbol;Acc:HGNC:8126] |
| ENSG00000108963 | HGLibA_13835 | 78 | -0.429156743 | DPH1 | diphthamide biosynthesis 1 [Source:HGNC Symbol;Acc:HGNC:3003] |
| ENSG00000109519 | HGLibA_20370 | 78 | -0.429156743 | GRPEL1 | mitochondrial [Source:HGNC Symbol;Acc:HGNC:19696]" |
| ENSG00000109794 | HGLibA_16150 | 78 | -0.429156743 | FAM149A | family with sequence similarity 149 member A [Source:HGNC Symbol;Acc:HGNC:24527] |
| ENSG00000110104 | HGLibA_07902 | 78 | -0.429156743 | CCDC86 | coiled-coil domain containing 86 [Source:HGNC Symbol;Acc:HGNC:28359] |
| ENSG00000110171 | HGLibA_51457 | 78 | -0.429156743 | TRIM3 | tripartite motif containing 3 [Source:HGNC Symbol;Acc:HGNC:10064] |
| ENSG00000110367 | HGLibA_12759 | 78 | -0.429156743 | DDX6 | DEAD-box helicase 6 [Source:HGNC Symbol;Acc:HGNC:2747] |
| ENSG00000110881 | HGLibA_03300 | 78 | -0.429156743 | ASIC1 | acid sensing ion channel subunit 1 [Source:HGNC Symbol;Acc:HGNC:100] |
| ENSG00000110944 | HGLibA_23098 | 78 | -0.429156743 | IL23A | interleukin 23 subunit alpha [Source:HGNC Symbol;Acc:HGNC:15488] |
| ENSG00000111404 | HGLibA_40863 | 78 | -0.429156743 | RERGL | RERG like [Source:HGNC Symbol;Acc:HGNC:26213] |
| ENSG00000111540 | HGLibA_39964 | 78 | -0.429156743 | RAB5B | member RAS oncogene family [Source:HGNC Symbol;Acc:HGNC:9784]" |
| ENSG00000111670 | HGLibA_19573 | 78 | -0.429156743 | GNPTAB | N-acetylglucosamine-1-phosphate transferase subunits alpha and beta [Source:HGNC Symbol;Acc:HGNC:29670] |
| ENSG00000111678 | HGLibA_05269 | 78 | -0.429156743 | C12orf57 | chromosome 12 open reading frame 57 [Source:HGNC Symbol;Acc:HGNC:29521] |
| ENSG00000111817 | HGLibA_14015 | 78 | -0.429156743 | DSE | dermatan sulfate epimerase [Source:HGNC Symbol;Acc:HGNC:21144] |
| ENSG00000111837 | HGLibA_28015 | 78 | -0.429156743 | MAK | male germ cell associated kinase [Source:HGNC Symbol;Acc:HGNC:6816] |
| ENSG00000111885 | HGLibA_28057 | 78 | -0.429156743 | MAN1A1 | mannosidase alpha class 1A member 1 [Source:HGNC Symbol;Acc:HGNC:6821] |
| ENSG00000111886 | HGLibA_18406 | 78 | -0.429156743 | GABRR2 | gamma-aminobutyric acid type A receptor rho2 subunit [Source:HGNC Symbol;Acc:HGNC:4091] |
| ENSG00000112294 | HGLibA_01578 | 78 | -0.429156743 | ALDH5A1 | aldehyde dehydrogenase 5 family member A1 [Source:HGNC Symbol;Acc:HGNC:408] |
| ENSG00000115616 | HGLibA_45404 | 78 | -0.429156743 | SLC9A2 | solute carrier family 9 member A2 [Source:HGNC Symbol;Acc:HGNC:11072] |
| ENSG00000116191 | HGLibA_40170 | 78 | -0.429156743 | RALGPS2 | Ral GEF with PH domain and SH3 binding motif 2 [Source:HGNC Symbol;Acc:HGNC:30279] |
| ENSG00000117472 | HGLibA_51891 | 78 | -0.429156743 | TSPAN1 | tetraspanin 1 [Source:HGNC Symbol;Acc:HGNC:20657] |
| ENSG00000119919 | HGLibA_32037 | 78 | -0.429156743 | NKX2-3 | NK2 homeobox 3 [Source:HGNC Symbol;Acc:HGNC:7836] |
| ENSG00000120370 | HGLibA_19674 | 78 | -0.429156743 | GORAB | RAB6 interacting [Source:HGNC Symbol;Acc:HGNC:25676]" |
| ENSG00000122574 | HGLibA_54310 | 78 | -0.429156743 | WIPF3 | WAS/WASL interacting protein family member 3 [Source:HGNC Symbol;Acc:HGNC:22004] |
| ENSG00000123131 | HGLibA_38417 | 78 | -0.429156743 | PRDX4 | peroxiredoxin 4 [Source:HGNC Symbol;Acc:HGNC:17169] |
| ENSG00000124155 | HGLibA_36589 | 78 | -0.429156743 | PIGT | phosphatidylinositol glycan anchor biosynthesis class T [Source:HGNC Symbol;Acc:HGNC:14938] |
| ENSG00000124249 | HGLibA_24306 | 78 | -0.429156743 | KCNK15 | potassium two pore domain channel subfamily K member 15 [Source:HGNC Symbol;Acc:HGNC:13814] |
| ENSG00000124260 | HGLibA_27894 | 78 | -0.429156743 | MAGEA10 | MAGE family member A10 [Source:HGNC Symbol;Acc:HGNC:6797] |
| ENSG00000124508 | HGLibA_04965 | 78 | -0.429156743 | BTN2A2 | butyrophilin subfamily 2 member A2 [Source:HGNC Symbol;Acc:HGNC:1137] |
| ENSG00000127954 | HGLibA_47431 | 78 | -0.429156743 | STEAP4 | STEAP4 metalloreductase [Source:HGNC Symbol;Acc:HGNC:21923] |
| ENSG00000128040 | HGLibA_46692 | 78 | -0.429156743 | SPINK2 | Kazal type 2 [Source:HGNC Symbol;Acc:HGNC:11245]" |
| ENSG00000128203 | HGLibA_03357 | 78 | -0.429156743 | ASPHD2 | aspartate beta-hydroxylase domain containing 2 [Source:HGNC Symbol;Acc:HGNC:30437] |
| ENSG00000129451 | HGLibA_25235 | 78 | -0.429156743 | KLK10 | kallikrein related peptidase 10 [Source:HGNC Symbol;Acc:HGNC:6358] |
| ENSG00000130294 | HGLibA_24882 | 78 | -0.429156743 | KIF1A | kinesin family member 1A [Source:HGNC Symbol;Acc:HGNC:888] |
| ENSG00000130717 | HGLibA_52811 | 78 | -0.429156743 | UCK1 | uridine-cytidine kinase 1 [Source:HGNC Symbol;Acc:HGNC:14859] |
| ENSG00000131242 | HGLibA_39801 | 78 | -0.429156743 | RAB11FIP4 | RAB11 family interacting protein 4 [Source:HGNC Symbol;Acc:HGNC:30267] |
| ENSG00000132323 | HGLibA_23222 | 78 | -0.429156743 | ILKAP | ILK associated serine/threonine phosphatase [Source:HGNC Symbol;Acc:HGNC:15566] |
| ENSG00000132517 | HGLibA_45243 | 78 | -0.429156743 | SLC52A1 | solute carrier family 52 member 1 [Source:HGNC Symbol;Acc:HGNC:30225] |
| ENSG00000132950 | HGLibA_55403 | 78 | -0.429156743 | ZMYM5 | zinc finger MYM-type containing 5 [Source:HGNC Symbol;Acc:HGNC:13029] |
| ENSG00000132975 | HGLibA_19869 | 78 | -0.429156743 | GPR12 | G protein-coupled receptor 12 [Source:HGNC Symbol;Acc:HGNC:4466] |
| ENSG00000133112 | HGLibA_51209 | 78 | -0.429156743 | TPT1 | translationally-controlled 1 [Source:HGNC Symbol;Acc:HGNC:12022]" |
| ENSG00000133477 | HGLibA_16755 | 78 | -0.429156743 | FAM83F | family with sequence similarity 83 member F [Source:HGNC Symbol;Acc:HGNC:25148] |
| ENSG00000133678 | HGLibA_50326 | 78 | -0.429156743 | TMEM254 | transmembrane protein 254 [Source:HGNC Symbol;Acc:HGNC:25804] |
| ENSG00000134255 | HGLibA_09186 | 78 | -0.429156743 | CEPT1 | choline/ethanolamine phosphotransferase 1 [Source:HGNC Symbol;Acc:HGNC:24289] |
| ENSG00000134802 | HGLibA_45155 | 78 | -0.429156743 | SLC43A3 | solute carrier family 43 member 3 [Source:HGNC Symbol;Acc:HGNC:17466] |
| ENSG00000134817 | HGLibA_02436 | 78 | -0.429156743 | APLNR | apelin receptor [Source:HGNC Symbol;Acc:HGNC:339] |
| ENSG00000136535 | HGLibA_48632 | 78 | -0.429156743 | TBR1 | brain 1 [Source:HGNC Symbol;Acc:HGNC:11590]" |
| ENSG00000136826 | HGLibA_25072 | 78 | -0.429156743 | KLF4 | Kruppel like factor 4 [Source:HGNC Symbol;Acc:HGNC:6348] |
| ENSG00000137449 | HGLibA_10919 | 78 | -0.429156743 | CPEB2 | cytoplasmic polyadenylation element binding protein 2 [Source:HGNC Symbol;Acc:HGNC:21745] |
| ENSG00000137831 | HGLibA_52505 | 78 | -0.429156743 | UACA | uveal autoantigen with coiled-coil domains and ankyrin repeats [Source:HGNC Symbol;Acc:HGNC:15947] |
| ENSG00000138078 | HGLibA_38440 | 78 | -0.429156743 | PREPL | prolyl endopeptidase like [Source:HGNC Symbol;Acc:HGNC:30228] |
| ENSG00000138430 | HGLibA_33338 | 78 | -0.429156743 | OLA1 | Obg like ATPase 1 [Source:HGNC Symbol;Acc:HGNC:28833] |
| ENSG00000139133 | HGLibA_01604 | 78 | -0.429156743 | ALG10 | 2-glucosyltransferase [Source:HGNC Symbol;Acc:HGNC:23162]" |
| ENSG00000140006 | HGLibA_54212 | 78 | -0.429156743 | WDR89 | WD repeat domain 89 [Source:HGNC Symbol;Acc:HGNC:20489] |
| ENSG00000140006 | HGLibA_54211 | 78 | -0.429156743 | WDR89 | WD repeat domain 89 [Source:HGNC Symbol;Acc:HGNC:20489] |
| ENSG00000140254 | HGLibA_14102 | 78 | -0.429156743 | DUOXA1 | dual oxidase maturation factor 1 [Source:HGNC Symbol;Acc:HGNC:26507] |
| ENSG00000140323 | HGLibA_13284 | 78 | -0.429156743 | DISP2 | dispatched RND transporter family member 2 [Source:HGNC Symbol;Acc:HGNC:19712] |
| ENSG00000141753 | HGLibA_22824 | 78 | -0.429156743 | IGFBP4 | insulin like growth factor binding protein 4 [Source:HGNC Symbol;Acc:HGNC:5473] |
| ENSG00000142733 | HGLibA_28193 | 78 | -0.429156743 | MAP3K6 | mitogen-activated protein kinase kinase kinase 6 [Source:HGNC Symbol;Acc:HGNC:6858] |
| ENSG00000143013 | HGLibA_26711 | 78 | -0.429156743 | LMO4 | LIM domain only 4 [Source:HGNC Symbol;Acc:HGNC:6644] |
| ENSG00000144199 | HGLibA_15960 | 78 | -0.429156743 | FAHD2B | fumarylacetoacetate hydrolase domain containing 2B [Source:HGNC Symbol;Acc:HGNC:25318] |
| ENSG00000147570 | HGLibA_13627 | 78 | -0.429156743 | DNAJC5B | DnaJ heat shock protein family (Hsp40) member C5 beta [Source:HGNC Symbol;Acc:HGNC:24138] |
| ENSG00000148604 | HGLibA_41046 | 78 | -0.429156743 | RGR | retinal G protein coupled receptor [Source:HGNC Symbol;Acc:HGNC:9990] |
| ENSG00000149972 | HGLibA_10414 | 78 | -0.429156743 | CNTN5 | contactin 5 [Source:HGNC Symbol;Acc:HGNC:2175] |
| ENSG00000151338 | HGLibA_29298 | 78 | -0.429156743 | MIPOL1 | mirror-image polydactyly 1 [Source:HGNC Symbol;Acc:HGNC:21460] |
| ENSG00000154096 | HGLibA_49424 | 78 | -0.429156743 | THY1 | Thy-1 cell surface antigen [Source:HGNC Symbol;Acc:HGNC:11801] |
| ENSG00000154269 | HGLibA_15174 | 78 | -0.429156743 | ENPP3 | ectonucleotide pyrophosphatase/phosphodiesterase 3 [Source:HGNC Symbol;Acc:HGNC:3358] |
| ENSG00000156052 | HGLibA_19465 | 78 | -0.429156743 | GNAQ | G protein subunit alpha q [Source:HGNC Symbol;Acc:HGNC:4390] |
| ENSG00000156261 | HGLibA_08217 | 78 | -0.429156743 | CCT8 | chaperonin containing TCP1 subunit 8 [Source:HGNC Symbol;Acc:HGNC:1623] |
| ENSG00000158022 | HGLibA_51572 | 78 | -0.429156743 | TRIM63 | tripartite motif containing 63 [Source:HGNC Symbol;Acc:HGNC:16007] |
| ENSG00000161634 | HGLibA_12477 | 78 | -0.429156743 | DCD | dermcidin [Source:HGNC Symbol;Acc:HGNC:14669] |
| ENSG00000163083 | HGLibA_23305 | 78 | -0.429156743 | INHBB | inhibin subunit beta B [Source:HGNC Symbol;Acc:HGNC:6067] |
| ENSG00000163239 | HGLibA_48898 | 78 | -0.429156743 | TDRD10 | tudor domain containing 10 [Source:HGNC Symbol;Acc:HGNC:25316] |
| ENSG00000163462 | HGLibA_51513 | 78 | -0.429156743 | TRIM46 | tripartite motif containing 46 [Source:HGNC Symbol;Acc:HGNC:19019] |
| ENSG00000164182 | HGLibA_31484 | 78 | -0.429156743 | NDUFAF2 | NADH:ubiquinone oxidoreductase complex assembly factor 2 [Source:HGNC Symbol;Acc:HGNC:28086] |
| ENSG00000164663 | HGLibA_53387 | 78 | -0.429156743 | USP49 | ubiquitin specific peptidase 49 [Source:HGNC Symbol;Acc:HGNC:20078] |
| ENSG00000164690 | HGLibA_44029 | 78 | -0.429156743 | SHH | sonic hedgehog [Source:HGNC Symbol;Acc:HGNC:10848] |
| ENSG00000164880 | HGLibA_23407 | 78 | -0.429156743 | INTS1 | integrator complex subunit 1 [Source:HGNC Symbol;Acc:HGNC:24555] |
| ENSG00000164896 | HGLibA_16899 | 78 | -0.429156743 | FASTK | Fas activated serine/threonine kinase [Source:HGNC Symbol;Acc:HGNC:24676] |
| ENSG00000165506 | HGLibA_13461 | 78 | -0.429156743 | DNAAF2 | dynein axonemal assembly factor 2 [Source:HGNC Symbol;Acc:HGNC:20188] |
| ENSG00000166664 | HGLibA_09545 | 78 | -0.429156743 | CHRFAM7A | CHRNA7 (exons 5-10) and FAM7A (exons A-E) fusion [Source:HGNC Symbol;Acc:HGNC:15781] |
| ENSG00000166831 | HGLibA_40655 | 78 | -0.429156743 | RBPMS2 | mRNA processing factor 2 [Source:HGNC Symbol;Acc:HGNC:19098]" |
| ENSG00000167716 | HGLibA_54188 | 78 | -0.429156743 | WDR81 | WD repeat domain 81 [Source:HGNC Symbol;Acc:HGNC:26600] |
| ENSG00000169031 | HGLibA_10571 | 78 | -0.429156743 | COL4A3 | collagen type IV alpha 3 chain [Source:HGNC Symbol;Acc:HGNC:2204] |
| ENSG00000169635 | HGLibA_21290 | 78 | -0.429156743 | HIC2 | HIC ZBTB transcriptional repressor 2 [Source:HGNC Symbol;Acc:HGNC:18595] |
| ENSG00000169762 | HGLibA_48326 | 78 | -0.429156743 | TAPT1 | transmembrane anterior posterior transformation 1 [Source:HGNC Symbol;Acc:HGNC:26887] |
| ENSG00000170860 | HGLibA_27540 | 78 | -0.429156743 | LSM3 | U6 small nuclear RNA and mRNA degradation associated [Source:HGNC Symbol;Acc:HGNC:17874]" |
| ENSG00000170890 | HGLibA_36882 | 78 | -0.429156743 | PLA2G1B | phospholipase A2 group IB [Source:HGNC Symbol;Acc:HGNC:9030] |
| ENSG00000170923 | HGLibA_34485 | 78 | -0.429156743 | OR7G2 | olfactory receptor family 7 subfamily G member 2 [Source:HGNC Symbol;Acc:HGNC:8466] |
| ENSG00000172239 | HGLibA_35029 | 78 | -0.429156743 | PAIP1 | poly(A) binding protein interacting protein 1 [Source:HGNC Symbol;Acc:HGNC:16945] |
| ENSG00000172575 | HGLibA_40344 | 78 | -0.429156743 | RASGRP1 | RAS guanyl releasing protein 1 [Source:HGNC Symbol;Acc:HGNC:9878] |
| ENSG00000172725 | HGLibA_10776 | 78 | -0.429156743 | CORO1B | coronin 1B [Source:HGNC Symbol;Acc:HGNC:2253] |
| ENSG00000172830 | HGLibA_47139 | 78 | -0.429156743 | SSH3 | slingshot protein phosphatase 3 [Source:HGNC Symbol;Acc:HGNC:30581] |
| ENSG00000175482 | HGLibA_37500 | 78 | -0.429156743 | POLD4 | accessory subunit [Source:HGNC Symbol;Acc:HGNC:14106]" |
| ENSG00000175879 | HGLibA_22005 | 78 | -0.429156743 | HOXD8 | homeobox D8 [Source:HGNC Symbol;Acc:HGNC:5139] |
| ENSG00000176177 | HGLibA_15190 | 78 | -0.429156743 | ENTHD1 | ENTH domain containing 1 [Source:HGNC Symbol;Acc:HGNC:26352] |
| ENSG00000176422 | HGLibA_46852 | 78 | -0.429156743 | SPRYD4 | SPRY domain containing 4 [Source:HGNC Symbol;Acc:HGNC:27468] |
| ENSG00000176971 | HGLibA_17502 | 78 | -0.429156743 | FIBIN | fin bud initiation factor homolog [Source:HGNC Symbol;Acc:HGNC:33747] |
| ENSG00000177697 | HGLibA_08236 | 78 | -0.429156743 | CD151 | CD151 molecule (Raph blood group) [Source:HGNC Symbol;Acc:HGNC:1630] |
| ENSG00000178802 | HGLibA_29733 | 78 | -0.429156743 | MPI | mannose phosphate isomerase [Source:HGNC Symbol;Acc:HGNC:7216] |

| | | | | | |
|---|---|---|---|---|---|
| ENSG00000180817 | HGLibA_37805 | 78 | -0.429156743 | PPA1 | pyrophosphatase (inorganic) 1 [Source:HGNC Symbol;Acc:HGNC:9226] |
| ENSG00000180855 | HGLibA_56010 | 78 | -0.429156743 | ZNF443 | zinc finger protein 443 [Source:HGNC Symbol;Acc:HGNC:20878] |
| ENSG00000180855 | HGLibA_56012 | 78 | -0.429156743 | ZNF443 | zinc finger protein 443 [Source:HGNC Symbol;Acc:HGNC:20878] |
| ENSG00000180878 | HGLibA_05140 | 78 | -0.429156743 | C11orf42 | chromosome 11 open reading frame 42 [Source:HGNC Symbol;Acc:HGNC:28541] |
| ENSG00000183010 | HGLibA_39672 | 78 | -0.429156743 | PYCR1 | pyrroline-5-carboxylate reductase 1 [Source:HGNC Symbol;Acc:HGNC:9721] |
| ENSG00000183230 | HGLibA_11643 | 78 | -0.429156743 | CTNNA3 | catenin alpha 3 [Source:HGNC Symbol;Acc:HGNC:2511] |
| ENSG00000183454 | HGLibA_20298 | 78 | -0.429156743 | GRIN2A | glutamate ionotropic receptor NMDA type subunit 2A [Source:HGNC Symbol;Acc:HGNC:4585] |
| ENSG00000183775 | HGLibA_24433 | 78 | -0.429156743 | KCTD16 | potassium channel tetramerization domain containing 16 [Source:HGNC Symbol;Acc:HGNC:29244] |
| ENSG00000184205 | HGLibA_51983 | 78 | -0.429156743 | TSPYL2 | TSPY like 2 [Source:HGNC Symbol;Acc:HGNC:24358] |
| ENSG00000184454 | HGLibA_31332 | 78 | -0.429156743 | NCMAP | non-compact myelin associated protein [Source:HGNC Symbol;Acc:HGNC:29332] |
| ENSG00000185010 | HGLibA_15884 | 78 | -0.429156743 | F8 | coagulation factor VIII [Source:HGNC Symbol;Acc:HGNC:3546] |
| ENSG00000185022 | HGLibA_27877 | 78 | -0.429156743 | MAFF | MAF bZIP transcription factor F [Source:HGNC Symbol;Acc:HGNC:6780] |
| ENSG00000185966 | HGLibA_26143 | 78 | -0.429156743 | LCE3E | late cornified envelope 3E [Source:HGNC Symbol;Acc:HGNC:29463] |
| ENSG00000186130 | HGLibA_54913 | 78 | -0.429156743 | ZBTB6 | zinc finger and BTB domain containing 6 [Source:HGNC Symbol;Acc:HGNC:16764] |
| ENSG00000186306 | HGLibA_33548 | 78 | -0.429156743 | OR10T2 | olfactory receptor family 10 subfamily T member 2 [Source:HGNC Symbol;Acc:HGNC:14816] |
| ENSG00000186364 | HGLibA_32957 | 78 | -0.429156743 | NUDT17 | nudix hydrolase 17 [Source:HGNC Symbol;Acc:HGNC:26618] |
| ENSG00000186446 | HGLibA_56110 | 78 | -0.429156743 | ZNF501 | zinc finger protein 501 [Source:HGNC Symbol;Acc:HGNC:23717] |
| ENSG00000186910 | HGLibA_43510 | 78 | -0.429156743 | SERPINA11 | serpin family A member 11 [Source:HGNC Symbol;Acc:HGNC:19193] |
| ENSG00000187005 | HGLibA_25709 | 78 | -0.429156743 | KRTAP21-1 | keratin associated protein 21-1 [Source:HGNC Symbol;Acc:HGNC:18945] |
| ENSG00000187902 | HGLibA_44049 | 78 | -0.429156743 | SHISA7 | shisa family member 7 [Source:HGNC Symbol;Acc:HGNC:35409] |
| ENSG00000188050 | HGLibA_41507 | 78 | -0.429156743 | RNF133 | ring finger protein 133 [Source:HGNC Symbol;Acc:HGNC:21154] |
| ENSG00000188629 | HGLibA_55530 | 78 | -0.429156743 | ZNF177 | zinc finger protein 177 [Source:HGNC Symbol;Acc:HGNC:12966] |
| ENSG00000188735 | HGLibA_49899 | 78 | -0.429156743 | TMEM120B | transmembrane protein 120B [Source:HGNC Symbol;Acc:HGNC:32008] |
| ENSG00000196466 | HGLibA_56776 | 78 | -0.429156743 | ZNF799 | zinc finger protein 799 [Source:HGNC Symbol;Acc:HGNC:28071] |
| ENSG00000196944 | HGLibA_33884 | 78 | -0.429156743 | OR2T4 | olfactory receptor family 2 subfamily T member 4 [Source:HGNC Symbol;Acc:HGNC:15016] |
| ENSG00000198077 | HGLibA_12108 | 78 | -0.429156743 | CYP2A7 | cytochrome P450 family 2 subfamily A member 7 [Source:HGNC Symbol;Acc:HGNC:2611] |
| ENSG00000198146 | HGLibA_56705 | 78 | -0.429156743 | ZNF770 | zinc finger protein 770 [Source:HGNC Symbol;Acc:HGNC:26061] |
| ENSG00000198203 | HGLibA_47746 | 78 | -0.429156743 | SULT1C2 | sulfotransferase family 1C member 2 [Source:HGNC Symbol;Acc:HGNC:11456] |
| ENSG00000198689 | HGLibA_45422 | 78 | -0.429156743 | SLC9A6 | solute carrier family 9 member A6 [Source:HGNC Symbol;Acc:HGNC:11079] |
| ENSG00000204007 | HGLibA_19335 | 78 | -0.429156743 | GLT6D1 | glycosyltransferase 6 domain containing 1 [Source:HGNC Symbol;Acc:HGNC:23671] |
| ENSG00000204175 | HGLibA_20142 | 78 | -0.429156743 | GPRIN2 | G protein regulated inducer of neurite outgrowth 2 [Source:HGNC Symbol;Acc:HGNC:23730] |
| ENSG00000204237 | HGLibA_34824 | 78 | -0.429156743 | OXLD1 | oxidoreductase like domain containing 1 [Source:HGNC Symbol;Acc:HGNC:27901] |
| ENSG00000204315 | HGLibA_17593 | 78 | -0.429156743 | FKBPL | FK506 binding protein like [Source:HGNC Symbol;Acc:HGNC:13949] |
| ENSG00000204524 | HGLibA_56795 | 78 | -0.429156743 | ZNF805 | zinc finger protein 805 [Source:HGNC Symbol;Acc:HGNC:23272] |
| ENSG00000204657 | HGLibA_33806 | 78 | -0.429156743 | OR2H2 | olfactory receptor family 2 subfamily H member 2 [Source:HGNC Symbol;Acc:HGNC:8253] |
| ENSG00000205758 | HGLibA_11297 | 78 | -0.429156743 | CRYZL1 | crystallin zeta like 1 [Source:HGNC Symbol;Acc:HGNC:2420] |
| ENSG00000205838 | HGLibA_52081 | 78 | -0.429156743 | TTC23L | tetratricopeptide repeat domain 23 like [Source:HGNC Symbol;Acc:HGNC:26355] |
| ENSG00000206531 | HGLibA_08288 | 78 | -0.429156743 | CD200R1L | CD200 receptor 1 like [Source:HGNC Symbol;Acc:HGNC:24665] |
| ENSG00000213139 | HGLibA_11284 | 78 | -0.429156743 | CRYGS | crystallin gamma S [Source:HGNC Symbol;Acc:HGNC:2417] |
| ENSG00000214026 | HGLibA_29933 | 78 | -0.429156743 | MRPL23 | mitochondrial ribosomal protein L23 [Source:HGNC Symbol;Acc:HGNC:10322] |
| ENSG00000215113 | HGLibA_11935 | 78 | -0.429156743 | CXorf49B | chromosome X open reading frame 49B [Source:HGNC Symbol;Acc:HGNC:34229] |
| ENSG00000229676 | HGLibA_56094 | 78 | -0.429156743 | ZNF492 | zinc finger protein 492 [Source:HGNC Symbol;Acc:HGNC:23707] |
| ENSG00000230178 | HGLibA_34004 | 78 | -0.429156743 | OR4F3 | olfactory receptor family 4 subfamily F member 3 [Source:HGNC Symbol;Acc:HGNC:8300] |
| ENSG00000232399 | HGLibA_53223 | 78 | -0.429156743 | USP17L13 | ubiquitin specific peptidase 17-like family member 13 [Source:HGNC Symbol;Acc:HGNC:44441] |
| ENSG00000239839 | HGLibA_12799 | 78 | -0.429156743 | DEFA3 | defensin alpha 3 [Source:HGNC Symbol;Acc:HGNC:2762] |
| ENSG00000244624 | HGLibA_25699 | 78 | -0.429156743 | KRTAP20-1 | keratin associated protein 20-1 [Source:HGNC Symbol;Acc:HGNC:18943] |
| ENSG00000247596 | HGLibA_52373 | 78 | -0.429156743 | TWF2 | twinfilin actin binding protein 2 [Source:HGNC Symbol;Acc:HGNC:9621] |
| ENSG00000254772 | HGLibA_14505 | 78 | -0.429156743 | EEF1G | eukaryotic translation elongation factor 1 gamma [Source:HGNC Symbol;Acc:HGNC:3213] |
| ENSG00000258986 | HGLibA_50087 | 78 | -0.429156743 | TMEM179 | transmembrane protein 179 [Source:HGNC Symbol;Acc:HGNC:20137] |
| ENSG00000260230 | HGLibA_18043 | 78 | -0.429156743 | FRRS1L | ferric chelate reductase 1 like [Source:HGNC Symbol;Acc:HGNC:1362] |
| ENSG00000265190 | HGLibA_02251 | 78 | -0.429156743 | ANXA8 | annexin A8 [Source:HGNC Symbol;Acc:HGNC:546] |
| ENSG00000268182 | HGLibA_45736 | 78 | -0.429156743 | SMIM17 | small integral membrane protein 17 [Source:HGNC Symbol;Acc:HGNC:27114] |
| ENSG00000269713 | HGLibA_31246 | 78 | -0.429156743 | NBPF9 | NBPF member 9 [Source:HGNC Symbol;Acc:HGNC:31991] |
| ENSG00000274611 | HGLibA_48530 | 78 | -0.429156743 | TBC1D3 | TBC1 domain family member 3 [Source:HGNC Symbol;Acc:HGNC:19031] |
| ENSG00000275111 | HGLibA_55564 | 78 | -0.429156743 | ZNF2 | zinc finger protein 2 [Source:HGNC Symbol;Acc:HGNC:12991] |
| ENSG00000275395 | HGLibA_17181 | 78 | -0.429156743 | FCGBP | Fc fragment of IgG binding protein [Source:HGNC Symbol;Acc:HGNC:13572] |
| ENSG00000279270 | HGLibA_34206 | 78 | -0.429156743 | OR52R1 | olfactory receptor family 52 subfamily R member 1 (gene/pseudogene) [Source:HGNC Symbol;Acc:HGNC:15235] |
| FAM129B | HGLibA_16099 | 78 | -0.429156743 | FAM129B | - |
| HFE2 | HGLibA_21228 | 78 | -0.429156743 | HFE2 | - |
| HIST1H2AA | HGLibA_21374 | 78 | -0.429156743 | HIST1H2AA | - |
| HIST1H3J | HGLibA_21483 | 78 | -0.429156743 | HIST1H3J | - |
| HIST1H4A | HGLibA_21486 | 78 | -0.429156743 | HIST1H4A | - |
| HRASLS | HGLibA_22076 | 78 | -0.429156743 | HRASLS | - |
| hsa-mir-1324 | HGLibA_57767 | 78 | -0.429156743 | hsa-mir-1324 | - |
| hsa-mir-19b-1 | HGLibA_58145 | 78 | -0.429156743 | hsa-mir-19b-1 | - |
| hsa-mir-3153 | HGLibA_58671 | 78 | -0.429156743 | hsa-mir-3153 | - |
| hsa-mir-4271 | HGLibA_59793 | 78 | -0.429156743 | hsa-mir-4271 | - |
| hsa-mir-4330 | HGLibA_60049 | 78 | -0.429156743 | hsa-mir-4330 | - |
| hsa-mir-4521 | HGLibA_60540 | 78 | -0.429156743 | hsa-mir-4521 | - |
| hsa-mir-4531 | HGLibA_60583 | 78 | -0.429156743 | hsa-mir-4531 | - |
| hsa-mir-4737 | HGLibA_61045 | 78 | -0.429156743 | hsa-mir-4737 | - |
| hsa-mir-4798 | HGLibA_61299 | 78 | -0.429156743 | hsa-mir-4798 | - |
| hsa-mir-4800 | HGLibA_61307 | 78 | -0.429156743 | hsa-mir-4800 | - |
| hsa-mir-509-3 | HGLibA_61515 | 78 | -0.429156743 | hsa-mir-509-3 | - |
| hsa-mir-518f | HGLibA_61675 | 78 | -0.429156743 | hsa-mir-518f | - |
| hsa-mir-520g | HGLibA_61759 | 78 | -0.429156743 | hsa-mir-520g | - |
| hsa-mir-548i-2 | HGLibA_62016 | 78 | -0.429156743 | hsa-mir-548i-2 | - |
| hsa-mir-5583-1 | HGLibA_62170 | 78 | -0.429156743 | hsa-mir-5583-1 | - |
| hsa-mir-5690 | HGLibA_62278 | 78 | -0.429156743 | hsa-mir-5690 | - |
| hsa-mir-580 | HGLibA_62418 | 78 | -0.429156743 | hsa-mir-580 | - |
| hsa-mir-609 | HGLibA_62622 | 78 | -0.429156743 | hsa-mir-609 | - |
| hsa-mir-6505 | HGLibA_62872 | 78 | -0.429156743 | hsa-mir-6505 | - |
| hsa-mir-6757 | HGLibA_63186 | 78 | -0.429156743 | hsa-mir-6757 | - |
| hsa-mir-6771 | HGLibA_63257 | 78 | -0.429156743 | hsa-mir-6771 | - |
| hsa-mir-6786 | HGLibA_63321 | 78 | -0.429156743 | hsa-mir-6786 | - |
| hsa-mir-6843 | HGLibA_63547 | 78 | -0.429156743 | hsa-mir-6843 | - |
| hsa-mir-7111 | HGLibA_63808 | 78 | -0.429156743 | hsa-mir-7111 | - |
| KIAA0247 | HGLibA_24613 | 78 | -0.429156743 | KIAA0247 | - |
| PRSS45 | HGLibA_38943 | 78 | -0.429156743 | PRSS45 | - |
| C19orf77 | HGLibA_05708 | 77 | -0.438202086 | C19orf77 | - |
| ENSG00000004948 | HGLibA_07004 | 77 | -0.438202086 | CALCR | calcitonin receptor [Source:HGNC Symbol;Acc:HGNC:1440] |
| ENSG00000009307 | HGLibA_11315 | 77 | -0.438202086 | CSDE1 | cold shock domain containing E1 [Source:HGNC Symbol;Acc:HGNC:29905] |
| ENSG00000013016 | HGLibA_14673 | 77 | -0.438202086 | EHD3 | EH domain containing 3 [Source:HGNC Symbol;Acc:HGNC:3244] |
| ENSG00000013810 | HGLibA_48165 | 77 | -0.438202086 | TACC3 | transforming acidic coiled-coil containing protein 3 [Source:HGNC Symbol;Acc:HGNC:11524] |
| ENSG00000019991 | HGLibA_21241 | 77 | -0.438202086 | HGF | hepatocyte growth factor [Source:HGNC Symbol;Acc:HGNC:4893] |
| ENSG00000034693 | HGLibA_36114 | 77 | -0.438202086 | PEX3 | peroxisomal biogenesis factor 3 [Source:HGNC Symbol;Acc:HGNC:8858] |
| ENSG00000053254 | HGLibA_17914 | 77 | -0.438202086 | FOXN3 | forkhead box N3 [Source:HGNC Symbol;Acc:HGNC:1928] |
| ENSG00000054654 | HGLibA_47980 | 77 | -0.438202086 | SYNE2 | spectrin repeat containing nuclear envelope protein 2 [Source:HGNC Symbol;Acc:HGNC:17084] |
| ENSG00000069974 | HGLibA_39856 | 77 | -0.438202086 | RAB27A | member RAS oncogene family [Source:HGNC Symbol;Acc:HGNC:9766]" |
| ENSG00000070159 | HGLibA_39465 | 77 | -0.438202086 | PTPN3 | non-receptor type 3 [Source:HGNC Symbol;Acc:HGNC:9655]" |
| ENSG00000070214 | HGLibA_45156 | 77 | -0.438202086 | SLC44A1 | solute carrier family 44 member 1 [Source:HGNC Symbol;Acc:HGNC:18798] |
| ENSG00000072682 | HGLibA_34904 | 77 | -0.438202086 | P4HA2 | prolyl 4-hydroxylase subunit alpha 2 [Source:HGNC Symbol;Acc:HGNC:8547] |
| ENSG00000074755 | HGLibA_57090 | 77 | -0.438202086 | ZZEF1 | zinc finger ZZ-type and EF-hand domain containing 1 [Source:HGNC Symbol;Acc:HGNC:29027] |
| ENSG00000077254 | HGLibA_53338 | 77 | -0.438202086 | USP33 | ubiquitin specific peptidase 33 [Source:HGNC Symbol;Acc:HGNC:20059] |
| ENSG00000082556 | HGLibA_33446 | 77 | -0.438202086 | OPRK1 | opioid receptor kappa 1 [Source:HGNC Symbol;Acc:HGNC:8154] |
| ENSG00000088387 | HGLibA_13761 | 77 | -0.438202086 | DOCK9 | dedicator of cytokinesis 9 [Source:HGNC Symbol;Acc:HGNC:14132] |
| ENSG00000100105 | HGLibA_35288 | 77 | -0.438202086 | PATZ1 | POZ/BTB and AT hook containing zinc finger 1 [Source:HGNC Symbol;Acc:HGNC:13071] |
| ENSG00000101470 | HGLibA_50867 | 77 | -0.438202086 | TNNC2 | fast skeletal type [Source:HGNC Symbol;Acc:HGNC:11944]" |
| ENSG00000101940 | HGLibA_54032 | 77 | -0.438202086 | WDR13 | WD repeat domain 13 [Source:HGNC Symbol;Acc:HGNC:14352] |
| ENSG00000102870 | HGLibA_56432 | 77 | -0.438202086 | ZNF629 | zinc finger protein 629 [Source:HGNC Symbol;Acc:HGNC:29008] |
| ENSG00000104047 | HGLibA_14073 | 77 | -0.438202086 | DTWD1 | DTW domain containing 1 [Source:HGNC Symbol;Acc:HGNC:30926] |
| ENSG00000105352 | HGLibA_08940 | 77 | -0.438202086 | CEACAM4 | carcinoembryonic antigen related cell adhesion molecule 4 [Source:HGNC Symbol;Acc:HGNC:1816] |
| ENSG00000105404 | HGLibA_39998 | 77 | -0.438202086 | RABAC1 | Rab acceptor 1 [Source:HGNC Symbol;Acc:HGNC:9794] |
| ENSG00000105656 | HGLibA_14920 | 77 | -0.438202086 | ELL | elongation factor for RNA polymerase II [Source:HGNC Symbol;Acc:HGNC:23114] |
| ENSG00000106258 | HGLibA_12161 | 77 | -0.438202086 | CYP3A5 | cytochrome P450 family 3 subfamily A member 5 [Source:HGNC Symbol;Acc:HGNC:2638] |
| ENSG00000106258 | HGLibA_12159 | 77 | -0.438202086 | CYP3A5 | cytochrome P450 family 3 subfamily A member 5 [Source:HGNC Symbol;Acc:HGNC:2638] |
| ENSG00000107438 | HGLibA_35908 | 77 | -0.438202086 | PDLIM1 | PDZ and LIM domain 1 [Source:HGNC Symbol;Acc:HGNC:2067] |
| ENSG00000108773 | HGLibA_24051 | 77 | -0.438202086 | KAT2A | lysine acetyltransferase 2A [Source:HGNC Symbol;Acc:HGNC:4201] |
| ENSG00000109674 | HGLibA_31635 | 77 | -0.438202086 | NEIL3 | nei like DNA glycosylase 3 [Source:HGNC Symbol;Acc:HGNC:24573] |
| ENSG00000110680 | HGLibA_06992 | 77 | -0.438202086 | CALCA | calcitonin related polypeptide alpha [Source:HGNC Symbol;Acc:HGNC:1437] |
| ENSG00000112214 | HGLibA_17489 | 77 | -0.438202086 | FHL5 | four and a half LIM domains 5 [Source:HGNC Symbol;Acc:HGNC:17371] |
| ENSG00000112309 | HGLibA_03974 | 77 | -0.438202086 | B3GAT2 | 3-glucuronyltransferase 2 [Source:HGNC Symbol;Acc:HGNC:922]" |
| ENSG00000113805 | HGLibA_10406 | 77 | -0.438202086 | CNTN3 | contactin 3 [Source:HGNC Symbol;Acc:HGNC:2173] |
| ENSG00000114744 | HGLibA_10655 | 77 | -0.438202086 | COMMD2 | COMM domain containing 2 [Source:HGNC Symbol;Acc:HGNC:24993] |
| ENSG00000115484 | HGLibA_08200 | 77 | -0.438202086 | CCT4 | chaperonin containing TCP1 subunit 4 [Source:HGNC Symbol;Acc:HGNC:1617] |
| ENSG00000116138 | HGLibA_13582 | 77 | -0.438202086 | DNAJC16 | DnaJ heat shock protein family (Hsp40) member C16 [Source:HGNC Symbol;Acc:HGNC:29157] |
| ENSG00000117461 | HGLibA_36653 | 77 | -0.438202086 | PIK3R3 | phosphoinositide-3-kinase regulatory subunit 3 [Source:HGNC Symbol;Acc:HGNC:8981] |
| ENSG00000120306 | HGLibA_12231 | 77 | -0.438202086 | CYSTM1 | cysteine rich transmembrane module containing 1 [Source:HGNC Symbol;Acc:HGNC:30239] |
| ENSG00000120669 | HGLibA_46164 | 77 | -0.438202086 | SOHLH2 | spermatogenesis and oogenesis specific basic helix-loop-helix 2 [Source:HGNC Symbol;Acc:HGNC:26026] |
| ENSG00000121377 | HGLibA_48425 | 77 | -0.438202086 | TAS2R7 | taste 2 receptor member 7 [Source:HGNC Symbol;Acc:HGNC:14913] |
| ENSG00000124116 | HGLibA_54264 | 77 | -0.438202086 | WFDC3 | WAP four-disulfide core domain 3 [Source:HGNC Symbol;Acc:HGNC:15957] |
| ENSG00000125846 | HGLibA_55465 | 77 | -0.438202086 | ZNF133 | zinc finger protein 133 [Source:HGNC Symbol;Acc:HGNC:12917] |
| ENSG00000127084 | HGLibA_17347 | 77 | -0.438202086 | FGD3 | RhoGEF and PH domain containing 3 [Source:HGNC Symbol;Acc:HGNC:16027]" |
| ENSG00000127419 | HGLibA_50069 | 77 | -0.438202086 | TMEM175 | transmembrane protein 175 [Source:HGNC Symbol;Acc:HGNC:28709] |
| ENSG00000129450 | HGLibA_44152 | 77 | -0.438202086 | SIGLEC9 | sialic acid binding Ig like lectin 9 [Source:HGNC Symbol;Acc:HGNC:10878] |

| | | | | | |
|---|---|---|---|---|---|
| ENSG00000130287 | HGLibA_31259 | 77 | -0.438202086 | NCAN | neurocan [Source:HGNC Symbol;Acc:HGNC:2465] |
| ENSG00000130544 | HGLibA_56231 | 77 | -0.438202086 | ZNF557 | zinc finger protein 557 [Source:HGNC Symbol;Acc:HGNC:28632] |
| ENSG00000132376 | HGLibA_23361 | 77 | -0.438202086 | INPP5K | inositol polyphosphate-5-phosphatase K [Source:HGNC Symbol;Acc:HGNC:33882] |
| ENSG00000133030 | HGLibA_29776 | 77 | -0.438202086 | MPRIP | myosin phosphatase Rho interacting protein [Source:HGNC Symbol;Acc:HGNC:30321] |
| ENSG00000133112 | HGLibA_51211 | 77 | -0.438202086 | TPT1 | translationally-controlled 1 [Source:HGNC Symbol;Acc:HGNC:12022]" |
| ENSG00000134827 | HGLibA_48822 | 77 | -0.438202086 | TCN1 | transcobalamin 1 [Source:HGNC Symbol;Acc:HGNC:11652] |
| ENSG00000135338 | HGLibA_26092 | 77 | -0.438202086 | LCA5 | lebercilin [Source:HGNC Symbol;Acc:HGNC:31923]" |
| ENSG00000135643 | HGLibA_24351 | 77 | -0.438202086 | KCNMB4 | potassium calcium-activated channel subfamily M regulatory beta subunit 4 [Source:HGNC Symbol;Acc:HGNC:6289] |
| ENSG00000135945 | HGLibA_40884 | 77 | -0.438202086 | REV1 | DNA directed polymerase [Source:HGNC Symbol;Acc:HGNC:14060]" |
| ENSG00000136698 | HGLibA_09247 | 77 | -0.438202086 | CFC1 | cryptic family 1 [Source:HGNC Symbol;Acc:HGNC:18292]" |
| ENSG00000137098 | HGLibA_46370 | 77 | -0.438202086 | SPAG8 | sperm associated antigen 8 [Source:HGNC Symbol;Acc:HGNC:14105] |
| ENSG00000137193 | HGLibA_36675 | 77 | -0.438202086 | PIM1 | serine/threonine kinase [Source:HGNC Symbol;Acc:HGNC:8986]" |
| ENSG00000138311 | HGLibA_55875 | 77 | -0.438202086 | ZNF365 | zinc finger protein 365 [Source:HGNC Symbol;Acc:HGNC:18194] |
| ENSG00000138442 | HGLibA_54028 | 77 | -0.438202086 | WDR12 | WD repeat domain 12 [Source:HGNC Symbol;Acc:HGNC:14098] |
| ENSG00000138792 | HGLibA_15164 | 77 | -0.438202086 | ENPEP | glutamyl aminopeptidase [Source:HGNC Symbol;Acc:HGNC:3355] |
| ENSG00000139055 | HGLibA_15525 | 77 | -0.438202086 | ERP27 | endoplasmic reticulum protein 27 [Source:HGNC Symbol;Acc:HGNC:26495] |
| ENSG00000139163 | HGLibA_15633 | 77 | -0.438202086 | ETNK1 | ethanolamine kinase 1 [Source:HGNC Symbol;Acc:HGNC:24649] |
| ENSG00000140876 | HGLibA_32988 | 77 | -0.438202086 | NUDT7 | nudix hydrolase 7 [Source:HGNC Symbol;Acc:HGNC:8054] |
| ENSG00000142910 | HGLibA_49551 | 77 | -0.438202086 | TINAGL1 | tubulointerstitial nephritis antigen like 1 [Source:HGNC Symbol;Acc:HGNC:19168] |
| ENSG00000143434 | HGLibA_43371 | 77 | -0.438202086 | SEMA6C | semaphorin 6C [Source:HGNC Symbol;Acc:HGNC:10740] |
| ENSG00000143786 | HGLibA_10290 | 77 | -0.438202086 | CNIH3 | cornichon family AMPA receptor auxiliary protein 3 [Source:HGNC Symbol;Acc:HGNC:26802] |
| ENSG00000143842 | HGLibA_46238 | 77 | -0.438202086 | SOX13 | SRY-box 13 [Source:HGNC Symbol;Acc:HGNC:11192] |
| ENSG00000144452 | HGLibA_00100 | 77 | -0.438202086 | ABCA12 | ATP binding cassette subfamily A member 12 [Source:HGNC Symbol;Acc:HGNC:14637] |
| ENSG00000145681 | HGLibA_20842 | 77 | -0.438202086 | HAPLN1 | hyaluronan and proteoglycan link protein 1 [Source:HGNC Symbol;Acc:HGNC:2380] |
| ENSG00000148572 | HGLibA_32652 | 77 | -0.438202086 | NRBF2 | nuclear receptor binding factor 2 [Source:HGNC Symbol;Acc:HGNC:19692] |
| ENSG00000149243 | HGLibA_25194 | 77 | -0.438202086 | KLHL35 | kelch like family member 35 [Source:HGNC Symbol;Acc:HGNC:26597] |
| ENSG00000149269 | HGLibA_35038 | 77 | -0.438202086 | PAK1 | p21 (RAC1) activated kinase 1 [Source:HGNC Symbol;Acc:HGNC:8590] |
| ENSG00000151929 | HGLibA_04081 | 77 | -0.438202086 | BAG3 | BCL2 associated athanogene 3 [Source:HGNC Symbol;Acc:HGNC:939] |
| ENSG00000152439 | HGLibA_56713 | 77 | -0.438202086 | ZNF773 | zinc finger protein 773 [Source:HGNC Symbol;Acc:HGNC:30487] |
| ENSG00000152910 | HGLibA_10430 | 77 | -0.438202086 | CNTNAP4 | contactin associated protein like 4 [Source:HGNC Symbol;Acc:HGNC:18747] |
| ENSG00000153347 | HGLibA_16737 | 77 | -0.438202086 | FAM81B | family with sequence similarity 81 member B [Source:HGNC Symbol;Acc:HGNC:26335] |
| ENSG00000155363 | HGLibA_29693 | 77 | -0.438202086 | MOV10 | Mov10 RISC complex RNA helicase [Source:HGNC Symbol;Acc:HGNC:7200] |
| ENSG00000155792 | HGLibA_12989 | 77 | -0.438202086 | DEPTOR | DEP domain containing MTOR interacting protein [Source:HGNC Symbol;Acc:HGNC:22953] |
| ENSG00000156273 | HGLibA_04065 | 77 | -0.438202086 | BACH1 | BTB domain and CNC homolog 1 [Source:HGNC Symbol;Acc:HGNC:935] |
| ENSG00000157429 | HGLibA_55556 | 77 | -0.438202086 | ZNF19 | zinc finger protein 19 [Source:HGNC Symbol;Acc:HGNC:12981] |
| ENSG00000157890 | HGLibA_28849 | 77 | -0.438202086 | MEGF11 | multiple EGF like domains 11 [Source:HGNC Symbol;Acc:HGNC:29635] |
| ENSG00000160213 | HGLibA_11479 | 77 | -0.438202086 | CSTB | cystatin B [Source:HGNC Symbol;Acc:HGNC:2482] |
| ENSG00000160323 | HGLibA_00800 | 77 | -0.438202086 | ADAMTS13 | ADAM metallopeptidase with thrombospondin type 1 motif 13 [Source:HGNC Symbol;Acc:HGNC:1366] |
| ENSG00000161267 | HGLibA_04374 | 77 | -0.438202086 | BDH1 | 3-hydroxybutyrate dehydrogenase 1 [Source:HGNC Symbol;Acc:HGNC:1027] |
| ENSG00000161653 | HGLibA_31074 | 77 | -0.438202086 | NAGS | N-acetylglutamate synthase [Source:HGNC Symbol;Acc:HGNC:17996] |
| ENSG00000162366 | HGLibA_35995 | 77 | -0.438202086 | PDZK1IP1 | PDZK1 interacting protein 1 [Source:HGNC Symbol;Acc:HGNC:16887] |
| ENSG00000162551 | HGLibA_01710 | 77 | -0.438202086 | ALPL | biomineralization associated [Source:HGNC Symbol;Acc:HGNC:438]" |
| ENSG00000162891 | HGLibA_23074 | 77 | -0.438202086 | IL20 | interleukin 20 [Source:HGNC Symbol;Acc:HGNC:6002] |
| ENSG00000163389 | HGLibA_37478 | 77 | -0.438202086 | POGLUT1 | protein O-glucosyltransferase 1 [Source:HGNC Symbol;Acc:HGNC:22954] |
| ENSG00000163558 | HGLibA_38554 | 77 | -0.438202086 | PRKCI | protein kinase C iota [Source:HGNC Symbol;Acc:HGNC:9404] |
| ENSG00000163817 | HGLibA_45321 | 77 | -0.438202086 | SLC6A20 | solute carrier family 6 member 20 [Source:HGNC Symbol;Acc:HGNC:30927] |
| ENSG00000163867 | HGLibA_55406 | 77 | -0.438202086 | ZMYM6 | zinc finger MYM-type containing 6 [Source:HGNC Symbol;Acc:HGNC:13050] |
| ENSG00000163888 | HGLibA_07082 | 77 | -0.438202086 | CAMK2N2 | calcium/calmodulin dependent protein kinase II inhibitor 2 [Source:HGNC Symbol;Acc:HGNC:24197] |
| ENSG00000163931 | HGLibA_49586 | 77 | -0.438202086 | TKT | transketolase [Source:HGNC Symbol;Acc:HGNC:11834] |
| ENSG00000164485 | HGLibA_23094 | 77 | -0.438202086 | IL22RA2 | interleukin 22 receptor subunit alpha 2 [Source:HGNC Symbol;Acc:HGNC:14901] |
| ENSG00000165449 | HGLibA_44457 | 77 | -0.438202086 | SLC16A9 | solute carrier family 16 member 9 [Source:HGNC Symbol;Acc:HGNC:23520] |
| ENSG00000165916 | HGLibA_39132 | 77 | -0.438202086 | PSMC3 | ATPase 3 [Source:HGNC Symbol;Acc:HGNC:9549]" |
| ENSG00000166148 | HGLibA_03904 | 77 | -0.438202086 | AVPR1A | arginine vasopressin receptor 1A [Source:HGNC Symbol;Acc:HGNC:895] |
| ENSG00000166477 | HGLibA_26306 | 77 | -0.438202086 | LEO1 | Paf1/RNA polymerase II complex component [Source:HGNC Symbol;Acc:HGNC:30401]" |
| ENSG00000167077 | HGLibA_28863 | 77 | -0.438202086 | MEI1 | meiotic double-stranded break formation protein 1 [Source:HGNC Symbol;Acc:HGNC:28613] |
| ENSG00000167139 | HGLibA_48500 | 77 | -0.438202086 | TBC1D21 | TBC1 domain family member 21 [Source:HGNC Symbol;Acc:HGNC:28536] |
| ENSG00000167515 | HGLibA_51314 | 77 | -0.438202086 | TRAPPC2L | trafficking protein particle complex 2 like [Source:HGNC Symbol;Acc:HGNC:30887] |
| ENSG00000167858 | HGLibA_48972 | 77 | -0.438202086 | TEKT1 | tektin 1 [Source:HGNC Symbol;Acc:HGNC:15534] |
| ENSG00000168386 | HGLibA_17531 | 77 | -0.438202086 | FILIP1L | filamin A interacting protein 1 like [Source:HGNC Symbol;Acc:HGNC:24589] |
| ENSG00000168393 | HGLibA_14095 | 77 | -0.438202086 | DTYMK | deoxythymidylate kinase [Source:HGNC Symbol;Acc:HGNC:3061] |
| ENSG00000169118 | HGLibA_11393 | 77 | -0.438202086 | CSNK1G1 | casein kinase 1 gamma 1 [Source:HGNC Symbol;Acc:HGNC:2454] |
| ENSG00000169327 | HGLibA_34252 | 77 | -0.438202086 | OR5AU1 | olfactory receptor family 5 subfamily AU member 1 [Source:HGNC Symbol;Acc:HGNC:15362] |
| ENSG00000169550 | HGLibA_30527 | 77 | -0.438202086 | MUC15 | cell surface associated [Source:HGNC Symbol;Acc:HGNC:14956]" |
| ENSG00000169760 | HGLibA_32066 | 77 | -0.438202086 | NLGN1 | neuroligin 1 [Source:HGNC Symbol;Acc:HGNC:14291] |
| ENSG00000170522 | HGLibA_14973 | 77 | -0.438202086 | ELOVL6 | ELOVL fatty acid elongase 6 [Source:HGNC Symbol;Acc:HGNC:15829] |
| ENSG00000170653 | HGLibA_03452 | 77 | -0.438202086 | ATF7 | activating transcription factor 7 [Source:HGNC Symbol;Acc:HGNC:792] |
| ENSG00000170776 | HGLibA_01416 | 77 | -0.438202086 | AKAP13 | A-kinase anchoring protein 13 [Source:HGNC Symbol;Acc:HGNC:371] |
| ENSG00000171051 | HGLibA_17962 | 77 | -0.438202086 | FPR1 | formyl peptide receptor 1 [Source:HGNC Symbol;Acc:HGNC:3826] |
| ENSG00000172464 | HGLibA_34245 | 77 | -0.438202086 | OR5AP2 | olfactory receptor family 5 subfamily AP member 2 [Source:HGNC Symbol;Acc:HGNC:15258] |
| ENSG00000173207 | HGLibA_09788 | 77 | -0.438202086 | CKS1B | CDC28 protein kinase regulatory subunit 1B [Source:HGNC Symbol;Acc:HGNC:19083] |
| ENSG00000174567 | HGLibA_19663 | 77 | -0.438202086 | GOLT1A | golgi transport 1A [Source:HGNC Symbol;Acc:HGNC:24766] |
| ENSG00000174957 | HGLibA_34308 | 77 | -0.438202086 | OR5J2 | olfactory receptor family 5 subfamily J member 2 [Source:HGNC Symbol;Acc:HGNC:19612] |
| ENSG00000176095 | HGLibA_23445 | 77 | -0.438202086 | IP6K1 | inositol hexakisphosphate kinase 1 [Source:HGNC Symbol;Acc:HGNC:18360] |
| ENSG00000176953 | HGLibA_31778 | 77 | -0.438202086 | NFATC2IP | nuclear factor of activated T cells 2 interacting protein [Source:HGNC Symbol;Acc:HGNC:25906] |
| ENSG00000177489 | HGLibA_33794 | 77 | -0.438202086 | OR2G2 | olfactory receptor family 2 subfamily G member 2 [Source:HGNC Symbol;Acc:HGNC:15007] |
| ENSG00000177576 | HGLibA_05612 | 77 | -0.438202086 | C18orf32 | chromosome 18 open reading frame 32 [Source:HGNC Symbol;Acc:HGNC:31690] |
| ENSG00000178217 | HGLibA_43901 | 77 | -0.438202086 | SH2D4B | SH2 domain containing 4B [Source:HGNC Symbol;Acc:HGNC:31440] |
| ENSG00000178449 | HGLibA_10808 | 77 | -0.438202086 | COX14 | cytochrome c oxidase assembly factor COX14 [Source:HGNC Symbol;Acc:HGNC:28216] |
| ENSG00000179912 | HGLibA_39776 | 77 | -0.438202086 | R3HDM2 | R3H domain containing 2 [Source:HGNC Symbol;Acc:HGNC:29167] |
| ENSG00000180644 | HGLibA_38449 | 77 | -0.438202086 | PRF1 | perforin 1 [Source:HGNC Symbol;Acc:HGNC:9360] |
| ENSG00000181035 | HGLibA_44740 | 77 | -0.438202086 | SLC25A42 | solute carrier family 25 member 42 [Source:HGNC Symbol;Acc:HGNC:28380] |
| ENSG00000182187 | HGLibA_11272 | 77 | -0.438202086 | CRYGB | crystallin gamma B [Source:HGNC Symbol;Acc:HGNC:2409] |
| ENSG00000182545 | HGLibA_41391 | 77 | -0.438202086 | RNASE10 | ribonuclease A family member 10 (inactive) [Source:HGNC Symbol;Acc:HGNC:19275] |
| ENSG00000182732 | HGLibA_41099 | 77 | -0.438202086 | RGS6 | regulator of G protein signaling 6 [Source:HGNC Symbol;Acc:HGNC:10002] |
| ENSG00000183153 | HGLibA_19189 | 77 | -0.438202086 | GJD3 | gap junction protein delta 3 [Source:HGNC Symbol;Acc:HGNC:19147] |
| ENSG00000183571 | HGLibA_36282 | 77 | -0.438202086 | PGPEP1L | pyroglutamyl-peptidase I like [Source:HGNC Symbol;Acc:HGNC:27080] |
| ENSG00000183580 | HGLibA_17018 | 77 | -0.438202086 | FBXL7 | F-box and leucine rich repeat protein 7 [Source:HGNC Symbol;Acc:HGNC:13604] |
| ENSG00000184012 | HGLibA_50635 | 77 | -0.438202086 | TMPRSS2 | transmembrane serine protease 2 [Source:HGNC Symbol;Acc:HGNC:11876] |
| ENSG00000184033 | HGLibA_11560 | 77 | -0.438202086 | CTAG1B | cancer/testis antigen 1B [Source:HGNC Symbol;Acc:HGNC:2491] |
| ENSG00000185615 | HGLibA_35874 | 77 | -0.438202086 | PDIA2 | protein disulfide isomerase family A member 2 [Source:HGNC Symbol;Acc:HGNC:14180] |
| ENSG00000185761 | HGLibA_00859 | 77 | -0.438202086 | ADAMTSL5 | ADAMTS like 5 [Source:HGNC Symbol;Acc:HGNC:27912] |
| ENSG00000185821 | HGLibA_34411 | 77 | -0.438202086 | OR6C76 | olfactory receptor family 6 subfamily C member 76 [Source:HGNC Symbol;Acc:HGNC:31305] |
| ENSG00000186184 | HGLibA_37558 | 77 | -0.438202086 | POLR1D | RNA polymerase I and III subunit D [Source:HGNC Symbol;Acc:HGNC:20422] |
| ENSG00000186340 | HGLibA_49325 | 77 | -0.438202086 | THBS2 | thrombospondin 2 [Source:HGNC Symbol;Acc:HGNC:11786] |
| ENSG00000186462 | HGLibA_31114 | 77 | -0.438202086 | NAP1L2 | nucleosome assembly protein 1 like 2 [Source:HGNC Symbol;Acc:HGNC:7638] |
| ENSG00000187024 | HGLibA_39550 | 77 | -0.438202086 | PTRH1 | peptidyl-tRNA hydrolase 1 homolog [Source:HGNC Symbol;Acc:HGNC:27039] |
| ENSG00000187555 | HGLibA_53409 | 77 | -0.438202086 | USP7 | ubiquitin specific peptidase 7 [Source:HGNC Symbol;Acc:HGNC:12630] |
| ENSG00000187634 | HGLibA_42664 | 77 | -0.438202086 | SAMD11 | sterile alpha motif domain containing 11 [Source:HGNC Symbol;Acc:HGNC:28706] |
| ENSG00000188938 | HGLibA_16057 | 77 | -0.438202086 | FAM120AOS | family with sequence similarity 120A opposite strand [Source:HGNC Symbol;Acc:HGNC:23389] |
| ENSG00000189007 | HGLibA_00882 | 77 | -0.438202086 | ADAT2 | tRNA specific 2 [Source:HGNC Symbol;Acc:HGNC:21172]" |
| ENSG00000189298 | HGLibA_55350 | 77 | -0.438202086 | ZKSCAN3 | zinc finger with KRAB and SCAN domains 3 [Source:HGNC Symbol;Acc:HGNC:13853] |
| ENSG00000196119 | HGLibA_34490 | 77 | -0.438202086 | OR8A1 | olfactory receptor family 8 subfamily A member 1 [Source:HGNC Symbol;Acc:HGNC:8469] |
| ENSG00000196208 | HGLibA_20237 | 77 | -0.438202086 | GREB1 | growth regulating estrogen receptor binding 1 [Source:HGNC Symbol;Acc:HGNC:24885] |
| ENSG00000196812 | HGLibA_56988 | 77 | -0.438202086 | ZSCAN16 | zinc finger and SCAN domain containing 16 [Source:HGNC Symbol;Acc:HGNC:20813] |
| ENSG00000197013 | HGLibA_55971 | 77 | -0.438202086 | ZNF429 | zinc finger protein 429 [Source:HGNC Symbol;Acc:HGNC:20817] |
| ENSG00000197261 | HGLibA_06449 | 77 | -0.438202086 | C6orf141 | chromosome 6 open reading frame 141 [Source:HGNC Symbol;Acc:HGNC:21351] |
| ENSG00000197380 | HGLibA_12287 | 77 | -0.438202086 | DACT3 | dishevelled binding antagonist of beta catenin 3 [Source:HGNC Symbol;Acc:HGNC:30745] |
| ENSG00000197714 | HGLibA_56036 | 77 | -0.438202086 | ZNF460 | zinc finger protein 460 [Source:HGNC Symbol;Acc:HGNC:21628] |
| ENSG00000197889 | HGLibA_28866 | 77 | -0.438202086 | MEIG1 | meiosis/spermiogenesis associated 1 [Source:HGNC Symbol;Acc:HGNC:23429] |
| ENSG00000198393 | HGLibA_55688 | 77 | -0.438202086 | ZNF26 | zinc finger protein 26 [Source:HGNC Symbol;Acc:HGNC:13053] |
| ENSG00000198576 | HGLibA_02633 | 77 | -0.438202086 | ARC | activity regulated cytoskeleton associated protein [Source:HGNC Symbol;Acc:HGNC:648] |
| ENSG00000198863 | HGLibA_42446 | 77 | -0.438202086 | RUNDC1 | RUN domain containing 1 [Source:HGNC Symbol;Acc:HGNC:25418] |
| ENSG00000203734 | HGLibA_14427 | 77 | -0.438202086 | ECT2L | epithelial cell transforming 2 like [Source:HGNC Symbol;Acc:HGNC:21118] |
| ENSG00000203880 | HGLibA_35630 | 77 | -0.438202086 | PCMTD2 | protein-L-isoaspartate (D-aspartate) O-methyltransferase domain containing 2 [Source:HGNC Symbol;Acc:HGNC:15882] |
| ENSG00000204130 | HGLibA_42440 | 77 | -0.438202086 | RUFY2 | RUN and FYVE domain containing 2 [Source:HGNC Symbol;Acc:HGNC:19761] |
| ENSG00000204175 | HGLibA_20143 | 77 | -0.438202086 | GPRIN2 | G protein regulated inducer of neurite outgrowth 2 [Source:HGNC Symbol;Acc:HGNC:23730] |
| ENSG00000204361 | HGLibA_33154 | 77 | -0.438202086 | NXPE2 | neurexophilin and PC-esterase domain family member 2 [Source:HGNC Symbol;Acc:HGNC:26331] |
| ENSG00000204571 | HGLibA_25789 | 77 | -0.438202086 | KRTAP5-11 | keratin associated protein 5-11 [Source:HGNC Symbol;Acc:HGNC:23606] |
| ENSG00000204571 | HGLibA_25788 | 77 | -0.438202086 | KRTAP5-11 | keratin associated protein 5-11 [Source:HGNC Symbol;Acc:HGNC:23606] |
| ENSG00000211445 | HGLibA_20176 | 77 | -0.438202086 | GPX3 | glutathione peroxidase 3 [Source:HGNC Symbol;Acc:HGNC:4555] |
| ENSG00000213930 | HGLibA_18597 | 77 | -0.438202086 | GALT | galactose-1-phosphate uridylyltransferase [Source:HGNC Symbol;Acc:HGNC:4135] |
| ENSG00000226929 | HGLibA_11521 | 77 | -0.438202086 | CT47A11 | member A11 [Source:HGNC Symbol;Acc:HGNC:27397]" |
| ENSG00000241563 | HGLibA_10797 | 77 | -0.438202086 | CORT | cortistatin [Source:HGNC Symbol;Acc:HGNC:2257] |
| ENSG00000241935 | HGLibA_21861 | 77 | -0.438202086 | HOGA1 | 4-hydroxy-2-oxoglutarate aldolase 1 [Source:HGNC Symbol;Acc:HGNC:25155] |
| ENSG00000242550 | HGLibA_43538 | 77 | -0.438202086 | SERPINB10 | serpin family B member 10 [Source:HGNC Symbol;Acc:HGNC:8942] |
| ENSG00000244414 | HGLibA_09264 | 77 | -0.438202086 | CFHR1 | complement factor H related 1 [Source:HGNC Symbol;Acc:HGNC:4888] |
| ENSG00000244617 | HGLibA_03361 | 77 | -0.438202086 | ASPRV1 | aspartic peptidase retroviral like 1 [Source:HGNC Symbol;Acc:HGNC:26321] |
| ENSG00000253873 | HGLibA_35522 | 77 | -0.438202086 | PCDHGA11 | 11 [Source:HGNC Symbol;Acc:HGNC:8698]" |
| ENSG00000256229 | HGLibA_56083 | 77 | -0.438202086 | ZNF486 | zinc finger protein 486 [Source:HGNC Symbol;Acc:HGNC:20807] |
| ENSG00000259384 | HGLibA_19029 | 77 | -0.438202086 | GH1 | growth hormone 1 [Source:HGNC Symbol;Acc:HGNC:4261] |
| ENSG00000259956 | HGLibA_40495 | 77 | -0.438202086 | RBM15B | RNA binding motif protein 15B [Source:HGNC Symbol;Acc:HGNC:24303] |
| ENSG00000263353 | HGLibA_37896 | 77 | -0.438202086 | PPIAL4A | peptidylprolyl isomerase A like 4A [Source:HGNC Symbol;Acc:HGNC:24369] |
| ENSG00000274226 | HGLibA_48554 | 77 | -0.438202086 | TBC1D3H | TBC1 domain family member 3H [Source:HGNC Symbol;Acc:HGNC:30708] |
| ENSG00000278299 | HGLibA_48545 | 77 | -0.438202086 | TBC1D3C | TBC1 domain family member 3C [Source:HGNC Symbol;Acc:HGNC:24889] |
| ENSG00000278311 | HGLibA_19003 | 77 | -0.438202086 | GGNBP2 | gametogenetin binding protein 2 [Source:HGNC Symbol;Acc:HGNC:19357] |

| | | | | | |
|---|---|---|---|---|---|
| FAM213A | HGLibA_16470 | 77 | -0.438202086 | FAM213A | - |
| FTSJD2 | HGLibA_18147 | 77 | -0.438202086 | FTSJD2 | - |
| hsa-mir-1278 | HGLibA_57566 | 77 | -0.438202086 | hsa-mir-1278 | - |
| hsa-mir-191 | HGLibA_58043 | 77 | -0.438202086 | hsa-mir-191 | - |
| hsa-mir-20b | HGLibA_58206 | 77 | -0.438202086 | hsa-mir-20b | - |
| hsa-mir-21 | HGLibA_58207 | 77 | -0.438202086 | hsa-mir-21 | - |
| hsa-mir-23a | HGLibA_58330 | 77 | -0.438202086 | hsa-mir-23a | - |
| hsa-mir-3613 | HGLibA_59082 | 77 | -0.438202086 | hsa-mir-3613 | - |
| hsa-mir-371b | HGLibA_59379 | 77 | -0.438202086 | hsa-mir-371b | - |
| hsa-mir-508 | HGLibA_61491 | 77 | -0.438202086 | hsa-mir-508 | - |
| hsa-mir-509-2 | HGLibA_61509 | 77 | -0.438202086 | hsa-mir-509-2 | - |
| hsa-mir-515-1 | HGLibA_61601 | 77 | -0.438202086 | hsa-mir-515-1 | - |
| hsa-mir-6077-1 | HGLibA_62562 | 77 | -0.438202086 | hsa-mir-6077-1 | - |
| hsa-mir-6756 | HGLibA_63181 | 77 | -0.438202086 | hsa-mir-6756 | - |
| hsa-mir-6758 | HGLibA_63191 | 77 | -0.438202086 | hsa-mir-6758 | - |
| hsa-mir-6770-1 | HGLibA_63246 | 77 | -0.438202086 | hsa-mir-6770-1 | - |
| hsa-mir-6816 | HGLibA_63442 | 77 | -0.438202086 | hsa-mir-6816 | - |
| hsa-mir-6883 | HGLibA_63716 | 77 | -0.438202086 | hsa-mir-6883 | - |
| hsa-mir-758 | HGLibA_63892 | 77 | -0.438202086 | hsa-mir-758 | - |
| hsa-mir-7853 | HGLibA_64001 | 77 | -0.438202086 | hsa-mir-7853 | - |
| hsa-mir-8075 | HGLibA_64144 | 77 | -0.438202086 | hsa-mir-8075 | - |
| hsa-mir-933 | HGLibA_64308 | 77 | -0.438202086 | hsa-mir-933 | - |
| NPIPL3 | HGLibA_32477 | 77 | -0.438202086 | NPIPL3 | - |
| WISP2 | HGLibA_54322 | 77 | -0.438202086 | WISP2 | - |
| C7orf65 | HGLibA_06575 | 76 | -0.447247429 | C7orf65 | - |
| CRAMP1L | HGLibA_11063 | 76 | -0.447247429 | CRAMP1L | - |
| ENSG00000006534 | HGLibA_01568 | 76 | -0.447247429 | ALDH3B1 | aldehyde dehydrogenase 3 family member B1 [Source:HGNC Symbol;Acc:HGNC:410] |
| ENSG00000007541 | HGLibA_36578 | 76 | -0.447247429 | PIGQ | phosphatidylinositol glycan anchor biosynthesis class Q [Source:HGNC Symbol;Acc:HGNC:14135] |
| ENSG00000008277 | HGLibA_00758 | 76 | -0.447247429 | ADAM22 | ADAM metallopeptidase domain 22 [Source:HGNC Symbol;Acc:HGNC:201] |
| ENSG00000010610 | HGLibA_08381 | 76 | -0.447247429 | CD4 | CD4 molecule [Source:HGNC Symbol;Acc:HGNC:1678] |
| ENSG00000047249 | HGLibA_03765 | 76 | -0.447247429 | ATP6V1H | ATPase H+ transporting V1 subunit H [Source:HGNC Symbol;Acc:HGNC:18303] |
| ENSG00000048392 | HGLibA_42257 | 76 | -0.447247429 | RRM2B | ribonucleotide reductase regulatory TP53 inducible subunit M2B [Source:HGNC Symbol;Acc:HGNC:17296] |
| ENSG00000048707 | HGLibA_53739 | 76 | -0.447247429 | VPS13D | vacuolar protein sorting 13 homolog D [Source:HGNC Symbol;Acc:HGNC:23595] |
| ENSG00000054356 | HGLibA_39519 | 76 | -0.447247429 | PTPRN | receptor type N [Source:HGNC Symbol;Acc:HGNC:9676]" |
| ENSG00000058453 | HGLibA_11211 | 76 | -0.447247429 | CROCC | rootletin [Source:HGNC Symbol;Acc:HGNC:21299]" |
| ENSG00000060138 | HGLibA_54649 | 76 | -0.447247429 | YBX3 | Y-box binding protein 3 [Source:HGNC Symbol;Acc:HGNC:2428] |
| ENSG00000064309 | HGLibA_08858 | 76 | -0.447247429 | CDON | oncogene regulated [Source:HGNC Symbol;Acc:HGNC:17104]" |
| ENSG00000064995 | HGLibA_48201 | 76 | -0.447247429 | TAF11 | TATA-box binding protein associated factor 11 [Source:HGNC Symbol;Acc:HGNC:11544] |
| ENSG00000068400 | HGLibA_20322 | 76 | -0.447247429 | GRIPAP1 | GRIP1 associated protein 1 [Source:HGNC Symbol;Acc:HGNC:18706] |
| ENSG00000069431 | HGLibA_00189 | 76 | -0.447247429 | ABCC9 | ATP binding cassette subfamily C member 9 [Source:HGNC Symbol;Acc:HGNC:60] |
| ENSG00000075618 | HGLibA_18069 | 76 | -0.447247429 | FSCN1 | fascin actin-bundling protein 1 [Source:HGNC Symbol;Acc:HGNC:11148] |
| ENSG00000077942 | HGLibA_16942 | 76 | -0.447247429 | FBLN1 | fibulin 1 [Source:HGNC Symbol;Acc:HGNC:3600] |
| ENSG00000078579 | HGLibA_17393 | 76 | -0.447247429 | FGF20 | fibroblast growth factor 20 [Source:HGNC Symbol;Acc:HGNC:3677] |
| ENSG00000079393 | HGLibA_14139 | 76 | -0.447247429 | DUSP13 | dual specificity phosphatase 13 [Source:HGNC Symbol;Acc:HGNC:19681] |
| ENSG00000082684 | HGLibA_43360 | 76 | -0.447247429 | SEMA5B | semaphorin 5B [Source:HGNC Symbol;Acc:HGNC:10737] |
| ENSG00000090060 | HGLibA_35135 | 76 | -0.447247429 | PAPOLA | poly(A) polymerase alpha [Source:HGNC Symbol;Acc:HGNC:14981] |
| ENSG00000099617 | HGLibA_14586 | 76 | -0.447247429 | EFNA2 | ephrin A2 [Source:HGNC Symbol;Acc:HGNC:3222] |
| ENSG00000100219 | HGLibA_54489 | 76 | -0.447247429 | XBP1 | X-box binding protein 1 [Source:HGNC Symbol;Acc:HGNC:12801] |
| ENSG00000100335 | HGLibA_29243 | 76 | -0.447247429 | MIEF1 | mitochondrial elongation factor 1 [Source:HGNC Symbol;Acc:HGNC:25979] |
| ENSG00000100360 | HGLibA_22755 | 76 | -0.447247429 | IFT27 | intraflagellar transport 27 [Source:HGNC Symbol;Acc:HGNC:18626] |
| ENSG00000100726 | HGLibA_48986 | 76 | -0.447247429 | TELO2 | telomere maintenance 2 [Source:HGNC Symbol;Acc:HGNC:29099] |
| ENSG00000100968 | HGLibA_31784 | 76 | -0.447247429 | NFATC4 | nuclear factor of activated T cells 4 [Source:HGNC Symbol;Acc:HGNC:7778] |
| ENSG00000101311 | HGLibA_17295 | 76 | -0.447247429 | FERMT1 | fermitin family member 1 [Source:HGNC Symbol;Acc:HGNC:15889] |
| ENSG00000101542 | HGLibA_08662 | 76 | -0.447247429 | CDH20 | cadherin 20 [Source:HGNC Symbol;Acc:HGNC:1760] |
| ENSG00000101974 | HGLibA_03561 | 76 | -0.447247429 | ATP11C | ATPase phospholipid transporting 11C [Source:HGNC Symbol;Acc:HGNC:13554] |
| ENSG00000102053 | HGLibA_54956 | 76 | -0.447247429 | ZC3H12B | zinc finger CCCH-type containing 12B [Source:HGNC Symbol;Acc:HGNC:17407] |
| ENSG00000103326 | HGLibA_07160 | 76 | -0.447247429 | CAPN15 | calpain 15 [Source:HGNC Symbol;Acc:HGNC:11182] |
| ENSG00000103528 | HGLibA_48069 | 76 | -0.447247429 | SYT17 | synaptotagmin 17 [Source:HGNC Symbol;Acc:HGNC:24119] |
| ENSG00000103671 | HGLibA_51644 | 76 | -0.447247429 | TRIP4 | thyroid hormone receptor interactor 4 [Source:HGNC Symbol;Acc:HGNC:12310] |
| ENSG00000104823 | HGLibA_14390 | 76 | -0.447247429 | ECH1 | enoyl-CoA hydratase 1 [Source:HGNC Symbol;Acc:HGNC:3149] |
| ENSG00000104872 | HGLibA_36608 | 76 | -0.447247429 | PIH1D1 | PIH1 domain containing 1 [Source:HGNC Symbol;Acc:HGNC:26075] |
| ENSG00000105290 | HGLibA_02439 | 76 | -0.447247429 | APLP1 | amyloid beta precursor like protein 1 [Source:HGNC Symbol;Acc:HGNC:597] |
| ENSG00000105538 | HGLibA_40355 | 76 | -0.447247429 | RASIP1 | Ras interacting protein 1 [Source:HGNC Symbol;Acc:HGNC:24716] |
| ENSG00000106299 | HGLibA_53965 | 76 | -0.447247429 | WASL | Wiskott-Aldrich syndrome like [Source:HGNC Symbol;Acc:HGNC:12735] |
| ENSG00000106333 | HGLibA_35651 | 76 | -0.447247429 | PCOLCE | procollagen C-endopeptidase enhancer [Source:HGNC Symbol;Acc:HGNC:8738] |
| ENSG00000106484 | HGLibA_28922 | 76 | -0.447247429 | MEST | mesoderm specific transcript [Source:HGNC Symbol;Acc:HGNC:7028] |
| ENSG00000111405 | HGLibA_15120 | 76 | -0.447247429 | ENDOU | poly(U) specific [Source:HGNC Symbol;Acc:HGNC:14369]" |
| ENSG00000111716 | HGLibA_26228 | 76 | -0.447247429 | LDHB | lactate dehydrogenase B [Source:HGNC Symbol;Acc:HGNC:6541] |
| ENSG00000111790 | HGLibA_17442 | 76 | -0.447247429 | FGFR1OP2 | FGFR1 oncogene partner 2 [Source:HGNC Symbol;Acc:HGNC:23098] |
| ENSG00000112146 | HGLibA_17133 | 76 | -0.447247429 | FBXO9 | F-box protein 9 [Source:HGNC Symbol;Acc:HGNC:13588] |
| ENSG00000112335 | HGLibA_46081 | 76 | -0.447247429 | SNX3 | sorting nexin 3 [Source:HGNC Symbol;Acc:HGNC:11174] |
| ENSG00000112667 | HGLibA_13714 | 76 | -0.447247429 | DNPH1 | 2'-deoxynucleoside 5'-phosphate N-hydrolase 1 [Source:HGNC Symbol;Acc:HGNC:21218] |
| ENSG00000113569 | HGLibA_33028 | 76 | -0.447247429 | NUP155 | nucleoporin 155 [Source:HGNC Symbol;Acc:HGNC:8063] |
| ENSG00000114544 | HGLibA_45145 | 76 | -0.447247429 | SLC41A3 | solute carrier family 41 member 3 [Source:HGNC Symbol;Acc:HGNC:31046] |
| ENSG00000116668 | HGLibA_47904 | 76 | -0.447247429 | SWT1 | RNA endoribonuclease homolog [Source:HGNC Symbol;Acc:HGNC:16785]" |
| ENSG00000116824 | HGLibA_08278 | 76 | -0.447247429 | CD2 | CD2 molecule [Source:HGNC Symbol;Acc:HGNC:1639] |
| ENSG00000116991 | HGLibA_44196 | 76 | -0.447247429 | SIPA1L2 | signal induced proliferation associated 1 like 2 [Source:HGNC Symbol;Acc:HGNC:23800] |
| ENSG00000117298 | HGLibA_14381 | 76 | -0.447247429 | ECE1 | endothelin converting enzyme 1 [Source:HGNC Symbol;Acc:HGNC:3146] |
| ENSG00000117322 | HGLibA_11050 | 76 | -0.447247429 | CR2 | complement C3d receptor 2 [Source:HGNC Symbol;Acc:HGNC:2336] |
| ENSG00000117335 | HGLibA_08394 | 76 | -0.447247429 | CD46 | CD46 molecule [Source:HGNC Symbol;Acc:HGNC:6953] |
| ENSG00000117682 | HGLibA_13098 | 76 | -0.447247429 | DHDDS | dehydrodolichyl diphosphate synthase subunit [Source:HGNC Symbol;Acc:HGNC:20603] |
| ENSG00000118515 | HGLibA_43817 | 76 | -0.447247429 | SGK1 | serum/glucocorticoid regulated kinase 1 [Source:HGNC Symbol;Acc:HGNC:10810] |
| ENSG00000121749 | HGLibA_48482 | 76 | -0.447247429 | TBC1D15 | TBC1 domain family member 15 [Source:HGNC Symbol;Acc:HGNC:25694] |
| ENSG00000122136 | HGLibA_33224 | 76 | -0.447247429 | OBP2A | odorant binding protein 2A [Source:HGNC Symbol;Acc:HGNC:23380] |
| ENSG00000122696 | HGLibA_44765 | 76 | -0.447247429 | SLC25A51 | solute carrier family 25 member 51 [Source:HGNC Symbol;Acc:HGNC:23323] |
| ENSG00000124003 | HGLibA_29625 | 76 | -0.447247429 | MOGAT1 | monoacylglycerol O-acyltransferase 1 [Source:HGNC Symbol;Acc:HGNC:18210] |
| ENSG00000128245 | HGLibA_54749 | 76 | -0.447247429 | YWHAH | tyrosine 3-monooxygenase/tryptophan 5-monooxygenase activation protein eta [Source:HGNC Symbol;Acc:HGNC:12853] |
| ENSG00000130294 | HGLibA_24884 | 76 | -0.447247429 | KIF1A | kinesin family member 1A [Source:HGNC Symbol;Acc:HGNC:888] |
| ENSG00000130856 | HGLibA_55650 | 76 | -0.447247429 | ZNF236 | zinc finger protein 236 [Source:HGNC Symbol;Acc:HGNC:13028] |
| ENSG00000131023 | HGLibA_26063 | 76 | -0.447247429 | LATS1 | large tumor suppressor kinase 1 [Source:HGNC Symbol;Acc:HGNC:6514] |
| ENSG00000131398 | HGLibA_24168 | 76 | -0.447247429 | KCNC3 | potassium voltage-gated channel subfamily C member 3 [Source:HGNC Symbol;Acc:HGNC:6235] |
| ENSG00000131504 | HGLibA_13217 | 76 | -0.447247429 | DIAPH1 | diaphanous related formin 1 [Source:HGNC Symbol;Acc:HGNC:2876] |
| ENSG00000134817 | HGLibA_02437 | 76 | -0.447247429 | APLNR | apelin receptor [Source:HGNC Symbol;Acc:HGNC:339] |
| ENSG00000135093 | HGLibA_53330 | 76 | -0.447247429 | USP30 | ubiquitin specific peptidase 30 [Source:HGNC Symbol;Acc:HGNC:20065] |
| ENSG00000137707 | HGLibA_04949 | 76 | -0.447247429 | BTG4 | BTG anti-proliferation factor 4 [Source:HGNC Symbol;Acc:HGNC:13862] |
| ENSG00000137936 | HGLibA_04248 | 76 | -0.447247429 | BCAR3 | NSP family adaptor protein [Source:HGNC Symbol;Acc:HGNC:973]" |
| ENSG00000138036 | HGLibA_14249 | 76 | -0.447247429 | DYNC2LI1 | dynein cytoplasmic 2 light intermediate chain 1 [Source:HGNC Symbol;Acc:HGNC:24595] |
| ENSG00000138496 | HGLibA_35247 | 76 | -0.447247429 | PARP9 | poly(ADP-ribose) polymerase family member 9 [Source:HGNC Symbol;Acc:HGNC:24118] |
| ENSG00000139636 | HGLibA_26673 | 76 | -0.447247429 | LMBR1L | limb development membrane protein 1 like [Source:HGNC Symbol;Acc:HGNC:18268] |
| ENSG00000139908 | HGLibA_52019 | 76 | -0.447247429 | TSSK4 | testis specific serine kinase 4 [Source:HGNC Symbol;Acc:HGNC:19825] |
| ENSG00000139921 | HGLibA_50688 | 76 | -0.447247429 | TMX1 | thioredoxin related transmembrane protein 1 [Source:HGNC Symbol;Acc:HGNC:15487] |
| ENSG00000140262 | HGLibA_48762 | 76 | -0.447247429 | TCF12 | transcription factor 12 [Source:HGNC Symbol;Acc:HGNC:11623] |
| ENSG00000141480 | HGLibA_03109 | 76 | -0.447247429 | ARRB2 | arrestin beta 2 [Source:HGNC Symbol;Acc:HGNC:712] |
| ENSG00000141582 | HGLibA_07453 | 76 | -0.447247429 | CBX4 | chromobox 4 [Source:HGNC Symbol;Acc:HGNC:1554] |
| ENSG00000142864 | HGLibA_43466 | 76 | -0.447247429 | SERBP1 | SERPINE1 mRNA binding protein 1 [Source:HGNC Symbol;Acc:HGNC:17860] |
| ENSG00000143126 | HGLibA_09024 | 76 | -0.447247429 | CELSR2 | cadherin EGF LAG seven-pass G-type receptor 2 [Source:HGNC Symbol;Acc:HGNC:3231] |
| ENSG00000143799 | HGLibA_35211 | 76 | -0.447247429 | PARP1 | poly(ADP-ribose) polymerase 1 [Source:HGNC Symbol;Acc:HGNC:270] |
| ENSG00000144834 | HGLibA_48276 | 76 | -0.447247429 | TAGLN3 | transgelin 3 [Source:HGNC Symbol;Acc:HGNC:29868] |
| ENSG00000146385 | HGLibA_48136 | 76 | -0.447247429 | TAAR8 | trace amine associated receptor 8 [Source:HGNC Symbol;Acc:HGNC:14964] |
| ENSG00000146416 | HGLibA_01347 | 76 | -0.447247429 | AIG1 | androgen induced 1 [Source:HGNC Symbol;Acc:HGNC:21607] |
| ENSG00000147183 | HGLibA_11034 | 76 | -0.447247429 | CPXCR1 | candidate 1 [Source:HGNC Symbol;Acc:HGNC:2332]" |
| ENSG00000148482 | HGLibA_45097 | 76 | -0.447247429 | SLC39A12 | solute carrier family 39 member 12 [Source:HGNC Symbol;Acc:HGNC:20860] |
| ENSG00000148842 | HGLibA_10318 | 76 | -0.447247429 | CNNM2 | cyclin and CBS domain divalent metal cation transport mediator 2 [Source:HGNC Symbol;Acc:HGNC:103] |
| ENSG00000149295 | HGLibA_13961 | 76 | -0.447247429 | DRD2 | dopamine receptor D2 [Source:HGNC Symbol;Acc:HGNC:3023] |
| ENSG00000154016 | HGLibA_20213 | 76 | -0.447247429 | GRAP | GRB2 related adaptor protein [Source:HGNC Symbol;Acc:HGNC:4562] |
| ENSG00000154845 | HGLibA_38204 | 76 | -0.447247429 | PPP4R1 | protein phosphatase 4 regulatory subunit 1 [Source:HGNC Symbol;Acc:HGNC:9320] |
| ENSG00000157064 | HGLibA_32187 | 76 | -0.447247429 | NMNAT2 | nicotinamide nucleotide adenylyltransferase 2 [Source:HGNC Symbol;Acc:HGNC:16789] |
| ENSG00000158793 | HGLibA_31983 | 76 | -0.447247429 | NIT1 | nitrilase 1 [Source:HGNC Symbol;Acc:HGNC:7828] |
| ENSG00000159882 | HGLibA_55635 | 76 | -0.447247429 | ZNF230 | zinc finger protein 230 [Source:HGNC Symbol;Acc:HGNC:13024] |
| ENSG00000160062 | HGLibA_54926 | 76 | -0.447247429 | ZBTB8A | zinc finger and BTB domain containing 8A [Source:HGNC Symbol;Acc:HGNC:24172] |
| ENSG00000160785 | HGLibA_44747 | 76 | -0.447247429 | SLC25A44 | solute carrier family 25 member 44 [Source:HGNC Symbol;Acc:HGNC:29036] |
| ENSG00000162222 | HGLibA_52166 | 76 | -0.447247429 | TTC9C | tetratricopeptide repeat domain 9C [Source:HGNC Symbol;Acc:HGNC:28432] |
| ENSG00000162441 | HGLibA_27798 | 76 | -0.447247429 | LZIC | leucine zipper and CTNNBIP1 domain containing [Source:HGNC Symbol;Acc:HGNC:17497] |
| ENSG00000162460 | HGLibA_50522 | 76 | -0.447247429 | TMEM82 | transmembrane protein 82 [Source:HGNC Symbol;Acc:HGNC:32350] |
| ENSG00000163453 | HGLibA_22833 | 76 | -0.447247429 | IGFBP7 | insulin like growth factor binding protein 7 [Source:HGNC Symbol;Acc:HGNC:5476] |
| ENSG00000164535 | HGLibA_12298 | 76 | -0.447247429 | DAGLB | diacylglycerol lipase beta [Source:HGNC Symbol;Acc:HGNC:28923] |
| ENSG00000164951 | HGLibA_35925 | 76 | -0.447247429 | PDP1 | pyruvate dehyrogenase phosphatase catalytic subunit 1 [Source:HGNC Symbol;Acc:HGNC:9279] |
| ENSG00000165076 | HGLibA_38935 | 76 | -0.447247429 | PRSS37 | serine protease 37 [Source:HGNC Symbol;Acc:HGNC:29211] |
| ENSG00000165118 | HGLibA_06748 | 76 | -0.447247429 | C9orf64 | chromosome 9 open reading frame 64 [Source:HGNC Symbol;Acc:HGNC:28144] |
| ENSG00000165879 | HGLibA_17978 | 76 | -0.447247429 | FRAT1 | WNT signaling pathway regulator [Source:HGNC Symbol;Acc:HGNC:3944]" |
| ENSG00000166188 | HGLibA_55789 | 76 | -0.447247429 | ZNF319 | zinc finger protein 319 [Source:HGNC Symbol;Acc:HGNC:13644] |
| ENSG00000166260 | HGLibA_10805 | 76 | -0.447247429 | COX11 | cytochrome c oxidase copper chaperone COX11 [Source:HGNC Symbol;Acc:HGNC:2261] |
| ENSG00000166289 | HGLibA_37094 | 76 | -0.447247429 | PLEKHF1 | pleckstrin homology and FYVE domain containing 1 [Source:HGNC Symbol;Acc:HGNC:20764] |
| ENSG00000166482 | HGLibA_29051 | 76 | -0.447247429 | MFAP4 | microfibril associated protein 4 [Source:HGNC Symbol;Acc:HGNC:7035] |
| ENSG00000167183 | HGLibA_38789 | 76 | -0.447247429 | PRR15L | proline rich 15 like [Source:HGNC Symbol;Acc:HGNC:28149] |

| | | | | | |
|---|---|---|---|---|---|
| ENSG00000167315 | HGLibA_00343 | 76 | -0.447247429 | ACAA2 | acetyl-CoA acyltransferase 2 [Source:HGNC Symbol;Acc:HGNC:83] |
| ENSG00000167513 | HGLibA_08889 | 76 | -0.447247429 | CDT1 | chromatin licensing and DNA replication factor 1 [Source:HGNC Symbol;Acc:HGNC:24576] |
| ENSG00000168152 | HGLibA_49315 | 76 | -0.447247429 | THAP9 | THAP domain containing 9 [Source:HGNC Symbol;Acc:HGNC:23192] |
| ENSG00000168564 | HGLibA_08832 | 76 | -0.447247429 | CDKN2AIP | CDKN2A interacting protein [Source:HGNC Symbol;Acc:HGNC:24325] |
| ENSG00000168619 | HGLibA_00742 | 76 | -0.447247429 | ADAM18 | ADAM metallopeptidase domain 18 [Source:HGNC Symbol;Acc:HGNC:196] |
| ENSG00000169297 | HGLibA_32569 | 76 | -0.447247429 | NR0B1 | nuclear receptor subfamily 0 group B member 1 [Source:HGNC Symbol;Acc:HGNC:7960] |
| ENSG00000170145 | HGLibA_44166 | 76 | -0.447247429 | SIK2 | salt inducible kinase 2 [Source:HGNC Symbol;Acc:HGNC:21680] |
| ENSG00000170638 | HGLibA_51229 | 76 | -0.447247429 | TRABD | TraB domain containing [Source:HGNC Symbol;Acc:HGNC:28805] |
| ENSG00000170837 | HGLibA_20011 | 76 | -0.447247429 | GPR27 | G protein-coupled receptor 27 [Source:HGNC Symbol;Acc:HGNC:4482] |
| ENSG00000171133 | HGLibA_33816 | 76 | -0.447247429 | OR2K2 | olfactory receptor family 2 subfamily K member 2 [Source:HGNC Symbol;Acc:HGNC:8264] |
| ENSG00000171450 | HGLibA_08778 | 76 | -0.447247429 | CDK5R2 | cyclin dependent kinase 5 regulatory subunit 2 [Source:HGNC Symbol;Acc:HGNC:1776] |
| ENSG00000171456 | HGLibA_03389 | 76 | -0.447247429 | ASXL1 | ASXL transcriptional regulator 1 [Source:HGNC Symbol;Acc:HGNC:18318] |
| ENSG00000171492 | HGLibA_27436 | 76 | -0.447247429 | LRRC8D | leucine rich repeat containing 8 VRAC subunit D [Source:HGNC Symbol;Acc:HGNC:16992] |
| ENSG00000174886 | HGLibA_31442 | 76 | -0.447247429 | NDUFA11 | NADH:ubiquinone oxidoreductase subunit A11 [Source:HGNC Symbol;Acc:HGNC:20371] |
| ENSG00000176049 | HGLibA_23926 | 76 | -0.447247429 | JAKMIP2 | janus kinase and microtubule interacting protein 2 [Source:HGNC Symbol;Acc:HGNC:29067] |
| ENSG00000176555 | HGLibA_34061 | 76 | -0.447247429 | OR4S1 | olfactory receptor family 4 subfamily S member 1 [Source:HGNC Symbol;Acc:HGNC:14705] |
| ENSG00000176853 | HGLibA_16794 | 76 | -0.447247429 | FAM91A1 | family with sequence similarity 91 member A1 [Source:HGNC Symbol;Acc:HGNC:26306] |
| ENSG00000176973 | HGLibA_16785 | 76 | -0.447247429 | FAM89B | family with sequence similarity 89 member B [Source:HGNC Symbol;Acc:HGNC:16708] |
| ENSG00000177201 | HGLibA_33861 | 76 | -0.447247429 | OR2T12 | olfactory receptor family 2 subfamily T member 12 [Source:HGNC Symbol;Acc:HGNC:19592] |
| ENSG00000178035 | HGLibA_23259 | 76 | -0.447247429 | IMPDH2 | inosine monophosphate dehydrogenase 2 [Source:HGNC Symbol;Acc:HGNC:6053] |
| ENSG00000178150 | HGLibA_55443 | 76 | -0.447247429 | ZNF114 | zinc finger protein 114 [Source:HGNC Symbol;Acc:HGNC:12894] |
| ENSG00000178229 | HGLibA_56194 | 76 | -0.447247429 | ZNF543 | zinc finger protein 543 [Source:HGNC Symbol;Acc:HGNC:25281] |
| ENSG00000178502 | HGLibA_25125 | 76 | -0.447247429 | KLHL11 | kelch like family member 11 [Source:HGNC Symbol;Acc:HGNC:19008] |
| ENSG00000179058 | HGLibA_06741 | 76 | -0.447247429 | C9orf50 | chromosome 9 open reading frame 50 [Source:HGNC Symbol;Acc:HGNC:23677] |
| ENSG00000179477 | HGLibA_01679 | 76 | -0.447247429 | ALOX12B | 12R type [Source:HGNC Symbol;Acc:HGNC:430]" |
| ENSG00000179813 | HGLibA_16485 | 76 | -0.447247429 | FAM216B | family with sequence similarity 216 member B [Source:HGNC Symbol;Acc:HGNC:26883] |
| ENSG00000180745 | HGLibA_10128 | 76 | -0.447247429 | CLRN3 | clarin 3 [Source:HGNC Symbol;Acc:HGNC:20795] |
| ENSG00000180901 | HGLibA_24445 | 76 | -0.447247429 | KCTD2 | potassium channel tetramerization domain containing 2 [Source:HGNC Symbol;Acc:HGNC:21294] |
| ENSG00000181718 | HGLibA_34357 | 76 | -0.447247429 | OR5T2 | olfactory receptor family 5 subfamily T member 2 [Source:HGNC Symbol;Acc:HGNC:15296] |
| ENSG00000182095 | HGLibA_50911 | 76 | -0.447247429 | TNRC18 | trinucleotide repeat containing 18 [Source:HGNC Symbol;Acc:HGNC:11962] |
| ENSG00000183691 | HGLibA_32253 | 76 | -0.447247429 | NOG | noggin [Source:HGNC Symbol;Acc:HGNC:7866] |
| ENSG00000183979 | HGLibA_32409 | 76 | -0.447247429 | NPB | neuropeptide B [Source:HGNC Symbol;Acc:HGNC:30099] |
| ENSG00000185056 | HGLibA_06393 | 76 | -0.447247429 | C5orf47 | chromosome 5 open reading frame 47 [Source:HGNC Symbol;Acc:HGNC:27026] |
| ENSG00000185924 | HGLibA_42417 | 76 | -0.447247429 | RTN4RL1 | reticulon 4 receptor like 1 [Source:HGNC Symbol;Acc:HGNC:21329] |
| ENSG00000186393 | HGLibA_25462 | 76 | -0.447247429 | KRT26 | keratin 26 [Source:HGNC Symbol;Acc:HGNC:30840] |
| ENSG00000187726 | HGLibA_13531 | 76 | -0.447247429 | DNAJB13 | DnaJ heat shock protein family (Hsp40) member B13 [Source:HGNC Symbol;Acc:HGNC:30718] |
| ENSG00000187806 | HGLibA_50182 | 76 | -0.447247429 | TMEM202 | transmembrane protein 202 [Source:HGNC Symbol;Acc:HGNC:33733] |
| ENSG00000187821 | HGLibA_21119 | 76 | -0.447247429 | HELT | helt bHLH transcription factor [Source:HGNC Symbol;Acc:HGNC:33783] |
| ENSG00000188215 | HGLibA_12571 | 76 | -0.447247429 | DCUN1D3 | defective in cullin neddylation 1 domain containing 3 [Source:HGNC Symbol;Acc:HGNC:28734] |
| ENSG00000189433 | HGLibA_19166 | 76 | -0.447247429 | GJB4 | gap junction protein beta 4 [Source:HGNC Symbol;Acc:HGNC:4286] |
| ENSG00000196136 | HGLibA_43516 | 76 | -0.447247429 | SERPINA3 | serpin family A member 3 [Source:HGNC Symbol;Acc:HGNC:16] |
| ENSG00000196470 | HGLibA_44105 | 76 | -0.447247429 | SIAH1 | siah E3 ubiquitin protein ligase 1 [Source:HGNC Symbol;Acc:HGNC:10857] |
| ENSG00000196961 | HGLibA_02305 | 76 | -0.447247429 | AP2A1 | adaptor related protein complex 2 subunit alpha 1 [Source:HGNC Symbol;Acc:HGNC:561] |
| ENSG00000198807 | HGLibA_35318 | 76 | -0.447247429 | PAX9 | paired box 9 [Source:HGNC Symbol;Acc:HGNC:8623] |
| ENSG00000198948 | HGLibA_29049 | 76 | -0.447247429 | MFAP3L | microfibril associated protein 3 like [Source:HGNC Symbol;Acc:HGNC:29083] |
| ENSG00000203952 | HGLibA_07669 | 76 | -0.447247429 | CCDC160 | coiled-coil domain containing 160 [Source:HGNC Symbol;Acc:HGNC:37286] |
| ENSG00000204779 | HGLibA_17852 | 76 | -0.447247429 | FOXD4L5 | forkhead box D4 like 5 [Source:HGNC Symbol;Acc:HGNC:18522] |
| ENSG00000204918 | HGLibA_38803 | 76 | -0.447247429 | PRR20B | proline rich 20B [Source:HGNC Symbol;Acc:HGNC:37220] |
| ENSG00000205022 | HGLibA_34944 | 76 | -0.447247429 | PABPN1L | cytoplasmic [Source:HGNC Symbol;Acc:HGNC:37237]" |
| ENSG00000205279 | HGLibA_11762 | 76 | -0.447247429 | CTXN3 | cortexin 3 [Source:HGNC Symbol;Acc:HGNC:31110] |
| ENSG00000214530 | HGLibA_47350 | 76 | -0.447247429 | STARD10 | StAR related lipid transfer domain containing 10 [Source:HGNC Symbol;Acc:HGNC:10666] |
| ENSG00000221874 | HGLibA_56815 | 76 | -0.447247429 | ZNF816-ZNF321P | ZNF816-ZNF321P readthrough [Source:HGNC Symbol;Acc:HGNC:38879] |
| ENSG00000223417 | HGLibA_51532 | 76 | -0.447247429 | TRIM49D1 | tripartite motif containing 49D1 [Source:HGNC Symbol;Acc:HGNC:43973] |
| ENSG00000227507 | HGLibA_27580 | 76 | -0.447247429 | LTB | lymphotoxin beta [Source:HGNC Symbol;Acc:HGNC:6711] |
| ENSG00000244405 | HGLibA_15662 | 76 | -0.447247429 | ETV5 | ETS variant 5 [Source:HGNC Symbol;Acc:HGNC:3494] |
| ENSG00000244623 | HGLibA_33745 | 76 | -0.447247429 | OR2AE1 | olfactory receptor family 2 subfamily AE member 1 [Source:HGNC Symbol;Acc:HGNC:15087] |
| ENSG00000250361 | HGLibA_20699 | 76 | -0.447247429 | GYPB | glycophorin B (MNS blood group) [Source:HGNC Symbol;Acc:HGNC:4703] |
| ENSG00000251349 | HGLibA_30179 | 76 | -0.447247429 | MSANTD3-TMEFF1 | MSANTD3-TMEFF1 readthrough [Source:HGNC Symbol;Acc:HGNC:38838] |
| ENSG00000253485 | HGLibA_35534 | 76 | -0.447247429 | PCDHGA5 | 5 [Source:HGNC Symbol;Acc:HGNC:8703]" |
| ENSG00000255012 | HGLibA_34327 | 76 | -0.447247429 | OR5M1 | olfactory receptor family 5 subfamily M member 1 [Source:HGNC Symbol;Acc:HGNC:8352] |
| ENSG00000255245 | HGLibA_18242 | 76 | -0.447247429 | FXYD6-FXYD2 | FXYD6-FXYD2 readthrough [Source:HGNC Symbol;Acc:HGNC:39978] |
| ENSG00000268629 | HGLibA_49058 | 76 | -0.447247429 | TEX13A | testis expressed 13A [Source:HGNC Symbol;Acc:HGNC:11735] |
| ENSG00000275489 | HGLibA_05601 | 76 | -0.447247429 | C17orf98 | chromosome 17 open reading frame 98 [Source:HGNC Symbol;Acc:HGNC:34492] |
| ENSG00000276380 | HGLibA_52640 | 76 | -0.447247429 | UBE2NL | ubiquitin conjugating enzyme E2 N like (gene/pseudogene) [Source:HGNC Symbol;Acc:HGNC:31710] |
| ENSG00000278311 | HGLibA_19004 | 76 | -0.447247429 | GGNBP2 | gametogenetin binding protein 2 [Source:HGNC Symbol;Acc:HGNC:19357] |
| FLJ22184 | HGLibA_17621 | 76 | -0.447247429 | FLJ22184 | - |
| HFE2 | HGLibA_21227 | 76 | -0.447247429 | HFE2 | - |
| hsa-let-7a-2 | HGLibA_57102 | 76 | -0.447247429 | hsa-let-7a-2 | - |
| hsa-let-7g | HGLibA_57133 | 76 | -0.447247429 | hsa-let-7g | - |
| hsa-mir-1255a | HGLibA_57422 | 76 | -0.447247429 | hsa-mir-1255a | - |
| hsa-mir-1283-2 | HGLibA_57594 | 76 | -0.447247429 | hsa-mir-1283-2 | - |
| hsa-mir-1912 | HGLibA_58054 | 76 | -0.447247429 | hsa-mir-1912 | - |
| hsa-mir-214 | HGLibA_58248 | 76 | -0.447247429 | hsa-mir-214 | - |
| hsa-mir-3137 | HGLibA_58606 | 76 | -0.447247429 | hsa-mir-3137 | - |
| hsa-mir-3180-5 | HGLibA_58817 | 76 | -0.447247429 | hsa-mir-3180-5 | - |
| hsa-mir-328 | HGLibA_58977 | 76 | -0.447247429 | hsa-mir-328 | - |
| hsa-mir-335 | HGLibA_58996 | 76 | -0.447247429 | hsa-mir-335 | - |
| hsa-mir-384 | HGLibA_59487 | 76 | -0.447247429 | hsa-mir-384 | - |
| hsa-mir-4418 | HGLibA_60057 | 76 | -0.447247429 | hsa-mir-4418 | - |
| hsa-mir-4460 | HGLibA_60246 | 76 | -0.447247429 | hsa-mir-4460 | - |
| hsa-mir-4460 | HGLibA_60248 | 76 | -0.447247429 | hsa-mir-4460 | - |
| hsa-mir-4647 | HGLibA_60691 | 76 | -0.447247429 | hsa-mir-4647 | - |
| hsa-mir-4654 | HGLibA_60724 | 76 | -0.447247429 | hsa-mir-4654 | - |
| hsa-mir-4722 | HGLibA_60987 | 76 | -0.447247429 | hsa-mir-4722 | - |
| hsa-mir-548ax | HGLibA_61941 | 76 | -0.447247429 | hsa-mir-548ax | - |
| hsa-mir-573 | HGLibA_62384 | 76 | -0.447247429 | hsa-mir-573 | - |
| hsa-mir-579 | HGLibA_62415 | 76 | -0.447247429 | hsa-mir-579 | - |
| hsa-mir-6850 | HGLibA_63572 | 76 | -0.447247429 | hsa-mir-6850 | - |
| hsa-mir-6850 | HGLibA_63571 | 76 | -0.447247429 | hsa-mir-6850 | - |
| hsa-mir-6855 | HGLibA_63592 | 76 | -0.447247429 | hsa-mir-6855 | - |
| hsa-mir-6867 | HGLibA_63651 | 76 | -0.447247429 | hsa-mir-6867 | - |
| hsa-mir-7974 | HGLibA_64024 | 76 | -0.447247429 | hsa-mir-7974 | - |
| hsa-mir-8054 | HGLibA_64056 | 76 | -0.447247429 | hsa-mir-8054 | - |
| hsa-mir-8065 | HGLibA_64100 | 76 | -0.447247429 | hsa-mir-8065 | - |
| hsa-mir-8076 | HGLibA_64148 | 76 | -0.447247429 | hsa-mir-8076 | - |
| hsa-mir-92a-2 | HGLibA_64294 | 76 | -0.447247429 | hsa-mir-92a-2 | - |
| LOC730183 | HGLibA_27004 | 76 | -0.447247429 | LOC730183 | - |
| NGFRAP1 | HGLibA_31877 | 76 | -0.447247429 | NGFRAP1 | - |
| PALM2-AKAP2 | HGLibA_35073 | 76 | -0.447247429 | PALM2-AKAP2 | - |
| PPAPDC2 | HGLibA_37830 | 76 | -0.447247429 | PPAPDC2 | - |
| UFD1L | HGLibA_52846 | 76 | -0.447247429 | UFD1L | - |
| ANXA8L2 | HGLibA_02259 | 75 | -0.456292772 | ANXA8L2 | - |
| C11orf30 | HGLibA_05126 | 75 | -0.456292772 | C11orf30 | - |
| C19orf57 | HGLibA_05685 | 75 | -0.456292772 | C19orf57 | - |
| C20orf197 | HGLibA_06008 | 75 | -0.456292772 | C20orf197 | - |
| C9orf89 | HGLibA_06770 | 75 | -0.456292772 | C9orf89 | - |
| ENSG00000005381 | HGLibA_29743 | 75 | -0.456292772 | MPO | myeloperoxidase [Source:HGNC Symbol;Acc:HGNC:7218] |
| ENSG00000024862 | HGLibA_07745 | 75 | -0.456292772 | CCDC28A | coiled-coil domain containing 28A [Source:HGNC Symbol;Acc:HGNC:21098] |
| ENSG00000039139 | HGLibA_13490 | 75 | -0.456292772 | DNAH5 | dynein axonemal heavy chain 5 [Source:HGNC Symbol;Acc:HGNC:2950] |
| ENSG00000049541 | HGLibA_40904 | 75 | -0.456292772 | RFC2 | replication factor C subunit 2 [Source:HGNC Symbol;Acc:HGNC:9970] |
| ENSG00000049768 | HGLibA_17935 | 75 | -0.456292772 | FOXP3 | forkhead box P3 [Source:HGNC Symbol;Acc:HGNC:6106] |
| ENSG00000054392 | HGLibA_21252 | 75 | -0.456292772 | HHAT | hedgehog acyltransferase [Source:HGNC Symbol;Acc:HGNC:18270] |
| ENSG00000054965 | HGLibA_16245 | 75 | -0.456292772 | FAM168A | family with sequence similarity 168 member A [Source:HGNC Symbol;Acc:HGNC:28999] |
| ENSG00000055609 | HGLibA_25327 | 75 | -0.456292772 | KMT2C | lysine methyltransferase 2C [Source:HGNC Symbol;Acc:HGNC:13726] |
| ENSG00000062194 | HGLibA_19755 | 75 | -0.456292772 | GPBP1 | GC-rich promoter binding protein 1 [Source:HGNC Symbol;Acc:HGNC:29520] |
| ENSG00000064787 | HGLibA_04250 | 75 | -0.456292772 | BCAS1 | breast carcinoma amplified sequence 1 [Source:HGNC Symbol;Acc:HGNC:974] |
| ENSG00000066629 | HGLibA_15056 | 75 | -0.456292772 | EML1 | echinoderm microtubule associated protein like 1 [Source:HGNC Symbol;Acc:HGNC:3330] |
| ENSG00000068885 | HGLibA_22773 | 75 | -0.456292772 | IFT80 | intraflagellar transport 80 [Source:HGNC Symbol;Acc:HGNC:29262] |
| ENSG00000073067 | HGLibA_12147 | 75 | -0.456292772 | CYP2W1 | cytochrome P450 family 2 subfamily W member 1 [Source:HGNC Symbol;Acc:HGNC:20243] |
| ENSG00000076201 | HGLibA_39462 | 75 | -0.456292772 | PTPN23 | non-receptor type 23 [Source:HGNC Symbol;Acc:HGNC:14406]" |
| ENSG00000076356 | HGLibA_37261 | 75 | -0.456292772 | PLXNA2 | plexin A2 [Source:HGNC Symbol;Acc:HGNC:9100] |
| ENSG00000079337 | HGLibA_40252 | 75 | -0.456292772 | RAPGEF3 | Rap guanine nucleotide exchange factor 3 [Source:HGNC Symbol;Acc:HGNC:16629] |
| ENSG00000082397 | HGLibA_15248 | 75 | -0.456292772 | EPB41L3 | erythrocyte membrane protein band 4.1 like 3 [Source:HGNC Symbol;Acc:HGNC:3380] |
| ENSG00000084092 | HGLibA_32230 | 75 | -0.456292772 | NOA1 | nitric oxide associated 1 [Source:HGNC Symbol;Acc:HGNC:28473] |
| ENSG00000091622 | HGLibA_36755 | 75 | -0.456292772 | PITPNM3 | PITPNM family member 3 [Source:HGNC Symbol;Acc:HGNC:21043] |
| ENSG00000093000 | HGLibA_33058 | 75 | -0.456292772 | NUP50 | nucleoporin 50 [Source:HGNC Symbol;Acc:HGNC:8065] |
| ENSG00000099999 | HGLibA_41627 | 75 | -0.456292772 | RNF215 | ring finger protein 215 [Source:HGNC Symbol;Acc:HGNC:33434] |
| ENSG00000100519 | HGLibA_39145 | 75 | -0.456292772 | PSMC6 | ATPase 6 [Source:HGNC Symbol;Acc:HGNC:9553]" |
| ENSG00000100564 | HGLibA_36559 | 75 | -0.456292772 | PIGH | phosphatidylinositol glycan anchor biosynthesis class H [Source:HGNC Symbol;Acc:HGNC:8964] |
| ENSG00000100938 | HGLibA_19431 | 75 | -0.456292772 | GMPR2 | guanosine monophosphate reductase 2 [Source:HGNC Symbol;Acc:HGNC:4377] |
| ENSG00000101605 | HGLibA_30890 | 75 | -0.456292772 | MYOM1 | myomesin 1 [Source:HGNC Symbol;Acc:HGNC:7613] |
| ENSG00000101670 | HGLibA_26612 | 75 | -0.456292772 | LIPG | endothelial type [Source:HGNC Symbol;Acc:HGNC:6623]" |
| ENSG00000102290 | HGLibA_35392 | 75 | -0.456292772 | PCDH11X | protocadherin 11 X-linked [Source:HGNC Symbol;Acc:HGNC:8656] |
| ENSG00000102384 | HGLibA_09059 | 75 | -0.456292772 | CENPI | centromere protein I [Source:HGNC Symbol;Acc:HGNC:3968] |
| ENSG00000102738 | HGLibA_30089 | 75 | -0.456292772 | MRPS31 | mitochondrial ribosomal protein S31 [Source:HGNC Symbol;Acc:HGNC:16632] |

| | | | | | |
|---|---|---|---|---|---|
| ENSG00000103599 | HGLibA_23521 | 75 | -0.456292772 | IQCH | IQ motif containing H [Source:HGNC Symbol;Acc:HGNC:25721] |
| ENSG00000104381 | HGLibA_18838 | 75 | -0.456292772 | GDAP1 | ganglioside induced differentiation associated protein 1 [Source:HGNC Symbol;Acc:HGNC:15968] |
| ENSG00000105401 | HGLibA_08545 | 75 | -0.456292772 | CDC37 | cell division cycle 37 [Source:HGNC Symbol;Acc:HGNC:1735] |
| ENSG00000105492 | HGLibA_44143 | 75 | -0.456292772 | SIGLEC6 | sialic acid binding Ig like lectin 6 [Source:HGNC Symbol;Acc:HGNC:10875] |
| ENSG00000107554 | HGLibA_13694 | 75 | -0.456292772 | DNMBP | dynamin binding protein [Source:HGNC Symbol;Acc:HGNC:30373] |
| ENSG00000107742 | HGLibA_46752 | 75 | -0.456292772 | SPOCK2 | cwcv and kazal like domains proteoglycan 2 [Source:HGNC Symbol;Acc:HGNC:13564]" |
| ENSG00000107863 | HGLibA_02726 | 75 | -0.456292772 | ARHGAP21 | Rho GTPase activating protein 21 [Source:HGNC Symbol;Acc:HGNC:23725] |
| ENSG00000110324 | HGLibA_22946 | 75 | -0.456292772 | IL10RA | interleukin 10 receptor subunit alpha [Source:HGNC Symbol;Acc:HGNC:5964] |
| ENSG00000110841 | HGLibA_37885 | 75 | -0.456292772 | PPFIBP1 | PPFIA binding protein 1 [Source:HGNC Symbol;Acc:HGNC:9249] |
| ENSG00000113594 | HGLibA_26476 | 75 | -0.456292772 | LIFR | LIF receptor alpha [Source:HGNC Symbol;Acc:HGNC:6597] |
| ENSG00000113719 | HGLibA_15468 | 75 | -0.456292772 | ERGIC1 | endoplasmic reticulum-golgi intermediate compartment 1 [Source:HGNC Symbol;Acc:HGNC:29205] |
| ENSG00000113916 | HGLibA_04333 | 75 | -0.456292772 | BCL6 | B cell CLL/lymphoma 6 [Source:HGNC Symbol;Acc:HGNC:1001] |
| ENSG00000115758 | HGLibA_33271 | 75 | -0.456292772 | ODC1 | ornithine decarboxylase 1 [Source:HGNC Symbol;Acc:HGNC:8109] |
| ENSG00000116044 | HGLibA_31795 | 75 | -0.456292772 | NFE2L2 | erythroid 2 like 2 [Source:HGNC Symbol;Acc:HGNC:7782]" |
| ENSG00000116157 | HGLibA_20187 | 75 | -0.456292772 | GPX7 | glutathione peroxidase 7 [Source:HGNC Symbol;Acc:HGNC:4559] |
| ENSG00000116497 | HGLibA_42587 | 75 | -0.456292772 | S100PBP | S100P binding protein [Source:HGNC Symbol;Acc:HGNC:25768] |
| ENSG00000116688 | HGLibA_29073 | 75 | -0.456292772 | MFN2 | mitofusin 2 [Source:HGNC Symbol;Acc:HGNC:16877] |
| ENSG00000116745 | HGLibA_41825 | 75 | -0.456292772 | RPE65 | retinoid isomerohydrolase [Source:HGNC Symbol;Acc:HGNC:10294]" |
| ENSG00000117472 | HGLibA_51892 | 75 | -0.456292772 | TSPAN1 | tetraspanin 1 [Source:HGNC Symbol;Acc:HGNC:20657] |
| ENSG00000118276 | HGLibA_04039 | 75 | -0.456292772 | B4GALT6 | 4-galactosyltransferase 6 [Source:HGNC Symbol;Acc:HGNC:929]" |
| ENSG00000118804 | HGLibA_47411 | 75 | -0.456292772 | STBD1 | starch binding domain 1 [Source:HGNC Symbol;Acc:HGNC:24854] |
| ENSG00000119522 | HGLibA_12930 | 75 | -0.456292772 | DENND1A | DENN domain containing 1A [Source:HGNC Symbol;Acc:HGNC:29324] |
| ENSG00000120057 | HGLibA_43744 | 75 | -0.456292772 | SFRP5 | secreted frizzled related protein 5 [Source:HGNC Symbol;Acc:HGNC:10779] |
| ENSG00000120519 | HGLibA_44348 | 75 | -0.456292772 | SLC10A7 | solute carrier family 10 member 7 [Source:HGNC Symbol;Acc:HGNC:23088] |
| ENSG00000120533 | HGLibA_15221 | 75 | -0.456292772 | ENY2 | transcription and export complex 2 subunit [Source:HGNC Symbol;Acc:HGNC:24449]" |
| ENSG00000121381 | HGLibA_48431 | 75 | -0.456292772 | TAS2R9 | taste 2 receptor member 9 [Source:HGNC Symbol;Acc:HGNC:14917] |
| ENSG00000122707 | HGLibA_40765 | 75 | -0.456292772 | RECK | reversion inducing cysteine rich protein with kazal motifs [Source:HGNC Symbol;Acc:HGNC:11345] |
| ENSG00000124102 | HGLibA_36488 | 75 | -0.456292772 | PI3 | peptidase inhibitor 3 [Source:HGNC Symbol;Acc:HGNC:8947] |
| ENSG00000124243 | HGLibA_04260 | 75 | -0.456292772 | BCAS4 | breast carcinoma amplified sequence 4 [Source:HGNC Symbol;Acc:HGNC:14367] |
| ENSG00000126337 | HGLibA_25493 | 75 | -0.456292772 | KRT36 | keratin 36 [Source:HGNC Symbol;Acc:HGNC:6454] |
| ENSG00000126453 | HGLibA_04312 | 75 | -0.456292772 | BCL2L12 | BCL2 like 12 [Source:HGNC Symbol;Acc:HGNC:13787] |
| ENSG00000127914 | HGLibA_01452 | 75 | -0.456292772 | AKAP9 | A-kinase anchoring protein 9 [Source:HGNC Symbol;Acc:HGNC:379] |
| ENSG00000129566 | HGLibA_49008 | 75 | -0.456292772 | TEP1 | telomerase associated protein 1 [Source:HGNC Symbol;Acc:HGNC:11726] |
| ENSG00000131591 | HGLibA_05782 | 75 | -0.456292772 | C1orf159 | chromosome 1 open reading frame 159 [Source:HGNC Symbol;Acc:HGNC:26062] |
| ENSG00000132000 | HGLibA_37455 | 75 | -0.456292772 | PODNL1 | podocan like 1 [Source:HGNC Symbol;Acc:HGNC:26275] |
| ENSG00000132510 | HGLibA_24537 | 75 | -0.456292772 | KDM6B | lysine demethylase 6B [Source:HGNC Symbol;Acc:HGNC:29012] |
| ENSG00000132681 | HGLibA_03591 | 75 | -0.456292772 | ATP1A4 | ATPase Na+/K+ transporting subunit alpha 4 [Source:HGNC Symbol;Acc:HGNC:14073] |
| ENSG00000135436 | HGLibA_16340 | 75 | -0.456292772 | FAM186B | family with sequence similarity 186 member B [Source:HGNC Symbol;Acc:HGNC:25296] |
| ENSG00000135845 | HGLibA_36549 | 75 | -0.456292772 | PIGC | phosphatidylinositol glycan anchor biosynthesis class C [Source:HGNC Symbol;Acc:HGNC:8960] |
| ENSG00000138185 | HGLibA_15198 | 75 | -0.456292772 | ENTPD1 | ectonucleoside triphosphate diphosphohydrolase 1 [Source:HGNC Symbol;Acc:HGNC:3363] |
| ENSG00000139192 | HGLibA_48325 | 75 | -0.456292772 | TAPBPL | TAP binding protein like [Source:HGNC Symbol;Acc:HGNC:30683] |
| ENSG00000139370 | HGLibA_44415 | 75 | -0.456292772 | SLC15A4 | solute carrier family 15 member 4 [Source:HGNC Symbol;Acc:HGNC:23090] |
| ENSG00000140299 | HGLibA_04653 | 75 | -0.456292772 | BNIP2 | BCL2 interacting protein 2 [Source:HGNC Symbol;Acc:HGNC:1083] |
| ENSG00000141424 | HGLibA_45118 | 75 | -0.456292772 | SLC39A6 | solute carrier family 39 member 6 [Source:HGNC Symbol;Acc:HGNC:18607] |
| ENSG00000141736 | HGLibA_15413 | 75 | -0.456292772 | ERBB2 | erb-b2 receptor tyrosine kinase 2 [Source:HGNC Symbol;Acc:HGNC:3430] |
| ENSG00000144043 | HGLibA_49083 | 75 | -0.456292772 | TEX261 | testis expressed 261 [Source:HGNC Symbol;Acc:HGNC:30712] |
| ENSG00000145439 | HGLibA_07423 | 75 | -0.456292772 | CBR4 | carbonyl reductase 4 [Source:HGNC Symbol;Acc:HGNC:25891] |
| ENSG00000145975 | HGLibA_16489 | 75 | -0.456292772 | FAM217A | family with sequence similarity 217 member A [Source:HGNC Symbol;Acc:HGNC:21362] |
| ENSG00000146072 | HGLibA_50777 | 75 | -0.456292772 | TNFRSF21 | TNF receptor superfamily member 21 [Source:HGNC Symbol;Acc:HGNC:13469] |
| ENSG00000147586 | HGLibA_30084 | 75 | -0.456292772 | MRPS28 | mitochondrial ribosomal protein S28 [Source:HGNC Symbol;Acc:HGNC:14513] |
| ENSG00000148308 | HGLibA_20592 | 75 | -0.456292772 | GTF3C5 | general transcription factor IIIC subunit 5 [Source:HGNC Symbol;Acc:HGNC:4668] |
| ENSG00000148358 | HGLibA_19844 | 75 | -0.456292772 | GPR107 | G protein-coupled receptor 107 [Source:HGNC Symbol;Acc:HGNC:17830] |
| ENSG00000148824 | HGLibA_30369 | 75 | -0.456292772 | MTG1 | mitochondrial ribosome associated GTPase 1 [Source:HGNC Symbol;Acc:HGNC:32159] |
| ENSG00000149596 | HGLibA_23983 | 75 | -0.456292772 | JPH2 | junctophilin 2 [Source:HGNC Symbol;Acc:HGNC:14202] |
| ENSG00000149823 | HGLibA_53804 | 75 | -0.456292772 | VPS51 | GARP complex subunit [Source:HGNC Symbol;Acc:HGNC:1172]" |
| ENSG00000150637 | HGLibA_08300 | 75 | -0.456292772 | CD226 | CD226 molecule [Source:HGNC Symbol;Acc:HGNC:16961] |
| ENSG00000151388 | HGLibA_00798 | 75 | -0.456292772 | ADAMTS12 | ADAM metallopeptidase with thrombospondin type 1 motif 12 [Source:HGNC Symbol;Acc:HGNC:14605] |
| ENSG00000151962 | HGLibA_40562 | 75 | -0.456292772 | RBM46 | RNA binding motif protein 46 [Source:HGNC Symbol;Acc:HGNC:28401] |
| ENSG00000152518 | HGLibA_55209 | 75 | -0.456292772 | ZFP36L2 | ZFP36 ring finger protein like 2 [Source:HGNC Symbol;Acc:HGNC:1108] |
| ENSG00000152818 | HGLibA_53457 | 75 | -0.456292772 | UTRN | utrophin [Source:HGNC Symbol;Acc:HGNC:12635] |
| ENSG00000153443 | HGLibA_52541 | 75 | -0.456292772 | UBALD1 | UBA like domain containing 1 [Source:HGNC Symbol;Acc:HGNC:29576] |
| ENSG00000156127 | HGLibA_04174 | 75 | -0.456292772 | BATF | basic leucine zipper ATF-like transcription factor [Source:HGNC Symbol;Acc:HGNC:958] |
| ENSG00000157764 | HGLibA_04753 | 75 | -0.456292772 | BRAF | serine/threonine kinase [Source:HGNC Symbol;Acc:HGNC:1097]" |
| ENSG00000159212 | HGLibA_10037 | 75 | -0.456292772 | CLIC6 | chloride intracellular channel 6 [Source:HGNC Symbol;Acc:HGNC:2065] |
| ENSG00000160013 | HGLibA_39346 | 75 | -0.456292772 | PTGIR | prostaglandin I2 receptor [Source:HGNC Symbol;Acc:HGNC:9602] |
| ENSG00000160318 | HGLibA_09918 | 75 | -0.456292772 | CLDND2 | claudin domain containing 2 [Source:HGNC Symbol;Acc:HGNC:28511] |
| ENSG00000160791 | HGLibA_08158 | 75 | -0.456292772 | CCR5 | C-C motif chemokine receptor 5 (gene/pseudogene) [Source:HGNC Symbol;Acc:HGNC:1606] |
| ENSG00000161960 | HGLibA_14805 | 75 | -0.456292772 | EIF4A1 | eukaryotic translation initiation factor 4A1 [Source:HGNC Symbol;Acc:HGNC:3282] |
| ENSG00000162959 | HGLibA_28883 | 75 | -0.456292772 | MEMO1 | mediator of cell motility 1 [Source:HGNC Symbol;Acc:HGNC:14014] |
| ENSG00000163110 | HGLibA_35918 | 75 | -0.456292772 | PDLIM5 | PDZ and LIM domain 5 [Source:HGNC Symbol;Acc:HGNC:17468] |
| ENSG00000164068 | HGLibA_41489 | 75 | -0.456292772 | RNF123 | ring finger protein 123 [Source:HGNC Symbol;Acc:HGNC:21148] |
| ENSG00000164303 | HGLibA_15182 | 75 | -0.456292772 | ENPP6 | ectonucleotide pyrophosphatase/phosphodiesterase 6 [Source:HGNC Symbol;Acc:HGNC:23409] |
| ENSG00000165028 | HGLibA_31978 | 75 | -0.456292772 | NIPSNAP3B | nipsnap homolog 3B [Source:HGNC Symbol;Acc:HGNC:23641] |
| ENSG00000166947 | HGLibA_15260 | 75 | -0.456292772 | EPB42 | erythrocyte membrane protein band 4.2 [Source:HGNC Symbol;Acc:HGNC:3381] |
| ENSG00000167136 | HGLibA_15117 | 75 | -0.456292772 | ENDOG | endonuclease G [Source:HGNC Symbol;Acc:HGNC:3346] |
| ENSG00000167549 | HGLibA_10788 | 75 | -0.456292772 | CORO6 | coronin 6 [Source:HGNC Symbol;Acc:HGNC:21356] |
| ENSG00000167613 | HGLibA_25931 | 75 | -0.456292772 | LAIR1 | leukocyte associated immunoglobulin like receptor 1 [Source:HGNC Symbol;Acc:HGNC:6477] |
| ENSG00000167721 | HGLibA_51993 | 75 | -0.456292772 | TSR1 | ribosome maturation factor [Source:HGNC Symbol;Acc:HGNC:25542]" |
| ENSG00000167797 | HGLibA_08763 | 75 | -0.456292772 | CDK2AP2 | cyclin dependent kinase 2 associated protein 2 [Source:HGNC Symbol;Acc:HGNC:30833] |
| ENSG00000168398 | HGLibA_04384 | 75 | -0.456292772 | BDKRB2 | bradykinin receptor B2 [Source:HGNC Symbol;Acc:HGNC:1030] |
| ENSG00000169398 | HGLibA_39378 | 75 | -0.456292772 | PTK2 | protein tyrosine kinase 2 [Source:HGNC Symbol;Acc:HGNC:9611] |
| ENSG00000169641 | HGLibA_27637 | 75 | -0.456292772 | LUZP1 | leucine zipper protein 1 [Source:HGNC Symbol;Acc:HGNC:14985] |
| ENSG00000169967 | HGLibA_28180 | 75 | -0.456292772 | MAP3K2 | mitogen-activated protein kinase kinase kinase 2 [Source:HGNC Symbol;Acc:HGNC:6854] |
| ENSG00000170113 | HGLibA_31949 | 75 | -0.456292772 | NIPA1 | NIPA magnesium transporter 1 [Source:HGNC Symbol;Acc:HGNC:17043] |
| ENSG00000170279 | HGLibA_06536 | 75 | -0.456292772 | C7orf33 | chromosome 7 open reading frame 33 [Source:HGNC Symbol;Acc:HGNC:21724] |
| ENSG00000170412 | HGLibA_20132 | 75 | -0.456292772 | GPRC5C | G protein-coupled receptor class C group 5 member C [Source:HGNC Symbol;Acc:HGNC:13309] |
| ENSG00000170465 | HGLibA_25518 | 75 | -0.456292772 | KRT6C | keratin 6C [Source:HGNC Symbol;Acc:HGNC:20406] |
| ENSG00000170549 | HGLibA_23629 | 75 | -0.456292772 | IRX1 | iroquois homeobox 1 [Source:HGNC Symbol;Acc:HGNC:14358] |
| ENSG00000171262 | HGLibA_16811 | 75 | -0.456292772 | FAM98B | family with sequence similarity 98 member B [Source:HGNC Symbol;Acc:HGNC:26773] |
| ENSG00000171475 | HGLibA_54306 | 75 | -0.456292772 | WIPF2 | WAS/WASL interacting protein family member 2 [Source:HGNC Symbol;Acc:HGNC:30923] |
| ENSG00000171631 | HGLibA_34890 | 75 | -0.456292772 | P2RY6 | pyrimidinergic receptor P2Y6 [Source:HGNC Symbol;Acc:HGNC:8543] |
| ENSG00000171824 | HGLibA_15771 | 75 | -0.456292772 | EXOSC10 | exosome component 10 [Source:HGNC Symbol;Acc:HGNC:9138] |
| ENSG00000172031 | HGLibA_15332 | 75 | -0.456292772 | EPHX4 | epoxide hydrolase 4 [Source:HGNC Symbol;Acc:HGNC:23758] |
| ENSG00000172468 | HGLibA_22242 | 75 | -0.456292772 | HSFY1 | heat shock transcription factor Y-linked 1 [Source:HGNC Symbol;Acc:HGNC:18568] |
| ENSG00000173349 | HGLibA_43757 | 75 | -0.456292772 | SFT2D3 | SFT2 domain containing 3 [Source:HGNC Symbol;Acc:HGNC:28767] |
| ENSG00000173917 | HGLibA_21932 | 75 | -0.456292772 | HOXB2 | homeobox B2 [Source:HGNC Symbol;Acc:HGNC:5113] |
| ENSG00000174938 | HGLibA_43685 | 75 | -0.456292772 | SEZ6L2 | seizure related 6 homolog like 2 [Source:HGNC Symbol;Acc:HGNC:30844] |
| ENSG00000175267 | HGLibA_53902 | 75 | -0.456292772 | VWA3A | von Willebrand factor A domain containing 3A [Source:HGNC Symbol;Acc:HGNC:27088] |
| ENSG00000175354 | HGLibA_39447 | 75 | -0.456292772 | PTPN2 | non-receptor type 2 [Source:HGNC Symbol;Acc:HGNC:9650]" |
| ENSG00000177243 | HGLibA_12815 | 75 | -0.456292772 | DEFB103B | defensin beta 103B [Source:HGNC Symbol;Acc:HGNC:31702] |
| ENSG00000177363 | HGLibA_27483 | 75 | -0.456292772 | LRRN4CL | LRRN4 C-terminal like [Source:HGNC Symbol;Acc:HGNC:33724] |
| ENSG00000177570 | HGLibA_42667 | 75 | -0.456292772 | SAMD12 | sterile alpha motif domain containing 12 [Source:HGNC Symbol;Acc:HGNC:31750] |
| ENSG00000178226 | HGLibA_38931 | 75 | -0.456292772 | PRSS36 | serine protease 36 [Source:HGNC Symbol;Acc:HGNC:26906] |
| ENSG00000179431 | HGLibA_17549 | 75 | -0.456292772 | FJX1 | four-jointed box kinase 1 [Source:HGNC Symbol;Acc:HGNC:17166] |
| ENSG00000179639 | HGLibA_17169 | 75 | -0.456292772 | FCER1A | Fc fragment of IgE receptor Ia [Source:HGNC Symbol;Acc:HGNC:3609] |
| ENSG00000180304 | HGLibA_33217 | 75 | -0.456292772 | OAZ2 | ornithine decarboxylase antizyme 2 [Source:HGNC Symbol;Acc:HGNC:8096] |
| ENSG00000180318 | HGLibA_01733 | 75 | -0.456292772 | ALX1 | ALX homeobox 1 [Source:HGNC Symbol;Acc:HGNC:1494] |
| ENSG00000180787 | HGLibA_55197 | 75 | -0.456292772 | ZFP3 | ZFP3 zinc finger protein [Source:HGNC Symbol;Acc:HGNC:12861] |
| ENSG00000181031 | HGLibA_41847 | 75 | -0.456292772 | RPH3AL | rabphilin 3A like (without C2 domains) [Source:HGNC Symbol;Acc:HGNC:10296] |
| ENSG00000181274 | HGLibA_17982 | 75 | -0.456292772 | FRAT2 | WNT signaling pathway regulator [Source:HGNC Symbol;Acc:HGNC:16048]" |
| ENSG00000181804 | HGLibA_45430 | 75 | -0.456292772 | SLC9A9 | solute carrier family 9 member A9 [Source:HGNC Symbol;Acc:HGNC:20653] |
| ENSG00000182481 | HGLibA_25357 | 75 | -0.456292772 | KPNA2 | karyopherin subunit alpha 2 [Source:HGNC Symbol;Acc:HGNC:6395] |
| ENSG00000182896 | HGLibA_50565 | 75 | -0.456292772 | TMEM95 | transmembrane protein 95 [Source:HGNC Symbol;Acc:HGNC:27898] |
| ENSG00000185101 | HGLibA_02196 | 75 | -0.456292772 | ANO9 | anoctamin 9 [Source:HGNC Symbol;Acc:HGNC:20679] |
| ENSG00000185262 | HGLibA_52543 | 75 | -0.456292772 | UBALD2 | UBA like domain containing 2 [Source:HGNC Symbol;Acc:HGNC:28438] |
| ENSG00000185344 | HGLibA_03705 | 75 | -0.456292772 | ATP6V0A2 | ATPase H+ transporting V0 subunit a2 [Source:HGNC Symbol;Acc:HGNC:18481] |
| ENSG00000185565 | HGLibA_27511 | 75 | -0.456292772 | LSAMP | limbic system associated membrane protein [Source:HGNC Symbol;Acc:HGNC:6705] |
| ENSG00000185666 | HGLibA_47961 | 75 | -0.456292772 | SYN3 | synapsin III [Source:HGNC Symbol;Acc:HGNC:11496] |
| ENSG00000185862 | HGLibA_15688 | 75 | -0.456292772 | EVI2B | ecotropic viral integration site 2B [Source:HGNC Symbol;Acc:HGNC:3500] |
| ENSG00000186687 | HGLibA_27758 | 75 | -0.456292772 | LYRM7 | LYR motif containing 7 [Source:HGNC Symbol;Acc:HGNC:28072] |
| ENSG00000187627 | HGLibA_41024 | 75 | -0.456292772 | RGPD1 | RANBP2-like and GRIP domain containing 1 [Source:HGNC Symbol;Acc:HGNC:32414] |
| ENSG00000188177 | HGLibA_54978 | 75 | -0.456292772 | ZC3H6 | zinc finger CCCH-type containing 6 [Source:HGNC Symbol;Acc:HGNC:24762] |
| ENSG00000189051 | HGLibA_41643 | 75 | -0.456292772 | RNF222 | ring finger protein 222 [Source:HGNC Symbol;Acc:HGNC:34517] |
| ENSG00000196228 | HGLibA_47749 | 75 | -0.456292772 | SULT1C3 | sulfotransferase family 1C member 3 [Source:HGNC Symbol;Acc:HGNC:33543] |
| ENSG00000196338 | HGLibA_32074 | 75 | -0.456292772 | NLGN3 | neuroligin 3 [Source:HGNC Symbol;Acc:HGNC:14289] |
| ENSG00000196655 | HGLibA_51323 | 75 | -0.456292772 | TRAPPC4 | trafficking protein particle complex 4 [Source:HGNC Symbol;Acc:HGNC:19943] |
| ENSG00000197150 | HGLibA_00153 | 75 | -0.456292772 | ABCB8 | ATP binding cassette subfamily B member 8 [Source:HGNC Symbol;Acc:HGNC:49] |
| ENSG00000197614 | HGLibA_29053 | 75 | -0.456292772 | MFAP5 | microfibril associated protein 5 [Source:HGNC Symbol;Acc:HGNC:29673] |
| ENSG00000197860 | HGLibA_43870 | 75 | -0.456292772 | SGTB | small glutamine rich tetratricopeptide repeat containing beta [Source:HGNC Symbol;Acc:HGNC:23567] |
| ENSG00000198648 | HGLibA_47507 | 75 | -0.456292772 | STK39 | serine/threonine kinase 39 [Source:HGNC Symbol;Acc:HGNC:17717] |
| ENSG00000203870 | HGLibA_45772 | 75 | -0.456292772 | SMIM9 | small integral membrane protein 9 [Source:HGNC Symbol;Acc:HGNC:41915] |
| ENSG00000203965 | HGLibA_14553 | 75 | -0.456292772 | EFCAB7 | EF-hand calcium binding domain 7 [Source:HGNC Symbol;Acc:HGNC:29379] |
| ENSG00000204128 | HGLibA_06176 | 75 | -0.456292772 | C2orf72 | chromosome 2 open reading frame 72 [Source:HGNC Symbol;Acc:HGNC:27418] |
| ENSG00000204386 | HGLibA_31711 | 75 | -0.456292772 | NEU1 | neuraminidase 1 [Source:HGNC Symbol;Acc:HGNC:7758] |
| ENSG00000212864 | HGLibA_41617 | 75 | -0.456292772 | RNF208 | ring finger protein 208 [Source:HGNC Symbol;Acc:HGNC:25420] |

| | | | | | |
|---|---|---|---|---|---|
| ENSG00000213145 | HGLibA_11149 | 75 | -0.456292772 | CRIP1 | cysteine rich protein 1 [Source:HGNC Symbol;Acc:HGNC:2360] |
| ENSG00000213186 | HGLibA_51554 | 75 | -0.456292772 | TRIM59 | tripartite motif containing 59 [Source:HGNC Symbol;Acc:HGNC:30834] |
| ENSG00000213516 | HGLibA_40602 | 75 | -0.456292772 | RBMXL1 | RBMX like 1 [Source:HGNC Symbol;Acc:HGNC:25073] |
| ENSG00000213619 | HGLibA_31551 | 75 | -0.456292772 | NDUFS3 | NADH:ubiquinone oxidoreductase core subunit S3 [Source:HGNC Symbol;Acc:HGNC:7710] |
| ENSG00000213937 | HGLibA_09913 | 75 | -0.456292772 | CLDN9 | claudin 9 [Source:HGNC Symbol;Acc:HGNC:2051] |
| ENSG00000214376 | HGLibA_53873 | 75 | -0.456292772 | VSTM5 | V-set and transmembrane domain containing 5 [Source:HGNC Symbol;Acc:HGNC:34443] |
| ENSG00000215041 | HGLibA_31731 | 75 | -0.456292772 | NEURL4 | neuralized E3 ubiquitin protein ligase 4 [Source:HGNC Symbol;Acc:HGNC:34410] |
| ENSG00000229665 | HGLibA_38806 | 75 | -0.456292772 | PRR20C | proline rich 20C [Source:HGNC Symbol;Acc:HGNC:37221] |
| ENSG00000235268 | HGLibA_24518 | 75 | -0.456292772 | KDM4E | lysine demethylase 4E [Source:HGNC Symbol;Acc:HGNC:37098] |
| ENSG00000240230 | HGLibA_10824 | 75 | -0.456292772 | COX19 | cytochrome c oxidase assembly factor COX19 [Source:HGNC Symbol;Acc:HGNC:28074] |
| ENSG00000240403 | HGLibA_24994 | 75 | -0.456292772 | KIR3DL2 | three Ig domains and long cytoplasmic tail 2 [Source:HGNC Symbol;Acc:HGNC:6339]" |
| ENSG00000243772 | HGLibA_24984 | 75 | -0.456292772 | KIR2DL3 | two Ig domains and long cytoplasmic tail 3 [Source:HGNC Symbol;Acc:HGNC:6331]" |
| ENSG00000256394 | HGLibA_03310 | 75 | -0.456292772 | ASIC5 | acid sensing ion channel subunit family member 5 [Source:HGNC Symbol;Acc:HGNC:17537] |
| ENSG00000260238 | HGLibA_37305 | 75 | -0.456292772 | PMF1-BGLAP | PMF1-BGLAP readthrough [Source:HGNC Symbol;Acc:HGNC:42953] |
| ENSG00000261787 | HGLibA_48779 | 75 | -0.456292772 | TCF24 | transcription factor 24 [Source:HGNC Symbol;Acc:HGNC:32275] |
| ENSG00000265817 | HGLibA_18063 | 75 | -0.456292772 | FSBP | fibrinogen silencer binding protein [Source:HGNC Symbol;Acc:HGNC:43653] |
| ENSG00000278289 | HGLibA_11514 | 75 | -0.456292772 | CT45A6 | cancer/testis antigen family 45 member A6 [Source:HGNC Symbol;Acc:HGNC:33271] |
| GSG2 | HGLibA_20426 | 75 | -0.456292772 | GSG2 | - |
| HIST1H2AM | HGLibA_21409 | 75 | -0.456292772 | HIST1H2AM | - |
| HIST1H3C | HGLibA_21461 | 75 | -0.456292772 | HIST1H3C | - |
| hsa-mir-124-3 | HGLibA_57353 | 75 | -0.456292772 | hsa-mir-124-3 | - |
| hsa-mir-1275 | HGLibA_57554 | 75 | -0.456292772 | hsa-mir-1275 | - |
| hsa-mir-1285-2 | HGLibA_57604 | 75 | -0.456292772 | hsa-mir-1285-2 | - |
| hsa-mir-181c | HGLibA_57976 | 75 | -0.456292772 | hsa-mir-181c | - |
| hsa-mir-1825 | HGLibA_57987 | 75 | -0.456292772 | hsa-mir-1825 | - |
| hsa-mir-18a | HGLibA_58018 | 75 | -0.456292772 | hsa-mir-18a | - |
| hsa-mir-190b | HGLibA_58040 | 75 | -0.456292772 | hsa-mir-190b | - |
| hsa-mir-19a | HGLibA_58141 | 75 | -0.456292772 | hsa-mir-19a | - |
| hsa-mir-3116-2 | HGLibA_58506 | 75 | -0.456292772 | hsa-mir-3116-2 | - |
| hsa-mir-3140 | HGLibA_58617 | 75 | -0.456292772 | hsa-mir-3140 | - |
| hsa-mir-3180-2 | HGLibA_58806 | 75 | -0.456292772 | hsa-mir-3180-2 | - |
| hsa-mir-34b | HGLibA_59038 | 75 | -0.456292772 | hsa-mir-34b | - |
| hsa-mir-3649 | HGLibA_59142 | 75 | -0.456292772 | hsa-mir-3649 | - |
| hsa-mir-374b | HGLibA_59393 | 75 | -0.456292772 | hsa-mir-374b | - |
| hsa-mir-4261 | HGLibA_59750 | 75 | -0.456292772 | hsa-mir-4261 | - |
| hsa-mir-4266 | HGLibA_59771 | 75 | -0.456292772 | hsa-mir-4266 | - |
| hsa-mir-4277 | HGLibA_59813 | 75 | -0.456292772 | hsa-mir-4277 | - |
| hsa-mir-4290 | HGLibA_59871 | 75 | -0.456292772 | hsa-mir-4290 | - |
| hsa-mir-4301 | HGLibA_59917 | 75 | -0.456292772 | hsa-mir-4301 | - |
| hsa-mir-495 | HGLibA_61382 | 75 | -0.456292772 | hsa-mir-495 | - |
| hsa-mir-5092 | HGLibA_61525 | 75 | -0.456292772 | hsa-mir-5092 | - |
| hsa-mir-518a-1 | HGLibA_61653 | 75 | -0.456292772 | hsa-mir-518a-1 | - |
| hsa-mir-520c | HGLibA_61743 | 75 | -0.456292772 | hsa-mir-520c | - |
| hsa-mir-597 | HGLibA_62482 | 75 | -0.456292772 | hsa-mir-597 | - |
| hsa-mir-647 | HGLibA_62831 | 75 | -0.456292772 | hsa-mir-647 | - |
| hsa-mir-6734 | HGLibA_63089 | 75 | -0.456292772 | hsa-mir-6734 | - |
| hsa-mir-6848 | HGLibA_63564 | 75 | -0.456292772 | hsa-mir-6848 | - |
| hsa-mir-8075 | HGLibA_64142 | 75 | -0.456292772 | hsa-mir-8075 | - |
| hsa-mir-8084 | HGLibA_64179 | 75 | -0.456292772 | hsa-mir-8084 | - |
| NARFL | HGLibA_31145 | 75 | -0.456292772 | NARFL | - |
| 2-Sep | HGLibA_43435 | 75 | -0.456292772 | 2-Sep | - |
| TMEM27 | HGLibA_50357 | 75 | -0.456292772 | TMEM27 | - |
| VAMP7_Y | HGLibA_53505 | 75 | -0.456292772 | VAMP7_Y | - |
| AGAP8 | HGLibA_01161 | 74 | -0.465338115 | AGAP8 | - |
| ATP5C1 | HGLibA_03643 | 74 | -0.465338115 | ATP5C1 | - |
| C6orf165 | HGLibA_06458 | 74 | -0.465338115 | C6orf165 | - |
| C7orf65 | HGLibA_06576 | 74 | -0.465338115 | C7orf65 | - |
| CCDC19 | HGLibA_07728 | 74 | -0.465338115 | CCDC19 | - |
| CCRL1 | HGLibA_08174 | 74 | -0.465338115 | CCRL1 | - |
| CXorf21 | HGLibA_11902 | 74 | -0.465338115 | CXorf21 | - |
| CXorf56 | HGLibA_11943 | 74 | -0.465338115 | CXorf56 | - |
| ENSG00000005882 | HGLibA_35899 | 74 | -0.465338115 | PDK2 | pyruvate dehydrogenase kinase 2 [Source:HGNC Symbol;Acc:HGNC:8810] |
| ENSG00000006831 | HGLibA_00984 | 74 | -0.465338115 | ADIPOR2 | adiponectin receptor 2 [Source:HGNC Symbol;Acc:HGNC:24041] |
| ENSG00000007968 | HGLibA_14310 | 74 | -0.465338115 | E2F2 | E2F transcription factor 2 [Source:HGNC Symbol;Acc:HGNC:3114] |
| ENSG00000023839 | HGLibA_00170 | 74 | -0.465338115 | ABCC2 | ATP binding cassette subfamily C member 2 [Source:HGNC Symbol;Acc:HGNC:53] |
| ENSG00000048991 | HGLibA_39772 | 74 | -0.465338115 | R3HDM1 | R3H domain containing 1 [Source:HGNC Symbol;Acc:HGNC:9757] |
| ENSG00000064932 | HGLibA_42809 | 74 | -0.465338115 | SBNO2 | strawberry notch homolog 2 [Source:HGNC Symbol;Acc:HGNC:29158] |
| ENSG00000065029 | HGLibA_56682 | 74 | -0.465338115 | ZNF76 | zinc finger protein 76 [Source:HGNC Symbol;Acc:HGNC:13149] |
| ENSG00000070526 | HGLibA_47265 | 74 | -0.465338115 | ST6GALNAC1 | 6-sialyltransferase 1 [Source:HGNC Symbol;Acc:HGNC:23614]" |
| ENSG00000075213 | HGLibA_43318 | 74 | -0.465338115 | SEMA3A | semaphorin 3A [Source:HGNC Symbol;Acc:HGNC:10723] |
| ENSG00000075413 | HGLibA_28369 | 74 | -0.465338115 | MARK3 | microtubule affinity regulating kinase 3 [Source:HGNC Symbol;Acc:HGNC:6897] |
| ENSG00000079387 | HGLibA_43386 | 74 | -0.465338115 | SENP1 | SUMO specific peptidase 1 [Source:HGNC Symbol;Acc:HGNC:17927] |
| ENSG00000087460 | HGLibA_19469 | 74 | -0.465338115 | GNAS | GNAS complex locus [Source:HGNC Symbol;Acc:HGNC:4392] |
| ENSG00000090975 | HGLibA_36753 | 74 | -0.465338115 | PITPNM2 | phosphatidylinositol transfer protein membrane associated 2 [Source:HGNC Symbol;Acc:HGNC:21044] |
| ENSG00000095380 | HGLibA_31109 | 74 | -0.465338115 | NANS | N-acetylneuraminate synthase [Source:HGNC Symbol;Acc:HGNC:19237] |
| ENSG00000095574 | HGLibA_22941 | 74 | -0.465338115 | IKZF5 | IKAROS family zinc finger 5 [Source:HGNC Symbol;Acc:HGNC:14283] |
| ENSG00000100664 | HGLibA_14852 | 74 | -0.465338115 | EIF5 | eukaryotic translation initiation factor 5 [Source:HGNC Symbol;Acc:HGNC:3299] |
| ENSG00000100865 | HGLibA_09716 | 74 | -0.465338115 | CINP | cyclin dependent kinase 2 interacting protein [Source:HGNC Symbol;Acc:HGNC:23789] |
| ENSG00000101162 | HGLibA_52273 | 74 | -0.465338115 | TUBB1 | tubulin beta 1 class VI [Source:HGNC Symbol;Acc:HGNC:16257] |
| ENSG00000103335 | HGLibA_36535 | 74 | -0.465338115 | PIEZO1 | piezo type mechanosensitive ion channel component 1 [Source:HGNC Symbol;Acc:HGNC:28993] |
| ENSG00000103540 | HGLibA_08137 | 74 | -0.465338115 | CCP110 | centriolar coiled-coil protein 110 [Source:HGNC Symbol;Acc:HGNC:24342] |
| ENSG00000103599 | HGLibA_23519 | 74 | -0.465338115 | IQCH | IQ motif containing H [Source:HGNC Symbol;Acc:HGNC:25721] |
| ENSG00000103647 | HGLibA_10784 | 74 | -0.465338115 | CORO2B | coronin 2B [Source:HGNC Symbol;Acc:HGNC:2256] |
| ENSG00000104313 | HGLibA_15818 | 74 | -0.465338115 | EYA1 | EYA transcriptional coactivator and phosphatase 1 [Source:HGNC Symbol;Acc:HGNC:3519] |
| ENSG00000104951 | HGLibA_23168 | 74 | -0.465338115 | IL4I1 | interleukin 4 induced 1 [Source:HGNC Symbol;Acc:HGNC:19094] |
| ENSG00000106823 | HGLibA_14414 | 74 | -0.465338115 | ECM2 | extracellular matrix protein 2 [Source:HGNC Symbol;Acc:HGNC:3154] |
| ENSG00000107614 | HGLibA_51345 | 74 | -0.465338115 | TRDMT1 | tRNA aspartic acid methyltransferase 1 [Source:HGNC Symbol;Acc:HGNC:2977] |
| ENSG00000108468 | HGLibA_07446 | 74 | -0.465338115 | CBX1 | chromobox 1 [Source:HGNC Symbol;Acc:HGNC:1551] |
| ENSG00000108592 | HGLibA_18139 | 74 | -0.465338115 | FTSJ3 | FtsJ RNA methyltransferase homolog 3 [Source:HGNC Symbol;Acc:HGNC:17136] |
| ENSG00000108671 | HGLibA_39153 | 74 | -0.465338115 | PSMD11 | non-ATPase 11 [Source:HGNC Symbol;Acc:HGNC:9556]" |
| ENSG00000108839 | HGLibA_01675 | 74 | -0.465338115 | ALOX12 | 12S type [Source:HGNC Symbol;Acc:HGNC:429]" |
| ENSG00000109016 | HGLibA_13148 | 74 | -0.465338115 | DHRS7B | dehydrogenase/reductase 7B [Source:HGNC Symbol;Acc:HGNC:24547] |
| ENSG00000110723 | HGLibA_15798 | 74 | -0.465338115 | EXPH5 | exophilin 5 [Source:HGNC Symbol;Acc:HGNC:30578] |
| ENSG00000111832 | HGLibA_42479 | 74 | -0.465338115 | RWDD1 | RWD domain containing 1 [Source:HGNC Symbol;Acc:HGNC:20993] |
| ENSG00000111907 | HGLibA_51115 | 74 | -0.465338115 | TPD52L1 | TPD52 like 1 [Source:HGNC Symbol;Acc:HGNC:12006] |
| ENSG00000112530 | HGLibA_34948 | 74 | -0.465338115 | PACRG | parkin coregulated [Source:HGNC Symbol;Acc:HGNC:19152] |
| ENSG00000113212 | HGLibA_35509 | 74 | -0.465338115 | PCDHB7 | protocadherin beta 7 [Source:HGNC Symbol;Acc:HGNC:8692] |
| ENSG00000113361 | HGLibA_08685 | 74 | -0.465338115 | CDH6 | cadherin 6 [Source:HGNC Symbol;Acc:HGNC:1765] |
| ENSG00000114982 | HGLibA_24044 | 74 | -0.465338115 | KANSL3 | KAT8 regulatory NSL complex subunit 3 [Source:HGNC Symbol;Acc:HGNC:25473] |
| ENSG00000115266 | HGLibA_02391 | 74 | -0.465338115 | APC2 | WNT signaling pathway regulator [Source:HGNC Symbol;Acc:HGNC:24036]" |
| ENSG00000115694 | HGLibA_47475 | 74 | -0.465338115 | STK25 | serine/threonine kinase 25 [Source:HGNC Symbol;Acc:HGNC:11404] |
| ENSG00000115750 | HGLibA_48215 | 74 | -0.465338115 | TAF1B | RNA polymerase I subunit B [Source:HGNC Symbol;Acc:HGNC:11533]" |
| ENSG00000116703 | HGLibA_35712 | 74 | -0.465338115 | PDC | phosducin [Source:HGNC Symbol;Acc:HGNC:8759] |
| ENSG00000117983 | HGLibA_30549 | 74 | -0.465338115 | MUC5B | oligomeric mucus/gel-forming [Source:HGNC Symbol;Acc:HGNC:7516]" |
| ENSG00000117984 | HGLibA_11697 | 74 | -0.465338115 | CTSD | cathepsin D [Source:HGNC Symbol;Acc:HGNC:2529] |
| ENSG00000118507 | HGLibA_01443 | 74 | -0.465338115 | AKAP7 | A-kinase anchoring protein 7 [Source:HGNC Symbol;Acc:HGNC:377] |
| ENSG00000118762 | HGLibA_36798 | 74 | -0.465338115 | PKD2 | transient receptor potential cation channel [Source:HGNC Symbol;Acc:HGNC:9009]" |
| ENSG00000120215 | HGLibA_29362 | 74 | -0.465338115 | MLANA | melan-A [Source:HGNC Symbol;Acc:HGNC:7124] |
| ENSG00000120289 | HGLibA_27949 | 74 | -0.465338115 | MAGEB4 | MAGE family member B4 [Source:HGNC Symbol;Acc:HGNC:6811] |
| ENSG00000120709 | HGLibA_16641 | 74 | -0.465338115 | FAM53C | family with sequence similarity 53 member C [Source:HGNC Symbol;Acc:HGNC:1336] |
| ENSG00000123119 | HGLibA_31585 | 74 | -0.465338115 | NECAB1 | N-terminal EF-hand calcium binding protein 1 [Source:HGNC Symbol;Acc:HGNC:20983] |
| ENSG00000123700 | HGLibA_24272 | 74 | -0.465338115 | KCNJ2 | potassium voltage-gated channel subfamily J member 2 [Source:HGNC Symbol;Acc:HGNC:6263] |
| ENSG00000124151 | HGLibA_31342 | 74 | -0.465338115 | NCOA3 | nuclear receptor coactivator 3 [Source:HGNC Symbol;Acc:HGNC:7670] |
| ENSG00000124232 | HGLibA_40651 | 74 | -0.465338115 | RBPJL | recombination signal binding protein for immunoglobulin kappa J region like [Source:HGNC Symbol;Acc:HGNC:13761] |
| ENSG00000125534 | HGLibA_37864 | 74 | -0.465338115 | PPDPF | pancreatic progenitor cell differentiation and proliferation factor [Source:HGNC Symbol;Acc:HGNC:16142] |
| ENSG00000125851 | HGLibA_35673 | 74 | -0.465338115 | PCSK2 | proprotein convertase subtilisin/kexin type 2 [Source:HGNC Symbol;Acc:HGNC:8744] |
| ENSG00000125895 | HGLibA_50514 | 74 | -0.465338115 | TMEM74B | transmembrane protein 74B [Source:HGNC Symbol;Acc:HGNC:15893] |
| ENSG00000125952 | HGLibA_28449 | 74 | -0.465338115 | MAX | MYC associated factor X [Source:HGNC Symbol;Acc:HGNC:6913] |
| ENSG00000127249 | HGLibA_03576 | 74 | -0.465338115 | ATP13A4 | ATPase 13A4 [Source:HGNC Symbol;Acc:HGNC:25422] |
| ENSG00000129128 | HGLibA_46563 | 74 | -0.465338115 | SPCS3 | signal peptidase complex subunit 3 [Source:HGNC Symbol;Acc:HGNC:26212] |
| ENSG00000129680 | HGLibA_28242 | 74 | -0.465338115 | MAP7D3 | MAP7 domain containing 3 [Source:HGNC Symbol;Acc:HGNC:25742] |
| ENSG00000130592 | HGLibA_27563 | 74 | -0.465338115 | LSP1 | lymphocyte specific protein 1 [Source:HGNC Symbol;Acc:HGNC:6707] |
| ENSG00000130726 | HGLibA_51450 | 74 | -0.465338115 | TRIM28 | tripartite motif containing 28 [Source:HGNC Symbol;Acc:HGNC:16384] |
| ENSG00000130826 | HGLibA_13292 | 74 | -0.465338115 | DKC1 | dyskerin pseudouridine synthase 1 [Source:HGNC Symbol;Acc:HGNC:2890] |
| ENSG00000130940 | HGLibA_07350 | 74 | -0.465338115 | CASZ1 | castor zinc finger 1 [Source:HGNC Symbol;Acc:HGNC:26002] |
| ENSG00000131386 | HGLibA_18545 | 74 | -0.465338115 | GALNT15 | polypeptide N-acetylgalactosaminyltransferase 15 [Source:HGNC Symbol;Acc:HGNC:21531] |
| ENSG00000132481 | HGLibA_51516 | 74 | -0.465338115 | TRIM47 | tripartite motif containing 47 [Source:HGNC Symbol;Acc:HGNC:19020] |
| ENSG00000132581 | HGLibA_43124 | 74 | -0.465338115 | SDF2 | stromal cell derived factor 2 [Source:HGNC Symbol;Acc:HGNC:10675] |
| ENSG00000133019 | HGLibA_09553 | 74 | -0.465338115 | CHRM3 | cholinergic receptor muscarinic 3 [Source:HGNC Symbol;Acc:HGNC:1952] |
| ENSG00000133315 | HGLibA_27835 | 74 | -0.465338115 | MACROD1 | MACRO domain containing 1 [Source:HGNC Symbol;Acc:HGNC:29598] |
| ENSG00000133657 | HGLibA_03571 | 74 | -0.465338115 | ATP13A3 | ATPase 13A3 [Source:HGNC Symbol;Acc:HGNC:24113] |
| ENSG00000134389 | HGLibA_09275 | 74 | -0.465338115 | CFHR5 | complement factor H related 5 [Source:HGNC Symbol;Acc:HGNC:24668] |
| ENSG00000134882 | HGLibA_52538 | 74 | -0.465338115 | UBAC2 | UBA domain containing 2 [Source:HGNC Symbol;Acc:HGNC:20486] |
| ENSG00000135637 | HGLibA_07618 | 74 | -0.465338115 | CCDC142 | coiled-coil domain containing 142 [Source:HGNC Symbol;Acc:HGNC:25889] |
| ENSG00000135953 | HGLibA_29120 | 74 | -0.465338115 | MFSD9 | major facilitator superfamily domain containing 9 [Source:HGNC Symbol;Acc:HGNC:28158] |

| | | | | | |
|---|---|---|---|---|---|
| ENSG00000136824 | HGLibA_45646 | 74 | -0.465338115 | SMC2 | structural maintenance of chromosomes 2 [Source:HGNC Symbol;Acc:HGNC:14011] |
| ENSG00000137270 | HGLibA_18799 | 74 | -0.465338115 | GCM1 | glial cells missing homolog 1 [Source:HGNC Symbol;Acc:HGNC:4197] |
| ENSG00000137502 | HGLibA_39872 | 74 | -0.465338115 | RAB30 | member RAS oncogene family [Source:HGNC Symbol;Acc:HGNC:9770]" |
| ENSG00000137601 | HGLibA_31638 | 74 | -0.465338115 | NEK1 | NIMA related kinase 1 [Source:HGNC Symbol;Acc:HGNC:7744] |
| ENSG00000137674 | HGLibA_29511 | 74 | -0.465338115 | MMP20 | matrix metallopeptidase 20 [Source:HGNC Symbol;Acc:HGNC:7167] |
| ENSG00000137726 | HGLibA_18239 | 74 | -0.465338115 | FXYD6 | FXYD domain containing ion transport regulator 6 [Source:HGNC Symbol;Acc:HGNC:4030] |
| ENSG00000137764 | HGLibA_28151 | 74 | -0.465338115 | MAP2K5 | mitogen-activated protein kinase kinase 5 [Source:HGNC Symbol;Acc:HGNC:6845] |
| ENSG00000138081 | HGLibA_17028 | 74 | -0.465338115 | FBXO11 | F-box protein 11 [Source:HGNC Symbol;Acc:HGNC:13590] |
| ENSG00000140386 | HGLibA_42859 | 74 | -0.465338115 | SCAPER | S-phase cyclin A associated protein in the ER [Source:HGNC Symbol;Acc:HGNC:13081] |
| ENSG00000142453 | HGLibA_07253 | 74 | -0.465338115 | CARM1 | coactivator associated arginine methyltransferase 1 [Source:HGNC Symbol;Acc:HGNC:23393] |
| ENSG00000143756 | HGLibA_17060 | 74 | -0.465338115 | FBXO28 | F-box protein 28 [Source:HGNC Symbol;Acc:HGNC:29046] |
| ENSG00000144285 | HGLibA_42974 | 74 | -0.465338115 | SCN1A | sodium voltage-gated channel alpha subunit 1 [Source:HGNC Symbol;Acc:HGNC:10585] |
| ENSG00000145949 | HGLibA_30793 | 74 | -0.465338115 | MYLK4 | myosin light chain kinase family member 4 [Source:HGNC Symbol;Acc:HGNC:27972] |
| ENSG00000146122 | HGLibA_12262 | 74 | -0.465338115 | DAAM2 | dishevelled associated activator of morphogenesis 2 [Source:HGNC Symbol;Acc:HGNC:18143] |
| ENSG00000146282 | HGLibA_40292 | 74 | -0.465338115 | RARS2 | mitochondrial [Source:HGNC Symbol;Acc:HGNC:21406]" |
| ENSG00000147041 | HGLibA_48109 | 74 | -0.465338115 | SYTL5 | synaptotagmin like 5 [Source:HGNC Symbol;Acc:HGNC:15589] |
| ENSG00000147160 | HGLibA_03915 | 74 | -0.465338115 | AWAT2 | acyl-CoA wax alcohol acyltransferase 2 [Source:HGNC Symbol;Acc:HGNC:23251] |
| ENSG00000147255 | HGLibA_22874 | 74 | -0.465338115 | IGSF1 | immunoglobulin superfamily member 1 [Source:HGNC Symbol;Acc:HGNC:5948] |
| ENSG00000147533 | HGLibA_19634 | 74 | -0.465338115 | GOLGA7 | golgin A7 [Source:HGNC Symbol;Acc:HGNC:24876] |
| ENSG00000147955 | HGLibA_44160 | 74 | -0.465338115 | SIGMAR1 | sigma non-opioid intracellular receptor 1 [Source:HGNC Symbol;Acc:HGNC:8157] |
| ENSG00000148450 | HGLibA_30245 | 74 | -0.465338115 | MSRB2 | methionine sulfoxide reductase B2 [Source:HGNC Symbol;Acc:HGNC:17061] |
| ENSG00000151651 | HGLibA_00781 | 74 | -0.465338115 | ADAM8 | ADAM metallopeptidase domain 8 [Source:HGNC Symbol;Acc:HGNC:215] |
| ENSG00000151778 | HGLibA_43501 | 74 | -0.465338115 | SERP2 | stress associated endoplasmic reticulum protein family member 2 [Source:HGNC Symbol;Acc:HGNC:20607] |
| ENSG00000153046 | HGLibA_08916 | 74 | -0.465338115 | CDYL | chromodomain Y like [Source:HGNC Symbol;Acc:HGNC:1811] |
| ENSG00000154144 | HGLibA_48635 | 74 | -0.465338115 | TBRG1 | transforming growth factor beta regulator 1 [Source:HGNC Symbol;Acc:HGNC:29551] |
| ENSG00000154263 | HGLibA_00098 | 74 | -0.465338115 | ABCA10 | ATP binding cassette subfamily A member 10 [Source:HGNC Symbol;Acc:HGNC:30] |
| ENSG00000154642 | HGLibA_06049 | 74 | -0.465338115 | C21orf91 | chromosome 21 open reading frame 91 [Source:HGNC Symbol;Acc:HGNC:16459] |
| ENSG00000155052 | HGLibA_10435 | 74 | -0.465338115 | CNTNAP5 | contactin associated protein like 5 [Source:HGNC Symbol;Acc:HGNC:18748] |
| ENSG00000156150 | HGLibA_01737 | 74 | -0.465338115 | ALX3 | ALX homeobox 3 [Source:HGNC Symbol;Acc:HGNC:449] |
| ENSG00000156222 | HGLibA_44830 | 74 | -0.465338115 | SLC28A1 | solute carrier family 28 member 1 [Source:HGNC Symbol;Acc:HGNC:11001] |
| ENSG00000156239 | HGLibA_30965 | 74 | -0.465338115 | N6AMT1 | N-6 adenine-specific DNA methyltransferase 1 [Source:HGNC Symbol;Acc:HGNC:16021] |
| ENSG00000157240 | HGLibA_18259 | 74 | -0.465338115 | FZD1 | frizzled class receptor 1 [Source:HGNC Symbol;Acc:HGNC:4038] |
| ENSG00000157734 | HGLibA_46065 | 74 | -0.465338115 | SNX22 | sorting nexin 22 [Source:HGNC Symbol;Acc:HGNC:16315] |
| ENSG00000160072 | HGLibA_03412 | 74 | -0.465338115 | ATAD3B | AAA domain containing 3B [Source:HGNC Symbol;Acc:HGNC:24007]" |
| ENSG00000160094 | HGLibA_55874 | 74 | -0.465338115 | ZNF362 | zinc finger protein 362 [Source:HGNC Symbol;Acc:HGNC:18079] |
| ENSG00000160710 | HGLibA_00868 | 74 | -0.465338115 | ADAR | RNA specific [Source:HGNC Symbol;Acc:HGNC:225]" |
| ENSG00000161281 | HGLibA_10856 | 74 | -0.465338115 | COX7A1 | cytochrome c oxidase subunit 7A1 [Source:HGNC Symbol;Acc:HGNC:2287] |
| ENSG00000161653 | HGLibA_31075 | 74 | -0.465338115 | NAGS | N-acetylglutamate synthase [Source:HGNC Symbol;Acc:HGNC:17996] |
| ENSG00000162129 | HGLibA_10098 | 74 | -0.465338115 | CLPB | mitochondrial AAA ATPase chaperonin [Source:HGNC Symbol;Acc:HGNC:30664]" |
| ENSG00000162591 | HGLibA_28854 | 74 | -0.465338115 | MEGF6 | multiple EGF like domains 6 [Source:HGNC Symbol;Acc:HGNC:3232] |
| ENSG00000162702 | HGLibA_55735 | 74 | -0.465338115 | ZNF281 | zinc finger protein 281 [Source:HGNC Symbol;Acc:HGNC:13075] |
| ENSG00000162782 | HGLibA_48907 | 74 | -0.465338115 | TDRD5 | tudor domain containing 5 [Source:HGNC Symbol;Acc:HGNC:20614] |
| ENSG00000164123 | HGLibA_06331 | 74 | -0.465338115 | C4orf45 | chromosome 4 open reading frame 45 [Source:HGNC Symbol;Acc:HGNC:26342] |
| ENSG00000164440 | HGLibA_52393 | 74 | -0.465338115 | TXLNB | taxilin beta [Source:HGNC Symbol;Acc:HGNC:21617] |
| ENSG00000164463 | HGLibA_11113 | 74 | -0.465338115 | CREBRF | CREB3 regulatory factor [Source:HGNC Symbol;Acc:HGNC:24050] |
| ENSG00000164483 | HGLibA_42680 | 74 | -0.465338115 | SAMD3 | sterile alpha motif domain containing 3 [Source:HGNC Symbol;Acc:HGNC:21574] |
| ENSG00000166128 | HGLibA_39989 | 74 | -0.465338115 | RAB8B | member RAS oncogene family [Source:HGNC Symbol;Acc:HGNC:30273]" |
| ENSG00000166689 | HGLibA_37079 | 74 | -0.465338115 | PLEKHA7 | pleckstrin homology domain containing A7 [Source:HGNC Symbol;Acc:HGNC:27049] |
| ENSG00000166965 | HGLibA_40690 | 74 | -0.465338115 | RCCD1 | RCC1 domain containing 1 [Source:HGNC Symbol;Acc:HGNC:30457] |
| ENSG00000167380 | HGLibA_55625 | 74 | -0.465338115 | ZNF226 | zinc finger protein 226 [Source:HGNC Symbol;Acc:HGNC:13019] |
| ENSG00000167632 | HGLibA_51338 | 74 | -0.465338115 | TRAPPC9 | trafficking protein particle complex 9 [Source:HGNC Symbol;Acc:HGNC:30832] |
| ENSG00000168036 | HGLibA_11651 | 74 | -0.465338115 | CTNNB1 | catenin beta 1 [Source:HGNC Symbol;Acc:HGNC:2514] |
| ENSG00000168412 | HGLibA_30446 | 74 | -0.465338115 | MTNR1A | melatonin receptor 1A [Source:HGNC Symbol;Acc:HGNC:7463] |
| ENSG00000169933 | HGLibA_18038 | 74 | -0.465338115 | FRMPD4 | FERM and PDZ domain containing 4 [Source:HGNC Symbol;Acc:HGNC:29007] |
| ENSG00000170836 | HGLibA_37970 | 74 | -0.465338115 | PPM1D | Mg2+/Mn2+ dependent 1D [Source:HGNC Symbol;Acc:HGNC:9277]" |
| ENSG00000171401 | HGLibA_25422 | 74 | -0.465338115 | KRT13 | keratin 13 [Source:HGNC Symbol;Acc:HGNC:6415] |
| ENSG00000171944 | HGLibA_34142 | 74 | -0.465338115 | OR52A5 | olfactory receptor family 52 subfamily A member 5 [Source:HGNC Symbol;Acc:HGNC:19580] |
| ENSG00000172262 | HGLibA_55458 | 74 | -0.465338115 | ZNF131 | zinc finger protein 131 [Source:HGNC Symbol;Acc:HGNC:12915] |
| ENSG00000173040 | HGLibA_15683 | 74 | -0.465338115 | EVC2 | EvC ciliary complex subunit 2 [Source:HGNC Symbol;Acc:HGNC:19747] |
| ENSG00000173302 | HGLibA_19921 | 74 | -0.465338115 | GPR148 | G protein-coupled receptor 148 [Source:HGNC Symbol;Acc:HGNC:23623] |
| ENSG00000173511 | HGLibA_53613 | 74 | -0.465338115 | VEGFB | vascular endothelial growth factor B [Source:HGNC Symbol;Acc:HGNC:12681] |
| ENSG00000174937 | HGLibA_34336 | 74 | -0.465338115 | OR5M3 | olfactory receptor family 5 subfamily M member 3 [Source:HGNC Symbol;Acc:HGNC:14806] |
| ENSG00000175899 | HGLibA_00009 | 74 | -0.465338115 | A2M | alpha-2-macroglobulin [Source:HGNC Symbol;Acc:HGNC:7] |
| ENSG00000176358 | HGLibA_48156 | 74 | -0.465338115 | TAC4 | tachykinin 4 [Source:HGNC Symbol;Acc:HGNC:16641] |
| ENSG00000176438 | HGLibA_47983 | 74 | -0.465338115 | SYNE3 | spectrin repeat containing nuclear envelope family member 3 [Source:HGNC Symbol;Acc:HGNC:19861] |
| ENSG00000176532 | HGLibA_38785 | 74 | -0.465338115 | PRR15 | proline rich 15 [Source:HGNC Symbol;Acc:HGNC:22310] |
| ENSG00000177558 | HGLibA_16343 | 74 | -0.465338115 | FAM187B | family with sequence similarity 187 member B [Source:HGNC Symbol;Acc:HGNC:26366] |
| ENSG00000177842 | HGLibA_56405 | 74 | -0.465338115 | ZNF620 | zinc finger protein 620 [Source:HGNC Symbol;Acc:HGNC:28742] |
| ENSG00000177943 | HGLibA_28040 | 74 | -0.465338115 | MAMDC4 | MAM domain containing 4 [Source:HGNC Symbol;Acc:HGNC:24083] |
| ENSG00000179144 | HGLibA_19089 | 74 | -0.465338115 | GIMAP7 | IMAP family member 7 [Source:HGNC Symbol;Acc:HGNC:22404]" |
| ENSG00000180979 | HGLibA_27394 | 74 | -0.465338115 | LRRC57 | leucine rich repeat containing 57 [Source:HGNC Symbol;Acc:HGNC:26719] |
| ENSG00000182208 | HGLibA_29590 | 74 | -0.465338115 | MOB2 | MOB kinase activator 2 [Source:HGNC Symbol;Acc:HGNC:24904] |
| ENSG00000182674 | HGLibA_24159 | 74 | -0.465338115 | KCNB2 | potassium voltage-gated channel subfamily B member 2 [Source:HGNC Symbol;Acc:HGNC:6232] |
| ENSG00000183066 | HGLibA_53980 | 74 | -0.465338115 | WBP2NL | WBP2 N-terminal like [Source:HGNC Symbol;Acc:HGNC:28389] |
| ENSG00000183831 | HGLibA_02105 | 74 | -0.465338115 | ANKRD45 | ankyrin repeat domain 45 [Source:HGNC Symbol;Acc:HGNC:24786] |
| ENSG00000184005 | HGLibA_47269 | 74 | -0.465338115 | ST6GALNAC3 | 6-sialyltransferase 3 [Source:HGNC Symbol;Acc:HGNC:19343]" |
| ENSG00000184110 | HGLibA_14767 | 74 | -0.465338115 | EIF3C | eukaryotic translation initiation factor 3 subunit C [Source:HGNC Symbol;Acc:HGNC:3279] |
| ENSG00000184985 | HGLibA_46185 | 74 | -0.465338115 | SORCS2 | sortilin related VPS10 domain containing receptor 2 [Source:HGNC Symbol;Acc:HGNC:16698] |
| ENSG00000185619 | HGLibA_35597 | 74 | -0.465338115 | PCGF3 | polycomb group ring finger 3 [Source:HGNC Symbol;Acc:HGNC:10066] |
| ENSG00000186318 | HGLibA_04059 | 74 | -0.465338115 | BACE1 | beta-secretase 1 [Source:HGNC Symbol;Acc:HGNC:933] |
| ENSG00000186827 | HGLibA_50784 | 74 | -0.465338115 | TNFRSF4 | TNF receptor superfamily member 4 [Source:HGNC Symbol;Acc:HGNC:11918] |
| ENSG00000186919 | HGLibA_54767 | 74 | -0.465338115 | ZACN | zinc activated ion channel [Source:HGNC Symbol;Acc:HGNC:29504] |
| ENSG00000188152 | HGLibA_33111 | 74 | -0.465338115 | NUTM2G | NUT family member 2G [Source:HGNC Symbol;Acc:HGNC:23449] |
| ENSG00000188554 | HGLibA_31249 | 74 | -0.465338115 | NBR1 | autophagy cargo receptor [Source:HGNC Symbol;Acc:HGNC:6746]" |
| ENSG00000188566 | HGLibA_31406 | 74 | -0.465338115 | NDOR1 | NADPH dependent diflavin oxidoreductase 1 [Source:HGNC Symbol;Acc:HGNC:29838] |
| ENSG00000188687 | HGLibA_45222 | 74 | -0.465338115 | SLC4A5 | solute carrier family 4 member 5 [Source:HGNC Symbol;Acc:HGNC:18168] |
| ENSG00000196110 | HGLibA_56566 | 74 | -0.465338115 | ZNF699 | zinc finger protein 699 [Source:HGNC Symbol;Acc:HGNC:24750] |
| ENSG00000196557 | HGLibA_06895 | 74 | -0.465338115 | CACNA1H | calcium voltage-gated channel subunit alpha1 H [Source:HGNC Symbol;Acc:HGNC:1395] |
| ENSG00000197106 | HGLibA_45310 | 74 | -0.465338115 | SLC6A17 | solute carrier family 6 member 17 [Source:HGNC Symbol;Acc:HGNC:31399] |
| ENSG00000197121 | HGLibA_36206 | 74 | -0.465338115 | PGAP1 | post-GPI attachment to proteins 1 [Source:HGNC Symbol;Acc:HGNC:25712] |
| ENSG00000197763 | HGLibA_52449 | 74 | -0.465338115 | TXNRD3 | thioredoxin reductase 3 [Source:HGNC Symbol;Acc:HGNC:20667] |
| ENSG00000198039 | HGLibA_55708 | 74 | -0.465338115 | ZNF273 | zinc finger protein 273 [Source:HGNC Symbol;Acc:HGNC:13067] |
| ENSG00000198286 | HGLibA_07221 | 74 | -0.465338115 | CARD11 | caspase recruitment domain family member 11 [Source:HGNC Symbol;Acc:HGNC:16393] |
| ENSG00000198336 | HGLibA_30762 | 74 | -0.465338115 | MYL4 | myosin light chain 4 [Source:HGNC Symbol;Acc:HGNC:7585] |
| ENSG00000198556 | HGLibA_56759 | 74 | -0.465338115 | ZNF789 | zinc finger protein 789 [Source:HGNC Symbol;Acc:HGNC:27801] |
| ENSG00000198753 | HGLibA_37277 | 74 | -0.465338115 | PLXNB3 | plexin B3 [Source:HGNC Symbol;Acc:HGNC:9105] |
| ENSG00000198924 | HGLibA_12509 | 74 | -0.465338115 | DCLRE1A | DNA cross-link repair 1A [Source:HGNC Symbol;Acc:HGNC:17660] |
| ENSG00000198934 | HGLibA_27979 | 74 | -0.465338115 | MAGEE1 | MAGE family member E1 [Source:HGNC Symbol;Acc:HGNC:24934] |
| ENSG00000203663 | HGLibA_33820 | 74 | -0.465338115 | OR2L2 | olfactory receptor family 2 subfamily L member 2 [Source:HGNC Symbol;Acc:HGNC:8266] |
| ENSG00000203757 | HGLibA_34420 | 74 | -0.465338115 | OR6K3 | olfactory receptor family 6 subfamily K member 3 [Source:HGNC Symbol;Acc:HGNC:15030] |
| ENSG00000203910 | HGLibA_05773 | 74 | -0.465338115 | C1orf146 | chromosome 1 open reading frame 146 [Source:HGNC Symbol;Acc:HGNC:24032] |
| ENSG00000204315 | HGLibA_17595 | 74 | -0.465338115 | FKBPL | FK506 binding protein like [Source:HGNC Symbol;Acc:HGNC:13949] |
| ENSG00000204568 | HGLibA_30054 | 74 | -0.465338115 | MRPS18B | mitochondrial ribosomal protein S18B [Source:HGNC Symbol;Acc:HGNC:14516] |
| ENSG00000212916 | HGLibA_28111 | 74 | -0.465338115 | MAP10 | microtubule associated protein 10 [Source:HGNC Symbol;Acc:HGNC:29265] |
| ENSG00000213626 | HGLibA_26077 | 74 | -0.465338115 | LBH | limb bud and heart development [Source:HGNC Symbol;Acc:HGNC:29532] |
| ENSG00000213949 | HGLibA_23715 | 74 | -0.465338115 | ITGA1 | integrin subunit alpha 1 [Source:HGNC Symbol;Acc:HGNC:6134] |
| ENSG00000215612 | HGLibA_21760 | 74 | -0.465338115 | HMX1 | H6 family homeobox 1 [Source:HGNC Symbol;Acc:HGNC:5017] |
| ENSG00000224470 | HGLibA_03834 | 74 | -0.465338115 | ATXN1L | ataxin 1 like [Source:HGNC Symbol;Acc:HGNC:33279] |
| ENSG00000232237 | HGLibA_03278 | 74 | -0.465338115 | ASCL5 | achaete-scute family bHLH transcription factor 5 [Source:HGNC Symbol;Acc:HGNC:33169] |
| ENSG00000232264 | HGLibA_53255 | 74 | -0.465338115 | USP17L24 | ubiquitin specific peptidase 17-like family member 24 [Source:HGNC Symbol;Acc:HGNC:44453] |
| ENSG00000250298 | HGLibA_19094 | 74 | -0.465338115 | GIMD1 | GIMAP family P-loop NTPase domain containing 1 [Source:HGNC Symbol;Acc:HGNC:44141] |
| ENSG00000251623 | HGLibA_16170 | 74 | -0.465338115 | FAM153B | - |
| ENSG00000253506 | HGLibA_31033 | 74 | -0.465338115 | NACA2 | nascent polypeptide associated complex subunit alpha 2 [Source:HGNC Symbol;Acc:HGNC:23290] |
| ENSG00000256797 | HGLibA_25305 | 74 | -0.465338115 | KLRF2 | killer cell lectin like receptor F2 [Source:HGNC Symbol;Acc:HGNC:37646] |
| ENSG00000259332 | HGLibA_47234 | 74 | -0.465338115 | ST20-MTHFS | ST20-MTHFS readthrough [Source:HGNC Symbol;Acc:HGNC:44655] |
| ENSG00000262406 | HGLibA_29485 | 74 | -0.465338115 | MMP12 | matrix metallopeptidase 12 [Source:HGNC Symbol;Acc:HGNC:7158] |
| ENSG00000276380 | HGLibA_52639 | 74 | -0.465338115 | UBE2NL | ubiquitin conjugating enzyme E2 N like (gene/pseudogene) [Source:HGNC Symbol;Acc:HGNC:31710] |
| ENSG00000284733 | HGLibA_34001 | 74 | -0.465338115 | OR4F29 | olfactory receptor family 4 subfamily F member 29 [Source:HGNC Symbol;Acc:HGNC:31275] |
| FAM19A2 | HGLibA_16400 | 74 | -0.465338115 | FAM19A2 | - |
| FAM57A | HGLibA_16643 | 74 | -0.465338115 | FAM57A | - |
| hsa-mir-1205 | HGLibA_57277 | 74 | -0.465338115 | hsa-mir-1205 | - |
| hsa-mir-1287 | HGLibA_57614 | 74 | -0.465338115 | hsa-mir-1287 | - |
| hsa-mir-18b | HGLibA_58022 | 74 | -0.465338115 | hsa-mir-18b | - |
| hsa-mir-203b | HGLibA_58171 | 74 | -0.465338115 | hsa-mir-203b | - |
| hsa-mir-216a | HGLibA_58258 | 74 | -0.465338115 | hsa-mir-216a | - |
| hsa-mir-26b | HGLibA_58372 | 74 | -0.465338115 | hsa-mir-26b | - |
| hsa-mir-26b | HGLibA_58371 | 74 | -0.465338115 | hsa-mir-26b | - |
| hsa-mir-3125 | HGLibA_58554 | 74 | -0.465338115 | hsa-mir-3125 | - |
| hsa-mir-375 | HGLibA_59403 | 74 | -0.465338115 | hsa-mir-375 | - |
| hsa-mir-3921 | HGLibA_59562 | 74 | -0.465338115 | hsa-mir-3921 | - |
| hsa-mir-3934 | HGLibA_59596 | 74 | -0.465338115 | hsa-mir-3934 | - |
| hsa-mir-4270 | HGLibA_59789 | 74 | -0.465338115 | hsa-mir-4270 | - |
| hsa-mir-4275 | HGLibA_59802 | 74 | -0.465338115 | hsa-mir-4275 | - |
| hsa-mir-4481 | HGLibA_60344 | 74 | -0.465338115 | hsa-mir-4481 | - |
| hsa-mir-4638 | HGLibA_60657 | 74 | -0.465338115 | hsa-mir-4638 | - |

| | | | | | |
|---|---|---|---|---|---|
| hsa-mir-4657 | HGLibA_60734 | 74 | -0.465338115 | hsa-mir-4657 | - |
| hsa-mir-4733 | HGLibA_61028 | 74 | -0.465338115 | hsa-mir-4733 | - |
| hsa-mir-585 | HGLibA_62438 | 74 | -0.465338115 | hsa-mir-585 | - |
| hsa-mir-623 | HGLibA_62730 | 74 | -0.465338115 | hsa-mir-623 | - |
| hsa-mir-631 | HGLibA_62764 | 74 | -0.465338115 | hsa-mir-631 | - |
| hsa-mir-651 | HGLibA_62888 | 74 | -0.465338115 | hsa-mir-651 | - |
| hsa-mir-6735 | HGLibA_63093 | 74 | -0.465338115 | hsa-mir-6735 | - |
| hsa-mir-6760 | HGLibA_63203 | 74 | -0.465338115 | hsa-mir-6760 | - |
| hsa-mir-6853 | HGLibA_63584 | 74 | -0.465338115 | hsa-mir-6853 | - |
| hsa-mir-922 | HGLibA_64284 | 74 | -0.465338115 | hsa-mir-922 | - |
| hsa-mir-941-3 | HGLibA_64347 | 74 | -0.465338115 | hsa-mir-941-3 | - |
| KIAA1432 | HGLibA_24756 | 74 | -0.465338115 | KIAA1432 | - |
| MINOS1 | HGLibA_29278 | 74 | -0.465338115 | MINOS1 | - |
| PALM2 | HGLibA_35070 | 74 | -0.465338115 | PALM2 | - |
| PIH1D3 | HGLibA_36616 | 74 | -0.465338115 | PIH1D3 | - |
| PRUNE | HGLibA_38985 | 74 | -0.465338115 | PRUNE | - |
| SGK110 | HGLibA_43820 | 74 | -0.465338115 | SGK110 | - |
| TCEB3C | HGLibA_48746 | 74 | -0.465338115 | TCEB3C | - |
| TMEM173 | HGLibA_50063 | 74 | -0.465338115 | TMEM173 | - |
| ACPT | HGLibA_00526 | 73 | -0.474383458 | ACPT | - |
| ADSS | HGLibA_01076 | 73 | -0.474383458 | ADSS | - |
| ATP5L2 | HGLibA_03683 | 73 | -0.474383458 | ATP5L2 | - |
| C14orf177 | HGLibA_05343 | 73 | -0.474383458 | C14orf177 | - |
| C5orf20 | HGLibA_06363 | 73 | -0.474383458 | C5orf20 | - |
| CCDC94 | HGLibA_07935 | 73 | -0.474383458 | CCDC94 | - |
| CSB-PGBD3 | HGLibA_11310 | 73 | -0.474383458 | CSB-PGBD3 | - |
| DYX1C1 | HGLibA_14293 | 73 | -0.474383458 | DYX1C1 | - |
| ENSG00000001626 | HGLibA_09292 | 73 | -0.474383458 | CFTR | cystic fibrosis transmembrane conductance regulator [Source:HGNC Symbol;Acc:HGNC:1884] |
| ENSG00000010810 | HGLibA_18254 | 73 | -0.474383458 | FYN | Src family tyrosine kinase [Source:HGNC Symbol;Acc:HGNC:4037]" |
| ENSG00000011275 | HGLibA_41631 | 73 | -0.474383458 | RNF216 | ring finger protein 216 [Source:HGNC Symbol;Acc:HGNC:21698] |
| ENSG00000032219 | HGLibA_02899 | 73 | -0.474383458 | ARID4A | AT-rich interaction domain 4A [Source:HGNC Symbol;Acc:HGNC:9885] |
| ENSG00000035862 | HGLibA_49539 | 73 | -0.474383458 | TIMP2 | TIMP metallopeptidase inhibitor 2 [Source:HGNC Symbol;Acc:HGNC:11821] |
| ENSG00000042813 | HGLibA_56962 | 73 | -0.474383458 | ZPBP | zona pellucida binding protein [Source:HGNC Symbol;Acc:HGNC:15662] |
| ENSG00000047634 | HGLibA_42960 | 73 | -0.474383458 | SCML1 | Scm polycomb group protein like 1 [Source:HGNC Symbol;Acc:HGNC:10580] |
| ENSG00000064687 | HGLibA_00122 | 73 | -0.474383458 | ABCA7 | ATP binding cassette subfamily A member 7 [Source:HGNC Symbol;Acc:HGNC:37] |
| ENSG00000065029 | HGLibA_56684 | 73 | -0.474383458 | ZNF76 | zinc finger protein 76 [Source:HGNC Symbol;Acc:HGNC:13149] |
| ENSG00000073861 | HGLibA_48663 | 73 | -0.474383458 | TBX21 | T-box 21 [Source:HGNC Symbol;Acc:HGNC:11599] |
| ENSG00000076382 | HGLibA_46359 | 73 | -0.474383458 | SPAG5 | sperm associated antigen 5 [Source:HGNC Symbol;Acc:HGNC:13452] |
| ENSG00000079931 | HGLibA_29699 | 73 | -0.474383458 | MOXD1 | monooxygenase DBH like 1 [Source:HGNC Symbol;Acc:HGNC:21063] |
| ENSG00000081853 | HGLibA_35529 | 73 | -0.474383458 | PCDHGA2 | 2 [Source:HGNC Symbol;Acc:HGNC:8700]" |
| ENSG00000082684 | HGLibA_43361 | 73 | -0.474383458 | SEMA5B | semaphorin 5B [Source:HGNC Symbol;Acc:HGNC:10737] |
| ENSG00000090013 | HGLibA_04578 | 73 | -0.474383458 | BLVRB | biliverdin reductase B [Source:HGNC Symbol;Acc:HGNC:1063] |
| ENSG00000090661 | HGLibA_09208 | 73 | -0.474383458 | CERS4 | ceramide synthase 4 [Source:HGNC Symbol;Acc:HGNC:23747] |
| ENSG00000091536 | HGLibA_30804 | 73 | -0.474383458 | MYO15A | myosin XVA [Source:HGNC Symbol;Acc:HGNC:7594] |
| ENSG00000099260 | HGLibA_35078 | 73 | -0.474383458 | PALMD | palmdelphin [Source:HGNC Symbol;Acc:HGNC:15846] |
| ENSG00000099875 | HGLibA_29346 | 73 | -0.474383458 | MKNK2 | MAP kinase interacting serine/threonine kinase 2 [Source:HGNC Symbol;Acc:HGNC:7111] |
| ENSG00000101182 | HGLibA_39088 | 73 | -0.474383458 | PSMA7 | proteasome subunit alpha 7 [Source:HGNC Symbol;Acc:HGNC:9536] |
| ENSG00000101204 | HGLibA_09574 | 73 | -0.474383458 | CHRNA4 | cholinergic receptor nicotinic alpha 4 subunit [Source:HGNC Symbol;Acc:HGNC:1958] |
| ENSG00000104231 | HGLibA_55145 | 73 | -0.474383458 | ZFAND1 | zinc finger AN1-type containing 1 [Source:HGNC Symbol;Acc:HGNC:25858] |
| ENSG00000104361 | HGLibA_31960 | 73 | -0.474383458 | NIPAL2 | NIPA like domain containing 2 [Source:HGNC Symbol;Acc:HGNC:25854] |
| ENSG00000104723 | HGLibA_52347 | 73 | -0.474383458 | TUSC3 | tumor suppressor candidate 3 [Source:HGNC Symbol;Acc:HGNC:30242] |
| ENSG00000104889 | HGLibA_41427 | 73 | -0.474383458 | RNASEH2A | ribonuclease H2 subunit A [Source:HGNC Symbol;Acc:HGNC:18518] |
| ENSG00000105366 | HGLibA_44150 | 73 | -0.474383458 | SIGLEC8 | sialic acid binding Ig like lectin 8 [Source:HGNC Symbol;Acc:HGNC:10877] |
| ENSG00000105640 | HGLibA_41888 | 73 | -0.474383458 | RPL18A | ribosomal protein L18a [Source:HGNC Symbol;Acc:HGNC:10311] |
| ENSG00000105698 | HGLibA_53171 | 73 | -0.474383458 | USF2 | c-fos interacting [Source:HGNC Symbol;Acc:HGNC:12594]" |
| ENSG00000106077 | HGLibA_00236 | 73 | -0.474383458 | ABHD11 | abhydrolase domain containing 11 [Source:HGNC Symbol;Acc:HGNC:16407] |
| ENSG00000108094 | HGLibA_11777 | 73 | -0.474383458 | CUL2 | cullin 2 [Source:HGNC Symbol;Acc:HGNC:2552] |
| ENSG00000108829 | HGLibA_27402 | 73 | -0.474383458 | LRRC59 | leucine rich repeat containing 59 [Source:HGNC Symbol;Acc:HGNC:28817] |
| ENSG00000109062 | HGLibA_45408 | 73 | -0.474383458 | SLC9A3R1 | SLC9A3 regulator 1 [Source:HGNC Symbol;Acc:HGNC:11075] |
| ENSG00000109255 | HGLibA_32212 | 73 | -0.474383458 | NMU | neuromedin U [Source:HGNC Symbol;Acc:HGNC:7859] |
| ENSG00000109390 | HGLibA_31535 | 73 | -0.474383458 | NDUFC1 | NADH:ubiquinone oxidoreductase subunit C1 [Source:HGNC Symbol;Acc:HGNC:7705] |
| ENSG00000110514 | HGLibA_27858 | 73 | -0.474383458 | MADD | MAP kinase activating death domain [Source:HGNC Symbol;Acc:HGNC:6766] |
| ENSG00000110583 | HGLibA_30998 | 73 | -0.474383458 | NAA40 | NatD catalytic subunit [Source:HGNC Symbol;Acc:HGNC:25845]" |
| ENSG00000110768 | HGLibA_20545 | 73 | -0.474383458 | GTF2H1 | general transcription factor IIH subunit 1 [Source:HGNC Symbol;Acc:HGNC:4655] |
| ENSG00000111335 | HGLibA_33201 | 73 | -0.474383458 | OAS2 | 2'-5'-oligoadenylate synthetase 2 [Source:HGNC Symbol;Acc:HGNC:8087] |
| ENSG00000111596 | HGLibA_10334 | 73 | -0.474383458 | CNOT2 | CCR4-NOT transcription complex subunit 2 [Source:HGNC Symbol;Acc:HGNC:7878] |
| ENSG00000112245 | HGLibA_39403 | 73 | -0.474383458 | PTP4A1 | member 1 [Source:HGNC Symbol;Acc:HGNC:9634]" |
| ENSG00000112763 | HGLibA_04963 | 73 | -0.474383458 | BTN2A1 | butyrophilin subfamily 2 member A1 [Source:HGNC Symbol;Acc:HGNC:1136] |
| ENSG00000114124 | HGLibA_20337 | 73 | -0.474383458 | GRK7 | G protein-coupled receptor kinase 7 [Source:HGNC Symbol;Acc:HGNC:17031] |
| ENSG00000115274 | HGLibA_23322 | 73 | -0.474383458 | INO80B | INO80 complex subunit B [Source:HGNC Symbol;Acc:HGNC:13324] |
| ENSG00000115993 | HGLibA_51278 | 73 | -0.474383458 | TRAK2 | trafficking kinesin protein 2 [Source:HGNC Symbol;Acc:HGNC:13206] |
| ENSG00000116721 | HGLibA_38278 | 73 | -0.474383458 | PRAMEF1 | PRAME family member 1 [Source:HGNC Symbol;Acc:HGNC:28840] |
| ENSG00000117151 | HGLibA_11590 | 73 | -0.474383458 | CTBS | chitobiase [Source:HGNC Symbol;Acc:HGNC:2496] |
| ENSG00000118434 | HGLibA_46324 | 73 | -0.474383458 | SPACA1 | sperm acrosome associated 1 [Source:HGNC Symbol;Acc:HGNC:14967] |
| ENSG00000118705 | HGLibA_42019 | 73 | -0.474383458 | RPN2 | ribophorin II [Source:HGNC Symbol;Acc:HGNC:10382] |
| ENSG00000119943 | HGLibA_39706 | 73 | -0.474383458 | PYROXD2 | pyridine nucleotide-disulphide oxidoreductase domain 2 [Source:HGNC Symbol;Acc:HGNC:23517] |
| ENSG00000120526 | HGLibA_32923 | 73 | -0.474383458 | NUDCD1 | NudC domain containing 1 [Source:HGNC Symbol;Acc:HGNC:24306] |
| ENSG00000120594 | HGLibA_37256 | 73 | -0.474383458 | PLXDC2 | plexin domain containing 2 [Source:HGNC Symbol;Acc:HGNC:21013] |
| ENSG00000120952 | HGLibA_38306 | 73 | -0.474383458 | PRAMEF2 | PRAME family member 2 [Source:HGNC Symbol;Acc:HGNC:28841] |
| ENSG00000121053 | HGLibA_15392 | 73 | -0.474383458 | EPX | eosinophil peroxidase [Source:HGNC Symbol;Acc:HGNC:3423] |
| ENSG00000121270 | HGLibA_00164 | 73 | -0.474383458 | ABCC11 | ATP binding cassette subfamily C member 11 [Source:HGNC Symbol;Acc:HGNC:14639] |
| ENSG00000122873 | HGLibA_09726 | 73 | -0.474383458 | CISD1 | CDGSH iron sulfur domain 1 [Source:HGNC Symbol;Acc:HGNC:30880] |
| ENSG00000123096 | HGLibA_47147 | 73 | -0.474383458 | SSPN | sarcospan [Source:HGNC Symbol;Acc:HGNC:11322] |
| ENSG00000124092 | HGLibA_11599 | 73 | -0.474383458 | CTCFL | CCCTC-binding factor like [Source:HGNC Symbol;Acc:HGNC:16234] |
| ENSG00000124103 | HGLibA_16438 | 73 | -0.474383458 | FAM209A | family with sequence similarity 209 member A [Source:HGNC Symbol;Acc:HGNC:16100] |
| ENSG00000124302 | HGLibA_09650 | 73 | -0.474383458 | CHST8 | carbohydrate sulfotransferase 8 [Source:HGNC Symbol;Acc:HGNC:15993] |
| ENSG00000124588 | HGLibA_32565 | 73 | -0.474383458 | NQO2 | N-ribosyldihydronicotinamide:quinone reductase 2 [Source:HGNC Symbol;Acc:HGNC:7856] |
| ENSG00000124812 | HGLibA_11164 | 73 | -0.474383458 | CRISP1 | cysteine rich secretory protein 1 [Source:HGNC Symbol;Acc:HGNC:304] |
| ENSG00000125352 | HGLibA_41473 | 73 | -0.474383458 | RNF113A | ring finger protein 113A [Source:HGNC Symbol;Acc:HGNC:12974] |
| ENSG00000125618 | HGLibA_35317 | 73 | -0.474383458 | PAX8 | paired box 8 [Source:HGNC Symbol;Acc:HGNC:8622] |
| ENSG00000125931 | HGLibA_09743 | 73 | -0.474383458 | CITED1 | Cbp/p300 interacting transactivator with Glu/Asp rich carboxy-terminal domain 1 [Source:HGNC Symbol;Acc:HGNC:1986] |
| ENSG00000126012 | HGLibA_24526 | 73 | -0.474383458 | KDM5C | lysine demethylase 5C [Source:HGNC Symbol;Acc:HGNC:11114] |
| ENSG00000126107 | HGLibA_21099 | 73 | -0.474383458 | HECTD3 | HECT domain E3 ubiquitin protein ligase 3 [Source:HGNC Symbol;Acc:HGNC:26117] |
| ENSG00000126895 | HGLibA_03910 | 73 | -0.474383458 | AVPR2 | arginine vasopressin receptor 2 [Source:HGNC Symbol;Acc:HGNC:897] |
| ENSG00000127377 | HGLibA_11282 | 73 | -0.474383458 | CRYGN | crystallin gamma N [Source:HGNC Symbol;Acc:HGNC:20458] |
| ENSG00000128228 | HGLibA_43128 | 73 | -0.474383458 | SDF2L1 | stromal cell derived factor 2 like 1 [Source:HGNC Symbol;Acc:HGNC:10676] |
| ENSG00000129472 | HGLibA_39868 | 73 | -0.474383458 | RAB2B | member RAS oncogene family [Source:HGNC Symbol;Acc:HGNC:20246]" |
| ENSG00000129911 | HGLibA_25060 | 73 | -0.474383458 | KLF16 | Kruppel like factor 16 [Source:HGNC Symbol;Acc:HGNC:16857] |
| ENSG00000130176 | HGLibA_10306 | 73 | -0.474383458 | CNN1 | calponin 1 [Source:HGNC Symbol;Acc:HGNC:2155] |
| ENSG00000130748 | HGLibA_50022 | 73 | -0.474383458 | TMEM160 | transmembrane protein 160 [Source:HGNC Symbol;Acc:HGNC:26042] |
| ENSG00000131721 | HGLibA_41217 | 73 | -0.474383458 | RHOXF2 | Rhox homeobox family member 2 [Source:HGNC Symbol;Acc:HGNC:30011] |
| ENSG00000133110 | HGLibA_37710 | 73 | -0.474383458 | POSTN | periostin [Source:HGNC Symbol;Acc:HGNC:16953] |
| ENSG00000133392 | HGLibA_30708 | 73 | -0.474383458 | MYH11 | myosin heavy chain 11 [Source:HGNC Symbol;Acc:HGNC:7569] |
| ENSG00000133731 | HGLibA_23245 | 73 | -0.474383458 | IMPA1 | inositol monophosphatase 1 [Source:HGNC Symbol;Acc:HGNC:6050] |
| ENSG00000133739 | HGLibA_27444 | 73 | -0.474383458 | LRRCC1 | leucine rich repeat and coiled-coil centrosomal protein 1 [Source:HGNC Symbol;Acc:HGNC:29373] |
| ENSG00000134571 | HGLibA_30654 | 73 | -0.474383458 | MYBPC3 | cardiac [Source:HGNC Symbol;Acc:HGNC:7551]" |
| ENSG00000134698 | HGLibA_01215 | 73 | -0.474383458 | AGO4 | RISC catalytic component [Source:HGNC Symbol;Acc:HGNC:18424]" |
| ENSG00000135766 | HGLibA_14640 | 73 | -0.474383458 | EGLN1 | egl-9 family hypoxia inducible factor 1 [Source:HGNC Symbol;Acc:HGNC:1232] |
| ENSG00000136044 | HGLibA_02569 | 73 | -0.474383458 | APPL2 | phosphotyrosine interacting with PH domain and leucine zipper 2 [Source:HGNC Symbol;Acc:HGNC:18242]" |
| ENSG00000136379 | HGLibA_00268 | 73 | -0.474383458 | ABHD17C | abhydrolase domain containing 17C [Source:HGNC Symbol;Acc:HGNC:26925] |
| ENSG00000136819 | HGLibA_06761 | 73 | -0.474383458 | C9orf78 | chromosome 9 open reading frame 78 [Source:HGNC Symbol;Acc:HGNC:24932] |
| ENSG00000137491 | HGLibA_45463 | 73 | -0.474383458 | SLCO2B1 | solute carrier organic anion transporter family member 2B1 [Source:HGNC Symbol;Acc:HGNC:10962] |
| ENSG00000137509 | HGLibA_38360 | 73 | -0.474383458 | PRCP | prolylcarboxypeptidase [Source:HGNC Symbol;Acc:HGNC:9344] |
| ENSG00000137727 | HGLibA_02722 | 73 | -0.474383458 | ARHGAP20 | Rho GTPase activating protein 20 [Source:HGNC Symbol;Acc:HGNC:18357] |
| ENSG00000137975 | HGLibA_09809 | 73 | -0.474383458 | CLCA2 | chloride channel accessory 2 [Source:HGNC Symbol;Acc:HGNC:2016] |
| ENSG00000138029 | HGLibA_20810 | 73 | -0.474383458 | HADHB | hydroxyacyl-CoA dehydrogenase trifunctional multienzyme complex subunit beta [Source:HGNC Symbol;Acc:HGNC:4803] |
| ENSG00000138231 | HGLibA_12402 | 73 | -0.474383458 | DBR1 | debranching RNA lariats 1 [Source:HGNC Symbol;Acc:HGNC:15594] |
| ENSG00000139154 | HGLibA_01087 | 73 | -0.474383458 | AEBP2 | AE binding protein 2 [Source:HGNC Symbol;Acc:HGNC:24051] |
| ENSG00000139531 | HGLibA_47799 | 73 | -0.474383458 | SUOX | sulfite oxidase [Source:HGNC Symbol;Acc:HGNC:11460] |
| ENSG00000139648 | HGLibA_25525 | 73 | -0.474383458 | KRT71 | keratin 71 [Source:HGNC Symbol;Acc:HGNC:28927] |
| ENSG00000141127 | HGLibA_38766 | 73 | -0.474383458 | PRPSAP2 | phosphoribosyl pyrophosphate synthetase associated protein 2 [Source:HGNC Symbol;Acc:HGNC:9467] |
| ENSG00000141385 | HGLibA_01118 | 73 | -0.474383458 | AFG3L2 | AFG3 like matrix AAA peptidase subunit 2 [Source:HGNC Symbol;Acc:HGNC:315] |
| ENSG00000143401 | HGLibA_02208 | 73 | -0.474383458 | ANP32E | acidic nuclear phosphoprotein 32 family member E [Source:HGNC Symbol;Acc:HGNC:16673] |
| ENSG00000143768 | HGLibA_26276 | 73 | -0.474383458 | LEFTY2 | left-right determination factor 2 [Source:HGNC Symbol;Acc:HGNC:3122] |
| ENSG00000144134 | HGLibA_40026 | 73 | -0.474383458 | RABL2A | member of RAS oncogene family like 2A [Source:HGNC Symbol;Acc:HGNC:9799]" |
| ENSG00000144339 | HGLibA_49834 | 73 | -0.474383458 | TMEFF2 | transmembrane protein with EGF like and two follistatin like domains 2 [Source:HGNC Symbol;Acc:HGNC:11867] |
| ENSG00000145824 | HGLibA_11856 | 73 | -0.474383458 | CXCL14 | C-X-C motif chemokine ligand 14 [Source:HGNC Symbol;Acc:HGNC:10640] |
| ENSG00000146205 | HGLibA_02188 | 73 | -0.474383458 | ANO7 | anoctamin 7 [Source:HGNC Symbol;Acc:HGNC:31677] |
| ENSG00000146221 | HGLibA_48858 | 73 | -0.474383458 | TCTE1 | t-complex-associated-testis-expressed 1 [Source:HGNC Symbol;Acc:HGNC:11693] |
| ENSG00000146399 | HGLibA_48122 | 73 | -0.474383458 | TAAR1 | trace amine associated receptor 1 [Source:HGNC Symbol;Acc:HGNC:17734] |
| ENSG00000147485 | HGLibA_39651 | 73 | -0.474383458 | PXDNL | peroxidasin like [Source:HGNC Symbol;Acc:HGNC:26359] |
| ENSG00000149256 | HGLibA_49003 | 73 | -0.474383458 | TENM4 | teneurin transmembrane protein 4 [Source:HGNC Symbol;Acc:HGNC:29945] |
| ENSG00000149483 | HGLibA_49956 | 73 | -0.474383458 | TMEM138 | transmembrane protein 138 [Source:HGNC Symbol;Acc:HGNC:26944] |
| ENSG00000151694 | HGLibA_00741 | 73 | -0.474383458 | ADAM17 | ADAM metallopeptidase domain 17 [Source:HGNC Symbol;Acc:HGNC:195] |
| ENSG00000152822 | HGLibA_20340 | 73 | -0.474383458 | GRM1 | glutamate metabotropic receptor 1 [Source:HGNC Symbol;Acc:HGNC:4593] |

| | | | | | |
|---|---|---|---|---|---|
| ENSG00000154310 | HGLibA_50833 | 73 | -0.474383458 | TNIK | TRAF2 and NCK interacting kinase [Source:HGNC Symbol;Acc:HGNC:30765] |
| ENSG00000156218 | HGLibA_00854 | 73 | -0.474383458 | ADAMTSL3 | ADAMTS like 3 [Source:HGNC Symbol;Acc:HGNC:14633] |
| ENSG00000156475 | HGLibA_38149 | 73 | -0.474383458 | PPP2R2B | protein phosphatase 2 regulatory subunit Bbeta [Source:HGNC Symbol;Acc:HGNC:9305] |
| ENSG00000156599 | HGLibA_55120 | 73 | -0.474383458 | ZDHHC5 | zinc finger DHHC-type containing 5 [Source:HGNC Symbol;Acc:HGNC:18472] |
| ENSG00000158793 | HGLibA_31984 | 73 | -0.474383458 | NIT1 | nitrilase 1 [Source:HGNC Symbol;Acc:HGNC:7828] |
| ENSG00000159708 | HGLibA_27324 | 73 | -0.474383458 | LRRC36 | leucine rich repeat containing 36 [Source:HGNC Symbol;Acc:HGNC:25615] |
| ENSG00000159921 | HGLibA_19505 | 73 | -0.474383458 | GNE | glucosamine (UDP-N-acetyl)-2-epimerase/N-acetylmannosamine kinase [Source:HGNC Symbol;Acc:HGNC:23657] |
| ENSG00000160408 | HGLibA_47279 | 73 | -0.474383458 | ST6GALNAC6 | 6-sialyltransferase 6 [Source:HGNC Symbol;Acc:HGNC:23364]" |
| ENSG00000160588 | HGLibA_29802 | 73 | -0.474383458 | MPZL3 | myelin protein zero like 3 [Source:HGNC Symbol;Acc:HGNC:27279] |
| ENSG00000160991 | HGLibA_34591 | 73 | -0.474383458 | ORAI2 | ORAI calcium release-activated calcium modulator 2 [Source:HGNC Symbol;Acc:HGNC:21667] |
| ENSG00000161618 | HGLibA_01534 | 73 | -0.474383458 | ALDH16A1 | aldehyde dehydrogenase 16 family member A1 [Source:HGNC Symbol;Acc:HGNC:28114] |
| ENSG00000161905 | HGLibA_01682 | 73 | -0.474383458 | ALOX15 | arachidonate 15-lipoxygenase [Source:HGNC Symbol;Acc:HGNC:433] |
| ENSG00000163069 | HGLibA_43798 | 73 | -0.474383458 | SGCB | sarcoglycan beta [Source:HGNC Symbol;Acc:HGNC:10806] |
| ENSG00000163202 | HGLibA_26138 | 73 | -0.474383458 | LCE3D | late cornified envelope 3D [Source:HGNC Symbol;Acc:HGNC:16615] |
| ENSG00000163485 | HGLibA_01009 | 73 | -0.474383458 | ADORA1 | adenosine A1 receptor [Source:HGNC Symbol;Acc:HGNC:262] |
| ENSG00000163655 | HGLibA_19433 | 73 | -0.474383458 | GMPS | guanine monophosphate synthase [Source:HGNC Symbol;Acc:HGNC:4378] |
| ENSG00000163960 | HGLibA_52794 | 73 | -0.474383458 | UBXN7 | UBX domain protein 7 [Source:HGNC Symbol;Acc:HGNC:29119] |
| ENSG00000165115 | HGLibA_24921 | 73 | -0.474383458 | KIF27 | kinesin family member 27 [Source:HGNC Symbol;Acc:HGNC:18632] |
| ENSG00000165443 | HGLibA_36478 | 73 | -0.474383458 | PHYHIPL | phytanoyl-CoA 2-hydroxylase interacting protein like [Source:HGNC Symbol;Acc:HGNC:29378] |
| ENSG00000165795 | HGLibA_31415 | 73 | -0.474383458 | NDRG2 | NDRG family member 2 [Source:HGNC Symbol;Acc:HGNC:14460] |
| ENSG00000166930 | HGLibA_30154 | 73 | -0.474383458 | MS4A5 | membrane spanning 4-domains A5 [Source:HGNC Symbol;Acc:HGNC:13374] |
| ENSG00000167081 | HGLibA_35350 | 73 | -0.474383458 | PBX3 | PBX homeobox 3 [Source:HGNC Symbol;Acc:HGNC:8634] |
| ENSG00000167767 | HGLibA_25555 | 73 | -0.474383458 | KRT80 | keratin 80 [Source:HGNC Symbol;Acc:HGNC:27056] |
| ENSG00000167900 | HGLibA_49581 | 73 | -0.474383458 | TK1 | thymidine kinase 1 [Source:HGNC Symbol;Acc:HGNC:11830] |
| ENSG00000167978 | HGLibA_47048 | 73 | -0.474383458 | SRRM2 | serine/arginine repetitive matrix 2 [Source:HGNC Symbol;Acc:HGNC:16639] |
| ENSG00000167985 | HGLibA_43139 | 73 | -0.474383458 | SDHAF2 | succinate dehydrogenase complex assembly factor 2 [Source:HGNC Symbol;Acc:HGNC:26034] |
| ENSG00000168038 | HGLibA_52974 | 73 | -0.474383458 | ULK4 | unc-51 like kinase 4 [Source:HGNC Symbol;Acc:HGNC:15784] |
| ENSG00000168528 | HGLibA_43487 | 73 | -0.474383458 | SERINC2 | serine incorporator 2 [Source:HGNC Symbol;Acc:HGNC:23231] |
| ENSG00000168906 | HGLibA_28419 | 73 | -0.474383458 | MAT2A | methionine adenosyltransferase 2A [Source:HGNC Symbol;Acc:HGNC:6904] |
| ENSG00000169692 | HGLibA_01219 | 73 | -0.474383458 | AGPAT2 | 1-acylglycerol-3-phosphate O-acyltransferase 2 [Source:HGNC Symbol;Acc:HGNC:325] |
| ENSG00000170899 | HGLibA_20461 | 73 | -0.474383458 | GSTA4 | glutathione S-transferase alpha 4 [Source:HGNC Symbol;Acc:HGNC:4629] |
| ENSG00000170903 | HGLibA_30180 | 73 | -0.474383458 | MSANTD4 | Myb/SANT DNA binding domain containing 4 with coiled-coils [Source:HGNC Symbol;Acc:HGNC:29383] |
| ENSG00000171150 | HGLibA_46135 | 73 | -0.474383458 | SOCS5 | suppressor of cytokine signaling 5 [Source:HGNC Symbol;Acc:HGNC:16852] |
| ENSG00000171522 | HGLibA_39323 | 73 | -0.474383458 | PTGER4 | prostaglandin E receptor 4 [Source:HGNC Symbol;Acc:HGNC:9596] |
| ENSG00000171928 | HGLibA_52359 | 73 | -0.474383458 | TVP23B | trans-golgi network vesicle protein 23 homolog B [Source:HGNC Symbol;Acc:HGNC:20399] |
| ENSG00000171987 | HGLibA_05135 | 73 | -0.474383458 | C11orf40 | chromosome 11 open reading frame 40 [Source:HGNC Symbol;Acc:HGNC:23986] |
| ENSG00000172339 | HGLibA_01618 | 73 | -0.474383458 | ALG14 | UDP-N-acetylglucosaminyltransferase subunit [Source:HGNC Symbol;Acc:HGNC:28287]" |
| ENSG00000172493 | HGLibA_01105 | 73 | -0.474383458 | AFF1 | AF4/FMR2 family member 1 [Source:HGNC Symbol;Acc:HGNC:7135] |
| ENSG00000172673 | HGLibA_49350 | 73 | -0.474383458 | THEMIS | thymocyte selection associated [Source:HGNC Symbol;Acc:HGNC:21569] |
| ENSG00000172731 | HGLibA_28281 | 73 | -0.474383458 | LRRC20 | leucine rich repeat containing 20 [Source:HGNC Symbol;Acc:HGNC:23421] |
| ENSG00000173744 | HGLibA_01185 | 73 | -0.474383458 | AGFG1 | ArfGAP with FG repeats 1 [Source:HGNC Symbol;Acc:HGNC:5175] |
| ENSG00000175206 | HGLibA_32498 | 73 | -0.474383458 | NPPA | natriuretic peptide A [Source:HGNC Symbol;Acc:HGNC:7939] |
| ENSG00000176230 | HGLibA_34027 | 73 | -0.474383458 | OR4K17 | olfactory receptor family 4 subfamily K member 17 [Source:HGNC Symbol;Acc:HGNC:15355] |
| ENSG00000176402 | HGLibA_19184 | 73 | -0.474383458 | GJC3 | gap junction protein gamma 3 [Source:HGNC Symbol;Acc:HGNC:17495] |
| ENSG00000176681 | HGLibA_27327 | 73 | -0.474383458 | LRRC37A | leucine rich repeat containing 37A [Source:HGNC Symbol;Acc:HGNC:29069] |
| ENSG00000177156 | HGLibA_48286 | 73 | -0.474383458 | TALDO1 | transaldolase 1 [Source:HGNC Symbol;Acc:HGNC:11559] |
| ENSG00000177508 | HGLibA_23634 | 73 | -0.474383458 | IRX3 | iroquois homeobox 3 [Source:HGNC Symbol;Acc:HGNC:14360] |
| ENSG00000178796 | HGLibA_41247 | 73 | -0.474383458 | RIIAD1 | regulatory subunit of type II PKA R-subunit (RIIa) domain containing 1 [Source:HGNC Symbol;Acc:HGNC:26686] |
| ENSG00000179104 | HGLibA_50674 | 73 | -0.474383458 | TMTC2 | transmembrane and tetratricopeptide repeat containing 2 [Source:HGNC Symbol;Acc:HGNC:25440] |
| ENSG00000179546 | HGLibA_22362 | 73 | -0.474383458 | HTR1D | 5-hydroxytryptamine receptor 1D [Source:HGNC Symbol;Acc:HGNC:5289] |
| ENSG00000181381 | HGLibA_12764 | 73 | -0.474383458 | DDX60L | DExD/H-box 60 like [Source:HGNC Symbol;Acc:HGNC:26429] |
| ENSG00000181588 | HGLibA_29036 | 73 | -0.474383458 | MEX3D | mex-3 RNA binding family member D [Source:HGNC Symbol;Acc:HGNC:16734] |
| ENSG00000181786 | HGLibA_00635 | 73 | -0.474383458 | ACTL9 | actin like 9 [Source:HGNC Symbol;Acc:HGNC:28494] |
| ENSG00000182318 | HGLibA_57004 | 73 | -0.474383458 | ZSCAN22 | zinc finger and SCAN domain containing 22 [Source:HGNC Symbol;Acc:HGNC:4929] |
| ENSG00000182670 | HGLibA_52101 | 73 | -0.474383458 | TTC3 | tetratricopeptide repeat domain 3 [Source:HGNC Symbol;Acc:HGNC:12393] |
| ENSG00000182712 | HGLibA_10192 | 73 | -0.474383458 | CMC4 | C-X9-C motif containing 4 [Source:HGNC Symbol;Acc:HGNC:35428] |
| ENSG00000182899 | HGLibA_41949 | 73 | -0.474383458 | RPL35A | ribosomal protein L35a [Source:HGNC Symbol;Acc:HGNC:10345] |
| ENSG00000183734 | HGLibA_03268 | 73 | -0.474383458 | ASCL2 | achaete-scute family bHLH transcription factor 2 [Source:HGNC Symbol;Acc:HGNC:739] |
| ENSG00000183785 | HGLibA_52265 | 73 | -0.474383458 | TUBA8 | tubulin alpha 8 [Source:HGNC Symbol;Acc:HGNC:12410] |
| ENSG00000184261 | HGLibA_24301 | 73 | -0.474383458 | KCNK12 | potassium two pore domain channel subfamily K member 12 [Source:HGNC Symbol;Acc:HGNC:6274] |
| ENSG00000185742 | HGLibA_05204 | 73 | -0.474383458 | C11orf87 | chromosome 11 open reading frame 87 [Source:HGNC Symbol;Acc:HGNC:33788] |
| ENSG00000185787 | HGLibA_29659 | 73 | -0.474383458 | MORF4L1 | mortality factor 4 like 1 [Source:HGNC Symbol;Acc:HGNC:16989] |
| ENSG00000186191 | HGLibA_04728 | 73 | -0.474383458 | BPIFB4 | BPI fold containing family B member 4 [Source:HGNC Symbol;Acc:HGNC:16179] |
| ENSG00000187123 | HGLibA_27728 | 73 | -0.474383458 | LYPD6 | LY6/PLAUR domain containing 6 [Source:HGNC Symbol;Acc:HGNC:28751] |
| ENSG00000187753 | HGLibA_06691 | 73 | -0.474383458 | C9orf153 | chromosome 9 open reading frame 153 [Source:HGNC Symbol;Acc:HGNC:31456] |
| ENSG00000187961 | HGLibA_25140 | 73 | -0.474383458 | KLHL17 | kelch like family member 17 [Source:HGNC Symbol;Acc:HGNC:24023] |
| ENSG00000188064 | HGLibA_54385 | 73 | -0.474383458 | WNT7B | Wnt family member 7B [Source:HGNC Symbol;Acc:HGNC:12787] |
| ENSG00000188655 | HGLibA_41421 | 73 | -0.474383458 | RNASE9 | ribonuclease A family member 9 (inactive) [Source:HGNC Symbol;Acc:HGNC:20673] |
| ENSG00000189430 | HGLibA_31362 | 73 | -0.474383458 | NCR1 | natural cytotoxicity triggering receptor 1 [Source:HGNC Symbol;Acc:HGNC:6731] |
| ENSG00000196396 | HGLibA_39429 | 73 | -0.474383458 | PTPN1 | non-receptor type 1 [Source:HGNC Symbol;Acc:HGNC:9642]" |
| ENSG00000196778 | HGLibA_34180 | 73 | -0.474383458 | OR52K1 | olfactory receptor family 52 subfamily K member 1 [Source:HGNC Symbol;Acc:HGNC:15222] |
| ENSG00000196839 | HGLibA_00715 | 73 | -0.474383458 | ADA | adenosine deaminase [Source:HGNC Symbol;Acc:HGNC:186] |
| ENSG00000196932 | HGLibA_50349 | 73 | -0.474383458 | TMEM26 | transmembrane protein 26 [Source:HGNC Symbol;Acc:HGNC:28550] |
| ENSG00000197147 | HGLibA_27432 | 73 | -0.474383458 | LRRC8B | leucine rich repeat containing 8 VRAC subunit B [Source:HGNC Symbol;Acc:HGNC:30692] |
| ENSG00000197651 | HGLibA_07943 | 73 | -0.474383458 | CCER1 | coiled-coil glutamate rich protein 1 [Source:HGNC Symbol;Acc:HGNC:28373] |
| ENSG00000198160 | HGLibA_29253 | 73 | -0.474383458 | MIER1 | MIER1 transcriptional regulator [Source:HGNC Symbol;Acc:HGNC:29657] |
| ENSG00000198218 | HGLibA_39741 | 73 | -0.474383458 | QRICH1 | glutamine rich 1 [Source:HGNC Symbol;Acc:HGNC:24713] |
| ENSG00000203666 | HGLibA_14536 | 73 | -0.474383458 | EFCAB2 | EF-hand calcium binding domain 2 [Source:HGNC Symbol;Acc:HGNC:28166] |
| ENSG00000203734 | HGLibA_14426 | 73 | -0.474383458 | ECT2L | epithelial cell transforming 2 like [Source:HGNC Symbol;Acc:HGNC:21118] |
| ENSG00000203933 | HGLibA_11963 | 73 | -0.474383458 | CXorf66 | chromosome X open reading frame 66 [Source:HGNC Symbol;Acc:HGNC:33743] |
| ENSG00000204291 | HGLibA_10514 | 73 | -0.474383458 | COL15A1 | collagen type XV alpha 1 chain [Source:HGNC Symbol;Acc:HGNC:2192] |
| ENSG00000204335 | HGLibA_46305 | 73 | -0.474383458 | SP5 | Sp5 transcription factor [Source:HGNC Symbol;Acc:HGNC:14529] |
| ENSG00000205426 | HGLibA_25557 | 73 | -0.474383458 | KRT81 | keratin 81 [Source:HGNC Symbol;Acc:HGNC:6458] |
| ENSG00000213185 | HGLibA_16550 | 73 | -0.474383458 | FAM24B | family with sequence similarity 24 member B [Source:HGNC Symbol;Acc:HGNC:23475] |
| ENSG00000213231 | HGLibA_48819 | 73 | -0.474383458 | TCL1B | T cell leukemia/lymphoma 1B [Source:HGNC Symbol;Acc:HGNC:11649] |
| ENSG00000214510 | HGLibA_46686 | 73 | -0.474383458 | SPINK13 | Kazal type 13 (putative) [Source:HGNC Symbol;Acc:HGNC:27200]" |
| ENSG00000218823 | HGLibA_35140 | 73 | -0.474383458 | PAPOLB | poly(A) polymerase beta [Source:HGNC Symbol;Acc:HGNC:15970] |
| ENSG00000221818 | HGLibA_14351 | 73 | -0.474383458 | EBF2 | EBF transcription factor 2 [Source:HGNC Symbol;Acc:HGNC:19090] |
| ENSG00000239382 | HGLibA_01661 | 73 | -0.474383458 | ALKBH6 | alkB homolog 6 [Source:HGNC Symbol;Acc:HGNC:28243] |
| ENSG00000243449 | HGLibA_06341 | 73 | -0.474383458 | C4orf48 | chromosome 4 open reading frame 48 [Source:HGNC Symbol;Acc:HGNC:34437] |
| ENSG00000244752 | HGLibA_11265 | 73 | -0.474383458 | CRYBB2 | crystallin beta B2 [Source:HGNC Symbol;Acc:HGNC:2398] |
| ENSG00000249693 | HGLibA_49338 | 73 | -0.474383458 | THEGL | theg spermatid protein like [Source:HGNC Symbol;Acc:HGNC:43771] |
| ENSG00000251664 | HGLibA_35433 | 73 | -0.474383458 | PCDHA12 | protocadherin alpha 12 [Source:HGNC Symbol;Acc:HGNC:8666] |
| ENSG00000257727 | HGLibA_10365 | 73 | -0.474383458 | CNPY2 | canopy FGF signaling regulator 2 [Source:HGNC Symbol;Acc:HGNC:13529] |
| ENSG00000268182 | HGLibA_45737 | 73 | -0.474383458 | SMIM17 | small integral membrane protein 17 [Source:HGNC Symbol;Acc:HGNC:27114] |
| ENSG00000268606 | HGLibA_27904 | 73 | -0.474383458 | MAGEA2 | MAGE family member A2 [Source:HGNC Symbol;Acc:HGNC:6800] |
| ENSG00000271321 | HGLibA_11579 | 73 | -0.474383458 | CTAGE6 | CTAGE family member 6 [Source:HGNC Symbol;Acc:HGNC:28644] |
| ENSG00000273111 | HGLibA_27721 | 73 | -0.474383458 | LYPD4 | LY6/PLAUR domain containing 4 [Source:HGNC Symbol;Acc:HGNC:28659] |
| ENSG00000275152 | HGLibA_07977 | 73 | -0.474383458 | CCL16 | C-C motif chemokine ligand 16 [Source:HGNC Symbol;Acc:HGNC:10614] |
| ENSG00000275969 | HGLibA_46475 | 73 | -0.474383458 | SPATA31A3 | SPATA31 subfamily A member 3 [Source:HGNC Symbol;Acc:HGNC:32003] |
| FAM21B | HGLibA_16507 | 73 | -0.474383458 | FAM21B | - |
| GPR124 | HGLibA_19875 | 73 | -0.474383458 | GPR124 | - |
| hsa-mir-1914 | HGLibA_58059 | 73 | -0.474383458 | hsa-mir-1914 | - |
| hsa-mir-22 | HGLibA_58287 | 73 | -0.474383458 | hsa-mir-22 | - |
| hsa-mir-3117 | HGLibA_58511 | 73 | -0.474383458 | hsa-mir-3117 | - |
| hsa-mir-3155b | HGLibA_58683 | 73 | -0.474383458 | hsa-mir-3155b | - |
| hsa-mir-3200 | HGLibA_58906 | 73 | -0.474383458 | hsa-mir-3200 | - |
| hsa-mir-33b | HGLibA_59014 | 73 | -0.474383458 | hsa-mir-33b | - |
| hsa-mir-3688-2 | HGLibA_59311 | 73 | -0.474383458 | hsa-mir-3688-2 | - |
| hsa-mir-374c | HGLibA_59399 | 73 | -0.474383458 | hsa-mir-374c | - |
| hsa-mir-4463 | HGLibA_60258 | 73 | -0.474383458 | hsa-mir-4463 | - |
| hsa-mir-4472-1 | HGLibA_60295 | 73 | -0.474383458 | hsa-mir-4472-1 | - |
| hsa-mir-4483 | HGLibA_60351 | 73 | -0.474383458 | hsa-mir-4483 | - |
| hsa-mir-4649 | HGLibA_60699 | 73 | -0.474383458 | hsa-mir-4649 | - |
| hsa-mir-4663 | HGLibA_60767 | 73 | -0.474383458 | hsa-mir-4663 | - |
| hsa-mir-4685 | HGLibA_60849 | 73 | -0.474383458 | hsa-mir-4685 | - |
| hsa-mir-4705 | HGLibA_60919 | 73 | -0.474383458 | hsa-mir-4705 | - |
| hsa-mir-4742 | HGLibA_61065 | 73 | -0.474383458 | hsa-mir-4742 | - |
| hsa-mir-550a-2 | HGLibA_62103 | 73 | -0.474383458 | hsa-mir-550a-2 | - |
| hsa-mir-596 | HGLibA_62478 | 73 | -0.474383458 | hsa-mir-596 | - |
| hsa-mir-6072 | HGLibA_62542 | 73 | -0.474383458 | hsa-mir-6072 | - |
| hsa-mir-643 | HGLibA_62817 | 73 | -0.474383458 | hsa-mir-643 | - |
| hsa-mir-6846 | HGLibA_63557 | 73 | -0.474383458 | hsa-mir-6846 | - |
| hsa-mir-7162 | HGLibA_63875 | 73 | -0.474383458 | hsa-mir-7162 | - |
| hsa-mir-8062 | HGLibA_64087 | 73 | -0.474383458 | hsa-mir-8062 | - |
| NOV | HGLibA_32362 | 73 | -0.474383458 | NOV | - |
| TMEM14E | HGLibA_49987 | 73 | -0.474383458 | TMEM14E | - |
| ATP5J | HGLibA_03670 | 72 | -0.483428801 | ATP5J | - |
| C10orf111 | HGLibA_05032 | 72 | -0.483428801 | C10orf111 | - |
| C4orf27 | HGLibA_06311 | 72 | -0.483428801 | C4orf27 | - |
| C6orf203 | HGLibA_06469 | 72 | -0.483428801 | C6orf203 | - |
| ENSG00000004866 | HGLibA_47283 | 72 | -0.483428801 | ST7 | suppression of tumorigenicity 7 [Source:HGNC Symbol;Acc:HGNC:11351] |
| ENSG00000005175 | HGLibA_41821 | 72 | -0.483428801 | RPAP3 | RNA polymerase II associated protein 3 [Source:HGNC Symbol;Acc:HGNC:26151] |
| ENSG00000006611 | HGLibA_53172 | 72 | -0.483428801 | USH1C | USH1 protein network component harmonin [Source:HGNC Symbol;Acc:HGNC:12597] |
| ENSG00000011465 | HGLibA_12518 | 72 | -0.483428801 | DCN | decorin [Source:HGNC Symbol;Acc:HGNC:2705] |

| | | | | | |
|---|---|---|---|---|---|
| ENSG00000018189 | HGLibA_42442 | 72 | -0.483428801 | RUFY3 | RUN and FYVE domain containing 3 [Source:HGNC Symbol;Acc:HGNC:30285] |
| ENSG00000029993 | HGLibA_21695 | 72 | -0.483428801 | HMGB3 | high mobility group box 3 [Source:HGNC Symbol;Acc:HGNC:5004] |
| ENSG00000030582 | HGLibA_20364 | 72 | -0.483428801 | GRN | granulin precursor [Source:HGNC Symbol;Acc:HGNC:4601] |
| ENSG00000037042 | HGLibA_52307 | 72 | -0.483428801 | TUBG2 | tubulin gamma 2 [Source:HGNC Symbol;Acc:HGNC:12419] |
| ENSG00000041515 | HGLibA_30807 | 72 | -0.483428801 | MYO16 | myosin XVI [Source:HGNC Symbol;Acc:HGNC:29822] |
| ENSG00000044574 | HGLibA_22289 | 72 | -0.483428801 | HSPA5 | heat shock protein family A (Hsp70) member 5 [Source:HGNC Symbol;Acc:HGNC:5238] |
| ENSG00000060709 | HGLibA_41260 | 72 | -0.483428801 | RIMBP2 | RIMS binding protein 2 [Source:HGNC Symbol;Acc:HGNC:30339] |
| ENSG00000066777 | HGLibA_02669 | 72 | -0.483428801 | ARFGEF1 | ADP ribosylation factor guanine nucleotide exchange factor 1 [Source:HGNC Symbol;Acc:HGNC:15772] |
| ENSG00000067048 | HGLibA_12705 | 72 | -0.483428801 | DDX3Y | DEAD-box helicase 3 Y-linked [Source:HGNC Symbol;Acc:HGNC:2699] |
| ENSG00000070018 | HGLibA_27230 | 72 | -0.483428801 | LRP6 | LDL receptor related protein 6 [Source:HGNC Symbol;Acc:HGNC:6698] |
| ENSG00000070831 | HGLibA_08555 | 72 | -0.483428801 | CDC42 | cell division cycle 42 [Source:HGNC Symbol;Acc:HGNC:1736] |
| ENSG00000072062 | HGLibA_38502 | 72 | -0.483428801 | PRKACA | protein kinase cAMP-activated catalytic subunit alpha [Source:HGNC Symbol;Acc:HGNC:9380] |
| ENSG00000073598 | HGLibA_17760 | 72 | -0.483428801 | FNDC8 | fibronectin type III domain containing 8 [Source:HGNC Symbol;Acc:HGNC:25286] |
| ENSG00000074211 | HGLibA_38152 | 72 | -0.483428801 | PPP2R2C | protein phosphatase 2 regulatory subunit Bgamma [Source:HGNC Symbol;Acc:HGNC:9306] |
| ENSG00000077157 | HGLibA_38023 | 72 | -0.483428801 | PPP1R12B | protein phosphatase 1 regulatory subunit 12B [Source:HGNC Symbol;Acc:HGNC:7619] |
| ENSG00000077585 | HGLibA_19900 | 72 | -0.483428801 | GPR137B | G protein-coupled receptor 137B [Source:HGNC Symbol;Acc:HGNC:11862] |
| ENSG00000078098 | HGLibA_16866 | 72 | -0.483428801 | FAP | fibroblast activation protein alpha [Source:HGNC Symbol;Acc:HGNC:3590] |
| ENSG00000078295 | HGLibA_00911 | 72 | -0.483428801 | ADCY2 | adenylate cyclase 2 [Source:HGNC Symbol;Acc:HGNC:233] |
| ENSG00000078699 | HGLibA_07384 | 72 | -0.483428801 | CBFA2T2 | CBFA2/RUNX1 translocation partner 2 [Source:HGNC Symbol;Acc:HGNC:1536] |
| ENSG00000079393 | HGLibA_14140 | 72 | -0.483428801 | DUSP13 | dual specificity phosphatase 13 [Source:HGNC Symbol;Acc:HGNC:19681] |
| ENSG00000079557 | HGLibA_01121 | 72 | -0.483428801 | AFM | afamin [Source:HGNC Symbol;Acc:HGNC:316] |
| ENSG00000081087 | HGLibA_34713 | 72 | -0.483428801 | OSTM1 | osteoclastogenesis associated transmembrane protein 1 [Source:HGNC Symbol;Acc:HGNC:21652] |
| ENSG00000082175 | HGLibA_36286 | 72 | -0.483428801 | PGR | progesterone receptor [Source:HGNC Symbol;Acc:HGNC:8910] |
| ENSG00000083812 | HGLibA_55802 | 72 | -0.483428801 | ZNF324 | zinc finger protein 324 [Source:HGNC Symbol;Acc:HGNC:14096] |
| ENSG00000086758 | HGLibA_22432 | 72 | -0.483428801 | HUWE1 | E3 ubiquitin protein ligase [Source:HGNC Symbol;Acc:HGNC:30892]" |
| ENSG00000089327 | HGLibA_18235 | 72 | -0.483428801 | FXYD5 | FXYD domain containing ion transport regulator 5 [Source:HGNC Symbol;Acc:HGNC:4029] |
| ENSG00000099904 | HGLibA_55129 | 72 | -0.483428801 | ZDHHC8 | zinc finger DHHC-type containing 8 [Source:HGNC Symbol;Acc:HGNC:18474] |
| ENSG00000100038 | HGLibA_51003 | 72 | -0.483428801 | TOP3B | DNA topoisomerase III beta [Source:HGNC Symbol;Acc:HGNC:11993] |
| ENSG00000100288 | HGLibA_09461 | 72 | -0.483428801 | CHKB | choline kinase beta [Source:HGNC Symbol;Acc:HGNC:1938] |
| ENSG00000100652 | HGLibA_44331 | 72 | -0.483428801 | SLC10A1 | solute carrier family 10 member 1 [Source:HGNC Symbol;Acc:HGNC:10905] |
| ENSG00000100802 | HGLibA_05376 | 72 | -0.483428801 | C14orf93 | chromosome 14 open reading frame 93 [Source:HGNC Symbol;Acc:HGNC:20162] |
| ENSG00000101190 | HGLibA_48800 | 72 | -0.483428801 | TCFL5 | transcription factor like 5 [Source:HGNC Symbol;Acc:HGNC:11646] |
| ENSG00000101871 | HGLibA_29230 | 72 | -0.483428801 | MID1 | midline 1 [Source:HGNC Symbol;Acc:HGNC:7095] |
| ENSG00000102078 | HGLibA_44649 | 72 | -0.483428801 | SLC25A14 | solute carrier family 25 member 14 [Source:HGNC Symbol;Acc:HGNC:10984] |
| ENSG00000104341 | HGLibA_26018 | 72 | -0.483428801 | LAPTM4B | lysosomal protein transmembrane 4 beta [Source:HGNC Symbol;Acc:HGNC:13646] |
| ENSG00000104427 | HGLibA_54936 | 72 | -0.483428801 | ZC2HC1A | zinc finger C2HC-type containing 1A [Source:HGNC Symbol;Acc:HGNC:24277] |
| ENSG00000104643 | HGLibA_30442 | 72 | -0.483428801 | MTMR9 | myotubularin related protein 9 [Source:HGNC Symbol;Acc:HGNC:14596] |
| ENSG00000104722 | HGLibA_31624 | 72 | -0.483428801 | NEFM | neurofilament medium [Source:HGNC Symbol;Acc:HGNC:7734] |
| ENSG00000104941 | HGLibA_42339 | 72 | -0.483428801 | RSPH6A | radial spoke head 6 homolog A [Source:HGNC Symbol;Acc:HGNC:14241] |
| ENSG00000104976 | HGLibA_45893 | 72 | -0.483428801 | SNAPC2 | small nuclear RNA activating complex polypeptide 2 [Source:HGNC Symbol;Acc:HGNC:11135] |
| ENSG00000105677 | HGLibA_49976 | 72 | -0.483428801 | TMEM147 | transmembrane protein 147 [Source:HGNC Symbol;Acc:HGNC:30414] |
| ENSG00000106686 | HGLibA_46524 | 72 | -0.483428801 | SPATA6L | spermatogenesis associated 6 like [Source:HGNC Symbol;Acc:HGNC:25472] |
| ENSG00000107957 | HGLibA_43963 | 72 | -0.483428801 | SH3PXD2A | SH3 and PX domains 2A [Source:HGNC Symbol;Acc:HGNC:23664] |
| ENSG00000110693 | HGLibA_46269 | 72 | -0.483428801 | SOX6 | SRY-box 6 [Source:HGNC Symbol;Acc:HGNC:16421] |
| ENSG00000110975 | HGLibA_48049 | 72 | -0.483428801 | SYT10 | synaptotagmin 10 [Source:HGNC Symbol;Acc:HGNC:19266] |
| ENSG00000111581 | HGLibA_33017 | 72 | -0.483428801 | NUP107 | nucleoporin 107 [Source:HGNC Symbol;Acc:HGNC:29914] |
| ENSG00000112033 | HGLibA_37839 | 72 | -0.483428801 | PPARD | peroxisome proliferator activated receptor delta [Source:HGNC Symbol;Acc:HGNC:9235] |
| ENSG00000113889 | HGLibA_25341 | 72 | -0.483428801 | KNG1 | kininogen 1 [Source:HGNC Symbol;Acc:HGNC:6383] |
| ENSG00000114054 | HGLibA_35381 | 72 | -0.483428801 | PCCB | propionyl-CoA carboxylase subunit beta [Source:HGNC Symbol;Acc:HGNC:8654] |
| ENSG00000115970 | HGLibA_49282 | 72 | -0.483428801 | THADA | armadillo repeat containing [Source:HGNC Symbol;Acc:HGNC:19217]" |
| ENSG00000116138 | HGLibA_13583 | 72 | -0.483428801 | DNAJC16 | DnaJ heat shock protein family (Hsp40) member C16 [Source:HGNC Symbol;Acc:HGNC:29157] |
| ENSG00000116983 | HGLibA_22021 | 72 | -0.483428801 | HPCAL4 | hippocalcin like 4 [Source:HGNC Symbol;Acc:HGNC:18212] |
| ENSG00000117450 | HGLibA_38406 | 72 | -0.483428801 | PRDX1 | peroxiredoxin 1 [Source:HGNC Symbol;Acc:HGNC:9352] |
| ENSG00000119125 | HGLibA_18837 | 72 | -0.483428801 | GDA | guanine deaminase [Source:HGNC Symbol;Acc:HGNC:4212] |
| ENSG00000120837 | HGLibA_31856 | 72 | -0.483428801 | NFYB | nuclear transcription factor Y subunit beta [Source:HGNC Symbol;Acc:HGNC:7805] |
| ENSG00000122304 | HGLibA_38611 | 72 | -0.483428801 | PRM2 | protamine 2 [Source:HGNC Symbol;Acc:HGNC:9448] |
| ENSG00000122386 | HGLibA_55576 | 72 | -0.483428801 | ZNF205 | zinc finger protein 205 [Source:HGNC Symbol;Acc:HGNC:12996] |
| ENSG00000123454 | HGLibA_12381 | 72 | -0.483428801 | DBH | dopamine beta-hydroxylase [Source:HGNC Symbol;Acc:HGNC:2689] |
| ENSG00000124608 | HGLibA_00072 | 72 | -0.483428801 | AARS2 | mitochondrial [Source:HGNC Symbol;Acc:HGNC:21022]" |
| ENSG00000125355 | HGLibA_50329 | 72 | -0.483428801 | TMEM255A | transmembrane protein 255A [Source:HGNC Symbol;Acc:HGNC:26086] |
| ENSG00000126461 | HGLibA_42821 | 72 | -0.483428801 | SCAF1 | SR-related CTD associated factor 1 [Source:HGNC Symbol;Acc:HGNC:30403] |
| ENSG00000127838 | HGLibA_37348 | 72 | -0.483428801 | PNKD | MBL domain containing [Source:HGNC Symbol;Acc:HGNC:9153]" |
| ENSG00000128789 | HGLibA_39207 | 72 | -0.483428801 | PSMG2 | proteasome assembly chaperone 2 [Source:HGNC Symbol;Acc:HGNC:24929] |
| ENSG00000129255 | HGLibA_29710 | 72 | -0.483428801 | MPDU1 | mannose-P-dolichol utilization defect 1 [Source:HGNC Symbol;Acc:HGNC:7207] |
| ENSG00000129535 | HGLibA_32704 | 72 | -0.483428801 | NRL | neural retina leucine zipper [Source:HGNC Symbol;Acc:HGNC:8002] |
| ENSG00000129667 | HGLibA_41137 | 72 | -0.483428801 | RHBDF2 | rhomboid 5 homolog 2 [Source:HGNC Symbol;Acc:HGNC:20788] |
| ENSG00000129864 | HGLibA_53592 | 72 | -0.483428801 | VCY | variable charge Y-linked [Source:HGNC Symbol;Acc:HGNC:12668] |
| ENSG00000130561 | HGLibA_42642 | 72 | -0.483428801 | SAG | S-antigen visual arrestin [Source:HGNC Symbol;Acc:HGNC:10521] |
| ENSG00000134595 | HGLibA_46257 | 72 | -0.483428801 | SOX3 | SRY-box 3 [Source:HGNC Symbol;Acc:HGNC:11199] |
| ENSG00000134755 | HGLibA_13992 | 72 | -0.483428801 | DSC2 | desmocollin 2 [Source:HGNC Symbol;Acc:HGNC:3036] |
| ENSG00000135457 | HGLibA_49146 | 72 | -0.483428801 | TFCP2 | transcription factor CP2 [Source:HGNC Symbol;Acc:HGNC:11748] |
| ENSG00000136521 | HGLibA_31521 | 72 | -0.483428801 | NDUFB5 | NADH:ubiquinone oxidoreductase subunit B5 [Source:HGNC Symbol;Acc:HGNC:7700] |
| ENSG00000136883 | HGLibA_24854 | 72 | -0.483428801 | KIF12 | kinesin family member 12 [Source:HGNC Symbol;Acc:HGNC:21495] |
| ENSG00000137040 | HGLibA_40208 | 72 | -0.483428801 | RANBP6 | RAN binding protein 6 [Source:HGNC Symbol;Acc:HGNC:9851] |
| ENSG00000138379 | HGLibA_30264 | 72 | -0.483428801 | MSTN | myostatin [Source:HGNC Symbol;Acc:HGNC:4223] |
| ENSG00000138674 | HGLibA_43240 | 72 | -0.483428801 | SEC31A | COPII coat complex component [Source:HGNC Symbol;Acc:HGNC:17052]" |
| ENSG00000138823 | HGLibA_30503 | 72 | -0.483428801 | MTTP | microsomal triglyceride transfer protein [Source:HGNC Symbol;Acc:HGNC:7467] |
| ENSG00000140067 | HGLibA_16319 | 72 | -0.483428801 | FAM181A | family with sequence similarity 181 member A [Source:HGNC Symbol;Acc:HGNC:20491] |
| ENSG00000140548 | HGLibA_56612 | 72 | -0.483428801 | ZNF710 | zinc finger protein 710 [Source:HGNC Symbol;Acc:HGNC:25352] |
| ENSG00000143157 | HGLibA_37475 | 72 | -0.483428801 | POGK | pogo transposable element derived with KRAB domain [Source:HGNC Symbol;Acc:HGNC:18800] |
| ENSG00000143641 | HGLibA_18553 | 72 | -0.483428801 | GALNT2 | polypeptide N-acetylgalactosaminyltransferase 2 [Source:HGNC Symbol;Acc:HGNC:4124] |
| ENSG00000144035 | HGLibA_31183 | 72 | -0.483428801 | NAT8 | N-acetyltransferase 8 (putative) [Source:HGNC Symbol;Acc:HGNC:18069] |
| ENSG00000145358 | HGLibA_12620 | 72 | -0.483428801 | DDIT4L | DNA damage inducible transcript 4 like [Source:HGNC Symbol;Acc:HGNC:30555] |
| ENSG00000145916 | HGLibA_41385 | 72 | -0.483428801 | RMND5B | required for meiotic nuclear division 5 homolog B [Source:HGNC Symbol;Acc:HGNC:26181] |
| ENSG00000146151 | HGLibA_21705 | 72 | -0.483428801 | HMGCLL1 | 3-hydroxymethyl-3-methylglutaryl-CoA lyase like 1 [Source:HGNC Symbol;Acc:HGNC:21359] |
| ENSG00000147601 | HGLibA_49012 | 72 | -0.483428801 | TERF1 | telomeric repeat binding factor 1 [Source:HGNC Symbol;Acc:HGNC:11728] |
| ENSG00000148483 | HGLibA_50274 | 72 | -0.483428801 | TMEM236 | transmembrane protein 236 [Source:HGNC Symbol;Acc:HGNC:23473] |
| ENSG00000148735 | HGLibA_37155 | 72 | -0.483428801 | PLEKHS1 | pleckstrin homology domain containing S1 [Source:HGNC Symbol;Acc:HGNC:26285] |
| ENSG00000149257 | HGLibA_43594 | 72 | -0.483428801 | SERPINH1 | serpin family H member 1 [Source:HGNC Symbol;Acc:HGNC:1546] |
| ENSG00000151164 | HGLibA_40110 | 72 | -0.483428801 | RAD9B | RAD9 checkpoint clamp component B [Source:HGNC Symbol;Acc:HGNC:21700] |
| ENSG00000152467 | HGLibA_56983 | 72 | -0.483428801 | ZSCAN1 | zinc finger and SCAN domain containing 1 [Source:HGNC Symbol;Acc:HGNC:23712] |
| ENSG00000153066 | HGLibA_52404 | 72 | -0.483428801 | TXNDC11 | thioredoxin domain containing 11 [Source:HGNC Symbol;Acc:HGNC:28030] |
| ENSG00000153822 | HGLibA_24268 | 72 | -0.483428801 | KCNJ16 | potassium voltage-gated channel subfamily J member 16 [Source:HGNC Symbol;Acc:HGNC:6262] |
| ENSG00000156026 | HGLibA_28661 | 72 | -0.483428801 | MCU | mitochondrial calcium uniporter [Source:HGNC Symbol;Acc:HGNC:23526] |
| ENSG00000157017 | HGLibA_19048 | 72 | -0.483428801 | GHRL | ghrelin and obestatin prepropeptide [Source:HGNC Symbol;Acc:HGNC:18129] |
| ENSG00000160208 | HGLibA_42276 | 72 | -0.483428801 | RRP1B | ribosomal RNA processing 1B [Source:HGNC Symbol;Acc:HGNC:23818] |
| ENSG00000160325 | HGLibA_06869 | 72 | -0.483428801 | CACFD1 | calcium channel flower domain containing 1 [Source:HGNC Symbol;Acc:HGNC:1365] |
| ENSG00000161533 | HGLibA_00497 | 72 | -0.483428801 | ACOX1 | acyl-CoA oxidase 1 [Source:HGNC Symbol;Acc:HGNC:119] |
| ENSG00000162654 | HGLibA_18738 | 72 | -0.483428801 | GBP4 | guanylate binding protein 4 [Source:HGNC Symbol;Acc:HGNC:20480] |
| ENSG00000162869 | HGLibA_38078 | 72 | -0.483428801 | PPP1R21 | protein phosphatase 1 regulatory subunit 21 [Source:HGNC Symbol;Acc:HGNC:30595] |
| ENSG00000163499 | HGLibA_11256 | 72 | -0.483428801 | CRYBA2 | crystallin beta A2 [Source:HGNC Symbol;Acc:HGNC:2395] |
| ENSG00000163531 | HGLibA_31767 | 72 | -0.483428801 | NFASC | neurofascin [Source:HGNC Symbol;Acc:HGNC:29866] |
| ENSG00000163684 | HGLibA_42023 | 72 | -0.483428801 | RPP14 | ribonuclease P/MRP subunit p14 [Source:HGNC Symbol;Acc:HGNC:30327] |
| ENSG00000164086 | HGLibA_14189 | 72 | -0.483428801 | DUSP7 | dual specificity phosphatase 7 [Source:HGNC Symbol;Acc:HGNC:3073] |
| ENSG00000164188 | HGLibA_40207 | 72 | -0.483428801 | RANBP3L | RAN binding protein 3 like [Source:HGNC Symbol;Acc:HGNC:26353] |
| ENSG00000164344 | HGLibA_25277 | 72 | -0.483428801 | KLKB1 | kallikrein B1 [Source:HGNC Symbol;Acc:HGNC:6371] |
| ENSG00000165059 | HGLibA_38510 | 72 | -0.483428801 | PRKACG | protein kinase cAMP-activated catalytic subunit gamma [Source:HGNC Symbol;Acc:HGNC:9382] |
| ENSG00000165119 | HGLibA_21830 | 72 | -0.483428801 | HNRNPK | heterogeneous nuclear ribonucleoprotein K [Source:HGNC Symbol;Acc:HGNC:5044] |
| ENSG00000165509 | HGLibA_27965 | 72 | -0.483428801 | MAGEC3 | MAGE family member C3 [Source:HGNC Symbol;Acc:HGNC:23798] |
| ENSG00000165555 | HGLibA_32388 | 72 | -0.483428801 | NOXRED1 | NADP dependent oxidoreductase domain containing 1 [Source:HGNC Symbol;Acc:HGNC:20487] |
| ENSG00000166670 | HGLibA_29481 | 72 | -0.483428801 | MMP10 | matrix metallopeptidase 10 [Source:HGNC Symbol;Acc:HGNC:7156] |
| ENSG00000166974 | HGLibA_28316 | 72 | -0.483428801 | MAPRE2 | microtubule associated protein RP/EB family member 2 [Source:HGNC Symbol;Acc:HGNC:6891] |
| ENSG00000167618 | HGLibA_25935 | 72 | -0.483428801 | LAIR2 | leukocyte associated immunoglobulin like receptor 2 [Source:HGNC Symbol;Acc:HGNC:6478] |
| ENSG00000167723 | HGLibA_51789 | 72 | -0.483428801 | TRPV3 | transient receptor potential cation channel subfamily V member 3 [Source:HGNC Symbol;Acc:HGNC:18084] |
| ENSG00000167797 | HGLibA_08764 | 72 | -0.483428801 | CDK2AP2 | cyclin dependent kinase 2 associated protein 2 [Source:HGNC Symbol;Acc:HGNC:30833] |
| ENSG00000168758 | HGLibA_43347 | 72 | -0.483428801 | SEMA4C | semaphorin 4C [Source:HGNC Symbol;Acc:HGNC:10731] |
| ENSG00000169059 | HGLibA_53586 | 72 | -0.483428801 | VCX3A | variable charge X-linked 3A [Source:HGNC Symbol;Acc:HGNC:18159] |
| ENSG00000169131 | HGLibA_55862 | 72 | -0.483428801 | ZNF354A | zinc finger protein 354A [Source:HGNC Symbol;Acc:HGNC:11628] |
| ENSG00000169876 | HGLibA_30531 | 72 | -0.483428801 | MUC17 | cell surface associated [Source:HGNC Symbol;Acc:HGNC:16800]" |
| ENSG00000170128 | HGLibA_20006 | 72 | -0.483428801 | GPR25 | G protein-coupled receptor 25 [Source:HGNC Symbol;Acc:HGNC:4480] |
| ENSG00000170456 | HGLibA_12961 | 72 | -0.483428801 | DENND5B | DENN domain containing 5B [Source:HGNC Symbol;Acc:HGNC:28338] |
| ENSG00000170624 | HGLibA_43801 | 72 | -0.483428801 | SGCD | sarcoglycan delta [Source:HGNC Symbol;Acc:HGNC:10807] |
| ENSG00000170689 | HGLibA_21954 | 72 | -0.483428801 | HOXB9 | homeobox B9 [Source:HGNC Symbol;Acc:HGNC:5120] |
| ENSG00000171396 | HGLibA_25763 | 72 | -0.483428801 | KRTAP4-4 | keratin associated protein 4-4 [Source:HGNC Symbol;Acc:HGNC:16928] |
| ENSG00000171735 | HGLibA_07116 | 72 | -0.483428801 | CAMTA1 | calmodulin binding transcription activator 1 [Source:HGNC Symbol;Acc:HGNC:18806] |
| ENSG00000172493 | HGLibA_01106 | 72 | -0.483428801 | AFF1 | AF4/FMR2 family member 1 [Source:HGNC Symbol;Acc:HGNC:7135] |
| ENSG00000172943 | HGLibA_36391 | 72 | -0.483428801 | PHF8 | PHD finger protein 8 [Source:HGNC Symbol;Acc:HGNC:20672] |
| ENSG00000173826 | HGLibA_24226 | 72 | -0.483428801 | KCNH6 | potassium voltage-gated channel subfamily H member 6 [Source:HGNC Symbol;Acc:HGNC:18862] |
| ENSG00000173908 | HGLibA_25468 | 72 | -0.483428801 | KRT28 | keratin 28 [Source:HGNC Symbol;Acc:HGNC:30842] |
| ENSG00000174371 | HGLibA_15724 | 72 | -0.483428801 | EXO1 | exonuclease 1 [Source:HGNC Symbol;Acc:HGNC:3511] |
| ENSG00000174776 | HGLibA_54112 | 72 | -0.483428801 | WDR49 | WD repeat domain 49 [Source:HGNC Symbol;Acc:HGNC:26587] |
| ENSG00000175324 | HGLibA_27519 | 72 | -0.483428801 | LSM1 | mRNA degradation associated [Source:HGNC Symbol;Acc:HGNC:20472]" |
| ENSG00000175575 | HGLibA_34917 | 72 | -0.483428801 | PAAF1 | proteasomal ATPase associated factor 1 [Source:HGNC Symbol;Acc:HGNC:25687] |
| ENSG00000176697 | HGLibA_04385 | 72 | -0.483428801 | BDNF | brain derived neurotrophic factor [Source:HGNC Symbol;Acc:HGNC:1033] |
| ENSG00000177272 | HGLibA_24133 | 72 | -0.483428801 | KCNA3 | potassium voltage-gated channel subfamily A member 3 [Source:HGNC Symbol;Acc:HGNC:6221] |

| | | | | | |
|---|---|---|---|---|---|
| ENSG00000177984 | HGLibA_26174 | 72 | -0.483428801 | LCN15 | lipocalin 15 [Source:HGNC Symbol;Acc:HGNC:33777] |
| ENSG00000178035 | HGLibA_23260 | 72 | -0.483428801 | IMPDH2 | inosine monophosphate dehydrogenase 2 [Source:HGNC Symbol;Acc:HGNC:6053] |
| ENSG00000178425 | HGLibA_32821 | 72 | -0.483428801 | NT5DC1 | 5'-nucleotidase domain containing 1 [Source:HGNC Symbol;Acc:HGNC:21556] |
| ENSG00000178761 | HGLibA_16500 | 72 | -0.483428801 | FAM219B | family with sequence similarity 219 member B [Source:HGNC Symbol;Acc:HGNC:24695] |
| ENSG00000179097 | HGLibA_22367 | 72 | -0.483428801 | HTR1F | 5-hydroxytryptamine receptor 1F [Source:HGNC Symbol;Acc:HGNC:5292] |
| ENSG00000180535 | HGLibA_04466 | 72 | -0.483428801 | BHLHA15 | basic helix-loop-helix family member a15 [Source:HGNC Symbol;Acc:HGNC:22265] |
| ENSG00000181135 | HGLibA_56600 | 72 | -0.483428801 | ZNF707 | zinc finger protein 707 [Source:HGNC Symbol;Acc:HGNC:27815] |
| ENSG00000181924 | HGLibA_10448 | 72 | -0.483428801 | COA4 | cytochrome c oxidase assembly factor 4 homolog [Source:HGNC Symbol;Acc:HGNC:24604] |
| ENSG00000182199 | HGLibA_44063 | 72 | -0.483428801 | SHMT2 | serine hydroxymethyltransferase 2 [Source:HGNC Symbol;Acc:HGNC:10852] |
| ENSG00000182446 | HGLibA_32485 | 72 | -0.483428801 | NPLOC4 | ubiquitin recognition factor [Source:HGNC Symbol;Acc:HGNC:18261]" |
| ENSG00000182541 | HGLibA_26542 | 72 | -0.483428801 | LIMK2 | LIM domain kinase 2 [Source:HGNC Symbol;Acc:HGNC:6614] |
| ENSG00000183166 | HGLibA_07043 | 72 | -0.483428801 | CALN1 | calneuron 1 [Source:HGNC Symbol;Acc:HGNC:13248] |
| ENSG00000183431 | HGLibA_43697 | 72 | -0.483428801 | SF3A3 | splicing factor 3a subunit 3 [Source:HGNC Symbol;Acc:HGNC:10767] |
| ENSG00000183621 | HGLibA_55992 | 72 | -0.483428801 | ZNF438 | zinc finger protein 438 [Source:HGNC Symbol;Acc:HGNC:21029] |
| ENSG00000184524 | HGLibA_09034 | 72 | -0.483428801 | CEND1 | cell cycle exit and neuronal differentiation 1 [Source:HGNC Symbol;Acc:HGNC:24153] |
| ENSG00000186952 | HGLibA_50262 | 72 | -0.483428801 | TMEM232 | transmembrane protein 232 [Source:HGNC Symbol;Acc:HGNC:37270] |
| ENSG00000188060 | HGLibA_39946 | 72 | -0.483428801 | RAB42 | member RAS oncogene family [Source:HGNC Symbol;Acc:HGNC:28702]" |
| ENSG00000189043 | HGLibA_31457 | 72 | -0.483428801 | NDUFA4 | mitochondrial complex associated [Source:HGNC Symbol;Acc:HGNC:7687]" |
| ENSG00000196182 | HGLibA_47513 | 72 | -0.483428801 | STK40 | serine/threonine kinase 40 [Source:HGNC Symbol;Acc:HGNC:21373] |
| ENSG00000196511 | HGLibA_51137 | 72 | -0.483428801 | TPK1 | thiamin pyrophosphokinase 1 [Source:HGNC Symbol;Acc:HGNC:17358] |
| ENSG00000196576 | HGLibA_37272 | 72 | -0.483428801 | PLXNB2 | plexin B2 [Source:HGNC Symbol;Acc:HGNC:9104] |
| ENSG00000196586 | HGLibA_30858 | 72 | -0.483428801 | MYO6 | myosin VI [Source:HGNC Symbol;Acc:HGNC:7605] |
| ENSG00000196954 | HGLibA_07313 | 72 | -0.483428801 | CASP4 | caspase 4 [Source:HGNC Symbol;Acc:HGNC:1505] |
| ENSG00000197093 | HGLibA_18505 | 72 | -0.483428801 | GAL3ST4 | galactose-3-O-sulfotransferase 4 [Source:HGNC Symbol;Acc:HGNC:24145] |
| ENSG00000197705 | HGLibA_25136 | 72 | -0.483428801 | KLHL14 | kelch like family member 14 [Source:HGNC Symbol;Acc:HGNC:29266] |
| ENSG00000197858 | HGLibA_19720 | 72 | -0.483428801 | GPAA1 | glycosylphosphatidylinositol anchor attachment 1 [Source:HGNC Symbol;Acc:HGNC:4446] |
| ENSG00000198601 | HGLibA_33834 | 72 | -0.483428801 | OR2M2 | olfactory receptor family 2 subfamily M member 2 [Source:HGNC Symbol;Acc:HGNC:8268] |
| ENSG00000198668 | HGLibA_07021 | 72 | -0.483428801 | CALM1 | calmodulin 1 [Source:HGNC Symbol;Acc:HGNC:1442] |
| ENSG00000198742 | HGLibA_45845 | 72 | -0.483428801 | SMURF1 | SMAD specific E3 ubiquitin protein ligase 1 [Source:HGNC Symbol;Acc:HGNC:16807] |
| ENSG00000198951 | HGLibA_31063 | 72 | -0.483428801 | NAGA | alpha-N-acetylgalactosaminidase [Source:HGNC Symbol;Acc:HGNC:7631] |
| ENSG00000203727 | HGLibA_42689 | 72 | -0.483428801 | SAMD5 | sterile alpha motif domain containing 5 [Source:HGNC Symbol;Acc:HGNC:21180] |
| ENSG00000205882 | HGLibA_12905 | 72 | -0.483428801 | DEFB134 | defensin beta 134 [Source:HGNC Symbol;Acc:HGNC:32399] |
| ENSG00000206047 | HGLibA_12792 | 72 | -0.483428801 | DEFA1 | defensin alpha 1 [Source:HGNC Symbol;Acc:HGNC:2761] |
| ENSG00000213199 | HGLibA_03306 | 72 | -0.483428801 | ASIC3 | acid sensing ion channel subunit 3 [Source:HGNC Symbol;Acc:HGNC:101] |
| ENSG00000213853 | HGLibA_15078 | 72 | -0.483428801 | EMP2 | epithelial membrane protein 2 [Source:HGNC Symbol;Acc:HGNC:3334] |
| ENSG00000215041 | HGLibA_31732 | 72 | -0.483428801 | NEURL4 | neuralized E3 ubiquitin protein ligase 4 [Source:HGNC Symbol;Acc:HGNC:34410] |
| ENSG00000226685 | HGLibA_11524 | 72 | -0.483428801 | CT47A12 | member A12 [Source:HGNC Symbol;Acc:HGNC:33292]" |
| ENSG00000228856 | HGLibA_53276 | 72 | -0.483428801 | USP17L30 | ubiquitin specific peptidase 17-like family member 30 [Source:HGNC Symbol;Acc:HGNC:44458] |
| ENSG00000235194 | HGLibA_38109 | 72 | -0.483428801 | PPP1R3E | protein phosphatase 1 regulatory subunit 3E [Source:HGNC Symbol;Acc:HGNC:14943] |
| ENSG00000242220 | HGLibA_48835 | 72 | -0.483428801 | TCP10L | t-complex 10 like [Source:HGNC Symbol;Acc:HGNC:11657] |
| ENSG00000267206 | HGLibA_26181 | 72 | -0.483428801 | LCN6 | lipocalin 6 [Source:HGNC Symbol;Acc:HGNC:17337] |
| ENSG00000272333 | HGLibA_25323 | 72 | -0.483428801 | KMT2B | lysine methyltransferase 2B [Source:HGNC Symbol;Acc:HGNC:15840] |
| ENSG00000285053 | HGLibA_48594 | 72 | -0.483428801 | TBCE | tubulin folding cofactor E [Source:NCBI gene;Acc:6905] |
| FAM160B2 | HGLibA_16212 | 72 | -0.483428801 | FAM160B2 | - |
| FAM92A1 | HGLibA_16796 | 72 | -0.483428801 | FAM92A1 | - |
| GYLTL1B | HGLibA_20694 | 72 | -0.483428801 | GYLTL1B | - |
| HIST1H2AH | HGLibA_21394 | 72 | -0.483428801 | HIST1H2AH | - |
| hsa-let-7d | HGLibA_57118 | 72 | -0.483428801 | hsa-let-7d | - |
| hsa-mir-1205 | HGLibA_57279 | 72 | -0.483428801 | hsa-mir-1205 | - |
| hsa-mir-1276 | HGLibA_57558 | 72 | -0.483428801 | hsa-mir-1276 | - |
| hsa-mir-135b | HGLibA_57801 | 72 | -0.483428801 | hsa-mir-135b | - |
| hsa-mir-204 | HGLibA_58176 | 72 | -0.483428801 | hsa-mir-204 | - |
| hsa-mir-214 | HGLibA_58249 | 72 | -0.483428801 | hsa-mir-214 | - |
| hsa-mir-3158-2 | HGLibA_58706 | 72 | -0.483428801 | hsa-mir-3158-2 | - |
| hsa-mir-3162 | HGLibA_58728 | 72 | -0.483428801 | hsa-mir-3162 | - |
| hsa-mir-3173 | HGLibA_58767 | 72 | -0.483428801 | hsa-mir-3173 | - |
| hsa-mir-3177 | HGLibA_58782 | 72 | -0.483428801 | hsa-mir-3177 | - |
| hsa-mir-34b | HGLibA_59040 | 72 | -0.483428801 | hsa-mir-34b | - |
| hsa-mir-34c | HGLibA_59042 | 72 | -0.483428801 | hsa-mir-34c | - |
| hsa-mir-3529 | HGLibA_59047 | 72 | -0.483428801 | hsa-mir-3529 | - |
| hsa-mir-3658 | HGLibA_59177 | 72 | -0.483428801 | hsa-mir-3658 | - |
| hsa-mir-3689b | HGLibA_59319 | 72 | -0.483428801 | hsa-mir-3689b | - |
| hsa-mir-425 | HGLibA_59709 | 72 | -0.483428801 | hsa-mir-425 | - |
| hsa-mir-4275 | HGLibA_59804 | 72 | -0.483428801 | hsa-mir-4275 | - |
| hsa-mir-4498 | HGLibA_60409 | 72 | -0.483428801 | hsa-mir-4498 | - |
| hsa-mir-4795 | HGLibA_61285 | 72 | -0.483428801 | hsa-mir-4795 | - |
| hsa-mir-5683 | HGLibA_62250 | 72 | -0.483428801 | hsa-mir-5683 | - |
| hsa-mir-6808 | HGLibA_63408 | 72 | -0.483428801 | hsa-mir-6808 | - |
| hsa-mir-6837 | HGLibA_63524 | 72 | -0.483428801 | hsa-mir-6837 | - |
| hsa-mir-6839 | HGLibA_63531 | 72 | -0.483428801 | hsa-mir-6839 | - |
| hsa-mir-6876 | HGLibA_63687 | 72 | -0.483428801 | hsa-mir-6876 | - |
| hsa-mir-7161 | HGLibA_63871 | 72 | -0.483428801 | hsa-mir-7161 | - |
| hsa-mir-7515 | HGLibA_63889 | 72 | -0.483428801 | hsa-mir-7515 | - |
| hsa-mir-759 | HGLibA_63897 | 72 | -0.483428801 | hsa-mir-759 | - |
| hsa-mir-8071-2 | HGLibA_64126 | 72 | -0.483428801 | hsa-mir-8071-2 | - |
| hsa-mir-8077 | HGLibA_64152 | 72 | -0.483428801 | hsa-mir-8077 | - |
| hsa-mir-940 | HGLibA_64334 | 72 | -0.483428801 | hsa-mir-940 | - |
| KIAA1033 | HGLibA_24686 | 72 | -0.483428801 | KIAA1033 | - |
| KIAA1704 | HGLibA_24806 | 72 | -0.483428801 | KIAA1704 | - |
| LECT1 | HGLibA_26264 | 72 | -0.483428801 | LECT1 | - |
| LOC286238 | HGLibA_26917 | 72 | -0.483428801 | LOC286238 | - |
| LOC646498 | HGLibA_26975 | 72 | -0.483428801 | LOC646498 | - |
| MKI67IP | HGLibA_29328 | 72 | -0.483428801 | MKI67IP | - |
| MLF1IP | HGLibA_29375 | 72 | -0.483428801 | MLF1IP | - |
| SLC35E2 | HGLibA_44989 | 72 | -0.483428801 | SLC35E2 | - |
| VIMP | HGLibA_53659 | 72 | -0.483428801 | VIMP | - |
| C17orf74 | HGLibA_05570 | 71 | -0.492474144 | C17orf74 | - |
| CCDC58 | HGLibA_07804 | 71 | -0.492474144 | CCDC58 | - |
| ELTD1 | HGLibA_14996 | 71 | -0.492474144 | ELTD1 | - |
| ENSG00000001630 | HGLibA_12209 | 71 | -0.492474144 | CYP51A1 | cytochrome P450 family 51 subfamily A member 1 [Source:HGNC Symbol;Acc:HGNC:2649] |
| ENSG00000002933 | HGLibA_50071 | 71 | -0.492474144 | TMEM176A | transmembrane protein 176A [Source:HGNC Symbol;Acc:HGNC:24930] |
| ENSG00000006283 | HGLibA_06893 | 71 | -0.492474144 | CACNA1G | calcium voltage-gated channel subunit alpha1 G [Source:HGNC Symbol;Acc:HGNC:1394] |
| ENSG00000021574 | HGLibA_46423 | 71 | -0.492474144 | SPAST | spastin [Source:HGNC Symbol;Acc:HGNC:11233] |
| ENSG00000023608 | HGLibA_45891 | 71 | -0.492474144 | SNAPC1 | small nuclear RNA activating complex polypeptide 1 [Source:HGNC Symbol;Acc:HGNC:11134] |
| ENSG00000052802 | HGLibA_30227 | 71 | -0.492474144 | MSMO1 | methylsterol monooxygenase 1 [Source:HGNC Symbol;Acc:HGNC:10545] |
| ENSG00000058091 | HGLibA_08736 | 71 | -0.492474144 | CDK14 | cyclin dependent kinase 14 [Source:HGNC Symbol;Acc:HGNC:8883] |
| ENSG00000063127 | HGLibA_45308 | 71 | -0.492474144 | SLC6A16 | solute carrier family 6 member 16 [Source:HGNC Symbol;Acc:HGNC:13622] |
| ENSG00000063241 | HGLibA_23686 | 71 | -0.492474144 | ISOC2 | isochorismatase domain containing 2 [Source:HGNC Symbol;Acc:HGNC:26278] |
| ENSG00000064787 | HGLibA_04251 | 71 | -0.492474144 | BCAS1 | breast carcinoma amplified sequence 1 [Source:HGNC Symbol;Acc:HGNC:974] |
| ENSG00000071575 | HGLibA_51392 | 71 | -0.492474144 | TRIB2 | tribbles pseudokinase 2 [Source:HGNC Symbol;Acc:HGNC:30809] |
| ENSG00000074527 | HGLibA_32858 | 71 | -0.492474144 | NTN4 | netrin 4 [Source:HGNC Symbol;Acc:HGNC:13658] |
| ENSG00000080839 | HGLibA_40466 | 71 | -0.492474144 | RBL1 | RB transcriptional corepressor like 1 [Source:HGNC Symbol;Acc:HGNC:9893] |
| ENSG00000081665 | HGLibA_56118 | 71 | -0.492474144 | ZNF506 | zinc finger protein 506 [Source:HGNC Symbol;Acc:HGNC:23780] |
| ENSG00000083720 | HGLibA_34814 | 71 | -0.492474144 | OXCT1 | 3-oxoacid CoA-transferase 1 [Source:HGNC Symbol;Acc:HGNC:8527] |
| ENSG00000089280 | HGLibA_18175 | 71 | -0.492474144 | FUS | FUS RNA binding protein [Source:HGNC Symbol;Acc:HGNC:4010] |
| ENSG00000089723 | HGLibA_34755 | 71 | -0.492474144 | OTUB2 | ubiquitin aldehyde binding 2 [Source:HGNC Symbol;Acc:HGNC:20351]" |
| ENSG00000090565 | HGLibA_39800 | 71 | -0.492474144 | RAB11FIP3 | RAB11 family interacting protein 3 [Source:HGNC Symbol;Acc:HGNC:17224] |
| ENSG00000095380 | HGLibA_31108 | 71 | -0.492474144 | NANS | N-acetylneuraminate synthase [Source:HGNC Symbol;Acc:HGNC:19237] |
| ENSG00000095539 | HGLibA_43354 | 71 | -0.492474144 | SEMA4G | semaphorin 4G [Source:HGNC Symbol;Acc:HGNC:10735] |
| ENSG00000099968 | HGLibA_04315 | 71 | -0.492474144 | BCL2L13 | BCL2 like 13 [Source:HGNC Symbol;Acc:HGNC:17164] |
| ENSG00000099995 | HGLibA_43690 | 71 | -0.492474144 | SF3A1 | splicing factor 3a subunit 1 [Source:HGNC Symbol;Acc:HGNC:10765] |
| ENSG00000100055 | HGLibA_12243 | 71 | -0.492474144 | CYTH4 | cytohesin 4 [Source:HGNC Symbol;Acc:HGNC:9505] |
| ENSG00000100055 | HGLibA_12244 | 71 | -0.492474144 | CYTH4 | cytohesin 4 [Source:HGNC Symbol;Acc:HGNC:9505] |
| ENSG00000101440 | HGLibA_03315 | 71 | -0.492474144 | ASIP | agouti signaling protein [Source:HGNC Symbol;Acc:HGNC:745] |
| ENSG00000102021 | HGLibA_27643 | 71 | -0.492474144 | LUZP4 | leucine zipper protein 4 [Source:HGNC Symbol;Acc:HGNC:24971] |
| ENSG00000103051 | HGLibA_10480 | 71 | -0.492474144 | COG4 | component of oligomeric golgi complex 4 [Source:HGNC Symbol;Acc:HGNC:18620] |
| ENSG00000103064 | HGLibA_45373 | 71 | -0.492474144 | SLC7A6 | solute carrier family 7 member 6 [Source:HGNC Symbol;Acc:HGNC:11064] |
| ENSG00000103326 | HGLibA_07159 | 71 | -0.492474144 | CAPN15 | calpain 15 [Source:HGNC Symbol;Acc:HGNC:11182] |
| ENSG00000104341 | HGLibA_26020 | 71 | -0.492474144 | LAPTM4B | lysosomal protein transmembrane 4 beta [Source:HGNC Symbol;Acc:HGNC:13646] |
| ENSG00000104728 | HGLibA_02808 | 71 | -0.492474144 | ARHGEF10 | Rho guanine nucleotide exchange factor 10 [Source:HGNC Symbol;Acc:HGNC:14103] |
| ENSG00000104760 | HGLibA_17462 | 71 | -0.492474144 | FGL1 | fibrinogen like 1 [Source:HGNC Symbol;Acc:HGNC:3695] |
| ENSG00000105707 | HGLibA_22036 | 71 | -0.492474144 | HPN | hepsin [Source:HGNC Symbol;Acc:HGNC:5155] |
| ENSG00000106052 | HGLibA_48448 | 71 | -0.492474144 | TAX1BP1 | Tax1 binding protein 1 [Source:HGNC Symbol;Acc:HGNC:11575] |
| ENSG00000106483 | HGLibA_43743 | 71 | -0.492474144 | SFRP4 | secreted frizzled related protein 4 [Source:HGNC Symbol;Acc:HGNC:10778] |
| ENSG00000107020 | HGLibA_37168 | 71 | -0.492474144 | PLGRKT | plasminogen receptor with a C-terminal lysine [Source:HGNC Symbol;Acc:HGNC:23633] |
| ENSG00000107362 | HGLibA_00265 | 71 | -0.492474144 | ABHD17B | abhydrolase domain containing 17B [Source:HGNC Symbol;Acc:HGNC:24278] |
| ENSG00000108825 | HGLibA_39338 | 71 | -0.492474144 | PTGES3L-AARSD1 | PTGES3L-AARSD1 readthrough [Source:HGNC Symbol;Acc:HGNC:43946] |
| ENSG00000109118 | HGLibA_36338 | 71 | -0.492474144 | PHF12 | PHD finger protein 12 [Source:HGNC Symbol;Acc:HGNC:20816] |
| ENSG00000109475 | HGLibA_41944 | 71 | -0.492474144 | RPL34 | ribosomal protein L34 [Source:HGNC Symbol;Acc:HGNC:10340] |
| ENSG00000109684 | HGLibA_10087 | 71 | -0.492474144 | CLNK | cytokine dependent hematopoietic cell linker [Source:HGNC Symbol;Acc:HGNC:17438] |
| ENSG00000110958 | HGLibA_39331 | 71 | -0.492474144 | PTGES3 | prostaglandin E synthase 3 [Source:HGNC Symbol;Acc:HGNC:16049] |
| ENSG00000111912 | HGLibA_31354 | 71 | -0.492474144 | NCOA7 | nuclear receptor coactivator 7 [Source:HGNC Symbol;Acc:HGNC:21081] |
| ENSG00000112038 | HGLibA_33452 | 71 | -0.492474144 | OPRM1 | opioid receptor mu 1 [Source:HGNC Symbol;Acc:HGNC:8156] |
| ENSG00000112559 | HGLibA_28671 | 71 | -0.492474144 | MDFI | MyoD family inhibitor [Source:HGNC Symbol;Acc:HGNC:6967] |
| ENSG00000112818 | HGLibA_28896 | 71 | -0.492474144 | MEP1A | meprin A subunit alpha [Source:HGNC Symbol;Acc:HGNC:7015] |

| | | | | | |
|---|---|---|---|---|---|
| ENSG00000113494 | HGLibA_38604 | 71 | -0.492474144 | PRLR | prolactin receptor [Source:HGNC Symbol;Acc:HGNC:9446] |
| ENSG00000113810 | HGLibA_45652 | 71 | -0.492474144 | SMC4 | structural maintenance of chromosomes 4 [Source:HGNC Symbol;Acc:HGNC:14013] |
| ENSG00000114544 | HGLibA_45146 | 71 | -0.492474144 | SLC41A3 | solute carrier family 41 member 3 [Source:HGNC Symbol;Acc:HGNC:31046] |
| ENSG00000114739 | HGLibA_00699 | 71 | -0.492474144 | ACVR2B | activin A receptor type 2B [Source:HGNC Symbol;Acc:HGNC:174] |
| ENSG00000114993 | HGLibA_42390 | 71 | -0.492474144 | RTKN | rhotekin [Source:HGNC Symbol;Acc:HGNC:10466] |
| ENSG00000118473 | HGLibA_43813 | 71 | -0.492474144 | SGIP1 | SH3 domain GRB2 like endophilin interacting protein 1 [Source:HGNC Symbol;Acc:HGNC:25412] |
| ENSG00000118733 | HGLibA_33350 | 71 | -0.492474144 | OLFM3 | olfactomedin 3 [Source:HGNC Symbol;Acc:HGNC:17990] |
| ENSG00000118898 | HGLibA_37959 | 71 | -0.492474144 | PPL | periplakin [Source:HGNC Symbol;Acc:HGNC:9273] |
| ENSG00000119321 | HGLibA_17562 | 71 | -0.492474144 | FKBP15 | FK506 binding protein 15 [Source:HGNC Symbol;Acc:HGNC:23397] |
| ENSG00000120549 | HGLibA_24721 | 71 | -0.492474144 | KIAA1217 | KIAA1217 [Source:HGNC Symbol;Acc:HGNC:25428] |
| ENSG00000120875 | HGLibA_14180 | 71 | -0.492474144 | DUSP4 | dual specificity phosphatase 4 [Source:HGNC Symbol;Acc:HGNC:3070] |
| ENSG00000121039 | HGLibA_40732 | 71 | -0.492474144 | RDH10 | retinol dehydrogenase 10 [Source:HGNC Symbol;Acc:HGNC:19975] |
| ENSG00000123358 | HGLibA_32625 | 71 | -0.492474144 | NR4A1 | nuclear receptor subfamily 4 group A member 1 [Source:HGNC Symbol;Acc:HGNC:7980] |
| ENSG00000124243 | HGLibA_04259 | 71 | -0.492474144 | BCAS4 | breast carcinoma amplified sequence 4 [Source:HGNC Symbol;Acc:HGNC:14367] |
| ENSG00000124766 | HGLibA_46265 | 71 | -0.492474144 | SOX4 | SRY-box 4 [Source:HGNC Symbol;Acc:HGNC:11200] |
| ENSG00000124882 | HGLibA_15459 | 71 | -0.492474144 | EREG | epiregulin [Source:HGNC Symbol;Acc:HGNC:3443] |
| ENSG00000125492 | HGLibA_04159 | 71 | -0.492474144 | BARHL1 | BarH like homeobox 1 [Source:HGNC Symbol;Acc:HGNC:953] |
| ENSG00000129646 | HGLibA_39744 | 71 | -0.492474144 | QRICH2 | glutamine rich 2 [Source:HGNC Symbol;Acc:HGNC:25326] |
| ENSG00000131018 | HGLibA_47975 | 71 | -0.492474144 | SYNE1 | spectrin repeat containing nuclear envelope protein 1 [Source:HGNC Symbol;Acc:HGNC:17089] |
| ENSG00000131981 | HGLibA_26363 | 71 | -0.492474144 | LGALS3 | galectin 3 [Source:HGNC Symbol;Acc:HGNC:6563] |
| ENSG00000132010 | HGLibA_55566 | 71 | -0.492474144 | ZNF20 | zinc finger protein 20 [Source:HGNC Symbol;Acc:HGNC:12992] |
| ENSG00000132698 | HGLibA_39850 | 71 | -0.492474144 | RAB25 | member RAS oncogene family [Source:HGNC Symbol;Acc:HGNC:18238]" |
| ENSG00000132846 | HGLibA_54796 | 71 | -0.492474144 | ZBED3 | zinc finger BED-type containing 3 [Source:HGNC Symbol;Acc:HGNC:20711] |
| ENSG00000134070 | HGLibA_23559 | 71 | -0.492474144 | IRAK2 | interleukin 1 receptor associated kinase 2 [Source:HGNC Symbol;Acc:HGNC:6113] |
| ENSG00000134864 | HGLibA_18990 | 71 | -0.492474144 | GGACT | gamma-glutamylamine cyclotransferase [Source:HGNC Symbol;Acc:HGNC:25100] |
| ENSG00000134917 | HGLibA_00842 | 71 | -0.492474144 | ADAMTS8 | ADAM metallopeptidase with thrombospondin type 1 motif 8 [Source:HGNC Symbol;Acc:HGNC:224] |
| ENSG00000135486 | HGLibA_21793 | 71 | -0.492474144 | HNRNPA1 | heterogeneous nuclear ribonucleoprotein A1 [Source:HGNC Symbol;Acc:HGNC:5031] |
| ENSG00000135709 | HGLibA_24641 | 71 | -0.492474144 | KIAA0513 | KIAA0513 [Source:HGNC Symbol;Acc:HGNC:29058] |
| ENSG00000135837 | HGLibA_09135 | 71 | -0.492474144 | CEP350 | centrosomal protein 350 [Source:HGNC Symbol;Acc:HGNC:24238] |
| ENSG00000136897 | HGLibA_30007 | 71 | -0.492474144 | MRPL50 | mitochondrial ribosomal protein L50 [Source:HGNC Symbol;Acc:HGNC:16654] |
| ENSG00000137288 | HGLibA_53101 | 71 | -0.492474144 | UQCC2 | ubiquinol-cytochrome c reductase complex assembly factor 2 [Source:HGNC Symbol;Acc:HGNC:21237] |
| ENSG00000138669 | HGLibA_38580 | 71 | -0.492474144 | PRKG2 | protein kinase cGMP-dependent 2 [Source:HGNC Symbol;Acc:HGNC:9416] |
| ENSG00000139151 | HGLibA_37033 | 71 | -0.492474144 | PLCZ1 | phospholipase C zeta 1 [Source:HGNC Symbol;Acc:HGNC:19218] |
| ENSG00000139437 | HGLibA_48810 | 71 | -0.492474144 | TCHP | trichoplein keratin filament binding [Source:HGNC Symbol;Acc:HGNC:28135] |
| ENSG00000140511 | HGLibA_20847 | 71 | -0.492474144 | HAPLN3 | hyaluronan and proteoglycan link protein 3 [Source:HGNC Symbol;Acc:HGNC:21446] |
| ENSG00000141293 | HGLibA_44271 | 71 | -0.492474144 | SKAP1 | src kinase associated phosphoprotein 1 [Source:HGNC Symbol;Acc:HGNC:15605] |
| ENSG00000143341 | HGLibA_21675 | 71 | -0.492474144 | HMCN1 | hemicentin 1 [Source:HGNC Symbol;Acc:HGNC:19194] |
| ENSG00000145741 | HGLibA_04935 | 71 | -0.492474144 | BTF3 | basic transcription factor 3 [Source:HGNC Symbol;Acc:HGNC:1125] |
| ENSG00000146352 | HGLibA_10169 | 71 | -0.492474144 | CLVS2 | clavesin 2 [Source:HGNC Symbol;Acc:HGNC:23046] |
| ENSG00000148229 | HGLibA_37515 | 71 | -0.492474144 | POLE3 | accessory subunit [Source:HGNC Symbol;Acc:HGNC:13546]" |
| ENSG00000148942 | HGLibA_45263 | 71 | -0.492474144 | SLC5A12 | solute carrier family 5 member 12 [Source:HGNC Symbol;Acc:HGNC:28750] |
| ENSG00000149639 | HGLibA_46153 | 71 | -0.492474144 | SOGA1 | autophagy associated 1 [Source:HGNC Symbol;Acc:HGNC:16111]" |
| ENSG00000150054 | HGLibA_29765 | 71 | -0.492474144 | MPP7 | membrane palmitoylated protein 7 [Source:HGNC Symbol;Acc:HGNC:26542] |
| ENSG00000150995 | HGLibA_23854 | 71 | -0.492474144 | ITPR1 | 5-trisphosphate receptor type 1 [Source:HGNC Symbol;Acc:HGNC:6180]" |
| ENSG00000151838 | HGLibA_07705 | 71 | -0.492474144 | CCDC175 | coiled-coil domain containing 175 [Source:HGNC Symbol;Acc:HGNC:19847] |
| ENSG00000152944 | HGLibA_28776 | 71 | -0.492474144 | MED21 | mediator complex subunit 21 [Source:HGNC Symbol;Acc:HGNC:11473] |
| ENSG00000155052 | HGLibA_10434 | 71 | -0.492474144 | CNTNAP5 | contactin associated protein like 5 [Source:HGNC Symbol;Acc:HGNC:18748] |
| ENSG00000157014 | HGLibA_48443 | 71 | -0.492474144 | TATDN2 | TatD DNase domain containing 2 [Source:HGNC Symbol;Acc:HGNC:28988] |
| ENSG00000157445 | HGLibA_06910 | 71 | -0.492474144 | CACNA2D3 | calcium voltage-gated channel auxiliary subunit alpha2delta 3 [Source:HGNC Symbol;Acc:HGNC:15460] |
| ENSG00000159256 | HGLibA_29654 | 71 | -0.492474144 | MORC3 | MORC family CW-type zinc finger 3 [Source:HGNC Symbol;Acc:HGNC:23572] |
| ENSG00000159387 | HGLibA_23643 | 71 | -0.492474144 | IRX6 | iroquois homeobox 6 [Source:HGNC Symbol;Acc:HGNC:14675] |
| ENSG00000160058 | HGLibA_04864 | 71 | -0.492474144 | BSDC1 | BSD domain containing 1 [Source:HGNC Symbol;Acc:HGNC:25501] |
| ENSG00000160408 | HGLibA_47280 | 71 | -0.492474144 | ST6GALNAC6 | 6-sialyltransferase 6 [Source:HGNC Symbol;Acc:HGNC:23364]" |
| ENSG00000160917 | HGLibA_11007 | 71 | -0.492474144 | CPSF4 | cleavage and polyadenylation specific factor 4 [Source:HGNC Symbol;Acc:HGNC:2327] |
| ENSG00000162598 | HGLibA_05903 | 71 | -0.492474144 | C1orf87 | chromosome 1 open reading frame 87 [Source:HGNC Symbol;Acc:HGNC:28547] |
| ENSG00000162881 | HGLibA_34818 | 71 | -0.492474144 | OXER1 | oxoeicosanoid receptor 1 [Source:HGNC Symbol;Acc:HGNC:24884] |
| ENSG00000163347 | HGLibA_09848 | 71 | -0.492474144 | CLDN1 | claudin 1 [Source:HGNC Symbol;Acc:HGNC:2032] |
| ENSG00000163565 | HGLibA_22593 | 71 | -0.492474144 | IFI16 | interferon gamma inducible protein 16 [Source:HGNC Symbol;Acc:HGNC:5395] |
| ENSG00000163630 | HGLibA_48026 | 71 | -0.492474144 | SYNPR | synaptoporin [Source:HGNC Symbol;Acc:HGNC:16507] |
| ENSG00000163820 | HGLibA_18251 | 71 | -0.492474144 | FYCO1 | FYVE and coiled-coil domain containing 1 [Source:HGNC Symbol;Acc:HGNC:14673] |
| ENSG00000163950 | HGLibA_44326 | 71 | -0.492474144 | SLBP | stem-loop binding protein [Source:HGNC Symbol;Acc:HGNC:10904] |
| ENSG00000165028 | HGLibA_31976 | 71 | -0.492474144 | NIPSNAP3B | nipsnap homolog 3B [Source:HGNC Symbol;Acc:HGNC:23641] |
| ENSG00000165269 | HGLibA_02606 | 71 | -0.492474144 | AQP7 | aquaporin 7 [Source:HGNC Symbol;Acc:HGNC:640] |
| ENSG00000165282 | HGLibA_36572 | 71 | -0.492474144 | PIGO | phosphatidylinositol glycan anchor biosynthesis class O [Source:HGNC Symbol;Acc:HGNC:23215] |
| ENSG00000166394 | HGLibA_12005 | 71 | -0.492474144 | CYB5R2 | cytochrome b5 reductase 2 [Source:HGNC Symbol;Acc:HGNC:24376] |
| ENSG00000166847 | HGLibA_12555 | 71 | -0.492474144 | DCTN5 | dynactin subunit 5 [Source:HGNC Symbol;Acc:HGNC:24594] |
| ENSG00000166965 | HGLibA_40688 | 71 | -0.492474144 | RCCD1 | RCC1 domain containing 1 [Source:HGNC Symbol;Acc:HGNC:30457] |
| ENSG00000167822 | HGLibA_34542 | 71 | -0.492474144 | OR8J3 | olfactory receptor family 8 subfamily J member 3 [Source:HGNC Symbol;Acc:HGNC:15312] |
| ENSG00000167971 | HGLibA_07290 | 71 | -0.492474144 | CASKIN1 | CASK interacting protein 1 [Source:HGNC Symbol;Acc:HGNC:20879] |
| ENSG00000167987 | HGLibA_53781 | 71 | -0.492474144 | VPS37C | ESCRT-I subunit [Source:HGNC Symbol;Acc:HGNC:26097]" |
| ENSG00000168016 | HGLibA_51292 | 71 | -0.492474144 | TRANK1 | tetratricopeptide repeat and ankyrin repeat containing 1 [Source:HGNC Symbol;Acc:HGNC:29011] |
| ENSG00000168843 | HGLibA_18109 | 71 | -0.492474144 | FSTL5 | follistatin like 5 [Source:HGNC Symbol;Acc:HGNC:21386] |
| ENSG00000169021 | HGLibA_53118 | 71 | -0.492474144 | UQCRFS1 | Rieske iron-sulfur polypeptide 1 [Source:HGNC Symbol;Acc:HGNC:12587]" |
| ENSG00000170788 | HGLibA_14219 | 71 | -0.492474144 | DYDC1 | DPY30 domain containing 1 [Source:HGNC Symbol;Acc:HGNC:23460] |
| ENSG00000171155 | HGLibA_05725 | 71 | -0.492474144 | C1GALT1C1 | C1GALT1 specific chaperone 1 [Source:HGNC Symbol;Acc:HGNC:24338] |
| ENSG00000171236 | HGLibA_27168 | 71 | -0.492474144 | LRG1 | leucine rich alpha-2-glycoprotein 1 [Source:HGNC Symbol;Acc:HGNC:29480] |
| ENSG00000171643 | HGLibA_42591 | 71 | -0.492474144 | S100Z | S100 calcium binding protein Z [Source:HGNC Symbol;Acc:HGNC:30367] |
| ENSG00000171681 | HGLibA_03455 | 71 | -0.492474144 | ATF7IP | activating transcription factor 7 interacting protein [Source:HGNC Symbol;Acc:HGNC:20092] |
| ENSG00000172922 | HGLibA_41433 | 71 | -0.492474144 | RNASEH2C | ribonuclease H2 subunit C [Source:HGNC Symbol;Acc:HGNC:24116] |
| ENSG00000173482 | HGLibA_39518 | 71 | -0.492474144 | PTPRM | receptor type M [Source:HGNC Symbol;Acc:HGNC:9675]" |
| ENSG00000173818 | HGLibA_15123 | 71 | -0.492474144 | ENDOV | endonuclease V [Source:HGNC Symbol;Acc:HGNC:26640] |
| ENSG00000173898 | HGLibA_46884 | 71 | -0.492474144 | SPTBN2 | non-erythrocytic 2 [Source:HGNC Symbol;Acc:HGNC:11276]" |
| ENSG00000173947 | HGLibA_36540 | 71 | -0.492474144 | PIFO | primary cilia formation [Source:HGNC Symbol;Acc:HGNC:27009] |
| ENSG00000174010 | HGLibA_25139 | 71 | -0.492474144 | KLHL15 | kelch like family member 15 [Source:HGNC Symbol;Acc:HGNC:29347] |
| ENSG00000174325 | HGLibA_13265 | 71 | -0.492474144 | DIRC1 | disrupted in renal carcinoma 1 [Source:HGNC Symbol;Acc:HGNC:15760] |
| ENSG00000174564 | HGLibA_23080 | 71 | -0.492474144 | IL20RB | interleukin 20 receptor subunit beta [Source:HGNC Symbol;Acc:HGNC:6004] |
| ENSG00000174946 | HGLibA_19965 | 71 | -0.492474144 | GPR171 | G protein-coupled receptor 171 [Source:HGNC Symbol;Acc:HGNC:30057] |
| ENSG00000176884 | HGLibA_20297 | 71 | -0.492474144 | GRIN1 | glutamate ionotropic receptor NMDA type subunit 1 [Source:HGNC Symbol;Acc:HGNC:4584] |
| ENSG00000177291 | HGLibA_19194 | 71 | -0.492474144 | GJD4 | gap junction protein delta 4 [Source:HGNC Symbol;Acc:HGNC:23296] |
| ENSG00000177324 | HGLibA_04401 | 71 | -0.492474144 | BEND2 | BEN domain containing 2 [Source:HGNC Symbol;Acc:HGNC:28509] |
| ENSG00000177426 | HGLibA_49237 | 71 | -0.492474144 | TGIF1 | TGFB induced factor homeobox 1 [Source:HGNC Symbol;Acc:HGNC:11776] |
| ENSG00000177873 | HGLibA_56400 | 71 | -0.492474144 | ZNF619 | zinc finger protein 619 [Source:HGNC Symbol;Acc:HGNC:26910] |
| ENSG00000178690 | HGLibA_14230 | 71 | -0.492474144 | DYNAP | dynactin associated protein [Source:HGNC Symbol;Acc:HGNC:26808] |
| ENSG00000179363 | HGLibA_50366 | 71 | -0.492474144 | TMEM31 | transmembrane protein 31 [Source:HGNC Symbol;Acc:HGNC:28601] |
| ENSG00000180801 | HGLibA_03152 | 71 | -0.492474144 | ARSJ | arylsulfatase family member J [Source:HGNC Symbol;Acc:HGNC:26286] |
| ENSG00000181019 | HGLibA_32561 | 71 | -0.492474144 | NQO1 | NAD(P)H quinone dehydrogenase 1 [Source:HGNC Symbol;Acc:HGNC:2874] |
| ENSG00000181785 | HGLibA_34249 | 71 | -0.492474144 | OR5AS1 | olfactory receptor family 5 subfamily AS member 1 [Source:HGNC Symbol;Acc:HGNC:15261] |
| ENSG00000182053 | HGLibA_51524 | 71 | -0.492474144 | TRIM49B | tripartite motif containing 49B [Source:HGNC Symbol;Acc:HGNC:42955] |
| ENSG00000182541 | HGLibA_26543 | 71 | -0.492474144 | LIMK2 | LIM domain kinase 2 [Source:HGNC Symbol;Acc:HGNC:6614] |
| ENSG00000182544 | HGLibA_29104 | 71 | -0.492474144 | MFSD5 | major facilitator superfamily domain containing 5 [Source:HGNC Symbol;Acc:HGNC:28156] |
| ENSG00000182809 | HGLibA_11153 | 71 | -0.492474144 | CRIP2 | cysteine rich protein 2 [Source:HGNC Symbol;Acc:HGNC:2361] |
| ENSG00000185885 | HGLibA_22639 | 71 | -0.492474144 | IFITM1 | interferon induced transmembrane protein 1 [Source:HGNC Symbol;Acc:HGNC:5412] |
| ENSG00000185933 | HGLibA_07013 | 71 | -0.492474144 | CALHM1 | calcium homeostasis modulator 1 [Source:HGNC Symbol;Acc:HGNC:23494] |
| ENSG00000187730 | HGLibA_18384 | 71 | -0.492474144 | GABRD | gamma-aminobutyric acid type A receptor delta subunit [Source:HGNC Symbol;Acc:HGNC:4084] |
| ENSG00000187792 | HGLibA_56571 | 71 | -0.492474144 | ZNF70 | zinc finger protein 70 [Source:HGNC Symbol;Acc:HGNC:13140] |
| ENSG00000187833 | HGLibA_06185 | 71 | -0.492474144 | C2orf78 | chromosome 2 open reading frame 78 [Source:HGNC Symbol;Acc:HGNC:34349] |
| ENSG00000188958 | HGLibA_53464 | 71 | -0.492474144 | UTS2B | urotensin 2B [Source:HGNC Symbol;Acc:HGNC:30894] |
| ENSG00000189108 | HGLibA_23057 | 71 | -0.492474144 | IL1RAPL2 | interleukin 1 receptor accessory protein like 2 [Source:HGNC Symbol;Acc:HGNC:5997] |
| ENSG00000196090 | HGLibA_39537 | 71 | -0.492474144 | PTPRT | receptor type T [Source:HGNC Symbol;Acc:HGNC:9682]" |
| ENSG00000196131 | HGLibA_53701 | 71 | -0.492474144 | VN1R2 | vomeronasal 1 receptor 2 [Source:HGNC Symbol;Acc:HGNC:19872] |
| ENSG00000196358 | HGLibA_32868 | 71 | -0.492474144 | NTNG2 | netrin G2 [Source:HGNC Symbol;Acc:HGNC:14288] |
| ENSG00000196365 | HGLibA_27015 | 71 | -0.492474144 | LONP1 | mitochondrial [Source:HGNC Symbol;Acc:HGNC:9479]" |
| ENSG00000196418 | HGLibA_55455 | 71 | -0.492474144 | ZNF124 | zinc finger protein 124 [Source:HGNC Symbol;Acc:HGNC:12907] |
| ENSG00000196834 | HGLibA_37741 | 71 | -0.492474144 | POTEI | POTE ankyrin domain family member I [Source:HGNC Symbol;Acc:HGNC:37093] |
| ENSG00000198369 | HGLibA_46793 | 71 | -0.492474144 | SPRED2 | sprouty related EVH1 domain containing 2 [Source:HGNC Symbol;Acc:HGNC:17722] |
| ENSG00000198853 | HGLibA_42471 | 71 | -0.492474144 | RUSC2 | RUN and SH3 domain containing 2 [Source:HGNC Symbol;Acc:HGNC:23625] |
| ENSG00000204852 | HGLibA_48871 | 71 | -0.492474144 | TCTN1 | tectonic family member 1 [Source:HGNC Symbol;Acc:HGNC:26113] |
| ENSG00000206560 | HGLibA_02055 | 71 | -0.492474144 | ANKRD28 | ankyrin repeat domain 28 [Source:HGNC Symbol;Acc:HGNC:29024] |
| ENSG00000213563 | HGLibA_06651 | 71 | -0.492474144 | C8orf82 | chromosome 8 open reading frame 82 [Source:HGNC Symbol;Acc:HGNC:33826] |
| ENSG00000214642 | HGLibA_12852 | 71 | -0.492474144 | DEFB113 | defensin beta 113 [Source:HGNC Symbol;Acc:HGNC:18094] |
| ENSG00000226023 | HGLibA_11540 | 71 | -0.492474144 | CT47A6 | member A6 [Source:HGNC Symbol;Acc:HGNC:33287]" |
| ENSG00000228607 | HGLibA_09892 | 71 | -0.492474144 | CLDN25 | claudin 25 [Source:HGNC Symbol;Acc:HGNC:37218] |
| ENSG00000235961 | HGLibA_37377 | 71 | -0.492474144 | PNMA6A | PNMA family member 6A [Source:HGNC Symbol;Acc:HGNC:28248] |
| ENSG00000236371 | HGLibA_11515 | 71 | -0.492474144 | CT47A1 | member A1 [Source:HGNC Symbol;Acc:HGNC:33282]" |
| ENSG00000239839 | HGLibA_12798 | 71 | -0.492474144 | DEFA3 | defensin alpha 3 [Source:HGNC Symbol;Acc:HGNC:2762] |
| ENSG00000248871 | HGLibA_50805 | 71 | -0.492474144 | TNFSF12-TNFSF13 | TNFSF12-TNFSF13 readthrough [Source:HGNC Symbol;Acc:HGNC:33537] |
| ENSG00000248920 | HGLibA_53237 | 71 | -0.492474144 | USP17L19 | ubiquitin specific peptidase 17-like family member 19 [Source:HGNC Symbol;Acc:HGNC:44447] |
| ENSG00000250506 | HGLibA_08768 | 71 | -0.492474144 | CDK3 | cyclin dependent kinase 3 [Source:HGNC Symbol;Acc:HGNC:1772] |
| ENSG00000254521 | HGLibA_44133 | 71 | -0.492474144 | SIGLEC12 | sialic acid binding Ig like lectin 12 (gene/pseudogene) [Source:HGNC Symbol;Acc:HGNC:15482] |
| ENSG00000255690 | HGLibA_51398 | 71 | -0.492474144 | TRIL | TLR4 interactor with leucine rich repeats [Source:HGNC Symbol;Acc:HGNC:22200] |
| ENSG00000259224 | HGLibA_45027 | 71 | -0.492474144 | SLC35G6 | solute carrier family 35 member G6 [Source:HGNC Symbol;Acc:HGNC:31351] |
| ENSG00000285053 | HGLibA_48592 | 71 | -0.492474144 | TBCE | tubulin folding cofactor E [Source:NCBI gene;Acc:6905] |
| FAM65C | HGLibA_16674 | 71 | -0.492474144 | FAM65C | - |
| FTSJD1 | HGLibA_18144 | 71 | -0.492474144 | FTSJD1 | - |
| GPR98 | HGLibA_20117 | 71 | -0.492474144 | GPR98 | - |

| | | | | | |
|---|---|---|---|---|---|
| hsa-mir-103b-2 | HGLibA_57174 | 71 | -0.492474144 | hsa-mir-103b-2 | - |
| hsa-mir-1254-2 | HGLibA_57418 | 71 | -0.492474144 | hsa-mir-1254-2 | - |
| hsa-mir-1273c | HGLibA_57537 | 71 | -0.492474144 | hsa-mir-1273c | - |
| hsa-mir-1302-7 | HGLibA_57717 | 71 | -0.492474144 | hsa-mir-1302-7 | - |
| hsa-mir-1539 | HGLibA_57924 | 71 | -0.492474144 | hsa-mir-1539 | - |
| hsa-mir-1825 | HGLibA_57985 | 71 | -0.492474144 | hsa-mir-1825 | - |
| hsa-mir-24-1 | HGLibA_58339 | 71 | -0.492474144 | hsa-mir-24-1 | - |
| hsa-mir-301a | HGLibA_58429 | 71 | -0.492474144 | hsa-mir-301a | - |
| hsa-mir-3161 | HGLibA_58722 | 71 | -0.492474144 | hsa-mir-3161 | - |
| hsa-mir-3179-2 | HGLibA_58795 | 71 | -0.492474144 | hsa-mir-3179-2 | - |
| hsa-mir-3202-1 | HGLibA_58917 | 71 | -0.492474144 | hsa-mir-3202-1 | - |
| hsa-mir-3683 | HGLibA_59284 | 71 | -0.492474144 | hsa-mir-3683 | - |
| hsa-mir-3913-2 | HGLibA_59525 | 71 | -0.492474144 | hsa-mir-3913-2 | - |
| hsa-mir-4294 | HGLibA_59886 | 71 | -0.492474144 | hsa-mir-4294 | - |
| hsa-mir-4432 | HGLibA_60114 | 71 | -0.492474144 | hsa-mir-4432 | - |
| hsa-mir-4701 | HGLibA_60913 | 71 | -0.492474144 | hsa-mir-4701 | - |
| hsa-mir-4722 | HGLibA_60985 | 71 | -0.492474144 | hsa-mir-4722 | - |
| hsa-mir-509-1 | HGLibA_61505 | 71 | -0.492474144 | hsa-mir-509-1 | - |
| hsa-mir-5589 | HGLibA_62194 | 71 | -0.492474144 | hsa-mir-5589 | - |
| hsa-mir-566 | HGLibA_62222 | 71 | -0.492474144 | hsa-mir-566 | - |
| hsa-mir-6508 | HGLibA_62882 | 71 | -0.492474144 | hsa-mir-6508 | - |
| hsa-mir-6734 | HGLibA_63090 | 71 | -0.492474144 | hsa-mir-6734 | - |
| hsa-mir-6747 | HGLibA_63143 | 71 | -0.492474144 | hsa-mir-6747 | - |
| hsa-mir-6817 | HGLibA_63447 | 71 | -0.492474144 | hsa-mir-6817 | - |
| hsa-mir-6832 | HGLibA_63504 | 71 | -0.492474144 | hsa-mir-6832 | - |
| hsa-mir-7157 | HGLibA_63858 | 71 | -0.492474144 | hsa-mir-7157 | - |
| hsa-mir-8057 | HGLibA_64068 | 71 | -0.492474144 | hsa-mir-8057 | - |
| hsa-mir-876 | HGLibA_64214 | 71 | -0.492474144 | hsa-mir-876 | - |
| hsa-mir-887 | HGLibA_64227 | 71 | -0.492474144 | hsa-mir-887 | - |
| KIAA1009 | HGLibA_24676 | 71 | -0.492474144 | KIAA1009 | - |
| KIAA1644 | HGLibA_24796 | 71 | -0.492474144 | KIAA1644 | - |
| LOC100652824 | HGLibA_26874 | 71 | -0.492474144 | LOC100652824 | - |
| LOC100653515 | HGLibA_26875 | 71 | -0.492474144 | LOC100653515 | - |
| LOC100862671 | HGLibA_26878 | 71 | -0.492474144 | LOC100862671 | - |
| LOC440243 | HGLibA_26941 | 71 | -0.492474144 | LOC440243 | - |
| MFI2 | HGLibA_29065 | 71 | -0.492474144 | MFI2 | - |
| MTERFD1 | HGLibA_30339 | 71 | -0.492474144 | MTERFD1 | - |
| NUTMG | HGLibA_33116 | 71 | -0.492474144 | NUTMG | - |
| PCNX | HGLibA_35643 | 71 | -0.492474144 | PCNX | - |
| TCTEX1D2 | HGLibA_48866 | 71 | -0.492474144 | TCTEX1D2 | - |
| C12orf49 | HGLibA_05252 | 70 | -0.501519486 | C12orf49 | - |
| C1orf86 | HGLibA_05899 | 70 | -0.501519486 | C1orf86 | - |
| C9orf3 | HGLibA_06722 | 70 | -0.501519486 | C9orf3 | - |
| CD99_Y | HGLibA_08491 | 70 | -0.501519486 | CD99_Y | - |
| ENSG00000005206 | HGLibA_46777 | 70 | -0.501519486 | SPPL2B | signal peptide peptidase like 2B [Source:HGNC Symbol;Acc:HGNC:30627] |
| ENSG00000007866 | HGLibA_48931 | 70 | -0.501519486 | TEAD3 | TEA domain transcription factor 3 [Source:HGNC Symbol;Acc:HGNC:11716] |
| ENSG00000011052 | HGLibA_32154 | 70 | -0.501519486 | NME1-NME2 | NME1-NME2 readthrough [Source:HGNC Symbol;Acc:HGNC:33531] |
| ENSG00000018280 | HGLibA_44352 | 70 | -0.501519486 | SLC11A1 | solute carrier family 11 member 1 [Source:HGNC Symbol;Acc:HGNC:10907] |
| ENSG00000023608 | HGLibA_45889 | 70 | -0.501519486 | SNAPC1 | small nuclear RNA activating complex polypeptide 1 [Source:HGNC Symbol;Acc:HGNC:11134] |
| ENSG00000024048 | HGLibA_52750 | 70 | -0.501519486 | UBR2 | ubiquitin protein ligase E3 component n-recognin 2 [Source:HGNC Symbol;Acc:HGNC:21289] |
| ENSG00000026025 | HGLibA_53657 | 70 | -0.501519486 | VIM | vimentin [Source:HGNC Symbol;Acc:HGNC:12692] |
| ENSG00000041880 | HGLibA_35235 | 70 | -0.501519486 | PARP3 | poly(ADP-ribose) polymerase family member 3 [Source:HGNC Symbol;Acc:HGNC:273] |
| ENSG00000046651 | HGLibA_33298 | 70 | -0.501519486 | OFD1 | centriole and centriolar satellite protein [Source:HGNC Symbol;Acc:HGNC:2567]" |
| ENSG00000047936 | HGLibA_41783 | 70 | -0.501519486 | ROS1 | receptor tyrosine kinase [Source:HGNC Symbol;Acc:HGNC:10261]" |
| ENSG00000049449 | HGLibA_40702 | 70 | -0.501519486 | RCN1 | reticulocalbin 1 [Source:HGNC Symbol;Acc:HGNC:9934] |
| ENSG00000066739 | HGLibA_03483 | 70 | -0.501519486 | ATG2B | autophagy related 2B [Source:HGNC Symbol;Acc:HGNC:20187] |
| ENSG00000067900 | HGLibA_41746 | 70 | -0.501519486 | ROCK1 | Rho associated coiled-coil containing protein kinase 1 [Source:HGNC Symbol;Acc:HGNC:10251] |
| ENSG00000069667 | HGLibA_41774 | 70 | -0.501519486 | RORA | RAR related orphan receptor A [Source:HGNC Symbol;Acc:HGNC:10258] |
| ENSG00000069966 | HGLibA_19502 | 70 | -0.501519486 | GNB5 | G protein subunit beta 5 [Source:HGNC Symbol;Acc:HGNC:4401] |
| ENSG00000073067 | HGLibA_12149 | 70 | -0.501519486 | CYP2W1 | cytochrome P450 family 2 subfamily W member 1 [Source:HGNC Symbol;Acc:HGNC:20243] |
| ENSG00000083635 | HGLibA_33001 | 70 | -0.501519486 | NUFIP1 | nuclear FMR1 interacting protein 1 [Source:HGNC Symbol;Acc:HGNC:8057] |
| ENSG00000083857 | HGLibA_16914 | 70 | -0.501519486 | FAT1 | FAT atypical cadherin 1 [Source:HGNC Symbol;Acc:HGNC:3595] |
| ENSG00000085433 | HGLibA_54105 | 70 | -0.501519486 | WDR47 | WD repeat domain 47 [Source:HGNC Symbol;Acc:HGNC:29141] |
| ENSG00000087303 | HGLibA_31926 | 70 | -0.501519486 | NID2 | nidogen 2 [Source:HGNC Symbol;Acc:HGNC:13389] |
| ENSG00000091428 | HGLibA_40255 | 70 | -0.501519486 | RAPGEF4 | Rap guanine nucleotide exchange factor 4 [Source:HGNC Symbol;Acc:HGNC:16626] |
| ENSG00000092607 | HGLibA_48646 | 70 | -0.501519486 | TBX15 | T-box 15 [Source:HGNC Symbol;Acc:HGNC:11594] |
| ENSG00000092929 | HGLibA_53003 | 70 | -0.501519486 | UNC13D | unc-13 homolog D [Source:HGNC Symbol;Acc:HGNC:23147] |
| ENSG00000095059 | HGLibA_13115 | 70 | -0.501519486 | DHPS | deoxyhypusine synthase [Source:HGNC Symbol;Acc:HGNC:2869] |
| ENSG00000100124 | HGLibA_02123 | 70 | -0.501519486 | ANKRD54 | ankyrin repeat domain 54 [Source:HGNC Symbol;Acc:HGNC:25185] |
| ENSG00000100368 | HGLibA_11336 | 70 | -0.501519486 | CSF2RB | colony stimulating factor 2 receptor beta common subunit [Source:HGNC Symbol;Acc:HGNC:2436] |
| ENSG00000100490 | HGLibA_08805 | 70 | -0.501519486 | CDKL1 | cyclin dependent kinase like 1 [Source:HGNC Symbol;Acc:HGNC:1781] |
| ENSG00000100888 | HGLibA_09408 | 70 | -0.501519486 | CHD8 | chromodomain helicase DNA binding protein 8 [Source:HGNC Symbol;Acc:HGNC:20153] |
| ENSG00000101213 | HGLibA_39384 | 70 | -0.501519486 | PTK6 | protein tyrosine kinase 6 [Source:HGNC Symbol;Acc:HGNC:9617] |
| ENSG00000101746 | HGLibA_32267 | 70 | -0.501519486 | NOL4 | nucleolar protein 4 [Source:HGNC Symbol;Acc:HGNC:7870] |
| ENSG00000102081 | HGLibA_17719 | 70 | -0.501519486 | FMR1 | fragile X mental retardation 1 [Source:HGNC Symbol;Acc:HGNC:3775] |
| ENSG00000102290 | HGLibA_35391 | 70 | -0.501519486 | PCDH11X | protocadherin 11 X-linked [Source:HGNC Symbol;Acc:HGNC:8656] |
| ENSG00000102390 | HGLibA_35327 | 70 | -0.501519486 | PBDC1 | polysaccharide biosynthesis domain containing 1 [Source:HGNC Symbol;Acc:HGNC:28790] |
| ENSG00000102524 | HGLibA_50810 | 70 | -0.501519486 | TNFSF13B | TNF superfamily member 13b [Source:HGNC Symbol;Acc:HGNC:11929] |
| ENSG00000103723 | HGLibA_02323 | 70 | -0.501519486 | AP3B2 | adaptor related protein complex 3 subunit beta 2 [Source:HGNC Symbol;Acc:HGNC:567] |
| ENSG00000105383 | HGLibA_08355 | 70 | -0.501519486 | CD33 | CD33 molecule [Source:HGNC Symbol;Acc:HGNC:1659] |
| ENSG00000105617 | HGLibA_26297 | 70 | -0.501519486 | LENG1 | leukocyte receptor cluster member 1 [Source:HGNC Symbol;Acc:HGNC:15502] |
| ENSG00000105737 | HGLibA_20293 | 70 | -0.501519486 | GRIK5 | glutamate ionotropic receptor kainate type subunit 5 [Source:HGNC Symbol;Acc:HGNC:4583] |
| ENSG00000107404 | HGLibA_14207 | 70 | -0.501519486 | DVL1 | dishevelled segment polarity protein 1 [Source:HGNC Symbol;Acc:HGNC:3084] |
| ENSG00000110492 | HGLibA_28690 | 70 | -0.501519486 | MDK | midkine [Source:HGNC Symbol;Acc:HGNC:6972] |
| ENSG00000111796 | HGLibA_25282 | 70 | -0.501519486 | KLRB1 | killer cell lectin like receptor B1 [Source:HGNC Symbol;Acc:HGNC:6373] |
| ENSG00000113248 | HGLibA_35488 | 70 | -0.501519486 | PCDHB15 | protocadherin beta 15 [Source:HGNC Symbol;Acc:HGNC:8686] |
| ENSG00000113273 | HGLibA_03131 | 70 | -0.501519486 | ARSB | arylsulfatase B [Source:HGNC Symbol;Acc:HGNC:714] |
| ENSG00000115268 | HGLibA_42076 | 70 | -0.501519486 | RPS15 | ribosomal protein S15 [Source:HGNC Symbol;Acc:HGNC:10388] |
| ENSG00000115295 | HGLibA_10051 | 70 | -0.501519486 | CLIP4 | CAP-Gly domain containing linker protein family member 4 [Source:HGNC Symbol;Acc:HGNC:26108] |
| ENSG00000115607 | HGLibA_23030 | 70 | -0.501519486 | IL18RAP | interleukin 18 receptor accessory protein [Source:HGNC Symbol;Acc:HGNC:5989] |
| ENSG00000116574 | HGLibA_41209 | 70 | -0.501519486 | RHOU | ras homolog family member U [Source:HGNC Symbol;Acc:HGNC:17794] |
| ENSG00000116584 | HGLibA_02833 | 70 | -0.501519486 | ARHGEF2 | Rho/Rac guanine nucleotide exchange factor 2 [Source:HGNC Symbol;Acc:HGNC:682] |
| ENSG00000116748 | HGLibA_01823 | 70 | -0.501519486 | AMPD1 | adenosine monophosphate deaminase 1 [Source:HGNC Symbol;Acc:HGNC:468] |
| ENSG00000117133 | HGLibA_41830 | 70 | -0.501519486 | RPF1 | ribosome production factor 1 homolog [Source:HGNC Symbol;Acc:HGNC:30350] |
| ENSG00000119280 | HGLibA_05820 | 70 | -0.501519486 | C1orf198 | chromosome 1 open reading frame 198 [Source:HGNC Symbol;Acc:HGNC:25900] |
| ENSG00000119669 | HGLibA_23582 | 70 | -0.501519486 | IRF2BPL | interferon regulatory factor 2 binding protein like [Source:HGNC Symbol;Acc:HGNC:14282] |
| ENSG00000119771 | HGLibA_25175 | 70 | -0.501519486 | KLHL29 | kelch like family member 29 [Source:HGNC Symbol;Acc:HGNC:29404] |
| ENSG00000120899 | HGLibA_39382 | 70 | -0.501519486 | PTK2B | protein tyrosine kinase 2 beta [Source:HGNC Symbol;Acc:HGNC:9612] |
| ENSG00000123545 | HGLibA_31490 | 70 | -0.501519486 | NDUFAF4 | NADH:ubiquinone oxidoreductase complex assembly factor 4 [Source:HGNC Symbol;Acc:HGNC:21034] |
| ENSG00000124205 | HGLibA_14474 | 70 | -0.501519486 | EDN3 | endothelin 3 [Source:HGNC Symbol;Acc:HGNC:3178] |
| ENSG00000124343 | HGLibA_54506 | 70 | -0.501519486 | XG | Xg glycoprotein (Xg blood group) [Source:HGNC Symbol;Acc:HGNC:12806] |
| ENSG00000124795 | HGLibA_12926 | 70 | -0.501519486 | DEK | DEK proto-oncogene [Source:HGNC Symbol;Acc:HGNC:2768] |
| ENSG00000124920 | HGLibA_30916 | 70 | -0.501519486 | MYRF | myelin regulatory factor [Source:HGNC Symbol;Acc:HGNC:1181] |
| ENSG00000126653 | HGLibA_32779 | 70 | -0.501519486 | NSRP1 | nuclear speckle splicing regulatory protein 1 [Source:HGNC Symbol;Acc:HGNC:25305] |
| ENSG00000130254 | HGLibA_42637 | 70 | -0.501519486 | SAFB2 | scaffold attachment factor B2 [Source:HGNC Symbol;Acc:HGNC:21605] |
| ENSG00000131844 | HGLibA_28568 | 70 | -0.501519486 | MCCC2 | methylcrotonoyl-CoA carboxylase 2 [Source:HGNC Symbol;Acc:HGNC:6937] |
| ENSG00000132561 | HGLibA_28429 | 70 | -0.501519486 | MATN2 | matrilin 2 [Source:HGNC Symbol;Acc:HGNC:6908] |
| ENSG00000133195 | HGLibA_45095 | 70 | -0.501519486 | SLC39A11 | solute carrier family 39 member 11 [Source:HGNC Symbol;Acc:HGNC:14463] |
| ENSG00000133816 | HGLibA_29203 | 70 | -0.501519486 | MICAL2 | calponin and LIM domain containing 2 [Source:HGNC Symbol;Acc:HGNC:24693]" |
| ENSG00000135549 | HGLibA_36822 | 70 | -0.501519486 | PKIB | cAMP-dependent protein kinase inhibitor beta [Source:HGNC Symbol;Acc:HGNC:9018] |
| ENSG00000136197 | HGLibA_06525 | 70 | -0.501519486 | C7orf25 | chromosome 7 open reading frame 25 [Source:HGNC Symbol;Acc:HGNC:21703] |
| ENSG00000138641 | HGLibA_21160 | 70 | -0.501519486 | HERC3 | HECT and RLD domain containing E3 ubiquitin protein ligase 3 [Source:HGNC Symbol;Acc:HGNC:4876] |
| ENSG00000140157 | HGLibA_31953 | 70 | -0.501519486 | NIPA2 | NIPA magnesium transporter 2 [Source:HGNC Symbol;Acc:HGNC:17044] |
| ENSG00000141002 | HGLibA_48783 | 70 | -0.501519486 | TCF25 | transcription factor 25 [Source:HGNC Symbol;Acc:HGNC:29181] |
| ENSG00000143473 | HGLibA_24211 | 70 | -0.501519486 | KCNH1 | potassium voltage-gated channel subfamily H member 1 [Source:HGNC Symbol;Acc:HGNC:6250] |
| ENSG00000143869 | HGLibA_18875 | 70 | -0.501519486 | GDF7 | growth differentiation factor 7 [Source:HGNC Symbol;Acc:HGNC:4222] |
| ENSG00000144028 | HGLibA_45942 | 70 | -0.501519486 | SNRNP200 | small nuclear ribonucleoprotein U5 subunit 200 [Source:HGNC Symbol;Acc:HGNC:30859] |
| ENSG00000144426 | HGLibA_31210 | 70 | -0.501519486 | NBEAL1 | neurobeachin like 1 [Source:HGNC Symbol;Acc:HGNC:20681] |
| ENSG00000145192 | HGLibA_01320 | 70 | -0.501519486 | AHSG | alpha 2-HS glycoprotein [Source:HGNC Symbol;Acc:HGNC:349] |
| ENSG00000145734 | HGLibA_04390 | 70 | -0.501519486 | BDP1 | subunit of RNA polymerase III transcription initiation factor IIIB [Source:HGNC Symbol;Acc:HGNC:13652]" |
| ENSG00000146373 | HGLibA_41633 | 70 | -0.501519486 | RNF217 | ring finger protein 217 [Source:HGNC Symbol;Acc:HGNC:21487] |
| ENSG00000147789 | HGLibA_56569 | 70 | -0.501519486 | ZNF7 | zinc finger protein 7 [Source:HGNC Symbol;Acc:HGNC:13139] |
| ENSG00000148840 | HGLibA_38229 | 70 | -0.501519486 | PPRC1 | coactivator-related 1 [Source:HGNC Symbol;Acc:HGNC:30025]" |
| ENSG00000149735 | HGLibA_19797 | 70 | -0.501519486 | GPHA2 | glycoprotein hormone alpha 2 [Source:HGNC Symbol;Acc:HGNC:18054] |
| ENSG00000151893 | HGLibA_06957 | 70 | -0.501519486 | CACUL1 | CDK2 associated cullin domain 1 [Source:HGNC Symbol;Acc:HGNC:23727] |
| ENSG00000152582 | HGLibA_46613 | 70 | -0.501519486 | SPEF2 | sperm flagellar 2 [Source:HGNC Symbol;Acc:HGNC:26293] |
| ENSG00000153015 | HGLibA_11821 | 70 | -0.501519486 | CWC27 | CWC27 spliceosome associated protein homolog [Source:HGNC Symbol;Acc:HGNC:10664] |
| ENSG00000153132 | HGLibA_10014 | 70 | -0.501519486 | CLGN | calmegin [Source:HGNC Symbol;Acc:HGNC:2060] |
| ENSG00000154227 | HGLibA_09206 | 70 | -0.501519486 | CERS3 | ceramide synthase 3 [Source:HGNC Symbol;Acc:HGNC:23752] |
| ENSG00000158077 | HGLibA_32113 | 70 | -0.501519486 | NLRP14 | NLR family pyrin domain containing 14 [Source:HGNC Symbol;Acc:HGNC:22939] |
| ENSG00000159335 | HGLibA_39395 | 70 | -0.501519486 | PTMS | parathymosin [Source:HGNC Symbol;Acc:HGNC:9629] |
| ENSG00000160703 | HGLibA_32140 | 70 | -0.501519486 | NLRX1 | NLR family member X1 [Source:HGNC Symbol;Acc:HGNC:29890] |
| ENSG00000161642 | HGLibA_55896 | 70 | -0.501519486 | ZNF385A | zinc finger protein 385A [Source:HGNC Symbol;Acc:HGNC:17521] |
| ENSG00000161920 | HGLibA_28738 | 70 | -0.501519486 | MED11 | mediator complex subunit 11 [Source:HGNC Symbol;Acc:HGNC:32687] |
| ENSG00000161996 | HGLibA_54213 | 70 | -0.501519486 | WDR90 | WD repeat domain 90 [Source:HGNC Symbol;Acc:HGNC:26960] |

| | | | | | |
|---|---|---|---|---|---|
| ENSG00000162302 | HGLibA_42168 | 70 | -0.501519486 | RPS6KA4 | ribosomal protein S6 kinase A4 [Source:HGNC Symbol;Acc:HGNC:10433] |
| ENSG00000163808 | HGLibA_24866 | 70 | -0.501519486 | KIF15 | kinesin family member 15 [Source:HGNC Symbol;Acc:HGNC:17273] |
| ENSG00000164163 | HGLibA_00203 | 70 | -0.501519486 | ABCE1 | ATP binding cassette subfamily E member 1 [Source:HGNC Symbol;Acc:HGNC:69] |
| ENSG00000164258 | HGLibA_31553 | 70 | -0.501519486 | NDUFS4 | NADH:ubiquinone oxidoreductase subunit S4 [Source:HGNC Symbol;Acc:HGNC:7711] |
| ENSG00000164442 | HGLibA_09746 | 70 | -0.501519486 | CITED2 | Cbp/p300 interacting transactivator with Glu/Asp rich carboxy-terminal domain 2 [Source:HGNC Symbol;Acc:HGNC:1987] |
| ENSG00000164825 | HGLibA_12811 | 70 | -0.501519486 | DEFB1 | defensin beta 1 [Source:HGNC Symbol;Acc:HGNC:2766] |
| ENSG00000165076 | HGLibA_38934 | 70 | -0.501519486 | PRSS37 | serine protease 37 [Source:HGNC Symbol;Acc:HGNC:29211] |
| ENSG00000165120 | HGLibA_47141 | 70 | -0.501519486 | SSMEM1 | serine rich single-pass membrane protein 1 [Source:HGNC Symbol;Acc:HGNC:29580] |
| ENSG00000165588 | HGLibA_34785 | 70 | -0.501519486 | OTX2 | orthodenticle homeobox 2 [Source:HGNC Symbol;Acc:HGNC:8522] |
| ENSG00000165646 | HGLibA_44489 | 70 | -0.501519486 | SLC18A2 | solute carrier family 18 member A2 [Source:HGNC Symbol;Acc:HGNC:10935] |
| ENSG00000165943 | HGLibA_29582 | 70 | -0.501519486 | MOAP1 | modulator of apoptosis 1 [Source:HGNC Symbol;Acc:HGNC:16658] |
| ENSG00000166845 | HGLibA_05617 | 70 | -0.501519486 | C18orf54 | chromosome 18 open reading frame 54 [Source:HGNC Symbol;Acc:HGNC:13796] |
| ENSG00000166908 | HGLibA_36710 | 70 | -0.501519486 | PIP4K2C | phosphatidylinositol-5-phosphate 4-kinase type 2 gamma [Source:HGNC Symbol;Acc:HGNC:23786] |
| ENSG00000167037 | HGLibA_43858 | 70 | -0.501519486 | SGSM1 | small G protein signaling modulator 1 [Source:HGNC Symbol;Acc:HGNC:29410] |
| ENSG00000167131 | HGLibA_07513 | 70 | -0.501519486 | CCDC103 | coiled-coil domain containing 103 [Source:HGNC Symbol;Acc:HGNC:32700] |
| ENSG00000167232 | HGLibA_56906 | 70 | -0.501519486 | ZNF91 | zinc finger protein 91 [Source:HGNC Symbol;Acc:HGNC:13166] |
| ENSG00000167549 | HGLibA_10789 | 70 | -0.501519486 | CORO6 | coronin 6 [Source:HGNC Symbol;Acc:HGNC:21356] |
| ENSG00000167555 | HGLibA_56170 | 70 | -0.501519486 | ZNF528 | zinc finger protein 528 [Source:HGNC Symbol;Acc:HGNC:29384] |
| ENSG00000167874 | HGLibA_50537 | 70 | -0.501519486 | TMEM88 | transmembrane protein 88 [Source:HGNC Symbol;Acc:HGNC:32371] |
| ENSG00000168214 | HGLibA_40647 | 70 | -0.501519486 | RBPJ | recombination signal binding protein for immunoglobulin kappa J region [Source:HGNC Symbol;Acc:HGNC:5724] |
| ENSG00000168615 | HGLibA_00785 | 70 | -0.501519486 | ADAM9 | ADAM metallopeptidase domain 9 [Source:HGNC Symbol;Acc:HGNC:216] |
| ENSG00000169432 | HGLibA_43007 | 70 | -0.501519486 | SCN9A | sodium voltage-gated channel alpha subunit 9 [Source:HGNC Symbol;Acc:HGNC:10597] |
| ENSG00000170175 | HGLibA_09590 | 70 | -0.501519486 | CHRNB1 | cholinergic receptor nicotinic beta 1 subunit [Source:HGNC Symbol;Acc:HGNC:1961] |
| ENSG00000170500 | HGLibA_27024 | 70 | -0.501519486 | LONRF2 | LON peptidase N-terminal domain and ring finger 2 [Source:HGNC Symbol;Acc:HGNC:24788] |
| ENSG00000171115 | HGLibA_19092 | 70 | -0.501519486 | GIMAP8 | IMAP family member 8 [Source:HGNC Symbol;Acc:HGNC:21792]" |
| ENSG00000171234 | HGLibA_52931 | 70 | -0.501519486 | UGT2B7 | UDP glucuronosyltransferase family 2 member B7 [Source:HGNC Symbol;Acc:HGNC:12554] |
| ENSG00000171357 | HGLibA_27632 | 70 | -0.501519486 | LURAP1 | leucine rich adaptor protein 1 [Source:HGNC Symbol;Acc:HGNC:32327] |
| ENSG00000171435 | HGLibA_25865 | 70 | -0.501519486 | KSR2 | kinase suppressor of ras 2 [Source:HGNC Symbol;Acc:HGNC:18610] |
| ENSG00000172548 | HGLibA_31966 | 70 | -0.501519486 | NIPAL4 | NIPA like domain containing 4 [Source:HGNC Symbol;Acc:HGNC:28018] |
| ENSG00000172728 | HGLibA_18182 | 70 | -0.501519486 | FUT10 | fucosyltransferase 10 [Source:HGNC Symbol;Acc:HGNC:19234] |
| ENSG00000173442 | HGLibA_14663 | 70 | -0.501519486 | EHBP1L1 | EH domain binding protein 1 like 1 [Source:HGNC Symbol;Acc:HGNC:30682] |
| ENSG00000175097 | HGLibA_40135 | 70 | -0.501519486 | RAG2 | recombination activating 2 [Source:HGNC Symbol;Acc:HGNC:9832] |
| ENSG00000175471 | HGLibA_28651 | 70 | -0.501519486 | MCTP1 | multiple C2 and transmembrane domain containing 1 [Source:HGNC Symbol;Acc:HGNC:26183] |
| ENSG00000176925 | HGLibA_34106 | 70 | -0.501519486 | OR51F2 | olfactory receptor family 51 subfamily F member 2 [Source:HGNC Symbol;Acc:HGNC:15197] |
| ENSG00000177576 | HGLibA_05613 | 70 | -0.501519486 | C18orf32 | chromosome 18 open reading frame 32 [Source:HGNC Symbol;Acc:HGNC:31690] |
| ENSG00000177693 | HGLibA_34007 | 70 | -0.501519486 | OR4F4 | olfactory receptor family 4 subfamily F member 4 [Source:HGNC Symbol;Acc:HGNC:8301] |
| ENSG00000178226 | HGLibA_38932 | 70 | -0.501519486 | PRSS36 | serine protease 36 [Source:HGNC Symbol;Acc:HGNC:26906] |
| ENSG00000178233 | HGLibA_50003 | 70 | -0.501519486 | TMEM151B | transmembrane protein 151B [Source:HGNC Symbol;Acc:HGNC:21315] |
| ENSG00000178997 | HGLibA_15715 | 70 | -0.501519486 | EXD1 | exonuclease 3'-5' domain containing 1 [Source:HGNC Symbol;Acc:HGNC:28507] |
| ENSG00000179761 | HGLibA_36726 | 70 | -0.501519486 | PIPOX | pipecolic acid and sarcosine oxidase [Source:HGNC Symbol;Acc:HGNC:17804] |
| ENSG00000180104 | HGLibA_15737 | 70 | -0.501519486 | EXOC3 | exocyst complex component 3 [Source:HGNC Symbol;Acc:HGNC:30378] |
| ENSG00000180116 | HGLibA_05239 | 70 | -0.501519486 | C12orf40 | chromosome 12 open reading frame 40 [Source:HGNC Symbol;Acc:HGNC:26846] |
| ENSG00000182149 | HGLibA_23689 | 70 | -0.501519486 | IST1 | ESCRT-III associated factor [Source:HGNC Symbol;Acc:HGNC:28977]" |
| ENSG00000182836 | HGLibA_37029 | 70 | -0.501519486 | PLCXD3 | phosphatidylinositol specific phospholipase C X domain containing 3 [Source:HGNC Symbol;Acc:HGNC:31822] |
| ENSG00000183723 | HGLibA_10218 | 70 | -0.501519486 | CMTM4 | CKLF like MARVEL transmembrane domain containing 4 [Source:HGNC Symbol;Acc:HGNC:19175] |
| ENSG00000184524 | HGLibA_09033 | 70 | -0.501519486 | CEND1 | cell cycle exit and neuronal differentiation 1 [Source:HGNC Symbol;Acc:HGNC:24153] |
| ENSG00000184788 | HGLibA_42783 | 70 | -0.501519486 | SATL1 | spermidine/spermine N1-acetyl transferase like 1 [Source:HGNC Symbol;Acc:HGNC:27992] |
| ENSG00000184860 | HGLibA_43166 | 70 | -0.501519486 | SDR42E1 | member 1 [Source:HGNC Symbol;Acc:HGNC:29834]" |
| ENSG00000185880 | HGLibA_51595 | 70 | -0.501519486 | TRIM69 | tripartite motif containing 69 [Source:HGNC Symbol;Acc:HGNC:17857] |
| ENSG00000186198 | HGLibA_45242 | 70 | -0.501519486 | SLC51B | solute carrier family 51 beta subunit [Source:HGNC Symbol;Acc:HGNC:29956] |
| ENSG00000186532 | HGLibA_45859 | 70 | -0.501519486 | SMYD4 | SET and MYND domain containing 4 [Source:HGNC Symbol;Acc:HGNC:21067] |
| ENSG00000187323 | HGLibA_12476 | 70 | -0.501519486 | DCC | DCC netrin 1 receptor [Source:HGNC Symbol;Acc:HGNC:2701] |
| ENSG00000187479 | HGLibA_05220 | 70 | -0.501519486 | C11orf96 | chromosome 11 open reading frame 96 [Source:HGNC Symbol;Acc:HGNC:38675] |
| ENSG00000187747 | HGLibA_34152 | 70 | -0.501519486 | OR52B6 | olfactory receptor family 52 subfamily B member 6 [Source:HGNC Symbol;Acc:HGNC:15211] |
| ENSG00000187944 | HGLibA_06160 | 70 | -0.501519486 | C2orf66 | chromosome 2 open reading frame 66 [Source:HGNC Symbol;Acc:HGNC:33809] |
| ENSG00000188389 | HGLibA_35715 | 70 | -0.501519486 | PDCD1 | programmed cell death 1 [Source:HGNC Symbol;Acc:HGNC:8760] |
| ENSG00000188848 | HGLibA_04408 | 70 | -0.501519486 | BEND4 | BEN domain containing 4 [Source:HGNC Symbol;Acc:HGNC:23815] |
| ENSG00000189152 | HGLibA_20218 | 70 | -0.501519486 | GRAPL | GRB2 related adaptor protein like [Source:HGNC Symbol;Acc:HGNC:37240] |
| ENSG00000196199 | HGLibA_29730 | 70 | -0.501519486 | MPHOSPH8 | M-phase phosphoprotein 8 [Source:HGNC Symbol;Acc:HGNC:29810] |
| ENSG00000196597 | HGLibA_56739 | 70 | -0.501519486 | ZNF782 | zinc finger protein 782 [Source:HGNC Symbol;Acc:HGNC:33110] |
| ENSG00000197125 | HGLibA_34506 | 70 | -0.501519486 | OR8B8 | olfactory receptor family 8 subfamily B member 8 [Source:HGNC Symbol;Acc:HGNC:8477] |
| ENSG00000197323 | HGLibA_51465 | 70 | -0.501519486 | TRIM33 | tripartite motif containing 33 [Source:HGNC Symbol;Acc:HGNC:16290] |
| ENSG00000197587 | HGLibA_13397 | 70 | -0.501519486 | DMBX1 | diencephalon/mesencephalon homeobox 1 [Source:HGNC Symbol;Acc:HGNC:19026] |
| ENSG00000197776 | HGLibA_25089 | 70 | -0.501519486 | KLHDC1 | kelch domain containing 1 [Source:HGNC Symbol;Acc:HGNC:19836] |
| ENSG00000198046 | HGLibA_56492 | 70 | -0.501519486 | ZNF667 | zinc finger protein 667 [Source:HGNC Symbol;Acc:HGNC:28854] |
| ENSG00000198792 | HGLibA_50114 | 70 | -0.501519486 | TMEM184B | transmembrane protein 184B [Source:HGNC Symbol;Acc:HGNC:1310] |
| ENSG00000198945 | HGLibA_25902 | 70 | -0.501519486 | L3MBTL3 | histone methyl-lysine binding protein [Source:HGNC Symbol;Acc:HGNC:23035]" |
| ENSG00000203485 | HGLibA_23281 | 70 | -0.501519486 | INF2 | FH2 and WH2 domain containing [Source:HGNC Symbol;Acc:HGNC:23791]" |
| ENSG00000203760 | HGLibA_09093 | 70 | -0.501519486 | CENPW | centromere protein W [Source:HGNC Symbol;Acc:HGNC:21488] |
| ENSG00000204842 | HGLibA_03836 | 70 | -0.501519486 | ATXN2 | ataxin 2 [Source:HGNC Symbol;Acc:HGNC:10555] |
| ENSG00000205464 | HGLibA_03697 | 70 | -0.501519486 | ATP6AP1L | ATPase H+ transporting accessory protein 1 like [Source:HGNC Symbol;Acc:HGNC:28091] |
| ENSG00000214491 | HGLibA_43205 | 70 | -0.501519486 | SEC14L6 | SEC14 like lipid binding 6 [Source:HGNC Symbol;Acc:HGNC:40047] |
| ENSG00000215218 | HGLibA_52651 | 70 | -0.501519486 | UBE2QL1 | ubiquitin conjugating enzyme E2 Q family like 1 [Source:HGNC Symbol;Acc:HGNC:37269] |
| ENSG00000221978 | HGLibA_08120 | 70 | -0.501519486 | CCNL2 | cyclin L2 [Source:HGNC Symbol;Acc:HGNC:20570] |
| ENSG00000231924 | HGLibA_39034 | 70 | -0.501519486 | PSG1 | pregnancy specific beta-1-glycoprotein 1 [Source:HGNC Symbol;Acc:HGNC:9514] |
| ENSG00000241563 | HGLibA_10798 | 70 | -0.501519486 | CORT | cortistatin [Source:HGNC Symbol;Acc:HGNC:2257] |
| ENSG00000242247 | HGLibA_02668 | 70 | -0.501519486 | ARFGAP3 | ADP ribosylation factor GTPase activating protein 3 [Source:HGNC Symbol;Acc:HGNC:661] |
| ENSG00000242550 | HGLibA_43537 | 70 | -0.501519486 | SERPINB10 | serpin family B member 10 [Source:HGNC Symbol;Acc:HGNC:8942] |
| ENSG00000244607 | HGLibA_07588 | 70 | -0.501519486 | CCDC13 | coiled-coil domain containing 13 [Source:HGNC Symbol;Acc:HGNC:26358] |
| ENSG00000250844 | HGLibA_53234 | 70 | -0.501519486 | USP17L18 | ubiquitin specific peptidase 17-like family member 18 [Source:HGNC Symbol;Acc:HGNC:44446] |
| ENSG00000253304 | HGLibA_50174 | 70 | -0.501519486 | TMEM200B | transmembrane protein 200B [Source:HGNC Symbol;Acc:HGNC:33785] |
| ENSG00000254999 | HGLibA_04828 | 70 | -0.501519486 | BRK1 | SCAR/WAVE actin nucleating complex subunit [Source:HGNC Symbol;Acc:HGNC:23057]" |
| ENSG00000265972 | HGLibA_52432 | 70 | -0.501519486 | TXNIP | thioredoxin interacting protein [Source:HGNC Symbol;Acc:HGNC:16952] |
| ENSG00000266412 | HGLibA_31343 | 70 | -0.501519486 | NCOA4 | nuclear receptor coactivator 4 [Source:HGNC Symbol;Acc:HGNC:7671] |
| ENSG00000268500 | HGLibA_44141 | 70 | -0.501519486 | SIGLEC5 | sense intronic to AC018755.18" |
| ENSG00000268651 | HGLibA_11558 | 70 | -0.501519486 | CTAG1A | cancer/testis antigen 1A [Source:HGNC Symbol;Acc:HGNC:24198] |
| ENSG00000272804 | HGLibA_25619 | 70 | -0.501519486 | KRTAP10-7 | keratin associated protein 10-7 [Source:HGNC Symbol;Acc:HGNC:22970] |
| ENSG00000274808 | HGLibA_48542 | 70 | -0.501519486 | TBC1D3B | TBC1 domain family member 3B [Source:HGNC Symbol;Acc:HGNC:27011] |
| FAM115C | HGLibA_16038 | 70 | -0.501519486 | FAM115C | - |
| FAM154A | HGLibA_16174 | 70 | -0.501519486 | FAM154A | - |
| GPR128 | HGLibA_19886 | 70 | -0.501519486 | GPR128 | - |
| HIST1H3H | HGLibA_21478 | 70 | -0.501519486 | HIST1H3H | - |
| hsa-mir-1197 | HGLibA_57255 | 70 | -0.501519486 | hsa-mir-1197 | - |
| hsa-mir-1244-3 | HGLibA_57370 | 70 | -0.501519486 | hsa-mir-1244-3 | - |
| hsa-mir-1538 | HGLibA_57920 | 70 | -0.501519486 | hsa-mir-1538 | - |
| hsa-mir-27a | HGLibA_58378 | 70 | -0.501519486 | hsa-mir-27a | - |
| hsa-mir-3155a | HGLibA_58679 | 70 | -0.501519486 | hsa-mir-3155a | - |
| hsa-mir-3165 | HGLibA_58741 | 70 | -0.501519486 | hsa-mir-3165 | - |
| hsa-mir-3190 | HGLibA_58858 | 70 | -0.501519486 | hsa-mir-3190 | - |
| hsa-mir-3196 | HGLibA_58881 | 70 | -0.501519486 | hsa-mir-3196 | - |
| hsa-mir-3688-1 | HGLibA_59307 | 70 | -0.501519486 | hsa-mir-3688-1 | - |
| hsa-mir-383 | HGLibA_59486 | 70 | -0.501519486 | hsa-mir-383 | - |
| hsa-mir-3916 | HGLibA_59542 | 70 | -0.501519486 | hsa-mir-3916 | - |
| hsa-mir-3926-2 | HGLibA_59580 | 70 | -0.501519486 | hsa-mir-3926-2 | - |
| hsa-mir-4280 | HGLibA_59822 | 70 | -0.501519486 | hsa-mir-4280 | - |
| hsa-mir-4326 | HGLibA_60026 | 70 | -0.501519486 | hsa-mir-4326 | - |
| hsa-mir-4657 | HGLibA_60736 | 70 | -0.501519486 | hsa-mir-4657 | - |
| hsa-mir-4705 | HGLibA_60921 | 70 | -0.501519486 | hsa-mir-4705 | - |
| hsa-mir-4714 | HGLibA_60958 | 70 | -0.501519486 | hsa-mir-4714 | - |
| hsa-mir-494 | HGLibA_61375 | 70 | -0.501519486 | hsa-mir-494 | - |
| hsa-mir-5000 | HGLibA_61408 | 70 | -0.501519486 | hsa-mir-5000 | - |
| hsa-mir-513a-1 | HGLibA_61572 | 70 | -0.501519486 | hsa-mir-513a-1 | - |
| hsa-mir-551b | HGLibA_62123 | 70 | -0.501519486 | hsa-mir-551b | - |
| hsa-mir-573 | HGLibA_62383 | 70 | -0.501519486 | hsa-mir-573 | - |
| hsa-mir-588 | HGLibA_62450 | 70 | -0.501519486 | hsa-mir-588 | - |
| hsa-mir-640 | HGLibA_62799 | 70 | -0.501519486 | hsa-mir-640 | - |
| hsa-mir-6723 | HGLibA_63054 | 70 | -0.501519486 | hsa-mir-6723 | - |
| hsa-mir-6789 | HGLibA_63333 | 70 | -0.501519486 | hsa-mir-6789 | - |
| hsa-mir-6840 | HGLibA_63537 | 70 | -0.501519486 | hsa-mir-6840 | - |
| hsa-mir-7854 | HGLibA_64007 | 70 | -0.501519486 | hsa-mir-7854 | - |
| hsa-mir-8057 | HGLibA_64065 | 70 | -0.501519486 | hsa-mir-8057 | - |
| hsa-mir-892b | HGLibA_64254 | 70 | -0.501519486 | hsa-mir-892b | - |
| hsa-mir-941-4 | HGLibA_64351 | 70 | -0.501519486 | hsa-mir-941-4 | - |
| KIAA1430 | HGLibA_24755 | 70 | -0.501519486 | KIAA1430 | - |
| KIAA1598 | HGLibA_24791 | 70 | -0.501519486 | KIAA1598 | - |
| WISP1 | HGLibA_54320 | 70 | -0.501519486 | WISP1 | - |
| 8-Mar | HGLibA_28350 | 69 | -0.510564829 | 8-Mar | - |
| ASMT_X | HGLibA_03320 | 69 | -0.510564829 | ASMT_X | - |
| ATP5D | HGLibA_03647 | 69 | -0.510564829 | ATP5D | - |
| ATPIF1 | HGLibA_03808 | 69 | -0.510564829 | ATPIF1 | - |
| C11orf73 | HGLibA_05181 | 69 | -0.510564829 | C11orf73 | - |
| C11orf84 | HGLibA_05196 | 69 | -0.510564829 | C11orf84 | - |
| C19orf45 | HGLibA_05665 | 69 | -0.510564829 | C19orf45 | - |
| C3orf72 | HGLibA_06267 | 69 | -0.510564829 | C3orf72 | - |
| C4orf26 | HGLibA_06307 | 69 | -0.510564829 | C4orf26 | - |

| | | | | | |
|---|---|---|---|---|---|
| C6orf10 | HGLibA_06432 | 69 | -0.510564829 | C6orf10 | - |
| ENSG00000003056 | HGLibA_27816 | 69 | -0.510564829 | M6PR | cation dependent [Source:HGNC Symbol;Acc:HGNC:6752]" |
| ENSG00000004975 | HGLibA_14212 | 69 | -0.510564829 | DVL2 | dishevelled segment polarity protein 2 [Source:HGNC Symbol;Acc:HGNC:3086] |
| ENSG00000006652 | HGLibA_22736 | 69 | -0.510564829 | IFRD1 | interferon related developmental regulator 1 [Source:HGNC Symbol;Acc:HGNC:5456] |
| ENSG00000009694 | HGLibA_48995 | 69 | -0.510564829 | TENM1 | teneurin transmembrane protein 1 [Source:HGNC Symbol;Acc:HGNC:8117] |
| ENSG00000013583 | HGLibA_21083 | 69 | -0.510564829 | HEBP1 | heme binding protein 1 [Source:HGNC Symbol;Acc:HGNC:17176] |
| ENSG00000016082 | HGLibA_23665 | 69 | -0.510564829 | ISL1 | ISL LIM homeobox 1 [Source:HGNC Symbol;Acc:HGNC:6132] |
| ENSG00000016864 | HGLibA_19338 | 69 | -0.510564829 | GLT8D1 | glycosyltransferase 8 domain containing 1 [Source:HGNC Symbol;Acc:HGNC:24870] |
| ENSG00000020577 | HGLibA_42683 | 69 | -0.510564829 | SAMD4A | sterile alpha motif domain containing 4A [Source:HGNC Symbol;Acc:HGNC:23023] |
| ENSG00000023228 | HGLibA_31544 | 69 | -0.510564829 | NDUFS1 | NADH:ubiquinone oxidoreductase core subunit S1 [Source:HGNC Symbol;Acc:HGNC:7707] |
| ENSG00000055483 | HGLibA_53346 | 69 | -0.510564829 | USP36 | ubiquitin specific peptidase 36 [Source:HGNC Symbol;Acc:HGNC:20062] |
| ENSG00000059915 | HGLibA_39014 | 69 | -0.510564829 | PSD | pleckstrin and Sec7 domain containing [Source:HGNC Symbol;Acc:HGNC:9507] |
| ENSG00000062038 | HGLibA_08676 | 69 | -0.510564829 | CDH3 | cadherin 3 [Source:HGNC Symbol;Acc:HGNC:1762] |
| ENSG00000066135 | HGLibA_24506 | 69 | -0.510564829 | KDM4A | lysine demethylase 4A [Source:HGNC Symbol;Acc:HGNC:22978] |
| ENSG00000067365 | HGLibA_28988 | 69 | -0.510564829 | METTL22 | methyltransferase like 22 [Source:HGNC Symbol;Acc:HGNC:28368] |
| ENSG00000068137 | HGLibA_37132 | 69 | -0.510564829 | PLEKHH3 | MyTH4 and FERM domain containing H3 [Source:HGNC Symbol;Acc:HGNC:26105]" |
| ENSG00000070785 | HGLibA_14742 | 69 | -0.510564829 | EIF2B3 | eukaryotic translation initiation factor 2B subunit gamma [Source:HGNC Symbol;Acc:HGNC:3259] |
| ENSG00000072518 | HGLibA_28367 | 69 | -0.510564829 | MARK2 | microtubule affinity regulating kinase 2 [Source:HGNC Symbol;Acc:HGNC:3332] |
| ENSG00000077327 | HGLibA_46363 | 69 | -0.510564829 | SPAG6 | sperm associated antigen 6 [Source:HGNC Symbol;Acc:HGNC:11215] |
| ENSG00000078369 | HGLibA_19483 | 69 | -0.510564829 | GNB1 | G protein subunit beta 1 [Source:HGNC Symbol;Acc:HGNC:4396] |
| ENSG00000079277 | HGLibA_29342 | 69 | -0.510564829 | MKNK1 | MAP kinase interacting serine/threonine kinase 1 [Source:HGNC Symbol;Acc:HGNC:7110] |
| ENSG00000080986 | HGLibA_31382 | 69 | -0.510564829 | NDC80 | kinetochore complex component [Source:HGNC Symbol;Acc:HGNC:16909]" |
| ENSG00000085117 | HGLibA_08460 | 69 | -0.510564829 | CD82 | CD82 molecule [Source:HGNC Symbol;Acc:HGNC:6210] |
| ENSG00000090932 | HGLibA_13363 | 69 | -0.510564829 | DLL3 | delta like canonical Notch ligand 3 [Source:HGNC Symbol;Acc:HGNC:2909] |
| ENSG00000092847 | HGLibA_01204 | 69 | -0.510564829 | AGO1 | RISC catalytic component [Source:HGNC Symbol;Acc:HGNC:3262]" |
| ENSG00000095203 | HGLibA_15254 | 69 | -0.510564829 | EPB41L4B | erythrocyte membrane protein band 4.1 like 4B [Source:HGNC Symbol;Acc:HGNC:19818] |
| ENSG00000099246 | HGLibA_39822 | 69 | -0.510564829 | RAB18 | member RAS oncogene family [Source:HGNC Symbol;Acc:HGNC:14244]" |
| ENSG00000099617 | HGLibA_14584 | 69 | -0.510564829 | EFNA2 | ephrin A2 [Source:HGNC Symbol;Acc:HGNC:3222] |
| ENSG00000100031 | HGLibA_19011 | 69 | -0.510564829 | GGT1 | gamma-glutamyltransferase 1 [Source:HGNC Symbol;Acc:HGNC:4250] |
| ENSG00000100206 | HGLibA_13398 | 69 | -0.510564829 | DMC1 | DNA meiotic recombinase 1 [Source:HGNC Symbol;Acc:HGNC:2927] |
| ENSG00000100519 | HGLibA_39146 | 69 | -0.510564829 | PSMC6 | ATPase 6 [Source:HGNC Symbol;Acc:HGNC:9553]" |
| ENSG00000101493 | HGLibA_56142 | 69 | -0.510564829 | ZNF516 | zinc finger protein 516 [Source:HGNC Symbol;Acc:HGNC:28990] |
| ENSG00000101596 | HGLibA_45661 | 69 | -0.510564829 | SMCHD1 | structural maintenance of chromosomes flexible hinge domain containing 1 [Source:HGNC Symbol;Acc:HGNC:29090] |
| ENSG00000101843 | HGLibA_39151 | 69 | -0.510564829 | PSMD10 | non-ATPase 10 [Source:HGNC Symbol;Acc:HGNC:9555]" |
| ENSG00000101850 | HGLibA_19914 | 69 | -0.510564829 | GPR143 | G protein-coupled receptor 143 [Source:HGNC Symbol;Acc:HGNC:20145] |
| ENSG00000102181 | HGLibA_08495 | 69 | -0.510564829 | CD99L2 | CD99 molecule like 2 [Source:HGNC Symbol;Acc:HGNC:18237] |
| ENSG00000102805 | HGLibA_10079 | 69 | -0.510564829 | CLN5 | intracellular trafficking protein [Source:HGNC Symbol;Acc:HGNC:2076]" |
| ENSG00000103489 | HGLibA_54618 | 69 | -0.510564829 | XYLT1 | xylosyltransferase 1 [Source:HGNC Symbol;Acc:HGNC:15516] |
| ENSG00000104626 | HGLibA_15480 | 69 | -0.510564829 | ERI1 | exoribonuclease 1 [Source:HGNC Symbol;Acc:HGNC:23994] |
| ENSG00000105058 | HGLibA_16570 | 69 | -0.510564829 | FAM32A | family with sequence similarity 32 member A [Source:HGNC Symbol;Acc:HGNC:24563] |
| ENSG00000105732 | HGLibA_56286 | 69 | -0.510564829 | ZNF574 | zinc finger protein 574 [Source:HGNC Symbol;Acc:HGNC:26166] |
| ENSG00000108405 | HGLibA_34847 | 69 | -0.510564829 | P2RX1 | purinergic receptor P2X 1 [Source:HGNC Symbol;Acc:HGNC:8533] |
| ENSG00000108599 | HGLibA_01407 | 69 | -0.510564829 | AKAP10 | A-kinase anchoring protein 10 [Source:HGNC Symbol;Acc:HGNC:368] |
| ENSG00000110497 | HGLibA_01755 | 69 | -0.510564829 | AMBRA1 | autophagy and beclin 1 regulator 1 [Source:HGNC Symbol;Acc:HGNC:25990] |
| ENSG00000110931 | HGLibA_07092 | 69 | -0.510564829 | CAMKK2 | calcium/calmodulin dependent protein kinase kinase 2 [Source:HGNC Symbol;Acc:HGNC:1470] |
| ENSG00000111786 | HGLibA_47094 | 69 | -0.510564829 | SRSF9 | serine and arginine rich splicing factor 9 [Source:HGNC Symbol;Acc:HGNC:10791] |
| ENSG00000112130 | HGLibA_41699 | 69 | -0.510564829 | RNF8 | ring finger protein 8 [Source:HGNC Symbol;Acc:HGNC:10071] |
| ENSG00000118922 | HGLibA_25049 | 69 | -0.510564829 | KLF12 | Kruppel like factor 12 [Source:HGNC Symbol;Acc:HGNC:6346] |
| ENSG00000120324 | HGLibA_35471 | 69 | -0.510564829 | PCDHB10 | protocadherin beta 10 [Source:HGNC Symbol;Acc:HGNC:8681] |
| ENSG00000120457 | HGLibA_24282 | 69 | -0.510564829 | KCNJ5 | potassium voltage-gated channel subfamily J member 5 [Source:HGNC Symbol;Acc:HGNC:6266] |
| ENSG00000120675 | HGLibA_13578 | 69 | -0.510564829 | DNAJC15 | DnaJ heat shock protein family (Hsp40) member C15 [Source:HGNC Symbol;Acc:HGNC:20325] |
| ENSG00000120685 | HGLibA_38694 | 69 | -0.510564829 | PROSER1 | proline and serine rich 1 [Source:HGNC Symbol;Acc:HGNC:20291] |
| ENSG00000120708 | HGLibA_49220 | 69 | -0.510564829 | TGFBI | transforming growth factor beta induced [Source:HGNC Symbol;Acc:HGNC:11771] |
| ENSG00000120727 | HGLibA_35031 | 69 | -0.510564829 | PAIP2 | poly(A) binding protein interacting protein 2 [Source:HGNC Symbol;Acc:HGNC:17970] |
| ENSG00000120805 | HGLibA_02922 | 69 | -0.510564829 | ARL1 | ADP ribosylation factor like GTPase 1 [Source:HGNC Symbol;Acc:HGNC:692] |
| ENSG00000123975 | HGLibA_09791 | 69 | -0.510564829 | CKS2 | CDC28 protein kinase regulatory subunit 2 [Source:HGNC Symbol;Acc:HGNC:2000] |
| ENSG00000123992 | HGLibA_13711 | 69 | -0.510564829 | DNPEP | aspartyl aminopeptidase [Source:HGNC Symbol;Acc:HGNC:2981] |
| ENSG00000124201 | HGLibA_56919 | 69 | -0.510564829 | ZNFX1 | zinc finger NFX1-type containing 1 [Source:HGNC Symbol;Acc:HGNC:29271] |
| ENSG00000124479 | HGLibA_31409 | 69 | -0.510564829 | NDP | norrin cystine knot growth factor [Source:HGNC Symbol;Acc:HGNC:7678]" |
| ENSG00000125798 | HGLibA_17811 | 69 | -0.510564829 | FOXA2 | forkhead box A2 [Source:HGNC Symbol;Acc:HGNC:5022] |
| ENSG00000125877 | HGLibA_23838 | 69 | -0.510564829 | ITPA | inosine triphosphatase [Source:HGNC Symbol;Acc:HGNC:6176] |
| ENSG00000126838 | HGLibA_39715 | 69 | -0.510564829 | PZP | alpha-2-macroglobulin like [Source:HGNC Symbol;Acc:HGNC:9750]" |
| ENSG00000126861 | HGLibA_33385 | 69 | -0.510564829 | OMG | oligodendrocyte myelin glycoprotein [Source:HGNC Symbol;Acc:HGNC:8135] |
| ENSG00000127366 | HGLibA_48418 | 69 | -0.510564829 | TAS2R5 | taste 2 receptor member 5 [Source:HGNC Symbol;Acc:HGNC:14912] |
| ENSG00000127529 | HGLibA_34469 | 69 | -0.510564829 | OR7C2 | olfactory receptor family 7 subfamily C member 2 [Source:HGNC Symbol;Acc:HGNC:8374] |
| ENSG00000127980 | HGLibA_36079 | 69 | -0.510564829 | PEX1 | peroxisomal biogenesis factor 1 [Source:HGNC Symbol;Acc:HGNC:8850] |
| ENSG00000128595 | HGLibA_07053 | 69 | -0.510564829 | CALU | calumenin [Source:HGNC Symbol;Acc:HGNC:1458] |
| ENSG00000129862 | HGLibA_53596 | 69 | -0.510564829 | VCY1B | variable charge Y-linked 1B [Source:HGNC Symbol;Acc:HGNC:31751] |
| ENSG00000130287 | HGLibA_31260 | 69 | -0.510564829 | NCAN | neurocan [Source:HGNC Symbol;Acc:HGNC:2465] |
| ENSG00000130876 | HGLibA_45348 | 69 | -0.510564829 | SLC7A10 | solute carrier family 7 member 10 [Source:HGNC Symbol;Acc:HGNC:11058] |
| ENSG00000131183 | HGLibA_44939 | 69 | -0.510564829 | SLC34A1 | solute carrier family 34 member 1 [Source:HGNC Symbol;Acc:HGNC:11019] |
| ENSG00000131507 | HGLibA_31391 | 69 | -0.510564829 | NDFIP1 | Nedd4 family interacting protein 1 [Source:HGNC Symbol;Acc:HGNC:17592] |
| ENSG00000132207 | HGLibA_45558 | 69 | -0.510564829 | SLX1A | structure-specific endonuclease subunit [Source:HGNC Symbol;Acc:HGNC:20922]" |
| ENSG00000134809 | HGLibA_49493 | 69 | -0.510564829 | TIMM10 | translocase of inner mitochondrial membrane 10 [Source:HGNC Symbol;Acc:HGNC:11814] |
| ENSG00000134824 | HGLibA_15937 | 69 | -0.510564829 | FADS2 | fatty acid desaturase 2 [Source:HGNC Symbol;Acc:HGNC:3575] |
| ENSG00000134853 | HGLibA_35854 | 69 | -0.510564829 | PDGFRA | platelet derived growth factor receptor alpha [Source:HGNC Symbol;Acc:HGNC:8803] |
| ENSG00000135046 | HGLibA_02218 | 69 | -0.510564829 | ANXA1 | annexin A1 [Source:HGNC Symbol;Acc:HGNC:533] |
| ENSG00000135297 | HGLibA_30450 | 69 | -0.510564829 | MTO1 | mitochondrial tRNA translation optimization 1 [Source:HGNC Symbol;Acc:HGNC:19261] |
| ENSG00000135974 | HGLibA_06139 | 69 | -0.510564829 | C2orf49 | chromosome 2 open reading frame 49 [Source:HGNC Symbol;Acc:HGNC:28772] |
| ENSG00000136143 | HGLibA_47695 | 69 | -0.510564829 | SUCLA2 | succinate-CoA ligase ADP-forming beta subunit [Source:HGNC Symbol;Acc:HGNC:11448] |
| ENSG00000136490 | HGLibA_26535 | 69 | -0.510564829 | LIMD2 | LIM domain containing 2 [Source:HGNC Symbol;Acc:HGNC:28142] |
| ENSG00000136874 | HGLibA_47626 | 69 | -0.510564829 | STX17 | syntaxin 17 [Source:HGNC Symbol;Acc:HGNC:11432] |
| ENSG00000137404 | HGLibA_32706 | 69 | -0.510564829 | NRM | nurim [Source:HGNC Symbol;Acc:HGNC:8003] |
| ENSG00000137501 | HGLibA_48098 | 69 | -0.510564829 | SYTL2 | synaptotagmin like 2 [Source:HGNC Symbol;Acc:HGNC:15585] |
| ENSG00000137842 | HGLibA_50471 | 69 | -0.510564829 | TMEM62 | transmembrane protein 62 [Source:HGNC Symbol;Acc:HGNC:26269] |
| ENSG00000139970 | HGLibA_42398 | 69 | -0.510564829 | RTN1 | reticulon 1 [Source:HGNC Symbol;Acc:HGNC:10467] |
| ENSG00000139998 | HGLibA_39818 | 69 | -0.510564829 | RAB15 | member RAS oncogene family [Source:HGNC Symbol;Acc:HGNC:20150]" |
| ENSG00000140280 | HGLibA_27768 | 69 | -0.510564829 | LYSMD2 | LysM domain containing 2 [Source:HGNC Symbol;Acc:HGNC:28571] |
| ENSG00000140829 | HGLibA_13196 | 69 | -0.510564829 | DHX38 | DEAH-box helicase 38 [Source:HGNC Symbol;Acc:HGNC:17211] |
| ENSG00000140988 | HGLibA_42102 | 69 | -0.510564829 | RPS2 | ribosomal protein S2 [Source:HGNC Symbol;Acc:HGNC:10404] |
| ENSG00000141027 | HGLibA_31357 | 69 | -0.510564829 | NCOR1 | nuclear receptor corepressor 1 [Source:HGNC Symbol;Acc:HGNC:7672] |
| ENSG00000141098 | HGLibA_18956 | 69 | -0.510564829 | GFOD2 | glucose-fructose oxidoreductase domain containing 2 [Source:HGNC Symbol;Acc:HGNC:28159] |
| ENSG00000142224 | HGLibA_23034 | 69 | -0.510564829 | IL19 | interleukin 19 [Source:HGNC Symbol;Acc:HGNC:5990] |
| ENSG00000142530 | HGLibA_16699 | 69 | -0.510564829 | FAM71E1 | family with sequence similarity 71 member E1 [Source:HGNC Symbol;Acc:HGNC:25107] |
| ENSG00000143067 | HGLibA_56564 | 69 | -0.510564829 | ZNF697 | zinc finger protein 697 [Source:HGNC Symbol;Acc:HGNC:32034] |
| ENSG00000143375 | HGLibA_09319 | 69 | -0.510564829 | CGN | cingulin [Source:HGNC Symbol;Acc:HGNC:17429] |
| ENSG00000143748 | HGLibA_33121 | 69 | -0.510564829 | NVL | nuclear VCP-like [Source:HGNC Symbol;Acc:HGNC:8070] |
| ENSG00000144036 | HGLibA_15756 | 69 | -0.510564829 | EXOC6B | exocyst complex component 6B [Source:HGNC Symbol;Acc:HGNC:17085] |
| ENSG00000144357 | HGLibA_52753 | 69 | -0.510564829 | UBR3 | ubiquitin protein ligase E3 component n-recognin 3 [Source:HGNC Symbol;Acc:HGNC:30467] |
| ENSG00000144619 | HGLibA_10411 | 69 | -0.510564829 | CNTN4 | contactin 4 [Source:HGNC Symbol;Acc:HGNC:2174] |
| ENSG00000144681 | HGLibA_47312 | 69 | -0.510564829 | STAC | SH3 and cysteine rich domain [Source:HGNC Symbol;Acc:HGNC:11353] |
| ENSG00000146006 | HGLibA_27489 | 69 | -0.510564829 | LRRTM2 | leucine rich repeat transmembrane neuronal 2 [Source:HGNC Symbol;Acc:HGNC:19409] |
| ENSG00000147224 | HGLibA_38754 | 69 | -0.510564829 | PRPS1 | phosphoribosyl pyrophosphate synthetase 1 [Source:HGNC Symbol;Acc:HGNC:9462] |
| ENSG00000148331 | HGLibA_03245 | 69 | -0.510564829 | ASB6 | ankyrin repeat and SOCS box containing 6 [Source:HGNC Symbol;Acc:HGNC:17181] |
| ENSG00000150551 | HGLibA_27713 | 69 | -0.510564829 | LYPD1 | LY6/PLAUR domain containing 1 [Source:HGNC Symbol;Acc:HGNC:28431] |
| ENSG00000151834 | HGLibA_18360 | 69 | -0.510564829 | GABRA2 | gamma-aminobutyric acid type A receptor alpha2 subunit [Source:HGNC Symbol;Acc:HGNC:4076] |
| ENSG00000152503 | HGLibA_51475 | 69 | -0.510564829 | TRIM36 | tripartite motif containing 36 [Source:HGNC Symbol;Acc:HGNC:16280] |
| ENSG00000152904 | HGLibA_19007 | 69 | -0.510564829 | GGPS1 | geranylgeranyl diphosphate synthase 1 [Source:HGNC Symbol;Acc:HGNC:4249] |
| ENSG00000154358 | HGLibA_33231 | 69 | -0.510564829 | OBSCN | cytoskeletal calmodulin and titin-interacting RhoGEF [Source:HGNC Symbol;Acc:HGNC:15719]" |
| ENSG00000156876 | HGLibA_42768 | 69 | -0.510564829 | SASS6 | SAS-6 centriolar assembly protein [Source:HGNC Symbol;Acc:HGNC:25403] |
| ENSG00000156983 | HGLibA_04839 | 69 | -0.510564829 | BRPF1 | bromodomain and PHD finger containing 1 [Source:HGNC Symbol;Acc:HGNC:14255] |
| ENSG00000159210 | HGLibA_45927 | 69 | -0.510564829 | SNF8 | ESCRT-II complex subunit [Source:HGNC Symbol;Acc:HGNC:17028]" |
| ENSG00000160188 | HGLibA_42322 | 69 | -0.510564829 | RSPH1 | radial spoke head component 1 [Source:HGNC Symbol;Acc:HGNC:12371] |
| ENSG00000160785 | HGLibA_44746 | 69 | -0.510564829 | SLC25A44 | solute carrier family 25 member 44 [Source:HGNC Symbol;Acc:HGNC:29036] |
| ENSG00000160867 | HGLibA_17450 | 69 | -0.510564829 | FGFR4 | fibroblast growth factor receptor 4 [Source:HGNC Symbol;Acc:HGNC:3691] |
| ENSG00000161921 | HGLibA_11860 | 69 | -0.510564829 | CXCL16 | C-X-C motif chemokine ligand 16 [Source:HGNC Symbol;Acc:HGNC:16642] |
| ENSG00000163221 | HGLibA_42538 | 69 | -0.510564829 | S100A12 | S100 calcium binding protein A12 [Source:HGNC Symbol;Acc:HGNC:10489] |
| ENSG00000163273 | HGLibA_32505 | 69 | -0.510564829 | NPPC | natriuretic peptide C [Source:HGNC Symbol;Acc:HGNC:7941] |
| ENSG00000163659 | HGLibA_49556 | 69 | -0.510564829 | TIPARP | TCDD inducible poly(ADP-ribose) polymerase [Source:HGNC Symbol;Acc:HGNC:23696] |
| ENSG00000164953 | HGLibA_50493 | 69 | -0.510564829 | TMEM67 | transmembrane protein 67 [Source:HGNC Symbol;Acc:HGNC:28396] |
| ENSG00000165271 | HGLibA_32271 | 69 | -0.510564829 | NOL6 | nucleolar protein 6 [Source:HGNC Symbol;Acc:HGNC:19910] |
| ENSG00000165410 | HGLibA_09285 | 69 | -0.510564829 | CFL2 | cofilin 2 [Source:HGNC Symbol;Acc:HGNC:1875] |
| ENSG00000165832 | HGLibA_51803 | 69 | -0.510564829 | TRUB1 | TruB pseudouridine synthase family member 1 [Source:HGNC Symbol;Acc:HGNC:16060] |
| ENSG00000167191 | HGLibA_20128 | 69 | -0.510564829 | GPRC5B | G protein-coupled receptor class C group 5 member B [Source:HGNC Symbol;Acc:HGNC:13308] |
| ENSG00000167397 | HGLibA_53676 | 69 | -0.510564829 | VKORC1 | vitamin K epoxide reductase complex subunit 1 [Source:HGNC Symbol;Acc:HGNC:23663] |
| ENSG00000167671 | HGLibA_52792 | 69 | -0.510564829 | UBXN6 | UBX domain protein 6 [Source:HGNC Symbol;Acc:HGNC:14928] |
| ENSG00000168079 | HGLibA_42865 | 69 | -0.510564829 | SCARA5 | scavenger receptor class A member 5 [Source:HGNC Symbol;Acc:HGNC:28701] |
| ENSG00000168918 | HGLibA_23348 | 69 | -0.510564829 | INPP5D | inositol polyphosphate-5-phosphatase D [Source:HGNC Symbol;Acc:HGNC:6079] |
| ENSG00000169946 | HGLibA_55255 | 69 | -0.510564829 | ZFPM2 | FOG family member 2 [Source:HGNC Symbol;Acc:HGNC:16700]" |
| ENSG00000170545 | HGLibA_45592 | 69 | -0.510564829 | SMAGP | small cell adhesion glycoprotein [Source:HGNC Symbol;Acc:HGNC:26918] |
| ENSG00000171100 | HGLibA_30406 | 69 | -0.510564829 | MTM1 | myotubularin 1 [Source:HGNC Symbol;Acc:HGNC:7448] |
| ENSG00000171360 | HGLibA_25498 | 69 | -0.510564829 | KRT38 | keratin 38 [Source:HGNC Symbol;Acc:HGNC:6456] |
| ENSG00000172365 | HGLibA_34262 | 69 | -0.510564829 | OR5B2 | olfactory receptor family 5 subfamily B member 2 [Source:HGNC Symbol;Acc:HGNC:8323] |
| ENSG00000172732 | HGLibA_30574 | 69 | -0.510564829 | MUS81 | MUS81 structure-specific endonuclease subunit [Source:HGNC Symbol;Acc:HGNC:29814] |
| ENSG00000172954 | HGLibA_26156 | 69 | -0.510564829 | LCLAT1 | lysocardiolipin acyltransferase 1 [Source:HGNC Symbol;Acc:HGNC:26756] |

| | | | | | |
|---|---|---|---|---|---|
| ENSG00000173258 | HGLibA_56074 | 69 | -0.510564829 | ZNF483 | zinc finger protein 483 [Source:HGNC Symbol;Acc:HGNC:23384] |
| ENSG00000173960 | HGLibA_52784 | 69 | -0.510564829 | UBXN2A | UBX domain protein 2A [Source:HGNC Symbol;Acc:HGNC:27265] |
| ENSG00000174099 | HGLibA_30246 | 69 | -0.510564829 | MSRB3 | methionine sulfoxide reductase B3 [Source:HGNC Symbol;Acc:HGNC:27375] |
| ENSG00000174306 | HGLibA_55318 | 69 | -0.510564829 | ZHX3 | zinc fingers and homeoboxes 3 [Source:HGNC Symbol;Acc:HGNC:15935] |
| ENSG00000174358 | HGLibA_45316 | 69 | -0.510564829 | SLC6A19 | solute carrier family 6 member 19 [Source:HGNC Symbol;Acc:HGNC:27960] |
| ENSG00000174483 | HGLibA_04206 | 69 | -0.510564829 | BBS1 | Bardet-Biedl syndrome 1 [Source:HGNC Symbol;Acc:HGNC:966] |
| ENSG00000174945 | HGLibA_01856 | 69 | -0.510564829 | AMZ1 | archaelysin family metallopeptidase 1 [Source:HGNC Symbol;Acc:HGNC:22231] |
| ENSG00000175416 | HGLibA_10146 | 69 | -0.510564829 | CLTB | clathrin light chain B [Source:HGNC Symbol;Acc:HGNC:2091] |
| ENSG00000176495 | HGLibA_34240 | 69 | -0.510564829 | OR5AN1 | olfactory receptor family 5 subfamily AN member 1 [Source:HGNC Symbol;Acc:HGNC:15255] |
| ENSG00000176597 | HGLibA_03993 | 69 | -0.510564829 | B3GNT5 | 3-N-acetylglucosaminyltransferase 5 [Source:HGNC Symbol;Acc:HGNC:15684]" |
| ENSG00000176681 | HGLibA_27326 | 69 | -0.510564829 | LRRC37A | leucine rich repeat containing 37A [Source:HGNC Symbol;Acc:HGNC:29069] |
| ENSG00000176749 | HGLibA_08776 | 69 | -0.510564829 | CDK5R1 | cyclin dependent kinase 5 regulatory subunit 1 [Source:HGNC Symbol;Acc:HGNC:1775] |
| ENSG00000177301 | HGLibA_24130 | 69 | -0.510564829 | KCNA2 | potassium voltage-gated channel subfamily A member 2 [Source:HGNC Symbol;Acc:HGNC:6220] |
| ENSG00000177409 | HGLibA_42702 | 69 | -0.510564829 | SAMD9L | sterile alpha motif domain containing 9 like [Source:HGNC Symbol;Acc:HGNC:1349] |
| ENSG00000177476 | HGLibA_33797 | 69 | -0.510564829 | OR2G3 | olfactory receptor family 2 subfamily G member 3 [Source:HGNC Symbol;Acc:HGNC:15008] |
| ENSG00000177752 | HGLibA_54689 | 69 | -0.510564829 | YIPF7 | Yip1 domain family member 7 [Source:HGNC Symbol;Acc:HGNC:26825] |
| ENSG00000178665 | HGLibA_56618 | 69 | -0.510564829 | ZNF713 | zinc finger protein 713 [Source:HGNC Symbol;Acc:HGNC:22043] |
| ENSG00000179002 | HGLibA_48357 | 69 | -0.510564829 | TAS1R2 | taste 1 receptor member 2 [Source:HGNC Symbol;Acc:HGNC:14905] |
| ENSG00000179715 | HGLibA_35585 | 69 | -0.510564829 | PCED1B | PC-esterase domain containing 1B [Source:HGNC Symbol;Acc:HGNC:28255] |
| ENSG00000179921 | HGLibA_19754 | 69 | -0.510564829 | GPBAR1 | G protein-coupled bile acid receptor 1 [Source:HGNC Symbol;Acc:HGNC:19680] |
| ENSG00000179943 | HGLibA_17547 | 69 | -0.510564829 | FIZ1 | FLT3 interacting zinc finger 1 [Source:HGNC Symbol;Acc:HGNC:25917] |
| ENSG00000181026 | HGLibA_01091 | 69 | -0.510564829 | AEN | apoptosis enhancing nuclease [Source:HGNC Symbol;Acc:HGNC:25722] |
| ENSG00000181220 | HGLibA_56665 | 69 | -0.510564829 | ZNF746 | zinc finger protein 746 [Source:HGNC Symbol;Acc:HGNC:21948] |
| ENSG00000182111 | HGLibA_56623 | 69 | -0.510564829 | ZNF716 | zinc finger protein 716 [Source:HGNC Symbol;Acc:HGNC:32458] |
| ENSG00000182362 | HGLibA_54639 | 69 | -0.510564829 | YBEY | ybeY metalloendoribonuclease [Source:HGNC Symbol;Acc:HGNC:1299] |
| ENSG00000182557 | HGLibA_46740 | 69 | -0.510564829 | SPNS3 | sphingolipid transporter 3 (putative) [Source:HGNC Symbol;Acc:HGNC:28433] |
| ENSG00000183020 | HGLibA_02308 | 69 | -0.510564829 | AP2A2 | adaptor related protein complex 2 subunit alpha 2 [Source:HGNC Symbol;Acc:HGNC:562] |
| ENSG00000183397 | HGLibA_05701 | 69 | -0.510564829 | C19orf71 | chromosome 19 open reading frame 71 [Source:HGNC Symbol;Acc:HGNC:34496] |
| ENSG00000183439 | HGLibA_51567 | 69 | -0.510564829 | TRIM61 | tripartite motif containing 61 [Source:HGNC Symbol;Acc:HGNC:24339] |
| ENSG00000183831 | HGLibA_02106 | 69 | -0.510564829 | ANKRD45 | ankyrin repeat domain 45 [Source:HGNC Symbol;Acc:HGNC:24786] |
| ENSG00000183856 | HGLibA_23538 | 69 | -0.510564829 | IQGAP3 | IQ motif containing GTPase activating protein 3 [Source:HGNC Symbol;Acc:HGNC:20669] |
| ENSG00000184203 | HGLibA_38073 | 69 | -0.510564829 | PPP1R2 | protein phosphatase 1 regulatory inhibitor subunit 2 [Source:HGNC Symbol;Acc:HGNC:9288] |
| ENSG00000184838 | HGLibA_38792 | 69 | -0.510564829 | PRR16 | proline rich 16 [Source:HGNC Symbol;Acc:HGNC:29654] |
| ENSG00000184867 | HGLibA_03042 | 69 | -0.510564829 | ARMCX2 | armadillo repeat containing X-linked 2 [Source:HGNC Symbol;Acc:HGNC:16869] |
| ENSG00000184986 | HGLibA_49900 | 69 | -0.510564829 | TMEM121 | transmembrane protein 121 [Source:HGNC Symbol;Acc:HGNC:20511] |
| ENSG00000185448 | HGLibA_16607 | 69 | -0.510564829 | FAM47A | family with sequence similarity 47 member A [Source:HGNC Symbol;Acc:HGNC:29962] |
| ENSG00000185652 | HGLibA_32837 | 69 | -0.510564829 | NTF3 | neurotrophin 3 [Source:HGNC Symbol;Acc:HGNC:8023] |
| ENSG00000186501 | HGLibA_50240 | 69 | -0.510564829 | TMEM222 | transmembrane protein 222 [Source:HGNC Symbol;Acc:HGNC:25363] |
| ENSG00000186866 | HGLibA_37471 | 69 | -0.510564829 | POFUT2 | protein O-fucosyltransferase 2 [Source:HGNC Symbol;Acc:HGNC:14683] |
| ENSG00000186868 | HGLibA_28322 | 69 | -0.510564829 | MAPT | microtubule associated protein tau [Source:HGNC Symbol;Acc:HGNC:6893] |
| ENSG00000187479 | HGLibA_05219 | 69 | -0.510564829 | C11orf96 | chromosome 11 open reading frame 96 [Source:HGNC Symbol;Acc:HGNC:38675] |
| ENSG00000187806 | HGLibA_50184 | 69 | -0.510564829 | TMEM202 | transmembrane protein 202 [Source:HGNC Symbol;Acc:HGNC:33733] |
| ENSG00000188522 | HGLibA_16758 | 69 | -0.510564829 | FAM83G | family with sequence similarity 83 member G [Source:HGNC Symbol;Acc:HGNC:32554] |
| ENSG00000188909 | HGLibA_04887 | 69 | -0.510564829 | BSX | brain specific homeobox [Source:HGNC Symbol;Acc:HGNC:20450] |
| ENSG00000188992 | HGLibA_26619 | 69 | -0.510564829 | LIPI | lipase I [Source:HGNC Symbol;Acc:HGNC:18821] |
| ENSG00000189143 | HGLibA_09897 | 69 | -0.510564829 | CLDN4 | claudin 4 [Source:HGNC Symbol;Acc:HGNC:2046] |
| ENSG00000189334 | HGLibA_42544 | 69 | -0.510564829 | S100A14 | S100 calcium binding protein A14 [Source:HGNC Symbol;Acc:HGNC:18901] |
| ENSG00000196166 | HGLibA_06653 | 69 | -0.510564829 | C8orf86 | chromosome 8 open reading frame 86 [Source:HGNC Symbol;Acc:HGNC:33774] |
| ENSG00000196363 | HGLibA_54114 | 69 | -0.510564829 | WDR5 | WD repeat domain 5 [Source:HGNC Symbol;Acc:HGNC:12757] |
| ENSG00000197591 | HGLibA_33584 | 69 | -0.510564829 | OR11L1 | olfactory receptor family 11 subfamily L member 1 [Source:HGNC Symbol;Acc:HGNC:14998] |
| ENSG00000197696 | HGLibA_32141 | 69 | -0.510564829 | NMB | neuromedin B [Source:HGNC Symbol;Acc:HGNC:7842] |
| ENSG00000197753 | HGLibA_26442 | 69 | -0.510564829 | LHFPL5 | LHFPL tetraspan subfamily member 5 [Source:HGNC Symbol;Acc:HGNC:21253] |
| ENSG00000197888 | HGLibA_52920 | 69 | -0.510564829 | UGT2B17 | UDP glucuronosyltransferase family 2 member B17 [Source:HGNC Symbol;Acc:HGNC:12547] |
| ENSG00000198205 | HGLibA_57072 | 69 | -0.510564829 | ZXDA | zinc finger X-linked duplicated A [Source:HGNC Symbol;Acc:HGNC:13198] |
| ENSG00000198513 | HGLibA_03515 | 69 | -0.510564829 | ATL1 | atlastin GTPase 1 [Source:HGNC Symbol;Acc:HGNC:11231] |
| ENSG00000198598 | HGLibA_29502 | 69 | -0.510564829 | MMP17 | matrix metallopeptidase 17 [Source:HGNC Symbol;Acc:HGNC:7163] |
| ENSG00000198728 | HGLibA_26210 | 69 | -0.510564829 | LDB1 | LIM domain binding 1 [Source:HGNC Symbol;Acc:HGNC:6532] |
| ENSG00000198793 | HGLibA_30453 | 69 | -0.510564829 | MTOR | mechanistic target of rapamycin kinase [Source:HGNC Symbol;Acc:HGNC:3942] |
| ENSG00000198814 | HGLibA_19195 | 69 | -0.510564829 | GK | glycerol kinase [Source:HGNC Symbol;Acc:HGNC:4289] |
| ENSG00000198963 | HGLibA_41777 | 69 | -0.510564829 | RORB | RAR related orphan receptor B [Source:HGNC Symbol;Acc:HGNC:10259] |
| ENSG00000203697 | HGLibA_07179 | 69 | -0.510564829 | CAPN8 | calpain 8 [Source:HGNC Symbol;Acc:HGNC:1485] |
| ENSG00000204033 | HGLibA_27187 | 69 | -0.510564829 | LRIT2 | Ig-like and transmembrane domains 2 [Source:HGNC Symbol;Acc:HGNC:23443]" |
| ENSG00000204616 | HGLibA_51458 | 69 | -0.510564829 | TRIM31 | tripartite motif containing 31 [Source:HGNC Symbol;Acc:HGNC:16289] |
| ENSG00000204954 | HGLibA_05291 | 69 | -0.510564829 | C12orf73 | chromosome 12 open reading frame 73 [Source:HGNC Symbol;Acc:HGNC:34450] |
| ENSG00000204977 | HGLibA_51408 | 69 | -0.510564829 | TRIM13 | tripartite motif containing 13 [Source:HGNC Symbol;Acc:HGNC:9976] |
| ENSG00000205362 | HGLibA_30277 | 69 | -0.510564829 | MT1A | metallothionein 1A [Source:HGNC Symbol;Acc:HGNC:7393] |
| ENSG00000205423 | HGLibA_10258 | 69 | -0.510564829 | CNEP1R1 | CTD nuclear envelope phosphatase 1 regulatory subunit 1 [Source:HGNC Symbol;Acc:HGNC:26759] |
| ENSG00000205495 | HGLibA_34177 | 69 | -0.510564829 | OR52J3 | olfactory receptor family 52 subfamily J member 3 [Source:HGNC Symbol;Acc:HGNC:14799] |
| ENSG00000211455 | HGLibA_47505 | 69 | -0.510564829 | STK38L | serine/threonine kinase 38 like [Source:HGNC Symbol;Acc:HGNC:17848] |
| ENSG00000213937 | HGLibA_09914 | 69 | -0.510564829 | CLDN9 | claudin 9 [Source:HGNC Symbol;Acc:HGNC:2051] |
| ENSG00000214643 | HGLibA_12903 | 69 | -0.510564829 | DEFB133 | defensin beta 133 [Source:HGNC Symbol;Acc:HGNC:31331] |
| ENSG00000214941 | HGLibA_57056 | 69 | -0.510564829 | ZSWIM7 | zinc finger SWIM-type containing 7 [Source:HGNC Symbol;Acc:HGNC:26993] |
| ENSG00000223501 | HGLibA_53807 | 69 | -0.510564829 | VPS52 | GARP complex subunit [Source:HGNC Symbol;Acc:HGNC:10518]" |
| ENSG00000229292 | HGLibA_40942 | 69 | -0.510564829 | RFPL4AL1 | ret finger protein like 4A like 1 [Source:HGNC Symbol;Acc:HGNC:45147] |
| ENSG00000236279 | HGLibA_09971 | 69 | -0.510564829 | CLEC2L | C-type lectin domain family 2 member L [Source:HGNC Symbol;Acc:HGNC:21969] |
| ENSG00000241935 | HGLibA_21862 | 69 | -0.510564829 | HOGA1 | 4-hydroxy-2-oxoglutarate aldolase 1 [Source:HGNC Symbol;Acc:HGNC:25155] |
| ENSG00000242265 | HGLibA_36027 | 69 | -0.510564829 | PEG10 | paternally expressed 10 [Source:HGNC Symbol;Acc:HGNC:14005] |
| ENSG00000242574 | HGLibA_21607 | 69 | -0.510564829 | HLA-DMB | DM beta [Source:HGNC Symbol;Acc:HGNC:4935]" |
| ENSG00000242802 | HGLibA_02362 | 69 | -0.510564829 | AP5Z1 | adaptor related protein complex 5 subunit zeta 1 [Source:HGNC Symbol;Acc:HGNC:22197] |
| ENSG00000257446 | HGLibA_56886 | 69 | -0.510564829 | ZNF878 | zinc finger protein 878 [Source:HGNC Symbol;Acc:HGNC:37246] |
| ENSG00000264424 | HGLibA_30728 | 69 | -0.510564829 | MYH4 | myosin heavy chain 4 [Source:HGNC Symbol;Acc:HGNC:7574] |
| ENSG00000273540 | HGLibA_01165 | 69 | -0.510564829 | AGBL1 | ATP/GTP binding protein like 1 [Source:HGNC Symbol;Acc:HGNC:26504] |
| ENSG00000275004 | HGLibA_55726 | 69 | -0.510564829 | ZNF280B | zinc finger protein 280B [Source:HGNC Symbol;Acc:HGNC:23022] |
| ENSG00000279169 | HGLibA_38290 | 69 | -0.510564829 | PRAMEF13 | PRAME family member 13 [Source:HGNC Symbol;Acc:HGNC:13262] |
| ENSG00000284662 | HGLibA_33991 | 69 | -0.510564829 | OR4F16 | olfactory receptor family 4 subfamily F member 16 [Source:HGNC Symbol;Acc:HGNC:15079] |
| FAM21A | HGLibA_16505 | 69 | -0.510564829 | FAM21A | - |
| FAM21C | HGLibA_16509 | 69 | -0.510564829 | FAM21C | - |
| FLJ25363 | HGLibA_17623 | 69 | -0.510564829 | FLJ25363 | - |
| hsa-mir-16-1 | HGLibA_57947 | 69 | -0.510564829 | hsa-mir-16-1 | - |
| hsa-mir-208b | HGLibA_58200 | 69 | -0.510564829 | hsa-mir-208b | - |
| hsa-mir-34a | HGLibA_59035 | 69 | -0.510564829 | hsa-mir-34a | - |
| hsa-mir-384 | HGLibA_59490 | 69 | -0.510564829 | hsa-mir-384 | - |
| hsa-mir-421 | HGLibA_59690 | 69 | -0.510564829 | hsa-mir-421 | - |
| hsa-mir-4264 | HGLibA_59764 | 69 | -0.510564829 | hsa-mir-4264 | - |
| hsa-mir-4278 | HGLibA_59816 | 69 | -0.510564829 | hsa-mir-4278 | - |
| hsa-mir-4459 | HGLibA_60243 | 69 | -0.510564829 | hsa-mir-4459 | - |
| hsa-mir-4536-2 | HGLibA_60606 | 69 | -0.510564829 | hsa-mir-4536-2 | - |
| hsa-mir-4691 | HGLibA_60874 | 69 | -0.510564829 | hsa-mir-4691 | - |
| hsa-mir-4749 | HGLibA_61095 | 69 | -0.510564829 | hsa-mir-4749 | - |
| hsa-mir-4758 | HGLibA_61129 | 69 | -0.510564829 | hsa-mir-4758 | - |
| hsa-mir-4776-1 | HGLibA_61207 | 69 | -0.510564829 | hsa-mir-4776-1 | - |
| hsa-mir-4786 | HGLibA_61252 | 69 | -0.510564829 | hsa-mir-4786 | - |
| hsa-mir-511 | HGLibA_61554 | 69 | -0.510564829 | hsa-mir-511 | - |
| hsa-mir-518c | HGLibA_61662 | 69 | -0.510564829 | hsa-mir-518c | - |
| hsa-mir-545 | HGLibA_61833 | 69 | -0.510564829 | hsa-mir-545 | - |
| hsa-mir-548f-2 | HGLibA_61978 | 69 | -0.510564829 | hsa-mir-548f-2 | - |
| hsa-mir-5692a-1 | HGLibA_62286 | 69 | -0.510564829 | hsa-mir-5692a-1 | - |
| hsa-mir-570 | HGLibA_62331 | 69 | -0.510564829 | hsa-mir-570 | - |
| hsa-mir-588 | HGLibA_62449 | 69 | -0.510564829 | hsa-mir-588 | - |
| hsa-mir-6080 | HGLibA_62581 | 69 | -0.510564829 | hsa-mir-6080 | - |
| hsa-mir-6084 | HGLibA_62596 | 69 | -0.510564829 | hsa-mir-6084 | - |
| hsa-mir-6728 | HGLibA_63065 | 69 | -0.510564829 | hsa-mir-6728 | - |
| hsa-mir-6729 | HGLibA_63068 | 69 | -0.510564829 | hsa-mir-6729 | - |
| hsa-mir-6821 | HGLibA_63460 | 69 | -0.510564829 | hsa-mir-6821 | - |
| hsa-mir-6845 | HGLibA_63551 | 69 | -0.510564829 | hsa-mir-6845 | - |
| hsa-mir-8072 | HGLibA_64130 | 69 | -0.510564829 | hsa-mir-8072 | - |
| LOC100289187 | HGLibA_26840 | 69 | -0.510564829 | LOC100289187 | - |
| NGFRAP1 | HGLibA_31879 | 69 | -0.510564829 | NGFRAP1 | - |
| PPP2R3B_Y | HGLibA_38162 | 69 | -0.510564829 | PPP2R3B_Y | - |
| TENC1 | HGLibA_48992 | 69 | -0.510564829 | TENC1 | - |
| ACPL2 | HGLibA_00522 | 68 | -0.519610172 | ACPL2 | - |
| ALS2CR8 | HGLibA_01729 | 68 | -0.519610172 | ALS2CR8 | - |
| C12orf10 | HGLibA_05222 | 68 | -0.519610172 | C12orf10 | - |
| C15orf38 | HGLibA_05386 | 68 | -0.519610172 | C15orf38 | - |
| C18orf8 | HGLibA_05625 | 68 | -0.519610172 | C18orf8 | - |
| C20orf195 | HGLibA_06003 | 68 | -0.519610172 | C20orf195 | - |
| CCDC58 | HGLibA_07806 | 68 | -0.519610172 | CCDC58 | - |
| ENSG00000001036 | HGLibA_18159 | 68 | -0.519610172 | FUCA2 | alpha-L-fucosidase 2 [Source:HGNC Symbol;Acc:HGNC:4008] |
| ENSG00000003989 | HGLibA_45361 | 68 | -0.519610172 | SLC7A2 | solute carrier family 7 member 2 [Source:HGNC Symbol;Acc:HGNC:11060] |
| ENSG00000004468 | HGLibA_08367 | 68 | -0.519610172 | CD38 | CD38 molecule [Source:HGNC Symbol;Acc:HGNC:1667] |
| ENSG00000011258 | HGLibA_28531 | 68 | -0.519610172 | MBTD1 | mbt domain containing 1 [Source:HGNC Symbol;Acc:HGNC:19866] |
| ENSG00000039650 | HGLibA_37352 | 68 | -0.519610172 | PNKP | polynucleotide kinase 3'-phosphatase [Source:HGNC Symbol;Acc:HGNC:9154] |
| ENSG00000040731 | HGLibA_08633 | 68 | -0.519610172 | CDH10 | cadherin 10 [Source:HGNC Symbol;Acc:HGNC:1749] |
| ENSG00000043591 | HGLibA_01056 | 68 | -0.519610172 | ADRB1 | adrenoceptor beta 1 [Source:HGNC Symbol;Acc:HGNC:285] |
| ENSG00000064703 | HGLibA_12668 | 68 | -0.519610172 | DDX20 | DEAD-box helicase 20 [Source:HGNC Symbol;Acc:HGNC:2743] |

| | | | | | |
|---|---|---|---|---|---|
| ENSG00000066583 | HGLibA_23683 | 68 | -0.519610172 | ISOC1 | isochorismatase domain containing 1 [Source:HGNC Symbol;Acc:HGNC:24254] |
| ENSG00000066697 | HGLibA_30175 | 68 | -0.519610172 | MSANTD3 | Myb/SANT DNA binding domain containing 3 [Source:HGNC Symbol;Acc:HGNC:23370] |
| ENSG00000075886 | HGLibA_52256 | 68 | -0.519610172 | TUBA3D | tubulin alpha 3d [Source:HGNC Symbol;Acc:HGNC:24071] |
| ENSG00000076924 | HGLibA_54461 | 68 | -0.519610172 | XAB2 | XPA binding protein 2 [Source:HGNC Symbol;Acc:HGNC:14089] |
| ENSG00000077274 | HGLibA_07173 | 68 | -0.519610172 | CAPN6 | calpain 6 [Source:HGNC Symbol;Acc:HGNC:1483] |
| ENSG00000077458 | HGLibA_16724 | 68 | -0.519610172 | FAM76B | family with sequence similarity 76 member B [Source:HGNC Symbol;Acc:HGNC:28492] |
| ENSG00000080845 | HGLibA_13347 | 68 | -0.519610172 | DLGAP4 | DLG associated protein 4 [Source:HGNC Symbol;Acc:HGNC:24476] |
| ENSG00000082996 | HGLibA_41502 | 68 | -0.519610172 | RNF13 | ring finger protein 13 [Source:HGNC Symbol;Acc:HGNC:10057] |
| ENSG00000083544 | HGLibA_48904 | 68 | -0.519610172 | TDRD3 | tudor domain containing 3 [Source:HGNC Symbol;Acc:HGNC:20612] |
| ENSG00000084110 | HGLibA_20820 | 68 | -0.519610172 | HAL | histidine ammonia-lyase [Source:HGNC Symbol;Acc:HGNC:4806] |
| ENSG00000084693 | HGLibA_01177 | 68 | -0.519610172 | AGBL5 | ATP/GTP binding protein like 5 [Source:HGNC Symbol;Acc:HGNC:26147] |
| ENSG00000085276 | HGLibA_28724 | 68 | -0.519610172 | MECOM | MDS1 and EVI1 complex locus [Source:HGNC Symbol;Acc:HGNC:3498] |
| ENSG00000087116 | HGLibA_00822 | 68 | -0.519610172 | ADAMTS2 | ADAM metallopeptidase with thrombospondin type 1 motif 2 [Source:HGNC Symbol;Acc:HGNC:218] |
| ENSG00000089356 | HGLibA_18230 | 68 | -0.519610172 | FXYD3 | FXYD domain containing ion transport regulator 3 [Source:HGNC Symbol;Acc:HGNC:4027] |
| ENSG00000091542 | HGLibA_01658 | 68 | -0.519610172 | ALKBH5 | RNA demethylase [Source:HGNC Symbol;Acc:HGNC:25996]" |
| ENSG00000095777 | HGLibA_30845 | 68 | -0.519610172 | MYO3A | myosin IIIA [Source:HGNC Symbol;Acc:HGNC:7601] |
| ENSG00000099940 | HGLibA_45882 | 68 | -0.519610172 | SNAP29 | synaptosome associated protein 29 [Source:HGNC Symbol;Acc:HGNC:11133] |
| ENSG00000100285 | HGLibA_31618 | 68 | -0.519610172 | NEFH | neurofilament heavy [Source:HGNC Symbol;Acc:HGNC:7737] |
| ENSG00000100300 | HGLibA_51958 | 68 | -0.519610172 | TSPO | translocator protein [Source:HGNC Symbol;Acc:HGNC:1158] |
| ENSG00000100528 | HGLibA_10285 | 68 | -0.519610172 | CNIH1 | cornichon family AMPA receptor auxiliary protein 1 [Source:HGNC Symbol;Acc:HGNC:19431] |
| ENSG00000100554 | HGLibA_03745 | 68 | -0.519610172 | ATP6V1D | ATPase H+ transporting V1 subunit D [Source:HGNC Symbol;Acc:HGNC:13527] |
| ENSG00000101213 | HGLibA_39386 | 68 | -0.519610172 | PTK6 | protein tyrosine kinase 6 [Source:HGNC Symbol;Acc:HGNC:9617] |
| ENSG00000101846 | HGLibA_47603 | 68 | -0.519610172 | STS | steroid sulfatase [Source:HGNC Symbol;Acc:HGNC:11425] |
| ENSG00000101888 | HGLibA_33177 | 68 | -0.519610172 | NXT2 | nuclear transport factor 2 like export factor 2 [Source:HGNC Symbol;Acc:HGNC:18151] |
| ENSG00000102053 | HGLibA_54954 | 68 | -0.519610172 | ZC3H12B | zinc finger CCCH-type containing 12B [Source:HGNC Symbol;Acc:HGNC:17407] |
| ENSG00000103047 | HGLibA_48300 | 68 | -0.519610172 | TANGO6 | transport and golgi organization 6 homolog [Source:HGNC Symbol;Acc:HGNC:25749] |
| ENSG00000103495 | HGLibA_28451 | 68 | -0.519610172 | MAZ | MYC associated zinc finger protein [Source:HGNC Symbol;Acc:HGNC:6914] |
| ENSG00000104164 | HGLibA_04573 | 68 | -0.519610172 | BLOC1S6 | biogenesis of lysosomal organelles complex 1 subunit 6 [Source:HGNC Symbol;Acc:HGNC:8549] |
| ENSG00000105880 | HGLibA_13383 | 68 | -0.519610172 | DLX5 | distal-less homeobox 5 [Source:HGNC Symbol;Acc:HGNC:2918] |
| ENSG00000107175 | HGLibA_11091 | 68 | -0.519610172 | CREB3 | cAMP responsive element binding protein 3 [Source:HGNC Symbol;Acc:HGNC:2347] |
| ENSG00000107882 | HGLibA_47714 | 68 | -0.519610172 | SUFU | SUFU negative regulator of hedgehog signaling [Source:HGNC Symbol;Acc:HGNC:16466] |
| ENSG00000108219 | HGLibA_51903 | 68 | -0.519610172 | TSPAN14 | tetraspanin 14 [Source:HGNC Symbol;Acc:HGNC:23303] |
| ENSG00000108231 | HGLibA_26394 | 68 | -0.519610172 | LGI1 | leucine rich glioma inactivated 1 [Source:HGNC Symbol;Acc:HGNC:6572] |
| ENSG00000108342 | HGLibA_11340 | 68 | -0.519610172 | CSF3 | colony stimulating factor 3 [Source:HGNC Symbol;Acc:HGNC:2438] |
| ENSG00000109501 | HGLibA_54286 | 68 | -0.519610172 | WFS1 | wolframin ER transmembrane glycoprotein [Source:HGNC Symbol;Acc:HGNC:12762] |
| ENSG00000114857 | HGLibA_32024 | 68 | -0.519610172 | NKTR | natural killer cell triggering receptor [Source:HGNC Symbol;Acc:HGNC:7833] |
| ENSG00000115137 | HGLibA_13609 | 68 | -0.519610172 | DNAJC27 | DnaJ heat shock protein family (Hsp40) member C27 [Source:HGNC Symbol;Acc:HGNC:30290] |
| ENSG00000115297 | HGLibA_49676 | 68 | -0.519610172 | TLX2 | T cell leukemia homeobox 2 [Source:HGNC Symbol;Acc:HGNC:5057] |
| ENSG00000115457 | HGLibA_22817 | 68 | -0.519610172 | IGFBP2 | insulin like growth factor binding protein 2 [Source:HGNC Symbol;Acc:HGNC:5471] |
| ENSG00000116176 | HGLibA_51202 | 68 | -0.519610172 | TPSG1 | tryptase gamma 1 [Source:HGNC Symbol;Acc:HGNC:14134] |
| ENSG00000117560 | HGLibA_16893 | 68 | -0.519610172 | FASLG | Fas ligand [Source:HGNC Symbol;Acc:HGNC:11936] |
| ENSG00000118160 | HGLibA_45390 | 68 | -0.519610172 | SLC8A2 | solute carrier family 8 member A2 [Source:HGNC Symbol;Acc:HGNC:11069] |
| ENSG00000118298 | HGLibA_06801 | 68 | -0.519610172 | CA14 | carbonic anhydrase 14 [Source:HGNC Symbol;Acc:HGNC:1372] |
| ENSG00000119729 | HGLibA_41199 | 68 | -0.519610172 | RHOQ | ras homolog family member Q [Source:HGNC Symbol;Acc:HGNC:17736] |
| ENSG00000122735 | HGLibA_13503 | 68 | -0.519610172 | DNAI1 | dynein axonemal intermediate chain 1 [Source:HGNC Symbol;Acc:HGNC:2954] |
| ENSG00000123154 | HGLibA_54193 | 68 | -0.519610172 | WDR83 | WD repeat domain 83 [Source:HGNC Symbol;Acc:HGNC:32672] |
| ENSG00000123737 | HGLibA_15795 | 68 | -0.519610172 | EXOSC9 | exosome component 9 [Source:HGNC Symbol;Acc:HGNC:9137] |
| ENSG00000125816 | HGLibA_32040 | 68 | -0.519610172 | NKX2-4 | NK2 homeobox 4 [Source:HGNC Symbol;Acc:HGNC:7837] |
| ENSG00000127241 | HGLibA_28395 | 68 | -0.519610172 | MASP1 | mannan binding lectin serine peptidase 1 [Source:HGNC Symbol;Acc:HGNC:6901] |
| ENSG00000128016 | HGLibA_55204 | 68 | -0.519610172 | ZFP36 | ZFP36 ring finger protein [Source:HGNC Symbol;Acc:HGNC:12862] |
| ENSG00000128641 | HGLibA_30824 | 68 | -0.519610172 | MYO1B | myosin IB [Source:HGNC Symbol;Acc:HGNC:7596] |
| ENSG00000130165 | HGLibA_14953 | 68 | -0.519610172 | ELOF1 | elongation factor 1 homolog [Source:HGNC Symbol;Acc:HGNC:28691] |
| ENSG00000130810 | HGLibA_37810 | 68 | -0.519610172 | PPAN | peter pan homolog [Source:HGNC Symbol;Acc:HGNC:9227] |
| ENSG00000131845 | HGLibA_55777 | 68 | -0.519610172 | ZNF304 | zinc finger protein 304 [Source:HGNC Symbol;Acc:HGNC:13505] |
| ENSG00000132153 | HGLibA_13176 | 68 | -0.519610172 | DHX30 | DExH-box helicase 30 [Source:HGNC Symbol;Acc:HGNC:16716] |
| ENSG00000132952 | HGLibA_53423 | 68 | -0.519610172 | USPL1 | ubiquitin specific peptidase like 1 [Source:HGNC Symbol;Acc:HGNC:20294] |
| ENSG00000133316 | HGLibA_54171 | 68 | -0.519610172 | WDR74 | WD repeat domain 74 [Source:HGNC Symbol;Acc:HGNC:25529] |
| ENSG00000133612 | HGLibA_01144 | 68 | -0.519610172 | AGAP3 | ankyrin repeat and PH domain 3 [Source:HGNC Symbol;Acc:HGNC:16923]" |
| ENSG00000133985 | HGLibA_52158 | 68 | -0.519610172 | TTC9 | tetratricopeptide repeat domain 9 [Source:HGNC Symbol;Acc:HGNC:20267] |
| ENSG00000134121 | HGLibA_09463 | 68 | -0.519610172 | CHL1 | cell adhesion molecule L1 like [Source:HGNC Symbol;Acc:HGNC:1939] |
| ENSG00000134369 | HGLibA_31194 | 68 | -0.519610172 | NAV1 | neuron navigator 1 [Source:HGNC Symbol;Acc:HGNC:15989] |
| ENSG00000134489 | HGLibA_22102 | 68 | -0.519610172 | HRH4 | histamine receptor H4 [Source:HGNC Symbol;Acc:HGNC:17383] |
| ENSG00000135503 | HGLibA_00689 | 68 | -0.519610172 | ACVR1B | activin A receptor type 1B [Source:HGNC Symbol;Acc:HGNC:172] |
| ENSG00000136810 | HGLibA_52398 | 68 | -0.519610172 | TXN | thioredoxin [Source:HGNC Symbol;Acc:HGNC:12435] |
| ENSG00000137251 | HGLibA_49548 | 68 | -0.519610172 | TINAG | tubulointerstitial nephritis antigen [Source:HGNC Symbol;Acc:HGNC:14599] |
| ENSG00000137491 | HGLibA_45464 | 68 | -0.519610172 | SLCO2B1 | solute carrier organic anion transporter family member 2B1 [Source:HGNC Symbol;Acc:HGNC:10962] |
| ENSG00000138430 | HGLibA_33337 | 68 | -0.519610172 | OLA1 | Obg like ATPase 1 [Source:HGNC Symbol;Acc:HGNC:28833] |
| ENSG00000138685 | HGLibA_17389 | 68 | -0.519610172 | FGF2 | fibroblast growth factor 2 [Source:HGNC Symbol;Acc:HGNC:3676] |
| ENSG00000139219 | HGLibA_10560 | 68 | -0.519610172 | COL2A1 | collagen type II alpha 1 chain [Source:HGNC Symbol;Acc:HGNC:2200] |
| ENSG00000140350 | HGLibA_02198 | 68 | -0.519610172 | ANP32A | acidic nuclear phosphoprotein 32 family member A [Source:HGNC Symbol;Acc:HGNC:13233] |
| ENSG00000140400 | HGLibA_28082 | 68 | -0.519610172 | MAN2C1 | mannosidase alpha class 2C member 1 [Source:HGNC Symbol;Acc:HGNC:6827] |
| ENSG00000140464 | HGLibA_37312 | 68 | -0.519610172 | PML | promyelocytic leukemia [Source:HGNC Symbol;Acc:HGNC:9113] |
| ENSG00000141748 | HGLibA_02981 | 68 | -0.519610172 | ARL5C | ADP ribosylation factor like GTPase 5C [Source:HGNC Symbol;Acc:HGNC:31111] |
| ENSG00000141837 | HGLibA_06876 | 68 | -0.519610172 | CACNA1A | calcium voltage-gated channel subunit alpha1 A [Source:HGNC Symbol;Acc:HGNC:1388] |
| ENSG00000142698 | HGLibA_05906 | 68 | -0.519610172 | C1orf94 | chromosome 1 open reading frame 94 [Source:HGNC Symbol;Acc:HGNC:28250] |
| ENSG00000143633 | HGLibA_05768 | 68 | -0.519610172 | C1orf131 | chromosome 1 open reading frame 131 [Source:HGNC Symbol;Acc:HGNC:25332] |
| ENSG00000143772 | HGLibA_23848 | 68 | -0.519610172 | ITPKB | inositol-trisphosphate 3-kinase B [Source:HGNC Symbol;Acc:HGNC:6179] |
| ENSG00000144224 | HGLibA_52790 | 68 | -0.519610172 | UBXN4 | UBX domain protein 4 [Source:HGNC Symbol;Acc:HGNC:14860] |
| ENSG00000144231 | HGLibA_37573 | 68 | -0.519610172 | POLR2D | RNA polymerase II subunit D [Source:HGNC Symbol;Acc:HGNC:9191] |
| ENSG00000145246 | HGLibA_03551 | 68 | -0.519610172 | ATP10D | ATPase phospholipid transporting 10D (putative) [Source:HGNC Symbol;Acc:HGNC:13549] |
| ENSG00000146166 | HGLibA_26416 | 68 | -0.519610172 | LGSN | lens protein with glutamine synthetase domain [Source:HGNC Symbol;Acc:HGNC:21016]" |
| ENSG00000147873 | HGLibA_22683 | 68 | -0.519610172 | IFNA5 | interferon alpha 5 [Source:HGNC Symbol;Acc:HGNC:5426] |
| ENSG00000148158 | HGLibA_46083 | 68 | -0.519610172 | SNX30 | sorting nexin family member 30 [Source:HGNC Symbol;Acc:HGNC:23685] |
| ENSG00000148737 | HGLibA_48798 | 68 | -0.519610172 | TCF7L2 | transcription factor 7 like 2 [Source:HGNC Symbol;Acc:HGNC:11641] |
| ENSG00000149311 | HGLibA_03525 | 68 | -0.519610172 | ATM | ATM serine/threonine kinase [Source:HGNC Symbol;Acc:HGNC:795] |
| ENSG00000150712 | HGLibA_30417 | 68 | -0.519610172 | MTMR12 | myotubularin related protein 12 [Source:HGNC Symbol;Acc:HGNC:18191] |
| ENSG00000151348 | HGLibA_15806 | 68 | -0.519610172 | EXT2 | exostosin glycosyltransferase 2 [Source:HGNC Symbol;Acc:HGNC:3513] |
| ENSG00000152332 | HGLibA_52943 | 68 | -0.519610172 | UHMK1 | U2AF homology motif kinase 1 [Source:HGNC Symbol;Acc:HGNC:19683] |
| ENSG00000152465 | HGLibA_32207 | 68 | -0.519610172 | NMT2 | N-myristoyltransferase 2 [Source:HGNC Symbol;Acc:HGNC:7858] |
| ENSG00000152492 | HGLibA_07794 | 68 | -0.519610172 | CCDC50 | coiled-coil domain containing 50 [Source:HGNC Symbol;Acc:HGNC:18111] |
| ENSG00000153208 | HGLibA_28907 | 68 | -0.519610172 | MERTK | tyrosine kinase [Source:HGNC Symbol;Acc:HGNC:7027]" |
| ENSG00000153561 | HGLibA_41382 | 68 | -0.519610172 | RMND5A | required for meiotic nuclear division 5 homolog A [Source:HGNC Symbol;Acc:HGNC:25850] |
| ENSG00000154478 | HGLibA_20007 | 68 | -0.519610172 | GPR26 | G protein-coupled receptor 26 [Source:HGNC Symbol;Acc:HGNC:4481] |
| ENSG00000155189 | HGLibA_01228 | 68 | -0.519610172 | AGPAT5 | 1-acylglycerol-3-phosphate O-acyltransferase 5 [Source:HGNC Symbol;Acc:HGNC:20886] |
| ENSG00000155659 | HGLibA_53849 | 68 | -0.519610172 | VSIG4 | V-set and immunoglobulin domain containing 4 [Source:HGNC Symbol;Acc:HGNC:17032] |
| ENSG00000156096 | HGLibA_52926 | 68 | -0.519610172 | UGT2B4 | UDP glucuronosyltransferase family 2 member B4 [Source:HGNC Symbol;Acc:HGNC:12553] |
| ENSG00000157014 | HGLibA_48442 | 68 | -0.519610172 | TATDN2 | TatD DNase domain containing 2 [Source:HGNC Symbol;Acc:HGNC:28988] |
| ENSG00000157326 | HGLibA_13135 | 68 | -0.519610172 | DHRS4 | dehydrogenase/reductase 4 [Source:HGNC Symbol;Acc:HGNC:16985] |
| ENSG00000157637 | HGLibA_45059 | 68 | -0.519610172 | SLC38A10 | solute carrier family 38 member 10 [Source:HGNC Symbol;Acc:HGNC:28237] |
| ENSG00000157873 | HGLibA_50760 | 68 | -0.519610172 | TNFRSF14 | TNF receptor superfamily member 14 [Source:HGNC Symbol;Acc:HGNC:11912] |
| ENSG00000158079 | HGLibA_39412 | 68 | -0.519610172 | PTPDC1 | protein tyrosine phosphatase domain containing 1 [Source:HGNC Symbol;Acc:HGNC:30184] |
| ENSG00000158163 | HGLibA_14300 | 68 | -0.519610172 | DZIP1L | DAZ interacting zinc finger protein 1 like [Source:HGNC Symbol;Acc:HGNC:26551] |
| ENSG00000158220 | HGLibA_15610 | 68 | -0.519610172 | ESYT3 | extended synaptotagmin 3 [Source:HGNC Symbol;Acc:HGNC:24295] |
| ENSG00000158477 | HGLibA_08265 | 68 | -0.519610172 | CD1A | CD1a molecule [Source:HGNC Symbol;Acc:HGNC:1634] |
| ENSG00000159069 | HGLibA_17151 | 68 | -0.519610172 | FBXW5 | F-box and WD repeat domain containing 5 [Source:HGNC Symbol;Acc:HGNC:13613] |
| ENSG00000160310 | HGLibA_38624 | 68 | -0.519610172 | PRMT2 | protein arginine methyltransferase 2 [Source:HGNC Symbol;Acc:HGNC:5186] |
| ENSG00000162461 | HGLibA_44713 | 68 | -0.519610172 | SLC25A34 | solute carrier family 25 member 34 [Source:HGNC Symbol;Acc:HGNC:27653] |
| ENSG00000162896 | HGLibA_36583 | 68 | -0.519610172 | PIGR | polymeric immunoglobulin receptor [Source:HGNC Symbol;Acc:HGNC:8968] |
| ENSG00000162980 | HGLibA_02974 | 68 | -0.519610172 | ARL5A | ADP ribosylation factor like GTPase 5A [Source:HGNC Symbol;Acc:HGNC:696] |
| ENSG00000163531 | HGLibA_31768 | 68 | -0.519610172 | NFASC | neurofascin [Source:HGNC Symbol;Acc:HGNC:29866] |
| ENSG00000163630 | HGLibA_48027 | 68 | -0.519610172 | SYNPR | synaptoporin [Source:HGNC Symbol;Acc:HGNC:16507] |
| ENSG00000164258 | HGLibA_31554 | 68 | -0.519610172 | NDUFS4 | NADH:ubiquinone oxidoreductase subunit S4 [Source:HGNC Symbol;Acc:HGNC:7711] |
| ENSG00000164326 | HGLibA_07265 | 68 | -0.519610172 | CARTPT | CART prepropeptide [Source:HGNC Symbol;Acc:HGNC:24323] |
| ENSG00000164756 | HGLibA_44921 | 68 | -0.519610172 | SLC30A8 | solute carrier family 30 member 8 [Source:HGNC Symbol;Acc:HGNC:20303] |
| ENSG00000164830 | HGLibA_34831 | 68 | -0.519610172 | OXR1 | oxidation resistance 1 [Source:HGNC Symbol;Acc:HGNC:15822] |
| ENSG00000164893 | HGLibA_45355 | 68 | -0.519610172 | SLC7A13 | solute carrier family 7 member 13 [Source:HGNC Symbol;Acc:HGNC:23092] |
| ENSG00000166794 | HGLibA_37919 | 68 | -0.519610172 | PPIB | peptidylprolyl isomerase B [Source:HGNC Symbol;Acc:HGNC:9255] |
| ENSG00000167104 | HGLibA_04732 | 68 | -0.519610172 | BPIFB6 | BPI fold containing family B member 6 [Source:HGNC Symbol;Acc:HGNC:16504] |
| ENSG00000167257 | HGLibA_41626 | 68 | -0.519610172 | RNF214 | ring finger protein 214 [Source:HGNC Symbol;Acc:HGNC:25335] |
| ENSG00000167323 | HGLibA_47440 | 68 | -0.519610172 | STIM1 | stromal interaction molecule 1 [Source:HGNC Symbol;Acc:HGNC:11386] |
| ENSG00000168386 | HGLibA_17532 | 68 | -0.519610172 | FILIP1L | filamin A interacting protein 1 like [Source:HGNC Symbol;Acc:HGNC:24589] |
| ENSG00000168702 | HGLibA_27208 | 68 | -0.519610172 | LRP1B | LDL receptor related protein 1B [Source:HGNC Symbol;Acc:HGNC:6693] |
| ENSG00000168899 | HGLibA_53500 | 68 | -0.519610172 | VAMP5 | vesicle associated membrane protein 5 [Source:HGNC Symbol;Acc:HGNC:12646] |
| ENSG00000169059 | HGLibA_53587 | 68 | -0.519610172 | VCX3A | variable charge X-linked 3A [Source:HGNC Symbol;Acc:HGNC:18159] |
| ENSG00000169442 | HGLibA_08404 | 68 | -0.519610172 | CD52 | CD52 molecule [Source:HGNC Symbol;Acc:HGNC:1804] |
| ENSG00000169469 | HGLibA_46804 | 68 | -0.519610172 | SPRR1B | small proline rich protein 1B [Source:HGNC Symbol;Acc:HGNC:11260] |
| ENSG00000170113 | HGLibA_31951 | 68 | -0.519610172 | NIPA1 | NIPA magnesium transporter 1 [Source:HGNC Symbol;Acc:HGNC:17043] |
| ENSG00000170471 | HGLibA_40162 | 68 | -0.519610172 | RALGAPB | Ral GTPase activating protein non-catalytic beta subunit [Source:HGNC Symbol;Acc:HGNC:29221] |
| ENSG00000170471 | HGLibA_40161 | 68 | -0.519610172 | RALGAPB | Ral GTPase activating protein non-catalytic beta subunit [Source:HGNC Symbol;Acc:HGNC:29221] |
| ENSG00000170516 | HGLibA_10870 | 68 | -0.519610172 | COX7B2 | cytochrome c oxidase subunit 7B2 [Source:HGNC Symbol;Acc:HGNC:24381] |
| ENSG00000170634 | HGLibA_00714 | 68 | -0.519610172 | ACYP2 | acylphosphatase 2 [Source:HGNC Symbol;Acc:HGNC:180] |
| ENSG00000171987 | HGLibA_05136 | 68 | -0.519610172 | C11orf40 | chromosome 11 open reading frame 40 [Source:HGNC Symbol;Acc:HGNC:23986] |
| ENSG00000172465 | HGLibA_48705 | 68 | -0.519610172 | TCEAL1 | transcription elongation factor A like 1 [Source:HGNC Symbol;Acc:HGNC:11616] |
| ENSG00000173261 | HGLibA_36945 | 68 | -0.519610172 | PLAC8L1 | PLAC8 like 1 [Source:HGNC Symbol;Acc:HGNC:31746] |
| ENSG00000173442 | HGLibA_14664 | 68 | -0.519610172 | EHBP1L1 | EH domain binding protein 1 like 1 [Source:HGNC Symbol;Acc:HGNC:30682] |

| | | | | | |
|---|---|---|---|---|---|
| ENSG00000175336 | HGLibA_02520 | 68 | -0.519610172 | APOF | apolipoprotein F [Source:HGNC Symbol;Acc:HGNC:615] |
| ENSG00000176695 | HGLibA_33996 | 68 | -0.519610172 | OR4F17 | olfactory receptor family 4 subfamily F member 17 [Source:HGNC Symbol;Acc:HGNC:15381] |
| ENSG00000176887 | HGLibA_46231 | 68 | -0.519610172 | SOX11 | SRY-box 11 [Source:HGNC Symbol;Acc:HGNC:11191] |
| ENSG00000177504 | HGLibA_53585 | 68 | -0.519610172 | VCX2 | variable charge X-linked 2 [Source:HGNC Symbol;Acc:HGNC:18158] |
| ENSG00000180083 | HGLibA_54253 | 68 | -0.519610172 | WFDC11 | WAP four-disulfide core domain 11 [Source:HGNC Symbol;Acc:HGNC:20478] |
| ENSG00000180269 | HGLibA_19906 | 68 | -0.519610172 | GPR139 | G protein-coupled receptor 139 [Source:HGNC Symbol;Acc:HGNC:19995] |
| ENSG00000181617 | HGLibA_17250 | 68 | -0.519610172 | FDCSP | follicular dendritic cell secreted protein [Source:HGNC Symbol;Acc:HGNC:19215] |
| ENSG00000182195 | HGLibA_26256 | 68 | -0.519610172 | LDOC1 | regulator of NFKB signaling [Source:HGNC Symbol;Acc:HGNC:6548]" |
| ENSG00000182447 | HGLibA_34731 | 68 | -0.519610172 | OTOL1 | otolin 1 [Source:HGNC Symbol;Acc:HGNC:34071] |
| ENSG00000182606 | HGLibA_51276 | 68 | -0.519610172 | TRAK1 | trafficking kinesin protein 1 [Source:HGNC Symbol;Acc:HGNC:29947] |
| ENSG00000183036 | HGLibA_35661 | 68 | -0.519610172 | PCP4 | Purkinje cell protein 4 [Source:HGNC Symbol;Acc:HGNC:8742] |
| ENSG00000183281 | HGLibA_37160 | 68 | -0.519610172 | PLGLB1 | plasminogen-like B1 [Source:HGNC Symbol;Acc:HGNC:9072] |
| ENSG00000184108 | HGLibA_51622 | 68 | -0.519610172 | TRIML1 | tripartite motif family like 1 [Source:HGNC Symbol;Acc:HGNC:26698] |
| ENSG00000184388 | HGLibA_34931 | 68 | -0.519610172 | PABPC1L2B | poly(A) binding protein cytoplasmic 1 like 2B [Source:HGNC Symbol;Acc:HGNC:31852] |
| ENSG00000184697 | HGLibA_09905 | 68 | -0.519610172 | CLDN6 | claudin 6 [Source:HGNC Symbol;Acc:HGNC:2048] |
| ENSG00000184954 | HGLibA_34402 | 68 | -0.519610172 | OR6C70 | olfactory receptor family 6 subfamily C member 70 [Source:HGNC Symbol;Acc:HGNC:31299] |
| ENSG00000184990 | HGLibA_44241 | 68 | -0.519610172 | SIVA1 | SIVA1 apoptosis inducing factor [Source:HGNC Symbol;Acc:HGNC:17712] |
| ENSG00000185163 | HGLibA_12736 | 68 | -0.519610172 | DDX51 | DEAD-box helicase 51 [Source:HGNC Symbol;Acc:HGNC:20082] |
| ENSG00000185338 | HGLibA_46122 | 68 | -0.519610172 | SOCS1 | suppressor of cytokine signaling 1 [Source:HGNC Symbol;Acc:HGNC:19383] |
| ENSG00000185972 | HGLibA_07949 | 68 | -0.519610172 | CCIN | calicin [Source:HGNC Symbol;Acc:HGNC:1568] |
| ENSG00000187908 | HGLibA_13394 | 68 | -0.519610172 | DMBT1 | deleted in malignant brain tumors 1 [Source:HGNC Symbol;Acc:HGNC:2926] |
| ENSG00000188092 | HGLibA_20109 | 68 | -0.519610172 | GPR89B | G protein-coupled receptor 89B [Source:HGNC Symbol;Acc:HGNC:13840] |
| ENSG00000188155 | HGLibA_25616 | 68 | -0.519610172 | KRTAP10-6 | keratin associated protein 10-6 [Source:HGNC Symbol;Acc:HGNC:20523] |
| ENSG00000188186 | HGLibA_25997 | 68 | -0.519610172 | LAMTOR4 | MAPK and MTOR activator 4 [Source:HGNC Symbol;Acc:HGNC:33772]" |
| ENSG00000188760 | HGLibA_50161 | 68 | -0.519610172 | TMEM198 | transmembrane protein 198 [Source:HGNC Symbol;Acc:HGNC:33704] |
| ENSG00000188994 | HGLibA_55760 | 68 | -0.519610172 | ZNF292 | zinc finger protein 292 [Source:HGNC Symbol;Acc:HGNC:18410] |
| ENSG00000196369 | HGLibA_46972 | 68 | -0.519610172 | SRGAP2B | SLIT-ROBO Rho GTPase activating protein 2B [Source:HGNC Symbol;Acc:HGNC:35237] |
| ENSG00000196937 | HGLibA_16581 | 68 | -0.519610172 | FAM3C | family with sequence similarity 3 member C [Source:HGNC Symbol;Acc:HGNC:18664] |
| ENSG00000197147 | HGLibA_27430 | 68 | -0.519610172 | LRRC8B | leucine rich repeat containing 8 VRAC subunit B [Source:HGNC Symbol;Acc:HGNC:30692] |
| ENSG00000197296 | HGLibA_17544 | 68 | -0.519610172 | FITM2 | fat storage inducing transmembrane protein 2 [Source:HGNC Symbol;Acc:HGNC:16135] |
| ENSG00000197548 | HGLibA_03504 | 68 | -0.519610172 | ATG7 | autophagy related 7 [Source:HGNC Symbol;Acc:HGNC:16935] |
| ENSG00000197568 | HGLibA_21274 | 68 | -0.519610172 | HHLA3 | HERV-H LTR-associating 3 [Source:HGNC Symbol;Acc:HGNC:4906] |
| ENSG00000197608 | HGLibA_56854 | 68 | -0.519610172 | ZNF841 | zinc finger protein 841 [Source:HGNC Symbol;Acc:HGNC:27611] |
| ENSG00000197953 | HGLibA_00032 | 68 | -0.519610172 | AADACL2 | arylacetamide deacetylase like 2 [Source:HGNC Symbol;Acc:HGNC:24427] |
| ENSG00000198018 | HGLibA_15216 | 68 | -0.519610172 | ENTPD7 | ectonucleoside triphosphate diphosphohydrolase 7 [Source:HGNC Symbol;Acc:HGNC:19745] |
| ENSG00000198046 | HGLibA_56490 | 68 | -0.519610172 | ZNF667 | zinc finger protein 667 [Source:HGNC Symbol;Acc:HGNC:28854] |
| ENSG00000198246 | HGLibA_44845 | 68 | -0.519610172 | SLC29A3 | solute carrier family 29 member 3 [Source:HGNC Symbol;Acc:HGNC:23096] |
| ENSG00000198336 | HGLibA_30763 | 68 | -0.519610172 | MYL4 | myosin light chain 4 [Source:HGNC Symbol;Acc:HGNC:7585] |
| ENSG00000203661 | HGLibA_33887 | 68 | -0.519610172 | OR2T5 | olfactory receptor family 2 subfamily T member 5 [Source:HGNC Symbol;Acc:HGNC:15017] |
| ENSG00000203722 | HGLibA_40124 | 68 | -0.519610172 | RAET1G | retinoic acid early transcript 1G [Source:HGNC Symbol;Acc:HGNC:16795] |
| ENSG00000204310 | HGLibA_01216 | 68 | -0.519610172 | AGPAT1 | 1-acylglycerol-3-phosphate O-acyltransferase 1 [Source:HGNC Symbol;Acc:HGNC:324] |
| ENSG00000204472 | HGLibA_01332 | 68 | -0.519610172 | AIF1 | allograft inflammatory factor 1 [Source:HGNC Symbol;Acc:HGNC:352] |
| ENSG00000204510 | HGLibA_38327 | 68 | -0.519610172 | PRAMEF7 | PRAME family member 7 [Source:HGNC Symbol;Acc:HGNC:28415] |
| ENSG00000204514 | HGLibA_56808 | 68 | -0.519610172 | ZNF814 | zinc finger protein 814 [Source:HGNC Symbol;Acc:HGNC:33258] |
| ENSG00000204919 | HGLibA_38801 | 68 | -0.519610172 | PRR20A | proline rich 20A [Source:HGNC Symbol;Acc:HGNC:24754] |
| ENSG00000205937 | HGLibA_41730 | 68 | -0.519610172 | RNPS1 | RNA binding protein with serine rich domain 1 [Source:HGNC Symbol;Acc:HGNC:10080] |
| ENSG00000206047 | HGLibA_12793 | 68 | -0.519610172 | DEFA1 | defensin alpha 1 [Source:HGNC Symbol;Acc:HGNC:2761] |
| ENSG00000213218 | HGLibA_11355 | 68 | -0.519610172 | CSH2 | chorionic somatomammotropin hormone 2 [Source:HGNC Symbol;Acc:HGNC:2441] |
| ENSG00000214510 | HGLibA_46685 | 68 | -0.519610172 | SPINK13 | Kazal type 13 (putative) [Source:HGNC Symbol;Acc:HGNC:27200]" |
| ENSG00000223638 | HGLibA_40939 | 68 | -0.519610172 | RFPL4A | ret finger protein like 4A [Source:HGNC Symbol;Acc:HGNC:16449] |
| ENSG00000227729 | HGLibA_40729 | 68 | -0.519610172 | RD3L | retinal degeneration 3 like [Source:HGNC Symbol;Acc:HGNC:40912] |
| ENSG00000235631 | HGLibA_41538 | 68 | -0.519610172 | RNF148 | ring finger protein 148 [Source:HGNC Symbol;Acc:HGNC:22411] |
| ENSG00000241399 | HGLibA_08347 | 68 | -0.519610172 | CD302 | CD302 molecule [Source:HGNC Symbol;Acc:HGNC:30843] |
| ENSG00000242019 | HGLibA_24998 | 68 | -0.519610172 | KIR3DL3 | three Ig domains and long cytoplasmic tail 3 [Source:HGNC Symbol;Acc:HGNC:16312]" |
| ENSG00000254087 | HGLibA_27708 | 68 | -0.519610172 | LYN | Src family tyrosine kinase [Source:HGNC Symbol;Acc:HGNC:6735]" |
| ENSG00000257138 | HGLibA_48393 | 68 | -0.519610172 | TAS2R38 | taste 2 receptor member 38 [Source:HGNC Symbol;Acc:HGNC:9584] |
| ENSG00000258986 | HGLibA_50086 | 68 | -0.519610172 | TMEM179 | transmembrane protein 179 [Source:HGNC Symbol;Acc:HGNC:20137] |
| ENSG00000269067 | HGLibA_56638 | 68 | -0.519610172 | ZNF728 | zinc finger protein 728 [Source:HGNC Symbol;Acc:HGNC:32463] |
| ENSG00000271449 | HGLibA_11501 | 68 | -0.519610172 | CT45A2 | cancer/testis antigen family 45 member A2 [Source:HGNC Symbol;Acc:HGNC:28400] |
| ENSG00000274391 | HGLibA_51214 | 68 | -0.519610172 | TPTE | transmembrane phosphatase with tensin homology [Source:HGNC Symbol;Acc:HGNC:12023] |
| ENSG00000275111 | HGLibA_55563 | 68 | -0.519610172 | ZNF2 | zinc finger protein 2 [Source:HGNC Symbol;Acc:HGNC:12991] |
| ENSG00000288867 | HGLibA_37902 | 68 | -0.519610172 | PPIAL4C | - |
| FAM5B | HGLibA_16649 | 68 | -0.519610172 | FAM5B | - |
| GPR124 | HGLibA_19877 | 68 | -0.519610172 | GPR124 | - |
| HIST2H4B | HGLibA_21551 | 68 | -0.519610172 | HIST2H4B | - |
| hsa-mir-103a-2 | HGLibA_57166 | 68 | -0.519610172 | hsa-mir-103a-2 | - |
| hsa-mir-106a | HGLibA_57186 | 68 | -0.519610172 | hsa-mir-106a | - |
| hsa-mir-1246 | HGLibA_57381 | 68 | -0.519610172 | hsa-mir-1246 | - |
| hsa-mir-1265 | HGLibA_57483 | 68 | -0.519610172 | hsa-mir-1265 | - |
| hsa-mir-1289-1 | HGLibA_57619 | 68 | -0.519610172 | hsa-mir-1289-1 | - |
| hsa-mir-155 | HGLibA_57931 | 68 | -0.519610172 | hsa-mir-155 | - |
| hsa-mir-15b | HGLibA_57944 | 68 | -0.519610172 | hsa-mir-15b | - |
| hsa-mir-3160-1 | HGLibA_58714 | 68 | -0.519610172 | hsa-mir-3160-1 | - |
| hsa-mir-3180-5 | HGLibA_58816 | 68 | -0.519610172 | hsa-mir-3180-5 | - |
| hsa-mir-320a | HGLibA_58925 | 68 | -0.519610172 | hsa-mir-320a | - |
| hsa-mir-320d-1 | HGLibA_58944 | 68 | -0.519610172 | hsa-mir-320d-1 | - |
| hsa-mir-340 | HGLibA_59019 | 68 | -0.519610172 | hsa-mir-340 | - |
| hsa-mir-4505 | HGLibA_60446 | 68 | -0.519610172 | hsa-mir-4505 | - |
| hsa-mir-4655 | HGLibA_60726 | 68 | -0.519610172 | hsa-mir-4655 | - |
| hsa-mir-4658 | HGLibA_60740 | 68 | -0.519610172 | hsa-mir-4658 | - |
| hsa-mir-4662a | HGLibA_60759 | 68 | -0.519610172 | hsa-mir-4662a | - |
| hsa-mir-5002 | HGLibA_61415 | 68 | -0.519610172 | hsa-mir-5002 | - |
| hsa-mir-501 | HGLibA_61450 | 68 | -0.519610172 | hsa-mir-501 | - |
| hsa-mir-5191 | HGLibA_61681 | 68 | -0.519610172 | hsa-mir-5191 | - |
| hsa-mir-520h | HGLibA_61764 | 68 | -0.519610172 | hsa-mir-520h | - |
| hsa-mir-562 | HGLibA_62213 | 68 | -0.519610172 | hsa-mir-562 | - |
| hsa-mir-6131 | HGLibA_62675 | 68 | -0.519610172 | hsa-mir-6131 | - |
| hsa-mir-614 | HGLibA_62692 | 68 | -0.519610172 | hsa-mir-614 | - |
| hsa-mir-630 | HGLibA_62758 | 68 | -0.519610172 | hsa-mir-630 | - |
| hsa-mir-660 | HGLibA_62972 | 68 | -0.519610172 | hsa-mir-660 | - |
| hsa-mir-6740 | HGLibA_63113 | 68 | -0.519610172 | hsa-mir-6740 | - |
| hsa-mir-6742 | HGLibA_63123 | 68 | -0.519610172 | hsa-mir-6742 | - |
| hsa-mir-6818 | HGLibA_63450 | 68 | -0.519610172 | hsa-mir-6818 | - |
| hsa-mir-6875 | HGLibA_63685 | 68 | -0.519610172 | hsa-mir-6875 | - |
| hsa-mir-6875 | HGLibA_63683 | 68 | -0.519610172 | hsa-mir-6875 | - |
| KIAA1244 | HGLibA_24727 | 68 | -0.519610172 | KIAA1244 | - |
| LOC100287177 | HGLibA_26819 | 68 | -0.519610172 | LOC100287177 | - |
| LOC440563 | HGLibA_26949 | 68 | -0.519610172 | LOC440563 | - |
| LOC643669 | HGLibA_26969 | 68 | -0.519610172 | LOC643669 | - |
| MKI67IP | HGLibA_29327 | 68 | -0.519610172 | MKI67IP | - |
| MLLT4 | HGLibA_29419 | 68 | -0.519610172 | MLLT4 | - |
| NRD1 | HGLibA_32664 | 68 | -0.519610172 | NRD1 | - |
| NUTMG | HGLibA_33117 | 68 | -0.519610172 | NUTMG | - |
| NUTMG | HGLibA_33118 | 68 | -0.519610172 | NUTMG | - |
| PHF17 | HGLibA_36354 | 68 | -0.519610172 | PHF17 | - |
| SGK223 | HGLibA_43829 | 68 | -0.519610172 | SGK223 | - |
| C3orf17 | HGLibA_06211 | 67 | -0.528655515 | C3orf17 | - |
| CCDC64 | HGLibA_07826 | 67 | -0.528655515 | CCDC64 | - |
| CCDC64B | HGLibA_07828 | 67 | -0.528655515 | CCDC64B | - |
| CRLF2_Y | HGLibA_11193 | 67 | -0.528655515 | CRLF2_Y | - |
| ENSG00000006555 | HGLibA_52076 | 67 | -0.528655515 | TTC22 | tetratricopeptide repeat domain 22 [Source:HGNC Symbol;Acc:HGNC:26067] |
| ENSG00000007341 | HGLibA_47286 | 67 | -0.528655515 | ST7L | suppression of tumorigenicity 7 like [Source:HGNC Symbol;Acc:HGNC:18441] |
| ENSG00000008952 | HGLibA_43259 | 67 | -0.528655515 | SEC62 | preprotein translocation factor [Source:HGNC Symbol;Acc:HGNC:11846]" |
| ENSG00000014824 | HGLibA_44922 | 67 | -0.528655515 | SLC30A9 | solute carrier family 30 member 9 [Source:HGNC Symbol;Acc:HGNC:1329] |
| ENSG00000023516 | HGLibA_01409 | 67 | -0.528655515 | AKAP11 | A-kinase anchoring protein 11 [Source:HGNC Symbol;Acc:HGNC:369] |
| ENSG00000033627 | HGLibA_03702 | 67 | -0.528655515 | ATP6V0A1 | ATPase H+ transporting V0 subunit a1 [Source:HGNC Symbol;Acc:HGNC:865] |
| ENSG00000051180 | HGLibA_40079 | 67 | -0.528655515 | RAD51 | RAD51 recombinase [Source:HGNC Symbol;Acc:HGNC:9817] |
| ENSG00000053371 | HGLibA_01498 | 67 | -0.528655515 | AKR7A2 | aldo-keto reductase family 7 member A2 [Source:HGNC Symbol;Acc:HGNC:389] |
| ENSG00000054793 | HGLibA_03793 | 67 | -0.528655515 | ATP9A | ATPase phospholipid transporting 9A (putative) [Source:HGNC Symbol;Acc:HGNC:13540] |
| ENSG00000061936 | HGLibA_43748 | 67 | -0.528655515 | SFSWAP | splicing factor SWAP [Source:HGNC Symbol;Acc:HGNC:10790] |
| ENSG00000062725 | HGLibA_02563 | 67 | -0.528655515 | APPBP2 | amyloid beta precursor protein binding protein 2 [Source:HGNC Symbol;Acc:HGNC:622] |
| ENSG00000064763 | HGLibA_16870 | 67 | -0.528655515 | FAR2 | fatty acyl-CoA reductase 2 [Source:HGNC Symbol;Acc:HGNC:25531] |
| ENSG00000064933 | HGLibA_37334 | 67 | -0.528655515 | PMS1 | mismatch repair system component [Source:HGNC Symbol;Acc:HGNC:9121]" |
| ENSG00000066136 | HGLibA_31860 | 67 | -0.528655515 | NFYC | nuclear transcription factor Y subunit gamma [Source:HGNC Symbol;Acc:HGNC:7806] |
| ENSG00000072042 | HGLibA_40733 | 67 | -0.528655515 | RDH11 | retinol dehydrogenase 11 [Source:HGNC Symbol;Acc:HGNC:17964] |
| ENSG00000075426 | HGLibA_17804 | 67 | -0.528655515 | FOSL2 | AP-1 transcription factor subunit [Source:HGNC Symbol;Acc:HGNC:3798]" |
| ENSG00000075785 | HGLibA_39979 | 67 | -0.528655515 | RAB7A | member RAS oncogene family [Source:HGNC Symbol;Acc:HGNC:9788]" |
| ENSG00000075884 | HGLibA_02712 | 67 | -0.528655515 | ARHGAP15 | Rho GTPase activating protein 15 [Source:HGNC Symbol;Acc:HGNC:21030] |
| ENSG00000077092 | HGLibA_40276 | 67 | -0.528655515 | RARB | retinoic acid receptor beta [Source:HGNC Symbol;Acc:HGNC:9865] |
| ENSG00000077232 | HGLibA_13563 | 67 | -0.528655515 | DNAJC10 | DnaJ heat shock protein family (Hsp40) member C10 [Source:HGNC Symbol;Acc:HGNC:24637] |
| ENSG00000079459 | HGLibA_17252 | 67 | -0.528655515 | FDFT1 | farnesyl-diphosphate farnesyltransferase 1 [Source:HGNC Symbol;Acc:HGNC:3629] |
| ENSG00000090863 | HGLibA_19245 | 67 | -0.528655515 | GLG1 | golgi glycoprotein 1 [Source:HGNC Symbol;Acc:HGNC:4316] |
| ENSG00000092054 | HGLibA_30732 | 67 | -0.528655515 | MYH7 | myosin heavy chain 7 [Source:HGNC Symbol;Acc:HGNC:7577] |
| ENSG00000102796 | HGLibA_13123 | 67 | -0.528655515 | DHRS12 | dehydrogenase/reductase 12 [Source:HGNC Symbol;Acc:HGNC:25832] |
| ENSG00000104921 | HGLibA_17173 | 67 | -0.528655515 | FCER2 | Fc fragment of IgE receptor II [Source:HGNC Symbol;Acc:HGNC:3612] |

| | | | | | |
|---|---|---|---|---|---|
| ENSG00000105223 | HGLibA_37042 | 67 | -0.528655515 | PLD3 | phospholipase D family member 3 [Source:HGNC Symbol;Acc:HGNC:17158] |
| ENSG00000105835 | HGLibA_31088 | 67 | -0.528655515 | NAMPT | nicotinamide phosphoribosyltransferase [Source:HGNC Symbol;Acc:HGNC:30092] |
| ENSG00000106278 | HGLibA_39543 | 67 | -0.528655515 | PTPRZ1 | receptor type Z1 [Source:HGNC Symbol;Acc:HGNC:9685]" |
| ENSG00000108061 | HGLibA_44065 | 67 | -0.528655515 | SHOC2 | leucine rich repeat scaffold protein [Source:HGNC Symbol;Acc:HGNC:15454]" |
| ENSG00000108854 | HGLibA_45848 | 67 | -0.528655515 | SMURF2 | SMAD specific E3 ubiquitin protein ligase 2 [Source:HGNC Symbol;Acc:HGNC:16809] |
| ENSG00000110492 | HGLibA_28691 | 67 | -0.528655515 | MDK | midkine [Source:HGNC Symbol;Acc:HGNC:6972] |
| ENSG00000112110 | HGLibA_29914 | 67 | -0.528655515 | MRPL18 | mitochondrial ribosomal protein L18 [Source:HGNC Symbol;Acc:HGNC:14477] |
| ENSG00000112212 | HGLibA_51961 | 67 | -0.528655515 | TSPO2 | translocator protein 2 [Source:HGNC Symbol;Acc:HGNC:21256] |
| ENSG00000113240 | HGLibA_10063 | 67 | -0.528655515 | CLK4 | CDC like kinase 4 [Source:HGNC Symbol;Acc:HGNC:13659] |
| ENSG00000114126 | HGLibA_49154 | 67 | -0.528655515 | TFDP2 | transcription factor Dp-2 [Source:HGNC Symbol;Acc:HGNC:11751] |
| ENSG00000114529 | HGLibA_06247 | 67 | -0.528655515 | C3orf52 | chromosome 3 open reading frame 52 [Source:HGNC Symbol;Acc:HGNC:26255] |
| ENSG00000115232 | HGLibA_23734 | 67 | -0.528655515 | ITGA4 | integrin subunit alpha 4 [Source:HGNC Symbol;Acc:HGNC:6140] |
| ENSG00000115648 | HGLibA_29432 | 67 | -0.528655515 | MLPH | melanophilin [Source:HGNC Symbol;Acc:HGNC:29643] |
| ENSG00000115756 | HGLibA_22018 | 67 | -0.528655515 | HPCAL1 | hippocalcin like 1 [Source:HGNC Symbol;Acc:HGNC:5145] |
| ENSG00000116663 | HGLibA_17124 | 67 | -0.528655515 | FBXO6 | F-box protein 6 [Source:HGNC Symbol;Acc:HGNC:13585] |
| ENSG00000117009 | HGLibA_25317 | 67 | -0.528655515 | KMO | kynurenine 3-monooxygenase [Source:HGNC Symbol;Acc:HGNC:6381] |
| ENSG00000118007 | HGLibA_47321 | 67 | -0.528655515 | STAG1 | stromal antigen 1 [Source:HGNC Symbol;Acc:HGNC:11354] |
| ENSG00000118690 | HGLibA_03015 | 67 | -0.528655515 | ARMC2 | armadillo repeat containing 2 [Source:HGNC Symbol;Acc:HGNC:23045] |
| ENSG00000120137 | HGLibA_35102 | 67 | -0.528655515 | PANK3 | pantothenate kinase 3 [Source:HGNC Symbol;Acc:HGNC:19365] |
| ENSG00000120217 | HGLibA_08319 | 67 | -0.528655515 | CD274 | CD274 molecule [Source:HGNC Symbol;Acc:HGNC:17635] |
| ENSG00000121741 | HGLibA_55392 | 67 | -0.528655515 | ZMYM2 | zinc finger MYM-type containing 2 [Source:HGNC Symbol;Acc:HGNC:12989] |
| ENSG00000122574 | HGLibA_54309 | 67 | -0.528655515 | WIPF3 | WAS/WASL interacting protein family member 3 [Source:HGNC Symbol;Acc:HGNC:22004] |
| ENSG00000122778 | HGLibA_24778 | 67 | -0.528655515 | KIAA1549 | KIAA1549 [Source:HGNC Symbol;Acc:HGNC:22219] |
| ENSG00000124440 | HGLibA_21303 | 67 | -0.528655515 | HIF3A | hypoxia inducible factor 3 subunit alpha [Source:HGNC Symbol;Acc:HGNC:15825] |
| ENSG00000125144 | HGLibA_30290 | 67 | -0.528655515 | MT1G | metallothionein 1G [Source:HGNC Symbol;Acc:HGNC:7399] |
| ENSG00000125347 | HGLibA_23570 | 67 | -0.528655515 | IRF1 | interferon regulatory factor 1 [Source:HGNC Symbol;Acc:HGNC:6116] |
| ENSG00000125450 | HGLibA_33070 | 67 | -0.528655515 | NUP85 | nucleoporin 85 [Source:HGNC Symbol;Acc:HGNC:8734] |
| ENSG00000125779 | HGLibA_35101 | 67 | -0.528655515 | PANK2 | pantothenate kinase 2 [Source:HGNC Symbol;Acc:HGNC:15894] |
| ENSG00000126934 | HGLibA_28141 | 67 | -0.528655515 | MAP2K2 | mitogen-activated protein kinase kinase 2 [Source:HGNC Symbol;Acc:HGNC:6842] |
| ENSG00000126953 | HGLibA_49522 | 67 | -0.528655515 | TIMM8A | translocase of inner mitochondrial membrane 8A [Source:HGNC Symbol;Acc:HGNC:11817] |
| ENSG00000128713 | HGLibA_21989 | 67 | -0.528655515 | HOXD11 | homeobox D11 [Source:HGNC Symbol;Acc:HGNC:5134] |
| ENSG00000130487 | HGLibA_25109 | 67 | -0.528655515 | KLHDC7B | kelch domain containing 7B [Source:HGNC Symbol;Acc:HGNC:25145] |
| ENSG00000131943 | HGLibA_05629 | 67 | -0.528655515 | C19orf12 | chromosome 19 open reading frame 12 [Source:HGNC Symbol;Acc:HGNC:25443] |
| ENSG00000134324 | HGLibA_27094 | 67 | -0.528655515 | LPIN1 | lipin 1 [Source:HGNC Symbol;Acc:HGNC:13345] |
| ENSG00000134375 | HGLibA_49501 | 67 | -0.528655515 | TIMM17A | translocase of inner mitochondrial membrane 17A [Source:HGNC Symbol;Acc:HGNC:17315] |
| ENSG00000134758 | HGLibA_41515 | 67 | -0.528655515 | RNF138 | ring finger protein 138 [Source:HGNC Symbol;Acc:HGNC:17765] |
| ENSG00000135519 | HGLibA_24215 | 67 | -0.528655515 | KCNH3 | potassium voltage-gated channel subfamily H member 3 [Source:HGNC Symbol;Acc:HGNC:6252] |
| ENSG00000136244 | HGLibA_23181 | 67 | -0.528655515 | IL6 | interleukin 6 [Source:HGNC Symbol;Acc:HGNC:6018] |
| ENSG00000136546 | HGLibA_43002 | 67 | -0.528655515 | SCN7A | sodium voltage-gated channel alpha subunit 7 [Source:HGNC Symbol;Acc:HGNC:10594] |
| ENSG00000136834 | HGLibA_33674 | 67 | -0.528655515 | OR1J1 | olfactory receptor family 1 subfamily J member 1 [Source:HGNC Symbol;Acc:HGNC:8208] |
| ENSG00000136842 | HGLibA_50593 | 67 | -0.528655515 | TMOD1 | tropomodulin 1 [Source:HGNC Symbol;Acc:HGNC:11871] |
| ENSG00000137265 | HGLibA_23589 | 67 | -0.528655515 | IRF4 | interferon regulatory factor 4 [Source:HGNC Symbol;Acc:HGNC:6119] |
| ENSG00000137310 | HGLibA_48767 | 67 | -0.528655515 | TCF19 | transcription factor 19 [Source:HGNC Symbol;Acc:HGNC:11629] |
| ENSG00000137764 | HGLibA_28150 | 67 | -0.528655515 | MAP2K5 | mitogen-activated protein kinase kinase 5 [Source:HGNC Symbol;Acc:HGNC:6845] |
| ENSG00000138092 | HGLibA_09077 | 67 | -0.528655515 | CENPO | centromere protein O [Source:HGNC Symbol;Acc:HGNC:28152] |
| ENSG00000138449 | HGLibA_45135 | 67 | -0.528655515 | SLC40A1 | solute carrier family 40 member 1 [Source:HGNC Symbol;Acc:HGNC:10909] |
| ENSG00000138617 | HGLibA_35228 | 67 | -0.528655515 | PARP16 | poly(ADP-ribose) polymerase family member 16 [Source:HGNC Symbol;Acc:HGNC:26040] |
| ENSG00000138675 | HGLibA_17412 | 67 | -0.528655515 | FGF5 | fibroblast growth factor 5 [Source:HGNC Symbol;Acc:HGNC:3683] |
| ENSG00000138795 | HGLibA_26270 | 67 | -0.528655515 | LEF1 | lymphoid enhancer binding factor 1 [Source:HGNC Symbol;Acc:HGNC:6551] |
| ENSG00000139269 | HGLibA_23310 | 67 | -0.528655515 | INHBE | inhibin subunit beta E [Source:HGNC Symbol;Acc:HGNC:24029] |
| ENSG00000139410 | HGLibA_43174 | 67 | -0.528655515 | SDSL | serine dehydratase like [Source:HGNC Symbol;Acc:HGNC:30404] |
| ENSG00000139508 | HGLibA_45189 | 67 | -0.528655515 | SLC46A3 | solute carrier family 46 member 3 [Source:HGNC Symbol;Acc:HGNC:27501] |
| ENSG00000140443 | HGLibA_22794 | 67 | -0.528655515 | IGF1R | insulin like growth factor 1 receptor [Source:HGNC Symbol;Acc:HGNC:5465] |
| ENSG00000142235 | HGLibA_26728 | 67 | -0.528655515 | LMTK3 | lemur tyrosine kinase 3 [Source:HGNC Symbol;Acc:HGNC:19295] |
| ENSG00000142409 | HGLibA_56756 | 67 | -0.528655515 | ZNF787 | zinc finger protein 787 [Source:HGNC Symbol;Acc:HGNC:26998] |
| ENSG00000143162 | HGLibA_11119 | 67 | -0.528655515 | CREG1 | cellular repressor of E1A stimulated genes 1 [Source:HGNC Symbol;Acc:HGNC:2351] |
| ENSG00000143412 | HGLibA_02261 | 67 | -0.528655515 | ANXA9 | annexin A9 [Source:HGNC Symbol;Acc:HGNC:547] |
| ENSG00000143494 | HGLibA_53532 | 67 | -0.528655515 | VASH2 | vasohibin 2 [Source:HGNC Symbol;Acc:HGNC:25723] |
| ENSG00000143768 | HGLibA_26278 | 67 | -0.528655515 | LEFTY2 | left-right determination factor 2 [Source:HGNC Symbol;Acc:HGNC:3122] |
| ENSG00000144227 | HGLibA_33164 | 67 | -0.528655515 | NXPH2 | neurexophilin 2 [Source:HGNC Symbol;Acc:HGNC:8076] |
| ENSG00000145242 | HGLibA_15299 | 67 | -0.528655515 | EPHA5 | EPH receptor A5 [Source:HGNC Symbol;Acc:HGNC:3389] |
| ENSG00000145283 | HGLibA_44344 | 67 | -0.528655515 | SLC10A6 | solute carrier family 10 member 6 [Source:HGNC Symbol;Acc:HGNC:30603] |
| ENSG00000145335 | HGLibA_45908 | 67 | -0.528655515 | SNCA | synuclein alpha [Source:HGNC Symbol;Acc:HGNC:11138] |
| ENSG00000145428 | HGLibA_41577 | 67 | -0.528655515 | RNF175 | ring finger protein 175 [Source:HGNC Symbol;Acc:HGNC:27735] |
| ENSG00000146399 | HGLibA_48123 | 67 | -0.528655515 | TAAR1 | trace amine associated receptor 1 [Source:HGNC Symbol;Acc:HGNC:17734] |
| ENSG00000146530 | HGLibA_53932 | 67 | -0.528655515 | VWDE | von Willebrand factor D and EGF domains [Source:HGNC Symbol;Acc:HGNC:21897] |
| ENSG00000147251 | HGLibA_13736 | 67 | -0.528655515 | DOCK11 | dedicator of cytokinesis 11 [Source:HGNC Symbol;Acc:HGNC:23483] |
| ENSG00000148180 | HGLibA_20438 | 67 | -0.528655515 | GSN | gelsolin [Source:HGNC Symbol;Acc:HGNC:4620] |
| ENSG00000148843 | HGLibA_35720 | 67 | -0.528655515 | PDCD11 | programmed cell death 11 [Source:HGNC Symbol;Acc:HGNC:13408] |
| ENSG00000149043 | HGLibA_48091 | 67 | -0.528655515 | SYT8 | synaptotagmin 8 [Source:HGNC Symbol;Acc:HGNC:19264] |
| ENSG00000149100 | HGLibA_14802 | 67 | -0.528655515 | EIF3M | eukaryotic translation initiation factor 3 subunit M [Source:HGNC Symbol;Acc:HGNC:24460] |
| ENSG00000149308 | HGLibA_32406 | 67 | -0.528655515 | NPAT | coactivator of histone transcription [Source:HGNC Symbol;Acc:HGNC:7896]" |
| ENSG00000150510 | HGLibA_16075 | 67 | -0.528655515 | FAM124A | family with sequence similarity 124 member A [Source:HGNC Symbol;Acc:HGNC:26413] |
| ENSG00000151379 | HGLibA_30187 | 67 | -0.528655515 | MSGN1 | mesogenin 1 [Source:HGNC Symbol;Acc:HGNC:14907] |
| ENSG00000151572 | HGLibA_02180 | 67 | -0.528655515 | ANO4 | anoctamin 4 [Source:HGNC Symbol;Acc:HGNC:23837] |
| ENSG00000152463 | HGLibA_33341 | 67 | -0.528655515 | OLAH | oleoyl-ACP hydrolase [Source:HGNC Symbol;Acc:HGNC:25625] |
| ENSG00000153283 | HGLibA_08483 | 67 | -0.528655515 | CD96 | CD96 molecule [Source:HGNC Symbol;Acc:HGNC:16892] |
| ENSG00000153779 | HGLibA_49247 | 67 | -0.528655515 | TGIF2LX | TGFB induced factor homeobox 2 like X-linked [Source:HGNC Symbol;Acc:HGNC:18570] |
| ENSG00000154027 | HGLibA_01387 | 67 | -0.528655515 | AK5 | adenylate kinase 5 [Source:HGNC Symbol;Acc:HGNC:365] |
| ENSG00000154856 | HGLibA_02394 | 67 | -0.528655515 | APCDD1 | APC down-regulated 1 [Source:HGNC Symbol;Acc:HGNC:15718] |
| ENSG00000154957 | HGLibA_55534 | 67 | -0.528655515 | ZNF18 | zinc finger protein 18 [Source:HGNC Symbol;Acc:HGNC:12969] |
| ENSG00000156011 | HGLibA_39018 | 67 | -0.528655515 | PSD3 | pleckstrin and Sec7 domain containing 3 [Source:HGNC Symbol;Acc:HGNC:19093] |
| ENSG00000156958 | HGLibA_18518 | 67 | -0.528655515 | GALK2 | galactokinase 2 [Source:HGNC Symbol;Acc:HGNC:4119] |
| ENSG00000157110 | HGLibA_40653 | 67 | -0.528655515 | RBPMS | mRNA processing factor [Source:HGNC Symbol;Acc:HGNC:19097]" |
| ENSG00000159885 | HGLibA_55613 | 67 | -0.528655515 | ZNF222 | zinc finger protein 222 [Source:HGNC Symbol;Acc:HGNC:13015] |
| ENSG00000161267 | HGLibA_04373 | 67 | -0.528655515 | BDH1 | 3-hydroxybutyrate dehydrogenase 1 [Source:HGNC Symbol;Acc:HGNC:1027] |
| ENSG00000162344 | HGLibA_17386 | 67 | -0.528655515 | FGF19 | fibroblast growth factor 19 [Source:HGNC Symbol;Acc:HGNC:3675] |
| ENSG00000163563 | HGLibA_29566 | 67 | -0.528655515 | MNDA | myeloid cell nuclear differentiation antigen [Source:HGNC Symbol;Acc:HGNC:7183] |
| ENSG00000164099 | HGLibA_38902 | 67 | -0.528655515 | PRSS12 | serine protease 12 [Source:HGNC Symbol;Acc:HGNC:9477] |
| ENSG00000164684 | HGLibA_56584 | 67 | -0.528655515 | ZNF704 | zinc finger protein 704 [Source:HGNC Symbol;Acc:HGNC:32291] |
| ENSG00000164744 | HGLibA_47795 | 67 | -0.528655515 | SUN3 | Sad1 and UNC84 domain containing 3 [Source:HGNC Symbol;Acc:HGNC:22429] |
| ENSG00000164825 | HGLibA_12809 | 67 | -0.528655515 | DEFB1 | defensin beta 1 [Source:HGNC Symbol;Acc:HGNC:2766] |
| ENSG00000165506 | HGLibA_13463 | 67 | -0.528655515 | DNAAF2 | dynein axonemal assembly factor 2 [Source:HGNC Symbol;Acc:HGNC:20188] |
| ENSG00000165801 | HGLibA_02867 | 67 | -0.528655515 | ARHGEF40 | Rho guanine nucleotide exchange factor 40 [Source:HGNC Symbol;Acc:HGNC:25516] |
| ENSG00000166272 | HGLibA_53973 | 67 | -0.528655515 | WBP1L | WW domain binding protein 1 like [Source:HGNC Symbol;Acc:HGNC:23510] |
| ENSG00000167065 | HGLibA_14151 | 67 | -0.528655515 | DUSP18 | dual specificity phosphatase 18 [Source:HGNC Symbol;Acc:HGNC:18484] |
| ENSG00000167081 | HGLibA_35349 | 67 | -0.528655515 | PBX3 | PBX homeobox 3 [Source:HGNC Symbol;Acc:HGNC:8634] |
| ENSG00000167523 | HGLibA_46509 | 67 | -0.528655515 | SPATA33 | spermatogenesis associated 33 [Source:HGNC Symbol;Acc:HGNC:26463] |
| ENSG00000167565 | HGLibA_43611 | 67 | -0.528655515 | SERTAD3 | SERTA domain containing 3 [Source:HGNC Symbol;Acc:HGNC:17931] |
| ENSG00000167676 | HGLibA_37179 | 67 | -0.528655515 | PLIN4 | perilipin 4 [Source:HGNC Symbol;Acc:HGNC:29393] |
| ENSG00000167772 | HGLibA_01920 | 67 | -0.528655515 | ANGPTL4 | angiopoietin like 4 [Source:HGNC Symbol;Acc:HGNC:16039] |
| ENSG00000167925 | HGLibA_19033 | 67 | -0.528655515 | GHDC | GH3 domain containing [Source:HGNC Symbol;Acc:HGNC:24438] |
| ENSG00000168096 | HGLibA_02157 | 67 | -0.528655515 | ANKS3 | ankyrin repeat and sterile alpha motif domain containing 3 [Source:HGNC Symbol;Acc:HGNC:29422] |
| ENSG00000168291 | HGLibA_35868 | 67 | -0.528655515 | PDHB | pyruvate dehydrogenase E1 beta subunit [Source:HGNC Symbol;Acc:HGNC:8808] |
| ENSG00000168453 | HGLibA_22070 | 67 | -0.528655515 | HR | lysine demethylase and nuclear receptor corepressor [Source:HGNC Symbol;Acc:HGNC:5172]" |
| ENSG00000169083 | HGLibA_02620 | 67 | -0.528655515 | AR | androgen receptor [Source:HGNC Symbol;Acc:HGNC:644] |
| ENSG00000170043 | HGLibA_51297 | 67 | -0.528655515 | TRAPPC1 | trafficking protein particle complex 1 [Source:HGNC Symbol;Acc:HGNC:19894] |
| ENSG00000170298 | HGLibA_26383 | 67 | -0.528655515 | LGALS9B | galectin 9B [Source:HGNC Symbol;Acc:HGNC:24842] |
| ENSG00000170606 | HGLibA_22284 | 67 | -0.528655515 | HSPA4 | heat shock protein family A (Hsp70) member 4 [Source:HGNC Symbol;Acc:HGNC:5237] |
| ENSG00000171044 | HGLibA_54525 | 67 | -0.528655515 | XKR6 | XK related 6 [Source:HGNC Symbol;Acc:HGNC:27806] |
| ENSG00000172482 | HGLibA_01271 | 67 | -0.528655515 | AGXT | alanine--glyoxylate and serine--pyruvate aminotransferase [Source:HGNC Symbol;Acc:HGNC:341] |
| ENSG00000173335 | HGLibA_11471 | 67 | -0.528655515 | CST9 | cystatin 9 [Source:HGNC Symbol;Acc:HGNC:13261] |
| ENSG00000174529 | HGLibA_50518 | 67 | -0.528655515 | TMEM81 | transmembrane protein 81 [Source:HGNC Symbol;Acc:HGNC:32349] |
| ENSG00000174667 | HGLibA_34474 | 67 | -0.528655515 | OR7D4 | olfactory receptor family 7 subfamily D member 4 [Source:HGNC Symbol;Acc:HGNC:8380] |
| ENSG00000175356 | HGLibA_43072 | 67 | -0.528655515 | SCUBE2 | CUB domain and EGF like domain containing 2 [Source:HGNC Symbol;Acc:HGNC:30425]" |
| ENSG00000176058 | HGLibA_51190 | 67 | -0.528655515 | TPRN | taperin [Source:HGNC Symbol;Acc:HGNC:26894] |
| ENSG00000176566 | HGLibA_12445 | 67 | -0.528655515 | DCAF4L2 | DDB1 and CUL4 associated factor 4 like 2 [Source:HGNC Symbol;Acc:HGNC:26657] |
| ENSG00000176595 | HGLibA_24093 | 67 | -0.528655515 | KBTBD11 | kelch repeat and BTB domain containing 11 [Source:HGNC Symbol;Acc:HGNC:29104] |
| ENSG00000176619 | HGLibA_26698 | 67 | -0.528655515 | LMNB2 | lamin B2 [Source:HGNC Symbol;Acc:HGNC:6638] |
| ENSG00000177311 | HGLibA_54871 | 67 | -0.528655515 | ZBTB38 | zinc finger and BTB domain containing 38 [Source:HGNC Symbol;Acc:HGNC:26636] |
| ENSG00000178919 | HGLibA_17859 | 67 | -0.528655515 | FOXE1 | forkhead box E1 [Source:HGNC Symbol;Acc:HGNC:3806] |
| ENSG00000179094 | HGLibA_36053 | 67 | -0.528655515 | PER1 | period circadian regulator 1 [Source:HGNC Symbol;Acc:HGNC:8845] |
| ENSG00000179546 | HGLibA_22363 | 67 | -0.528655515 | HTR1D | 5-hydroxytryptamine receptor 1D [Source:HGNC Symbol;Acc:HGNC:5289] |
| ENSG00000180644 | HGLibA_38450 | 67 | -0.528655515 | PRF1 | perforin 1 [Source:HGNC Symbol;Acc:HGNC:9360] |
| ENSG00000180658 | HGLibA_33734 | 67 | -0.528655515 | OR2A4 | olfactory receptor family 2 subfamily A member 4 [Source:HGNC Symbol;Acc:HGNC:14729] |
| ENSG00000181333 | HGLibA_21147 | 67 | -0.528655515 | HEPHL1 | hephaestin like 1 [Source:HGNC Symbol;Acc:HGNC:30477] |
| ENSG00000181481 | HGLibA_41510 | 67 | -0.528655515 | RNF135 | ring finger protein 135 [Source:HGNC Symbol;Acc:HGNC:21158] |
| ENSG00000182050 | HGLibA_29147 | 67 | -0.528655515 | MGAT4C | MGAT4 family member C [Source:HGNC Symbol;Acc:HGNC:30871] |
| ENSG00000182261 | HGLibA_32099 | 67 | -0.528655515 | NLRP10 | NLR family pyrin domain containing 10 [Source:HGNC Symbol;Acc:HGNC:21464] |
| ENSG00000183087 | HGLibA_18653 | 67 | -0.528655515 | GAS6 | growth arrest specific 6 [Source:HGNC Symbol;Acc:HGNC:4168] |
| ENSG00000183347 | HGLibA_18742 | 67 | -0.528655515 | GBP6 | guanylate binding protein family member 6 [Source:HGNC Symbol;Acc:HGNC:25395] |
| ENSG00000185246 | HGLibA_38727 | 67 | -0.528655515 | PRPF39 | pre-mRNA processing factor 39 [Source:HGNC Symbol;Acc:HGNC:20314] |
| ENSG00000185972 | HGLibA_07948 | 67 | -0.528655515 | CCIN | calicin [Source:HGNC Symbol;Acc:HGNC:1568] |
| ENSG00000186532 | HGLibA_45860 | 67 | -0.528655515 | SMYD4 | SET and MYND domain containing 4 [Source:HGNC Symbol;Acc:HGNC:21067] |
| ENSG00000186583 | HGLibA_46536 | 67 | -0.528655515 | SPATC1 | spermatogenesis and centriole associated 1 [Source:HGNC Symbol;Acc:HGNC:30510] |

| | | | | | |
|---|---|---|---|---|---|
| ENSG00000186834 | HGLibA_21210 | 67 | -0.528655515 | HEXIM1 | hexamethylene bisacetamide inducible 1 [Source:HGNC Symbol;Acc:HGNC:24953] |
| ENSG00000188404 | HGLibA_43294 | 67 | -0.528655515 | SELL | selectin L [Source:HGNC Symbol;Acc:HGNC:10720] |
| ENSG00000196235 | HGLibA_47819 | 67 | -0.528655515 | SUPT5H | DSIF elongation factor subunit [Source:HGNC Symbol;Acc:HGNC:11469]" |
| ENSG00000197329 | HGLibA_36033 | 67 | -0.528655515 | PELI1 | pellino E3 ubiquitin protein ligase 1 [Source:HGNC Symbol;Acc:HGNC:8827] |
| ENSG00000197361 | HGLibA_17004 | 67 | -0.528655515 | FBXL22 | F-box and leucine rich repeat protein 22 [Source:HGNC Symbol;Acc:HGNC:27537] |
| ENSG00000197403 | HGLibA_34430 | 67 | -0.528655515 | OR6N1 | olfactory receptor family 6 subfamily N member 1 [Source:HGNC Symbol;Acc:HGNC:15034] |
| ENSG00000197841 | HGLibA_55539 | 67 | -0.528655515 | ZNF181 | zinc finger protein 181 [Source:HGNC Symbol;Acc:HGNC:12971] |
| ENSG00000198081 | HGLibA_54824 | 67 | -0.528655515 | ZBTB14 | zinc finger and BTB domain containing 14 [Source:HGNC Symbol;Acc:HGNC:12860] |
| ENSG00000198841 | HGLibA_25868 | 67 | -0.528655515 | KTI12 | KTI12 chromatin associated homolog [Source:HGNC Symbol;Acc:HGNC:25160] |
| ENSG00000205642 | HGLibA_53591 | 67 | -0.528655515 | VCX3B | variable charge X-linked 3B [Source:HGNC Symbol;Acc:HGNC:31838] |
| ENSG00000205795 | HGLibA_12223 | 67 | -0.528655515 | CYS1 | cystin 1 [Source:HGNC Symbol;Acc:HGNC:18525] |
| ENSG00000205856 | HGLibA_06078 | 67 | -0.528655515 | C22orf42 | chromosome 22 open reading frame 42 [Source:HGNC Symbol;Acc:HGNC:27160] |
| ENSG00000212657 | HGLibA_25658 | 67 | -0.528655515 | KRTAP16-1 | keratin associated protein 16-1 [Source:HGNC Symbol;Acc:HGNC:18916] |
| ENSG00000226600 | HGLibA_11548 | 67 | -0.528655515 | CT47A9 | member A9 [Source:HGNC Symbol;Acc:HGNC:33290]" |
| ENSG00000242110 | HGLibA_01744 | 67 | -0.528655515 | AMACR | alpha-methylacyl-CoA racemase [Source:HGNC Symbol;Acc:HGNC:451] |
| ENSG00000243566 | HGLibA_53081 | 67 | -0.528655515 | UPK3B | uroplakin 3B [Source:HGNC Symbol;Acc:HGNC:21444] |
| ENSG00000248933 | HGLibA_53252 | 67 | -0.528655515 | USP17L22 | ubiquitin specific peptidase 17-like family member 22 [Source:HGNC Symbol;Acc:HGNC:44450] |
| ENSG00000249104 | HGLibA_53231 | 67 | -0.528655515 | USP17L17 | ubiquitin specific peptidase 17-like family member 17 [Source:HGNC Symbol;Acc:HGNC:44445] |
| ENSG00000253731 | HGLibA_35537 | 67 | -0.528655515 | PCDHGA6 | 6 [Source:HGNC Symbol;Acc:HGNC:8704]" |
| ENSG00000254440 | HGLibA_35336 | 67 | -0.528655515 | PBOV1 | prostate and breast cancer overexpressed 1 [Source:HGNC Symbol;Acc:HGNC:21079] |
| ENSG00000254656 | HGLibA_42394 | 67 | -0.528655515 | RTL1 | retrotransposon Gag like 1 [Source:HGNC Symbol;Acc:HGNC:14665] |
| ENSG00000255072 | HGLibA_36602 | 67 | -0.528655515 | PIGY | phosphatidylinositol glycan anchor biosynthesis class Y [Source:HGNC Symbol;Acc:HGNC:28213] |
| ENSG00000255582 | HGLibA_33488 | 67 | -0.528655515 | OR10G2 | olfactory receptor family 10 subfamily G member 2 [Source:HGNC Symbol;Acc:HGNC:8170] |
| ENSG00000271503 | HGLibA_08035 | 67 | -0.528655515 | CCL5 | C-C motif chemokine ligand 5 [Source:HGNC Symbol;Acc:HGNC:10632] |
| ENSG00000276040 | HGLibA_46485 | 67 | -0.528655515 | SPATA31A7 | SPATA31 subfamily A member 7 [Source:HGNC Symbol;Acc:HGNC:32007] |
| ENSG00000278057 | HGLibA_49087 | 67 | -0.528655515 | TEX28 | testis expressed 28 [Source:HGNC Symbol;Acc:HGNC:2563] |
| ENSG00000279486 | HGLibA_33749 | 67 | -0.528655515 | OR2AG1 | olfactory receptor family 2 subfamily AG member 1 (gene/pseudogene) [Source:HGNC Symbol;Acc:HGNC:15142] |
| ENSG00000289549 | HGLibA_37905 | 67 | -0.528655515 | PPIAL4D | - |
| GATS | HGLibA_18699 | 67 | -0.528655515 | GATS | - |
| HIST1H2BL | HGLibA_21444 | 67 | -0.528655515 | HIST1H2BL | - |
| HIST1H3H | HGLibA_21476 | 67 | -0.528655515 | HIST1H3H | - |
| hsa-let-7f-2 | HGLibA_57128 | 67 | -0.528655515 | hsa-let-7f-2 | - |
| hsa-mir-1185-1 | HGLibA_57243 | 67 | -0.528655515 | hsa-mir-1185-1 | - |
| hsa-mir-1272 | HGLibA_57528 | 67 | -0.528655515 | hsa-mir-1272 | - |
| hsa-mir-1288 | HGLibA_57617 | 67 | -0.528655515 | hsa-mir-1288 | - |
| hsa-mir-1302-3 | HGLibA_57698 | 67 | -0.528655515 | hsa-mir-1302-3 | - |
| hsa-mir-1306 | HGLibA_57740 | 67 | -0.528655515 | hsa-mir-1306 | - |
| hsa-mir-190a | HGLibA_58035 | 67 | -0.528655515 | hsa-mir-190a | - |
| hsa-mir-1972-2 | HGLibA_58113 | 67 | -0.528655515 | hsa-mir-1972-2 | - |
| hsa-mir-2053 | HGLibA_58186 | 67 | -0.528655515 | hsa-mir-2053 | - |
| hsa-mir-219a-1 | HGLibA_58278 | 67 | -0.528655515 | hsa-mir-219a-1 | - |
| hsa-mir-3119-2 | HGLibA_58530 | 67 | -0.528655515 | hsa-mir-3119-2 | - |
| hsa-mir-329-1 | HGLibA_58978 | 67 | -0.528655515 | hsa-mir-329-1 | - |
| hsa-mir-3690-1 | HGLibA_59345 | 67 | -0.528655515 | hsa-mir-3690-1 | - |
| hsa-mir-374c | HGLibA_59397 | 67 | -0.528655515 | hsa-mir-374c | - |
| hsa-mir-3941 | HGLibA_59624 | 67 | -0.528655515 | hsa-mir-3941 | - |
| hsa-mir-4260 | HGLibA_59747 | 67 | -0.528655515 | hsa-mir-4260 | - |
| hsa-mir-4277 | HGLibA_59811 | 67 | -0.528655515 | hsa-mir-4277 | - |
| hsa-mir-4289 | HGLibA_59864 | 67 | -0.528655515 | hsa-mir-4289 | - |
| hsa-mir-4424 | HGLibA_60085 | 67 | -0.528655515 | hsa-mir-4424 | - |
| hsa-mir-4436a | HGLibA_60138 | 67 | -0.528655515 | hsa-mir-4436a | - |
| hsa-mir-4462 | HGLibA_60255 | 67 | -0.528655515 | hsa-mir-4462 | - |
| hsa-mir-4497 | HGLibA_60403 | 67 | -0.528655515 | hsa-mir-4497 | - |
| hsa-mir-4666b | HGLibA_60780 | 67 | -0.528655515 | hsa-mir-4666b | - |
| hsa-mir-500b | HGLibA_61447 | 67 | -0.528655515 | hsa-mir-500b | - |
| hsa-mir-5187 | HGLibA_61641 | 67 | -0.528655515 | hsa-mir-5187 | - |
| hsa-mir-518e | HGLibA_61671 | 67 | -0.528655515 | hsa-mir-518e | - |
| hsa-mir-548ah | HGLibA_61881 | 67 | -0.528655515 | hsa-mir-548ah | - |
| hsa-mir-548f-3 | HGLibA_61980 | 67 | -0.528655515 | hsa-mir-548f-3 | - |
| hsa-mir-550b-1 | HGLibA_62110 | 67 | -0.528655515 | hsa-mir-550b-1 | - |
| hsa-mir-557 | HGLibA_62142 | 67 | -0.528655515 | hsa-mir-557 | - |
| hsa-mir-5685 | HGLibA_62259 | 67 | -0.528655515 | hsa-mir-5685 | - |
| hsa-mir-6079 | HGLibA_62573 | 67 | -0.528655515 | hsa-mir-6079 | - |
| hsa-mir-6133 | HGLibA_62682 | 67 | -0.528655515 | hsa-mir-6133 | - |
| hsa-mir-6499 | HGLibA_62845 | 67 | -0.528655515 | hsa-mir-6499 | - |
| hsa-mir-650 | HGLibA_62846 | 67 | -0.528655515 | hsa-mir-650 | - |
| hsa-mir-6748 | HGLibA_63147 | 67 | -0.528655515 | hsa-mir-6748 | - |
| hsa-mir-6778 | HGLibA_63287 | 67 | -0.528655515 | hsa-mir-6778 | - |
| hsa-mir-6831 | HGLibA_63500 | 67 | -0.528655515 | hsa-mir-6831 | - |
| hsa-mir-6846 | HGLibA_63556 | 67 | -0.528655515 | hsa-mir-6846 | - |
| hsa-mir-7111 | HGLibA_63807 | 67 | -0.528655515 | hsa-mir-7111 | - |
| hsa-mir-7114 | HGLibA_63825 | 67 | -0.528655515 | hsa-mir-7114 | - |
| hsa-mir-7150 | HGLibA_63828 | 67 | -0.528655515 | hsa-mir-7150 | - |
| hsa-mir-761 | HGLibA_63904 | 67 | -0.528655515 | hsa-mir-761 | - |
| ORAOV1 | HGLibA_34598 | 67 | -0.528655515 | ORAOV1 | - |
| PPP2R3B_X | HGLibA_38159 | 67 | -0.528655515 | PPP2R3B_X | - |
| RNMTL1 | HGLibA_41720 | 67 | -0.528655515 | RNMTL1 | - |
| TENC1 | HGLibA_48993 | 67 | -0.528655515 | TENC1 | - |
| WDYHV1 | HGLibA_54236 | 67 | -0.528655515 | WDYHV1 | - |
| XAGE1C | HGLibA_54472 | 67 | -0.528655515 | XAGE1C | - |
| AQPEP | HGLibA_02614 | 66 | -0.537700858 | AQPEP | - |
| CXXC11 | HGLibA_11968 | 66 | -0.537700858 | CXXC11 | - |
| DYX1C1 | HGLibA_14292 | 66 | -0.537700858 | DYX1C1 | - |
| ENSG00000002726 | HGLibA_02268 | 66 | -0.537700858 | AOC1 | copper containing 1 [Source:HGNC Symbol;Acc:HGNC:80]" |
| ENSG00000008130 | HGLibA_31045 | 66 | -0.537700858 | NADK | NAD kinase [Source:HGNC Symbol;Acc:HGNC:29831] |
| ENSG00000010671 | HGLibA_04952 | 66 | -0.537700858 | BTK | Bruton tyrosine kinase [Source:HGNC Symbol;Acc:HGNC:1133] |
| ENSG00000011332 | HGLibA_13827 | 66 | -0.537700858 | DPF1 | double PHD fingers 1 [Source:HGNC Symbol;Acc:HGNC:20225] |
| ENSG00000023892 | HGLibA_12787 | 66 | -0.537700858 | DEF6 | guanine nucleotide exchange factor [Source:HGNC Symbol;Acc:HGNC:2760]" |
| ENSG00000040199 | HGLibA_36438 | 66 | -0.537700858 | PHLPP2 | PH domain and leucine rich repeat protein phosphatase 2 [Source:HGNC Symbol;Acc:HGNC:29149] |
| ENSG00000051596 | HGLibA_49371 | 66 | -0.537700858 | THOC3 | THO complex 3 [Source:HGNC Symbol;Acc:HGNC:19072] |
| ENSG00000056972 | HGLibA_51252 | 66 | -0.537700858 | TRAF3IP2 | TRAF3 interacting protein 2 [Source:HGNC Symbol;Acc:HGNC:1343] |
| ENSG00000066248 | HGLibA_31868 | 66 | -0.537700858 | NGEF | neuronal guanine nucleotide exchange factor [Source:HGNC Symbol;Acc:HGNC:7807] |
| ENSG00000068024 | HGLibA_21013 | 66 | -0.537700858 | HDAC4 | histone deacetylase 4 [Source:HGNC Symbol;Acc:HGNC:14063] |
| ENSG00000075420 | HGLibA_17750 | 66 | -0.537700858 | FNDC3B | fibronectin type III domain containing 3B [Source:HGNC Symbol;Acc:HGNC:24670] |
| ENSG00000076258 | HGLibA_17712 | 66 | -0.537700858 | FMO4 | flavin containing monooxygenase 4 [Source:HGNC Symbol;Acc:HGNC:3772] |
| ENSG00000079257 | HGLibA_27651 | 66 | -0.537700858 | LXN | latexin [Source:HGNC Symbol;Acc:HGNC:13347] |
| ENSG00000079308 | HGLibA_50923 | 66 | -0.537700858 | TNS1 | tensin 1 [Source:HGNC Symbol;Acc:HGNC:11973] |
| ENSG00000082212 | HGLibA_28711 | 66 | -0.537700858 | ME2 | malic enzyme 2 [Source:HGNC Symbol;Acc:HGNC:6984] |
| ENSG00000082497 | HGLibA_43613 | 66 | -0.537700858 | SERTAD4 | SERTA domain containing 4 [Source:HGNC Symbol;Acc:HGNC:25236] |
| ENSG00000086712 | HGLibA_52396 | 66 | -0.537700858 | TXLNG | taxilin gamma [Source:HGNC Symbol;Acc:HGNC:18578] |
| ENSG00000089234 | HGLibA_04755 | 66 | -0.537700858 | BRAP | BRCA1 associated protein [Source:HGNC Symbol;Acc:HGNC:1099] |
| ENSG00000090020 | HGLibA_45401 | 66 | -0.537700858 | SLC9A1 | solute carrier family 9 member A1 [Source:HGNC Symbol;Acc:HGNC:11071] |
| ENSG00000091164 | HGLibA_52435 | 66 | -0.537700858 | TXNL1 | thioredoxin like 1 [Source:HGNC Symbol;Acc:HGNC:12436] |
| ENSG00000092853 | HGLibA_10131 | 66 | -0.537700858 | CLSPN | claspin [Source:HGNC Symbol;Acc:HGNC:19715] |
| ENSG00000093183 | HGLibA_43218 | 66 | -0.537700858 | SEC22C | vesicle trafficking protein [Source:HGNC Symbol;Acc:HGNC:16828]" |
| ENSG00000096093 | HGLibA_14571 | 66 | -0.537700858 | EFHC1 | EF-hand domain containing 1 [Source:HGNC Symbol;Acc:HGNC:16406] |
| ENSG00000099385 | HGLibA_04344 | 66 | -0.537700858 | BCL7C | BCL tumor suppressor 7C [Source:HGNC Symbol;Acc:HGNC:1006] |
| ENSG00000100151 | HGLibA_36524 | 66 | -0.537700858 | PICK1 | protein interacting with PRKCA 1 [Source:HGNC Symbol;Acc:HGNC:9394] |
| ENSG00000100285 | HGLibA_31617 | 66 | -0.537700858 | NEFH | neurofilament heavy [Source:HGNC Symbol;Acc:HGNC:7737] |
| ENSG00000100320 | HGLibA_40457 | 66 | -0.537700858 | RBFOX2 | RNA binding fox-1 homolog 2 [Source:HGNC Symbol;Acc:HGNC:9906] |
| ENSG00000100353 | HGLibA_14773 | 66 | -0.537700858 | EIF3D | eukaryotic translation initiation factor 3 subunit D [Source:HGNC Symbol;Acc:HGNC:3278] |
| ENSG00000100441 | HGLibA_24579 | 66 | -0.537700858 | KHNYN | KH and NYN domain containing [Source:HGNC Symbol;Acc:HGNC:20166] |
| ENSG00000100596 | HGLibA_46895 | 66 | -0.537700858 | SPTLC2 | serine palmitoyltransferase long chain base subunit 2 [Source:HGNC Symbol;Acc:HGNC:11278] |
| ENSG00000100982 | HGLibA_35611 | 66 | -0.537700858 | PCIF1 | PDX1 C-terminal inhibiting factor 1 [Source:HGNC Symbol;Acc:HGNC:16200] |
| ENSG00000101638 | HGLibA_47300 | 66 | -0.537700858 | ST8SIA5 | 8-sialyltransferase 5 [Source:HGNC Symbol;Acc:HGNC:17827]" |
| ENSG00000102043 | HGLibA_30440 | 66 | -0.537700858 | MTMR8 | myotubularin related protein 8 [Source:HGNC Symbol;Acc:HGNC:16825] |
| ENSG00000102385 | HGLibA_13985 | 66 | -0.537700858 | DRP2 | dystrophin related protein 2 [Source:HGNC Symbol;Acc:HGNC:3032] |
| ENSG00000103599 | HGLibA_23520 | 66 | -0.537700858 | IQCH | IQ motif containing H [Source:HGNC Symbol;Acc:HGNC:25721] |
| ENSG00000105048 | HGLibA_50884 | 66 | -0.537700858 | TNNT1 | slow skeletal type [Source:HGNC Symbol;Acc:HGNC:11948]" |
| ENSG00000105497 | HGLibA_55527 | 66 | -0.537700858 | ZNF175 | zinc finger protein 175 [Source:HGNC Symbol;Acc:HGNC:12964] |
| ENSG00000105697 | HGLibA_20824 | 66 | -0.537700858 | HAMP | hepcidin antimicrobial peptide [Source:HGNC Symbol;Acc:HGNC:15598] |
| ENSG00000106089 | HGLibA_47637 | 66 | -0.537700858 | STX1A | syntaxin 1A [Source:HGNC Symbol;Acc:HGNC:11433] |
| ENSG00000107317 | HGLibA_39310 | 66 | -0.537700858 | PTGDS | prostaglandin D2 synthase [Source:HGNC Symbol;Acc:HGNC:9592] |
| ENSG00000108106 | HGLibA_52658 | 66 | -0.537700858 | UBE2S | ubiquitin conjugating enzyme E2 S [Source:HGNC Symbol;Acc:HGNC:17895] |
| ENSG00000108953 | HGLibA_54742 | 66 | -0.537700858 | YWHAE | tyrosine 3-monooxygenase/tryptophan 5-monooxygenase activation protein epsilon [Source:HGNC Symbol;Acc:HGNC:12851] |
| ENSG00000109047 | HGLibA_40722 | 66 | -0.537700858 | RCVRN | recoverin [Source:HGNC Symbol;Acc:HGNC:9937] |
| ENSG00000110237 | HGLibA_02824 | 66 | -0.537700858 | ARHGEF17 | Rho guanine nucleotide exchange factor 17 [Source:HGNC Symbol;Acc:HGNC:21726] |
| ENSG00000111641 | HGLibA_32309 | 66 | -0.537700858 | NOP2 | NOP2 nucleolar protein [Source:HGNC Symbol;Acc:HGNC:7867] |
| ENSG00000112149 | HGLibA_08462 | 66 | -0.537700858 | CD83 | CD83 molecule [Source:HGNC Symbol;Acc:HGNC:1703] |
| ENSG00000112511 | HGLibA_36330 | 66 | -0.537700858 | PHF1 | PHD finger protein 1 [Source:HGNC Symbol;Acc:HGNC:8919] |
| ENSG00000112640 | HGLibA_38180 | 66 | -0.537700858 | PPP2R5D | protein phosphatase 2 regulatory subunit B'delta [Source:HGNC Symbol;Acc:HGNC:9312] |
| ENSG00000112706 | HGLibA_23261 | 66 | -0.537700858 | IMPG1 | interphotoreceptor matrix proteoglycan 1 [Source:HGNC Symbol;Acc:HGNC:6055] |
| ENSG00000112936 | HGLibA_06518 | 66 | -0.537700858 | C7 | complement C7 [Source:HGNC Symbol;Acc:HGNC:1346] |
| ENSG00000113312 | HGLibA_52045 | 66 | -0.537700858 | TTC1 | tetratricopeptide repeat domain 1 [Source:HGNC Symbol;Acc:HGNC:12391] |
| ENSG00000113492 | HGLibA_01274 | 66 | -0.537700858 | AGXT2 | alanine--glyoxylate aminotransferase 2 [Source:HGNC Symbol;Acc:HGNC:14412] |

| | | | | | |
|---|---|---|---|---|---|
| ENSG00000115084 | HGLibA_45011 | 66 | -0.537700858 | SLC35F5 | solute carrier family 35 member F5 [Source:HGNC Symbol;Acc:HGNC:23617] |
| ENSG00000115155 | HGLibA_34725 | 66 | -0.537700858 | OTOF | otoferlin [Source:HGNC Symbol;Acc:HGNC:8515] |
| ENSG00000115325 | HGLibA_13767 | 66 | -0.537700858 | DOK1 | docking protein 1 [Source:HGNC Symbol;Acc:HGNC:2990] |
| ENSG00000115652 | HGLibA_53480 | 66 | -0.537700858 | UXS1 | UDP-glucuronate decarboxylase 1 [Source:HGNC Symbol;Acc:HGNC:17729] |
| ENSG00000115977 | HGLibA_00050 | 66 | -0.537700858 | AAK1 | AP2 associated kinase 1 [Source:HGNC Symbol;Acc:HGNC:19679] |
| ENSG00000116133 | HGLibA_13090 | 66 | -0.537700858 | DHCR24 | 24-dehydrocholesterol reductase [Source:HGNC Symbol;Acc:HGNC:2859] |
| ENSG00000119125 | HGLibA_18835 | 66 | -0.537700858 | GDA | guanine deaminase [Source:HGNC Symbol;Acc:HGNC:4212] |
| ENSG00000119973 | HGLibA_38602 | 66 | -0.537700858 | PRLHR | prolactin releasing hormone receptor [Source:HGNC Symbol;Acc:HGNC:4464] |
| ENSG00000120963 | HGLibA_56597 | 66 | -0.537700858 | ZNF706 | zinc finger protein 706 [Source:HGNC Symbol;Acc:HGNC:24992] |
| ENSG00000121390 | HGLibA_39222 | 66 | -0.537700858 | PSPC1 | paraspeckle component 1 [Source:HGNC Symbol;Acc:HGNC:20320] |
| ENSG00000122034 | HGLibA_20578 | 66 | -0.537700858 | GTF3A | general transcription factor IIIA [Source:HGNC Symbol;Acc:HGNC:4662] |
| ENSG00000122912 | HGLibA_44654 | 66 | -0.537700858 | SLC25A16 | solute carrier family 25 member 16 [Source:HGNC Symbol;Acc:HGNC:10986] |
| ENSG00000123130 | HGLibA_00495 | 66 | -0.537700858 | ACOT9 | acyl-CoA thioesterase 9 [Source:HGNC Symbol;Acc:HGNC:17152] |
| ENSG00000124479 | HGLibA_31410 | 66 | -0.537700858 | NDP | norrin cystine knot growth factor [Source:HGNC Symbol;Acc:HGNC:7678]" |
| ENSG00000126391 | HGLibA_18028 | 66 | -0.537700858 | FRMD8 | FERM domain containing 8 [Source:HGNC Symbol;Acc:HGNC:25462] |
| ENSG00000127129 | HGLibA_14472 | 66 | -0.537700858 | EDN2 | endothelin 2 [Source:HGNC Symbol;Acc:HGNC:3177] |
| ENSG00000127774 | HGLibA_15016 | 66 | -0.537700858 | EMC6 | ER membrane protein complex subunit 6 [Source:HGNC Symbol;Acc:HGNC:28430] |
| ENSG00000129682 | HGLibA_17372 | 66 | -0.537700858 | FGF13 | fibroblast growth factor 13 [Source:HGNC Symbol;Acc:HGNC:3670] |
| ENSG00000130159 | HGLibA_14422 | 66 | -0.537700858 | ECSIT | ECSIT signalling integrator [Source:HGNC Symbol;Acc:HGNC:29548] |
| ENSG00000130307 | HGLibA_53181 | 66 | -0.537700858 | USHBP1 | USH1 protein network component harmonin binding protein 1 [Source:HGNC Symbol;Acc:HGNC:24058] |
| ENSG00000130433 | HGLibA_06945 | 66 | -0.537700858 | CACNG6 | calcium voltage-gated channel auxiliary subunit gamma 6 [Source:HGNC Symbol;Acc:HGNC:13625] |
| ENSG00000130829 | HGLibA_14195 | 66 | -0.537700858 | DUSP9 | dual specificity phosphatase 9 [Source:HGNC Symbol;Acc:HGNC:3076] |
| ENSG00000131116 | HGLibA_55969 | 66 | -0.537700858 | ZNF428 | zinc finger protein 428 [Source:HGNC Symbol;Acc:HGNC:20804] |
| ENSG00000131848 | HGLibA_57027 | 66 | -0.537700858 | ZSCAN5A | zinc finger and SCAN domain containing 5A [Source:HGNC Symbol;Acc:HGNC:23710] |
| ENSG00000132872 | HGLibA_48079 | 66 | -0.537700858 | SYT4 | synaptotagmin 4 [Source:HGNC Symbol;Acc:HGNC:11512] |
| ENSG00000135723 | HGLibA_17493 | 66 | -0.537700858 | FHOD1 | formin homology 2 domain containing 1 [Source:HGNC Symbol;Acc:HGNC:17905] |
| ENSG00000135966 | HGLibA_49234 | 66 | -0.537700858 | TGFBRAP1 | transforming growth factor beta receptor associated protein 1 [Source:HGNC Symbol;Acc:HGNC:16836] |
| ENSG00000136098 | HGLibA_31649 | 66 | -0.537700858 | NEK3 | NIMA related kinase 3 [Source:HGNC Symbol;Acc:HGNC:7746] |
| ENSG00000136156 | HGLibA_23833 | 66 | -0.537700858 | ITM2B | integral membrane protein 2B [Source:HGNC Symbol;Acc:HGNC:6174] |
| ENSG00000136478 | HGLibA_49073 | 66 | -0.537700858 | TEX2 | testis expressed 2 [Source:HGNC Symbol;Acc:HGNC:30884] |
| ENSG00000137106 | HGLibA_20256 | 66 | -0.537700858 | GRHPR | glyoxylate and hydroxypyruvate reductase [Source:HGNC Symbol;Acc:HGNC:4570] |
| ENSG00000138821 | HGLibA_45125 | 66 | -0.537700858 | SLC39A8 | solute carrier family 39 member 8 [Source:HGNC Symbol;Acc:HGNC:20862] |
| ENSG00000139289 | HGLibA_36418 | 66 | -0.537700858 | PHLDA1 | pleckstrin homology like domain family A member 1 [Source:HGNC Symbol;Acc:HGNC:8933] |
| ENSG00000139515 | HGLibA_35954 | 66 | -0.537700858 | PDX1 | pancreatic and duodenal homeobox 1 [Source:HGNC Symbol;Acc:HGNC:6107] |
| ENSG00000141560 | HGLibA_17732 | 66 | -0.537700858 | FN3KRP | fructosamine 3 kinase related protein [Source:HGNC Symbol;Acc:HGNC:25700] |
| ENSG00000142684 | HGLibA_56342 | 66 | -0.537700858 | ZNF593 | zinc finger protein 593 [Source:HGNC Symbol;Acc:HGNC:30943] |
| ENSG00000143797 | HGLibA_28521 | 66 | -0.537700858 | MBOAT2 | membrane bound O-acyltransferase domain containing 2 [Source:HGNC Symbol;Acc:HGNC:25193] |
| ENSG00000145741 | HGLibA_04934 | 66 | -0.537700858 | BTF3 | basic transcription factor 3 [Source:HGNC Symbol;Acc:HGNC:1125] |
| ENSG00000146834 | HGLibA_28902 | 66 | -0.537700858 | MEPCE | methylphosphate capping enzyme [Source:HGNC Symbol;Acc:HGNC:20247] |
| ENSG00000148153 | HGLibA_23312 | 66 | -0.537700858 | INIP | INTS3 and NABP interacting protein [Source:HGNC Symbol;Acc:HGNC:24994] |
| ENSG00000150401 | HGLibA_12568 | 66 | -0.537700858 | DCUN1D2 | defective in cullin neddylation 1 domain containing 2 [Source:HGNC Symbol;Acc:HGNC:20328] |
| ENSG00000150764 | HGLibA_13289 | 66 | -0.537700858 | DIXDC1 | DIX domain containing 1 [Source:HGNC Symbol;Acc:HGNC:23695] |
| ENSG00000151079 | HGLibA_24142 | 66 | -0.537700858 | KCNA6 | potassium voltage-gated channel subfamily A member 6 [Source:HGNC Symbol;Acc:HGNC:6225] |
| ENSG00000151490 | HGLibA_39526 | 66 | -0.537700858 | PTPRO | receptor type O [Source:HGNC Symbol;Acc:HGNC:9678]" |
| ENSG00000152154 | HGLibA_50081 | 66 | -0.537700858 | TMEM178A | transmembrane protein 178A [Source:HGNC Symbol;Acc:HGNC:28517] |
| ENSG00000152430 | HGLibA_04692 | 66 | -0.537700858 | BOLL | RNA binding protein [Source:HGNC Symbol;Acc:HGNC:14273]" |
| ENSG00000152952 | HGLibA_37211 | 66 | -0.537700858 | PLOD2 | 2-oxoglutarate 5-dioxygenase 2 [Source:HGNC Symbol;Acc:HGNC:9082]" |
| ENSG00000154127 | HGLibA_52560 | 66 | -0.537700858 | UBASH3B | ubiquitin associated and SH3 domain containing B [Source:HGNC Symbol;Acc:HGNC:29884] |
| ENSG00000156531 | HGLibA_36385 | 66 | -0.537700858 | PHF6 | PHD finger protein 6 [Source:HGNC Symbol;Acc:HGNC:18145] |
| ENSG00000157119 | HGLibA_25207 | 66 | -0.537700858 | KLHL40 | kelch like family member 40 [Source:HGNC Symbol;Acc:HGNC:30372] |
| ENSG00000157470 | HGLibA_16733 | 66 | -0.537700858 | FAM81A | family with sequence similarity 81 member A [Source:HGNC Symbol;Acc:HGNC:28379] |
| ENSG00000157933 | HGLibA_44277 | 66 | -0.537700858 | SKI | SKI proto-oncogene [Source:HGNC Symbol;Acc:HGNC:10896] |
| ENSG00000158077 | HGLibA_32112 | 66 | -0.537700858 | NLRP14 | NLR family pyrin domain containing 14 [Source:HGNC Symbol;Acc:HGNC:22939] |
| ENSG00000158747 | HGLibA_31214 | 66 | -0.537700858 | NBL1 | DAN family BMP antagonist [Source:HGNC Symbol;Acc:HGNC:7650]" |
| ENSG00000158748 | HGLibA_22401 | 66 | -0.537700858 | HTR6 | 5-hydroxytryptamine receptor 6 [Source:HGNC Symbol;Acc:HGNC:5301] |
| ENSG00000161905 | HGLibA_01681 | 66 | -0.537700858 | ALOX15 | arachidonate 15-lipoxygenase [Source:HGNC Symbol;Acc:HGNC:433] |
| ENSG00000162300 | HGLibA_55248 | 66 | -0.537700858 | ZFPL1 | zinc finger protein like 1 [Source:HGNC Symbol;Acc:HGNC:12868] |
| ENSG00000163217 | HGLibA_04592 | 66 | -0.537700858 | BMP10 | bone morphogenetic protein 10 [Source:HGNC Symbol;Acc:HGNC:20869] |
| ENSG00000163499 | HGLibA_11255 | 66 | -0.537700858 | CRYBA2 | crystallin beta A2 [Source:HGNC Symbol;Acc:HGNC:2395] |
| ENSG00000163518 | HGLibA_17235 | 66 | -0.537700858 | FCRL4 | Fc receptor like 4 [Source:HGNC Symbol;Acc:HGNC:18507] |
| ENSG00000163590 | HGLibA_37989 | 66 | -0.537700858 | PPM1L | Mg2+/Mn2+ dependent 1L [Source:HGNC Symbol;Acc:HGNC:16381]" |
| ENSG00000165644 | HGLibA_10688 | 66 | -0.537700858 | COMTD1 | catechol-O-methyltransferase domain containing 1 [Source:HGNC Symbol;Acc:HGNC:26309] |
| ENSG00000165929 | HGLibA_48687 | 66 | -0.537700858 | TC2N | nuclear [Source:HGNC Symbol;Acc:HGNC:19859]" |
| ENSG00000166265 | HGLibA_12252 | 66 | -0.537700858 | CYYR1 | cysteine and tyrosine rich 1 [Source:HGNC Symbol;Acc:HGNC:16274] |
| ENSG00000167208 | HGLibA_46061 | 66 | -0.537700858 | SNX20 | sorting nexin 20 [Source:HGNC Symbol;Acc:HGNC:30390] |
| ENSG00000167535 | HGLibA_06923 | 66 | -0.537700858 | CACNB3 | calcium voltage-gated channel auxiliary subunit beta 3 [Source:HGNC Symbol;Acc:HGNC:1403] |
| ENSG00000167755 | HGLibA_25263 | 66 | -0.537700858 | KLK6 | kallikrein related peptidase 6 [Source:HGNC Symbol;Acc:HGNC:6367] |
| ENSG00000168032 | HGLibA_15203 | 66 | -0.537700858 | ENTPD3 | ectonucleoside triphosphate diphosphohydrolase 3 [Source:HGNC Symbol;Acc:HGNC:3365] |
| ENSG00000168124 | HGLibA_33664 | 66 | -0.537700858 | OR1F1 | olfactory receptor family 1 subfamily F member 1 [Source:HGNC Symbol;Acc:HGNC:8194] |
| ENSG00000168268 | HGLibA_32824 | 66 | -0.537700858 | NT5DC2 | 5'-nucleotidase domain containing 2 [Source:HGNC Symbol;Acc:HGNC:25717] |
| ENSG00000168811 | HGLibA_22960 | 66 | -0.537700858 | IL12A | interleukin 12A [Source:HGNC Symbol;Acc:HGNC:5969] |
| ENSG00000169599 | HGLibA_31845 | 66 | -0.537700858 | NFU1 | NFU1 iron-sulfur cluster scaffold [Source:HGNC Symbol;Acc:HGNC:16287] |
| ENSG00000169629 | HGLibA_41044 | 66 | -0.537700858 | RGPD8 | RANBP2-like and GRIP domain containing 8 [Source:HGNC Symbol;Acc:HGNC:9849] |
| ENSG00000170471 | HGLibA_40163 | 66 | -0.537700858 | RALGAPB | Ral GTPase activating protein non-catalytic beta subunit [Source:HGNC Symbol;Acc:HGNC:29221] |
| ENSG00000170871 | HGLibA_24612 | 66 | -0.537700858 | KIAA0232 | KIAA0232 [Source:HGNC Symbol;Acc:HGNC:28992] |
| ENSG00000171402 | HGLibA_54484 | 66 | -0.537700858 | XAGE3 | X antigen family member 3 [Source:HGNC Symbol;Acc:HGNC:14618] |
| ENSG00000171634 | HGLibA_04740 | 66 | -0.537700858 | BPTF | bromodomain PHD finger transcription factor [Source:HGNC Symbol;Acc:HGNC:3581] |
| ENSG00000171790 | HGLibA_45500 | 66 | -0.537700858 | SLFNL1 | schlafen like 1 [Source:HGNC Symbol;Acc:HGNC:26313] |
| ENSG00000172061 | HGLibA_27261 | 66 | -0.537700858 | LRRC15 | leucine rich repeat containing 15 [Source:HGNC Symbol;Acc:HGNC:20818] |
| ENSG00000172071 | HGLibA_14728 | 66 | -0.537700858 | EIF2AK3 | eukaryotic translation initiation factor 2 alpha kinase 3 [Source:HGNC Symbol;Acc:HGNC:3255] |
| ENSG00000172795 | HGLibA_12523 | 66 | -0.537700858 | DCP2 | decapping mRNA 2 [Source:HGNC Symbol;Acc:HGNC:24452] |
| ENSG00000172938 | HGLibA_29836 | 66 | -0.537700858 | MRGPRD | MAS related GPR family member D [Source:HGNC Symbol;Acc:HGNC:29626] |
| ENSG00000174059 | HGLibA_08358 | 66 | -0.537700858 | CD34 | CD34 molecule [Source:HGNC Symbol;Acc:HGNC:1662] |
| ENSG00000174332 | HGLibA_19270 | 66 | -0.537700858 | GLIS1 | GLIS family zinc finger 1 [Source:HGNC Symbol;Acc:HGNC:29525] |
| ENSG00000174343 | HGLibA_09585 | 66 | -0.537700858 | CHRNA9 | cholinergic receptor nicotinic alpha 9 subunit [Source:HGNC Symbol;Acc:HGNC:14079] |
| ENSG00000174748 | HGLibA_41876 | 66 | -0.537700858 | RPL15 | ribosomal protein L15 [Source:HGNC Symbol;Acc:HGNC:10306] |
| ENSG00000174791 | HGLibA_41289 | 66 | -0.537700858 | RIN1 | Ras and Rab interactor 1 [Source:HGNC Symbol;Acc:HGNC:18749] |
| ENSG00000174990 | HGLibA_06813 | 66 | -0.537700858 | CA5A | carbonic anhydrase 5A [Source:HGNC Symbol;Acc:HGNC:1377] |
| ENSG00000175311 | HGLibA_02159 | 66 | -0.537700858 | ANKS4B | ankyrin repeat and sterile alpha motif domain containing 4B [Source:HGNC Symbol;Acc:HGNC:26795] |
| ENSG00000175471 | HGLibA_28652 | 66 | -0.537700858 | MCTP1 | multiple C2 and transmembrane domain containing 1 [Source:HGNC Symbol;Acc:HGNC:26183] |
| ENSG00000175544 | HGLibA_06854 | 66 | -0.537700858 | CABP4 | calcium binding protein 4 [Source:HGNC Symbol;Acc:HGNC:1386] |
| ENSG00000176294 | HGLibA_34046 | 66 | -0.537700858 | OR4N2 | olfactory receptor family 4 subfamily N member 2 [Source:HGNC Symbol;Acc:HGNC:14742] |
| ENSG00000177103 | HGLibA_14001 | 66 | -0.537700858 | DSCAML1 | DS cell adhesion molecule like 1 [Source:HGNC Symbol;Acc:HGNC:14656] |
| ENSG00000177257 | HGLibA_12917 | 66 | -0.537700858 | DEFB4B | defensin beta 4B [Source:HGNC Symbol;Acc:HGNC:30193] |
| ENSG00000177628 | HGLibA_18711 | 66 | -0.537700858 | GBA | glucosylceramidase beta [Source:HGNC Symbol;Acc:HGNC:4177] |
| ENSG00000177853 | HGLibA_56149 | 66 | -0.537700858 | ZNF518A | zinc finger protein 518A [Source:HGNC Symbol;Acc:HGNC:29009] |
| ENSG00000178719 | HGLibA_20316 | 66 | -0.537700858 | GRINA | glutamate ionotropic receptor NMDA type subunit associated protein 1 [Source:HGNC Symbol;Acc:HGNC:4589] |
| ENSG00000178809 | HGLibA_51607 | 66 | -0.537700858 | TRIM73 | tripartite motif containing 73 [Source:HGNC Symbol;Acc:HGNC:18162] |
| ENSG00000179058 | HGLibA_06739 | 66 | -0.537700858 | C9orf50 | chromosome 9 open reading frame 50 [Source:HGNC Symbol;Acc:HGNC:23677] |
| ENSG00000179178 | HGLibA_49906 | 66 | -0.537700858 | TMEM125 | transmembrane protein 125 [Source:HGNC Symbol;Acc:HGNC:28275] |
| ENSG00000181610 | HGLibA_30068 | 66 | -0.537700858 | MRPS23 | mitochondrial ribosomal protein S23 [Source:HGNC Symbol;Acc:HGNC:14509] |
| ENSG00000181915 | HGLibA_01008 | 66 | -0.537700858 | ADO | 2-aminoethanethiol dioxygenase [Source:HGNC Symbol;Acc:HGNC:23506] |
| ENSG00000181963 | HGLibA_34183 | 66 | -0.537700858 | OR52K2 | olfactory receptor family 52 subfamily K member 2 [Source:HGNC Symbol;Acc:HGNC:15223] |
| ENSG00000182040 | HGLibA_53175 | 66 | -0.537700858 | USH1G | USH1 protein network component sans [Source:HGNC Symbol;Acc:HGNC:16356] |
| ENSG00000182631 | HGLibA_42499 | 66 | -0.537700858 | RXFP3 | relaxin family peptide receptor 3 [Source:HGNC Symbol;Acc:HGNC:24883] |
| ENSG00000182854 | HGLibA_33989 | 66 | -0.537700858 | OR4F15 | olfactory receptor family 4 subfamily F member 15 [Source:HGNC Symbol;Acc:HGNC:15078] |
| ENSG00000184999 | HGLibA_44536 | 66 | -0.537700858 | SLC22A10 | solute carrier family 22 member 10 [Source:HGNC Symbol;Acc:HGNC:18057] |
| ENSG00000185567 | HGLibA_01303 | 66 | -0.537700858 | AHNAK2 | AHNAK nucleoprotein 2 [Source:HGNC Symbol;Acc:HGNC:20125] |
| ENSG00000185634 | HGLibA_44008 | 66 | -0.537700858 | SHC4 | SHC adaptor protein 4 [Source:HGNC Symbol;Acc:HGNC:16743] |
| ENSG00000185674 | HGLibA_27701 | 66 | -0.537700858 | LYG2 | lysozyme g2 [Source:HGNC Symbol;Acc:HGNC:29615] |
| ENSG00000185803 | HGLibA_45248 | 66 | -0.537700858 | SLC52A2 | solute carrier family 52 member 2 [Source:HGNC Symbol;Acc:HGNC:30224] |
| ENSG00000187608 | HGLibA_23655 | 66 | -0.537700858 | ISG15 | ISG15 ubiquitin-like modifier [Source:HGNC Symbol;Acc:HGNC:4053] |
| ENSG00000188000 | HGLibA_34471 | 66 | -0.537700858 | OR7D2 | olfactory receptor family 7 subfamily D member 2 [Source:HGNC Symbol;Acc:HGNC:8378] |
| ENSG00000188021 | HGLibA_52734 | 66 | -0.537700858 | UBQLN2 | ubiquilin 2 [Source:HGNC Symbol;Acc:HGNC:12509] |
| ENSG00000188620 | HGLibA_21764 | 66 | -0.537700858 | HMX3 | H6 family homeobox 3 [Source:HGNC Symbol;Acc:HGNC:5019] |
| ENSG00000196099 | HGLibA_34426 | 66 | -0.537700858 | OR6M1 | olfactory receptor family 6 subfamily M member 1 [Source:HGNC Symbol;Acc:HGNC:14711] |
| ENSG00000196166 | HGLibA_06654 | 66 | -0.537700858 | C8orf86 | chromosome 8 open reading frame 86 [Source:HGNC Symbol;Acc:HGNC:33774] |
| ENSG00000197532 | HGLibA_34453 | 66 | -0.537700858 | OR6Y1 | olfactory receptor family 6 subfamily Y member 1 [Source:HGNC Symbol;Acc:HGNC:14823] |
| ENSG00000197721 | HGLibA_11049 | 66 | -0.537700858 | CR1L | complement C3b/C4b receptor 1 like [Source:HGNC Symbol;Acc:HGNC:2335] |
| ENSG00000197785 | HGLibA_03409 | 66 | -0.537700858 | ATAD3A | AAA domain containing 3A [Source:HGNC Symbol;Acc:HGNC:25567]" |
| ENSG00000198569 | HGLibA_44945 | 66 | -0.537700858 | SLC34A3 | solute carrier family 34 member 3 [Source:HGNC Symbol;Acc:HGNC:20305] |
| ENSG00000198768 | HGLibA_02398 | 66 | -0.537700858 | APCDD1L | APC down-regulated 1 like [Source:HGNC Symbol;Acc:HGNC:26892] |
| ENSG00000204323 | HGLibA_45761 | 66 | -0.537700858 | SMIM5 | small integral membrane protein 5 [Source:HGNC Symbol;Acc:HGNC:40030] |
| ENSG00000204634 | HGLibA_48565 | 66 | -0.537700858 | TBC1D8 | TBC1 domain family member 8 [Source:HGNC Symbol;Acc:HGNC:17791] |
| ENSG00000204642 | HGLibA_21646 | 66 | -0.537700858 | HLA-F | F [Source:HGNC Symbol;Acc:HGNC:4963]" |
| ENSG00000205497 | HGLibA_34076 | 66 | -0.537700858 | OR51A4 | olfactory receptor family 51 subfamily A member 4 [Source:HGNC Symbol;Acc:HGNC:14795] |
| ENSG00000205765 | HGLibA_06402 | 66 | -0.537700858 | C5orf51 | chromosome 5 open reading frame 51 [Source:HGNC Symbol;Acc:HGNC:27750] |
| ENSG00000213022 | HGLibA_25272 | 66 | -0.537700858 | KLK9 | kallikrein related peptidase 9 [Source:HGNC Symbol;Acc:HGNC:6370] |
| ENSG00000213906 | HGLibA_27587 | 66 | -0.537700858 | LTB4R2 | leukotriene B4 receptor 2 [Source:HGNC Symbol;Acc:HGNC:19260] |
| ENSG00000218336 | HGLibA_49000 | 66 | -0.537700858 | TENM3 | teneurin transmembrane protein 3 [Source:HGNC Symbol;Acc:HGNC:29944] |
| ENSG00000219073 | HGLibA_09002 | 66 | -0.537700858 | CELA3B | chymotrypsin like elastase family member 3B [Source:HGNC Symbol;Acc:HGNC:15945] |
| ENSG00000229676 | HGLibA_56095 | 66 | -0.537700858 | ZNF492 | zinc finger protein 492 [Source:HGNC Symbol;Acc:HGNC:23707] |
| ENSG00000232125 | HGLibA_14288 | 66 | -0.537700858 | DYTN | dystrotelin [Source:HGNC Symbol;Acc:HGNC:23279] |
| ENSG00000236637 | HGLibA_22680 | 66 | -0.537700858 | IFNA4 | interferon alpha 4 [Source:HGNC Symbol;Acc:HGNC:5425] |
| ENSG00000249709 | HGLibA_56255 | 66 | -0.537700858 | ZNF564 | zinc finger protein 564 [Source:HGNC Symbol;Acc:HGNC:31106] |

| | | | | | |
|---|---|---|---|---|---|
| ENSG00000269743 | HGLibA_44772 | 66 | -0.537700858 | SLC25A53 | solute carrier family 25 member 53 [Source:HGNC Symbol;Acc:HGNC:31894] |
| ENSG00000278535 | HGLibA_13121 | 66 | -0.537700858 | DHRS11 | dehydrogenase/reductase 11 [Source:HGNC Symbol;Acc:HGNC:28639] |
| ENSG00000278662 | HGLibA_19623 | 66 | -0.537700858 | GOLGA6L10 | golgin A6 family-like 10 [Source:HGNC Symbol;Acc:HGNC:37228] |
| FAM160B1 | HGLibA_16210 | 66 | -0.537700858 | FAM160B1 | - |
| FAM175A | HGLibA_16288 | 66 | -0.537700858 | FAM175A | - |
| HDHD1 | HGLibA_21052 | 66 | -0.537700858 | HDHD1 | - |
| HIST1H4J | HGLibA_21513 | 66 | -0.537700858 | HIST1H4J | - |
| hsa-mir-1286 | HGLibA_57608 | 66 | -0.537700858 | hsa-mir-1286 | - |
| hsa-mir-1302-2 | HGLibA_57694 | 66 | -0.537700858 | hsa-mir-1302-2 | - |
| hsa-mir-1537 | HGLibA_57916 | 66 | -0.537700858 | hsa-mir-1537 | - |
| hsa-mir-3074 | HGLibA_58466 | 66 | -0.537700858 | hsa-mir-3074 | - |
| hsa-mir-3116-1 | HGLibA_58505 | 66 | -0.537700858 | hsa-mir-3116-1 | - |
| hsa-mir-3163 | HGLibA_58733 | 66 | -0.537700858 | hsa-mir-3163 | - |
| hsa-mir-3191 | HGLibA_58862 | 66 | -0.537700858 | hsa-mir-3191 | - |
| hsa-mir-34a | HGLibA_59037 | 66 | -0.537700858 | hsa-mir-34a | - |
| hsa-mir-3688-2 | HGLibA_59310 | 66 | -0.537700858 | hsa-mir-3688-2 | - |
| hsa-mir-3913-1 | HGLibA_59521 | 66 | -0.537700858 | hsa-mir-3913-1 | - |
| hsa-mir-4255 | HGLibA_59727 | 66 | -0.537700858 | hsa-mir-4255 | - |
| hsa-mir-4260 | HGLibA_59749 | 66 | -0.537700858 | hsa-mir-4260 | - |
| hsa-mir-4285 | HGLibA_59849 | 66 | -0.537700858 | hsa-mir-4285 | - |
| hsa-mir-4488 | HGLibA_60369 | 66 | -0.537700858 | hsa-mir-4488 | - |
| hsa-mir-4708 | HGLibA_60932 | 66 | -0.537700858 | hsa-mir-4708 | - |
| hsa-mir-4720 | HGLibA_60976 | 66 | -0.537700858 | hsa-mir-4720 | - |
| hsa-mir-4728 | HGLibA_61008 | 66 | -0.537700858 | hsa-mir-4728 | - |
| hsa-mir-4791 | HGLibA_61269 | 66 | -0.537700858 | hsa-mir-4791 | - |
| hsa-mir-5004 | HGLibA_61423 | 66 | -0.537700858 | hsa-mir-5004 | - |
| hsa-mir-515-2 | HGLibA_61604 | 66 | -0.537700858 | hsa-mir-515-2 | - |
| hsa-mir-551b | HGLibA_62122 | 66 | -0.537700858 | hsa-mir-551b | - |
| hsa-mir-583 | HGLibA_62430 | 66 | -0.537700858 | hsa-mir-583 | - |
| hsa-mir-585 | HGLibA_62439 | 66 | -0.537700858 | hsa-mir-585 | - |
| hsa-mir-6080 | HGLibA_62580 | 66 | -0.537700858 | hsa-mir-6080 | - |
| hsa-mir-632 | HGLibA_62768 | 66 | -0.537700858 | hsa-mir-632 | - |
| hsa-mir-635 | HGLibA_62781 | 66 | -0.537700858 | hsa-mir-635 | - |
| hsa-mir-6726 | HGLibA_63058 | 66 | -0.537700858 | hsa-mir-6726 | - |
| hsa-mir-6727 | HGLibA_63060 | 66 | -0.537700858 | hsa-mir-6727 | - |
| hsa-mir-765 | HGLibA_63923 | 66 | -0.537700858 | hsa-mir-765 | - |
| hsa-mir-8070 | HGLibA_64118 | 66 | -0.537700858 | hsa-mir-8070 | - |
| LOC283710 | HGLibA_26911 | 66 | -0.537700858 | LOC283710 | - |
| PRKCDBP | HGLibA_38543 | 66 | -0.537700858 | PRKCDBP | - |
| SHOX_X | HGLibA_44069 | 66 | -0.537700858 | SHOX_X | - |
| APOA1BP | HGLibA_02453 | 65 | -0.546746201 | APOA1BP | - |
| C2orf27B | HGLibA_06120 | 65 | -0.546746201 | C2orf27B | - |
| C2orf47 | HGLibA_06134 | 65 | -0.546746201 | C2orf47 | - |
| C5orf55 | HGLibA_06411 | 65 | -0.546746201 | C5orf55 | - |
| CCDC108 | HGLibA_07530 | 65 | -0.546746201 | CCDC108 | - |
| CCDC42B | HGLibA_07785 | 65 | -0.546746201 | CCDC42B | - |
| CXorf23 | HGLibA_11907 | 65 | -0.546746201 | CXorf23 | - |
| EFCAB4B | HGLibA_14544 | 65 | -0.546746201 | EFCAB4B | - |
| ENSG00000004777 | HGLibA_02763 | 65 | -0.546746201 | ARHGAP33 | Rho GTPase activating protein 33 [Source:HGNC Symbol;Acc:HGNC:23085] |
| ENSG00000006611 | HGLibA_53174 | 65 | -0.546746201 | USH1C | USH1 protein network component harmonin [Source:HGNC Symbol;Acc:HGNC:12597] |
| ENSG00000008516 | HGLibA_29521 | 65 | -0.546746201 | MMP25 | matrix metallopeptidase 25 [Source:HGNC Symbol;Acc:HGNC:14246] |
| ENSG00000033800 | HGLibA_36507 | 65 | -0.546746201 | PIAS1 | protein inhibitor of activated STAT 1 [Source:HGNC Symbol;Acc:HGNC:2752] |
| ENSG00000040933 | HGLibA_23338 | 65 | -0.546746201 | INPP4A | inositol polyphosphate-4-phosphatase type I A [Source:HGNC Symbol;Acc:HGNC:6074] |
| ENSG00000052795 | HGLibA_17769 | 65 | -0.546746201 | FNIP2 | folliculin interacting protein 2 [Source:HGNC Symbol;Acc:HGNC:29280] |
| ENSG00000054983 | HGLibA_18508 | 65 | -0.546746201 | GALC | galactosylceramidase [Source:HGNC Symbol;Acc:HGNC:4115] |
| ENSG00000058056 | HGLibA_53204 | 65 | -0.546746201 | USP13 | ubiquitin specific peptidase 13 [Source:HGNC Symbol;Acc:HGNC:12611] |
| ENSG00000060982 | HGLibA_04262 | 65 | -0.546746201 | BCAT1 | branched chain amino acid transaminase 1 [Source:HGNC Symbol;Acc:HGNC:976] |
| ENSG00000061273 | HGLibA_21022 | 65 | -0.546746201 | HDAC7 | histone deacetylase 7 [Source:HGNC Symbol;Acc:HGNC:14067] |
| ENSG00000063245 | HGLibA_15340 | 65 | -0.546746201 | EPN1 | epsin 1 [Source:HGNC Symbol;Acc:HGNC:21604] |
| ENSG00000068400 | HGLibA_20323 | 65 | -0.546746201 | GRIPAP1 | GRIP1 associated protein 1 [Source:HGNC Symbol;Acc:HGNC:18706] |
| ENSG00000071282 | HGLibA_26681 | 65 | -0.546746201 | LMCD1 | LIM and cysteine rich domains 1 [Source:HGNC Symbol;Acc:HGNC:6633] |
| ENSG00000077984 | HGLibA_11464 | 65 | -0.546746201 | CST7 | cystatin F [Source:HGNC Symbol;Acc:HGNC:2479] |
| ENSG00000086506 | HGLibA_20937 | 65 | -0.546746201 | HBQ1 | hemoglobin subunit theta 1 [Source:HGNC Symbol;Acc:HGNC:4833] |
| ENSG00000087903 | HGLibA_40965 | 65 | -0.546746201 | RFX2 | regulatory factor X2 [Source:HGNC Symbol;Acc:HGNC:9983] |
| ENSG00000089558 | HGLibA_24220 | 65 | -0.546746201 | KCNH4 | potassium voltage-gated channel subfamily H member 4 [Source:HGNC Symbol;Acc:HGNC:6253] |
| ENSG00000090372 | HGLibA_47599 | 65 | -0.546746201 | STRN4 | striatin 4 [Source:HGNC Symbol;Acc:HGNC:15721] |
| ENSG00000090621 | HGLibA_34937 | 65 | -0.546746201 | PABPC4 | poly(A) binding protein cytoplasmic 4 [Source:HGNC Symbol;Acc:HGNC:8557] |
| ENSG00000096070 | HGLibA_04843 | 65 | -0.546746201 | BRPF3 | bromodomain and PHD finger containing 3 [Source:HGNC Symbol;Acc:HGNC:14256] |
| ENSG00000099954 | HGLibA_08976 | 65 | -0.546746201 | CECR2 | histone acetyl-lysine reader [Source:HGNC Symbol;Acc:HGNC:1840]" |
| ENSG00000100376 | HGLibA_16049 | 65 | -0.546746201 | FAM118A | family with sequence similarity 118 member A [Source:HGNC Symbol;Acc:HGNC:1313] |
| ENSG00000101280 | HGLibA_01906 | 65 | -0.546746201 | ANGPT4 | angiopoietin 4 [Source:HGNC Symbol;Acc:HGNC:487] |
| ENSG00000101654 | HGLibA_41719 | 65 | -0.546746201 | RNMT | RNA guanine-7 methyltransferase [Source:HGNC Symbol;Acc:HGNC:10075] |
| ENSG00000102239 | HGLibA_04845 | 65 | -0.546746201 | BRS3 | bombesin receptor subtype 3 [Source:HGNC Symbol;Acc:HGNC:1113] |
| ENSG00000102780 | HGLibA_13073 | 65 | -0.546746201 | DGKH | diacylglycerol kinase eta [Source:HGNC Symbol;Acc:HGNC:2854] |
| ENSG00000103034 | HGLibA_31423 | 65 | -0.546746201 | NDRG4 | NDRG family member 4 [Source:HGNC Symbol;Acc:HGNC:14466] |
| ENSG00000103168 | HGLibA_48218 | 65 | -0.546746201 | TAF1C | RNA polymerase I subunit C [Source:HGNC Symbol;Acc:HGNC:11534]" |
| ENSG00000105135 | HGLibA_23227 | 65 | -0.546746201 | ILVBL | ilvB acetolactate synthase like [Source:HGNC Symbol;Acc:HGNC:6041] |
| ENSG00000105278 | HGLibA_55262 | 65 | -0.546746201 | ZFR2 | zinc finger RNA binding protein 2 [Source:HGNC Symbol;Acc:HGNC:29189] |
| ENSG00000105429 | HGLibA_28855 | 65 | -0.546746201 | MEGF8 | multiple EGF like domains 8 [Source:HGNC Symbol;Acc:HGNC:3233] |
| ENSG00000105784 | HGLibA_42452 | 65 | -0.546746201 | RUNDC3B | RUN domain containing 3B [Source:HGNC Symbol;Acc:HGNC:30286] |
| ENSG00000105889 | HGLibA_47422 | 65 | -0.546746201 | STEAP1B | STEAP family member 1B [Source:HGNC Symbol;Acc:HGNC:41907] |
| ENSG00000107890 | HGLibA_02047 | 65 | -0.546746201 | ANKRD26 | ankyrin repeat domain 26 [Source:HGNC Symbol;Acc:HGNC:29186] |
| ENSG00000108187 | HGLibA_35333 | 65 | -0.546746201 | PBLD | phenazine biosynthesis like protein domain containing [Source:HGNC Symbol;Acc:HGNC:23301] |
| ENSG00000108813 | HGLibA_13380 | 65 | -0.546746201 | DLX4 | distal-less homeobox 4 [Source:HGNC Symbol;Acc:HGNC:2917] |
| ENSG00000110031 | HGLibA_27128 | 65 | -0.546746201 | LPXN | leupaxin [Source:HGNC Symbol;Acc:HGNC:14061] |
| ENSG00000110680 | HGLibA_06991 | 65 | -0.546746201 | CALCA | calcitonin related polypeptide alpha [Source:HGNC Symbol;Acc:HGNC:1437] |
| ENSG00000111906 | HGLibA_21033 | 65 | -0.546746201 | HDDC2 | HD domain containing 2 [Source:HGNC Symbol;Acc:HGNC:21078] |
| ENSG00000112039 | HGLibA_16844 | 65 | -0.546746201 | FANCE | FA complementation group E [Source:HGNC Symbol;Acc:HGNC:3586] |
| ENSG00000112972 | HGLibA_21710 | 65 | -0.546746201 | HMGCS1 | 3-hydroxy-3-methylglutaryl-CoA synthase 1 [Source:HGNC Symbol;Acc:HGNC:5007] |
| ENSG00000112996 | HGLibA_30086 | 65 | -0.546746201 | MRPS30 | mitochondrial ribosomal protein S30 [Source:HGNC Symbol;Acc:HGNC:8769] |
| ENSG00000114349 | HGLibA_19473 | 65 | -0.546746201 | GNAT1 | G protein subunit alpha transducin 1 [Source:HGNC Symbol;Acc:HGNC:4393] |
| ENSG00000114735 | HGLibA_21131 | 65 | -0.546746201 | HEMK1 | HemK methyltransferase family member 1 [Source:HGNC Symbol;Acc:HGNC:24923] |
| ENSG00000115267 | HGLibA_22620 | 65 | -0.546746201 | IFIH1 | interferon induced with helicase C domain 1 [Source:HGNC Symbol;Acc:HGNC:18873] |
| ENSG00000115363 | HGLibA_15672 | 65 | -0.546746201 | EVA1A | regulator of programmed cell death [Source:HGNC Symbol;Acc:HGNC:25816]" |
| ENSG00000115504 | HGLibA_14659 | 65 | -0.546746201 | EHBP1 | EH domain binding protein 1 [Source:HGNC Symbol;Acc:HGNC:29144] |
| ENSG00000115590 | HGLibA_23048 | 65 | -0.546746201 | IL1R2 | interleukin 1 receptor type 2 [Source:HGNC Symbol;Acc:HGNC:5994] |
| ENSG00000120992 | HGLibA_27737 | 65 | -0.546746201 | LYPLA1 | lysophospholipase I [Source:HGNC Symbol;Acc:HGNC:6737] |
| ENSG00000123358 | HGLibA_32626 | 65 | -0.546746201 | NR4A1 | nuclear receptor subfamily 4 group A member 1 [Source:HGNC Symbol;Acc:HGNC:7980] |
| ENSG00000123384 | HGLibA_27196 | 65 | -0.546746201 | LRP1 | LDL receptor related protein 1 [Source:HGNC Symbol;Acc:HGNC:6692] |
| ENSG00000124217 | HGLibA_29617 | 65 | -0.546746201 | MOCS3 | molybdenum cofactor synthesis 3 [Source:HGNC Symbol;Acc:HGNC:15765] |
| ENSG00000125046 | HGLibA_47192 | 65 | -0.546746201 | SSUH2 | ssu-2 homolog (C. elegans) [Source:HGNC Symbol;Acc:HGNC:24809] |
| ENSG00000125207 | HGLibA_36771 | 65 | -0.546746201 | PIWIL1 | piwi like RNA-mediated gene silencing 1 [Source:HGNC Symbol;Acc:HGNC:9007] |
| ENSG00000125498 | HGLibA_24982 | 65 | -0.546746201 | KIR2DL1 | two Ig domains and long cytoplasmic tail 1 [Source:HGNC Symbol;Acc:HGNC:6329]" |
| ENSG00000125879 | HGLibA_34743 | 65 | -0.546746201 | OTOR | otoraplin [Source:HGNC Symbol;Acc:HGNC:8517] |
| ENSG00000128694 | HGLibA_34685 | 65 | -0.546746201 | OSGEPL1 | O-sialoglycoprotein endopeptidase like 1 [Source:HGNC Symbol;Acc:HGNC:23075] |
| ENSG00000128849 | HGLibA_09322 | 65 | -0.546746201 | CGNL1 | cingulin like 1 [Source:HGNC Symbol;Acc:HGNC:25931] |
| ENSG00000129009 | HGLibA_23669 | 65 | -0.546746201 | ISLR | immunoglobulin superfamily containing leucine rich repeat [Source:HGNC Symbol;Acc:HGNC:6133] |
| ENSG00000129204 | HGLibA_53404 | 65 | -0.546746201 | USP6 | ubiquitin specific peptidase 6 [Source:HGNC Symbol;Acc:HGNC:12629] |
| ENSG00000129250 | HGLibA_24890 | 65 | -0.546746201 | KIF1C | kinesin family member 1C [Source:HGNC Symbol;Acc:HGNC:6317] |
| ENSG00000129691 | HGLibA_03295 | 65 | -0.546746201 | ASH2L | histone lysine methyltransferase complex subunit [Source:HGNC Symbol;Acc:HGNC:744]" |
| ENSG00000130635 | HGLibA_10587 | 65 | -0.546746201 | COL5A1 | collagen type V alpha 1 chain [Source:HGNC Symbol;Acc:HGNC:2209] |
| ENSG00000131323 | HGLibA_51247 | 65 | -0.546746201 | TRAF3 | TNF receptor associated factor 3 [Source:HGNC Symbol;Acc:HGNC:12033] |
| ENSG00000131379 | HGLibA_06217 | 65 | -0.546746201 | C3orf20 | chromosome 3 open reading frame 20 [Source:HGNC Symbol;Acc:HGNC:25320] |
| ENSG00000131788 | HGLibA_36514 | 65 | -0.546746201 | PIAS3 | protein inhibitor of activated STAT 3 [Source:HGNC Symbol;Acc:HGNC:16861] |
| ENSG00000132631 | HGLibA_43038 | 65 | -0.546746201 | SCP2D1 | SCP2 sterol binding domain containing 1 [Source:HGNC Symbol;Acc:HGNC:16211] |
| ENSG00000132821 | HGLibA_53865 | 65 | -0.546746201 | VSTM2L | V-set and transmembrane domain containing 2 like [Source:HGNC Symbol;Acc:HGNC:16096] |
| ENSG00000133069 | HGLibA_49776 | 65 | -0.546746201 | TMCC2 | transmembrane and coiled-coil domain family 2 [Source:HGNC Symbol;Acc:HGNC:24239] |
| ENSG00000133169 | HGLibA_04439 | 65 | -0.546746201 | BEX1 | brain expressed X-linked 1 [Source:HGNC Symbol;Acc:HGNC:1036] |
| ENSG00000133216 | HGLibA_15313 | 65 | -0.546746201 | EPHB2 | EPH receptor B2 [Source:HGNC Symbol;Acc:HGNC:3393] |
| ENSG00000133980 | HGLibA_53834 | 65 | -0.546746201 | VRTN | vertebrae development associated [Source:HGNC Symbol;Acc:HGNC:20223] |
| ENSG00000135912 | HGLibA_52204 | 65 | -0.546746201 | TTLL4 | tubulin tyrosine ligase like 4 [Source:HGNC Symbol;Acc:HGNC:28976] |
| ENSG00000136014 | HGLibA_53370 | 65 | -0.546746201 | USP44 | ubiquitin specific peptidase 44 [Source:HGNC Symbol;Acc:HGNC:20064] |
| ENSG00000136159 | HGLibA_32949 | 65 | -0.546746201 | NUDT15 | nudix hydrolase 15 [Source:HGNC Symbol;Acc:HGNC:23063] |
| ENSG00000136250 | HGLibA_02265 | 65 | -0.546746201 | AOAH | acyloxyacyl hydrolase [Source:HGNC Symbol;Acc:HGNC:548] |
| ENSG00000136709 | HGLibA_54066 | 65 | -0.546746201 | WDR33 | WD repeat domain 33 [Source:HGNC Symbol;Acc:HGNC:25651] |
| ENSG00000136758 | HGLibA_54700 | 65 | -0.546746201 | YME1L1 | YME1 like 1 ATPase [Source:HGNC Symbol;Acc:HGNC:12843] |
| ENSG00000137055 | HGLibA_36930 | 65 | -0.546746201 | PLAA | phospholipase A2 activating protein [Source:HGNC Symbol;Acc:HGNC:9043] |
| ENSG00000137509 | HGLibA_38359 | 65 | -0.546746201 | PRCP | prolylcarboxypeptidase [Source:HGNC Symbol;Acc:HGNC:9344] |
| ENSG00000137699 | HGLibA_51453 | 65 | -0.546746201 | TRIM29 | tripartite motif containing 29 [Source:HGNC Symbol;Acc:HGNC:17274] |
| ENSG00000137860 | HGLibA_44833 | 65 | -0.546746201 | SLC28A2 | solute carrier family 28 member 2 [Source:HGNC Symbol;Acc:HGNC:11002] |
| ENSG00000138119 | HGLibA_30882 | 65 | -0.546746201 | MYOF | myoferlin [Source:HGNC Symbol;Acc:HGNC:3656] |
| ENSG00000138172 | HGLibA_07017 | 65 | -0.546746201 | CALHM2 | calcium homeostasis modulator family member 2 [Source:HGNC Symbol;Acc:HGNC:23493] |
| ENSG00000138459 | HGLibA_44958 | 65 | -0.546746201 | SLC35A5 | solute carrier family 35 member A5 [Source:HGNC Symbol;Acc:HGNC:20792] |
| ENSG00000138821 | HGLibA_45124 | 65 | -0.546746201 | SLC39A8 | solute carrier family 39 member 8 [Source:HGNC Symbol;Acc:HGNC:20862] |
| ENSG00000139218 | HGLibA_42825 | 65 | -0.546746201 | SCAF11 | SR-related CTD associated factor 11 [Source:HGNC Symbol;Acc:HGNC:10784] |

| | | | | | |
|---|---|---|---|---|---|
| ENSG00000139324 | HGLibA_50676 | 65 | -0.546746201 | TMTC3 | transmembrane and tetratricopeptide repeat containing 3 [Source:HGNC Symbol;Acc:HGNC:26899] |
| ENSG00000140988 | HGLibA_42100 | 65 | -0.546746201 | RPS2 | ribosomal protein S2 [Source:HGNC Symbol;Acc:HGNC:10404] |
| ENSG00000141367 | HGLibA_10150 | 65 | -0.546746201 | CLTC | clathrin heavy chain [Source:HGNC Symbol;Acc:HGNC:2092] |
| ENSG00000142856 | HGLibA_23784 | 65 | -0.546746201 | ITGB3BP | integrin subunit beta 3 binding protein [Source:HGNC Symbol;Acc:HGNC:6157] |
| ENSG00000143067 | HGLibA_56562 | 65 | -0.546746201 | ZNF697 | zinc finger protein 697 [Source:HGNC Symbol;Acc:HGNC:32034] |
| ENSG00000143365 | HGLibA_41781 | 65 | -0.546746201 | RORC | RAR related orphan receptor C [Source:HGNC Symbol;Acc:HGNC:10260] |
| ENSG00000143437 | HGLibA_03061 | 65 | -0.546746201 | ARNT | aryl hydrocarbon receptor nuclear translocator [Source:HGNC Symbol;Acc:HGNC:700] |
| ENSG00000143499 | HGLibA_45855 | 65 | -0.546746201 | SMYD2 | SET and MYND domain containing 2 [Source:HGNC Symbol;Acc:HGNC:20982] |
| ENSG00000143839 | HGLibA_40835 | 65 | -0.546746201 | REN | renin [Source:HGNC Symbol;Acc:HGNC:9958] |
| ENSG00000143952 | HGLibA_53812 | 65 | -0.546746201 | VPS54 | GARP complex subunit [Source:HGNC Symbol;Acc:HGNC:18652]" |
| ENSG00000147010 | HGLibA_43961 | 65 | -0.546746201 | SH3KBP1 | SH3 domain containing kinase binding protein 1 [Source:HGNC Symbol;Acc:HGNC:13867] |
| ENSG00000147050 | HGLibA_24534 | 65 | -0.546746201 | KDM6A | lysine demethylase 6A [Source:HGNC Symbol;Acc:HGNC:12637] |
| ENSG00000147140 | HGLibA_32299 | 65 | -0.546746201 | NONO | non-POU domain containing octamer binding [Source:HGNC Symbol;Acc:HGNC:7871] |
| ENSG00000147533 | HGLibA_19633 | 65 | -0.546746201 | GOLGA7 | golgin A7 [Source:HGNC Symbol;Acc:HGNC:24876] |
| ENSG00000148484 | HGLibA_42365 | 65 | -0.546746201 | RSU1 | Ras suppressor protein 1 [Source:HGNC Symbol;Acc:HGNC:10464] |
| ENSG00000150630 | HGLibA_53618 | 65 | -0.546746201 | VEGFC | vascular endothelial growth factor C [Source:HGNC Symbol;Acc:HGNC:12682] |
| ENSG00000150722 | HGLibA_38072 | 65 | -0.546746201 | PPP1R1C | protein phosphatase 1 regulatory inhibitor subunit 1C [Source:HGNC Symbol;Acc:HGNC:14940] |
| ENSG00000151033 | HGLibA_27789 | 65 | -0.546746201 | LYZL2 | lysozyme like 2 [Source:HGNC Symbol;Acc:HGNC:29613] |
| ENSG00000151093 | HGLibA_34833 | 65 | -0.546746201 | OXSM | mitochondrial [Source:HGNC Symbol;Acc:HGNC:26063]" |
| ENSG00000151445 | HGLibA_53664 | 65 | -0.546746201 | VIPAS39 | spe-39 homolog [Source:HGNC Symbol;Acc:HGNC:20347]" |
| ENSG00000151623 | HGLibA_32621 | 65 | -0.546746201 | NR3C2 | nuclear receptor subfamily 3 group C member 2 [Source:HGNC Symbol;Acc:HGNC:7979] |
| ENSG00000152076 | HGLibA_07866 | 65 | -0.546746201 | CCDC74B | coiled-coil domain containing 74B [Source:HGNC Symbol;Acc:HGNC:25267] |
| ENSG00000152454 | HGLibA_55677 | 65 | -0.546746201 | ZNF256 | zinc finger protein 256 [Source:HGNC Symbol;Acc:HGNC:13049] |
| ENSG00000153820 | HGLibA_46650 | 65 | -0.546746201 | SPHKAP | AKAP domain containing [Source:HGNC Symbol;Acc:HGNC:30619]" |
| ENSG00000154889 | HGLibA_29768 | 65 | -0.546746201 | MPPE1 | metallophosphoesterase 1 [Source:HGNC Symbol;Acc:HGNC:15988] |
| ENSG00000155659 | HGLibA_53848 | 65 | -0.546746201 | VSIG4 | V-set and immunoglobulin domain containing 4 [Source:HGNC Symbol;Acc:HGNC:17032] |
| ENSG00000156269 | HGLibA_30972 | 65 | -0.546746201 | NAA11 | NatA catalytic subunit [Source:HGNC Symbol;Acc:HGNC:28125]" |
| ENSG00000157111 | HGLibA_50060 | 65 | -0.546746201 | TMEM171 | transmembrane protein 171 [Source:HGNC Symbol;Acc:HGNC:27031] |
| ENSG00000158477 | HGLibA_08264 | 65 | -0.546746201 | CD1A | CD1a molecule [Source:HGNC Symbol;Acc:HGNC:1634] |
| ENSG00000159527 | HGLibA_36255 | 65 | -0.546746201 | PGLYRP3 | peptidoglycan recognition protein 3 [Source:HGNC Symbol;Acc:HGNC:30014] |
| ENSG00000159873 | HGLibA_07558 | 65 | -0.546746201 | CCDC117 | coiled-coil domain containing 117 [Source:HGNC Symbol;Acc:HGNC:26599] |
| ENSG00000160683 | HGLibA_11893 | 65 | -0.546746201 | CXCR5 | C-X-C motif chemokine receptor 5 [Source:HGNC Symbol;Acc:HGNC:1060] |
| ENSG00000163618 | HGLibA_06978 | 65 | -0.546746201 | CADPS | calcium dependent secretion activator [Source:HGNC Symbol;Acc:HGNC:1426] |
| ENSG00000164404 | HGLibA_18879 | 65 | -0.546746201 | GDF9 | growth differentiation factor 9 [Source:HGNC Symbol;Acc:HGNC:4224] |
| ENSG00000165275 | HGLibA_51662 | 65 | -0.546746201 | TRMT10B | tRNA methyltransferase 10B [Source:HGNC Symbol;Acc:HGNC:26454] |
| ENSG00000165525 | HGLibA_31689 | 65 | -0.546746201 | NEMF | nuclear export mediator factor [Source:HGNC Symbol;Acc:HGNC:10663] |
| ENSG00000165661 | HGLibA_39756 | 65 | -0.546746201 | QSOX2 | quiescin sulfhydryl oxidase 2 [Source:HGNC Symbol;Acc:HGNC:30249] |
| ENSG00000166225 | HGLibA_18046 | 65 | -0.546746201 | FRS2 | fibroblast growth factor receptor substrate 2 [Source:HGNC Symbol;Acc:HGNC:16971] |
| ENSG00000166226 | HGLibA_08196 | 65 | -0.546746201 | CCT2 | chaperonin containing TCP1 subunit 2 [Source:HGNC Symbol;Acc:HGNC:1615] |
| ENSG00000167536 | HGLibA_13126 | 65 | -0.546746201 | DHRS13 | dehydrogenase/reductase 13 [Source:HGNC Symbol;Acc:HGNC:28326] |
| ENSG00000167895 | HGLibA_49770 | 65 | -0.546746201 | TMC8 | transmembrane channel like 8 [Source:HGNC Symbol;Acc:HGNC:20474] |
| ENSG00000168591 | HGLibA_50685 | 65 | -0.546746201 | TMUB2 | transmembrane and ubiquitin like domain containing 2 [Source:HGNC Symbol;Acc:HGNC:28459] |
| ENSG00000169062 | HGLibA_53063 | 65 | -0.546746201 | UPF3A | regulator of nonsense mediated mRNA decay [Source:HGNC Symbol;Acc:HGNC:20332]" |
| ENSG00000170615 | HGLibA_44799 | 65 | -0.546746201 | SLC26A5 | solute carrier family 26 member 5 [Source:HGNC Symbol;Acc:HGNC:9359] |
| ENSG00000170775 | HGLibA_20031 | 65 | -0.546746201 | GPR37 | G protein-coupled receptor 37 [Source:HGNC Symbol;Acc:HGNC:4494] |
| ENSG00000171105 | HGLibA_23400 | 65 | -0.546746201 | INSR | insulin receptor [Source:HGNC Symbol;Acc:HGNC:6091] |
| ENSG00000171928 | HGLibA_52360 | 65 | -0.546746201 | TVP23B | trans-golgi network vesicle protein 23 homolog B [Source:HGNC Symbol;Acc:HGNC:20399] |
| ENSG00000172137 | HGLibA_06989 | 65 | -0.546746201 | CALB2 | calbindin 2 [Source:HGNC Symbol;Acc:HGNC:1435] |
| ENSG00000172175 | HGLibA_28035 | 65 | -0.546746201 | MALT1 | MALT1 paracaspase [Source:HGNC Symbol;Acc:HGNC:6819] |
| ENSG00000172772 | HGLibA_33555 | 65 | -0.546746201 | OR10W1 | olfactory receptor family 10 subfamily W member 1 [Source:HGNC Symbol;Acc:HGNC:15139] |
| ENSG00000172869 | HGLibA_13454 | 65 | -0.546746201 | DMXL1 | Dmx like 1 [Source:HGNC Symbol;Acc:HGNC:2937] |
| ENSG00000173262 | HGLibA_44866 | 65 | -0.546746201 | SLC2A14 | solute carrier family 2 member 14 [Source:HGNC Symbol;Acc:HGNC:18301] |
| ENSG00000173908 | HGLibA_25467 | 65 | -0.546746201 | KRT28 | keratin 28 [Source:HGNC Symbol;Acc:HGNC:30842] |
| ENSG00000174576 | HGLibA_32402 | 65 | -0.546746201 | NPAS4 | neuronal PAS domain protein 4 [Source:HGNC Symbol;Acc:HGNC:18983] |
| ENSG00000175106 | HGLibA_52363 | 65 | -0.546746201 | TVP23C | trans-golgi network vesicle protein 23 homolog C [Source:HGNC Symbol;Acc:HGNC:30453] |
| ENSG00000177082 | HGLibA_54168 | 65 | -0.546746201 | WDR73 | WD repeat domain 73 [Source:HGNC Symbol;Acc:HGNC:25928] |
| ENSG00000177098 | HGLibA_42995 | 65 | -0.546746201 | SCN4B | sodium voltage-gated channel beta subunit 4 [Source:HGNC Symbol;Acc:HGNC:10592] |
| ENSG00000177483 | HGLibA_40558 | 65 | -0.546746201 | RBM44 | RNA binding motif protein 44 [Source:HGNC Symbol;Acc:HGNC:24756] |
| ENSG00000177917 | HGLibA_02993 | 65 | -0.546746201 | ARL6IP6 | ADP ribosylation factor like GTPase 6 interacting protein 6 [Source:HGNC Symbol;Acc:HGNC:24048] |
| ENSG00000178028 | HGLibA_13390 | 65 | -0.546746201 | DMAP1 | DNA methyltransferase 1 associated protein 1 [Source:HGNC Symbol;Acc:HGNC:18291] |
| ENSG00000178295 | HGLibA_18928 | 65 | -0.546746201 | GEN1 | Holliday junction 5' flap endonuclease [Source:HGNC Symbol;Acc:HGNC:26881]" |
| ENSG00000178685 | HGLibA_35214 | 65 | -0.546746201 | PARP10 | poly(ADP-ribose) polymerase family member 10 [Source:HGNC Symbol;Acc:HGNC:25895] |
| ENSG00000179833 | HGLibA_43607 | 65 | -0.546746201 | SERTAD2 | SERTA domain containing 2 [Source:HGNC Symbol;Acc:HGNC:30784] |
| ENSG00000179909 | HGLibA_55500 | 65 | -0.546746201 | ZNF154 | zinc finger protein 154 [Source:HGNC Symbol;Acc:HGNC:12939] |
| ENSG00000179912 | HGLibA_39775 | 65 | -0.546746201 | R3HDM2 | R3H domain containing 2 [Source:HGNC Symbol;Acc:HGNC:29167] |
| ENSG00000181029 | HGLibA_51328 | 65 | -0.546746201 | TRAPPC5 | trafficking protein particle complex 5 [Source:HGNC Symbol;Acc:HGNC:23067] |
| ENSG00000181074 | HGLibA_34198 | 65 | -0.546746201 | OR52N4 | olfactory receptor family 52 subfamily N member 4 (gene/pseudogene) [Source:HGNC Symbol;Acc:HGNC:15230] |
| ENSG00000181433 | HGLibA_42643 | 65 | -0.546746201 | SAGE1 | sarcoma antigen 1 [Source:HGNC Symbol;Acc:HGNC:30369] |
| ENSG00000181704 | HGLibA_54684 | 65 | -0.546746201 | YIPF6 | Yip1 domain family member 6 [Source:HGNC Symbol;Acc:HGNC:28304] |
| ENSG00000181961 | HGLibA_33926 | 65 | -0.546746201 | OR4A16 | olfactory receptor family 4 subfamily A member 16 [Source:HGNC Symbol;Acc:HGNC:15153] |
| ENSG00000182132 | HGLibA_24235 | 65 | -0.546746201 | KCNIP1 | potassium voltage-gated channel interacting protein 1 [Source:HGNC Symbol;Acc:HGNC:15521] |
| ENSG00000182150 | HGLibA_15453 | 65 | -0.546746201 | ERCC6L2 | ERCC excision repair 6 like 2 [Source:HGNC Symbol;Acc:HGNC:26922] |
| ENSG00000182552 | HGLibA_42491 | 65 | -0.546746201 | RWDD4 | RWD domain containing 4 [Source:HGNC Symbol;Acc:HGNC:23750] |
| ENSG00000183605 | HGLibA_43789 | 65 | -0.546746201 | SFXN4 | sideroflexin 4 [Source:HGNC Symbol;Acc:HGNC:16088] |
| ENSG00000183655 | HGLibA_25165 | 65 | -0.546746201 | KLHL25 | kelch like family member 25 [Source:HGNC Symbol;Acc:HGNC:25732] |
| ENSG00000183684 | HGLibA_01741 | 65 | -0.546746201 | ALYREF | Aly/REF export factor [Source:HGNC Symbol;Acc:HGNC:19071] |
| ENSG00000183770 | HGLibA_17903 | 65 | -0.546746201 | FOXL2 | forkhead box L2 [Source:HGNC Symbol;Acc:HGNC:1092] |
| ENSG00000184207 | HGLibA_36275 | 65 | -0.546746201 | PGP | phosphoglycolate phosphatase [Source:HGNC Symbol;Acc:HGNC:8909] |
| ENSG00000184227 | HGLibA_00467 | 65 | -0.546746201 | ACOT1 | acyl-CoA thioesterase 1 [Source:HGNC Symbol;Acc:HGNC:33128] |
| ENSG00000184381 | HGLibA_36920 | 65 | -0.546746201 | PLA2G6 | phospholipase A2 group VI [Source:HGNC Symbol;Acc:HGNC:9039] |
| ENSG00000184635 | HGLibA_56912 | 65 | -0.546746201 | ZNF93 | zinc finger protein 93 [Source:HGNC Symbol;Acc:HGNC:13169] |
| ENSG00000185436 | HGLibA_22730 | 65 | -0.546746201 | IFNLR1 | interferon lambda receptor 1 [Source:HGNC Symbol;Acc:HGNC:18584] |
| ENSG00000185591 | HGLibA_46283 | 65 | -0.546746201 | SP1 | Sp1 transcription factor [Source:HGNC Symbol;Acc:HGNC:11205] |
| ENSG00000185873 | HGLibA_50615 | 65 | -0.546746201 | TMPRSS11B | transmembrane serine protease 11B [Source:HGNC Symbol;Acc:HGNC:25398] |
| ENSG00000185909 | HGLibA_25113 | 65 | -0.546746201 | KLHDC8B | kelch domain containing 8B [Source:HGNC Symbol;Acc:HGNC:28557] |
| ENSG00000186474 | HGLibA_25241 | 65 | -0.546746201 | KLK12 | kallikrein related peptidase 12 [Source:HGNC Symbol;Acc:HGNC:6360] |
| ENSG00000187764 | HGLibA_43348 | 65 | -0.546746201 | SEMA4D | semaphorin 4D [Source:HGNC Symbol;Acc:HGNC:10732] |
| ENSG00000188015 | HGLibA_42554 | 65 | -0.546746201 | S100A3 | S100 calcium binding protein A3 [Source:HGNC Symbol;Acc:HGNC:10493] |
| ENSG00000188177 | HGLibA_54979 | 65 | -0.546746201 | ZC3H6 | zinc finger CCCH-type containing 6 [Source:HGNC Symbol;Acc:HGNC:24762] |
| ENSG00000188828 | HGLibA_19308 | 65 | -0.546746201 | GLRA4 | glycine receptor alpha 4 [Source:HGNC Symbol;Acc:HGNC:31715] |
| ENSG00000189091 | HGLibA_43708 | 65 | -0.546746201 | SF3B3 | splicing factor 3b subunit 3 [Source:HGNC Symbol;Acc:HGNC:10770] |
| ENSG00000189253 | HGLibA_51580 | 65 | -0.546746201 | TRIM64B | tripartite motif containing 64B [Source:HGNC Symbol;Acc:HGNC:37147] |
| ENSG00000196407 | HGLibA_49344 | 65 | -0.546746201 | THEM5 | thioesterase superfamily member 5 [Source:HGNC Symbol;Acc:HGNC:26755] |
| ENSG00000196440 | HGLibA_03047 | 65 | -0.546746201 | ARMCX4 | armadillo repeat containing X-linked 4 [Source:HGNC Symbol;Acc:HGNC:28615] |
| ENSG00000196550 | HGLibA_16707 | 65 | -0.546746201 | FAM72A | family with sequence similarity 72 member A [Source:HGNC Symbol;Acc:HGNC:24044] |
| ENSG00000197114 | HGLibA_55305 | 65 | -0.546746201 | ZGPAT | zinc finger CCCH-type and G-patch domain containing [Source:HGNC Symbol;Acc:HGNC:15948] |
| ENSG00000197170 | HGLibA_39156 | 65 | -0.546746201 | PSMD12 | non-ATPase 12 [Source:HGNC Symbol;Acc:HGNC:9557]" |
| ENSG00000197245 | HGLibA_16020 | 65 | -0.546746201 | FAM110D | family with sequence similarity 110 member D [Source:HGNC Symbol;Acc:HGNC:25860] |
| ENSG00000198398 | HGLibA_50198 | 65 | -0.546746201 | TMEM207 | transmembrane protein 207 [Source:HGNC Symbol;Acc:HGNC:33705] |
| ENSG00000198576 | HGLibA_02634 | 65 | -0.546746201 | ARC | activity regulated cytoskeleton associated protein [Source:HGNC Symbol;Acc:HGNC:648] |
| ENSG00000198720 | HGLibA_02003 | 65 | -0.546746201 | ANKRD13B | ankyrin repeat domain 13B [Source:HGNC Symbol;Acc:HGNC:26363] |
| ENSG00000204356 | HGLibA_31679 | 65 | -0.546746201 | NELFE | negative elongation factor complex member E [Source:HGNC Symbol;Acc:HGNC:13974] |
| ENSG00000205442 | HGLibA_23898 | 65 | -0.546746201 | IZUMO3 | IZUMO family member 3 [Source:HGNC Symbol;Acc:HGNC:31421] |
| ENSG00000213171 | HGLibA_26594 | 65 | -0.546746201 | LINGO4 | leucine rich repeat and Ig domain containing 4 [Source:HGNC Symbol;Acc:HGNC:31814] |
| ENSG00000222038 | HGLibA_37743 | 65 | -0.546746201 | POTEJ | POTE ankyrin domain family member J [Source:HGNC Symbol;Acc:HGNC:37094] |
| ENSG00000226600 | HGLibA_11549 | 65 | -0.546746201 | CT47A9 | member A9 [Source:HGNC Symbol;Acc:HGNC:33290]" |
| ENSG00000232119 | HGLibA_28659 | 65 | -0.546746201 | MCTS1 | re-initiation and release factor [Source:HGNC Symbol;Acc:HGNC:23357]" |
| ENSG00000233838 | HGLibA_13844 | 65 | -0.546746201 | DPH3P1 | diphthamide biosynthesis 3 pseudogene 1 [Source:HGNC Symbol;Acc:HGNC:16136] |
| ENSG00000239305 | HGLibA_41458 | 65 | -0.546746201 | RNF103 | ring finger protein 103 [Source:HGNC Symbol;Acc:HGNC:12859] |
| ENSG00000243207 | HGLibA_37813 | 65 | -0.546746201 | PPAN-P2RY11 | PPAN-P2RY11 readthrough [Source:HGNC Symbol;Acc:HGNC:33526] |
| ENSG00000243207 | HGLibA_37812 | 65 | -0.546746201 | PPAN-P2RY11 | PPAN-P2RY11 readthrough [Source:HGNC Symbol;Acc:HGNC:33526] |
| ENSG00000250151 | HGLibA_03089 | 65 | -0.546746201 | ARPC4-TTLL3 | ARPC4-TTLL3 readthrough [Source:HGNC Symbol;Acc:HGNC:38830] |
| ENSG00000275718 | HGLibA_07972 | 65 | -0.546746201 | CCL15 | C-C motif chemokine ligand 15 [Source:HGNC Symbol;Acc:HGNC:10613] |
| FAM115A | HGLibA_16037 | 65 | -0.546746201 | FAM115A | - |
| FAM178A | HGLibA_16299 | 65 | -0.546746201 | FAM178A | - |
| hsa-mir-1182 | HGLibA_57220 | 65 | -0.546746201 | hsa-mir-1182 | - |
| hsa-mir-124-2 | HGLibA_57352 | 65 | -0.546746201 | hsa-mir-124-2 | - |
| hsa-mir-1295a | HGLibA_57656 | 65 | -0.546746201 | hsa-mir-1295a | - |
| hsa-mir-1302-11 | HGLibA_57690 | 65 | -0.546746201 | hsa-mir-1302-11 | - |
| hsa-mir-1302-4 | HGLibA_57704 | 65 | -0.546746201 | hsa-mir-1302-4 | - |
| hsa-mir-3614 | HGLibA_59085 | 65 | -0.546746201 | hsa-mir-3614 | - |
| hsa-mir-3912 | HGLibA_59516 | 65 | -0.546746201 | hsa-mir-3912 | - |
| hsa-mir-3927 | HGLibA_59584 | 65 | -0.546746201 | hsa-mir-3927 | - |
| hsa-mir-4283-1 | HGLibA_59836 | 65 | -0.546746201 | hsa-mir-4283-1 | - |
| hsa-mir-4306 | HGLibA_59937 | 65 | -0.546746201 | hsa-mir-4306 | - |
| hsa-mir-4312 | HGLibA_59965 | 65 | -0.546746201 | hsa-mir-4312 | - |
| hsa-mir-4459 | HGLibA_60242 | 65 | -0.546746201 | hsa-mir-4459 | - |
| hsa-mir-5088 | HGLibA_61497 | 65 | -0.546746201 | hsa-mir-5088 | - |
| hsa-mir-575 | HGLibA_62396 | 65 | -0.546746201 | hsa-mir-575 | - |
| hsa-mir-580 | HGLibA_62420 | 65 | -0.546746201 | hsa-mir-580 | - |
| hsa-mir-595 | HGLibA_62472 | 65 | -0.546746201 | hsa-mir-595 | - |
| hsa-mir-642a | HGLibA_62808 | 65 | -0.546746201 | hsa-mir-642a | - |
| hsa-mir-6715b | HGLibA_63022 | 65 | -0.546746201 | hsa-mir-6715b | - |
| hsa-mir-6776 | HGLibA_63276 | 65 | -0.546746201 | hsa-mir-6776 | - |
| hsa-mir-6777 | HGLibA_63280 | 65 | -0.546746201 | hsa-mir-6777 | - |

| | | | | | |
|---|---|---|---|---|---|
| hsa-mir-6886 | HGLibA_63728 | 65 | -0.546746201 | hsa-mir-6886 | - |
| hsa-mir-6886 | HGLibA_63727 | 65 | -0.546746201 | hsa-mir-6886 | - |
| hsa-mir-8085 | HGLibA_64182 | 65 | -0.546746201 | hsa-mir-8085 | - |
| hsa-mir-8089 | HGLibA_64200 | 65 | -0.546746201 | hsa-mir-8089 | - |
| hsa-mir-942 | HGLibA_64354 | 65 | -0.546746201 | hsa-mir-942 | - |
| LOC100505549 | HGLibA_26848 | 65 | -0.546746201 | LOC100505549 | - |
| LRRC16A | HGLibA_27262 | 65 | -0.546746201 | LRRC16A | - |
| PRR21 | HGLibA_38814 | 65 | -0.546746201 | PRR21 | - |
| PRR25 | HGLibA_38832 | 65 | -0.546746201 | PRR25 | - |
| SELO | HGLibA_43302 | 65 | -0.546746201 | SELO | - |
| 6-Sep | HGLibA_43449 | 65 | -0.546746201 | 6-Sep | - |
| TMEM27 | HGLibA_50356 | 65 | -0.546746201 | TMEM27 | - |
| WDR65 | HGLibA_54150 | 65 | -0.546746201 | WDR65 | - |
| ZCCHC5 | HGLibA_55039 | 65 | -0.546746201 | ZCCHC5 | - |
| ASMT_X | HGLibA_03319 | 64 | -0.555791544 | ASMT_X | - |
| C11orf70 | HGLibA_05175 | 64 | -0.555791544 | C11orf70 | - |
| C16orf80 | HGLibA_05487 | 64 | -0.555791544 | C16orf80 | - |
| C22orf24 | HGLibA_06058 | 64 | -0.555791544 | C22orf24 | - |
| C8orf59 | HGLibA_06640 | 64 | -0.555791544 | C8orf59 | - |
| CXorf64 | HGLibA_11955 | 64 | -0.555791544 | CXorf64 | - |
| ENSG00000002726 | HGLibA_02266 | 64 | -0.555791544 | AOC1 | copper containing 1 [Source:HGNC Symbol;Acc:HGNC:80]" |
| ENSG00000004399 | HGLibA_37282 | 64 | -0.555791544 | PLXND1 | plexin D1 [Source:HGNC Symbol;Acc:HGNC:9107] |
| ENSG00000005339 | HGLibA_11107 | 64 | -0.555791544 | CREBBP | CREB binding protein [Source:HGNC Symbol;Acc:HGNC:2348] |
| ENSG00000007255 | HGLibA_51330 | 64 | -0.555791544 | TRAPPC6A | trafficking protein particle complex 6A [Source:HGNC Symbol;Acc:HGNC:23069] |
| ENSG00000019549 | HGLibA_45870 | 64 | -0.555791544 | SNAI2 | snail family transcriptional repressor 2 [Source:HGNC Symbol;Acc:HGNC:11094] |
| ENSG00000019582 | HGLibA_08443 | 64 | -0.555791544 | CD74 | CD74 molecule [Source:HGNC Symbol;Acc:HGNC:1697] |
| ENSG00000024526 | HGLibA_12973 | 64 | -0.555791544 | DEPDC1 | DEP domain containing 1 [Source:HGNC Symbol;Acc:HGNC:22949] |
| ENSG00000048028 | HGLibA_53320 | 64 | -0.555791544 | USP28 | ubiquitin specific peptidase 28 [Source:HGNC Symbol;Acc:HGNC:12625] |
| ENSG00000059728 | HGLibA_30611 | 64 | -0.555791544 | MXD1 | MAX dimerization protein 1 [Source:HGNC Symbol;Acc:HGNC:6761] |
| ENSG00000068394 | HGLibA_19813 | 64 | -0.555791544 | GPKOW | G-patch domain and KOW motifs [Source:HGNC Symbol;Acc:HGNC:30677] |
| ENSG00000079246 | HGLibA_54594 | 64 | -0.555791544 | XRCC5 | X-ray repair cross complementing 5 [Source:HGNC Symbol;Acc:HGNC:12833] |
| ENSG00000081181 | HGLibA_02689 | 64 | -0.555791544 | ARG2 | arginase 2 [Source:HGNC Symbol;Acc:HGNC:664] |
| ENSG00000083750 | HGLibA_42226 | 64 | -0.555791544 | RRAGB | Ras related GTP binding B [Source:HGNC Symbol;Acc:HGNC:19901] |
| ENSG00000102934 | HGLibA_37202 | 64 | -0.555791544 | PLLP | plasmolipin [Source:HGNC Symbol;Acc:HGNC:18553] |
| ENSG00000104442 | HGLibA_03004 | 64 | -0.555791544 | ARMC1 | armadillo repeat containing 1 [Source:HGNC Symbol;Acc:HGNC:17684] |
| ENSG00000104804 | HGLibA_52332 | 64 | -0.555791544 | TULP2 | tubby like protein 2 [Source:HGNC Symbol;Acc:HGNC:12424] |
| ENSG00000105141 | HGLibA_07304 | 64 | -0.555791544 | CASP14 | caspase 14 [Source:HGNC Symbol;Acc:HGNC:1502] |
| ENSG00000105173 | HGLibA_08083 | 64 | -0.555791544 | CCNE1 | cyclin E1 [Source:HGNC Symbol;Acc:HGNC:1589] |
| ENSG00000105486 | HGLibA_26480 | 64 | -0.555791544 | LIG1 | DNA ligase 1 [Source:HGNC Symbol;Acc:HGNC:6598] |
| ENSG00000106034 | HGLibA_10930 | 64 | -0.555791544 | CPED1 | cadherin like and PC-esterase domain containing 1 [Source:HGNC Symbol;Acc:HGNC:26159] |
| ENSG00000106804 | HGLibA_06350 | 64 | -0.555791544 | C5 | complement C5 [Source:HGNC Symbol;Acc:HGNC:1331] |
| ENSG00000108018 | HGLibA_46184 | 64 | -0.555791544 | SORCS1 | sortilin related VPS10 domain containing receptor 1 [Source:HGNC Symbol;Acc:HGNC:16697] |
| ENSG00000109452 | HGLibA_23340 | 64 | -0.555791544 | INPP4B | inositol polyphosphate-4-phosphatase type II B [Source:HGNC Symbol;Acc:HGNC:6075] |
| ENSG00000109534 | HGLibA_18622 | 64 | -0.555791544 | GAR1 | GAR1 ribonucleoprotein [Source:HGNC Symbol;Acc:HGNC:14264] |
| ENSG00000110074 | HGLibA_17952 | 64 | -0.555791544 | FOXRED1 | FAD dependent oxidoreductase domain containing 1 [Source:HGNC Symbol;Acc:HGNC:26927] |
| ENSG00000111144 | HGLibA_27579 | 64 | -0.555791544 | LTA4H | leukotriene A4 hydrolase [Source:HGNC Symbol;Acc:HGNC:6710] |
| ENSG00000112096 | HGLibA_46147 | 64 | -0.555791544 | SOD2 | superoxide dismutase 2 [Source:HGNC Symbol;Acc:HGNC:11180] |
| ENSG00000112599 | HGLibA_20638 | 64 | -0.555791544 | GUCA1B | guanylate cyclase activator 1B [Source:HGNC Symbol;Acc:HGNC:4679] |
| ENSG00000112902 | HGLibA_43358 | 64 | -0.555791544 | SEMA5A | semaphorin 5A [Source:HGNC Symbol;Acc:HGNC:10736] |
| ENSG00000112996 | HGLibA_30087 | 64 | -0.555791544 | MRPS30 | mitochondrial ribosomal protein S30 [Source:HGNC Symbol;Acc:HGNC:8769] |
| ENSG00000114166 | HGLibA_24055 | 64 | -0.555791544 | KAT2B | lysine acetyltransferase 2B [Source:HGNC Symbol;Acc:HGNC:8638] |
| ENSG00000114547 | HGLibA_41763 | 64 | -0.555791544 | ROPN1B | rhophilin associated tail protein 1B [Source:HGNC Symbol;Acc:HGNC:31927] |
| ENSG00000115718 | HGLibA_38651 | 64 | -0.555791544 | PROC | inactivator of coagulation factors Va and VIIIa [Source:HGNC Symbol;Acc:HGNC:9451]" |
| ENSG00000115808 | HGLibA_47595 | 64 | -0.555791544 | STRN | striatin [Source:HGNC Symbol;Acc:HGNC:11424] |
| ENSG00000115827 | HGLibA_12435 | 64 | -0.555791544 | DCAF17 | DDB1 and CUL4 associated factor 17 [Source:HGNC Symbol;Acc:HGNC:25784] |
| ENSG00000116678 | HGLibA_26311 | 64 | -0.555791544 | LEPR | leptin receptor [Source:HGNC Symbol;Acc:HGNC:6554] |
| ENSG00000116785 | HGLibA_09270 | 64 | -0.555791544 | CFHR3 | complement factor H related 3 [Source:HGNC Symbol;Acc:HGNC:16980] |
| ENSG00000117152 | HGLibA_41095 | 64 | -0.555791544 | RGS4 | regulator of G protein signaling 4 [Source:HGNC Symbol;Acc:HGNC:10000] |
| ENSG00000118137 | HGLibA_02450 | 64 | -0.555791544 | APOA1 | apolipoprotein A1 [Source:HGNC Symbol;Acc:HGNC:600] |
| ENSG00000118263 | HGLibA_25082 | 64 | -0.555791544 | KLF7 | Kruppel like factor 7 [Source:HGNC Symbol;Acc:HGNC:6350] |
| ENSG00000118515 | HGLibA_43816 | 64 | -0.555791544 | SGK1 | serum/glucocorticoid regulated kinase 1 [Source:HGNC Symbol;Acc:HGNC:10810] |
| ENSG00000121680 | HGLibA_36103 | 64 | -0.555791544 | PEX16 | peroxisomal biogenesis factor 16 [Source:HGNC Symbol;Acc:HGNC:8857] |
| ENSG00000121864 | HGLibA_56440 | 64 | -0.555791544 | ZNF639 | zinc finger protein 639 [Source:HGNC Symbol;Acc:HGNC:30950] |
| ENSG00000121871 | HGLibA_45519 | 64 | -0.555791544 | SLITRK3 | SLIT and NTRK like family member 3 [Source:HGNC Symbol;Acc:HGNC:23501] |
| ENSG00000122257 | HGLibA_40434 | 64 | -0.555791544 | RBBP6 | ubiquitin ligase [Source:HGNC Symbol;Acc:HGNC:9889]" |
| ENSG00000123496 | HGLibA_22976 | 64 | -0.555791544 | IL13RA2 | interleukin 13 receptor subunit alpha 2 [Source:HGNC Symbol;Acc:HGNC:5975] |
| ENSG00000124733 | HGLibA_28717 | 64 | -0.555791544 | MEA1 | male-enhanced antigen 1 [Source:HGNC Symbol;Acc:HGNC:6986] |
| ENSG00000127241 | HGLibA_28393 | 64 | -0.555791544 | MASP1 | mannan binding lectin serine peptidase 1 [Source:HGNC Symbol;Acc:HGNC:6901] |
| ENSG00000127314 | HGLibA_40223 | 64 | -0.555791544 | RAP1B | member of RAS oncogene family [Source:HGNC Symbol;Acc:HGNC:9857]" |
| ENSG00000129204 | HGLibA_53405 | 64 | -0.555791544 | USP6 | ubiquitin specific peptidase 6 [Source:HGNC Symbol;Acc:HGNC:12629] |
| ENSG00000129214 | HGLibA_43996 | 64 | -0.555791544 | SHBG | sex hormone binding globulin [Source:HGNC Symbol;Acc:HGNC:10839] |
| ENSG00000129474 | HGLibA_01373 | 64 | -0.555791544 | AJUBA | ajuba LIM protein [Source:HGNC Symbol;Acc:HGNC:20250] |
| ENSG00000130005 | HGLibA_18600 | 64 | -0.555791544 | GAMT | guanidinoacetate N-methyltransferase [Source:HGNC Symbol;Acc:HGNC:4136] |
| ENSG00000130656 | HGLibA_20944 | 64 | -0.555791544 | HBZ | hemoglobin subunit zeta [Source:HGNC Symbol;Acc:HGNC:4835] |
| ENSG00000131067 | HGLibA_19018 | 64 | -0.555791544 | GGT7 | gamma-glutamyltransferase 7 [Source:HGNC Symbol;Acc:HGNC:4259] |
| ENSG00000132128 | HGLibA_27355 | 64 | -0.555791544 | LRRC41 | leucine rich repeat containing 41 [Source:HGNC Symbol;Acc:HGNC:16917] |
| ENSG00000133103 | HGLibA_10486 | 64 | -0.555791544 | COG6 | component of oligomeric golgi complex 6 [Source:HGNC Symbol;Acc:HGNC:18621] |
| ENSG00000133134 | HGLibA_04442 | 64 | -0.555791544 | BEX2 | brain expressed X-linked 2 [Source:HGNC Symbol;Acc:HGNC:30933] |
| ENSG00000133985 | HGLibA_52160 | 64 | -0.555791544 | TTC9 | tetratricopeptide repeat domain 9 [Source:HGNC Symbol;Acc:HGNC:20267] |
| ENSG00000134516 | HGLibA_13741 | 64 | -0.555791544 | DOCK2 | dedicator of cytokinesis 2 [Source:HGNC Symbol;Acc:HGNC:2988] |
| ENSG00000135002 | HGLibA_40922 | 64 | -0.555791544 | RFK | riboflavin kinase [Source:HGNC Symbol;Acc:HGNC:30324] |
| ENSG00000135473 | HGLibA_35090 | 64 | -0.555791544 | PAN2 | poly(A) specific ribonuclease subunit PAN2 [Source:HGNC Symbol;Acc:HGNC:20074] |
| ENSG00000136861 | HGLibA_08784 | 64 | -0.555791544 | CDK5RAP2 | CDK5 regulatory subunit associated protein 2 [Source:HGNC Symbol;Acc:HGNC:18672] |
| ENSG00000136868 | HGLibA_44926 | 64 | -0.555791544 | SLC31A1 | solute carrier family 31 member 1 [Source:HGNC Symbol;Acc:HGNC:11016] |
| ENSG00000137076 | HGLibA_49635 | 64 | -0.555791544 | TLN1 | talin 1 [Source:HGNC Symbol;Acc:HGNC:11845] |
| ENSG00000137563 | HGLibA_18997 | 64 | -0.555791544 | GGH | gamma-glutamyl hydrolase [Source:HGNC Symbol;Acc:HGNC:4248] |
| ENSG00000137713 | HGLibA_38142 | 64 | -0.555791544 | PPP2R1B | protein phosphatase 2 scaffold subunit Abeta [Source:HGNC Symbol;Acc:HGNC:9303] |
| ENSG00000138074 | HGLibA_45276 | 64 | -0.555791544 | SLC5A6 | solute carrier family 5 member 6 [Source:HGNC Symbol;Acc:HGNC:11041] |
| ENSG00000138162 | HGLibA_48163 | 64 | -0.555791544 | TACC2 | transforming acidic coiled-coil containing protein 2 [Source:HGNC Symbol;Acc:HGNC:11523] |
| ENSG00000138271 | HGLibA_20102 | 64 | -0.555791544 | GPR87 | G protein-coupled receptor 87 [Source:HGNC Symbol;Acc:HGNC:4538] |
| ENSG00000139973 | HGLibA_48067 | 64 | -0.555791544 | SYT16 | synaptotagmin 16 [Source:HGNC Symbol;Acc:HGNC:23142] |
| ENSG00000141380 | HGLibA_47103 | 64 | -0.555791544 | SS18 | nBAF chromatin remodeling complex subunit [Source:HGNC Symbol;Acc:HGNC:11340]" |
| ENSG00000141404 | HGLibA_19459 | 64 | -0.555791544 | GNAL | G protein subunit alpha L [Source:HGNC Symbol;Acc:HGNC:4388] |
| ENSG00000141510 | HGLibA_51047 | 64 | -0.555791544 | TP53 | tumor protein p53 [Source:HGNC Symbol;Acc:HGNC:11998] |
| ENSG00000141644 | HGLibA_28462 | 64 | -0.555791544 | MBD1 | methyl-CpG binding domain protein 1 [Source:HGNC Symbol;Acc:HGNC:6916] |
| ENSG00000142025 | HGLibA_13441 | 64 | -0.555791544 | DMRTC2 | DMRT like family C2 [Source:HGNC Symbol;Acc:HGNC:13911] |
| ENSG00000142611 | HGLibA_38384 | 64 | -0.555791544 | PRDM16 | PR/SET domain 16 [Source:HGNC Symbol;Acc:HGNC:14000] |
| ENSG00000142619 | HGLibA_34975 | 64 | -0.555791544 | PADI3 | peptidyl arginine deiminase 3 [Source:HGNC Symbol;Acc:HGNC:18337] |
| ENSG00000142748 | HGLibA_17222 | 64 | -0.555791544 | FCN3 | ficolin 3 [Source:HGNC Symbol;Acc:HGNC:3625] |
| ENSG00000143355 | HGLibA_26468 | 64 | -0.555791544 | LHX9 | LIM homeobox 9 [Source:HGNC Symbol;Acc:HGNC:14222] |
| ENSG00000144331 | HGLibA_55901 | 64 | -0.555791544 | ZNF385B | zinc finger protein 385B [Source:HGNC Symbol;Acc:HGNC:26332] |
| ENSG00000144908 | HGLibA_01553 | 64 | -0.555791544 | ALDH1L1 | aldehyde dehydrogenase 1 family member L1 [Source:HGNC Symbol;Acc:HGNC:3978] |
| ENSG00000145216 | HGLibA_17534 | 64 | -0.555791544 | FIP1L1 | factor interacting with PAPOLA and CPSF1 [Source:HGNC Symbol;Acc:HGNC:19124] |
| ENSG00000146232 | HGLibA_31831 | 64 | -0.555791544 | NFKBIE | NFKB inhibitor epsilon [Source:HGNC Symbol;Acc:HGNC:7799] |
| ENSG00000146555 | HGLibA_43150 | 64 | -0.555791544 | SDK1 | sidekick cell adhesion molecule 1 [Source:HGNC Symbol;Acc:HGNC:19307] |
| ENSG00000146576 | HGLibA_06527 | 64 | -0.555791544 | C7orf26 | chromosome 7 open reading frame 26 [Source:HGNC Symbol;Acc:HGNC:21702] |
| ENSG00000146618 | HGLibA_17290 | 64 | -0.555791544 | FERD3L | Fer3 like bHLH transcription factor [Source:HGNC Symbol;Acc:HGNC:16660] |
| ENSG00000147099 | HGLibA_21025 | 64 | -0.555791544 | HDAC8 | histone deacetylase 8 [Source:HGNC Symbol;Acc:HGNC:13315] |
| ENSG00000147465 | HGLibA_47349 | 64 | -0.555791544 | STAR | steroidogenic acute regulatory protein [Source:HGNC Symbol;Acc:HGNC:11359] |
| ENSG00000148411 | HGLibA_31042 | 64 | -0.555791544 | NACC2 | NACC family member 2 [Source:HGNC Symbol;Acc:HGNC:23846] |
| ENSG00000149311 | HGLibA_03524 | 64 | -0.555791544 | ATM | ATM serine/threonine kinase [Source:HGNC Symbol;Acc:HGNC:795] |
| ENSG00000149428 | HGLibA_22460 | 64 | -0.555791544 | HYOU1 | hypoxia up-regulated 1 [Source:HGNC Symbol;Acc:HGNC:16931] |
| ENSG00000150403 | HGLibA_49786 | 64 | -0.555791544 | TMCO3 | transmembrane and coiled-coil domains 3 [Source:HGNC Symbol;Acc:HGNC:20329] |
| ENSG00000150556 | HGLibA_27732 | 64 | -0.555791544 | LYPD6B | LY6/PLAUR domain containing 6B [Source:HGNC Symbol;Acc:HGNC:27018] |
| ENSG00000151033 | HGLibA_27787 | 64 | -0.555791544 | LYZL2 | lysozyme like 2 [Source:HGNC Symbol;Acc:HGNC:29613] |
| ENSG00000151475 | HGLibA_44704 | 64 | -0.555791544 | SLC25A31 | solute carrier family 25 member 31 [Source:HGNC Symbol;Acc:HGNC:25319] |
| ENSG00000152240 | HGLibA_20873 | 64 | -0.555791544 | HAUS1 | HAUS augmin like complex subunit 1 [Source:HGNC Symbol;Acc:HGNC:25174] |
| ENSG00000153233 | HGLibA_39533 | 64 | -0.555791544 | PTPRR | receptor type R [Source:HGNC Symbol;Acc:HGNC:9680]" |
| ENSG00000153823 | HGLibA_36528 | 64 | -0.555791544 | PID1 | phosphotyrosine interaction domain containing 1 [Source:HGNC Symbol;Acc:HGNC:26084] |
| ENSG00000154065 | HGLibA_02056 | 64 | -0.555791544 | ANKRD29 | ankyrin repeat domain 29 [Source:HGNC Symbol;Acc:HGNC:27110] |
| ENSG00000156515 | HGLibA_21579 | 64 | -0.555791544 | HK1 | hexokinase 1 [Source:HGNC Symbol;Acc:HGNC:4922] |
| ENSG00000159200 | HGLibA_40669 | 64 | -0.555791544 | RCAN1 | regulator of calcineurin 1 [Source:HGNC Symbol;Acc:HGNC:3040] |
| ENSG00000159714 | HGLibA_55067 | 64 | -0.555791544 | ZDHHC1 | zinc finger DHHC-type containing 1 [Source:HGNC Symbol;Acc:HGNC:17916] |
| ENSG00000160050 | HGLibA_07747 | 64 | -0.555791544 | CCDC28B | coiled-coil domain containing 28B [Source:HGNC Symbol;Acc:HGNC:28163] |
| ENSG00000160321 | HGLibA_55581 | 64 | -0.555791544 | ZNF208 | zinc finger protein 208 [Source:HGNC Symbol;Acc:HGNC:12999] |
| ENSG00000160352 | HGLibA_56619 | 64 | -0.555791544 | ZNF714 | zinc finger protein 714 [Source:HGNC Symbol;Acc:HGNC:27124] |
| ENSG00000162413 | HGLibA_25152 | 64 | -0.555791544 | KLHL21 | kelch like family member 21 [Source:HGNC Symbol;Acc:HGNC:29041] |
| ENSG00000162526 | HGLibA_52015 | 64 | -0.555791544 | TSSK3 | testis specific serine kinase 3 [Source:HGNC Symbol;Acc:HGNC:15473] |
| ENSG00000163380 | HGLibA_26722 | 64 | -0.555791544 | LMOD3 | leiomodin 3 [Source:HGNC Symbol;Acc:HGNC:6649] |
| ENSG00000163449 | HGLibA_50048 | 64 | -0.555791544 | TMEM169 | transmembrane protein 169 [Source:HGNC Symbol;Acc:HGNC:25130] |
| ENSG00000163792 | HGLibA_48775 | 64 | -0.555791544 | TCF23 | transcription factor 23 [Source:HGNC Symbol;Acc:HGNC:18602] |
| ENSG00000163808 | HGLibA_24864 | 64 | -0.555791544 | KIF15 | kinesin family member 15 [Source:HGNC Symbol;Acc:HGNC:17273] |
| ENSG00000163956 | HGLibA_27237 | 64 | -0.555791544 | LRPAP1 | LDL receptor related protein associated protein 1 [Source:HGNC Symbol;Acc:HGNC:6701] |
| ENSG00000164050 | HGLibA_37271 | 64 | -0.555791544 | PLXNB1 | plexin B1 [Source:HGNC Symbol;Acc:HGNC:9103] |
| ENSG00000164068 | HGLibA_41490 | 64 | -0.555791544 | RNF123 | ring finger protein 123 [Source:HGNC Symbol;Acc:HGNC:21148] |

| | | | | | |
|---|---|---|---|---|---|
| ENSG00000164118 | HGLibA_09141 | 64 | -0.555791544 | CEP44 | centrosomal protein 44 [Source:HGNC Symbol;Acc:HGNC:29356] |
| ENSG00000164185 | HGLibA_56063 | 64 | -0.555791544 | ZNF474 | zinc finger protein 474 [Source:HGNC Symbol;Acc:HGNC:23245] |
| ENSG00000164292 | HGLibA_41177 | 64 | -0.555791544 | RHOBTB3 | Rho related BTB domain containing 3 [Source:HGNC Symbol;Acc:HGNC:18757] |
| ENSG00000164647 | HGLibA_47419 | 64 | -0.555791544 | STEAP1 | STEAP family member 1 [Source:HGNC Symbol;Acc:HGNC:11378] |
| ENSG00000164741 | HGLibA_13312 | 64 | -0.555791544 | DLC1 | DLC1 Rho GTPase activating protein [Source:HGNC Symbol;Acc:HGNC:2897] |
| ENSG00000164741 | HGLibA_13313 | 64 | -0.555791544 | DLC1 | DLC1 Rho GTPase activating protein [Source:HGNC Symbol;Acc:HGNC:2897] |
| ENSG00000164754 | HGLibA_40062 | 64 | -0.555791544 | RAD21 | RAD21 cohesin complex component [Source:HGNC Symbol;Acc:HGNC:9811] |
| ENSG00000165168 | HGLibA_12019 | 64 | -0.555791544 | CYBB | cytochrome b-245 beta chain [Source:HGNC Symbol;Acc:HGNC:2578] |
| ENSG00000165309 | HGLibA_03018 | 64 | -0.555791544 | ARMC3 | armadillo repeat containing 3 [Source:HGNC Symbol;Acc:HGNC:30964] |
| ENSG00000166479 | HGLibA_50695 | 64 | -0.555791544 | TMX3 | thioredoxin related transmembrane protein 3 [Source:HGNC Symbol;Acc:HGNC:24718] |
| ENSG00000166866 | HGLibA_30819 | 64 | -0.555791544 | MYO1A | myosin IA [Source:HGNC Symbol;Acc:HGNC:7595] |
| ENSG00000167323 | HGLibA_47441 | 64 | -0.555791544 | STIM1 | stromal interaction molecule 1 [Source:HGNC Symbol;Acc:HGNC:11386] |
| ENSG00000167822 | HGLibA_34540 | 64 | -0.555791544 | OR8J3 | olfactory receptor family 8 subfamily J member 3 [Source:HGNC Symbol;Acc:HGNC:15312] |
| ENSG00000168887 | HGLibA_06165 | 64 | -0.555791544 | C2orf68 | chromosome 2 open reading frame 68 [Source:HGNC Symbol;Acc:HGNC:34353] |
| ENSG00000169188 | HGLibA_02411 | 64 | -0.555791544 | APEX2 | apurinic/apyrimidinic endodeoxyribonuclease 2 [Source:HGNC Symbol;Acc:HGNC:17889] |
| ENSG00000169507 | HGLibA_45062 | 64 | -0.555791544 | SLC38A11 | solute carrier family 38 member 11 [Source:HGNC Symbol;Acc:HGNC:26836] |
| ENSG00000170485 | HGLibA_32398 | 64 | -0.555791544 | NPAS2 | neuronal PAS domain protein 2 [Source:HGNC Symbol;Acc:HGNC:7895] |
| ENSG00000170920 | HGLibA_34487 | 64 | -0.555791544 | OR7G3 | olfactory receptor family 7 subfamily G member 3 [Source:HGNC Symbol;Acc:HGNC:8467] |
| ENSG00000171160 | HGLibA_29675 | 64 | -0.555791544 | MORN4 | MORN repeat containing 4 [Source:HGNC Symbol;Acc:HGNC:24001] |
| ENSG00000171209 | HGLibA_11378 | 64 | -0.555791544 | CSN3 | casein kappa [Source:HGNC Symbol;Acc:HGNC:2446] |
| ENSG00000171885 | HGLibA_02595 | 64 | -0.555791544 | AQP4 | aquaporin 4 [Source:HGNC Symbol;Acc:HGNC:637] |
| ENSG00000172273 | HGLibA_21320 | 64 | -0.555791544 | HINFP | histone H4 transcription factor [Source:HGNC Symbol;Acc:HGNC:17850] |
| ENSG00000172752 | HGLibA_10605 | 64 | -0.555791544 | COL6A5 | collagen type VI alpha 5 chain [Source:HGNC Symbol;Acc:HGNC:26674] |
| ENSG00000172878 | HGLibA_28930 | 64 | -0.555791544 | METAP1D | mitochondrial [Source:HGNC Symbol;Acc:HGNC:32583]" |
| ENSG00000173120 | HGLibA_24493 | 64 | -0.555791544 | KDM2A | lysine demethylase 2A [Source:HGNC Symbol;Acc:HGNC:13606] |
| ENSG00000173372 | HGLibA_05911 | 64 | -0.555791544 | C1QA | complement C1q A chain [Source:HGNC Symbol;Acc:HGNC:1241] |
| ENSG00000173431 | HGLibA_41419 | 64 | -0.555791544 | RNASE8 | ribonuclease A family member 8 [Source:HGNC Symbol;Acc:HGNC:19277] |
| ENSG00000174950 | HGLibA_08251 | 64 | -0.555791544 | CD164L2 | CD164 molecule like 2 [Source:HGNC Symbol;Acc:HGNC:32043] |
| ENSG00000175792 | HGLibA_42474 | 64 | -0.555791544 | RUVBL1 | RuvB like AAA ATPase 1 [Source:HGNC Symbol;Acc:HGNC:10474] |
| ENSG00000176438 | HGLibA_47981 | 64 | -0.555791544 | SYNE3 | spectrin repeat containing nuclear envelope family member 3 [Source:HGNC Symbol;Acc:HGNC:19861] |
| ENSG00000176714 | HGLibA_07568 | 64 | -0.555791544 | CCDC121 | coiled-coil domain containing 121 [Source:HGNC Symbol;Acc:HGNC:25833] |
| ENSG00000176723 | HGLibA_56856 | 64 | -0.555791544 | ZNF843 | zinc finger protein 843 [Source:HGNC Symbol;Acc:HGNC:28710] |
| ENSG00000176845 | HGLibA_28940 | 64 | -0.555791544 | METRNL | glial cell differentiation regulator [Source:HGNC Symbol;Acc:HGNC:27584]" |
| ENSG00000176986 | HGLibA_43236 | 64 | -0.555791544 | SEC24C | COPII coat complex component [Source:HGNC Symbol;Acc:HGNC:10705]" |
| ENSG00000177462 | HGLibA_33894 | 64 | -0.555791544 | OR2T8 | olfactory receptor family 2 subfamily T member 8 [Source:HGNC Symbol;Acc:HGNC:15020] |
| ENSG00000178057 | HGLibA_31489 | 64 | -0.555791544 | NDUFAF3 | NADH:ubiquinone oxidoreductase complex assembly factor 3 [Source:HGNC Symbol;Acc:HGNC:29918] |
| ENSG00000178694 | HGLibA_32783 | 64 | -0.555791544 | NSUN3 | NOP2/Sun RNA methyltransferase family member 3 [Source:HGNC Symbol;Acc:HGNC:26208] |
| ENSG00000178934 | HGLibA_26374 | 64 | -0.555791544 | LGALS7B | galectin 7B [Source:HGNC Symbol;Acc:HGNC:34447] |
| ENSG00000179055 | HGLibA_33614 | 64 | -0.555791544 | OR13D1 | olfactory receptor family 13 subfamily D member 1 [Source:HGNC Symbol;Acc:HGNC:14695] |
| ENSG00000179526 | HGLibA_43990 | 64 | -0.555791544 | SHARPIN | SHANK associated RH domain interactor [Source:HGNC Symbol;Acc:HGNC:25321] |
| ENSG00000180818 | HGLibA_21956 | 64 | -0.555791544 | HOXC10 | homeobox C10 [Source:HGNC Symbol;Acc:HGNC:5122] |
| ENSG00000181625 | HGLibA_45561 | 64 | -0.555791544 | SLX1B | structure-specific endonuclease subunit [Source:HGNC Symbol;Acc:HGNC:28748]" |
| ENSG00000182798 | HGLibA_27938 | 64 | -0.555791544 | MAGEB17 | MAGE family member B17 [Source:HGNC Symbol;Acc:HGNC:17418] |
| ENSG00000183020 | HGLibA_02307 | 64 | -0.555791544 | AP2A2 | adaptor related protein complex 2 subunit alpha 2 [Source:HGNC Symbol;Acc:HGNC:562] |
| ENSG00000183067 | HGLibA_22895 | 64 | -0.555791544 | IGSF5 | immunoglobulin superfamily member 5 [Source:HGNC Symbol;Acc:HGNC:5952] |
| ENSG00000183305 | HGLibA_27908 | 64 | -0.555791544 | MAGEA2B | MAGE family member A2B [Source:HGNC Symbol;Acc:HGNC:19340] |
| ENSG00000183396 | HGLibA_50542 | 64 | -0.555791544 | TMEM89 | transmembrane protein 89 [Source:HGNC Symbol;Acc:HGNC:32372] |
| ENSG00000183733 | HGLibA_17515 | 64 | -0.555791544 | FIGLA | folliculogenesis specific bHLH transcription factor [Source:HGNC Symbol;Acc:HGNC:24669] |
| ENSG00000184110 | HGLibA_14769 | 64 | -0.555791544 | EIF3C | eukaryotic translation initiation factor 3 subunit C [Source:HGNC Symbol;Acc:HGNC:3279] |
| ENSG00000184156 | HGLibA_24372 | 64 | -0.555791544 | KCNQ3 | potassium voltage-gated channel subfamily Q member 3 [Source:HGNC Symbol;Acc:HGNC:6297] |
| ENSG00000184611 | HGLibA_24227 | 64 | -0.555791544 | KCNH7 | potassium voltage-gated channel subfamily H member 7 [Source:HGNC Symbol;Acc:HGNC:18863] |
| ENSG00000184675 | HGLibA_01772 | 64 | -0.555791544 | AMER1 | APC membrane recruitment protein 1 [Source:HGNC Symbol;Acc:HGNC:26837] |
| ENSG00000185666 | HGLibA_47960 | 64 | -0.555791544 | SYN3 | synapsin III [Source:HGNC Symbol;Acc:HGNC:11496] |
| ENSG00000186010 | HGLibA_31449 | 64 | -0.555791544 | NDUFA13 | NADH:ubiquinone oxidoreductase subunit A13 [Source:HGNC Symbol;Acc:HGNC:17194] |
| ENSG00000186119 | HGLibA_34282 | 64 | -0.555791544 | OR5D18 | olfactory receptor family 5 subfamily D member 18 [Source:HGNC Symbol;Acc:HGNC:15285] |
| ENSG00000187045 | HGLibA_50648 | 64 | -0.555791544 | TMPRSS6 | transmembrane serine protease 6 [Source:HGNC Symbol;Acc:HGNC:16517] |
| ENSG00000187187 | HGLibA_56200 | 64 | -0.555791544 | ZNF546 | zinc finger protein 546 [Source:HGNC Symbol;Acc:HGNC:28671] |
| ENSG00000187527 | HGLibA_03577 | 64 | -0.555791544 | ATP13A5 | ATPase 13A5 [Source:HGNC Symbol;Acc:HGNC:31789] |
| ENSG00000187678 | HGLibA_46848 | 64 | -0.555791544 | SPRY4 | sprouty RTK signaling antagonist 4 [Source:HGNC Symbol;Acc:HGNC:15533] |
| ENSG00000188408 | HGLibA_27952 | 64 | -0.555791544 | MAGEB5 | MAGE family member B5 [Source:HGNC Symbol;Acc:HGNC:23795] |
| ENSG00000188779 | HGLibA_44291 | 64 | -0.555791544 | SKOR1 | SKI family transcriptional corepressor 1 [Source:HGNC Symbol;Acc:HGNC:21326] |
| ENSG00000189013 | HGLibA_24987 | 64 | -0.555791544 | KIR2DL4 | two Ig domains and long cytoplasmic tail 4 [Source:HGNC Symbol;Acc:HGNC:6332]" |
| ENSG00000189023 | HGLibA_27935 | 64 | -0.555791544 | MAGEB16 | MAGE family member B16 [Source:HGNC Symbol;Acc:HGNC:21188] |
| ENSG00000189099 | HGLibA_38950 | 64 | -0.555791544 | PRSS48 | serine protease 48 [Source:HGNC Symbol;Acc:HGNC:24635] |
| ENSG00000196388 | HGLibA_23274 | 64 | -0.555791544 | INCA1 | cyclin A1 interacting protein 1 [Source:HGNC Symbol;Acc:HGNC:32224]" |
| ENSG00000196465 | HGLibA_30772 | 64 | -0.555791544 | MYL6B | myosin light chain 6B [Source:HGNC Symbol;Acc:HGNC:29823] |
| ENSG00000197020 | HGLibA_55431 | 64 | -0.555791544 | ZNF100 | zinc finger protein 100 [Source:HGNC Symbol;Acc:HGNC:12880] |
| ENSG00000197479 | HGLibA_35475 | 64 | -0.555791544 | PCDHB11 | protocadherin beta 11 [Source:HGNC Symbol;Acc:HGNC:8682] |
| ENSG00000197540 | HGLibA_20731 | 64 | -0.555791544 | GZMM | granzyme M [Source:HGNC Symbol;Acc:HGNC:4712] |
| ENSG00000197863 | HGLibA_56763 | 64 | -0.555791544 | ZNF790 | zinc finger protein 790 [Source:HGNC Symbol;Acc:HGNC:33114] |
| ENSG00000198001 | HGLibA_23564 | 64 | -0.555791544 | IRAK4 | interleukin 1 receptor associated kinase 4 [Source:HGNC Symbol;Acc:HGNC:17967] |
| ENSG00000198417 | HGLibA_30287 | 64 | -0.555791544 | MT1F | metallothionein 1F [Source:HGNC Symbol;Acc:HGNC:7398] |
| ENSG00000198574 | HGLibA_43886 | 64 | -0.555791544 | SH2D1B | SH2 domain containing 1B [Source:HGNC Symbol;Acc:HGNC:30416] |
| ENSG00000204592 | HGLibA_21643 | 64 | -0.555791544 | HLA-E | E [Source:HGNC Symbol;Acc:HGNC:4962]" |
| ENSG00000204701 | HGLibA_33813 | 64 | -0.555791544 | OR2J3 | olfactory receptor family 2 subfamily J member 3 [Source:HGNC Symbol;Acc:HGNC:8261] |
| ENSG00000205029 | HGLibA_34281 | 64 | -0.555791544 | OR5D16 | olfactory receptor family 5 subfamily D member 16 [Source:HGNC Symbol;Acc:HGNC:15283] |
| ENSG00000221926 | HGLibA_51416 | 64 | -0.555791544 | TRIM16 | tripartite motif containing 16 [Source:HGNC Symbol;Acc:HGNC:17241] |
| ENSG00000221968 | HGLibA_15940 | 64 | -0.555791544 | FADS3 | fatty acid desaturase 3 [Source:HGNC Symbol;Acc:HGNC:3576] |
| ENSG00000221983 | HGLibA_52525 | 64 | -0.555791544 | UBA52 | ubiquitin A-52 residue ribosomal protein fusion product 1 [Source:HGNC Symbol;Acc:HGNC:12458] |
| ENSG00000229579 | HGLibA_53261 | 64 | -0.555791544 | USP17L26 | ubiquitin specific peptidase 17-like family member 26 [Source:HGNC Symbol;Acc:HGNC:44454] |
| ENSG00000244482 | HGLibA_26504 | 64 | -0.555791544 | LILRA6 | leukocyte immunoglobulin like receptor A6 [Source:HGNC Symbol;Acc:HGNC:15495] |
| ENSG00000253958 | HGLibA_09887 | 64 | -0.555791544 | CLDN23 | claudin 23 [Source:HGNC Symbol;Acc:HGNC:17591] |
| ENSG00000265241 | HGLibA_40583 | 64 | -0.555791544 | RBM8A | RNA binding motif protein 8A [Source:HGNC Symbol;Acc:HGNC:9905] |
| ENSG00000266560 | HGLibA_27896 | 64 | -0.555791544 | MAGEA10-MAGEA5 | MAGEA10-MAGEA5 readthrough [Source:NCBI gene;Acc:100533997] |
| ENSG00000266964 | HGLibA_18224 | 64 | -0.555791544 | FXYD1 | FXYD domain containing ion transport regulator 1 [Source:HGNC Symbol;Acc:HGNC:4025] |
| ENSG00000271043 | HGLibA_30476 | 64 | -0.555791544 | MTRNR2L2 | MT-RNR2 like 2 [Source:HGNC Symbol;Acc:HGNC:37156] |
| ENSG00000272920 | HGLibA_57411 | 64 | -0.555791544 | hsa-mir-1253 | hsa-mir-1253 [Source:miRBase;Acc:MI0006387] |
| ENSG00000277494 | HGLibA_19811 | 64 | -0.555791544 | GPIHBP1 | glycosylphosphatidylinositol anchored high density lipoprotein binding protein 1 [Source:HGNC Symbol;Acc:HGNC:24945] |
| ENSG00000277972 | HGLibA_09732 | 64 | -0.555791544 | CISD3 | CDGSH iron sulfur domain 3 [Source:HGNC Symbol;Acc:HGNC:27578] |
| FAIM3 | HGLibA_15969 | 64 | -0.555791544 | FAIM3 | - |
| FAM45A | HGLibA_16592 | 64 | -0.555791544 | FAM45A | - |
| HIST1H3F | HGLibA_21471 | 64 | -0.555791544 | HIST1H3F | - |
| hsa-mir-101-2 | HGLibA_57157 | 64 | -0.555791544 | hsa-mir-101-2 | - |
| hsa-mir-1268b | HGLibA_57498 | 64 | -0.555791544 | hsa-mir-1268b | - |
| hsa-mir-147a | HGLibA_57871 | 64 | -0.555791544 | hsa-mir-147a | - |
| hsa-mir-1908 | HGLibA_58026 | 64 | -0.555791544 | hsa-mir-1908 | - |
| hsa-mir-24-2 | HGLibA_58345 | 64 | -0.555791544 | hsa-mir-24-2 | - |
| hsa-mir-302f | HGLibA_58457 | 64 | -0.555791544 | hsa-mir-302f | - |
| hsa-mir-3149 | HGLibA_58653 | 64 | -0.555791544 | hsa-mir-3149 | - |
| hsa-mir-3178 | HGLibA_58785 | 64 | -0.555791544 | hsa-mir-3178 | - |
| hsa-mir-3666 | HGLibA_59216 | 64 | -0.555791544 | hsa-mir-3666 | - |
| hsa-mir-3688-1 | HGLibA_59306 | 64 | -0.555791544 | hsa-mir-3688-1 | - |
| hsa-mir-371b | HGLibA_59378 | 64 | -0.555791544 | hsa-mir-371b | - |
| hsa-mir-4302 | HGLibA_59918 | 64 | -0.555791544 | hsa-mir-4302 | - |
| hsa-mir-4472-1 | HGLibA_60294 | 64 | -0.555791544 | hsa-mir-4472-1 | - |
| hsa-mir-4487 | HGLibA_60367 | 64 | -0.555791544 | hsa-mir-4487 | - |
| hsa-mir-4516 | HGLibA_60508 | 64 | -0.555791544 | hsa-mir-4516 | - |
| hsa-mir-4522 | HGLibA_60547 | 64 | -0.555791544 | hsa-mir-4522 | - |
| hsa-mir-4692 | HGLibA_60877 | 64 | -0.555791544 | hsa-mir-4692 | - |
| hsa-mir-4713 | HGLibA_60952 | 64 | -0.555791544 | hsa-mir-4713 | - |
| hsa-mir-4765 | HGLibA_61155 | 64 | -0.555791544 | hsa-mir-4765 | - |
| hsa-mir-5193 | HGLibA_61689 | 64 | -0.555791544 | hsa-mir-5193 | - |
| hsa-mir-5195 | HGLibA_61699 | 64 | -0.555791544 | hsa-mir-5195 | - |
| hsa-mir-548az | HGLibA_61949 | 64 | -0.555791544 | hsa-mir-548az | - |
| hsa-mir-550a-1 | HGLibA_62098 | 64 | -0.555791544 | hsa-mir-550a-1 | - |
| hsa-mir-5696 | HGLibA_62317 | 64 | -0.555791544 | hsa-mir-5696 | - |
| hsa-mir-5701-1 | HGLibA_62342 | 64 | -0.555791544 | hsa-mir-5701-1 | - |
| hsa-mir-617 | HGLibA_62709 | 64 | -0.555791544 | hsa-mir-617 | - |
| hsa-mir-6719 | HGLibA_63039 | 64 | -0.555791544 | hsa-mir-6719 | - |
| hsa-mir-6732 | HGLibA_63082 | 64 | -0.555791544 | hsa-mir-6732 | - |
| hsa-mir-6736 | HGLibA_63098 | 64 | -0.555791544 | hsa-mir-6736 | - |
| hsa-mir-6830 | HGLibA_63498 | 64 | -0.555791544 | hsa-mir-6830 | - |
| hsa-mir-8070 | HGLibA_64119 | 64 | -0.555791544 | hsa-mir-8070 | - |
| KIAA1429 | HGLibA_24750 | 64 | -0.555791544 | KIAA1429 | - |
| N6AMT2 | HGLibA_30967 | 64 | -0.555791544 | N6AMT2 | - |
| PTPLAD1 | HGLibA_39417 | 64 | -0.555791544 | PTPLAD1 | - |
| SPG20 | HGLibA_46633 | 64 | -0.555791544 | SPG20 | - |
| ZFP106 | HGLibA_55184 | 64 | -0.555791544 | ZFP106 | - |
| ADCK4 | HGLibA_00898 | 63 | -0.564836887 | ADCK4 | - |
| ANKRD20A2 | HGLibA_02031 | 63 | -0.564836887 | ANKRD20A2 | - |
| C17orf77 | HGLibA_05577 | 63 | -0.564836887 | C17orf77 | - |
| C7orf43 | HGLibA_06545 | 63 | -0.564836887 | C7orf43 | - |
| CCDC41 | HGLibA_07778 | 63 | -0.564836887 | CCDC41 | - |
| ENSG00000001629 | HGLibA_01971 | 63 | -0.564836887 | ANKIB1 | ankyrin repeat and IBR domain containing 1 [Source:HGNC Symbol;Acc:HGNC:22215] |
| ENSG00000002016 | HGLibA_40097 | 63 | -0.564836887 | RAD52 | DNA repair protein [Source:HGNC Symbol;Acc:HGNC:9824]" |

| | | | | | |
|---|---|---|---|---|---|
| ENSG00000008324 | HGLibA_47108 | 63 | -0.564836887 | SS18L2 | SS18 like 2 [Source:HGNC Symbol;Acc:HGNC:15593] |
| ENSG00000011347 | HGLibA_48086 | 63 | -0.564836887 | SYT7 | synaptotagmin 7 [Source:HGNC Symbol;Acc:HGNC:11514] |
| ENSG00000014216 | HGLibA_07143 | 63 | -0.564836887 | CAPN1 | calpain 1 [Source:HGNC Symbol;Acc:HGNC:1476] |
| ENSG00000017260 | HGLibA_03627 | 63 | -0.564836887 | ATP2C1 | ATPase secretory pathway Ca2+ transporting 1 [Source:HGNC Symbol;Acc:HGNC:13211] |
| ENSG00000025293 | HGLibA_36364 | 63 | -0.564836887 | PHF20 | PHD finger protein 20 [Source:HGNC Symbol;Acc:HGNC:16098] |
| ENSG00000050405 | HGLibA_26524 | 63 | -0.564836887 | LIMA1 | LIM domain and actin binding 1 [Source:HGNC Symbol;Acc:HGNC:24636] |
| ENSG00000053770 | HGLibA_02357 | 63 | -0.564836887 | AP5M1 | adaptor related protein complex 5 subunit mu 1 [Source:HGNC Symbol;Acc:HGNC:20192] |
| ENSG00000063176 | HGLibA_46647 | 63 | -0.564836887 | SPHK2 | sphingosine kinase 2 [Source:HGNC Symbol;Acc:HGNC:18859] |
| ENSG00000064607 | HGLibA_47721 | 63 | -0.564836887 | SUGP2 | SURP and G-patch domain containing 2 [Source:HGNC Symbol;Acc:HGNC:18641] |
| ENSG00000066405 | HGLibA_09872 | 63 | -0.564836887 | CLDN18 | claudin 18 [Source:HGNC Symbol;Acc:HGNC:2039] |
| ENSG00000071564 | HGLibA_48786 | 63 | -0.564836887 | TCF3 | transcription factor 3 [Source:HGNC Symbol;Acc:HGNC:11633] |
| ENSG00000072571 | HGLibA_21744 | 63 | -0.564836887 | HMMR | hyaluronan mediated motility receptor [Source:HGNC Symbol;Acc:HGNC:5012] |
| ENSG00000072756 | HGLibA_51714 | 63 | -0.564836887 | TRNT1 | tRNA nucleotidyl transferase 1 [Source:HGNC Symbol;Acc:HGNC:17341] |
| ENSG00000072954 | HGLibA_50379 | 63 | -0.564836887 | TMEM38A | transmembrane protein 38A [Source:HGNC Symbol;Acc:HGNC:28462] |
| ENSG00000079931 | HGLibA_29698 | 63 | -0.564836887 | MOXD1 | monooxygenase DBH like 1 [Source:HGNC Symbol;Acc:HGNC:21063] |
| ENSG00000085433 | HGLibA_54106 | 63 | -0.564836887 | WDR47 | WD repeat domain 47 [Source:HGNC Symbol;Acc:HGNC:29141] |
| ENSG00000085999 | HGLibA_40102 | 63 | -0.564836887 | RAD54L | RAD54 like [Source:HGNC Symbol;Acc:HGNC:9826] |
| ENSG00000087266 | HGLibA_43927 | 63 | -0.564836887 | SH3BP2 | SH3 domain binding protein 2 [Source:HGNC Symbol;Acc:HGNC:10825] |
| ENSG00000088298 | HGLibA_14458 | 63 | -0.564836887 | EDEM2 | ER degradation enhancing alpha-mannosidase like protein 2 [Source:HGNC Symbol;Acc:HGNC:15877] |
| ENSG00000090097 | HGLibA_35375 | 63 | -0.564836887 | PCBP4 | poly(rC) binding protein 4 [Source:HGNC Symbol;Acc:HGNC:8652] |
| ENSG00000091482 | HGLibA_45821 | 63 | -0.564836887 | SMPX | small muscle protein X-linked [Source:HGNC Symbol;Acc:HGNC:11122] |
| ENSG00000095059 | HGLibA_13116 | 63 | -0.564836887 | DHPS | deoxyhypusine synthase [Source:HGNC Symbol;Acc:HGNC:2869] |
| ENSG00000096006 | HGLibA_11170 | 63 | -0.564836887 | CRISP3 | cysteine rich secretory protein 3 [Source:HGNC Symbol;Acc:HGNC:16904] |
| ENSG00000099810 | HGLibA_30320 | 63 | -0.564836887 | MTAP | methylthioadenosine phosphorylase [Source:HGNC Symbol;Acc:HGNC:7413] |
| ENSG00000100121 | HGLibA_19025 | 63 | -0.564836887 | GGTLC2 | gamma-glutamyltransferase light chain 2 [Source:HGNC Symbol;Acc:HGNC:18596] |
| ENSG00000100249 | HGLibA_06072 | 63 | -0.564836887 | C22orf31 | chromosome 22 open reading frame 31 [Source:HGNC Symbol;Acc:HGNC:26931] |
| ENSG00000100276 | HGLibA_40360 | 63 | -0.564836887 | RASL10A | RAS like family 10 member A [Source:HGNC Symbol;Acc:HGNC:16954] |
| ENSG00000100345 | HGLibA_30741 | 63 | -0.564836887 | MYH9 | myosin heavy chain 9 [Source:HGNC Symbol;Acc:HGNC:7579] |
| ENSG00000101180 | HGLibA_22099 | 63 | -0.564836887 | HRH3 | histamine receptor H3 [Source:HGNC Symbol;Acc:HGNC:5184] |
| ENSG00000101347 | HGLibA_42704 | 63 | -0.564836887 | SAMHD1 | SAM and HD domain containing deoxynucleoside triphosphate triphosphohydrolase 1 [Source:HGNC Symbol;Acc:HGNC:15925] |
| ENSG00000101367 | HGLibA_28314 | 63 | -0.564836887 | MAPRE1 | microtubule associated protein RP/EB family member 1 [Source:HGNC Symbol;Acc:HGNC:6890] |
| ENSG00000101407 | HGLibA_52174 | 63 | -0.564836887 | TTI1 | TELO2 interacting protein 1 [Source:HGNC Symbol;Acc:HGNC:29029] |
| ENSG00000101745 | HGLibA_01996 | 63 | -0.564836887 | ANKRD12 | ankyrin repeat domain 12 [Source:HGNC Symbol;Acc:HGNC:29135] |
| ENSG00000101751 | HGLibA_37531 | 63 | -0.564836887 | POLI | DNA polymerase iota [Source:HGNC Symbol;Acc:HGNC:9182] |
| ENSG00000102034 | HGLibA_14900 | 63 | -0.564836887 | ELF4 | E74 like ETS transcription factor 4 [Source:HGNC Symbol;Acc:HGNC:3319] |
| ENSG00000102898 | HGLibA_33098 | 63 | -0.564836887 | NUTF2 | nuclear transport factor 2 [Source:HGNC Symbol;Acc:HGNC:13722] |
| ENSG00000104814 | HGLibA_28212 | 63 | -0.564836887 | MAP4K1 | mitogen-activated protein kinase kinase kinase kinase 1 [Source:HGNC Symbol;Acc:HGNC:6863] |
| ENSG00000105171 | HGLibA_37689 | 63 | -0.564836887 | POP4 | ribonuclease P/MRP subunit [Source:HGNC Symbol;Acc:HGNC:30081]" |
| ENSG00000105323 | HGLibA_21849 | 63 | -0.564836887 | HNRNPUL1 | heterogeneous nuclear ribonucleoprotein U like 1 [Source:HGNC Symbol;Acc:HGNC:17011] |
| ENSG00000105329 | HGLibA_49207 | 63 | -0.564836887 | TGFB1 | transforming growth factor beta 1 [Source:HGNC Symbol;Acc:HGNC:11766] |
| ENSG00000105507 | HGLibA_06857 | 63 | -0.564836887 | CABP5 | calcium binding protein 5 [Source:HGNC Symbol;Acc:HGNC:13714] |
| ENSG00000105879 | HGLibA_07403 | 63 | -0.564836887 | CBLL1 | Cbl proto-oncogene like 1 [Source:HGNC Symbol;Acc:HGNC:21225] |
| ENSG00000106305 | HGLibA_01357 | 63 | -0.564836887 | AIMP2 | aminoacyl tRNA synthetase complex interacting multifunctional protein 2 [Source:HGNC Symbol;Acc:HGNC:20609] |
| ENSG00000106355 | HGLibA_27546 | 63 | -0.564836887 | LSM5 | U6 small nuclear RNA and mRNA degradation associated [Source:HGNC Symbol;Acc:HGNC:17162]" |
| ENSG00000107874 | HGLibA_11771 | 63 | -0.564836887 | CUEDC2 | CUE domain containing 2 [Source:HGNC Symbol;Acc:HGNC:28352] |
| ENSG00000108389 | HGLibA_30429 | 63 | -0.564836887 | MTMR4 | myotubularin related protein 4 [Source:HGNC Symbol;Acc:HGNC:7452] |
| ENSG00000108773 | HGLibA_24050 | 63 | -0.564836887 | KAT2A | lysine acetyltransferase 2A [Source:HGNC Symbol;Acc:HGNC:4201] |
| ENSG00000109920 | HGLibA_17740 | 63 | -0.564836887 | FNBP4 | formin binding protein 4 [Source:HGNC Symbol;Acc:HGNC:19752] |
| ENSG00000111490 | HGLibA_48534 | 63 | -0.564836887 | TBC1D30 | TBC1 domain family member 30 [Source:HGNC Symbol;Acc:HGNC:29164] |
| ENSG00000111653 | HGLibA_23292 | 63 | -0.564836887 | ING4 | inhibitor of growth family member 4 [Source:HGNC Symbol;Acc:HGNC:19423] |
| ENSG00000111799 | HGLibA_10507 | 63 | -0.564836887 | COL12A1 | collagen type XII alpha 1 chain [Source:HGNC Symbol;Acc:HGNC:2188] |
| ENSG00000112137 | HGLibA_36296 | 63 | -0.564836887 | PHACTR1 | phosphatase and actin regulator 1 [Source:HGNC Symbol;Acc:HGNC:20990] |
| ENSG00000113300 | HGLibA_10344 | 63 | -0.564836887 | CNOT6 | CCR4-NOT transcription complex subunit 6 [Source:HGNC Symbol;Acc:HGNC:14099] |
| ENSG00000113578 | HGLibA_17359 | 63 | -0.564836887 | FGF1 | fibroblast growth factor 1 [Source:HGNC Symbol;Acc:HGNC:3665] |
| ENSG00000113621 | HGLibA_52411 | 63 | -0.564836887 | TXNDC15 | thioredoxin domain containing 15 [Source:HGNC Symbol;Acc:HGNC:20652] |
| ENSG00000114923 | HGLibA_45218 | 63 | -0.564836887 | SLC4A3 | solute carrier family 4 member 3 [Source:HGNC Symbol;Acc:HGNC:11029] |
| ENSG00000115009 | HGLibA_07990 | 63 | -0.564836887 | CCL20 | C-C motif chemokine ligand 20 [Source:HGNC Symbol;Acc:HGNC:10619] |
| ENSG00000117394 | HGLibA_44852 | 63 | -0.564836887 | SLC2A1 | solute carrier family 2 member 1 [Source:HGNC Symbol;Acc:HGNC:11005] |
| ENSG00000118231 | HGLibA_11279 | 63 | -0.564836887 | CRYGD | crystallin gamma D [Source:HGNC Symbol;Acc:HGNC:2411] |
| ENSG00000119203 | HGLibA_11000 | 63 | -0.564836887 | CPSF3 | cleavage and polyadenylation specific factor 3 [Source:HGNC Symbol;Acc:HGNC:2326] |
| ENSG00000119318 | HGLibA_40071 | 63 | -0.564836887 | RAD23B | nucleotide excision repair protein [Source:HGNC Symbol;Acc:HGNC:9813]" |
| ENSG00000119616 | HGLibA_17177 | 63 | -0.564836887 | FCF1 | rRNA-processing protein [Source:HGNC Symbol;Acc:HGNC:20220]" |
| ENSG00000120160 | HGLibA_15398 | 63 | -0.564836887 | EQTN | equatorin [Source:HGNC Symbol;Acc:HGNC:1359] |
| ENSG00000120907 | HGLibA_01036 | 63 | -0.564836887 | ADRA1A | adrenoceptor alpha 1A [Source:HGNC Symbol;Acc:HGNC:277] |
| ENSG00000121318 | HGLibA_48366 | 63 | -0.564836887 | TAS2R10 | taste 2 receptor member 10 [Source:HGNC Symbol;Acc:HGNC:14918] |
| ENSG00000122257 | HGLibA_40433 | 63 | -0.564836887 | RBBP6 | ubiquitin ligase [Source:HGNC Symbol;Acc:HGNC:9889]" |
| ENSG00000123594 | HGLibA_03847 | 63 | -0.564836887 | ATXN3L | ataxin 3 like [Source:HGNC Symbol;Acc:HGNC:24173] |
| ENSG00000124467 | HGLibA_39056 | 63 | -0.564836887 | PSG8 | pregnancy specific beta-1-glycoprotein 8 [Source:HGNC Symbol;Acc:HGNC:9525] |
| ENSG00000124812 | HGLibA_11165 | 63 | -0.564836887 | CRISP1 | cysteine rich secretory protein 1 [Source:HGNC Symbol;Acc:HGNC:304] |
| ENSG00000125285 | HGLibA_46256 | 63 | -0.564836887 | SOX21 | SRY-box 21 [Source:HGNC Symbol;Acc:HGNC:11197] |
| ENSG00000125520 | HGLibA_44880 | 63 | -0.564836887 | SLC2A4RG | SLC2A4 regulator [Source:HGNC Symbol;Acc:HGNC:15930] |
| ENSG00000125651 | HGLibA_20537 | 63 | -0.564836887 | GTF2F1 | general transcription factor IIF subunit 1 [Source:HGNC Symbol;Acc:HGNC:4652] |
| ENSG00000125965 | HGLibA_18869 | 63 | -0.564836887 | GDF5 | growth differentiation factor 5 [Source:HGNC Symbol;Acc:HGNC:4220] |
| ENSG00000126226 | HGLibA_35606 | 63 | -0.564836887 | PCID2 | PCI domain containing 2 [Source:HGNC Symbol;Acc:HGNC:25653] |
| ENSG00000126353 | HGLibA_08165 | 63 | -0.564836887 | CCR7 | C-C motif chemokine receptor 7 [Source:HGNC Symbol;Acc:HGNC:1608] |
| ENSG00000128714 | HGLibA_21995 | 63 | -0.564836887 | HOXD13 | homeobox D13 [Source:HGNC Symbol;Acc:HGNC:5136] |
| ENSG00000130158 | HGLibA_13752 | 63 | -0.564836887 | DOCK6 | dedicator of cytokinesis 6 [Source:HGNC Symbol;Acc:HGNC:19189] |
| ENSG00000130203 | HGLibA_02518 | 63 | -0.564836887 | APOE | apolipoprotein E [Source:HGNC Symbol;Acc:HGNC:613] |
| ENSG00000130312 | HGLibA_29956 | 63 | -0.564836887 | MRPL34 | mitochondrial ribosomal protein L34 [Source:HGNC Symbol;Acc:HGNC:14488] |
| ENSG00000130592 | HGLibA_27562 | 63 | -0.564836887 | LSP1 | lymphocyte specific protein 1 [Source:HGNC Symbol;Acc:HGNC:6707] |
| ENSG00000131584 | HGLibA_00389 | 63 | -0.564836887 | ACAP3 | ankyrin repeat and PH domains 3 [Source:HGNC Symbol;Acc:HGNC:16754]" |
| ENSG00000131966 | HGLibA_00646 | 63 | -0.564836887 | ACTR10 | actin related protein 10 homolog [Source:HGNC Symbol;Acc:HGNC:17372] |
| ENSG00000132467 | HGLibA_53453 | 63 | -0.564836887 | UTP3 | small subunit processome component [Source:HGNC Symbol;Acc:HGNC:24477]" |
| ENSG00000132478 | HGLibA_53047 | 63 | -0.564836887 | UNK | unkempt family zinc finger [Source:HGNC Symbol;Acc:HGNC:29369] |
| ENSG00000134255 | HGLibA_09185 | 63 | -0.564836887 | CEPT1 | choline/ethanolamine phosphotransferase 1 [Source:HGNC Symbol;Acc:HGNC:24289] |
| ENSG00000135119 | HGLibA_41705 | 63 | -0.564836887 | RNFT2 | transmembrane 2 [Source:HGNC Symbol;Acc:HGNC:25905]" |
| ENSG00000135469 | HGLibA_10742 | 63 | -0.564836887 | COQ10A | coenzyme Q10A [Source:HGNC Symbol;Acc:HGNC:26515] |
| ENSG00000136048 | HGLibA_13945 | 63 | -0.564836887 | DRAM1 | DNA damage regulated autophagy modulator 1 [Source:HGNC Symbol;Acc:HGNC:25645] |
| ENSG00000136718 | HGLibA_23240 | 63 | -0.564836887 | IMP4 | U3 small nucleolar ribonucleoprotein [Source:HGNC Symbol;Acc:HGNC:30856]" |
| ENSG00000136754 | HGLibA_00289 | 63 | -0.564836887 | ABI1 | abl interactor 1 [Source:HGNC Symbol;Acc:HGNC:11320] |
| ENSG00000136854 | HGLibA_47664 | 63 | -0.564836887 | STXBP1 | syntaxin binding protein 1 [Source:HGNC Symbol;Acc:HGNC:11444] |
| ENSG00000136891 | HGLibA_49046 | 63 | -0.564836887 | TEX10 | testis expressed 10 [Source:HGNC Symbol;Acc:HGNC:25988] |
| ENSG00000137261 | HGLibA_24619 | 63 | -0.564836887 | KIAA0319 | KIAA0319 [Source:HGNC Symbol;Acc:HGNC:21580] |
| ENSG00000137821 | HGLibA_27378 | 63 | -0.564836887 | LRRC49 | leucine rich repeat containing 49 [Source:HGNC Symbol;Acc:HGNC:25965] |
| ENSG00000137959 | HGLibA_22615 | 63 | -0.564836887 | IFI44L | interferon induced protein 44 like [Source:HGNC Symbol;Acc:HGNC:17817] |
| ENSG00000137975 | HGLibA_09807 | 63 | -0.564836887 | CLCA2 | chloride channel accessory 2 [Source:HGNC Symbol;Acc:HGNC:2016] |
| ENSG00000139990 | HGLibA_12449 | 63 | -0.564836887 | DCAF5 | DDB1 and CUL4 associated factor 5 [Source:HGNC Symbol;Acc:HGNC:20224] |
| ENSG00000140332 | HGLibA_49612 | 63 | -0.564836887 | TLE3 | transducin like enhancer of split 3 [Source:HGNC Symbol;Acc:HGNC:11839] |
| ENSG00000140398 | HGLibA_31629 | 63 | -0.564836887 | NEIL1 | nei like DNA glycosylase 1 [Source:HGNC Symbol;Acc:HGNC:18448] |
| ENSG00000140983 | HGLibA_41206 | 63 | -0.564836887 | RHOT2 | ras homolog family member T2 [Source:HGNC Symbol;Acc:HGNC:21169] |
| ENSG00000140987 | HGLibA_57022 | 63 | -0.564836887 | ZSCAN32 | zinc finger and SCAN domain containing 32 [Source:HGNC Symbol;Acc:HGNC:20812] |
| ENSG00000141376 | HGLibA_04258 | 63 | -0.564836887 | BCAS3 | microtubule associated cell migration factor [Source:HGNC Symbol;Acc:HGNC:14347]" |
| ENSG00000141968 | HGLibA_53547 | 63 | -0.564836887 | VAV1 | vav guanine nucleotide exchange factor 1 [Source:HGNC Symbol;Acc:HGNC:12657] |
| ENSG00000144395 | HGLibA_07642 | 63 | -0.564836887 | CCDC150 | coiled-coil domain containing 150 [Source:HGNC Symbol;Acc:HGNC:26834] |
| ENSG00000145703 | HGLibA_23534 | 63 | -0.564836887 | IQGAP2 | IQ motif containing GTPase activating protein 2 [Source:HGNC Symbol;Acc:HGNC:6111] |
| ENSG00000146670 | HGLibA_08612 | 63 | -0.564836887 | CDCA5 | cell division cycle associated 5 [Source:HGNC Symbol;Acc:HGNC:14626] |
| ENSG00000147316 | HGLibA_28646 | 63 | -0.564836887 | MCPH1 | microcephalin 1 [Source:HGNC Symbol;Acc:HGNC:6954] |
| ENSG00000147408 | HGLibA_11345 | 63 | -0.564836887 | CSGALNACT1 | chondroitin sulfate N-acetylgalactosaminyltransferase 1 [Source:HGNC Symbol;Acc:HGNC:24290] |
| ENSG00000148291 | HGLibA_47834 | 63 | -0.564836887 | SURF2 | surfeit 2 [Source:HGNC Symbol;Acc:HGNC:11475] |
| ENSG00000149428 | HGLibA_22461 | 63 | -0.564836887 | HYOU1 | hypoxia up-regulated 1 [Source:HGNC Symbol;Acc:HGNC:16931] |
| ENSG00000151640 | HGLibA_13933 | 63 | -0.564836887 | DPYSL4 | dihydropyrimidinase like 4 [Source:HGNC Symbol;Acc:HGNC:3016] |
| ENSG00000152380 | HGLibA_16165 | 63 | -0.564836887 | FAM151B | family with sequence similarity 151 member B [Source:HGNC Symbol;Acc:HGNC:33716] |
| ENSG00000152492 | HGLibA_07793 | 63 | -0.564836887 | CCDC50 | coiled-coil domain containing 50 [Source:HGNC Symbol;Acc:HGNC:18111] |
| ENSG00000152670 | HGLibA_12708 | 63 | -0.564836887 | DDX4 | DEAD-box helicase 4 [Source:HGNC Symbol;Acc:HGNC:18700] |
| ENSG00000153093 | HGLibA_00505 | 63 | -0.564836887 | ACOXL | acyl-CoA oxidase like [Source:HGNC Symbol;Acc:HGNC:25621] |
| ENSG00000153815 | HGLibA_10193 | 63 | -0.564836887 | CMIP | c-Maf inducing protein [Source:HGNC Symbol;Acc:HGNC:24319] |
| ENSG00000153944 | HGLibA_30209 | 63 | -0.564836887 | MSI2 | musashi RNA binding protein 2 [Source:HGNC Symbol;Acc:HGNC:18585] |
| ENSG00000154646 | HGLibA_50634 | 63 | -0.564836887 | TMPRSS15 | transmembrane serine protease 15 [Source:HGNC Symbol;Acc:HGNC:9490] |
| ENSG00000155066 | HGLibA_38683 | 63 | -0.564836887 | PROM2 | prominin 2 [Source:HGNC Symbol;Acc:HGNC:20685] |
| ENSG00000155659 | HGLibA_53847 | 63 | -0.564836887 | VSIG4 | V-set and immunoglobulin domain containing 4 [Source:HGNC Symbol;Acc:HGNC:17032] |
| ENSG00000156113 | HGLibA_24340 | 63 | -0.564836887 | KCNMA1 | potassium calcium-activated channel subfamily M alpha 1 [Source:HGNC Symbol;Acc:HGNC:6284] |
| ENSG00000156414 | HGLibA_48916 | 63 | -0.564836887 | TDRD9 | tudor domain containing 9 [Source:HGNC Symbol;Acc:HGNC:20122] |
| ENSG00000157214 | HGLibA_47427 | 63 | -0.564836887 | STEAP2 | STEAP2 metalloreductase [Source:HGNC Symbol;Acc:HGNC:17885] |
| ENSG00000157388 | HGLibA_06885 | 63 | -0.564836887 | CACNA1D | calcium voltage-gated channel subunit alpha1 D [Source:HGNC Symbol;Acc:HGNC:1391] |
| ENSG00000157625 | HGLibA_48147 | 63 | -0.564836887 | TAB3 | TGF-beta activated kinase 1 (MAP3K7) binding protein 3 [Source:HGNC Symbol;Acc:HGNC:30681] |
| ENSG00000159433 | HGLibA_47378 | 63 | -0.564836887 | STARD9 | StAR related lipid transfer domain containing 9 [Source:HGNC Symbol;Acc:HGNC:19162] |
| ENSG00000160886 | HGLibA_27676 | 63 | -0.564836887 | LY6K | lymphocyte antigen 6 family member K [Source:HGNC Symbol;Acc:HGNC:24225] |
| ENSG00000160957 | HGLibA_40770 | 63 | -0.564836887 | RECQL4 | RecQ like helicase 4 [Source:HGNC Symbol;Acc:HGNC:9949] |
| ENSG00000161618 | HGLibA_01536 | 63 | -0.564836887 | ALDH16A1 | aldehyde dehydrogenase 16 family member A1 [Source:HGNC Symbol;Acc:HGNC:28114] |
| ENSG00000162227 | HGLibA_48248 | 63 | -0.564836887 | TAF6L | TATA-box binding protein associated factor 6 like [Source:HGNC Symbol;Acc:HGNC:17305] |
| ENSG00000162738 | HGLibA_53515 | 63 | -0.564836887 | VANGL2 | VANGL planar cell polarity protein 2 [Source:HGNC Symbol;Acc:HGNC:15511] |
| ENSG00000163629 | HGLibA_39438 | 63 | -0.564836887 | PTPN13 | non-receptor type 13 [Source:HGNC Symbol;Acc:HGNC:9646]" |
| ENSG00000163701 | HGLibA_23016 | 63 | -0.564836887 | IL17RE | interleukin 17 receptor E [Source:HGNC Symbol;Acc:HGNC:18439] |
| ENSG00000163738 | HGLibA_30384 | 63 | -0.564836887 | MTHFD2L | methylenetetrahydrofolate dehydrogenase (NADP+ dependent) 2 like [Source:HGNC Symbol;Acc:HGNC:31865] |
| ENSG00000163939 | HGLibA_35339 | 63 | -0.564836887 | PBRM1 | polybromo 1 [Source:HGNC Symbol;Acc:HGNC:30064] |

| | | | | | |
|---|---|---|---|---|---|
| ENSG00000163982 | HGLibA_34734 | 63 | -0.564836887 | OTOP1 | otopetrin 1 [Source:HGNC Symbol;Acc:HGNC:19656] |
| ENSG00000164309 | HGLibA_10239 | 63 | -0.564836887 | CMYA5 | cardiomyopathy associated 5 [Source:HGNC Symbol;Acc:HGNC:14305] |
| ENSG00000164405 | HGLibA_53127 | 63 | -0.564836887 | UQCRQ | ubiquinol-cytochrome c reductase complex III subunit VII [Source:HGNC Symbol;Acc:HGNC:29594] |
| ENSG00000164588 | HGLibA_20974 | 63 | -0.564836887 | HCN1 | hyperpolarization activated cyclic nucleotide gated potassium channel 1 [Source:HGNC Symbol;Acc:HGNC:4845] |
| ENSG00000165071 | HGLibA_50503 | 63 | -0.564836887 | TMEM71 | transmembrane protein 71 [Source:HGNC Symbol;Acc:HGNC:26572] |
| ENSG00000165512 | HGLibA_55607 | 63 | -0.564836887 | ZNF22 | zinc finger protein 22 [Source:HGNC Symbol;Acc:HGNC:13012] |
| ENSG00000165934 | HGLibA_10997 | 63 | -0.564836887 | CPSF2 | cleavage and polyadenylation specific factor 2 [Source:HGNC Symbol;Acc:HGNC:2325] |
| ENSG00000166337 | HGLibA_48197 | 63 | -0.564836887 | TAF10 | TATA-box binding protein associated factor 10 [Source:HGNC Symbol;Acc:HGNC:11543] |
| ENSG00000166477 | HGLibA_26307 | 63 | -0.564836887 | LEO1 | Paf1/RNA polymerase II complex component [Source:HGNC Symbol;Acc:HGNC:30401]" |
| ENSG00000166582 | HGLibA_09089 | 63 | -0.564836887 | CENPV | centromere protein V [Source:HGNC Symbol;Acc:HGNC:29920] |
| ENSG00000166828 | HGLibA_43023 | 63 | -0.564836887 | SCNN1G | sodium channel epithelial 1 gamma subunit [Source:HGNC Symbol;Acc:HGNC:10602] |
| ENSG00000166925 | HGLibA_51827 | 63 | -0.564836887 | TSC22D4 | TSC22 domain family member 4 [Source:HGNC Symbol;Acc:HGNC:21696] |
| ENSG00000167657 | HGLibA_12328 | 63 | -0.564836887 | DAPK3 | death associated protein kinase 3 [Source:HGNC Symbol;Acc:HGNC:2676] |
| ENSG00000168158 | HGLibA_33776 | 63 | -0.564836887 | OR2C1 | olfactory receptor family 2 subfamily C member 1 [Source:HGNC Symbol;Acc:HGNC:8242] |
| ENSG00000168575 | HGLibA_44530 | 63 | -0.564836887 | SLC20A2 | solute carrier family 20 member 2 [Source:HGNC Symbol;Acc:HGNC:10947] |
| ENSG00000168671 | HGLibA_52937 | 63 | -0.564836887 | UGT3A2 | UDP glycosyltransferase family 3 member A2 [Source:HGNC Symbol;Acc:HGNC:27266] |
| ENSG00000169241 | HGLibA_45234 | 63 | -0.564836887 | SLC50A1 | solute carrier family 50 member 1 [Source:HGNC Symbol;Acc:HGNC:30657] |
| ENSG00000169247 | HGLibA_43981 | 63 | -0.564836887 | SH3TC2 | SH3 domain and tetratricopeptide repeats 2 [Source:HGNC Symbol;Acc:HGNC:29427] |
| ENSG00000169302 | HGLibA_47482 | 63 | -0.564836887 | STK32A | serine/threonine kinase 32A [Source:HGNC Symbol;Acc:HGNC:28317] |
| ENSG00000169359 | HGLibA_44935 | 63 | -0.564836887 | SLC33A1 | solute carrier family 33 member 1 [Source:HGNC Symbol;Acc:HGNC:95] |
| ENSG00000169826 | HGLibA_11348 | 63 | -0.564836887 | CSGALNACT2 | chondroitin sulfate N-acetylgalactosaminyltransferase 2 [Source:HGNC Symbol;Acc:HGNC:24292] |
| ENSG00000169964 | HGLibA_50399 | 63 | -0.564836887 | TMEM42 | transmembrane protein 42 [Source:HGNC Symbol;Acc:HGNC:28444] |
| ENSG00000170390 | HGLibA_12502 | 63 | -0.564836887 | DCLK2 | doublecortin like kinase 2 [Source:HGNC Symbol;Acc:HGNC:19002] |
| ENSG00000170516 | HGLibA_10868 | 63 | -0.564836887 | COX7B2 | cytochrome c oxidase subunit 7B2 [Source:HGNC Symbol;Acc:HGNC:24381] |
| ENSG00000170703 | HGLibA_52210 | 63 | -0.564836887 | TTLL6 | tubulin tyrosine ligase like 6 [Source:HGNC Symbol;Acc:HGNC:26664] |
| ENSG00000170835 | HGLibA_08986 | 63 | -0.564836887 | CEL | carboxyl ester lipase [Source:HGNC Symbol;Acc:HGNC:1848] |
| ENSG00000172046 | HGLibA_53292 | 63 | -0.564836887 | USP19 | ubiquitin specific peptidase 19 [Source:HGNC Symbol;Acc:HGNC:12617] |
| ENSG00000172345 | HGLibA_47367 | 63 | -0.564836887 | STARD5 | StAR related lipid transfer domain containing 5 [Source:HGNC Symbol;Acc:HGNC:18065] |
| ENSG00000172377 | HGLibA_34576 | 63 | -0.564836887 | OR9I1 | olfactory receptor family 9 subfamily I member 1 [Source:HGNC Symbol;Acc:HGNC:14718] |
| ENSG00000172531 | HGLibA_38004 | 63 | -0.564836887 | PPP1CA | protein phosphatase 1 catalytic subunit alpha [Source:HGNC Symbol;Acc:HGNC:9281] |
| ENSG00000173080 | HGLibA_42504 | 63 | -0.564836887 | RXFP4 | relaxin family peptide/INSL5 receptor 4 [Source:HGNC Symbol;Acc:HGNC:14666] |
| ENSG00000173163 | HGLibA_10651 | 63 | -0.564836887 | COMMD1 | copper metabolism domain containing 1 [Source:HGNC Symbol;Acc:HGNC:23024] |
| ENSG00000173715 | HGLibA_05187 | 63 | -0.564836887 | C11orf80 | chromosome 11 open reading frame 80 [Source:HGNC Symbol;Acc:HGNC:26197] |
| ENSG00000173930 | HGLibA_45472 | 63 | -0.564836887 | SLCO4C1 | solute carrier organic anion transporter family member 4C1 [Source:HGNC Symbol;Acc:HGNC:23612] |
| ENSG00000174370 | HGLibA_05141 | 63 | -0.564836887 | C11orf45 | chromosome 11 open reading frame 45 [Source:HGNC Symbol;Acc:HGNC:28584] |
| ENSG00000174775 | HGLibA_22072 | 63 | -0.564836887 | HRAS | GTPase [Source:HGNC Symbol;Acc:HGNC:5173]" |
| ENSG00000175414 | HGLibA_02923 | 63 | -0.564836887 | ARL10 | ADP ribosylation factor like GTPase 10 [Source:HGNC Symbol;Acc:HGNC:22042] |
| ENSG00000176087 | HGLibA_44955 | 63 | -0.564836887 | SLC35A4 | solute carrier family 35 member A4 [Source:HGNC Symbol;Acc:HGNC:20753] |
| ENSG00000177932 | HGLibA_55867 | 63 | -0.564836887 | ZNF354C | zinc finger protein 354C [Source:HGNC Symbol;Acc:HGNC:16736] |
| ENSG00000179111 | HGLibA_21194 | 63 | -0.564836887 | HES7 | hes family bHLH transcription factor 7 [Source:HGNC Symbol;Acc:HGNC:15977] |
| ENSG00000180263 | HGLibA_17356 | 63 | -0.564836887 | FGD6 | RhoGEF and PH domain containing 6 [Source:HGNC Symbol;Acc:HGNC:21740]" |
| ENSG00000180875 | HGLibA_20244 | 63 | -0.564836887 | GREM2 | DAN family BMP antagonist [Source:HGNC Symbol;Acc:HGNC:17655]" |
| ENSG00000182718 | HGLibA_02230 | 63 | -0.564836887 | ANXA2 | annexin A2 [Source:HGNC Symbol;Acc:HGNC:537] |
| ENSG00000182993 | HGLibA_05273 | 63 | -0.564836887 | C12orf60 | chromosome 12 open reading frame 60 [Source:HGNC Symbol;Acc:HGNC:28726] |
| ENSG00000183137 | HGLibA_09150 | 63 | -0.564836887 | CEP57L1 | centrosomal protein 57 like 1 [Source:HGNC Symbol;Acc:HGNC:21561] |
| ENSG00000183495 | HGLibA_15233 | 63 | -0.564836887 | EP400 | E1A binding protein p400 [Source:HGNC Symbol;Acc:HGNC:11958] |
| ENSG00000183735 | HGLibA_48602 | 63 | -0.564836887 | TBK1 | TANK binding kinase 1 [Source:HGNC Symbol;Acc:HGNC:11584] |
| ENSG00000183814 | HGLibA_26581 | 63 | -0.564836887 | LIN9 | lin-9 DREAM MuvB core complex component [Source:HGNC Symbol;Acc:HGNC:30830] |
| ENSG00000183826 | HGLibA_04925 | 63 | -0.564836887 | BTBD9 | BTB domain containing 9 [Source:HGNC Symbol;Acc:HGNC:21228] |
| ENSG00000184014 | HGLibA_12960 | 63 | -0.564836887 | DENND5A | DENN domain containing 5A [Source:HGNC Symbol;Acc:HGNC:19344] |
| ENSG00000184305 | HGLibA_08189 | 63 | -0.564836887 | CCSER1 | coiled-coil serine rich protein 1 [Source:HGNC Symbol;Acc:HGNC:29349] |
| ENSG00000184459 | HGLibA_04733 | 63 | -0.564836887 | BPIFC | BPI fold containing family C [Source:HGNC Symbol;Acc:HGNC:16503] |
| ENSG00000184895 | HGLibA_47100 | 63 | -0.564836887 | SRY | sex determining region Y [Source:HGNC Symbol;Acc:HGNC:11311] |
| ENSG00000185101 | HGLibA_02195 | 63 | -0.564836887 | ANO9 | anoctamin 9 [Source:HGNC Symbol;Acc:HGNC:20679] |
| ENSG00000186026 | HGLibA_55743 | 63 | -0.564836887 | ZNF284 | zinc finger protein 284 [Source:HGNC Symbol;Acc:HGNC:13078] |
| ENSG00000186081 | HGLibA_25510 | 63 | -0.564836887 | KRT5 | keratin 5 [Source:HGNC Symbol;Acc:HGNC:6442] |
| ENSG00000186153 | HGLibA_54447 | 63 | -0.564836887 | WWOX | WW domain containing oxidoreductase [Source:HGNC Symbol;Acc:HGNC:12799] |
| ENSG00000186635 | HGLibA_02626 | 63 | -0.564836887 | ARAP1 | ankyrin repeat and PH domain 1 [Source:HGNC Symbol;Acc:HGNC:16925]" |
| ENSG00000187566 | HGLibA_31895 | 63 | -0.564836887 | NHLRC1 | NHL repeat containing E3 ubiquitin protein ligase 1 [Source:HGNC Symbol;Acc:HGNC:21576] |
| ENSG00000187730 | HGLibA_18383 | 63 | -0.564836887 | GABRD | gamma-aminobutyric acid type A receptor delta subunit [Source:HGNC Symbol;Acc:HGNC:4084] |
| ENSG00000188373 | HGLibA_05113 | 63 | -0.564836887 | C10orf99 | chromosome 10 open reading frame 99 [Source:HGNC Symbol;Acc:HGNC:31428] |
| ENSG00000188385 | HGLibA_23929 | 63 | -0.564836887 | JAKMIP3 | Janus kinase and microtubule interacting protein 3 [Source:HGNC Symbol;Acc:HGNC:23523] |
| ENSG00000188554 | HGLibA_31248 | 63 | -0.564836887 | NBR1 | autophagy cargo receptor [Source:HGNC Symbol;Acc:HGNC:6746]" |
| ENSG00000188558 | HGLibA_33799 | 63 | -0.564836887 | OR2G6 | olfactory receptor family 2 subfamily G member 6 [Source:HGNC Symbol;Acc:HGNC:27019] |
| ENSG00000188976 | HGLibA_32236 | 63 | -0.564836887 | NOC2L | NOC2 like nucleolar associated transcriptional repressor [Source:HGNC Symbol;Acc:HGNC:24517] |
| ENSG00000189180 | HGLibA_55830 | 63 | -0.564836887 | ZNF33A | zinc finger protein 33A [Source:HGNC Symbol;Acc:HGNC:13096] |
| ENSG00000196196 | HGLibA_22087 | 63 | -0.564836887 | HRCT1 | histidine rich carboxyl terminus 1 [Source:HGNC Symbol;Acc:HGNC:33872] |
| ENSG00000196248 | HGLibA_33544 | 63 | -0.564836887 | OR10S1 | olfactory receptor family 10 subfamily S member 1 [Source:HGNC Symbol;Acc:HGNC:14807] |
| ENSG00000196381 | HGLibA_56736 | 63 | -0.564836887 | ZNF781 | zinc finger protein 781 [Source:HGNC Symbol;Acc:HGNC:26745] |
| ENSG00000196449 | HGLibA_54720 | 63 | -0.564836887 | YRDC | yrdC N6-threonylcarbamoyltransferase domain containing [Source:HGNC Symbol;Acc:HGNC:28905] |
| ENSG00000196476 | HGLibA_06032 | 63 | -0.564836887 | C20orf96 | chromosome 20 open reading frame 96 [Source:HGNC Symbol;Acc:HGNC:16227] |
| ENSG00000196600 | HGLibA_44579 | 63 | -0.564836887 | SLC22A25 | solute carrier family 22 member 25 [Source:HGNC Symbol;Acc:HGNC:32935] |
| ENSG00000197114 | HGLibA_55306 | 63 | -0.564836887 | ZGPAT | zinc finger CCCH-type and G-patch domain containing [Source:HGNC Symbol;Acc:HGNC:15948] |
| ENSG00000197272 | HGLibA_23113 | 63 | -0.564836887 | IL27 | interleukin 27 [Source:HGNC Symbol;Acc:HGNC:19157] |
| ENSG00000197566 | HGLibA_56417 | 63 | -0.564836887 | ZNF624 | zinc finger protein 624 [Source:HGNC Symbol;Acc:HGNC:29254] |
| ENSG00000197753 | HGLibA_26441 | 63 | -0.564836887 | LHFPL5 | LHFPL tetraspan subfamily member 5 [Source:HGNC Symbol;Acc:HGNC:21253] |
| ENSG00000198205 | HGLibA_57073 | 63 | -0.564836887 | ZXDA | zinc finger X-linked duplicated A [Source:HGNC Symbol;Acc:HGNC:13198] |
| ENSG00000198873 | HGLibA_20333 | 63 | -0.564836887 | GRK5 | G protein-coupled receptor kinase 5 [Source:HGNC Symbol;Acc:HGNC:4544] |
| ENSG00000204392 | HGLibA_27536 | 63 | -0.564836887 | LSM2 | U6 small nuclear RNA and mRNA degradation associated [Source:HGNC Symbol;Acc:HGNC:13940]" |
| ENSG00000204882 | HGLibA_19996 | 63 | -0.564836887 | GPR20 | G protein-coupled receptor 20 [Source:HGNC Symbol;Acc:HGNC:4475] |
| ENSG00000205029 | HGLibA_34279 | 63 | -0.564836887 | OR5D16 | olfactory receptor family 5 subfamily D member 16 [Source:HGNC Symbol;Acc:HGNC:15283] |
| ENSG00000205084 | HGLibA_50259 | 63 | -0.564836887 | TMEM231 | transmembrane protein 231 [Source:HGNC Symbol;Acc:HGNC:37234] |
| ENSG00000205220 | HGLibA_39096 | 63 | -0.564836887 | PSMB10 | proteasome subunit beta 10 [Source:HGNC Symbol;Acc:HGNC:9538] |
| ENSG00000205496 | HGLibA_34073 | 63 | -0.564836887 | OR51A2 | olfactory receptor family 51 subfamily A member 2 [Source:HGNC Symbol;Acc:HGNC:14764] |
| ENSG00000206203 | HGLibA_52011 | 63 | -0.564836887 | TSSK2 | testis specific serine kinase 2 [Source:HGNC Symbol;Acc:HGNC:11401] |
| ENSG00000213171 | HGLibA_26593 | 63 | -0.564836887 | LINGO4 | leucine rich repeat and Ig domain containing 4 [Source:HGNC Symbol;Acc:HGNC:31814] |
| ENSG00000213390 | HGLibA_02721 | 63 | -0.564836887 | ARHGAP19 | Rho GTPase activating protein 19 [Source:HGNC Symbol;Acc:HGNC:23724] |
| ENSG00000213512 | HGLibA_18745 | 63 | -0.564836887 | GBP7 | guanylate binding protein 7 [Source:HGNC Symbol;Acc:HGNC:29606] |
| ENSG00000215193 | HGLibA_36112 | 63 | -0.564836887 | PEX26 | peroxisomal biogenesis factor 26 [Source:HGNC Symbol;Acc:HGNC:22965] |
| ENSG00000221909 | HGLibA_16412 | 63 | -0.564836887 | FAM200A | family with sequence similarity 200 member A [Source:HGNC Symbol;Acc:HGNC:25401] |
| ENSG00000223865 | HGLibA_21618 | 63 | -0.564836887 | HLA-DPB1 | DP beta 1 [Source:HGNC Symbol;Acc:HGNC:4940]" |
| ENSG00000226929 | HGLibA_11522 | 63 | -0.564836887 | CT47A11 | member A11 [Source:HGNC Symbol;Acc:HGNC:27397]" |
| ENSG00000231637 | HGLibA_53270 | 63 | -0.564836887 | USP17L29 | ubiquitin specific peptidase 17-like family member 29 [Source:HGNC Symbol;Acc:HGNC:44457] |
| ENSG00000235780 | HGLibA_53264 | 63 | -0.564836887 | USP17L27 | ubiquitin specific peptidase 17-like family member 27 [Source:HGNC Symbol;Acc:HGNC:44455] |
| ENSG00000240432 | HGLibA_25649 | 63 | -0.564836887 | KRTAP13-3 | keratin associated protein 13-3 [Source:HGNC Symbol;Acc:HGNC:18925] |
| ENSG00000241186 | HGLibA_48883 | 63 | -0.564836887 | TDGF1 | teratocarcinoma-derived growth factor 1 [Source:HGNC Symbol;Acc:HGNC:11701] |
| ENSG00000242114 | HGLibA_30358 | 63 | -0.564836887 | MTFP1 | mitochondrial fission process 1 [Source:HGNC Symbol;Acc:HGNC:26945] |
| ENSG00000244045 | HGLibA_50164 | 63 | -0.564836887 | TMEM199 | transmembrane protein 199 [Source:HGNC Symbol;Acc:HGNC:18085] |
| ENSG00000244274 | HGLibA_12394 | 63 | -0.564836887 | DBNDD2 | dysbindin domain containing 2 [Source:HGNC Symbol;Acc:HGNC:15881] |
| ENSG00000253159 | HGLibA_35525 | 63 | -0.564836887 | PCDHGA12 | 12 [Source:HGNC Symbol;Acc:HGNC:8699]" |
| ENSG00000253276 | HGLibA_07857 | 63 | -0.564836887 | CCDC71L | coiled-coil domain containing 71 like [Source:HGNC Symbol;Acc:HGNC:26685] |
| ENSG00000254122 | HGLibA_35567 | 63 | -0.564836887 | PCDHGB7 | 7 [Source:HGNC Symbol;Acc:HGNC:8714]" |
| ENSG00000255819 | HGLibA_25296 | 63 | -0.564836887 | KLRC4-KLRK1 | KLRC4-KLRK1 readthrough [Source:HGNC Symbol;Acc:HGNC:48357] |
| ENSG00000258223 | HGLibA_38972 | 63 | -0.564836887 | PRSS58 | serine protease 58 [Source:HGNC Symbol;Acc:HGNC:39125] |
| ENSG00000258839 | HGLibA_28542 | 63 | -0.564836887 | MC1R | melanocortin 1 receptor [Source:HGNC Symbol;Acc:HGNC:6929] |
| ENSG00000259384 | HGLibA_19027 | 63 | -0.564836887 | GH1 | growth hormone 1 [Source:HGNC Symbol;Acc:HGNC:4261] |
| ENSG00000265241 | HGLibA_40585 | 63 | -0.564836887 | RBM8A | RNA binding motif protein 8A [Source:HGNC Symbol;Acc:HGNC:9905] |
| ENSG00000269791 | HGLibA_47214 | 63 | -0.564836887 | SSX4B | SSX family member 4B [Source:HGNC Symbol;Acc:HGNC:16880] |
| ENSG00000270181 | HGLibA_04536 | 63 | -0.564836887 | BIVM-ERCC5 | BIVM-ERCC5 readthrough [Source:HGNC Symbol;Acc:HGNC:43690] |
| ENSG00000270647 | HGLibA_48211 | 63 | -0.564836887 | TAF15 | TATA-box binding protein associated factor 15 [Source:HGNC Symbol;Acc:HGNC:11547] |
| ENSG00000280165 | HGLibA_35413 | 63 | -0.564836887 | PCDH20 | protocadherin 20 [Source:HGNC Symbol;Acc:HGNC:14257] |
| FAM63A | HGLibA_16660 | 63 | -0.564836887 | FAM63A | - |
| FAM69C | HGLibA_16684 | 63 | -0.564836887 | FAM69C | - |
| GARS | HGLibA_18636 | 63 | -0.564836887 | GARS | - |
| GPR124 | HGLibA_19876 | 63 | -0.564836887 | GPR124 | - |
| HIST2H4A | HGLibA_21549 | 63 | -0.564836887 | HIST2H4A | - |
| hsa-mir-106a | HGLibA_57184 | 63 | -0.564836887 | hsa-mir-106a | - |
| hsa-mir-1270-1 | HGLibA_57515 | 63 | -0.564836887 | hsa-mir-1270-1 | - |
| hsa-mir-1284 | HGLibA_57598 | 63 | -0.564836887 | hsa-mir-1284 | - |
| hsa-mir-130b | HGLibA_57751 | 63 | -0.564836887 | hsa-mir-130b | - |
| hsa-mir-135a-1 | HGLibA_57794 | 63 | -0.564836887 | hsa-mir-135a-1 | - |
| hsa-mir-192 | HGLibA_58070 | 63 | -0.564836887 | hsa-mir-192 | - |
| hsa-mir-196a-1 | HGLibA_58092 | 63 | -0.564836887 | hsa-mir-196a-1 | - |
| hsa-mir-1972-1 | HGLibA_58107 | 63 | -0.564836887 | hsa-mir-1972-1 | - |
| hsa-mir-2681 | HGLibA_58356 | 63 | -0.564836887 | hsa-mir-2681 | - |
| hsa-mir-29b-1 | HGLibA_58414 | 63 | -0.564836887 | hsa-mir-29b-1 | - |
| hsa-mir-3136 | HGLibA_58602 | 63 | -0.564836887 | hsa-mir-3136 | - |
| hsa-mir-3138 | HGLibA_58611 | 63 | -0.564836887 | hsa-mir-3138 | - |
| hsa-mir-375 | HGLibA_59400 | 63 | -0.564836887 | hsa-mir-375 | - |
| hsa-mir-3974 | HGLibA_59655 | 63 | -0.564836887 | hsa-mir-3974 | - |
| hsa-mir-4256 | HGLibA_59733 | 63 | -0.564836887 | hsa-mir-4256 | - |
| hsa-mir-4425 | HGLibA_60087 | 63 | -0.564836887 | hsa-mir-4425 | - |
| hsa-mir-4499 | HGLibA_60414 | 63 | -0.564836887 | hsa-mir-4499 | - |
| hsa-mir-4532 | HGLibA_60586 | 63 | -0.564836887 | hsa-mir-4532 | - |
| hsa-mir-4646 | HGLibA_60688 | 63 | -0.564836887 | hsa-mir-4646 | - |

| | | | | | |
|---|---|---|---|---|---|
| hsa-mir-516a-2 | HGLibA_61613 | 63 | -0.564836887 | hsa-mir-516a-2 | - |
| hsa-mir-548au | HGLibA_61929 | 63 | -0.564836887 | hsa-mir-548au | - |
| hsa-mir-6074 | HGLibA_62548 | 63 | -0.564836887 | hsa-mir-6074 | - |
| hsa-mir-670 | HGLibA_63008 | 63 | -0.564836887 | hsa-mir-670 | - |
| hsa-mir-6745 | HGLibA_63132 | 63 | -0.564836887 | hsa-mir-6745 | - |
| hsa-mir-6806 | HGLibA_63400 | 63 | -0.564836887 | hsa-mir-6806 | - |
| hsa-mir-6835 | HGLibA_63517 | 63 | -0.564836887 | hsa-mir-6835 | - |
| hsa-mir-7155 | HGLibA_63847 | 63 | -0.564836887 | hsa-mir-7155 | - |
| hsa-mir-941-1 | HGLibA_64339 | 63 | -0.564836887 | hsa-mir-941-1 | - |
| IL3RA_X | HGLibA_23160 | 63 | -0.564836887 | IL3RA_X | - |
| MTL5 | HGLibA_30404 | 63 | -0.564836887 | MTL5 | - |
| SGK196 | HGLibA_43823 | 63 | -0.564836887 | SGK196 | - |
| TMEM133 | HGLibA_49943 | 63 | -0.564836887 | TMEM133 | - |
| TMEM35 | HGLibA_50372 | 63 | -0.564836887 | TMEM35 | - |
| WBP5 | HGLibA_53986 | 63 | -0.564836887 | WBP5 | - |
| ZFYVE20 | HGLibA_55279 | 63 | -0.564836887 | ZFYVE20 | - |
| C11orf35 | HGLibA_05134 | 62 | -0.57388223 | C11orf35 | - |
| C12orf61 | HGLibA_05275 | 62 | -0.57388223 | C12orf61 | - |
| CXorf21 | HGLibA_11903 | 62 | -0.57388223 | CXorf21 | - |
| DARS | HGLibA_12341 | 62 | -0.57388223 | DARS | - |
| DOM3Z | HGLibA_13794 | 62 | -0.57388223 | DOM3Z | - |
| DOPEY1 | HGLibA_13799 | 62 | -0.57388223 | DOPEY1 | - |
| ENSG00000003137 | HGLibA_12089 | 62 | -0.57388223 | CYP26B1 | cytochrome P450 family 26 subfamily B member 1 [Source:HGNC Symbol;Acc:HGNC:20581] |
| ENSG00000005020 | HGLibA_44272 | 62 | -0.57388223 | SKAP2 | src kinase associated phosphoprotein 2 [Source:HGNC Symbol;Acc:HGNC:15687] |
| ENSG00000005075 | HGLibA_37591 | 62 | -0.57388223 | POLR2J | RNA polymerase II subunit J [Source:HGNC Symbol;Acc:HGNC:9197] |
| ENSG00000006837 | HGLibA_08811 | 62 | -0.57388223 | CDKL3 | cyclin dependent kinase like 3 [Source:HGNC Symbol;Acc:HGNC:15483] |
| ENSG00000011566 | HGLibA_28216 | 62 | -0.57388223 | MAP4K3 | mitogen-activated protein kinase kinase kinase kinase 3 [Source:HGNC Symbol;Acc:HGNC:6865] |
| ENSG00000013588 | HGLibA_20125 | 62 | -0.57388223 | GPRC5A | G protein-coupled receptor class C group 5 member A [Source:HGNC Symbol;Acc:HGNC:9836] |
| ENSG00000013810 | HGLibA_48164 | 62 | -0.57388223 | TACC3 | transforming acidic coiled-coil containing protein 3 [Source:HGNC Symbol;Acc:HGNC:11524] |
| ENSG00000014824 | HGLibA_44924 | 62 | -0.57388223 | SLC30A9 | solute carrier family 30 member 9 [Source:HGNC Symbol;Acc:HGNC:1329] |
| ENSG00000023839 | HGLibA_00171 | 62 | -0.57388223 | ABCC2 | ATP binding cassette subfamily C member 2 [Source:HGNC Symbol;Acc:HGNC:53] |
| ENSG00000039650 | HGLibA_37351 | 62 | -0.57388223 | PNKP | polynucleotide kinase 3'-phosphatase [Source:HGNC Symbol;Acc:HGNC:9154] |
| ENSG00000057704 | HGLibA_49778 | 62 | -0.57388223 | TMCC3 | transmembrane and coiled-coil domain family 3 [Source:HGNC Symbol;Acc:HGNC:29199] |
| ENSG00000066629 | HGLibA_15055 | 62 | -0.57388223 | EML1 | echinoderm microtubule associated protein like 1 [Source:HGNC Symbol;Acc:HGNC:3330] |
| ENSG00000070367 | HGLibA_15752 | 62 | -0.57388223 | EXOC5 | exocyst complex component 5 [Source:HGNC Symbol;Acc:HGNC:10696] |
| ENSG00000071282 | HGLibA_26680 | 62 | -0.57388223 | LMCD1 | LIM and cysteine rich domains 1 [Source:HGNC Symbol;Acc:HGNC:6633] |
| ENSG00000074181 | HGLibA_32349 | 62 | -0.57388223 | NOTCH3 | notch 3 [Source:HGNC Symbol;Acc:HGNC:7883] |
| ENSG00000077097 | HGLibA_50996 | 62 | -0.57388223 | TOP2B | DNA topoisomerase II beta [Source:HGNC Symbol;Acc:HGNC:11990] |
| ENSG00000077585 | HGLibA_19899 | 62 | -0.57388223 | GPR137B | G protein-coupled receptor 137B [Source:HGNC Symbol;Acc:HGNC:11862] |
| ENSG00000078549 | HGLibA_00938 | 62 | -0.57388223 | ADCYAP1R1 | ADCYAP receptor type I [Source:HGNC Symbol;Acc:HGNC:242] |
| ENSG00000080189 | HGLibA_44974 | 62 | -0.57388223 | SLC35C2 | solute carrier family 35 member C2 [Source:HGNC Symbol;Acc:HGNC:17117] |
| ENSG00000080815 | HGLibA_39026 | 62 | -0.57388223 | PSEN1 | presenilin 1 [Source:HGNC Symbol;Acc:HGNC:9508] |
| ENSG00000081138 | HGLibA_08688 | 62 | -0.57388223 | CDH7 | cadherin 7 [Source:HGNC Symbol;Acc:HGNC:1766] |
| ENSG00000083457 | HGLibA_23755 | 62 | -0.57388223 | ITGAE | integrin subunit alpha E [Source:HGNC Symbol;Acc:HGNC:6147] |
| ENSG00000086506 | HGLibA_20936 | 62 | -0.57388223 | HBQ1 | hemoglobin subunit theta 1 [Source:HGNC Symbol;Acc:HGNC:4833] |
| ENSG00000087253 | HGLibA_27073 | 62 | -0.57388223 | LPCAT2 | lysophosphatidylcholine acyltransferase 2 [Source:HGNC Symbol;Acc:HGNC:26032] |
| ENSG00000091127 | HGLibA_39603 | 62 | -0.57388223 | PUS7 | pseudouridine synthase 7 [Source:HGNC Symbol;Acc:HGNC:26033] |
| ENSG00000091138 | HGLibA_44793 | 62 | -0.57388223 | SLC26A3 | solute carrier family 26 member 3 [Source:HGNC Symbol;Acc:HGNC:3018] |
| ENSG00000092847 | HGLibA_01205 | 62 | -0.57388223 | AGO1 | RISC catalytic component [Source:HGNC Symbol;Acc:HGNC:3262]" |
| ENSG00000095794 | HGLibA_11132 | 62 | -0.57388223 | CREM | cAMP responsive element modulator [Source:HGNC Symbol;Acc:HGNC:2352] |
| ENSG00000096968 | HGLibA_23917 | 62 | -0.57388223 | JAK2 | Janus kinase 2 [Source:HGNC Symbol;Acc:HGNC:6192] |
| ENSG00000100373 | HGLibA_53078 | 62 | -0.57388223 | UPK3A | uroplakin 3A [Source:HGNC Symbol;Acc:HGNC:12580] |
| ENSG00000101391 | HGLibA_08782 | 62 | -0.57388223 | CDK5RAP1 | CDK5 regulatory subunit associated protein 1 [Source:HGNC Symbol;Acc:HGNC:15880] |
| ENSG00000101446 | HGLibA_46718 | 62 | -0.57388223 | SPINT3 | Kunitz type 3 [Source:HGNC Symbol;Acc:HGNC:11248]" |
| ENSG00000101974 | HGLibA_03559 | 62 | -0.57388223 | ATP11C | ATPase phospholipid transporting 11C [Source:HGNC Symbol;Acc:HGNC:13554] |
| ENSG00000102900 | HGLibA_33074 | 62 | -0.57388223 | NUP93 | nucleoporin 93 [Source:HGNC Symbol;Acc:HGNC:28958] |
| ENSG00000103540 | HGLibA_08138 | 62 | -0.57388223 | CCP110 | centriolar coiled-coil protein 110 [Source:HGNC Symbol;Acc:HGNC:24342] |
| ENSG00000105176 | HGLibA_53145 | 62 | -0.57388223 | URI1 | prefoldin like chaperone [Source:HGNC Symbol;Acc:HGNC:13236]" |
| ENSG00000105220 | HGLibA_19808 | 62 | -0.57388223 | GPI | glucose-6-phosphate isomerase [Source:HGNC Symbol;Acc:HGNC:4458] |
| ENSG00000105810 | HGLibA_08792 | 62 | -0.57388223 | CDK6 | cyclin dependent kinase 6 [Source:HGNC Symbol;Acc:HGNC:1777] |
| ENSG00000106070 | HGLibA_20224 | 62 | -0.57388223 | GRB10 | growth factor receptor bound protein 10 [Source:HGNC Symbol;Acc:HGNC:4564] |
| ENSG00000107643 | HGLibA_28283 | 62 | -0.57388223 | MAPK8 | mitogen-activated protein kinase 8 [Source:HGNC Symbol;Acc:HGNC:6881] |
| ENSG00000107779 | HGLibA_04628 | 62 | -0.57388223 | BMPR1A | bone morphogenetic protein receptor type 1A [Source:HGNC Symbol;Acc:HGNC:1076] |
| ENSG00000107829 | HGLibA_17147 | 62 | -0.57388223 | FBXW4 | F-box and WD repeat domain containing 4 [Source:HGNC Symbol;Acc:HGNC:10847] |
| ENSG00000107854 | HGLibA_50856 | 62 | -0.57388223 | TNKS2 | tankyrase 2 [Source:HGNC Symbol;Acc:HGNC:15677] |
| ENSG00000108342 | HGLibA_11339 | 62 | -0.57388223 | CSF3 | colony stimulating factor 3 [Source:HGNC Symbol;Acc:HGNC:2438] |
| ENSG00000109111 | HGLibA_47823 | 62 | -0.57388223 | SUPT6H | histone chaperone [Source:HGNC Symbol;Acc:HGNC:11470]" |
| ENSG00000109576 | HGLibA_00040 | 62 | -0.57388223 | AADAT | aminoadipate aminotransferase [Source:HGNC Symbol;Acc:HGNC:17929] |
| ENSG00000109762 | HGLibA_46073 | 62 | -0.57388223 | SNX25 | sorting nexin 25 [Source:HGNC Symbol;Acc:HGNC:21883] |
| ENSG00000110092 | HGLibA_08071 | 62 | -0.57388223 | CCND1 | cyclin D1 [Source:HGNC Symbol;Acc:HGNC:1582] |
| ENSG00000111885 | HGLibA_28058 | 62 | -0.57388223 | MAN1A1 | mannosidase alpha class 1A member 1 [Source:HGNC Symbol;Acc:HGNC:6821] |
| ENSG00000113083 | HGLibA_27032 | 62 | -0.57388223 | LOX | lysyl oxidase [Source:HGNC Symbol;Acc:HGNC:6664] |
| ENSG00000113387 | HGLibA_47692 | 62 | -0.57388223 | SUB1 | transcriptional regulator [Source:HGNC Symbol;Acc:HGNC:19985]" |
| ENSG00000113790 | HGLibA_14681 | 62 | -0.57388223 | EHHADH | enoyl-CoA hydratase and 3-hydroxyacyl CoA dehydrogenase [Source:HGNC Symbol;Acc:HGNC:3247] |
| ENSG00000114270 | HGLibA_10611 | 62 | -0.57388223 | COL7A1 | collagen type VII alpha 1 chain [Source:HGNC Symbol;Acc:HGNC:2214] |
| ENSG00000115137 | HGLibA_13608 | 62 | -0.57388223 | DNAJC27 | DnaJ heat shock protein family (Hsp40) member C27 [Source:HGNC Symbol;Acc:HGNC:30290] |
| ENSG00000116198 | HGLibA_09094 | 62 | -0.57388223 | CEP104 | centrosomal protein 104 [Source:HGNC Symbol;Acc:HGNC:24866] |
| ENSG00000116539 | HGLibA_03292 | 62 | -0.57388223 | ASH1L | ASH1 like histone lysine methyltransferase [Source:HGNC Symbol;Acc:HGNC:19088] |
| ENSG00000116586 | HGLibA_25993 | 62 | -0.57388223 | LAMTOR2 | MAPK and MTOR activator 2 [Source:HGNC Symbol;Acc:HGNC:29796]" |
| ENSG00000116741 | HGLibA_41079 | 62 | -0.57388223 | RGS2 | regulator of G protein signaling 2 [Source:HGNC Symbol;Acc:HGNC:9998] |
| ENSG00000118640 | HGLibA_53510 | 62 | -0.57388223 | VAMP8 | vesicle associated membrane protein 8 [Source:HGNC Symbol;Acc:HGNC:12647] |
| ENSG00000118849 | HGLibA_40282 | 62 | -0.57388223 | RARRES1 | retinoic acid receptor responder 1 [Source:HGNC Symbol;Acc:HGNC:9867] |
| ENSG00000120129 | HGLibA_14127 | 62 | -0.57388223 | DUSP1 | dual specificity phosphatase 1 [Source:HGNC Symbol;Acc:HGNC:3064] |
| ENSG00000120149 | HGLibA_30275 | 62 | -0.57388223 | MSX2 | msh homeobox 2 [Source:HGNC Symbol;Acc:HGNC:7392] |
| ENSG00000124164 | HGLibA_53520 | 62 | -0.57388223 | VAPB | VAMP associated protein B and C [Source:HGNC Symbol;Acc:HGNC:12649] |
| ENSG00000124228 | HGLibA_12686 | 62 | -0.57388223 | DDX27 | DEAD-box helicase 27 [Source:HGNC Symbol;Acc:HGNC:15837] |
| ENSG00000124406 | HGLibA_03775 | 62 | -0.57388223 | ATP8A1 | ATPase phospholipid transporting 8A1 [Source:HGNC Symbol;Acc:HGNC:13531] |
| ENSG00000126246 | HGLibA_22851 | 62 | -0.57388223 | IGFLR1 | IGF like family receptor 1 [Source:HGNC Symbol;Acc:HGNC:23620] |
| ENSG00000127824 | HGLibA_52260 | 62 | -0.57388223 | TUBA4A | tubulin alpha 4a [Source:HGNC Symbol;Acc:HGNC:12407] |
| ENSG00000129317 | HGLibA_39608 | 62 | -0.57388223 | PUS7L | pseudouridine synthase 7 like [Source:HGNC Symbol;Acc:HGNC:25276] |
| ENSG00000129625 | HGLibA_40789 | 62 | -0.57388223 | REEP5 | receptor accessory protein 5 [Source:HGNC Symbol;Acc:HGNC:30077] |
| ENSG00000130222 | HGLibA_18421 | 62 | -0.57388223 | GADD45G | growth arrest and DNA damage inducible gamma [Source:HGNC Symbol;Acc:HGNC:4097] |
| ENSG00000130287 | HGLibA_31261 | 62 | -0.57388223 | NCAN | neurocan [Source:HGNC Symbol;Acc:HGNC:2465] |
| ENSG00000130707 | HGLibA_03371 | 62 | -0.57388223 | ASS1 | argininosuccinate synthase 1 [Source:HGNC Symbol;Acc:HGNC:758] |
| ENSG00000131669 | HGLibA_31937 | 62 | -0.57388223 | NINJ1 | ninjurin 1 [Source:HGNC Symbol;Acc:HGNC:7824] |
| ENSG00000131773 | HGLibA_24571 | 62 | -0.57388223 | KHDRBS3 | signal transduction associated 3 [Source:HGNC Symbol;Acc:HGNC:18117]" |
| ENSG00000132002 | HGLibA_13523 | 62 | -0.57388223 | DNAJB1 | DnaJ heat shock protein family (Hsp40) member B1 [Source:HGNC Symbol;Acc:HGNC:5270] |
| ENSG00000132294 | HGLibA_14606 | 62 | -0.57388223 | EFR3A | EFR3 homolog A [Source:HGNC Symbol;Acc:HGNC:28970] |
| ENSG00000132470 | HGLibA_23786 | 62 | -0.57388223 | ITGB4 | integrin subunit beta 4 [Source:HGNC Symbol;Acc:HGNC:6158] |
| ENSG00000132763 | HGLibA_29456 | 62 | -0.57388223 | MMACHC | metabolism of cobalamin associated C [Source:HGNC Symbol;Acc:HGNC:24525] |
| ENSG00000132854 | HGLibA_24034 | 62 | -0.57388223 | KANK4 | KN motif and ankyrin repeat domains 4 [Source:HGNC Symbol;Acc:HGNC:27263] |
| ENSG00000133627 | HGLibA_00662 | 62 | -0.57388223 | ACTR3B | ARP3 actin related protein 3 homolog B [Source:HGNC Symbol;Acc:HGNC:17256] |
| ENSG00000134897 | HGLibA_04532 | 62 | -0.57388223 | BIVM | immunoglobulin-like variable motif containing [Source:HGNC Symbol;Acc:HGNC:16034]" |
| ENSG00000135116 | HGLibA_22105 | 62 | -0.57388223 | HRK | BCL2 interacting protein [Source:HGNC Symbol;Acc:HGNC:5185]" |
| ENSG00000135373 | HGLibA_14677 | 62 | -0.57388223 | EHF | ETS homologous factor [Source:HGNC Symbol;Acc:HGNC:3246] |
| ENSG00000135506 | HGLibA_34635 | 62 | -0.57388223 | OS9 | endoplasmic reticulum lectin [Source:HGNC Symbol;Acc:HGNC:16994]" |
| ENSG00000135801 | HGLibA_48244 | 62 | -0.57388223 | TAF5L | TATA-box binding protein associated factor 5 like [Source:HGNC Symbol;Acc:HGNC:17304] |
| ENSG00000136122 | HGLibA_04699 | 62 | -0.57388223 | BORA | aurora kinase A activator [Source:HGNC Symbol;Acc:HGNC:24724]" |
| ENSG00000136237 | HGLibA_40256 | 62 | -0.57388223 | RAPGEF5 | Rap guanine nucleotide exchange factor 5 [Source:HGNC Symbol;Acc:HGNC:16862] |
| ENSG00000136689 | HGLibA_23066 | 62 | -0.57388223 | IL1RN | interleukin 1 receptor antagonist [Source:HGNC Symbol;Acc:HGNC:6000] |
| ENSG00000136731 | HGLibA_52867 | 62 | -0.57388223 | UGGT1 | UDP-glucose glycoprotein glucosyltransferase 1 [Source:HGNC Symbol;Acc:HGNC:15663] |
| ENSG00000137842 | HGLibA_50472 | 62 | -0.57388223 | TMEM62 | transmembrane protein 62 [Source:HGNC Symbol;Acc:HGNC:26269] |
| ENSG00000138785 | HGLibA_23413 | 62 | -0.57388223 | INTS12 | integrator complex subunit 12 [Source:HGNC Symbol;Acc:HGNC:25067] |
| ENSG00000139572 | HGLibA_20096 | 62 | -0.57388223 | GPR84 | G protein-coupled receptor 84 [Source:HGNC Symbol;Acc:HGNC:4535] |
| ENSG00000140478 | HGLibA_19617 | 62 | -0.57388223 | GOLGA6D | golgin A6 family member D [Source:HGNC Symbol;Acc:HGNC:32204] |
| ENSG00000141469 | HGLibA_44400 | 62 | -0.57388223 | SLC14A1 | solute carrier family 14 member 1 (Kidd blood group) [Source:HGNC Symbol;Acc:HGNC:10918] |
| ENSG00000141668 | HGLibA_07409 | 62 | -0.57388223 | CBLN2 | cerebellin 2 precursor [Source:HGNC Symbol;Acc:HGNC:1544] |
| ENSG00000142798 | HGLibA_22341 | 62 | -0.57388223 | HSPG2 | heparan sulfate proteoglycan 2 [Source:HGNC Symbol;Acc:HGNC:5273] |
| ENSG00000143158 | HGLibA_29709 | 62 | -0.57388223 | MPC2 | mitochondrial pyruvate carrier 2 [Source:HGNC Symbol;Acc:HGNC:24515] |
| ENSG00000143320 | HGLibA_11058 | 62 | -0.57388223 | CRABP2 | cellular retinoic acid binding protein 2 [Source:HGNC Symbol;Acc:HGNC:2339] |
| ENSG00000143322 | HGLibA_00304 | 62 | -0.57388223 | ABL2 | non-receptor tyrosine kinase [Source:HGNC Symbol;Acc:HGNC:77]" |
| ENSG00000143630 | HGLibA_20980 | 62 | -0.57388223 | HCN3 | hyperpolarization activated cyclic nucleotide gated potassium channel 3 [Source:HGNC Symbol;Acc:HGNC:19183] |
| ENSG00000144713 | HGLibA_41940 | 62 | -0.57388223 | RPL32 | ribosomal protein L32 [Source:HGNC Symbol;Acc:HGNC:10336] |
| ENSG00000145391 | HGLibA_43658 | 62 | -0.57388223 | SETD7 | SET domain containing lysine methyltransferase 7 [Source:HGNC Symbol;Acc:HGNC:30412] |
| ENSG00000147677 | HGLibA_14785 | 62 | -0.57388223 | EIF3H | eukaryotic translation initiation factor 3 subunit H [Source:HGNC Symbol;Acc:HGNC:3273] |
| ENSG00000148459 | HGLibA_35949 | 62 | -0.57388223 | PDSS1 | decaprenyl diphosphate synthase subunit 1 [Source:HGNC Symbol;Acc:HGNC:17759] |
| ENSG00000149201 | HGLibA_07881 | 62 | -0.57388223 | CCDC81 | coiled-coil domain containing 81 [Source:HGNC Symbol;Acc:HGNC:26281] |
| ENSG00000151532 | HGLibA_53886 | 62 | -0.57388223 | VTI1A | vesicle transport through interaction with t-SNAREs 1A [Source:HGNC Symbol;Acc:HGNC:17792] |
| ENSG00000151789 | HGLibA_55903 | 62 | -0.57388223 | ZNF385D | zinc finger protein 385D [Source:HGNC Symbol;Acc:HGNC:26191] |
| ENSG00000153048 | HGLibA_07248 | 62 | -0.57388223 | CARHSP1 | calcium regulated heat stable protein 1 [Source:HGNC Symbol;Acc:HGNC:17150] |
| ENSG00000153291 | HGLibA_44690 | 62 | -0.57388223 | SLC25A27 | solute carrier family 25 member 27 [Source:HGNC Symbol;Acc:HGNC:21065] |
| ENSG00000153391 | HGLibA_23325 | 62 | -0.57388223 | INO80C | INO80 complex subunit C [Source:HGNC Symbol;Acc:HGNC:26994] |
| ENSG00000154227 | HGLibA_09205 | 62 | -0.57388223 | CERS3 | ceramide synthase 3 [Source:HGNC Symbol;Acc:HGNC:23752] |
| ENSG00000154727 | HGLibA_18348 | 62 | -0.57388223 | GABPA | GA binding protein transcription factor subunit alpha [Source:HGNC Symbol;Acc:HGNC:4071] |
| ENSG00000157368 | HGLibA_23142 | 62 | -0.57388223 | IL34 | interleukin 34 [Source:HGNC Symbol;Acc:HGNC:28529] |

| | | | | | |
|---|---|---|---|---|---|
| ENSG00000157851 | HGLibA_13936 | 62 | -0.57388223 | DPYSL5 | dihydropyrimidinase like 5 [Source:HGNC Symbol;Acc:HGNC:20637] |
| ENSG00000159840 | HGLibA_57088 | 62 | -0.57388223 | ZYX | zyxin [Source:HGNC Symbol;Acc:HGNC:13200] |
| ENSG00000161040 | HGLibA_16977 | 62 | -0.57388223 | FBXL13 | F-box and leucine rich repeat protein 13 [Source:HGNC Symbol;Acc:HGNC:21658] |
| ENSG00000162735 | HGLibA_36106 | 62 | -0.57388223 | PEX19 | peroxisomal biogenesis factor 19 [Source:HGNC Symbol;Acc:HGNC:9713] |
| ENSG00000163040 | HGLibA_07861 | 62 | -0.57388223 | CCDC74A | coiled-coil domain containing 74A [Source:HGNC Symbol;Acc:HGNC:25197] |
| ENSG00000163218 | HGLibA_36257 | 62 | -0.57388223 | PGLYRP4 | peptidoglycan recognition protein 4 [Source:HGNC Symbol;Acc:HGNC:30015] |
| ENSG00000163810 | HGLibA_49263 | 62 | -0.57388223 | TGM4 | transglutaminase 4 [Source:HGNC Symbol;Acc:HGNC:11780] |
| ENSG00000163930 | HGLibA_04153 | 62 | -0.57388223 | BAP1 | BRCA1 associated protein 1 [Source:HGNC Symbol;Acc:HGNC:950] |
| ENSG00000164031 | HGLibA_13534 | 62 | -0.57388223 | DNAJB14 | DnaJ heat shock protein family (Hsp40) member B14 [Source:HGNC Symbol;Acc:HGNC:25881] |
| ENSG00000164100 | HGLibA_31432 | 62 | -0.57388223 | NDST3 | N-deacetylase and N-sulfotransferase 3 [Source:HGNC Symbol;Acc:HGNC:7682] |
| ENSG00000164442 | HGLibA_09744 | 62 | -0.57388223 | CITED2 | Cbp/p300 interacting transactivator with Glu/Asp rich carboxy-terminal domain 2 [Source:HGNC Symbol;Acc:HGNC:1987] |
| ENSG00000164975 | HGLibA_45896 | 62 | -0.57388223 | SNAPC3 | small nuclear RNA activating complex polypeptide 3 [Source:HGNC Symbol;Acc:HGNC:11136] |
| ENSG00000165271 | HGLibA_32270 | 62 | -0.57388223 | NOL6 | nucleolar protein 6 [Source:HGNC Symbol;Acc:HGNC:19910] |
| ENSG00000165288 | HGLibA_04856 | 62 | -0.57388223 | BRWD3 | bromodomain and WD repeat domain containing 3 [Source:HGNC Symbol;Acc:HGNC:17342] |
| ENSG00000165338 | HGLibA_21096 | 62 | -0.57388223 | HECTD2 | HECT domain E3 ubiquitin protein ligase 2 [Source:HGNC Symbol;Acc:HGNC:26736] |
| ENSG00000166069 | HGLibA_49792 | 62 | -0.57388223 | TMCO5A | transmembrane and coiled-coil domains 5A [Source:HGNC Symbol;Acc:HGNC:28558] |
| ENSG00000167114 | HGLibA_44821 | 62 | -0.57388223 | SLC27A4 | solute carrier family 27 member 4 [Source:HGNC Symbol;Acc:HGNC:10998] |
| ENSG00000167656 | HGLibA_27653 | 62 | -0.57388223 | LY6D | lymphocyte antigen 6 family member D [Source:HGNC Symbol;Acc:HGNC:13348] |
| ENSG00000167825 | HGLibA_34305 | 62 | -0.57388223 | OR5I1 | olfactory receptor family 5 subfamily I member 1 [Source:HGNC Symbol;Acc:HGNC:8347] |
| ENSG00000168959 | HGLibA_20354 | 62 | -0.57388223 | GRM5 | glutamate metabotropic receptor 5 [Source:HGNC Symbol;Acc:HGNC:4597] |
| ENSG00000169241 | HGLibA_45235 | 62 | -0.57388223 | SLC50A1 | solute carrier family 50 member 1 [Source:HGNC Symbol;Acc:HGNC:30657] |
| ENSG00000170777 | HGLibA_51120 | 62 | -0.57388223 | TPD52L3 | TPD52 like 3 [Source:HGNC Symbol;Acc:HGNC:23382] |
| ENSG00000171148 | HGLibA_48193 | 62 | -0.57388223 | TADA3 | transcriptional adaptor 3 [Source:HGNC Symbol;Acc:HGNC:19422] |
| ENSG00000171365 | HGLibA_09832 | 62 | -0.57388223 | CLCN5 | chloride voltage-gated channel 5 [Source:HGNC Symbol;Acc:HGNC:2023] |
| ENSG00000172594 | HGLibA_45816 | 62 | -0.57388223 | SMPDL3A | sphingomyelin phosphodiesterase acid like 3A [Source:HGNC Symbol;Acc:HGNC:17389] |
| ENSG00000172987 | HGLibA_22064 | 62 | -0.57388223 | HPSE2 | heparanase 2 (inactive) [Source:HGNC Symbol;Acc:HGNC:18374] |
| ENSG00000173389 | HGLibA_23502 | 62 | -0.57388223 | IQCF1 | IQ motif containing F1 [Source:HGNC Symbol;Acc:HGNC:28607] |
| ENSG00000174840 | HGLibA_35766 | 62 | -0.57388223 | PDE12 | phosphodiesterase 12 [Source:HGNC Symbol;Acc:HGNC:25386] |
| ENSG00000175048 | HGLibA_55077 | 62 | -0.57388223 | ZDHHC14 | zinc finger DHHC-type containing 14 [Source:HGNC Symbol;Acc:HGNC:20341] |
| ENSG00000175189 | HGLibA_23308 | 62 | -0.57388223 | INHBC | inhibin subunit beta C [Source:HGNC Symbol;Acc:HGNC:6068] |
| ENSG00000176209 | HGLibA_45742 | 62 | -0.57388223 | SMIM19 | small integral membrane protein 19 [Source:HGNC Symbol;Acc:HGNC:25166] |
| ENSG00000176396 | HGLibA_14696 | 62 | -0.57388223 | EID2 | EP300 interacting inhibitor of differentiation 2 [Source:HGNC Symbol;Acc:HGNC:28292] |
| ENSG00000176920 | HGLibA_18189 | 62 | -0.57388223 | FUT2 | fucosyltransferase 2 [Source:HGNC Symbol;Acc:HGNC:4013] |
| ENSG00000178913 | HGLibA_48251 | 62 | -0.57388223 | TAF7 | TATA-box binding protein associated factor 7 [Source:HGNC Symbol;Acc:HGNC:11541] |
| ENSG00000179909 | HGLibA_55501 | 62 | -0.57388223 | ZNF154 | zinc finger protein 154 [Source:HGNC Symbol;Acc:HGNC:12939] |
| ENSG00000179913 | HGLibA_03988 | 62 | -0.57388223 | B3GNT3 | 3-N-acetylglucosaminyltransferase 3 [Source:HGNC Symbol;Acc:HGNC:13528]" |
| ENSG00000180155 | HGLibA_27710 | 62 | -0.57388223 | LYNX1 | Ly6/neurotoxin 1 [Source:HGNC Symbol;Acc:HGNC:29604] |
| ENSG00000180481 | HGLibA_19266 | 62 | -0.57388223 | GLIPR1L2 | GLIPR1 like 2 [Source:HGNC Symbol;Acc:HGNC:28592] |
| ENSG00000180708 | HGLibA_33533 | 62 | -0.57388223 | OR10K2 | olfactory receptor family 10 subfamily K member 2 [Source:HGNC Symbol;Acc:HGNC:14826] |
| ENSG00000180878 | HGLibA_05139 | 62 | -0.57388223 | C11orf42 | chromosome 11 open reading frame 42 [Source:HGNC Symbol;Acc:HGNC:28541] |
| ENSG00000182742 | HGLibA_21937 | 62 | -0.57388223 | HOXB4 | homeobox B4 [Source:HGNC Symbol;Acc:HGNC:5115] |
| ENSG00000183454 | HGLibA_20299 | 62 | -0.57388223 | GRIN2A | glutamate ionotropic receptor NMDA type subunit 2A [Source:HGNC Symbol;Acc:HGNC:4585] |
| ENSG00000183569 | HGLibA_43481 | 62 | -0.57388223 | SERHL2 | serine hydrolase like 2 [Source:HGNC Symbol;Acc:HGNC:29446] |
| ENSG00000183690 | HGLibA_14572 | 62 | -0.57388223 | EFHC2 | EF-hand domain containing 2 [Source:HGNC Symbol;Acc:HGNC:26233] |
| ENSG00000183778 | HGLibA_03964 | 62 | -0.57388223 | B3GALT5 | 3-galactosyltransferase 5 [Source:HGNC Symbol;Acc:HGNC:920]" |
| ENSG00000184271 | HGLibA_37795 | 62 | -0.57388223 | POU6F1 | POU class 6 homeobox 1 [Source:HGNC Symbol;Acc:HGNC:9224] |
| ENSG00000184349 | HGLibA_14595 | 62 | -0.57388223 | EFNA5 | ephrin A5 [Source:HGNC Symbol;Acc:HGNC:3225] |
| ENSG00000184924 | HGLibA_39557 | 62 | -0.57388223 | PTRHD1 | peptidyl-tRNA hydrolase domain containing 1 [Source:HGNC Symbol;Acc:HGNC:33782] |
| ENSG00000185339 | HGLibA_48825 | 62 | -0.57388223 | TCN2 | transcobalamin 2 [Source:HGNC Symbol;Acc:HGNC:11653] |
| ENSG00000185897 | HGLibA_17329 | 62 | -0.57388223 | FFAR3 | free fatty acid receptor 3 [Source:HGNC Symbol;Acc:HGNC:4499] |
| ENSG00000185958 | HGLibA_16337 | 62 | -0.57388223 | FAM186A | family with sequence similarity 186 member A [Source:HGNC Symbol;Acc:HGNC:26980] |
| ENSG00000186075 | HGLibA_56966 | 62 | -0.57388223 | ZPBP2 | zona pellucida binding protein 2 [Source:HGNC Symbol;Acc:HGNC:20678] |
| ENSG00000186113 | HGLibA_34276 | 62 | -0.57388223 | OR5D14 | olfactory receptor family 5 subfamily D member 14 [Source:HGNC Symbol;Acc:HGNC:15281] |
| ENSG00000186207 | HGLibA_26148 | 62 | -0.57388223 | LCE5A | late cornified envelope 5A [Source:HGNC Symbol;Acc:HGNC:16614] |
| ENSG00000186468 | HGLibA_42109 | 62 | -0.57388223 | RPS23 | ribosomal protein S23 [Source:HGNC Symbol;Acc:HGNC:10410] |
| ENSG00000186509 | HGLibA_34582 | 62 | -0.57388223 | OR9Q1 | olfactory receptor family 9 subfamily Q member 1 [Source:HGNC Symbol;Acc:HGNC:14724] |
| ENSG00000187140 | HGLibA_17835 | 62 | -0.57388223 | FOXD3 | forkhead box D3 [Source:HGNC Symbol;Acc:HGNC:3804] |
| ENSG00000187581 | HGLibA_10878 | 62 | -0.57388223 | COX8C | cytochrome c oxidase subunit 8C [Source:HGNC Symbol;Acc:HGNC:24382] |
| ENSG00000188026 | HGLibA_41252 | 62 | -0.57388223 | RILPL1 | Rab interacting lysosomal protein like 1 [Source:HGNC Symbol;Acc:HGNC:26814] |
| ENSG00000188385 | HGLibA_23927 | 62 | -0.57388223 | JAKMIP3 | Janus kinase and microtubule interacting protein 3 [Source:HGNC Symbol;Acc:HGNC:23523] |
| ENSG00000189420 | HGLibA_55245 | 62 | -0.57388223 | ZFP92 | ZFP92 zinc finger protein [Source:HGNC Symbol;Acc:HGNC:12865] |
| ENSG00000196151 | HGLibA_54228 | 62 | -0.57388223 | WDSUB1 | sterile alpha motif and U-box domain containing 1 [Source:HGNC Symbol;Acc:HGNC:26697]" |
| ENSG00000196196 | HGLibA_22089 | 62 | -0.57388223 | HRCT1 | histidine rich carboxyl terminus 1 [Source:HGNC Symbol;Acc:HGNC:33872] |
| ENSG00000196427 | HGLibA_31237 | 62 | -0.57388223 | NBPF4 | NBPF member 4 [Source:HGNC Symbol;Acc:HGNC:26550] |
| ENSG00000196569 | HGLibA_25943 | 62 | -0.57388223 | LAMA2 | laminin subunit alpha 2 [Source:HGNC Symbol;Acc:HGNC:6482] |
| ENSG00000196604 | HGLibA_37733 | 62 | -0.57388223 | POTEF | POTE ankyrin domain family member F [Source:HGNC Symbol;Acc:HGNC:33905] |
| ENSG00000196844 | HGLibA_35273 | 62 | -0.57388223 | PATE2 | prostate and testis expressed 2 [Source:HGNC Symbol;Acc:HGNC:32249] |
| ENSG00000196859 | HGLibA_25502 | 62 | -0.57388223 | KRT39 | keratin 39 [Source:HGNC Symbol;Acc:HGNC:32971] |
| ENSG00000197601 | HGLibA_16868 | 62 | -0.57388223 | FAR1 | fatty acyl-CoA reductase 1 [Source:HGNC Symbol;Acc:HGNC:26222] |
| ENSG00000197713 | HGLibA_41823 | 62 | -0.57388223 | RPE | ribulose-5-phosphate-3-epimerase [Source:HGNC Symbol;Acc:HGNC:10293] |
| ENSG00000198642 | HGLibA_25228 | 62 | -0.57388223 | KLHL9 | kelch like family member 9 [Source:HGNC Symbol;Acc:HGNC:18732] |
| ENSG00000198677 | HGLibA_52125 | 62 | -0.57388223 | TTC37 | tetratricopeptide repeat domain 37 [Source:HGNC Symbol;Acc:HGNC:23639] |
| ENSG00000203737 | HGLibA_20050 | 62 | -0.57388223 | GPR52 | G protein-coupled receptor 52 [Source:HGNC Symbol;Acc:HGNC:4508] |
| ENSG00000204450 | HGLibA_51577 | 62 | -0.57388223 | TRIM64 | tripartite motif containing 64 [Source:HGNC Symbol;Acc:HGNC:14663] |
| ENSG00000204673 | HGLibA_01507 | 62 | -0.57388223 | AKT1S1 | AKT1 substrate 1 [Source:HGNC Symbol;Acc:HGNC:28426] |
| ENSG00000204688 | HGLibA_33802 | 62 | -0.57388223 | OR2H1 | olfactory receptor family 2 subfamily H member 1 [Source:HGNC Symbol;Acc:HGNC:8252] |
| ENSG00000204872 | HGLibA_31186 | 62 | -0.57388223 | NAT8B | gene/pseudogene) [Source:HGNC Symbol;Acc:HGNC:30235]" |
| ENSG00000204899 | HGLibA_30940 | 62 | -0.57388223 | MZT1 | mitotic spindle organizing protein 1 [Source:HGNC Symbol;Acc:HGNC:33830] |
| ENSG00000205143 | HGLibA_02897 | 62 | -0.57388223 | ARID3C | AT-rich interaction domain 3C [Source:HGNC Symbol;Acc:HGNC:21209] |
| ENSG00000205858 | HGLibA_27423 | 62 | -0.57388223 | LRRC72 | leucine rich repeat containing 72 [Source:HGNC Symbol;Acc:HGNC:42972] |
| ENSG00000211448 | HGLibA_13241 | 62 | -0.57388223 | DIO2 | iodothyronine deiodinase 2 [Source:HGNC Symbol;Acc:HGNC:2884] |
| ENSG00000213689 | HGLibA_51373 | 62 | -0.57388223 | TREX1 | three prime repair exonuclease 1 [Source:HGNC Symbol;Acc:HGNC:12269] |
| ENSG00000221829 | HGLibA_16850 | 62 | -0.57388223 | FANCG | FA complementation group G [Source:HGNC Symbol;Acc:HGNC:3588] |
| ENSG00000230594 | HGLibA_11534 | 62 | -0.57388223 | CT47A4 | member A4 [Source:HGNC Symbol;Acc:HGNC:33285]" |
| ENSG00000230657 | HGLibA_38346 | 62 | -0.57388223 | PRB4 | proline rich protein BstNI subfamily 4 [Source:HGNC Symbol;Acc:HGNC:9340] |
| ENSG00000234616 | HGLibA_23990 | 62 | -0.57388223 | JRK | Jrk helix-turn-helix protein [Source:HGNC Symbol;Acc:HGNC:6199] |
| ENSG00000240654 | HGLibA_05961 | 62 | -0.57388223 | C1QTNF9 | C1q and TNF related 9 [Source:HGNC Symbol;Acc:HGNC:28732] |
| ENSG00000241935 | HGLibA_21860 | 62 | -0.57388223 | HOGA1 | 4-hydroxy-2-oxoglutarate aldolase 1 [Source:HGNC Symbol;Acc:HGNC:25155] |
| ENSG00000242875 | HGLibA_40615 | 62 | -0.57388223 | RBMY1B | member B [Source:HGNC Symbol;Acc:HGNC:23914]" |
| ENSG00000245680 | HGLibA_56324 | 62 | -0.57388223 | ZNF585B | zinc finger protein 585B [Source:HGNC Symbol;Acc:HGNC:30948] |
| ENSG00000253159 | HGLibA_35526 | 62 | -0.57388223 | PCDHGA12 | 12 [Source:HGNC Symbol;Acc:HGNC:8699]" |
| ENSG00000256892 | HGLibA_30489 | 62 | -0.57388223 | MTRNR2L7 | MT-RNR2 like 7 [Source:HGNC Symbol;Acc:HGNC:37164] |
| ENSG00000258890 | HGLibA_09180 | 62 | -0.57388223 | CEP95 | centrosomal protein 95 [Source:HGNC Symbol;Acc:HGNC:25141] |
| ENSG00000260916 | HGLibA_08140 | 62 | -0.57388223 | CCPG1 | cell cycle progression 1 [Source:HGNC Symbol;Acc:HGNC:24227] |
| ENSG00000262655 | HGLibA_46756 | 62 | -0.57388223 | SPON1 | spondin 1 [Source:HGNC Symbol;Acc:HGNC:11252] |
| ENSG00000262664 | HGLibA_34790 | 62 | -0.57388223 | OVCA2 | serine hydrolase domain containing [Source:HGNC Symbol;Acc:HGNC:24203]" |
| ENSG00000265817 | HGLibA_18061 | 62 | -0.57388223 | FSBP | fibrinogen silencer binding protein [Source:HGNC Symbol;Acc:HGNC:43653] |
| ENSG00000270872 | HGLibA_46978 | 62 | -0.57388223 | SRGAP2D | SLIT-ROBO Rho GTPase activating protein 2D (pseudogene) [Source:HGNC Symbol;Acc:HGNC:43932] |
| ENSG00000273173 | HGLibA_46017 | 62 | -0.57388223 | SNURF | SNRPN upstream reading frame [Source:HGNC Symbol;Acc:HGNC:11171] |
| ENSG00000276023 | HGLibA_14143 | 62 | -0.57388223 | DUSP14 | dual specificity phosphatase 14 [Source:HGNC Symbol;Acc:HGNC:17007] |
| ENSG00000277258 | HGLibA_35594 | 62 | -0.57388223 | PCGF2 | polycomb group ring finger 2 [Source:HGNC Symbol;Acc:HGNC:12929] |
| ENSG00000280204 | HGLibA_33712 | 62 | -0.57388223 | OR1S1 | olfactory receptor family 1 subfamily S member 1 (gene/pseudogene) [Source:HGNC Symbol;Acc:HGNC:8227] |
| FAM188A | HGLibA_16345 | 62 | -0.57388223 | FAM188A | - |
| FAM19A5 | HGLibA_16408 | 62 | -0.57388223 | FAM19A5 | - |
| FAM211A | HGLibA_16460 | 62 | -0.57388223 | FAM211A | - |
| FAM211B | HGLibA_16463 | 62 | -0.57388223 | FAM211B | - |
| FAM63B | HGLibA_16661 | 62 | -0.57388223 | FAM63B | - |
| GATS | HGLibA_18698 | 62 | -0.57388223 | GATS | - |
| HN1L | HGLibA_21770 | 62 | -0.57388223 | HN1L | - |
| hsa-let-7f-1 | HGLibA_57124 | 62 | -0.57388223 | hsa-let-7f-1 | - |
| hsa-mir-10b | HGLibA_57202 | 62 | -0.57388223 | hsa-mir-10b | - |
| hsa-mir-1302-6 | HGLibA_57710 | 62 | -0.57388223 | hsa-mir-1302-6 | - |
| hsa-mir-1304 | HGLibA_57730 | 62 | -0.57388223 | hsa-mir-1304 | - |
| hsa-mir-2682 | HGLibA_58362 | 62 | -0.57388223 | hsa-mir-2682 | - |
| hsa-mir-2861 | HGLibA_58387 | 62 | -0.57388223 | hsa-mir-2861 | - |
| hsa-mir-3116-2 | HGLibA_58509 | 62 | -0.57388223 | hsa-mir-3116-2 | - |
| hsa-mir-3119-2 | HGLibA_58529 | 62 | -0.57388223 | hsa-mir-3119-2 | - |
| hsa-mir-3140 | HGLibA_58619 | 62 | -0.57388223 | hsa-mir-3140 | - |
| hsa-mir-3169 | HGLibA_58754 | 62 | -0.57388223 | hsa-mir-3169 | - |
| hsa-mir-3193 | HGLibA_58871 | 62 | -0.57388223 | hsa-mir-3193 | - |
| hsa-mir-3193 | HGLibA_58868 | 62 | -0.57388223 | hsa-mir-3193 | - |
| hsa-mir-365a | HGLibA_59186 | 62 | -0.57388223 | hsa-mir-365a | - |
| hsa-mir-3674 | HGLibA_59251 | 62 | -0.57388223 | hsa-mir-3674 | - |
| hsa-mir-3935 | HGLibA_59602 | 62 | -0.57388223 | hsa-mir-3935 | - |
| hsa-mir-3974 | HGLibA_59654 | 62 | -0.57388223 | hsa-mir-3974 | - |
| hsa-mir-421 | HGLibA_59693 | 62 | -0.57388223 | hsa-mir-421 | - |
| hsa-mir-4306 | HGLibA_59934 | 62 | -0.57388223 | hsa-mir-4306 | - |
| hsa-mir-4309 | HGLibA_59949 | 62 | -0.57388223 | hsa-mir-4309 | - |
| hsa-mir-4317 | HGLibA_59986 | 62 | -0.57388223 | hsa-mir-4317 | - |
| hsa-mir-4471 | HGLibA_60291 | 62 | -0.57388223 | hsa-mir-4471 | - |
| hsa-mir-4644 | HGLibA_60681 | 62 | -0.57388223 | hsa-mir-4644 | - |
| hsa-mir-4751 | HGLibA_61101 | 62 | -0.57388223 | hsa-mir-4751 | - |
| hsa-mir-4768 | HGLibA_61170 | 62 | -0.57388223 | hsa-mir-4768 | - |
| hsa-mir-510 | HGLibA_61548 | 62 | -0.57388223 | hsa-mir-510 | - |
| hsa-mir-519a-2 | HGLibA_61714 | 62 | -0.57388223 | hsa-mir-519a-2 | - |

| | | | | | |
|---|---|---|---|---|---|
| hsa-mir-526a-1 | HGLibA_61790 | 62 | -0.57388223 | hsa-mir-526a-1 | - |
| hsa-mir-550b-1 | HGLibA_62111 | 62 | -0.57388223 | hsa-mir-550b-1 | - |
| hsa-mir-576 | HGLibA_62400 | 62 | -0.57388223 | hsa-mir-576 | - |
| hsa-mir-599 | HGLibA_62487 | 62 | -0.57388223 | hsa-mir-599 | - |
| hsa-mir-6087 | HGLibA_62609 | 62 | -0.57388223 | hsa-mir-6087 | - |
| hsa-mir-642b | HGLibA_62811 | 62 | -0.57388223 | hsa-mir-642b | - |
| hsa-mir-6770-3 | HGLibA_63252 | 62 | -0.57388223 | hsa-mir-6770-3 | - |
| hsa-mir-7856 | HGLibA_64015 | 62 | -0.57388223 | hsa-mir-7856 | - |
| hsa-mir-8079 | HGLibA_64160 | 62 | -0.57388223 | hsa-mir-8079 | - |
| LOC100506688 | HGLibA_26864 | 62 | -0.57388223 | LOC100506688 | - |
| LOC388849 | HGLibA_26927 | 62 | -0.57388223 | LOC388849 | - |
| LOC391322 | HGLibA_26934 | 62 | -0.57388223 | LOC391322 | - |
| LOC402160 | HGLibA_26938 | 62 | -0.57388223 | LOC402160 | - |
| PHOSPHO2-KLHL23 | HGLibA_36446 | 62 | -0.57388223 | PHOSPHO2-KLHL23 | - |
| PRAMEF16 | HGLibA_38296 | 62 | -0.57388223 | PRAMEF16 | - |
| PRMT10 | HGLibA_38619 | 62 | -0.57388223 | PRMT10 | - |
| 4-Sep | HGLibA_43441 | 62 | -0.57388223 | 4-Sep | - |
| SPERT | HGLibA_46625 | 62 | -0.57388223 | SPERT | - |
| SSFA2 | HGLibA_47128 | 62 | -0.57388223 | SSFA2 | - |
| TMEM2 | HGLibA_50169 | 62 | -0.57388223 | TMEM2 | - |
| WARS | HGLibA_53944 | 62 | -0.57388223 | WARS | - |
| C11orf48 | HGLibA_05146 | 61 | -0.582927573 | C11orf48 | - |
| C15orf54 | HGLibA_05414 | 61 | -0.582927573 | C15orf54 | - |
| C20orf111 | HGLibA_05980 | 61 | -0.582927573 | C20orf111 | - |
| C4orf22 | HGLibA_06306 | 61 | -0.582927573 | C4orf22 | - |
| ENSG00000004059 | HGLibA_02655 | 61 | -0.582927573 | ARF5 | ADP ribosylation factor 5 [Source:HGNC Symbol;Acc:HGNC:658] |
| ENSG00000004776 | HGLibA_22312 | 61 | -0.582927573 | HSPB6 | heat shock protein family B (small) member 6 [Source:HGNC Symbol;Acc:HGNC:26511] |
| ENSG00000012232 | HGLibA_15813 | 61 | -0.582927573 | EXTL3 | exostosin like glycosyltransferase 3 [Source:HGNC Symbol;Acc:HGNC:3518] |
| ENSG00000035928 | HGLibA_40903 | 61 | -0.582927573 | RFC1 | replication factor C subunit 1 [Source:HGNC Symbol;Acc:HGNC:9969] |
| ENSG00000042753 | HGLibA_02317 | 61 | -0.582927573 | AP2S1 | adaptor related protein complex 2 subunit sigma 1 [Source:HGNC Symbol;Acc:HGNC:565] |
| ENSG00000046889 | HGLibA_38446 | 61 | -0.582927573 | PREX2 | 5-trisphosphate dependent Rac exchange factor 2 [Source:HGNC Symbol;Acc:HGNC:22950]" |
| ENSG00000050344 | HGLibA_31796 | 61 | -0.582927573 | NFE2L3 | erythroid 2 like 3 [Source:HGNC Symbol;Acc:HGNC:7783]" |
| ENSG00000058056 | HGLibA_53203 | 61 | -0.582927573 | USP13 | ubiquitin specific peptidase 13 [Source:HGNC Symbol;Acc:HGNC:12611] |
| ENSG00000065485 | HGLibA_35882 | 61 | -0.582927573 | PDIA5 | protein disulfide isomerase family A member 5 [Source:HGNC Symbol;Acc:HGNC:24811] |
| ENSG00000076826 | HGLibA_07112 | 61 | -0.582927573 | CAMSAP3 | calmodulin regulated spectrin associated protein family member 3 [Source:HGNC Symbol;Acc:HGNC:29307] |
| ENSG00000078053 | HGLibA_01831 | 61 | -0.582927573 | AMPH | amphiphysin [Source:HGNC Symbol;Acc:HGNC:471] |
| ENSG00000084110 | HGLibA_20819 | 61 | -0.582927573 | HAL | histidine ammonia-lyase [Source:HGNC Symbol;Acc:HGNC:4806] |
| ENSG00000089177 | HGLibA_24868 | 61 | -0.582927573 | KIF16B | kinesin family member 16B [Source:HGNC Symbol;Acc:HGNC:15869] |
| ENSG00000092841 | HGLibA_30770 | 61 | -0.582927573 | MYL6 | myosin light chain 6 [Source:HGNC Symbol;Acc:HGNC:7587] |
| ENSG00000095587 | HGLibA_49632 | 61 | -0.582927573 | TLL2 | tolloid like 2 [Source:HGNC Symbol;Acc:HGNC:11844] |
| ENSG00000100336 | HGLibA_02535 | 61 | -0.582927573 | APOL4 | apolipoprotein L4 [Source:HGNC Symbol;Acc:HGNC:14867] |
| ENSG00000100949 | HGLibA_40019 | 61 | -0.582927573 | RABGGTA | Rab geranylgeranyltransferase subunit alpha [Source:HGNC Symbol;Acc:HGNC:9795] |
| ENSG00000101187 | HGLibA_45470 | 61 | -0.582927573 | SLCO4A1 | solute carrier organic anion transporter family member 4A1 [Source:HGNC Symbol;Acc:HGNC:10953] |
| ENSG00000101188 | HGLibA_32887 | 61 | -0.582927573 | NTSR1 | neurotensin receptor 1 [Source:HGNC Symbol;Acc:HGNC:8039] |
| ENSG00000102531 | HGLibA_17748 | 61 | -0.582927573 | FNDC3A | fibronectin type III domain containing 3A [Source:HGNC Symbol;Acc:HGNC:20296] |
| ENSG00000102921 | HGLibA_30950 | 61 | -0.582927573 | N4BP1 | NEDD4 binding protein 1 [Source:HGNC Symbol;Acc:HGNC:29850] |
| ENSG00000102962 | HGLibA_07998 | 61 | -0.582927573 | CCL22 | C-C motif chemokine ligand 22 [Source:HGNC Symbol;Acc:HGNC:10621] |
| ENSG00000103723 | HGLibA_02322 | 61 | -0.582927573 | AP3B2 | adaptor related protein complex 3 subunit beta 2 [Source:HGNC Symbol;Acc:HGNC:567] |
| ENSG00000104691 | HGLibA_52798 | 61 | -0.582927573 | UBXN8 | UBX domain protein 8 [Source:HGNC Symbol;Acc:HGNC:30307] |
| ENSG00000105426 | HGLibA_39534 | 61 | -0.582927573 | PTPRS | receptor type S [Source:HGNC Symbol;Acc:HGNC:9681]" |
| ENSG00000105647 | HGLibA_36652 | 61 | -0.582927573 | PIK3R2 | phosphoinositide-3-kinase regulatory subunit 2 [Source:HGNC Symbol;Acc:HGNC:8980] |
| ENSG00000105855 | HGLibA_23798 | 61 | -0.582927573 | ITGB8 | integrin subunit beta 8 [Source:HGNC Symbol;Acc:HGNC:6163] |
| ENSG00000106346 | HGLibA_53366 | 61 | -0.582927573 | USP42 | ubiquitin specific peptidase 42 [Source:HGNC Symbol;Acc:HGNC:20068] |
| ENSG00000106537 | HGLibA_51901 | 61 | -0.582927573 | TSPAN13 | tetraspanin 13 [Source:HGNC Symbol;Acc:HGNC:21643] |
| ENSG00000106723 | HGLibA_46668 | 61 | -0.582927573 | SPIN1 | spindlin 1 [Source:HGNC Symbol;Acc:HGNC:11243] |
| ENSG00000106992 | HGLibA_01377 | 61 | -0.582927573 | AK1 | adenylate kinase 1 [Source:HGNC Symbol;Acc:HGNC:361] |
| ENSG00000107742 | HGLibA_46750 | 61 | -0.582927573 | SPOCK2 | cwcv and kazal like domains proteoglycan 2 [Source:HGNC Symbol;Acc:HGNC:13564]" |
| ENSG00000107771 | HGLibA_08192 | 61 | -0.582927573 | CCSER2 | coiled-coil serine rich protein 2 [Source:HGNC Symbol;Acc:HGNC:29197] |
| ENSG00000108306 | HGLibA_16999 | 61 | -0.582927573 | FBXL20 | F-box and leucine rich repeat protein 20 [Source:HGNC Symbol;Acc:HGNC:24679] |
| ENSG00000108582 | HGLibA_10910 | 61 | -0.582927573 | CPD | carboxypeptidase D [Source:HGNC Symbol;Acc:HGNC:2301] |
| ENSG00000109101 | HGLibA_17908 | 61 | -0.582927573 | FOXN1 | forkhead box N1 [Source:HGNC Symbol;Acc:HGNC:12765] |
| ENSG00000109854 | HGLibA_22345 | 61 | -0.582927573 | HTATIP2 | HIV-1 Tat interactive protein 2 [Source:HGNC Symbol;Acc:HGNC:16637] |
| ENSG00000110711 | HGLibA_01360 | 61 | -0.582927573 | AIP | aryl hydrocarbon receptor interacting protein [Source:HGNC Symbol;Acc:HGNC:358] |
| ENSG00000112308 | HGLibA_06502 | 61 | -0.582927573 | C6orf62 | chromosome 6 open reading frame 62 [Source:HGNC Symbol;Acc:HGNC:20998] |
| ENSG00000112695 | HGLibA_10861 | 61 | -0.582927573 | COX7A2 | cytochrome c oxidase subunit 7A2 [Source:HGNC Symbol;Acc:HGNC:2288] |
| ENSG00000112695 | HGLibA_10860 | 61 | -0.582927573 | COX7A2 | cytochrome c oxidase subunit 7A2 [Source:HGNC Symbol;Acc:HGNC:2288] |
| ENSG00000114698 | HGLibA_37240 | 61 | -0.582927573 | PLSCR4 | phospholipid scramblase 4 [Source:HGNC Symbol;Acc:HGNC:16497] |
| ENSG00000115042 | HGLibA_15959 | 61 | -0.582927573 | FAHD2A | fumarylacetoacetate hydrolase domain containing 2A [Source:HGNC Symbol;Acc:HGNC:24252] |
| ENSG00000115159 | HGLibA_19789 | 61 | -0.582927573 | GPD2 | glycerol-3-phosphate dehydrogenase 2 [Source:HGNC Symbol;Acc:HGNC:4456] |
| ENSG00000115808 | HGLibA_47594 | 61 | -0.582927573 | STRN | striatin [Source:HGNC Symbol;Acc:HGNC:11424] |
| ENSG00000116679 | HGLibA_23884 | 61 | -0.582927573 | IVNS1ABP | influenza virus NS1A binding protein [Source:HGNC Symbol;Acc:HGNC:16951] |
| ENSG00000117601 | HGLibA_43570 | 61 | -0.582927573 | SERPINC1 | serpin family C member 1 [Source:HGNC Symbol;Acc:HGNC:775] |
| ENSG00000117862 | HGLibA_52409 | 61 | -0.582927573 | TXNDC12 | thioredoxin domain containing 12 [Source:HGNC Symbol;Acc:HGNC:24626] |
| ENSG00000118816 | HGLibA_08102 | 61 | -0.582927573 | CCNI | cyclin I [Source:HGNC Symbol;Acc:HGNC:1595] |
| ENSG00000118922 | HGLibA_25047 | 61 | -0.582927573 | KLF12 | Kruppel like factor 12 [Source:HGNC Symbol;Acc:HGNC:6346] |
| ENSG00000121067 | HGLibA_46764 | 61 | -0.582927573 | SPOP | speckle type BTB/POZ protein [Source:HGNC Symbol;Acc:HGNC:11254] |
| ENSG00000121858 | HGLibA_50797 | 61 | -0.582927573 | TNFSF10 | TNF superfamily member 10 [Source:HGNC Symbol;Acc:HGNC:11925] |
| ENSG00000121931 | HGLibA_27173 | 61 | -0.582927573 | LRIF1 | ligand dependent nuclear receptor interacting factor 1 [Source:HGNC Symbol;Acc:HGNC:30299] |
| ENSG00000122299 | HGLibA_54983 | 61 | -0.582927573 | ZC3H7A | zinc finger CCCH-type containing 7A [Source:HGNC Symbol;Acc:HGNC:30959] |
| ENSG00000124564 | HGLibA_44466 | 61 | -0.582927573 | SLC17A3 | solute carrier family 17 member 3 [Source:HGNC Symbol;Acc:HGNC:10931] |
| ENSG00000125122 | HGLibA_27301 | 61 | -0.582927573 | LRRC29 | leucine rich repeat containing 29 [Source:HGNC Symbol;Acc:HGNC:13605] |
| ENSG00000125355 | HGLibA_50331 | 61 | -0.582927573 | TMEM255A | transmembrane protein 255A [Source:HGNC Symbol;Acc:HGNC:26086] |
| ENSG00000125571 | HGLibA_23156 | 61 | -0.582927573 | IL37 | interleukin 37 [Source:HGNC Symbol;Acc:HGNC:15563] |
| ENSG00000126709 | HGLibA_22617 | 61 | -0.582927573 | IFI6 | interferon alpha inducible protein 6 [Source:HGNC Symbol;Acc:HGNC:4054] |
| ENSG00000127419 | HGLibA_50070 | 61 | -0.582927573 | TMEM175 | transmembrane protein 175 [Source:HGNC Symbol;Acc:HGNC:28709] |
| ENSG00000127481 | HGLibA_52756 | 61 | -0.582927573 | UBR4 | ubiquitin protein ligase E3 component n-recognin 4 [Source:HGNC Symbol;Acc:HGNC:30313] |
| ENSG00000127824 | HGLibA_52262 | 61 | -0.582927573 | TUBA4A | tubulin alpha 4a [Source:HGNC Symbol;Acc:HGNC:12407] |
| ENSG00000127870 | HGLibA_41693 | 61 | -0.582927573 | RNF6 | ring finger protein 6 [Source:HGNC Symbol;Acc:HGNC:10069] |
| ENSG00000129167 | HGLibA_51130 | 61 | -0.582927573 | TPH1 | tryptophan hydroxylase 1 [Source:HGNC Symbol;Acc:HGNC:12008] |
| ENSG00000129221 | HGLibA_01365 | 61 | -0.582927573 | AIPL1 | aryl hydrocarbon receptor interacting protein like 1 [Source:HGNC Symbol;Acc:HGNC:359] |
| ENSG00000130311 | HGLibA_12585 | 61 | -0.582927573 | DDA1 | DET1 and DDB1 associated 1 [Source:HGNC Symbol;Acc:HGNC:28360] |
| ENSG00000133884 | HGLibA_13829 | 61 | -0.582927573 | DPF2 | double PHD fingers 2 [Source:HGNC Symbol;Acc:HGNC:9964] |
| ENSG00000134072 | HGLibA_07059 | 61 | -0.582927573 | CAMK1 | calcium/calmodulin dependent protein kinase I [Source:HGNC Symbol;Acc:HGNC:1459] |
| ENSG00000134138 | HGLibA_28874 | 61 | -0.582927573 | MEIS2 | Meis homeobox 2 [Source:HGNC Symbol;Acc:HGNC:7001] |
| ENSG00000134294 | HGLibA_45063 | 61 | -0.582927573 | SLC38A2 | solute carrier family 38 member 2 [Source:HGNC Symbol;Acc:HGNC:13448] |
| ENSG00000135355 | HGLibA_19140 | 61 | -0.582927573 | GJA10 | gap junction protein alpha 10 [Source:HGNC Symbol;Acc:HGNC:16995] |
| ENSG00000135482 | HGLibA_54946 | 61 | -0.582927573 | ZC3H10 | zinc finger CCCH-type containing 10 [Source:HGNC Symbol;Acc:HGNC:25893] |
| ENSG00000135521 | HGLibA_27617 | 61 | -0.582927573 | LTV1 | LTV1 ribosome biogenesis factor [Source:HGNC Symbol;Acc:HGNC:21173] |
| ENSG00000136052 | HGLibA_45141 | 61 | -0.582927573 | SLC41A2 | solute carrier family 41 member 2 [Source:HGNC Symbol;Acc:HGNC:31045] |
| ENSG00000136213 | HGLibA_09618 | 61 | -0.582927573 | CHST12 | carbohydrate sulfotransferase 12 [Source:HGNC Symbol;Acc:HGNC:17423] |
| ENSG00000136237 | HGLibA_40257 | 61 | -0.582927573 | RAPGEF5 | Rap guanine nucleotide exchange factor 5 [Source:HGNC Symbol;Acc:HGNC:16862] |
| ENSG00000136286 | HGLibA_30839 | 61 | -0.582927573 | MYO1G | myosin IG [Source:HGNC Symbol;Acc:HGNC:13880] |
| ENSG00000136925 | HGLibA_52032 | 61 | -0.582927573 | TSTD2 | thiosulfate sulfurtransferase like domain containing 2 [Source:HGNC Symbol;Acc:HGNC:30087] |
| ENSG00000137221 | HGLibA_49567 | 61 | -0.582927573 | TJAP1 | tight junction associated protein 1 [Source:HGNC Symbol;Acc:HGNC:17949] |
| ENSG00000137449 | HGLibA_10920 | 61 | -0.582927573 | CPEB2 | cytoplasmic polyadenylation element binding protein 2 [Source:HGNC Symbol;Acc:HGNC:21745] |
| ENSG00000138468 | HGLibA_43399 | 61 | -0.582927573 | SENP7 | SUMO specific peptidase 7 [Source:HGNC Symbol;Acc:HGNC:30402] |
| ENSG00000138757 | HGLibA_18303 | 61 | -0.582927573 | G3BP2 | G3BP stress granule assembly factor 2 [Source:HGNC Symbol;Acc:HGNC:30291] |
| ENSG00000138780 | HGLibA_20465 | 61 | -0.582927573 | GSTCD | glutathione S-transferase C-terminal domain containing [Source:HGNC Symbol;Acc:HGNC:25806] |
| ENSG00000138829 | HGLibA_16958 | 61 | -0.582927573 | FBN2 | fibrillin 2 [Source:HGNC Symbol;Acc:HGNC:3604] |
| ENSG00000139200 | HGLibA_36504 | 61 | -0.582927573 | PIANP | PILR alpha associated neural protein [Source:HGNC Symbol;Acc:HGNC:25338] |
| ENSG00000139547 | HGLibA_40746 | 61 | -0.582927573 | RDH16 | retinol dehydrogenase 16 [Source:HGNC Symbol;Acc:HGNC:29674] |
| ENSG00000140396 | HGLibA_31339 | 61 | -0.582927573 | NCOA2 | nuclear receptor coactivator 2 [Source:HGNC Symbol;Acc:HGNC:7669] |
| ENSG00000140835 | HGLibA_09636 | 61 | -0.582927573 | CHST4 | carbohydrate sulfotransferase 4 [Source:HGNC Symbol;Acc:HGNC:1972] |
| ENSG00000140939 | HGLibA_32264 | 61 | -0.582927573 | NOL3 | nucleolar protein 3 [Source:HGNC Symbol;Acc:HGNC:7869] |
| ENSG00000141574 | HGLibA_43270 | 61 | -0.582927573 | SECTM1 | secreted and transmembrane 1 [Source:HGNC Symbol;Acc:HGNC:10707] |
| ENSG00000142623 | HGLibA_34969 | 61 | -0.582927573 | PADI1 | peptidyl arginine deiminase 1 [Source:HGNC Symbol;Acc:HGNC:18367] |
| ENSG00000143032 | HGLibA_04162 | 61 | -0.582927573 | BARHL2 | BarH like homeobox 2 [Source:HGNC Symbol;Acc:HGNC:954] |
| ENSG00000143119 | HGLibA_08408 | 61 | -0.582927573 | CD53 | CD53 molecule [Source:HGNC Symbol;Acc:HGNC:1686] |
| ENSG00000144407 | HGLibA_39374 | 61 | -0.582927573 | PTH2R | parathyroid hormone 2 receptor [Source:HGNC Symbol;Acc:HGNC:9609] |
| ENSG00000146090 | HGLibA_40335 | 61 | -0.582927573 | RASGEF1C | RasGEF domain family member 1C [Source:HGNC Symbol;Acc:HGNC:27400] |
| ENSG00000147596 | HGLibA_38377 | 61 | -0.582927573 | PRDM14 | PR/SET domain 14 [Source:HGNC Symbol;Acc:HGNC:14001] |
| ENSG00000147869 | HGLibA_09189 | 61 | -0.582927573 | CER1 | DAN family BMP antagonist [Source:HGNC Symbol;Acc:HGNC:1862]" |
| ENSG00000148288 | HGLibA_18725 | 61 | -0.582927573 | GBGT1 | 3-N-acetylgalactosaminyltransferase 1 (FORS blood group) [Source:HGNC Symbol;Acc:HGNC:20460]" |
| ENSG00000148339 | HGLibA_44684 | 61 | -0.582927573 | SLC25A25 | solute carrier family 25 member 25 [Source:HGNC Symbol;Acc:HGNC:20663] |
| ENSG00000149269 | HGLibA_35036 | 61 | -0.582927573 | PAK1 | p21 (RAC1) activated kinase 1 [Source:HGNC Symbol;Acc:HGNC:8590] |
| ENSG00000151208 | HGLibA_13335 | 61 | -0.582927573 | DLG5 | discs large MAGUK scaffold protein 5 [Source:HGNC Symbol;Acc:HGNC:2904] |
| ENSG00000151208 | HGLibA_13337 | 61 | -0.582927573 | DLG5 | discs large MAGUK scaffold protein 5 [Source:HGNC Symbol;Acc:HGNC:2904] |
| ENSG00000151952 | HGLibA_49938 | 61 | -0.582927573 | TMEM132D | transmembrane protein 132D [Source:HGNC Symbol;Acc:HGNC:29411] |
| ENSG00000152056 | HGLibA_02302 | 61 | -0.582927573 | AP1S3 | adaptor related protein complex 1 subunit sigma 3 [Source:HGNC Symbol;Acc:HGNC:18971] |
| ENSG00000152683 | HGLibA_44913 | 61 | -0.582927573 | SLC30A6 | solute carrier family 30 member 6 [Source:HGNC Symbol;Acc:HGNC:19305] |
| ENSG00000153976 | HGLibA_22127 | 61 | -0.582927573 | HS3ST3A1 | heparan sulfate-glucosamine 3-sulfotransferase 3A1 [Source:HGNC Symbol;Acc:HGNC:5196] |
| ENSG00000154027 | HGLibA_01389 | 61 | -0.582927573 | AK5 | adenylate kinase 5 [Source:HGNC Symbol;Acc:HGNC:365] |
| ENSG00000154814 | HGLibA_34827 | 61 | -0.582927573 | OXNAD1 | oxidoreductase NAD binding domain containing 1 [Source:HGNC Symbol;Acc:HGNC:25128] |
| ENSG00000155275 | HGLibA_51688 | 61 | -0.582927573 | TRMT44 | tRNA methyltransferase 44 homolog [Source:HGNC Symbol;Acc:HGNC:26653] |
| ENSG00000155744 | HGLibA_16085 | 61 | -0.582927573 | FAM126B | family with sequence similarity 126 member B [Source:HGNC Symbol;Acc:HGNC:28593] |

| | | | | | |
|---|---|---|---|---|---|
| ENSG00000158195 | HGLibA_53957 | 61 | -0.582927573 | WASF2 | WAS protein family member 2 [Source:HGNC Symbol;Acc:HGNC:12733] |
| ENSG00000158402 | HGLibA_08532 | 61 | -0.582927573 | CDC25C | cell division cycle 25C [Source:HGNC Symbol;Acc:HGNC:1727] |
| ENSG00000158488 | HGLibA_08277 | 61 | -0.582927573 | CD1E | CD1e molecule [Source:HGNC Symbol;Acc:HGNC:1638] |
| ENSG00000158714 | HGLibA_44320 | 61 | -0.582927573 | SLAMF8 | SLAM family member 8 [Source:HGNC Symbol;Acc:HGNC:21391] |
| ENSG00000159433 | HGLibA_47379 | 61 | -0.582927573 | STARD9 | StAR related lipid transfer domain containing 9 [Source:HGNC Symbol;Acc:HGNC:19162] |
| ENSG00000160145 | HGLibA_24020 | 61 | -0.582927573 | KALRN | kalirin RhoGEF kinase [Source:HGNC Symbol;Acc:HGNC:4814] |
| ENSG00000162144 | HGLibA_11981 | 61 | -0.582927573 | CYB561A3 | cytochrome b561 family member A3 [Source:HGNC Symbol;Acc:HGNC:23014] |
| ENSG00000162302 | HGLibA_42167 | 61 | -0.582927573 | RPS6KA4 | ribosomal protein S6 kinase A4 [Source:HGNC Symbol;Acc:HGNC:10433] |
| ENSG00000162616 | HGLibA_13544 | 61 | -0.582927573 | DNAJB4 | DnaJ heat shock protein family (Hsp40) member B4 [Source:HGNC Symbol;Acc:HGNC:14886] |
| ENSG00000162616 | HGLibA_13543 | 61 | -0.582927573 | DNAJB4 | DnaJ heat shock protein family (Hsp40) member B4 [Source:HGNC Symbol;Acc:HGNC:14886] |
| ENSG00000162745 | HGLibA_33362 | 61 | -0.582927573 | OLFML2B | olfactomedin like 2B [Source:HGNC Symbol;Acc:HGNC:24558] |
| ENSG00000163257 | HGLibA_12434 | 61 | -0.582927573 | DCAF16 | DDB1 and CUL4 associated factor 16 [Source:HGNC Symbol;Acc:HGNC:25987] |
| ENSG00000163440 | HGLibA_35752 | 61 | -0.582927573 | PDCL2 | phosducin like 2 [Source:HGNC Symbol;Acc:HGNC:29524] |
| ENSG00000163527 | HGLibA_47608 | 61 | -0.582927573 | STT3B | catalytic subunit of the oligosaccharyltransferase complex [Source:HGNC Symbol;Acc:HGNC:30611]" |
| ENSG00000163958 | HGLibA_55093 | 61 | -0.582927573 | ZDHHC19 | zinc finger DHHC-type containing 19 [Source:HGNC Symbol;Acc:HGNC:20713] |
| ENSG00000163959 | HGLibA_45239 | 61 | -0.582927573 | SLC51A | solute carrier family 51 alpha subunit [Source:HGNC Symbol;Acc:HGNC:29955] |
| ENSG00000164266 | HGLibA_46683 | 61 | -0.582927573 | SPINK1 | Kazal type 1 [Source:HGNC Symbol;Acc:HGNC:11244]" |
| ENSG00000164619 | HGLibA_04625 | 61 | -0.582927573 | BMPER | BMP binding endothelial regulator [Source:HGNC Symbol;Acc:HGNC:24154] |
| ENSG00000164620 | HGLibA_40820 | 61 | -0.582927573 | RELL2 | RELT like 2 [Source:HGNC Symbol;Acc:HGNC:26902] |
| ENSG00000164729 | HGLibA_45022 | 61 | -0.582927573 | SLC35G3 | solute carrier family 35 member G3 [Source:HGNC Symbol;Acc:HGNC:26848] |
| ENSG00000164853 | HGLibA_53040 | 61 | -0.582927573 | UNCX | UNC homeobox [Source:HGNC Symbol;Acc:HGNC:33194] |
| ENSG00000165458 | HGLibA_23363 | 61 | -0.582927573 | INPPL1 | inositol polyphosphate phosphatase like 1 [Source:HGNC Symbol;Acc:HGNC:6080] |
| ENSG00000165684 | HGLibA_45900 | 61 | -0.582927573 | SNAPC4 | small nuclear RNA activating complex polypeptide 4 [Source:HGNC Symbol;Acc:HGNC:11137] |
| ENSG00000165685 | HGLibA_50434 | 61 | -0.582927573 | TMEM52B | transmembrane protein 52B [Source:HGNC Symbol;Acc:HGNC:26438] |
| ENSG00000165699 | HGLibA_51810 | 61 | -0.582927573 | TSC1 | TSC complex subunit 1 [Source:HGNC Symbol;Acc:HGNC:12362] |
| ENSG00000166228 | HGLibA_35360 | 61 | -0.582927573 | PCBD1 | pterin-4 alpha-carbinolamine dehydratase 1 [Source:HGNC Symbol;Acc:HGNC:8646] |
| ENSG00000166704 | HGLibA_56368 | 61 | -0.582927573 | ZNF606 | zinc finger protein 606 [Source:HGNC Symbol;Acc:HGNC:25879] |
| ENSG00000167414 | HGLibA_19535 | 61 | -0.582927573 | GNG8 | G protein subunit gamma 8 [Source:HGNC Symbol;Acc:HGNC:19664] |
| ENSG00000167968 | HGLibA_13658 | 61 | -0.582927573 | DNASE1L2 | deoxyribonuclease 1 like 2 [Source:HGNC Symbol;Acc:HGNC:2958] |
| ENSG00000167981 | HGLibA_56354 | 61 | -0.582927573 | ZNF597 | zinc finger protein 597 [Source:HGNC Symbol;Acc:HGNC:26573] |
| ENSG00000168228 | HGLibA_55037 | 61 | -0.582927573 | ZCCHC4 | zinc finger CCHC-type containing 4 [Source:HGNC Symbol;Acc:HGNC:22917] |
| ENSG00000168477 | HGLibA_50932 | 61 | -0.582927573 | TNXB | tenascin XB [Source:HGNC Symbol;Acc:HGNC:11976] |
| ENSG00000168875 | HGLibA_46240 | 61 | -0.582927573 | SOX14 | SRY-box 14 [Source:HGNC Symbol;Acc:HGNC:11193] |
| ENSG00000170439 | HGLibA_29022 | 61 | -0.582927573 | METTL7B | methyltransferase like 7B [Source:HGNC Symbol;Acc:HGNC:28276] |
| ENSG00000171711 | HGLibA_12914 | 61 | -0.582927573 | DEFB4A | defensin beta 4A [Source:HGNC Symbol;Acc:HGNC:2767] |
| ENSG00000172071 | HGLibA_14729 | 61 | -0.582927573 | EIF2AK3 | eukaryotic translation initiation factor 2 alpha kinase 3 [Source:HGNC Symbol;Acc:HGNC:3255] |
| ENSG00000172232 | HGLibA_03941 | 61 | -0.582927573 | AZU1 | azurocidin 1 [Source:HGNC Symbol;Acc:HGNC:913] |
| ENSG00000172500 | HGLibA_17504 | 61 | -0.582927573 | FIBP | FGF1 intracellular binding protein [Source:HGNC Symbol;Acc:HGNC:3705] |
| ENSG00000172794 | HGLibA_39894 | 61 | -0.582927573 | RAB37 | member RAS oncogene family [Source:HGNC Symbol;Acc:HGNC:30268]" |
| ENSG00000173262 | HGLibA_44868 | 61 | -0.582927573 | SLC2A14 | solute carrier family 2 member 14 [Source:HGNC Symbol;Acc:HGNC:18301] |
| ENSG00000173535 | HGLibA_50739 | 61 | -0.582927573 | TNFRSF10C | TNF receptor superfamily member 10c [Source:HGNC Symbol;Acc:HGNC:11906] |
| ENSG00000174521 | HGLibA_52161 | 61 | -0.582927573 | TTC9B | tetratricopeptide repeat domain 9B [Source:HGNC Symbol;Acc:HGNC:26395] |
| ENSG00000174721 | HGLibA_17431 | 61 | -0.582927573 | FGFBP3 | fibroblast growth factor binding protein 3 [Source:HGNC Symbol;Acc:HGNC:23428] |
| ENSG00000176182 | HGLibA_30914 | 61 | -0.582927573 | MYPOP | partner of profilin [Source:HGNC Symbol;Acc:HGNC:20178]" |
| ENSG00000176194 | HGLibA_09696 | 61 | -0.582927573 | CIDEA | cell death-inducing DFFA-like effector a [Source:HGNC Symbol;Acc:HGNC:1976] |
| ENSG00000176294 | HGLibA_34047 | 61 | -0.582927573 | OR4N2 | olfactory receptor family 4 subfamily N member 2 [Source:HGNC Symbol;Acc:HGNC:14742] |
| ENSG00000176473 | HGLibA_54052 | 61 | -0.582927573 | WDR25 | WD repeat domain 25 [Source:HGNC Symbol;Acc:HGNC:21064] |
| ENSG00000176624 | HGLibA_29034 | 61 | -0.582927573 | MEX3C | mex-3 RNA binding family member C [Source:HGNC Symbol;Acc:HGNC:28040] |
| ENSG00000176641 | HGLibA_41550 | 61 | -0.582927573 | RNF152 | ring finger protein 152 [Source:HGNC Symbol;Acc:HGNC:26811] |
| ENSG00000177694 | HGLibA_31014 | 61 | -0.582927573 | NAALADL2 | N-acetylated alpha-linked acidic dipeptidase like 2 [Source:HGNC Symbol;Acc:HGNC:23219] |
| ENSG00000177721 | HGLibA_02235 | 61 | -0.582927573 | ANXA2R | annexin A2 receptor [Source:HGNC Symbol;Acc:HGNC:33463] |
| ENSG00000178287 | HGLibA_46344 | 61 | -0.582927573 | SPAG11A | sperm associated antigen 11A [Source:HGNC Symbol;Acc:HGNC:33342] |
| ENSG00000178573 | HGLibA_27865 | 61 | -0.582927573 | MAF | MAF bZIP transcription factor [Source:HGNC Symbol;Acc:HGNC:6776] |
| ENSG00000179520 | HGLibA_44481 | 61 | -0.582927573 | SLC17A8 | solute carrier family 17 member 8 [Source:HGNC Symbol;Acc:HGNC:20151] |
| ENSG00000179528 | HGLibA_26089 | 61 | -0.582927573 | LBX2 | ladybird homeobox 2 [Source:HGNC Symbol;Acc:HGNC:15525] |
| ENSG00000180113 | HGLibA_48911 | 61 | -0.582927573 | TDRD6 | tudor domain containing 6 [Source:HGNC Symbol;Acc:HGNC:21339] |
| ENSG00000180269 | HGLibA_19907 | 61 | -0.582927573 | GPR139 | G protein-coupled receptor 139 [Source:HGNC Symbol;Acc:HGNC:19995] |
| ENSG00000180357 | HGLibA_56377 | 61 | -0.582927573 | ZNF609 | zinc finger protein 609 [Source:HGNC Symbol;Acc:HGNC:29003] |
| ENSG00000180801 | HGLibA_03151 | 61 | -0.582927573 | ARSJ | arylsulfatase family member J [Source:HGNC Symbol;Acc:HGNC:26286] |
| ENSG00000181061 | HGLibA_21306 | 61 | -0.582927573 | HIGD1A | HIG1 hypoxia inducible domain family member 1A [Source:HGNC Symbol;Acc:HGNC:29527] |
| ENSG00000181767 | HGLibA_34530 | 61 | -0.582927573 | OR8H2 | olfactory receptor family 8 subfamily H member 2 [Source:HGNC Symbol;Acc:HGNC:15308] |
| ENSG00000182545 | HGLibA_41390 | 61 | -0.582927573 | RNASE10 | ribonuclease A family member 10 (inactive) [Source:HGNC Symbol;Acc:HGNC:19275] |
| ENSG00000183255 | HGLibA_39564 | 61 | -0.582927573 | PTTG1IP | PTTG1 interacting protein [Source:HGNC Symbol;Acc:HGNC:13524] |
| ENSG00000183513 | HGLibA_10452 | 61 | -0.582927573 | COA5 | cytochrome c oxidase assembly factor 5 [Source:HGNC Symbol;Acc:HGNC:33848] |
| ENSG00000183742 | HGLibA_27829 | 61 | -0.582927573 | MACC1 | MET transcriptional regulator MACC1 [Source:HGNC Symbol;Acc:HGNC:30215] |
| ENSG00000184557 | HGLibA_46130 | 61 | -0.582927573 | SOCS3 | suppressor of cytokine signaling 3 [Source:HGNC Symbol;Acc:HGNC:19391] |
| ENSG00000184908 | HGLibA_09844 | 61 | -0.582927573 | CLCNKB | chloride voltage-gated channel Kb [Source:HGNC Symbol;Acc:HGNC:2027] |
| ENSG00000186086 | HGLibA_31238 | 61 | -0.582927573 | NBPF6 | NBPF member 6 [Source:HGNC Symbol;Acc:HGNC:31988] |
| ENSG00000186510 | HGLibA_09840 | 61 | -0.582927573 | CLCNKA | chloride voltage-gated channel Ka [Source:HGNC Symbol;Acc:HGNC:2026] |
| ENSG00000186517 | HGLibA_02754 | 61 | -0.582927573 | ARHGAP30 | Rho GTPase activating protein 30 [Source:HGNC Symbol;Acc:HGNC:27414] |
| ENSG00000187021 | HGLibA_37356 | 61 | -0.582927573 | PNLIPRP1 | pancreatic lipase related protein 1 [Source:HGNC Symbol;Acc:HGNC:9156] |
| ENSG00000187510 | HGLibA_37123 | 61 | -0.582927573 | PLEKHG7 | pleckstrin homology and RhoGEF domain containing G7 [Source:HGNC Symbol;Acc:HGNC:33829] |
| ENSG00000188263 | HGLibA_23019 | 61 | -0.582927573 | IL17REL | interleukin 17 receptor E like [Source:HGNC Symbol;Acc:HGNC:33808] |
| ENSG00000188581 | HGLibA_25580 | 61 | -0.582927573 | KRTAP1-1 | keratin associated protein 1-1 [Source:HGNC Symbol;Acc:HGNC:16772] |
| ENSG00000196353 | HGLibA_10961 | 61 | -0.582927573 | CPNE4 | copine 4 [Source:HGNC Symbol;Acc:HGNC:2317] |
| ENSG00000196415 | HGLibA_38983 | 61 | -0.582927573 | PRTN3 | proteinase 3 [Source:HGNC Symbol;Acc:HGNC:9495] |
| ENSG00000196460 | HGLibA_40984 | 61 | -0.582927573 | RFX8 | lacking RFX DNA binding domain [Source:HGNC Symbol;Acc:HGNC:37253]" |
| ENSG00000196705 | HGLibA_55981 | 61 | -0.582927573 | ZNF431 | zinc finger protein 431 [Source:HGNC Symbol;Acc:HGNC:20809] |
| ENSG00000197070 | HGLibA_03113 | 61 | -0.582927573 | ARRDC1 | arrestin domain containing 1 [Source:HGNC Symbol;Acc:HGNC:28633] |
| ENSG00000197226 | HGLibA_48576 | 61 | -0.582927573 | TBC1D9B | TBC1 domain family member 9B [Source:HGNC Symbol;Acc:HGNC:29097] |
| ENSG00000197345 | HGLibA_29927 | 61 | -0.582927573 | MRPL21 | mitochondrial ribosomal protein L21 [Source:HGNC Symbol;Acc:HGNC:14479] |
| ENSG00000197887 | HGLibA_33715 | 61 | -0.582927573 | OR1S2 | olfactory receptor family 1 subfamily S member 2 [Source:HGNC Symbol;Acc:HGNC:15141] |
| ENSG00000198133 | HGLibA_50252 | 61 | -0.582927573 | TMEM229B | transmembrane protein 229B [Source:HGNC Symbol;Acc:HGNC:20130] |
| ENSG00000203697 | HGLibA_07177 | 61 | -0.582927573 | CAPN8 | calpain 8 [Source:HGNC Symbol;Acc:HGNC:1485] |
| ENSG00000205038 | HGLibA_36816 | 61 | -0.582927573 | PKHD1L1 | PKHD1 like 1 [Source:HGNC Symbol;Acc:HGNC:20313] |
| ENSG00000206560 | HGLibA_02054 | 61 | -0.582927573 | ANKRD28 | ankyrin repeat domain 28 [Source:HGNC Symbol;Acc:HGNC:29024] |
| ENSG00000225556 | HGLibA_06104 | 61 | -0.582927573 | C2CD4D | C2 calcium dependent domain containing 4D [Source:HGNC Symbol;Acc:HGNC:37210] |
| ENSG00000226023 | HGLibA_11539 | 61 | -0.582927573 | CT47A6 | member A6 [Source:HGNC Symbol;Acc:HGNC:33287]" |
| ENSG00000228836 | HGLibA_11510 | 61 | -0.582927573 | CT45A5 | cancer/testis antigen family 45 member A5 [Source:HGNC Symbol;Acc:HGNC:33270] |
| ENSG00000228836 | HGLibA_11509 | 61 | -0.582927573 | CT45A5 | cancer/testis antigen family 45 member A5 [Source:HGNC Symbol;Acc:HGNC:33270] |
| ENSG00000229833 | HGLibA_36068 | 61 | -0.582927573 | PET100 | PET100 homolog [Source:HGNC Symbol;Acc:HGNC:40038] |
| ENSG00000232119 | HGLibA_28657 | 61 | -0.582927573 | MCTS1 | re-initiation and release factor [Source:HGNC Symbol;Acc:HGNC:23357]" |
| ENSG00000240247 | HGLibA_12795 | 61 | -0.582927573 | DEFA1B | defensin alpha 1B [Source:HGNC Symbol;Acc:HGNC:33596] |
| ENSG00000244187 | HGLibA_49964 | 61 | -0.582927573 | TMEM141 | transmembrane protein 141 [Source:HGNC Symbol;Acc:HGNC:28211] |
| ENSG00000248485 | HGLibA_35663 | 61 | -0.582927573 | PCP4L1 | Purkinje cell protein 4 like 1 [Source:HGNC Symbol;Acc:HGNC:20448] |
| ENSG00000256977 | HGLibA_26553 | 61 | -0.582927573 | LIMS3 | LIM zinc finger domain containing 3 [Source:HGNC Symbol;Acc:HGNC:30047] |
| ENSG00000257591 | HGLibA_56420 | 61 | -0.582927573 | ZNF625 | zinc finger protein 625 [Source:HGNC Symbol;Acc:HGNC:30571] |
| ENSG00000261787 | HGLibA_48780 | 61 | -0.582927573 | TCF24 | transcription factor 24 [Source:HGNC Symbol;Acc:HGNC:32275] |
| ENSG00000280987 | HGLibA_28438 | 61 | -0.582927573 | MATR3 | matrin 3 [Source:NCBI gene;Acc:9782] |
| ENSG00000284701 | HGLibA_50306 | 61 | -0.582927573 | TMEM247 | transmembrane protein 247 [Source:HGNC Symbol;Acc:HGNC:42967] |
| ENSG00000287151 | HGLibA_06116 | 61 | -0.582927573 | C2orf27A | - |
| FAM21B | HGLibA_16508 | 61 | -0.582927573 | FAM21B | - |
| FAM26E | HGLibA_16564 | 61 | -0.582927573 | FAM26E | - |
| FAM57A | HGLibA_16645 | 61 | -0.582927573 | FAM57A | - |
| FLJ45513 | HGLibA_17635 | 61 | -0.582927573 | FLJ45513 | - |
| HIST1H2AG | HGLibA_21391 | 61 | -0.582927573 | HIST1H2AG | - |
| hsa-mir-1204 | HGLibA_57274 | 61 | -0.582927573 | hsa-mir-1204 | - |
| hsa-mir-139 | HGLibA_57820 | 61 | -0.582927573 | hsa-mir-139 | - |
| hsa-mir-3168 | HGLibA_58750 | 61 | -0.582927573 | hsa-mir-3168 | - |
| hsa-mir-3179-1 | HGLibA_58788 | 61 | -0.582927573 | hsa-mir-3179-1 | - |
| hsa-mir-3180-4 | HGLibA_58812 | 61 | -0.582927573 | hsa-mir-3180-4 | - |
| hsa-mir-328 | HGLibA_58974 | 61 | -0.582927573 | hsa-mir-328 | - |
| hsa-mir-433 | HGLibA_60044 | 61 | -0.582927573 | hsa-mir-433 | - |
| hsa-mir-4422 | HGLibA_60074 | 61 | -0.582927573 | hsa-mir-4422 | - |
| hsa-mir-4438 | HGLibA_60156 | 61 | -0.582927573 | hsa-mir-4438 | - |
| hsa-mir-4444-2 | HGLibA_60184 | 61 | -0.582927573 | hsa-mir-4444-2 | - |
| hsa-mir-4455 | HGLibA_60227 | 61 | -0.582927573 | hsa-mir-4455 | - |
| hsa-mir-4497 | HGLibA_60404 | 61 | -0.582927573 | hsa-mir-4497 | - |
| hsa-mir-4536-1 | HGLibA_60604 | 61 | -0.582927573 | hsa-mir-4536-1 | - |
| hsa-mir-4643 | HGLibA_60677 | 61 | -0.582927573 | hsa-mir-4643 | - |
| hsa-mir-4663 | HGLibA_60766 | 61 | -0.582927573 | hsa-mir-4663 | - |
| hsa-mir-518c | HGLibA_61664 | 61 | -0.582927573 | hsa-mir-518c | - |
| hsa-mir-5194 | HGLibA_61696 | 61 | -0.582927573 | hsa-mir-5194 | - |
| hsa-mir-550a-3 | HGLibA_62108 | 61 | -0.582927573 | hsa-mir-550a-3 | - |
| hsa-mir-558 | HGLibA_62159 | 61 | -0.582927573 | hsa-mir-558 | - |
| hsa-mir-587 | HGLibA_62447 | 61 | -0.582927573 | hsa-mir-587 | - |
| hsa-mir-6089-2 | HGLibA_62621 | 61 | -0.582927573 | hsa-mir-6089-2 | - |
| hsa-mir-638 | HGLibA_62793 | 61 | -0.582927573 | hsa-mir-638 | - |
| hsa-mir-6508 | HGLibA_62881 | 61 | -0.582927573 | hsa-mir-6508 | - |
| hsa-mir-6774 | HGLibA_63271 | 61 | -0.582927573 | hsa-mir-6774 | - |
| hsa-mir-6816 | HGLibA_63440 | 61 | -0.582927573 | hsa-mir-6816 | - |
| hsa-mir-6868 | HGLibA_63657 | 61 | -0.582927573 | hsa-mir-6868 | - |
| hsa-mir-6885 | HGLibA_63723 | 61 | -0.582927573 | hsa-mir-6885 | - |
| hsa-mir-8067 | HGLibA_64105 | 61 | -0.582927573 | hsa-mir-8067 | - |
| KIAA1432 | HGLibA_24758 | 61 | -0.582927573 | KIAA1432 | - |

| | | | | | |
|---|---|---|---|---|---|
| LOC100505478 | HGLibA_26847 | 61 | -0.582927573 | LOC100505478 | - |
| MFSD7 | HGLibA_29113 | 61 | -0.582927573 | MFSD7 | - |
| MYEOV2 | HGLibA_30695 | 61 | -0.582927573 | MYEOV2 | - |
| PPAP2A | HGLibA_37815 | 61 | -0.582927573 | PPAP2A | - |
| PROSC | HGLibA_38692 | 61 | -0.582927573 | PROSC | - |
| SPERT | HGLibA_46624 | 61 | -0.582927573 | SPERT | - |
| TTC18 | HGLibA_52062 | 61 | -0.582927573 | TTC18 | - |
| VAMP7_X | HGLibA_53503 | 61 | -0.582927573 | VAMP7_X | - |
| C2orf43 | HGLibA_06128 | 60 | -0.591972916 | C2orf43 | - |
| CCDC19 | HGLibA_07727 | 60 | -0.591972916 | CCDC19 | - |
| CD97 | HGLibA_08487 | 60 | -0.591972916 | CD97 | - |
| CXorf57 | HGLibA_11948 | 60 | -0.591972916 | CXorf57 | - |
| CYR61 | HGLibA_12221 | 60 | -0.591972916 | CYR61 | - |
| ENSG00000018699 | HGLibA_52094 | 60 | -0.591972916 | TTC27 | tetratricopeptide repeat domain 27 [Source:HGNC Symbol;Acc:HGNC:25986] |
| ENSG00000019995 | HGLibA_56971 | 60 | -0.591972916 | ZRANB1 | zinc finger RANBP2-type containing 1 [Source:HGNC Symbol;Acc:HGNC:18224] |
| ENSG00000025156 | HGLibA_22222 | 60 | -0.591972916 | HSF2 | heat shock transcription factor 2 [Source:HGNC Symbol;Acc:HGNC:5225] |
| ENSG00000050165 | HGLibA_13300 | 60 | -0.591972916 | DKK3 | dickkopf WNT signaling pathway inhibitor 3 [Source:HGNC Symbol;Acc:HGNC:2893] |
| ENSG00000057663 | HGLibA_03500 | 60 | -0.591972916 | ATG5 | autophagy related 5 [Source:HGNC Symbol;Acc:HGNC:589] |
| ENSG00000064201 | HGLibA_51930 | 60 | -0.591972916 | TSPAN32 | tetraspanin 32 [Source:HGNC Symbol;Acc:HGNC:13410] |
| ENSG00000067177 | HGLibA_36401 | 60 | -0.591972916 | PHKA1 | phosphorylase kinase regulatory subunit alpha 1 [Source:HGNC Symbol;Acc:HGNC:8925] |
| ENSG00000069482 | HGLibA_18495 | 60 | -0.591972916 | GAL | galanin and GMAP prepropeptide [Source:HGNC Symbol;Acc:HGNC:4114] |
| ENSG00000072210 | HGLibA_01565 | 60 | -0.591972916 | ALDH3A2 | aldehyde dehydrogenase 3 family member A2 [Source:HGNC Symbol;Acc:HGNC:403] |
| ENSG00000075043 | HGLibA_24370 | 60 | -0.591972916 | KCNQ2 | potassium voltage-gated channel subfamily Q member 2 [Source:HGNC Symbol;Acc:HGNC:6296] |
| ENSG00000076053 | HGLibA_40581 | 60 | -0.591972916 | RBM7 | RNA binding motif protein 7 [Source:HGNC Symbol;Acc:HGNC:9904] |
| ENSG00000078898 | HGLibA_04722 | 60 | -0.591972916 | BPIFB2 | BPI fold containing family B member 2 [Source:HGNC Symbol;Acc:HGNC:16177] |
| ENSG00000080910 | HGLibA_09265 | 60 | -0.591972916 | CFHR2 | complement factor H related 2 [Source:HGNC Symbol;Acc:HGNC:4890] |
| ENSG00000086062 | HGLibA_04023 | 60 | -0.591972916 | B4GALT1 | 4-galactosyltransferase 1 [Source:HGNC Symbol;Acc:HGNC:924]" |
| ENSG00000087077 | HGLibA_51648 | 60 | -0.591972916 | TRIP6 | thyroid hormone receptor interactor 6 [Source:HGNC Symbol;Acc:HGNC:12311] |
| ENSG00000087460 | HGLibA_19470 | 60 | -0.591972916 | GNAS | GNAS complex locus [Source:HGNC Symbol;Acc:HGNC:4392] |
| ENSG00000088387 | HGLibA_13760 | 60 | -0.591972916 | DOCK9 | dedicator of cytokinesis 9 [Source:HGNC Symbol;Acc:HGNC:14132] |
| ENSG00000100104 | HGLibA_47043 | 60 | -0.591972916 | SRRD | SRR1 domain containing [Source:HGNC Symbol;Acc:HGNC:33910] |
| ENSG00000100146 | HGLibA_46229 | 60 | -0.591972916 | SOX10 | SRY-box 10 [Source:HGNC Symbol;Acc:HGNC:11190] |
| ENSG00000100312 | HGLibA_00530 | 60 | -0.591972916 | ACR | acrosin [Source:HGNC Symbol;Acc:HGNC:126] |
| ENSG00000100380 | HGLibA_47222 | 60 | -0.591972916 | ST13 | Hsp70 interacting protein [Source:HGNC Symbol;Acc:HGNC:11343]" |
| ENSG00000101940 | HGLibA_54030 | 60 | -0.591972916 | WDR13 | WD repeat domain 13 [Source:HGNC Symbol;Acc:HGNC:14352] |
| ENSG00000104131 | HGLibA_14793 | 60 | -0.591972916 | EIF3J | eukaryotic translation initiation factor 3 subunit J [Source:HGNC Symbol;Acc:HGNC:3270] |
| ENSG00000105227 | HGLibA_38993 | 60 | -0.591972916 | PRX | periaxin [Source:HGNC Symbol;Acc:HGNC:13797] |
| ENSG00000105518 | HGLibA_50193 | 60 | -0.591972916 | TMEM205 | transmembrane protein 205 [Source:HGNC Symbol;Acc:HGNC:29631] |
| ENSG00000105642 | HGLibA_24353 | 60 | -0.591972916 | KCNN1 | potassium calcium-activated channel subfamily N member 1 [Source:HGNC Symbol;Acc:HGNC:6290] |
| ENSG00000106341 | HGLibA_38060 | 60 | -0.591972916 | PPP1R17 | protein phosphatase 1 regulatory subunit 17 [Source:HGNC Symbol;Acc:HGNC:16973] |
| ENSG00000106565 | HGLibA_50076 | 60 | -0.591972916 | TMEM176B | transmembrane protein 176B [Source:HGNC Symbol;Acc:HGNC:29596] |
| ENSG00000106714 | HGLibA_10426 | 60 | -0.591972916 | CNTNAP3 | contactin associated protein like 3 [Source:HGNC Symbol;Acc:HGNC:13834] |
| ENSG00000107099 | HGLibA_13757 | 60 | -0.591972916 | DOCK8 | dedicator of cytokinesis 8 [Source:HGNC Symbol;Acc:HGNC:19191] |
| ENSG00000108262 | HGLibA_19130 | 60 | -0.591972916 | GIT1 | GIT ArfGAP 1 [Source:HGNC Symbol;Acc:HGNC:4272] |
| ENSG00000109133 | HGLibA_50370 | 60 | -0.591972916 | TMEM33 | transmembrane protein 33 [Source:HGNC Symbol;Acc:HGNC:25541] |
| ENSG00000110079 | HGLibA_30150 | 60 | -0.591972916 | MS4A4A | membrane spanning 4-domains A4A [Source:HGNC Symbol;Acc:HGNC:13371] |
| ENSG00000110944 | HGLibA_23097 | 60 | -0.591972916 | IL23A | interleukin 23 subunit alpha [Source:HGNC Symbol;Acc:HGNC:15488] |
| ENSG00000111206 | HGLibA_17907 | 60 | -0.591972916 | FOXM1 | forkhead box M1 [Source:HGNC Symbol;Acc:HGNC:3818] |
| ENSG00000112782 | HGLibA_10034 | 60 | -0.591972916 | CLIC5 | chloride intracellular channel 5 [Source:HGNC Symbol;Acc:HGNC:13517] |
| ENSG00000113205 | HGLibA_35497 | 60 | -0.591972916 | PCDHB3 | protocadherin beta 3 [Source:HGNC Symbol;Acc:HGNC:8688] |
| ENSG00000114503 | HGLibA_31283 | 60 | -0.591972916 | NCBP2 | nuclear cap binding protein subunit 2 [Source:HGNC Symbol;Acc:HGNC:7659] |
| ENSG00000114631 | HGLibA_37463 | 60 | -0.591972916 | PODXL2 | podocalyxin like 2 [Source:HGNC Symbol;Acc:HGNC:17936] |
| ENSG00000115705 | HGLibA_51156 | 60 | -0.591972916 | TPO | thyroid peroxidase [Source:HGNC Symbol;Acc:HGNC:12015] |
| ENSG00000116127 | HGLibA_01674 | 60 | -0.591972916 | ALMS1 | centrosome and basal body associated protein [Source:HGNC Symbol;Acc:HGNC:428]" |
| ENSG00000116353 | HGLibA_28729 | 60 | -0.591972916 | MECR | mitochondrial trans-2-enoyl-CoA reductase [Source:HGNC Symbol;Acc:HGNC:19691] |
| ENSG00000116996 | HGLibA_56960 | 60 | -0.591972916 | ZP4 | zona pellucida glycoprotein 4 [Source:HGNC Symbol;Acc:HGNC:15770] |
| ENSG00000119397 | HGLibA_10436 | 60 | -0.591972916 | CNTRL | centriolin [Source:HGNC Symbol;Acc:HGNC:1858] |
| ENSG00000120149 | HGLibA_30273 | 60 | -0.591972916 | MSX2 | msh homeobox 2 [Source:HGNC Symbol;Acc:HGNC:7392] |
| ENSG00000120253 | HGLibA_33055 | 60 | -0.591972916 | NUP43 | nucleoporin 43 [Source:HGNC Symbol;Acc:HGNC:21182] |
| ENSG00000120705 | HGLibA_15617 | 60 | -0.591972916 | ETF1 | eukaryotic translation termination factor 1 [Source:HGNC Symbol;Acc:HGNC:3477] |
| ENSG00000122966 | HGLibA_09739 | 60 | -0.591972916 | CIT | citron rho-interacting serine/threonine kinase [Source:HGNC Symbol;Acc:HGNC:1985] |
| ENSG00000123200 | HGLibA_54962 | 60 | -0.591972916 | ZC3H13 | zinc finger CCCH-type containing 13 [Source:HGNC Symbol;Acc:HGNC:20368] |
| ENSG00000123349 | HGLibA_36148 | 60 | -0.591972916 | PFDN5 | prefoldin subunit 5 [Source:HGNC Symbol;Acc:HGNC:8869] |
| ENSG00000123374 | HGLibA_08756 | 60 | -0.591972916 | CDK2 | cyclin dependent kinase 2 [Source:HGNC Symbol;Acc:HGNC:1771] |
| ENSG00000123643 | HGLibA_45031 | 60 | -0.591972916 | SLC36A1 | solute carrier family 36 member 1 [Source:HGNC Symbol;Acc:HGNC:18761] |
| ENSG00000124160 | HGLibA_31348 | 60 | -0.591972916 | NCOA5 | nuclear receptor coactivator 5 [Source:HGNC Symbol;Acc:HGNC:15909] |
| ENSG00000124216 | HGLibA_45866 | 60 | -0.591972916 | SNAI1 | snail family transcriptional repressor 1 [Source:HGNC Symbol;Acc:HGNC:11128] |
| ENSG00000125409 | HGLibA_48977 | 60 | -0.591972916 | TEKT3 | tektin 3 [Source:HGNC Symbol;Acc:HGNC:14293] |
| ENSG00000125970 | HGLibA_40173 | 60 | -0.591972916 | RALY | RALY heterogeneous nuclear ribonucleoprotein [Source:HGNC Symbol;Acc:HGNC:15921] |
| ENSG00000128283 | HGLibA_08567 | 60 | -0.591972916 | CDC42EP1 | CDC42 effector protein 1 [Source:HGNC Symbol;Acc:HGNC:17014] |
| ENSG00000128422 | HGLibA_25436 | 60 | -0.591972916 | KRT17 | keratin 17 [Source:HGNC Symbol;Acc:HGNC:6427] |
| ENSG00000129910 | HGLibA_08644 | 60 | -0.591972916 | CDH15 | cadherin 15 [Source:HGNC Symbol;Acc:HGNC:1754] |
| ENSG00000130368 | HGLibA_28387 | 60 | -0.591972916 | MAS1 | G protein-coupled receptor [Source:HGNC Symbol;Acc:HGNC:6899]" |
| ENSG00000130522 | HGLibA_24008 | 60 | -0.591972916 | JUND | AP-1 transcription factor subunit [Source:HGNC Symbol;Acc:HGNC:6206]" |
| ENSG00000130653 | HGLibA_37424 | 60 | -0.591972916 | PNPLA7 | patatin like phospholipase domain containing 7 [Source:HGNC Symbol;Acc:HGNC:24768] |
| ENSG00000130811 | HGLibA_14784 | 60 | -0.591972916 | EIF3G | eukaryotic translation initiation factor 3 subunit G [Source:HGNC Symbol;Acc:HGNC:3274] |
| ENSG00000130826 | HGLibA_13291 | 60 | -0.591972916 | DKC1 | dyskerin pseudouridine synthase 1 [Source:HGNC Symbol;Acc:HGNC:2890] |
| ENSG00000131653 | HGLibA_51268 | 60 | -0.591972916 | TRAF7 | TNF receptor associated factor 7 [Source:HGNC Symbol;Acc:HGNC:20456] |
| ENSG00000132286 | HGLibA_49495 | 60 | -0.591972916 | TIMM10B | translocase of inner mitochondrial membrane 10B [Source:HGNC Symbol;Acc:HGNC:4022] |
| ENSG00000134109 | HGLibA_14454 | 60 | -0.591972916 | EDEM1 | ER degradation enhancing alpha-mannosidase like protein 1 [Source:HGNC Symbol;Acc:HGNC:18967] |
| ENSG00000134490 | HGLibA_50288 | 60 | -0.591972916 | TMEM241 | transmembrane protein 241 [Source:HGNC Symbol;Acc:HGNC:31723] |
| ENSG00000134910 | HGLibA_47605 | 60 | -0.591972916 | STT3A | catalytic subunit of the oligosaccharyltransferase complex [Source:HGNC Symbol;Acc:HGNC:6172]" |
| ENSG00000134940 | HGLibA_00538 | 60 | -0.591972916 | ACRV1 | acrosomal vesicle protein 1 [Source:HGNC Symbol;Acc:HGNC:127] |
| ENSG00000135439 | HGLibA_01143 | 60 | -0.591972916 | AGAP2 | ankyrin repeat and PH domain 2 [Source:HGNC Symbol;Acc:HGNC:16921]" |
| ENSG00000136014 | HGLibA_53371 | 60 | -0.591972916 | USP44 | ubiquitin specific peptidase 44 [Source:HGNC Symbol;Acc:HGNC:20064] |
| ENSG00000136478 | HGLibA_49072 | 60 | -0.591972916 | TEX2 | testis expressed 2 [Source:HGNC Symbol;Acc:HGNC:30884] |
| ENSG00000136696 | HGLibA_23149 | 60 | -0.591972916 | IL36B | interleukin 36 beta [Source:HGNC Symbol;Acc:HGNC:15564] |
| ENSG00000136856 | HGLibA_44890 | 60 | -0.591972916 | SLC2A8 | solute carrier family 2 member 8 [Source:HGNC Symbol;Acc:HGNC:13812] |
| ENSG00000137343 | HGLibA_03422 | 60 | -0.591972916 | ATAT1 | alpha tubulin acetyltransferase 1 [Source:HGNC Symbol;Acc:HGNC:21186] |
| ENSG00000137714 | HGLibA_17257 | 60 | -0.591972916 | FDX1 | ferredoxin 1 [Source:HGNC Symbol;Acc:HGNC:3638] |
| ENSG00000139675 | HGLibA_21795 | 60 | -0.591972916 | HNRNPA1L2 | heterogeneous nuclear ribonucleoprotein A1-like 2 [Source:HGNC Symbol;Acc:HGNC:27067] |
| ENSG00000141429 | HGLibA_18526 | 60 | -0.591972916 | GALNT1 | polypeptide N-acetylgalactosaminyltransferase 1 [Source:HGNC Symbol;Acc:HGNC:4123] |
| ENSG00000141580 | HGLibA_54100 | 60 | -0.591972916 | WDR45B | WD repeat domain 45B [Source:HGNC Symbol;Acc:HGNC:25072] |
| ENSG00000143801 | HGLibA_39028 | 60 | -0.591972916 | PSEN2 | presenilin 2 [Source:HGNC Symbol;Acc:HGNC:9509] |
| ENSG00000145022 | HGLibA_48854 | 60 | -0.591972916 | TCTA | T cell leukemia translocation altered [Source:HGNC Symbol;Acc:HGNC:11692] |
| ENSG00000145675 | HGLibA_36649 | 60 | -0.591972916 | PIK3R1 | phosphoinositide-3-kinase regulatory subunit 1 [Source:HGNC Symbol;Acc:HGNC:8979] |
| ENSG00000145819 | HGLibA_02742 | 60 | -0.591972916 | ARHGAP26 | Rho GTPase activating protein 26 [Source:HGNC Symbol;Acc:HGNC:17073] |
| ENSG00000145879 | HGLibA_46702 | 60 | -0.591972916 | SPINK7 | Kazal type 7 (putative) [Source:HGNC Symbol;Acc:HGNC:24643]" |
| ENSG00000145996 | HGLibA_08802 | 60 | -0.591972916 | CDKAL1 | CDK5 regulatory subunit associated protein 1 like 1 [Source:HGNC Symbol;Acc:HGNC:21050] |
| ENSG00000146250 | HGLibA_38928 | 60 | -0.591972916 | PRSS35 | serine protease 35 [Source:HGNC Symbol;Acc:HGNC:21387] |
| ENSG00000148215 | HGLibA_34270 | 60 | -0.591972916 | OR5C1 | olfactory receptor family 5 subfamily C member 1 [Source:HGNC Symbol;Acc:HGNC:8331] |
| ENSG00000148339 | HGLibA_44685 | 60 | -0.591972916 | SLC25A25 | solute carrier family 25 member 25 [Source:HGNC Symbol;Acc:HGNC:20663] |
| ENSG00000149782 | HGLibA_36985 | 60 | -0.591972916 | PLCB3 | phospholipase C beta 3 [Source:HGNC Symbol;Acc:HGNC:9056] |
| ENSG00000151006 | HGLibA_38956 | 60 | -0.591972916 | PRSS53 | serine protease 53 [Source:HGNC Symbol;Acc:HGNC:34407] |
| ENSG00000151418 | HGLibA_03763 | 60 | -0.591972916 | ATP6V1G3 | ATPase H+ transporting V1 subunit G3 [Source:HGNC Symbol;Acc:HGNC:18265] |
| ENSG00000151657 | HGLibA_24978 | 60 | -0.591972916 | KIN | Kin17 DNA and RNA binding protein [Source:HGNC Symbol;Acc:HGNC:6327] |
| ENSG00000151694 | HGLibA_00740 | 60 | -0.591972916 | ADAM17 | ADAM metallopeptidase domain 17 [Source:HGNC Symbol;Acc:HGNC:195] |
| ENSG00000153086 | HGLibA_00456 | 60 | -0.591972916 | ACMSD | aminocarboxymuconate semialdehyde decarboxylase [Source:HGNC Symbol;Acc:HGNC:19288] |
| ENSG00000153902 | HGLibA_26403 | 60 | -0.591972916 | LGI4 | leucine rich repeat LGI family member 4 [Source:HGNC Symbol;Acc:HGNC:18712] |
| ENSG00000154133 | HGLibA_41741 | 60 | -0.591972916 | ROBO4 | roundabout guidance receptor 4 [Source:HGNC Symbol;Acc:HGNC:17985] |
| ENSG00000156711 | HGLibA_28259 | 60 | -0.591972916 | MAPK13 | mitogen-activated protein kinase 13 [Source:HGNC Symbol;Acc:HGNC:6875] |
| ENSG00000158435 | HGLibA_10332 | 60 | -0.591972916 | CNOT11 | CCR4-NOT transcription complex subunit 11 [Source:HGNC Symbol;Acc:HGNC:25217] |
| ENSG00000158615 | HGLibA_38049 | 60 | -0.591972916 | PPP1R15B | protein phosphatase 1 regulatory subunit 15B [Source:HGNC Symbol;Acc:HGNC:14951] |
| ENSG00000159685 | HGLibA_09378 | 60 | -0.591972916 | CHCHD6 | coiled-coil-helix-coiled-coil-helix domain containing 6 [Source:HGNC Symbol;Acc:HGNC:28184] |
| ENSG00000159763 | HGLibA_36703 | 60 | -0.591972916 | PIP | prolactin induced protein [Source:HGNC Symbol;Acc:HGNC:8993] |
| ENSG00000159788 | HGLibA_41058 | 60 | -0.591972916 | RGS12 | regulator of G protein signaling 12 [Source:HGNC Symbol;Acc:HGNC:9994] |
| ENSG00000160606 | HGLibA_49596 | 60 | -0.591972916 | TLCD1 | TLC domain containing 1 [Source:HGNC Symbol;Acc:HGNC:25177] |
| ENSG00000160796 | HGLibA_31211 | 60 | -0.591972916 | NBEAL2 | neurobeachin like 2 [Source:HGNC Symbol;Acc:HGNC:31928] |
| ENSG00000161664 | HGLibA_03224 | 60 | -0.591972916 | ASB16 | ankyrin repeat and SOCS box containing 16 [Source:HGNC Symbol;Acc:HGNC:19768] |
| ENSG00000162086 | HGLibA_56677 | 60 | -0.591972916 | ZNF75A | zinc finger protein 75a [Source:HGNC Symbol;Acc:HGNC:13146] |
| ENSG00000162598 | HGLibA_05902 | 60 | -0.591972916 | C1orf87 | chromosome 1 open reading frame 87 [Source:HGNC Symbol;Acc:HGNC:28547] |
| ENSG00000163521 | HGLibA_19220 | 60 | -0.591972916 | GLB1L | galactosidase beta 1 like [Source:HGNC Symbol;Acc:HGNC:28129] |
| ENSG00000163807 | HGLibA_24697 | 60 | -0.591972916 | KIAA1143 | KIAA1143 [Source:HGNC Symbol;Acc:HGNC:29198] |
| ENSG00000164346 | HGLibA_32742 | 60 | -0.591972916 | NSA2 | ribosome biogenesis homolog [Source:HGNC Symbol;Acc:HGNC:30728]" |
| ENSG00000164379 | HGLibA_17943 | 60 | -0.591972916 | FOXQ1 | forkhead box Q1 [Source:HGNC Symbol;Acc:HGNC:20951] |
| ENSG00000165030 | HGLibA_31810 | 60 | -0.591972916 | NFIL3 | interleukin 3 regulated [Source:HGNC Symbol;Acc:HGNC:7787]" |
| ENSG00000165591 | HGLibA_15904 | 60 | -0.591972916 | FAAH2 | fatty acid amide hydrolase 2 [Source:HGNC Symbol;Acc:HGNC:26440] |
| ENSG00000165915 | HGLibA_45099 | 60 | -0.591972916 | SLC39A13 | solute carrier family 39 member 13 [Source:HGNC Symbol;Acc:HGNC:20859] |
| ENSG00000166046 | HGLibA_48849 | 60 | -0.591972916 | TCP11L2 | t-complex 11 like 2 [Source:HGNC Symbol;Acc:HGNC:28627] |
| ENSG00000166401 | HGLibA_43564 | 60 | -0.591972916 | SERPINB8 | serpin family B member 8 [Source:HGNC Symbol;Acc:HGNC:8952] |
| ENSG00000166448 | HGLibA_49924 | 60 | -0.591972916 | TMEM130 | transmembrane protein 130 [Source:HGNC Symbol;Acc:HGNC:25429] |
| ENSG00000166578 | HGLibA_23497 | 60 | -0.591972916 | IQCD | IQ motif containing D [Source:HGNC Symbol;Acc:HGNC:25168] |
| ENSG00000166822 | HGLibA_50053 | 60 | -0.591972916 | TMEM170A | transmembrane protein 170A [Source:HGNC Symbol;Acc:HGNC:29577] |
| ENSG00000167791 | HGLibA_06852 | 60 | -0.591972916 | CABP2 | calcium binding protein 2 [Source:HGNC Symbol;Acc:HGNC:1385] |
| ENSG00000168481 | HGLibA_26398 | 60 | -0.591972916 | LGI3 | leucine rich repeat LGI family member 3 [Source:HGNC Symbol;Acc:HGNC:18711] |

| | | | | | |
|---|---|---|---|---|---|
| ENSG00000169105 | HGLibA_09626 | 60 | -0.591972916 | CHST14 | carbohydrate sulfotransferase 14 [Source:HGNC Symbol;Acc:HGNC:24464] |
| ENSG00000169427 | HGLibA_24337 | 60 | -0.591972916 | KCNK9 | potassium two pore domain channel subfamily K member 9 [Source:HGNC Symbol;Acc:HGNC:6283] |
| ENSG00000170222 | HGLibA_01033 | 60 | -0.591972916 | ADPRM | manganese dependent [Source:HGNC Symbol;Acc:HGNC:30925]" |
| ENSG00000170340 | HGLibA_03983 | 60 | -0.591972916 | B3GNT2 | 3-N-acetylglucosaminyltransferase 2 [Source:HGNC Symbol;Acc:HGNC:15629]" |
| ENSG00000171189 | HGLibA_20280 | 60 | -0.591972916 | GRIK1 | glutamate ionotropic receptor kainate type subunit 1 [Source:HGNC Symbol;Acc:HGNC:4579] |
| ENSG00000171631 | HGLibA_34891 | 60 | -0.591972916 | P2RY6 | pyrimidinergic receptor P2Y6 [Source:HGNC Symbol;Acc:HGNC:8543] |
| ENSG00000171824 | HGLibA_15773 | 60 | -0.591972916 | EXOSC10 | exosome component 10 [Source:HGNC Symbol;Acc:HGNC:9138] |
| ENSG00000171903 | HGLibA_12182 | 60 | -0.591972916 | CYP4F11 | cytochrome P450 family 4 subfamily F member 11 [Source:HGNC Symbol;Acc:HGNC:13265] |
| ENSG00000172046 | HGLibA_53293 | 60 | -0.591972916 | USP19 | ubiquitin specific peptidase 19 [Source:HGNC Symbol;Acc:HGNC:12617] |
| ENSG00000172167 | HGLibA_30322 | 60 | -0.591972916 | MTBP | MDM2 binding protein [Source:HGNC Symbol;Acc:HGNC:7417] |
| ENSG00000172650 | HGLibA_01151 | 60 | -0.591972916 | AGAP5 | ankyrin repeat and PH domain 5 [Source:HGNC Symbol;Acc:HGNC:23467]" |
| ENSG00000173406 | HGLibA_12266 | 60 | -0.591972916 | DAB1 | reelin adaptor protein [Source:HGNC Symbol;Acc:HGNC:2661]" |
| ENSG00000174325 | HGLibA_13266 | 60 | -0.591972916 | DIRC1 | disrupted in renal carcinoma 1 [Source:HGNC Symbol;Acc:HGNC:15760] |
| ENSG00000174448 | HGLibA_47369 | 60 | -0.591972916 | STARD6 | StAR related lipid transfer domain containing 6 [Source:HGNC Symbol;Acc:HGNC:18066] |
| ENSG00000175505 | HGLibA_09818 | 60 | -0.591972916 | CLCF1 | cardiotrophin like cytokine factor 1 [Source:HGNC Symbol;Acc:HGNC:17412] |
| ENSG00000176597 | HGLibA_03994 | 60 | -0.591972916 | B3GNT5 | 3-N-acetylglucosaminyltransferase 5 [Source:HGNC Symbol;Acc:HGNC:15684]" |
| ENSG00000177138 | HGLibA_16821 | 60 | -0.591972916 | FAM9B | family with sequence similarity 9 member B [Source:HGNC Symbol;Acc:HGNC:18404] |
| ENSG00000177879 | HGLibA_02335 | 60 | -0.591972916 | AP3S1 | adaptor related protein complex 3 subunit sigma 1 [Source:HGNC Symbol;Acc:HGNC:2013] |
| ENSG00000178591 | HGLibA_12879 | 60 | -0.591972916 | DEFB125 | defensin beta 125 [Source:HGNC Symbol;Acc:HGNC:18105] |
| ENSG00000178662 | HGLibA_11430 | 60 | -0.591972916 | CSRNP3 | cysteine and serine rich nuclear protein 3 [Source:HGNC Symbol;Acc:HGNC:30729] |
| ENSG00000178913 | HGLibA_48252 | 60 | -0.591972916 | TAF7 | TATA-box binding protein associated factor 7 [Source:HGNC Symbol;Acc:HGNC:11541] |
| ENSG00000178951 | HGLibA_54916 | 60 | -0.591972916 | ZBTB7A | zinc finger and BTB domain containing 7A [Source:HGNC Symbol;Acc:HGNC:18078] |
| ENSG00000179241 | HGLibA_26246 | 60 | -0.591972916 | LDLRAD3 | low density lipoprotein receptor class A domain containing 3 [Source:HGNC Symbol;Acc:HGNC:27046] |
| ENSG00000179304 | HGLibA_16189 | 60 | -0.591972916 | FAM156B | family with sequence similarity 156 member B [Source:HGNC Symbol;Acc:HGNC:31962] |
| ENSG00000179632 | HGLibA_27868 | 60 | -0.591972916 | MAF1 | negative regulator of RNA polymerase III [Source:HGNC Symbol;Acc:HGNC:24966]" |
| ENSG00000181009 | HGLibA_34202 | 60 | -0.591972916 | OR52N5 | olfactory receptor family 52 subfamily N member 5 [Source:HGNC Symbol;Acc:HGNC:15231] |
| ENSG00000183067 | HGLibA_22897 | 60 | -0.591972916 | IGSF5 | immunoglobulin superfamily member 5 [Source:HGNC Symbol;Acc:HGNC:5952] |
| ENSG00000183092 | HGLibA_04398 | 60 | -0.591972916 | BEGAIN | brain enriched guanylate kinase associated [Source:HGNC Symbol;Acc:HGNC:24163] |
| ENSG00000183484 | HGLibA_19887 | 60 | -0.591972916 | GPR132 | G protein-coupled receptor 132 [Source:HGNC Symbol;Acc:HGNC:17482] |
| ENSG00000184378 | HGLibA_00684 | 60 | -0.591972916 | ACTRT3 | actin related protein T3 [Source:HGNC Symbol;Acc:HGNC:24022] |
| ENSG00000185697 | HGLibA_30642 | 60 | -0.591972916 | MYBL1 | MYB proto-oncogene like 1 [Source:HGNC Symbol;Acc:HGNC:7547] |
| ENSG00000186185 | HGLibA_24878 | 60 | -0.591972916 | KIF18B | kinesin family member 18B [Source:HGNC Symbol;Acc:HGNC:27102] |
| ENSG00000187537 | HGLibA_37736 | 60 | -0.591972916 | POTEG | POTE ankyrin domain family member G [Source:HGNC Symbol;Acc:HGNC:33896] |
| ENSG00000187871 | HGLibA_18978 | 60 | -0.591972916 | GFRAL | GDNF family receptor alpha like [Source:HGNC Symbol;Acc:HGNC:32789] |
| ENSG00000188107 | HGLibA_15828 | 60 | -0.591972916 | EYS | eyes shut homolog [Source:HGNC Symbol;Acc:HGNC:21555] |
| ENSG00000188215 | HGLibA_12572 | 60 | -0.591972916 | DCUN1D3 | defective in cullin neddylation 1 domain containing 3 [Source:HGNC Symbol;Acc:HGNC:28734] |
| ENSG00000188687 | HGLibA_45223 | 60 | -0.591972916 | SLC4A5 | solute carrier family 4 member 5 [Source:HGNC Symbol;Acc:HGNC:18168] |
| ENSG00000189068 | HGLibA_53858 | 60 | -0.591972916 | VSTM1 | V-set and transmembrane domain containing 1 [Source:HGNC Symbol;Acc:HGNC:29455] |
| ENSG00000196132 | HGLibA_30924 | 60 | -0.591972916 | MYT1 | myelin transcription factor 1 [Source:HGNC Symbol;Acc:HGNC:7622] |
| ENSG00000196664 | HGLibA_49661 | 60 | -0.591972916 | TLR7 | toll like receptor 7 [Source:HGNC Symbol;Acc:HGNC:15631] |
| ENSG00000197168 | HGLibA_31657 | 60 | -0.591972916 | NEK5 | NIMA related kinase 5 [Source:HGNC Symbol;Acc:HGNC:7748] |
| ENSG00000197594 | HGLibA_15168 | 60 | -0.591972916 | ENPP1 | ectonucleotide pyrophosphatase/phosphodiesterase 1 [Source:HGNC Symbol;Acc:HGNC:3356] |
| ENSG00000198183 | HGLibA_04710 | 60 | -0.591972916 | BPIFA1 | BPI fold containing family A member 1 [Source:HGNC Symbol;Acc:HGNC:15749] |
| ENSG00000198346 | HGLibA_56806 | 60 | -0.591972916 | ZNF813 | zinc finger protein 813 [Source:HGNC Symbol;Acc:HGNC:33257] |
| ENSG00000198844 | HGLibA_02819 | 60 | -0.591972916 | ARHGEF15 | Rho guanine nucleotide exchange factor 15 [Source:HGNC Symbol;Acc:HGNC:15590] |
| ENSG00000203668 | HGLibA_09468 | 60 | -0.591972916 | CHML | Rab escort protein 2 [Source:HGNC Symbol;Acc:HGNC:1941]" |
| ENSG00000204138 | HGLibA_36307 | 60 | -0.591972916 | PHACTR4 | phosphatase and actin regulator 4 [Source:HGNC Symbol;Acc:HGNC:25793] |
| ENSG00000204390 | HGLibA_22278 | 60 | -0.591972916 | HSPA1L | heat shock protein family A (Hsp70) member 1 like [Source:HGNC Symbol;Acc:HGNC:5234] |
| ENSG00000204887 | HGLibA_25585 | 60 | -0.591972916 | KRTAP1-4 | keratin associated protein 1-4 [Source:HGNC Symbol;Acc:HGNC:18904] |
| ENSG00000204919 | HGLibA_38799 | 60 | -0.591972916 | PRR20A | proline rich 20A [Source:HGNC Symbol;Acc:HGNC:24754] |
| ENSG00000212724 | HGLibA_25693 | 60 | -0.591972916 | KRTAP2-3 | keratin associated protein 2-3 [Source:HGNC Symbol;Acc:HGNC:18906] |
| ENSG00000213214 | HGLibA_02853 | 60 | -0.591972916 | ARHGEF35 | Rho guanine nucleotide exchange factor 35 [Source:HGNC Symbol;Acc:HGNC:33846] |
| ENSG00000213619 | HGLibA_31550 | 60 | -0.591972916 | NDUFS3 | NADH:ubiquinone oxidoreductase core subunit S3 [Source:HGNC Symbol;Acc:HGNC:7710] |
| ENSG00000213918 | HGLibA_13654 | 60 | -0.591972916 | DNASE1 | deoxyribonuclease 1 [Source:HGNC Symbol;Acc:HGNC:2956] |
| ENSG00000213983 | HGLibA_02286 | 60 | -0.591972916 | AP1G2 | adaptor related protein complex 1 subunit gamma 2 [Source:HGNC Symbol;Acc:HGNC:556] |
| ENSG00000214946 | HGLibA_48518 | 60 | -0.591972916 | TBC1D26 | TBC1 domain family member 26 [Source:HGNC Symbol;Acc:HGNC:28745] |
| ENSG00000221916 | HGLibA_05705 | 60 | -0.591972916 | C19orf73 | chromosome 19 open reading frame 73 [Source:HGNC Symbol;Acc:HGNC:25534] |
| ENSG00000228517 | HGLibA_11543 | 60 | -0.591972916 | CT47A7 | member A7 [Source:HGNC Symbol;Acc:HGNC:33288]" |
| ENSG00000234616 | HGLibA_23992 | 60 | -0.591972916 | JRK | Jrk helix-turn-helix protein [Source:HGNC Symbol;Acc:HGNC:6199] |
| ENSG00000234906 | HGLibA_02505 | 60 | -0.591972916 | APOC2 | apolipoprotein C2 [Source:HGNC Symbol;Acc:HGNC:609] |
| ENSG00000242114 | HGLibA_30359 | 60 | -0.591972916 | MTFP1 | mitochondrial fission process 1 [Source:HGNC Symbol;Acc:HGNC:26945] |
| ENSG00000253719 | HGLibA_03862 | 60 | -0.591972916 | ATXN7L3B | ataxin 7 like 3B [Source:HGNC Symbol;Acc:HGNC:37931] |
| ENSG00000254834 | HGLibA_34330 | 60 | -0.591972916 | OR5M10 | olfactory receptor family 5 subfamily M member 10 [Source:HGNC Symbol;Acc:HGNC:15290] |
| ENSG00000255221 | HGLibA_07228 | 60 | -0.591972916 | CARD17 | caspase recruitment domain family member 17 [Source:HGNC Symbol;Acc:HGNC:33827] |
| ENSG00000258818 | HGLibA_41410 | 60 | -0.591972916 | RNASE4 | ribonuclease A family member 4 [Source:HGNC Symbol;Acc:HGNC:10047] |
| ENSG00000260287 | HGLibA_48551 | 60 | -0.591972916 | TBC1D3G | TBC1 domain family member 3G [Source:HGNC Symbol;Acc:HGNC:29860] |
| ENSG00000267795 | HGLibA_45753 | 60 | -0.591972916 | SMIM22 | small integral membrane protein 22 [Source:HGNC Symbol;Acc:HGNC:48329] |
| ENSG00000268182 | HGLibA_45738 | 60 | -0.591972916 | SMIM17 | small integral membrane protein 17 [Source:HGNC Symbol;Acc:HGNC:27114] |
| ENSG00000270181 | HGLibA_04535 | 60 | -0.591972916 | BIVM-ERCC5 | BIVM-ERCC5 readthrough [Source:HGNC Symbol;Acc:HGNC:43690] |
| ENSG00000272573 | HGLibA_30580 | 60 | -0.591972916 | MUSTN1 | embryonic nuclear protein 1 [Source:HGNC Symbol;Acc:HGNC:22144]" |
| ENSG00000272920 | HGLibA_57410 | 60 | -0.591972916 | hsa-mir-1253 | hsa-mir-1253 [Source:miRBase;Acc:MI0006387] |
| ENSG00000279170 | HGLibA_52036 | 60 | -0.591972916 | TSTD3 | TEC |
| FAM73A | HGLibA_16715 | 60 | -0.591972916 | FAM73A | - |
| GAREML | HGLibA_18628 | 60 | -0.591972916 | GAREML | - |
| HIST1H2BO | HGLibA_21452 | 60 | -0.591972916 | HIST1H2BO | - |
| hsa-mir-1244-2 | HGLibA_57367 | 60 | -0.591972916 | hsa-mir-1244-2 | - |
| hsa-mir-1265 | HGLibA_57485 | 60 | -0.591972916 | hsa-mir-1265 | - |
| hsa-mir-1973 | HGLibA_58117 | 60 | -0.591972916 | hsa-mir-1973 | - |
| hsa-mir-2278 | HGLibA_58315 | 60 | -0.591972916 | hsa-mir-2278 | - |
| hsa-mir-3147 | HGLibA_58644 | 60 | -0.591972916 | hsa-mir-3147 | - |
| hsa-mir-33b | HGLibA_59017 | 60 | -0.591972916 | hsa-mir-33b | - |
| hsa-mir-3661 | HGLibA_59196 | 60 | -0.591972916 | hsa-mir-3661 | - |
| hsa-mir-4265 | HGLibA_59768 | 60 | -0.591972916 | hsa-mir-4265 | - |
| hsa-mir-4295 | HGLibA_59890 | 60 | -0.591972916 | hsa-mir-4295 | - |
| hsa-mir-4425 | HGLibA_60086 | 60 | -0.591972916 | hsa-mir-4425 | - |
| hsa-mir-4435-1 | HGLibA_60129 | 60 | -0.591972916 | hsa-mir-4435-1 | - |
| hsa-mir-4667 | HGLibA_60787 | 60 | -0.591972916 | hsa-mir-4667 | - |
| hsa-mir-4676 | HGLibA_60820 | 60 | -0.591972916 | hsa-mir-4676 | - |
| hsa-mir-4803 | HGLibA_61317 | 60 | -0.591972916 | hsa-mir-4803 | - |
| hsa-mir-527 | HGLibA_61803 | 60 | -0.591972916 | hsa-mir-527 | - |
| hsa-mir-550a-1 | HGLibA_62099 | 60 | -0.591972916 | hsa-mir-550a-1 | - |
| hsa-mir-5572 | HGLibA_62150 | 60 | -0.591972916 | hsa-mir-5572 | - |
| hsa-mir-5702 | HGLibA_62350 | 60 | -0.591972916 | hsa-mir-5702 | - |
| hsa-mir-5705 | HGLibA_62360 | 60 | -0.591972916 | hsa-mir-5705 | - |
| hsa-mir-574 | HGLibA_62392 | 60 | -0.591972916 | hsa-mir-574 | - |
| hsa-mir-575 | HGLibA_62394 | 60 | -0.591972916 | hsa-mir-575 | - |
| hsa-mir-586 | HGLibA_62442 | 60 | -0.591972916 | hsa-mir-586 | - |
| hsa-mir-629 | HGLibA_62757 | 60 | -0.591972916 | hsa-mir-629 | - |
| hsa-mir-6506 | HGLibA_62874 | 60 | -0.591972916 | hsa-mir-6506 | - |
| hsa-mir-6511a-4 | HGLibA_62911 | 60 | -0.591972916 | hsa-mir-6511a-4 | - |
| hsa-mir-6719 | HGLibA_63037 | 60 | -0.591972916 | hsa-mir-6719 | - |
| hsa-mir-6726 | HGLibA_63056 | 60 | -0.591972916 | hsa-mir-6726 | - |
| hsa-mir-6792 | HGLibA_63347 | 60 | -0.591972916 | hsa-mir-6792 | - |
| hsa-mir-6835 | HGLibA_63516 | 60 | -0.591972916 | hsa-mir-6835 | - |
| ICT1 | HGLibA_22521 | 60 | -0.591972916 | ICT1 | - |
| LOC100287534 | HGLibA_26824 | 60 | -0.591972916 | LOC100287534 | - |
| LOC728819 | HGLibA_26993 | 60 | -0.591972916 | LOC728819 | - |
| NRD1 | HGLibA_32665 | 60 | -0.591972916 | NRD1 | - |
| TMEM35 | HGLibA_50373 | 60 | -0.591972916 | TMEM35 | - |
| ZAK | HGLibA_54773 | 60 | -0.591972916 | ZAK | - |
| ZNF664-FAM101A | HGLibA_56486 | 60 | -0.591972916 | ZNF664-FAM101A | - |
| ATP5D | HGLibA_03648 | 59 | -0.601018258 | ATP5D | - |
| C11orf63 | HGLibA_05167 | 59 | -0.601018258 | C11orf63 | - |
| C14orf105 | HGLibA_05317 | 59 | -0.601018258 | C14orf105 | - |
| C19orf35 | HGLibA_05652 | 59 | -0.601018258 | C19orf35 | - |
| C5orf54 | HGLibA_06407 | 59 | -0.601018258 | C5orf54 | - |
| CD99_Y | HGLibA_08492 | 59 | -0.601018258 | CD99_Y | - |
| ENSG00000004866 | HGLibA_47282 | 59 | -0.601018258 | ST7 | suppression of tumorigenicity 7 [Source:HGNC Symbol;Acc:HGNC:11351] |
| ENSG00000006194 | HGLibA_55693 | 59 | -0.601018258 | ZNF263 | zinc finger protein 263 [Source:HGNC Symbol;Acc:HGNC:13056] |
| ENSG00000006555 | HGLibA_52075 | 59 | -0.601018258 | TTC22 | tetratricopeptide repeat domain 22 [Source:HGNC Symbol;Acc:HGNC:26067] |
| ENSG00000006659 | HGLibA_26353 | 59 | -0.601018258 | LGALS14 | galectin 14 [Source:HGNC Symbol;Acc:HGNC:30054] |
| ENSG00000011485 | HGLibA_38211 | 59 | -0.601018258 | PPP5C | protein phosphatase 5 catalytic subunit [Source:HGNC Symbol;Acc:HGNC:9322] |
| ENSG00000014919 | HGLibA_10813 | 59 | -0.601018258 | COX15 | cytochrome c oxidase assembly homolog COX15 [Source:HGNC Symbol;Acc:HGNC:2263] |
| ENSG00000025434 | HGLibA_32584 | 59 | -0.601018258 | NR1H3 | nuclear receptor subfamily 1 group H member 3 [Source:HGNC Symbol;Acc:HGNC:7966] |
| ENSG00000035862 | HGLibA_49538 | 59 | -0.601018258 | TIMP2 | TIMP metallopeptidase inhibitor 2 [Source:HGNC Symbol;Acc:HGNC:11821] |
| ENSG00000041982 | HGLibA_50700 | 59 | -0.601018258 | TNC | tenascin C [Source:HGNC Symbol;Acc:HGNC:5318] |
| ENSG00000056291 | HGLibA_32442 | 59 | -0.601018258 | NPFFR2 | neuropeptide FF receptor 2 [Source:HGNC Symbol;Acc:HGNC:4525] |
| ENSG00000059728 | HGLibA_30610 | 59 | -0.601018258 | MXD1 | MAX dimerization protein 1 [Source:HGNC Symbol;Acc:HGNC:6761] |
| ENSG00000063046 | HGLibA_14813 | 59 | -0.601018258 | EIF4B | eukaryotic translation initiation factor 4B [Source:HGNC Symbol;Acc:HGNC:3285] |
| ENSG00000067836 | HGLibA_41752 | 59 | -0.601018258 | ROGDI | rogdi homolog [Source:HGNC Symbol;Acc:HGNC:29478] |
| ENSG00000070019 | HGLibA_20661 | 59 | -0.601018258 | GUCY2C | guanylate cyclase 2C [Source:HGNC Symbol;Acc:HGNC:4688] |
| ENSG00000070831 | HGLibA_08554 | 59 | -0.601018258 | CDC42 | cell division cycle 42 [Source:HGNC Symbol;Acc:HGNC:1736] |
| ENSG00000072310 | HGLibA_46950 | 59 | -0.601018258 | SREBF1 | sterol regulatory element binding transcription factor 1 [Source:HGNC Symbol;Acc:HGNC:11289] |
| ENSG00000079999 | HGLibA_24547 | 59 | -0.601018258 | KEAP1 | kelch like ECH associated protein 1 [Source:HGNC Symbol;Acc:HGNC:23177] |
| ENSG00000080493 | HGLibA_45221 | 59 | -0.601018258 | SLC4A4 | solute carrier family 4 member 4 [Source:HGNC Symbol;Acc:HGNC:11030] |

| | | | | | |
|---|---|---|---|---|---|
| ENSG00000080709 | HGLibA_24358 | 59 | -0.601018258 | KCNN2 | potassium calcium-activated channel subfamily N member 2 [Source:HGNC Symbol;Acc:HGNC:6291] |
| ENSG00000080823 | HGLibA_29636 | 59 | -0.601018258 | MOK | MOK protein kinase [Source:HGNC Symbol;Acc:HGNC:9833] |
| ENSG00000085840 | HGLibA_34602 | 59 | -0.601018258 | ORC1 | origin recognition complex subunit 1 [Source:HGNC Symbol;Acc:HGNC:8487] |
| ENSG00000090266 | HGLibA_31513 | 59 | -0.601018258 | NDUFB2 | NADH:ubiquinone oxidoreductase subunit B2 [Source:HGNC Symbol;Acc:HGNC:7697] |
| ENSG00000092140 | HGLibA_18295 | 59 | -0.601018258 | G2E3 | G2/M-phase specific E3 ubiquitin protein ligase [Source:HGNC Symbol;Acc:HGNC:20338] |
| ENSG00000092871 | HGLibA_40919 | 59 | -0.601018258 | RFFL | ring finger and FYVE like domain containing E3 ubiquitin protein ligase [Source:HGNC Symbol;Acc:HGNC:24821] |
| ENSG00000100065 | HGLibA_07217 | 59 | -0.601018258 | CARD10 | caspase recruitment domain family member 10 [Source:HGNC Symbol;Acc:HGNC:16422] |
| ENSG00000100246 | HGLibA_13648 | 59 | -0.601018258 | DNAL4 | dynein axonemal light chain 4 [Source:HGNC Symbol;Acc:HGNC:2955] |
| ENSG00000101079 | HGLibA_31418 | 59 | -0.601018258 | NDRG3 | NDRG family member 3 [Source:HGNC Symbol;Acc:HGNC:14462] |
| ENSG00000101194 | HGLibA_44483 | 59 | -0.601018258 | SLC17A9 | solute carrier family 17 member 9 [Source:HGNC Symbol;Acc:HGNC:16192] |
| ENSG00000102174 | HGLibA_36326 | 59 | -0.601018258 | PHEX | phosphate regulating endopeptidase homolog X-linked [Source:HGNC Symbol;Acc:HGNC:8918] |
| ENSG00000102309 | HGLibA_36686 | 59 | -0.601018258 | PIN4 | NIMA-interacting 4 [Source:HGNC Symbol;Acc:HGNC:8992]" |
| ENSG00000104419 | HGLibA_31413 | 59 | -0.601018258 | NDRG1 | N-myc downstream regulated 1 [Source:HGNC Symbol;Acc:HGNC:7679] |
| ENSG00000105372 | HGLibA_42095 | 59 | -0.601018258 | RPS19 | ribosomal protein S19 [Source:HGNC Symbol;Acc:HGNC:10402] |
| ENSG00000106128 | HGLibA_19045 | 59 | -0.601018258 | GHRHR | growth hormone releasing hormone receptor [Source:HGNC Symbol;Acc:HGNC:4266] |
| ENSG00000106823 | HGLibA_14416 | 59 | -0.601018258 | ECM2 | extracellular matrix protein 2 [Source:HGNC Symbol;Acc:HGNC:3154] |
| ENSG00000106976 | HGLibA_13684 | 59 | -0.601018258 | DNM1 | dynamin 1 [Source:HGNC Symbol;Acc:HGNC:2972] |
| ENSG00000107262 | HGLibA_04074 | 59 | -0.601018258 | BAG1 | BCL2 associated athanogene 1 [Source:HGNC Symbol;Acc:HGNC:937] |
| ENSG00000109065 | HGLibA_31191 | 59 | -0.601018258 | NAT9 | N-acetyltransferase 9 (putative) [Source:HGNC Symbol;Acc:HGNC:23133] |
| ENSG00000109099 | HGLibA_37323 | 59 | -0.601018258 | PMP22 | peripheral myelin protein 22 [Source:HGNC Symbol;Acc:HGNC:9118] |
| ENSG00000109101 | HGLibA_17909 | 59 | -0.601018258 | FOXN1 | forkhead box N1 [Source:HGNC Symbol;Acc:HGNC:12765] |
| ENSG00000109576 | HGLibA_00041 | 59 | -0.601018258 | AADAT | aminoadipate aminotransferase [Source:HGNC Symbol;Acc:HGNC:17929] |
| ENSG00000111266 | HGLibA_14149 | 59 | -0.601018258 | DUSP16 | dual specificity phosphatase 16 [Source:HGNC Symbol;Acc:HGNC:17909] |
| ENSG00000111424 | HGLibA_53609 | 59 | -0.601018258 | VDR | vitamin D receptor [Source:HGNC Symbol;Acc:HGNC:12679] |
| ENSG00000111665 | HGLibA_08605 | 59 | -0.601018258 | CDCA3 | cell division cycle associated 3 [Source:HGNC Symbol;Acc:HGNC:14624] |
| ENSG00000111834 | HGLibA_42336 | 59 | -0.601018258 | RSPH4A | radial spoke head component 4A [Source:HGNC Symbol;Acc:HGNC:21558] |
| ENSG00000112494 | HGLibA_53035 | 59 | -0.601018258 | UNC93A | unc-93 homolog A [Source:HGNC Symbol;Acc:HGNC:12570] |
| ENSG00000112659 | HGLibA_11795 | 59 | -0.601018258 | CUL9 | cullin 9 [Source:HGNC Symbol;Acc:HGNC:15982] |
| ENSG00000113580 | HGLibA_32620 | 59 | -0.601018258 | NR3C1 | nuclear receptor subfamily 3 group C member 1 [Source:HGNC Symbol;Acc:HGNC:7978] |
| ENSG00000114933 | HGLibA_23329 | 59 | -0.601018258 | INO80D | INO80 complex subunit D [Source:HGNC Symbol;Acc:HGNC:25997] |
| ENSG00000115112 | HGLibA_49149 | 59 | -0.601018258 | TFCP2L1 | transcription factor CP2 like 1 [Source:HGNC Symbol;Acc:HGNC:17925] |
| ENSG00000115241 | HGLibA_37977 | 59 | -0.601018258 | PPM1G | Mg2+/Mn2+ dependent 1G [Source:HGNC Symbol;Acc:HGNC:9278]" |
| ENSG00000115541 | HGLibA_22335 | 59 | -0.601018258 | HSPE1 | heat shock protein family E (Hsp10) member 1 [Source:HGNC Symbol;Acc:HGNC:5269] |
| ENSG00000115806 | HGLibA_19680 | 59 | -0.601018258 | GORASP2 | golgi reassembly stacking protein 2 [Source:HGNC Symbol;Acc:HGNC:17500] |
| ENSG00000116353 | HGLibA_28730 | 59 | -0.601018258 | MECR | mitochondrial trans-2-enoyl-CoA reductase [Source:HGNC Symbol;Acc:HGNC:19691] |
| ENSG00000116473 | HGLibA_40222 | 59 | -0.601018258 | RAP1A | member of RAS oncogene family [Source:HGNC Symbol;Acc:HGNC:9855]" |
| ENSG00000120279 | HGLibA_30682 | 59 | -0.601018258 | MYCT1 | MYC target 1 [Source:HGNC Symbol;Acc:HGNC:23172] |
| ENSG00000120903 | HGLibA_09567 | 59 | -0.601018258 | CHRNA2 | cholinergic receptor nicotinic alpha 2 subunit [Source:HGNC Symbol;Acc:HGNC:1956] |
| ENSG00000122863 | HGLibA_09635 | 59 | -0.601018258 | CHST3 | carbohydrate sulfotransferase 3 [Source:HGNC Symbol;Acc:HGNC:1971] |
| ENSG00000123415 | HGLibA_45843 | 59 | -0.601018258 | SMUG1 | single-strand-selective monofunctional uracil-DNA glycosylase 1 [Source:HGNC Symbol;Acc:HGNC:17148] |
| ENSG00000124767 | HGLibA_19282 | 59 | -0.601018258 | GLO1 | glyoxalase I [Source:HGNC Symbol;Acc:HGNC:4323] |
| ENSG00000125841 | HGLibA_32727 | 59 | -0.601018258 | NRSN2 | neurensin 2 [Source:HGNC Symbol;Acc:HGNC:16229] |
| ENSG00000125861 | HGLibA_18975 | 59 | -0.601018258 | GFRA4 | GDNF family receptor alpha 4 [Source:HGNC Symbol;Acc:HGNC:13821] |
| ENSG00000125885 | HGLibA_28624 | 59 | -0.601018258 | MCM8 | minichromosome maintenance 8 homologous recombination repair factor [Source:HGNC Symbol;Acc:HGNC:16147] |
| ENSG00000126759 | HGLibA_09290 | 59 | -0.601018258 | CFP | complement factor properdin [Source:HGNC Symbol;Acc:HGNC:8864] |
| ENSG00000127325 | HGLibA_04424 | 59 | -0.601018258 | BEST3 | bestrophin 3 [Source:HGNC Symbol;Acc:HGNC:17105] |
| ENSG00000127993 | HGLibA_40568 | 59 | -0.601018258 | RBM48 | RNA binding motif protein 48 [Source:HGNC Symbol;Acc:HGNC:21785] |
| ENSG00000128271 | HGLibA_01014 | 59 | -0.601018258 | ADORA2A | adenosine A2a receptor [Source:HGNC Symbol;Acc:HGNC:263] |
| ENSG00000129028 | HGLibA_49290 | 59 | -0.601018258 | THAP10 | THAP domain containing 10 [Source:HGNC Symbol;Acc:HGNC:23193] |
| ENSG00000129355 | HGLibA_08845 | 59 | -0.601018258 | CDKN2D | cyclin dependent kinase inhibitor 2D [Source:HGNC Symbol;Acc:HGNC:1790] |
| ENSG00000129535 | HGLibA_32702 | 59 | -0.601018258 | NRL | neural retina leucine zipper [Source:HGNC Symbol;Acc:HGNC:8002] |
| ENSG00000130147 | HGLibA_43931 | 59 | -0.601018258 | SH3BP4 | SH3 domain binding protein 4 [Source:HGNC Symbol;Acc:HGNC:10826] |
| ENSG00000130303 | HGLibA_04884 | 59 | -0.601018258 | BST2 | bone marrow stromal cell antigen 2 [Source:HGNC Symbol;Acc:HGNC:1119] |
| ENSG00000130943 | HGLibA_36810 | 59 | -0.601018258 | PKDREJ | polycystin family receptor for egg jelly [Source:HGNC Symbol;Acc:HGNC:9015] |
| ENSG00000131373 | HGLibA_20802 | 59 | -0.601018258 | HACL1 | 2-hydroxyacyl-CoA lyase 1 [Source:HGNC Symbol;Acc:HGNC:17856] |
| ENSG00000131711 | HGLibA_28118 | 59 | -0.601018258 | MAP1B | microtubule associated protein 1B [Source:HGNC Symbol;Acc:HGNC:6836] |
| ENSG00000131725 | HGLibA_54094 | 59 | -0.601018258 | WDR44 | WD repeat domain 44 [Source:HGNC Symbol;Acc:HGNC:30512] |
| ENSG00000132464 | HGLibA_15107 | 59 | -0.601018258 | ENAM | enamelin [Source:HGNC Symbol;Acc:HGNC:3344] |
| ENSG00000133114 | HGLibA_19723 | 59 | -0.601018258 | GPALPP1 | GPALPP motifs containing 1 [Source:HGNC Symbol;Acc:HGNC:20298] |
| ENSG00000133433 | HGLibA_20501 | 59 | -0.601018258 | GSTT2B | glutathione S-transferase theta 2B (gene/pseudogene) [Source:HGNC Symbol;Acc:HGNC:33437] |
| ENSG00000133466 | HGLibA_05952 | 59 | -0.601018258 | C1QTNF6 | C1q and TNF related 6 [Source:HGNC Symbol;Acc:HGNC:14343] |
| ENSG00000135097 | HGLibA_30204 | 59 | -0.601018258 | MSI1 | musashi RNA binding protein 1 [Source:HGNC Symbol;Acc:HGNC:7330] |
| ENSG00000135604 | HGLibA_47619 | 59 | -0.601018258 | STX11 | syntaxin 11 [Source:HGNC Symbol;Acc:HGNC:11429] |
| ENSG00000135643 | HGLibA_24352 | 59 | -0.601018258 | KCNMB4 | potassium calcium-activated channel subfamily M regulatory beta subunit 4 [Source:HGNC Symbol;Acc:HGNC:6289] |
| ENSG00000135736 | HGLibA_07509 | 59 | -0.601018258 | CCDC102A | coiled-coil domain containing 102A [Source:HGNC Symbol;Acc:HGNC:28097] |
| ENSG00000136155 | HGLibA_42889 | 59 | -0.601018258 | SCEL | sciellin [Source:HGNC Symbol;Acc:HGNC:10573] |
| ENSG00000136205 | HGLibA_50925 | 59 | -0.601018258 | TNS3 | tensin 3 [Source:HGNC Symbol;Acc:HGNC:21616] |
| ENSG00000136811 | HGLibA_33277 | 59 | -0.601018258 | ODF2 | outer dense fiber of sperm tails 2 [Source:HGNC Symbol;Acc:HGNC:8114] |
| ENSG00000138119 | HGLibA_30883 | 59 | -0.601018258 | MYOF | myoferlin [Source:HGNC Symbol;Acc:HGNC:3656] |
| ENSG00000138594 | HGLibA_50600 | 59 | -0.601018258 | TMOD3 | tropomodulin 3 [Source:HGNC Symbol;Acc:HGNC:11873] |
| ENSG00000138646 | HGLibA_21165 | 59 | -0.601018258 | HERC5 | HECT and RLD domain containing E3 ubiquitin protein ligase 5 [Source:HGNC Symbol;Acc:HGNC:24368] |
| ENSG00000138735 | HGLibA_35802 | 59 | -0.601018258 | PDE5A | phosphodiesterase 5A [Source:HGNC Symbol;Acc:HGNC:8784] |
| ENSG00000139218 | HGLibA_42824 | 59 | -0.601018258 | SCAF11 | SR-related CTD associated factor 11 [Source:HGNC Symbol;Acc:HGNC:10784] |
| ENSG00000139343 | HGLibA_45989 | 59 | -0.601018258 | SNRPF | small nuclear ribonucleoprotein polypeptide F [Source:HGNC Symbol;Acc:HGNC:11162] |
| ENSG00000141552 | HGLibA_01867 | 59 | -0.601018258 | ANAPC11 | anaphase promoting complex subunit 11 [Source:HGNC Symbol;Acc:HGNC:14452] |
| ENSG00000142173 | HGLibA_10599 | 59 | -0.601018258 | COL6A2 | collagen type VI alpha 2 chain [Source:HGNC Symbol;Acc:HGNC:2212] |
| ENSG00000143119 | HGLibA_08407 | 59 | -0.601018258 | CD53 | CD53 molecule [Source:HGNC Symbol;Acc:HGNC:1686] |
| ENSG00000143442 | HGLibA_37480 | 59 | -0.601018258 | POGZ | pogo transposable element derived with ZNF domain [Source:HGNC Symbol;Acc:HGNC:18801] |
| ENSG00000143545 | HGLibA_39810 | 59 | -0.601018258 | RAB13 | member RAS oncogene family [Source:HGNC Symbol;Acc:HGNC:9762]" |
| ENSG00000144677 | HGLibA_11613 | 59 | -0.601018258 | CTDSPL | CTD small phosphatase like [Source:HGNC Symbol;Acc:HGNC:16890] |
| ENSG00000145375 | HGLibA_46515 | 59 | -0.601018258 | SPATA5 | spermatogenesis associated 5 [Source:HGNC Symbol;Acc:HGNC:18119] |
| ENSG00000146374 | HGLibA_42349 | 59 | -0.601018258 | RSPO3 | R-spondin 3 [Source:HGNC Symbol;Acc:HGNC:20866] |
| ENSG00000146425 | HGLibA_14265 | 59 | -0.601018258 | DYNLT1 | dynein light chain Tctex-type 1 [Source:HGNC Symbol;Acc:HGNC:11697] |
| ENSG00000146857 | HGLibA_47570 | 59 | -0.601018258 | STRA8 | stimulated by retinoic acid 8 [Source:HGNC Symbol;Acc:HGNC:30653] |
| ENSG00000146909 | HGLibA_32286 | 59 | -0.601018258 | NOM1 | nucleolar protein with MIF4G domain 1 [Source:HGNC Symbol;Acc:HGNC:13244] |
| ENSG00000147081 | HGLibA_01433 | 59 | -0.601018258 | AKAP4 | A-kinase anchoring protein 4 [Source:HGNC Symbol;Acc:HGNC:374] |
| ENSG00000147138 | HGLibA_19972 | 59 | -0.601018258 | GPR174 | G protein-coupled receptor 174 [Source:HGNC Symbol;Acc:HGNC:30245] |
| ENSG00000148488 | HGLibA_47303 | 59 | -0.601018258 | ST8SIA6 | 8-sialyltransferase 6 [Source:HGNC Symbol;Acc:HGNC:23317]" |
| ENSG00000149091 | HGLibA_13084 | 59 | -0.601018258 | DGKZ | diacylglycerol kinase zeta [Source:HGNC Symbol;Acc:HGNC:2857] |
| ENSG00000149451 | HGLibA_00775 | 59 | -0.601018258 | ADAM33 | ADAM metallopeptidase domain 33 [Source:HGNC Symbol;Acc:HGNC:15478] |
| ENSG00000149483 | HGLibA_49954 | 59 | -0.601018258 | TMEM138 | transmembrane protein 138 [Source:HGNC Symbol;Acc:HGNC:26944] |
| ENSG00000151611 | HGLibA_29449 | 59 | -0.601018258 | MMAA | metabolism of cobalamin associated A [Source:HGNC Symbol;Acc:HGNC:18871] |
| ENSG00000155115 | HGLibA_20595 | 59 | -0.601018258 | GTF3C6 | general transcription factor IIIC subunit 6 [Source:HGNC Symbol;Acc:HGNC:20872] |
| ENSG00000155530 | HGLibA_27170 | 59 | -0.601018258 | LRGUK | leucine rich repeats and guanylate kinase domain containing [Source:HGNC Symbol;Acc:HGNC:21964] |
| ENSG00000156966 | HGLibA_03998 | 59 | -0.601018258 | B3GNT7 | 3-N-acetylglucosaminyltransferase 7 [Source:HGNC Symbol;Acc:HGNC:18811]" |
| ENSG00000157999 | HGLibA_02133 | 59 | -0.601018258 | ANKRD61 | ankyrin repeat domain 61 [Source:HGNC Symbol;Acc:HGNC:22467] |
| ENSG00000158042 | HGLibA_29912 | 59 | -0.601018258 | MRPL17 | mitochondrial ribosomal protein L17 [Source:HGNC Symbol;Acc:HGNC:14053] |
| ENSG00000158553 | HGLibA_37651 | 59 | -0.601018258 | POM121L2 | POM121 transmembrane nucleoporin like 2 [Source:HGNC Symbol;Acc:HGNC:13973] |
| ENSG00000158683 | HGLibA_36792 | 59 | -0.601018258 | PKD1L1 | transient receptor potential channel interacting [Source:HGNC Symbol;Acc:HGNC:18053]" |
| ENSG00000158850 | HGLibA_04028 | 59 | -0.601018258 | B4GALT3 | 4-galactosyltransferase 3 [Source:HGNC Symbol;Acc:HGNC:926]" |
| ENSG00000159140 | HGLibA_46172 | 59 | -0.601018258 | SON | SON DNA binding protein [Source:HGNC Symbol;Acc:HGNC:11183] |
| ENSG00000160211 | HGLibA_18313 | 59 | -0.601018258 | G6PD | glucose-6-phosphate dehydrogenase [Source:HGNC Symbol;Acc:HGNC:4057] |
| ENSG00000161057 | HGLibA_39129 | 59 | -0.601018258 | PSMC2 | ATPase 2 [Source:HGNC Symbol;Acc:HGNC:9548]" |
| ENSG00000161509 | HGLibA_20305 | 59 | -0.601018258 | GRIN2C | glutamate ionotropic receptor NMDA type subunit 2C [Source:HGNC Symbol;Acc:HGNC:4587] |
| ENSG00000162676 | HGLibA_18940 | 59 | -0.601018258 | GFI1 | growth factor independent 1 transcriptional repressor [Source:HGNC Symbol;Acc:HGNC:4237] |
| ENSG00000162924 | HGLibA_40810 | 59 | -0.601018258 | REL | NF-kB subunit [Source:HGNC Symbol;Acc:HGNC:9954]" |
| ENSG00000163141 | HGLibA_04662 | 59 | -0.601018258 | BNIPL | BCL2 interacting protein like [Source:HGNC Symbol;Acc:HGNC:16976] |
| ENSG00000163145 | HGLibA_05955 | 59 | -0.601018258 | C1QTNF7 | C1q and TNF related 7 [Source:HGNC Symbol;Acc:HGNC:14342] |
| ENSG00000163291 | HGLibA_35156 | 59 | -0.601018258 | PAQR3 | progestin and adipoQ receptor family member 3 [Source:HGNC Symbol;Acc:HGNC:30130] |
| ENSG00000163348 | HGLibA_39697 | 59 | -0.601018258 | PYGO2 | pygopus family PHD finger 2 [Source:HGNC Symbol;Acc:HGNC:30257] |
| ENSG00000163378 | HGLibA_15224 | 59 | -0.601018258 | EOGT | EGF domain specific O-linked N-acetylglucosamine transferase [Source:HGNC Symbol;Acc:HGNC:28526] |
| ENSG00000163444 | HGLibA_50104 | 59 | -0.601018258 | TMEM183A | transmembrane protein 183A [Source:HGNC Symbol;Acc:HGNC:20173] |
| ENSG00000163599 | HGLibA_11636 | 59 | -0.601018258 | CTLA4 | cytotoxic T-lymphocyte associated protein 4 [Source:HGNC Symbol;Acc:HGNC:2505] |
| ENSG00000163785 | HGLibA_42517 | 59 | -0.601018258 | RYK | receptor-like tyrosine kinase [Source:HGNC Symbol;Acc:HGNC:10481] |
| ENSG00000163832 | HGLibA_14990 | 59 | -0.601018258 | ELP6 | elongator acetyltransferase complex subunit 6 [Source:HGNC Symbol;Acc:HGNC:25976] |
| ENSG00000163877 | HGLibA_45930 | 59 | -0.601018258 | SNIP1 | Smad nuclear interacting protein 1 [Source:HGNC Symbol;Acc:HGNC:30587] |
| ENSG00000164062 | HGLibA_02404 | 59 | -0.601018258 | APEH | acylaminoacyl-peptide hydrolase [Source:HGNC Symbol;Acc:HGNC:586] |
| ENSG00000164066 | HGLibA_23438 | 59 | -0.601018258 | INTU | inturned planar cell polarity protein [Source:HGNC Symbol;Acc:HGNC:29239] |
| ENSG00000164091 | HGLibA_54191 | 59 | -0.601018258 | WDR82 | WD repeat domain 82 [Source:HGNC Symbol;Acc:HGNC:28826] |
| ENSG00000164190 | HGLibA_31967 | 59 | -0.601018258 | NIPBL | cohesin loading factor [Source:HGNC Symbol;Acc:HGNC:28862]" |
| ENSG00000164332 | HGLibA_52716 | 59 | -0.601018258 | UBLCP1 | ubiquitin like domain containing CTD phosphatase 1 [Source:HGNC Symbol;Acc:HGNC:28110] |
| ENSG00000165269 | HGLibA_02605 | 59 | -0.601018258 | AQP7 | aquaporin 7 [Source:HGNC Symbol;Acc:HGNC:640] |
| ENSG00000165457 | HGLibA_17787 | 59 | -0.601018258 | FOLR2 | folate receptor beta [Source:HGNC Symbol;Acc:HGNC:3793] |
| ENSG00000165807 | HGLibA_38091 | 59 | -0.601018258 | PPP1R36 | protein phosphatase 1 regulatory subunit 36 [Source:HGNC Symbol;Acc:HGNC:20097] |
| ENSG00000165891 | HGLibA_14326 | 59 | -0.601018258 | E2F7 | E2F transcription factor 7 [Source:HGNC Symbol;Acc:HGNC:23820] |
| ENSG00000166337 | HGLibA_48198 | 59 | -0.601018258 | TAF10 | TATA-box binding protein associated factor 10 [Source:HGNC Symbol;Acc:HGNC:11543] |
| ENSG00000166813 | HGLibA_24960 | 59 | -0.601018258 | KIF7 | kinesin family member 7 [Source:HGNC Symbol;Acc:HGNC:30497] |
| ENSG00000166839 | HGLibA_01945 | 59 | -0.601018258 | ANKDD1A | ankyrin repeat and death domain containing 1A [Source:HGNC Symbol;Acc:HGNC:28002] |
| ENSG00000168280 | HGLibA_24955 | 59 | -0.601018258 | KIF5C | kinesin family member 5C [Source:HGNC Symbol;Acc:HGNC:6325] |
| ENSG00000168427 | HGLibA_25180 | 59 | -0.601018258 | KLHL30 | kelch like family member 30 [Source:HGNC Symbol;Acc:HGNC:24770] |
| ENSG00000168610 | HGLibA_47388 | 59 | -0.601018258 | STAT3 | signal transducer and activator of transcription 3 [Source:HGNC Symbol;Acc:HGNC:11364] |
| ENSG00000168724 | HGLibA_13596 | 59 | -0.601018258 | DNAJC21 | DnaJ heat shock protein family (Hsp40) member C21 [Source:HGNC Symbol;Acc:HGNC:27030] |
| ENSG00000169193 | HGLibA_07580 | 59 | -0.601018258 | CCDC126 | coiled-coil domain containing 126 [Source:HGNC Symbol;Acc:HGNC:22398] |
| ENSG00000169217 | HGLibA_08330 | 59 | -0.601018258 | CD2BP2 | CD2 cytoplasmic tail binding protein 2 [Source:HGNC Symbol;Acc:HGNC:1656] |
| ENSG00000170122 | HGLibA_17836 | 59 | -0.601018258 | FOXD4 | forkhead box D4 [Source:HGNC Symbol;Acc:HGNC:3805] |

| | | | | | |
|---|---|---|---|---|---|
| ENSG00000170166 | HGLibA_22000 | 59 | -0.601018258 | HOXD4 | homeobox D4 [Source:HGNC Symbol;Acc:HGNC:5138] |
| ENSG00000170426 | HGLibA_43168 | 59 | -0.601018258 | SDR9C7 | short chain dehydrogenase/reductase family 9C member 7 [Source:HGNC Symbol;Acc:HGNC:29958] |
| ENSG00000171100 | HGLibA_30407 | 59 | -0.601018258 | MTM1 | myotubularin 1 [Source:HGNC Symbol;Acc:HGNC:7448] |
| ENSG00000171116 | HGLibA_22234 | 59 | -0.601018258 | HSFX1 | X-linked 1 [Source:HGNC Symbol;Acc:HGNC:29603]" |
| ENSG00000171302 | HGLibA_07126 | 59 | -0.601018258 | CANT1 | calcium activated nucleotidase 1 [Source:HGNC Symbol;Acc:HGNC:19721] |
| ENSG00000172459 | HGLibA_34248 | 59 | -0.601018258 | OR5AR1 | olfactory receptor family 5 subfamily AR member 1 (gene/pseudogene) [Source:HGNC Symbol;Acc:HGNC:15260] |
| ENSG00000172468 | HGLibA_22240 | 59 | -0.601018258 | HSFY1 | heat shock transcription factor Y-linked 1 [Source:HGNC Symbol;Acc:HGNC:18568] |
| ENSG00000174156 | HGLibA_20458 | 59 | -0.601018258 | GSTA3 | glutathione S-transferase alpha 3 [Source:HGNC Symbol;Acc:HGNC:4628] |
| ENSG00000174695 | HGLibA_50039 | 59 | -0.601018258 | TMEM167A | transmembrane protein 167A [Source:HGNC Symbol;Acc:HGNC:28330] |
| ENSG00000174943 | HGLibA_24422 | 59 | -0.601018258 | KCTD13 | potassium channel tetramerization domain containing 13 [Source:HGNC Symbol;Acc:HGNC:22234] |
| ENSG00000175536 | HGLibA_26635 | 59 | -0.601018258 | LIPT2 | lipoyl(octanoyl) transferase 2 [Source:HGNC Symbol;Acc:HGNC:37216] |
| ENSG00000176281 | HGLibA_34035 | 59 | -0.601018258 | OR4K5 | olfactory receptor family 4 subfamily K member 5 [Source:HGNC Symbol;Acc:HGNC:14745] |
| ENSG00000176658 | HGLibA_30830 | 59 | -0.601018258 | MYO1D | myosin ID [Source:HGNC Symbol;Acc:HGNC:7598] |
| ENSG00000177558 | HGLibA_16342 | 59 | -0.601018258 | FAM187B | family with sequence similarity 187 member B [Source:HGNC Symbol;Acc:HGNC:26366] |
| ENSG00000177971 | HGLibA_23238 | 59 | -0.601018258 | IMP3 | U3 small nucleolar ribonucleoprotein [Source:HGNC Symbol;Acc:HGNC:14497]" |
| ENSG00000179213 | HGLibA_44157 | 59 | -0.601018258 | SIGLECL1 | SIGLEC family like 1 [Source:HGNC Symbol;Acc:HGNC:26856] |
| ENSG00000179241 | HGLibA_26247 | 59 | -0.601018258 | LDLRAD3 | low density lipoprotein receptor class A domain containing 3 [Source:HGNC Symbol;Acc:HGNC:27046] |
| ENSG00000179933 | HGLibA_05321 | 59 | -0.601018258 | C14orf119 | chromosome 14 open reading frame 119 [Source:HGNC Symbol;Acc:HGNC:20270] |
| ENSG00000180660 | HGLibA_27820 | 59 | -0.601018258 | MAB21L1 | mab-21 like 1 [Source:HGNC Symbol;Acc:HGNC:6757] |
| ENSG00000180694 | HGLibA_50484 | 59 | -0.601018258 | TMEM64 | transmembrane protein 64 [Source:HGNC Symbol;Acc:HGNC:25441] |
| ENSG00000182218 | HGLibA_21265 | 59 | -0.601018258 | HHIPL1 | HHIP like 1 [Source:HGNC Symbol;Acc:HGNC:19710] |
| ENSG00000182330 | HGLibA_38328 | 59 | -0.601018258 | PRAMEF8 | PRAME family member 8 [Source:HGNC Symbol;Acc:HGNC:24074] |
| ENSG00000182359 | HGLibA_24106 | 59 | -0.601018258 | KBTBD3 | kelch repeat and BTB domain containing 3 [Source:HGNC Symbol;Acc:HGNC:22934] |
| ENSG00000183111 | HGLibA_02854 | 59 | -0.601018258 | ARHGEF37 | Rho guanine nucleotide exchange factor 37 [Source:HGNC Symbol;Acc:HGNC:34430] |
| ENSG00000184613 | HGLibA_31685 | 59 | -0.601018258 | NELL2 | neural EGFL like 2 [Source:HGNC Symbol;Acc:HGNC:7751] |
| ENSG00000185304 | HGLibA_41029 | 59 | -0.601018258 | RGPD2 | RANBP2-like and GRIP domain containing 2 [Source:HGNC Symbol;Acc:HGNC:32415] |
| ENSG00000185619 | HGLibA_35598 | 59 | -0.601018258 | PCGF3 | polycomb group ring finger 3 [Source:HGNC Symbol;Acc:HGNC:10066] |
| ENSG00000185974 | HGLibA_20325 | 59 | -0.601018258 | GRK1 | G protein-coupled receptor kinase 1 [Source:HGNC Symbol;Acc:HGNC:10013] |
| ENSG00000187627 | HGLibA_41025 | 59 | -0.601018258 | RGPD1 | RANBP2-like and GRIP domain containing 1 [Source:HGNC Symbol;Acc:HGNC:32414] |
| ENSG00000188033 | HGLibA_56088 | 59 | -0.601018258 | ZNF490 | zinc finger protein 490 [Source:HGNC Symbol;Acc:HGNC:23705] |
| ENSG00000188219 | HGLibA_37728 | 59 | -0.601018258 | POTEE | POTE ankyrin domain family member E [Source:HGNC Symbol;Acc:HGNC:33895] |
| ENSG00000188334 | HGLibA_04874 | 59 | -0.601018258 | BSPH1 | binder of sperm protein homolog 1 [Source:HGNC Symbol;Acc:HGNC:33906] |
| ENSG00000189144 | HGLibA_56285 | 59 | -0.601018258 | ZNF573 | zinc finger protein 573 [Source:HGNC Symbol;Acc:HGNC:26420] |
| ENSG00000189308 | HGLibA_26570 | 59 | -0.601018258 | LIN54 | lin-54 DREAM MuvB core complex component [Source:HGNC Symbol;Acc:HGNC:25397] |
| ENSG00000196228 | HGLibA_47750 | 59 | -0.601018258 | SULT1C3 | sulfotransferase family 1C member 3 [Source:HGNC Symbol;Acc:HGNC:33543] |
| ENSG00000196337 | HGLibA_09310 | 59 | -0.601018258 | CGB7 | chorionic gonadotropin subunit beta 7 [Source:HGNC Symbol;Acc:HGNC:16451] |
| ENSG00000196420 | HGLibA_42560 | 59 | -0.601018258 | S100A5 | S100 calcium binding protein A5 [Source:HGNC Symbol;Acc:HGNC:10495] |
| ENSG00000196876 | HGLibA_43005 | 59 | -0.601018258 | SCN8A | sodium voltage-gated channel alpha subunit 8 [Source:HGNC Symbol;Acc:HGNC:10596] |
| ENSG00000197790 | HGLibA_34191 | 59 | -0.601018258 | OR52M1 | olfactory receptor family 52 subfamily M member 1 [Source:HGNC Symbol;Acc:HGNC:15225] |
| ENSG00000197894 | HGLibA_00959 | 59 | -0.601018258 | ADH5 | chi polypeptide [Source:HGNC Symbol;Acc:HGNC:253]" |
| ENSG00000198077 | HGLibA_12110 | 59 | -0.601018258 | CYP2A7 | cytochrome P450 family 2 subfamily A member 7 [Source:HGNC Symbol;Acc:HGNC:2611] |
| ENSG00000198517 | HGLibA_27885 | 59 | -0.601018258 | MAFK | MAF bZIP transcription factor K [Source:HGNC Symbol;Acc:HGNC:6782] |
| ENSG00000198814 | HGLibA_19196 | 59 | -0.601018258 | GK | glycerol kinase [Source:HGNC Symbol;Acc:HGNC:4289] |
| ENSG00000203747 | HGLibA_17195 | 59 | -0.601018258 | FCGR3A | Fc fragment of IgG receptor IIIa [Source:HGNC Symbol;Acc:HGNC:3619] |
| ENSG00000204403 | HGLibA_07302 | 59 | -0.601018258 | CASP12 | caspase 12 (gene/pseudogene) [Source:HGNC Symbol;Acc:HGNC:19004] |
| ENSG00000204472 | HGLibA_01331 | 59 | -0.601018258 | AIF1 | allograft inflammatory factor 1 [Source:HGNC Symbol;Acc:HGNC:352] |
| ENSG00000205209 | HGLibA_42928 | 59 | -0.601018258 | SCGB2B2 | secretoglobin family 2B member 2 [Source:HGNC Symbol;Acc:HGNC:27616] |
| ENSG00000205531 | HGLibA_31119 | 59 | -0.601018258 | NAP1L4 | nucleosome assembly protein 1 like 4 [Source:HGNC Symbol;Acc:HGNC:7640] |
| ENSG00000205669 | HGLibA_00485 | 59 | -0.601018258 | ACOT6 | acyl-CoA thioesterase 6 [Source:HGNC Symbol;Acc:HGNC:33159] |
| ENSG00000205718 | HGLibA_28481 | 59 | -0.601018258 | MBD3L4 | methyl-CpG binding domain protein 3 like 4 [Source:HGNC Symbol;Acc:HGNC:37206] |
| ENSG00000205981 | HGLibA_13592 | 59 | -0.601018258 | DNAJC19 | DnaJ heat shock protein family (Hsp40) member C19 [Source:HGNC Symbol;Acc:HGNC:30528] |
| ENSG00000213064 | HGLibA_43754 | 59 | -0.601018258 | SFT2D2 | SFT2 domain containing 2 [Source:HGNC Symbol;Acc:HGNC:25140] |
| ENSG00000213413 | HGLibA_39620 | 59 | -0.601018258 | PVRIG | PVR related immunoglobulin domain containing [Source:HGNC Symbol;Acc:HGNC:32190] |
| ENSG00000213996 | HGLibA_49712 | 59 | -0.601018258 | TM6SF2 | transmembrane 6 superfamily member 2 [Source:HGNC Symbol;Acc:HGNC:11861] |
| ENSG00000215375 | HGLibA_30765 | 59 | -0.601018258 | MYL5 | myosin light chain 5 [Source:HGNC Symbol;Acc:HGNC:7586] |
| ENSG00000221818 | HGLibA_14352 | 59 | -0.601018258 | EBF2 | EBF transcription factor 2 [Source:HGNC Symbol;Acc:HGNC:19090] |
| ENSG00000226685 | HGLibA_11525 | 59 | -0.601018258 | CT47A12 | member A12 [Source:HGNC Symbol;Acc:HGNC:33292]" |
| ENSG00000229665 | HGLibA_38805 | 59 | -0.601018258 | PRR20C | proline rich 20C [Source:HGNC Symbol;Acc:HGNC:37221] |
| ENSG00000229972 | HGLibA_23509 | 59 | -0.601018258 | IQCF3 | IQ motif containing F3 [Source:HGNC Symbol;Acc:HGNC:31816] |
| ENSG00000230347 | HGLibA_11546 | 59 | -0.601018258 | CT47A8 | member A8 [Source:HGNC Symbol;Acc:HGNC:33289]" |
| ENSG00000236126 | HGLibA_11531 | 59 | -0.601018258 | CT47A3 | member A3 [Source:HGNC Symbol;Acc:HGNC:33284]" |
| ENSG00000244362 | HGLibA_25682 | 59 | -0.601018258 | KRTAP19-7 | keratin associated protein 19-7 [Source:HGNC Symbol;Acc:HGNC:18942] |
| ENSG00000250745 | HGLibA_53246 | 59 | -0.601018258 | USP17L20 | ubiquitin specific peptidase 17-like family member 20 [Source:HGNC Symbol;Acc:HGNC:44448] |
| ENSG00000283632 | HGLibA_15742 | 59 | -0.601018258 | EXOC3L2 | exocyst complex component 3 like 2 [Source:HGNC Symbol;Acc:HGNC:30162] |
| FAM69B | HGLibA_16679 | 59 | -0.601018258 | FAM69B | - |
| HIST1H2AH | HGLibA_21392 | 59 | -0.601018258 | HIST1H2AH | - |
| HIST1H4C | HGLibA_21491 | 59 | -0.601018258 | HIST1H4C | - |
| HMHA1 | HGLibA_21738 | 59 | -0.601018258 | HMHA1 | - |
| hsa-mir-1202 | HGLibA_57264 | 59 | -0.601018258 | hsa-mir-1202 | - |
| hsa-mir-1238 | HGLibA_57342 | 59 | -0.601018258 | hsa-mir-1238 | - |
| hsa-mir-1291 | HGLibA_57642 | 59 | -0.601018258 | hsa-mir-1291 | - |
| hsa-mir-1469 | HGLibA_57852 | 59 | -0.601018258 | hsa-mir-1469 | - |
| hsa-mir-147b | HGLibA_57876 | 59 | -0.601018258 | hsa-mir-147b | - |
| hsa-mir-26a-1 | HGLibA_58366 | 59 | -0.601018258 | hsa-mir-26a-1 | - |
| hsa-mir-3179-1 | HGLibA_58791 | 59 | -0.601018258 | hsa-mir-3179-1 | - |
| hsa-mir-3183 | HGLibA_58830 | 59 | -0.601018258 | hsa-mir-3183 | - |
| hsa-mir-3942 | HGLibA_59627 | 59 | -0.601018258 | hsa-mir-3942 | - |
| hsa-mir-4444-1 | HGLibA_60180 | 59 | -0.601018258 | hsa-mir-4444-1 | - |
| hsa-mir-4452 | HGLibA_60216 | 59 | -0.601018258 | hsa-mir-4452 | - |
| hsa-mir-4634 | HGLibA_60641 | 59 | -0.601018258 | hsa-mir-4634 | - |
| hsa-mir-4692 | HGLibA_60880 | 59 | -0.601018258 | hsa-mir-4692 | - |
| hsa-mir-4732 | HGLibA_61027 | 59 | -0.601018258 | hsa-mir-4732 | - |
| hsa-mir-539 | HGLibA_61812 | 59 | -0.601018258 | hsa-mir-539 | - |
| hsa-mir-548ae-1 | HGLibA_61868 | 59 | -0.601018258 | hsa-mir-548ae-1 | - |
| hsa-mir-5692a-2 | HGLibA_62292 | 59 | -0.601018258 | hsa-mir-5692a-2 | - |
| hsa-mir-6756 | HGLibA_63182 | 59 | -0.601018258 | hsa-mir-6756 | - |
| hsa-mir-6789 | HGLibA_63335 | 59 | -0.601018258 | hsa-mir-6789 | - |
| hsa-mir-6797 | HGLibA_63364 | 59 | -0.601018258 | hsa-mir-6797 | - |
| hsa-mir-6854 | HGLibA_63588 | 59 | -0.601018258 | hsa-mir-6854 | - |
| hsa-mir-8088 | HGLibA_64194 | 59 | -0.601018258 | hsa-mir-8088 | - |
| hsa-mir-891a | HGLibA_64241 | 59 | -0.601018258 | hsa-mir-891a | - |
| LOC100289561 | HGLibA_26842 | 59 | -0.601018258 | LOC100289561 | - |
| MGEA5 | HGLibA_29156 | 59 | -0.601018258 | MGEA5 | - |
| PNMAL1 | HGLibA_37385 | 59 | -0.601018258 | PNMAL1 | - |
| SMCR7 | HGLibA_45677 | 59 | -0.601018258 | SMCR7 | - |
| TMEM55B | HGLibA_50446 | 59 | -0.601018258 | TMEM55B | - |
| WBSCR28 | HGLibA_54002 | 59 | -0.601018258 | WBSCR28 | - |
| ADPRHL2 | HGLibA_01032 | 58 | -0.610063601 | ADPRHL2 | - |
| ADRBK2 | HGLibA_01067 | 58 | -0.610063601 | ADRBK2 | - |
| ATP5J2-PTCD1 | HGLibA_03676 | 58 | -0.610063601 | ATP5J2-PTCD1 | - |
| C10orf54 | HGLibA_05076 | 58 | -0.610063601 | C10orf54 | - |
| C10orf54 | HGLibA_05075 | 58 | -0.610063601 | C10orf54 | - |
| C12orf61 | HGLibA_05277 | 58 | -0.610063601 | C12orf61 | - |
| C19orf52 | HGLibA_05675 | 58 | -0.610063601 | C19orf52 | - |
| C1orf110 | HGLibA_05743 | 58 | -0.610063601 | C1orf110 | - |
| CD99_X | HGLibA_08488 | 58 | -0.610063601 | CD99_X | - |
| CSF2RA_X | HGLibA_11330 | 58 | -0.610063601 | CSF2RA_X | - |
| ENSG00000003509 | HGLibA_31500 | 58 | -0.610063601 | NDUFAF7 | NADH:ubiquinone oxidoreductase complex assembly factor 7 [Source:HGNC Symbol;Acc:HGNC:28816] |
| ENSG00000004534 | HGLibA_40578 | 58 | -0.610063601 | RBM6 | RNA binding motif protein 6 [Source:HGNC Symbol;Acc:HGNC:9903] |
| ENSG00000038945 | HGLibA_30234 | 58 | -0.610063601 | MSR1 | macrophage scavenger receptor 1 [Source:HGNC Symbol;Acc:HGNC:7376] |
| ENSG00000047644 | HGLibA_54446 | 58 | -0.610063601 | WWC3 | WWC family member 3 [Source:HGNC Symbol;Acc:HGNC:29237] |
| ENSG00000048028 | HGLibA_53321 | 58 | -0.610063601 | USP28 | ubiquitin specific peptidase 28 [Source:HGNC Symbol;Acc:HGNC:12625] |
| ENSG00000051128 | HGLibA_21871 | 58 | -0.610063601 | HOMER3 | homer scaffold protein 3 [Source:HGNC Symbol;Acc:HGNC:17514] |
| ENSG00000060971 | HGLibA_00340 | 58 | -0.610063601 | ACAA1 | acetyl-CoA acyltransferase 1 [Source:HGNC Symbol;Acc:HGNC:82] |
| ENSG00000064763 | HGLibA_16871 | 58 | -0.610063601 | FAR2 | fatty acyl-CoA reductase 2 [Source:HGNC Symbol;Acc:HGNC:25531] |
| ENSG00000064932 | HGLibA_42810 | 58 | -0.610063601 | SBNO2 | strawberry notch homolog 2 [Source:HGNC Symbol;Acc:HGNC:29158] |
| ENSG00000065615 | HGLibA_12009 | 58 | -0.610063601 | CYB5R4 | cytochrome b5 reductase 4 [Source:HGNC Symbol;Acc:HGNC:20147] |
| ENSG00000066468 | HGLibA_17445 | 58 | -0.610063601 | FGFR2 | fibroblast growth factor receptor 2 [Source:HGNC Symbol;Acc:HGNC:3689] |
| ENSG00000069275 | HGLibA_32916 | 58 | -0.610063601 | NUCKS1 | nuclear casein kinase and cyclin dependent kinase substrate 1 [Source:HGNC Symbol;Acc:HGNC:29923] |
| ENSG00000070950 | HGLibA_40061 | 58 | -0.610063601 | RAD18 | E3 ubiquitin protein ligase [Source:HGNC Symbol;Acc:HGNC:18278]" |
| ENSG00000071677 | HGLibA_38599 | 58 | -0.610063601 | PRLH | prolactin releasing hormone [Source:HGNC Symbol;Acc:HGNC:17945] |
| ENSG00000078081 | HGLibA_25984 | 58 | -0.610063601 | LAMP3 | lysosomal associated membrane protein 3 [Source:HGNC Symbol;Acc:HGNC:14582] |
| ENSG00000079308 | HGLibA_50922 | 58 | -0.610063601 | TNS1 | tensin 1 [Source:HGNC Symbol;Acc:HGNC:11973] |
| ENSG00000080822 | HGLibA_09916 | 58 | -0.610063601 | CLDND1 | claudin domain containing 1 [Source:HGNC Symbol;Acc:HGNC:1322] |
| ENSG00000085662 | HGLibA_01471 | 58 | -0.610063601 | AKR1B1 | aldo-keto reductase family 1 member B [Source:HGNC Symbol;Acc:HGNC:381] |
| ENSG00000085760 | HGLibA_30396 | 58 | -0.610063601 | MTIF2 | mitochondrial translational initiation factor 2 [Source:HGNC Symbol;Acc:HGNC:7441] |
| ENSG00000085840 | HGLibA_34601 | 58 | -0.610063601 | ORC1 | origin recognition complex subunit 1 [Source:HGNC Symbol;Acc:HGNC:8487] |
| ENSG00000087258 | HGLibA_19462 | 58 | -0.610063601 | GNAO1 | G protein subunit alpha o1 [Source:HGNC Symbol;Acc:HGNC:4389] |
| ENSG00000089558 | HGLibA_24218 | 58 | -0.610063601 | KCNH4 | potassium voltage-gated channel subfamily H member 4 [Source:HGNC Symbol;Acc:HGNC:6253] |
| ENSG00000089775 | HGLibA_54851 | 58 | -0.610063601 | ZBTB25 | zinc finger and BTB domain containing 25 [Source:HGNC Symbol;Acc:HGNC:13112] |
| ENSG00000089818 | HGLibA_31594 | 58 | -0.610063601 | NECAP1 | NECAP endocytosis associated 1 [Source:HGNC Symbol;Acc:HGNC:24539] |
| ENSG00000096093 | HGLibA_14569 | 58 | -0.610063601 | EFHC1 | EF-hand domain containing 1 [Source:HGNC Symbol;Acc:HGNC:16406] |
| ENSG00000100207 | HGLibA_48771 | 58 | -0.610063601 | TCF20 | transcription factor 20 [Source:HGNC Symbol;Acc:HGNC:11631] |
| ENSG00000100242 | HGLibA_47790 | 58 | -0.610063601 | SUN2 | Sad1 and UNC84 domain containing 2 [Source:HGNC Symbol;Acc:HGNC:14210] |
| ENSG00000100604 | HGLibA_09432 | 58 | -0.610063601 | CHGA | chromogranin A [Source:HGNC Symbol;Acc:HGNC:1929] |

| | | | | | |
|---|---|---|---|---|---|
| ENSG00000100605 | HGLibA_23840 | 58 | -0.610063601 | ITPK1 | inositol-tetrakisphosphate 1-kinase [Source:HGNC Symbol;Acc:HGNC:6177] |
| ENSG00000100906 | HGLibA_31822 | 58 | -0.610063601 | NFKBIA | NFKB inhibitor alpha [Source:HGNC Symbol;Acc:HGNC:7797] |
| ENSG00000101883 | HGLibA_41214 | 58 | -0.610063601 | RHOXF1 | Rhox homeobox family member 1 [Source:HGNC Symbol;Acc:HGNC:29993] |
| ENSG00000102109 | HGLibA_35671 | 58 | -0.610063601 | PCSK1N | proprotein convertase subtilisin/kexin type 1 inhibitor [Source:HGNC Symbol;Acc:HGNC:17301] |
| ENSG00000102935 | HGLibA_55959 | 58 | -0.610063601 | ZNF423 | zinc finger protein 423 [Source:HGNC Symbol;Acc:HGNC:16762] |
| ENSG00000102962 | HGLibA_07996 | 58 | -0.610063601 | CCL22 | C-C motif chemokine ligand 22 [Source:HGNC Symbol;Acc:HGNC:10621] |
| ENSG00000103310 | HGLibA_56952 | 58 | -0.610063601 | ZP2 | zona pellucida glycoprotein 2 [Source:HGNC Symbol;Acc:HGNC:13188] |
| ENSG00000103490 | HGLibA_39671 | 58 | -0.610063601 | PYCARD | PYD and CARD domain containing [Source:HGNC Symbol;Acc:HGNC:16608] |
| ENSG00000105793 | HGLibA_20601 | 58 | -0.610063601 | GTPBP10 | GTP binding protein 10 [Source:HGNC Symbol;Acc:HGNC:25106] |
| ENSG00000107338 | HGLibA_43993 | 58 | -0.610063601 | SHB | SH2 domain containing adaptor protein B [Source:HGNC Symbol;Acc:HGNC:10838] |
| ENSG00000108821 | HGLibA_10531 | 58 | -0.610063601 | COL1A1 | collagen type I alpha 1 chain [Source:HGNC Symbol;Acc:HGNC:2197] |
| ENSG00000109083 | HGLibA_22751 | 58 | -0.610063601 | IFT20 | intraflagellar transport 20 [Source:HGNC Symbol;Acc:HGNC:30989] |
| ENSG00000110717 | HGLibA_31566 | 58 | -0.610063601 | NDUFS8 | NADH:ubiquinone oxidoreductase core subunit S8 [Source:HGNC Symbol;Acc:HGNC:7715] |
| ENSG00000110917 | HGLibA_29368 | 58 | -0.610063601 | MLEC | malectin [Source:HGNC Symbol;Acc:HGNC:28973] |
| ENSG00000112039 | HGLibA_16846 | 58 | -0.610063601 | FANCE | FA complementation group E [Source:HGNC Symbol;Acc:HGNC:3586] |
| ENSG00000112541 | HGLibA_35759 | 58 | -0.610063601 | PDE10A | phosphodiesterase 10A [Source:HGNC Symbol;Acc:HGNC:8772] |
| ENSG00000113734 | HGLibA_04649 | 58 | -0.610063601 | BNIP1 | BCL2 interacting protein 1 [Source:HGNC Symbol;Acc:HGNC:1082] |
| ENSG00000114120 | HGLibA_44720 | 58 | -0.610063601 | SLC25A36 | solute carrier family 25 member 36 [Source:HGNC Symbol;Acc:HGNC:25554] |
| ENSG00000114455 | HGLibA_21270 | 58 | -0.610063601 | HHLA2 | HERV-H LTR-associating 2 [Source:HGNC Symbol;Acc:HGNC:4905] |
| ENSG00000114902 | HGLibA_46559 | 58 | -0.610063601 | SPCS1 | signal peptidase complex subunit 1 [Source:HGNC Symbol;Acc:HGNC:23401] |
| ENSG00000115507 | HGLibA_34783 | 58 | -0.610063601 | OTX1 | orthodenticle homeobox 1 [Source:HGNC Symbol;Acc:HGNC:8521] |
| ENSG00000115604 | HGLibA_23027 | 58 | -0.610063601 | IL18R1 | interleukin 18 receptor 1 [Source:HGNC Symbol;Acc:HGNC:5988] |
| ENSG00000115657 | HGLibA_00146 | 58 | -0.610063601 | ABCB6 | ATP binding cassette subfamily B member 6 (Langereis blood group) [Source:HGNC Symbol;Acc:HGNC:47] |
| ENSG00000116690 | HGLibA_38458 | 58 | -0.610063601 | PRG4 | proteoglycan 4 [Source:HGNC Symbol;Acc:HGNC:9364] |
| ENSG00000116786 | HGLibA_37141 | 58 | -0.610063601 | PLEKHM2 | pleckstrin homology and RUN domain containing M2 [Source:HGNC Symbol;Acc:HGNC:29131] |
| ENSG00000119431 | HGLibA_21056 | 58 | -0.610063601 | HDHD3 | haloacid dehalogenase like hydrolase domain containing 3 [Source:HGNC Symbol;Acc:HGNC:28171] |
| ENSG00000120094 | HGLibA_21927 | 58 | -0.610063601 | HOXB1 | homeobox B1 [Source:HGNC Symbol;Acc:HGNC:5111] |
| ENSG00000120235 | HGLibA_22686 | 58 | -0.610063601 | IFNA6 | interferon alpha 6 [Source:HGNC Symbol;Acc:HGNC:5427] |
| ENSG00000122254 | HGLibA_22125 | 58 | -0.610063601 | HS3ST2 | heparan sulfate-glucosamine 3-sulfotransferase 2 [Source:HGNC Symbol;Acc:HGNC:5195] |
| ENSG00000122861 | HGLibA_36963 | 58 | -0.610063601 | PLAU | urokinase [Source:HGNC Symbol;Acc:HGNC:9052]" |
| ENSG00000126233 | HGLibA_45555 | 58 | -0.610063601 | SLURP1 | secreted LY6/PLAUR domain containing 1 [Source:HGNC Symbol;Acc:HGNC:18746] |
| ENSG00000126456 | HGLibA_23586 | 58 | -0.610063601 | IRF3 | interferon regulatory factor 3 [Source:HGNC Symbol;Acc:HGNC:6118] |
| ENSG00000126878 | HGLibA_01334 | 58 | -0.610063601 | AIF1L | allograft inflammatory factor 1 like [Source:HGNC Symbol;Acc:HGNC:28904] |
| ENSG00000127995 | HGLibA_07283 | 58 | -0.610063601 | CASD1 | CAS1 domain containing 1 [Source:HGNC Symbol;Acc:HGNC:16014] |
| ENSG00000129566 | HGLibA_49006 | 58 | -0.610063601 | TEP1 | telomerase associated protein 1 [Source:HGNC Symbol;Acc:HGNC:11726] |
| ENSG00000129595 | HGLibA_15250 | 58 | -0.610063601 | EPB41L4A | erythrocyte membrane protein band 4.1 like 4A [Source:HGNC Symbol;Acc:HGNC:13278] |
| ENSG00000130638 | HGLibA_03830 | 58 | -0.610063601 | ATXN10 | ataxin 10 [Source:HGNC Symbol;Acc:HGNC:10549] |
| ENSG00000131467 | HGLibA_39197 | 58 | -0.610063601 | PSME3 | proteasome activator subunit 3 [Source:HGNC Symbol;Acc:HGNC:9570] |
| ENSG00000132911 | HGLibA_32218 | 58 | -0.610063601 | NMUR2 | neuromedin U receptor 2 [Source:HGNC Symbol;Acc:HGNC:16454] |
| ENSG00000133131 | HGLibA_29656 | 58 | -0.610063601 | MORC4 | MORC family CW-type zinc finger 4 [Source:HGNC Symbol;Acc:HGNC:23485] |
| ENSG00000135052 | HGLibA_19653 | 58 | -0.610063601 | GOLM1 | golgi membrane protein 1 [Source:HGNC Symbol;Acc:HGNC:15451] |
| ENSG00000135387 | HGLibA_07191 | 58 | -0.610063601 | CAPRIN1 | cell cycle associated protein 1 [Source:HGNC Symbol;Acc:HGNC:6743] |
| ENSG00000135392 | HGLibA_13575 | 58 | -0.610063601 | DNAJC14 | DnaJ heat shock protein family (Hsp40) member C14 [Source:HGNC Symbol;Acc:HGNC:24581] |
| ENSG00000135775 | HGLibA_10473 | 58 | -0.610063601 | COG2 | component of oligomeric golgi complex 2 [Source:HGNC Symbol;Acc:HGNC:6546] |
| ENSG00000137522 | HGLibA_41483 | 58 | -0.610063601 | RNF121 | ring finger protein 121 [Source:HGNC Symbol;Acc:HGNC:21070] |
| ENSG00000137815 | HGLibA_42383 | 58 | -0.610063601 | RTF1 | Paf1/RNA polymerase II complex component [Source:HGNC Symbol;Acc:HGNC:28996]" |
| ENSG00000138604 | HGLibA_19232 | 58 | -0.610063601 | GLCE | glucuronic acid epimerase [Source:HGNC Symbol;Acc:HGNC:17855] |
| ENSG00000138639 | HGLibA_02736 | 58 | -0.610063601 | ARHGAP24 | Rho GTPase activating protein 24 [Source:HGNC Symbol;Acc:HGNC:25361] |
| ENSG00000139209 | HGLibA_45071 | 58 | -0.610063601 | SLC38A4 | solute carrier family 38 member 4 [Source:HGNC Symbol;Acc:HGNC:14679] |
| ENSG00000140090 | HGLibA_44625 | 58 | -0.610063601 | SLC24A4 | solute carrier family 24 member 4 [Source:HGNC Symbol;Acc:HGNC:10978] |
| ENSG00000140398 | HGLibA_31630 | 58 | -0.610063601 | NEIL1 | nei like DNA glycosylase 1 [Source:HGNC Symbol;Acc:HGNC:18448] |
| ENSG00000142046 | HGLibA_50558 | 58 | -0.610063601 | TMEM91 | transmembrane protein 91 [Source:HGNC Symbol;Acc:HGNC:32393] |
| ENSG00000142227 | HGLibA_15081 | 58 | -0.610063601 | EMP3 | epithelial membrane protein 3 [Source:HGNC Symbol;Acc:HGNC:3335] |
| ENSG00000142319 | HGLibA_45326 | 58 | -0.610063601 | SLC6A3 | solute carrier family 6 member 3 [Source:HGNC Symbol;Acc:HGNC:11049] |
| ENSG00000142867 | HGLibA_04286 | 58 | -0.610063601 | BCL10 | B cell CLL/lymphoma 10 [Source:HGNC Symbol;Acc:HGNC:989] |
| ENSG00000143183 | HGLibA_49780 | 58 | -0.610063601 | TMCO1 | transmembrane and coiled-coil domains 1 [Source:HGNC Symbol;Acc:HGNC:18188] |
| ENSG00000143194 | HGLibA_27862 | 58 | -0.610063601 | MAEL | maelstrom spermatogenic transposon silencer [Source:HGNC Symbol;Acc:HGNC:25929] |
| ENSG00000143933 | HGLibA_07026 | 58 | -0.610063601 | CALM2 | calmodulin 2 [Source:HGNC Symbol;Acc:HGNC:1445] |
| ENSG00000145626 | HGLibA_52932 | 58 | -0.610063601 | UGT3A1 | UDP glycosyltransferase family 3 member A1 [Source:HGNC Symbol;Acc:HGNC:26625] |
| ENSG00000145817 | HGLibA_54682 | 58 | -0.610063601 | YIPF5 | Yip1 domain family member 5 [Source:HGNC Symbol;Acc:HGNC:24877] |
| ENSG00000146378 | HGLibA_48126 | 58 | -0.610063601 | TAAR2 | trace amine associated receptor 2 (gene/pseudogene) [Source:HGNC Symbol;Acc:HGNC:4514] |
| ENSG00000147642 | HGLibA_47911 | 58 | -0.610063601 | SYBU | syntabulin [Source:HGNC Symbol;Acc:HGNC:26011] |
| ENSG00000148218 | HGLibA_01519 | 58 | -0.610063601 | ALAD | aminolevulinate dehydratase [Source:HGNC Symbol;Acc:HGNC:395] |
| ENSG00000151365 | HGLibA_49396 | 58 | -0.610063601 | THRSP | thyroid hormone responsive [Source:HGNC Symbol;Acc:HGNC:11800] |
| ENSG00000152242 | HGLibA_05608 | 58 | -0.610063601 | C18orf25 | chromosome 18 open reading frame 25 [Source:HGNC Symbol;Acc:HGNC:28172] |
| ENSG00000152642 | HGLibA_19785 | 58 | -0.610063601 | GPD1L | glycerol-3-phosphate dehydrogenase 1 like [Source:HGNC Symbol;Acc:HGNC:28956] |
| ENSG00000154611 | HGLibA_39091 | 58 | -0.610063601 | PSMA8 | proteasome subunit alpha 8 [Source:HGNC Symbol;Acc:HGNC:22985] |
| ENSG00000155229 | HGLibA_29553 | 58 | -0.610063601 | MMS19 | cytosolic iron-sulfur assembly component [Source:HGNC Symbol;Acc:HGNC:13824]" |
| ENSG00000156050 | HGLibA_16220 | 58 | -0.610063601 | FAM161B | centrosomal protein [Source:HGNC Symbol;Acc:HGNC:19854]" |
| ENSG00000156508 | HGLibA_14490 | 58 | -0.610063601 | EEF1A1 | eukaryotic translation elongation factor 1 alpha 1 [Source:HGNC Symbol;Acc:HGNC:3189] |
| ENSG00000156521 | HGLibA_52478 | 58 | -0.610063601 | TYSND1 | trypsin domain containing 1 [Source:HGNC Symbol;Acc:HGNC:28531] |
| ENSG00000157224 | HGLibA_09855 | 58 | -0.610063601 | CLDN12 | claudin 12 [Source:HGNC Symbol;Acc:HGNC:2034] |
| ENSG00000157557 | HGLibA_15644 | 58 | -0.610063601 | ETS2 | transcription factor [Source:HGNC Symbol;Acc:HGNC:3489]" |
| ENSG00000160208 | HGLibA_42274 | 58 | -0.610063601 | RRP1B | ribosomal RNA processing 1B [Source:HGNC Symbol;Acc:HGNC:23818] |
| ENSG00000160991 | HGLibA_34593 | 58 | -0.610063601 | ORAI2 | ORAI calcium release-activated calcium modulator 2 [Source:HGNC Symbol;Acc:HGNC:21667] |
| ENSG00000161243 | HGLibA_17058 | 58 | -0.610063601 | FBXO27 | F-box protein 27 [Source:HGNC Symbol;Acc:HGNC:18753] |
| ENSG00000161904 | HGLibA_26289 | 58 | -0.610063601 | LEMD2 | LEM domain containing 2 [Source:HGNC Symbol;Acc:HGNC:21244] |
| ENSG00000162413 | HGLibA_25153 | 58 | -0.610063601 | KLHL21 | kelch like family member 21 [Source:HGNC Symbol;Acc:HGNC:29041] |
| ENSG00000162526 | HGLibA_52016 | 58 | -0.610063601 | TSSK3 | testis specific serine kinase 3 [Source:HGNC Symbol;Acc:HGNC:15473] |
| ENSG00000162885 | HGLibA_03950 | 58 | -0.610063601 | B3GALNT2 | 3-N-acetylgalactosaminyltransferase 2 [Source:HGNC Symbol;Acc:HGNC:28596]" |
| ENSG00000163114 | HGLibA_35864 | 58 | -0.610063601 | PDHA2 | pyruvate dehydrogenase E1 alpha 2 subunit [Source:HGNC Symbol;Acc:HGNC:8807] |
| ENSG00000163166 | HGLibA_23887 | 58 | -0.610063601 | IWS1 | SUPT6H interacting protein [Source:HGNC Symbol;Acc:HGNC:25467]" |
| ENSG00000163631 | HGLibA_01530 | 58 | -0.610063601 | ALB | albumin [Source:HGNC Symbol;Acc:HGNC:399] |
| ENSG00000164134 | HGLibA_30976 | 58 | -0.610063601 | NAA15 | NatA auxiliary subunit [Source:HGNC Symbol;Acc:HGNC:30782]" |
| ENSG00000164334 | HGLibA_16258 | 58 | -0.610063601 | FAM170A | family with sequence similarity 170 member A [Source:HGNC Symbol;Acc:HGNC:27963] |
| ENSG00000164871 | HGLibA_46348 | 58 | -0.610063601 | SPAG11B | sperm associated antigen 11B [Source:HGNC Symbol;Acc:HGNC:14534] |
| ENSG00000165091 | HGLibA_49747 | 58 | -0.610063601 | TMC1 | transmembrane channel like 1 [Source:HGNC Symbol;Acc:HGNC:16513] |
| ENSG00000165685 | HGLibA_50436 | 58 | -0.610063601 | TMEM52B | transmembrane protein 52B [Source:HGNC Symbol;Acc:HGNC:26438] |
| ENSG00000165810 | HGLibA_04983 | 58 | -0.610063601 | BTNL9 | butyrophilin like 9 [Source:HGNC Symbol;Acc:HGNC:24176] |
| ENSG00000165915 | HGLibA_45101 | 58 | -0.610063601 | SLC39A13 | solute carrier family 39 member 13 [Source:HGNC Symbol;Acc:HGNC:20859] |
| ENSG00000166266 | HGLibA_11789 | 58 | -0.610063601 | CUL5 | cullin 5 [Source:HGNC Symbol;Acc:HGNC:2556] |
| ENSG00000167618 | HGLibA_25936 | 58 | -0.610063601 | LAIR2 | leukocyte associated immunoglobulin like receptor 2 [Source:HGNC Symbol;Acc:HGNC:6478] |
| ENSG00000167642 | HGLibA_46715 | 58 | -0.610063601 | SPINT2 | Kunitz type 2 [Source:HGNC Symbol;Acc:HGNC:11247]" |
| ENSG00000168060 | HGLibA_31012 | 58 | -0.610063601 | NAALADL1 | N-acetylated alpha-linked acidic dipeptidase like 1 [Source:HGNC Symbol;Acc:HGNC:23536] |
| ENSG00000168763 | HGLibA_10319 | 58 | -0.610063601 | CNNM3 | cyclin and CBS domain divalent metal cation transport mediator 3 [Source:HGNC Symbol;Acc:HGNC:104] |
| ENSG00000170178 | HGLibA_21992 | 58 | -0.610063601 | HOXD12 | homeobox D12 [Source:HGNC Symbol;Acc:HGNC:5135] |
| ENSG00000170385 | HGLibA_44898 | 58 | -0.610063601 | SLC30A1 | solute carrier family 30 member 1 [Source:HGNC Symbol;Acc:HGNC:11012] |
| ENSG00000170571 | HGLibA_14998 | 58 | -0.610063601 | EMB | embigin [Source:HGNC Symbol;Acc:HGNC:30465] |
| ENSG00000171307 | HGLibA_55085 | 58 | -0.610063601 | ZDHHC16 | zinc finger DHHC-type containing 16 [Source:HGNC Symbol;Acc:HGNC:20714] |
| ENSG00000171501 | HGLibA_33708 | 58 | -0.610063601 | OR1N2 | olfactory receptor family 1 subfamily N member 2 [Source:HGNC Symbol;Acc:HGNC:15111] |
| ENSG00000171792 | HGLibA_41160 | 58 | -0.610063601 | RHNO1 | RAD9-HUS1-RAD1 interacting nuclear orphan 1 [Source:HGNC Symbol;Acc:HGNC:28206] |
| ENSG00000171848 | HGLibA_42255 | 58 | -0.610063601 | RRM2 | ribonucleotide reductase regulatory subunit M2 [Source:HGNC Symbol;Acc:HGNC:10452] |
| ENSG00000172139 | HGLibA_45439 | 58 | -0.610063601 | SLC9C1 | solute carrier family 9 member C1 [Source:HGNC Symbol;Acc:HGNC:31401] |
| ENSG00000172336 | HGLibA_37696 | 58 | -0.610063601 | POP7 | ribonuclease P/MRP subunit [Source:HGNC Symbol;Acc:HGNC:19949]" |
| ENSG00000172716 | HGLibA_45482 | 58 | -0.610063601 | SLFN11 | schlafen family member 11 [Source:HGNC Symbol;Acc:HGNC:26633] |
| ENSG00000173193 | HGLibA_35224 | 58 | -0.610063601 | PARP14 | poly(ADP-ribose) polymerase family member 14 [Source:HGNC Symbol;Acc:HGNC:29232] |
| ENSG00000176422 | HGLibA_46854 | 58 | -0.610063601 | SPRYD4 | SPRY domain containing 4 [Source:HGNC Symbol;Acc:HGNC:27468] |
| ENSG00000177885 | HGLibA_20231 | 58 | -0.610063601 | GRB2 | growth factor receptor bound protein 2 [Source:HGNC Symbol;Acc:HGNC:4566] |
| ENSG00000178201 | HGLibA_53698 | 58 | -0.610063601 | VN1R1 | vomeronasal 1 receptor 1 [Source:HGNC Symbol;Acc:HGNC:13548] |
| ENSG00000180433 | HGLibA_34423 | 58 | -0.610063601 | OR6K6 | olfactory receptor family 6 subfamily K member 6 [Source:HGNC Symbol;Acc:HGNC:15033] |
| ENSG00000183137 | HGLibA_09148 | 58 | -0.610063601 | CEP57L1 | centrosomal protein 57 like 1 [Source:HGNC Symbol;Acc:HGNC:21561] |
| ENSG00000184451 | HGLibA_08147 | 58 | -0.610063601 | CCR10 | C-C motif chemokine receptor 10 [Source:HGNC Symbol;Acc:HGNC:4474] |
| ENSG00000184709 | HGLibA_27294 | 58 | -0.610063601 | LRRC26 | leucine rich repeat containing 26 [Source:HGNC Symbol;Acc:HGNC:31409] |
| ENSG00000184949 | HGLibA_16529 | 58 | -0.610063601 | FAM227A | family with sequence similarity 227 member A [Source:HGNC Symbol;Acc:HGNC:44197] |
| ENSG00000185056 | HGLibA_06391 | 58 | -0.610063601 | C5orf47 | chromosome 5 open reading frame 47 [Source:HGNC Symbol;Acc:HGNC:27026] |
| ENSG00000185610 | HGLibA_12409 | 58 | -0.610063601 | DBX2 | developing brain homeobox 2 [Source:HGNC Symbol;Acc:HGNC:33186] |
| ENSG00000185730 | HGLibA_56561 | 58 | -0.610063601 | ZNF696 | zinc finger protein 696 [Source:HGNC Symbol;Acc:HGNC:25872] |
| ENSG00000185873 | HGLibA_50614 | 58 | -0.610063601 | TMPRSS11B | transmembrane serine protease 11B [Source:HGNC Symbol;Acc:HGNC:25398] |
| ENSG00000186092 | HGLibA_34011 | 58 | -0.610063601 | OR4F5 | olfactory receptor family 4 subfamily F member 5 [Source:HGNC Symbol;Acc:HGNC:14825] |
| ENSG00000186448 | HGLibA_55562 | 58 | -0.610063601 | ZNF197 | zinc finger protein 197 [Source:HGNC Symbol;Acc:HGNC:12988] |
| ENSG00000186970 | HGLibA_25654 | 58 | -0.610063601 | KRTAP15-1 | keratin associated protein 15-1 [Source:HGNC Symbol;Acc:HGNC:18927] |
| ENSG00000187003 | HGLibA_00626 | 58 | -0.610063601 | ACTL7A | actin like 7A [Source:HGNC Symbol;Acc:HGNC:161] |
| ENSG00000188340 | HGLibA_34432 | 58 | -0.610063601 | OR6N2 | olfactory receptor family 6 subfamily N member 2 [Source:HGNC Symbol;Acc:HGNC:15035] |
| ENSG00000188610 | HGLibA_16710 | 58 | -0.610063601 | FAM72B | family with sequence similarity 72 member B [Source:HGNC Symbol;Acc:HGNC:24805] |
| ENSG00000196350 | HGLibA_56642 | 58 | -0.610063601 | ZNF729 | zinc finger protein 729 [Source:HGNC Symbol;Acc:HGNC:32464] |
| ENSG00000196405 | HGLibA_15698 | 58 | -0.610063601 | EVL | Enah/Vasp-like [Source:HGNC Symbol;Acc:HGNC:20234] |
| ENSG00000196562 | HGLibA_47728 | 58 | -0.610063601 | SULF2 | sulfatase 2 [Source:HGNC Symbol;Acc:HGNC:20392] |
| ENSG00000197037 | HGLibA_57011 | 58 | -0.610063601 | ZSCAN25 | zinc finger and SCAN domain containing 25 [Source:HGNC Symbol;Acc:HGNC:21961] |
| ENSG00000197056 | HGLibA_55391 | 58 | -0.610063601 | ZMYM1 | zinc finger MYM-type containing 1 [Source:HGNC Symbol;Acc:HGNC:26253] |
| ENSG00000197818 | HGLibA_45428 | 58 | -0.610063601 | SLC9A8 | solute carrier family 9 member A8 [Source:HGNC Symbol;Acc:HGNC:20728] |
| ENSG00000197937 | HGLibA_55851 | 58 | -0.610063601 | ZNF347 | zinc finger protein 347 [Source:HGNC Symbol;Acc:HGNC:16447] |
| ENSG00000198455 | HGLibA_57075 | 58 | -0.610063601 | ZXDB | zinc finger X-linked duplicated B [Source:HGNC Symbol;Acc:HGNC:13199] |
| ENSG00000198466 | HGLibA_56329 | 58 | -0.610063601 | ZNF587 | zinc finger protein 587 [Source:HGNC Symbol;Acc:HGNC:30955] |
| ENSG00000198598 | HGLibA_29500 | 58 | -0.610063601 | MMP17 | matrix metallopeptidase 17 [Source:HGNC Symbol;Acc:HGNC:7163] |
| ENSG00000198642 | HGLibA_25227 | 58 | -0.610063601 | KLHL9 | kelch like family member 9 [Source:HGNC Symbol;Acc:HGNC:18732] |

| | | | | | |
|---|---|---|---|---|---|
| ENSG00000198825 | HGLibA_23356 | 58 | -0.610063601 | INPP5F | inositol polyphosphate-5-phosphatase F [Source:HGNC Symbol;Acc:HGNC:17054] |
| ENSG00000198865 | HGLibA_07649 | 58 | -0.610063601 | CCDC152 | coiled-coil domain containing 152 [Source:HGNC Symbol;Acc:HGNC:34438] |
| ENSG00000198937 | HGLibA_07675 | 58 | -0.610063601 | CCDC167 | coiled-coil domain containing 167 [Source:HGNC Symbol;Acc:HGNC:21239] |
| ENSG00000204001 | HGLibA_26185 | 58 | -0.610063601 | LCN8 | lipocalin 8 [Source:HGNC Symbol;Acc:HGNC:27038] |
| ENSG00000204348 | HGLibA_14216 | 58 | -0.610063601 | DXO | decapping exoribonuclease [Source:HGNC Symbol;Acc:HGNC:2992] |
| ENSG00000204379 | HGLibA_54465 | 58 | -0.610063601 | XAGE1A | X antigen family member 1A [Source:HGNC Symbol;Acc:HGNC:4111] |
| ENSG00000204574 | HGLibA_00207 | 58 | -0.610063601 | ABCF1 | ATP binding cassette subfamily F member 1 [Source:HGNC Symbol;Acc:HGNC:70] |
| ENSG00000205809 | HGLibA_25289 | 58 | -0.610063601 | KLRC2 | killer cell lectin like receptor C2 [Source:HGNC Symbol;Acc:HGNC:6375] |
| ENSG00000213999 | HGLibA_28830 | 58 | -0.610063601 | MEF2B | myocyte enhancer factor 2B [Source:HGNC Symbol;Acc:HGNC:6995] |
| ENSG00000215397 | HGLibA_43060 | 58 | -0.610063601 | SCRT2 | scratch family transcriptional repressor 2 [Source:HGNC Symbol;Acc:HGNC:15952] |
| ENSG00000221931 | HGLibA_34450 | 58 | -0.610063601 | OR6X1 | olfactory receptor family 6 subfamily X member 1 [Source:HGNC Symbol;Acc:HGNC:14737] |
| ENSG00000221954 | HGLibA_33941 | 58 | -0.610063601 | OR4C12 | olfactory receptor family 4 subfamily C member 12 [Source:HGNC Symbol;Acc:HGNC:15168] |
| ENSG00000221963 | HGLibA_02541 | 58 | -0.610063601 | APOL6 | apolipoprotein L6 [Source:HGNC Symbol;Acc:HGNC:14870] |
| ENSG00000223547 | HGLibA_56859 | 58 | -0.610063601 | ZNF844 | zinc finger protein 844 [Source:HGNC Symbol;Acc:HGNC:25932] |
| ENSG00000224089 | HGLibA_11519 | 58 | -0.610063601 | CT47A10 | member A10 [Source:HGNC Symbol;Acc:HGNC:33291]" |
| ENSG00000229665 | HGLibA_38807 | 58 | -0.610063601 | PRR20C | proline rich 20C [Source:HGNC Symbol;Acc:HGNC:37221] |
| ENSG00000234284 | HGLibA_56891 | 58 | -0.610063601 | ZNF879 | zinc finger protein 879 [Source:HGNC Symbol;Acc:HGNC:37273] |
| ENSG00000236371 | HGLibA_11516 | 58 | -0.610063601 | CT47A1 | member A1 [Source:HGNC Symbol;Acc:HGNC:33282]" |
| ENSG00000244395 | HGLibA_40618 | 58 | -0.610063601 | RBMY1D | member D [Source:HGNC Symbol;Acc:HGNC:23915]" |
| ENSG00000253506 | HGLibA_31032 | 58 | -0.610063601 | NACA2 | nascent polypeptide associated complex subunit alpha 2 [Source:HGNC Symbol;Acc:HGNC:23290] |
| ENSG00000258405 | HGLibA_56298 | 58 | -0.610063601 | ZNF578 | zinc finger protein 578 [Source:HGNC Symbol;Acc:HGNC:26449] |
| ENSG00000266265 | HGLibA_25053 | 58 | -0.610063601 | KLF14 | Kruppel like factor 14 [Source:HGNC Symbol;Acc:HGNC:23025] |
| ENSG00000267796 | HGLibA_26565 | 58 | -0.610063601 | LIN37 | lin-37 DREAM MuvB core complex component [Source:HGNC Symbol;Acc:HGNC:33234] |
| ENSG00000268629 | HGLibA_49057 | 58 | -0.610063601 | TEX13A | testis expressed 13A [Source:HGNC Symbol;Acc:HGNC:11735] |
| ENSG00000276070 | HGLibA_08034 | 58 | -0.610063601 | CCL4L2 | C-C motif chemokine ligand 4 like 2 [Source:HGNC Symbol;Acc:HGNC:24066] |
| ENSG00000280109 | HGLibA_36938 | 58 | -0.610063601 | PLAC4 | placenta specific 4 [Source:HGNC Symbol;Acc:HGNC:14616] |
| ENSG00000288649 | HGLibA_00618 | 58 | -0.610063601 | ACTL10 | - |
| FAM188A | HGLibA_16346 | 58 | -0.610063601 | FAM188A | - |
| HDGFRP2 | HGLibA_21044 | 58 | -0.610063601 | HDGFRP2 | - |
| HIST1H4F | HGLibA_21501 | 58 | -0.610063601 | HIST1H4F | - |
| hsa-let-7a-3 | HGLibA_57106 | 58 | -0.610063601 | hsa-let-7a-3 | - |
| hsa-let-7g | HGLibA_57135 | 58 | -0.610063601 | hsa-let-7g | - |
| hsa-mir-1246 | HGLibA_57383 | 58 | -0.610063601 | hsa-mir-1246 | - |
| hsa-mir-1270-2 | HGLibA_57519 | 58 | -0.610063601 | hsa-mir-1270-2 | - |
| hsa-mir-1283-1 | HGLibA_57590 | 58 | -0.610063601 | hsa-mir-1283-1 | - |
| hsa-mir-1302-9 | HGLibA_57722 | 58 | -0.610063601 | hsa-mir-1302-9 | - |
| hsa-mir-132 | HGLibA_57754 | 58 | -0.610063601 | hsa-mir-132 | - |
| hsa-mir-202 | HGLibA_58163 | 58 | -0.610063601 | hsa-mir-202 | - |
| hsa-mir-212 | HGLibA_58243 | 58 | -0.610063601 | hsa-mir-212 | - |
| hsa-mir-219b | HGLibA_58284 | 58 | -0.610063601 | hsa-mir-219b | - |
| hsa-mir-298 | HGLibA_58402 | 58 | -0.610063601 | hsa-mir-298 | - |
| hsa-mir-31 | HGLibA_58497 | 58 | -0.610063601 | hsa-mir-31 | - |
| hsa-mir-3147 | HGLibA_58646 | 58 | -0.610063601 | hsa-mir-3147 | - |
| hsa-mir-3179-2 | HGLibA_58792 | 58 | -0.610063601 | hsa-mir-3179-2 | - |
| hsa-mir-361 | HGLibA_59070 | 58 | -0.610063601 | hsa-mir-361 | - |
| hsa-mir-3619 | HGLibA_59104 | 58 | -0.610063601 | hsa-mir-3619 | - |
| hsa-mir-3678 | HGLibA_59262 | 58 | -0.610063601 | hsa-mir-3678 | - |
| hsa-mir-3685 | HGLibA_59292 | 58 | -0.610063601 | hsa-mir-3685 | - |
| hsa-mir-3689d-2 | HGLibA_59328 | 58 | -0.610063601 | hsa-mir-3689d-2 | - |
| hsa-mir-377 | HGLibA_59420 | 58 | -0.610063601 | hsa-mir-377 | - |
| hsa-mir-378g | HGLibA_59453 | 58 | -0.610063601 | hsa-mir-378g | - |
| hsa-mir-4265 | HGLibA_59767 | 58 | -0.610063601 | hsa-mir-4265 | - |
| hsa-mir-4438 | HGLibA_60154 | 58 | -0.610063601 | hsa-mir-4438 | - |
| hsa-mir-4451 | HGLibA_60212 | 58 | -0.610063601 | hsa-mir-4451 | - |
| hsa-mir-4463 | HGLibA_60260 | 58 | -0.610063601 | hsa-mir-4463 | - |
| hsa-mir-4761 | HGLibA_61141 | 58 | -0.610063601 | hsa-mir-4761 | - |
| hsa-mir-5095 | HGLibA_61540 | 58 | -0.610063601 | hsa-mir-5095 | - |
| hsa-mir-5100 | HGLibA_61549 | 58 | -0.610063601 | hsa-mir-5100 | - |
| hsa-mir-548j | HGLibA_62028 | 58 | -0.610063601 | hsa-mir-548j | - |
| hsa-mir-5681b | HGLibA_62245 | 58 | -0.610063601 | hsa-mir-5681b | - |
| hsa-mir-6071 | HGLibA_62537 | 58 | -0.610063601 | hsa-mir-6071 | - |
| hsa-mir-6090 | HGLibA_62629 | 58 | -0.610063601 | hsa-mir-6090 | - |
| hsa-mir-627 | HGLibA_62746 | 58 | -0.610063601 | hsa-mir-627 | - |
| hsa-mir-640 | HGLibA_62800 | 58 | -0.610063601 | hsa-mir-640 | - |
| hsa-mir-644a | HGLibA_62818 | 58 | -0.610063601 | hsa-mir-644a | - |
| hsa-mir-6504 | HGLibA_62868 | 58 | -0.610063601 | hsa-mir-6504 | - |
| hsa-mir-6785 | HGLibA_63316 | 58 | -0.610063601 | hsa-mir-6785 | - |
| hsa-mir-6827 | HGLibA_63484 | 58 | -0.610063601 | hsa-mir-6827 | - |
| hsa-mir-6828 | HGLibA_63488 | 58 | -0.610063601 | hsa-mir-6828 | - |
| hsa-mir-6838 | HGLibA_63528 | 58 | -0.610063601 | hsa-mir-6838 | - |
| hsa-mir-6886 | HGLibA_63729 | 58 | -0.610063601 | hsa-mir-6886 | - |
| hsa-mir-6892 | HGLibA_63754 | 58 | -0.610063601 | hsa-mir-6892 | - |
| hsa-mir-7-2 | HGLibA_63773 | 58 | -0.610063601 | hsa-mir-7-2 | - |
| hsa-mir-764 | HGLibA_63912 | 58 | -0.610063601 | hsa-mir-764 | - |
| hsa-mir-770 | HGLibA_63939 | 58 | -0.610063601 | hsa-mir-770 | - |
| hsa-mir-96 | HGLibA_64369 | 58 | -0.610063601 | hsa-mir-96 | - |
| KARS | HGLibA_24047 | 58 | -0.610063601 | KARS | - |
| LOC441155 | HGLibA_26950 | 58 | -0.610063601 | LOC441155 | - |
| LPHN2 | HGLibA_27088 | 58 | -0.610063601 | LPHN2 | - |
| MUM1L1 | HGLibA_30569 | 58 | -0.610063601 | MUM1L1 | - |
| RARRES3 | HGLibA_40288 | 58 | -0.610063601 | RARRES3 | - |
| WDR63 | HGLibA_54147 | 58 | -0.610063601 | WDR63 | - |
| ZCCHC6 | HGLibA_55041 | 58 | -0.610063601 | ZCCHC6 | - |
| ASMTL_Y | HGLibA_03330 | 57 | -0.619108944 | ASMTL_Y | - |
| C11orf63 | HGLibA_05165 | 57 | -0.619108944 | C11orf63 | - |
| C5orf42 | HGLibA_06382 | 57 | -0.619108944 | C5orf42 | - |
| C5orf55 | HGLibA_06409 | 57 | -0.619108944 | C5orf55 | - |
| C6orf25 | HGLibA_06485 | 57 | -0.619108944 | C6orf25 | - |
| ENSG00000003436 | HGLibA_49183 | 57 | -0.619108944 | TFPI | tissue factor pathway inhibitor [Source:HGNC Symbol;Acc:HGNC:11760] |
| ENSG00000005001 | HGLibA_38914 | 57 | -0.619108944 | PRSS22 | serine protease 22 [Source:HGNC Symbol;Acc:HGNC:14368] |
| ENSG00000007933 | HGLibA_17707 | 57 | -0.619108944 | FMO3 | flavin containing monooxygenase 3 [Source:HGNC Symbol;Acc:HGNC:3771] |
| ENSG00000009335 | HGLibA_52685 | 57 | -0.619108944 | UBE3C | ubiquitin protein ligase E3C [Source:HGNC Symbol;Acc:HGNC:16803] |
| ENSG00000015413 | HGLibA_13818 | 57 | -0.619108944 | DPEP1 | dipeptidase 1 [Source:HGNC Symbol;Acc:HGNC:3002] |
| ENSG00000016490 | HGLibA_09804 | 57 | -0.619108944 | CLCA1 | chloride channel accessory 1 [Source:HGNC Symbol;Acc:HGNC:2015] |
| ENSG00000044446 | HGLibA_36406 | 57 | -0.619108944 | PHKA2 | phosphorylase kinase regulatory subunit alpha 2 [Source:HGNC Symbol;Acc:HGNC:8926] |
| ENSG00000047410 | HGLibA_51174 | 57 | -0.619108944 | TPR | nuclear basket protein [Source:HGNC Symbol;Acc:HGNC:12017]" |
| ENSG00000057657 | HGLibA_38361 | 57 | -0.619108944 | PRDM1 | PR/SET domain 1 [Source:HGNC Symbol;Acc:HGNC:9346] |
| ENSG00000059588 | HGLibA_48329 | 57 | -0.619108944 | TARBP1 | TAR (HIV-1) RNA binding protein 1 [Source:HGNC Symbol;Acc:HGNC:11568] |
| ENSG00000065357 | HGLibA_13057 | 57 | -0.619108944 | DGKA | diacylglycerol kinase alpha [Source:HGNC Symbol;Acc:HGNC:2849] |
| ENSG00000065371 | HGLibA_41761 | 57 | -0.619108944 | ROPN1 | rhophilin associated tail protein 1 [Source:HGNC Symbol;Acc:HGNC:17692] |
| ENSG00000065613 | HGLibA_45531 | 57 | -0.619108944 | SLK | STE20 like kinase [Source:HGNC Symbol;Acc:HGNC:11088] |
| ENSG00000068028 | HGLibA_40374 | 57 | -0.619108944 | RASSF1 | Ras association domain family member 1 [Source:HGNC Symbol;Acc:HGNC:9882] |
| ENSG00000068097 | HGLibA_21081 | 57 | -0.619108944 | HEATR6 | HEAT repeat containing 6 [Source:HGNC Symbol;Acc:HGNC:24076] |
| ENSG00000068745 | HGLibA_23449 | 57 | -0.619108944 | IP6K2 | inositol hexakisphosphate kinase 2 [Source:HGNC Symbol;Acc:HGNC:17313] |
| ENSG00000070476 | HGLibA_57078 | 57 | -0.619108944 | ZXDC | ZXD family zinc finger C [Source:HGNC Symbol;Acc:HGNC:28160] |
| ENSG00000070808 | HGLibA_07068 | 57 | -0.619108944 | CAMK2A | calcium/calmodulin dependent protein kinase II alpha [Source:HGNC Symbol;Acc:HGNC:1460] |
| ENSG00000071189 | HGLibA_46037 | 57 | -0.619108944 | SNX13 | sorting nexin 13 [Source:HGNC Symbol;Acc:HGNC:21335] |
| ENSG00000075340 | HGLibA_00945 | 57 | -0.619108944 | ADD2 | adducin 2 [Source:HGNC Symbol;Acc:HGNC:244] |
| ENSG00000076944 | HGLibA_47666 | 57 | -0.619108944 | STXBP2 | syntaxin binding protein 2 [Source:HGNC Symbol;Acc:HGNC:11445] |
| ENSG00000078124 | HGLibA_00435 | 57 | -0.619108944 | ACER3 | alkaline ceramidase 3 [Source:HGNC Symbol;Acc:HGNC:16066] |
| ENSG00000079150 | HGLibA_17586 | 57 | -0.619108944 | FKBP7 | FK506 binding protein 7 [Source:HGNC Symbol;Acc:HGNC:3723] |
| ENSG00000080618 | HGLibA_10908 | 57 | -0.619108944 | CPB2 | carboxypeptidase B2 [Source:HGNC Symbol;Acc:HGNC:2300] |
| ENSG00000080709 | HGLibA_24356 | 57 | -0.619108944 | KCNN2 | potassium calcium-activated channel subfamily N member 2 [Source:HGNC Symbol;Acc:HGNC:6291] |
| ENSG00000088543 | HGLibA_06216 | 57 | -0.619108944 | C3orf18 | chromosome 3 open reading frame 18 [Source:HGNC Symbol;Acc:HGNC:24837] |
| ENSG00000088832 | HGLibA_17565 | 57 | -0.619108944 | FKBP1A | FK506 binding protein 1A [Source:HGNC Symbol;Acc:HGNC:3711] |
| ENSG00000090565 | HGLibA_39799 | 57 | -0.619108944 | RAB11FIP3 | RAB11 family interacting protein 3 [Source:HGNC Symbol;Acc:HGNC:17224] |
| ENSG00000092931 | HGLibA_29088 | 57 | -0.619108944 | MFSD11 | major facilitator superfamily domain containing 11 [Source:HGNC Symbol;Acc:HGNC:25458] |
| ENSG00000099260 | HGLibA_35079 | 57 | -0.619108944 | PALMD | palmdelphin [Source:HGNC Symbol;Acc:HGNC:15846] |
| ENSG00000099385 | HGLibA_04343 | 57 | -0.619108944 | BCL7C | BCL tumor suppressor 7C [Source:HGNC Symbol;Acc:HGNC:1006] |
| ENSG00000100823 | HGLibA_02407 | 57 | -0.619108944 | APEX1 | apurinic/apyrimidinic endodeoxyribonuclease 1 [Source:HGNC Symbol;Acc:HGNC:587] |
| ENSG00000101004 | HGLibA_31944 | 57 | -0.619108944 | NINL | ninein like [Source:HGNC Symbol;Acc:HGNC:29163] |
| ENSG00000101146 | HGLibA_40117 | 57 | -0.619108944 | RAE1 | ribonucleic acid export 1 [Source:HGNC Symbol;Acc:HGNC:9828] |
| ENSG00000101222 | HGLibA_46610 | 57 | -0.619108944 | SPEF1 | sperm flagellar 1 [Source:HGNC Symbol;Acc:HGNC:15874] |
| ENSG00000101333 | HGLibA_36988 | 57 | -0.619108944 | PLCB4 | phospholipase C beta 4 [Source:HGNC Symbol;Acc:HGNC:9059] |
| ENSG00000101333 | HGLibA_36986 | 57 | -0.619108944 | PLCB4 | phospholipase C beta 4 [Source:HGNC Symbol;Acc:HGNC:9059] |
| ENSG00000101955 | HGLibA_47033 | 57 | -0.619108944 | SRPX | sushi repeat containing protein X-linked [Source:HGNC Symbol;Acc:HGNC:11309] |
| ENSG00000102001 | HGLibA_06889 | 57 | -0.619108944 | CACNA1F | calcium voltage-gated channel subunit alpha1 F [Source:HGNC Symbol;Acc:HGNC:1393] |
| ENSG00000104881 | HGLibA_38032 | 57 | -0.619108944 | PPP1R13L | protein phosphatase 1 regulatory subunit 13 like [Source:HGNC Symbol;Acc:HGNC:18838] |
| ENSG00000104936 | HGLibA_13415 | 57 | -0.619108944 | DMPK | DM1 protein kinase [Source:HGNC Symbol;Acc:HGNC:2933] |
| ENSG00000105173 | HGLibA_08085 | 57 | -0.619108944 | CCNE1 | cyclin E1 [Source:HGNC Symbol;Acc:HGNC:1589] |
| ENSG00000105193 | HGLibA_42083 | 57 | -0.619108944 | RPS16 | ribosomal protein S16 [Source:HGNC Symbol;Acc:HGNC:10396] |
| ENSG00000105298 | HGLibA_06953 | 57 | -0.619108944 | CACTIN | spliceosome C complex subunit [Source:HGNC Symbol;Acc:HGNC:29938]" |
| ENSG00000105669 | HGLibA_10700 | 57 | -0.619108944 | COPE | coatomer protein complex subunit epsilon [Source:HGNC Symbol;Acc:HGNC:2234] |
| ENSG00000106236 | HGLibA_32534 | 57 | -0.619108944 | NPTX2 | neuronal pentraxin 2 [Source:HGNC Symbol;Acc:HGNC:7953] |
| ENSG00000106772 | HGLibA_38989 | 57 | -0.619108944 | PRUNE2 | prune homolog 2 [Source:HGNC Symbol;Acc:HGNC:25209] |

| | | | | | |
|---|---|---|---|---|---|
| ENSG00000107614 | HGLibA_51346 | 57 | -0.619108944 | TRDMT1 | tRNA aspartic acid methyltransferase 1 [Source:HGNC Symbol;Acc:HGNC:2977] |
| ENSG00000108179 | HGLibA_37929 | 57 | -0.619108944 | PPIF | peptidylprolyl isomerase F [Source:HGNC Symbol;Acc:HGNC:9259] |
| ENSG00000108799 | HGLibA_15833 | 57 | -0.619108944 | EZH1 | enhancer of zeste 1 polycomb repressive complex 2 subunit [Source:HGNC Symbol;Acc:HGNC:3526] |
| ENSG00000108840 | HGLibA_21015 | 57 | -0.619108944 | HDAC5 | histone deacetylase 5 [Source:HGNC Symbol;Acc:HGNC:14068] |
| ENSG00000109046 | HGLibA_54415 | 57 | -0.619108944 | WSB1 | WD repeat and SOCS box containing 1 [Source:HGNC Symbol;Acc:HGNC:19221] |
| ENSG00000109118 | HGLibA_36339 | 57 | -0.619108944 | PHF12 | PHD finger protein 12 [Source:HGNC Symbol;Acc:HGNC:20816] |
| ENSG00000109320 | HGLibA_31814 | 57 | -0.619108944 | NFKB1 | nuclear factor kappa B subunit 1 [Source:HGNC Symbol;Acc:HGNC:7794] |
| ENSG00000109680 | HGLibA_48492 | 57 | -0.619108944 | TBC1D19 | TBC1 domain family member 19 [Source:HGNC Symbol;Acc:HGNC:25624] |
| ENSG00000110944 | HGLibA_23096 | 57 | -0.619108944 | IL23A | interleukin 23 subunit alpha [Source:HGNC Symbol;Acc:HGNC:15488] |
| ENSG00000111653 | HGLibA_23291 | 57 | -0.619108944 | ING4 | inhibitor of growth family member 4 [Source:HGNC Symbol;Acc:HGNC:19423] |
| ENSG00000111790 | HGLibA_17441 | 57 | -0.619108944 | FGFR1OP2 | FGFR1 oncogene partner 2 [Source:HGNC Symbol;Acc:HGNC:23098] |
| ENSG00000111912 | HGLibA_31352 | 57 | -0.619108944 | NCOA7 | nuclear receptor coactivator 7 [Source:HGNC Symbol;Acc:HGNC:21081] |
| ENSG00000112304 | HGLibA_00477 | 57 | -0.619108944 | ACOT13 | acyl-CoA thioesterase 13 [Source:HGNC Symbol;Acc:HGNC:20999] |
| ENSG00000112782 | HGLibA_10033 | 57 | -0.619108944 | CLIC5 | chloride intracellular channel 5 [Source:HGNC Symbol;Acc:HGNC:13517] |
| ENSG00000113492 | HGLibA_01275 | 57 | -0.619108944 | AGXT2 | alanine--glyoxylate aminotransferase 2 [Source:HGNC Symbol;Acc:HGNC:14412] |
| ENSG00000116132 | HGLibA_38894 | 57 | -0.619108944 | PRRX1 | paired related homeobox 1 [Source:HGNC Symbol;Acc:HGNC:9142] |
| ENSG00000116871 | HGLibA_28234 | 57 | -0.619108944 | MAP7D1 | MAP7 domain containing 1 [Source:HGNC Symbol;Acc:HGNC:25514] |
| ENSG00000117620 | HGLibA_44954 | 57 | -0.619108944 | SLC35A3 | solute carrier family 35 member A3 [Source:HGNC Symbol;Acc:HGNC:11023] |
| ENSG00000118246 | HGLibA_16904 | 57 | -0.619108944 | FASTKD2 | FAST kinase domains 2 [Source:HGNC Symbol;Acc:HGNC:29160] |
| ENSG00000121743 | HGLibA_19141 | 57 | -0.619108944 | GJA3 | gap junction protein alpha 3 [Source:HGNC Symbol;Acc:HGNC:4277] |
| ENSG00000122986 | HGLibA_22435 | 57 | -0.619108944 | HVCN1 | hydrogen voltage gated channel 1 [Source:HGNC Symbol;Acc:HGNC:28240] |
| ENSG00000123342 | HGLibA_29503 | 57 | -0.619108944 | MMP19 | matrix metallopeptidase 19 [Source:HGNC Symbol;Acc:HGNC:7165] |
| ENSG00000124140 | HGLibA_44369 | 57 | -0.619108944 | SLC12A5 | solute carrier family 12 member 5 [Source:HGNC Symbol;Acc:HGNC:13818] |
| ENSG00000125386 | HGLibA_16363 | 57 | -0.619108944 | FAM193A | family with sequence similarity 193 member A [Source:HGNC Symbol;Acc:HGNC:16822] |
| ENSG00000125551 | HGLibA_37163 | 57 | -0.619108944 | PLGLB2 | plasminogen-like B2 [Source:HGNC Symbol;Acc:HGNC:9073] |
| ENSG00000127415 | HGLibA_22568 | 57 | -0.619108944 | IDUA | alpha-L- [Source:HGNC Symbol;Acc:HGNC:5391]" |
| ENSG00000128309 | HGLibA_29780 | 57 | -0.619108944 | MPST | mercaptopyruvate sulfurtransferase [Source:HGNC Symbol;Acc:HGNC:7223] |
| ENSG00000128342 | HGLibA_26473 | 57 | -0.619108944 | LIF | interleukin 6 family cytokine [Source:HGNC Symbol;Acc:HGNC:6596]" |
| ENSG00000128524 | HGLibA_03754 | 57 | -0.619108944 | ATP6V1F | ATPase H+ transporting V1 subunit F [Source:HGNC Symbol;Acc:HGNC:16832] |
| ENSG00000129450 | HGLibA_44153 | 57 | -0.619108944 | SIGLEC9 | sialic acid binding Ig like lectin 9 [Source:HGNC Symbol;Acc:HGNC:10878] |
| ENSG00000130182 | HGLibA_56987 | 57 | -0.619108944 | ZSCAN10 | zinc finger and SCAN domain containing 10 [Source:HGNC Symbol;Acc:HGNC:12997] |
| ENSG00000131471 | HGLibA_02274 | 57 | -0.619108944 | AOC3 | copper containing 3 [Source:HGNC Symbol;Acc:HGNC:550]" |
| ENSG00000132326 | HGLibA_36057 | 57 | -0.619108944 | PER2 | period circadian regulator 2 [Source:HGNC Symbol;Acc:HGNC:8846] |
| ENSG00000132463 | HGLibA_20381 | 57 | -0.619108944 | GRSF1 | G-rich RNA sequence binding factor 1 [Source:HGNC Symbol;Acc:HGNC:4610] |
| ENSG00000134186 | HGLibA_38724 | 57 | -0.619108944 | PRPF38B | pre-mRNA processing factor 38B [Source:HGNC Symbol;Acc:HGNC:25512] |
| ENSG00000134716 | HGLibA_12136 | 57 | -0.619108944 | CYP2J2 | cytochrome P450 family 2 subfamily J member 2 [Source:HGNC Symbol;Acc:HGNC:2634] |
| ENSG00000135423 | HGLibA_19329 | 57 | -0.619108944 | GLS2 | glutaminase 2 [Source:HGNC Symbol;Acc:HGNC:29570] |
| ENSG00000135525 | HGLibA_28231 | 57 | -0.619108944 | MAP7 | microtubule associated protein 7 [Source:HGNC Symbol;Acc:HGNC:6869] |
| ENSG00000136371 | HGLibA_30391 | 57 | -0.619108944 | MTHFS | methenyltetrahydrofolate synthetase [Source:HGNC Symbol;Acc:HGNC:7437] |
| ENSG00000137507 | HGLibA_27315 | 57 | -0.619108944 | LRRC32 | leucine rich repeat containing 32 [Source:HGNC Symbol;Acc:HGNC:4161] |
| ENSG00000137764 | HGLibA_28152 | 57 | -0.619108944 | MAP2K5 | mitogen-activated protein kinase kinase 5 [Source:HGNC Symbol;Acc:HGNC:6845] |
| ENSG00000137801 | HGLibA_49323 | 57 | -0.619108944 | THBS1 | thrombospondin 1 [Source:HGNC Symbol;Acc:HGNC:11785] |
| ENSG00000137871 | HGLibA_55731 | 57 | -0.619108944 | ZNF280D | zinc finger protein 280D [Source:HGNC Symbol;Acc:HGNC:25953] |
| ENSG00000138138 | HGLibA_03401 | 57 | -0.619108944 | ATAD1 | AAA domain containing 1 [Source:HGNC Symbol;Acc:HGNC:25903]" |
| ENSG00000138172 | HGLibA_07016 | 57 | -0.619108944 | CALHM2 | calcium homeostasis modulator family member 2 [Source:HGNC Symbol;Acc:HGNC:23493] |
| ENSG00000138600 | HGLibA_46776 | 57 | -0.619108944 | SPPL2A | signal peptide peptidase like 2A [Source:HGNC Symbol;Acc:HGNC:30227] |
| ENSG00000138642 | HGLibA_21169 | 57 | -0.619108944 | HERC6 | HECT and RLD domain containing E3 ubiquitin protein ligase family member 6 [Source:HGNC Symbol;Acc:HGNC:26072] |
| ENSG00000138867 | HGLibA_20650 | 57 | -0.619108944 | GUCD1 | guanylyl cyclase domain containing 1 [Source:HGNC Symbol;Acc:HGNC:14237] |
| ENSG00000140459 | HGLibA_12051 | 57 | -0.619108944 | CYP11A1 | cytochrome P450 family 11 subfamily A member 1 [Source:HGNC Symbol;Acc:HGNC:2590] |
| ENSG00000140553 | HGLibA_53004 | 57 | -0.619108944 | UNC45A | unc-45 myosin chaperone A [Source:HGNC Symbol;Acc:HGNC:30594] |
| ENSG00000141642 | HGLibA_14871 | 57 | -0.619108944 | ELAC1 | elaC ribonuclease Z 1 [Source:HGNC Symbol;Acc:HGNC:14197] |
| ENSG00000141994 | HGLibA_14121 | 57 | -0.619108944 | DUS3L | dihydrouridine synthase 3 like [Source:HGNC Symbol;Acc:HGNC:26920] |
| ENSG00000143315 | HGLibA_36567 | 57 | -0.619108944 | PIGM | phosphatidylinositol glycan anchor biosynthesis class M [Source:HGNC Symbol;Acc:HGNC:18858] |
| ENSG00000144331 | HGLibA_55899 | 57 | -0.619108944 | ZNF385B | zinc finger protein 385B [Source:HGNC Symbol;Acc:HGNC:26332] |
| ENSG00000144455 | HGLibA_47771 | 57 | -0.619108944 | SUMF1 | sulfatase modifying factor 1 [Source:HGNC Symbol;Acc:HGNC:20376] |
| ENSG00000144848 | HGLibA_03484 | 57 | -0.619108944 | ATG3 | autophagy related 3 [Source:HGNC Symbol;Acc:HGNC:20962] |
| ENSG00000145113 | HGLibA_30547 | 57 | -0.619108944 | MUC4 | cell surface associated [Source:HGNC Symbol;Acc:HGNC:7514]" |
| ENSG00000145287 | HGLibA_36943 | 57 | -0.619108944 | PLAC8 | placenta specific 8 [Source:HGNC Symbol;Acc:HGNC:19254] |
| ENSG00000145623 | HGLibA_34695 | 57 | -0.619108944 | OSMR | oncostatin M receptor [Source:HGNC Symbol;Acc:HGNC:8507] |
| ENSG00000145817 | HGLibA_54681 | 57 | -0.619108944 | YIPF5 | Yip1 domain family member 5 [Source:HGNC Symbol;Acc:HGNC:24877] |
| ENSG00000147604 | HGLibA_41994 | 57 | -0.619108944 | RPL7 | ribosomal protein L7 [Source:HGNC Symbol;Acc:HGNC:10363] |
| ENSG00000148337 | HGLibA_09750 | 57 | -0.619108944 | CIZ1 | CDKN1A interacting zinc finger protein 1 [Source:HGNC Symbol;Acc:HGNC:16744] |
| ENSG00000148600 | HGLibA_08699 | 57 | -0.619108944 | CDHR1 | cadherin related family member 1 [Source:HGNC Symbol;Acc:HGNC:14550] |
| ENSG00000151923 | HGLibA_49436 | 57 | -0.619108944 | TIAL1 | TIA1 cytotoxic granule associated RNA binding protein like 1 [Source:HGNC Symbol;Acc:HGNC:11804] |
| ENSG00000152661 | HGLibA_19135 | 57 | -0.619108944 | GJA1 | gap junction protein alpha 1 [Source:HGNC Symbol;Acc:HGNC:4274] |
| ENSG00000153487 | HGLibA_23282 | 57 | -0.619108944 | ING1 | inhibitor of growth family member 1 [Source:HGNC Symbol;Acc:HGNC:6062] |
| ENSG00000153498 | HGLibA_46340 | 57 | -0.619108944 | SPACA7 | sperm acrosome associated 7 [Source:HGNC Symbol;Acc:HGNC:29575] |
| ENSG00000155265 | HGLibA_19636 | 57 | -0.619108944 | GOLGA7B | golgin A7 family member B [Source:HGNC Symbol;Acc:HGNC:31668] |
| ENSG00000156140 | HGLibA_00828 | 57 | -0.619108944 | ADAMTS3 | ADAM metallopeptidase with thrombospondin type 1 motif 3 [Source:HGNC Symbol;Acc:HGNC:219] |
| ENSG00000157483 | HGLibA_30833 | 57 | -0.619108944 | MYO1E | myosin IE [Source:HGNC Symbol;Acc:HGNC:7599] |
| ENSG00000157600 | HGLibA_50033 | 57 | -0.619108944 | TMEM164 | transmembrane protein 164 [Source:HGNC Symbol;Acc:HGNC:26217] |
| ENSG00000157954 | HGLibA_54317 | 57 | -0.619108944 | WIPI2 | phosphoinositide interacting 2 [Source:HGNC Symbol;Acc:HGNC:32225]" |
| ENSG00000158716 | HGLibA_14167 | 57 | -0.619108944 | DUSP23 | dual specificity phosphatase 23 [Source:HGNC Symbol;Acc:HGNC:21480] |
| ENSG00000158825 | HGLibA_08497 | 57 | -0.619108944 | CDA | cytidine deaminase [Source:HGNC Symbol;Acc:HGNC:1712] |
| ENSG00000159263 | HGLibA_44176 | 57 | -0.619108944 | SIM2 | SIM bHLH transcription factor 2 [Source:HGNC Symbol;Acc:HGNC:10883] |
| ENSG00000160161 | HGLibA_09712 | 57 | -0.619108944 | CILP2 | cartilage intermediate layer protein 2 [Source:HGNC Symbol;Acc:HGNC:24213] |
| ENSG00000160972 | HGLibA_38052 | 57 | -0.619108944 | PPP1R16A | protein phosphatase 1 regulatory subunit 16A [Source:HGNC Symbol;Acc:HGNC:14941] |
| ENSG00000162032 | HGLibA_46864 | 57 | -0.619108944 | SPSB3 | splA/ryanodine receptor domain and SOCS box containing 3 [Source:HGNC Symbol;Acc:HGNC:30629] |
| ENSG00000162373 | HGLibA_04411 | 57 | -0.619108944 | BEND5 | BEN domain containing 5 [Source:HGNC Symbol;Acc:HGNC:25668] |
| ENSG00000163154 | HGLibA_50725 | 57 | -0.619108944 | TNFAIP8L2 | TNF alpha induced protein 8 like 2 [Source:HGNC Symbol;Acc:HGNC:26277] |
| ENSG00000163344 | HGLibA_37340 | 57 | -0.619108944 | PMVK | phosphomevalonate kinase [Source:HGNC Symbol;Acc:HGNC:9141] |
| ENSG00000163513 | HGLibA_49225 | 57 | -0.619108944 | TGFBR2 | transforming growth factor beta receptor 2 [Source:HGNC Symbol;Acc:HGNC:11773] |
| ENSG00000163565 | HGLibA_22594 | 57 | -0.619108944 | IFI16 | interferon gamma inducible protein 16 [Source:HGNC Symbol;Acc:HGNC:5395] |
| ENSG00000163644 | HGLibA_37986 | 57 | -0.619108944 | PPM1K | Mg2+/Mn2+ dependent 1K [Source:HGNC Symbol;Acc:HGNC:25415]" |
| ENSG00000164053 | HGLibA_03815 | 57 | -0.619108944 | ATRIP | ATR interacting protein [Source:HGNC Symbol;Acc:HGNC:33499] |
| ENSG00000164631 | HGLibA_55450 | 57 | -0.619108944 | ZNF12 | zinc finger protein 12 [Source:HGNC Symbol;Acc:HGNC:12902] |
| ENSG00000164930 | HGLibA_18279 | 57 | -0.619108944 | FZD6 | frizzled class receptor 6 [Source:HGNC Symbol;Acc:HGNC:4044] |
| ENSG00000165462 | HGLibA_36450 | 57 | -0.619108944 | PHOX2A | paired like homeobox 2A [Source:HGNC Symbol;Acc:HGNC:691] |
| ENSG00000166143 | HGLibA_38044 | 57 | -0.619108944 | PPP1R14D | protein phosphatase 1 regulatory inhibitor subunit 14D [Source:HGNC Symbol;Acc:HGNC:14953] |
| ENSG00000166159 | HGLibA_27503 | 57 | -0.619108944 | LRTM2 | leucine rich repeats and transmembrane domains 2 [Source:HGNC Symbol;Acc:HGNC:32443] |
| ENSG00000166900 | HGLibA_47644 | 57 | -0.619108944 | STX3 | syntaxin 3 [Source:HGNC Symbol;Acc:HGNC:11438] |
| ENSG00000166930 | HGLibA_30155 | 57 | -0.619108944 | MS4A5 | membrane spanning 4-domains A5 [Source:HGNC Symbol;Acc:HGNC:13374] |
| ENSG00000167771 | HGLibA_40714 | 57 | -0.619108944 | RCOR2 | REST corepressor 2 [Source:HGNC Symbol;Acc:HGNC:27455] |
| ENSG00000168032 | HGLibA_15202 | 57 | -0.619108944 | ENTPD3 | ectonucleoside triphosphate diphosphohydrolase 3 [Source:HGNC Symbol;Acc:HGNC:3365] |
| ENSG00000169217 | HGLibA_08329 | 57 | -0.619108944 | CD2BP2 | CD2 cytoplasmic tail binding protein 2 [Source:HGNC Symbol;Acc:HGNC:1656] |
| ENSG00000169504 | HGLibA_10031 | 57 | -0.619108944 | CLIC4 | chloride intracellular channel 4 [Source:HGNC Symbol;Acc:HGNC:13518] |
| ENSG00000169946 | HGLibA_55254 | 57 | -0.619108944 | ZFPM2 | FOG family member 2 [Source:HGNC Symbol;Acc:HGNC:16700]" |
| ENSG00000170442 | HGLibA_25572 | 57 | -0.619108944 | KRT86 | keratin 86 [Source:HGNC Symbol;Acc:HGNC:6463] |
| ENSG00000170482 | HGLibA_44607 | 57 | -0.619108944 | SLC23A1 | solute carrier family 23 member 1 [Source:HGNC Symbol;Acc:HGNC:10974] |
| ENSG00000170500 | HGLibA_27022 | 57 | -0.619108944 | LONRF2 | LON peptidase N-terminal domain and ring finger 2 [Source:HGNC Symbol;Acc:HGNC:24788] |
| ENSG00000170549 | HGLibA_23627 | 57 | -0.619108944 | IRX1 | iroquois homeobox 1 [Source:HGNC Symbol;Acc:HGNC:14358] |
| ENSG00000171033 | HGLibA_36819 | 57 | -0.619108944 | PKIA | cAMP-dependent protein kinase inhibitor alpha [Source:HGNC Symbol;Acc:HGNC:9017] |
| ENSG00000171161 | HGLibA_56507 | 57 | -0.619108944 | ZNF672 | zinc finger protein 672 [Source:HGNC Symbol;Acc:HGNC:26179] |
| ENSG00000171448 | HGLibA_54852 | 57 | -0.619108944 | ZBTB26 | zinc finger and BTB domain containing 26 [Source:HGNC Symbol;Acc:HGNC:23383] |
| ENSG00000171724 | HGLibA_53546 | 57 | -0.619108944 | VAT1L | vesicle amine transport 1 like [Source:HGNC Symbol;Acc:HGNC:29315] |
| ENSG00000171773 | HGLibA_33145 | 57 | -0.619108944 | NXNL1 | nucleoredoxin like 1 [Source:HGNC Symbol;Acc:HGNC:25179] |
| ENSG00000172175 | HGLibA_28033 | 57 | -0.619108944 | MALT1 | MALT1 paracaspase [Source:HGNC Symbol;Acc:HGNC:6819] |
| ENSG00000173992 | HGLibA_08183 | 57 | -0.619108944 | CCS | copper chaperone for superoxide dismutase [Source:HGNC Symbol;Acc:HGNC:1613] |
| ENSG00000175766 | HGLibA_14818 | 57 | -0.619108944 | EIF4E1B | eukaryotic translation initiation factor 4E family member 1B [Source:HGNC Symbol;Acc:HGNC:33179] |
| ENSG00000176219 | HGLibA_33581 | 57 | -0.619108944 | OR11H6 | olfactory receptor family 11 subfamily H member 6 [Source:HGNC Symbol;Acc:HGNC:15349] |
| ENSG00000176428 | HGLibA_53784 | 57 | -0.619108944 | VPS37D | ESCRT-I subunit [Source:HGNC Symbol;Acc:HGNC:18287]" |
| ENSG00000177354 | HGLibA_05090 | 57 | -0.619108944 | C10orf71 | chromosome 10 open reading frame 71 [Source:HGNC Symbol;Acc:HGNC:26973] |
| ENSG00000178726 | HGLibA_49318 | 57 | -0.619108944 | THBD | thrombomodulin [Source:HGNC Symbol;Acc:HGNC:11784] |
| ENSG00000179163 | HGLibA_18156 | 57 | -0.619108944 | FUCA1 | alpha-L-fucosidase 1 [Source:HGNC Symbol;Acc:HGNC:4006] |
| ENSG00000179361 | HGLibA_02893 | 57 | -0.619108944 | ARID3B | AT-rich interaction domain 3B [Source:HGNC Symbol;Acc:HGNC:14350] |
| ENSG00000179832 | HGLibA_29868 | 57 | -0.619108944 | MROH1 | maestro heat like repeat family member 1 [Source:HGNC Symbol;Acc:HGNC:26958] |
| ENSG00000179914 | HGLibA_23823 | 57 | -0.619108944 | ITLN1 | intelectin 1 [Source:HGNC Symbol;Acc:HGNC:18259] |
| ENSG00000180921 | HGLibA_16762 | 57 | -0.619108944 | FAM83H | family with sequence similarity 83 member H [Source:HGNC Symbol;Acc:HGNC:24797] |
| ENSG00000180998 | HGLibA_19903 | 57 | -0.619108944 | GPR137C | G protein-coupled receptor 137C [Source:HGNC Symbol;Acc:HGNC:25445] |
| ENSG00000182551 | HGLibA_00971 | 57 | -0.619108944 | ADI1 | acireductone dioxygenase 1 [Source:HGNC Symbol;Acc:HGNC:30576] |
| ENSG00000185499 | HGLibA_30521 | 57 | -0.619108944 | MUC1 | cell surface associated [Source:HGNC Symbol;Acc:HGNC:7508]" |
| ENSG00000186300 | HGLibA_56225 | 57 | -0.619108944 | ZNF555 | zinc finger protein 555 [Source:HGNC Symbol;Acc:HGNC:28382] |
| ENSG00000186814 | HGLibA_57015 | 57 | -0.619108944 | ZSCAN30 | zinc finger and SCAN domain containing 30 [Source:HGNC Symbol;Acc:HGNC:33517] |
| ENSG00000186998 | HGLibA_15043 | 57 | -0.619108944 | EMID1 | EMI domain containing 1 [Source:HGNC Symbol;Acc:HGNC:18036] |
| ENSG00000187082 | HGLibA_12835 | 57 | -0.619108944 | DEFB106B | defensin beta 106B [Source:HGNC Symbol;Acc:HGNC:28879] |
| ENSG00000187566 | HGLibA_31896 | 57 | -0.619108944 | NHLRC1 | NHL repeat containing E3 ubiquitin protein ligase 1 [Source:HGNC Symbol;Acc:HGNC:21576] |
| ENSG00000187733 | HGLibA_01846 | 57 | -0.619108944 | AMY1C | alpha 1C (salivary) [Source:HGNC Symbol;Acc:HGNC:476]" |
| ENSG00000187902 | HGLibA_44048 | 57 | -0.619108944 | SHISA7 | shisa family member 7 [Source:HGNC Symbol;Acc:HGNC:35409] |
| ENSG00000188859 | HGLibA_16732 | 57 | -0.619108944 | FAM78B | family with sequence similarity 78 member B [Source:HGNC Symbol;Acc:HGNC:13495] |
| ENSG00000188981 | HGLibA_30168 | 57 | -0.619108944 | MSANTD1 | Myb/SANT DNA binding domain containing 1 [Source:HGNC Symbol;Acc:HGNC:33741] |
| ENSG00000189091 | HGLibA_43709 | 57 | -0.619108944 | SF3B3 | splicing factor 3b subunit 3 [Source:HGNC Symbol;Acc:HGNC:10770] |
| ENSG00000196350 | HGLibA_56641 | 57 | -0.619108944 | ZNF729 | zinc finger protein 729 [Source:HGNC Symbol;Acc:HGNC:32464] |
| ENSG00000197008 | HGLibA_55478 | 57 | -0.619108944 | ZNF138 | zinc finger protein 138 [Source:HGNC Symbol;Acc:HGNC:12922] |
| ENSG00000197043 | HGLibA_02247 | 57 | -0.619108944 | ANXA6 | annexin A6 [Source:HGNC Symbol;Acc:HGNC:544] |
| ENSG00000197386 | HGLibA_22420 | 57 | -0.619108944 | HTT | huntingtin [Source:HGNC Symbol;Acc:HGNC:4851] |

| | | | | | |
|---|---|---|---|---|---|
| ENSG00000198053 | HGLibA_44201 | 57 | -0.619108944 | SIRPA | signal regulatory protein alpha [Source:HGNC Symbol;Acc:HGNC:9662] |
| ENSG00000198062 | HGLibA_37739 | 57 | -0.619108944 | POTEH | POTE ankyrin domain family member H [Source:HGNC Symbol;Acc:HGNC:133] |
| ENSG00000198083 | HGLibA_25852 | 57 | -0.619108944 | KRTAP9-9 | keratin associated protein 9-9 [Source:HGNC Symbol;Acc:HGNC:16773] |
| ENSG00000198482 | HGLibA_56798 | 57 | -0.619108944 | ZNF808 | zinc finger protein 808 [Source:HGNC Symbol;Acc:HGNC:33230] |
| ENSG00000203757 | HGLibA_34421 | 57 | -0.619108944 | OR6K3 | olfactory receptor family 6 subfamily K member 3 [Source:HGNC Symbol;Acc:HGNC:15030] |
| ENSG00000203972 | HGLibA_19380 | 57 | -0.619108944 | GLYATL3 | glycine-N-acyltransferase like 3 [Source:HGNC Symbol;Acc:HGNC:21349] |
| ENSG00000204612 | HGLibA_17820 | 57 | -0.619108944 | FOXB2 | forkhead box B2 [Source:HGNC Symbol;Acc:HGNC:23315] |
| ENSG00000204918 | HGLibA_38804 | 57 | -0.619108944 | PRR20B | proline rich 20B [Source:HGNC Symbol;Acc:HGNC:37220] |
| ENSG00000204983 | HGLibA_38900 | 57 | -0.619108944 | PRSS1 | serine protease 1 [Source:HGNC Symbol;Acc:HGNC:9475] |
| ENSG00000205002 | HGLibA_00065 | 57 | -0.619108944 | AARD | alanine and arginine rich domain containing protein [Source:HGNC Symbol;Acc:HGNC:33842] |
| ENSG00000205560 | HGLibA_11020 | 57 | -0.619108944 | CPT1B | carnitine palmitoyltransferase 1B [Source:HGNC Symbol;Acc:HGNC:2329] |
| ENSG00000206474 | HGLibA_33486 | 57 | -0.619108944 | OR10C1 | olfactory receptor family 10 subfamily C member 1 (gene/pseudogene) [Source:HGNC Symbol;Acc:HGNC:8165] |
| ENSG00000214078 | HGLibA_10953 | 57 | -0.619108944 | CPNE1 | copine 1 [Source:HGNC Symbol;Acc:HGNC:2314] |
| ENSG00000214357 | HGLibA_31724 | 57 | -0.619108944 | NEURL1B | neuralized E3 ubiquitin protein ligase 1B [Source:HGNC Symbol;Acc:HGNC:35422] |
| ENSG00000215545 | HGLibA_12860 | 57 | -0.619108944 | DEFB116 | defensin beta 116 [Source:HGNC Symbol;Acc:HGNC:18097] |
| ENSG00000215704 | HGLibA_08995 | 57 | -0.619108944 | CELA2B | chymotrypsin like elastase family member 2B [Source:HGNC Symbol;Acc:HGNC:29995] |
| ENSG00000225899 | HGLibA_17998 | 57 | -0.619108944 | FRG2B | FSHD region gene 2 family member B [Source:HGNC Symbol;Acc:HGNC:33518] |
| ENSG00000227151 | HGLibA_38810 | 57 | -0.619108944 | PRR20D | proline rich 20D [Source:HGNC Symbol;Acc:HGNC:37222] |
| ENSG00000248405 | HGLibA_38844 | 57 | -0.619108944 | PRR5-ARHGAP8 | PRR5-ARHGAP8 readthrough [Source:HGNC Symbol;Acc:HGNC:34512] |
| ENSG00000253117 | HGLibA_33238 | 57 | -0.619108944 | OC90 | otoconin 90 [Source:HGNC Symbol;Acc:HGNC:8100] |
| ENSG00000261678 | HGLibA_43059 | 57 | -0.619108944 | SCRT1 | scratch family transcriptional repressor 1 [Source:HGNC Symbol;Acc:HGNC:15950] |
| ENSG00000265491 | HGLibA_41480 | 57 | -0.619108944 | RNF115 | ring finger protein 115 [Source:HGNC Symbol;Acc:HGNC:18154] |
| ENSG00000266200 | HGLibA_37361 | 57 | -0.619108944 | PNLIPRP2 | pancreatic lipase related protein 2 (gene/pseudogene) [Source:HGNC Symbol;Acc:HGNC:9157] |
| ENSG00000268738 | HGLibA_22238 | 57 | -0.619108944 | HSFX2 | X-linked 2 [Source:HGNC Symbol;Acc:HGNC:32701]" |
| ENSG00000269897 | HGLibA_10662 | 57 | -0.619108944 | COMMD3-BMI1 | COMMD3-BMI1 readthrough [Source:HGNC Symbol;Acc:HGNC:48326] |
| ENSG00000275835 | HGLibA_52317 | 57 | -0.619108944 | TUBGCP5 | tubulin gamma complex associated protein 5 [Source:HGNC Symbol;Acc:HGNC:18600] |
| ENSG00000276380 | HGLibA_52638 | 57 | -0.619108944 | UBE2NL | ubiquitin conjugating enzyme E2 N like (gene/pseudogene) [Source:HGNC Symbol;Acc:HGNC:31710] |
| ENSG00000288709 | HGLibA_15890 | 57 | -0.619108944 | F8A2 | - |
| FOPNL | HGLibA_17793 | 57 | -0.619108944 | FOPNL | - |
| hsa-mir-126 | HGLibA_57457 | 57 | -0.619108944 | hsa-mir-126 | - |
| hsa-mir-1271 | HGLibA_57523 | 57 | -0.619108944 | hsa-mir-1271 | - |
| hsa-mir-18b | HGLibA_58024 | 57 | -0.619108944 | hsa-mir-18b | - |
| hsa-mir-208a | HGLibA_58197 | 57 | -0.619108944 | hsa-mir-208a | - |
| hsa-mir-3115 | HGLibA_58498 | 57 | -0.619108944 | hsa-mir-3115 | - |
| hsa-mir-3163 | HGLibA_58730 | 57 | -0.619108944 | hsa-mir-3163 | - |
| hsa-mir-3165 | HGLibA_58739 | 57 | -0.619108944 | hsa-mir-3165 | - |
| hsa-mir-3174 | HGLibA_58771 | 57 | -0.619108944 | hsa-mir-3174 | - |
| hsa-mir-339 | HGLibA_59006 | 57 | -0.619108944 | hsa-mir-339 | - |
| hsa-mir-3667 | HGLibA_59221 | 57 | -0.619108944 | hsa-mir-3667 | - |
| hsa-mir-3688-1 | HGLibA_59304 | 57 | -0.619108944 | hsa-mir-3688-1 | - |
| hsa-mir-3689e | HGLibA_59332 | 57 | -0.619108944 | hsa-mir-3689e | - |
| hsa-mir-411 | HGLibA_59685 | 57 | -0.619108944 | hsa-mir-411 | - |
| hsa-mir-4252 | HGLibA_59714 | 57 | -0.619108944 | hsa-mir-4252 | - |
| hsa-mir-4304 | HGLibA_59929 | 57 | -0.619108944 | hsa-mir-4304 | - |
| hsa-mir-450a-2 | HGLibA_60478 | 57 | -0.619108944 | hsa-mir-450a-2 | - |
| hsa-mir-4513 | HGLibA_60496 | 57 | -0.619108944 | hsa-mir-4513 | - |
| hsa-mir-4641 | HGLibA_60668 | 57 | -0.619108944 | hsa-mir-4641 | - |
| hsa-mir-4685 | HGLibA_60850 | 57 | -0.619108944 | hsa-mir-4685 | - |
| hsa-mir-4720 | HGLibA_60978 | 57 | -0.619108944 | hsa-mir-4720 | - |
| hsa-mir-4735 | HGLibA_61039 | 57 | -0.619108944 | hsa-mir-4735 | - |
| hsa-mir-4740 | HGLibA_61058 | 57 | -0.619108944 | hsa-mir-4740 | - |
| hsa-mir-5094 | HGLibA_61535 | 57 | -0.619108944 | hsa-mir-5094 | - |
| hsa-mir-517a | HGLibA_61623 | 57 | -0.619108944 | hsa-mir-517a | - |
| hsa-mir-542 | HGLibA_61818 | 57 | -0.619108944 | hsa-mir-542 | - |
| hsa-mir-548c | HGLibA_61956 | 57 | -0.619108944 | hsa-mir-548c | - |
| hsa-mir-5583-2 | HGLibA_62174 | 57 | -0.619108944 | hsa-mir-5583-2 | - |
| hsa-mir-5706 | HGLibA_62366 | 57 | -0.619108944 | hsa-mir-5706 | - |
| hsa-mir-582 | HGLibA_62428 | 57 | -0.619108944 | hsa-mir-582 | - |
| hsa-mir-6086 | HGLibA_62604 | 57 | -0.619108944 | hsa-mir-6086 | - |
| hsa-mir-611 | HGLibA_62634 | 57 | -0.619108944 | hsa-mir-611 | - |
| hsa-mir-6124 | HGLibA_62642 | 57 | -0.619108944 | hsa-mir-6124 | - |
| hsa-mir-642a | HGLibA_62809 | 57 | -0.619108944 | hsa-mir-642a | - |
| hsa-mir-6503 | HGLibA_62861 | 57 | -0.619108944 | hsa-mir-6503 | - |
| hsa-mir-657 | HGLibA_62961 | 57 | -0.619108944 | hsa-mir-657 | - |
| hsa-mir-660 | HGLibA_62975 | 57 | -0.619108944 | hsa-mir-660 | - |
| hsa-mir-6805 | HGLibA_63396 | 57 | -0.619108944 | hsa-mir-6805 | - |
